# Supplementary material for: Effect of implant abutment surface treatments on bacterial biofilm composition and structure
Source: J Oral Microbiol. 2025 Feb 5;17(1):2459922. doi: 10.1080/20002297.2025.2459922 (PMC11800344; doi:10.1080/20002297.2025.2459922)
Supplement: Supplementary material.zip [file Supplementary material 3.pdf › Supplementary material 3.pdf]

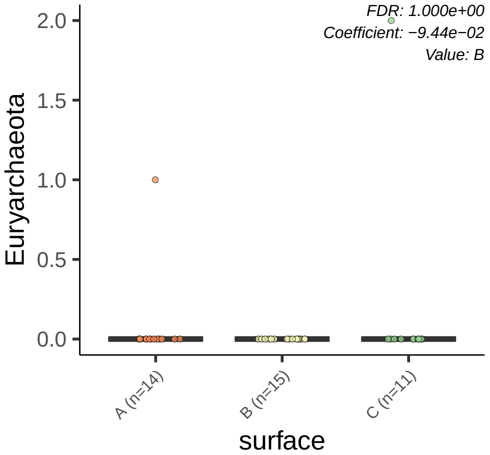

FDR: 1.000e+00
Coefficient: −9.44e−02
Value: B
Euryarchaeota
2.0
1.5
1.0
0.5
0.0
A (n=14)
B (n=15)
C (n=11)
surface

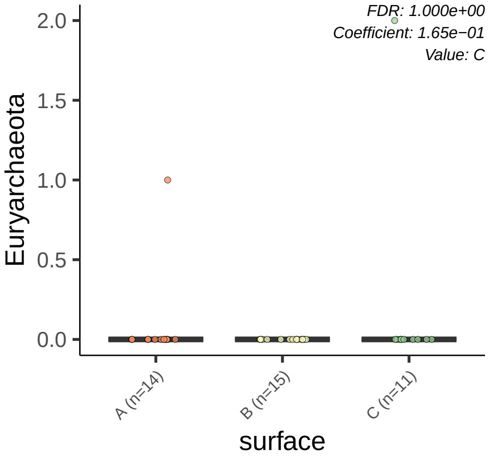

FDR: 1.000e+00
Coefficient: 1.65e−01
Value: C
Euryarchaeota
2.0
1.5
1.0
0.5
0.0
A (n=14)
B (n=15)
C (n=11)
surface

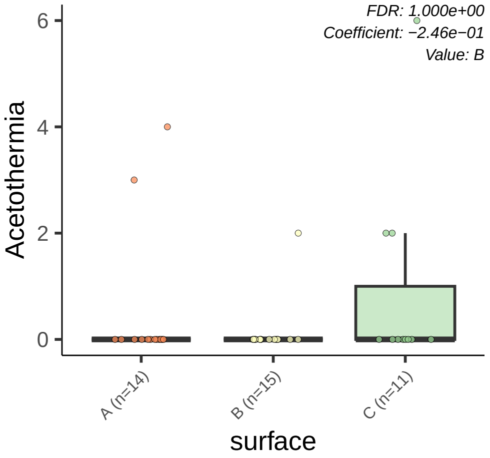
FDR: 1.000e+00
Coefficient: −2.46e−01
Value: B
Acetothermia
6
4
2
0
A (n=14)
B (n=15)
C (n=11)
surface

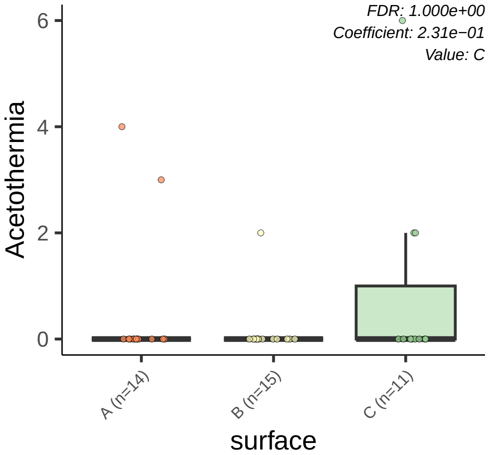

FDR: 1.000e+00
Coefficient: 2.31e−01
Value: C
Acetothermia
6
4
2
0
A (n=14)
B (n=15)
C (n=11)
surface

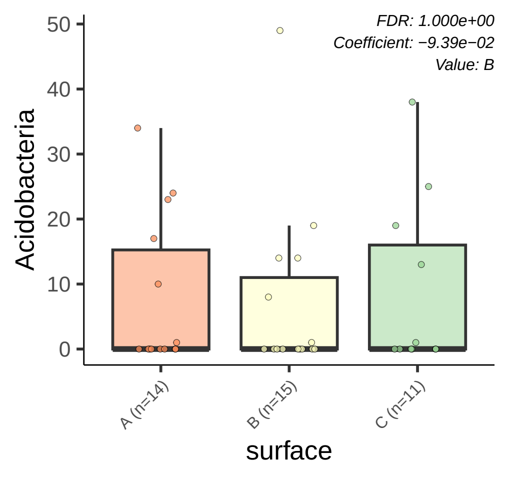
FDR: 1.000e+00
Coefficient: −9.39e−02
Value: B
Acidobacteria
0
10
20
30
40
50
A (n=14)
B (n=15)
C (n=11)
surface

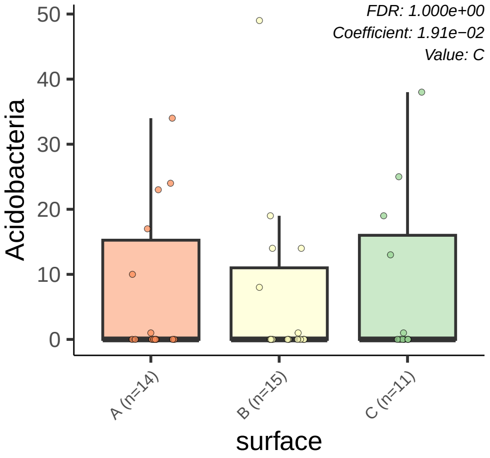
FDR: 1.000e+00
Coefficient: 1.91e−02
Value: C
Acidobacteria
0
10
20
30
40
50
A (n=14)
B (n=15)
C (n=11)
surface

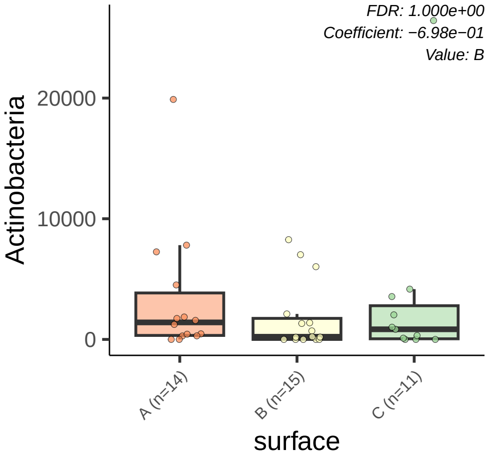

FDR: 1.000e+00
Coefficient: −6.98e−01
Value: B
Actinobacteria
20000
10000
0
A (n=14)
B (n=15)
C (n=11)
surface

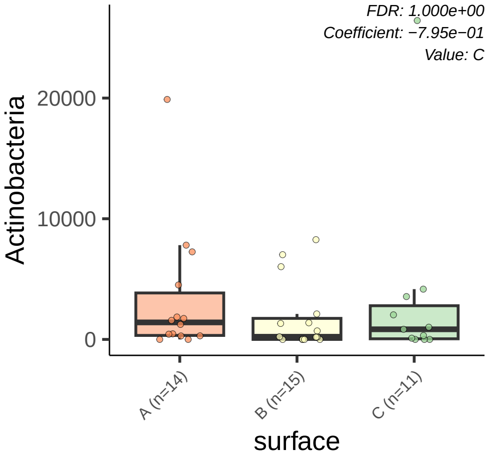

FDR: 1.000e+00
Coefficient: −7.95e−01
Value: C
Actinobacteria
20000
10000
0
A (n=14)
B (n=15)
C (n=11)
surface

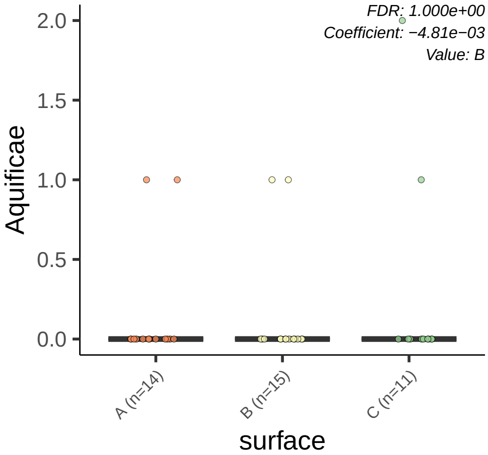

FDR: 1.000e+00
Coefficient: −4.81e−03
Value: B
Aquificae
2.0
1.5
1.0
0.5
0.0
A (n=14)
B (n=15)
C (n=11)
surface

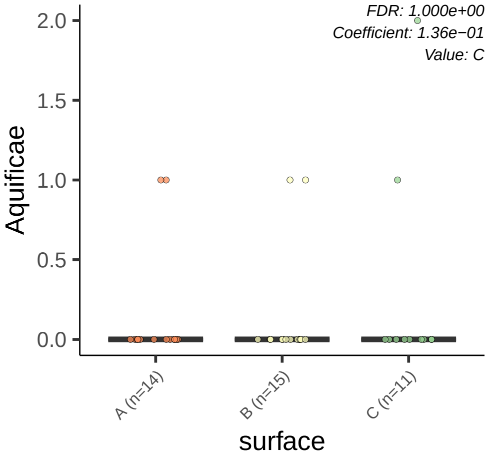
FDR: 1.000e+00
Coefficient: 1.36e−01
Value: C
Aquificae
2.0
1.5
1.0
0.5
0.0
A (n=14)
B (n=15)
C (n=11)
surface

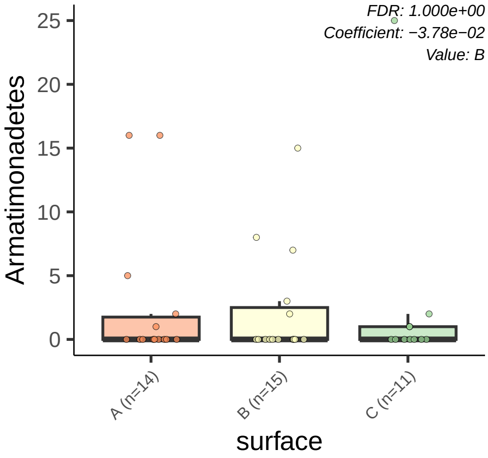
FDR: 1.000e+00
Coefficient: −3.78e−02
Value: B
Armatimonadetes
25
20
15
10
5
0
A (n=14)
B (n=15)
C (n=11)
surface

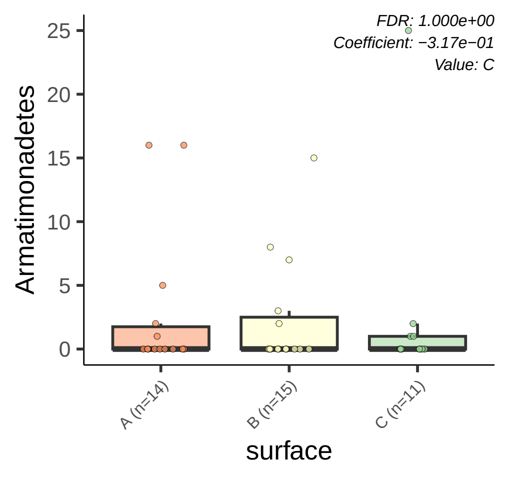
FDR: 1.000e+00
Coefficient: −3.17e−01
Value: C
Armatimonadetes
0
5
10
15
20
25
A (n=14)
B (n=15)
C (n=11)
surface

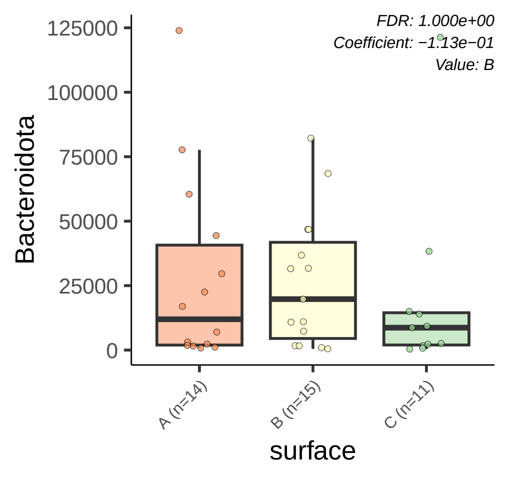
FDR: 1.000e+00
Coefficient: −1.13e−01
Value: B
Bacteroidota
125000
100000
75000
50000
25000
0
A (n=14)
B (n=15)
C (n=11)
surface

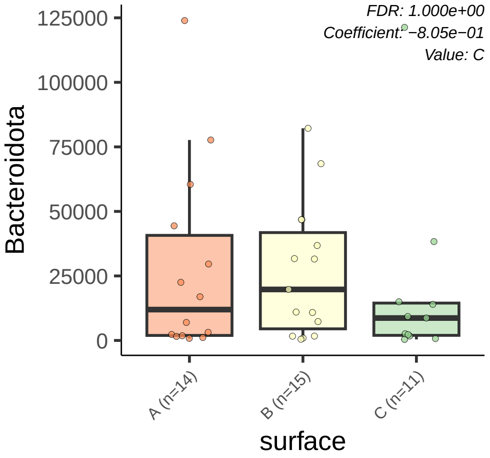
FDR: 1.000e+00
Coefficient: −8.05e−01
Value: C
Bacteroidota
125000
100000
75000
50000
25000
0
A (n=14)
B (n=15)
C (n=11)
surface

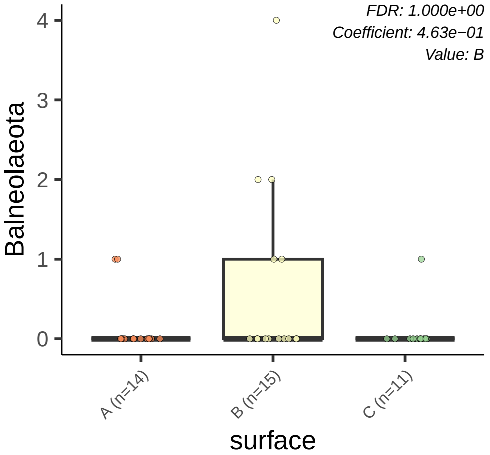
FDR: 1.000e+00
Coefficient: 4.63e−01
Value: B
Balneolaeota
0
1
2
3
4
A (n=14)
B (n=15)
C (n=11)
surface

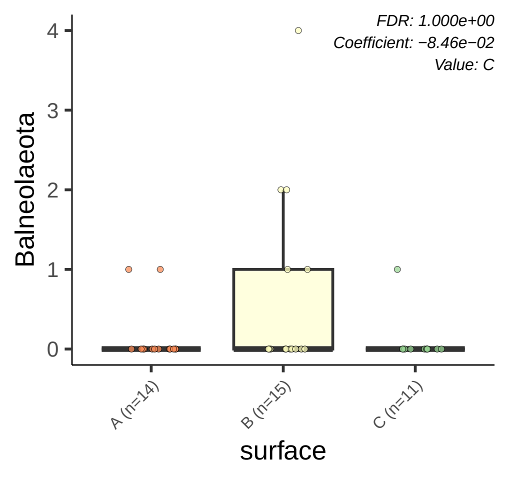
FDR: 1.000e+00
Coefficient: −8.46e−02
Value: C
Balneolaeota
0
1
2
3
4
A (n=14)
B (n=15)
C (n=11)
surface

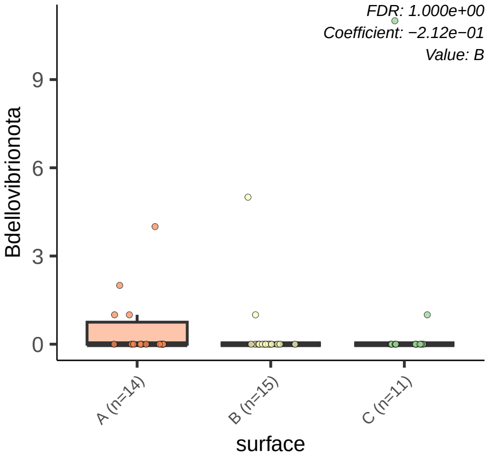
FDR: 1.000e+00
Coefficient: −2.12e−01
Value: B
Bdellovibrionota
9
6
3
0
A (n=14)
B (n=15)
C (n=11)
surface

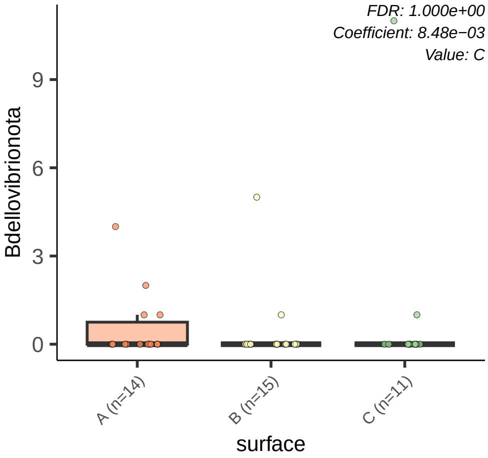
FDR: 1.000e+00
Coefficient: 8.48e−03
Value: C
Bdellovibrionota
9
6
3
0
A (n=14)
B (n=15)
C (n=11)
surface

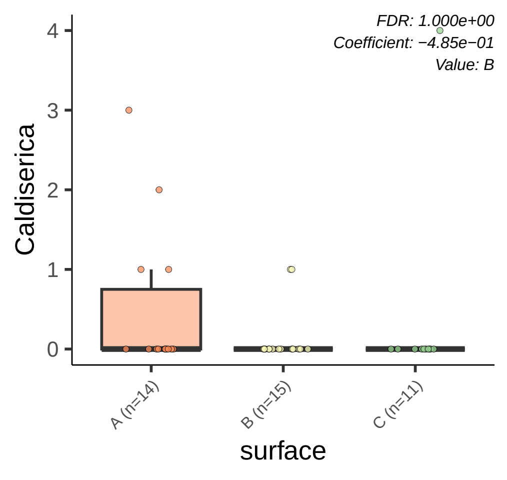

FDR: 1.000e+00
Coefficient: −4.85e−01
Value: B
Caldiserica
4
3
2
1
0
A (n=14)
B (n=15)
C (n=11)
surface

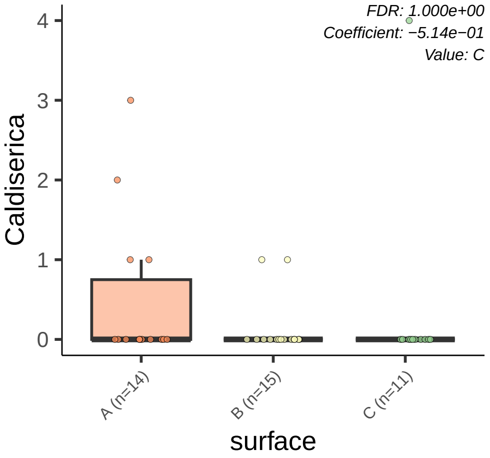
FDR: 1.000e+00
Coefficient: −5.14e−01
Value: C
Caldiserica
4
3
2
1
0
A (n=14)
B (n=15)
C (n=11)
surface

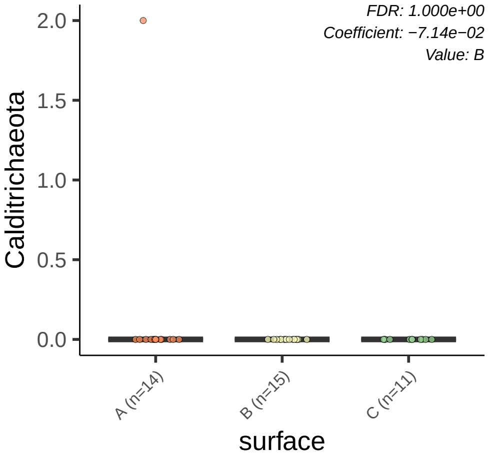
FDR: 1.000e+00
Coefficient: −7.14e−02
Value: B
Calditrichaeota
2.0
1.5
1.0
0.5
0.0
A (n=14)
B (n=15)
C (n=11)
surface

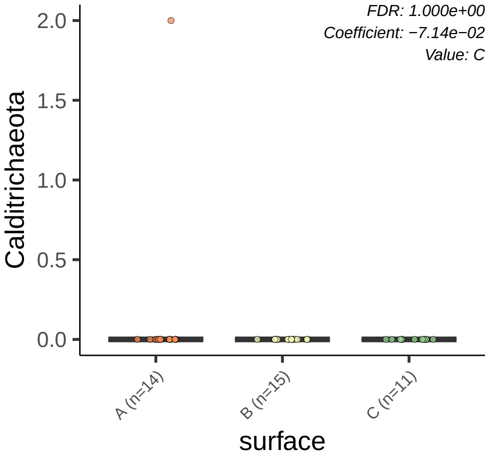

FDR: 1.000e+00
Coefficient: −7.14e−02
Value: C
Calditrichaeota
2.0
1.5
1.0
0.5
0.0
A (n=14)
B (n=15)
C (n=11)
surface

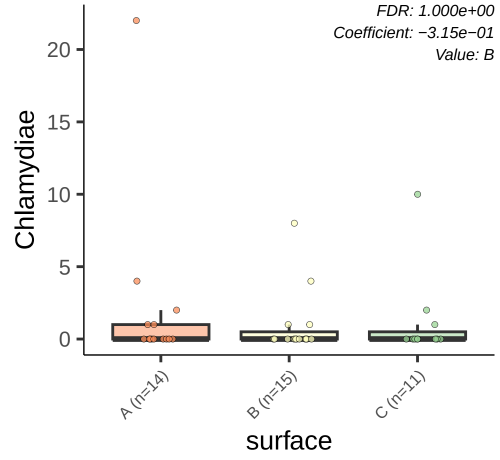

FDR: 1.000e+00
Coefficient: −3.15e−01
Value: B
Chlamydiae
0
5
10
15
20
A (n=14)
B (n=15)
C (n=11)
surface

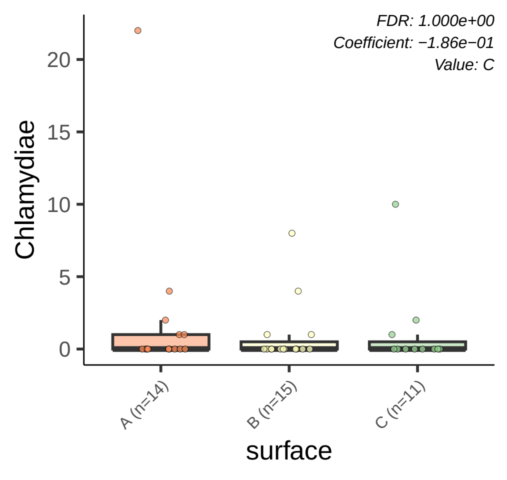

FDR: 1.000e+00
Coefficient: −1.86e−01
Value: C
Chlamydiae
0
5
10
15
20
A (n=14)
B (n=15)
C (n=11)
surface

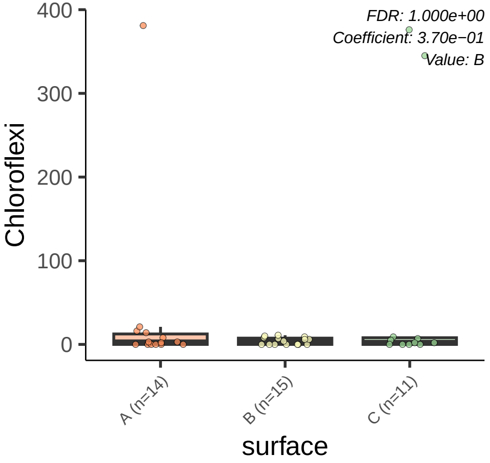
FDR: 1.000e+00
Coefficient: 3.70e−01
Value: B
Chloroflexi
400
300
200
100
0
A (n=14)
B (n=15)
C (n=11)
surface

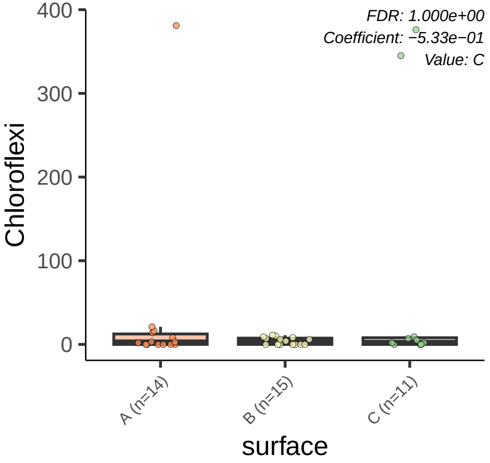

FDR: 1.000e+00
Coefficient: −5.33e−01
Value: C
Chloroflexi
400
300
200
100
0
A (n=14)
B (n=15)
C (n=11)
surface

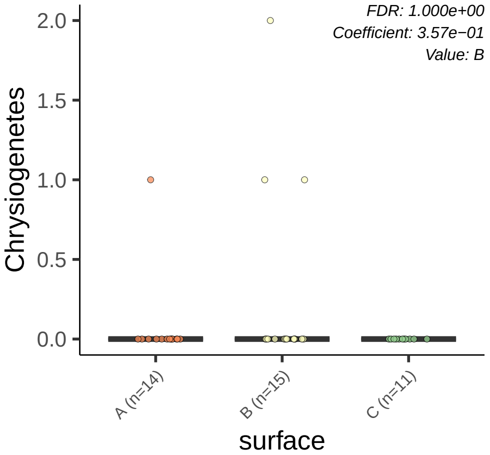

FDR: 1.000e+00
Coefficient: 3.57e−01
Value: B
Chrysiogenetes
2.0
1.5
1.0
0.5
0.0
A (n=14)
B (n=15)
C (n=11)
surface

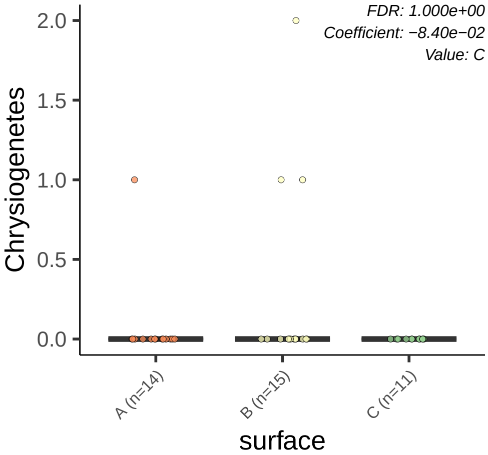
FDR: 1.000e+00
Coefficient: −8.40e−02
Value: C
Chrysiogenetes
2.0
1.5
1.0
0.5
0.0
A (n=14)
B (n=15)
C (n=11)
surface

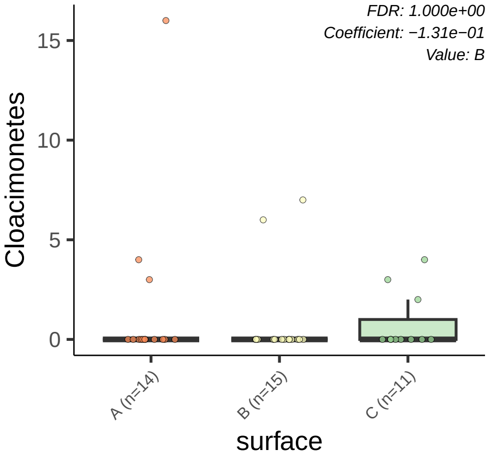
FDR: 1.000e+00
Coefficient: −1.31e−01
Value: B
Cloacimonetes
15
10
5
0
A (n=14)
B (n=15)
C (n=11)
surface

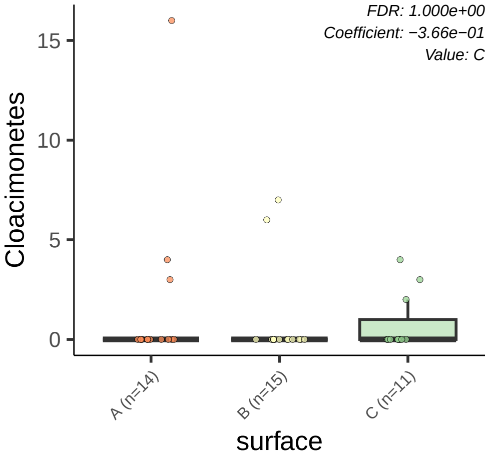

FDR: 1.000e+00
Coefficient: −3.66e−01
Value: C
Cloacimonetes
15
10
5
0
A (n=14)
B (n=15)
C (n=11)
surface

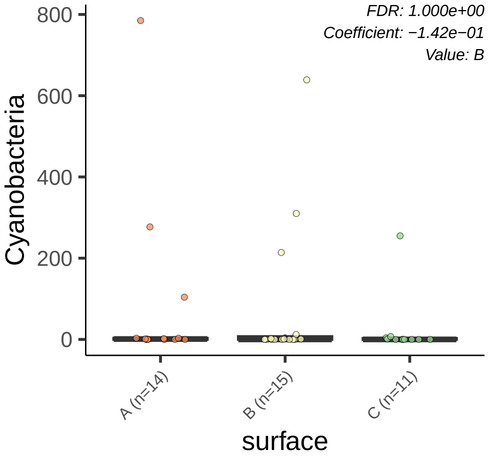

FDR: 1.000e+00
Coefficient: −1.42e−01
Value: B
Cyanobacteria
800
600
400
200
0
A (n=14)
B (n=15)
C (n=11)
surface

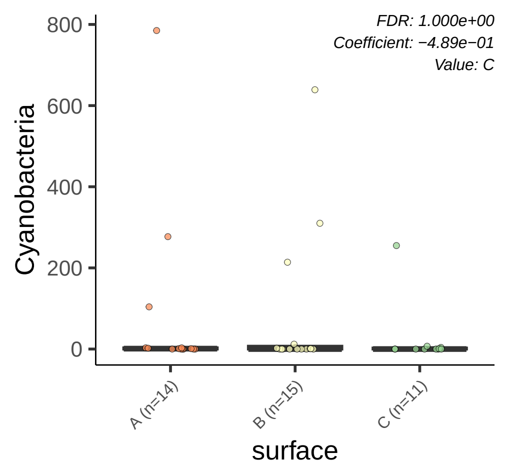

FDR: 1.000e+00
Coefficient: −4.89e−01
Value: C
Cyanobacteria
800
600
400
200
0
A (n=14)
B (n=15)
C (n=11)
surface

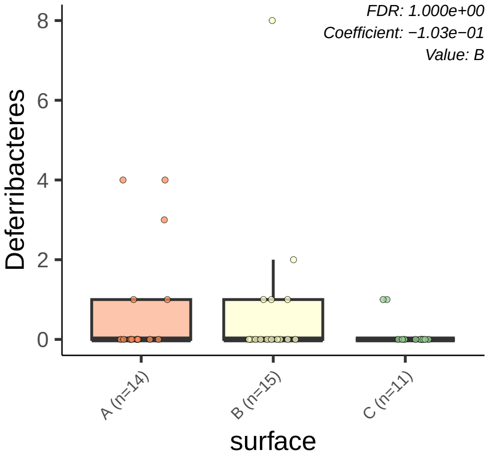
FDR: 1.000e+00
Coefficient: −1.03e−01
Value: B
Deferribacteres
8
6
4
2
0
A (n=14)
B (n=15)
C (n=11)
surface

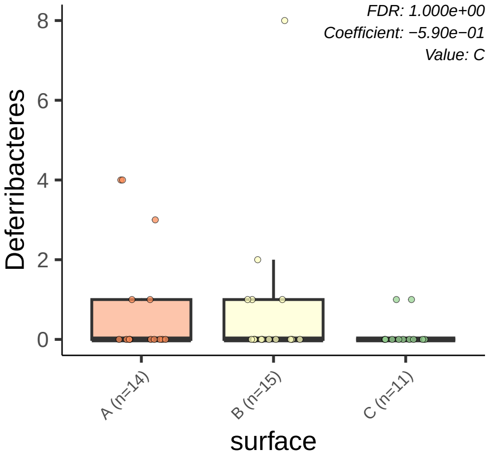

FDR: 1.000e+00
Coefficient: −5.90e−01
Value: C
Deferribacteres
0
2
4
6
8
A (n=14)
B (n=15)
C (n=11)
surface

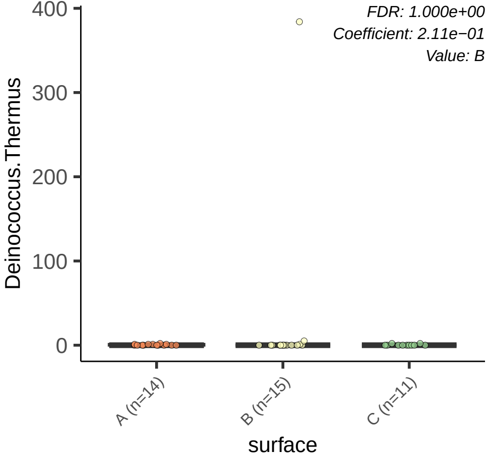
FDR: 1.000e+00
Coefficient: 2.11e−01
Value: B
Deinococcus.Thermus
400
300
200
100
0
A (n=14)
B (n=15)
C (n=11)
surface

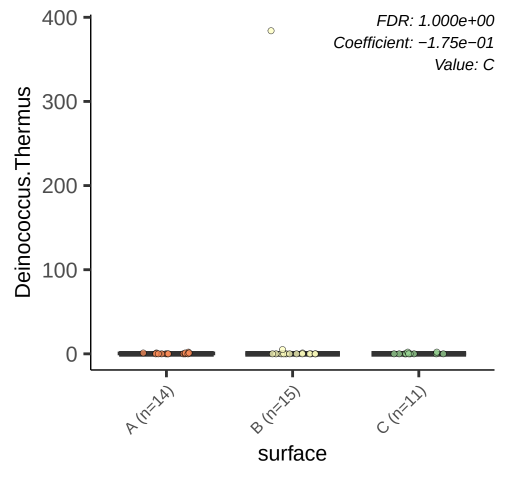

FDR: 1.000e+00
Coefficient: −1.75e−01
Value: C
Deinococcus.Thermus
400
300
200
100
0
A (n=14)
B (n=15)
C (n=11)
surface

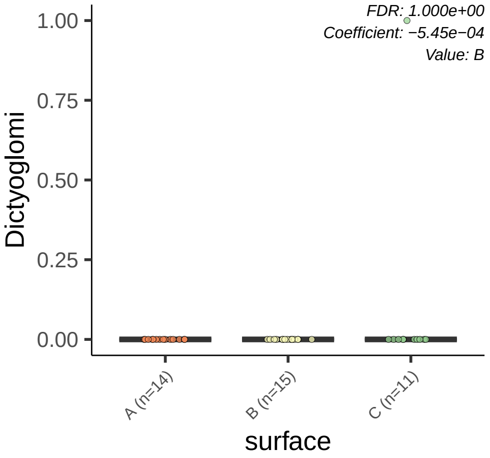
FDR: 1.000e+00
Coefficient: −5.45e−04
Value: B
Dictyoglomi
1.00
0.75
0.50
0.25
0.00
A (n=14)
B (n=15)
C (n=11)
surface

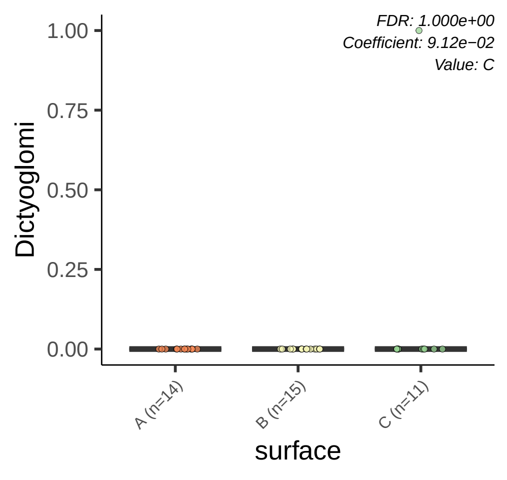
FDR: 1.000e+00
Coefficient: 9.12e−02
Value: C
1.00
0.75
0.50
0.25
0.00
Dictyoglomi
A (n=14)
B (n=15)
C (n=11)
surface

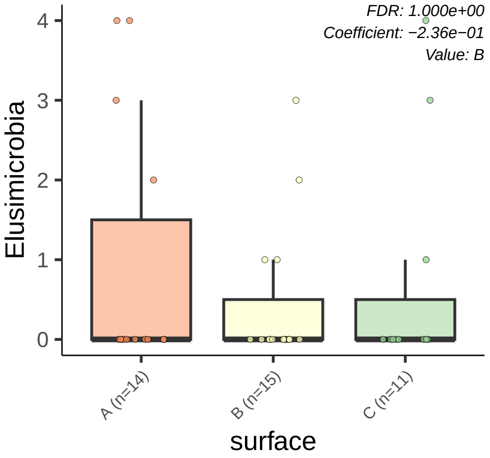

FDR: 1.000e+00
Coefficient: −2.36e−01
Value: B
Elusimicrobia
0
1
2
3
4
A (n=14)
B (n=15)
C (n=11)
surface

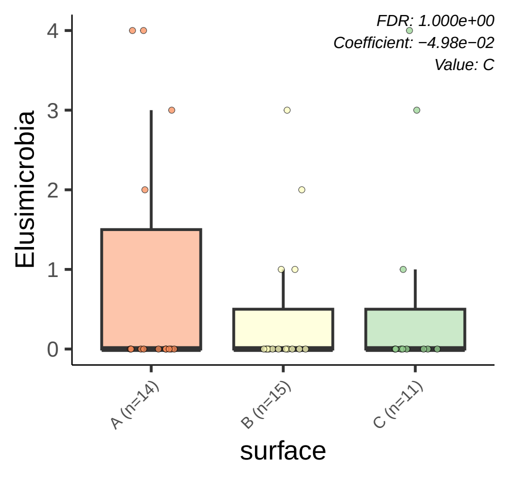
FDR: 1.000e+00
Coefficient: −4.98e−02
Value: C
Elusimicrobia
4
3
2
1
0
A (n=14)
B (n=15)
C (n=11)
surface

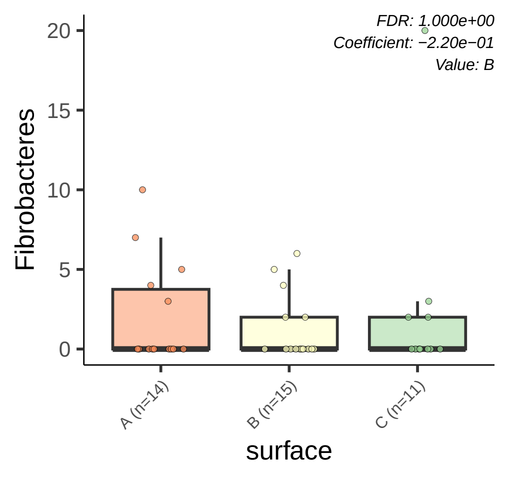

FDR: 1.000e+00
Coefficient: −2.20e−01
Value: B
Fibrobacteres
20
15
10
5
0
A (n=14)
B (n=15)
C (n=11)
surface

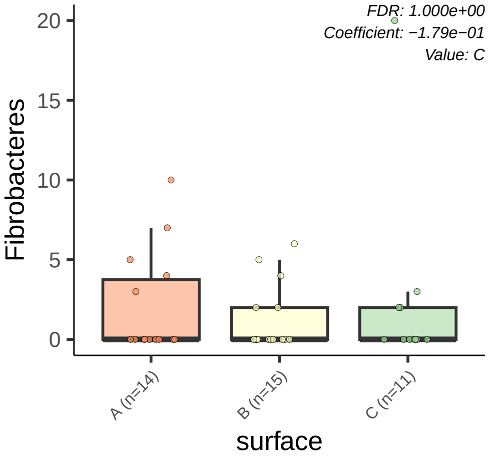

FDR: 1.000e+00
Coefficient: −1.79e−01
Value: C
Fibrobacteres
20
15
10
5
0
A (n=14)
B (n=15)
C (n=11)
surface

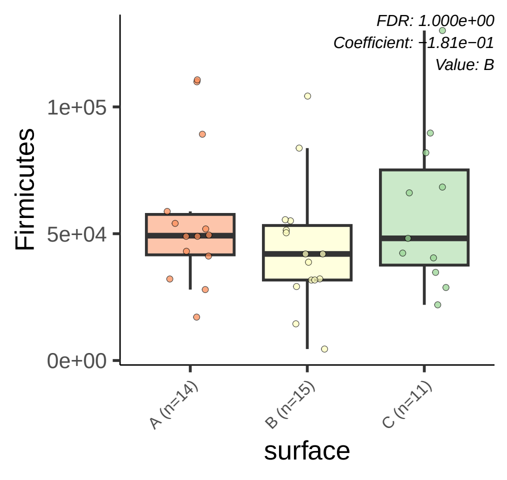
FDR: 1.000e+00
Coefficient: −1.81e−01
Value: B
Firmicutes
1e+05
5e+04
0e+00
A (n=14)
B (n=15)
C (n=11)
surface

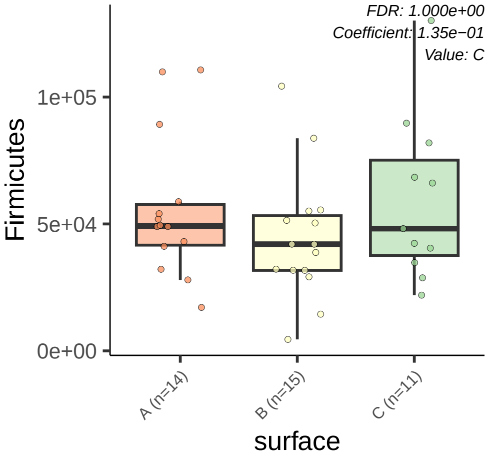

FDR: 1.000e+00
Coefficient: 1.35e−01
Value: C
Firmicutes
1e+05
5e+04
0e+00
A (n=14)
B (n=15)
C (n=11)
surface

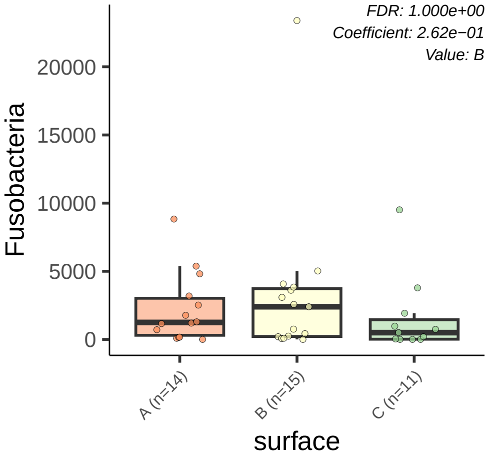
FDR: 1.000e+00
Coefficient: 2.62e−01
Value: B
Fusobacteria
20000
15000
10000
5000
0
A (n=14)
B (n=15)
C (n=11)
surface

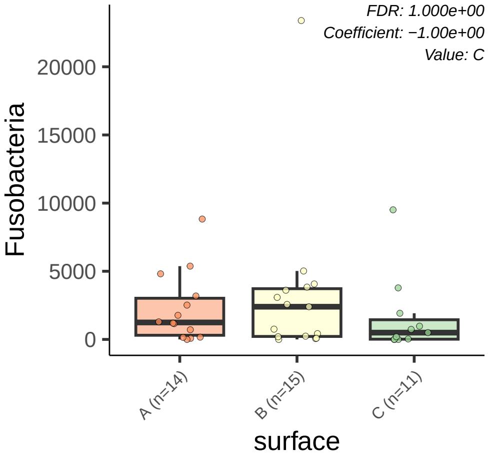
FDR: 1.000e+00
Coefficient: −1.00e+00
Value: C
Fusobacteria
20000
15000
10000
5000
0
A (n=14)
B (n=15)
C (n=11)
surface

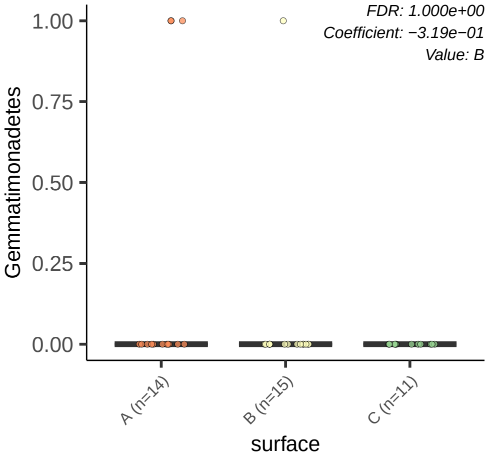

FDR: 1.000e+00
Coefficient: −3.19e−01
Value: B
Gemmatimonadetes
1.00
0.75
0.50
0.25
0.00
A (n=14)
B (n=15)
C (n=11)
surface

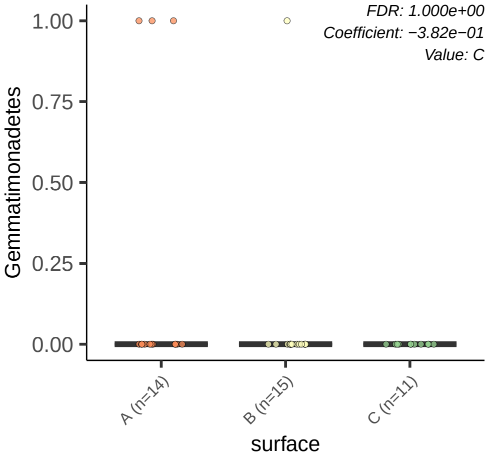
FDR: 1.000e+00
Coefficient: −3.82e−01
Value: C
Gemmatimonadetes
1.00
0.75
0.50
0.25
0.00
A (n=14)
B (n=15)
C (n=11)
surface

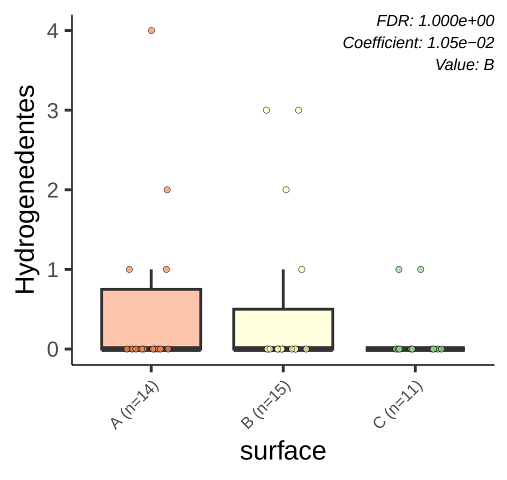
FDR: 1.000e+00
Coefficient: 1.05e−02
Value: B
Hydrogenedentes
4
3
2
1
0
A (n=14)
B (n=15)
C (n=11)
surface

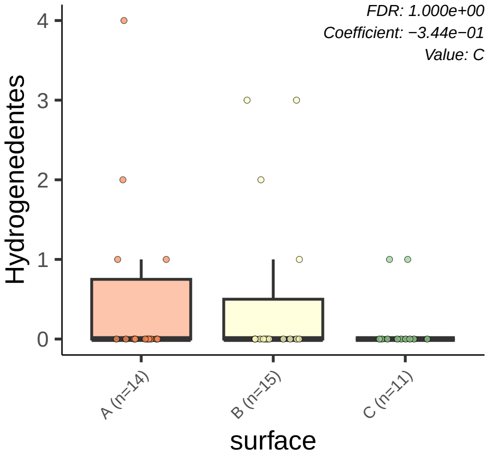
FDR: 1.000e+00
Coefficient: −3.44e−01
Value: C
Hydrogenedentes
4
3
2
1
0
A (n=14)
B (n=15)
C (n=11)
surface

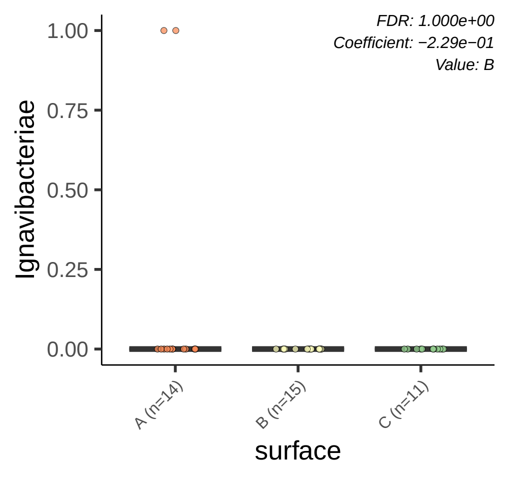
FDR: 1.000e+00
Coefficient: −2.29e−01
Value: B
Ignavibacteriae
1.00
0.75
0.50
0.25
0.00
A (n=14)
B (n=15)
C (n=11)
surface

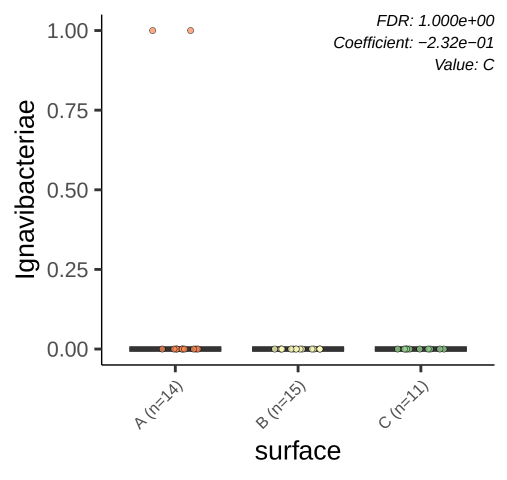
FDR: 1.000e+00
Coefficient: −2.32e−01
Value: C
Ignavibacteriae
1.00
0.75
0.50
0.25
0.00
A (n=14)
B (n=15)
C (n=11)
surface

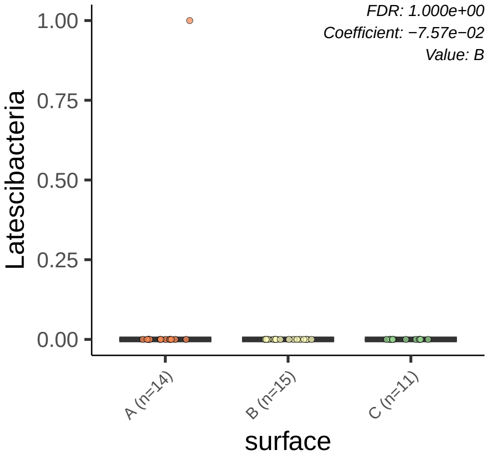
FDR: 1.000e+00
Coefficient: −7.57e−02
Value: B
Latescibacteria
1.00
0.75
0.50
0.25
0.00
A (n=14)
B (n=15)
C (n=11)
surface

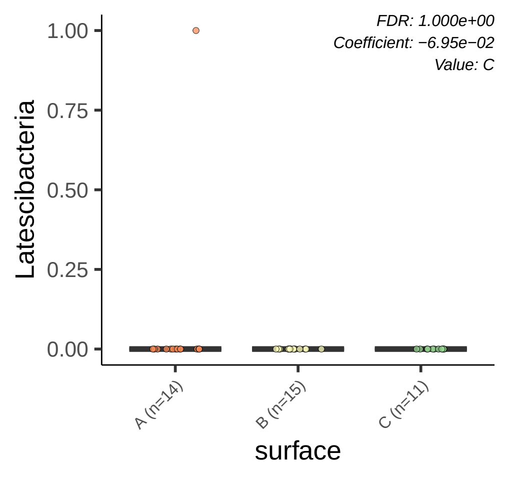
FDR: 1.000e+00
Coefficient: −6.95e−02
Value: C
Latescibacteria
1.00
0.75
0.50
0.25
0.00
A (n=14)
B (n=15)
C (n=11)
surface

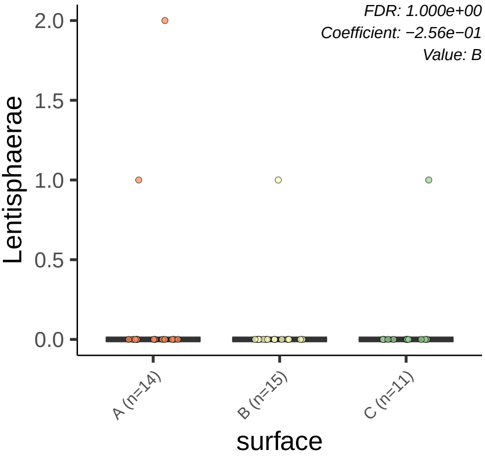
FDR: 1.000e+00
Coefficient: −2.56e−01
Value: B
Lentisphaerae
2.0
1.5
1.0
0.5
0.0
A (n=14)
B (n=15)
C (n=11)
surface

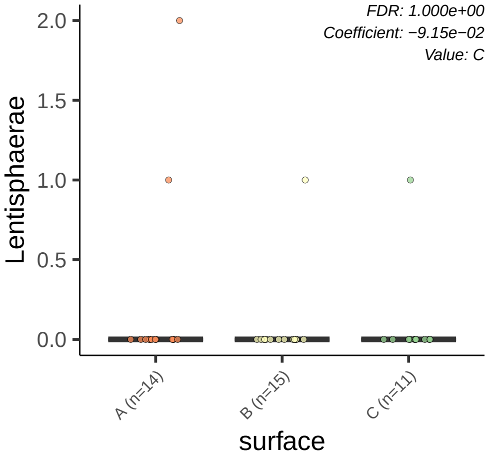
FDR: 1.000e+00
Coefficient: −9.15e−02
Value: C
Lentisphaerae
2.0
1.5
1.0
0.5
0.0
A (n=14)
B (n=15)
C (n=11)
surface

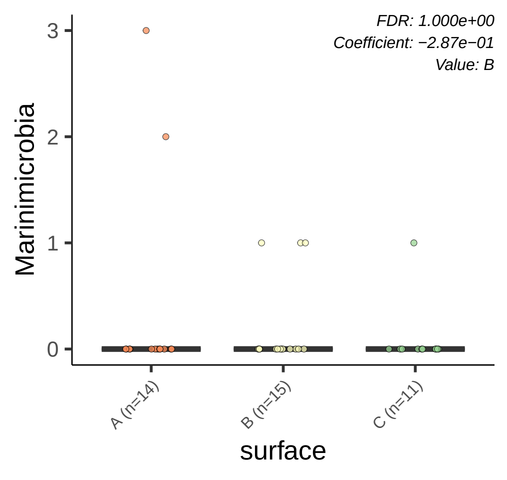

FDR: 1.000e+00
Coefficient: −2.87e−01
Value: B
Marinimicrobia
3
2
1
0
A (n=14)
B (n=15)
C (n=11)
surface

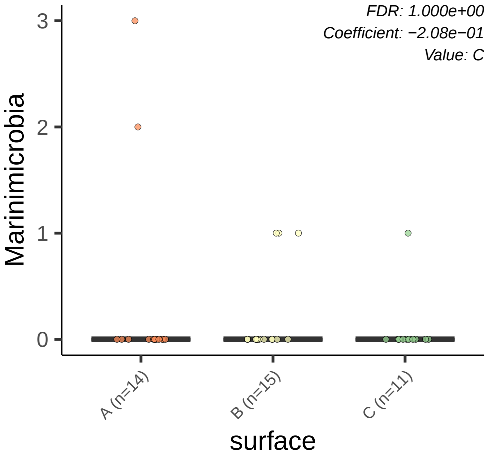
FDR: 1.000e+00
Coefficient: −2.08e−01
Value: C
Marinimicrobia
0
1
2
3
A (n=14)
B (n=15)
C (n=11)
surface

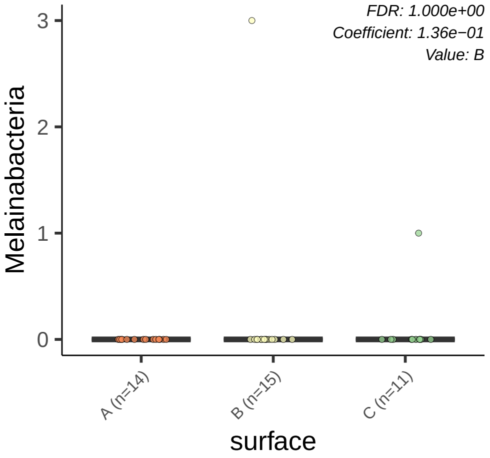

FDR: 1.000e+00
Coefficient: 1.36e−01
Value: B
Melainabacteria
3
2
1
0
A (n=14)
B (n=15)
C (n=11)
surface

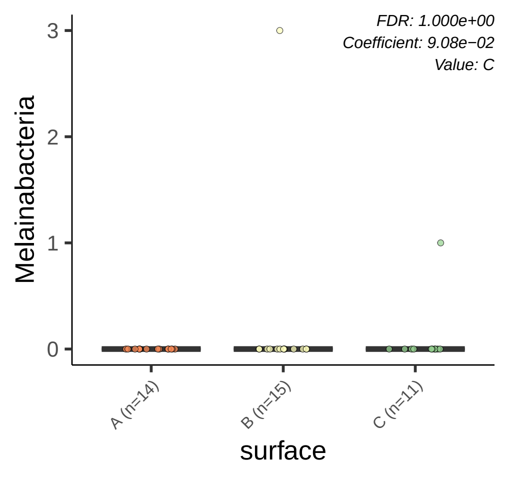

FDR: 1.000e+00
Coefficient: 9.08e−02
Value: C
Melainabacteria
3
2
1
0
A (n=14)
B (n=15)
C (n=11)
surface

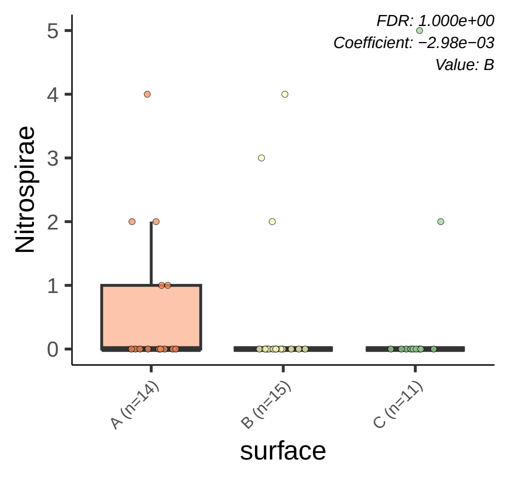
FDR: 1.000e+00
Coefficient: −2.98e−03
Value: B
Nitrospirae
5
4
3
2
1
0
A (n=14)
B (n=15)
C (n=11)
surface

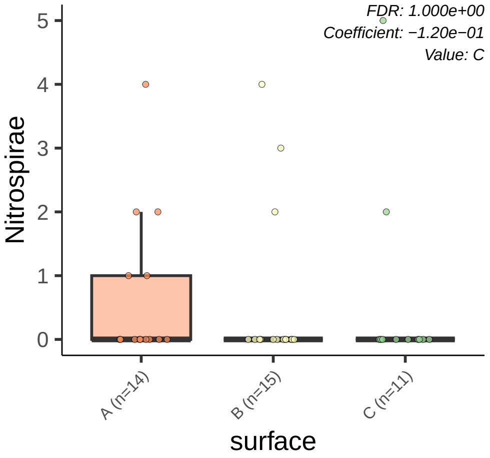

FDR: 1.000e+00
Coefficient: −1.20e−01
Value: C
Nitrospirae
0
1
2
3
4
5
A (n=14)
B (n=15)
C (n=11)
surface

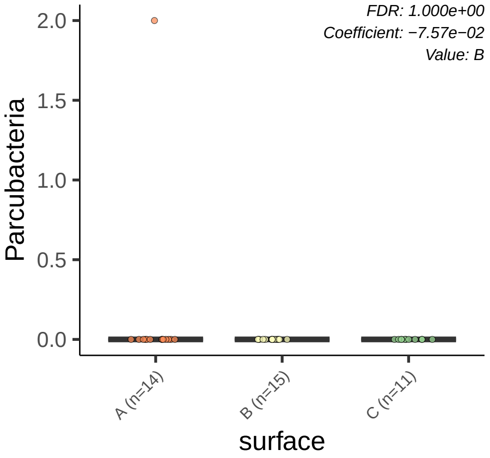
FDR: 1.000e+00
Coefficient: −7.57e−02
Value: B
Parcubacteria
2.0
1.5
1.0
0.5
0.0
A (n=14)
B (n=15)
C (n=11)
surface

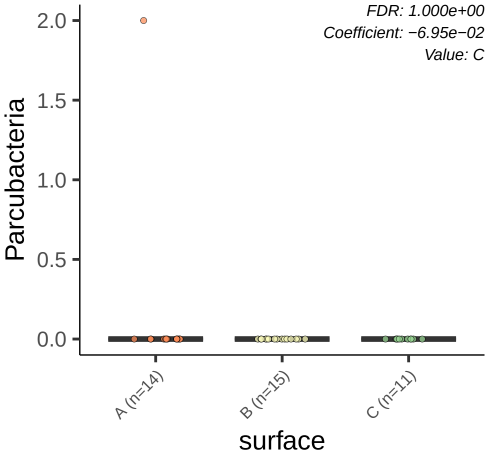

FDR: 1.000e+00
Coefficient: −6.95e−02
Value: C
Parcubacteria
2.0
1.5
1.0
0.5
0.0
A (n=14)
B (n=15)
C (n=11)
surface

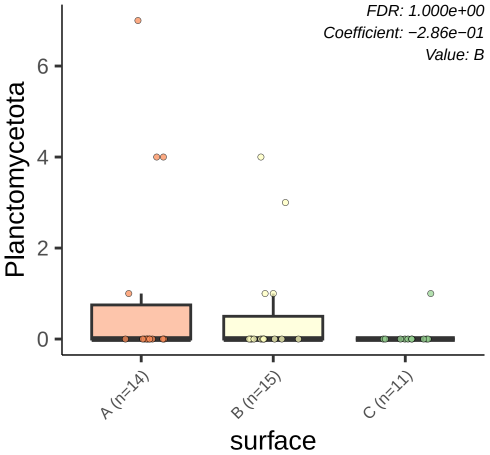

FDR: 1.000e+00
Coefficient: −2.86e−01
Value: B
Planctomycetota
0
2
4
6
A (n=14)
B (n=15)
C (n=11)
surface

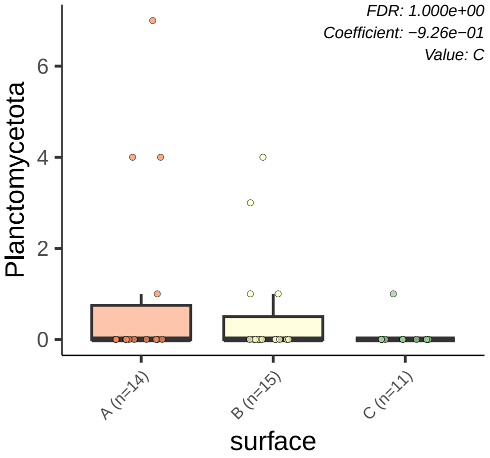
FDR: 1.000e+00
Coefficient: −9.26e−01
Value: C
Planctomycetota
0
2
4
6
A (n=14)
B (n=15)
C (n=11)
surface

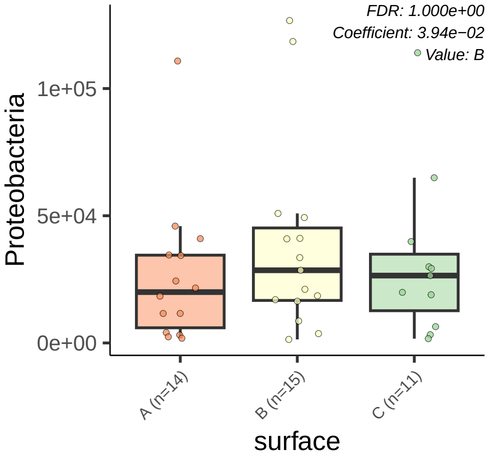

FDR: 1.000e+00
Coefficient: 3.94e−02
Value: B
Proteobacteria
1e+05
5e+04
0e+00
A (n=14)
B (n=15)
C (n=11)
surface

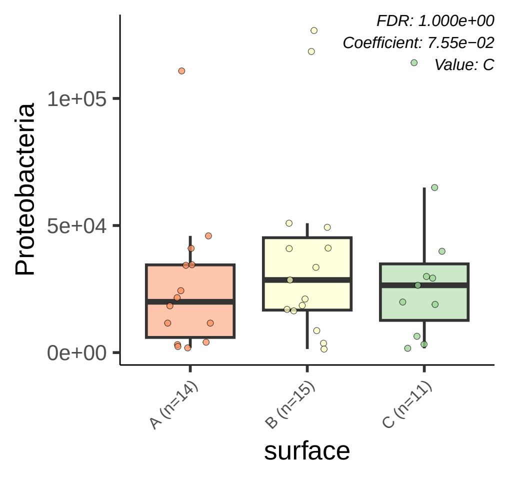
FDR: 1.000e+00
Coefficient: 7.55e−02
Value: C
Proteobacteria
1e+05
5e+04
0e+00
A (n=14)
B (n=15)
C (n=11)
surface

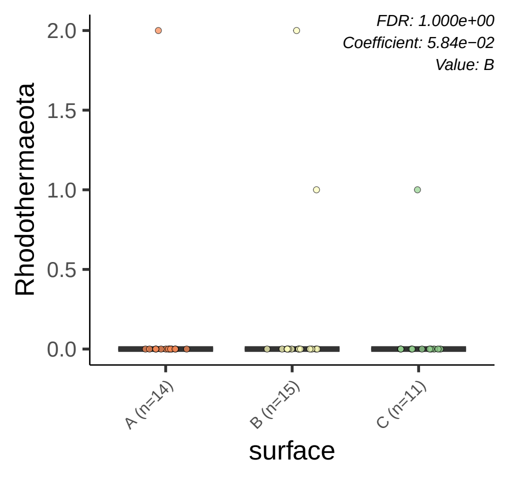

FDR: 1.000e+00
Coefficient: 5.84e−02
Value: B
Rhodothermaeota
2.0
1.5
1.0
0.5
0.0
A (n=14)
B (n=15)
C (n=11)
surface

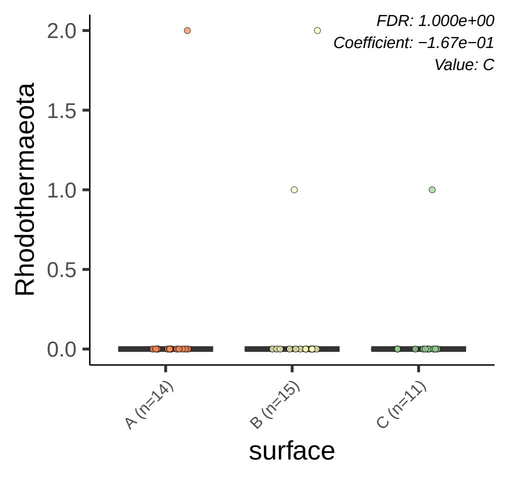
FDR: 1.000e+00
Coefficient: −1.67e−01
Value: C
Rhodothermaeota
2.0
1.5
1.0
0.5
0.0
A (n=14)
B (n=15)
C (n=11)
surface

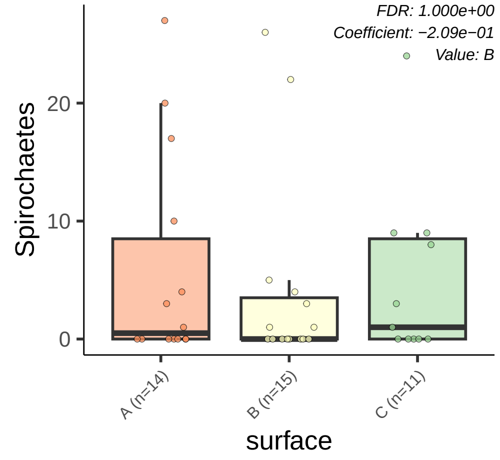

FDR: 1.000e+00
Coefficient: −2.09e−01
Value: B
Spirochaetes
0
10
20
A (n=14)
B (n=15)
C (n=11)
surface

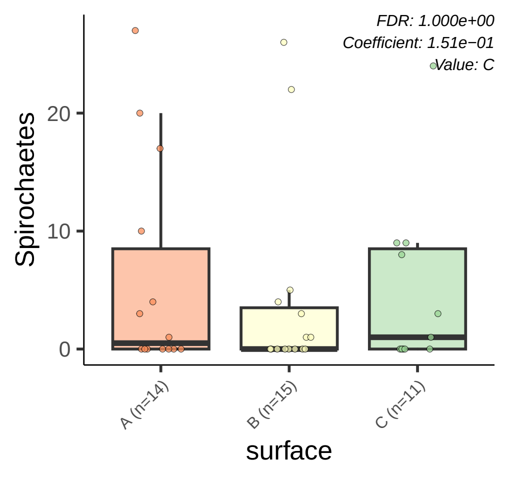
FDR: 1.000e+00
Coefficient: 1.51e−01
Value: C
Spirochaetes
0
10
20
A (n=14)
B (n=15)
C (n=11)
surface

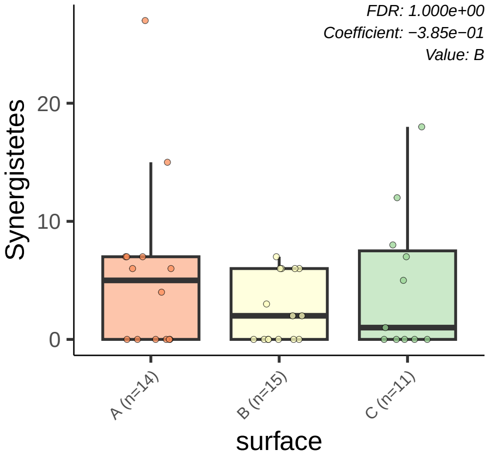

FDR: 1.000e+00
Coefficient: −3.85e−01
Value: B
Synergistetes
20
10
0
A (n=14)
B (n=15)
C (n=11)
surface

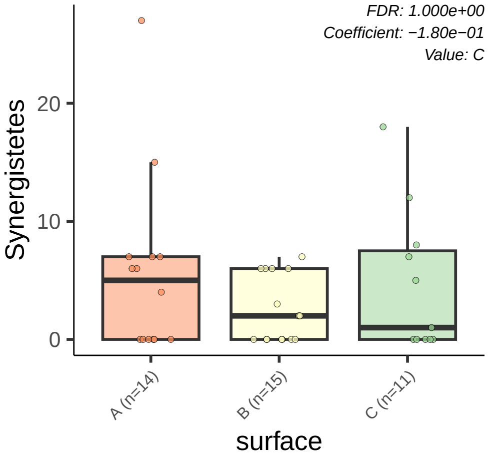
FDR: 1.000e+00
Coefficient: −1.80e−01
Value: C
Synergistetes
20
10
0
A (n=14)
B (n=15)
C (n=11)
surface

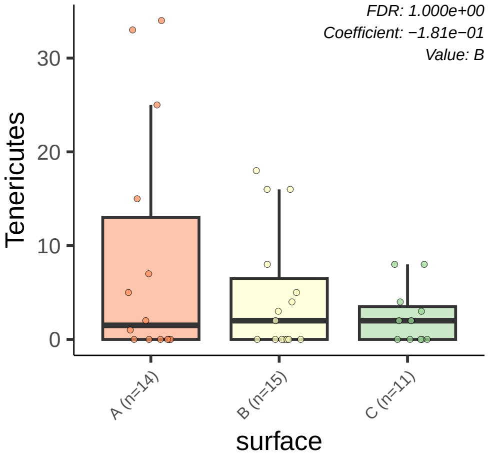

FDR: 1.000e+00
Coefficient: −1.81e−01
Value: B
Tenericutes
30
20
10
0
A (n=14)
B (n=15)
C (n=11)
surface

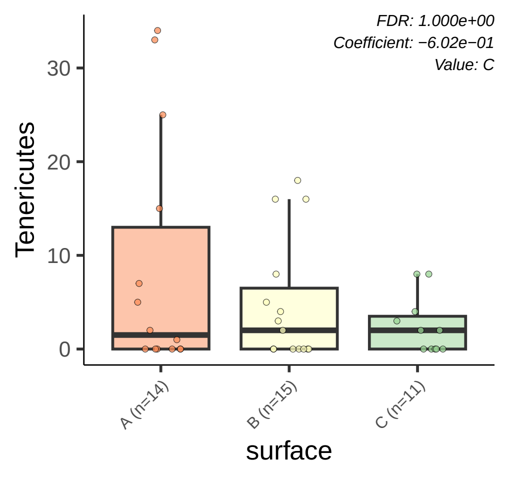

FDR: 1.000e+00
Coefficient: −6.02e−01
Value: C
Tenericutes
30
20
10
0
A (n=14)
B (n=15)
C (n=11)
surface

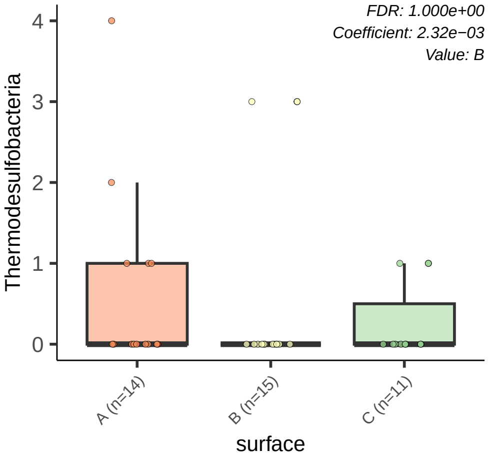
FDR: 1.000e+00
Coefficient: 2.32e−03
Value: B
Thermodesulfobacteria
0
1
2
3
4
A (n=14)
B (n=15)
C (n=11)
surface

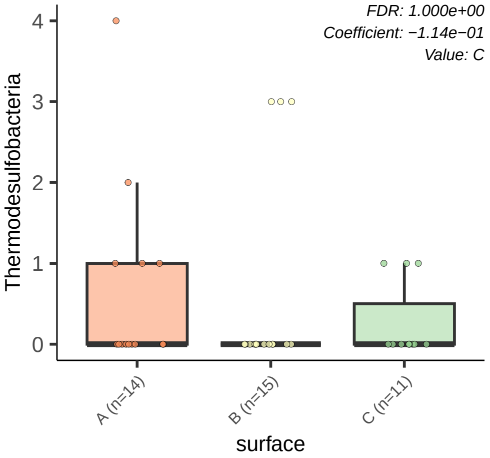
FDR: 1.000e+00
Coefficient: −1.14e−01
Value: C
Thermodesulfobacteria
0
1
2
3
4
A (n=14)
B (n=15)
C (n=11)
surface

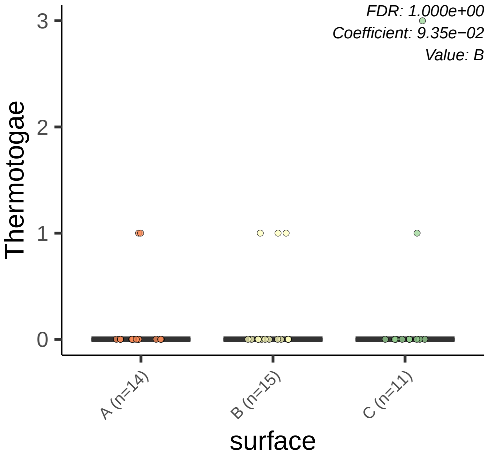
FDR: 1.000e+00
Coefficient: 9.35e−02
Value: B
Thermotogae
3
2
1
0
A (n=14)
B (n=15)
C (n=11)
surface

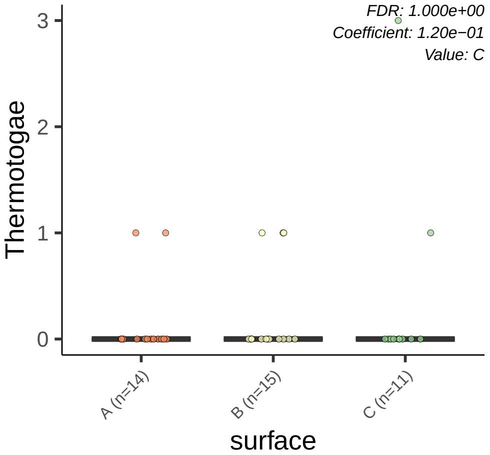

FDR: 1.000e+00
Coefficient: 1.20e−01
Value: C
Thermotogae
3
2
1
0
A (n=14)
B (n=15)
C (n=11)
surface

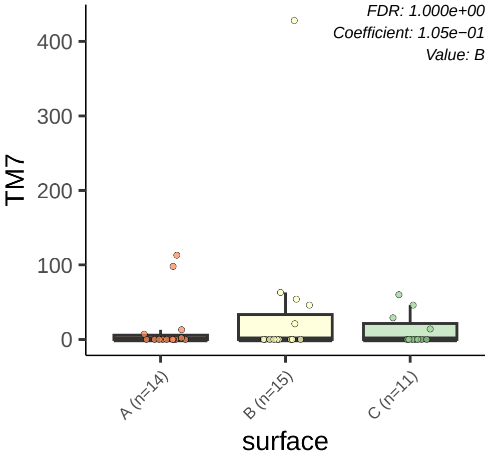

FDR: 1.000e+00
Coefficient: 1.05e−01
Value: B
TM7
400
300
200
100
0
A (n=14)
B (n=15)
C (n=11)
surface

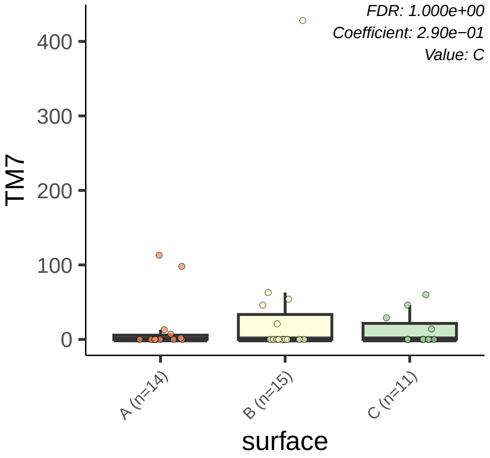
FDR: 1.000e+00
Coefficient: 2.90e−01
Value: C
TM7
400
300
200
100
0
A (n=14)
B (n=15)
C (n=11)
surface

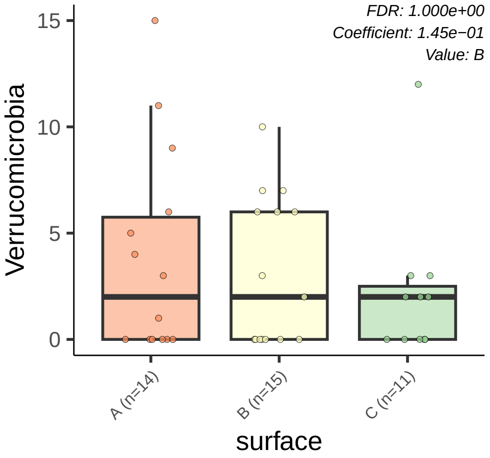

FDR: 1.000e+00
Coefficient: 1.45e−01
Value: B
Verrucomicrobia
15
10
5
0
A (n=14)
B (n=15)
C (n=11)
surface

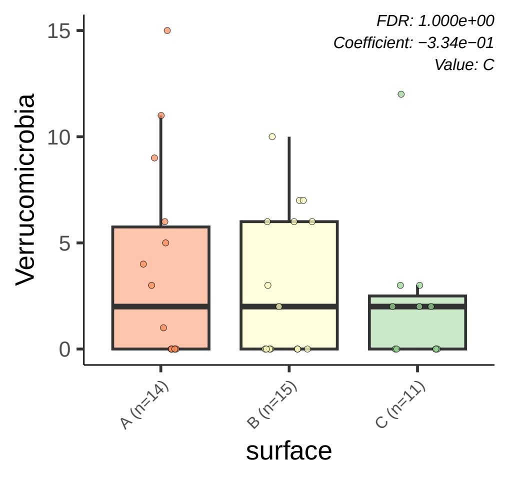

FDR: 1.000e+00
Coefficient: −3.34e−01
Value: C
Verrucomicrobia
15
10
5
0
A (n=14)
B (n=15)
C (n=11)
surface

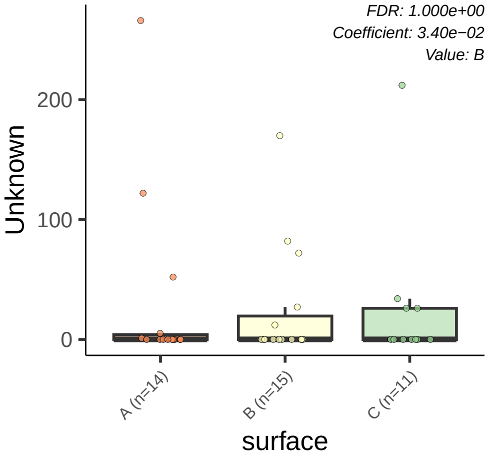
FDR: 1.000e+00
Coefficient: 3.40e−02
Value: B
Unknown
200
100
0
A (n=14)
B (n=15)
C (n=11)
surface

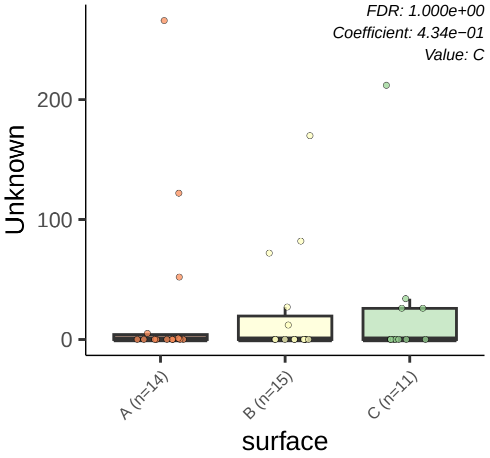

FDR: 1.000e+00
Coefficient: 4.34e−01
Value: C
Unknown
200
100
0
A (n=14)
B (n=15)
C (n=11)
surface

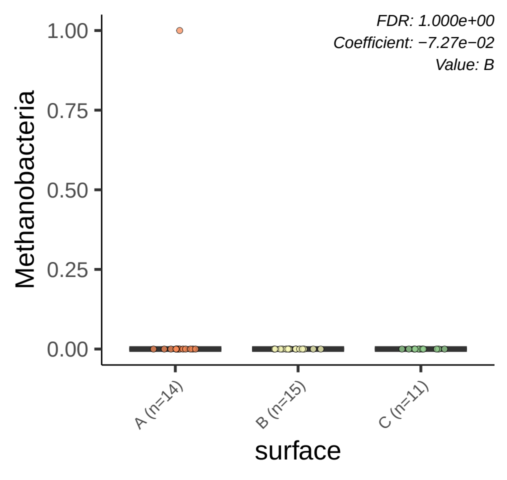
FDR: 1.000e+00
Coefficient: −7.27e−02
Value: B
Methanobacteria
1.00
0.75
0.50
0.25
0.00
A (n=14)
B (n=15)
C (n=11)
surface

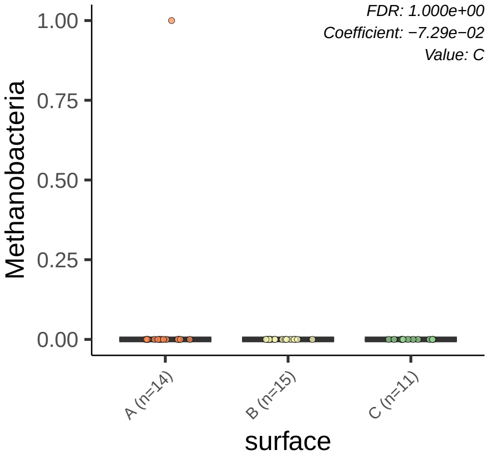

FDR: 1.000e+00
Coefficient: −7.29e−02
Value: C
Methanobacteria
1.00
0.75
0.50
0.25
0.00
A (n=14)
B (n=15)
C (n=11)
surface

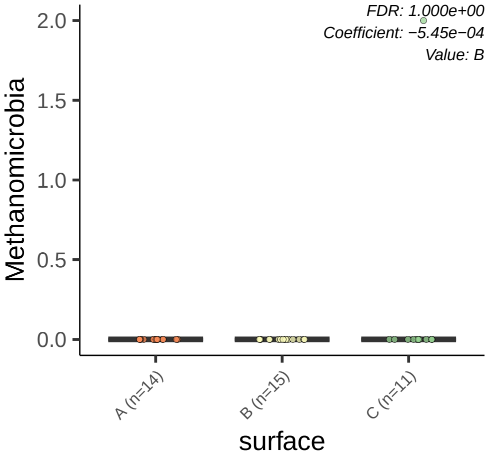
FDR: 1.000e+00
Coefficient: −5.45e−04
Value: B
Methanomicrobia
2.0
1.5
1.0
0.5
0.0
A (n=14)
B (n=15)
C (n=11)
surface

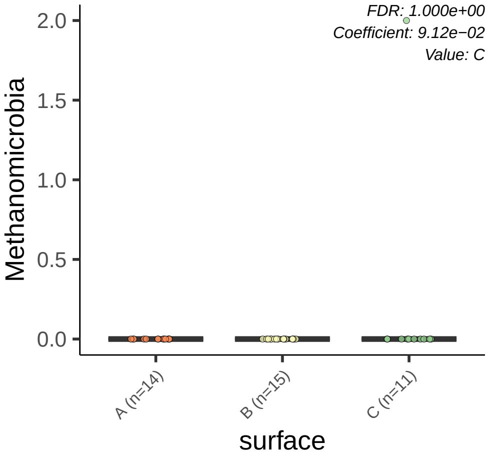
FDR: 1.000e+00
Coefficient: 9.12e−02
Value: C
Methanomicrobia
2.0
1.5
1.0
0.5
0.0
A (n=14)
B (n=15)
C (n=11)
surface

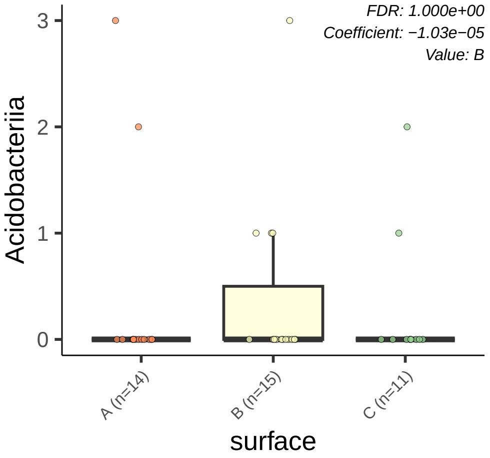
FDR: 1.000e+00
Coefficient: −1.03e−05
Value: B
Acidobacteriia
0
1
2
3
A (n=14)
B (n=15)
C (n=11)
surface

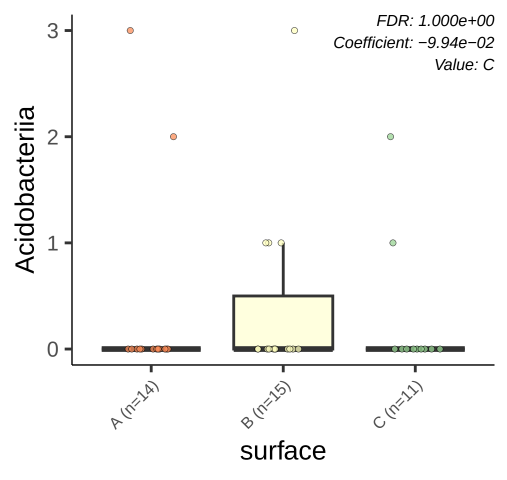

FDR: 1.000e+00
Coefficient: −9.94e−02
Value: C
Acidobacteriia
3
2
1
0
A (n=14)
B (n=15)
C (n=11)
surface

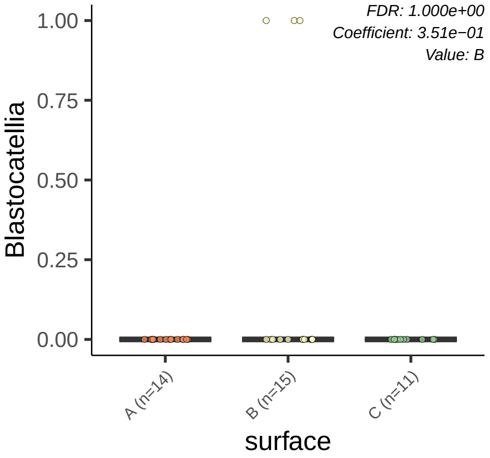

FDR: 1.000e+00
Coefficient: 3.51e−01
Value: B
1.00
0.75
0.50
0.25
0.00
Blastocatellia
A (n=14)
B (n=15)
C (n=11)
surface

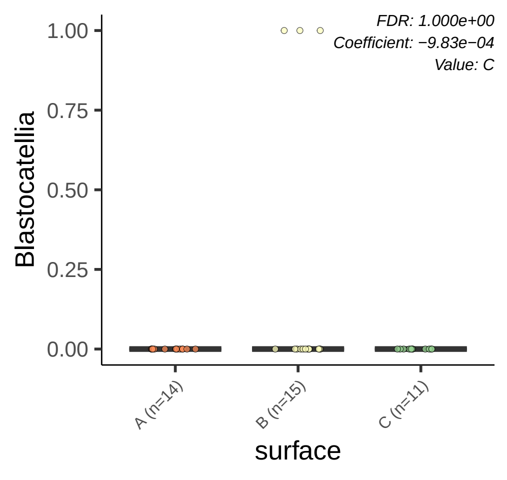

FDR: 1.000e+00
Coefficient: −9.83e−04
Value: C
Blastocatellia
1.00
0.75
0.50
0.25
0.00
A (n=14)
B (n=15)
C (n=11)
surface

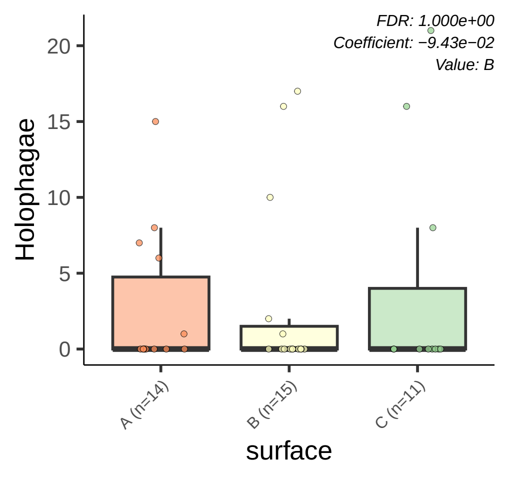
FDR: 1.000e+00
Coefficient: −9.43e−02
Value: B
Holophagae
20
15
10
5
0
A (n=14)
B (n=15)
C (n=11)
surface

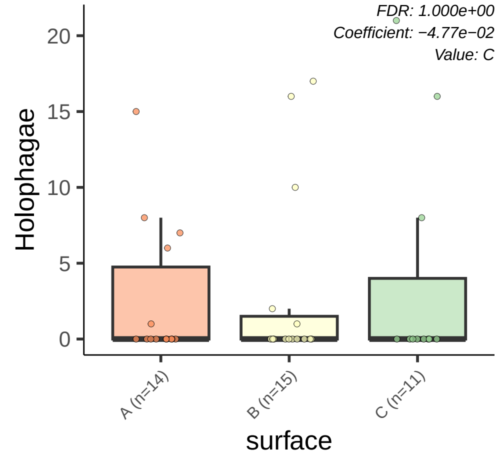

FDR: 1.000e+00
Coefficient: −4.77e−02
Value: C
Holophagae
0
5
10
15
20
A (n=14)
B (n=15)
C (n=11)
surface

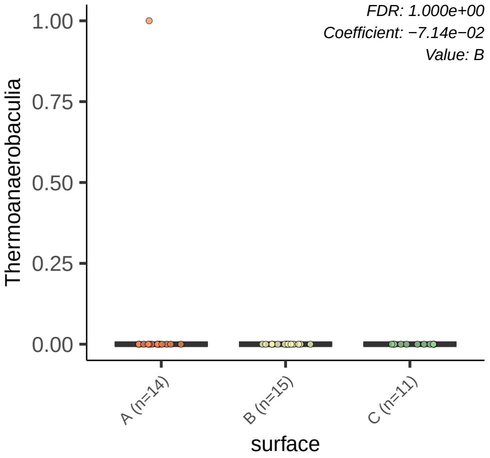

FDR: 1.000e+00
Coefficient: −7.14e−02
Value: B
Thermoanaerobaculia
1.00
0.75
0.50
0.25
0.00
A (n=14)
B (n=15)
C (n=11)
surface

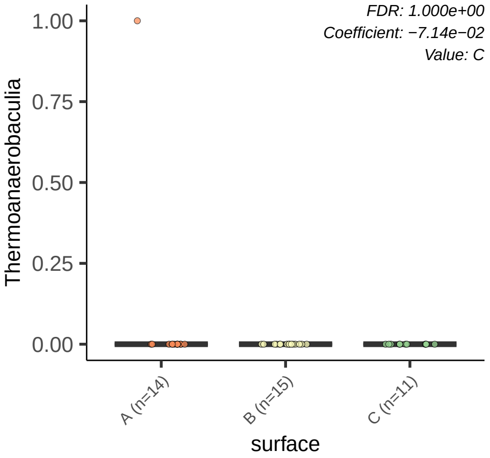
FDR: 1.000e+00
Coefficient: −7.14e−02
Value: C
Thermoanaerobaculia
1.00
0.75
0.50
0.25
0.00
A (n=14)
B (n=15)
C (n=11)
surface

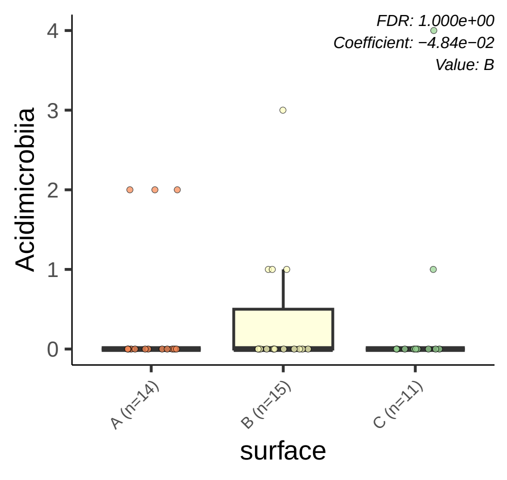

FDR: 1.000e+00
Coefficient: −4.84e−02
Value: B
Acidimicrobiia
0
1
2
3
4
A (n=14)
B (n=15)
C (n=11)
surface

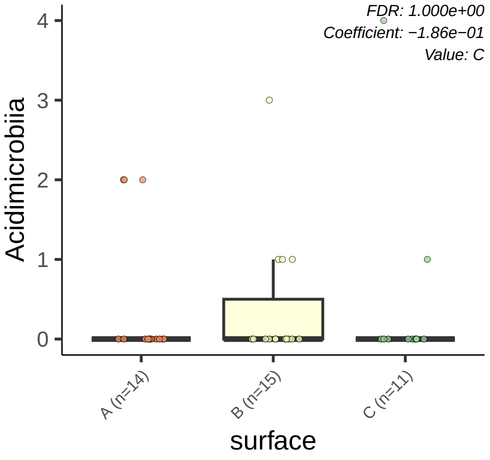

FDR: 1.000e+00
Coefficient: −1.86e−01
Value: C
Acidimicrobiia
0
1
2
3
4
A (n=14)
B (n=15)
C (n=11)
surface

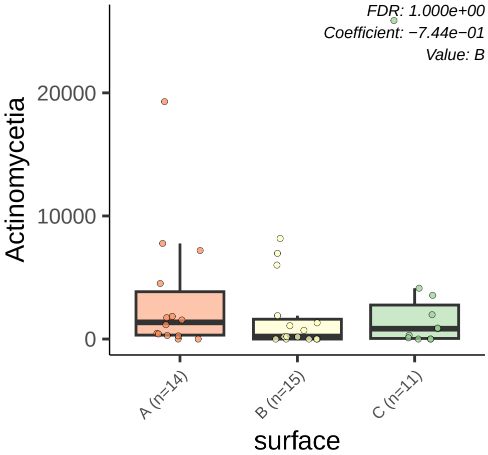
FDR: 1.000e+00
Coefficient: −7.44e−01
Value: B
Actinomycetia
20000
10000
0
A (n=14)
B (n=15)
C (n=11)
surface

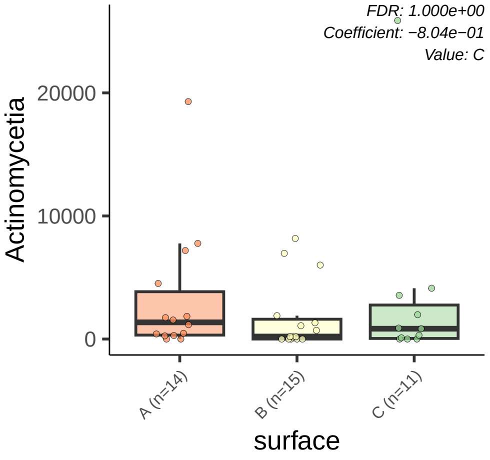
FDR: 1.000e+00
Coefficient: −8.04e−01
Value: C
20000
10000
0
Actinomycetia
A (n=14)
B (n=15)
C (n=11)
surface

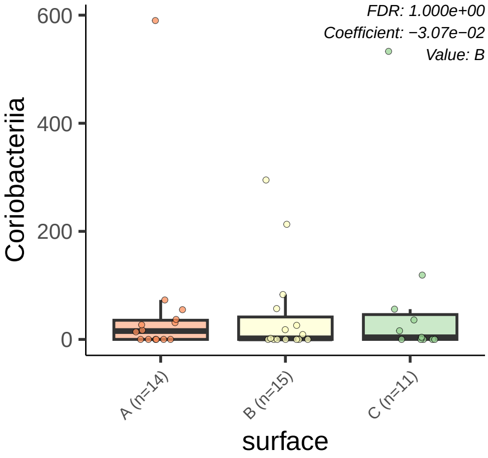

FDR: 1.000e+00
Coefficient: −3.07e−02
Value: B
Coriobacteriia
600
400
200
0
A (n=14)
B (n=15)
C (n=11)
surface

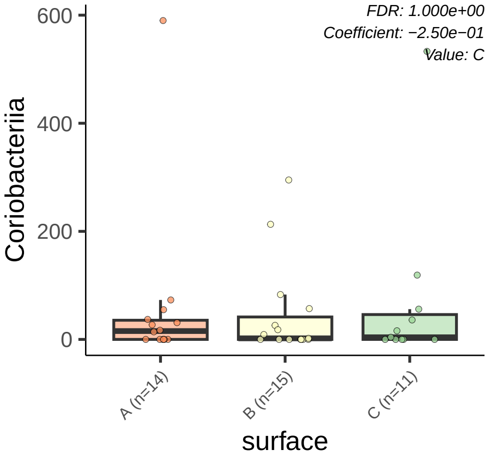

FDR: 1.000e+00
Coefficient: −2.50e−01
Value: C
Coriobacteriia
600
400
200
0
A (n=14)
B (n=15)
C (n=11)
surface

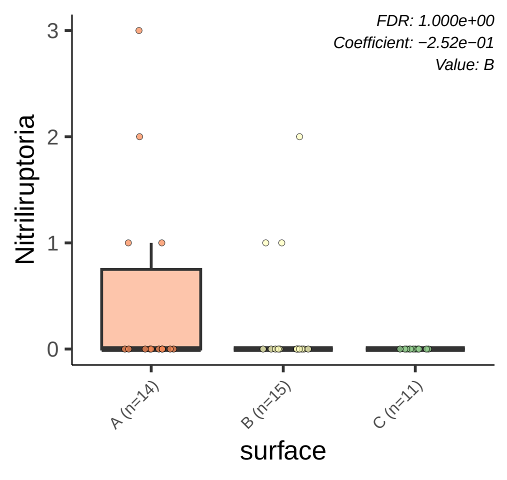

FDR: 1.000e+00
Coefficient: −2.52e−01
Value: B
Nitriliruptoria
3
2
1
0
A (n=14)
B (n=15)
C (n=11)
surface

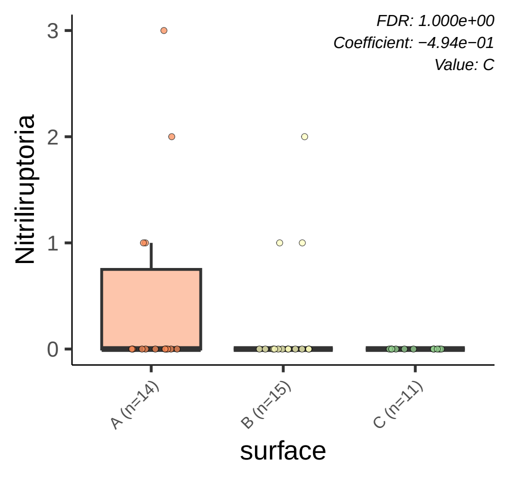

FDR: 1.000e+00
Coefficient: −4.94e−01
Value: C
Nitriliruptoria
3
2
1
0
A (n=14)
B (n=15)
C (n=11)
surface

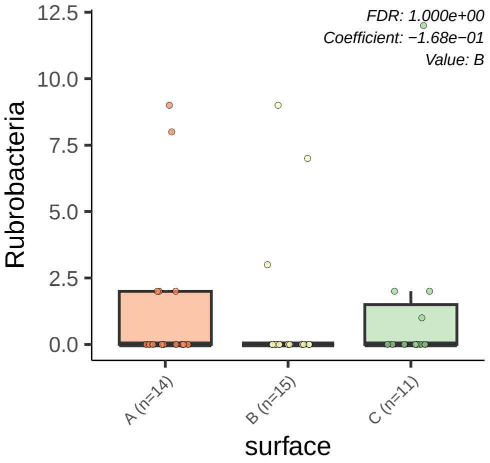

FDR: 1.000e+00
Coefficient: −1.68e−01
Value: B
Rubrobacteria
12.5
10.0
7.5
5.0
2.5
0.0
A (n=14)
B (n=15)
C (n=11)
surface

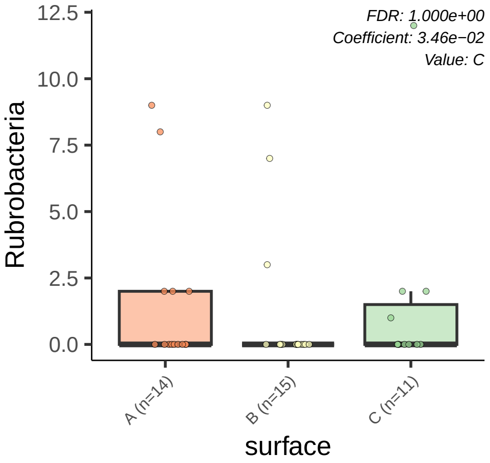
FDR: 1.000e+00
Coefficient: 3.46e−02
Value: C
Rubrobacteria
12.5
10.0
7.5
5.0
2.5
0.0
A (n=14)
B (n=15)
C (n=11)
surface

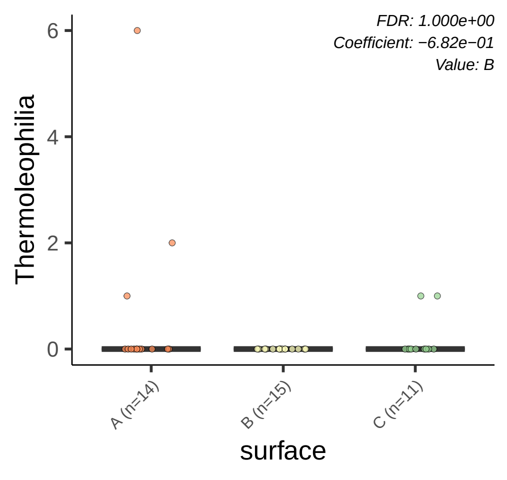
FDR: 1.000e+00
Coefficient: −6.82e−01
Value: B
Thermoleophilia
6
4
2
0
A (n=14)
B (n=15)
C (n=11)
surface

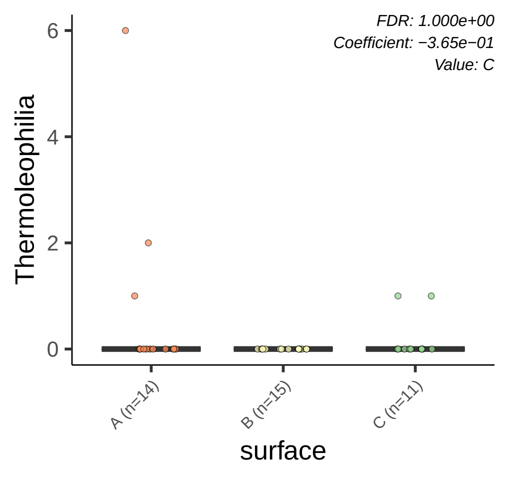

FDR: 1.000e+00
Coefficient: −3.65e−01
Value: C
Thermoleophilia
6
4
2
0
A (n=14)
B (n=15)
C (n=11)
surface

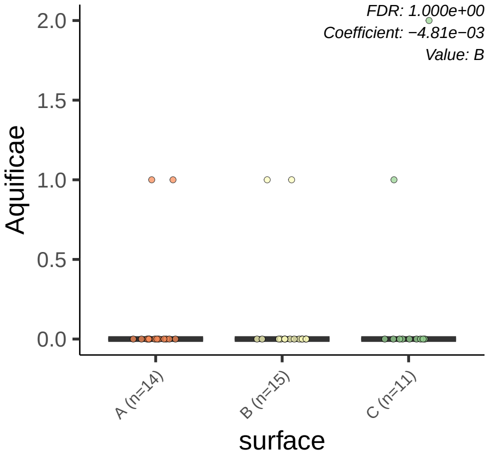
FDR: 1.000e+00
Coefficient: −4.81e−03
Value: B
Aquificae
2.0
1.5
1.0
0.5
0.0
A (n=14)
B (n=15)
C (n=11)
surface

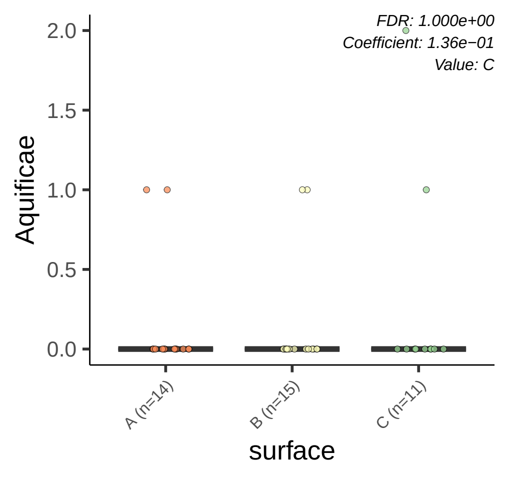

FDR: 1.000e+00
Coefficient: 1.36e−01
Value: C
Aquificae
2.0
1.5
1.0
0.5
0.0
A (n=14)
B (n=15)
C (n=11)
surface

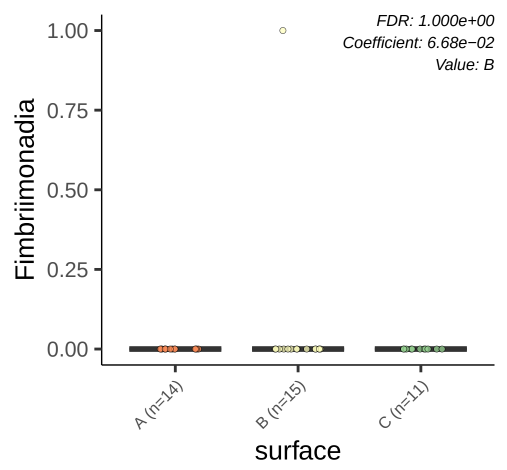

FDR: 1.000e+00
Coefficient: 6.68e−02
Value: B
Fimbriimonadia
1.00
0.75
0.50
0.25
0.00
A (n=14)
B (n=15)
C (n=11)
surface

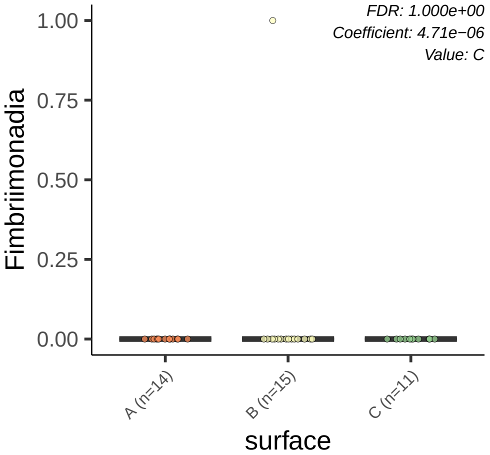

FDR: 1.000e+00
Coefficient: 4.71e−06
Value: C
Fimbriimonadia
1.00
0.75
0.50
0.25
0.00
A (n=14)
B (n=15)
C (n=11)
surface

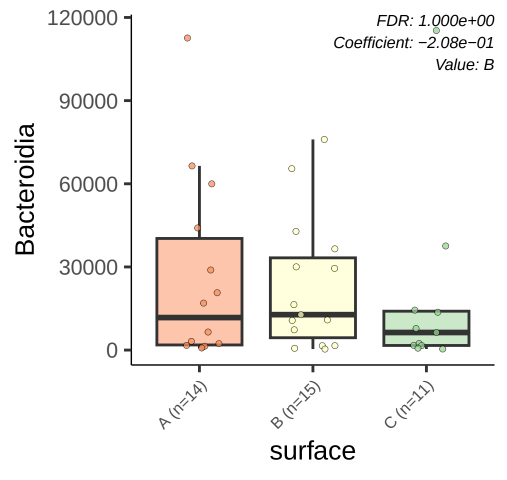

FDR: 1.000e+00
Coefficient: −2.08e−01
Value: B
120000
90000
60000
30000
0
Bacteroidia
A (n=14)
B (n=15)
C (n=11)
surface

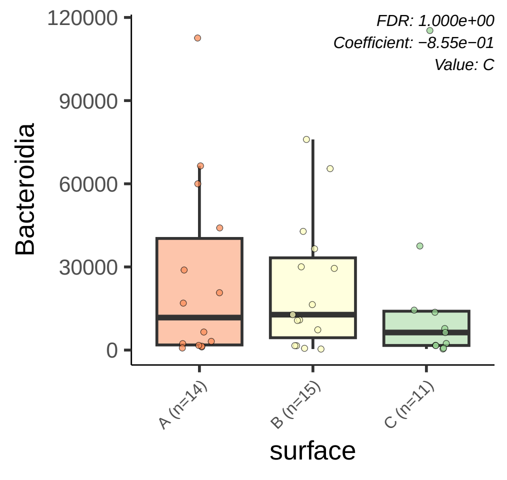

FDR: 1.000e+00
Coefficient: −8.55e−01
Value: C
120000
90000
60000
30000
0
Bacteroidia
A (n=14)
B (n=15)
C (n=11)
surface

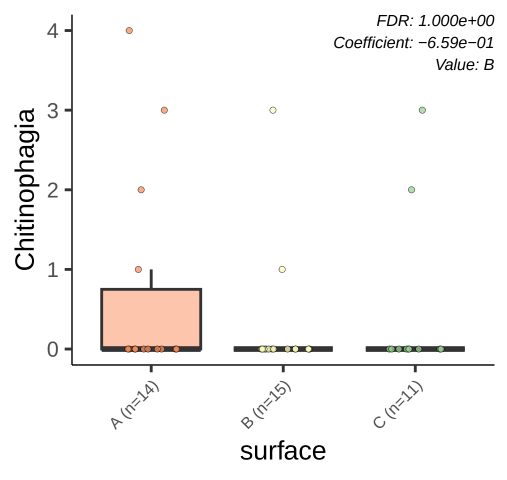

FDR: 1.000e+00
Coefficient: −6.59e−01
Value: B
Chitinophagia
4
3
2
1
0
A (n=14)
B (n=15)
C (n=11)
surface

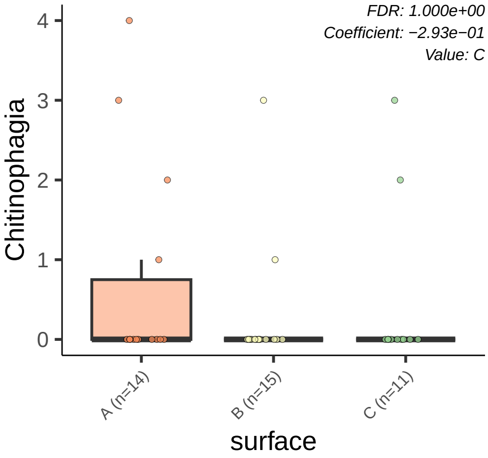

FDR: 1.000e+00
Coefficient: −2.93e−01
Value: C
Chitinophagia
4
3
2
1
0
A (n=14)
B (n=15)
C (n=11)
surface

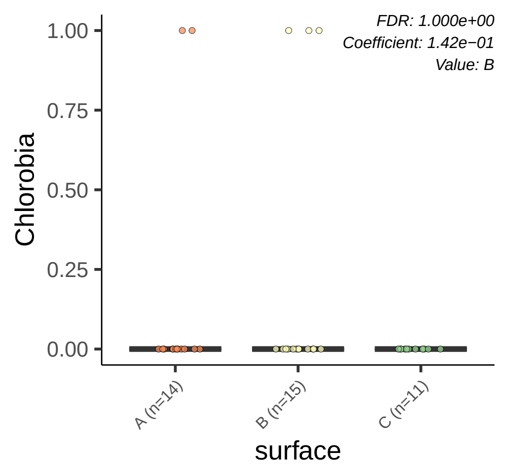

FDR: 1.000e+00
Coefficient: 1.42e−01
Value: B
Chlorobia
1.00
0.75
0.50
0.25
0.00
A (n=14)
B (n=15)
C (n=11)
surface

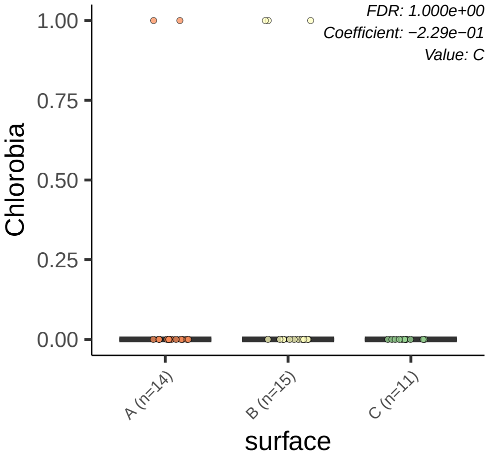
FDR: 1.000e+00
Coefficient: −2.29e−01
Value: C
Chlorobia
1.00
0.75
0.50
0.25
0.00
A (n=14)
B (n=15)
C (n=11)
surface

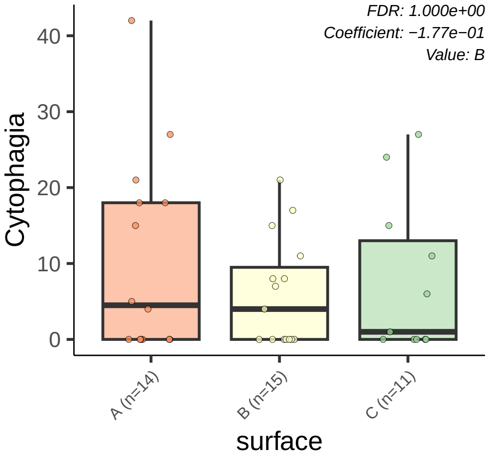
FDR: 1.000e+00
Coefficient: −1.77e−01
Value: B
Cytophagia
0
10
20
30
40
A (n=14)
B (n=15)
C (n=11)
surface

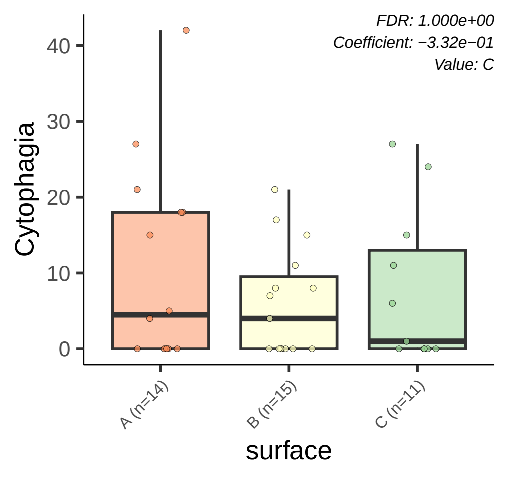

FDR: 1.000e+00
Coefficient: −3.32e−01
Value: C
Cytophagia
0
10
20
30
40
A (n=14)
B (n=15)
C (n=11)
surface

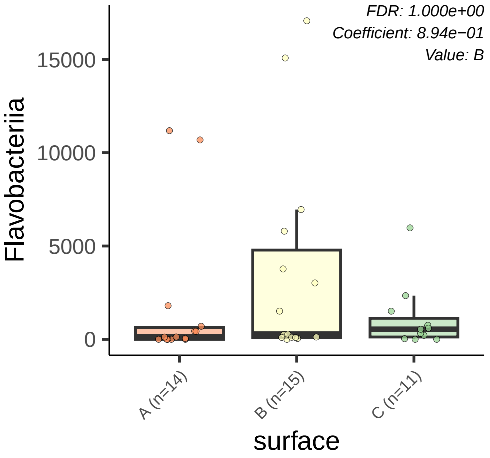

FDR: 1.000e+00
Coefficient: 8.94e−01
Value: B
Flavobacteriia
15000
10000
5000
0
A (n=14)
B (n=15)
C (n=11)
surface

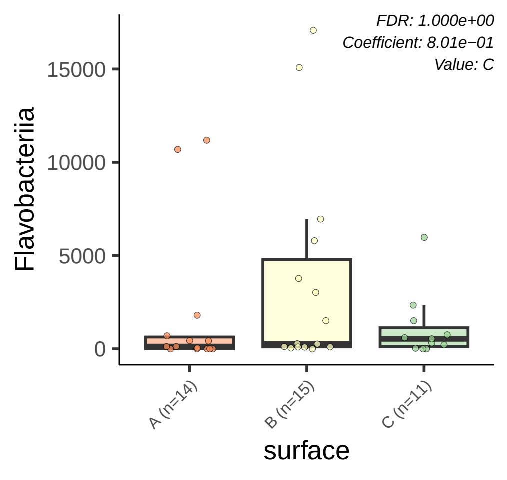

FDR: 1.000e+00
Coefficient: 8.01e−01
Value: C
Flavobacteriia
15000
10000
5000
0
A (n=14)
B (n=15)
C (n=11)
surface

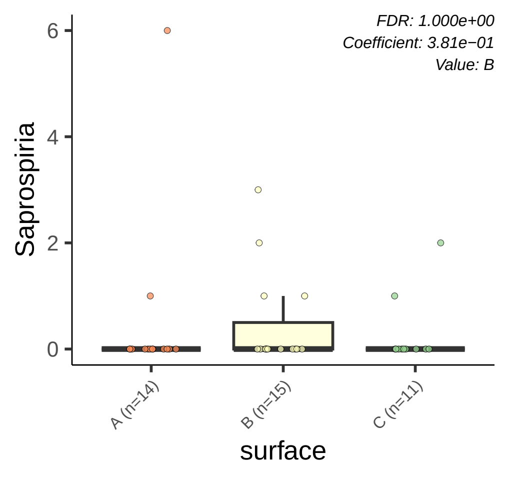

FDR: 1.000e+00
Coefficient: 3.81e−01
Value: B
Saprospiria
6
4
2
0
A (n=14)
B (n=15)
C (n=11)
surface

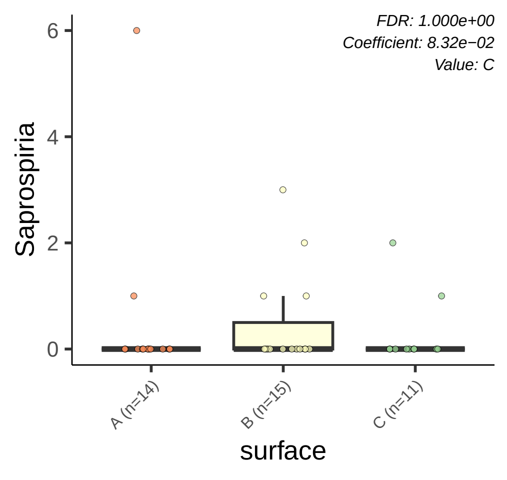

FDR: 1.000e+00
Coefficient: 8.32e−02
Value: C
Saprospiria
6
4
2
0
A (n=14)
B (n=15)
C (n=11)
surface

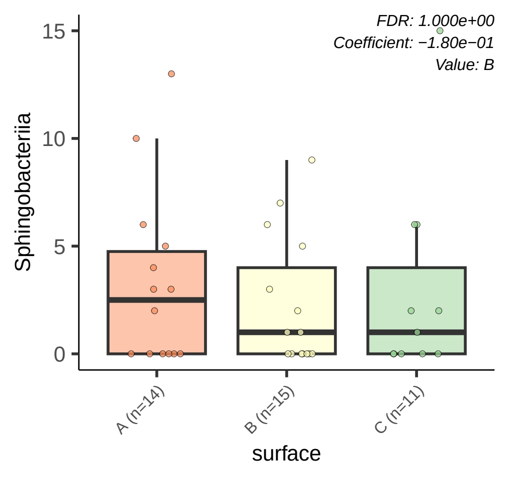

FDR: 1.000e+00
Coefficient: −1.80e−01
Value: B
Sphingobacteriia
15
10
5
0
A (n=14)
B (n=15)
C (n=11)
surface

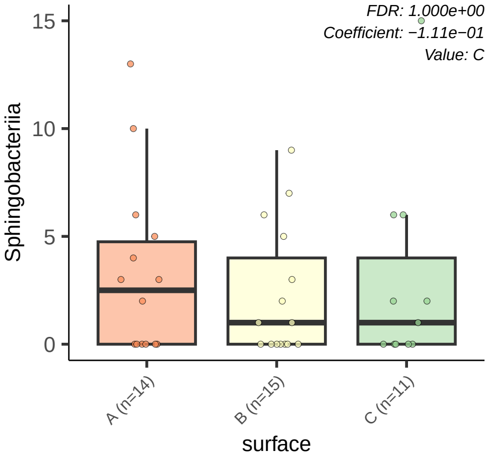
FDR: 1.000e+00
Coefficient: −1.11e−01
Value: C
Sphingobacteriia
15
10
5
0
A (n=14)
B (n=15)
C (n=11)
surface

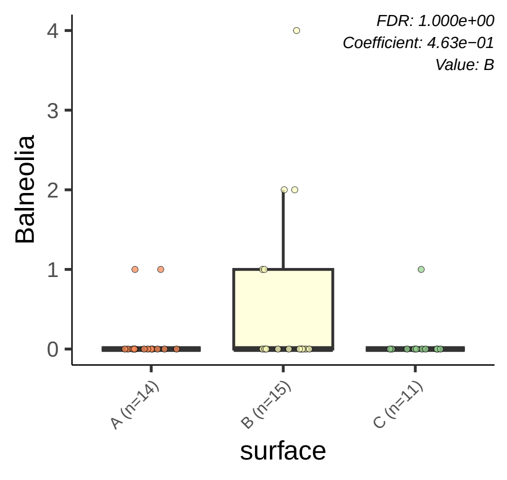

FDR: 1.000e+00
Coefficient: 4.63e−01
Value: B
Balneolia
0
1
2
3
4
A (n=14)
B (n=15)
C (n=11)
surface

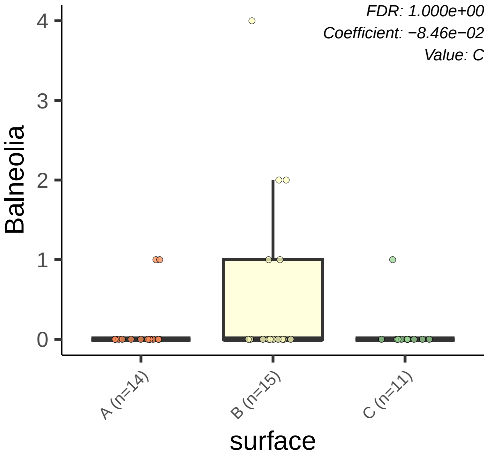

FDR: 1.000e+00
Coefficient: −8.46e−02
Value: C
Balneolia
0
1
2
3
4
A (n=14)
B (n=15)
C (n=11)
surface

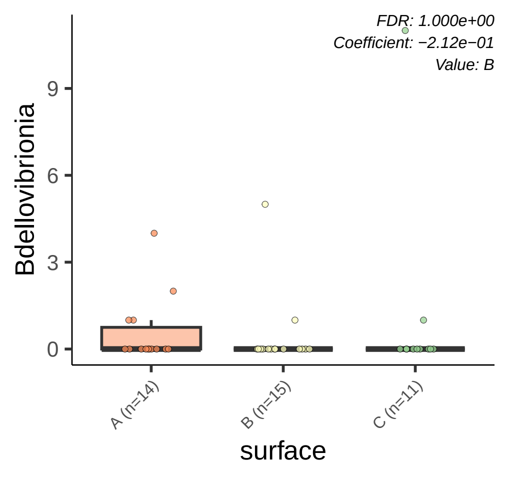
FDR: 1.000e+00
Coefficient: −2.12e−01
Value: B
Bdellovibrionia
9
6
3
0
A (n=14)
B (n=15)
C (n=11)
surface

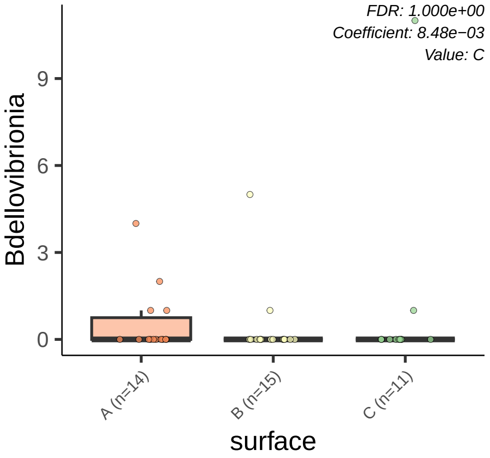

FDR: 1.000e+00
Coefficient: 8.48e−03
Value: C
Bdellovibrionia
9
6
3
0
A (n=14)
B (n=15)
C (n=11)
surface

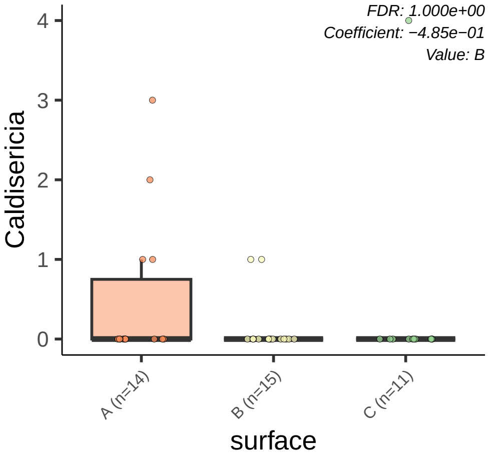

FDR: 1.000e+00
Coefficient: −4.85e−01
Value: B
Caldisericia
0
1
2
3
4
A (n=14)
B (n=15)
C (n=11)
surface

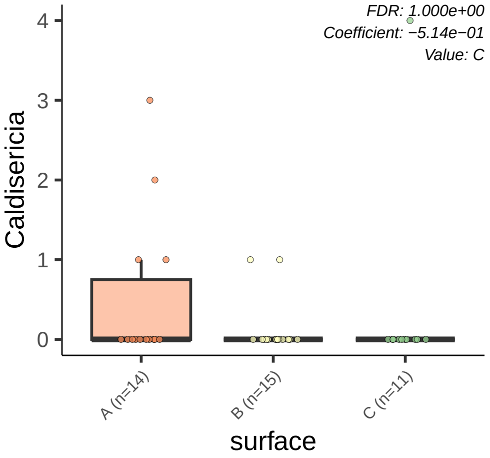

FDR: 1.000e+00
Coefficient: −5.14e−01
Value: C
Caldisericia
0
1
2
3
4
A (n=14)
B (n=15)
C (n=11)
surface

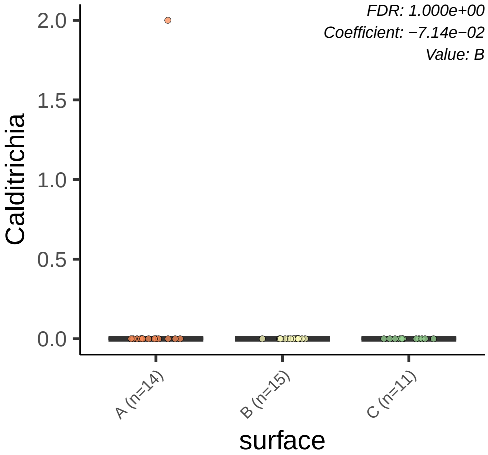

FDR: 1.000e+00
Coefficient: −7.14e−02
Value: B
Calditrichia
2.0
1.5
1.0
0.5
0.0
A (n=14)
B (n=15)
C (n=11)
surface

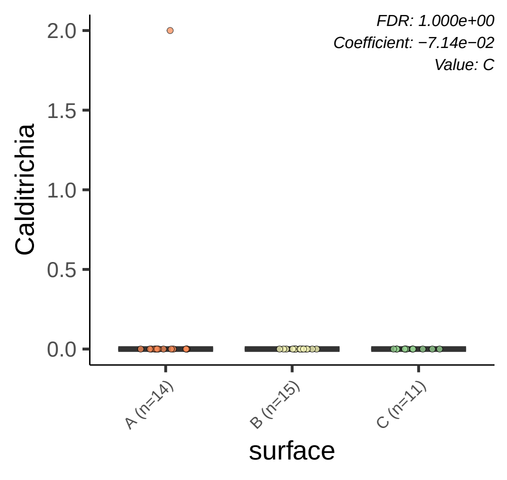

FDR: 1.000e+00
Coefficient: −7.14e−02
Value: C
2.0
1.5
1.0
0.5
0.0
Calditrichia
A (n=14)
B (n=15)
C (n=11)
surface

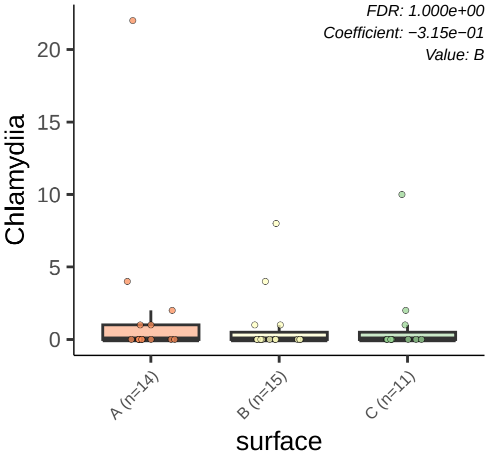

FDR: 1.000e+00
Coefficient: −3.15e−01
Value: B
Chlamydiia
0
5
10
15
20
A (n=14)
B (n=15)
C (n=11)
surface

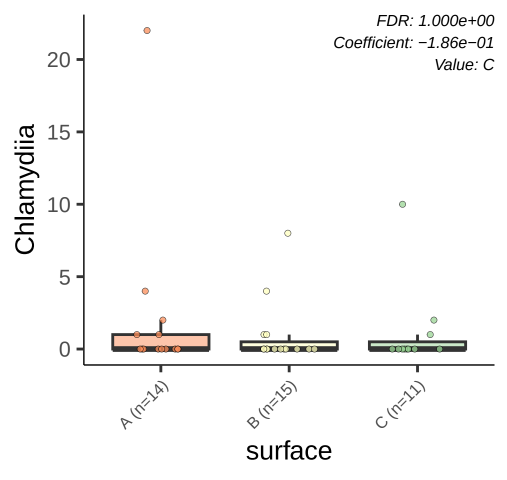

FDR: 1.000e+00
Coefficient: −1.86e−01
Value: C
Chlamydiia
0
5
10
15
20
A (n=14)
B (n=15)
C (n=11)
surface

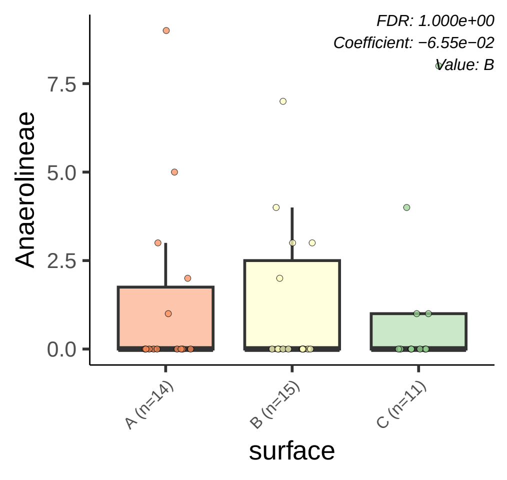

FDR: 1.000e+00
Coefficient: −6.55e−02
Value: B
Anaerolineae
0.0
2.5
5.0
7.5
A (n=14)
B (n=15)
C (n=11)
surface

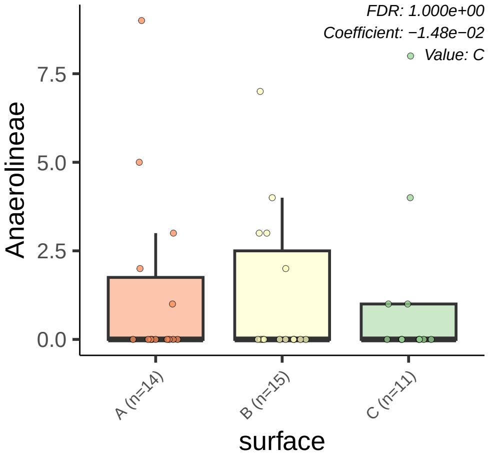

FDR: 1.000e+00
Coefficient: −1.48e−02
Value: C
Anaerolineae
7.5
5.0
2.5
0.0
A (n=14)
B (n=15)
C (n=11)
surface

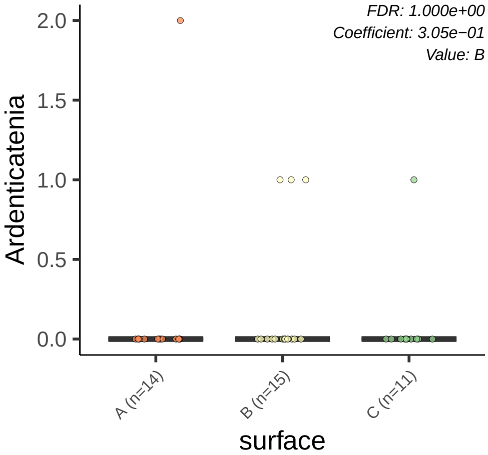

FDR: 1.000e+00
Coefficient: 3.05e−01
Value: B
Ardenticatenia
2.0
1.5
1.0
0.5
0.0
A (n=14)
B (n=15)
C (n=11)
surface

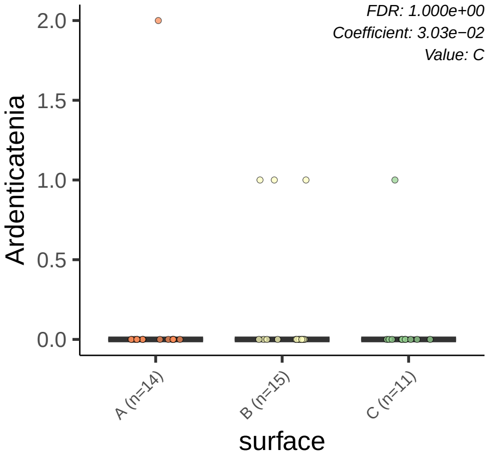

FDR: 1.000e+00
Coefficient: 3.03e−02
Value: C
Ardenticatenia
2.0
1.5
1.0
0.5
0.0
A (n=14)
B (n=15)
C (n=11)
surface

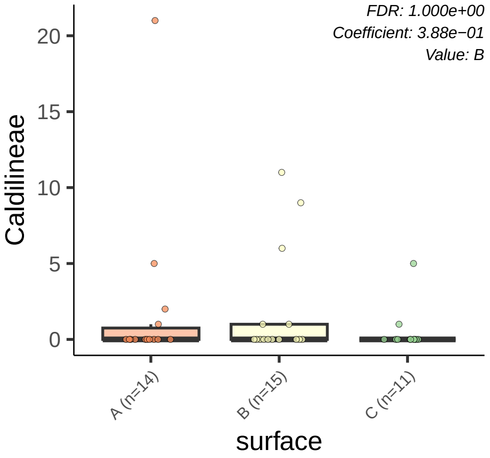

FDR: 1.000e+00
Coefficient: 3.88e−01
Value: B
Caldilineae
20
15
10
5
0
A (n=14)
B (n=15)
C (n=11)
surface

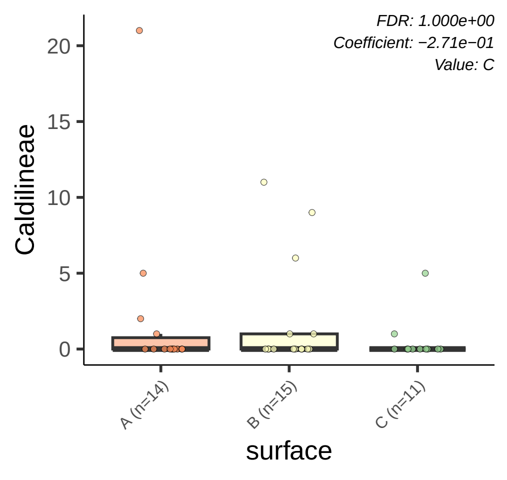
FDR: 1.000e+00
Coefficient: −2.71e−01
Value: C
Caldilineae
20
15
10
5
0
A (n=14)
B (n=15)
C (n=11)
surface

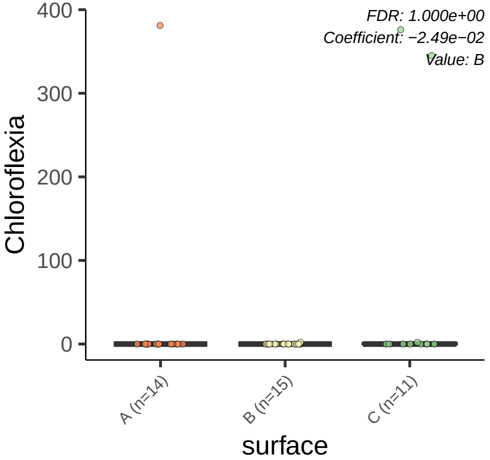
FDR: 1.000e+00
Coefficient: −2.49e−02
Value: B
Chloroflexia
400
300
200
100
0
A (n=14)
B (n=15)
C (n=11)
surface

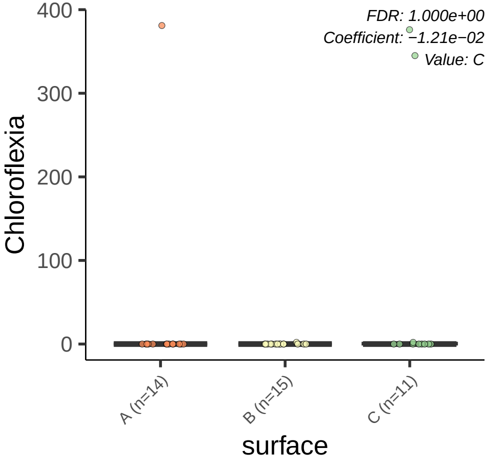
FDR: 1.000e+00
Coefficient: −1.21e−02
Value: C
Chloroflexia
400
300
200
100
0
A (n=14)
B (n=15)
C (n=11)
surface

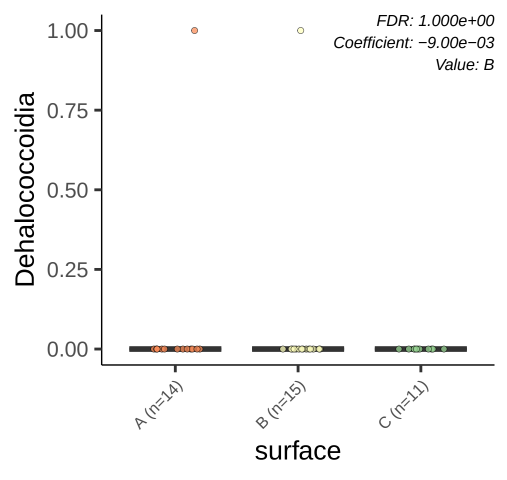

FDR: 1.000e+00
Coefficient: −9.00e−03
Value: B
Dehalococcoidia
1.00
0.75
0.50
0.25
0.00
A (n=14)
B (n=15)
C (n=11)
surface

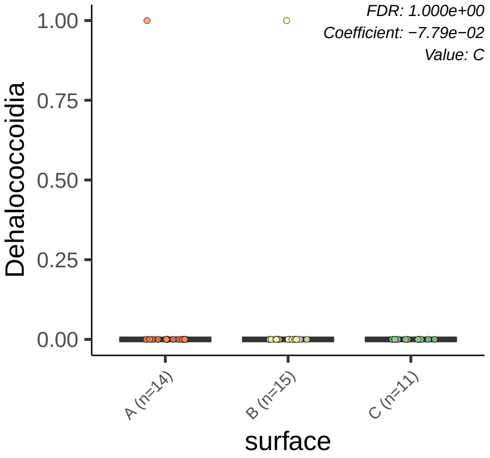
FDR: 1.000e+00
Coefficient: −7.79e−02
Value: C
Dehalococcoidia
1.00
0.75
0.50
0.25
0.00
A (n=14)
B (n=15)
C (n=11)
surface

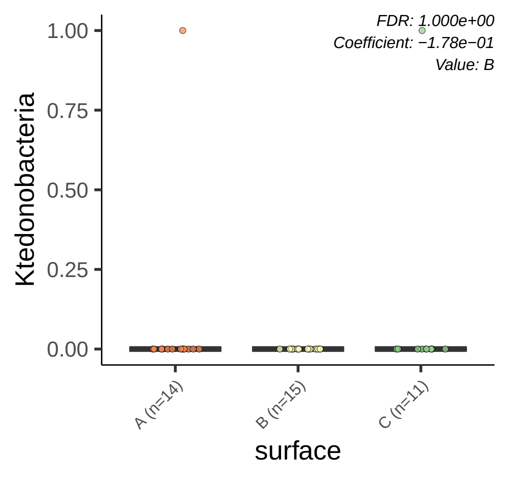
FDR: 1.000e+00
Coefficient: −1.78e−01
Value: B
Ktedonobacteria
1.00
0.75
0.50
0.25
0.00
A (n=14)
B (n=15)
C (n=11)
surface

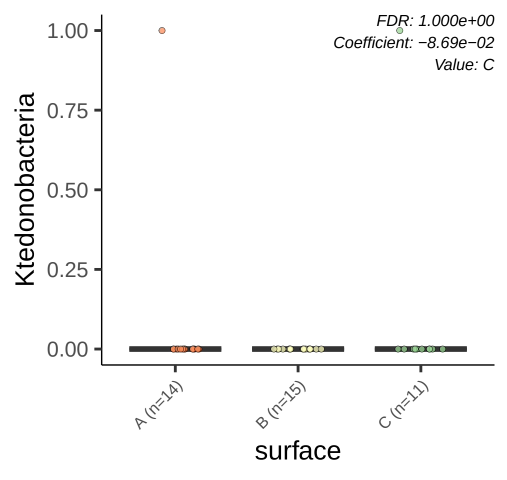
FDR: 1.000e+00
Coefficient: −8.69e−02
Value: C
Ktedonobacteria
1.00
0.75
0.50
0.25
0.00
A (n=14)
B (n=15)
C (n=11)
surface

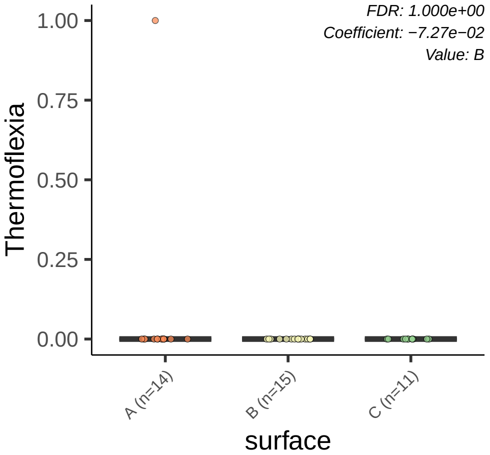

FDR: 1.000e+00
Coefficient: −7.27e−02
Value: B
Thermoflexia
1.00
0.75
0.50
0.25
0.00
A (n=14)
B (n=15)
C (n=11)
surface

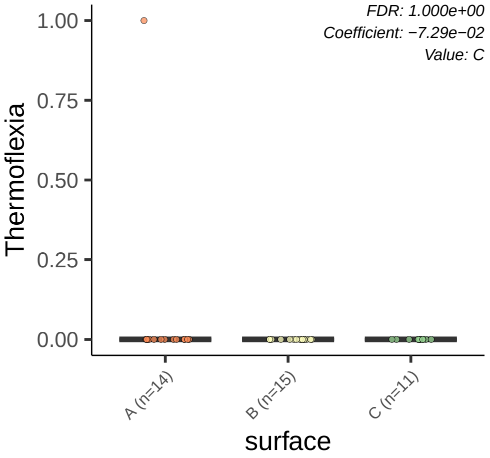
FDR: 1.000e+00
Coefficient: −7.29e−02
Value: C
Thermoflexia
1.00
0.75
0.50
0.25
0.00
A (n=14)
B (n=15)
C (n=11)
surface

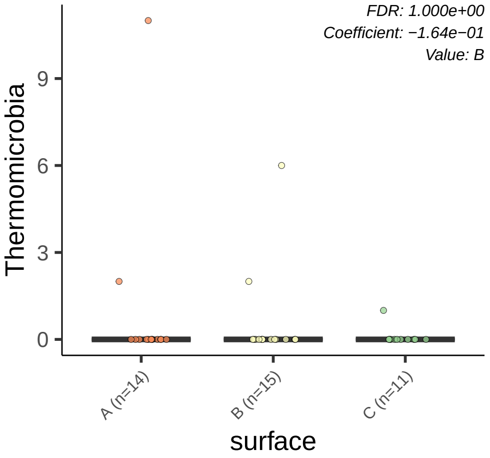
FDR: 1.000e+00
Coefficient: −1.64e−01
Value: B
Thermomicrobia
0
3
6
9
A (n=14)
B (n=15)
C (n=11)
surface

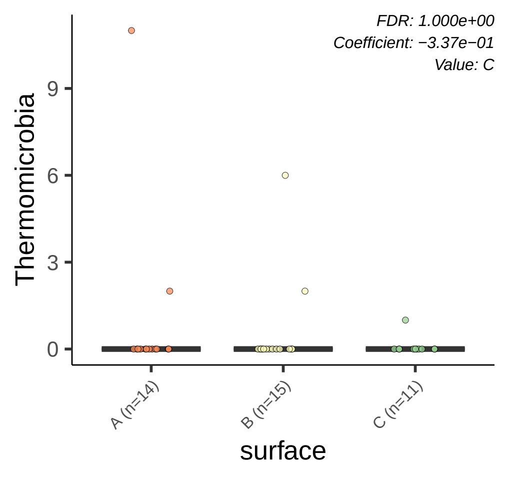

FDR: 1.000e+00
Coefficient: −3.37e−01
Value: C
Thermomicrobia
9
6
3
0
A (n=14)
B (n=15)
C (n=11)
surface

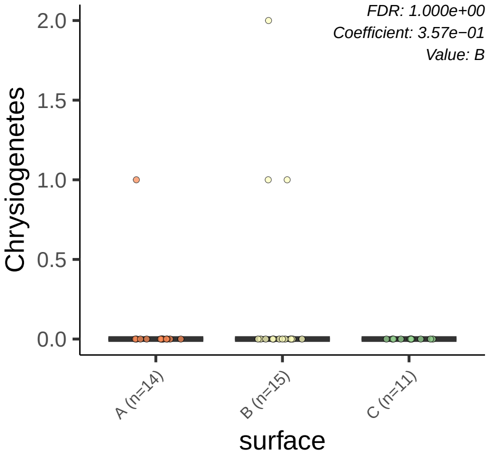

FDR: 1.000e+00
Coefficient: 3.57e−01
Value: B
Chrysiogenetes
2.0
1.5
1.0
0.5
0.0
A (n=14)
B (n=15)
C (n=11)
surface

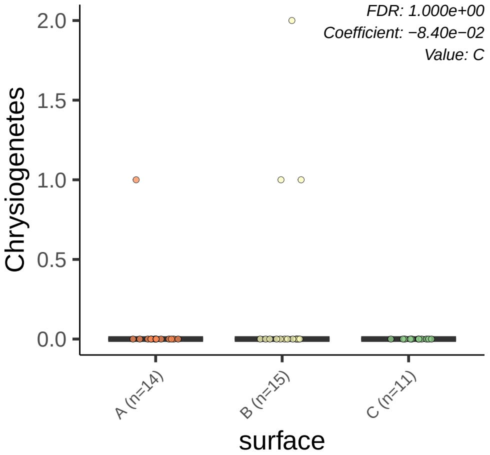

FDR: 1.000e+00
Coefficient: −8.40e−02
Value: C
Chrysiogenetes
2.0
1.5
1.0
0.5
0.0
A (n=14)
B (n=15)
C (n=11)
surface

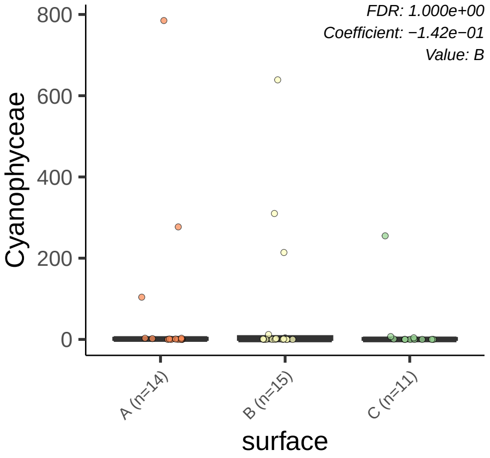

FDR: 1.000e+00
Coefficient: −1.42e−01
Value: B
800
600
400
200
0
Cyanophyceae
A (n=14)
B (n=15)
C (n=11)
surface

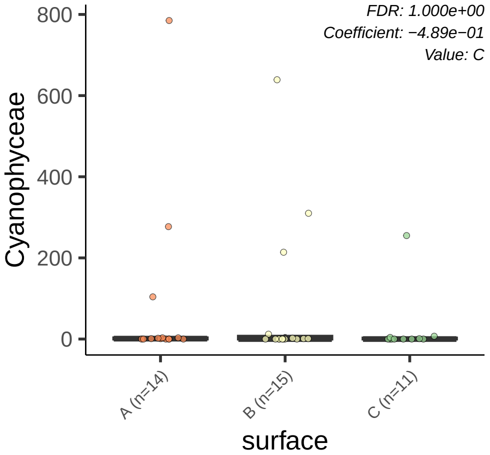
FDR: 1.000e+00
Coefficient: −4.89e−01
Value: C
Cyanophyceae
800
600
400
200
0
A (n=14)
B (n=15)
C (n=11)
surface

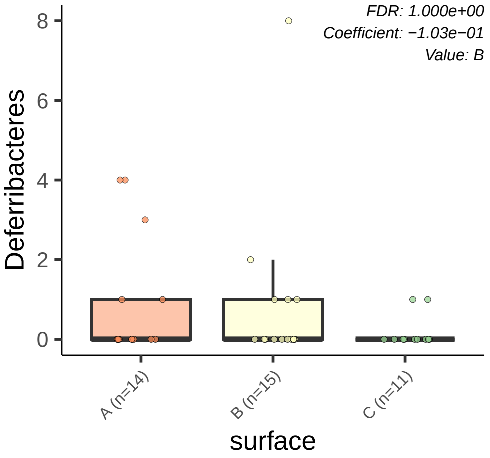

FDR: 1.000e+00
Coefficient: −1.03e−01
Value: B
Deferribacteres
0
2
4
6
8
A (n=14)
B (n=15)
C (n=11)
surface

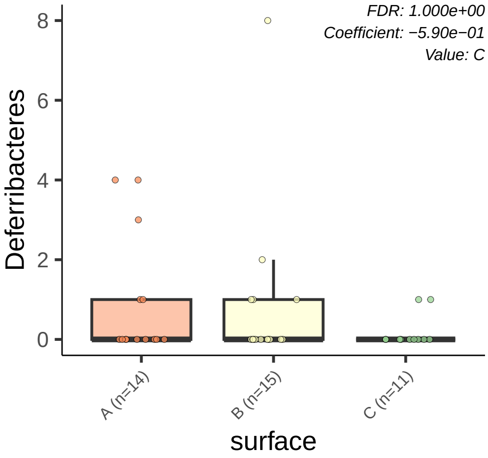
FDR: 1.000e+00
Coefficient: −5.90e−01
Value: C
Deferribacteres
8
6
4
2
0
A (n=14)
B (n=15)
C (n=11)
surface

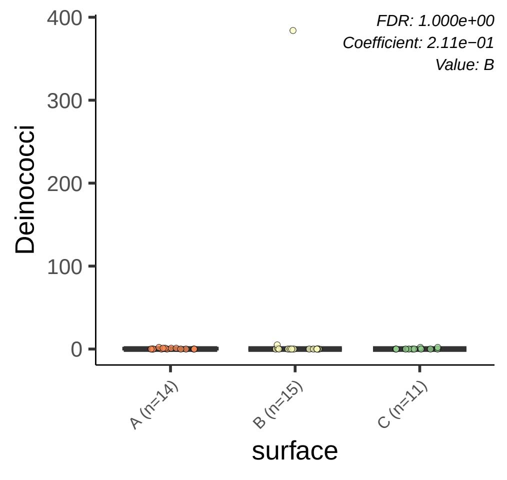

FDR: 1.000e+00
Coefficient: 2.11e−01
Value: B
400
300
200
100
0
Deinococci
A (n=14)
B (n=15)
C (n=11)
surface

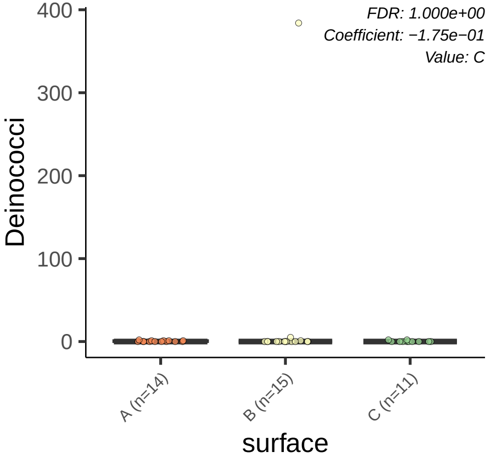
FDR: 1.000e+00
Coefficient: −1.75e−01
Value: C
Deinococci
400
300
200
100
0
A (n=14)
B (n=15)
C (n=11)
surface

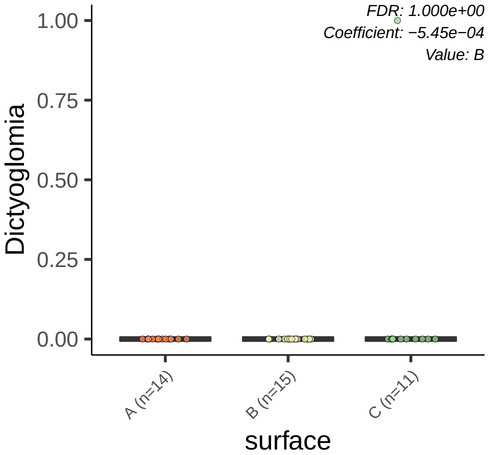
FDR: 1.000e+00
Coefficient: −5.45e−04
Value: B
1.00
0.75
0.50
0.25
0.00
Dictyoglomia
A (n=14)
B (n=15)
C (n=11)
surface

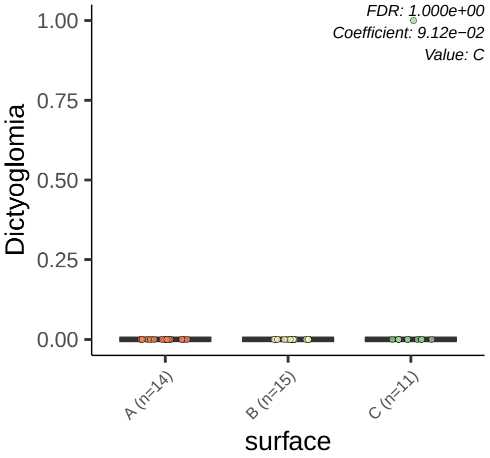

FDR: 1.000e+00
Coefficient: 9.12e−02
Value: C
Dictyoglomia
1.00
0.75
0.50
0.25
0.00
A (n=14)
B (n=15)
C (n=11)
surface

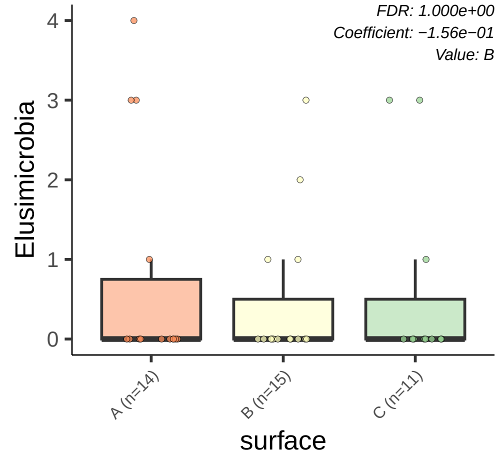
FDR: 1.000e+00
Coefficient: −1.56e−01
Value: B
Elusimicrobia
4
3
2
1
0
A (n=14)
B (n=15)
C (n=11)
surface

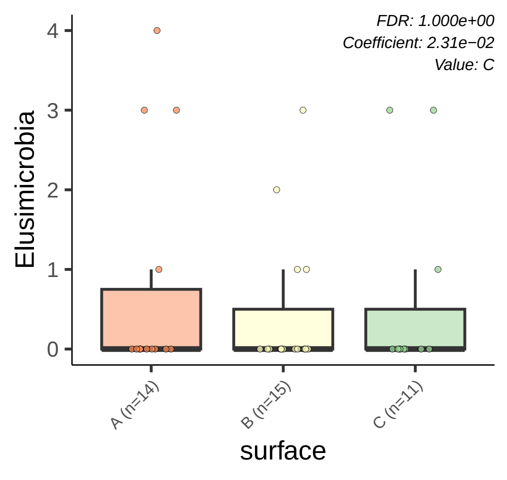
FDR: 1.000e+00
Coefficient: 2.31e−02
Value: C
Elusimicrobia
0
1
2
3
4
A (n=14)
B (n=15)
C (n=11)
surface

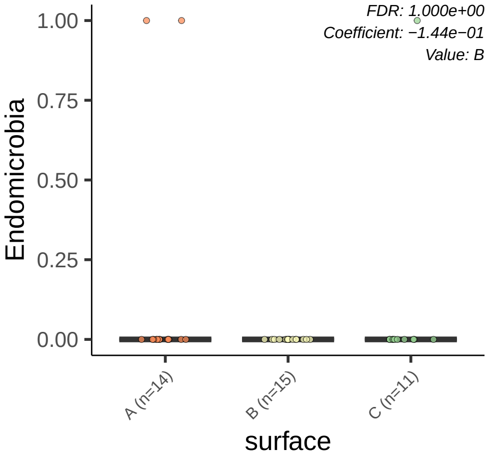

FDR: 1.000e+00
Coefficient: −1.44e−01
Value: B
Endomicrobia
1.00
0.75
0.50
0.25
0.00
A (n=14)
B (n=15)
C (n=11)
surface

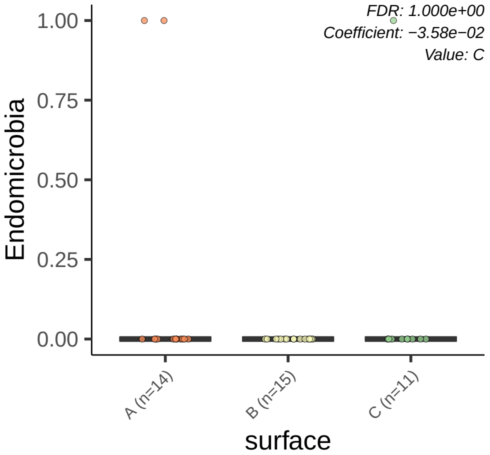

FDR: 1.000e+00
Coefficient: −3.58e−02
Value: C
Endomicrobia
1.00
0.75
0.50
0.25
0.00
A (n=14)
B (n=15)
C (n=11)
surface

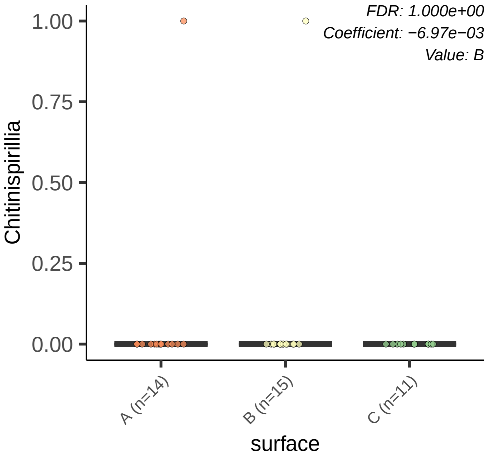
FDR: 1.000e+00
Coefficient: −6.97e−03
Value: B
Chitinispirillia
1.00
0.75
0.50
0.25
0.00
A (n=14)
B (n=15)
C (n=11)
surface

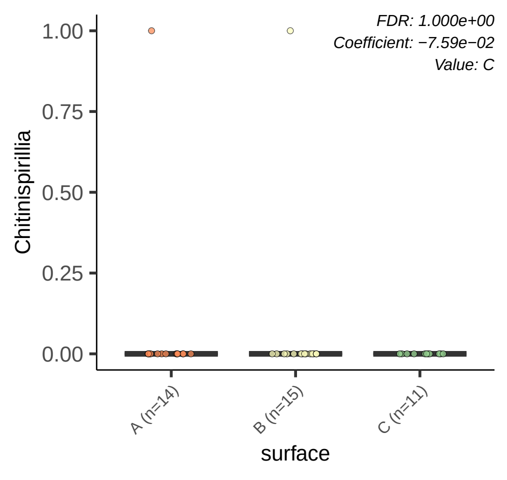

FDR: 1.000e+00
Coefficient: −7.59e−02
Value: C
Chitinispirillia
1.00
0.75
0.50
0.25
0.00
A (n=14)
B (n=15)
C (n=11)
surface

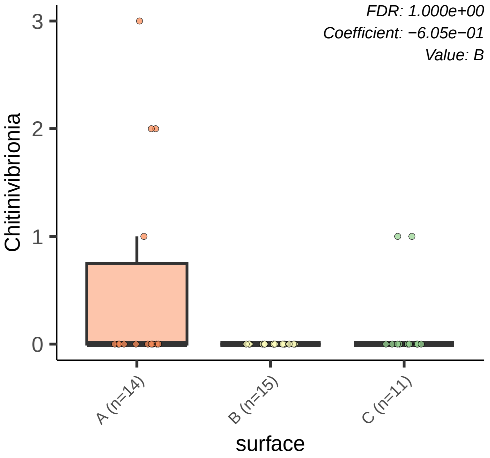

FDR: 1.000e+00
Coefficient: −6.05e−01
Value: B
Chitinivibrionia
3
2
1
0
A (n=14)
B (n=15)
C (n=11)
surface

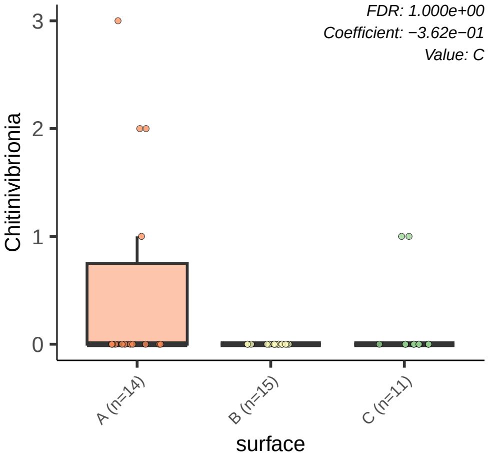

FDR: 1.000e+00
Coefficient: −3.62e−01
Value: C
Chitinivibrionia
3
2
1
0
A (n=14)
B (n=15)
C (n=11)
surface

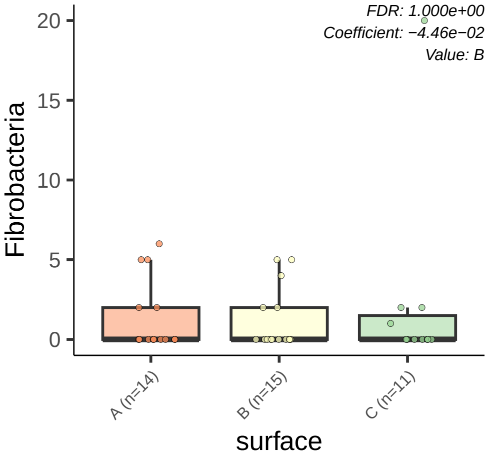
FDR: 1.000e+00
Coefficient: −4.46e−02
Value: B
Fibrobacteria
20
15
10
5
0
A (n=14)
B (n=15)
C (n=11)
surface

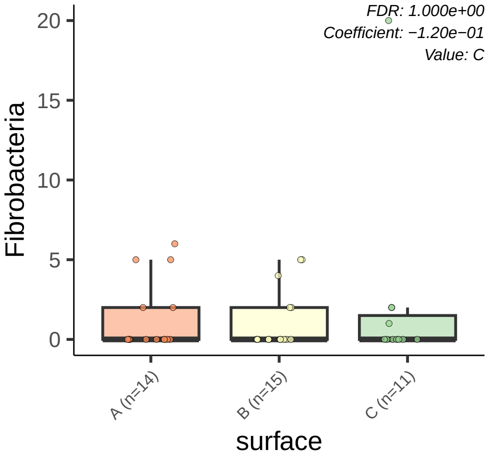
FDR: 1.000e+00
Coefficient: −1.20e−01
Value: C
Fibrobacteria
20
15
10
5
0
A (n=14)
B (n=15)
C (n=11)
surface

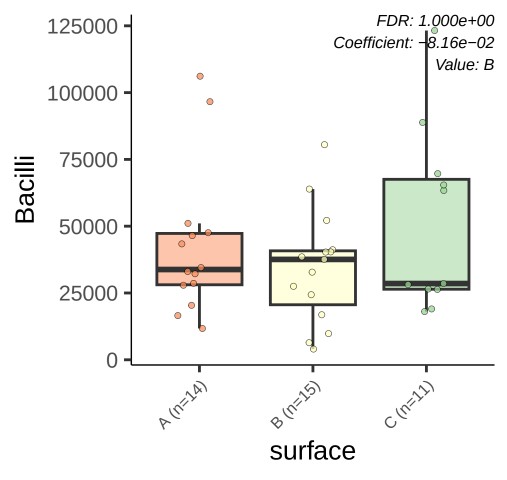

FDR: 1.000e+00
Coefficient: −8.16e−02
Value: B
Bacilli
125000
100000
75000
50000
25000
0
A (n=14)
B (n=15)
C (n=11)
surface

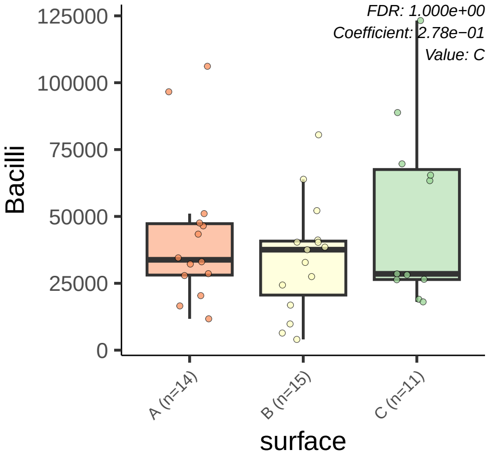
FDR: 1.000e+00
Coefficient: 2.78e−01
Value: C
125000
100000
75000
50000
25000
0
Bacilli
A (n=14)
B (n=15)
C (n=11)
surface

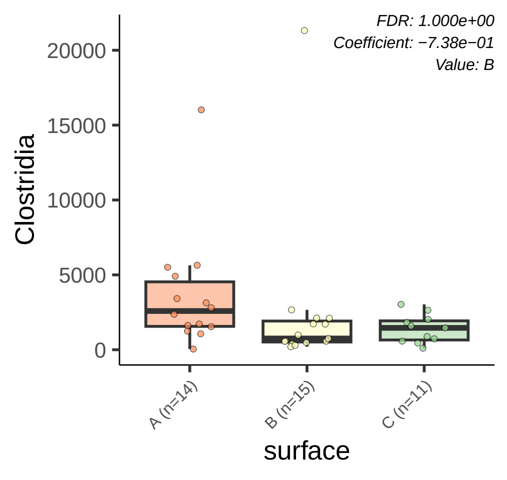

FDR: 1.000e+00
Coefficient: −7.38e−01
Value: B
Clostridia
20000
15000
10000
5000
0
A (n=14)
B (n=15)
C (n=11)
surface

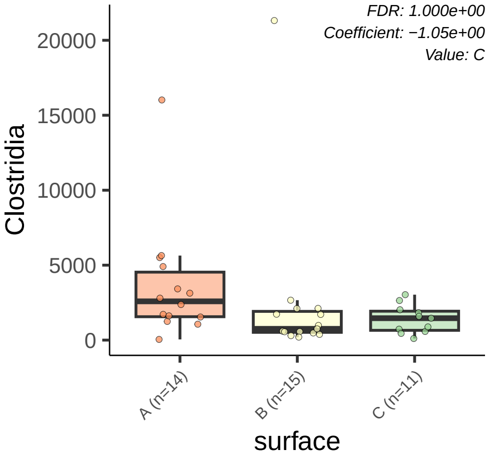
FDR: 1.000e+00
Coefficient: −1.05e+00
Value: C
Clostridia
20000
15000
10000
5000
0
A (n=14)
B (n=15)
C (n=11)
surface

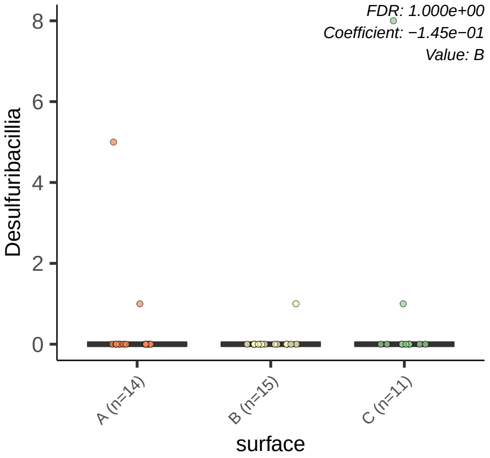
FDR: 1.000e+00
Coefficient: −1.45e−01
Value: B
Desulfuribacillia
8
6
4
2
0
A (n=14)
B (n=15)
C (n=11)
surface

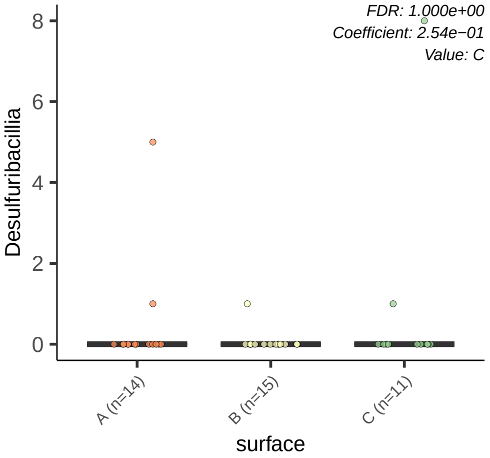

FDR: 1.000e+00
Coefficient: 2.54e−01
Value: C
Desulfuribacillia
8
6
4
2
0
A (n=14)
B (n=15)
C (n=11)
surface

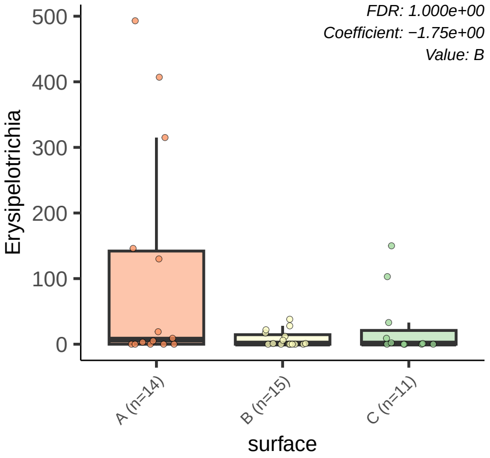

FDR: 1.000e+00
Coefficient: −1.75e+00
Value: B
Erysipelotrichia
500
400
300
200
100
0
A (n=14)
B (n=15)
C (n=11)
surface

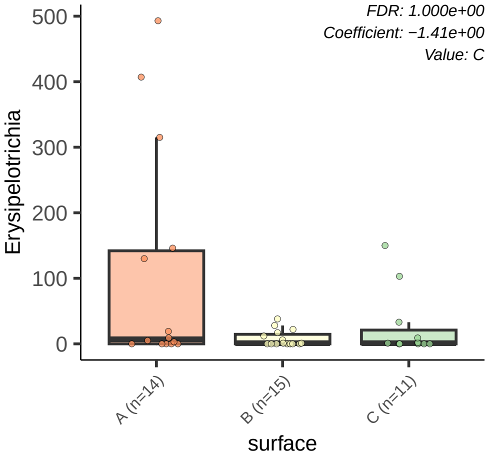

FDR: 1.000e+00
Coefficient: −1.41e+00
Value: C
Erysipelotrichia
500
400
300
200
100
0
A (n=14)
B (n=15)
C (n=11)
surface

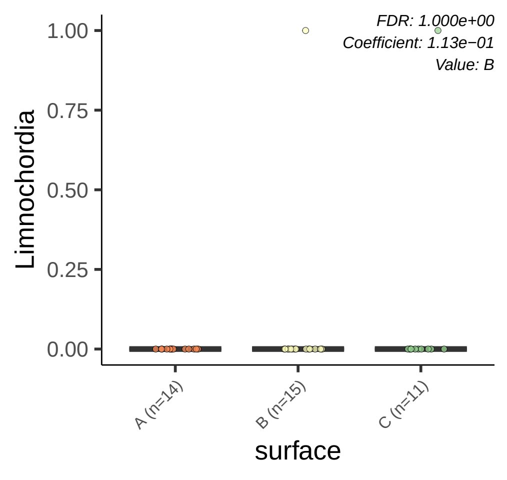

FDR: 1.000e+00
Coefficient: 1.13e−01
Value: B
Limnochordia
1.00
0.75
0.50
0.25
0.00
A (n=14)
B (n=15)
C (n=11)
surface

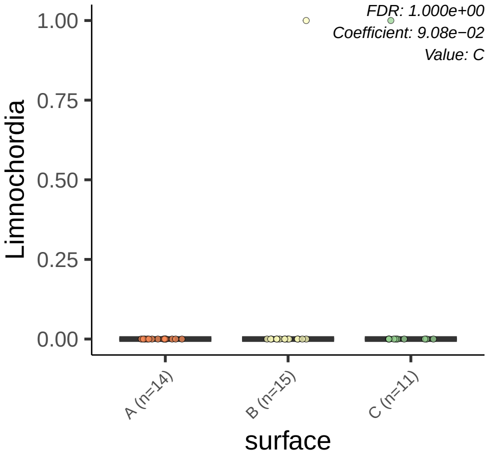

FDR: 1.000e+00
Coefficient: 9.08e−02
Value: C
1.00
0.75
0.50
0.25
0.00
Limnochordia
A (n=14)
B (n=15)
C (n=11)
surface

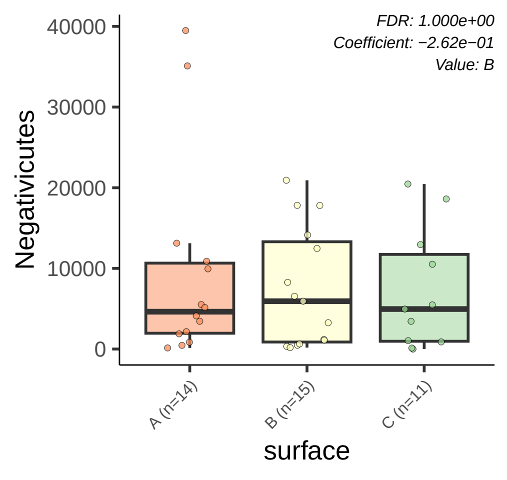

FDR: 1.000e+00
Coefficient: −2.62e−01
Value: B
Negativicutes
40000
30000
20000
10000
0
A (n=14)
B (n=15)
C (n=11)
surface

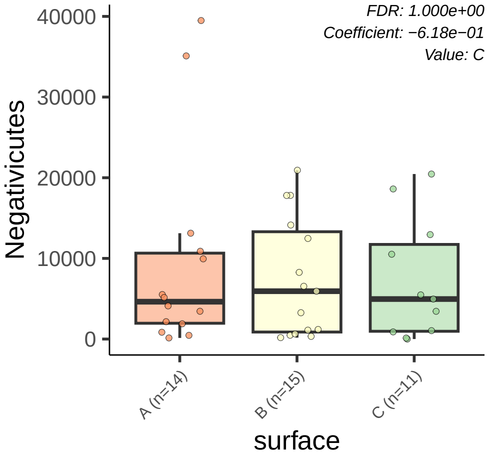

FDR: 1.000e+00
Coefficient: −6.18e−01
Value: C
Negativicutes
40000
30000
20000
10000
0
A (n=14)
B (n=15)
C (n=11)
surface

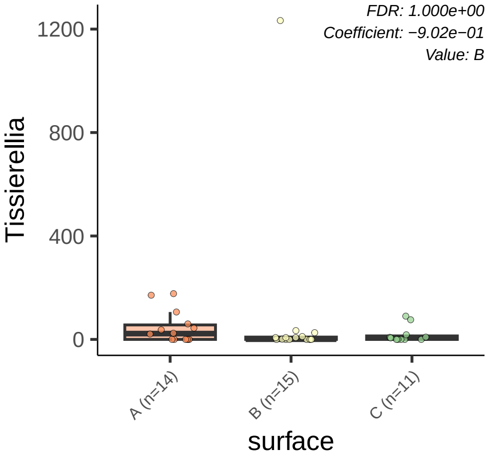

FDR: 1.000e+00
Coefficient: −9.02e−01
Value: B
1200
800
400
0
Tissierellia
A (n=14)
B (n=15)
C (n=11)
surface

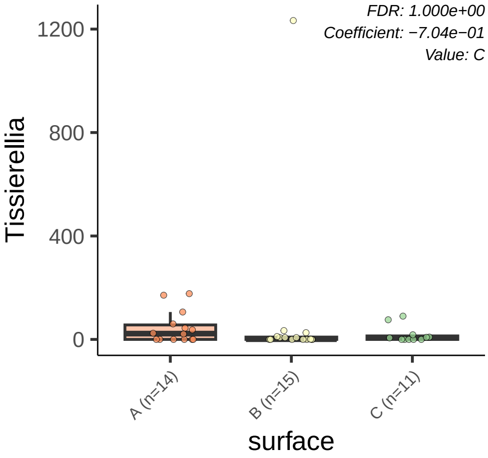

FDR: 1.000e+00
Coefficient: −7.04e−01
Value: C
Tissierellia
1200
800
400
0
A (n=14)
B (n=15)
C (n=11)
surface

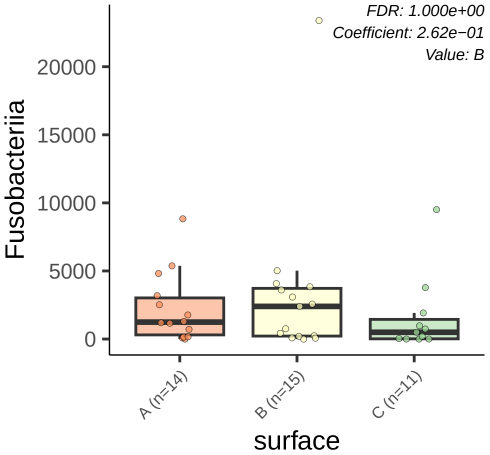
FDR: 1.000e+00
Coefficient: 2.62e−01
Value: B
Fusobacteriia
20000
15000
10000
5000
0
A (n=14)
B (n=15)
C (n=11)
surface

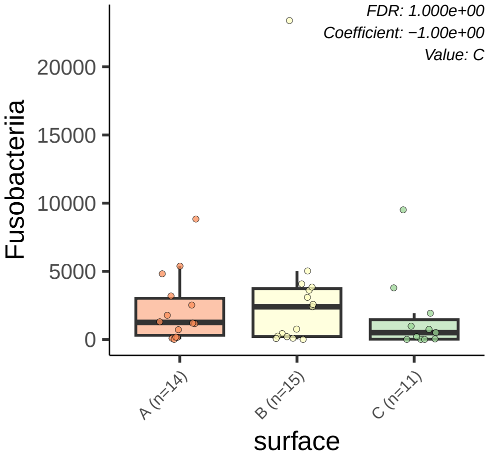

FDR: 1.000e+00
Coefficient: −1.00e+00
Value: C
20000
15000
10000
5000
0
Fusobacteriia
A (n=14)
B (n=15)
C (n=11)
surface

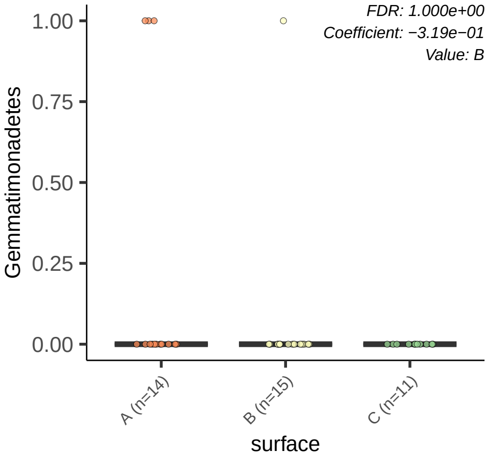
FDR: 1.000e+00
Coefficient: −3.19e−01
Value: B
Gemmatimonadetes
1.00
0.75
0.50
0.25
0.00
A (n=14)
B (n=15)
C (n=11)
surface

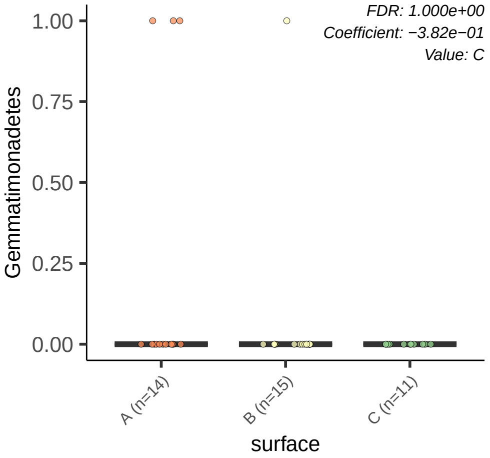

FDR: 1.000e+00
Coefficient: −3.82e−01
Value: C
Gemmatimonadetes
1.00
0.75
0.50
0.25
0.00
A (n=14)
B (n=15)
C (n=11)
surface

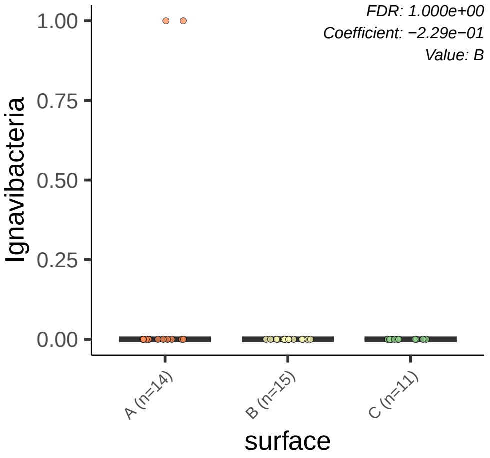

FDR: 1.000e+00
Coefficient: −2.29e−01
Value: B
Ignavibacteria
1.00
0.75
0.50
0.25
0.00
A (n=14)
B (n=15)
C (n=11)
surface

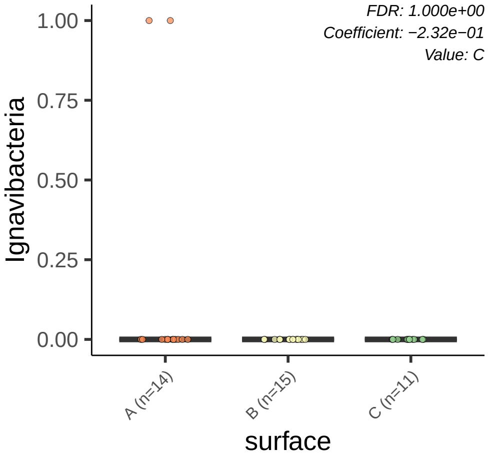

FDR: 1.000e+00
Coefficient: −2.32e−01
Value: C
Ignavibacteria
1.00
0.75
0.50
0.25
0.00
A (n=14)
B (n=15)
C (n=11)
surface

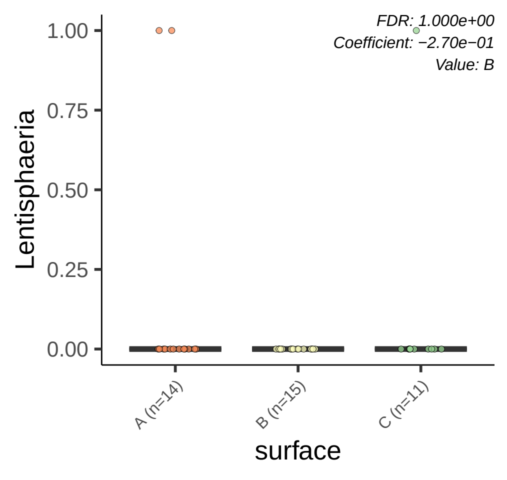

FDR: 1.000e+00
Coefficient: −2.70e−01
Value: B
Lentisphaeria
1.00
0.75
0.50
0.25
0.00
A (n=14)
B (n=15)
C (n=11)
surface

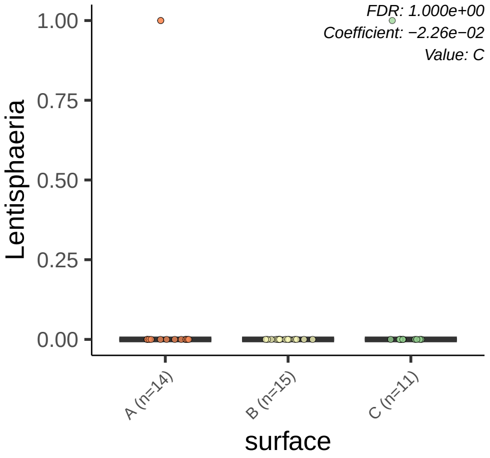
FDR: 1.000e+00
Coefficient: −2.26e−02
Value: C
Lentisphaeria
1.00
0.75
0.50
0.25
0.00
A (n=14)
B (n=15)
C (n=11)
surface

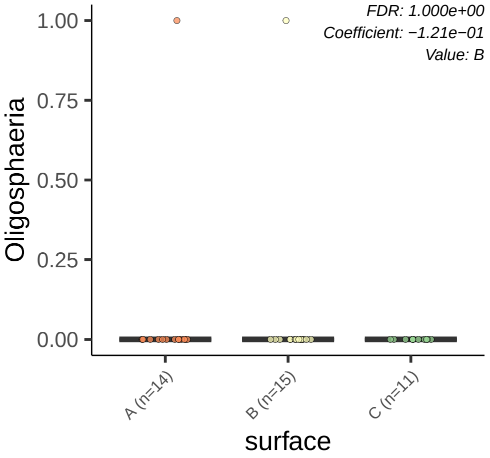

FDR: 1.000e+00
Coefficient: −1.21e−01
Value: B
Oligosphaeria
1.00
0.75
0.50
0.25
0.00
A (n=14)
B (n=15)
C (n=11)
surface

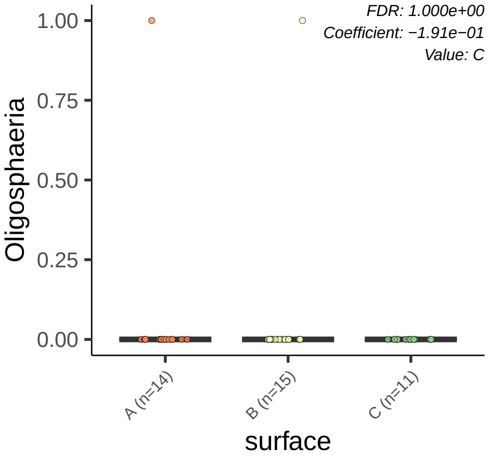
FDR: 1.000e+00
Coefficient: −1.91e−01
Value: C
Oligosphaeria
1.00
0.75
0.50
0.25
0.00
A (n=14)
B (n=15)
C (n=11)
surface

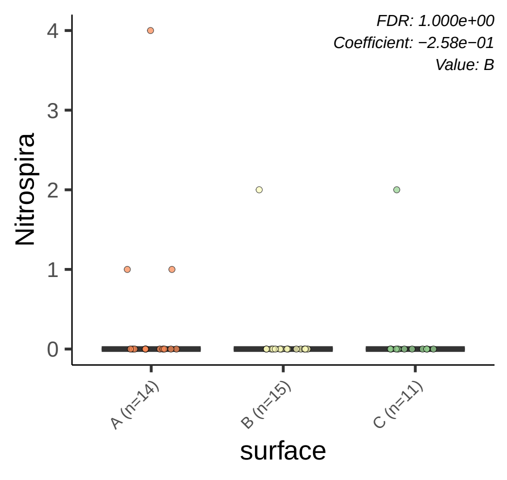

FDR: 1.000e+00
Coefficient: −2.58e−01
Value: B
Nitrospira
4
3
2
1
0
A (n=14)
B (n=15)
C (n=11)
surface

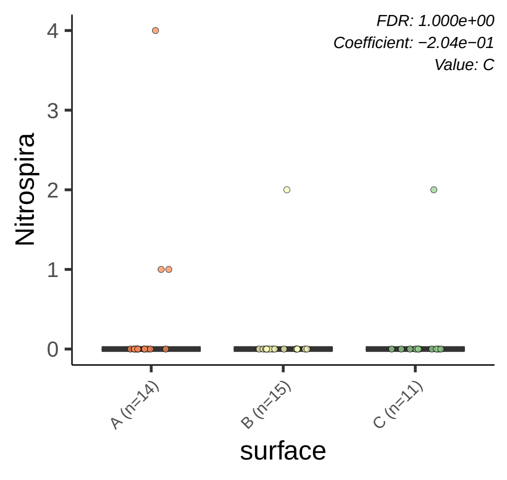

FDR: 1.000e+00
Coefficient: −2.04e−01
Value: C
Nitrospira
4
3
2
1
0
A (n=14)
B (n=15)
C (n=11)
surface

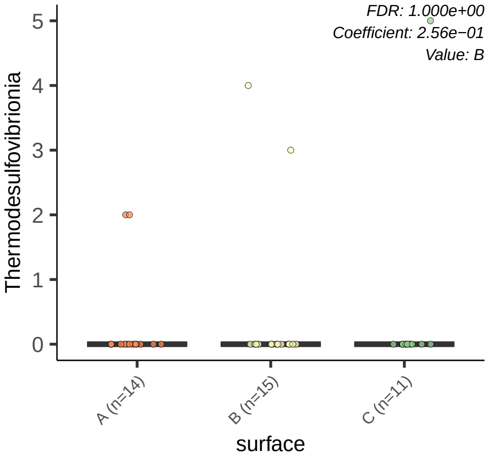

FDR: 1.000e+00
Coefficient: 2.56e−01
Value: B
Thermodesulfovibrionia
0
1
2
3
4
5
A (n=14)
B (n=15)
C (n=11)
surface

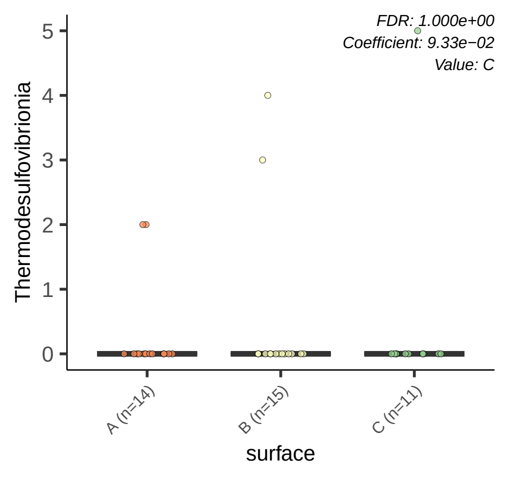
FDR: 1.000e+00
Coefficient: 9.33e−02
Value: C
Thermodesulfovibrionia
0
1
2
3
4
5
A (n=14)
B (n=15)
C (n=11)
surface

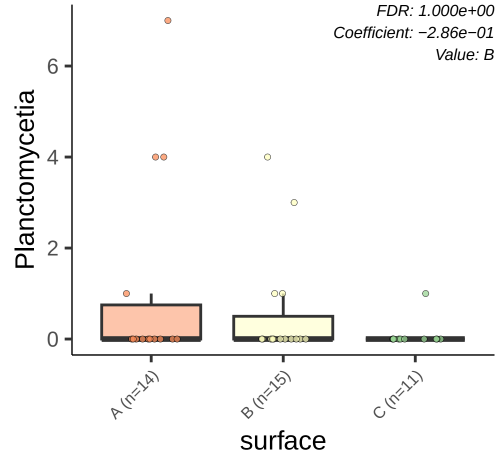
FDR: 1.000e+00
Coefficient: −2.86e−01
Value: B
Planctomycetia
0
2
4
6
A (n=14)
B (n=15)
C (n=11)
surface

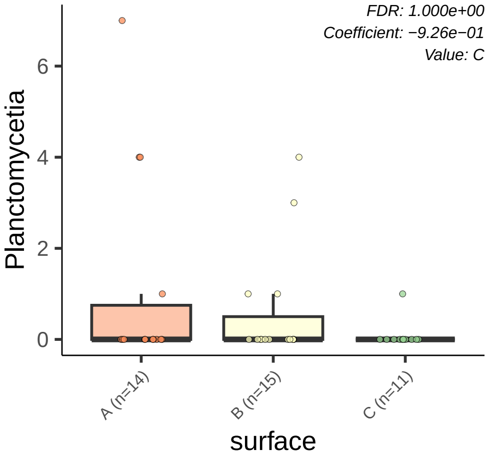

FDR: 1.000e+00
Coefficient: −9.26e−01
Value: C
Planctomycetia
0
2
4
6
A (n=14)
B (n=15)
C (n=11)
surface

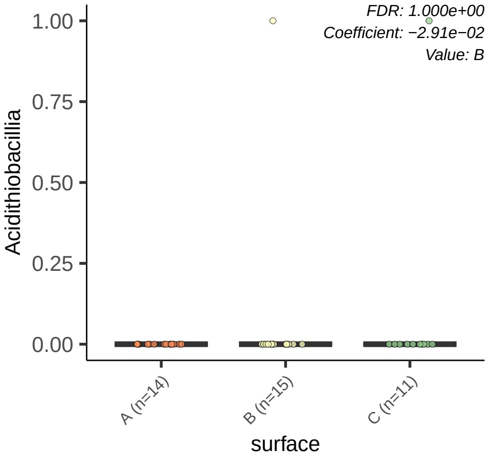

FDR: 1.000e+00
Coefficient: −2.91e−02
Value: B
Acidithiobacillia
1.00
0.75
0.50
0.25
0.00
A (n=14)
B (n=15)
C (n=11)
surface

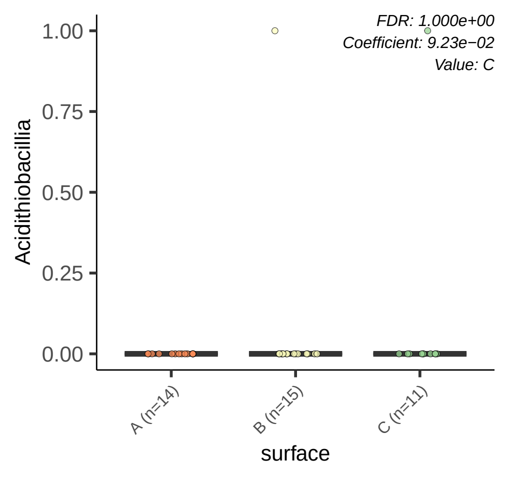
FDR: 1.000e+00
Coefficient: 9.23e−02
Value: C
Acidithiobacillia
1.00
0.75
0.50
0.25
0.00
A (n=14)
B (n=15)
C (n=11)
surface

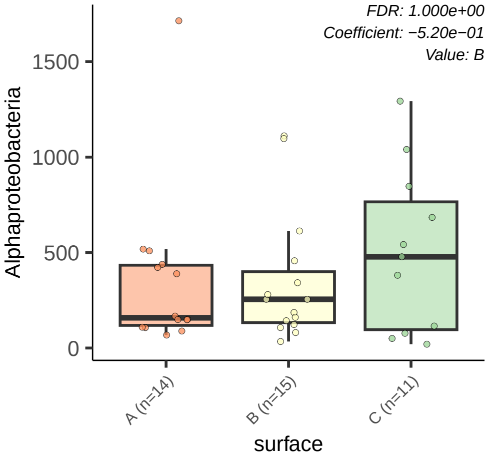

FDR: 1.000e+00
Coefficient: −5.20e−01
Value: B
1500
1000
500
0
Alphaproteobacteria
A (n=14)
B (n=15)
C (n=11)
surface

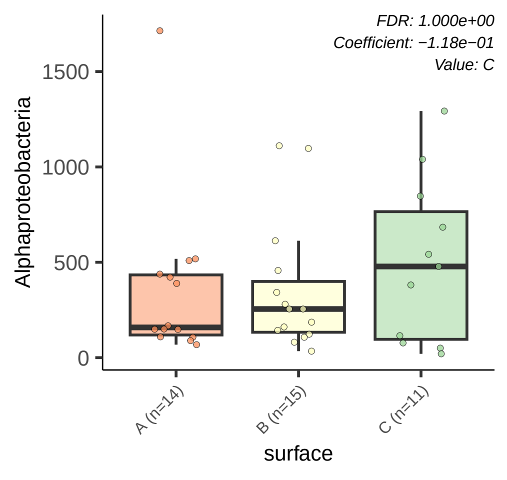
FDR: 1.000e+00
Coefficient: −1.18e−01
Value: C
Alphaproteobacteria
1500
1000
500
0
A (n=14)
B (n=15)
C (n=11)
surface

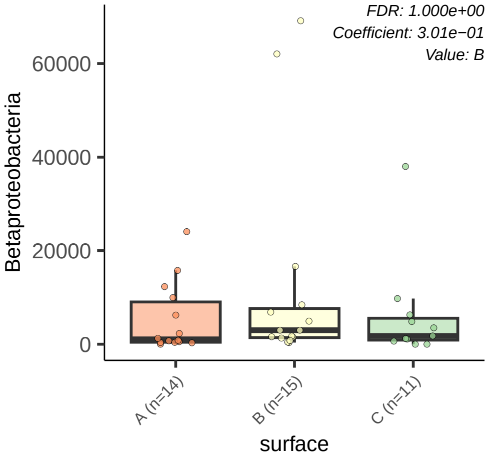

FDR: 1.000e+00
Coefficient: 3.01e−01
Value: B
Betaproteobacteria
60000
40000
20000
0
A (n=14)
B (n=15)
C (n=11)
surface

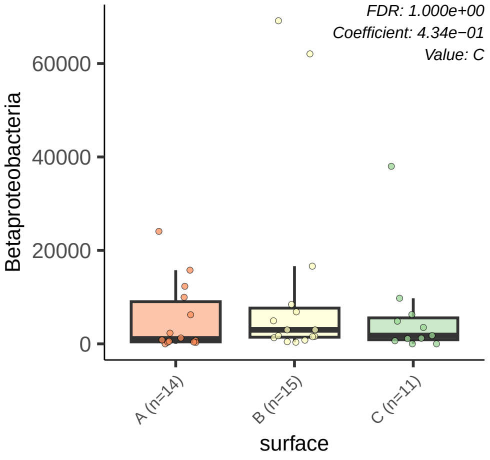

FDR: 1.000e+00
Coefficient: 4.34e−01
Value: C
Betaproteobacteria
60000
40000
20000
0
A (n=14)
B (n=15)
C (n=11)
surface

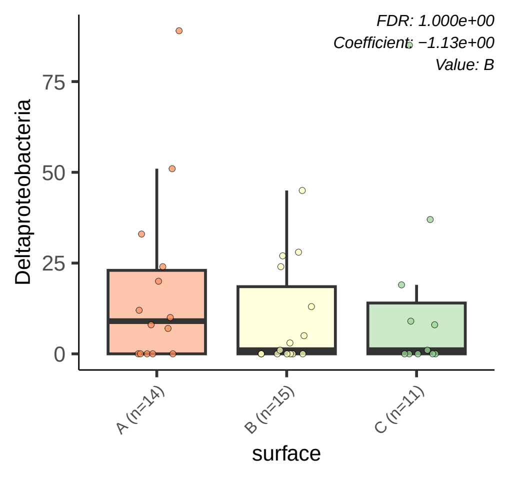
FDR: 1.000e+00
Coefficient: −1.13e+00
Value: B
Deltaproteobacteria
75
50
25
0
A (n=14)
B (n=15)
C (n=11)
surface

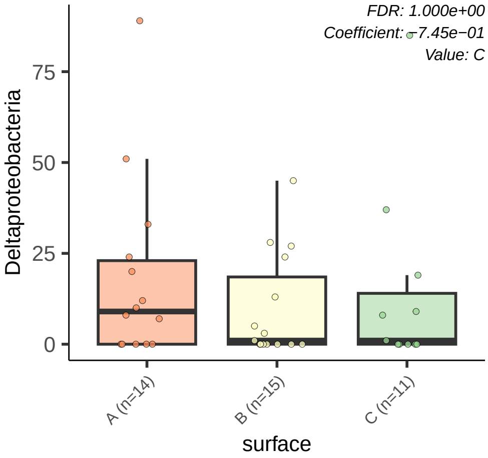
FDR: 1.000e+00
Coefficient: −7.45e−01
Value: C
Deltaproteobacteria
75
50
25
0
A (n=14)
B (n=15)
C (n=11)
surface

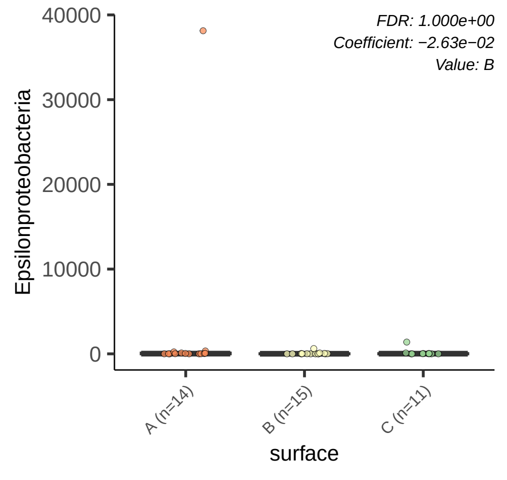

FDR: 1.000e+00
Coefficient: −2.63e−02
Value: B
Epsilonproteobacteria
40000
30000
20000
10000
0
A (n=14)
B (n=15)
C (n=11)
surface

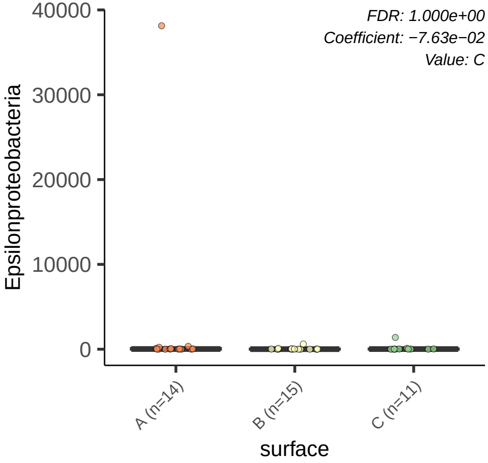

FDR: 1.000e+00
Coefficient: −7.63e−02
Value: C
Epsilonproteobacteria
40000
30000
20000
10000
0
A (n=14)
B (n=15)
C (n=11)
surface

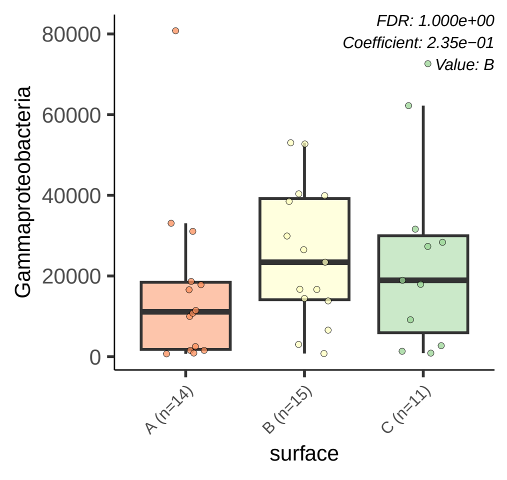

FDR: 1.000e+00
Coefficient: 2.35e−01
Value: B
80000
60000
40000
20000
0
Gammaproteobacteria
A (n=14)
B (n=15)
C (n=11)
surface

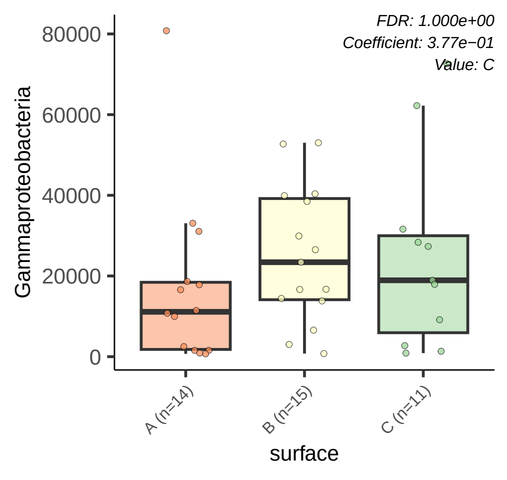

FDR: 1.000e+00
Coefficient: 3.77e−01
Value: C
Gammaproteobacteria
80000
60000
40000
20000
0
A (n=14)
B (n=15)
C (n=11)
surface

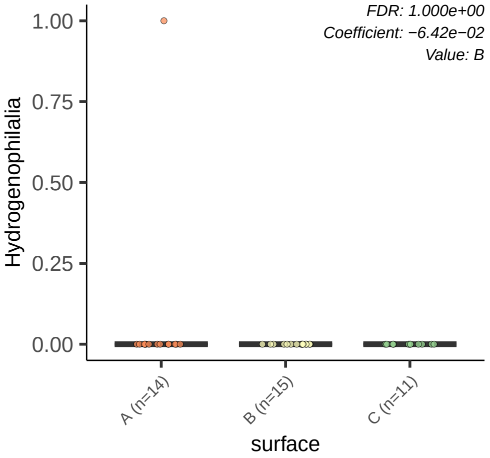

FDR: 1.000e+00
Coefficient: −6.42e−02
Value: B
Hydrogenophilalia
1.00
0.75
0.50
0.25
0.00
A (n=14)
B (n=15)
C (n=11)
surface

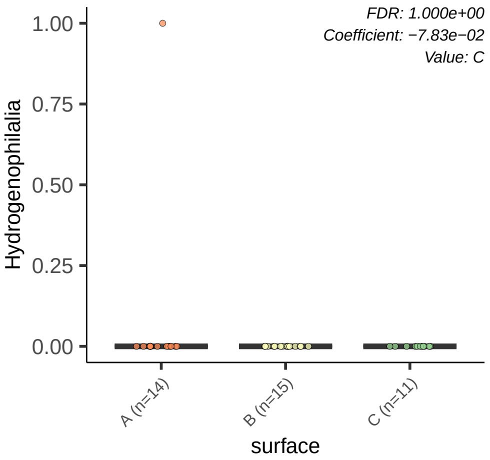
FDR: 1.000e+00
Coefficient: −7.83e−02
Value: C
Hydrogenophilalia
1.00
0.75
0.50
0.25
0.00
A (n=14)
B (n=15)
C (n=11)
surface

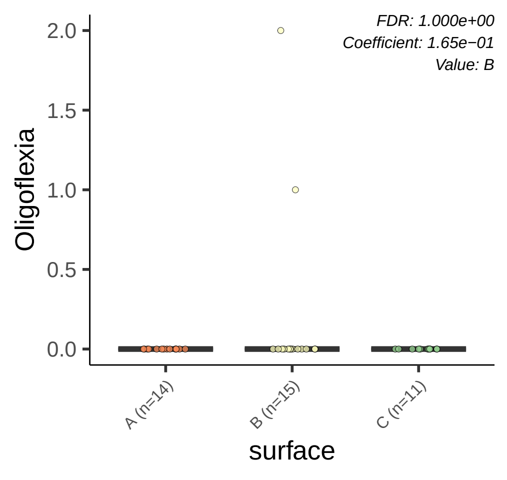
FDR: 1.000e+00
Coefficient: 1.65e−01
Value: B
Oligoflexia
2.0
1.5
1.0
0.5
0.0
A (n=14)
B (n=15)
C (n=11)
surface

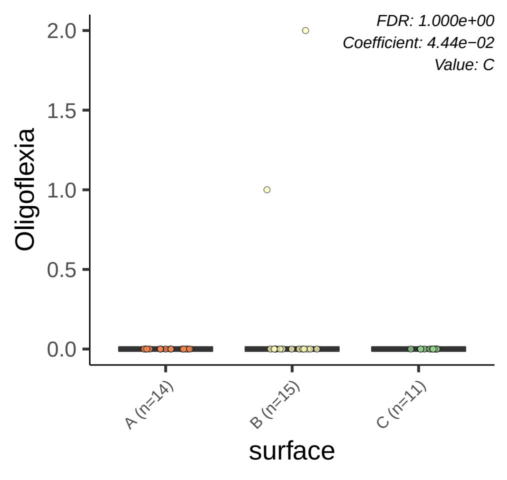
FDR: 1.000e+00
Coefficient: 4.44e−02
Value: C
Oligoflexia
2.0
1.5
1.0
0.5
0.0
A (n=14)
B (n=15)
C (n=11)
surface

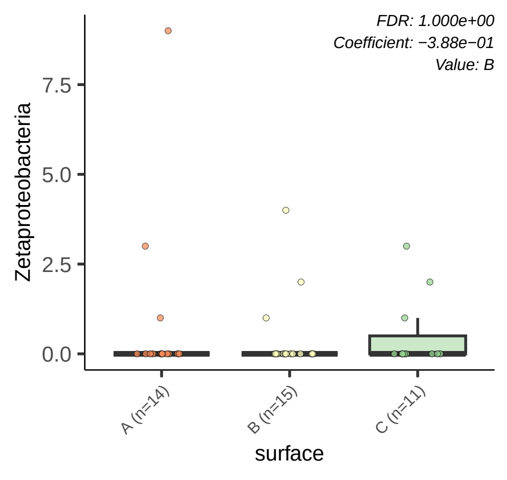

FDR: 1.000e+00
Coefficient: −3.88e−01
Value: B
Zetaproteobacteria
7.5
5.0
2.5
0.0
A (n=14)
B (n=15)
C (n=11)
surface

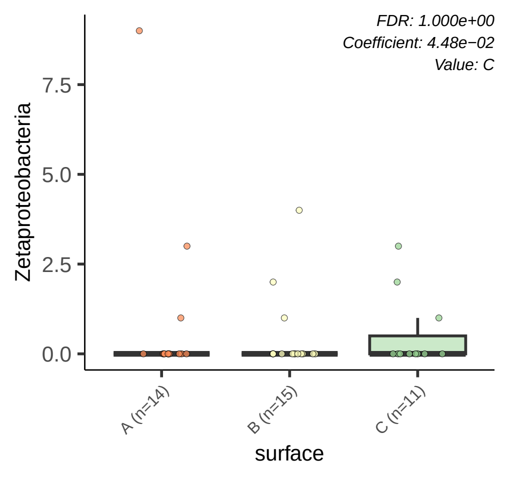
FDR: 1.000e+00
Coefficient: 4.48e−02
Value: C
Zetaproteobacteria
7.5
5.0
2.5
0.0
A (n=14)
B (n=15)
C (n=11)
surface

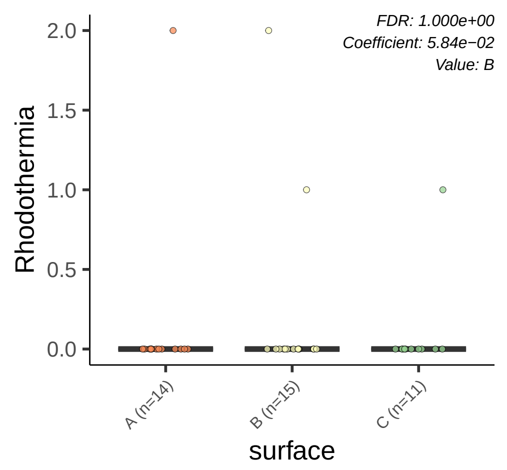

FDR: 1.000e+00
Coefficient: 5.84e−02
Value: B
Rhodothermia
2.0
1.5
1.0
0.5
0.0
A (n=14)
B (n=15)
C (n=11)
surface

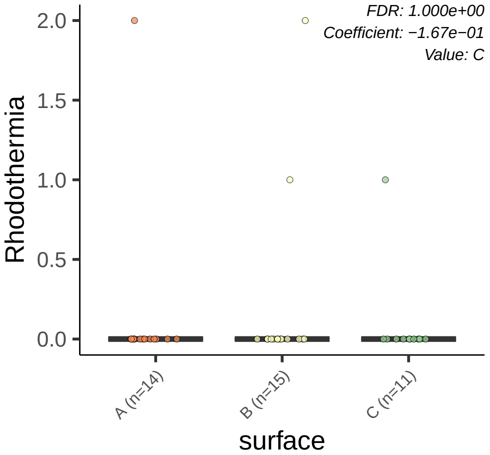

FDR: 1.000e+00
Coefficient: −1.67e−01
Value: C
Rhodothermia
2.0
1.5
1.0
0.5
0.0
A (n=14)
B (n=15)
C (n=11)
surface

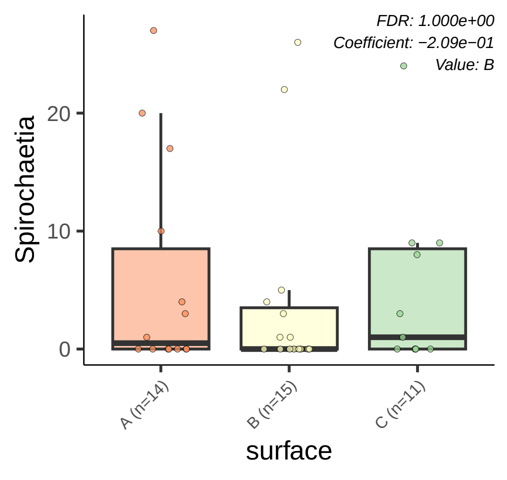
FDR: 1.000e+00
Coefficient: −2.09e−01
Value: B
Spirochaetia
20
10
0
A (n=14)
B (n=15)
C (n=11)
surface

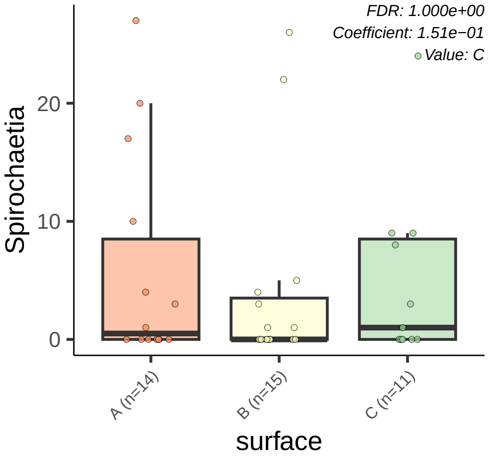
FDR: 1.000e+00
Coefficient: 1.51e−01
Value: C
Spirochaetia
0
10
20
A (n=14)
B (n=15)
C (n=11)
surface

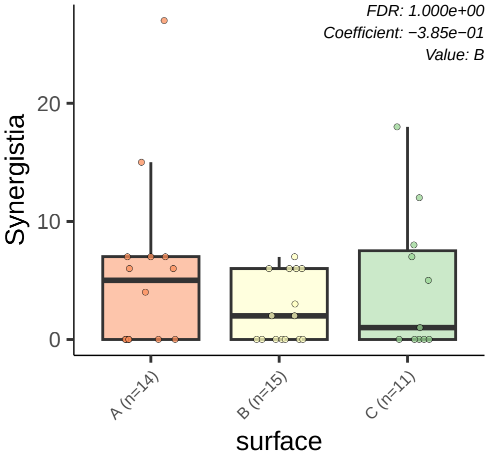

FDR: 1.000e+00
Coefficient: −3.85e−01
Value: B
Synergistia
20
10
0
A (n=14)
B (n=15)
C (n=11)
surface

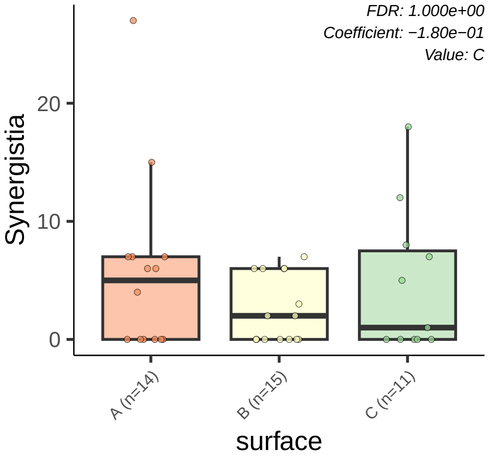

FDR: 1.000e+00
Coefficient: −1.80e−01
Value: C
Synergistia
20
10
0
A (n=14)
B (n=15)
C (n=11)
surface

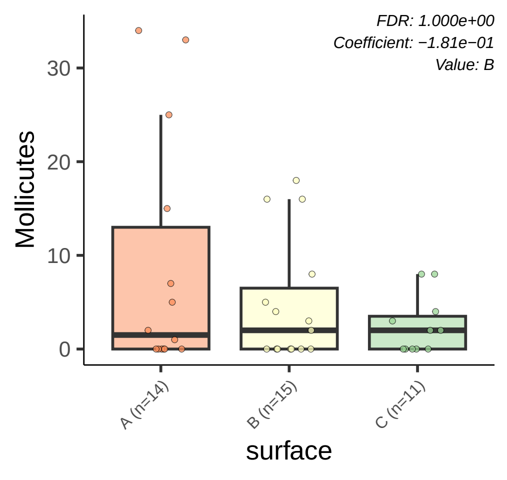
FDR: 1.000e+00
Coefficient: −1.81e−01
Value: B
Mollicutes
0
10
20
30
A (n=14)
B (n=15)
C (n=11)
surface

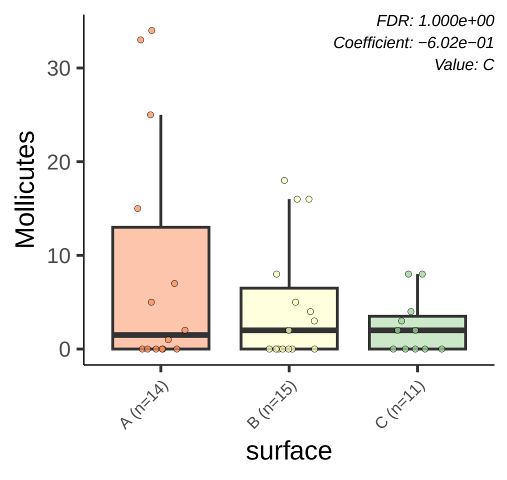

FDR: 1.000e+00
Coefficient: −6.02e−01
Value: C
Mollicutes
0
10
20
30
A (n=14)
B (n=15)
C (n=11)
surface

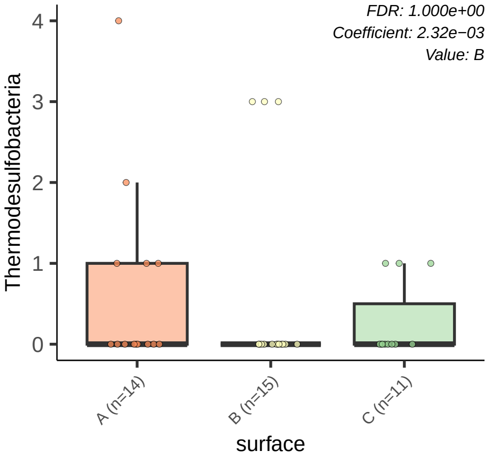

FDR: 1.000e+00
Coefficient: 2.32e−03
Value: B
Thermodesulfobacteria
0
1
2
3
4
A (n=14)
B (n=15)
C (n=11)
surface

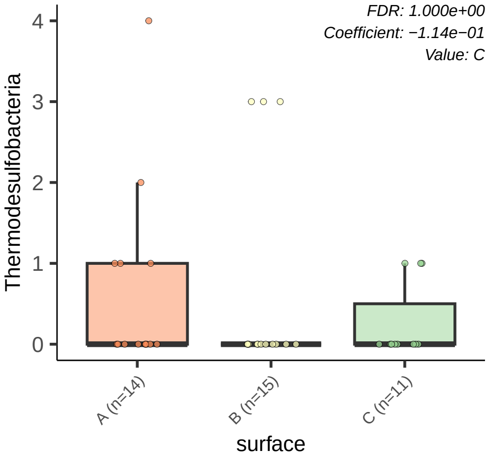

FDR: 1.000e+00
Coefficient: −1.14e−01
Value: C
Thermodesulfobacteria
4
3
2
1
0
A (n=14)
B (n=15)
C (n=11)
surface

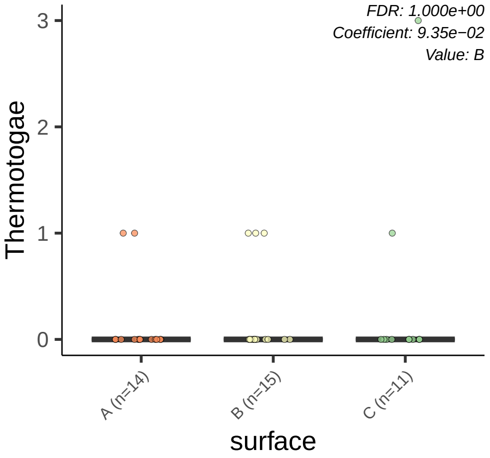
FDR: 1.000e+00
Coefficient: 9.35e−02
Value: B
Thermotogae
3
2
1
0
A (n=14)
B (n=15)
C (n=11)
surface

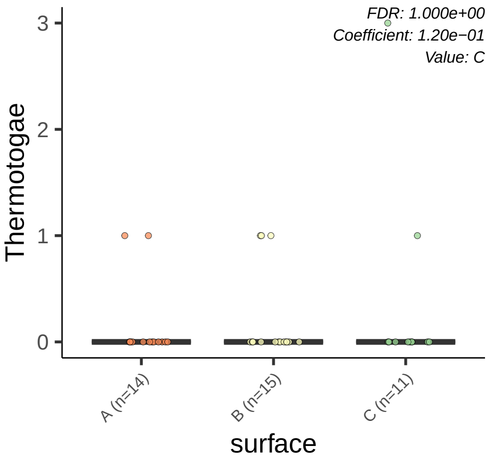
FDR: 1.000e+00
Coefficient: 1.20e−01
Value: C
Thermotogae
3
2
1
0
A (n=14)
B (n=15)
C (n=11)
surface

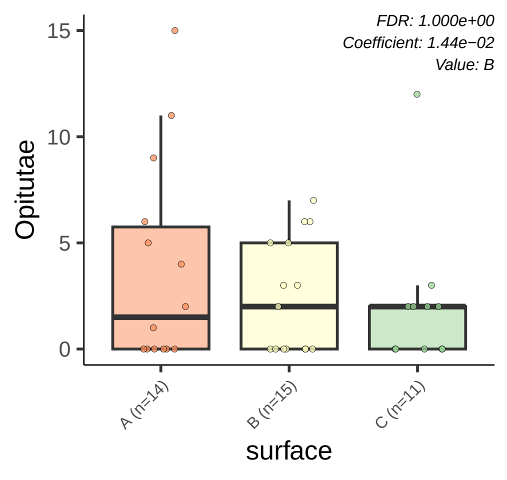

FDR: 1.000e+00
Coefficient: 1.44e−02
Value: B
Opitutae
15
10
5
0
A (n=14)
B (n=15)
C (n=11)
surface

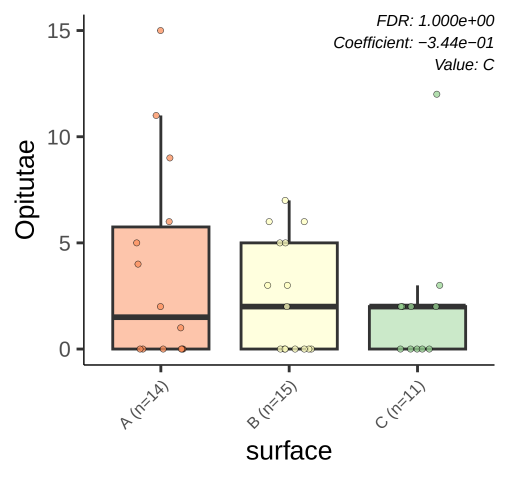
FDR: 1.000e+00
Coefficient: −3.44e−01
Value: C
Opitutae
15
10
5
0
A (n=14)
B (n=15)
C (n=11)
surface

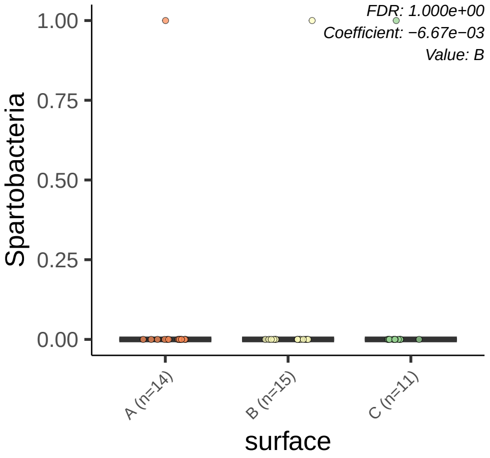

FDR: 1.000e+00
Coefficient: −6.67e−03
Value: B
Spartobacteria
1.00
0.75
0.50
0.25
0.00
A (n=14)
B (n=15)
C (n=11)
surface

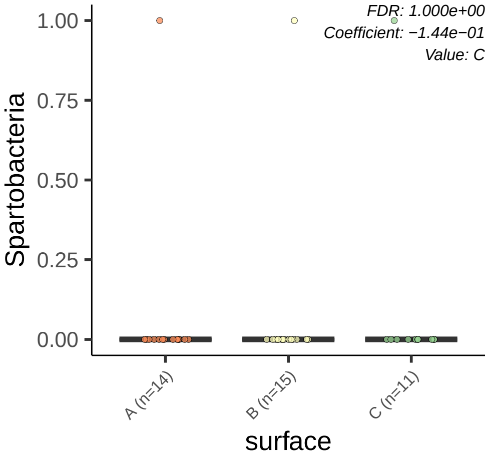

FDR: 1.000e+00
Coefficient: −1.44e−01
Value: C
Spartobacteria
1.00
0.75
0.50
0.25
0.00
A (n=14)
B (n=15)
C (n=11)
surface

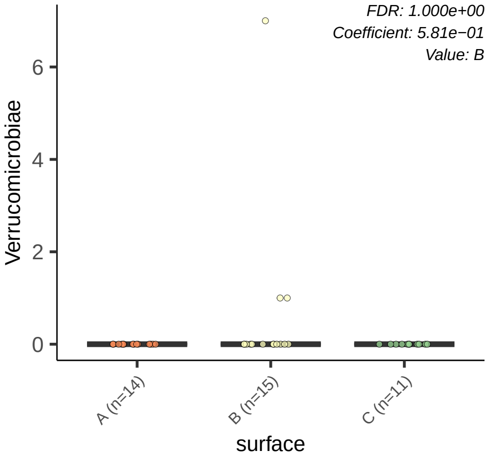

FDR: 1.000e+00
Coefficient: 5.81e−01
Value: B
Verrucomicrobiae
0
2
4
6
A (n=14)
B (n=15)
C (n=11)
surface

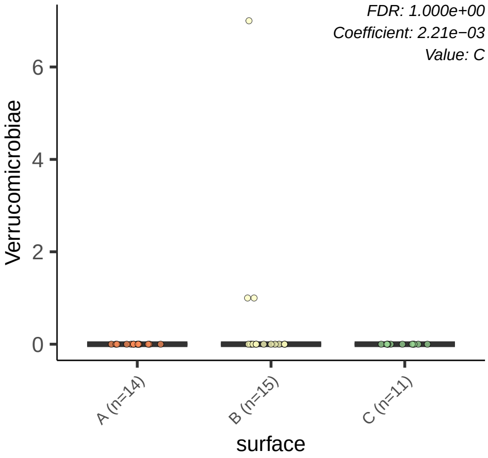
FDR: 1.000e+00
Coefficient: 2.21e−03
Value: C
Verrucomicrobiae
0
2
4
6
A (n=14)
B (n=15)
C (n=11)
surface

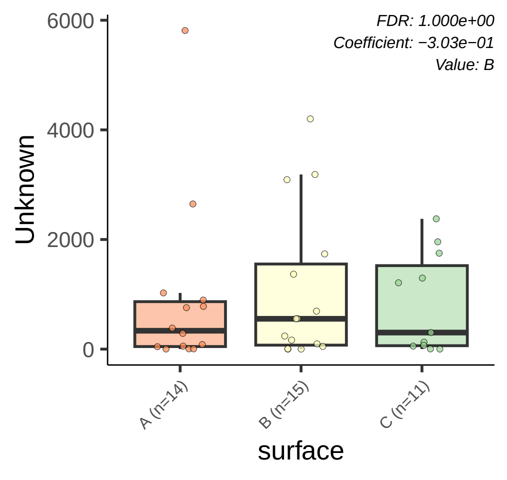

FDR: 1.000e+00
Coefficient: −3.03e−01
Value: B
6000
4000
2000
0
Unknown
A (n=14)
B (n=15)
C (n=11)
surface

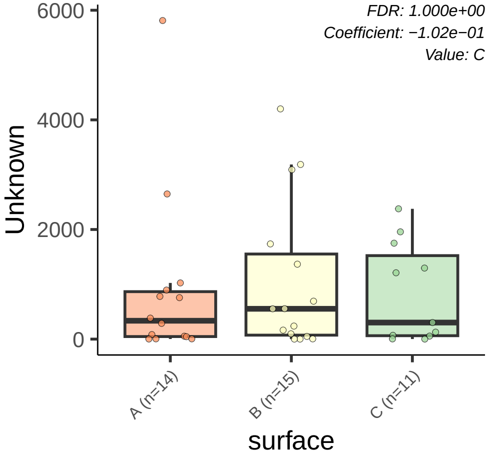
FDR: 1.000e+00
Coefficient: −1.02e−01
Value: C
6000
4000
2000
0
Unknown
A (n=14)
B (n=15)
C (n=11)
surface

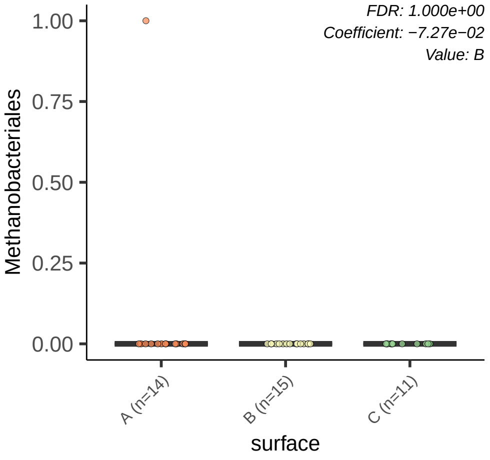
FDR: 1.000e+00
Coefficient: −7.27e−02
Value: B
Methanobacteriales
1.00
0.75
0.50
0.25
0.00
A (n=14)
B (n=15)
C (n=11)
surface

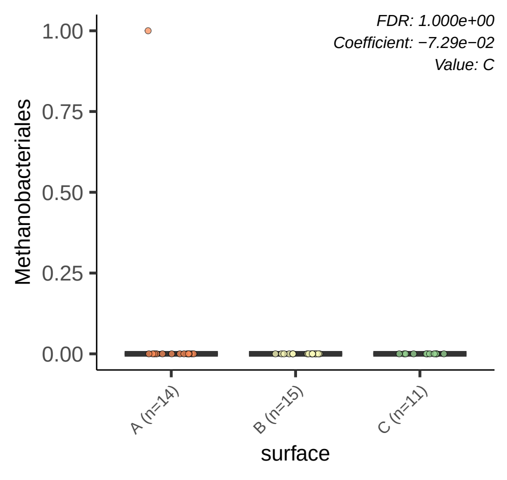

FDR: 1.000e+00
Coefficient: −7.29e−02
Value: C
Methanobacteriales
1.00
0.75
0.50
0.25
0.00
A (n=14)
B (n=15)
C (n=11)
surface

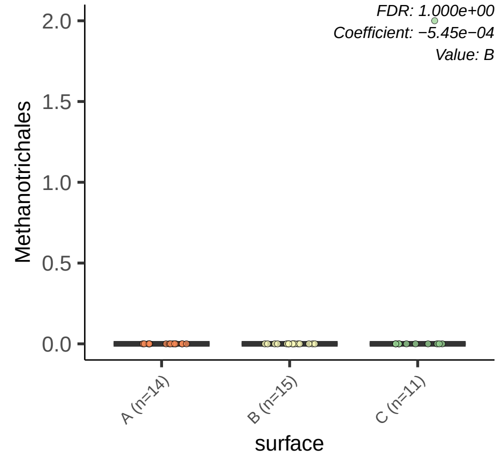

FDR: 1.000e+00
Coefficient: −5.45e−04
Value: B
Methanotrichales
2.0
1.5
1.0
0.5
0.0
A (n=14)
B (n=15)
C (n=11)
surface

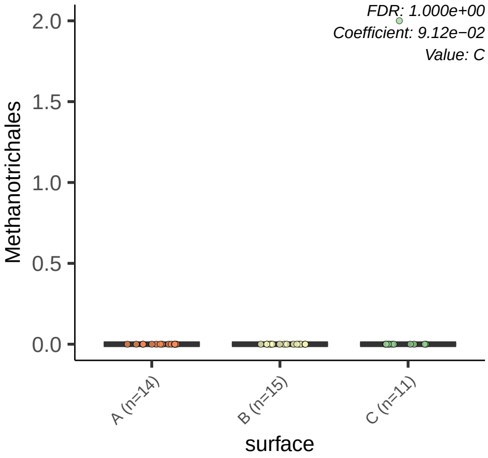
FDR: 1.000e+00
Coefficient: 9.12e−02
Value: C
Methanotrichales
2.0
1.5
1.0
0.5
0.0
A (n=14)
B (n=15)
C (n=11)
surface

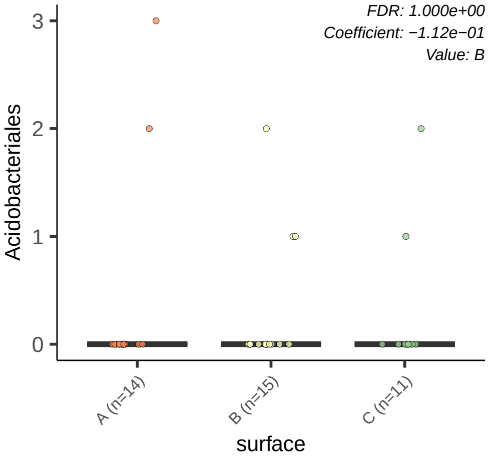

FDR: 1.000e+00
Coefficient: −1.12e−01
Value: B
Acidobacteriales
3
2
1
0
A (n=14)
B (n=15)
C (n=11)
surface

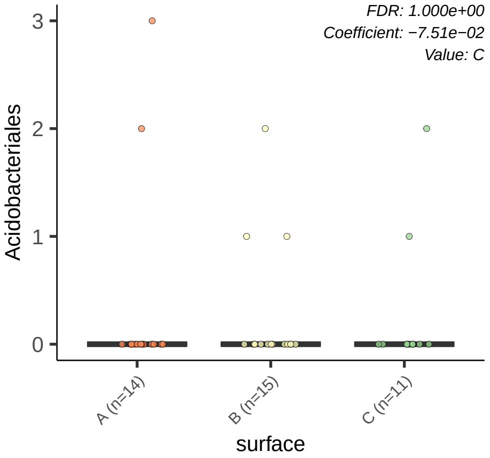
FDR: 1.000e+00
Coefficient: −7.51e−02
Value: C
Acidobacteriales
0
1
2
3
A (n=14)
B (n=15)
C (n=11)
surface

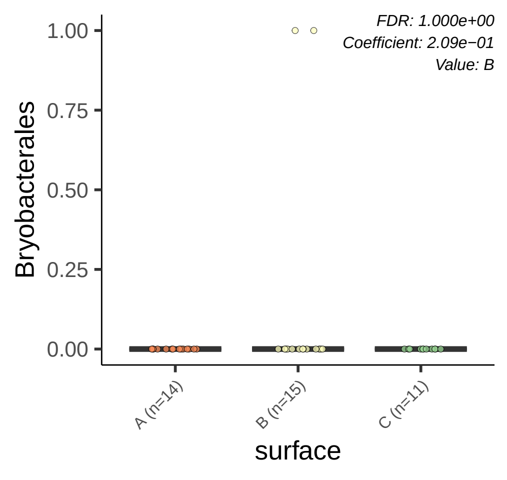

FDR: 1.000e+00
Coefficient: 2.09e−01
Value: B
Bryobacterales
1.00
0.75
0.50
0.25
0.00
A (n=14)
B (n=15)
C (n=11)
surface

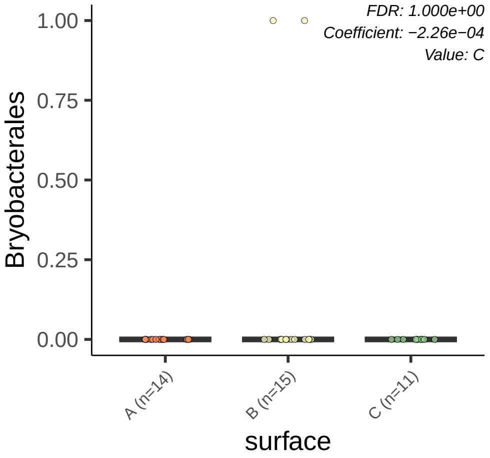

FDR: 1.000e+00
Coefficient: −2.26e−04
Value: C
Bryobacterales
1.00
0.75
0.50
0.25
0.00
A (n=14)
B (n=15)
C (n=11)
surface

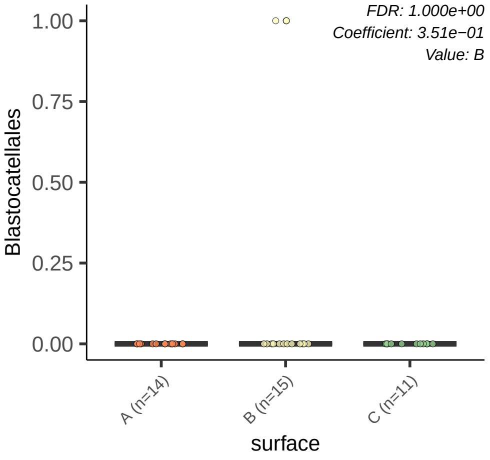
FDR: 1.000e+00
Coefficient: 3.51e−01
Value: B
1.00
0.75
0.50
0.25
0.00
Blastocatellales
A (n=14)
B (n=15)
C (n=11)
surface

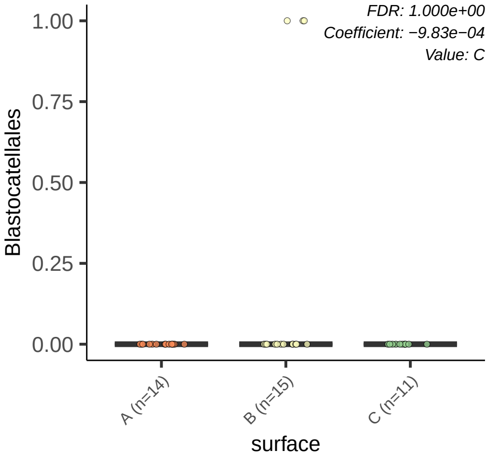

FDR: 1.000e+00
Coefficient: −9.83e−04
Value: C
Blastocatellales
1.00
0.75
0.50
0.25
0.00
A (n=14)
B (n=15)
C (n=11)
surface

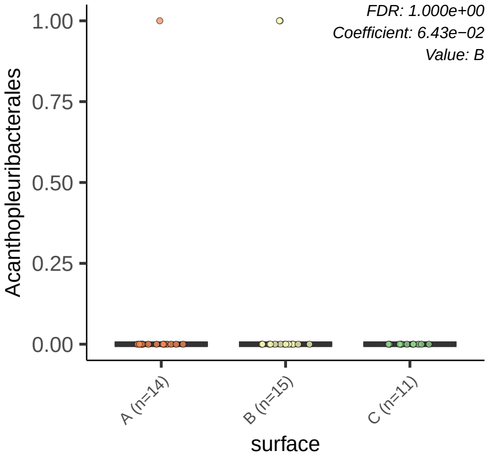
FDR: 1.000e+00
Coefficient: 6.43e−02
Value: B
Acanthopleuribacterales
1.00
0.75
0.50
0.25
0.00
A (n=14)
B (n=15)
C (n=11)
surface

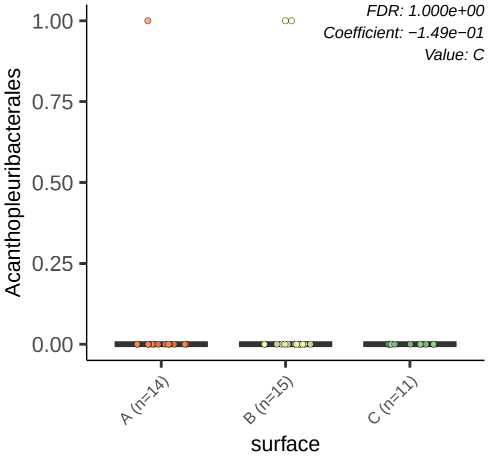

FDR: 1.000e+00
Coefficient: −1.49e−01
Value: C
Acanthopleuribacterales
1.00
0.75
0.50
0.25
0.00
A (n=14)
B (n=15)
C (n=11)
surface

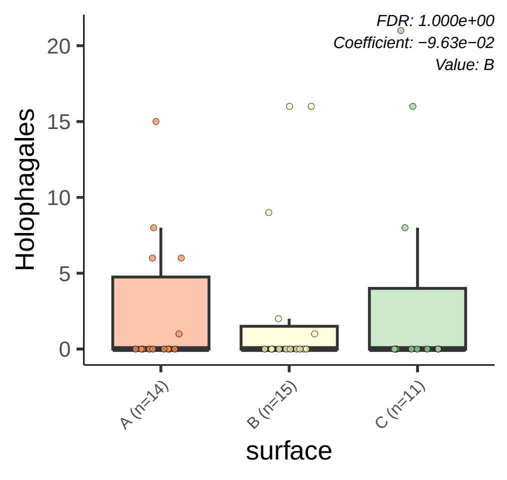

FDR: 1.000e+00
Coefficient: −9.63e−02
Value: B
Holophagales
0
5
10
15
20
A (n=14)
B (n=15)
C (n=11)
surface

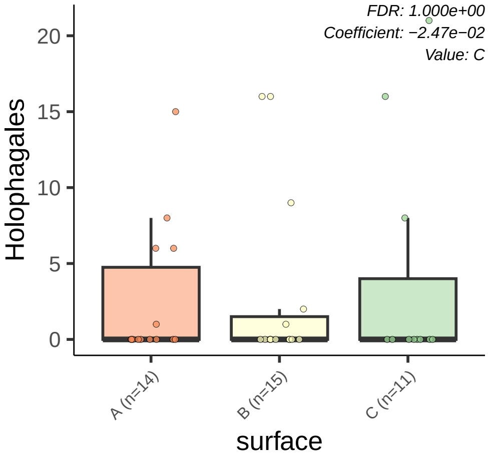
FDR: 1.000e+00
Coefficient: −2.47e−02
Value: C
Holophagales
0
5
10
15
20
A (n=14)
B (n=15)
C (n=11)
surface

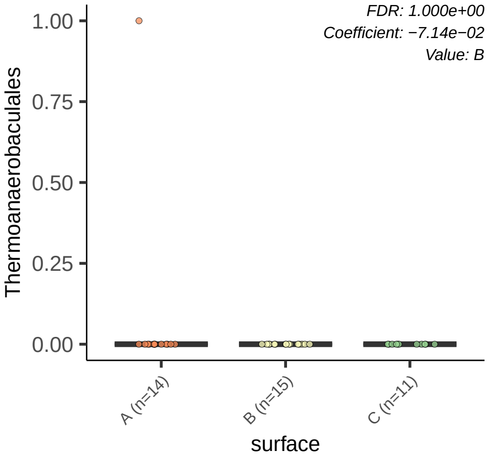
FDR: 1.000e+00
Coefficient: −7.14e−02
Value: B
Thermoanaerobaculales
1.00
0.75
0.50
0.25
0.00
A (n=14)
B (n=15)
C (n=11)
surface

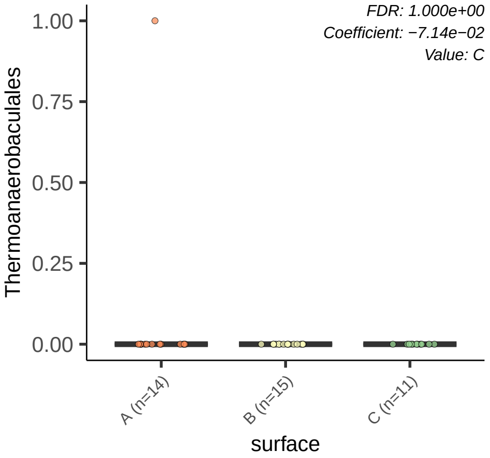
FDR: 1.000e+00
Coefficient: −7.14e−02
Value: C
Thermoanaerobaculales
1.00
0.75
0.50
0.25
0.00
A (n=14)
B (n=15)
C (n=11)
surface

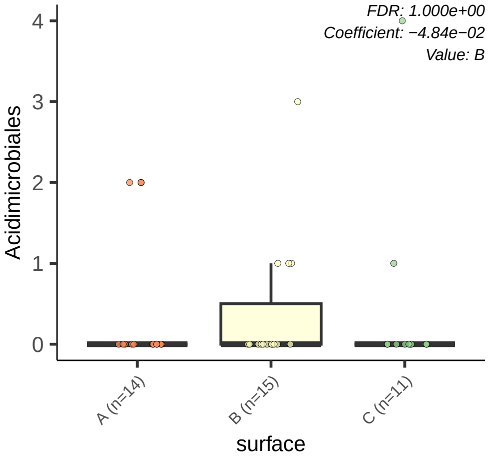
FDR: 1.000e+00
Coefficient: −4.84e−02
Value: B
Acidimicrobiales
4
3
2
1
0
A (n=14)
B (n=15)
C (n=11)
surface

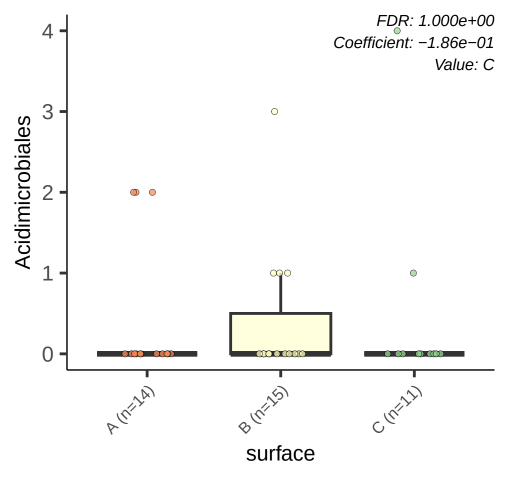

FDR: 1.000e+00
Coefficient: −1.86e−01
Value: C
Acidimicrobiales
4
3
2
1
0
A (n=14)
B (n=15)
C (n=11)
surface

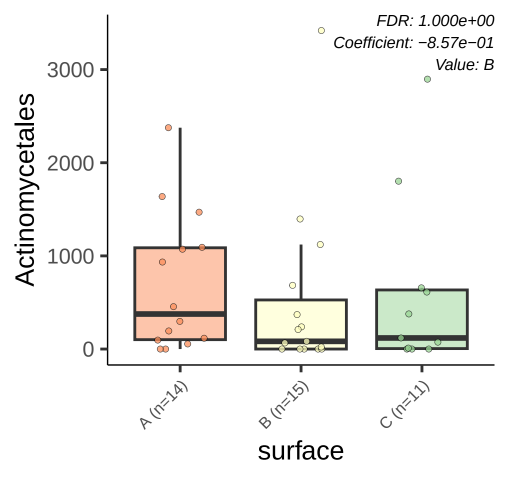
FDR: 1.000e+00
Coefficient: −8.57e−01
Value: B
Actinomycetales
3000
2000
1000
0
A (n=14)
B (n=15)
C (n=11)
surface

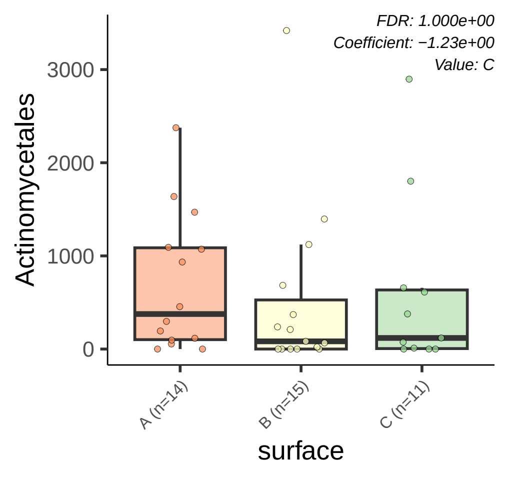
FDR: 1.000e+00
Coefficient: −1.23e+00
Value: C
Actinomycetales
3000
2000
1000
0
A (n=14)
B (n=15)
C (n=11)
surface

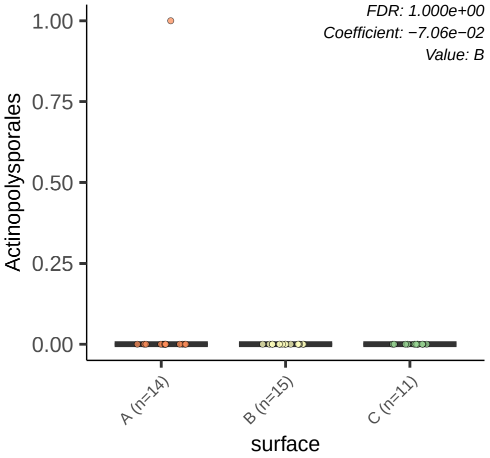
FDR: 1.000e+00
Coefficient: −7.06e−02
Value: B
Actinopolysporales
1.00
0.75
0.50
0.25
0.00
A (n=14)
B (n=15)
C (n=11)
surface

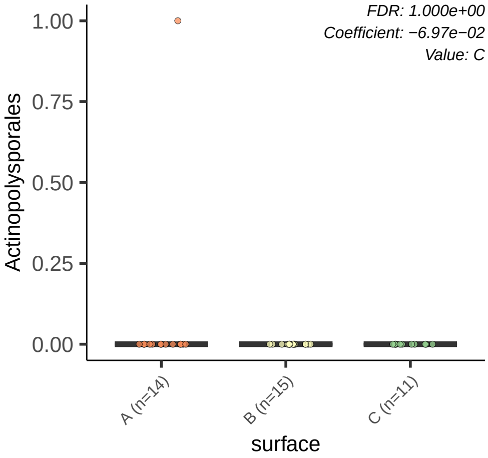
FDR: 1.000e+00
Coefficient: −6.97e−02
Value: C
Actinopolysporales
1.00
0.75
0.50
0.25
0.00
A (n=14)
B (n=15)
C (n=11)
surface

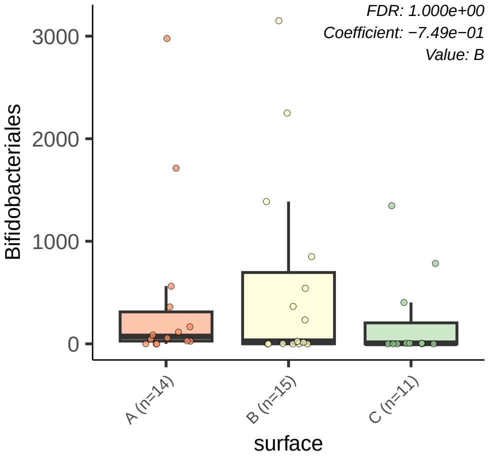

FDR: 1.000e+00
Coefficient: −7.49e−01
Value: B
3000
2000
1000
0
Bifidobacteriales
A (n=14)
B (n=15)
C (n=11)
surface

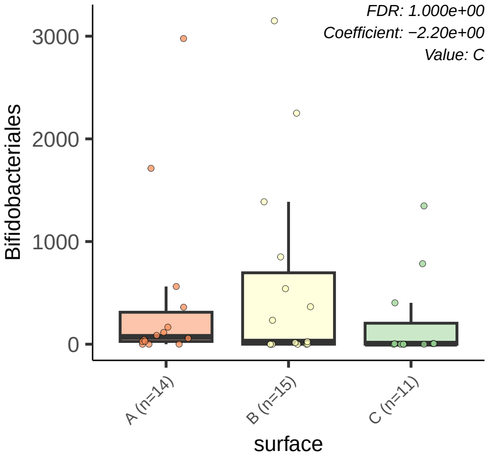
FDR: 1.000e+00
Coefficient: −2.20e+00
Value: C
Bifidobacteriales
3000
2000
1000
0
A (n=14)
B (n=15)
C (n=11)
surface

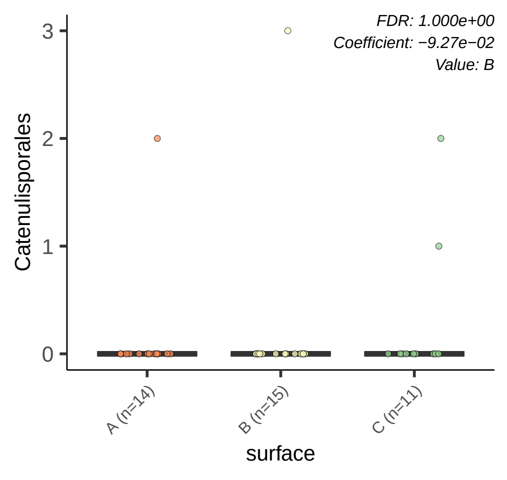

FDR: 1.000e+00
Coefficient: −9.27e−02
Value: B
Catenulisporales
3
2
1
0
A (n=14)
B (n=15)
C (n=11)
surface

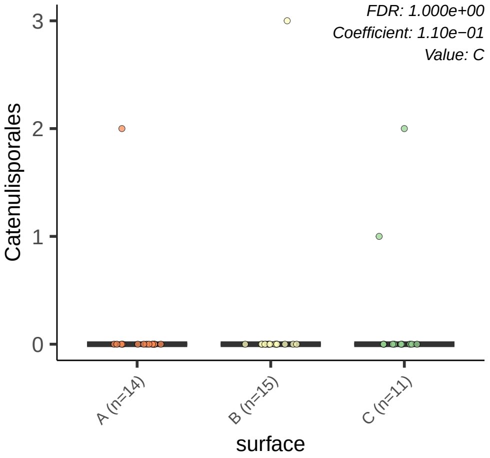
FDR: 1.000e+00
Coefficient: 1.10e−01
Value: C
Catenulisporales
3
2
1
0
A (n=14)
B (n=15)
C (n=11)
surface

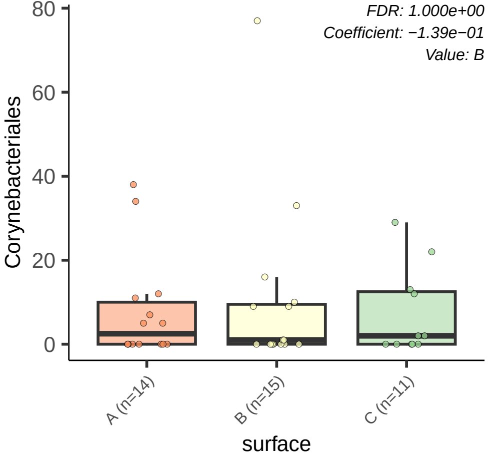
FDR: 1.000e+00
Coefficient: −1.39e−01
Value: B
Corynebacteriales
80
60
40
20
0
A (n=14)
B (n=15)
C (n=11)
surface

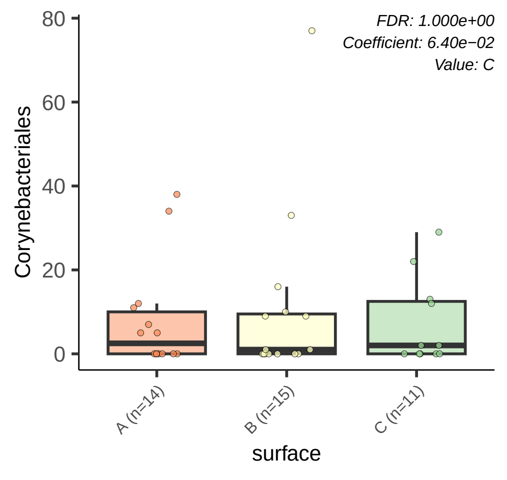

FDR: 1.000e+00
Coefficient: 6.40e−02
Value: C
Corynebacteriales
80
60
40
20
0
A (n=14)
B (n=15)
C (n=11)
surface

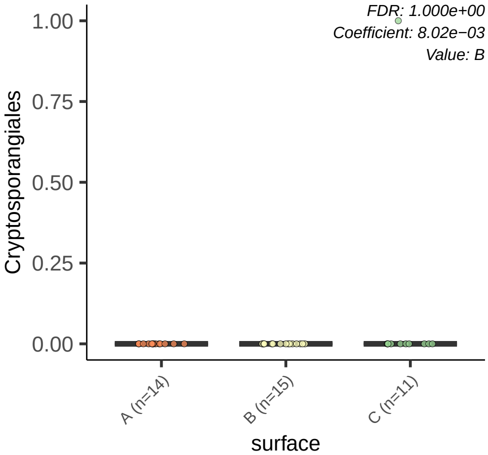
FDR: 1.000e+00
Coefficient: 8.02e−03
Value: B
Cryptosporangiales
1.00
0.75
0.50
0.25
0.00
A (n=14)
B (n=15)
C (n=11)
surface

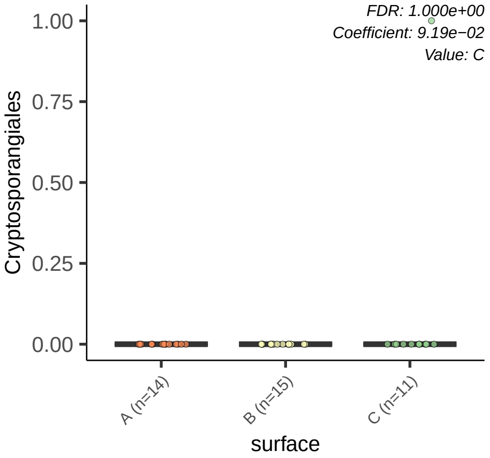
FDR: 1.000e+00
Coefficient: 9.19e−02
Value: C
Cryptosporangiales
1.00
0.75
0.50
0.25
0.00
A (n=14)
B (n=15)
C (n=11)
surface

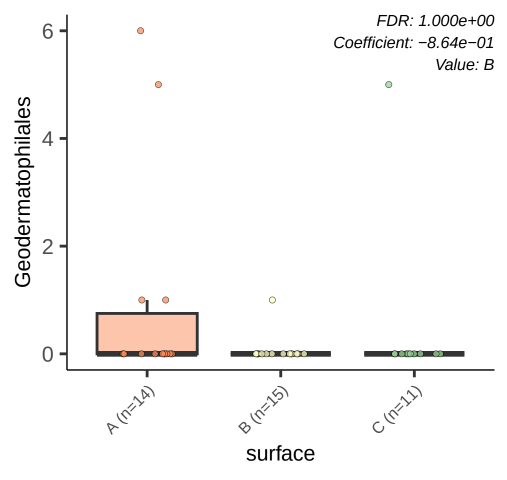
FDR: 1.000e+00
Coefficient: −8.64e−01
Value: B
Geodermatophilales
6
4
2
0
A (n=14)
B (n=15)
C (n=11)
surface

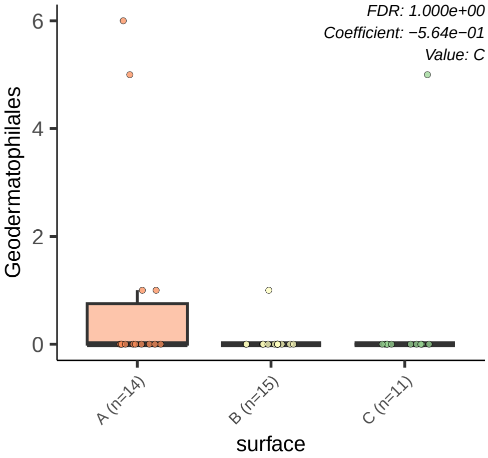

FDR: 1.000e+00
Coefficient: −5.64e−01
Value: C
Geodermatophilales
6
4
2
0
A (n=14)
B (n=15)
C (n=11)
surface

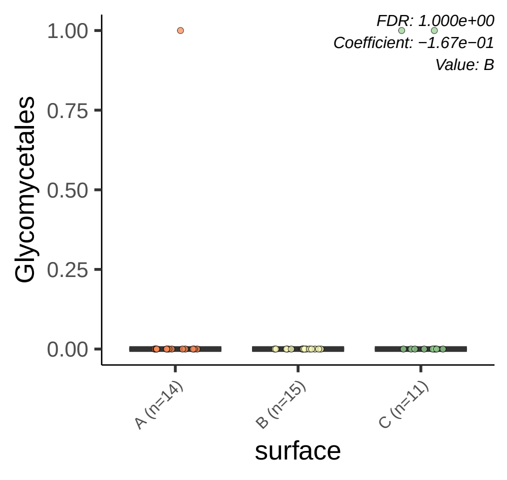

FDR: 1.000e+00
Coefficient: −1.67e−01
Value: B
Glycomycetales
1.00
0.75
0.50
0.25
0.00
A (n=14)
B (n=15)
C (n=11)
surface

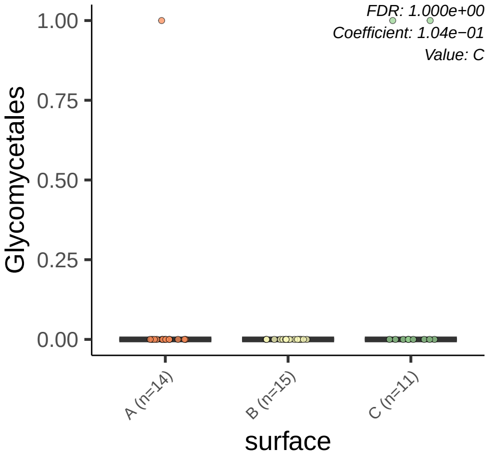

FDR: 1.000e+00
Coefficient: 1.04e−01
Value: C
Glycomycetales
1.00
0.75
0.50
0.25
0.00
A (n=14)
B (n=15)
C (n=11)
surface

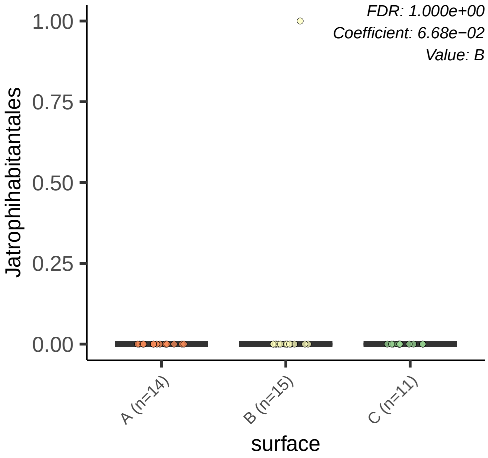
FDR: 1.000e+00
Coefficient: 6.68e−02
Value: B
Jatrophihabitantales
1.00
0.75
0.50
0.25
0.00
A (n=14)
B (n=15)
C (n=11)
surface

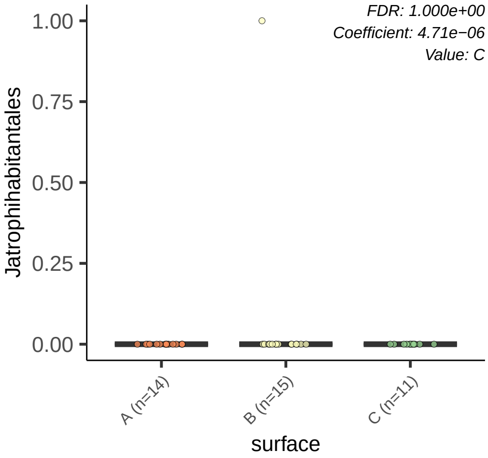
FDR: 1.000e+00
Coefficient: 4.71e−06
Value: C
Jatrophihabitantales
1.00
0.75
0.50
0.25
0.00
A (n=14)
B (n=15)
C (n=11)
surface

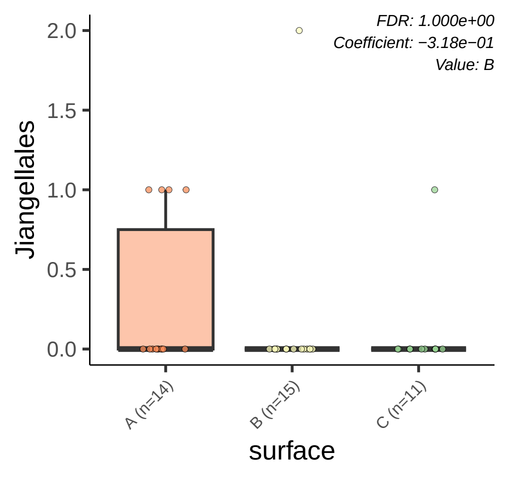

FDR: 1.000e+00
Coefficient: −3.18e−01
Value: B
Jiangellales
2.0
1.5
1.0
0.5
0.0
A (n=14)
B (n=15)
C (n=11)
surface

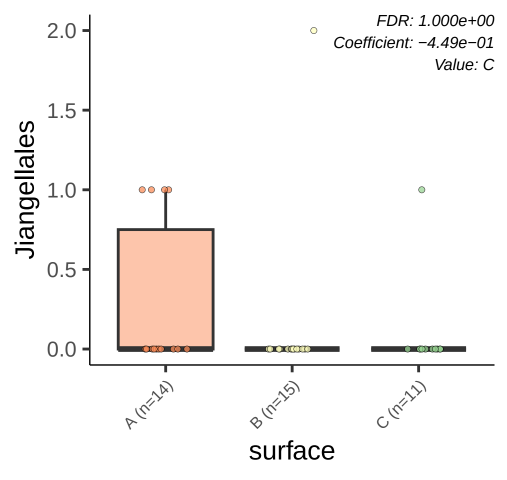

FDR: 1.000e+00
Coefficient: −4.49e−01
Value: C
Jiangellales
2.0
1.5
1.0
0.5
0.0
A (n=14)
B (n=15)
C (n=11)
surface

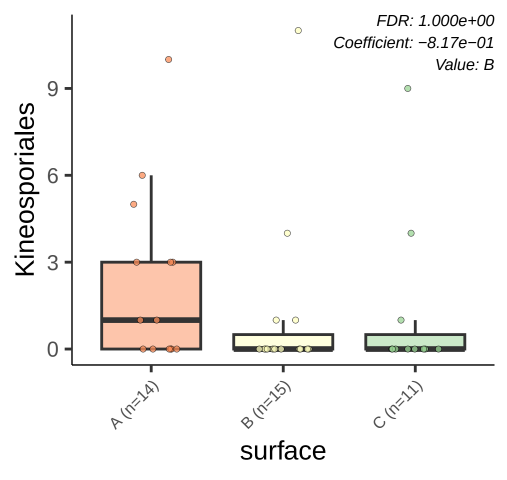

FDR: 1.000e+00
Coefficient: −8.17e−01
Value: B
Kineosporiales
0
3
6
9
A (n=14)
B (n=15)
C (n=11)
surface

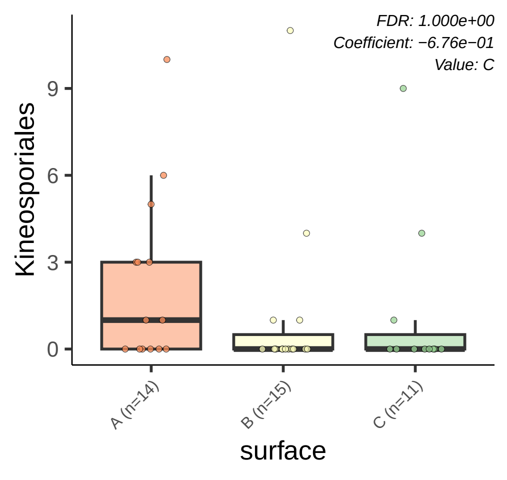
FDR: 1.000e+00
Coefficient: −6.76e−01
Value: C
Kineosporiales
0
3
6
9
A (n=14)
B (n=15)
C (n=11)
surface

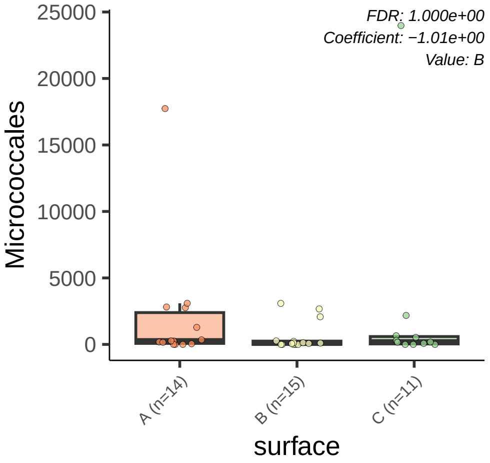

FDR: 1.000e+00
Coefficient: −1.01e+00
Value: B
Micrococcales
25000
20000
15000
10000
5000
0
A (n=14)
B (n=15)
C (n=11)
surface

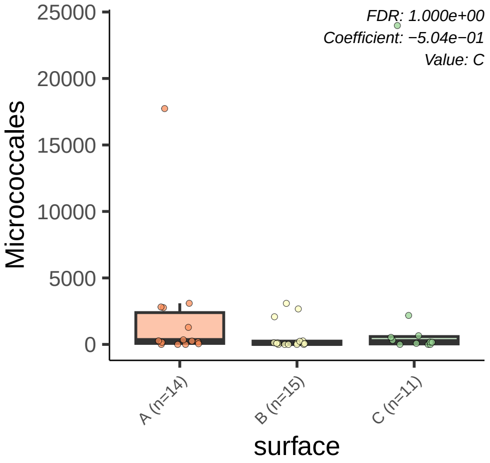

FDR: 1.000e+00
Coefficient: −5.04e−01
Value: C
Microccocales
25000
20000
15000
10000
5000
0
A (n=14)
B (n=15)
C (n=11)
surface

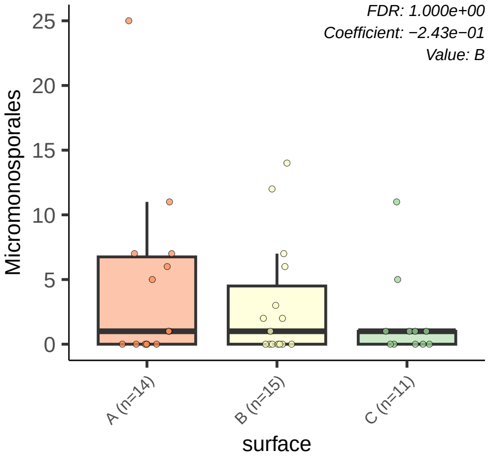
FDR: 1.000e+00
Coefficient: −2.43e−01
Value: B
Micromonosporales
0
5
10
15
20
25
A (n=14)
B (n=15)
C (n=11)
surface

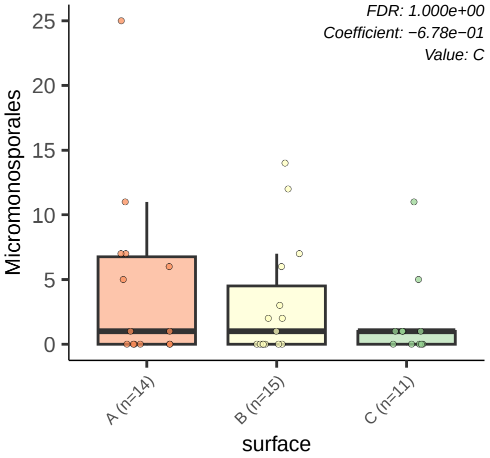
FDR: 1.000e+00
Coefficient: −6.78e−01
Value: C
Micromonosporales
25
20
15
10
5
0
A (n=14)
B (n=15)
C (n=11)
surface

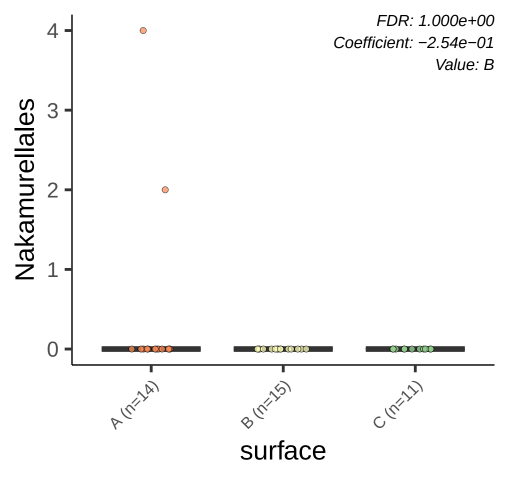
FDR: 1.000e+00
Coefficient: −2.54e−01
Value: B
Nakamurellales
4
3
2
1
0
A (n=14)
B (n=15)
C (n=11)
surface

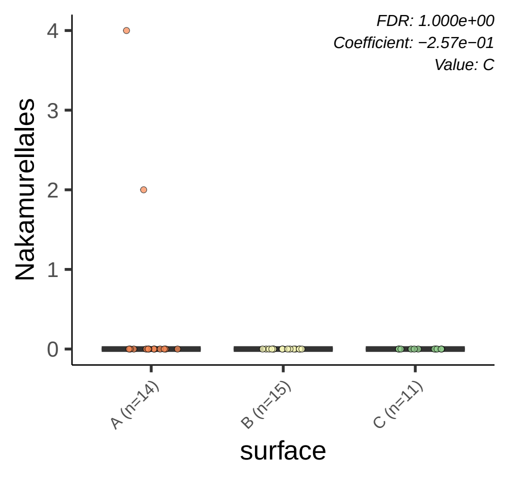

FDR: 1.000e+00
Coefficient: −2.57e−01
Value: C
Nakamurellales
4
3
2
1
0
A (n=14)
B (n=15)
C (n=11)
surface

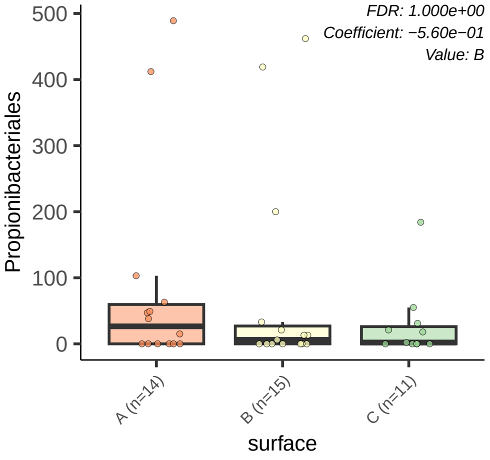

FDR: 1.000e+00
Coefficient: −5.60e−01
Value: B
Propionibacteriales
500
400
300
200
100
0
A (n=14)
B (n=15)
C (n=11)
surface

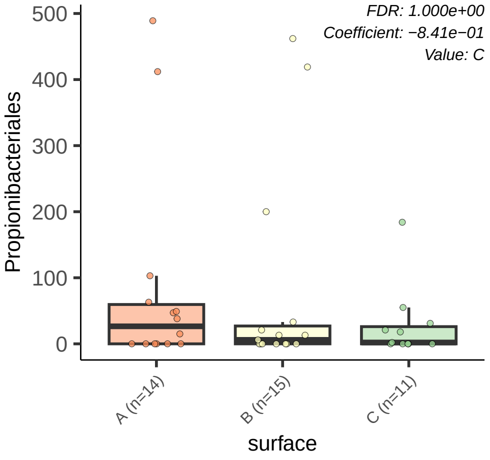
FDR: 1.000e+00
Coefficient: −8.41e−01
Value: C
Propionibacteriales
500
400
300
200
100
0
A (n=14)
B (n=15)
C (n=11)
surface

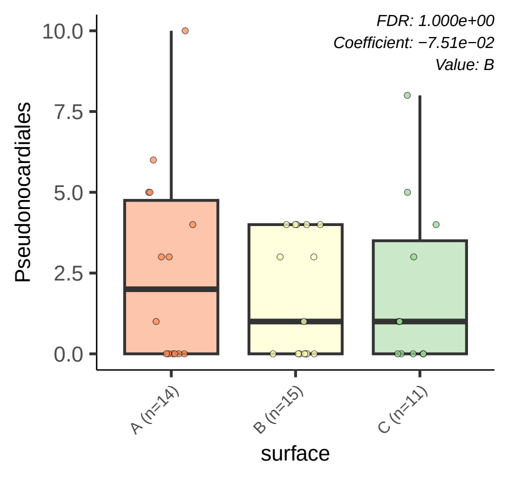
FDR: 1.000e+00
Coefficient: −7.51e−02
Value: B
Pseudonocardiales
10.0
7.5
5.0
2.5
0.0
A (n=14)
B (n=15)
C (n=11)
surface

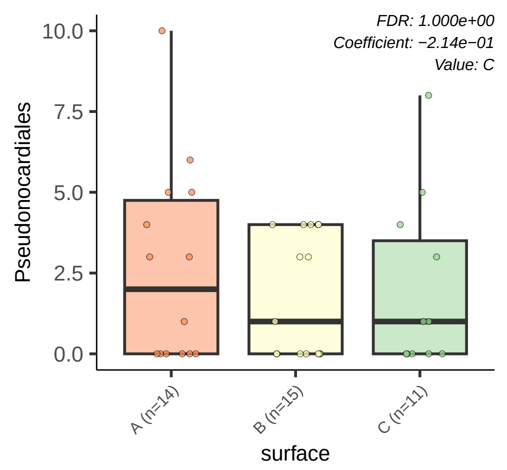

FDR: 1.000e+00
Coefficient: −2.14e−01
Value: C
Pseudonocardiales
10.0
7.5
5.0
2.5
0.0
A (n=14)
B (n=15)
C (n=11)
surface

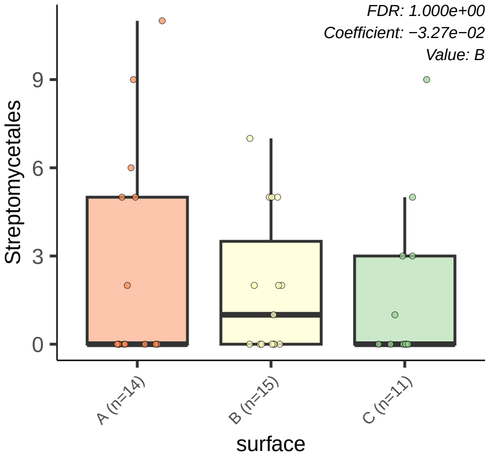
FDR: 1.000e+00
Coefficient: −3.27e−02
Value: B
Streptomycetales
0
3
6
9
A (n=14)
B (n=15)
C (n=11)
surface

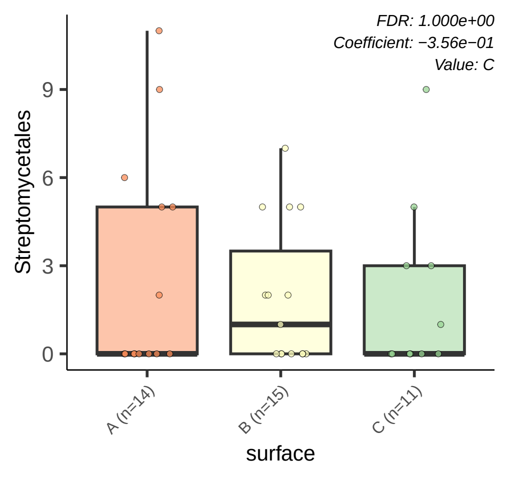

FDR: 1.000e+00
Coefficient: −3.56e−01
Value: C
Streptomycetales
0
3
6
9
A (n=14)
B (n=15)
C (n=11)
surface

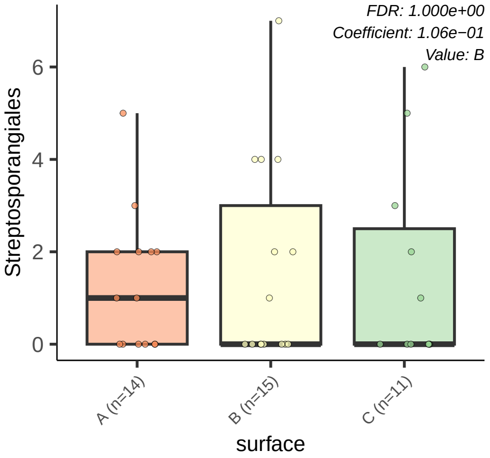

FDR: 1.000e+00
Coefficient: 1.06e−01
Value: B
Streptosporangiales
0
2
4
6
A (n=14)
B (n=15)
C (n=11)
surface

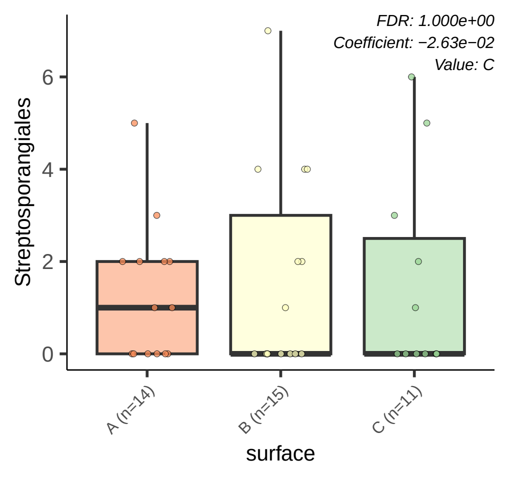

FDR: 1.000e+00
Coefficient: −2.63e−02
Value: C
Streptosporangiales
0
2
4
6
A (n=14)
B (n=15)
C (n=11)
surface

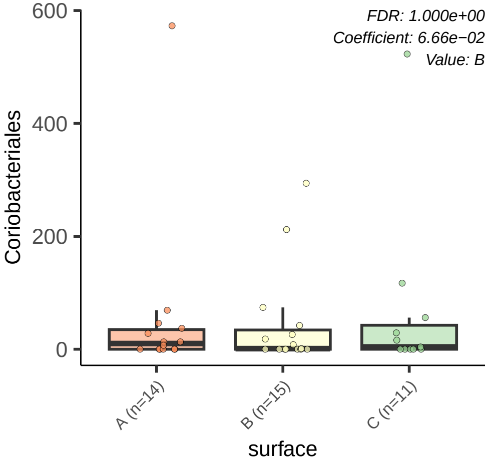

FDR: 1.000e+00
Coefficient: 6.66e−02
Value: B
Coriobacteriales
600
400
200
0
A (n=14)
B (n=15)
C (n=11)
surface

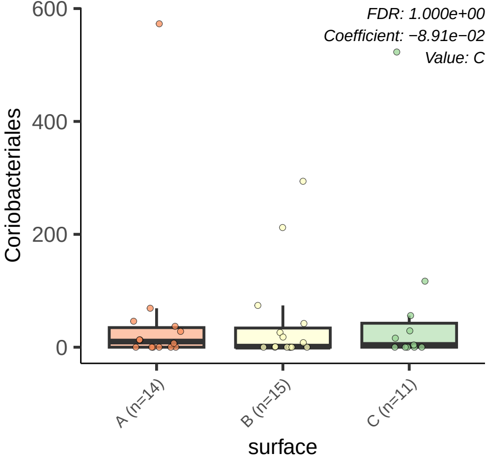
FDR: 1.000e+00
Coefficient: −8.91e−02
Value: C
Coriobacteriales
600
400
200
0
A (n=14)
B (n=15)
C (n=11)
surface

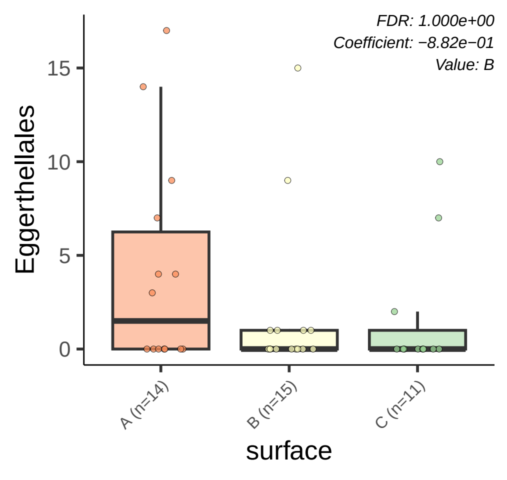

FDR: 1.000e+00
Coefficient: −8.82e−01
Value: B
Eggerthellales
0
5
10
15
A (n=14)
B (n=15)
C (n=11)
surface

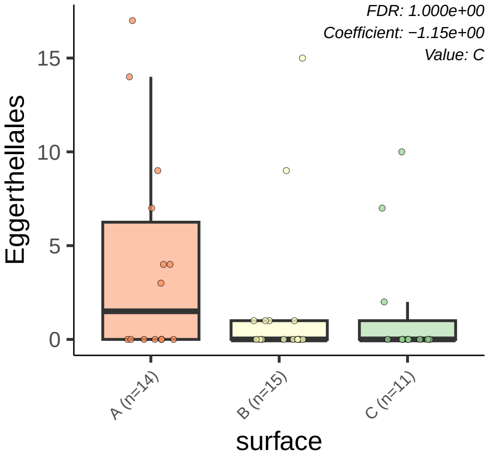

FDR: 1.000e+00
Coefficient: −1.15e+00
Value: C
Eggerthellales
15
10
5
0
A (n=14)
B (n=15)
C (n=11)
surface

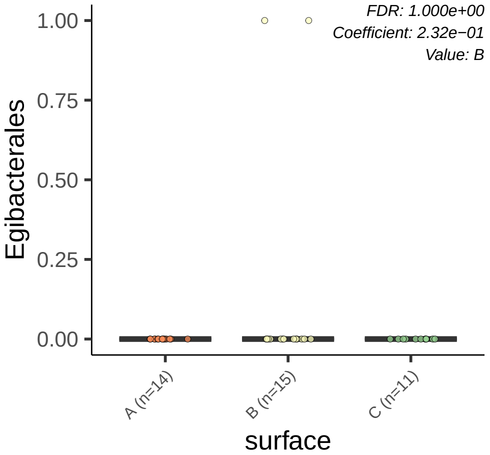

FDR: 1.000e+00
Coefficient: 2.32e−01
Value: B
Egibacterales
1.00
0.75
0.50
0.25
0.00
A (n=14)
B (n=15)
C (n=11)
surface

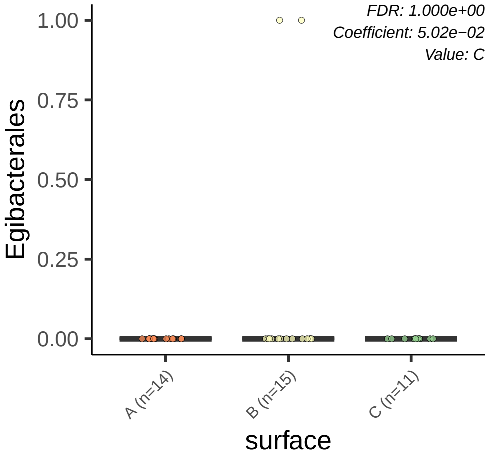
FDR: 1.000e+00
Coefficient: 5.02e−02
Value: C
Egibacterales
1.00
0.75
0.50
0.25
0.00
A (n=14)
B (n=15)
C (n=11)
surface

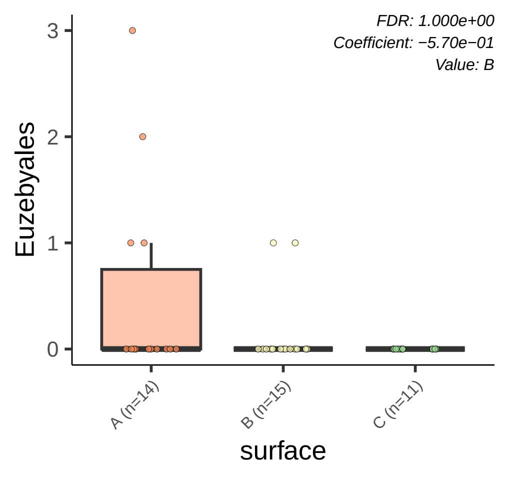

FDR: 1.000e+00
Coefficient: −5.70e−01
Value: B
Euzebyales
3
2
1
0
A (n=14)
B (n=15)
C (n=11)
surface

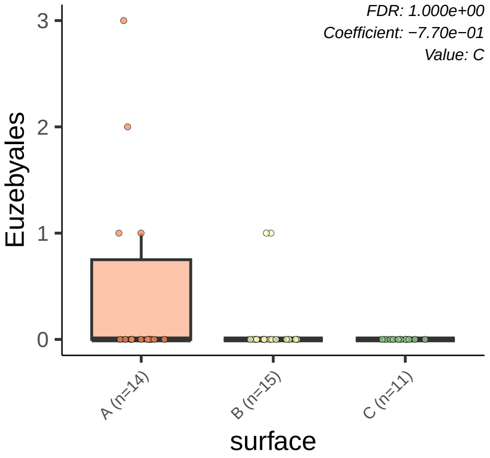
FDR: 1.000e+00
Coefficient: −7.70e−01
Value: C
Euzebyales
3
2
1
0
A (n=14)
B (n=15)
C (n=11)
surface

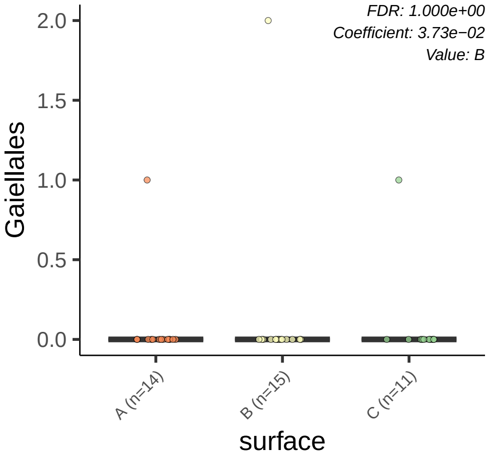
FDR: 1.000e+00
Coefficient: 3.73e−02
Value: B
Gaiellales
2.0
1.5
1.0
0.5
0.0
A (n=14)
B (n=15)
C (n=11)
surface

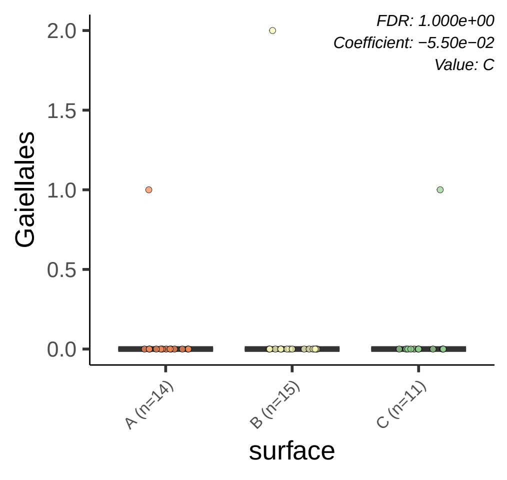

FDR: 1.000e+00
Coefficient: −5.50e−02
Value: C
Gaiellales
2.0
1.5
1.0
0.5
0.0
A (n=14)
B (n=15)
C (n=11)
surface

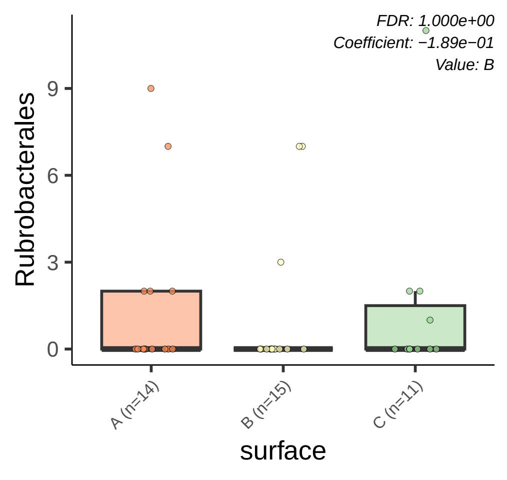
FDR: 1.000e+00
Coefficient: −1.89e−01
Value: B
Rubrobacterales
9
6
3
0
A (n=14)
B (n=15)
C (n=11)
surface

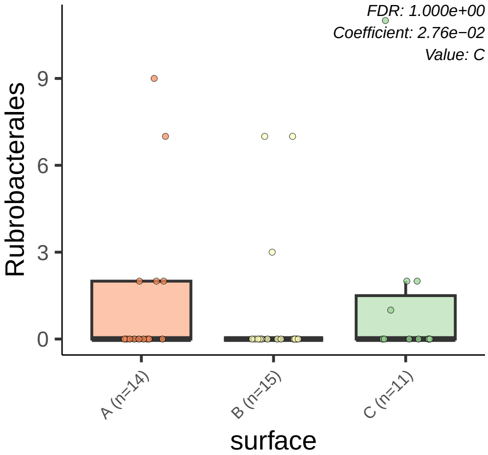

FDR: 1.000e+00
Coefficient: 2.76e−02
Value: C
Rubrobacterales
0
3
6
9
A (n=14)
B (n=15)
C (n=11)
surface

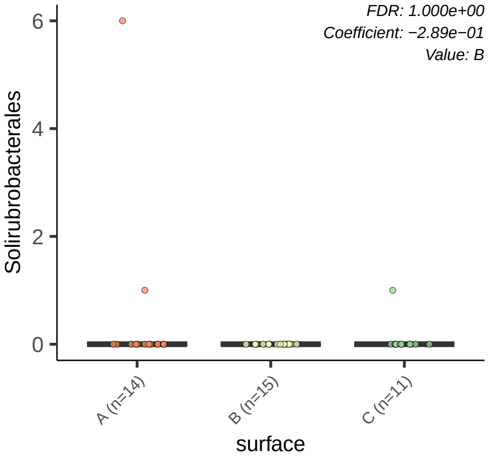
FDR: 1.000e+00
Coefficient: −2.89e−01
Value: B
Solirubrobacterales
6
4
2
0
A (n=14)
B (n=15)
C (n=11)
surface

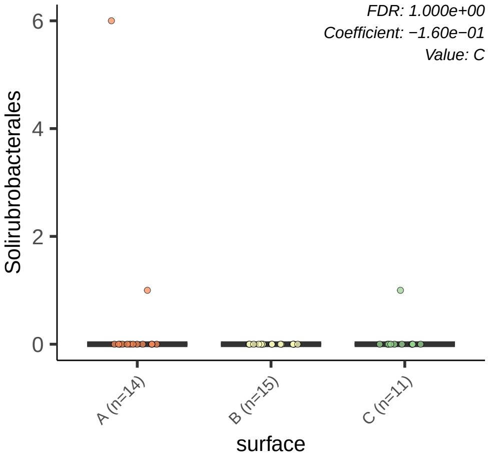

FDR: 1.000e+00
Coefficient: −1.60e−01
Value: C
Solirubrobacterales
6
4
2
0
A (n=14)
B (n=15)
C (n=11)
surface

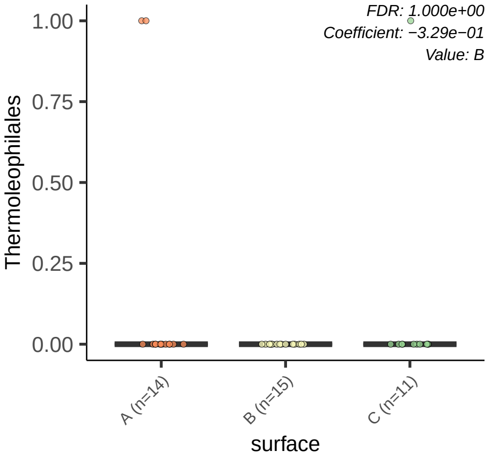
FDR: 1.000e+00
Coefficient: −3.29e−01
Value: B
Thermoleophilales
1.00
0.75
0.50
0.25
0.00
A (n=14)
B (n=15)
C (n=11)
surface

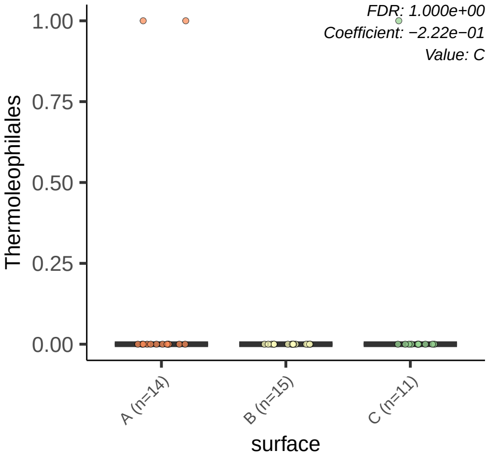

FDR: 1.000e+00
Coefficient: −2.22e−01
Value: C
Thermoleophilales
1.00
0.75
0.50
0.25
0.00
A (n=14)
B (n=15)
C (n=11)
surface

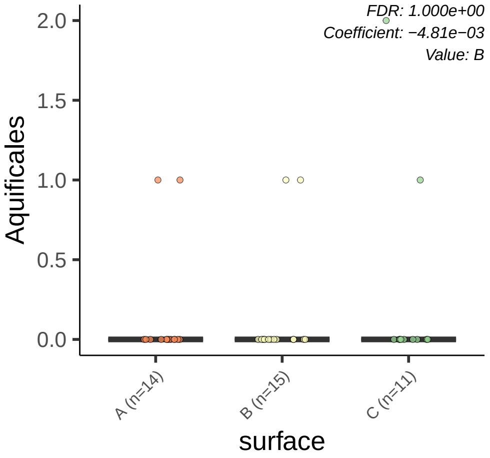

FDR: 1.000e+00
Coefficient: −4.81e−03
Value: B
Aquificales
2.0
1.5
1.0
0.5
0.0
A (n=14)
B (n=15)
C (n=11)
surface

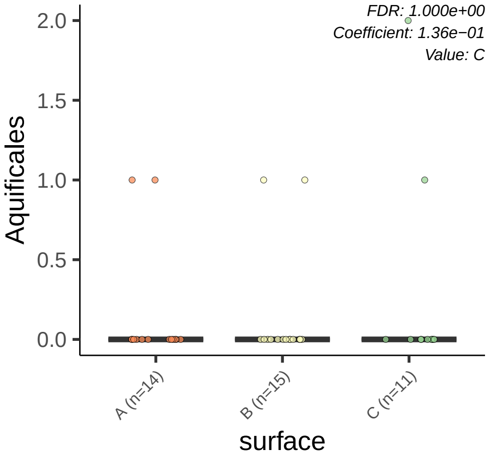

FDR: 1.000e+00
Coefficient: 1.36e−01
Value: C
2.0
1.5
1.0
0.5
0.0
Aquificales
A (n=14)
B (n=15)
C (n=11)
surface

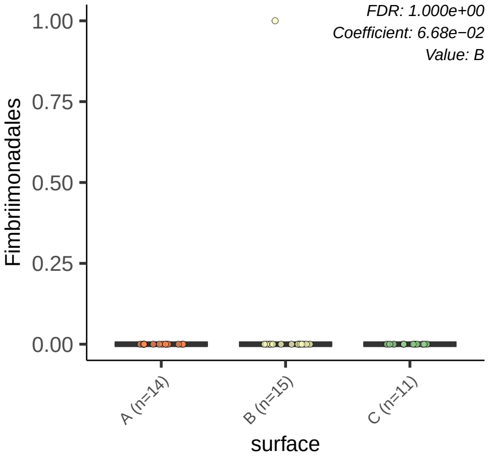
FDR: 1.000e+00
Coefficient: 6.68e−02
Value: B
Fimbriimonadales
1.00
0.75
0.50
0.25
0.00
A (n=14)
B (n=15)
C (n=11)
surface

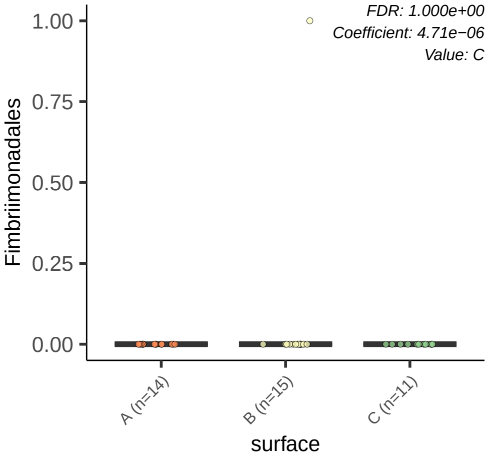
FDR: 1.000e+00
Coefficient: 4.71e−06
Value: C
1.00
0.75
0.50
0.25
0.00
Fimbriimonadales
A (n=14)
B (n=15)
C (n=11)
surface

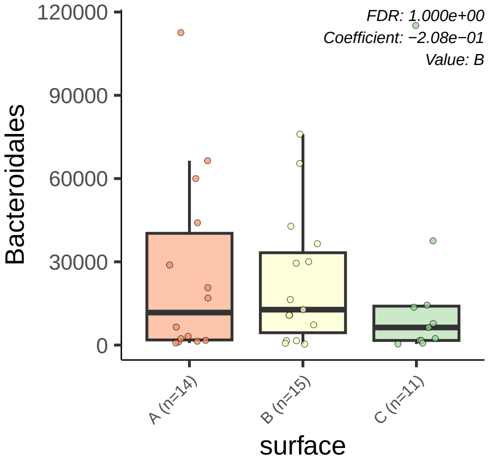

FDR: 1.000e+00
Coefficient: −2.08e−01
Value: B
120000
90000
60000
30000
0
Bacteroidales
A (n=14)
B (n=15)
C (n=11)
surface

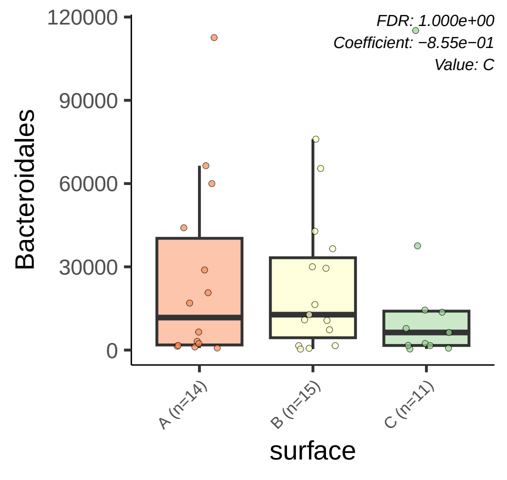
FDR: 1.000e+00
Coefficient: −8.55e−01
Value: C
Bacteroidales
120000
90000
60000
30000
0
A (n=14)
B (n=15)
C (n=11)
surface

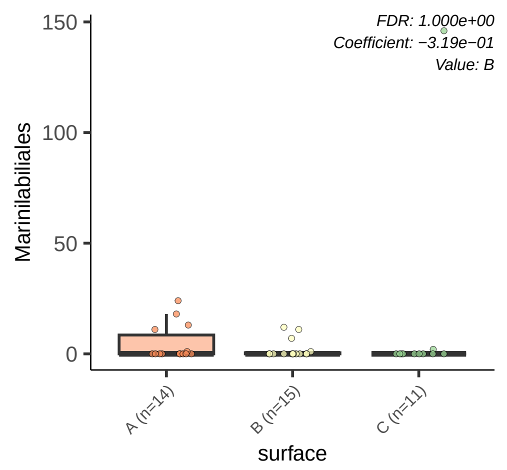
FDR: 1.000e+00
Coefficient: −3.19e−01
Value: B
Marinilabiliales
150
100
50
0
A (n=14)
B (n=15)
C (n=11)
surface

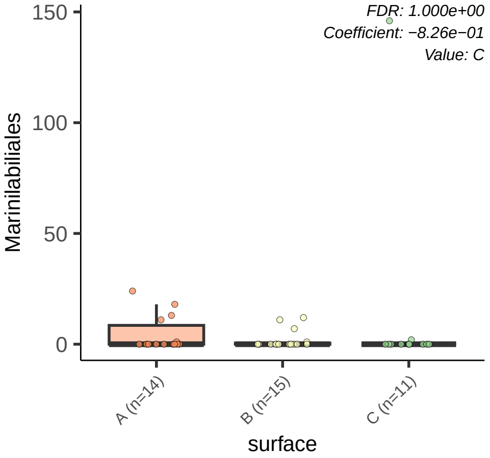
FDR: 1.000e+00
Coefficient: −8.26e−01
Value: C
150
100
50
0
Marinilabiliales
A (n=14)
B (n=15)
C (n=11)
surface

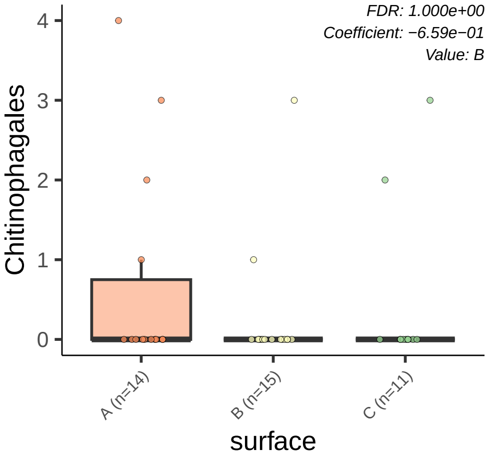

FDR: 1.000e+00
Coefficient: −6.59e−01
Value: B
Chitinophagales
0
1
2
3
4
A (n=14)
B (n=15)
C (n=11)
surface

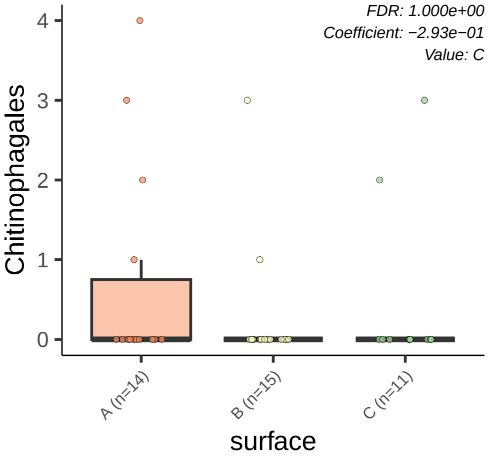
FDR: 1.000e+00
Coefficient: −2.93e−01
Value: C
Chitinophagales
0
1
2
3
4
A (n=14)
B (n=15)
C (n=11)
surface

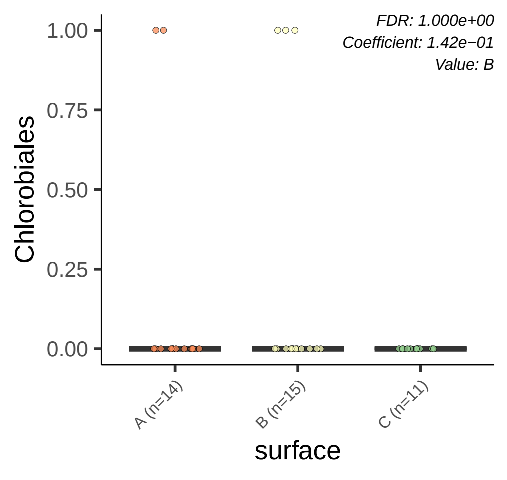
FDR: 1.000e+00
Coefficient: 1.42e−01
Value: B
Chlorobiales
1.00
0.75
0.50
0.25
0.00
A (n=14)
B (n=15)
C (n=11)
surface

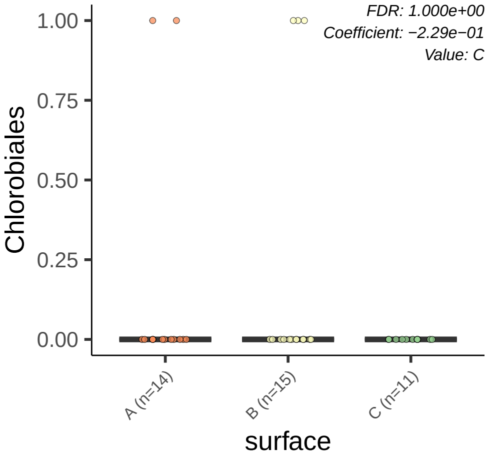

FDR: 1.000e+00
Coefficient: −2.29e−01
Value: C
Chlorobiales
1.00
0.75
0.50
0.25
0.00
A (n=14)
B (n=15)
C (n=11)
surface

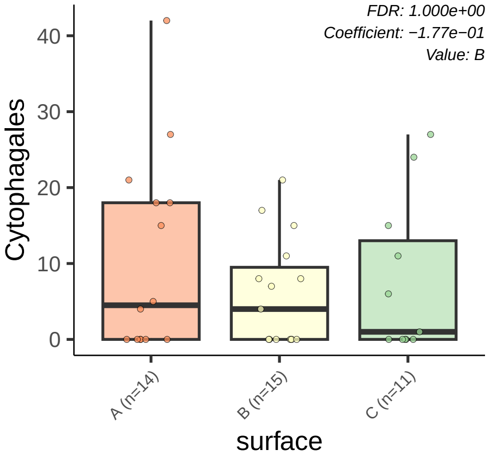

FDR: 1.000e+00
Coefficient: −1.77e−01
Value: B
Cytophagales
0
10
20
30
40
A (n=14)
B (n=15)
C (n=11)
surface

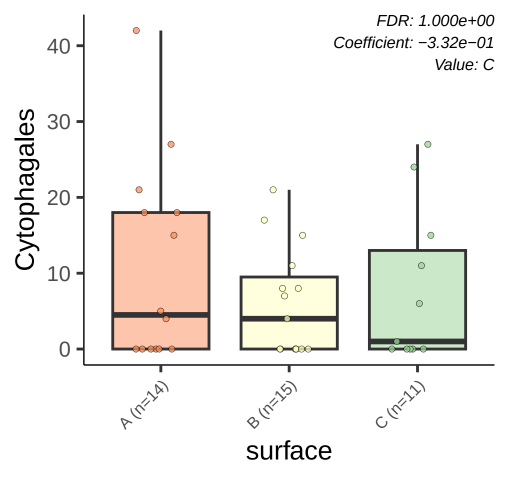
FDR: 1.000e+00
Coefficient: −3.32e−01
Value: C
Cytophagales
40
30
20
10
0
A (n=14)
B (n=15)
C (n=11)
surface

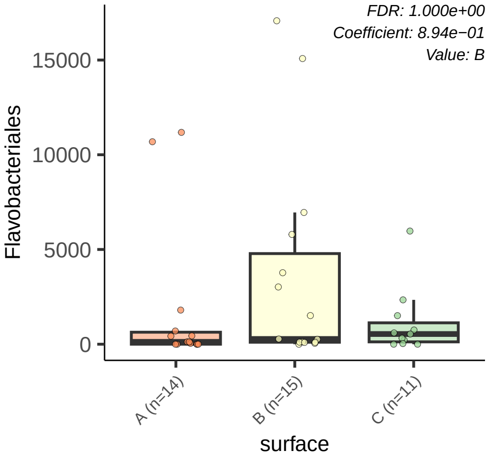

FDR: 1.000e+00
Coefficient: 8.94e−01
Value: B
Flavobacteriales
15000
10000
5000
0
A (n=14)
B (n=15)
C (n=11)
surface

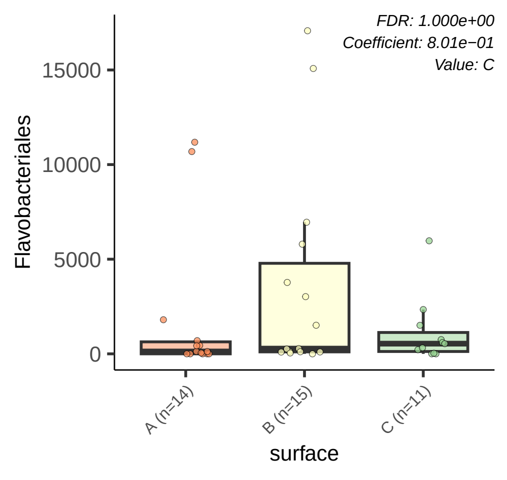

FDR: 1.000e+00
Coefficient: 8.01e−01
Value: C
Flavobacteriales
15000
10000
5000
0
A (n=14)
B (n=15)
C (n=11)
surface

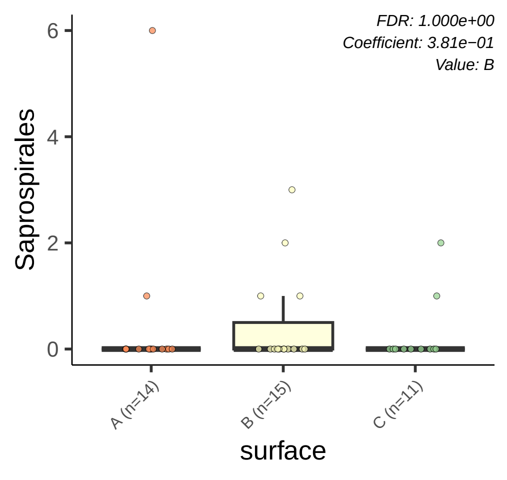
FDR: 1.000e+00
Coefficient: 3.81e−01
Value: B
Saprospirales
0
2
4
6
A (n=14)
B (n=15)
C (n=11)
surface

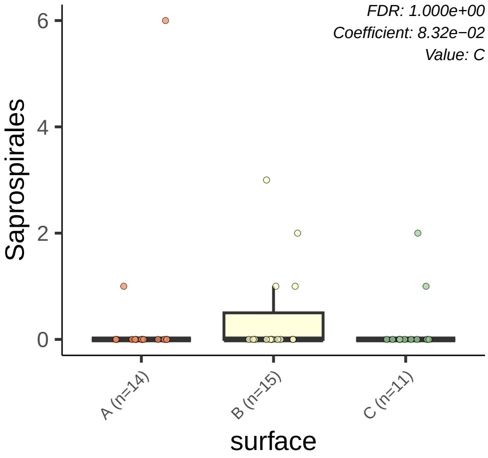

FDR: 1.000e+00
Coefficient: 8.32e−02
Value: C
Saprospirales
6
4
2
0
A (n=14)
B (n=15)
C (n=11)
surface

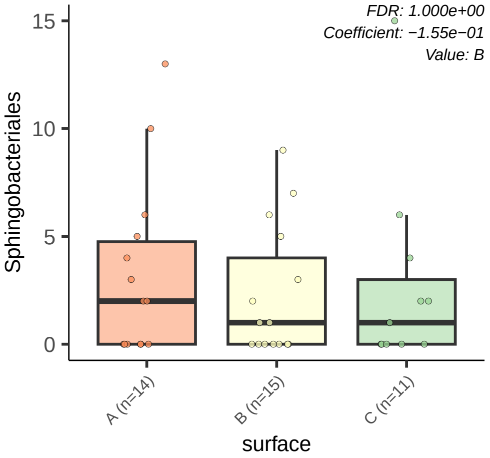

FDR: 1.000e+00
Coefficient: −1.55e−01
Value: B
Sphingobacteriales
15
10
5
0
A (n=14)
B (n=15)
C (n=11)
surface

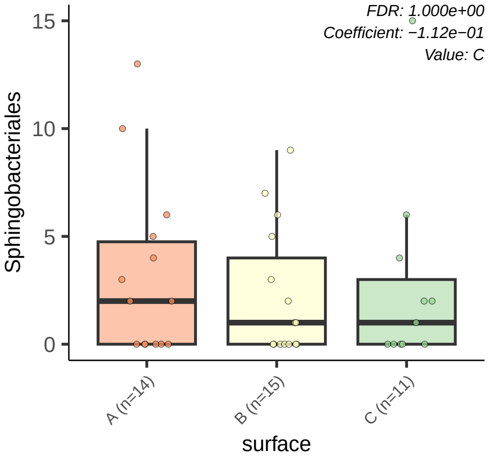
FDR: 1.000e+00
Coefficient: −1.12e−01
Value: C
Sphingobacteriales
15
10
5
0
A (n=14)
B (n=15)
C (n=11)
surface

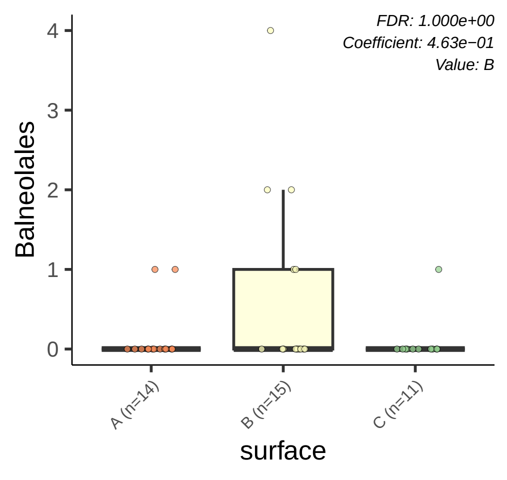

FDR: 1.000e+00
Coefficient: 4.63e−01
Value: B
Balneolales
0
1
2
3
4
A (n=14)
B (n=15)
C (n=11)
surface

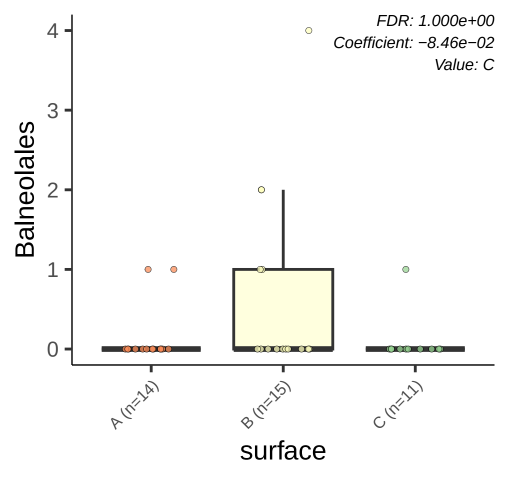

FDR: 1.000e+00
Coefficient: −8.46e−02
Value: C
Balneolales
0
1
2
3
4
A (n=14)
B (n=15)
C (n=11)
surface

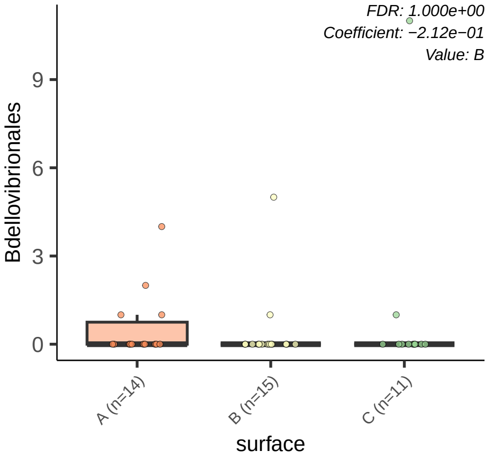

FDR: 1.000e+00
Coefficient: −2.12e−01
Value: B
Bdellovibrionales
9
6
3
0
A (n=14)
B (n=15)
C (n=11)
surface

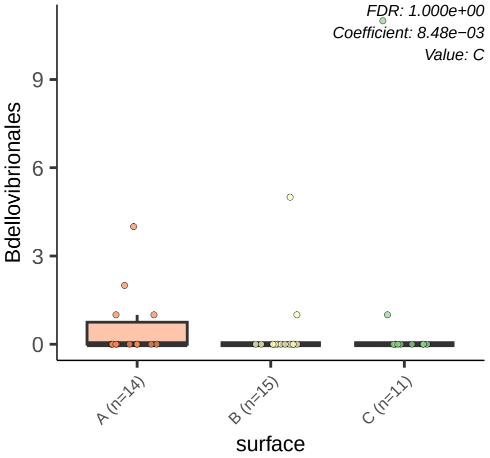

FDR: 1.000e+00
Coefficient: 8.48e−03
Value: C
Bdellovibrionales
0
3
6
9
A (n=14)
B (n=15)
C (n=11)
surface

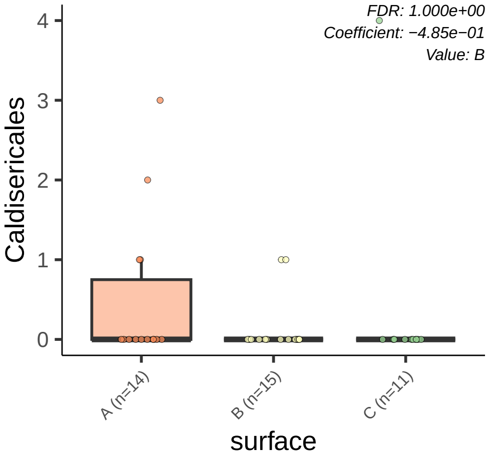
FDR: 1.000e+00
Coefficient: −4.85e−01
Value: B
Caldisericales
4
3
2
1
0
A (n=14)
B (n=15)
C (n=11)
surface

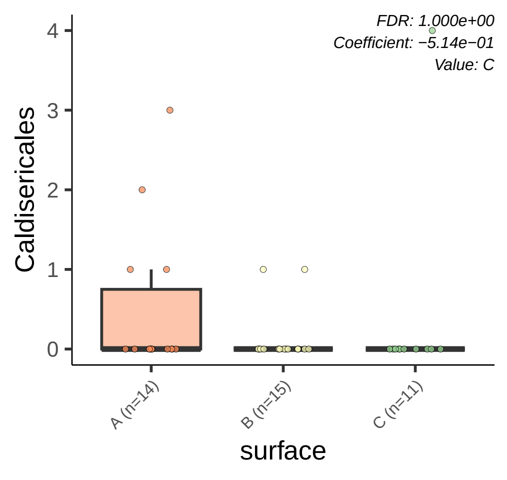

FDR: 1.000e+00
Coefficient: −5.14e−01
Value: C
Caldisericales
4
3
2
1
0
A (n=14)
B (n=15)
C (n=11)
surface

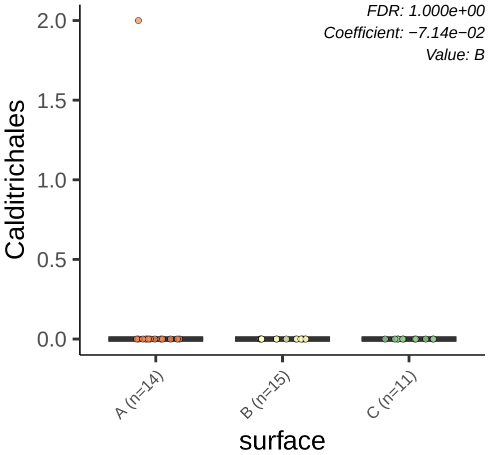

FDR: 1.000e+00
Coefficient: −7.14e−02
Value: B
Calditrichales
2.0
1.5
1.0
0.5
0.0
A (n=14)
B (n=15)
C (n=11)
surface

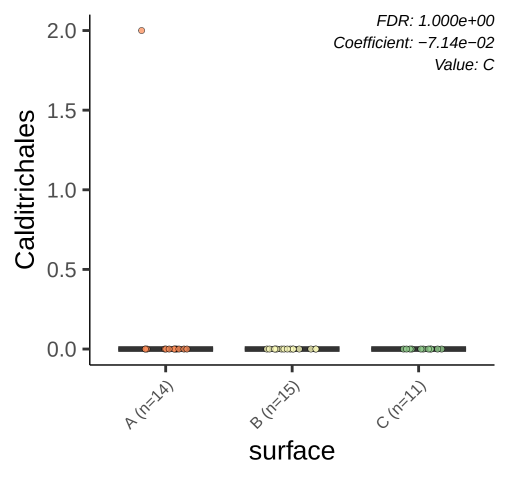

FDR: 1.000e+00
Coefficient: −7.14e−02
Value: C
Calditrichales
2.0
1.5
1.0
0.5
0.0
A (n=14)
B (n=15)
C (n=11)
surface

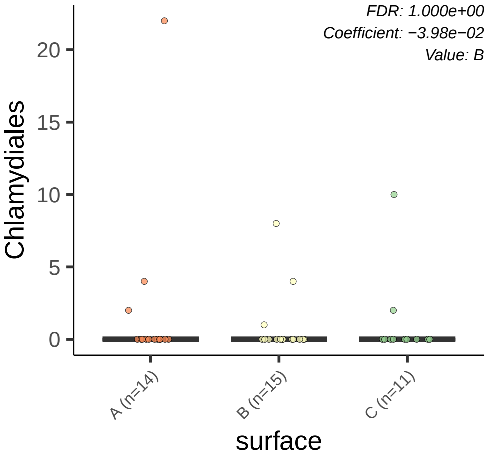
FDR: 1.000e+00
Coefficient: −3.98e−02
Value: B
Chlamydiales
0
5
10
15
20
A (n=14)
B (n=15)
C (n=11)
surface

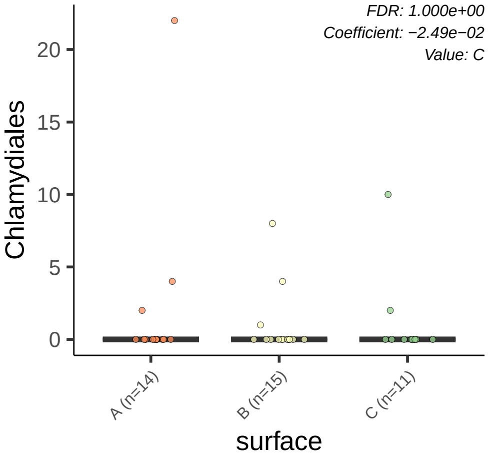

FDR: 1.000e+00
Coefficient: −2.49e−02
Value: C
Chlamydiales
0
5
10
15
20
A (n=14)
B (n=15)
C (n=11)
surface

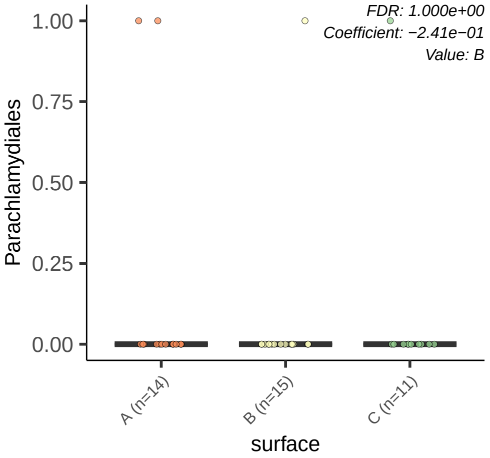

FDR: 1.000e+00
Coefficient: −2.41e−01
Value: B
Parachlamydiales
1.00
0.75
0.50
0.25
0.00
A (n=14)
B (n=15)
C (n=11)
surface

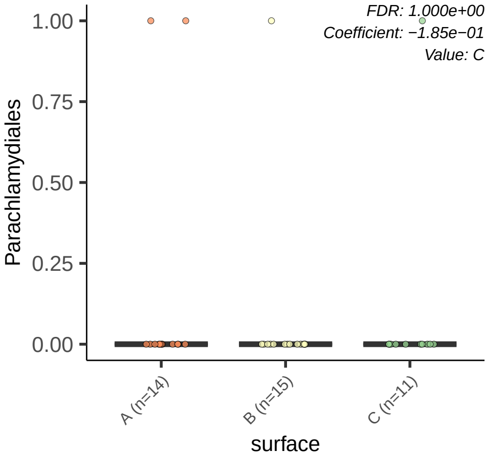
FDR: 1.000e+00
Coefficient: −1.85e−01
Value: C
Parachlamydiales
1.00
0.75
0.50
0.25
0.00
A (n=14)
B (n=15)
C (n=11)
surface

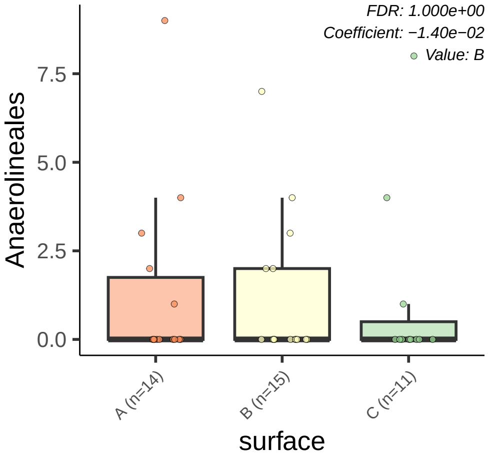
FDR: 1.000e+00
Coefficient: −1.40e−02
Value: B
Anaerolineales
7.5
5.0
2.5
0.0
A (n=14)
B (n=15)
C (n=11)
surface

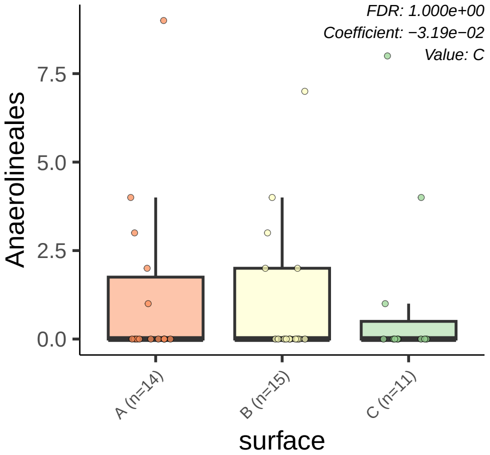
FDR: 1.000e+00
Coefficient: −3.19e−02
Value: C
Anaerolineales
0.0
2.5
5.0
7.5
A (n=14)
B (n=15)
C (n=11)
surface

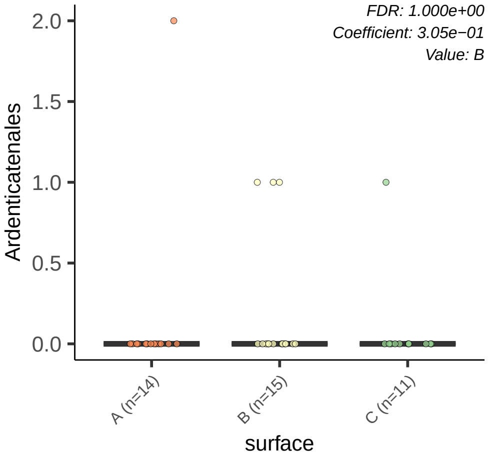

FDR: 1.000e+00
Coefficient: 3.05e−01
Value: B
Ardenticatenales
2.0
1.5
1.0
0.5
0.0
A (n=14)
B (n=15)
C (n=11)
surface

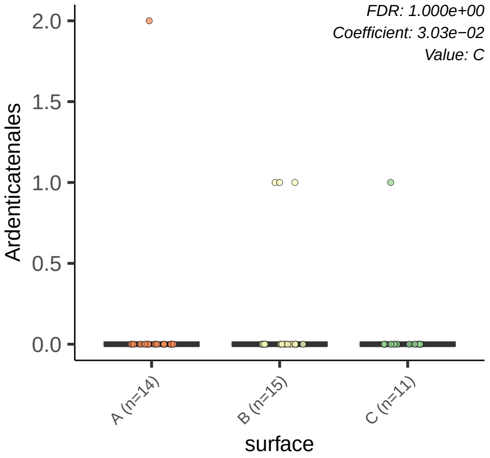
FDR: 1.000e+00
Coefficient: 3.03e−02
Value: C
Ardenticatenales
2.0
1.5
1.0
0.5
0.0
A (n=14)
B (n=15)
C (n=11)
surface

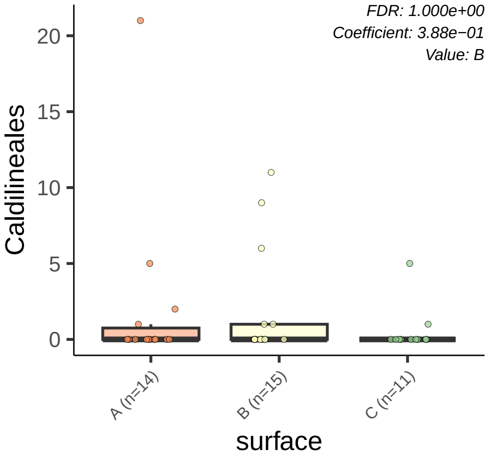
FDR: 1.000e+00
Coefficient: 3.88e−01
Value: B
Caldilineales
20
15
10
5
0
A (n=14)
B (n=15)
C (n=11)
surface

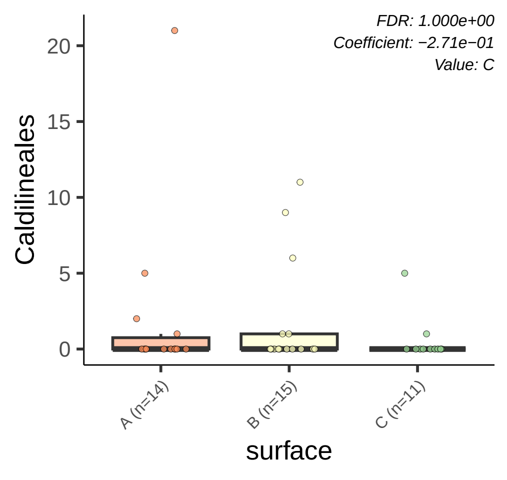

FDR: 1.000e+00
Coefficient: −2.71e−01
Value: C
Caldilineales
0
5
10
15
20
A (n=14)
B (n=15)
C (n=11)
surface

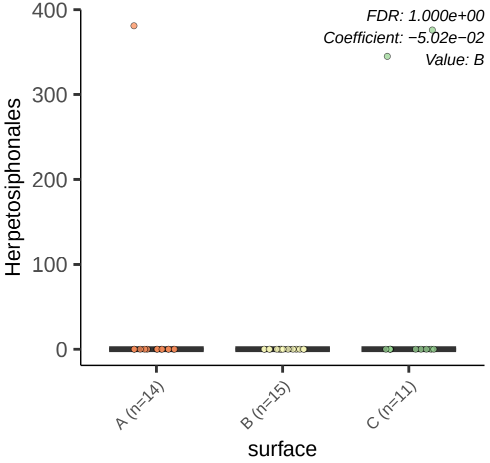

FDR: 1.000e+00
Coefficient: −5.02e−02
Value: B
Herpetosiphonales
400
300
200
100
0
A (n=14)
B (n=15)
C (n=11)
surface

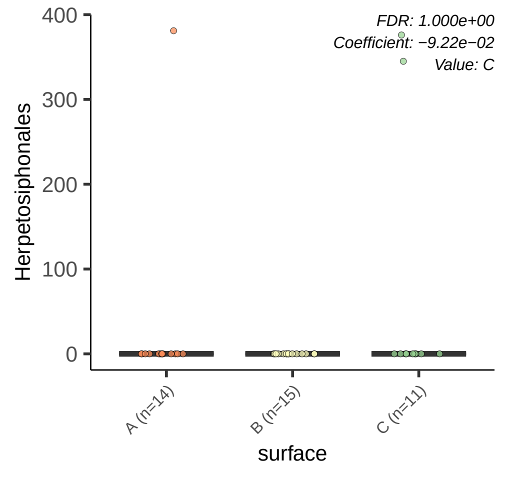
FDR: 1.000e+00
Coefficient: −9.22e−02
Value: C
Herpetosiphonales
400
300
200
100
0
A (n=14)
B (n=15)
C (n=11)
surface

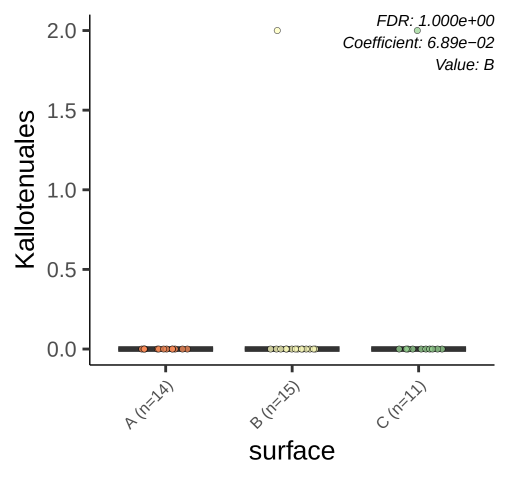

FDR: 1.000e+00
Coefficient: 6.89e−02
Value: B
Kallotenuales
2.0
1.5
1.0
0.5
0.0
A (n=14)
B (n=15)
C (n=11)
surface

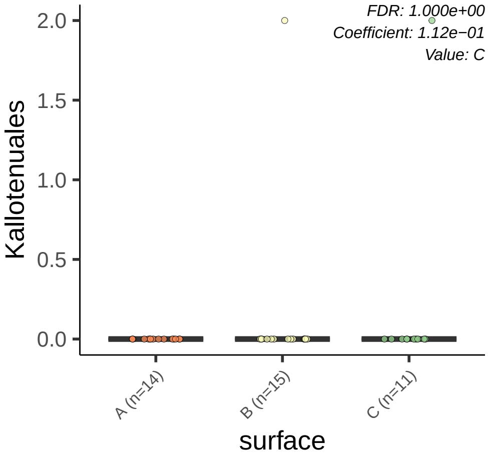
FDR: 1.000e+00
Coefficient: 1.12e−01
Value: C
Kallotenuales
2.0
1.5
1.0
0.5
0.0
A (n=14)
B (n=15)
C (n=11)
surface

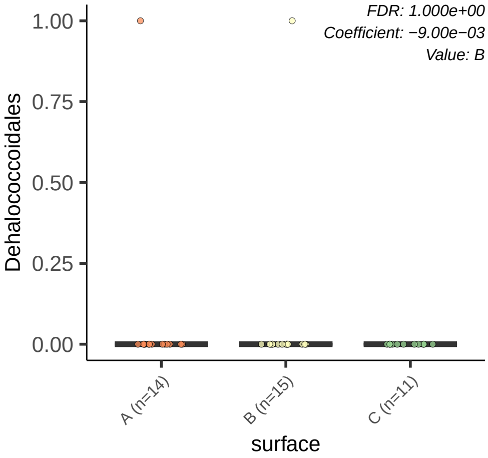

FDR: 1.000e+00
Coefficient: −9.00e−03
Value: B
Dehalococcoidales
1.00
0.75
0.50
0.25
0.00
A (n=14)
B (n=15)
C (n=11)
surface

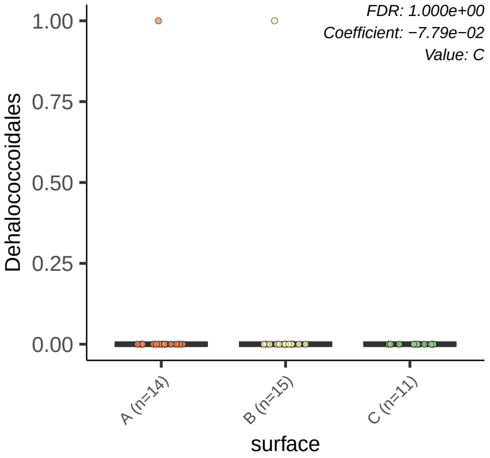
FDR: 1.000e+00
Coefficient: −7.79e−02
Value: C
Dehalococcoidales
1.00
0.75
0.50
0.25
0.00
A (n=14)
B (n=15)
C (n=11)
surface

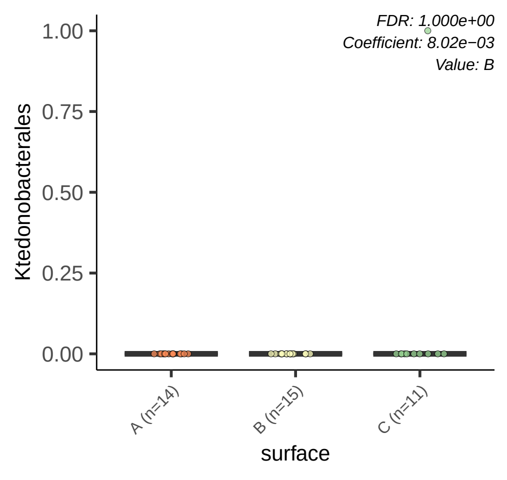

FDR: 1.000e+00
Coefficient: 8.02e−03
Value: B
Ktedonobacterales
1.00
0.75
0.50
0.25
0.00
A (n=14)
B (n=15)
C (n=11)
surface

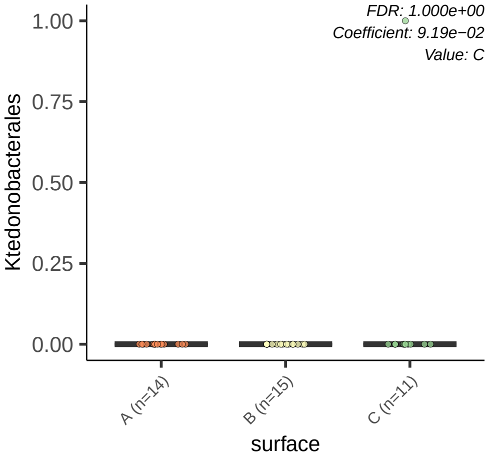
FDR: 1.000e+00
Coefficient: 9.19e−02
Value: C
Ktedonobacterales
1.00
0.75
0.50
0.25
0.00
A (n=14)
B (n=15)
C (n=11)
surface

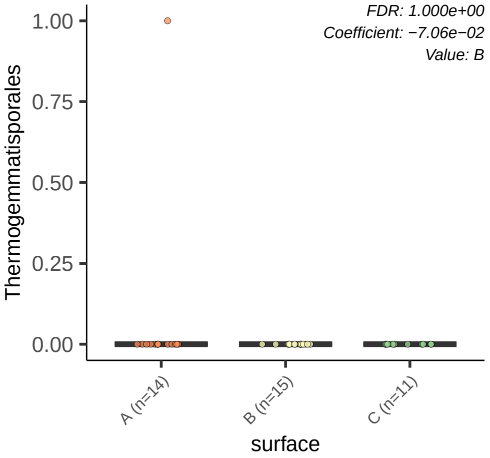

FDR: 1.000e+00
Coefficient: −7.06e−02
Value: B
Thermogemmatisporales
1.00
0.75
0.50
0.25
0.00
A (n=14)
B (n=15)
C (n=11)
surface

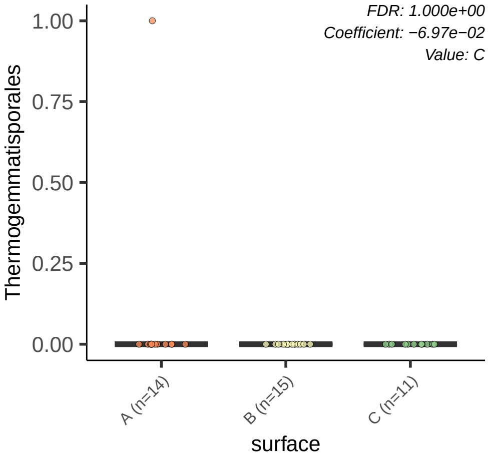
FDR: 1.000e+00
Coefficient: −6.97e−02
Value: C
Thermogemmatisporales
1.00
0.75
0.50
0.25
0.00
A (n=14)
B (n=15)
C (n=11)
surface

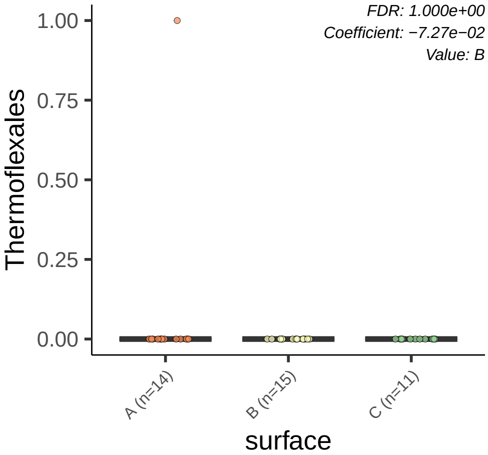

FDR: 1.000e+00
Coefficient: −7.27e−02
Value: B
Thermoflexales
1.00
0.75
0.50
0.25
0.00
A (n=14)
B (n=15)
C (n=11)
surface

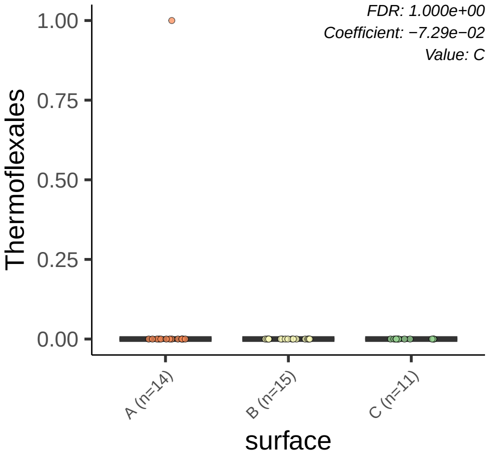

FDR: 1.000e+00
Coefficient: −7.29e−02
Value: C
Thermoflexales
1.00
0.75
0.50
0.25
0.00
A (n=14)
B (n=15)
C (n=11)
surface

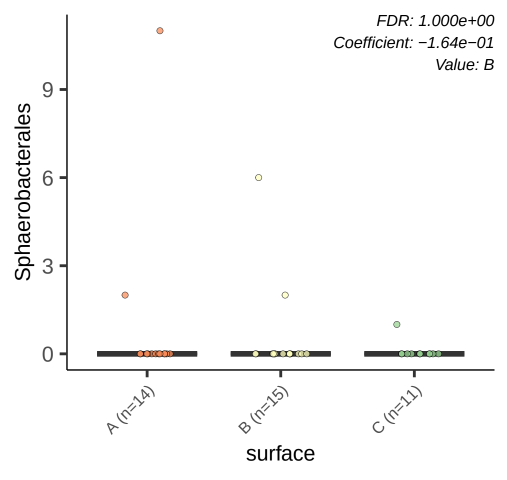

FDR: 1.000e+00
Coefficient: −1.64e−01
Value: B
Sphaerobacterales
0
3
6
9
A (n=14)
B (n=15)
C (n=11)
surface

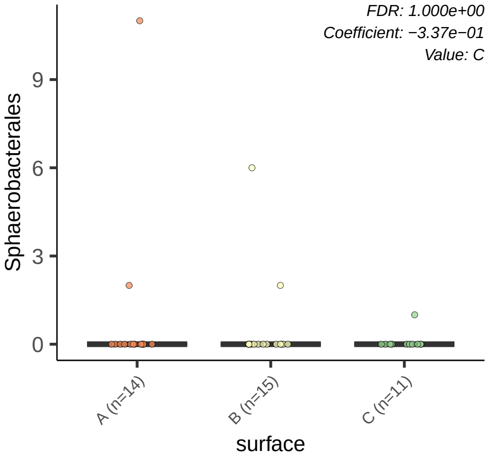
FDR: 1.000e+00
Coefficient: −3.37e−01
Value: C
Sphaerobacterales
0
3
6
9
A (n=14)
B (n=15)
C (n=11)
surface

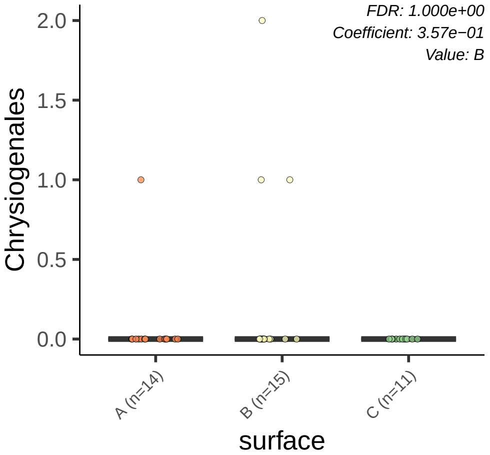
FDR: 1.000e+00
Coefficient: 3.57e−01
Value: B
Chrysiogenales
2.0
1.5
1.0
0.5
0.0
A (n=14)
B (n=15)
C (n=11)
surface

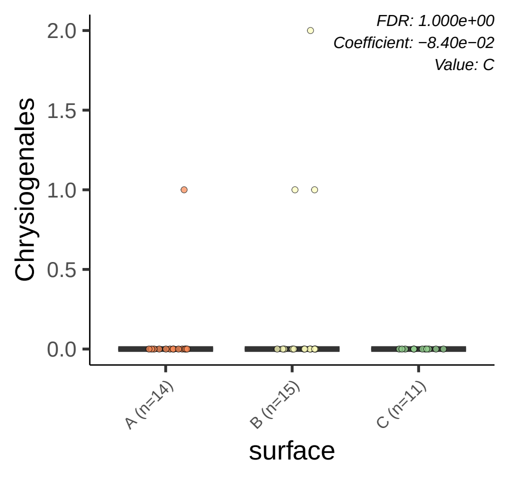

FDR: 1.000e+00
Coefficient: −8.40e−02
Value: C
Chrysiogenales
2.0
1.5
1.0
0.5
0.0
A (n=14)
B (n=15)
C (n=11)
surface

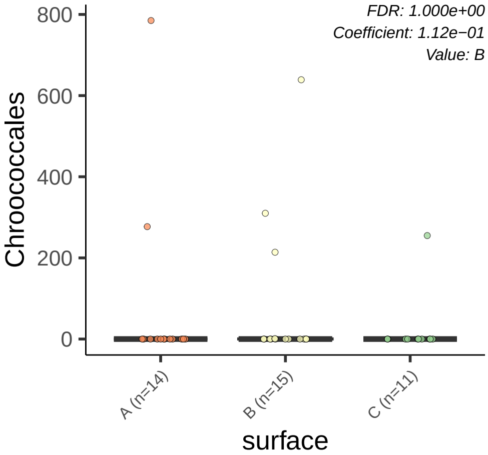

FDR: 1.000e+00
Coefficient: 1.12e−01
Value: B
Chroococcales
800
600
400
200
0
A (n=14)
B (n=15)
C (n=11)
surface

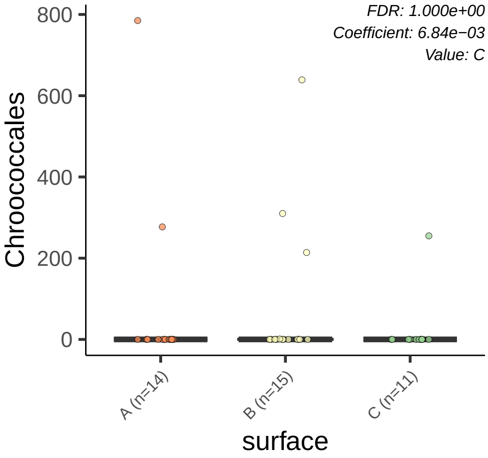
FDR: 1.000e+00
Coefficient: 6.84e−03
Value: C
Chroococcales
800
600
400
200
0
A (n=14)
B (n=15)
C (n=11)
surface

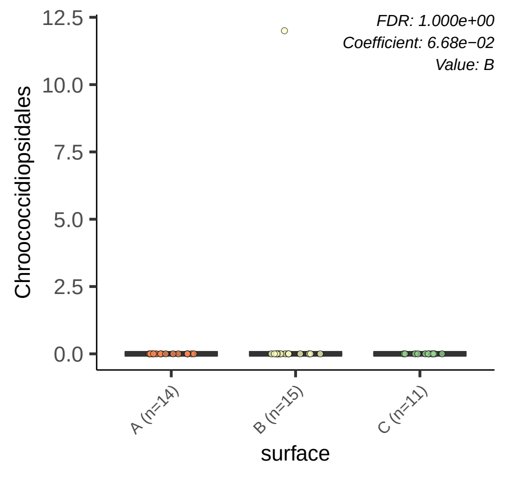

FDR: 1.000e+00
Coefficient: 6.68e−02
Value: B
Chroococcidiopsidales
12.5
10.0
7.5
5.0
2.5
0.0
A (n=14)
B (n=15)
C (n=11)
surface

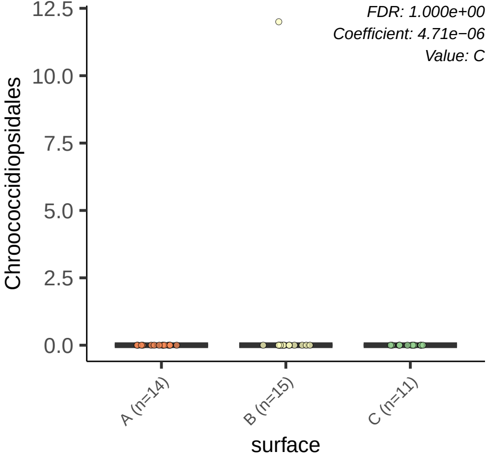

FDR: 1.000e+00
Coefficient: 4.71e−06
Value: C
Chroococcidiopsidales
12.5
10.0
7.5
5.0
2.5
0.0
A (n=14)
B (n=15)
C (n=11)
surface

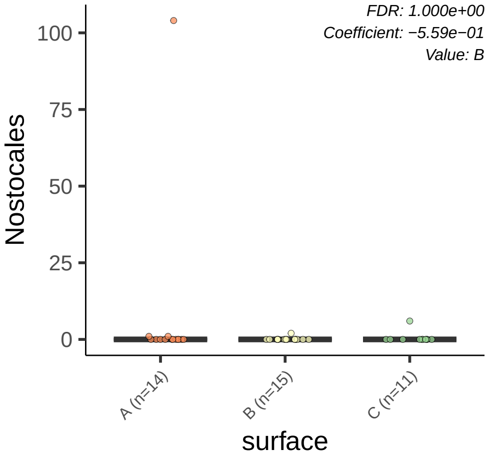

FDR: 1.000e+00
Coefficient: −5.59e−01
Value: B
Nostocales
100
75
50
25
0
A (n=14)
B (n=15)
C (n=11)
surface

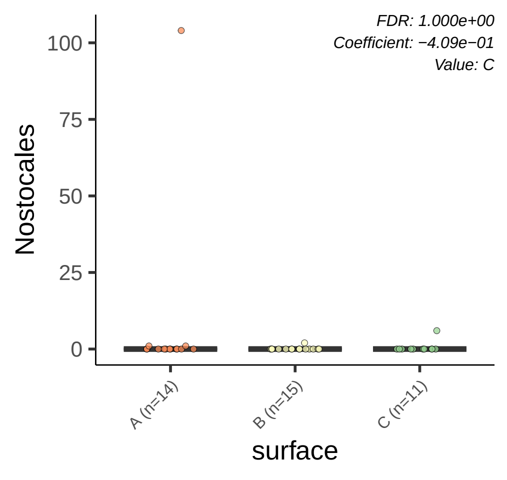

FDR: 1.000e+00
Coefficient: −4.09e−01
Value: C
Nostocales
100
75
50
25
0
A (n=14)
B (n=15)
C (n=11)
surface

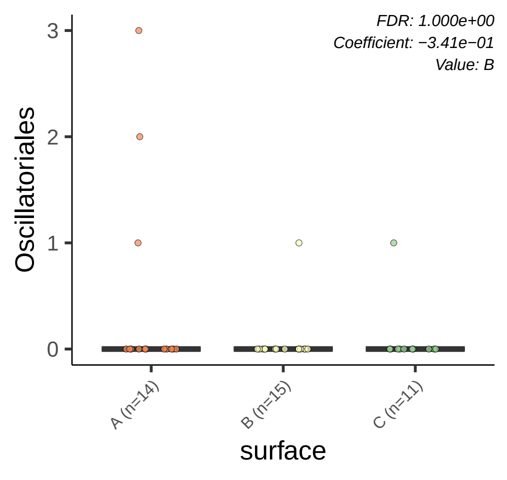

FDR: 1.000e+00
Coefficient: −3.41e−01
Value: B
Oscillatoriales
3
2
1
0
A (n=14)
B (n=15)
C (n=11)
surface

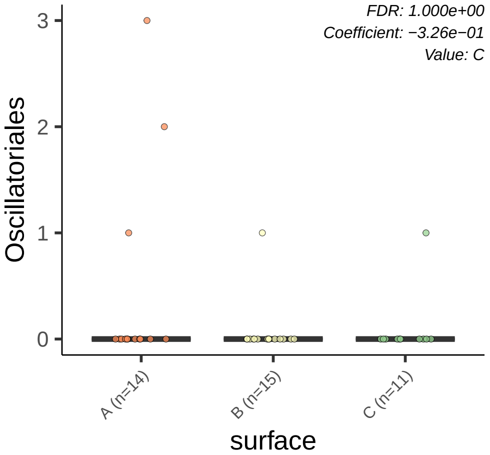

FDR: 1.000e+00
Coefficient: −3.26e−01
Value: C
Oscillatoriales
3
2
1
0
A (n=14)
B (n=15)
C (n=11)
surface

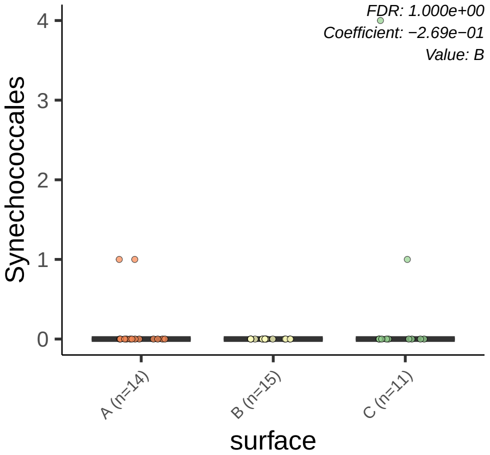
FDR: 1.000e+00
Coefficient: −2.69e−01
Value: B
Synechococcales
4
3
2
1
0
A (n=14)
B (n=15)
C (n=11)
surface

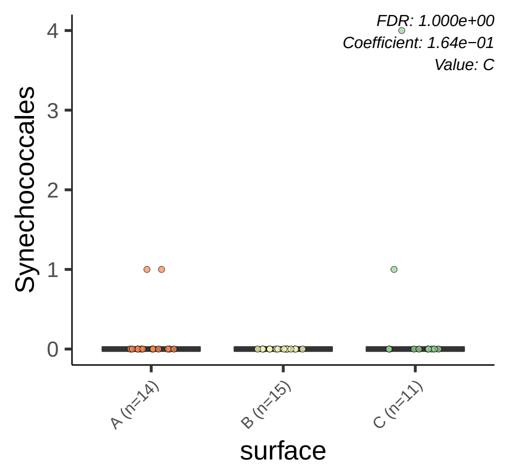

FDR: 1.000e+00
Coefficient: 1.64e−01
Value: C
Synechococcales
4
3
2
1
0
A (n=14)
B (n=15)
C (n=11)
surface

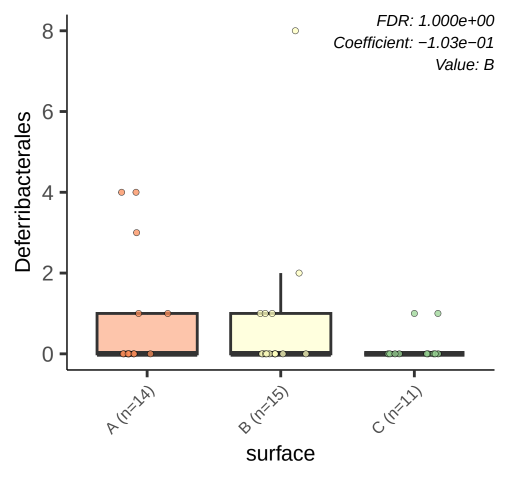

FDR: 1.000e+00
Coefficient: −1.03e−01
Value: B
Deferribacterales
0
2
4
6
8
A (n=14)
B (n=15)
C (n=11)
surface

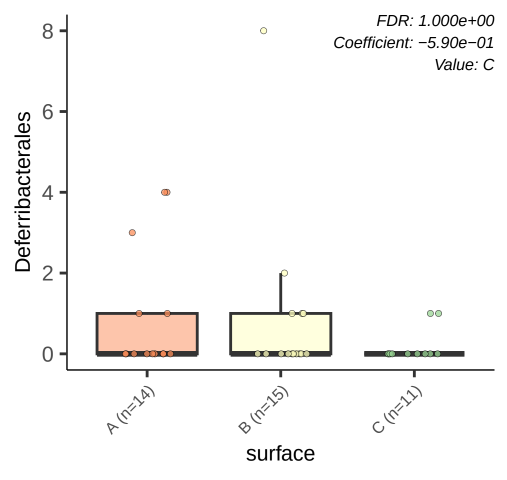

FDR: 1.000e+00
Coefficient: −5.90e−01
Value: C
Deferribacterales
0
2
4
6
8
A (n=14)
B (n=15)
C (n=11)
surface

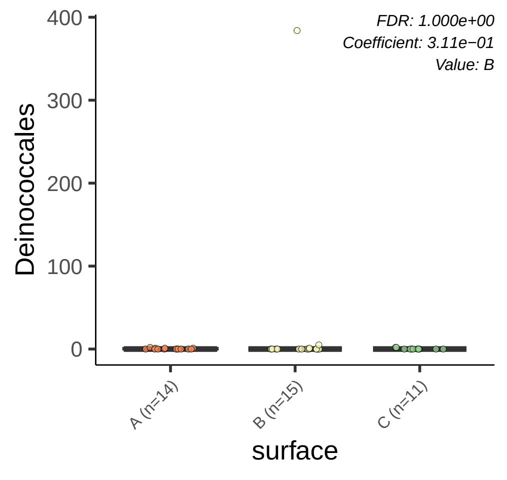

FDR: 1.000e+00
Coefficient: 3.11e−01
Value: B
Deinococcales
400
300
200
100
0
A (n=14)
B (n=15)
C (n=11)
surface

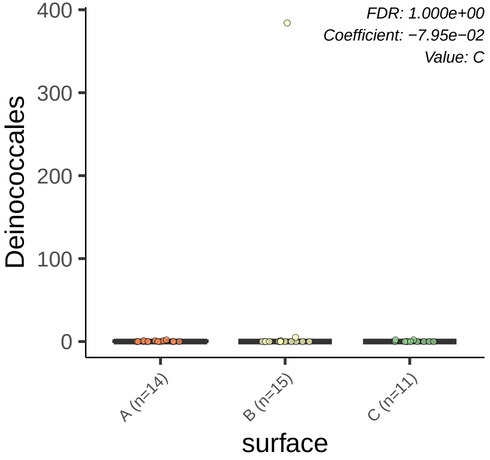

FDR: 1.000e+00
Coefficient: −7.95e−02
Value: C
Deinococcales
400
300
200
100
0
A (n=14)
B (n=15)
C (n=11)
surface

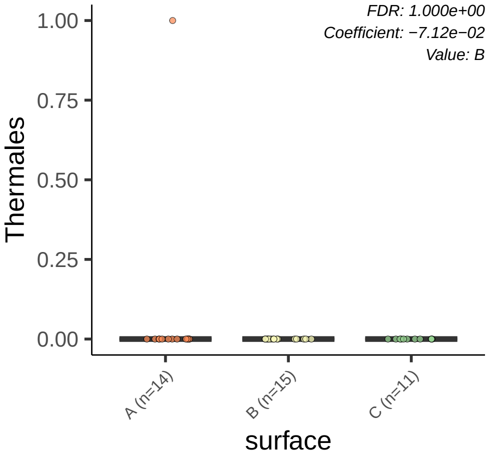

FDR: 1.000e+00
Coefficient: −7.12e−02
Value: B
1.00
0.75
0.50
0.25
0.00
Thermales
A (n=14)
B (n=15)
C (n=11)
surface

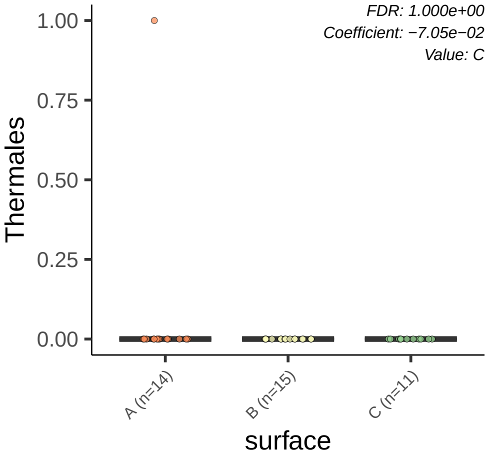
FDR: 1.000e+00
Coefficient: −7.05e−02
Value: C
Thermales
1.00
0.75
0.50
0.25
0.00
A (n=14)
B (n=15)
C (n=11)
surface

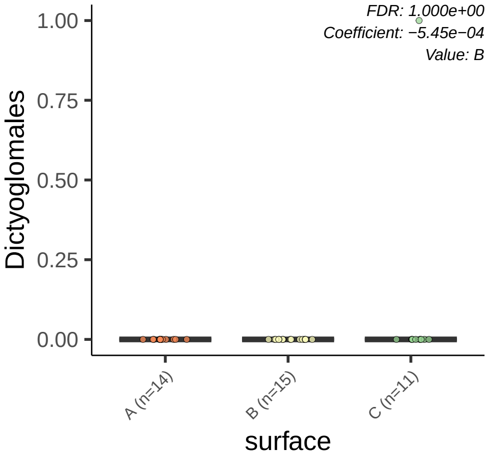

FDR: 1.000e+00
Coefficient: −5.45e−04
Value: B
Dictyoglomales
1.00
0.75
0.50
0.25
0.00
A (n=14)
B (n=15)
C (n=11)
surface

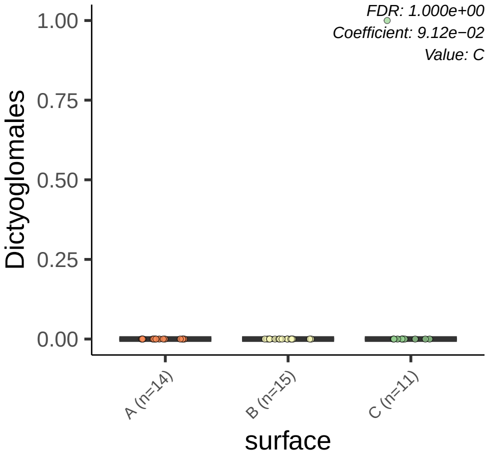
FDR: 1.000e+00
Coefficient: 9.12e−02
Value: C
Dictyoglomales
1.00
0.75
0.50
0.25
0.00
A (n=14)
B (n=15)
C (n=11)
surface

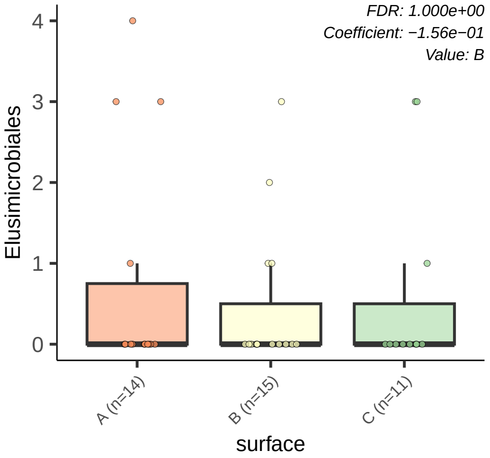

FDR: 1.000e+00
Coefficient: −1.56e−01
Value: B
Elusimicrobiales
4
3
2
1
0
A (n=14)
B (n=15)
C (n=11)
surface

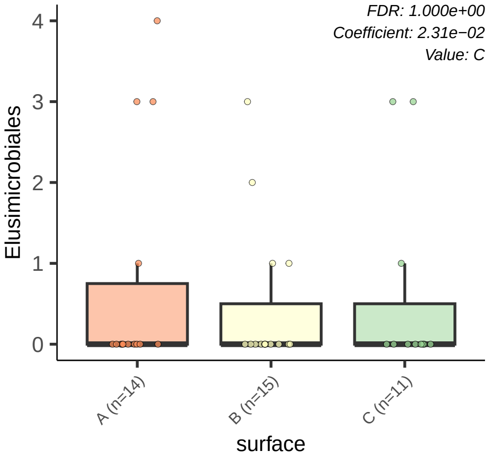

FDR: 1.000e+00
Coefficient: 2.31e−02
Value: C
Elusimicrobiales
4
3
2
1
0
A (n=14)
B (n=15)
C (n=11)
surface

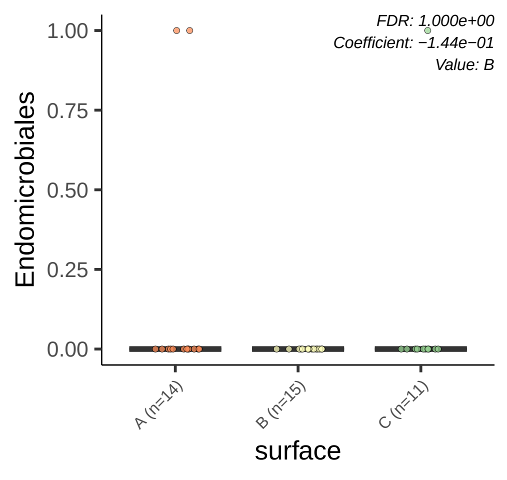

FDR: 1.000e+00
Coefficient: −1.44e−01
Value: B
Endomicrobiales
1.00
0.75
0.50
0.25
0.00
A (n=14)
B (n=15)
C (n=11)
surface

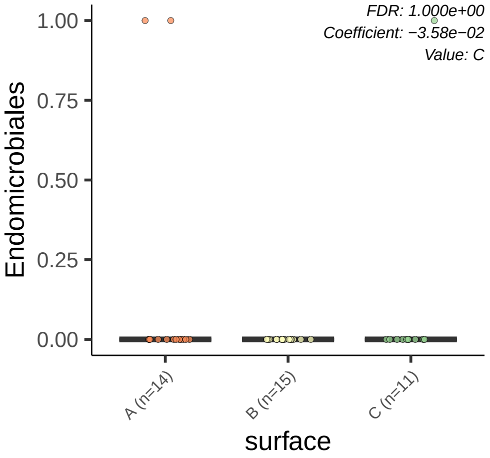
FDR: 1.000e+00
Coefficient: −3.58e−02
Value: C
Endomicrobiales
1.00
0.75
0.50
0.25
0.00
A (n=14)
B (n=15)
C (n=11)
surface

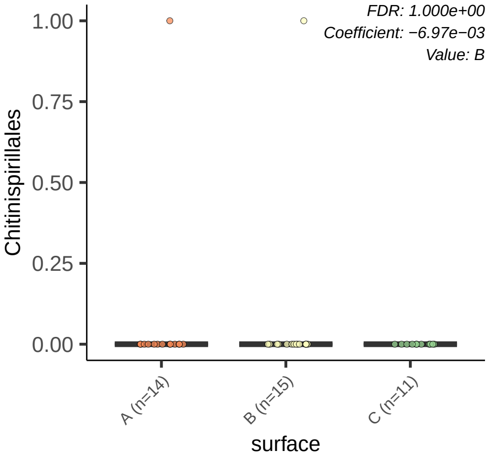
FDR: 1.000e+00
Coefficient: −6.97e−03
Value: B
Chitinispirillales
1.00
0.75
0.50
0.25
0.00
A (n=14)
B (n=15)
C (n=11)
surface

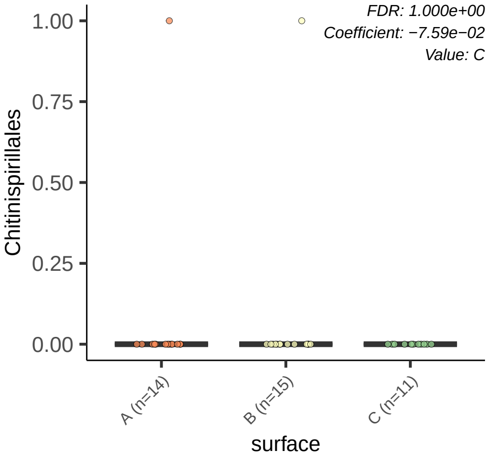
FDR: 1.000e+00
Coefficient: −7.59e−02
Value: C
Chitinispirillales
1.00
0.75
0.50
0.25
0.00
A (n=14)
B (n=15)
C (n=11)
surface

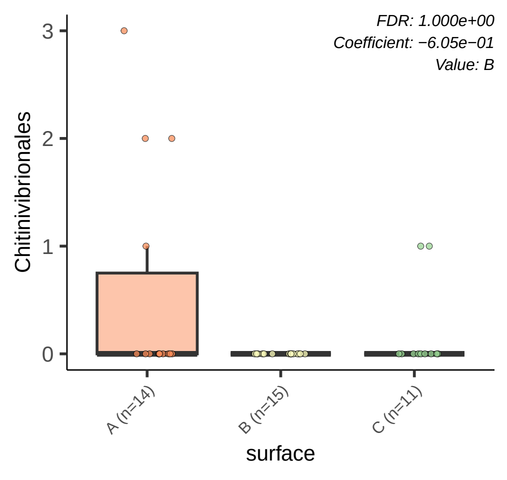
FDR: 1.000e+00
Coefficient: −6.05e−01
Value: B
Chitinivibrionales
3
2
1
0
A (n=14)
B (n=15)
C (n=11)
surface

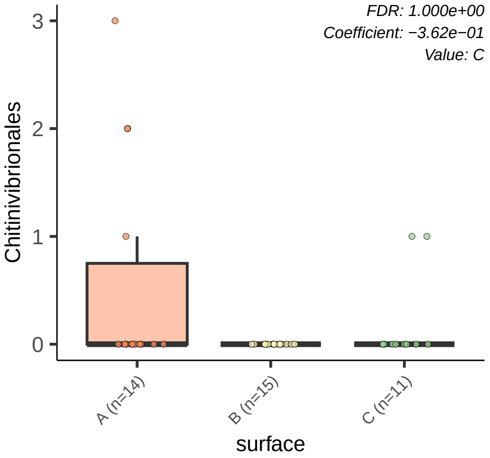
FDR: 1.000e+00
Coefficient: −3.62e−01
Value: C
Chitinivibrionales
3
2
1
0
A (n=14)
B (n=15)
C (n=11)
surface

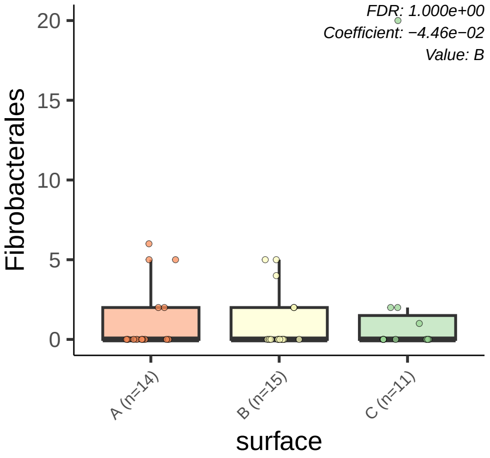

FDR: 1.000e+00
Coefficient: −4.46e−02
Value: B
Fibrobacterales
20
15
10
5
0
A (n=14)
B (n=15)
C (n=11)
surface

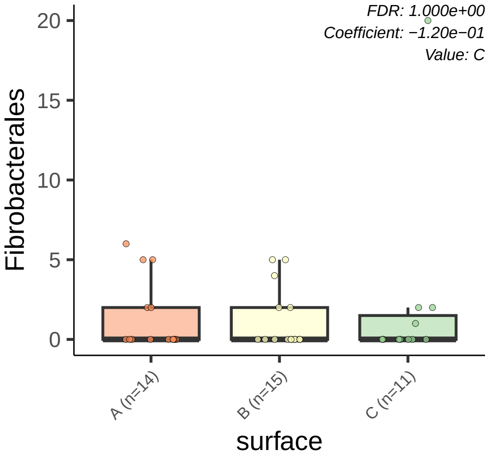

FDR: 1.000e+00
Coefficient: −1.20e−01
Value: C
Fibrobacterales
20
15
10
5
0
A (n=14)
B (n=15)
C (n=11)
surface

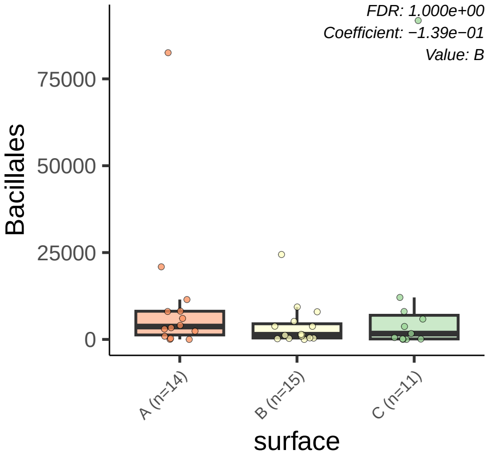
FDR: 1.000e+00
Coefficient: −1.39e−01
Value: B
Bacillales
75000
50000
25000
0
A (n=14)
B (n=15)
C (n=11)
surface

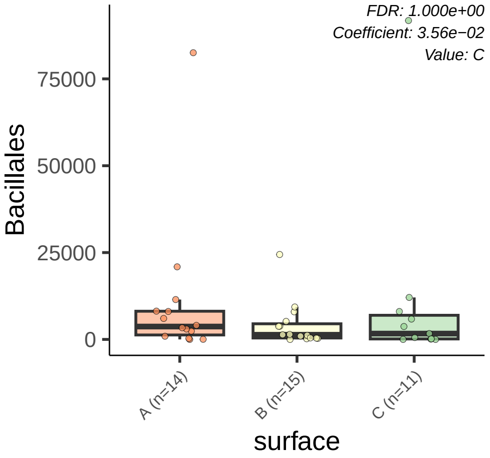

FDR: 1.000e+00
Coefficient: 3.56e−02
Value: C
Bacillales
75000
50000
25000
0
A (n=14)
B (n=15)
C (n=11)
surface

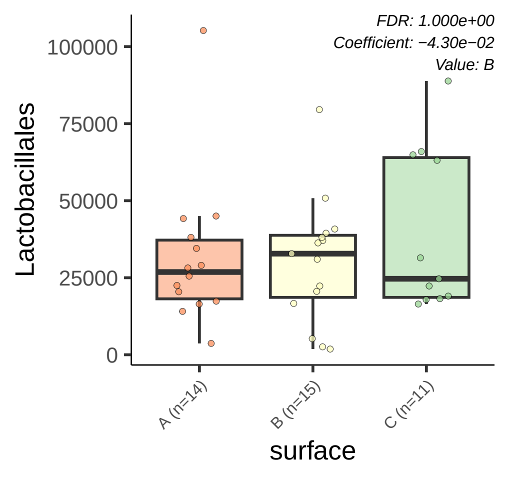
FDR: 1.000e+00
Coefficient: −4.30e−02
Value: B
Lactobacillales
100000
75000
50000
25000
0
A (n=14)
B (n=15)
C (n=11)
surface

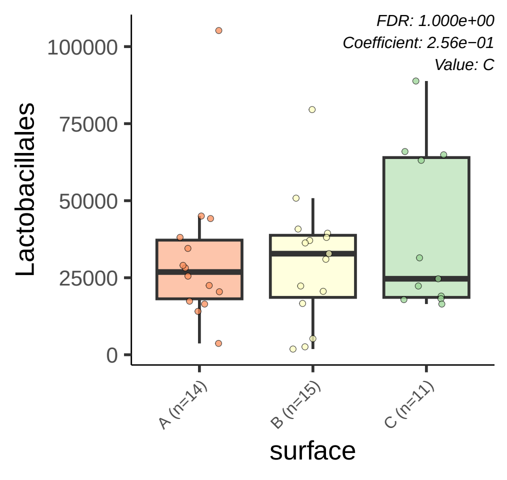
FDR: 1.000e+00
Coefficient: 2.56e−01
Value: C
Lactobacillales
100000
75000
50000
25000
0
A (n=14)
B (n=15)
C (n=11)
surface

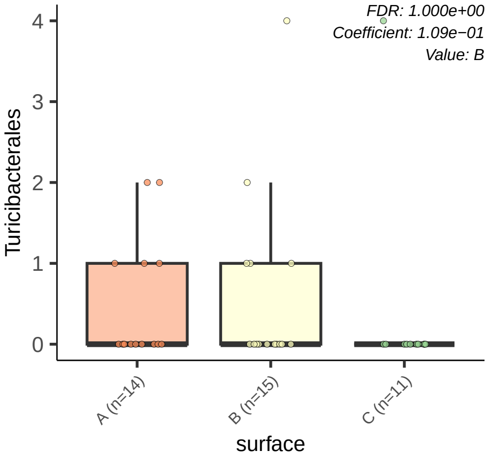
FDR: 1.000e+00
Coefficient: 1.09e−01
Value: B
Turicibacterales
4
3
2
1
0
A (n=14)
B (n=15)
C (n=11)
surface

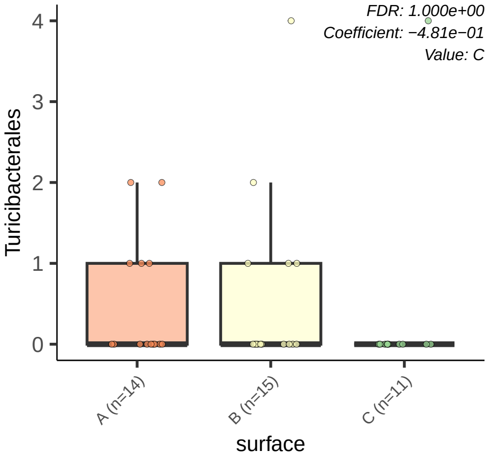

FDR: 1.000e+00
Coefficient: −4.81e−01
Value: C
Turicibacterales
4
3
2
1
0
A (n=14)
B (n=15)
C (n=11)
surface

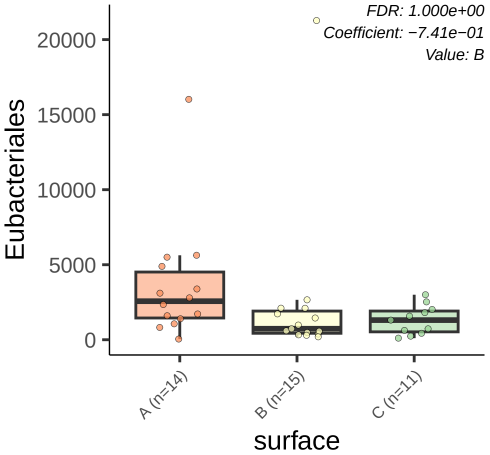

FDR: 1.000e+00
Coefficient: −7.41e−01
Value: B
Eubacteriales
20000
15000
10000
5000
0
A (n=14)
B (n=15)
C (n=11)
surface

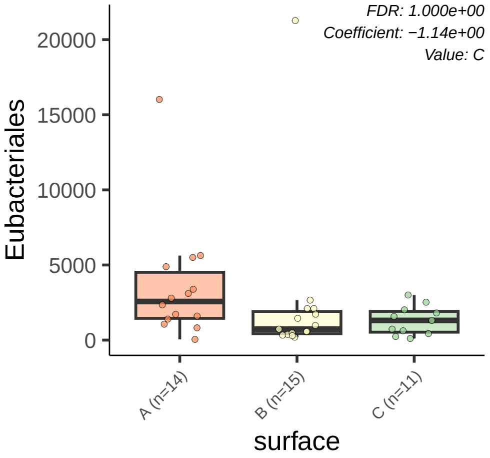
FDR: 1.000e+00
Coefficient: −1.14e+00
Value: C
Eubacteriales
20000
15000
10000
5000
0
A (n=14)
B (n=15)
C (n=11)
surface

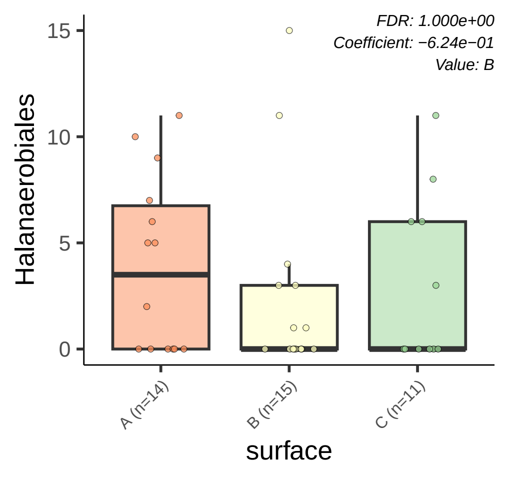
FDR: 1.000e+00
Coefficient: −6.24e−01
Value: B
Halanaerobiales
15
10
5
0
A (n=14)
B (n=15)
C (n=11)
surface

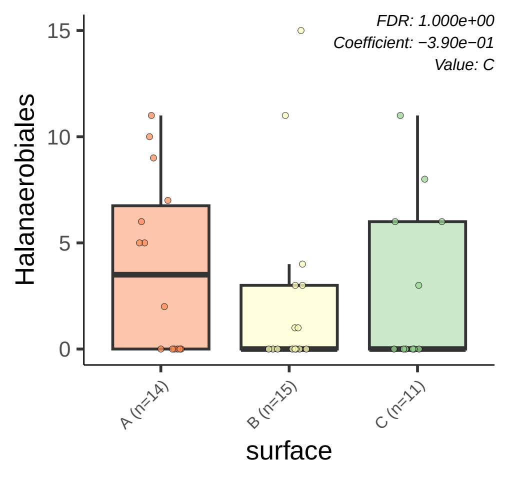
FDR: 1.000e+00
Coefficient: −3.90e−01
Value: C
Halanaerobiales
15
10
5
0
A (n=14)
B (n=15)
C (n=11)
surface

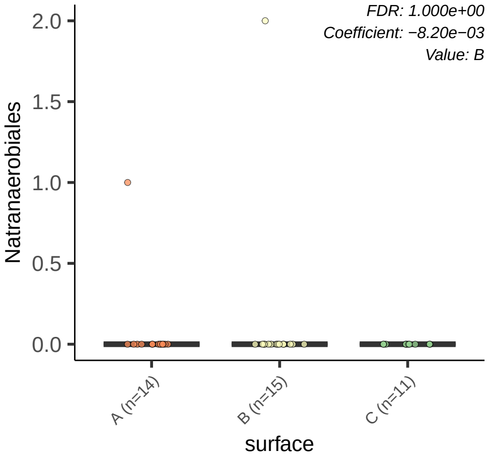
FDR: 1.000e+00
Coefficient: −8.20e−03
Value: B
Natranaerobiales
2.0
1.5
1.0
0.5
0.0
A (n=14)
B (n=15)
C (n=11)
surface

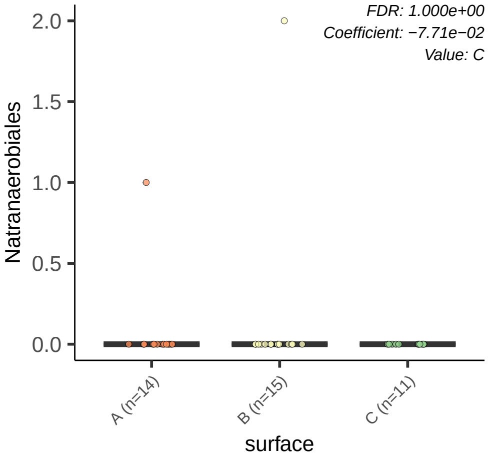

FDR: 1.000e+00
Coefficient: −7.71e−02
Value: C
Natranaerobiales
2.0
1.5
1.0
0.5
0.0
A (n=14)
B (n=15)
C (n=11)
surface

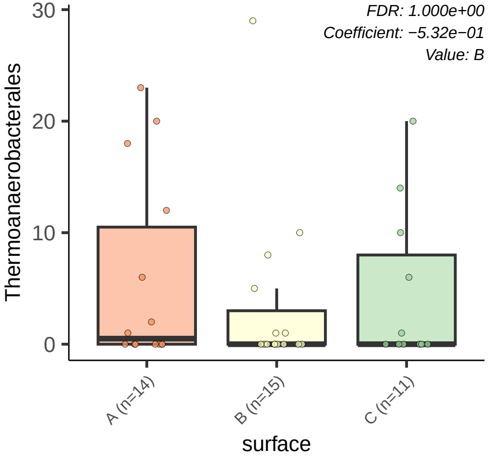
FDR: 1.000e+00
Coefficient: −5.32e−01
Value: B
Thermoanaerobacterales
30
20
10
0
A (n=14)
B (n=15)
C (n=11)
surface

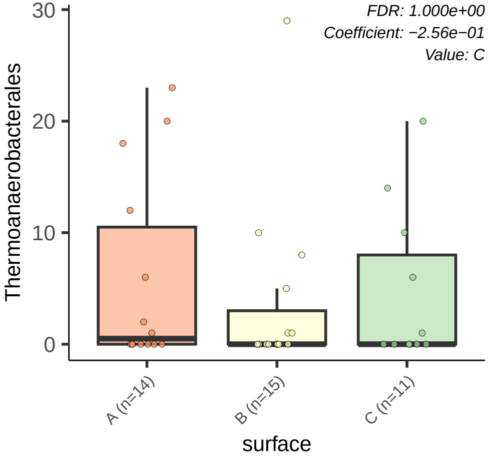

FDR: 1.000e+00
Coefficient: −2.56e−01
Value: C
Thermoanaerobacterales
30
20
10
0
A (n=14)
B (n=15)
C (n=11)
surface

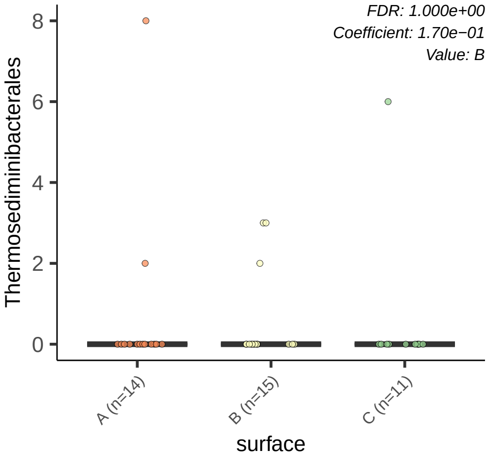

FDR: 1.000e+00
Coefficient: 1.70e−01
Value: B
Thermosediminibacterales
0
2
4
6
8
A (n=14)
B (n=15)
C (n=11)
surface

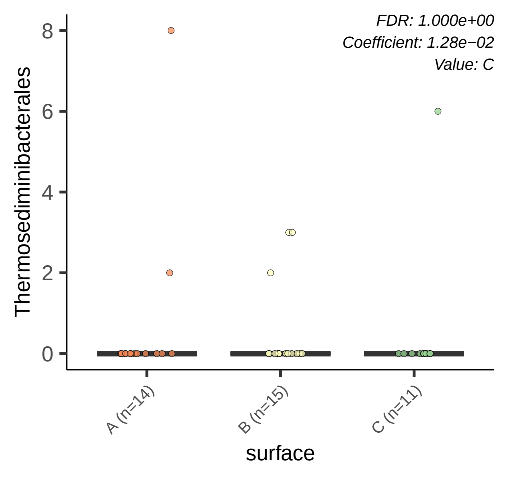

FDR: 1.000e+00
Coefficient: 1.28e−02
Value: C
Thermosediminibacterales
8
6
4
2
0
A (n=14)
B (n=15)
C (n=11)
surface

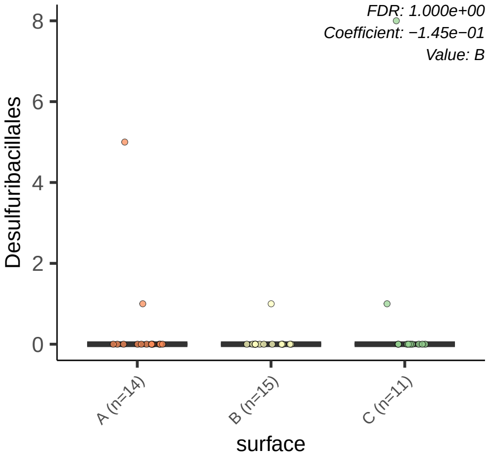
FDR: 1.000e+00
Coefficient: −1.45e−01
Value: B
Desulfuribacillales
8
6
4
2
0
A (n=14)
B (n=15)
C (n=11)
surface

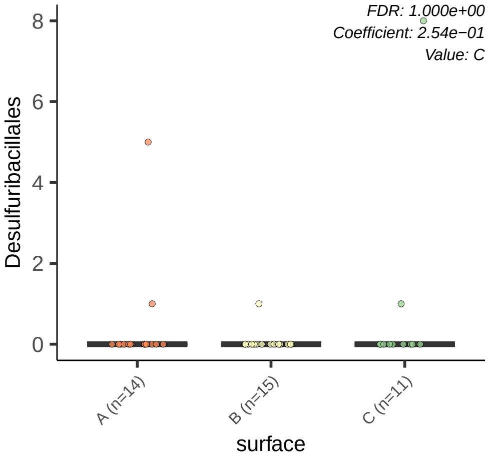

FDR: 1.000e+00
Coefficient: 2.54e−01
Value: C
Desulfuribacillales
8
6
4
2
0
A (n=14)
B (n=15)
C (n=11)
surface

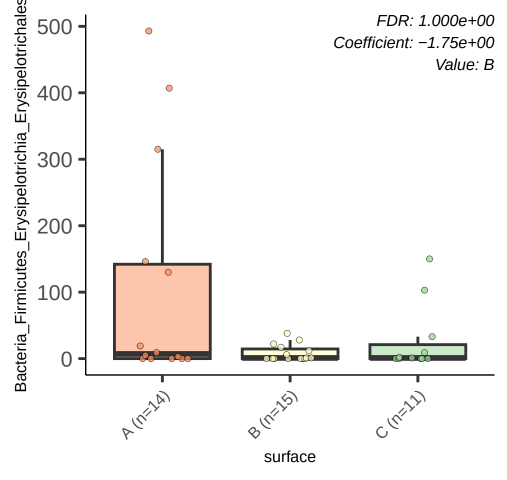

FDR: 1.000e+00
Coefficient: −1.75e+00
Value: B
Bacteria_Firmicutes_Erysipelotrichia_Erysipelotrichales
500
400
300
200
100
0
A (n=14)
B (n=15)
C (n=11)
surface

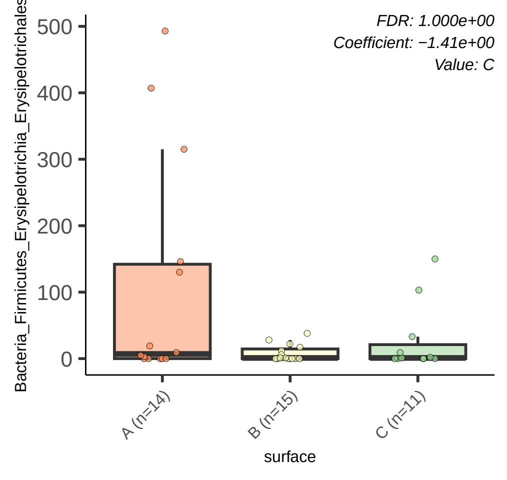

FDR: 1.000e+00
Coefficient: −1.41e+00
Value: C
Bacteria_Firmicutes_Erysipelotrichia_Erysipelotrichales
0
100
200
300
400
500
A (n=14)
B (n=15)
C (n=11)
surface

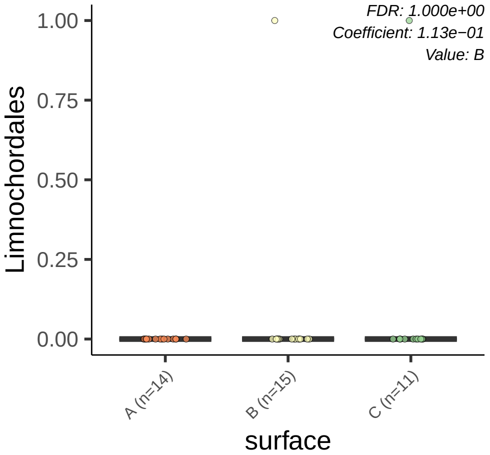

FDR: 1.000e+00
Coefficient: 1.13e−01
Value: B
Limnochordales
1.00
0.75
0.50
0.25
0.00
A (n=14)
B (n=15)
C (n=11)
surface

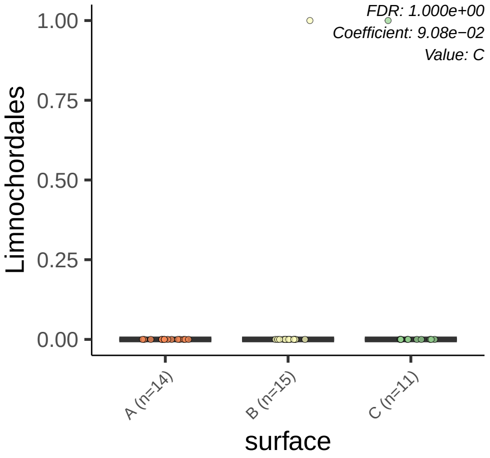

FDR: 1.000e+00
Coefficient: 9.08e−02
Value: C
Limnochordales
1.00
0.75
0.50
0.25
0.00
A (n=14)
B (n=15)
C (n=11)
surface

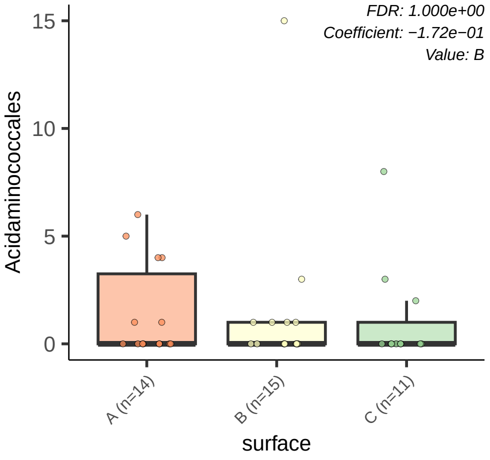

FDR: 1.000e+00
Coefficient: −1.72e−01
Value: B
Acidaminococcales
15
10
5
0
A (n=14)
B (n=15)
C (n=11)
surface

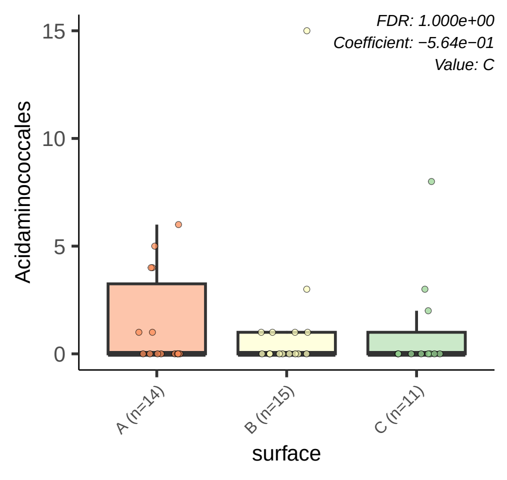

FDR: 1.000e+00
Coefficient: −5.64e−01
Value: C
Acidaminococcales
15
10
5
0
A (n=14)
B (n=15)
C (n=11)
surface

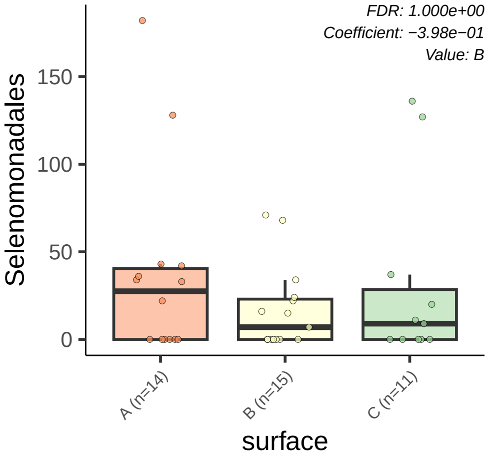

FDR: 1.000e+00
Coefficient: −3.98e−01
Value: B
Selenomonadales
150
100
50
0
A (n=14)
B (n=15)
C (n=11)
surface

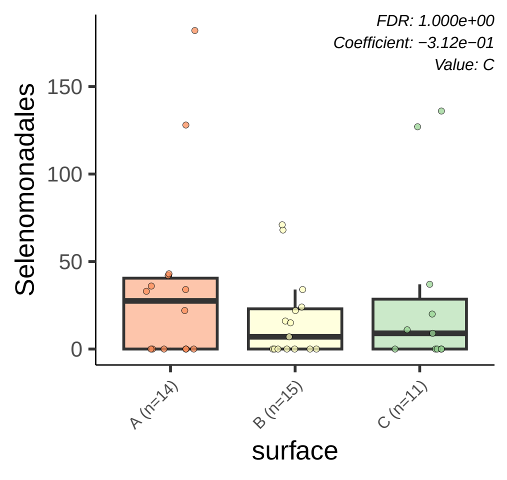
FDR: 1.000e+00
Coefficient: −3.12e−01
Value: C
Selenomonadales
150
100
50
0
A (n=14)
B (n=15)
C (n=11)
surface

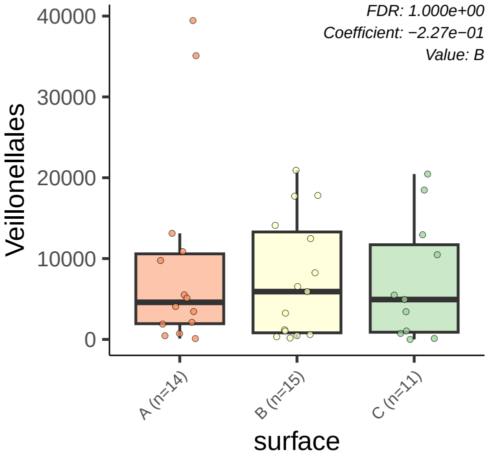
FDR: 1.000e+00
Coefficient: −2.27e−01
Value: B
Veillonellales
40000
30000
20000
10000
0
A (n=14)
B (n=15)
C (n=11)
surface

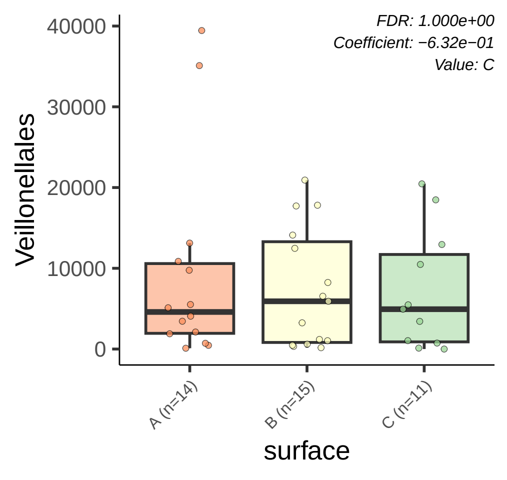

FDR: 1.000e+00
Coefficient: −6.32e−01
Value: C
40000
30000
20000
10000
0
Veillonellales
A (n=14)
B (n=15)
C (n=11)
surface

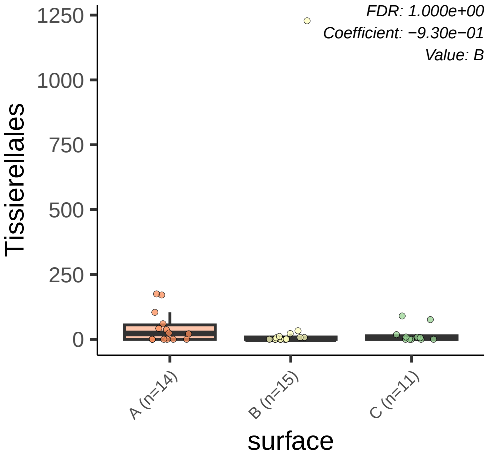

FDR: 1.000e+00
Coefficient: −9.30e−01
Value: B
Tissierellales
1250
1000
750
500
250
0
A (n=14)
B (n=15)
C (n=11)
surface

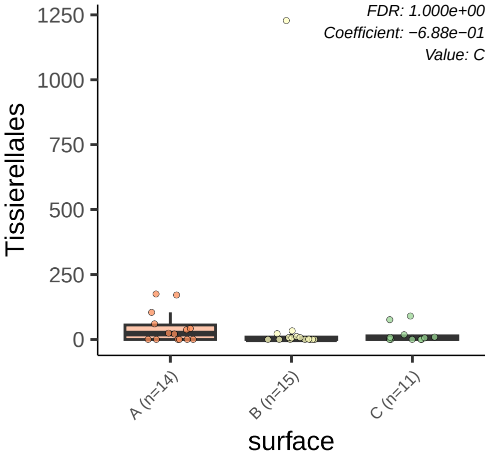

FDR: 1.000e+00
Coefficient: −6.88e−01
Value: C
Tissierellales
1250
1000
750
500
250
0
A (n=14)
B (n=15)
C (n=11)
surface

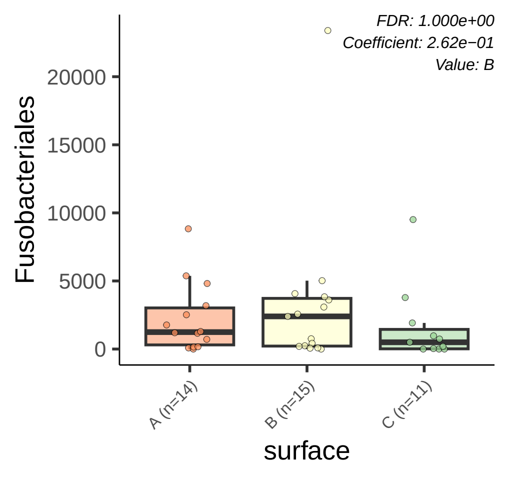

FDR: 1.000e+00
Coefficient: 2.62e−01
Value: B
Fusobacteriales
20000
15000
10000
5000
0
A (n=14)
B (n=15)
C (n=11)
surface

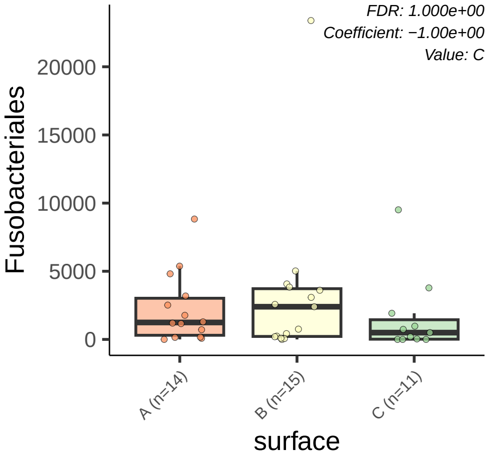

FDR: 1.000e+00
Coefficient: −1.00e+00
Value: C
Fusobacteriales
20000
15000
10000
5000
0
A (n=14)
B (n=15)
C (n=11)
surface

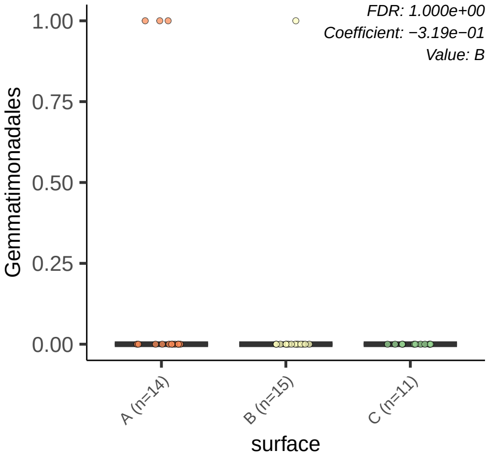
FDR: 1.000e+00
Coefficient: −3.19e−01
Value: B
Gemmatimonadales
1.00
0.75
0.50
0.25
0.00
A (n=14)
B (n=15)
C (n=11)
surface

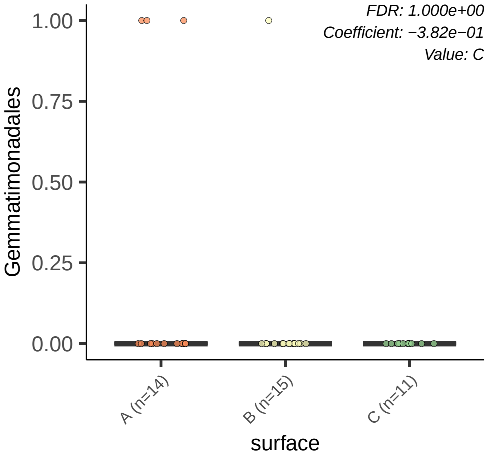

FDR: 1.000e+00
Coefficient: −3.82e−01
Value: C
Gemmatimonadales
1.00
0.75
0.50
0.25
0.00
A (n=14)
B (n=15)
C (n=11)
surface

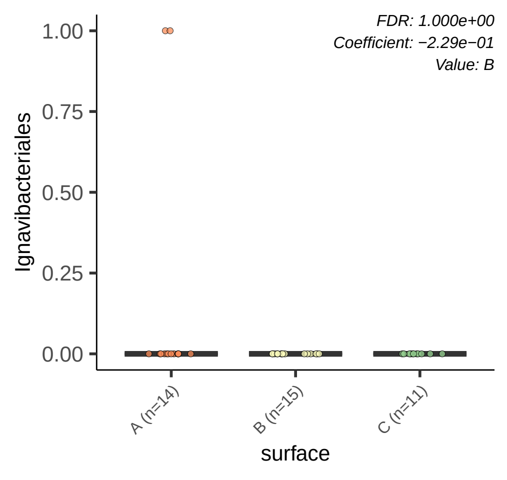

FDR: 1.000e+00
Coefficient: −2.29e−01
Value: B
Ignavibacteriales
1.00
0.75
0.50
0.25
0.00
A (n=14)
B (n=15)
C (n=11)
surface

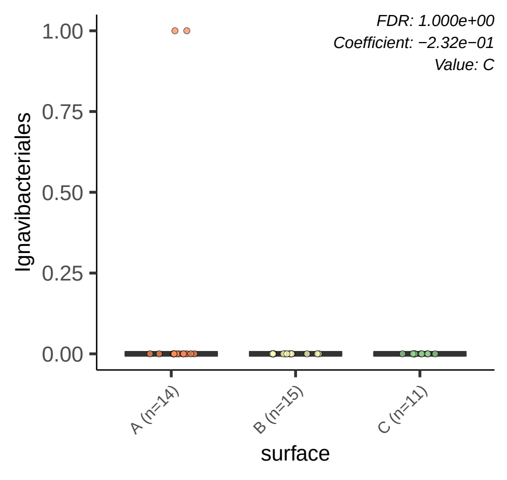

FDR: 1.000e+00
Coefficient: −2.32e−01
Value: C
Ignavibacteriales
1.00
0.75
0.50
0.25
0.00
A (n=14)
B (n=15)
C (n=11)
surface

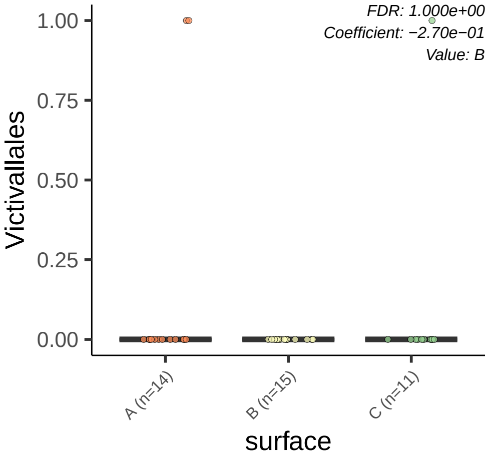
FDR: 1.000e+00
Coefficient: −2.70e−01
Value: B
Victivallales
1.00
0.75
0.50
0.25
0.00
A (n=14)
B (n=15)
C (n=11)
surface

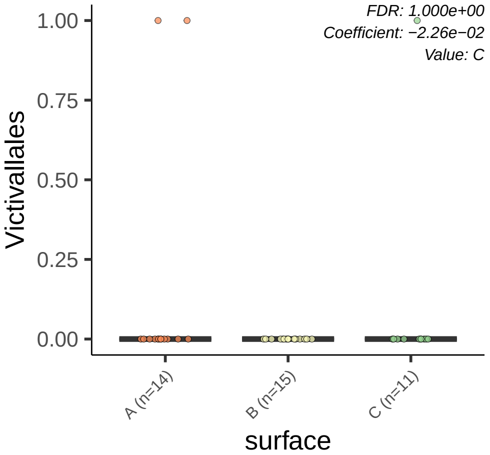
FDR: 1.000e+00
Coefficient: −2.26e−02
Value: C
1.00
0.75
0.50
0.25
0.00
Victivallales
A (n=14)
B (n=15)
C (n=11)
surface

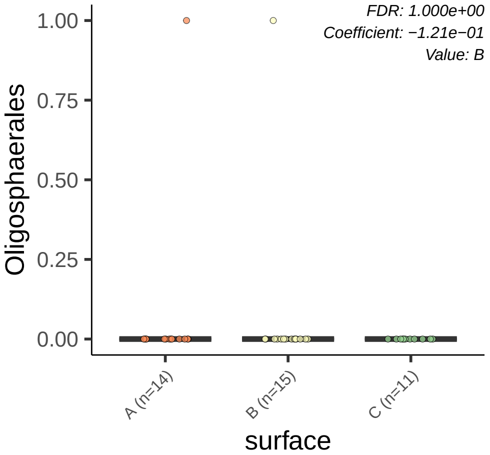

FDR: 1.000e+00
Coefficient: −1.21e−01
Value: B
Oligosphaerales
1.00
0.75
0.50
0.25
0.00
A (n=14)
B (n=15)
C (n=11)
surface

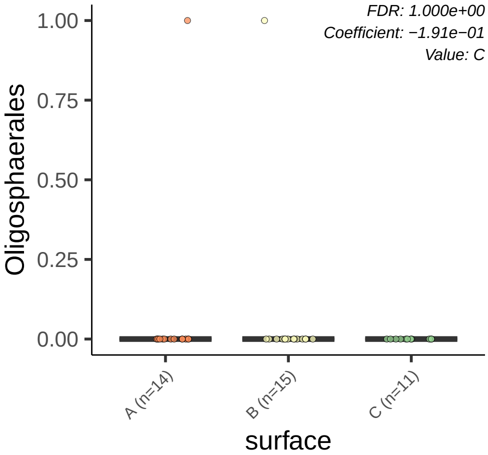
FDR: 1.000e+00
Coefficient: −1.91e−01
Value: C
Oligosphaerales
1.00
0.75
0.50
0.25
0.00
A (n=14)
B (n=15)
C (n=11)
surface

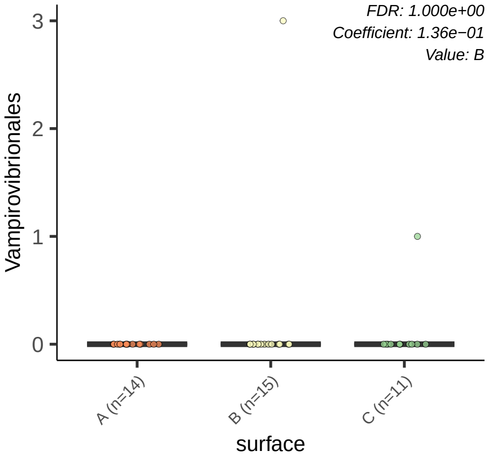
FDR: 1.000e+00
Coefficient: 1.36e−01
Value: B
Vampirovibrionales
3
2
1
0
A (n=14)
B (n=15)
C (n=11)
surface

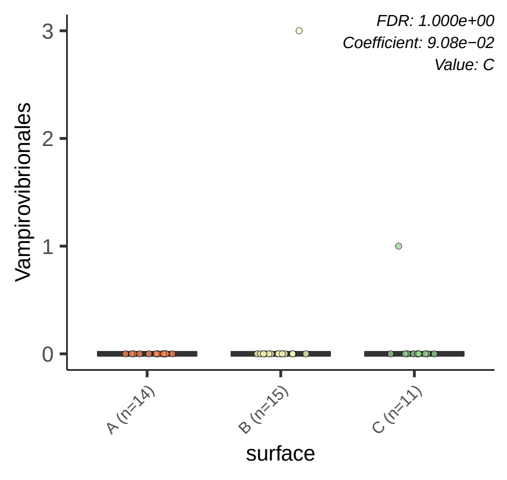

FDR: 1.000e+00
Coefficient: 9.08e−02
Value: C
Vampirovibrionales
3
2
1
0
A (n=14)
B (n=15)
C (n=11)
surface

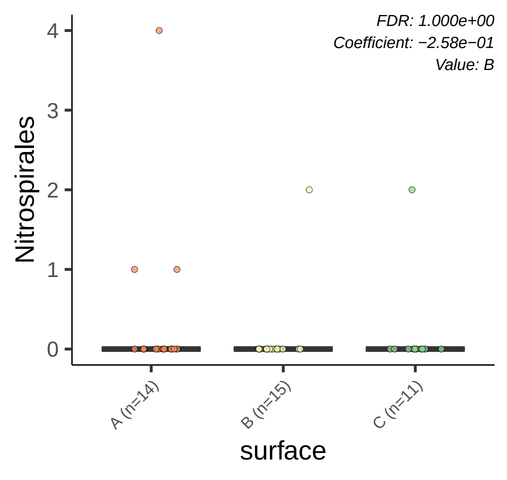

FDR: 1.000e+00
Coefficient: −2.58e−01
Value: B
Nitrospirales
4
3
2
1
0
A (n=14)
B (n=15)
C (n=11)
surface

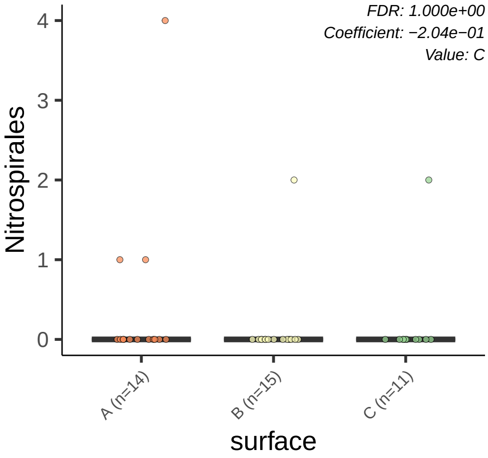

FDR: 1.000e+00
Coefficient: −2.04e−01
Value: C
Nitrospirales
0
1
2
3
4
A (n=14)
B (n=15)
C (n=11)
surface

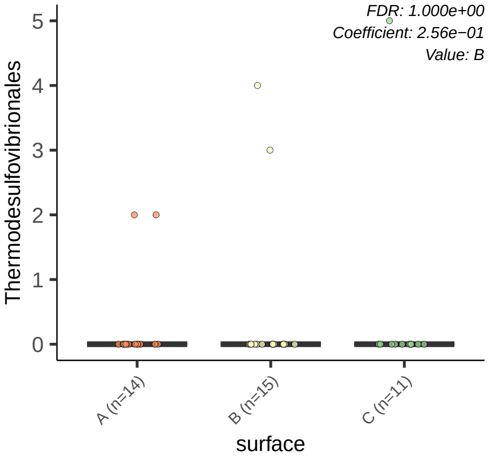
FDR: 1.000e+00
Coefficient: 2.56e−01
Value: B
Thermodesulfovibrionales
0
1
2
3
4
5
A (n=14)
B (n=15)
C (n=11)
surface

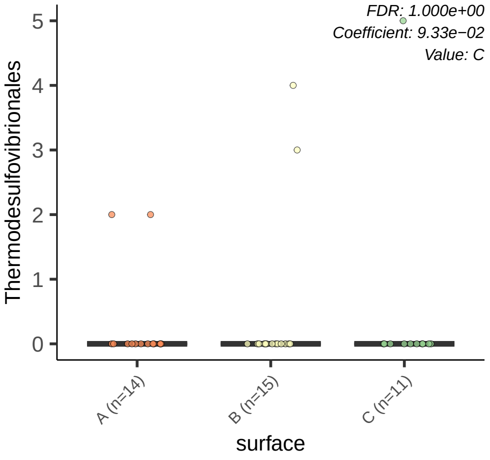

FDR: 1.000e+00
Coefficient: 9.33e−02
Value: C
Thermodesulfovibrionales
0
1
2
3
4
5
A (n=14)
B (n=15)
C (n=11)
surface

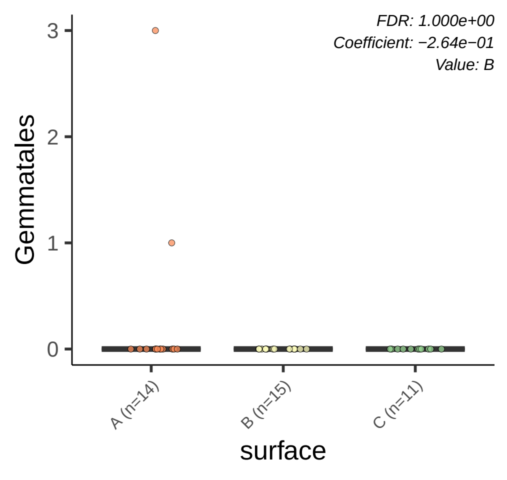

FDR: 1.000e+00
Coefficient: −2.64e−01
Value: B
Gemmatales
3
2
1
0
A (n=14)
B (n=15)
C (n=11)
surface

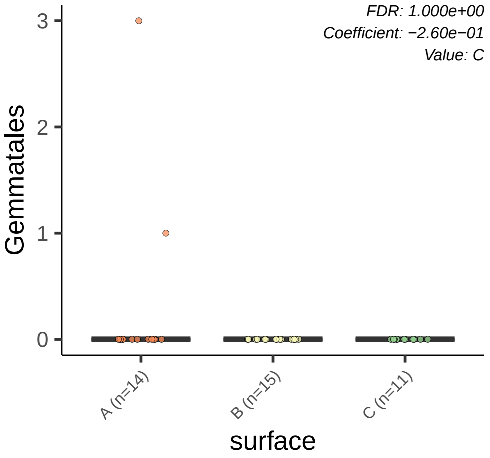

FDR: 1.000e+00
Coefficient: −2.60e−01
Value: C
Gemmatales
3
2
1
0
A (n=14)
B (n=15)
C (n=11)
surface

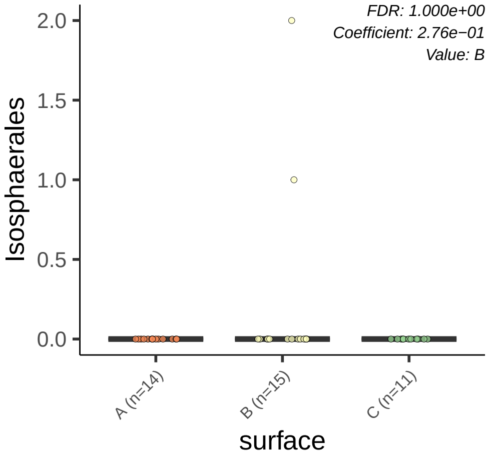
FDR: 1.000e+00
Coefficient: 2.76e−01
Value: B
Isosphaerales
2.0
1.5
1.0
0.5
0.0
A (n=14)
B (n=15)
C (n=11)
surface

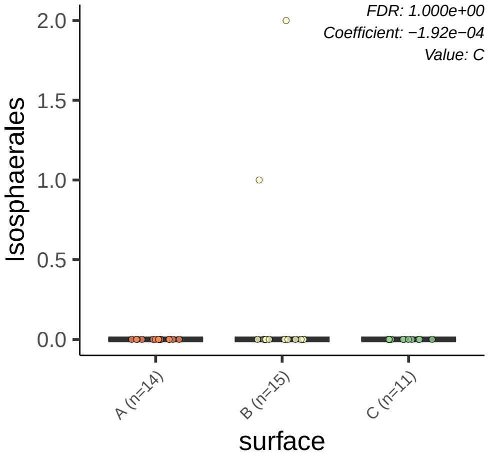

FDR: 1.000e+00
Coefficient: −1.92e−04
Value: C
Isosphaerales
2.0
1.5
1.0
0.5
0.0
A (n=14)
B (n=15)
C (n=11)
surface

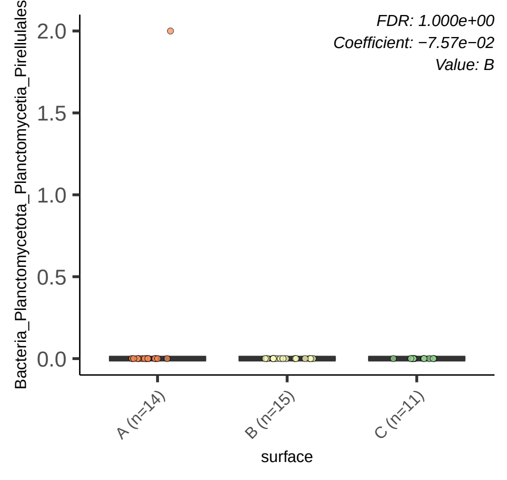

FDR: 1.000e+00
Coefficient: −7.57e−02
Value: B
Bacteria_Planctomycetota_Planctomycetia_Pirellulales
2.0
1.5
1.0
0.5
0.0
A (n=14)
B (n=15)
C (n=11)
surface

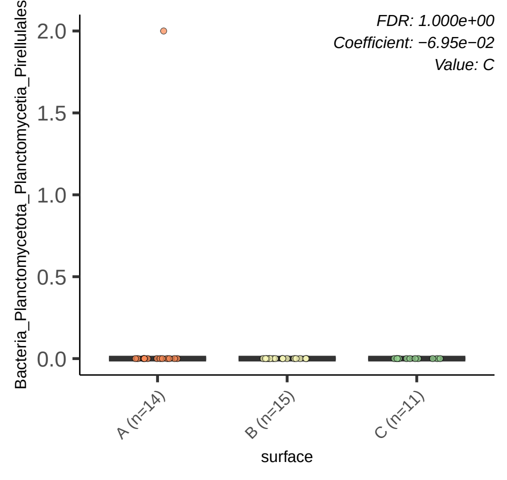

FDR: 1.000e+00
Coefficient: −6.95e−02
Value: C
Bacteria_Planctomycetota_Planctomycetia_Pirellulales
2.0
1.5
1.0
0.5
0.0
A (n=14)
B (n=15)
C (n=11)
surface

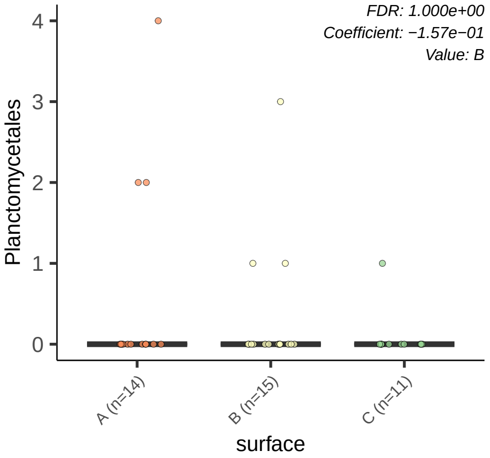
FDR: 1.000e+00
Coefficient: −1.57e−01
Value: B
Planctomycetales
4
3
2
1
0
A (n=14)
B (n=15)
C (n=11)
surface

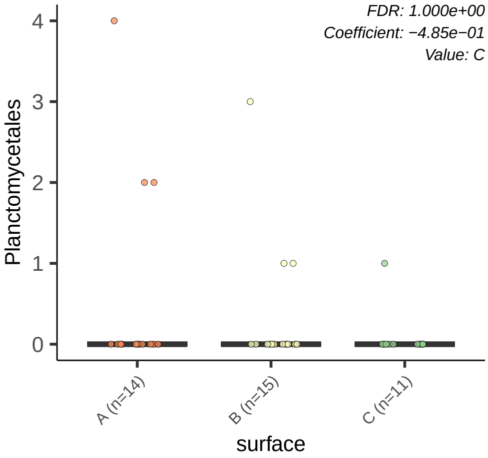

FDR: 1.000e+00
Coefficient: −4.85e−01
Value: C
Planctomycetales
4
3
2
1
0
A (n=14)
B (n=15)
C (n=11)
surface

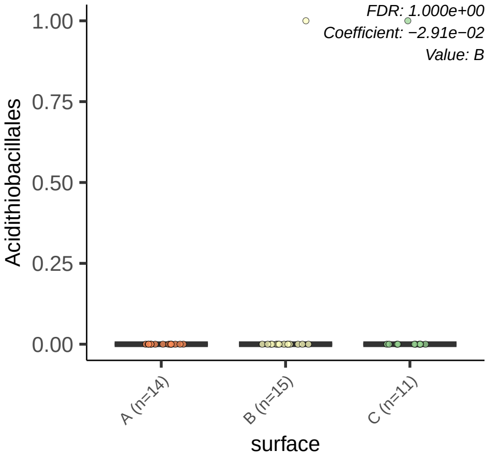
FDR: 1.000e+00
Coefficient: −2.91e−02
Value: B
Acidithiobacillales
1.00
0.75
0.50
0.25
0.00
A (n=14)
B (n=15)
C (n=11)
surface

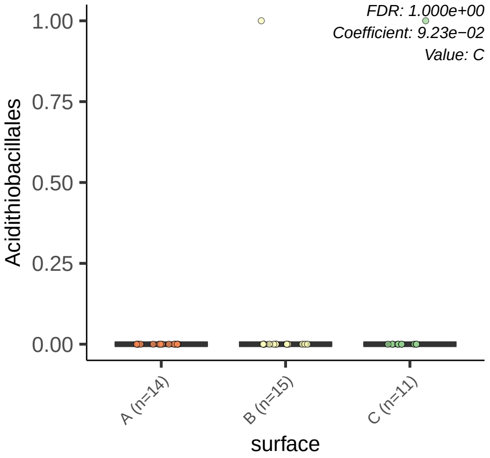

FDR: 1.000e+00
Coefficient: 9.23e−02
Value: C
1.00
0.75
0.50
0.25
0.00
Acidithiobacillales
A (n=14)
B (n=15)
C (n=11)
surface

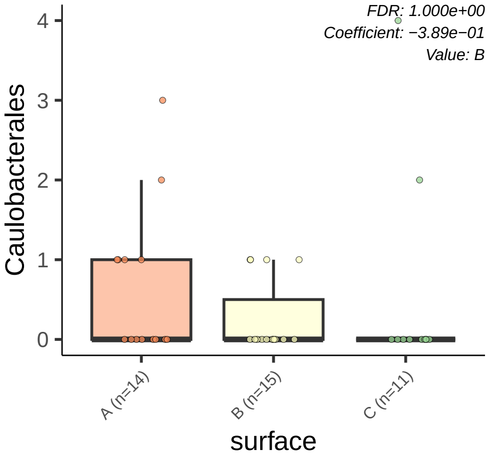

FDR: 1.000e+00
Coefficient: −3.89e−01
Value: B
Caulobacterales
0
1
2
3
4
A (n=14)
B (n=15)
C (n=11)
surface

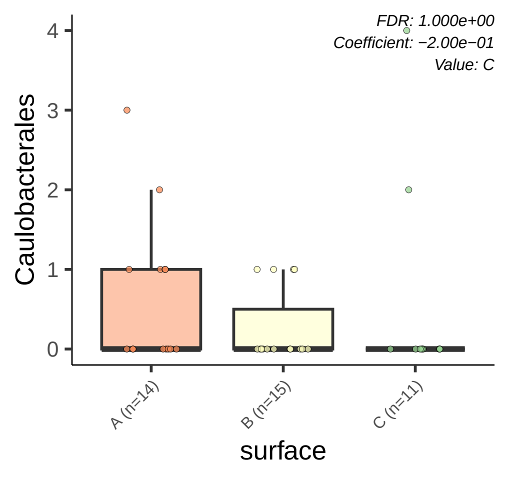
FDR: 1.000e+00
Coefficient: −2.00e−01
Value: C
Caulobacterales
0
1
2
3
4
A (n=14)
B (n=15)
C (n=11)
surface

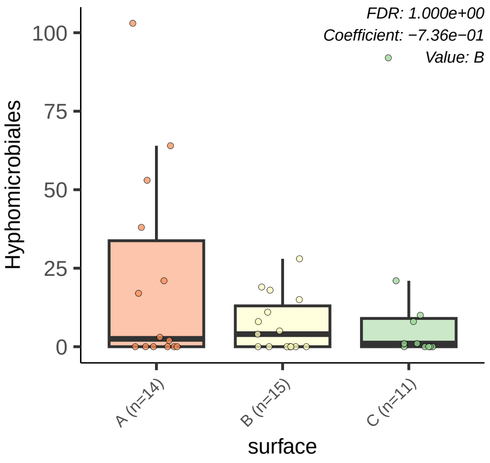

FDR: 1.000e+00
Coefficient: −7.36e−01
Value: B
100
75
50
25
0
Hyphomicrobiales
A (n=14)
B (n=15)
C (n=11)
surface

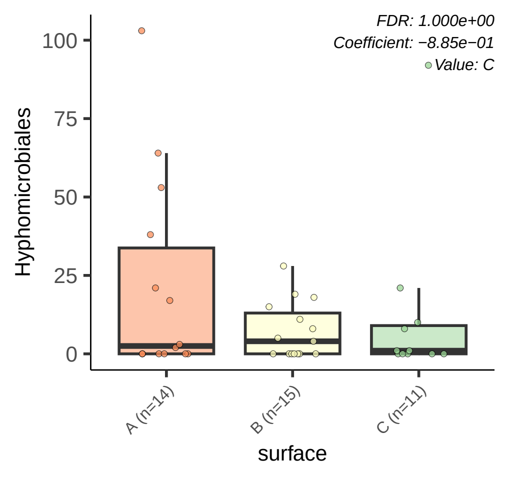

FDR: 1.000e+00
Coefficient: −8.85e−01
Value: C
100
75
50
25
0
Hyphomicrobiales
A (n=14)
B (n=15)
C (n=11)
surface

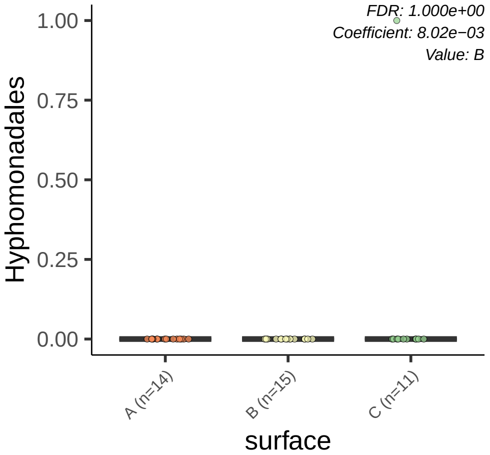

FDR: 1.000e+00
Coefficient: 8.02e−03
Value: B
Hyphomonadales
1.00
0.75
0.50
0.25
0.00
A (n=14)
B (n=15)
C (n=11)
surface

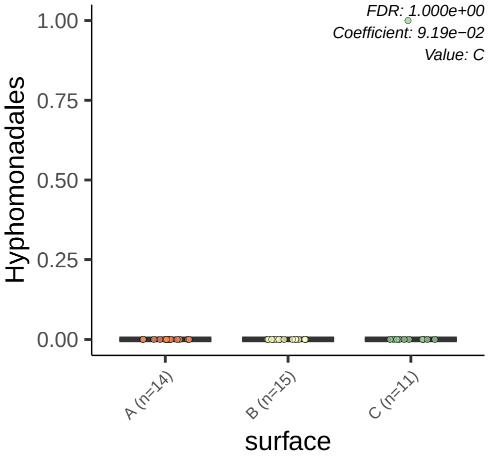
FDR: 1.000e+00
Coefficient: 9.19e−02
Value: C
Hyphomonadales
1.00
0.75
0.50
0.25
0.00
A (n=14)
B (n=15)
C (n=11)
surface

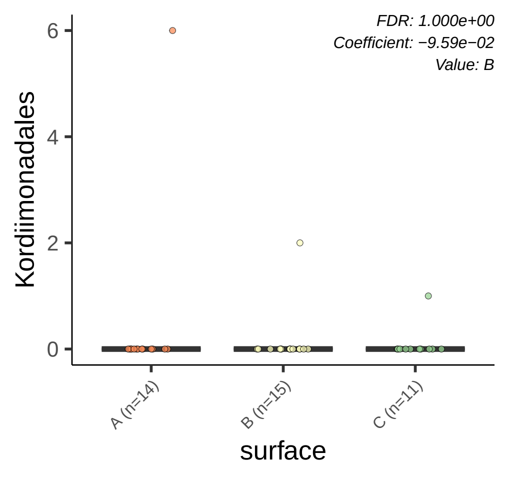
FDR: 1.000e+00
Coefficient: −9.59e−02
Value: B
Kordiimonadales
6
4
2
0
A (n=14)
B (n=15)
C (n=11)
surface

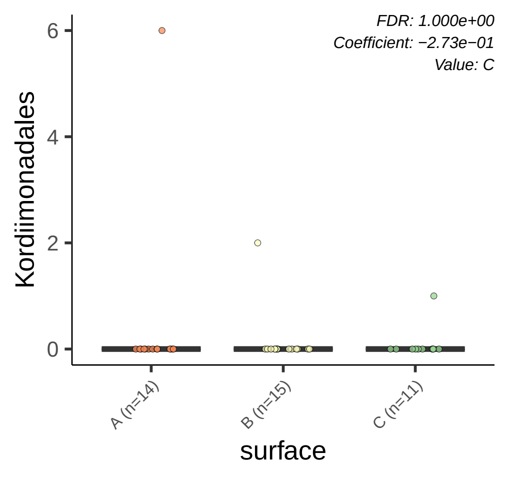
FDR: 1.000e+00
Coefficient: −2.73e−01
Value: C
Kordiimonadales
6
4
2
0
A (n=14)
B (n=15)
C (n=11)
surface

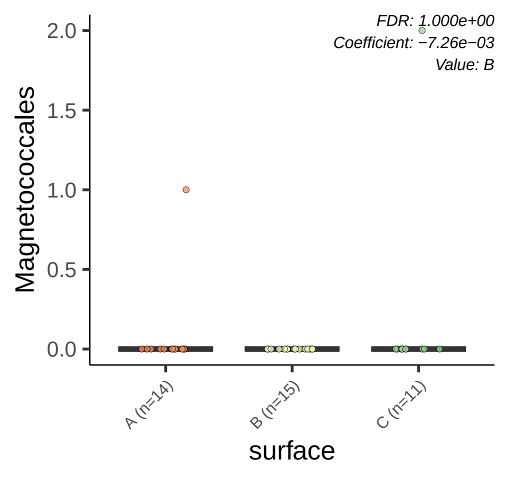

FDR: 1.000e+00
Coefficient: −7.26e−03
Value: B
Magnetococcales
2.0
1.5
1.0
0.5
0.0
A (n=14)
B (n=15)
C (n=11)
surface

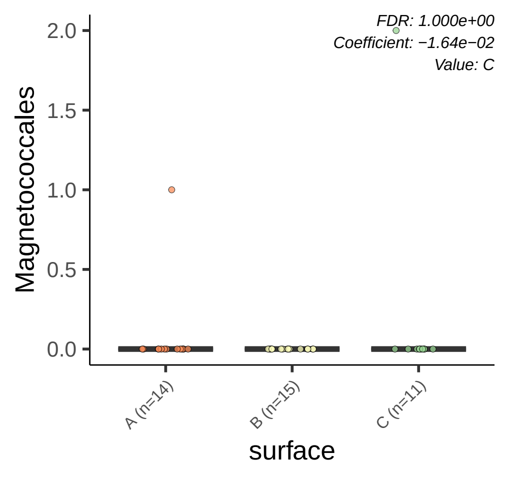
FDR: 1.000e+00
Coefficient: −1.64e−02
Value: C
Magnetococcales
2.0
1.5
1.0
0.5
0.0
A (n=14)
B (n=15)
C (n=11)
surface

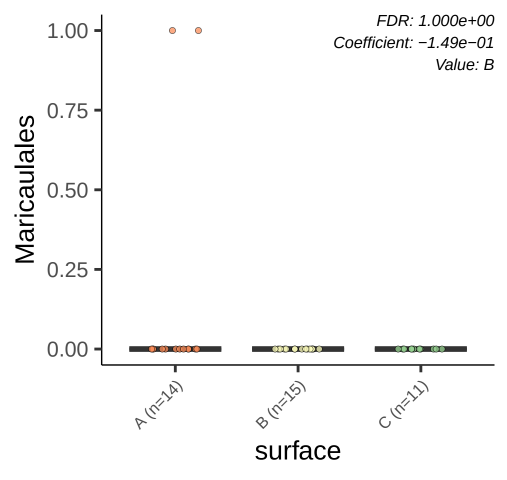

FDR: 1.000e+00
Coefficient: −1.49e−01
Value: B
Maricaulales
1.00
0.75
0.50
0.25
0.00
A (n=14)
B (n=15)
C (n=11)
surface

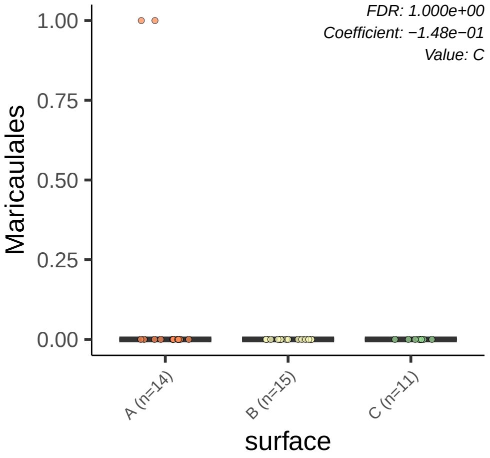

FDR: 1.000e+00
Coefficient: −1.48e−01
Value: C
Maricaulales
1.00
0.75
0.50
0.25
0.00
A (n=14)
B (n=15)
C (n=11)
surface

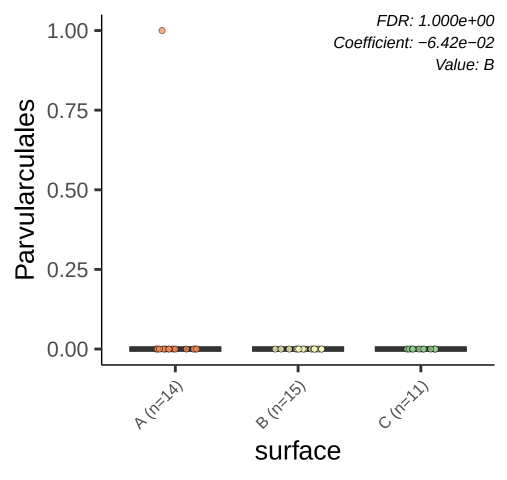

FDR: 1.000e+00
Coefficient: −6.42e−02
Value: B
Parvularculales
1.00
0.75
0.50
0.25
0.00
A (n=14)
B (n=15)
C (n=11)
surface

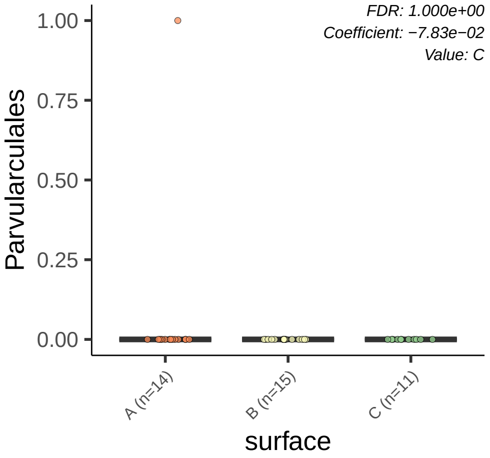
FDR: 1.000e+00
Coefficient: −7.83e−02
Value: C
Parvularculales
1.00
0.75
0.50
0.25
0.00
A (n=14)
B (n=15)
C (n=11)
surface

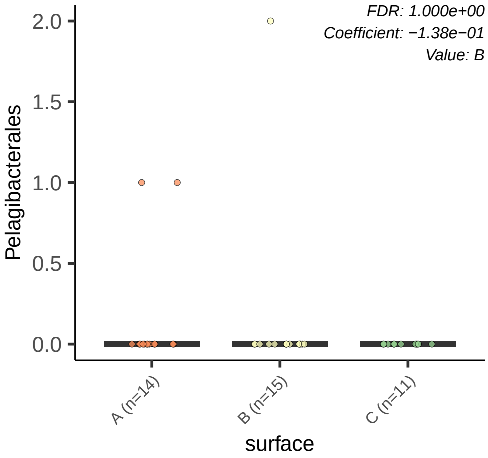

FDR: 1.000e+00
Coefficient: −1.38e−01
Value: B
Pelagibacterales
2.0
1.5
1.0
0.5
0.0
A (n=14)
B (n=15)
C (n=11)
surface

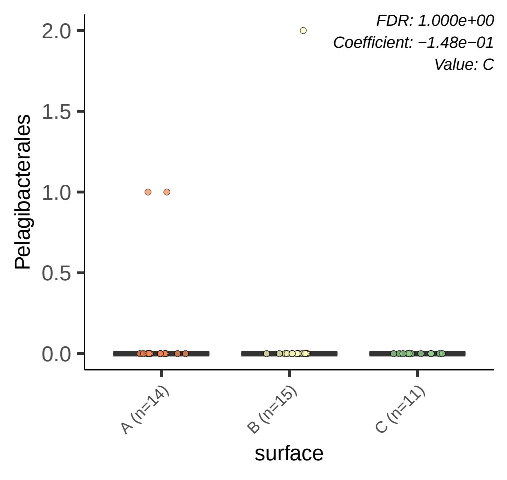
FDR: 1.000e+00
Coefficient: −1.48e−01
Value: C
Pelagibacterales
2.0
1.5
1.0
0.5
0.0
A (n=14)
B (n=15)
C (n=11)
surface

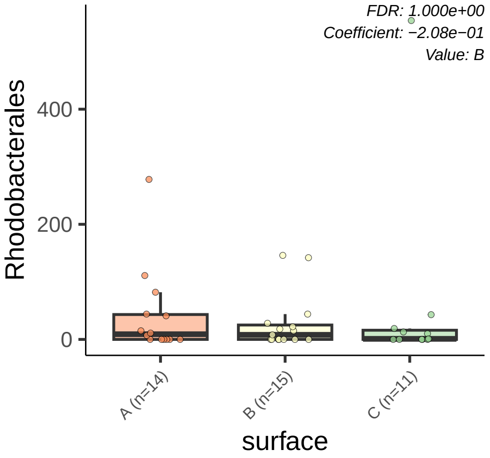
FDR: 1.000e+00
Coefficient: −2.08e−01
Value: B
Rhodobacterales
400
200
0
A (n=14)
B (n=15)
C (n=11)
surface

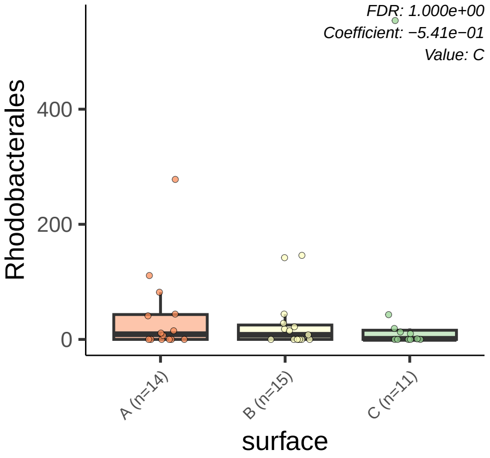
FDR: 1.000e+00
Coefficient: −5.41e−01
Value: C
Rhodobacterales
400
200
0
A (n=14)
B (n=15)
C (n=11)
surface

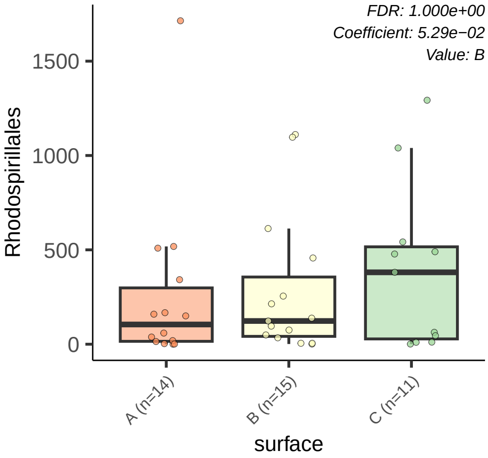

FDR: 1.000e+00
Coefficient: 5.29e−02
Value: B
Rhodospirillales
1500
1000
500
0
A (n=14)
B (n=15)
C (n=11)
surface

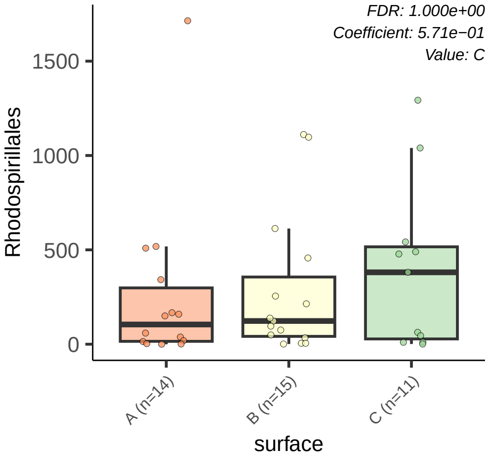
FDR: 1.000e+00
Coefficient: 5.71e−01
Value: C
Rhodospirillales
1500
1000
500
0
A (n=14)
B (n=15)
C (n=11)
surface

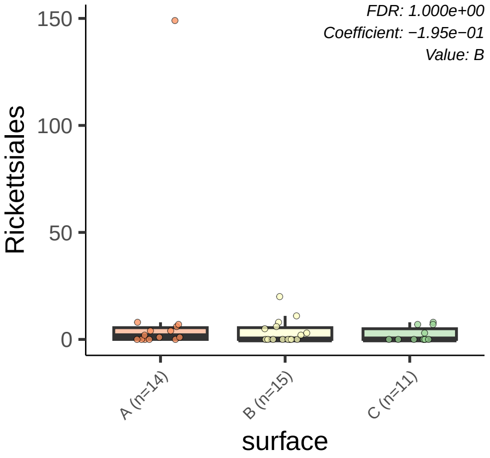

FDR: 1.000e+00
Coefficient: −1.95e−01
Value: B
Rickettsiales
150
100
50
0
A (n=14)
B (n=15)
C (n=11)
surface

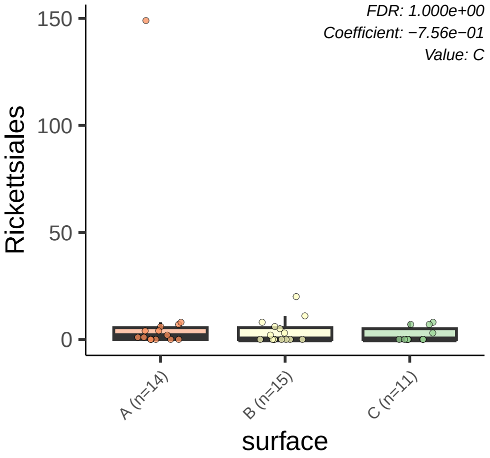
FDR: 1.000e+00
Coefficient: −7.56e−01
Value: C
Rickettsiales
150
100
50
0
A (n=14)
B (n=15)
C (n=11)
surface

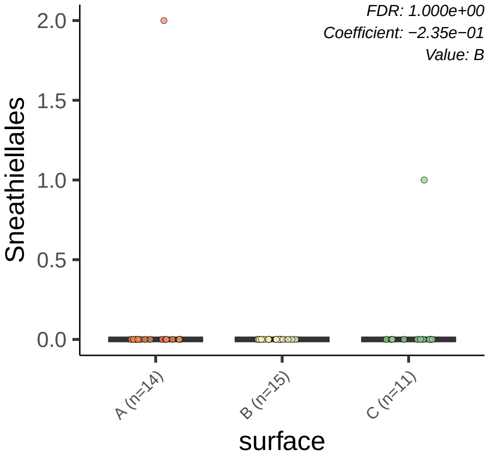

FDR: 1.000e+00
Coefficient: −2.35e−01
Value: B
Sneathiellales
2.0
1.5
1.0
0.5
0.0
A (n=14)
B (n=15)
C (n=11)
surface

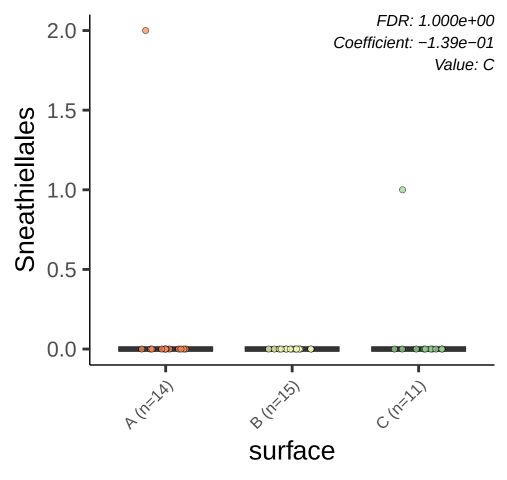
FDR: 1.000e+00
Coefficient: −1.39e−01
Value: C
Sneathiellales
2.0
1.5
1.0
0.5
0.0
A (n=14)
B (n=15)
C (n=11)
surface

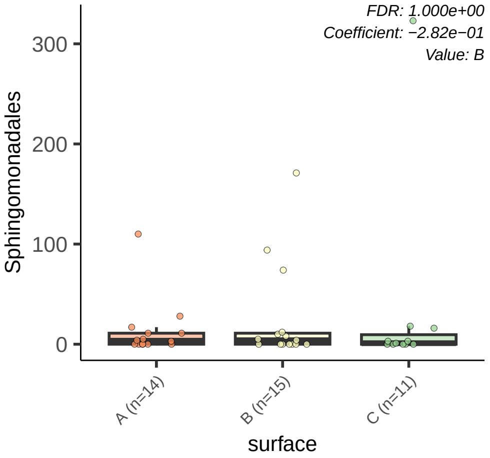

FDR: 1.000e+00
Coefficient: −2.82e−01
Value: B
Sphingomonadales
300
200
100
0
A (n=14)
B (n=15)
C (n=11)
surface

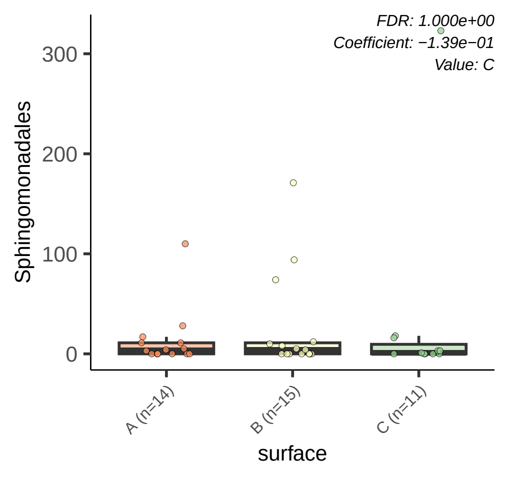
FDR: 1.000e+00
Coefficient: −1.39e−01
Value: C
Sphingomonadales
300
200
100
0
A (n=14)
B (n=15)
C (n=11)
surface

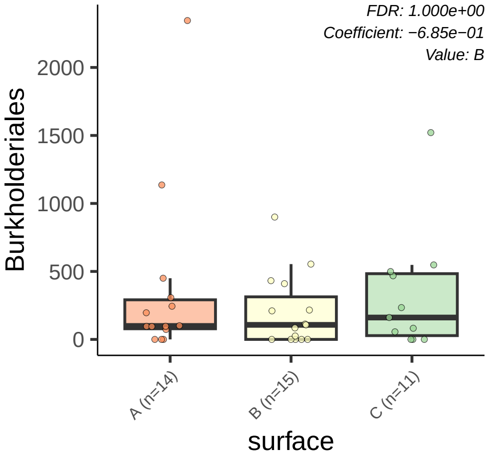
FDR: 1.000e+00
Coefficient: −6.85e−01
Value: B
Burkholderiales
2000
1500
1000
500
0
A (n=14)
B (n=15)
C (n=11)
surface

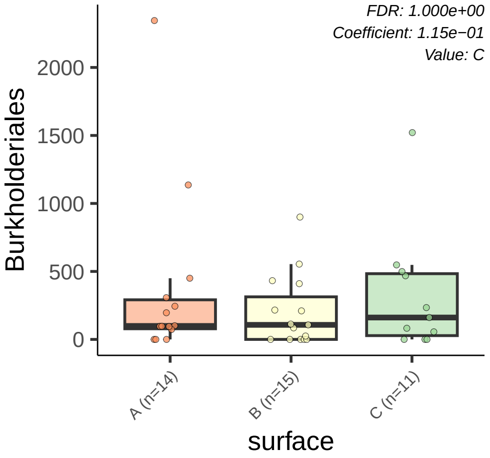
FDR: 1.000e+00
Coefficient: 1.15e−01
Value: C
Burkholderiales
2000
1500
1000
500
0
A (n=14)
B (n=15)
C (n=11)
surface

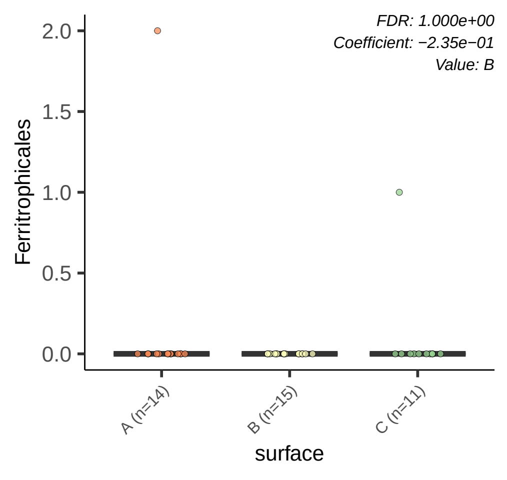
FDR: 1.000e+00
Coefficient: −2.35e−01
Value: B
Ferritrophicales
2.0
1.5
1.0
0.5
0.0
A (n=14)
B (n=15)
C (n=11)
surface

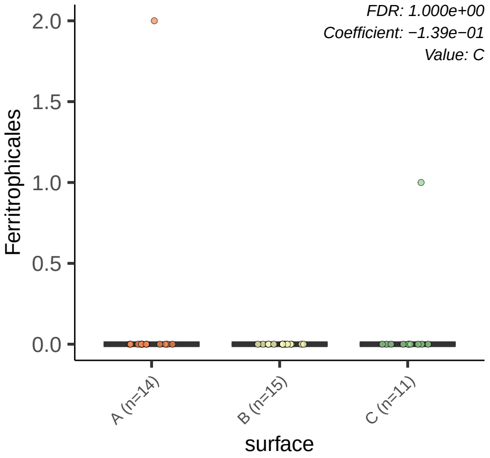
FDR: 1.000e+00
Coefficient: −1.39e−01
Value: C
Ferritrophicales
2.0
1.5
1.0
0.5
0.0
A (n=14)
B (n=15)
C (n=11)
surface

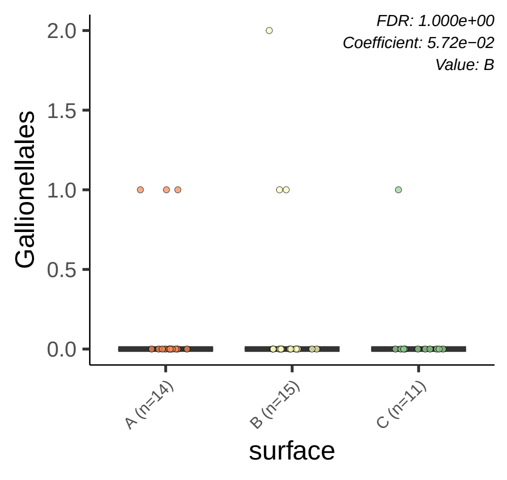
FDR: 1.000e+00
Coefficient: 5.72e−02
Value: B
Gallionellales
2.0
1.5
1.0
0.5
0.0
A (n=14)
B (n=15)
C (n=11)
surface

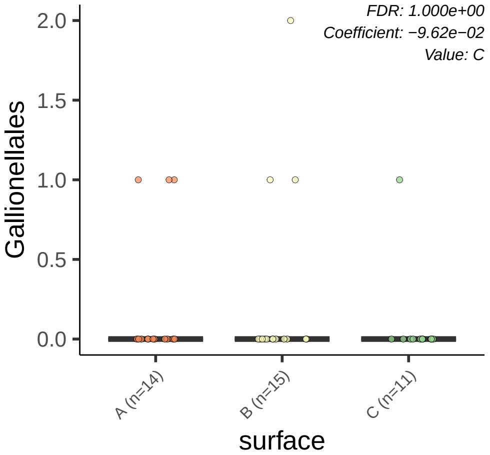

FDR: 1.000e+00
Coefficient: −9.62e−02
Value: C
Gallionellales
2.0
1.5
1.0
0.5
0.0
A (n=14)
B (n=15)
C (n=11)
surface

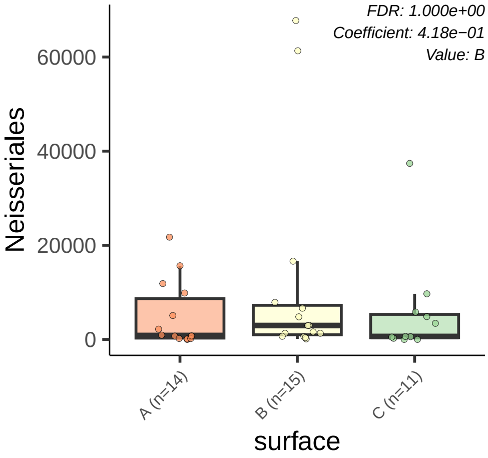

FDR: 1.000e+00
Coefficient: 4.18e−01
Value: B
Neisseriales
60000
40000
20000
0
A (n=14)
B (n=15)
C (n=11)
surface

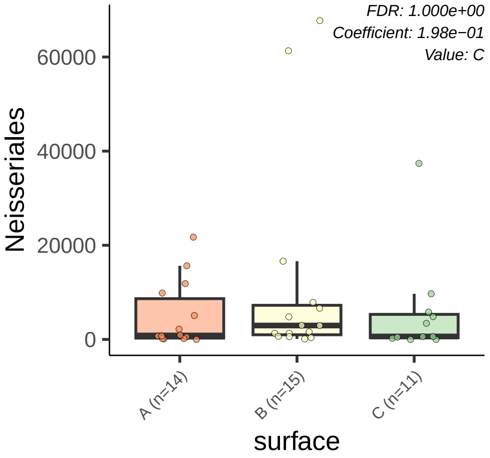
FDR: 1.000e+00
Coefficient: 1.98e−01
Value: C
Neisseriales
60000
40000
20000
0
A (n=14)
B (n=15)
C (n=11)
surface

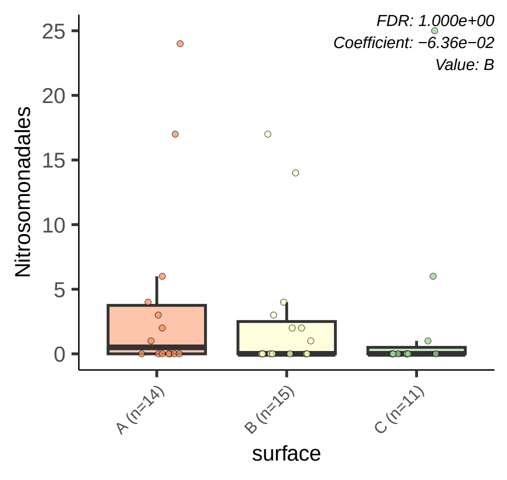

FDR: 1.000e+00
Coefficient: −6.36e−02
Value: B
Nitrosomonadales
25
20
15
10
5
0
A (n=14)
B (n=15)
C (n=11)
surface

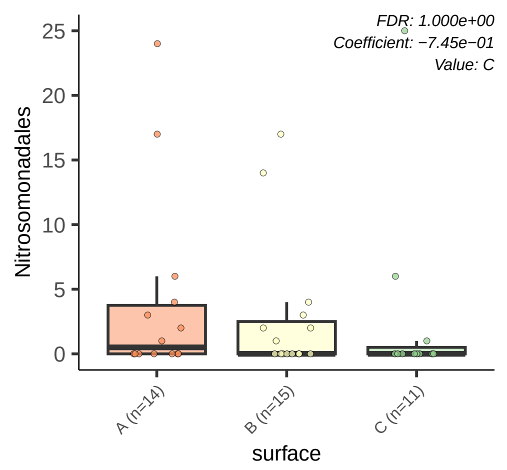

FDR: 1.000e+00
Coefficient: −7.45e−01
Value: C
Nitrosomonadales
25
20
15
10
5
0
A (n=14)
B (n=15)
C (n=11)
surface

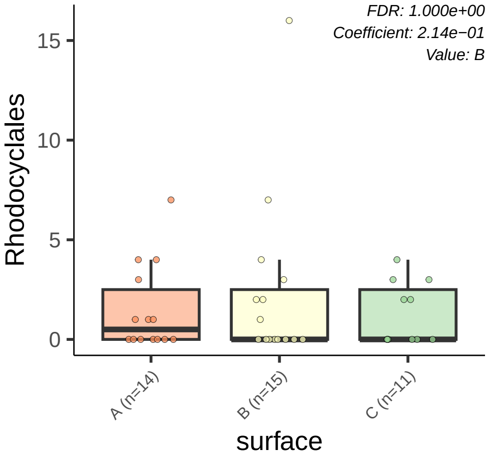
FDR: 1.000e+00
Coefficient: 2.14e−01
Value: B
Rhodocyclales
15
10
5
0
A (n=14)
B (n=15)
C (n=11)
surface

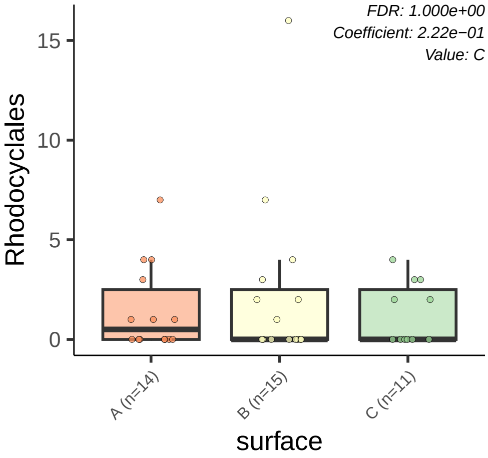
FDR: 1.000e+00
Coefficient: 2.22e−01
Value: C
Rhodocyclales
15
10
5
0
A (n=14)
B (n=15)
C (n=11)
surface

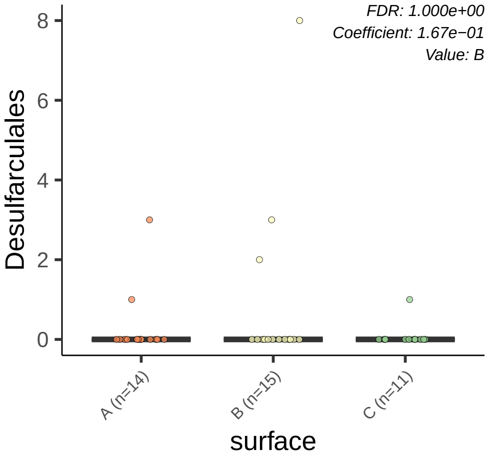

FDR: 1.000e+00
Coefficient: 1.67e−01
Value: B
Desulfarculales
8
6
4
2
0
A (n=14)
B (n=15)
C (n=11)
surface

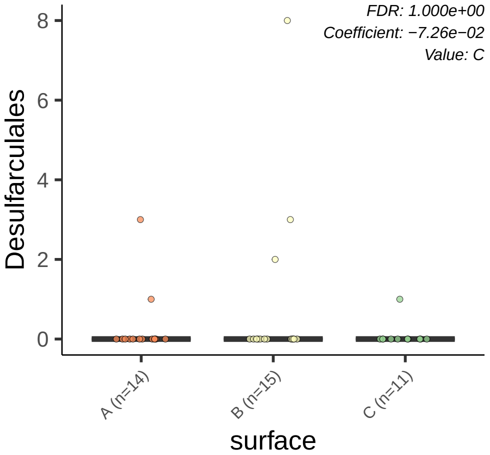

FDR: 1.000e+00
Coefficient: −7.26e−02
Value: C
Desulfarculales
8
6
4
2
0
A (n=14)
B (n=15)
C (n=11)
surface

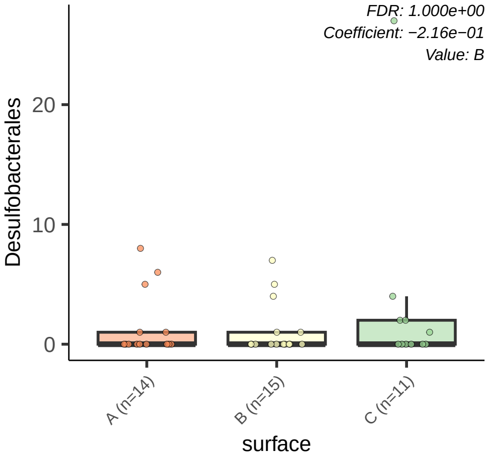

FDR: 1.000e+00
Coefficient: −2.16e−01
Value: B
Desulfobacterales
0
10
20
A (n=14)
B (n=15)
C (n=11)
surface

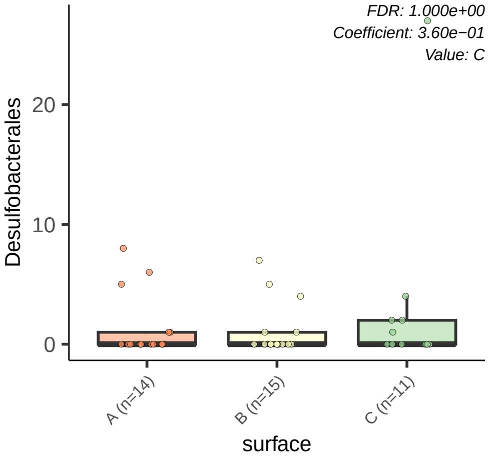
FDR: 1.000e+00
Coefficient: 3.60e−01
Value: C
Desulfobacterales
20
10
0
A (n=14)
B (n=15)
C (n=11)
surface

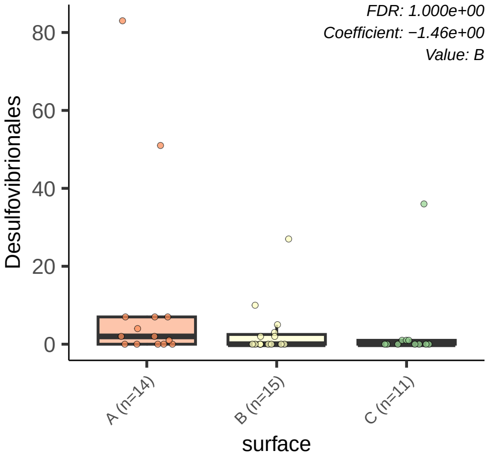
FDR: 1.000e+00
Coefficient: −1.46e+00
Value: B
Desulfovibrionales
80
60
40
20
0
A (n=14)
B (n=15)
C (n=11)
surface

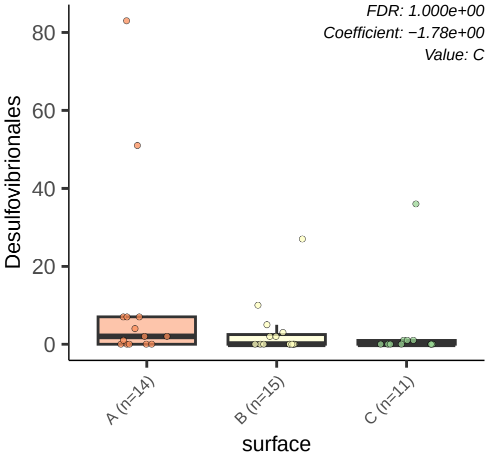

FDR: 1.000e+00
Coefficient: −1.78e+00
Value: C
Desulfovibrionales
0
20
40
60
80
A (n=14)
B (n=15)
C (n=11)
surface

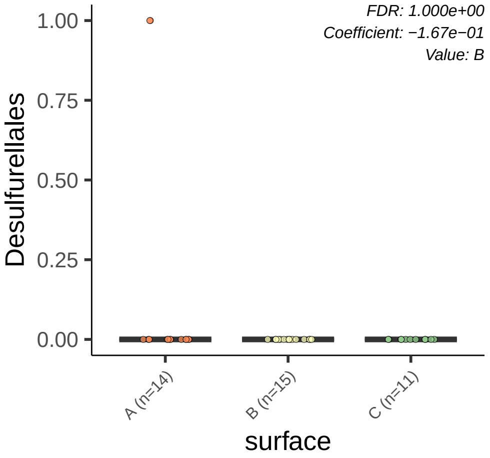

FDR: 1.000e+00
Coefficient: −1.67e−01
Value: B
Desulfurellales
1.00
0.75
0.50
0.25
0.00
A (n=14)
B (n=15)
C (n=11)
surface

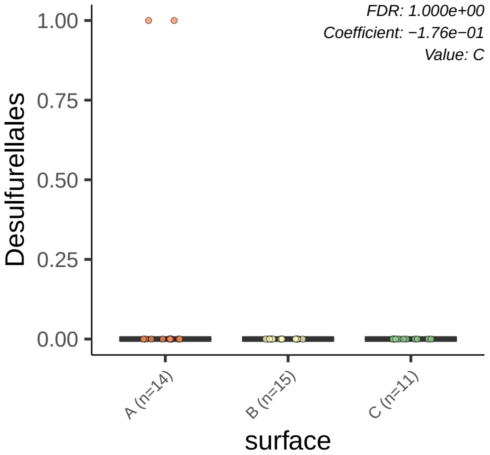

FDR: 1.000e+00
Coefficient: −1.76e−01
Value: C
Desulfurellales
1.00
0.75
0.50
0.25
0.00
A (n=14)
B (n=15)
C (n=11)
surface

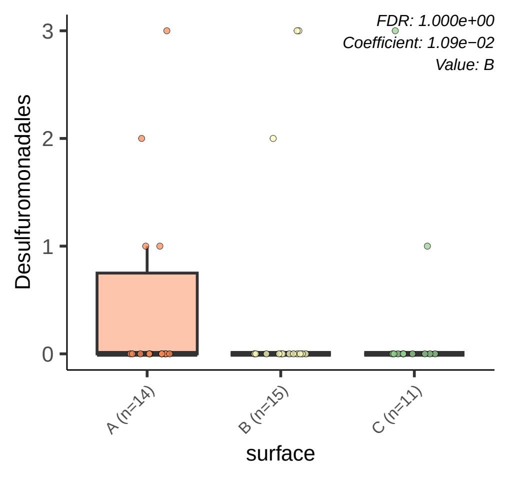

FDR: 1.000e+00
Coefficient: 1.09e−02
Value: B
Desulfuromonadales
3
2
1
0
A (n=14)
B (n=15)
C (n=11)
surface

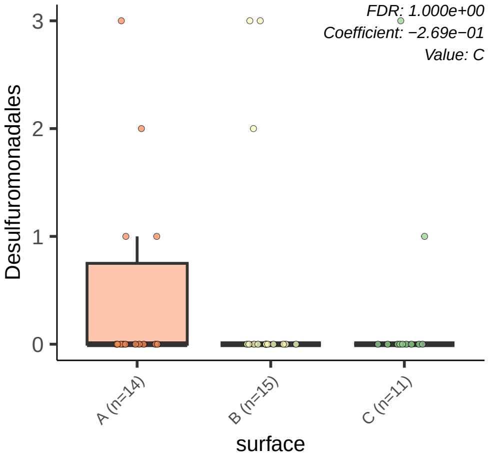

FDR: 1.000e+00
Coefficient: −2.69e−01
Value: C
Desulfuromonadales
3
2
1
0
A (n=14)
B (n=15)
C (n=11)
surface

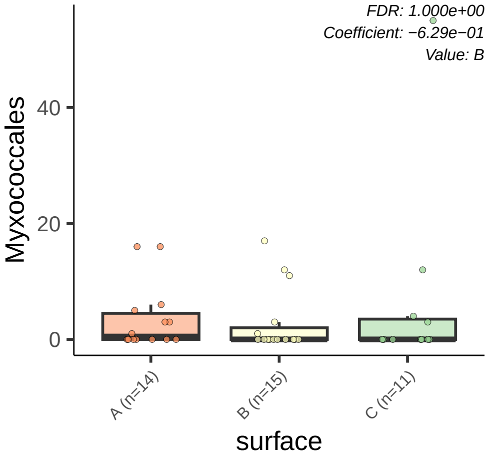
FDR: 1.000e+00
Coefficient: −6.29e−01
Value: B
Myxococcales
40
20
0
A (n=14)
B (n=15)
C (n=11)
surface

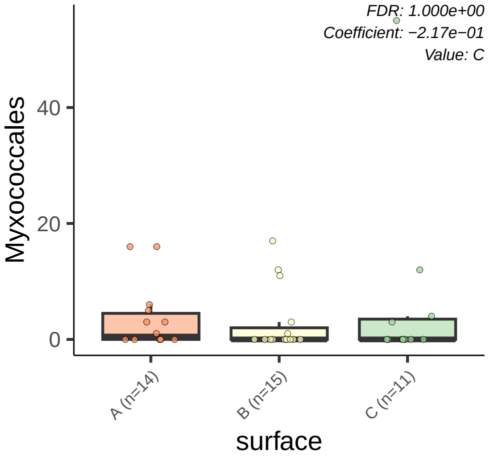

FDR: 1.000e+00
Coefficient: −2.17e−01
Value: C
Myxococcales
40
20
0
A (n=14)
B (n=15)
C (n=11)
surface

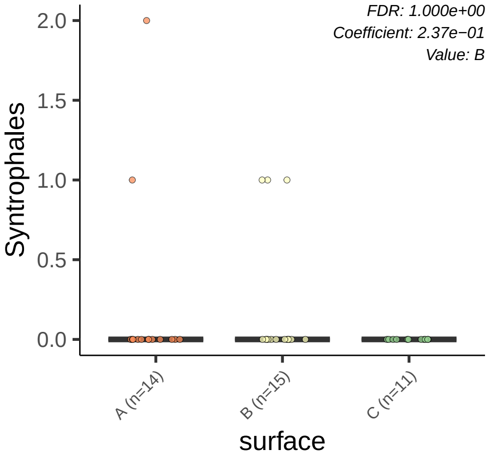
FDR: 1.000e+00
Coefficient: 2.37e−01
Value: B
Syntrophales
2.0
1.5
1.0
0.5
0.0
A (n=14)
B (n=15)
C (n=11)
surface

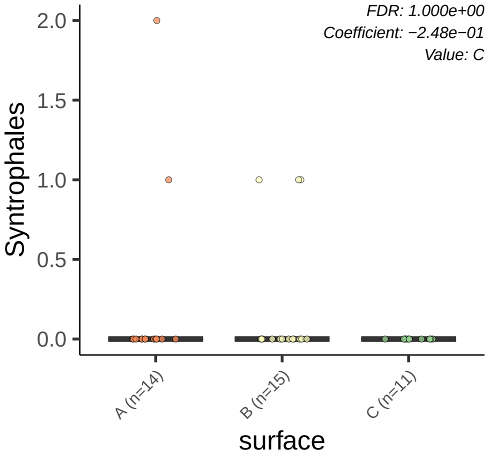
FDR: 1.000e+00
Coefficient: −2.48e−01
Value: C
Syntrophales
2.0
1.5
1.0
0.5
0.0
A (n=14)
B (n=15)
C (n=11)
surface

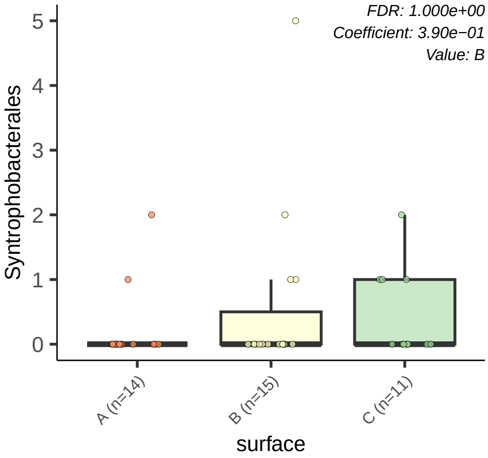

FDR: 1.000e+00
Coefficient: 3.90e−01
Value: B
Syntrophobacterales
0
1
2
3
4
5
A (n=14)
B (n=15)
C (n=11)
surface

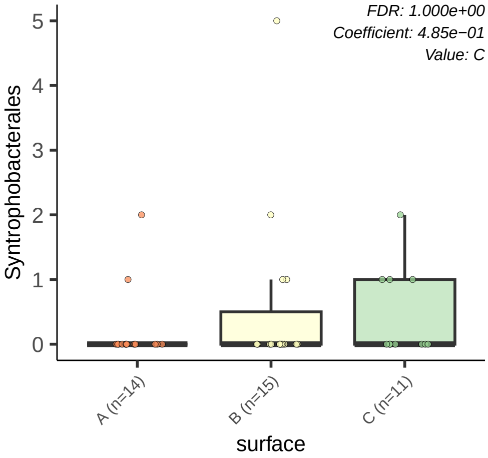

FDR: 1.000e+00
Coefficient: 4.85e−01
Value: C
Syntrophobacterales
0
1
2
3
4
5
A (n=14)
B (n=15)
C (n=11)
surface

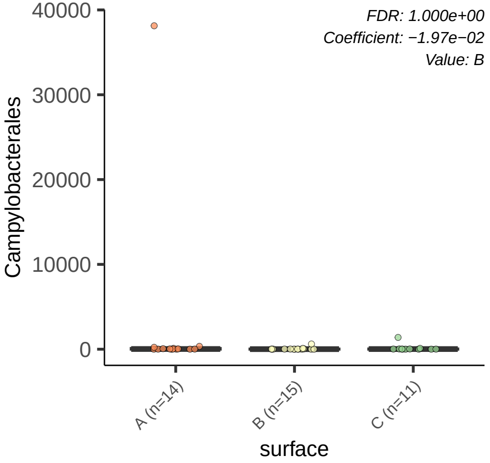

FDR: 1.000e+00
Coefficient: −1.97e−02
Value: B
Campylobacterales
40000
30000
20000
10000
0
A (n=14)
B (n=15)
C (n=11)
surface

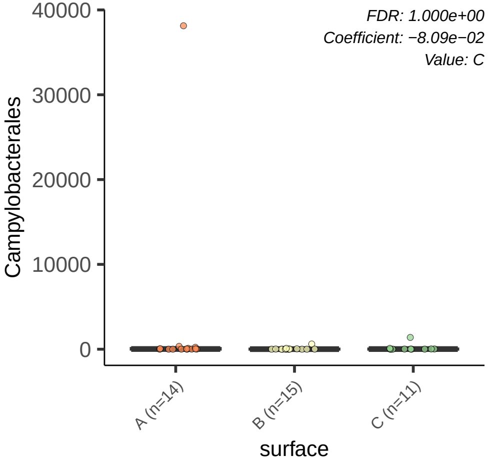

FDR: 1.000e+00
Coefficient: −8.09e−02
Value: C
Campylobacterales
40000
30000
20000
10000
0
A (n=14)
B (n=15)
C (n=11)
surface

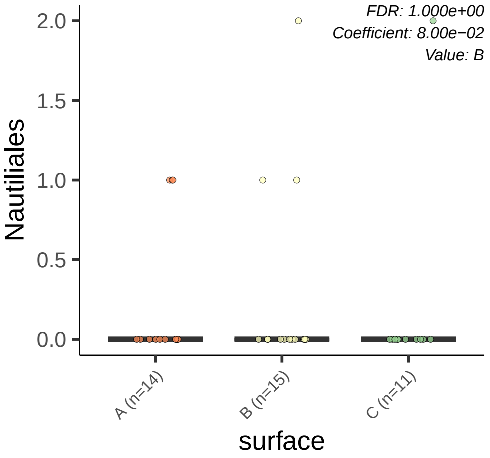

FDR: 1.000e+00
Coefficient: 8.00e−02
Value: B
Nautiliales
2.0
1.5
1.0
0.5
0.0
A (n=14)
B (n=15)
C (n=11)
surface

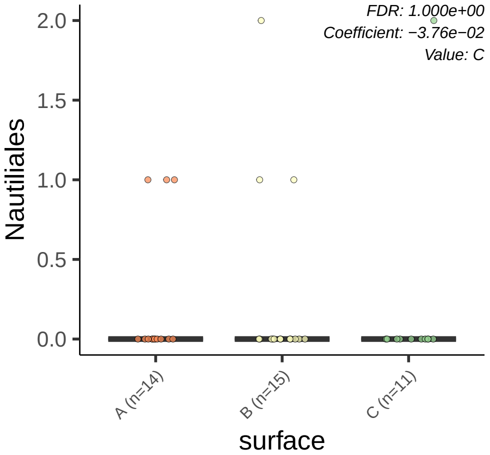
FDR: 1.000e+00
Coefficient: −3.76e−02
Value: C
Nautiliales
2.0
1.5
1.0
0.5
0.0
A (n=14)
B (n=15)
C (n=11)
surface

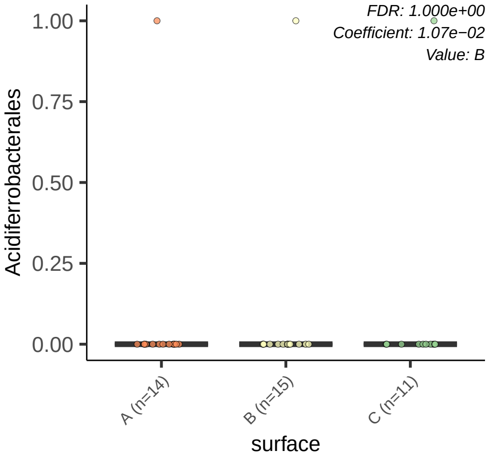

FDR: 1.000e+00
Coefficient: 1.07e−02
Value: B
Acidiferrobacterales
1.00
0.75
0.50
0.25
0.00
A (n=14)
B (n=15)
C (n=11)
surface

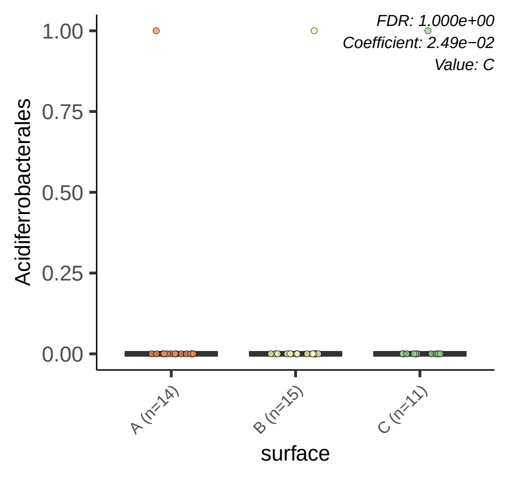
FDR: 1.000e+00
Coefficient: 2.49e−02
Value: C
Acidiferrobacterales
1.00
0.75
0.50
0.25
0.00
A (n=14)
B (n=15)
C (n=11)
surface

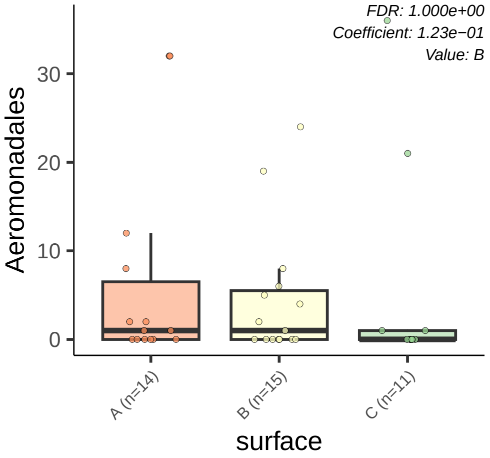
FDR: 1.000e+00
Coefficient: 1.23e−01
Value: B
Aeromonadales
30
20
10
0
A (n=14)
B (n=15)
C (n=11)
surface

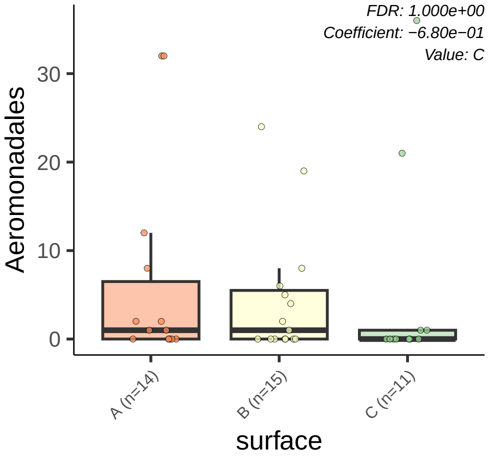

FDR: 1.000e+00
Coefficient: −6.80e−01
Value: C
Aeromonadales
30
20
10
0
A (n=14)
B (n=15)
C (n=11)
surface

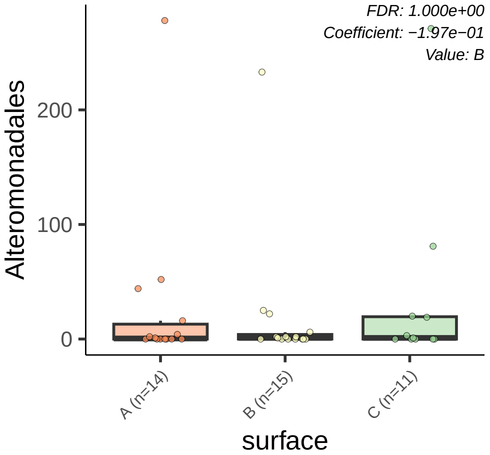

FDR: 1.000e+00
Coefficient: −1.97e−01
Value: B
Alteromonadales
200
100
0
A (n=14)
B (n=15)
C (n=11)
surface

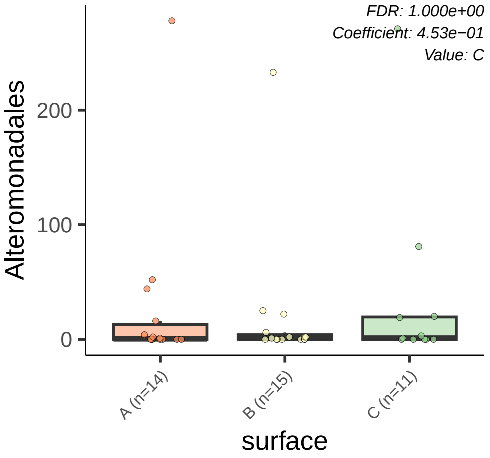

FDR: 1.000e+00
Coefficient: 4.53e−01
Value: C
Alteromonadales
200
100
0
A (n=14)
B (n=15)
C (n=11)
surface

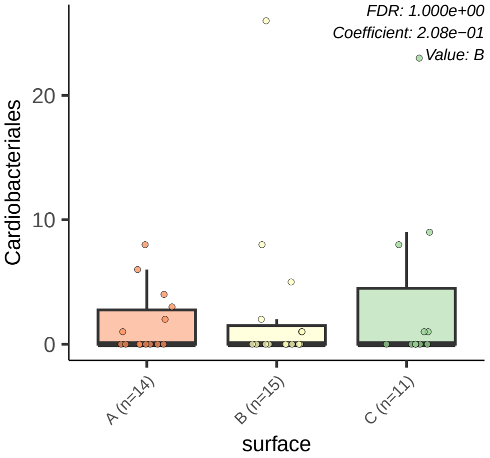

FDR: 1.000e+00
Coefficient: 2.08e−01
Value: B
Cardiobacteriales
0
10
20
A (n=14)
B (n=15)
C (n=11)
surface

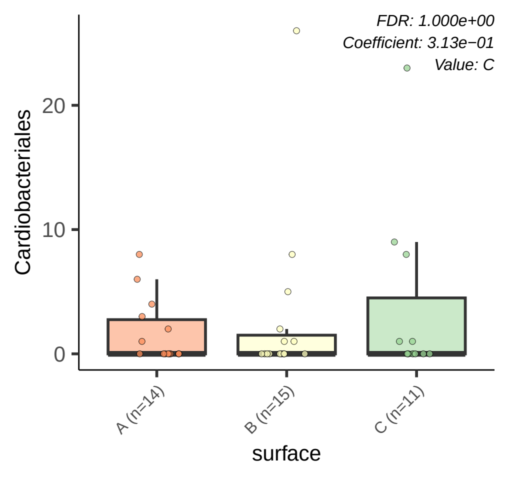
FDR: 1.000e+00
Coefficient: 3.13e−01
Value: C
Cardiobacteriales
0
10
20
A (n=14)
B (n=15)
C (n=11)
surface

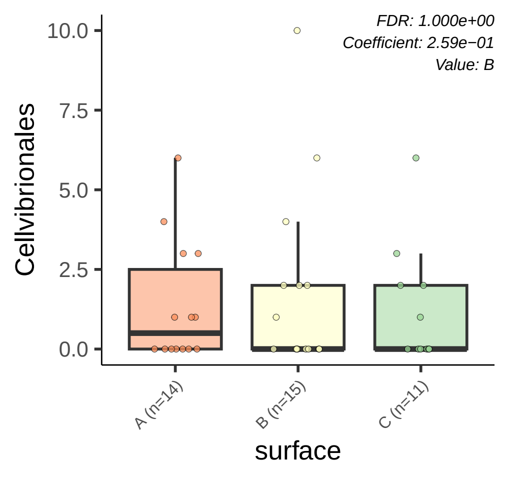
FDR: 1.000e+00
Coefficient: 2.59e−01
Value: B
Cellvibrionales
10.0
7.5
5.0
2.5
0.0
A (n=14)
B (n=15)
C (n=11)
surface

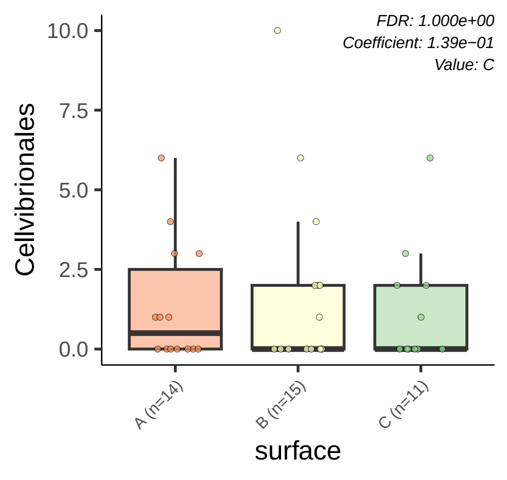

FDR: 1.000e+00
Coefficient: 1.39e−01
Value: C
Cellvibrionales
10.0
7.5
5.0
2.5
0.0
A (n=14)
B (n=15)
C (n=11)
surface

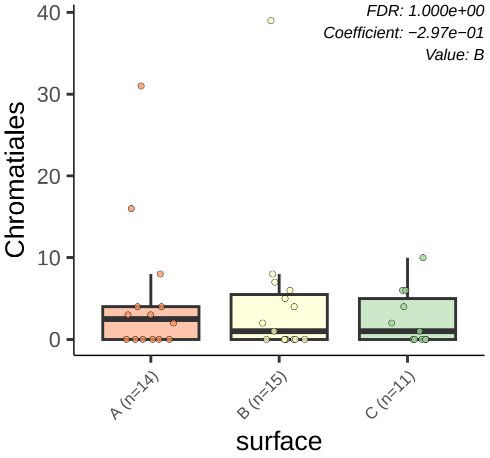

FDR: 1.000e+00
Coefficient: −2.97e−01
Value: B
Chromatiales
40
30
20
10
0
A (n=14)
B (n=15)
C (n=11)
surface

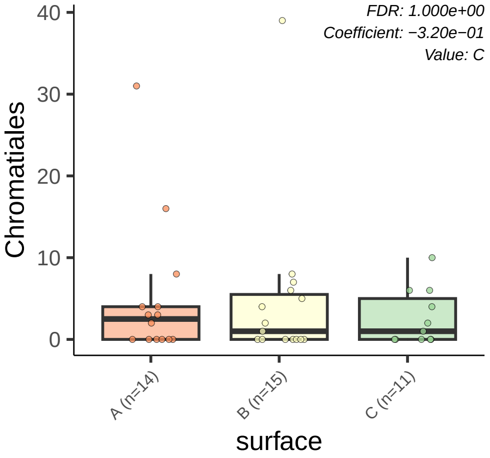
FDR: 1.000e+00
Coefficient: −3.20e−01
Value: C
Chromatiales
40
30
20
10
0
A (n=14)
B (n=15)
C (n=11)
surface

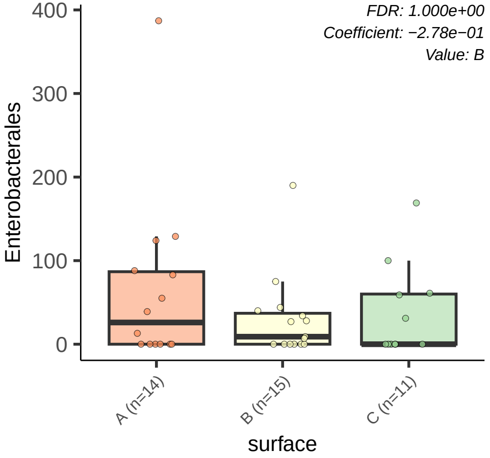

FDR: 1.000e+00
Coefficient: −2.78e−01
Value: B
400
300
200
100
0
Enterobacterales
A (n=14)
B (n=15)
C (n=11)
surface

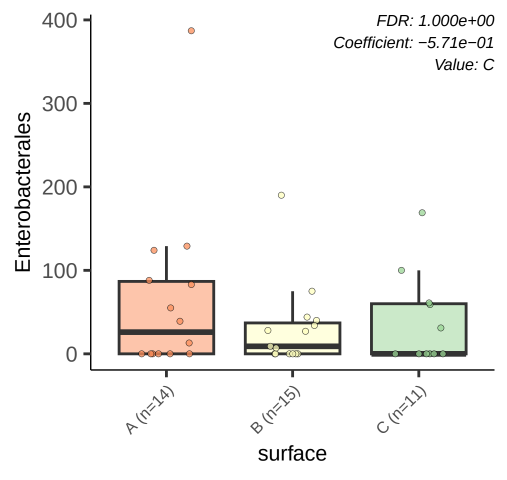

FDR: 1.000e+00
Coefficient: −5.71e−01
Value: C
Enterobacterales
400
300
200
100
0
A (n=14)
B (n=15)
C (n=11)
surface

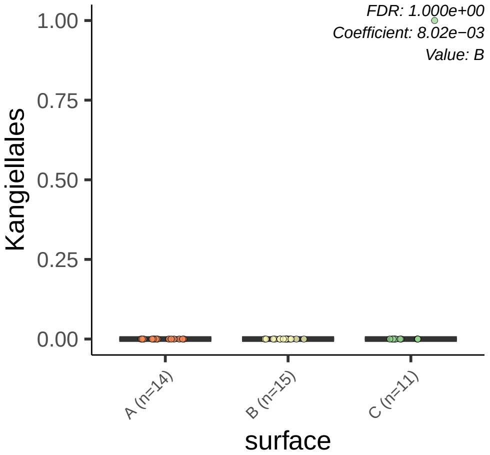
FDR: 1.000e+00
Coefficient: 8.02e−03
Value: B
Kangiellales
1.00
0.75
0.50
0.25
0.00
A (n=14)
B (n=15)
C (n=11)
surface

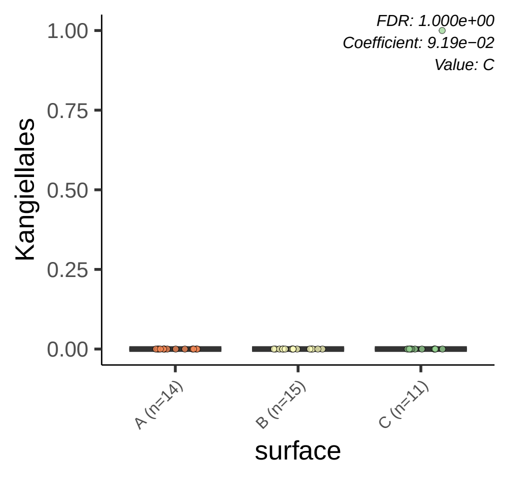

FDR: 1.000e+00
Coefficient: 9.19e−02
Value: C
Kangiellales
1.00
0.75
0.50
0.25
0.00
A (n=14)
B (n=15)
C (n=11)
surface

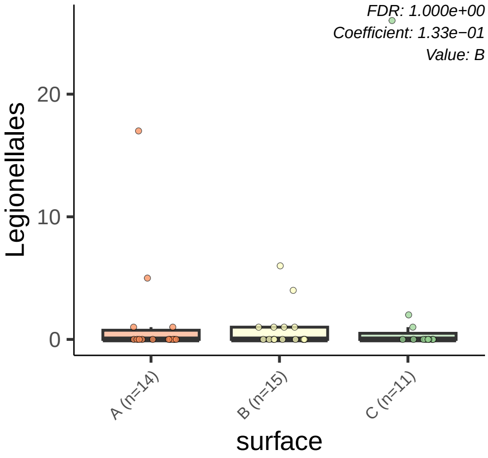

FDR: 1.000e+00
Coefficient: 1.33e−01
Value: B
Legionellales
20
10
0
A (n=14)
B (n=15)
C (n=11)
surface

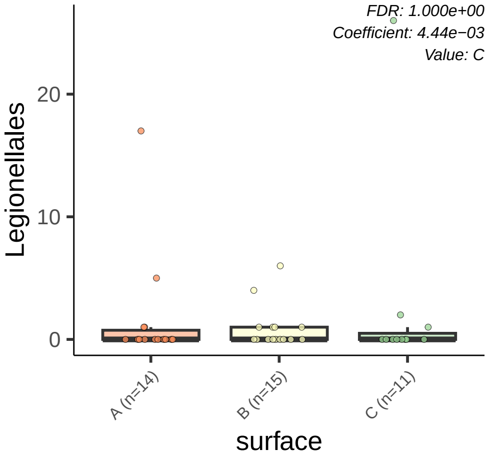
FDR: 1.000e+00
Coefficient: 4.44e−03
Value: C
Legionellales
20
10
0
A (n=14)
B (n=15)
C (n=11)
surface

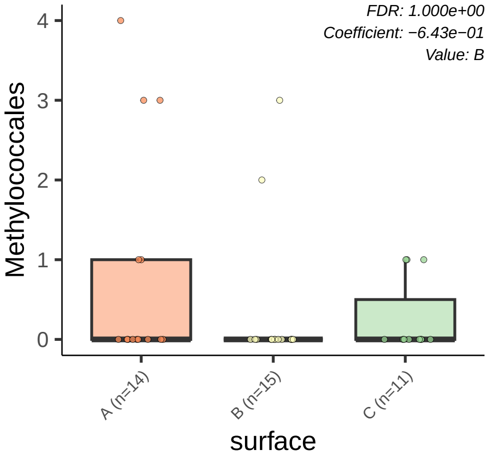

FDR: 1.000e+00
Coefficient: −6.43e−01
Value: B
Methylococcales
0
1
2
3
4
A (n=14)
B (n=15)
C (n=11)
surface

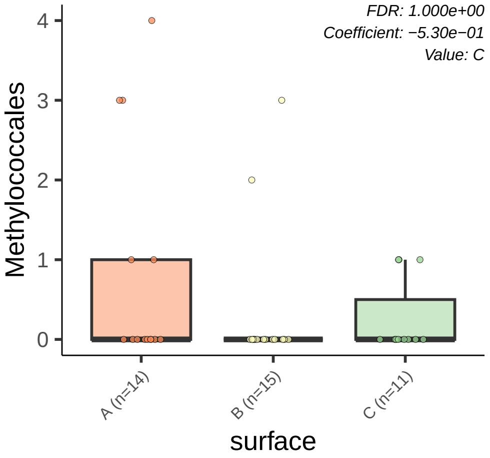
FDR: 1.000e+00
Coefficient: −5.30e−01
Value: C
Methylococcales
0
1
2
3
4
A (n=14)
B (n=15)
C (n=11)
surface

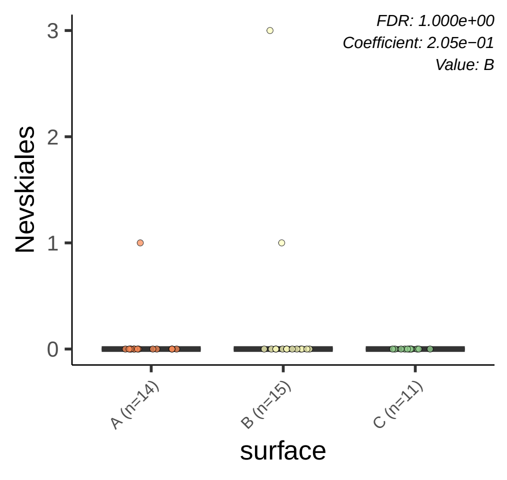

FDR: 1.000e+00
Coefficient: 2.05e−01
Value: B
Nevskiales
3
2
1
0
A (n=14)
B (n=15)
C (n=11)
surface

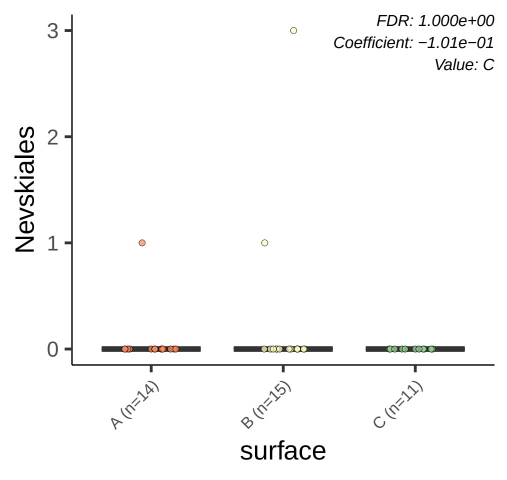

FDR: 1.000e+00
Coefficient: −1.01e−01
Value: C
Nevskiales
3
2
1
0
A (n=14)
B (n=15)
C (n=11)
surface

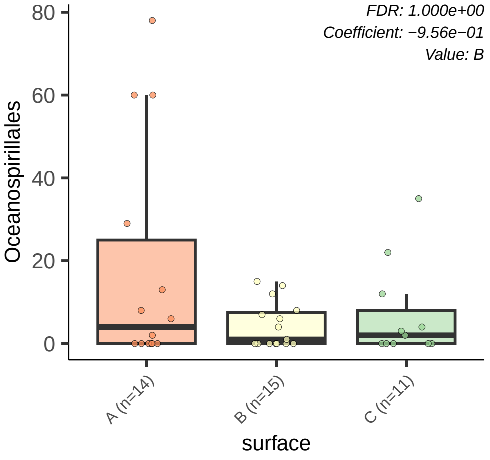

FDR: 1.000e+00
Coefficient: −9.56e−01
Value: B
Oceanospirillales
0
20
40
60
80
A (n=14)
B (n=15)
C (n=11)
surface

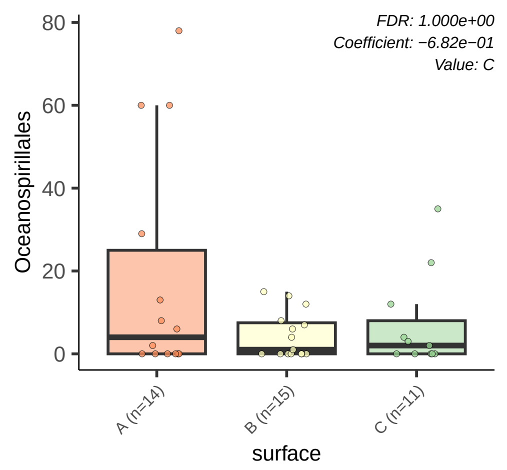

FDR: 1.000e+00
Coefficient: −6.82e−01
Value: C
Oceanospirillales
80
60
40
20
0
A (n=14)
B (n=15)
C (n=11)
surface

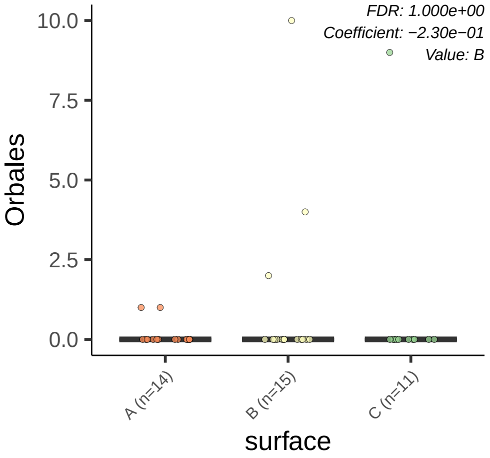
FDR: 1.000e+00
Coefficient: −2.30e−01
Value: B
Orbales
10.0
7.5
5.0
2.5
0.0
A (n=14)
B (n=15)
C (n=11)
surface

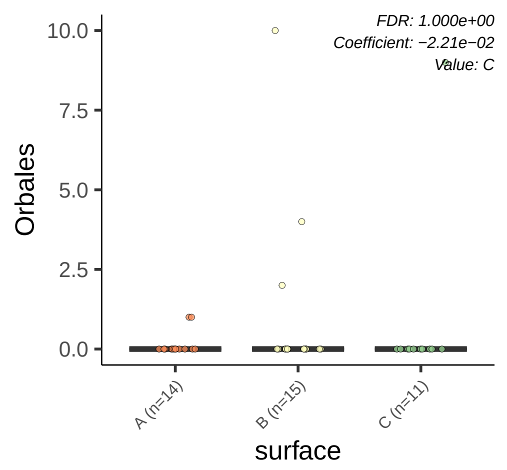

FDR: 1.000e+00
Coefficient: −2.21e−02
Value: C
10.0
7.5
5.0
2.5
0.0
Orbales
A (n=14)
B (n=15)
C (n=11)
surface

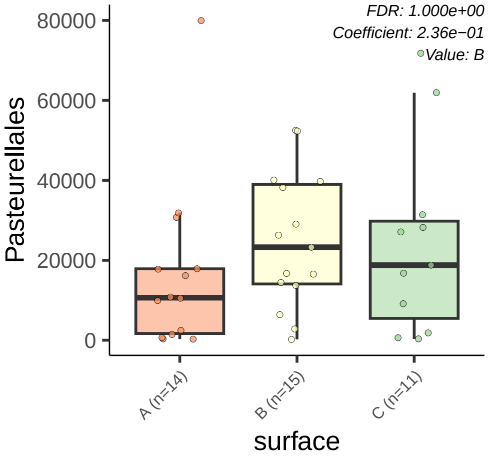

FDR: 1.000e+00
Coefficient: 2.36e−01
Value: B
Pasteurellales
80000
60000
40000
20000
0
A (n=14)
B (n=15)
C (n=11)
surface

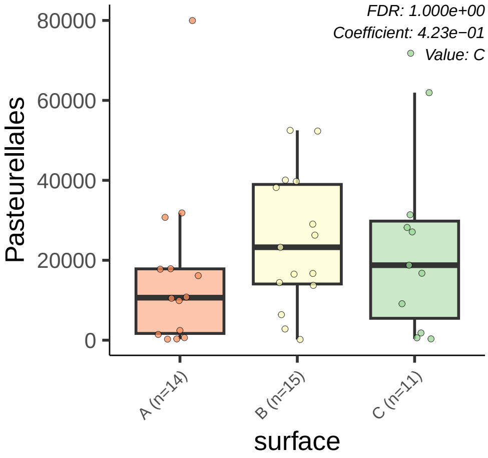
FDR: 1.000e+00
Coefficient: 4.23e−01
Value: C
Pasteurellales
80000
60000
40000
20000
0
A (n=14)
B (n=15)
C (n=11)
surface

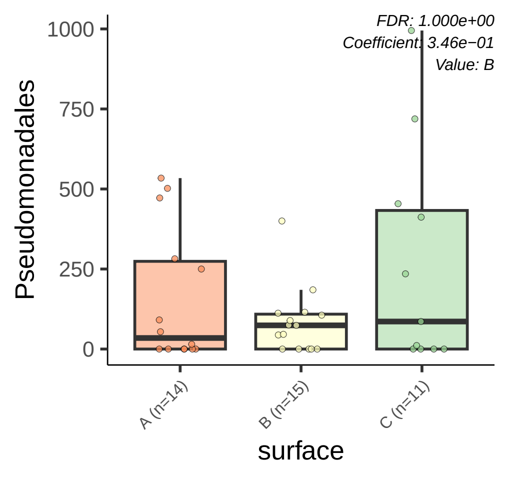

FDR: 1.000e+00
Coefficient: 3.46e−01
Value: B
Pseudomonadales
1000
750
500
250
0
A (n=14)
B (n=15)
C (n=11)
surface

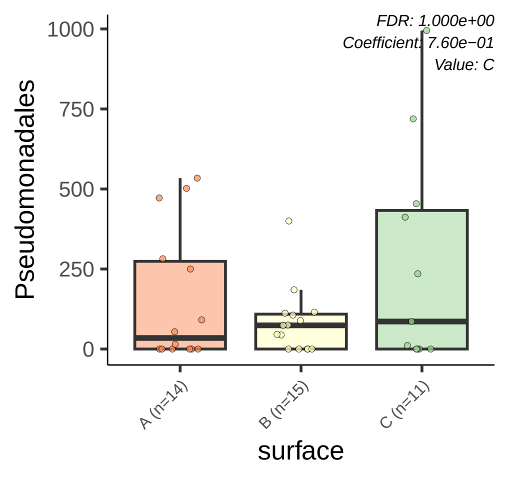
FDR: 1.000e+00
Coefficient: 7.60e−01
Value: C
Pseudomonadales
1000
750
500
250
0
A (n=14)
B (n=15)
C (n=11)
surface

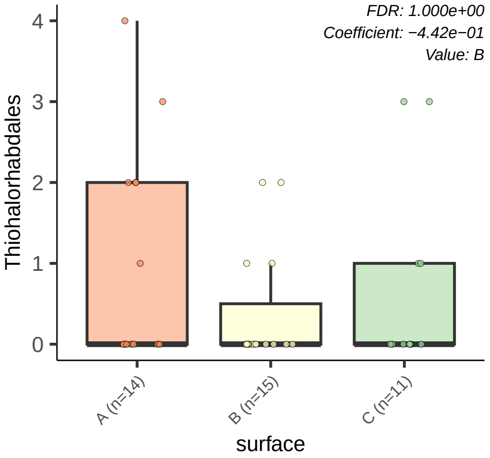
FDR: 1.000e+00
Coefficient: −4.42e−01
Value: B
Thiohalorhabdales
0
1
2
3
4
A (n=14)
B (n=15)
C (n=11)
surface

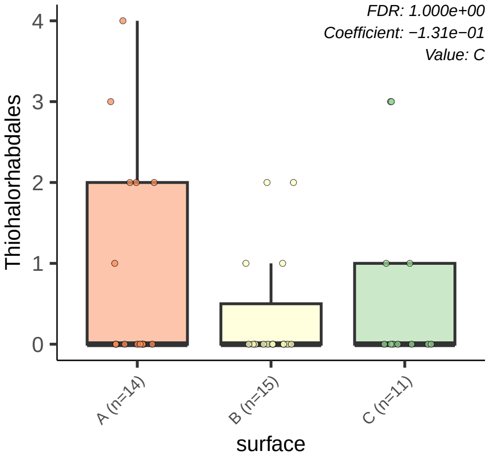

FDR: 1.000e+00
Coefficient: −1.31e−01
Value: C
Thiohalorhabdales
4
3
2
1
0
A (n=14)
B (n=15)
C (n=11)
surface

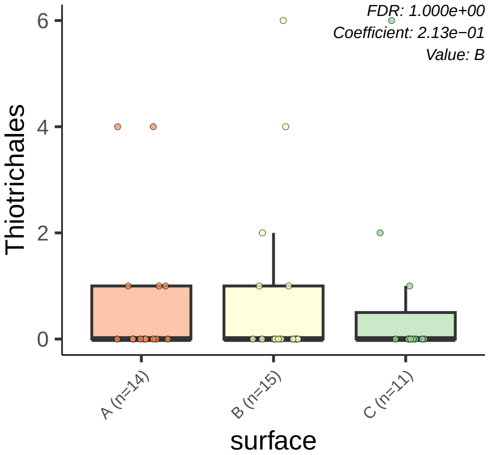

FDR: 1.000e+00
Coefficient: 2.13e−01
Value: B
Thiotrichales
0
2
4
6
A (n=14)
B (n=15)
C (n=11)
surface

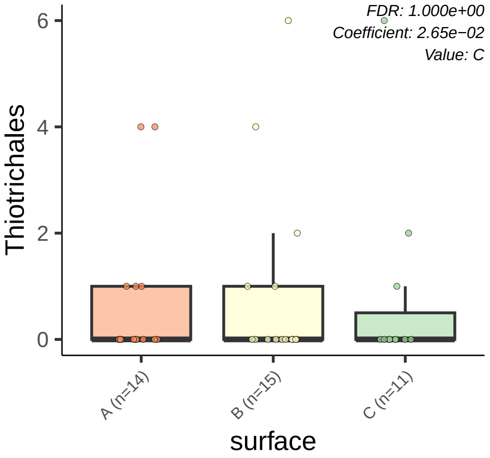

FDR: 1.000e+00
Coefficient: 2.65e−02
Value: C
Thiotrichales
0
2
4
6
A (n=14)
B (n=15)
C (n=11)
surface

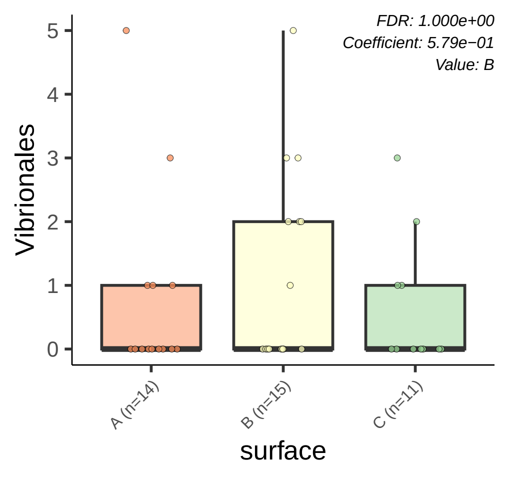
FDR: 1.000e+00
Coefficient: 5.79e−01
Value: B
Vibrionales
0
1
2
3
4
5
A (n=14)
B (n=15)
C (n=11)
surface

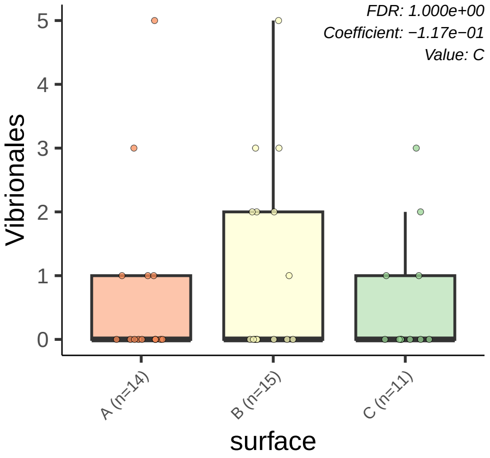
FDR: 1.000e+00
Coefficient: −1.17e−01
Value: C
Vibrionales
0
1
2
3
4
5
A (n=14)
B (n=15)
C (n=11)
surface

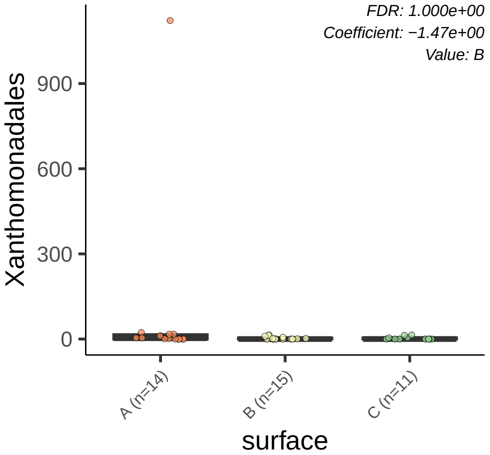
FDR: 1.000e+00
Coefficient: −1.47e+00
Value: B
Xanthomonadales
900
600
300
0
A (n=14)
B (n=15)
C (n=11)
surface

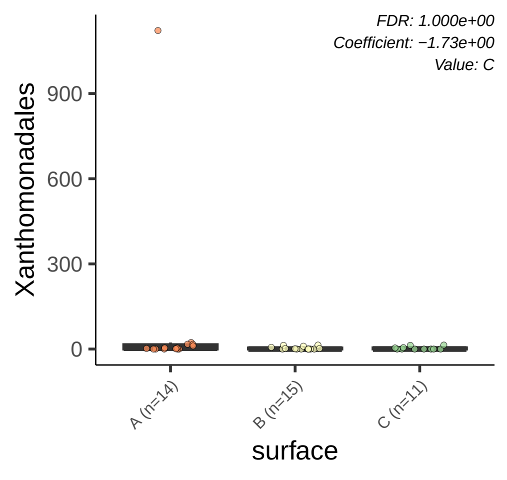
FDR: 1.000e+00
Coefficient: −1.73e+00
Value: C
Xanthomonadales
900
600
300
0
A (n=14)
B (n=15)
C (n=11)
surface

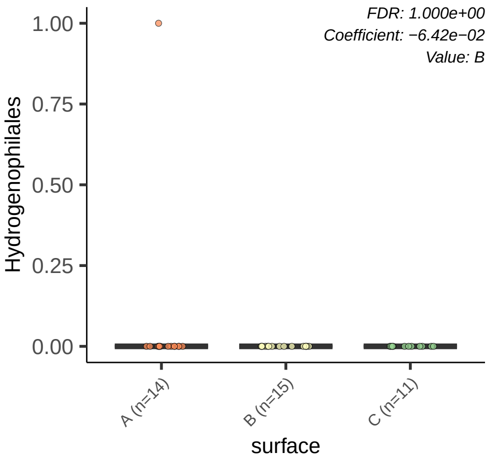

FDR: 1.000e+00
Coefficient: −6.42e−02
Value: B
Hydrogenophilales
1.00
0.75
0.50
0.25
0.00
A (n=14)
B (n=15)
C (n=11)
surface

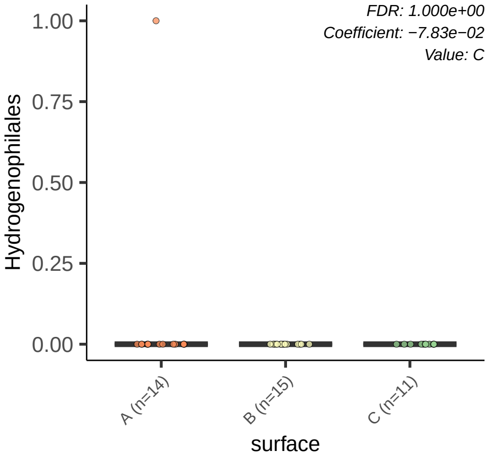

FDR: 1.000e+00
Coefficient: −7.83e−02
Value: C
Hydrogenophilales
1.00
0.75
0.50
0.25
0.00
A (n=14)
B (n=15)
C (n=11)
surface

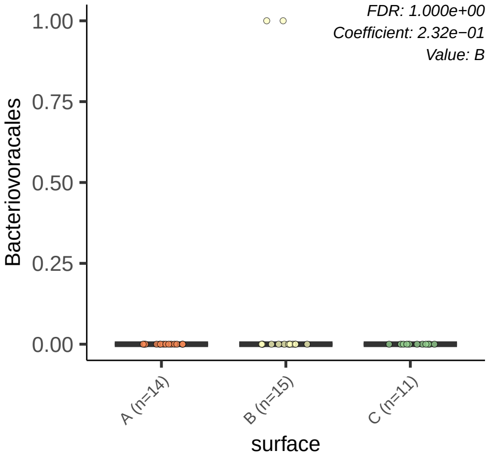
FDR: 1.000e+00
Coefficient: 2.32e−01
Value: B
1.00
0.75
0.50
0.25
0.00
Bacteriovoracales
A (n=14)
B (n=15)
C (n=11)
surface

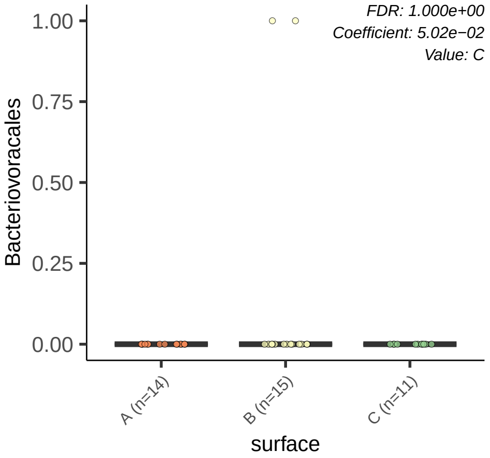

FDR: 1.000e+00
Coefficient: 5.02e−02
Value: C
Bacteriovoracales
1.00
0.75
0.50
0.25
0.00
A (n=14)
B (n=15)
C (n=11)
surface

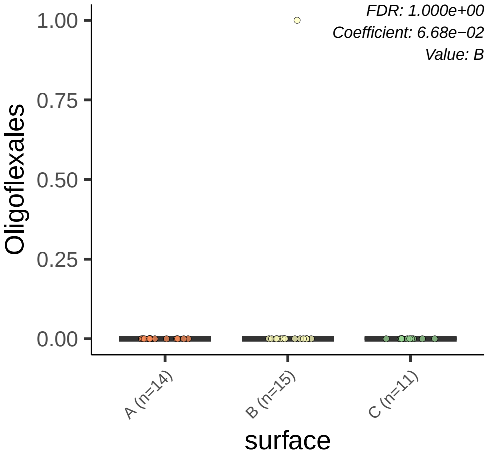
FDR: 1.000e+00
Coefficient: 6.68e−02
Value: B
Oligoflexales
1.00
0.75
0.50
0.25
0.00
A (n=14)
B (n=15)
C (n=11)
surface

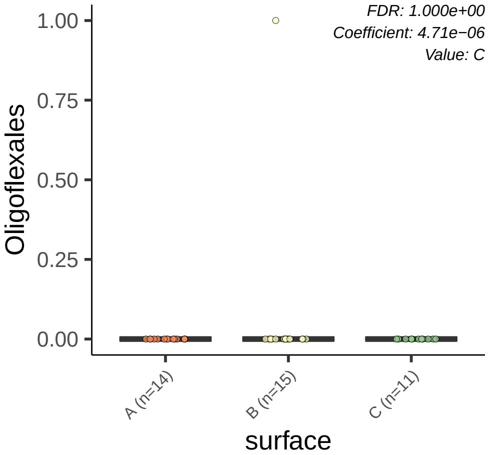
FDR: 1.000e+00
Coefficient: 4.71e−06
Value: C
Oligoflexales
1.00
0.75
0.50
0.25
0.00
A (n=14)
B (n=15)
C (n=11)
surface

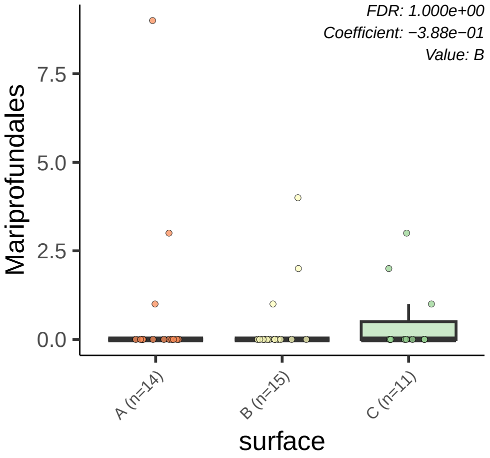

FDR: 1.000e+00
Coefficient: −3.88e−01
Value: B
Mariprofundales
7.5
5.0
2.5
0.0
A (n=14)
B (n=15)
C (n=11)
surface

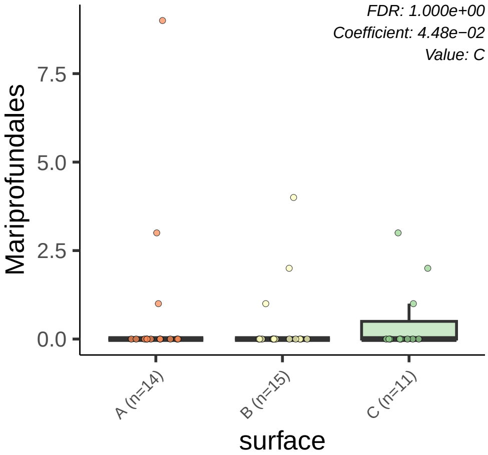

FDR: 1.000e+00
Coefficient: 4.48e−02
Value: C
Mariprofundales
7.5
5.0
2.5
0.0
A (n=14)
B (n=15)
C (n=11)
surface

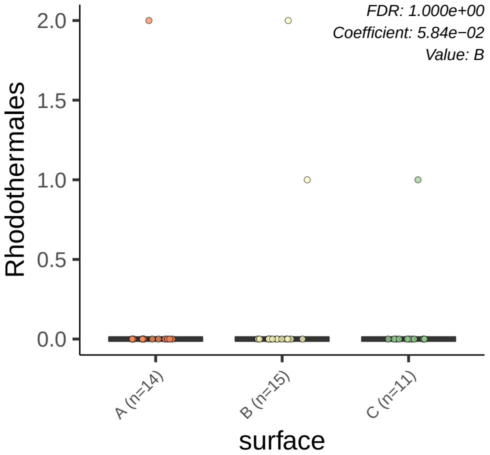
FDR: 1.000e+00
Coefficient: 5.84e−02
Value: B
Rhodothermales
2.0
1.5
1.0
0.5
0.0
A (n=14)
B (n=15)
C (n=11)
surface

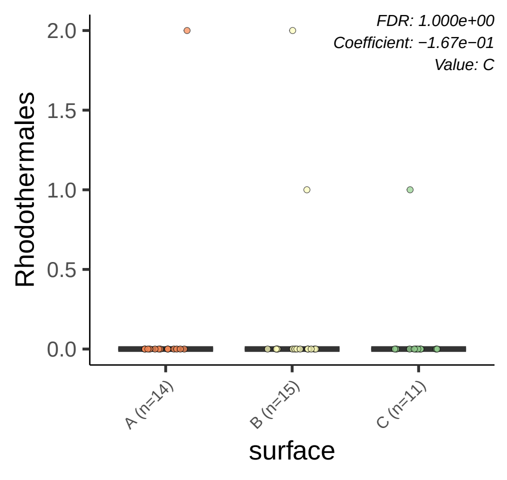

FDR: 1.000e+00
Coefficient: −1.67e−01
Value: C
Rhodothermales
2.0
1.5
1.0
0.5
0.0
A (n=14)
B (n=15)
C (n=11)
surface

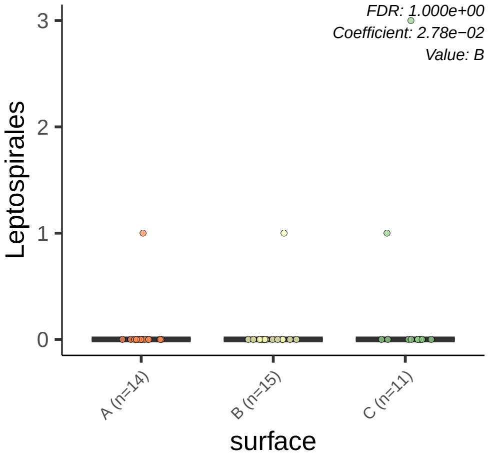

FDR: 1.000e+00
Coefficient: 2.78e−02
Value: B
Leptospirales
0
1
2
3
A (n=14)
B (n=15)
C (n=11)
surface

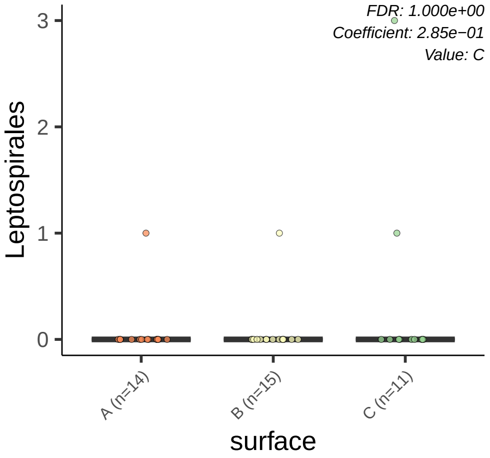

FDR: 1.000e+00
Coefficient: 2.85e−01
Value: C
Leptospirales
0
1
2
3
A (n=14)
B (n=15)
C (n=11)
surface

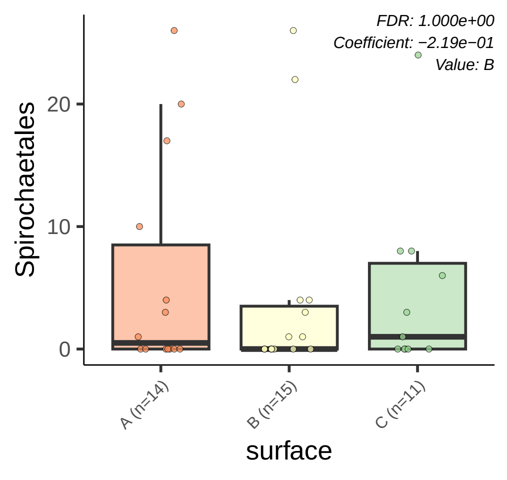

FDR: 1.000e+00
Coefficient: −2.19e−01
Value: B
Spirochaetales
0
10
20
A (n=14)
B (n=15)
C (n=11)
surface

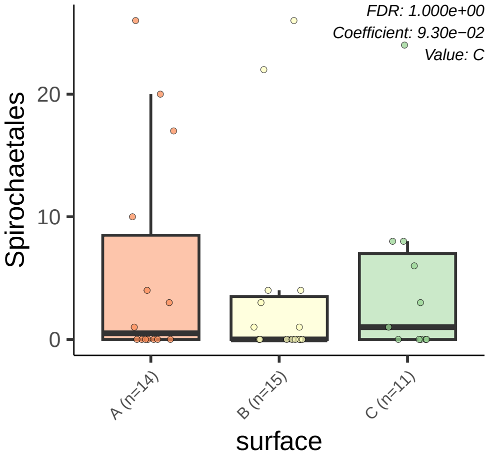

FDR: 1.000e+00
Coefficient: 9.30e−02
Value: C
Spirochaetales
20
10
0
A (n=14)
B (n=15)
C (n=11)
surface

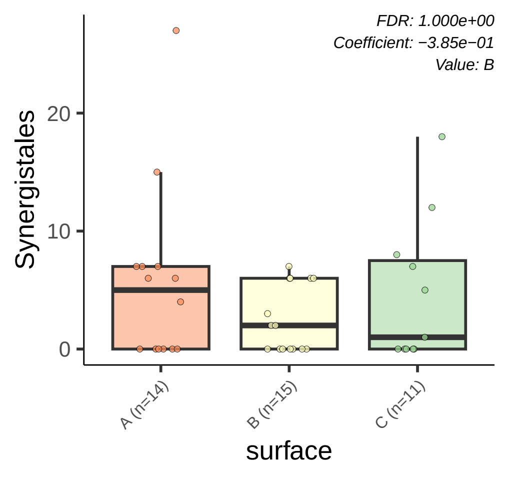
FDR: 1.000e+00
Coefficient: −3.85e−01
Value: B
Synergistales
20
10
0
A (n=14)
B (n=15)
C (n=11)
surface

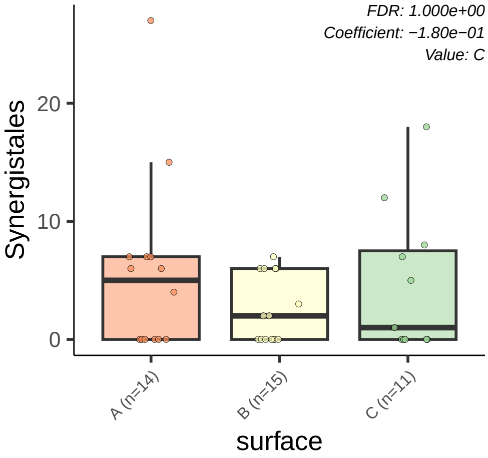

FDR: 1.000e+00
Coefficient: −1.80e−01
Value: C
Synergistales
20
10
0
A (n=14)
B (n=15)
C (n=11)
surface

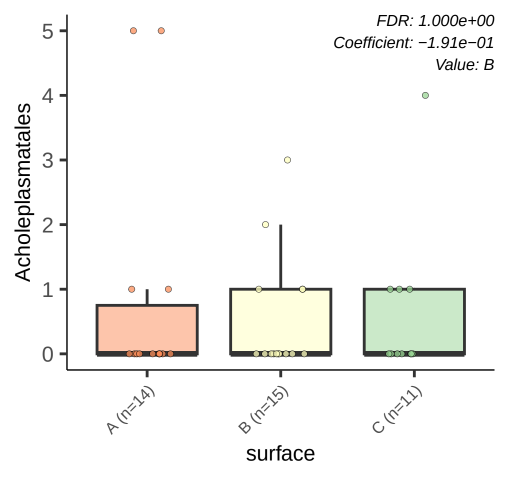

FDR: 1.000e+00
Coefficient: −1.91e−01
Value: B
Acholeplasmatales
5
4
3
2
1
0
A (n=14)
B (n=15)
C (n=11)
surface

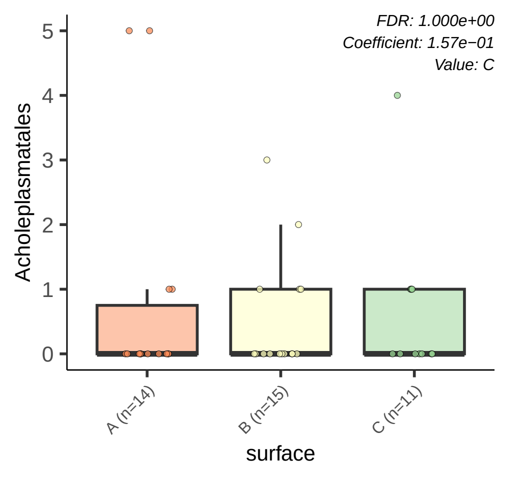

FDR: 1.000e+00
Coefficient: 1.57e−01
Value: C
Acholeplasmatales
0
1
2
3
4
5
A (n=14)
B (n=15)
C (n=11)
surface

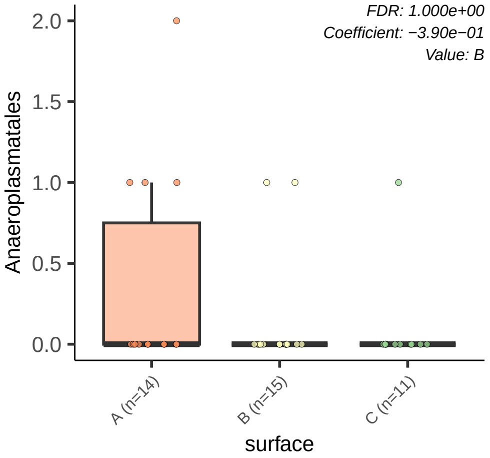

FDR: 1.000e+00
Coefficient: −3.90e−01
Value: B
Anaeroplasmatales
2.0
1.5
1.0
0.5
0.0
A (n=14)
B (n=15)
C (n=11)
surface

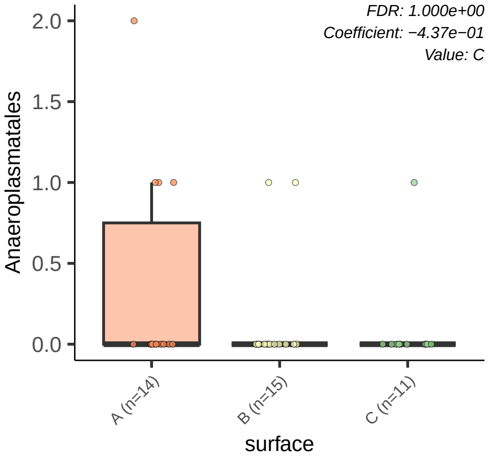
FDR: 1.000e+00
Coefficient: −4.37e−01
Value: C
Anaeroplasmatales
2.0
1.5
1.0
0.5
0.0
A (n=14)
B (n=15)
C (n=11)
surface

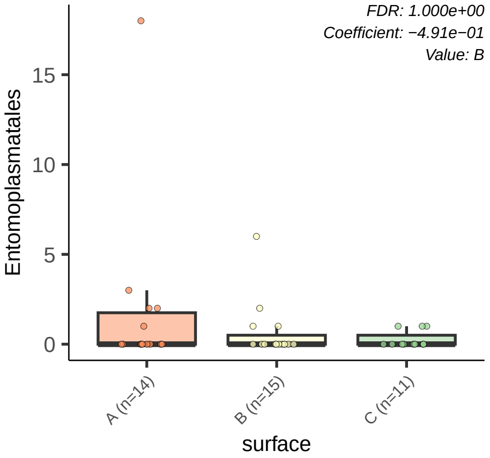

FDR: 1.000e+00
Coefficient: −4.91e−01
Value: B
Entomoplasmatales
15
10
5
0
A (n=14)
B (n=15)
C (n=11)
surface

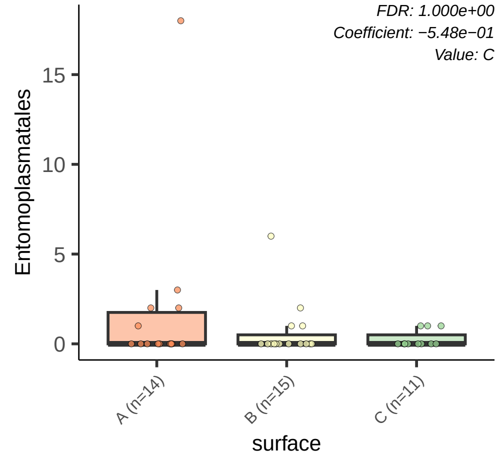

FDR: 1.000e+00
Coefficient: −5.48e−01
Value: C
Entomoplasmatales
0
5
10
15
A (n=14)
B (n=15)
C (n=11)
surface

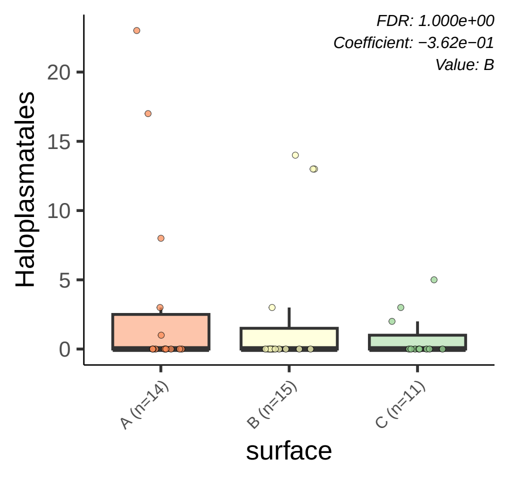

FDR: 1.000e+00
Coefficient: −3.62e−01
Value: B
Haloplasmatales
0
5
10
15
20
A (n=14)
B (n=15)
C (n=11)
surface

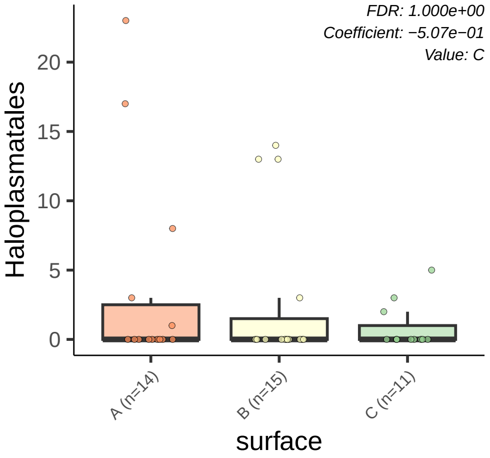
FDR: 1.000e+00
Coefficient: −5.07e−01
Value: C
Haloplasmatales
20
15
10
5
0
A (n=14)
B (n=15)
C (n=11)
surface

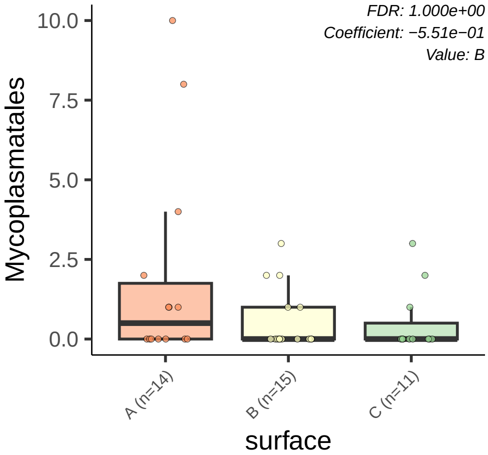
FDR: 1.000e+00
Coefficient: −5.51e−01
Value: B
Mycoplasmatales
10.0
7.5
5.0
2.5
0.0
A (n=14)
B (n=15)
C (n=11)
surface

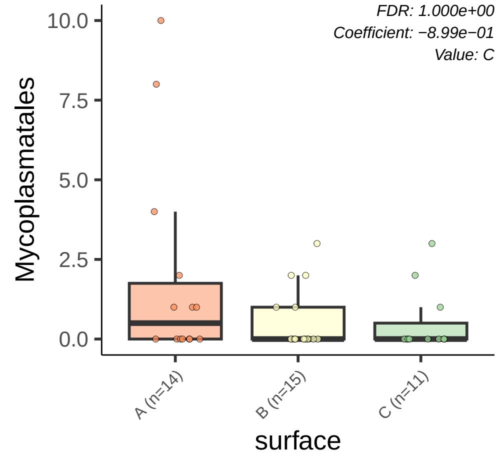

FDR: 1.000e+00
Coefficient: −8.99e−01
Value: C
Mycoplasmatales
10.0
7.5
5.0
2.5
0.0
A (n=14)
B (n=15)
C (n=11)
surface

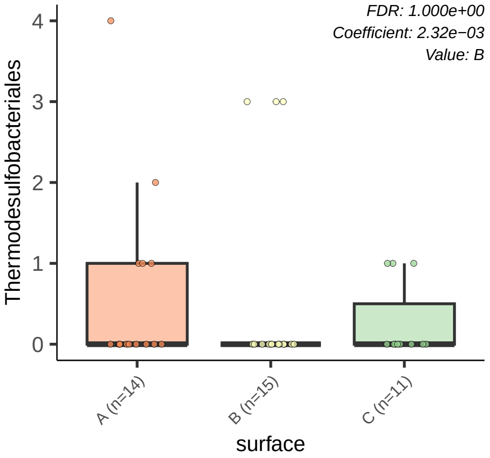

FDR: 1.000e+00
Coefficient: 2.32e−03
Value: B
Thermodesulfobacteriales
0
1
2
3
4
A (n=14)
B (n=15)
C (n=11)
surface

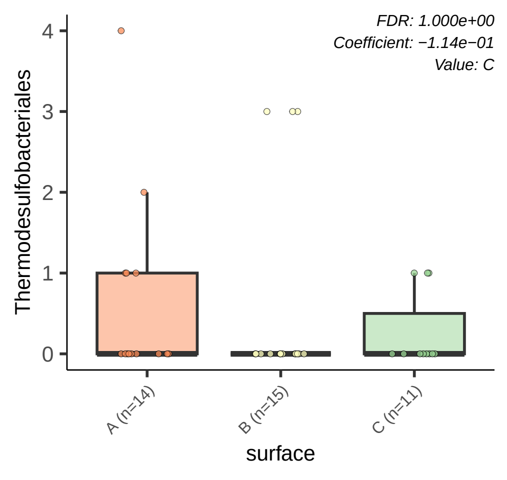

FDR: 1.000e+00
Coefficient: −1.14e−01
Value: C
Thermodesulfobacteriales
0
1
2
3
4
A (n=14)
B (n=15)
C (n=11)
surface

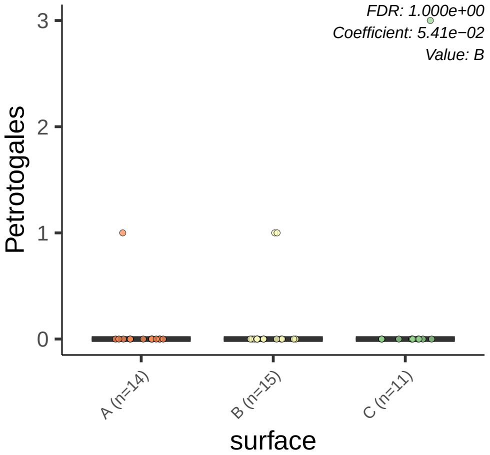
FDR: 1.000e+00
Coefficient: 5.41e−02
Value: B
Petrotogales
0
1
2
3
A (n=14)
B (n=15)
C (n=11)
surface

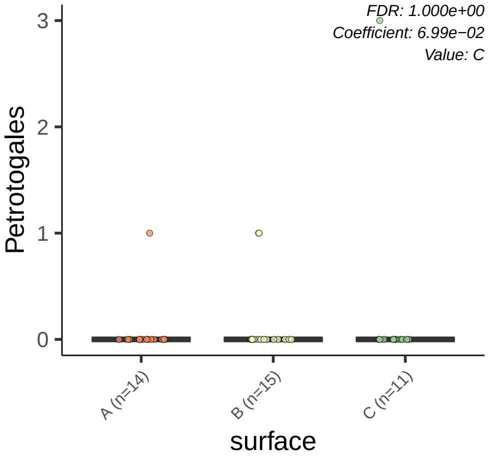

FDR: 1.000e+00
Coefficient: 6.99e−02
Value: C
Petrotogales
3
2
1
0
A (n=14)
B (n=15)
C (n=11)
surface

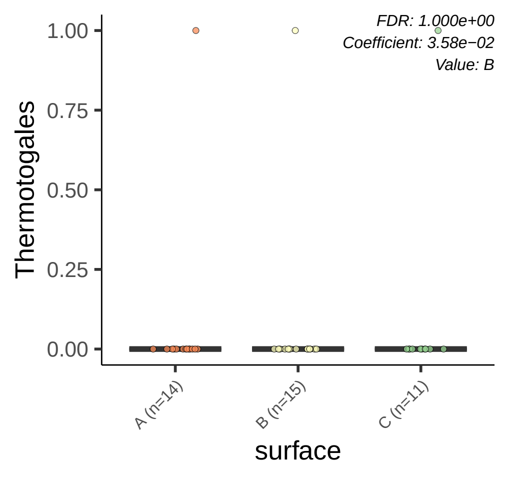
FDR: 1.000e+00
Coefficient: 3.58e−02
Value: B
Thermotogales
1.00
0.75
0.50
0.25
0.00
A (n=14)
B (n=15)
C (n=11)
surface

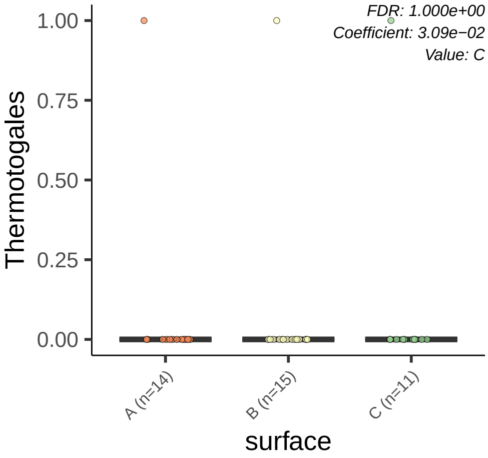

FDR: 1.000e+00
Coefficient: 3.09e−02
Value: C
Thermotogales
1.00
0.75
0.50
0.25
0.00
A (n=14)
B (n=15)
C (n=11)
surface

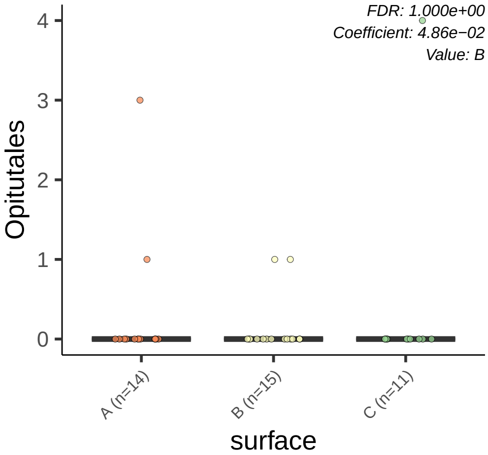

FDR: 1.000e+00
Coefficient: 4.86e−02
Value: B
Opitutales
4
3
2
1
0
A (n=14)
B (n=15)
C (n=11)
surface

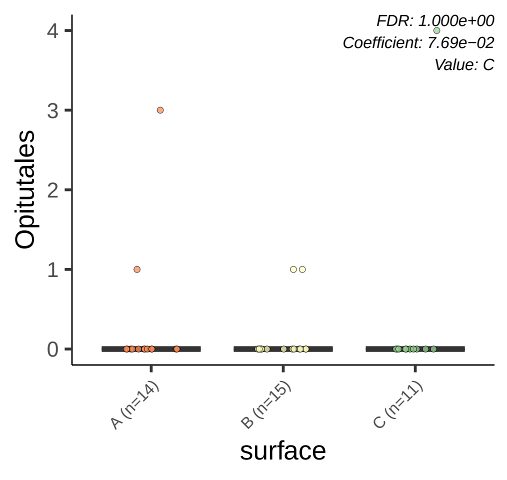
FDR: 1.000e+00
Coefficient: 7.69e−02
Value: C
Opitutales
4
3
2
1
0
A (n=14)
B (n=15)
C (n=11)
surface

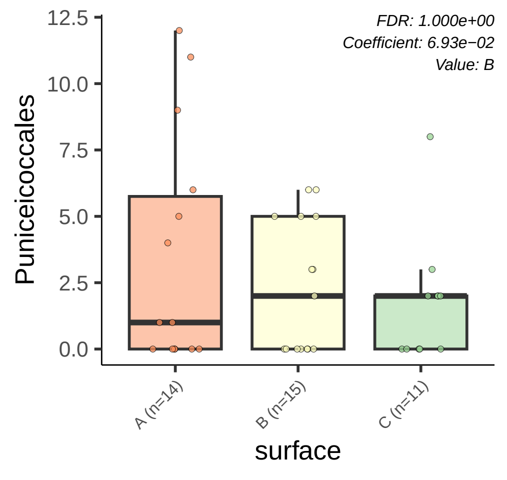

FDR: 1.000e+00
Coefficient: 6.93e−02
Value: B
Puniceicoccales
12.5
10.0
7.5
5.0
2.5
0.0
A (n=14)
B (n=15)
C (n=11)
surface

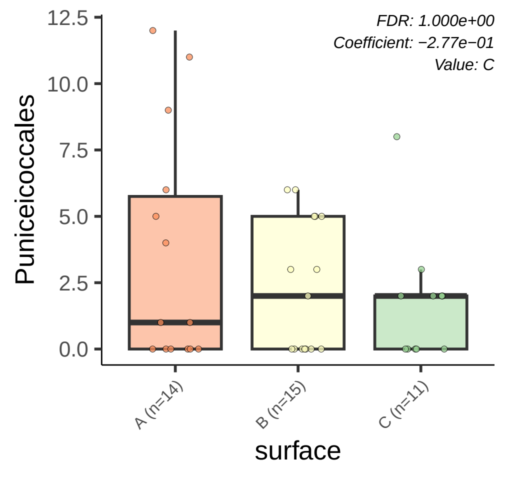

FDR: 1.000e+00
Coefficient: −2.77e−01
Value: C
Puniceicoccales
12.5
10.0
7.5
5.0
2.5
0.0
A (n=14)
B (n=15)
C (n=11)
surface

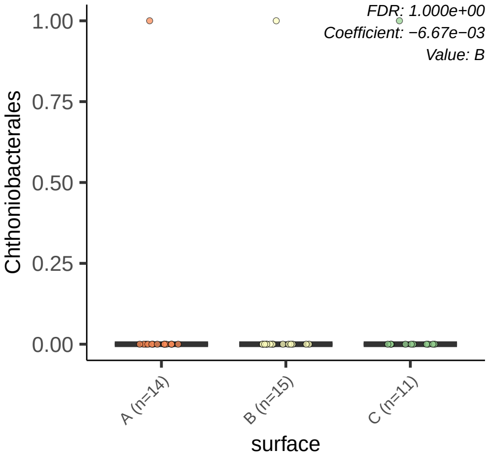

FDR: 1.000e+00
Coefficient: −6.67e−03
Value: B
1.00
0.75
0.50
0.25
0.00
Chthoniobacterales
A (n=14)
B (n=15)
C (n=11)
surface

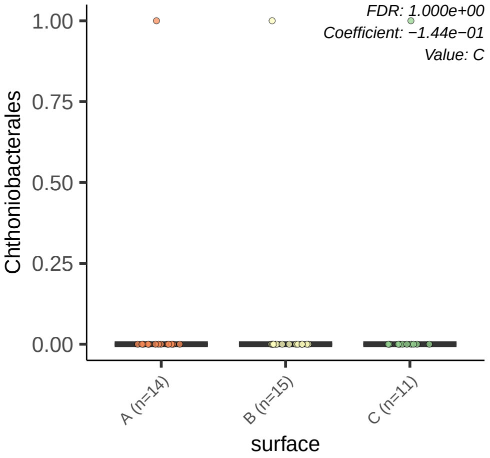
FDR: 1.000e+00
Coefficient: −1.44e−01
Value: C
Chthoniobacterales
1.00
0.75
0.50
0.25
0.00
A (n=14)
B (n=15)
C (n=11)
surface

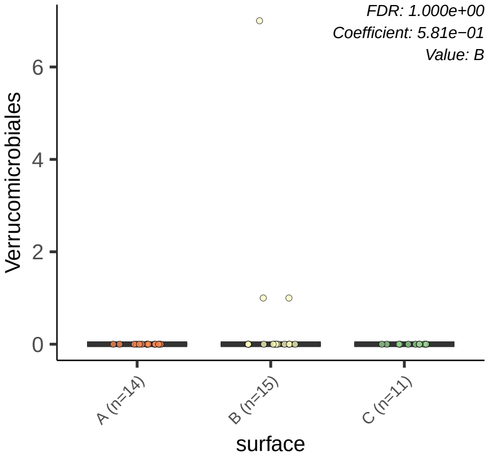

FDR: 1.000e+00
Coefficient: 5.81e−01
Value: B
Verrucomicrobiales
0
2
4
6
A (n=14)
B (n=15)
C (n=11)
surface

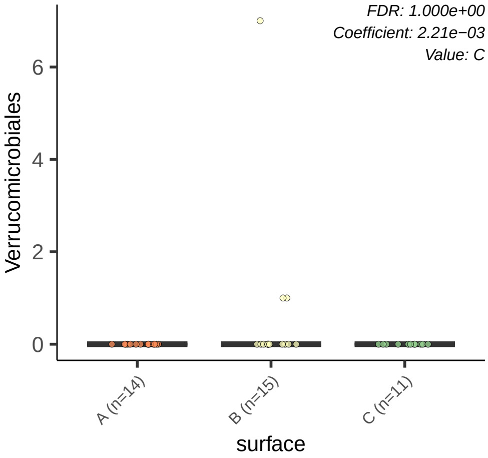

FDR: 1.000e+00
Coefficient: 2.21e−03
Value: C
Verrucomicrobiales
0
2
4
6
A (n=14)
B (n=15)
C (n=11)
surface

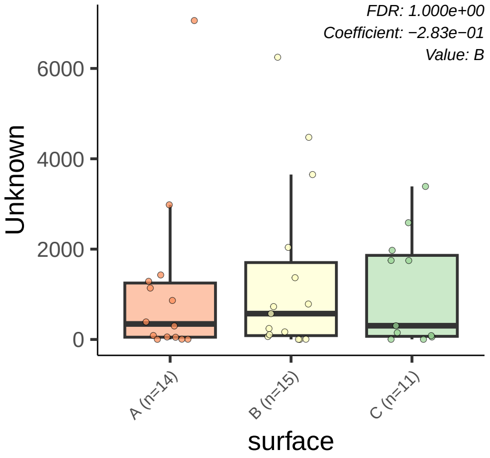
FDR: 1.000e+00
Coefficient: −2.83e−01
Value: B
Unknown
6000
4000
2000
0
A (n=14)
B (n=15)
C (n=11)
surface

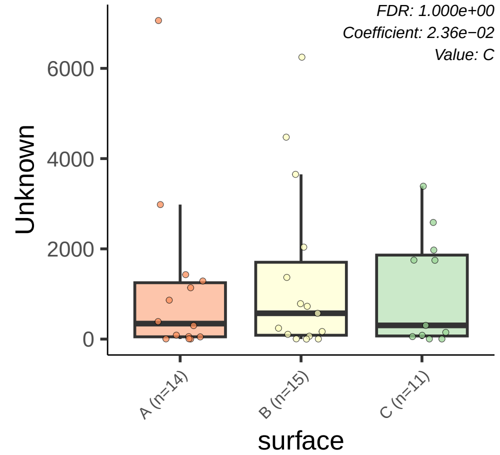
FDR: 1.000e+00
Coefficient: 2.36e−02
Value: C
Unknown
6000
4000
2000
0
A (n=14)
B (n=15)
C (n=11)
surface

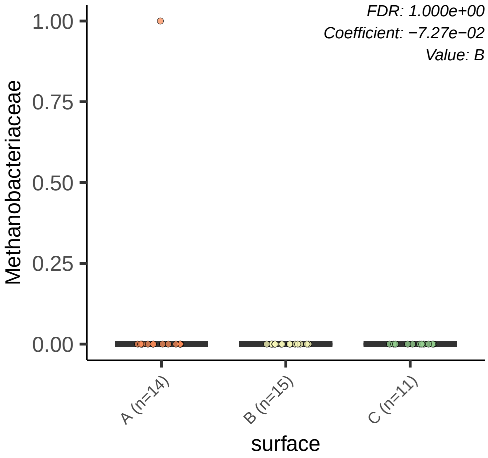

FDR: 1.000e+00
Coefficient: −7.27e−02
Value: B
Methanobacteriaceae
1.00
0.75
0.50
0.25
0.00
A (n=14)
B (n=15)
C (n=11)
surface

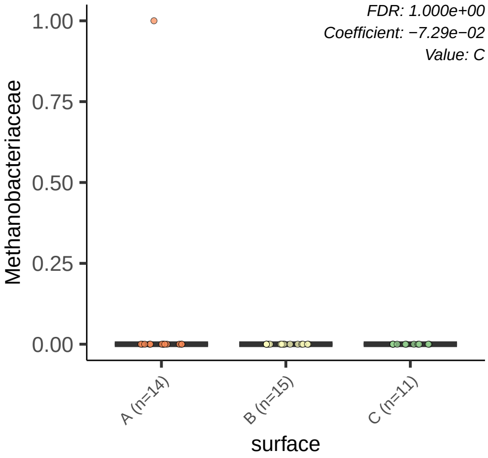

FDR: 1.000e+00
Coefficient: −7.29e−02
Value: C
Methanobacteriaceae
1.00
0.75
0.50
0.25
0.00
A (n=14)
B (n=15)
C (n=11)
surface

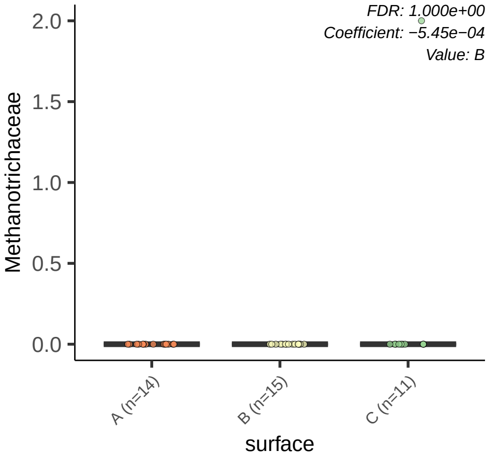

FDR: 1.000e+00
Coefficient: −5.45e−04
Value: B
Methanotrichaceae
2.0
1.5
1.0
0.5
0.0
A (n=14)
B (n=15)
C (n=11)
surface

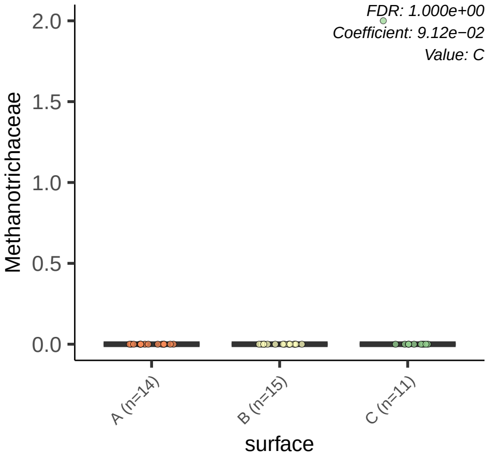

FDR: 1.000e+00
Coefficient: 9.12e−02
Value: C
Methanotrichaceae
2.0
1.5
1.0
0.5
0.0
A (n=14)
B (n=15)
C (n=11)
surface

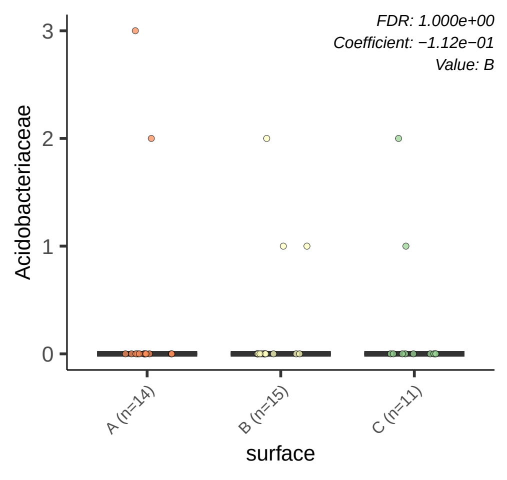

FDR: 1.000e+00
Coefficient: −1.12e−01
Value: B
Acidobacteriaceae
3
2
1
0
A (n=14)
B (n=15)
C (n=11)
surface

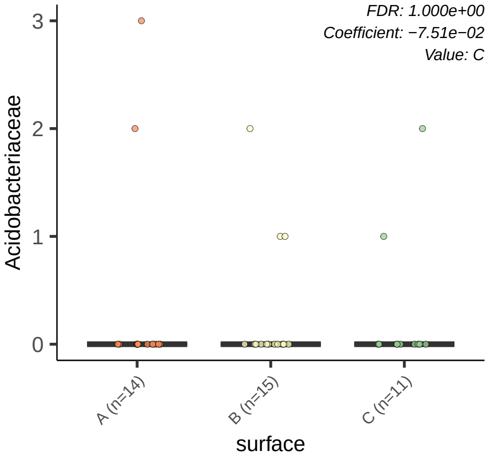

FDR: 1.000e+00
Coefficient: −7.51e−02
Value: C
Acidobacteriaceae
3
2
1
0
A (n=14)
B (n=15)
C (n=11)
surface

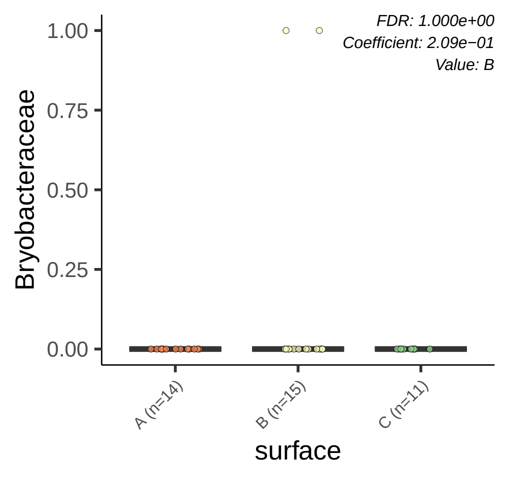
FDR: 1.000e+00
Coefficient: 2.09e−01
Value: B
Bryobacteraceae
1.00
0.75
0.50
0.25
0.00
A (n=14)
B (n=15)
C (n=11)
surface

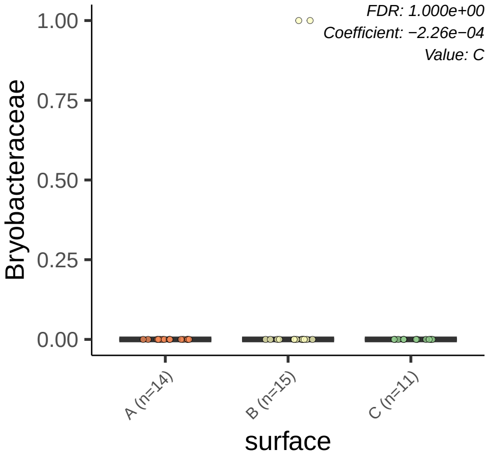

FDR: 1.000e+00
Coefficient: −2.26e−04
Value: C
Bryobacteraceae
1.00
0.75
0.50
0.25
0.00
A (n=14)
B (n=15)
C (n=11)
surface

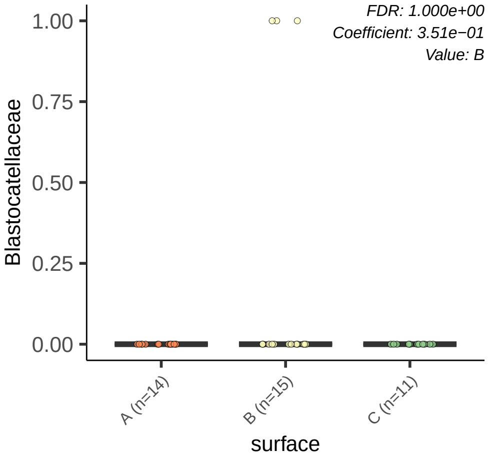
FDR: 1.000e+00
Coefficient: 3.51e−01
Value: B
Blastocatellaceae
1.00
0.75
0.50
0.25
0.00
A (n=14)
B (n=15)
C (n=11)
surface

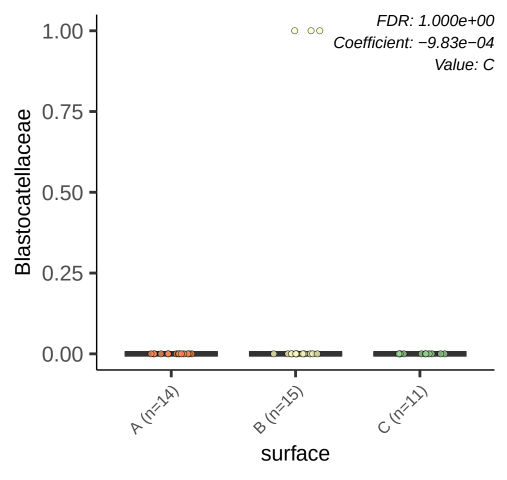

FDR: 1.000e+00
Coefficient: −9.83e−04
Value: C
Blastocatellaceae
1.00
0.75
0.50
0.25
0.00
A (n=14)
B (n=15)
C (n=11)
surface

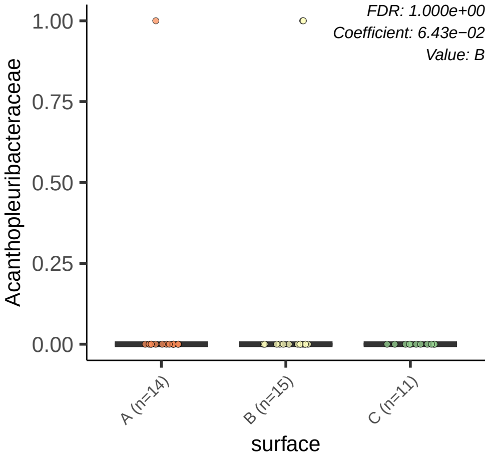

FDR: 1.000e+00
Coefficient: 6.43e−02
Value: B
Acanthopleuribacteraceae
1.00
0.75
0.50
0.25
0.00
A (n=14)
B (n=15)
C (n=11)
surface

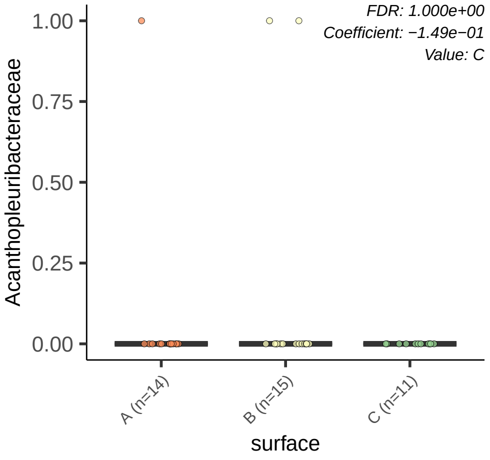

FDR: 1.000e+00
Coefficient: −1.49e−01
Value: C
Acanthopleuribacteraceae
1.00
0.75
0.50
0.25
0.00
A (n=14)
B (n=15)
C (n=11)
surface

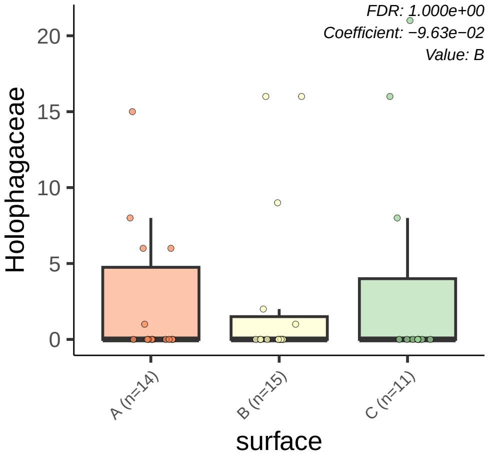

FDR: 1.000e+00
Coefficient: −9.63e−02
Value: B
Holophagaceae
20
15
10
5
0
A (n=14)
B (n=15)
C (n=11)
surface

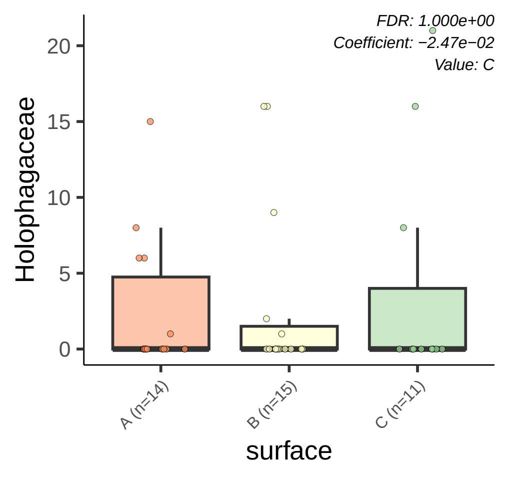

FDR: 1.000e+00
Coefficient: −2.47e−02
Value: C
Holophagaceae
0
5
10
15
20
A (n=14)
B (n=15)
C (n=11)
surface

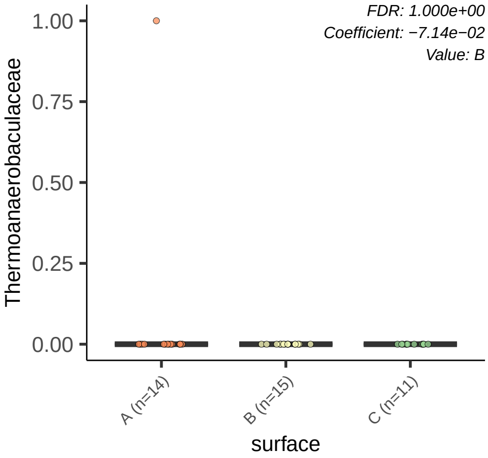
FDR: 1.000e+00
Coefficient: −7.14e−02
Value: B
Thermoanaerobaculaceae
1.00
0.75
0.50
0.25
0.00
A (n=14)
B (n=15)
C (n=11)
surface

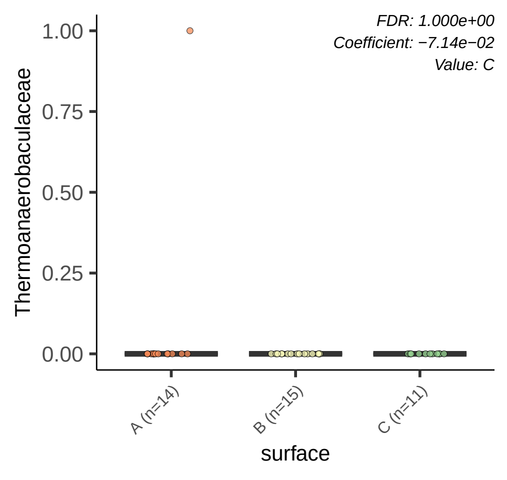
FDR: 1.000e+00
Coefficient: −7.14e−02
Value: C
Thermoanaerobaculaceae
1.00
0.75
0.50
0.25
0.00
A (n=14)
B (n=15)
C (n=11)
surface

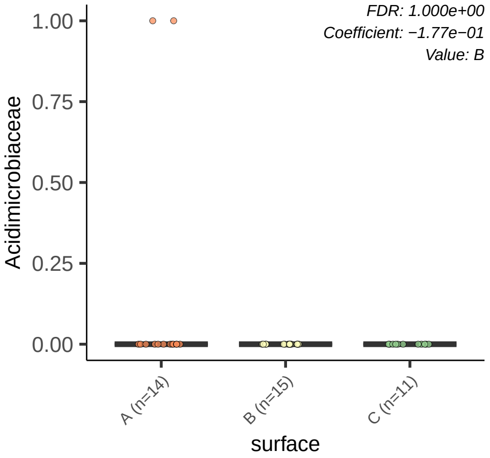

FDR: 1.000e+00
Coefficient: −1.77e−01
Value: B
Acidimicrobiaceae
1.00
0.75
0.50
0.25
0.00
A (n=14)
B (n=15)
C (n=11)
surface

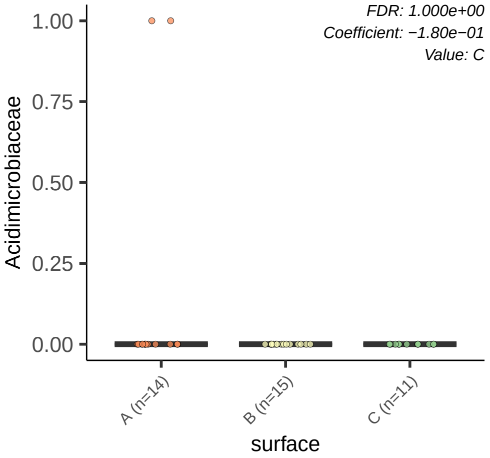

FDR: 1.000e+00
Coefficient: −1.80e−01
Value: C
1.00
0.75
0.50
0.25
0.00
Acidimicrobiaceae
A (n=14)
B (n=15)
C (n=11)
surface

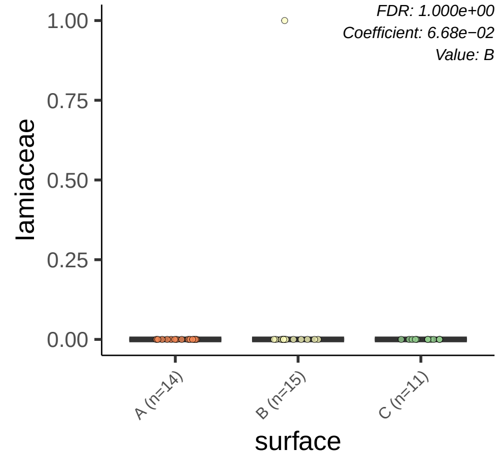
FDR: 1.000e+00
Coefficient: 6.68e−02
Value: B
1.00
0.75
0.50
0.25
0.00
lamiaceae
A (n=14)
B (n=15)
C (n=11)
surface

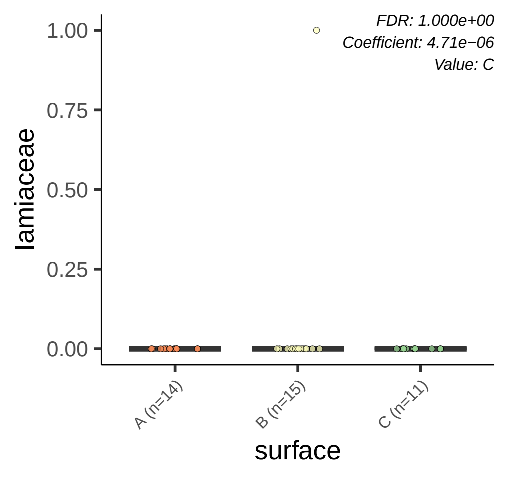

FDR: 1.000e+00
Coefficient: 4.71e−06
Value: C
1.00
0.75
0.50
0.25
0.00
lamiaceae
A (n=14)
B (n=15)
C (n=11)
surface

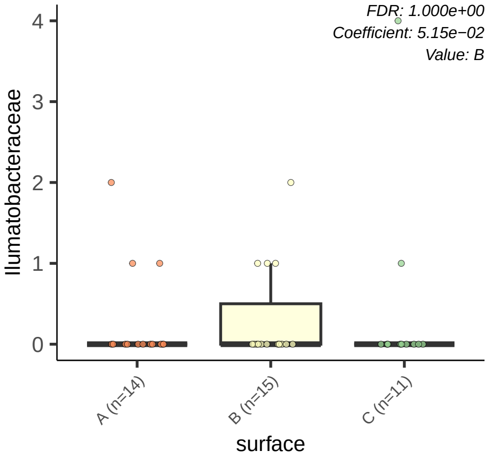

FDR: 1.000e+00
Coefficient: 5.15e−02
Value: B
Ilumatobacteraceae
0
1
2
3
4
A (n=14)
B (n=15)
C (n=11)
surface

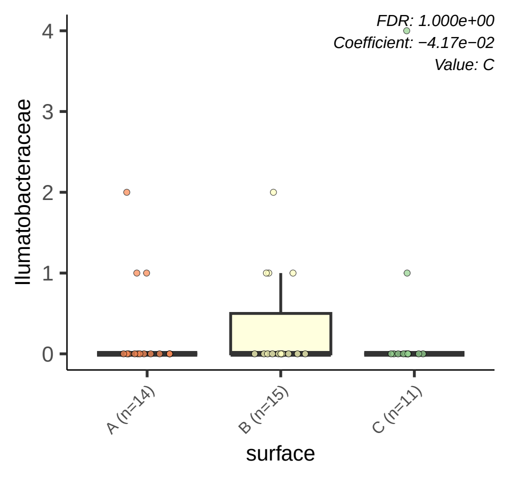

FDR: 1.000e+00
Coefficient: −4.17e−02
Value: C
Ilumatobacteraceae
4
3
2
1
0
A (n=14)
B (n=15)
C (n=11)
surface

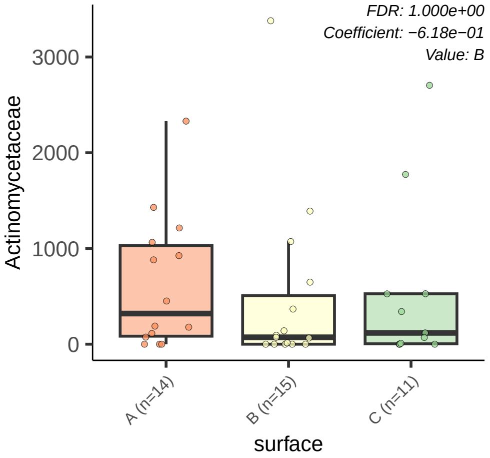

FDR: 1.000e+00
Coefficient: −6.18e−01
Value: B
Actinomycetaceae
3000
2000
1000
0
A (n=14)
B (n=15)
C (n=11)
surface

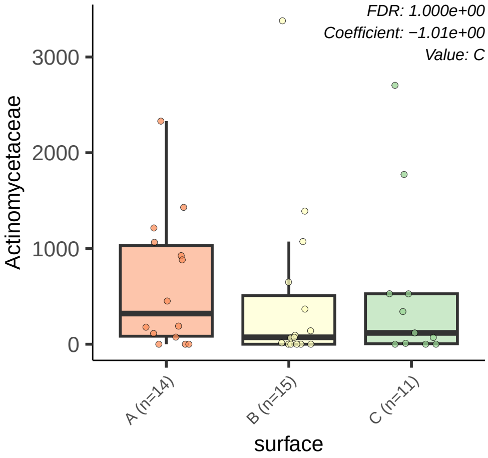

FDR: 1.000e+00
Coefficient: −1.01e+00
Value: C
Actinomycetaceae
3000
2000
1000
0
A (n=14)
B (n=15)
C (n=11)
surface

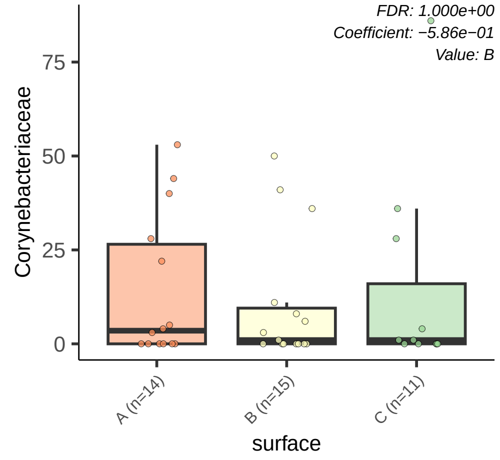

FDR: 1.000e+00
Coefficient: −5.86e−01
Value: B
Corynebacteriaceae
75
50
25
0
A (n=14)
B (n=15)
C (n=11)
surface

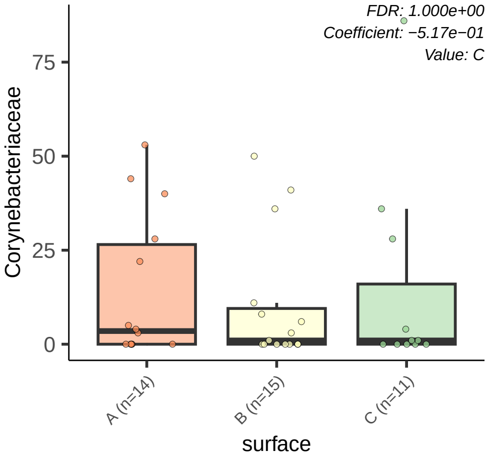
FDR: 1.000e+00
Coefficient: −5.17e−01
Value: C
Corynebacteriaceae
75
50
25
0
A (n=14)
B (n=15)
C (n=11)
surface

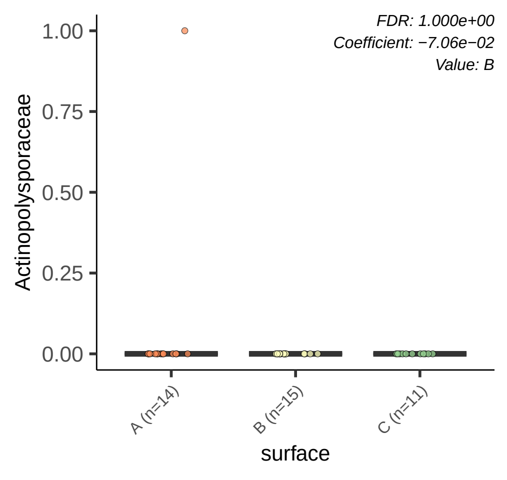

FDR: 1.000e+00
Coefficient: −7.06e−02
Value: B
Actinopolysporaceae
1.00
0.75
0.50
0.25
0.00
A (n=14)
B (n=15)
C (n=11)
surface

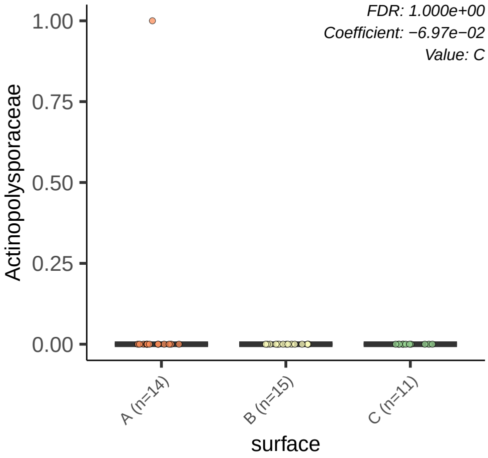

FDR: 1.000e+00
Coefficient: −6.97e−02
Value: C
Actinopolysporaceae
1.00
0.75
0.50
0.25
0.00
A (n=14)
B (n=15)
C (n=11)
surface

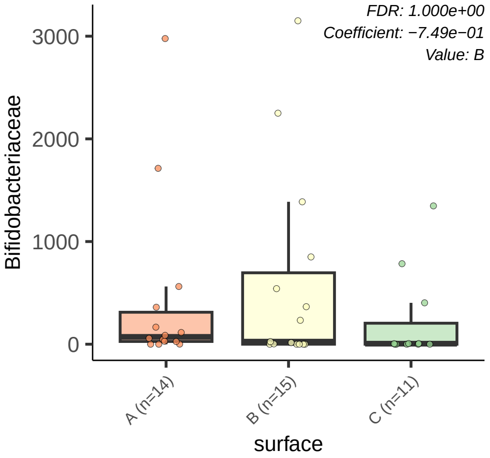

FDR: 1.000e+00
Coefficient: −7.49e−01
Value: B
Bifidobacteriaceae
3000
2000
1000
0
A (n=14)
B (n=15)
C (n=11)
surface

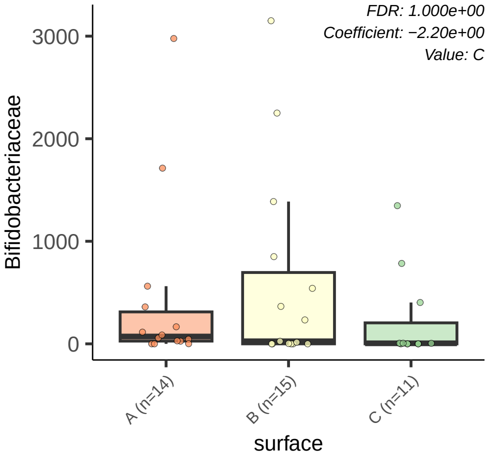
FDR: 1.000e+00
Coefficient: −2.20e+00
Value: C
Bifidobacteriaceae
3000
2000
1000
0
A (n=14)
B (n=15)
C (n=11)
surface

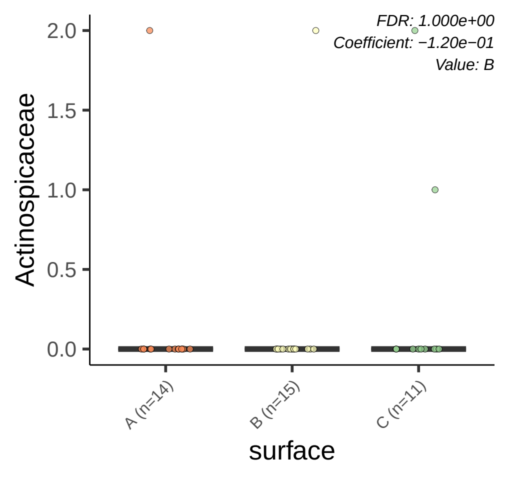
FDR: 1.000e+00
Coefficient: −1.20e−01
Value: B
Actinospicaceae
2.0
1.5
1.0
0.5
0.0
A (n=14)
B (n=15)
C (n=11)
surface

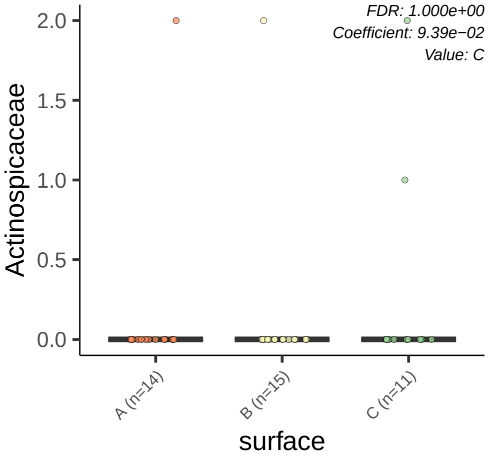
FDR: 1.000e+00
Coefficient: 9.39e−02
Value: C
Actinospicaceae
2.0
1.5
1.0
0.5
0.0
A (n=14)
B (n=15)
C (n=11)
surface

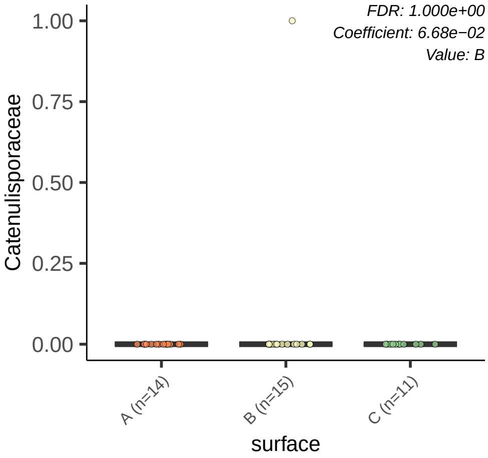

FDR: 1.000e+00
Coefficient: 6.68e−02
Value: B
Catenulisporaceae
1.00
0.75
0.50
0.25
0.00
A (n=14)
B (n=15)
C (n=11)
surface

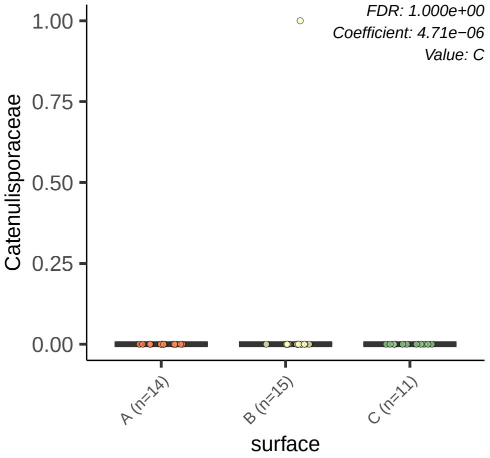

FDR: 1.000e+00
Coefficient: 4.71e−06
Value: C
Catenulisporaceae
1.00
0.75
0.50
0.25
0.00
A (n=14)
B (n=15)
C (n=11)
surface

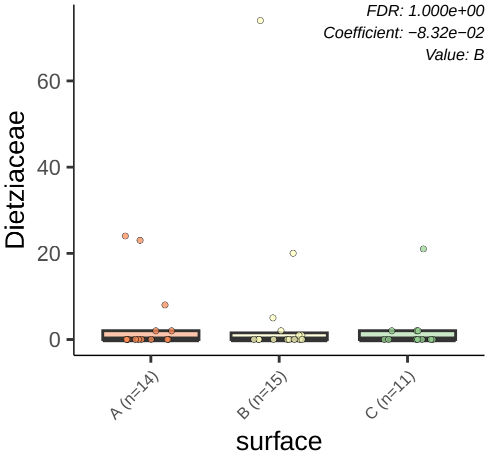
FDR: 1.000e+00
Coefficient: −8.32e−02
Value: B
Dietziaceae
60
40
20
0
A (n=14)
B (n=15)
C (n=11)
surface

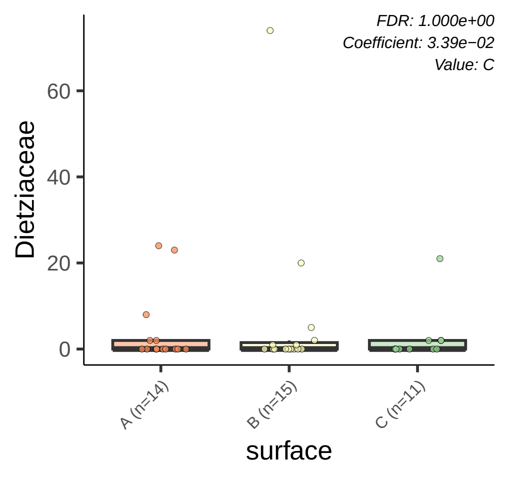

FDR: 1.000e+00
Coefficient: 3.39e−02
Value: C
Dietziaceae
60
40
20
0
A (n=14)
B (n=15)
C (n=11)
surface

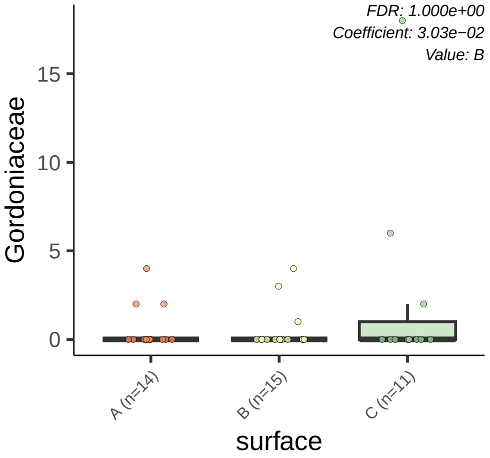

FDR: 1.000e+00
Coefficient: 3.03e−02
Value: B
Gordoniaceae
15
10
5
0
A (n=14)
B (n=15)
C (n=11)
surface

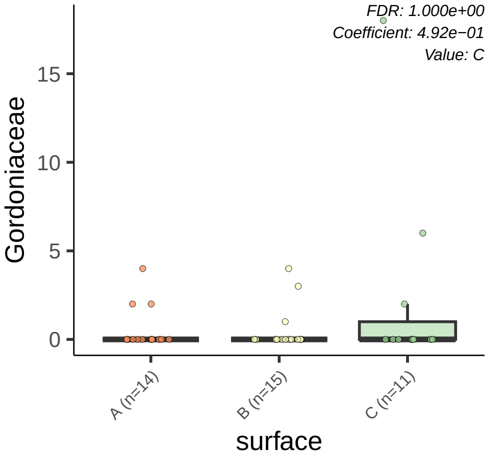
FDR: 1.000e+00
Coefficient: 4.92e−01
Value: C
Gordoniaceae
15
10
5
0
A (n=14)
B (n=15)
C (n=11)
surface

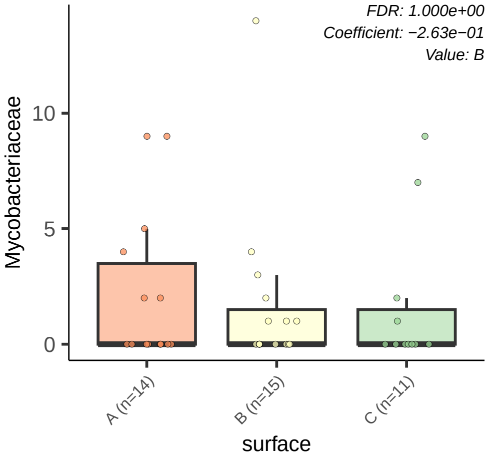
FDR: 1.000e+00
Coefficient: −2.63e−01
Value: B
Mycobacteriaceae
10
5
0
A (n=14)
B (n=15)
C (n=11)
surface

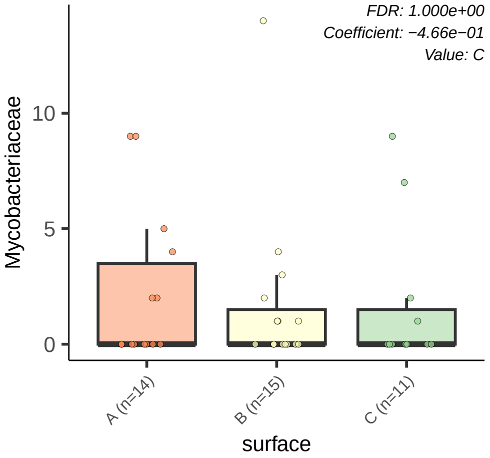
FDR: 1.000e+00
Coefficient: −4.66e−01
Value: C
Mycobacteriaceae
10
5
0
A (n=14)
B (n=15)
C (n=11)
surface

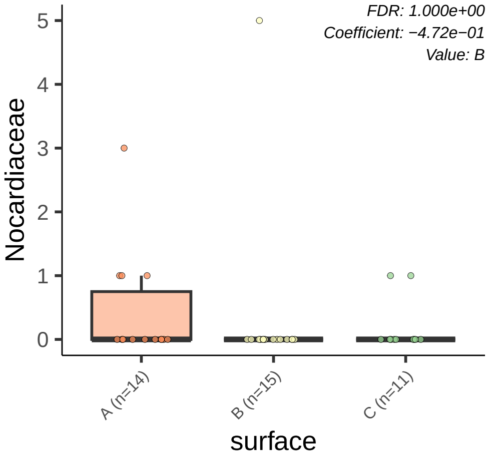
FDR: 1.000e+00
Coefficient: −4.72e−01
Value: B
Nocardiaceae
5
4
3
2
1
0
A (n=14)
B (n=15)
C (n=11)
surface

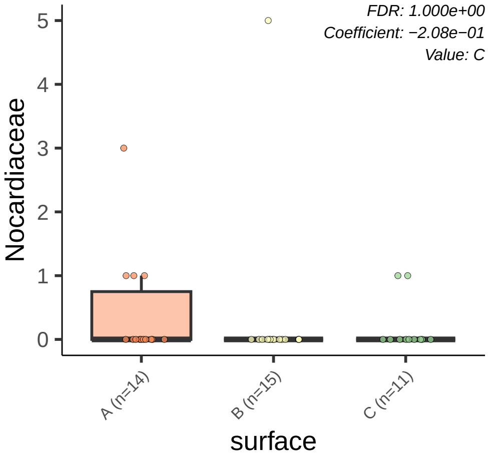

FDR: 1.000e+00
Coefficient: −2.08e−01
Value: C
Nocardiaceae
5
4
3
2
1
0
A (n=14)
B (n=15)
C (n=11)
surface

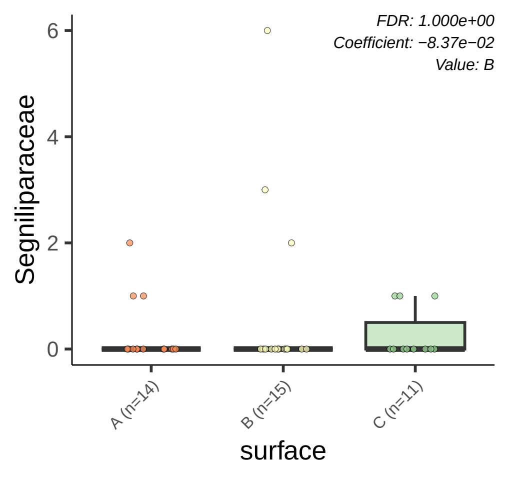
FDR: 1.000e+00
Coefficient: −8.37e−02
Value: B
Segniliparaceae
0
2
4
6
A (n=14)
B (n=15)
C (n=11)
surface

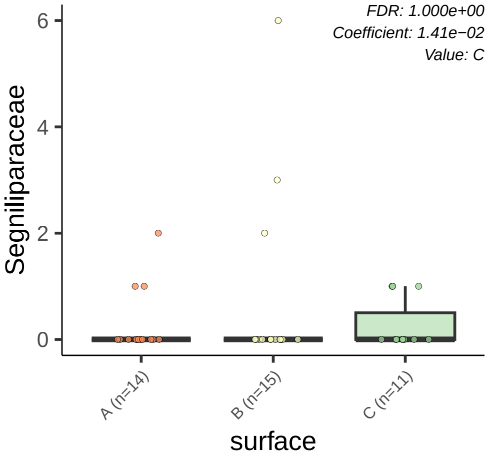

FDR: 1.000e+00
Coefficient: 1.41e−02
Value: C
Segniliparaceae
6
4
2
0
A (n=14)
B (n=15)
C (n=11)
surface

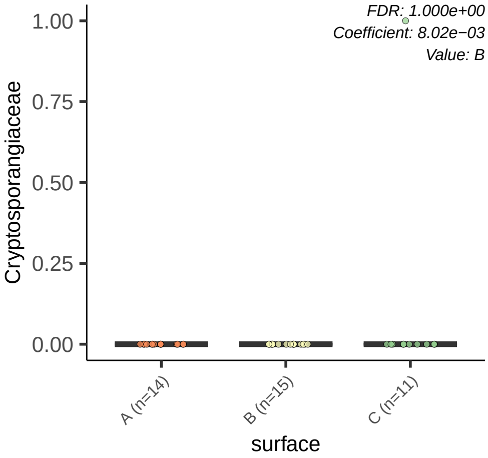
FDR: 1.000e+00
Coefficient: 8.02e−03
Value: B
Cryptosporangiaceae
1.00
0.75
0.50
0.25
0.00
A (n=14)
B (n=15)
C (n=11)
surface

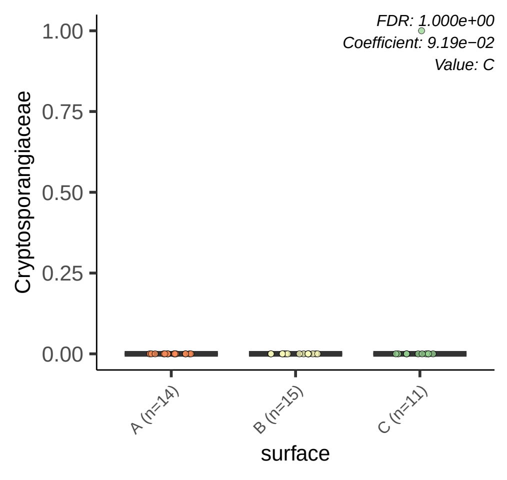
FDR: 1.000e+00
Coefficient: 9.19e−02
Value: C
Cryptosporangiaceae
1.00
0.75
0.50
0.25
0.00
A (n=14)
B (n=15)
C (n=11)
surface

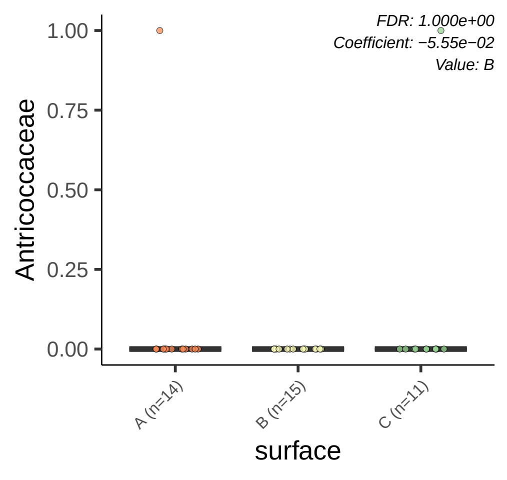

FDR: 1.000e+00
Coefficient: −5.55e−02
Value: B
Antricoccaceae
1.00
0.75
0.50
0.25
0.00
A (n=14)
B (n=15)
C (n=11)
surface

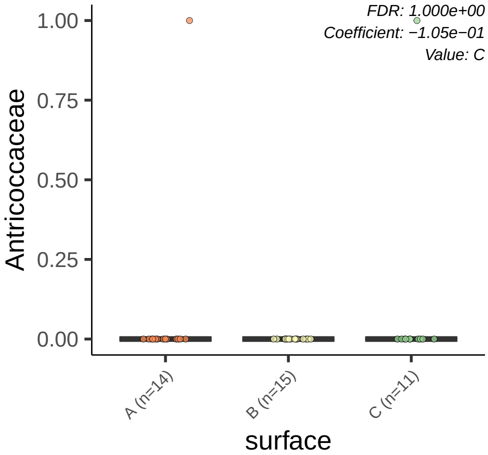

FDR: 1.000e+00
Coefficient: −1.05e−01
Value: C
Antricoccaceae
1.00
0.75
0.50
0.25
0.00
A (n=14)
B (n=15)
C (n=11)
surface

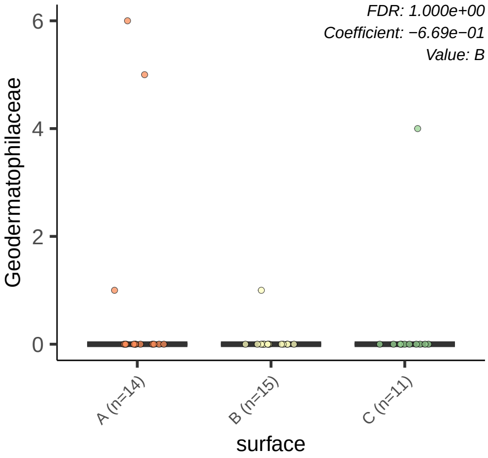

FDR: 1.000e+00
Coefficient: −6.69e−01
Value: B
Geodermatophilaceae
6
4
2
0
A (n=14)
B (n=15)
C (n=11)
surface

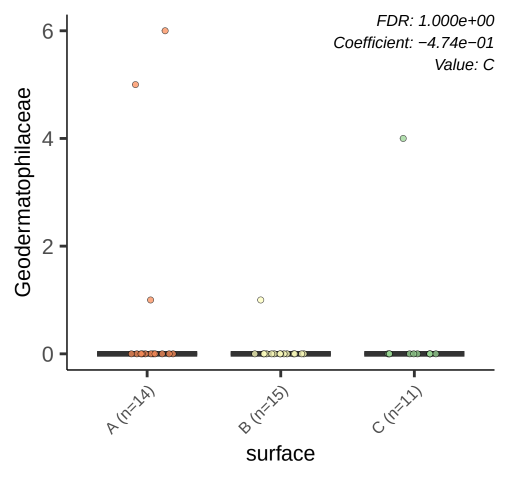
FDR: 1.000e+00
Coefficient: −4.74e−01
Value: C
Geodermatophilaceae
6
4
2
0
A (n=14)
B (n=15)
C (n=11)
surface

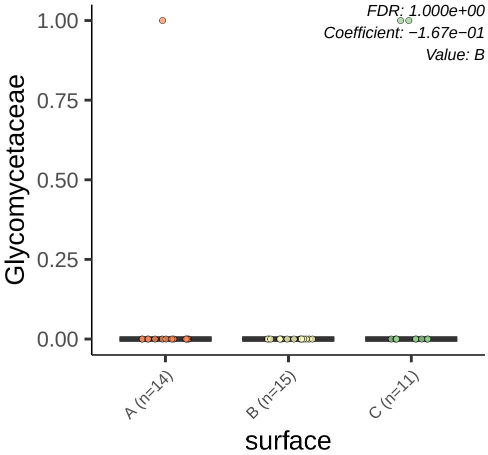

FDR: 1.000e+00
Coefficient: −1.67e−01
Value: B
Glycomycetaceae
1.00
0.75
0.50
0.25
0.00
A (n=14)
B (n=15)
C (n=11)
surface

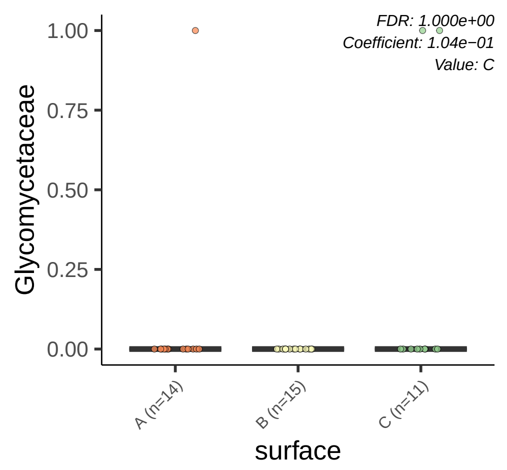
FDR: 1.000e+00
Coefficient: 1.04e−01
Value: C
Glycomycetaceae
1.00
0.75
0.50
0.25
0.00
A (n=14)
B (n=15)
C (n=11)
surface

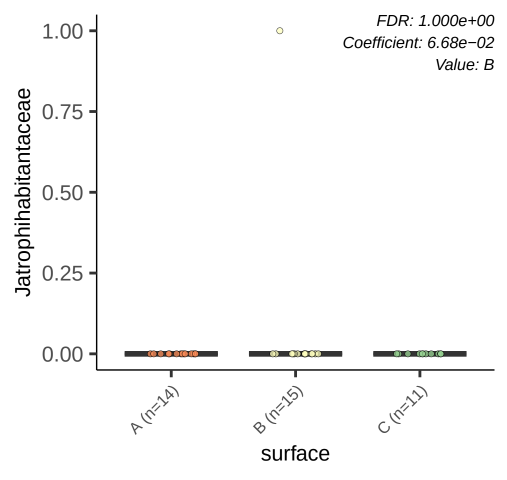

FDR: 1.000e+00
Coefficient: 6.68e−02
Value: B
Jatrophihabitantaceae
1.00
0.75
0.50
0.25
0.00
A (n=14)
B (n=15)
C (n=11)
surface

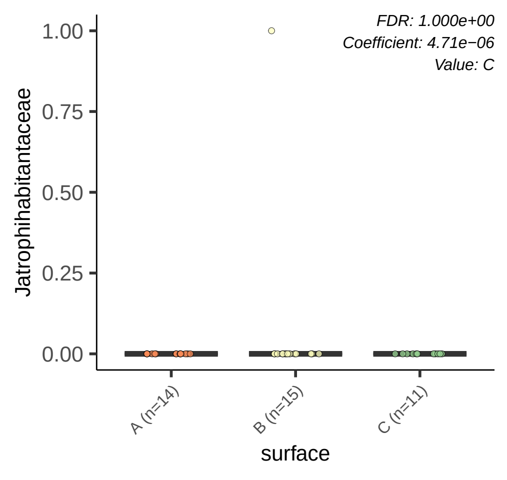

FDR: 1.000e+00
Coefficient: 4.71e−06
Value: C
Jatrophihabitantaceae
1.00
0.75
0.50
0.25
0.00
A (n=14)
B (n=15)
C (n=11)
surface

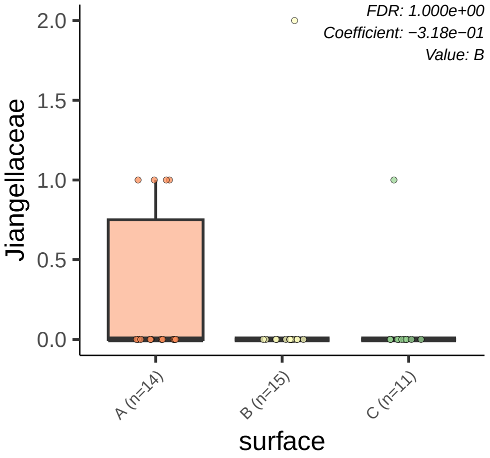

FDR: 1.000e+00
Coefficient: −3.18e−01
Value: B
Jiangellaceae
2.0
1.5
1.0
0.5
0.0
A (n=14)
B (n=15)
C (n=11)
surface

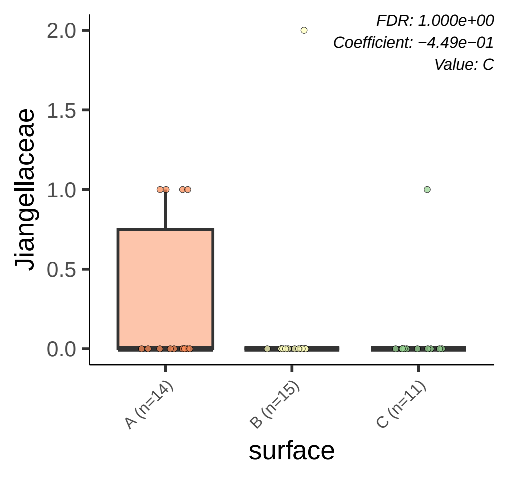

FDR: 1.000e+00
Coefficient: −4.49e−01
Value: C
2.0
1.5
1.0
0.5
0.0
Jiangellaceae
A (n=14)
B (n=15)
C (n=11)
surface

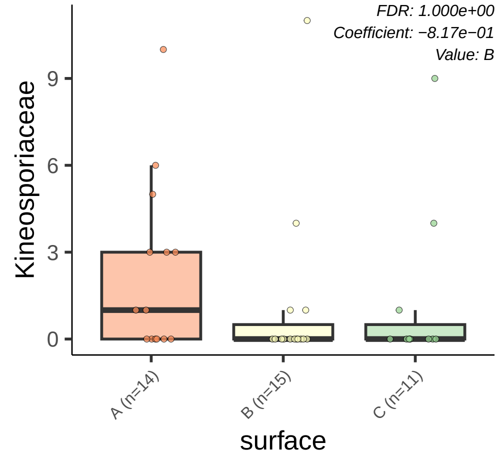

FDR: 1.000e+00
Coefficient: −8.17e−01
Value: B
Kineosporiaceae
0
3
6
9
A (n=14)
B (n=15)
C (n=11)
surface

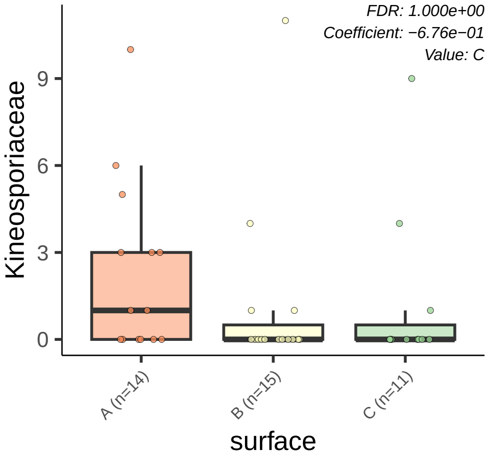

FDR: 1.000e+00
Coefficient: −6.76e−01
Value: C
Kineosporiaceae
0
3
6
9
A (n=14)
B (n=15)
C (n=11)
surface

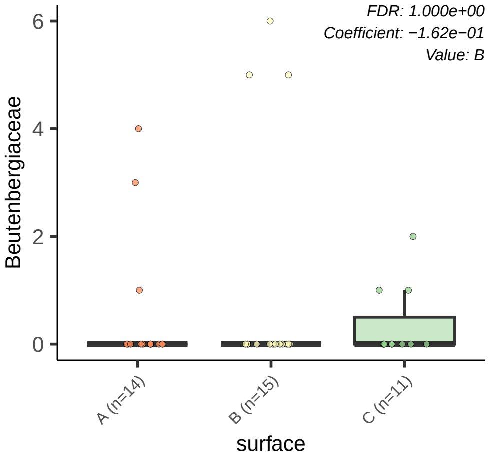

FDR: 1.000e+00
Coefficient: −1.62e−01
Value: B
Beutenbergiaceae
6
4
2
0
A (n=14)
B (n=15)
C (n=11)
surface

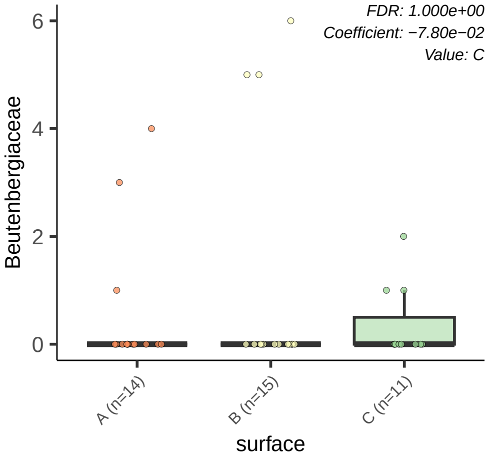

FDR: 1.000e+00
Coefficient: −7.80e−02
Value: C
Beutenbergiaceae
6
4
2
0
A (n=14)
B (n=15)
C (n=11)
surface

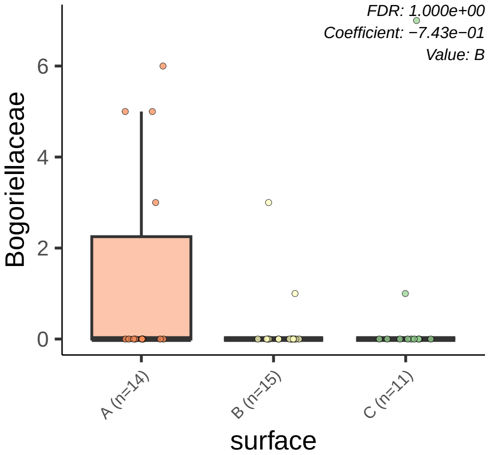

FDR: 1.000e+00
Coefficient: −7.43e−01
Value: B
Bogoriellaceae
6
4
2
0
A (n=14)
B (n=15)
C (n=11)
surface

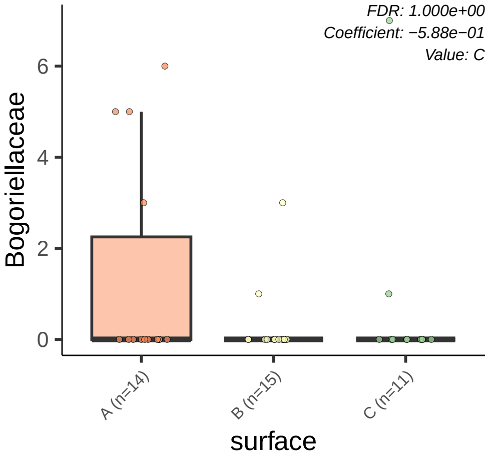
FDR: 1.000e+00
Coefficient: −5.88e−01
Value: C
Bogoriellaceae
0
2
4
6
A (n=14)
B (n=15)
C (n=11)
surface

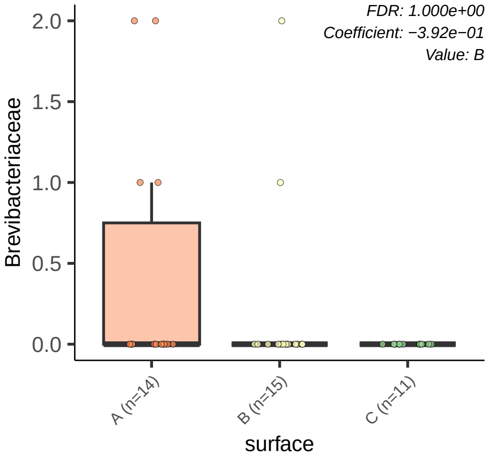
FDR: 1.000e+00
Coefficient: −3.92e−01
Value: B
Brevibacteriaceae
2.0
1.5
1.0
0.5
0.0
A (n=14)
B (n=15)
C (n=11)
surface

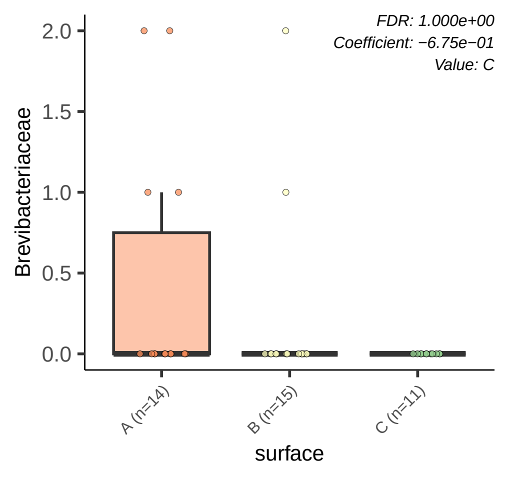

FDR: 1.000e+00
Coefficient: −6.75e−01
Value: C
Brevibacteriaceae
2.0
1.5
1.0
0.5
0.0
A (n=14)
B (n=15)
C (n=11)
surface

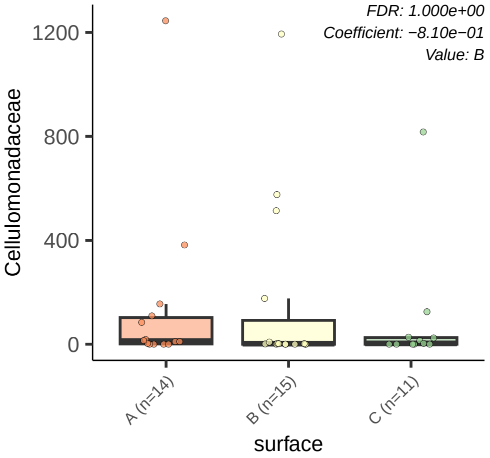
FDR: 1.000e+00
Coefficient: −8.10e−01
Value: B
Cellulomonadaceae
1200
800
400
0
A (n=14)
B (n=15)
C (n=11)
surface

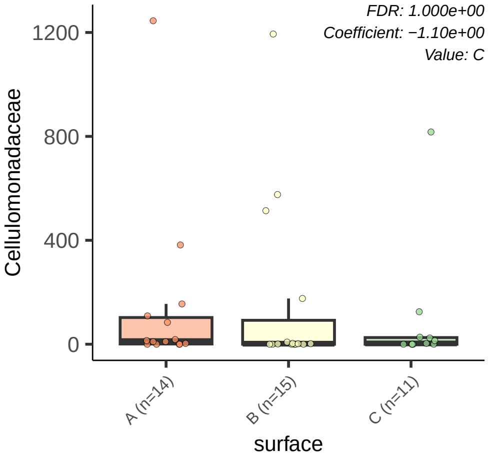

FDR: 1.000e+00
Coefficient: −1.10e+00
Value: C
1200
800
400
0
Cellulomonadaceae
A (n=14)
B (n=15)
C (n=11)
surface

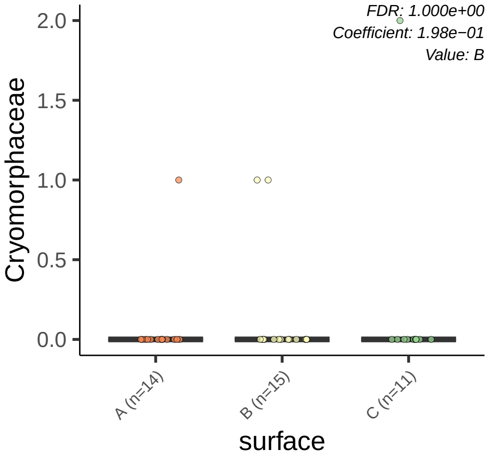
FDR: 1.000e+00
Coefficient: 1.98e−01
Value: B
Cryomorphaceae
2.0
1.5
1.0
0.5
0.0
A (n=14)
B (n=15)
C (n=11)
surface

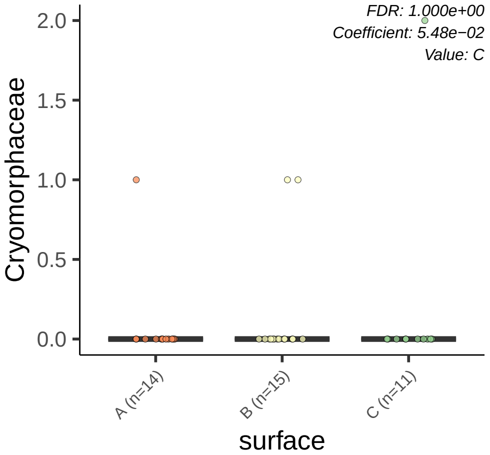
FDR: 1.000e+00
Coefficient: 5.48e−02
Value: C
Cryomorphaceae
2.0
1.5
1.0
0.5
0.0
A (n=14)
B (n=15)
C (n=11)
surface

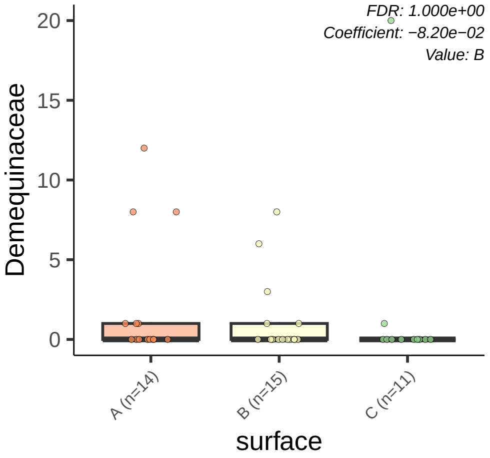

FDR: 1.000e+00
Coefficient: −8.20e−02
Value: B
Demequinaceae
20
15
10
5
0
A (n=14)
B (n=15)
C (n=11)
surface

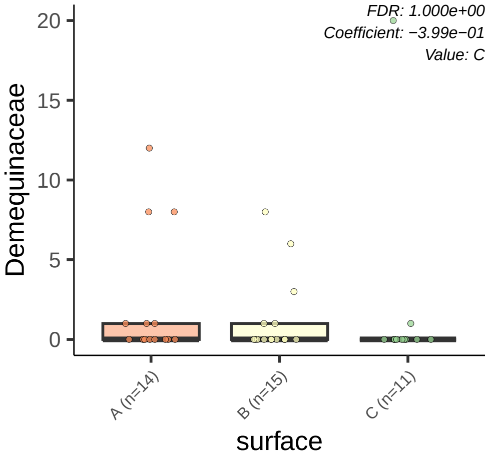
FDR: 1.000e+00
Coefficient: −3.99e−01
Value: C
Demequinaceae
20
15
10
5
0
A (n=14)
B (n=15)
C (n=11)
surface

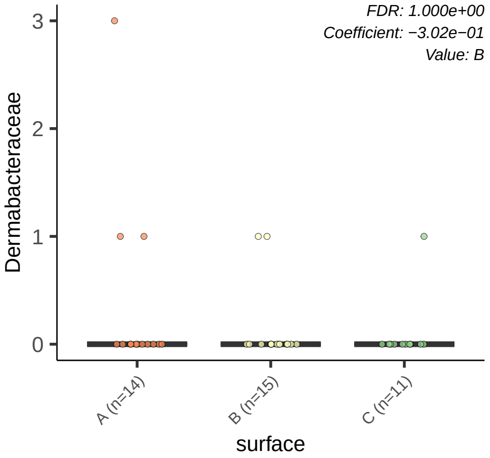

FDR: 1.000e+00
Coefficient: −3.02e−01
Value: B
Dermabacteraceae
3
2
1
0
A (n=14)
B (n=15)
C (n=11)
surface

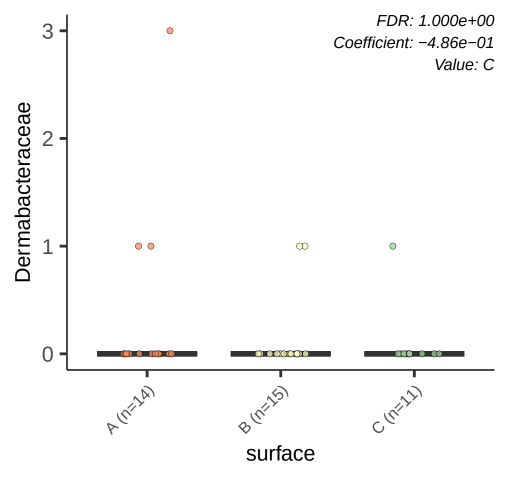
FDR: 1.000e+00
Coefficient: −4.86e−01
Value: C
Dermabacteraceae
3
2
1
0
A (n=14)
B (n=15)
C (n=11)
surface

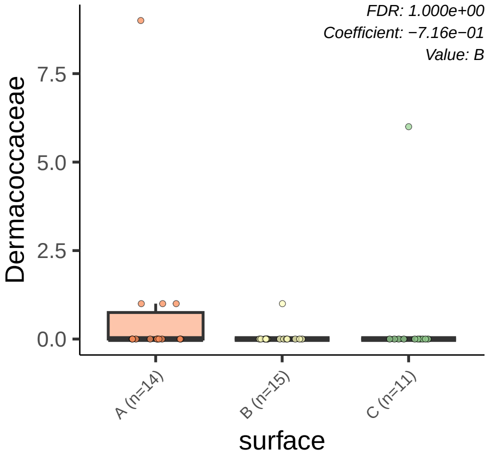
FDR: 1.000e+00
Coefficient: −7.16e−01
Value: B
Dermacoccaceae
7.5
5.0
2.5
0.0
A (n=14)
B (n=15)
C (n=11)
surface

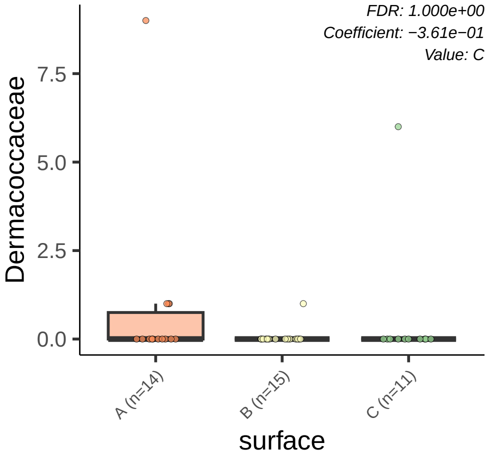
FDR: 1.000e+00
Coefficient: −3.61e−01
Value: C
Dermacoccaceae
7.5
5.0
2.5
0.0
A (n=14)
B (n=15)
C (n=11)
surface

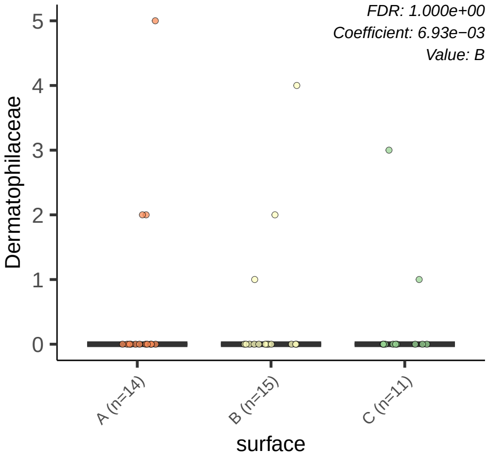

FDR: 1.000e+00
Coefficient: 6.93e−03
Value: B
Dermatophilaceae
0
1
2
3
4
5
A (n=14)
B (n=15)
C (n=11)
surface

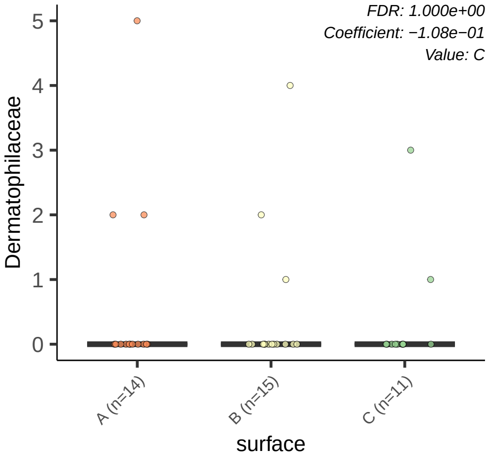

FDR: 1.000e+00
Coefficient: −1.08e−01
Value: C
Dermatophilaceae
0
1
2
3
4
5
A (n=14)
B (n=15)
C (n=11)
surface

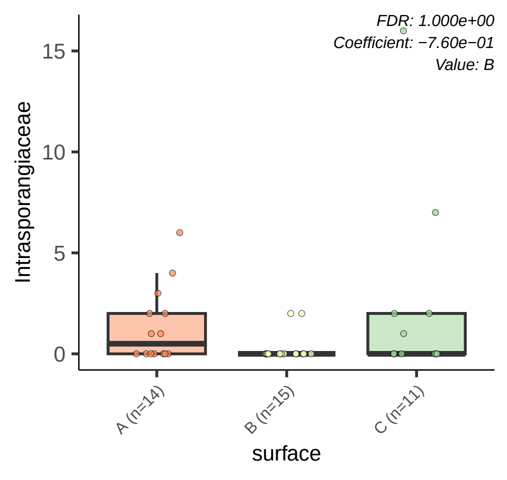

FDR: 1.000e+00
Coefficient: −7.60e−01
Value: B
Intrasporangiaceae
0
5
10
15
A (n=14)
B (n=15)
C (n=11)
surface

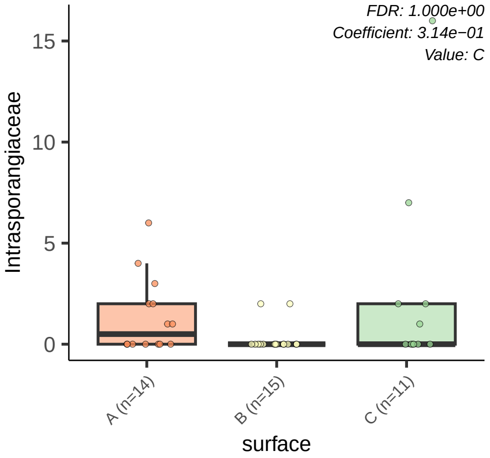
FDR: 1.000e+00
Coefficient: 3.14e−01
Value: C
Intrasporangiaceae
0
5
10
15
A (n=14)
B (n=15)
C (n=11)
surface

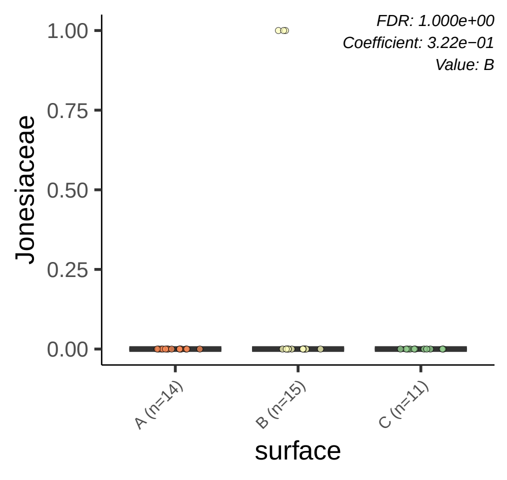

FDR: 1.000e+00
Coefficient: 3.22e−01
Value: B
1.00
0.75
0.50
0.25
0.00
Jonesiaceae
A (n=14)
B (n=15)
C (n=11)
surface

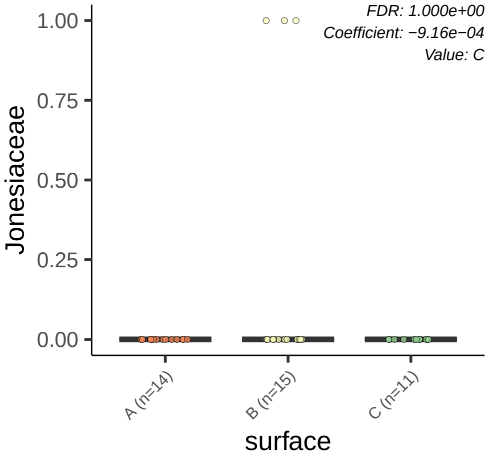

FDR: 1.000e+00
Coefficient: −9.16e−04
Value: C
1.00
0.75
0.50
0.25
0.00
Jonesiaceae
A (n=14)
B (n=15)
C (n=11)
surface

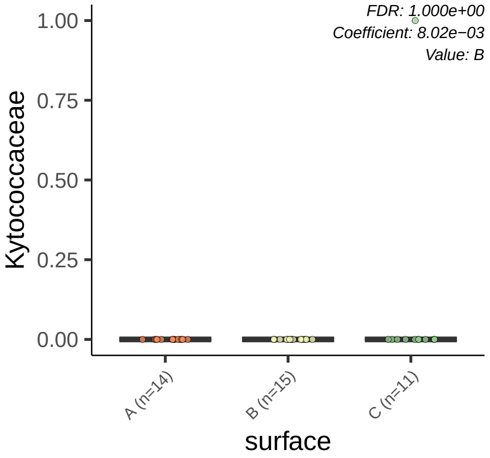

FDR: 1.000e+00
Coefficient: 8.02e−03
Value: B
Kytococcaceae
1.00
0.75
0.50
0.25
0.00
A (n=14)
B (n=15)
C (n=11)
surface

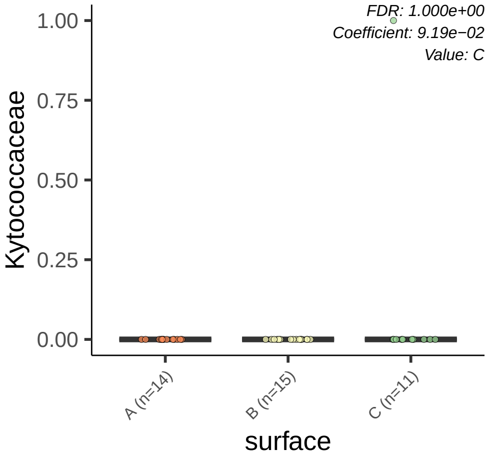
FDR: 1.000e+00
Coefficient: 9.19e−02
Value: C
Kytococcaceae
1.00
0.75
0.50
0.25
0.00
A (n=14)
B (n=15)
C (n=11)
surface

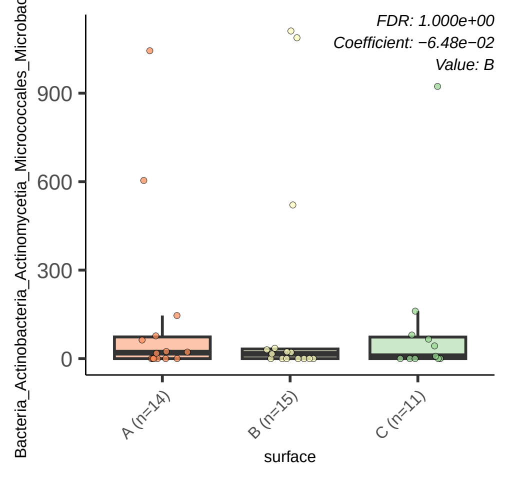

FDR: 1.000e+00
Coefficient: −6.48e−02
Value: B
Bacteria_Actinobacteria_Actinomycetia_Micrococcales_Microbac
900
600
300
0
A (n=14)
B (n=15)
C (n=11)
surface

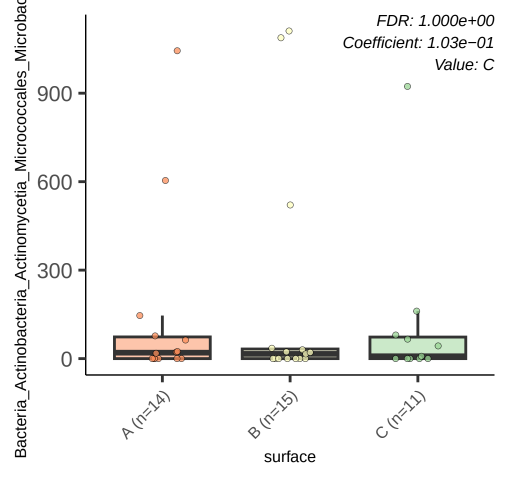

FDR: 1.000e+00
Coefficient: 1.03e−01
Value: C
Bacteria_Actinobacteria_Actinomycetia_Micrococcales_Microbac
900
600
300
0
A (n=14)
B (n=15)
C (n=11)
surface

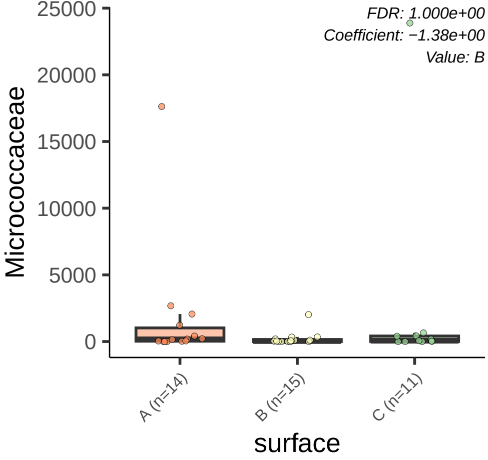

FDR: 1.000e+00
Coefficient: −1.38e+00
Value: B
Micrococcaceae
25000
20000
15000
10000
5000
0
A (n=14)
B (n=15)
C (n=11)
surface

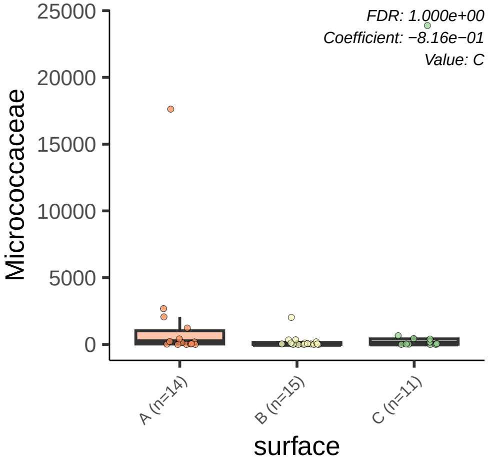
FDR: 1.000e+00
Coefficient: −8.16e−01
Value: C
Micrococcaceae
25000
20000
15000
10000
5000
0
A (n=14)
B (n=15)
C (n=11)
surface

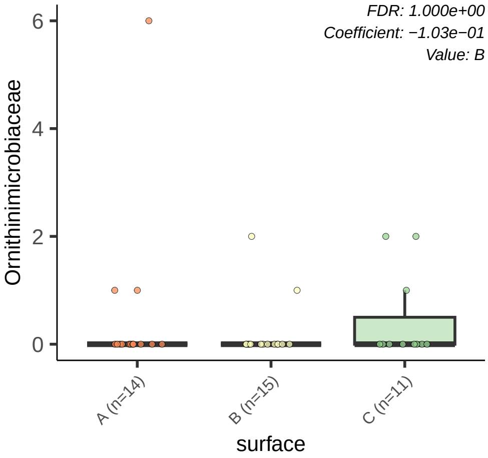
FDR: 1.000e+00
Coefficient: −1.03e−01
Value: B
Ornithinimicrobiaceae
6
4
2
0
A (n=14)
B (n=15)
C (n=11)
surface

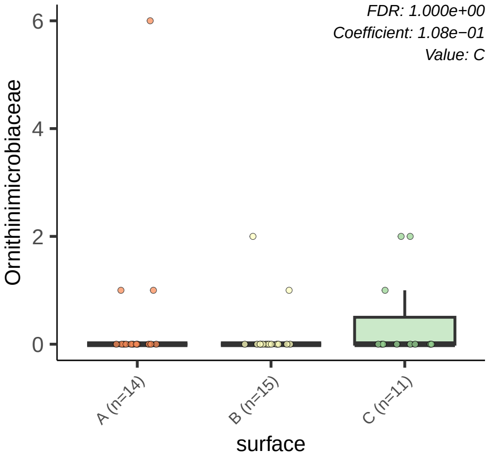

FDR: 1.000e+00
Coefficient: 1.08e−01
Value: C
Ornithinimicrobiaceae
6
4
2
0
A (n=14)
B (n=15)
C (n=11)
surface

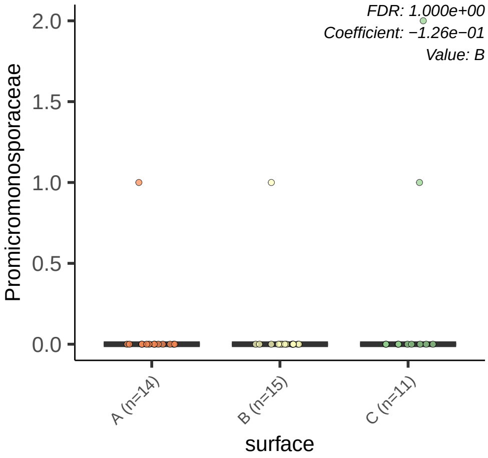
FDR: 1.000e+00
Coefficient: −1.26e−01
Value: B
2.0
1.5
1.0
0.5
0.0
Promicromonosporaceae
A (n=14)
B (n=15)
C (n=11)
surface

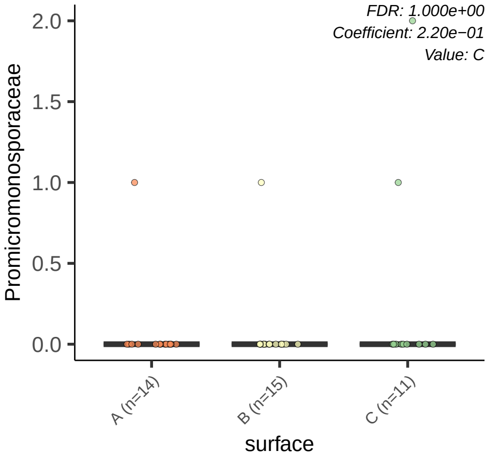
FDR: 1.000e+00
Coefficient: 2.20e−01
Value: C
Promicromonosporaceae
2.0
1.5
1.0
0.5
0.0
A (n=14)
B (n=15)
C (n=11)
surface

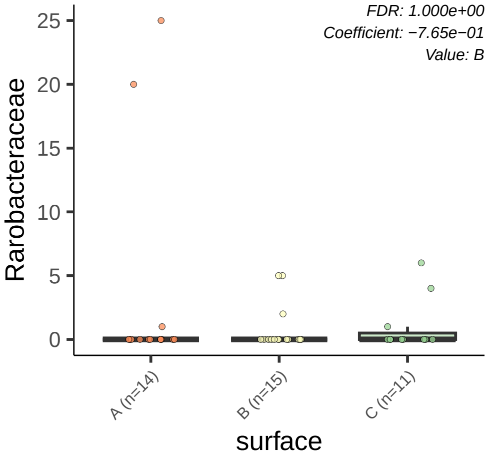
FDR: 1.000e+00
Coefficient: −7.65e−01
Value: B
Rarobacteraceae
25
20
15
10
5
0
A (n=14)
B (n=15)
C (n=11)
surface

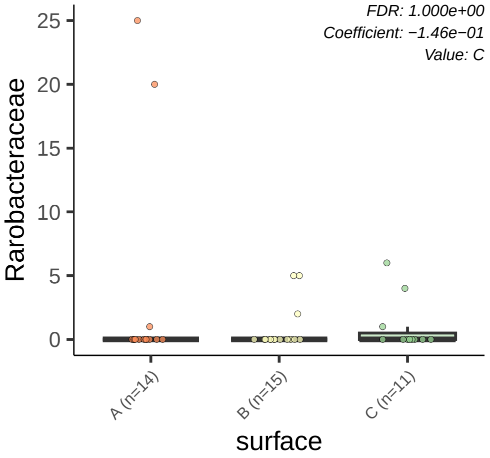
FDR: 1.000e+00
Coefficient: −1.46e−01
Value: C
Rarobacteraceae
25
20
15
10
5
0
A (n=14)
B (n=15)
C (n=11)
surface

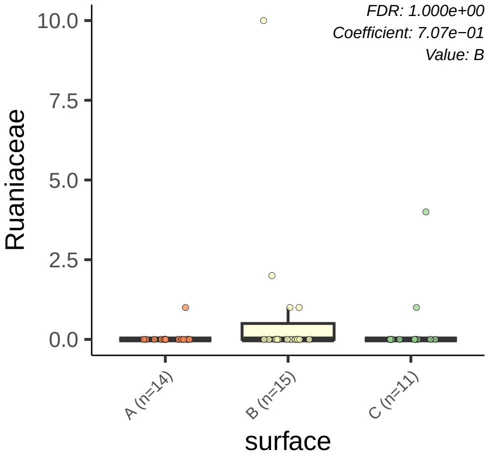
FDR: 1.000e+00
Coefficient: 7.07e−01
Value: B
Ruaniaceae
10.0
7.5
5.0
2.5
0.0
A (n=14)
B (n=15)
C (n=11)
surface

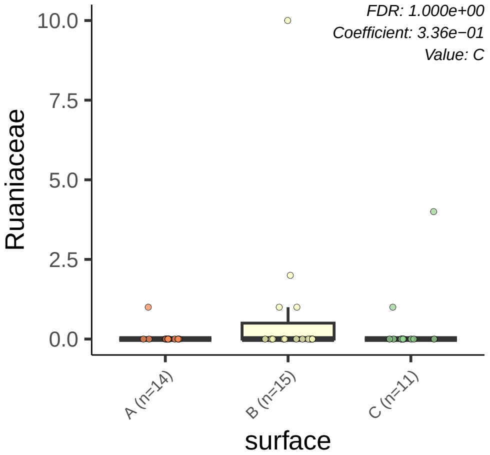

FDR: 1.000e+00
Coefficient: 3.36e−01
Value: C
Ruaniaceae
10.0
7.5
5.0
2.5
0.0
A (n=14)
B (n=15)
C (n=11)
surface

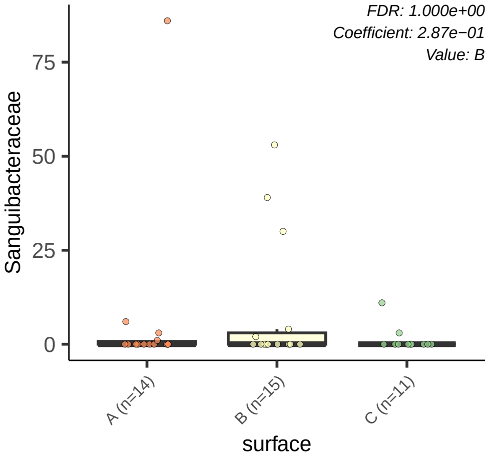

FDR: 1.000e+00
Coefficient: 2.87e−01
Value: B
Sanguibacteraceae
0
25
50
75
A (n=14)
B (n=15)
C (n=11)
surface

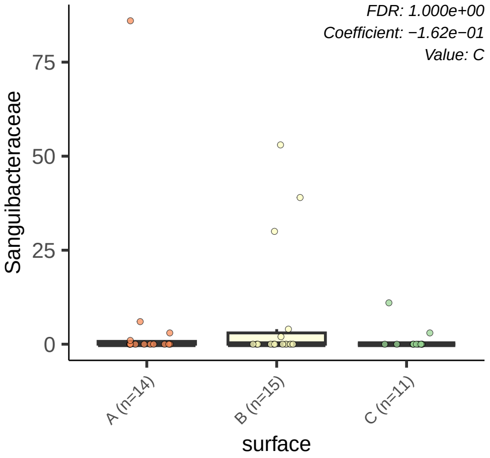
FDR: 1.000e+00
Coefficient: −1.62e−01
Value: C
Sanguibacteraceae
75
50
25
0
A (n=14)
B (n=15)
C (n=11)
surface

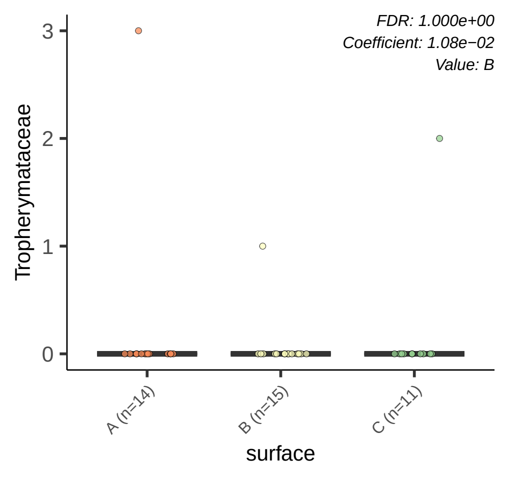

FDR: 1.000e+00
Coefficient: 1.08e−02
Value: B
Tropherymataceae
3
2
1
0
A (n=14)
B (n=15)
C (n=11)
surface

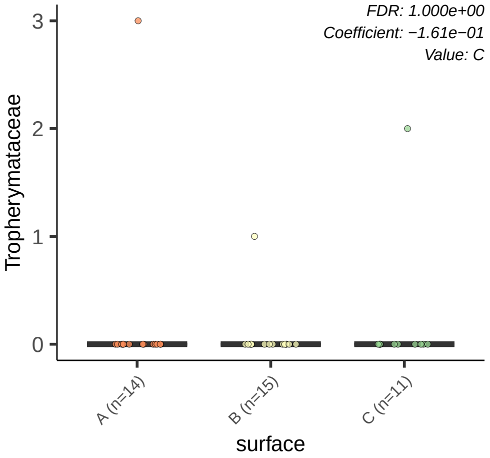

FDR: 1.000e+00
Coefficient: −1.61e−01
Value: C
Tropherymataceae
3
2
1
0
A (n=14)
B (n=15)
C (n=11)
surface

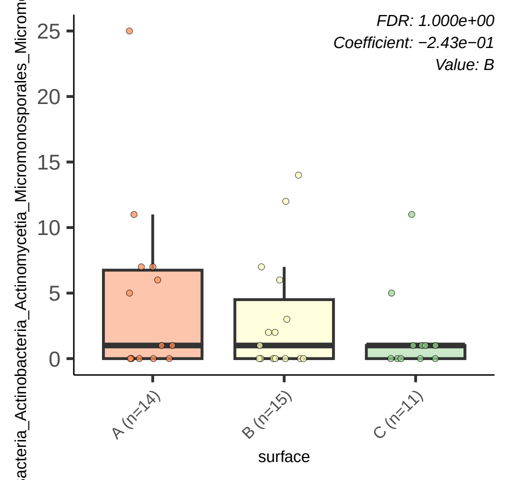
FDR: 1.000e+00
Coefficient: −2.43e−01
Value: B
25
20
15
10
5
0
A (n=14)
B (n=15)
C (n=11)
surface

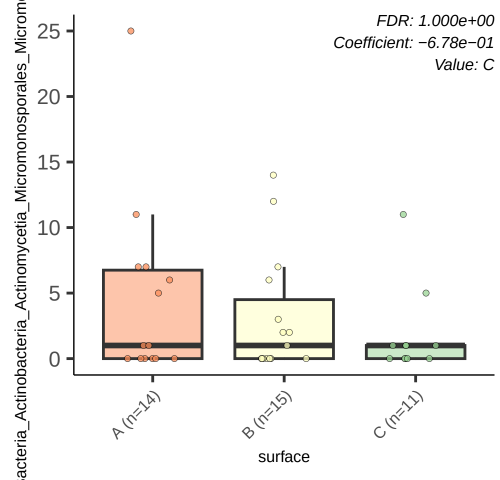

FDR: 1.000e+00
Coefficient: −6.78e−01
Value: C
0
5
10
15
20
25
A (n=14)
B (n=15)
C (n=11)
surface
acteria_Actinobacteria_Actinomycetia_Micromonosporales_Micromo

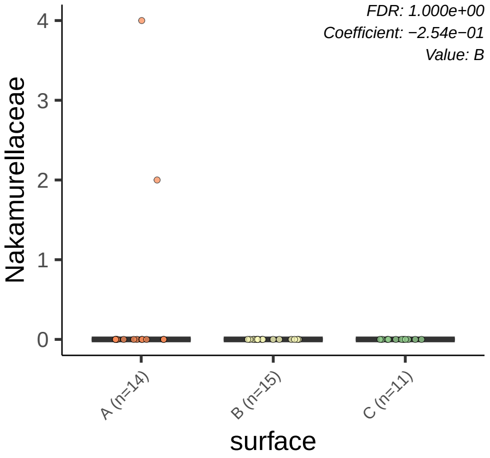

FDR: 1.000e+00
Coefficient: −2.54e−01
Value: B
Nakamurellaceae
0
1
2
3
4
A (n=14)
B (n=15)
C (n=11)
surface

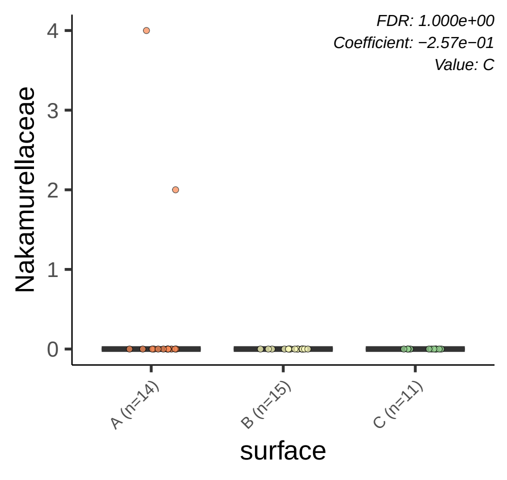

FDR: 1.000e+00
Coefficient: −2.57e−01
Value: C
Nakamurellaceae
4
3
2
1
0
A (n=14)
B (n=15)
C (n=11)
surface

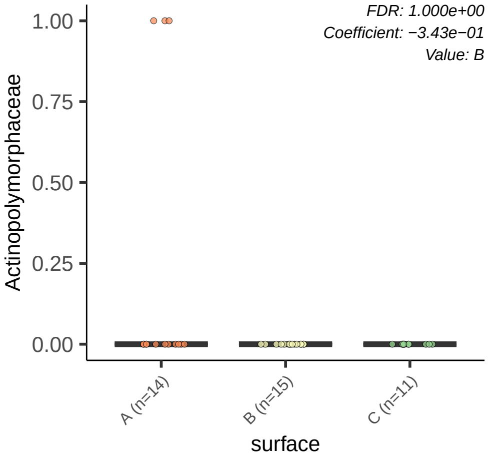

FDR: 1.000e+00
Coefficient: −3.43e−01
Value: B
Actinopolymorphaceae
1.00
0.75
0.50
0.25
0.00
A (n=14)
B (n=15)
C (n=11)
surface

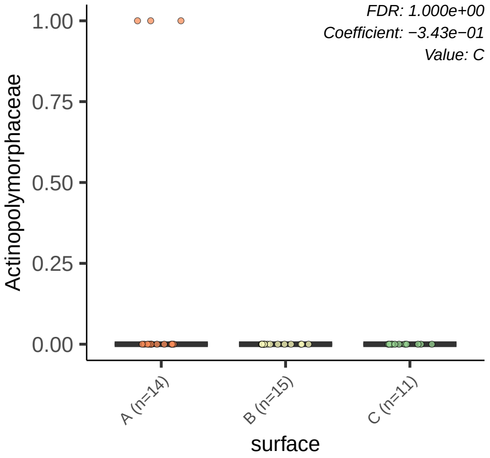
FDR: 1.000e+00
Coefficient: −3.43e−01
Value: C
Actinopolymorphaceae
1.00
0.75
0.50
0.25
0.00
A (n=14)
B (n=15)
C (n=11)
surface

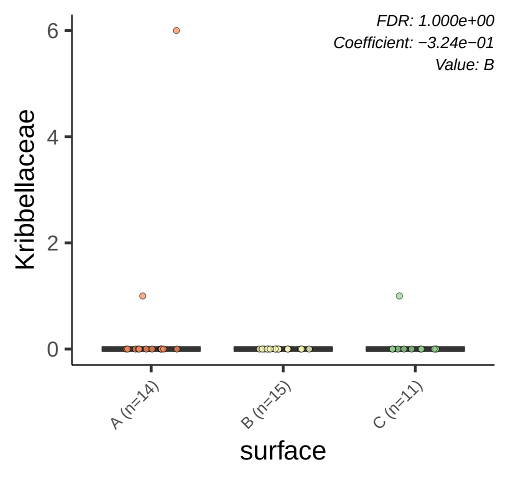

FDR: 1.000e+00
Coefficient: −3.24e−01
Value: B
Kribbellaceae
6
4
2
0
A (n=14)
B (n=15)
C (n=11)
surface

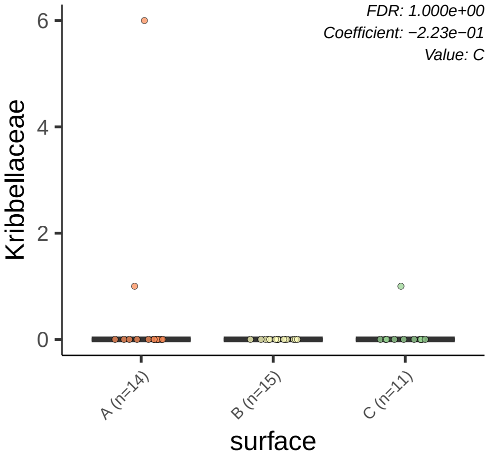

FDR: 1.000e+00
Coefficient: −2.23e−01
Value: C
Kribbellaceae
6
4
2
0
A (n=14)
B (n=15)
C (n=11)
surface

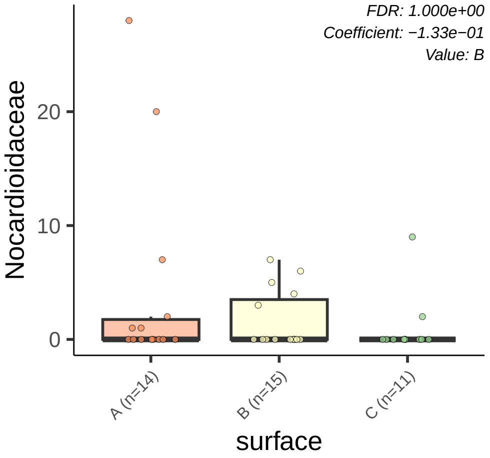

FDR: 1.000e+00
Coefficient: −1.33e−01
Value: B
Nocardioidaceae
20
10
0
A (n=14)
B (n=15)
C (n=11)
surface

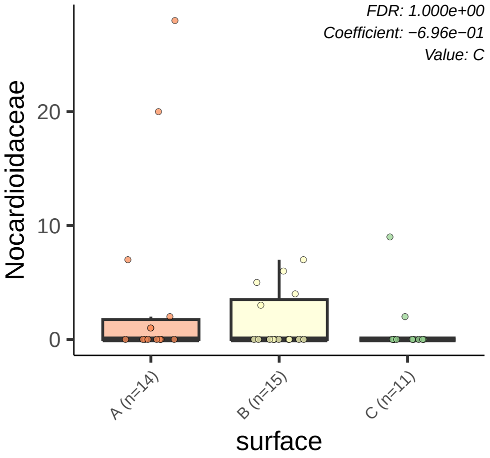
FDR: 1.000e+00
Coefficient: −6.96e−01
Value: C
Nocardioidaceae
20
10
0
A (n=14)
B (n=15)
C (n=11)
surface

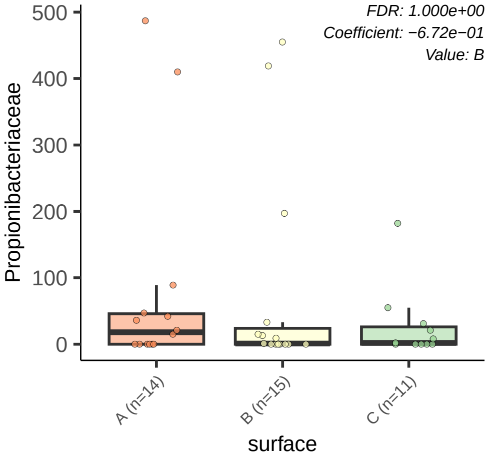
FDR: 1.000e+00
Coefficient: −6.72e−01
Value: B
Propionibacteriaceae
500
400
300
200
100
0
A (n=14)
B (n=15)
C (n=11)
surface

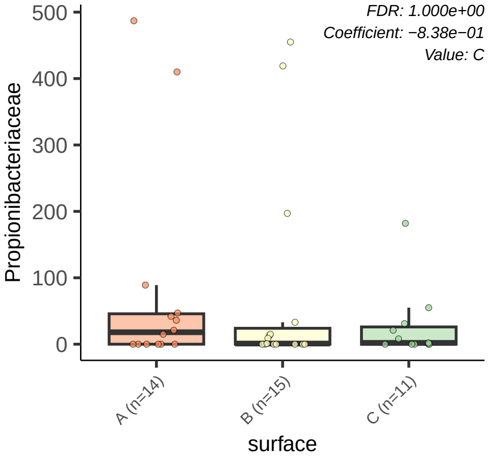

FDR: 1.000e+00
Coefficient: −8.38e−01
Value: C
Propionibacteriaceae
500
400
300
200
100
0
A (n=14)
B (n=15)
C (n=11)
surface

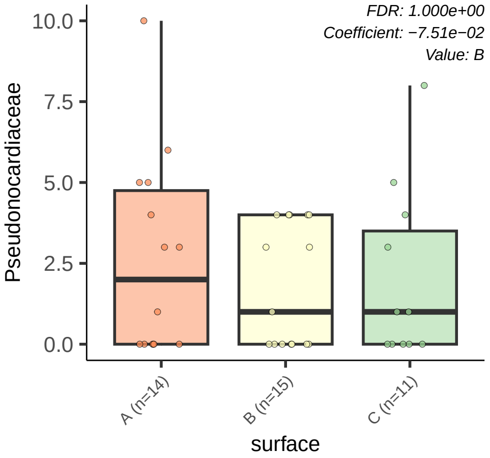
FDR: 1.000e+00
Coefficient: −7.51e−02
Value: B
Pseudonocardiaceae
10.0
7.5
5.0
2.5
0.0
A (n=14)
B (n=15)
C (n=11)
surface

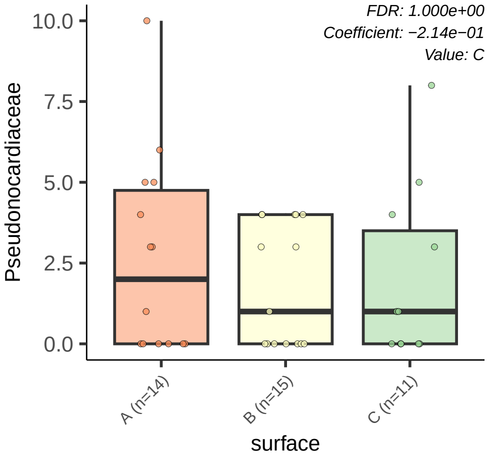

FDR: 1.000e+00
Coefficient: −2.14e−01
Value: C
Pseudonocardiaceae
10.0
7.5
5.0
2.5
0.0
A (n=14)
B (n=15)
C (n=11)
surface

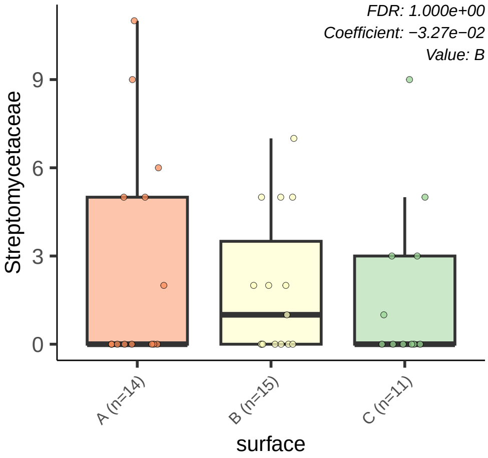

FDR: 1.000e+00
Coefficient: −3.27e−02
Value: B
Streptomycetaceae
9
6
3
0
A (n=14)
B (n=15)
C (n=11)
surface

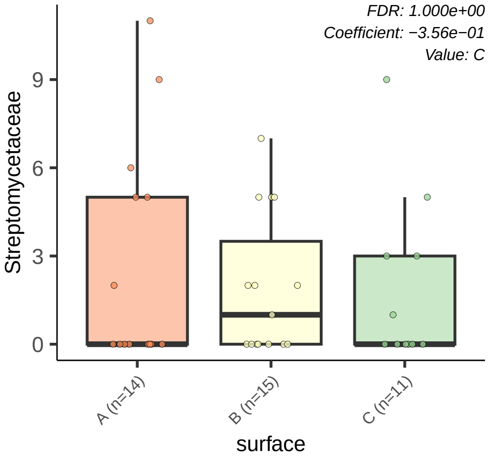

FDR: 1.000e+00
Coefficient: −3.56e−01
Value: C
Streptomycetaceae
0
3
6
9
A (n=14)
B (n=15)
C (n=11)
surface

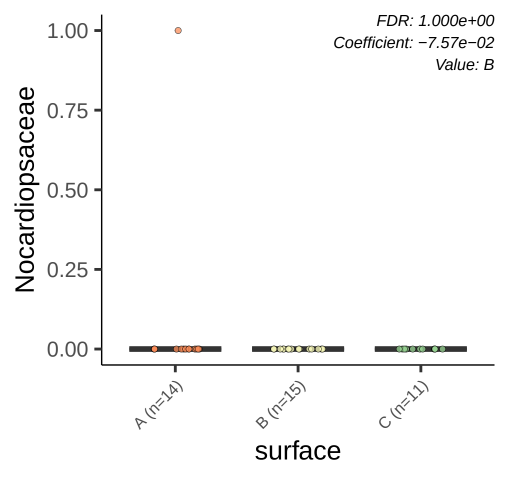

FDR: 1.000e+00
Coefficient: −7.57e−02
Value: B
Nocardiopsaceae
1.00
0.75
0.50
0.25
0.00
A (n=14)
B (n=15)
C (n=11)
surface

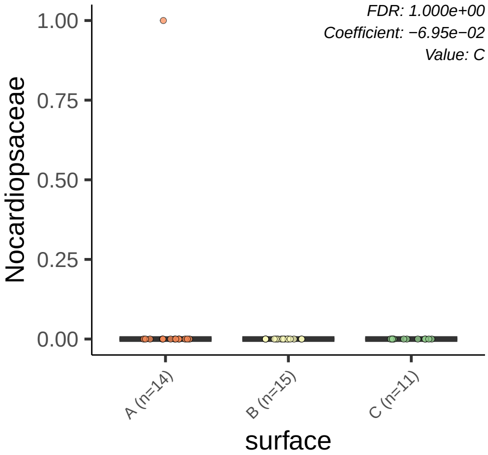

FDR: 1.000e+00
Coefficient: −6.95e−02
Value: C
Nocardiopsaceae
1.00
0.75
0.50
0.25
0.00
A (n=14)
B (n=15)
C (n=11)
surface

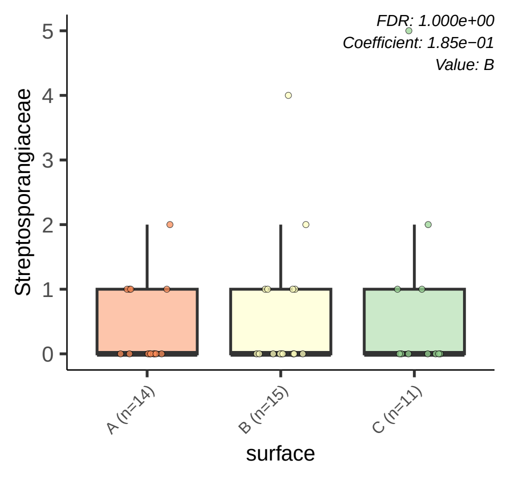

FDR: 1.000e+00
Coefficient: 1.85e−01
Value: B
Streptosporangiaceae
0
1
2
3
4
5
A (n=14)
B (n=15)
C (n=11)
surface

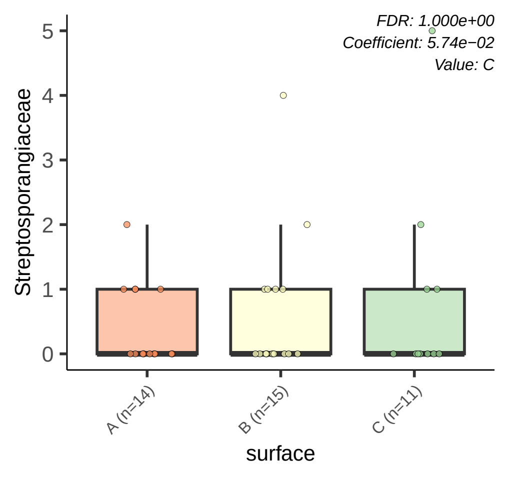

FDR: 1.000e+00
Coefficient: 5.74e−02
Value: C
Streptosporangiaceae
0
1
2
3
4
5
A (n=14)
B (n=15)
C (n=11)
surface

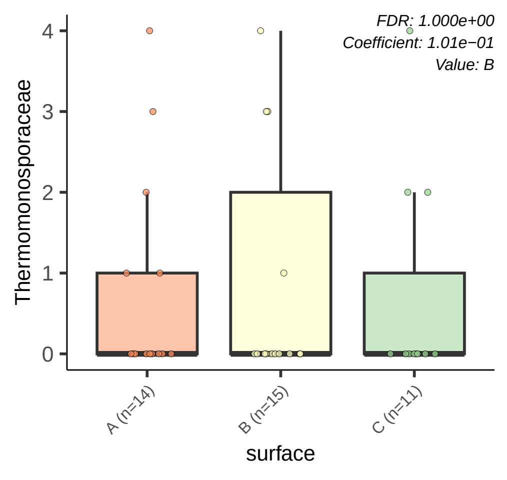

FDR: 1.000e+00
Coefficient: 1.01e−01
Value: B
Thermomonosporaceae
0
1
2
3
4
A (n=14)
B (n=15)
C (n=11)
surface

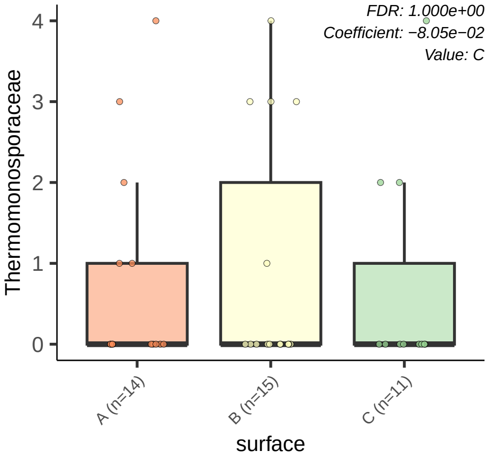

FDR: 1.000e+00
Coefficient: −8.05e−02
Value: C
Thermomonosporaceae
4
3
2
1
0
A (n=14)
B (n=15)
C (n=11)
surface

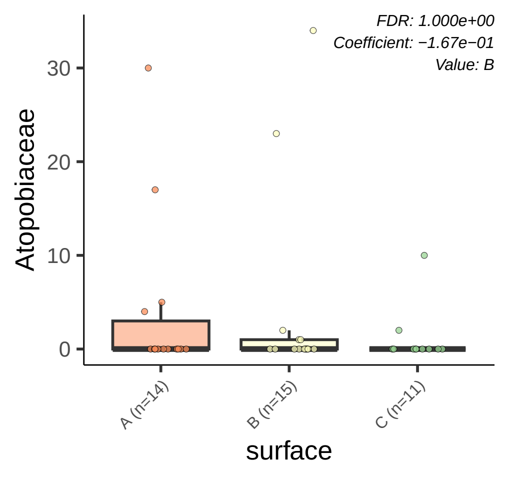

FDR: 1.000e+00
Coefficient: −1.67e−01
Value: B
Atopobiaceae
30
20
10
0
A (n=14)
B (n=15)
C (n=11)
surface

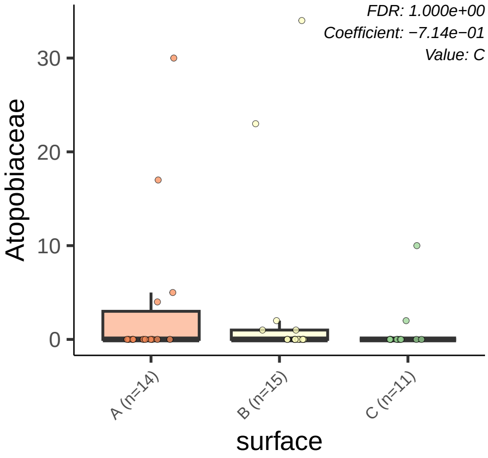
FDR: 1.000e+00
Coefficient: −7.14e−01
Value: C
Atopobiaceae
30
20
10
0
A (n=14)
B (n=15)
C (n=11)
surface

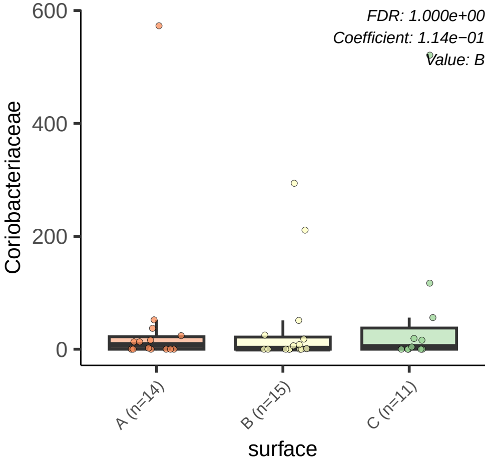
FDR: 1.000e+00
Coefficient: 1.14e−01
Value: B
Coriobacteriaceae
0
200
400
600
A (n=14)
B (n=15)
C (n=11)
surface

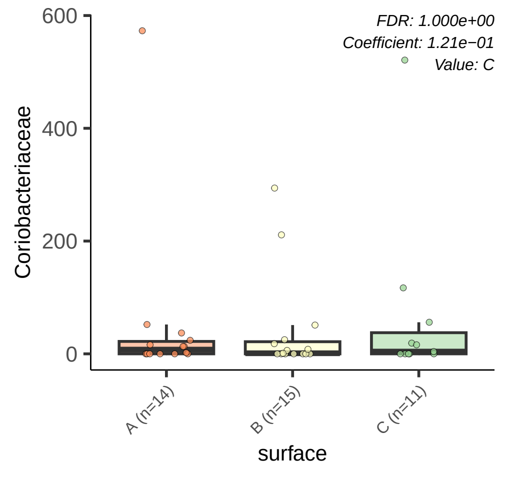
FDR: 1.000e+00
Coefficient: 1.21e−01
Value: C
Coriobacteriaceae
600
400
200
0
A (n=14)
B (n=15)
C (n=11)
surface

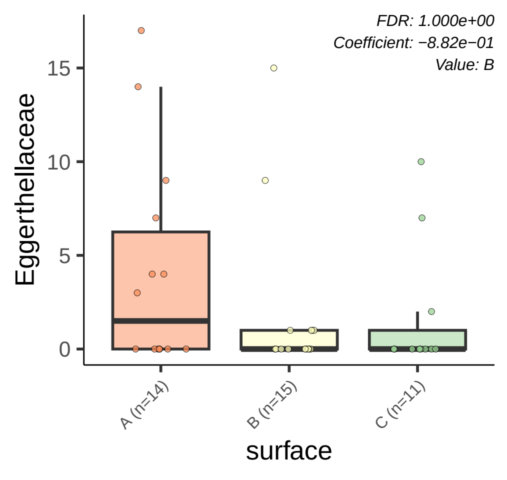

FDR: 1.000e+00
Coefficient: −8.82e−01
Value: B
Eggerthellaceae
0
5
10
15
A (n=14)
B (n=15)
C (n=11)
surface

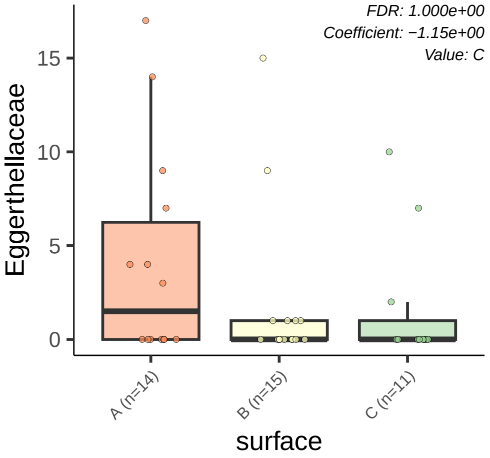

FDR: 1.000e+00
Coefficient: −1.15e+00
Value: C
Eggerthellaceae
15
10
5
0
A (n=14)
B (n=15)
C (n=11)
surface

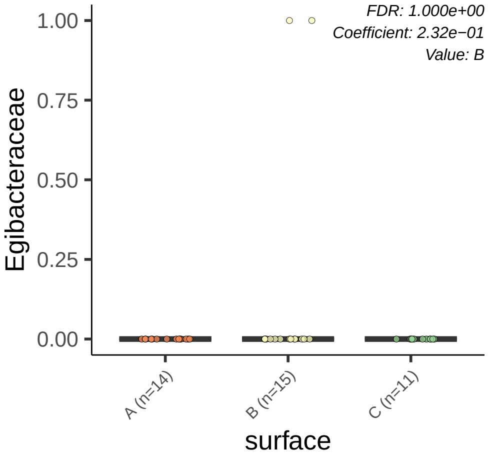

FDR: 1.000e+00
Coefficient: 2.32e−01
Value: B
Egibacteraceae
1.00
0.75
0.50
0.25
0.00
A (n=14)
B (n=15)
C (n=11)
surface

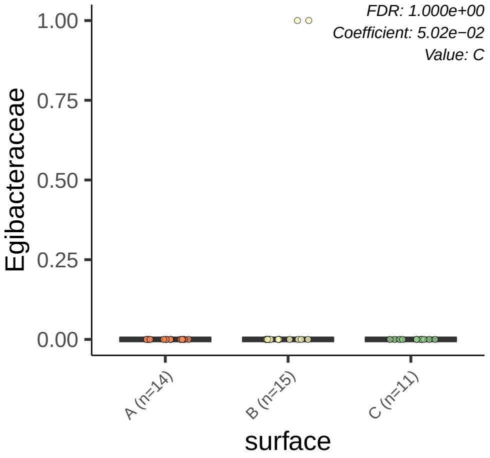

FDR: 1.000e+00
Coefficient: 5.02e−02
Value: C
Egibacteraceae
1.00
0.75
0.50
0.25
0.00
A (n=14)
B (n=15)
C (n=11)
surface

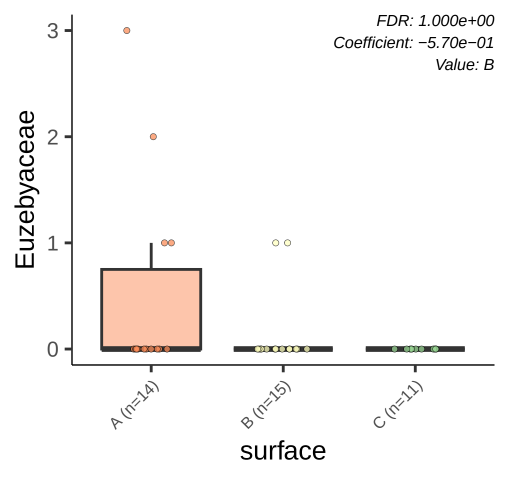

FDR: 1.000e+00
Coefficient: −5.70e−01
Value: B
Euzebyaceae
0
1
2
3
A (n=14)
B (n=15)
C (n=11)
surface

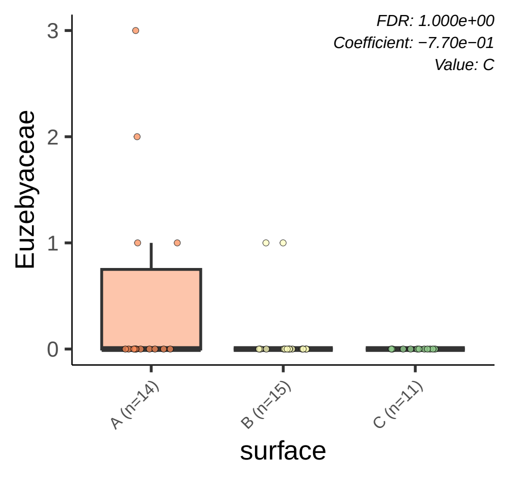

FDR: 1.000e+00
Coefficient: −7.70e−01
Value: C
Euzebyaceae
3
2
1
0
A (n=14)
B (n=15)
C (n=11)
surface

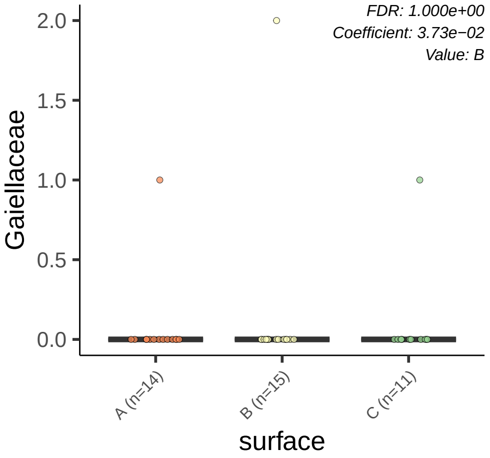

FDR: 1.000e+00
Coefficient: 3.73e−02
Value: B
Gaiellaceae
2.0
1.5
1.0
0.5
0.0
A (n=14)
B (n=15)
C (n=11)
surface

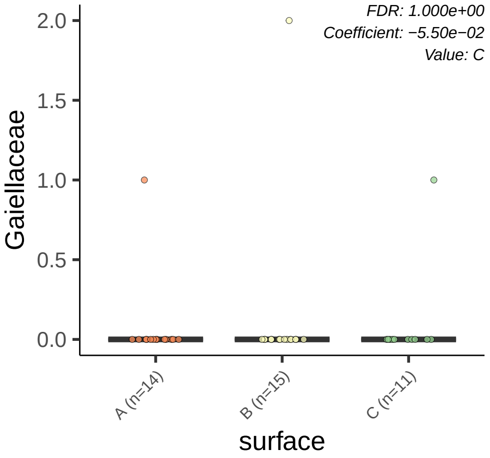
FDR: 1.000e+00
Coefficient: −5.50e−02
Value: C
Gaiellaceae
2.0
1.5
1.0
0.5
0.0
A (n=14)
B (n=15)
C (n=11)
surface

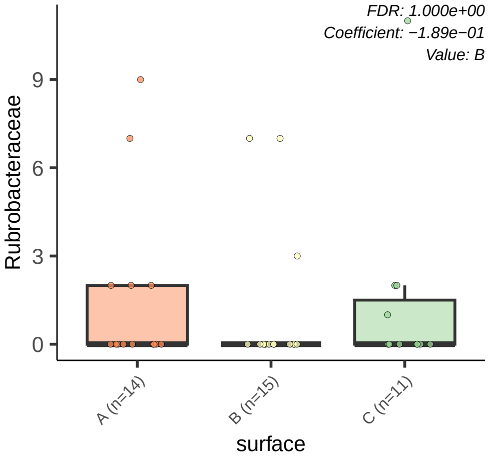
FDR: 1.000e+00
Coefficient: −1.89e−01
Value: B
Rubrobacteraceae
0
3
6
9
A (n=14)
B (n=15)
C (n=11)
surface

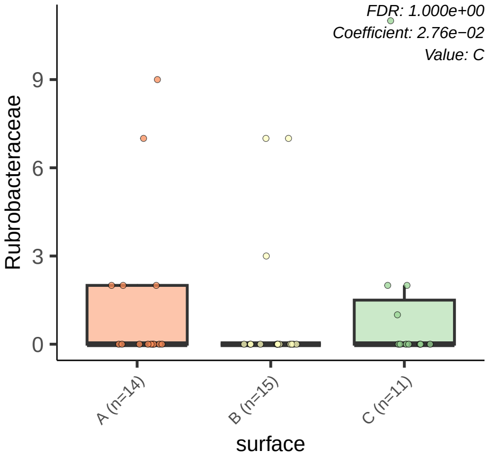
FDR: 1.000e+00
Coefficient: 2.76e−02
Value: C
Rubrobacteraceae
0
3
6
9
A (n=14)
B (n=15)
C (n=11)
surface

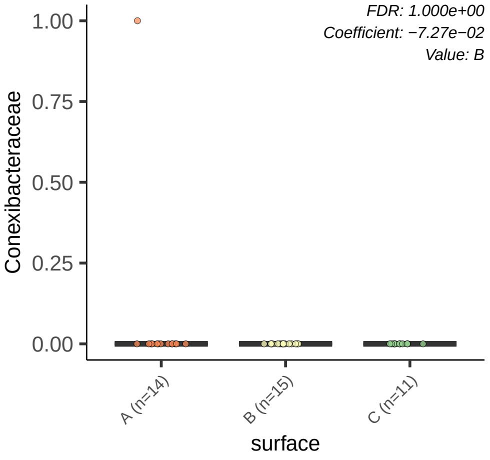
FDR: 1.000e+00
Coefficient: −7.27e−02
Value: B
1.00
0.75
0.50
0.25
0.00
Conexibacteraceae
A (n=14)
B (n=15)
C (n=11)
surface

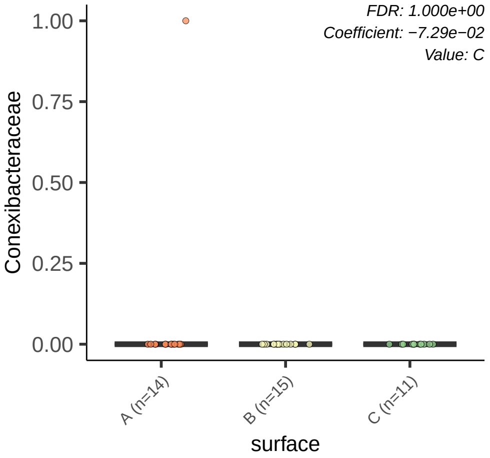

FDR: 1.000e+00
Coefficient: −7.29e−02
Value: C
Conexibacteraceae
1.00
0.75
0.50
0.25
0.00
A (n=14)
B (n=15)
C (n=11)
surface

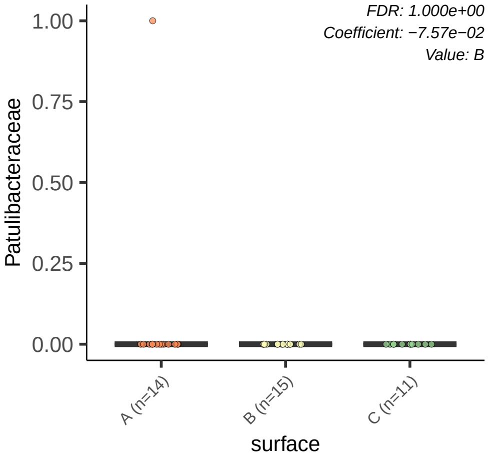
FDR: 1.000e+00
Coefficient: −7.57e−02
Value: B
Patulibacteraceae
1.00
0.75
0.50
0.25
0.00
A (n=14)
B (n=15)
C (n=11)
surface

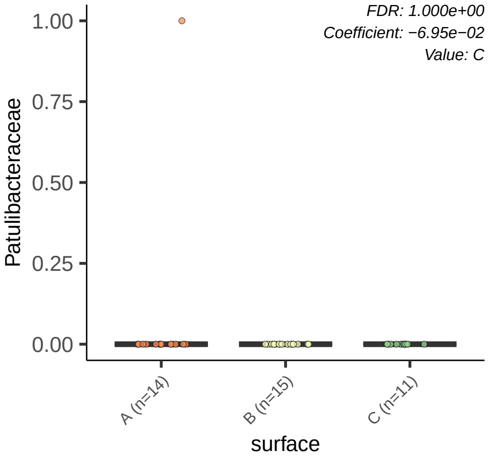
FDR: 1.000e+00
Coefficient: −6.95e−02
Value: C
Patulibacteraceae
1.00
0.75
0.50
0.25
0.00
A (n=14)
B (n=15)
C (n=11)
surface

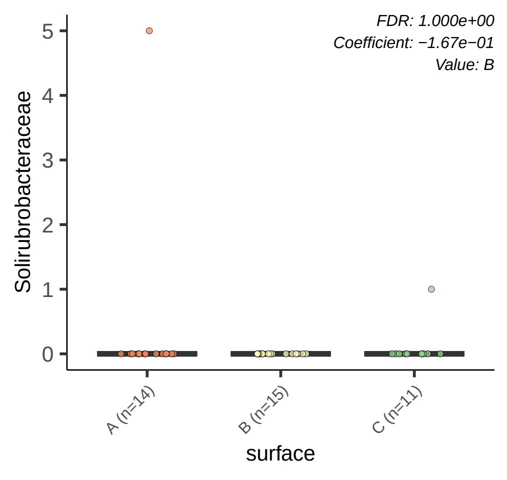
FDR: 1.000e+00
Coefficient: −1.67e−01
Value: B
Solirubrobacteraceae
5
4
3
2
1
0
A (n=14)
B (n=15)
C (n=11)
surface

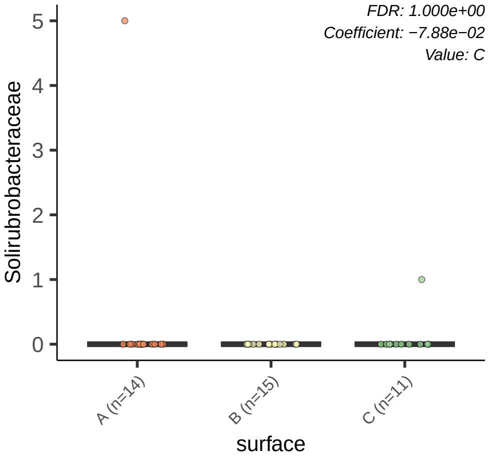

FDR: 1.000e+00
Coefficient: −7.88e−02
Value: C
Solirubrobacteraceae
0
1
2
3
4
5
A (n=14)
B (n=15)
C (n=11)
surface

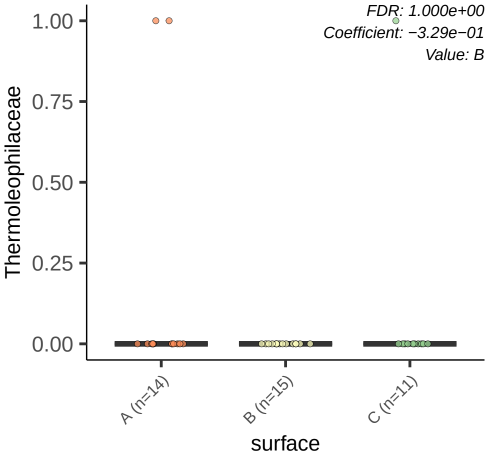
FDR: 1.000e+00
Coefficient: −3.29e−01
Value: B
Thermoleophilaceae
1.00
0.75
0.50
0.25
0.00
A (n=14)
B (n=15)
C (n=11)
surface

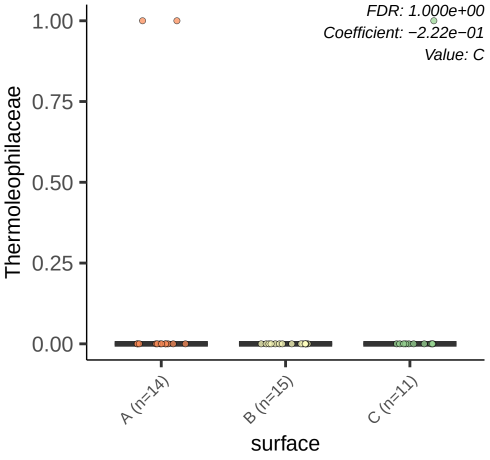
FDR: 1.000e+00
Coefficient: −2.22e−01
Value: C
Thermoleophilaceae
1.00
0.75
0.50
0.25
0.00
A (n=14)
B (n=15)
C (n=11)
surface

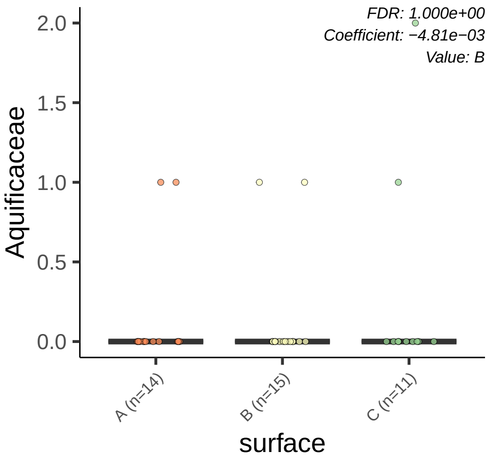
FDR: 1.000e+00
Coefficient: −4.81e−03
Value: B
Aquificaceae
2.0
1.5
1.0
0.5
0.0
A (n=14)
B (n=15)
C (n=11)
surface

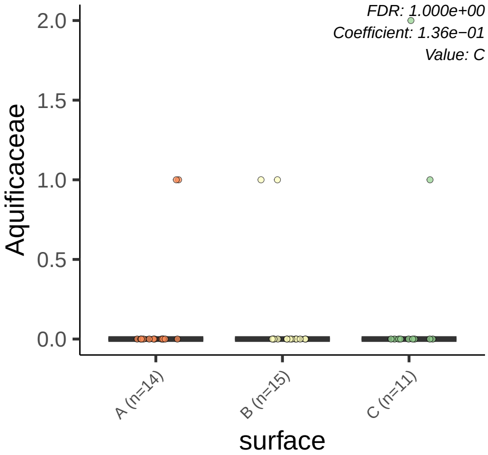
FDR: 1.000e+00
Coefficient: 1.36e−01
Value: C
Aquificaceae
2.0
1.5
1.0
0.5
0.0
A (n=14)
B (n=15)
C (n=11)
surface

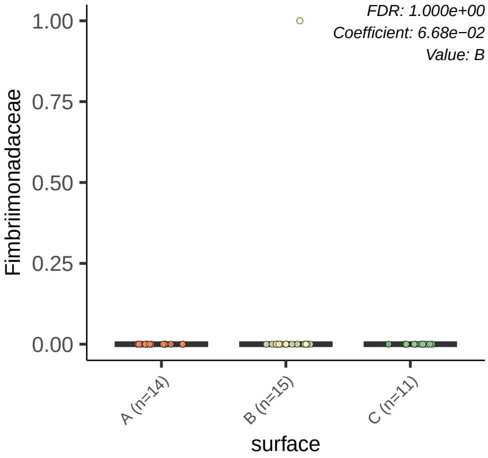

FDR: 1.000e+00
Coefficient: 6.68e−02
Value: B
Fimbriimonadaceae
1.00
0.75
0.50
0.25
0.00
A (n=14)
B (n=15)
C (n=11)
surface

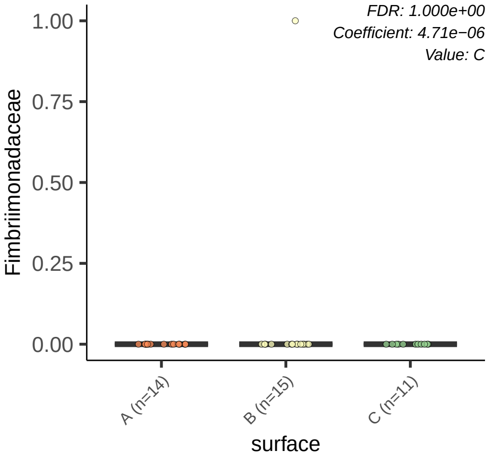
FDR: 1.000e+00
Coefficient: 4.71e−06
Value: C
Fimbriimonadaceae
1.00
0.75
0.50
0.25
0.00
A (n=14)
B (n=15)
C (n=11)
surface

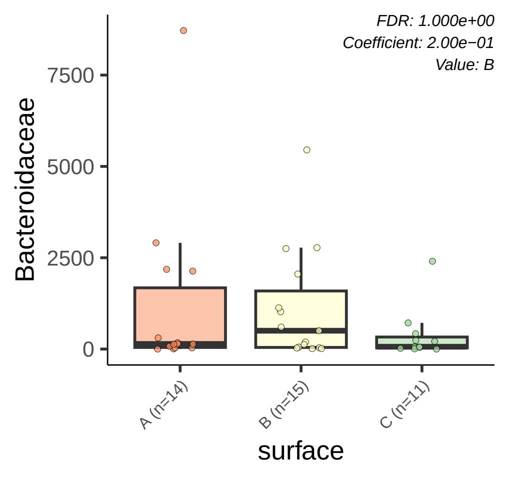
FDR: 1.000e+00
Coefficient: 2.00e−01
Value: B
Bacteroidaceae
7500
5000
2500
0
A (n=14)
B (n=15)
C (n=11)
surface

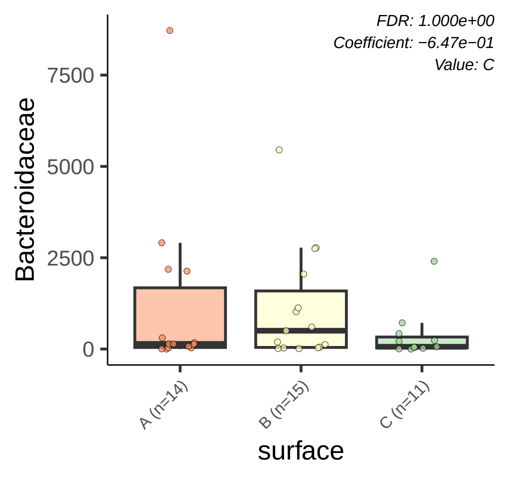
FDR: 1.000e+00
Coefficient: −6.47e−01
Value: C
Bacteroidaceae
7500
5000
2500
0
A (n=14)
B (n=15)
C (n=11)
surface

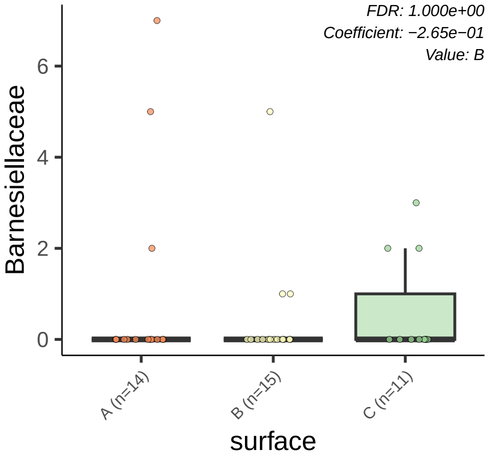

FDR: 1.000e+00
Coefficient: −2.65e−01
Value: B
Barnesiellaceae
0
2
4
6
A (n=14)
B (n=15)
C (n=11)
surface

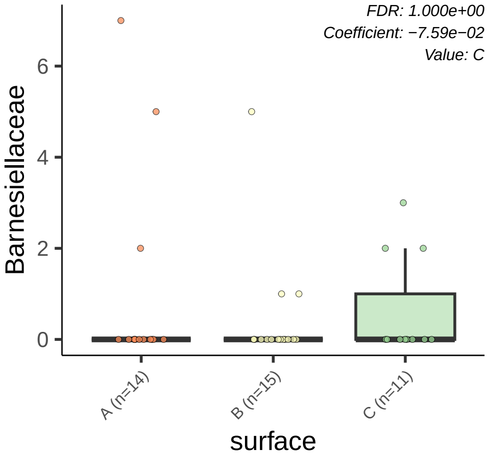
FDR: 1.000e+00
Coefficient: −7.59e−02
Value: C
Barnesiellaceae
0
2
4
6
A (n=14)
B (n=15)
C (n=11)
surface

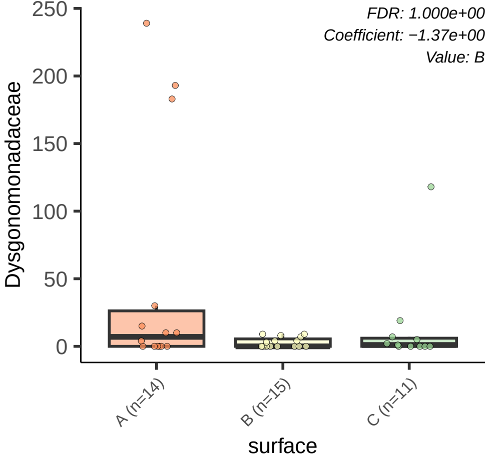

FDR: 1.000e+00
Coefficient: −1.37e+00
Value: B
Dysgonomonadaceae
250
200
150
100
50
0
A (n=14)
B (n=15)
C (n=11)
surface

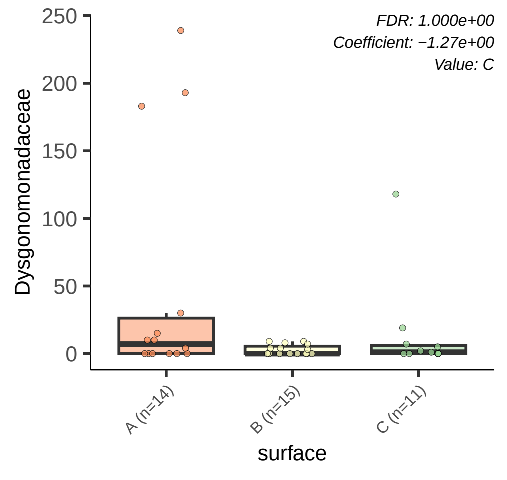

FDR: 1.000e+00
Coefficient: −1.27e+00
Value: C
Dysgonomonadaceae
250
200
150
100
50
0
A (n=14)
B (n=15)
C (n=11)
surface

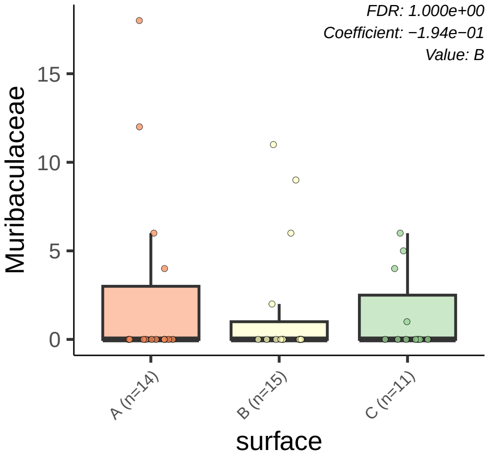
FDR: 1.000e+00
Coefficient: −1.94e−01
Value: B
Muribaculaceae
15
10
5
0
A (n=14)
B (n=15)
C (n=11)
surface

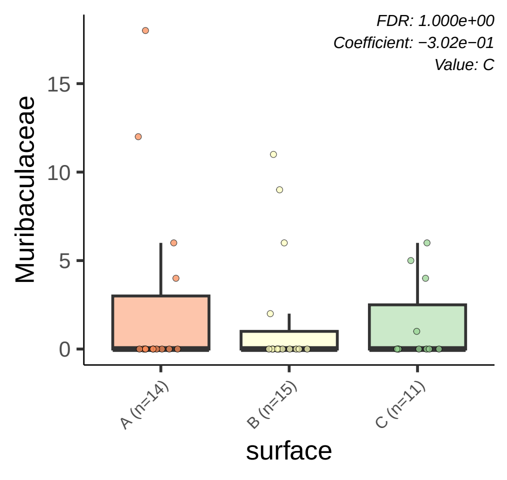

FDR: 1.000e+00
Coefficient: −3.02e−01
Value: C
Muribaculaceae
0
5
10
15
A (n=14)
B (n=15)
C (n=11)
surface

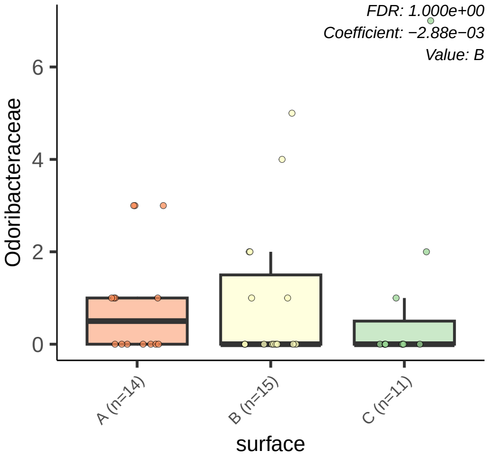
FDR: 1.000e+00
Coefficient: −2.88e−03
Value: B
Odoribacteraceae
0
2
4
6
A (n=14)
B (n=15)
C (n=11)
surface

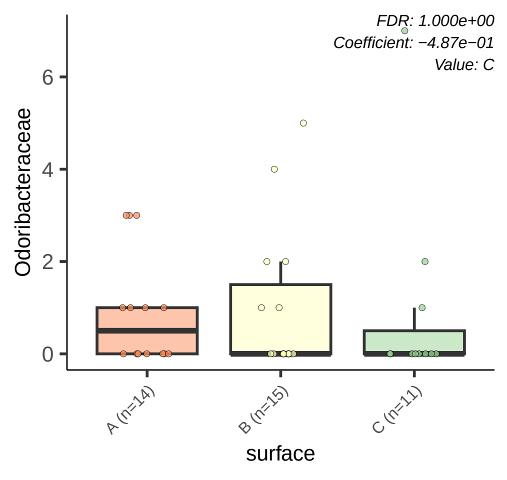

FDR: 1.000e+00
Coefficient: −4.87e−01
Value: C
Odoribacteraceae
0
2
4
6
A (n=14)
B (n=15)
C (n=11)
surface

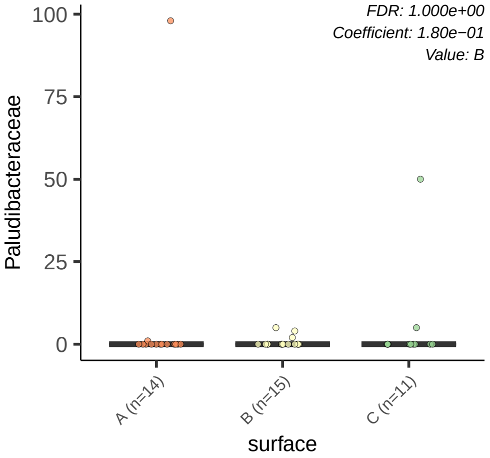

FDR: 1.000e+00
Coefficient: 1.80e−01
Value: B
Paludibacteraceae
100
75
50
25
0
A (n=14)
B (n=15)
C (n=11)
surface

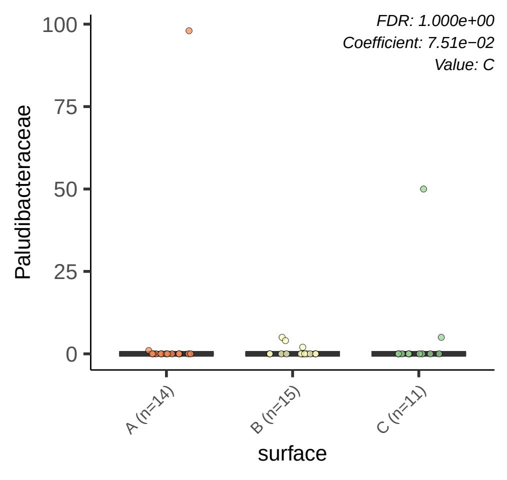

FDR: 1.000e+00
Coefficient: 7.51e−02
Value: C
Paludibacteraceae
100
75
50
25
0
A (n=14)
B (n=15)
C (n=11)
surface

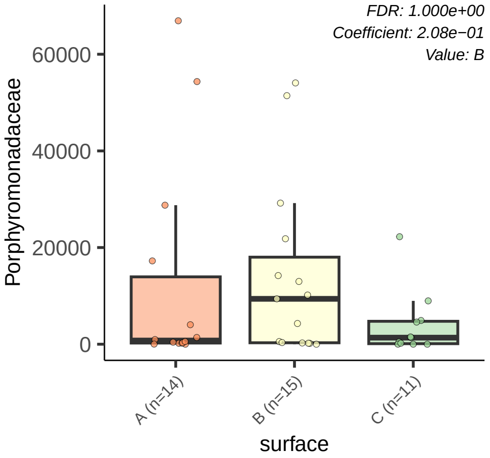

FDR: 1.000e+00
Coefficient: 2.08e−01
Value: B
Porphyromonadaceae
60000
40000
20000
0
A (n=14)
B (n=15)
C (n=11)
surface

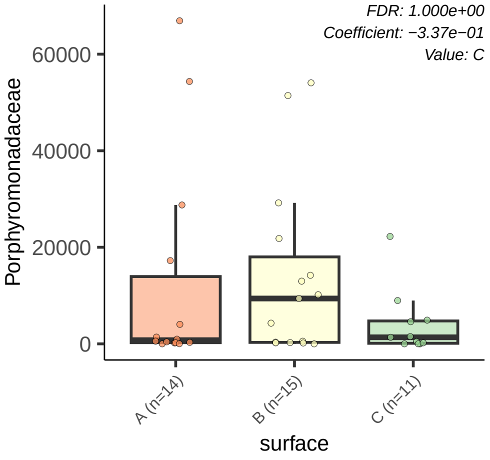

FDR: 1.000e+00
Coefficient: −3.37e−01
Value: C
Porphyromonadaceae
60000
40000
20000
0
A (n=14)
B (n=15)
C (n=11)
surface

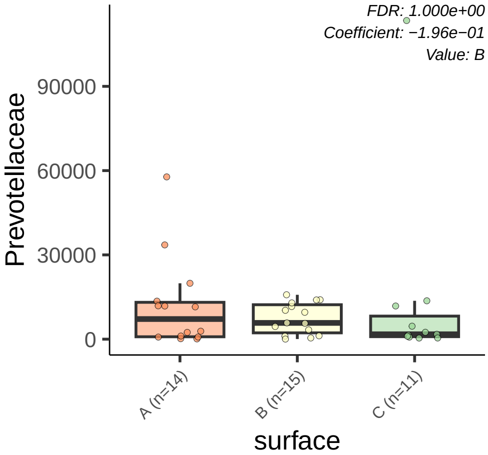

FDR: 1.000e+00
Coefficient: −1.96e−01
Value: B
Prevotellaceae
90000
60000
30000
0
A (n=14)
B (n=15)
C (n=11)
surface

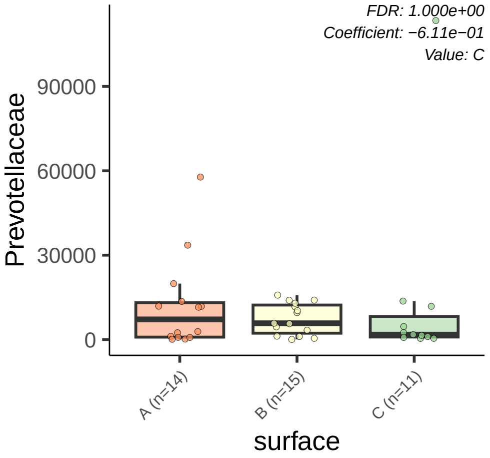

FDR: 1.000e+00
Coefficient: −6.11e−01
Value: C
Prevotellaceae
90000
60000
30000
0
A (n=14)
B (n=15)
C (n=11)
surface

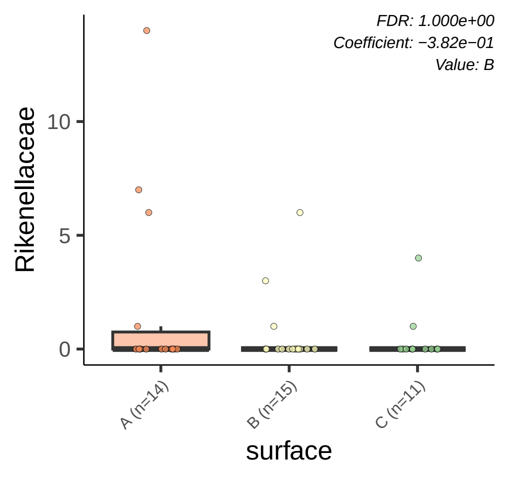
FDR: 1.000e+00
Coefficient: −3.82e−01
Value: B
Rikenellaceae
10
5
0
A (n=14)
B (n=15)
C (n=11)
surface

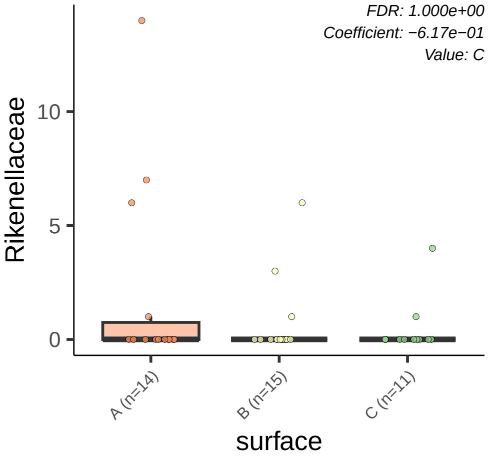

FDR: 1.000e+00
Coefficient: −6.17e−01
Value: C
Rikenellaceae
10
5
0
A (n=14)
B (n=15)
C (n=11)
surface

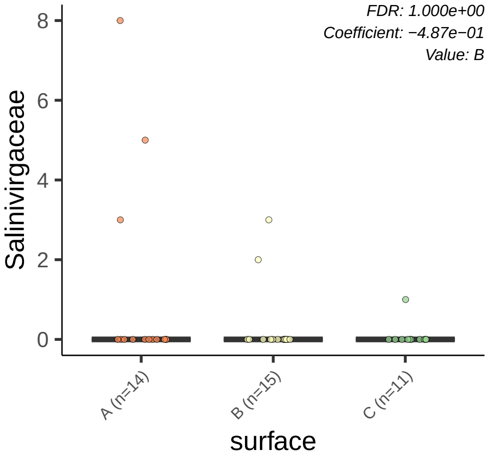
FDR: 1.000e+00
Coefficient: −4.87e−01
Value: B
Salinivirgaceae
0
2
4
6
8
A (n=14)
B (n=15)
C (n=11)
surface

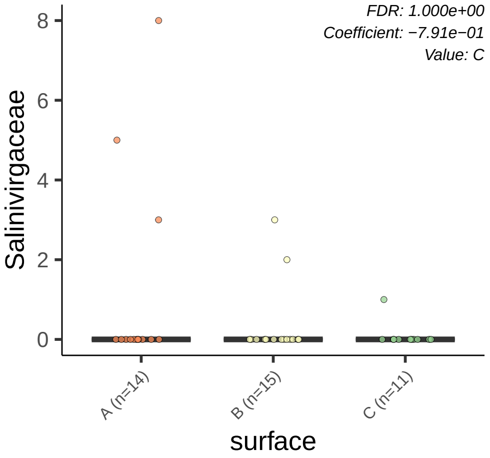

FDR: 1.000e+00
Coefficient: −7.91e−01
Value: C
Salinivirgaceae
8
6
4
2
0
A (n=14)
B (n=15)
C (n=11)
surface

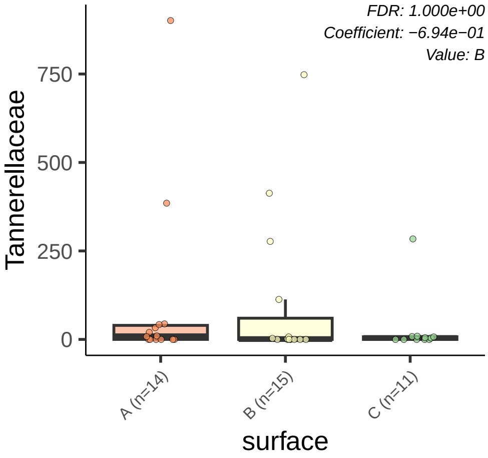

FDR: 1.000e+00
Coefficient: −6.94e−01
Value: B
Tannerellaceae
750
500
250
0
A (n=14)
B (n=15)
C (n=11)
surface

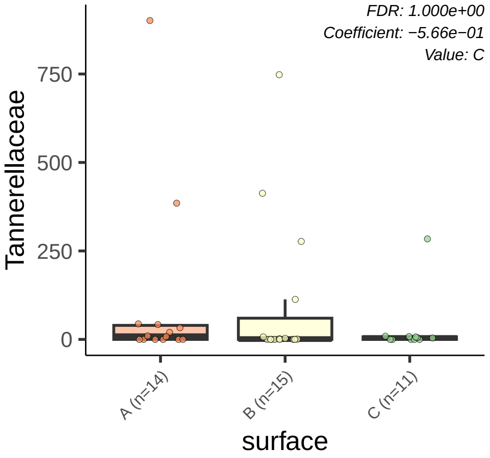
FDR: 1.000e+00
Coefficient: −5.66e−01
Value: C
Tannerellaceae
750
500
250
0
A (n=14)
B (n=15)
C (n=11)
surface

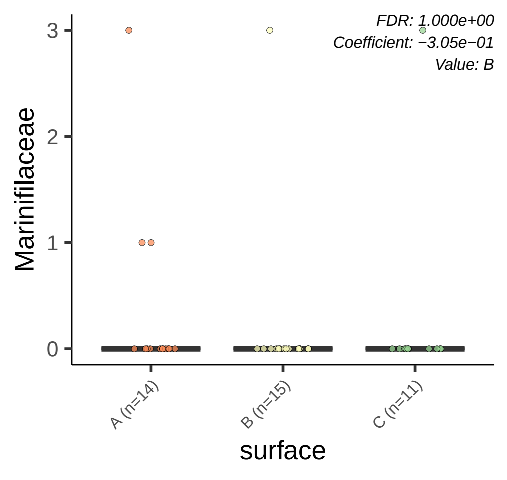

FDR: 1.000e+00
Coefficient: −3.05e−01
Value: B
Marinifilaceae
3
2
1
0
A (n=14)
B (n=15)
C (n=11)
surface

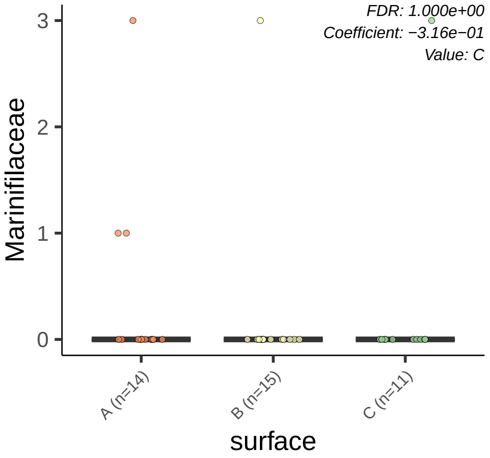
FDR: 1.000e+00
Coefficient: −3.16e−01
Value: C
Marinifilaceae
0
1
2
3
A (n=14)
B (n=15)
C (n=11)
surface

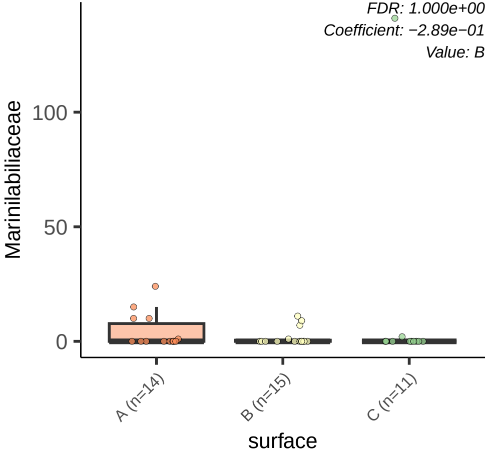
FDR: 1.000e+00
Coefficient: −2.89e−01
Value: B
Marinilabiliaceae
100
50
0
A (n=14)
B (n=15)
C (n=11)
surface

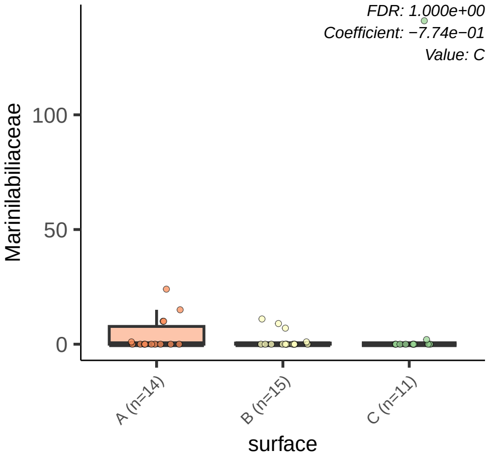
FDR: 1.000e+00
Coefficient: −7.74e−01
Value: C
Marinilabiliaceae
100
50
0
A (n=14)
B (n=15)
C (n=11)
surface

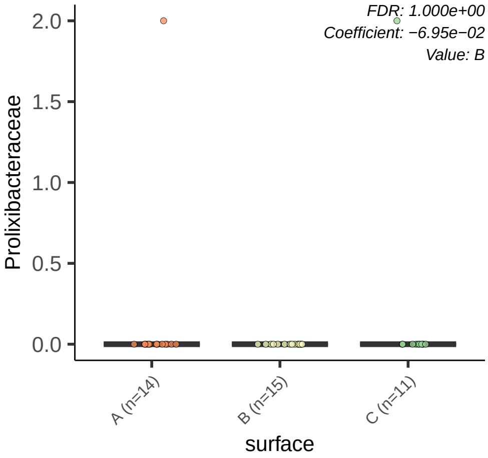
FDR: 1.000e+00
Coefficient: −6.95e−02
Value: B
Prolixibacteraceae
2.0
1.5
1.0
0.5
0.0
A (n=14)
B (n=15)
C (n=11)
surface

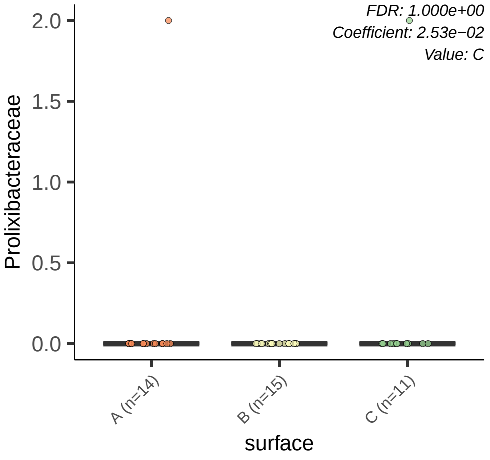
FDR: 1.000e+00
Coefficient: 2.53e−02
Value: C
Prolixibacteraceae
2.0
1.5
1.0
0.5
0.0
A (n=14)
B (n=15)
C (n=11)
surface

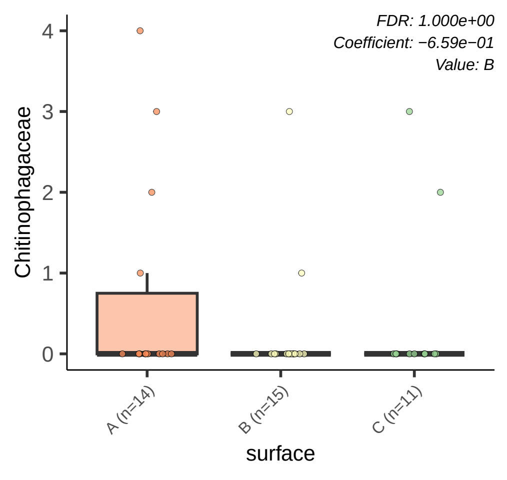
FDR: 1.000e+00
Coefficient: −6.59e−01
Value: B
Chitinophagaceae
0
1
2
3
4
A (n=14)
B (n=15)
C (n=11)
surface

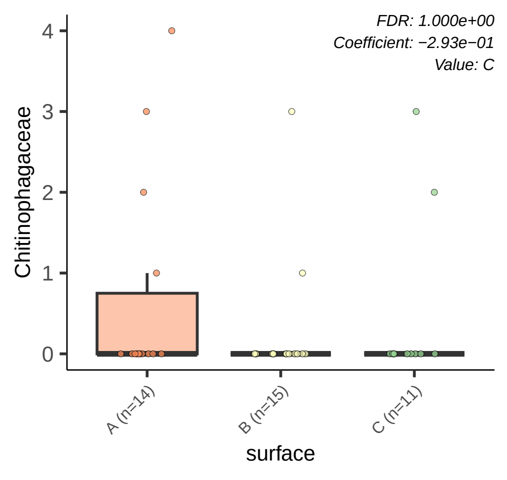
FDR: 1.000e+00
Coefficient: −2.93e−01
Value: C
Chitinophagaceae
4
3
2
1
0
A (n=14)
B (n=15)
C (n=11)
surface

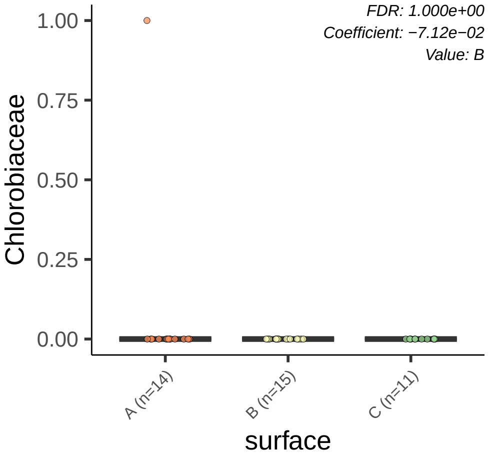
FDR: 1.000e+00
Coefficient: −7.12e−02
Value: B
Chlorobiaceae
1.00
0.75
0.50
0.25
0.00
A (n=14)
B (n=15)
C (n=11)
surface

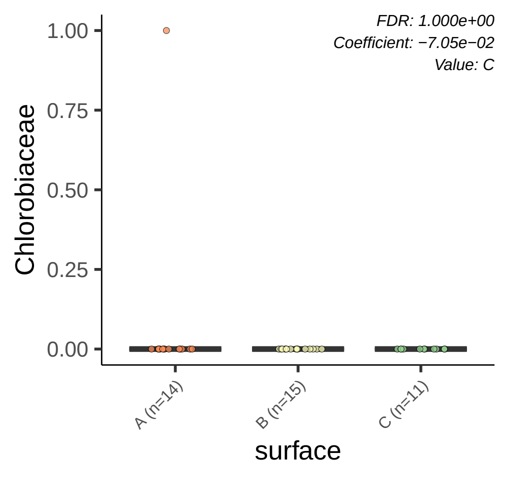
FDR: 1.000e+00
Coefficient: −7.05e−02
Value: C
Chlorobiaceae
1.00
0.75
0.50
0.25
0.00
A (n=14)
B (n=15)
C (n=11)
surface

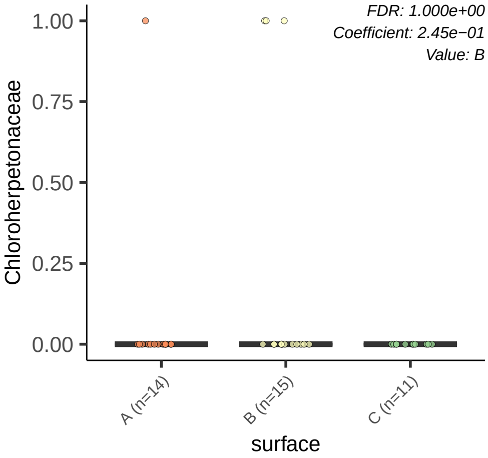

FDR: 1.000e+00
Coefficient: 2.45e−01
Value: B
Chloroherpetonaceae
1.00
0.75
0.50
0.25
0.00
A (n=14)
B (n=15)
C (n=11)
surface

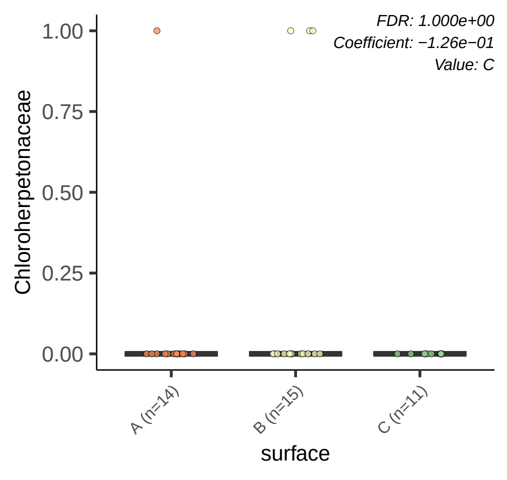
FDR: 1.000e+00
Coefficient: −1.26e−01
Value: C
Chloroherpetonaceae
1.00
0.75
0.50
0.25
0.00
A (n=14)
B (n=15)
C (n=11)
surface

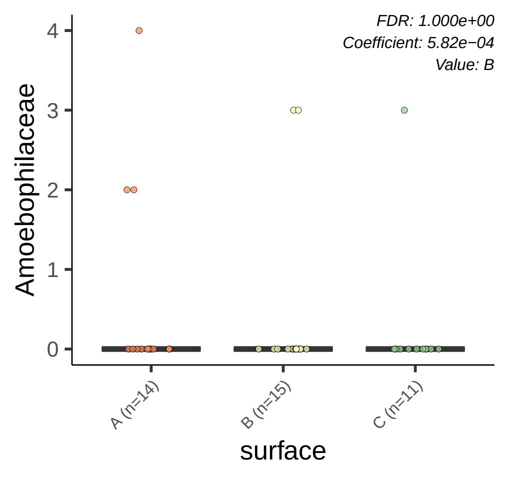
FDR: 1.000e+00
Coefficient: 5.82e−04
Value: B
Amoebophilaceae
4
3
2
1
0
A (n=14)
B (n=15)
C (n=11)
surface

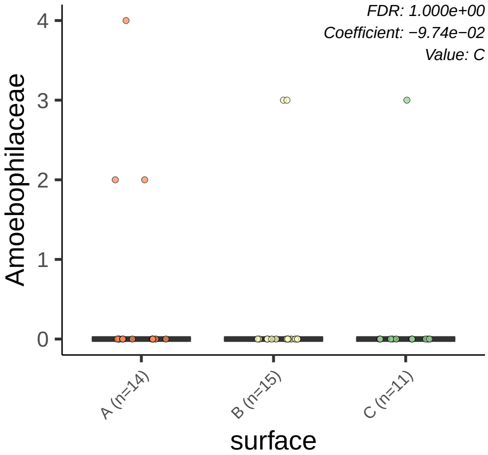

FDR: 1.000e+00
Coefficient: −9.74e−02
Value: C
Amoebophilaceae
4
3
2
1
0
A (n=14)
B (n=15)
C (n=11)
surface

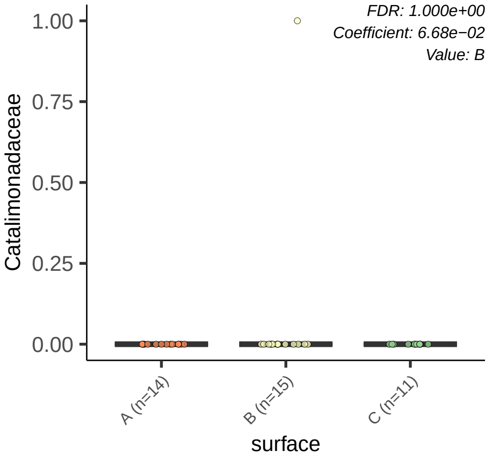
FDR: 1.000e+00
Coefficient: 6.68e−02
Value: B
1.00
0.75
0.50
0.25
0.00
Catalimonadaceae
A (n=14)
B (n=15)
C (n=11)
surface

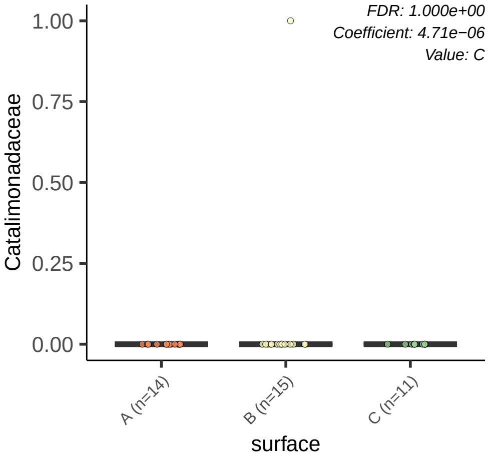
FDR: 1.000e+00
Coefficient: 4.71e−06
Value: C
Catalimonadaceae
1.00
0.75
0.50
0.25
0.00
A (n=14)
B (n=15)
C (n=11)
surface

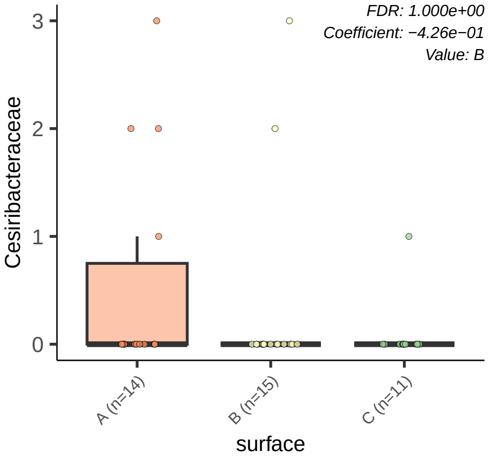
FDR: 1.000e+00
Coefficient: −4.26e−01
Value: B
Cesiribacteraceae
3
2
1
0
A (n=14)
B (n=15)
C (n=11)
surface

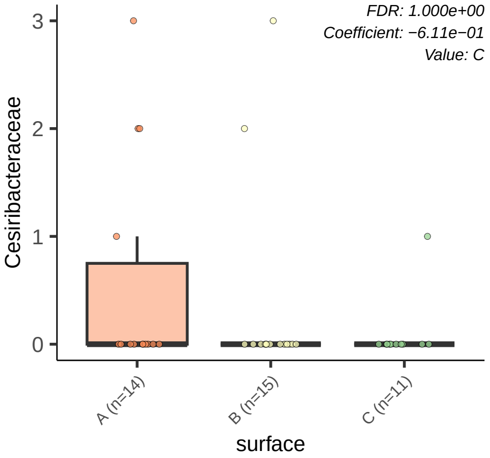

FDR: 1.000e+00
Coefficient: −6.11e−01
Value: C
Cesiribacteraceae
3
2
1
0
A (n=14)
B (n=15)
C (n=11)
surface

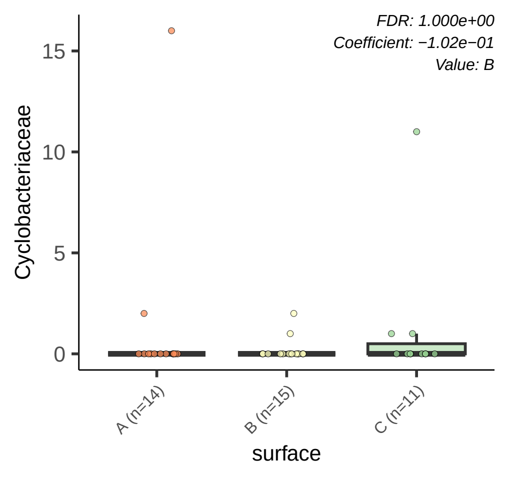
FDR: 1.000e+00
Coefficient: −1.02e−01
Value: B
Cyclobacteriaceae
0
5
10
15
A (n=14)
B (n=15)
C (n=11)
surface

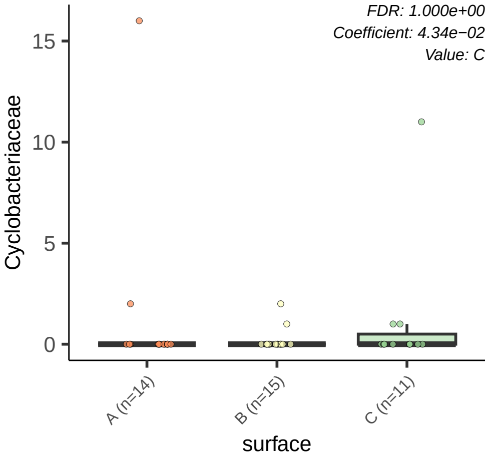

FDR: 1.000e+00
Coefficient: 4.34e−02
Value: C
Cyclobacteriaceae
0
5
10
15
A (n=14)
B (n=15)
C (n=11)
surface

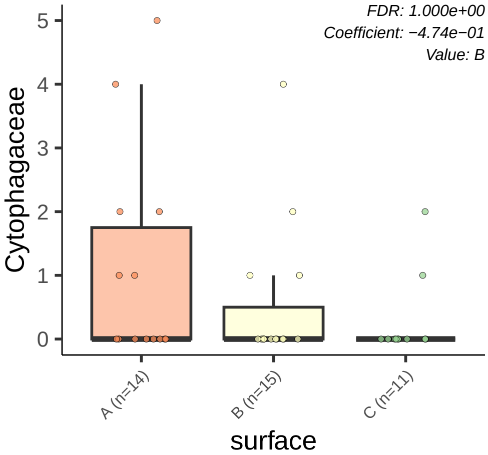

FDR: 1.000e+00
Coefficient: −4.74e−01
Value: B
Cytophagaceae
0
1
2
3
4
5
A (n=14)
B (n=15)
C (n=11)
surface

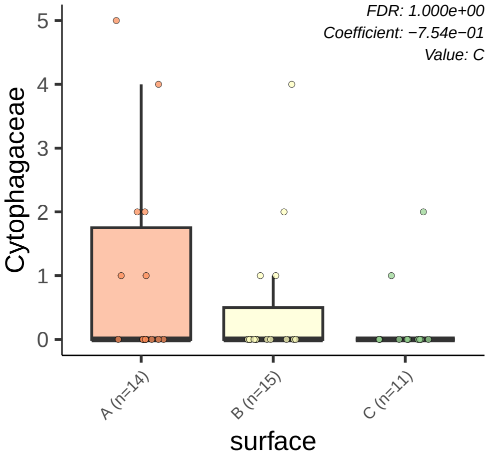
FDR: 1.000e+00
Coefficient: −7.54e−01
Value: C
Cytophagaceae
0
1
2
3
4
5
A (n=14)
B (n=15)
C (n=11)
surface

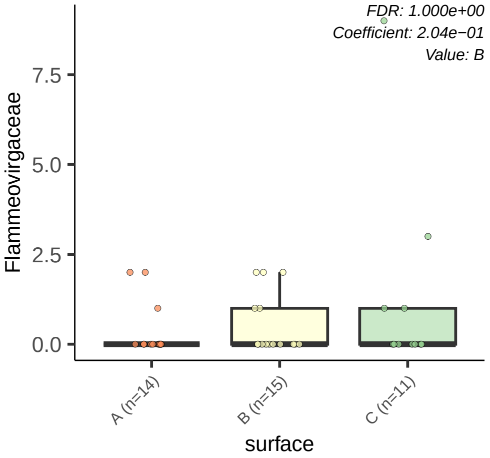

FDR: 1.000e+00
Coefficient: 2.04e−01
Value: B
Flammeovirgaceae
7.5
5.0
2.5
0.0
A (n=14)
B (n=15)
C (n=11)
surface

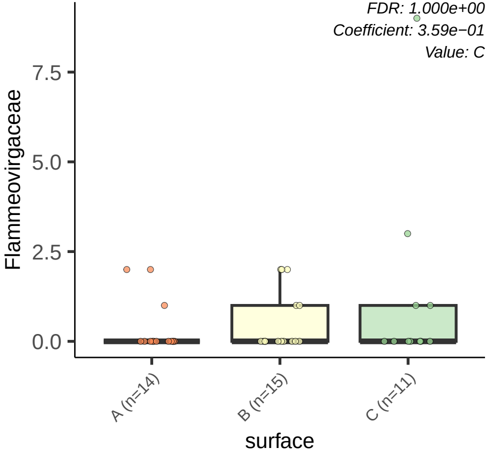

FDR: 1.000e+00
Coefficient: 3.59e−01
Value: C
Flammeovirgaceae
7.5
5.0
2.5
0.0
A (n=14)
B (n=15)
C (n=11)
surface

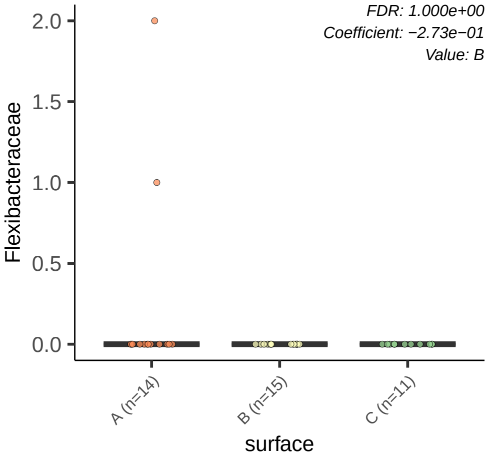

FDR: 1.000e+00
Coefficient: −2.73e−01
Value: B
Flexibacteraceae
2.0
1.5
1.0
0.5
0.0
A (n=14)
B (n=15)
C (n=11)
surface

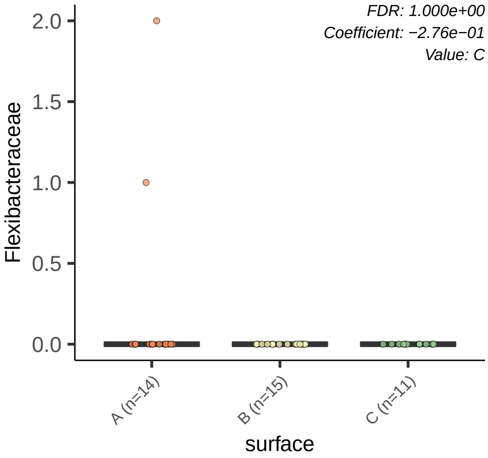

FDR: 1.000e+00
Coefficient: −2.76e−01
Value: C
Flexibacteraceae
2.0
1.5
1.0
0.5
0.0
A (n=14)
B (n=15)
C (n=11)
surface

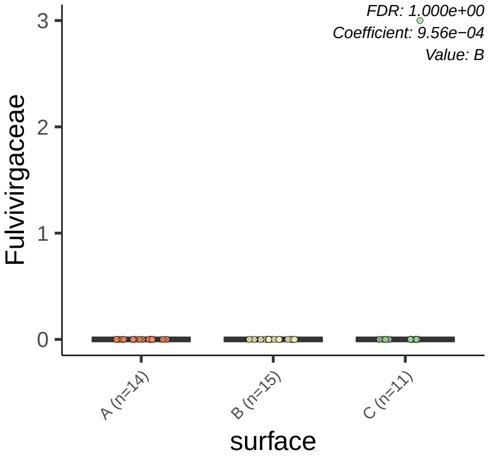

FDR: 1.000e+00
Coefficient: 9.56e−04
Value: B
Fulvivirgaceae
3
2
1
0
A (n=14)
B (n=15)
C (n=11)
surface

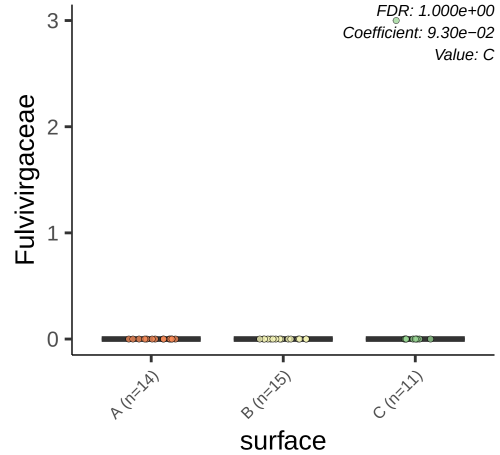

FDR: 1.000e+00
Coefficient: 9.30e−02
Value: C
Fulvivirgaceae
3
2
1
0
A (n=14)
B (n=15)
C (n=11)
surface

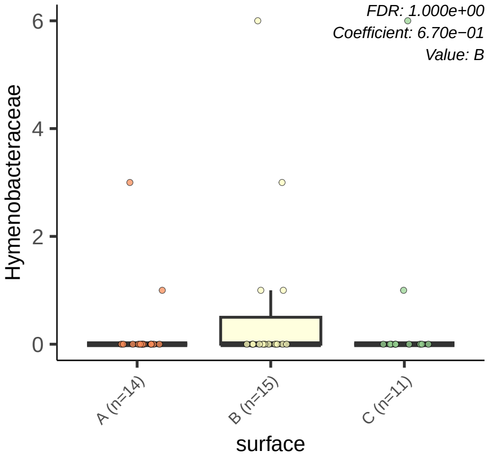
FDR: 1.000e+00
Coefficient: 6.70e−01
Value: B
Hymenobacteraceae
0
2
4
6
A (n=14)
B (n=15)
C (n=11)
surface

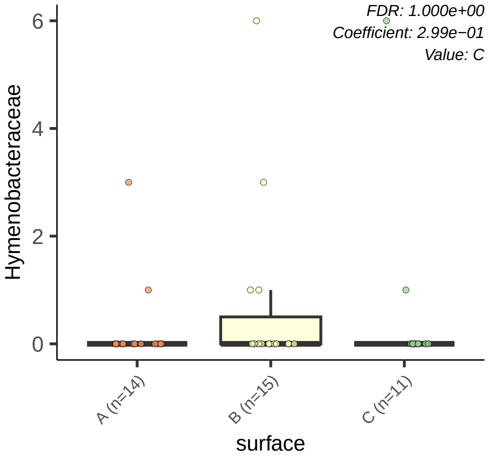

FDR: 1.000e+00
Coefficient: 2.99e−01
Value: C
Hymenobacteraceae
0
2
4
6
A (n=14)
B (n=15)
C (n=11)
surface

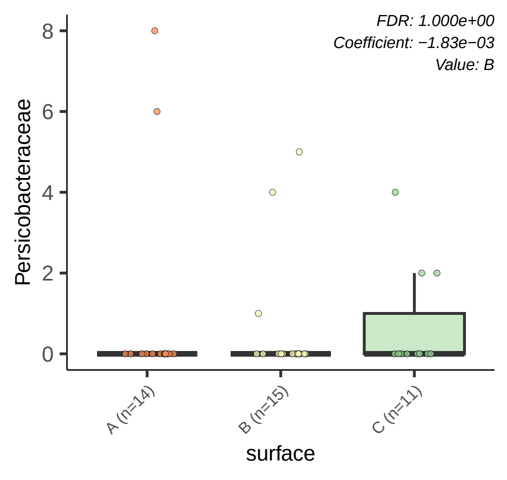

FDR: 1.000e+00
Coefficient: −1.83e−03
Value: B
Persicobacteraceae
0
2
4
6
8
A (n=14)
B (n=15)
C (n=11)
surface

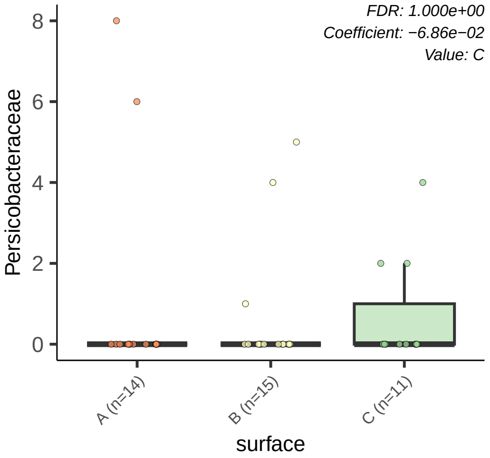
FDR: 1.000e+00
Coefficient: −6.86e−02
Value: C
Persicobacteraceae
8
6
4
2
0
A (n=14)
B (n=15)
C (n=11)
surface

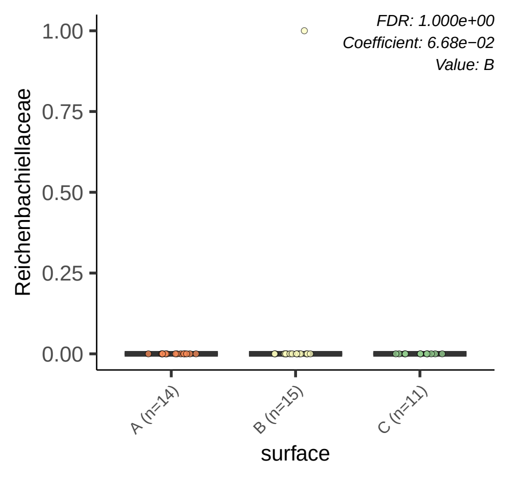
FDR: 1.000e+00
Coefficient: 6.68e−02
Value: B
Reichenbachiellaceae
1.00
0.75
0.50
0.25
0.00
A (n=14)
B (n=15)
C (n=11)
surface

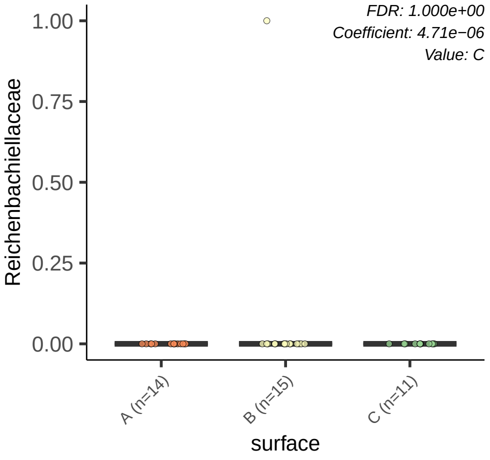

FDR: 1.000e+00
Coefficient: 4.71e−06
Value: C
Reichenbachiellaceae
1.00
0.75
0.50
0.25
0.00
A (n=14)
B (n=15)
C (n=11)
surface

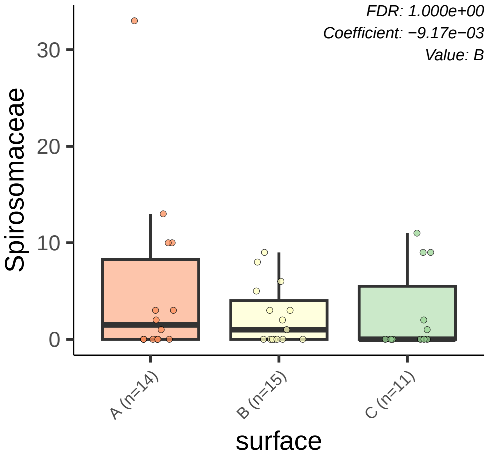

FDR: 1.000e+00
Coefficient: −9.17e−03
Value: B
Spirosomaceae
0
10
20
30
A (n=14)
B (n=15)
C (n=11)
surface

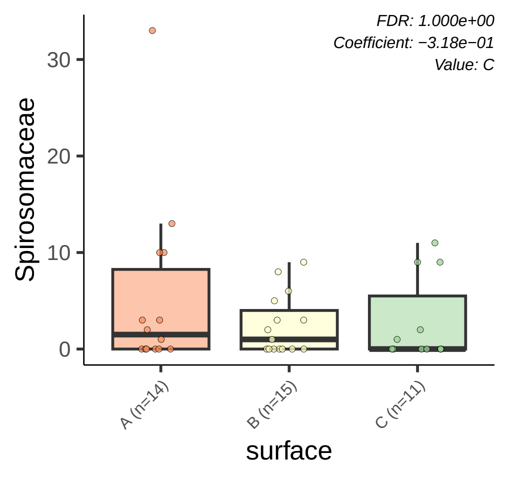
FDR: 1.000e+00
Coefficient: −3.18e−01
Value: C
Spirosomaceae
0
10
20
30
A (n=14)
B (n=15)
C (n=11)
surface

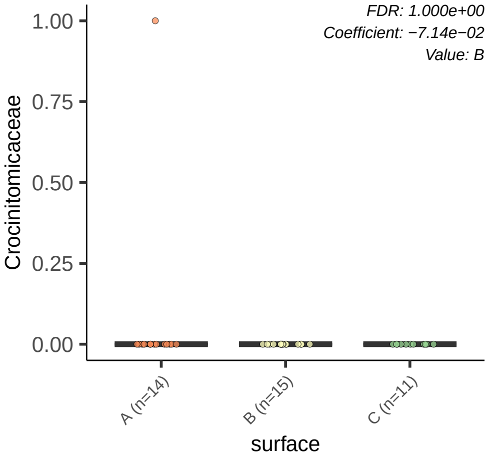

FDR: 1.000e+00
Coefficient: −7.14e−02
Value: B
Crocinitomicaceae
1.00
0.75
0.50
0.25
0.00
A (n=14)
B (n=15)
C (n=11)
surface

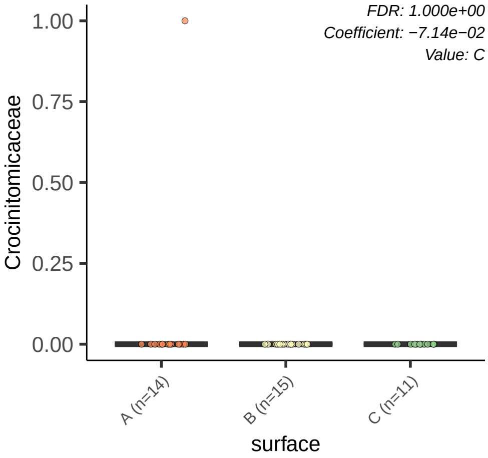

FDR: 1.000e+00
Coefficient: −7.14e−02
Value: C
Crocinitomicaceae
1.00
0.75
0.50
0.25
0.00
A (n=14)
B (n=15)
C (n=11)
surface

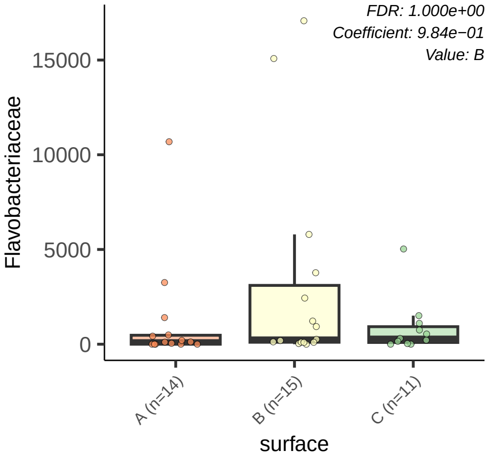

FDR: 1.000e+00
Coefficient: 9.84e−01
Value: B
Flavobacteriaceae
15000
10000
5000
0
A (n=14)
B (n=15)
C (n=11)
surface

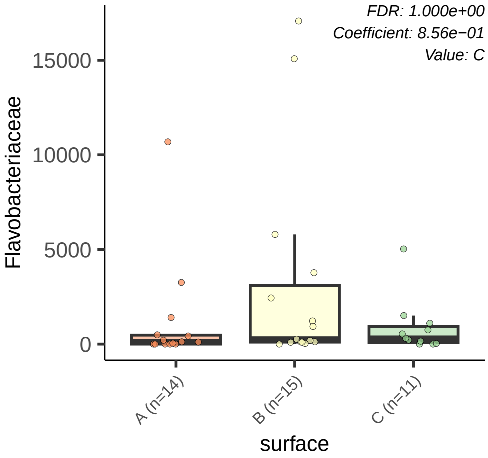

FDR: 1.000e+00
Coefficient: 8.56e−01
Value: C
Flavobacteriaceae
15000
10000
5000
0
A (n=14)
B (n=15)
C (n=11)
surface

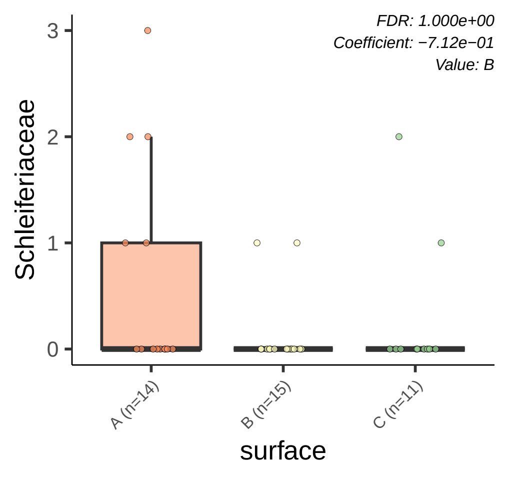

FDR: 1.000e+00
Coefficient: −7.12e−01
Value: B
Schleiferiaceae
3
2
1
0
A (n=14)
B (n=15)
C (n=11)
surface

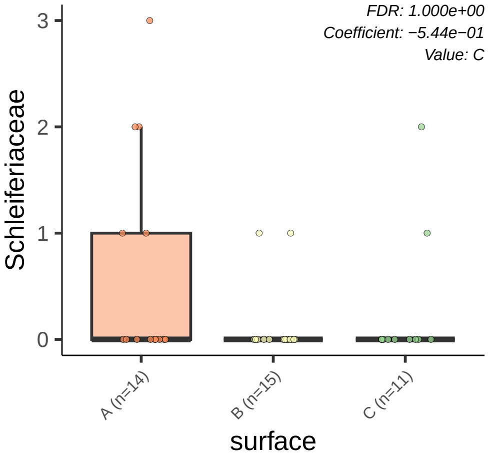

FDR: 1.000e+00
Coefficient: −5.44e−01
Value: C
Schleiferiaceae
3
2
1
0
A (n=14)
B (n=15)
C (n=11)
surface

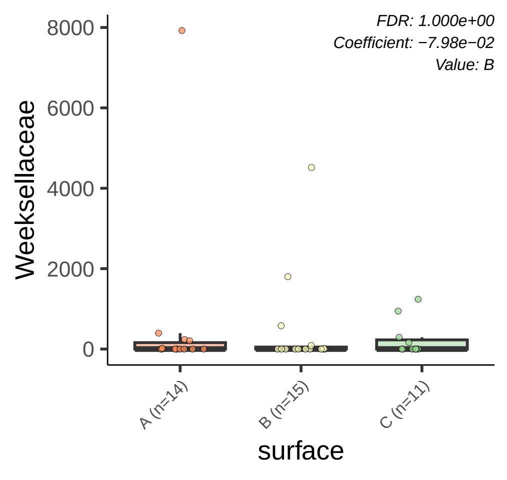
FDR: 1.000e+00
Coefficient: −7.98e−02
Value: B
Weeksellaceae
8000
6000
4000
2000
0
A (n=14)
B (n=15)
C (n=11)
surface

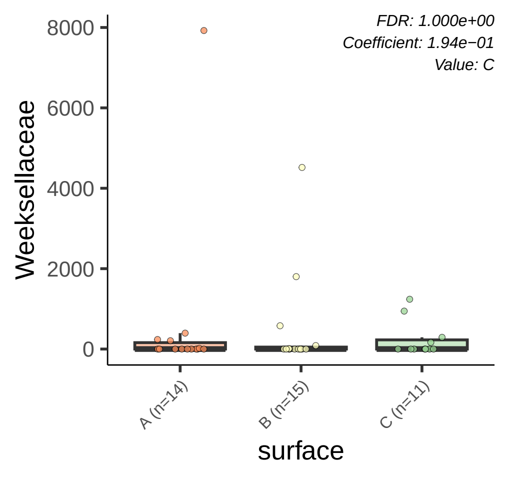

FDR: 1.000e+00
Coefficient: 1.94e−01
Value: C
Weeksellaceae
8000
6000
4000
2000
0
A (n=14)
B (n=15)
C (n=11)
surface

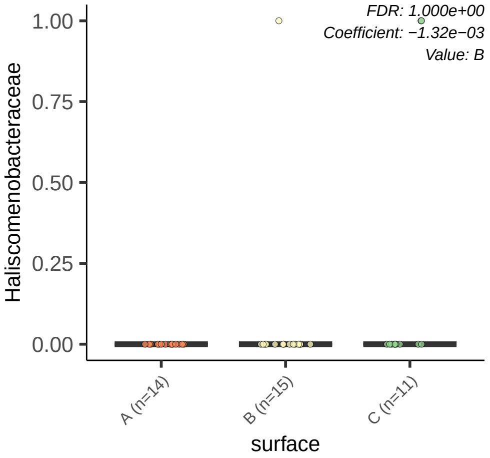
FDR: 1.000e+00
Coefficient: −1.32e−03
Value: B
Haliscomenobacteraceae
1.00
0.75
0.50
0.25
0.00
A (n=14)
B (n=15)
C (n=11)
surface

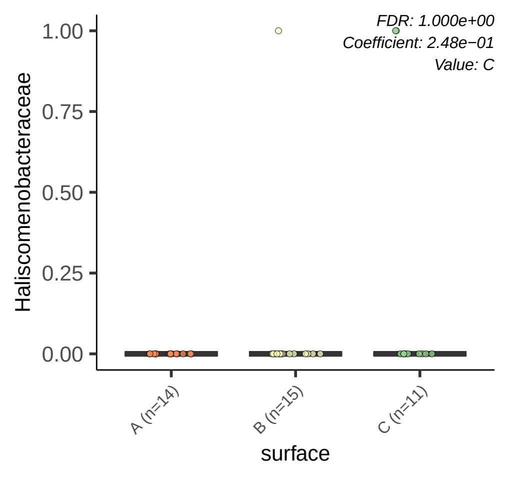
FDR: 1.000e+00
Coefficient: 2.48e−01
Value: C
Haliscomenobacteraceae
1.00
0.75
0.50
0.25
0.00
A (n=14)
B (n=15)
C (n=11)
surface

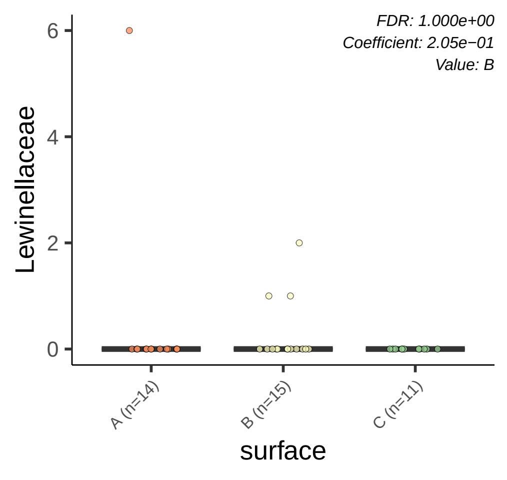
FDR: 1.000e+00
Coefficient: 2.05e−01
Value: B
Lewinellaceae
6
4
2
0
A (n=14)
B (n=15)
C (n=11)
surface

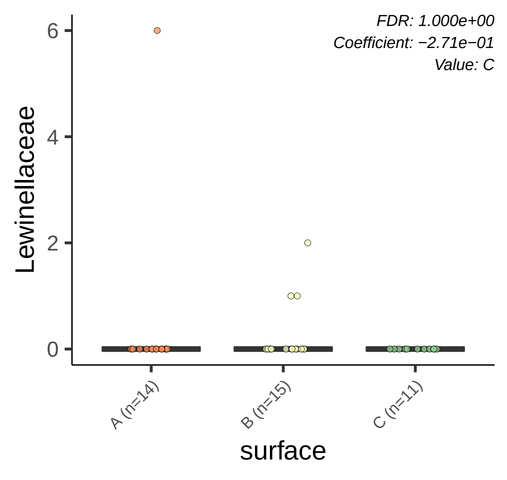

FDR: 1.000e+00
Coefficient: −2.71e−01
Value: C
Lewinellaceae
6
4
2
0
A (n=14)
B (n=15)
C (n=11)
surface

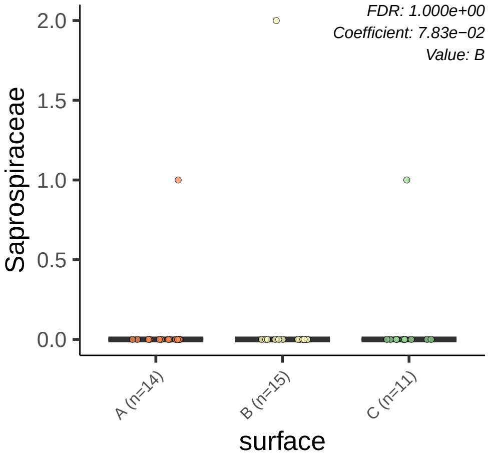

FDR: 1.000e+00
Coefficient: 7.83e−02
Value: B
Saprospiraceae
2.0
1.5
1.0
0.5
0.0
A (n=14)
B (n=15)
C (n=11)
surface

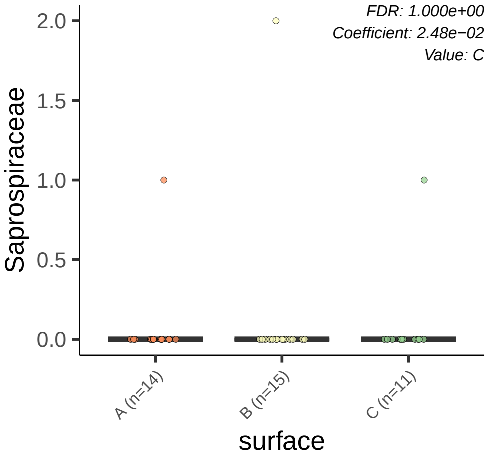
FDR: 1.000e+00
Coefficient: 2.48e−02
Value: C
Saprospiraceae
2.0
1.5
1.0
0.5
0.0
A (n=14)
B (n=15)
C (n=11)
surface

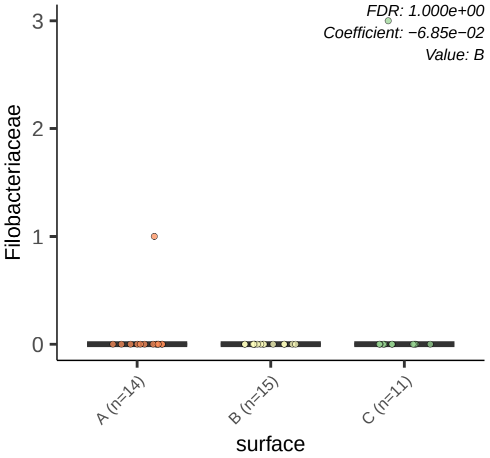

FDR: 1.000e+00
Coefficient: −6.85e−02
Value: B
Filobacteriaceae
3
2
1
0
A (n=14)
B (n=15)
C (n=11)
surface

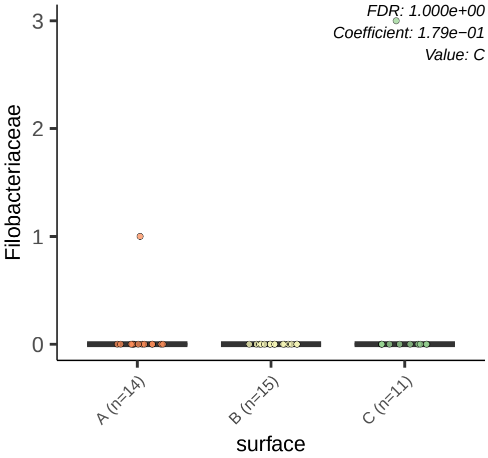

FDR: 1.000e+00
Coefficient: 1.79e−01
Value: C
Filobacteriaceae
3
2
1
0
A (n=14)
B (n=15)
C (n=11)
surface

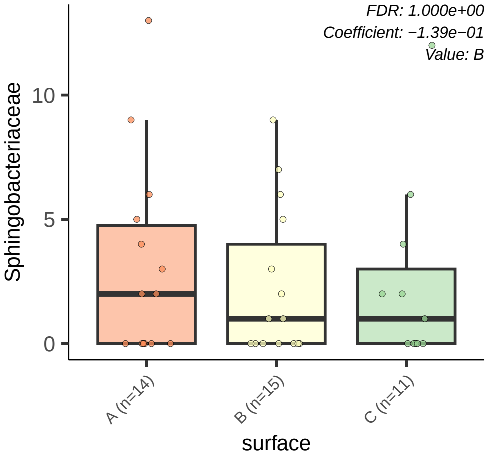

FDR: 1.000e+00
Coefficient: −1.39e−01
Value: B
Sphingobacteriaceae
10
5
0
A (n=14)
B (n=15)
C (n=11)
surface

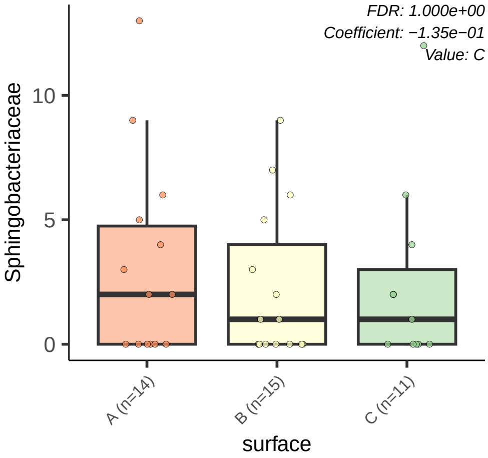
FDR: 1.000e+00
Coefficient: −1.35e−01
Value: C
Sphingobacteriaceae
10
5
0
A (n=14)
B (n=15)
C (n=11)
surface

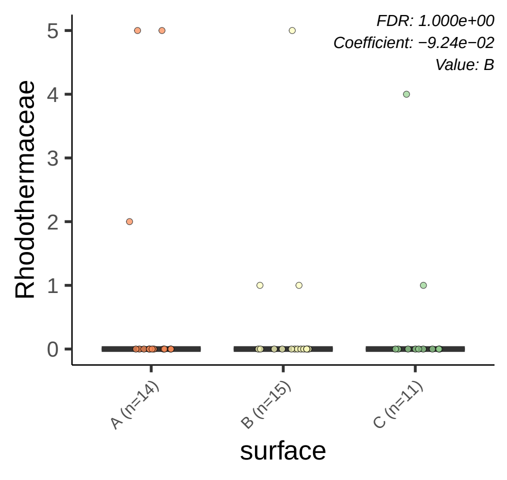

FDR: 1.000e+00
Coefficient: −9.24e−02
Value: B
Rhodothermaceae
0
1
2
3
4
5
A (n=14)
B (n=15)
C (n=11)
surface

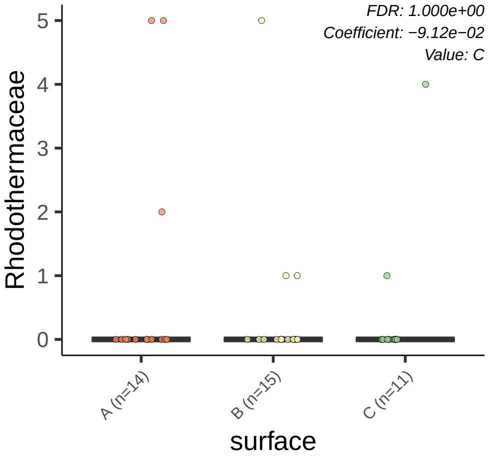

FDR: 1.000e+00
Coefficient: −9.12e−02
Value: C
Rhodothermaceae
5
4
3
2
1
0
A (n=14)
B (n=15)
C (n=11)
surface

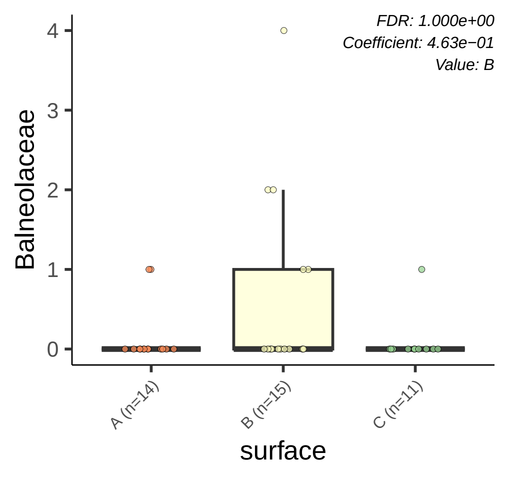

FDR: 1.000e+00
Coefficient: 4.63e−01
Value: B
Balneolaceae
0
1
2
3
4
A (n=14)
B (n=15)
C (n=11)
surface

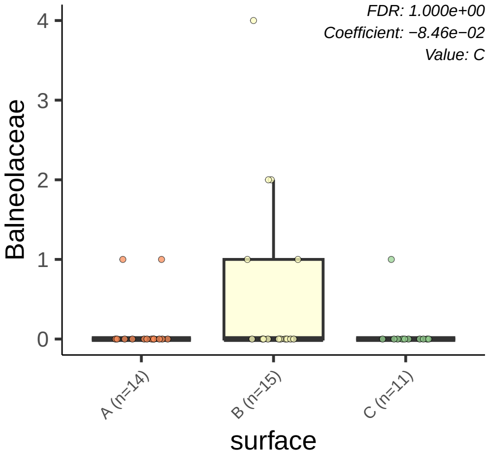

FDR: 1.000e+00
Coefficient: −8.46e−02
Value: C
Balneolaceae
4
3
2
1
0
A (n=14)
B (n=15)
C (n=11)
surface

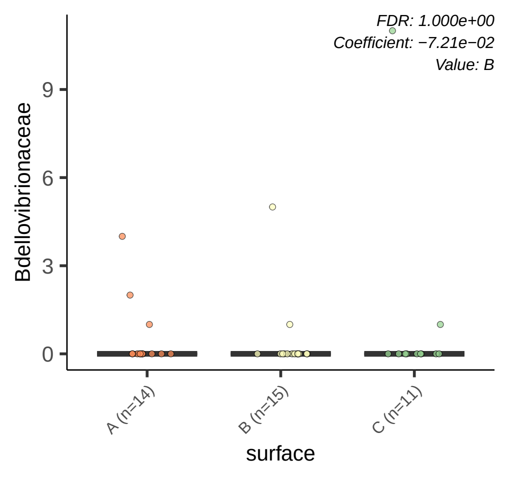
FDR: 1.000e+00
Coefficient: −7.21e−02
Value: B
Bdellovibrionaceae
9
6
3
0
A (n=14)
B (n=15)
C (n=11)
surface

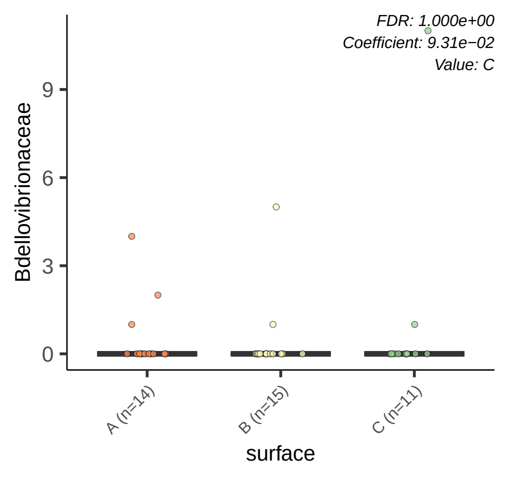
FDR: 1.000e+00
Coefficient: 9.31e−02
Value: C
Bdellovibrionaceae
9
6
3
0
A (n=14)
B (n=15)
C (n=11)
surface

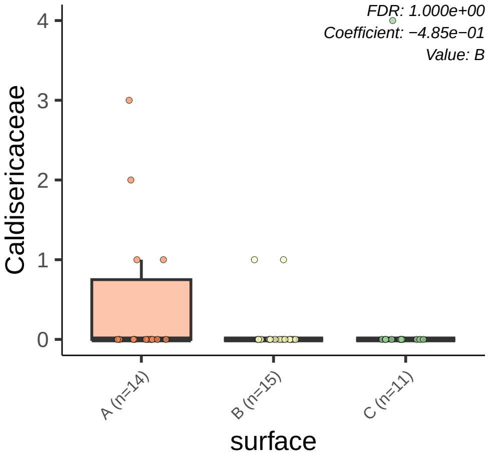

FDR: 1.000e+00
Coefficient: −4.85e−01
Value: B
Caldisericaceae
0
1
2
3
4
A (n=14)
B (n=15)
C (n=11)
surface

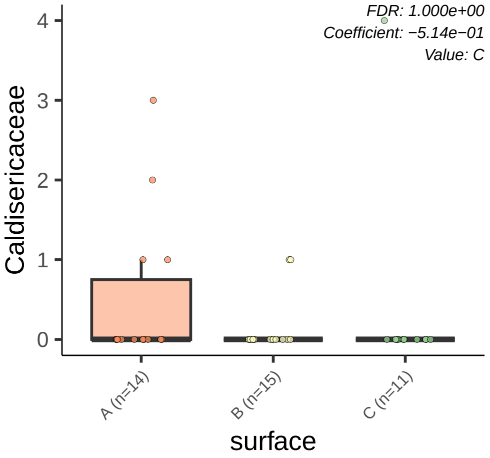
FDR: 1.000e+00
Coefficient: −5.14e−01
Value: C
Caldisericaceae
4
3
2
1
0
A (n=14)
B (n=15)
C (n=11)
surface

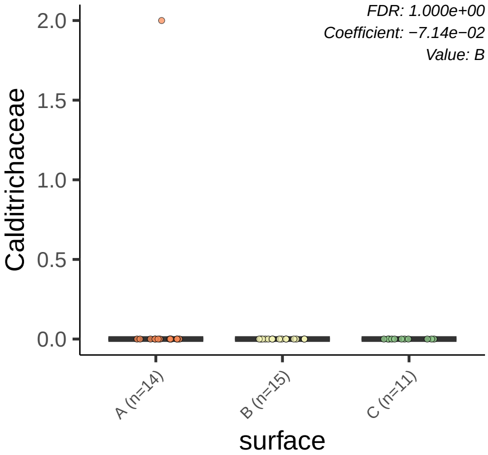
FDR: 1.000e+00
Coefficient: −7.14e−02
Value: B
Calditrichaceae
2.0
1.5
1.0
0.5
0.0
A (n=14)
B (n=15)
C (n=11)
surface

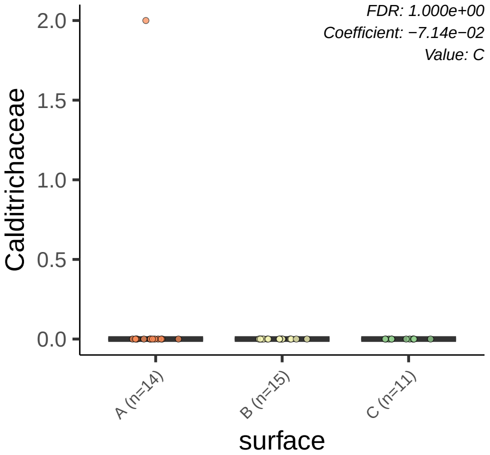
FDR: 1.000e+00
Coefficient: −7.14e−02
Value: C
Calditrichaceae
2.0
1.5
1.0
0.5
0.0
A (n=14)
B (n=15)
C (n=11)
surface

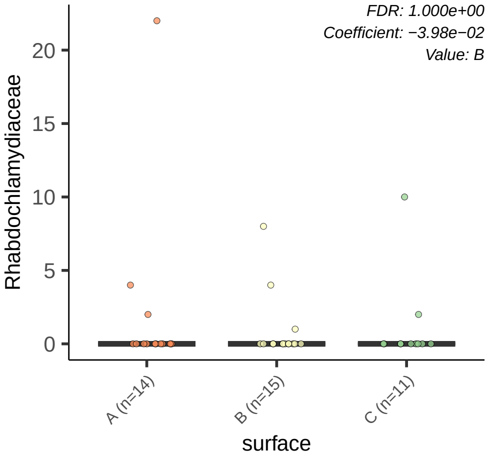

FDR: 1.000e+00
Coefficient: −3.98e−02
Value: B
Rhabdochlamydiaceae
0
5
10
15
20
A (n=14)
B (n=15)
C (n=11)
surface

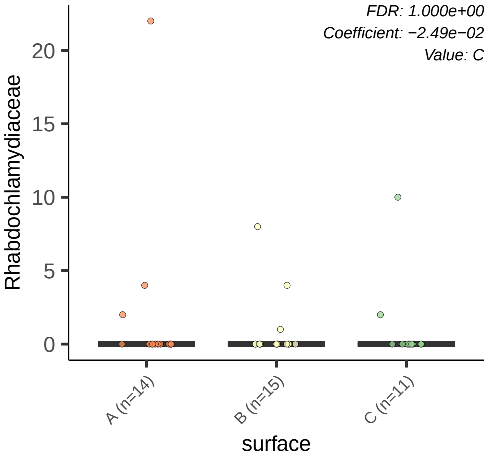

FDR: 1.000e+00
Coefficient: −2.49e−02
Value: C
Rhabdochlamydiaceae
20
15
10
5
0
A (n=14)
B (n=15)
C (n=11)
surface

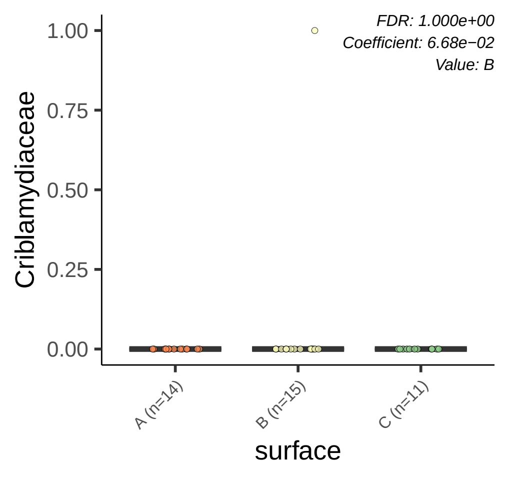
FDR: 1.000e+00
Coefficient: 6.68e−02
Value: B
Criblamydiaceae
1.00
0.75
0.50
0.25
0.00
A (n=14)
B (n=15)
C (n=11)
surface

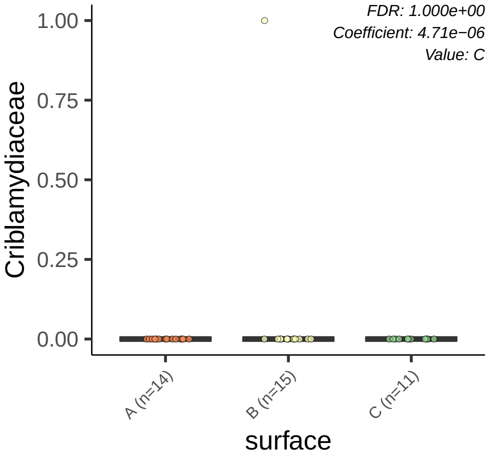

FDR: 1.000e+00
Coefficient: 4.71e−06
Value: C
Criblamydiaceae
1.00
0.75
0.50
0.25
0.00
A (n=14)
B (n=15)
C (n=11)
surface

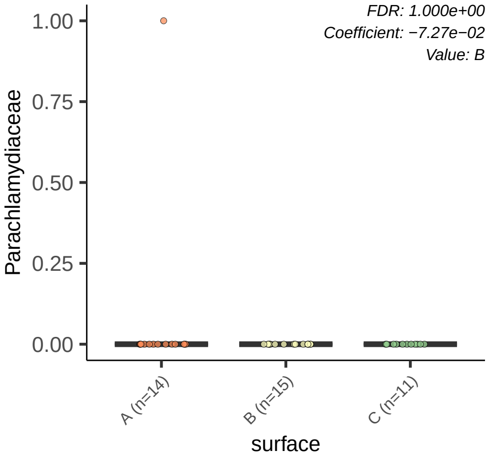
FDR: 1.000e+00
Coefficient: −7.27e−02
Value: B
Parachlamydiaceae
1.00
0.75
0.50
0.25
0.00
A (n=14)
B (n=15)
C (n=11)
surface

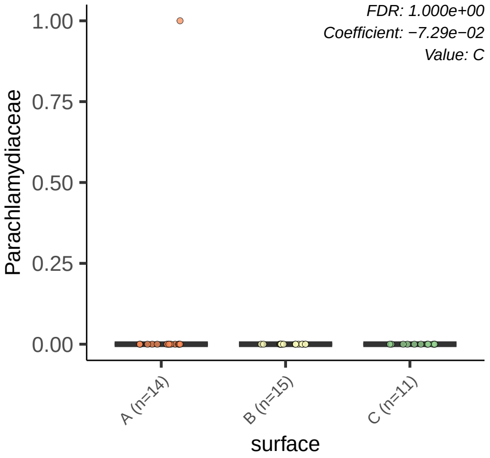

FDR: 1.000e+00
Coefficient: −7.29e−02
Value: C
Parachlamydiaceae
1.00
0.75
0.50
0.25
0.00
A (n=14)
B (n=15)
C (n=11)
surface

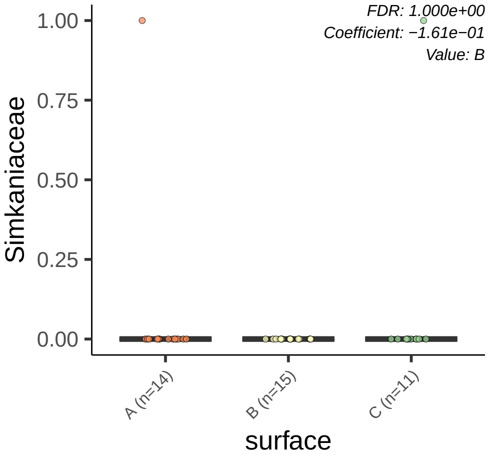

FDR: 1.000e+00
Coefficient: −1.61e−01
Value: B
Simkaniaceae
1.00
0.75
0.50
0.25
0.00
A (n=14)
B (n=15)
C (n=11)
surface

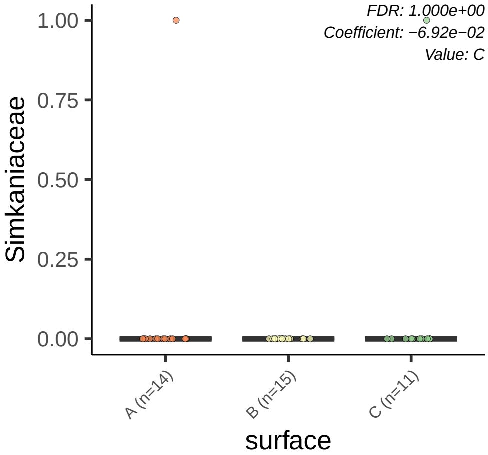

FDR: 1.000e+00
Coefficient: −6.92e−02
Value: C
Simkaniaceae
1.00
0.75
0.50
0.25
0.00
A (n=14)
B (n=15)
C (n=11)
surface

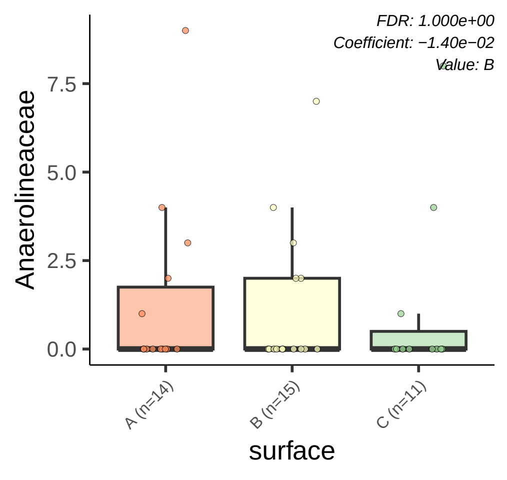
FDR: 1.000e+00
Coefficient: −1.40e−02
Value: B
Anaerolineaceae
7.5
5.0
2.5
0.0
A (n=14)
B (n=15)
C (n=11)
surface

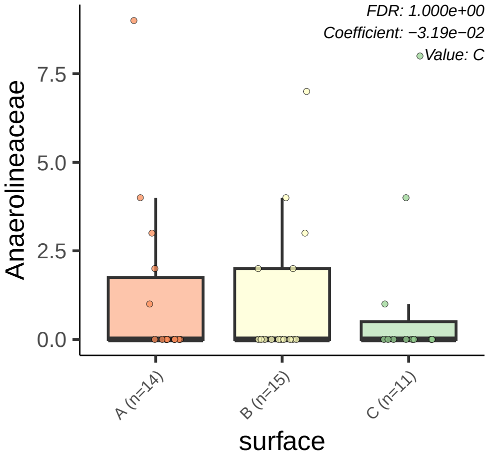
FDR: 1.000e+00
Coefficient: −3.19e−02
Value: C
Anaerolineaceae
7.5
5.0
2.5
0.0
A (n=14)
B (n=15)
C (n=11)
surface

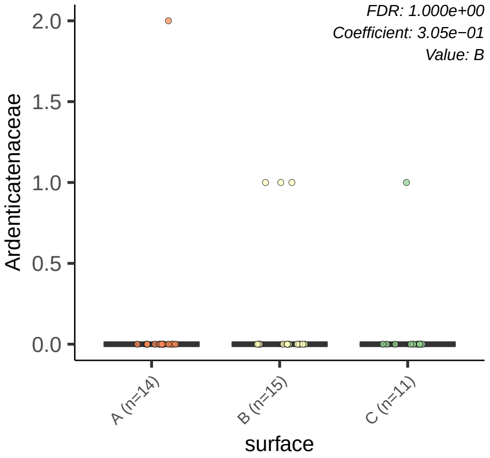

FDR: 1.000e+00
Coefficient: 3.05e−01
Value: B
Ardenticatenaceae
2.0
1.5
1.0
0.5
0.0
A (n=14)
B (n=15)
C (n=11)
surface

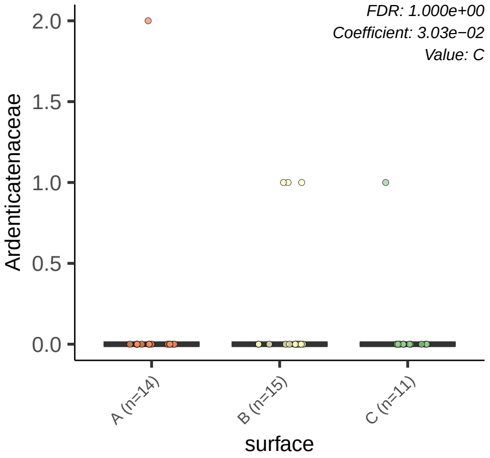
FDR: 1.000e+00
Coefficient: 3.03e−02
Value: C
Ardenticatenaceae
2.0
1.5
1.0
0.5
0.0
A (n=14)
B (n=15)
C (n=11)
surface

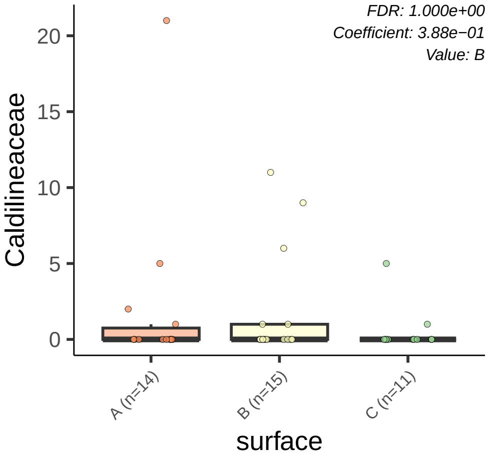
FDR: 1.000e+00
Coefficient: 3.88e−01
Value: B
Caldilineaceae
0
5
10
15
20
A (n=14)
B (n=15)
C (n=11)
surface

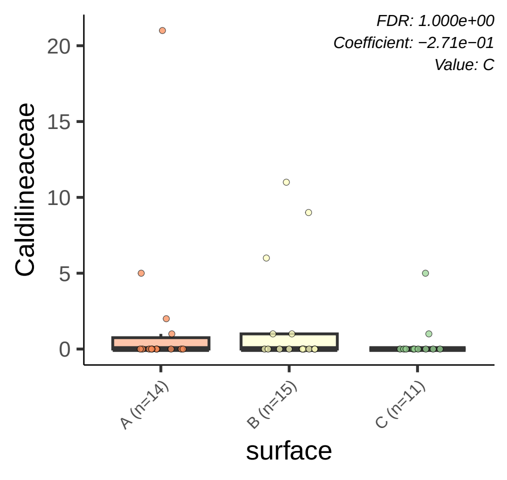
FDR: 1.000e+00
Coefficient: −2.71e−01
Value: C
Caldilineaceae
20
15
10
5
0
A (n=14)
B (n=15)
C (n=11)
surface

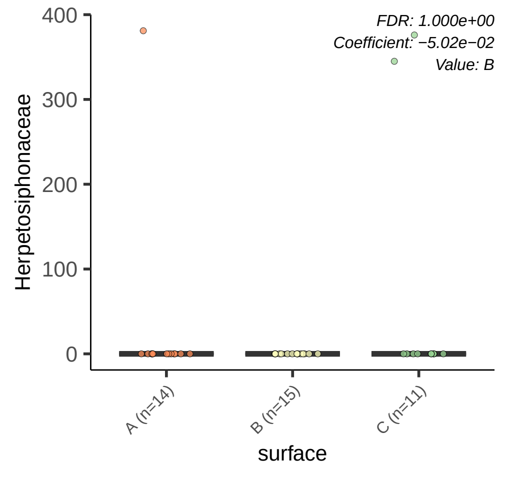
FDR: 1.000e+00
Coefficient: −5.02e−02
Value: B
Herpetosiphonaceae
400
300
200
100
0
A (n=14)
B (n=15)
C (n=11)
surface

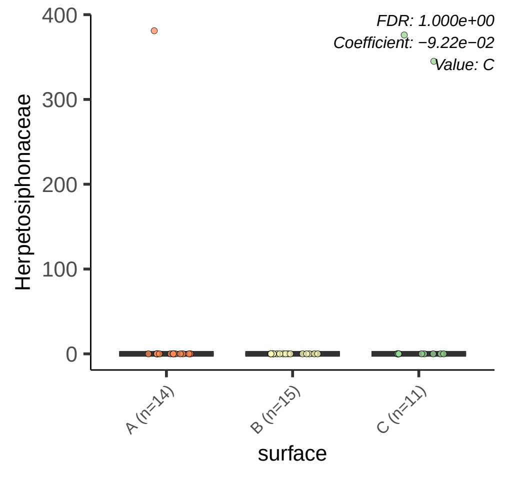

FDR: 1.000e+00
Coefficient: −9.22e−02
Value: C
400
300
200
100
0
Herpetosiphonaceae
A (n=14)
B (n=15)
C (n=11)
surface

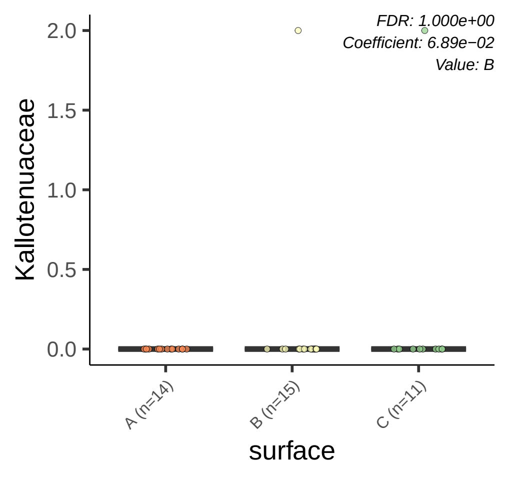
FDR: 1.000e+00
Coefficient: 6.89e−02
Value: B
Kallotenuaceae
2.0
1.5
1.0
0.5
0.0
A (n=14)
B (n=15)
C (n=11)
surface

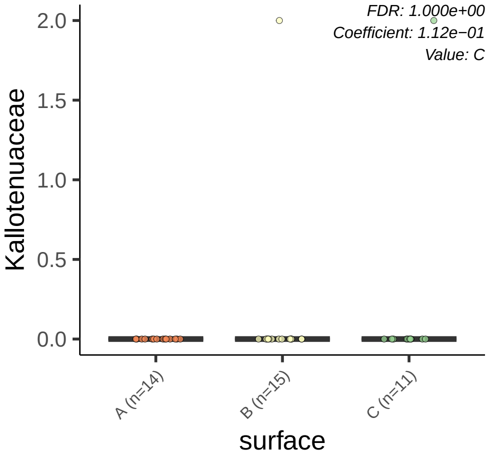

FDR: 1.000e+00
Coefficient: 1.12e−01
Value: C
Kallotenuaceae
2.0
1.5
1.0
0.5
0.0
A (n=14)
B (n=15)
C (n=11)
surface

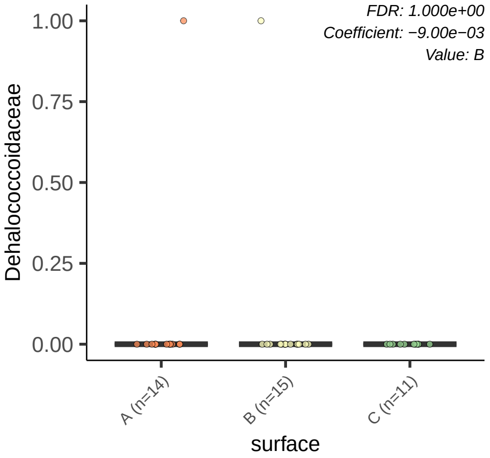
FDR: 1.000e+00
Coefficient: −9.00e−03
Value: B
Dehalococcoidaceae
1.00
0.75
0.50
0.25
0.00
A (n=14)
B (n=15)
C (n=11)
surface

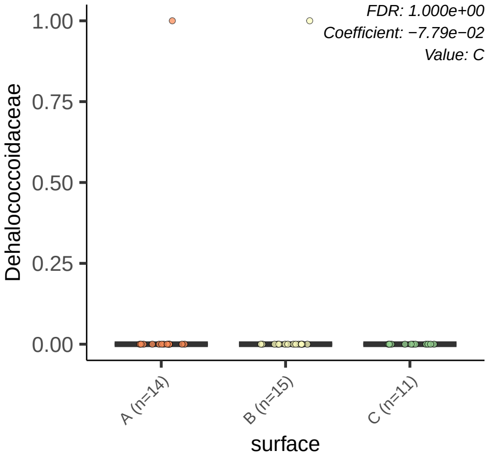
FDR: 1.000e+00
Coefficient: −7.79e−02
Value: C
Dehalococcoidaceae
1.00
0.75
0.50
0.25
0.00
A (n=14)
B (n=15)
C (n=11)
surface

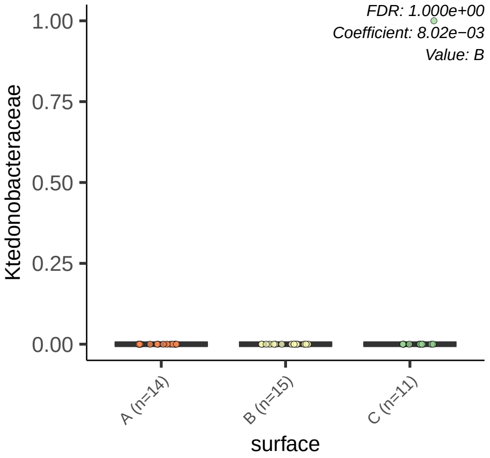
FDR: 1.000e+00
Coefficient: 8.02e−03
Value: B
Ktedonobacteraceae
1.00
0.75
0.50
0.25
0.00
A (n=14)
B (n=15)
C (n=11)
surface

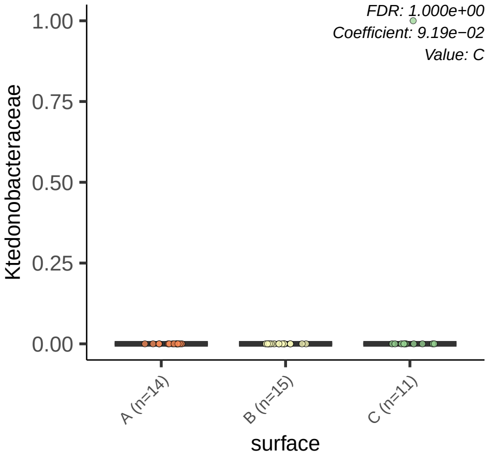

FDR: 1.000e+00
Coefficient: 9.19e−02
Value: C
Ktedonobacteraceae
1.00
0.75
0.50
0.25
0.00
A (n=14)
B (n=15)
C (n=11)
surface

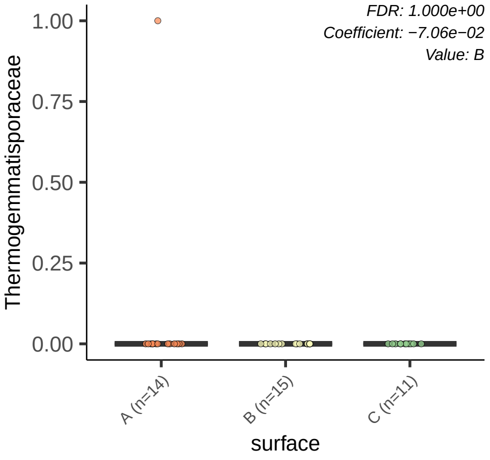

FDR: 1.000e+00
Coefficient: −7.06e−02
Value: B
Thermogemmatisporaceae
1.00
0.75
0.50
0.25
0.00
A (n=14)
B (n=15)
C (n=11)
surface

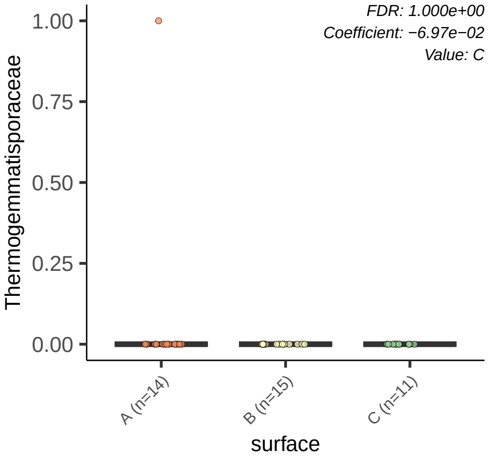

FDR: 1.000e+00
Coefficient: −6.97e−02
Value: C
Thermogemmatisporaceae
1.00
0.75
0.50
0.25
0.00
A (n=14)
B (n=15)
C (n=11)
surface

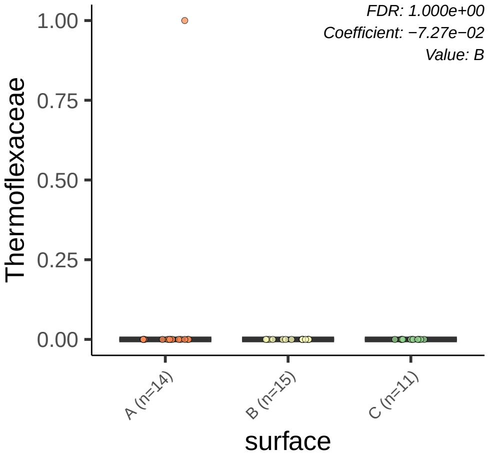
FDR: 1.000e+00
Coefficient: −7.27e−02
Value: B
Thermoflexaceae
1.00
0.75
0.50
0.25
0.00
A (n=14)
B (n=15)
C (n=11)
surface

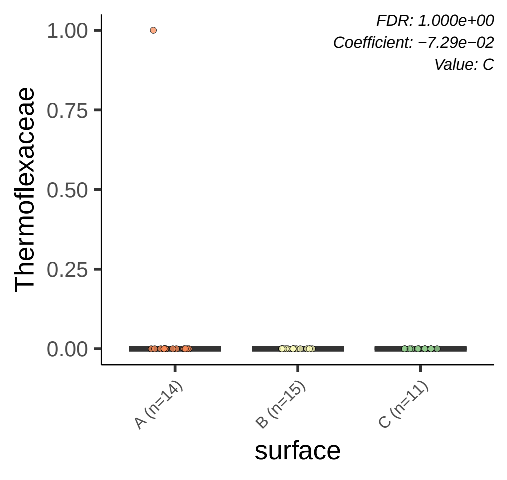
FDR: 1.000e+00
Coefficient: −7.29e−02
Value: C
Thermoflexaceae
1.00
0.75
0.50
0.25
0.00
A (n=14)
B (n=15)
C (n=11)
surface

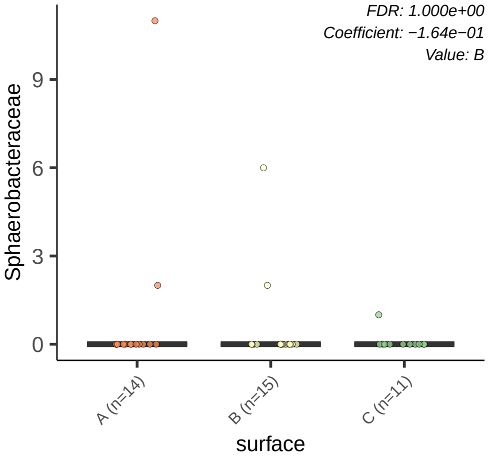

FDR: 1.000e+00
Coefficient: −1.64e−01
Value: B
Sphaerobacteraceae
9
6
3
0
A (n=14)
B (n=15)
C (n=11)
surface

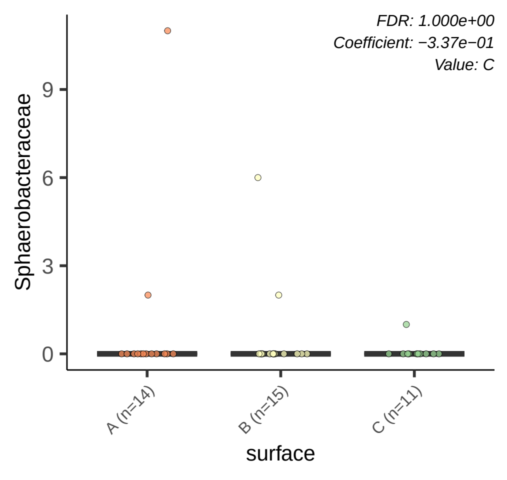
FDR: 1.000e+00
Coefficient: −3.37e−01
Value: C
Sphaerobacteraceae
9
6
3
0
A (n=14)
B (n=15)
C (n=11)
surface

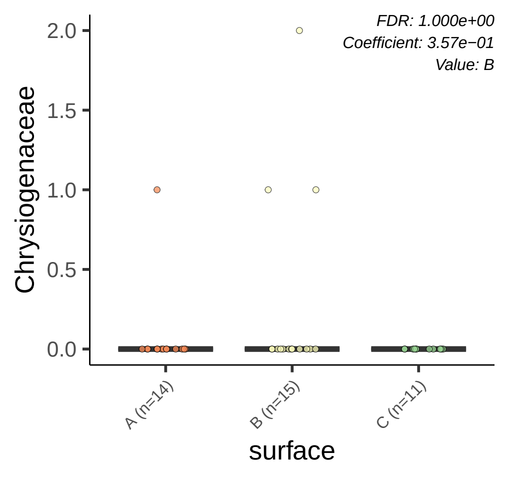
FDR: 1.000e+00
Coefficient: 3.57e−01
Value: B
Chrysiogenaceae
2.0
1.5
1.0
0.5
0.0
A (n=14)
B (n=15)
C (n=11)
surface

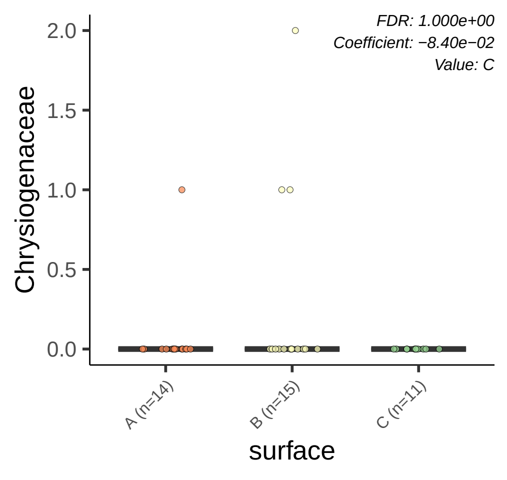

FDR: 1.000e+00
Coefficient: −8.40e−02
Value: C
Chrysiogenaceae
2.0
1.5
1.0
0.5
0.0
A (n=14)
B (n=15)
C (n=11)
surface

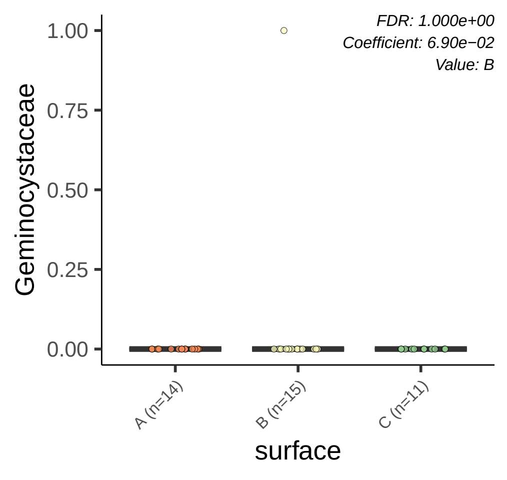

FDR: 1.000e+00
Coefficient: 6.90e−02
Value: B
Geminocystaceae
1.00
0.75
0.50
0.25
0.00
A (n=14)
B (n=15)
C (n=11)
surface

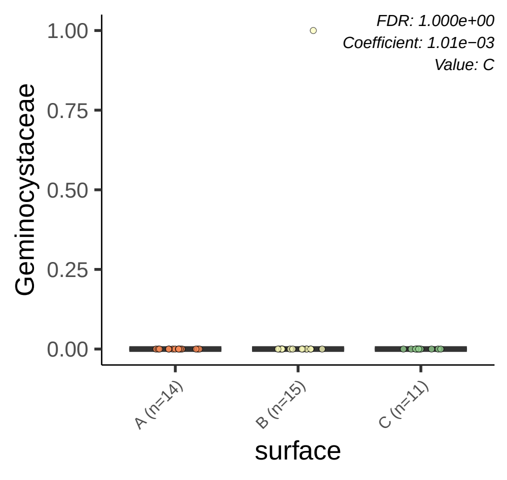

FDR: 1.000e+00
Coefficient: 1.01e−03
Value: C
Geminocystaceae
1.00
0.75
0.50
0.25
0.00
A (n=14)
B (n=15)
C (n=11)
surface

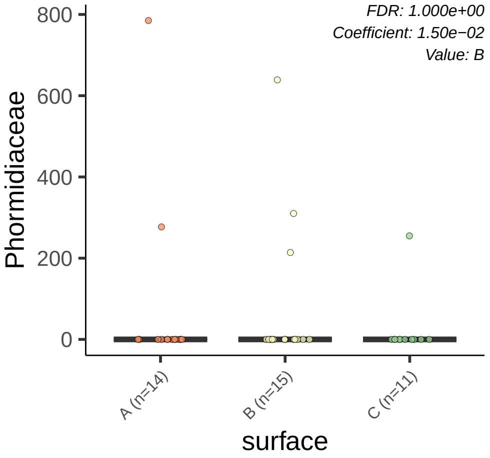
FDR: 1.000e+00
Coefficient: 1.50e−02
Value: B
Phormidiaceae
800
600
400
200
0
A (n=14)
B (n=15)
C (n=11)
surface

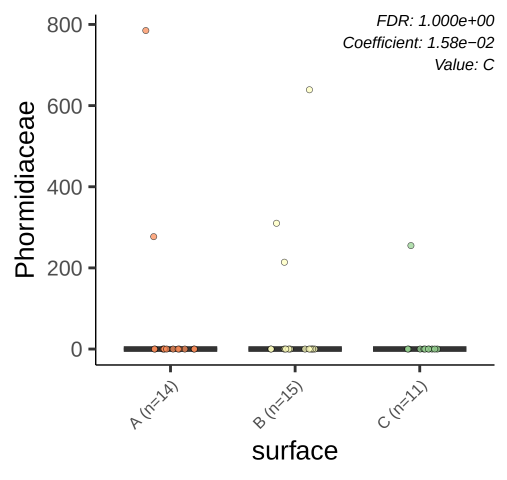

FDR: 1.000e+00
Coefficient: 1.58e−02
Value: C
Phormidiaceae
800
600
400
200
0
A (n=14)
B (n=15)
C (n=11)
surface

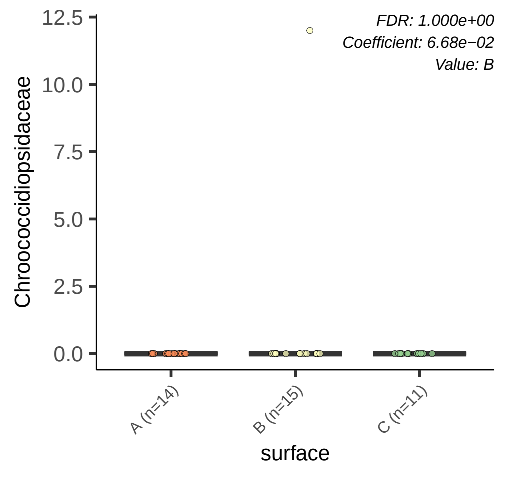
FDR: 1.000e+00
Coefficient: 6.68e−02
Value: B
Chroococcidiopsidaceae
12.5
10.0
7.5
5.0
2.5
0.0
A (n=14)
B (n=15)
C (n=11)
surface

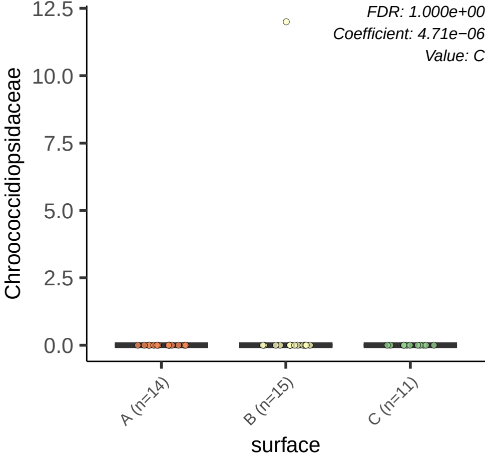

FDR: 1.000e+00
Coefficient: 4.71e−06
Value: C
Chroococcidiopsidaceae
12.5
10.0
7.5
5.0
2.5
0.0
A (n=14)
B (n=15)
C (n=11)
surface

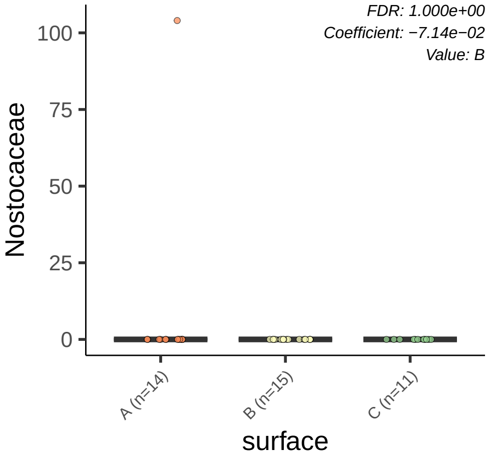
FDR: 1.000e+00
Coefficient: −7.14e−02
Value: B
Nostocaceae
100
75
50
25
0
A (n=14)
B (n=15)
C (n=11)
surface

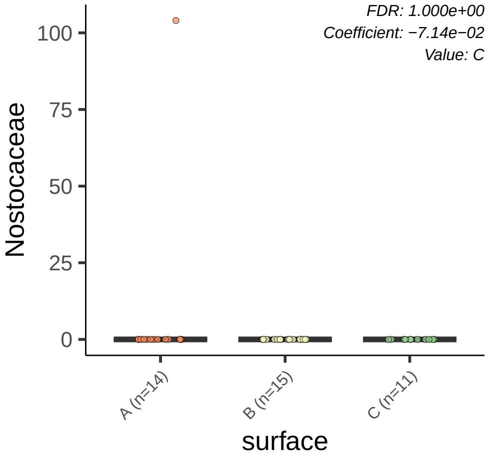

FDR: 1.000e+00
Coefficient: −7.14e−02
Value: C
Nostocaceae
100
75
50
25
0
A (n=14)
B (n=15)
C (n=11)
surface

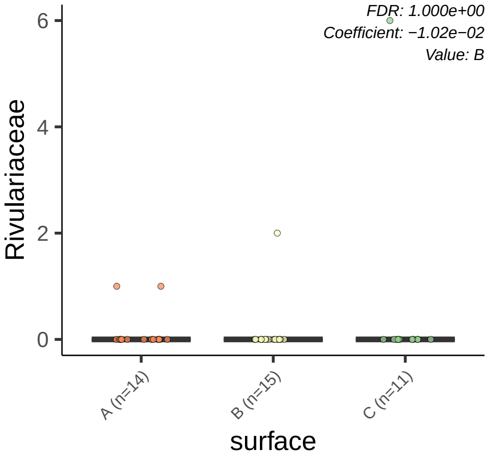
FDR: 1.000e+00
Coefficient: −1.02e−02
Value: B
Rivulariaceae
6
4
2
0
A (n=14)
B (n=15)
C (n=11)
surface

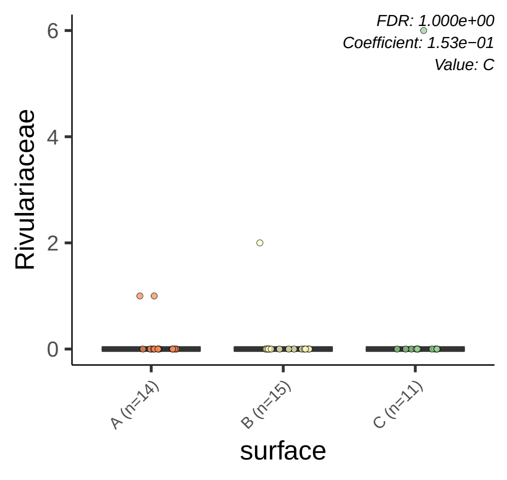

FDR: 1.000e+00
Coefficient: 1.53e−01
Value: C
Rivulariaceae
6
4
2
0
A (n=14)
B (n=15)
C (n=11)
surface

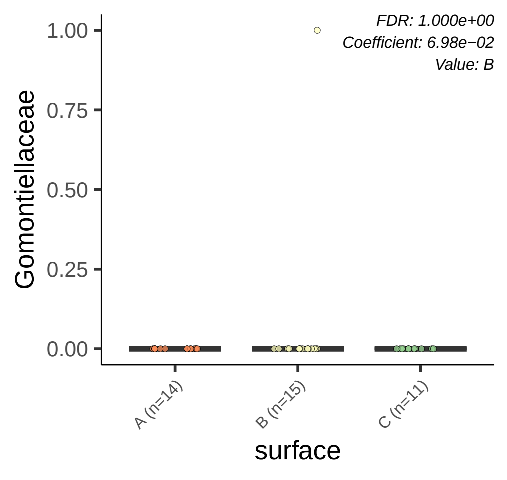
FDR: 1.000e+00
Coefficient: 6.98e−02
Value: B
Gomontiellaceae
1.00
0.75
0.50
0.25
0.00
A (n=14)
B (n=15)
C (n=11)
surface

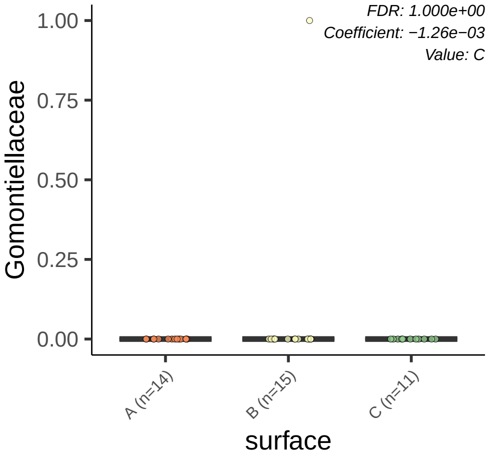

FDR: 1.000e+00
Coefficient: −1.26e−03
Value: C
1.00
0.75
0.50
0.25
0.00
Gomontiellaceae
A (n=14)
B (n=15)
C (n=11)
surface

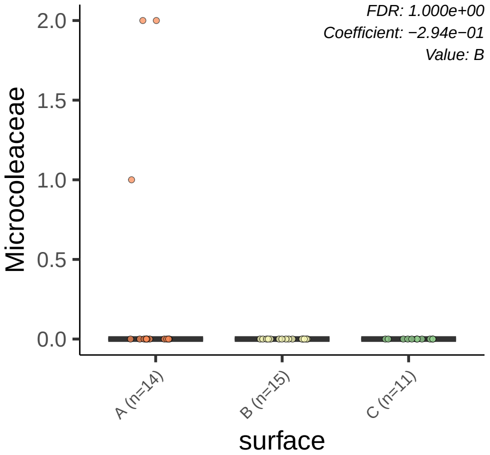
FDR: 1.000e+00
Coefficient: −2.94e−01
Value: B
Microcoleaceae
2.0
1.5
1.0
0.5
0.0
A (n=14)
B (n=15)
C (n=11)
surface

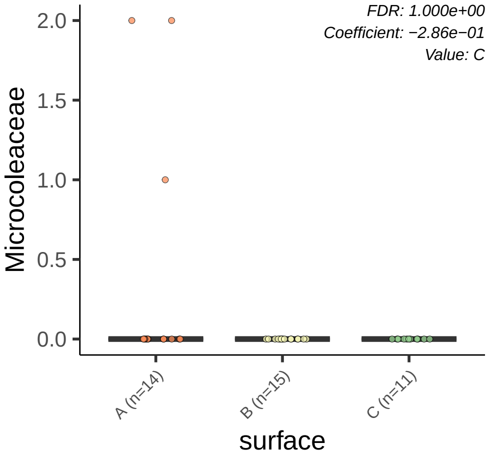
FDR: 1.000e+00
Coefficient: −2.86e−01
Value: C
Microcoleaceae
2.0
1.5
1.0
0.5
0.0
A (n=14)
B (n=15)
C (n=11)
surface

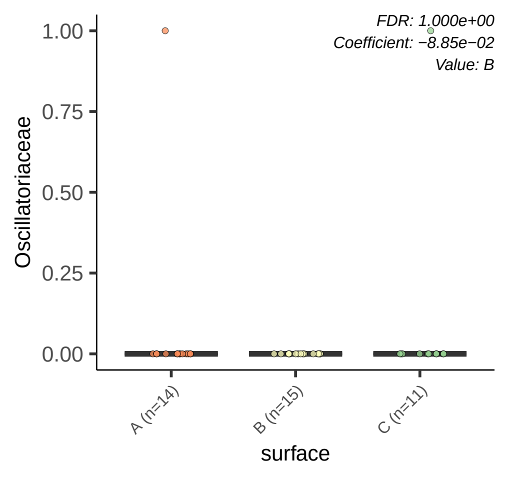

FDR: 1.000e+00
Coefficient: −8.85e−02
Value: B
Oscillatoriaceae
1.00
0.75
0.50
0.25
0.00
A (n=14)
B (n=15)
C (n=11)
surface

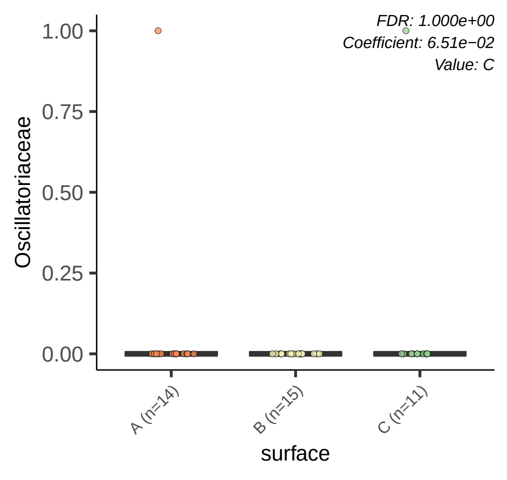

FDR: 1.000e+00
Coefficient: 6.51e−02
Value: C
Oscillatoriaceae
1.00
0.75
0.50
0.25
0.00
A (n=14)
B (n=15)
C (n=11)
surface

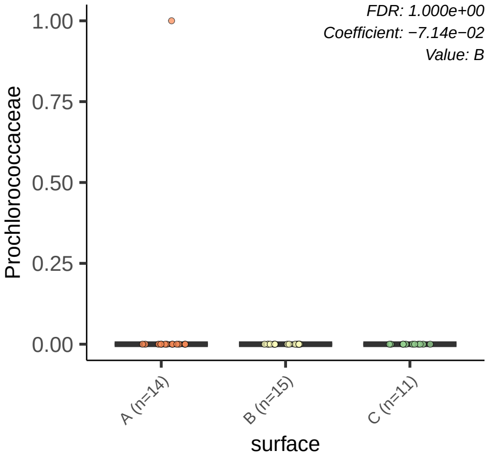

FDR: 1.000e+00
Coefficient: −7.14e−02
Value: B
Prochlorococcaceae
1.00
0.75
0.50
0.25
0.00
A (n=14)
B (n=15)
C (n=11)
surface

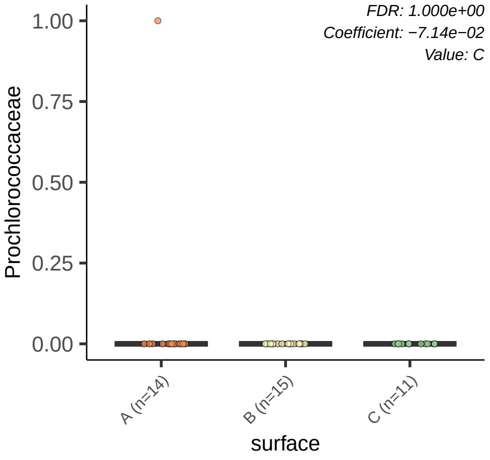

FDR: 1.000e+00
Coefficient: −7.14e−02
Value: C
Prochlorococcaceae
1.00
0.75
0.50
0.25
0.00
A (n=14)
B (n=15)
C (n=11)
surface

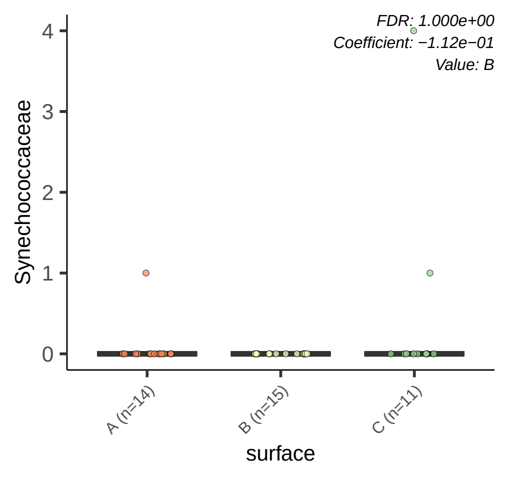

FDR: 1.000e+00
Coefficient: −1.12e−01
Value: B
Synechococcaceae
4
3
2
1
0
A (n=14)
B (n=15)
C (n=11)
surface

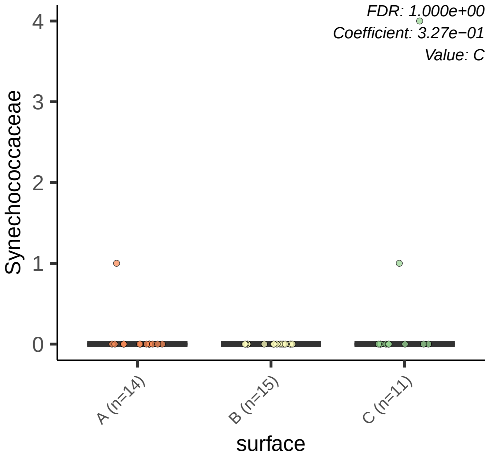

FDR: 1.000e+00
Coefficient: 3.27e−01
Value: C
Synechococcaceae
0
1
2
3
4
A (n=14)
B (n=15)
C (n=11)
surface

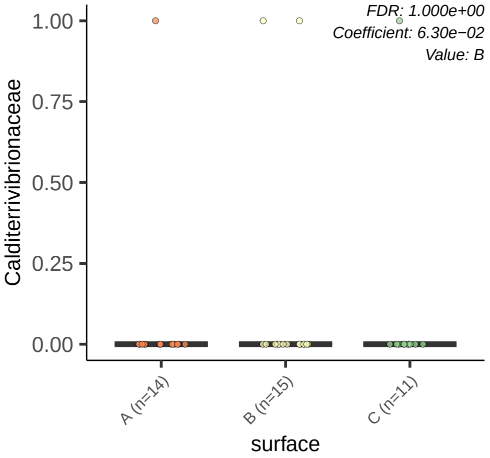
FDR: 1.000e+00
Coefficient: 6.30e−02
Value: B
Calditerrivibrionaceae
1.00
0.75
0.50
0.25
0.00
A (n=14)
B (n=15)
C (n=11)
surface

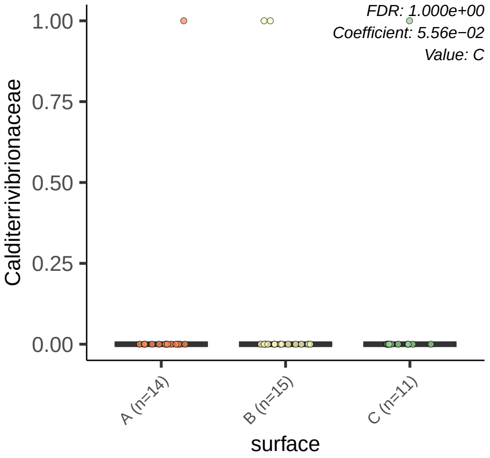
FDR: 1.000e+00
Coefficient: 5.56e−02
Value: C
Calditerrivibrionaceae
1.00
0.75
0.50
0.25
0.00
A (n=14)
B (n=15)
C (n=11)
surface

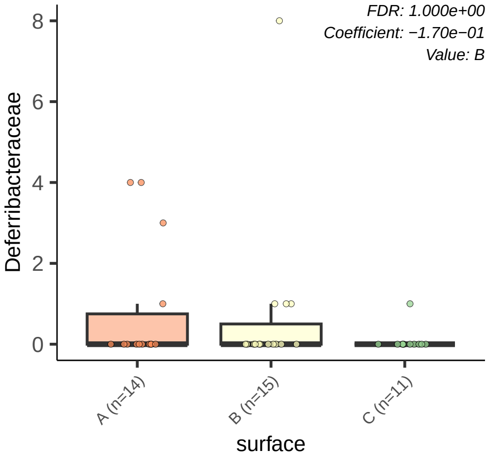

FDR: 1.000e+00
Coefficient: −1.70e−01
Value: B
Deferribacteraceae
0
2
4
6
8
A (n=14)
B (n=15)
C (n=11)
surface

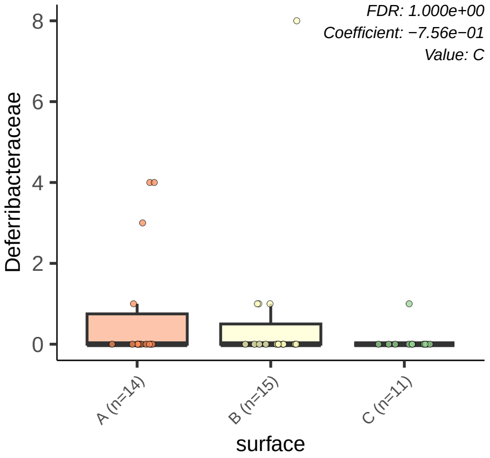

FDR: 1.000e+00
Coefficient: −7.56e−01
Value: C
Deferribacteraceae
0
2
4
6
8
A (n=14)
B (n=15)
C (n=11)
surface

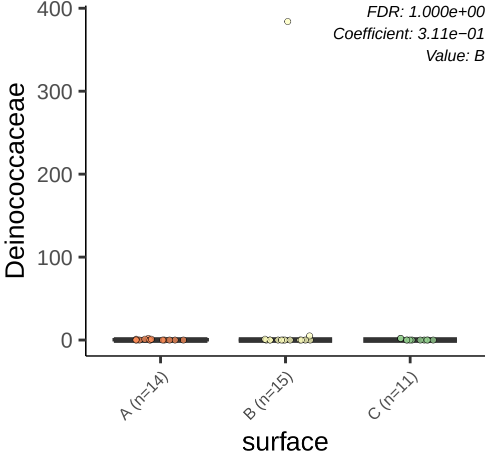

FDR: 1.000e+00
Coefficient: 3.11e−01
Value: B
Deinococcaceae
400
300
200
100
0
A (n=14)
B (n=15)
C (n=11)
surface

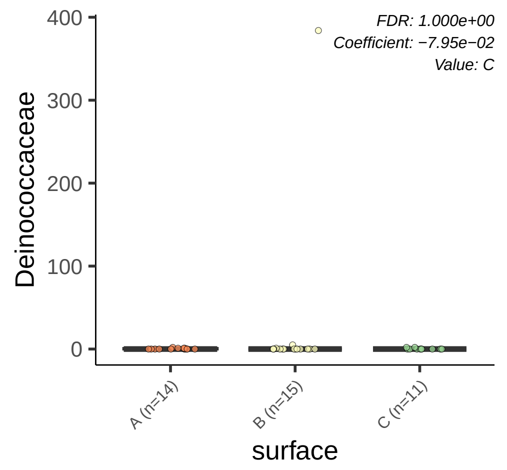

FDR: 1.000e+00
Coefficient: −7.95e−02
Value: C
Deinococcaceae
400
300
200
100
0
A (n=14)
B (n=15)
C (n=11)
surface

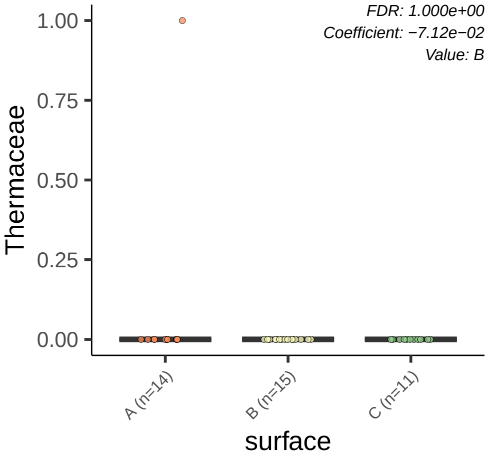
FDR: 1.000e+00
Coefficient: −7.12e−02
Value: B
1.00
0.75
0.50
0.25
0.00
Thermaceae
A (n=14)
B (n=15)
C (n=11)
surface

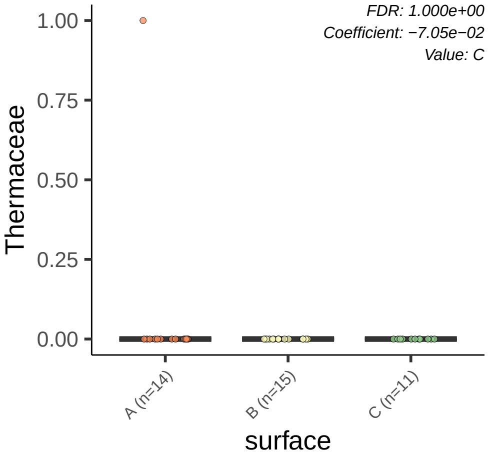
FDR: 1.000e+00
Coefficient: −7.05e−02
Value: C
Thermaceae
1.00
0.75
0.50
0.25
0.00
A (n=14)
B (n=15)
C (n=11)
surface

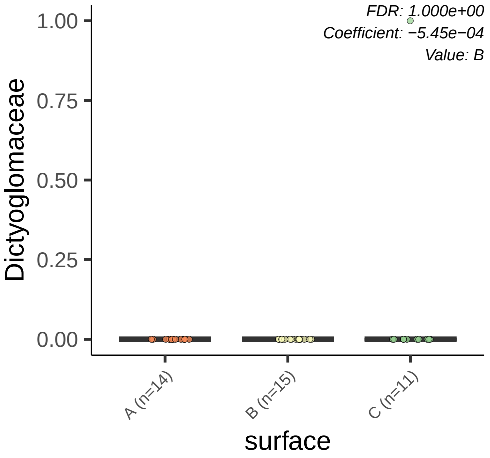

FDR: 1.000e+00
Coefficient: −5.45e−04
Value: B
Dictyoglomaceae
1.00
0.75
0.50
0.25
0.00
A (n=14)
B (n=15)
C (n=11)
surface

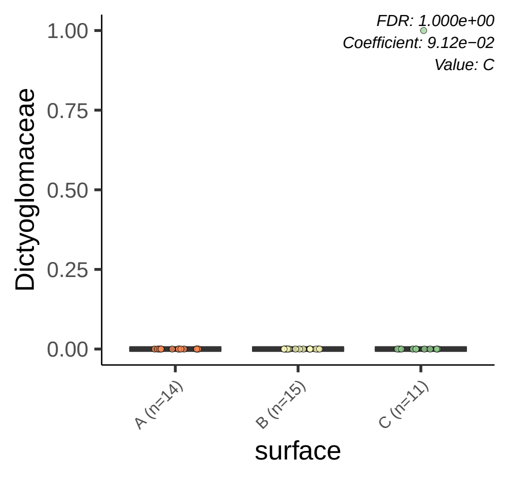

FDR: 1.000e+00
Coefficient: 9.12e−02
Value: C
Dictyoglomaceae
1.00
0.75
0.50
0.25
0.00
A (n=14)
B (n=15)
C (n=11)
surface

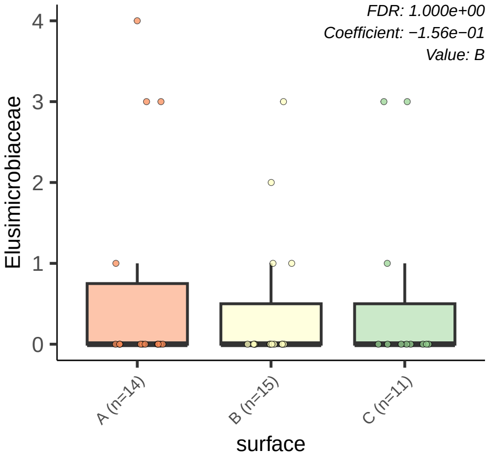
FDR: 1.000e+00
Coefficient: −1.56e−01
Value: B
Elusimicrobiaceae
4
3
2
1
0
A (n=14)
B (n=15)
C (n=11)
surface

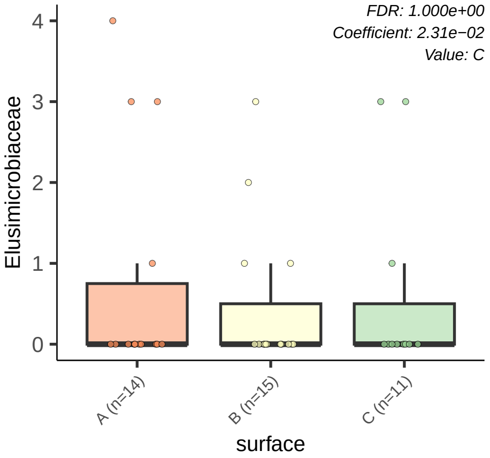

FDR: 1.000e+00
Coefficient: 2.31e−02
Value: C
Elusimicrobiaceae
0
1
2
3
4
A (n=14)
B (n=15)
C (n=11)
surface

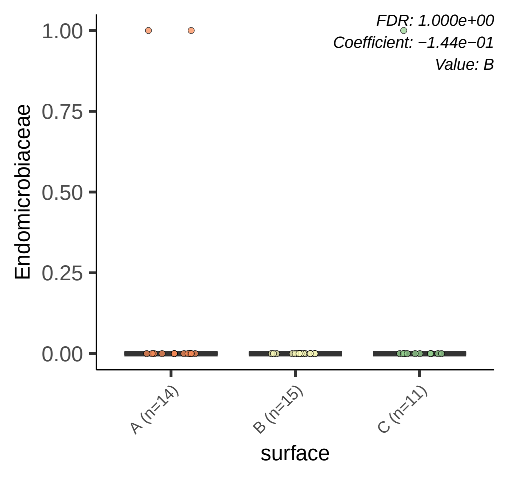
FDR: 1.000e+00
Coefficient: −1.44e−01
Value: B
Endomicrobiaceae
1.00
0.75
0.50
0.25
0.00
A (n=14)
B (n=15)
C (n=11)
surface

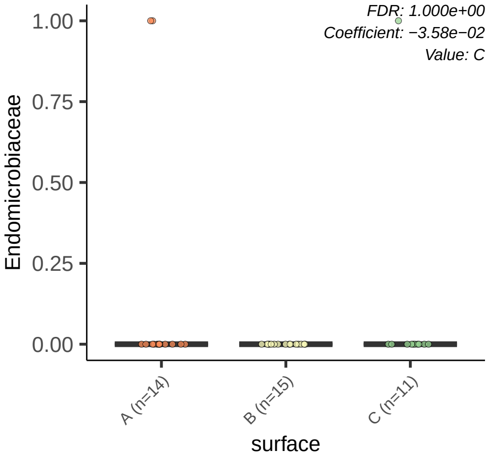

FDR: 1.000e+00
Coefficient: −3.58e−02
Value: C
Endomicrobiaceae
1.00
0.75
0.50
0.25
0.00
A (n=14)
B (n=15)
C (n=11)
surface

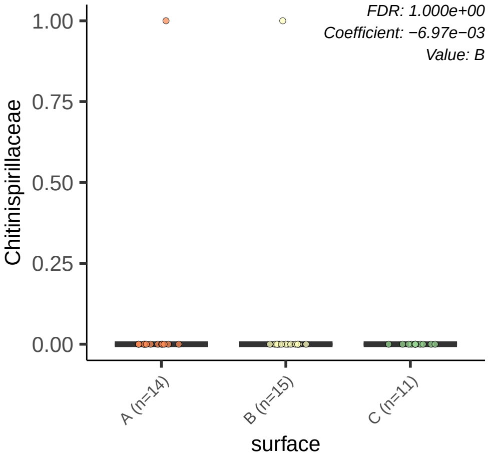
FDR: 1.000e+00
Coefficient: −6.97e−03
Value: B
Chitinispirillaceae
1.00
0.75
0.50
0.25
0.00
A (n=14)
B (n=15)
C (n=11)
surface

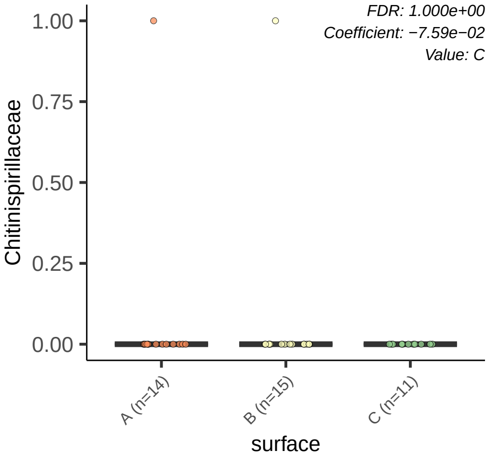
FDR: 1.000e+00
Coefficient: −7.59e−02
Value: C
Chitinispirillaceae
1.00
0.75
0.50
0.25
0.00
A (n=14)
B (n=15)
C (n=11)
surface

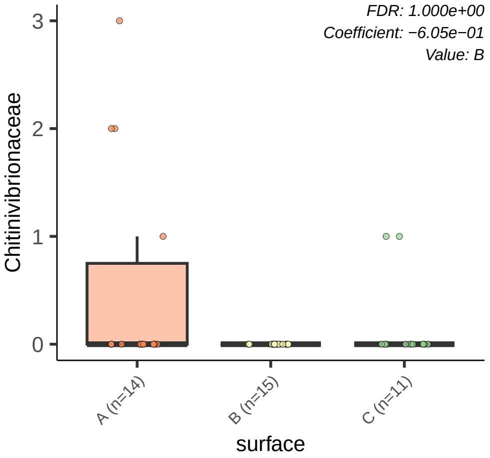
FDR: 1.000e+00
Coefficient: −6.05e−01
Value: B
Chitinivibrionaceae
3
2
1
0
A (n=14)
B (n=15)
C (n=11)
surface

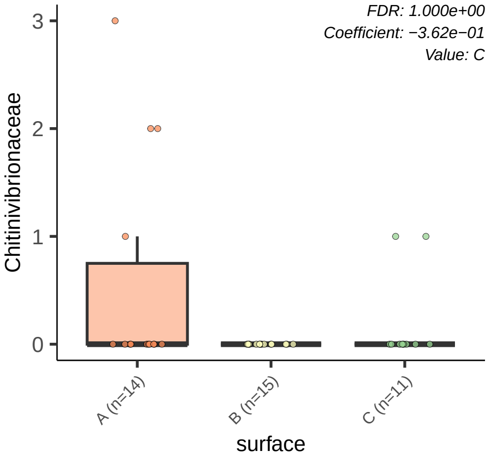

FDR: 1.000e+00
Coefficient: −3.62e−01
Value: C
Chitinivibrionaceae
3
2
1
0
A (n=14)
B (n=15)
C (n=11)
surface

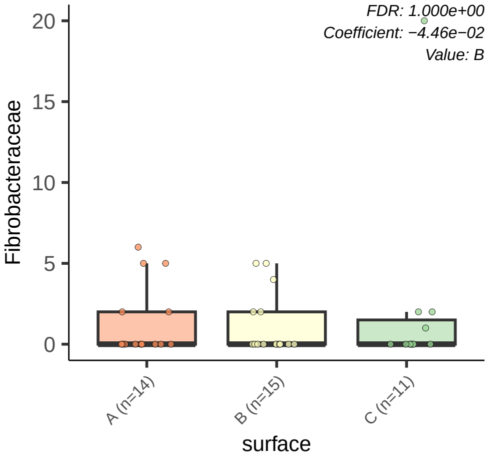

FDR: 1.000e+00
Coefficient: −4.46e−02
Value: B
Fibrobacteraceae
20
15
10
5
0
A (n=14)
B (n=15)
C (n=11)
surface

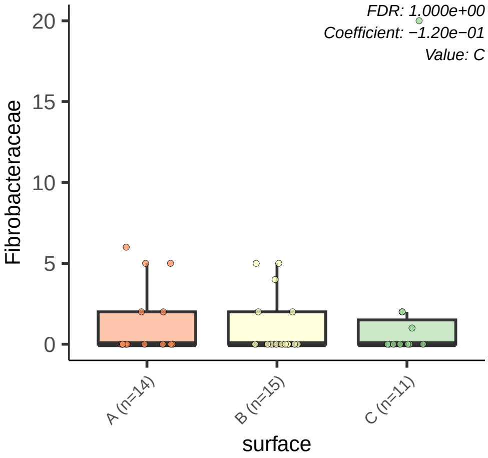
FDR: 1.000e+00
Coefficient: −1.20e−01
Value: C
Fibrobacteraceae
20
15
10
5
0
A (n=14)
B (n=15)
C (n=11)
surface

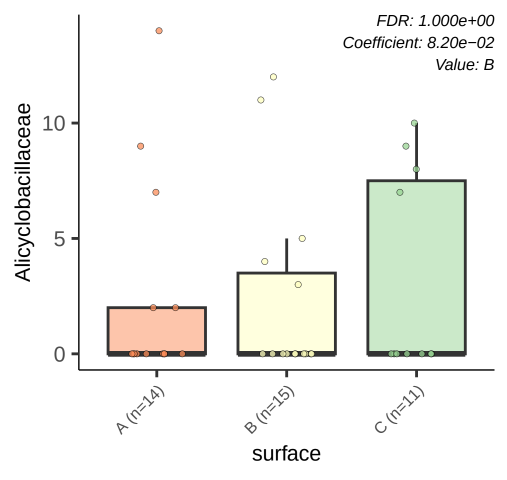
FDR: 1.000e+00
Coefficient: 8.20e−02
Value: B
Alicyclobacillaceae
10
5
0
A (n=14)
B (n=15)
C (n=11)
surface

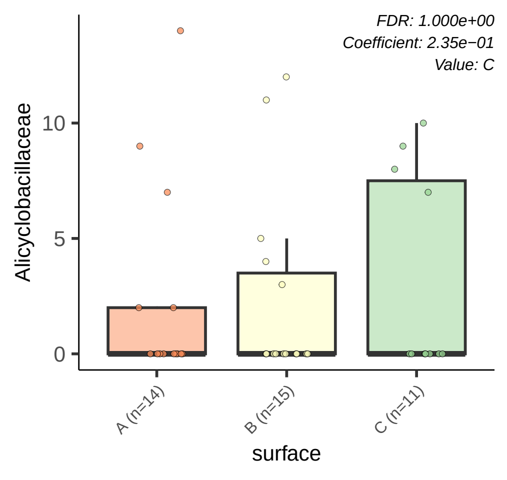
FDR: 1.000e+00
Coefficient: 2.35e−01
Value: C
Alicyclobacillaceae
0
5
10
A (n=14)
B (n=15)
C (n=11)
surface

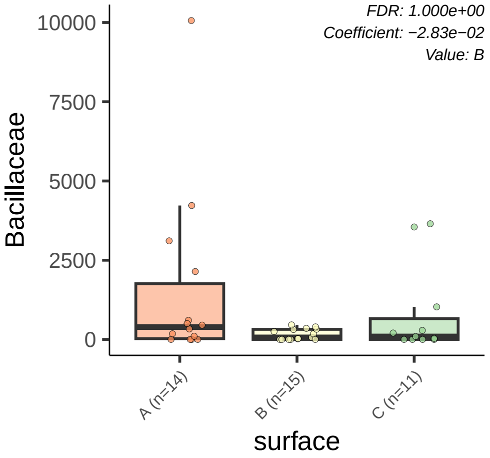

FDR: 1.000e+00
Coefficient: −2.83e−02
Value: B
Bacillaceae
10000
7500
5000
2500
0
A (n=14)
B (n=15)
C (n=11)
surface

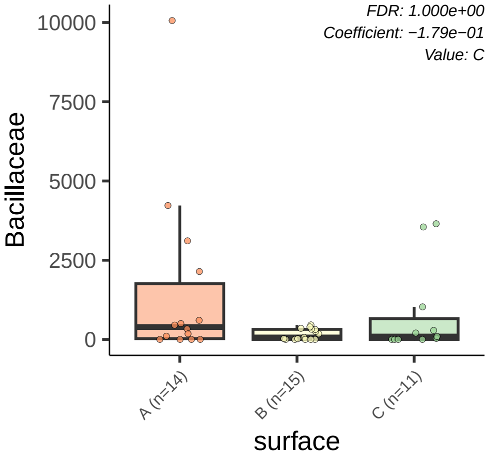
FDR: 1.000e+00
Coefficient: −1.79e−01
Value: C
Bacillaceae
10000
7500
5000
2500
0
A (n=14)
B (n=15)
C (n=11)
surface

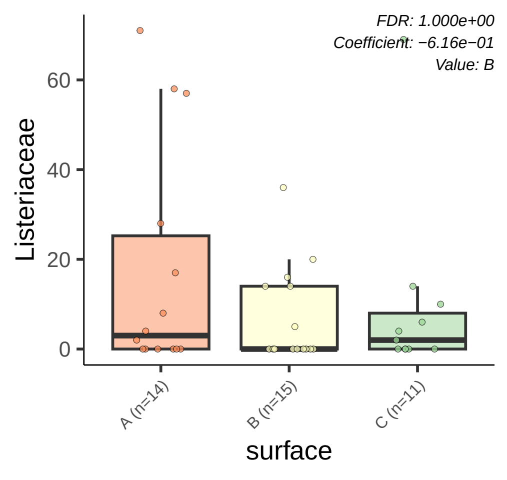

FDR: 1.000e+00
Coefficient: −6.16e−01
Value: B
Listeriaceae
0
20
40
60
A (n=14)
B (n=15)
C (n=11)
surface

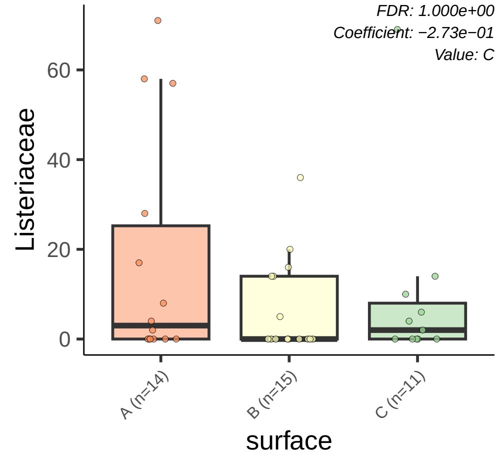
FDR: 1.000e+00
Coefficient: −2.73e−01
Value: C
Listeriaceae
60
40
20
0
A (n=14)
B (n=15)
C (n=11)
surface

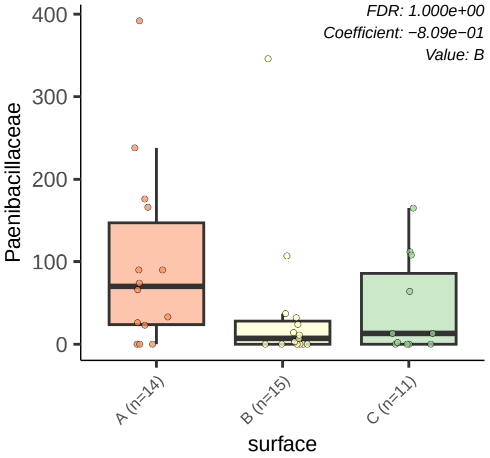
FDR: 1.000e+00
Coefficient: −8.09e−01
Value: B
Paenibacillaceae
400
300
200
100
0
A (n=14)
B (n=15)
C (n=11)
surface

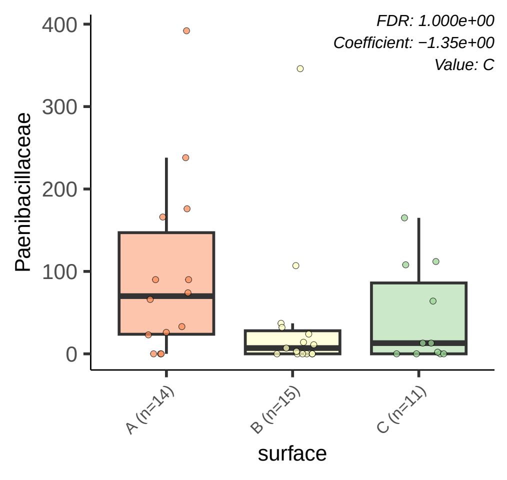

FDR: 1.000e+00
Coefficient: −1.35e+00
Value: C
Paenibacillaceae
400
300
200
100
0
A (n=14)
B (n=15)
C (n=11)
surface

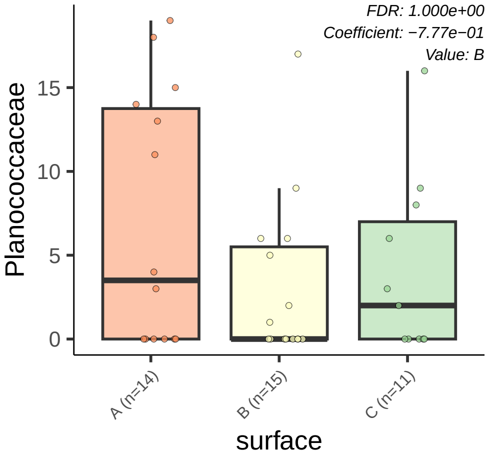
FDR: 1.000e+00
Coefficient: −7.77e−01
Value: B
Planococcaceae
15
10
5
0
A (n=14)
B (n=15)
C (n=11)
surface

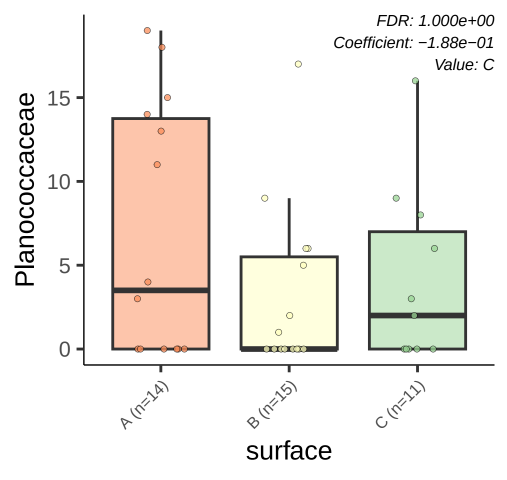

FDR: 1.000e+00
Coefficient: −1.88e−01
Value: C
Planococcaceae
0
5
10
15
A (n=14)
B (n=15)
C (n=11)
surface

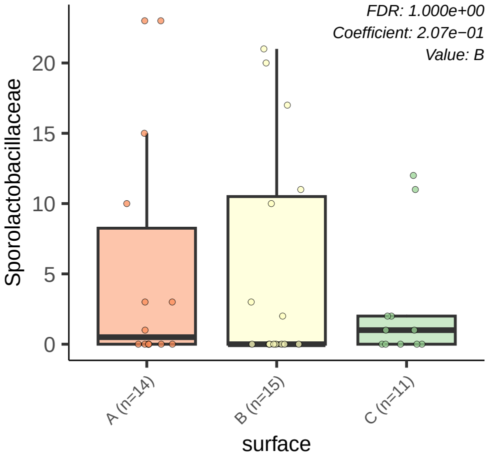
FDR: 1.000e+00
Coefficient: 2.07e−01
Value: B
Sporolactobacillaceae
0
5
10
15
20
A (n=14)
B (n=15)
C (n=11)
surface

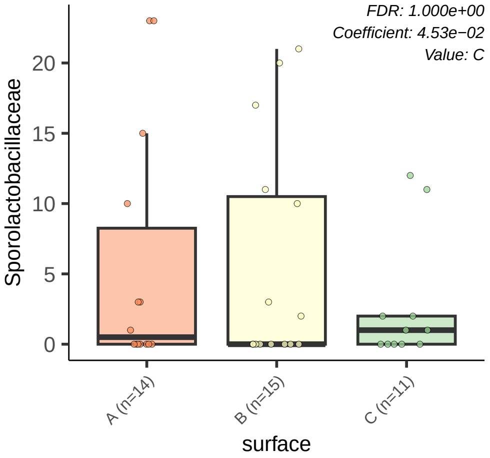
FDR: 1.000e+00
Coefficient: 4.53e−02
Value: C
Sporolactobacillaceae
0
5
10
15
20
A (n=14)
B (n=15)
C (n=11)
surface

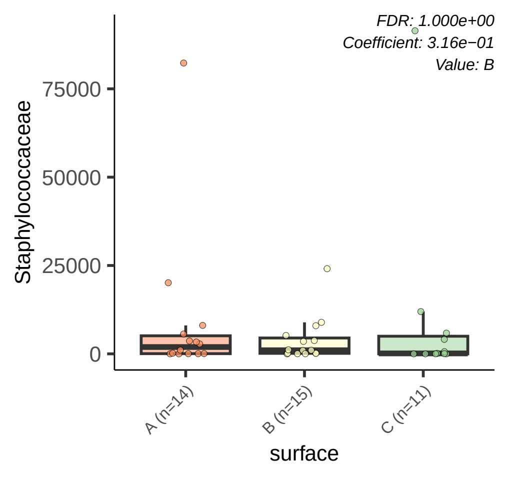

FDR: 1.000e+00
Coefficient: 3.16e−01
Value: B
Staphylococcaceae
75000
50000
25000
0
A (n=14)
B (n=15)
C (n=11)
surface

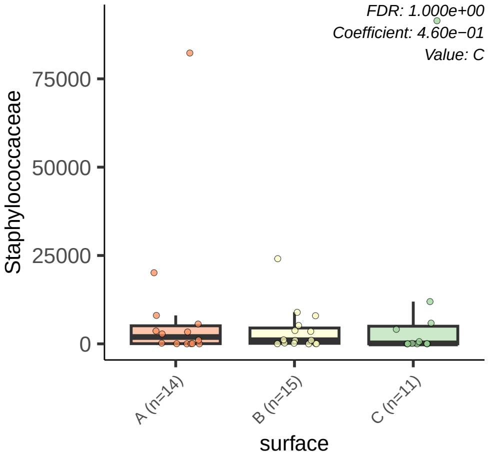

FDR: 1.000e+00
Coefficient: 4.60e−01
Value: C
Staphylococcaceae
75000
50000
25000
0
A (n=14)
B (n=15)
C (n=11)
surface

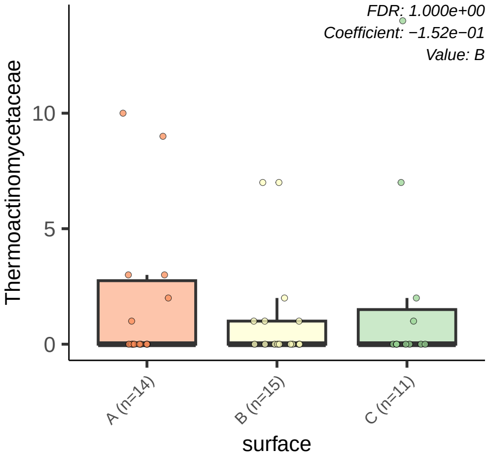

FDR: 1.000e+00
Coefficient: −1.52e−01
Value: B
Thermoactinomycetaceae
10
5
0
A (n=14)
B (n=15)
C (n=11)
surface

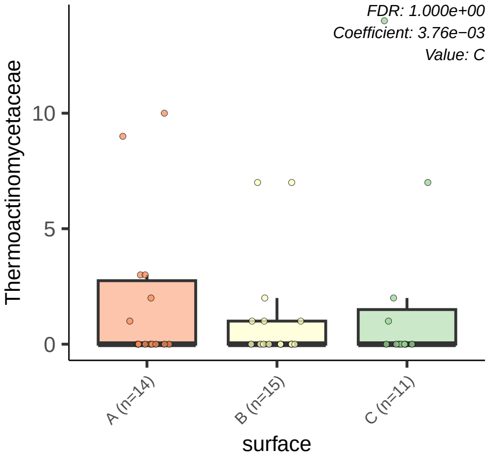

FDR: 1.000e+00
Coefficient: 3.76e−03
Value: C
Thermoactinomycetaceae
10
5
0
A (n=14)
B (n=15)
C (n=11)
surface

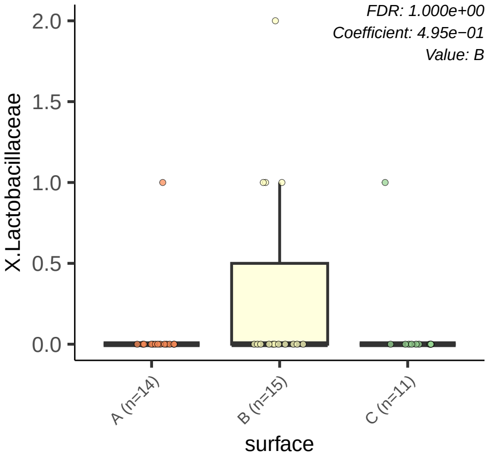
FDR: 1.000e+00
Coefficient: 4.95e−01
Value: B
X.Lactobacillaceae
2.0
1.5
1.0
0.5
0.0
A (n=14)
B (n=15)
C (n=11)
surface

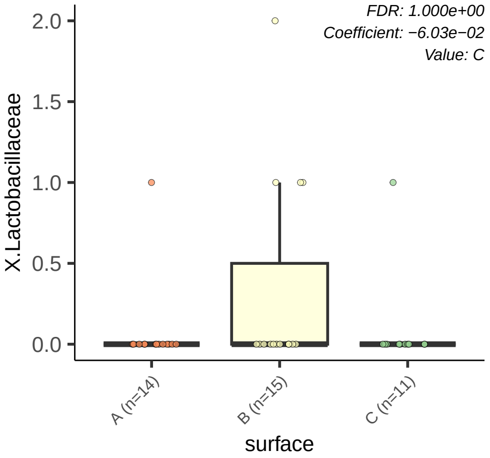
FDR: 1.000e+00
Coefficient: −6.03e−02
Value: C
X.Lactobacillaceae
2.0
1.5
1.0
0.5
0.0
A (n=14)
B (n=15)
C (n=11)
surface

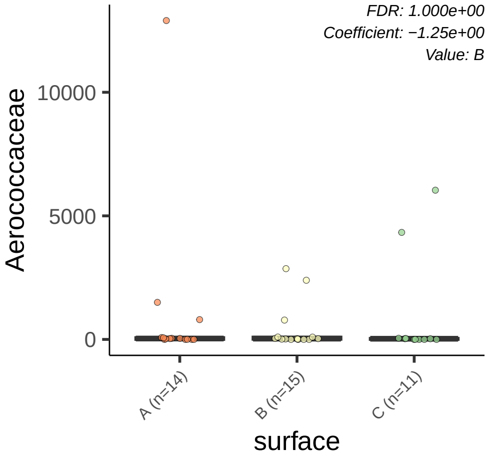

FDR: 1.000e+00
Coefficient: −1.25e+00
Value: B
Aerococcaceae
10000
5000
0
A (n=14)
B (n=15)
C (n=11)
surface

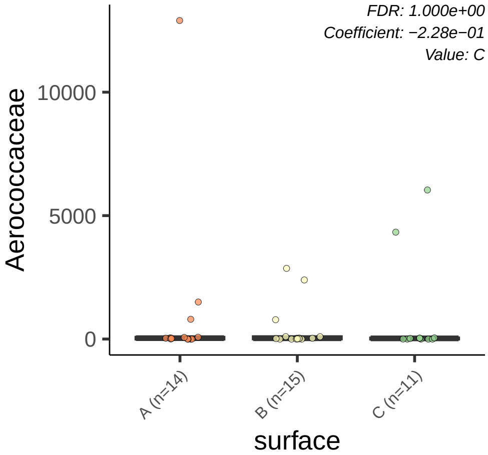
FDR: 1.000e+00
Coefficient: −2.28e−01
Value: C
Aerococcaceae
10000
5000
0
A (n=14)
B (n=15)
C (n=11)
surface

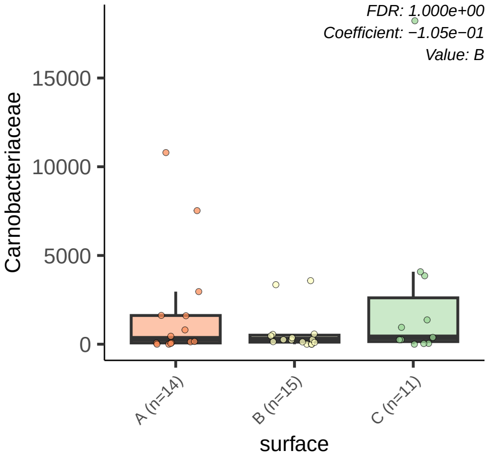

FDR: 1.000e+00
Coefficient: −1.05e−01
Value: B
15000
10000
5000
0
Carnobacteriaceae
A (n=14)
B (n=15)
C (n=11)
surface

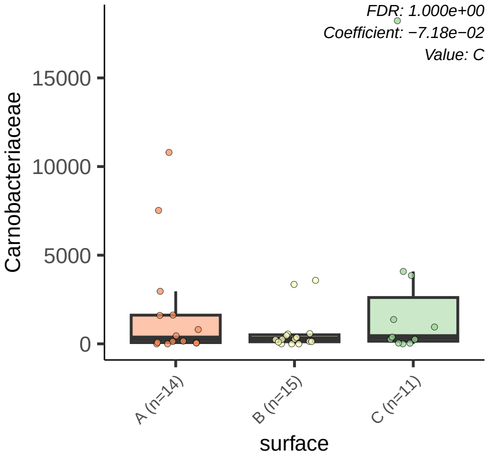

FDR: 1.000e+00
Coefficient: −7.18e−02
Value: C
Carnobacteriaceae
15000
10000
5000
0
A (n=14)
B (n=15)
C (n=11)
surface

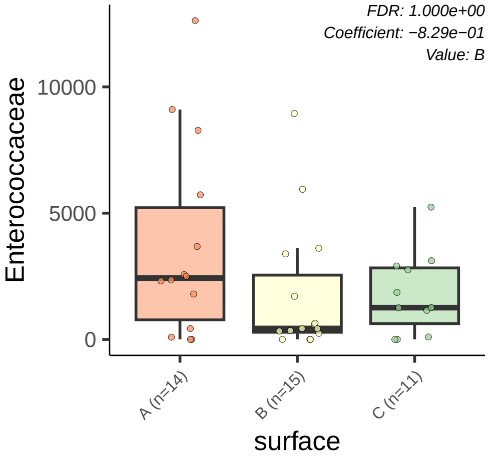

FDR: 1.000e+00
Coefficient: −8.29e−01
Value: B
Enterococcaceae
10000
5000
0
A (n=14)
B (n=15)
C (n=11)
surface

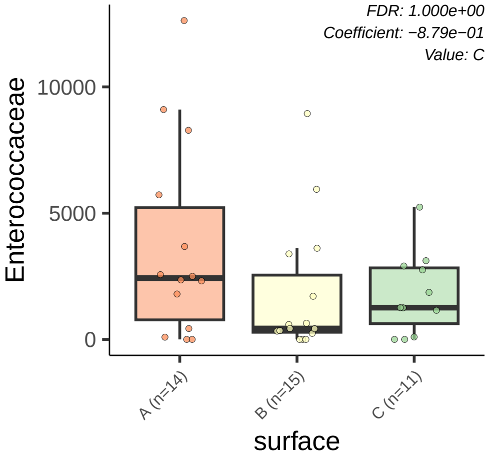
FDR: 1.000e+00
Coefficient: −8.79e−01
Value: C
Enterococcaceae
10000
5000
0
A (n=14)
B (n=15)
C (n=11)
surface

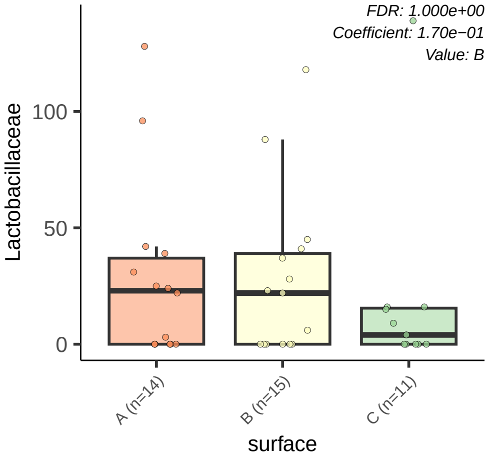

FDR: 1.000e+00
Coefficient: 1.70e−01
Value: B
Lactobacillaceae
100
50
0
A (n=14)
B (n=15)
C (n=11)
surface

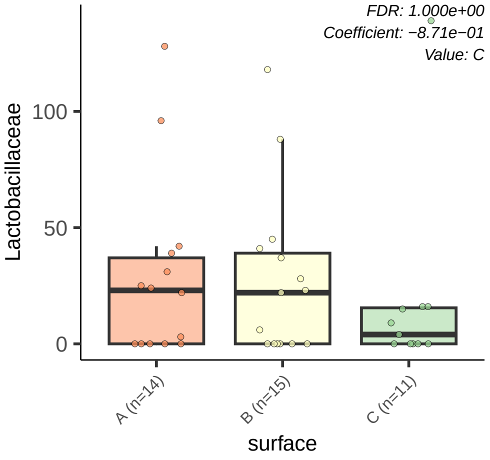

FDR: 1.000e+00
Coefficient: −8.71e−01
Value: C
Lactobacillaceae
100
50
0
A (n=14)
B (n=15)
C (n=11)
surface

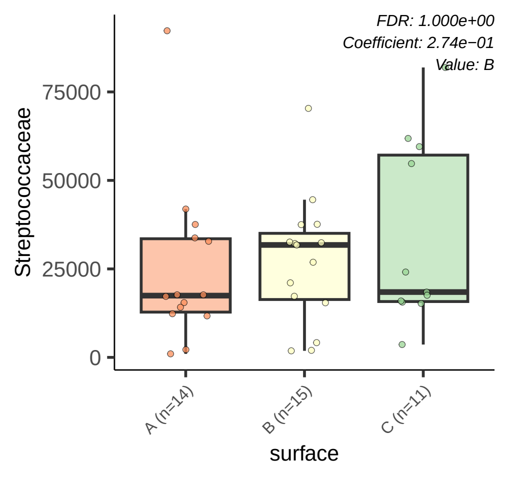
FDR: 1.000e+00
Coefficient: 2.74e−01
Value: B
Streptococcaceae
75000
50000
25000
0
A (n=14)
B (n=15)
C (n=11)
surface

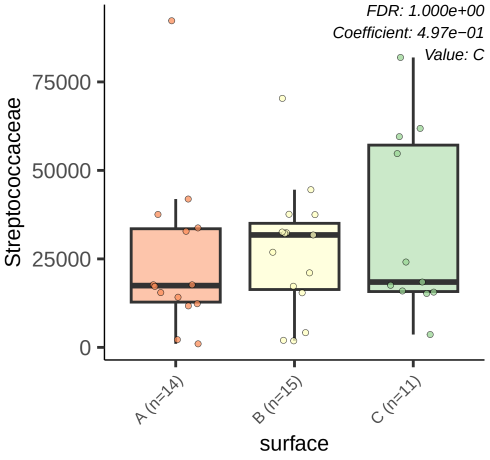

FDR: 1.000e+00
Coefficient: 4.97e−01
Value: C
Streptococcaceae
75000
50000
25000
0
A (n=14)
B (n=15)
C (n=11)
surface

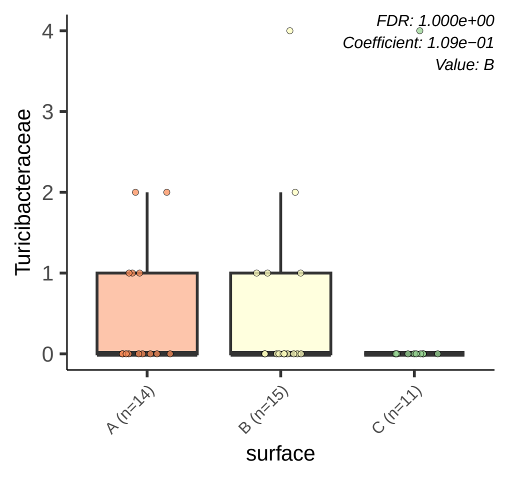

FDR: 1.000e+00
Coefficient: 1.09e−01
Value: B
Turicibacteraceae
0
1
2
3
4
A (n=14)
B (n=15)
C (n=11)
surface

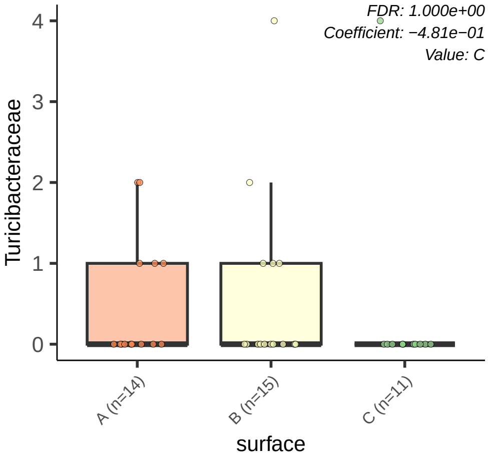
FDR: 1.000e+00
Coefficient: −4.81e−01
Value: C
Turicibacteraceae
4
3
2
1
0
A (n=14)
B (n=15)
C (n=11)
surface

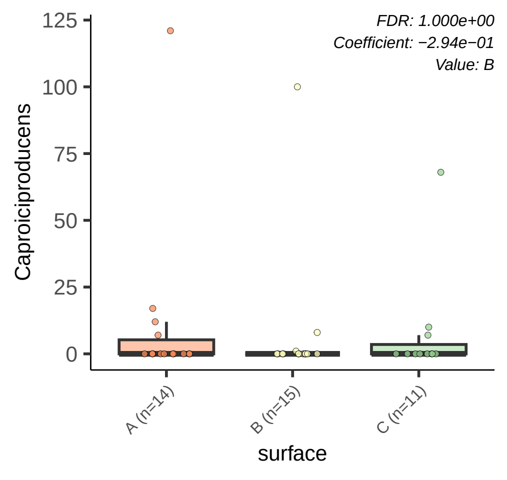

FDR: 1.000e+00
Coefficient: −2.94e−01
Value: B
Caproiciproducens
125
100
75
50
25
0
A (n=14)
B (n=15)
C (n=11)
surface

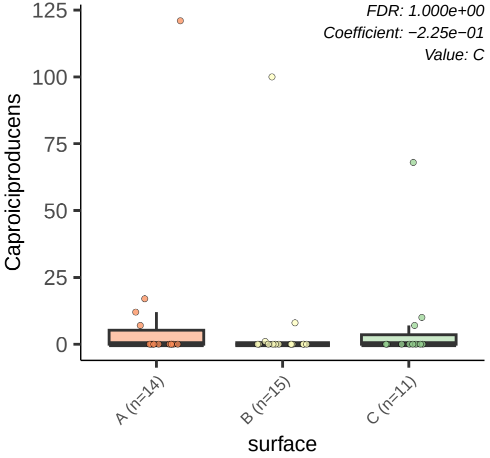
FDR: 1.000e+00
Coefficient: −2.25e−01
Value: C
125
100
75
50
25
0
Caproiciproducens
A (n=14)
B (n=15)
C (n=11)
surface

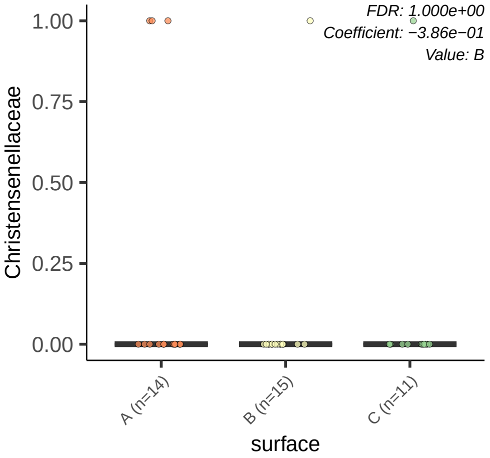

FDR: 1.000e+00
Coefficient: −3.86e−01
Value: B
Christensenellaceae
1.00
0.75
0.50
0.25
0.00
A (n=14)
B (n=15)
C (n=11)
surface

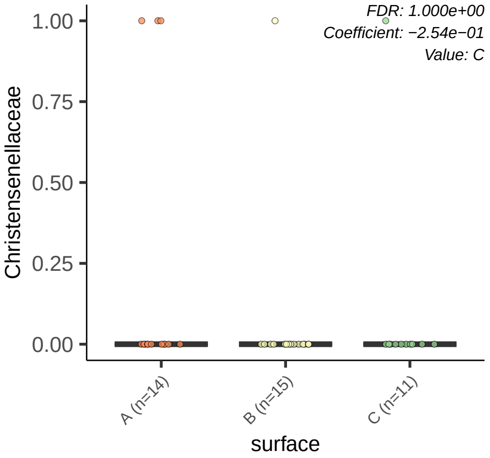
FDR: 1.000e+00
Coefficient: −2.54e−01
Value: C
Christensenellaceae
1.00
0.75
0.50
0.25
0.00
A (n=14)
B (n=15)
C (n=11)
surface

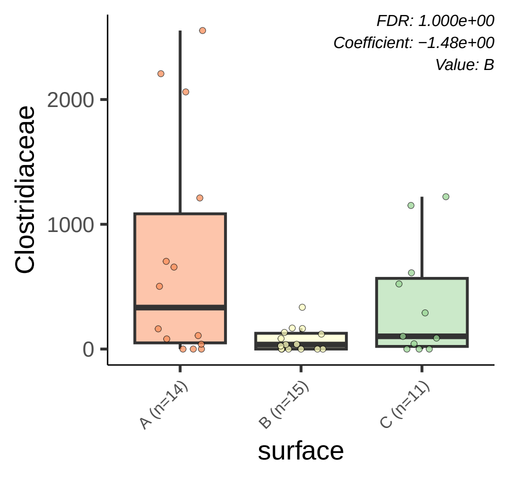
FDR: 1.000e+00
Coefficient: −1.48e+00
Value: B
Clostridiaceae
2000
1000
0
A (n=14)
B (n=15)
C (n=11)
surface

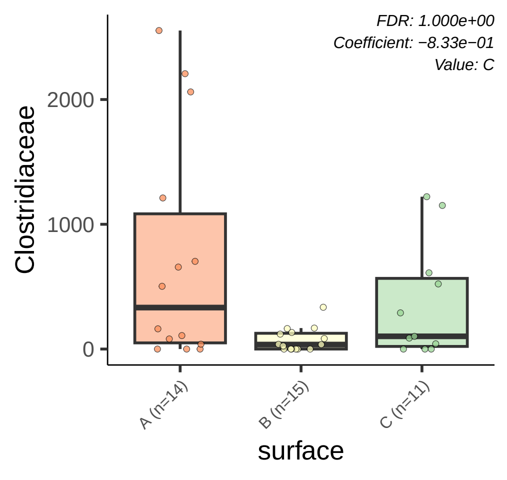
FDR: 1.000e+00
Coefficient: −8.33e−01
Value: C
Clostridiaceae
2000
1000
0
A (n=14)
B (n=15)
C (n=11)
surface

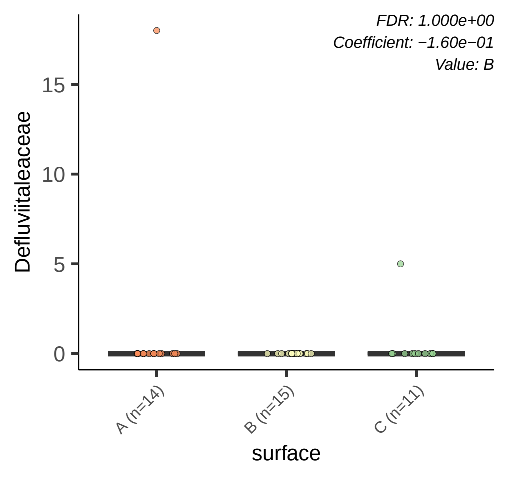
FDR: 1.000e+00
Coefficient: −1.60e−01
Value: B
Defluviitaleaceae
15
10
5
0
A (n=14)
B (n=15)
C (n=11)
surface

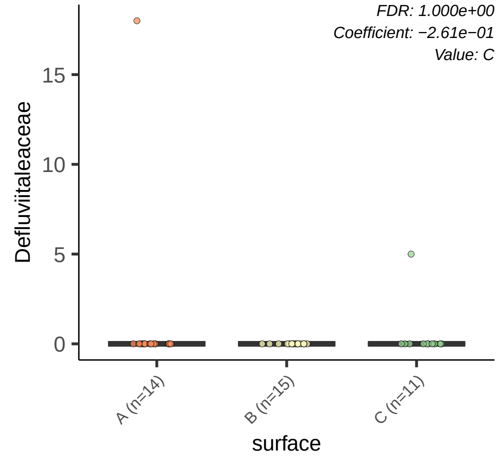

FDR: 1.000e+00
Coefficient: −2.61e−01
Value: C
Defluviitaleaceae
15
10
5
0
A (n=14)
B (n=15)
C (n=11)
surface

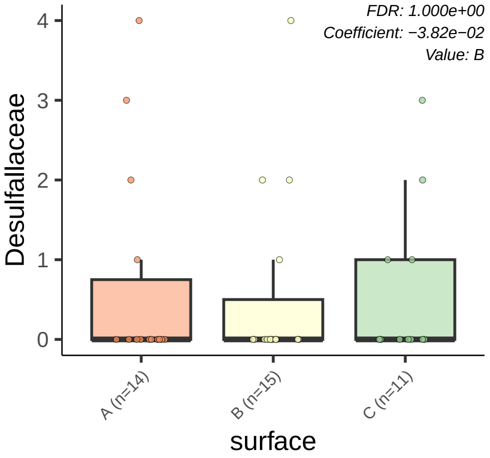
FDR: 1.000e+00
Coefficient: −3.82e−02
Value: B
Desulfallaceae
4
3
2
1
0
A (n=14)
B (n=15)
C (n=11)
surface

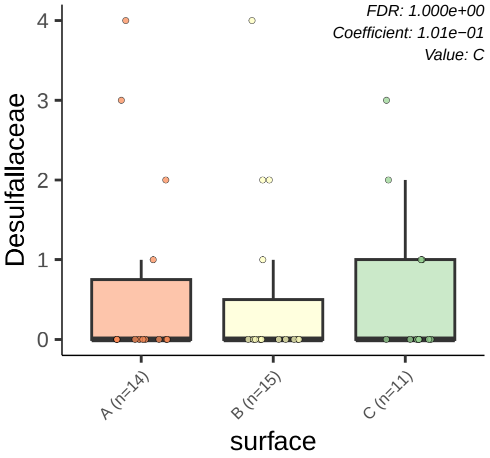

FDR: 1.000e+00
Coefficient: 1.01e−01
Value: C
Desulfallaceae
4
3
2
1
0
A (n=14)
B (n=15)
C (n=11)
surface

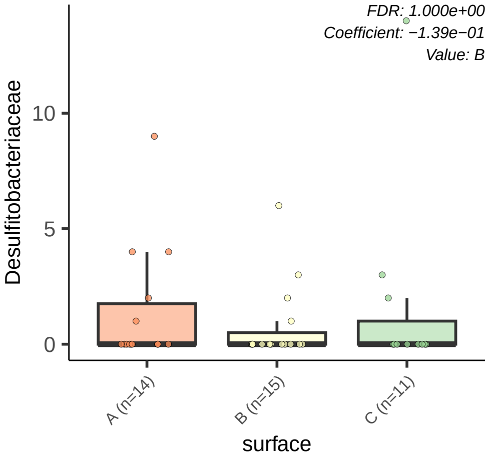

FDR: 1.000e+00
Coefficient: −1.39e−01
Value: B
Desulfitobacteriaceae
10
5
0
A (n=14)
B (n=15)
C (n=11)
surface

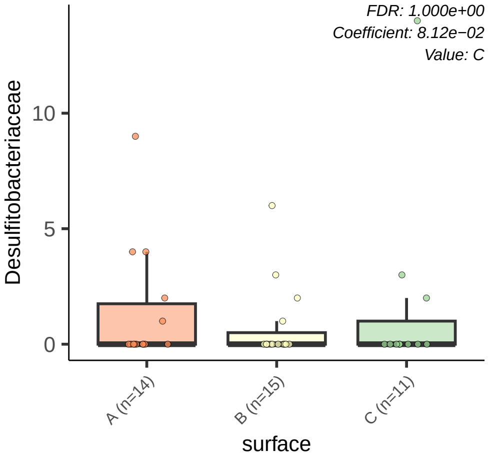
FDR: 1.000e+00
Coefficient: 8.12e−02
Value: C
Desulfitobacteriaceae
10
5
0
A (n=14)
B (n=15)
C (n=11)
surface

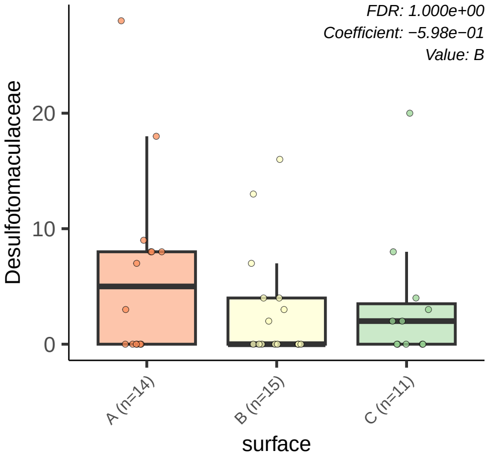

FDR: 1.000e+00
Coefficient: −5.98e−01
Value: B
Desulfotomaculaceae
0
10
20
A (n=14)
B (n=15)
C (n=11)
surface

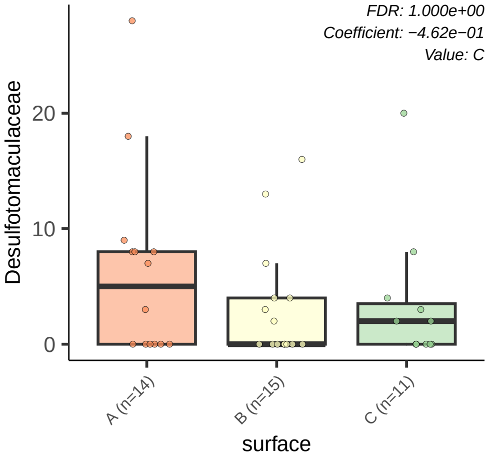
FDR: 1.000e+00
Coefficient: −4.62e−01
Value: C
Desulfotomaculaceae
0
10
20
A (n=14)
B (n=15)
C (n=11)
surface

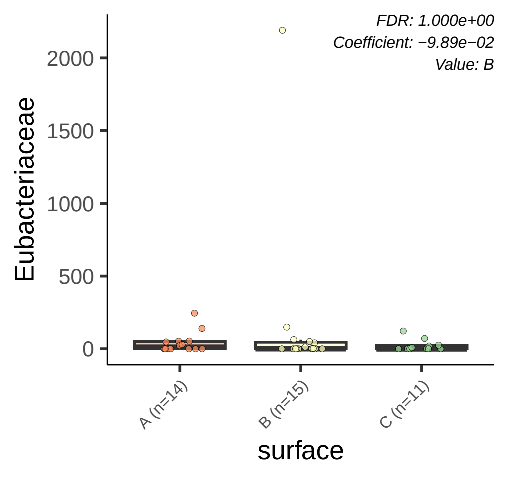

FDR: 1.000e+00
Coefficient: −9.89e−02
Value: B
Eubacteriaceae
2000
1500
1000
500
0
A (n=14)
B (n=15)
C (n=11)
surface

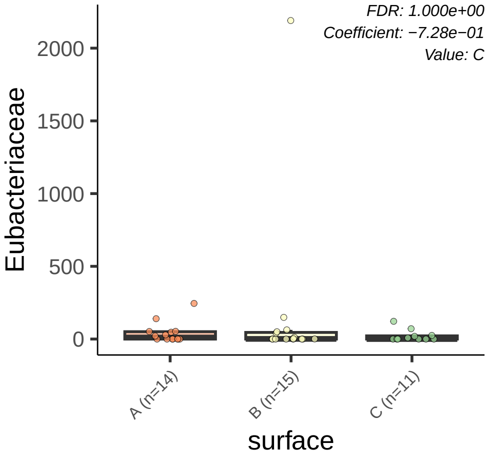

FDR: 1.000e+00
Coefficient: −7.28e−01
Value: C
Eubacteriaceae
2000
1500
1000
500
0
A (n=14)
B (n=15)
C (n=11)
surface

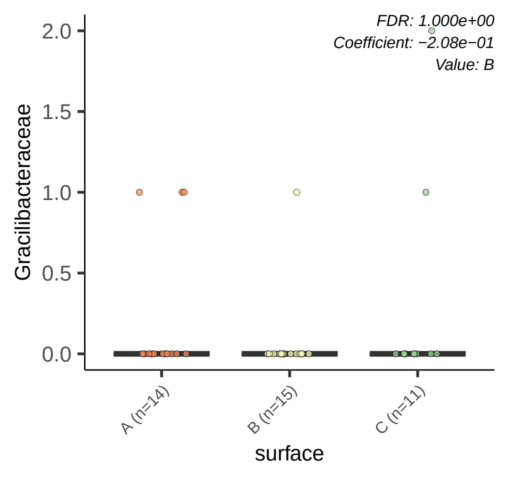

FDR: 1.000e+00
Coefficient: −2.08e−01
Value: B
Gracilibacteraceae
2.0
1.5
1.0
0.5
0.0
A (n=14)
B (n=15)
C (n=11)
surface

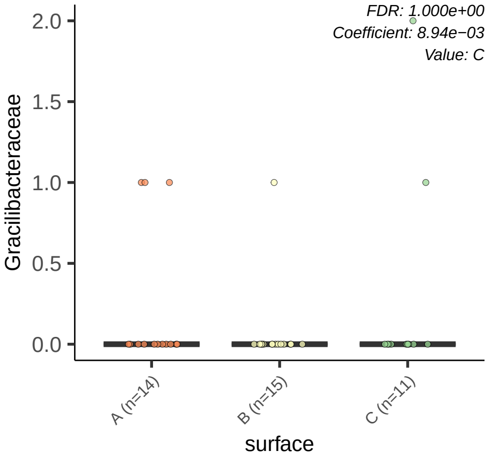

FDR: 1.000e+00
Coefficient: 8.94e−03
Value: C
Gracilibacteraceae
2.0
1.5
1.0
0.5
0.0
A (n=14)
B (n=15)
C (n=11)
surface

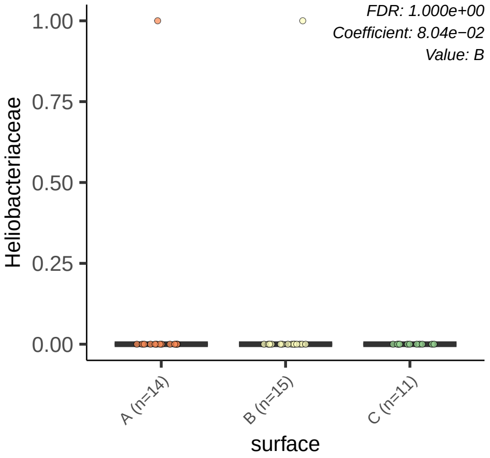

FDR: 1.000e+00
Coefficient: 8.04e−02
Value: B
Heliobacteriaceae
1.00
0.75
0.50
0.25
0.00
A (n=14)
B (n=15)
C (n=11)
surface

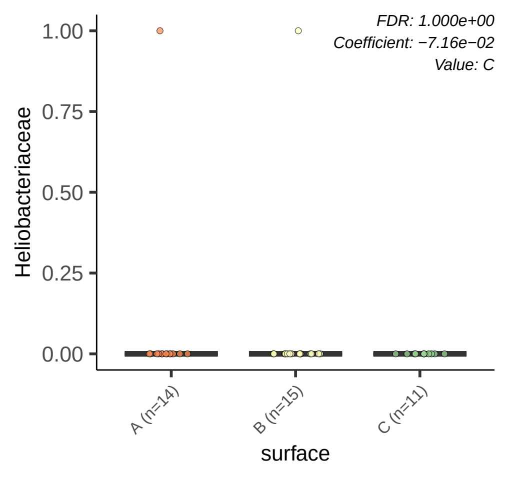
FDR: 1.000e+00
Coefficient: −7.16e−02
Value: C
Heliobacteriaceae
1.00
0.75
0.50
0.25
0.00
A (n=14)
B (n=15)
C (n=11)
surface

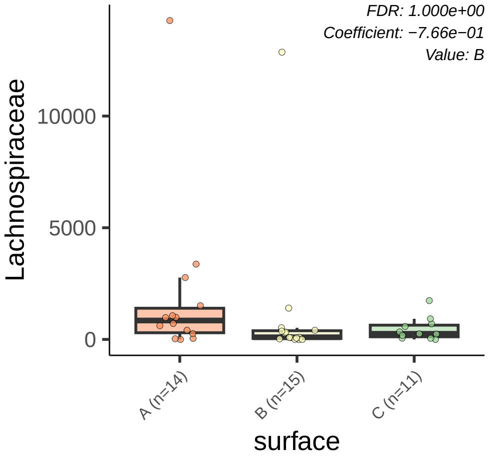

FDR: 1.000e+00
Coefficient: −7.66e−01
Value: B
Lachnospiraceae
10000
5000
0
A (n=14)
B (n=15)
C (n=11)
surface

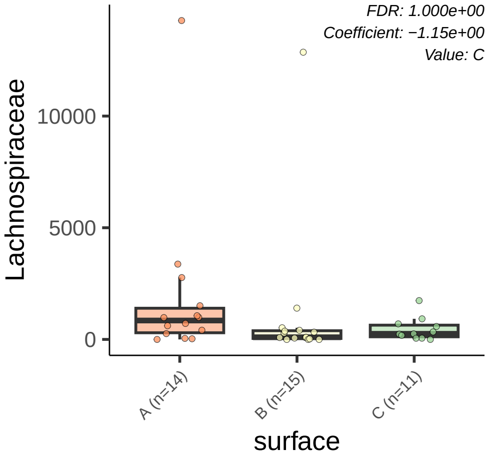

FDR: 1.000e+00
Coefficient: −1.15e+00
Value: C
Lachnospiraceae
10000
5000
0
A (n=14)
B (n=15)
C (n=11)
surface

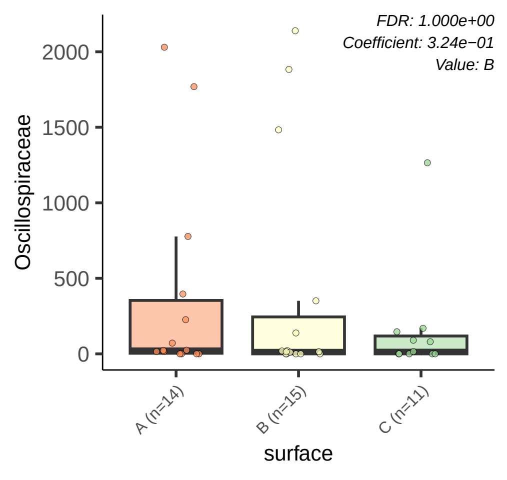

FDR: 1.000e+00
Coefficient: 3.24e−01
Value: B
Oscillospiraceae
2000
1500
1000
500
0
A (n=14)
B (n=15)
C (n=11)
surface

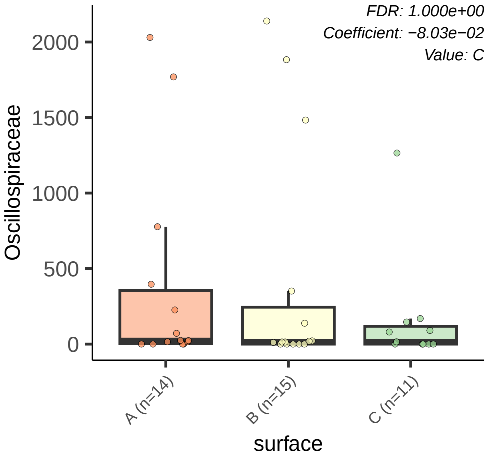
FDR: 1.000e+00
Coefficient: −8.03e−02
Value: C
Oscillospiraceae
0
500
1000
1500
2000
A (n=14)
B (n=15)
C (n=11)
surface

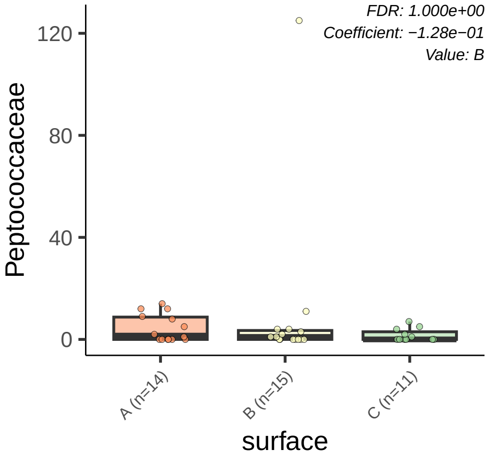
FDR: 1.000e+00
Coefficient: −1.28e−01
Value: B
Peptococcaceae
120
80
40
0
A (n=14)
B (n=15)
C (n=11)
surface

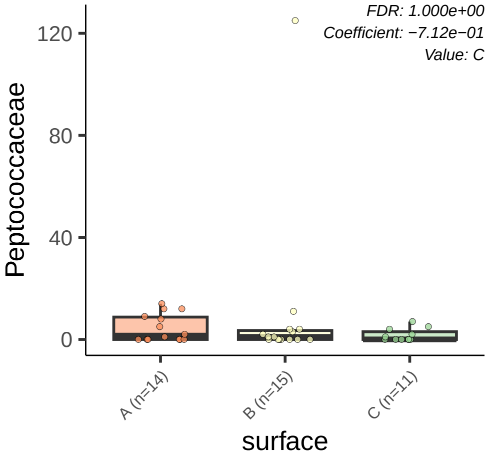

FDR: 1.000e+00
Coefficient: −7.12e−01
Value: C
Peptococcaceae
120
80
40
0
A (n=14)
B (n=15)
C (n=11)
surface

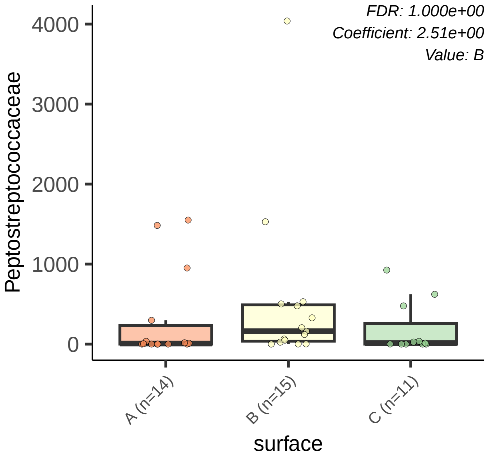
FDR: 1.000e+00
Coefficient: 2.51e+00
Value: B
Peptostreptococcaceae
4000
3000
2000
1000
0
A (n=14)
B (n=15)
C (n=11)
surface

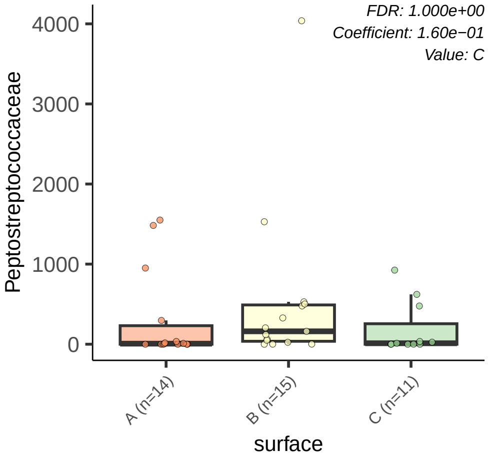

FDR: 1.000e+00
Coefficient: 1.60e−01
Value: C
Peptostreptococcaceae
4000
3000
2000
1000
0
A (n=14)
B (n=15)
C (n=11)
surface

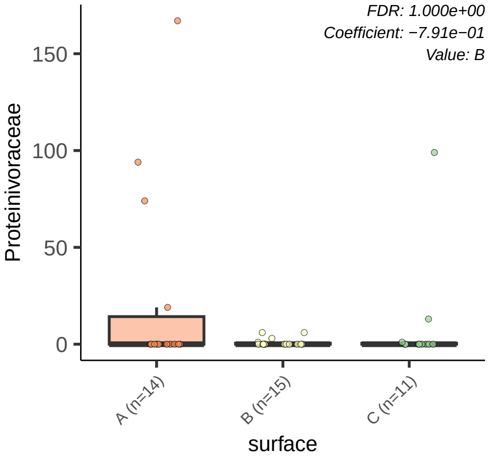

FDR: 1.000e+00
Coefficient: −7.91e−01
Value: B
Proteinivoraceae
150
100
50
0
A (n=14)
B (n=15)
C (n=11)
surface

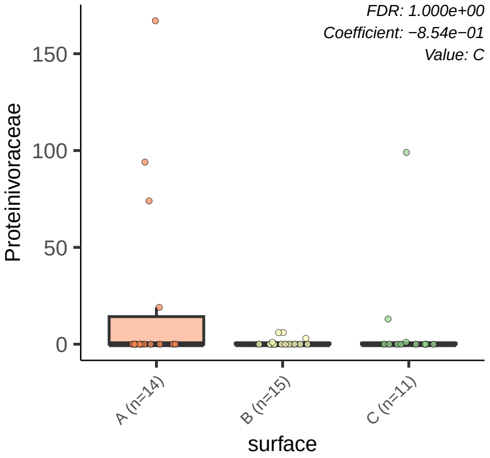

FDR: 1.000e+00
Coefficient: −8.54e−01
Value: C
Proteinivoraceae
150
100
50
0
A (n=14)
B (n=15)
C (n=11)
surface

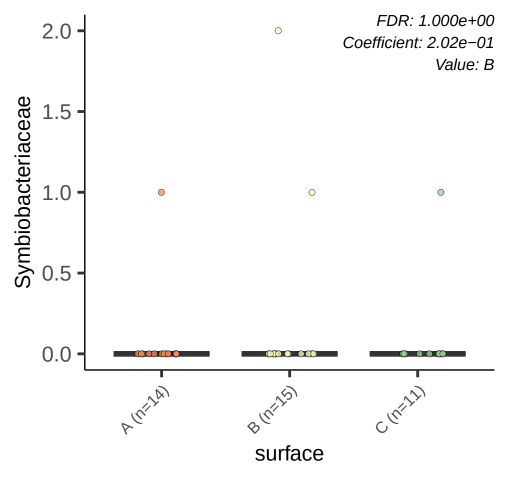
FDR: 1.000e+00
Coefficient: 2.02e−01
Value: B
Symbiobacteriaceae
2.0
1.5
1.0
0.5
0.0
A (n=14)
B (n=15)
C (n=11)
surface

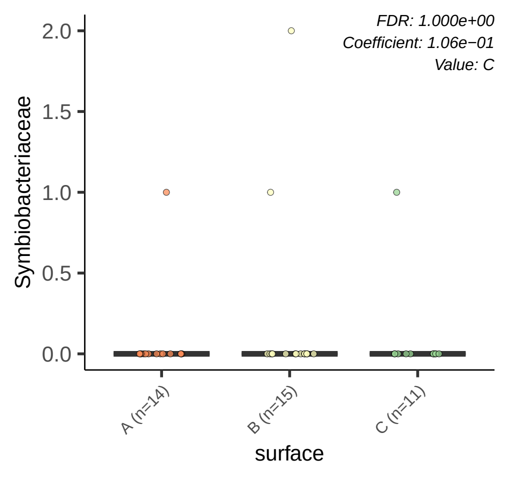

FDR: 1.000e+00
Coefficient: 1.06e−01
Value: C
Symbiobacteriaceae
2.0
1.5
1.0
0.5
0.0
A (n=14)
B (n=15)
C (n=11)
surface

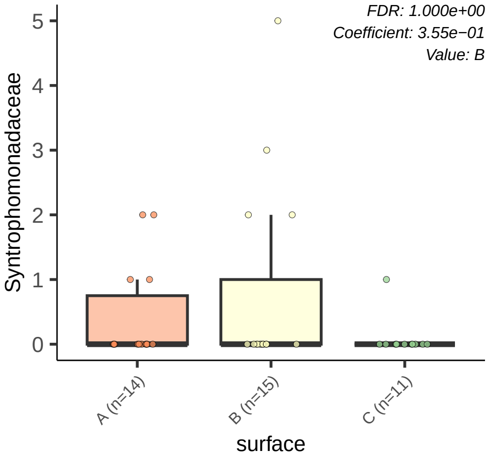

FDR: 1.000e+00
Coefficient: 3.55e−01
Value: B
Syntrophomonadaceae
0
1
2
3
4
5
A (n=14)
B (n=15)
C (n=11)
surface

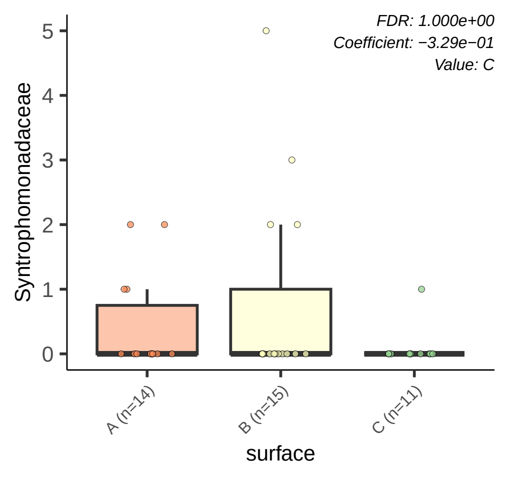

FDR: 1.000e+00
Coefficient: −3.29e−01
Value: C
Syntrophomonadaceae
0
1
2
3
4
5
A (n=14)
B (n=15)
C (n=11)
surface

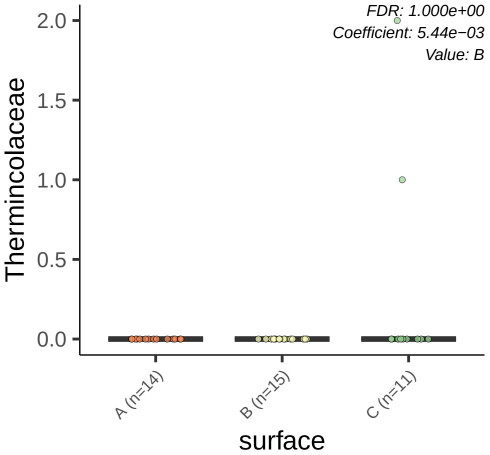

FDR: 1.000e+00
Coefficient: 5.44e−03
Value: B
Thermincolaceae
2.0
1.5
1.0
0.5
0.0
A (n=14)
B (n=15)
C (n=11)
surface

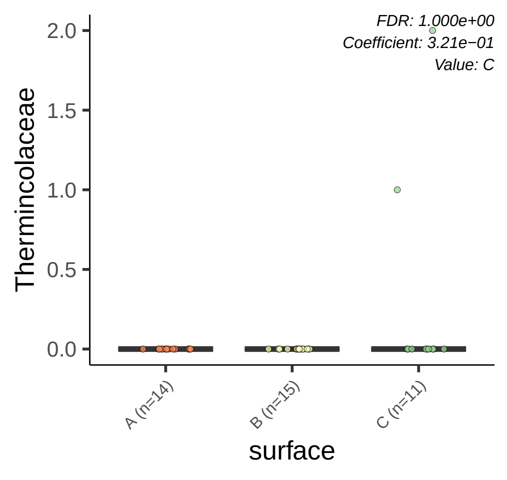
FDR: 1.000e+00
Coefficient: 3.21e−01
Value: C
Thermincolaceae
2.0
1.5
1.0
0.5
0.0
A (n=14)
B (n=15)
C (n=11)
surface

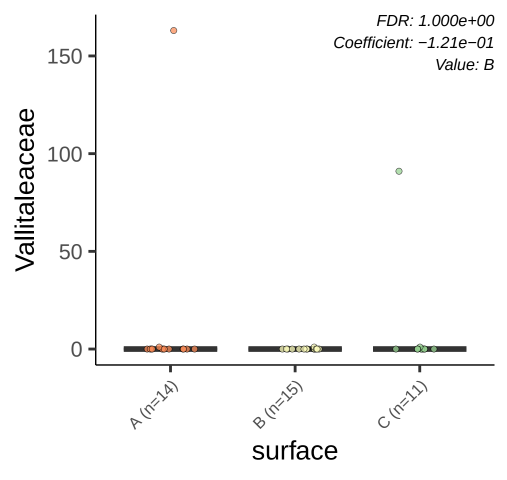

FDR: 1.000e+00
Coefficient: −1.21e−01
Value: B
Vallitaleaceae
150
100
50
0
A (n=14)
B (n=15)
C (n=11)
surface

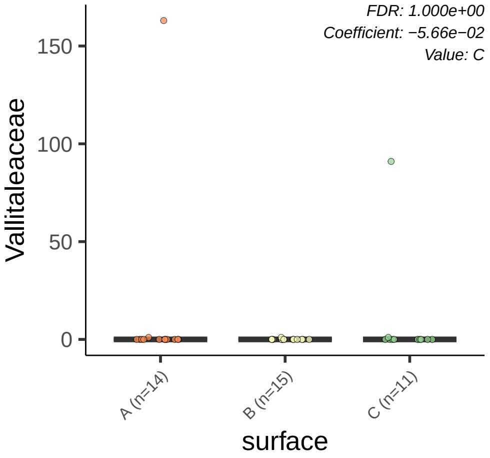
FDR: 1.000e+00
Coefficient: −5.66e−02
Value: C
Vallitaleaceae
150
100
50
0
A (n=14)
B (n=15)
C (n=11)
surface

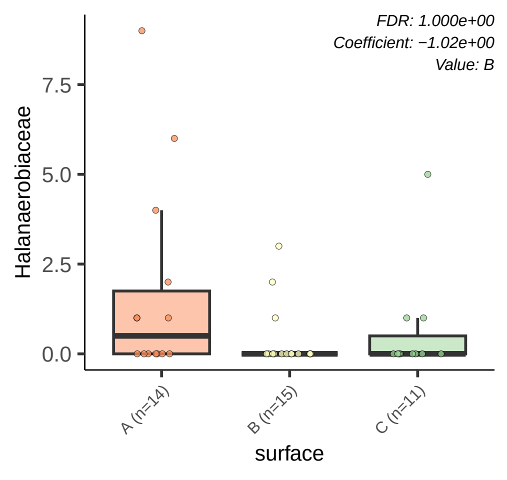

FDR: 1.000e+00
Coefficient: −1.02e+00
Value: B
Halanaerobiaceae
7.5
5.0
2.5
0.0
A (n=14)
B (n=15)
C (n=11)
surface

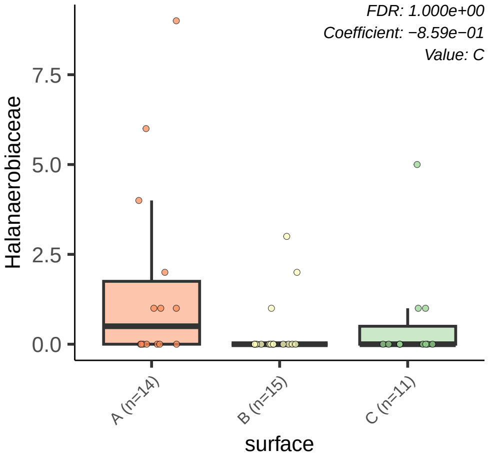

FDR: 1.000e+00
Coefficient: −8.59e−01
Value: C
Halanaerobiaceae
7.5
5.0
2.5
0.0
A (n=14)
B (n=15)
C (n=11)
surface

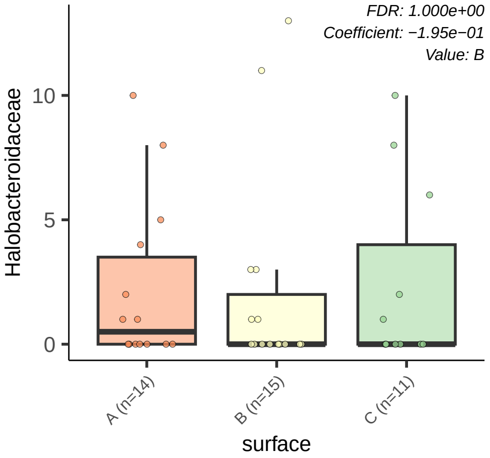

FDR: 1.000e+00
Coefficient: −1.95e−01
Value: B
Halobacteroidaceae
10
5
0
A (n=14)
B (n=15)
C (n=11)
surface

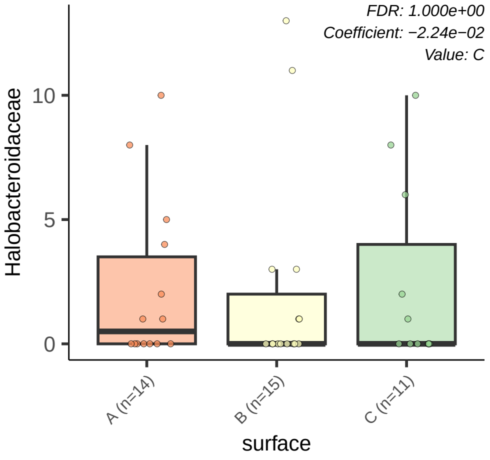

FDR: 1.000e+00
Coefficient: −2.24e−02
Value: C
Halobacteroidaceae
10
5
0
A (n=14)
B (n=15)
C (n=11)
surface

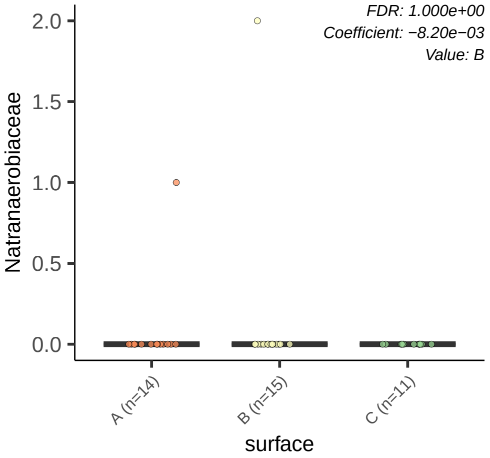
FDR: 1.000e+00
Coefficient: −8.20e−03
Value: B
2.0
1.5
1.0
0.5
0.0
Natranaerobiaceae
A (n=14)
B (n=15)
C (n=11)
surface

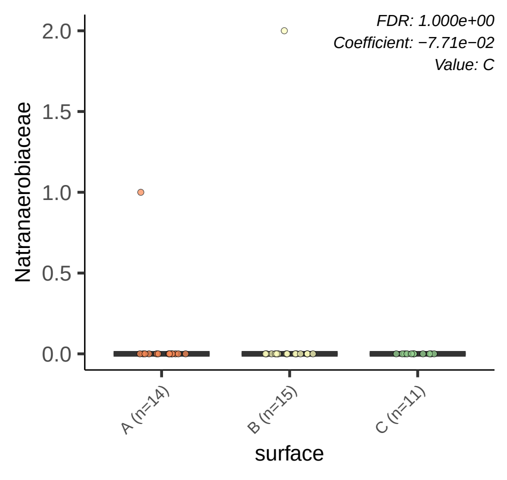

FDR: 1.000e+00
Coefficient: −7.71e−02
Value: C
Natranaerobiaceae
2.0
1.5
1.0
0.5
0.0
A (n=14)
B (n=15)
C (n=11)
surface

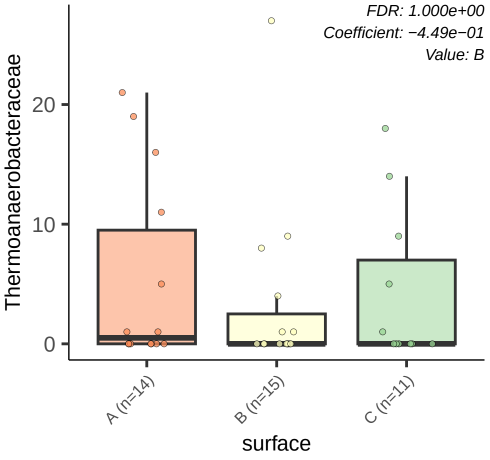

FDR: 1.000e+00
Coefficient: −4.49e−01
Value: B
Thermoanaerobacteraceae
0
10
20
A (n=14)
B (n=15)
C (n=11)
surface

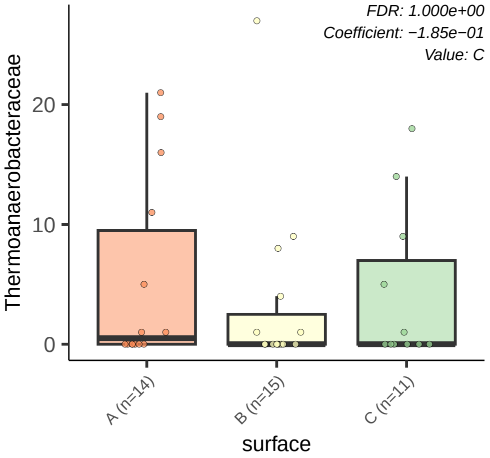

FDR: 1.000e+00
Coefficient: −1.85e−01
Value: C
Thermoanaerobacteraceae
20
10
0
A (n=14)
B (n=15)
C (n=11)
surface

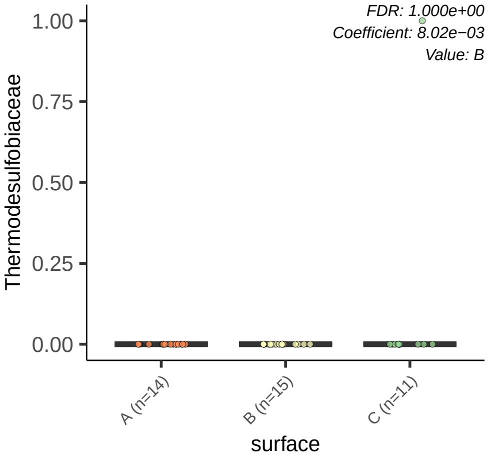
FDR: 1.000e+00
Coefficient: 8.02e−03
Value: B
1.00
0.75
0.50
0.25
0.00
Thermodesulfobiaceae
A (n=14)
B (n=15)
C (n=11)
surface

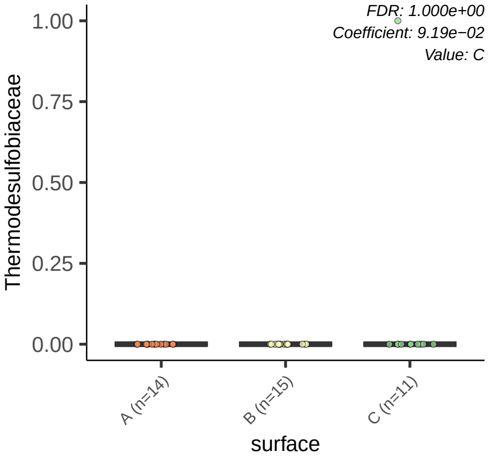
FDR: 1.000e+00
Coefficient: 9.19e−02
Value: C
Thermodesulfobiaceae
1.00
0.75
0.50
0.25
0.00
A (n=14)
B (n=15)
C (n=11)
surface

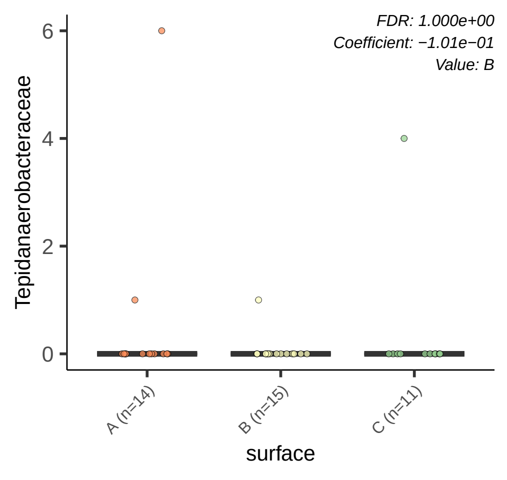
FDR: 1.000e+00
Coefficient: −1.01e−01
Value: B
Tepidanaerobacteraceae
0
2
4
6
A (n=14)
B (n=15)
C (n=11)
surface

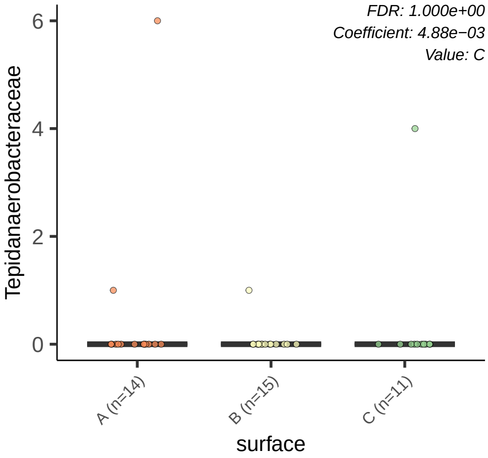
FDR: 1.000e+00
Coefficient: 4.88e−03
Value: C
Tepidanaerobacteraceae
0
2
4
6
A (n=14)
B (n=15)
C (n=11)
surface

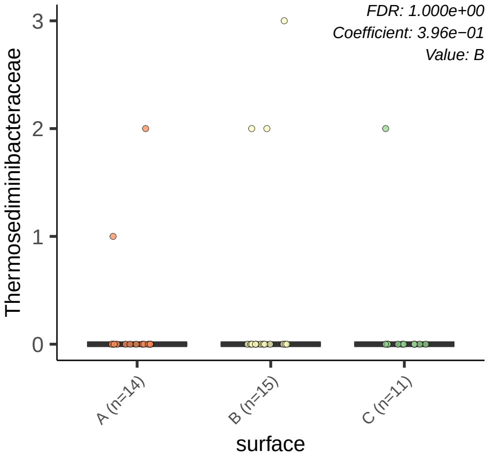

FDR: 1.000e+00
Coefficient: 3.96e−01
Value: B
Thermosediminibacteraceae
0
1
2
3
A (n=14)
B (n=15)
C (n=11)
surface

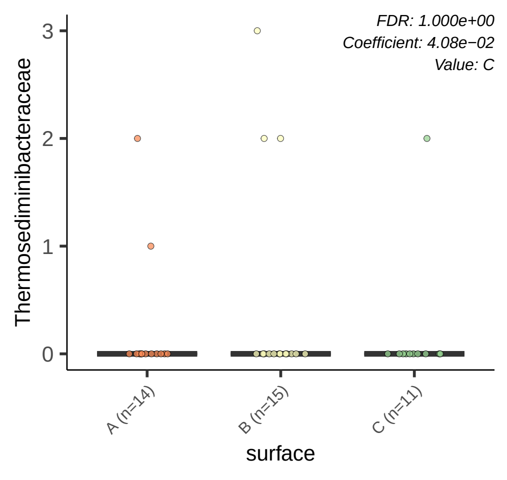

FDR: 1.000e+00
Coefficient: 4.08e−02
Value: C
Thermosediminibacteraceae
3
2
1
0
A (n=14)
B (n=15)
C (n=11)
surface

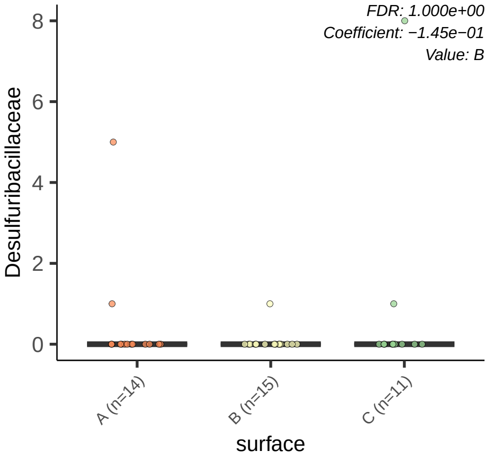
FDR: 1.000e+00
Coefficient: −1.45e−01
Value: B
Desulfuribacillaceae
0
2
4
6
8
A (n=14)
B (n=15)
C (n=11)
surface

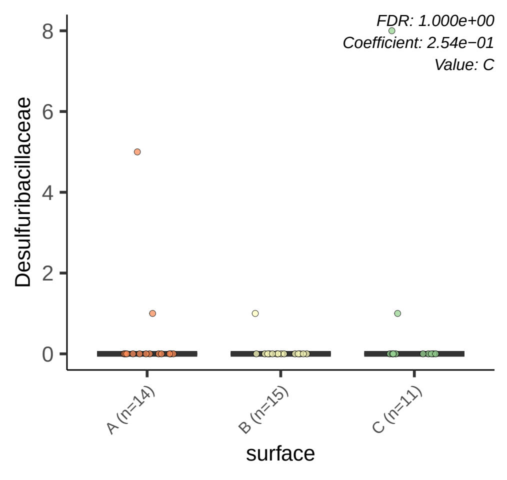
FDR: 1.000e+00
Coefficient: 2.54e−01
Value: C
Desulfuribacillaceae
8
6
4
2
0
A (n=14)
B (n=15)
C (n=11)
surface

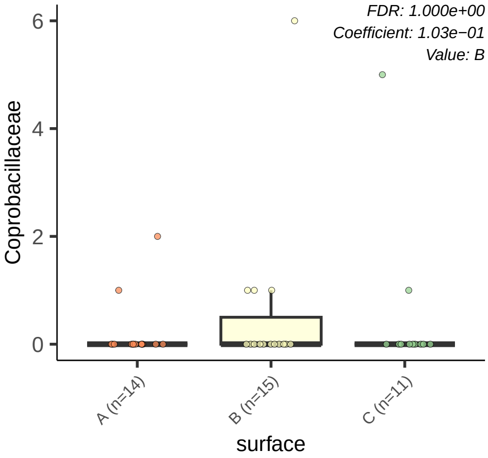

FDR: 1.000e+00
Coefficient: 1.03e−01
Value: B
Coprobacillaceae
0
2
4
6
A (n=14)
B (n=15)
C (n=11)
surface

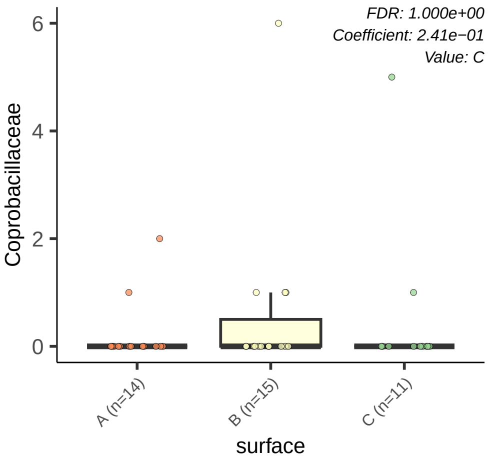

FDR: 1.000e+00
Coefficient: 2.41e−01
Value: C
Coprobacillaceae
6
4
2
0
A (n=14)
B (n=15)
C (n=11)
surface

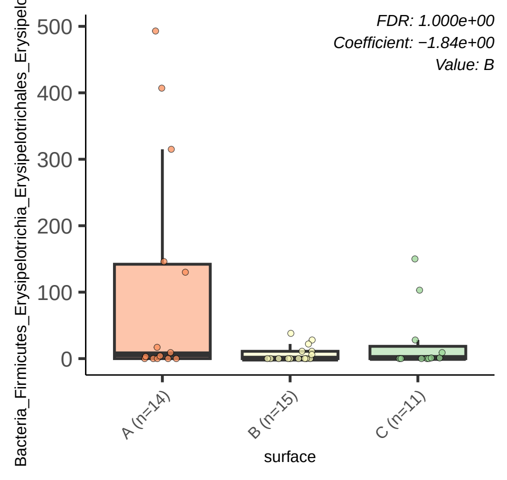
FDR: 1.000e+00
Coefficient: −1.84e+00
Value: B
Bacteria_Firmicutes_Erysipelotrichia_Erysipelotrichales_Erysipelo
500
400
300
200
100
0
A (n=14)
B (n=15)
C (n=11)
surface

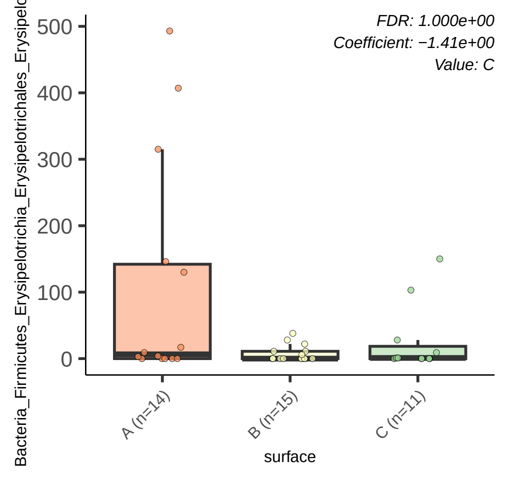

FDR: 1.000e+00
Coefficient: −1.41e+00
Value: C
Bacteria_Firmicutes_Erysipelotrichia_Erysipelotrichales_Erysipelo
500
400
300
200
100
0
A (n=14)
B (n=15)
C (n=11)
surface

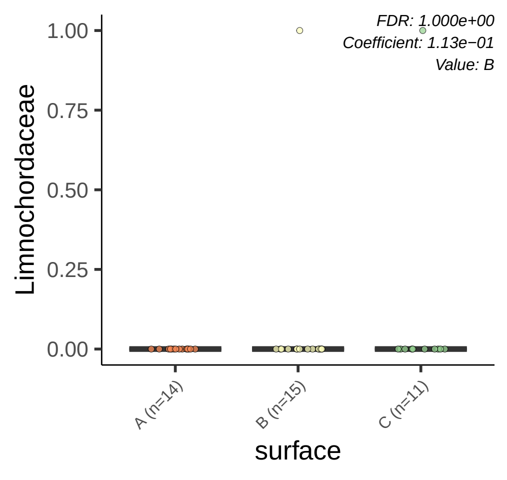

FDR: 1.000e+00
Coefficient: 1.13e−01
Value: B
Limnochordaceae
1.00
0.75
0.50
0.25
0.00
A (n=14)
B (n=15)
C (n=11)
surface

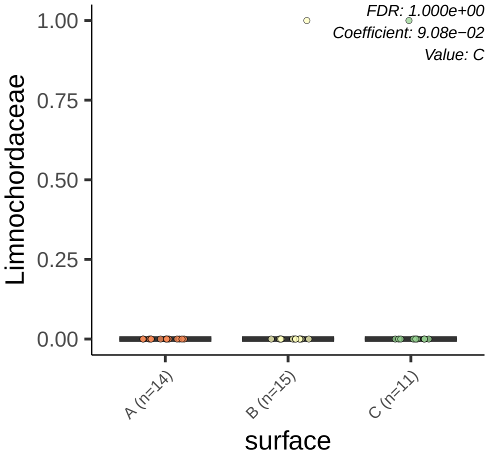

FDR: 1.000e+00
Coefficient: 9.08e−02
Value: C
Limnochordaceae
1.00
0.75
0.50
0.25
0.00
A (n=14)
B (n=15)
C (n=11)
surface

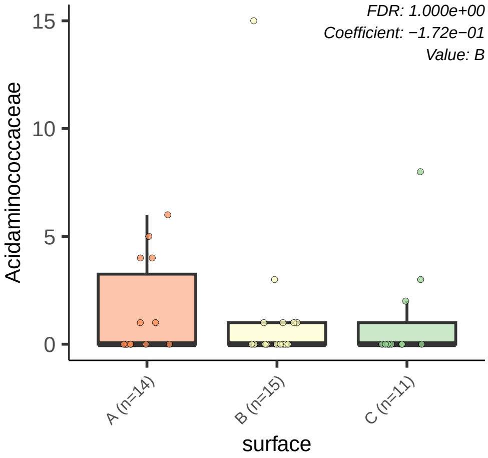
FDR: 1.000e+00
Coefficient: −1.72e−01
Value: B
Acidaminococcaceae
15
10
5
0
A (n=14)
B (n=15)
C (n=11)
surface

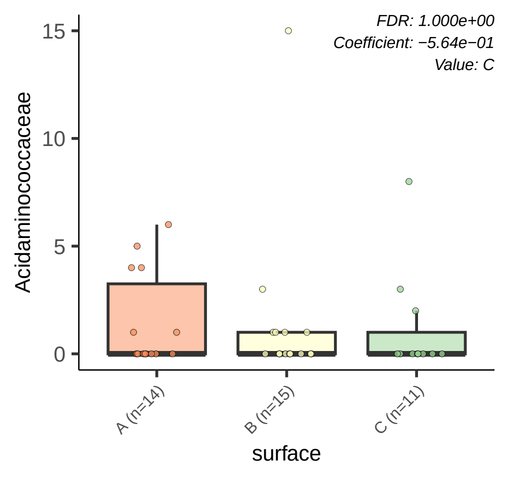
FDR: 1.000e+00
Coefficient: −5.64e−01
Value: C
Acidaminococcaceae
15
10
5
0
A (n=14)
B (n=15)
C (n=11)
surface

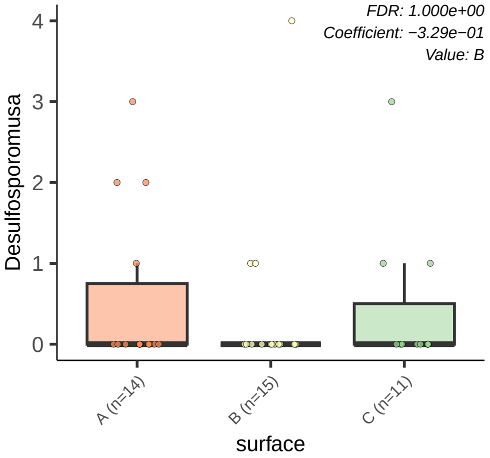
FDR: 1.000e+00
Coefficient: −3.29e−01
Value: B
Desulfosporomusa
0
1
2
3
4
A (n=14)
B (n=15)
C (n=11)
surface

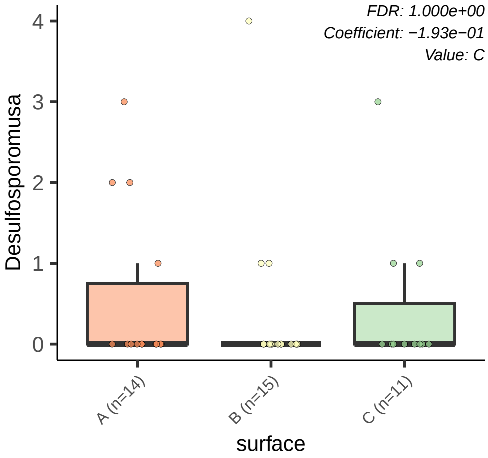
FDR: 1.000e+00
Coefficient: −1.93e−01
Value: C
Desulfosporomusa
4
3
2
1
0
A (n=14)
B (n=15)
C (n=11)
surface

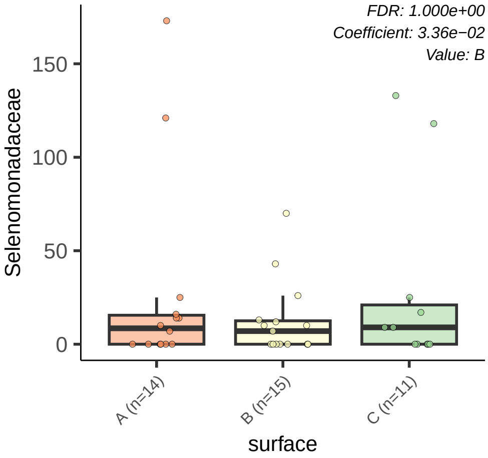

FDR: 1.000e+00
Coefficient: 3.36e−02
Value: B
Selenomonadaceae
150
100
50
0
A (n=14)
B (n=15)
C (n=11)
surface

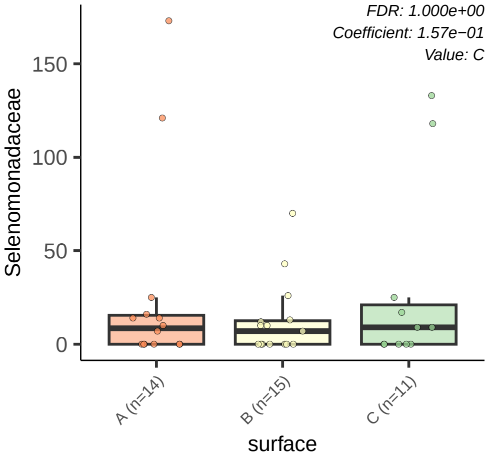

FDR: 1.000e+00
Coefficient: 1.57e−01
Value: C
Selenomonadaceae
150
100
50
0
A (n=14)
B (n=15)
C (n=11)
surface

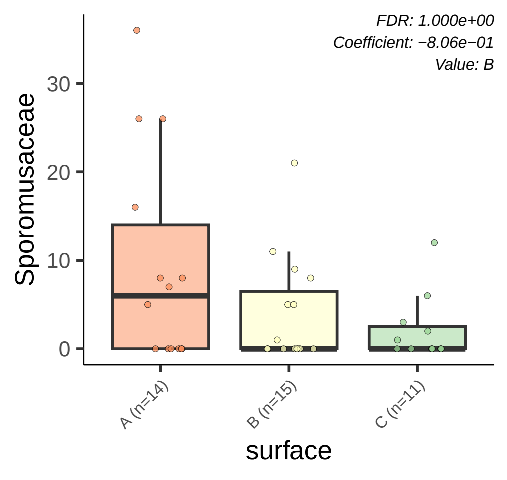

FDR: 1.000e+00
Coefficient: −8.06e−01
Value: B
Sporomusaceae
0
10
20
30
A (n=14)
B (n=15)
C (n=11)
surface

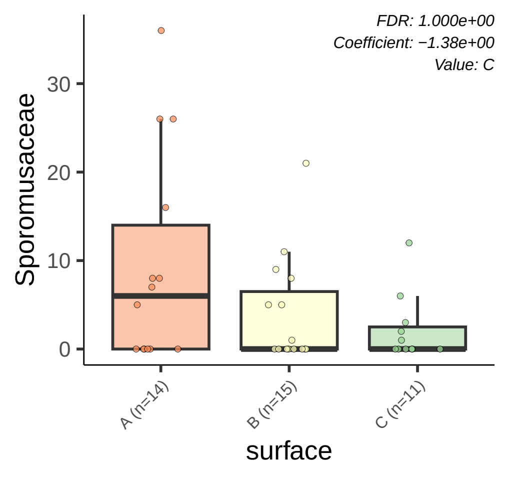
FDR: 1.000e+00
Coefficient: −1.38e+00
Value: C
Sporomusaceae
0
10
20
30
A (n=14)
B (n=15)
C (n=11)
surface

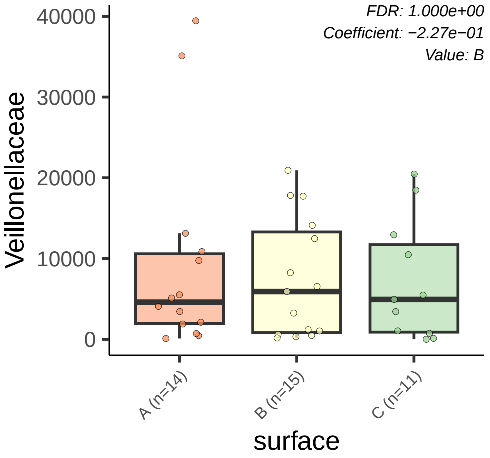

FDR: 1.000e+00
Coefficient: −2.27e−01
Value: B
Veillonellaceae
40000
30000
20000
10000
0
A (n=14)
B (n=15)
C (n=11)
surface

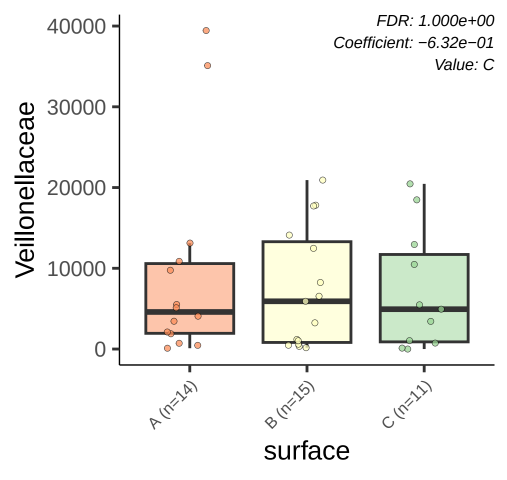

FDR: 1.000e+00
Coefficient: −6.32e−01
Value: C
Veillonellaceae
40000
30000
20000
10000
0
A (n=14)
B (n=15)
C (n=11)
surface

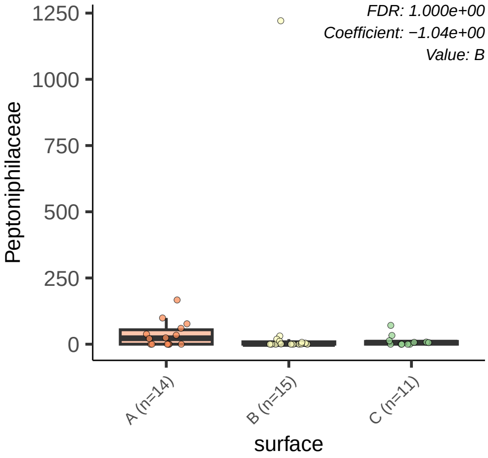

FDR: 1.000e+00
Coefficient: −1.04e+00
Value: B
Peptoniphilaceae
1250
1000
750
500
250
0
A (n=14)
B (n=15)
C (n=11)
surface

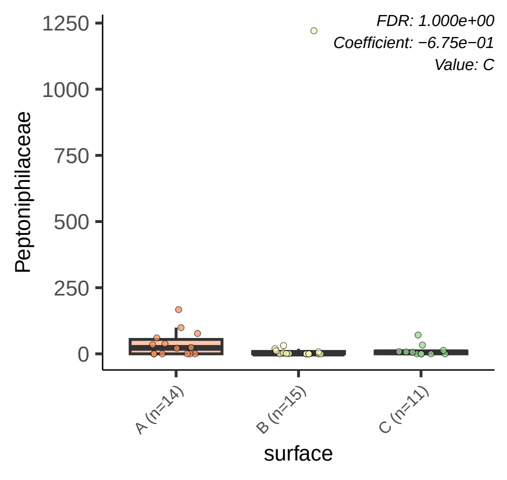
FDR: 1.000e+00
Coefficient: −6.75e−01
Value: C
Peptoniphilaceae
1250
1000
750
500
250
0
A (n=14)
B (n=15)
C (n=11)
surface

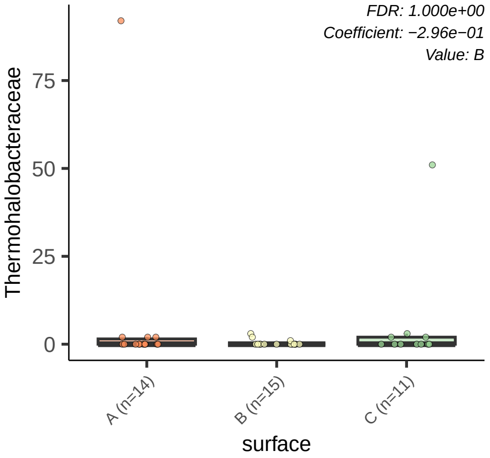

FDR: 1.000e+00
Coefficient: −2.96e−01
Value: B
Thermohalobacteraceae
75
50
25
0
A (n=14)
B (n=15)
C (n=11)
surface

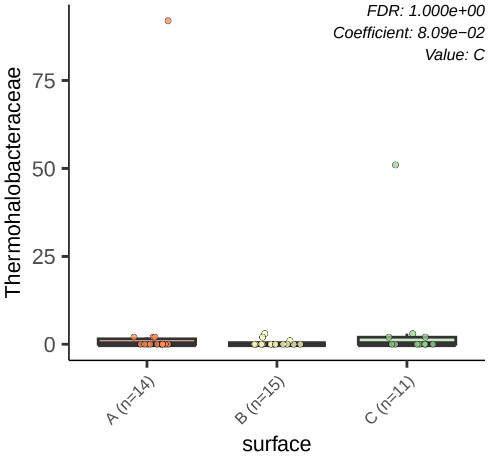

FDR: 1.000e+00
Coefficient: 8.09e−02
Value: C
Thermohalobacteraceae
75
50
25
0
A (n=14)
B (n=15)
C (n=11)
surface

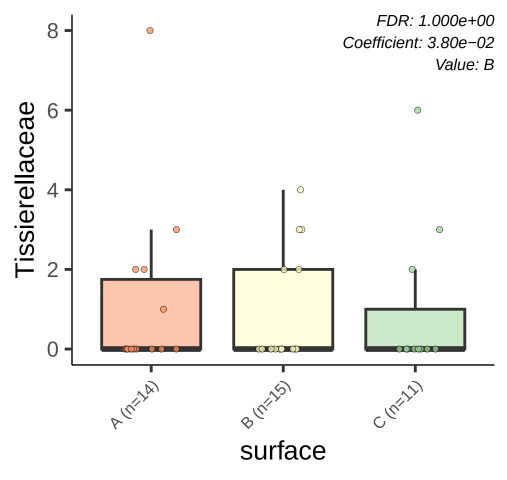
FDR: 1.000e+00
Coefficient: 3.80e−02
Value: B
Tissierellaceae
0
2
4
6
8
A (n=14)
B (n=15)
C (n=11)
surface

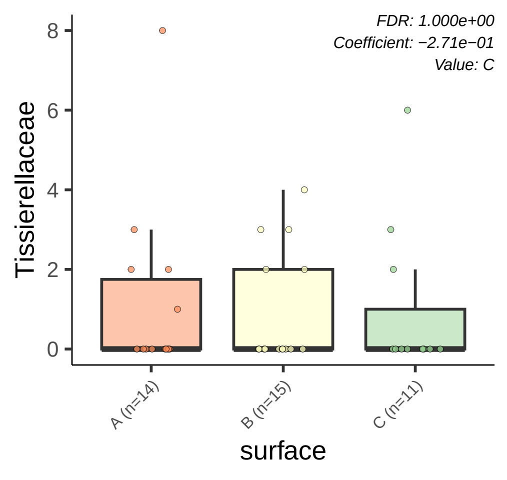

FDR: 1.000e+00
Coefficient: −2.71e−01
Value: C
Tissierellaceae
0
2
4
6
8
A (n=14)
B (n=15)
C (n=11)
surface

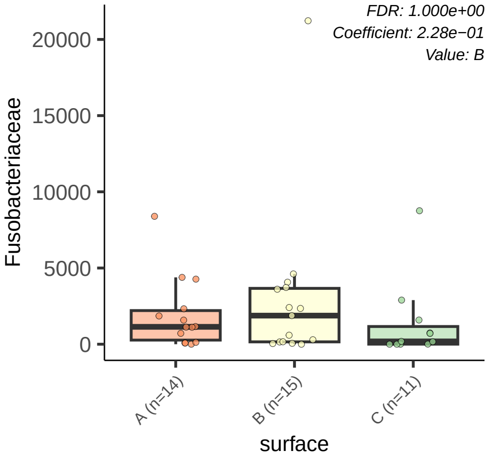

FDR: 1.000e+00
Coefficient: 2.28e−01
Value: B
Fusobacteriaceae
20000
15000
10000
5000
0
A (n=14)
B (n=15)
C (n=11)
surface

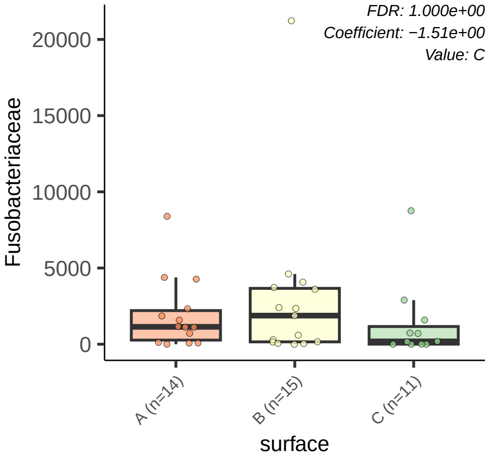
FDR: 1.000e+00
Coefficient: −1.51e+00
Value: C
Fusobacteriaceae
20000
15000
10000
5000
0
A (n=14)
B (n=15)
C (n=11)
surface

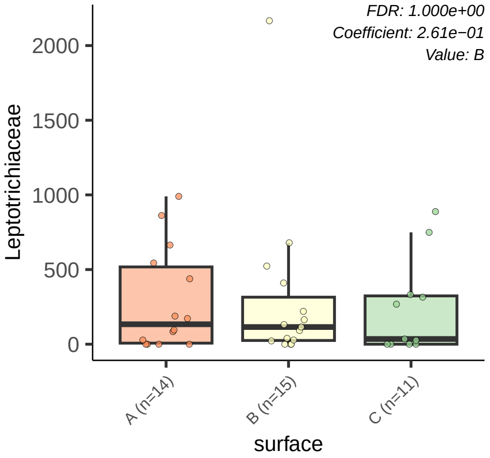
FDR: 1.000e+00
Coefficient: 2.61e−01
Value: B
Leptotrichiaceae
2000
1500
1000
500
0
A (n=14)
B (n=15)
C (n=11)
surface

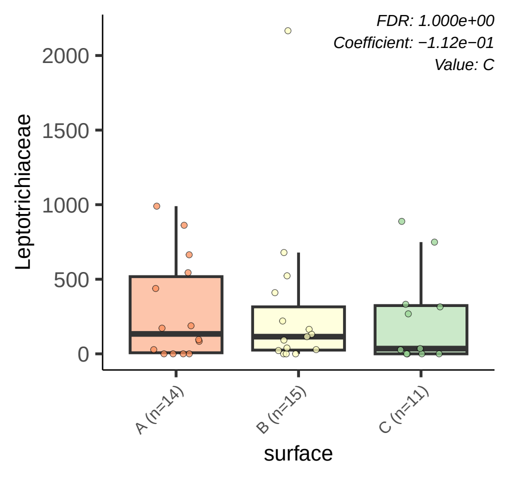
FDR: 1.000e+00
Coefficient: −1.12e−01
Value: C
Leptotrichiaceae
2000
1500
1000
500
0
A (n=14)
B (n=15)
C (n=11)
surface

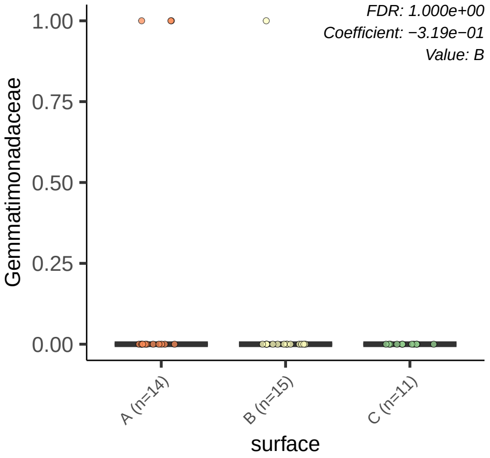

FDR: 1.000e+00
Coefficient: −3.19e−01
Value: B
Gemmatimonadaceae
1.00
0.75
0.50
0.25
0.00
A (n=14)
B (n=15)
C (n=11)
surface

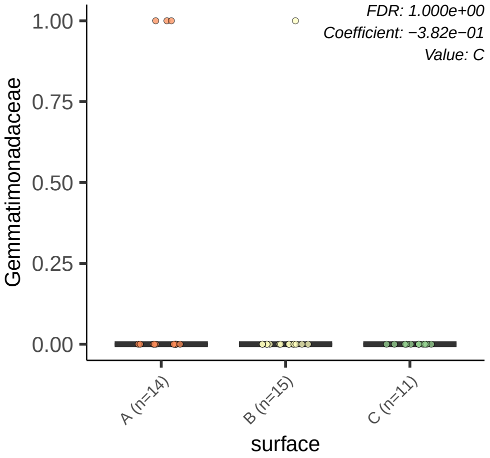

FDR: 1.000e+00
Coefficient: −3.82e−01
Value: C
Gemmatimonadaceae
1.00
0.75
0.50
0.25
0.00
A (n=14)
B (n=15)
C (n=11)
surface

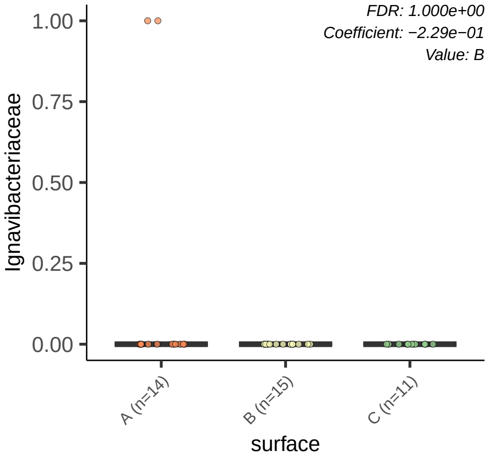

FDR: 1.000e+00
Coefficient: −2.29e−01
Value: B
Ignavibacteriaceae
1.00
0.75
0.50
0.25
0.00
A (n=14)
B (n=15)
C (n=11)
surface

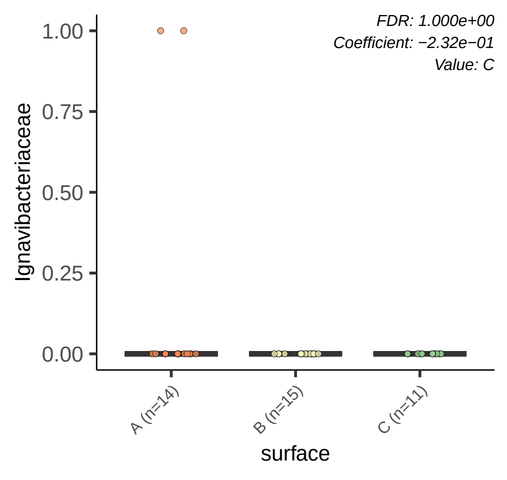
FDR: 1.000e+00
Coefficient: −2.32e−01
Value: C
Ignavibacteriaceae
1.00
0.75
0.50
0.25
0.00
A (n=14)
B (n=15)
C (n=11)
surface

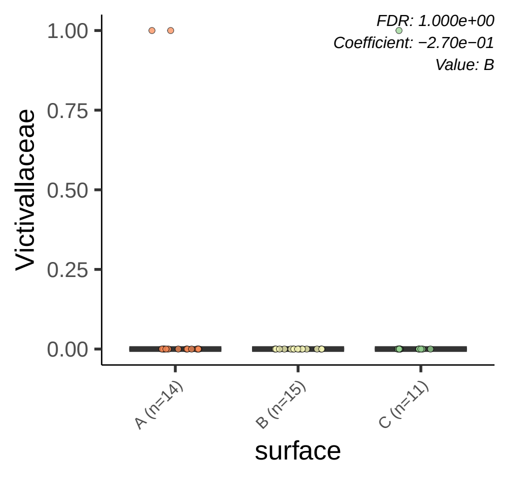

FDR: 1.000e+00
Coefficient: −2.70e−01
Value: B
Victivallaceae
1.00
0.75
0.50
0.25
0.00
A (n=14)
B (n=15)
C (n=11)
surface

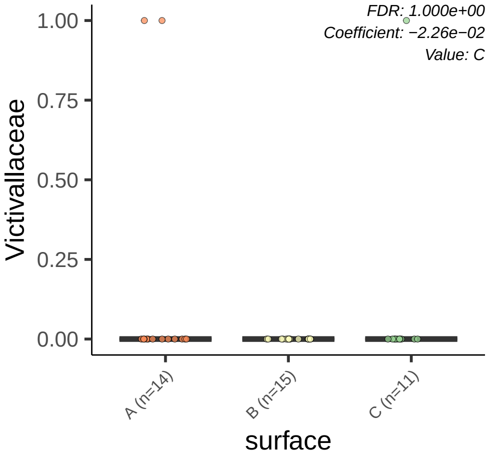

FDR: 1.000e+00
Coefficient: −2.26e−02
Value: C
Victivallaceae
1.00
0.75
0.50
0.25
0.00
A (n=14)
B (n=15)
C (n=11)
surface

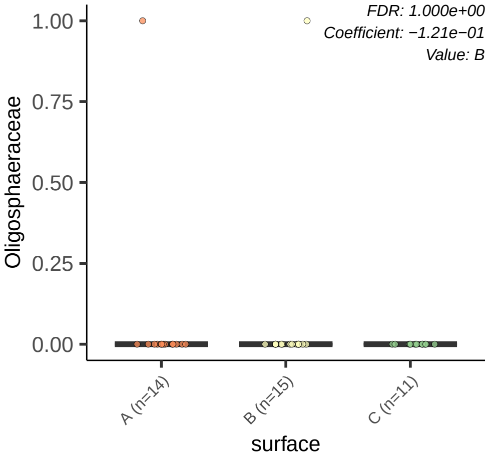

FDR: 1.000e+00
Coefficient: −1.21e−01
Value: B
Oligosphaeraceae
1.00
0.75
0.50
0.25
0.00
A (n=14)
B (n=15)
C (n=11)
surface

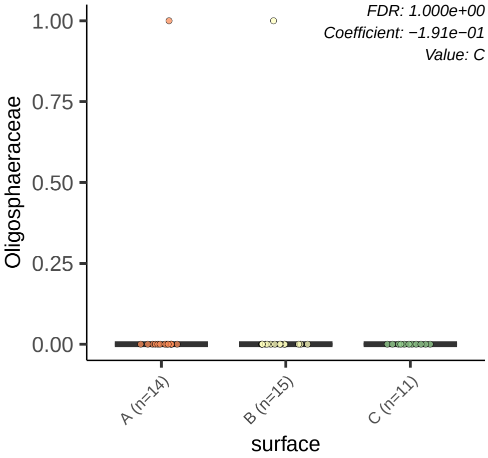
FDR: 1.000e+00
Coefficient: −1.91e−01
Value: C
Oligosphaeraceae
1.00
0.75
0.50
0.25
0.00
A (n=14)
B (n=15)
C (n=11)
surface

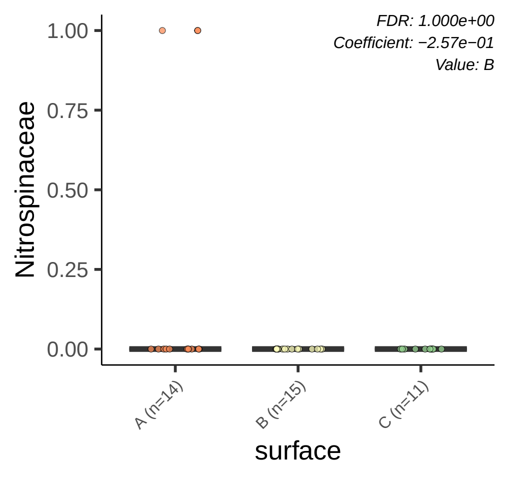
FDR: 1.000e+00
Coefficient: −2.57e−01
Value: B
Nitrospinaceae
1.00
0.75
0.50
0.25
0.00
A (n=14)
B (n=15)
C (n=11)
surface

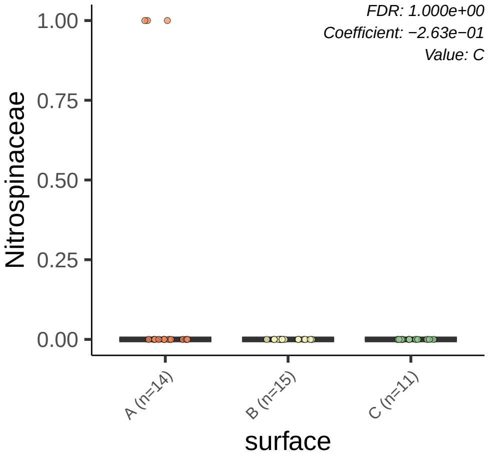

FDR: 1.000e+00
Coefficient: −2.63e−01
Value: C
Nitrospinaceae
1.00
0.75
0.50
0.25
0.00
A (n=14)
B (n=15)
C (n=11)
surface

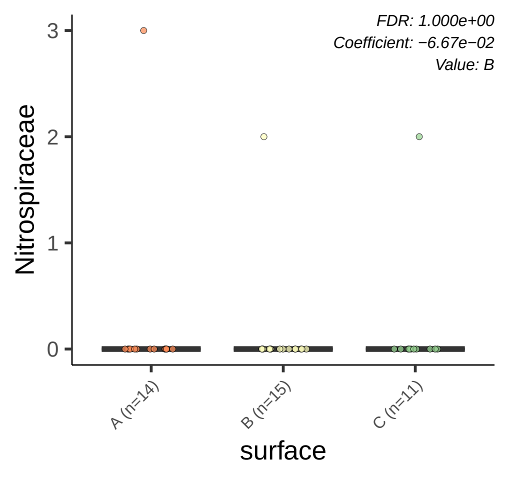

FDR: 1.000e+00
Coefficient: −6.67e−02
Value: B
Nitrospiraceae
3
2
1
0
A (n=14)
B (n=15)
C (n=11)
surface

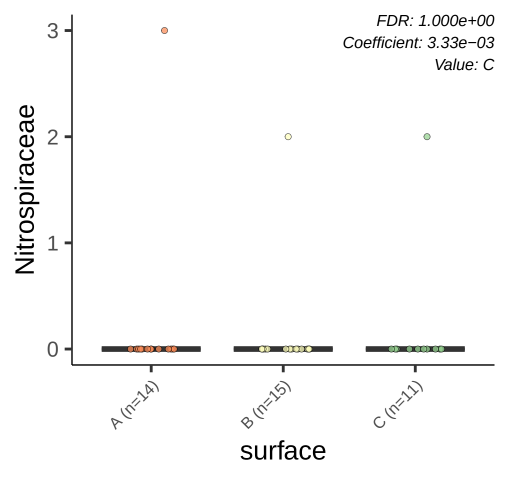

FDR: 1.000e+00
Coefficient: 3.33e−03
Value: C
Nitrospiraceae
3
2
1
0
A (n=14)
B (n=15)
C (n=11)
surface

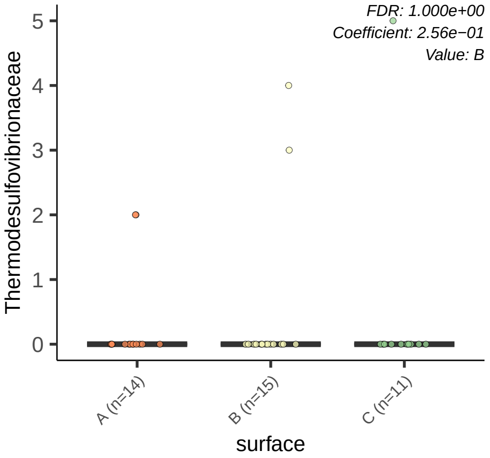

FDR: 1.000e+00
Coefficient: 2.56e−01
Value: B
Thermodesulfovibrionaceae
5
4
3
2
1
0
A (n=14)
B (n=15)
C (n=11)
surface

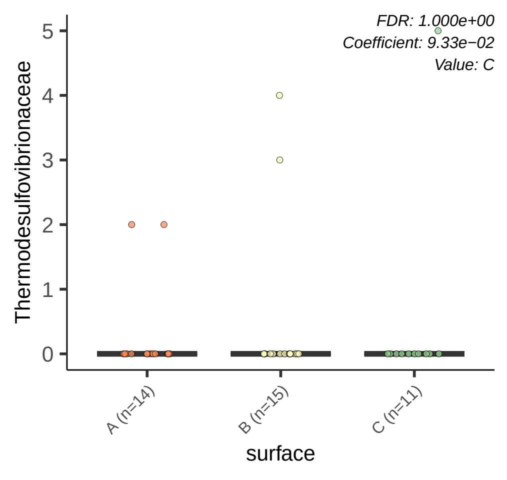
FDR: 1.000e+00
Coefficient: 9.33e−02
Value: C
Thermodesulfovibrionaceae
0
1
2
3
4
5
A (n=14)
B (n=15)
C (n=11)
surface

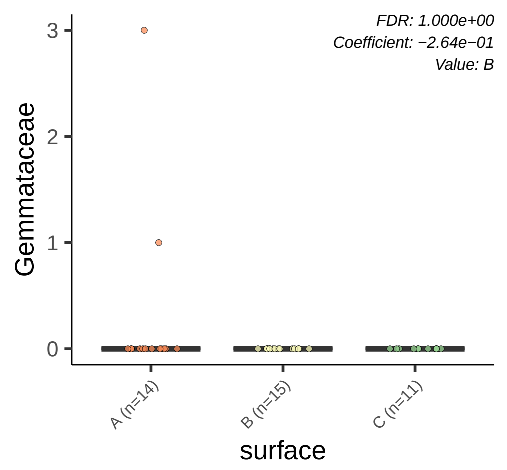
FDR: 1.000e+00
Coefficient: −2.64e−01
Value: B
Gemmataceae
3
2
1
0
A (n=14)
B (n=15)
C (n=11)
surface

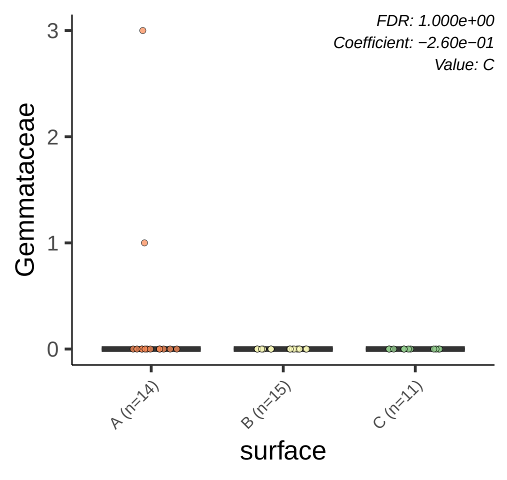

FDR: 1.000e+00
Coefficient: −2.60e−01
Value: C
Gemmataceae
3
2
1
0
A (n=14)
B (n=15)
C (n=11)
surface

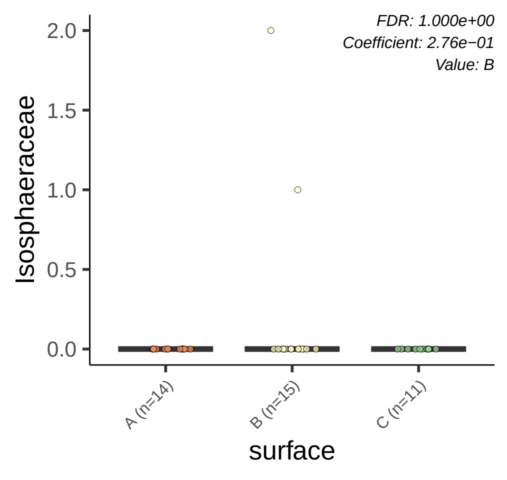
FDR: 1.000e+00
Coefficient: 2.76e−01
Value: B
Isosphaeraceae
2.0
1.5
1.0
0.5
0.0
A (n=14)
B (n=15)
C (n=11)
surface

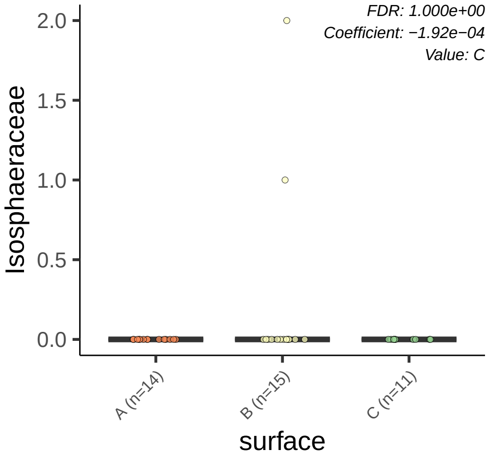
FDR: 1.000e+00
Coefficient: −1.92e−04
Value: C
Isosphaeraceae
2.0
1.5
1.0
0.5
0.0
A (n=14)
B (n=15)
C (n=11)
surface

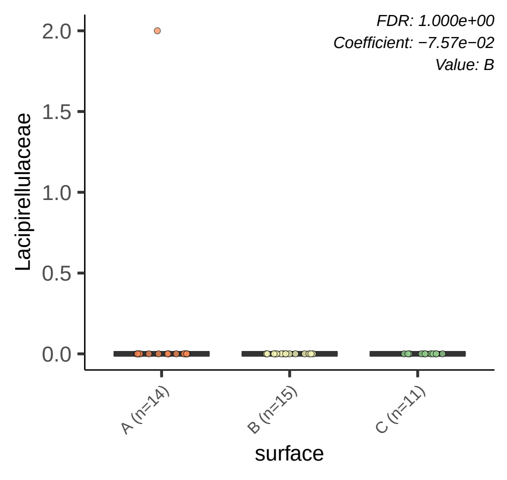
FDR: 1.000e+00
Coefficient: −7.57e−02
Value: B
Lacipirellulaceae
2.0
1.5
1.0
0.5
0.0
A (n=14)
B (n=15)
C (n=11)
surface

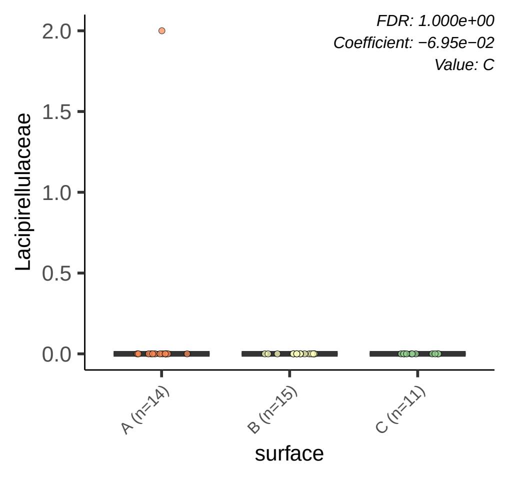
FDR: 1.000e+00
Coefficient: −6.95e−02
Value: C
Lacipirellulaceae
2.0
1.5
1.0
0.5
0.0
A (n=14)
B (n=15)
C (n=11)
surface

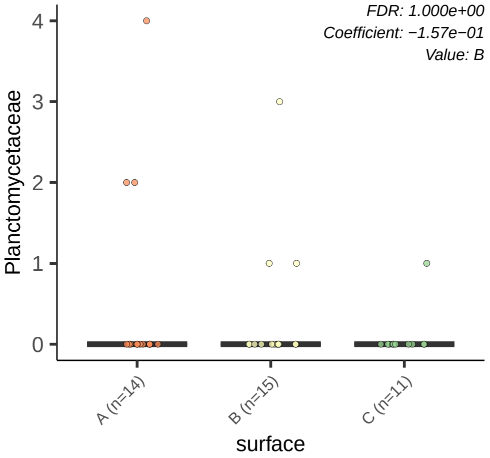
FDR: 1.000e+00
Coefficient: −1.57e−01
Value: B
Planctomycetaceae
0
1
2
3
4
A (n=14)
B (n=15)
C (n=11)
surface

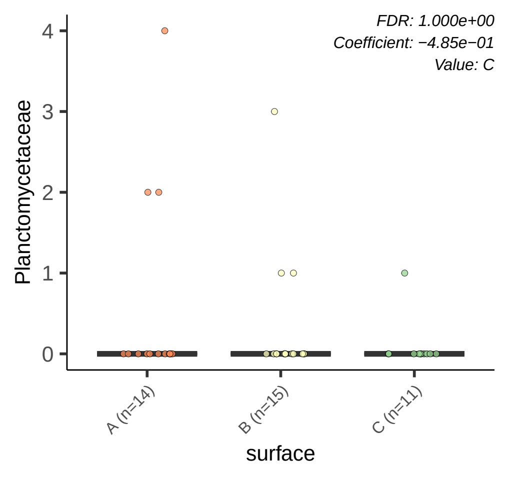
FDR: 1.000e+00
Coefficient: −4.85e−01
Value: C
Planctomycetaceae
4
3
2
1
0
A (n=14)
B (n=15)
C (n=11)
surface

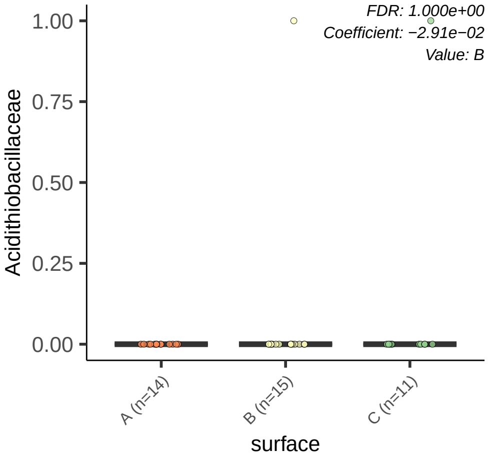
FDR: 1.000e+00
Coefficient: −2.91e−02
Value: B
Acidithiobacillaceae
1.00
0.75
0.50
0.25
0.00
A (n=14)
B (n=15)
C (n=11)
surface

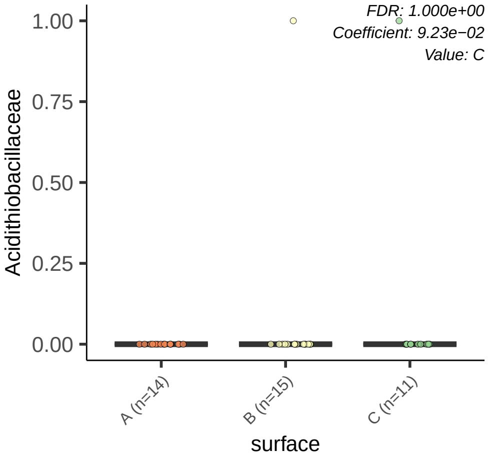

FDR: 1.000e+00
Coefficient: 9.23e−02
Value: C
1.00
0.75
0.50
0.25
0.00
Acidithiobacillaceae
A (n=14)
B (n=15)
C (n=11)
surface

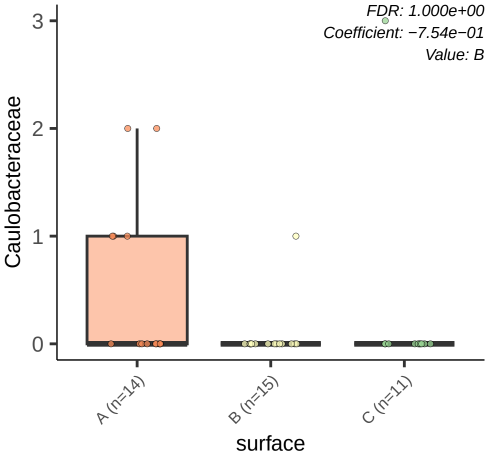

FDR: 1.000e+00
Coefficient: −7.54e−01
Value: B
Caulobacteraceae
3
2
1
0
A (n=14)
B (n=15)
C (n=11)
surface

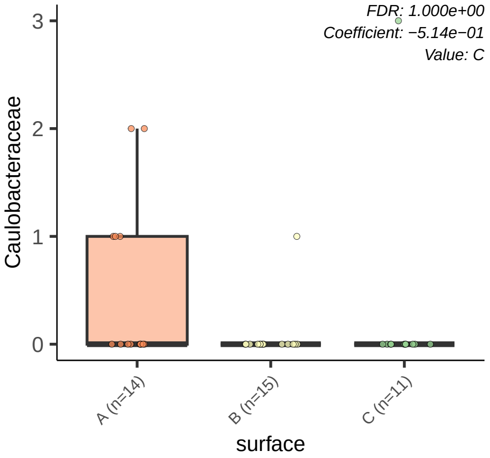
FDR: 1.000e+00
Coefficient: −5.14e−01
Value: C
Caulobacteraceae
3
2
1
0
A (n=14)
B (n=15)
C (n=11)
surface

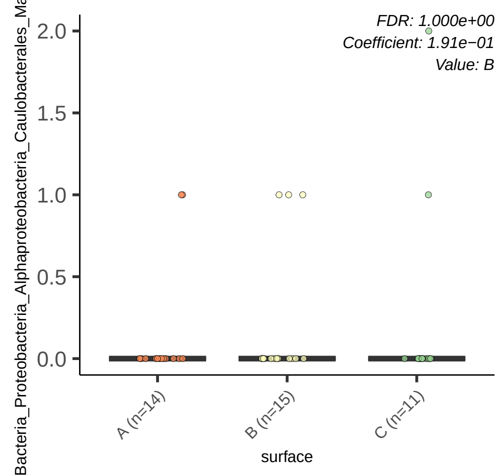

FDR: 1.000e+00
Coefficient: 1.91e−01
Value: B
Bacteria_Proteobacteria_Alphaproteobacteria_Caulobacterales_Ma
2.0
1.5
1.0
0.5
0.0
A (n=14)
B (n=15)
C (n=11)
surface

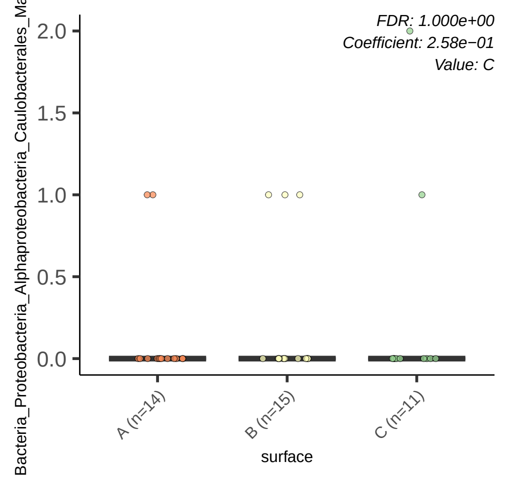
FDR: 1.000e+00
Coefficient: 2.58e−01
Value: C
Bacteria_Proteobacteria_Alphaproteobacteria_Caulobacterales_Ma
2.0
1.5
1.0
0.5
0.0
A (n=14)
B (n=15)
C (n=11)
surface

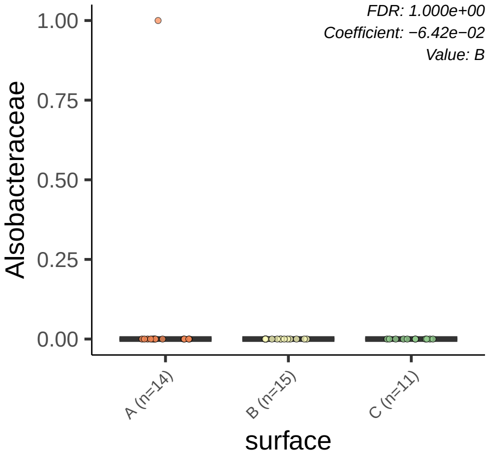
FDR: 1.000e+00
Coefficient: −6.42e−02
Value: B
Alsobacteraceae
1.00
0.75
0.50
0.25
0.00
A (n=14)
B (n=15)
C (n=11)
surface

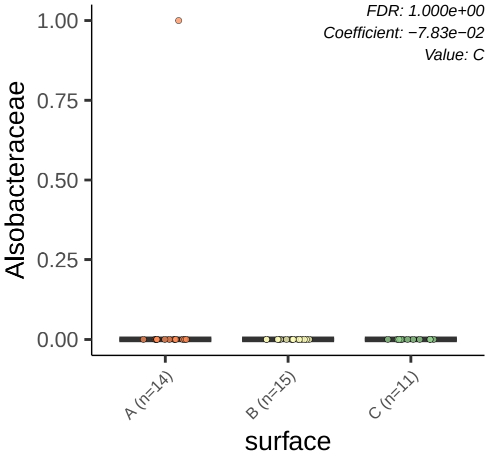
FDR: 1.000e+00
Coefficient: −7.83e−02
Value: C
Alsobacteraceae
1.00
0.75
0.50
0.25
0.00
A (n=14)
B (n=15)
C (n=11)
surface

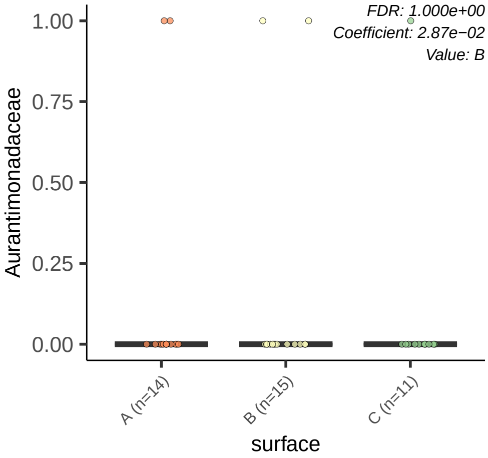

FDR: 1.000e+00
Coefficient: 2.87e−02
Value: B
Aurantimonadaceae
1.00
0.75
0.50
0.25
0.00
A (n=14)
B (n=15)
C (n=11)
surface

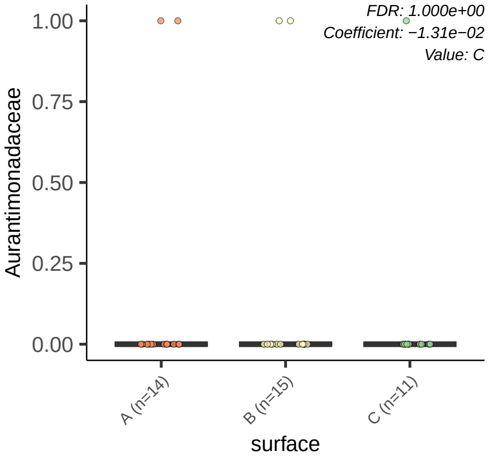
FDR: 1.000e+00
Coefficient: −1.31e−02
Value: C
Aurantimonadaceae
1.00
0.75
0.50
0.25
0.00
A (n=14)
B (n=15)
C (n=11)
surface

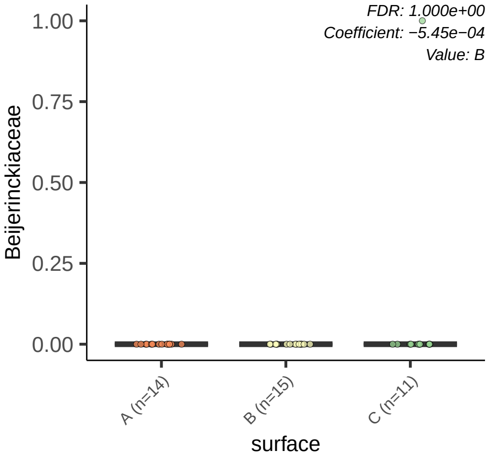
FDR: 1.000e+00
Coefficient: −5.45e−04
Value: B
Beijerinckiaceae
1.00
0.75
0.50
0.25
0.00
A (n=14)
B (n=15)
C (n=11)
surface

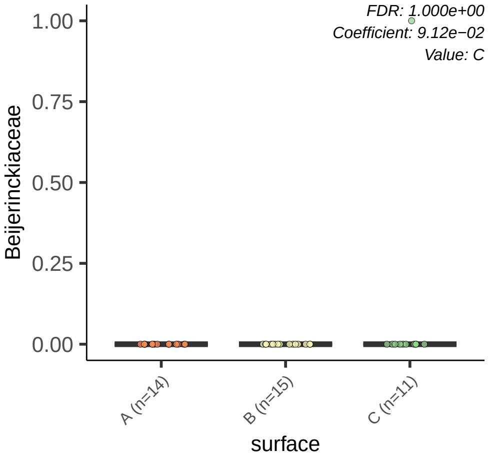
FDR: 1.000e+00
Coefficient: 9.12e−02
Value: C
Beijerinckiaceae
1.00
0.75
0.50
0.25
0.00
A (n=14)
B (n=15)
C (n=11)
surface

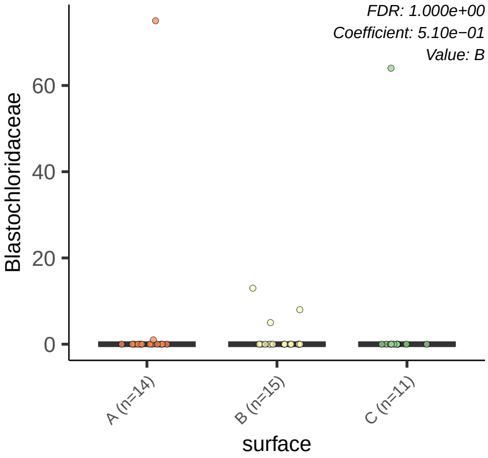

FDR: 1.000e+00
Coefficient: 5.10e−01
Value: B
Blastochloridaceae
0
20
40
60
A (n=14)
B (n=15)
C (n=11)
surface

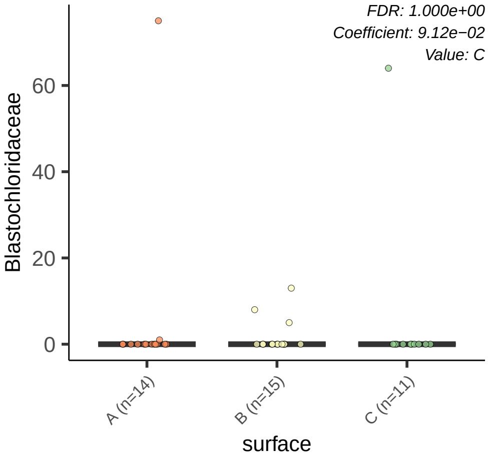
FDR: 1.000e+00
Coefficient: 9.12e−02
Value: C
Blastochloridaceae
60
40
20
0
A (n=14)
B (n=15)
C (n=11)
surface

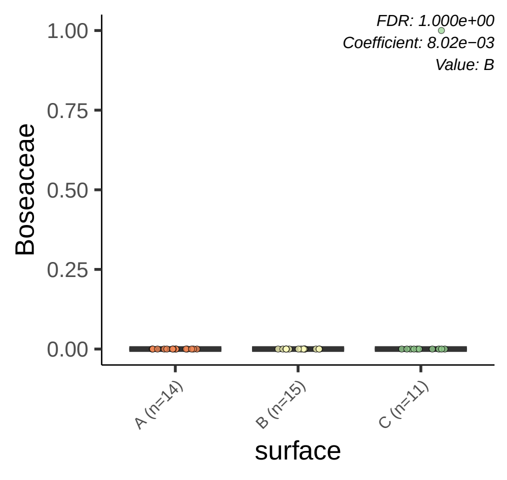
FDR: 1.000e+00
Coefficient: 8.02e−03
Value: B
1.00
0.75
0.50
0.25
0.00
Boseaceae
A (n=14)
B (n=15)
C (n=11)
surface

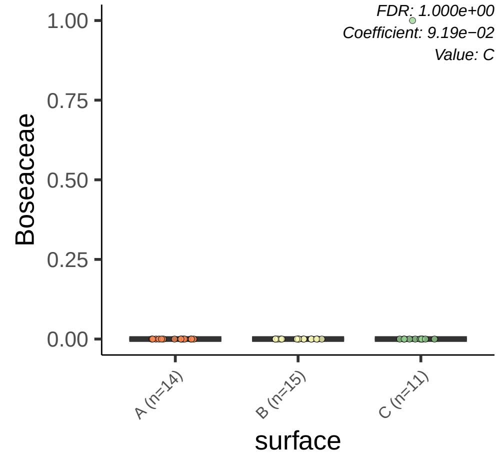

FDR: 1.000e+00
Coefficient: 9.19e−02
Value: C
1.00
0.75
0.50
0.25
0.00
Boseaceae
A (n=14)
B (n=15)
C (n=11)
surface

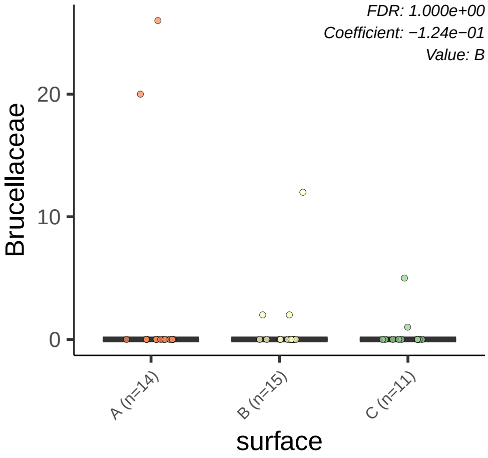
FDR: 1.000e+00
Coefficient: −1.24e−01
Value: B
Brucellaceae
20
10
0
A (n=14)
B (n=15)
C (n=11)
surface

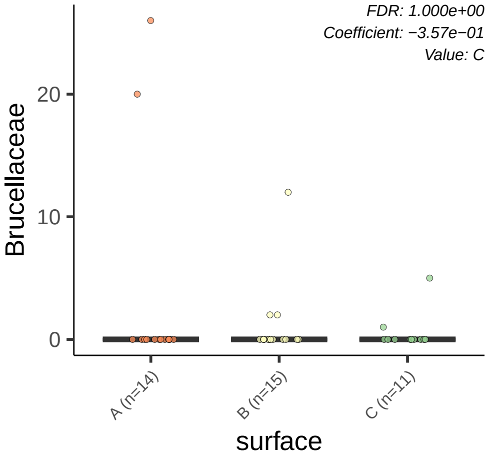

FDR: 1.000e+00
Coefficient: −3.57e−01
Value: C
Brucellaceae
20
10
0
A (n=14)
B (n=15)
C (n=11)
surface

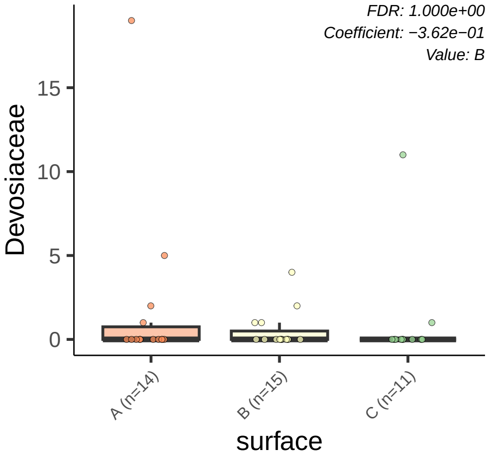
FDR: 1.000e+00
Coefficient: −3.62e−01
Value: B
Devosiaceae
0
5
10
15
A (n=14)
B (n=15)
C (n=11)
surface

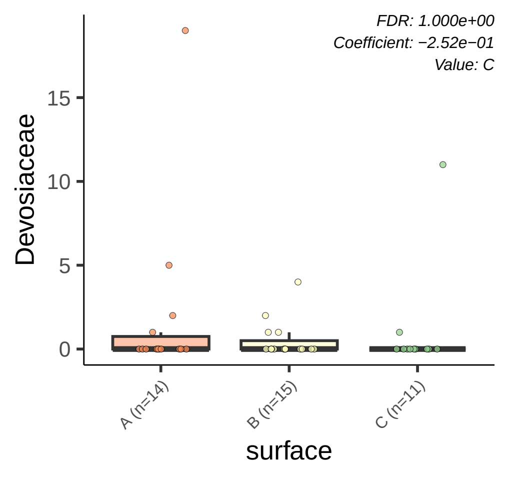
FDR: 1.000e+00
Coefficient: −2.52e−01
Value: C
Devosiaceae
0
5
10
15
A (n=14)
B (n=15)
C (n=11)
surface

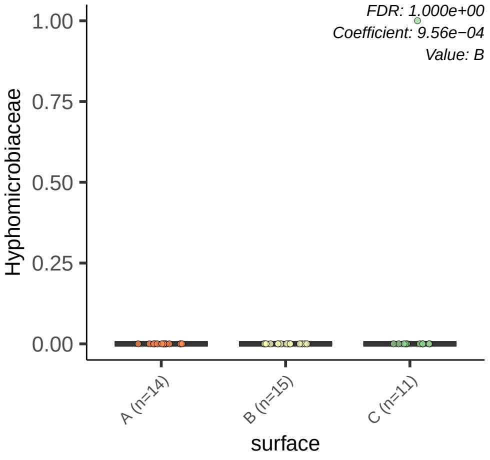
FDR: 1.000e+00
Coefficient: 9.56e−04
Value: B
Hyphomicrobiaceae
1.00
0.75
0.50
0.25
0.00
A (n=14)
B (n=15)
C (n=11)
surface

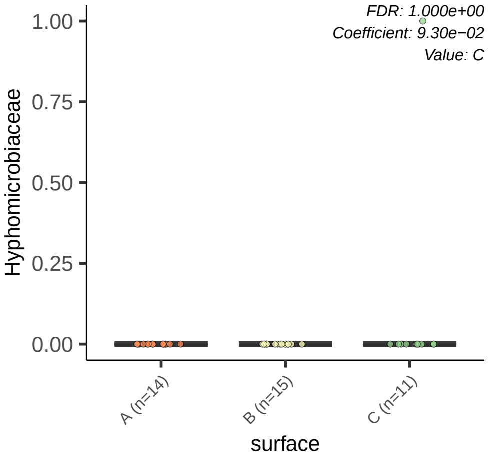
FDR: 1.000e+00
Coefficient: 9.30e−02
Value: C
Hyphomicrobiaceae
1.00
0.75
0.50
0.25
0.00
A (n=14)
B (n=15)
C (n=11)
surface

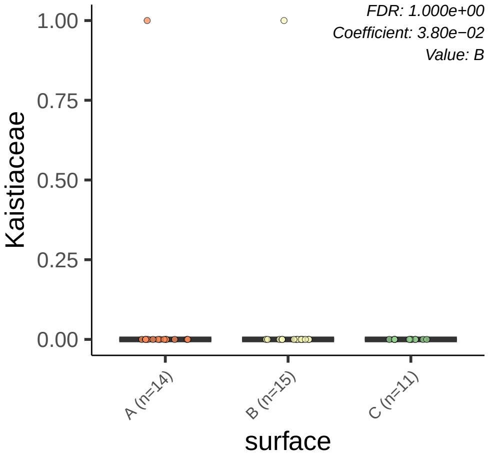
FDR: 1.000e+00
Coefficient: 3.80e−02
Value: B
Kaistiaceae
1.00
0.75
0.50
0.25
0.00
A (n=14)
B (n=15)
C (n=11)
surface

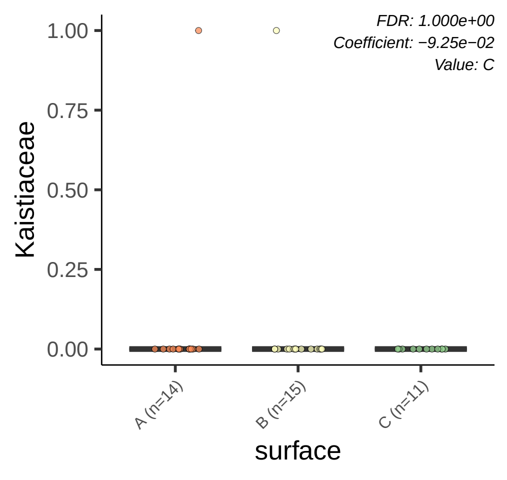

FDR: 1.000e+00
Coefficient: −9.25e−02
Value: C
Kaistiaceae
1.00
0.75
0.50
0.25
0.00
A (n=14)
B (n=15)
C (n=11)
surface

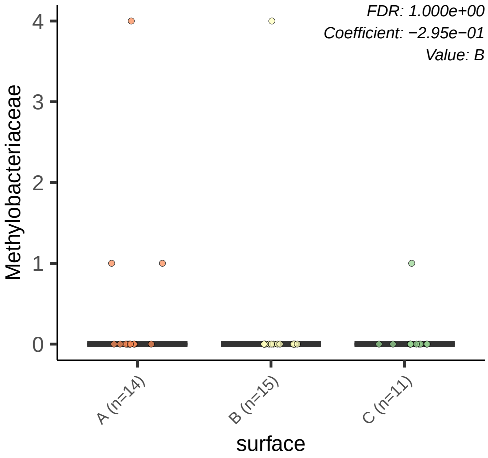
FDR: 1.000e+00
Coefficient: −2.95e−01
Value: B
Methylobacteriaceae
0
1
2
3
4
A (n=14)
B (n=15)
C (n=11)
surface

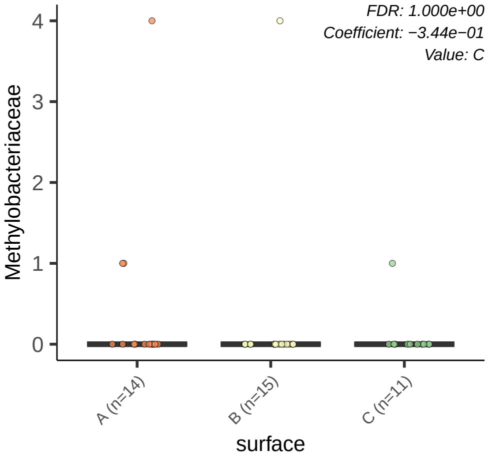

FDR: 1.000e+00
Coefficient: −3.44e−01
Value: C
Methylobacteriaceae
4
3
2
1
0
A (n=14)
B (n=15)
C (n=11)
surface

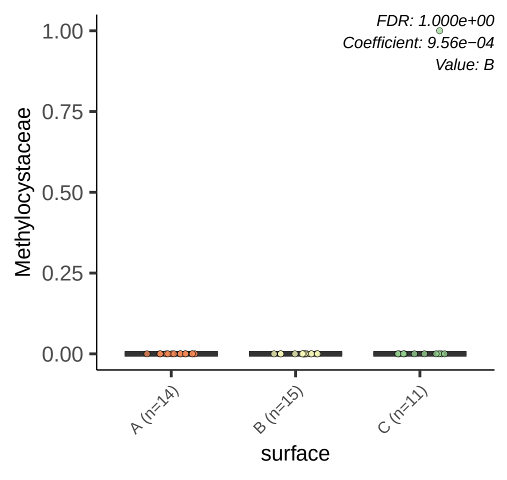

FDR: 1.000e+00
Coefficient: 9.56e−04
Value: B
1.00
0.75
0.50
0.25
0.00
Methylocystaceae
A (n=14)
B (n=15)
C (n=11)
surface

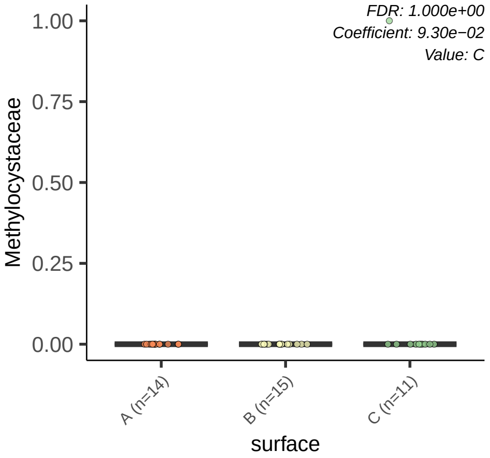

FDR: 1.000e+00
Coefficient: 9.30e−02
Value: C
Methylocystaceae
1.00
0.75
0.50
0.25
0.00
A (n=14)
B (n=15)
C (n=11)
surface

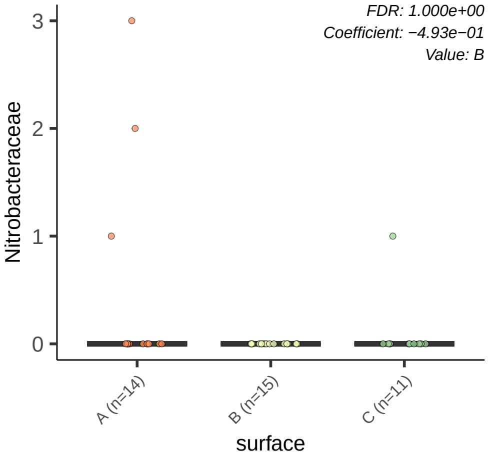
FDR: 1.000e+00
Coefficient: −4.93e−01
Value: B
Nitrobacteraceae
0
1
2
3
A (n=14)
B (n=15)
C (n=11)
surface

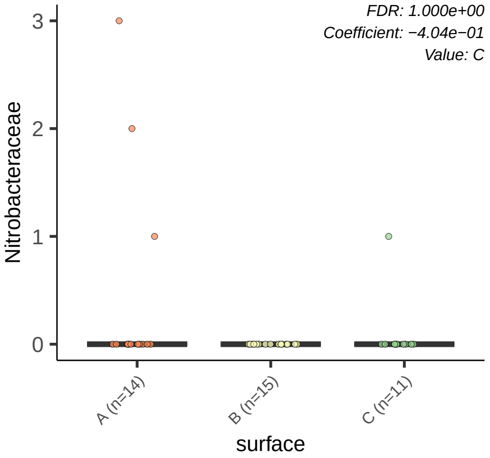
FDR: 1.000e+00
Coefficient: −4.04e−01
Value: C
Nitrobacteraceae
3
2
1
0
A (n=14)
B (n=15)
C (n=11)
surface

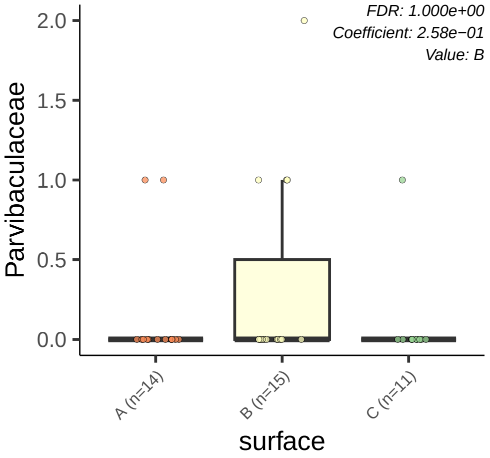
FDR: 1.000e+00
Coefficient: 2.58e−01
Value: B
Parvibaculaceae
2.0
1.5
1.0
0.5
0.0
A (n=14)
B (n=15)
C (n=11)
surface

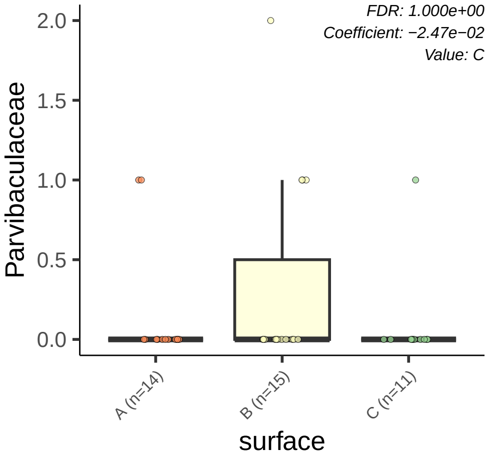
FDR: 1.000e+00
Coefficient: −2.47e−02
Value: C
Parvibaculaceae
2.0
1.5
1.0
0.5
0.0
A (n=14)
B (n=15)
C (n=11)
surface

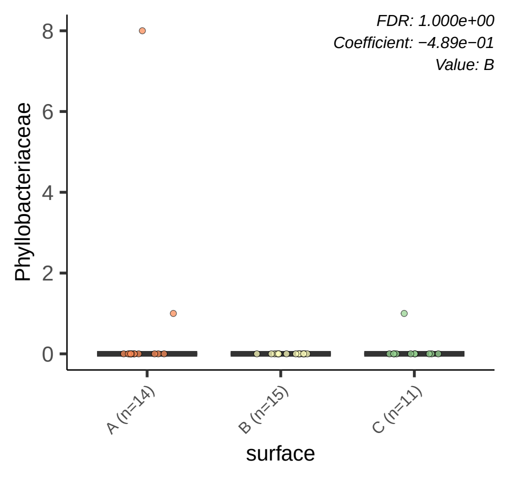

FDR: 1.000e+00
Coefficient: −4.89e−01
Value: B
Phyllobacteriaceae
8
6
4
2
0
A (n=14)
B (n=15)
C (n=11)
surface

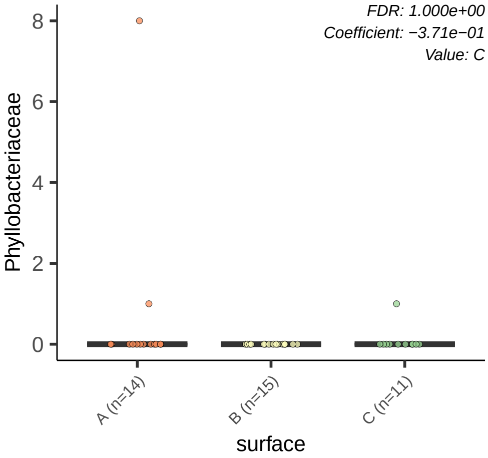
FDR: 1.000e+00
Coefficient: −3.71e−01
Value: C
Phyllobacteriaceae
8
6
4
2
0
A (n=14)
B (n=15)
C (n=11)
surface

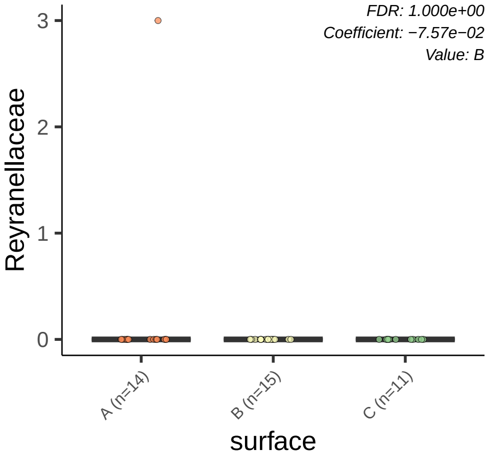

FDR: 1.000e+00
Coefficient: −7.57e−02
Value: B
Reyranellaceae
0
1
2
3
A (n=14)
B (n=15)
C (n=11)
surface

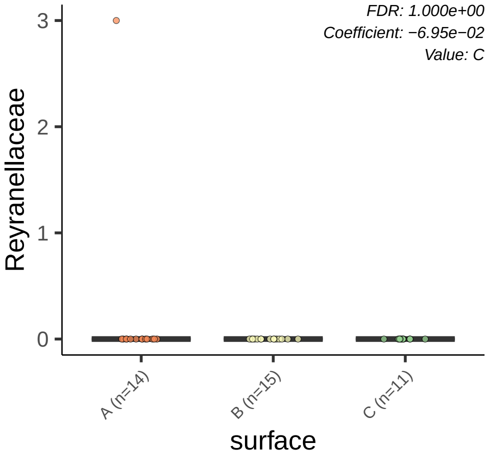
FDR: 1.000e+00
Coefficient: −6.95e−02
Value: C
Reyranellaceae
3
2
1
0
A (n=14)
B (n=15)
C (n=11)
surface

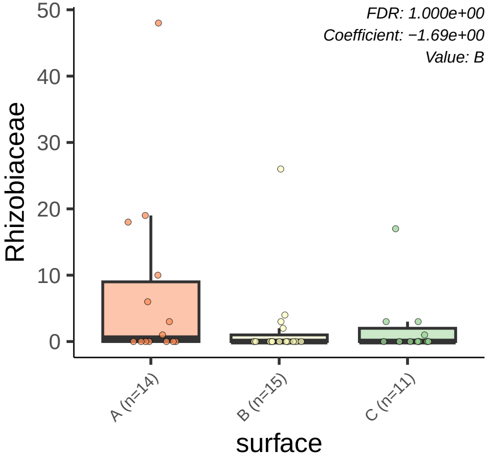
FDR: 1.000e+00
Coefficient: −1.69e+00
Value: B
Rhizobiaceae
50
40
30
20
10
0
A (n=14)
B (n=15)
C (n=11)
surface

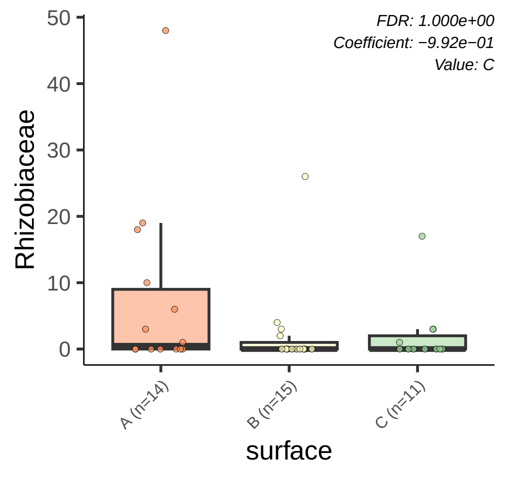
FDR: 1.000e+00
Coefficient: −9.92e−01
Value: C
Rhizobiaceae
0
10
20
30
40
50
A (n=14)
B (n=15)
C (n=11)
surface

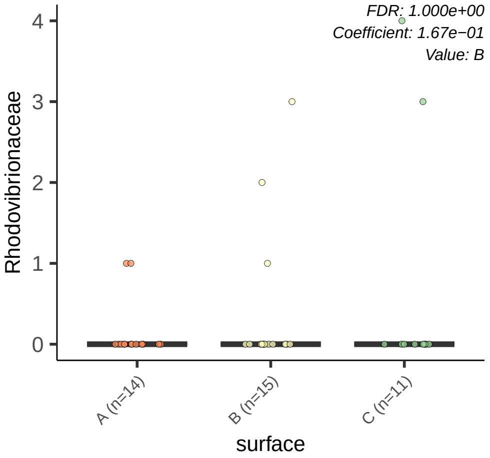

FDR: 1.000e+00
Coefficient: 1.67e−01
Value: B
Rhodovibrionaceae
0
1
2
3
4
A (n=14)
B (n=15)
C (n=11)
surface

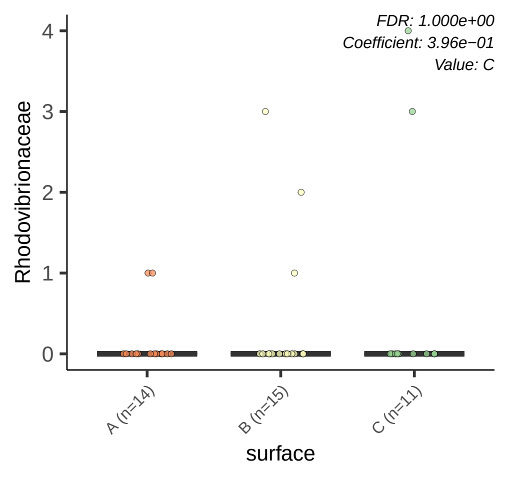

FDR: 1.000e+00
Coefficient: 3.96e−01
Value: C
Rhodovibrionaceae
0
1
2
3
4
A (n=14)
B (n=15)
C (n=11)
surface

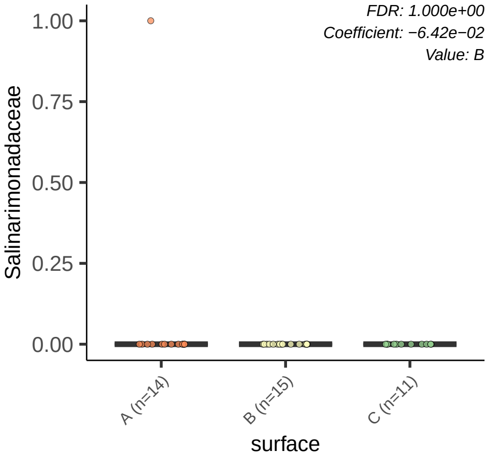
FDR: 1.000e+00
Coefficient: −6.42e−02
Value: B
Salinarimonadaceae
1.00
0.75
0.50
0.25
0.00
A (n=14)
B (n=15)
C (n=11)
surface

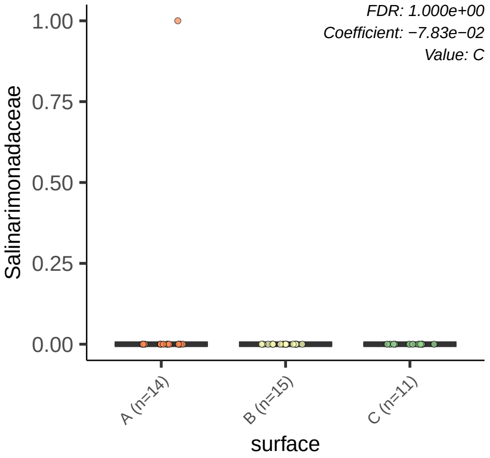
FDR: 1.000e+00
Coefficient: −7.83e−02
Value: C
Salinarimonadaceae
1.00
0.75
0.50
0.25
0.00
A (n=14)
B (n=15)
C (n=11)
surface

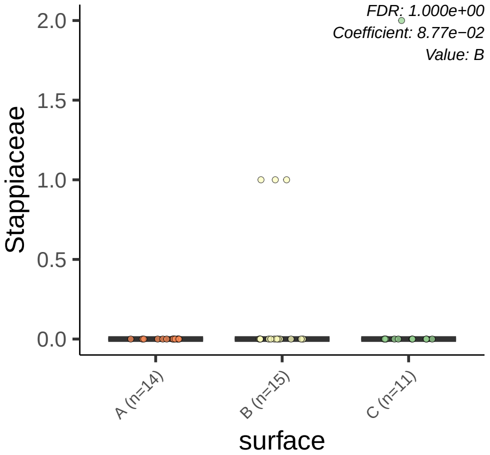

FDR: 1.000e+00
Coefficient: 8.77e−02
Value: B
Stappiaceae
2.0
1.5
1.0
0.5
0.0
A (n=14)
B (n=15)
C (n=11)
surface

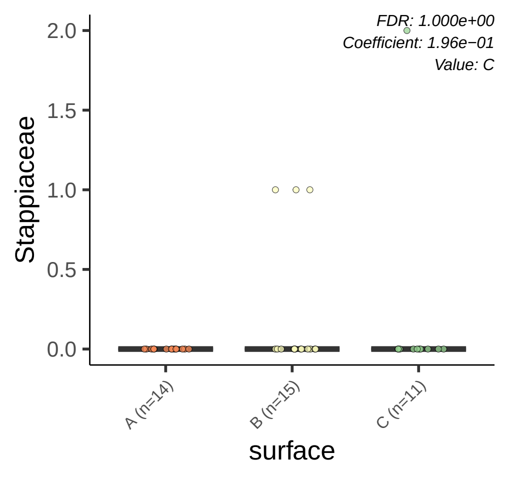
FDR: 1.000e+00
Coefficient: 1.96e−01
Value: C
Stappiaceae
2.0
1.5
1.0
0.5
0.0
A (n=14)
B (n=15)
C (n=11)
surface

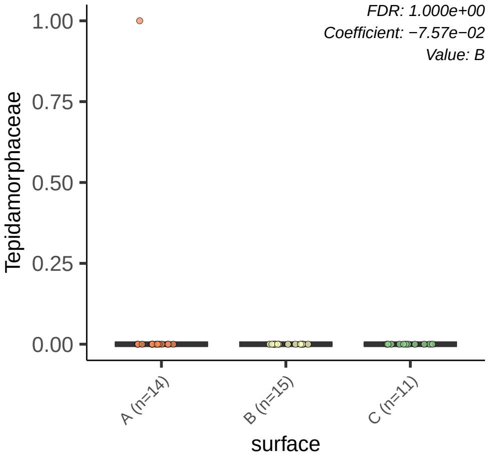
FDR: 1.000e+00
Coefficient: −7.57e−02
Value: B
Tepidamorphaceae
1.00
0.75
0.50
0.25
0.00
A (n=14)
B (n=15)
C (n=11)
surface

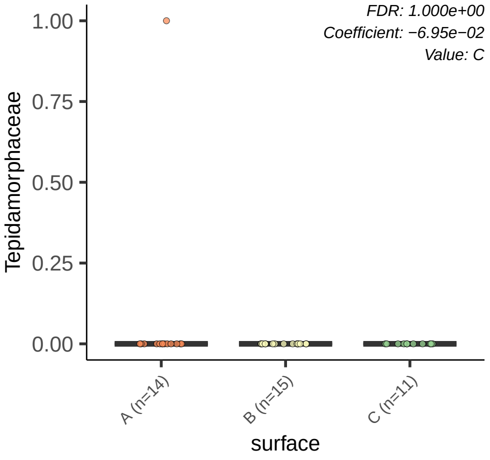
FDR: 1.000e+00
Coefficient: −6.95e−02
Value: C
Tepidamorphaceae
1.00
0.75
0.50
0.25
0.00
A (n=14)
B (n=15)
C (n=11)
surface

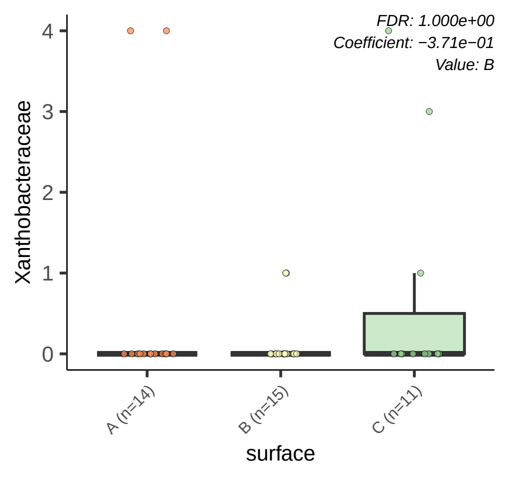
FDR: 1.000e+00
Coefficient: −3.71e−01
Value: B
Xanthobacteraceae
4
3
2
1
0
A (n=14)
B (n=15)
C (n=11)
surface

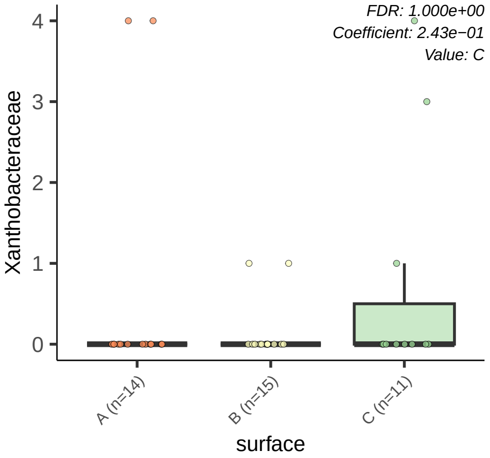

FDR: 1.000e+00
Coefficient: 2.43e−01
Value: C
Xanthobacteraceae
4
3
2
1
0
A (n=14)
B (n=15)
C (n=11)
surface

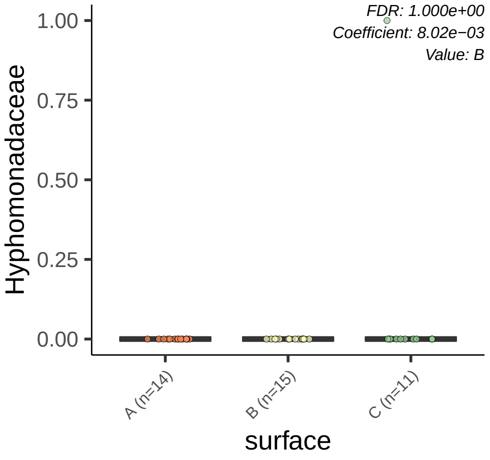
FDR: 1.000e+00
Coefficient: 8.02e−03
Value: B
Hyphomonadaceae
1.00
0.75
0.50
0.25
0.00
A (n=14)
B (n=15)
C (n=11)
surface

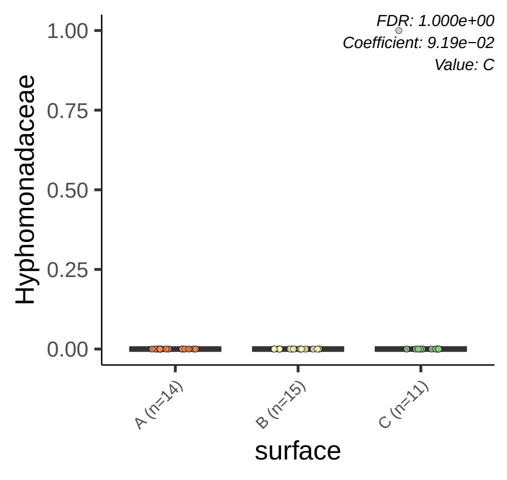
FDR: 1.000e+00
Coefficient: 9.19e−02
Value: C
Hyphomonadaceae
1.00
0.75
0.50
0.25
0.00
A (n=14)
B (n=15)
C (n=11)
surface

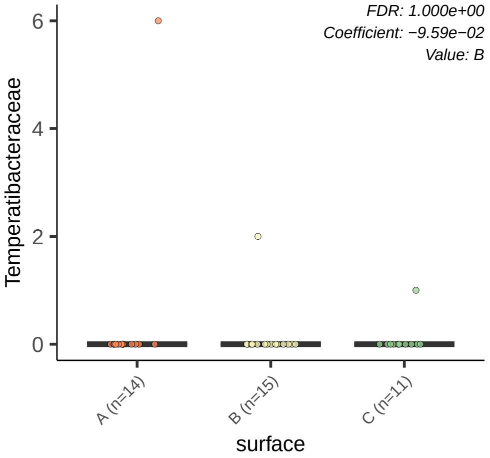
FDR: 1.000e+00
Coefficient: −9.59e−02
Value: B
Temperatibacteraceae
6
4
2
0
A (n=14)
B (n=15)
C (n=11)
surface

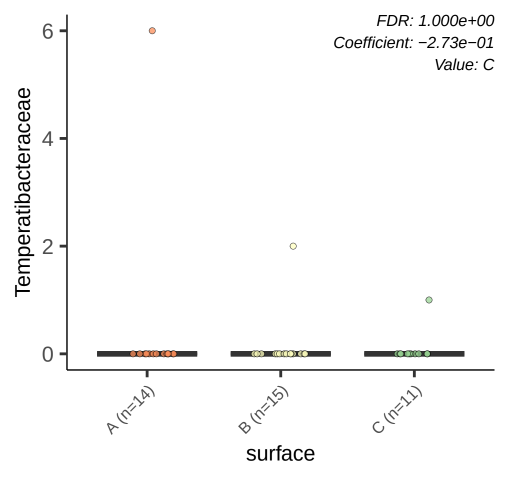
FDR: 1.000e+00
Coefficient: −2.73e−01
Value: C
Temperatibacteraceae
6
4
2
0
A (n=14)
B (n=15)
C (n=11)
surface

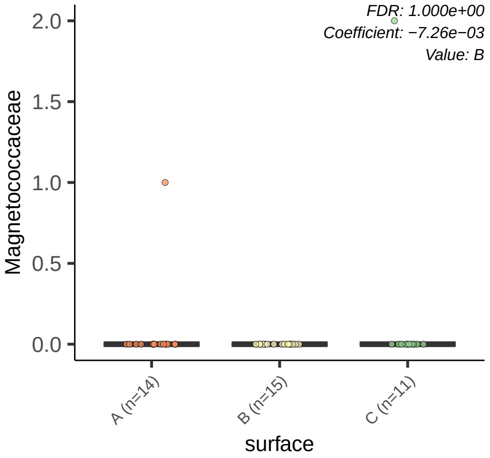
FDR: 1.000e+00
Coefficient: −7.26e−03
Value: B
Magnetococcaceae
2.0
1.5
1.0
0.5
0.0
A (n=14)
B (n=15)
C (n=11)
surface

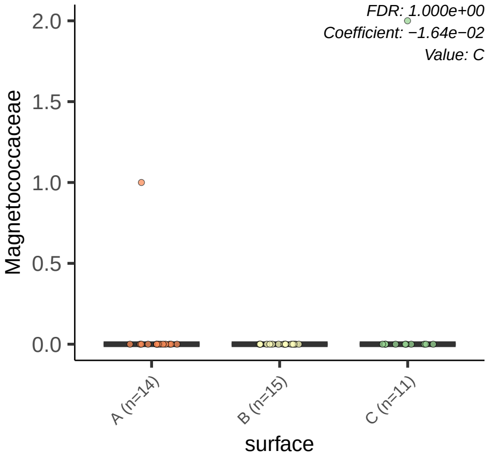
FDR: 1.000e+00
Coefficient: −1.64e−02
Value: C
Magnetococcaceae
2.0
1.5
1.0
0.5
0.0
A (n=14)
B (n=15)
C (n=11)
surface

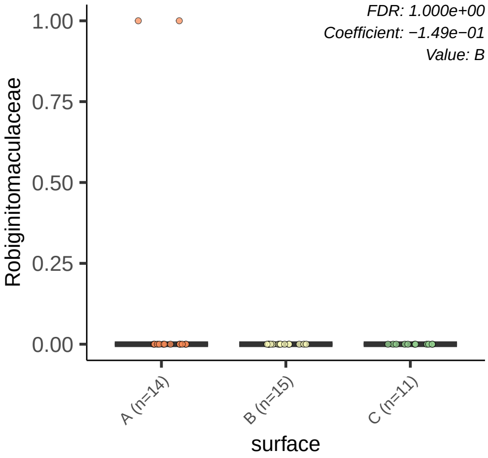
FDR: 1.000e+00
Coefficient: −1.49e−01
Value: B
Robiginitomaculaceae
1.00
0.75
0.50
0.25
0.00
A (n=14)
B (n=15)
C (n=11)
surface

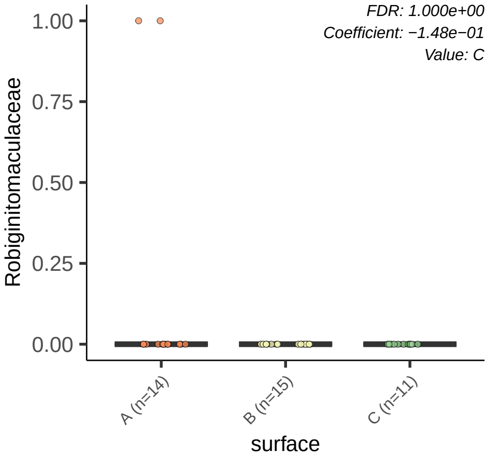

FDR: 1.000e+00
Coefficient: −1.48e−01
Value: C
Robiginitomaculaceae
1.00
0.75
0.50
0.25
0.00
A (n=14)
B (n=15)
C (n=11)
surface

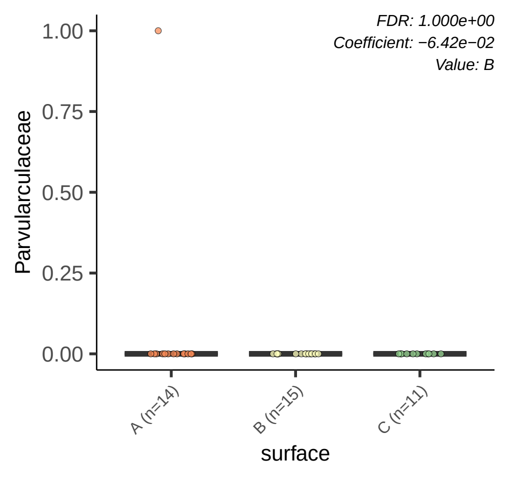
FDR: 1.000e+00
Coefficient: −6.42e−02
Value: B
Parvularculaceae
1.00
0.75
0.50
0.25
0.00
A (n=14)
B (n=15)
C (n=11)
surface

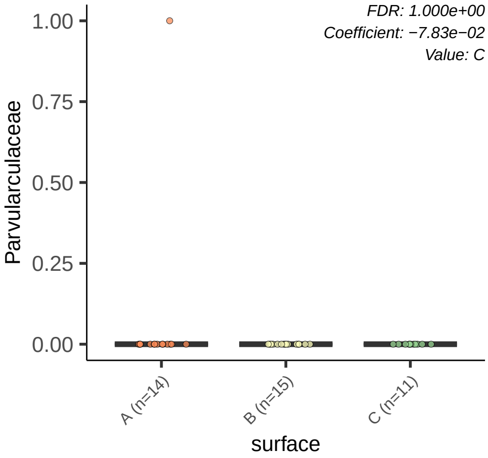
FDR: 1.000e+00
Coefficient: −7.83e−02
Value: C
Parvularculaceae
1.00
0.75
0.50
0.25
0.00
A (n=14)
B (n=15)
C (n=11)
surface

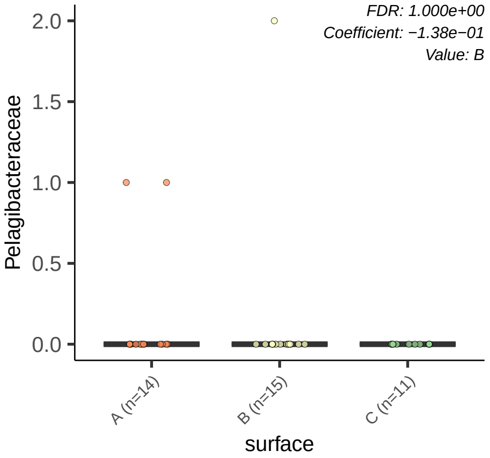

FDR: 1.000e+00
Coefficient: −1.38e−01
Value: B
2.0
1.5
1.0
0.5
0.0
Pelagibacteraceae
A (n=14)
B (n=15)
C (n=11)
surface

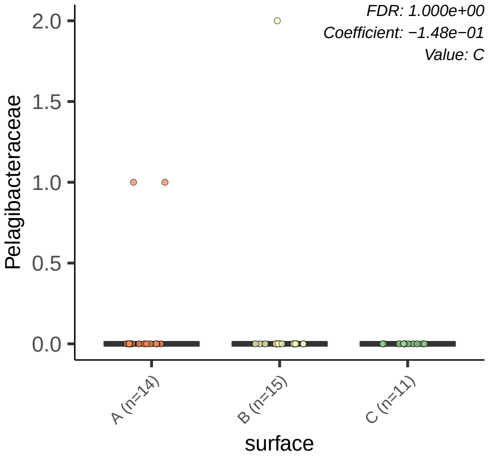

FDR: 1.000e+00
Coefficient: −1.48e−01
Value: C
Pelagibacteraceae
2.0
1.5
1.0
0.5
0.0
A (n=14)
B (n=15)
C (n=11)
surface

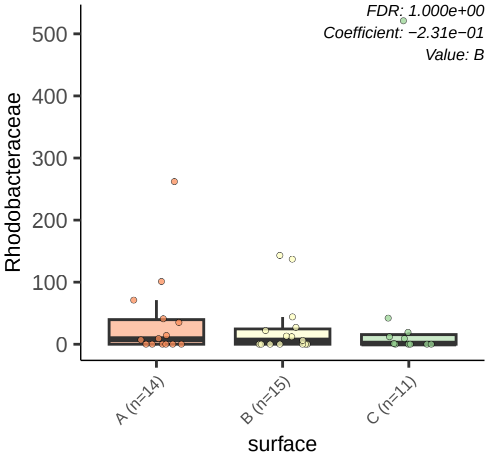

FDR: 1.000e+00
Coefficient: −2.31e−01
Value: B
Rhodobacteraceae
500
400
300
200
100
0
A (n=14)
B (n=15)
C (n=11)
surface

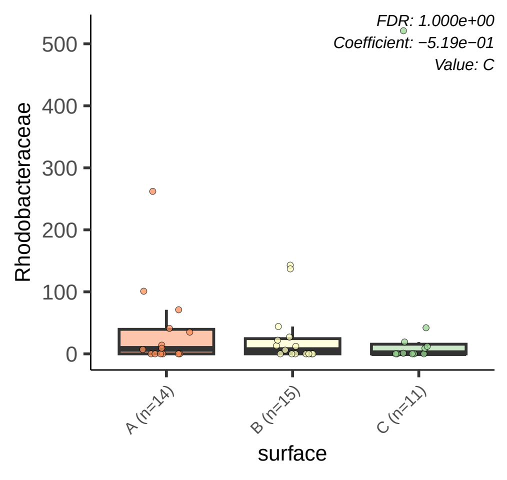

FDR: 1.000e+00
Coefficient: −5.19e−01
Value: C
Rhodobacteraceae
0
100
200
300
400
500
A (n=14)
B (n=15)
C (n=11)
surface

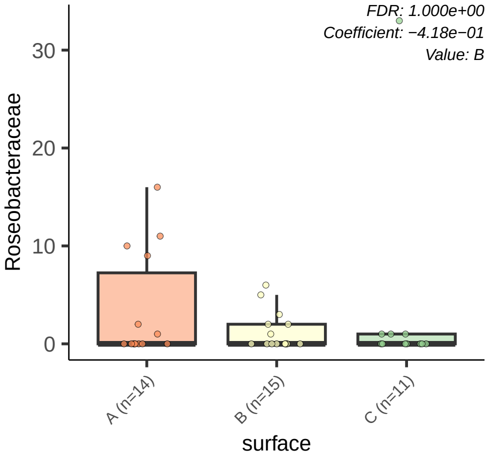
FDR: 1.000e+00
Coefficient: −4.18e−01
Value: B
Roseobacteraceae
0
10
20
30
A (n=14)
B (n=15)
C (n=11)
surface

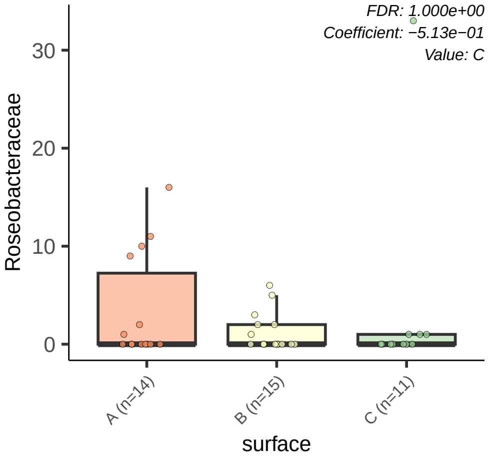

FDR: 1.000e+00
Coefficient: −5.13e−01
Value: C
Roseobacteraceae
0
10
20
30
A (n=14)
B (n=15)
C (n=11)
surface

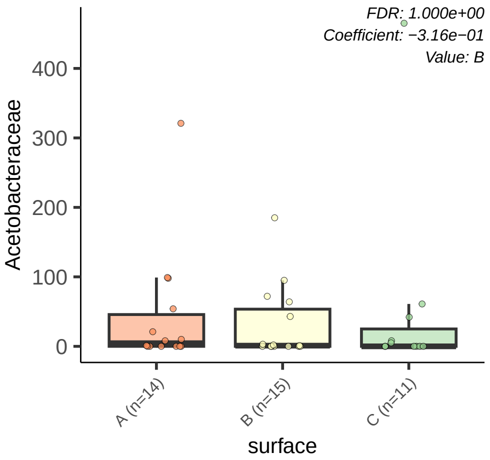

FDR: 1.000e+00
Coefficient: −3.16e−01
Value: B
Acetobacteraceae
0
100
200
300
400
A (n=14)
B (n=15)
C (n=11)
surface

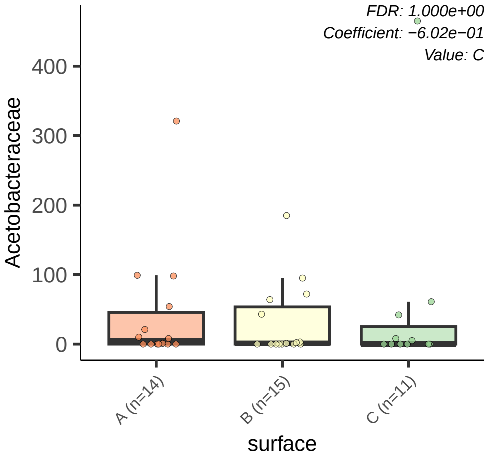
FDR: 1.000e+00
Coefficient: −6.02e−01
Value: C
Acetobacteraceae
400
300
200
100
0
A (n=14)
B (n=15)
C (n=11)
surface

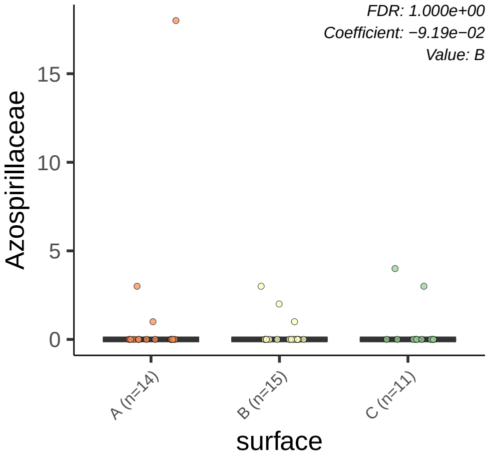
FDR: 1.000e+00
Coefficient: −9.19e−02
Value: B
Azospirillaceae
0
5
10
15
A (n=14)
B (n=15)
C (n=11)
surface

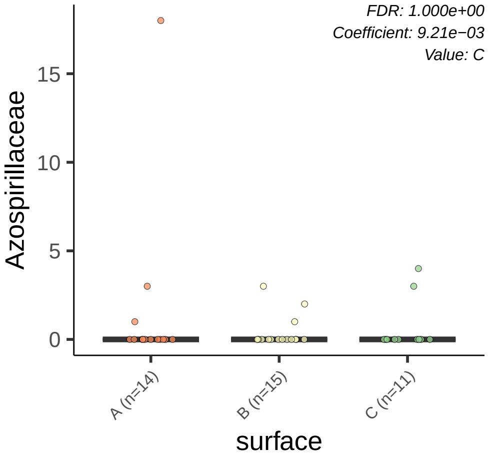

FDR: 1.000e+00
Coefficient: 9.21e−03
Value: C
Azospirillaceae
0
5
10
15
A (n=14)
B (n=15)
C (n=11)
surface

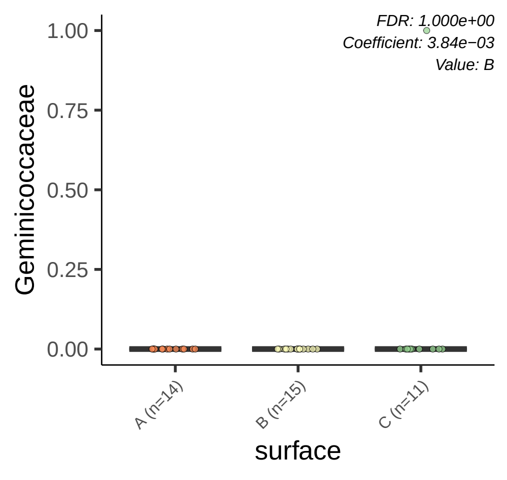

FDR: 1.000e+00
Coefficient: 3.84e−03
Value: B
Geminicoccaceae
1.00
0.75
0.50
0.25
0.00
A (n=14)
B (n=15)
C (n=11)
surface

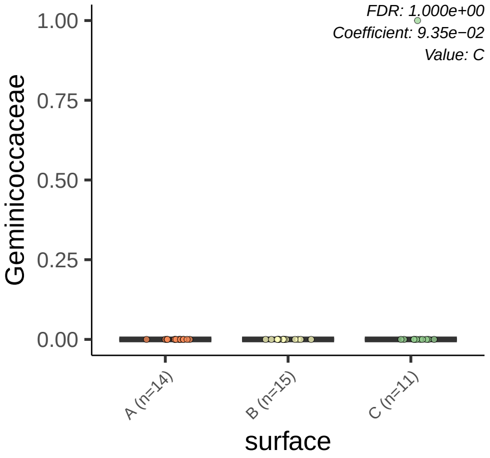
FDR: 1.000e+00
Coefficient: 9.35e−02
Value: C
Geminicoccaceae
1.00
0.75
0.50
0.25
0.00
A (n=14)
B (n=15)
C (n=11)
surface

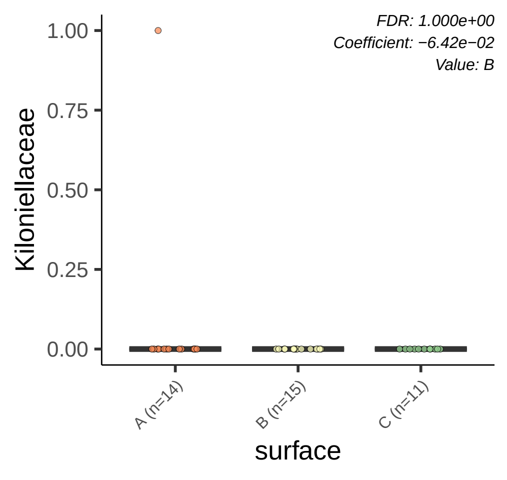
FDR: 1.000e+00
Coefficient: −6.42e−02
Value: B
Kiloniellaceae
1.00
0.75
0.50
0.25
0.00
A (n=14)
B (n=15)
C (n=11)
surface

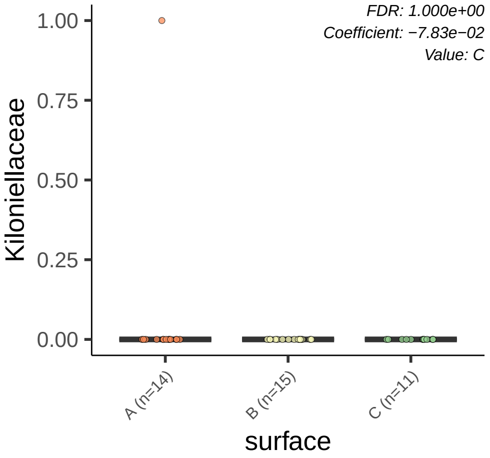
FDR: 1.000e+00
Coefficient: −7.83e−02
Value: C
Kiloniellaceae
1.00
0.75
0.50
0.25
0.00
A (n=14)
B (n=15)
C (n=11)
surface

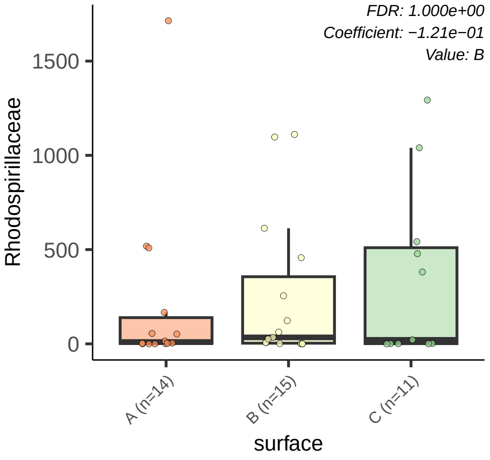

FDR: 1.000e+00
Coefficient: −1.21e−01
Value: B
Rhodospirillaceae
0
500
1000
1500
A (n=14)
B (n=15)
C (n=11)
surface

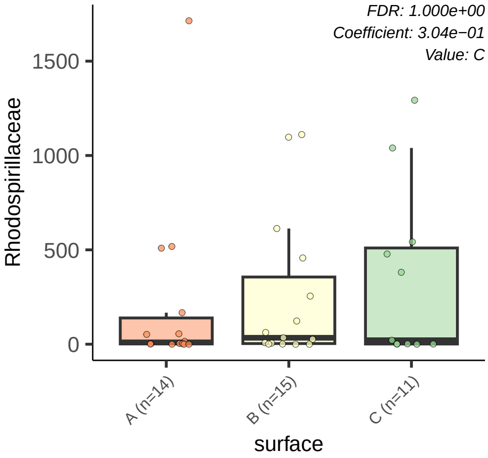
FDR: 1.000e+00
Coefficient: 3.04e−01
Value: C
Rhodospirillaceae
1500
1000
500
0
A (n=14)
B (n=15)
C (n=11)
surface

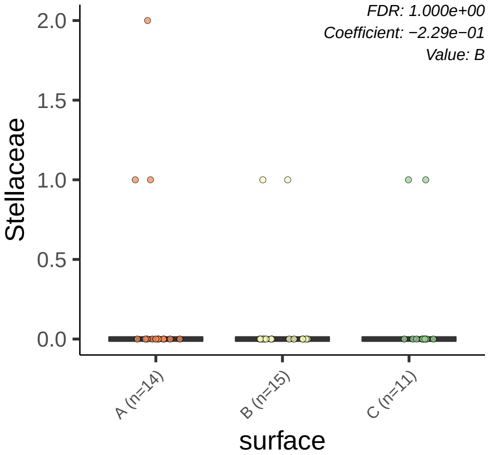

FDR: 1.000e+00
Coefficient: −2.29e−01
Value: B
Stellaceae
2.0
1.5
1.0
0.5
0.0
A (n=14)
B (n=15)
C (n=11)
surface

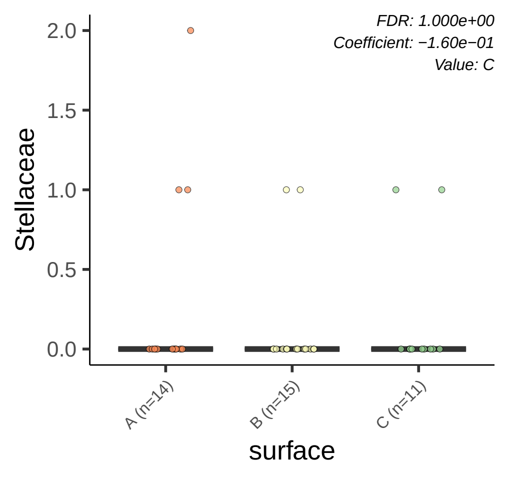

FDR: 1.000e+00
Coefficient: −1.60e−01
Value: C
Stellaceae
2.0
1.5
1.0
0.5
0.0
A (n=14)
B (n=15)
C (n=11)
surface

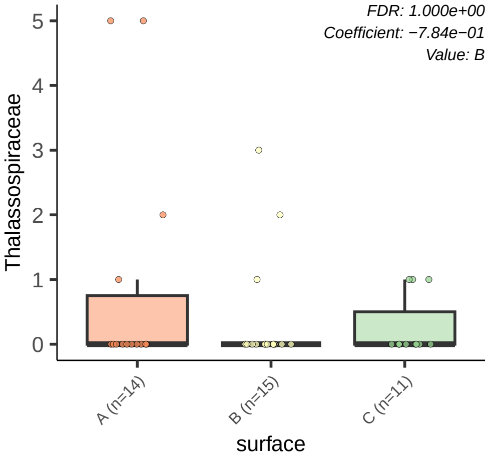
FDR: 1.000e+00
Coefficient: −7.84e−01
Value: B
Thalassospiraceae
5
4
3
2
1
0
A (n=14)
B (n=15)
C (n=11)
surface

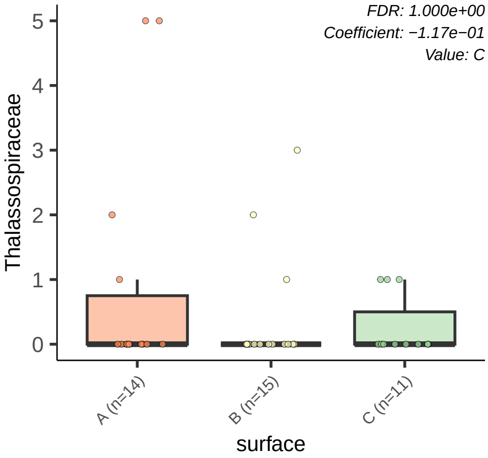

FDR: 1.000e+00
Coefficient: −1.17e−01
Value: C
Thalassospiraceae
0
1
2
3
4
5
A (n=14)
B (n=15)
C (n=11)
surface

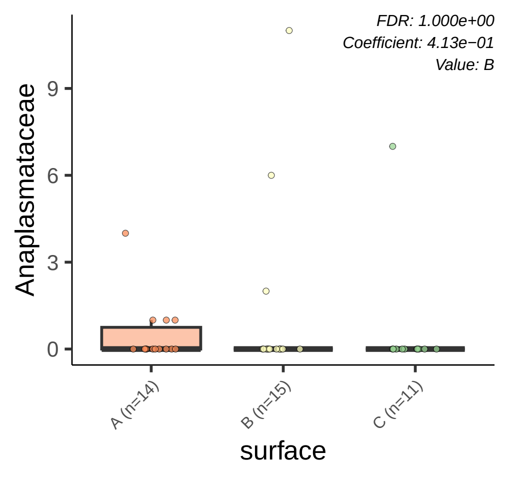
FDR: 1.000e+00
Coefficient: 4.13e−01
Value: B
Anaplasmataceae
0
3
6
9
A (n=14)
B (n=15)
C (n=11)
surface

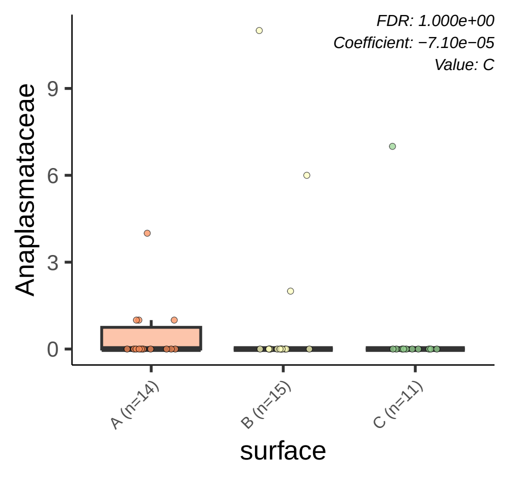
FDR: 1.000e+00
Coefficient: −7.10e−05
Value: C
Anaplasmataceae
0
3
6
9
A (n=14)
B (n=15)
C (n=11)
surface

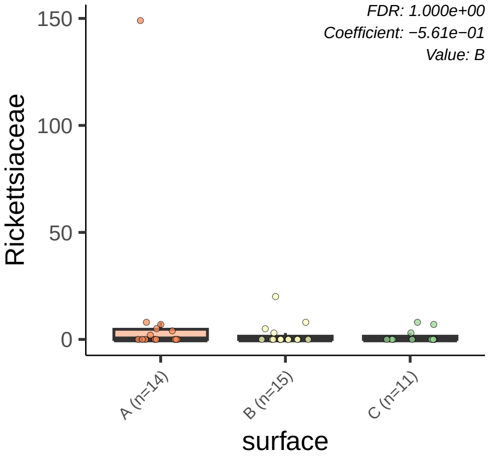
FDR: 1.000e+00
Coefficient: −5.61e−01
Value: B
Rickettsiaceae
150
100
50
0
A (n=14)
B (n=15)
C (n=11)
surface

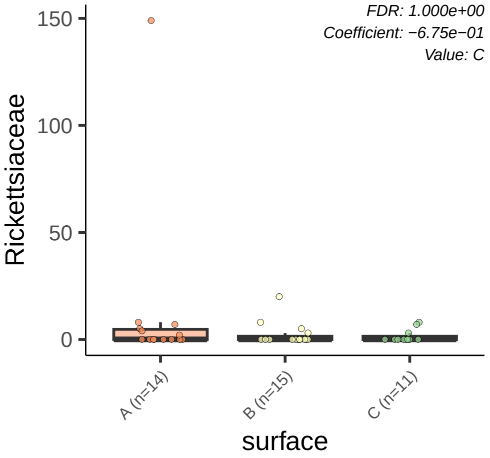
FDR: 1.000e+00
Coefficient: −6.75e−01
Value: C
Rickettsiaceae
150
100
50
0
A (n=14)
B (n=15)
C (n=11)
surface

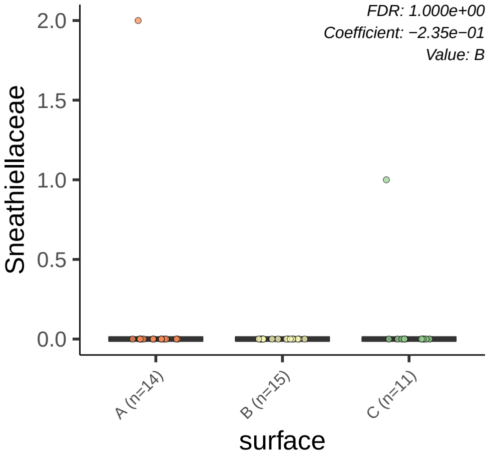
FDR: 1.000e+00
Coefficient: −2.35e−01
Value: B
Sneathiellaceae
2.0
1.5
1.0
0.5
0.0
A (n=14)
B (n=15)
C (n=11)
surface

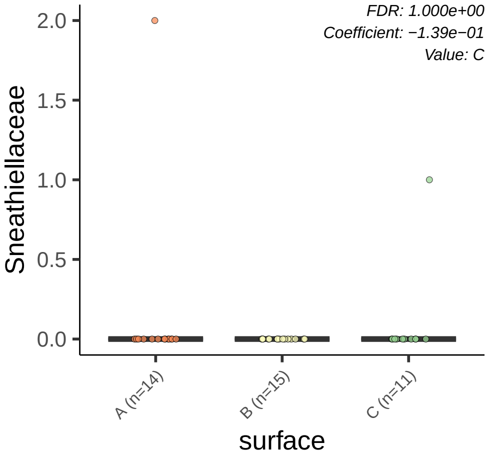

FDR: 1.000e+00
Coefficient: −1.39e−01
Value: C
Sneathiellaceae
2.0
1.5
1.0
0.5
0.0
A (n=14)
B (n=15)
C (n=11)
surface

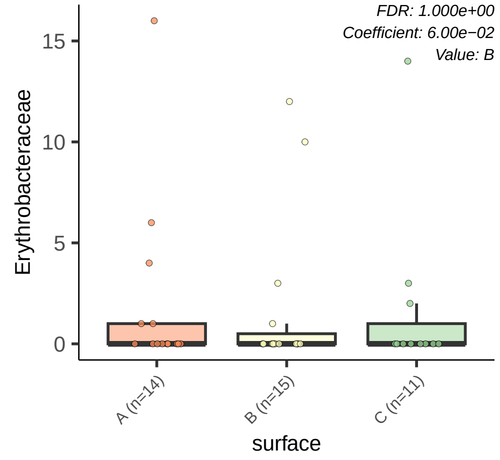
FDR: 1.000e+00
Coefficient: 6.00e−02
Value: B
Erythrobacteraceae
0
5
10
15
A (n=14)
B (n=15)
C (n=11)
surface

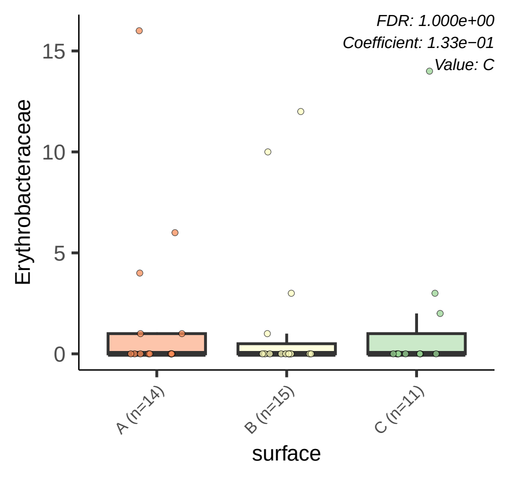
FDR: 1.000e+00
Coefficient: 1.33e−01
Value: C
Erythrobacteraceae
15
10
5
0
A (n=14)
B (n=15)
C (n=11)
surface

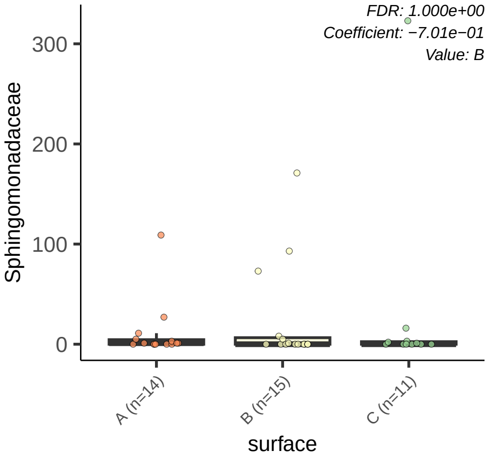

FDR: 1.000e+00
Coefficient: −7.01e−01
Value: B
Sphingomonadaceae
300
200
100
0
A (n=14)
B (n=15)
C (n=11)
surface

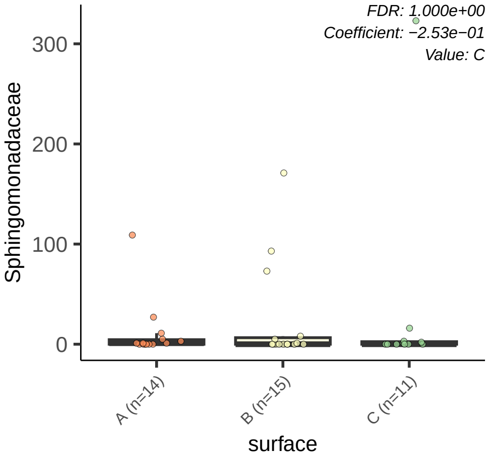
FDR: 1.000e+00
Coefficient: −2.53e−01
Value: C
Sphingomonadaceae
300
200
100
0
A (n=14)
B (n=15)
C (n=11)
surface

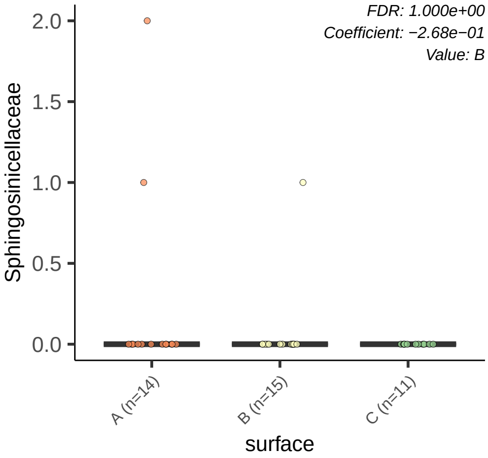
FDR: 1.000e+00
Coefficient: −2.68e−01
Value: B
Sphingosinicellaceae
2.0
1.5
1.0
0.5
0.0
A (n=14)
B (n=15)
C (n=11)
surface

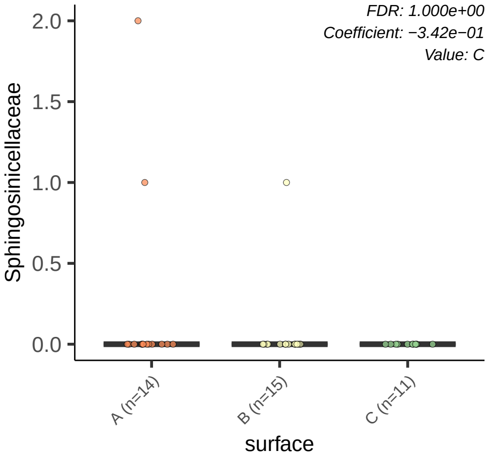
FDR: 1.000e+00
Coefficient: −3.42e−01
Value: C
Sphingosinicellaceae
2.0
1.5
1.0
0.5
0.0
A (n=14)
B (n=15)
C (n=11)
surface

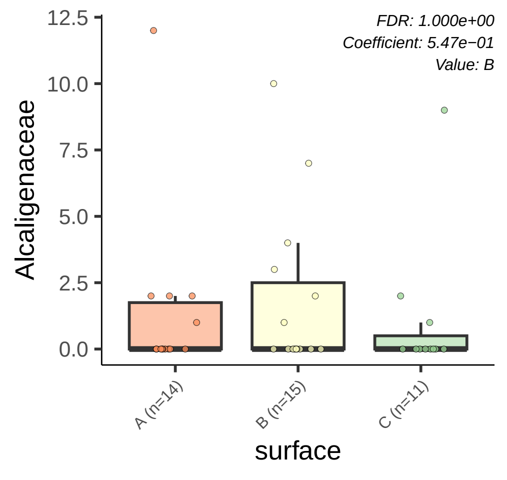

FDR: 1.000e+00
Coefficient: 5.47e−01
Value: B
Alcaligenaceae
12.5
10.0
7.5
5.0
2.5
0.0
A (n=14)
B (n=15)
C (n=11)
surface

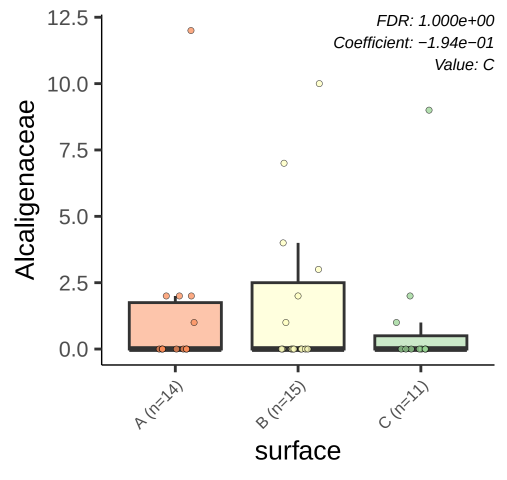
FDR: 1.000e+00
Coefficient: −1.94e−01
Value: C
Alcaligenaceae
12.5
10.0
7.5
5.0
2.5
0.0
A (n=14)
B (n=15)
C (n=11)
surface

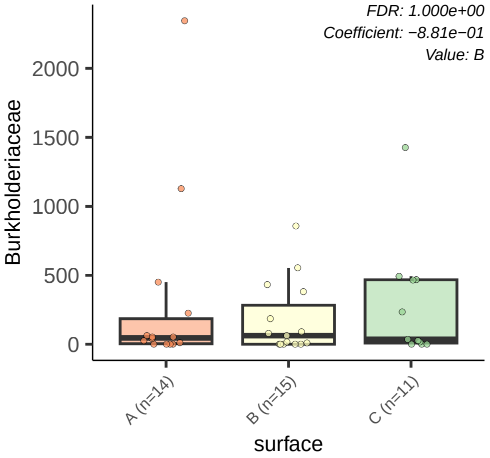
FDR: 1.000e+00
Coefficient: −8.81e−01
Value: B
Burkholderiaceae
2000
1500
1000
500
0
A (n=14)
B (n=15)
C (n=11)
surface

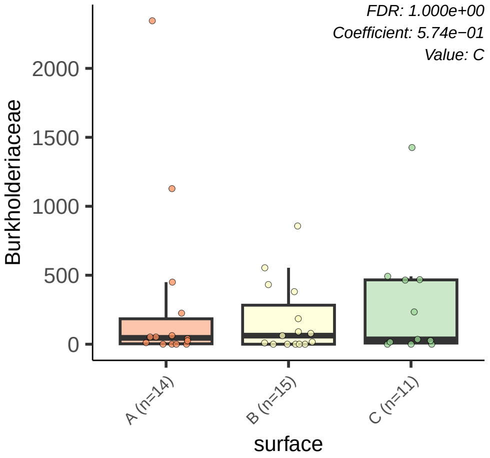
FDR: 1.000e+00
Coefficient: 5.74e−01
Value: C
Burkholderiaceae
2000
1500
1000
500
0
A (n=14)
B (n=15)
C (n=11)
surface

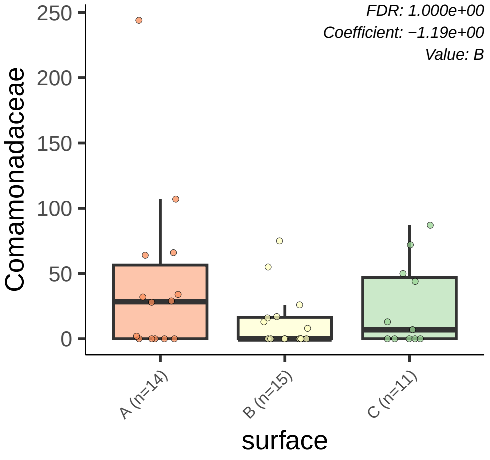
FDR: 1.000e+00
Coefficient: −1.19e+00
Value: B
Comamonadaceae
250
200
150
100
50
0
A (n=14)
B (n=15)
C (n=11)
surface

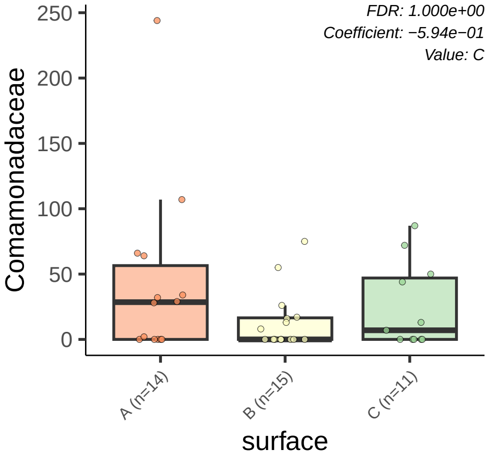

FDR: 1.000e+00
Coefficient: −5.94e−01
Value: C
Comamonadaceae
250
200
150
100
50
0
A (n=14)
B (n=15)
C (n=11)
surface

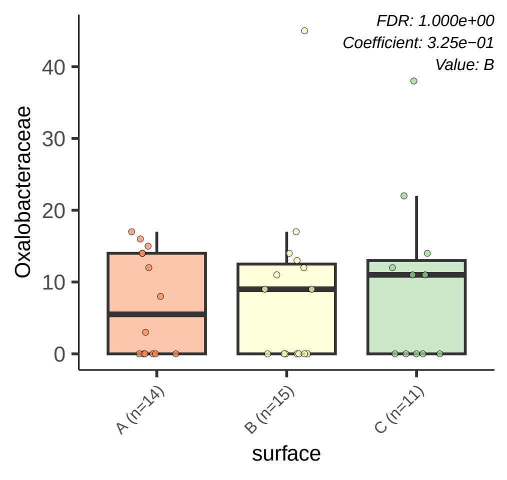
FDR: 1.000e+00
Coefficient: 3.25e−01
Value: B
Oxalobacteraceae
0
10
20
30
40
A (n=14)
B (n=15)
C (n=11)
surface

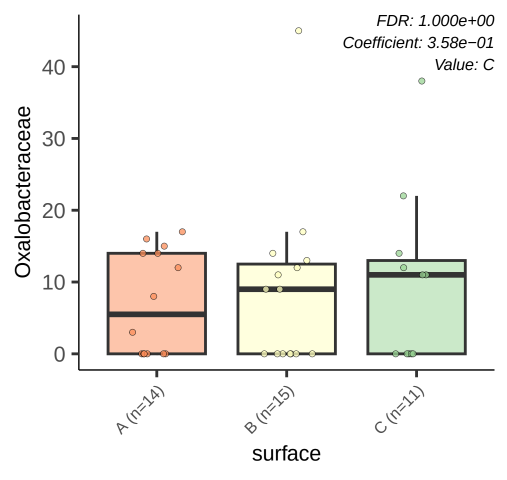
FDR: 1.000e+00
Coefficient: 3.58e−01
Value: C
Oxalobacteraceae
0
10
20
30
40
A (n=14)
B (n=15)
C (n=11)
surface

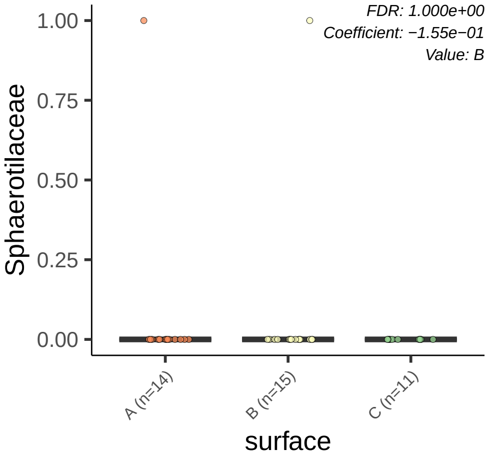
FDR: 1.000e+00
Coefficient: −1.55e−01
Value: B
Sphaerotilaceae
1.00
0.75
0.50
0.25
0.00
A (n=14)
B (n=15)
C (n=11)
surface

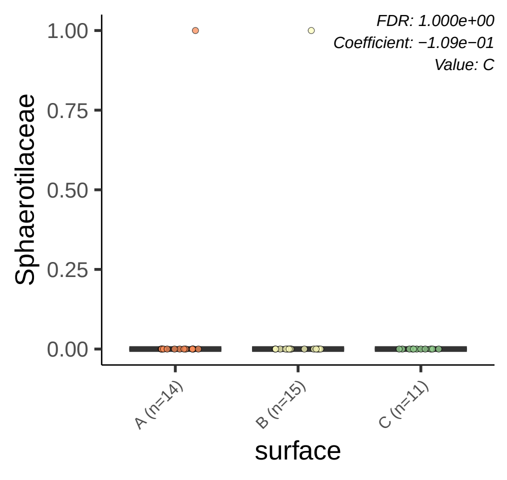
FDR: 1.000e+00
Coefficient: −1.09e−01
Value: C
Sphaerotilaceae
1.00
0.75
0.50
0.25
0.00
A (n=14)
B (n=15)
C (n=11)
surface

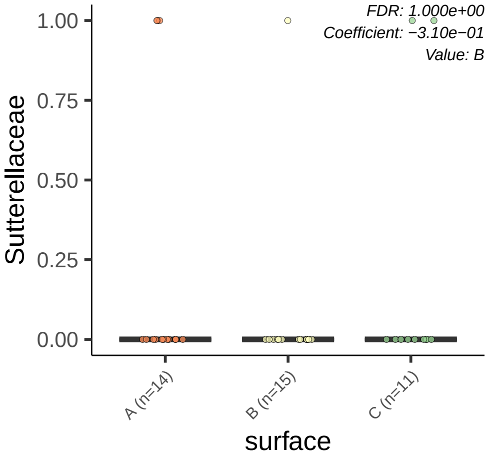
FDR: 1.000e+00
Coefficient: −3.10e−01
Value: B
Sutterellaceae
1.00
0.75
0.50
0.25
0.00
A (n=14)
B (n=15)
C (n=11)
surface

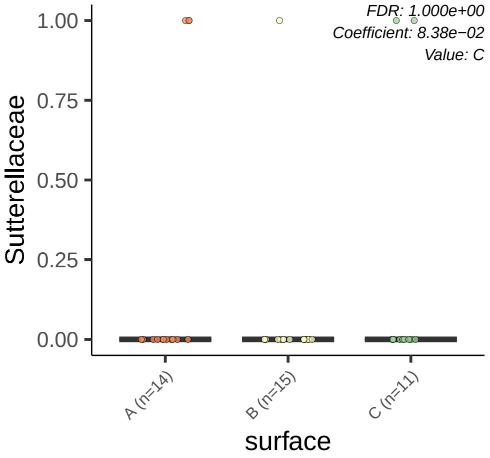
FDR: 1.000e+00
Coefficient: 8.38e−02
Value: C
Sutterellaceae
1.00
0.75
0.50
0.25
0.00
A (n=14)
B (n=15)
C (n=11)
surface

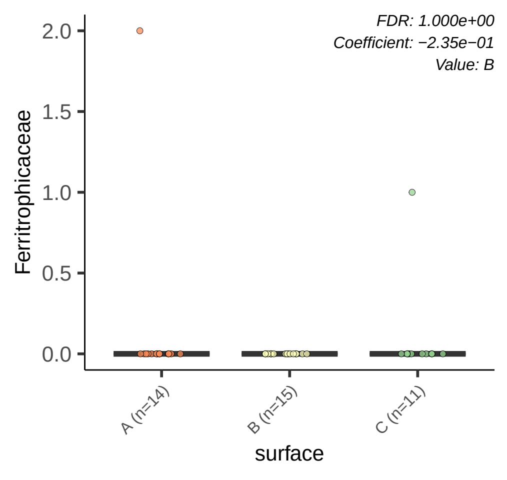
FDR: 1.000e+00
Coefficient: −2.35e−01
Value: B
Ferritrophicaceae
2.0
1.5
1.0
0.5
0.0
A (n=14)
B (n=15)
C (n=11)
surface

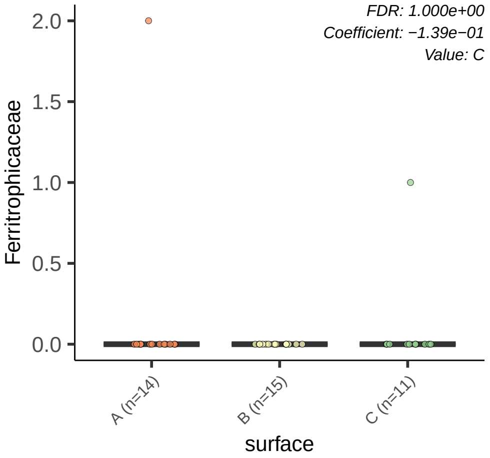

FDR: 1.000e+00
Coefficient: −1.39e−01
Value: C
Ferritrophicaceae
2.0
1.5
1.0
0.5
0.0
A (n=14)
B (n=15)
C (n=11)
surface

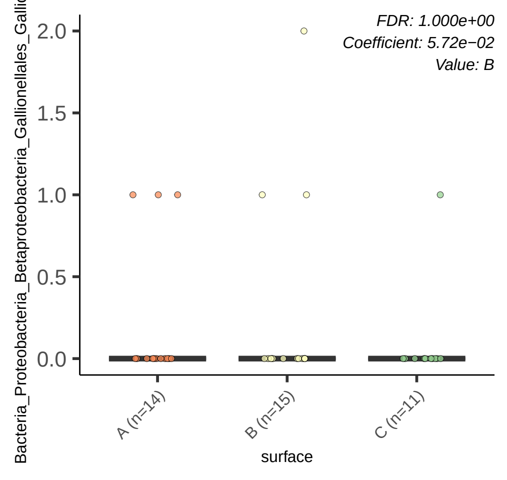

FDR: 1.000e+00
Coefficient: 5.72e−02
Value: B
Bacteria_Proteobacteria_Betaproteobacteria_Gallionellales_Gallio
2.0
1.5
1.0
0.5
0.0
A (n=14)
B (n=15)
C (n=11)
surface

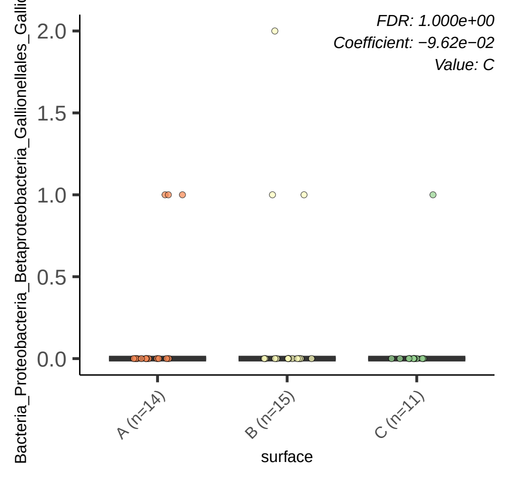
FDR: 1.000e+00
Coefficient: −9.62e−02
Value: C
Bacteria_Proteobacteria_Betaproteobacteria_Gallionellales_Galli
2.0
1.5
1.0
0.5
0.0
A (n=14)
B (n=15)
C (n=11)
surface

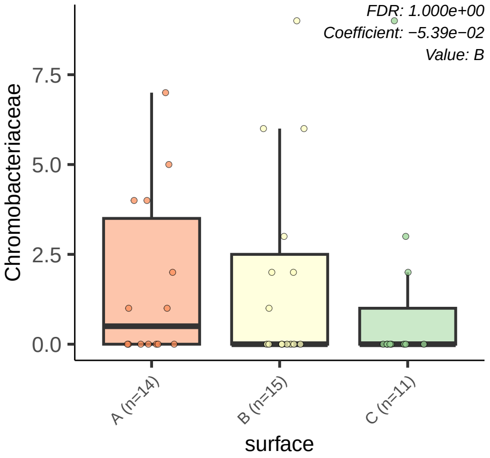
FDR: 1.000e+00
Coefficient: −5.39e−02
Value: B
Chromobacteriaceae
7.5
5.0
2.5
0.0
A (n=14)
B (n=15)
C (n=11)
surface

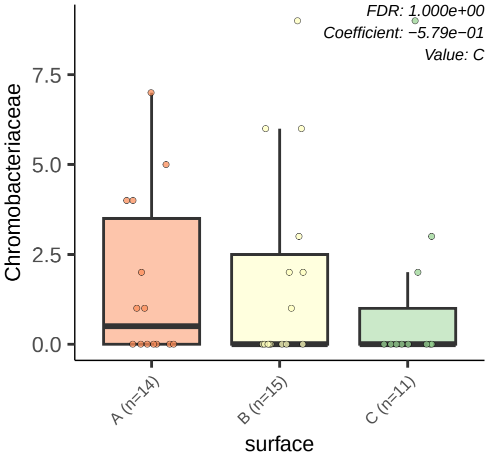
FDR: 1.000e+00
Coefficient: −5.79e−01
Value: C
Chromobacteriaceae
0.0
2.5
5.0
7.5
A (n=14)
B (n=15)
C (n=11)
surface

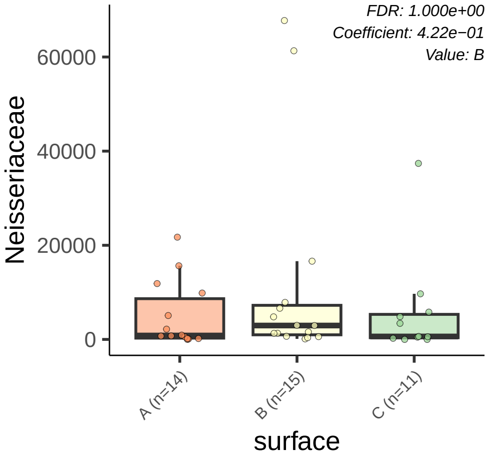
FDR: 1.000e+00
Coefficient: 4.22e−01
Value: B
Neisseriaceae
60000
40000
20000
0
A (n=14)
B (n=15)
C (n=11)
surface

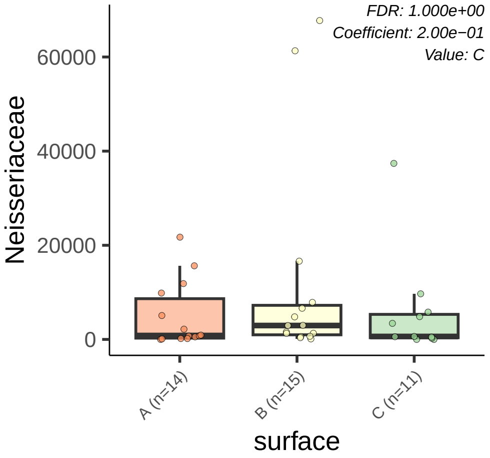
FDR: 1.000e+00
Coefficient: 2.00e−01
Value: C
Neisseriaceae
60000
40000
20000
0
A (n=14)
B (n=15)
C (n=11)
surface

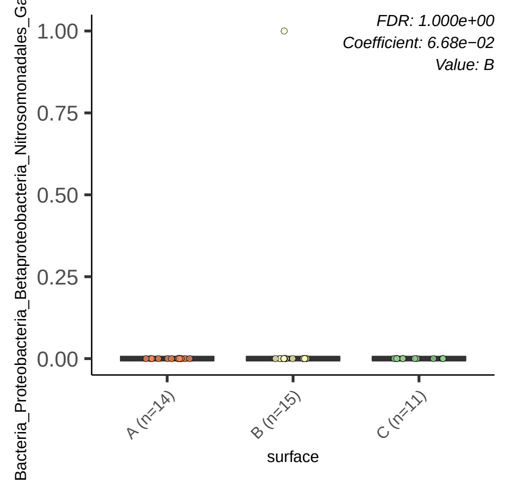
FDR: 1.000e+00
Coefficient: 6.68e−02
Value: B
Bacteria_Proteobacteria_Betaproteobacteria_Nitrosomonadales_Ga
1.00
0.75
0.50
0.25
0.00
A (n=14)
B (n=15)
C (n=11)
surface

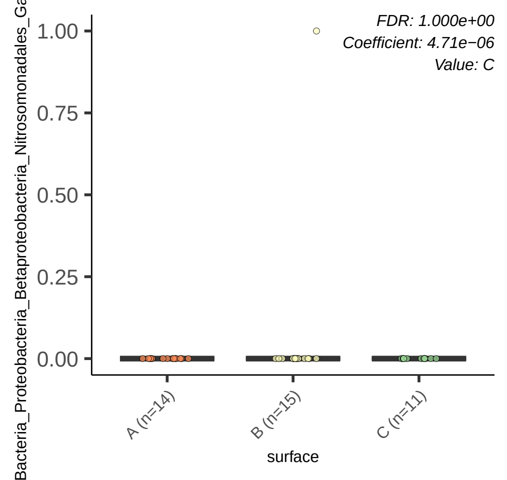
FDR: 1.000e+00
Coefficient: 4.71e−06
Value: C
1.00
0.75
0.50
0.25
0.00
Bacteria_Proteobacteria_Betaproteobacteria_Nitrosomonadales_Ga
A (n=14)
B (n=15)
C (n=11)
surface

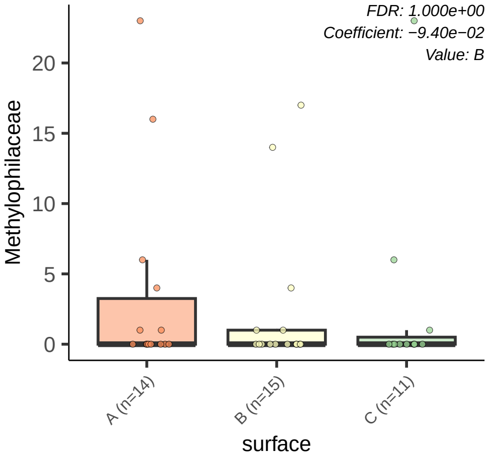

FDR: 1.000e+00
Coefficient: −9.40e−02
Value: B
Methylophilaceae
0
5
10
15
20
A (n=14)
B (n=15)
C (n=11)
surface

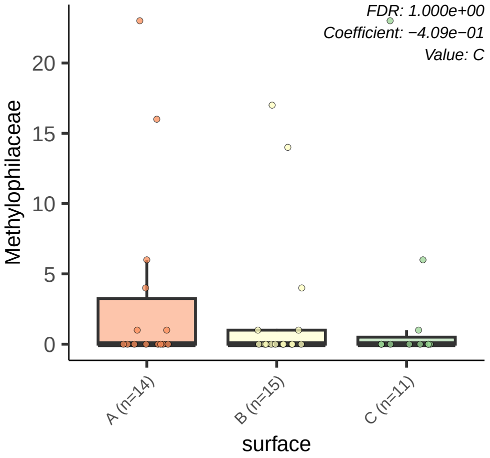

FDR: 1.000e+00
Coefficient: −4.09e−01
Value: C
Methylophilaceae
0
5
10
15
20
A (n=14)
B (n=15)
C (n=11)
surface

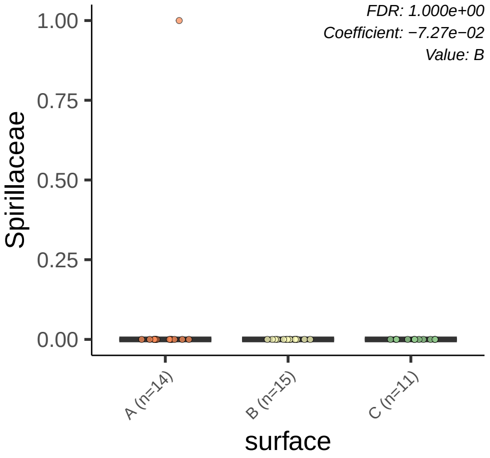
FDR: 1.000e+00
Coefficient: −7.27e−02
Value: B
Spirillaceae
1.00
0.75
0.50
0.25
0.00
A (n=14)
B (n=15)
C (n=11)
surface

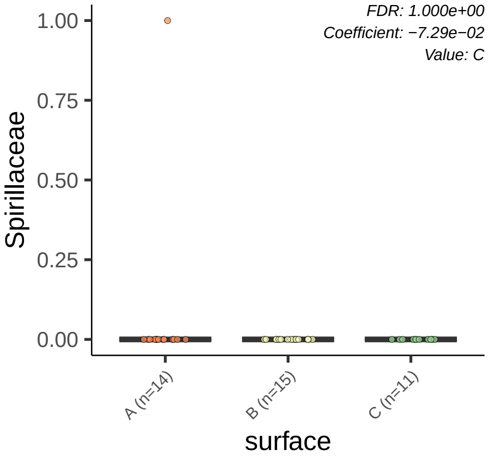
FDR: 1.000e+00
Coefficient: −7.29e−02
Value: C
Spirillaceae
1.00
0.75
0.50
0.25
0.00
A (n=14)
B (n=15)
C (n=11)
surface

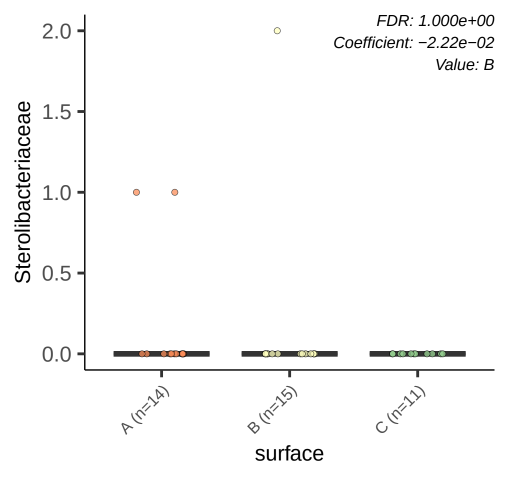

FDR: 1.000e+00
Coefficient: −2.22e−02
Value: B
Sterolibacteriaceae
2.0
1.5
1.0
0.5
0.0
A (n=14)
B (n=15)
C (n=11)
surface

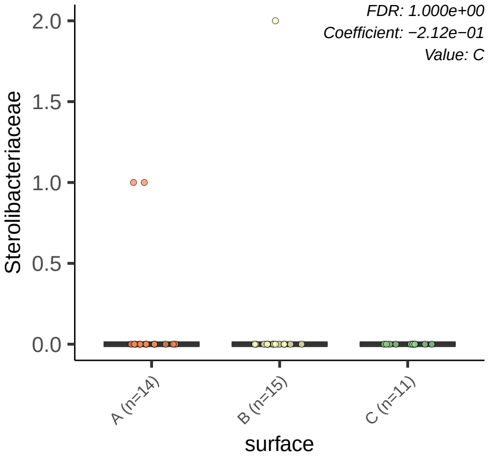
FDR: 1.000e+00
Coefficient: −2.12e−01
Value: C
Sterolibacteriaceae
2.0
1.5
1.0
0.5
0.0
A (n=14)
B (n=15)
C (n=11)
surface

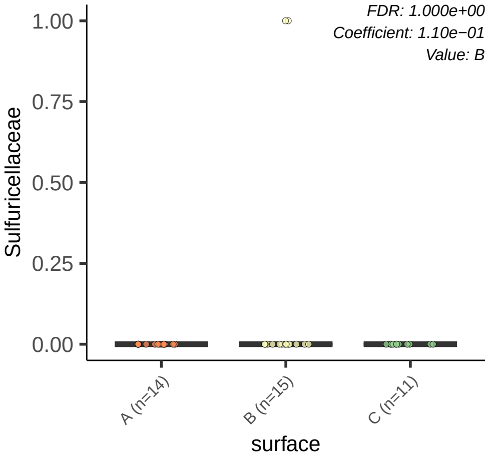
FDR: 1.000e+00
Coefficient: 1.10e−01
Value: B
Sulfuricellaceae
1.00
0.75
0.50
0.25
0.00
A (n=14)
B (n=15)
C (n=11)
surface

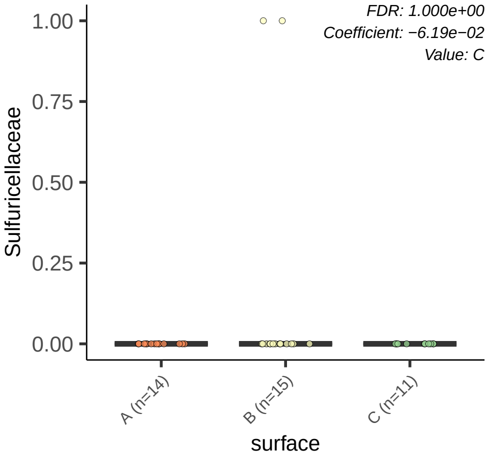

FDR: 1.000e+00
Coefficient: −6.19e−02
Value: C
Sulfuricellaceae
1.00
0.75
0.50
0.25
0.00
A (n=14)
B (n=15)
C (n=11)
surface

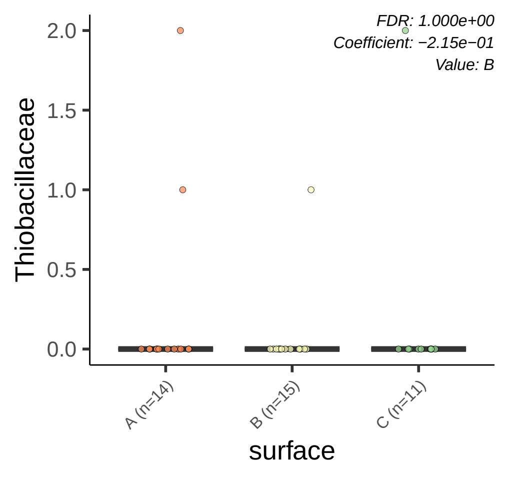
FDR: 1.000e+00
Coefficient: −2.15e−01
Value: B
Thiobacillaceae
2.0
1.5
1.0
0.5
0.0
A (n=14)
B (n=15)
C (n=11)
surface

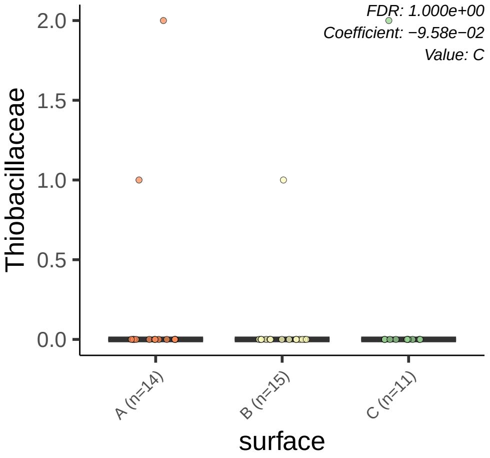
FDR: 1.000e+00
Coefficient: −9.58e−02
Value: C
Thiobacillaceae
2.0
1.5
1.0
0.5
0.0
A (n=14)
B (n=15)
C (n=11)
surface

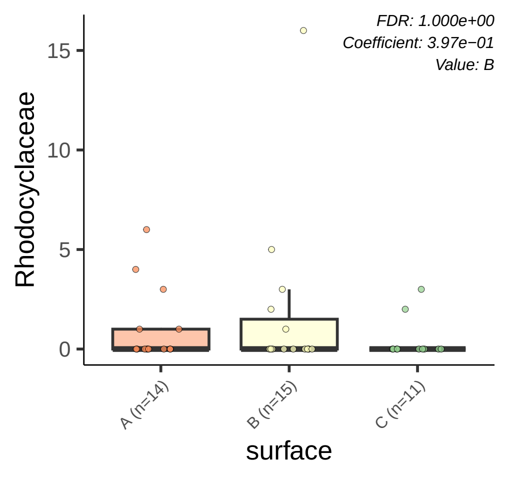

FDR: 1.000e+00
Coefficient: 3.97e−01
Value: B
Rhodocyclaceae
15
10
5
0
A (n=14)
B (n=15)
C (n=11)
surface

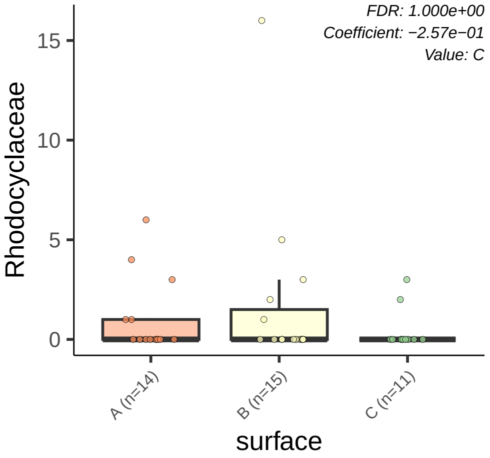

FDR: 1.000e+00
Coefficient: −2.57e−01
Value: C
Rhodocyclaceae
0
5
10
15
A (n=14)
B (n=15)
C (n=11)
surface

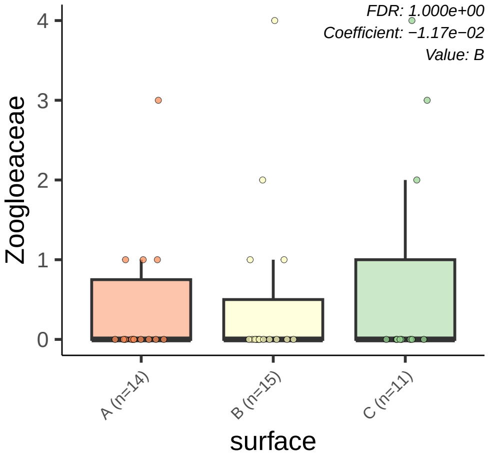

FDR: 1.000e+00
Coefficient: −1.17e−02
Value: B
Zoogloeaceae
0
1
2
3
4
A (n=14)
B (n=15)
C (n=11)
surface

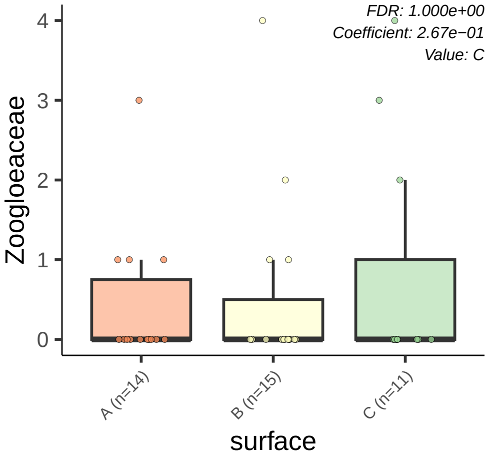
FDR: 1.000e+00
Coefficient: 2.67e−01
Value: C
Zoogloeaceae
0
1
2
3
4
A (n=14)
B (n=15)
C (n=11)
surface

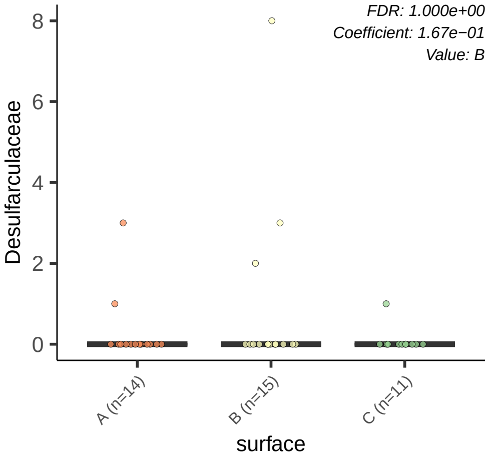

FDR: 1.000e+00
Coefficient: 1.67e−01
Value: B
Desulfarculaceae
0
2
4
6
8
A (n=14)
B (n=15)
C (n=11)
surface

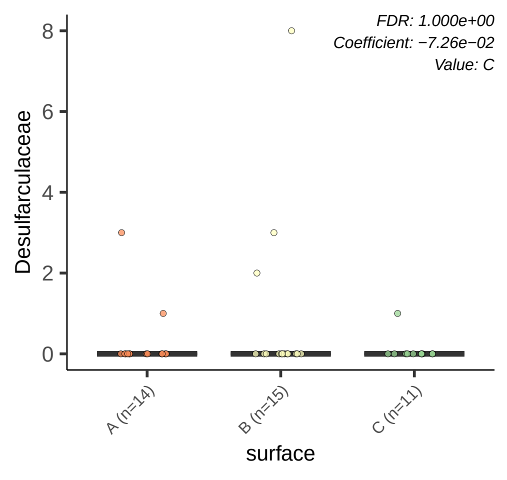
FDR: 1.000e+00
Coefficient: −7.26e−02
Value: C
Desulfarculaceae
0
2
4
6
8
A (n=14)
B (n=15)
C (n=11)
surface

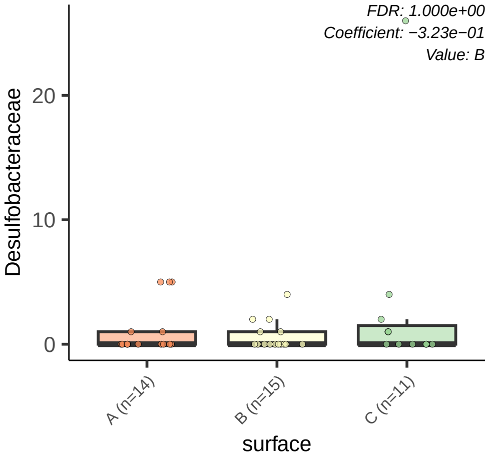
FDR: 1.000e+00
Coefficient: −3.23e−01
Value: B
Desulfobacteraceae
20
10
0
A (n=14)
B (n=15)
C (n=11)
surface

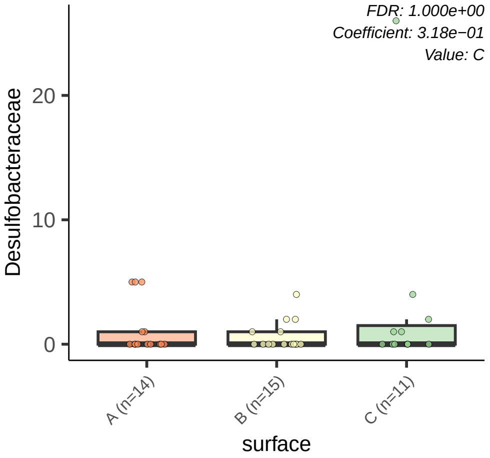
FDR: 1.000e+00
Coefficient: 3.18e−01
Value: C
Desulfobacteraceae
20
10
0
A (n=14)
B (n=15)
C (n=11)
surface

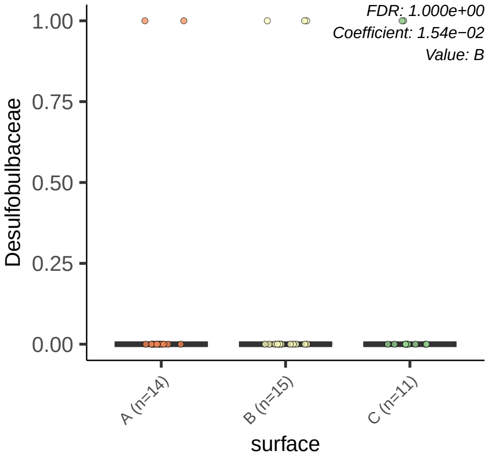
FDR: 1.000e+00
Coefficient: 1.54e−02
Value: B
Desulfobulbaceae
1.00
0.75
0.50
0.25
0.00
A (n=14)
B (n=15)
C (n=11)
surface

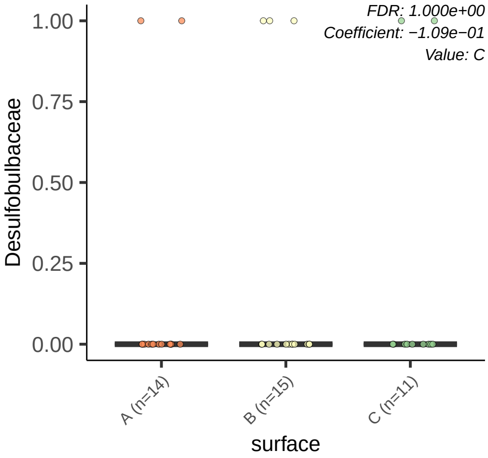

FDR: 1.000e+00
Coefficient: −1.09e−01
Value: C
Desulfobulbaceae
1.00
0.75
0.50
0.25
0.00
A (n=14)
B (n=15)
C (n=11)
surface

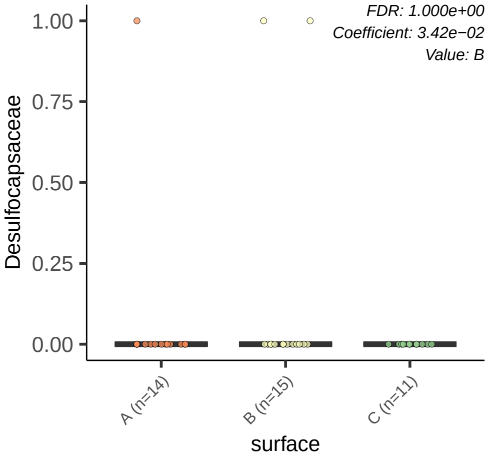

FDR: 1.000e+00
Coefficient: 3.42e−02
Value: B
Desulfocapsaceae
1.00
0.75
0.50
0.25
0.00
A (n=14)
B (n=15)
C (n=11)
surface

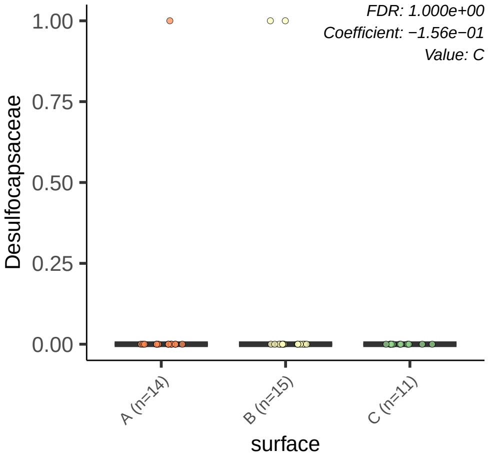

FDR: 1.000e+00
Coefficient: −1.56e−01
Value: C
Desulfocapsaceae
1.00
0.75
0.50
0.25
0.00
A (n=14)
B (n=15)
C (n=11)
surface

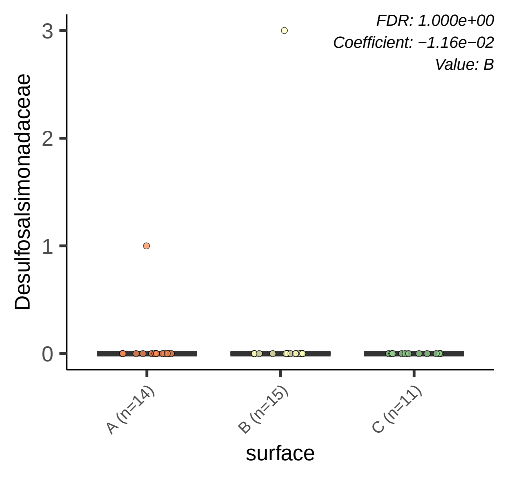

FDR: 1.000e+00
Coefficient: −1.16e−02
Value: B
Desulfosalsimonadaceae
0
1
2
3
A (n=14)
B (n=15)
C (n=11)
surface

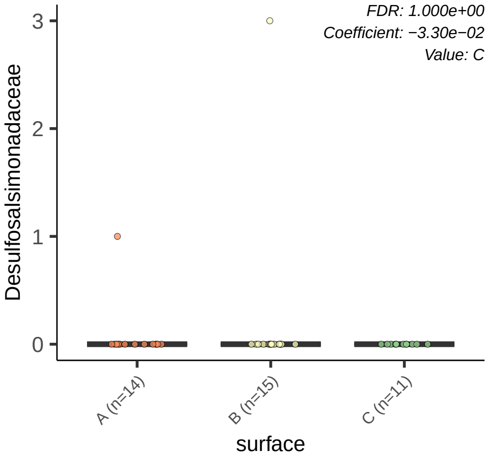

FDR: 1.000e+00
Coefficient: −3.30e−02
Value: C
Desulfosalsimonadaceae
3
2
1
0
A (n=14)
B (n=15)
C (n=11)
surface

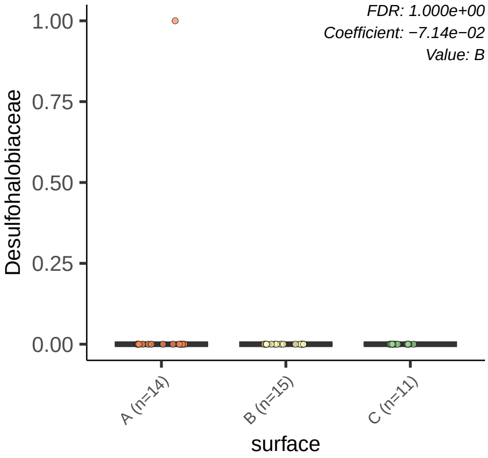

FDR: 1.000e+00
Coefficient: −7.14e−02
Value: B
Desulfohalobiaceae
1.00
0.75
0.50
0.25
0.00
A (n=14)
B (n=15)
C (n=11)
surface

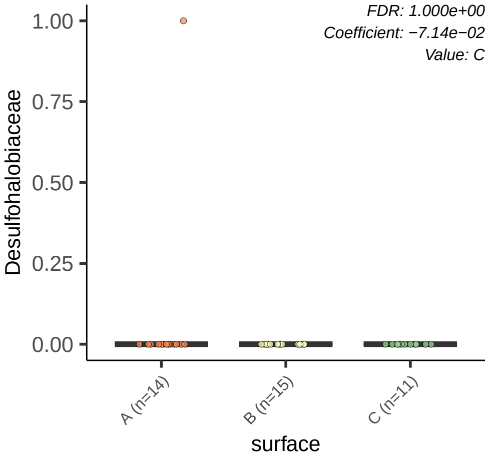
FDR: 1.000e+00
Coefficient: −7.14e−02
Value: C
Desulfohalobiaceae
1.00
0.75
0.50
0.25
0.00
A (n=14)
B (n=15)
C (n=11)
surface

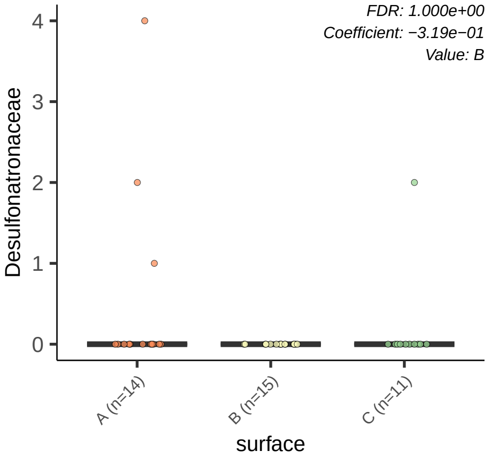
FDR: 1.000e+00
Coefficient: −3.19e−01
Value: B
Desulfonatronaceae
4
3
2
1
0
A (n=14)
B (n=15)
C (n=11)
surface

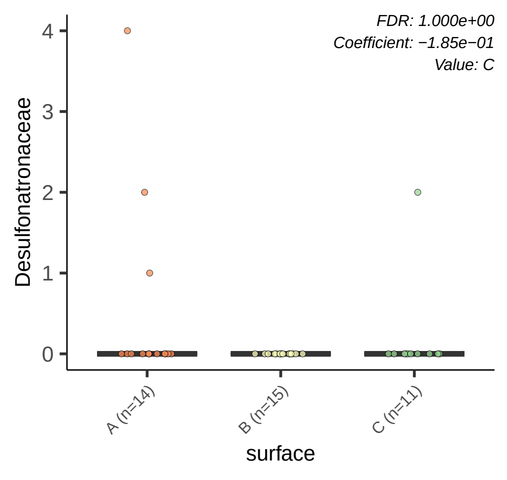
FDR: 1.000e+00
Coefficient: −1.85e−01
Value: C
Desulfonatronaceae
4
3
2
1
0
A (n=14)
B (n=15)
C (n=11)
surface

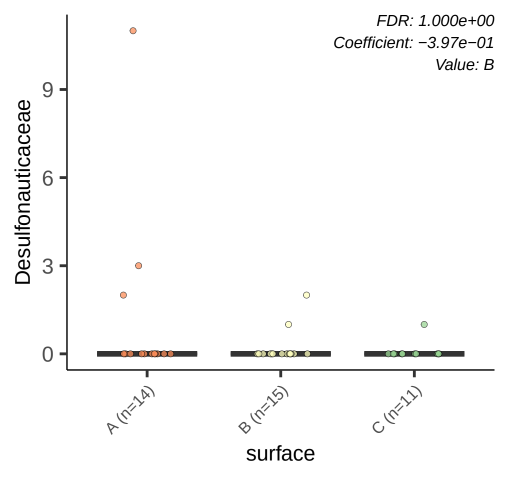
FDR: 1.000e+00
Coefficient: −3.97e−01
Value: B
Desulfonauticaceae
0
3
6
9
A (n=14)
B (n=15)
C (n=11)
surface

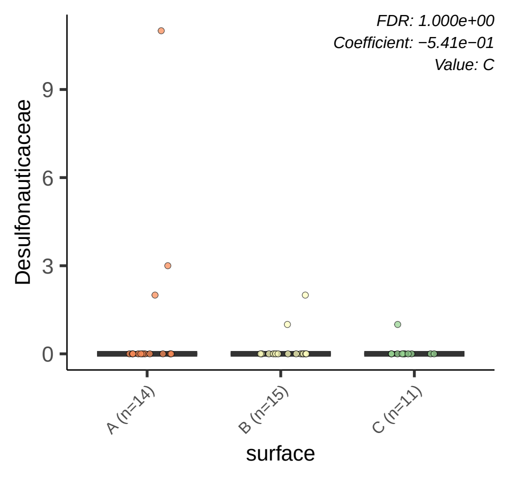
FDR: 1.000e+00
Coefficient: −5.41e−01
Value: C
Desulfonauticaceae
0
3
6
9
A (n=14)
B (n=15)
C (n=11)
surface

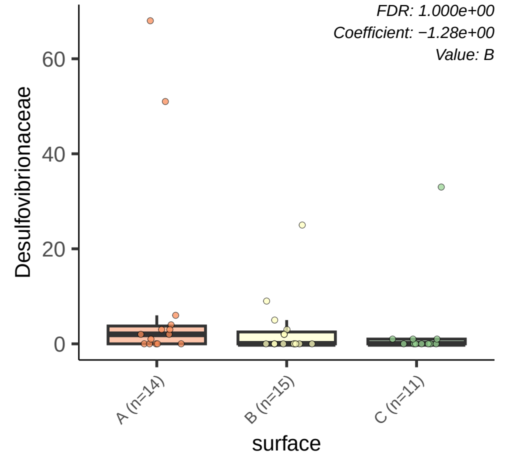
FDR: 1.000e+00
Coefficient: −1.28e+00
Value: B
Desulfovibrionaceae
60
40
20
0
A (n=14)
B (n=15)
C (n=11)
surface

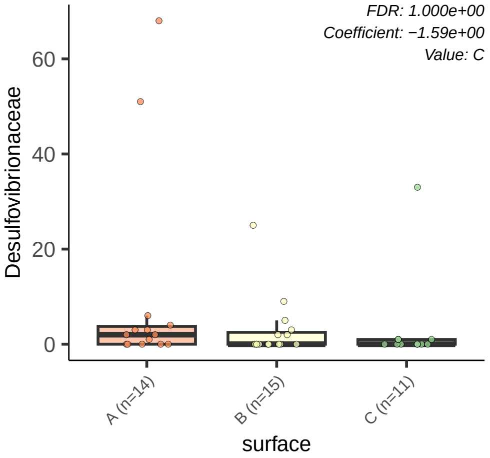

FDR: 1.000e+00
Coefficient: −1.59e+00
Value: C
Desulfovibrionaceae
0
20
40
60
A (n=14)
B (n=15)
C (n=11)
surface

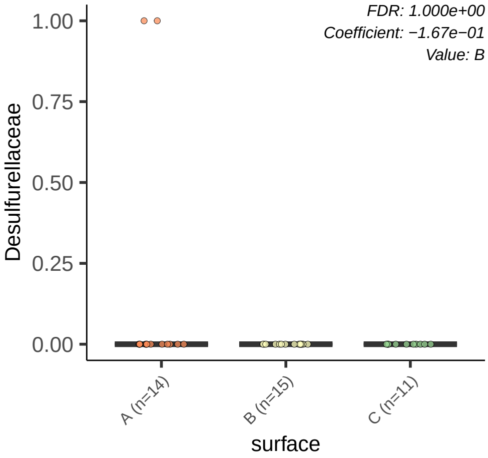
FDR: 1.000e+00
Coefficient: −1.67e−01
Value: B
Desulfurellaceae
1.00
0.75
0.50
0.25
0.00
A (n=14)
B (n=15)
C (n=11)
surface

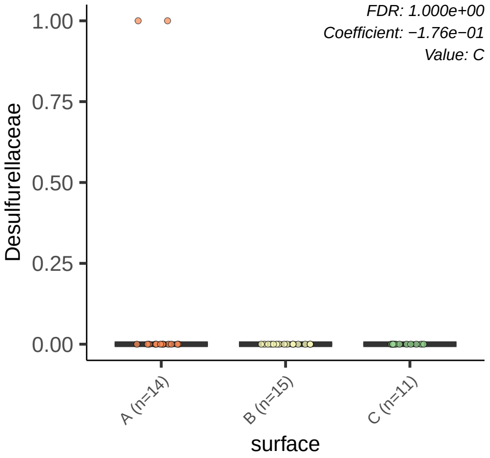
FDR: 1.000e+00
Coefficient: −1.76e−01
Value: C
Desulfurellaceae
1.00
0.75
0.50
0.25
0.00
A (n=14)
B (n=15)
C (n=11)
surface

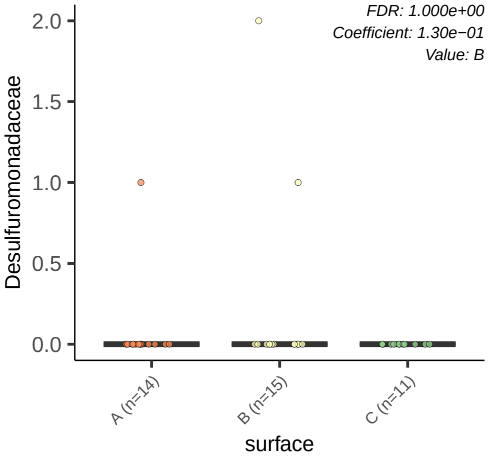

FDR: 1.000e+00
Coefficient: 1.30e−01
Value: B
Desulfuromonadaceae
2.0
1.5
1.0
0.5
0.0
A (n=14)
B (n=15)
C (n=11)
surface

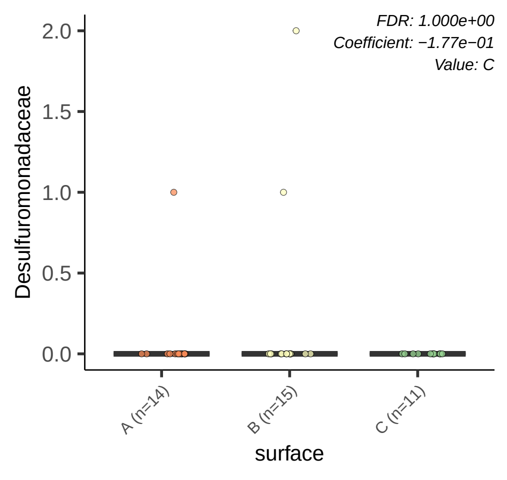
FDR: 1.000e+00
Coefficient: −1.77e−01
Value: C
Desulfuromonadaceae
2.0
1.5
1.0
0.5
0.0
A (n=14)
B (n=15)
C (n=11)
surface

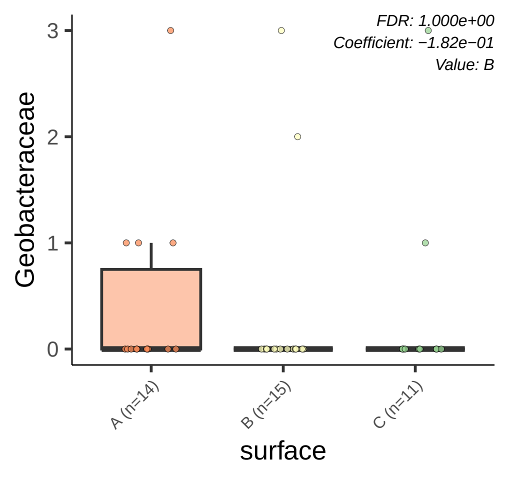

FDR: 1.000e+00
Coefficient: −1.82e−01
Value: B
Geobacteraceae
0
1
2
3
A (n=14)
B (n=15)
C (n=11)
surface

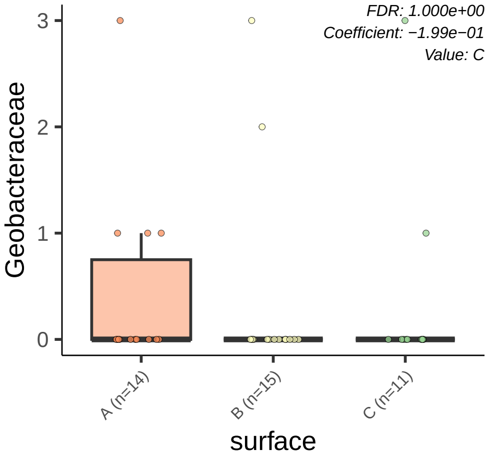
FDR: 1.000e+00
Coefficient: −1.99e−01
Value: C
Geobacteraceae
0
1
2
3
A (n=14)
B (n=15)
C (n=11)
surface

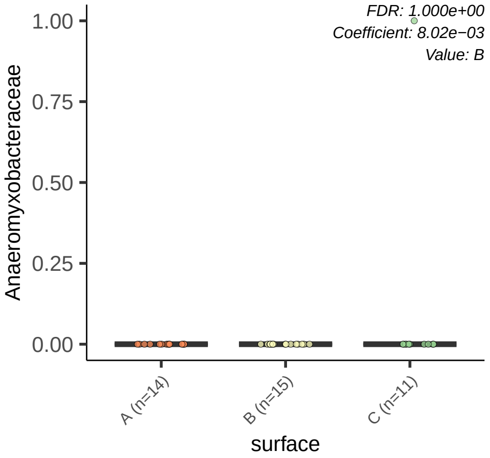
FDR: 1.000e+00
Coefficient: 8.02e−03
Value: B
Anaeromyxobacteraceae
1.00
0.75
0.50
0.25
0.00
A (n=14)
B (n=15)
C (n=11)
surface

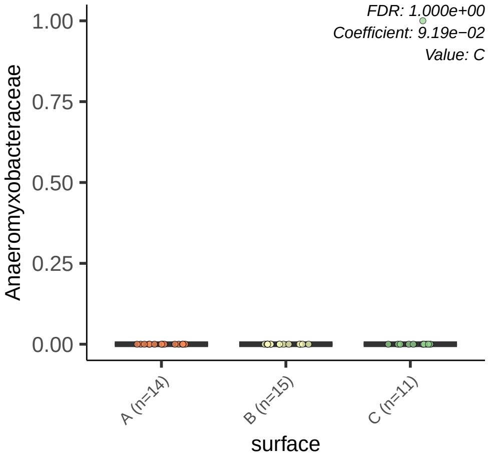

FDR: 1.000e+00
Coefficient: 9.19e−02
Value: C
Anaeromyxobacteraceae
1.00
0.75
0.50
0.25
0.00
A (n=14)
B (n=15)
C (n=11)
surface

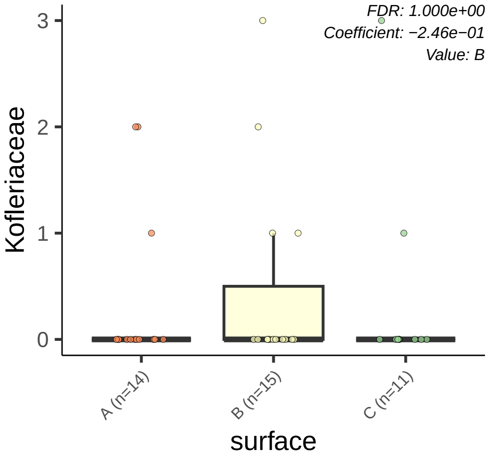

FDR: 1.000e+00
Coefficient: −2.46e−01
Value: B
Kofleriaceae
0
1
2
3
A (n=14)
B (n=15)
C (n=11)
surface

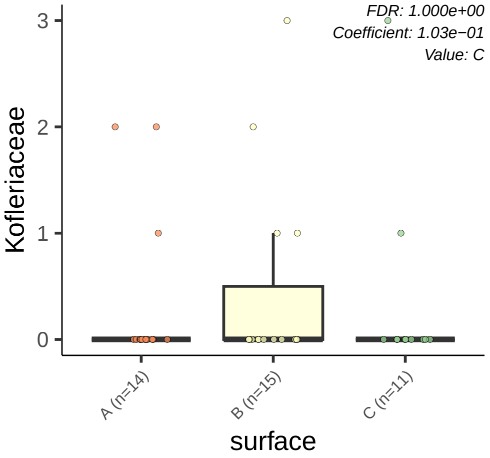

FDR: 1.000e+00
Coefficient: 1.03e−01
Value: C
Kofleriaceae
3
2
1
0
A (n=14)
B (n=15)
C (n=11)
surface

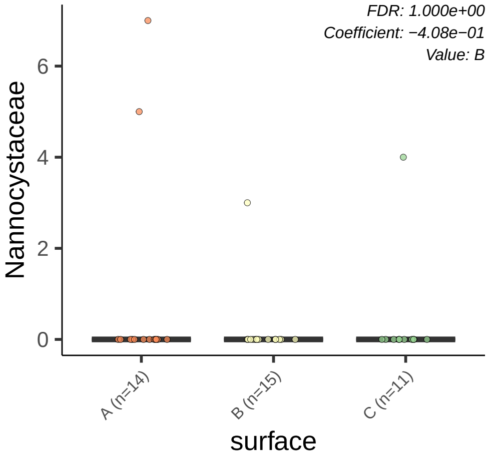

FDR: 1.000e+00
Coefficient: −4.08e−01
Value: B
Nannocystaceae
0
2
4
6
A (n=14)
B (n=15)
C (n=11)
surface

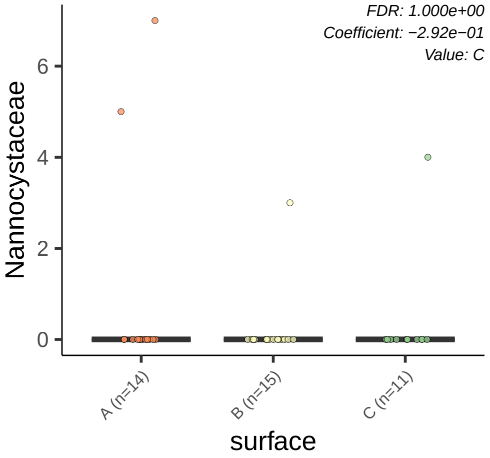
FDR: 1.000e+00
Coefficient: −2.92e−01
Value: C
Nannocystaceae
0
2
4
6
A (n=14)
B (n=15)
C (n=11)
surface

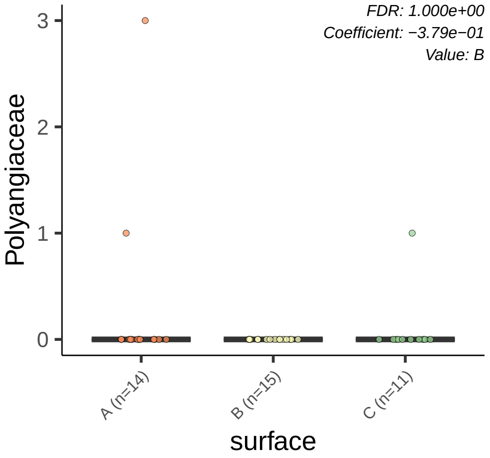
FDR: 1.000e+00
Coefficient: −3.79e−01
Value: B
Polyangiaceae
0
1
2
3
A (n=14)
B (n=15)
C (n=11)
surface

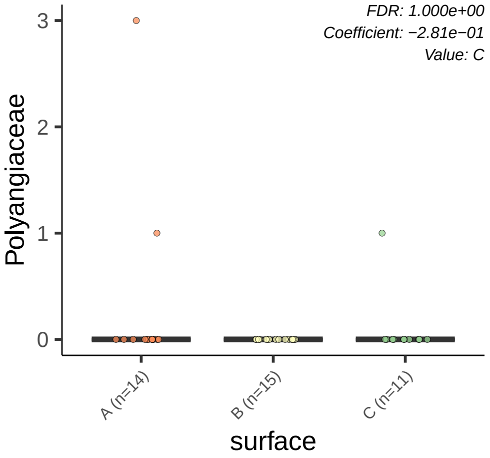
FDR: 1.000e+00
Coefficient: −2.81e−01
Value: C
Polyangiaceae
3
2
1
0
A (n=14)
B (n=15)
C (n=11)
surface

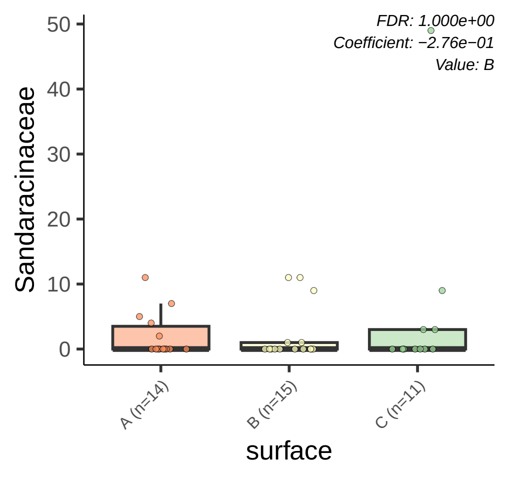
FDR: 1.000e+00
Coefficient: −2.76e−01
Value: B
Sandaracinaceae
50
40
30
20
10
0
A (n=14)
B (n=15)
C (n=11)
surface

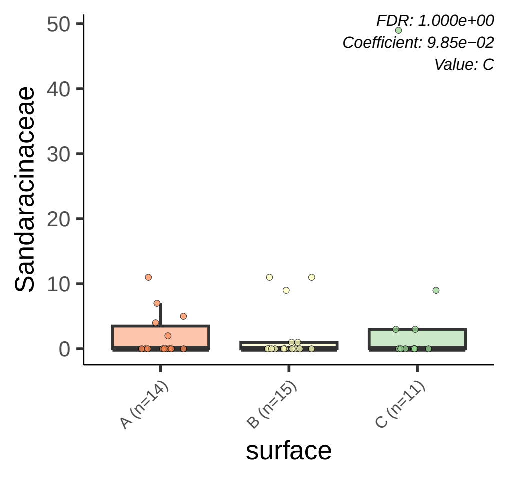

FDR: 1.000e+00
Coefficient: 9.85e−02
Value: C
Sandaracinaceae
0
10
20
30
40
50
A (n=14)
B (n=15)
C (n=11)
surface

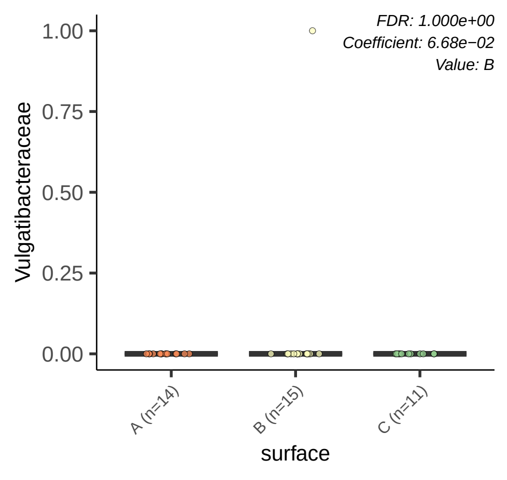
FDR: 1.000e+00
Coefficient: 6.68e−02
Value: B
Vulgatibacteraceae
1.00
0.75
0.50
0.25
0.00
A (n=14)
B (n=15)
C (n=11)
surface

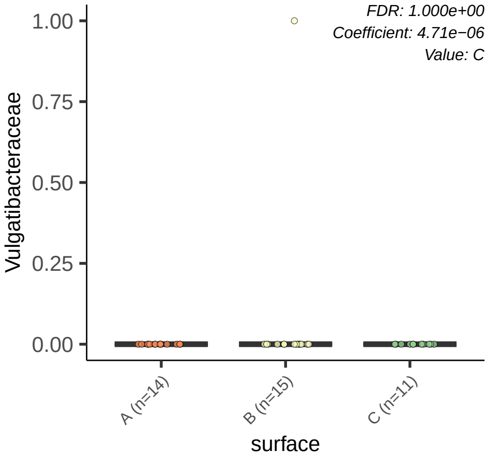
FDR: 1.000e+00
Coefficient: 4.71e−06
Value: C
Vulgatibacteraceae
1.00
0.75
0.50
0.25
0.00
A (n=14)
B (n=15)
C (n=11)
surface

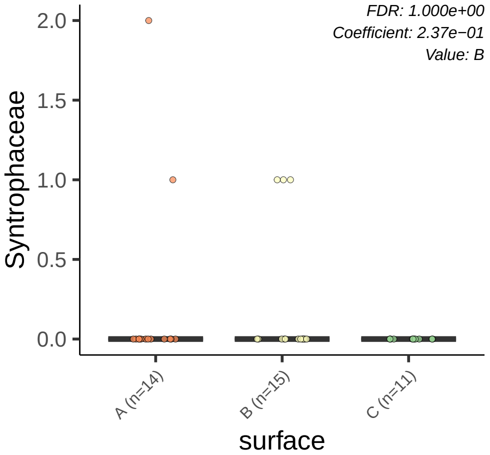

FDR: 1.000e+00
Coefficient: 2.37e−01
Value: B
Syntrophaceae
2.0
1.5
1.0
0.5
0.0
A (n=14)
B (n=15)
C (n=11)
surface

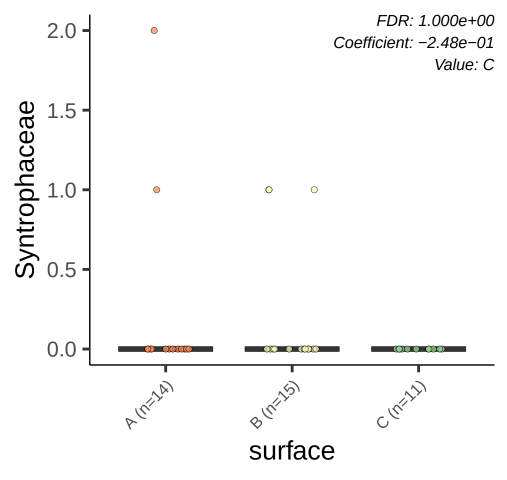
FDR: 1.000e+00
Coefficient: −2.48e−01
Value: C
Syntrophaceae
2.0
1.5
1.0
0.5
0.0
A (n=14)
B (n=15)
C (n=11)
surface

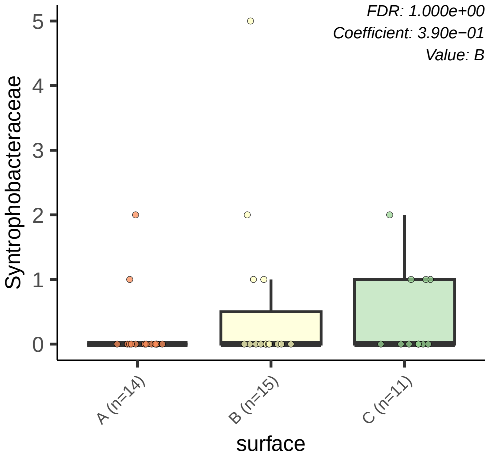

FDR: 1.000e+00
Coefficient: 3.90e−01
Value: B
Syntrophobacteraceae
0
1
2
3
4
5
A (n=14)
B (n=15)
C (n=11)
surface

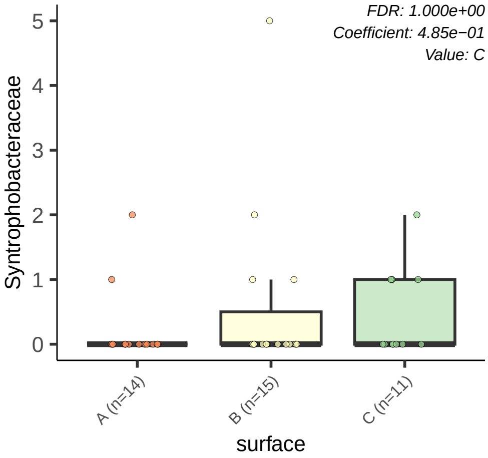

FDR: 1.000e+00
Coefficient: 4.85e−01
Value: C
Syntrophobacteraceae
0
1
2
3
4
5
A (n=14)
B (n=15)
C (n=11)
surface

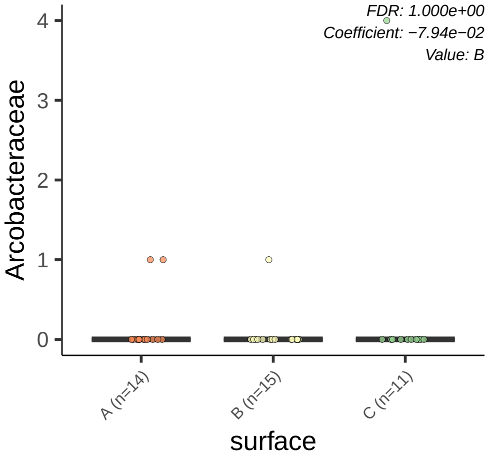
FDR: 1.000e+00
Coefficient: −7.94e−02
Value: B
Arcobacteraceae
0
1
2
3
4
A (n=14)
B (n=15)
C (n=11)
surface

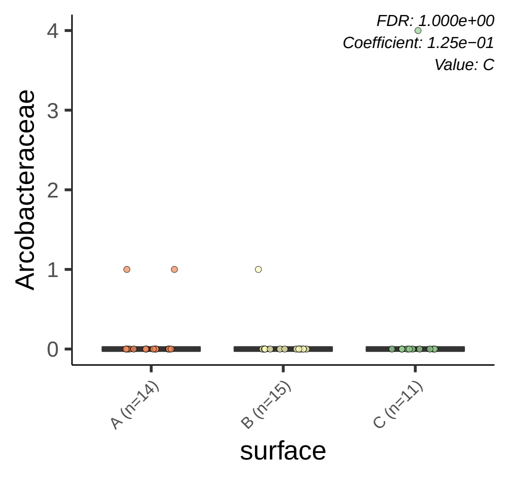

FDR: 1.000e+00
Coefficient: 1.25e−01
Value: C
Arcobacteraceae
4
3
2
1
0
A (n=14)
B (n=15)
C (n=11)
surface

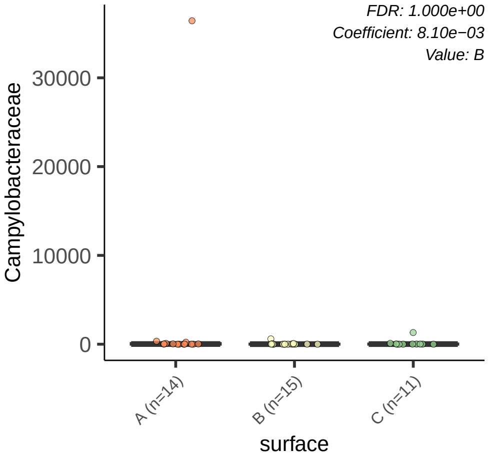

FDR: 1.000e+00
Coefficient: 8.10e−03
Value: B
Campylobacteraceae
30000
20000
10000
0
A (n=14)
B (n=15)
C (n=11)
surface

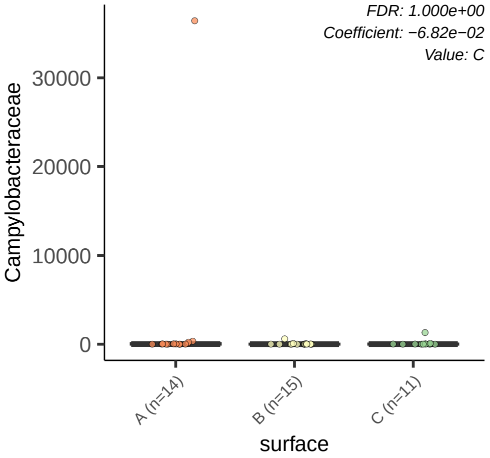
FDR: 1.000e+00
Coefficient: −6.82e−02
Value: C
Campylobacteraceae
30000
20000
10000
0
A (n=14)
B (n=15)
C (n=11)
surface

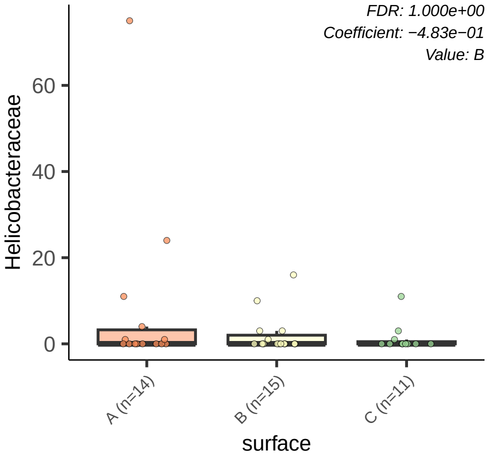
FDR: 1.000e+00
Coefficient: −4.83e−01
Value: B
Helicobacteraceae
0
20
40
60
A (n=14)
B (n=15)
C (n=11)
surface

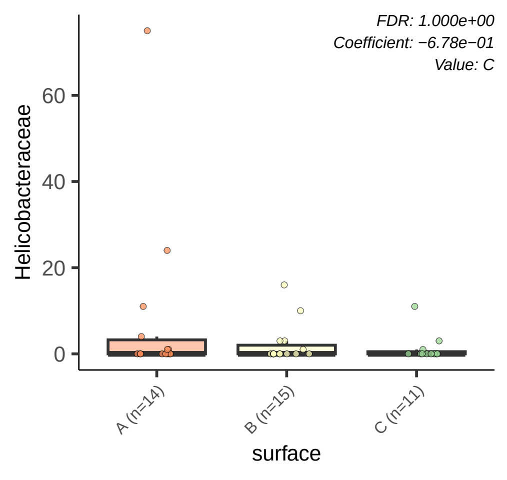
FDR: 1.000e+00
Coefficient: −6.78e−01
Value: C
Helicobacteraceae
0
20
40
60
A (n=14)
B (n=15)
C (n=11)
surface

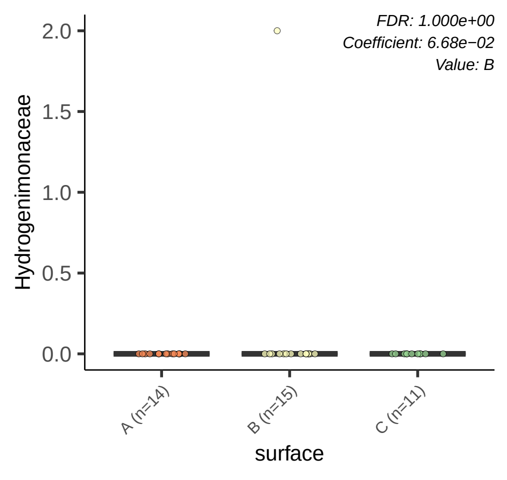
FDR: 1.000e+00
Coefficient: 6.68e−02
Value: B
Hydrogenimonaceae
2.0
1.5
1.0
0.5
0.0
A (n=14)
B (n=15)
C (n=11)
surface

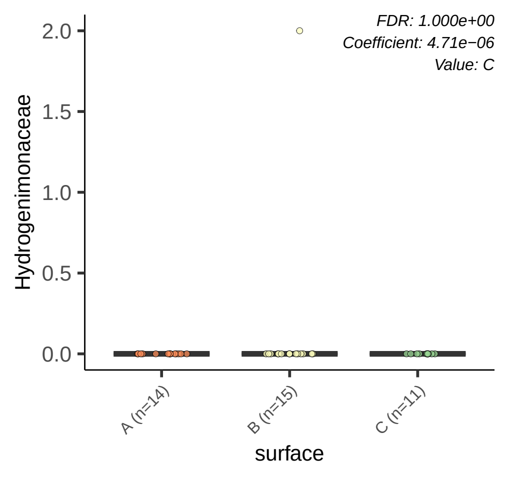
FDR: 1.000e+00
Coefficient: 4.71e−06
Value: C
Hydrogenimonaceae
2.0
1.5
1.0
0.5
0.0
A (n=14)
B (n=15)
C (n=11)
surface

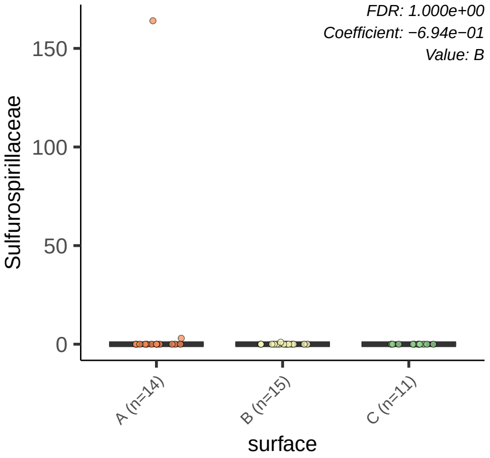
FDR: 1.000e+00
Coefficient: −6.94e−01
Value: B
Sulfurospirillaceae
150
100
50
0
A (n=14)
B (n=15)
C (n=11)
surface

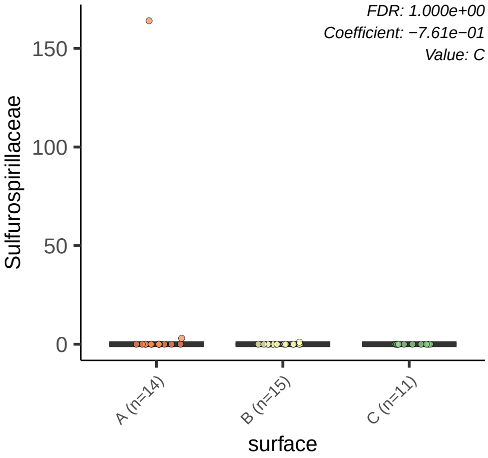

FDR: 1.000e+00
Coefficient: −7.61e−01
Value: C
Sulfurospirillaceae
150
100
50
0
A (n=14)
B (n=15)
C (n=11)
surface

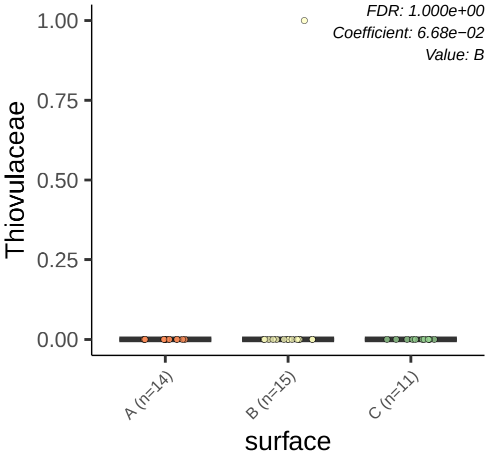

FDR: 1.000e+00
Coefficient: 6.68e−02
Value: B
1.00
0.75
0.50
0.25
0.00
Thiovulaceae
A (n=14)
B (n=15)
C (n=11)
surface

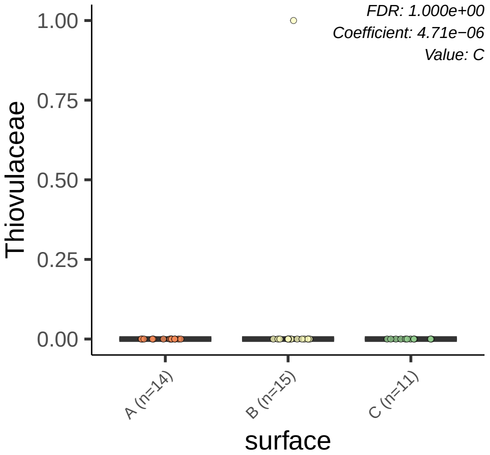
FDR: 1.000e+00
Coefficient: 4.71e−06
Value: C
Thiovulaceae
1.00
0.75
0.50
0.25
0.00
A (n=14)
B (n=15)
C (n=11)
surface

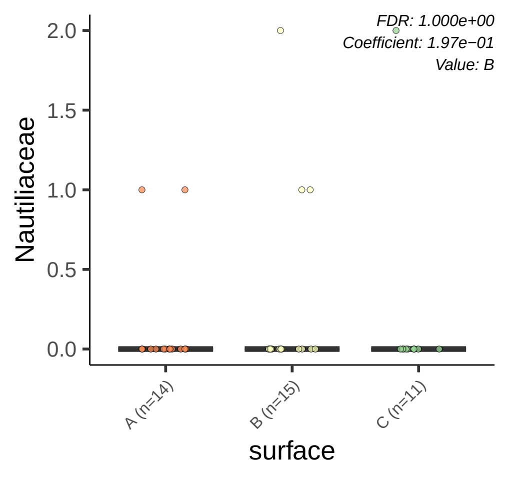
FDR: 1.000e+00
Coefficient: 1.97e−01
Value: B
Nautiliaceae
2.0
1.5
1.0
0.5
0.0
A (n=14)
B (n=15)
C (n=11)
surface

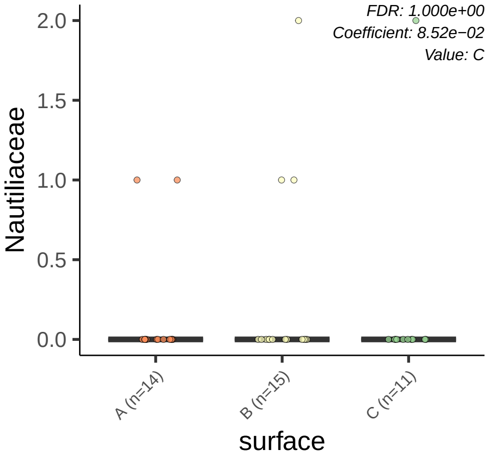
FDR: 1.000e+00
Coefficient: 8.52e−02
Value: C
Nautiliaceae
2.0
1.5
1.0
0.5
0.0
A (n=14)
B (n=15)
C (n=11)
surface

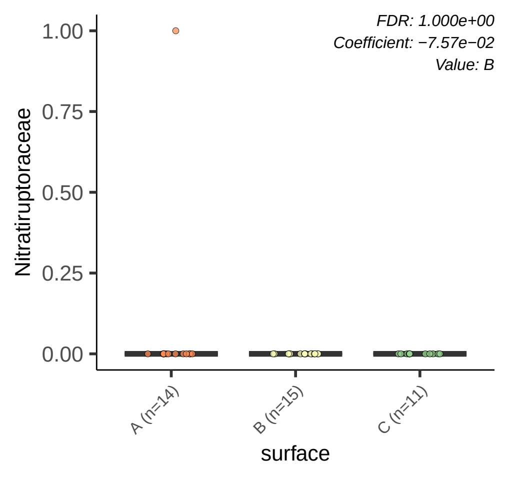
FDR: 1.000e+00
Coefficient: −7.57e−02
Value: B
Nitratiruptoraceae
1.00
0.75
0.50
0.25
0.00
A (n=14)
B (n=15)
C (n=11)
surface

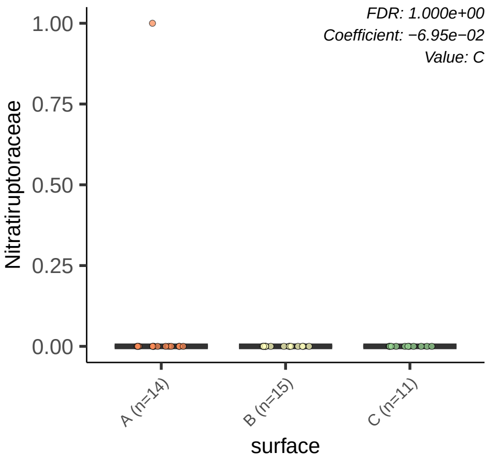
FDR: 1.000e+00
Coefficient: −6.95e−02
Value: C
Nitratiruptoraceae
1.00
0.75
0.50
0.25
0.00
A (n=14)
B (n=15)
C (n=11)
surface

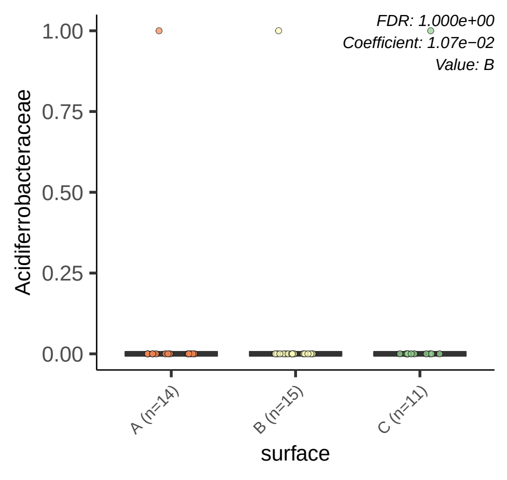

FDR: 1.000e+00
Coefficient: 1.07e−02
Value: B
Acidiferrobacteraceae
1.00
0.75
0.50
0.25
0.00
A (n=14)
B (n=15)
C (n=11)
surface

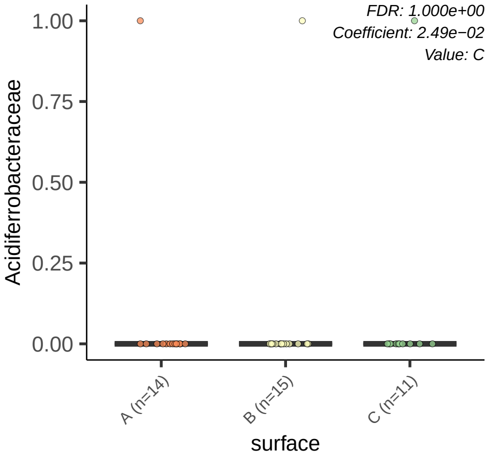
FDR: 1.000e+00
Coefficient: 2.49e−02
Value: C
Acidiferrobacteraceae
1.00
0.75
0.50
0.25
0.00
A (n=14)
B (n=15)
C (n=11)
surface

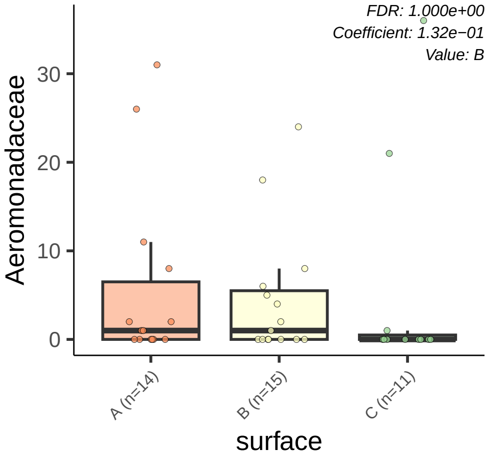
FDR: 1.000e+00
Coefficient: 1.32e−01
Value: B
Aeromonadaceae
0
10
20
30
A (n=14)
B (n=15)
C (n=11)
surface

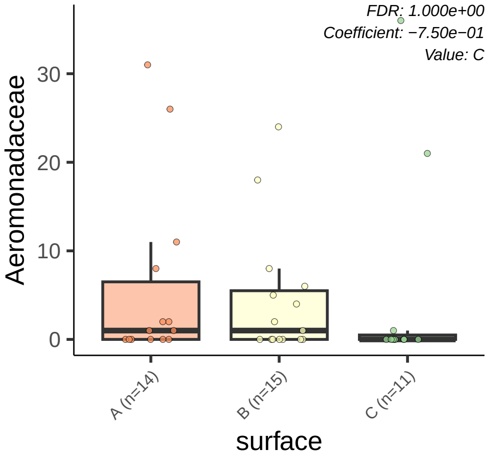
FDR: 1.000e+00
Coefficient: −7.50e−01
Value: C
Aeromonadaceae
0
10
20
30
A (n=14)
B (n=15)
C (n=11)
surface

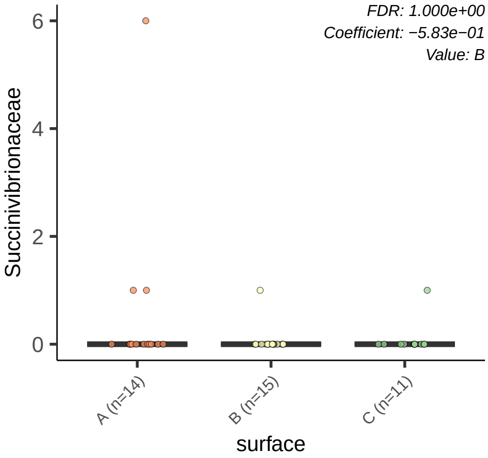

FDR: 1.000e+00
Coefficient: −5.83e−01
Value: B
Succinivibrionaceae
6
4
2
0
A (n=14)
B (n=15)
C (n=11)
surface

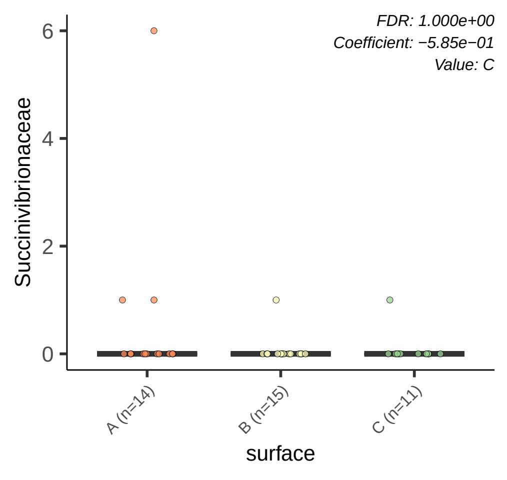
FDR: 1.000e+00
Coefficient: −5.85e−01
Value: C
Succinivibrionaceae
6
4
2
0
A (n=14)
B (n=15)
C (n=11)
surface

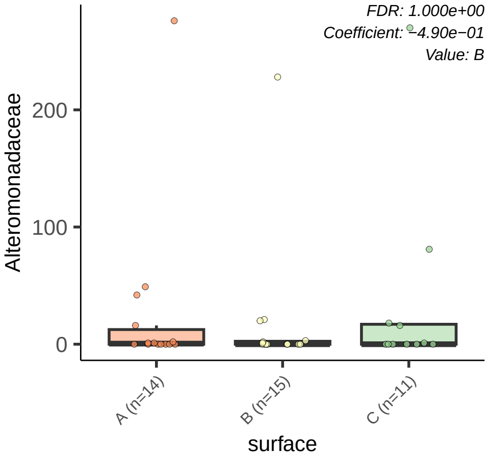
FDR: 1.000e+00
Coefficient: −4.90e−01
Value: B
Alteromonadaceae
200
100
0
A (n=14)
B (n=15)
C (n=11)
surface

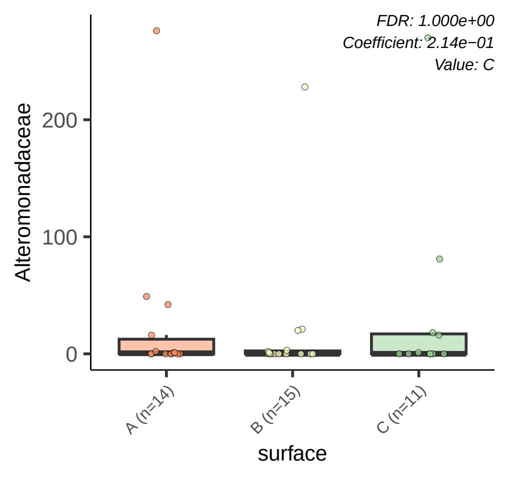

FDR: 1.000e+00
Coefficient: 2.14e−01
Value: C
Alteromonadaceae
200
100
0
A (n=14)
B (n=15)
C (n=11)
surface

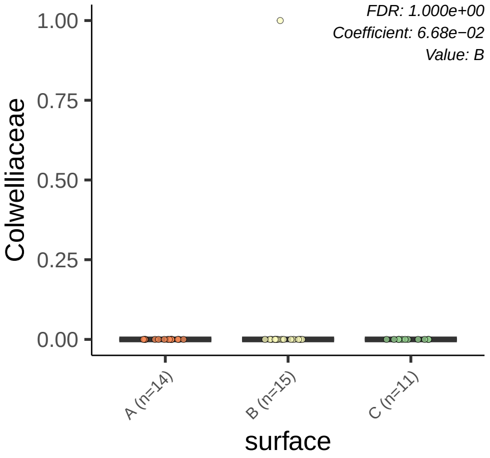
FDR: 1.000e+00
Coefficient: 6.68e−02
Value: B
Colwelliaceae
1.00
0.75
0.50
0.25
0.00
A (n=14)
B (n=15)
C (n=11)
surface

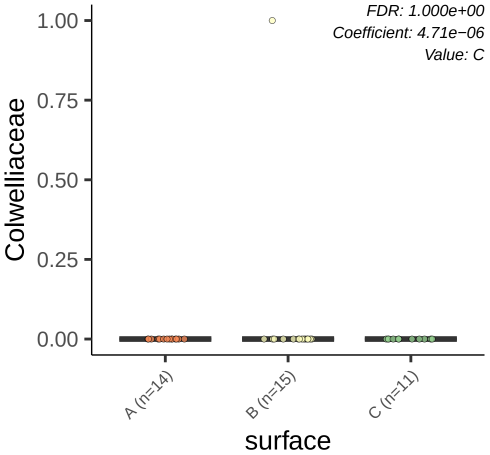
FDR: 1.000e+00
Coefficient: 4.71e−06
Value: C
Colwelliaceae
1.00
0.75
0.50
0.25
0.00
A (n=14)
B (n=15)
C (n=11)
surface

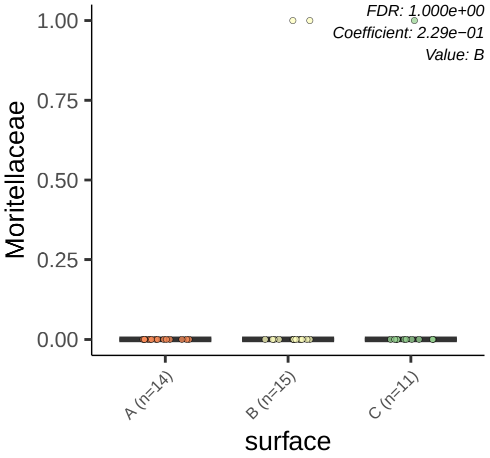
FDR: 1.000e+00
Coefficient: 2.29e−01
Value: B
1.00
0.75
0.50
0.25
0.00
Moritellaceae
A (n=14)
B (n=15)
C (n=11)
surface

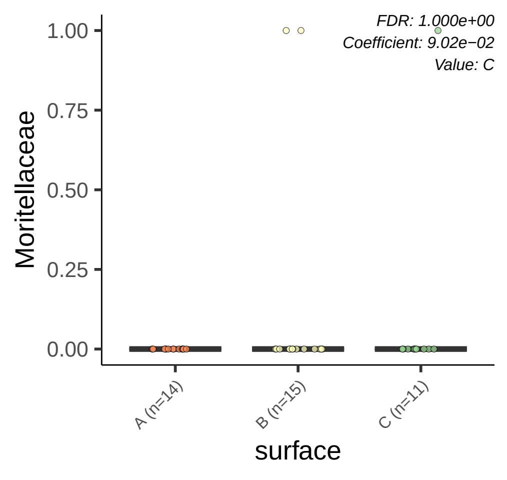

FDR: 1.000e+00
Coefficient: 9.02e−02
Value: C
Moritellaceae
1.00
0.75
0.50
0.25
0.00
A (n=14)
B (n=15)
C (n=11)
surface

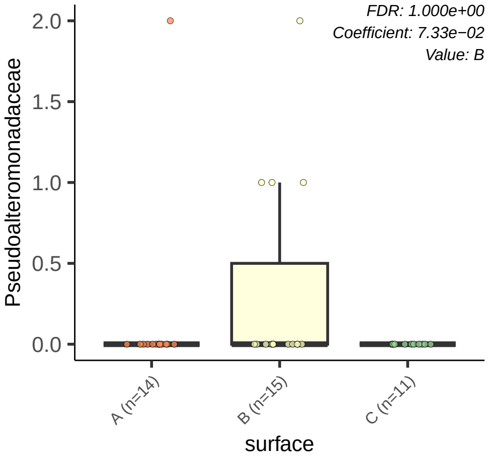
FDR: 1.000e+00
Coefficient: 7.33e−02
Value: B
Pseudoalteromonadaceae
2.0
1.5
1.0
0.5
0.0
A (n=14)
B (n=15)
C (n=11)
surface

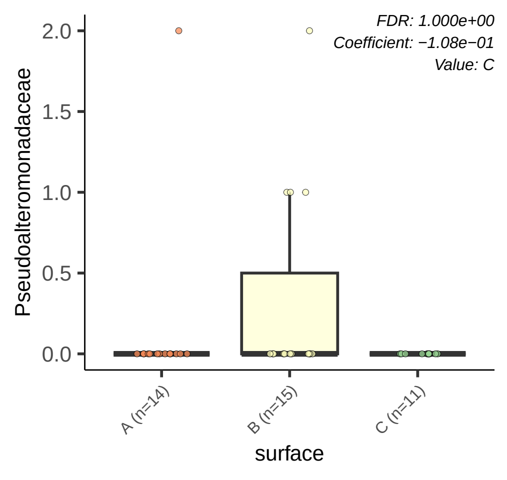
FDR: 1.000e+00
Coefficient: −1.08e−01
Value: C
Pseudoalteromonadaceae
2.0
1.5
1.0
0.5
0.0
A (n=14)
B (n=15)
C (n=11)
surface

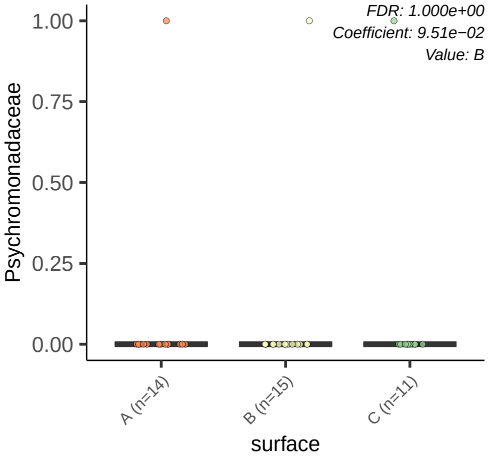
FDR: 1.000e+00
Coefficient: 9.51e−02
Value: B
Psychromonadaceae
1.00
0.75
0.50
0.25
0.00
A (n=14)
B (n=15)
C (n=11)
surface

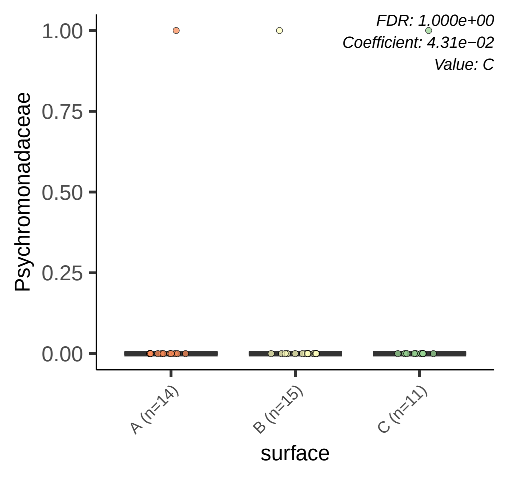
FDR: 1.000e+00
Coefficient: 4.31e−02
Value: C
Psychromonadaceae
1.00
0.75
0.50
0.25
0.00
A (n=14)
B (n=15)
C (n=11)
surface

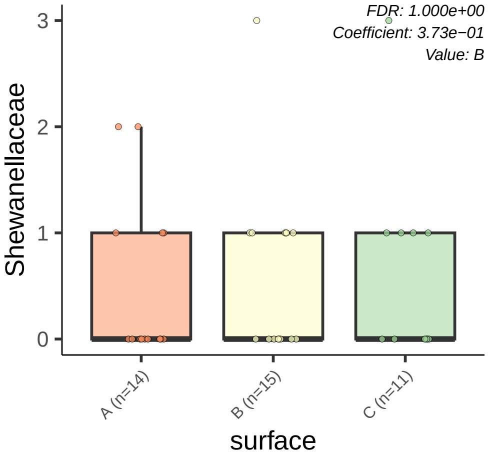
FDR: 1.000e+00
Coefficient: 3.73e−01
Value: B
Shewanellaceae
0
1
2
3
A (n=14)
B (n=15)
C (n=11)
surface

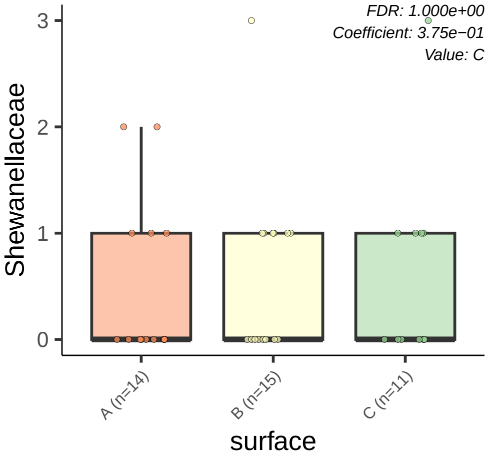

FDR: 1.000e+00
Coefficient: 3.75e−01
Value: C
Shewanellaceae
0
1
2
3
A (n=14)
B (n=15)
C (n=11)
surface

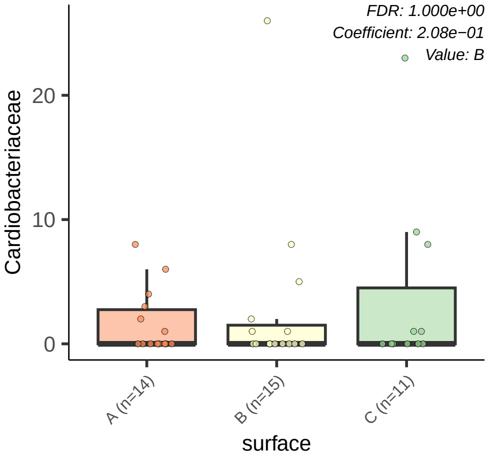

FDR: 1.000e+00
Coefficient: 2.08e−01
Value: B
Cardiobacteriaceae
0
10
20
A (n=14)
B (n=15)
C (n=11)
surface

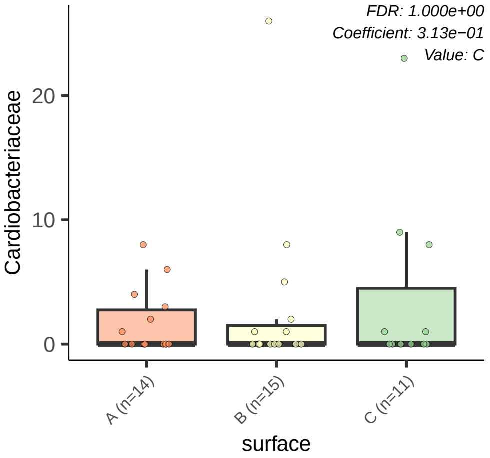
FDR: 1.000e+00
Coefficient: 3.13e−01
Value: C
Cardiobacteriaceae
20
10
0
A (n=14)
B (n=15)
C (n=11)
surface

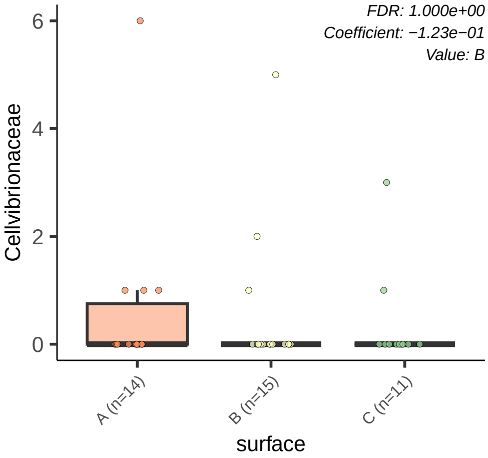
FDR: 1.000e+00
Coefficient: −1.23e−01
Value: B
Cellvibrionaceae
0
2
4
6
A (n=14)
B (n=15)
C (n=11)
surface

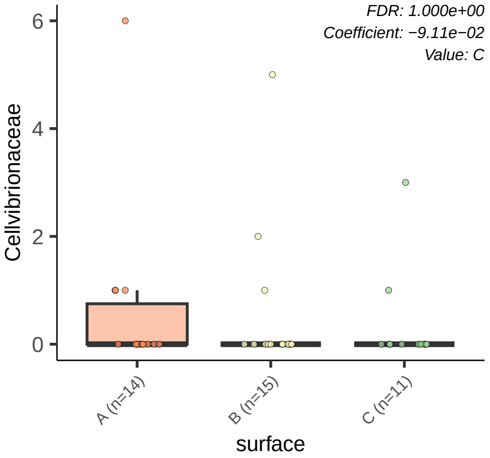
FDR: 1.000e+00
Coefficient: −9.11e−02
Value: C
Cellvibrionaceae
6
4
2
0
A (n=14)
B (n=15)
C (n=11)
surface

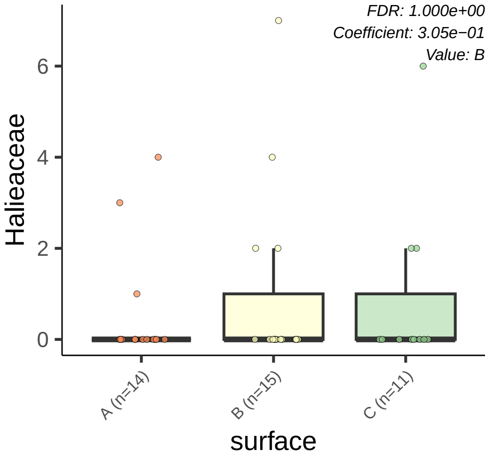
FDR: 1.000e+00
Coefficient: 3.05e−01
Value: B
Halieaceae
0
2
4
6
A (n=14)
B (n=15)
C (n=11)
surface

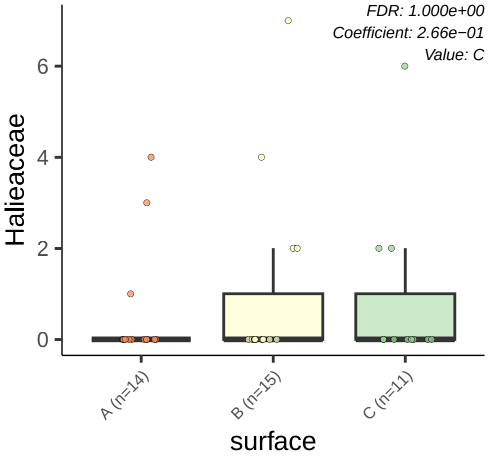

FDR: 1.000e+00
Coefficient: 2.66e−01
Value: C
Halieaceae
0
2
4
6
A (n=14)
B (n=15)
C (n=11)
surface

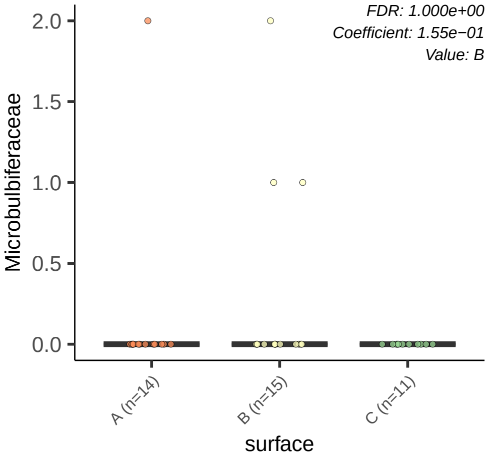

FDR: 1.000e+00
Coefficient: 1.55e−01
Value: B
Microbulbiferaceae
2.0
1.5
1.0
0.5
0.0
A (n=14)
B (n=15)
C (n=11)
surface

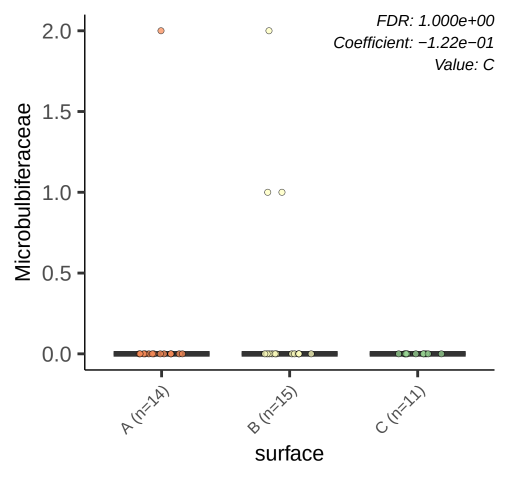

FDR: 1.000e+00
Coefficient: −1.22e−01
Value: C
Microbulbiferaceae
2.0
1.5
1.0
0.5
0.0
A (n=14)
B (n=15)
C (n=11)
surface

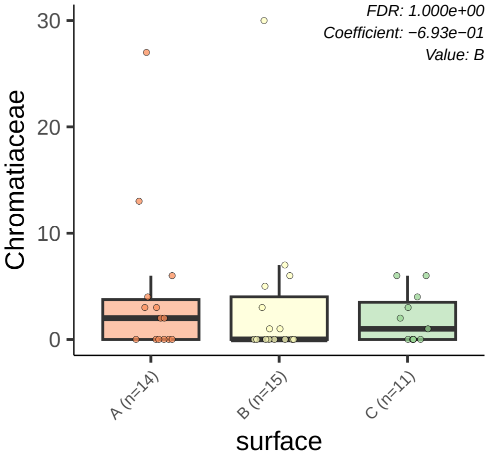

FDR: 1.000e+00
Coefficient: −6.93e−01
Value: B
Chromatiaceae
30
20
10
0
A (n=14)
B (n=15)
C (n=11)
surface

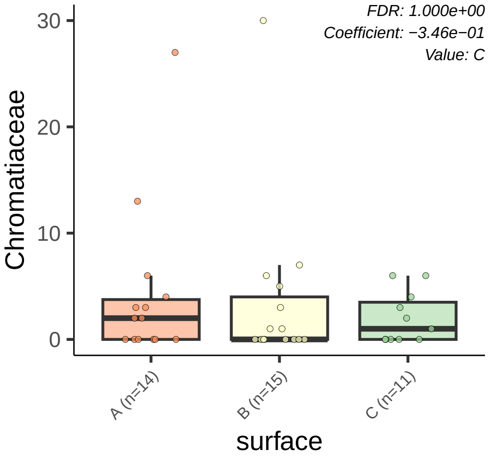
FDR: 1.000e+00
Coefficient: −3.46e−01
Value: C
Chromatiaceae
30
20
10
0
A (n=14)
B (n=15)
C (n=11)
surface

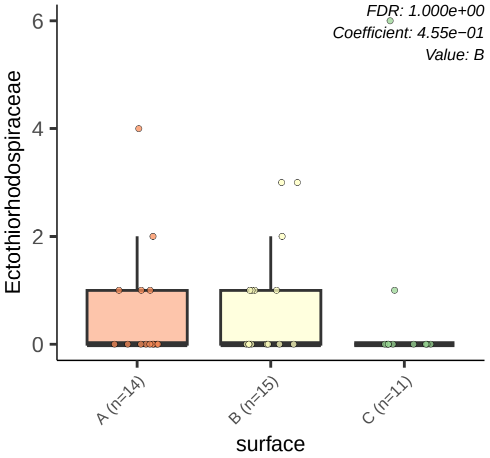

FDR: 1.000e+00
Coefficient: 4.55e−01
Value: B
Ectothiorhodospiraceae
6
4
2
0
A (n=14)
B (n=15)
C (n=11)
surface

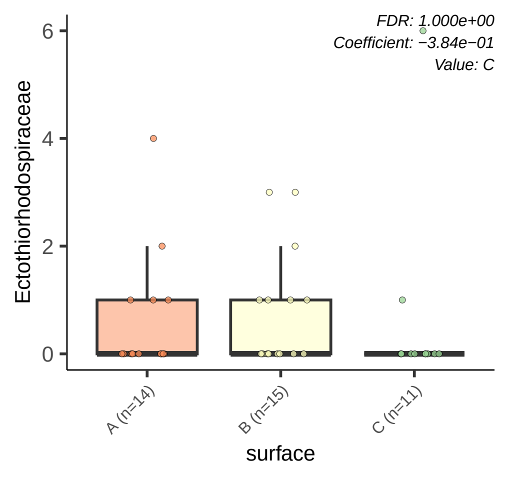
FDR: 1.000e+00
Coefficient: −3.84e−01
Value: C
Ectothiorhodospiraceae
0
2
4
6
A (n=14)
B (n=15)
C (n=11)
surface

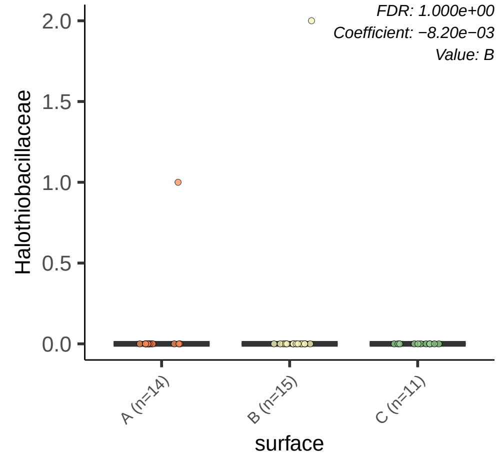
FDR: 1.000e+00
Coefficient: −8.20e−03
Value: B
Halothiobacillaceae
2.0
1.5
1.0
0.5
0.0
A (n=14)
B (n=15)
C (n=11)
surface

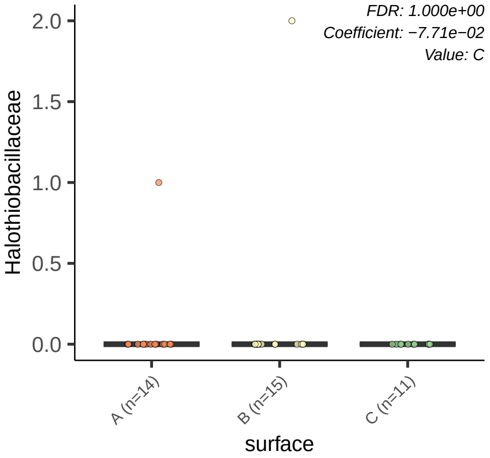

FDR: 1.000e+00
Coefficient: −7.71e−02
Value: C
Halothiobacillaceae
2.0
1.5
1.0
0.5
0.0
A (n=14)
B (n=15)
C (n=11)
surface

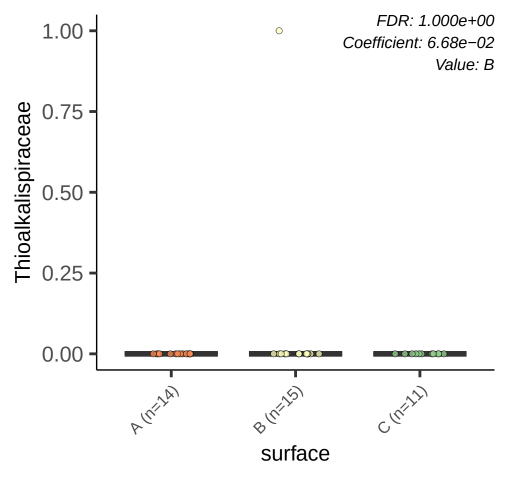
FDR: 1.000e+00
Coefficient: 6.68e−02
Value: B
Thioalkalispiraceae
1.00
0.75
0.50
0.25
0.00
A (n=14)
B (n=15)
C (n=11)
surface

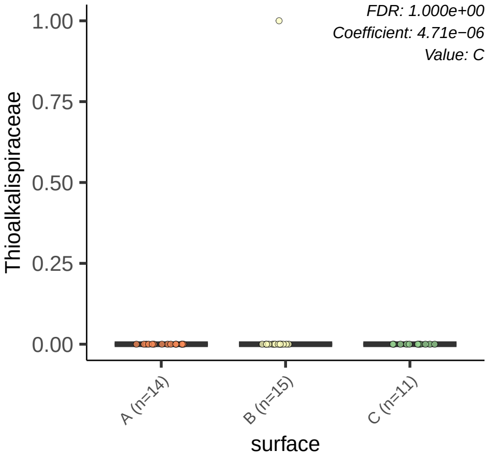
FDR: 1.000e+00
Coefficient: 4.71e−06
Value: C
1.00
0.75
0.50
0.25
0.00
Thioalkalispiraceae
A (n=14)
B (n=15)
C (n=11)
surface

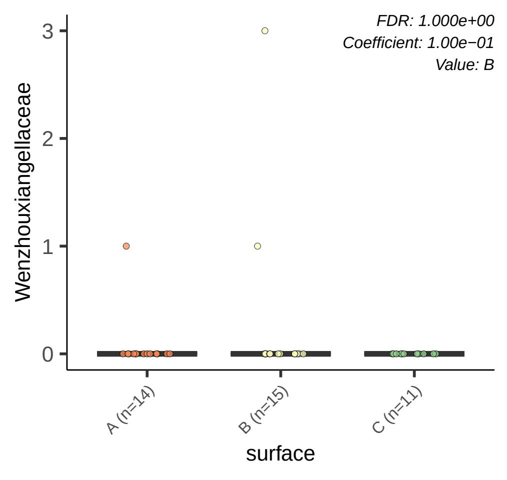

FDR: 1.000e+00
Coefficient: 1.00e−01
Value: B
Wenzhouxiangellaceae
0
1
2
3
A (n=14)
B (n=15)
C (n=11)
surface

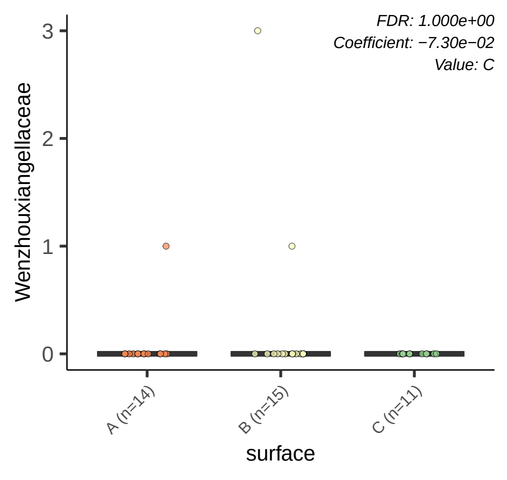
FDR: 1.000e+00
Coefficient: −7.30e−02
Value: C
Wenzhouxiangellaceae
3
2
1
0
A (n=14)
B (n=15)
C (n=11)
surface

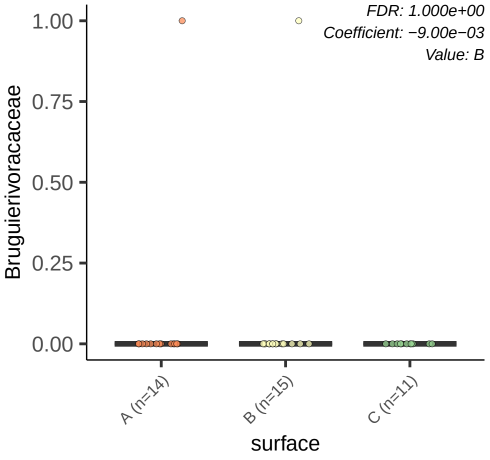

FDR: 1.000e+00
Coefficient: −9.00e−03
Value: B
Bruguierivoracaceae
1.00
0.75
0.50
0.25
0.00
A (n=14)
B (n=15)
C (n=11)
surface

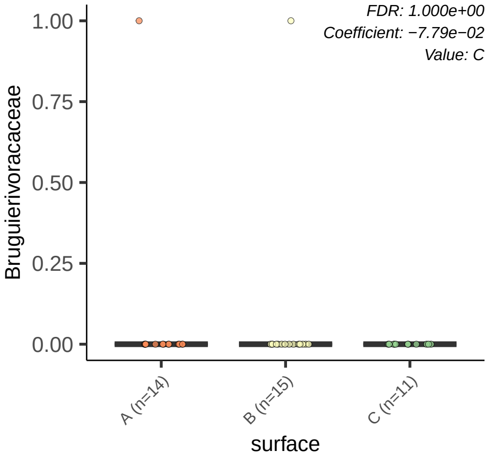
FDR: 1.000e+00
Coefficient: −7.79e−02
Value: C
Bruguierivoracaceae
1.00
0.75
0.50
0.25
0.00
A (n=14)
B (n=15)
C (n=11)
surface

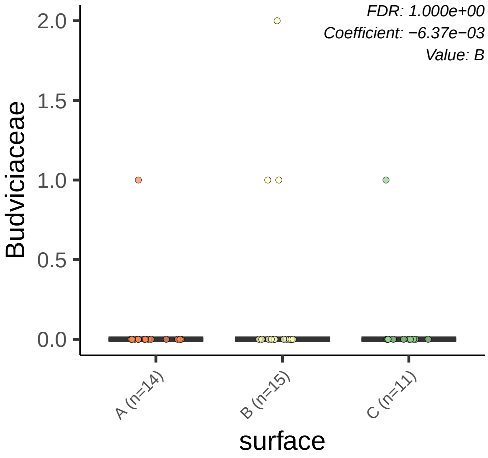

FDR: 1.000e+00
Coefficient: −6.37e−03
Value: B
Budviciaceae
2.0
1.5
1.0
0.5
0.0
A (n=14)
B (n=15)
C (n=11)
surface

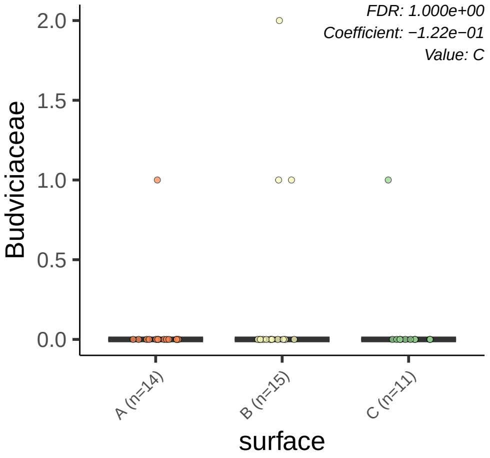
FDR: 1.000e+00
Coefficient: −1.22e−01
Value: C
Budviciaceae
2.0
1.5
1.0
0.5
0.0
A (n=14)
B (n=15)
C (n=11)
surface

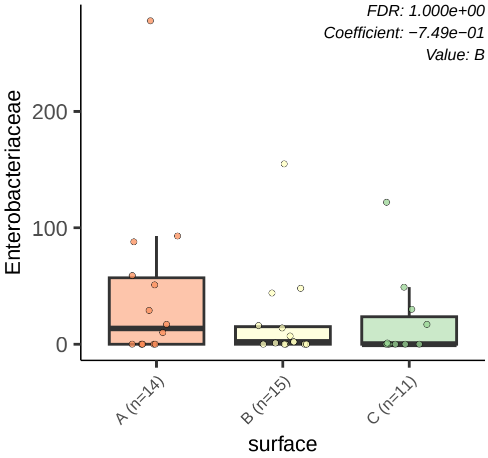

FDR: 1.000e+00
Coefficient: −7.49e−01
Value: B
Enterobacteriaceae
200
100
0
A (n=14)
B (n=15)
C (n=11)
surface

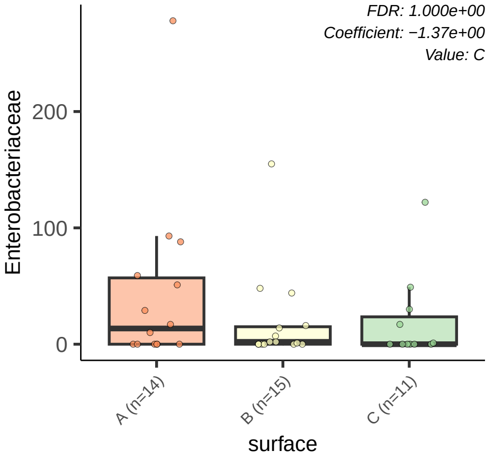
FDR: 1.000e+00
Coefficient: −1.37e+00
Value: C
Enterobacteriaceae
200
100
0
A (n=14)
B (n=15)
C (n=11)
surface

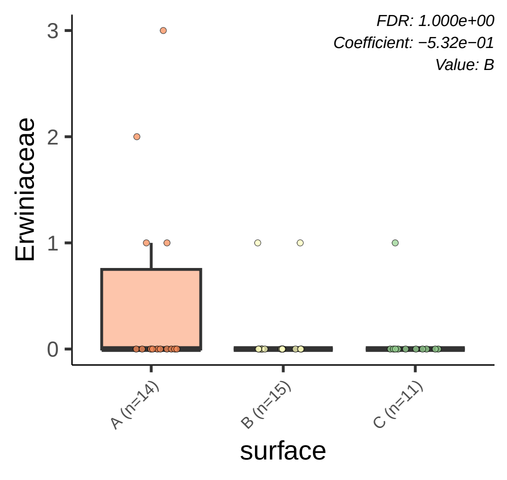

FDR: 1.000e+00
Coefficient: −5.32e−01
Value: B
3
2
1
0
Erwiniaceae
A (n=14)
B (n=15)
C (n=11)
surface

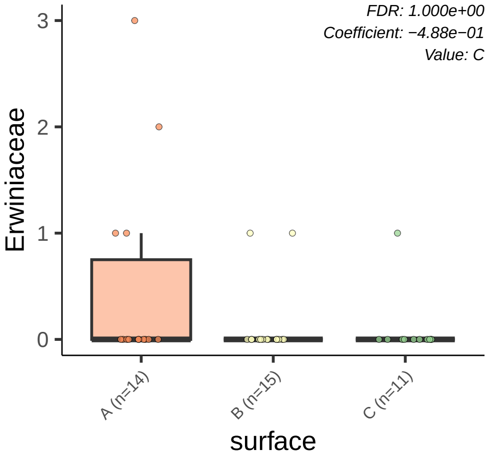

FDR: 1.000e+00
Coefficient: −4.88e−01
Value: C
Erwiniaceae
3
2
1
0
A (n=14)
B (n=15)
C (n=11)
surface

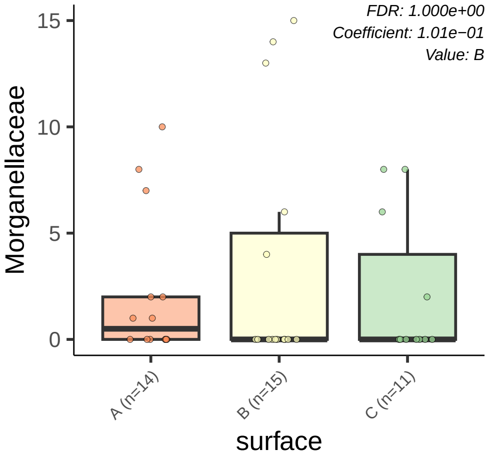
FDR: 1.000e+00
Coefficient: 1.01e−01
Value: B
Morganellaceae
15
10
5
0
A (n=14)
B (n=15)
C (n=11)
surface

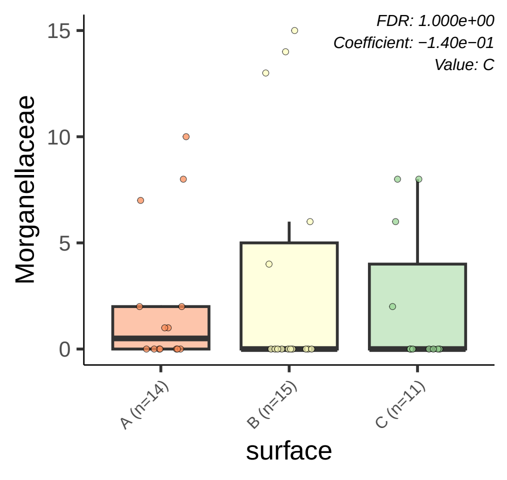
FDR: 1.000e+00
Coefficient: −1.40e−01
Value: C
Morganellaceae
15
10
5
0
A (n=14)
B (n=15)
C (n=11)
surface

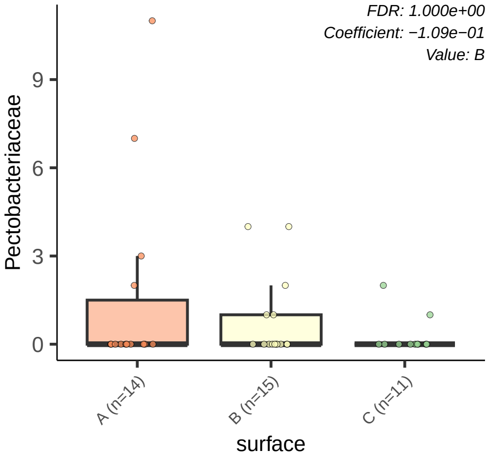

FDR: 1.000e+00
Coefficient: −1.09e−01
Value: B
Pectobacteriaceae
9
6
3
0
A (n=14)
B (n=15)
C (n=11)
surface

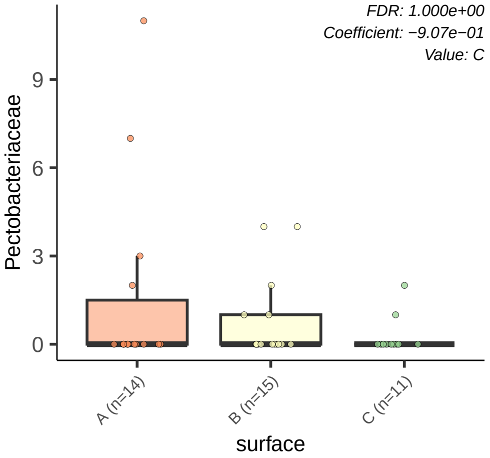
FDR: 1.000e+00
Coefficient: −9.07e−01
Value: C
Pectobacteriaceae
0
3
6
9
A (n=14)
B (n=15)
C (n=11)
surface

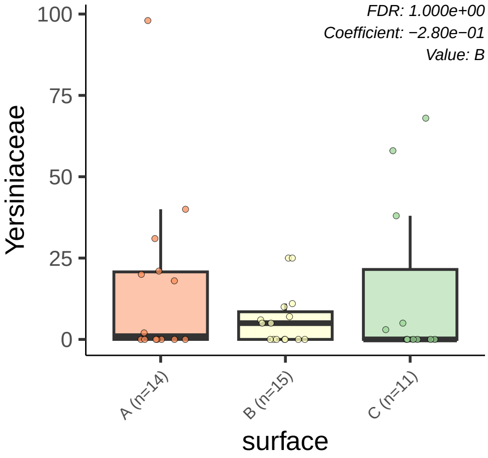

FDR: 1.000e+00
Coefficient: −2.80e−01
Value: B
Yersiniaceae
100
75
50
25
0
A (n=14)
B (n=15)
C (n=11)
surface

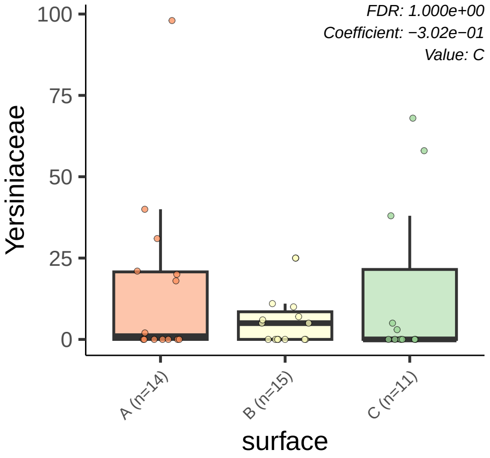
FDR: 1.000e+00
Coefficient: −3.02e−01
Value: C
Yersiniaceae
100
75
50
25
0
A (n=14)
B (n=15)
C (n=11)
surface

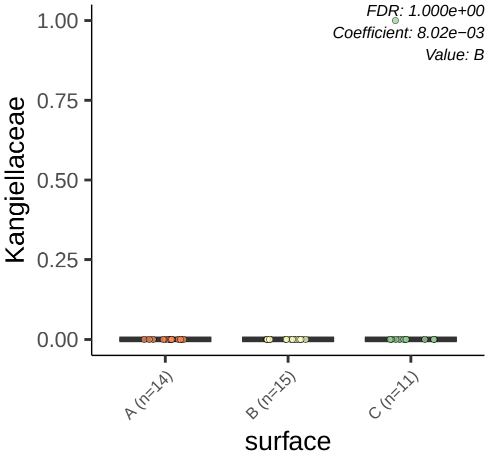

FDR: 1.000e+00
Coefficient: 8.02e−03
Value: B
1.00
0.75
0.50
0.25
0.00
Kangiellaceae
A (n=14)
B (n=15)
C (n=11)
surface

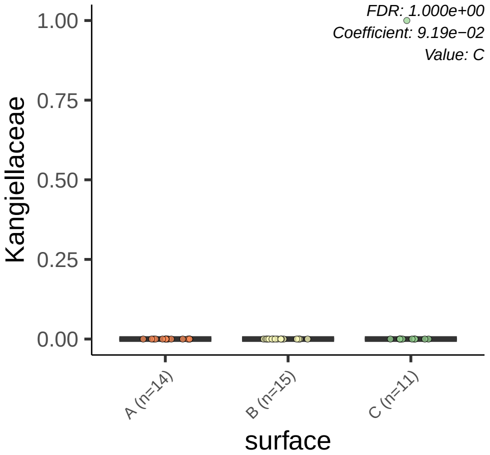

FDR: 1.000e+00
Coefficient: 9.19e−02
Value: C
Kangiellaceae
1.00
0.75
0.50
0.25
0.00
A (n=14)
B (n=15)
C (n=11)
surface

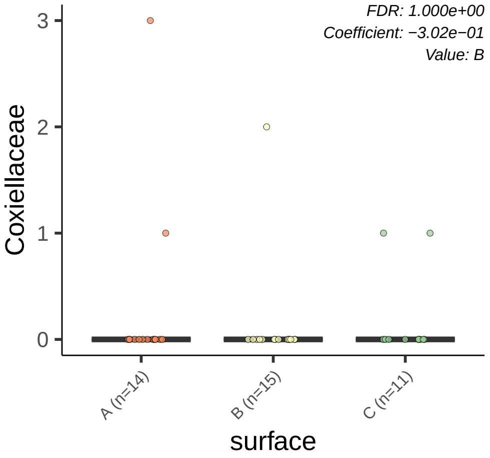
FDR: 1.000e+00
Coefficient: −3.02e−01
Value: B
Coxiellaceae
3
2
1
0
A (n=14)
B (n=15)
C (n=11)
surface

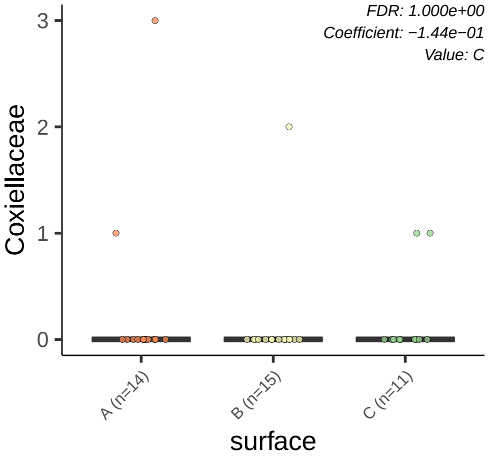

FDR: 1.000e+00
Coefficient: −1.44e−01
Value: C
Coxiellaceae
3
2
1
0
A (n=14)
B (n=15)
C (n=11)
surface

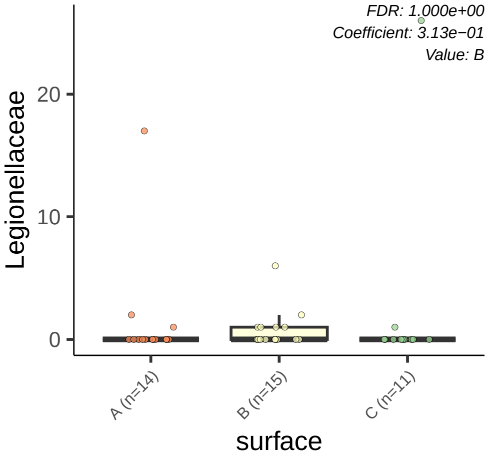

FDR: 1.000e+00
Coefficient: 3.13e−01
Value: B
Legionellaceae
20
10
0
A (n=14)
B (n=15)
C (n=11)
surface

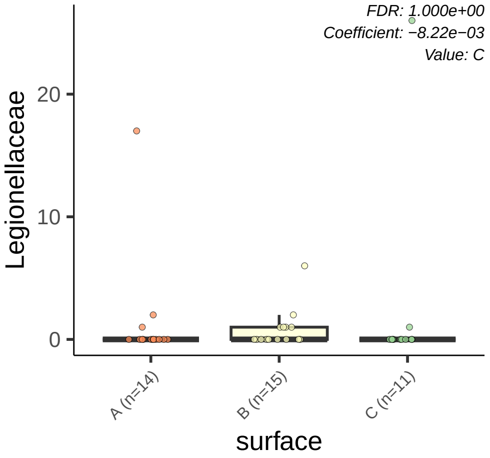

FDR: 1.000e+00
Coefficient: −8.22e−03
Value: C
Legionellaceae
20
10
0
A (n=14)
B (n=15)
C (n=11)
surface

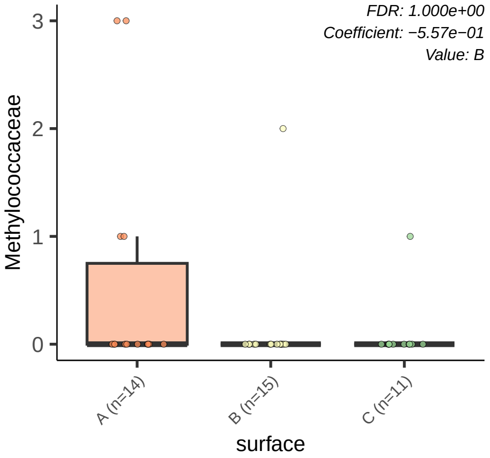
FDR: 1.000e+00
Coefficient: −5.57e−01
Value: B
Methylococcaceae
3
2
1
0
A (n=14)
B (n=15)
C (n=11)
surface

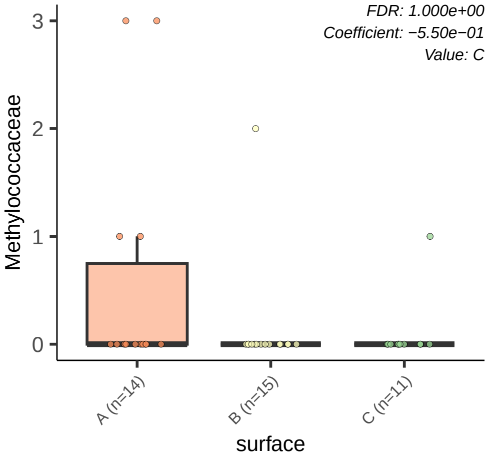
FDR: 1.000e+00
Coefficient: −5.50e−01
Value: C
Methylococcaceae
3
2
1
0
A (n=14)
B (n=15)
C (n=11)
surface

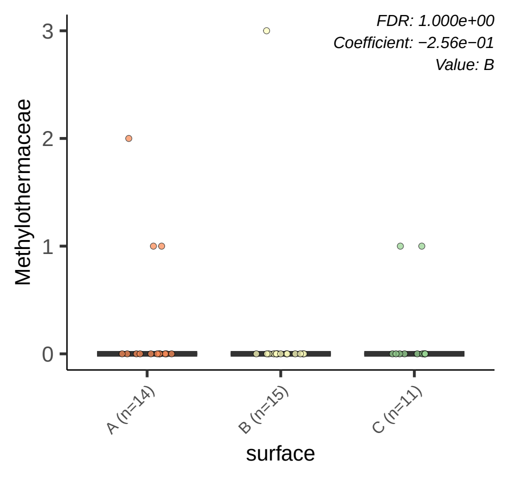
FDR: 1.000e+00
Coefficient: −2.56e−01
Value: B
Methylothermaceae
3
2
1
0
A (n=14)
B (n=15)
C (n=11)
surface

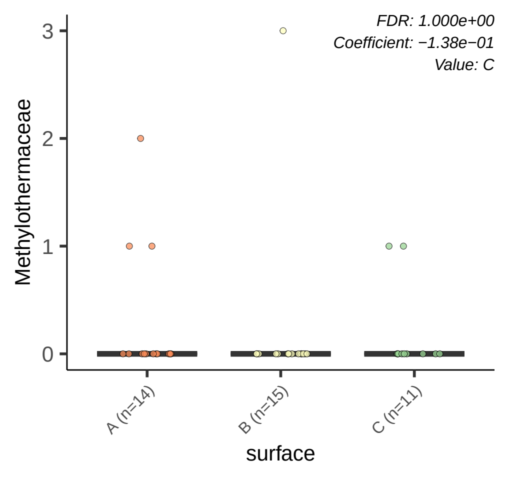

FDR: 1.000e+00
Coefficient: −1.38e−01
Value: C
Methylothermaceae
0
1
2
3
A (n=14)
B (n=15)
C (n=11)
surface

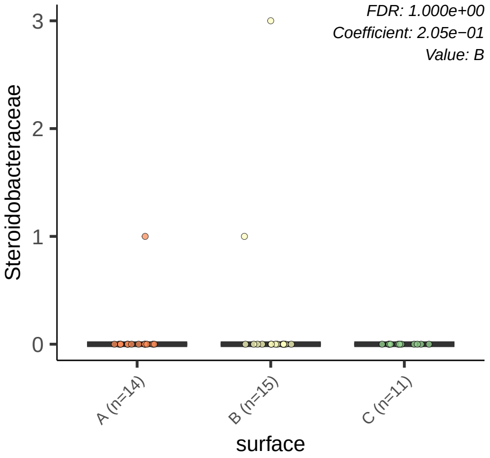

FDR: 1.000e+00
Coefficient: 2.05e−01
Value: B
Steroidobacteraceae
3
2
1
0
A (n=14)
B (n=15)
C (n=11)
surface

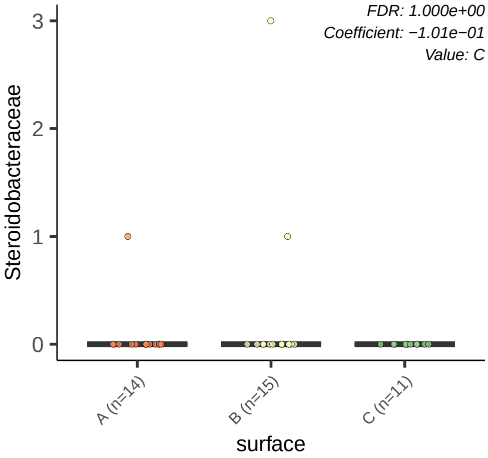

FDR: 1.000e+00
Coefficient: −1.01e−01
Value: C
Steroidobacteraceae
3
2
1
0
A (n=14)
B (n=15)
C (n=11)
surface

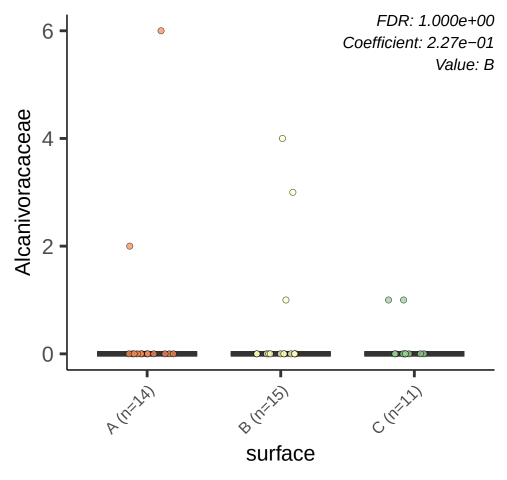
FDR: 1.000e+00
Coefficient: 2.27e−01
Value: B
Alcanivoracaceae
0
2
4
6
A (n=14)
B (n=15)
C (n=11)
surface

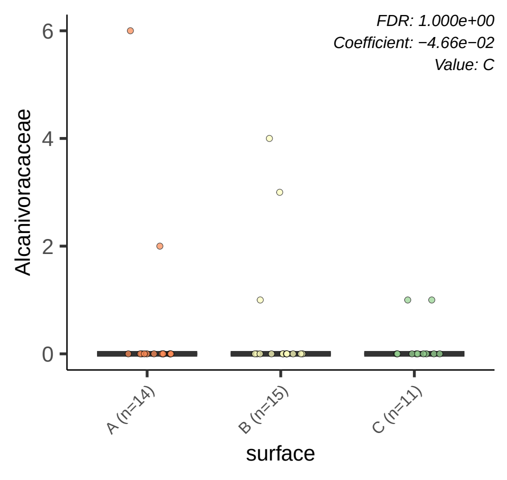

FDR: 1.000e+00
Coefficient: −4.66e−02
Value: C
Alcanivoracaceae
0
2
4
6
A (n=14)
B (n=15)
C (n=11)
surface

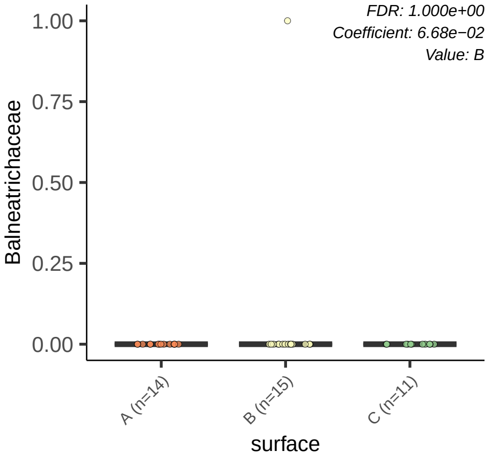
FDR: 1.000e+00
Coefficient: 6.68e−02
Value: B
Balneatrichaceae
1.00
0.75
0.50
0.25
0.00
A (n=14)
B (n=15)
C (n=11)
surface

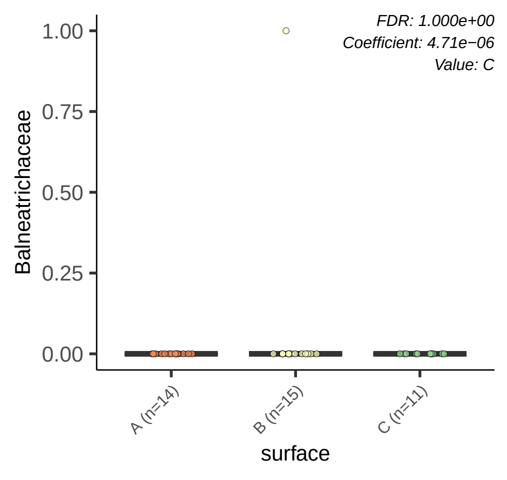
FDR: 1.000e+00
Coefficient: 4.71e−06
Value: C
Balneatrichaceae
1.00
0.75
0.50
0.25
0.00
A (n=14)
B (n=15)
C (n=11)
surface

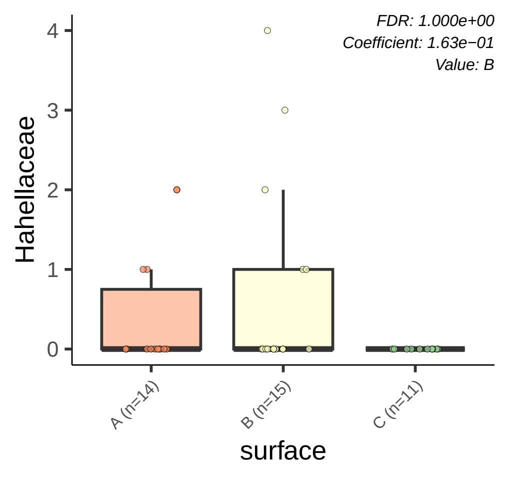
FDR: 1.000e+00
Coefficient: 1.63e−01
Value: B
Hahellaceae
4
3
2
1
0
A (n=14)
B (n=15)
C (n=11)
surface

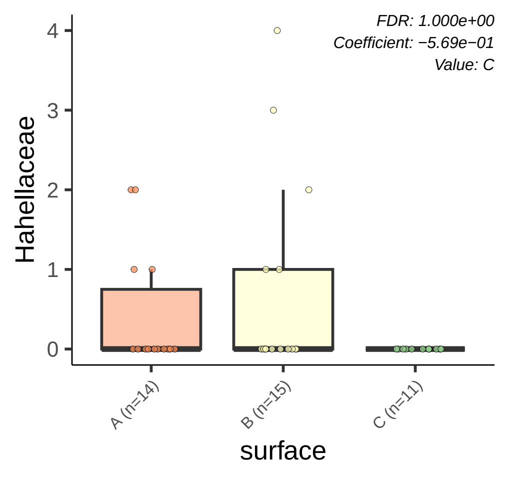
FDR: 1.000e+00
Coefficient: −5.69e−01
Value: C
Hahellaceae
0
1
2
3
4
A (n=14)
B (n=15)
C (n=11)
surface

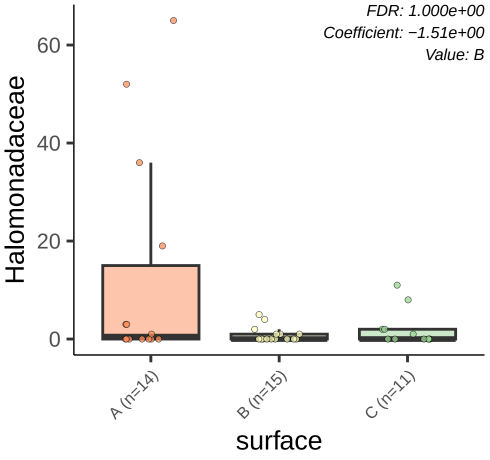
FDR: 1.000e+00
Coefficient: −1.51e+00
Value: B
Halomonadaceae
60
40
20
0
A (n=14)
B (n=15)
C (n=11)
surface

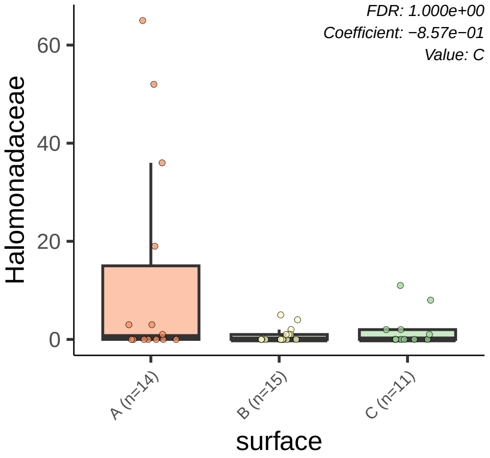
FDR: 1.000e+00
Coefficient: −8.57e−01
Value: C
Halomonadaceae
60
40
20
0
A (n=14)
B (n=15)
C (n=11)
surface

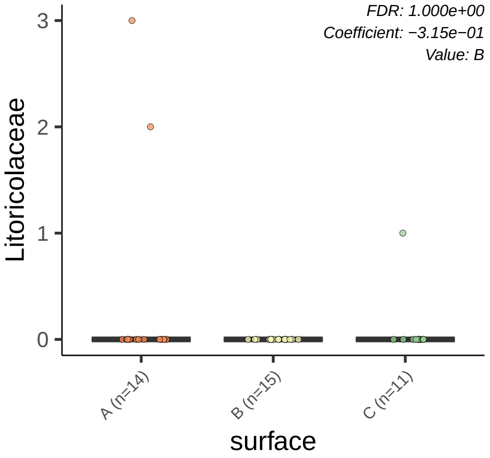
FDR: 1.000e+00
Coefficient: −3.15e−01
Value: B
Litoricolaceae
3
2
1
0
A (n=14)
B (n=15)
C (n=11)
surface

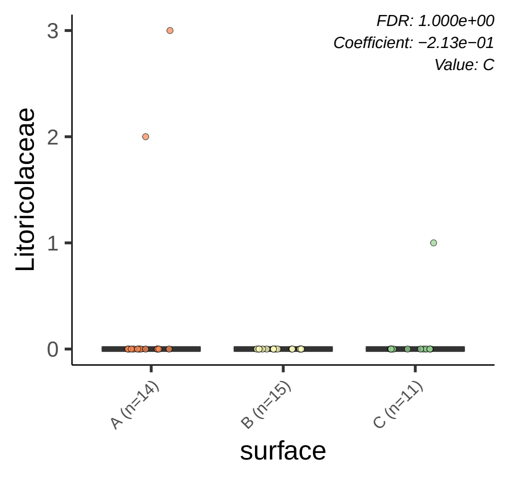

FDR: 1.000e+00
Coefficient: −2.13e−01
Value: C
Litoricolaceae
3
2
1
0
A (n=14)
B (n=15)
C (n=11)
surface

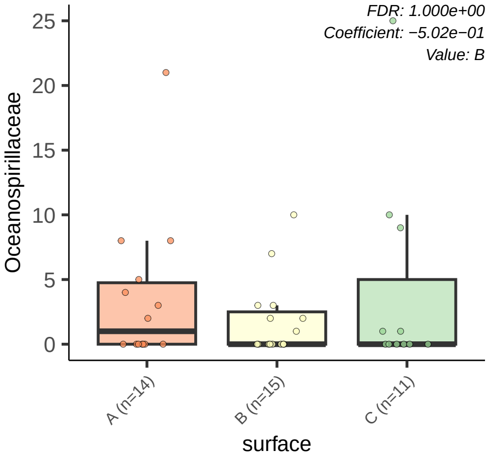

FDR: 1.000e+00
Coefficient: −5.02e−01
Value: B
Oceanospirillaceae
25
20
15
10
5
0
A (n=14)
B (n=15)
C (n=11)
surface

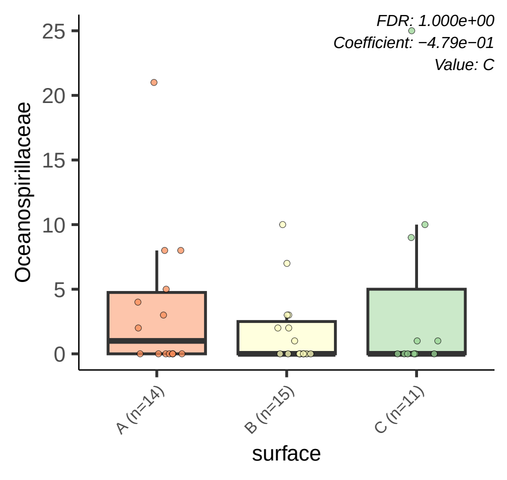

FDR: 1.000e+00
Coefficient: −4.79e−01
Value: C
Oceanospirillaceae
25
20
15
10
5
0
A (n=14)
B (n=15)
C (n=11)
surface

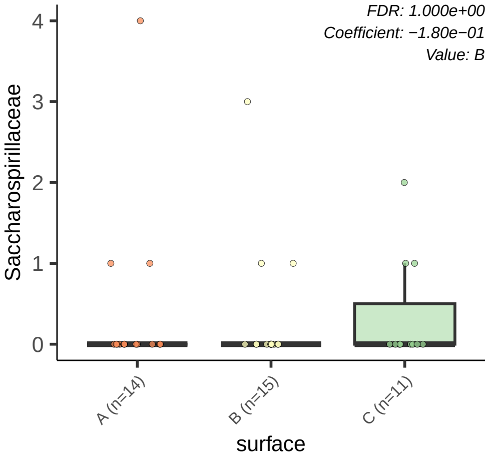

FDR: 1.000e+00
Coefficient: −1.80e−01
Value: B
Saccharospirillaceae
0
1
2
3
4
A (n=14)
B (n=15)
C (n=11)
surface

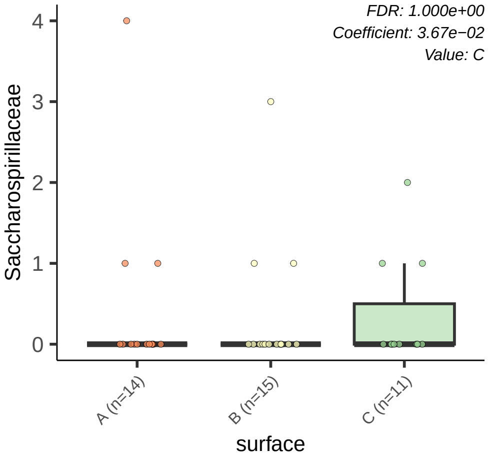

FDR: 1.000e+00
Coefficient: 3.67e−02
Value: C
Saccharospirillaceae
4
3
2
1
0
A (n=14)
B (n=15)
C (n=11)
surface

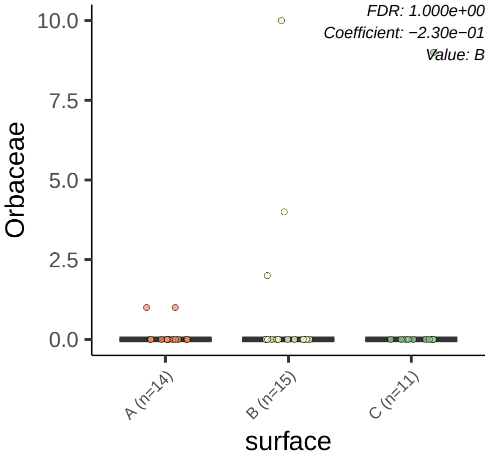

FDR: 1.000e+00
Coefficient: −2.30e−01
Value: B
Orbaceae
10.0
7.5
5.0
2.5
0.0
A (n=14)
B (n=15)
C (n=11)
surface

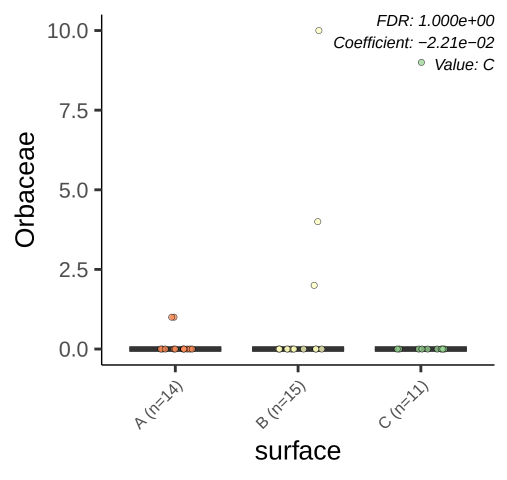

FDR: 1.000e+00
Coefficient: −2.21e−02
Value: C
Orbaceae
10.0
7.5
5.0
2.5
0.0
A (n=14)
B (n=15)
C (n=11)
surface

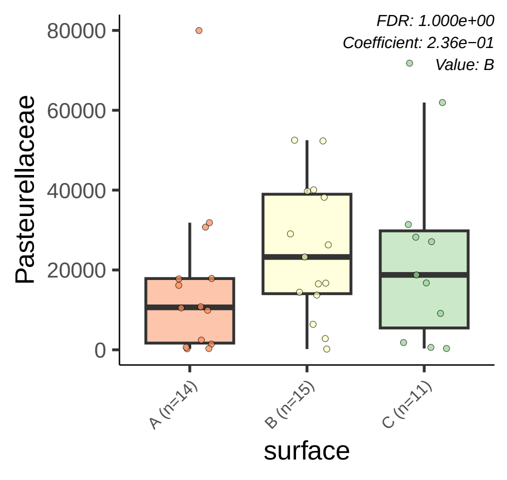
FDR: 1.000e+00
Coefficient: 2.36e−01
Value: B
Pasteurellaceae
80000
60000
40000
20000
0
A (n=14)
B (n=15)
C (n=11)
surface

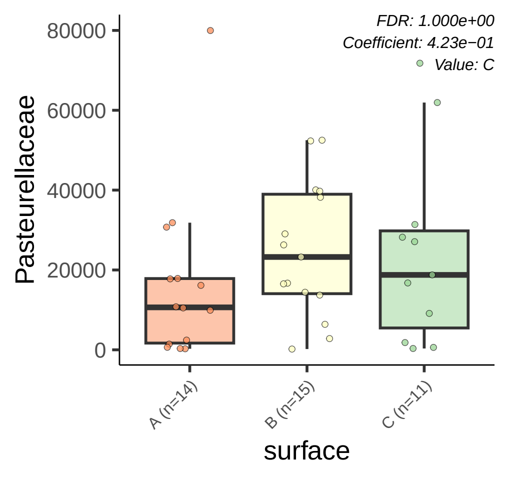

FDR: 1.000e+00
Coefficient: 4.23e−01
Value: C
80000
60000
40000
20000
0
Pasteurellaceae
A (n=14)
B (n=15)
C (n=11)
surface

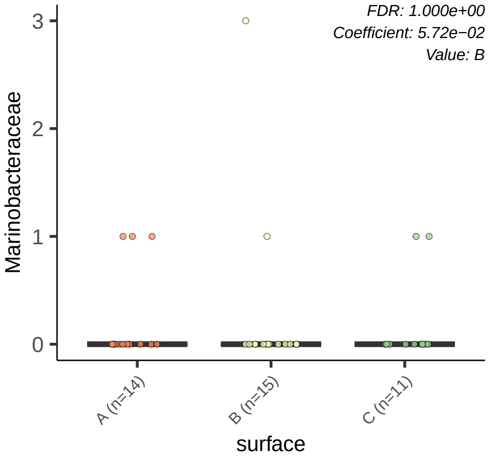

FDR: 1.000e+00
Coefficient: 5.72e−02
Value: B
Marinobacteraceae
0
1
2
3
A (n=14)
B (n=15)
C (n=11)
surface

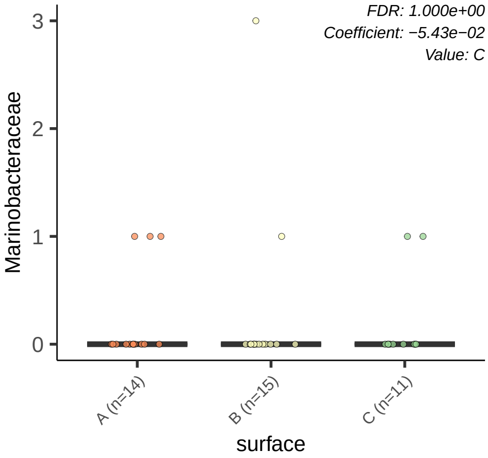

FDR: 1.000e+00
Coefficient: −5.43e−02
Value: C
Marinobacteraceae
3
2
1
0
A (n=14)
B (n=15)
C (n=11)
surface

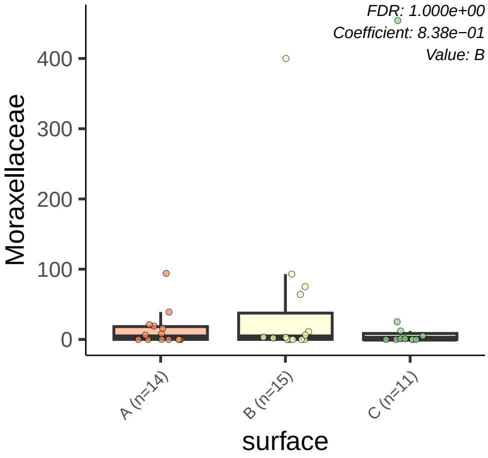

FDR: 1.000e+00
Coefficient: 8.38e−01
Value: B
Moraxellaceae
400
300
200
100
0
A (n=14)
B (n=15)
C (n=11)
surface

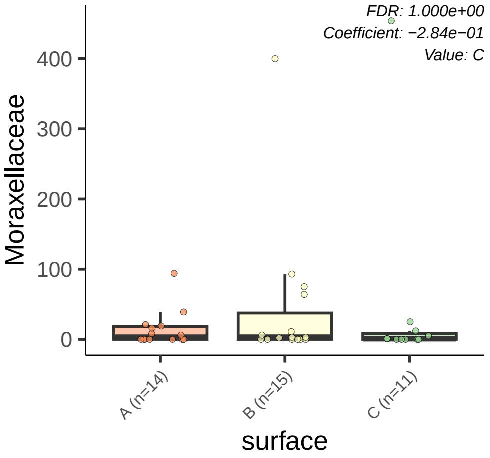

FDR: 1.000e+00
Coefficient: −2.84e−01
Value: C
Moraxellaceae
400
300
200
100
0
A (n=14)
B (n=15)
C (n=11)
surface

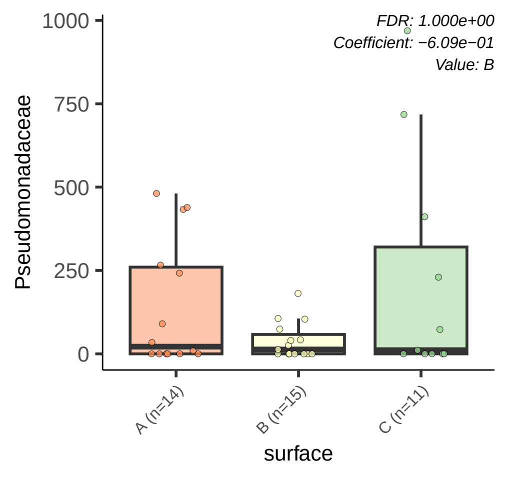

FDR: 1.000e+00
Coefficient: −6.09e−01
Value: B
1000
750
500
250
0
Pseudomonadaceae
A (n=14)
B (n=15)
C (n=11)
surface

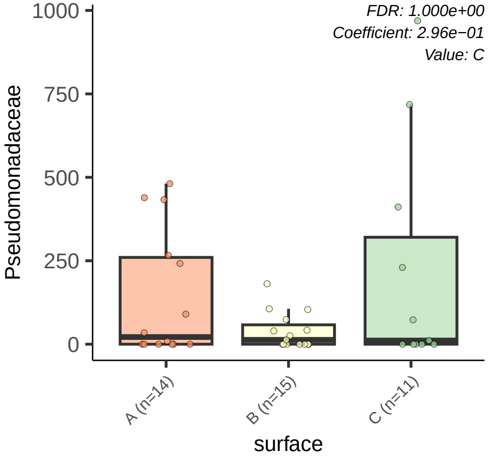

FDR: 1.000e+00
Coefficient: 2.96e−01
Value: C
Pseudomonadaceae
1000
750
500
250
0
A (n=14)
B (n=15)
C (n=11)
surface

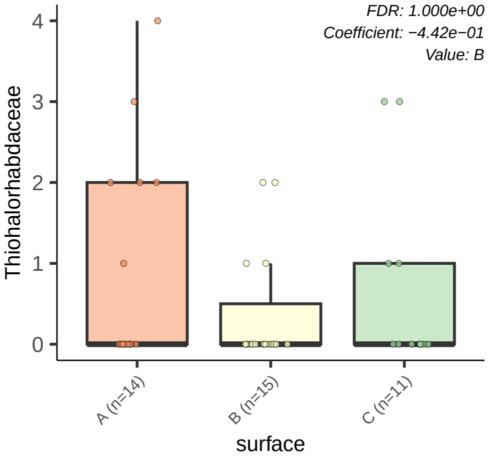

FDR: 1.000e+00
Coefficient: −4.42e−01
Value: B
Thiohalorhabdaceae
4
3
2
1
0
A (n=14)
B (n=15)
C (n=11)
surface

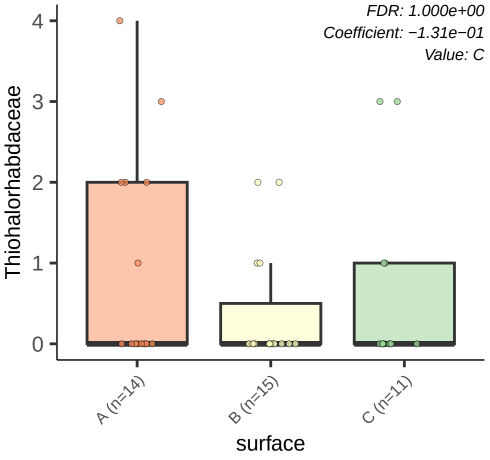

FDR: 1.000e+00
Coefficient: −1.31e−01
Value: C
Thiohalorhabdaceae
0
1
2
3
4
A (n=14)
B (n=15)
C (n=11)
surface

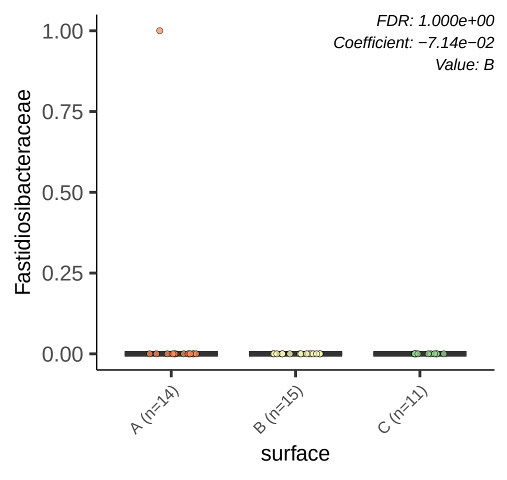
FDR: 1.000e+00
Coefficient: −7.14e−02
Value: B
Fastidiosibacteraceae
1.00
0.75
0.50
0.25
0.00
A (n=14)
B (n=15)
C (n=11)
surface

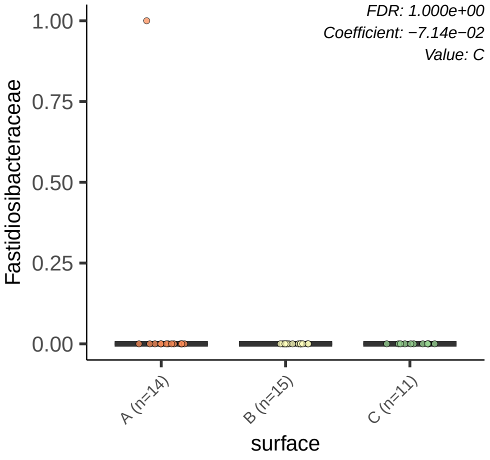
FDR: 1.000e+00
Coefficient: −7.14e−02
Value: C
Fastidiosibacteraceae
1.00
0.75
0.50
0.25
0.00
A (n=14)
B (n=15)
C (n=11)
surface

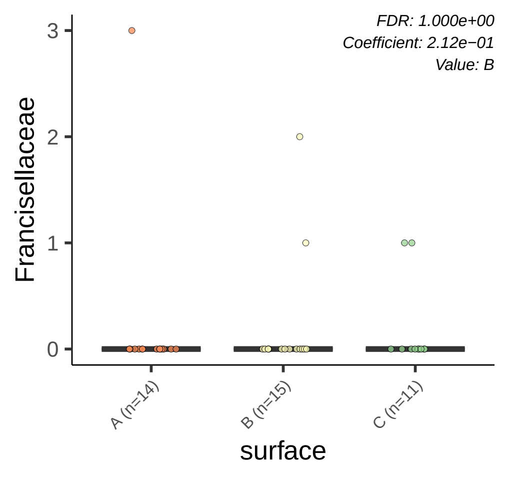
FDR: 1.000e+00
Coefficient: 2.12e−01
Value: B
Francisellaceae
3
2
1
0
A (n=14)
B (n=15)
C (n=11)
surface

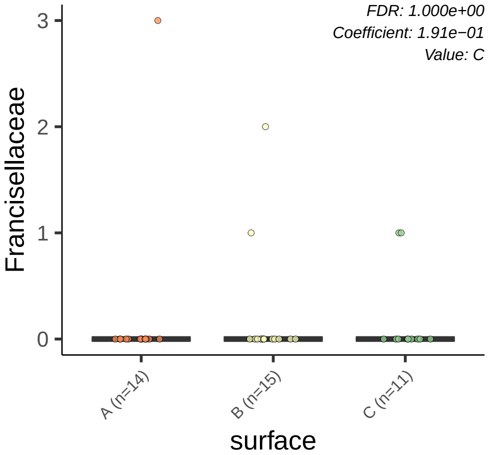

FDR: 1.000e+00
Coefficient: 1.91e−01
Value: C
Francisellaceae
3
2
1
0
A (n=14)
B (n=15)
C (n=11)
surface

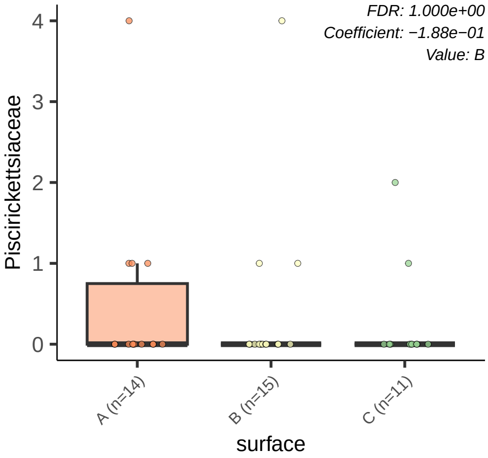
FDR: 1.000e+00
Coefficient: −1.88e−01
Value: B
Piscirickettsiaceae
0
1
2
3
4
A (n=14)
B (n=15)
C (n=11)
surface

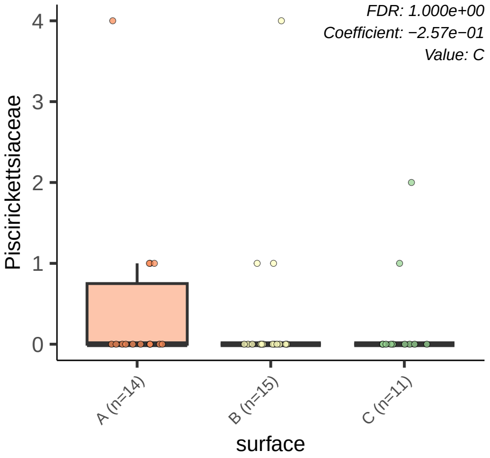
FDR: 1.000e+00
Coefficient: −2.57e−01
Value: C
Piscirickettsiaceae
4
3
2
1
0
A (n=14)
B (n=15)
C (n=11)
surface

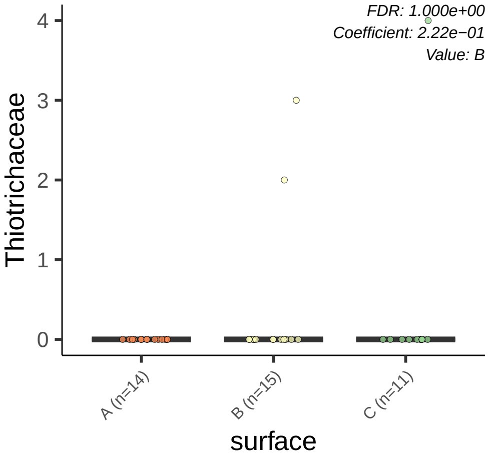
FDR: 1.000e+00
Coefficient: 2.22e−01
Value: B
Thiotrichaceae
4
3
2
1
0
A (n=14)
B (n=15)
C (n=11)
surface

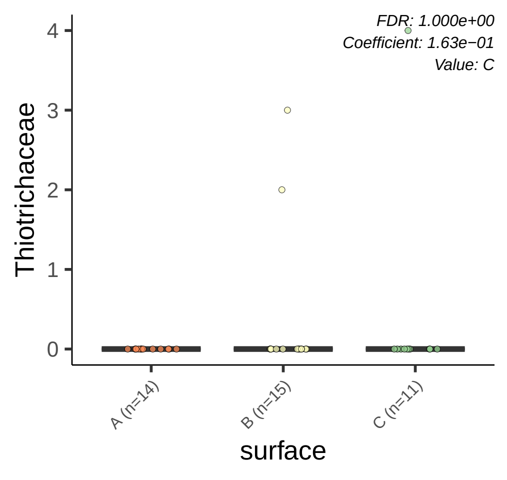
FDR: 1.000e+00
Coefficient: 1.63e−01
Value: C
Thiotrichaceae
0
1
2
3
4
A (n=14)
B (n=15)
C (n=11)
surface

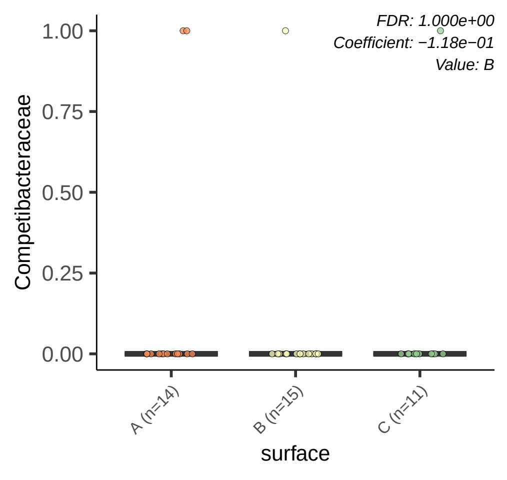

FDR: 1.000e+00
Coefficient: −1.18e−01
Value: B
Competibacteraceae
1.00
0.75
0.50
0.25
0.00
A (n=14)
B (n=15)
C (n=11)
surface

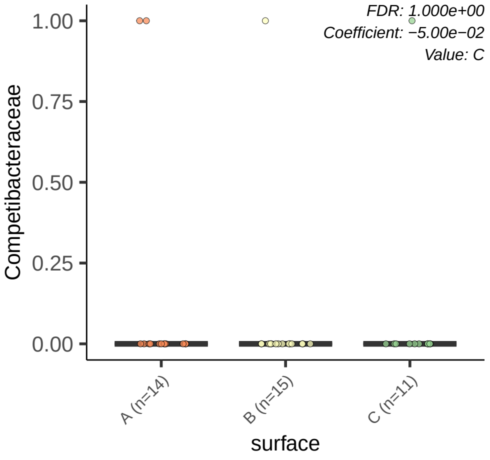
FDR: 1.000e+00
Coefficient: −5.00e−02
Value: C
Competibacteraceae
1.00
0.75
0.50
0.25
0.00
A (n=14)
B (n=15)
C (n=11)
surface

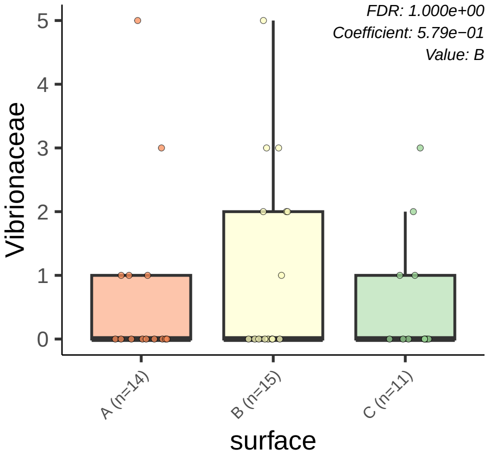

FDR: 1.000e+00
Coefficient: 5.79e−01
Value: B
Vibrionaceae
0
1
2
3
4
5
A (n=14)
B (n=15)
C (n=11)
surface

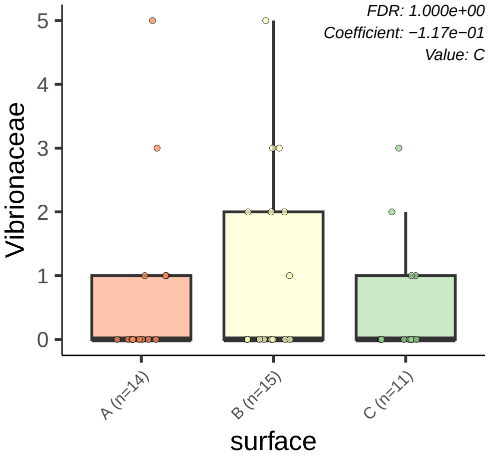

FDR: 1.000e+00
Coefficient: −1.17e−01
Value: C
Vibrionaceae
0
1
2
3
4
5
A (n=14)
B (n=15)
C (n=11)
surface

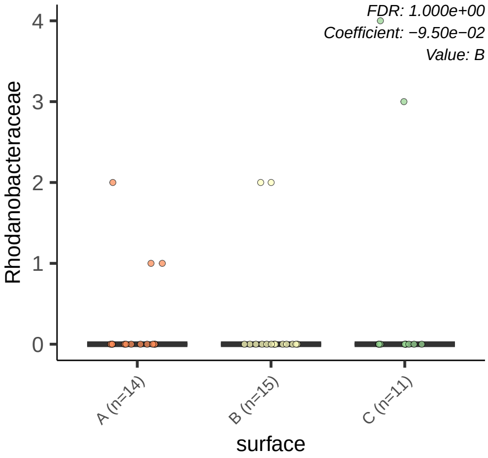

FDR: 1.000e+00
Coefficient: −9.50e−02
Value: B
Rhodanobacteraceae
4
3
2
1
0
A (n=14)
B (n=15)
C (n=11)
surface

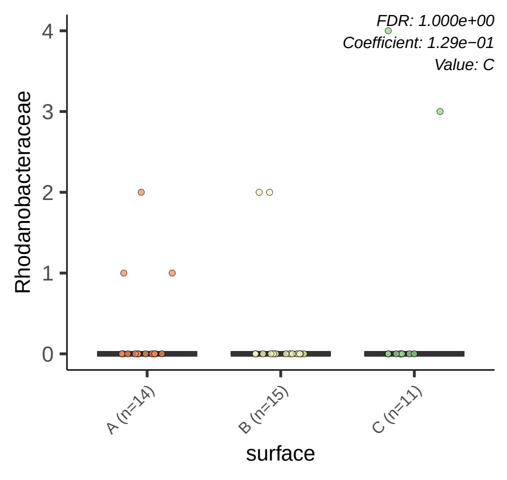

FDR: 1.000e+00
Coefficient: 1.29e−01
Value: C
Rhodanobacteraceae
0
1
2
3
4
A (n=14)
B (n=15)
C (n=11)
surface

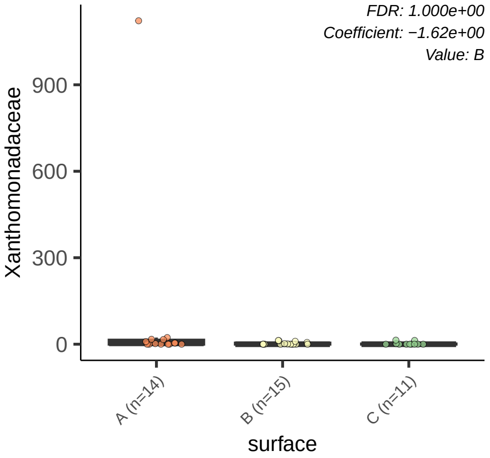

FDR: 1.000e+00
Coefficient: −1.62e+00
Value: B
Xanthomonadaceae
900
600
300
0
A (n=14)
B (n=15)
C (n=11)
surface

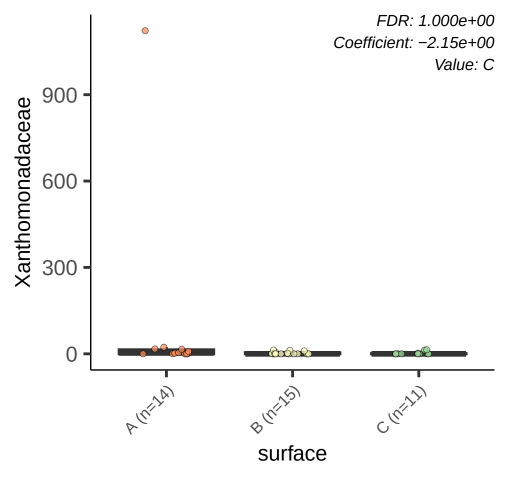
FDR: 1.000e+00
Coefficient: −2.15e+00
Value: C
Xanthomonadaceae
900
600
300
0
A (n=14)
B (n=15)
C (n=11)
surface

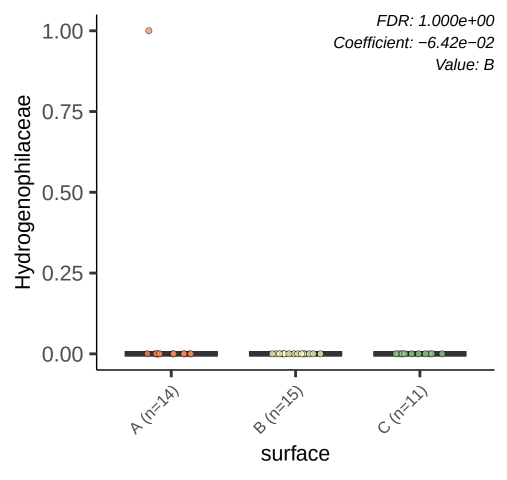
FDR: 1.000e+00
Coefficient: −6.42e−02
Value: B
Hydrogenophilaceae
1.00
0.75
0.50
0.25
0.00
A (n=14)
B (n=15)
C (n=11)
surface

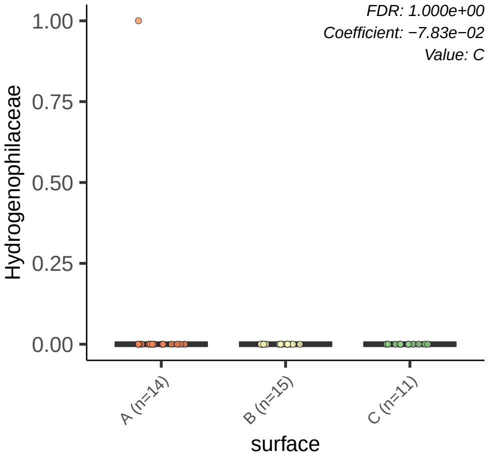

FDR: 1.000e+00
Coefficient: −7.83e−02
Value: C
Hydrogenophilaceae
1.00
0.75
0.50
0.25
0.00
A (n=14)
B (n=15)
C (n=11)
surface

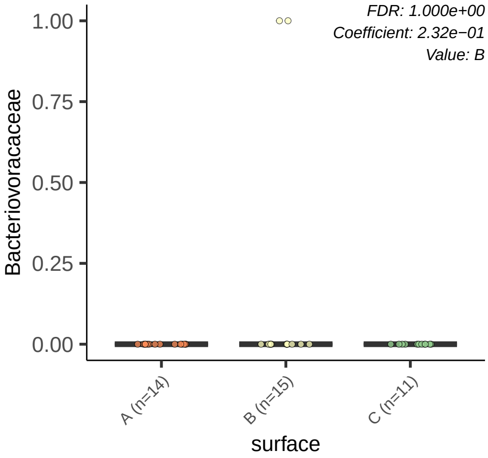
FDR: 1.000e+00
Coefficient: 2.32e−01
Value: B
Bacteriovoracaceae
1.00
0.75
0.50
0.25
0.00
A (n=14)
B (n=15)
C (n=11)
surface

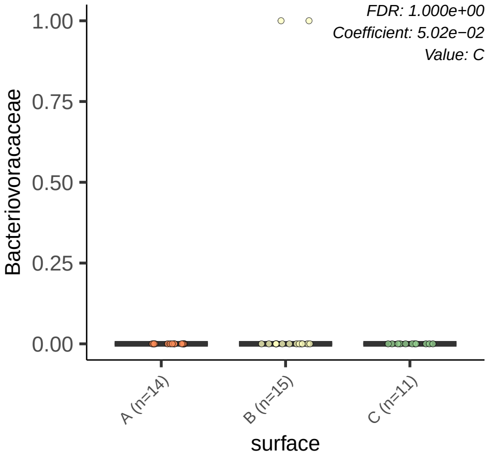
FDR: 1.000e+00
Coefficient: 5.02e−02
Value: C
Bacteriovoracaceae
1.00
0.75
0.50
0.25
0.00
A (n=14)
B (n=15)
C (n=11)
surface

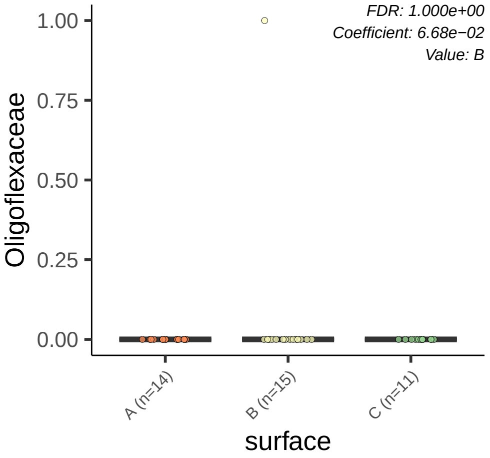
FDR: 1.000e+00
Coefficient: 6.68e−02
Value: B
Oligoflexaceae
1.00
0.75
0.50
0.25
0.00
A (n=14)
B (n=15)
C (n=11)
surface

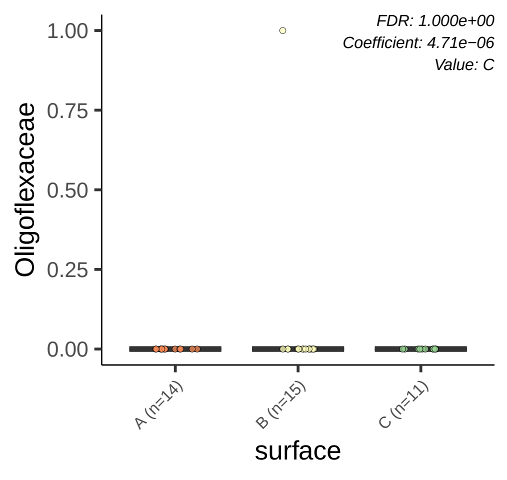
FDR: 1.000e+00
Coefficient: 4.71e−06
Value: C
Oligoflexaceae
1.00
0.75
0.50
0.25
0.00
A (n=14)
B (n=15)
C (n=11)
surface

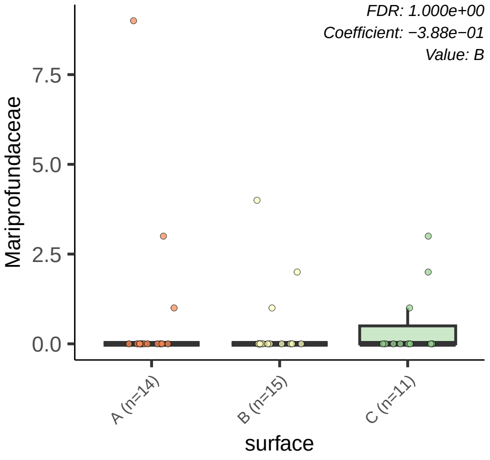
FDR: 1.000e+00
Coefficient: −3.88e−01
Value: B
Mariprofundaceae
0.0
2.5
5.0
7.5
A (n=14)
B (n=15)
C (n=11)
surface

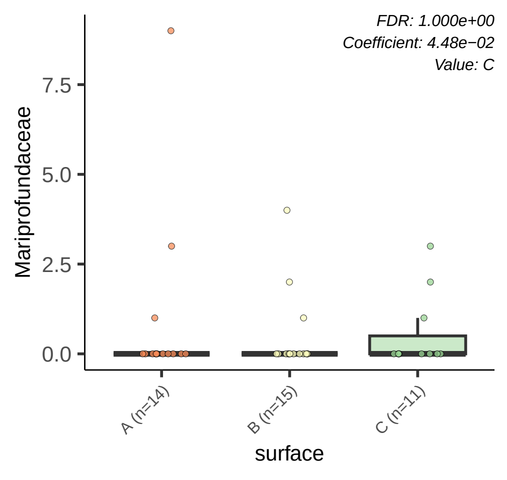
FDR: 1.000e+00
Coefficient: 4.48e−02
Value: C
Mariprofundaceae
7.5
5.0
2.5
0.0
A (n=14)
B (n=15)
C (n=11)
surface

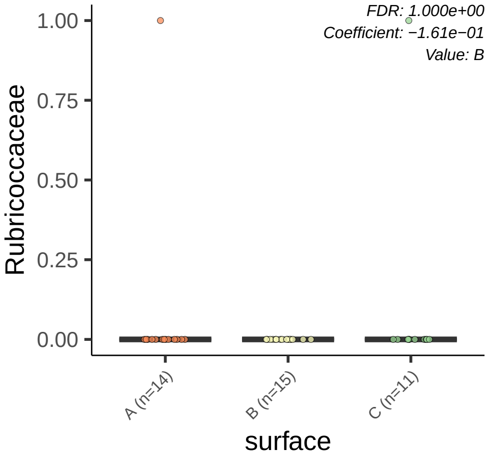
FDR: 1.000e+00
Coefficient: −1.61e−01
Value: B
Rubricoccaceae
1.00
0.75
0.50
0.25
0.00
A (n=14)
B (n=15)
C (n=11)
surface

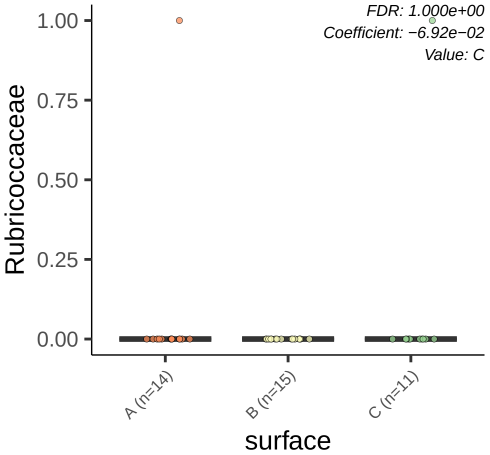
FDR: 1.000e+00
Coefficient: −6.92e−02
Value: C
Rubricoccaceae
1.00
0.75
0.50
0.25
0.00
A (n=14)
B (n=15)
C (n=11)
surface

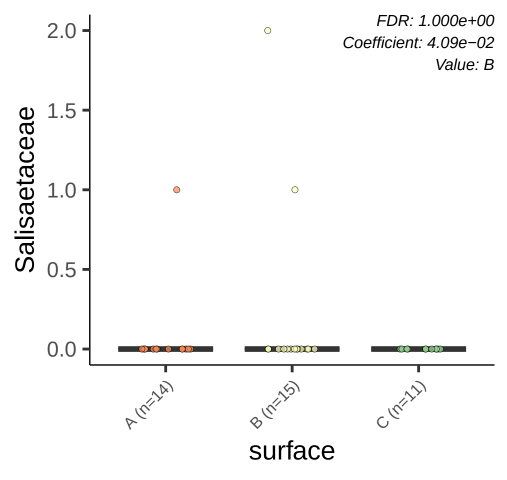
FDR: 1.000e+00
Coefficient: 4.09e−02
Value: B
Salisaetaceae
2.0
1.5
1.0
0.5
0.0
A (n=14)
B (n=15)
C (n=11)
surface

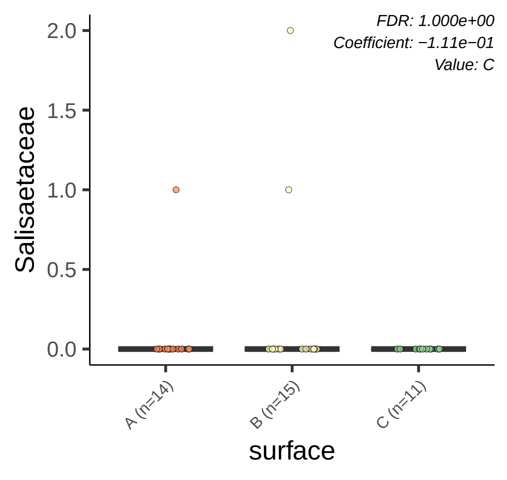
FDR: 1.000e+00
Coefficient: −1.11e−01
Value: C
Salisaetaceae
2.0
1.5
1.0
0.5
0.0
A (n=14)
B (n=15)
C (n=11)
surface

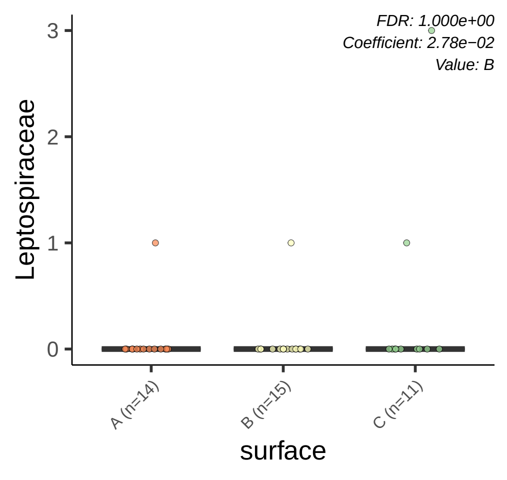
FDR: 1.000e+00
Coefficient: 2.78e−02
Value: B
Leptospiraceae
3
2
1
0
A (n=14)
B (n=15)
C (n=11)
surface

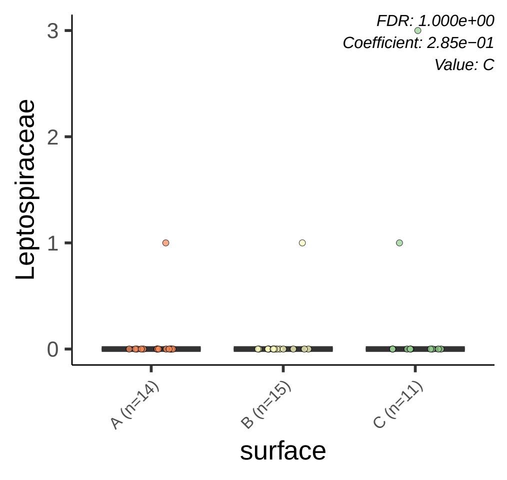

FDR: 1.000e+00
Coefficient: 2.85e−01
Value: C
Leptospiraceae
0
1
2
3
A (n=14)
B (n=15)
C (n=11)
surface

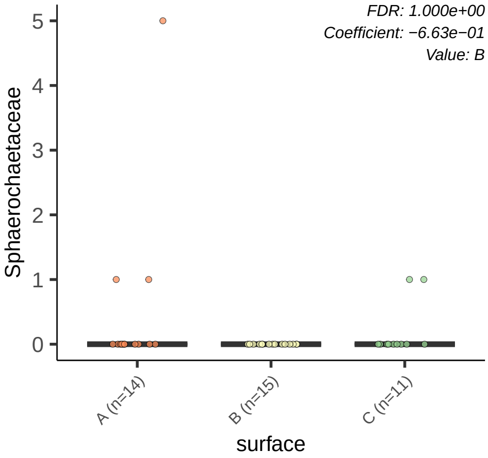
FDR: 1.000e+00
Coefficient: −6.63e−01
Value: B
Sphaerochaetaceae
0
1
2
3
4
5
A (n=14)
B (n=15)
C (n=11)
surface

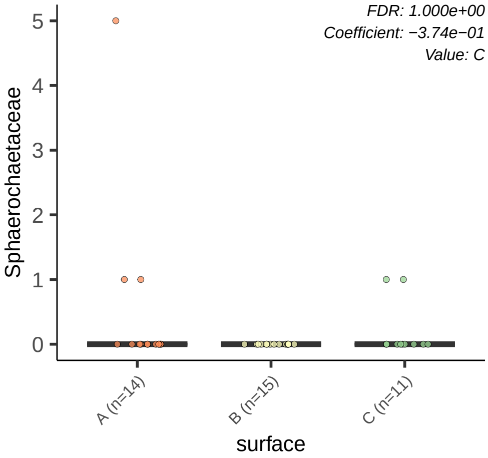
FDR: 1.000e+00
Coefficient: −3.74e−01
Value: C
Sphaerochaetaceae
0
1
2
3
4
5
A (n=14)
B (n=15)
C (n=11)
surface

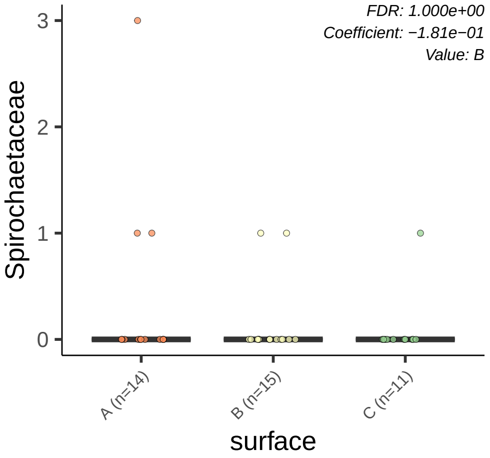
FDR: 1.000e+00
Coefficient: −1.81e−01
Value: B
Spirochaetaceae
3
2
1
0
A (n=14)
B (n=15)
C (n=11)
surface

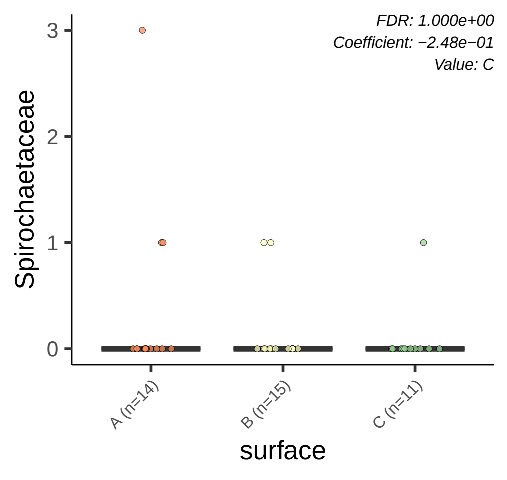

FDR: 1.000e+00
Coefficient: −2.48e−01
Value: C
Spirochaetaceae
3
2
1
0
A (n=14)
B (n=15)
C (n=11)
surface

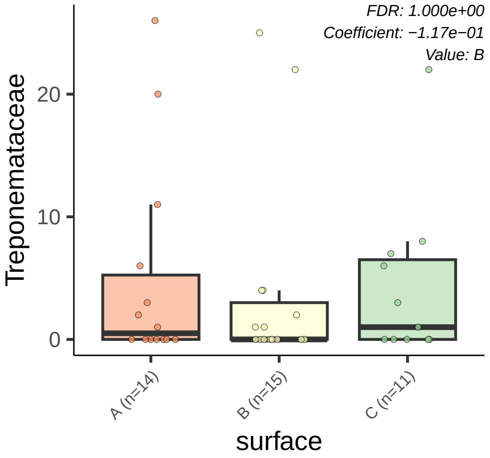
FDR: 1.000e+00
Coefficient: −1.17e−01
Value: B
Treponemataceae
20
10
0
A (n=14)
B (n=15)
C (n=11)
surface

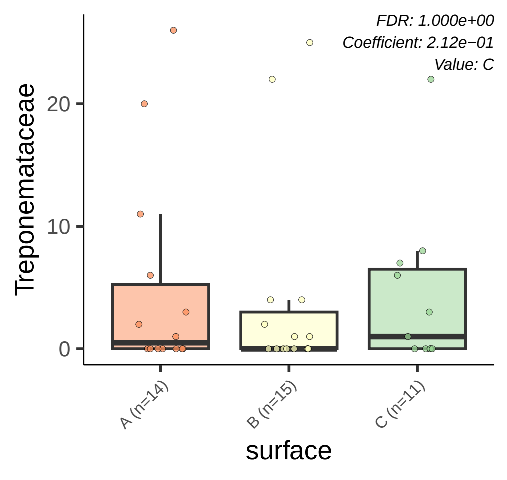
FDR: 1.000e+00
Coefficient: 2.12e−01
Value: C
Treponemataceae
0
10
20
A (n=14)
B (n=15)
C (n=11)
surface

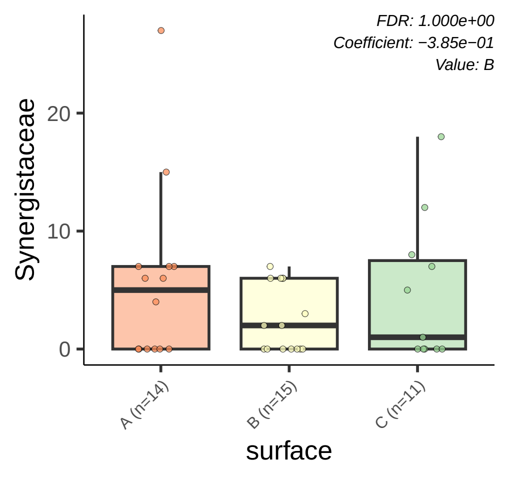
FDR: 1.000e+00
Coefficient: −3.85e−01
Value: B
Synergistaceae
20
10
0
A (n=14)
B (n=15)
C (n=11)
surface

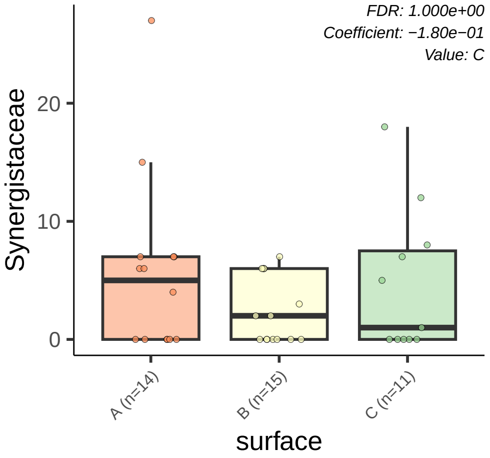
FDR: 1.000e+00
Coefficient: −1.80e−01
Value: C
Synergistaceae
20
10
0
A (n=14)
B (n=15)
C (n=11)
surface

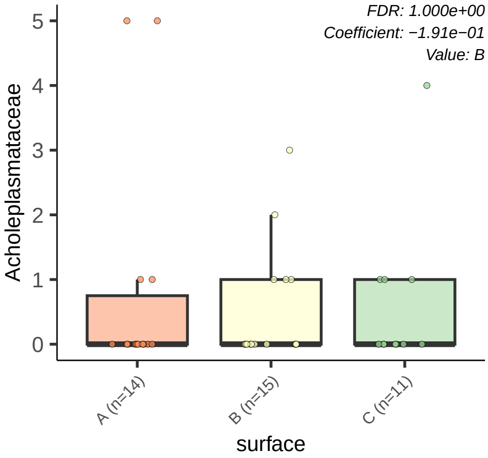

FDR: 1.000e+00
Coefficient: −1.91e−01
Value: B
Acholeplasmataceae
5
4
3
2
1
0
A (n=14)
B (n=15)
C (n=11)
surface

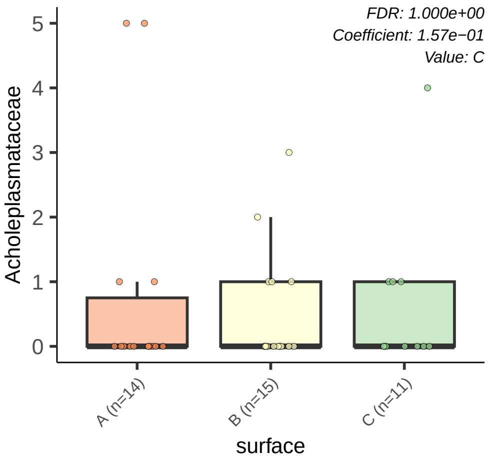
FDR: 1.000e+00
Coefficient: 1.57e−01
Value: C
Acholeplasmataceae
0
1
2
3
4
5
A (n=14)
B (n=15)
C (n=11)
surface

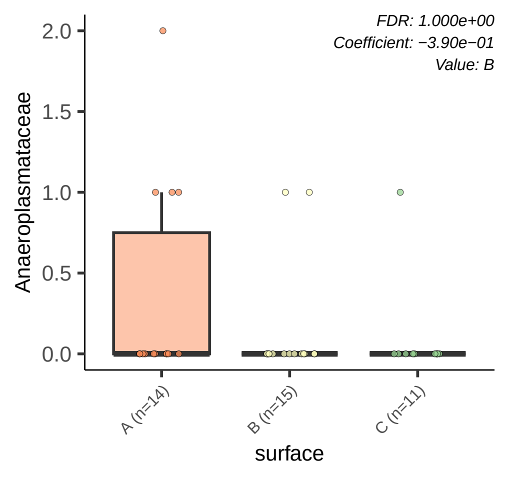
FDR: 1.000e+00
Coefficient: −3.90e−01
Value: B
Anaeroplasmataceae
2.0
1.5
1.0
0.5
0.0
A (n=14)
B (n=15)
C (n=11)
surface

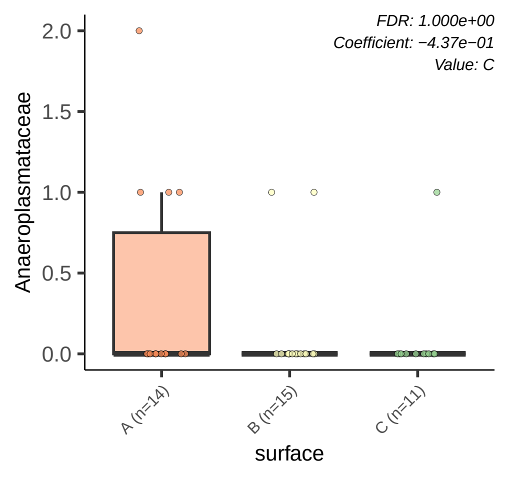
FDR: 1.000e+00
Coefficient: −4.37e−01
Value: C
Anaeroplasmataceae
2.0
1.5
1.0
0.5
0.0
A (n=14)
B (n=15)
C (n=11)
surface

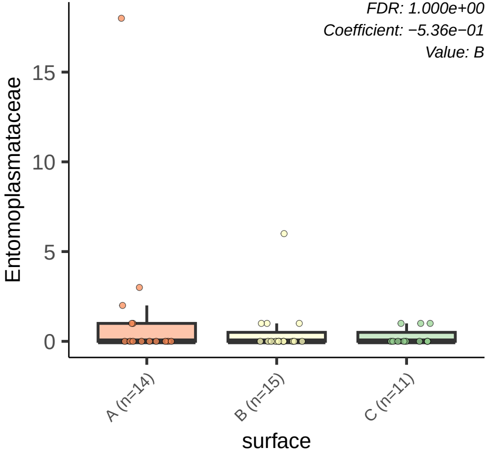

FDR: 1.000e+00
Coefficient: −5.36e−01
Value: B
Entomoplasmataceae
0
5
10
15
A (n=14)
B (n=15)
C (n=11)
surface

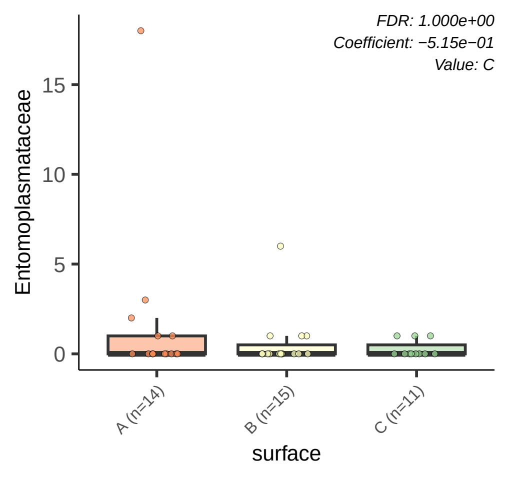

FDR: 1.000e+00
Coefficient: −5.15e−01
Value: C
Entomoplasmataceae
0
5
10
15
A (n=14)
B (n=15)
C (n=11)
surface

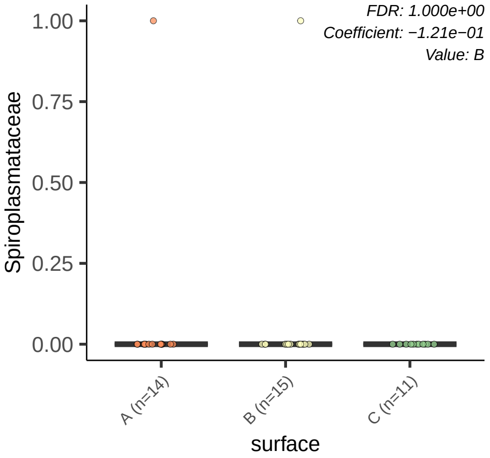

FDR: 1.000e+00
Coefficient: −1.21e−01
Value: B
Spiroplasmataceae
1.00
0.75
0.50
0.25
0.00
A (n=14)
B (n=15)
C (n=11)
surface

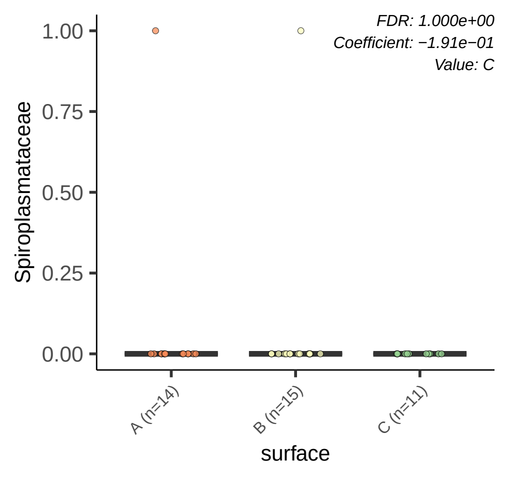
FDR: 1.000e+00
Coefficient: −1.91e−01
Value: C
Spiroplasmataceae
1.00
0.75
0.50
0.25
0.00
A (n=14)
B (n=15)
C (n=11)
surface

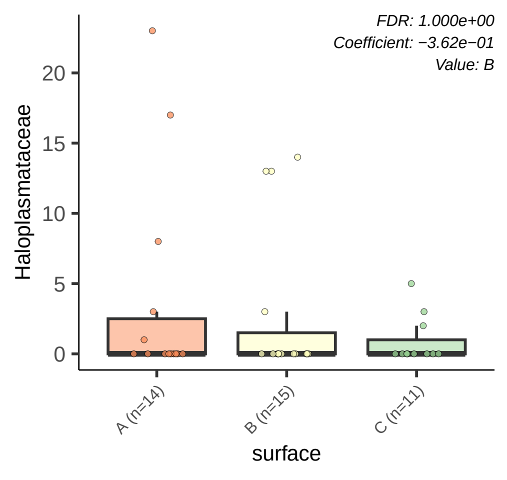

FDR: 1.000e+00
Coefficient: −3.62e−01
Value: B
Haloplasmataceae
0
5
10
15
20
A (n=14)
B (n=15)
C (n=11)
surface

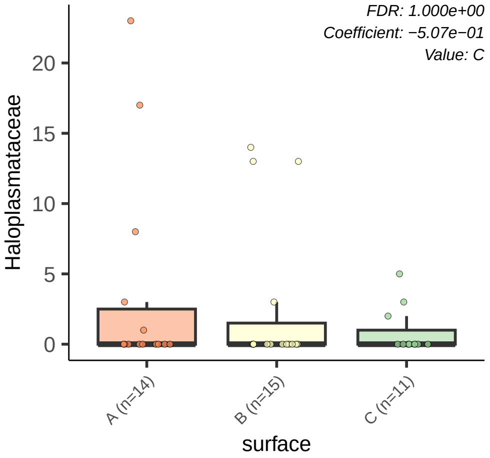

FDR: 1.000e+00
Coefficient: −5.07e−01
Value: C
Haloplasmataceae
20
15
10
5
0
A (n=14)
B (n=15)
C (n=11)
surface

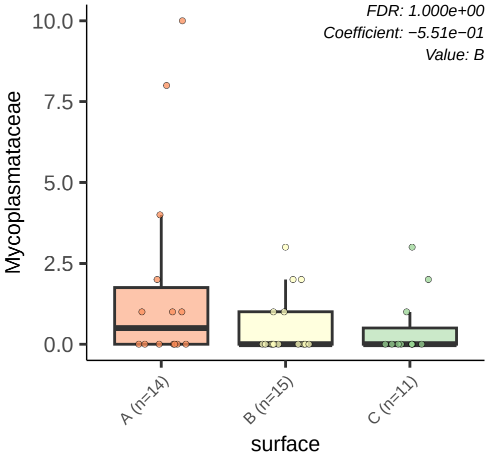

FDR: 1.000e+00
Coefficient: −5.51e−01
Value: B
Mycoplasmataceae
10.0
7.5
5.0
2.5
0.0
A (n=14)
B (n=15)
C (n=11)
surface

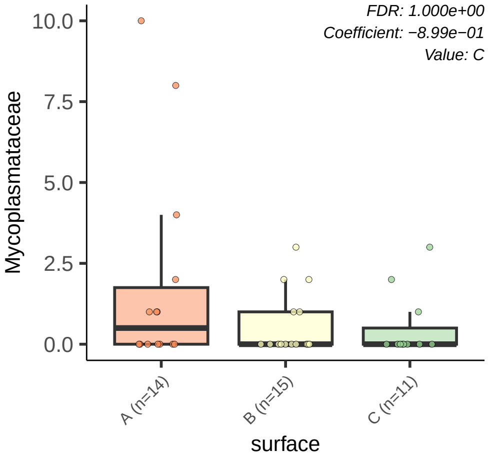

FDR: 1.000e+00
Coefficient: −8.99e−01
Value: C
Mycoplasmataceae
10.0
7.5
5.0
2.5
0.0
A (n=14)
B (n=15)
C (n=11)
surface

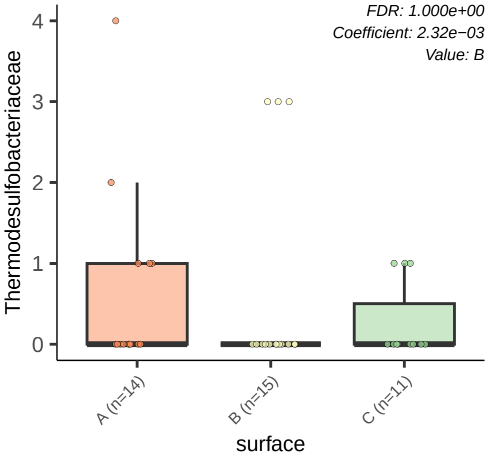
FDR: 1.000e+00
Coefficient: 2.32e−03
Value: B
Thermodesulfobacteriaceae
0
1
2
3
4
A (n=14)
B (n=15)
C (n=11)
surface

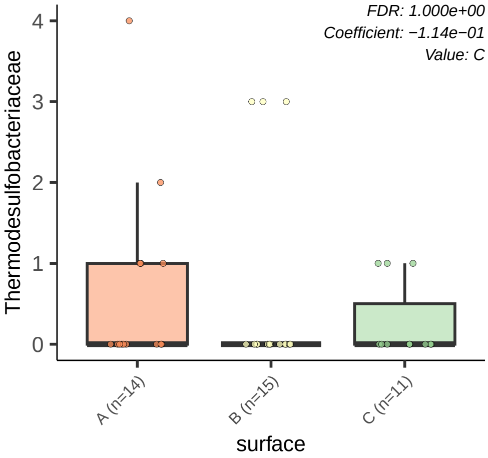
FDR: 1.000e+00
Coefficient: −1.14e−01
Value: C
Thermodesulfobacteriaceae
4
3
2
1
0
A (n=14)
B (n=15)
C (n=11)
surface

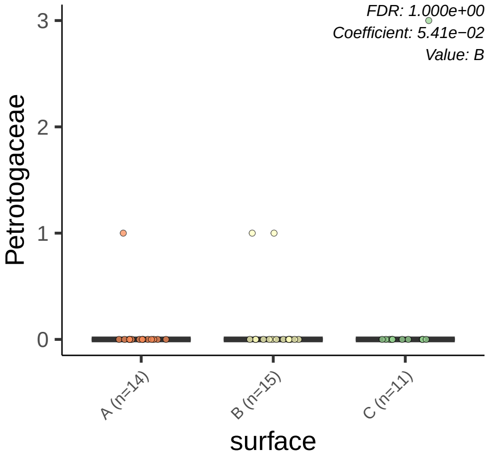

FDR: 1.000e+00
Coefficient: 5.41e−02
Value: B
Petrotogaceae
3
2
1
0
A (n=14)
B (n=15)
C (n=11)
surface

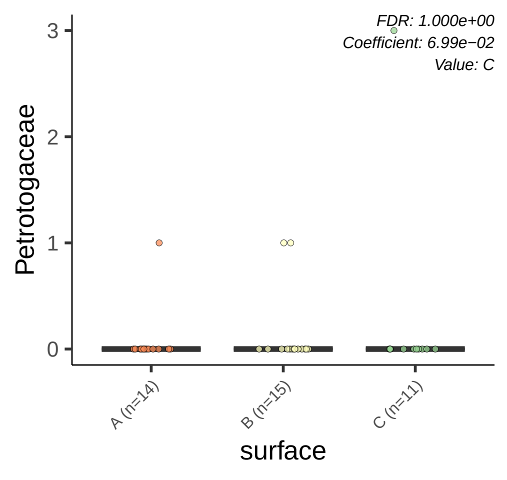
FDR: 1.000e+00
Coefficient: 6.99e−02
Value: C
Petrotogaceae
3
2
1
0
A (n=14)
B (n=15)
C (n=11)
surface

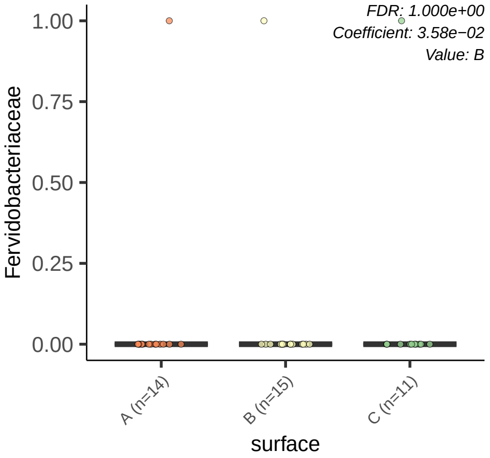

FDR: 1.000e+00
Coefficient: 3.58e−02
Value: B
Fervidobacteriaceae
1.00
0.75
0.50
0.25
0.00
A (n=14)
B (n=15)
C (n=11)
surface

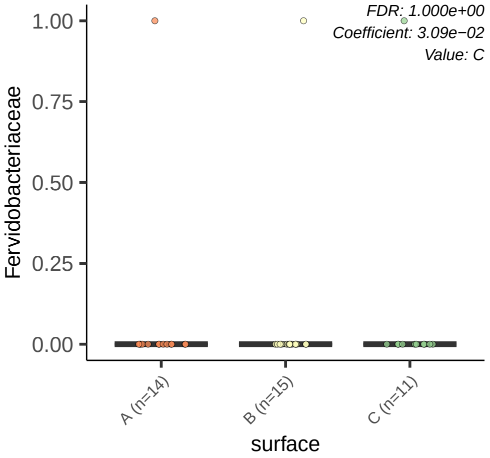
FDR: 1.000e+00
Coefficient: 3.09e−02
Value: C
Fervidobacteriaceae
1.00
0.75
0.50
0.25
0.00
A (n=14)
B (n=15)
C (n=11)
surface

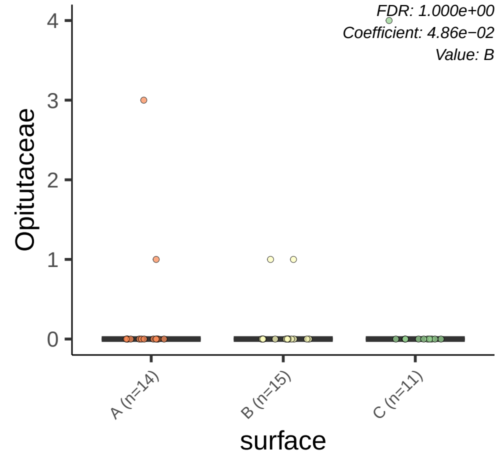

FDR: 1.000e+00
Coefficient: 4.86e−02
Value: B
Opitutaceae
4
3
2
1
0
A (n=14)
B (n=15)
C (n=11)
surface

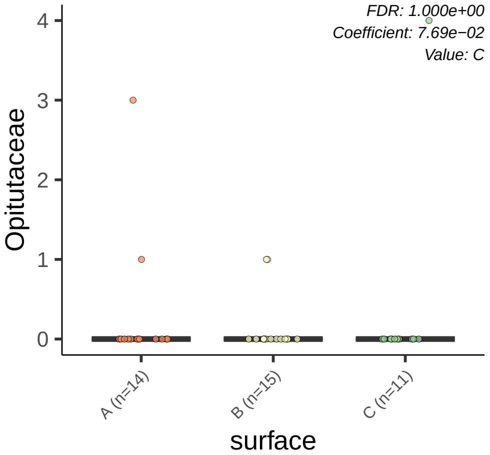

FDR: 1.000e+00
Coefficient: 7.69e−02
Value: C
Opitutaceae
0
1
2
3
4
A (n=14)
B (n=15)
C (n=11)
surface

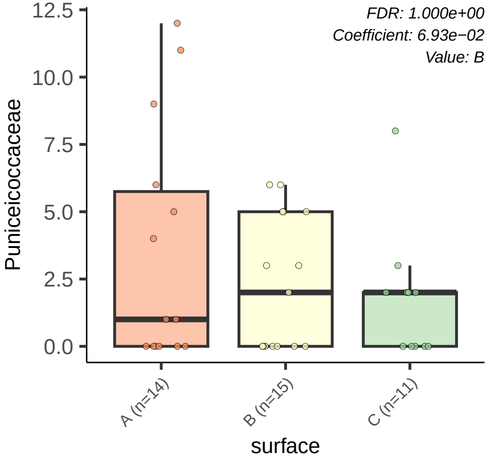

FDR: 1.000e+00
Coefficient: 6.93e−02
Value: B
Puniceicoccaceae
12.5
10.0
7.5
5.0
2.5
0.0
A (n=14)
B (n=15)
C (n=11)
surface

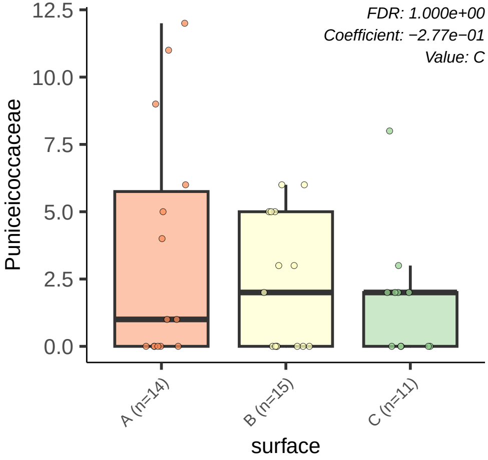

FDR: 1.000e+00
Coefficient: −2.77e−01
Value: C
Puniceicoccaceae
12.5
10.0
7.5
5.0
2.5
0.0
A (n=14)
B (n=15)
C (n=11)
surface

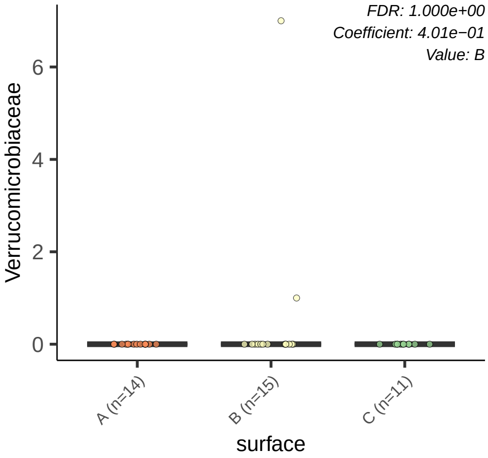

FDR: 1.000e+00
Coefficient: 4.01e−01
Value: B
Verrucomicrobiaceae
0
2
4
6
A (n=14)
B (n=15)
C (n=11)
surface

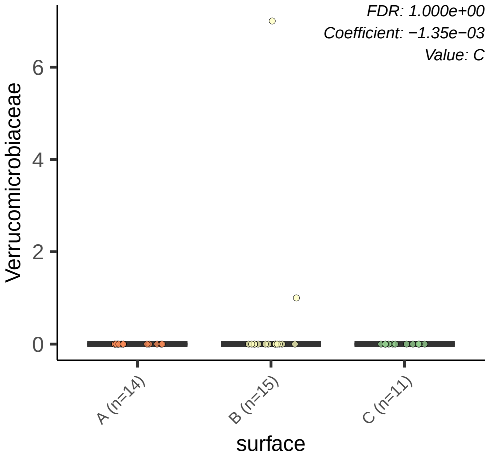
FDR: 1.000e+00
Coefficient: −1.35e−03
Value: C
Verrucomicrobiaceae
0
2
4
6
A (n=14)
B (n=15)
C (n=11)
surface

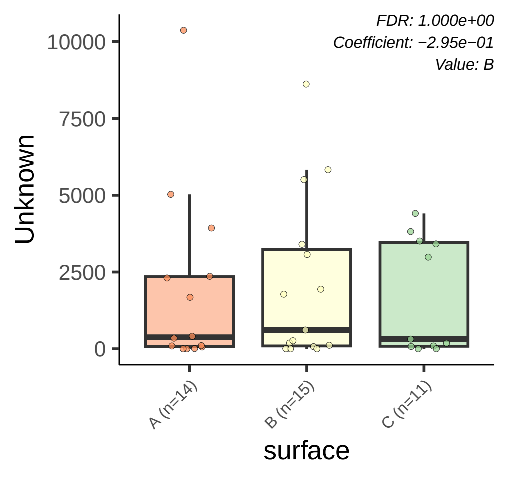
FDR: 1.000e+00
Coefficient: −2.95e−01
Value: B
Unknown
10000
7500
5000
2500
0
A (n=14)
B (n=15)
C (n=11)
surface

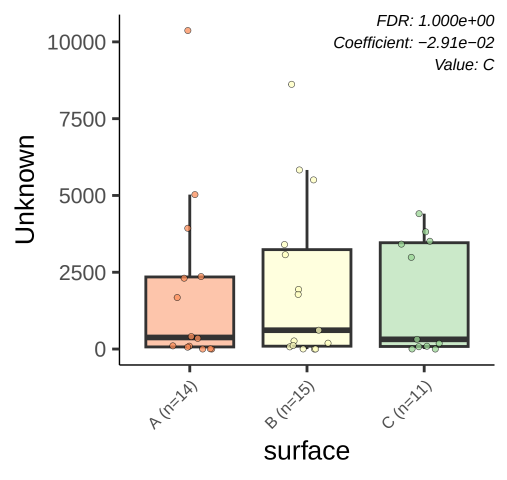
FDR: 1.000e+00
Coefficient: −2.91e−02
Value: C
Unknown
10000
7500
5000
2500
0
A (n=14)
B (n=15)
C (n=11)
surface

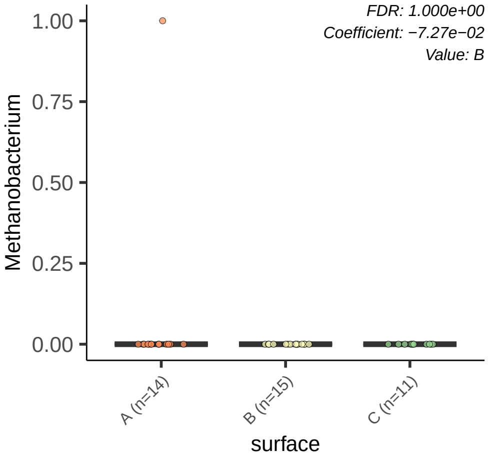

FDR: 1.000e+00
Coefficient: −7.27e−02
Value: B
Methanobacterium
1.00
0.75
0.50
0.25
0.00
A (n=14)
B (n=15)
C (n=11)
surface

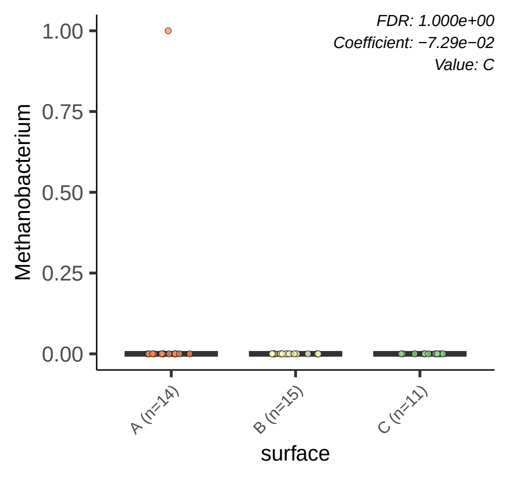
FDR: 1.000e+00
Coefficient: −7.29e−02
Value: C
Methanobacterium
1.00
0.75
0.50
0.25
0.00
A (n=14)
B (n=15)
C (n=11)
surface

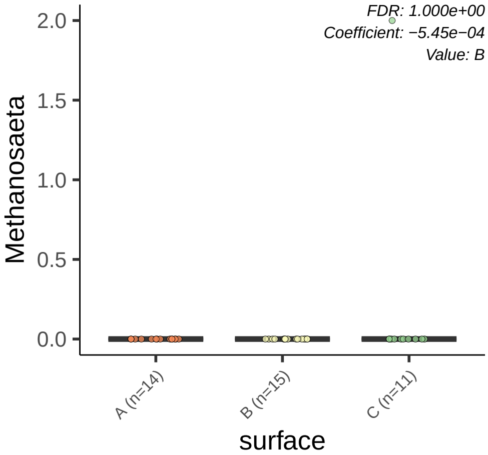

FDR: 1.000e+00
Coefficient: −5.45e−04
Value: B
Methanosaeta
2.0
1.5
1.0
0.5
0.0
A (n=14)
B (n=15)
C (n=11)
surface

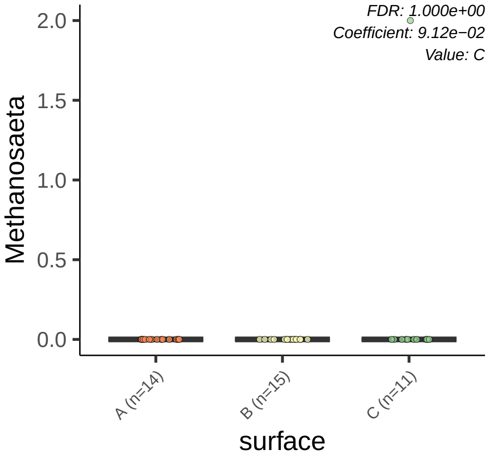

FDR: 1.000e+00
Coefficient: 9.12e−02
Value: C
Methanosaeta
2.0
1.5
1.0
0.5
0.0
A (n=14)
B (n=15)
C (n=11)
surface

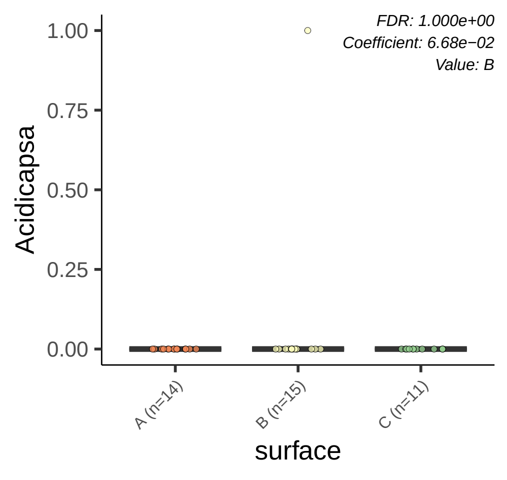
FDR: 1.000e+00
Coefficient: 6.68e−02
Value: B
Acidicapsa
1.00
0.75
0.50
0.25
0.00
A (n=14)
B (n=15)
C (n=11)
surface

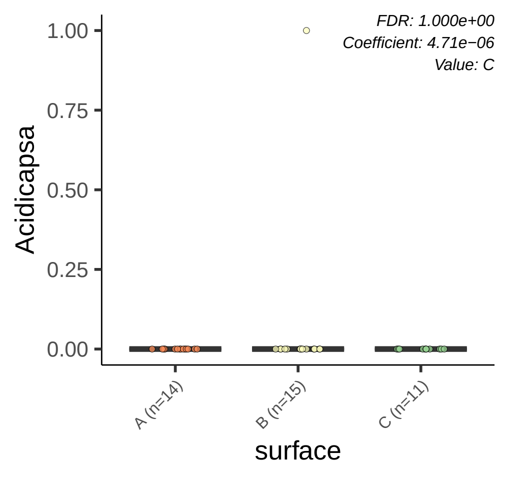
FDR: 1.000e+00
Coefficient: 4.71e−06
Value: C
Acidicapsa
1.00
0.75
0.50
0.25
0.00
A (n=14)
B (n=15)
C (n=11)
surface

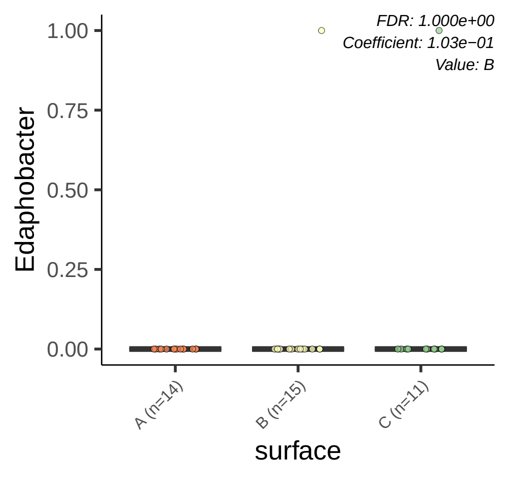

FDR: 1.000e+00
Coefficient: 1.03e−01
Value: B
Edaphobacter
1.00
0.75
0.50
0.25
0.00
A (n=14)
B (n=15)
C (n=11)
surface

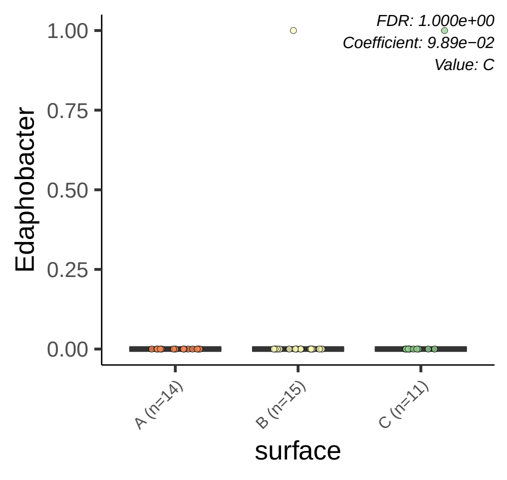
FDR: 1.000e+00
Coefficient: 9.89e−02
Value: C
Edaphobacter
1.00
0.75
0.50
0.25
0.00
A (n=14)
B (n=15)
C (n=11)
surface

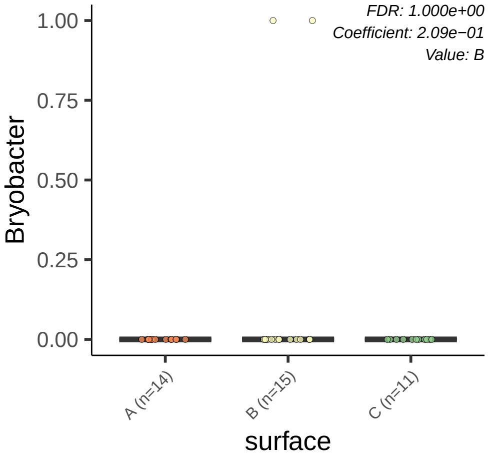

FDR: 1.000e+00
Coefficient: 2.09e−01
Value: B
Bryobacter
1.00
0.75
0.50
0.25
0.00
A (n=14)
B (n=15)
C (n=11)
surface

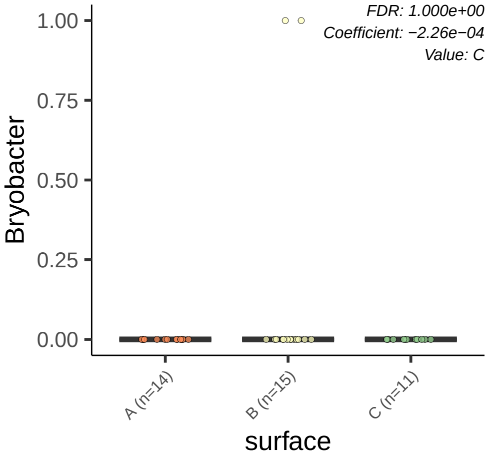

FDR: 1.000e+00
Coefficient: −2.26e−04
Value: C
Bryobacter
1.00
0.75
0.50
0.25
0.00
A (n=14)
B (n=15)
C (n=11)
surface

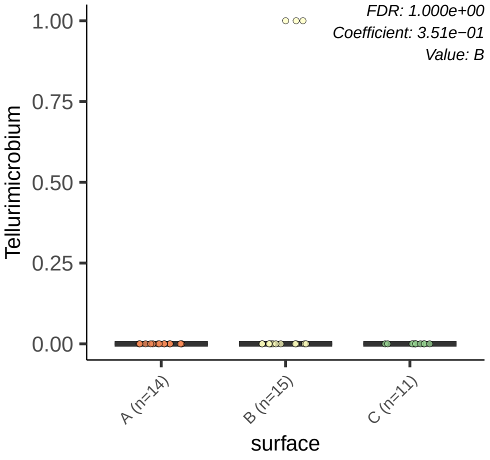
FDR: 1.000e+00
Coefficient: 3.51e−01
Value: B
Tellurimicrobium
1.00
0.75
0.50
0.25
0.00
A (n=14)
B (n=15)
C (n=11)
surface

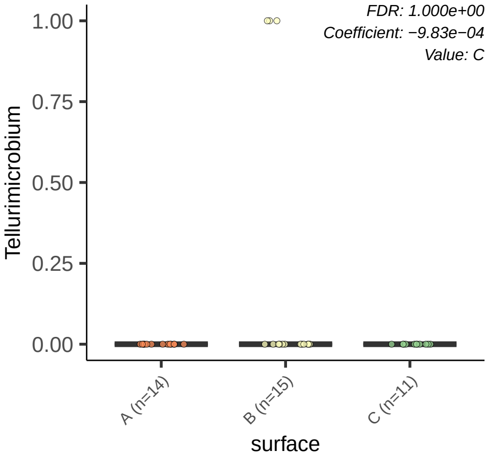
FDR: 1.000e+00
Coefficient: −9.83e−04
Value: C
Tellurimicrobium
1.00
0.75
0.50
0.25
0.00
A (n=14)
B (n=15)
C (n=11)
surface

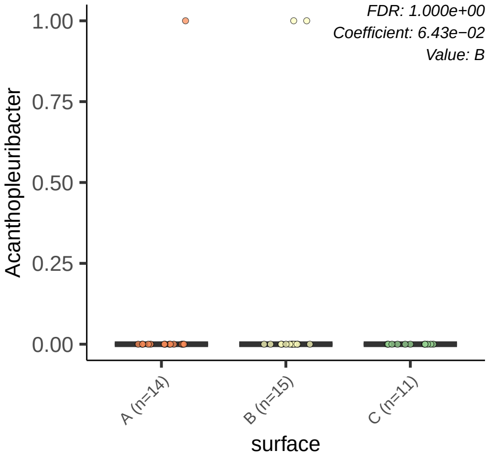

FDR: 1.000e+00
Coefficient: 6.43e−02
Value: B
Acanthopleuribacter
1.00
0.75
0.50
0.25
0.00
A (n=14)
B (n=15)
C (n=11)
surface

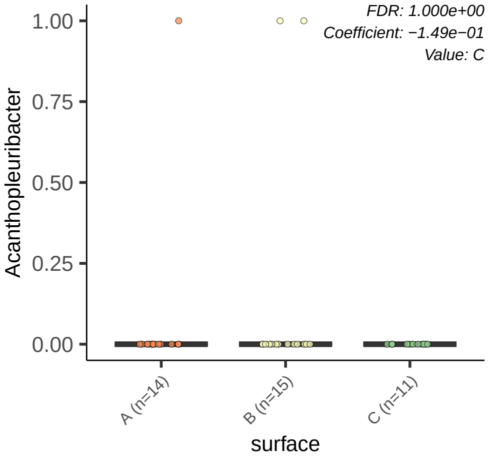
FDR: 1.000e+00
Coefficient: −1.49e−01
Value: C
Acanthopleuribacter
1.00
0.75
0.50
0.25
0.00
A (n=14)
B (n=15)
C (n=11)
surface

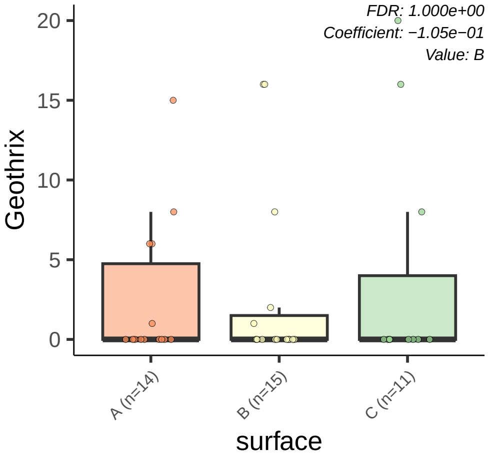
FDR: 1.000e+00
Coefficient: −1.05e−01
Value: B
Geothrix
0
5
10
15
20
A (n=14)
B (n=15)
C (n=11)
surface

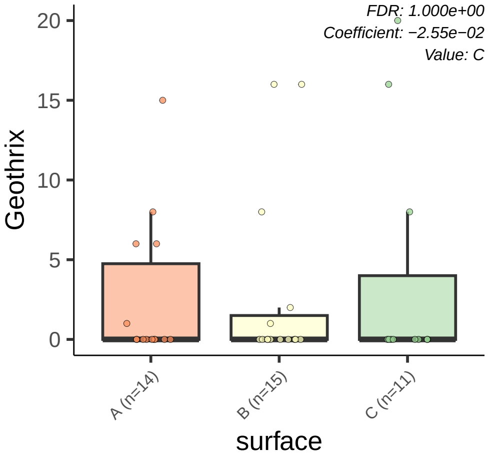

FDR: 1.000e+00
Coefficient: −2.55e−02
Value: C
Geothrix
0
5
10
15
20
A (n=14)
B (n=15)
C (n=11)
surface

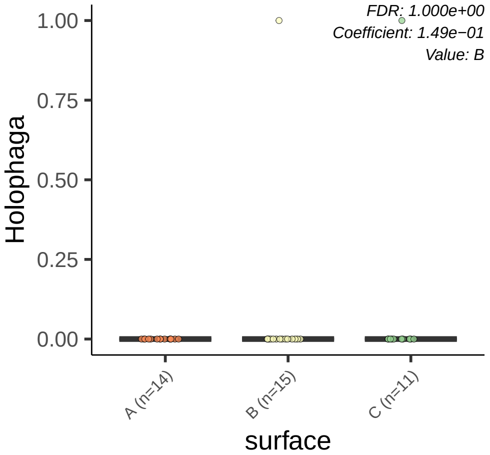
FDR: 1.000e+00
Coefficient: 1.49e−01
Value: B
Holophaga
1.00
0.75
0.50
0.25
0.00
A (n=14)
B (n=15)
C (n=11)
surface

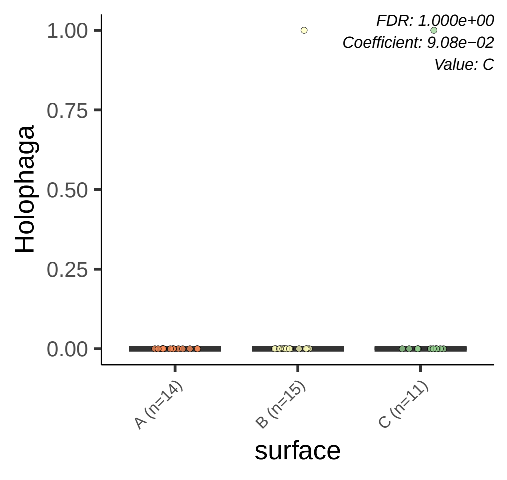
FDR: 1.000e+00
Coefficient: 9.08e−02
Value: C
Holophaga
1.00
0.75
0.50
0.25
0.00
A (n=14)
B (n=15)
C (n=11)
surface

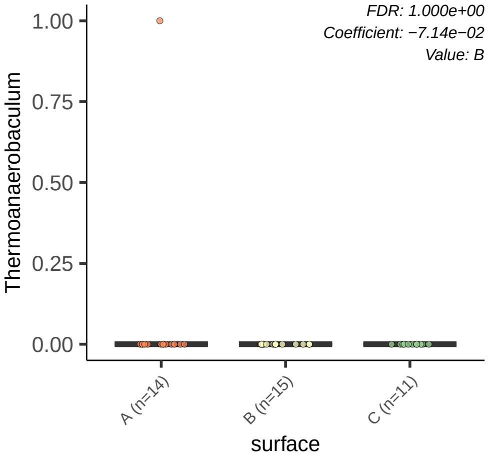
FDR: 1.000e+00
Coefficient: −7.14e−02
Value: B
Thermoanaerobaculum
1.00
0.75
0.50
0.25
0.00
A (n=14)
B (n=15)
C (n=11)
surface

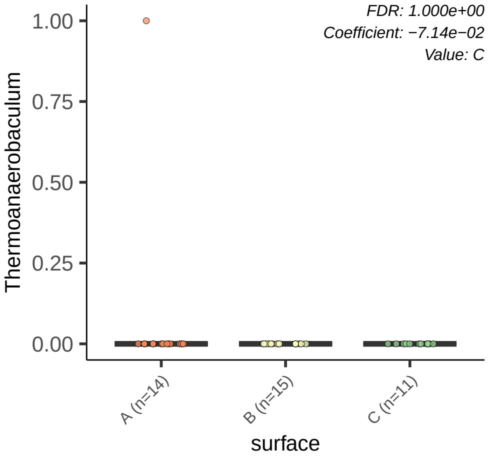
FDR: 1.000e+00
Coefficient: −7.14e−02
Value: C
Thermoanaerobaculum
1.00
0.75
0.50
0.25
0.00
A (n=14)
B (n=15)
C (n=11)
surface

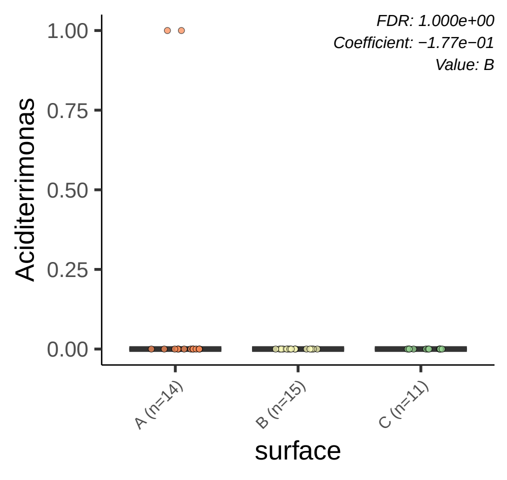
FDR: 1.000e+00
Coefficient: −1.77e−01
Value: B
Aciditerrimonas
1.00
0.75
0.50
0.25
0.00
A (n=14)
B (n=15)
C (n=11)
surface

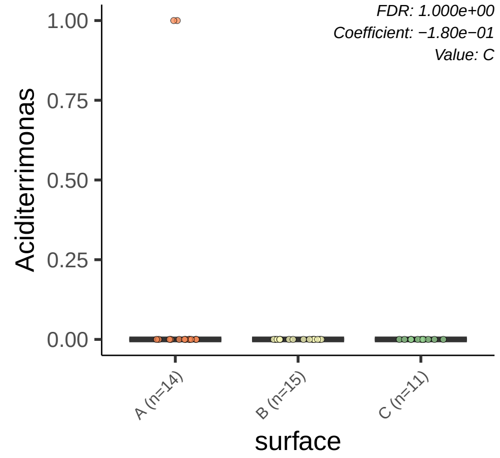

FDR: 1.000e+00
Coefficient: −1.80e−01
Value: C
Aciditerrimonas
1.00
0.75
0.50
0.25
0.00
A (n=14)
B (n=15)
C (n=11)
surface

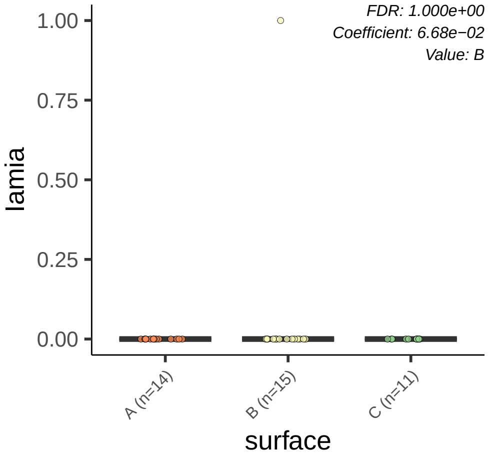

FDR: 1.000e+00
Coefficient: 6.68e−02
Value: B
lamia
1.00
0.75
0.50
0.25
0.00
A (n=14)
B (n=15)
C (n=11)
surface

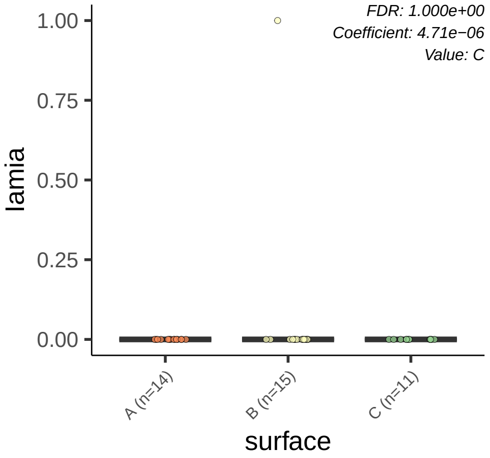
FDR: 1.000e+00
Coefficient: 4.71e−06
Value: C
lamia
1.00
0.75
0.50
0.25
0.00
A (n=14)
B (n=15)
C (n=11)
surface

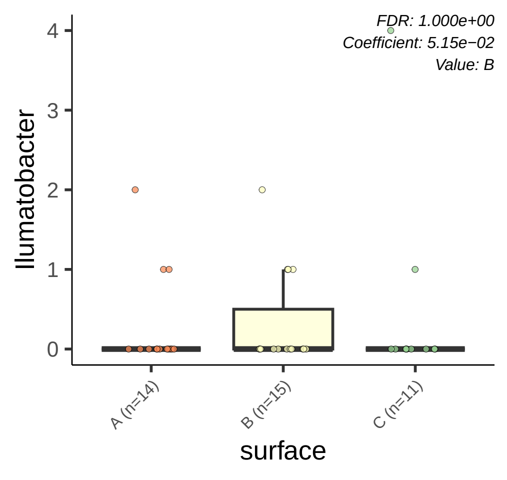
FDR: 1.000e+00
Coefficient: 5.15e−02
Value: B
Ilumatobacter
0
1
2
3
4
A (n=14)
B (n=15)
C (n=11)
surface

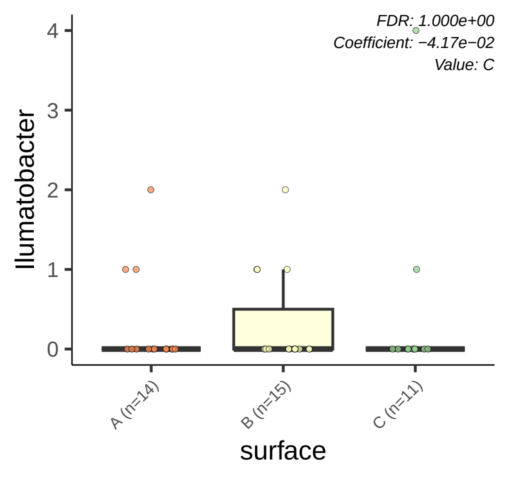

FDR: 1.000e+00
Coefficient: −4.17e−02
Value: C
Ilumatobacter
0
1
2
3
4
A (n=14)
B (n=15)
C (n=11)
surface

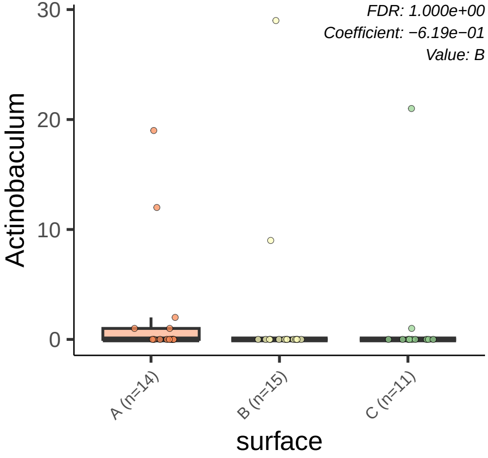

FDR: 1.000e+00
Coefficient: −6.19e−01
Value: B
Actinobaculum
30
20
10
0
A (n=14)
B (n=15)
C (n=11)
surface

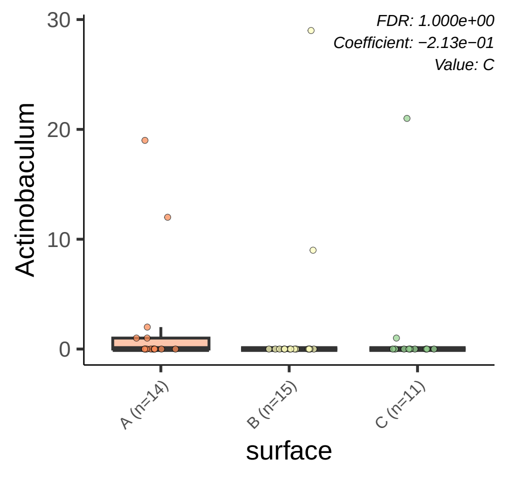
FDR: 1.000e+00
Coefficient: −2.13e−01
Value: C
Actinobaculum
30
20
10
0
A (n=14)
B (n=15)
C (n=11)
surface

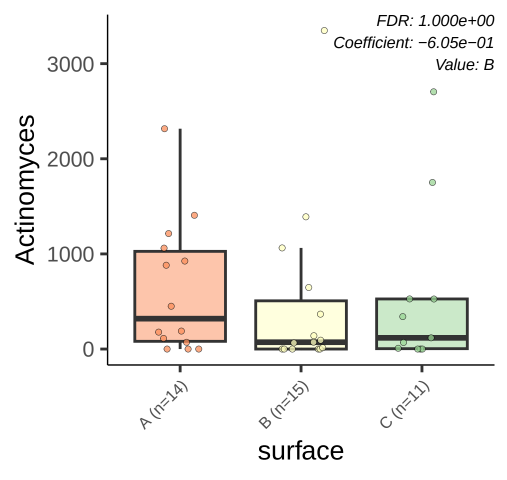

FDR: 1.000e+00
Coefficient: −6.05e−01
Value: B
Actinomyces
3000
2000
1000
0
A (n=14)
B (n=15)
C (n=11)
surface

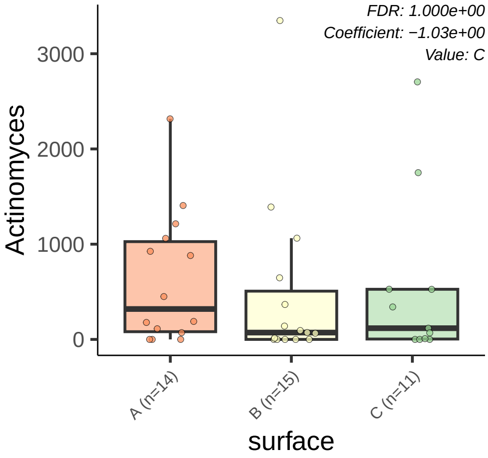
FDR: 1.000e+00
Coefficient: −1.03e+00
Value: C
Actinomyces
3000
2000
1000
0
A (n=14)
B (n=15)
C (n=11)
surface

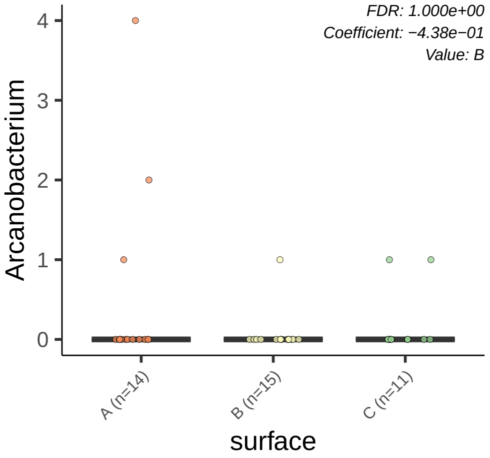
FDR: 1.000e+00
Coefficient: −4.38e−01
Value: B
Arcanobacterium
0
1
2
3
4
A (n=14)
B (n=15)
C (n=11)
surface

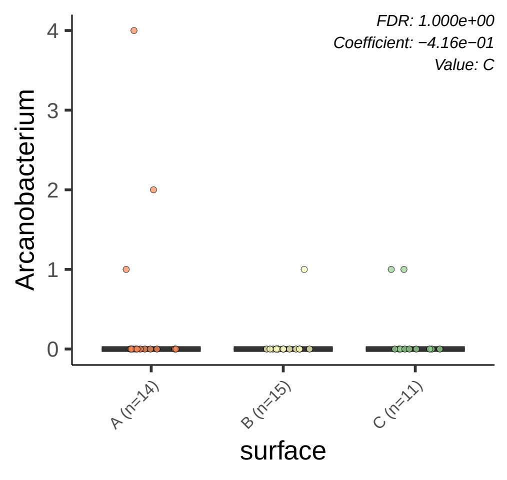
FDR: 1.000e+00
Coefficient: −4.16e−01
Value: C
Arcanobacterium
0
1
2
3
4
A (n=14)
B (n=15)
C (n=11)
surface

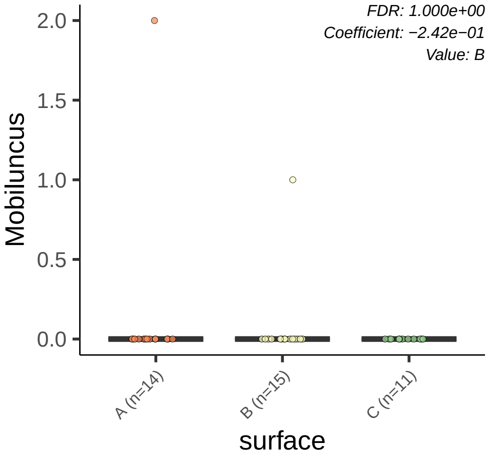

FDR: 1.000e+00
Coefficient: −2.42e−01
Value: B
Mobiluncus
2.0
1.5
1.0
0.5
0.0
A (n=14)
B (n=15)
C (n=11)
surface

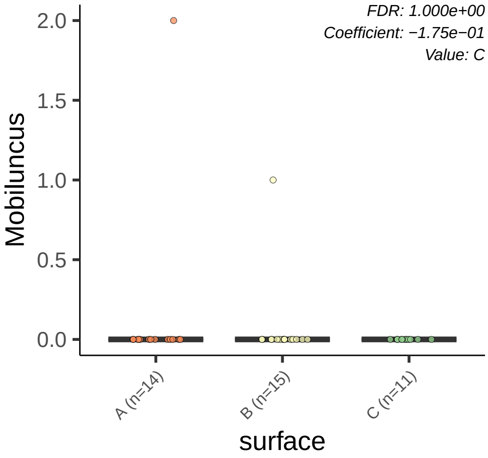
FDR: 1.000e+00
Coefficient: −1.75e−01
Value: C
Mobiluncus
2.0
1.5
1.0
0.5
0.0
A (n=14)
B (n=15)
C (n=11)
surface

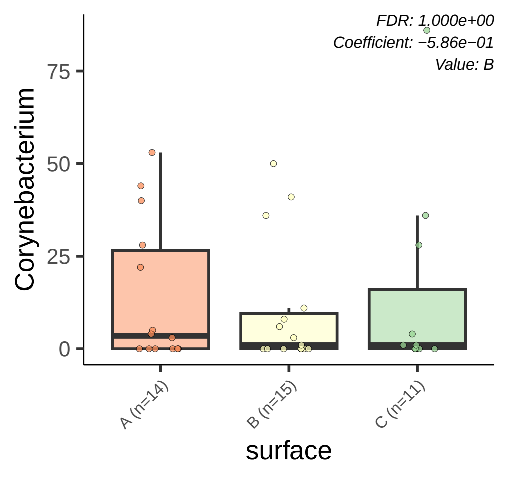

FDR: 1.000e+00
Coefficient: −5.86e−01
Value: B
Corynebacterium
0
25
50
75
A (n=14)
B (n=15)
C (n=11)
surface

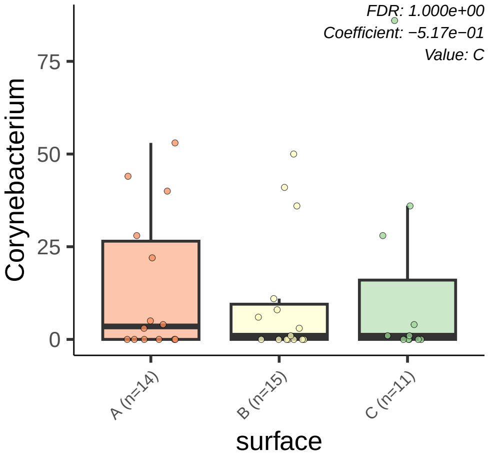

FDR: 1.000e+00
Coefficient: −5.17e−01
Value: C
Corynebacterium
75
50
25
0
A (n=14)
B (n=15)
C (n=11)
surface

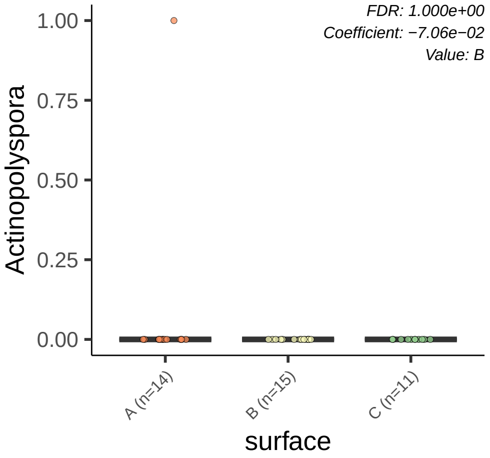
FDR: 1.000e+00
Coefficient: −7.06e−02
Value: B
Actinopolyspora
1.00
0.75
0.50
0.25
0.00
A (n=14)
B (n=15)
C (n=11)
surface

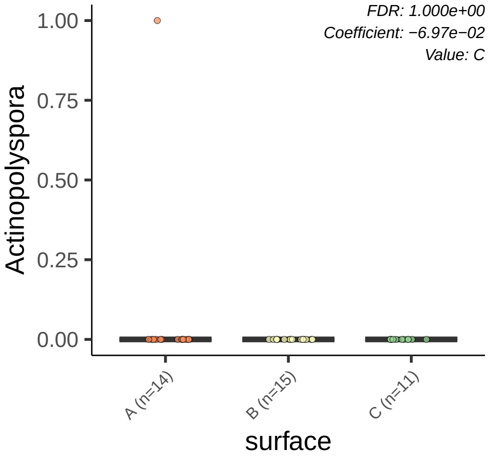

FDR: 1.000e+00
Coefficient: −6.97e−02
Value: C
Actinopolyspora
1.00
0.75
0.50
0.25
0.00
A (n=14)
B (n=15)
C (n=11)
surface

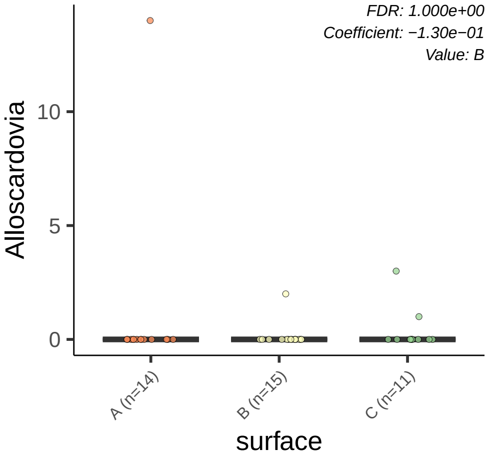
FDR: 1.000e+00
Coefficient: −1.30e−01
Value: B
Alloscardovia
10
5
0
A (n=14)
B (n=15)
C (n=11)
surface

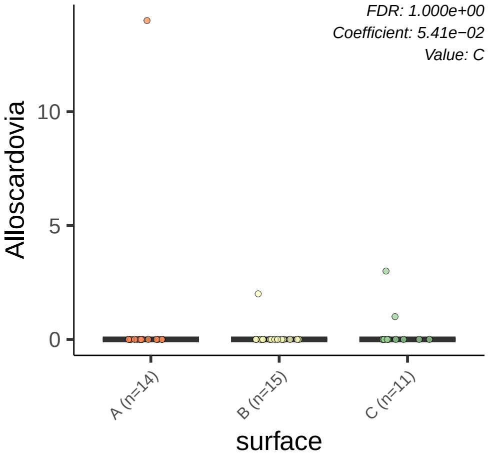
FDR: 1.000e+00
Coefficient: 5.41e−02
Value: C
Alloscardovia
0
5
10
A (n=14)
B (n=15)
C (n=11)
surface

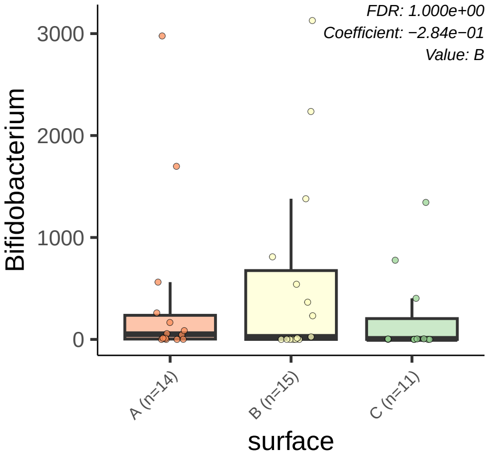

FDR: 1.000e+00
Coefficient: −2.84e−01
Value: B
Bifidobacterium
3000
2000
1000
0
A (n=14)
B (n=15)
C (n=11)
surface

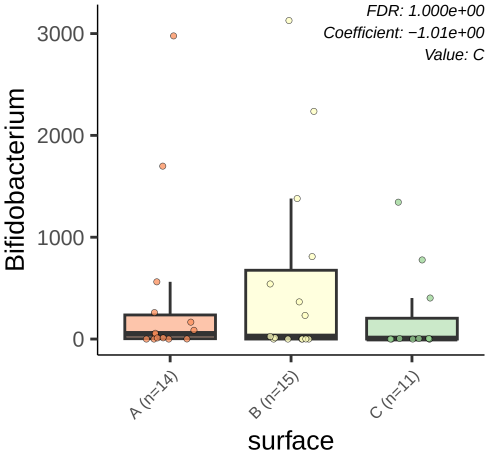
FDR: 1.000e+00
Coefficient: −1.01e+00
Value: C
Bifidobacterium
3000
2000
1000
0
A (n=14)
B (n=15)
C (n=11)
surface

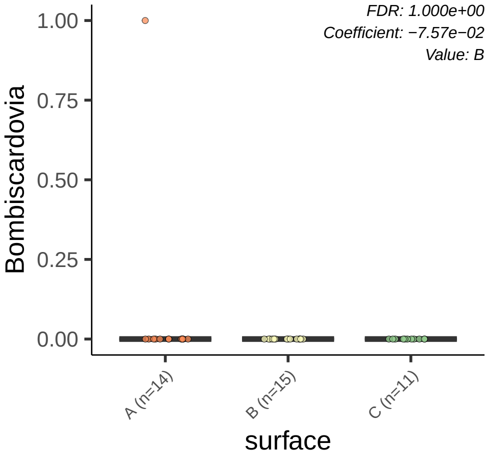
FDR: 1.000e+00
Coefficient: −7.57e−02
Value: B
Bombiscardovia
1.00
0.75
0.50
0.25
0.00
A (n=14)
B (n=15)
C (n=11)
surface

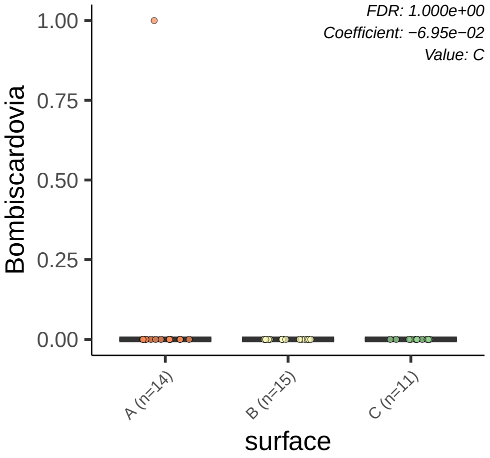
FDR: 1.000e+00
Coefficient: −6.95e−02
Value: C
Bombiscardovia
1.00
0.75
0.50
0.25
0.00
A (n=14)
B (n=15)
C (n=11)
surface

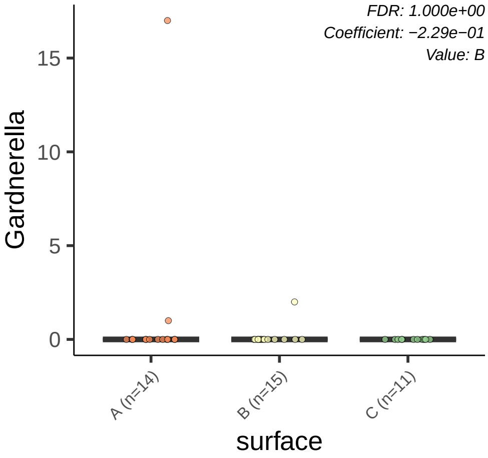
FDR: 1.000e+00
Coefficient: −2.29e−01
Value: B
Gardnerella
0
5
10
15
A (n=14)
B (n=15)
C (n=11)
surface

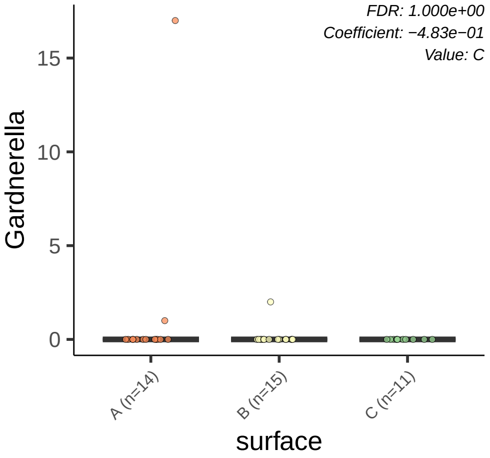
FDR: 1.000e+00
Coefficient: −4.83e−01
Value: C
Gardnerella
15
10
5
0
A (n=14)
B (n=15)
C (n=11)
surface

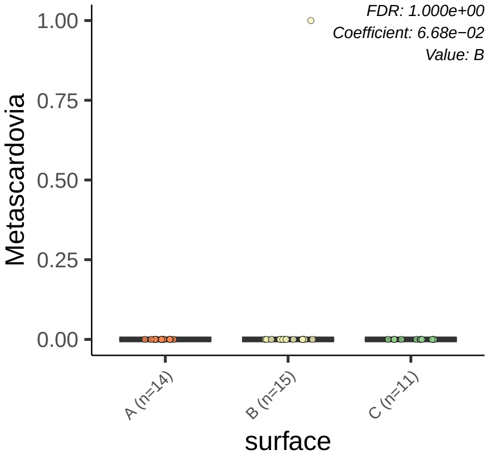
FDR: 1.000e+00
Coefficient: 6.68e−02
Value: B
Metascardovia
1.00
0.75
0.50
0.25
0.00
A (n=14)
B (n=15)
C (n=11)
surface

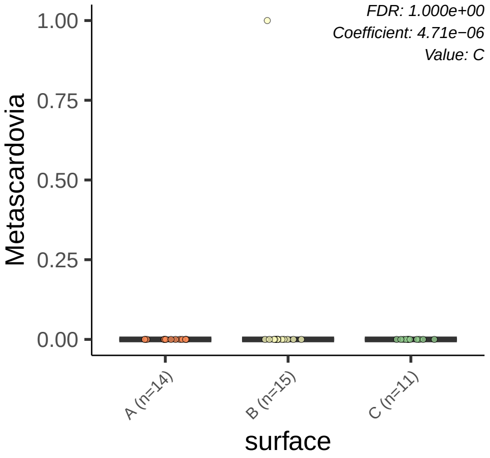
FDR: 1.000e+00
Coefficient: 4.71e−06
Value: C
Metascardovia
1.00
0.75
0.50
0.25
0.00
A (n=14)
B (n=15)
C (n=11)
surface

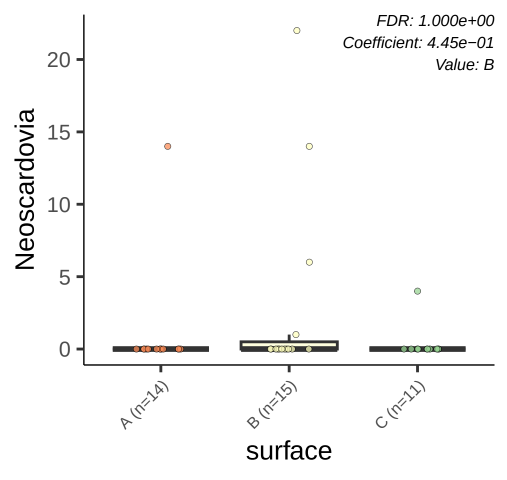

FDR: 1.000e+00
Coefficient: 4.45e−01
Value: B
Neoscardovia
0
5
10
15
20
A (n=14)
B (n=15)
C (n=11)
surface

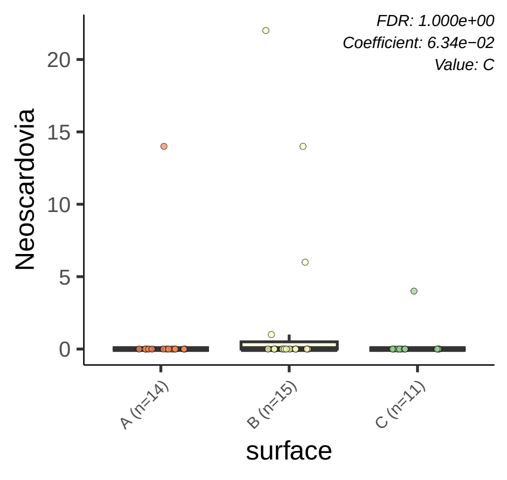

FDR: 1.000e+00
Coefficient: 6.34e−02
Value: C
Neoscardovia
20
15
10
5
0
A (n=14)
B (n=15)
C (n=11)
surface

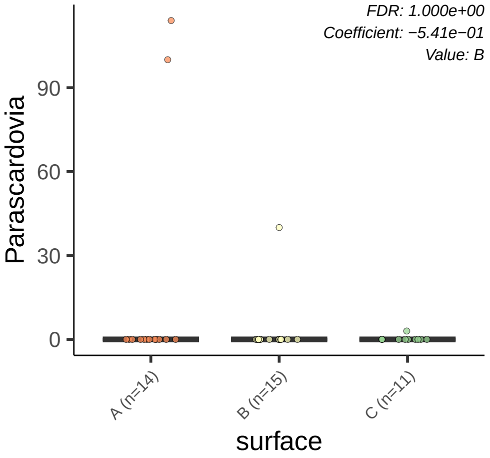
FDR: 1.000e+00
Coefficient: −5.41e−01
Value: B
Parascardovia
90
60
30
0
A (n=14)
B (n=15)
C (n=11)
surface

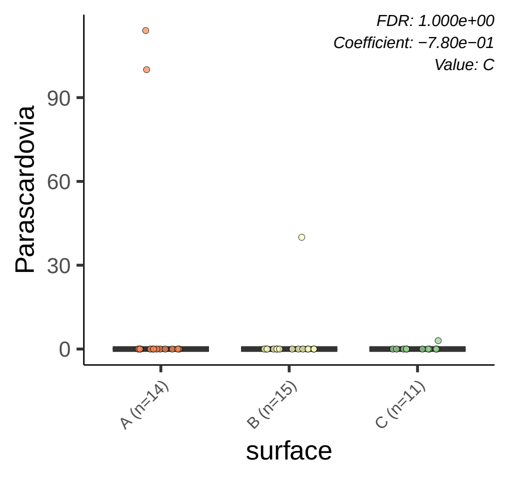
FDR: 1.000e+00
Coefficient: −7.80e−01
Value: C
Parascardovia
90
60
30
0
A (n=14)
B (n=15)
C (n=11)
surface

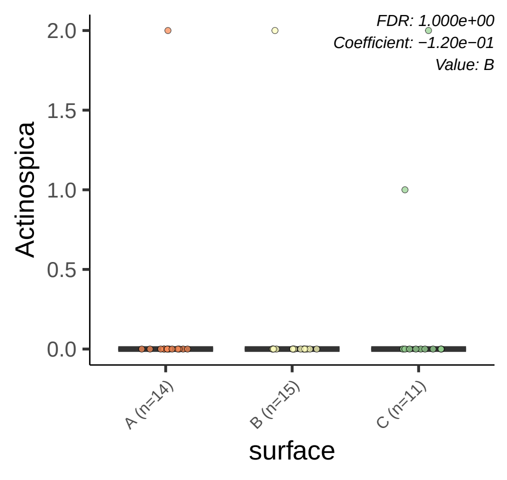

FDR: 1.000e+00
Coefficient: −1.20e−01
Value: B
Actinospica
2.0
1.5
1.0
0.5
0.0
A (n=14)
B (n=15)
C (n=11)
surface

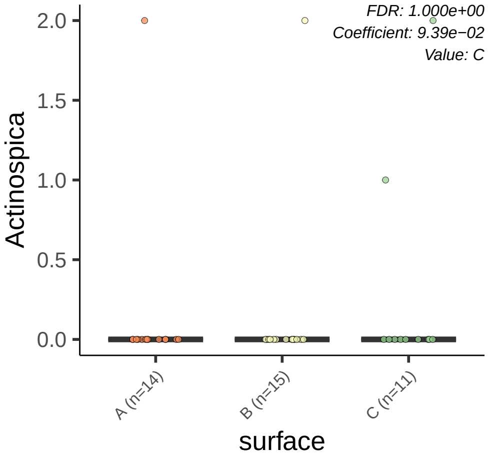
FDR: 1.000e+00
Coefficient: 9.39e−02
Value: C
Actinospica
2.0
1.5
1.0
0.5
0.0
A (n=14)
B (n=15)
C (n=11)
surface

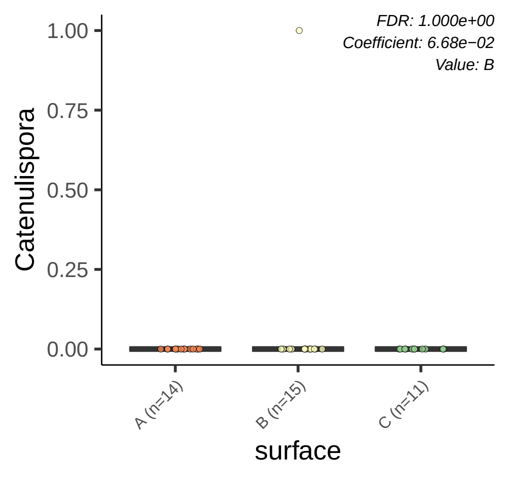
FDR: 1.000e+00
Coefficient: 6.68e−02
Value: B
Catenulispora
1.00
0.75
0.50
0.25
0.00
A (n=14)
B (n=15)
C (n=11)
surface

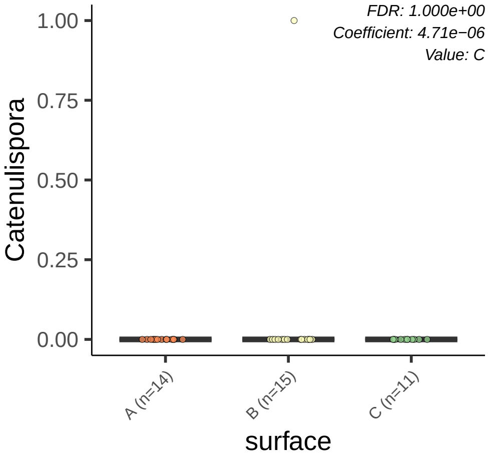
FDR: 1.000e+00
Coefficient: 4.71e−06
Value: C
Catenulispora
1.00
0.75
0.50
0.25
0.00
A (n=14)
B (n=15)
C (n=11)
surface

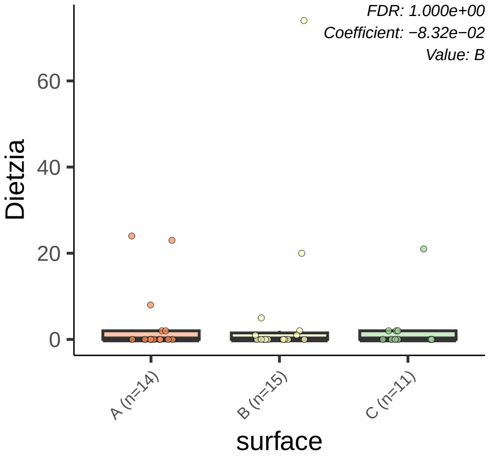
FDR: 1.000e+00
Coefficient: −8.32e−02
Value: B
Dietzia
0
20
40
60
A (n=14)
B (n=15)
C (n=11)
surface

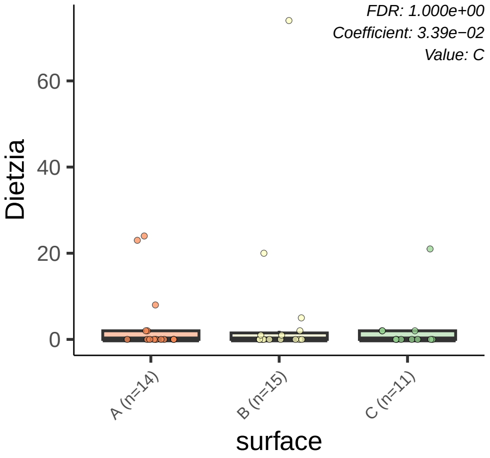
FDR: 1.000e+00
Coefficient: 3.39e−02
Value: C
Dietzia
0
20
40
60
A (n=14)
B (n=15)
C (n=11)
surface

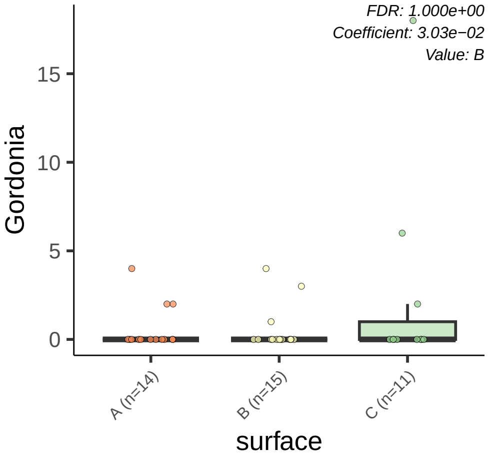
FDR: 1.000e+00
Coefficient: 3.03e−02
Value: B
Gordonia
15
10
5
0
A (n=14)
B (n=15)
C (n=11)
surface

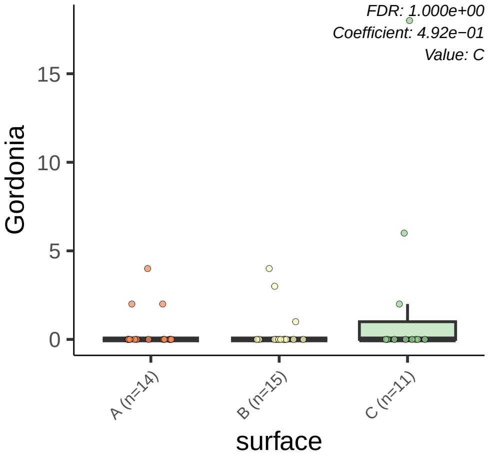

FDR: 1.000e+00
Coefficient: 4.92e−01
Value: C
Gordonia
0
5
10
15
A (n=14)
B (n=15)
C (n=11)
surface

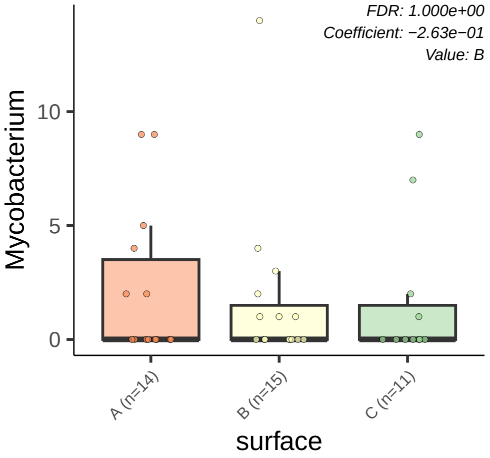
FDR: 1.000e+00
Coefficient: −2.63e−01
Value: B
Mycobacterium
10
5
0
A (n=14)
B (n=15)
C (n=11)
surface

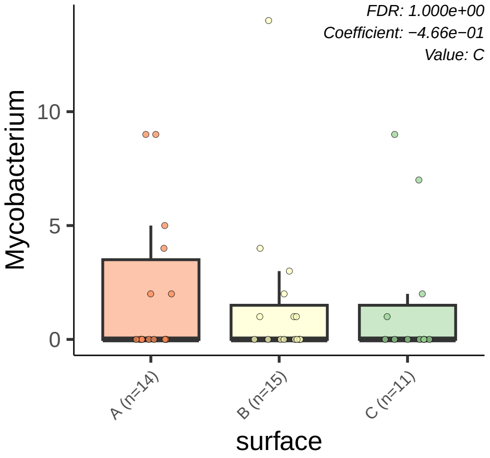
FDR: 1.000e+00
Coefficient: −4.66e−01
Value: C
Mycobacterium
10
5
0
A (n=14)
B (n=15)
C (n=11)
surface

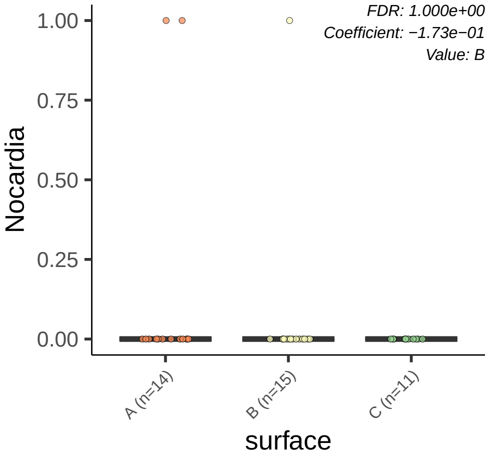
FDR: 1.000e+00
Coefficient: −1.73e−01
Value: B
Nocardia
1.00
0.75
0.50
0.25
0.00
A (n=14)
B (n=15)
C (n=11)
surface

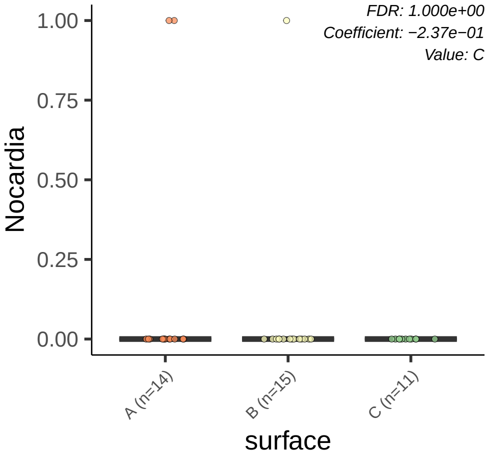
FDR: 1.000e+00
Coefficient: −2.37e−01
Value: C
Nocardia
1.00
0.75
0.50
0.25
0.00
A (n=14)
B (n=15)
C (n=11)
surface

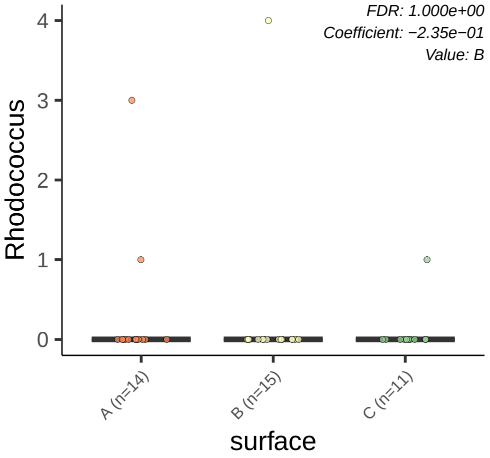
FDR: 1.000e+00
Coefficient: −2.35e−01
Value: B
Rhodococcus
0
1
2
3
4
A (n=14)
B (n=15)
C (n=11)
surface

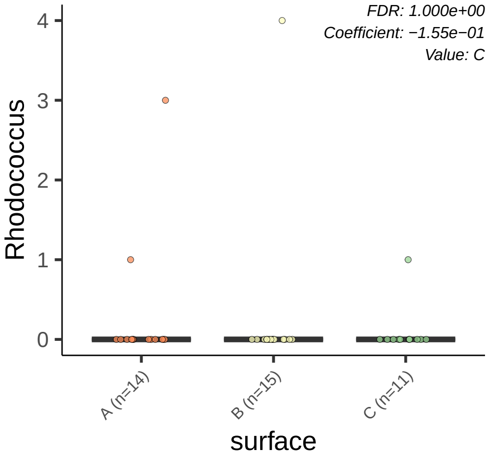
FDR: 1.000e+00
Coefficient: −1.55e−01
Value: C
Rhodococcus
0
1
2
3
4
A (n=14)
B (n=15)
C (n=11)
surface

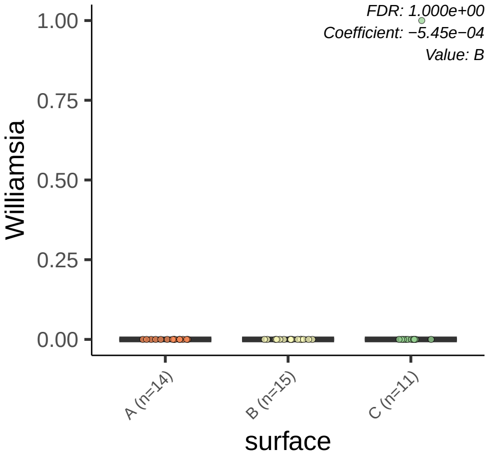
FDR: 1.000e+00
Coefficient: −5.45e−04
Value: B
Williamsia
1.00
0.75
0.50
0.25
0.00
A (n=14)
B (n=15)
C (n=11)
surface

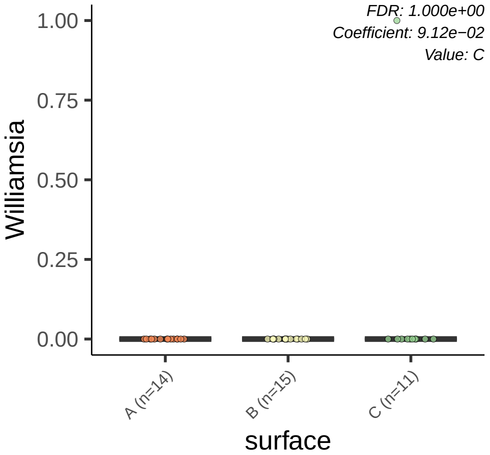
FDR: 1.000e+00
Coefficient: 9.12e−02
Value: C
Williamsia
1.00
0.75
0.50
0.25
0.00
A (n=14)
B (n=15)
C (n=11)
surface

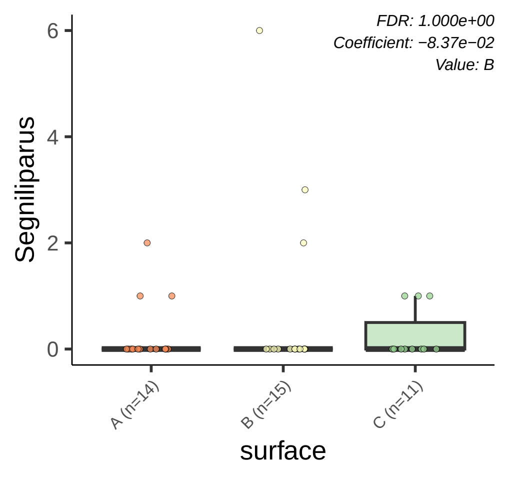

FDR: 1.000e+00
Coefficient: −8.37e−02
Value: B
Segniliparus
0
2
4
6
A (n=14)
B (n=15)
C (n=11)
surface

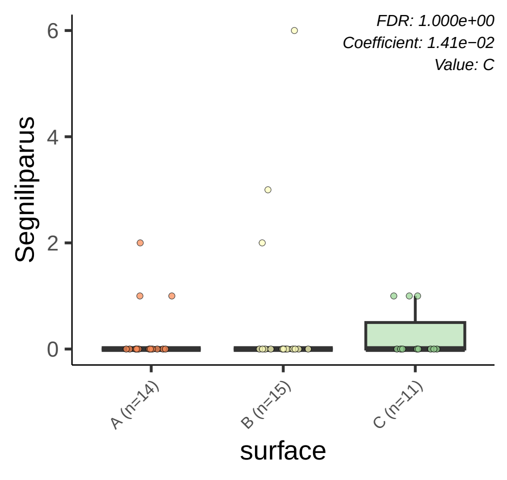
FDR: 1.000e+00
Coefficient: 1.41e−02
Value: C
Segniliparus
0
2
4
6
A (n=14)
B (n=15)
C (n=11)
surface

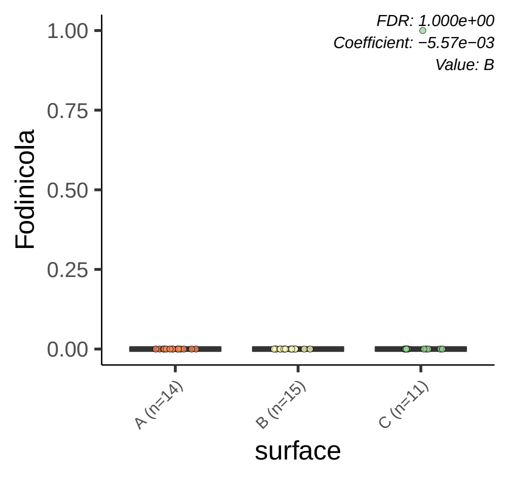

FDR: 1.000e+00
Coefficient: −5.57e−03
Value: B
Fodinicola
1.00
0.75
0.50
0.25
0.00
A (n=14)
B (n=15)
C (n=11)
surface

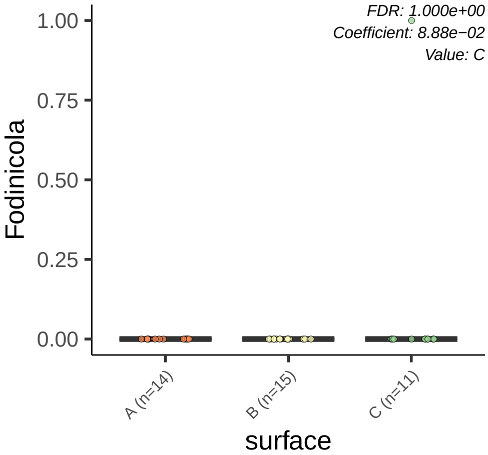

FDR: 1.000e+00
Coefficient: 8.88e−02
Value: C
Fodinicola
1.00
0.75
0.50
0.25
0.00
A (n=14)
B (n=15)
C (n=11)
surface

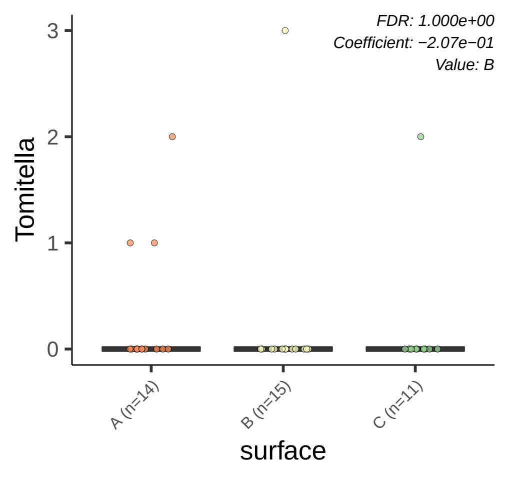
FDR: 1.000e+00
Coefficient: −2.07e−01
Value: B
Tomitella
3
2
1
0
A (n=14)
B (n=15)
C (n=11)
surface

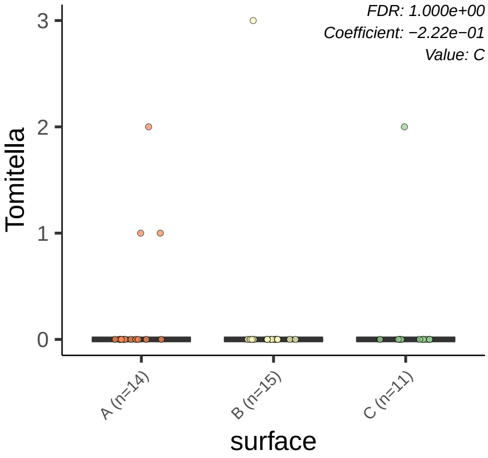
FDR: 1.000e+00
Coefficient: −2.22e−01
Value: C
Tomitella
3
2
1
0
A (n=14)
B (n=15)
C (n=11)
surface

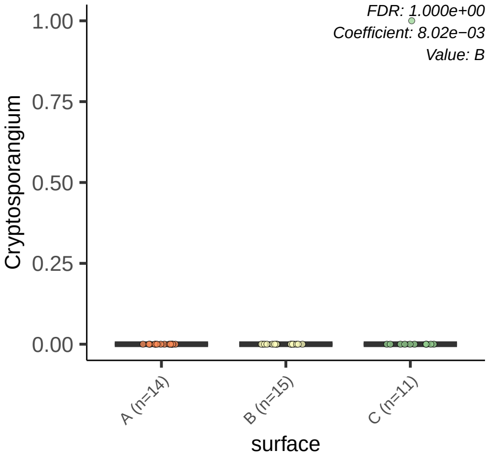

FDR: 1.000e+00
Coefficient: 8.02e−03
Value: B
Cryptosporangium
1.00
0.75
0.50
0.25
0.00
A (n=14)
B (n=15)
C (n=11)
surface

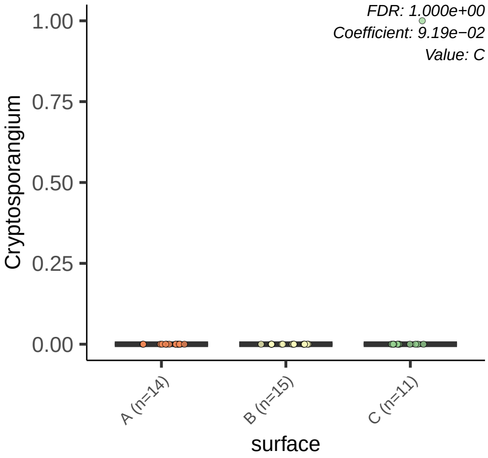

FDR: 1.000e+00
Coefficient: 9.19e−02
Value: C
Cryptosporangium
1.00
0.75
0.50
0.25
0.00
A (n=14)
B (n=15)
C (n=11)
surface

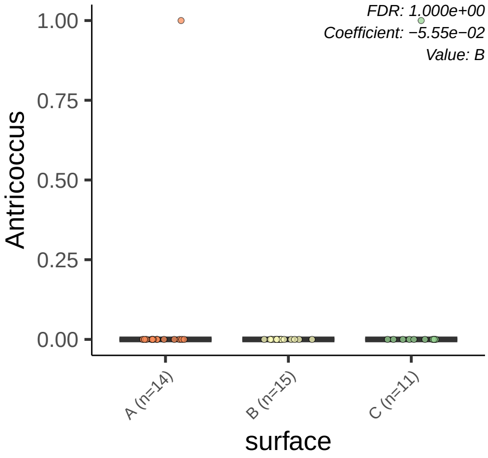

FDR: 1.000e+00
Coefficient: −5.55e−02
Value: B
Antricoccus
1.00
0.75
0.50
0.25
0.00
A (n=14)
B (n=15)
C (n=11)
surface

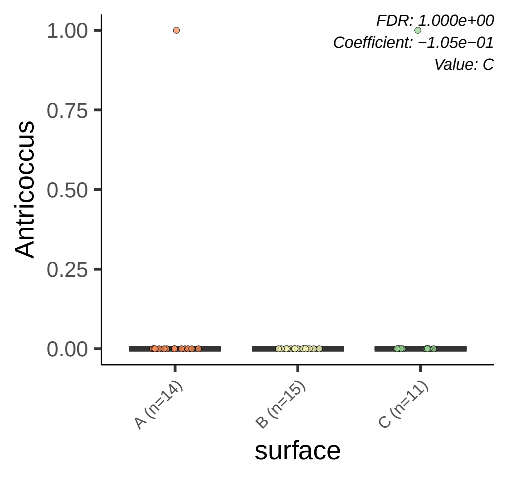
FDR: 1.000e+00
Coefficient: −1.05e−01
Value: C
Antricoccus
1.00
0.75
0.50
0.25
0.00
A (n=14)
B (n=15)
C (n=11)
surface

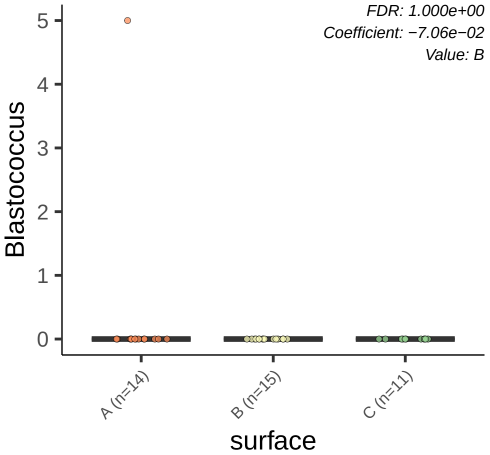
FDR: 1.000e+00
Coefficient: −7.06e−02
Value: B
Blastococcus
0
1
2
3
4
5
A (n=14)
B (n=15)
C (n=11)
surface

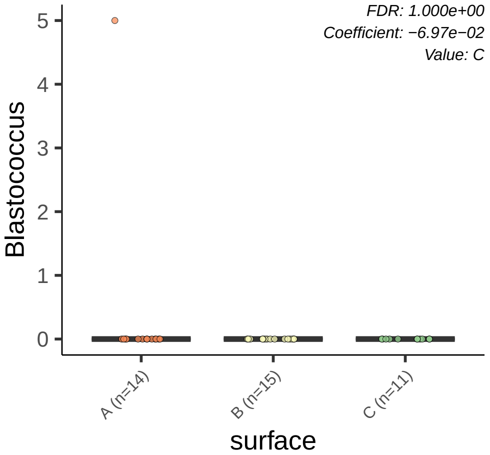
FDR: 1.000e+00
Coefficient: −6.97e−02
Value: C
Blastococcus
0
1
2
3
4
5
A (n=14)
B (n=15)
C (n=11)
surface

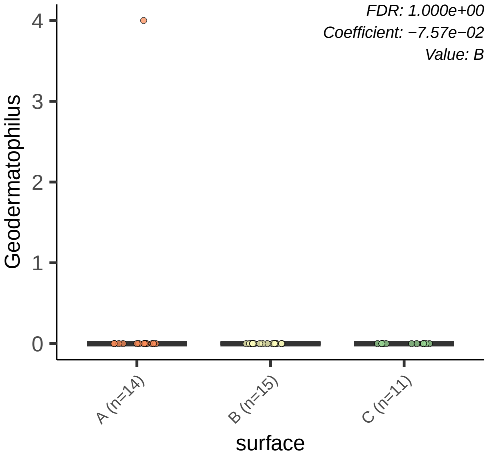
FDR: 1.000e+00
Coefficient: −7.57e−02
Value: B
Geodermatophilus
0
1
2
3
4
A (n=14)
B (n=15)
C (n=11)
surface

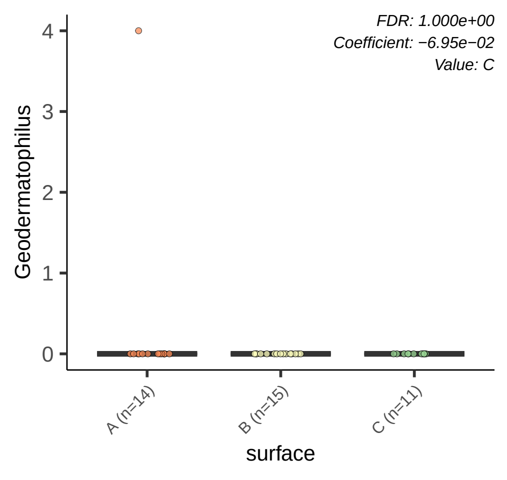

FDR: 1.000e+00
Coefficient: −6.95e−02
Value: C
Geodermatophilus
0
1
2
3
4
A (n=14)
B (n=15)
C (n=11)
surface

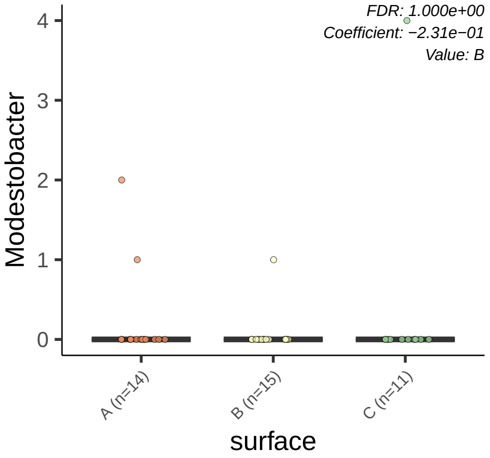
FDR: 1.000e+00
Coefficient: −2.31e−01
Value: B
Modestobacter
0
1
2
3
4
A (n=14)
B (n=15)
C (n=11)
surface

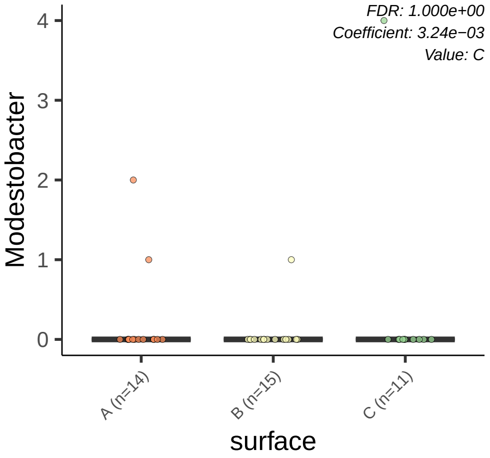

FDR: 1.000e+00
Coefficient: 3.24e−03
Value: C
Modestobacter
0
1
2
3
4
A (n=14)
B (n=15)
C (n=11)
surface

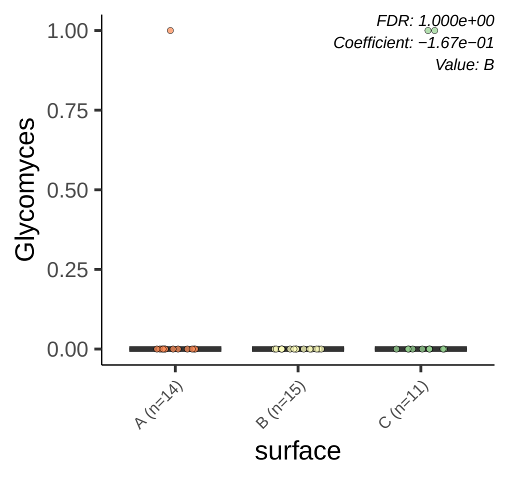

FDR: 1.000e+00
Coefficient: −1.67e−01
Value: B
Glycomyces
1.00
0.75
0.50
0.25
0.00
A (n=14)
B (n=15)
C (n=11)
surface

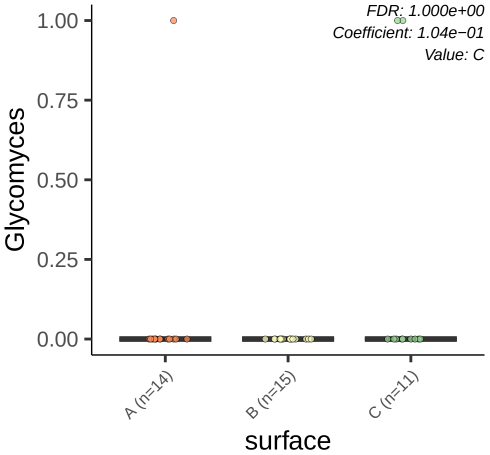
FDR: 1.000e+00
Coefficient: 1.04e−01
Value: C
Glycomyces
1.00
0.75
0.50
0.25
0.00
A (n=14)
B (n=15)
C (n=11)
surface

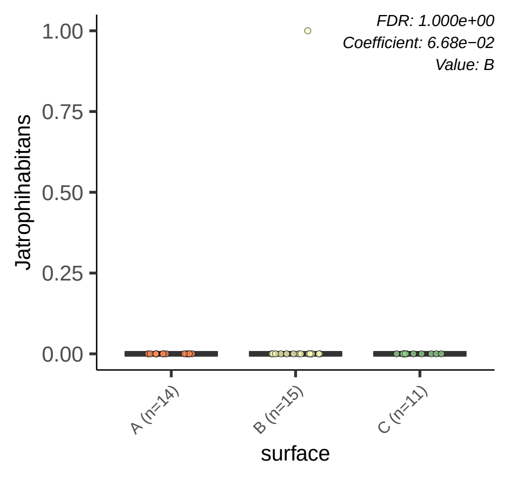

FDR: 1.000e+00
Coefficient: 6.68e−02
Value: B
Jatrophihabitans
1.00
0.75
0.50
0.25
0.00
A (n=14)
B (n=15)
C (n=11)
surface

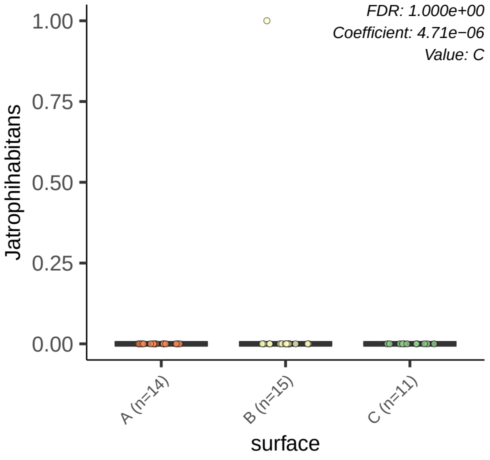
FDR: 1.000e+00
Coefficient: 4.71e−06
Value: C
Jatrophihabitans
1.00
0.75
0.50
0.25
0.00
A (n=14)
B (n=15)
C (n=11)
surface

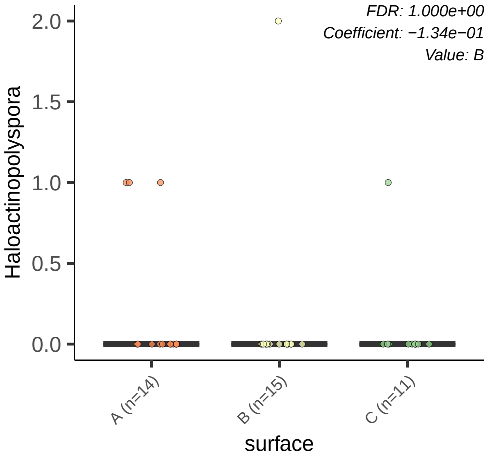

FDR: 1.000e+00
Coefficient: −1.34e−01
Value: B
Haloactinopolyspora
2.0
1.5
1.0
0.5
0.0
A (n=14)
B (n=15)
C (n=11)
surface

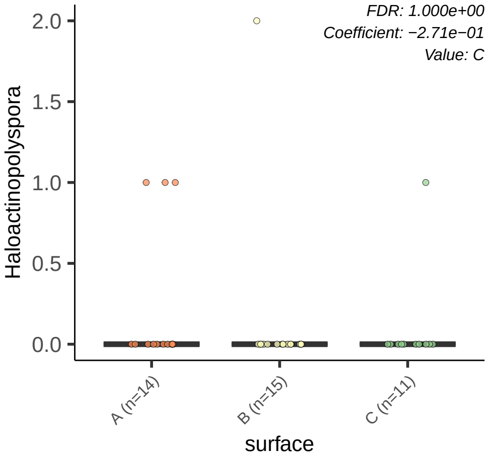
FDR: 1.000e+00
Coefficient: −2.71e−01
Value: C
Haloactinopolyspora
2.0
1.5
1.0
0.5
0.0
A (n=14)
B (n=15)
C (n=11)
surface

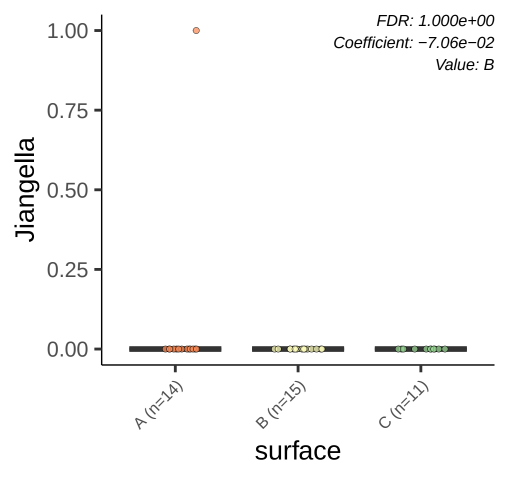
FDR: 1.000e+00
Coefficient: −7.06e−02
Value: B
Jiangella
1.00
0.75
0.50
0.25
0.00
A (n=14)
B (n=15)
C (n=11)
surface

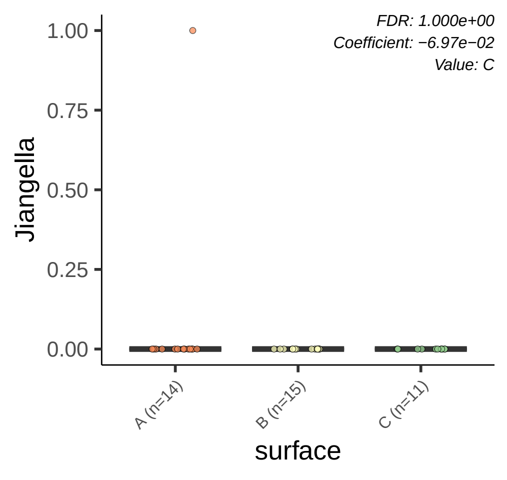
FDR: 1.000e+00
Coefficient: −6.97e−02
Value: C
Jiangella
1.00
0.75
0.50
0.25
0.00
A (n=14)
B (n=15)
C (n=11)
surface

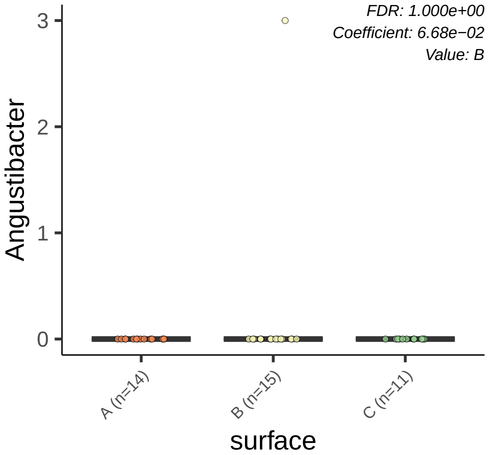

FDR: 1.000e+00
Coefficient: 6.68e−02
Value: B
Angustibacter
3
2
1
0
A (n=14)
B (n=15)
C (n=11)
surface

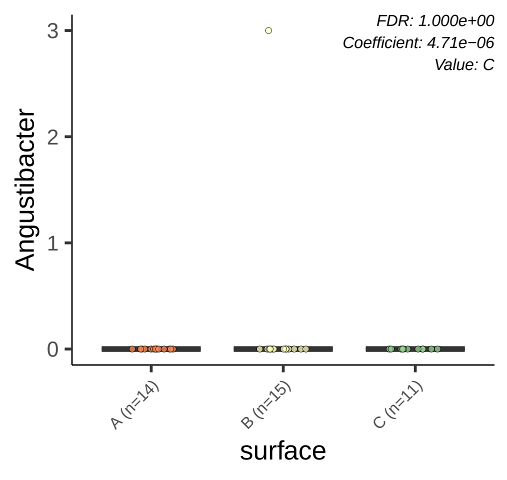
FDR: 1.000e+00
Coefficient: 4.71e−06
Value: C
Angustibacter
3
2
1
0
A (n=14)
B (n=15)
C (n=11)
surface

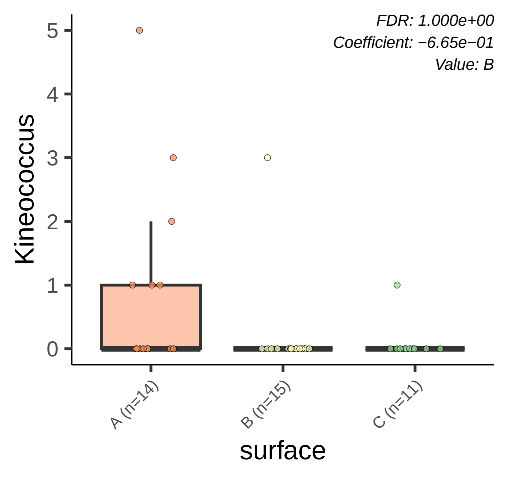
FDR: 1.000e+00
Coefficient: −6.65e−01
Value: B
Kineococcus
0
1
2
3
4
5
A (n=14)
B (n=15)
C (n=11)
surface

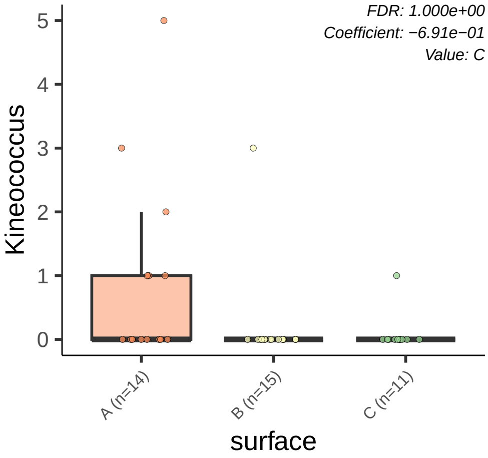

FDR: 1.000e+00
Coefficient: −6.91e−01
Value: C
Kineococcus
5
4
3
2
1
0
A (n=14)
B (n=15)
C (n=11)
surface

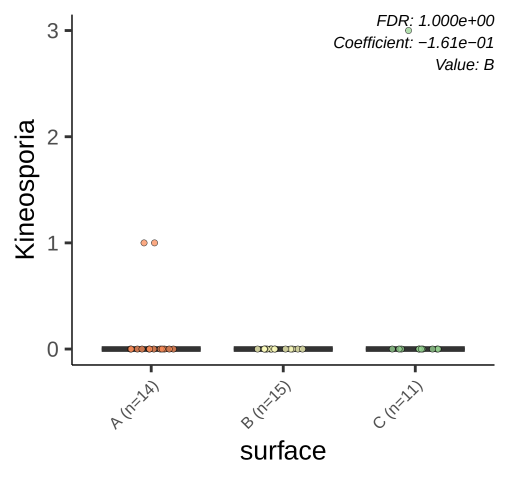
FDR: 1.000e+00
Coefficient: −1.61e−01
Value: B
Kineosporia
3
2
1
0
A (n=14)
B (n=15)
C (n=11)
surface

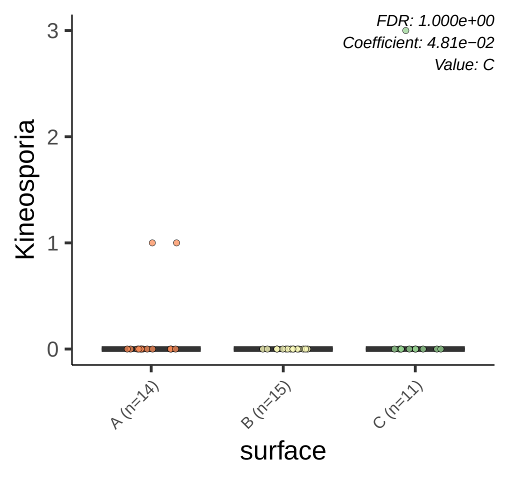

FDR: 1.000e+00
Coefficient: 4.81e−02
Value: C
Kineosporia
3
2
1
0
A (n=14)
B (n=15)
C (n=11)
surface

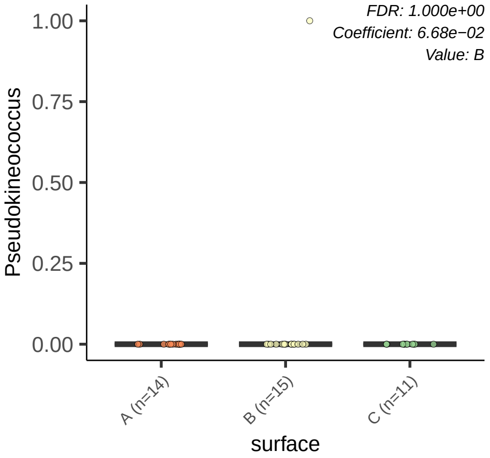

FDR: 1.000e+00
Coefficient: 6.68e−02
Value: B
Pseudokineococcus
1.00
0.75
0.50
0.25
0.00
A (n=14)
B (n=15)
C (n=11)
surface

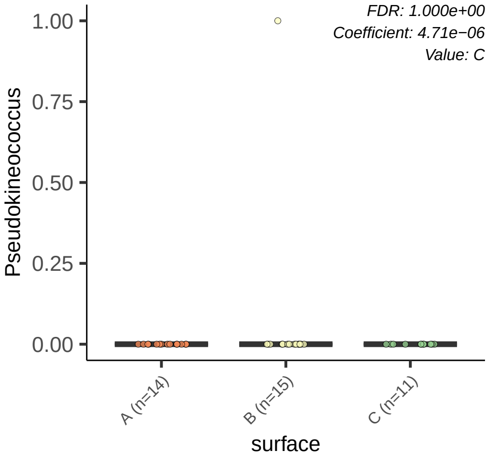

FDR: 1.000e+00
Coefficient: 4.71e−06
Value: C
Pseudokineococcus
1.00
0.75
0.50
0.25
0.00
A (n=14)
B (n=15)
C (n=11)
surface

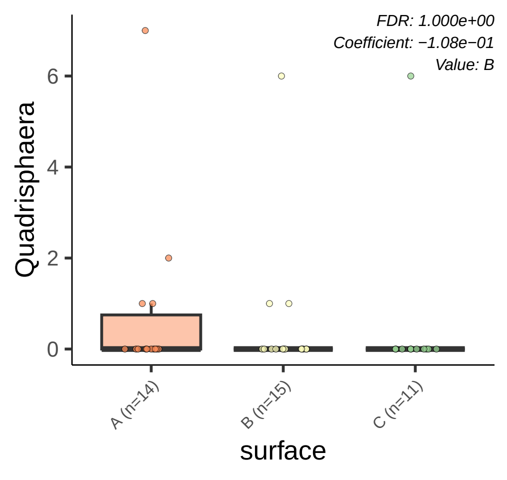
FDR: 1.000e+00
Coefficient: −1.08e−01
Value: B
Quadrisphaera
0
2
4
6
A (n=14)
B (n=15)
C (n=11)
surface

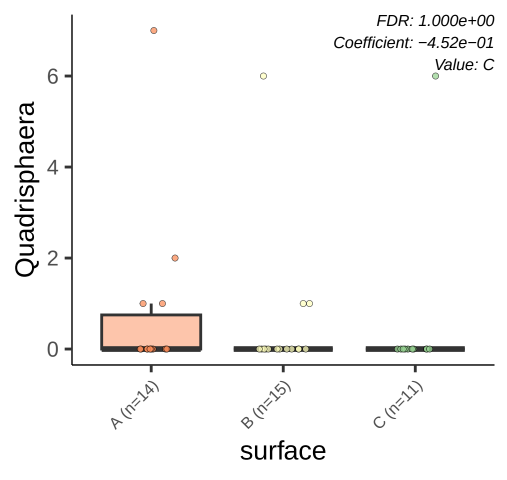

FDR: 1.000e+00
Coefficient: −4.52e−01
Value: C
Quadrisphaera
0
2
4
6
A (n=14)
B (n=15)
C (n=11)
surface

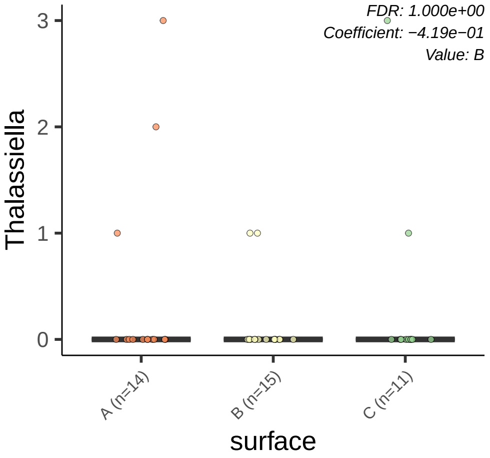
FDR: 1.000e+00
Coefficient: −4.19e−01
Value: B
Thalassiella
3
2
1
0
A (n=14)
B (n=15)
C (n=11)
surface

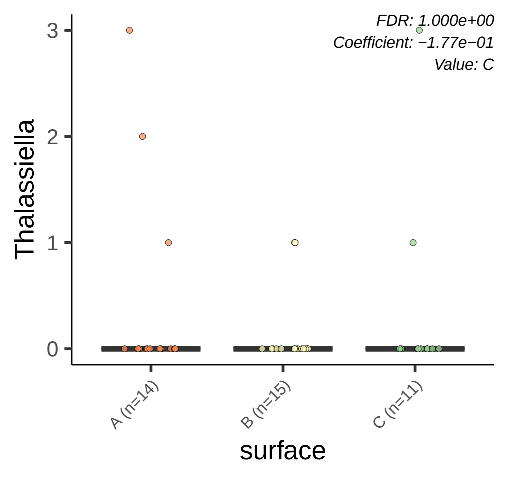
FDR: 1.000e+00
Coefficient: −1.77e−01
Value: C
Thalassiella
3
2
1
0
A (n=14)
B (n=15)
C (n=11)
surface

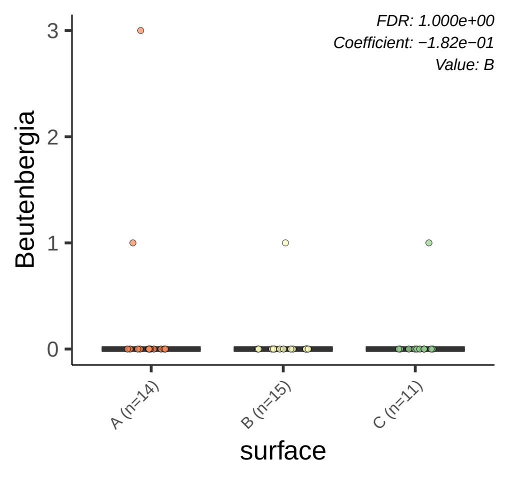
FDR: 1.000e+00
Coefficient: −1.82e−01
Value: B
Beutenbergia
3
2
1
0
A (n=14)
B (n=15)
C (n=11)
surface

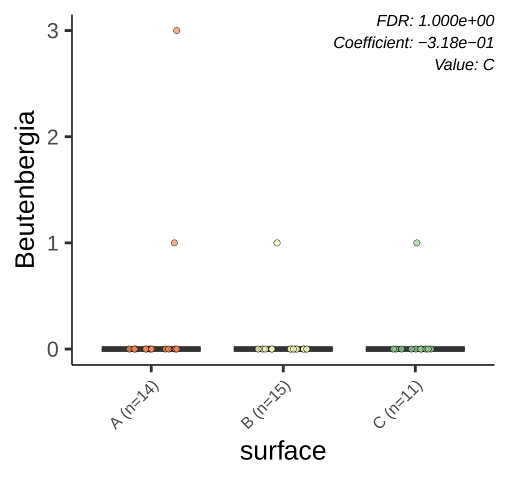
FDR: 1.000e+00
Coefficient: −3.18e−01
Value: C
Beutenbergia
3
2
1
0
A (n=14)
B (n=15)
C (n=11)
surface

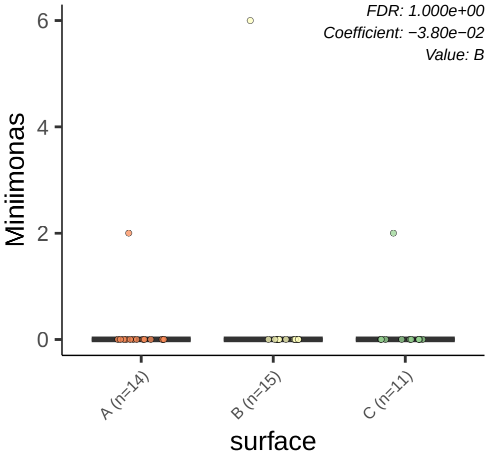
FDR: 1.000e+00
Coefficient: −3.80e−02
Value: B
Miniimonas
6
4
2
0
A (n=14)
B (n=15)
C (n=11)
surface

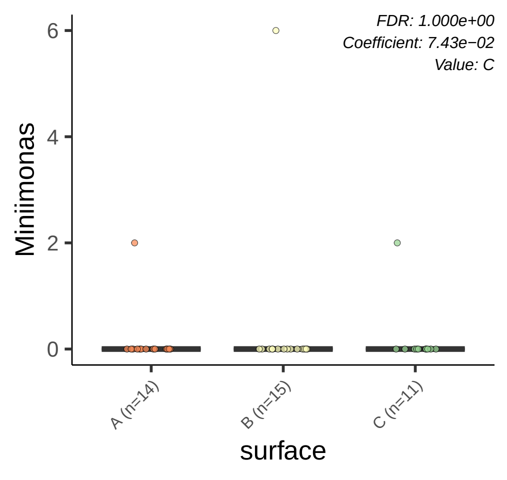
FDR: 1.000e+00
Coefficient: 7.43e−02
Value: C
Miniimonas
0
2
4
6
A (n=14)
B (n=15)
C (n=11)
surface

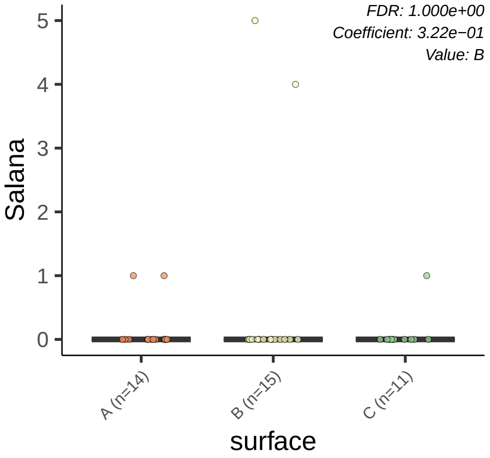

FDR: 1.000e+00
Coefficient: 3.22e−01
Value: B
Salana
5
4
3
2
1
0
A (n=14)
B (n=15)
C (n=11)
surface

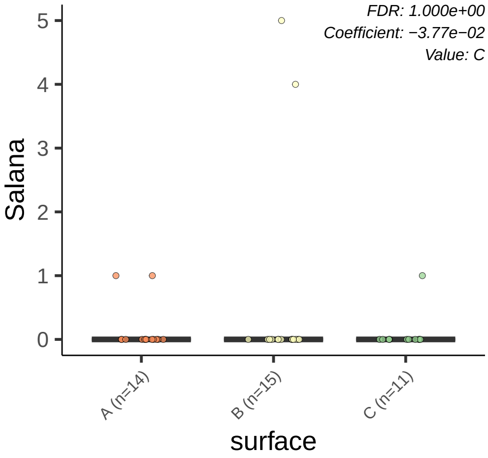

FDR: 1.000e+00
Coefficient: −3.77e−02
Value: C
Salana
5
4
3
2
1
0
A (n=14)
B (n=15)
C (n=11)
surface

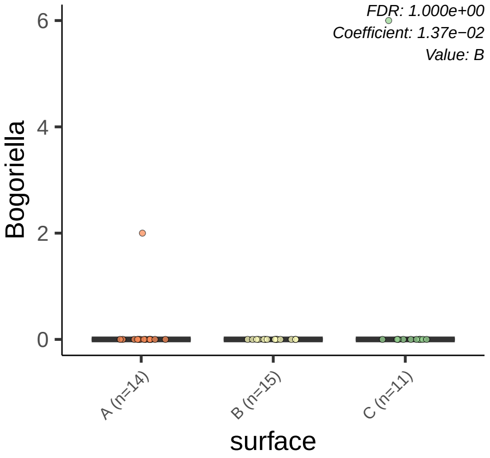
FDR: 1.000e+00
Coefficient: 1.37e−02
Value: B
Bogoriella
0
2
4
6
A (n=14)
B (n=15)
C (n=11)
surface

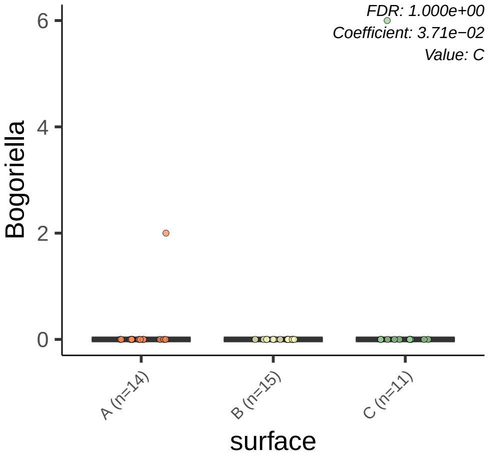
FDR: 1.000e+00
Coefficient: 3.71e−02
Value: C
Bogoriella
6
4
2
0
A (n=14)
B (n=15)
C (n=11)
surface

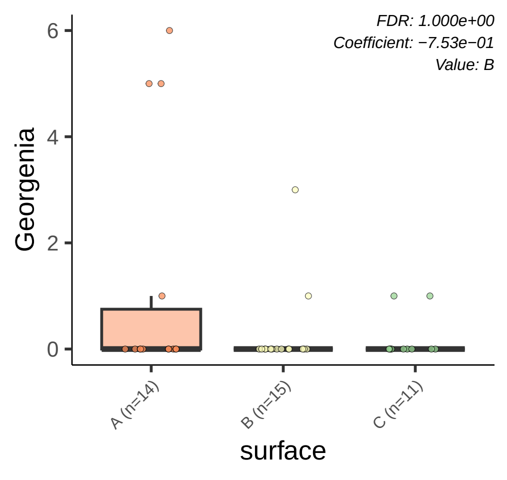
FDR: 1.000e+00
Coefficient: −7.53e−01
Value: B
Georgenia
0
2
4
6
A (n=14)
B (n=15)
C (n=11)
surface

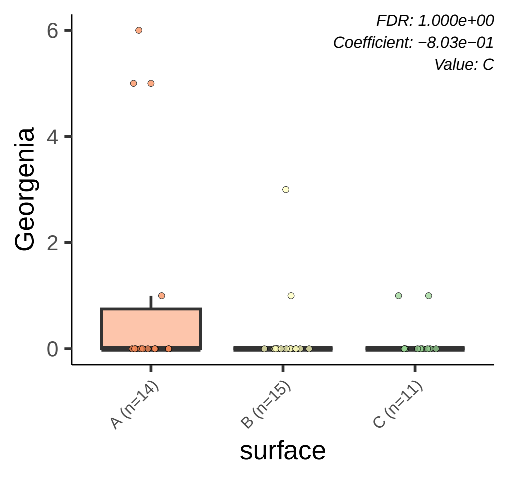
FDR: 1.000e+00
Coefficient: −8.03e−01
Value: C
Georgenia
0
2
4
6
A (n=14)
B (n=15)
C (n=11)
surface

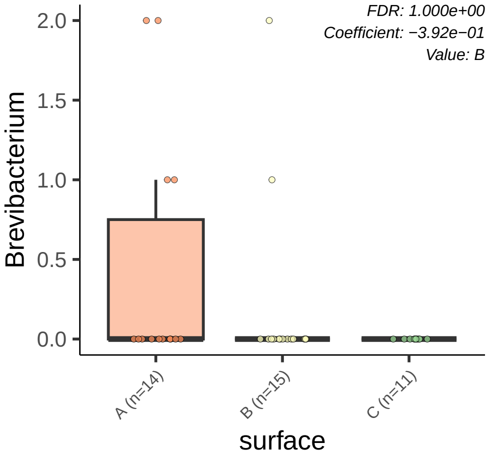
FDR: 1.000e+00
Coefficient: −3.92e−01
Value: B
Brevibacterium
2.0
1.5
1.0
0.5
0.0
A (n=14)
B (n=15)
C (n=11)
surface

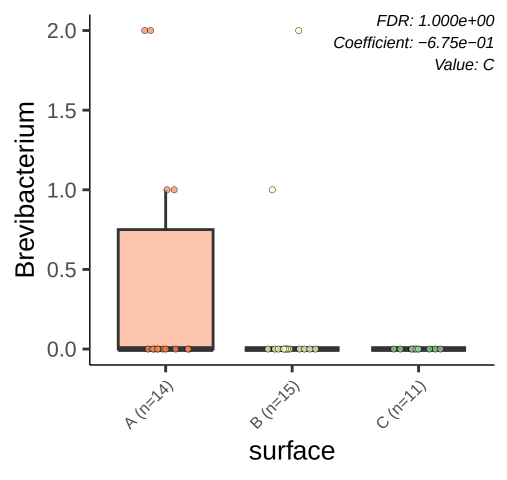
FDR: 1.000e+00
Coefficient: −6.75e−01
Value: C
Brevibacterium
2.0
1.5
1.0
0.5
0.0
A (n=14)
B (n=15)
C (n=11)
surface

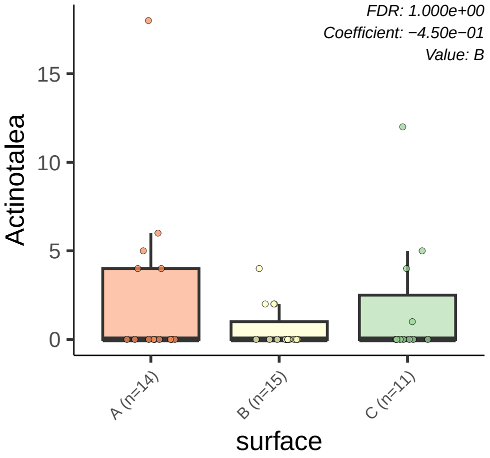

FDR: 1.000e+00
Coefficient: −4.50e−01
Value: B
Actinotalea
15
10
5
0
A (n=14)
B (n=15)
C (n=11)
surface

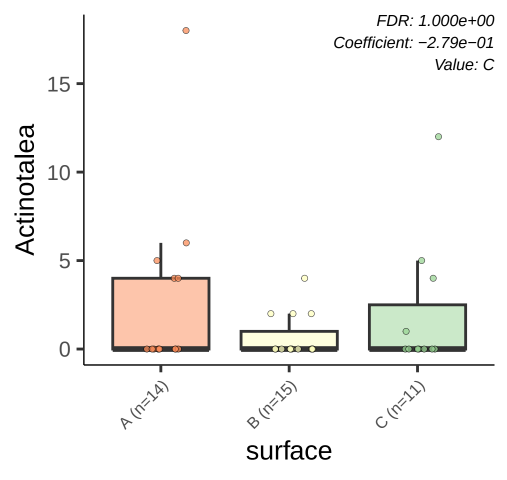
FDR: 1.000e+00
Coefficient: −2.79e−01
Value: C
Actinotalea
15
10
5
0
A (n=14)
B (n=15)
C (n=11)
surface

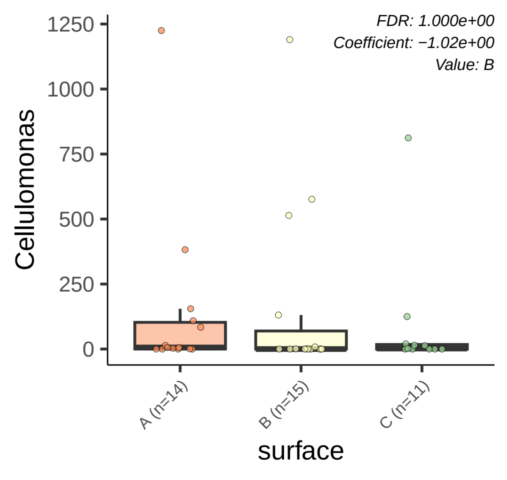

FDR: 1.000e+00
Coefficient: −1.02e+00
Value: B
Cellulomonas
1250
1000
750
500
250
0
A (n=14)
B (n=15)
C (n=11)
surface

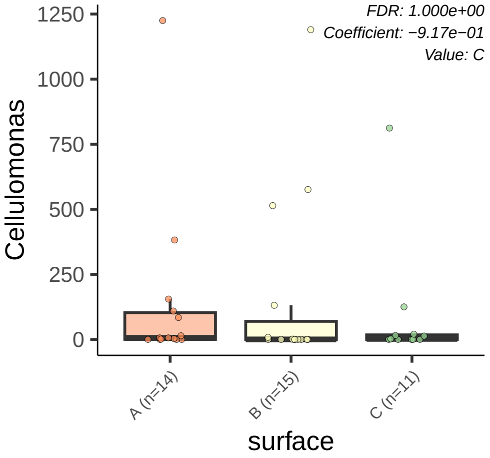

FDR: 1.000e+00
Coefficient: −9.17e−01
Value: C
Cellulomonas
1250
1000
750
500
250
0
A (n=14)
B (n=15)
C (n=11)
surface

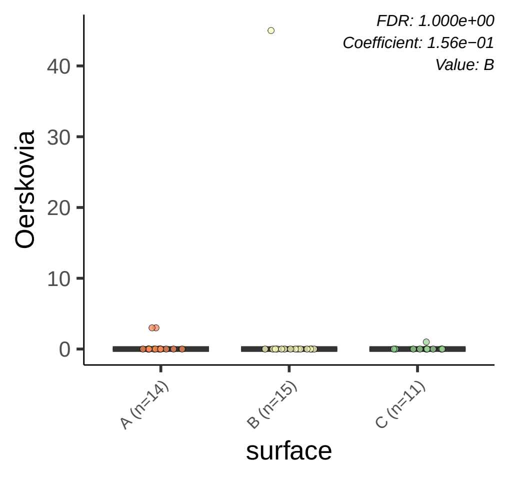
FDR: 1.000e+00
Coefficient: 1.56e−01
Value: B
Oerskovia
0
10
20
30
40
A (n=14)
B (n=15)
C (n=11)
surface

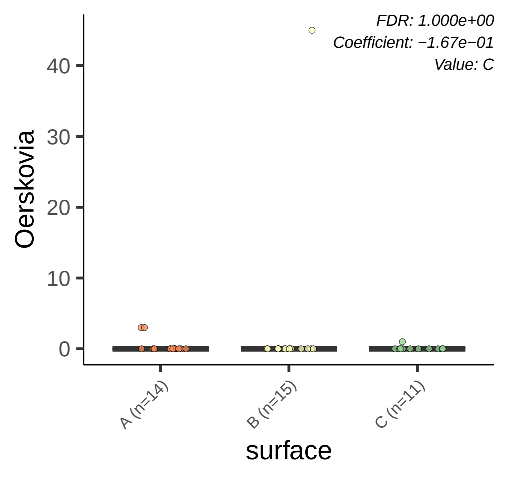

FDR: 1.000e+00
Coefficient: −1.67e−01
Value: C
Oerskovia
0
10
20
30
40
A (n=14)
B (n=15)
C (n=11)
surface

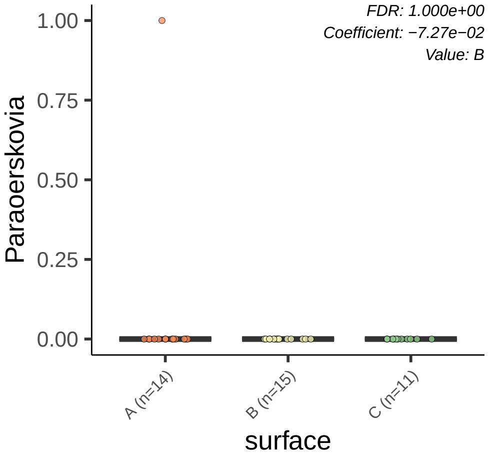
FDR: 1.000e+00
Coefficient: −7.27e−02
Value: B
Paraoerskovia
1.00
0.75
0.50
0.25
0.00
A (n=14)
B (n=15)
C (n=11)
surface

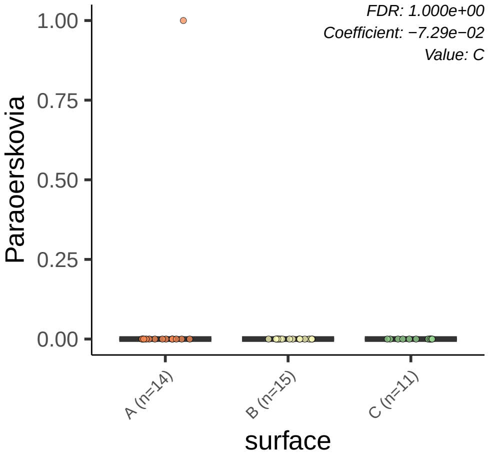

FDR: 1.000e+00
Coefficient: −7.29e−02
Value: C
Paraoerskovia
1.00
0.75
0.50
0.25
0.00
A (n=14)
B (n=15)
C (n=11)
surface

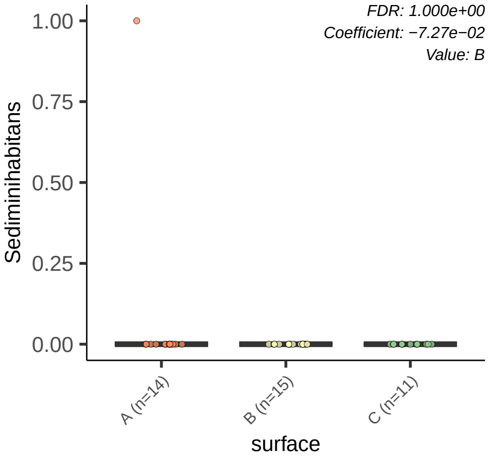

FDR: 1.000e+00
Coefficient: −7.27e−02
Value: B
Sediminihabitans
1.00
0.75
0.50
0.25
0.00
A (n=14)
B (n=15)
C (n=11)
surface

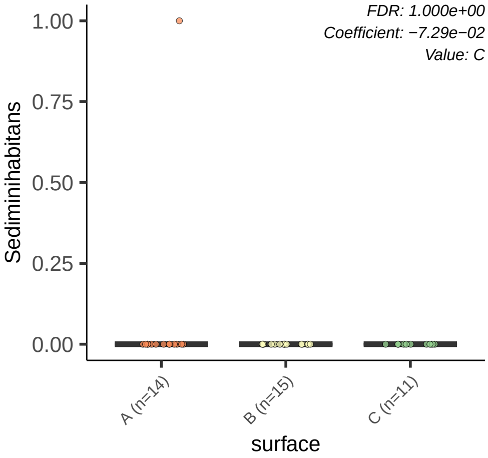
FDR: 1.000e+00
Coefficient: −7.29e−02
Value: C
Sediminihabitans
1.00
0.75
0.50
0.25
0.00
A (n=14)
B (n=15)
C (n=11)
surface

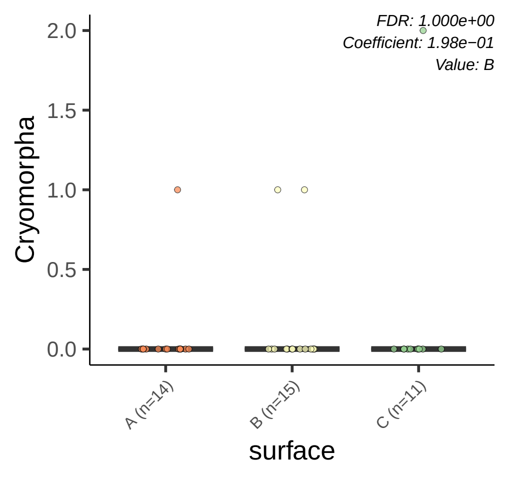

FDR: 1.000e+00
Coefficient: 1.98e−01
Value: B
Cryomorpha
2.0
1.5
1.0
0.5
0.0
A (n=14)
B (n=15)
C (n=11)
surface

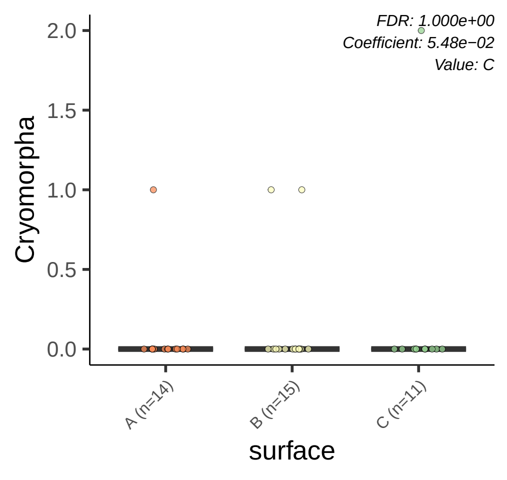

FDR: 1.000e+00
Coefficient: 5.48e−02
Value: C
Cryomorpha
2.0
1.5
1.0
0.5
0.0
A (n=14)
B (n=15)
C (n=11)
surface

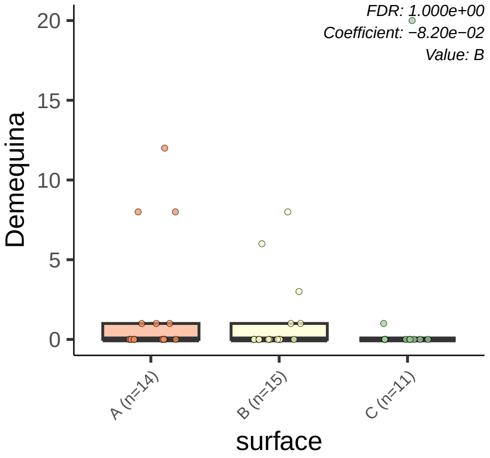
FDR: 1.000e+00
Coefficient: −8.20e−02
Value: B
Demequina
20
15
10
5
0
A (n=14)
B (n=15)
C (n=11)
surface

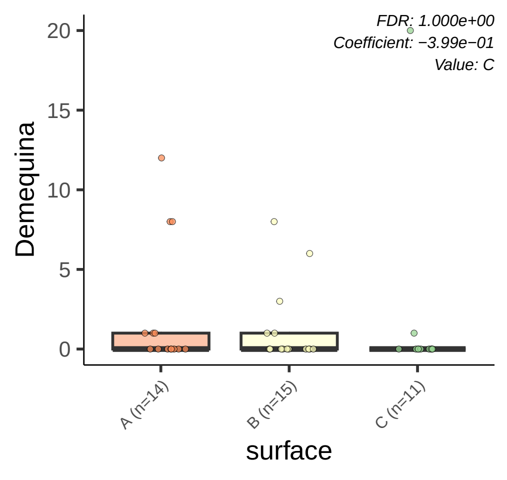

FDR: 1.000e+00
Coefficient: −3.99e−01
Value: C
Demequina
20
15
10
5
0
A (n=14)
B (n=15)
C (n=11)
surface

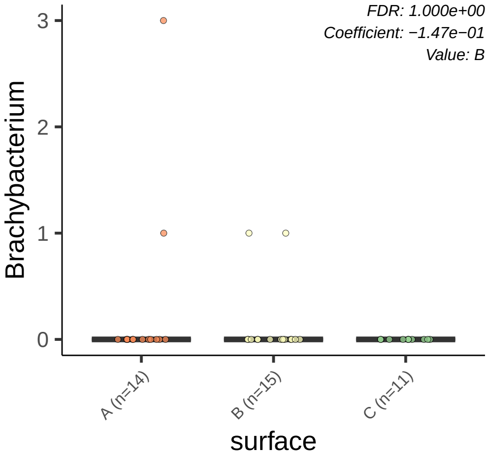
FDR: 1.000e+00
Coefficient: −1.47e−01
Value: B
Brachybacterium
3
2
1
0
A (n=14)
B (n=15)
C (n=11)
surface

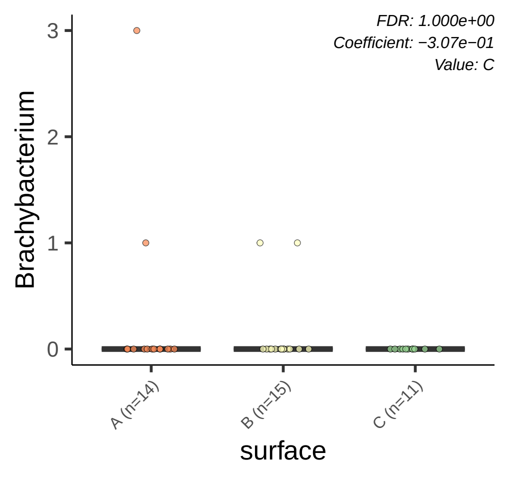
FDR: 1.000e+00
Coefficient: −3.07e−01
Value: C
Brachybacterium
3
2
1
0
A (n=14)
B (n=15)
C (n=11)
surface

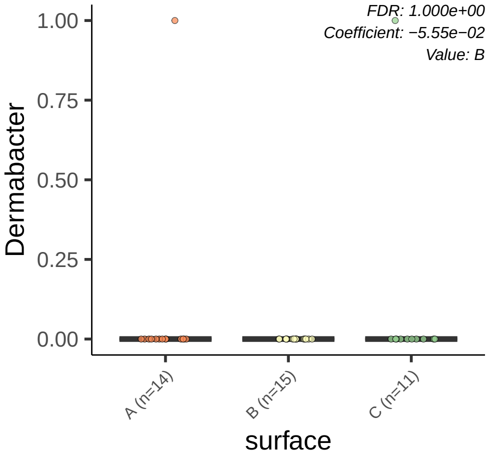

FDR: 1.000e+00
Coefficient: −5.55e−02
Value: B
Dermabacter
1.00
0.75
0.50
0.25
0.00
A (n=14)
B (n=15)
C (n=11)
surface

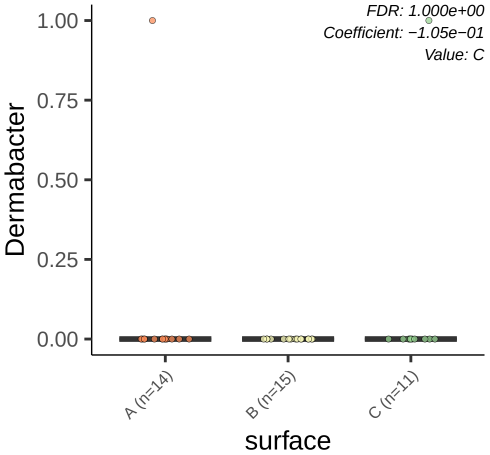
FDR: 1.000e+00
Coefficient: −1.05e−01
Value: C
1.00
0.75
0.50
0.25
0.00
Dermabacter
A (n=14)
B (n=15)
C (n=11)
surface

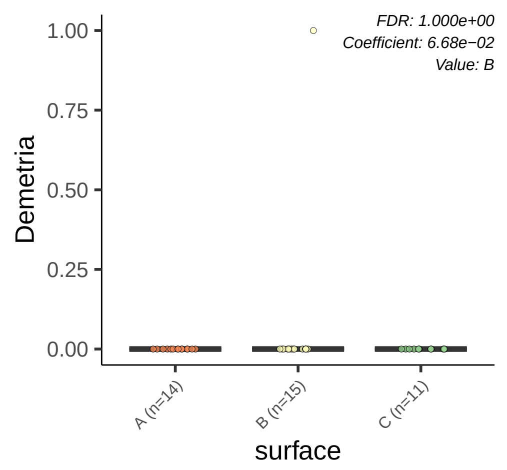
FDR: 1.000e+00
Coefficient: 6.68e−02
Value: B
Demetria
1.00
0.75
0.50
0.25
0.00
A (n=14)
B (n=15)
C (n=11)
surface

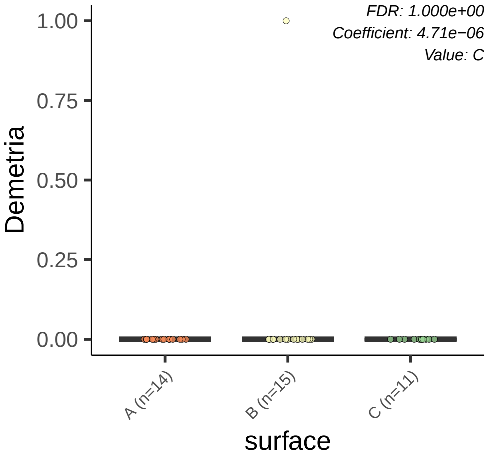
FDR: 1.000e+00
Coefficient: 4.71e−06
Value: C
Demetria
1.00
0.75
0.50
0.25
0.00
A (n=14)
B (n=15)
C (n=11)
surface

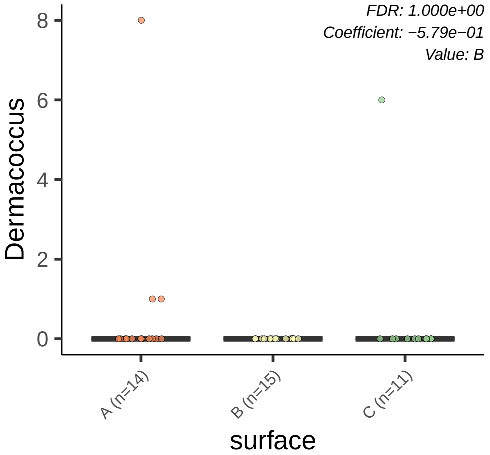
FDR: 1.000e+00
Coefficient: −5.79e−01
Value: B
Dermacoccus
8
6
4
2
0
A (n=14)
B (n=15)
C (n=11)
surface

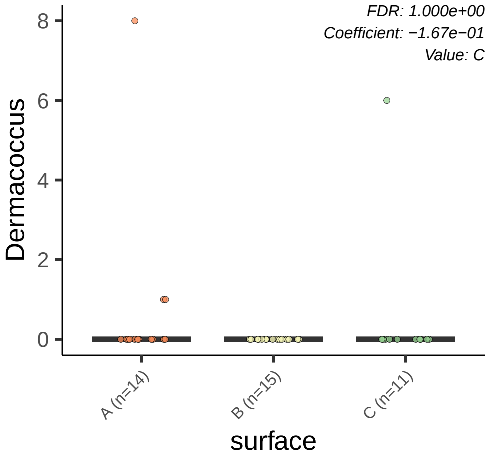

FDR: 1.000e+00
Coefficient: −1.67e−01
Value: C
Dermacoccus
8
6
4
2
0
A (n=14)
B (n=15)
C (n=11)
surface

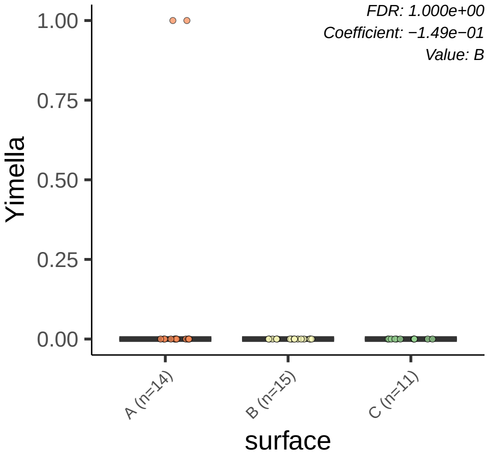

FDR: 1.000e+00
Coefficient: −1.49e−01
Value: B
Yimella
1.00
0.75
0.50
0.25
0.00
A (n=14)
B (n=15)
C (n=11)
surface

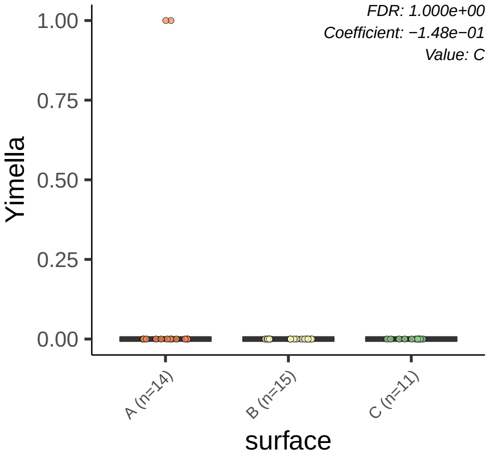
FDR: 1.000e+00
Coefficient: −1.48e−01
Value: C
Yimella
1.00
0.75
0.50
0.25
0.00
A (n=14)
B (n=15)
C (n=11)
surface

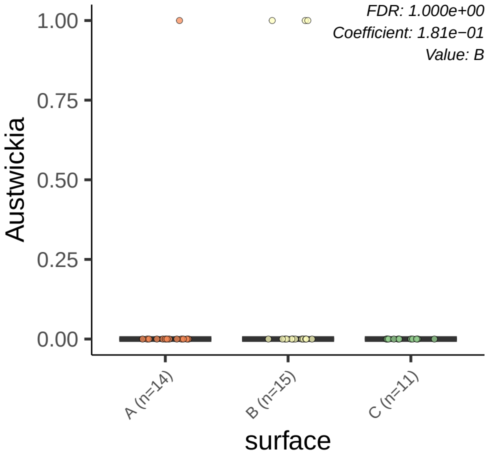
FDR: 1.000e+00
Coefficient: 1.81e−01
Value: B
Austwickia
1.00
0.75
0.50
0.25
0.00
A (n=14)
B (n=15)
C (n=11)
surface

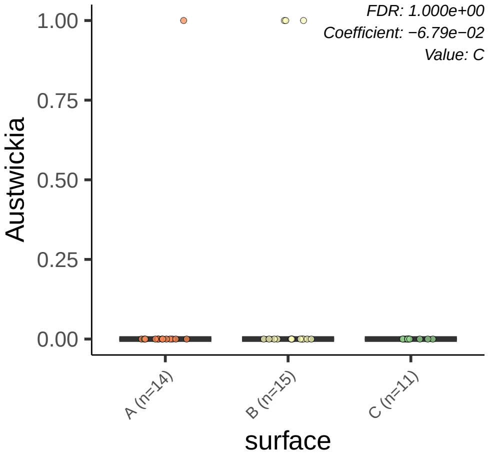

FDR: 1.000e+00
Coefficient: −6.79e−02
Value: C
Austwickia
1.00
0.75
0.50
0.25
0.00
A (n=14)
B (n=15)
C (n=11)
surface

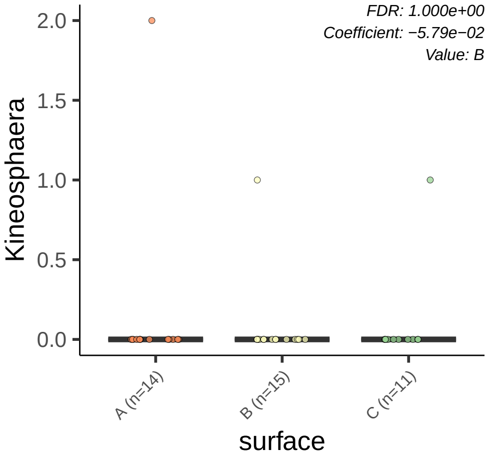
FDR: 1.000e+00
Coefficient: −5.79e−02
Value: B
Kineosphaera
2.0
1.5
1.0
0.5
0.0
A (n=14)
B (n=15)
C (n=11)
surface

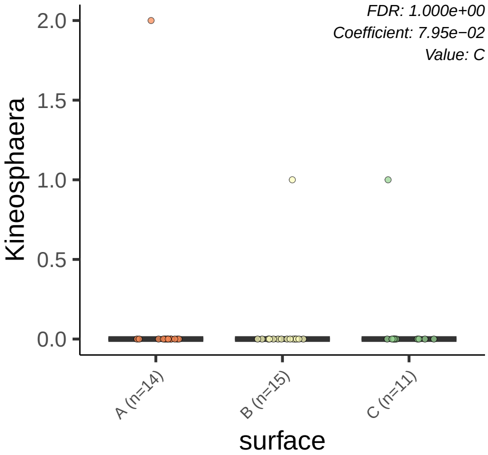
FDR: 1.000e+00
Coefficient: 7.95e−02
Value: C
Kineosphaera
2.0
1.5
1.0
0.5
0.0
A (n=14)
B (n=15)
C (n=11)
surface

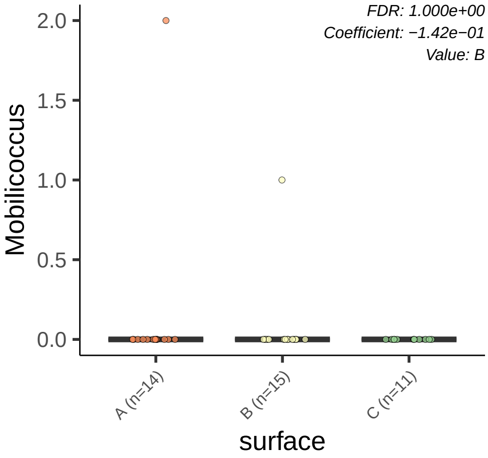
FDR: 1.000e+00
Coefficient: −1.42e−01
Value: B
Mobilicoccus
2.0
1.5
1.0
0.5
0.0
A (n=14)
B (n=15)
C (n=11)
surface

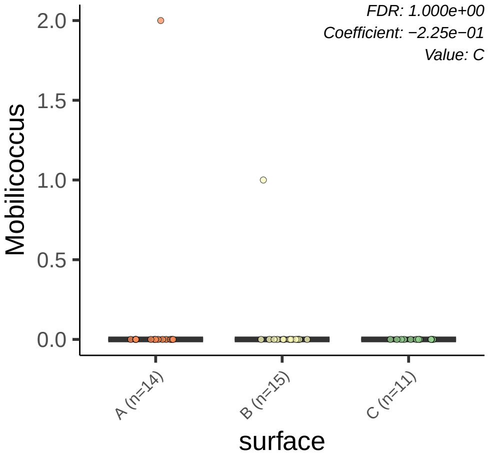
FDR: 1.000e+00
Coefficient: −2.25e−01
Value: C
Mobilicoccus
2.0
1.5
1.0
0.5
0.0
A (n=14)
B (n=15)
C (n=11)
surface

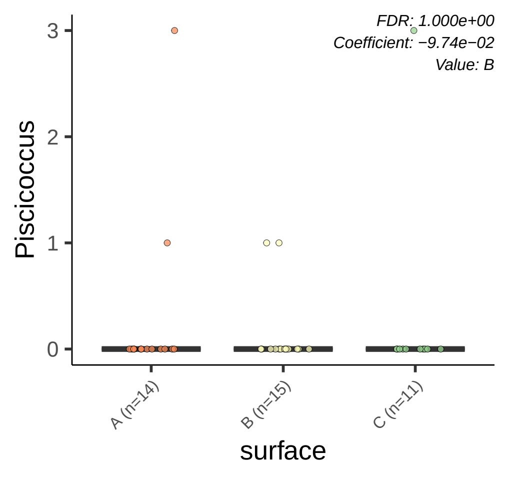

FDR: 1.000e+00
Coefficient: −9.74e−02
Value: B
Piscicoccus
3
2
1
0
A (n=14)
B (n=15)
C (n=11)
surface

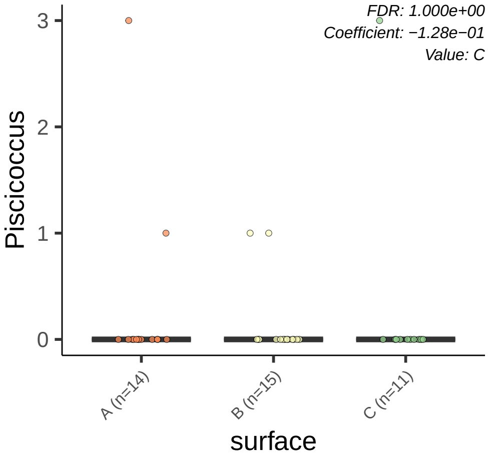

FDR: 1.000e+00
Coefficient: −1.28e−01
Value: C
Piscicoccus
3
2
1
0
A (n=14)
B (n=15)
C (n=11)
surface

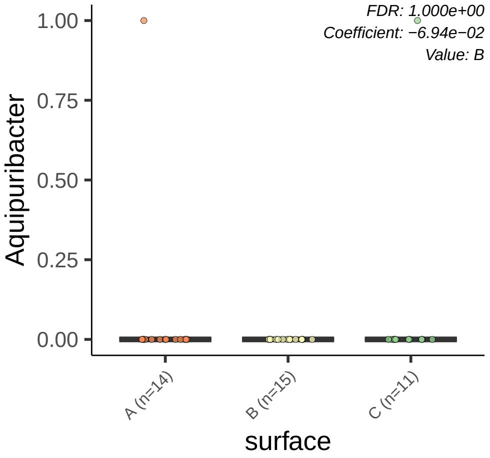

FDR: 1.000e+00
Coefficient: −6.94e−02
Value: B
Aquipuribacter
1.00
0.75
0.50
0.25
0.00
A (n=14)
B (n=15)
C (n=11)
surface

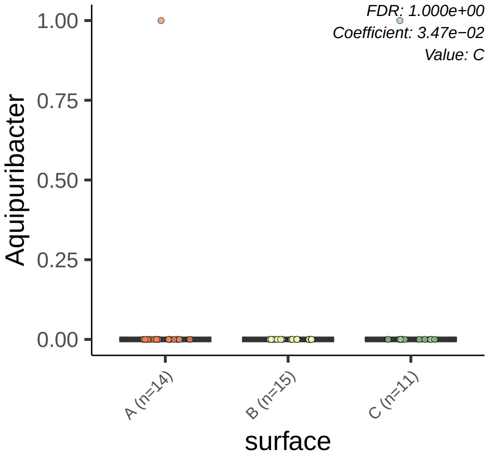
FDR: 1.000e+00
Coefficient: 3.47e−02
Value: C
Aquipuribacter
1.00
0.75
0.50
0.25
0.00
A (n=14)
B (n=15)
C (n=11)
surface

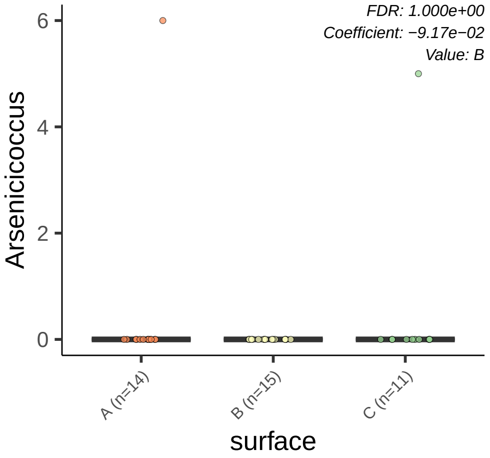

FDR: 1.000e+00
Coefficient: −9.17e−02
Value: B
Arsenicicoccus
0
2
4
6
A (n=14)
B (n=15)
C (n=11)
surface

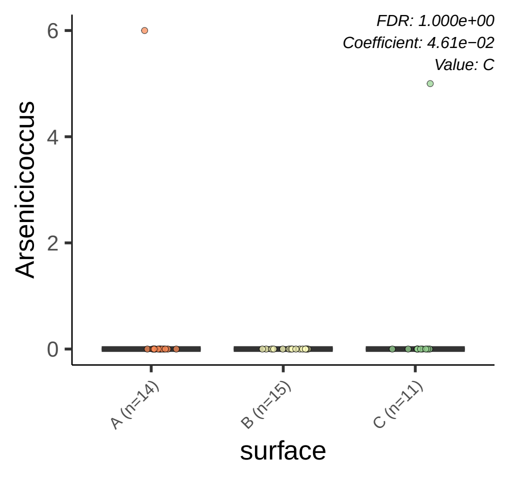
FDR: 1.000e+00
Coefficient: 4.61e−02
Value: C
Arsenicicoccus
6
4
2
0
A (n=14)
B (n=15)
C (n=11)
surface

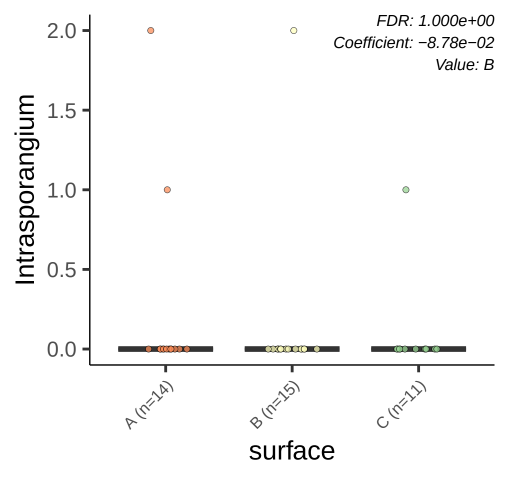

FDR: 1.000e+00
Coefficient: −8.78e−02
Value: B
Intrasporangium
2.0
1.5
1.0
0.5
0.0
A (n=14)
B (n=15)
C (n=11)
surface

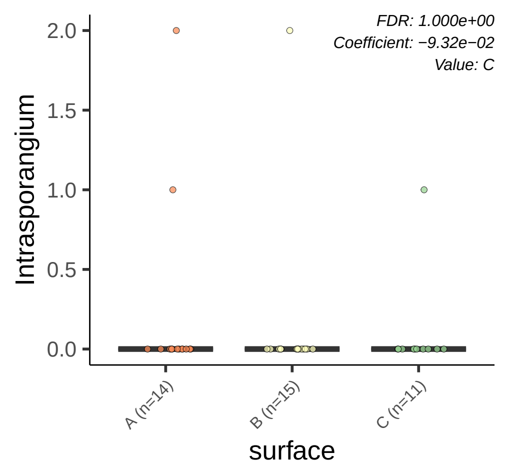

FDR: 1.000e+00
Coefficient: −9.32e−02
Value: C
Intrasporangium
2.0
1.5
1.0
0.5
0.0
A (n=14)
B (n=15)
C (n=11)
surface

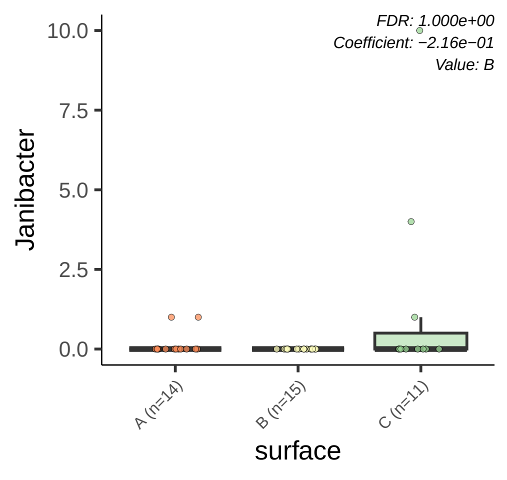

FDR: 1.000e+00
Coefficient: −2.16e−01
Value: B
Janibacter
10.0
7.5
5.0
2.5
0.0
A (n=14)
B (n=15)
C (n=11)
surface

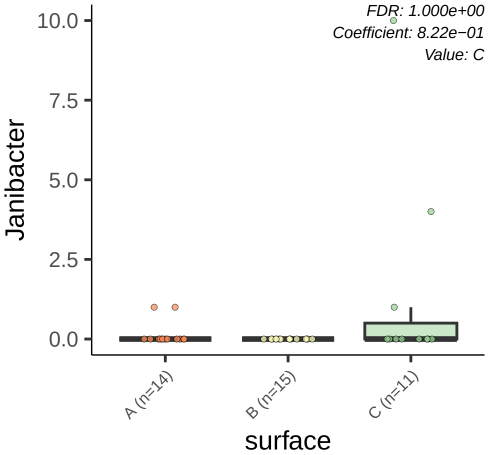
FDR: 1.000e+00
Coefficient: 8.22e−01
Value: C
Janibacter
10.0
7.5
5.0
2.5
0.0
A (n=14)
B (n=15)
C (n=11)
surface

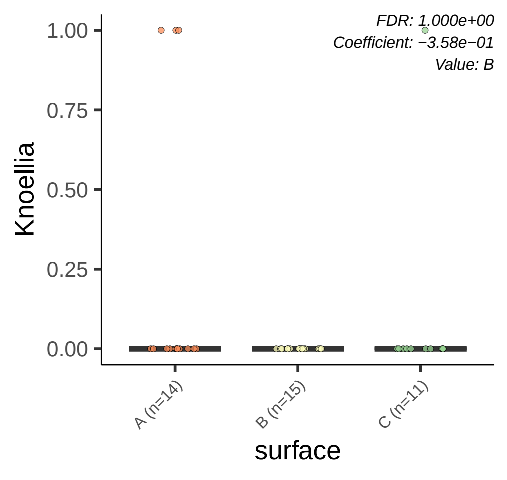

FDR: 1.000e+00
Coefficient: −3.58e−01
Value: B
Knoellia
1.00
0.75
0.50
0.25
0.00
A (n=14)
B (n=15)
C (n=11)
surface

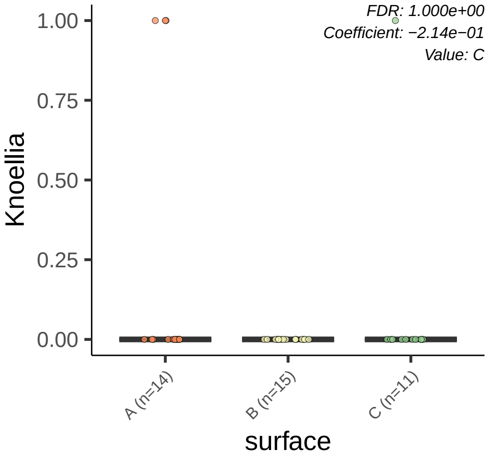

FDR: 1.000e+00
Coefficient: −2.14e−01
Value: C
Knoellia
1.00
0.75
0.50
0.25
0.00
A (n=14)
B (n=15)
C (n=11)
surface

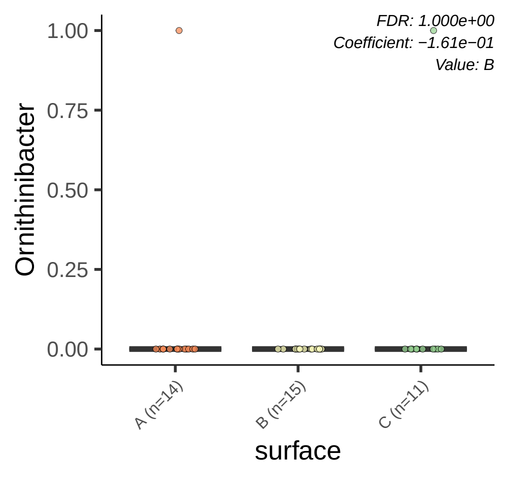
FDR: 1.000e+00
Coefficient: −1.61e−01
Value: B
Ornithinibacter
1.00
0.75
0.50
0.25
0.00
A (n=14)
B (n=15)
C (n=11)
surface

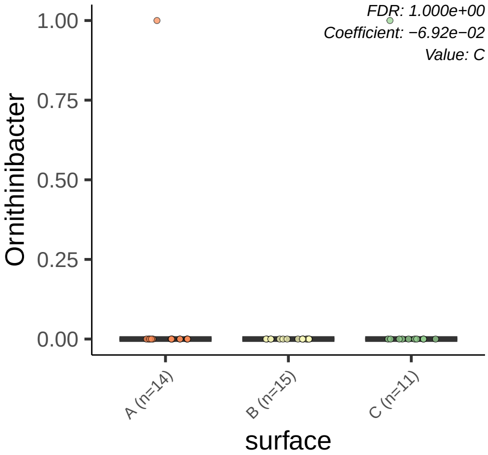
FDR: 1.000e+00
Coefficient: −6.92e−02
Value: C
Ornithinibacter
1.00
0.75
0.50
0.25
0.00
A (n=14)
B (n=15)
C (n=11)
surface

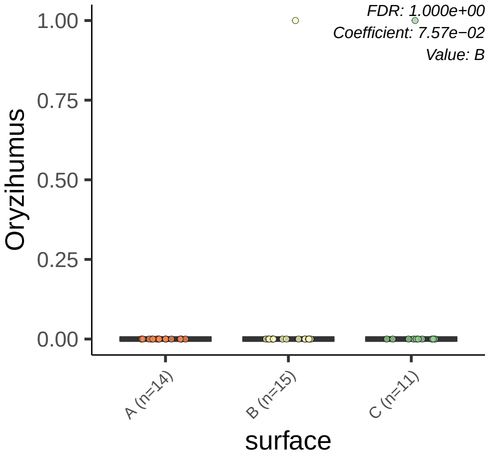
FDR: 1.000e+00
Coefficient: 7.57e−02
Value: B
1.00
0.75
0.50
0.25
0.00
Oryzihumus
A (n=14)
B (n=15)
C (n=11)
surface

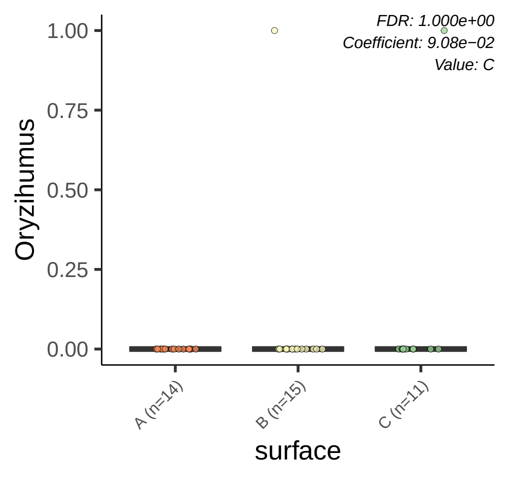

FDR: 1.000e+00
Coefficient: 9.08e−02
Value: C
Oryzihumus
1.00
0.75
0.50
0.25
0.00
A (n=14)
B (n=15)
C (n=11)
surface

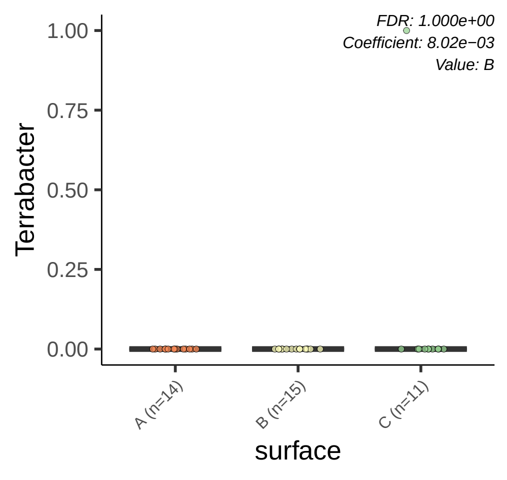
FDR: 1.000e+00
Coefficient: 8.02e−03
Value: B
Terrabacter
1.00
0.75
0.50
0.25
0.00
A (n=14)
B (n=15)
C (n=11)
surface

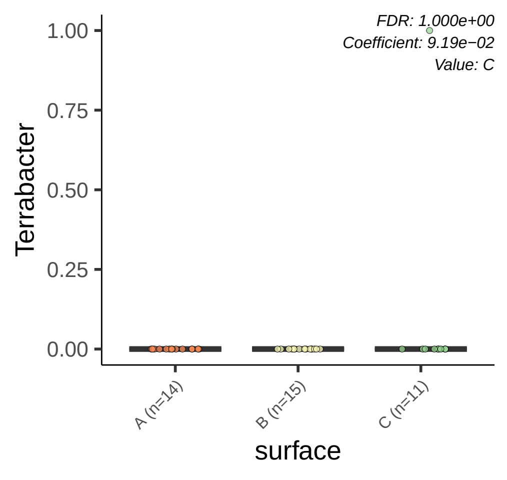
FDR: 1.000e+00
Coefficient: 9.19e−02
Value: C
Terrabacter
1.00
0.75
0.50
0.25
0.00
A (n=14)
B (n=15)
C (n=11)
surface

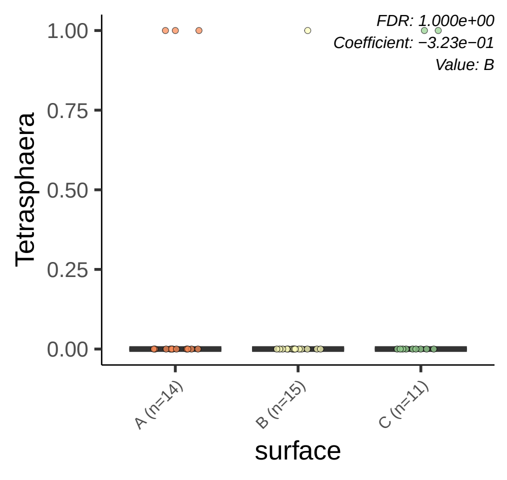
FDR: 1.000e+00
Coefficient: −3.23e−01
Value: B
Tetrasphaera
1.00
0.75
0.50
0.25
0.00
A (n=14)
B (n=15)
C (n=11)
surface

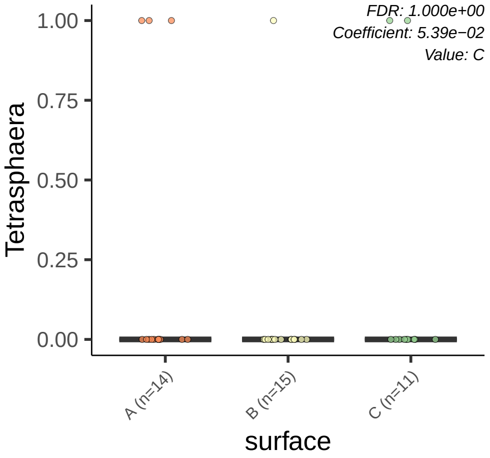

FDR: 1.000e+00
Coefficient: 5.39e−02
Value: C
Tetrasphaera
1.00
0.75
0.50
0.25
0.00
A (n=14)
B (n=15)
C (n=11)
surface

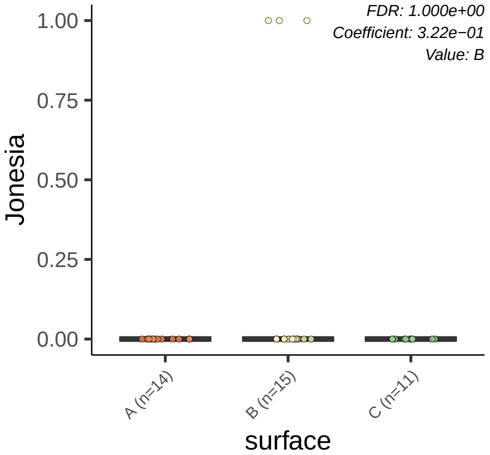
FDR: 1.000e+00
Coefficient: 3.22e−01
Value: B
Jonesia
1.00
0.75
0.50
0.25
0.00
A (n=14)
B (n=15)
C (n=11)
surface

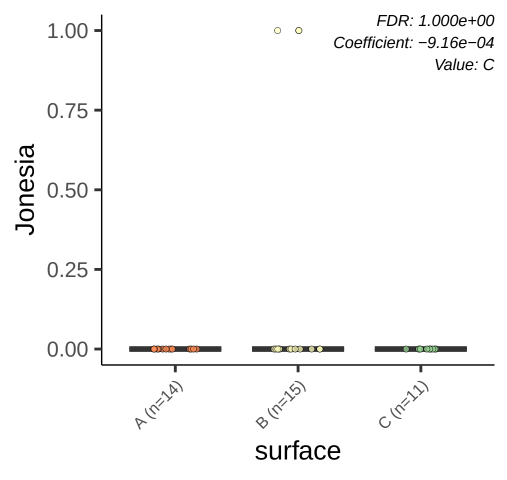
FDR: 1.000e+00
Coefficient: −9.16e−04
Value: C
Jonesia
1.00
0.75
0.50
0.25
0.00
A (n=14)
B (n=15)
C (n=11)
surface

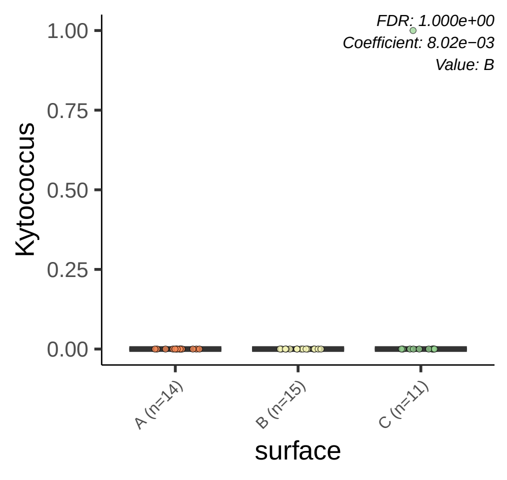
FDR: 1.000e+00
Coefficient: 8.02e−03
Value: B
Kytococcus
1.00
0.75
0.50
0.25
0.00
A (n=14)
B (n=15)
C (n=11)
surface

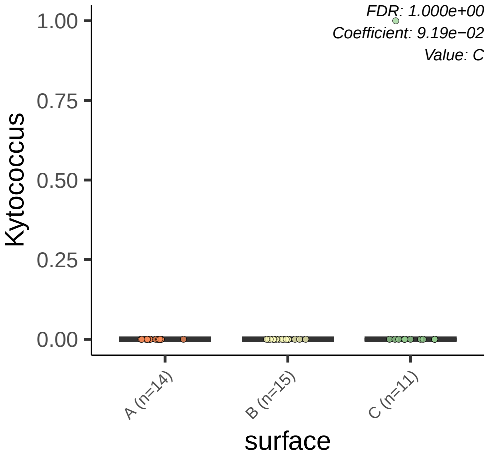

FDR: 1.000e+00
Coefficient: 9.19e−02
Value: C
Kytococcus
1.00
0.75
0.50
0.25
0.00
A (n=14)
B (n=15)
C (n=11)
surface

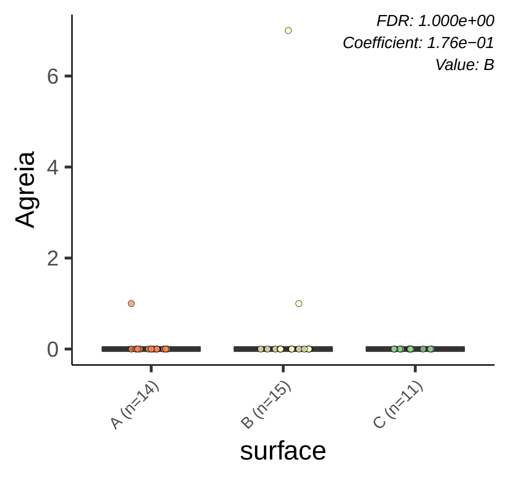

FDR: 1.000e+00
Coefficient: 1.76e−01
Value: B
Agreia
0
2
4
6
A (n=14)
B (n=15)
C (n=11)
surface

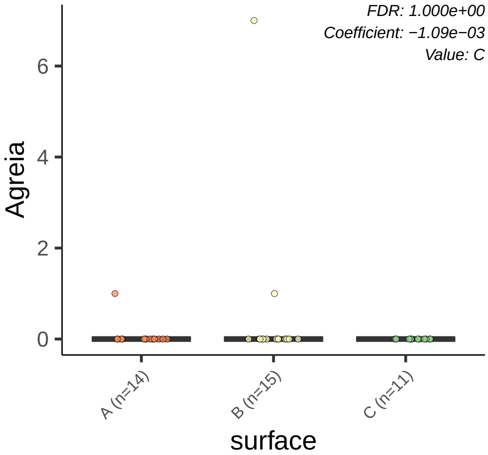
FDR: 1.000e+00
Coefficient: −1.09e−03
Value: C
Agreia
0
2
4
6
A (n=14)
B (n=15)
C (n=11)
surface

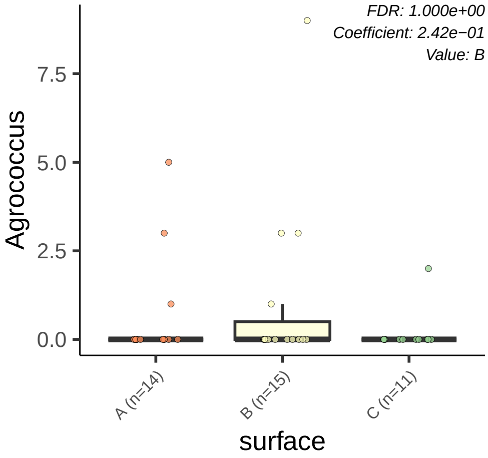
FDR: 1.000e+00
Coefficient: 2.42e−01
Value: B
Agrococcus
7.5
5.0
2.5
0.0
A (n=14)
B (n=15)
C (n=11)
surface

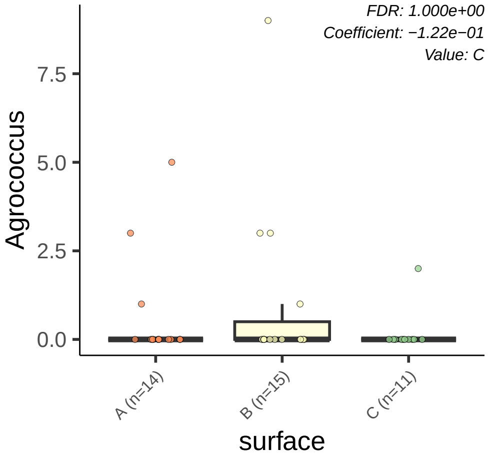

FDR: 1.000e+00
Coefficient: −1.22e−01
Value: C
Agrococcus
7.5
5.0
2.5
0.0
A (n=14)
B (n=15)
C (n=11)
surface

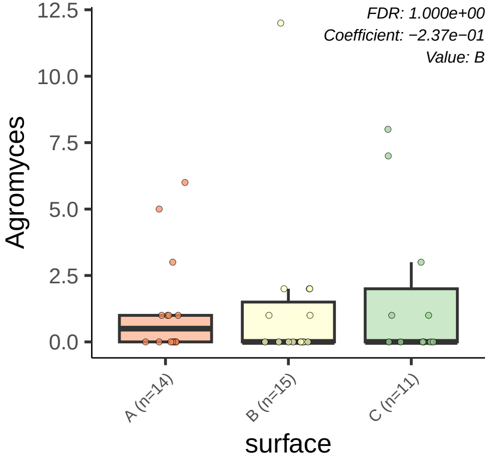

FDR: 1.000e+00
Coefficient: −2.37e−01
Value: B
Agromyces
12.5
10.0
7.5
5.0
2.5
0.0
A (n=14)
B (n=15)
C (n=11)
surface

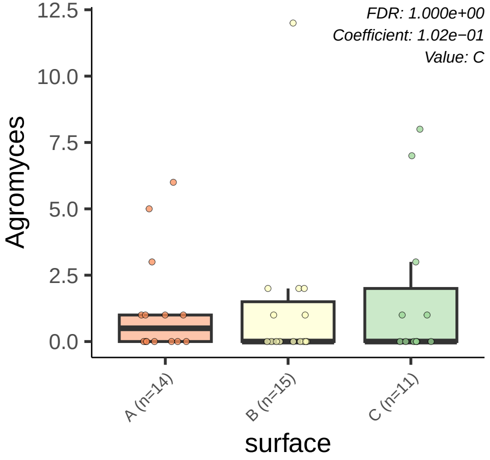
FDR: 1.000e+00
Coefficient: 1.02e−01
Value: C
Agromyces
12.5
10.0
7.5
5.0
2.5
0.0
A (n=14)
B (n=15)
C (n=11)
surface

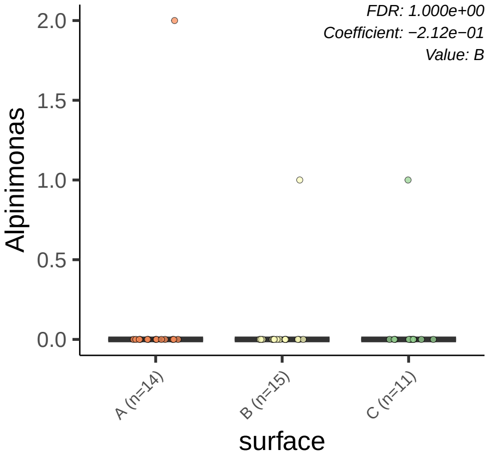
FDR: 1.000e+00
Coefficient: −2.12e−01
Value: B
Alpinimonas
2.0
1.5
1.0
0.5
0.0
A (n=14)
B (n=15)
C (n=11)
surface

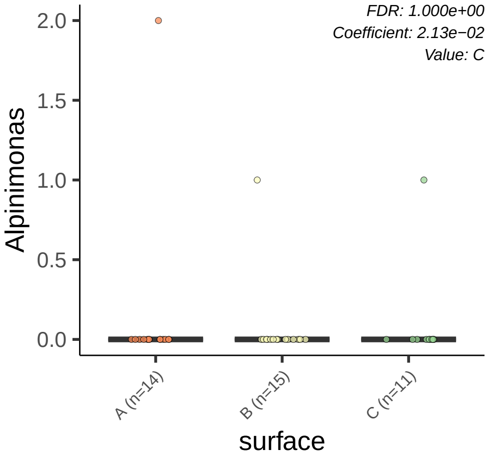
FDR: 1.000e+00
Coefficient: 2.13e−02
Value: C
Alpinimonas
2.0
1.5
1.0
0.5
0.0
A (n=14)
B (n=15)
C (n=11)
surface

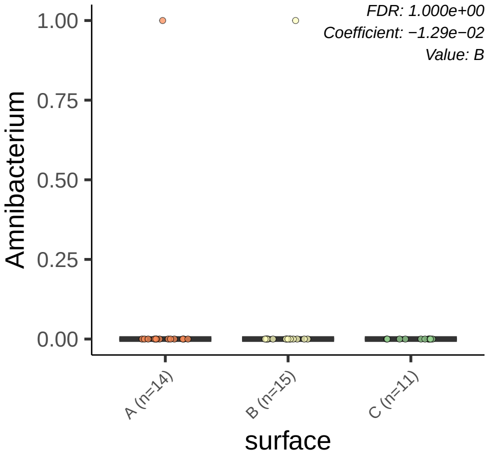
FDR: 1.000e+00
Coefficient: −1.29e−02
Value: B
Amnibacterium
1.00
0.75
0.50
0.25
0.00
A (n=14)
B (n=15)
C (n=11)
surface

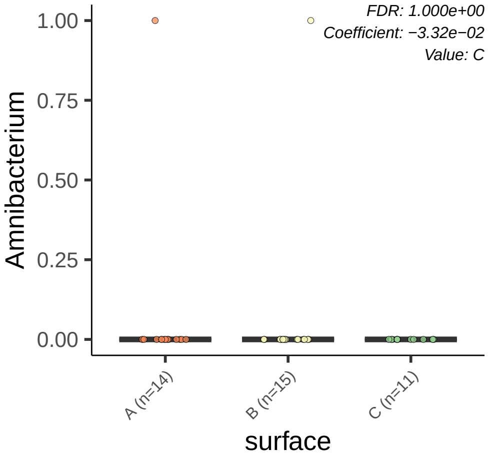

FDR: 1.000e+00
Coefficient: −3.32e−02
Value: C
Amnibacterium
1.00
0.75
0.50
0.25
0.00
A (n=14)
B (n=15)
C (n=11)
surface

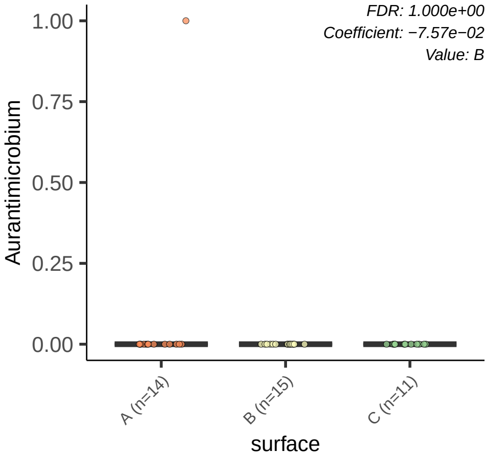
FDR: 1.000e+00
Coefficient: −7.57e−02
Value: B
Aurantimicrobium
1.00
0.75
0.50
0.25
0.00
A (n=14)
B (n=15)
C (n=11)
surface

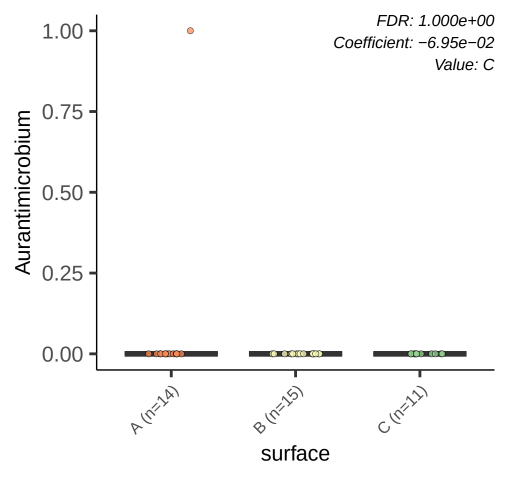
FDR: 1.000e+00
Coefficient: −6.95e−02
Value: C
Aurantimicrobium
1.00
0.75
0.50
0.25
0.00
A (n=14)
B (n=15)
C (n=11)
surface

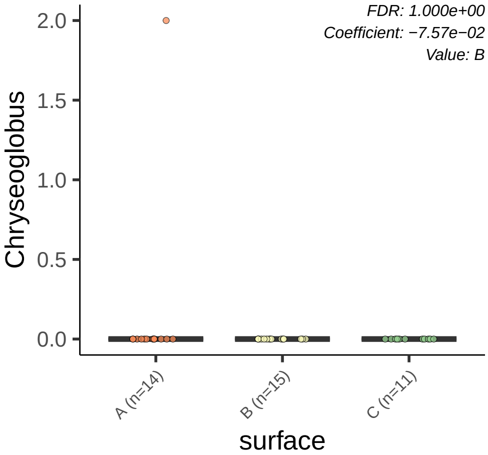
FDR: 1.000e+00
Coefficient: −7.57e−02
Value: B
Chryseoglobus
2.0
1.5
1.0
0.5
0.0
A (n=14)
B (n=15)
C (n=11)
surface

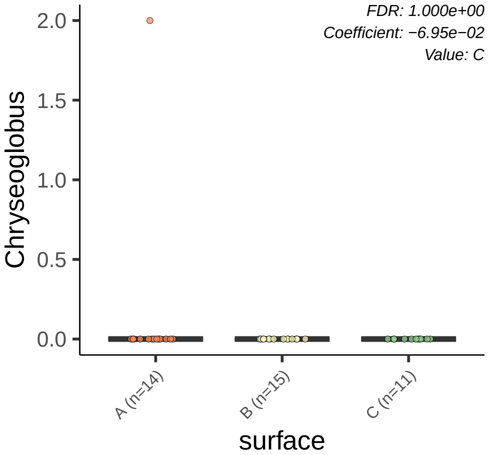

FDR: 1.000e+00
Coefficient: −6.95e−02
Value: C
Chryseoglobus
2.0
1.5
1.0
0.5
0.0
A (n=14)
B (n=15)
C (n=11)
surface

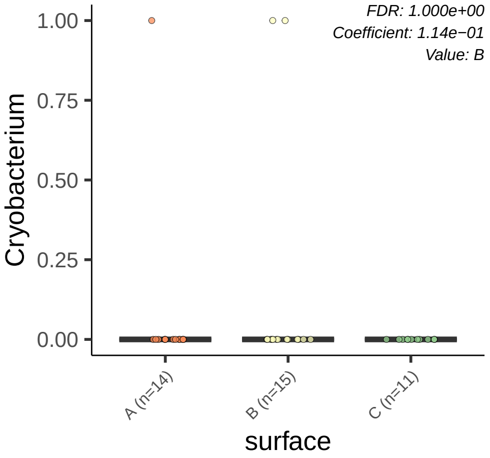
FDR: 1.000e+00
Coefficient: 1.14e−01
Value: B
Cryobacterium
1.00
0.75
0.50
0.25
0.00
A (n=14)
B (n=15)
C (n=11)
surface

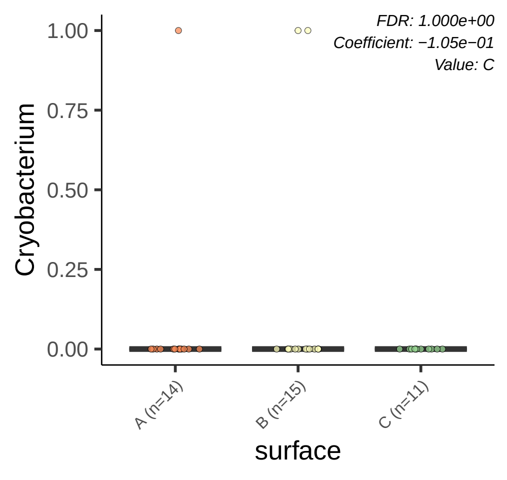
FDR: 1.000e+00
Coefficient: −1.05e−01
Value: C
Cryobacterium
1.00
0.75
0.50
0.25
0.00
A (n=14)
B (n=15)
C (n=11)
surface

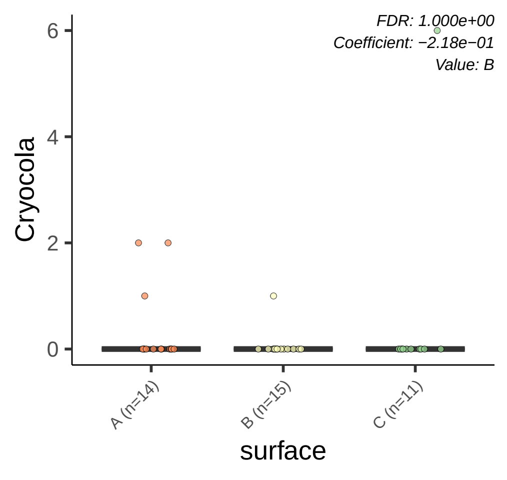
FDR: 1.000e+00
Coefficient: −2.18e−01
Value: B
Cryocola
0
2
4
6
A (n=14)
B (n=15)
C (n=11)
surface

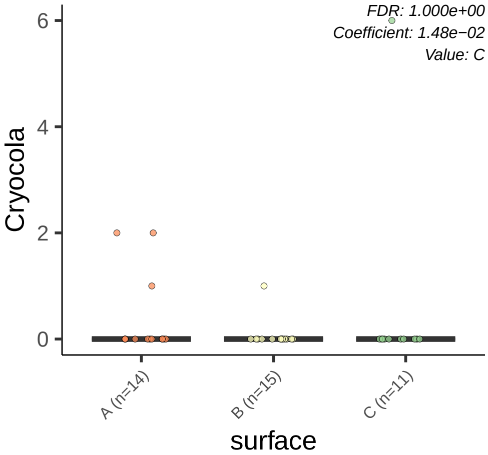

FDR: 1.000e+00
Coefficient: 1.48e−02
Value: C
Cryocola
6
4
2
0
A (n=14)
B (n=15)
C (n=11)
surface

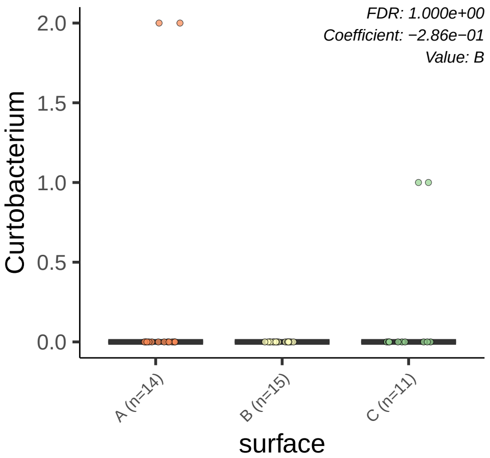
FDR: 1.000e+00
Coefficient: −2.86e−01
Value: B
Curtobacterium
2.0
1.5
1.0
0.5
0.0
A (n=14)
B (n=15)
C (n=11)
surface

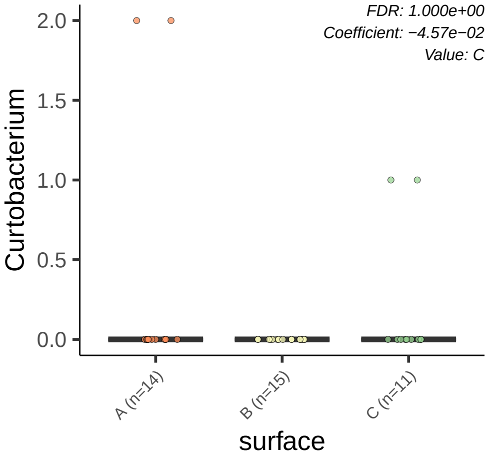
FDR: 1.000e+00
Coefficient: −4.57e−02
Value: C
Curtobacterium
2.0
1.5
1.0
0.5
0.0
A (n=14)
B (n=15)
C (n=11)
surface

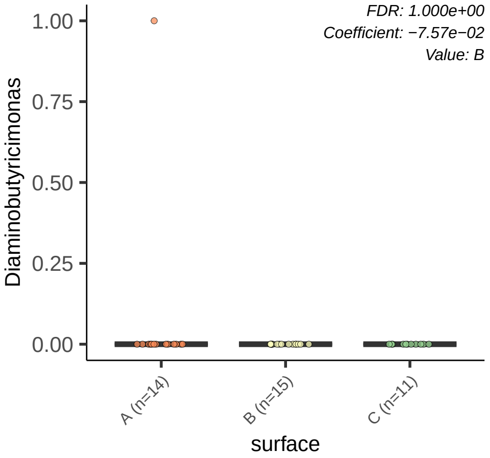
FDR: 1.000e+00
Coefficient: −7.57e−02
Value: B
Diaminobutyricimonas
1.00
0.75
0.50
0.25
0.00
A (n=14)
B (n=15)
C (n=11)
surface

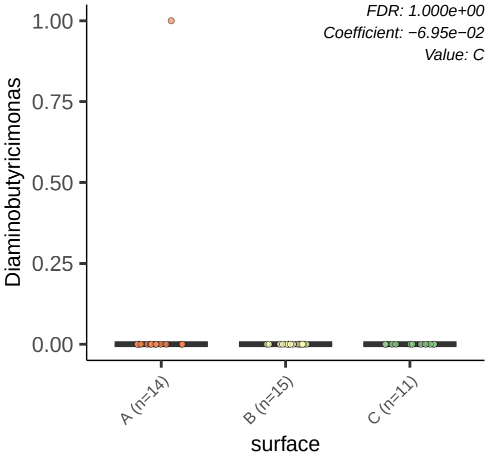

FDR: 1.000e+00
Coefficient: −6.95e−02
Value: C
Diaminobutyricimonas
1.00
0.75
0.50
0.25
0.00
A (n=14)
B (n=15)
C (n=11)
surface

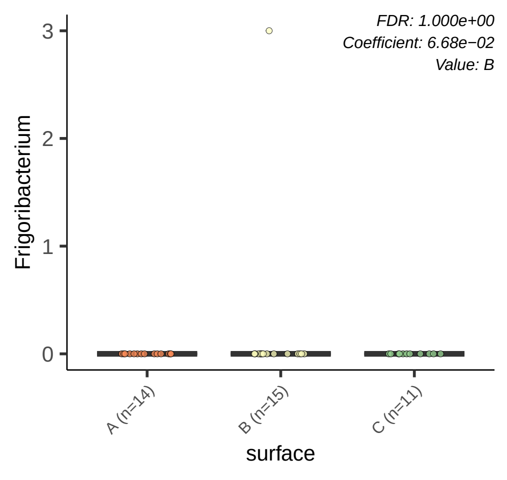
FDR: 1.000e+00
Coefficient: 6.68e−02
Value: B
Frigoribacterium
0
1
2
3
A (n=14)
B (n=15)
C (n=11)
surface

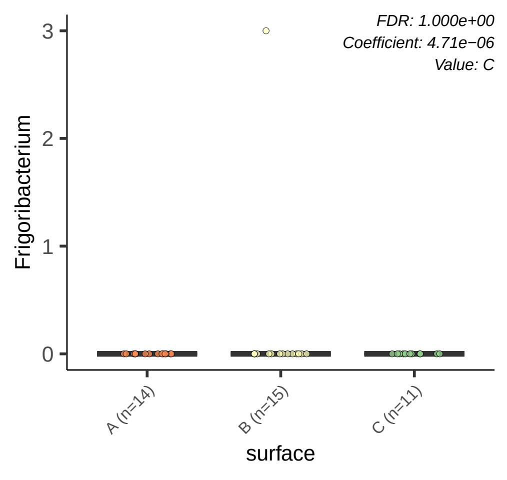
FDR: 1.000e+00
Coefficient: 4.71e−06
Value: C
Frigoribacterium
3
2
1
0
A (n=14)
B (n=15)
C (n=11)
surface

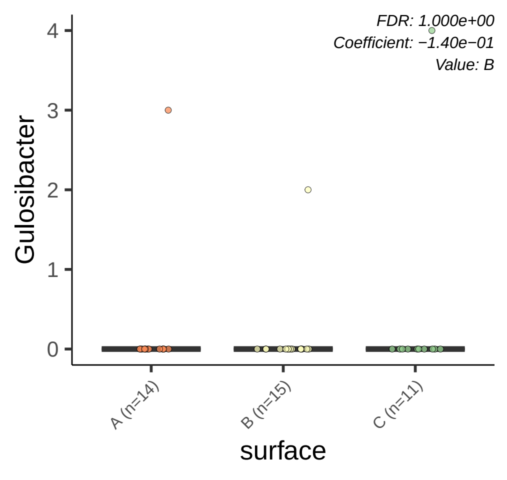

FDR: 1.000e+00
Coefficient: −1.40e−01
Value: B
Gulosibacter
4
3
2
1
0
A (n=14)
B (n=15)
C (n=11)
surface

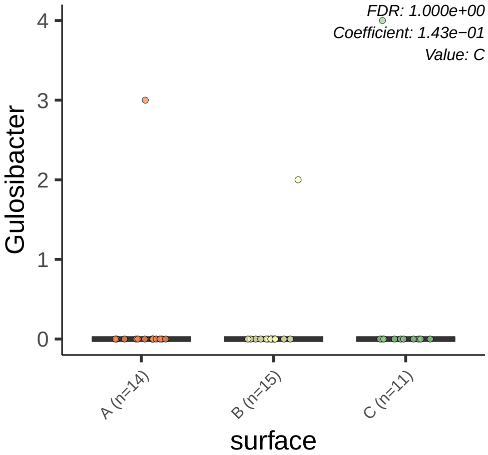
FDR: 1.000e+00
Coefficient: 1.43e−01
Value: C
Gulosibacter
4
3
2
1
0
A (n=14)
B (n=15)
C (n=11)
surface

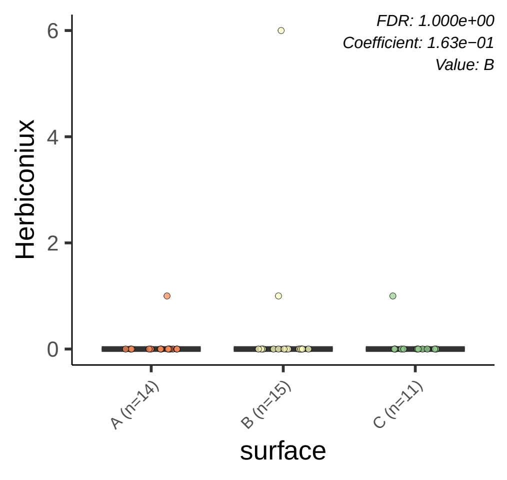
FDR: 1.000e+00
Coefficient: 1.63e−01
Value: B
Herbiconiux
0
2
4
6
A (n=14)
B (n=15)
C (n=11)
surface

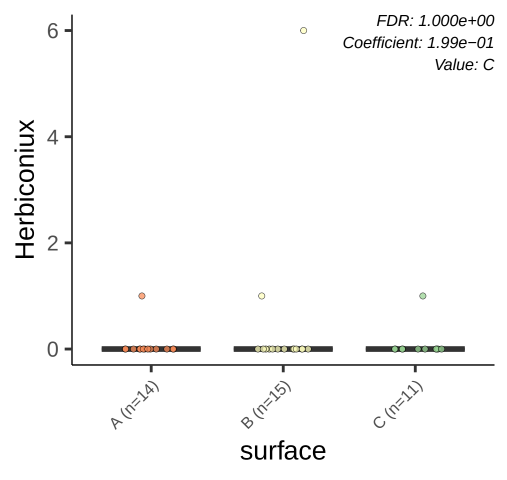

FDR: 1.000e+00
Coefficient: 1.99e−01
Value: C
Herbiconiux
0
2
4
6
A (n=14)
B (n=15)
C (n=11)
surface

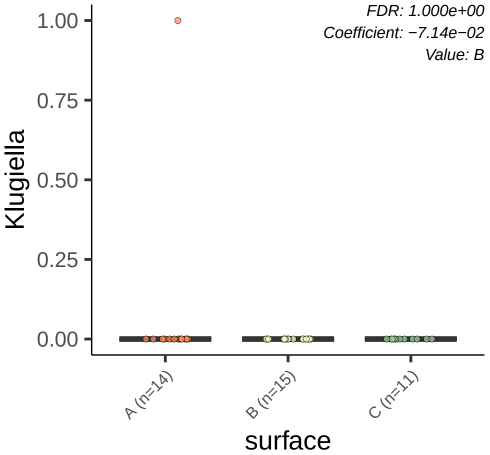

FDR: 1.000e+00
Coefficient: −7.14e−02
Value: B
Klugiella
1.00
0.75
0.50
0.25
0.00
A (n=14)
B (n=15)
C (n=11)
surface

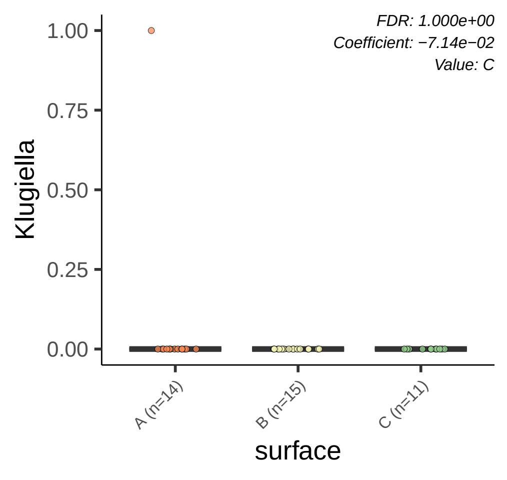
FDR: 1.000e+00
Coefficient: −7.14e−02
Value: C
Klugiella
1.00
0.75
0.50
0.25
0.00
A (n=14)
B (n=15)
C (n=11)
surface

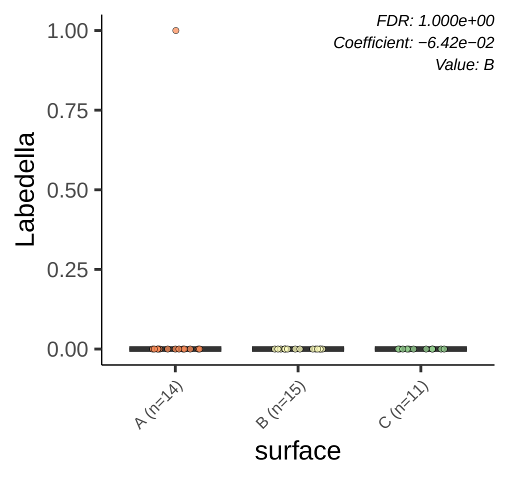
FDR: 1.000e+00
Coefficient: −6.42e−02
Value: B
1.00
0.75
0.50
0.25
0.00
Labedella
A (n=14)
B (n=15)
C (n=11)
surface

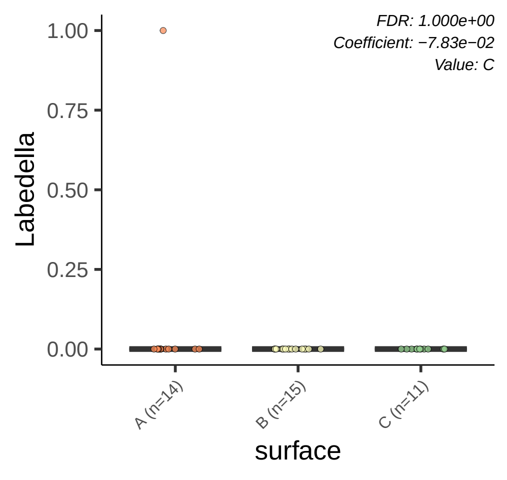
FDR: 1.000e+00
Coefficient: −7.83e−02
Value: C
Labedella
1.00
0.75
0.50
0.25
0.00
A (n=14)
B (n=15)
C (n=11)
surface

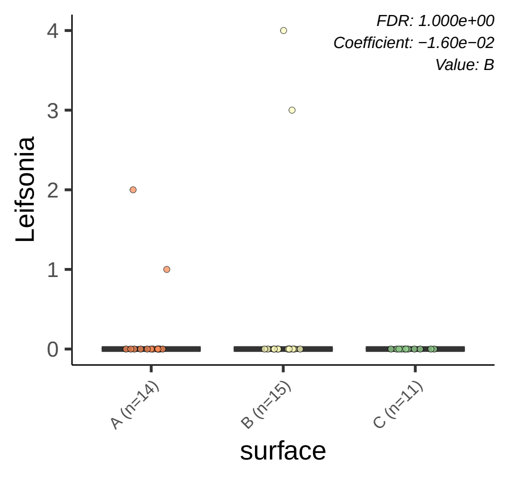
FDR: 1.000e+00
Coefficient: −1.60e−02
Value: B
Leifsonia
4
3
2
1
0
A (n=14)
B (n=15)
C (n=11)
surface

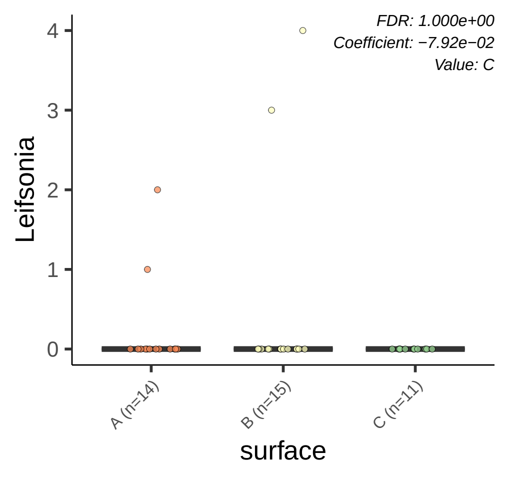
FDR: 1.000e+00
Coefficient: −7.92e−02
Value: C
Leifsonia
0
1
2
3
4
A (n=14)
B (n=15)
C (n=11)
surface

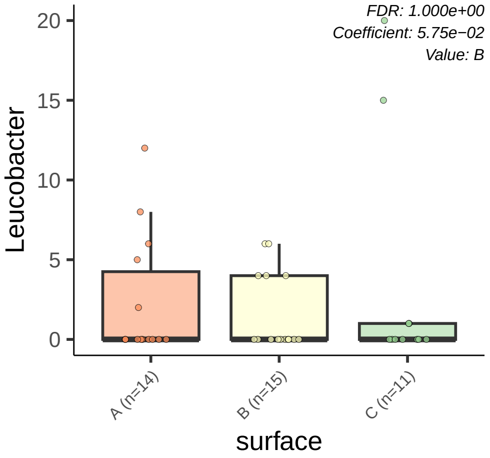
FDR: 1.000e+00
Coefficient: 5.75e−02
Value: B
Leucobacter
20
15
10
5
0
A (n=14)
B (n=15)
C (n=11)
surface

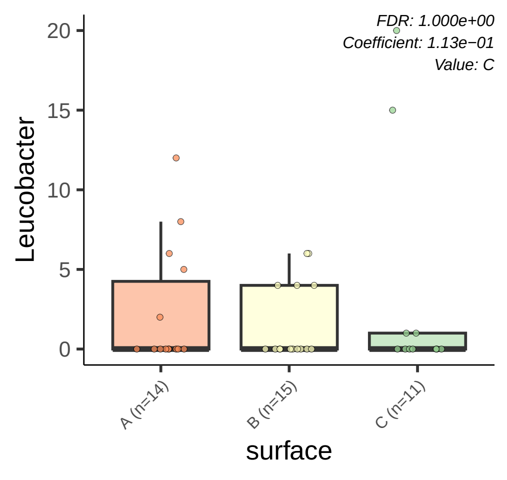
FDR: 1.000e+00
Coefficient: 1.13e−01
Value: C
Leucobacter
20
15
10
5
0
A (n=14)
B (n=15)
C (n=11)
surface

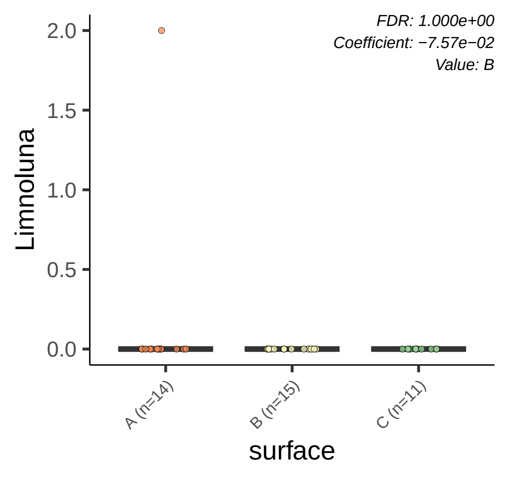

FDR: 1.000e+00
Coefficient: −7.57e−02
Value: B
Limnoluna
2.0
1.5
1.0
0.5
0.0
A (n=14)
B (n=15)
C (n=11)
surface

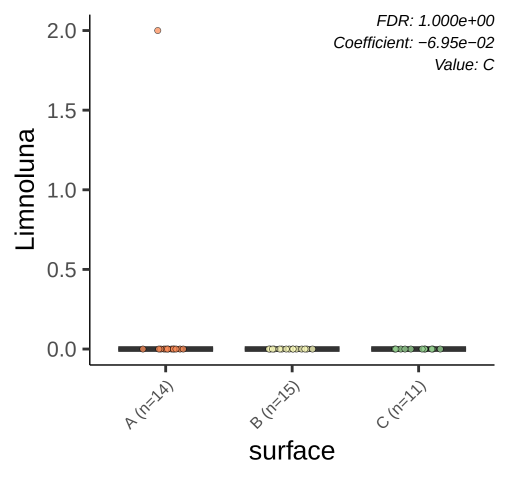
FDR: 1.000e+00
Coefficient: −6.95e−02
Value: C
Limnoluna
2.0
1.5
1.0
0.5
0.0
A (n=14)
B (n=15)
C (n=11)
surface

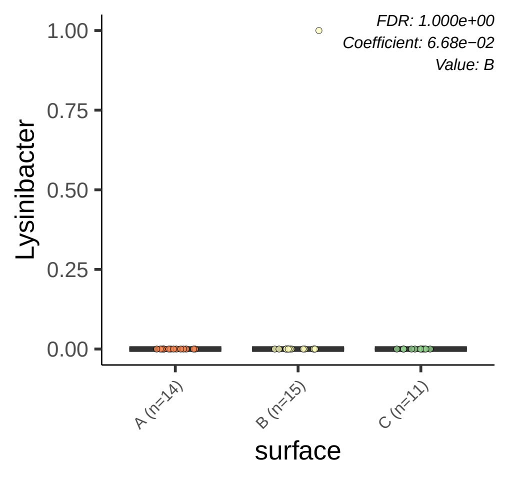

FDR: 1.000e+00
Coefficient: 6.68e−02
Value: B
Lysinibacter
1.00
0.75
0.50
0.25
0.00
A (n=14)
B (n=15)
C (n=11)
surface

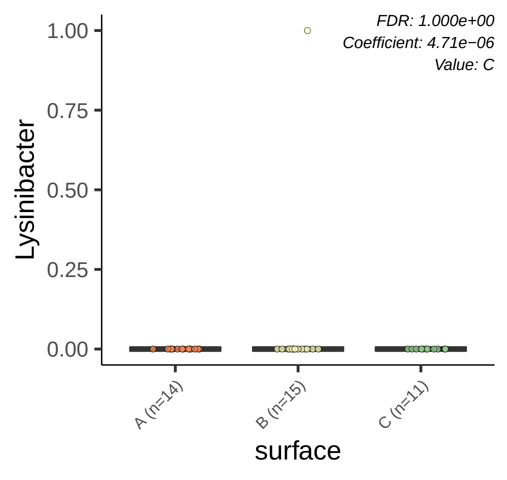
FDR: 1.000e+00
Coefficient: 4.71e−06
Value: C
Lysinibacter
1.00
0.75
0.50
0.25
0.00
A (n=14)
B (n=15)
C (n=11)
surface

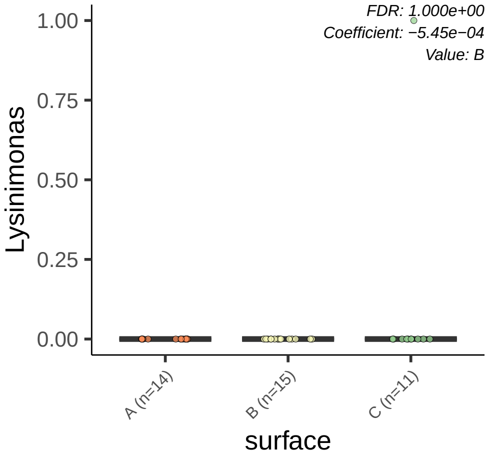
FDR: 1.000e+00
Coefficient: −5.45e−04
Value: B
Lysinimonas
1.00
0.75
0.50
0.25
0.00
A (n=14)
B (n=15)
C (n=11)
surface

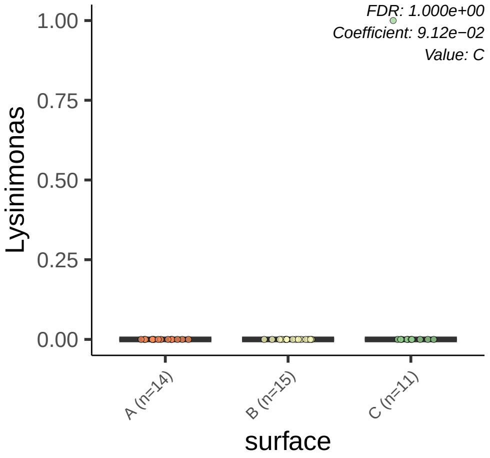
FDR: 1.000e+00
Coefficient: 9.12e−02
Value: C
1.00
0.75
0.50
0.25
0.00
Lysinimonas
A (n=14)
B (n=15)
C (n=11)
surface

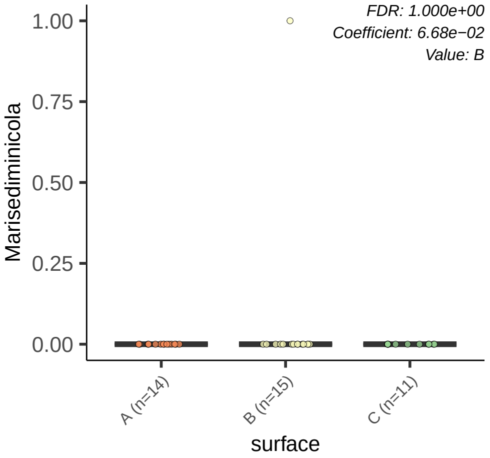
FDR: 1.000e+00
Coefficient: 6.68e−02
Value: B
Marisediminicola
1.00
0.75
0.50
0.25
0.00
A (n=14)
B (n=15)
C (n=11)
surface

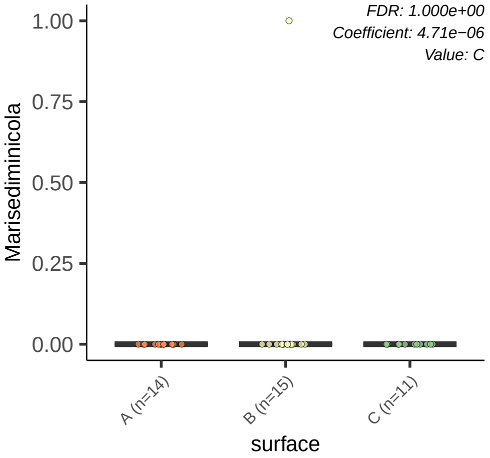

FDR: 1.000e+00
Coefficient: 4.71e−06
Value: C
Marisediminicola
1.00
0.75
0.50
0.25
0.00
A (n=14)
B (n=15)
C (n=11)
surface

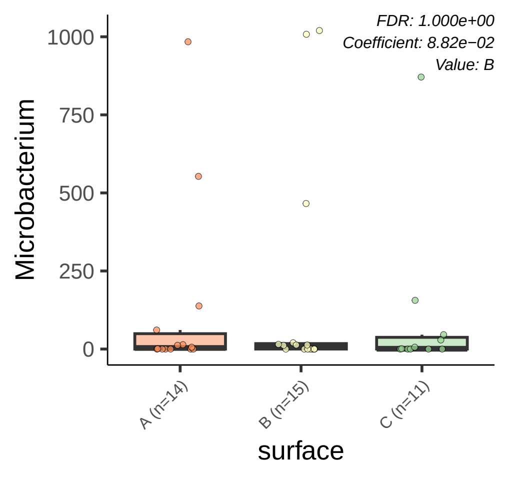

FDR: 1.000e+00
Coefficient: 8.82e−02
Value: B
Microbacterium
1000
750
500
250
0
A (n=14)
B (n=15)
C (n=11)
surface

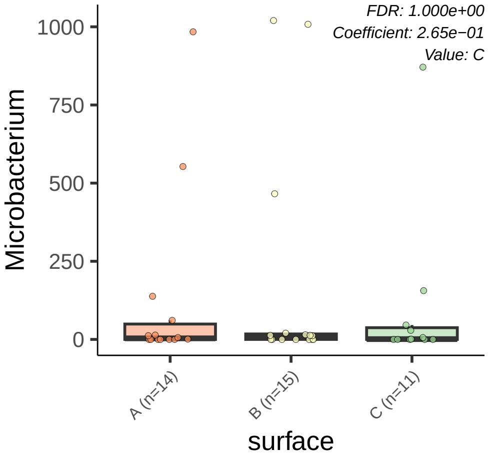

FDR: 1.000e+00
Coefficient: 2.65e−01
Value: C
Microbacterium
1000
750
500
250
0
A (n=14)
B (n=15)
C (n=11)
surface

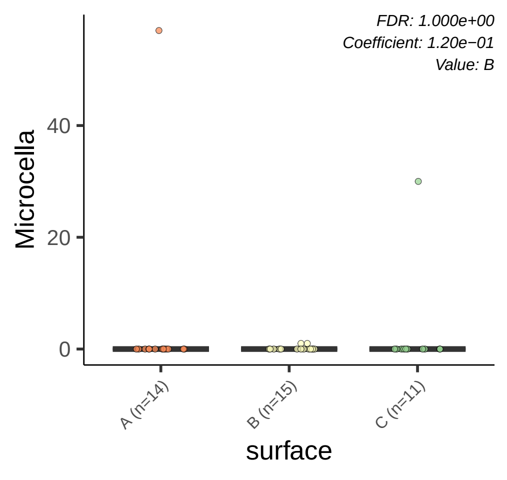
FDR: 1.000e+00
Coefficient: 1.20e−01
Value: B
Microcella
40
20
0
A (n=14)
B (n=15)
C (n=11)
surface

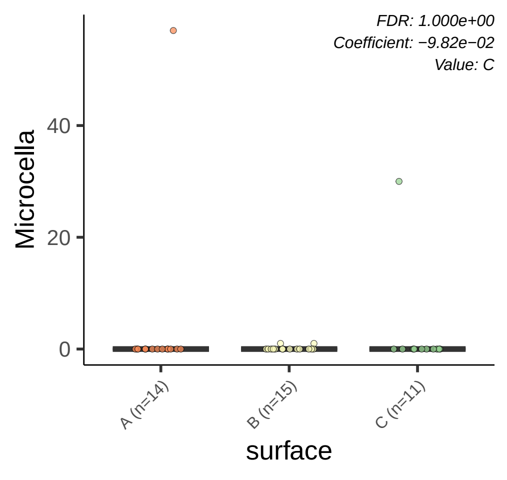
FDR: 1.000e+00
Coefficient: −9.82e−02
Value: C
Microcella
0
20
40
A (n=14)
B (n=15)
C (n=11)
surface

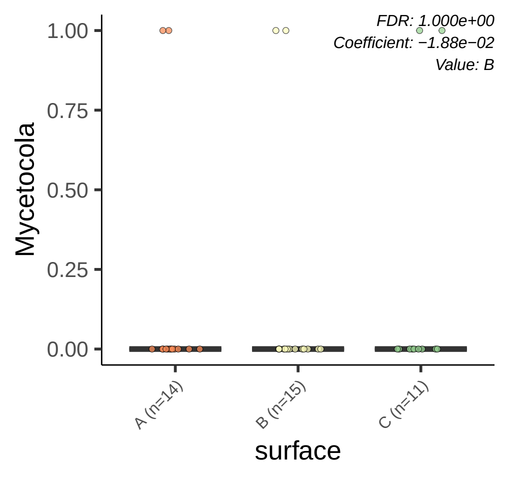
FDR: 1.000e+00
Coefficient: −1.88e−02
Value: B
Mycetocola
1.00
0.75
0.50
0.25
0.00
A (n=14)
B (n=15)
C (n=11)
surface

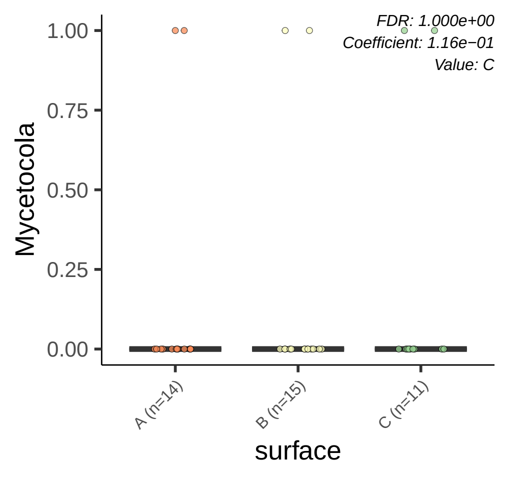

FDR: 1.000e+00
Coefficient: 1.16e−01
Value: C
Mycetocola
1.00
0.75
0.50
0.25
0.00
A (n=14)
B (n=15)
C (n=11)
surface

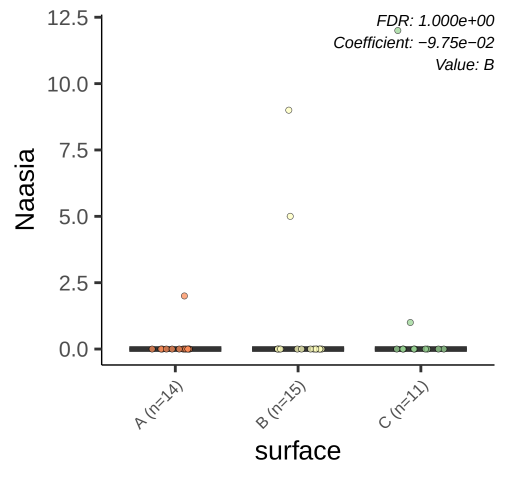
FDR: 1.000e+00
Coefficient: −9.75e−02
Value: B
12.5
10.0
7.5
5.0
2.5
0.0
Naasia
A (n=14)
B (n=15)
C (n=11)
surface

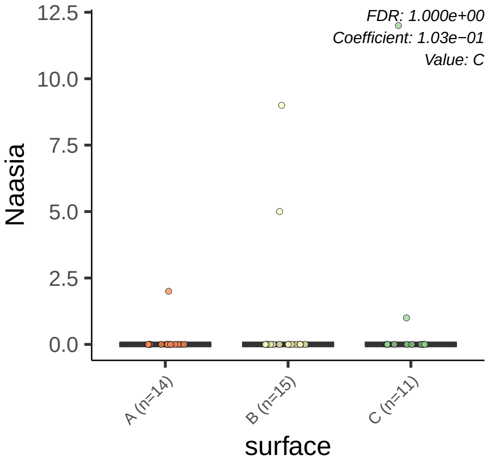

FDR: 1.000e+00
Coefficient: 1.03e−01
Value: C
12.5
10.0
7.5
5.0
2.5
0.0
Naasia
A (n=14)
B (n=15)
C (n=11)
surface

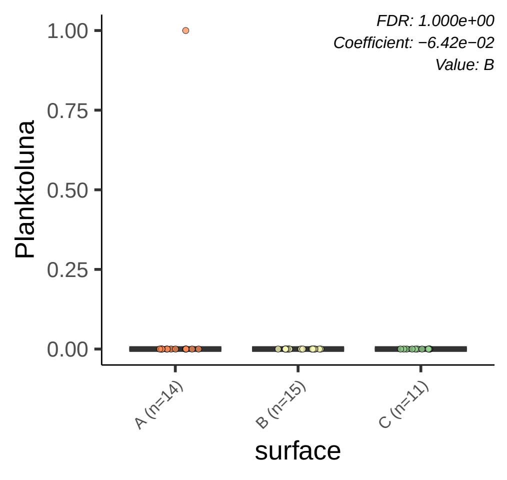

FDR: 1.000e+00
Coefficient: −6.42e−02
Value: B
Planktoluna
1.00
0.75
0.50
0.25
0.00
A (n=14)
B (n=15)
C (n=11)
surface

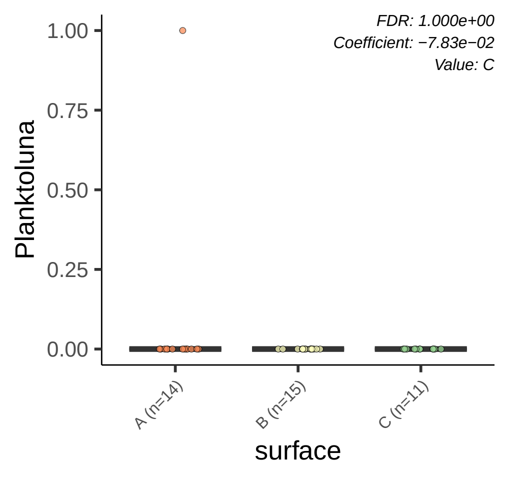
FDR: 1.000e+00
Coefficient: −7.83e−02
Value: C
Planktoluna
1.00
0.75
0.50
0.25
0.00
A (n=14)
B (n=15)
C (n=11)
surface

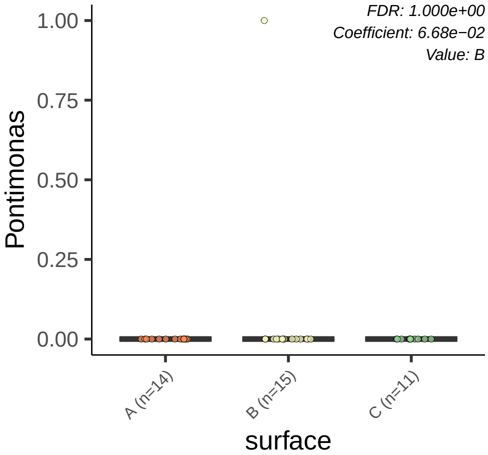

FDR: 1.000e+00
Coefficient: 6.68e−02
Value: B
Pontimonas
1.00
0.75
0.50
0.25
0.00
A (n=14)
B (n=15)
C (n=11)
surface

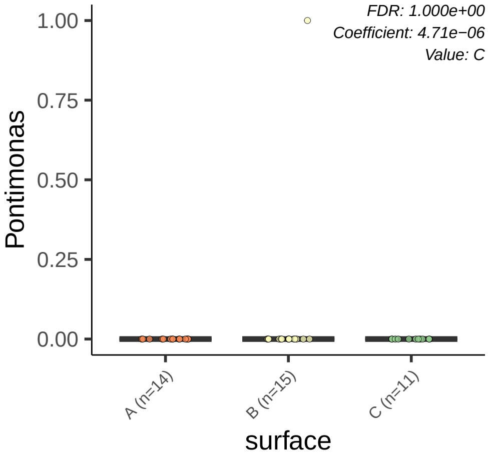
FDR: 1.000e+00
Coefficient: 4.71e−06
Value: C
Pontimonas
1.00
0.75
0.50
0.25
0.00
A (n=14)
B (n=15)
C (n=11)
surface

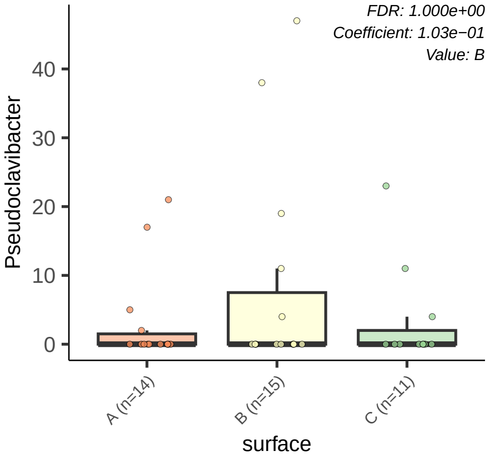
FDR: 1.000e+00
Coefficient: 1.03e−01
Value: B
Pseudoclavibacter
40
30
20
10
0
A (n=14)
B (n=15)
C (n=11)
surface

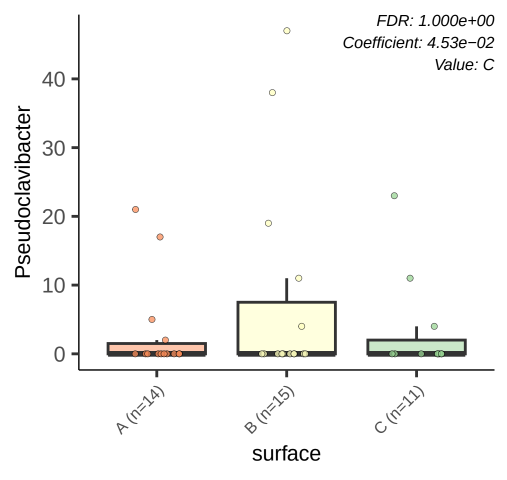
FDR: 1.000e+00
Coefficient: 4.53e−02
Value: C
Pseudoclavibacter
0
10
20
30
40
A (n=14)
B (n=15)
C (n=11)
surface

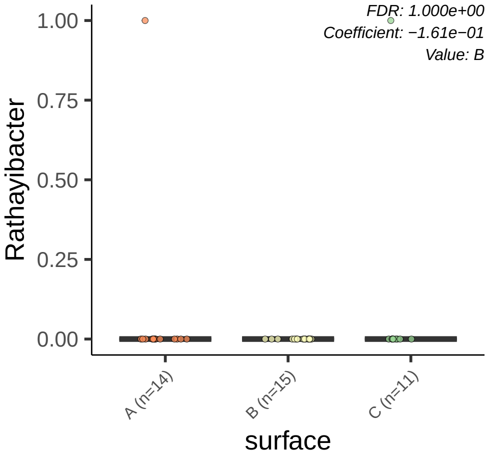
FDR: 1.000e+00
Coefficient: −1.61e−01
Value: B
Rathayibacter
1.00
0.75
0.50
0.25
0.00
A (n=14)
B (n=15)
C (n=11)
surface

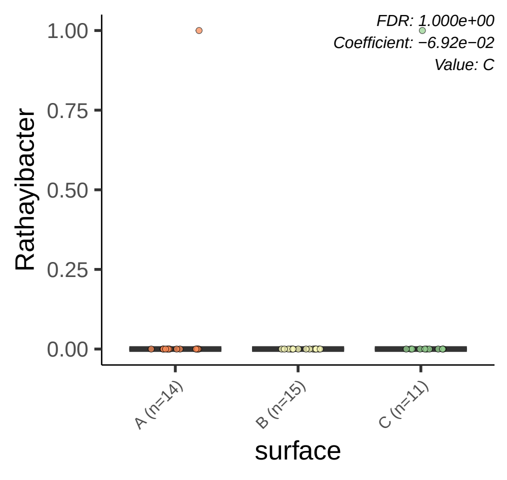
FDR: 1.000e+00
Coefficient: −6.92e−02
Value: C
Rathayibacter
1.00
0.75
0.50
0.25
0.00
A (n=14)
B (n=15)
C (n=11)
surface

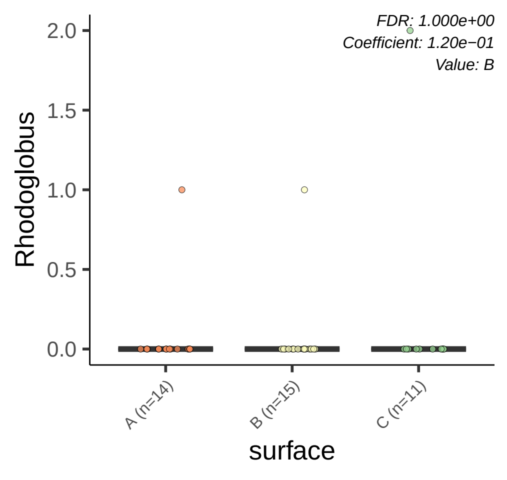
FDR: 1.000e+00
Coefficient: 1.20e−01
Value: B
Rhodoglobus
2.0
1.5
1.0
0.5
0.0
A (n=14)
B (n=15)
C (n=11)
surface

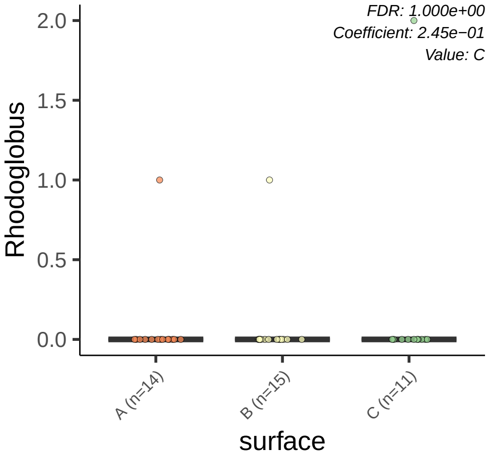
FDR: 1.000e+00
Coefficient: 2.45e−01
Value: C
Rhodoglobus
2.0
1.5
1.0
0.5
0.0
A (n=14)
B (n=15)
C (n=11)
surface

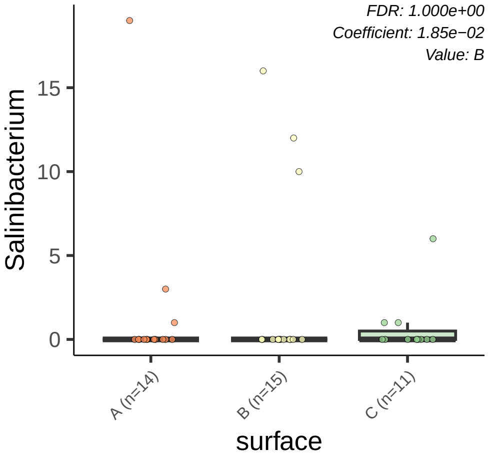

FDR: 1.000e+00
Coefficient: 1.85e−02
Value: B
Salinibacterium
15
10
5
0
A (n=14)
B (n=15)
C (n=11)
surface

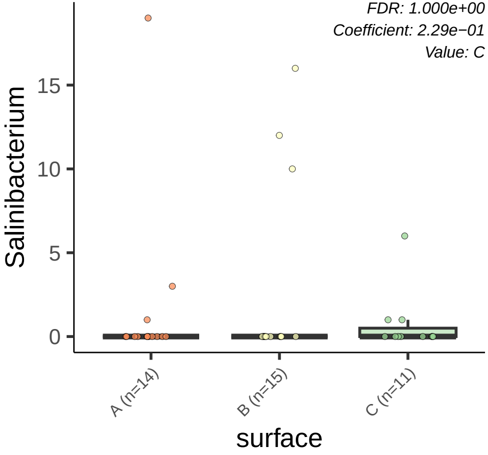
FDR: 1.000e+00
Coefficient: 2.29e−01
Value: C
Salinibacterium
15
10
5
0
A (n=14)
B (n=15)
C (n=11)
surface

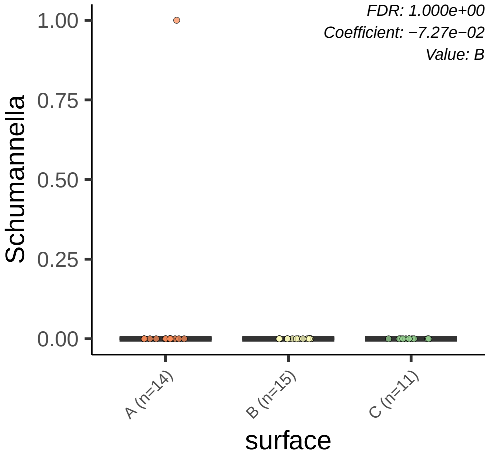
FDR: 1.000e+00
Coefficient: −7.27e−02
Value: B
Schumannella
1.00
0.75
0.50
0.25
0.00
A (n=14)
B (n=15)
C (n=11)
surface

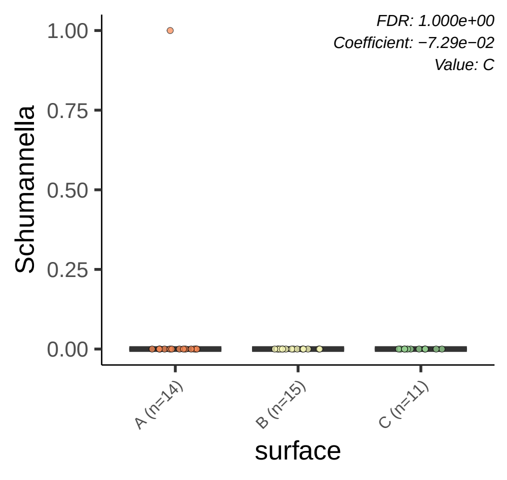

FDR: 1.000e+00
Coefficient: −7.29e−02
Value: C
Schumannella
1.00
0.75
0.50
0.25
0.00
A (n=14)
B (n=15)
C (n=11)
surface

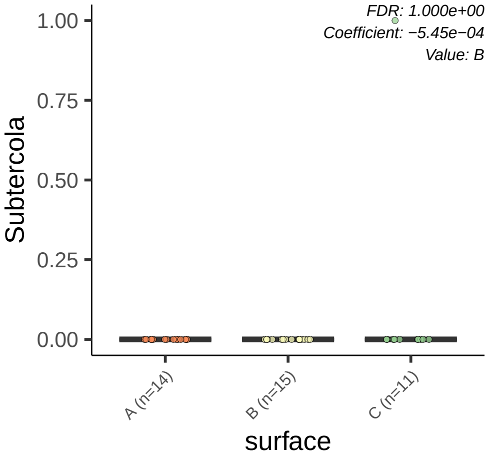

FDR: 1.000e+00
Coefficient: −5.45e−04
Value: B
Subtercola
1.00
0.75
0.50
0.25
0.00
A (n=14)
B (n=15)
C (n=11)
surface

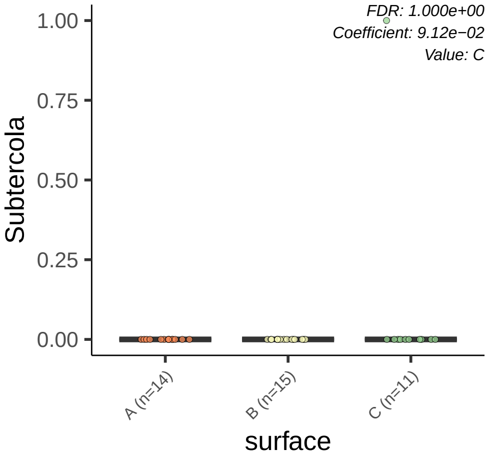
FDR: 1.000e+00
Coefficient: 9.12e−02
Value: C
1.00
0.75
0.50
0.25
0.00
Subtercola
A (n=14)
B (n=15)
C (n=11)
surface

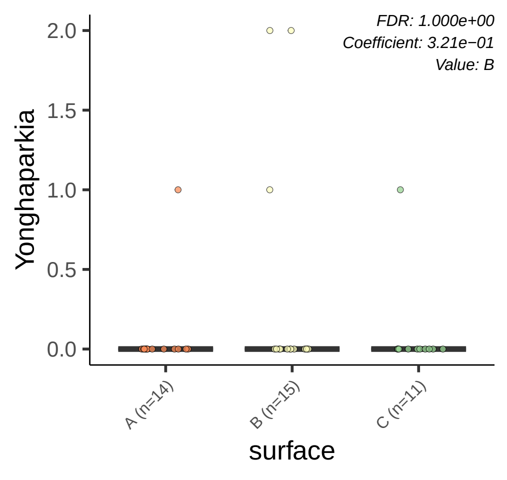

FDR: 1.000e+00
Coefficient: 3.21e−01
Value: B
Yonghaparkia
2.0
1.5
1.0
0.5
0.0
A (n=14)
B (n=15)
C (n=11)
surface

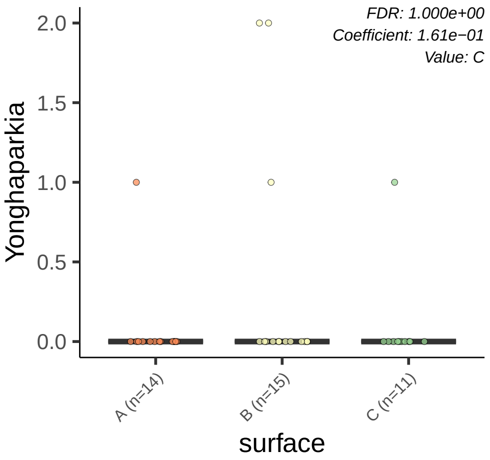
FDR: 1.000e+00
Coefficient: 1.61e−01
Value: C
Yonghaparkia
2.0
1.5
1.0
0.5
0.0
A (n=14)
B (n=15)
C (n=11)
surface

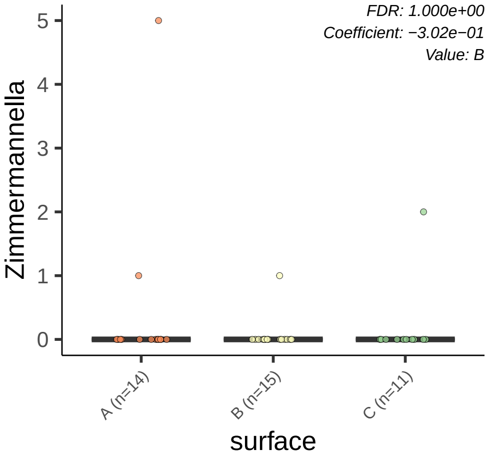
FDR: 1.000e+00
Coefficient: −3.02e−01
Value: B
Zimmermannella
5
4
3
2
1
0
A (n=14)
B (n=15)
C (n=11)
surface

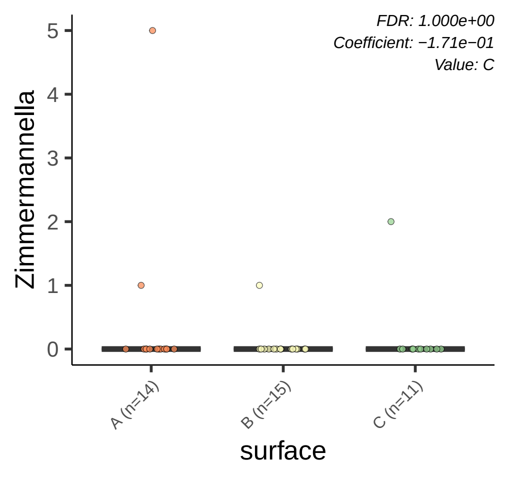
FDR: 1.000e+00
Coefficient: −1.71e−01
Value: C
Zimmermannella
0
1
2
3
4
5
A (n=14)
B (n=15)
C (n=11)
surface

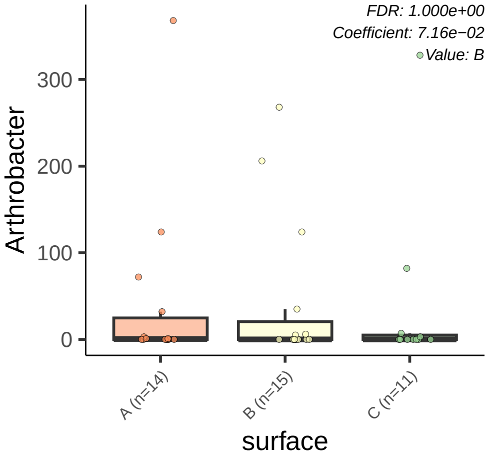
FDR: 1.000e+00
Coefficient: 7.16e−02
Value: B
Arthrobacter
300
200
100
0
A (n=14)
B (n=15)
C (n=11)
surface

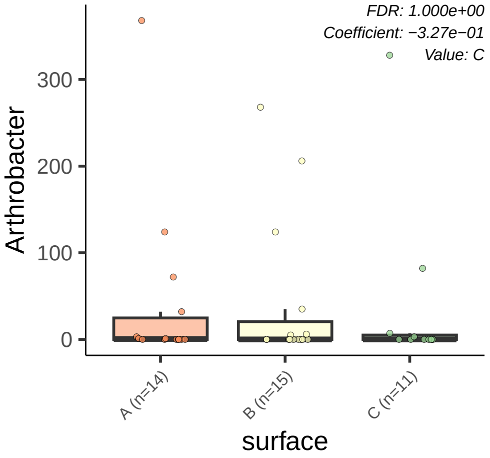
FDR: 1.000e+00
Coefficient: −3.27e−01
Value: C
Arthrobacter
300
200
100
0
A (n=14)
B (n=15)
C (n=11)
surface

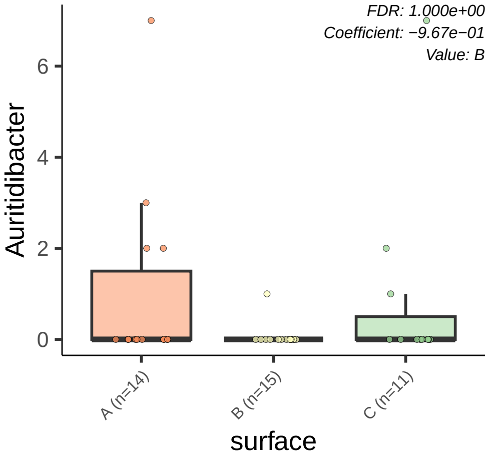
FDR: 1.000e+00
Coefficient: −9.67e−01
Value: B
Auritidibacter
0
2
4
6
A (n=14)
B (n=15)
C (n=11)
surface

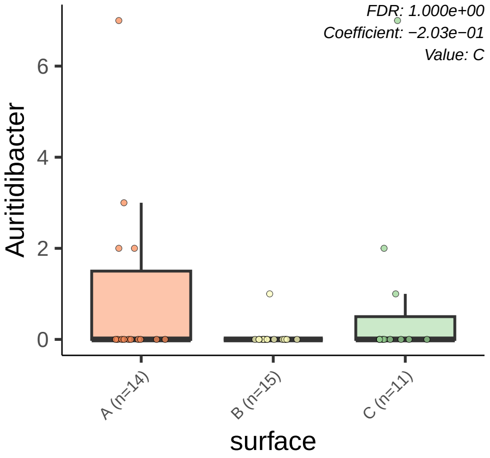
FDR: 1.000e+00
Coefficient: −2.03e−01
Value: C
Auritidibacter
0
2
4
6
A (n=14)
B (n=15)
C (n=11)
surface

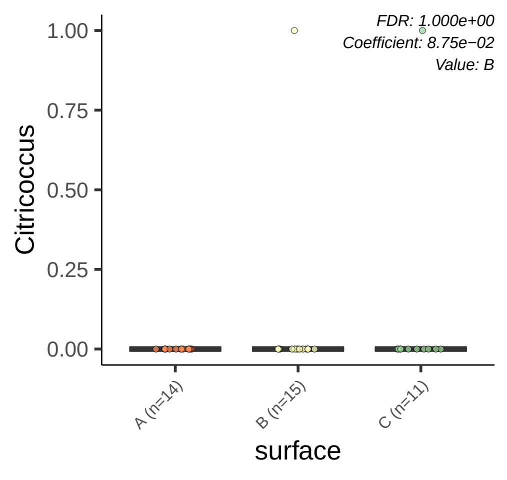
FDR: 1.000e+00
Coefficient: 8.75e−02
Value: B
Citricoccus
1.00
0.75
0.50
0.25
0.00
A (n=14)
B (n=15)
C (n=11)
surface

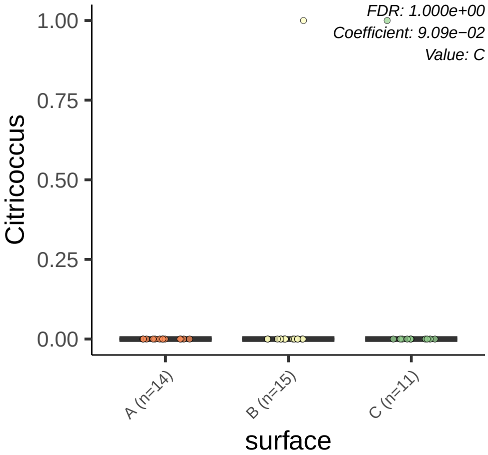

FDR: 1.000e+00
Coefficient: 9.09e−02
Value: C
Citricoccus
1.00
0.75
0.50
0.25
0.00
A (n=14)
B (n=15)
C (n=11)
surface

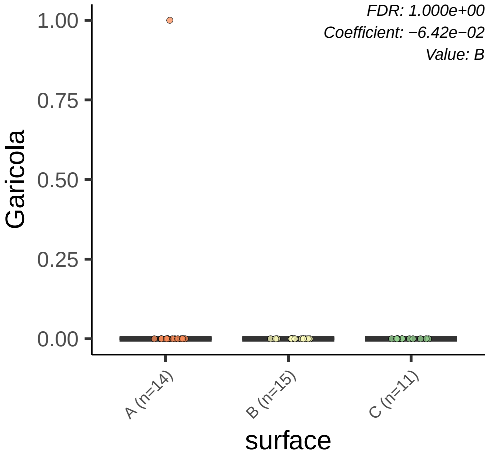
FDR: 1.000e+00
Coefficient: −6.42e−02
Value: B
Garicola
1.00
0.75
0.50
0.25
0.00
A (n=14)
B (n=15)
C (n=11)
surface

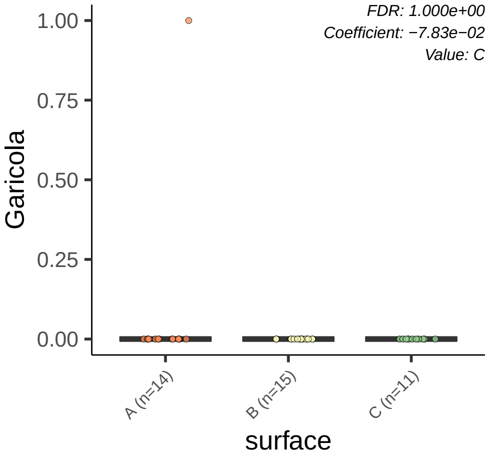
FDR: 1.000e+00
Coefficient: −7.83e−02
Value: C
Garicola
1.00
0.75
0.50
0.25
0.00
A (n=14)
B (n=15)
C (n=11)
surface

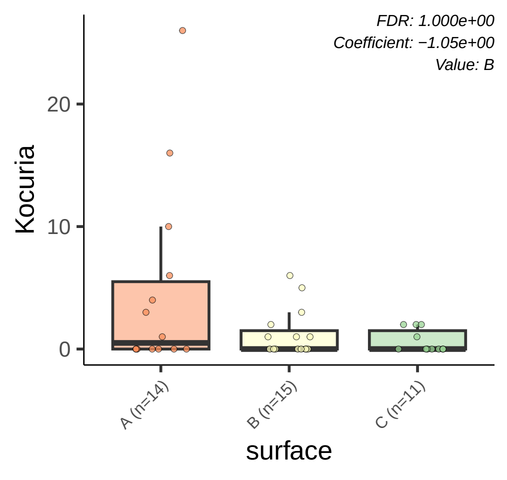

FDR: 1.000e+00
Coefficient: −1.05e+00
Value: B
Kocuria
20
10
0
A (n=14)
B (n=15)
C (n=11)
surface

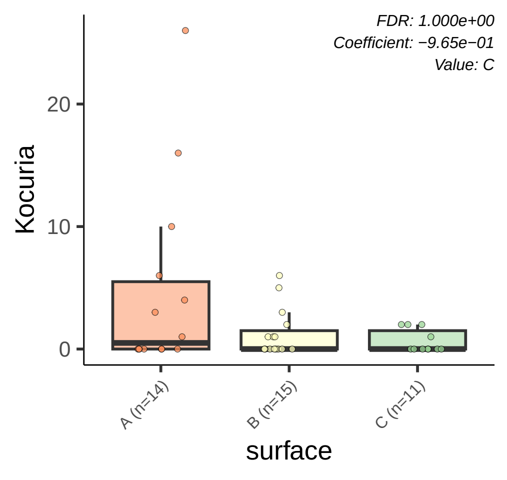

FDR: 1.000e+00
Coefficient: −9.65e−01
Value: C
Kocuria
20
10
0
A (n=14)
B (n=15)
C (n=11)
surface

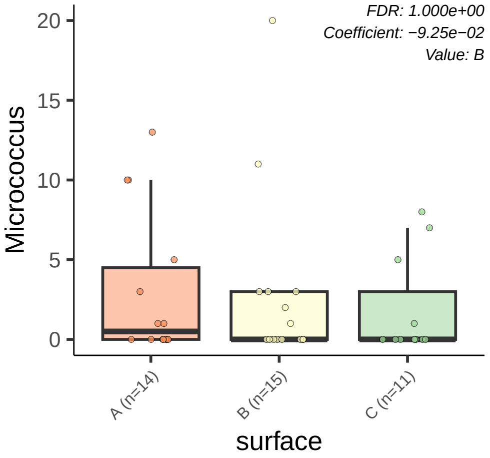
FDR: 1.000e+00
Coefficient: −9.25e−02
Value: B
Micrococcus
20
15
10
5
0
A (n=14)
B (n=15)
C (n=11)
surface

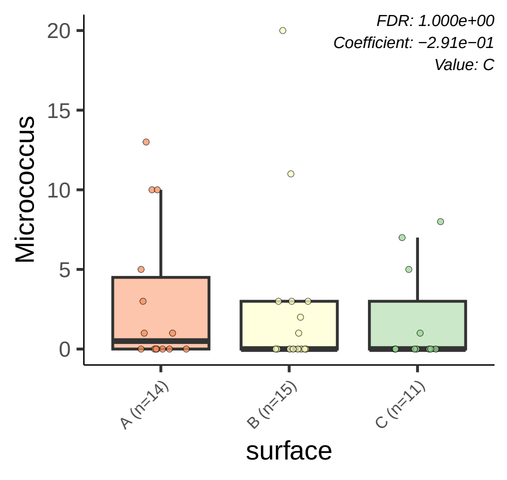
FDR: 1.000e+00
Coefficient: −2.91e−01
Value: C
Micrococcus
0
5
10
15
20
A (n=14)
B (n=15)
C (n=11)
surface

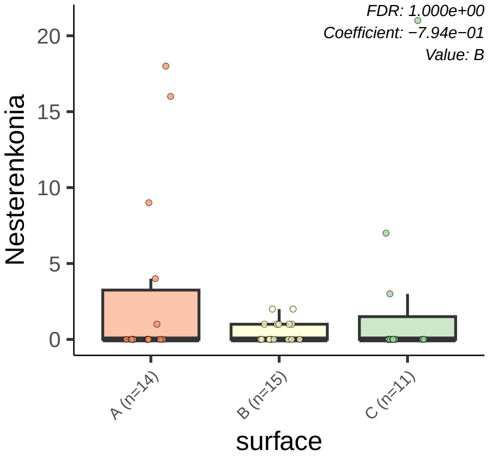

FDR: 1.000e+00
Coefficient: −7.94e−01
Value: B
Nesterenkonia
0
5
10
15
20
A (n=14)
B (n=15)
C (n=11)
surface

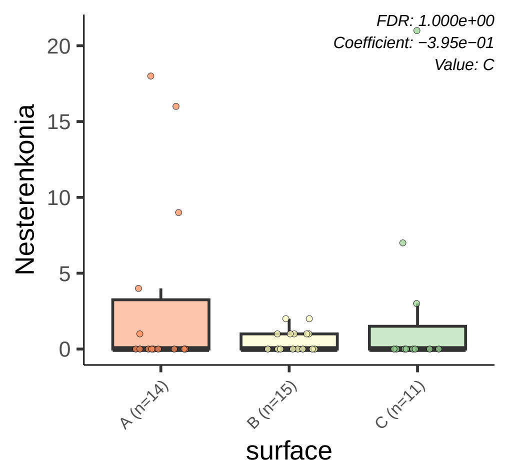
FDR: 1.000e+00
Coefficient: −3.95e−01
Value: C
Nesterenkonia
20
15
10
5
0
A (n=14)
B (n=15)
C (n=11)
surface

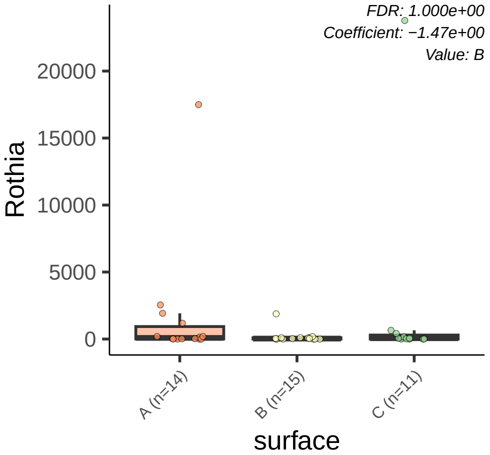
FDR: 1.000e+00
Coefficient: −1.47e+00
Value: B
Rothia
20000
15000
10000
5000
0
A (n=14)
B (n=15)
C (n=11)
surface

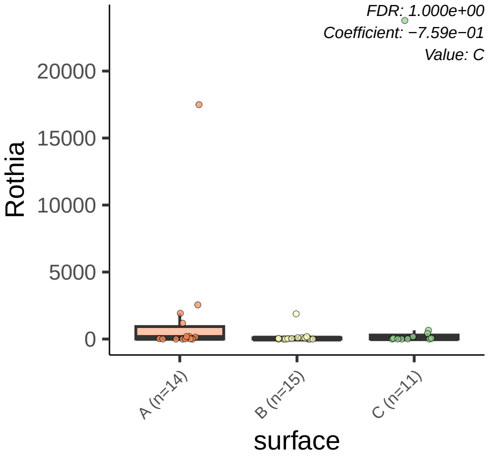

FDR: 1.000e+00
Coefficient: −7.59e−01
Value: C
Rothia
20000
15000
10000
5000
0
A (n=14)
B (n=15)
C (n=11)
surface

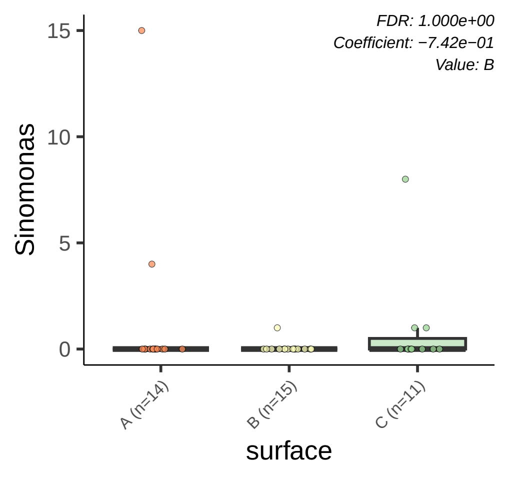
FDR: 1.000e+00
Coefficient: −7.42e−01
Value: B
Sinomonas
15
10
5
0
A (n=14)
B (n=15)
C (n=11)
surface

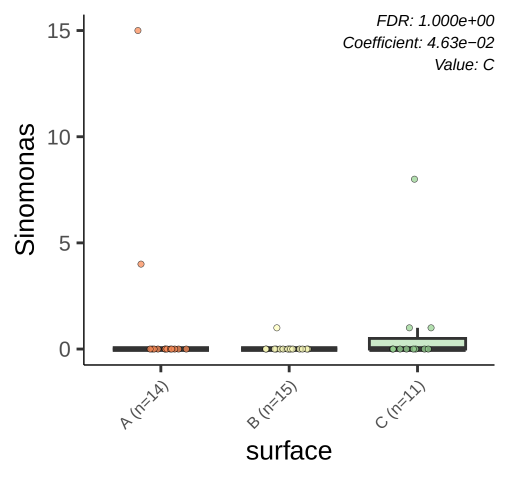
FDR: 1.000e+00
Coefficient: 4.63e−02
Value: C
Sinomonas
15
10
5
0
A (n=14)
B (n=15)
C (n=11)
surface

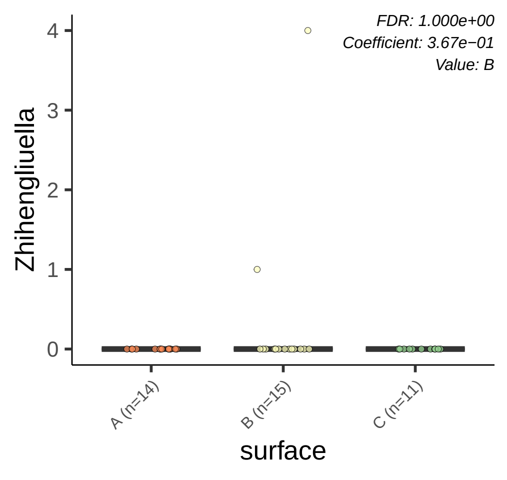
FDR: 1.000e+00
Coefficient: 3.67e−01
Value: B
Zhihengliuella
0
1
2
3
4
A (n=14)
B (n=15)
C (n=11)
surface

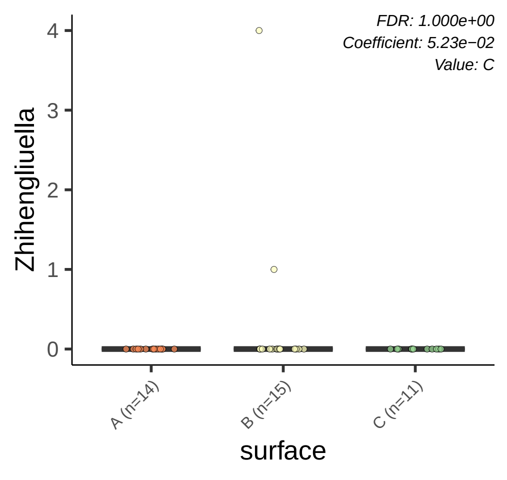

FDR: 1.000e+00
Coefficient: 5.23e−02
Value: C
Zhihengliuella
4
3
2
1
0
A (n=14)
B (n=15)
C (n=11)
surface

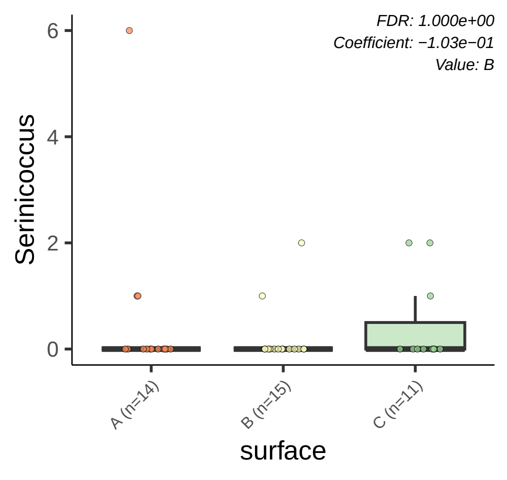
FDR: 1.000e+00
Coefficient: −1.03e−01
Value: B
Serinicoccus
6
4
2
0
A (n=14)
B (n=15)
C (n=11)
surface

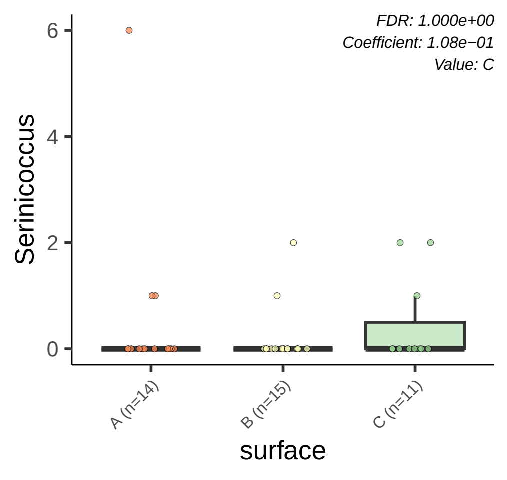
FDR: 1.000e+00
Coefficient: 1.08e−01
Value: C
Serinicoccus
0
2
4
6
A (n=14)
B (n=15)
C (n=11)
surface

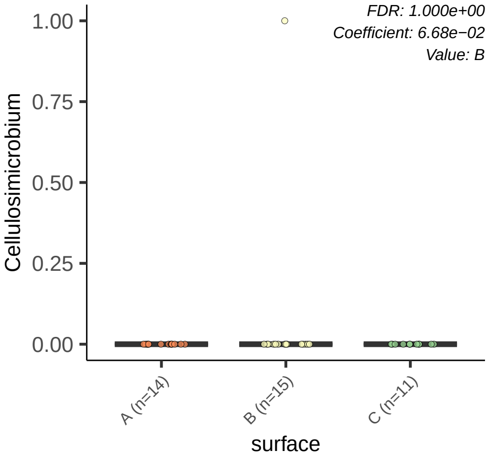

FDR: 1.000e+00
Coefficient: 6.68e−02
Value: B
Cellulosimicrobium
1.00
0.75
0.50
0.25
0.00
A (n=14)
B (n=15)
C (n=11)
surface

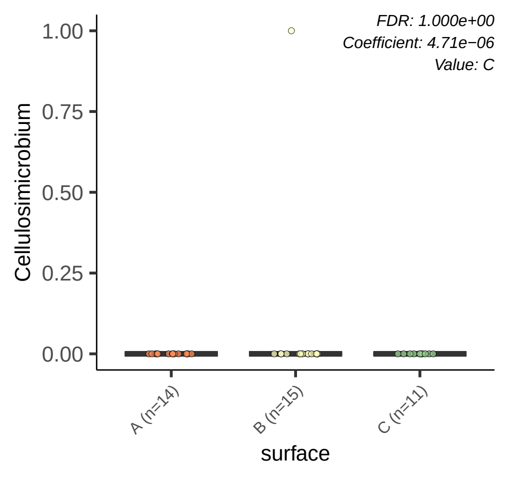
FDR: 1.000e+00
Coefficient: 4.71e−06
Value: C
Cellulosimicrobium
1.00
0.75
0.50
0.25
0.00
A (n=14)
B (n=15)
C (n=11)
surface

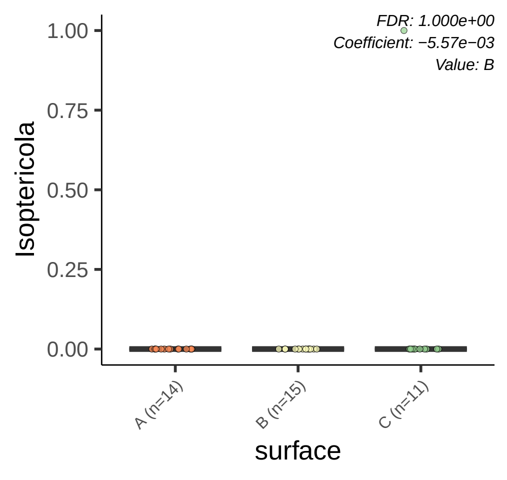

FDR: 1.000e+00
Coefficient: −5.57e−03
Value: B
Isoptericola
1.00
0.75
0.50
0.25
0.00
A (n=14)
B (n=15)
C (n=11)
surface

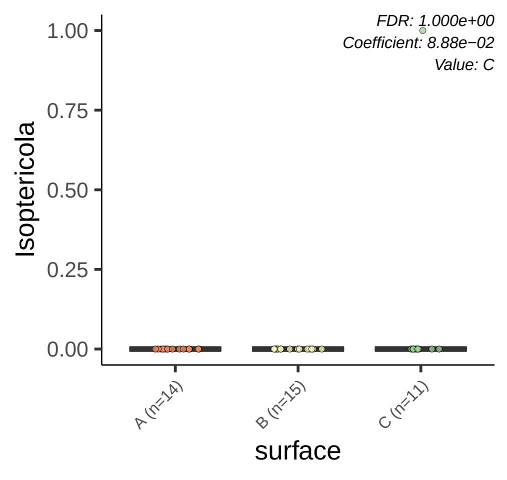
FDR: 1.000e+00
Coefficient: 8.88e−02
Value: C
Isoptericola
1.00
0.75
0.50
0.25
0.00
A (n=14)
B (n=15)
C (n=11)
surface

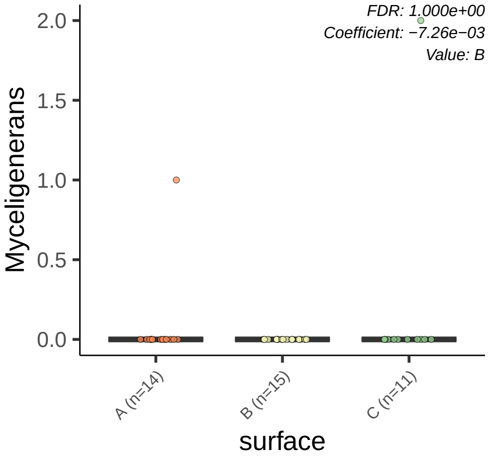

FDR: 1.000e+00
Coefficient: −7.26e−03
Value: B
Myceligenerans
2.0
1.5
1.0
0.5
0.0
A (n=14)
B (n=15)
C (n=11)
surface

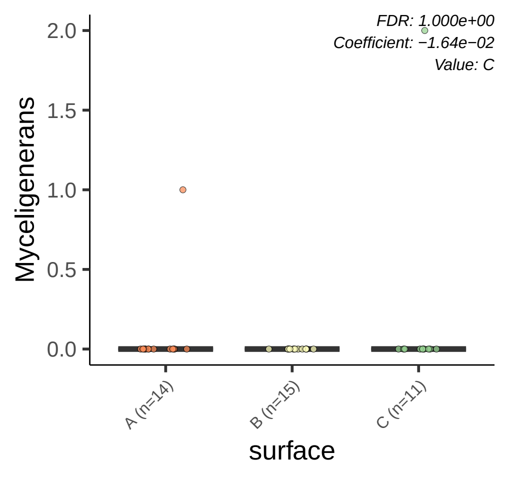
FDR: 1.000e+00
Coefficient: −1.64e−02
Value: C
Myceligenerans
2.0
1.5
1.0
0.5
0.0
A (n=14)
B (n=15)
C (n=11)
surface

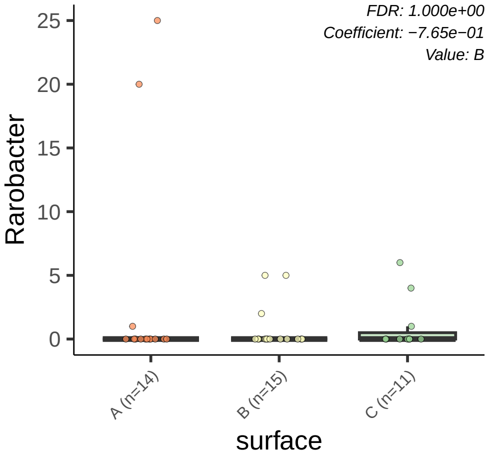

FDR: 1.000e+00
Coefficient: −7.65e−01
Value: B
Rarobacter
25
20
15
10
5
0
A (n=14)
B (n=15)
C (n=11)
surface

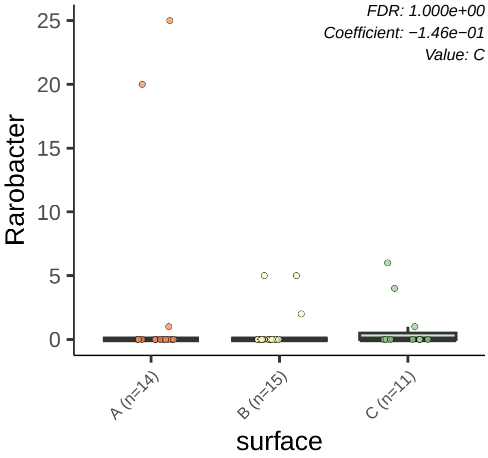

FDR: 1.000e+00
Coefficient: −1.46e−01
Value: C
Rarobacter
0
5
10
15
20
25
A (n=14)
B (n=15)
C (n=11)
surface

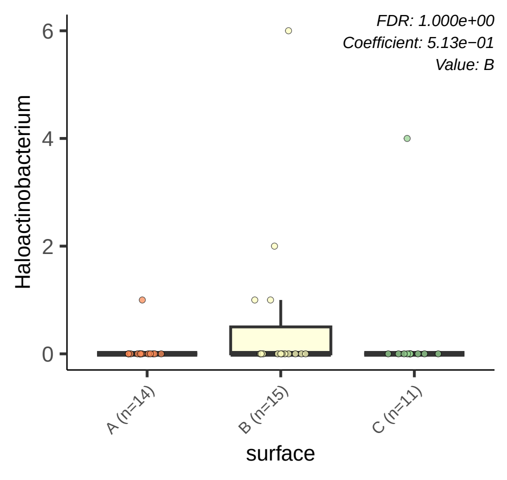
FDR: 1.000e+00
Coefficient: 5.13e−01
Value: B
Haloactinobacterium
0
2
4
6
A (n=14)
B (n=15)
C (n=11)
surface

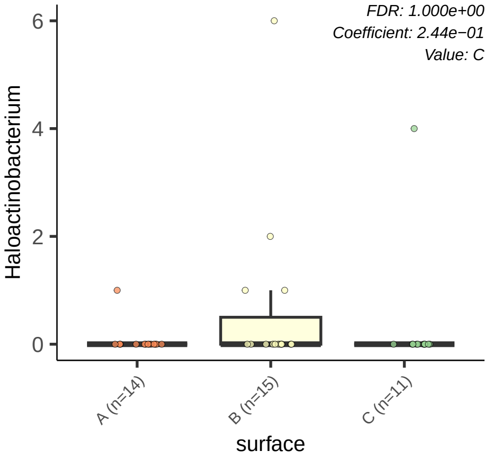
FDR: 1.000e+00
Coefficient: 2.44e−01
Value: C
Haloactinobacterium
0
2
4
6
A (n=14)
B (n=15)
C (n=11)
surface

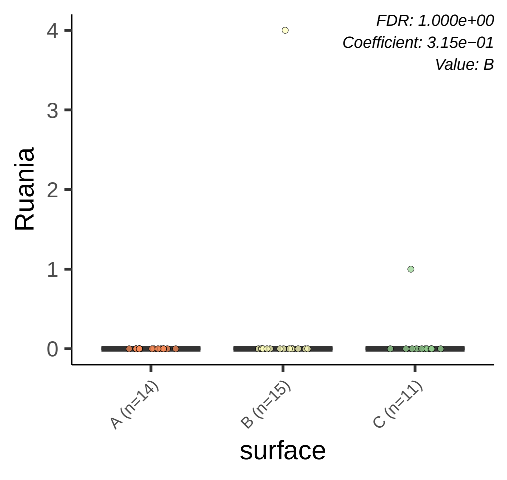

FDR: 1.000e+00
Coefficient: 3.15e−01
Value: B
Ruania
0
1
2
3
4
A (n=14)
B (n=15)
C (n=11)
surface

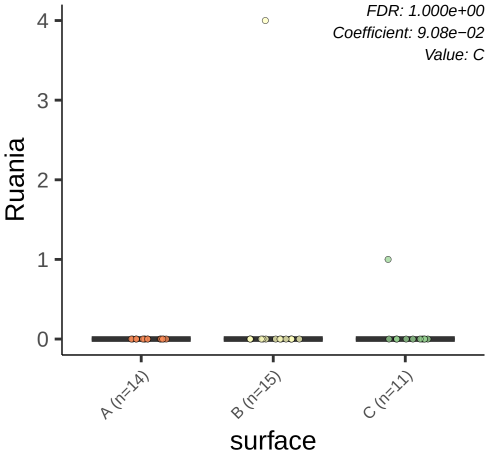
FDR: 1.000e+00
Coefficient: 9.08e−02
Value: C
Ruania
4
3
2
1
0
A (n=14)
B (n=15)
C (n=11)
surface

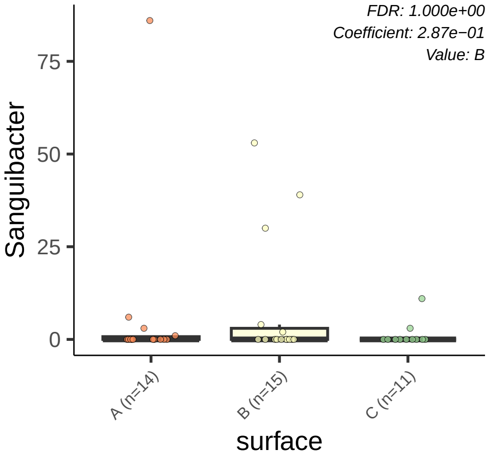
FDR: 1.000e+00
Coefficient: 2.87e−01
Value: B
Sanguibacter
75
50
25
0
A (n=14)
B (n=15)
C (n=11)
surface

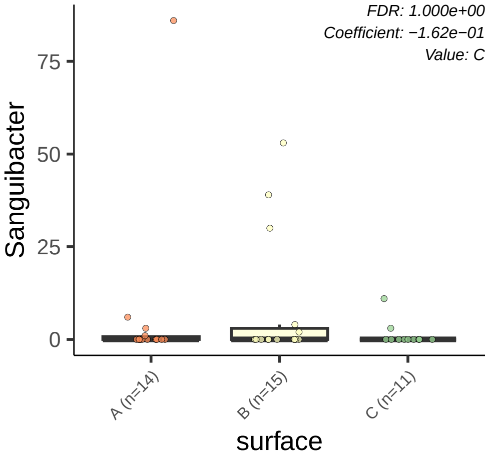

FDR: 1.000e+00
Coefficient: −1.62e−01
Value: C
Sanguibacter
75
50
25
0
A (n=14)
B (n=15)
C (n=11)
surface

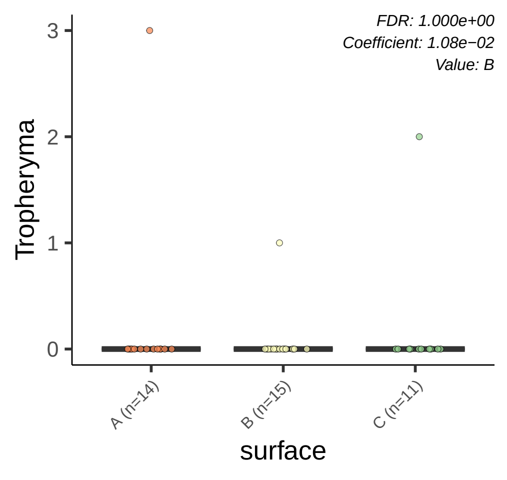

FDR: 1.000e+00
Coefficient: 1.08e−02
Value: B
Tropheryma
3
2
1
0
A (n=14)
B (n=15)
C (n=11)
surface

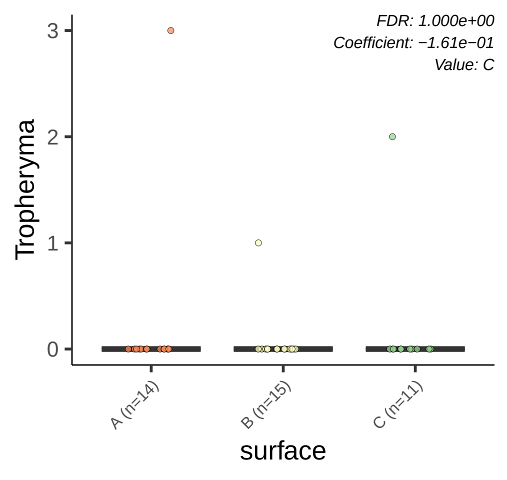
FDR: 1.000e+00
Coefficient: −1.61e−01
Value: C
Tropheryma
3
2
1
0
A (n=14)
B (n=15)
C (n=11)
surface

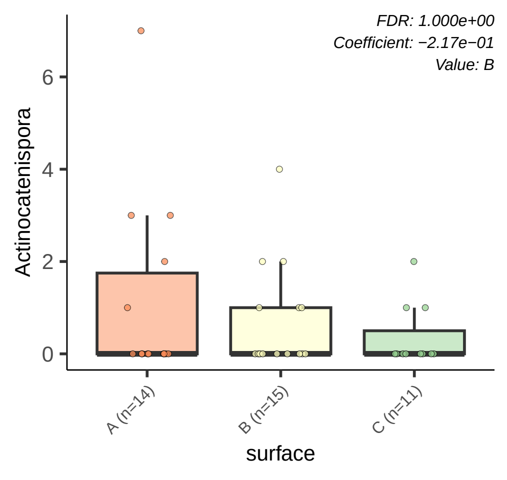
FDR: 1.000e+00
Coefficient: −2.17e−01
Value: B
Actinocatenispora
0
2
4
6
A (n=14)
B (n=15)
C (n=11)
surface

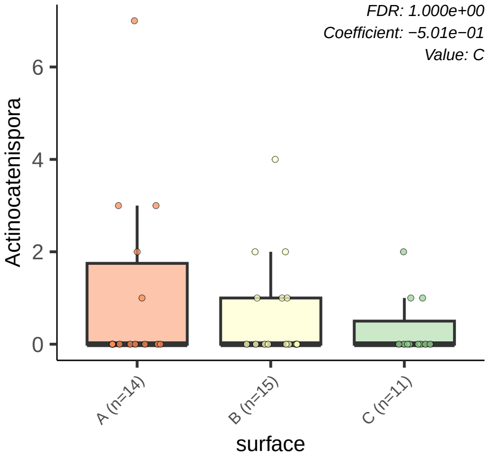
FDR: 1.000e+00
Coefficient: −5.01e−01
Value: C
Actinocatenispora
0
2
4
6
A (n=14)
B (n=15)
C (n=11)
surface

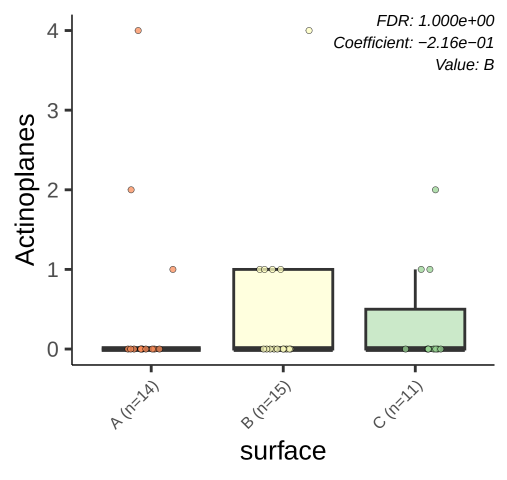
FDR: 1.000e+00
Coefficient: −2.16e−01
Value: B
Actinoplanes
0
1
2
3
4
A (n=14)
B (n=15)
C (n=11)
surface

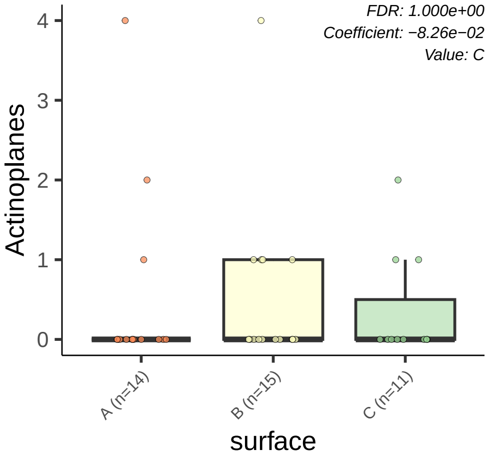

FDR: 1.000e+00
Coefficient: −8.26e−02
Value: C
Actinoplanes
0
1
2
3
4
A (n=14)
B (n=15)
C (n=11)
surface

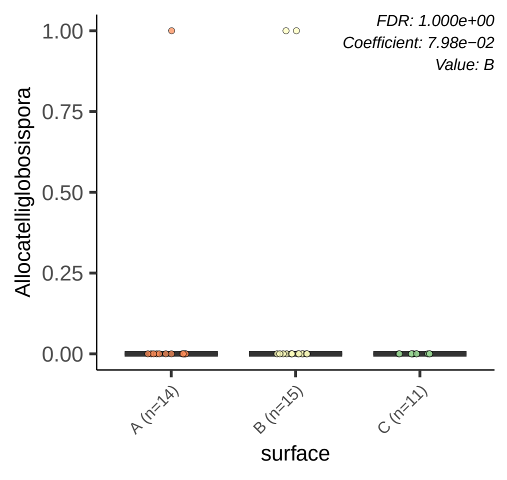

FDR: 1.000e+00
Coefficient: 7.98e−02
Value: B
Allocatelliglobosispora
1.00
0.75
0.50
0.25
0.00
A (n=14)
B (n=15)
C (n=11)
surface

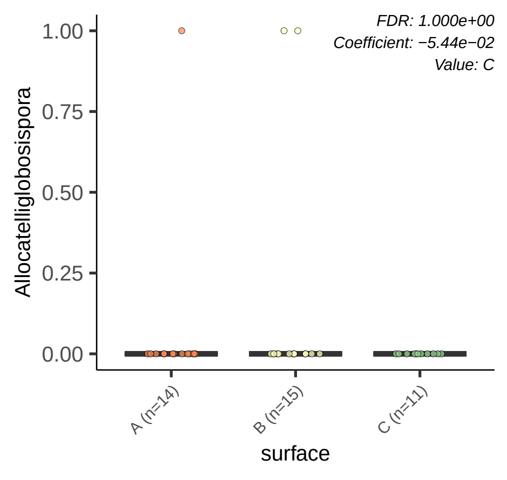
FDR: 1.000e+00
Coefficient: −5.44e−02
Value: C
Allocatelliglobosispora
1.00
0.75
0.50
0.25
0.00
A (n=14)
B (n=15)
C (n=11)
surface

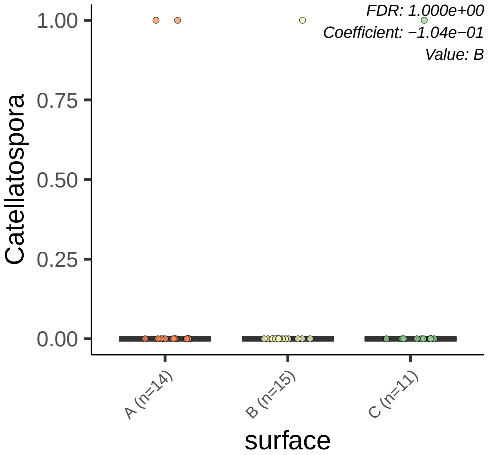
FDR: 1.000e+00
Coefficient: −1.04e−01
Value: B
Catellatospora
1.00
0.75
0.50
0.25
0.00
A (n=14)
B (n=15)
C (n=11)
surface

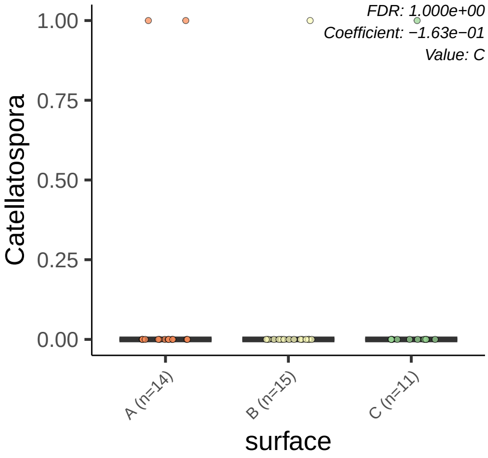
FDR: 1.000e+00
Coefficient: −1.63e−01
Value: C
Catellatospora
1.00
0.75
0.50
0.25
0.00
A (n=14)
B (n=15)
C (n=11)
surface

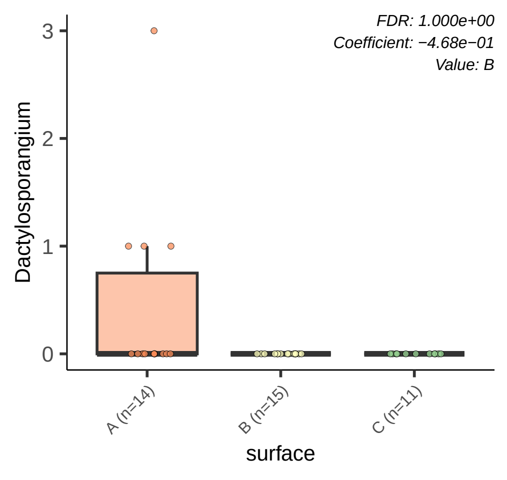
FDR: 1.000e+00
Coefficient: −4.68e−01
Value: B
Dactylosporangium
3
2
1
0
A (n=14)
B (n=15)
C (n=11)
surface

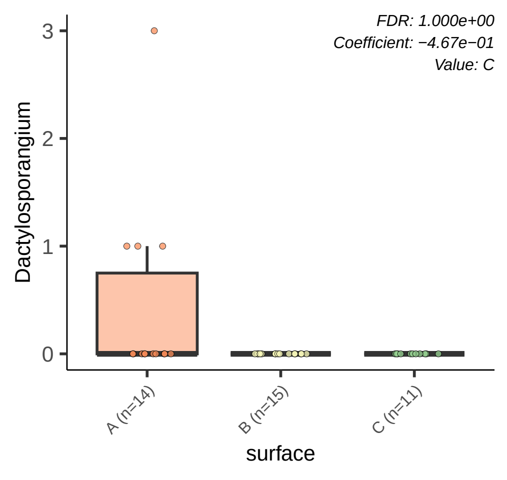
FDR: 1.000e+00
Coefficient: −4.67e−01
Value: C
Dactylosporangium
0
1
2
3
A (n=14)
B (n=15)
C (n=11)
surface

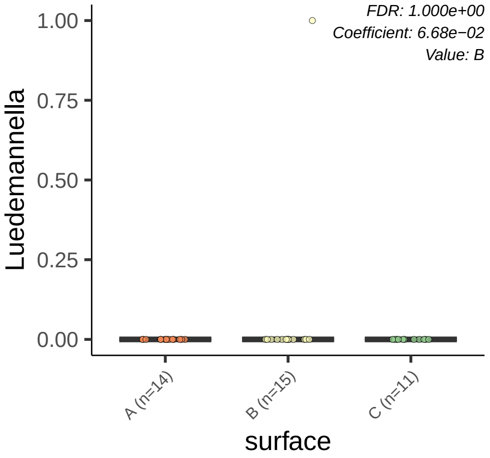
FDR: 1.000e+00
Coefficient: 6.68e−02
Value: B
Luedemannella
1.00
0.75
0.50
0.25
0.00
A (n=14)
B (n=15)
C (n=11)
surface

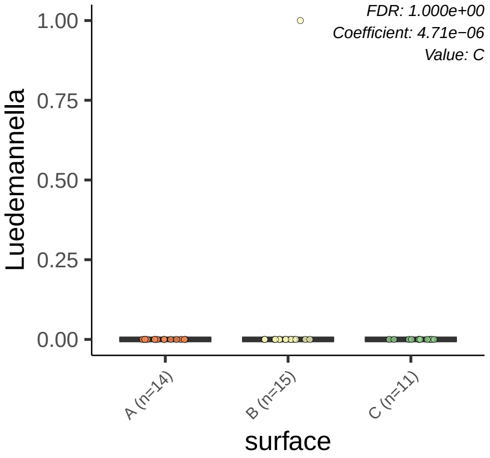
FDR: 1.000e+00
Coefficient: 4.71e−06
Value: C
Luedemannella
1.00
0.75
0.50
0.25
0.00
A (n=14)
B (n=15)
C (n=11)
surface

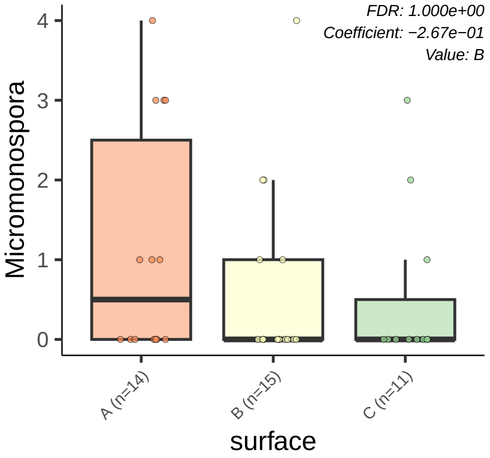
FDR: 1.000e+00
Coefficient: −2.67e−01
Value: B
Micromonospora
0
1
2
3
4
A (n=14)
B (n=15)
C (n=11)
surface

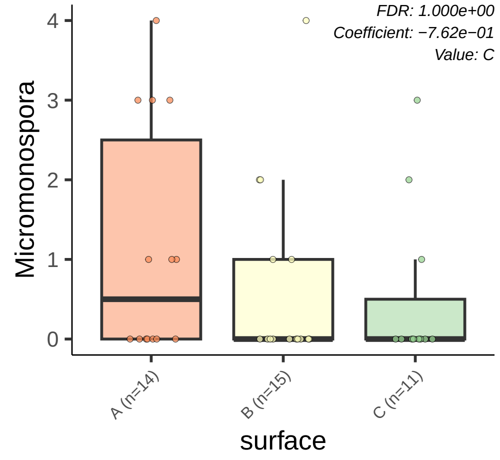

FDR: 1.000e+00
Coefficient: −7.62e−01
Value: C
Micromonospora
0
1
2
3
4
A (n=14)
B (n=15)
C (n=11)
surface

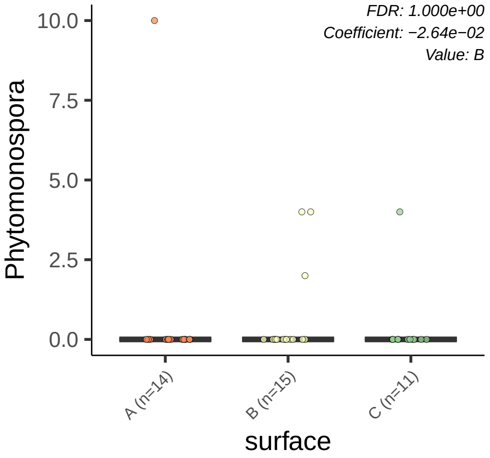
FDR: 1.000e+00
Coefficient: −2.64e−02
Value: B
Phytomonospora
10.0
7.5
5.0
2.5
0.0
A (n=14)
B (n=15)
C (n=11)
surface

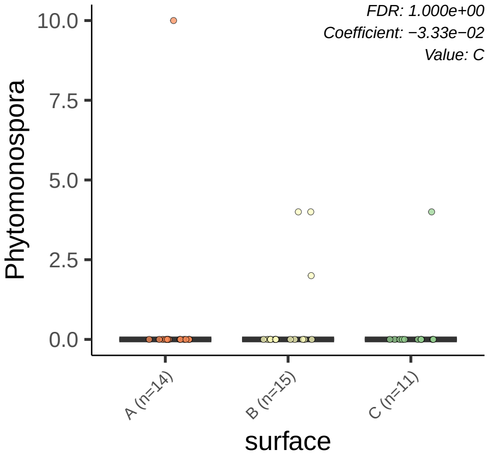
FDR: 1.000e+00
Coefficient: −3.33e−02
Value: C
Phytomonospora
10.0
7.5
5.0
2.5
0.0
A (n=14)
B (n=15)
C (n=11)
surface

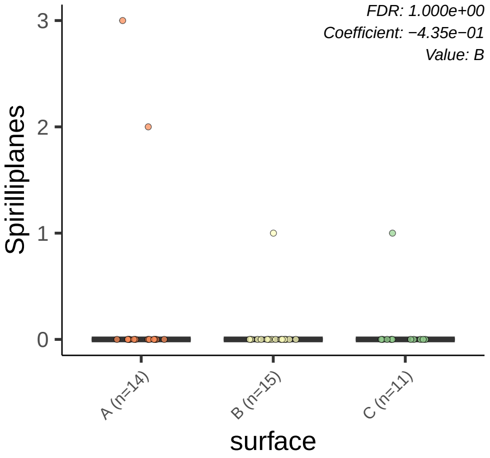
FDR: 1.000e+00
Coefficient: −4.35e−01
Value: B
Spirilliplanes
3
2
1
0
A (n=14)
B (n=15)
C (n=11)
surface

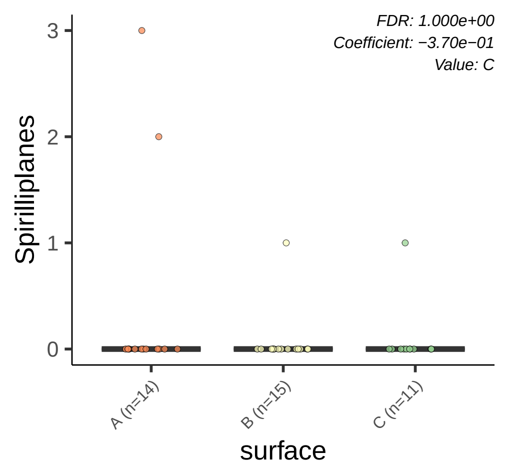
FDR: 1.000e+00
Coefficient: −3.70e−01
Value: C
Spirilliplanes
3
2
1
0
A (n=14)
B (n=15)
C (n=11)
surface

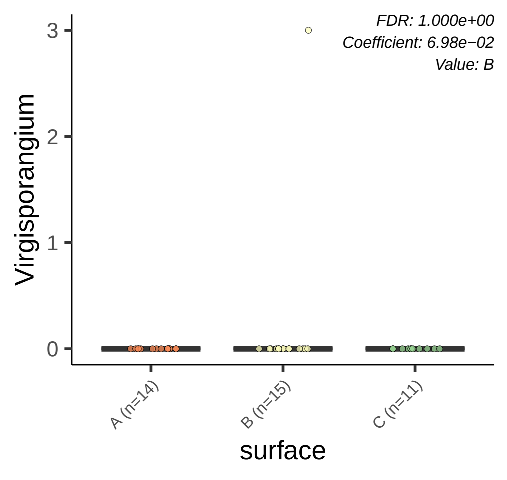
FDR: 1.000e+00
Coefficient: 6.98e−02
Value: B
Virgisporangium
3
2
1
0
A (n=14)
B (n=15)
C (n=11)
surface

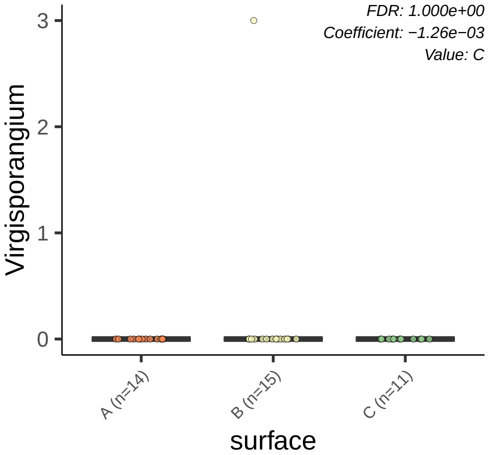
FDR: 1.000e+00
Coefficient: −1.26e−03
Value: C
Virgisporangium
3
2
1
0
A (n=14)
B (n=15)
C (n=11)
surface

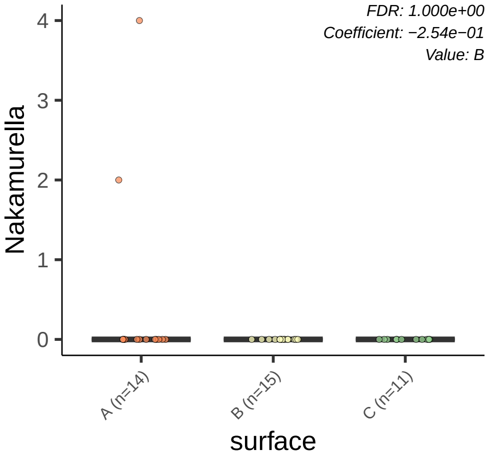

FDR: 1.000e+00
Coefficient: −2.54e−01
Value: B
Nakamurella
4
3
2
1
0
A (n=14)
B (n=15)
C (n=11)
surface

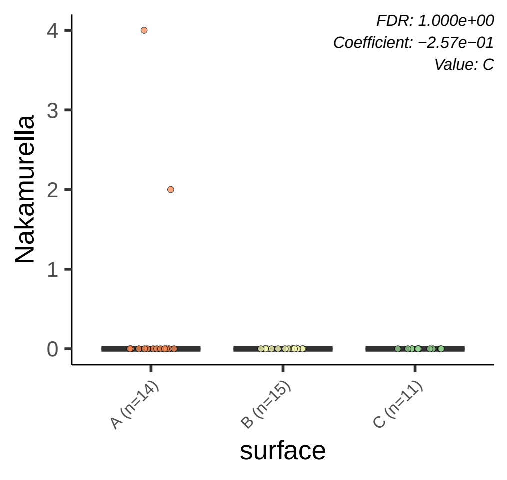

FDR: 1.000e+00
Coefficient: −2.57e−01
Value: C
Nakamurella
4
3
2
1
0
A (n=14)
B (n=15)
C (n=11)
surface

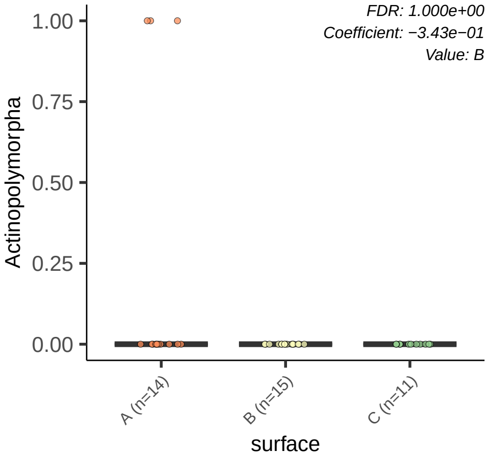

FDR: 1.000e+00
Coefficient: −3.43e−01
Value: B
Actinopolymorpha
1.00
0.75
0.50
0.25
0.00
A (n=14)
B (n=15)
C (n=11)
surface

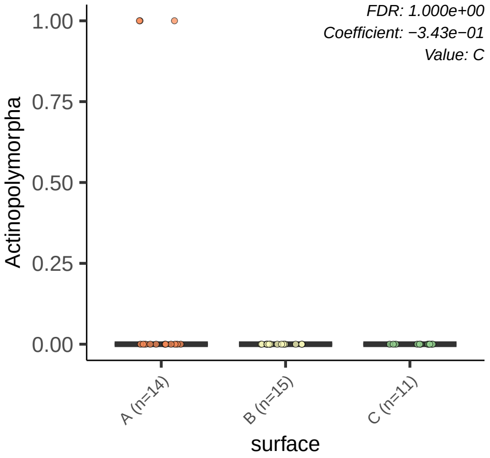
FDR: 1.000e+00
Coefficient: −3.43e−01
Value: C
Actinopolymorpha
1.00
0.75
0.50
0.25
0.00
A (n=14)
B (n=15)
C (n=11)
surface

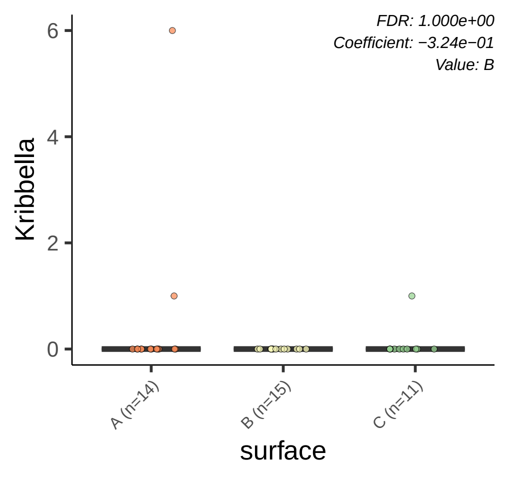
FDR: 1.000e+00
Coefficient: −3.24e−01
Value: B
Kribbella
6
4
2
0
A (n=14)
B (n=15)
C (n=11)
surface

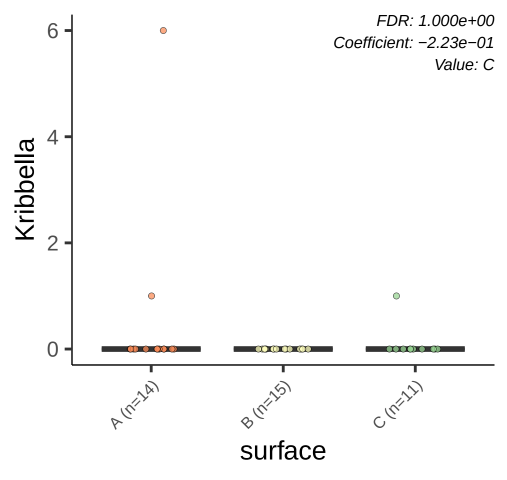
FDR: 1.000e+00
Coefficient: −2.23e−01
Value: C
Kribbella
6
4
2
0
A (n=14)
B (n=15)
C (n=11)
surface

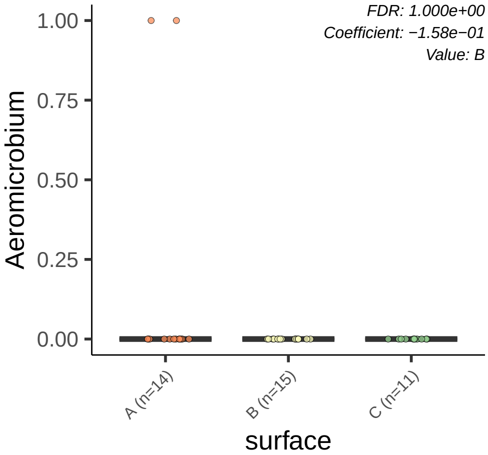

FDR: 1.000e+00
Coefficient: −1.58e−01
Value: B
Aeromicrobium
1.00
0.75
0.50
0.25
0.00
A (n=14)
B (n=15)
C (n=11)
surface

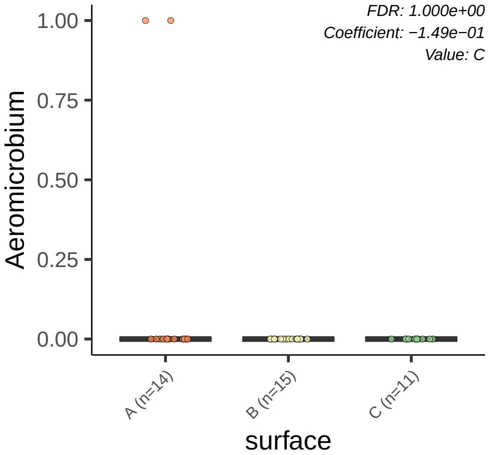

FDR: 1.000e+00
Coefficient: −1.49e−01
Value: C
Aeromicrobium
1.00
0.75
0.50
0.25
0.00
A (n=14)
B (n=15)
C (n=11)
surface

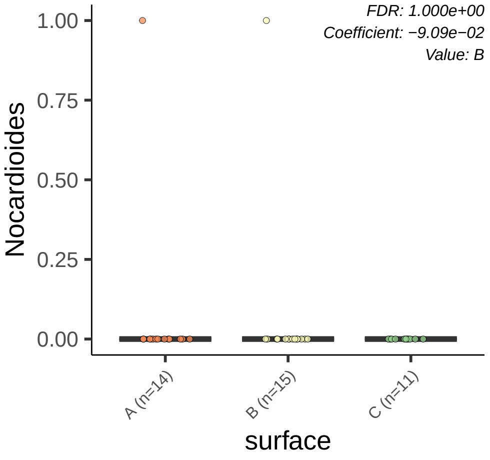
FDR: 1.000e+00
Coefficient: −9.09e−02
Value: B
Nocardioides
1.00
0.75
0.50
0.25
0.00
A (n=14)
B (n=15)
C (n=11)
surface

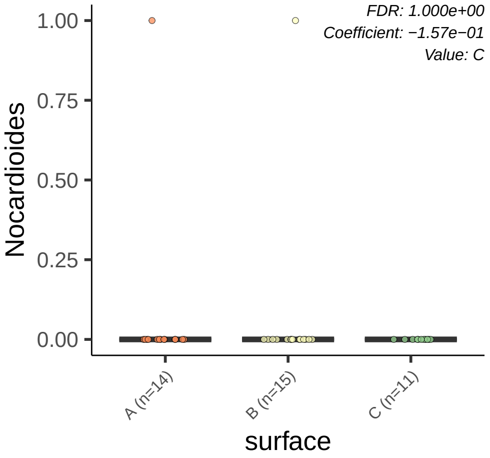
FDR: 1.000e+00
Coefficient: −1.57e−01
Value: C
Nocardioides
1.00
0.75
0.50
0.25
0.00
A (n=14)
B (n=15)
C (n=11)
surface

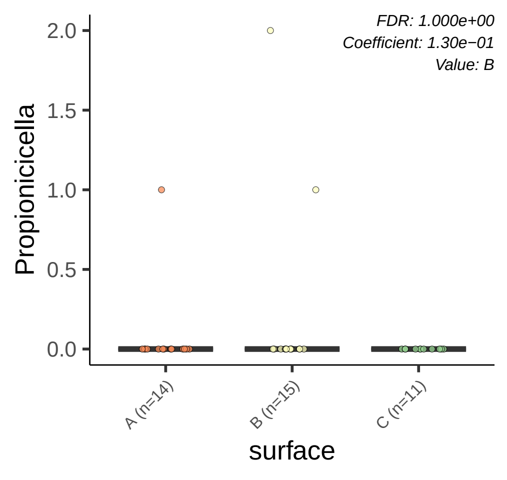

FDR: 1.000e+00
Coefficient: 1.30e−01
Value: B
Propionicicella
2.0
1.5
1.0
0.5
0.0
A (n=14)
B (n=15)
C (n=11)
surface

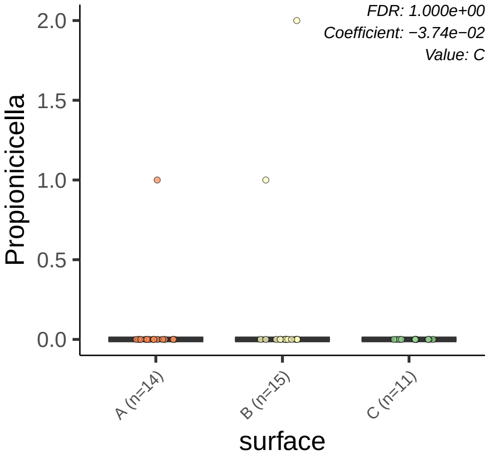

FDR: 1.000e+00
Coefficient: −3.74e−02
Value: C
Propionicicella
2.0
1.5
1.0
0.5
0.0
A (n=14)
B (n=15)
C (n=11)
surface

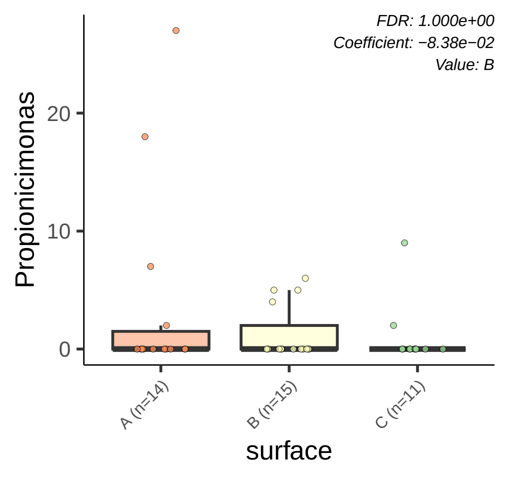

FDR: 1.000e+00
Coefficient: −8.38e−02
Value: B
Propionicimonas
20
10
0
A (n=14)
B (n=15)
C (n=11)
surface

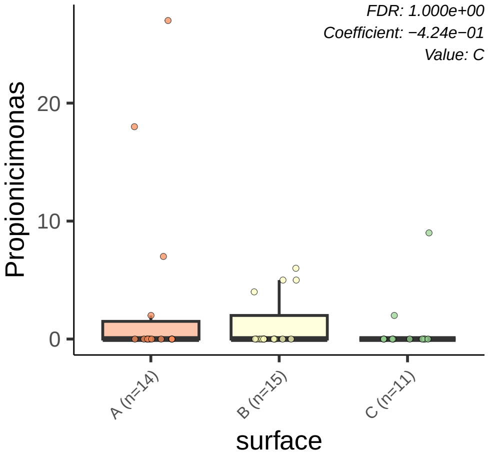

FDR: 1.000e+00
Coefficient: −4.24e−01
Value: C
Propionicimonas
20
10
0
A (n=14)
B (n=15)
C (n=11)
surface

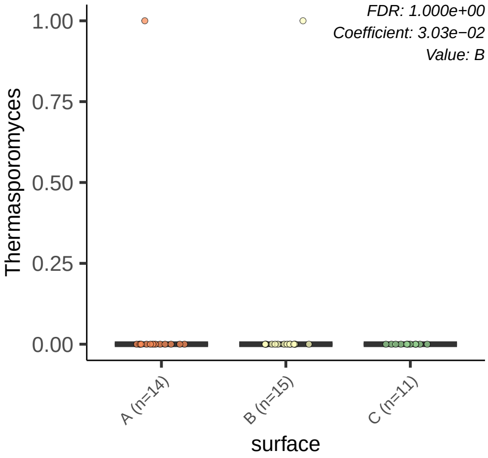
FDR: 1.000e+00
Coefficient: 3.03e−02
Value: B
Thermasporomyces
1.00
0.75
0.50
0.25
0.00
A (n=14)
B (n=15)
C (n=11)
surface

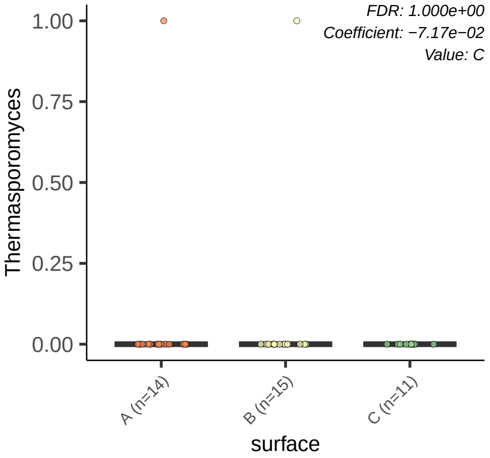
FDR: 1.000e+00
Coefficient: −7.17e−02
Value: C
Thermasporomyces
1.00
0.75
0.50
0.25
0.00
A (n=14)
B (n=15)
C (n=11)
surface

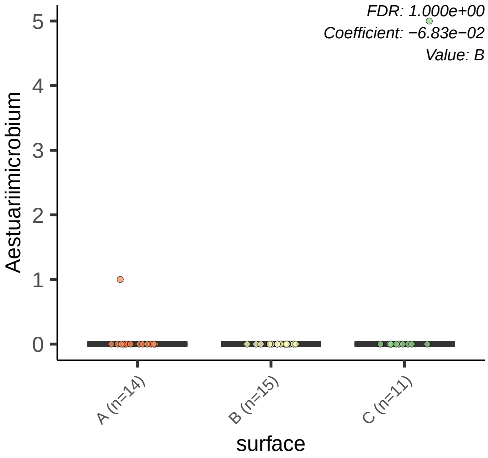

FDR: 1.000e+00
Coefficient: −6.83e−02
Value: B
Aestuariimicrobium
0
1
2
3
4
5
A (n=14)
B (n=15)
C (n=11)
surface

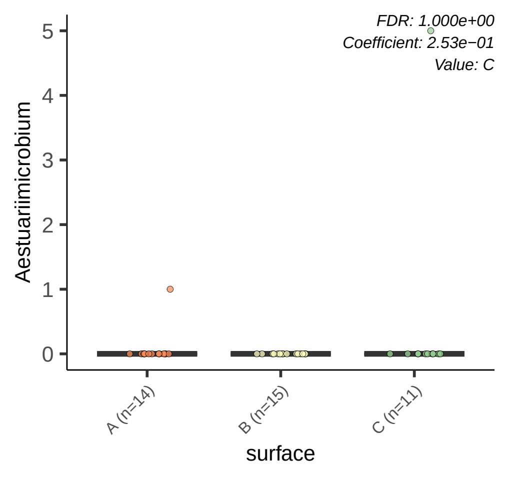

FDR: 1.000e+00
Coefficient: 2.53e−01
Value: C
Aestuariimicrobium
5
4
3
2
1
0
A (n=14)
B (n=15)
C (n=11)
surface

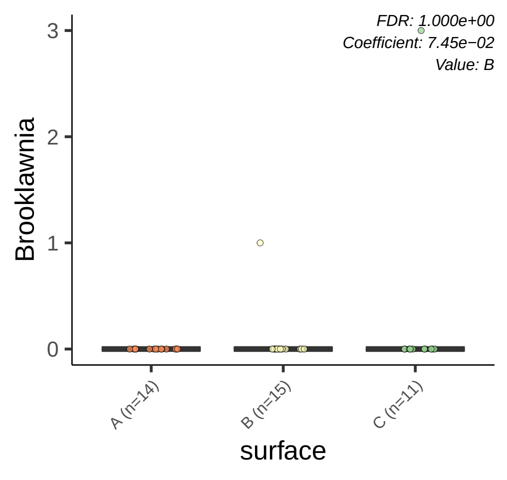
FDR: 1.000e+00
Coefficient: 7.45e−02
Value: B
Brooklawnia
3
2
1
0
A (n=14)
B (n=15)
C (n=11)
surface

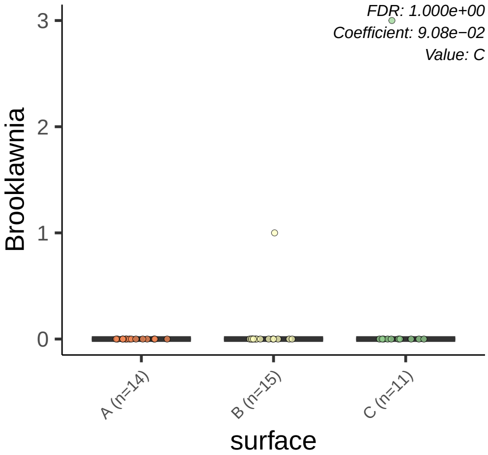
FDR: 1.000e+00
Coefficient: 9.08e−02
Value: C
Brooklawnia
0
1
2
3
A (n=14)
B (n=15)
C (n=11)
surface

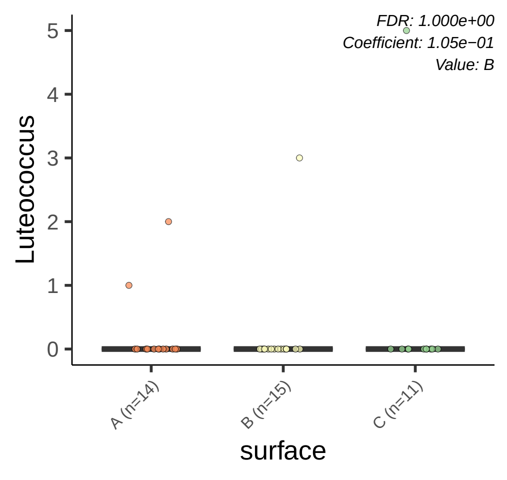
FDR: 1.000e+00
Coefficient: 1.05e−01
Value: B
Luteococcus
0
1
2
3
4
5
A (n=14)
B (n=15)
C (n=11)
surface

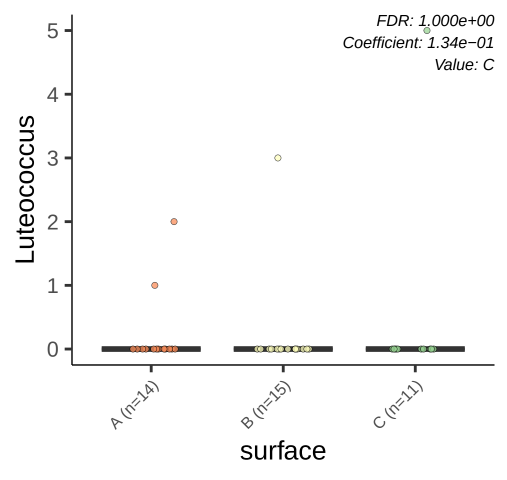

FDR: 1.000e+00
Coefficient: 1.34e−01
Value: C
Luteococcus
0
1
2
3
4
5
A (n=14)
B (n=15)
C (n=11)
surface

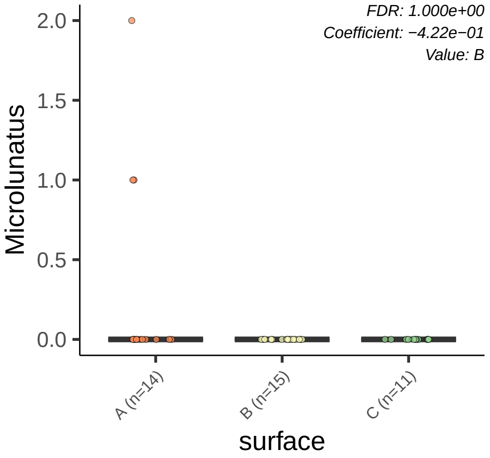

FDR: 1.000e+00
Coefficient: −4.22e−01
Value: B
Microlunatus
2.0
1.5
1.0
0.5
0.0
A (n=14)
B (n=15)
C (n=11)
surface

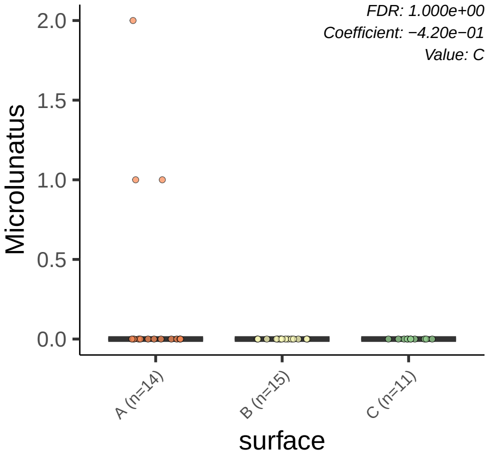
FDR: 1.000e+00
Coefficient: −4.20e−01
Value: C
Microlunatus
2.0
1.5
1.0
0.5
0.0
A (n=14)
B (n=15)
C (n=11)
surface

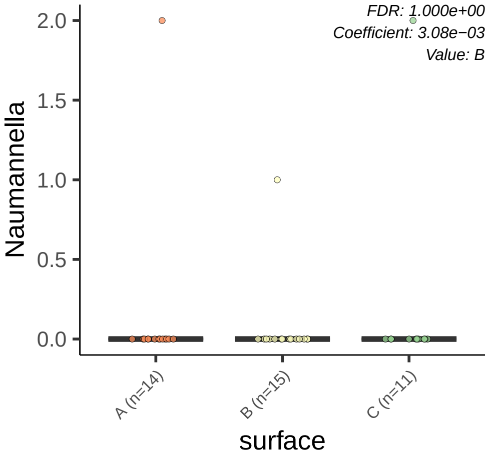
FDR: 1.000e+00
Coefficient: 3.08e−03
Value: B
Naumannella
2.0
1.5
1.0
0.5
0.0
A (n=14)
B (n=15)
C (n=11)
surface

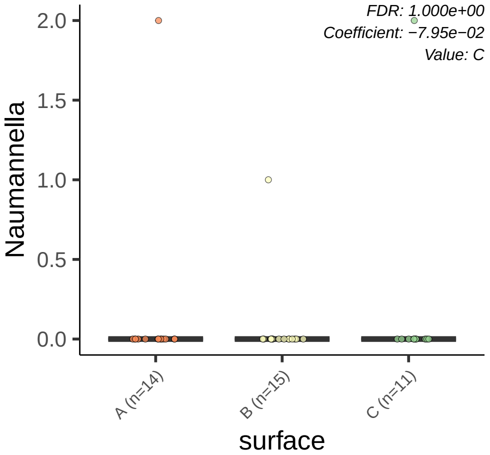

FDR: 1.000e+00
Coefficient: −7.95e−02
Value: C
Naumannella
2.0
1.5
1.0
0.5
0.0
A (n=14)
B (n=15)
C (n=11)
surface

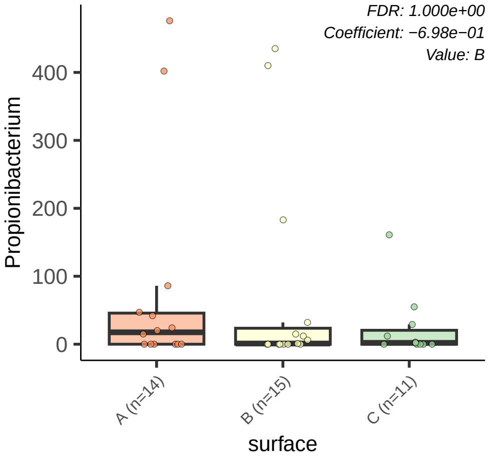

FDR: 1.000e+00
Coefficient: −6.98e−01
Value: B
Propionibacterium
400
300
200
100
0
A (n=14)
B (n=15)
C (n=11)
surface

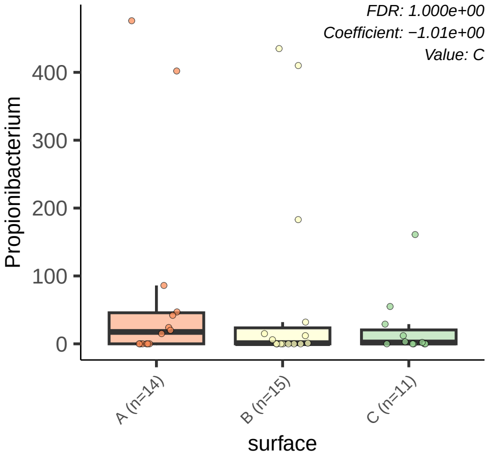

FDR: 1.000e+00
Coefficient: −1.01e+00
Value: C
Propionibacterium
0
100
200
300
400
A (n=14)
B (n=15)
C (n=11)
surface

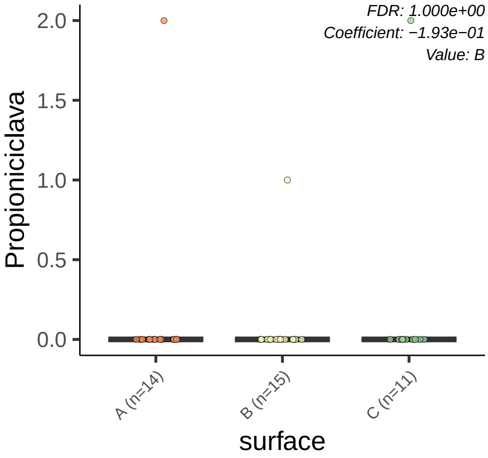

FDR: 1.000e+00
Coefficient: −1.93e−01
Value: B
Propioniciclava
2.0
1.5
1.0
0.5
0.0
A (n=14)
B (n=15)
C (n=11)
surface

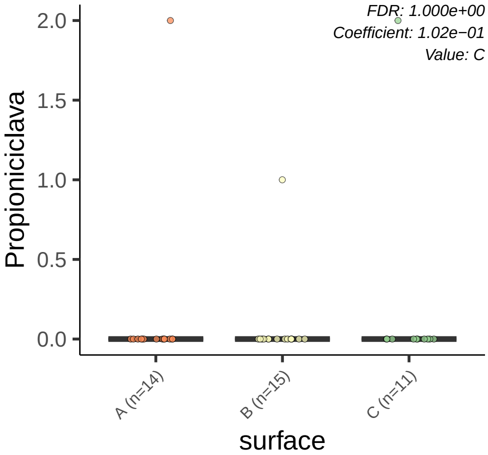

FDR: 1.000e+00
Coefficient: 1.02e−01
Value: C
Propioniciclava
2.0
1.5
1.0
0.5
0.0
A (n=14)
B (n=15)
C (n=11)
surface

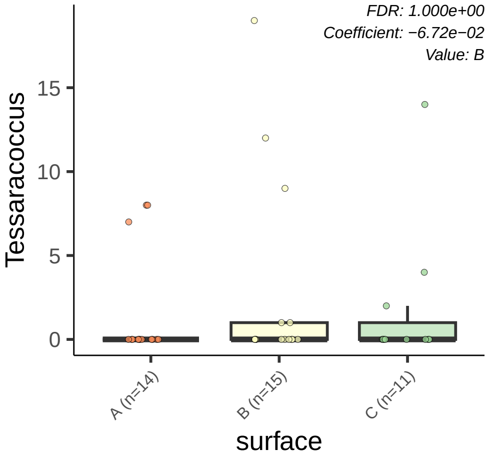

FDR: 1.000e+00
Coefficient: −6.72e−02
Value: B
Tessaracoccus
15
10
5
0
A (n=14)
B (n=15)
C (n=11)
surface

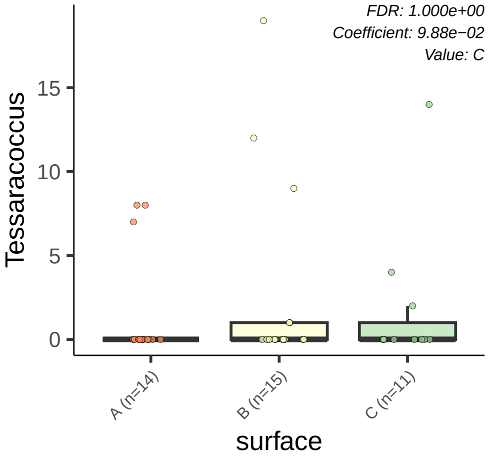
FDR: 1.000e+00
Coefficient: 9.88e−02
Value: C
Tessaracoccus
0
5
10
15
A (n=14)
B (n=15)
C (n=11)
surface

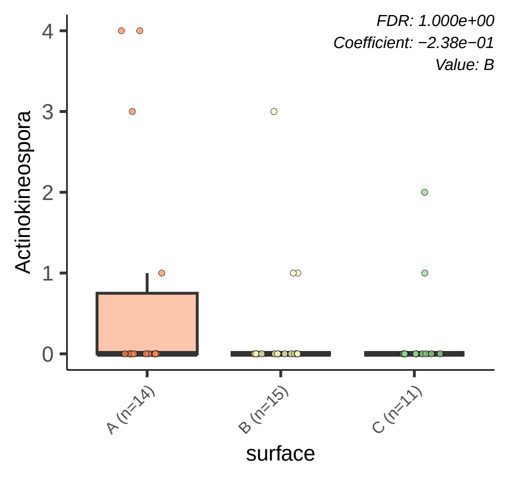

FDR: 1.000e+00
Coefficient: −2.38e−01
Value: B
Actinokineospora
4
3
2
1
0
A (n=14)
B (n=15)
C (n=11)
surface

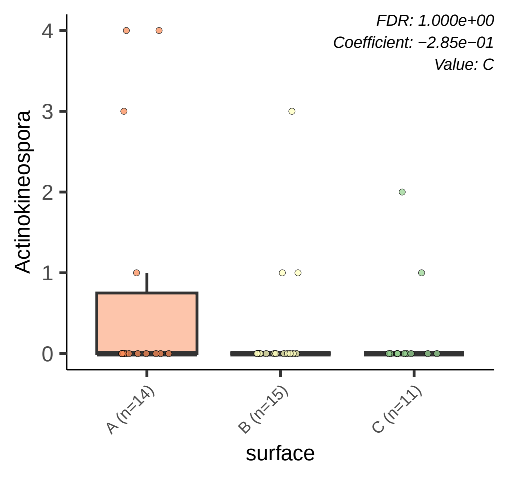
FDR: 1.000e+00
Coefficient: −2.85e−01
Value: C
Actinokineospora
0
1
2
3
4
A (n=14)
B (n=15)
C (n=11)
surface

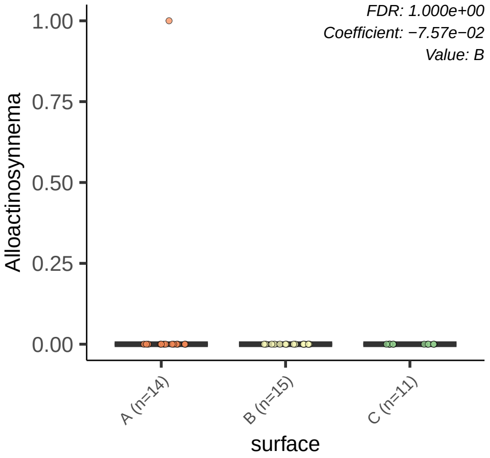

FDR: 1.000e+00
Coefficient: −7.57e−02
Value: B
Alloactinosynnema
1.00
0.75
0.50
0.25
0.00
A (n=14)
B (n=15)
C (n=11)
surface

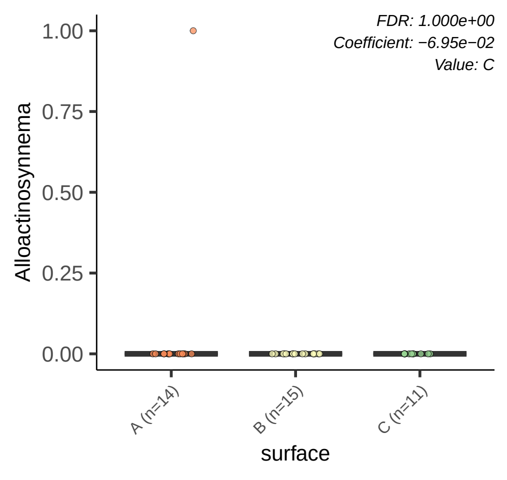

FDR: 1.000e+00
Coefficient: −6.95e−02
Value: C
Alloactinosynnema
1.00
0.75
0.50
0.25
0.00
A (n=14)
B (n=15)
C (n=11)
surface

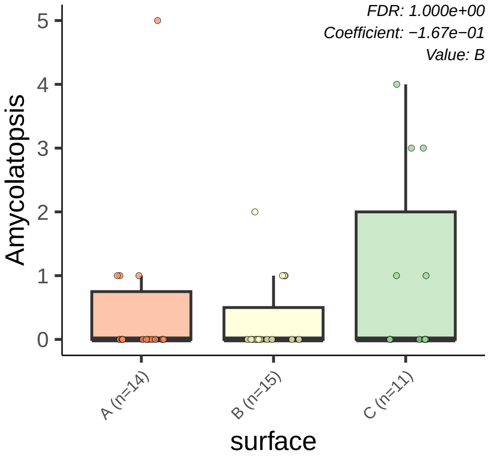
FDR: 1.000e+00
Coefficient: −1.67e−01
Value: B
Amycolatopsis
0
1
2
3
4
5
A (n=14)
B (n=15)
C (n=11)
surface

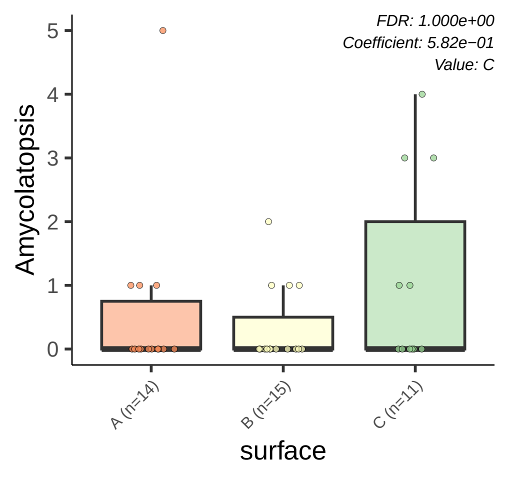

FDR: 1.000e+00
Coefficient: 5.82e−01
Value: C
Amycolatopsis
0
1
2
3
4
5
A (n=14)
B (n=15)
C (n=11)
surface

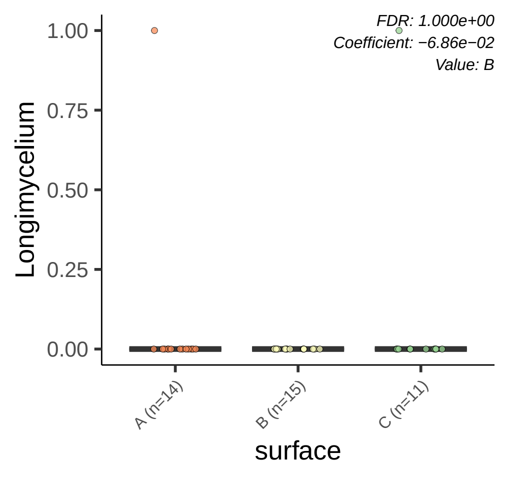
FDR: 1.000e+00
Coefficient: −6.86e−02
Value: B
Longimycelium
1.00
0.75
0.50
0.25
0.00
A (n=14)
B (n=15)
C (n=11)
surface

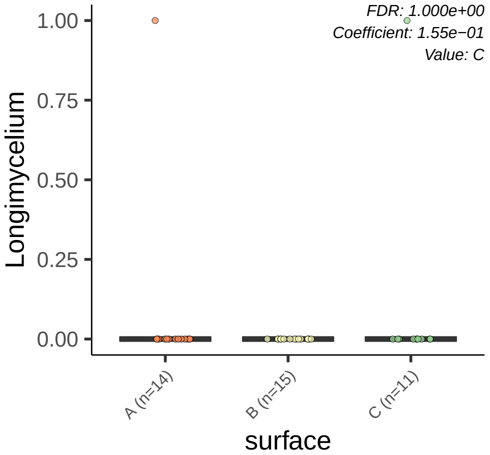

FDR: 1.000e+00
Coefficient: 1.55e−01
Value: C
Longimycelium
1.00
0.75
0.50
0.25
0.00
A (n=14)
B (n=15)
C (n=11)
surface

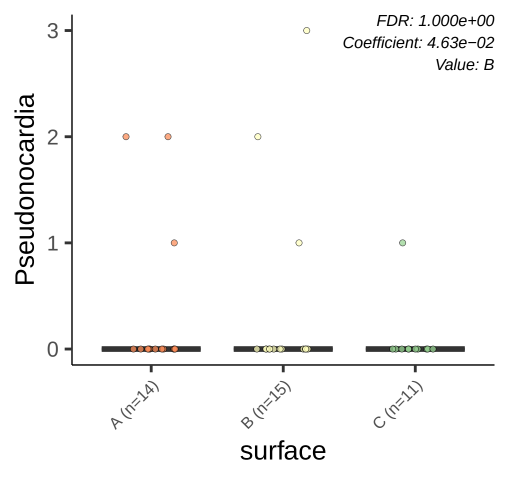

FDR: 1.000e+00
Coefficient: 4.63e−02
Value: B
Pseudonocardia
3
2
1
0
A (n=14)
B (n=15)
C (n=11)
surface

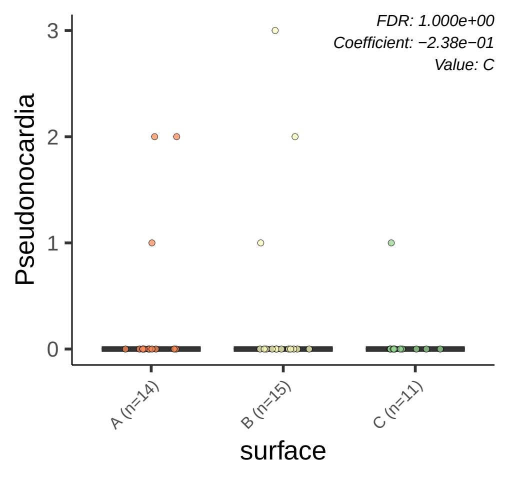
FDR: 1.000e+00
Coefficient: −2.38e−01
Value: C
Pseudonocardia
3
2
1
0
A (n=14)
B (n=15)
C (n=11)
surface

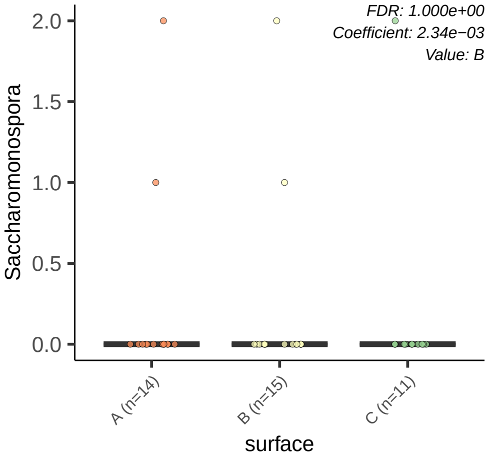

FDR: 1.000e+00
Coefficient: 2.34e−03
Value: B
Saccharomonospora
2.0
1.5
1.0
0.5
0.0
A (n=14)
B (n=15)
C (n=11)
surface

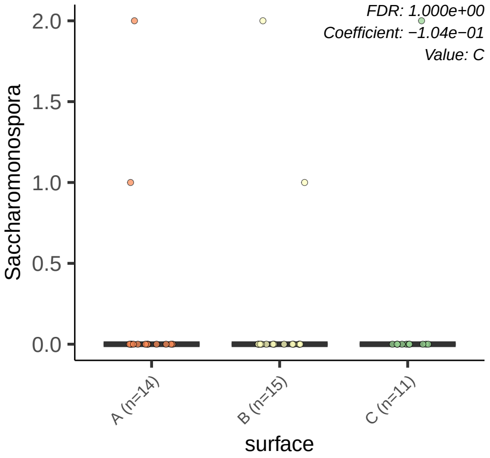

FDR: 1.000e+00
Coefficient: −1.04e−01
Value: C
Saccharomonospora
2.0
1.5
1.0
0.5
0.0
A (n=14)
B (n=15)
C (n=11)
surface

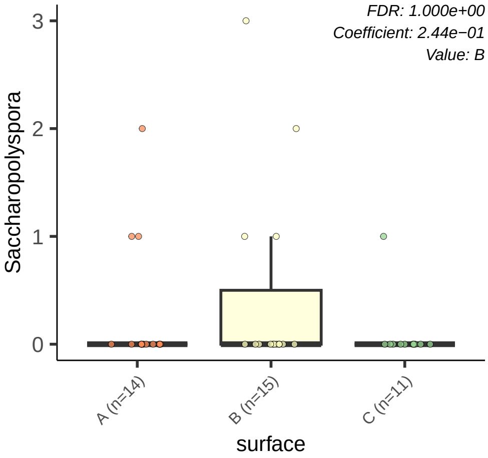

FDR: 1.000e+00
Coefficient: 2.44e−01
Value: B
Saccharopolyspora
0
1
2
3
A (n=14)
B (n=15)
C (n=11)
surface

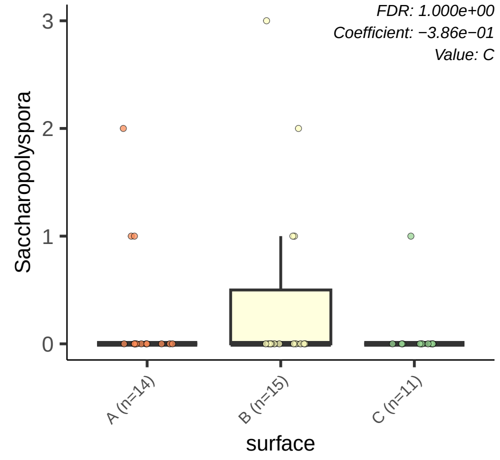

FDR: 1.000e+00
Coefficient: −3.86e−01
Value: C
Saccharopolyspora
3
2
1
0
A (n=14)
B (n=15)
C (n=11)
surface

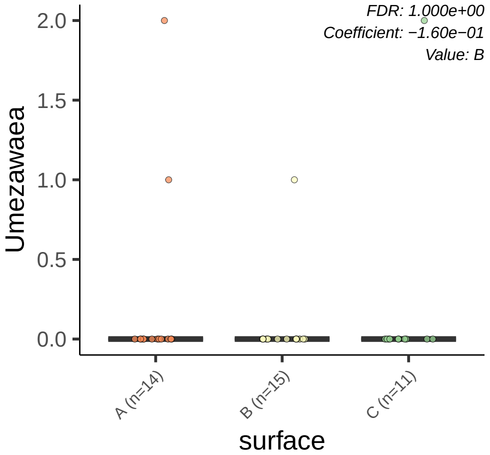
FDR: 1.000e+00
Coefficient: −1.60e−01
Value: B
Umezawaea
2.0
1.5
1.0
0.5
0.0
A (n=14)
B (n=15)
C (n=11)
surface

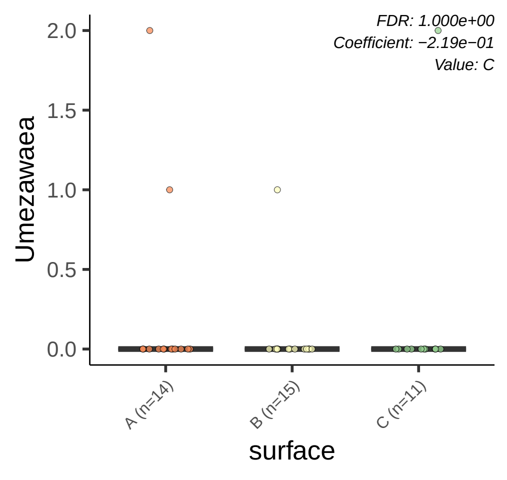
FDR: 1.000e+00
Coefficient: −2.19e−01
Value: C
Umezawaea
2.0
1.5
1.0
0.5
0.0
A (n=14)
B (n=15)
C (n=11)
surface

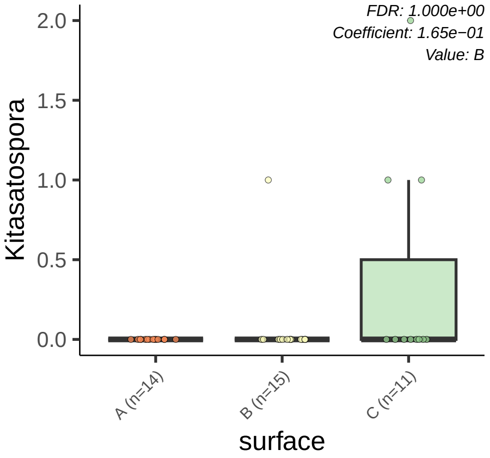
FDR: 1.000e+00
Coefficient: 1.65e−01
Value: B
Kitasatospora
2.0
1.5
1.0
0.5
0.0
A (n=14)
B (n=15)
C (n=11)
surface

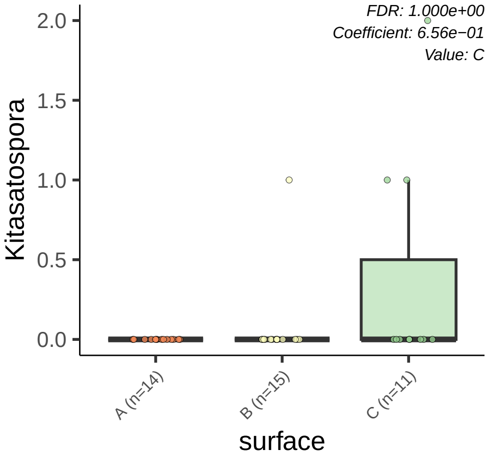

FDR: 1.000e+00
Coefficient: 6.56e−01
Value: C
Kitasatospora
2.0
1.5
1.0
0.5
0.0
A (n=14)
B (n=15)
C (n=11)
surface

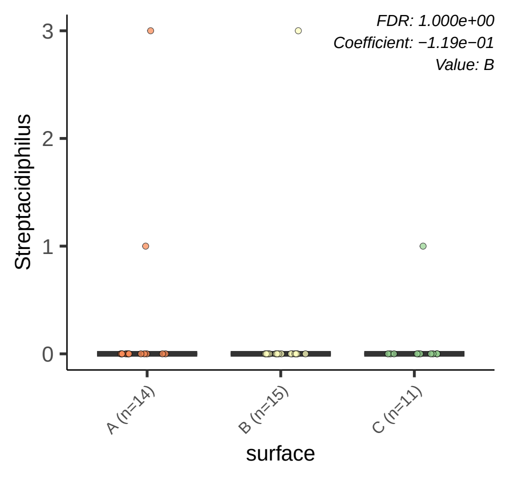
FDR: 1.000e+00
Coefficient: −1.19e−01
Value: B
Streptacidiphilus
0
1
2
3
A (n=14)
B (n=15)
C (n=11)
surface

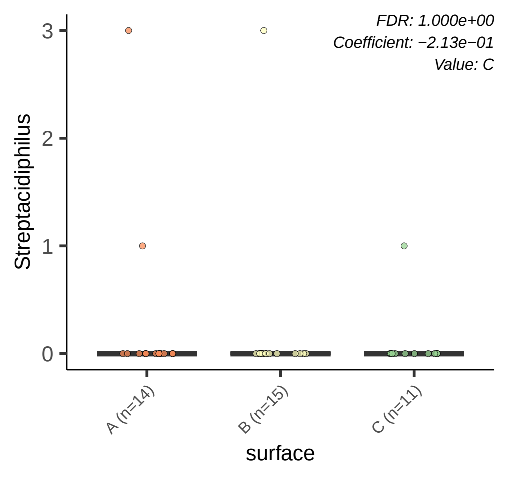

FDR: 1.000e+00
Coefficient: −2.13e−01
Value: C
Streptacidiphilus
0
1
2
3
A (n=14)
B (n=15)
C (n=11)
surface

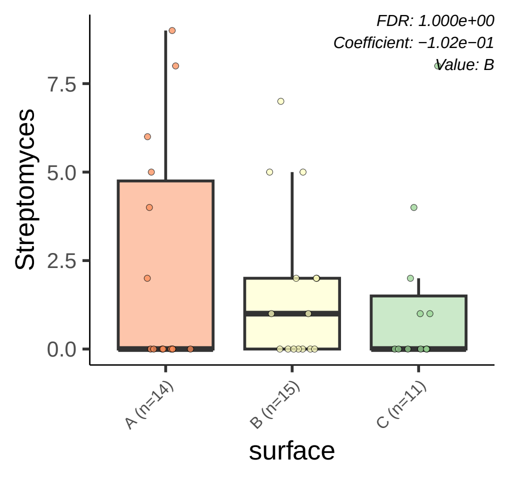

FDR: 1.000e+00
Coefficient: −1.02e−01
Value: B
Streptomyces
7.5
5.0
2.5
0.0
A (n=14)
B (n=15)
C (n=11)
surface

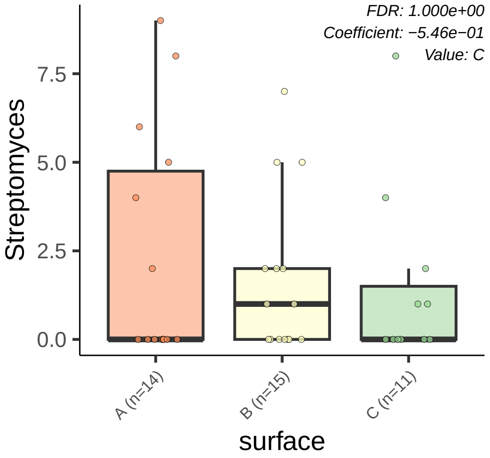

FDR: 1.000e+00
Coefficient: −5.46e−01
Value: C
Streptomyces
7.5
5.0
2.5
0.0
A (n=14)
B (n=15)
C (n=11)
surface

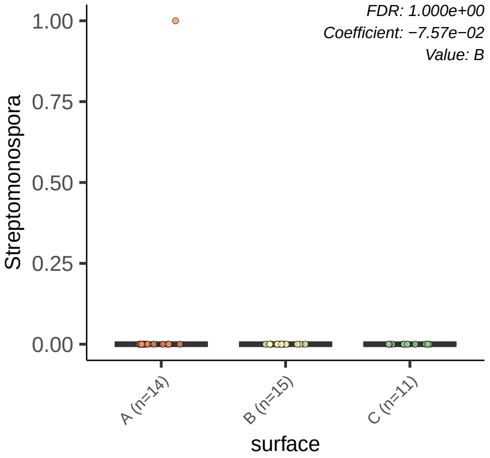
FDR: 1.000e+00
Coefficient: −7.57e−02
Value: B
Streptomonospora
1.00
0.75
0.50
0.25
0.00
A (n=14)
B (n=15)
C (n=11)
surface

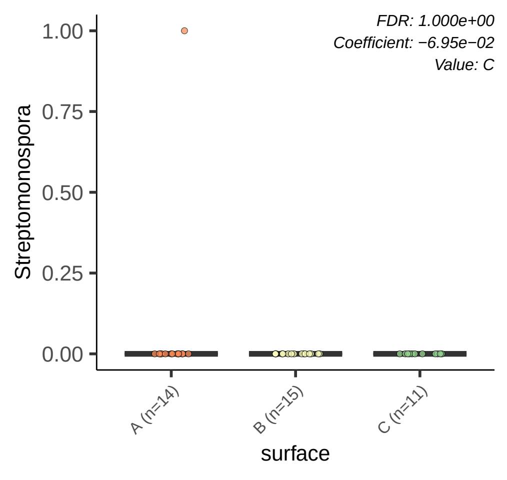
FDR: 1.000e+00
Coefficient: −6.95e−02
Value: C
1.00
0.75
0.50
0.25
0.00
Streptomonospora
A (n=14)
B (n=15)
C (n=11)
surface

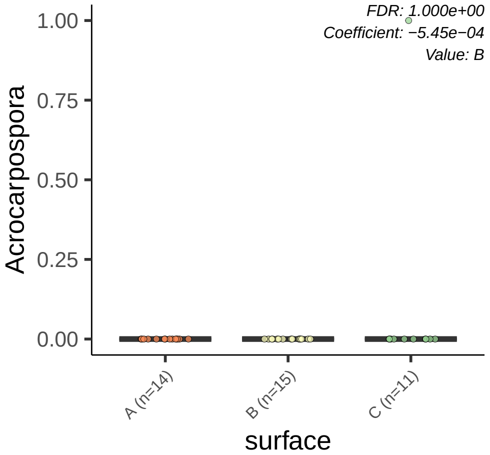

FDR: 1.000e+00
Coefficient: −5.45e−04
Value: B
Acrocarpospora
1.00
0.75
0.50
0.25
0.00
A (n=14)
B (n=15)
C (n=11)
surface

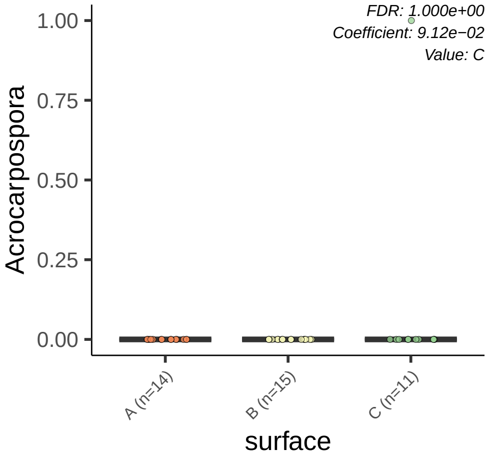
FDR: 1.000e+00
Coefficient: 9.12e−02
Value: C
Acrocarpospora
1.00
0.75
0.50
0.25
0.00
A (n=14)
B (n=15)
C (n=11)
surface

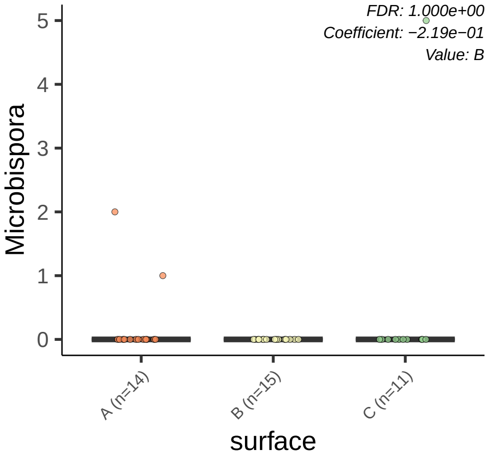

FDR: 1.000e+00
Coefficient: −2.19e−01
Value: B
Microbispora
0
1
2
3
4
5
A (n=14)
B (n=15)
C (n=11)
surface

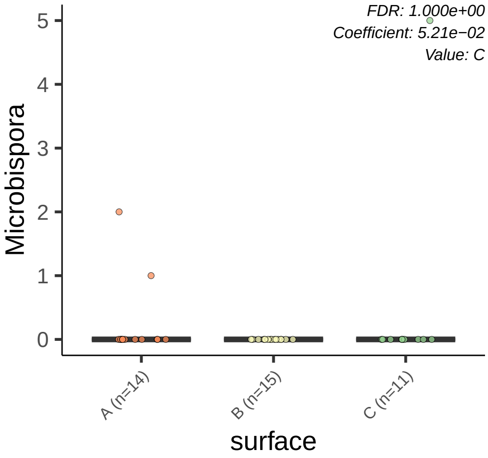

FDR: 1.000e+00
Coefficient: 5.21e−02
Value: C
Microbispora
5
4
3
2
1
0
A (n=14)
B (n=15)
C (n=11)
surface

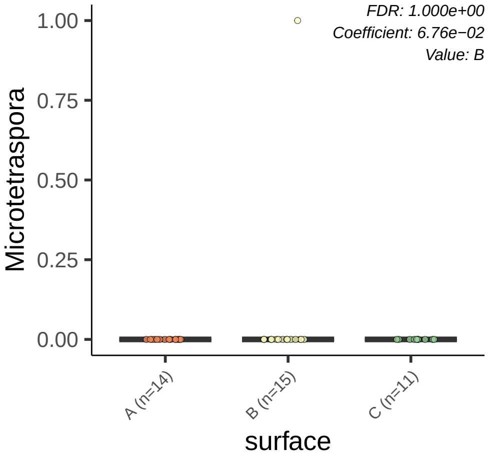
FDR: 1.000e+00
Coefficient: 6.76e−02
Value: B
Microtetraspora
1.00
0.75
0.50
0.25
0.00
A (n=14)
B (n=15)
C (n=11)
surface

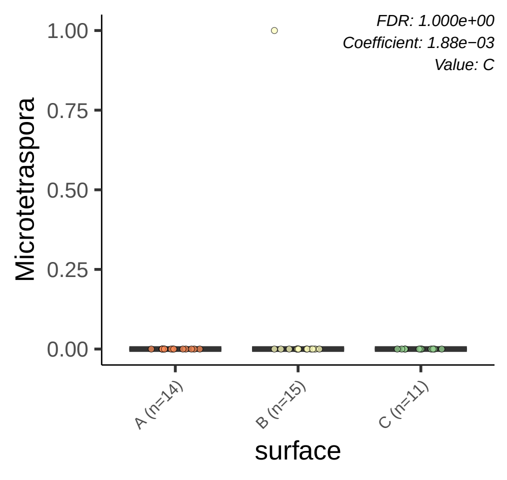
FDR: 1.000e+00
Coefficient: 1.88e−03
Value: C
Microtetraspora
1.00
0.75
0.50
0.25
0.00
A (n=14)
B (n=15)
C (n=11)
surface

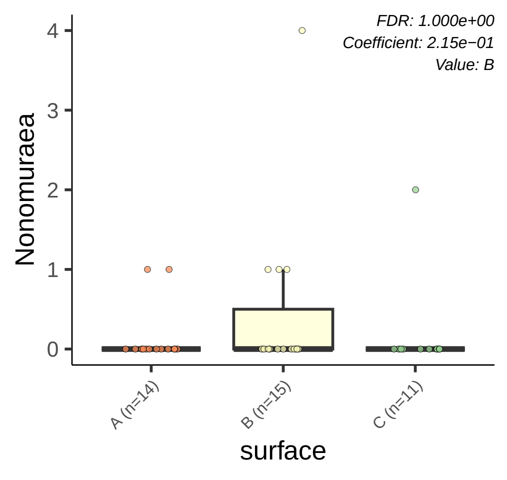

FDR: 1.000e+00
Coefficient: 2.15e−01
Value: B
Nonomuraea
0
1
2
3
4
A (n=14)
B (n=15)
C (n=11)
surface

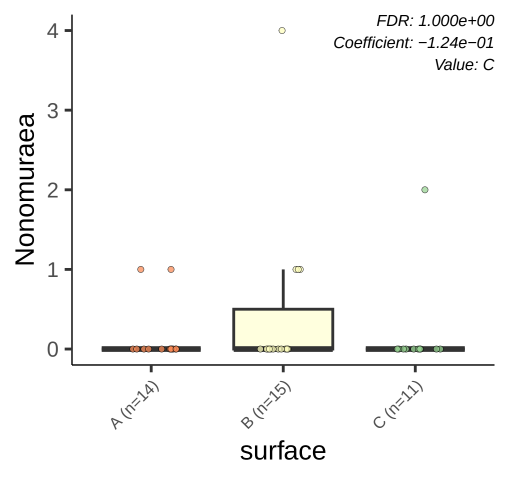

FDR: 1.000e+00
Coefficient: −1.24e−01
Value: C
Nonomuraea
0
1
2
3
4
A (n=14)
B (n=15)
C (n=11)
surface

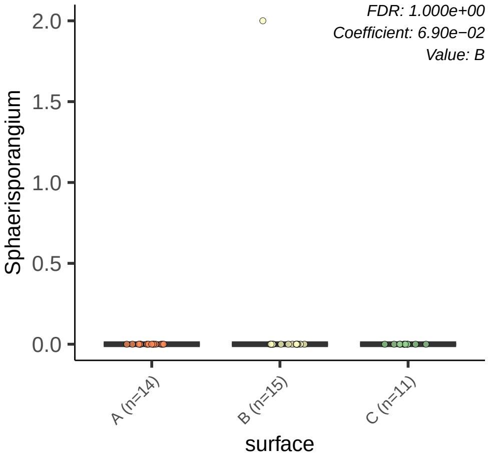

FDR: 1.000e+00
Coefficient: 6.90e−02
Value: B
Sphaerisporangium
2.0
1.5
1.0
0.5
0.0
A (n=14)
B (n=15)
C (n=11)
surface

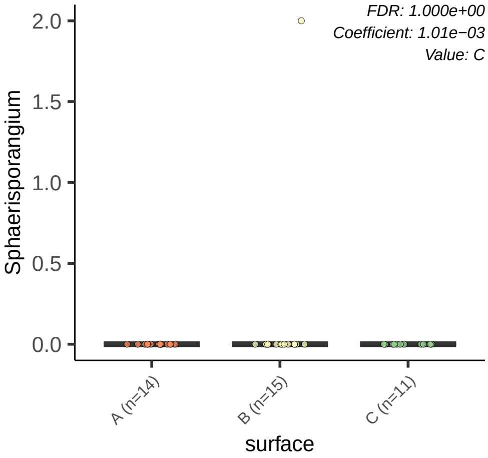

FDR: 1.000e+00
Coefficient: 1.01e−03
Value: C
Sphaerisporangium
2.0
1.5
1.0
0.5
0.0
A (n=14)
B (n=15)
C (n=11)
surface

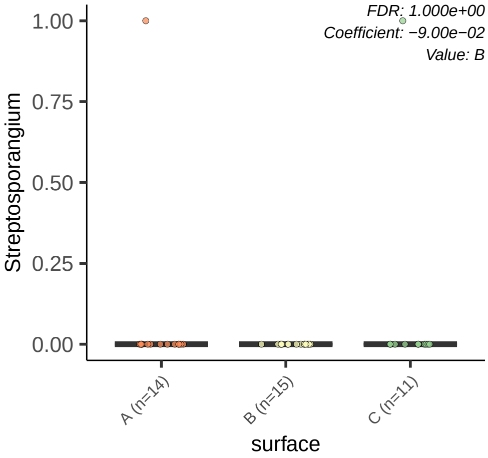
FDR: 1.000e+00
Coefficient: −9.00e−02
Value: B
Streptosporangium
1.00
0.75
0.50
0.25
0.00
A (n=14)
B (n=15)
C (n=11)
surface

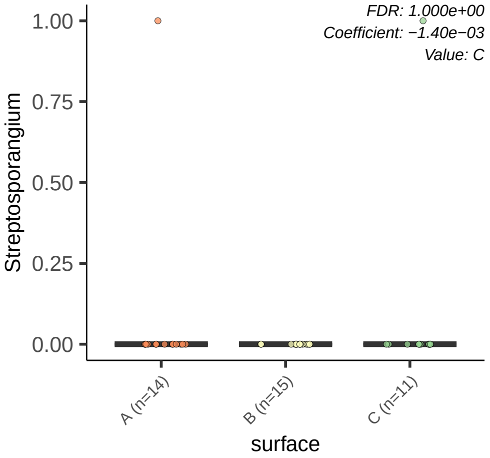
FDR: 1.000e+00
Coefficient: −1.40e−03
Value: C
Streptosporangium
1.00
0.75
0.50
0.25
0.00
A (n=14)
B (n=15)
C (n=11)
surface

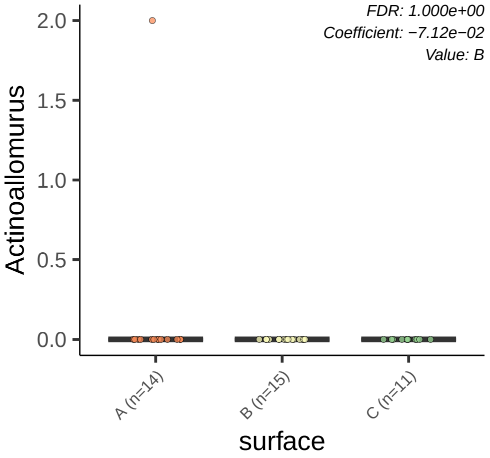
FDR: 1.000e+00
Coefficient: −7.12e−02
Value: B
Actinoallomurus
2.0
1.5
1.0
0.5
0.0
A (n=14)
B (n=15)
C (n=11)
surface

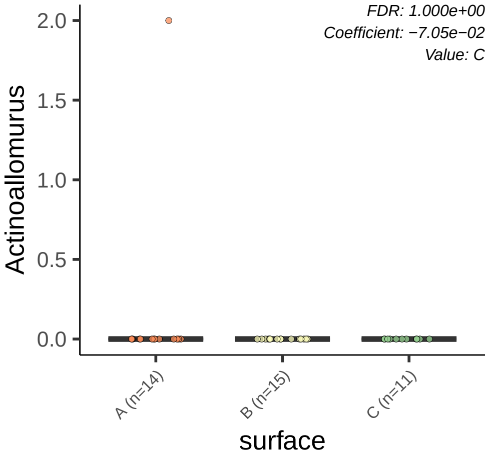

FDR: 1.000e+00
Coefficient: −7.05e−02
Value: C
Actinoallomurus
2.0
1.5
1.0
0.5
0.0
A (n=14)
B (n=15)
C (n=11)
surface

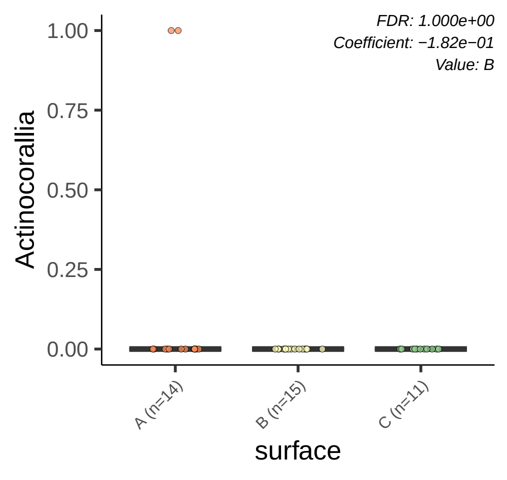
FDR: 1.000e+00
Coefficient: −1.82e−01
Value: B
Actinocorallia
1.00
0.75
0.50
0.25
0.00
A (n=14)
B (n=15)
C (n=11)
surface

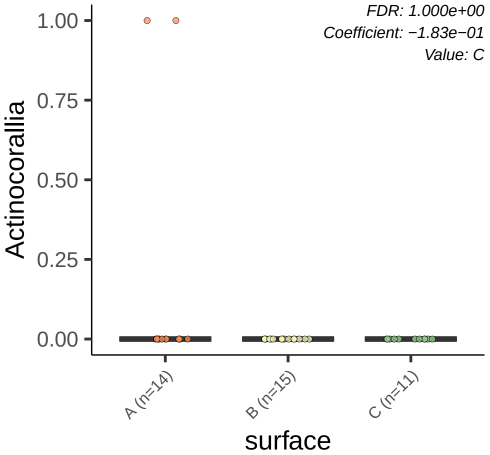
FDR: 1.000e+00
Coefficient: −1.83e−01
Value: C
Actinocorallia
1.00
0.75
0.50
0.25
0.00
A (n=14)
B (n=15)
C (n=11)
surface

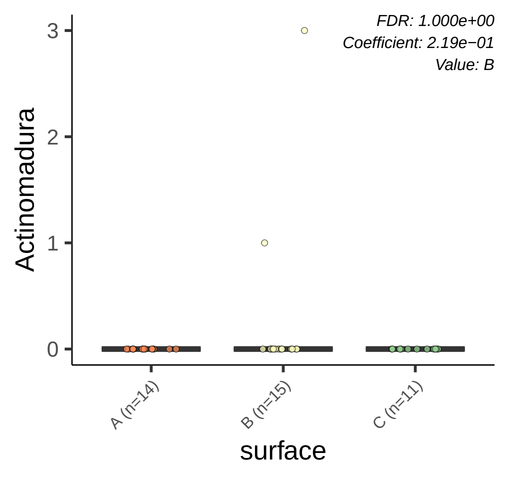

FDR: 1.000e+00
Coefficient: 2.19e−01
Value: B
Actinomadura
3
2
1
0
A (n=14)
B (n=15)
C (n=11)
surface

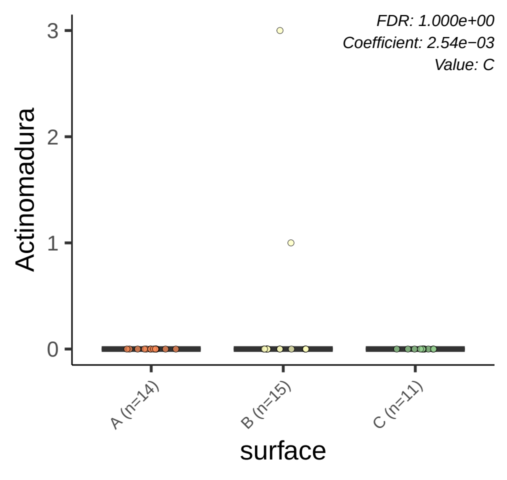

FDR: 1.000e+00
Coefficient: 2.54e−03
Value: C
Actinomadura
3
2
1
0
A (n=14)
B (n=15)
C (n=11)
surface

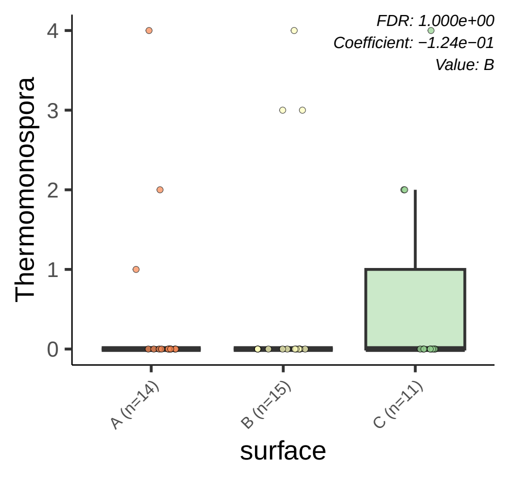
FDR: 1.000e+00
Coefficient: −1.24e−01
Value: B
Thermomonospora
4
3
2
1
0
A (n=14)
B (n=15)
C (n=11)
surface

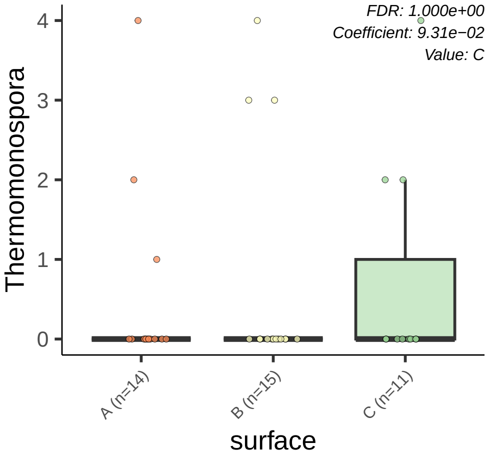
FDR: 1.000e+00
Coefficient: 9.31e−02
Value: C
Thermomonospora
0
1
2
3
4
A (n=14)
B (n=15)
C (n=11)
surface

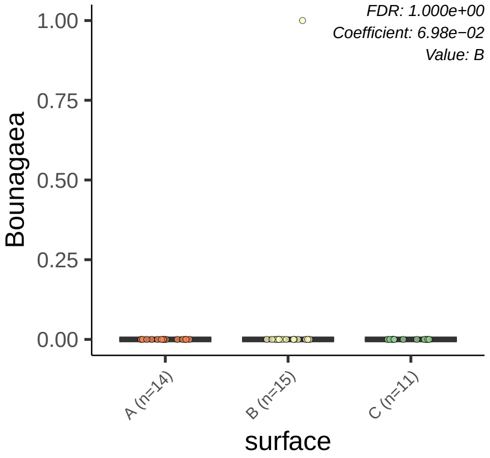
FDR: 1.000e+00
Coefficient: 6.98e−02
Value: B
Bounagaea
1.00
0.75
0.50
0.25
0.00
A (n=14)
B (n=15)
C (n=11)
surface

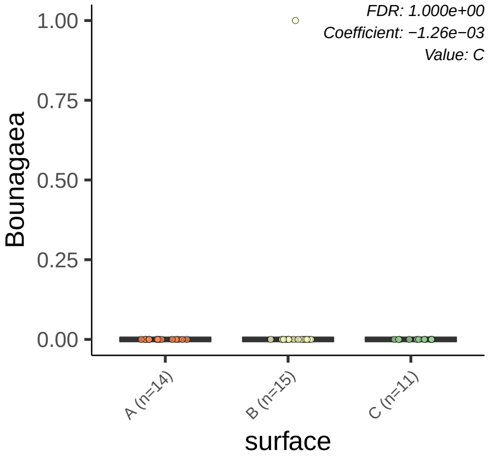
FDR: 1.000e+00
Coefficient: −1.26e−03
Value: C
Bounagaea
1.00
0.75
0.50
0.25
0.00
A (n=14)
B (n=15)
C (n=11)
surface

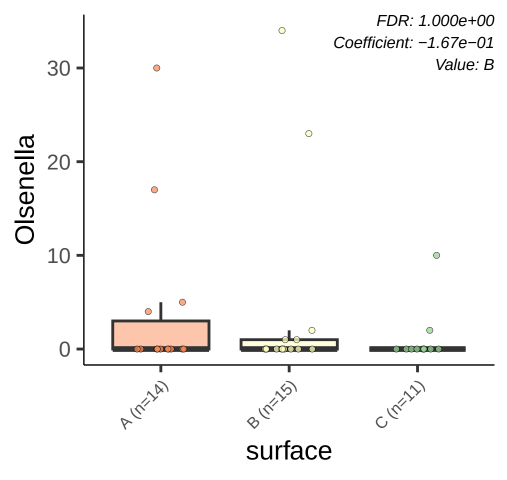
FDR: 1.000e+00
Coefficient: −1.67e−01
Value: B
Olsenella
30
20
10
0
A (n=14)
B (n=15)
C (n=11)
surface

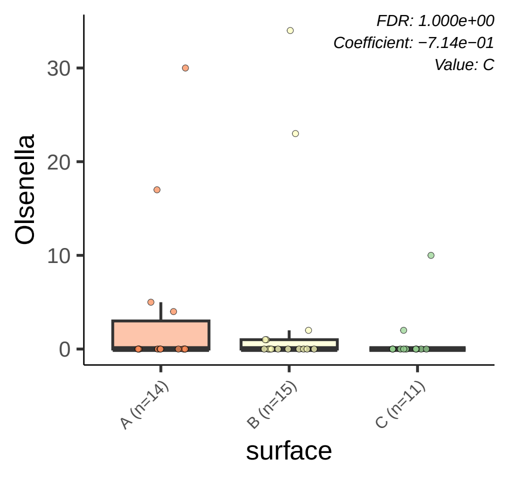

FDR: 1.000e+00
Coefficient: −7.14e−01
Value: C
Olsenella
30
20
10
0
A (n=14)
B (n=15)
C (n=11)
surface

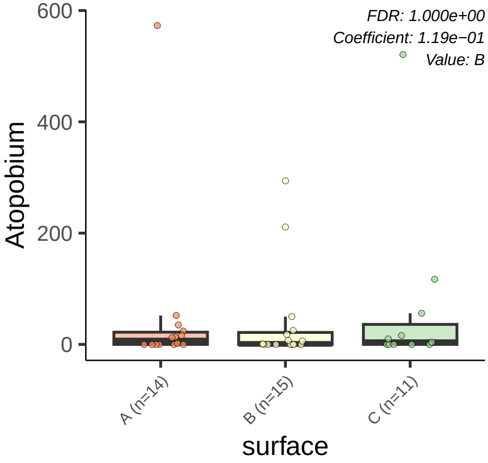

FDR: 1.000e+00
Coefficient: 1.19e−01
Value: B
600
400
200
0
Atopobium
A (n=14)
B (n=15)
C (n=11)
surface

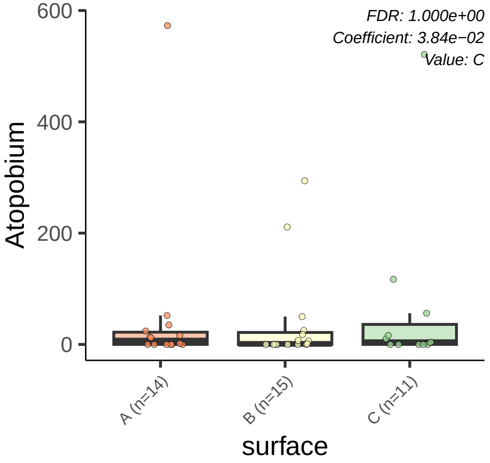

FDR: 1.000e+00
Coefficient: 3.84e−02
Value: C
600
400
200
0
Atopobium
A (n=14)
B (n=15)
C (n=11)
surface

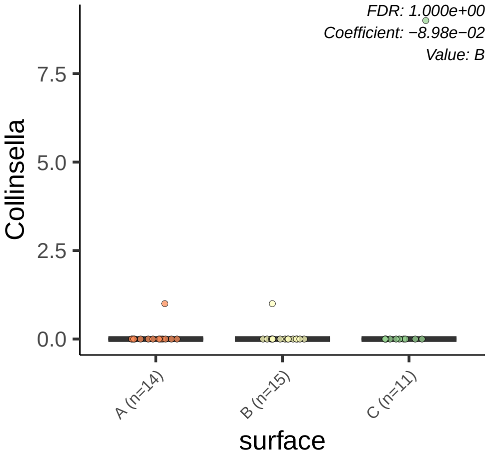

FDR: 1.000e+00
Coefficient: −8.98e−02
Value: B
Collinsella
7.5
5.0
2.5
0.0
A (n=14)
B (n=15)
C (n=11)
surface

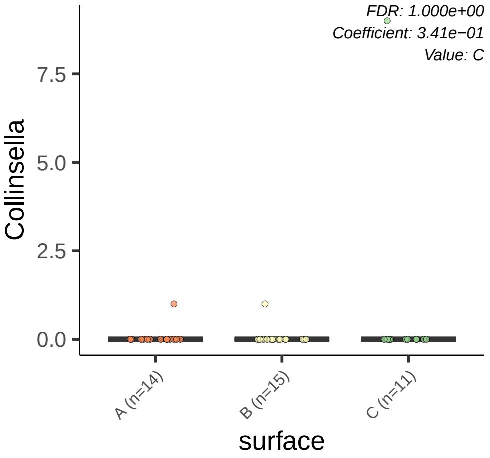

FDR: 1.000e+00
Coefficient: 3.41e−01
Value: C
Collinsella
7.5
5.0
2.5
0.0
A (n=14)
B (n=15)
C (n=11)
surface

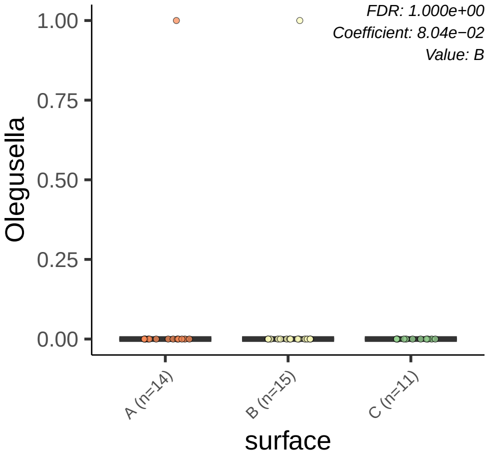

FDR: 1.000e+00
Coefficient: 8.04e−02
Value: B
Olegusella
1.00
0.75
0.50
0.25
0.00
A (n=14)
B (n=15)
C (n=11)
surface

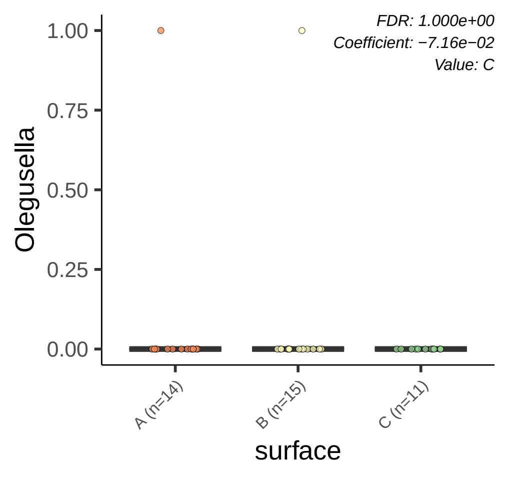

FDR: 1.000e+00
Coefficient: −7.16e−02
Value: C
Olegusella
1.00
0.75
0.50
0.25
0.00
A (n=14)
B (n=15)
C (n=11)
surface

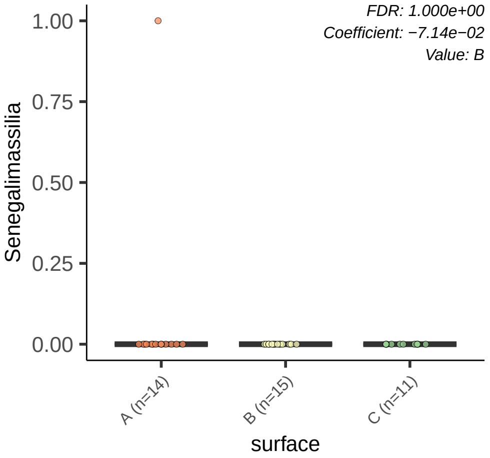

FDR: 1.000e+00
Coefficient: −7.14e−02
Value: B
Senegalimassilia
1.00
0.75
0.50
0.25
0.00
A (n=14)
B (n=15)
C (n=11)
surface

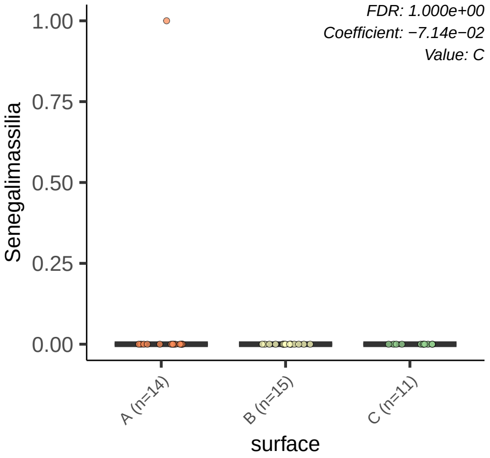
FDR: 1.000e+00
Coefficient: −7.14e−02
Value: C
Senegalimassilia
1.00
0.75
0.50
0.25
0.00
A (n=14)
B (n=15)
C (n=11)
surface

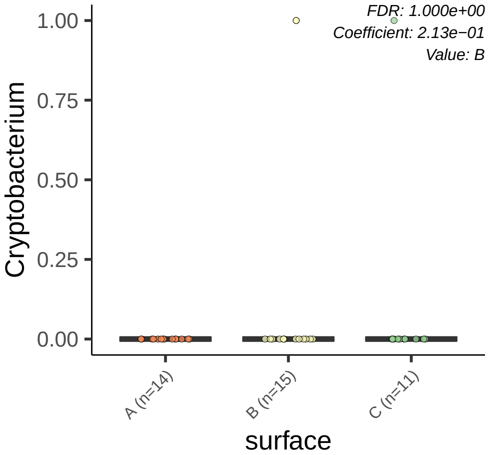
FDR: 1.000e+00
Coefficient: 2.13e−01
Value: B
Cryptobacterium
1.00
0.75
0.50
0.25
0.00
A (n=14)
B (n=15)
C (n=11)
surface

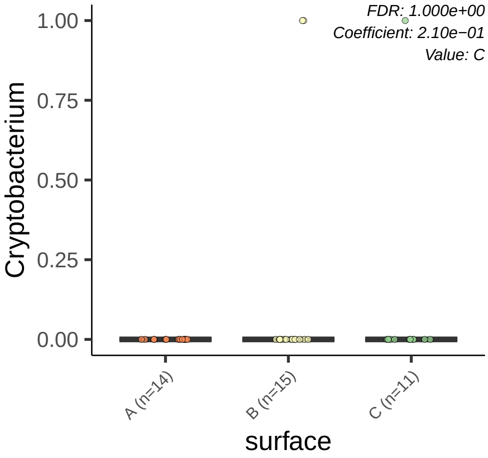
FDR: 1.000e+00
Coefficient: 2.10e−01
Value: C
Cryptobacterium
1.00
0.75
0.50
0.25
0.00
A (n=14)
B (n=15)
C (n=11)
surface

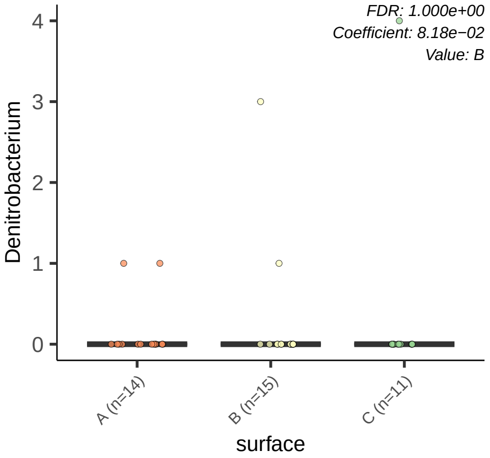

FDR: 1.000e+00
Coefficient: 8.18e−02
Value: B
Denitrobacterium
0
1
2
3
4
A (n=14)
B (n=15)
C (n=11)
surface

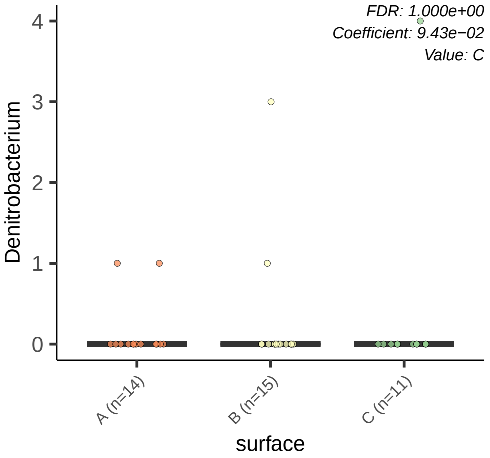
FDR: 1.000e+00
Coefficient: 9.43e−02
Value: C
Denitrobacterium
0
1
2
3
4
A (n=14)
B (n=15)
C (n=11)
surface

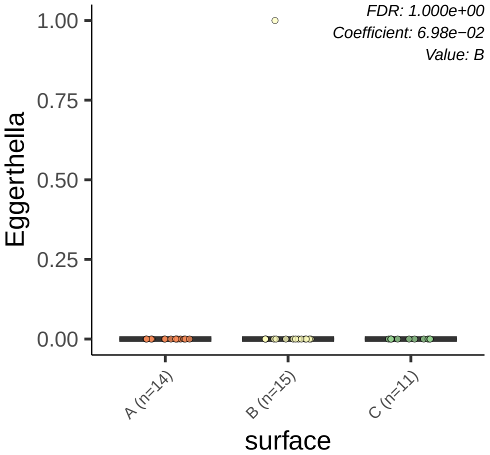
FDR: 1.000e+00
Coefficient: 6.98e−02
Value: B
Eggerthella
1.00
0.75
0.50
0.25
0.00
A (n=14)
B (n=15)
C (n=11)
surface

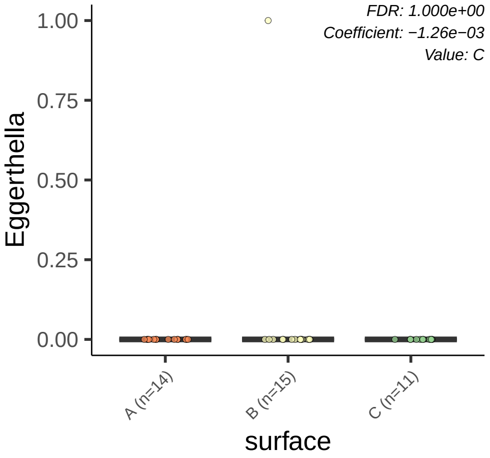

FDR: 1.000e+00
Coefficient: −1.26e−03
Value: C
1.00
0.75
0.50
0.25
0.00
Eggerthella
A (n=14)
B (n=15)
C (n=11)
surface

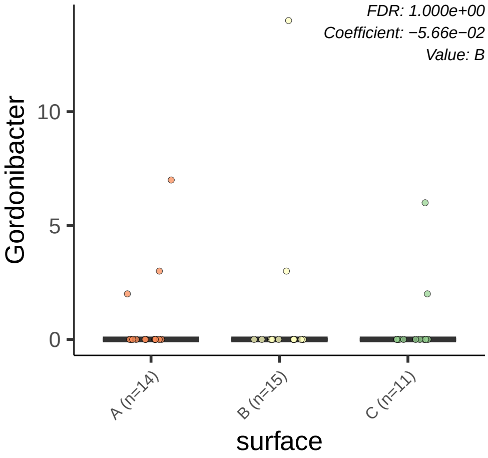

FDR: 1.000e+00
Coefficient: −5.66e−02
Value: B
Gordonibacter
10
5
0
A (n=14)
B (n=15)
C (n=11)
surface

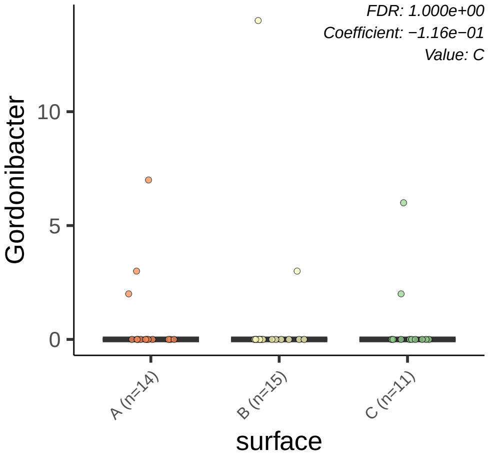

FDR: 1.000e+00
Coefficient: −1.16e−01
Value: C
Gordonibacter
10
5
0
A (n=14)
B (n=15)
C (n=11)
surface

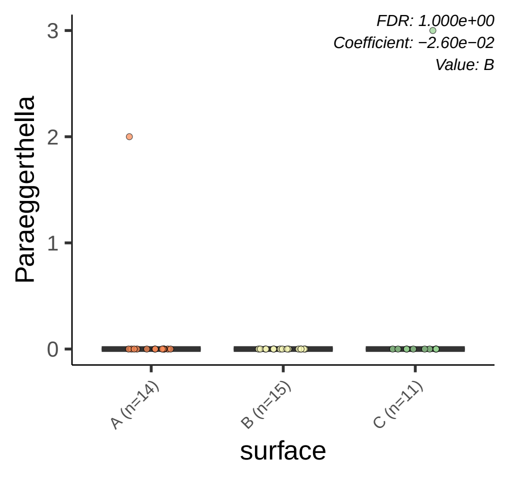
FDR: 1.000e+00
Coefficient: −2.60e−02
Value: B
Paraeggerthella
3
2
1
0
A (n=14)
B (n=15)
C (n=11)
surface

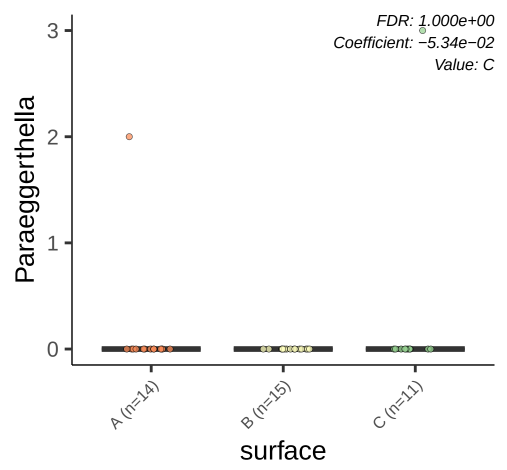

FDR: 1.000e+00
Coefficient: −5.34e−02
Value: C
Paraeggerthella
0
1
2
3
A (n=14)
B (n=15)
C (n=11)
surface

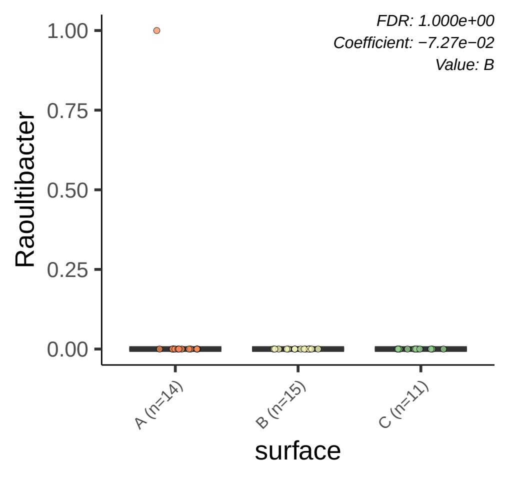
FDR: 1.000e+00
Coefficient: −7.27e−02
Value: B
Raoultibacter
1.00
0.75
0.50
0.25
0.00
A (n=14)
B (n=15)
C (n=11)
surface

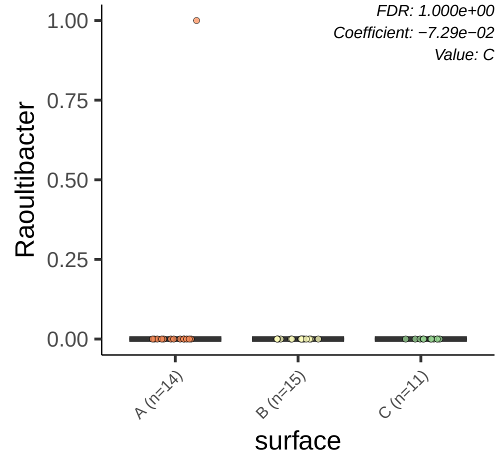
FDR: 1.000e+00
Coefficient: −7.29e−02
Value: C
Raoultibacter
1.00
0.75
0.50
0.25
0.00
A (n=14)
B (n=15)
C (n=11)
surface

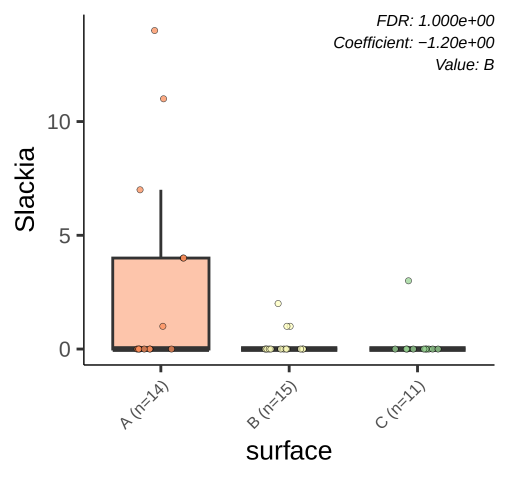

FDR: 1.000e+00
Coefficient: −1.20e+00
Value: B
Slackia
0
5
10
A (n=14)
B (n=15)
C (n=11)
surface

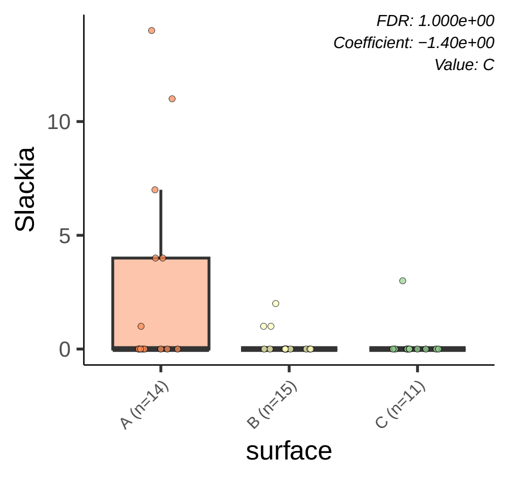
FDR: 1.000e+00
Coefficient: −1.40e+00
Value: C
Slackia
10
5
0
A (n=14)
B (n=15)
C (n=11)
surface

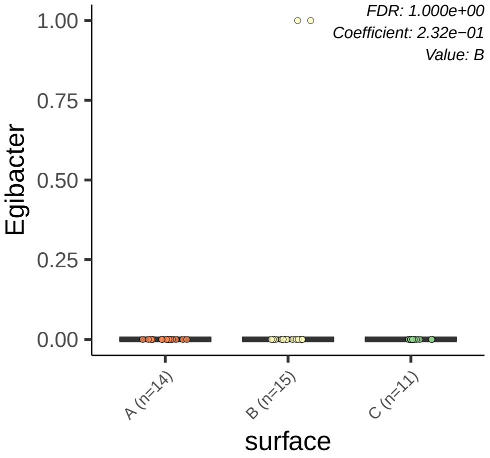
FDR: 1.000e+00
Coefficient: 2.32e−01
Value: B
Egibacter
1.00
0.75
0.50
0.25
0.00
A (n=14)
B (n=15)
C (n=11)
surface

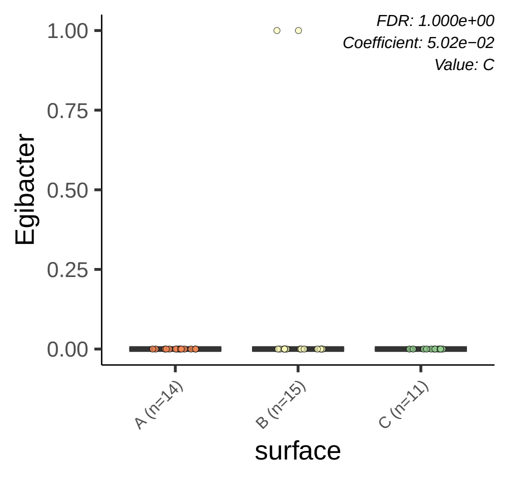

FDR: 1.000e+00
Coefficient: 5.02e−02
Value: C
1.00
0.75
0.50
0.25
0.00
Egibacter
A (n=14)
B (n=15)
C (n=11)
surface

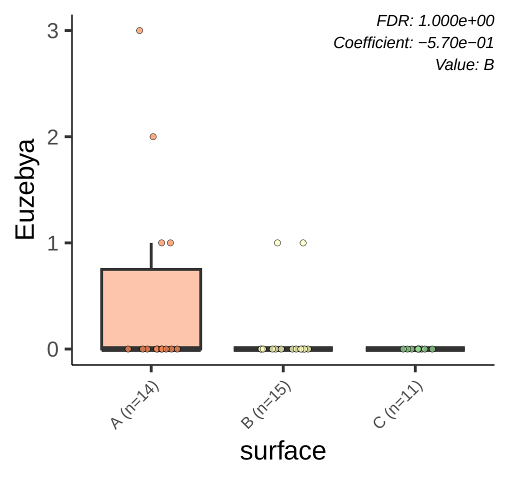

FDR: 1.000e+00
Coefficient: −5.70e−01
Value: B
Euzebya
3
2
1
0
A (n=14)
B (n=15)
C (n=11)
surface

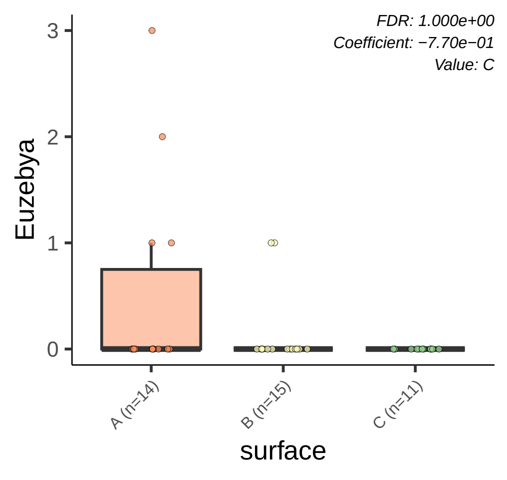
FDR: 1.000e+00
Coefficient: −7.70e−01
Value: C
Euzebya
3
2
1
0
A (n=14)
B (n=15)
C (n=11)
surface

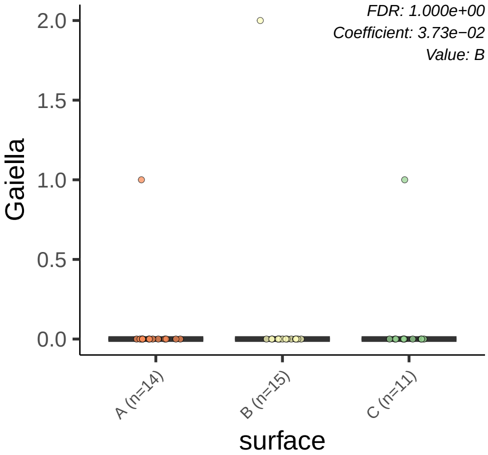
FDR: 1.000e+00
Coefficient: 3.73e−02
Value: B
Gaiella
2.0
1.5
1.0
0.5
0.0
A (n=14)
B (n=15)
C (n=11)
surface

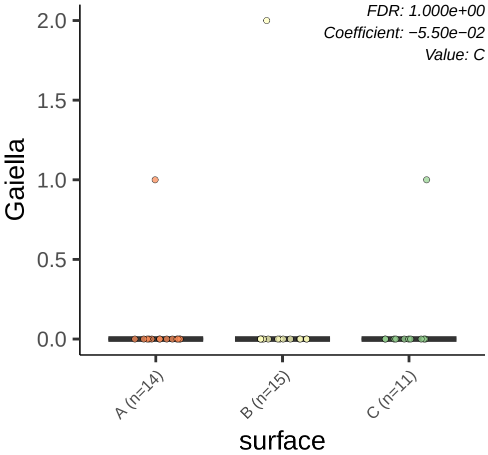
FDR: 1.000e+00
Coefficient: −5.50e−02
Value: C
Gaiella
2.0
1.5
1.0
0.5
0.0
A (n=14)
B (n=15)
C (n=11)
surface

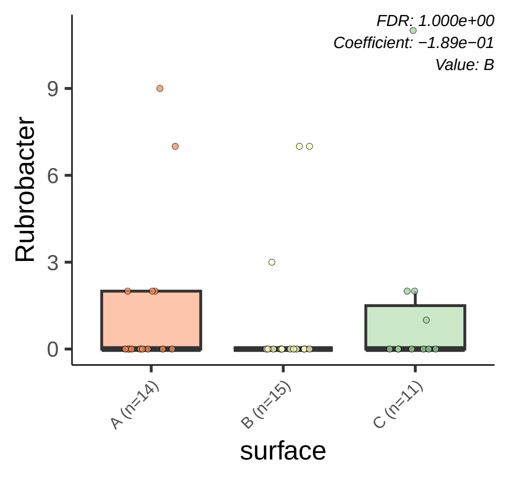
FDR: 1.000e+00
Coefficient: −1.89e−01
Value: B
Rubrobacter
9
6
3
0
A (n=14)
B (n=15)
C (n=11)
surface

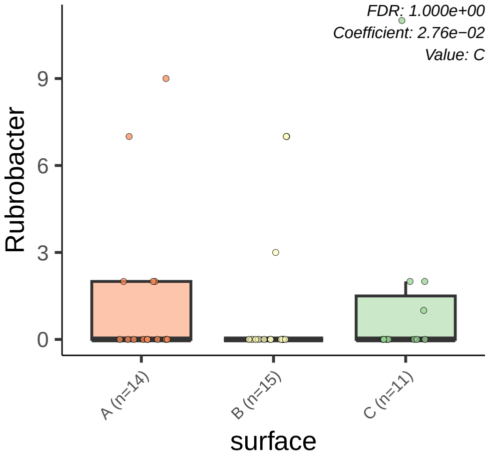

FDR: 1.000e+00
Coefficient: 2.76e−02
Value: C
Rubrobacter
0
3
6
9
A (n=14)
B (n=15)
C (n=11)
surface

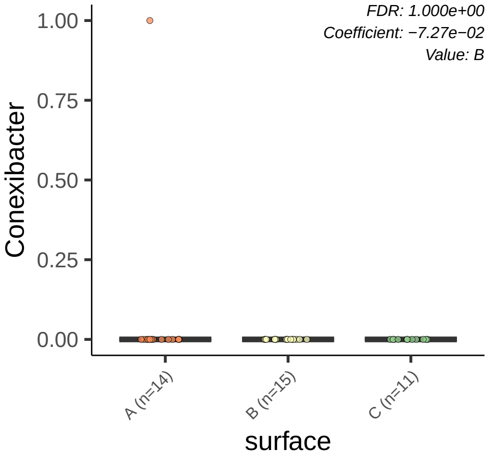
FDR: 1.000e+00
Coefficient: −7.27e−02
Value: B
Conexibacter
1.00
0.75
0.50
0.25
0.00
A (n=14)
B (n=15)
C (n=11)
surface

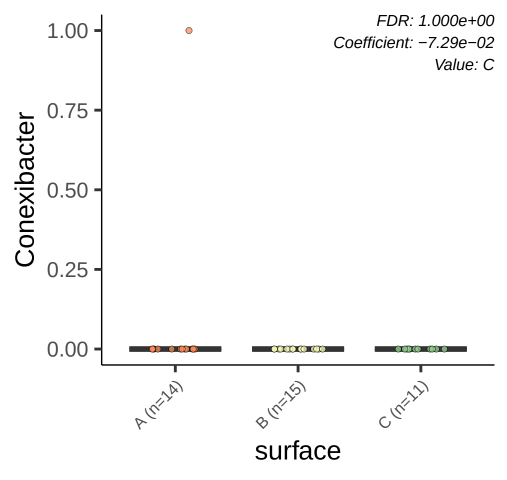
FDR: 1.000e+00
Coefficient: −7.29e−02
Value: C
Conexibacter
1.00
0.75
0.50
0.25
0.00
A (n=14)
B (n=15)
C (n=11)
surface

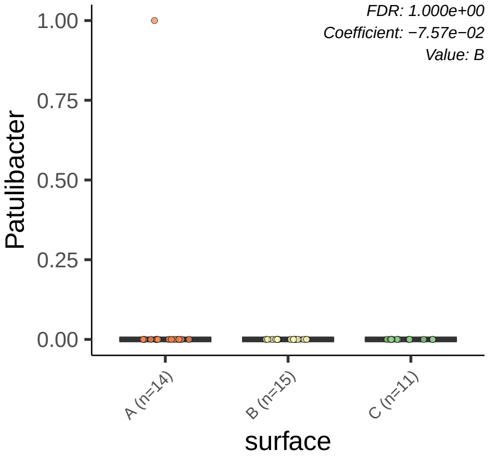

FDR: 1.000e+00
Coefficient: −7.57e−02
Value: B
Patulibacter
1.00
0.75
0.50
0.25
0.00
A (n=14)
B (n=15)
C (n=11)
surface

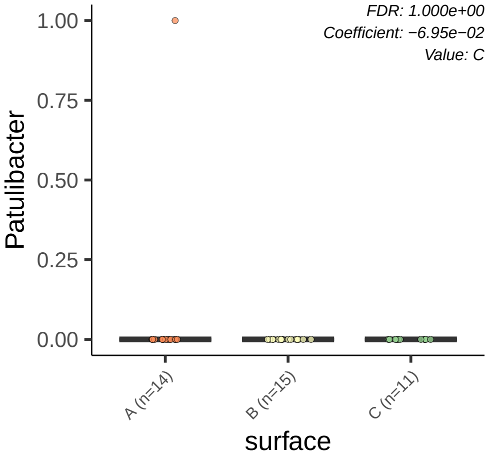
FDR: 1.000e+00
Coefficient: −6.95e−02
Value: C
Patulibacter
1.00
0.75
0.50
0.25
0.00
A (n=14)
B (n=15)
C (n=11)
surface

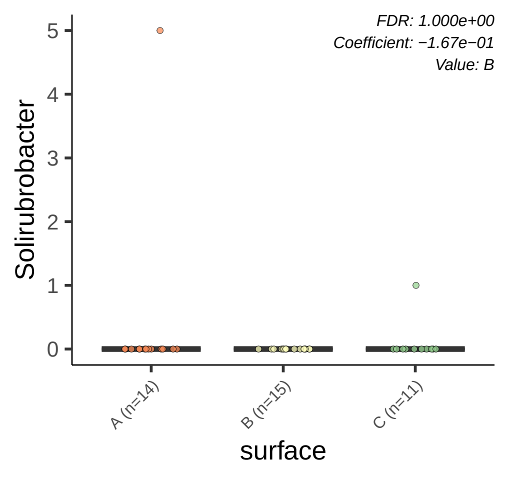

FDR: 1.000e+00
Coefficient: −1.67e−01
Value: B
Solirubrobacter
5
4
3
2
1
0
A (n=14)
B (n=15)
C (n=11)
surface

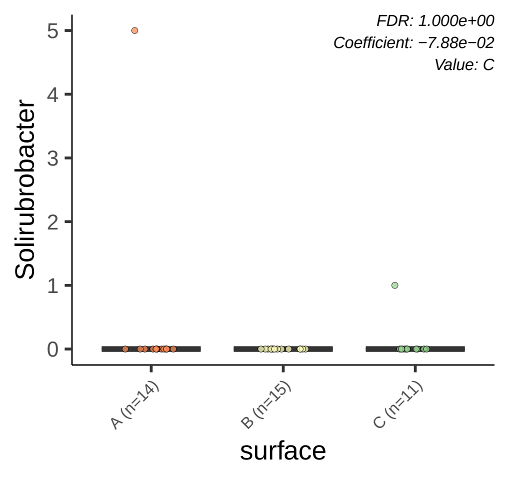
FDR: 1.000e+00
Coefficient: −7.88e−02
Value: C
Solirubrobacter
0
1
2
3
4
5
A (n=14)
B (n=15)
C (n=11)
surface

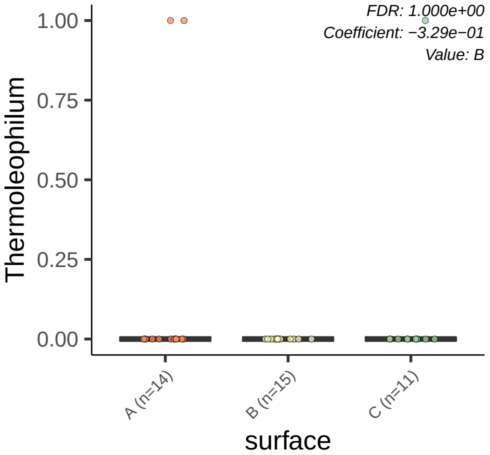
FDR: 1.000e+00
Coefficient: −3.29e−01
Value: B
Thermoleophilum
1.00
0.75
0.50
0.25
0.00
A (n=14)
B (n=15)
C (n=11)
surface

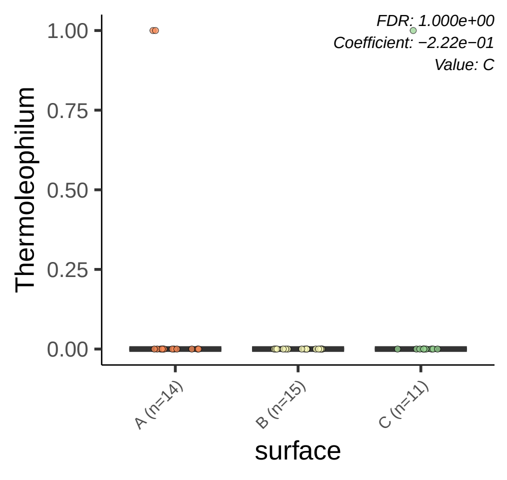
FDR: 1.000e+00
Coefficient: −2.22e−01
Value: C
Thermoleophilum
1.00
0.75
0.50
0.25
0.00
A (n=14)
B (n=15)
C (n=11)
surface

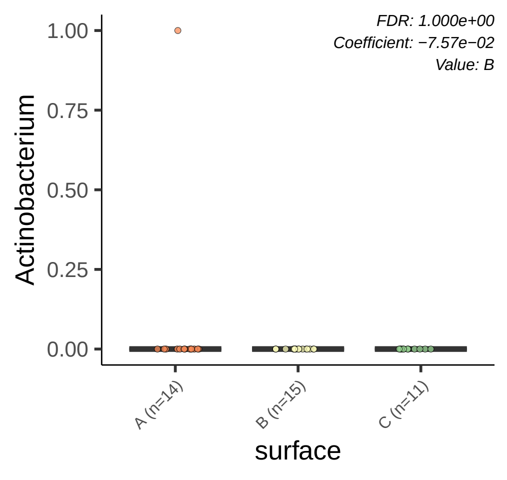
FDR: 1.000e+00
Coefficient: −7.57e−02
Value: B
Actinobacterium
1.00
0.75
0.50
0.25
0.00
A (n=14)
B (n=15)
C (n=11)
surface

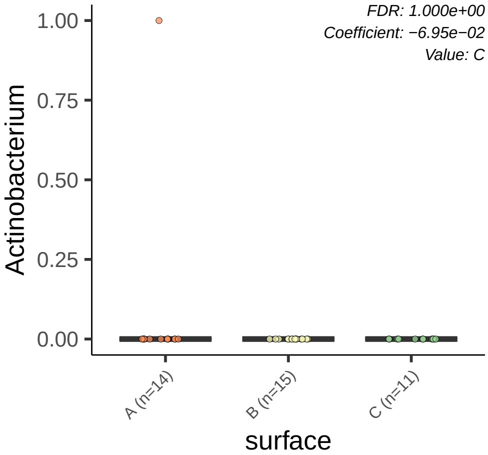
FDR: 1.000e+00
Coefficient: −6.95e−02
Value: C
Actinobacterium
1.00
0.75
0.50
0.25
0.00
A (n=14)
B (n=15)
C (n=11)
surface

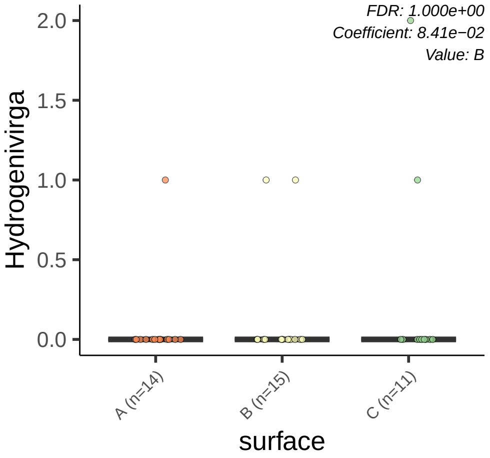
FDR: 1.000e+00
Coefficient: 8.41e−02
Value: B
Hydrogenivirga
2.0
1.5
1.0
0.5
0.0
A (n=14)
B (n=15)
C (n=11)
surface

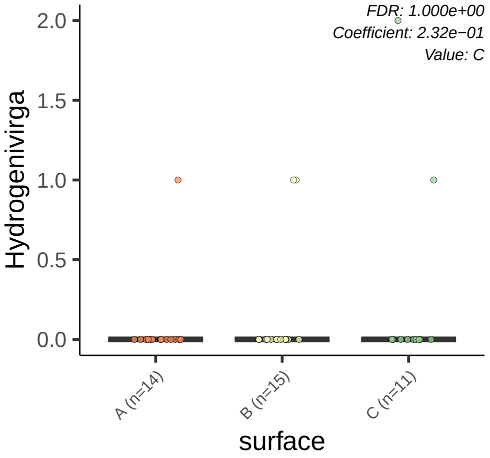

FDR: 1.000e+00
Coefficient: 2.32e−01
Value: C
Hydrogenivirga
2.0
1.5
1.0
0.5
0.0
A (n=14)
B (n=15)
C (n=11)
surface

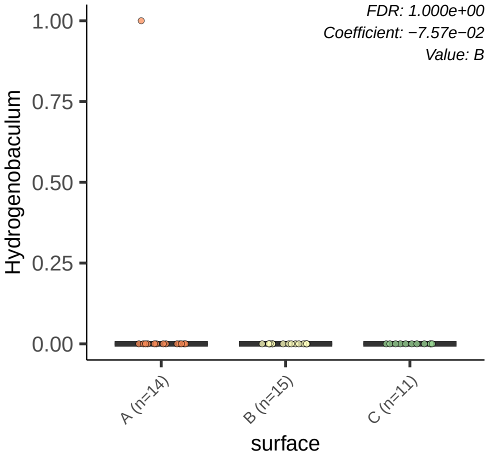

FDR: 1.000e+00
Coefficient: −7.57e−02
Value: B
Hydrogenobaculum
1.00
0.75
0.50
0.25
0.00
A (n=14)
B (n=15)
C (n=11)
surface

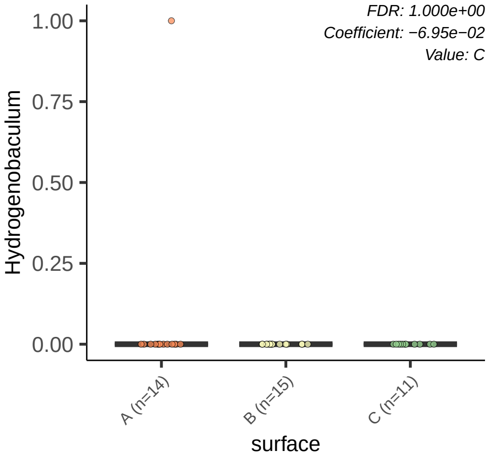
FDR: 1.000e+00
Coefficient: −6.95e−02
Value: C
1.00
0.75
0.50
0.25
0.00
Hydrogenobaculum
A (n=14)
B (n=15)
C (n=11)
surface

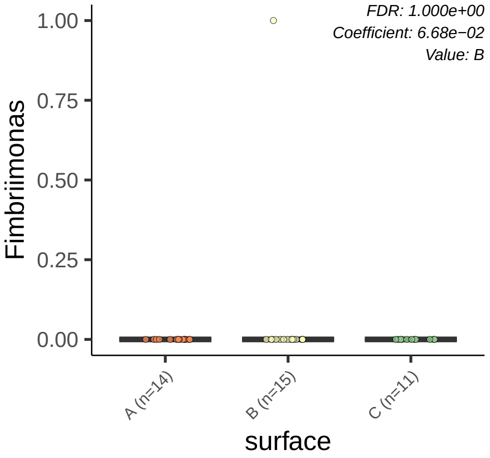
FDR: 1.000e+00
Coefficient: 6.68e−02
Value: B
Fimbriimonas
1.00
0.75
0.50
0.25
0.00
A (n=14)
B (n=15)
C (n=11)
surface

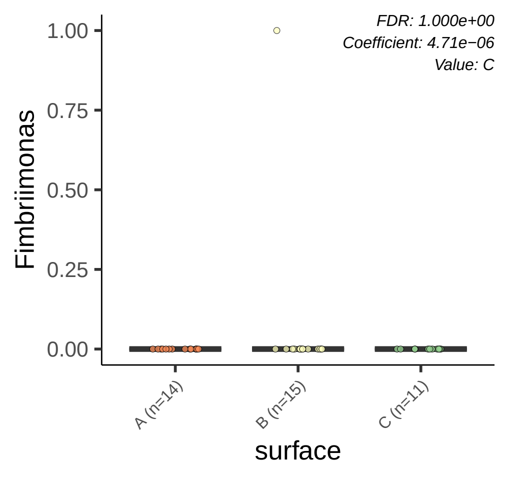
FDR: 1.000e+00
Coefficient: 4.71e−06
Value: C
Fimbriimonas
1.00
0.75
0.50
0.25
0.00
A (n=14)
B (n=15)
C (n=11)
surface

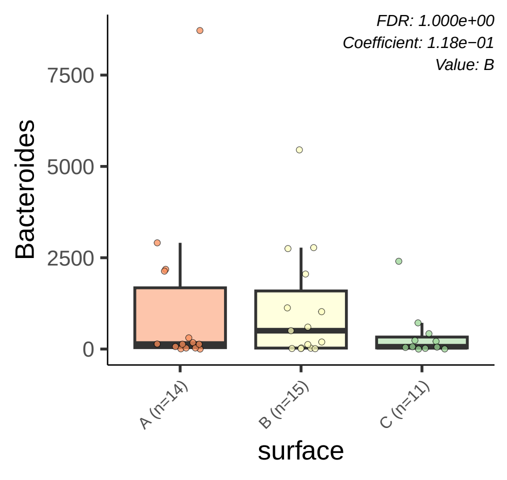
FDR: 1.000e+00
Coefficient: 1.18e−01
Value: B
Bacteroides
7500
5000
2500
0
A (n=14)
B (n=15)
C (n=11)
surface

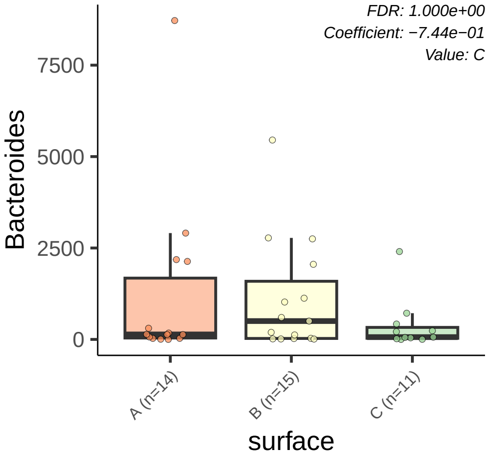

FDR: 1.000e+00
Coefficient: −7.44e−01
Value: C
Bacteroides
7500
5000
2500
0
A (n=14)
B (n=15)
C (n=11)
surface

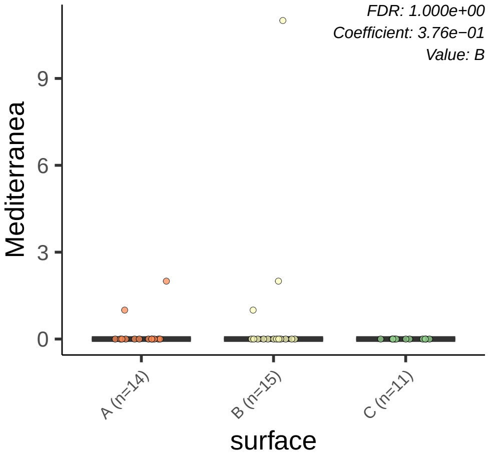
FDR: 1.000e+00
Coefficient: 3.76e−01
Value: B
Mediterranea
0
3
6
9
A (n=14)
B (n=15)
C (n=11)
surface

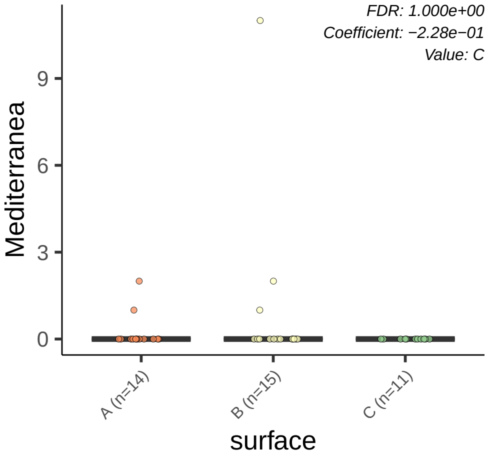

FDR: 1.000e+00
Coefficient: −2.28e−01
Value: C
Mediterranea
0
3
6
9
A (n=14)
B (n=15)
C (n=11)
surface

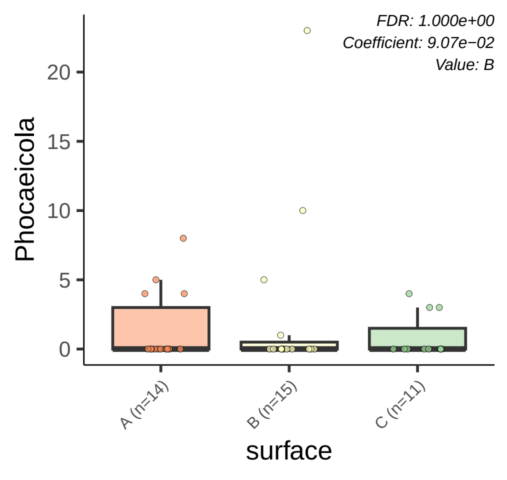
FDR: 1.000e+00
Coefficient: 9.07e−02
Value: B
Phocaeicola
0
5
10
15
20
A (n=14)
B (n=15)
C (n=11)
surface

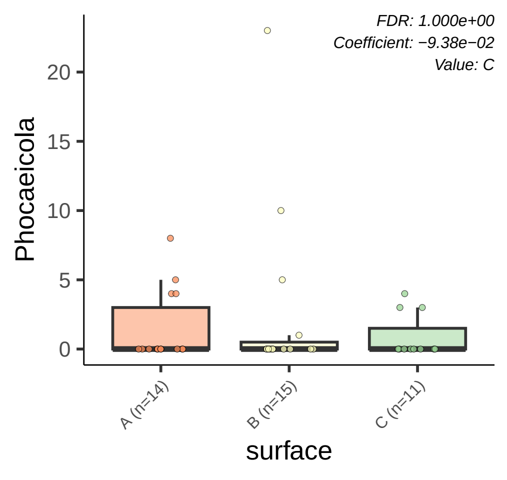
FDR: 1.000e+00
Coefficient: −9.38e−02
Value: C
Phocaeicola
0
5
10
15
20
A (n=14)
B (n=15)
C (n=11)
surface

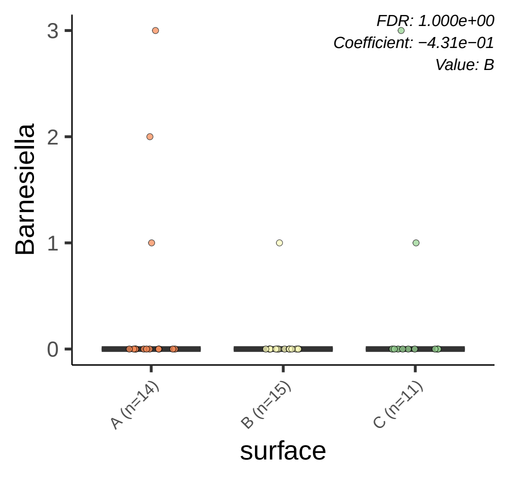

FDR: 1.000e+00
Coefficient: −4.31e−01
Value: B
Barnesiella
3
2
1
0
A (n=14)
B (n=15)
C (n=11)
surface

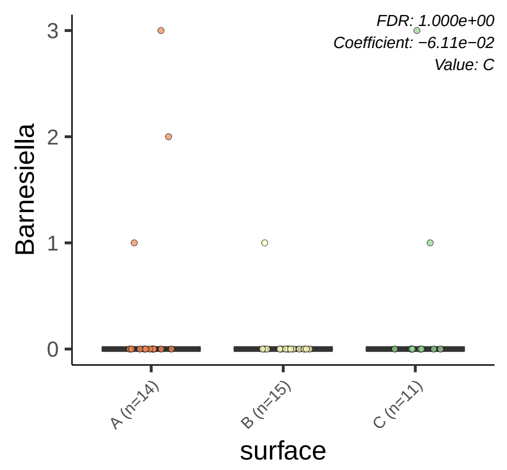

FDR: 1.000e+00
Coefficient: −6.11e−02
Value: C
Barnesiella
0
1
2
3
A (n=14)
B (n=15)
C (n=11)
surface

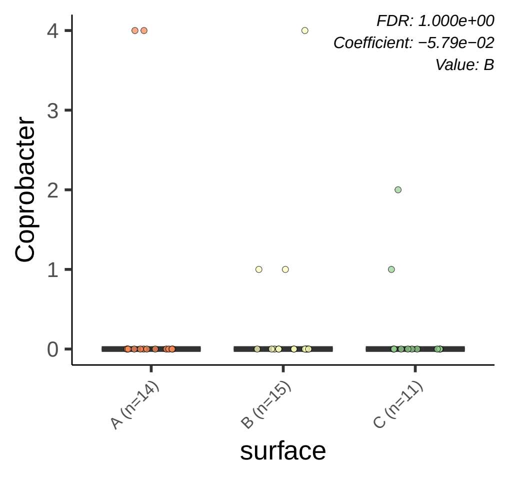
FDR: 1.000e+00
Coefficient: −5.79e−02
Value: B
Coprobacter
4
3
2
1
0
A (n=14)
B (n=15)
C (n=11)
surface

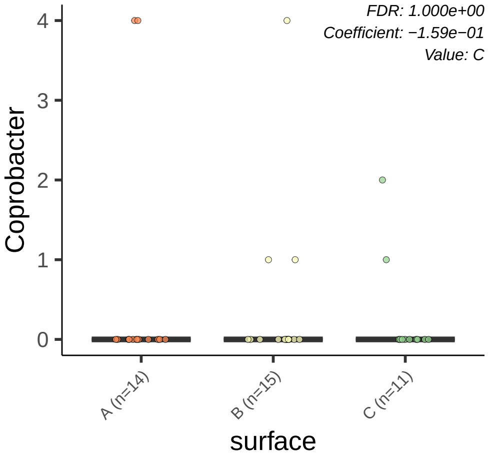
FDR: 1.000e+00
Coefficient: −1.59e−01
Value: C
Coprobacter
0
1
2
3
4
A (n=14)
B (n=15)
C (n=11)
surface

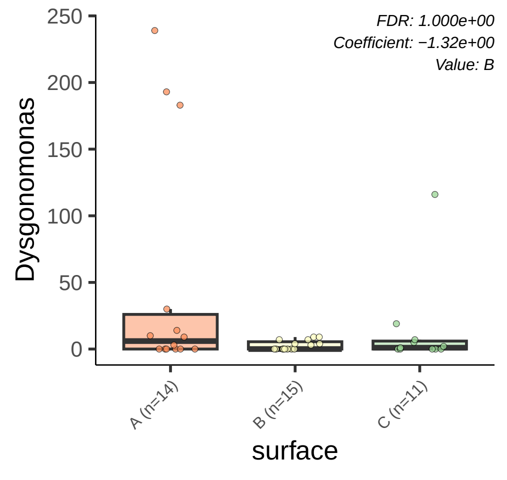
FDR: 1.000e+00
Coefficient: −1.32e+00
Value: B
Dysgonomonas
250
200
150
100
50
0
A (n=14)
B (n=15)
C (n=11)
surface

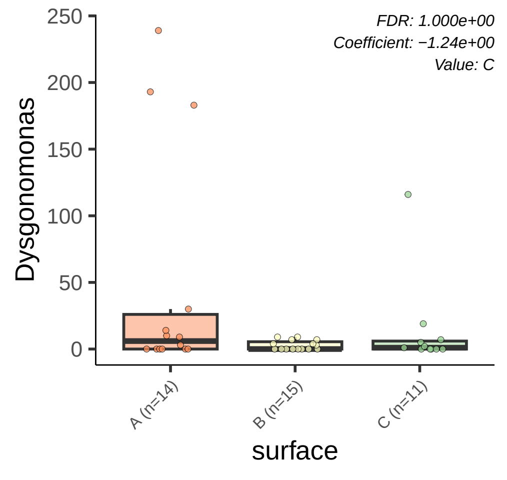
FDR: 1.000e+00
Coefficient: −1.24e+00
Value: C
Dysgonomonas
250
200
150
100
50
0
A (n=14)
B (n=15)
C (n=11)
surface

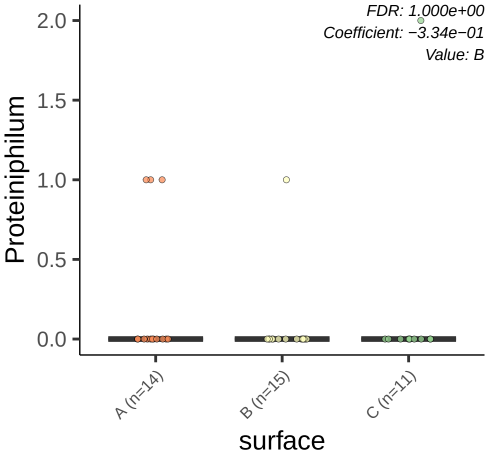
FDR: 1.000e+00
Coefficient: −3.34e−01
Value: B
Proteiniphilum
2.0
1.5
1.0
0.5
0.0
A (n=14)
B (n=15)
C (n=11)
surface

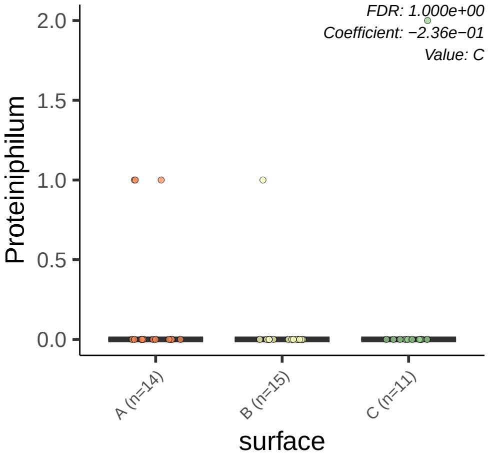
FDR: 1.000e+00
Coefficient: −2.36e−01
Value: C
Proteiniphilum
2.0
1.5
1.0
0.5
0.0
A (n=14)
B (n=15)
C (n=11)
surface

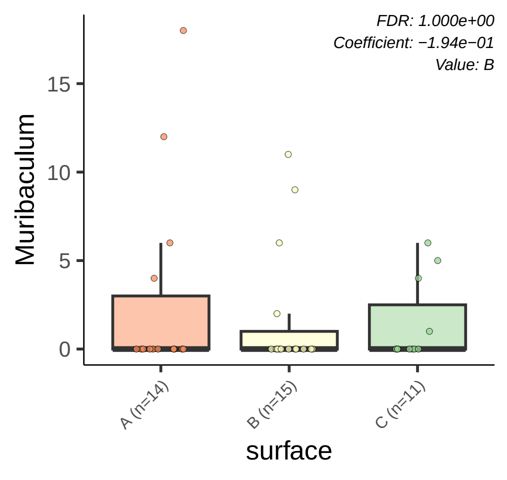
FDR: 1.000e+00
Coefficient: −1.94e−01
Value: B
Muribaculum
15
10
5
0
A (n=14)
B (n=15)
C (n=11)
surface

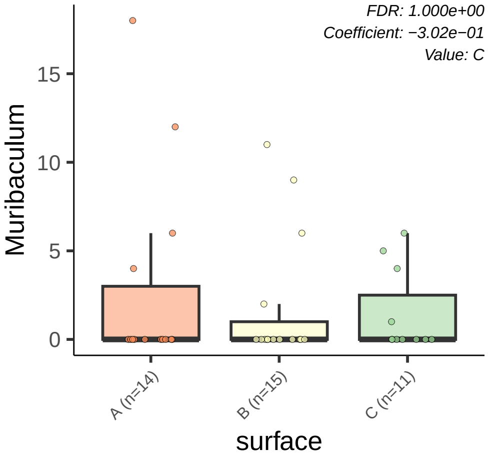
FDR: 1.000e+00
Coefficient: −3.02e−01
Value: C
Muribaculum
15
10
5
0
A (n=14)
B (n=15)
C (n=11)
surface

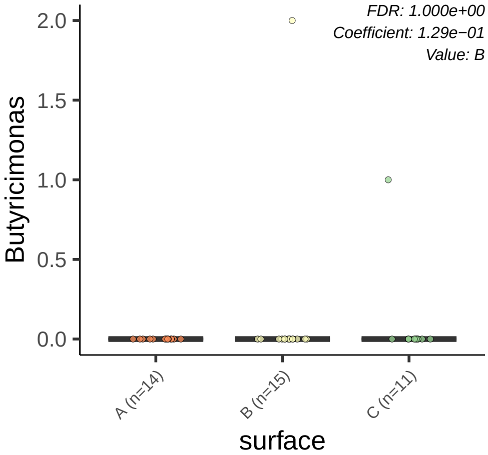

FDR: 1.000e+00
Coefficient: 1.29e−01
Value: B
Butyricimonas
2.0
1.5
1.0
0.5
0.0
A (n=14)
B (n=15)
C (n=11)
surface

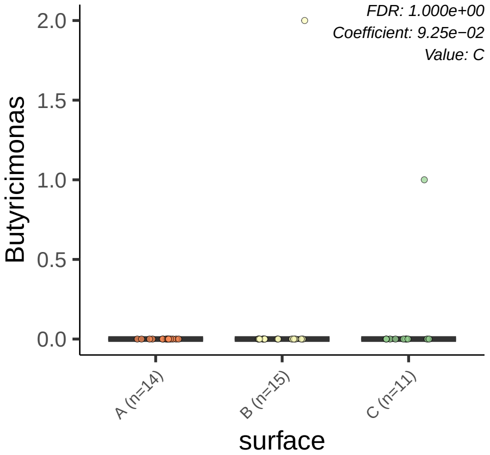
FDR: 1.000e+00
Coefficient: 9.25e−02
Value: C
Butyricimonas
2.0
1.5
1.0
0.5
0.0
A (n=14)
B (n=15)
C (n=11)
surface

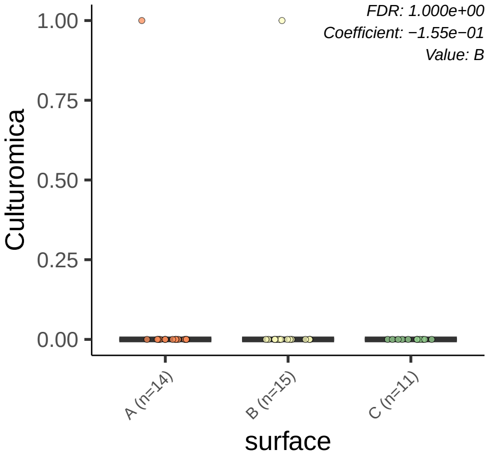

FDR: 1.000e+00
Coefficient: −1.55e−01
Value: B
1.00
0.75
0.50
0.25
0.00
Culturomica
A (n=14)
B (n=15)
C (n=11)
surface

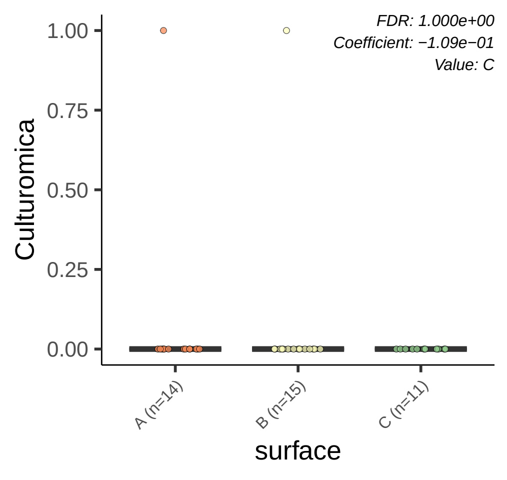
FDR: 1.000e+00
Coefficient: −1.09e−01
Value: C
Culturomica
1.00
0.75
0.50
0.25
0.00
A (n=14)
B (n=15)
C (n=11)
surface

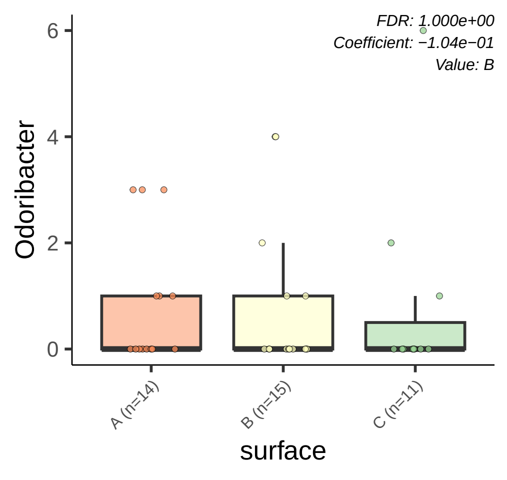
FDR: 1.000e+00
Coefficient: −1.04e−01
Value: B
Odoribacter
0
2
4
6
A (n=14)
B (n=15)
C (n=11)
surface

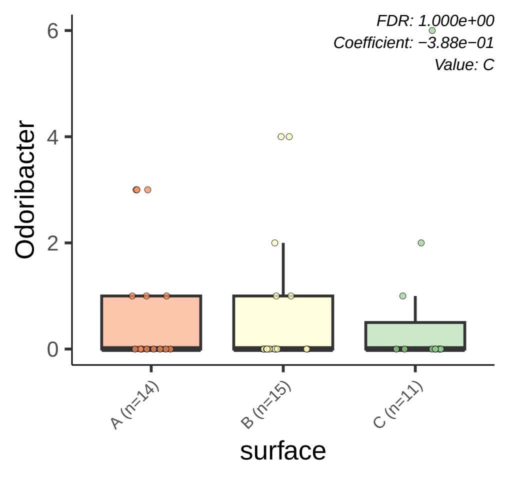

FDR: 1.000e+00
Coefficient: −3.88e−01
Value: C
Odoribacter
6
4
2
0
A (n=14)
B (n=15)
C (n=11)
surface

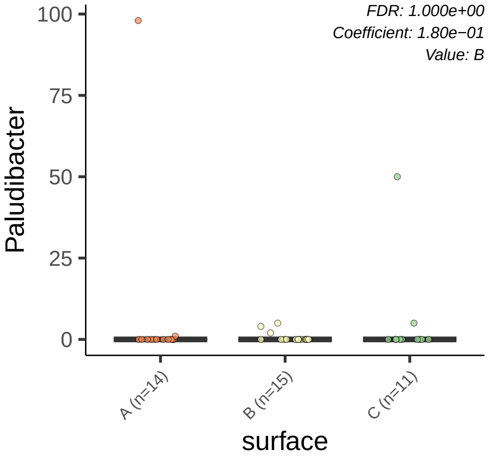
FDR: 1.000e+00
Coefficient: 1.80e−01
Value: B
Paludibacter
100
75
50
25
0
A (n=14)
B (n=15)
C (n=11)
surface

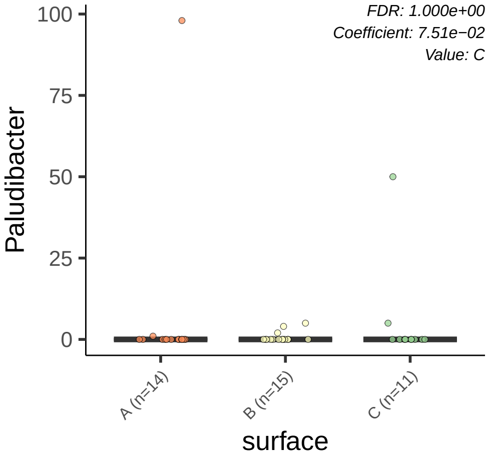

FDR: 1.000e+00
Coefficient: 7.51e−02
Value: C
Paludibacter
100
75
50
25
0
A (n=14)
B (n=15)
C (n=11)
surface

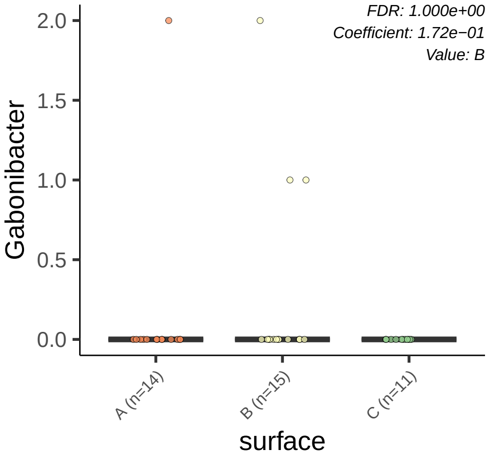

FDR: 1.000e+00
Coefficient: 1.72e−01
Value: B
Gabonibacter
2.0
1.5
1.0
0.5
0.0
A (n=14)
B (n=15)
C (n=11)
surface

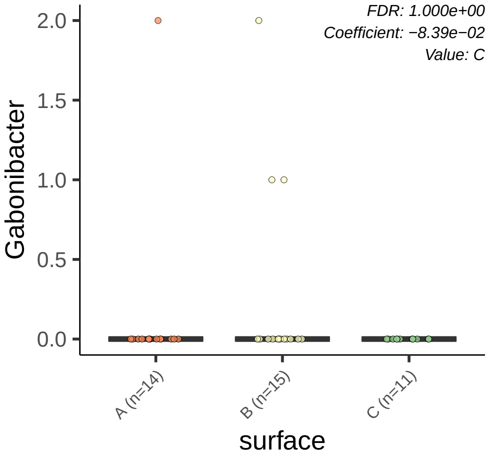

FDR: 1.000e+00
Coefficient: −8.39e−02
Value: C
Gabonibacter
2.0
1.5
1.0
0.5
0.0
A (n=14)
B (n=15)
C (n=11)
surface

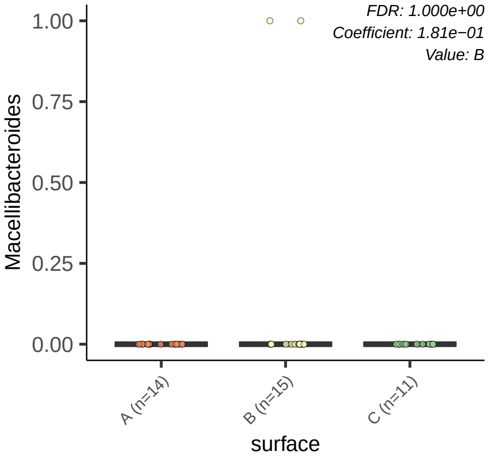
FDR: 1.000e+00
Coefficient: 1.81e−01
Value: B
Macellibacteroides
1.00
0.75
0.50
0.25
0.00
A (n=14)
B (n=15)
C (n=11)
surface

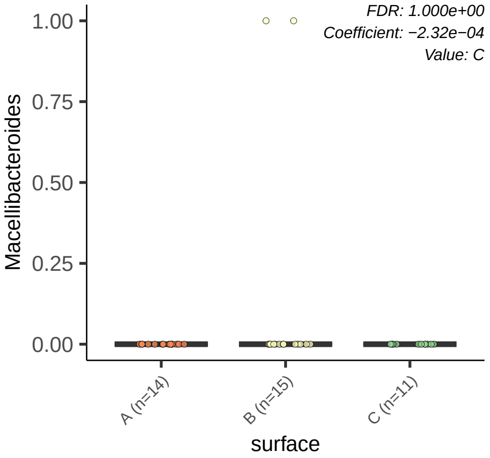
FDR: 1.000e+00
Coefficient: −2.32e−04
Value: C
Macellibacteroides
1.00
0.75
0.50
0.25
0.00
A (n=14)
B (n=15)
C (n=11)
surface

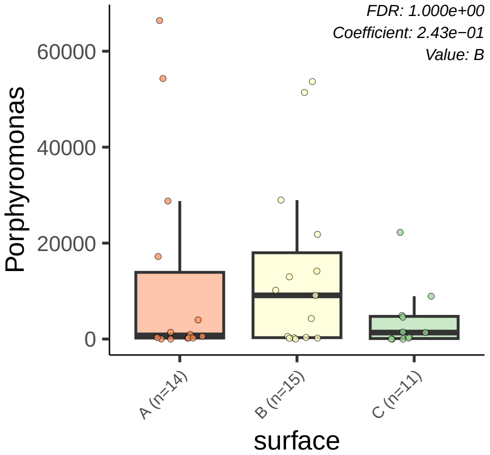
FDR: 1.000e+00
Coefficient: 2.43e−01
Value: B
Porphyromonas
60000
40000
20000
0
A (n=14)
B (n=15)
C (n=11)
surface

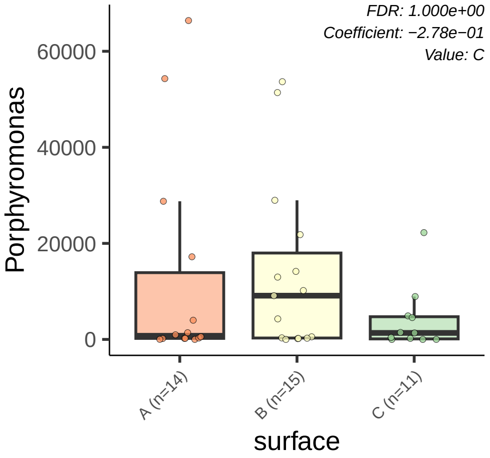
FDR: 1.000e+00
Coefficient: −2.78e−01
Value: C
Porphyromonas
60000
40000
20000
0
A (n=14)
B (n=15)
C (n=11)
surface

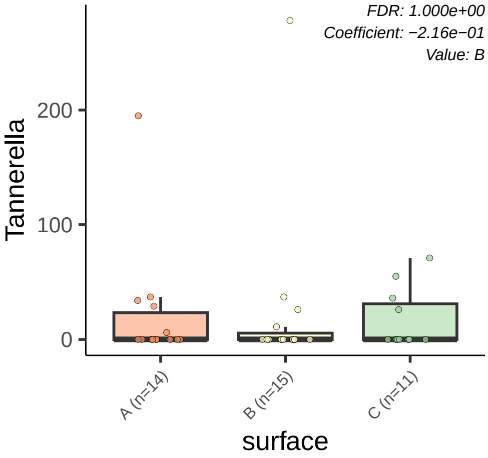
FDR: 1.000e+00
Coefficient: −2.16e−01
Value: B
Tannerella
200
100
0
A (n=14)
B (n=15)
C (n=11)
surface

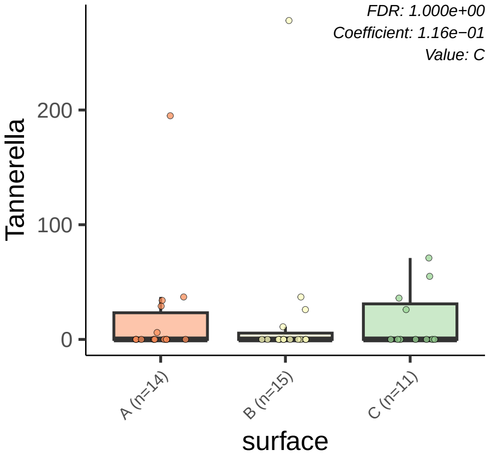
FDR: 1.000e+00
Coefficient: 1.16e−01
Value: C
Tannerella
200
100
0
A (n=14)
B (n=15)
C (n=11)
surface

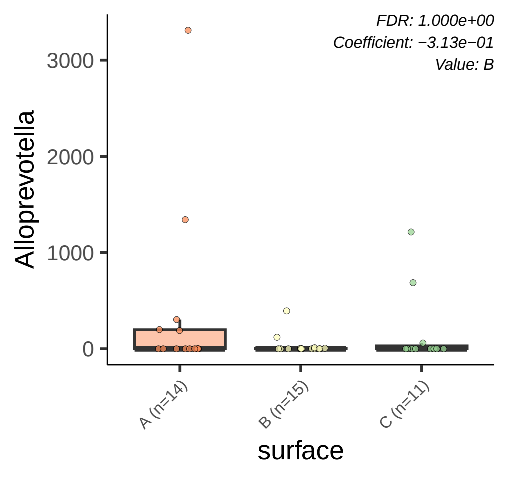
FDR: 1.000e+00
Coefficient: −3.13e−01
Value: B
Alloprevotella
3000
2000
1000
0
A (n=14)
B (n=15)
C (n=11)
surface

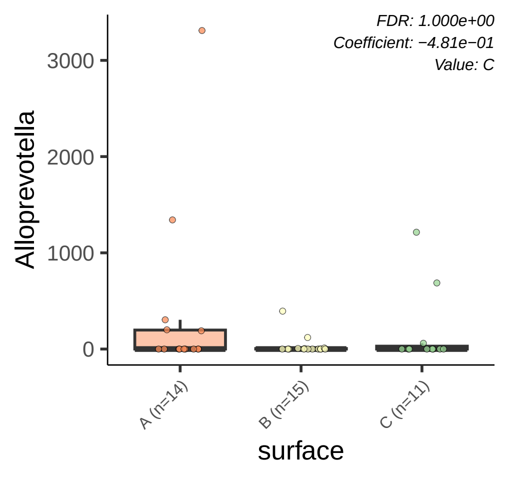

FDR: 1.000e+00
Coefficient: −4.81e−01
Value: C
Alloprevotella
3000
2000
1000
0
A (n=14)
B (n=15)
C (n=11)
surface

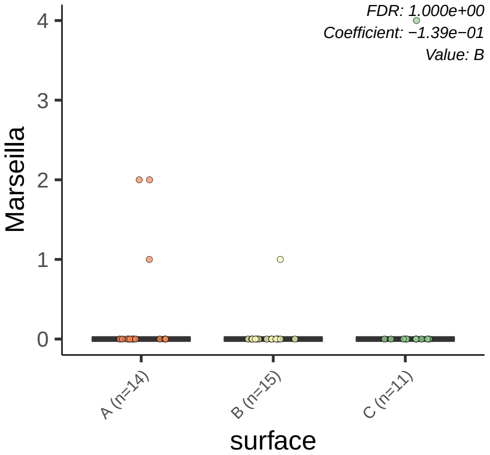

FDR: 1.000e+00
Coefficient: −1.39e−01
Value: B
Marseilla
0
1
2
3
4
A (n=14)
B (n=15)
C (n=11)
surface

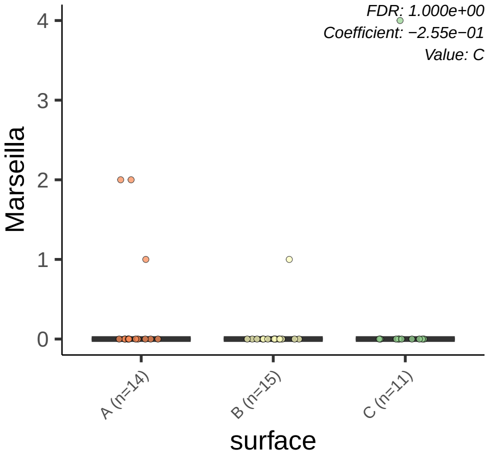

FDR: 1.000e+00
Coefficient: −2.55e−01
Value: C
Marseilla
4
3
2
1
0
A (n=14)
B (n=15)
C (n=11)
surface

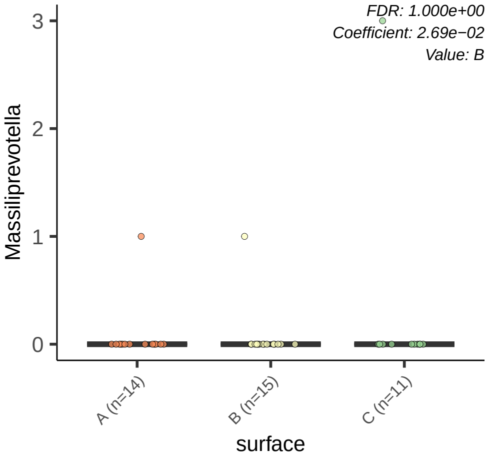
FDR: 1.000e+00
Coefficient: 2.69e−02
Value: B
Massiliprevotella
0
1
2
3
A (n=14)
B (n=15)
C (n=11)
surface

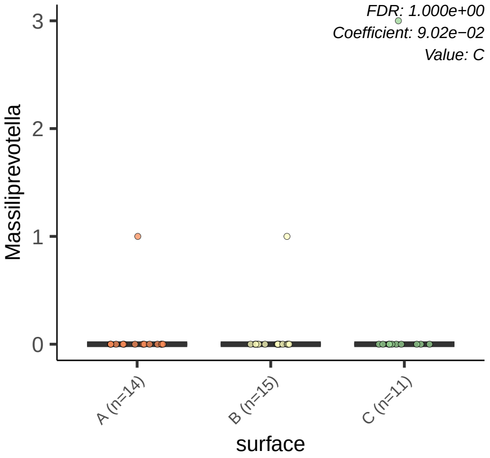

FDR: 1.000e+00
Coefficient: 9.02e−02
Value: C
Massiliprevotella
3
2
1
0
A (n=14)
B (n=15)
C (n=11)
surface

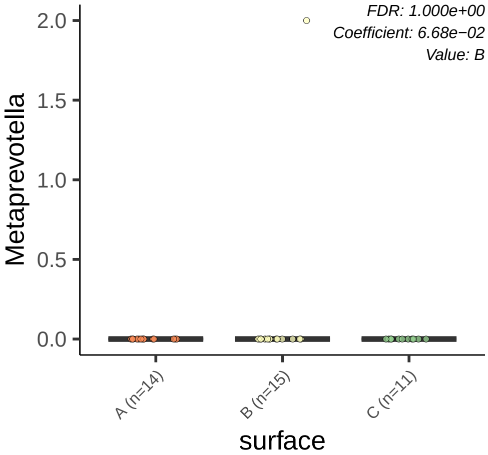
FDR: 1.000e+00
Coefficient: 6.68e−02
Value: B
Metaprevotella
2.0
1.5
1.0
0.5
0.0
A (n=14)
B (n=15)
C (n=11)
surface

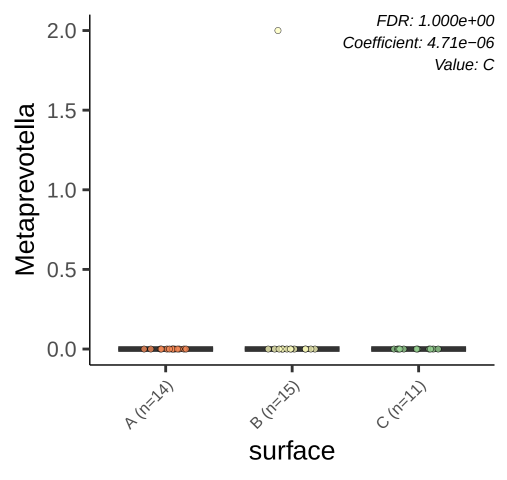
FDR: 1.000e+00
Coefficient: 4.71e−06
Value: C
Metaprevotella
2.0
1.5
1.0
0.5
0.0
A (n=14)
B (n=15)
C (n=11)
surface

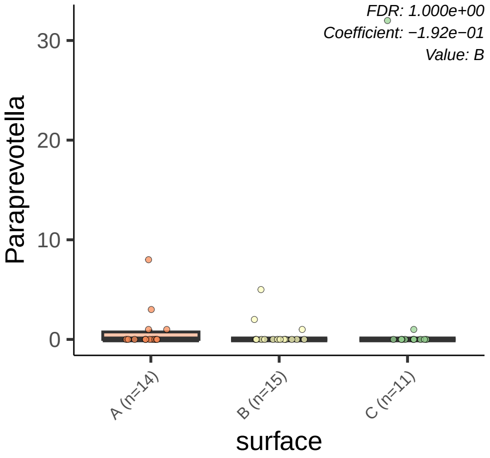

FDR: 1.000e+00
Coefficient: −1.92e−01
Value: B
Paraprevotella
30
20
10
0
A (n=14)
B (n=15)
C (n=11)
surface

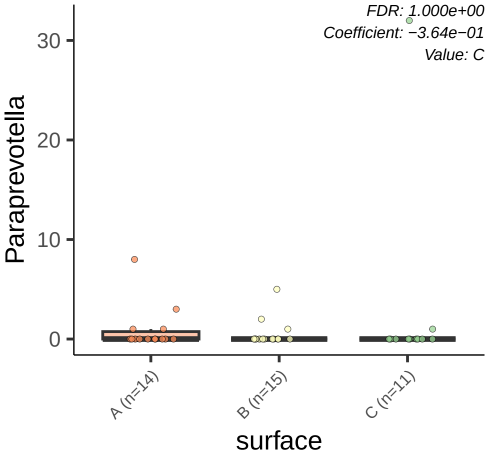
FDR: 1.000e+00
Coefficient: −3.64e−01
Value: C
Paraprevotella
30
20
10
0
A (n=14)
B (n=15)
C (n=11)
surface

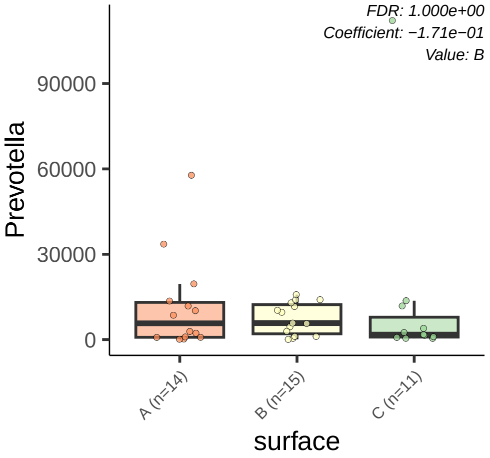
FDR: 1.000e+00
Coefficient: −1.71e−01
Value: B
Prevotella
90000
60000
30000
0
A (n=14)
B (n=15)
C (n=11)
surface

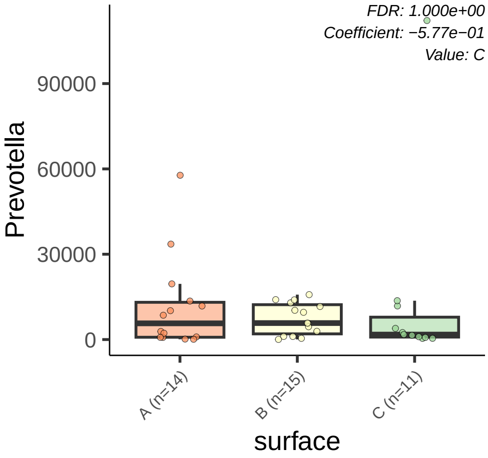
FDR: 1.000e+00
Coefficient: −5.77e−01
Value: C
Prevotella
90000
60000
30000
0
A (n=14)
B (n=15)
C (n=11)
surface

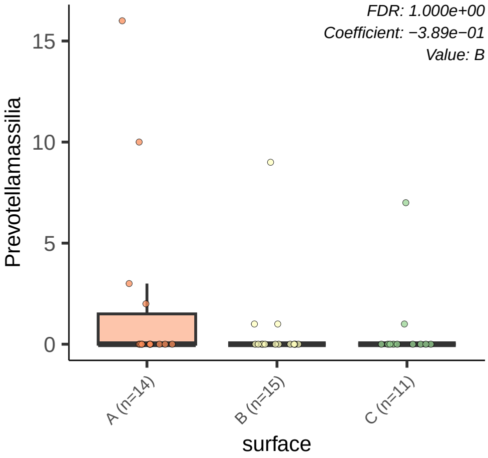

FDR: 1.000e+00
Coefficient: −3.89e−01
Value: B
Prevotellamassilia
15
10
5
0
A (n=14)
B (n=15)
C (n=11)
surface

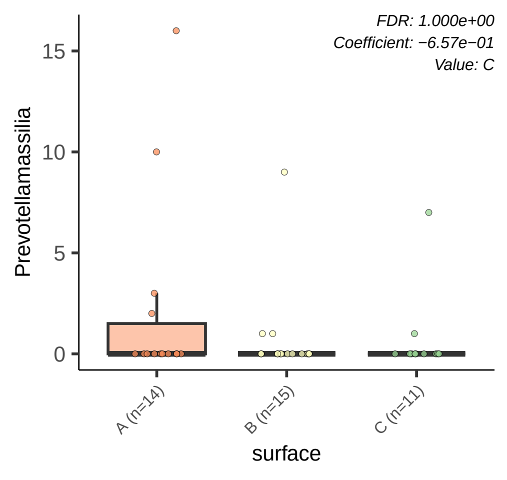
FDR: 1.000e+00
Coefficient: −6.57e−01
Value: C
Prevotellamassilia
0
5
10
15
A (n=14)
B (n=15)
C (n=11)
surface

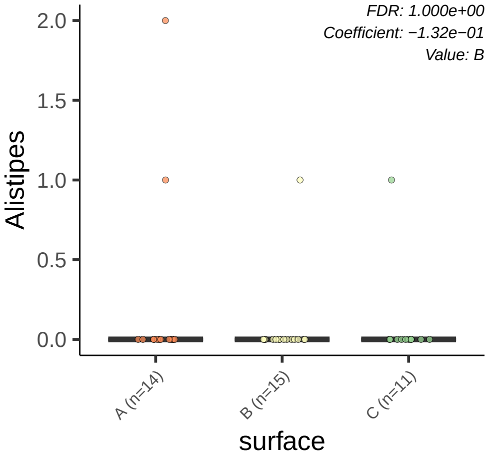
FDR: 1.000e+00
Coefficient: −1.32e−01
Value: B
Alistipes
2.0
1.5
1.0
0.5
0.0
A (n=14)
B (n=15)
C (n=11)
surface

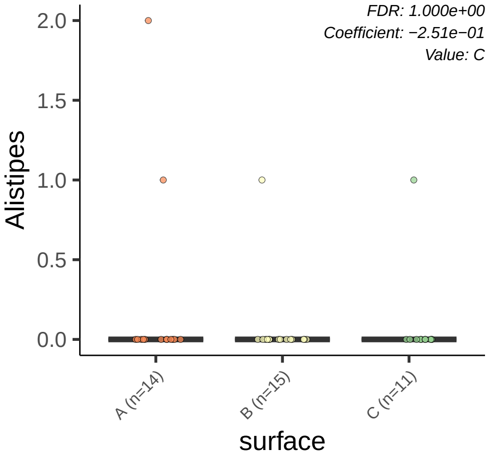
FDR: 1.000e+00
Coefficient: −2.51e−01
Value: C
Alistipes
2.0
1.5
1.0
0.5
0.0
A (n=14)
B (n=15)
C (n=11)
surface

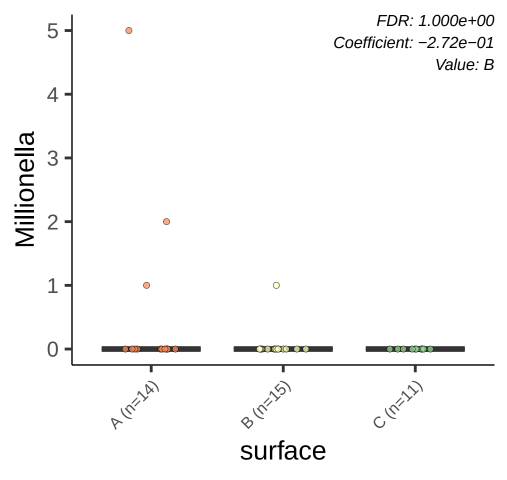
FDR: 1.000e+00
Coefficient: −2.72e−01
Value: B
Millionella
0
1
2
3
4
5
A (n=14)
B (n=15)
C (n=11)
surface

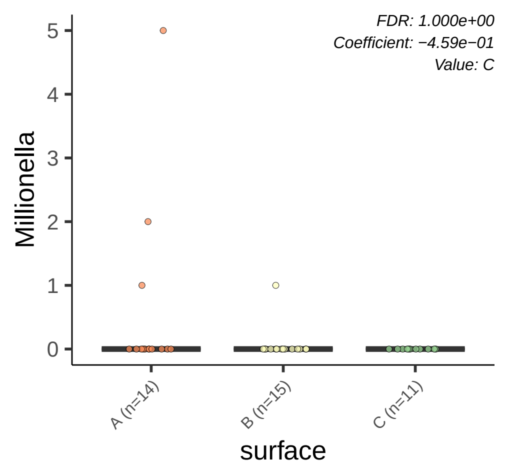

FDR: 1.000e+00
Coefficient: −4.59e−01
Value: C
Millionella
5
4
3
2
1
0
A (n=14)
B (n=15)
C (n=11)
surface

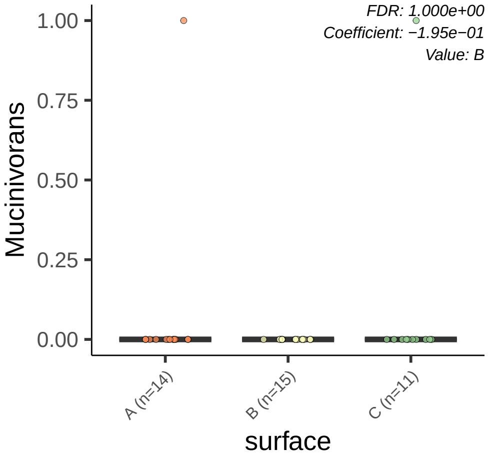
FDR: 1.000e+00
Coefficient: −1.95e−01
Value: B
Mucinivorans
1.00
0.75
0.50
0.25
0.00
A (n=14)
B (n=15)
C (n=11)
surface

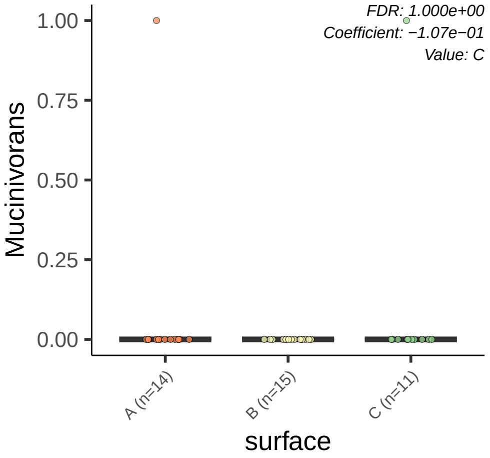
FDR: 1.000e+00
Coefficient: −1.07e−01
Value: C
Mucinivorans
1.00
0.75
0.50
0.25
0.00
A (n=14)
B (n=15)
C (n=11)
surface

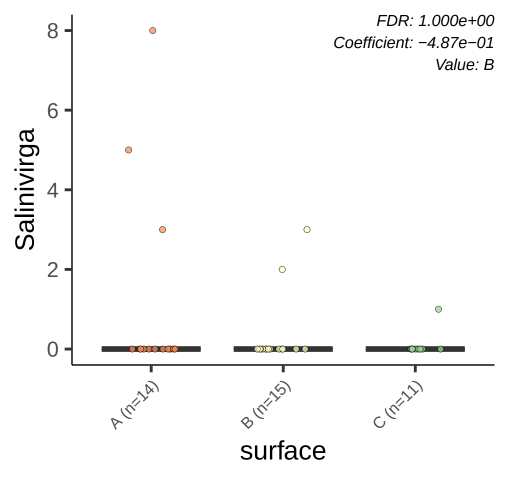

FDR: 1.000e+00
Coefficient: −4.87e−01
Value: B
Salinivirga
8
6
4
2
0
A (n=14)
B (n=15)
C (n=11)
surface

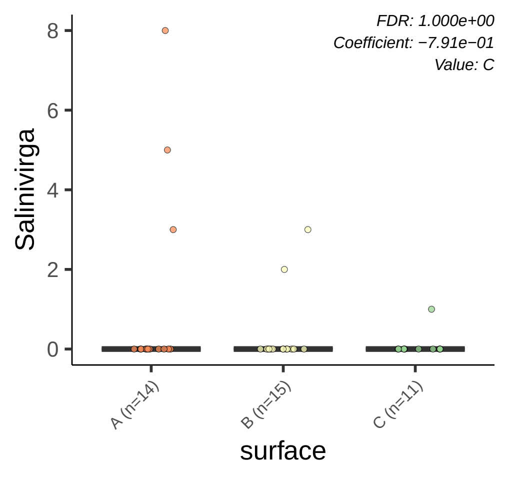
FDR: 1.000e+00
Coefficient: −7.91e−01
Value: C
Salinivirga
8
6
4
2
0
A (n=14)
B (n=15)
C (n=11)
surface

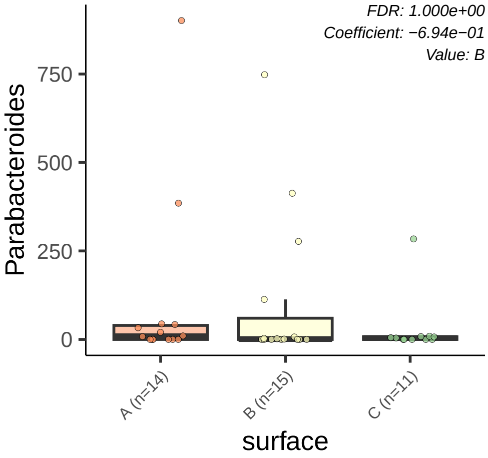

FDR: 1.000e+00
Coefficient: −6.94e−01
Value: B
Parabacteroides
750
500
250
0
A (n=14)
B (n=15)
C (n=11)
surface

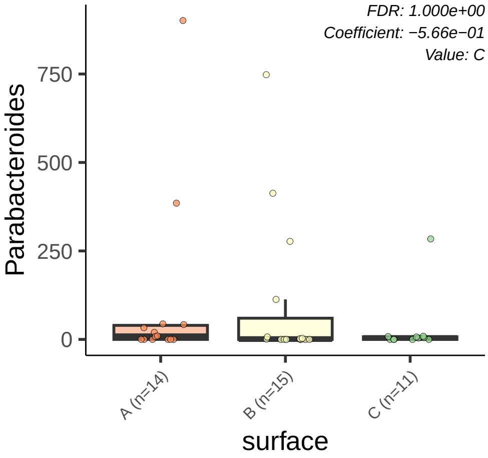
FDR: 1.000e+00
Coefficient: −5.66e−01
Value: C
Parabacteroides
750
500
250
0
A (n=14)
B (n=15)
C (n=11)
surface

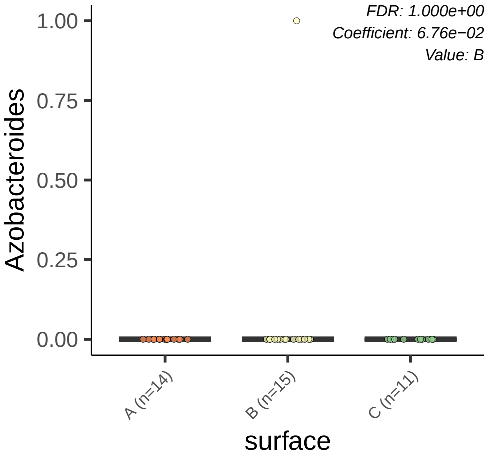
FDR: 1.000e+00
Coefficient: 6.76e−02
Value: B
Azobacteroides
1.00
0.75
0.50
0.25
0.00
A (n=14)
B (n=15)
C (n=11)
surface

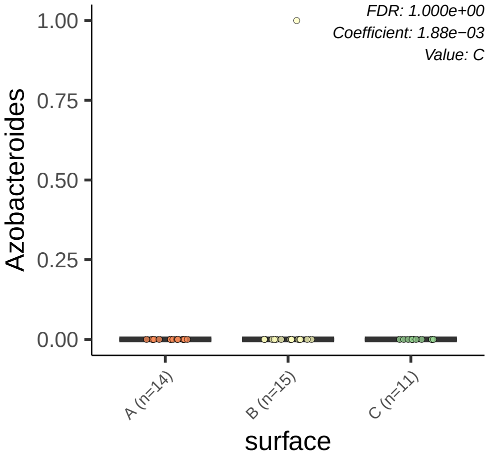

FDR: 1.000e+00
Coefficient: 1.88e−03
Value: C
Azobacteroides
1.00
0.75
0.50
0.25
0.00
A (n=14)
B (n=15)
C (n=11)
surface

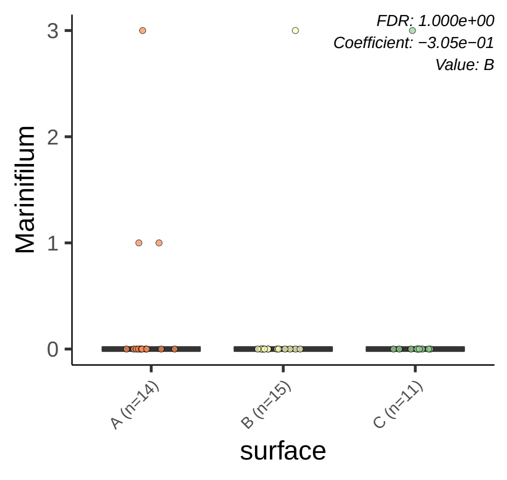
FDR: 1.000e+00
Coefficient: −3.05e−01
Value: B
Marinifilum
0
1
2
3
A (n=14)
B (n=15)
C (n=11)
surface

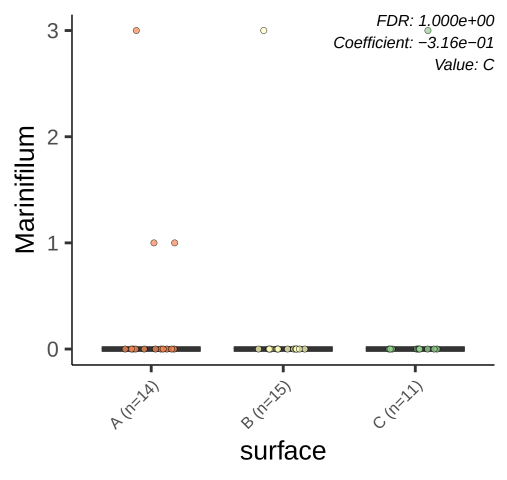

FDR: 1.000e+00
Coefficient: −3.16e−01
Value: C
Marinifilum
3
2
1
0
A (n=14)
B (n=15)
C (n=11)
surface

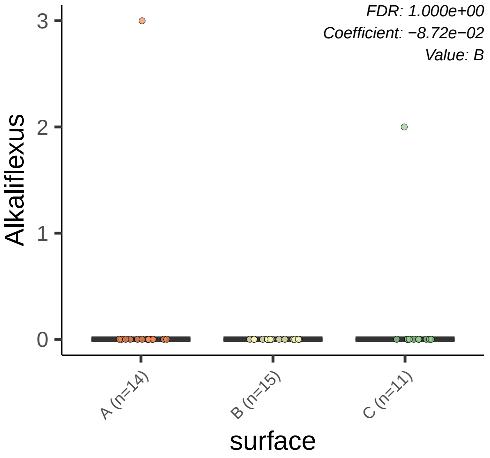
FDR: 1.000e+00
Coefficient: −8.72e−02
Value: B
Alkaliflexus
3
2
1
0
A (n=14)
B (n=15)
C (n=11)
surface

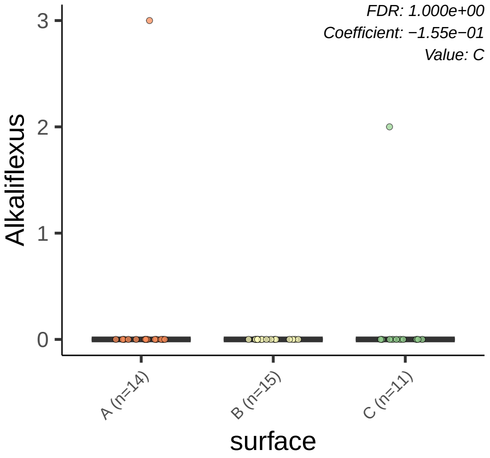
FDR: 1.000e+00
Coefficient: −1.55e−01
Value: C
Alkaliflexus
3
2
1
0
A (n=14)
B (n=15)
C (n=11)
surface

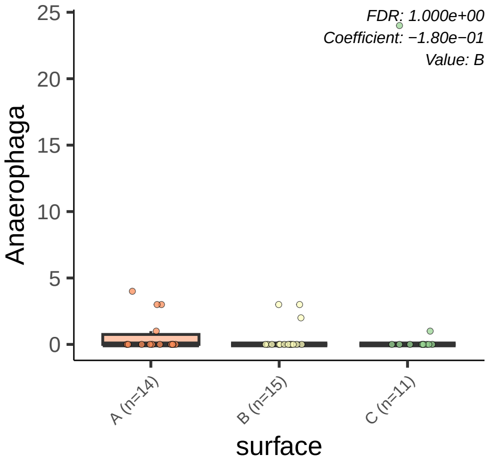
FDR: 1.000e+00
Coefficient: −1.80e−01
Value: B
Anaerophaga
25
20
15
10
5
0
A (n=14)
B (n=15)
C (n=11)
surface

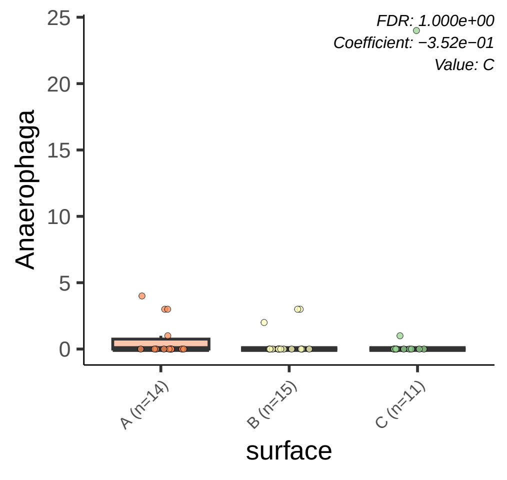
FDR: 1.000e+00
Coefficient: −3.52e−01
Value: C
Anaerophaga
0
5
10
15
20
25
A (n=14)
B (n=15)
C (n=11)
surface

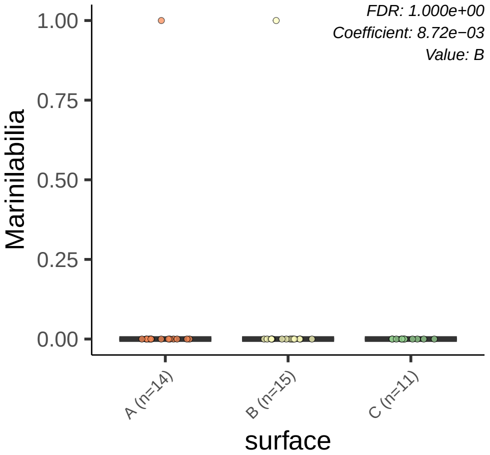
FDR: 1.000e+00
Coefficient: 8.72e−03
Value: B
Marinilabilia
1.00
0.75
0.50
0.25
0.00
A (n=14)
B (n=15)
C (n=11)
surface

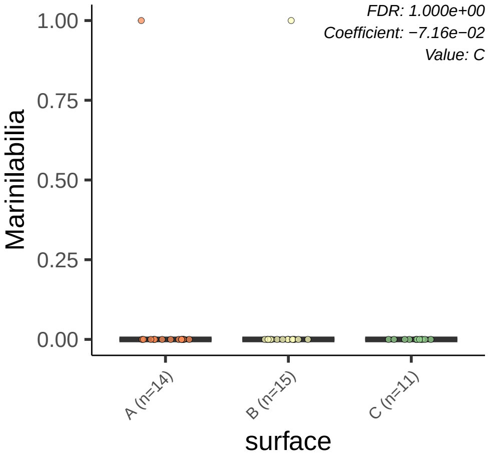
FDR: 1.000e+00
Coefficient: −7.16e−02
Value: C
Marinilabilia
1.00
0.75
0.50
0.25
0.00
A (n=14)
B (n=15)
C (n=11)
surface

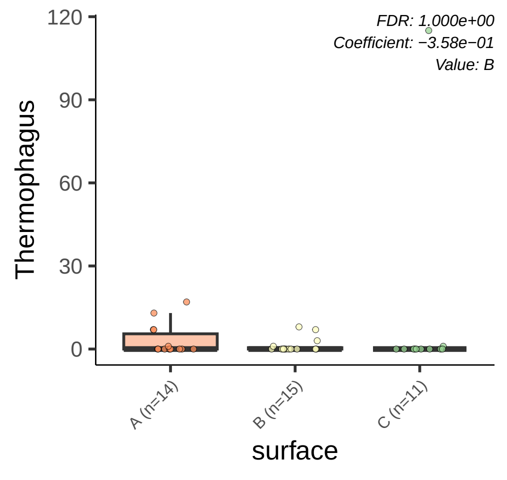

FDR: 1.000e+00
Coefficient: −3.58e−01
Value: B
Thermophagus
120
90
60
30
0
A (n=14)
B (n=15)
C (n=11)
surface

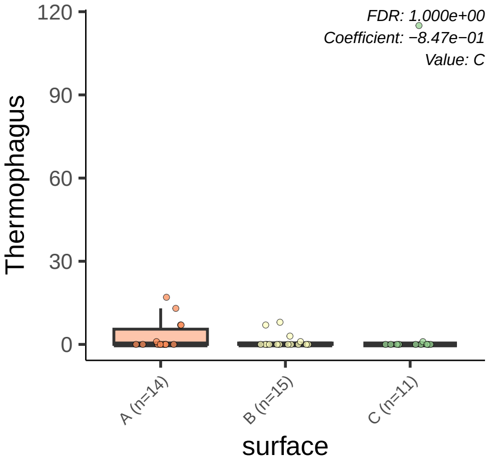
FDR: 1.000e+00
Coefficient: −8.47e−01
Value: C
Thermophagus
120
90
60
30
0
A (n=14)
B (n=15)
C (n=11)
surface

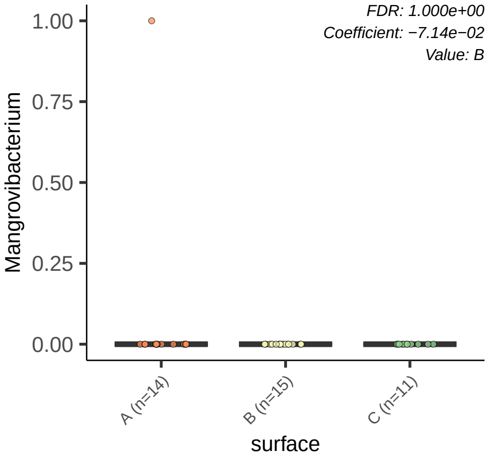

FDR: 1.000e+00
Coefficient: −7.14e−02
Value: B
Mangrovibacterium
1.00
0.75
0.50
0.25
0.00
A (n=14)
B (n=15)
C (n=11)
surface

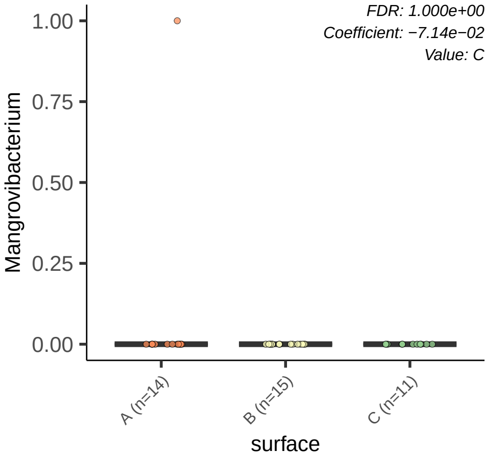

FDR: 1.000e+00
Coefficient: −7.14e−02
Value: C
Mangrovibacterium
1.00
0.75
0.50
0.25
0.00
A (n=14)
B (n=15)
C (n=11)
surface

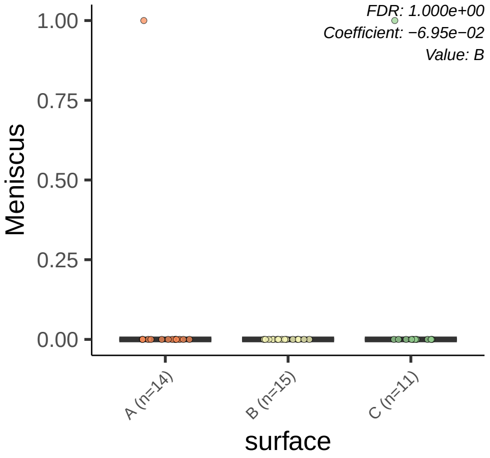
FDR: 1.000e+00
Coefficient: −6.95e−02
Value: B
Meniscus
1.00
0.75
0.50
0.25
0.00
A (n=14)
B (n=15)
C (n=11)
surface

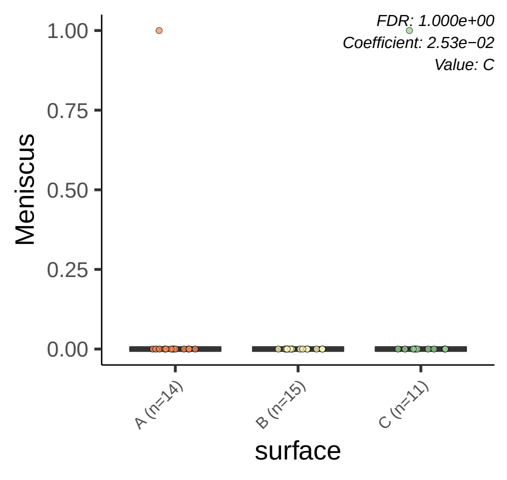

FDR: 1.000e+00
Coefficient: 2.53e−02
Value: C
Meniscus
1.00
0.75
0.50
0.25
0.00
A (n=14)
B (n=15)
C (n=11)
surface

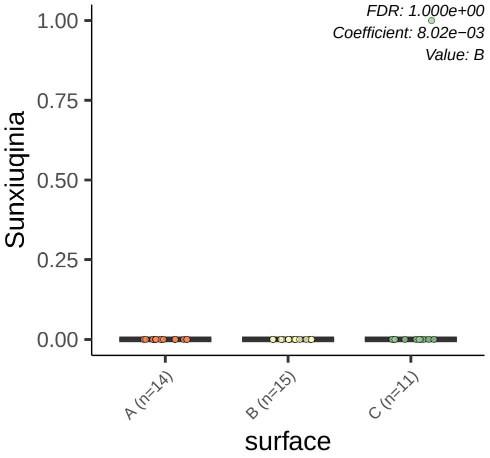
FDR: 1.000e+00
Coefficient: 8.02e−03
Value: B
Sunxiuqinia
1.00
0.75
0.50
0.25
0.00
A (n=14)
B (n=15)
C (n=11)
surface

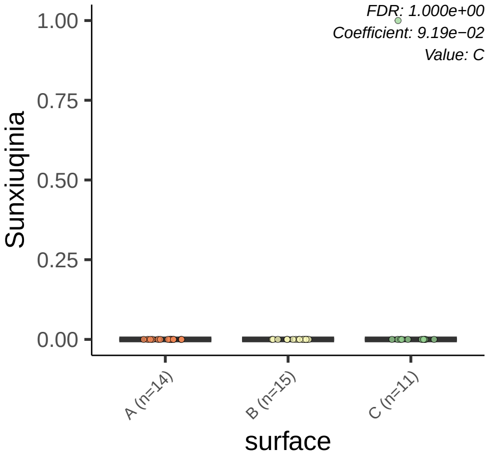
FDR: 1.000e+00
Coefficient: 9.19e−02
Value: C
Sunxiuqinia
1.00
0.75
0.50
0.25
0.00
A (n=14)
B (n=15)
C (n=11)
surface

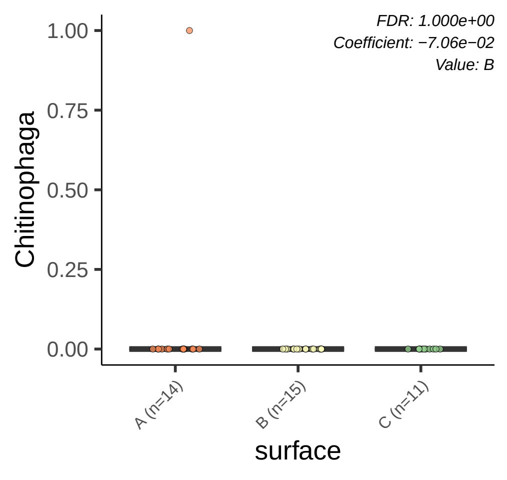
FDR: 1.000e+00
Coefficient: −7.06e−02
Value: B
Chitinophaga
1.00
0.75
0.50
0.25
0.00
A (n=14)
B (n=15)
C (n=11)
surface

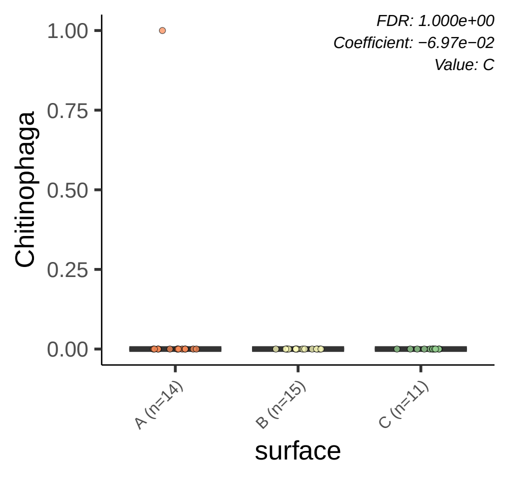
FDR: 1.000e+00
Coefficient: −6.97e−02
Value: C
Chitinophaga
1.00
0.75
0.50
0.25
0.00
A (n=14)
B (n=15)
C (n=11)
surface

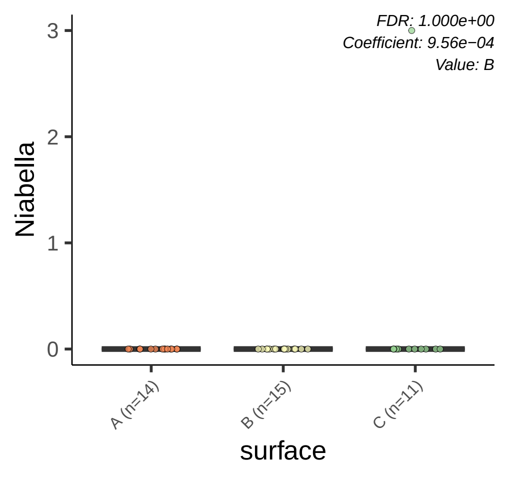
FDR: 1.000e+00
Coefficient: 9.56e−04
Value: B
Niabella
3
2
1
0
A (n=14)
B (n=15)
C (n=11)
surface

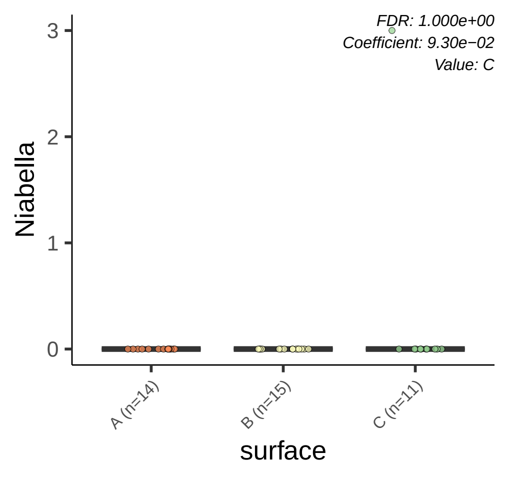
FDR: 1.000e+00
Coefficient: 9.30e−02
Value: C
Niabella
0
1
2
3
A (n=14)
B (n=15)
C (n=11)
surface

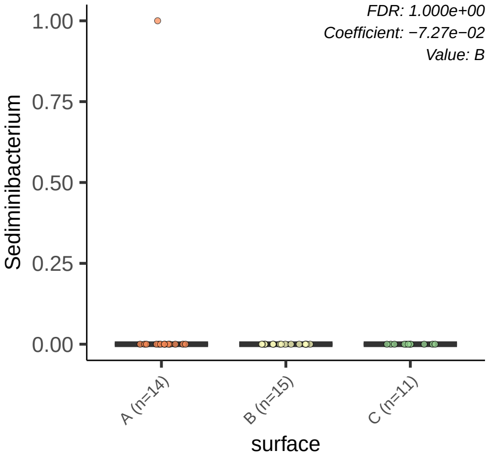

FDR: 1.000e+00
Coefficient: −7.27e−02
Value: B
Sediminibacterium
1.00
0.75
0.50
0.25
0.00
A (n=14)
B (n=15)
C (n=11)
surface

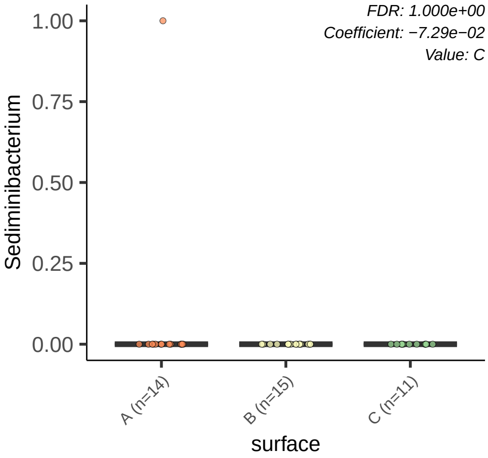
FDR: 1.000e+00
Coefficient: −7.29e−02
Value: C
1.00
0.75
0.50
0.25
0.00
Sediminibacterium
A (n=14)
B (n=15)
C (n=11)
surface

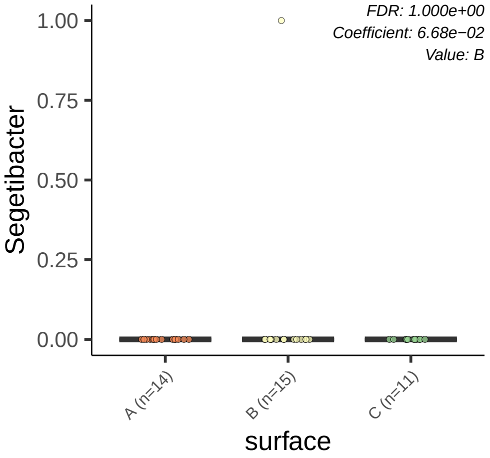
FDR: 1.000e+00
Coefficient: 6.68e−02
Value: B
Segetibacter
1.00
0.75
0.50
0.25
0.00
A (n=14)
B (n=15)
C (n=11)
surface

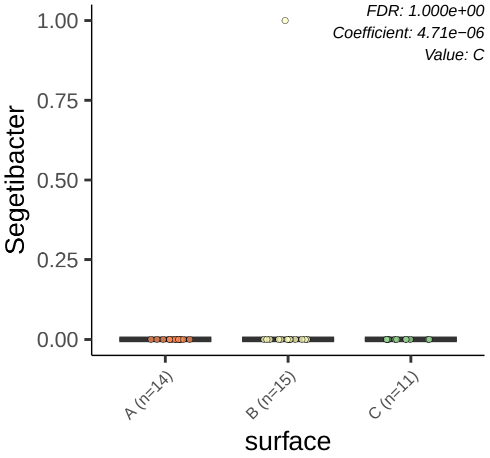

FDR: 1.000e+00
Coefficient: 4.71e−06
Value: C
Segetibacter
1.00
0.75
0.50
0.25
0.00
A (n=14)
B (n=15)
C (n=11)
surface

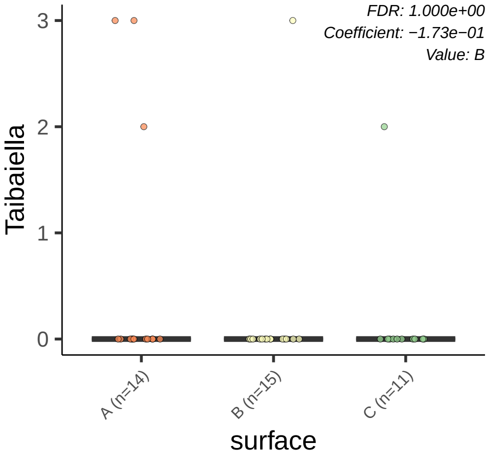

FDR: 1.000e+00
Coefficient: −1.73e−01
Value: B
Taibaiella
3
2
1
0
A (n=14)
B (n=15)
C (n=11)
surface

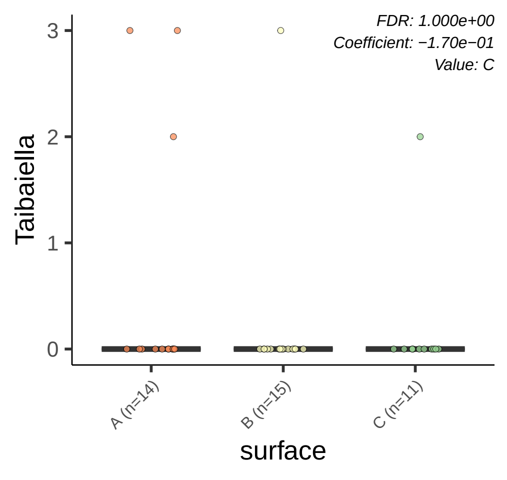
FDR: 1.000e+00
Coefficient: −1.70e−01
Value: C
Taibaiella
3
2
1
0
A (n=14)
B (n=15)
C (n=11)
surface

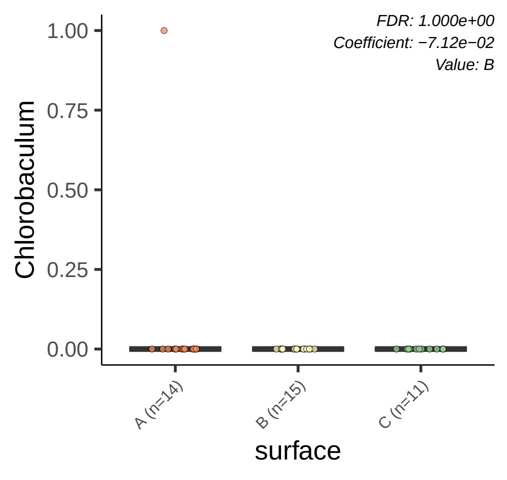

FDR: 1.000e+00
Coefficient: −7.12e−02
Value: B
Chlorobaculum
1.00
0.75
0.50
0.25
0.00
A (n=14)
B (n=15)
C (n=11)
surface

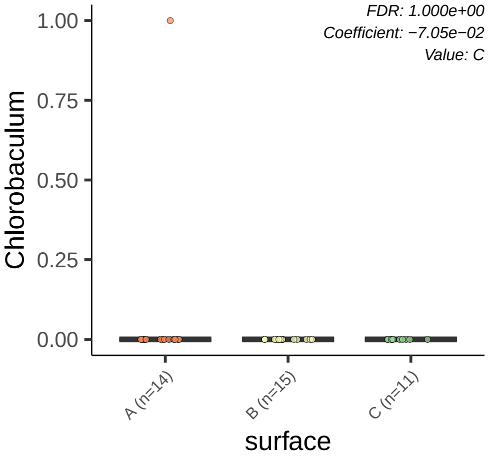
FDR: 1.000e+00
Coefficient: −7.05e−02
Value: C
Chlorobaculum
1.00
0.75
0.50
0.25
0.00
A (n=14)
B (n=15)
C (n=11)
surface

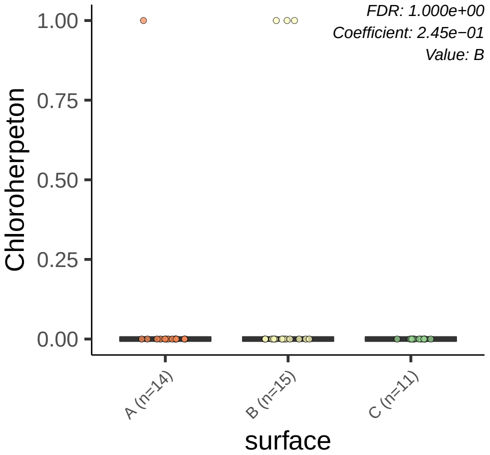
FDR: 1.000e+00
Coefficient: 2.45e−01
Value: B
Chloroherpeton
1.00
0.75
0.50
0.25
0.00
A (n=14)
B (n=15)
C (n=11)
surface

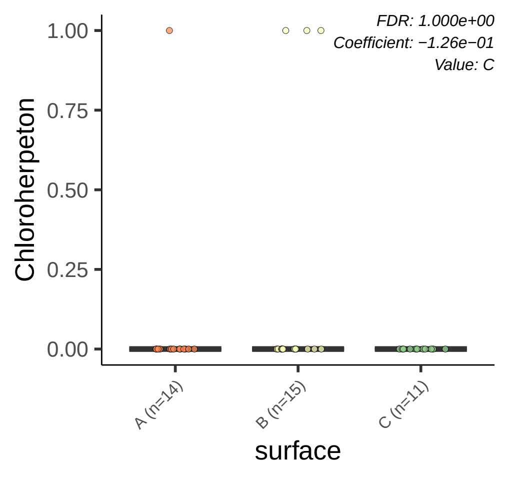
FDR: 1.000e+00
Coefficient: −1.26e−01
Value: C
Chloroherpeton
1.00
0.75
0.50
0.25
0.00
A (n=14)
B (n=15)
C (n=11)
surface

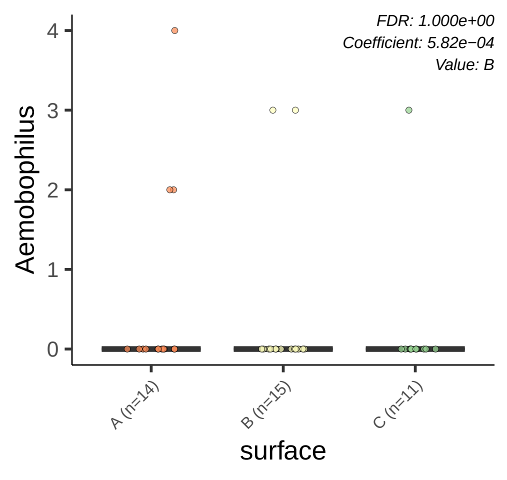
FDR: 1.000e+00
Coefficient: 5.82e−04
Value: B
Aemobophilus
4
3
2
1
0
A (n=14)
B (n=15)
C (n=11)
surface

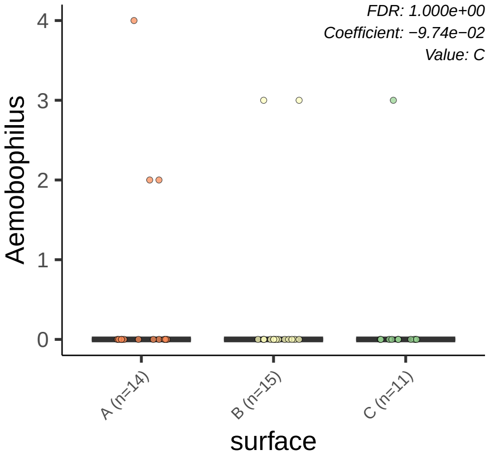
FDR: 1.000e+00
Coefficient: −9.74e−02
Value: C
Aemobophilus
4
3
2
1
0
A (n=14)
B (n=15)
C (n=11)
surface

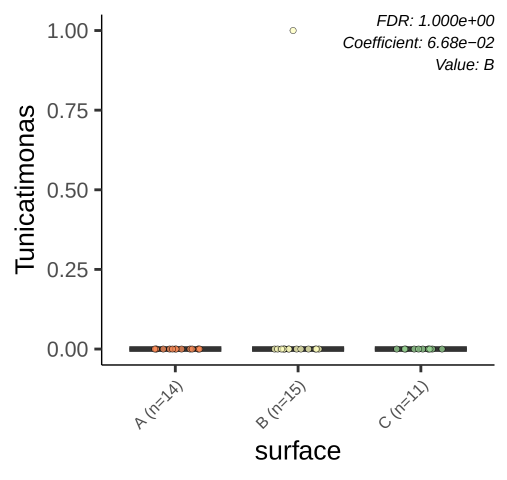

FDR: 1.000e+00
Coefficient: 6.68e−02
Value: B
Tunicatimonas
1.00
0.75
0.50
0.25
0.00
A (n=14)
B (n=15)
C (n=11)
surface

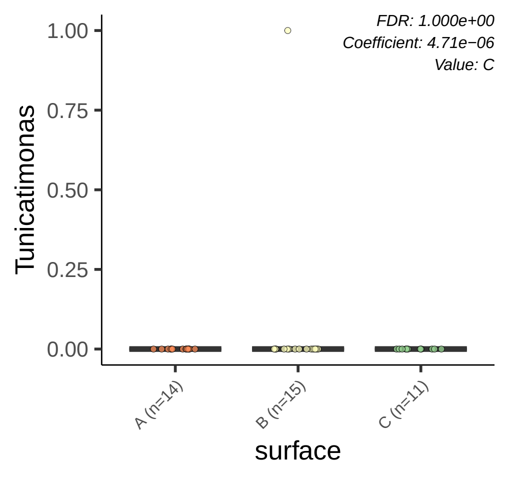

FDR: 1.000e+00
Coefficient: 4.71e−06
Value: C
Tunicatimonas
1.00
0.75
0.50
0.25
0.00
A (n=14)
B (n=15)
C (n=11)
surface

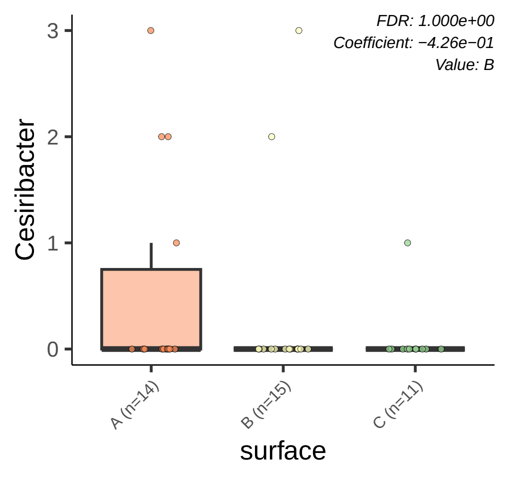
FDR: 1.000e+00
Coefficient: −4.26e−01
Value: B
Cesiribacter
3
2
1
0
A (n=14)
B (n=15)
C (n=11)
surface

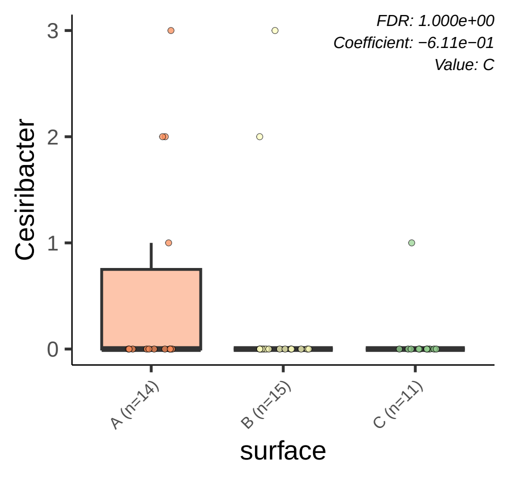

FDR: 1.000e+00
Coefficient: −6.11e−01
Value: C
Cesiribacter
0
1
2
3
A (n=14)
B (n=15)
C (n=11)
surface

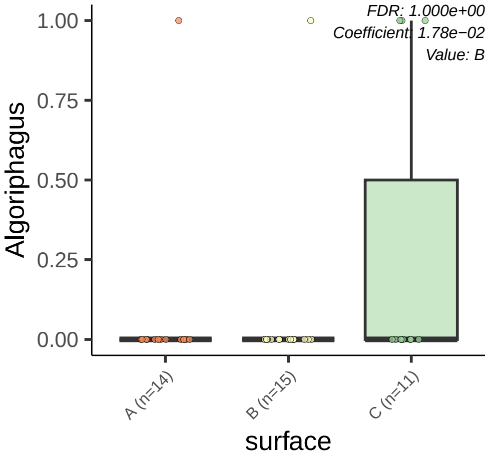
FDR: 1.000e+00
Coefficient: 1.78e−02
Value: B
1.00
0.75
0.50
0.25
0.00
Algoriphagus
A (n=14)
B (n=15)
C (n=11)
surface

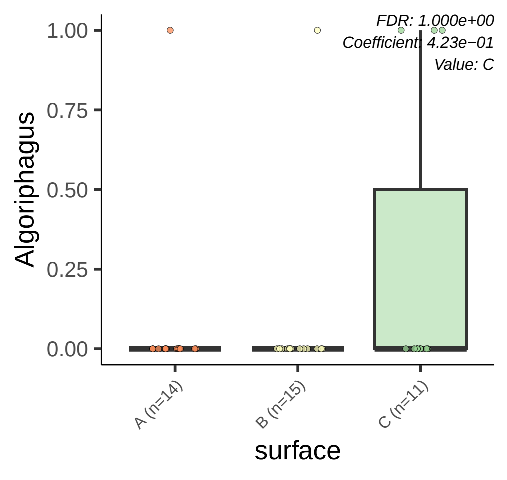
FDR: 1.000e+00
Coefficient: 4.23e−01
Value: C
Algoriphagus
1.00
0.75
0.50
0.25
0.00
A (n=14)
B (n=15)
C (n=11)
surface

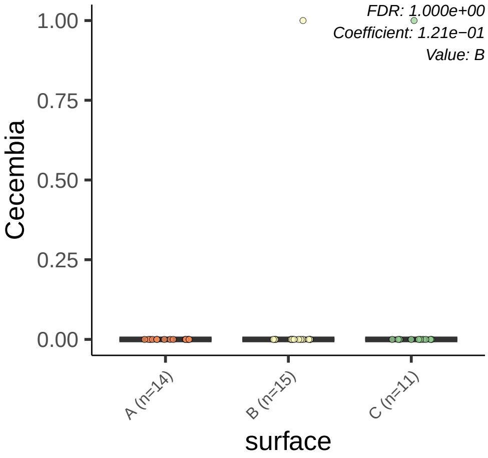
FDR: 1.000e+00
Coefficient: 1.21e−01
Value: B
Cecembia
1.00
0.75
0.50
0.25
0.00
A (n=14)
B (n=15)
C (n=11)
surface

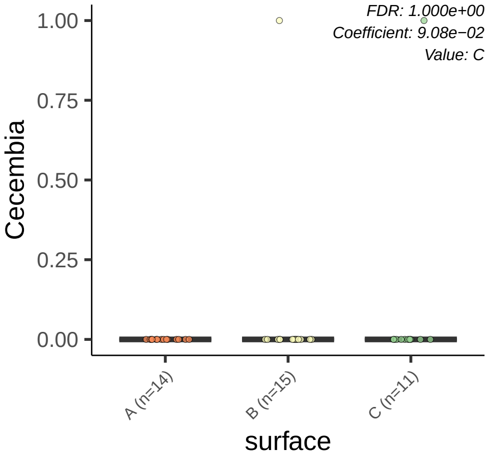

FDR: 1.000e+00
Coefficient: 9.08e−02
Value: C
Cecembia
1.00
0.75
0.50
0.25
0.00
A (n=14)
B (n=15)
C (n=11)
surface

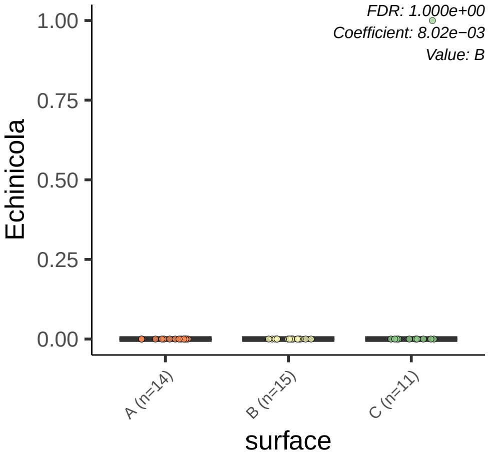
FDR: 1.000e+00
Coefficient: 8.02e−03
Value: B
1.00
0.75
0.50
0.25
0.00
Echinicola
A (n=14)
B (n=15)
C (n=11)
surface

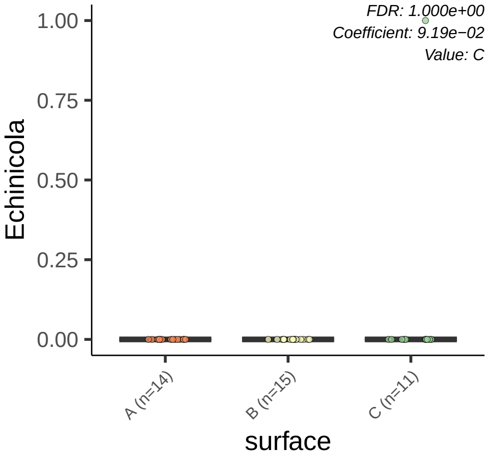

FDR: 1.000e+00
Coefficient: 9.19e−02
Value: C
Echinicola
1.00
0.75
0.50
0.25
0.00
A (n=14)
B (n=15)
C (n=11)
surface

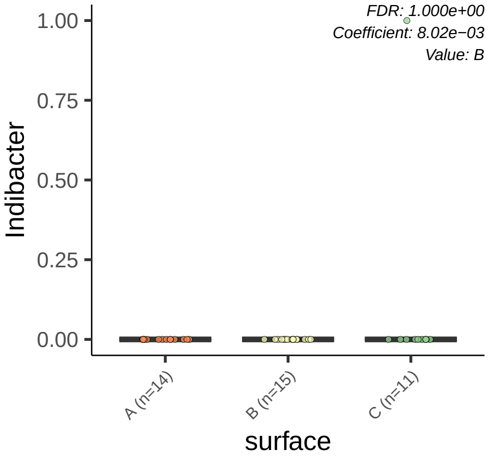
FDR: 1.000e+00
Coefficient: 8.02e−03
Value: B
Indibacter
1.00
0.75
0.50
0.25
0.00
A (n=14)
B (n=15)
C (n=11)
surface

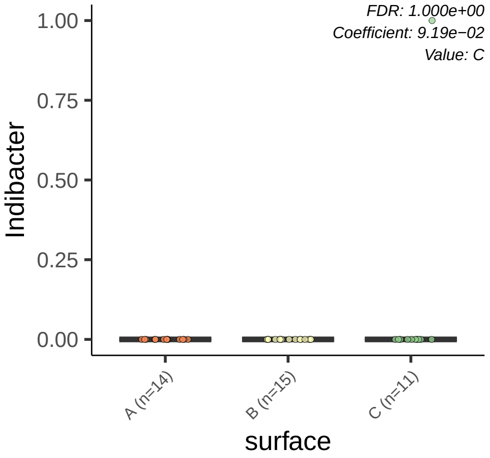
FDR: 1.000e+00
Coefficient: 9.19e−02
Value: C
Indibacter
1.00
0.75
0.50
0.25
0.00
A (n=14)
B (n=15)
C (n=11)
surface

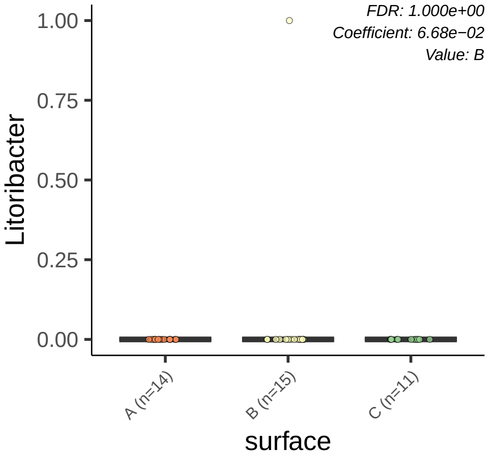
FDR: 1.000e+00
Coefficient: 6.68e−02
Value: B
Litoribacter
1.00
0.75
0.50
0.25
0.00
A (n=14)
B (n=15)
C (n=11)
surface

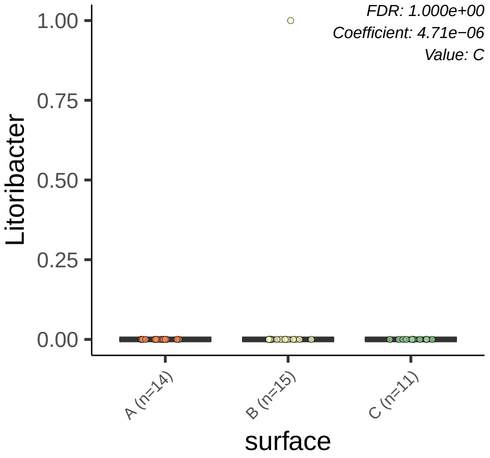
FDR: 1.000e+00
Coefficient: 4.71e−06
Value: C
Litoribacter
1.00
0.75
0.50
0.25
0.00
A (n=14)
B (n=15)
C (n=11)
surface

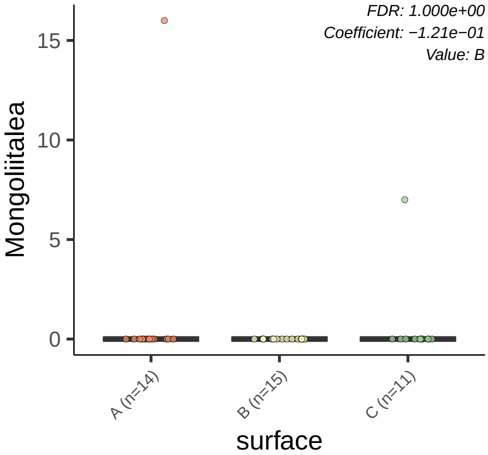
FDR: 1.000e+00
Coefficient: −1.21e−01
Value: B
Mongoliitalea
15
10
5
0
A (n=14)
B (n=15)
C (n=11)
surface

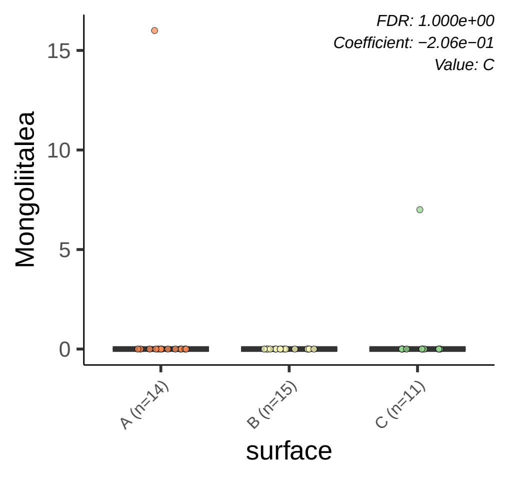
FDR: 1.000e+00
Coefficient: −2.06e−01
Value: C
Mongoliitalea
15
10
5
0
A (n=14)
B (n=15)
C (n=11)
surface

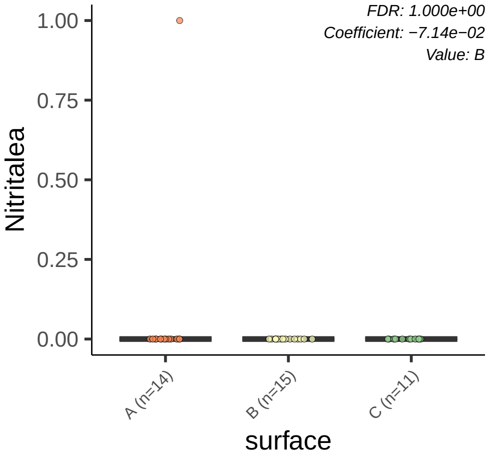

FDR: 1.000e+00
Coefficient: −7.14e−02
Value: B
Nitritalea
1.00
0.75
0.50
0.25
0.00
A (n=14)
B (n=15)
C (n=11)
surface

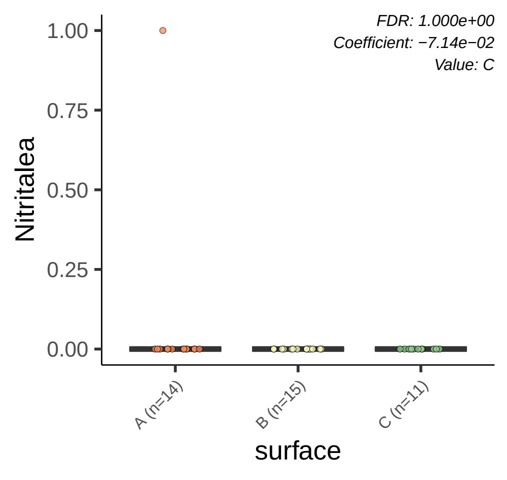
FDR: 1.000e+00
Coefficient: −7.14e−02
Value: C
1.00
0.75
0.50
0.25
0.00
Nitritalea
A (n=14)
B (n=15)
C (n=11)
surface

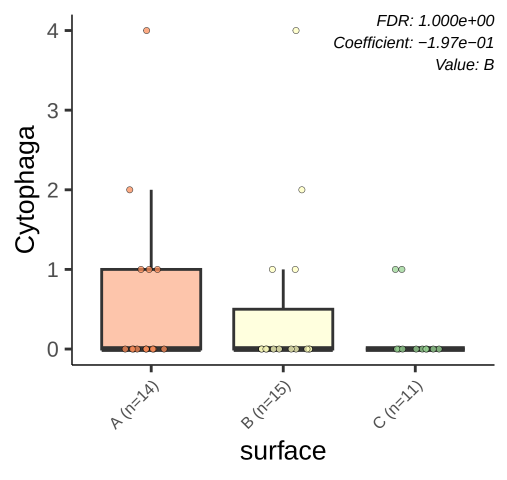
FDR: 1.000e+00
Coefficient: −1.97e−01
Value: B
Cytophaga
0
1
2
3
4
A (n=14)
B (n=15)
C (n=11)
surface

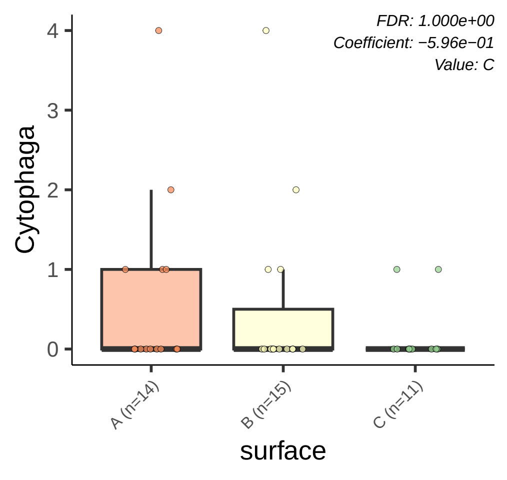
FDR: 1.000e+00
Coefficient: −5.96e−01
Value: C
Cytophaga
0
1
2
3
4
A (n=14)
B (n=15)
C (n=11)
surface

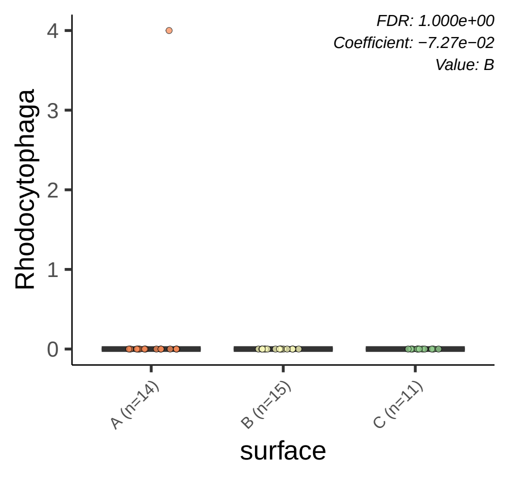
FDR: 1.000e+00
Coefficient: −7.27e−02
Value: B
Rhodocytophaga
4
3
2
1
0
A (n=14)
B (n=15)
C (n=11)
surface

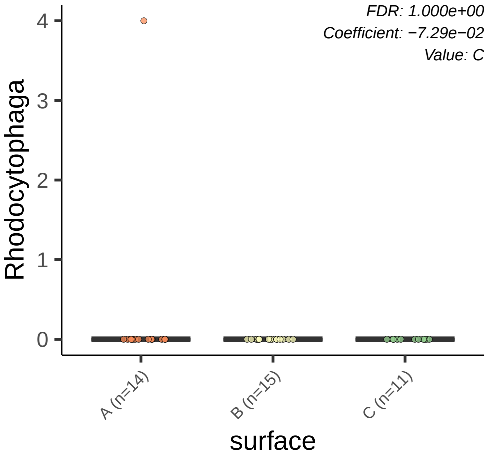

FDR: 1.000e+00
Coefficient: −7.29e−02
Value: C
Rhodocytophaga
4
3
2
1
0
A (n=14)
B (n=15)
C (n=11)
surface

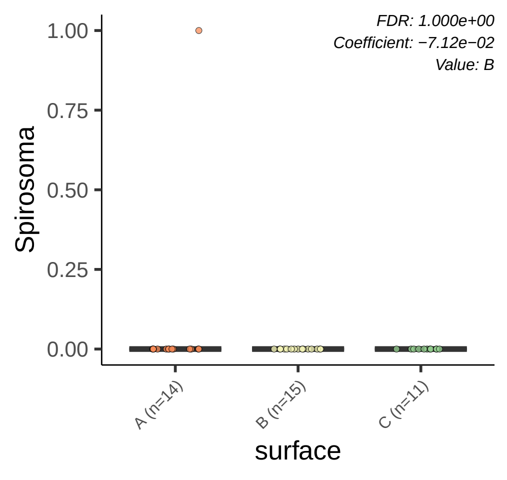
FDR: 1.000e+00
Coefficient: −7.12e−02
Value: B
1.00
0.75
0.50
0.25
0.00
Spirosoma
A (n=14)
B (n=15)
C (n=11)
surface

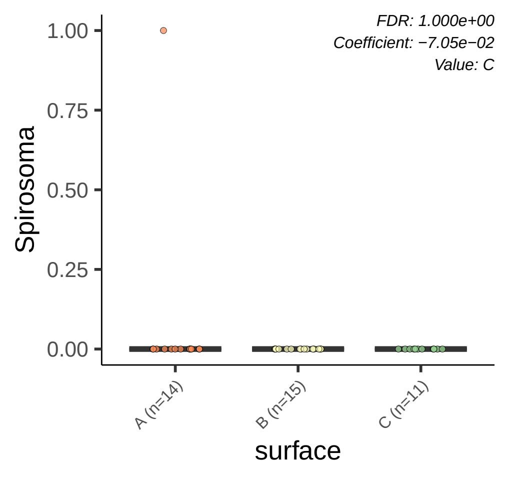
FDR: 1.000e+00
Coefficient: −7.05e−02
Value: C
Spirosoma
1.00
0.75
0.50
0.25
0.00
A (n=14)
B (n=15)
C (n=11)
surface

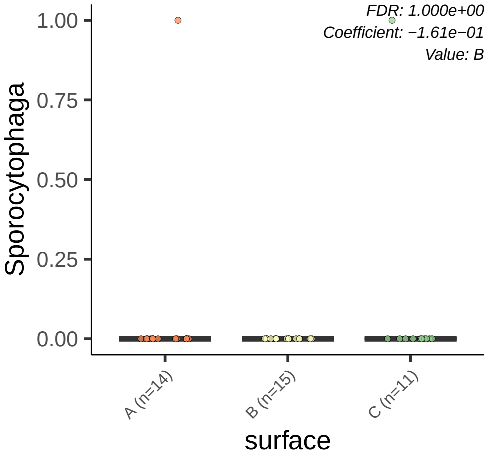

FDR: 1.000e+00
Coefficient: −1.61e−01
Value: B
Sporocytophaga
1.00
0.75
0.50
0.25
0.00
A (n=14)
B (n=15)
C (n=11)
surface

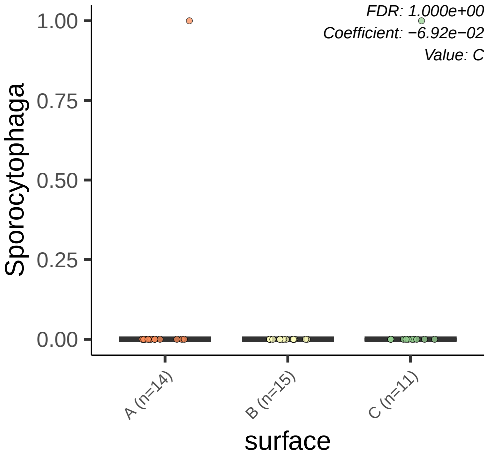
FDR: 1.000e+00
Coefficient: −6.92e−02
Value: C
Sporocytophaga
1.00
0.75
0.50
0.25
0.00
A (n=14)
B (n=15)
C (n=11)
surface

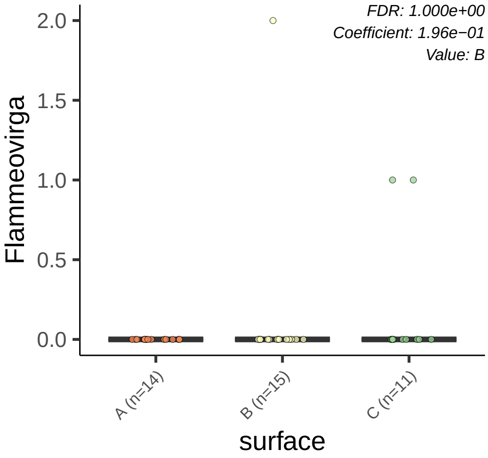
FDR: 1.000e+00
Coefficient: 1.96e−01
Value: B
Flammeovirga
2.0
1.5
1.0
0.5
0.0
A (n=14)
B (n=15)
C (n=11)
surface

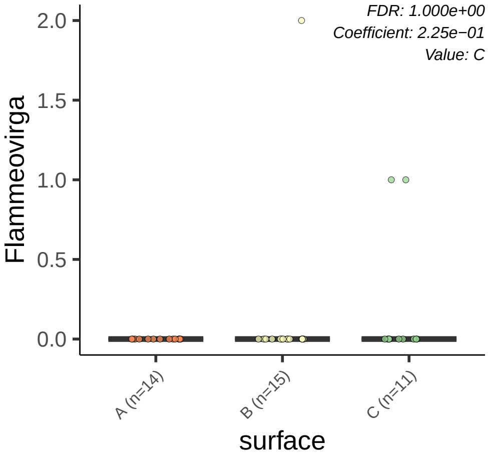
FDR: 1.000e+00
Coefficient: 2.25e−01
Value: C
Flammeovirga
2.0
1.5
1.0
0.5
0.0
A (n=14)
B (n=15)
C (n=11)
surface

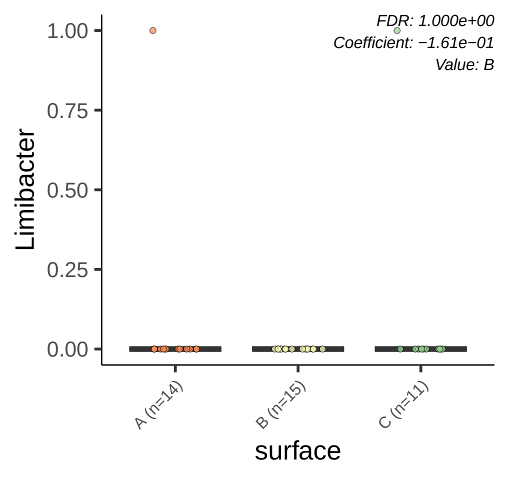
FDR: 1.000e+00
Coefficient: −1.61e−01
Value: B
Limibacter
1.00
0.75
0.50
0.25
0.00
A (n=14)
B (n=15)
C (n=11)
surface

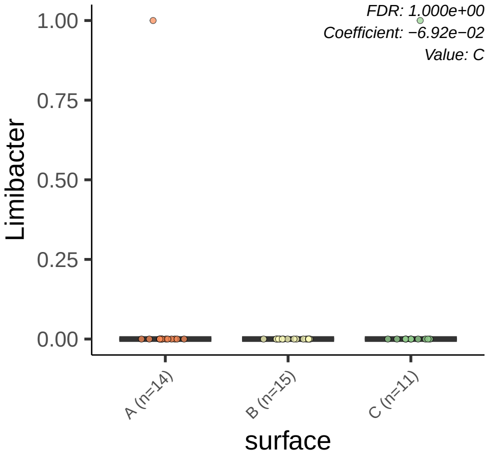

FDR: 1.000e+00
Coefficient: −6.92e−02
Value: C
Limibacter
1.00
0.75
0.50
0.25
0.00
A (n=14)
B (n=15)
C (n=11)
surface

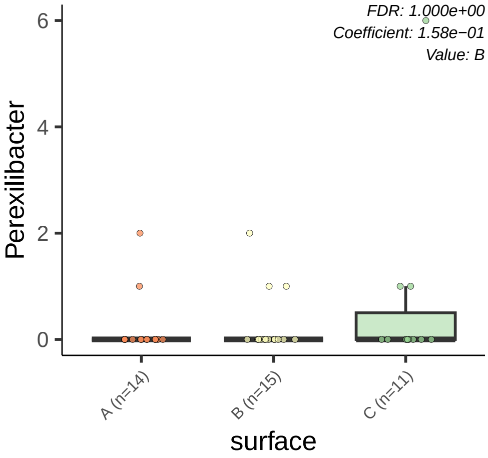

FDR: 1.000e+00
Coefficient: 1.58e−01
Value: B
Perexilibacter
6
4
2
0
A (n=14)
B (n=15)
C (n=11)
surface

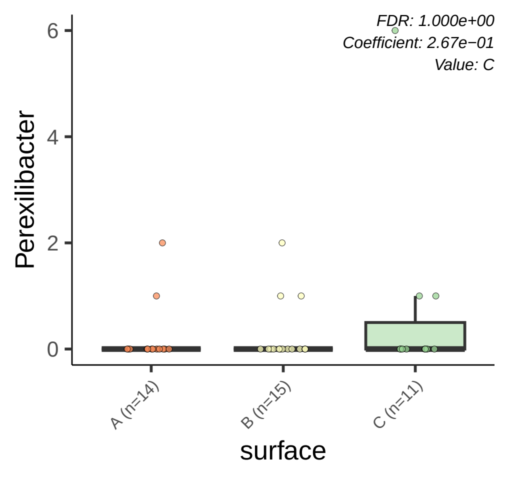

FDR: 1.000e+00
Coefficient: 2.67e−01
Value: C
Perexilibacter
6
4
2
0
A (n=14)
B (n=15)
C (n=11)
surface

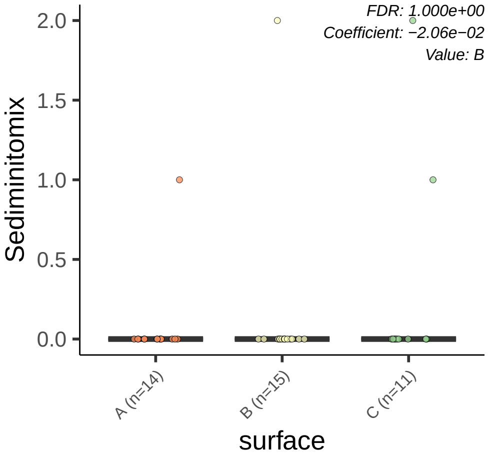

FDR: 1.000e+00
Coefficient: −2.06e−02
Value: B
Sedimintomix
2.0
1.5
1.0
0.5
0.0
A (n=14)
B (n=15)
C (n=11)
surface

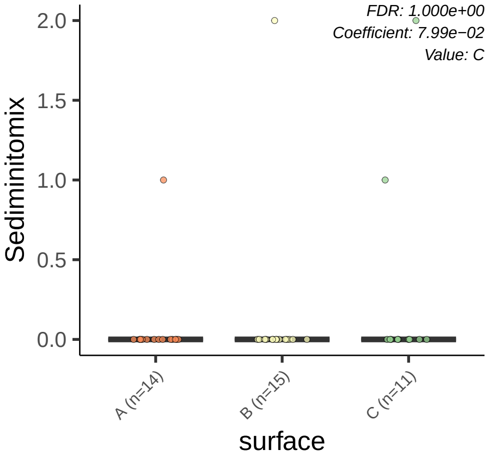
FDR: 1.000e+00
Coefficient: 7.99e−02
Value: C
Sedimínitomix
2.0
1.5
1.0
0.5
0.0
A (n=14)
B (n=15)
C (n=11)
surface

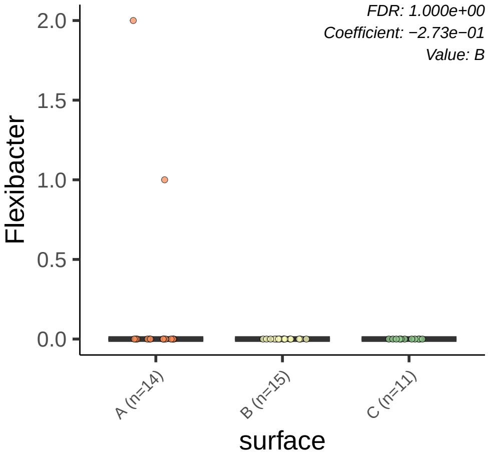

FDR: 1.000e+00
Coefficient: −2.73e−01
Value: B
Flexibacter
2.0
1.5
1.0
0.5
0.0
A (n=14)
B (n=15)
C (n=11)
surface

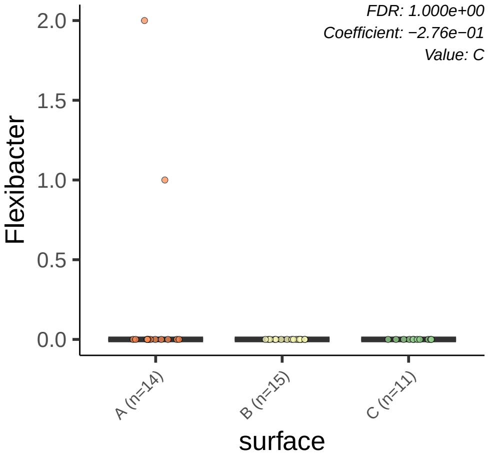

FDR: 1.000e+00
Coefficient: −2.76e−01
Value: C
Flexibacter
2.0
1.5
1.0
0.5
0.0
A (n=14)
B (n=15)
C (n=11)
surface

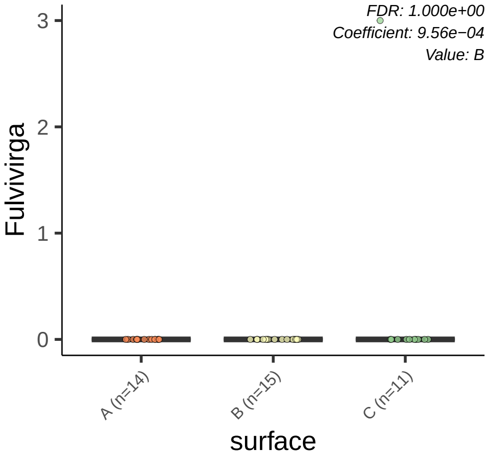

FDR: 1.000e+00
Coefficient: 9.56e−04
Value: B
Fulvivirga
3
2
1
0
A (n=14)
B (n=15)
C (n=11)
surface

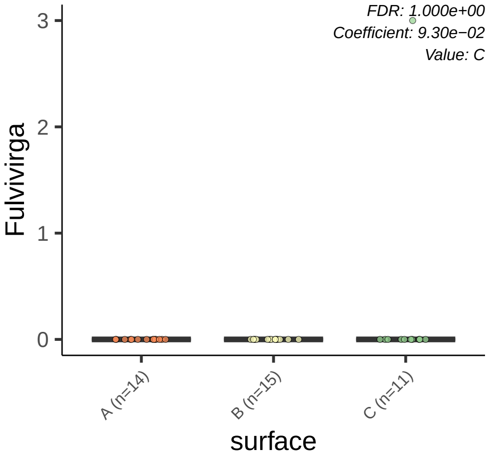
FDR: 1.000e+00
Coefficient: 9.30e−02
Value: C
Fulvivirga
0
1
2
3
A (n=14)
B (n=15)
C (n=11)
surface

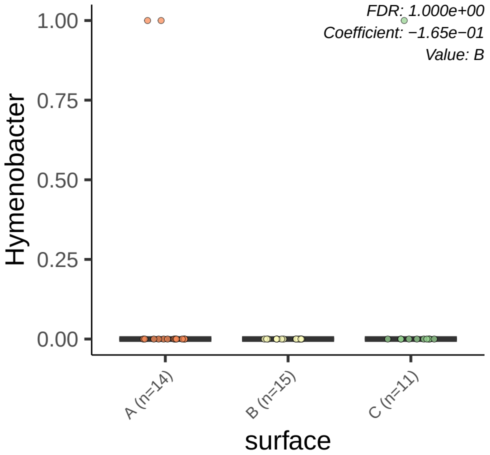
FDR: 1.000e+00
Coefficient: −1.65e−01
Value: B
Hymenobacter
1.00
0.75
0.50
0.25
0.00
A (n=14)
B (n=15)
C (n=11)
surface

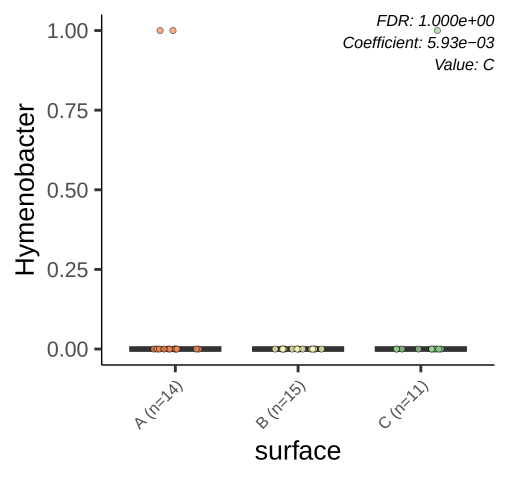

FDR: 1.000e+00
Coefficient: 5.93e−03
Value: C
Hymenobacter
1.00
0.75
0.50
0.25
0.00
A (n=14)
B (n=15)
C (n=11)
surface

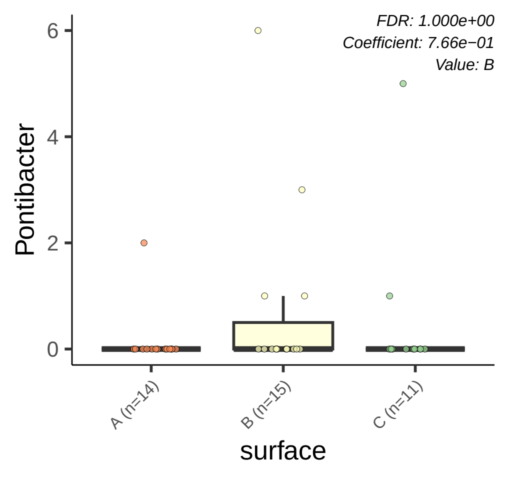

FDR: 1.000e+00
Coefficient: 7.66e−01
Value: B
Pontibacter
6
4
2
0
A (n=14)
B (n=15)
C (n=11)
surface

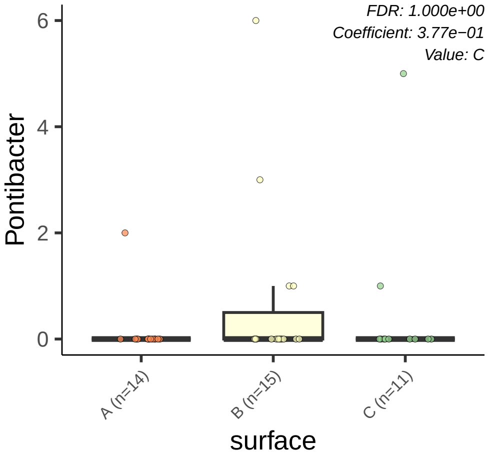

FDR: 1.000e+00
Coefficient: 3.77e−01
Value: C
Pontibacter
6
4
2
0
A (n=14)
B (n=15)
C (n=11)
surface

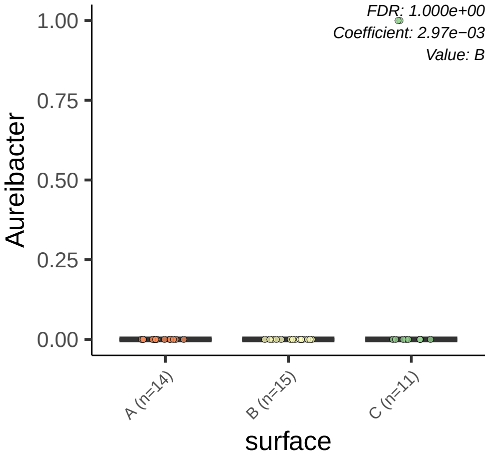

FDR: 1.000e+00
Coefficient: 2.97e−03
Value: B
Aureibacter
1.00
0.75
0.50
0.25
0.00
A (n=14)
B (n=15)
C (n=11)
surface

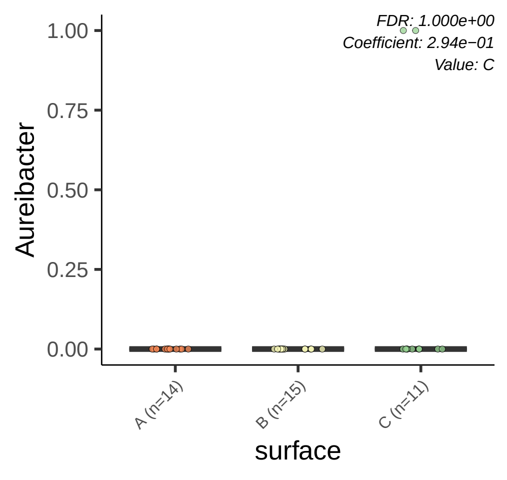

FDR: 1.000e+00
Coefficient: 2.94e−01
Value: C
1.00
0.75
0.50
0.25
0.00
Aureibacter
A (n=14)
B (n=15)
C (n=11)
surface

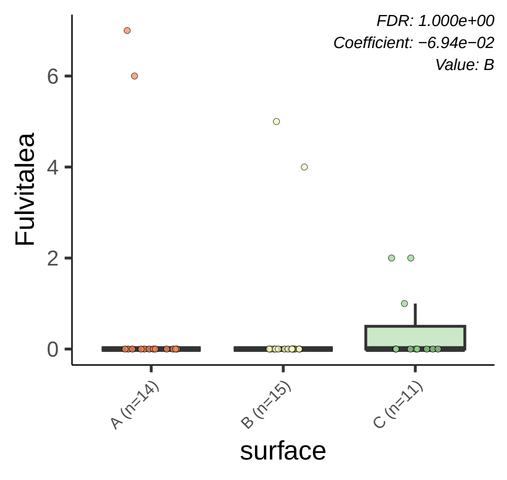
FDR: 1.000e+00
Coefficient: −6.94e−02
Value: B
Fulvitalea
0
2
4
6
A (n=14)
B (n=15)
C (n=11)
surface

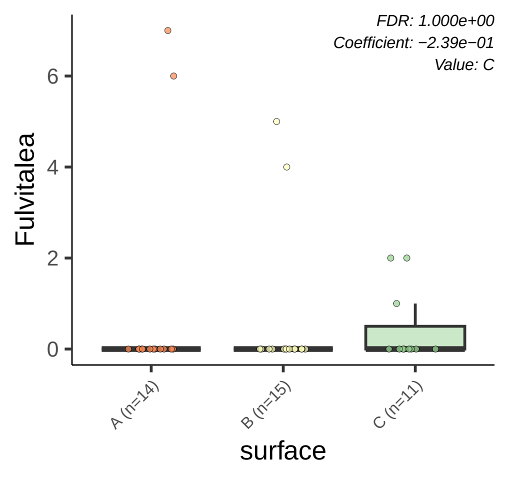
FDR: 1.000e+00
Coefficient: −2.39e−01
Value: C
Fulvitalea
0
2
4
6
A (n=14)
B (n=15)
C (n=11)
surface

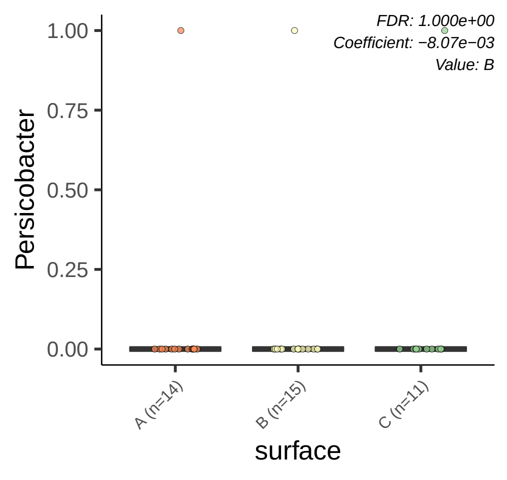
FDR: 1.000e+00
Coefficient: −8.07e−03
Value: B
Persicobacter
1.00
0.75
0.50
0.25
0.00
A (n=14)
B (n=15)
C (n=11)
surface

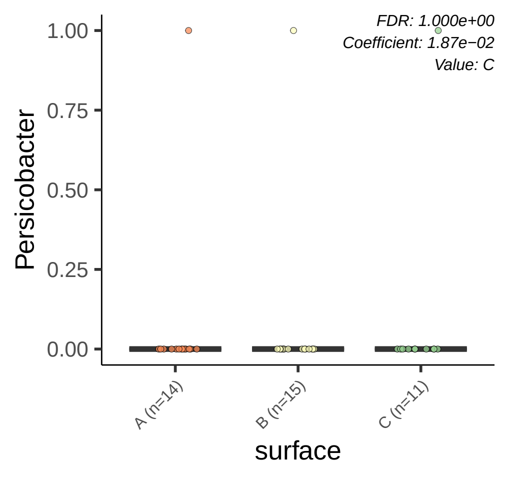
FDR: 1.000e+00
Coefficient: 1.87e−02
Value: C
Persicobacter
1.00
0.75
0.50
0.25
0.00
A (n=14)
B (n=15)
C (n=11)
surface

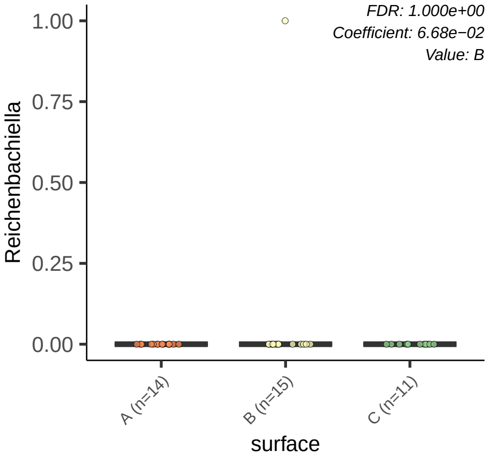

FDR: 1.000e+00
Coefficient: 6.68e−02
Value: B
Reichenbachiella
1.00
0.75
0.50
0.25
0.00
A (n=14)
B (n=15)
C (n=11)
surface

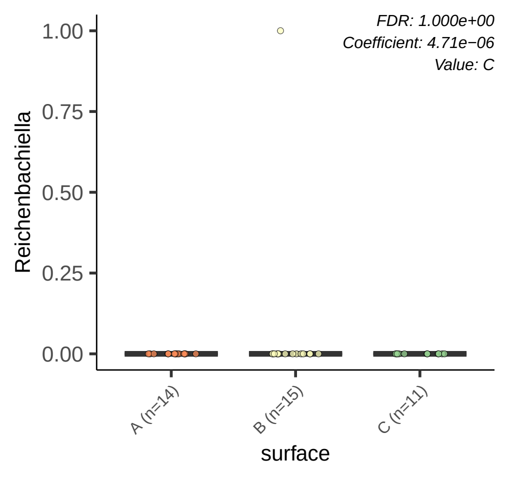
FDR: 1.000e+00
Coefficient: 4.71e−06
Value: C
Reichenbachiella
1.00
0.75
0.50
0.25
0.00
A (n=14)
B (n=15)
C (n=11)
surface

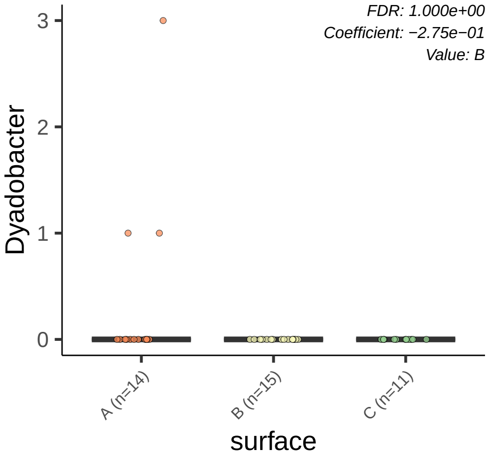

FDR: 1.000e+00
Coefficient: −2.75e−01
Value: B
Dyadobacter
3
2
1
0
A (n=14)
B (n=15)
C (n=11)
surface

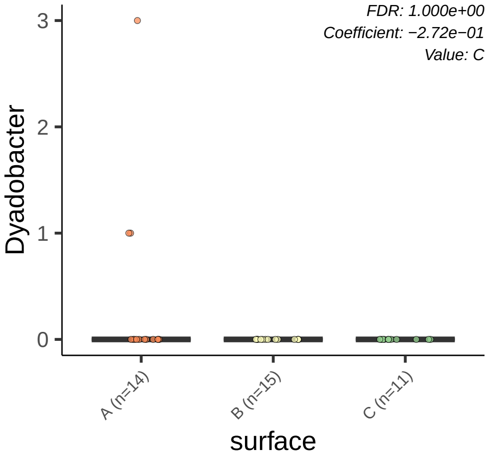
FDR: 1.000e+00
Coefficient: −2.72e−01
Value: C
Dyadobacter
3
2
1
0
A (n=14)
B (n=15)
C (n=11)
surface

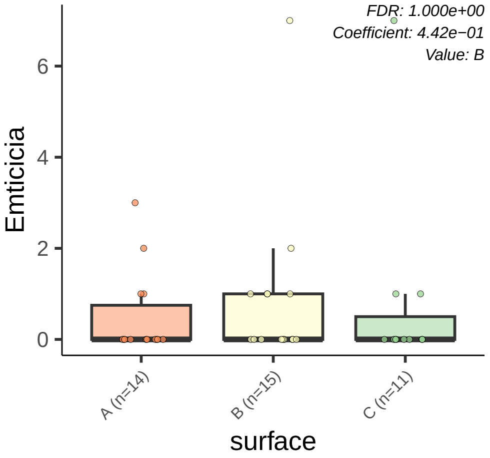
FDR: 1.000e+00
Coefficient: 4.42e−01
Value: B
Emticicia
0
2
4
6
A (n=14)
B (n=15)
C (n=11)
surface

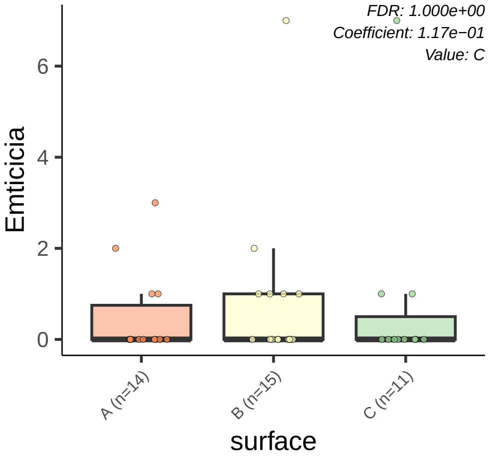

FDR: 1.000e+00
Coefficient: 1.17e−01
Value: C
Emticicia
0
2
4
6
A (n=14)
B (n=15)
C (n=11)
surface

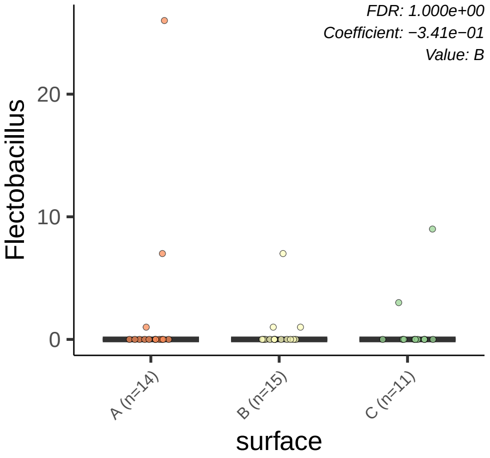
FDR: 1.000e+00
Coefficient: −3.41e−01
Value: B
Flectobacillus
0
10
20
A (n=14)
B (n=15)
C (n=11)
surface

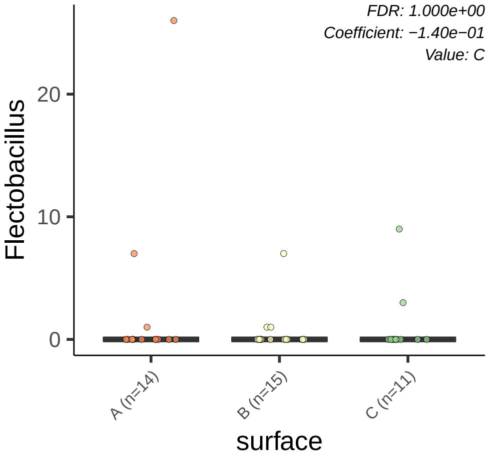

FDR: 1.000e+00
Coefficient: −1.40e−01
Value: C
Flectobacillus
20
10
0
A (n=14)
B (n=15)
C (n=11)
surface

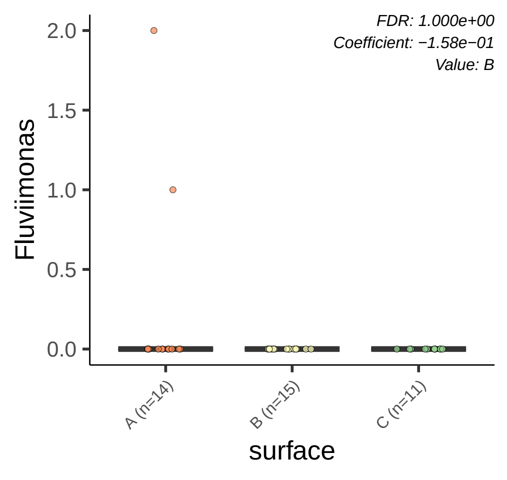

FDR: 1.000e+00
Coefficient: −1.58e−01
Value: B
Fluviimonas
2.0
1.5
1.0
0.5
0.0
A (n=14)
B (n=15)
C (n=11)
surface

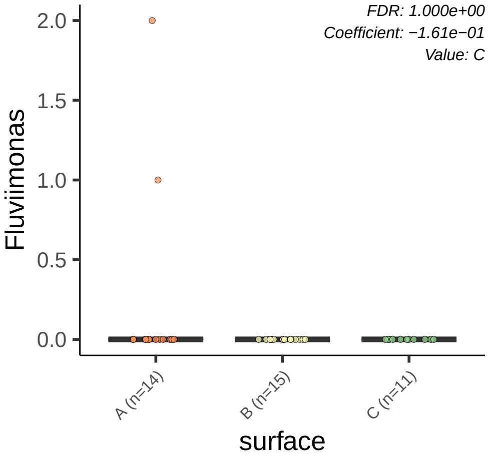
FDR: 1.000e+00
Coefficient: −1.61e−01
Value: C
Fluviimonas
2.0
1.5
1.0
0.5
0.0
A (n=14)
B (n=15)
C (n=11)
surface

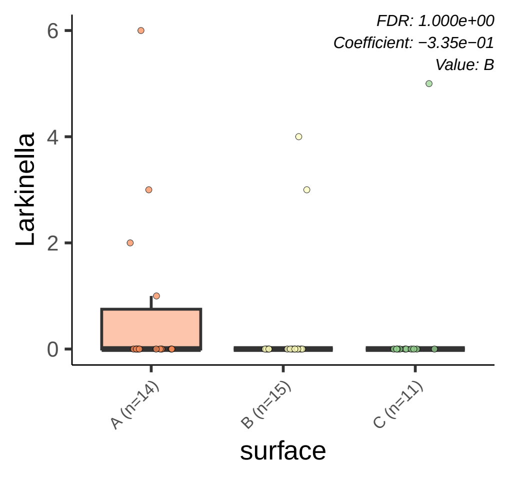
FDR: 1.000e+00
Coefficient: −3.35e−01
Value: B
Larkinella
0
2
4
6
A (n=14)
B (n=15)
C (n=11)
surface

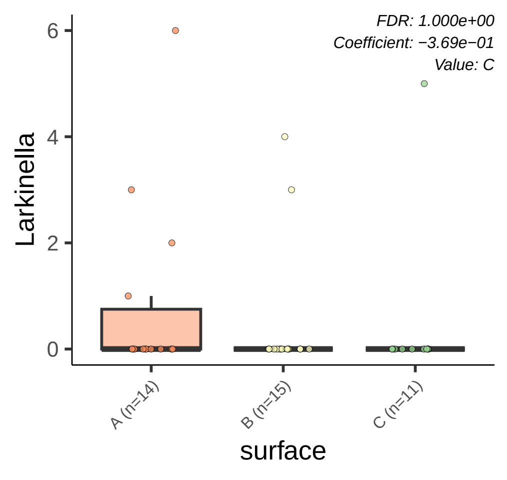

FDR: 1.000e+00
Coefficient: −3.69e−01
Value: C
Larkinella
0
2
4
6
A (n=14)
B (n=15)
C (n=11)
surface

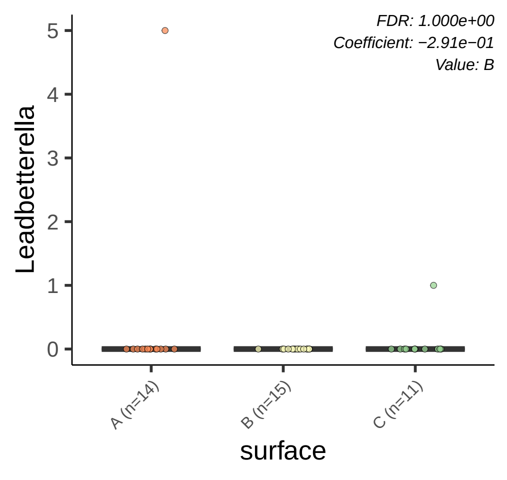
FDR: 1.000e+00
Coefficient: −2.91e−01
Value: B
Leadbetterella
0
1
2
3
4
5
A (n=14)
B (n=15)
C (n=11)
surface

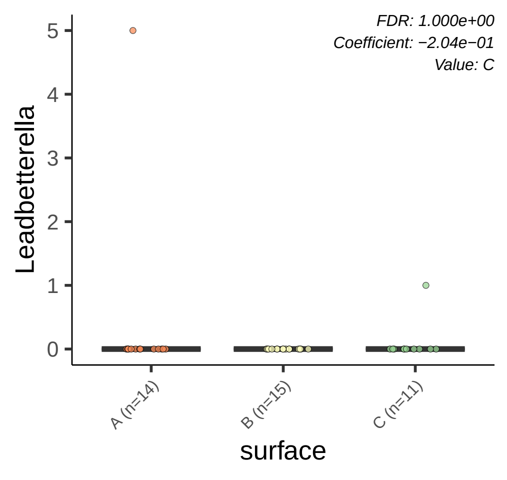

FDR: 1.000e+00
Coefficient: −2.04e−01
Value: C
Leadbetterella
0
1
2
3
4
5
A (n=14)
B (n=15)
C (n=11)
surface

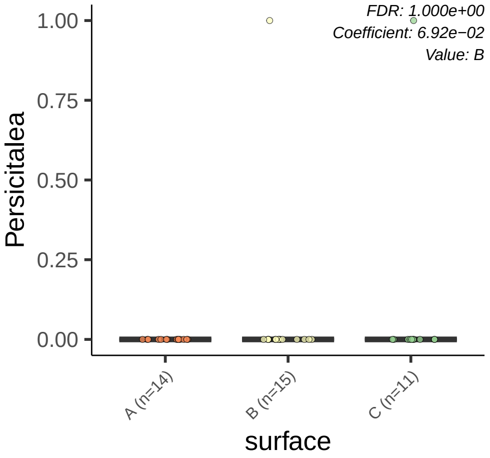

FDR: 1.000e+00
Coefficient: 6.92e−02
Value: B
Persicitalea
1.00
0.75
0.50
0.25
0.00
A (n=14)
B (n=15)
C (n=11)
surface

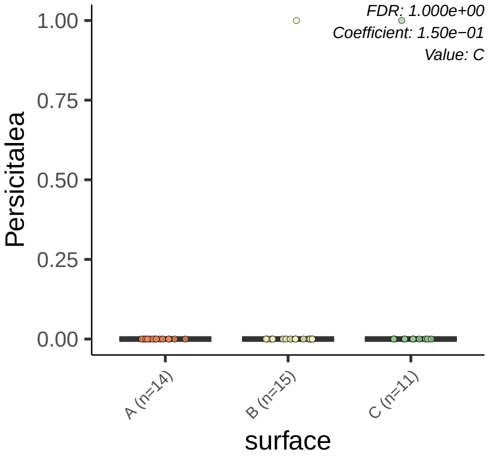
FDR: 1.000e+00
Coefficient: 1.50e−01
Value: C
Persicitalea
1.00
0.75
0.50
0.25
0.00
A (n=14)
B (n=15)
C (n=11)
surface

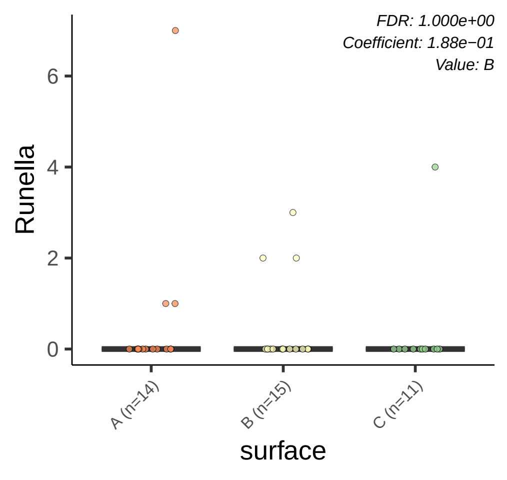
FDR: 1.000e+00
Coefficient: 1.88e−01
Value: B
Runella
0
2
4
6
A (n=14)
B (n=15)
C (n=11)
surface

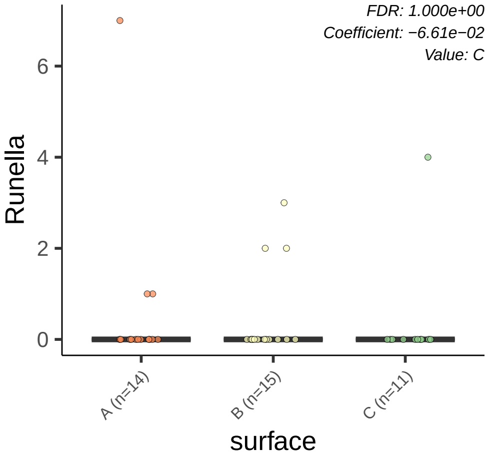
FDR: 1.000e+00
Coefficient: −6.61e−02
Value: C
Runella
0
2
4
6
A (n=14)
B (n=15)
C (n=11)
surface

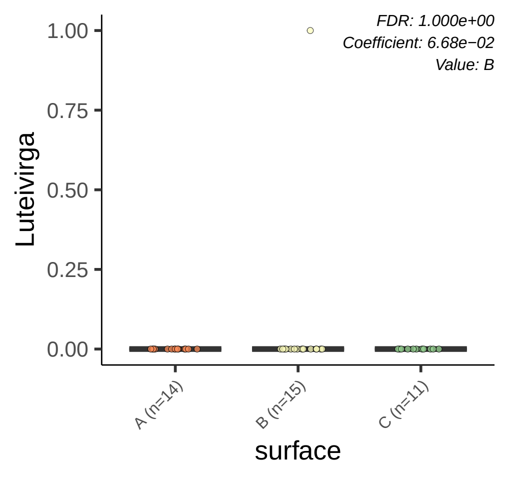
FDR: 1.000e+00
Coefficient: 6.68e−02
Value: B
Luteivirga
1.00
0.75
0.50
0.25
0.00
A (n=14)
B (n=15)
C (n=11)
surface

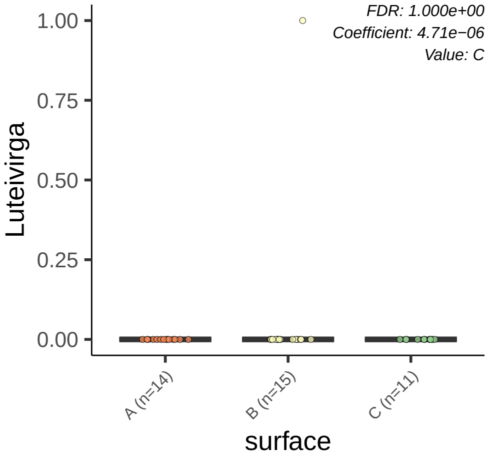
FDR: 1.000e+00
Coefficient: 4.71e−06
Value: C
1.00
0.75
0.50
0.25
0.00
Luteivirga
A (n=14)
B (n=15)
C (n=11)
surface

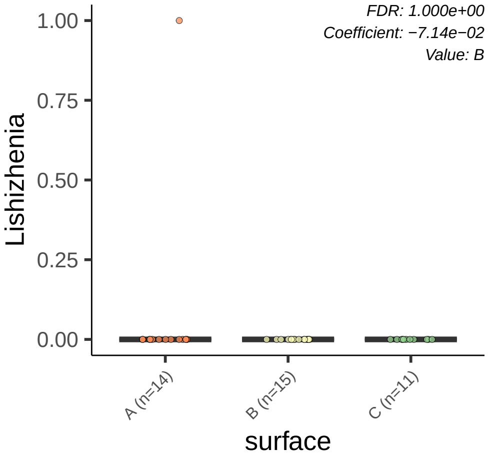

FDR: 1.000e+00
Coefficient: −7.14e−02
Value: B
Lishizhenia
1.00
0.75
0.50
0.25
0.00
A (n=14)
B (n=15)
C (n=11)
surface

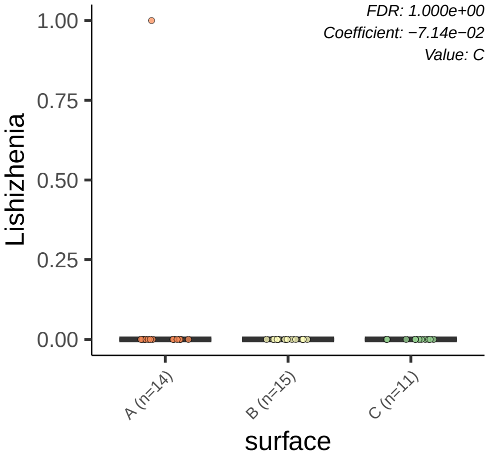
FDR: 1.000e+00
Coefficient: −7.14e−02
Value: C
Lishizhenia
1.00
0.75
0.50
0.25
0.00
A (n=14)
B (n=15)
C (n=11)
surface

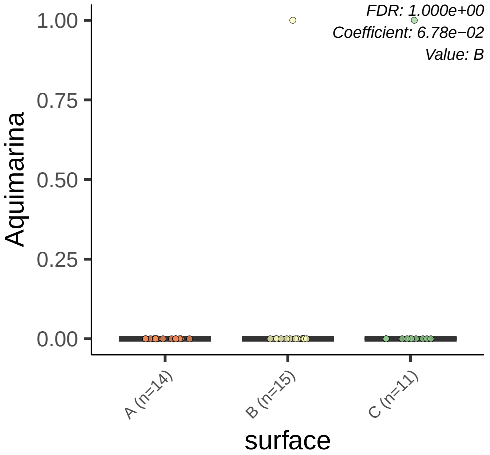
FDR: 1.000e+00
Coefficient: 6.78e−02
Value: B
Aquimarina
1.00
0.75
0.50
0.25
0.00
A (n=14)
B (n=15)
C (n=11)
surface

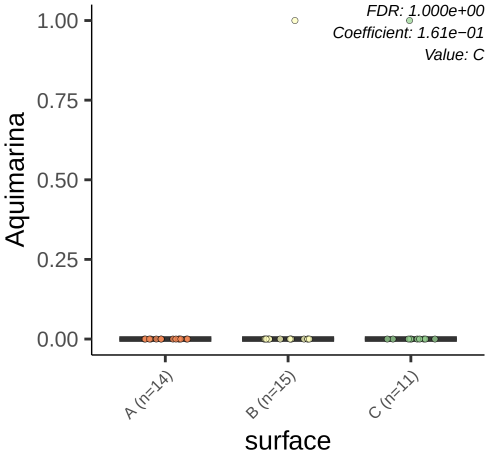
FDR: 1.000e+00
Coefficient: 1.61e−01
Value: C
Aquimarina
1.00
0.75
0.50
0.25
0.00
A (n=14)
B (n=15)
C (n=11)
surface

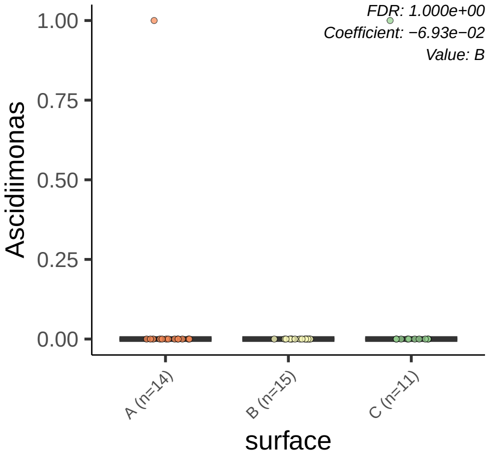
FDR: 1.000e+00
Coefficient: −6.93e−02
Value: B
Ascidimonas
1.00
0.75
0.50
0.25
0.00
A (n=14)
B (n=15)
C (n=11)
surface

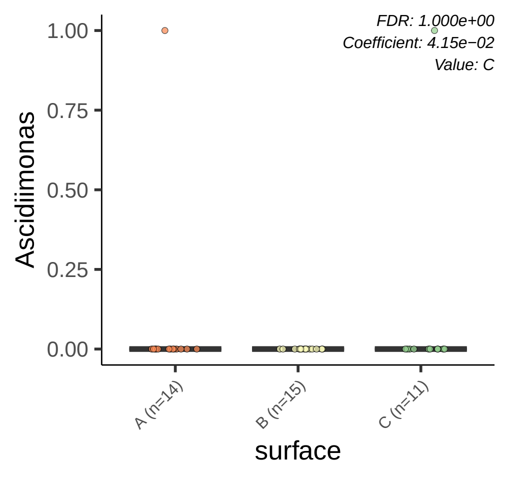

FDR: 1.000e+00
Coefficient: 4.15e−02
Value: C
Ascidimonas
1.00
0.75
0.50
0.25
0.00
A (n=14)
B (n=15)
C (n=11)
surface

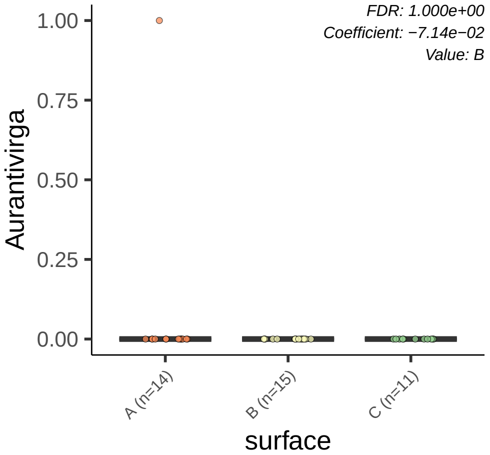

FDR: 1.000e+00
Coefficient: −7.14e−02
Value: B
Aurantivirga
1.00
0.75
0.50
0.25
0.00
A (n=14)
B (n=15)
C (n=11)
surface

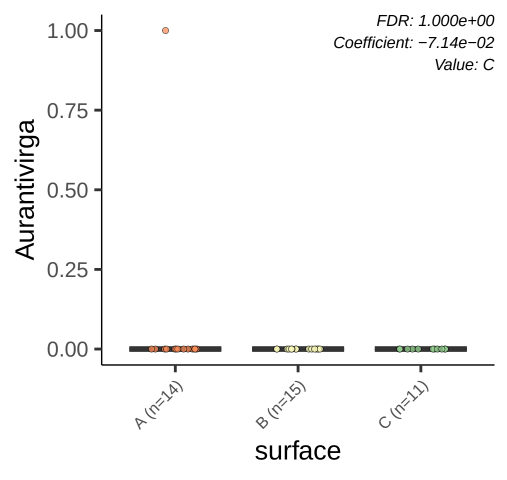

FDR: 1.000e+00
Coefficient: −7.14e−02
Value: C
Aurantivirga
1.00
0.75
0.50
0.25
0.00
A (n=14)
B (n=15)
C (n=11)
surface

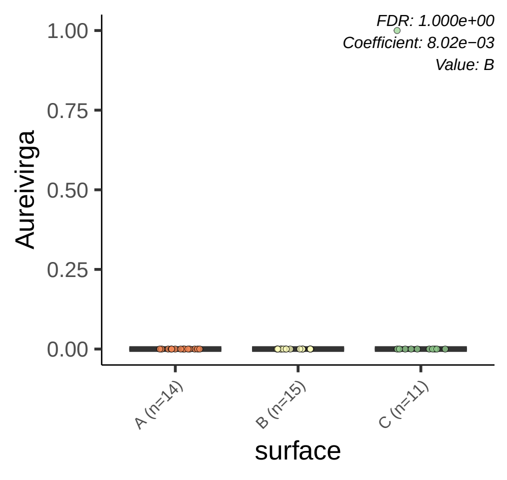
FDR: 1.000e+00
Coefficient: 8.02e−03
Value: B
Aureivirga
1.00
0.75
0.50
0.25
0.00
A (n=14)
B (n=15)
C (n=11)
surface

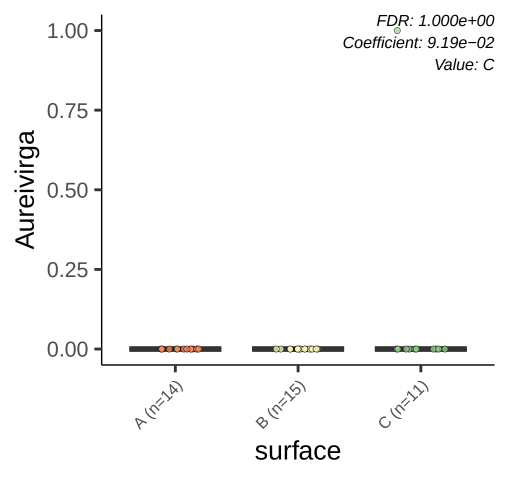
FDR: 1.000e+00
Coefficient: 9.19e−02
Value: C
Aureivirga
1.00
0.75
0.50
0.25
0.00
A (n=14)
B (n=15)
C (n=11)
surface

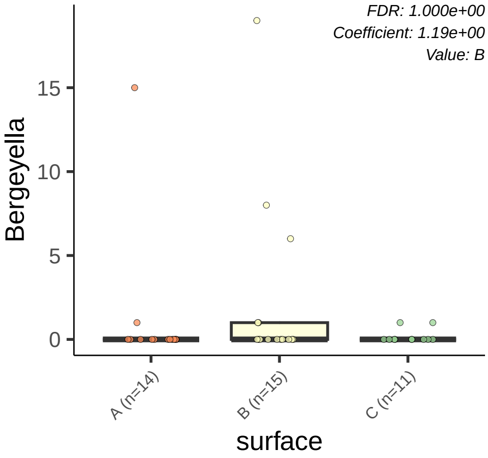
FDR: 1.000e+00
Coefficient: 1.19e+00
Value: B
Bergeyella
0
5
10
15
A (n=14)
B (n=15)
C (n=11)
surface

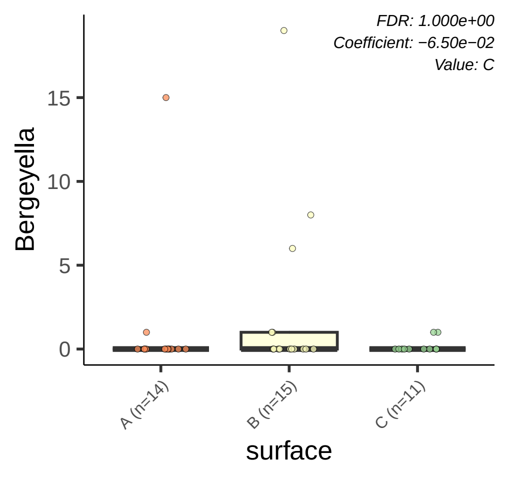
FDR: 1.000e+00
Coefficient: −6.50e−02
Value: C
Bergeyella
0
5
10
15
A (n=14)
B (n=15)
C (n=11)
surface

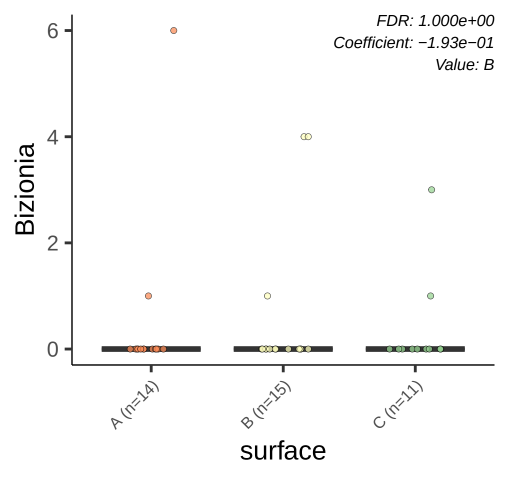
FDR: 1.000e+00
Coefficient: −1.93e−01
Value: B
Bizionia
0
2
4
6
A (n=14)
B (n=15)
C (n=11)
surface

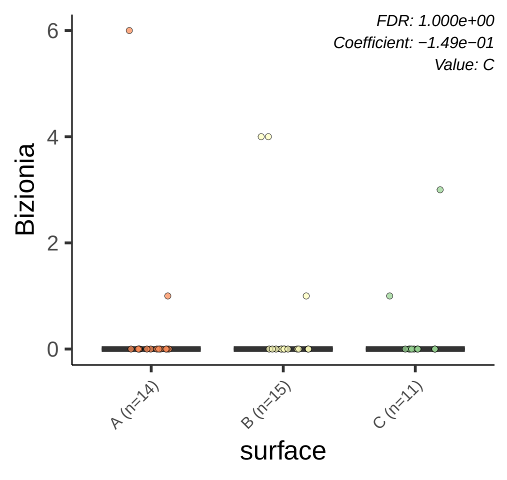

FDR: 1.000e+00
Coefficient: −1.49e−01
Value: C
Bizionia
6
4
2
0
A (n=14)
B (n=15)
C (n=11)
surface

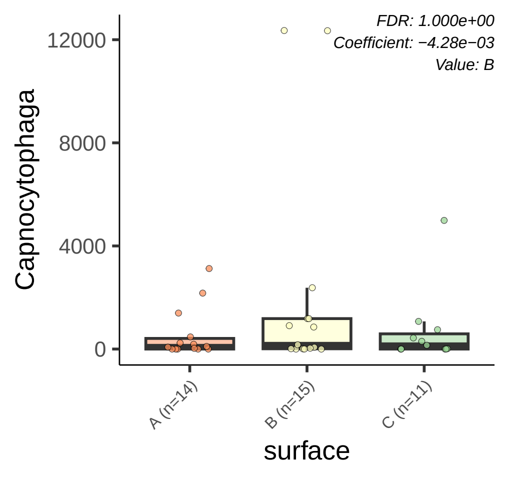

FDR: 1.000e+00
Coefficient: −4.28e−03
Value: B
Capnocytophaga
12000
8000
4000
0
A (n=14)
B (n=15)
C (n=11)
surface

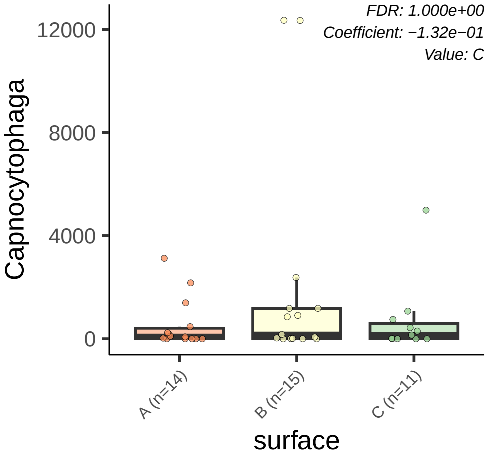

FDR: 1.000e+00
Coefficient: −1.32e−01
Value: C
Capnocytophaga
12000
8000
4000
0
A (n=14)
B (n=15)
C (n=11)
surface

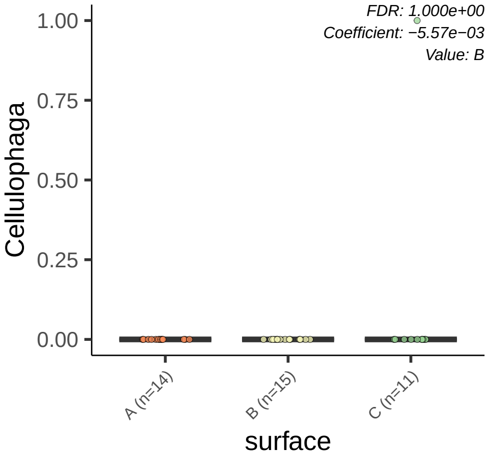
FDR: 1.000e+00
Coefficient: −5.57e−03
Value: B
1.00
0.75
0.50
0.25
0.00
Cellulophaga
A (n=14)
B (n=15)
C (n=11)
surface

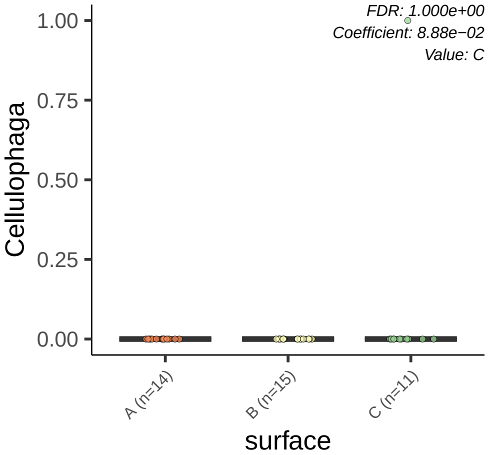
FDR: 1.000e+00
Coefficient: 8.88e−02
Value: C
Cellulophaga
1.00
0.75
0.50
0.25
0.00
A (n=14)
B (n=15)
C (n=11)
surface

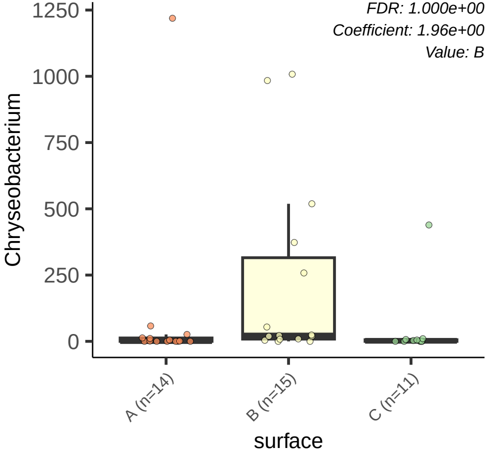
FDR: 1.000e+00
Coefficient: 1.96e+00
Value: B
Chryseobacterium
1250
1000
750
500
250
0
A (n=14)
B (n=15)
C (n=11)
surface

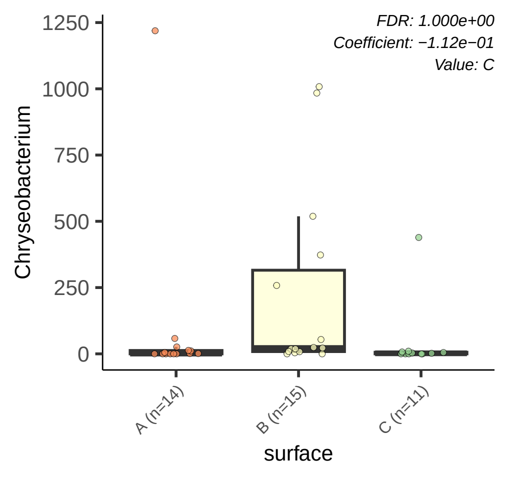
FDR: 1.000e+00
Coefficient: −1.12e−01
Value: C
Chryseobacterium
1250
1000
750
500
250
0
A (n=14)
B (n=15)
C (n=11)
surface

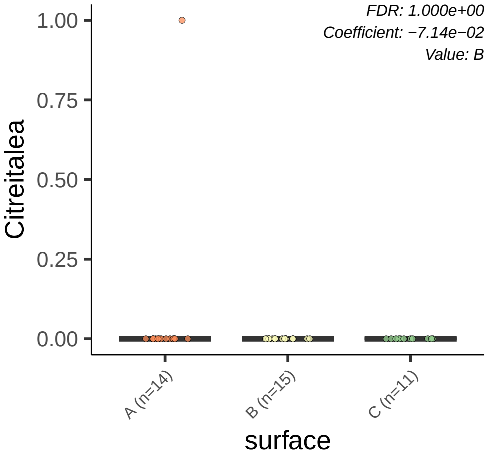
FDR: 1.000e+00
Coefficient: −7.14e−02
Value: B
Citreitalea
1.00
0.75
0.50
0.25
0.00
A (n=14)
B (n=15)
C (n=11)
surface

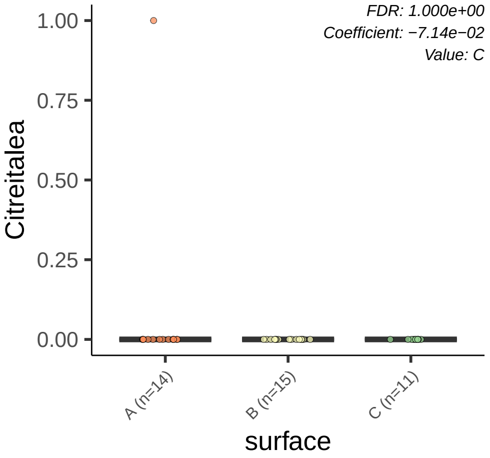

FDR: 1.000e+00
Coefficient: −7.14e−02
Value: C
Citreitalea
1.00
0.75
0.50
0.25
0.00
A (n=14)
B (n=15)
C (n=11)
surface

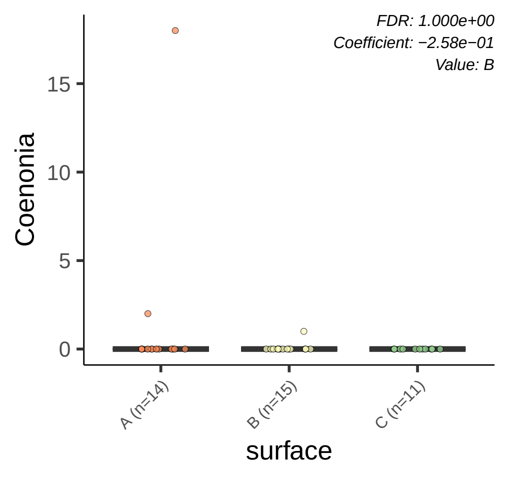
FDR: 1.000e+00
Coefficient: −2.58e−01
Value: B
Coenonia
0
5
10
15
A (n=14)
B (n=15)
C (n=11)
surface

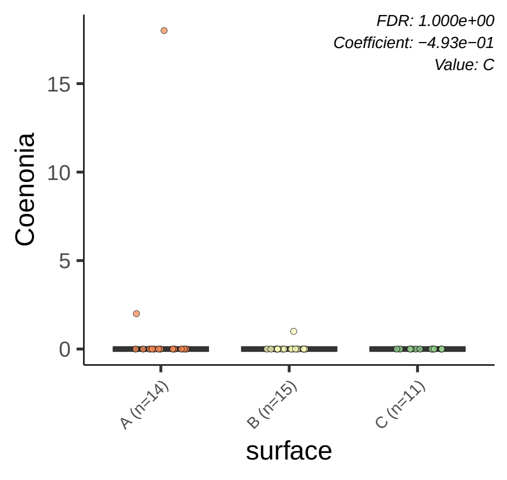

FDR: 1.000e+00
Coefficient: −4.93e−01
Value: C
Coenonia
15
10
5
0
A (n=14)
B (n=15)
C (n=11)
surface

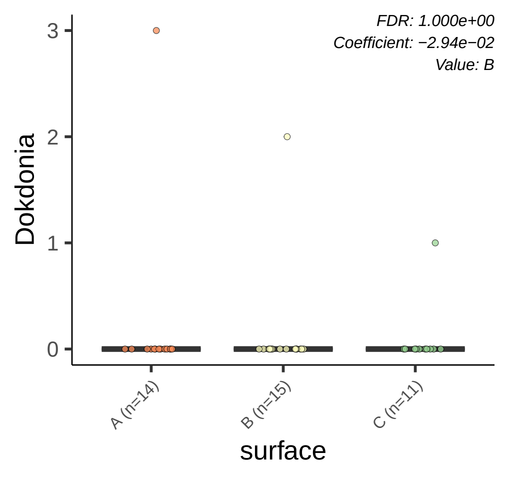

FDR: 1.000e+00
Coefficient: −2.94e−02
Value: B
Dokdonia
3
2
1
0
A (n=14)
B (n=15)
C (n=11)
surface

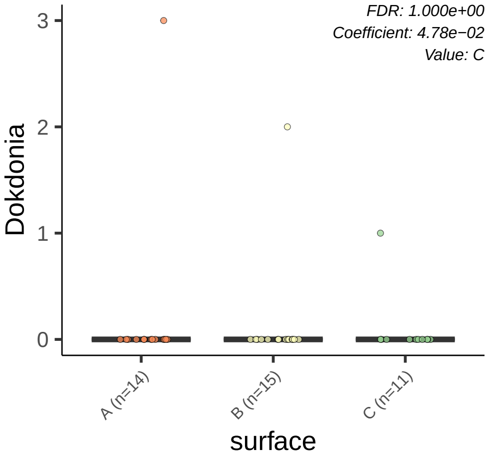
FDR: 1.000e+00
Coefficient: 4.78e−02
Value: C
Dokdonia
0
1
2
3
A (n=14)
B (n=15)
C (n=11)
surface

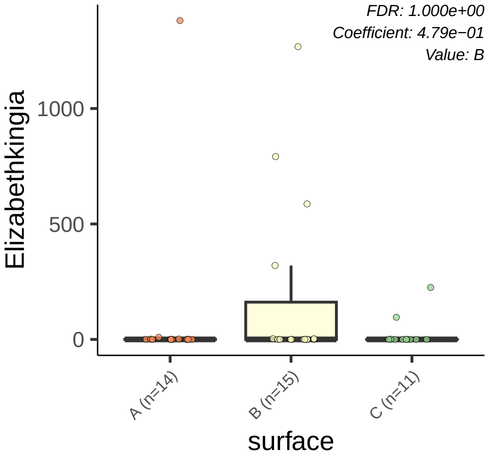

FDR: 1.000e+00
Coefficient: 4.79e−01
Value: B
Elizabethkingia
1000
500
0
A (n=14)
B (n=15)
C (n=11)
surface

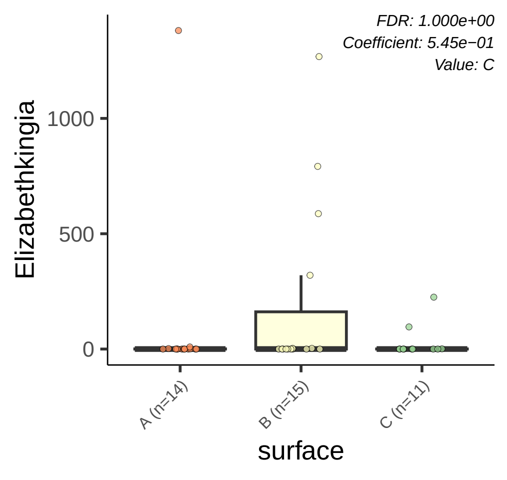
FDR: 1.000e+00
Coefficient: 5.45e−01
Value: C
Elizabethkingia
1000
500
0
A (n=14)
B (n=15)
C (n=11)
surface

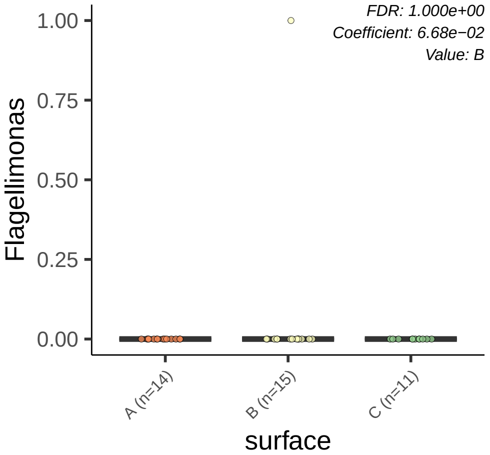

FDR: 1.000e+00
Coefficient: 6.68e−02
Value: B
Flagellimonas
1.00
0.75
0.50
0.25
0.00
A (n=14)
B (n=15)
C (n=11)
surface

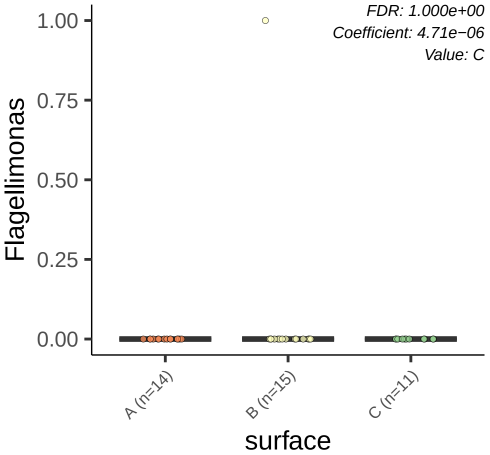
FDR: 1.000e+00
Coefficient: 4.71e−06
Value: C
Flagellimonas
1.00
0.75
0.50
0.25
0.00
A (n=14)
B (n=15)
C (n=11)
surface

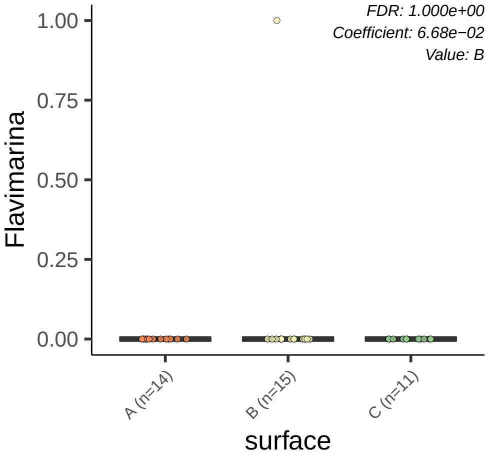
FDR: 1.000e+00
Coefficient: 6.68e−02
Value: B
Flavimarina
1.00
0.75
0.50
0.25
0.00
A (n=14)
B (n=15)
C (n=11)
surface

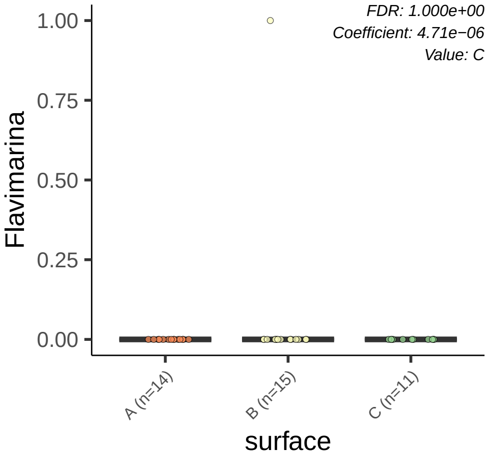

FDR: 1.000e+00
Coefficient: 4.71e−06
Value: C
Flavimarina
1.00
0.75
0.50
0.25
0.00
A (n=14)
B (n=15)
C (n=11)
surface

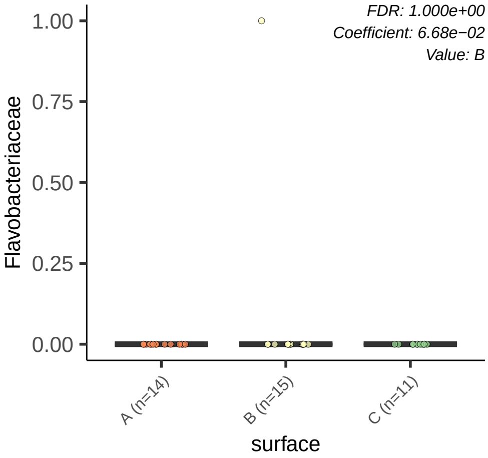

FDR: 1.000e+00
Coefficient: 6.68e−02
Value: B
Flavobacteriaceae
1.00
0.75
0.50
0.25
0.00
A (n=14)
B (n=15)
C (n=11)
surface

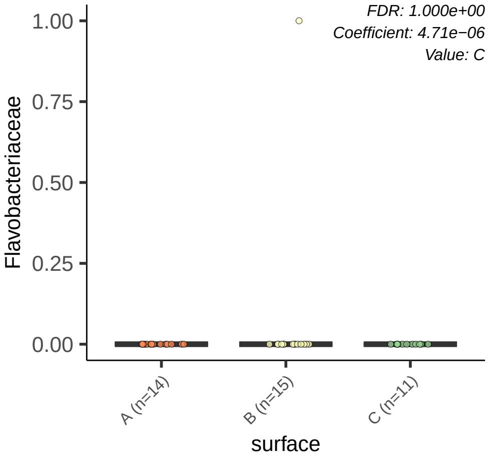
FDR: 1.000e+00
Coefficient: 4.71e−06
Value: C
Flavobacteriaceae
1.00
0.75
0.50
0.25
0.00
A (n=14)
B (n=15)
C (n=11)
surface

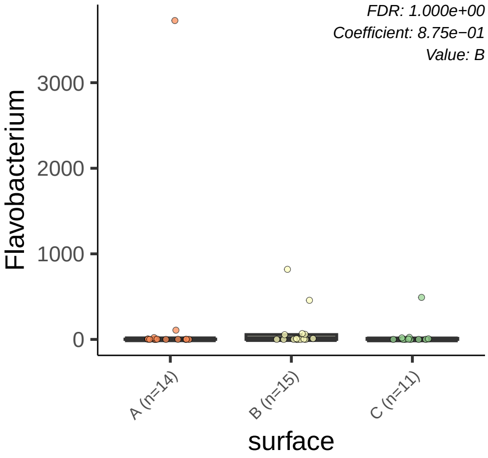
FDR: 1.000e+00
Coefficient: 8.75e−01
Value: B
Flavobacterium
3000
2000
1000
0
A (n=14)
B (n=15)
C (n=11)
surface

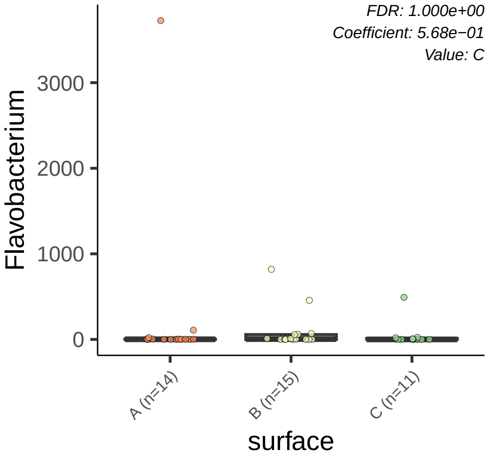
FDR: 1.000e+00
Coefficient: 5.68e−01
Value: C
Flavobacterium
3000
2000
1000
0
A (n=14)
B (n=15)
C (n=11)
surface

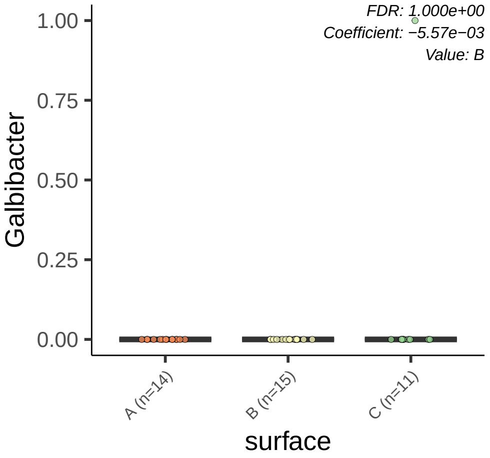

FDR: 1.000e+00
Coefficient: −5.57e−03
Value: B
1.00
0.75
0.50
0.25
0.00
Galbibacter
A (n=14)
B (n=15)
C (n=11)
surface

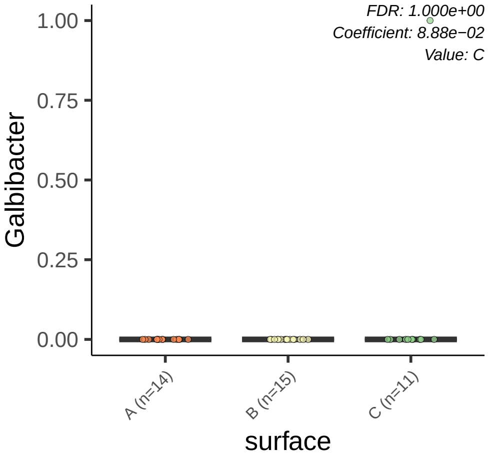

FDR: 1.000e+00
Coefficient: 8.88e−02
Value: C
Galbibacter
1.00
0.75
0.50
0.25
0.00
A (n=14)
B (n=15)
C (n=11)
surface

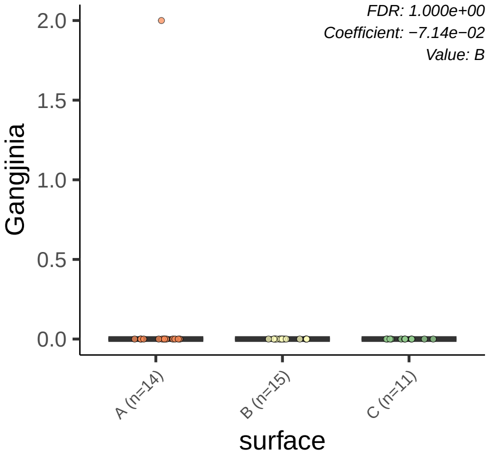
FDR: 1.000e+00
Coefficient: −7.14e−02
Value: B
2.0
1.5
1.0
0.5
0.0
Gangjinia
A (n=14)
B (n=15)
C (n=11)
surface

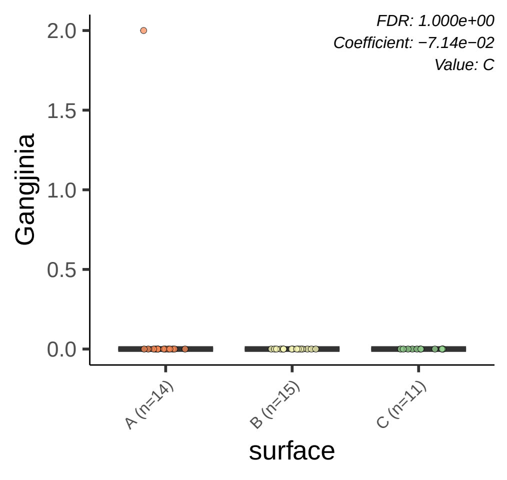
FDR: 1.000e+00
Coefficient: −7.14e−02
Value: C
Gangjinia
2.0
1.5
1.0
0.5
0.0
A (n=14)
B (n=15)
C (n=11)
surface

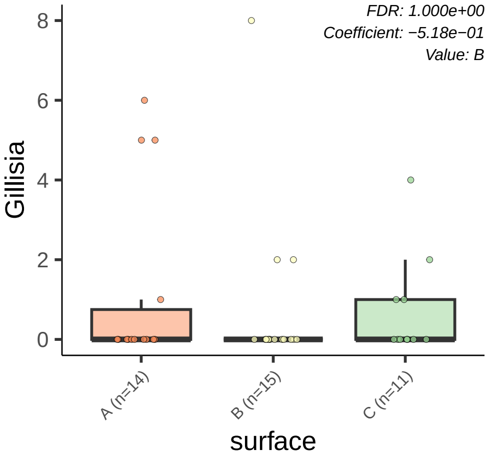
FDR: 1.000e+00
Coefficient: −5.18e−01
Value: B
Gillisia
8
6
4
2
0
A (n=14)
B (n=15)
C (n=11)
surface

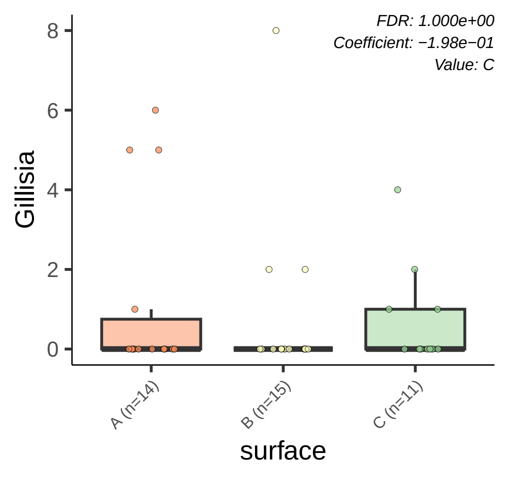
FDR: 1.000e+00
Coefficient: −1.98e−01
Value: C
Gillisia
8
6
4
2
0
A (n=14)
B (n=15)
C (n=11)
surface

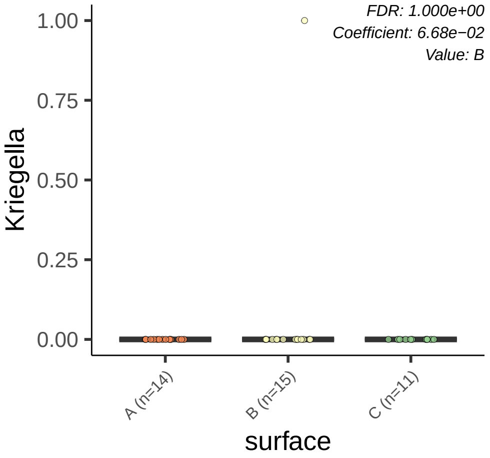
FDR: 1.000e+00
Coefficient: 6.68e−02
Value: B
Kriegella
1.00
0.75
0.50
0.25
0.00
A (n=14)
B (n=15)
C (n=11)
surface

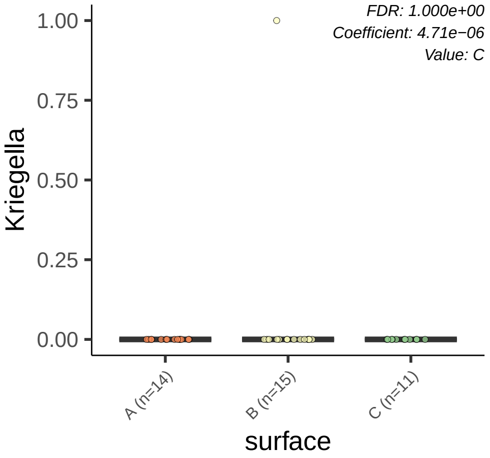
FDR: 1.000e+00
Coefficient: 4.71e−06
Value: C
Kriegella
1.00
0.75
0.50
0.25
0.00
A (n=14)
B (n=15)
C (n=11)
surface

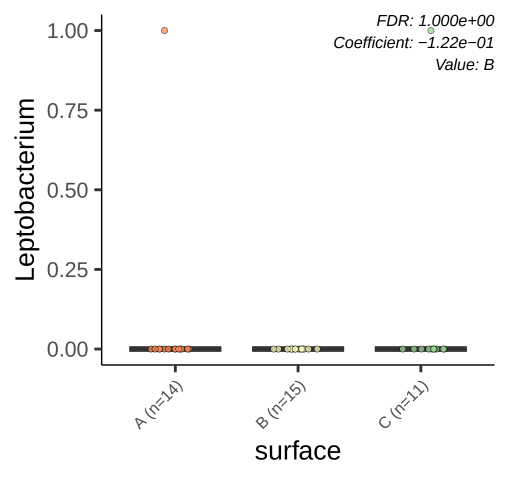
FDR: 1.000e+00
Coefficient: −1.22e−01
Value: B
Leptobacterium
1.00
0.75
0.50
0.25
0.00
A (n=14)
B (n=15)
C (n=11)
surface

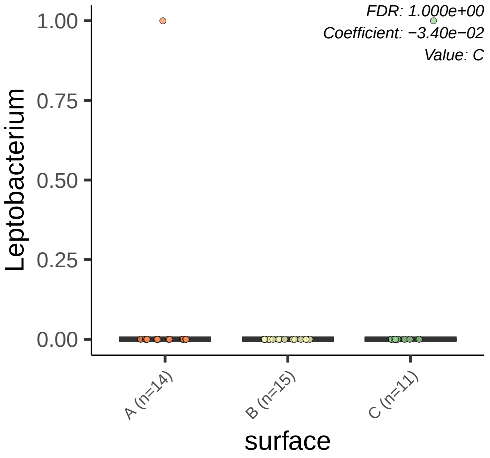
FDR: 1.000e+00
Coefficient: −3.40e−02
Value: C
Leptobacterium
1.00
0.75
0.50
0.25
0.00
A (n=14)
B (n=15)
C (n=11)
surface

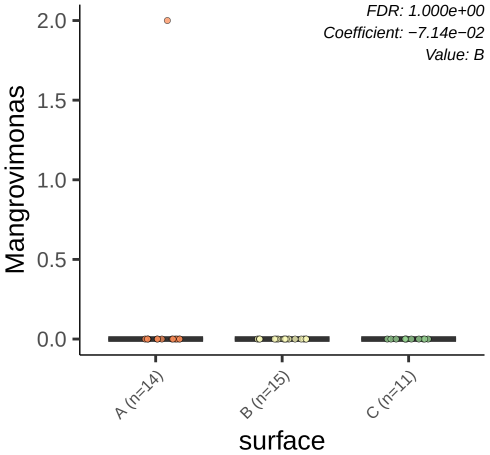

FDR: 1.000e+00
Coefficient: −7.14e−02
Value: B
Mangrovimonas
2.0
1.5
1.0
0.5
0.0
A (n=14)
B (n=15)
C (n=11)
surface

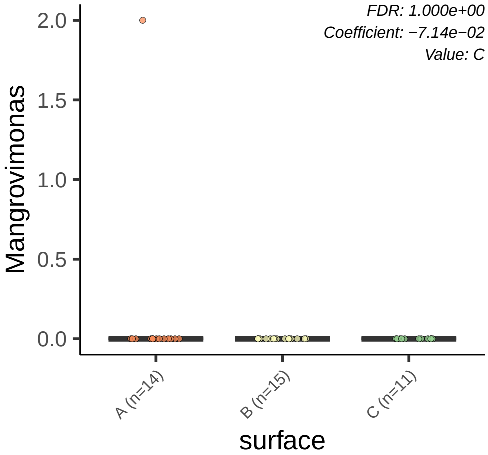

FDR: 1.000e+00
Coefficient: −7.14e−02
Value: C
Mangrovimonas
2.0
1.5
1.0
0.5
0.0
A (n=14)
B (n=15)
C (n=11)
surface

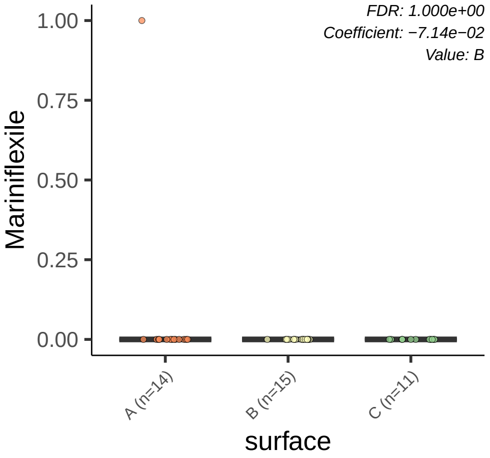
FDR: 1.000e+00
Coefficient: −7.14e−02
Value: B
Mariniflexile
1.00
0.75
0.50
0.25
0.00
A (n=14)
B (n=15)
C (n=11)
surface

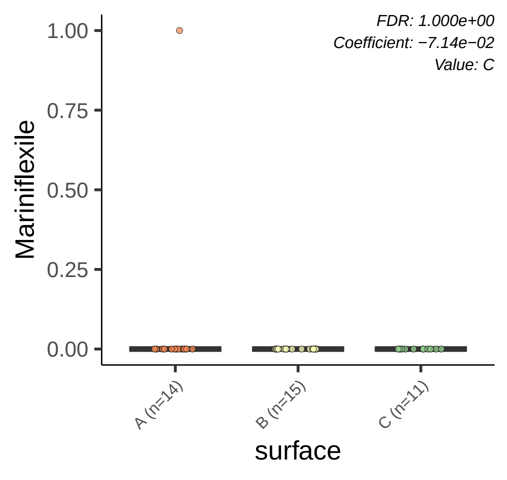
FDR: 1.000e+00
Coefficient: −7.14e−02
Value: C
Mariniflexile
1.00
0.75
0.50
0.25
0.00
A (n=14)
B (n=15)
C (n=11)
surface

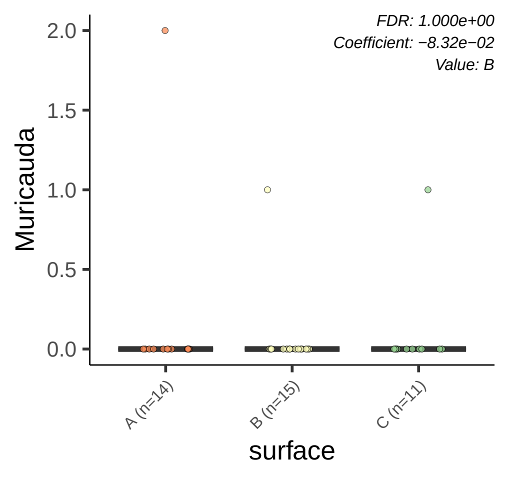
FDR: 1.000e+00
Coefficient: −8.32e−02
Value: B
Muricauda
2.0
1.5
1.0
0.5
0.0
A (n=14)
B (n=15)
C (n=11)
surface

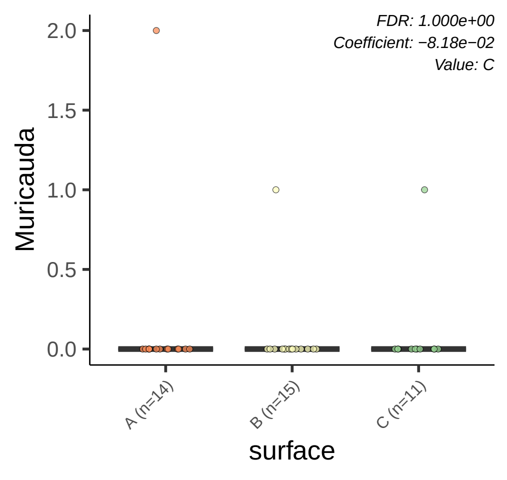
FDR: 1.000e+00
Coefficient: −8.18e−02
Value: C
Muricauda
2.0
1.5
1.0
0.5
0.0
A (n=14)
B (n=15)
C (n=11)
surface

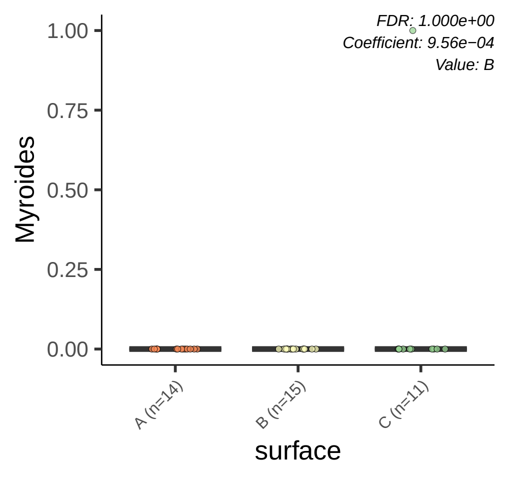
FDR: 1.000e+00
Coefficient: 9.56e−04
Value: B
Myroides
1.00
0.75
0.50
0.25
0.00
A (n=14)
B (n=15)
C (n=11)
surface

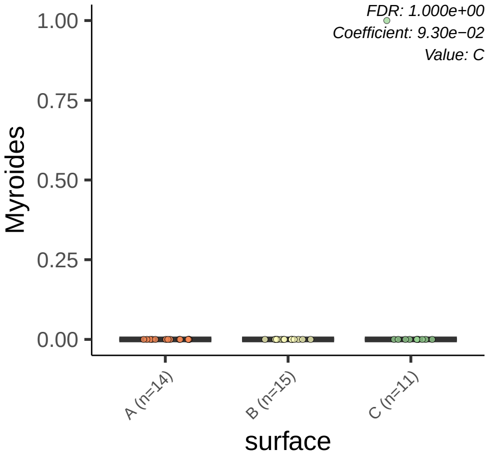

FDR: 1.000e+00
Coefficient: 9.30e−02
Value: C
1.00
0.75
0.50
0.25
0.00
Myroides
A (n=14)
B (n=15)
C (n=11)
surface

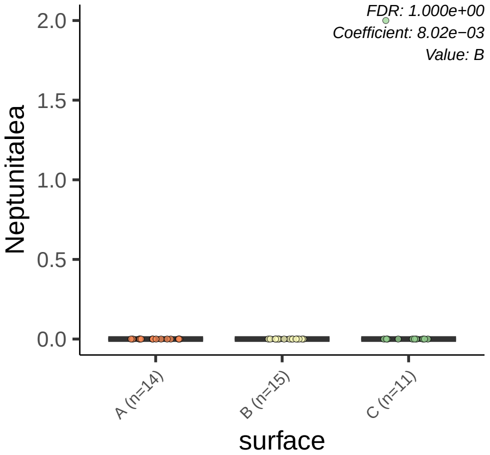
FDR: 1.000e+00
Coefficient: 8.02e−03
Value: B
Neptunitalea
2.0
1.5
1.0
0.5
0.0
A (n=14)
B (n=15)
C (n=11)
surface

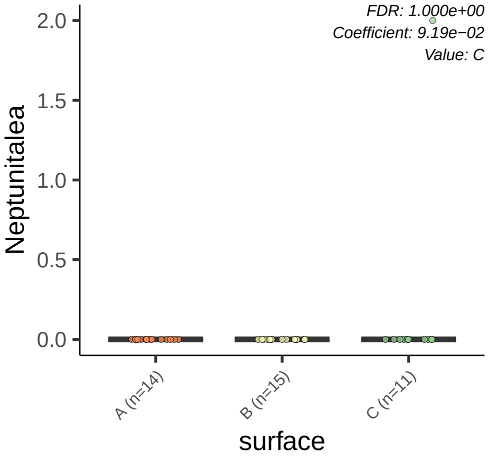

FDR: 1.000e+00
Coefficient: 9.19e−02
Value: C
Neptunitalea
2.0
1.5
1.0
0.5
0.0
A (n=14)
B (n=15)
C (n=11)
surface

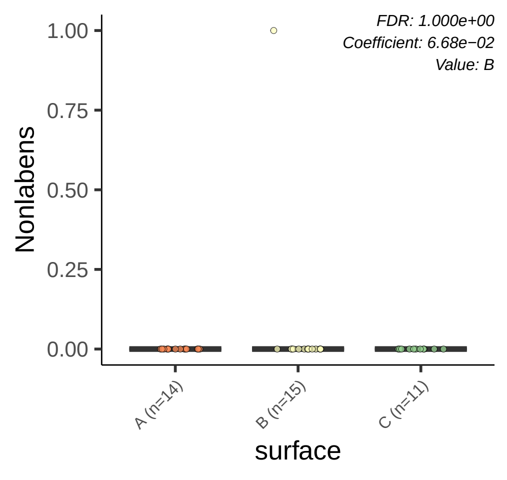
FDR: 1.000e+00
Coefficient: 6.68e−02
Value: B
Nonlabens
1.00
0.75
0.50
0.25
0.00
A (n=14)
B (n=15)
C (n=11)
surface

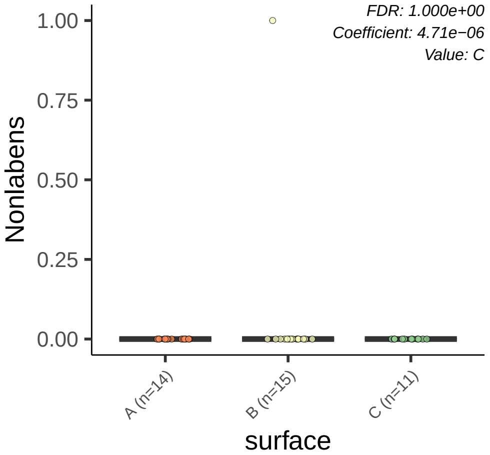
FDR: 1.000e+00
Coefficient: 4.71e−06
Value: C
Nonlabens
1.00
0.75
0.50
0.25
0.00
A (n=14)
B (n=15)
C (n=11)
surface

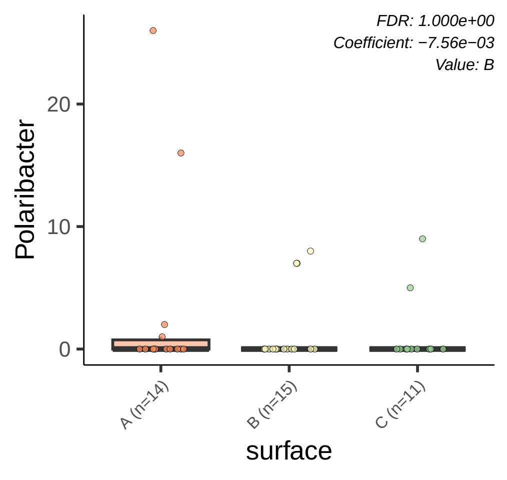

FDR: 1.000e+00
Coefficient: −7.56e−03
Value: B
Polaribacter
20
10
0
A (n=14)
B (n=15)
C (n=11)
surface

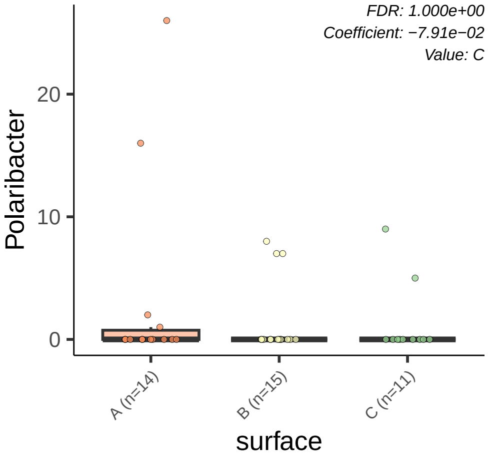

FDR: 1.000e+00
Coefficient: −7.91e−02
Value: C
Polaribacter
20
10
0
A (n=14)
B (n=15)
C (n=11)
surface

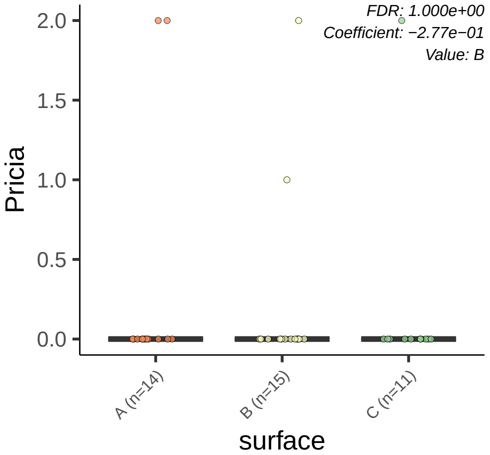

FDR: 1.000e+00
Coefficient: −2.77e−01
Value: B
Pricia
2.0
1.5
1.0
0.5
0.0
A (n=14)
B (n=15)
C (n=11)
surface

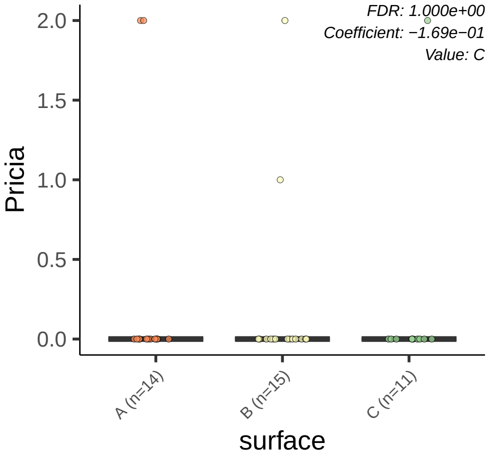
FDR: 1.000e+00
Coefficient: −1.69e−01
Value: C
Pricia
2.0
1.5
1.0
0.5
0.0
A (n=14)
B (n=15)
C (n=11)
surface

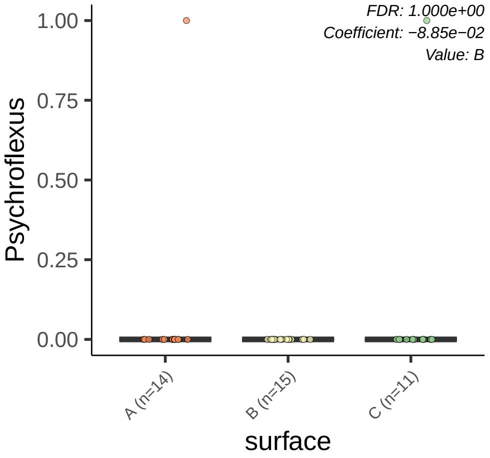

FDR: 1.000e+00
Coefficient: −8.85e−02
Value: B
Psychroflexus
1.00
0.75
0.50
0.25
0.00
A (n=14)
B (n=15)
C (n=11)
surface

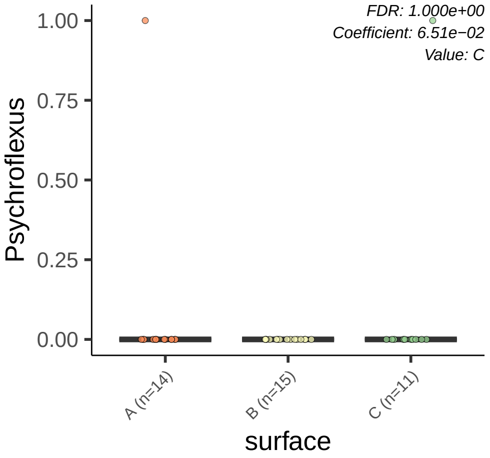
FDR: 1.000e+00
Coefficient: 6.51e−02
Value: C
Psychroflexus
1.00
0.75
0.50
0.25
0.00
A (n=14)
B (n=15)
C (n=11)
surface

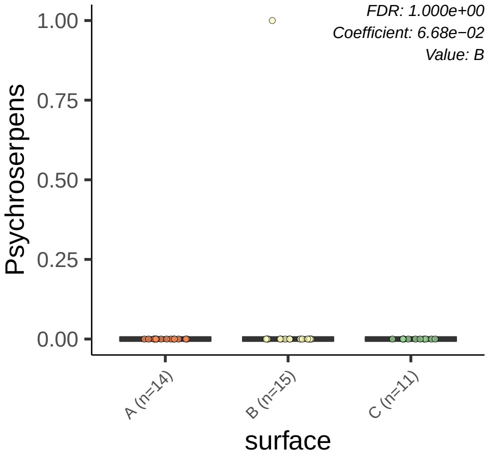

FDR: 1.000e+00
Coefficient: 6.68e−02
Value: B
Psychroserpens
1.00
0.75
0.50
0.25
0.00
A (n=14)
B (n=15)
C (n=11)
surface

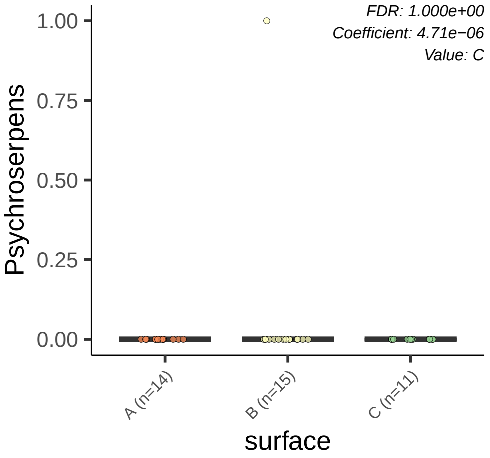

FDR: 1.000e+00
Coefficient: 4.71e−06
Value: C
Psychroserpens
1.00
0.75
0.50
0.25
0.00
A (n=14)
B (n=15)
C (n=11)
surface

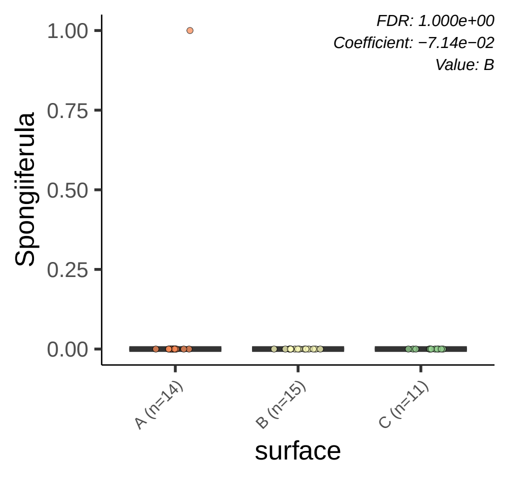

FDR: 1.000e+00
Coefficient: −7.14e−02
Value: B
Spongiiferula
1.00
0.75
0.50
0.25
0.00
A (n=14)
B (n=15)
C (n=11)
surface

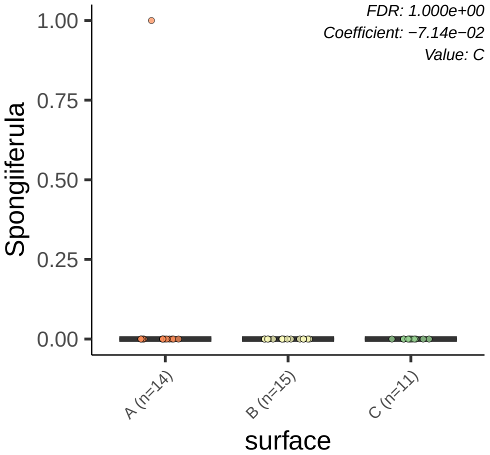

FDR: 1.000e+00
Coefficient: −7.14e−02
Value: C
Spongiiferula
1.00
0.75
0.50
0.25
0.00
A (n=14)
B (n=15)
C (n=11)
surface

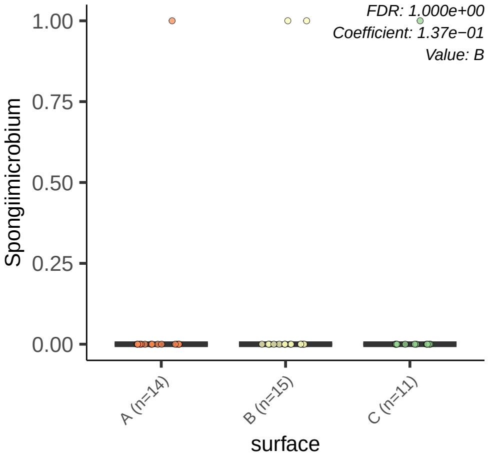

FDR: 1.000e+00
Coefficient: 1.37e−01
Value: B
Spongiimicrobium
1.00
0.75
0.50
0.25
0.00
A (n=14)
B (n=15)
C (n=11)
surface

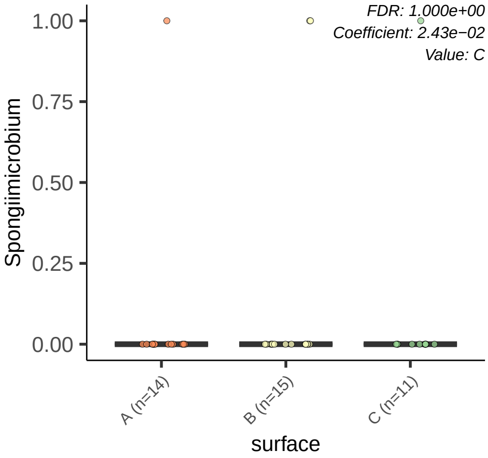
FDR: 1.000e+00
Coefficient: 2.43e−02
Value: C
Spongiimicrobium
1.00
0.75
0.50
0.25
0.00
A (n=14)
B (n=15)
C (n=11)
surface

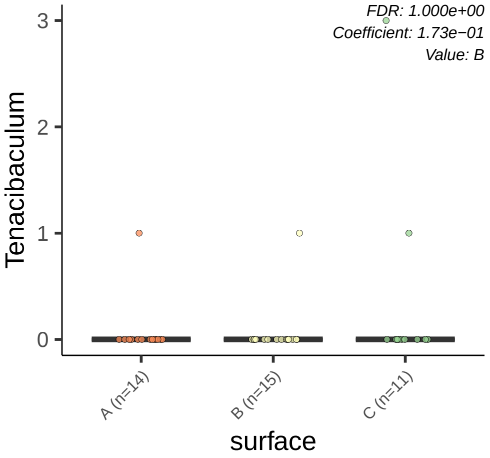
FDR: 1.000e+00
Coefficient: 1.73e−01
Value: B
Tenacibaculum
3
2
1
0
A (n=14)
B (n=15)
C (n=11)
surface

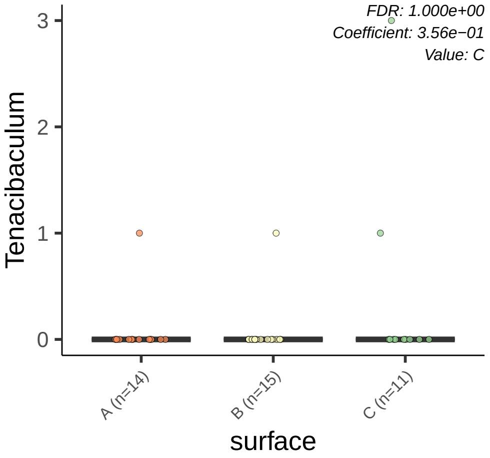
FDR: 1.000e+00
Coefficient: 3.56e−01
Value: C
Tenacibaculum
3
2
1
0
A (n=14)
B (n=15)
C (n=11)
surface

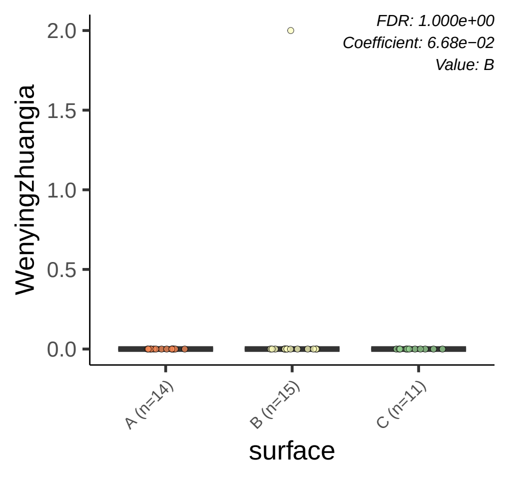
FDR: 1.000e+00
Coefficient: 6.68e−02
Value: B
Wenyingzhuangia
2.0
1.5
1.0
0.5
0.0
A (n=14)
B (n=15)
C (n=11)
surface

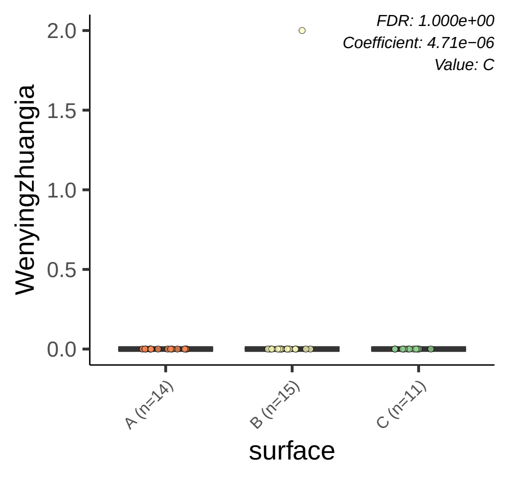

FDR: 1.000e+00
Coefficient: 4.71e−06
Value: C
Wenyingzhuangia
2.0
1.5
1.0
0.5
0.0
A (n=14)
B (n=15)
C (n=11)
surface

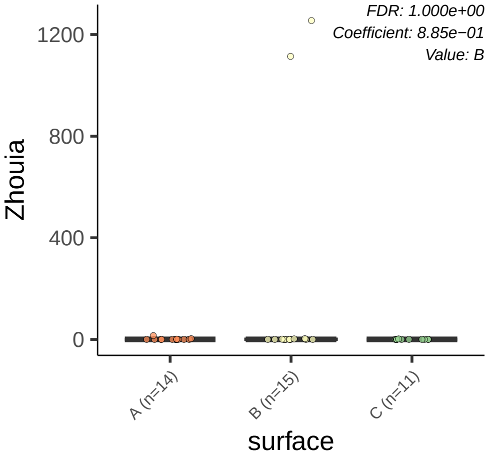

FDR: 1.000e+00
Coefficient: 8.85e−01
Value: B
Zhouia
1200
800
400
0
A (n=14)
B (n=15)
C (n=11)
surface

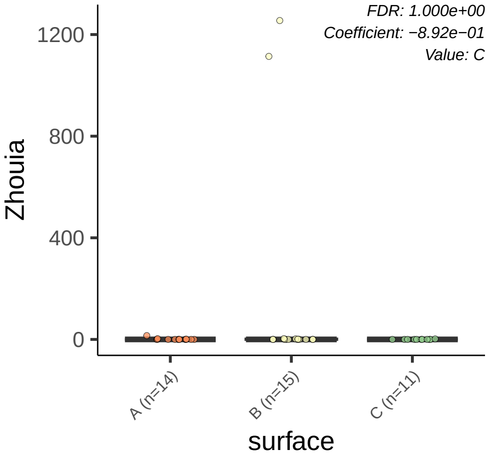

FDR: 1.000e+00
Coefficient: −8.92e−01
Value: C
Zhouia
1200
800
400
0
A (n=14)
B (n=15)
C (n=11)
surface

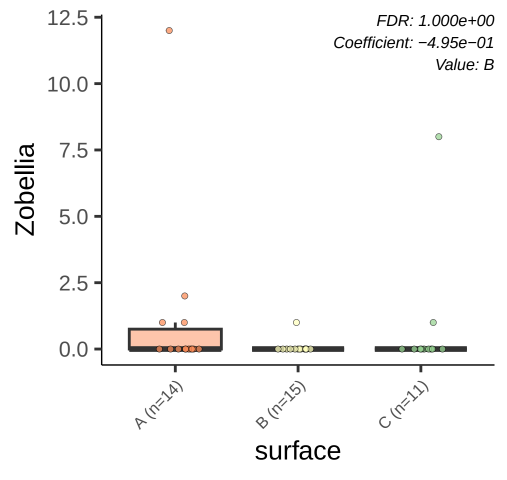
FDR: 1.000e+00
Coefficient: −4.95e−01
Value: B
Zobellia
12.5
10.0
7.5
5.0
2.5
0.0
A (n=14)
B (n=15)
C (n=11)
surface

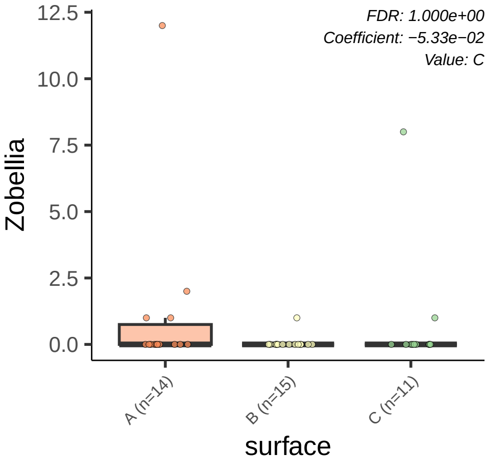
FDR: 1.000e+00
Coefficient: −5.33e−02
Value: C
Zobellia
12.5
10.0
7.5
5.0
2.5
0.0
A (n=14)
B (n=15)
C (n=11)
surface

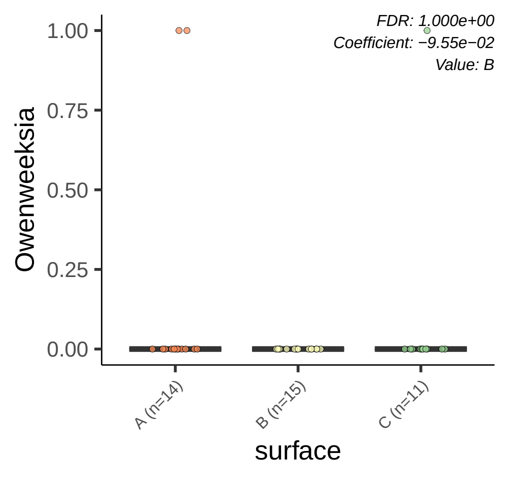
FDR: 1.000e+00
Coefficient: −9.55e−02
Value: B
Owenweeksia
1.00
0.75
0.50
0.25
0.00
A (n=14)
B (n=15)
C (n=11)
surface

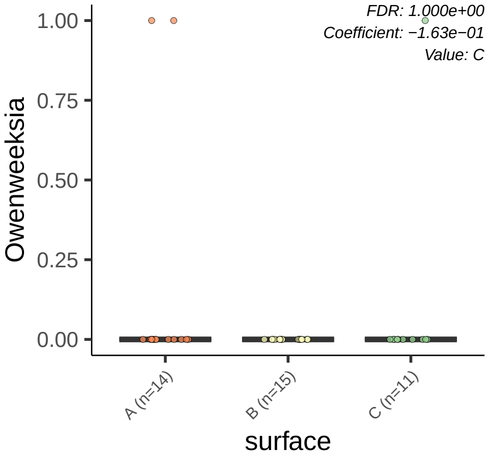

FDR: 1.000e+00
Coefficient: −1.63e−01
Value: C
Owenweeksia
1.00
0.75
0.50
0.25
0.00
A (n=14)
B (n=15)
C (n=11)
surface

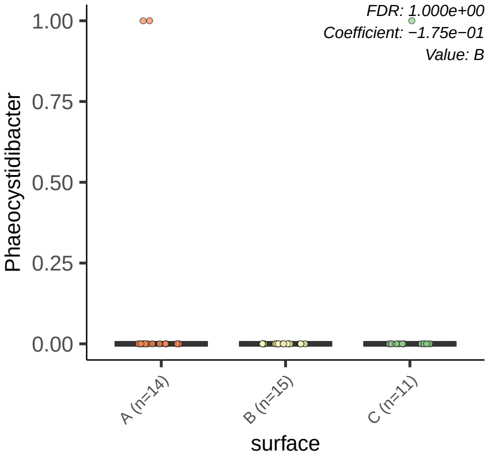

FDR: 1.000e+00
Coefficient: −1.75e−01
Value: B
Phaeocystidibacter
1.00
0.75
0.50
0.25
0.00
A (n=14)
B (n=15)
C (n=11)
surface

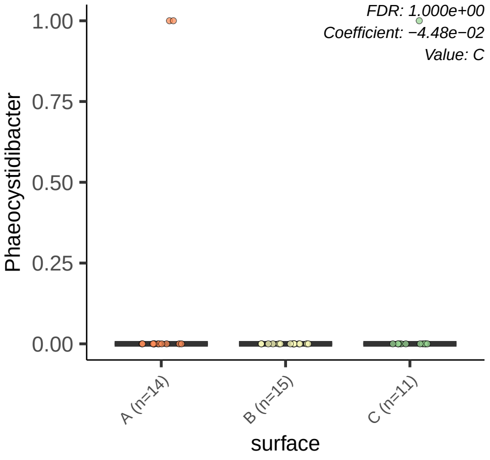
FDR: 1.000e+00
Coefficient: −4.48e−02
Value: C
Phaeocystidibacter
1.00
0.75
0.50
0.25
0.00
A (n=14)
B (n=15)
C (n=11)
surface

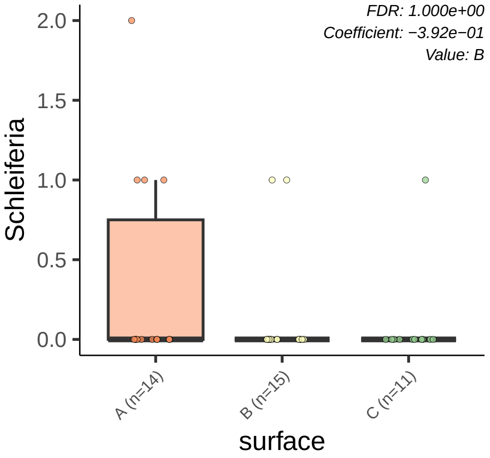
FDR: 1.000e+00
Coefficient: −3.92e−01
Value: B
Schleiferia
2.0
1.5
1.0
0.5
0.0
A (n=14)
B (n=15)
C (n=11)
surface

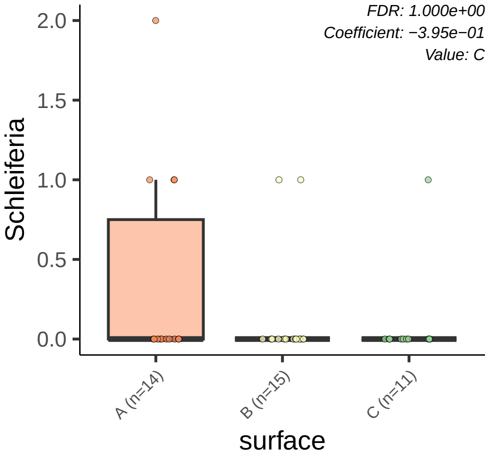

FDR: 1.000e+00
Coefficient: −3.95e−01
Value: C
Schleiferia
2.0
1.5
1.0
0.5
0.0
A (n=14)
B (n=15)
C (n=11)
surface

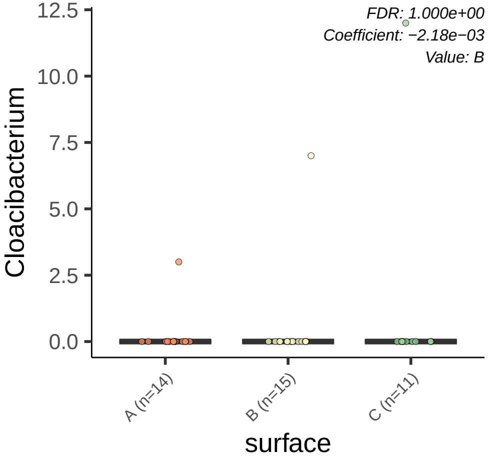
FDR: 1.000e+00
Coefficient: −2.18e−03
Value: B
Cloacibacterium
12.5
10.0
7.5
5.0
2.5
0.0
A (n=14)
B (n=15)
C (n=11)
surface

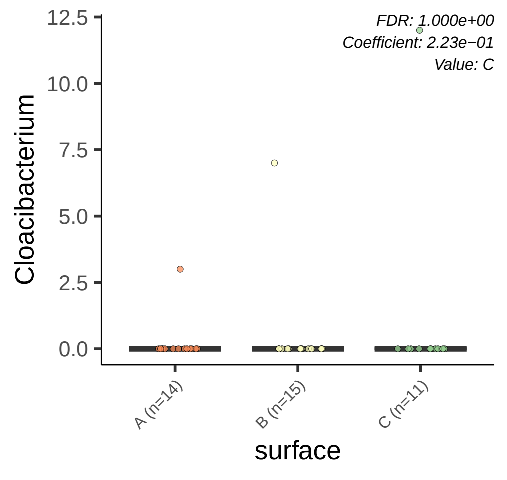
FDR: 1.000e+00
Coefficient: 2.23e−01
Value: C
Cloacibacterium
12.5
10.0
7.5
5.0
2.5
0.0
A (n=14)
B (n=15)
C (n=11)
surface

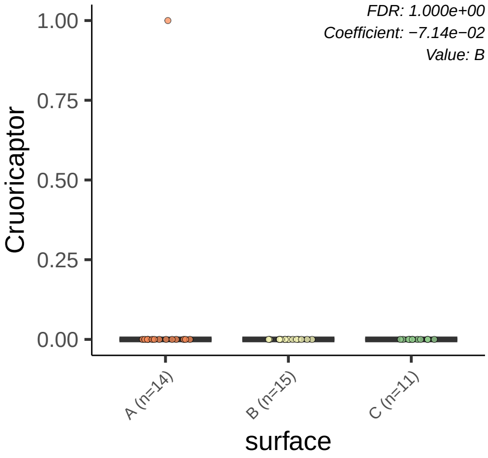
FDR: 1.000e+00
Coefficient: −7.14e−02
Value: B
Cruoricaptor
1.00
0.75
0.50
0.25
0.00
A (n=14)
B (n=15)
C (n=11)
surface

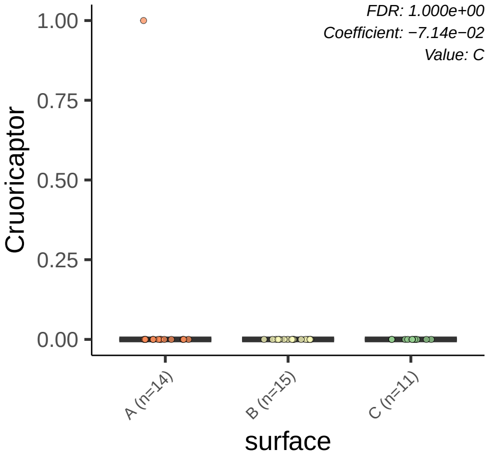
FDR: 1.000e+00
Coefficient: −7.14e−02
Value: C
Cruoricaptor
1.00
0.75
0.50
0.25
0.00
A (n=14)
B (n=15)
C (n=11)
surface

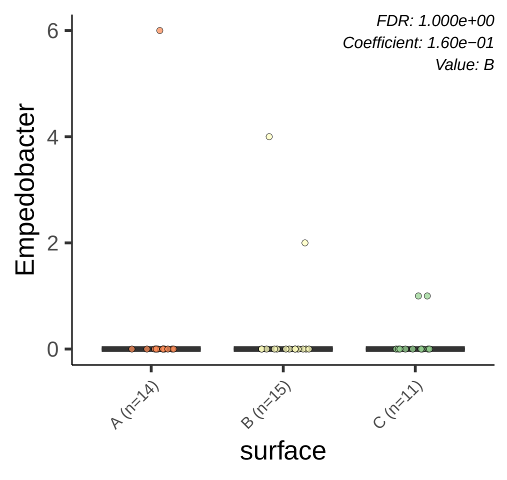
FDR: 1.000e+00
Coefficient: 1.60e−01
Value: B
Empedobacter
6
4
2
0
A (n=14)
B (n=15)
C (n=11)
surface

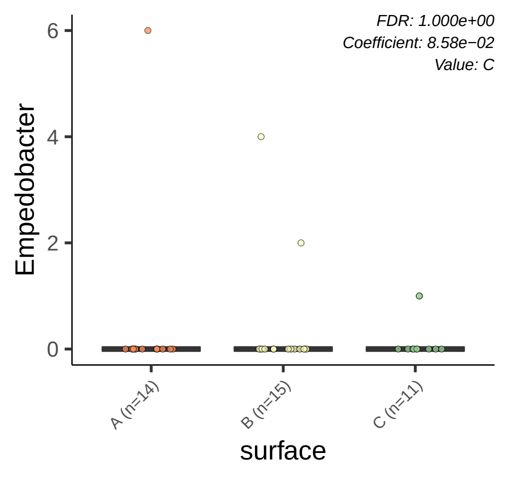

FDR: 1.000e+00
Coefficient: 8.58e−02
Value: C
Empedobacter
6
4
2
0
A (n=14)
B (n=15)
C (n=11)
surface

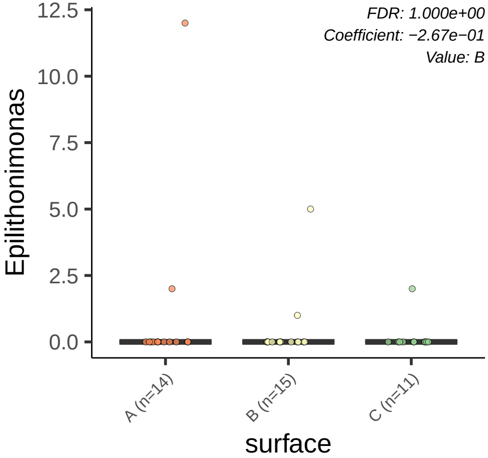
FDR: 1.000e+00
Coefficient: −2.67e−01
Value: B
Epilithonimonas
12.5
10.0
7.5
5.0
2.5
0.0
A (n=14)
B (n=15)
C (n=11)
surface

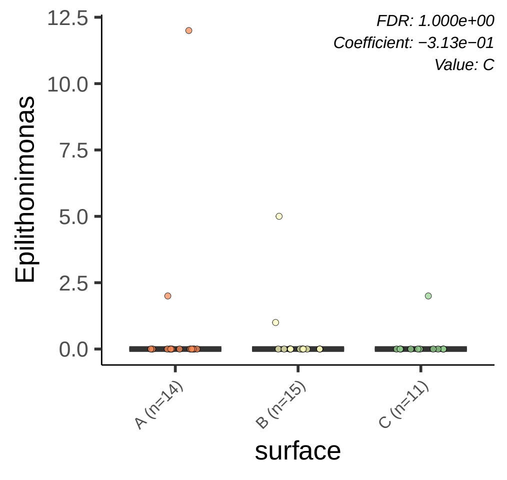
FDR: 1.000e+00
Coefficient: −3.13e−01
Value: C
Epilithonimonas
12.5
10.0
7.5
5.0
2.5
0.0
A (n=14)
B (n=15)
C (n=11)
surface

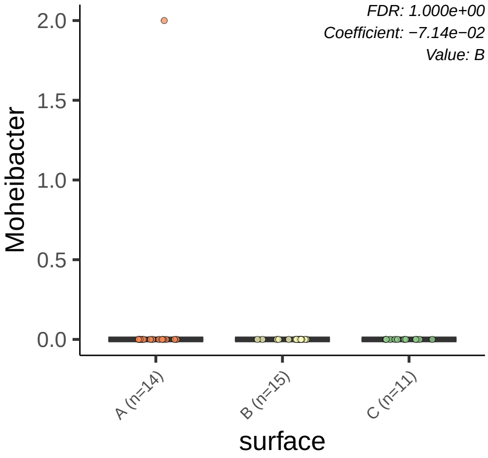
FDR: 1.000e+00
Coefficient: −7.14e−02
Value: B
Moheibacter
2.0
1.5
1.0
0.5
0.0
A (n=14)
B (n=15)
C (n=11)
surface

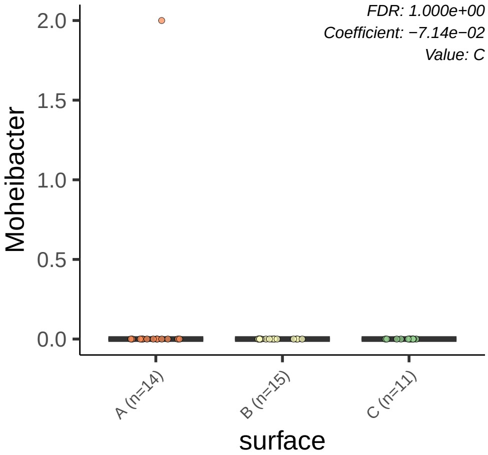

FDR: 1.000e+00
Coefficient: −7.14e−02
Value: C
Moheibacter
2.0
1.5
1.0
0.5
0.0
A (n=14)
B (n=15)
C (n=11)
surface

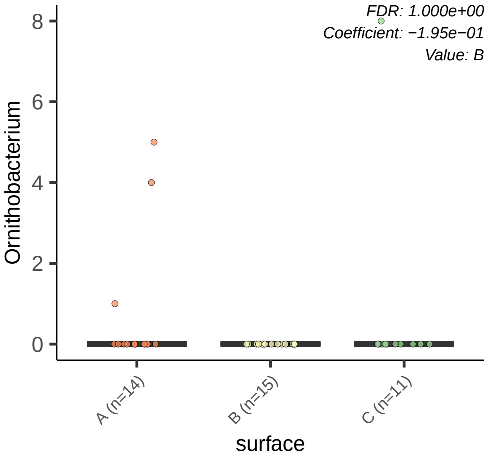
FDR: 1.000e+00
Coefficient: −1.95e−01
Value: B
Ornithobacterium
8
6
4
2
0
A (n=14)
B (n=15)
C (n=11)
surface

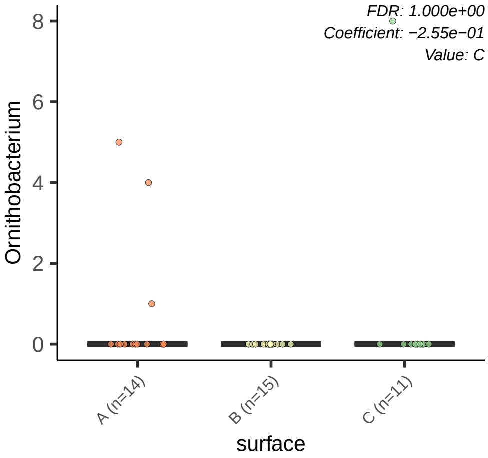
FDR: 1.000e+00
Coefficient: −2.55e−01
Value: C
Ornithobacterium
0
2
4
6
8
A (n=14)
B (n=15)
C (n=11)
surface

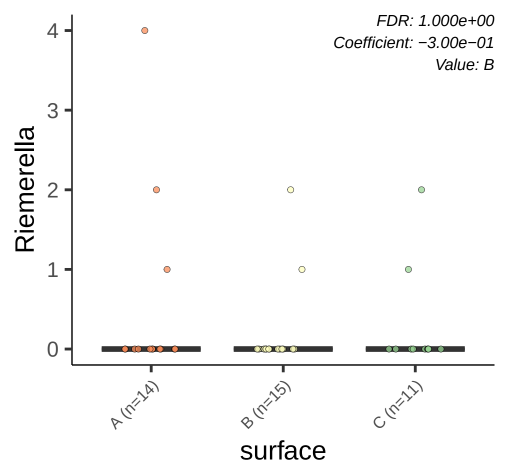
FDR: 1.000e+00
Coefficient: −3.00e−01
Value: B
Riemerella
0
1
2
3
4
A (n=14)
B (n=15)
C (n=11)
surface

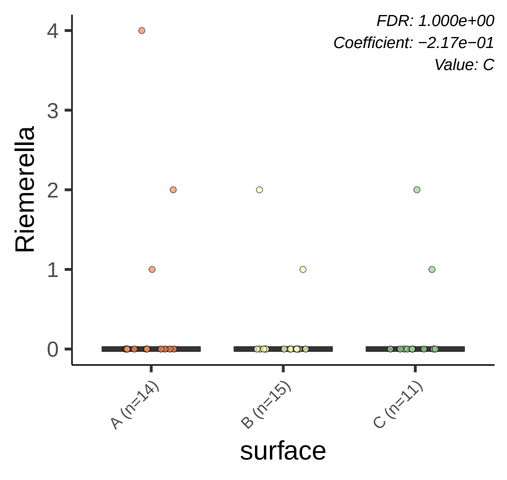
FDR: 1.000e+00
Coefficient: −2.17e−01
Value: C
Riemerella
0
1
2
3
4
A (n=14)
B (n=15)
C (n=11)
surface

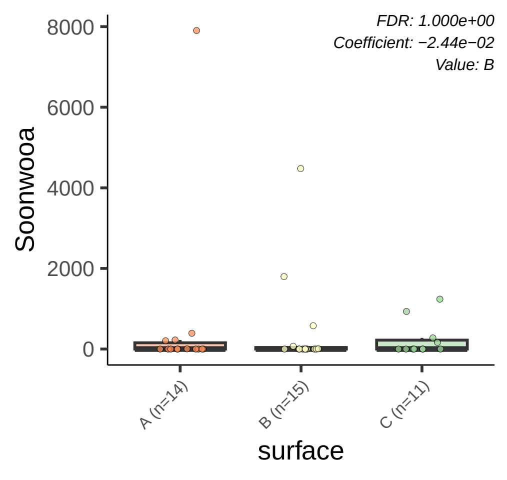

FDR: 1.000e+00
Coefficient: −2.44e−02
Value: B
Soonwooa
8000
6000
4000
2000
0
A (n=14)
B (n=15)
C (n=11)
surface

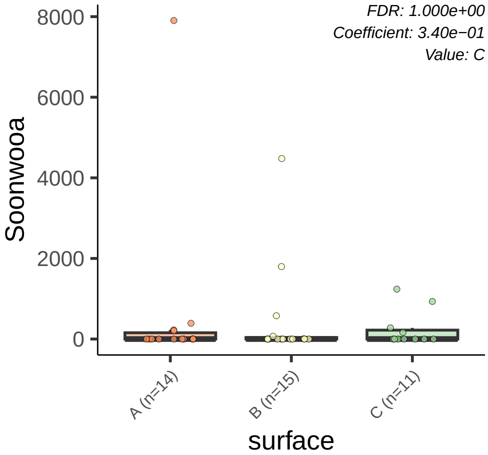
FDR: 1.000e+00
Coefficient: 3.40e−01
Value: C
Soonwooa
8000
6000
4000
2000
0
A (n=14)
B (n=15)
C (n=11)
surface

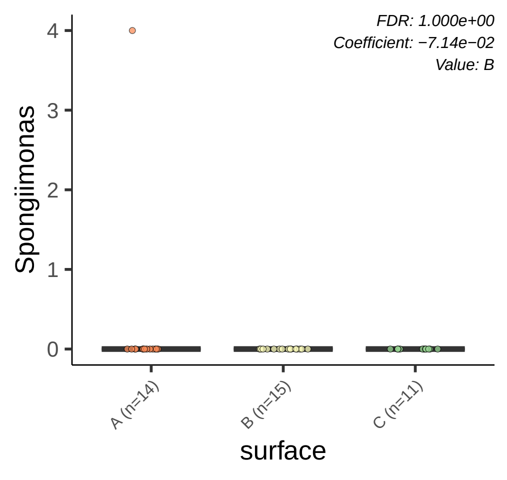
FDR: 1.000e+00
Coefficient: −7.14e−02
Value: B
Spongiimonas
0
1
2
3
4
A (n=14)
B (n=15)
C (n=11)
surface

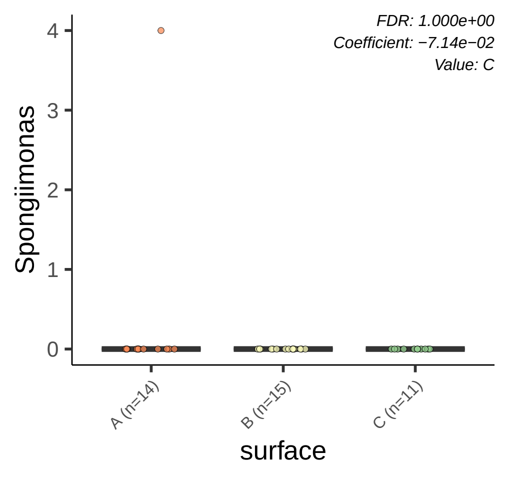
FDR: 1.000e+00
Coefficient: −7.14e−02
Value: C
Spongiimonas
surface
A (n=14)
B (n=15)
C (n=11)
0
1
2
3
4

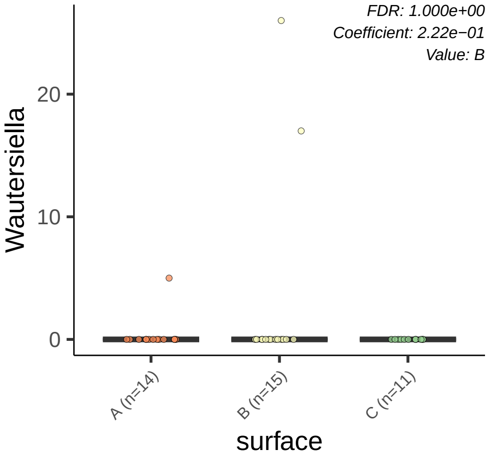
FDR: 1.000e+00
Coefficient: 2.22e−01
Value: B
Wautersiella
20
10
0
A (n=14)
B (n=15)
C (n=11)
surface

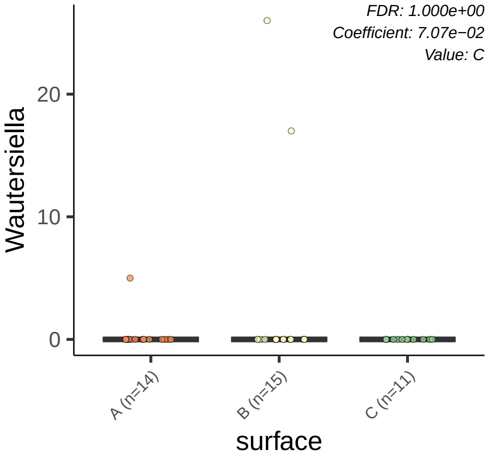
FDR: 1.000e+00
Coefficient: 7.07e−02
Value: C
Wautersiella
20
10
0
A (n=14)
B (n=15)
C (n=11)
surface

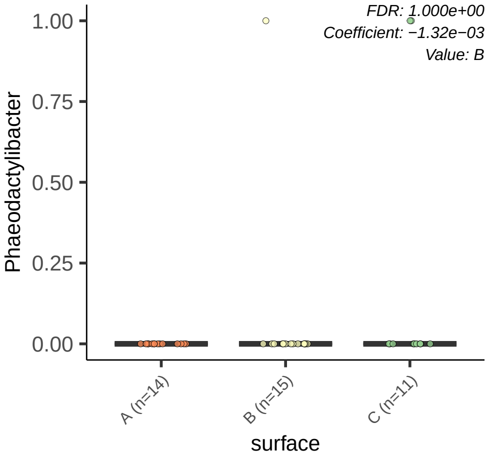

FDR: 1.000e+00
Coefficient: −1.32e−03
Value: B
Phaeodactylibacter
1.00
0.75
0.50
0.25
0.00
A (n=14)
B (n=15)
C (n=11)
surface

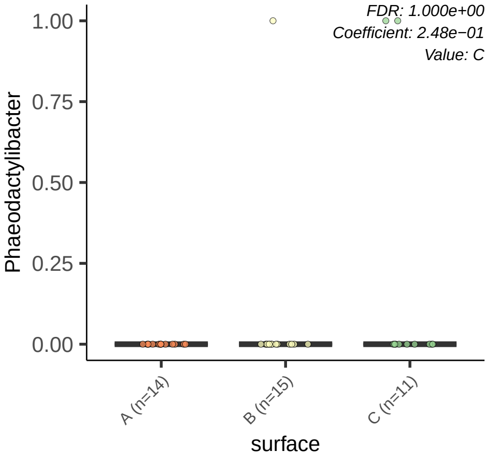

FDR: 1.000e+00
Coefficient: 2.48e−01
Value: C
Phaeodactylibacter
1.00
0.75
0.50
0.25
0.00
A (n=14)
B (n=15)
C (n=11)
surface

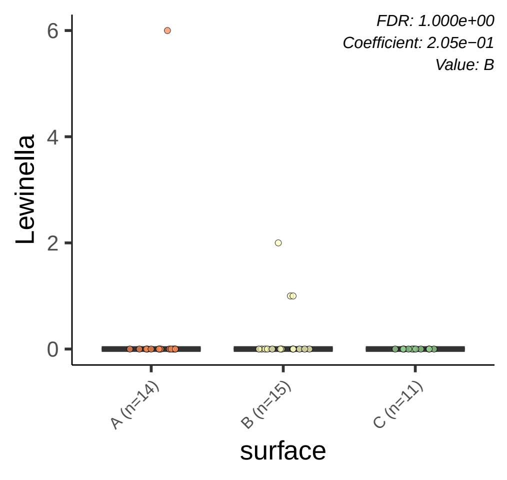

FDR: 1.000e+00
Coefficient: 2.05e−01
Value: B
Lewinella
6
4
2
0
A (n=14)
B (n=15)
C (n=11)
surface

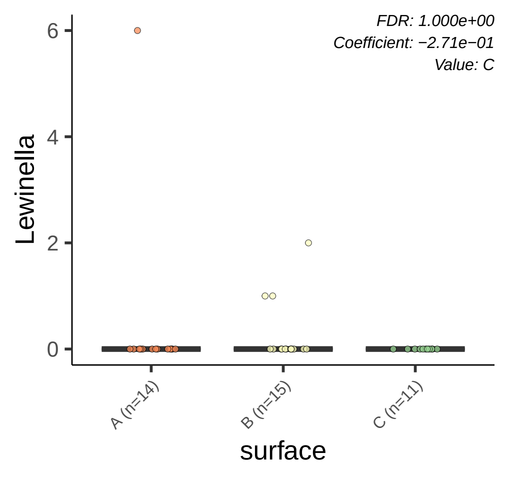
FDR: 1.000e+00
Coefficient: −2.71e−01
Value: C
Lewinella
6
4
2
0
A (n=14)
B (n=15)
C (n=11)
surface

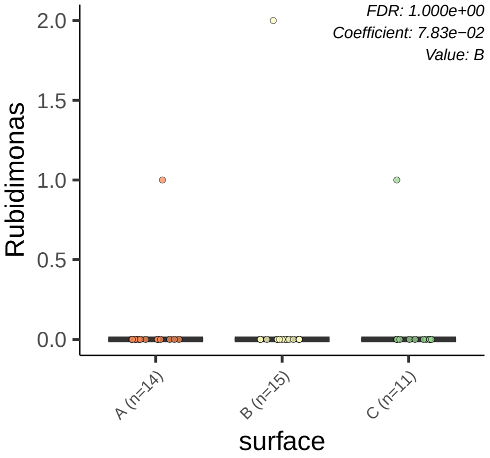
FDR: 1.000e+00
Coefficient: 7.83e−02
Value: B
Rubidimonas
2.0
1.5
1.0
0.5
0.0
A (n=14)
B (n=15)
C (n=11)
surface

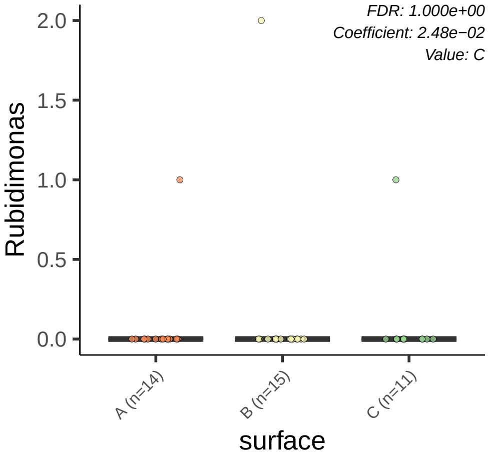
FDR: 1.000e+00
Coefficient: 2.48e−02
Value: C
Rubidimonas
2.0
1.5
1.0
0.5
0.0
A (n=14)
B (n=15)
C (n=11)
surface

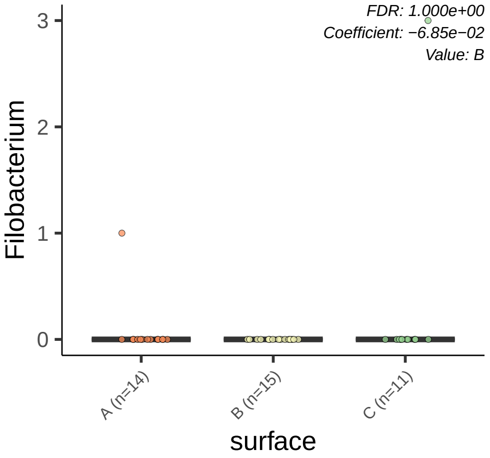
FDR: 1.000e+00
Coefficient: −6.85e−02
Value: B
Filobacterium
3
2
1
0
A (n=14)
B (n=15)
C (n=11)
surface

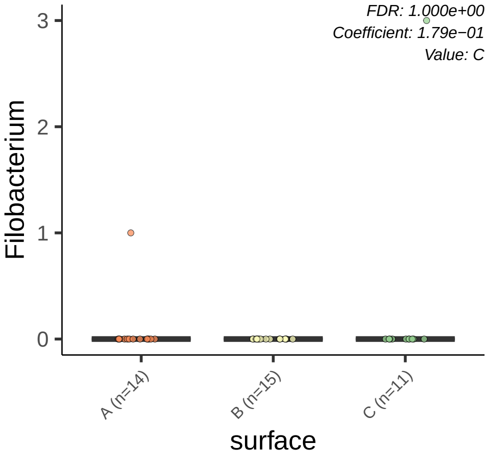
FDR: 1.000e+00
Coefficient: 1.79e−01
Value: C
Filobacterium
0
1
2
3
A (n=14)
B (n=15)
C (n=11)
surface

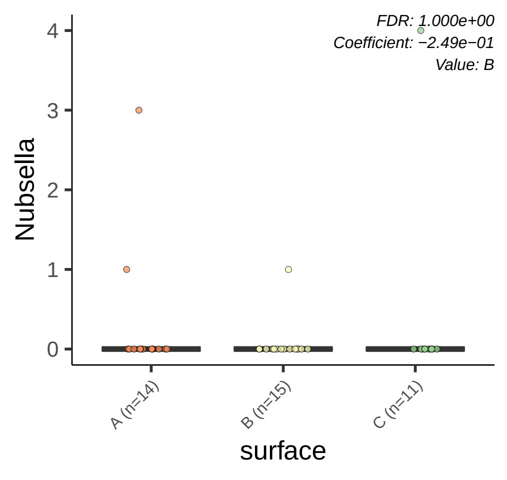
FDR: 1.000e+00
Coefficient: −2.49e−01
Value: B
Nubsella
4
3
2
1
0
A (n=14)
B (n=15)
C (n=11)
surface

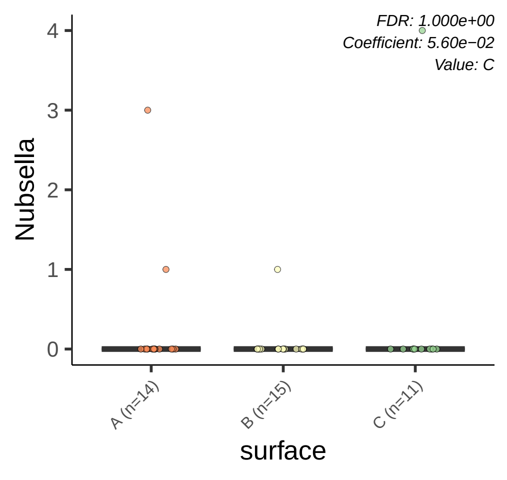

FDR: 1.000e+00
Coefficient: 5.60e−02
Value: C
Nubsella
4
3
2
1
0
A (n=14)
B (n=15)
C (n=11)
surface

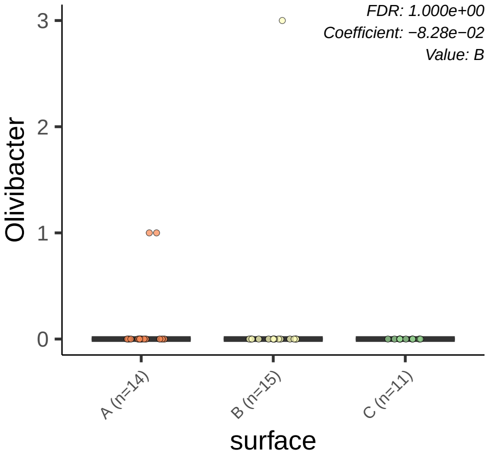

FDR: 1.000e+00
Coefficient: −8.28e−02
Value: B
Olivibacter
3
2
1
0
A (n=14)
B (n=15)
C (n=11)
surface

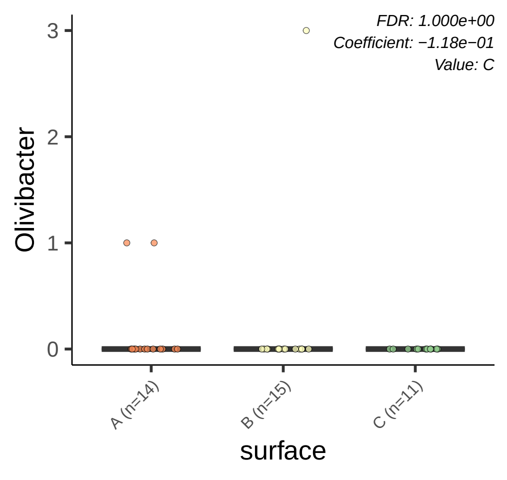

FDR: 1.000e+00
Coefficient: −1.18e−01
Value: C
Olivibacter
3
2
1
0
A (n=14)
B (n=15)
C (n=11)
surface

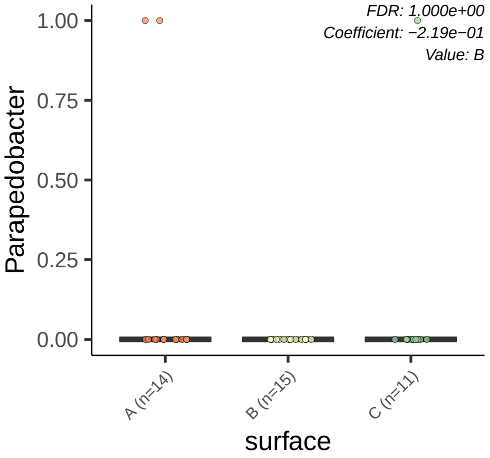
FDR: 1.000e+00
Coefficient: −2.19e−01
Value: B
Parapedobacter
1.00
0.75
0.50
0.25
0.00
A (n=14)
B (n=15)
C (n=11)
surface

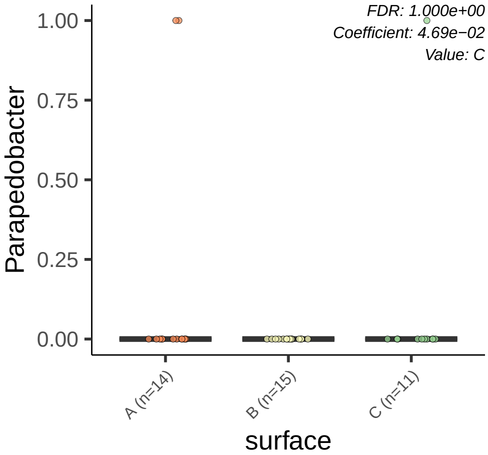

FDR: 1.000e+00
Coefficient: 4.69e−02
Value: C
1.00
0.75
0.50
0.25
0.00
Parapedobacter
A (n=14)
B (n=15)
C (n=11)
surface

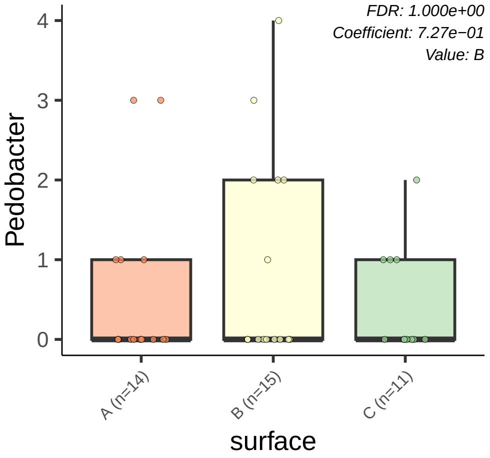

FDR: 1.000e+00
Coefficient: 7.27e−01
Value: B
Pedobacter
0
1
2
3
4
A (n=14)
B (n=15)
C (n=11)
surface

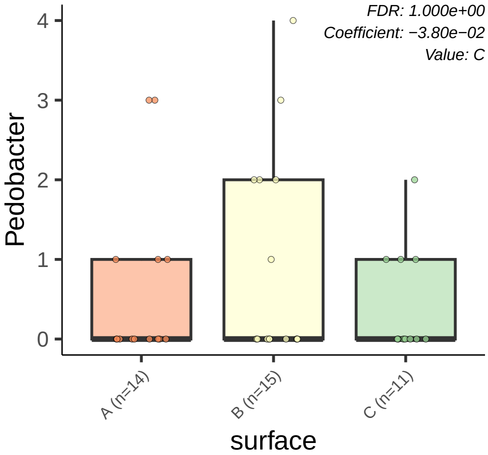
FDR: 1.000e+00
Coefficient: −3.80e−02
Value: C
Pedobacter
0
1
2
3
4
A (n=14)
B (n=15)
C (n=11)
surface

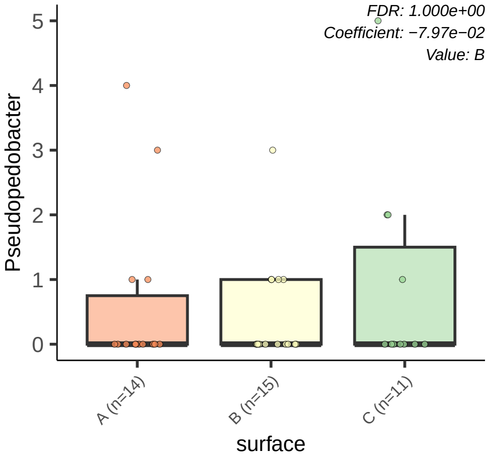

FDR: 1.000e+00
Coefficient: −7.97e−02
Value: B
Pseudopedobacter
0
1
2
3
4
5
A (n=14)
B (n=15)
C (n=11)
surface

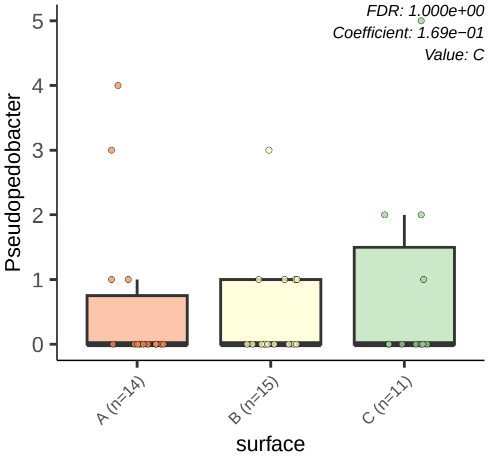

FDR: 1.000e+00
Coefficient: 1.69e−01
Value: C
Pseudopedobacter
0
1
2
3
4
5
A (n=14)
B (n=15)
C (n=11)
surface

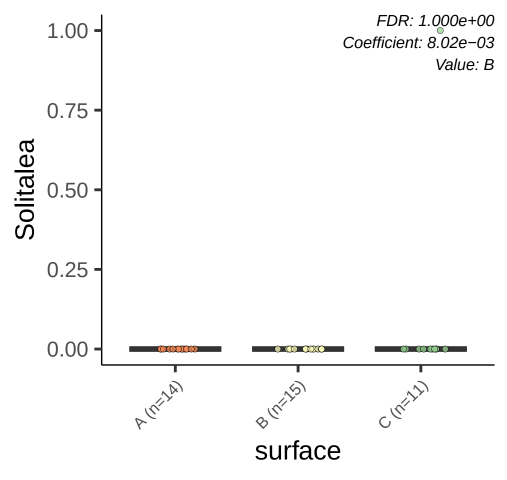
FDR: 1.000e+00
Coefficient: 8.02e−03
Value: B
Solitalea
1.00
0.75
0.50
0.25
0.00
A (n=14)
B (n=15)
C (n=11)
surface

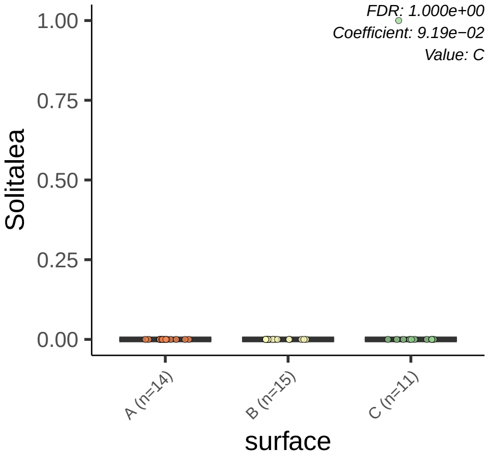
FDR: 1.000e+00
Coefficient: 9.19e−02
Value: C
1.00
0.75
0.50
0.25
0.00
Solitalea
A (n=14)
B (n=15)
C (n=11)
surface

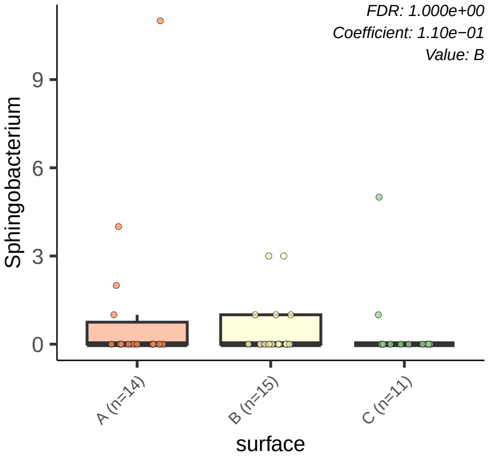
FDR: 1.000e+00
Coefficient: 1.10e−01
Value: B
Sphingobacterium
0
3
6
9
A (n=14)
B (n=15)
C (n=11)
surface

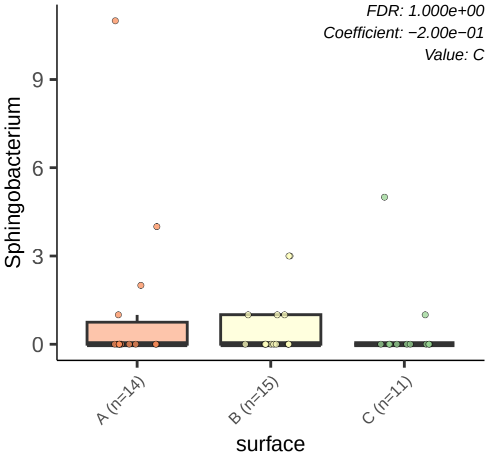
FDR: 1.000e+00
Coefficient: −2.00e−01
Value: C
Sphingobacterium
9
6
3
0
A (n=14)
B (n=15)
C (n=11)
surface

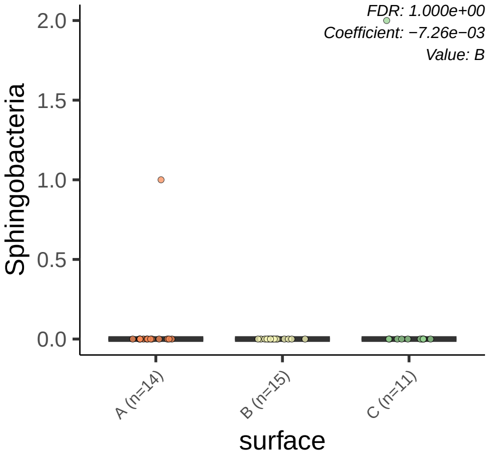
FDR: 1.000e+00
Coefficient: −7.26e−03
Value: B
Sphingobacteria
2.0
1.5
1.0
0.5
0.0
A (n=14)
B (n=15)
C (n=11)
surface

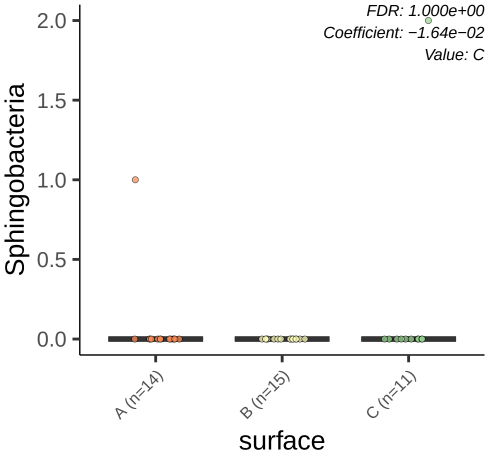
FDR: 1.000e+00
Coefficient: −1.64e−02
Value: C
Sphingobacteria
2.0
1.5
1.0
0.5
0.0
A (n=14)
B (n=15)
C (n=11)
surface

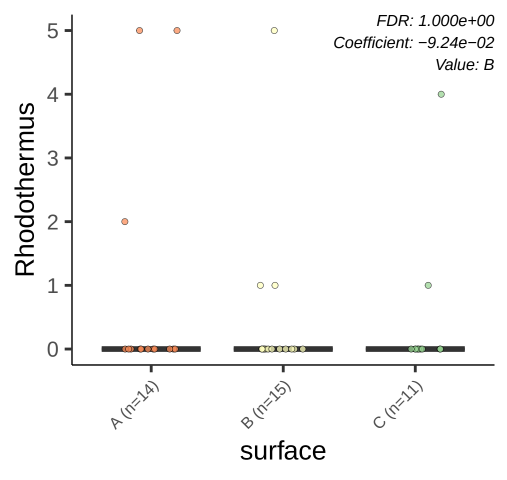
FDR: 1.000e+00
Coefficient: −9.24e−02
Value: B
Rhodothermus
0
1
2
3
4
5
A (n=14)
B (n=15)
C (n=11)
surface

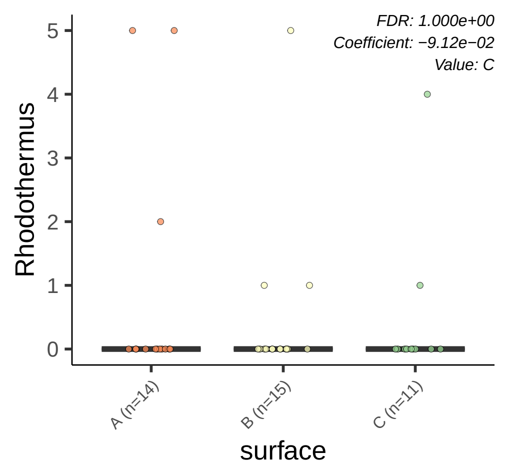

FDR: 1.000e+00
Coefficient: −9.12e−02
Value: C
Rhodothermus
5
4
3
2
1
0
A (n=14)
B (n=15)
C (n=11)
surface

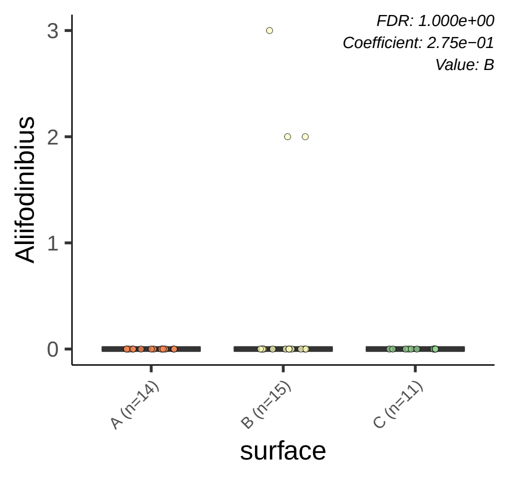
FDR: 1.000e+00
Coefficient: 2.75e−01
Value: B
Aliifodinibius
3
2
1
0
A (n=14)
B (n=15)
C (n=11)
surface

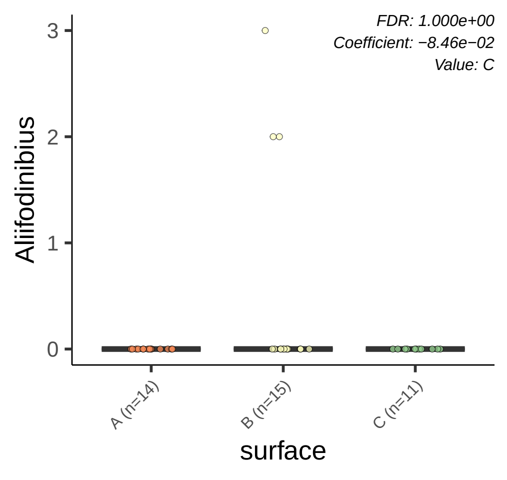

FDR: 1.000e+00
Coefficient: −8.46e−02
Value: C
Aliifodinibius
3
2
1
0
A (n=14)
B (n=15)
C (n=11)
surface

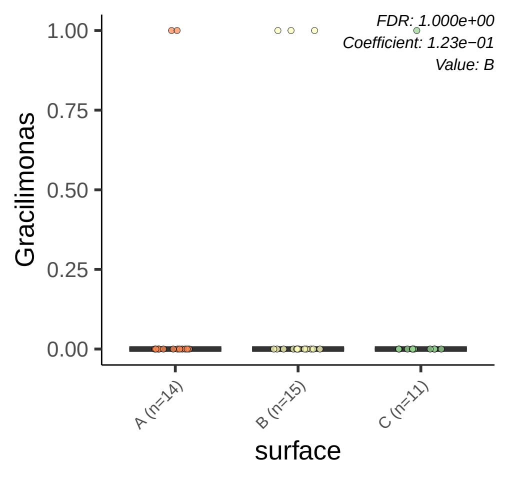

FDR: 1.000e+00
Coefficient: 1.23e−01
Value: B
Gracilimonas
1.00
0.75
0.50
0.25
0.00
A (n=14)
B (n=15)
C (n=11)
surface

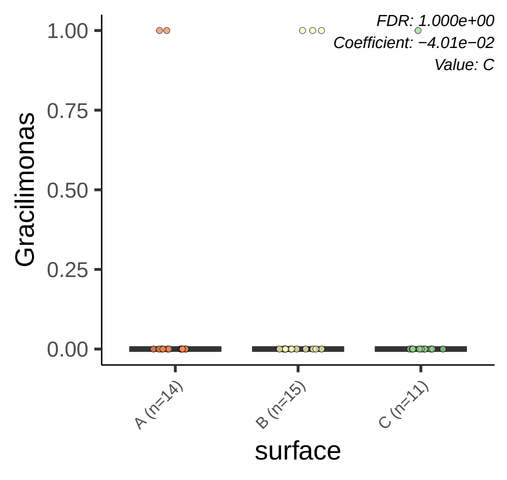
FDR: 1.000e+00
Coefficient: −4.01e−02
Value: C
Gracilimonas
1.00
0.75
0.50
0.25
0.00
A (n=14)
B (n=15)
C (n=11)
surface

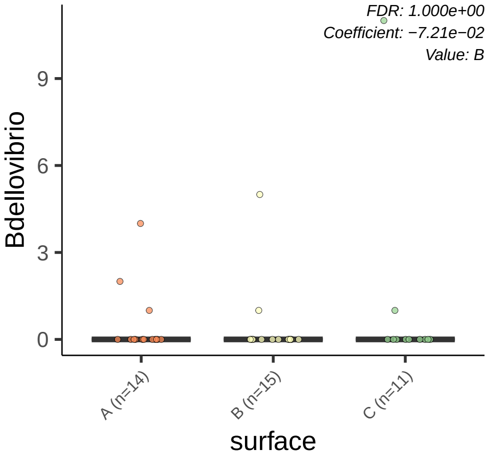
FDR: 1.000e+00
Coefficient: −7.21e−02
Value: B
Bdellovibrio
9
6
3
0
A (n=14)
B (n=15)
C (n=11)
surface

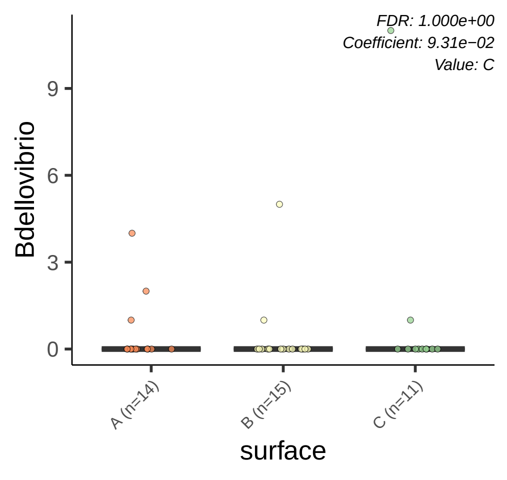
FDR: 1.000e+00
Coefficient: 9.31e−02
Value: C
Bdellovibrio
0
3
6
9
A (n=14)
B (n=15)
C (n=11)
surface

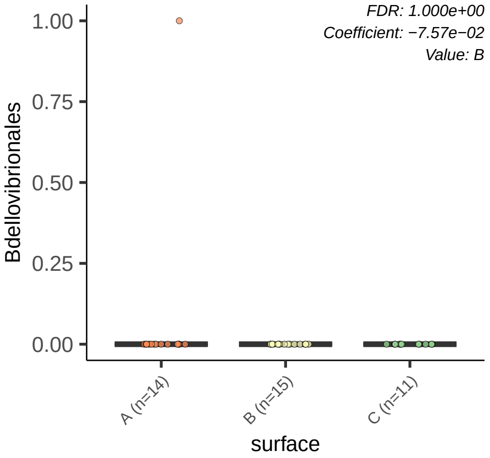
FDR: 1.000e+00
Coefficient: −7.57e−02
Value: B
Bdellovibrionales
1.00
0.75
0.50
0.25
0.00
A (n=14)
B (n=15)
C (n=11)
surface

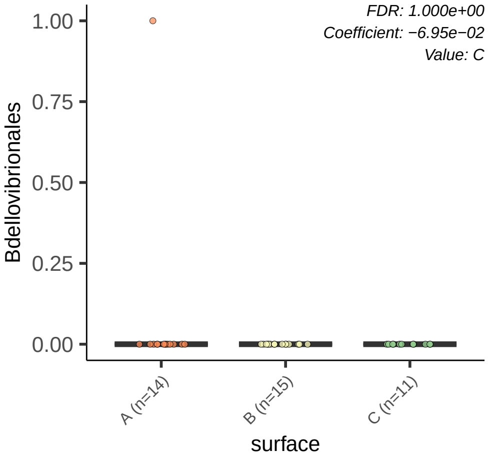
FDR: 1.000e+00
Coefficient: −6.95e−02
Value: C
Bdellovibrionales
1.00
0.75
0.50
0.25
0.00
A (n=14)
B (n=15)
C (n=11)
surface

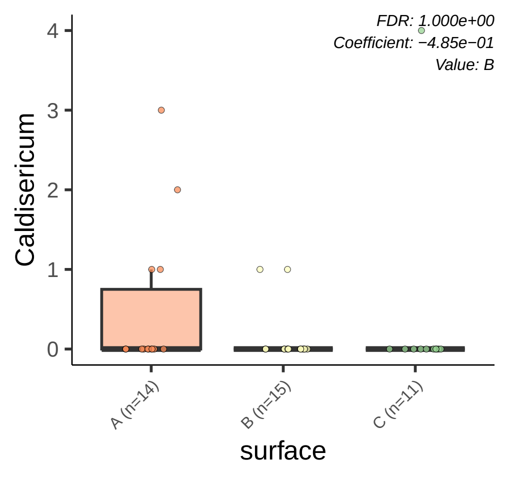

FDR: 1.000e+00
Coefficient: −4.85e−01
Value: B
Caldisericum
4
3
2
1
0
A (n=14)
B (n=15)
C (n=11)
surface

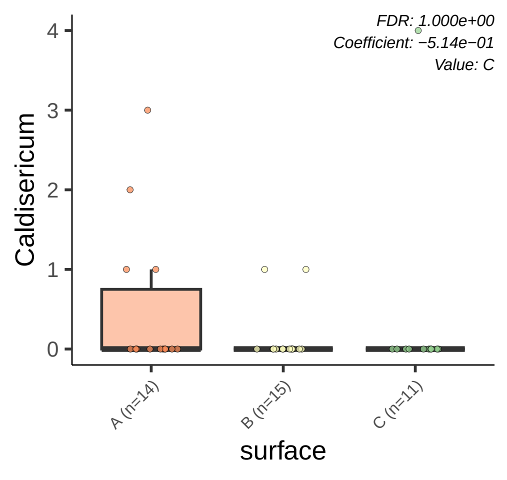

FDR: 1.000e+00
Coefficient: −5.14e−01
Value: C
Caldisericum
0
1
2
3
4
A (n=14)
B (n=15)
C (n=11)
surface

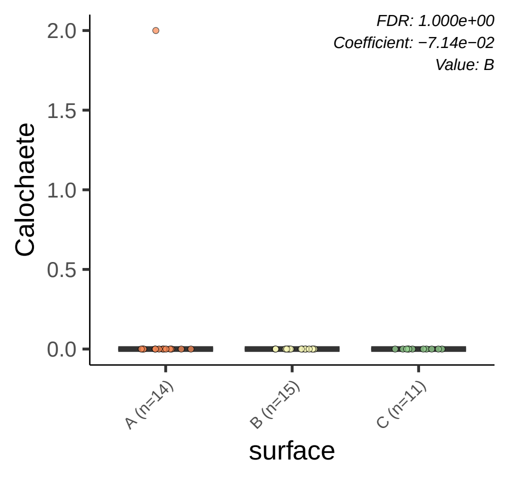
FDR: 1.000e+00
Coefficient: −7.14e−02
Value: B
Calochaete
2.0
1.5
1.0
0.5
0.0
A (n=14)
B (n=15)
C (n=11)
surface

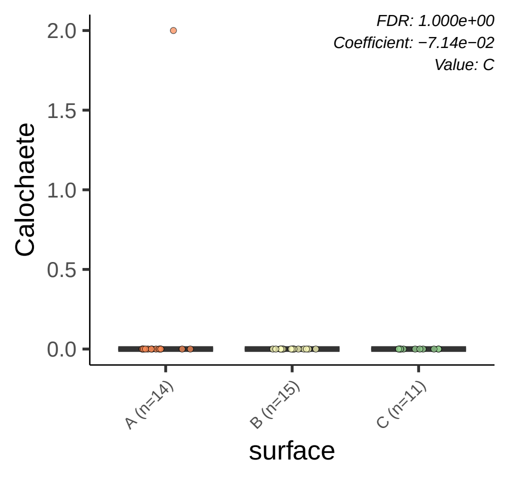
FDR: 1.000e+00
Coefficient: −7.14e−02
Value: C
2.0
1.5
1.0
0.5
0.0
Calochaete
A (n=14)
B (n=15)
C (n=11)
surface

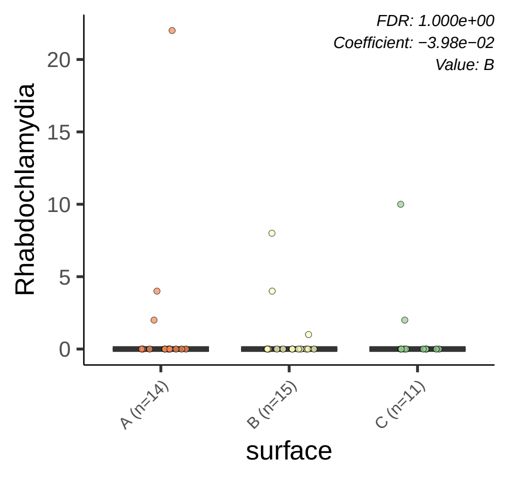
FDR: 1.000e+00
Coefficient: −3.98e−02
Value: B
Rhabdochlamydia
0
5
10
15
20
A (n=14)
B (n=15)
C (n=11)
surface

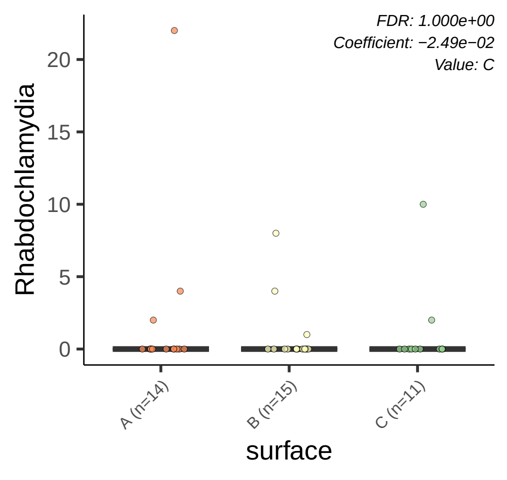
FDR: 1.000e+00
Coefficient: −2.49e−02
Value: C
Rhabdochlamydia
0
5
10
15
20
A (n=14)
B (n=15)
C (n=11)
surface

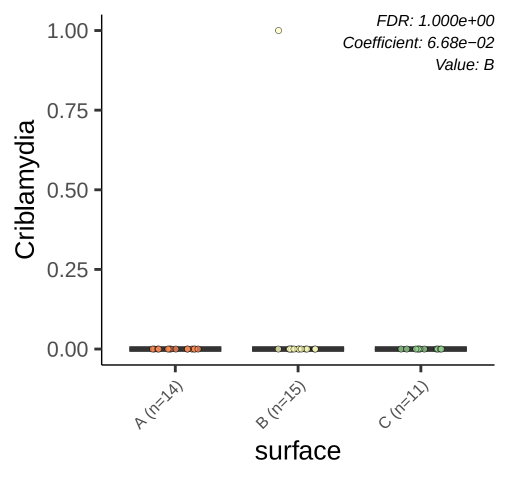
FDR: 1.000e+00
Coefficient: 6.68e−02
Value: B
Criblamydia
1.00
0.75
0.50
0.25
0.00
A (n=14)
B (n=15)
C (n=11)
surface

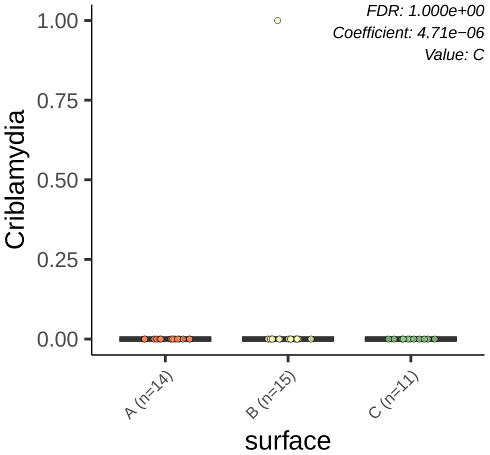
FDR: 1.000e+00
Coefficient: 4.71e−06
Value: C
1.00
0.75
0.50
0.25
0.00
Criblamydia
A (n=14)
B (n=15)
C (n=11)
surface

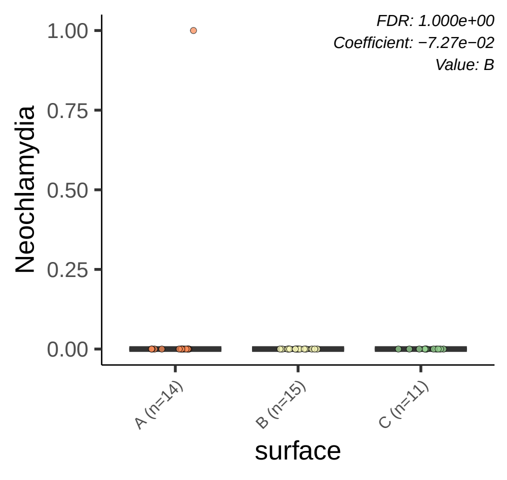
FDR: 1.000e+00
Coefficient: −7.27e−02
Value: B
Neochlamydia
1.00
0.75
0.50
0.25
0.00
A (n=14)
B (n=15)
C (n=11)
surface

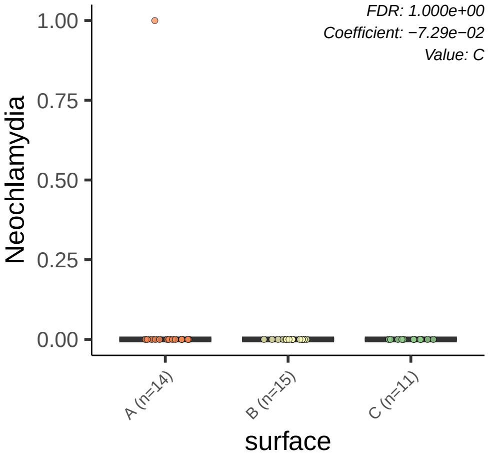
FDR: 1.000e+00
Coefficient: −7.29e−02
Value: C
Neochlamydia
1.00
0.75
0.50
0.25
0.00
A (n=14)
B (n=15)
C (n=11)
surface

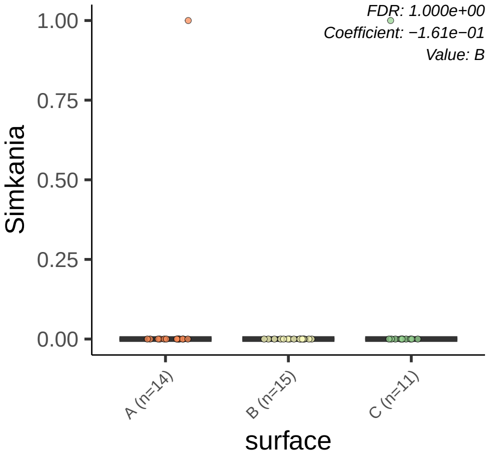
FDR: 1.000e+00
Coefficient: −1.61e−01
Value: B
Simkania
1.00
0.75
0.50
0.25
0.00
A (n=14)
B (n=15)
C (n=11)
surface

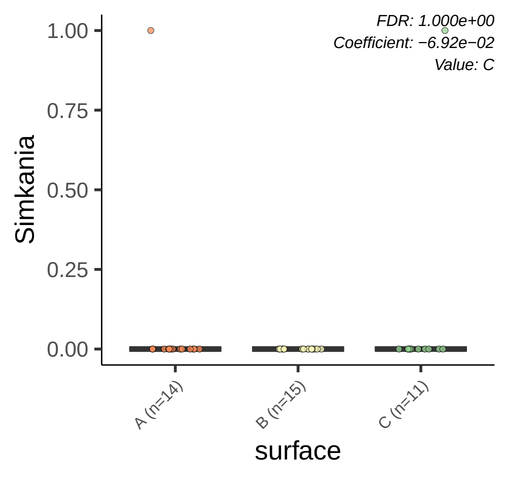
FDR: 1.000e+00
Coefficient: −6.92e−02
Value: C
Simkania
1.00
0.75
0.50
0.25
0.00
A (n=14)
B (n=15)
C (n=11)
surface

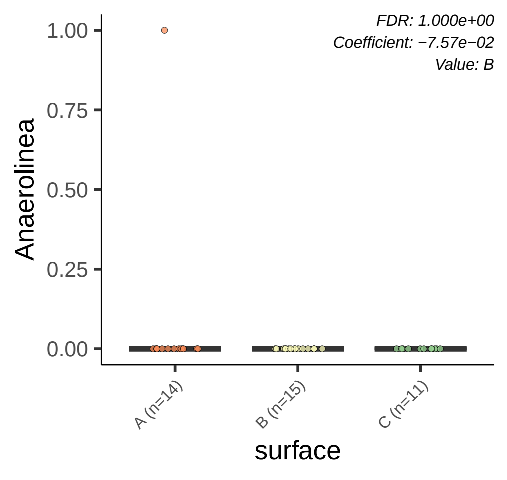
FDR: 1.000e+00
Coefficient: −7.57e−02
Value: B
Anaerolinea
1.00
0.75
0.50
0.25
0.00
A (n=14)
B (n=15)
C (n=11)
surface

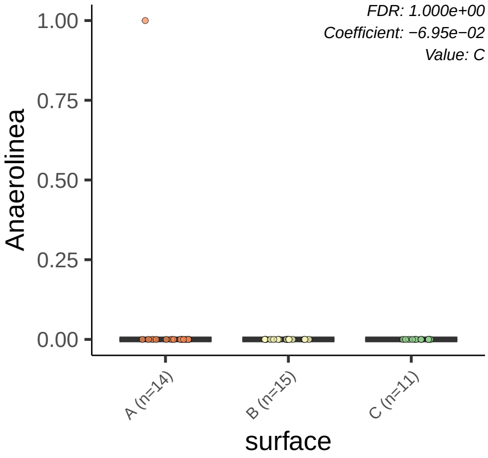
FDR: 1.000e+00
Coefficient: −6.95e−02
Value: C
Anaerolinea
1.00
0.75
0.50
0.25
0.00
A (n=14)
B (n=15)
C (n=11)
surface

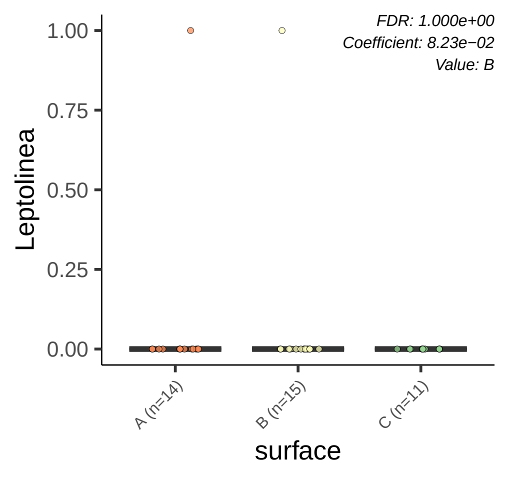
FDR: 1.000e+00
Coefficient: 8.23e−02
Value: B
Leptolinea
1.00
0.75
0.50
0.25
0.00
A (n=14)
B (n=15)
C (n=11)
surface

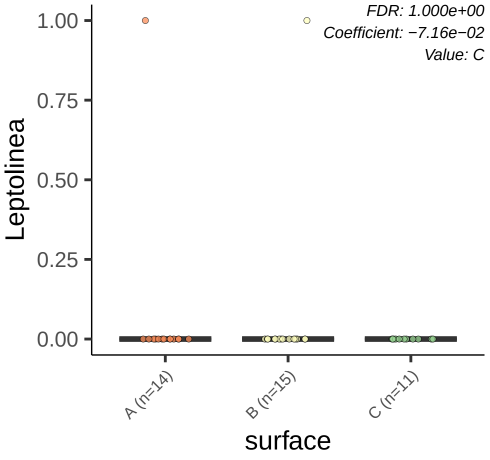
FDR: 1.000e+00
Coefficient: −7.16e−02
Value: C
Leptolinea
1.00
0.75
0.50
0.25
0.00
A (n=14)
B (n=15)
C (n=11)
surface

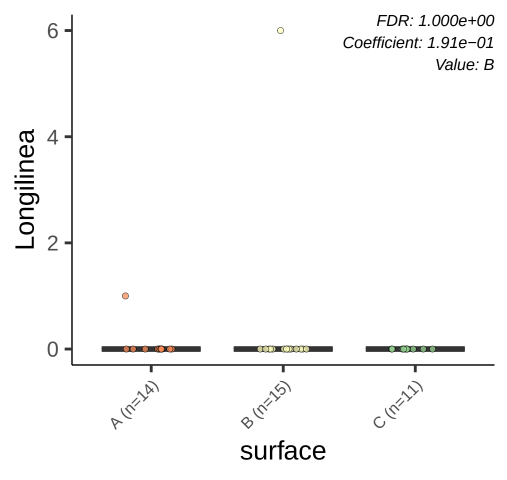

FDR: 1.000e+00
Coefficient: 1.91e−01
Value: B
Longilinea
0
2
4
6
A (n=14)
B (n=15)
C (n=11)
surface

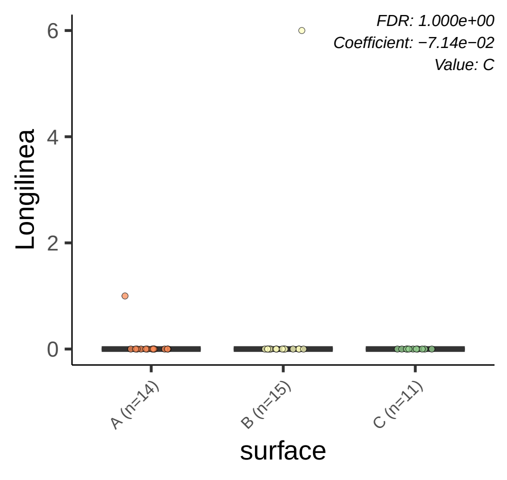
FDR: 1.000e+00
Coefficient: −7.14e−02
Value: C
Longilinea
0
2
4
6
A (n=14)
B (n=15)
C (n=11)
surface

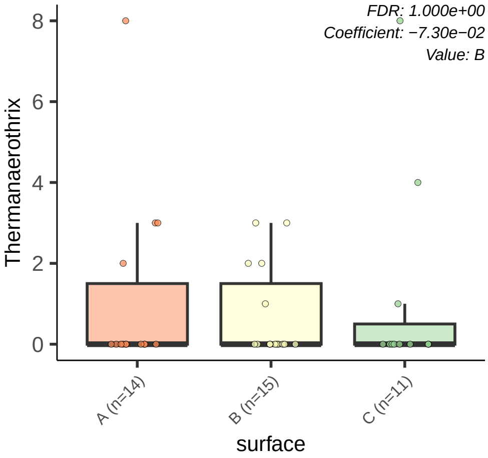
FDR: 1.000e+00
Coefficient: −7.30e−02
Value: B
Thermanaerothrix
0
2
4
6
8
A (n=14)
B (n=15)
C (n=11)
surface

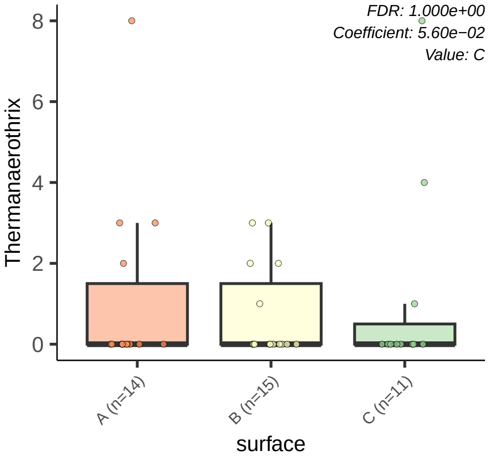

FDR: 1.000e+00
Coefficient: 5.60e−02
Value: C
Thermanaerothrix
0
2
4
6
8
A (n=14)
B (n=15)
C (n=11)
surface

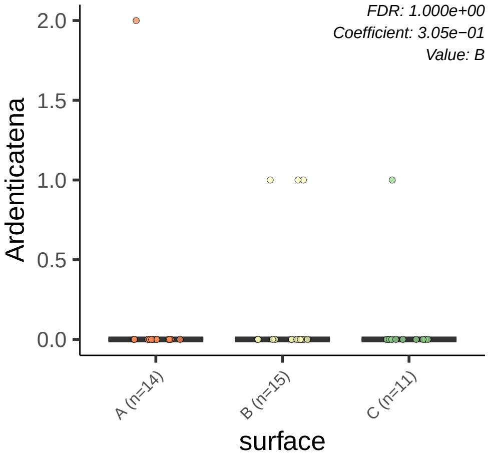

FDR: 1.000e+00
Coefficient: 3.05e−01
Value: B
Ardenticatena
2.0
1.5
1.0
0.5
0.0
A (n=14)
B (n=15)
C (n=11)
surface

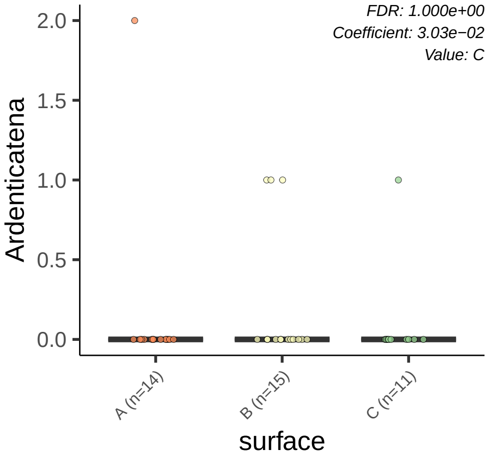

FDR: 1.000e+00
Coefficient: 3.03e−02
Value: C
Ardenticatena
2.0
1.5
1.0
0.5
0.0
A (n=14)
B (n=15)
C (n=11)
surface

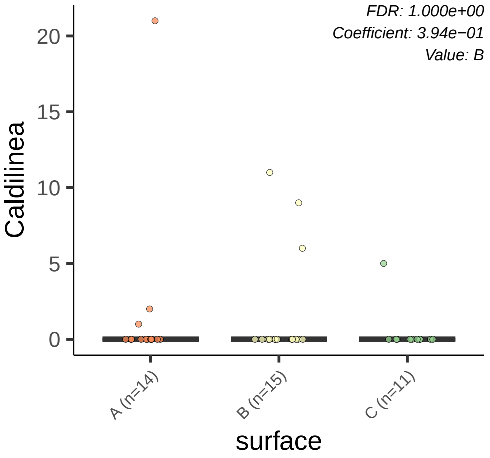

FDR: 1.000e+00
Coefficient: 3.94e−01
Value: B
Caldilinea
20
15
10
5
0
A (n=14)
B (n=15)
C (n=11)
surface

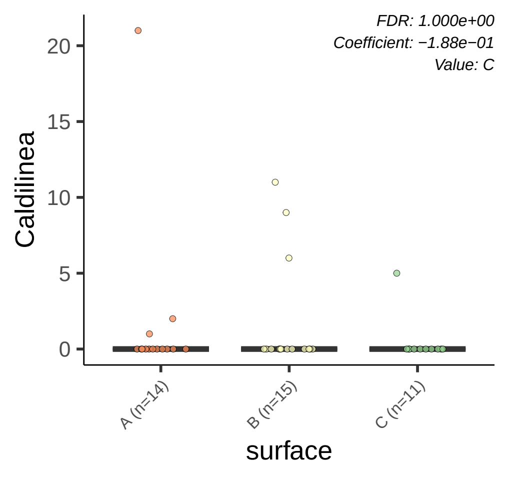

FDR: 1.000e+00
Coefficient: −1.88e−01
Value: C
Caldilinea
20
15
10
5
0
A (n=14)
B (n=15)
C (n=11)
surface

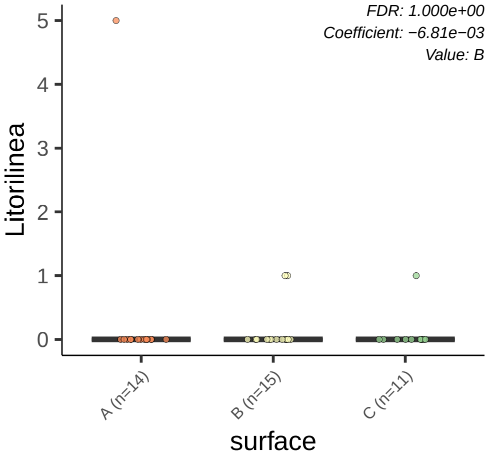

FDR: 1.000e+00
Coefficient: −6.81e−03
Value: B
Litorilinea
5
4
3
2
1
0
A (n=14)
B (n=15)
C (n=11)
surface

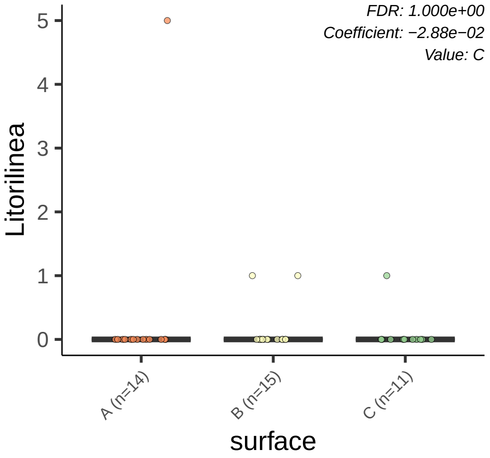

FDR: 1.000e+00
Coefficient: −2.88e−02
Value: C
Litorilinea
5
4
3
2
1
0
A (n=14)
B (n=15)
C (n=11)
surface

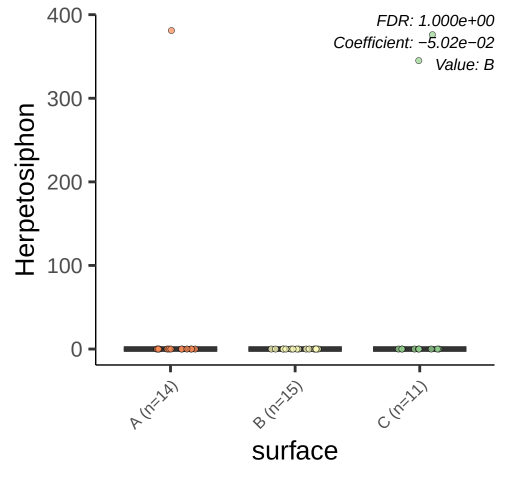
FDR: 1.000e+00
Coefficient: −5.02e−02
Value: B
Herpetosiphon
400
300
200
100
0
A (n=14)
B (n=15)
C (n=11)
surface

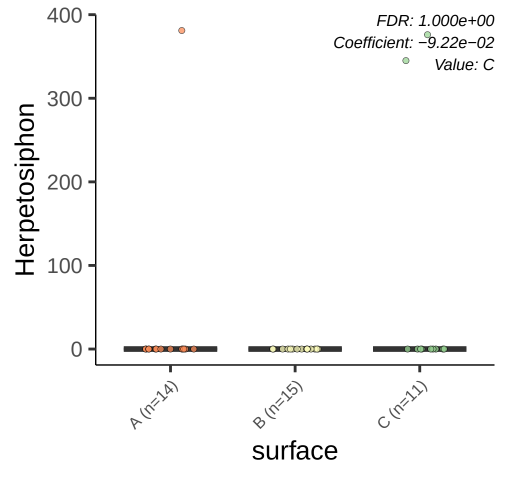

FDR: 1.000e+00
Coefficient: −9.22e−02
Value: C
400
300
200
100
0
Herpetosiphon
A (n=14)
B (n=15)
C (n=11)
surface

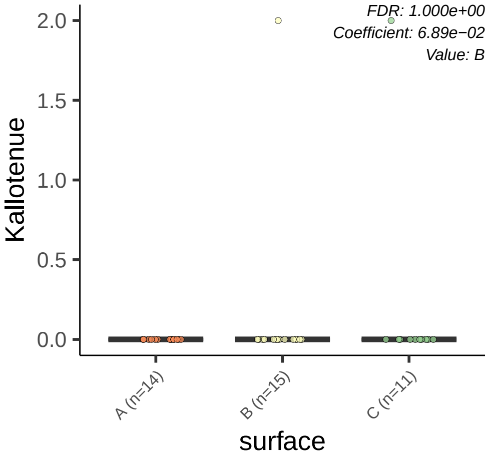
FDR: 1.000e+00
Coefficient: 6.89e−02
Value: B
Kallotenue
2.0
1.5
1.0
0.5
0.0
A (n=14)
B (n=15)
C (n=11)
surface

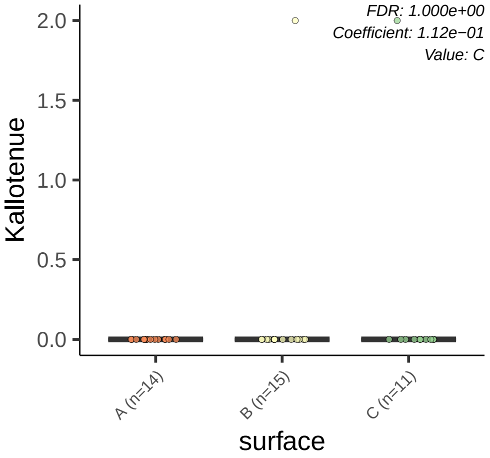
FDR: 1.000e+00
Coefficient: 1.12e−01
Value: C
Kallotenue
2.0
1.5
1.0
0.5
0.0
A (n=14)
B (n=15)
C (n=11)
surface

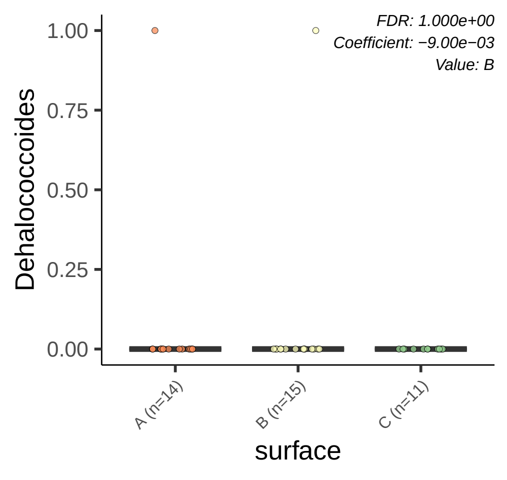
FDR: 1.000e+00
Coefficient: −9.00e−03
Value: B
Dehalococcoides
1.00
0.75
0.50
0.25
0.00
A (n=14)
B (n=15)
C (n=11)
surface

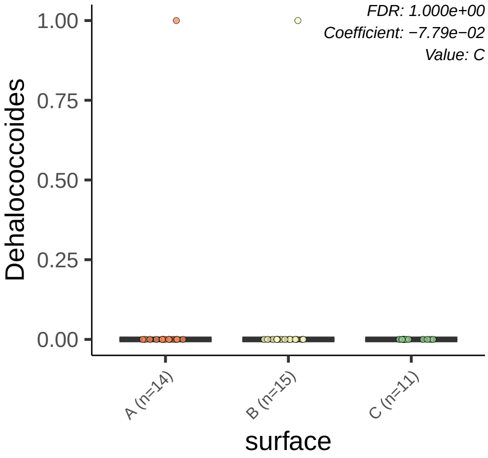
FDR: 1.000e+00
Coefficient: −7.79e−02
Value: C
Dehalococcoides
1.00
0.75
0.50
0.25
0.00
A (n=14)
B (n=15)
C (n=11)
surface

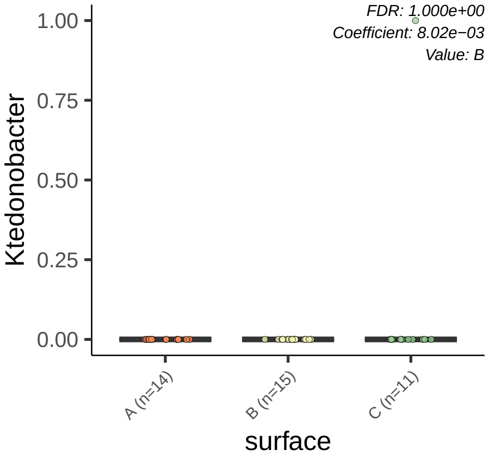
FDR: 1.000e+00
Coefficient: 8.02e−03
Value: B
Ktedonobacter
1.00
0.75
0.50
0.25
0.00
A (n=14)
B (n=15)
C (n=11)
surface

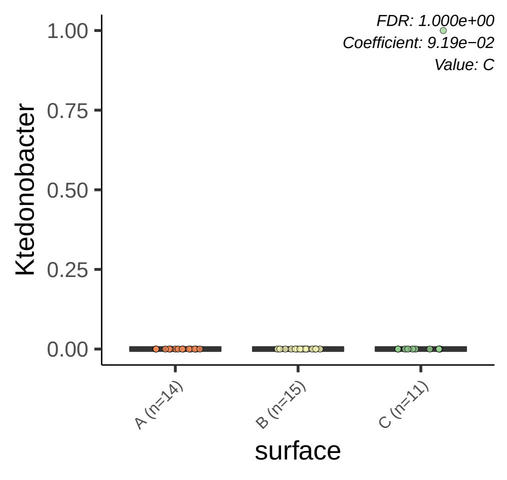

FDR: 1.000e+00
Coefficient: 9.19e−02
Value: C
Ktedonobacter
1.00
0.75
0.50
0.25
0.00
A (n=14)
B (n=15)
C (n=11)
surface

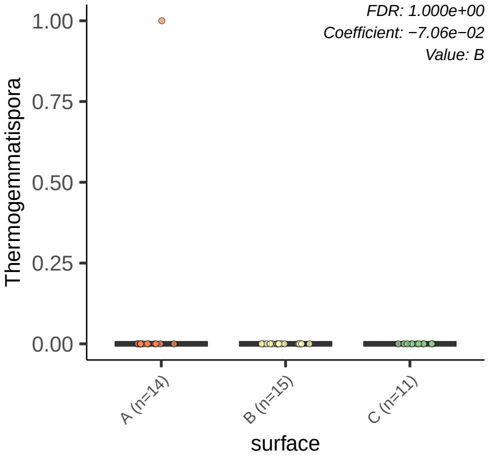

FDR: 1.000e+00
Coefficient: −7.06e−02
Value: B
Thermogemmatispora
1.00
0.75
0.50
0.25
0.00
A (n=14)
B (n=15)
C (n=11)
surface

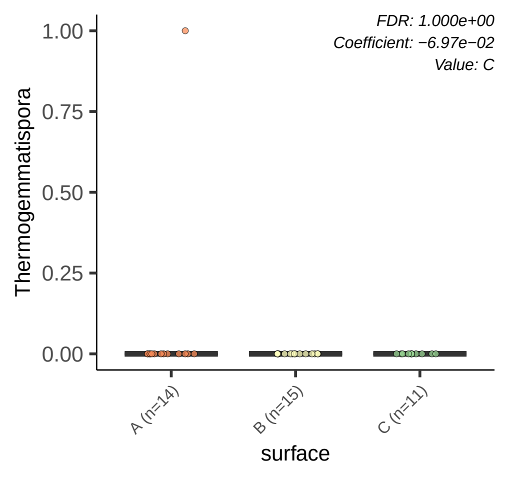
FDR: 1.000e+00
Coefficient: −6.97e−02
Value: C
Thermogemmatispora
1.00
0.75
0.50
0.25
0.00
A (n=14)
B (n=15)
C (n=11)
surface

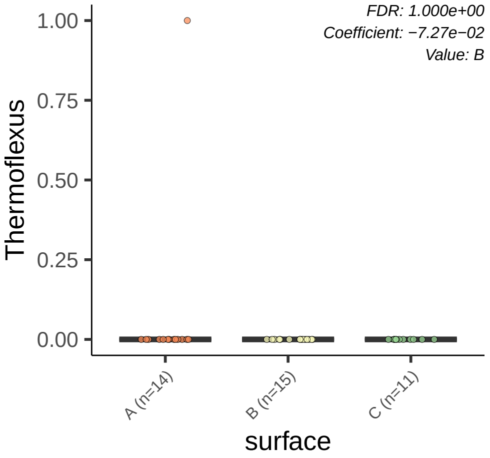
FDR: 1.000e+00
Coefficient: −7.27e−02
Value: B
Thermoflexus
1.00
0.75
0.50
0.25
0.00
A (n=14)
B (n=15)
C (n=11)
surface

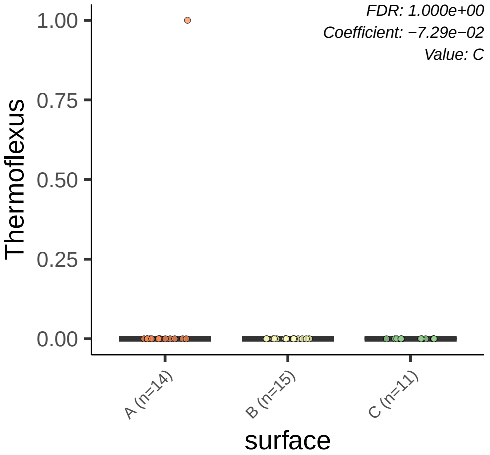
FDR: 1.000e+00
Coefficient: −7.29e−02
Value: C
Thermoflexus
1.00
0.75
0.50
0.25
0.00
A (n=14)
B (n=15)
C (n=11)
surface

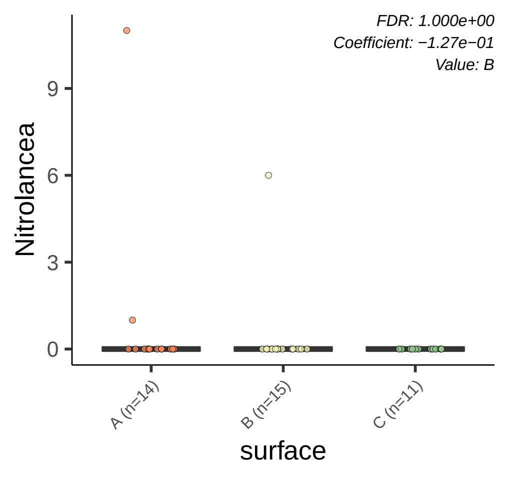
FDR: 1.000e+00
Coefficient: −1.27e−01
Value: B
Nitrolancea
9
6
3
0
A (n=14)
B (n=15)
C (n=11)
surface

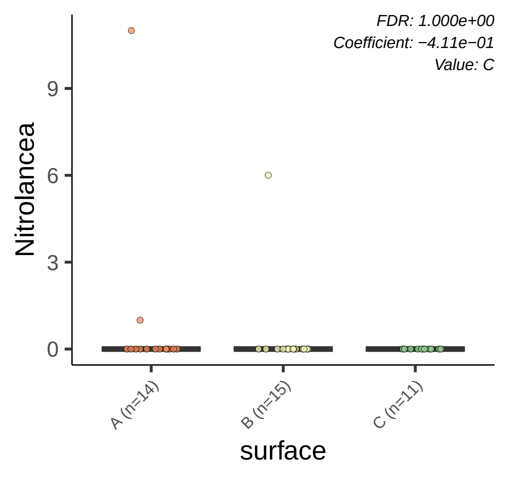

FDR: 1.000e+00
Coefficient: −4.11e−01
Value: C
Nitrolancea
0
3
6
9
A (n=14)
B (n=15)
C (n=11)
surface

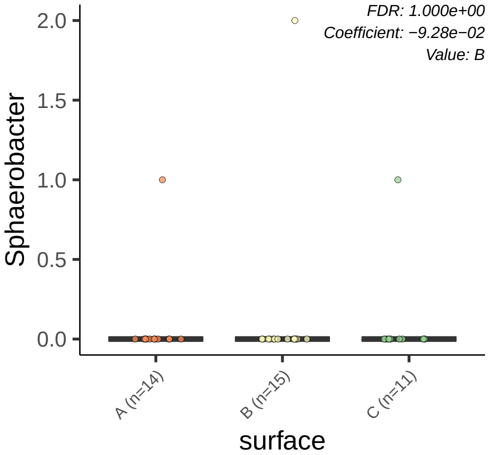
FDR: 1.000e+00
Coefficient: −9.28e−02
Value: B
Sphaerobacter
2.0
1.5
1.0
0.5
0.0
A (n=14)
B (n=15)
C (n=11)
surface

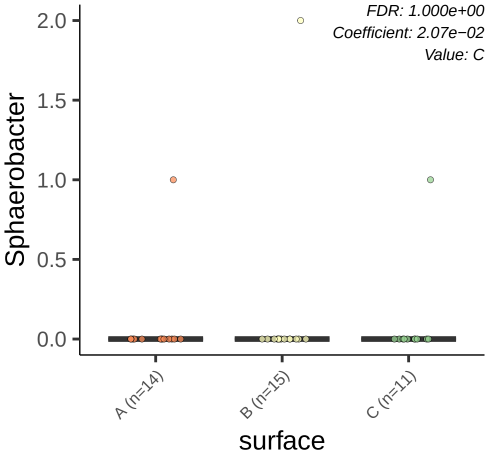
FDR: 1.000e+00
Coefficient: 2.07e−02
Value: C
Sphaerobacter
2.0
1.5
1.0
0.5
0.0
A (n=14)
B (n=15)
C (n=11)
surface

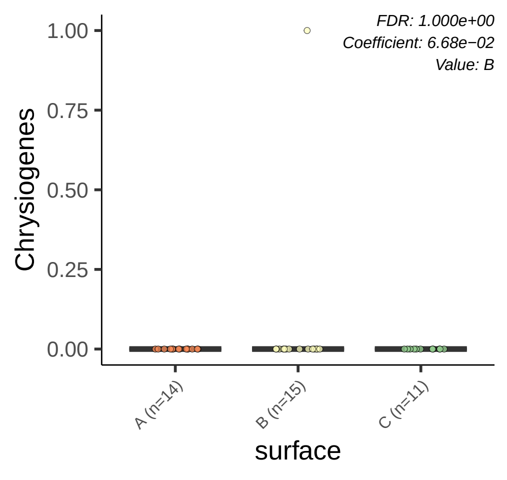
FDR: 1.000e+00
Coefficient: 6.68e−02
Value: B
1.00
0.75
0.50
0.25
0.00
Chrysiogenes
A (n=14)
B (n=15)
C (n=11)
surface

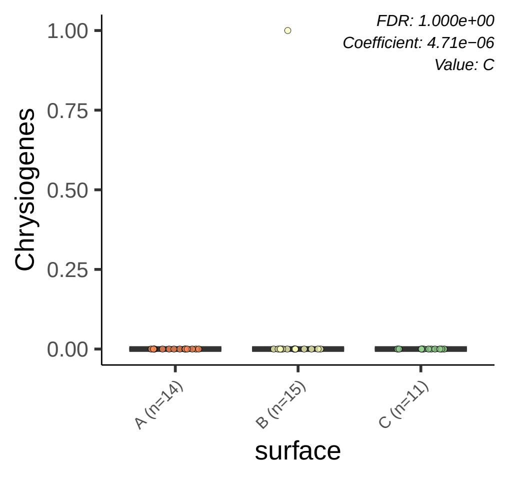
FDR: 1.000e+00
Coefficient: 4.71e−06
Value: C
1.00
0.75
0.50
0.25
0.00
Chrysiogenes
A (n=14)
B (n=15)
C (n=11)
surface

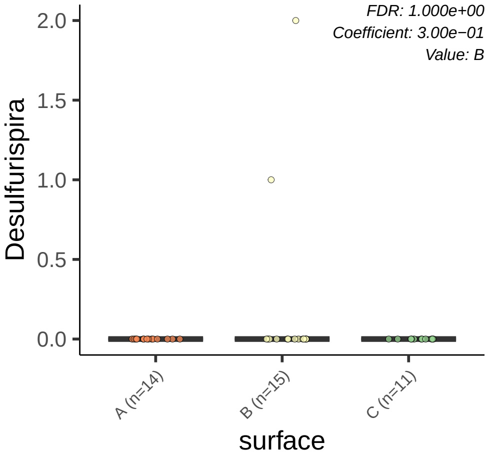
FDR: 1.000e+00
Coefficient: 3.00e−01
Value: B
Desulfurispira
2.0
1.5
1.0
0.5
0.0
A (n=14)
B (n=15)
C (n=11)
surface

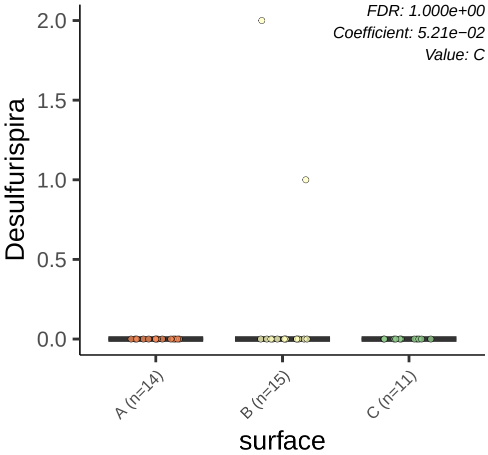
FDR: 1.000e+00
Coefficient: 5.21e−02
Value: C
Desulfurispira
2.0
1.5
1.0
0.5
0.0
A (n=14)
B (n=15)
C (n=11)
surface

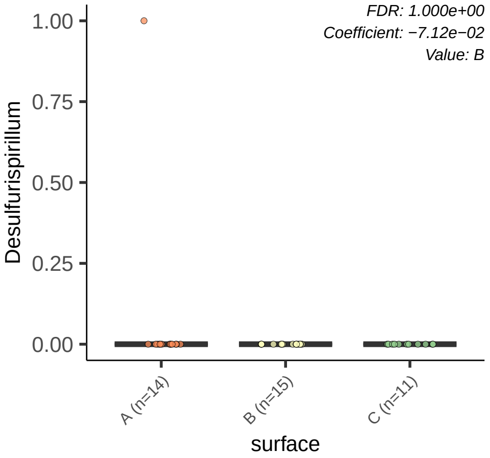
FDR: 1.000e+00
Coefficient: −7.12e−02
Value: B
Desulfurispirillum
1.00
0.75
0.50
0.25
0.00
A (n=14)
B (n=15)
C (n=11)
surface

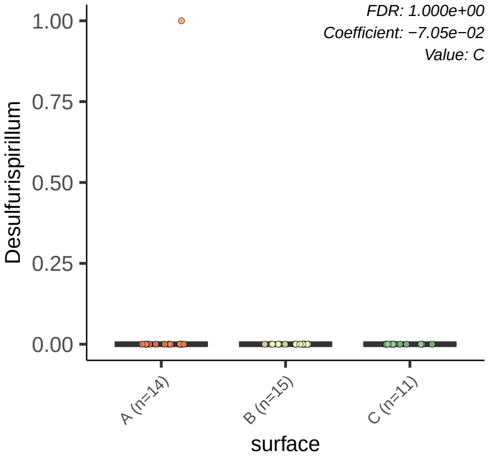
FDR: 1.000e+00
Coefficient: −7.05e−02
Value: C
Desulfurispirillum
1.00
0.75
0.50
0.25
0.00
A (n=14)
B (n=15)
C (n=11)
surface

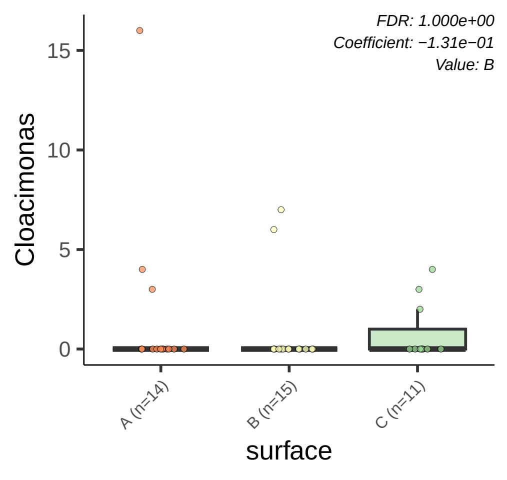

FDR: 1.000e+00
Coefficient: −1.31e−01
Value: B
Cloacimonas
15
10
5
0
A (n=14)
B (n=15)
C (n=11)
surface

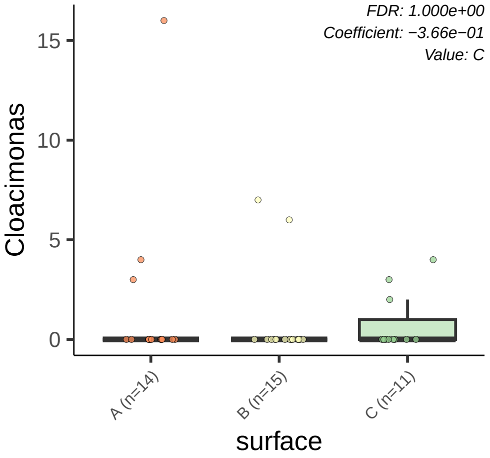

FDR: 1.000e+00
Coefficient: −3.66e−01
Value: C
Cloacimonas
15
10
5
0
A (n=14)
B (n=15)
C (n=11)
surface

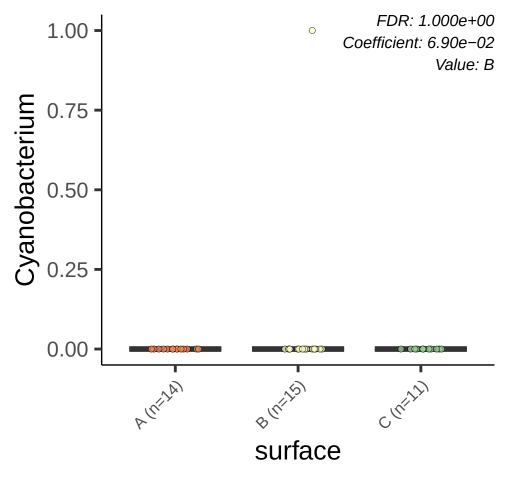

FDR: 1.000e+00
Coefficient: 6.90e−02
Value: B
Cyanobacterium
1.00
0.75
0.50
0.25
0.00
A (n=14)
B (n=15)
C (n=11)
surface

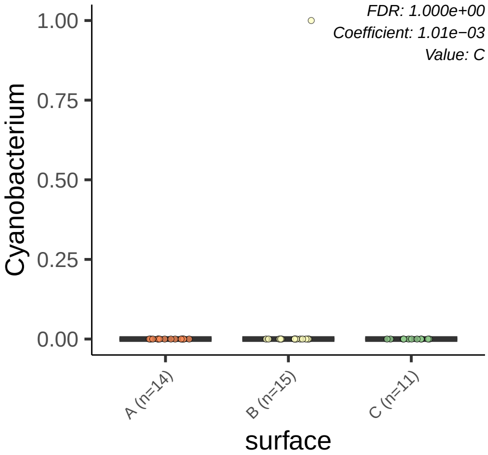

FDR: 1.000e+00
Coefficient: 1.01e−03
Value: C
Cyanobacterium
1.00
0.75
0.50
0.25
0.00
A (n=14)
B (n=15)
C (n=11)
surface

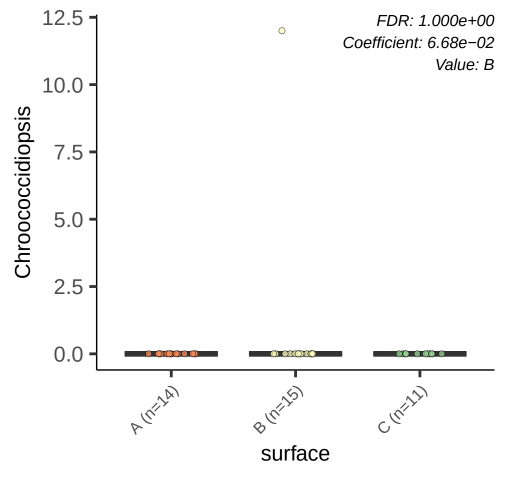
FDR: 1.000e+00
Coefficient: 6.68e−02
Value: B
Chroococcidiopsis
12.5
10.0
7.5
5.0
2.5
0.0
A (n=14)
B (n=15)
C (n=11)
surface

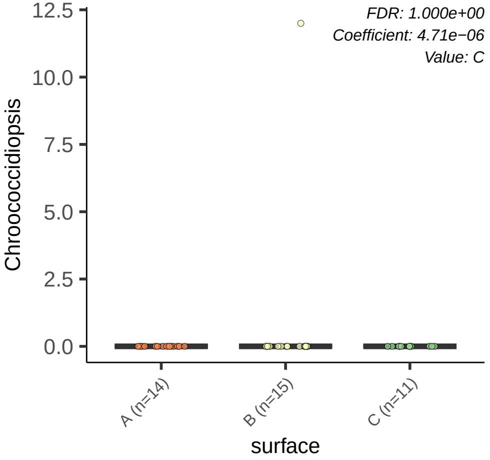
FDR: 1.000e+00
Coefficient: 4.71e−06
Value: C
Chroococcidiopsis
12.5
10.0
7.5
5.0
2.5
0.0
A (n=14)
B (n=15)
C (n=11)
surface

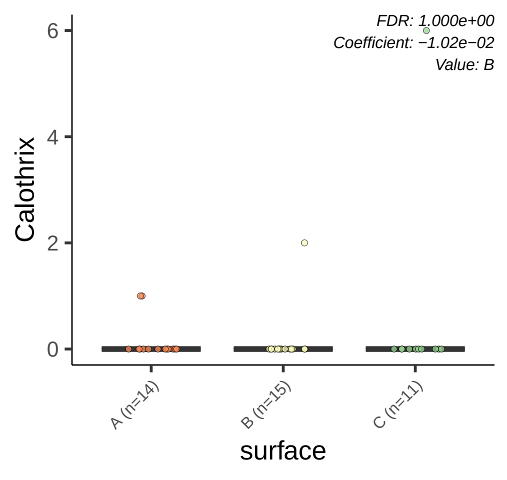

FDR: 1.000e+00
Coefficient: −1.02e−02
Value: B
Calothrix
6
4
2
0
A (n=14)
B (n=15)
C (n=11)
surface

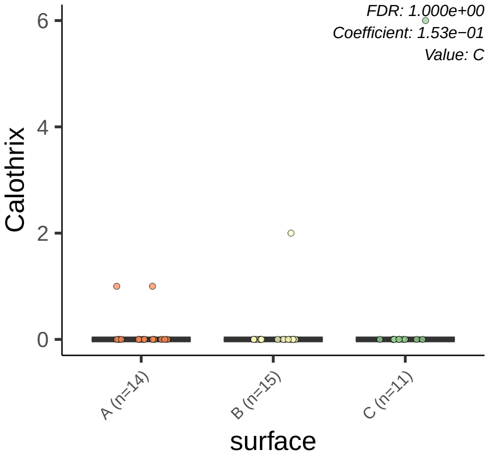
FDR: 1.000e+00
Coefficient: 1.53e−01
Value: C
Calothrix
6
4
2
0
A (n=14)
B (n=15)
C (n=11)
surface

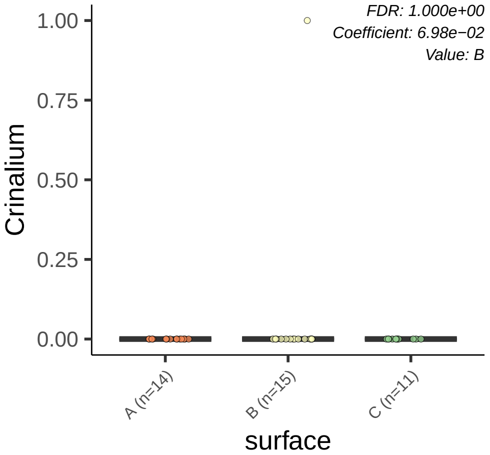

FDR: 1.000e+00
Coefficient: 6.98e−02
Value: B
Crinalium
1.00
0.75
0.50
0.25
0.00
A (n=14)
B (n=15)
C (n=11)
surface

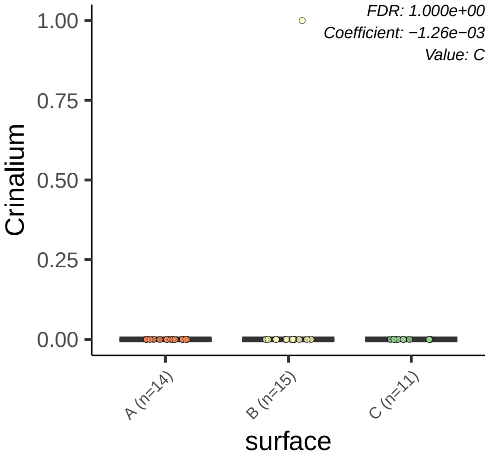
FDR: 1.000e+00
Coefficient: −1.26e−03
Value: C
1.00
0.75
0.50
0.25
0.00
Crinalium
A (n=14)
B (n=15)
C (n=11)
surface

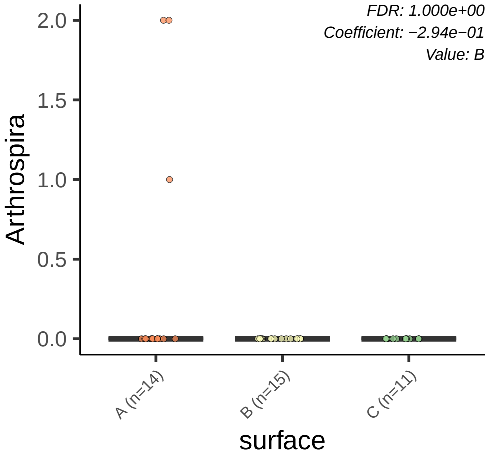

FDR: 1.000e+00
Coefficient: −2.94e−01
Value: B
Arthrospira
2.0
1.5
1.0
0.5
0.0
A (n=14)
B (n=15)
C (n=11)
surface

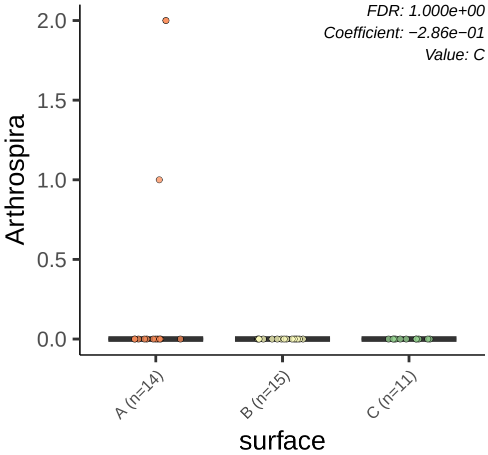

FDR: 1.000e+00
Coefficient: −2.86e−01
Value: C
Arthrospira
2.0
1.5
1.0
0.5
0.0
A (n=14)
B (n=15)
C (n=11)
surface

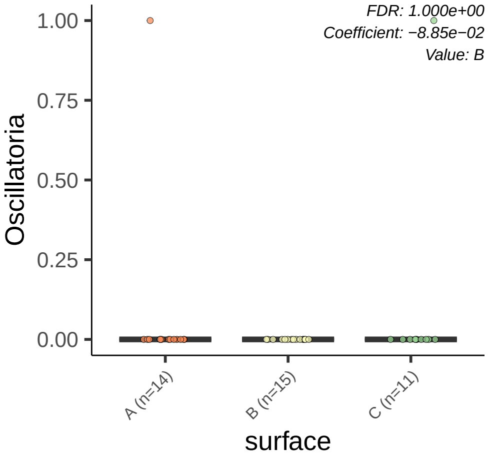
FDR: 1.000e+00
Coefficient: −8.85e−02
Value: B
1.00
0.75
0.50
0.25
0.00
Oscillatoria
A (n=14)
B (n=15)
C (n=11)
surface

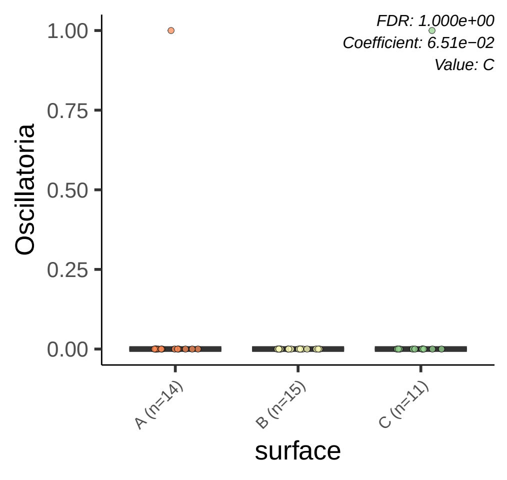

FDR: 1.000e+00
Coefficient: 6.51e−02
Value: C
Oscillatoria
1.00
0.75
0.50
0.25
0.00
A (n=14)
B (n=15)
C (n=11)
surface

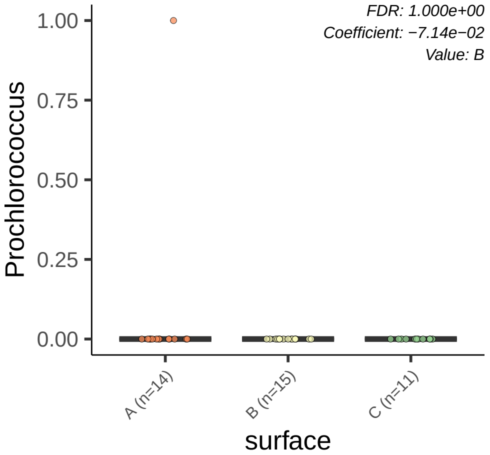
FDR: 1.000e+00
Coefficient: −7.14e−02
Value: B
Prochlorococcus
1.00
0.75
0.50
0.25
0.00
A (n=14)
B (n=15)
C (n=11)
surface

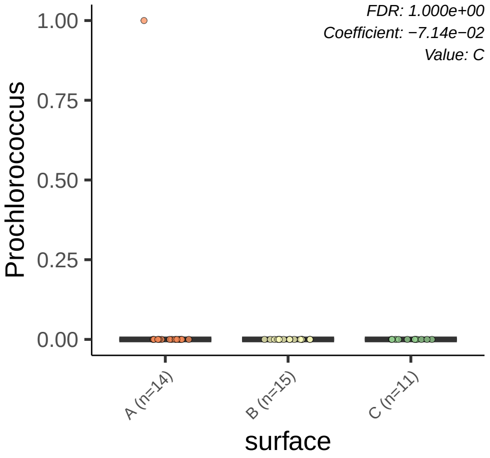
FDR: 1.000e+00
Coefficient: −7.14e−02
Value: C
Prochlorococcus
1.00
0.75
0.50
0.25
0.00
A (n=14)
B (n=15)
C (n=11)
surface

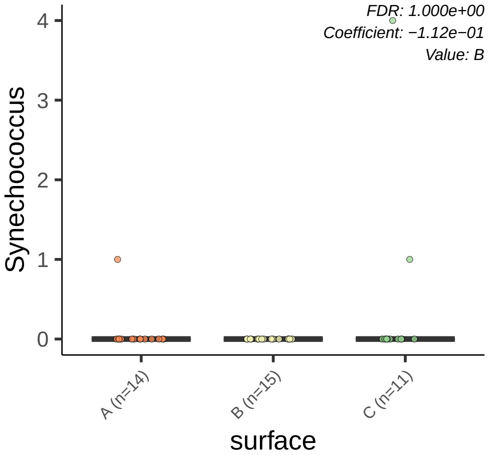

FDR: 1.000e+00
Coefficient: −1.12e−01
Value: B
Synechococcus
0
1
2
3
4
A (n=14)
B (n=15)
C (n=11)
surface

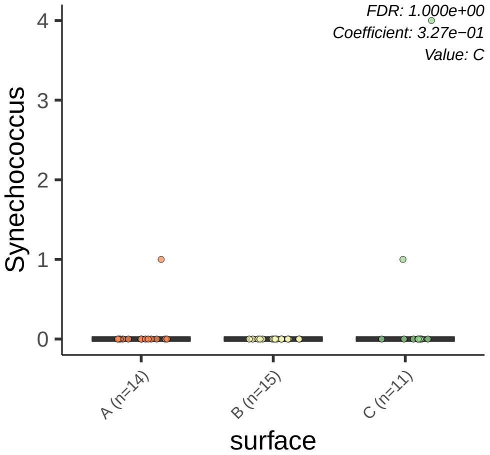
FDR: 1.000e+00
Coefficient: 3.27e−01
Value: C
Synechococcus
4
3
2
1
0
A (n=14)
B (n=15)
C (n=11)
surface

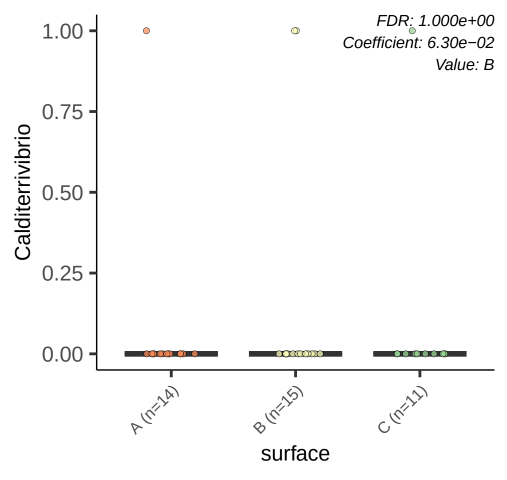
FDR: 1.000e+00
Coefficient: 6.30e−02
Value: B
Calditerrivibrio
1.00
0.75
0.50
0.25
0.00
A (n=14)
B (n=15)
C (n=11)
surface

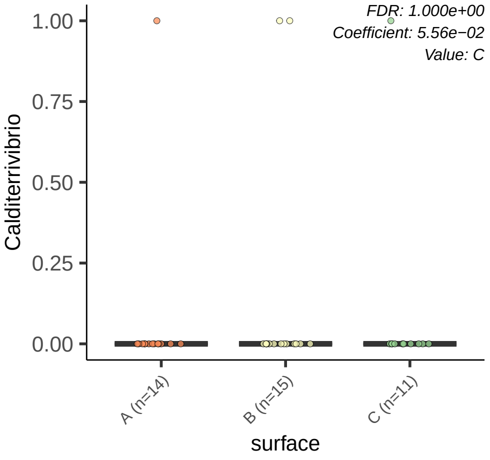

FDR: 1.000e+00
Coefficient: 5.56e−02
Value: C
1.00
0.75
0.50
0.25
0.00
Calditerrivibrio
A (n=14)
B (n=15)
C (n=11)
surface

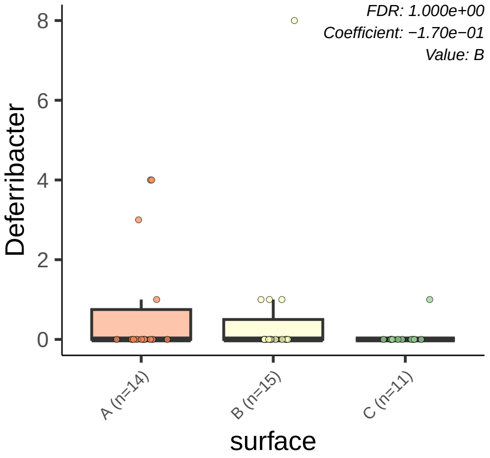
FDR: 1.000e+00
Coefficient: −1.70e−01
Value: B
Deferribacter
8
6
4
2
0
A (n=14)
B (n=15)
C (n=11)
surface

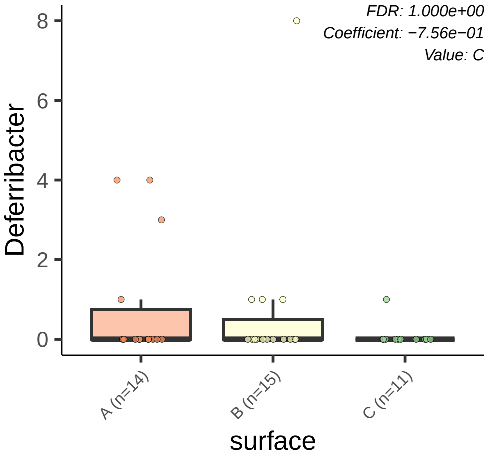
FDR: 1.000e+00
Coefficient: −7.56e−01
Value: C
Deferribacter
0
2
4
6
8
A (n=14)
B (n=15)
C (n=11)
surface

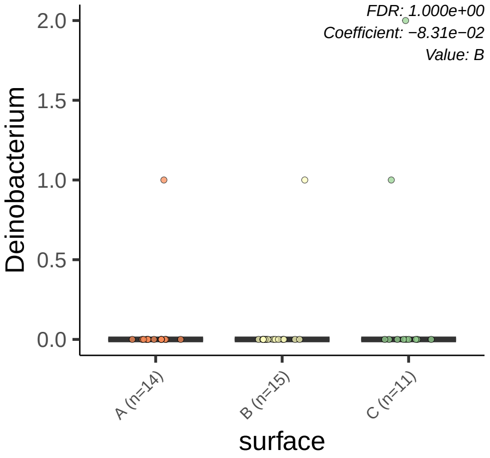

FDR: 1.000e+00
Coefficient: −8.31e−02
Value: B
Deinobacterium
2.0
1.5
1.0
0.5
0.0
A (n=14)
B (n=15)
C (n=11)
surface

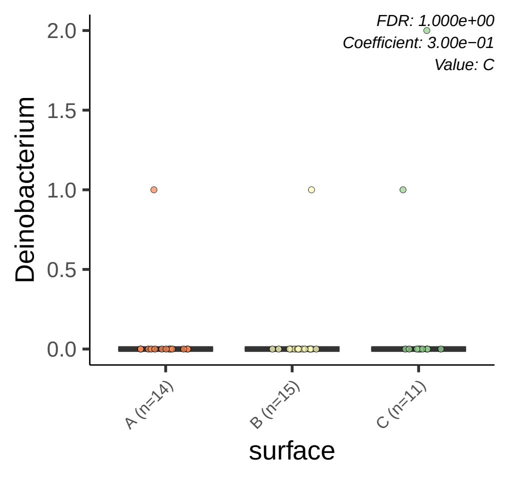
FDR: 1.000e+00
Coefficient: 3.00e−01
Value: C
Deinobacterium
2.0
1.5
1.0
0.5
0.0
A (n=14)
B (n=15)
C (n=11)
surface

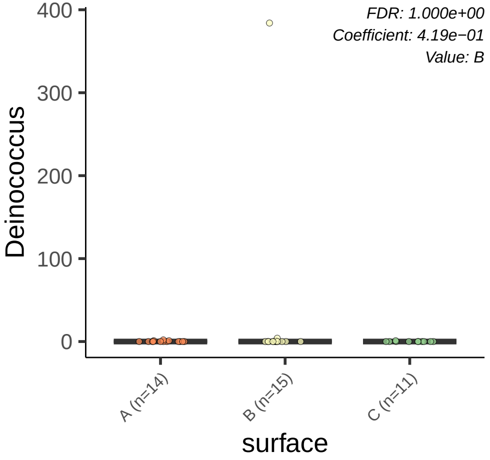
FDR: 1.000e+00
Coefficient: 4.19e−01
Value: B
400
300
200
100
0
Deinococcus
A (n=14)
B (n=15)
C (n=11)
surface

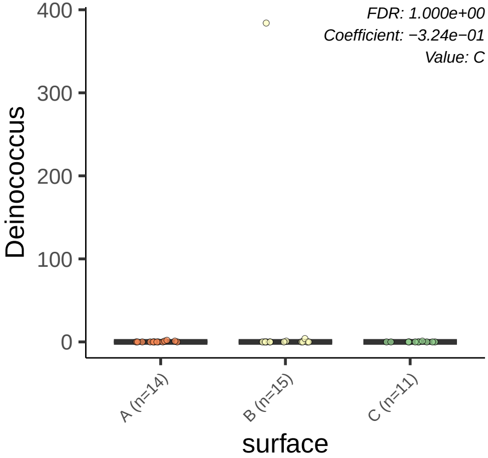
FDR: 1.000e+00
Coefficient: −3.24e−01
Value: C
Deinococcus
400
300
200
100
0
A (n=14)
B (n=15)
C (n=11)
surface

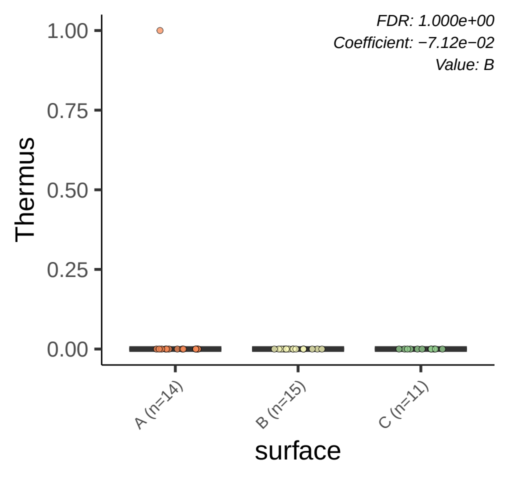
FDR: 1.000e+00
Coefficient: −7.12e−02
Value: B
Thermus
1.00
0.75
0.50
0.25
0.00
A (n=14)
B (n=15)
C (n=11)
surface

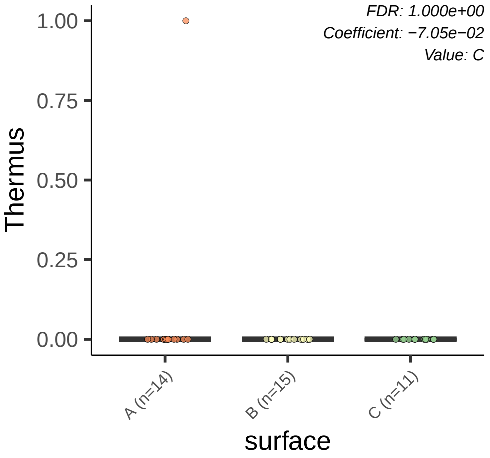
FDR: 1.000e+00
Coefficient: −7.05e−02
Value: C
1.00
0.75
0.50
0.25
0.00
Thermus
A (n=14)
B (n=15)
C (n=11)
surface

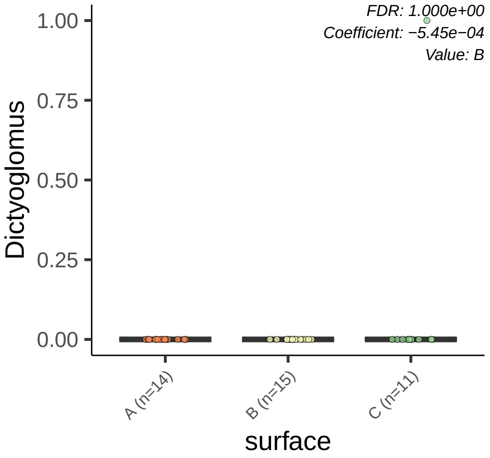

FDR: 1.000e+00
Coefficient: −5.45e−04
Value: B
Dictyoglomus
1.00
0.75
0.50
0.25
0.00
A (n=14)
B (n=15)
C (n=11)
surface

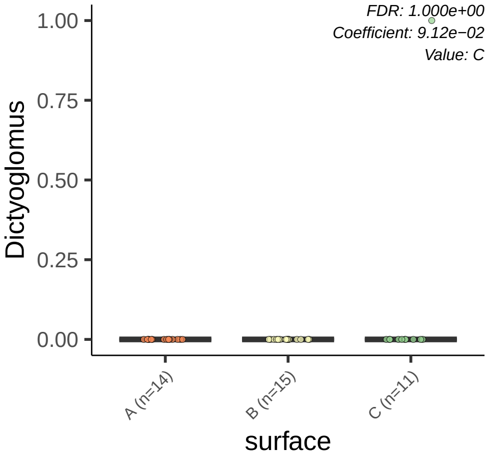
FDR: 1.000e+00
Coefficient: 9.12e−02
Value: C
Dictyoglomus
1.00
0.75
0.50
0.25
0.00
A (n=14)
B (n=15)
C (n=11)
surface

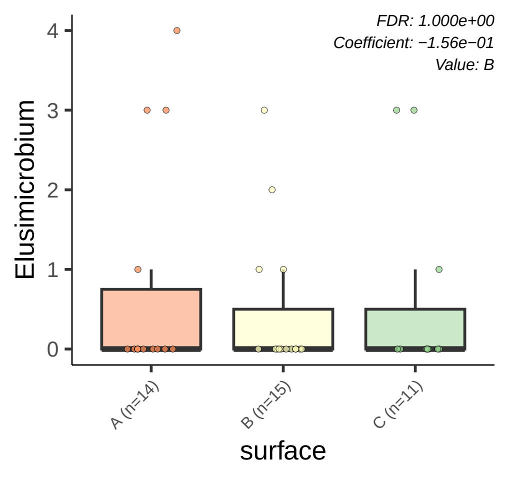
FDR: 1.000e+00
Coefficient: −1.56e−01
Value: B
Elusimicrobium
0
1
2
3
4
A (n=14)
B (n=15)
C (n=11)
surface

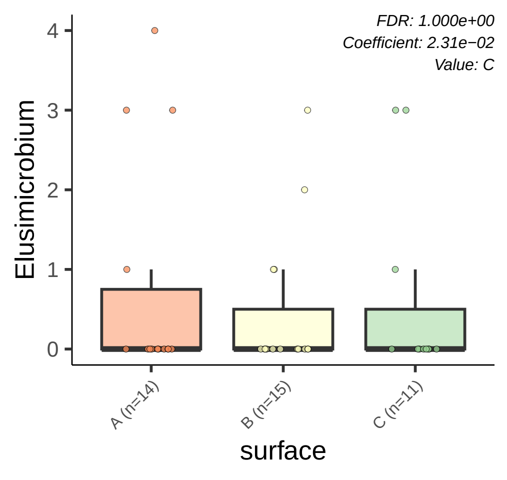
FDR: 1.000e+00
Coefficient: 2.31e−02
Value: C
Elusimicrobium
0
1
2
3
4
A (n=14)
B (n=15)
C (n=11)
surface

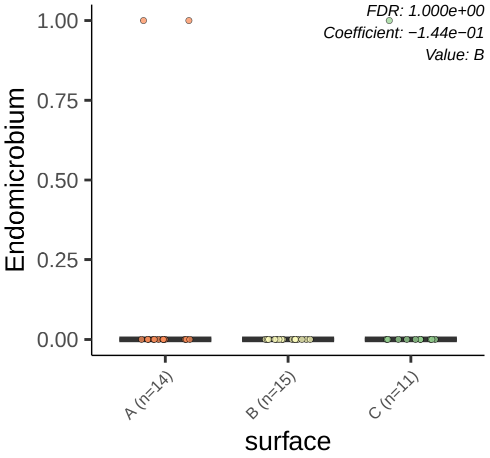
FDR: 1.000e+00
Coefficient: −1.44e−01
Value: B
Endomicrobium
1.00
0.75
0.50
0.25
0.00
A (n=14)
B (n=15)
C (n=11)
surface

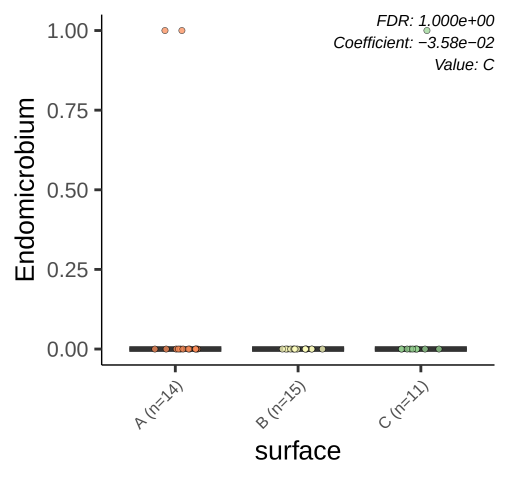

FDR: 1.000e+00
Coefficient: −3.58e−02
Value: C
Endomicrobium
1.00
0.75
0.50
0.25
0.00
A (n=14)
B (n=15)
C (n=11)
surface

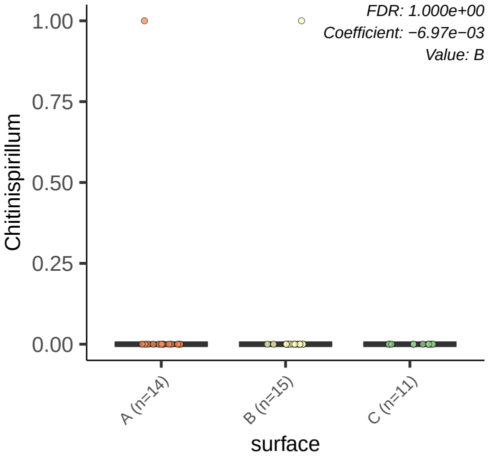

FDR: 1.000e+00
Coefficient: −6.97e−03
Value: B
1.00
0.75
0.50
0.25
0.00
Chitinispirillum
A (n=14)
B (n=15)
C (n=11)
surface

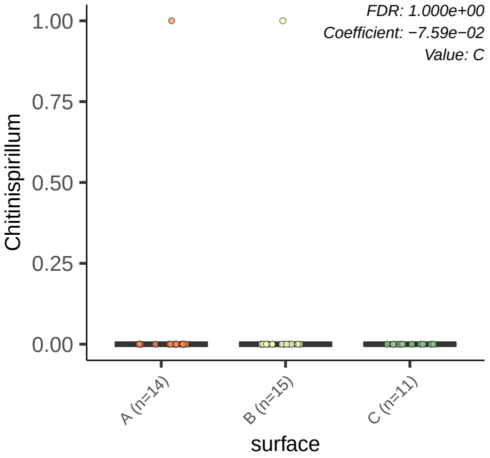

FDR: 1.000e+00
Coefficient: −7.59e−02
Value: C
Chitinispirillum
1.00
0.75
0.50
0.25
0.00
A (n=14)
B (n=15)
C (n=11)
surface

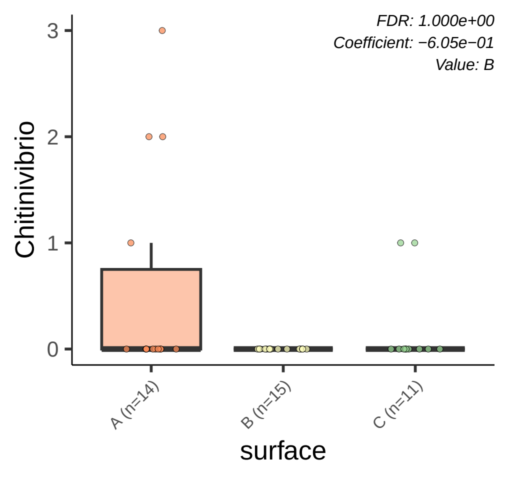
FDR: 1.000e+00
Coefficient: −6.05e−01
Value: B
Chitinivibrio
3
2
1
0
A (n=14)
B (n=15)
C (n=11)
surface

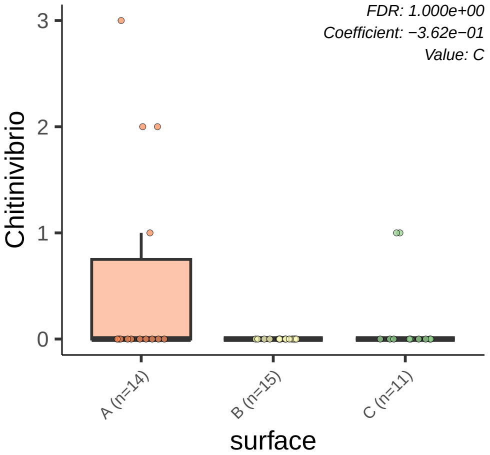
FDR: 1.000e+00
Coefficient: −3.62e−01
Value: C
Chitinivibrio
3
2
1
0
A (n=14)
B (n=15)
C (n=11)
surface

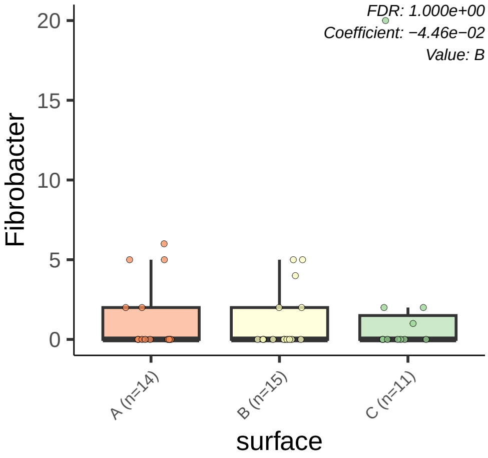
FDR: 1.000e+00
Coefficient: −4.46e−02
Value: B
Fibrobacter
0
5
10
15
20
A (n=14)
B (n=15)
C (n=11)
surface

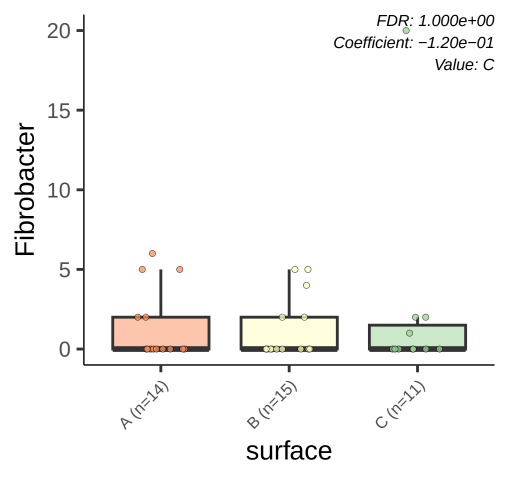

FDR: 1.000e+00
Coefficient: −1.20e−01
Value: C
Fibrobacter
20
15
10
5
0
A (n=14)
B (n=15)
C (n=11)
surface

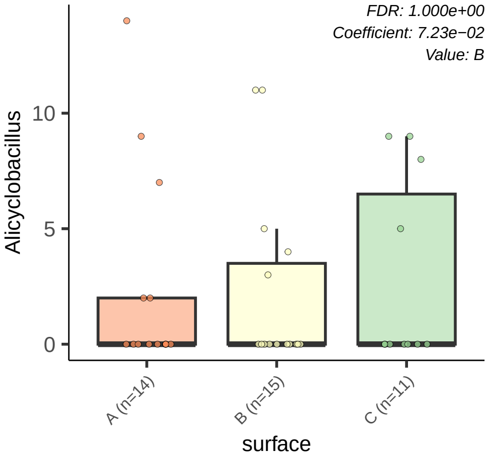
FDR: 1.000e+00
Coefficient: 7.23e−02
Value: B
Alicyclobacillus
10
5
0
A (n=14)
B (n=15)
C (n=11)
surface

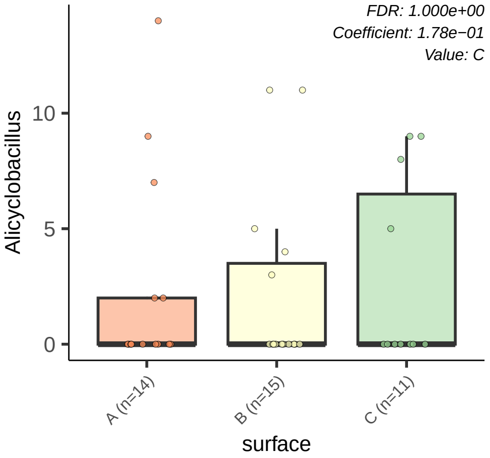
FDR: 1.000e+00
Coefficient: 1.78e−01
Value: C
Alicyclobacillus
10
5
0
A (n=14)
B (n=15)
C (n=11)
surface

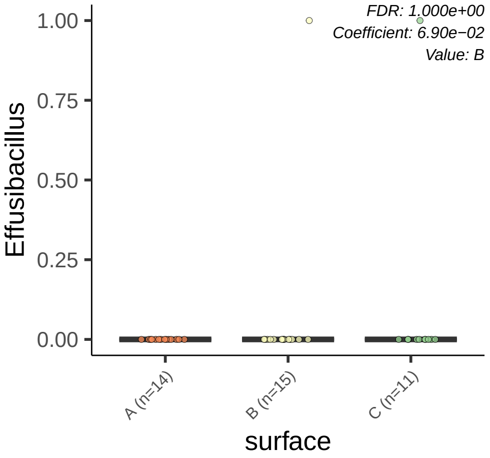
FDR: 1.000e+00
Coefficient: 6.90e−02
Value: B
Effusibacillus
1.00
0.75
0.50
0.25
0.00
A (n=14)
B (n=15)
C (n=11)
surface

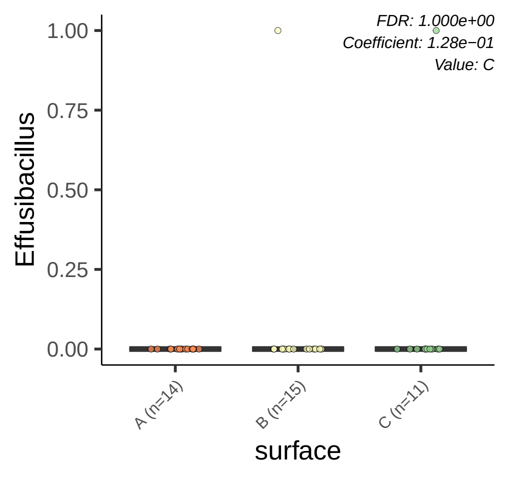
FDR: 1.000e+00
Coefficient: 1.28e−01
Value: C
1.00
0.75
0.50
0.25
0.00
Effusibacillus
A (n=14)
B (n=15)
C (n=11)
surface

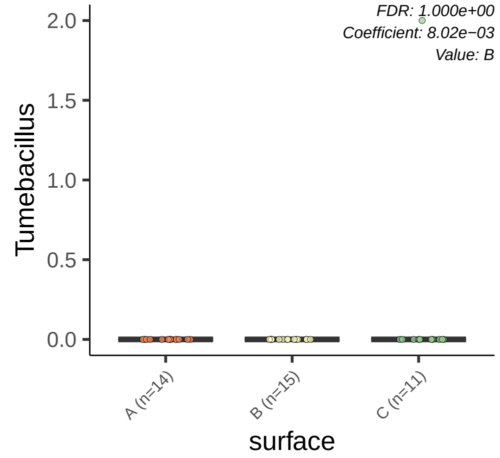

FDR: 1.000e+00
Coefficient: 8.02e−03
Value: B
Tumebacillus
2.0
1.5
1.0
0.5
0.0
A (n=14)
B (n=15)
C (n=11)
surface

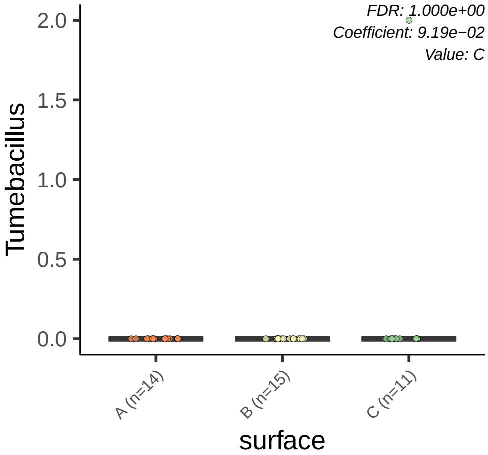
FDR: 1.000e+00
Coefficient: 9.19e−02
Value: C
Tumebacillus
2.0
1.5
1.0
0.5
0.0
A (n=14)
B (n=15)
C (n=11)
surface

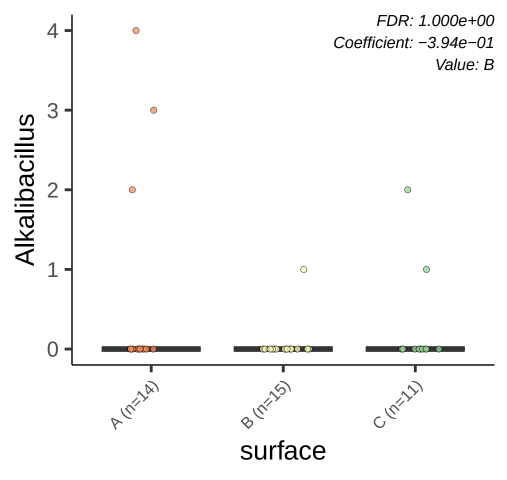

FDR: 1.000e+00
Coefficient: −3.94e−01
Value: B
Alkalibacillus
0
1
2
3
4
A (n=14)
B (n=15)
C (n=11)
surface

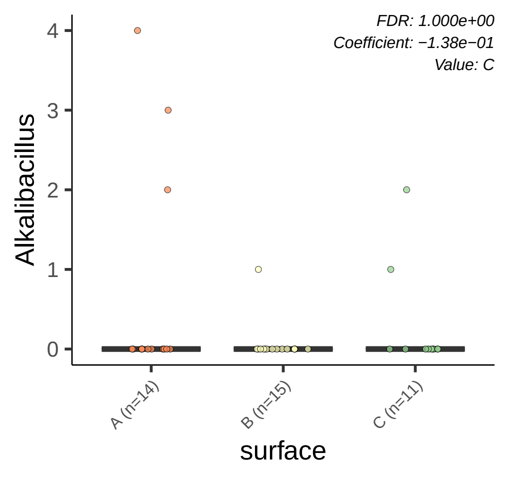

FDR: 1.000e+00
Coefficient: −1.38e−01
Value: C
Alkalibacillus
4
3
2
1
0
A (n=14)
B (n=15)
C (n=11)
surface

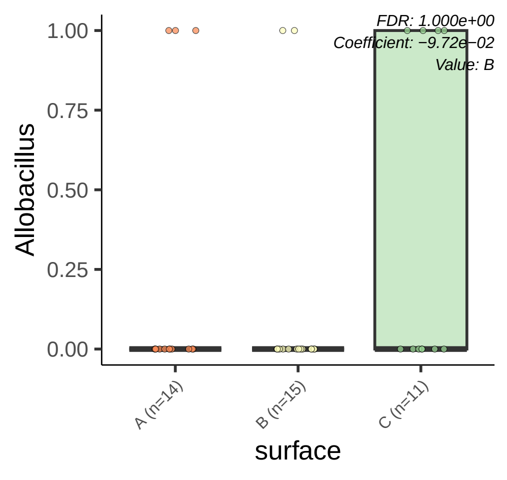
FDR: 1.000e+00
Coefficient: −9.72e−02
Value: B
Allobacillus
1.00
0.75
0.50
0.25
0.00
A (n=14)
B (n=15)
C (n=11)
surface

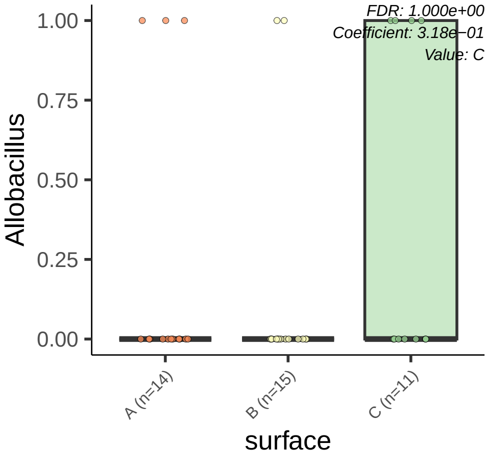
FDR: 1.000e+00
Coefficient: 3.18e−01
Value: C
Allobacillus
1.00
0.75
0.50
0.25
0.00
A (n=14)
B (n=15)
C (n=11)
surface

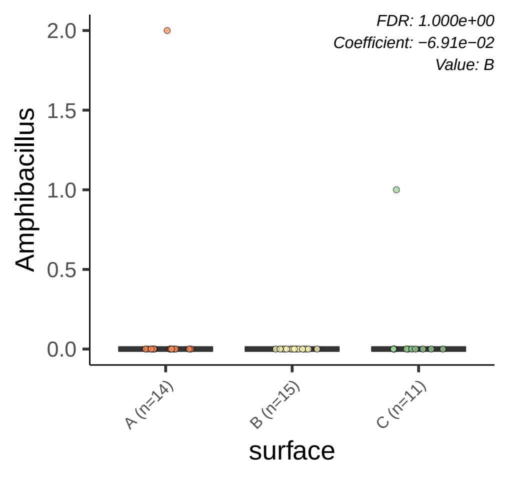
FDR: 1.000e+00
Coefficient: −6.91e−02
Value: B
Amphibacillus
2.0
1.5
1.0
0.5
0.0
A (n=14)
B (n=15)
C (n=11)
surface

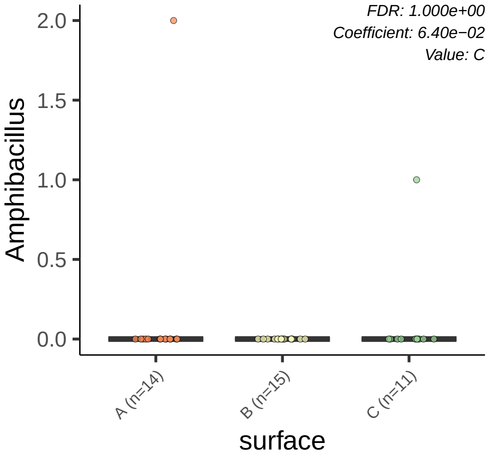
FDR: 1.000e+00
Coefficient: 6.40e−02
Value: C
Amphibacillus
2.0
1.5
1.0
0.5
0.0
A (n=14)
B (n=15)
C (n=11)
surface

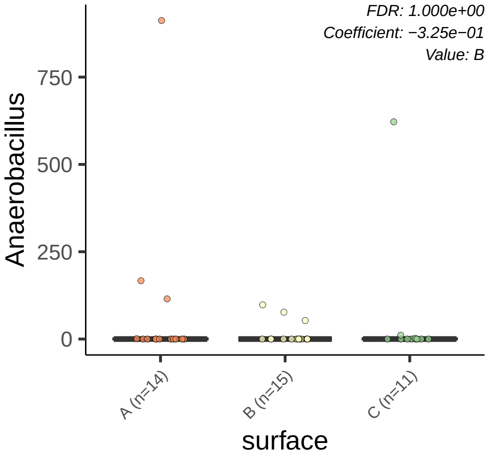

FDR: 1.000e+00
Coefficient: −3.25e−01
Value: B
Anaerobacillus
750
500
250
0
A (n=14)
B (n=15)
C (n=11)
surface

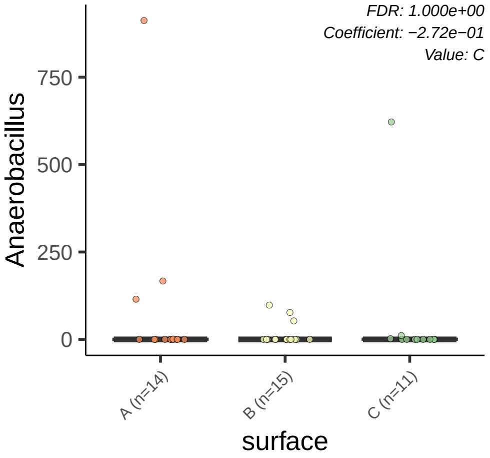
FDR: 1.000e+00
Coefficient: −2.72e−01
Value: C
Anaerobacillus
750
500
250
0
A (n=14)
B (n=15)
C (n=11)
surface

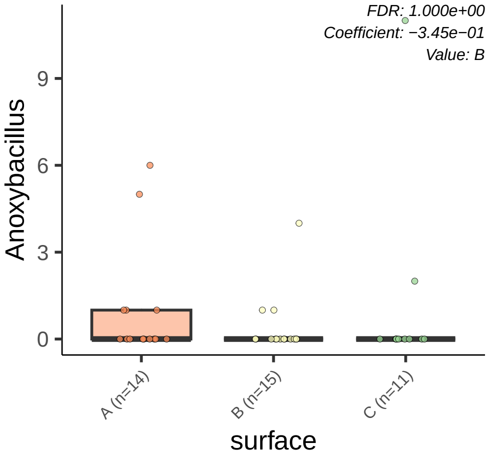
FDR: 1.000e+00
Coefficient: −3.45e−01
Value: B
Anoxybacillus
9
6
3
0
A (n=14)
B (n=15)
C (n=11)
surface

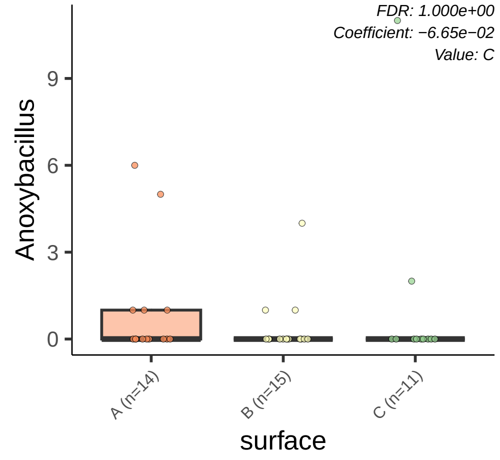
FDR: 1.000e+00
Coefficient: −6.65e−02
Value: C
Anoxybacillus
0
3
6
9
A (n=14)
B (n=15)
C (n=11)
surface

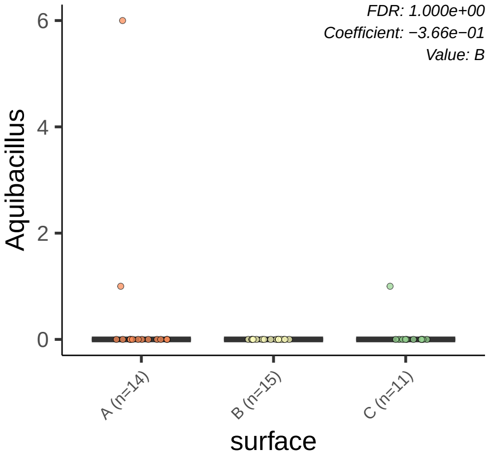
FDR: 1.000e+00
Coefficient: −3.66e−01
Value: B
Aquibacillus
6
4
2
0
A (n=14)
B (n=15)
C (n=11)
surface

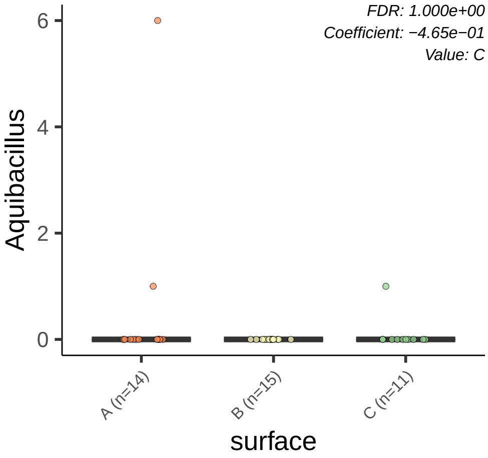
FDR: 1.000e+00
Coefficient: −4.65e−01
Value: C
Aquibacillus
6
4
2
0
A (n=14)
B (n=15)
C (n=11)
surface

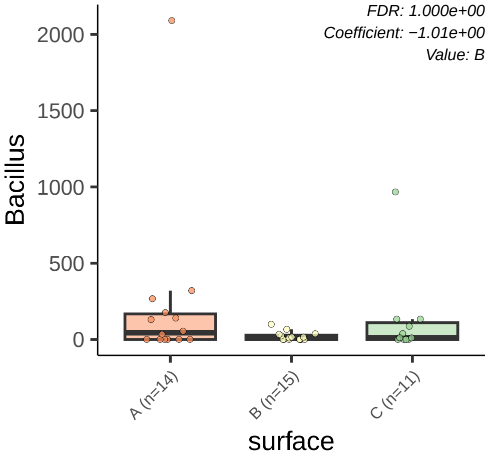
FDR: 1.000e+00
Coefficient: −1.01e+00
Value: B
Bacillus
2000
1500
1000
500
0
A (n=14)
B (n=15)
C (n=11)
surface

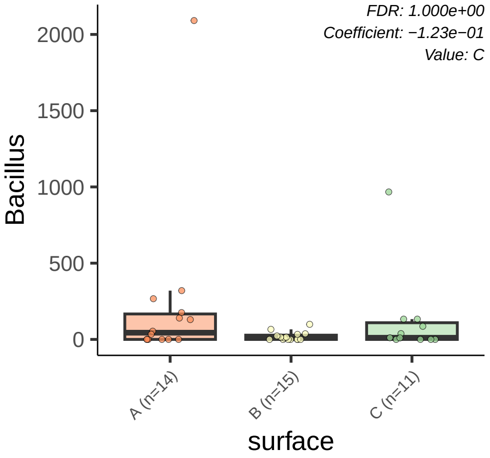
FDR: 1.000e+00
Coefficient: −1.23e−01
Value: C
Bacillus
2000
1500
1000
500
0
A (n=14)
B (n=15)
C (n=11)
surface

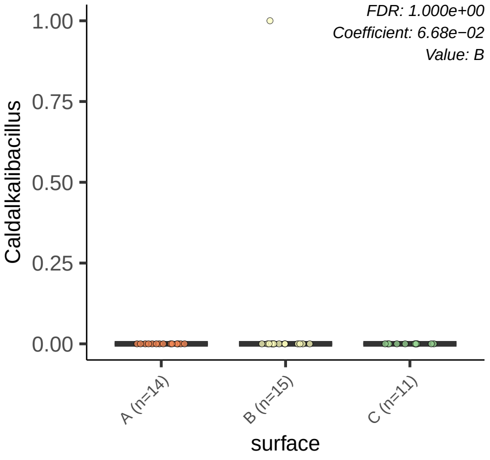
FDR: 1.000e+00
Coefficient: 6.68e−02
Value: B
Caldalkalibacillus
1.00
0.75
0.50
0.25
0.00
A (n=14)
B (n=15)
C (n=11)
surface

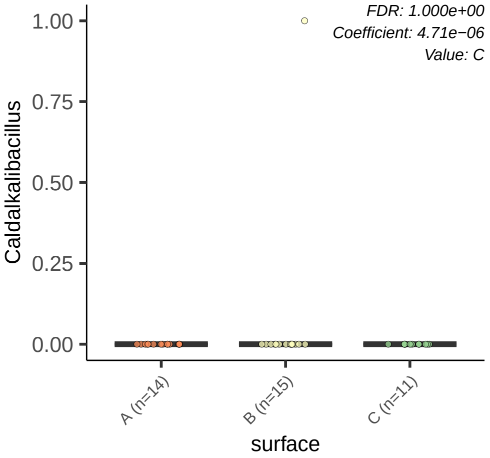
FDR: 1.000e+00
Coefficient: 4.71e−06
Value: C
Caldalkalibacillus
1.00
0.75
0.50
0.25
0.00
A (n=14)
B (n=15)
C (n=11)
surface

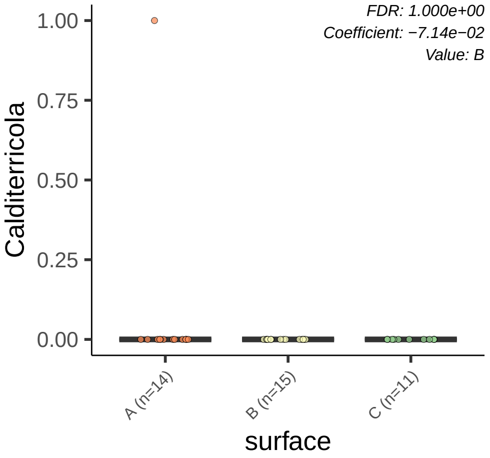
FDR: 1.000e+00
Coefficient: −7.14e−02
Value: B
Calditerricola
1.00
0.75
0.50
0.25
0.00
A (n=14)
B (n=15)
C (n=11)
surface

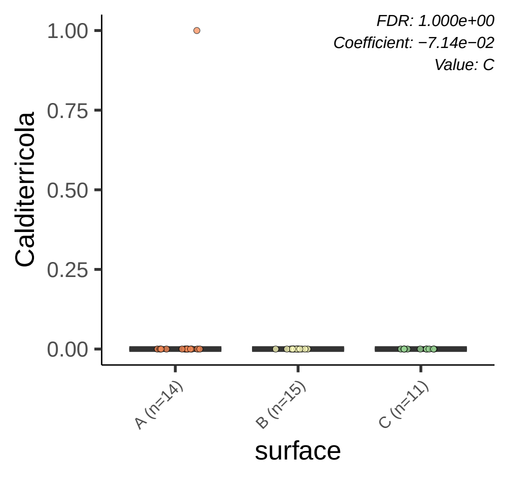

FDR: 1.000e+00
Coefficient: −7.14e−02
Value: C
Calditerricola
1.00
0.75
0.50
0.25
0.00
A (n=14)
B (n=15)
C (n=11)
surface

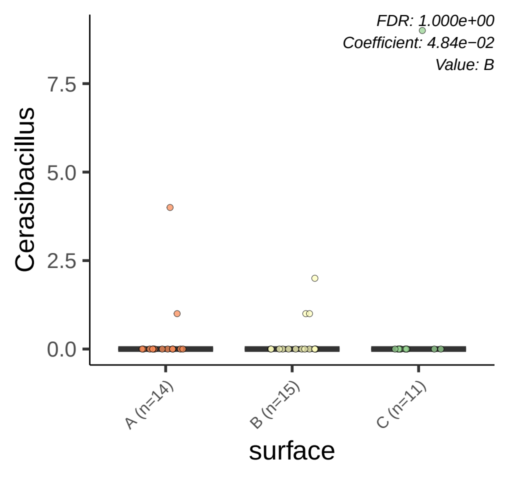
FDR: 1.000e+00
Coefficient: 4.84e−02
Value: B
Cerasibacillus
7.5
5.0
2.5
0.0
A (n=14)
B (n=15)
C (n=11)
surface

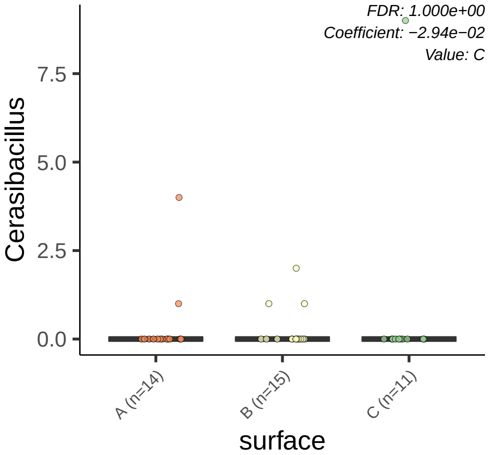
FDR: 1.000e+00
Coefficient: −2.94e−02
Value: C
Cerasibacillus
7.5
5.0
2.5
0.0
A (n=14)
B (n=15)
C (n=11)
surface

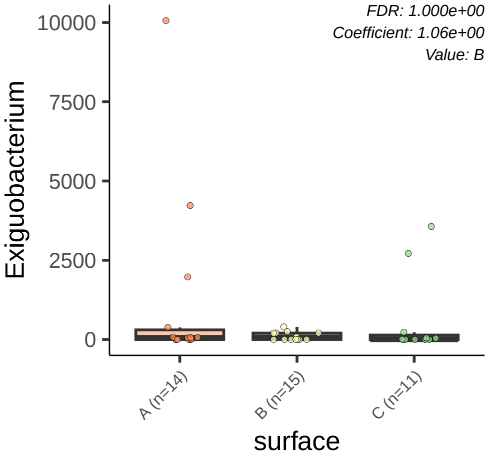

FDR: 1.000e+00
Coefficient: 1.06e+00
Value: B
Exiguobacterium
10000
7500
5000
2500
0
A (n=14)
B (n=15)
C (n=11)
surface

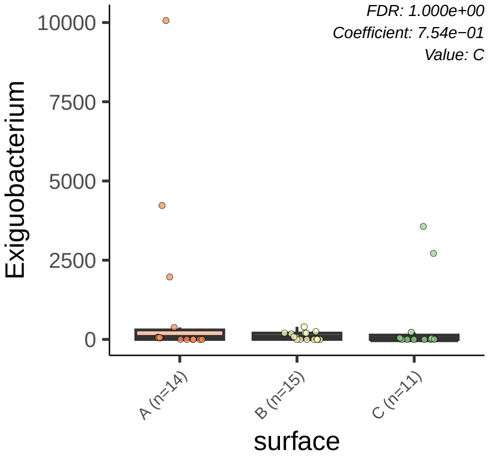
FDR: 1.000e+00
Coefficient: 7.54e−01
Value: C
Exiguobacterium
10000
7500
5000
2500
0
A (n=14)
B (n=15)
C (n=11)
surface

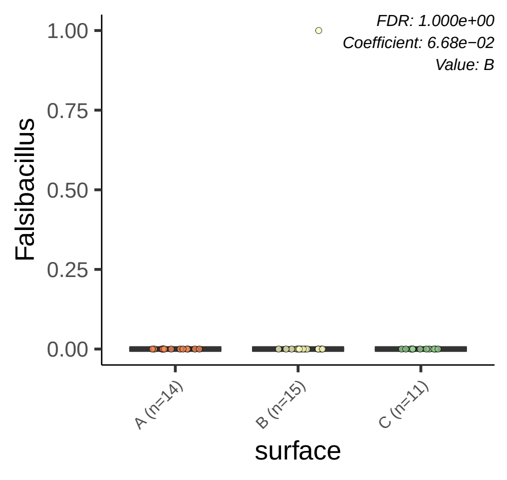
FDR: 1.000e+00
Coefficient: 6.68e−02
Value: B
Falsibacillus
1.00
0.75
0.50
0.25
0.00
A (n=14)
B (n=15)
C (n=11)
surface

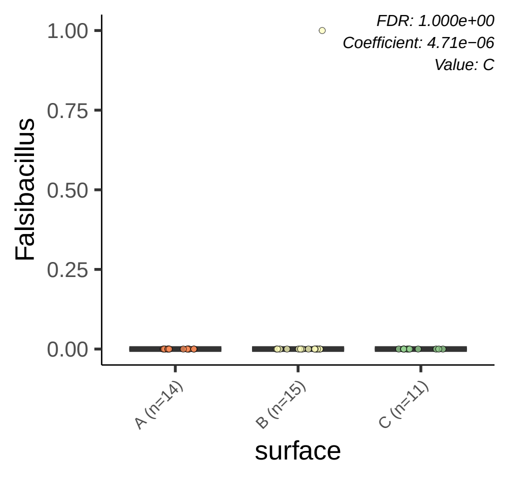
FDR: 1.000e+00
Coefficient: 4.71e−06
Value: C
Falsibacillus
1.00
0.75
0.50
0.25
0.00
A (n=14)
B (n=15)
C (n=11)
surface

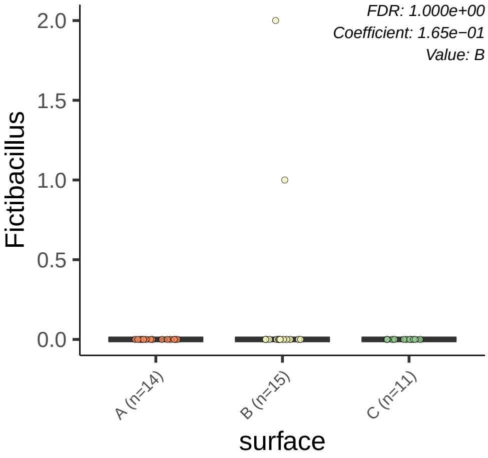

FDR: 1.000e+00
Coefficient: 1.65e−01
Value: B
2.0
1.5
1.0
0.5
0.0
Fictibacillus
A (n=14)
B (n=15)
C (n=11)
surface

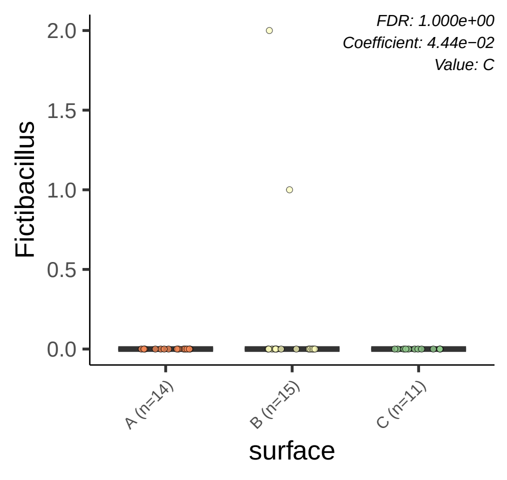
FDR: 1.000e+00
Coefficient: 4.44e−02
Value: C
Fictibacillus
2.0
1.5
1.0
0.5
0.0
A (n=14)
B (n=15)
C (n=11)
surface

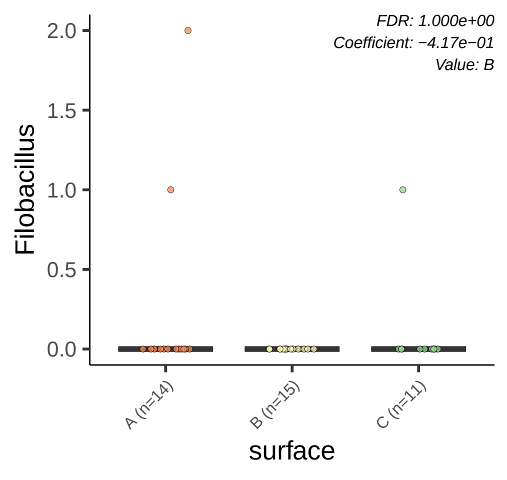
FDR: 1.000e+00
Coefficient: −4.17e−01
Value: B
Filobacillus
2.0
1.5
1.0
0.5
0.0
A (n=14)
B (n=15)
C (n=11)
surface

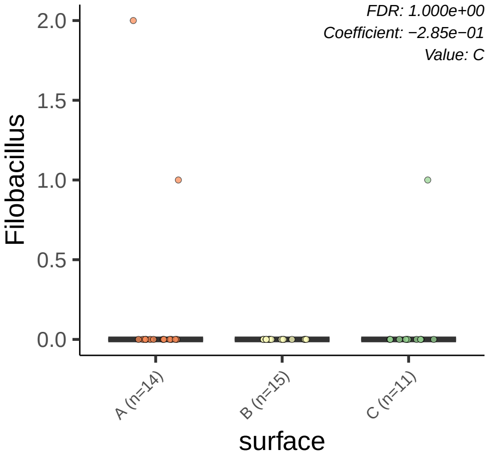
FDR: 1.000e+00
Coefficient: −2.85e−01
Value: C
Filobacillus
2.0
1.5
1.0
0.5
0.0
A (n=14)
B (n=15)
C (n=11)
surface

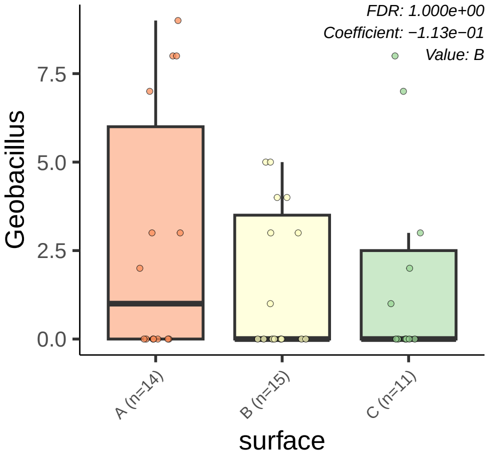
FDR: 1.000e+00
Coefficient: −1.13e−01
Value: B
Geobacillus
7.5
5.0
2.5
0.0
A (n=14)
B (n=15)
C (n=11)
surface

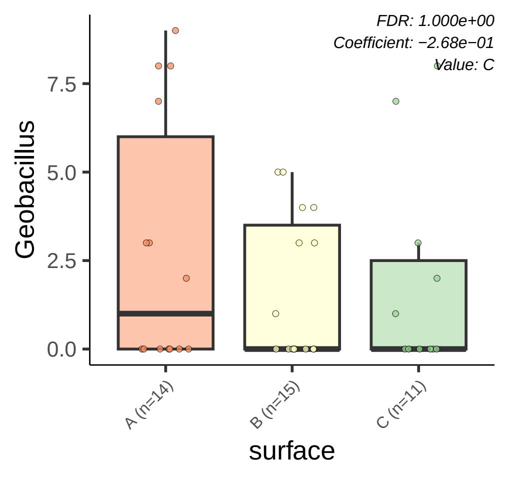

FDR: 1.000e+00
Coefficient: −2.68e−01
Value: C
Geobacillus
7.5
5.0
2.5
0.0
A (n=14)
B (n=15)
C (n=11)
surface

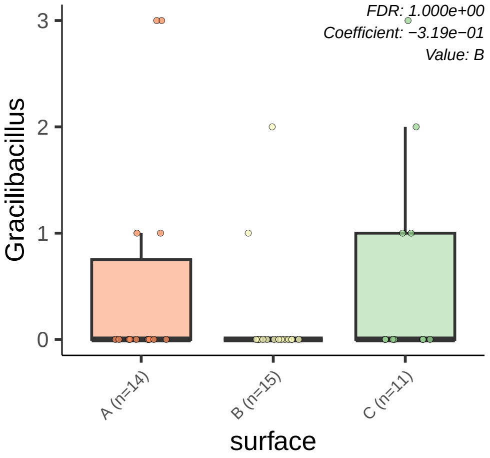
FDR: 1.000e+00
Coefficient: −3.19e−01
Value: B
Gracilibacillus
3
2
1
0
A (n=14)
B (n=15)
C (n=11)
surface

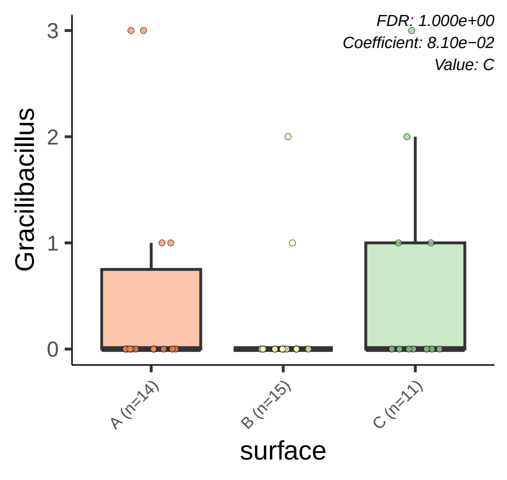

FDR: 1.000e+00
Coefficient: 8.10e−02
Value: C
Gracilibacillus
3
2
1
0
A (n=14)
B (n=15)
C (n=11)
surface

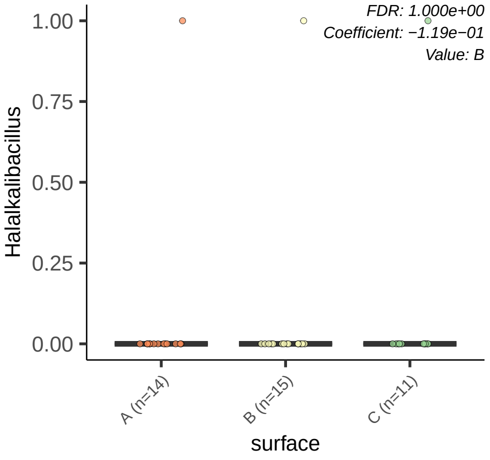

FDR: 1.000e+00
Coefficient: −1.19e−01
Value: B
Halalkalibacillus
1.00
0.75
0.50
0.25
0.00
A (n=14)
B (n=15)
C (n=11)
surface

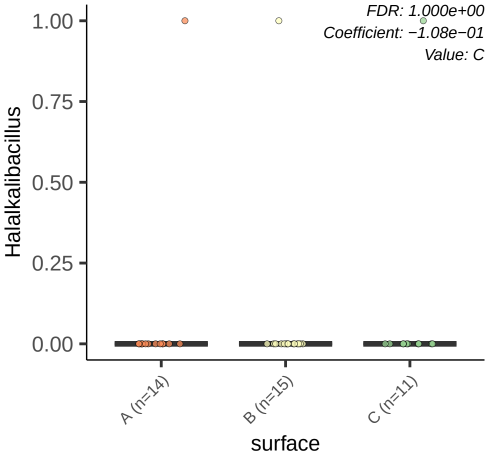

FDR: 1.000e+00
Coefficient: −1.08e−01
Value: C
Halalkalibacillus
1.00
0.75
0.50
0.25
0.00
A (n=14)
B (n=15)
C (n=11)
surface

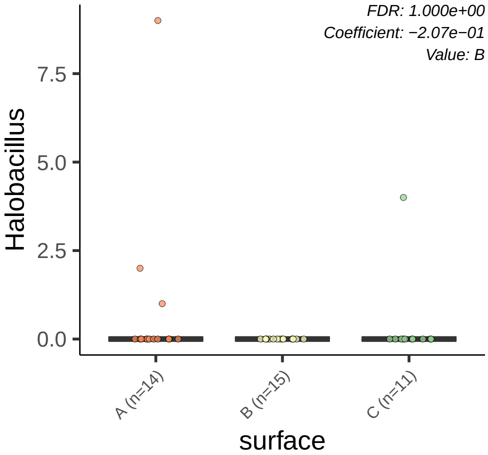

FDR: 1.000e+00
Coefficient: −2.07e−01
Value: B
Halobacillus
7.5
5.0
2.5
0.0
A (n=14)
B (n=15)
C (n=11)
surface

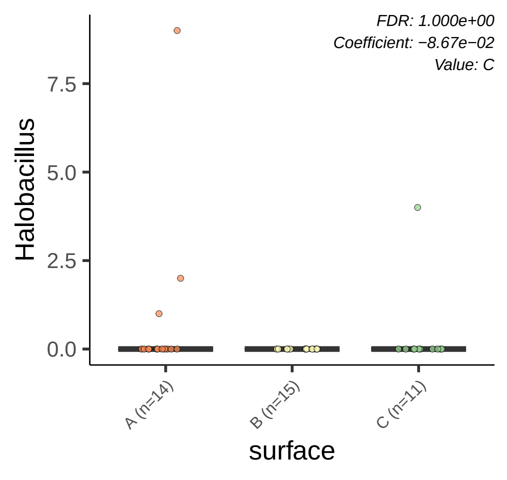
FDR: 1.000e+00
Coefficient: −8.67e−02
Value: C
Halobacillus
7.5
5.0
2.5
0.0
A (n=14)
B (n=15)
C (n=11)
surface

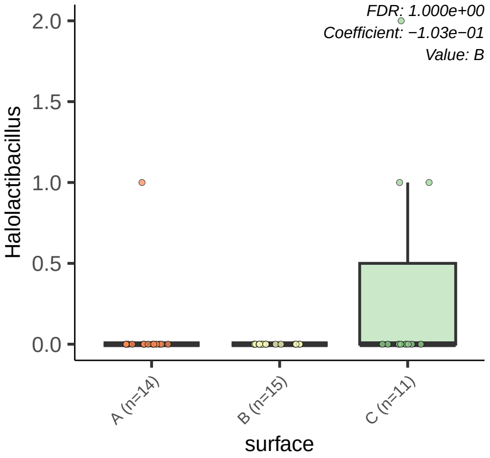

FDR: 1.000e+00
Coefficient: −1.03e−01
Value: B
2.0
1.5
1.0
0.5
0.0
Halolactibacillus
A (n=14)
B (n=15)
C (n=11)
surface

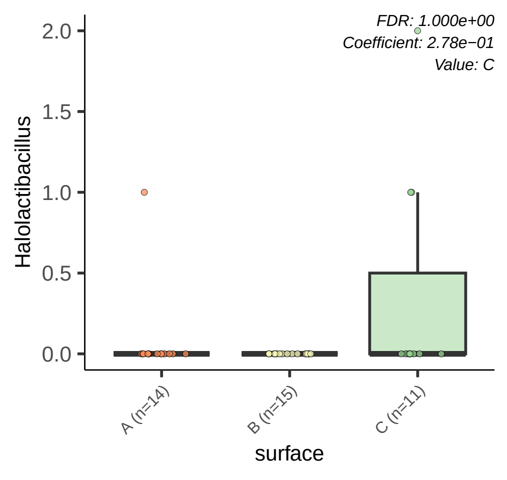

FDR: 1.000e+00
Coefficient: 2.78e−01
Value: C
Halolactibacillus
2.0
1.5
1.0
0.5
0.0
A (n=14)
B (n=15)
C (n=11)
surface

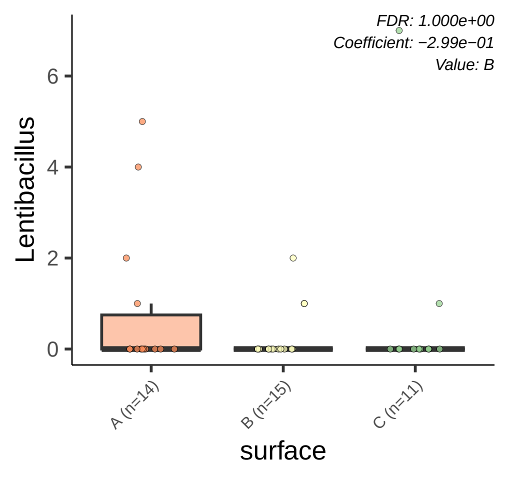

FDR: 1.000e+00
Coefficient: −2.99e−01
Value: B
Lentibacillus
0
2
4
6
A (n=14)
B (n=15)
C (n=11)
surface

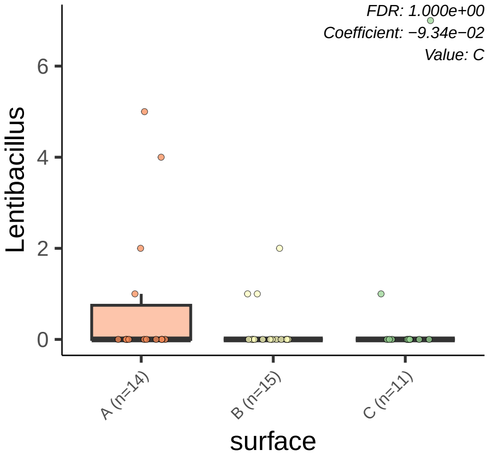

FDR: 1.000e+00
Coefficient: −9.34e−02
Value: C
Lentibacillus
6
4
2
0
A (n=14)
B (n=15)
C (n=11)
surface

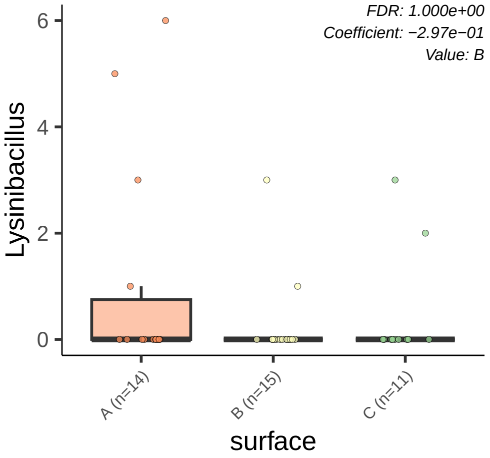

FDR: 1.000e+00
Coefficient: −2.97e−01
Value: B
Lysinibacillus
0
2
4
6
A (n=14)
B (n=15)
C (n=11)
surface

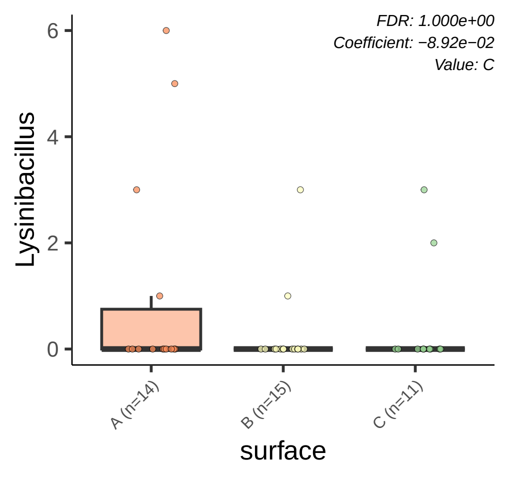
FDR: 1.000e+00
Coefficient: −8.92e−02
Value: C
Lysinibacillus
6
4
2
0
A (n=14)
B (n=15)
C (n=11)
surface

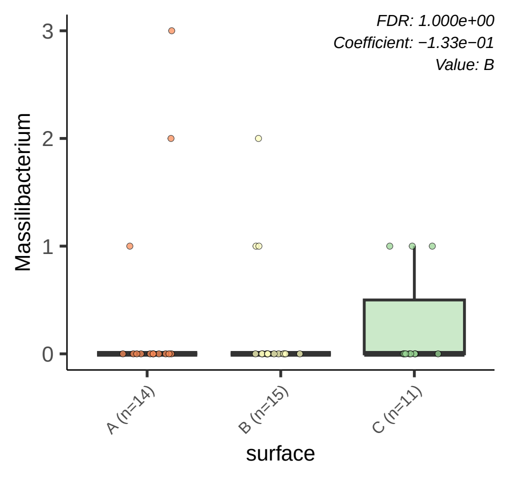

FDR: 1.000e+00
Coefficient: −1.33e−01
Value: B
Massilibacterium
3
2
1
0
A (n=14)
B (n=15)
C (n=11)
surface

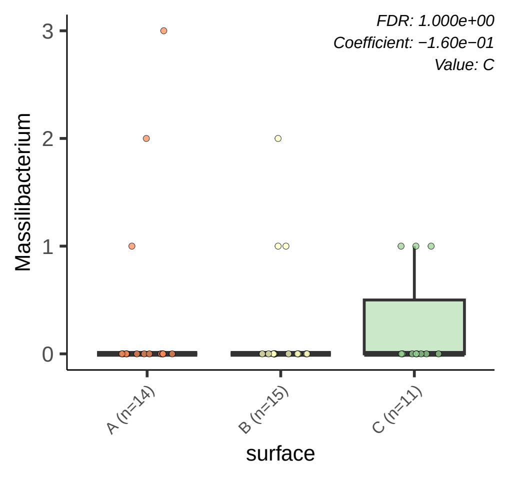
FDR: 1.000e+00
Coefficient: −1.60e−01
Value: C
Massilibacterium
3
2
1
0
A (n=14)
B (n=15)
C (n=11)
surface

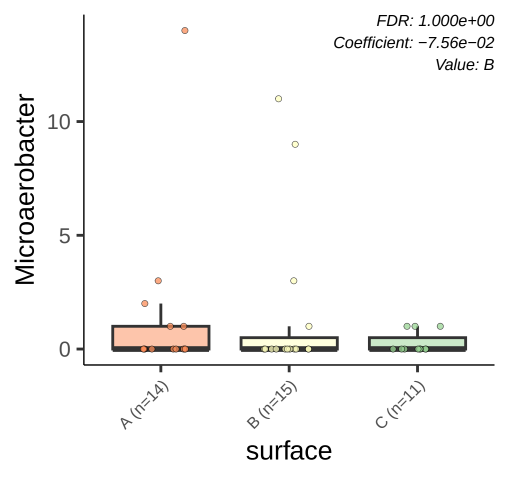

FDR: 1.000e+00
Coefficient: −7.56e−02
Value: B
Microaerobacter
10
5
0
A (n=14)
B (n=15)
C (n=11)
surface

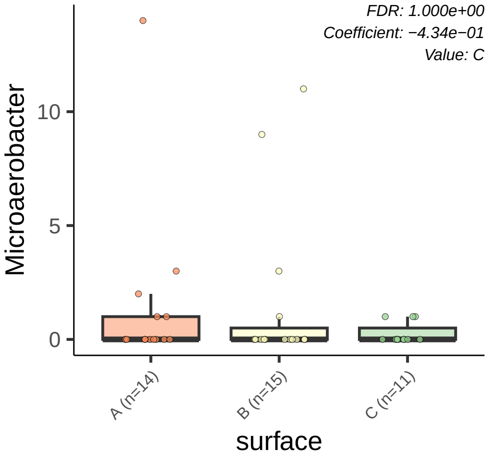
FDR: 1.000e+00
Coefficient: −4.34e−01
Value: C
Microaerobacter
10
5
0
A (n=14)
B (n=15)
C (n=11)
surface

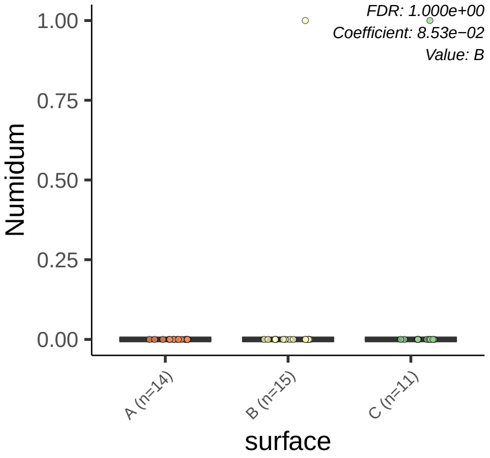
FDR: 1.000e+00
Coefficient: 8.53e−02
Value: B
Numidum
1.00
0.75
0.50
0.25
0.00
A (n=14)
B (n=15)
C (n=11)
surface

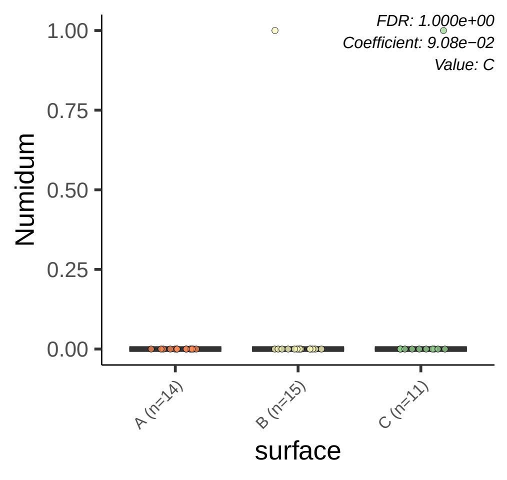
FDR: 1.000e+00
Coefficient: 9.08e−02
Value: C
Numidum
1.00
0.75
0.50
0.25
0.00
A (n=14)
B (n=15)
C (n=11)
surface

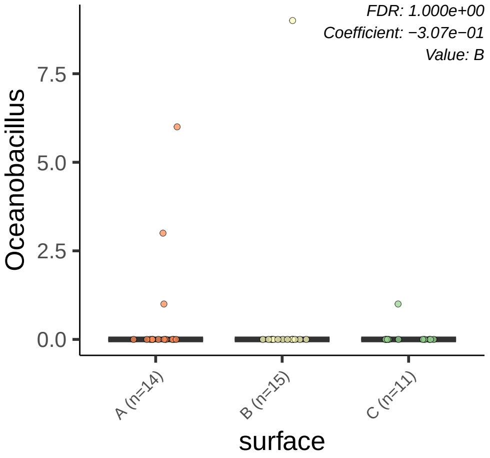
FDR: 1.000e+00
Coefficient: −3.07e−01
Value: B
Oceanobacillus
7.5
5.0
2.5
0.0
A (n=14)
B (n=15)
C (n=11)
surface

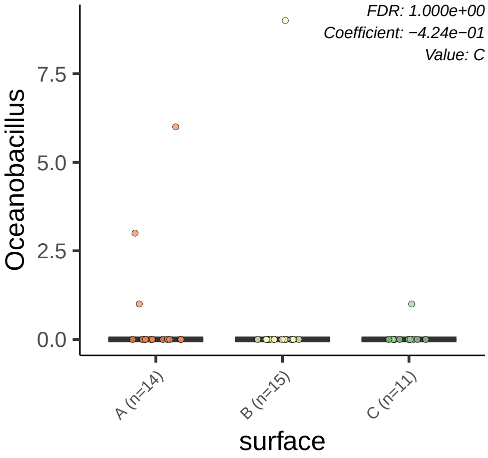

FDR: 1.000e+00
Coefficient: −4.24e−01
Value: C
Oceanobacillus
7.5
5.0
2.5
0.0
A (n=14)
B (n=15)
C (n=11)
surface

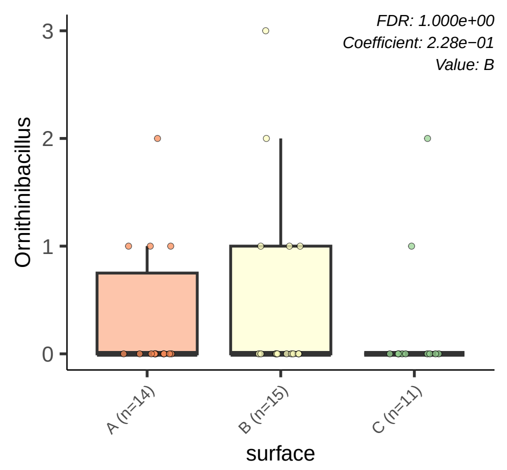

FDR: 1.000e+00
Coefficient: 2.28e−01
Value: B
Ornithinibacillus
3
2
1
0
A (n=14)
B (n=15)
C (n=11)
surface

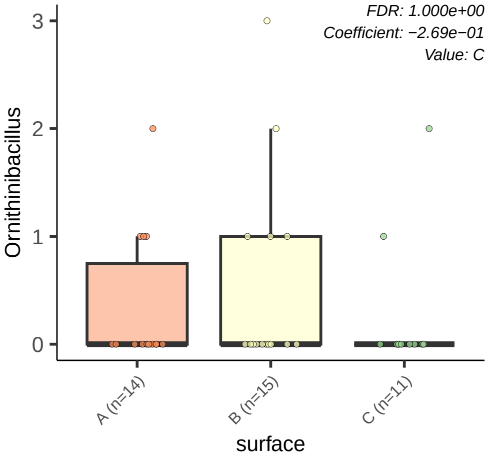
FDR: 1.000e+00
Coefficient: −2.69e−01
Value: C
Ornithinibacillus
3
2
1
0
A (n=14)
B (n=15)
C (n=11)
surface

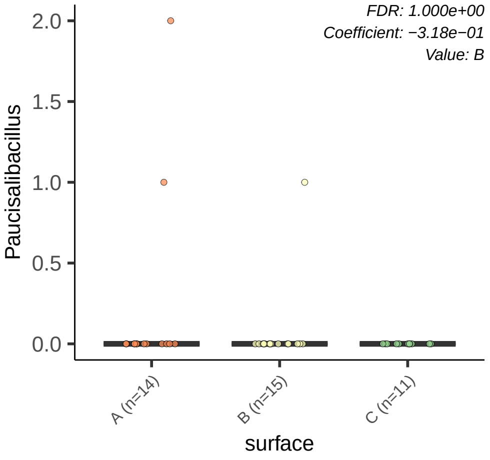
FDR: 1.000e+00
Coefficient: −3.18e−01
Value: B
Paucisalibacillus
2.0
1.5
1.0
0.5
0.0
A (n=14)
B (n=15)
C (n=11)
surface

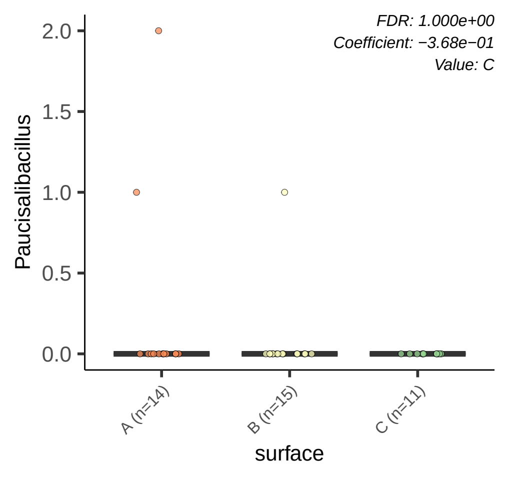
FDR: 1.000e+00
Coefficient: −3.68e−01
Value: C
Paucisalibacillus
2.0
1.5
1.0
0.5
0.0
A (n=14)
B (n=15)
C (n=11)
surface

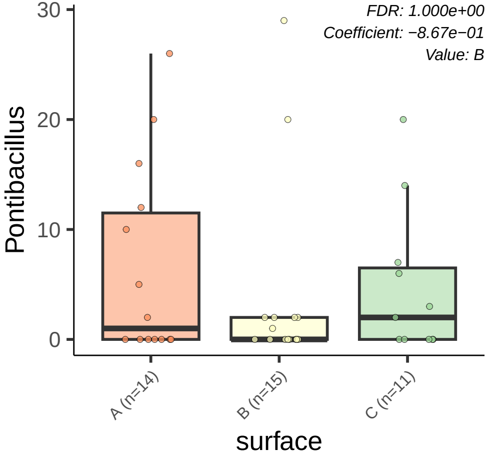
FDR: 1.000e+00
Coefficient: −8.67e−01
Value: B
Pontibacillus
30
20
10
0
A (n=14)
B (n=15)
C (n=11)
surface

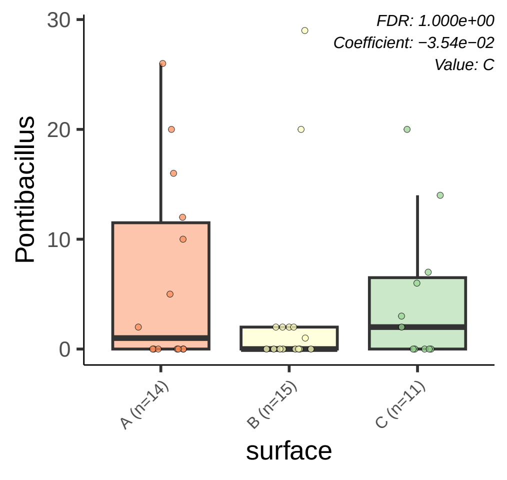
FDR: 1.000e+00
Coefficient: −3.54e−02
Value: C
Pontibacillus
30
20
10
0
A (n=14)
B (n=15)
C (n=11)
surface

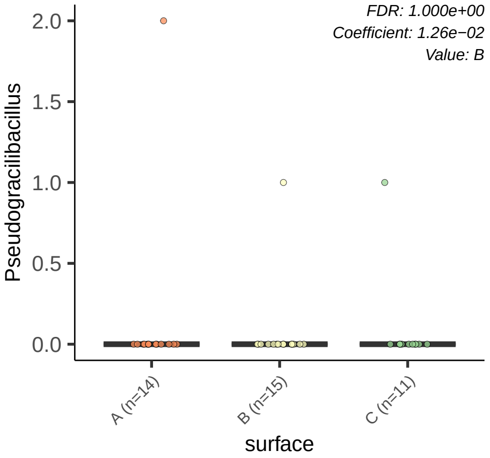

FDR: 1.000e+00
Coefficient: 1.26e−02
Value: B
Pseudogracilibacillus
2.0
1.5
1.0
0.5
0.0
A (n=14)
B (n=15)
C (n=11)
surface

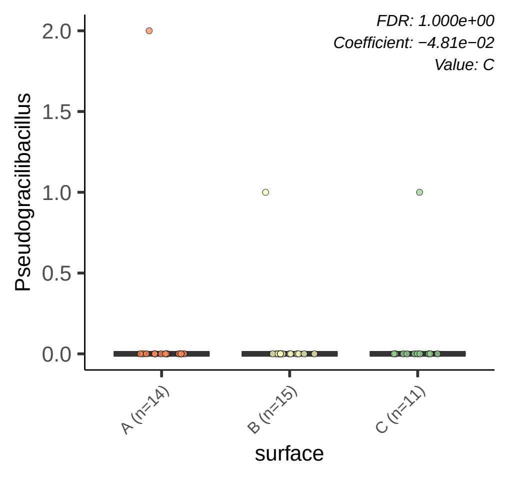
FDR: 1.000e+00
Coefficient: −4.81e−02
Value: C
Pseudogracilibacillus
2.0
1.5
1.0
0.5
0.0
A (n=14)
B (n=15)
C (n=11)
surface

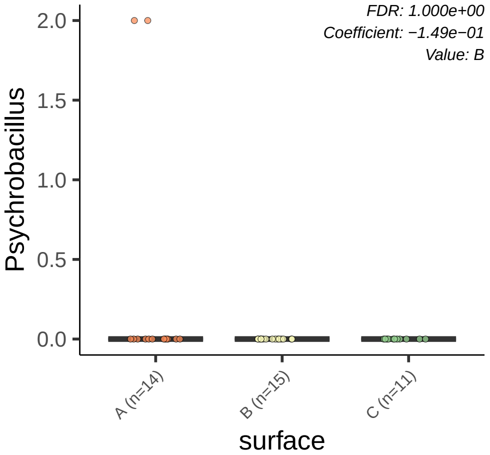

FDR: 1.000e+00
Coefficient: −1.49e−01
Value: B
Psychrobacillus
2.0
1.5
1.0
0.5
0.0
A (n=14)
B (n=15)
C (n=11)
surface

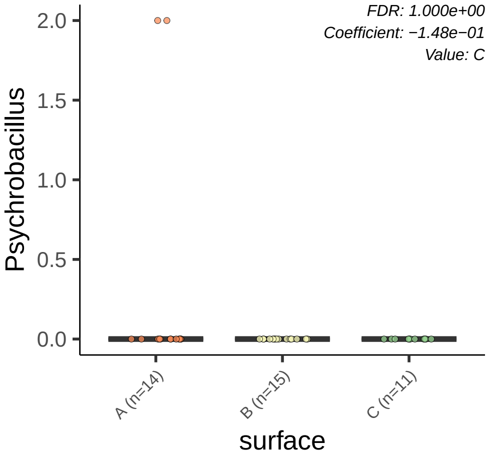

FDR: 1.000e+00
Coefficient: −1.48e−01
Value: C
Psychrobacillus
2.0
1.5
1.0
0.5
0.0
A (n=14)
B (n=15)
C (n=11)
surface

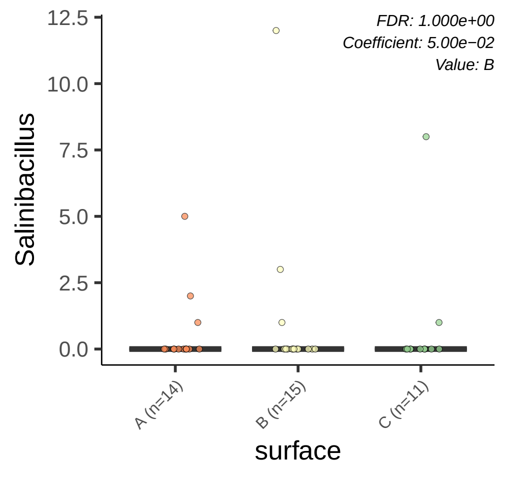
FDR: 1.000e+00
Coefficient: 5.00e−02
Value: B
Salinibacillus
12.5
10.0
7.5
5.0
2.5
0.0
A (n=14)
B (n=15)
C (n=11)
surface

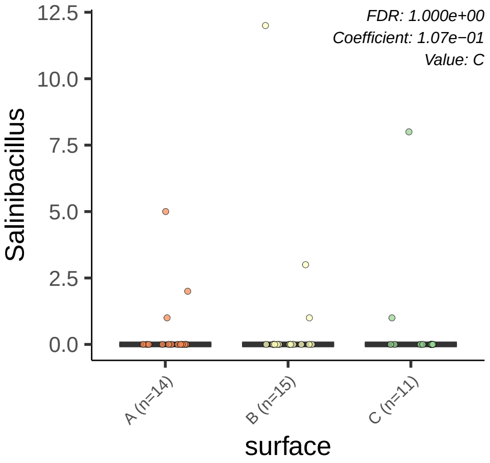

FDR: 1.000e+00
Coefficient: 1.07e−01
Value: C
Salinibacillus
12.5
10.0
7.5
5.0
2.5
0.0
A (n=14)
B (n=15)
C (n=11)
surface

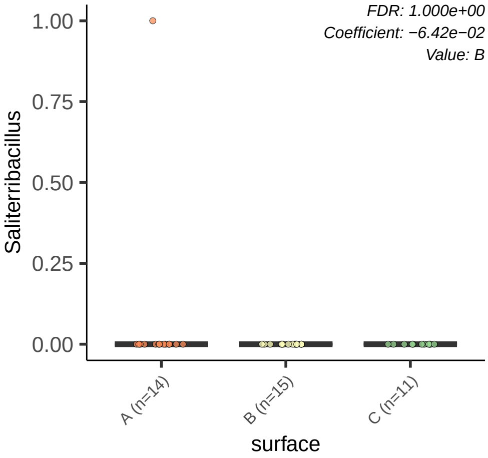

FDR: 1.000e+00
Coefficient: −6.42e−02
Value: B
Saliterribacillus
1.00
0.75
0.50
0.25
0.00
A (n=14)
B (n=15)
C (n=11)
surface

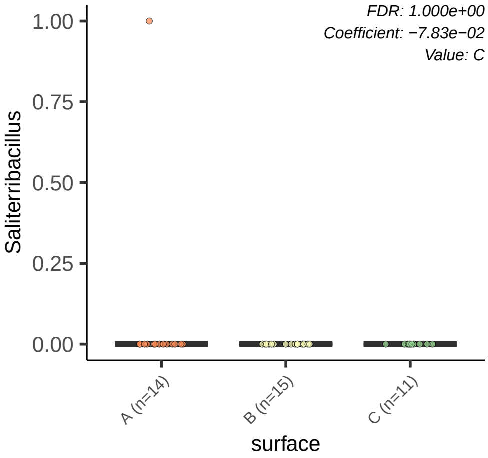
FDR: 1.000e+00
Coefficient: −7.83e−02
Value: C
Saliterribacillus
1.00
0.75
0.50
0.25
0.00
A (n=14)
B (n=15)
C (n=11)
surface

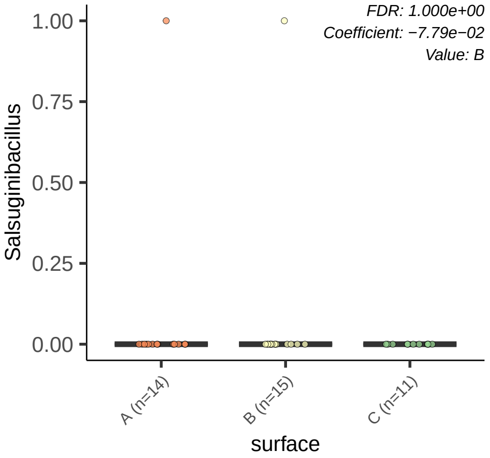

FDR: 1.000e+00
Coefficient: −7.79e−02
Value: B
Salsuginibacillus
1.00
0.75
0.50
0.25
0.00
A (n=14)
B (n=15)
C (n=11)
surface

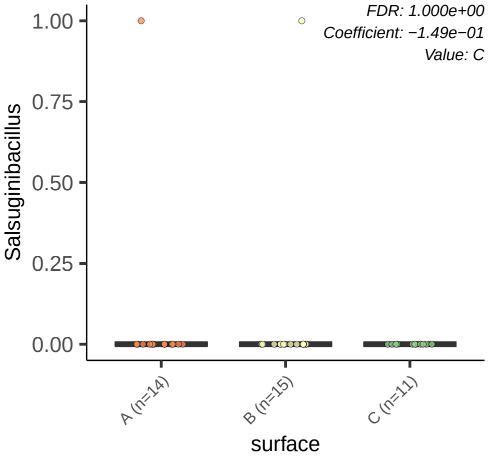
FDR: 1.000e+00
Coefficient: −1.49e−01
Value: C
Salsuginibacillus
1.00
0.75
0.50
0.25
0.00
A (n=14)
B (n=15)
C (n=11)
surface

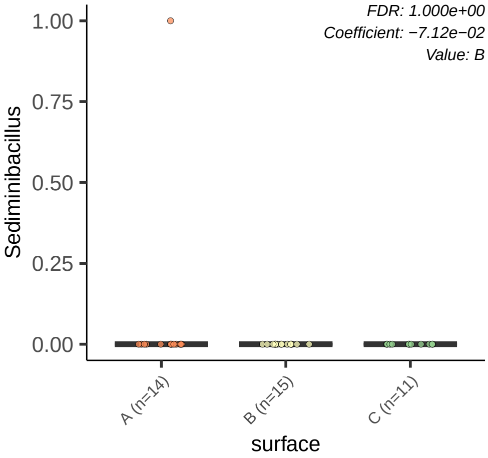
FDR: 1.000e+00
Coefficient: −7.12e−02
Value: B
Sediminibacillus
1.00
0.75
0.50
0.25
0.00
A (n=14)
B (n=15)
C (n=11)
surface

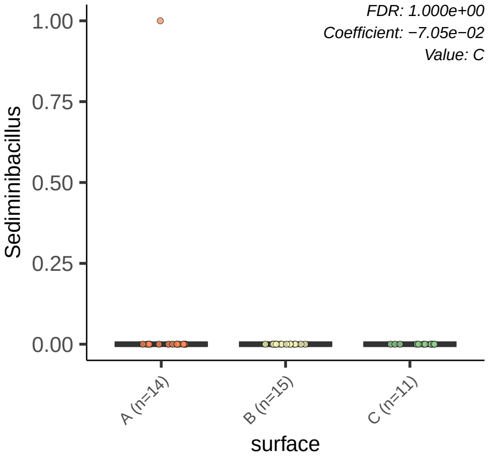
FDR: 1.000e+00
Coefficient: −7.05e−02
Value: C
Sediminibacillus
1.00
0.75
0.50
0.25
0.00
A (n=14)
B (n=15)
C (n=11)
surface

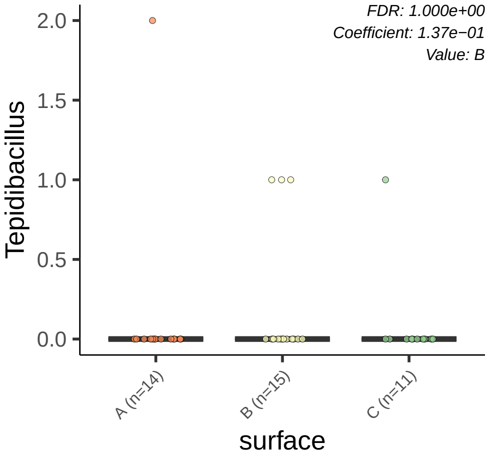
FDR: 1.000e+00
Coefficient: 1.37e−01
Value: B
Tepidibacillus
2.0
1.5
1.0
0.5
0.0
A (n=14)
B (n=15)
C (n=11)
surface

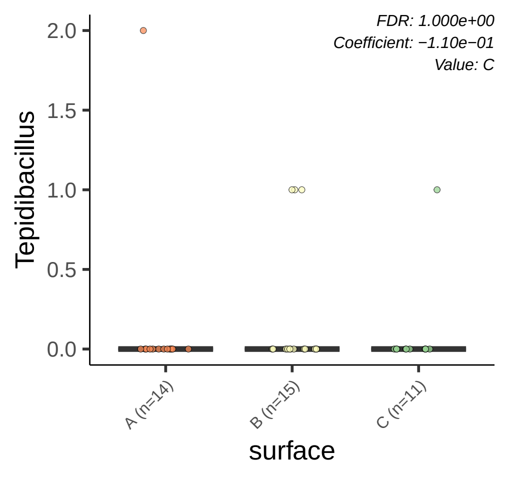
FDR: 1.000e+00
Coefficient: −1.10e−01
Value: C
Tepidibacillus
2.0
1.5
1.0
0.5
0.0
A (n=14)
B (n=15)
C (n=11)
surface

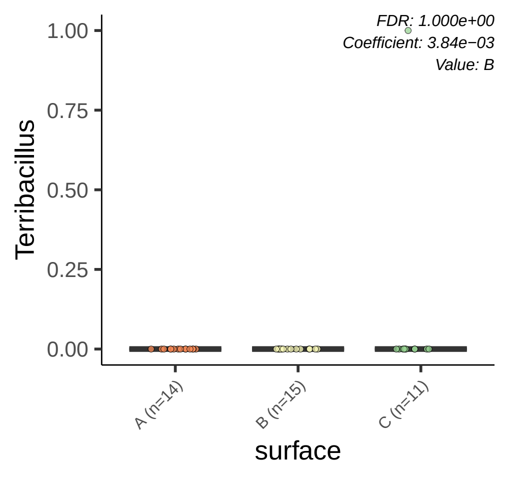

FDR: 1.000e+00
Coefficient: 3.84e−03
Value: B
Terribacillus
1.00
0.75
0.50
0.25
0.00
A (n=14)
B (n=15)
C (n=11)
surface

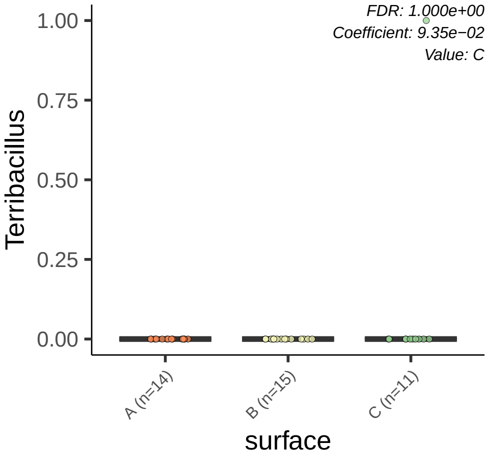
FDR: 1.000e+00
Coefficient: 9.35e−02
Value: C
Terribacillus
1.00
0.75
0.50
0.25
0.00
A (n=14)
B (n=15)
C (n=11)
surface

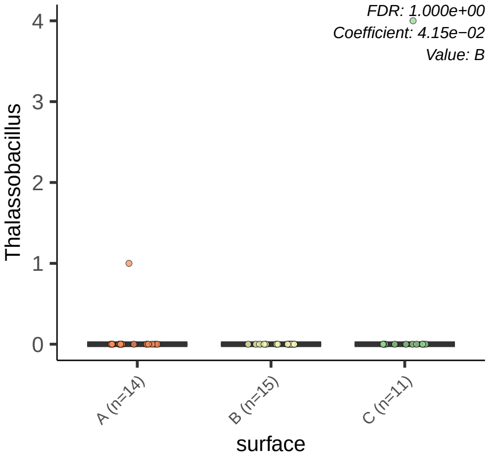
FDR: 1.000e+00
Coefficient: 4.15e−02
Value: B
Thalassobacillus
0
1
2
3
4
A (n=14)
B (n=15)
C (n=11)
surface

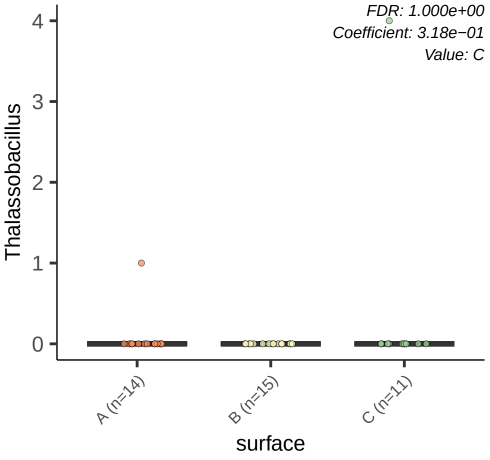

FDR: 1.000e+00
Coefficient: 3.18e−01
Value: C
Thalassobacillus
4
3
2
1
0
A (n=14)
B (n=15)
C (n=11)
surface

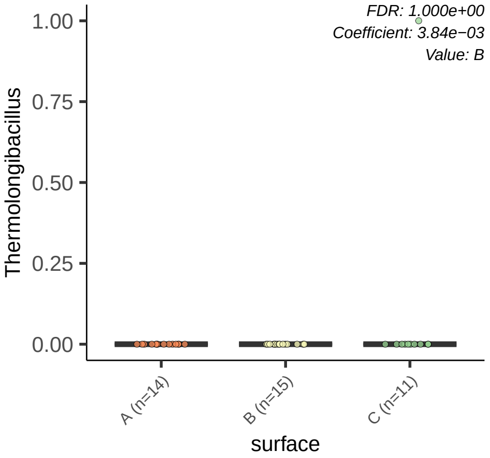

FDR: 1.000e+00
Coefficient: 3.84e−03
Value: B
Thermolongibacillus
1.00
0.75
0.50
0.25
0.00
A (n=14)
B (n=15)
C (n=11)
surface

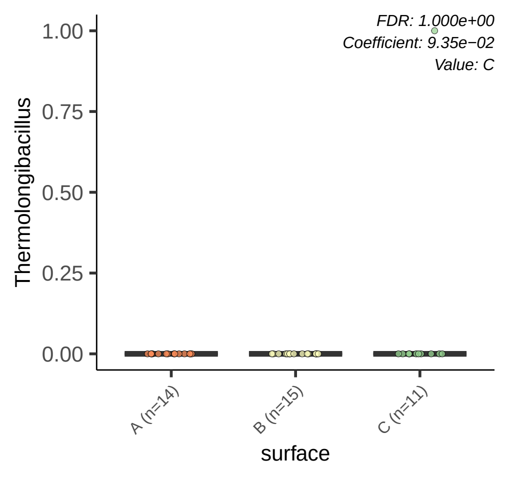
FDR: 1.000e+00
Coefficient: 9.35e−02
Value: C
Thermolongibacillus
1.00
0.75
0.50
0.25
0.00
A (n=14)
B (n=15)
C (n=11)
surface

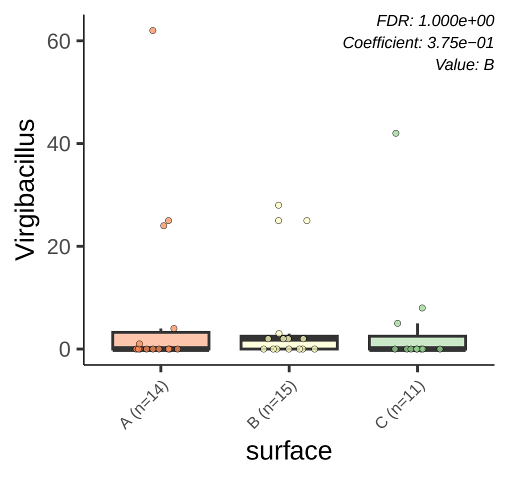
FDR: 1.000e+00
Coefficient: 3.75e−01
Value: B
Virgibacillus
60
40
20
0
A (n=14)
B (n=15)
C (n=11)
surface

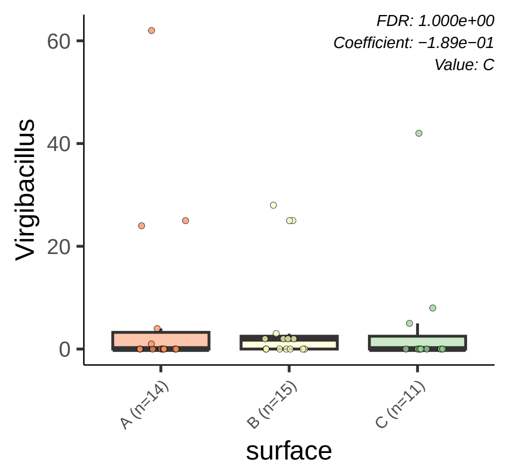
FDR: 1.000e+00
Coefficient: −1.89e−01
Value: C
Virgibacillus
60
40
20
0
A (n=14)
B (n=15)
C (n=11)
surface

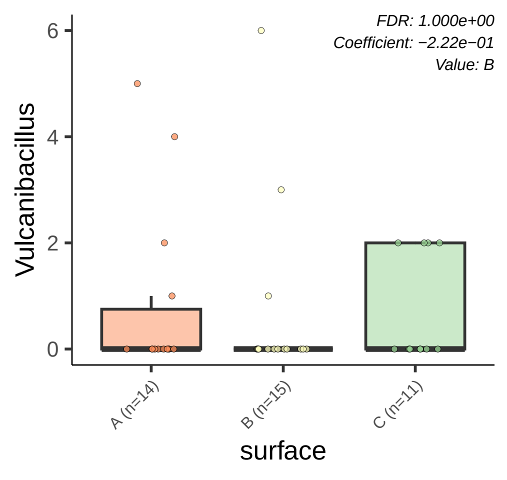

FDR: 1.000e+00
Coefficient: −2.22e−01
Value: B
Vulcanibacillus
0
2
4
6
A (n=14)
B (n=15)
C (n=11)
surface

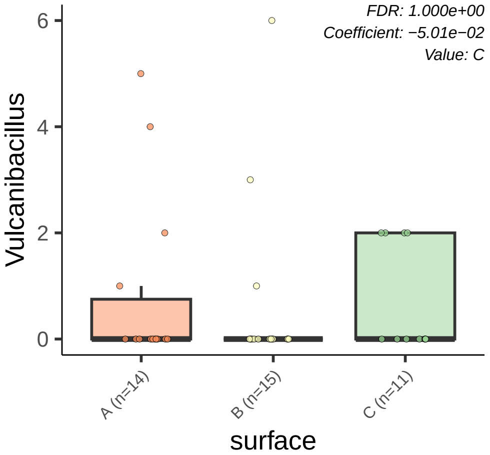

FDR: 1.000e+00
Coefficient: −5.01e−02
Value: C
Vulcanibacillus
6
4
2
0
A (n=14)
B (n=15)
C (n=11)
surface

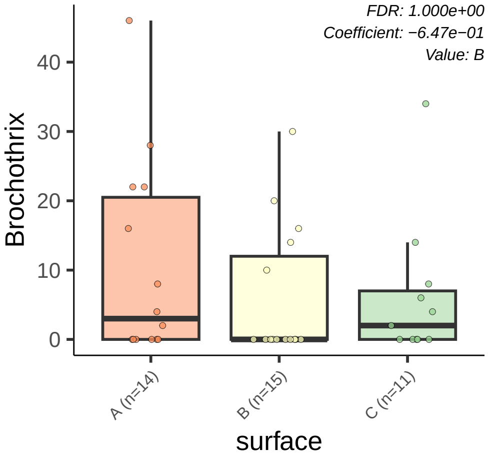
FDR: 1.000e+00
Coefficient: −6.47e−01
Value: B
Brochothrix
40
30
20
10
0
A (n=14)
B (n=15)
C (n=11)
surface

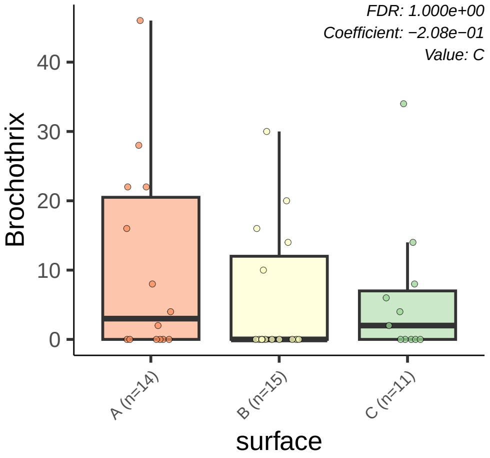

FDR: 1.000e+00
Coefficient: −2.08e−01
Value: C
Brochothrix
0
10
20
30
40
A (n=14)
B (n=15)
C (n=11)
surface

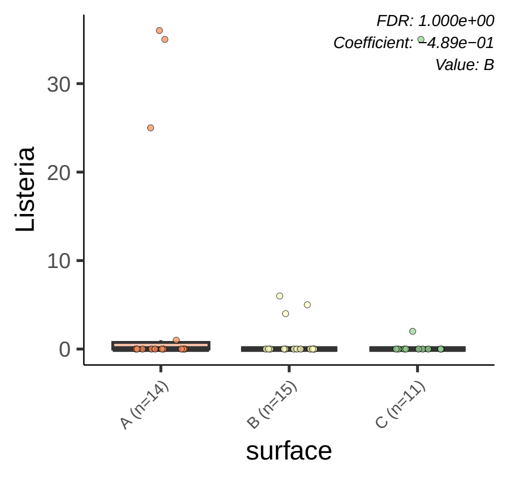

FDR: 1.000e+00
Coefficient: −4.89e−01
Value: B
Listeria
0
10
20
30
A (n=14)
B (n=15)
C (n=11)
surface

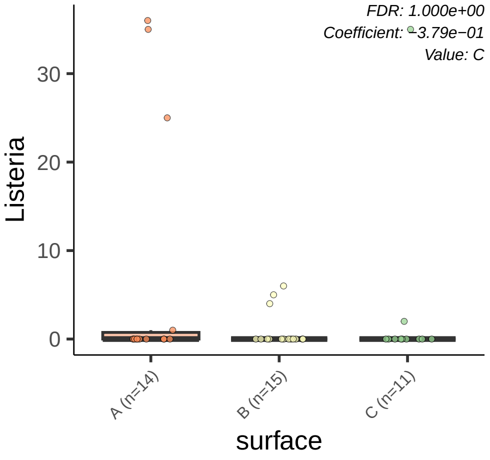
FDR: 1.000e+00
Coefficient: −3.79e−01
Value: C
Listeria
30
20
10
0
A (n=14)
B (n=15)
C (n=11)
surface

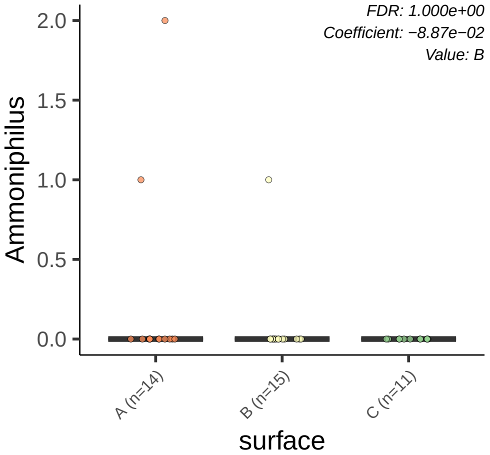
FDR: 1.000e+00
Coefficient: −8.87e−02
Value: B
Ammoniphilus
2.0
1.5
1.0
0.5
0.0
A (n=14)
B (n=15)
C (n=11)
surface

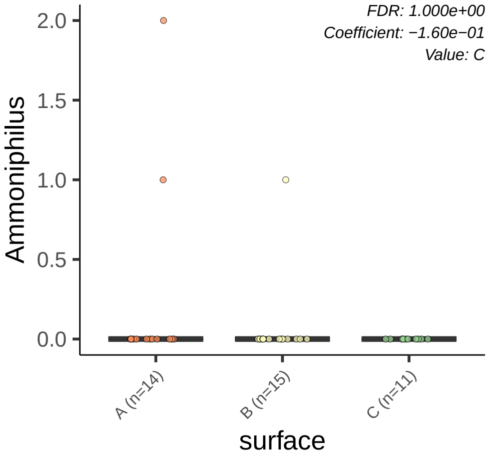
FDR: 1.000e+00
Coefficient: −1.60e−01
Value: C
Ammoniphilus
2.0
1.5
1.0
0.5
0.0
A (n=14)
B (n=15)
C (n=11)
surface

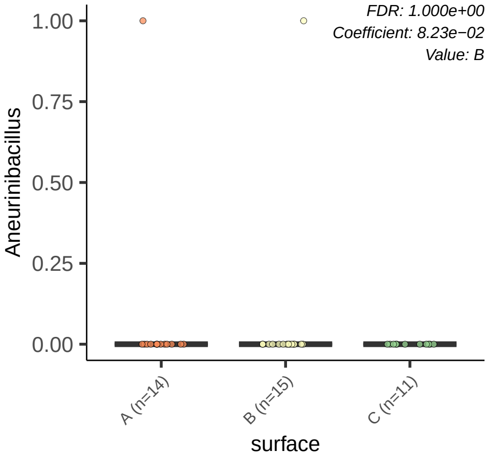
FDR: 1.000e+00
Coefficient: 8.23e−02
Value: B
1.00
0.75
0.50
0.25
0.00
Aneurinibacillus
A (n=14)
B (n=15)
C (n=11)
surface

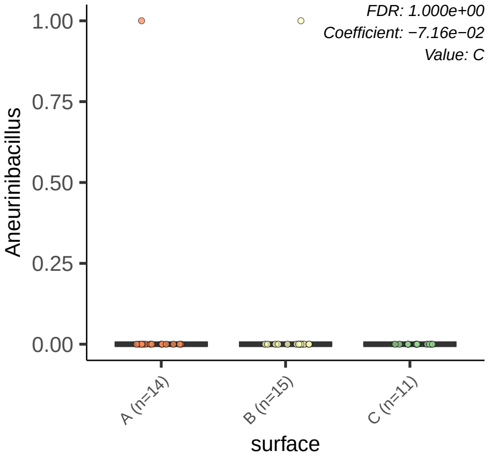
FDR: 1.000e+00
Coefficient: −7.16e−02
Value: C
Aneurinibacillus
1.00
0.75
0.50
0.25
0.00
A (n=14)
B (n=15)
C (n=11)
surface

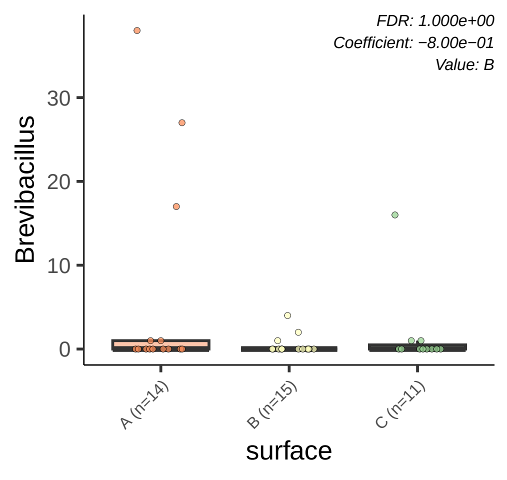

FDR: 1.000e+00
Coefficient: −8.00e−01
Value: B
Brevibacillus
0
10
20
30
A (n=14)
B (n=15)
C (n=11)
surface

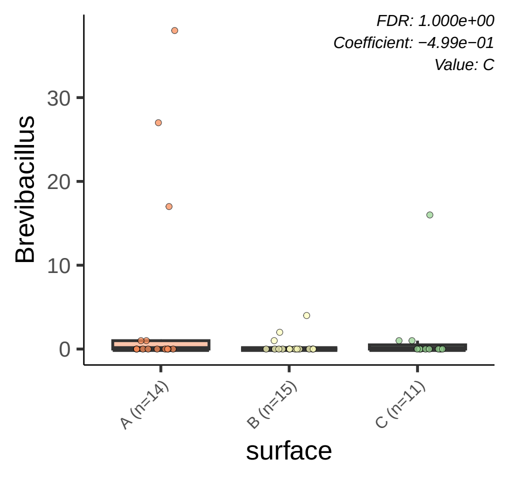
FDR: 1.000e+00
Coefficient: −4.99e−01
Value: C
Brevibacillus
30
20
10
0
A (n=14)
B (n=15)
C (n=11)
surface

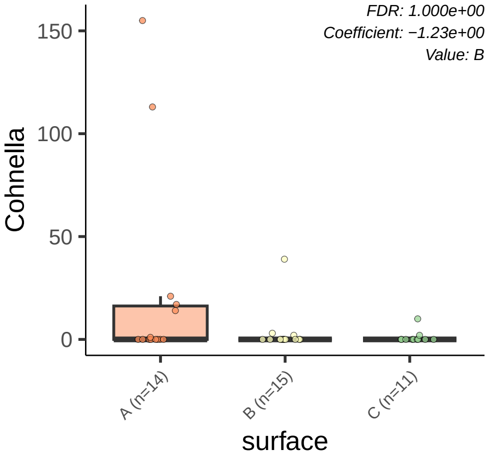
FDR: 1.000e+00
Coefficient: −1.23e+00
Value: B
Cohnella
150
100
50
0
A (n=14)
B (n=15)
C (n=11)
surface

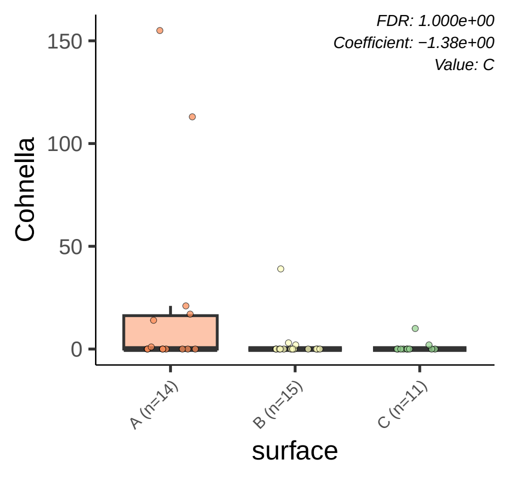
FDR: 1.000e+00
Coefficient: −1.38e+00
Value: C
Cohnella
150
100
50
0
A (n=14)
B (n=15)
C (n=11)
surface

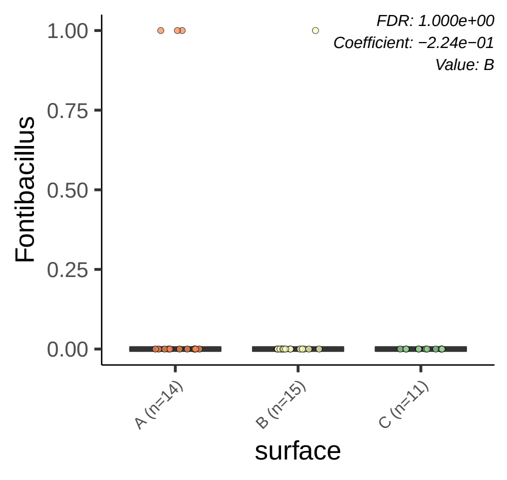

FDR: 1.000e+00
Coefficient: −2.24e−01
Value: B
1.00
0.75
0.50
0.25
0.00
Fontibacillus
A (n=14)
B (n=15)
C (n=11)
surface

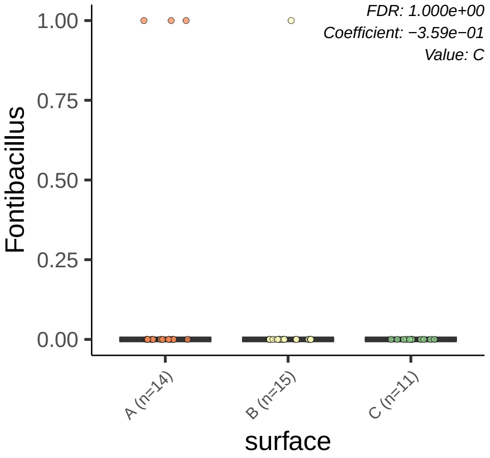

FDR: 1.000e+00
Coefficient: −3.59e−01
Value: C
Fontibacillus
1.00
0.75
0.50
0.25
0.00
A (n=14)
B (n=15)
C (n=11)
surface

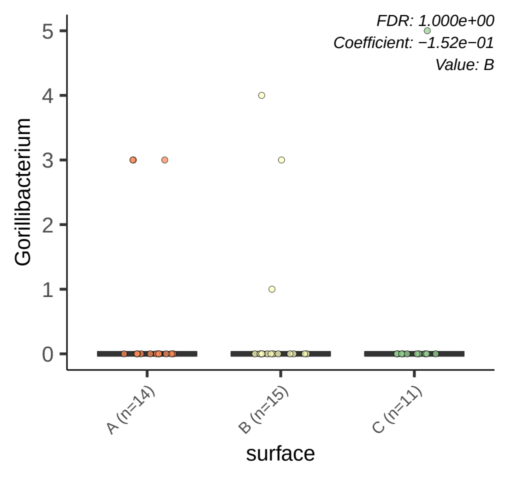

FDR: 1.000e+00
Coefficient: −1.52e−01
Value: B
Gorillibacterium
0
1
2
3
4
5
A (n=14)
B (n=15)
C (n=11)
surface

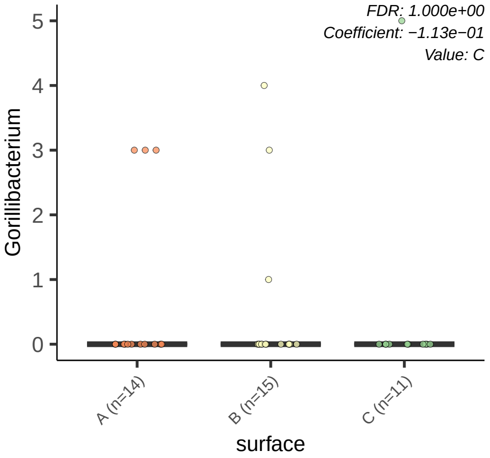
FDR: 1.000e+00
Coefficient: −1.13e−01
Value: C
Gorillibacterium
0
1
2
3
4
5
A (n=14)
B (n=15)
C (n=11)
surface

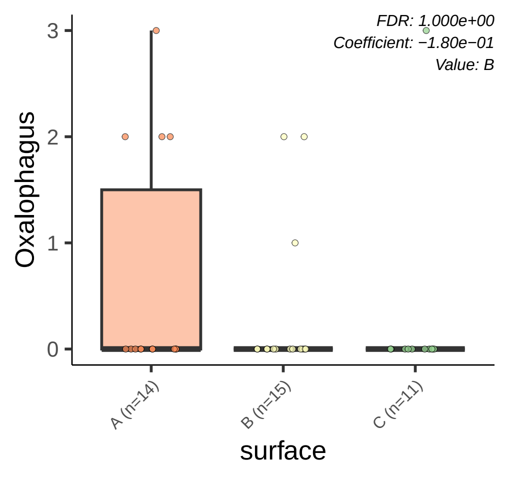
FDR: 1.000e+00
Coefficient: −1.80e−01
Value: B
Oxalophagus
3
2
1
0
A (n=14)
B (n=15)
C (n=11)
surface

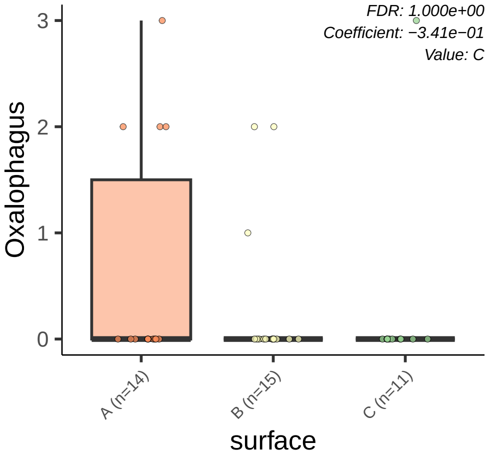
FDR: 1.000e+00
Coefficient: −3.41e−01
Value: C
Oxalophagus
3
2
1
0
A (n=14)
B (n=15)
C (n=11)
surface

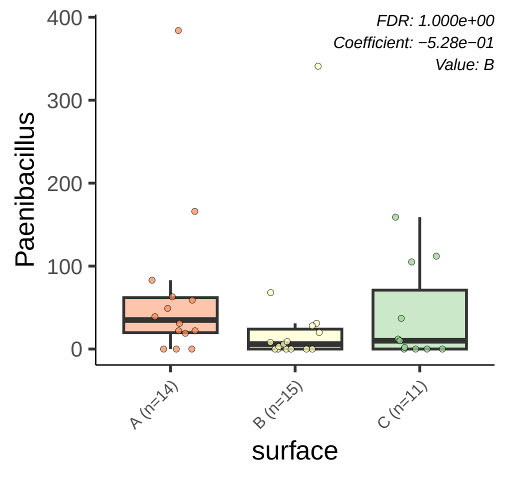
FDR: 1.000e+00
Coefficient: −5.28e−01
Value: B
Paenibacillus
400
300
200
100
0
A (n=14)
B (n=15)
C (n=11)
surface

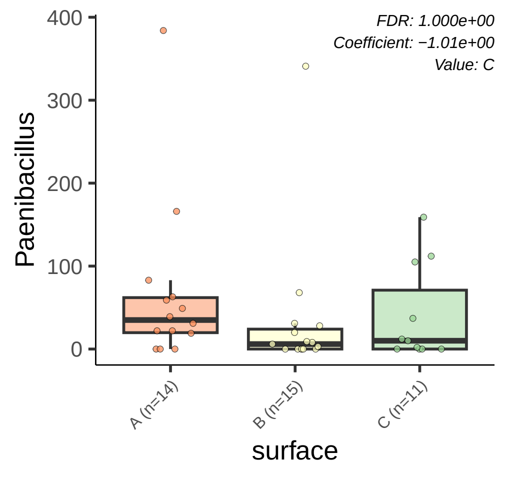

FDR: 1.000e+00
Coefficient: −1.01e+00
Value: C
Paenibacillus
400
300
200
100
0
A (n=14)
B (n=15)
C (n=11)
surface

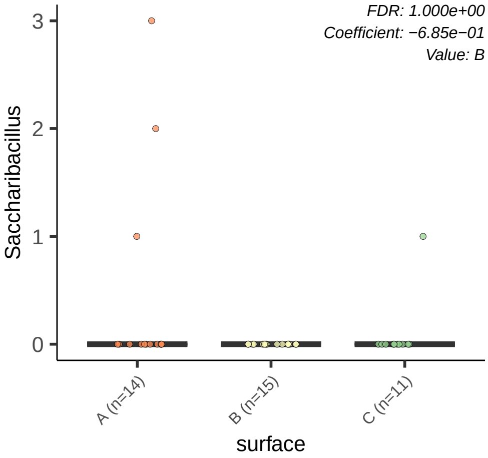
FDR: 1.000e+00
Coefficient: −6.85e−01
Value: B
Saccharibacillus
3
2
1
0
A (n=14)
B (n=15)
C (n=11)
surface

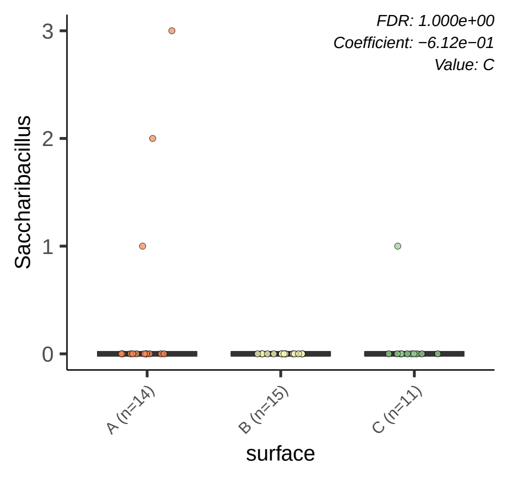

FDR: 1.000e+00
Coefficient: −6.12e−01
Value: C
Saccharibacillus
3
2
1
0
A (n=14)
B (n=15)
C (n=11)
surface

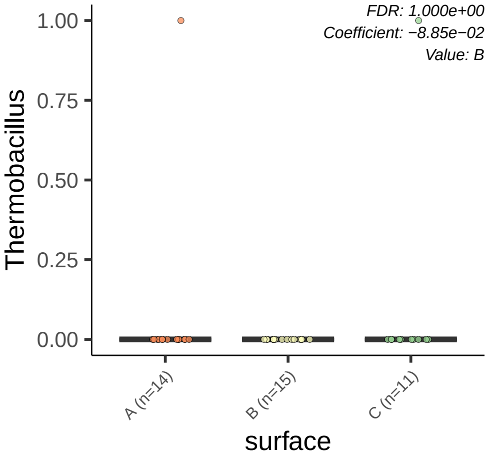

FDR: 1.000e+00
Coefficient: −8.85e−02
Value: B
Thermobacillus
1.00
0.75
0.50
0.25
0.00
A (n=14)
B (n=15)
C (n=11)
surface

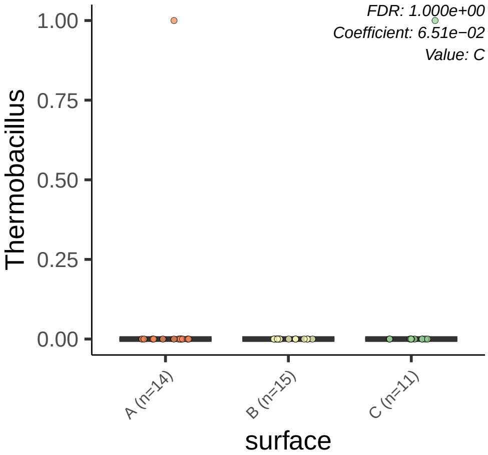
FDR: 1.000e+00
Coefficient: 6.51e−02
Value: C
Thermobacillus
1.00
0.75
0.50
0.25
0.00
A (n=14)
B (n=15)
C (n=11)
surface

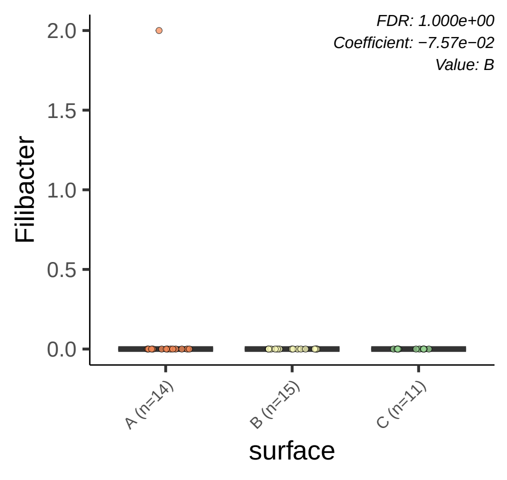

FDR: 1.000e+00
Coefficient: −7.57e−02
Value: B
Filibacter
2.0
1.5
1.0
0.5
0.0
A (n=14)
B (n=15)
C (n=11)
surface

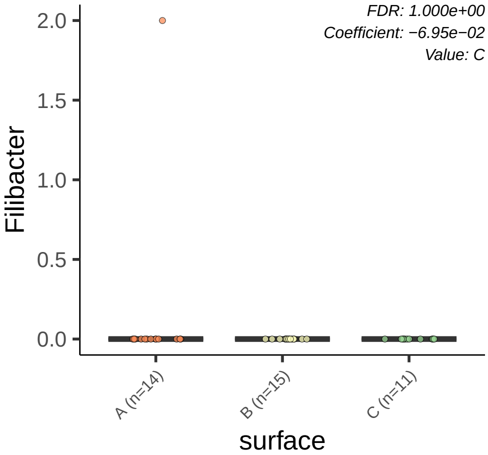
FDR: 1.000e+00
Coefficient: −6.95e−02
Value: C
Filibacter
2.0
1.5
1.0
0.5
0.0
A (n=14)
B (n=15)
C (n=11)
surface

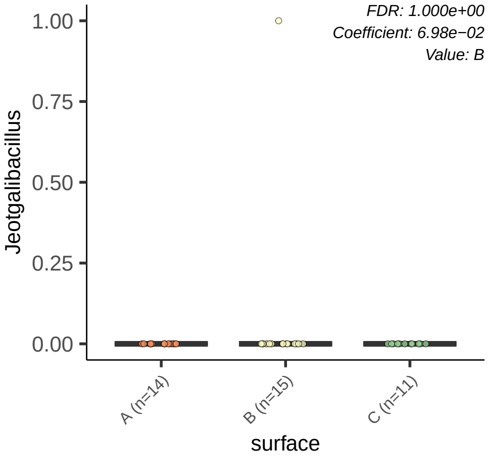
FDR: 1.000e+00
Coefficient: 6.98e−02
Value: B
1.00
0.75
0.50
0.25
0.00
Jeotgalibacillus
A (n=14)
B (n=15)
C (n=11)
surface

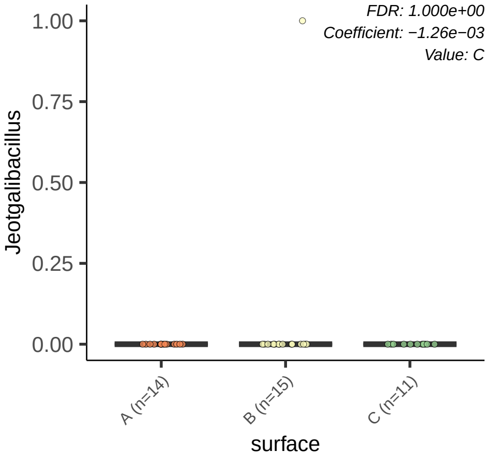
FDR: 1.000e+00
Coefficient: −1.26e−03
Value: C
Jeotgalibacillus
1.00
0.75
0.50
0.25
0.00
A (n=14)
B (n=15)
C (n=11)
surface

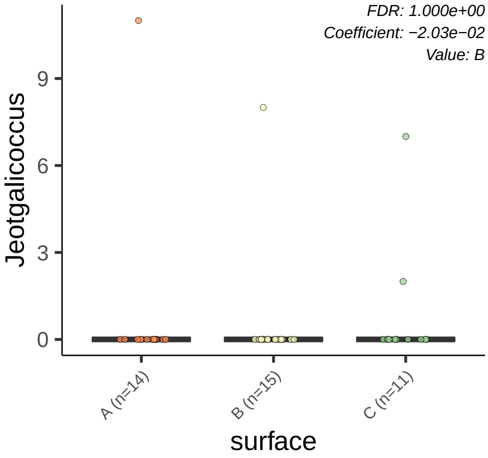

FDR: 1.000e+00
Coefficient: −2.03e−02
Value: B
Jeotgalicoccus
9
6
3
0
A (n=14)
B (n=15)
C (n=11)
surface

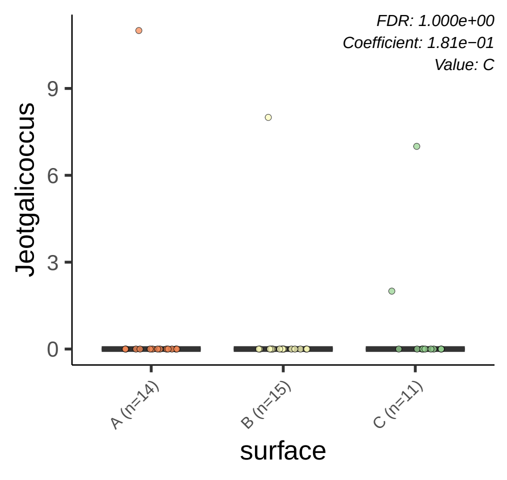

FDR: 1.000e+00
Coefficient: 1.81e−01
Value: C
Jeotgalicoccus
0
3
6
9
A (n=14)
B (n=15)
C (n=11)
surface

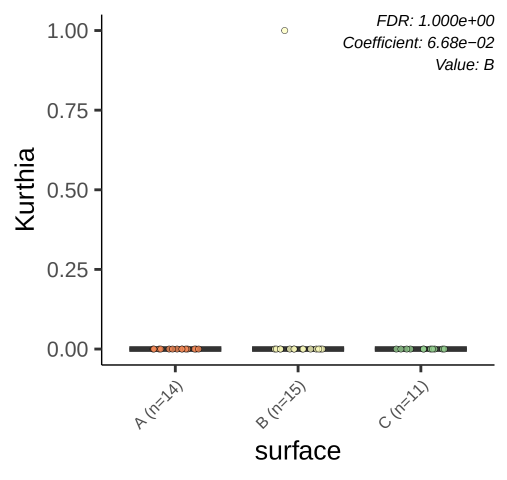

FDR: 1.000e+00
Coefficient: 6.68e−02
Value: B
Kurthia
1.00
0.75
0.50
0.25
0.00
A (n=14)
B (n=15)
C (n=11)
surface

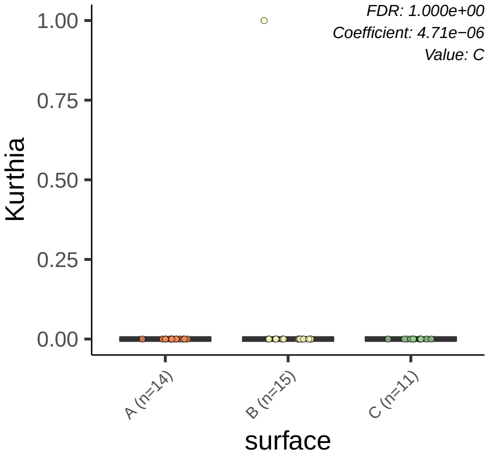
FDR: 1.000e+00
Coefficient: 4.71e−06
Value: C
Kurthia
1.00
0.75
0.50
0.25
0.00
A (n=14)
B (n=15)
C (n=11)
surface

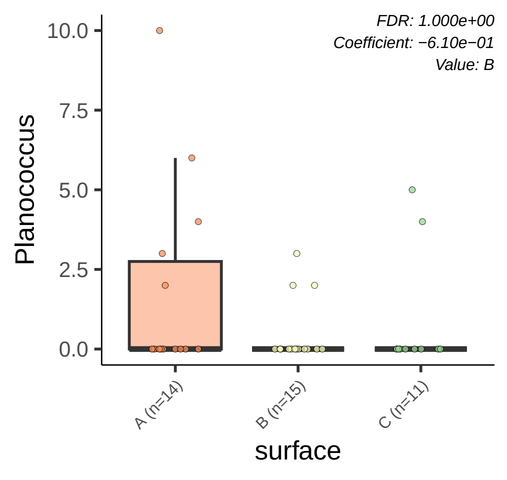

FDR: 1.000e+00
Coefficient: −6.10e−01
Value: B
Planococcus
10.0
7.5
5.0
2.5
0.0
A (n=14)
B (n=15)
C (n=11)
surface

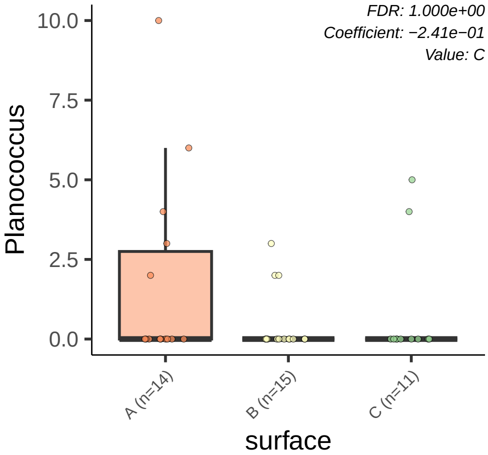
FDR: 1.000e+00
Coefficient: −2.41e−01
Value: C
Planococcus
10.0
7.5
5.0
2.5
0.0
A (n=14)
B (n=15)
C (n=11)
surface

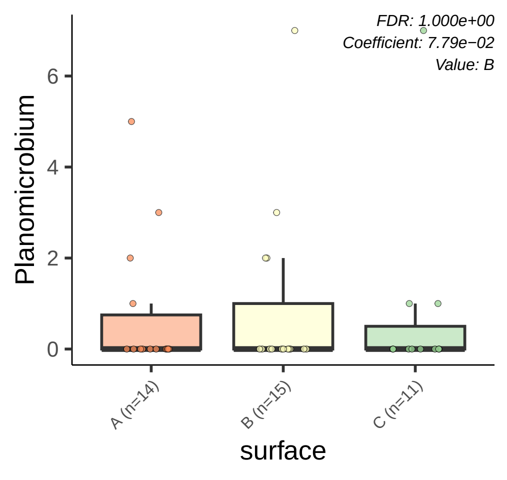

FDR: 1.000e+00
Coefficient: 7.79e−02
Value: B
Planomicrobium
0
2
4
6
A (n=14)
B (n=15)
C (n=11)
surface

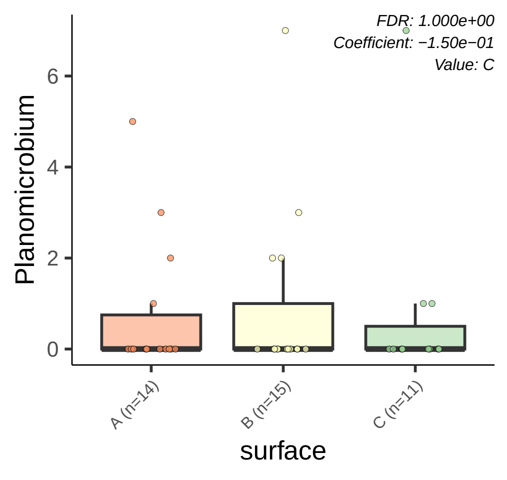
FDR: 1.000e+00
Coefficient: −1.50e−01
Value: C
Planomicrobium
0
2
4
6
A (n=14)
B (n=15)
C (n=11)
surface

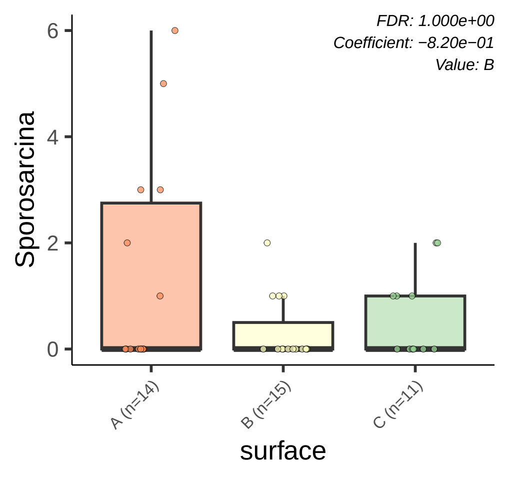

FDR: 1.000e+00
Coefficient: −8.20e−01
Value: B
Sporosarcina
0
2
4
6
A (n=14)
B (n=15)
C (n=11)
surface

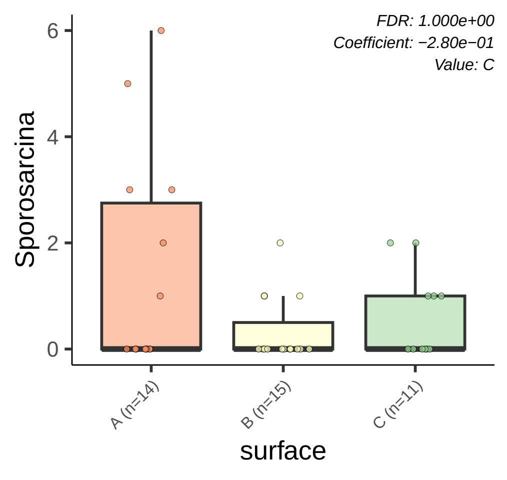
FDR: 1.000e+00
Coefficient: −2.80e−01
Value: C
Sporosarcina
0
2
4
6
A (n=14)
B (n=15)
C (n=11)
surface

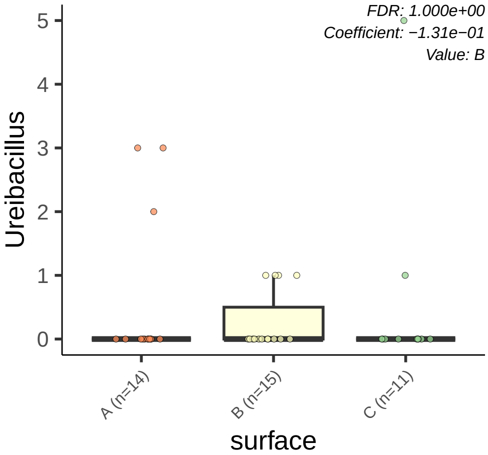
FDR: 1.000e+00
Coefficient: −1.31e−01
Value: B
Ureibacillus
5
4
3
2
1
0
A (n=14)
B (n=15)
C (n=11)
surface

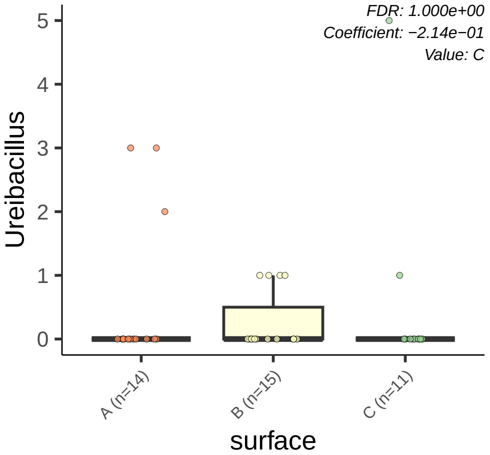
FDR: 1.000e+00
Coefficient: −2.14e−01
Value: C
Ureibacillus
0
1
2
3
4
5
A (n=14)
B (n=15)
C (n=11)
surface

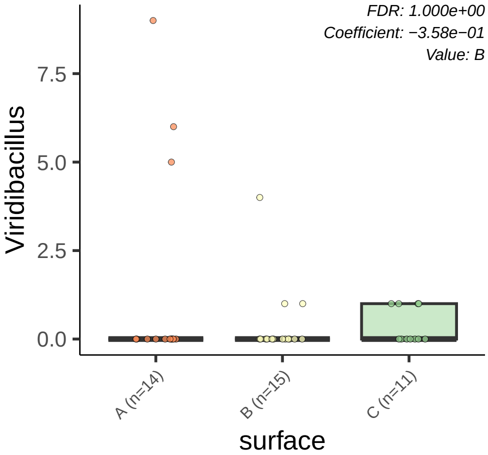

FDR: 1.000e+00
Coefficient: −3.58e−01
Value: B
Viridibacillus
7.5
5.0
2.5
0.0
A (n=14)
B (n=15)
C (n=11)
surface

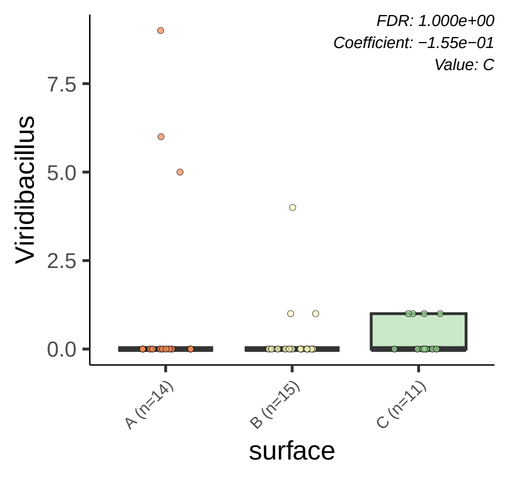

FDR: 1.000e+00
Coefficient: −1.55e−01
Value: C
Viridibacillus
7.5
5.0
2.5
0.0
A (n=14)
B (n=15)
C (n=11)
surface

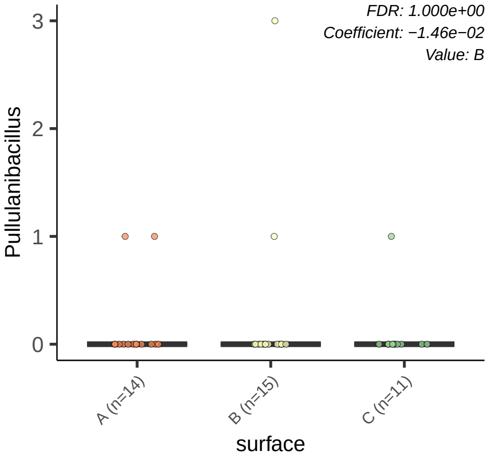

FDR: 1.000e+00
Coefficient: −1.46e−02
Value: B
Pullulanibacillus
0
1
2
3
A (n=14)
B (n=15)
C (n=11)
surface

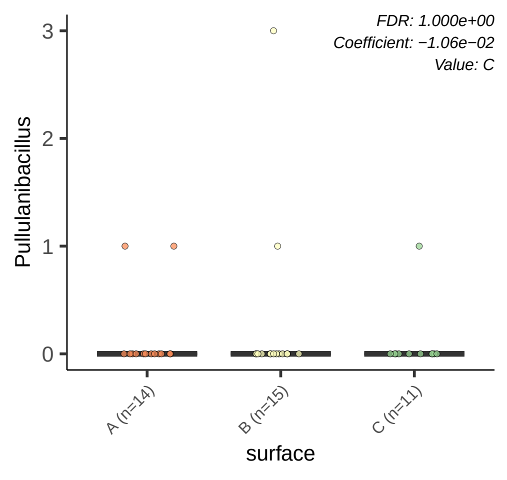
FDR: 1.000e+00
Coefficient: −1.06e−02
Value: C
Pullulanibacillus
3
2
1
0
A (n=14)
B (n=15)
C (n=11)
surface

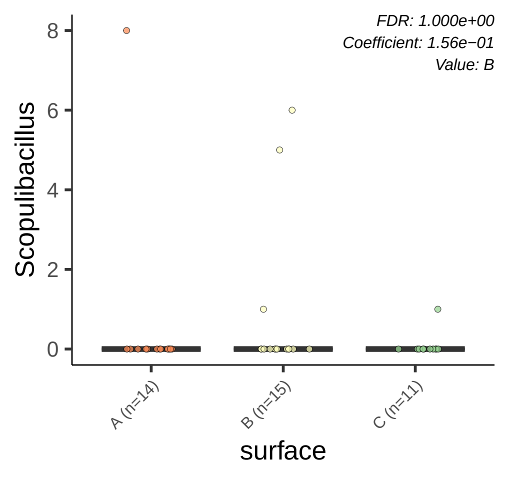
FDR: 1.000e+00
Coefficient: 1.56e−01
Value: B
Scopulibacillus
8
6
4
2
0
A (n=14)
B (n=15)
C (n=11)
surface

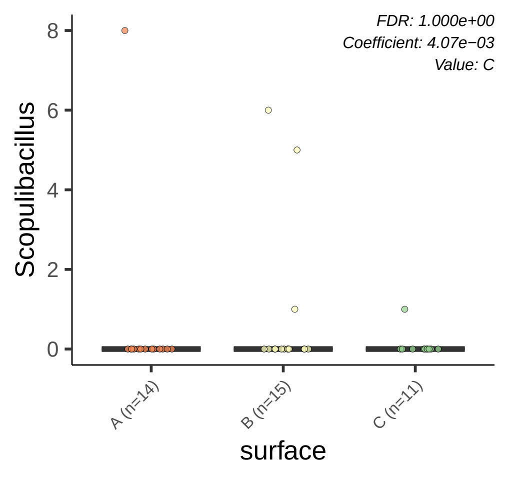

FDR: 1.000e+00
Coefficient: 4.07e−03
Value: C
Scopulibacillus
0
2
4
6
8
A (n=14)
B (n=15)
C (n=11)
surface

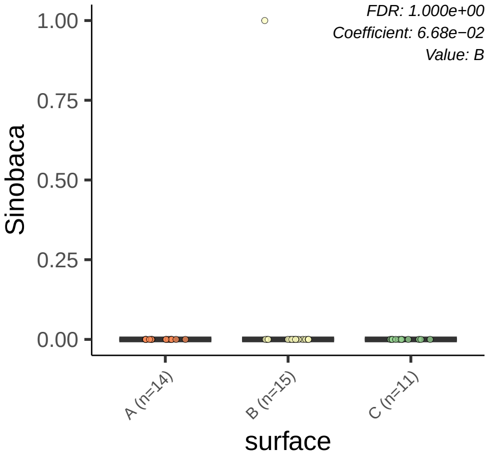

FDR: 1.000e+00
Coefficient: 6.68e−02
Value: B
Sinobaca
1.00
0.75
0.50
0.25
0.00
A (n=14)
B (n=15)
C (n=11)
surface

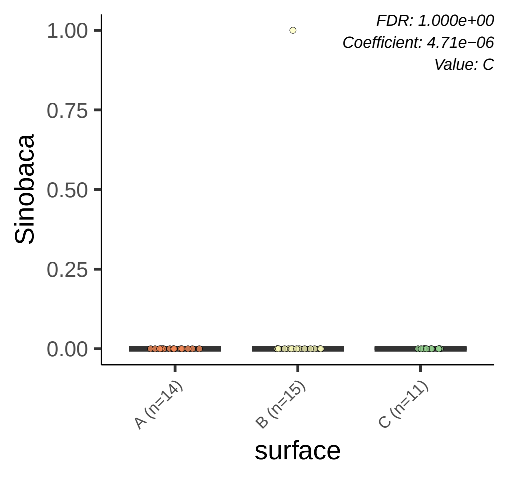
FDR: 1.000e+00
Coefficient: 4.71e−06
Value: C
Sinobaca
1.00
0.75
0.50
0.25
0.00
A (n=14)
B (n=15)
C (n=11)
surface

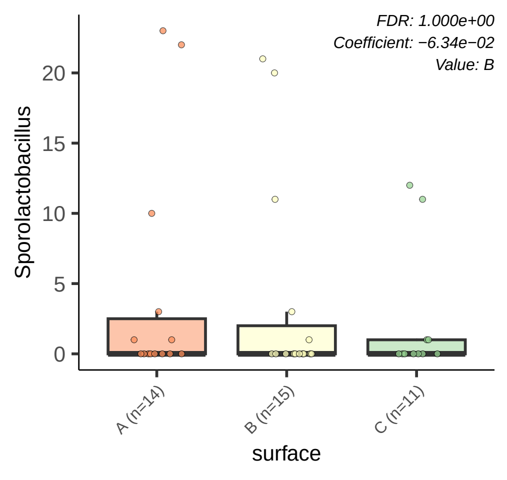
FDR: 1.000e+00
Coefficient: −6.34e−02
Value: B
Sporolactobacillus
0
5
10
15
20
A (n=14)
B (n=15)
C (n=11)
surface

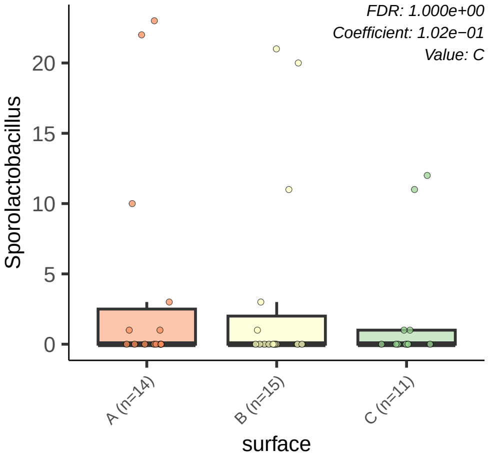

FDR: 1.000e+00
Coefficient: 1.02e−01
Value: C
Sporolactobacillus
0
5
10
15
20
A (n=14)
B (n=15)
C (n=11)
surface

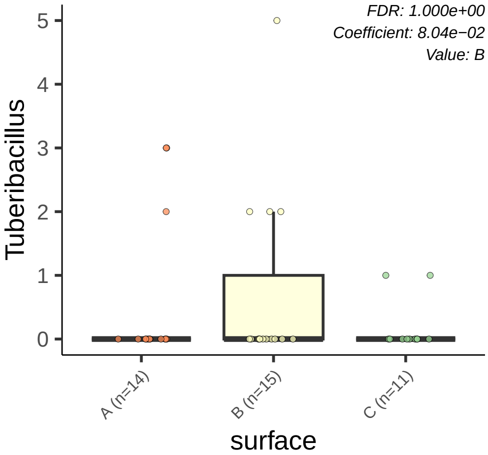
FDR: 1.000e+00
Coefficient: 8.04e−02
Value: B
Tuberibacillus
5
4
3
2
1
0
A (n=14)
B (n=15)
C (n=11)
surface

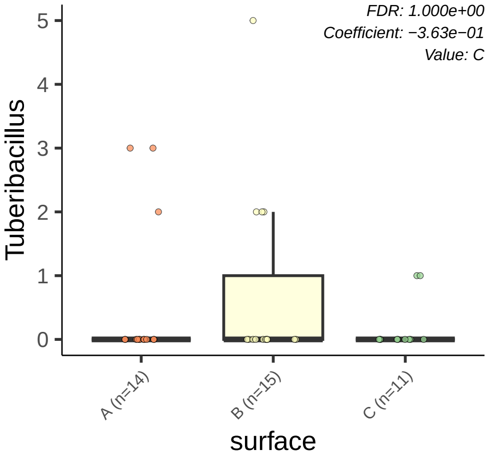
FDR: 1.000e+00
Coefficient: −3.63e−01
Value: C
Tuberibacillus
0
1
2
3
4
5
A (n=14)
B (n=15)
C (n=11)
surface

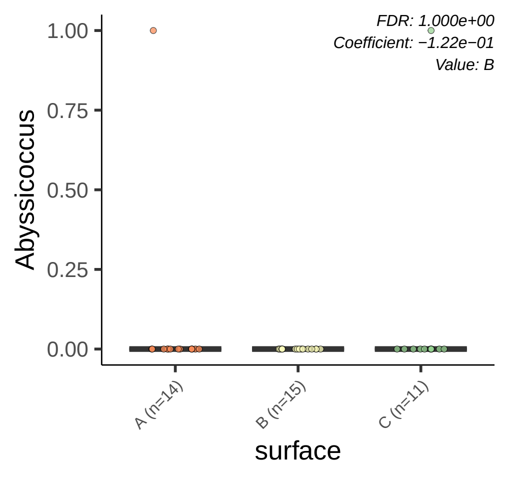
FDR: 1.000e+00
Coefficient: −1.22e−01
Value: B
Abyssicoccus
1.00
0.75
0.50
0.25
0.00
A (n=14)
B (n=15)
C (n=11)
surface

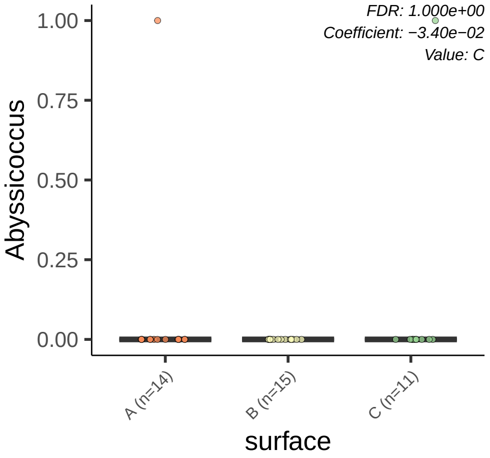

FDR: 1.000e+00
Coefficient: −3.40e−02
Value: C
Abyssicoccus
1.00
0.75
0.50
0.25
0.00
A (n=14)
B (n=15)
C (n=11)
surface

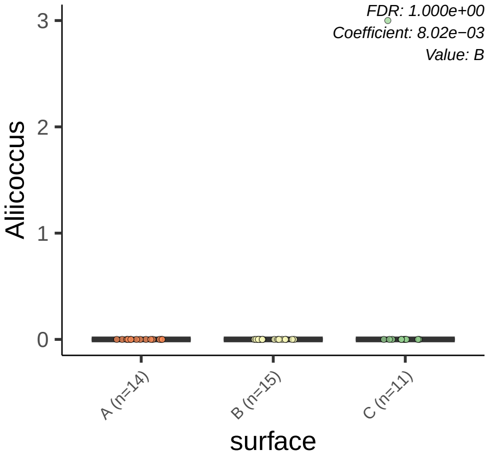
FDR: 1.000e+00
Coefficient: 8.02e−03
Value: B
Aliicoccus
3
2
1
0
A (n=14)
B (n=15)
C (n=11)
surface

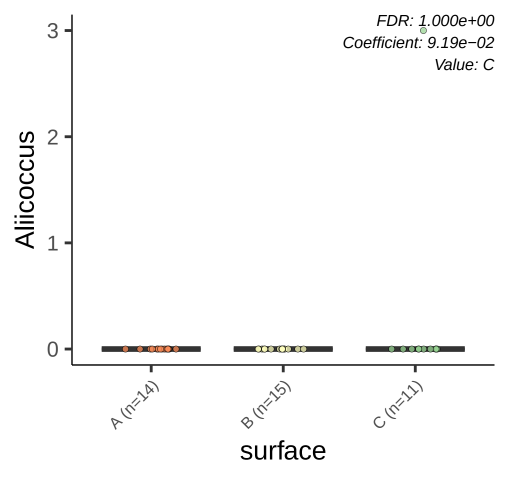
FDR: 1.000e+00
Coefficient: 9.19e−02
Value: C
Aliicoccus
3
2
1
0
A (n=14)
B (n=15)
C (n=11)
surface

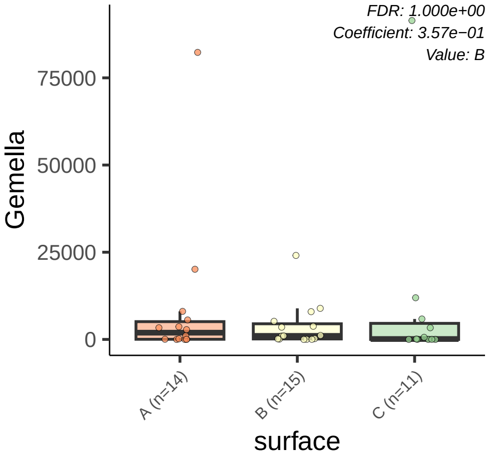

FDR: 1.000e+00
Coefficient: 3.57e−01
Value: B
Gemella
75000
50000
25000
0
A (n=14)
B (n=15)
C (n=11)
surface

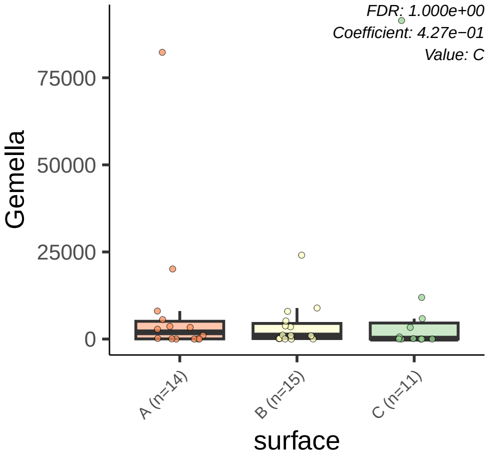
FDR: 1.000e+00
Coefficient: 4.27e−01
Value: C
Gemella
75000
50000
25000
0
A (n=14)
B (n=15)
C (n=11)
surface

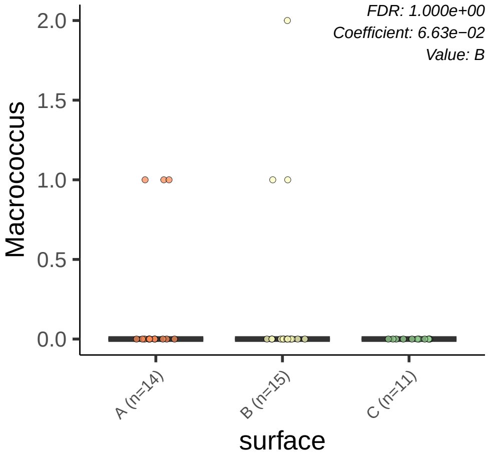

FDR: 1.000e+00
Coefficient: 6.63e−02
Value: B
Macrococcus
2.0
1.5
1.0
0.5
0.0
A (n=14)
B (n=15)
C (n=11)
surface

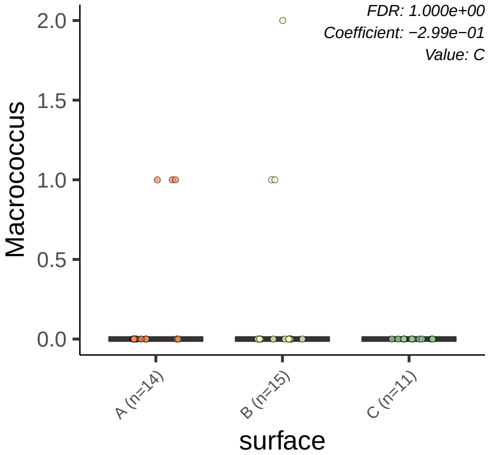
FDR: 1.000e+00
Coefficient: −2.99e−01
Value: C
Macrococcus
2.0
1.5
1.0
0.5
0.0
A (n=14)
B (n=15)
C (n=11)
surface

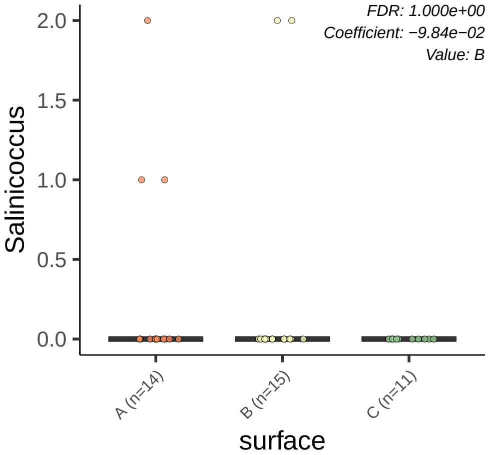
FDR: 1.000e+00
Coefficient: −9.84e−02
Value: B
Salinicoccus
2.0
1.5
1.0
0.5
0.0
A (n=14)
B (n=15)
C (n=11)
surface

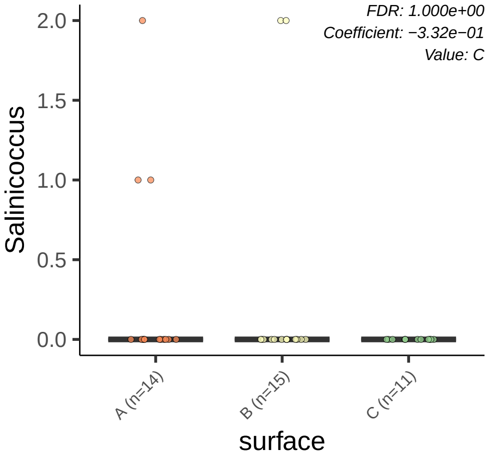
FDR: 1.000e+00
Coefficient: −3.32e−01
Value: C
Salinicoccus
2.0
1.5
1.0
0.5
0.0
A (n=14)
B (n=15)
C (n=11)
surface

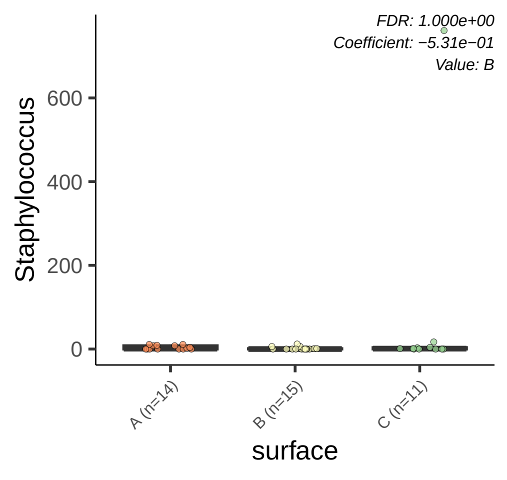
FDR: 1.000e+00
Coefficient: −5.31e−01
Value: B
Staphylococcus
600
400
200
0
A (n=14)
B (n=15)
C (n=11)
surface

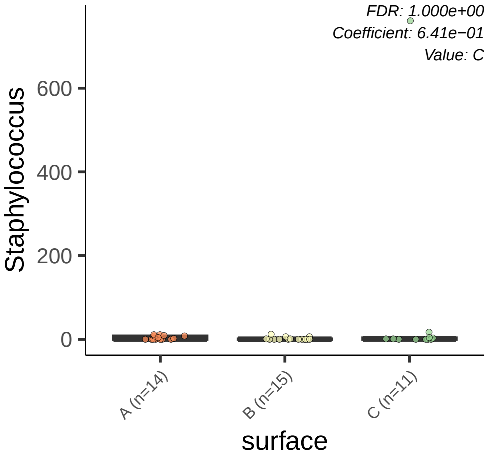

FDR: 1.000e+00
Coefficient: 6.41e−01
Value: C
Staphylococcus
600
400
200
0
A (n=14)
B (n=15)
C (n=11)
surface

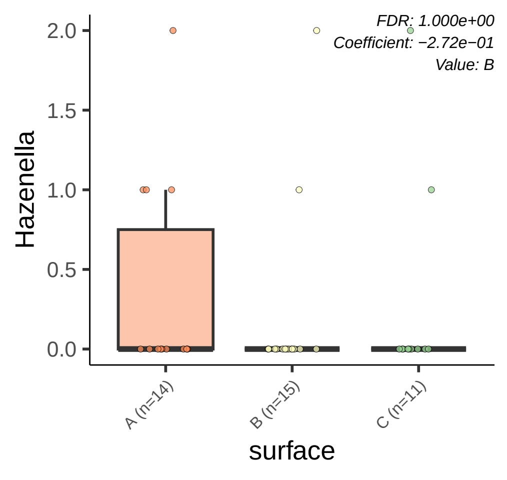
FDR: 1.000e+00
Coefficient: −2.72e−01
Value: B
Hazenella
2.0
1.5
1.0
0.5
0.0
A (n=14)
B (n=15)
C (n=11)
surface

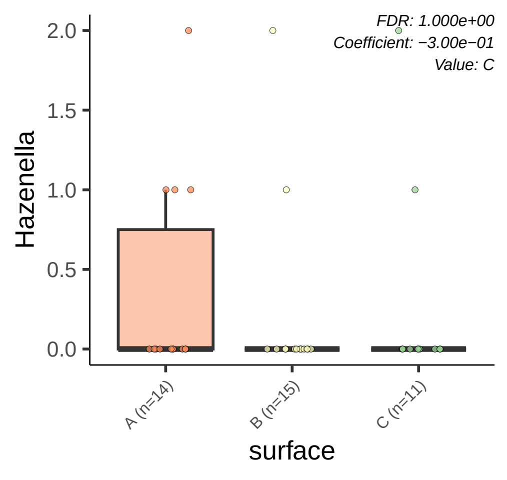
FDR: 1.000e+00
Coefficient: −3.00e−01
Value: C
Hazenella
2.0
1.5
1.0
0.5
0.0
A (n=14)
B (n=15)
C (n=11)
surface

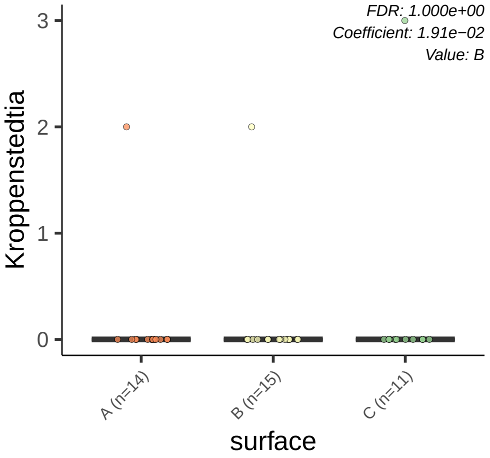

FDR: 1.000e+00
Coefficient: 1.91e−02
Value: B
Kroppenstedtia
3
2
1
0
A (n=14)
B (n=15)
C (n=11)
surface

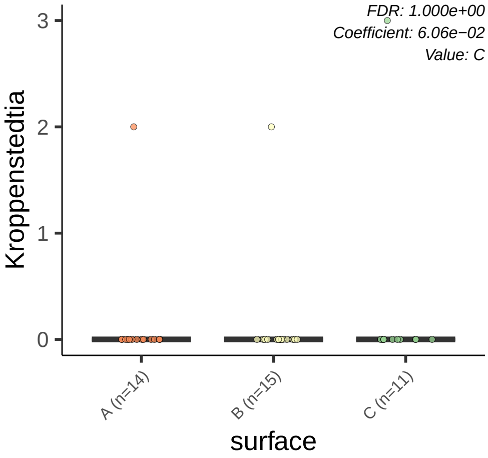
FDR: 1.000e+00
Coefficient: 6.06e−02
Value: C
3
2
1
0
Kroppenstedtia
A (n=14)
B (n=15)
C (n=11)
surface

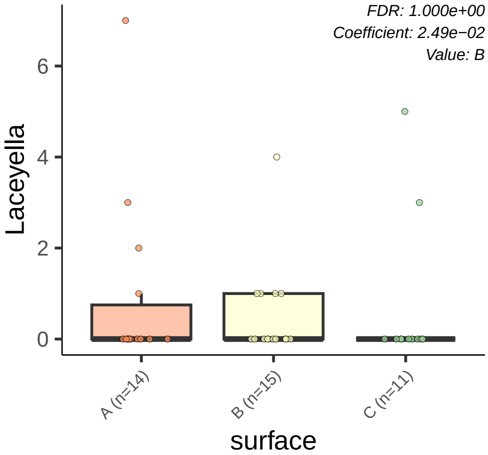
FDR: 1.000e+00
Coefficient: 2.49e−02
Value: B
Laceyella
0
2
4
6
A (n=14)
B (n=15)
C (n=11)
surface

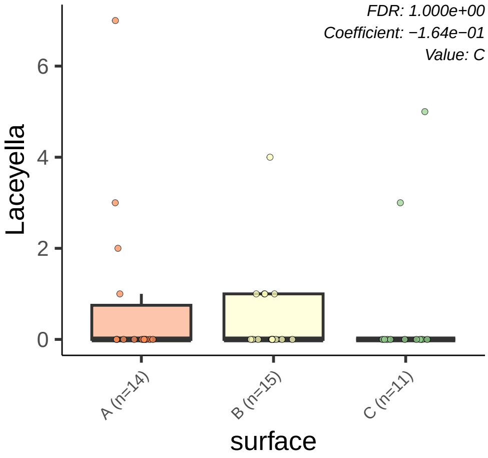
FDR: 1.000e+00
Coefficient: −1.64e−01
Value: C
Laceyella
0
2
4
6
A (n=14)
B (n=15)
C (n=11)
surface

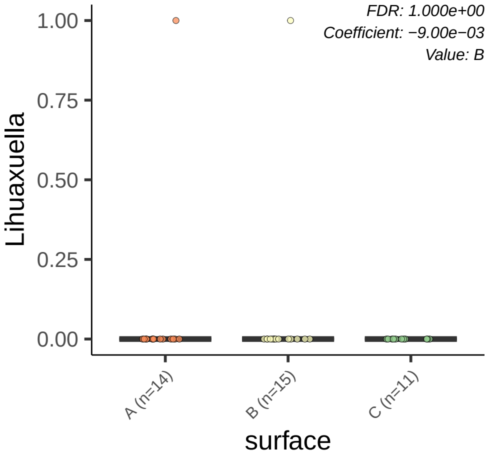
FDR: 1.000e+00
Coefficient: −9.00e−03
Value: B
Lihuaxuella
1.00
0.75
0.50
0.25
0.00
A (n=14)
B (n=15)
C (n=11)
surface

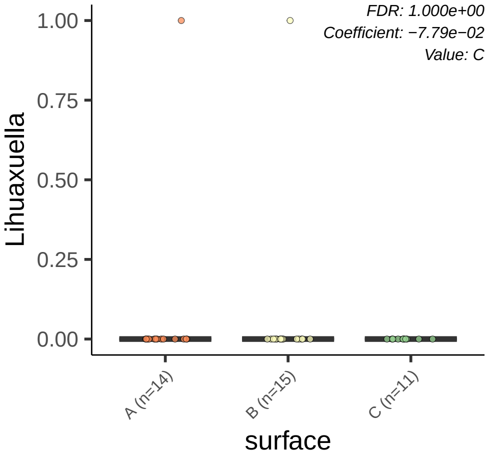

FDR: 1.000e+00
Coefficient: −7.79e−02
Value: C
Lihuaxuella
1.00
0.75
0.50
0.25
0.00
A (n=14)
B (n=15)
C (n=11)
surface

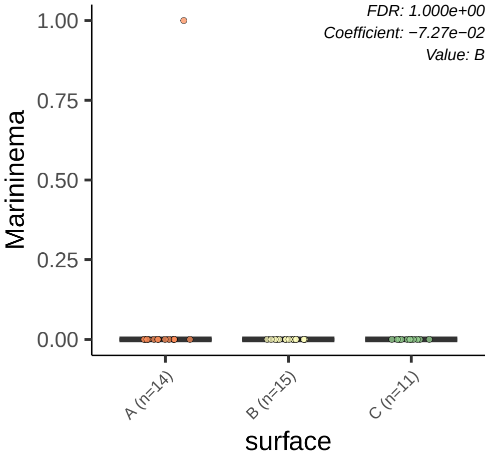

FDR: 1.000e+00
Coefficient: −7.27e−02
Value: B
Marininema
1.00
0.75
0.50
0.25
0.00
A (n=14)
B (n=15)
C (n=11)
surface

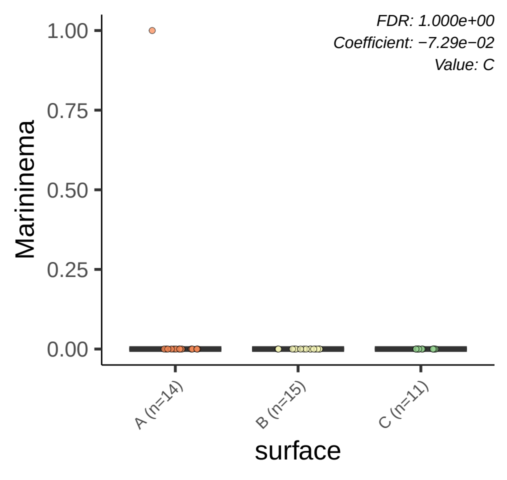
FDR: 1.000e+00
Coefficient: −7.29e−02
Value: C
Marininema
1.00
0.75
0.50
0.25
0.00
A (n=14)
B (n=15)
C (n=11)
surface

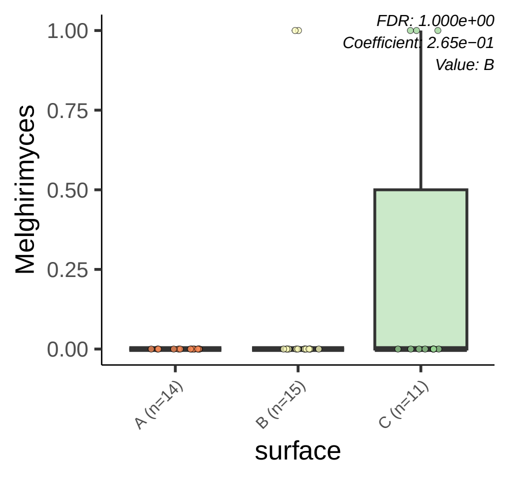

FDR: 1.000e+00
Coefficient: 2.65e−01
Value: B
Melghirimyces
1.00
0.75
0.50
0.25
0.00
A (n=14)
B (n=15)
C (n=11)
surface

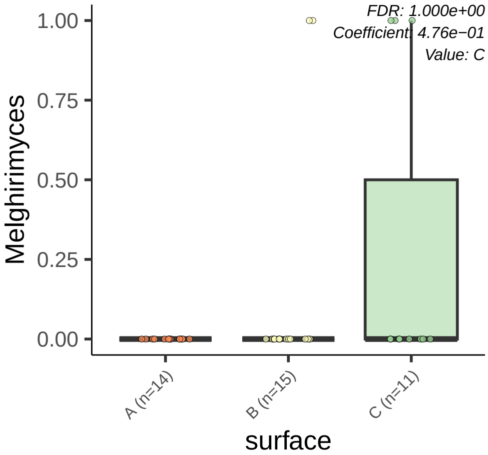
FDR: 1.000e+00
Coefficient: 4.76e−01
Value: C
Melghirimyces
1.00
0.75
0.50
0.25
0.00
A (n=14)
B (n=15)
C (n=11)
surface

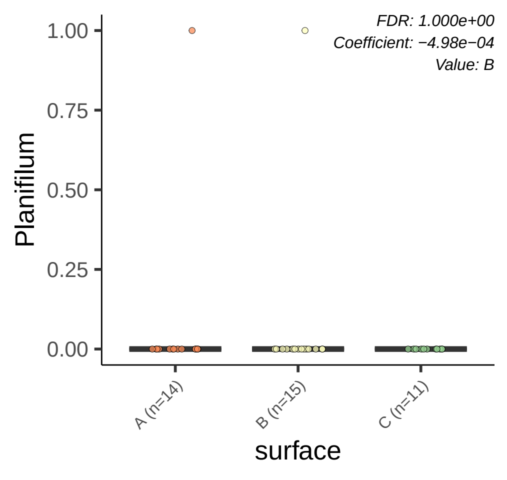

FDR: 1.000e+00
Coefficient: −4.98e−04
Value: B
Planifilum
1.00
0.75
0.50
0.25
0.00
A (n=14)
B (n=15)
C (n=11)
surface

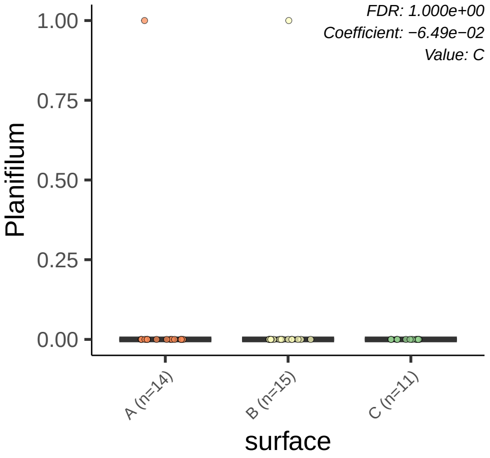
FDR: 1.000e+00
Coefficient: −6.49e−02
Value: C
Planifilum
1.00
0.75
0.50
0.25
0.00
A (n=14)
B (n=15)
C (n=11)
surface

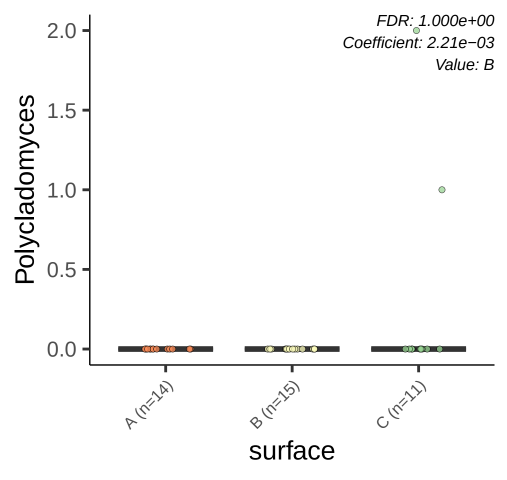
FDR: 1.000e+00
Coefficient: 2.21e−03
Value: B
Polycladomyces
2.0
1.5
1.0
0.5
0.0
A (n=14)
B (n=15)
C (n=11)
surface

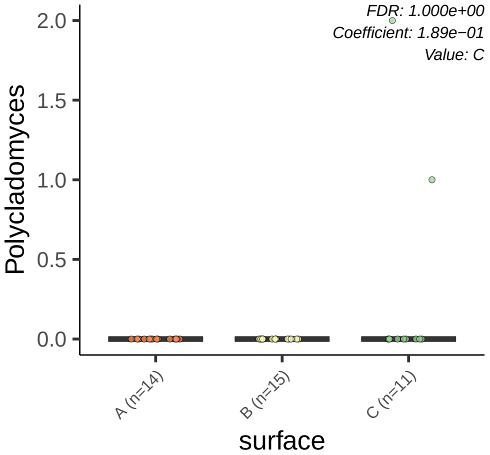
FDR: 1.000e+00
Coefficient: 1.89e−01
Value: C
Polycladomyces
2.0
1.5
1.0
0.5
0.0
A (n=14)
B (n=15)
C (n=11)
surface

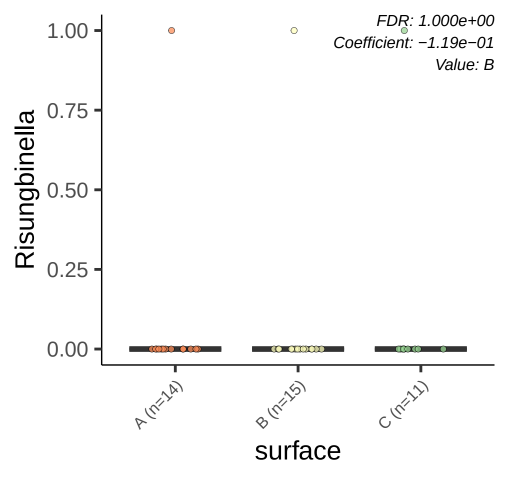

FDR: 1.000e+00
Coefficient: −1.19e−01
Value: B
Risungbinella
1.00
0.75
0.50
0.25
0.00
A (n=14)
B (n=15)
C (n=11)
surface

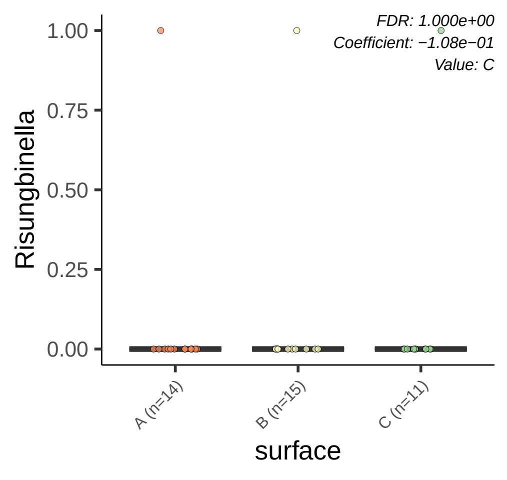

FDR: 1.000e+00
Coefficient: −1.08e−01
Value: C
Risungbinella
1.00
0.75
0.50
0.25
0.00
A (n=14)
B (n=15)
C (n=11)
surface

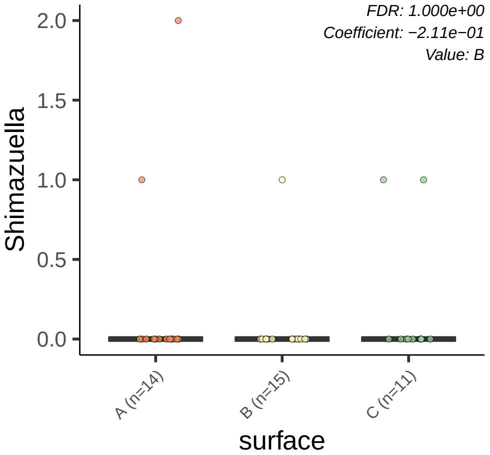
FDR: 1.000e+00
Coefficient: −2.11e−01
Value: B
Shimazuella
2.0
1.5
1.0
0.5
0.0
A (n=14)
B (n=15)
C (n=11)
surface

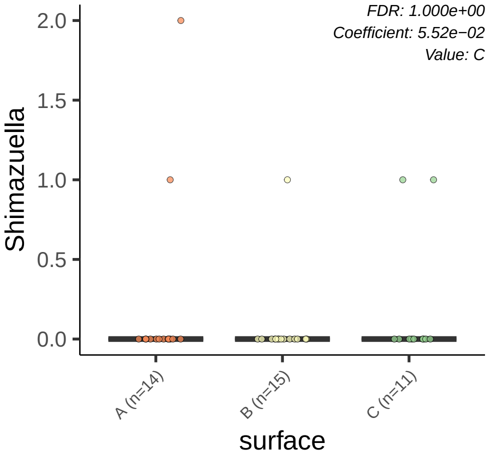
FDR: 1.000e+00
Coefficient: 5.52e−02
Value: C
Shimazuella
2.0
1.5
1.0
0.5
0.0
A (n=14)
B (n=15)
C (n=11)
surface

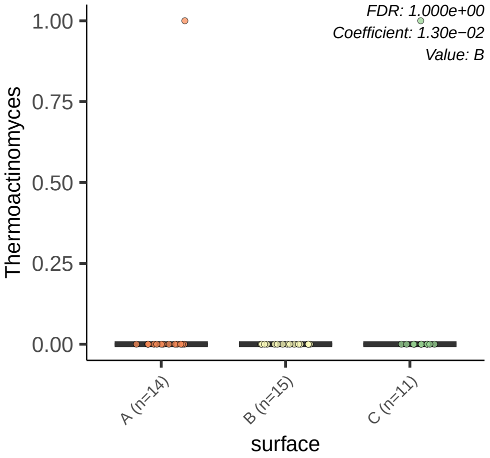
FDR: 1.000e+00
Coefficient: 1.30e−02
Value: B
Thermoactinomyces
1.00
0.75
0.50
0.25
0.00
A (n=14)
B (n=15)
C (n=11)
surface

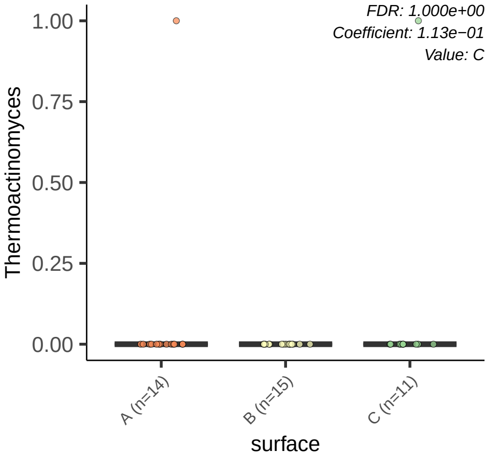
FDR: 1.000e+00
Coefficient: 1.13e−01
Value: C
Thermoactinomyces
1.00
0.75
0.50
0.25
0.00
A (n=14)
B (n=15)
C (n=11)
surface

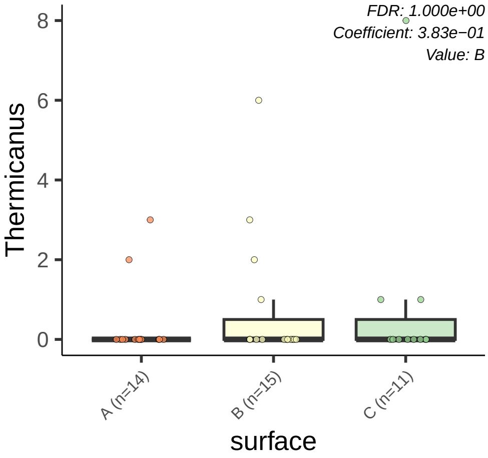
FDR: 1.000e+00
Coefficient: 3.83e−01
Value: B
Thermicanus
0
2
4
6
8
A (n=14)
B (n=15)
C (n=11)
surface

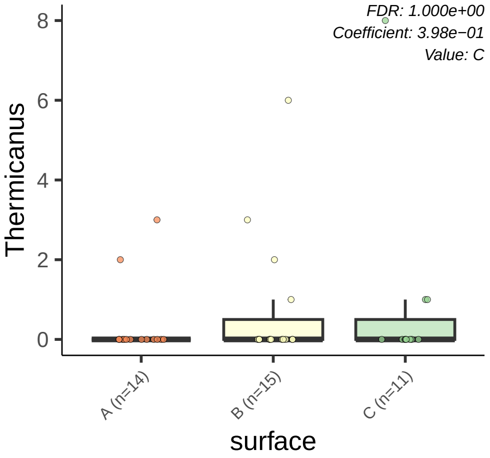
FDR: 1.000e+00
Coefficient: 3.98e−01
Value: C
Thermicanus
0
2
4
6
8
A (n=14)
B (n=15)
C (n=11)
surface

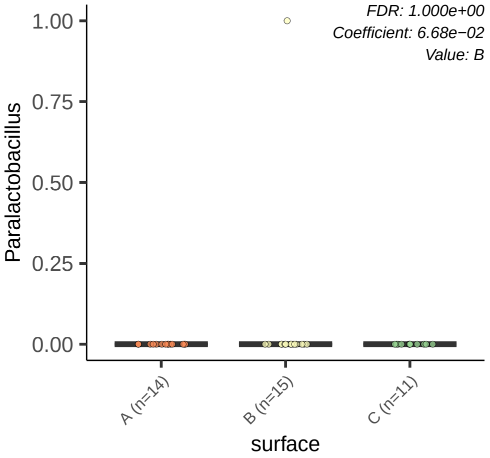

FDR: 1.000e+00
Coefficient: 6.68e−02
Value: B
Paralactobacillus
1.00
0.75
0.50
0.25
0.00
A (n=14)
B (n=15)
C (n=11)
surface

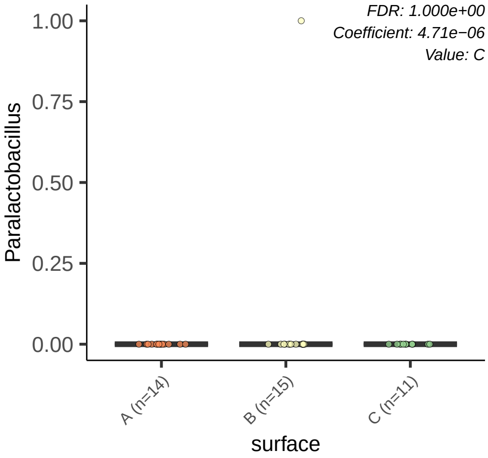

FDR: 1.000e+00
Coefficient: 4.71e−06
Value: C
Paralactobacillus
1.00
0.75
0.50
0.25
0.00
A (n=14)
B (n=15)
C (n=11)
surface

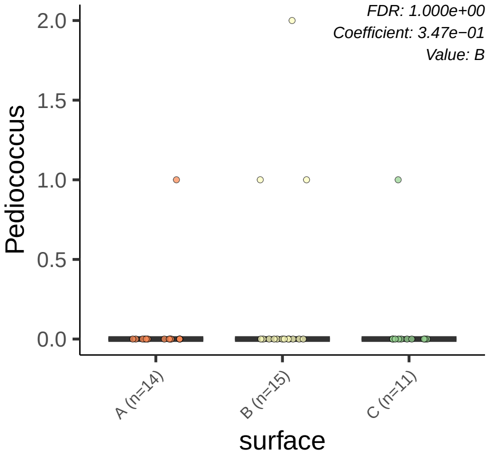
FDR: 1.000e+00
Coefficient: 3.47e−01
Value: B
Pediococcus
2.0
1.5
1.0
0.5
0.0
A (n=14)
B (n=15)
C (n=11)
surface

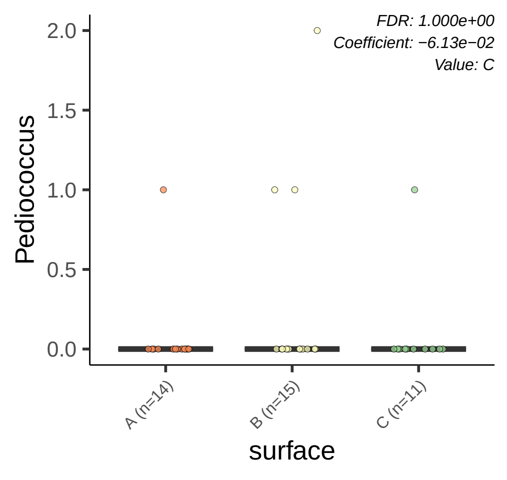
FDR: 1.000e+00
Coefficient: −6.13e−02
Value: C
Pediococcus
2.0
1.5
1.0
0.5
0.0
A (n=14)
B (n=15)
C (n=11)
surface

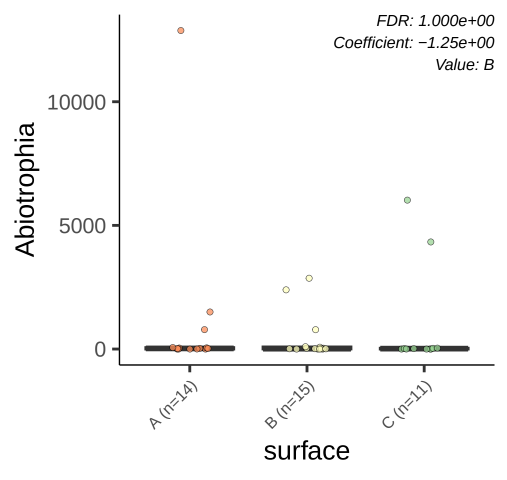

FDR: 1.000e+00
Coefficient: −1.25e+00
Value: B
Abiotrophia
10000
5000
0
A (n=14)
B (n=15)
C (n=11)
surface

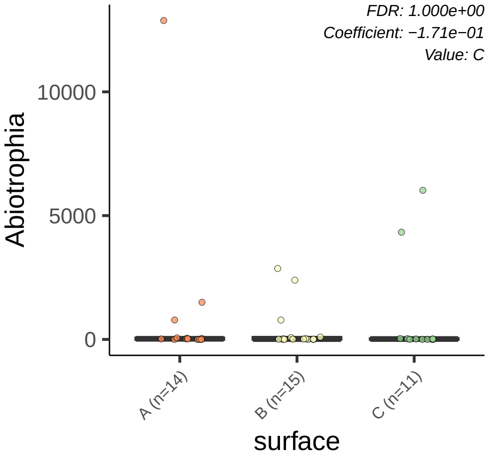
FDR: 1.000e+00
Coefficient: −1.71e−01
Value: C
Abiotrophia
10000
5000
0
A (n=14)
B (n=15)
C (n=11)
surface

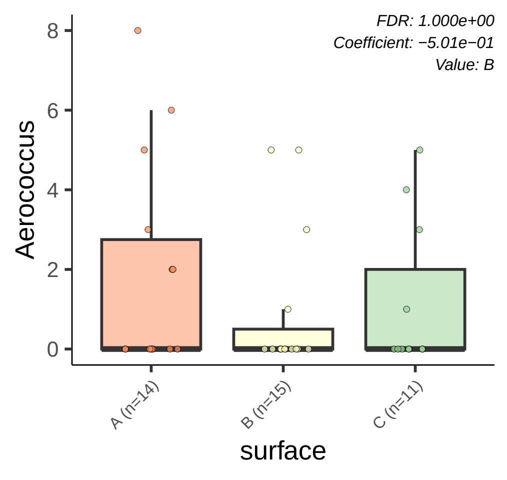

FDR: 1.000e+00
Coefficient: −5.01e−01
Value: B
Aerococcus
8
6
4
2
0
A (n=14)
B (n=15)
C (n=11)
surface

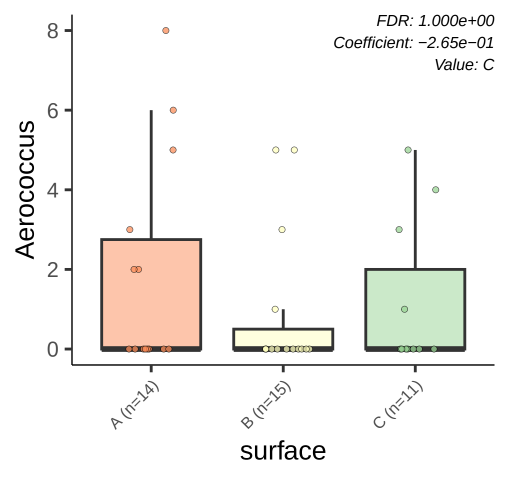

FDR: 1.000e+00
Coefficient: −2.65e−01
Value: C
Aerococcus
0
2
4
6
8
A (n=14)
B (n=15)
C (n=11)
surface

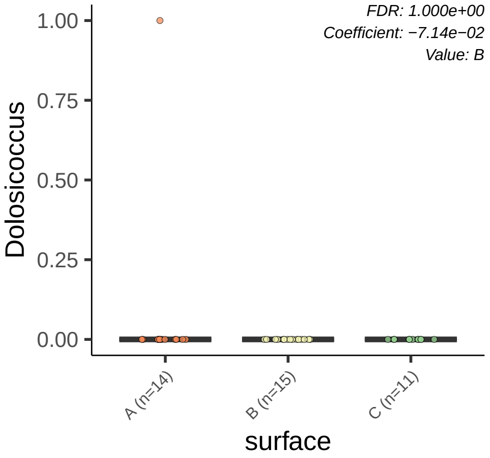
FDR: 1.000e+00
Coefficient: −7.14e−02
Value: B
Dolosicoccus
1.00
0.75
0.50
0.25
0.00
A (n=14)
B (n=15)
C (n=11)
surface

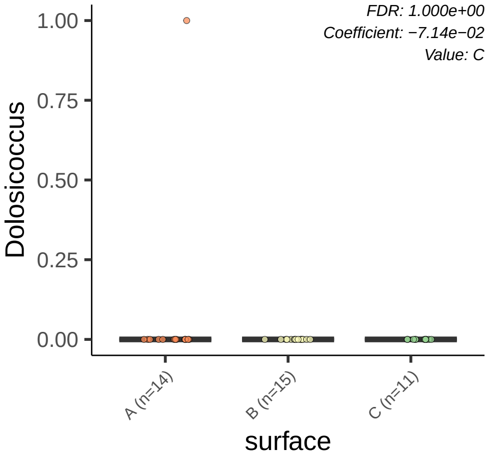

FDR: 1.000e+00
Coefficient: −7.14e−02
Value: C
Dolosicoccus
1.00
0.75
0.50
0.25
0.00
A (n=14)
B (n=15)
C (n=11)
surface

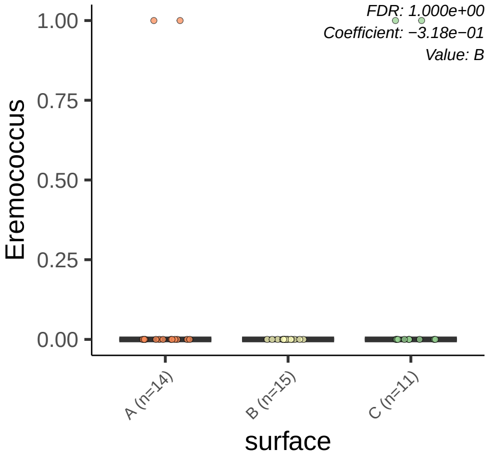
FDR: 1.000e+00
Coefficient: −3.18e−01
Value: B
Eremococcus
1.00
0.75
0.50
0.25
0.00
A (n=14)
B (n=15)
C (n=11)
surface

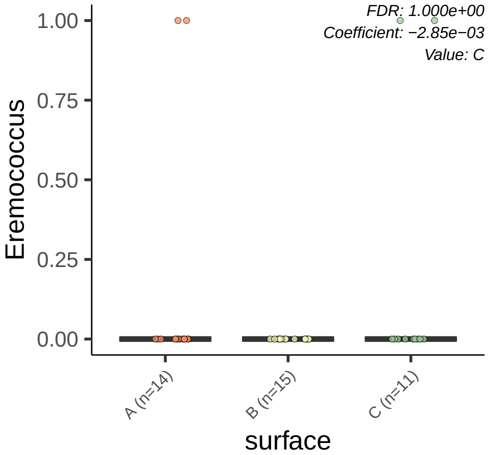
FDR: 1.000e+00
Coefficient: −2.85e−03
Value: C
Eremococcus
1.00
0.75
0.50
0.25
0.00
A (n=14)
B (n=15)
C (n=11)
surface

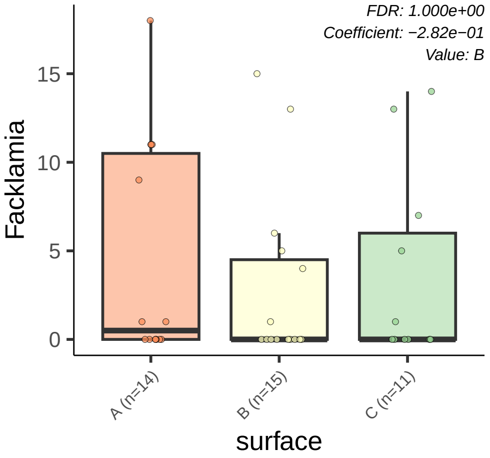
FDR: 1.000e+00
Coefficient: −2.82e−01
Value: B
Facklamia
0
5
10
15
A (n=14)
B (n=15)
C (n=11)
surface

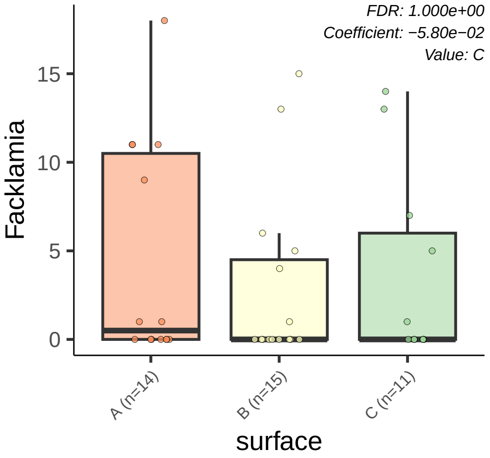

FDR: 1.000e+00
Coefficient: −5.80e−02
Value: C
Facklamia
15
10
5
0
A (n=14)
B (n=15)
C (n=11)
surface

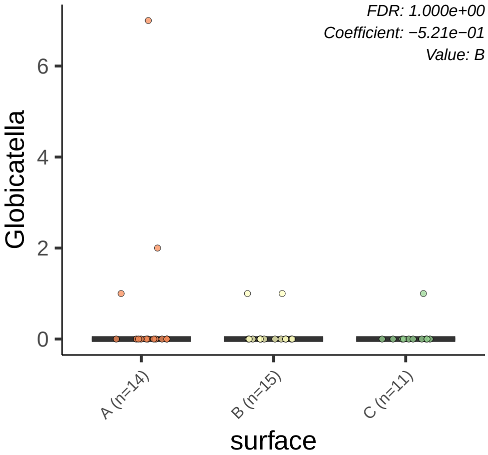

FDR: 1.000e+00
Coefficient: −5.21e−01
Value: B
Globicatella
0
2
4
6
A (n=14)
B (n=15)
C (n=11)
surface

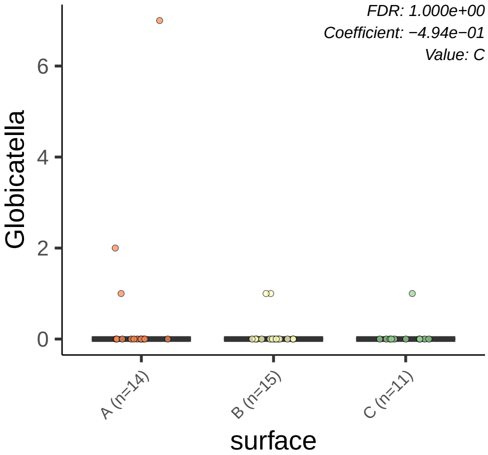

FDR: 1.000e+00
Coefficient: −4.94e−01
Value: C
Globicatella
0
2
4
6
A (n=14)
B (n=15)
C (n=11)
surface

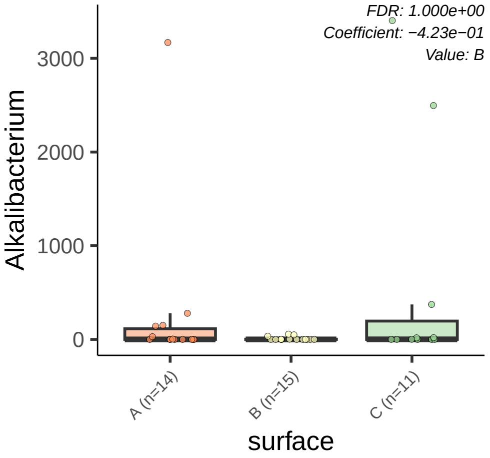

FDR: 1.000e+00
Coefficient: −4.23e−01
Value: B
Alkalibacterium
3000
2000
1000
0
A (n=14)
B (n=15)
C (n=11)
surface

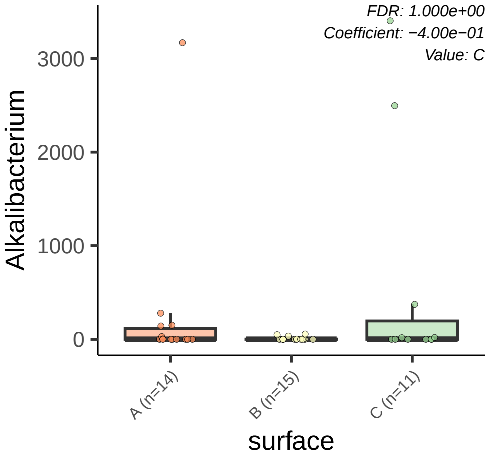

FDR: 1.000e+00
Coefficient: −4.00e−01
Value: C
Alkalibacterium
3000
2000
1000
0
A (n=14)
B (n=15)
C (n=11)
surface

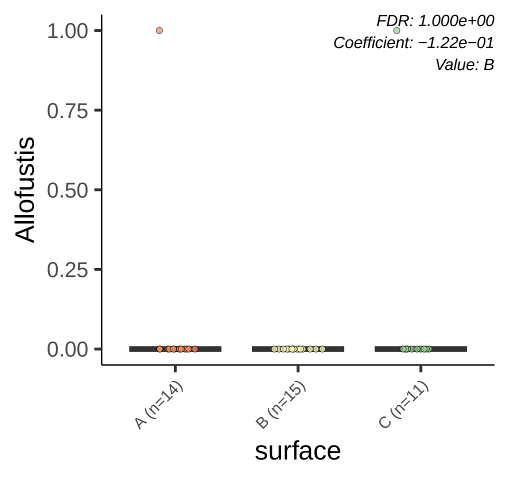

FDR: 1.000e+00
Coefficient: −1.22e−01
Value: B
Allofustis
1.00
0.75
0.50
0.25
0.00
A (n=14)
B (n=15)
C (n=11)
surface

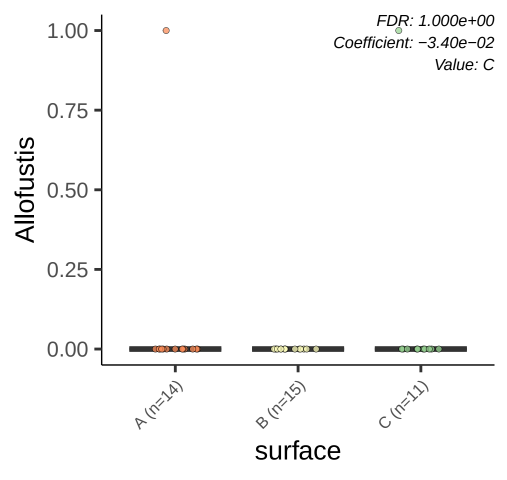

FDR: 1.000e+00
Coefficient: −3.40e−02
Value: C
Allofustis
1.00
0.75
0.50
0.25
0.00
A (n=14)
B (n=15)
C (n=11)
surface

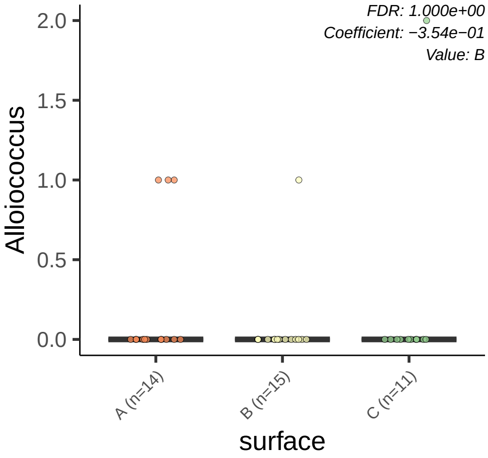
FDR: 1.000e+00
Coefficient: −3.54e−01
Value: B
2.0
1.5
1.0
0.5
0.0
Alloiococcus
A (n=14)
B (n=15)
C (n=11)
surface

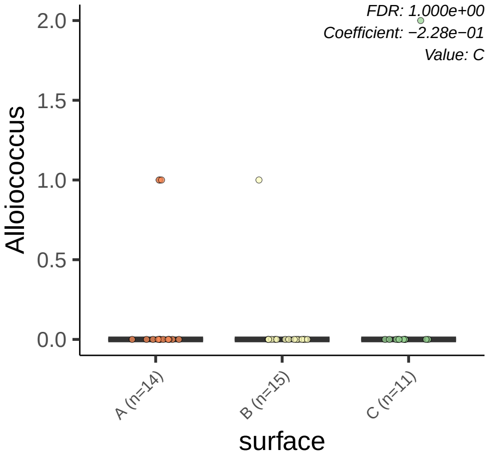

FDR: 1.000e+00
Coefficient: −2.28e−01
Value: C
Alloiococcus
2.0
1.5
1.0
0.5
0.0
A (n=14)
B (n=15)
C (n=11)
surface

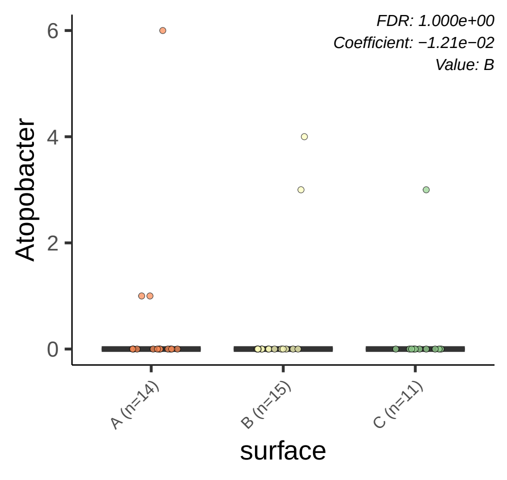
FDR: 1.000e+00
Coefficient: −1.21e−02
Value: B
Atopobacter
6
4
2
0
A (n=14)
B (n=15)
C (n=11)
surface

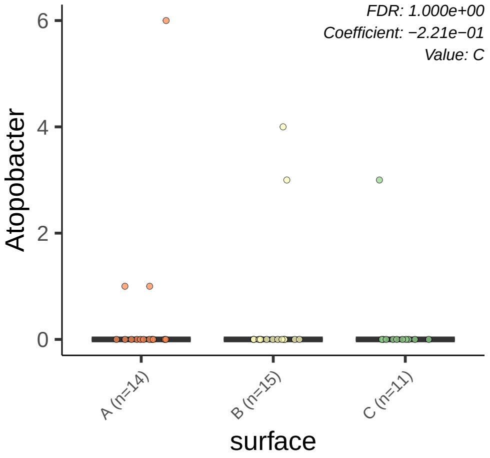
FDR: 1.000e+00
Coefficient: −2.21e−01
Value: C
Atopobacter
6
4
2
0
A (n=14)
B (n=15)
C (n=11)
surface

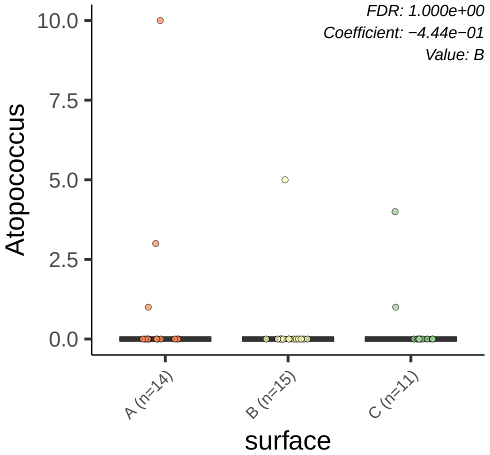
FDR: 1.000e+00
Coefficient: −4.44e−01
Value: B
Atopococcus
10.0
7.5
5.0
2.5
0.0
A (n=14)
B (n=15)
C (n=11)
surface

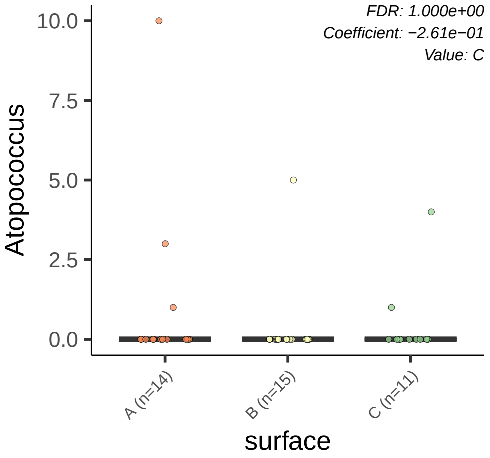
FDR: 1.000e+00
Coefficient: −2.61e−01
Value: C
Atopococcus
10.0
7.5
5.0
2.5
0.0
A (n=14)
B (n=15)
C (n=11)
surface

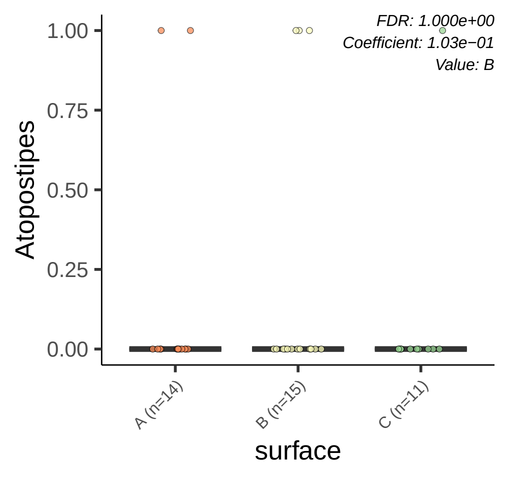
FDR: 1.000e+00
Coefficient: 1.03e−01
Value: B
1.00
0.75
0.50
0.25
0.00
Atopostipes
A (n=14)
B (n=15)
C (n=11)
surface

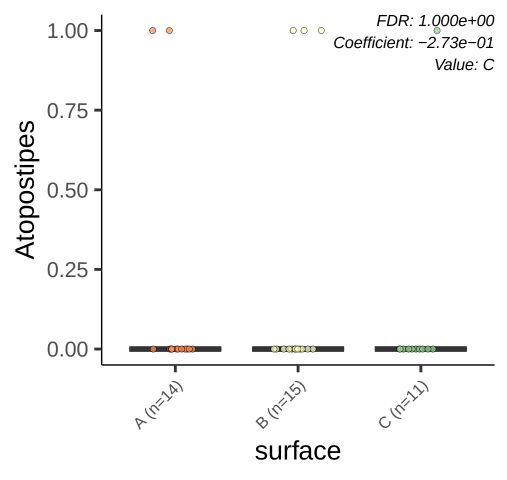
FDR: 1.000e+00
Coefficient: −2.73e−01
Value: C
Atopostipes
1.00
0.75
0.50
0.25
0.00
A (n=14)
B (n=15)
C (n=11)
surface

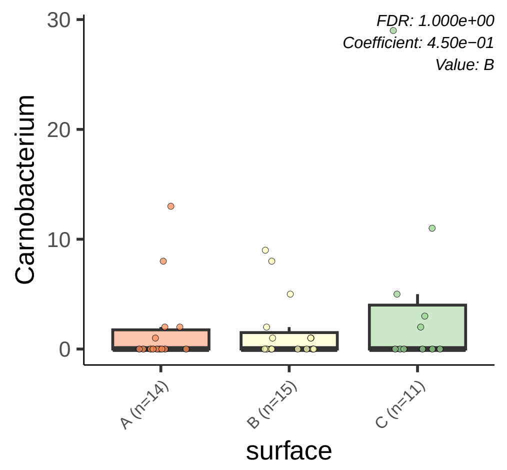

FDR: 1.000e+00
Coefficient: 4.50e−01
Value: B
Carnobacterium
0
10
20
30
A (n=14)
B (n=15)
C (n=11)
surface

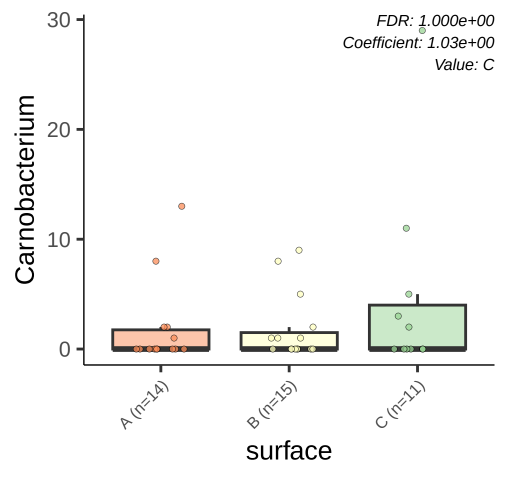

FDR: 1.000e+00
Coefficient: 1.03e+00
Value: C
Carnobacterium
30
20
10
0
A (n=14)
B (n=15)
C (n=11)
surface

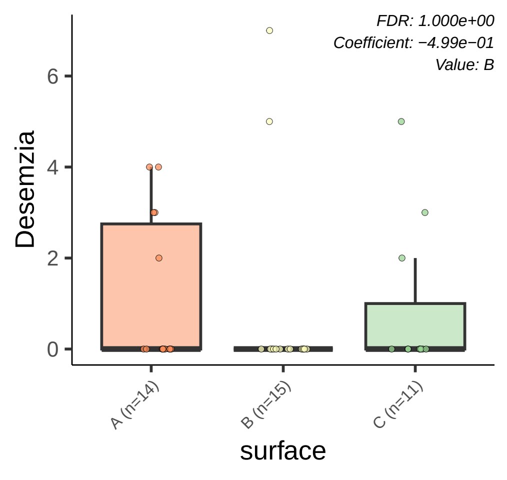
FDR: 1.000e+00
Coefficient: −4.99e−01
Value: B
Desemzia
0
2
4
6
A (n=14)
B (n=15)
C (n=11)
surface

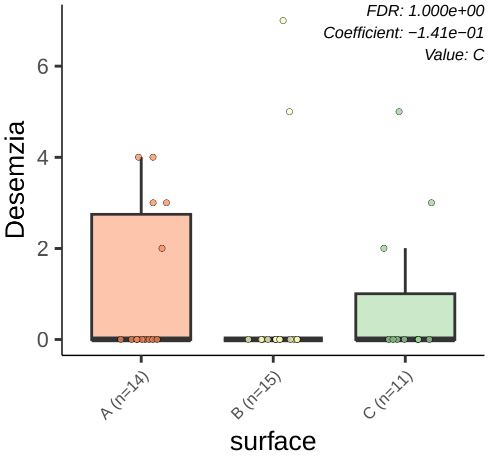
FDR: 1.000e+00
Coefficient: −1.41e−01
Value: C
Desemzia
0
2
4
6
A (n=14)
B (n=15)
C (n=11)
surface

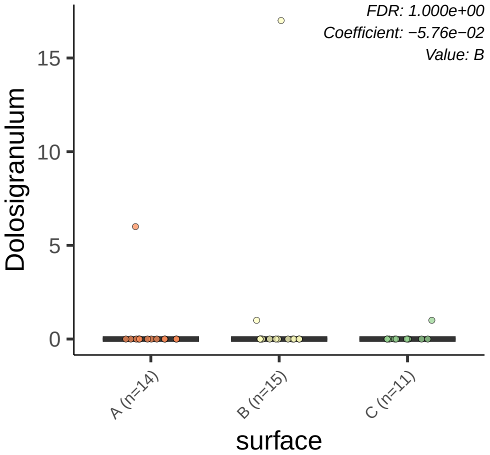

FDR: 1.000e+00
Coefficient: −5.76e−02
Value: B
Dolosigranulum
0
5
10
15
A (n=14)
B (n=15)
C (n=11)
surface

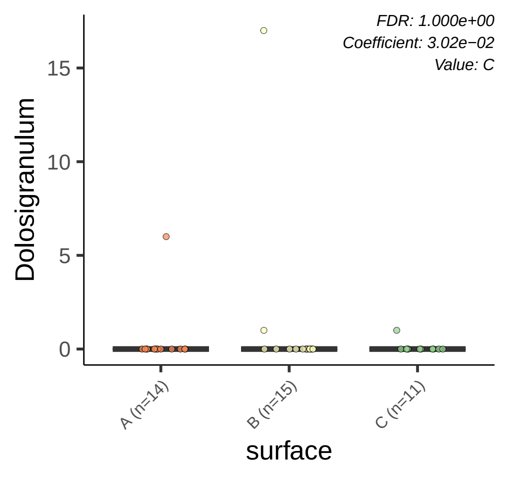
FDR: 1.000e+00
Coefficient: 3.02e−02
Value: C
Dolosigranulum
0
5
10
15
A (n=14)
B (n=15)
C (n=11)
surface

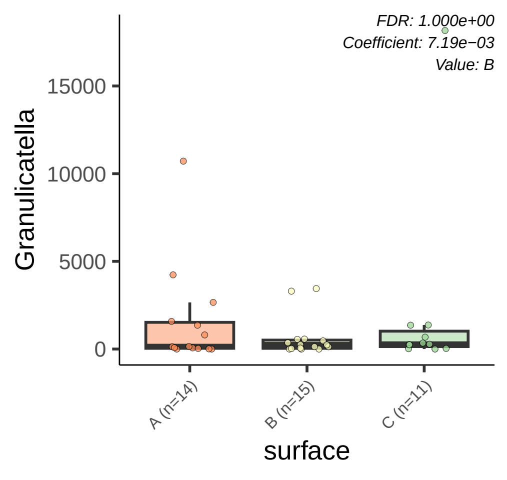
FDR: 1.000e+00
Coefficient: 7.19e−03
Value: B
Granulicatella
15000
10000
5000
0
A (n=14)
B (n=15)
C (n=11)
surface

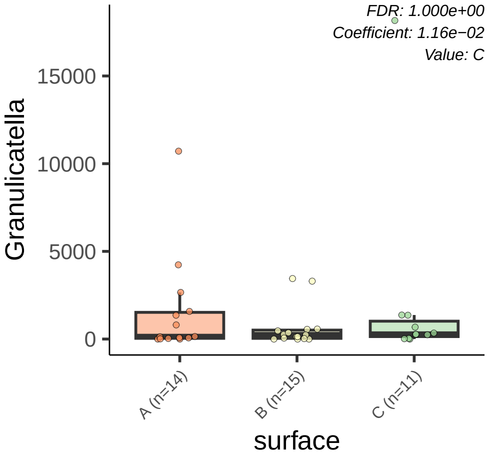

FDR: 1.000e+00
Coefficient: 1.16e−02
Value: C
Granulicatella
15000
10000
5000
0
A (n=14)
B (n=15)
C (n=11)
surface

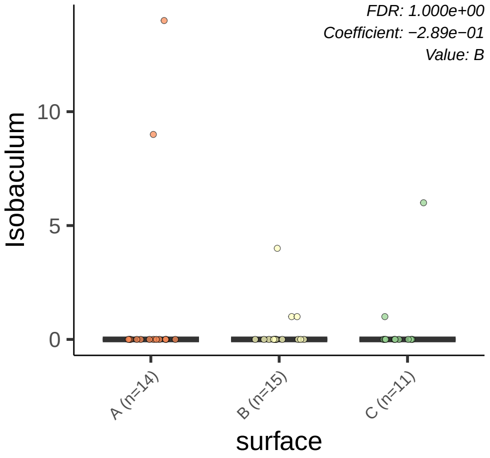
FDR: 1.000e+00
Coefficient: −2.89e−01
Value: B
Isobaculum
10
5
0
A (n=14)
B (n=15)
C (n=11)
surface

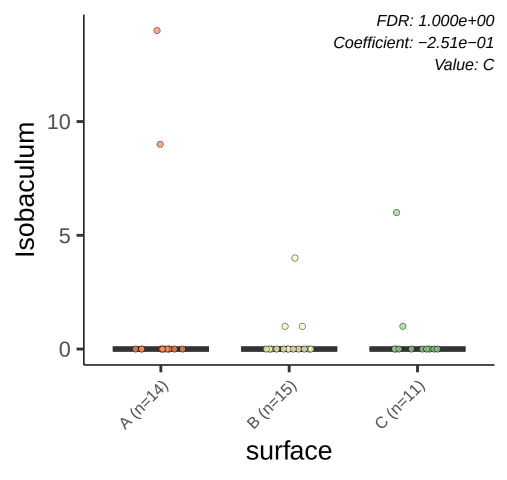

FDR: 1.000e+00
Coefficient: −2.51e−01
Value: C
Isobaculum
10
5
0
A (n=14)
B (n=15)
C (n=11)
surface

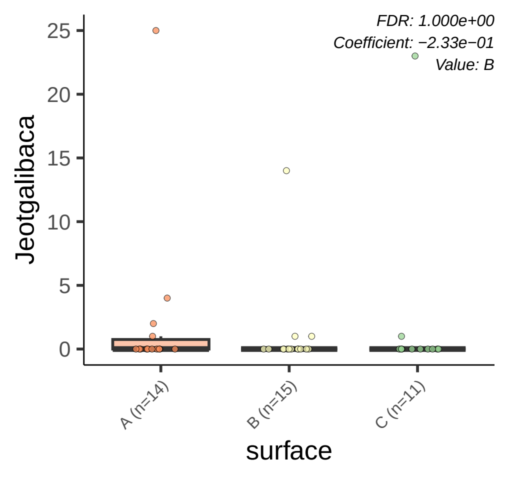

FDR: 1.000e+00
Coefficient: −2.33e−01
Value: B
Jeotgalibaca
0
5
10
15
20
25
A (n=14)
B (n=15)
C (n=11)
surface

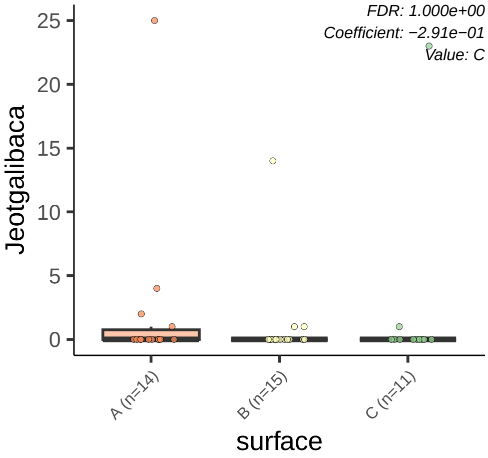

FDR: 1.000e+00
Coefficient: −2.91e−01
Value: C
Jeotgalibaca
0
5
10
15
20
25
A (n=14)
B (n=15)
C (n=11)
surface

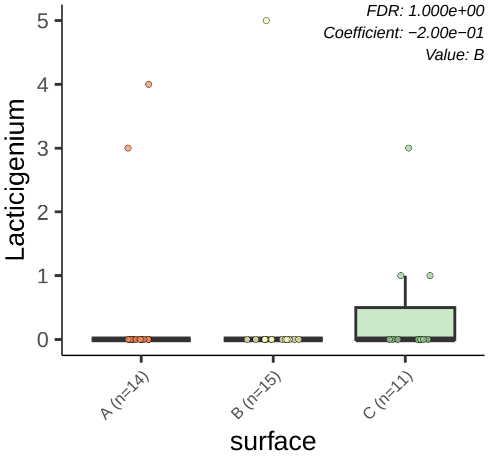

FDR: 1.000e+00
Coefficient: −2.00e−01
Value: B
Lacticigenium
0
1
2
3
4
5
A (n=14)
B (n=15)
C (n=11)
surface

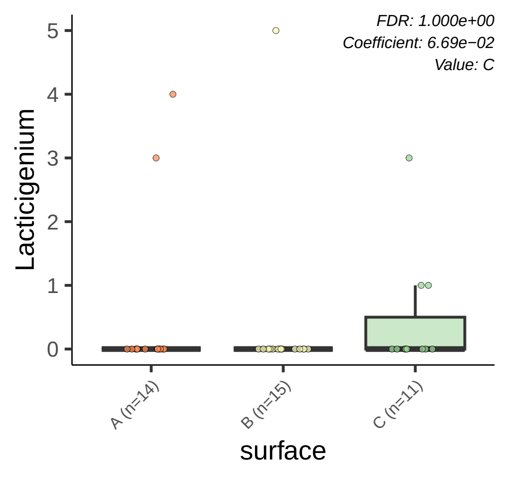

FDR: 1.000e+00
Coefficient: 6.69e−02
Value: C
Lacticigenium
5
4
3
2
1
0
A (n=14)
B (n=15)
C (n=11)
surface

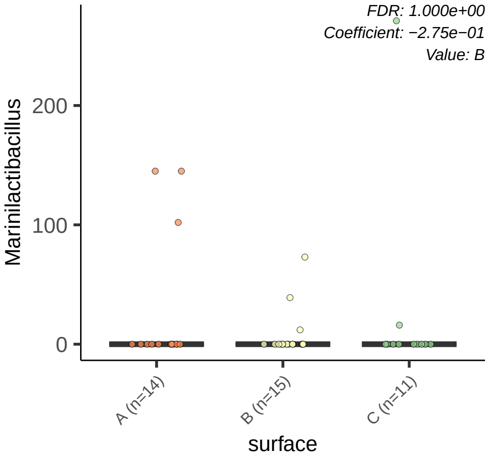

FDR: 1.000e+00
Coefficient: −2.75e−01
Value: B
Marinilactibacillus
200
100
0
A (n=14)
B (n=15)
C (n=11)
surface

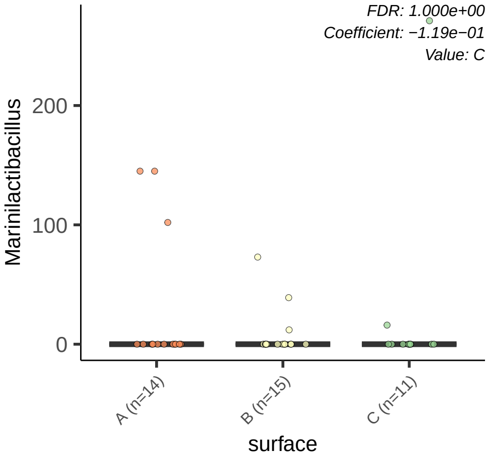
FDR: 1.000e+00
Coefficient: −1.19e−01
Value: C
Marinilactibacillus
200
100
0
A (n=14)
B (n=15)
C (n=11)
surface

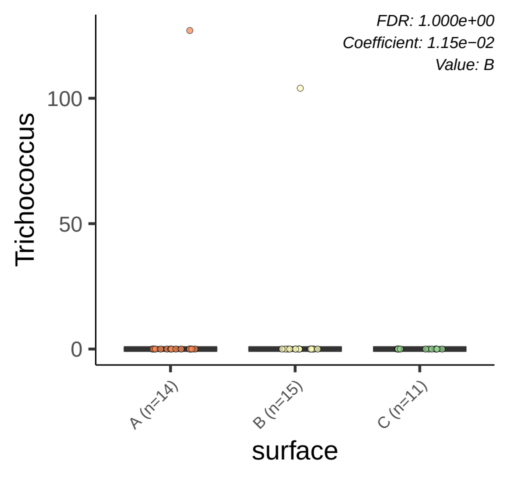
FDR: 1.000e+00
Coefficient: 1.15e−02
Value: B
Trichococcus
100
50
0
A (n=14)
B (n=15)
C (n=11)
surface

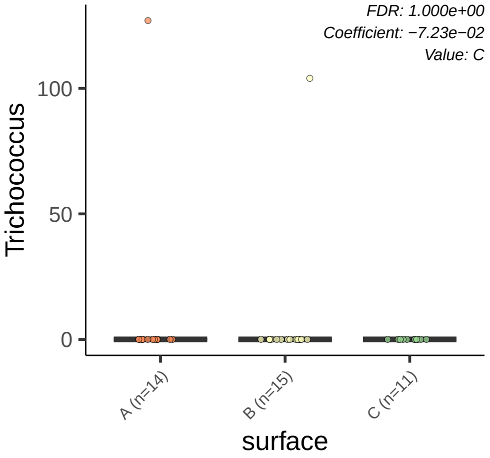

FDR: 1.000e+00
Coefficient: −7.23e−02
Value: C
Trichococcus
100
50
0
A (n=14)
B (n=15)
C (n=11)
surface

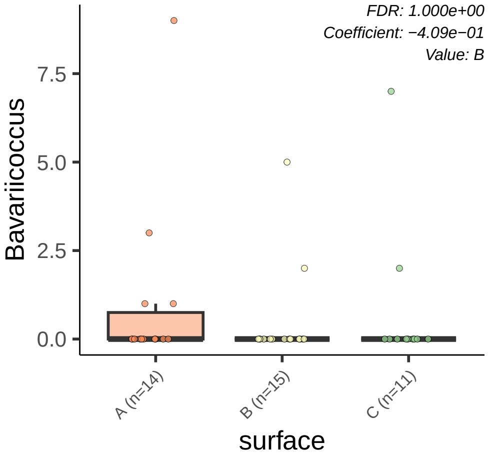
FDR: 1.000e+00
Coefficient: −4.09e−01
Value: B
Bavariicoccus
7.5
5.0
2.5
0.0
A (n=14)
B (n=15)
C (n=11)
surface

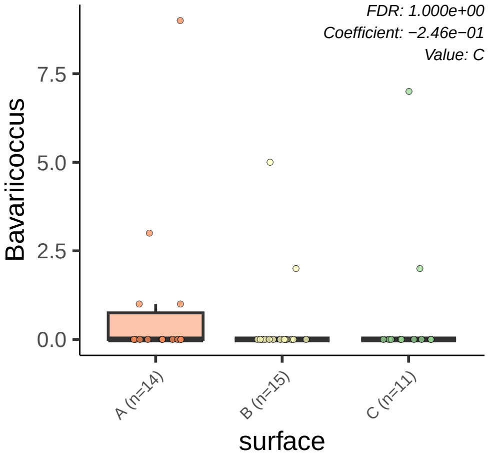
FDR: 1.000e+00
Coefficient: −2.46e−01
Value: C
Bavariicoccus
7.5
5.0
2.5
0.0
A (n=14)
B (n=15)
C (n=11)
surface

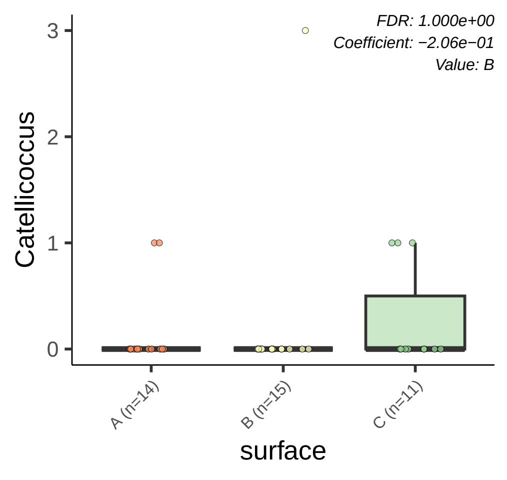
FDR: 1.000e+00
Coefficient: −2.06e−01
Value: B
Catellicoccus
3
2
1
0
A (n=14)
B (n=15)
C (n=11)
surface

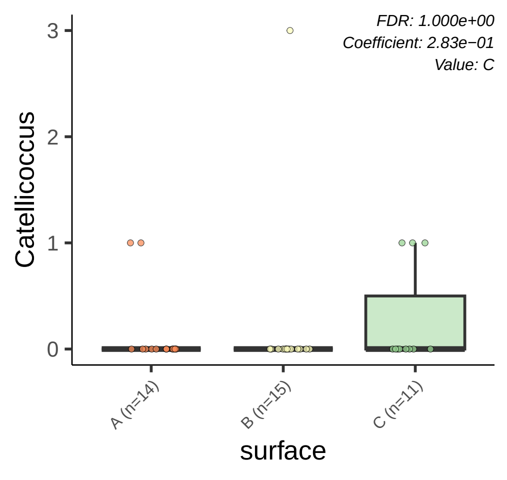

FDR: 1.000e+00
Coefficient: 2.83e−01
Value: C
Catellicoccus
0
1
2
3
A (n=14)
B (n=15)
C (n=11)
surface

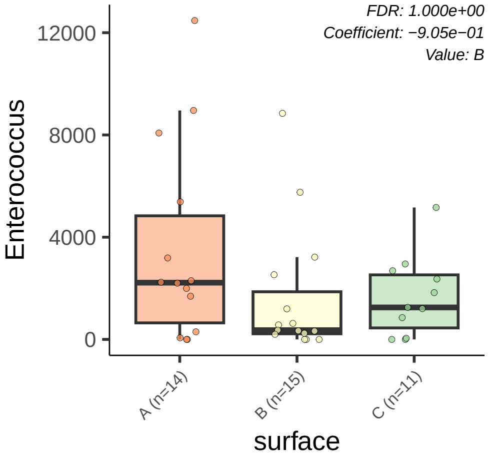

FDR: 1.000e+00
Coefficient: −9.05e−01
Value: B
12000
8000
4000
0
Enterococcus
A (n=14)
B (n=15)
C (n=11)
surface

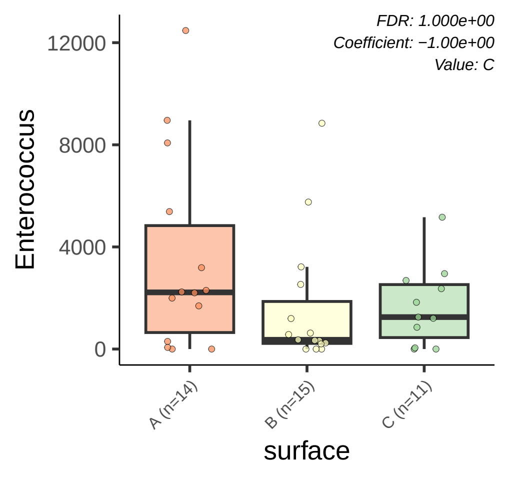
FDR: 1.000e+00
Coefficient: −1.00e+00
Value: C
Enterococcus
12000
8000
4000
0
A (n=14)
B (n=15)
C (n=11)
surface

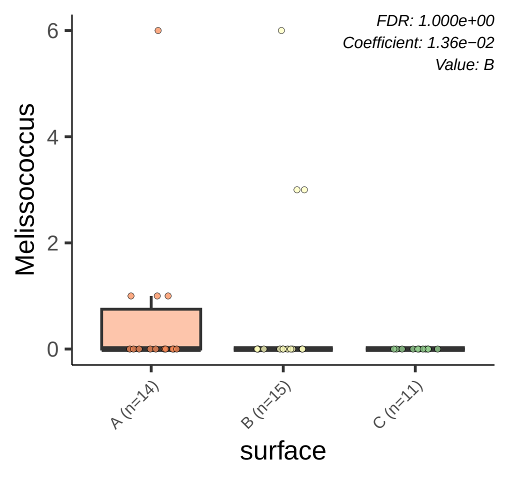

FDR: 1.000e+00
Coefficient: 1.36e−02
Value: B
Melissococcus
6
4
2
0
A (n=14)
B (n=15)
C (n=11)
surface

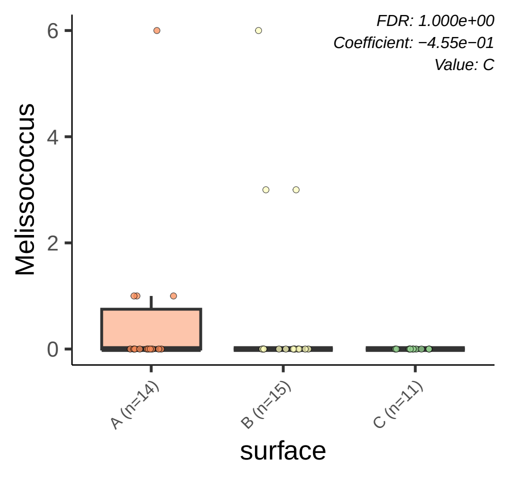
FDR: 1.000e+00
Coefficient: −4.55e−01
Value: C
Melissococcus
6
4
2
0
A (n=14)
B (n=15)
C (n=11)
surface

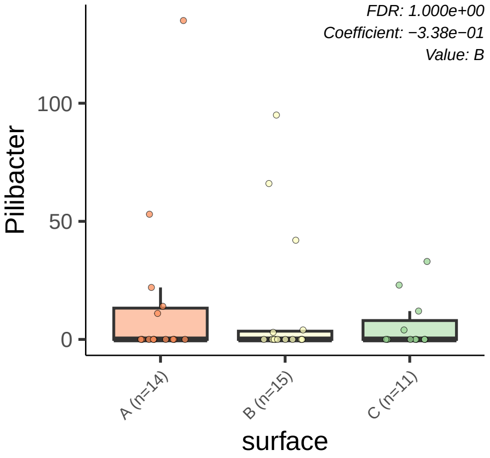
FDR: 1.000e+00
Coefficient: −3.38e−01
Value: B
Pilibacter
100
50
0
A (n=14)
B (n=15)
C (n=11)
surface

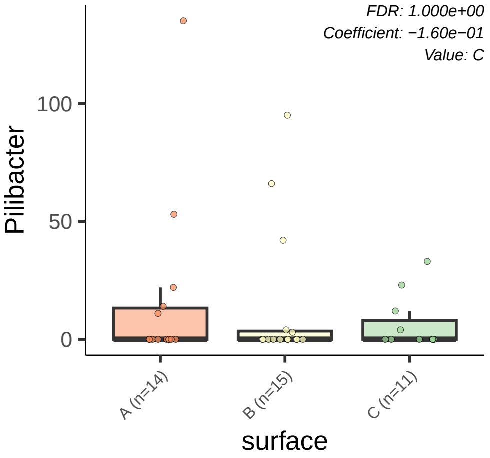
FDR: 1.000e+00
Coefficient: −1.60e−01
Value: C
Pilibacter
100
50
0
A (n=14)
B (n=15)
C (n=11)
surface

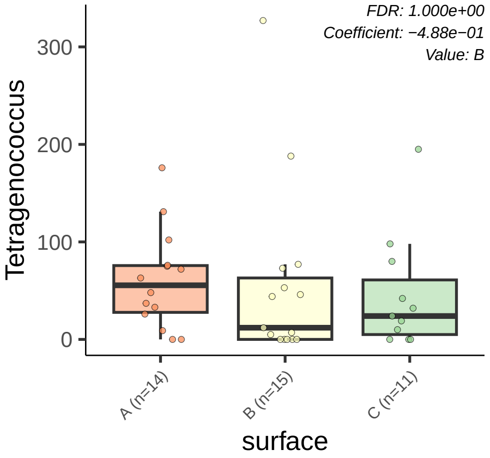

FDR: 1.000e+00
Coefficient: −4.88e−01
Value: B
Tetragenococcus
300
200
100
0
A (n=14)
B (n=15)
C (n=11)
surface

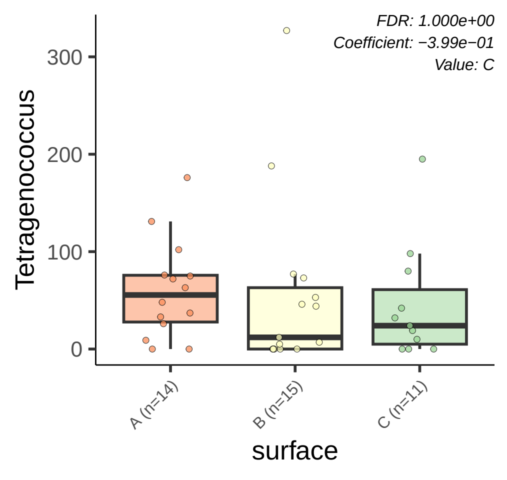

FDR: 1.000e+00
Coefficient: −3.99e−01
Value: C
Tetragenococcus
300
200
100
0
A (n=14)
B (n=15)
C (n=11)
surface

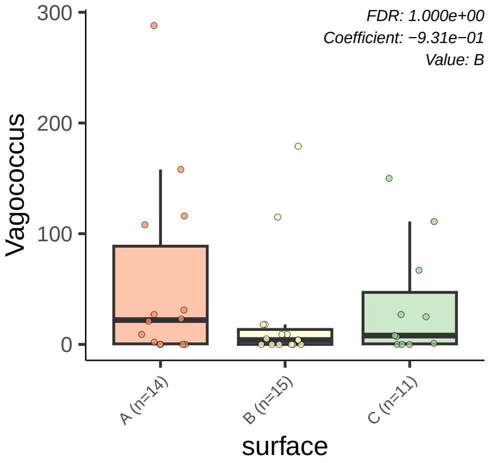
FDR: 1.000e+00
Coefficient: −9.31e−01
Value: B
Vagococcus
300
200
100
0
A (n=14)
B (n=15)
C (n=11)
surface

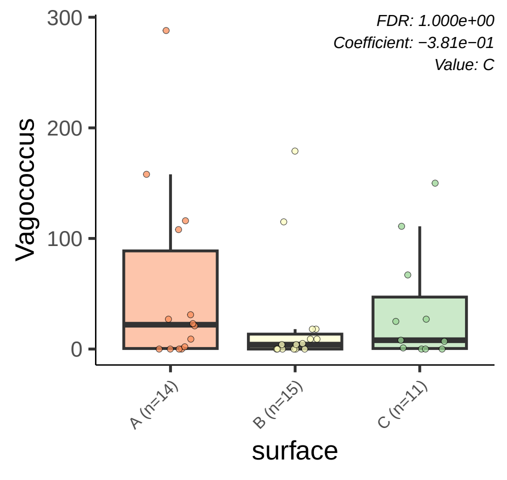

FDR: 1.000e+00
Coefficient: −3.81e−01
Value: C
Vagococcus
300
200
100
0
A (n=14)
B (n=15)
C (n=11)
surface

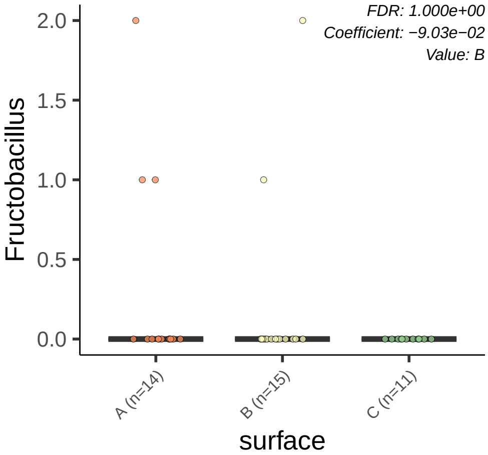

FDR: 1.000e+00
Coefficient: −9.03e−02
Value: B
Fructobacillus
2.0
1.5
1.0
0.5
0.0
A (n=14)
B (n=15)
C (n=11)
surface

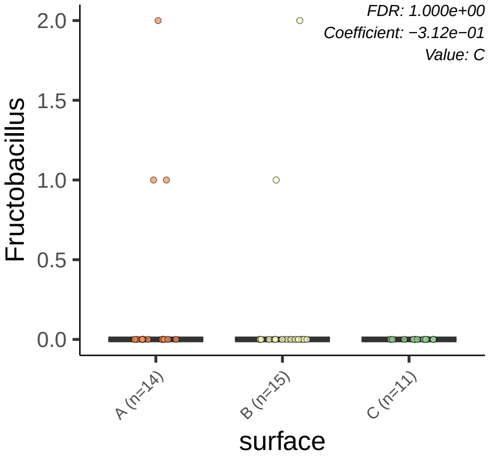

FDR: 1.000e+00
Coefficient: −3.12e−01
Value: C
Fructobacillus
2.0
1.5
1.0
0.5
0.0
A (n=14)
B (n=15)
C (n=11)
surface

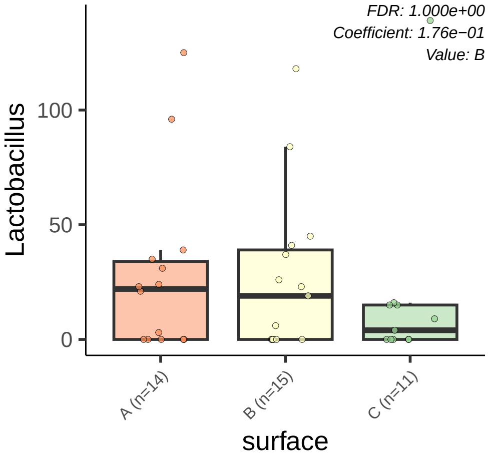

FDR: 1.000e+00
Coefficient: 1.76e−01
Value: B
Lactobacillus
100
50
0
A (n=14)
B (n=15)
C (n=11)
surface

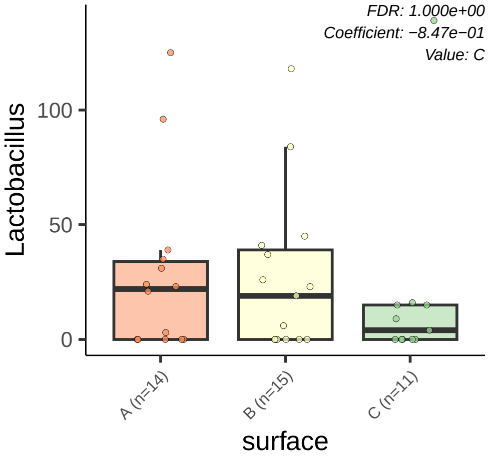

FDR: 1.000e+00
Coefficient: −8.47e−01
Value: C
Lactobacillus
100
50
0
A (n=14)
B (n=15)
C (n=11)
surface

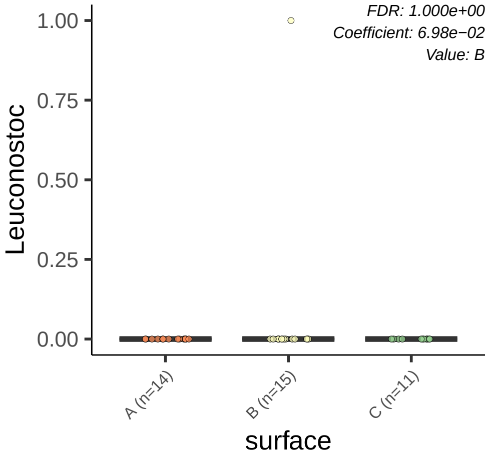

FDR: 1.000e+00
Coefficient: 6.98e−02
Value: B
1.00
0.75
0.50
0.25
0.00
Leuconostoc
A (n=14)
B (n=15)
C (n=11)
surface

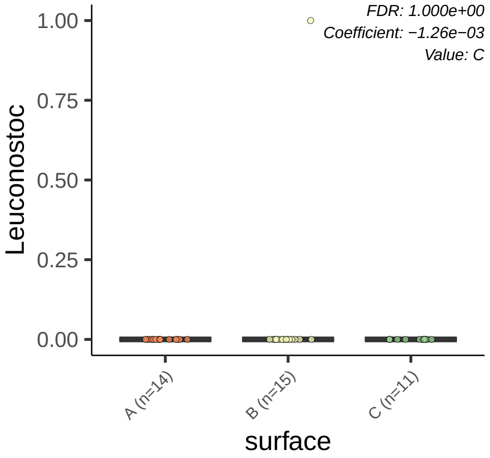

FDR: 1.000e+00
Coefficient: −1.26e−03
Value: C
Leuconostoc
1.00
0.75
0.50
0.25
0.00
A (n=14)
B (n=15)
C (n=11)
surface

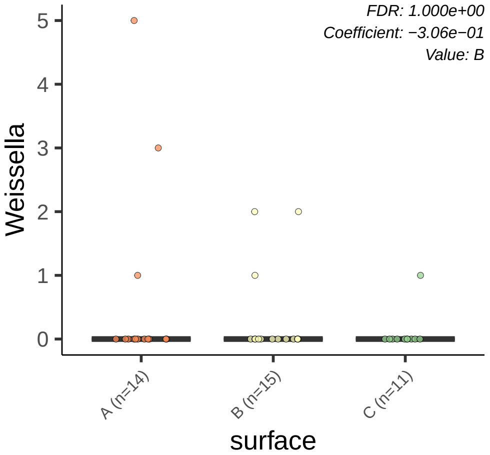
FDR: 1.000e+00
Coefficient: −3.06e−01
Value: B
Weissella
0
1
2
3
4
5
A (n=14)
B (n=15)
C (n=11)
surface

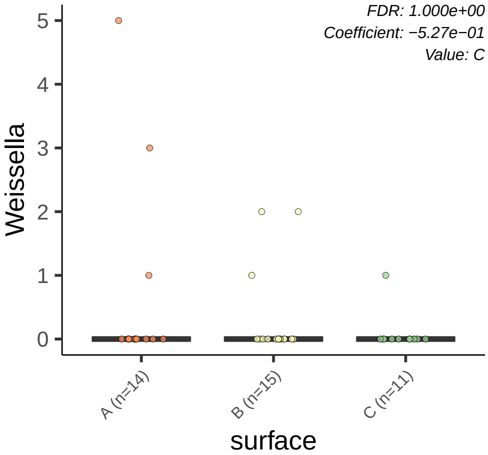

FDR: 1.000e+00
Coefficient: −5.27e−01
Value: C
Weissella
0
1
2
3
4
5
A (n=14)
B (n=15)
C (n=11)
surface

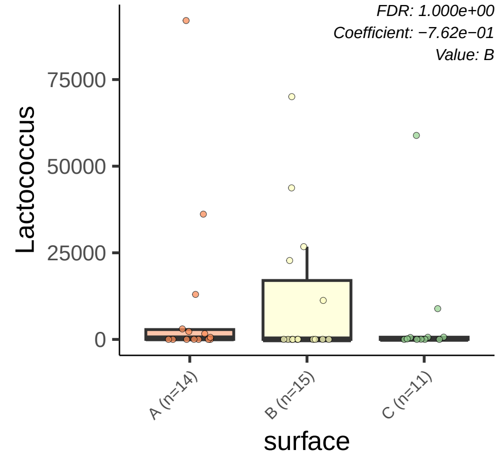

FDR: 1.000e+00
Coefficient: −7.62e−01
Value: B
Lactococcus
75000
50000
25000
0
A (n=14)
B (n=15)
C (n=11)
surface

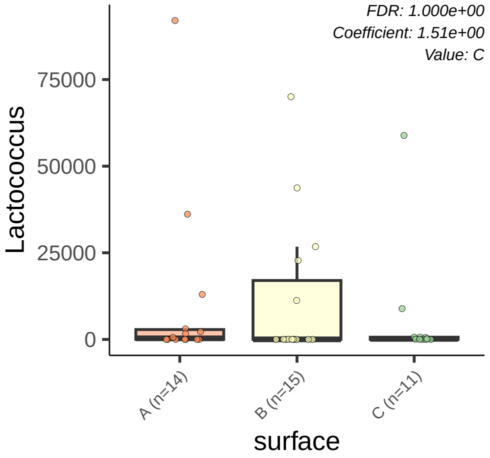

FDR: 1.000e+00
Coefficient: 1.51e+00
Value: C
Lactococcus
75000
50000
25000
0
A (n=14)
B (n=15)
C (n=11)
surface

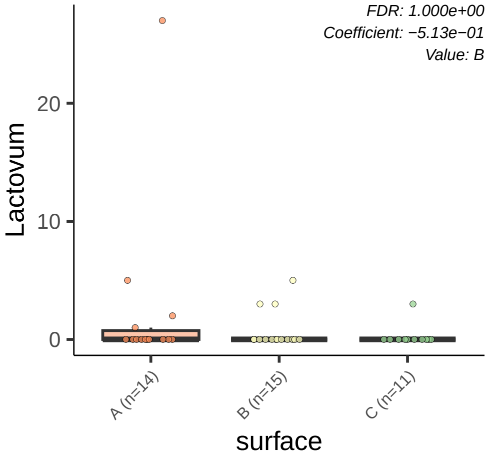
FDR: 1.000e+00
Coefficient: −5.13e−01
Value: B
Lactovum
0
10
20
A (n=14)
B (n=15)
C (n=11)
surface

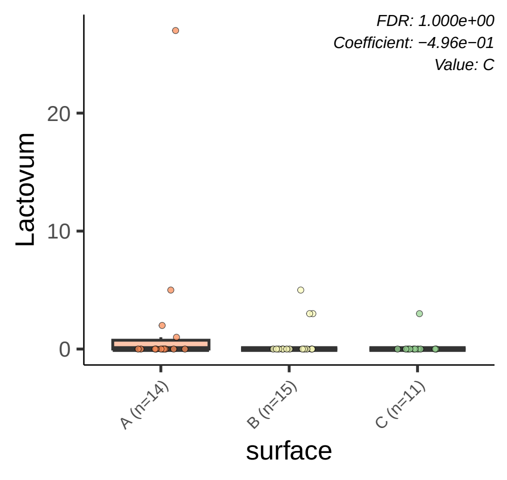

FDR: 1.000e+00
Coefficient: −4.96e−01
Value: C
Lactovum
20
10
0
A (n=14)
B (n=15)
C (n=11)
surface

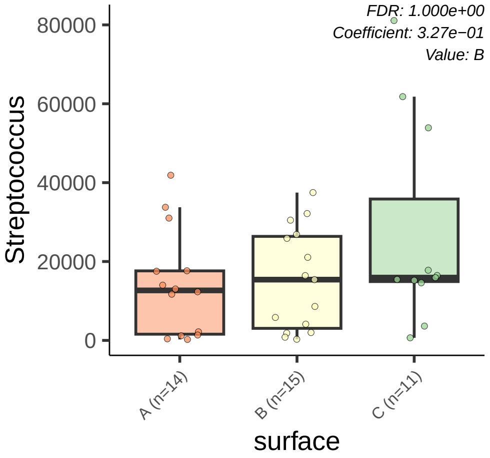

FDR: 1.000e+00
Coefficient: 3.27e−01
Value: B
80000
60000
40000
20000
0
Streptococcus
A (n=14)
B (n=15)
C (n=11)
surface

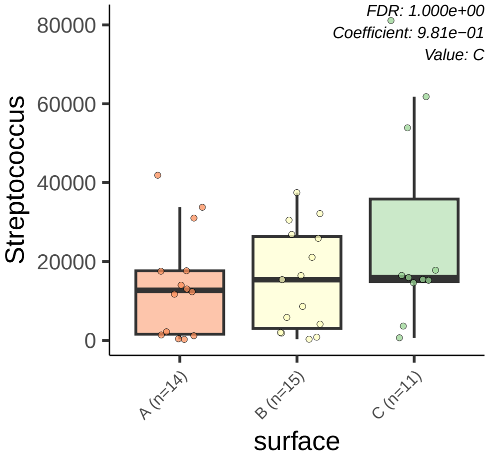
FDR: 1.000e+00
Coefficient: 9.81e−01
Value: C
Streptococcus
80000
60000
40000
20000
0
A (n=14)
B (n=15)
C (n=11)
surface

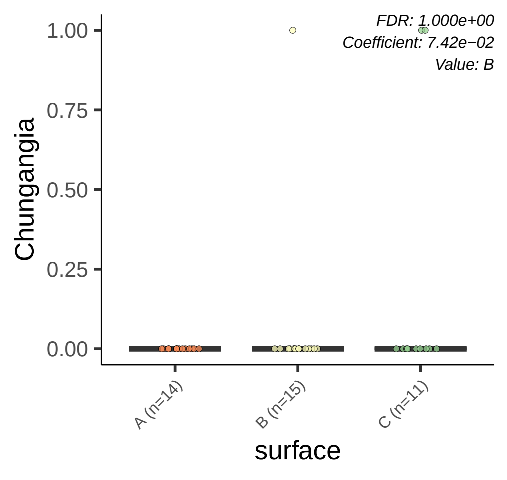

FDR: 1.000e+00
Coefficient: 7.42e−02
Value: B
1.00
0.75
0.50
0.25
0.00
Chungangia
A (n=14)
B (n=15)
C (n=11)
surface

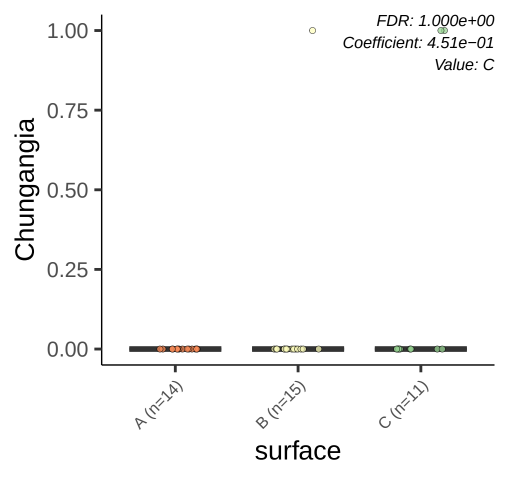

FDR: 1.000e+00
Coefficient: 4.51e−01
Value: C
Chungangia
1.00
0.75
0.50
0.25
0.00
A (n=14)
B (n=15)
C (n=11)
surface

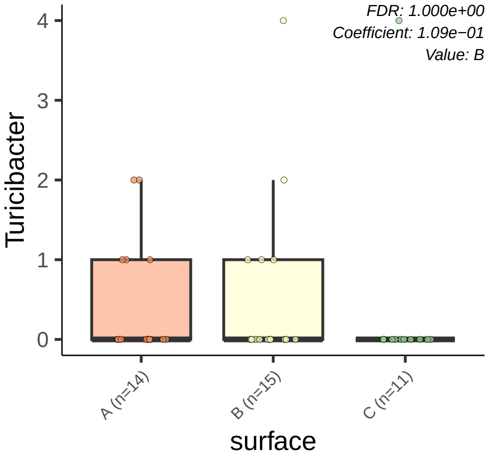

FDR: 1.000e+00
Coefficient: 1.09e−01
Value: B
Turicibacter
0
1
2
3
4
A (n=14)
B (n=15)
C (n=11)
surface

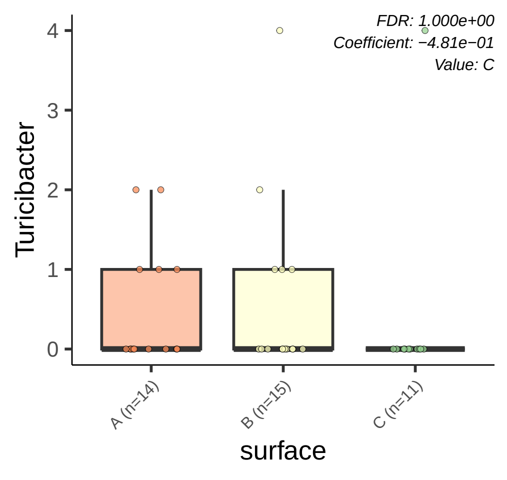

FDR: 1.000e+00
Coefficient: −4.81e−01
Value: C
Turicibacter
0
1
2
3
4
A (n=14)
B (n=15)
C (n=11)
surface

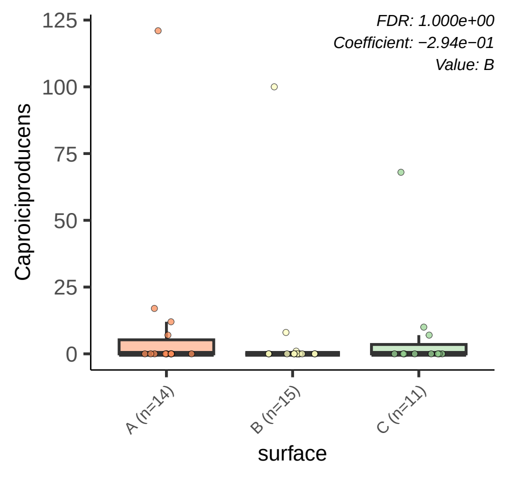
FDR: 1.000e+00
Coefficient: −2.94e−01
Value: B
Caproiciproducens
125
100
75
50
25
0
A (n=14)
B (n=15)
C (n=11)
surface

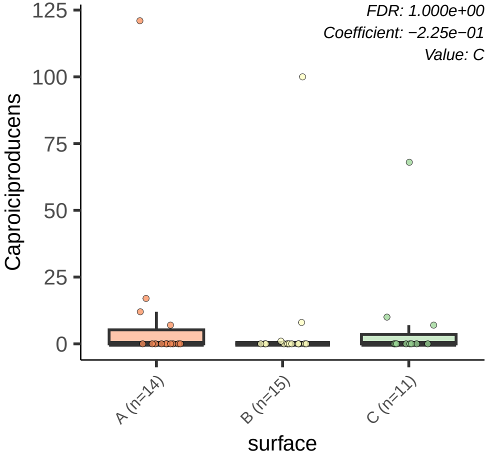

FDR: 1.000e+00
Coefficient: −2.25e−01
Value: C
Caproiciproducens
125
100
75
50
25
0
A (n=14)
B (n=15)
C (n=11)
surface

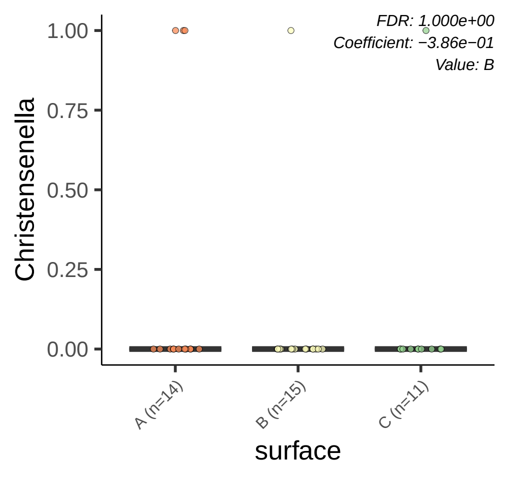
FDR: 1.000e+00
Coefficient: −3.86e−01
Value: B
Christensenella
1.00
0.75
0.50
0.25
0.00
A (n=14)
B (n=15)
C (n=11)
surface

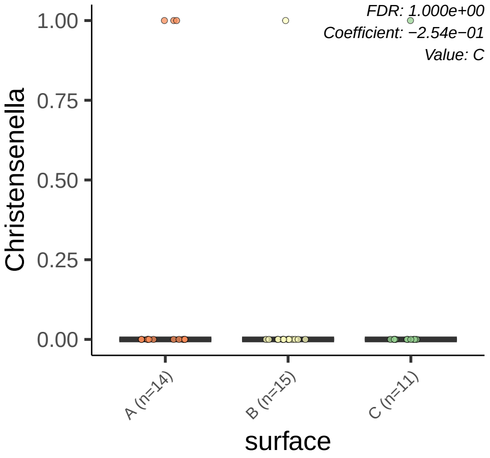

FDR: 1.000e+00
Coefficient: −2.54e−01
Value: C
Christensenella
1.00
0.75
0.50
0.25
0.00
A (n=14)
B (n=15)
C (n=11)
surface

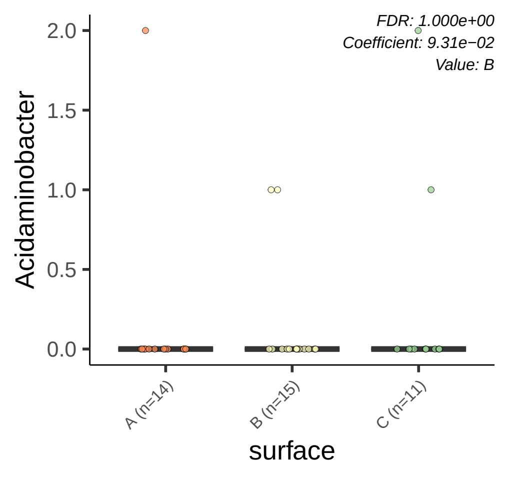
FDR: 1.000e+00
Coefficient: 9.31e−02
Value: B
Acidaminobacter
2.0
1.5
1.0
0.5
0.0
A (n=14)
B (n=15)
C (n=11)
surface

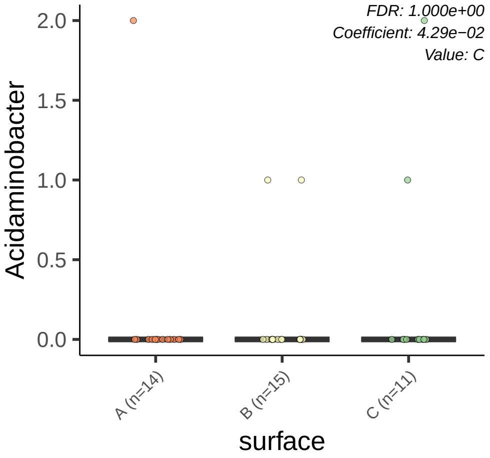
FDR: 1.000e+00
Coefficient: 4.29e−02
Value: C
Acidaminobacter
2.0
1.5
1.0
0.5
0.0
A (n=14)
B (n=15)
C (n=11)
surface

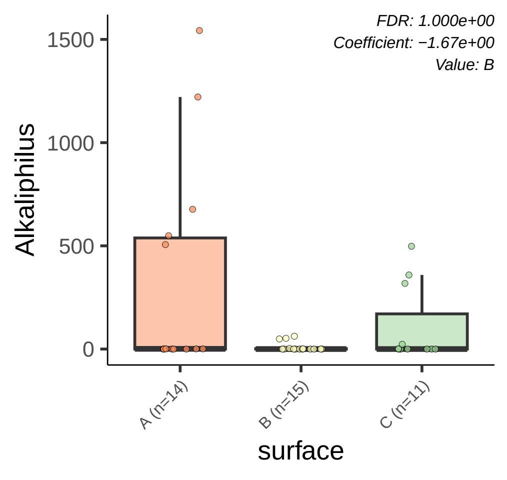
FDR: 1.000e+00
Coefficient: −1.67e+00
Value: B
Alkaliphilus
1500
1000
500
0
A (n=14)
B (n=15)
C (n=11)
surface

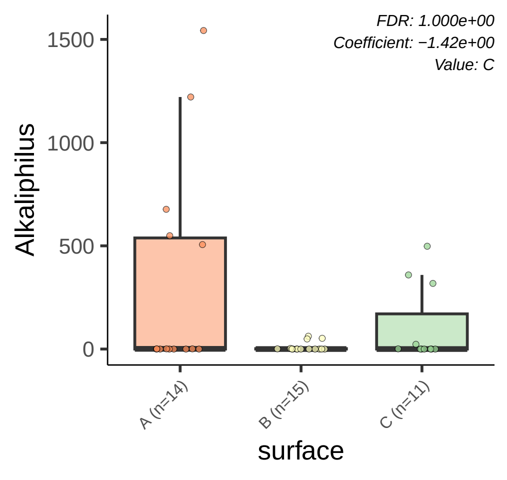

FDR: 1.000e+00
Coefficient: −1.42e+00
Value: C
Alkaliphilus
1500
1000
500
0
A (n=14)
B (n=15)
C (n=11)
surface

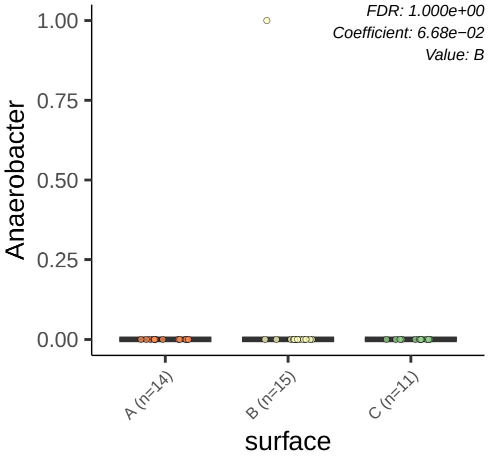
FDR: 1.000e+00
Coefficient: 6.68e−02
Value: B
Anaerobacter
1.00
0.75
0.50
0.25
0.00
A (n=14)
B (n=15)
C (n=11)
surface

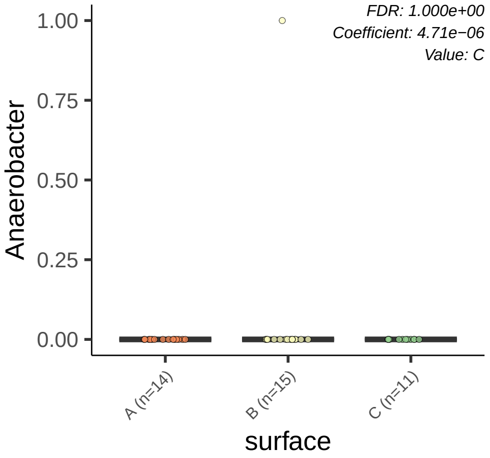

FDR: 1.000e+00
Coefficient: 4.71e−06
Value: C
Anaerobacter
1.00
0.75
0.50
0.25
0.00
A (n=14)
B (n=15)
C (n=11)
surface

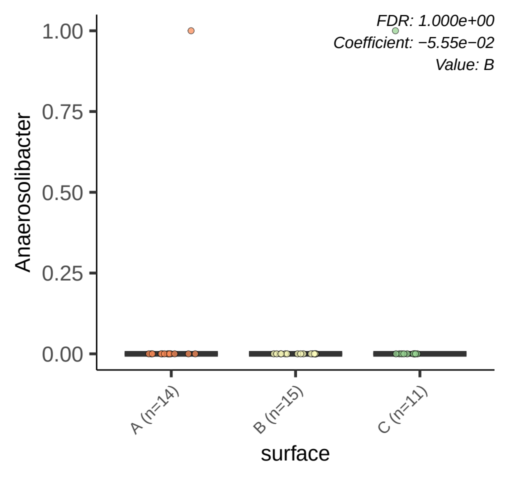
FDR: 1.000e+00
Coefficient: −5.55e−02
Value: B
Anaerosolibacter
1.00
0.75
0.50
0.25
0.00
A (n=14)
B (n=15)
C (n=11)
surface

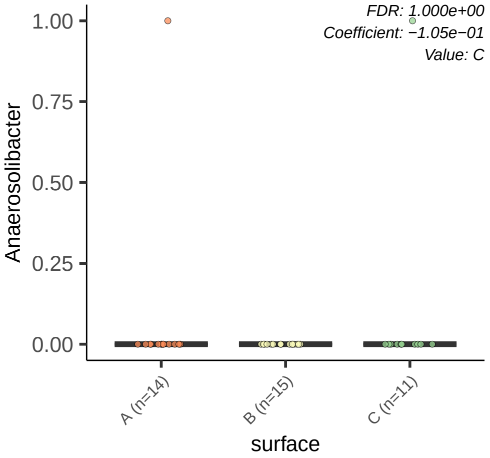

FDR: 1.000e+00
Coefficient: −1.05e−01
Value: C
Anaerosolibacter
1.00
0.75
0.50
0.25
0.00
A (n=14)
B (n=15)
C (n=11)
surface

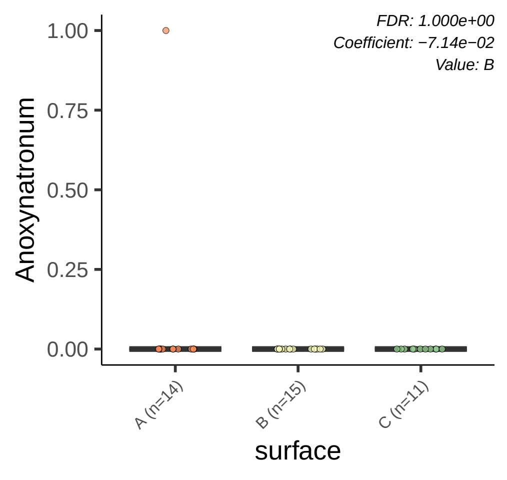
FDR: 1.000e+00
Coefficient: −7.14e−02
Value: B
Anoxynatronum
1.00
0.75
0.50
0.25
0.00
A (n=14)
B (n=15)
C (n=11)
surface

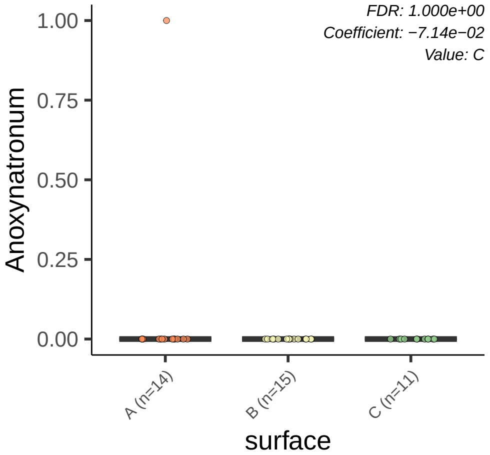

FDR: 1.000e+00
Coefficient: −7.14e−02
Value: C
Anoxynatronum
1.00
0.75
0.50
0.25
0.00
A (n=14)
B (n=15)
C (n=11)
surface

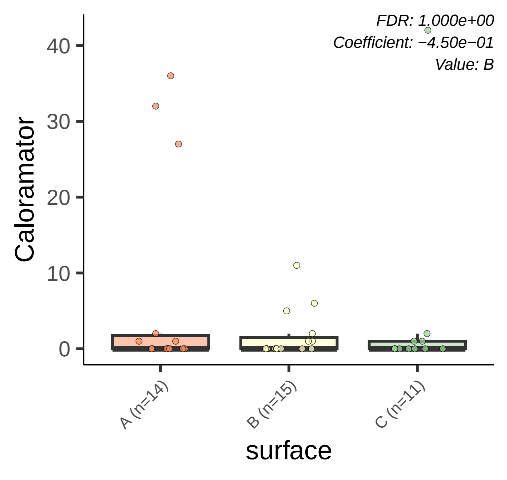
FDR: 1.000e+00
Coefficient: −4.50e−01
Value: B
Caloramator
40
30
20
10
0
A (n=14)
B (n=15)
C (n=11)
surface

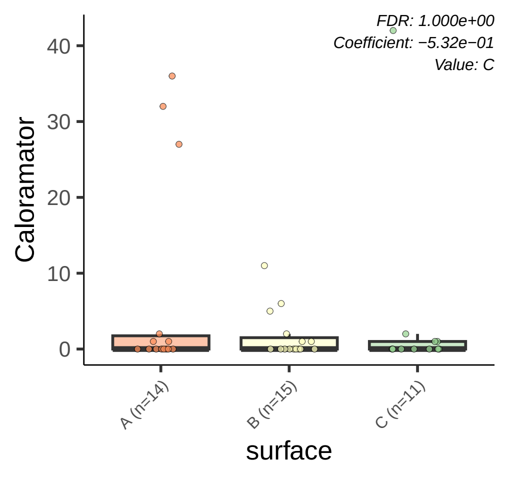

FDR: 1.000e+00
Coefficient: −5.32e−01
Value: C
Caloramator
0
10
20
30
40
A (n=14)
B (n=15)
C (n=11)
surface

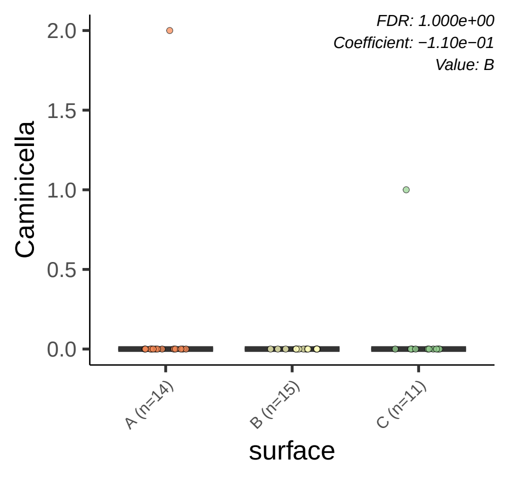

FDR: 1.000e+00
Coefficient: −1.10e−01
Value: B
Caminicella
2.0
1.5
1.0
0.5
0.0
A (n=14)
B (n=15)
C (n=11)
surface

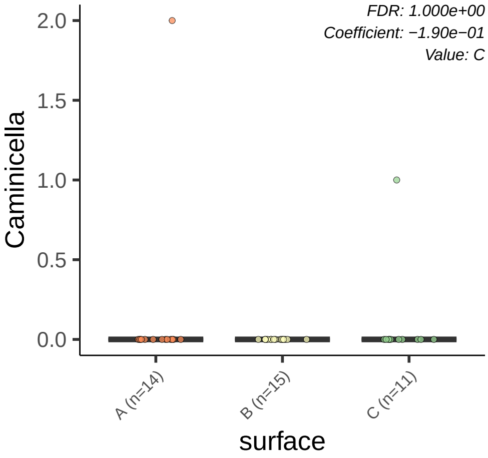
FDR: 1.000e+00
Coefficient: −1.90e−01
Value: C
Caminicella
2.0
1.5
1.0
0.5
0.0
A (n=14)
B (n=15)
C (n=11)
surface

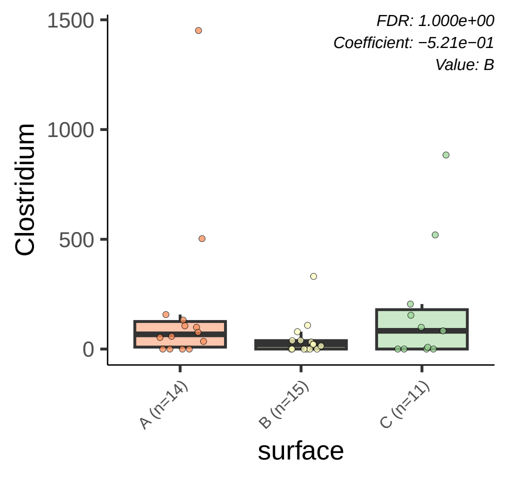
FDR: 1.000e+00
Coefficient: −5.21e−01
Value: B
1500
1000
500
0
Clostridium
A (n=14)
B (n=15)
C (n=11)
surface

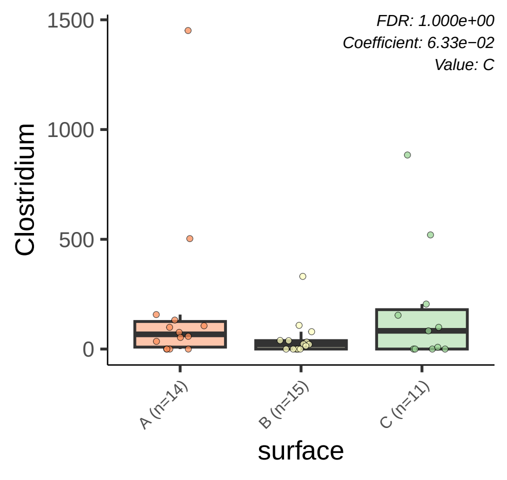
FDR: 1.000e+00
Coefficient: 6.33e−02
Value: C
Clostridium
1500
1000
500
0
A (n=14)
B (n=15)
C (n=11)
surface

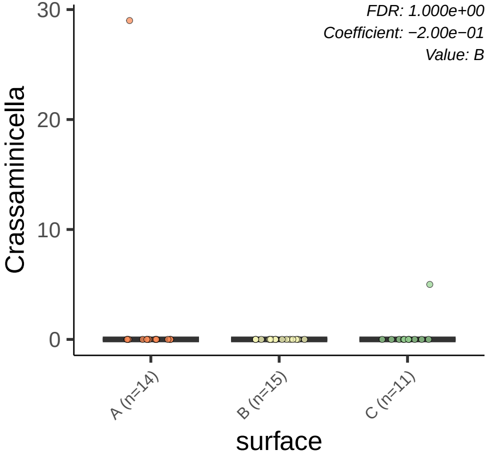
FDR: 1.000e+00
Coefficient: −2.00e−01
Value: B
Crassaminicella
30
20
10
0
A (n=14)
B (n=15)
C (n=11)
surface

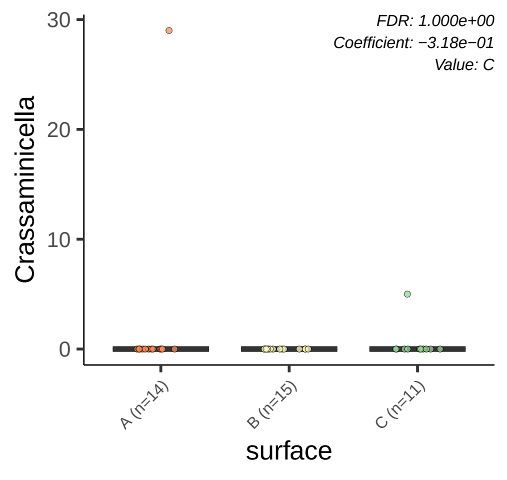
FDR: 1.000e+00
Coefficient: −3.18e−01
Value: C
Crassaminicella
30
20
10
0
A (n=14)
B (n=15)
C (n=11)
surface

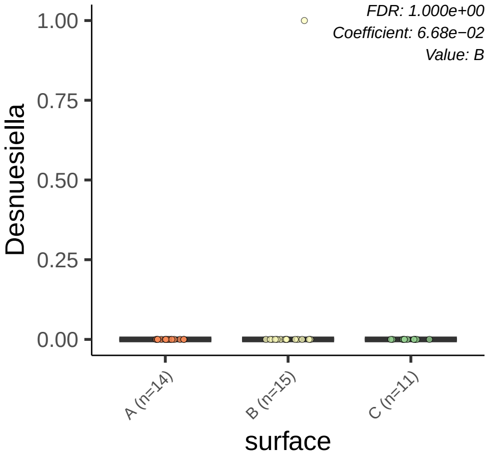
FDR: 1.000e+00
Coefficient: 6.68e−02
Value: B
Desnuesiella
1.00
0.75
0.50
0.25
0.00
A (n=14)
B (n=15)
C (n=11)
surface

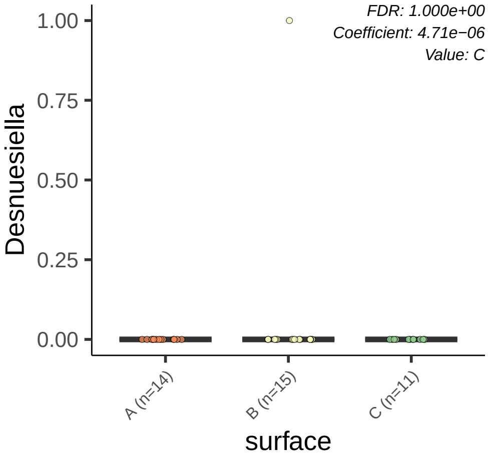
FDR: 1.000e+00
Coefficient: 4.71e−06
Value: C
Desnuesiella
1.00
0.75
0.50
0.25
0.00
A (n=14)
B (n=15)
C (n=11)
surface

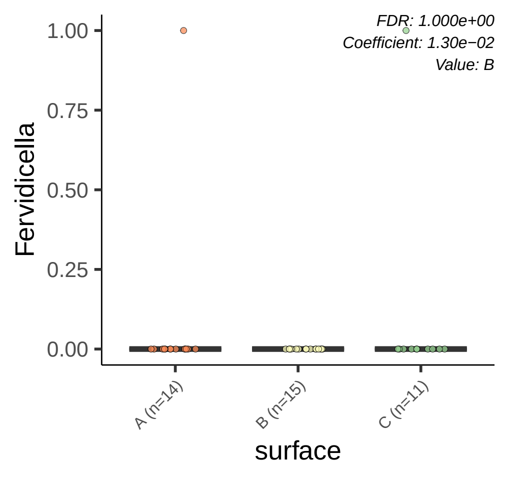
FDR: 1.000e+00
Coefficient: 1.30e−02
Value: B
Fervidicella
1.00
0.75
0.50
0.25
0.00
A (n=14)
B (n=15)
C (n=11)
surface

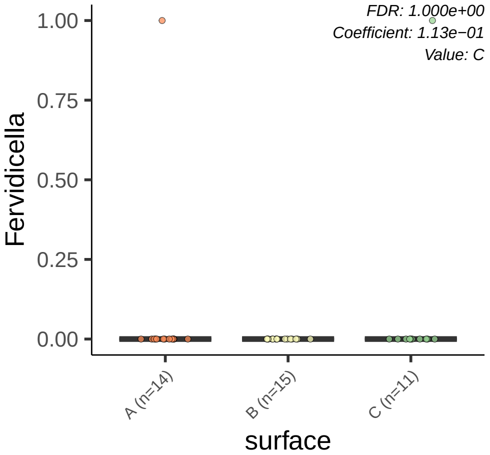

FDR: 1.000e+00
Coefficient: 1.13e−01
Value: C
Fervidicella
1.00
0.75
0.50
0.25
0.00
A (n=14)
B (n=15)
C (n=11)
surface

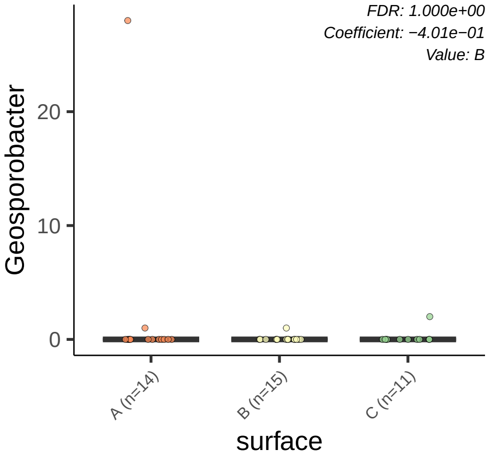
FDR: 1.000e+00
Coefficient: −4.01e−01
Value: B
Geosporobacter
0
10
20
A (n=14)
B (n=15)
C (n=11)
surface

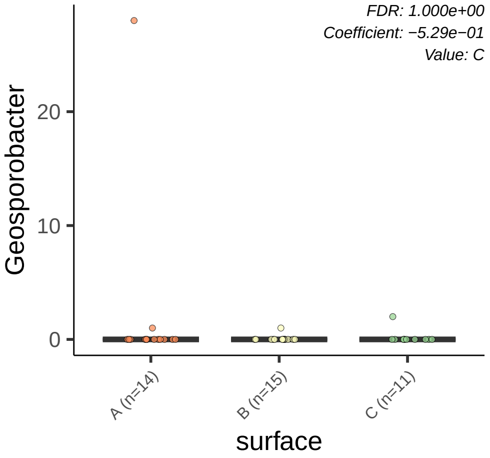

FDR: 1.000e+00
Coefficient: −5.29e−01
Value: C
Geosporobacter
0
10
20
A (n=14)
B (n=15)
C (n=11)
surface

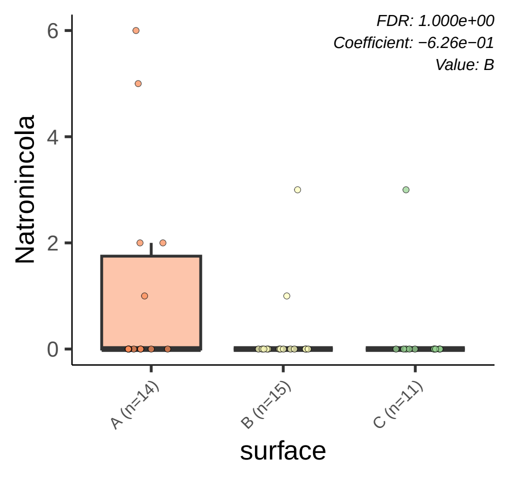

FDR: 1.000e+00
Coefficient: −6.26e−01
Value: B
Natronincola
6
4
2
0
A (n=14)
B (n=15)
C (n=11)
surface

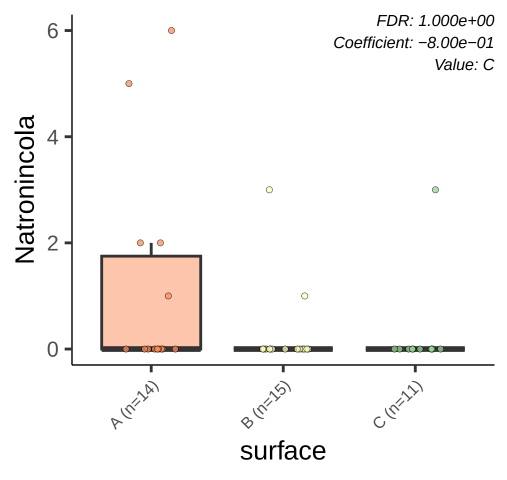

FDR: 1.000e+00
Coefficient: −8.00e−01
Value: C
Natronincola
0
2
4
6
A (n=14)
B (n=15)
C (n=11)
surface

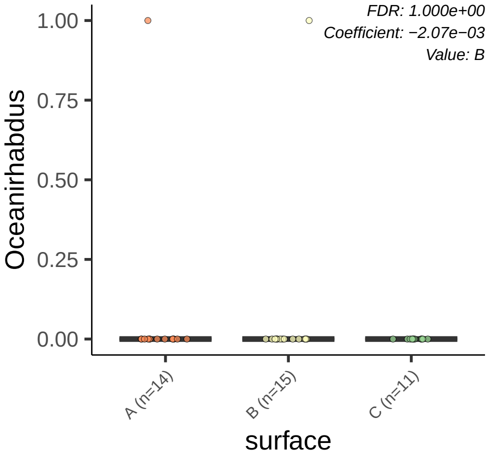
FDR: 1.000e+00
Coefficient: −2.07e−03
Value: B
Oceanirhabdus
1.00
0.75
0.50
0.25
0.00
A (n=14)
B (n=15)
C (n=11)
surface

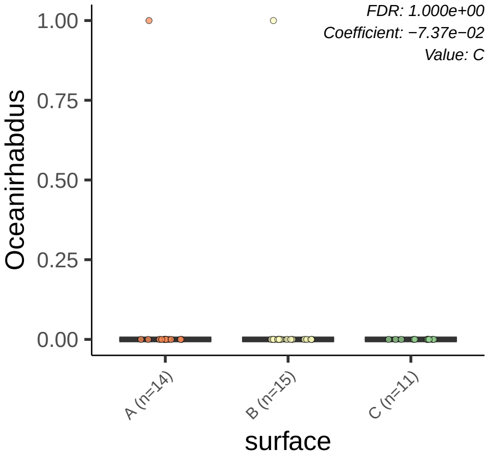
FDR: 1.000e+00
Coefficient: −7.37e−02
Value: C
Oceanirhabdus
1.00
0.75
0.50
0.25
0.00
A (n=14)
B (n=15)
C (n=11)
surface

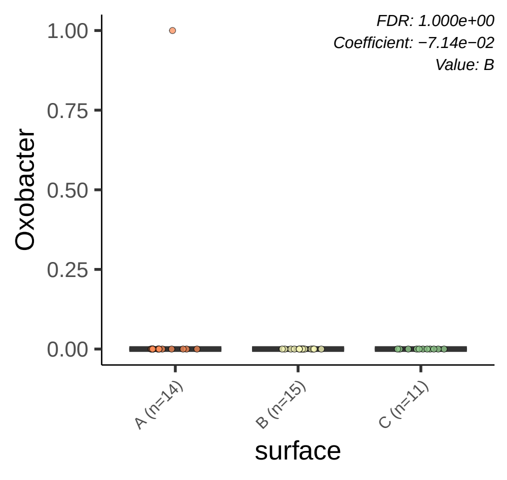
FDR: 1.000e+00
Coefficient: −7.14e−02
Value: B
Oxobacter
1.00
0.75
0.50
0.25
0.00
A (n=14)
B (n=15)
C (n=11)
surface

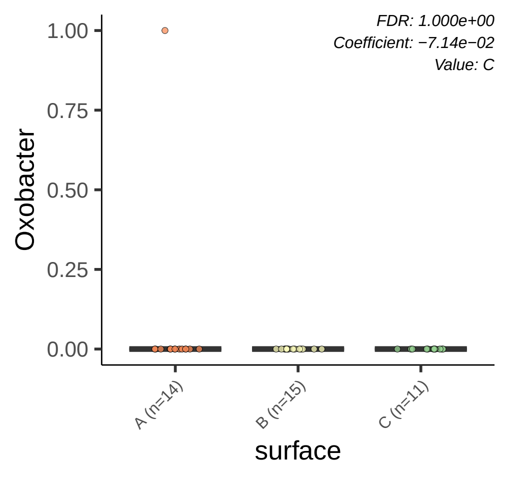
FDR: 1.000e+00
Coefficient: −7.14e−02
Value: C
Oxobacter
1.00
0.75
0.50
0.25
0.00
A (n=14)
B (n=15)
C (n=11)
surface

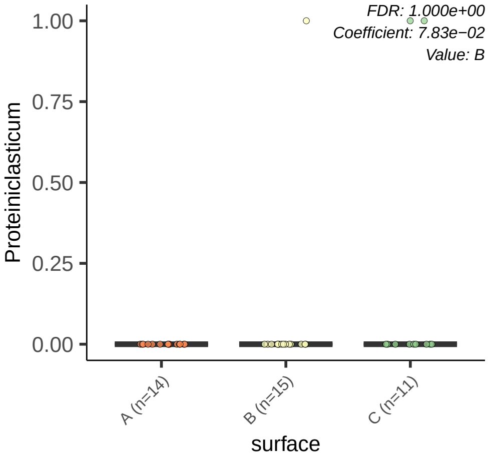

FDR: 1.000e+00
Coefficient: 7.83e−02
Value: B
Proteiniclasticum
1.00
0.75
0.50
0.25
0.00
A (n=14)
B (n=15)
C (n=11)
surface

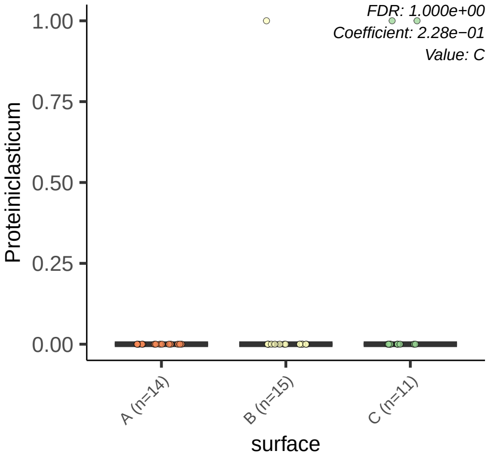

FDR: 1.000e+00
Coefficient: 2.28e−01
Value: C
1.00
0.75
0.50
0.25
0.00
Proteiniclasticum
A (n=14)
B (n=15)
C (n=11)
surface

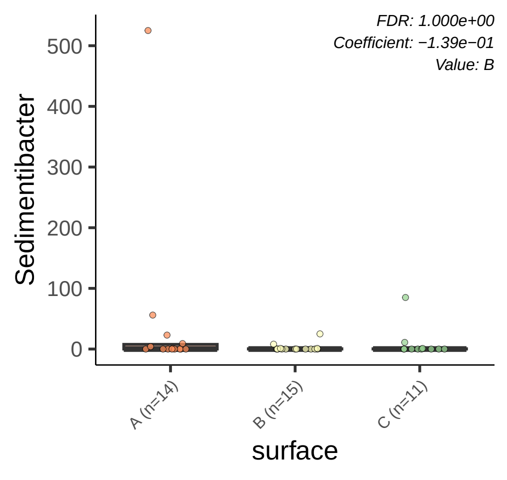

FDR: 1.000e+00
Coefficient: −1.39e−01
Value: B
Sedimentibacter
0
100
200
300
400
500
A (n=14)
B (n=15)
C (n=11)
surface

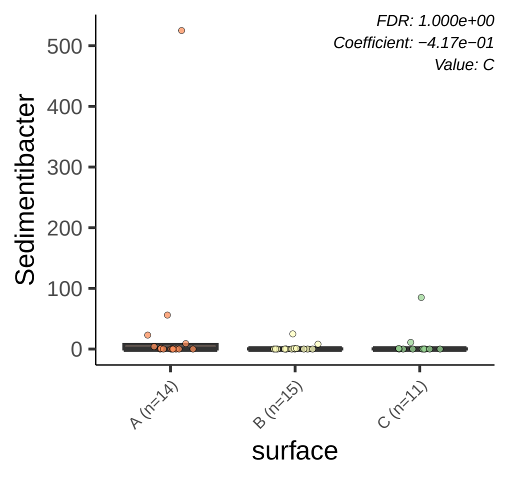

FDR: 1.000e+00
Coefficient: −4.17e−01
Value: C
Sedimentibacter
500
400
300
200
100
0
A (n=14)
B (n=15)
C (n=11)
surface

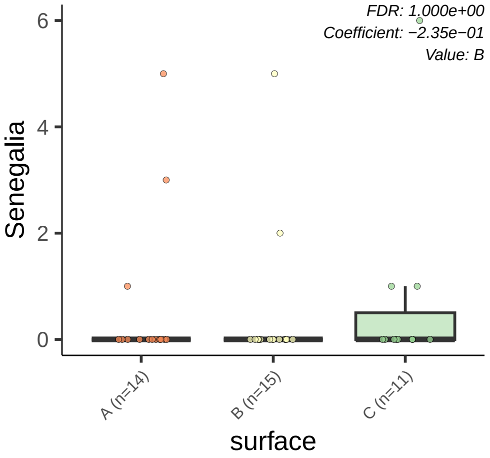
FDR: 1.000e+00
Coefficient: −2.35e−01
Value: B
Senegalia
0
2
4
6
A (n=14)
B (n=15)
C (n=11)
surface

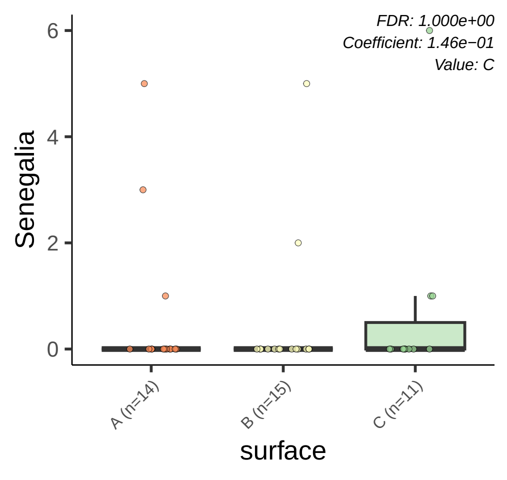
FDR: 1.000e+00
Coefficient: 1.46e−01
Value: C
Senegalia
6
4
2
0
A (n=14)
B (n=15)
C (n=11)
surface

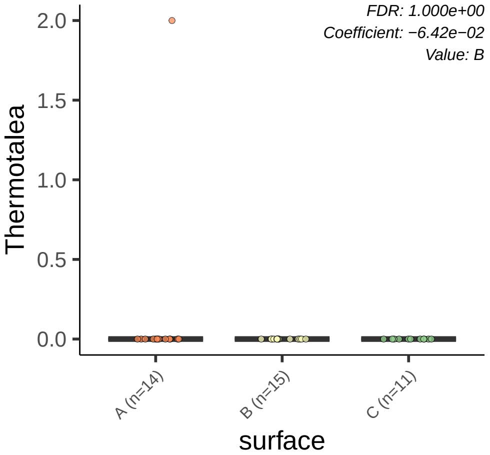
FDR: 1.000e+00
Coefficient: −6.42e−02
Value: B
Thermotalea
2.0
1.5
1.0
0.5
0.0
A (n=14)
B (n=15)
C (n=11)
surface

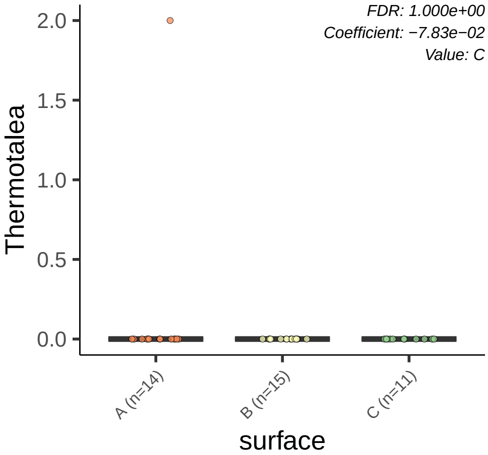

FDR: 1.000e+00
Coefficient: −7.83e−02
Value: C
Thermotalea
2.0
1.5
1.0
0.5
0.0
A (n=14)
B (n=15)
C (n=11)
surface

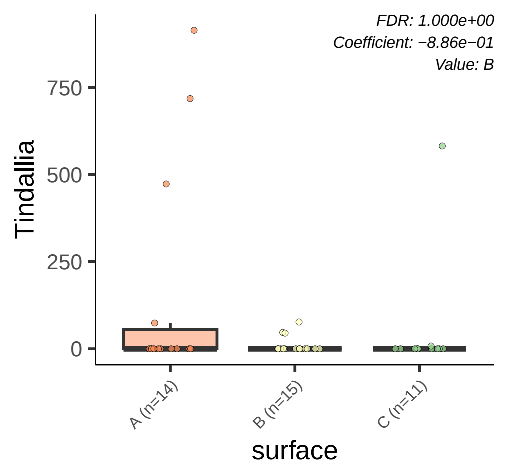
FDR: 1.000e+00
Coefficient: −8.86e−01
Value: B
Tindallia
750
500
250
0
A (n=14)
B (n=15)
C (n=11)
surface

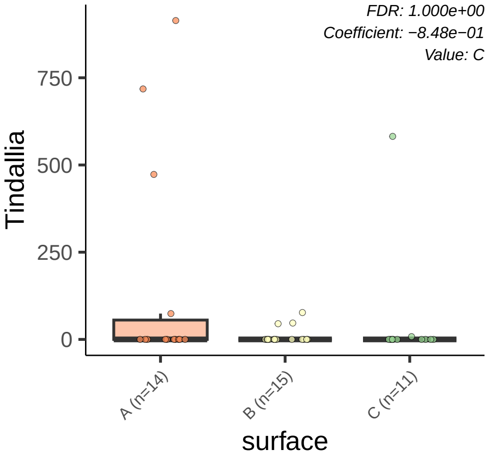

FDR: 1.000e+00
Coefficient: −8.48e−01
Value: C
Tindallia
750
500
250
0
A (n=14)
B (n=15)
C (n=11)
surface

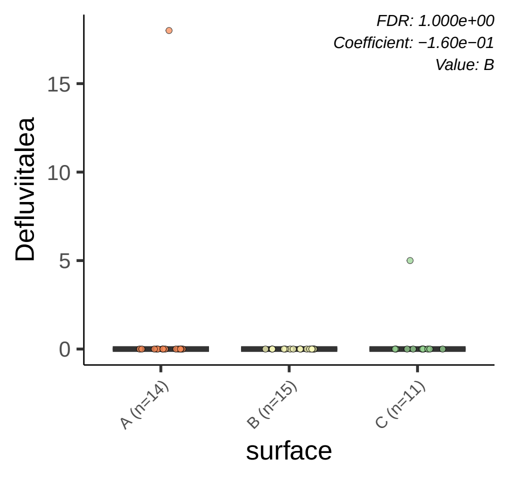

FDR: 1.000e+00
Coefficient: −1.60e−01
Value: B
Defluviitalea
15
10
5
0
A (n=14)
B (n=15)
C (n=11)
surface

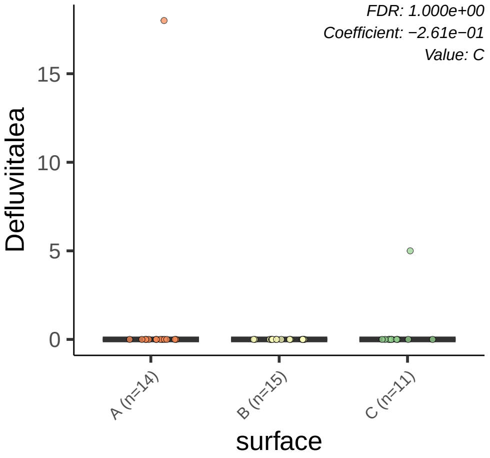

FDR: 1.000e+00
Coefficient: −2.61e−01
Value: C
Defluviitalea
15
10
5
0
A (n=14)
B (n=15)
C (n=11)
surface

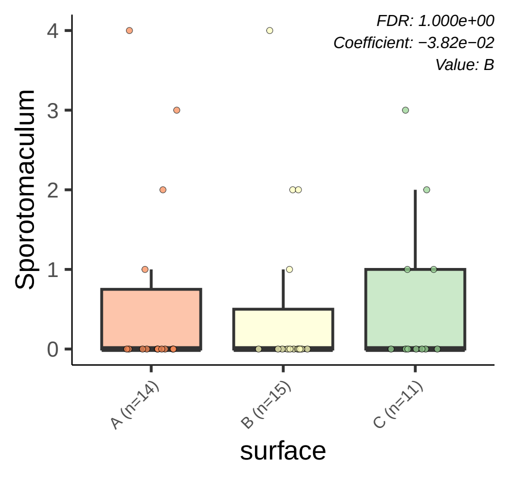

FDR: 1.000e+00
Coefficient: −3.82e−02
Value: B
Sporotomaculum
0
1
2
3
4
A (n=14)
B (n=15)
C (n=11)
surface

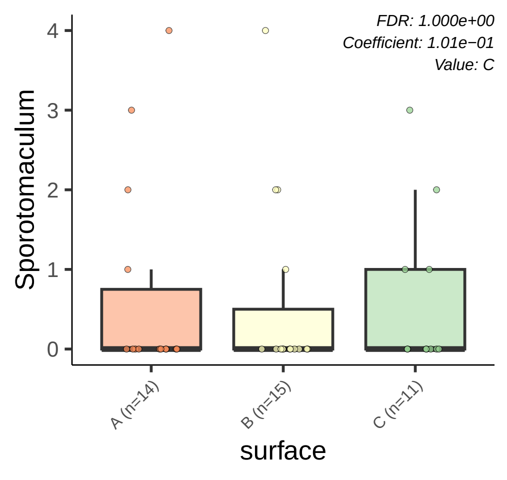
FDR: 1.000e+00
Coefficient: 1.01e−01
Value: C
Sporotomaculum
0
1
2
3
4
A (n=14)
B (n=15)
C (n=11)
surface

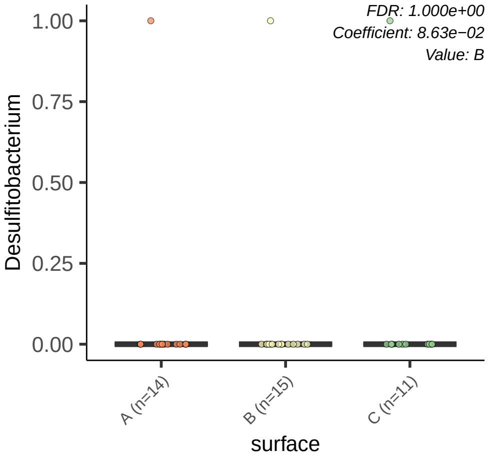
FDR: 1.000e+00
Coefficient: 8.63e−02
Value: B
Desulfitobacterium
1.00
0.75
0.50
0.25
0.00
A (n=14)
B (n=15)
C (n=11)
surface

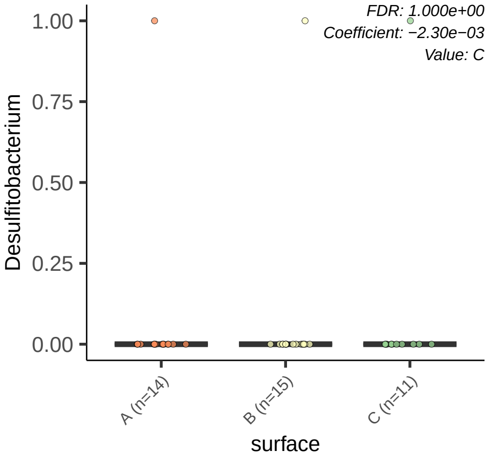
FDR: 1.000e+00
Coefficient: −2.30e−03
Value: C
Desulfitobacterium
1.00
0.75
0.50
0.25
0.00
A (n=14)
B (n=15)
C (n=11)
surface

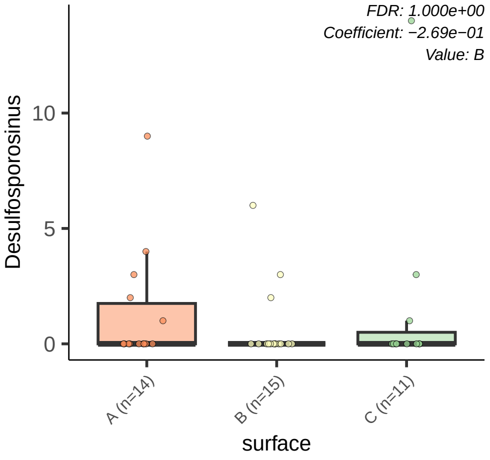
FDR: 1.000e+00
Coefficient: −2.69e−01
Value: B
Desulfosporosinus
10
5
0
A (n=14)
B (n=15)
C (n=11)
surface

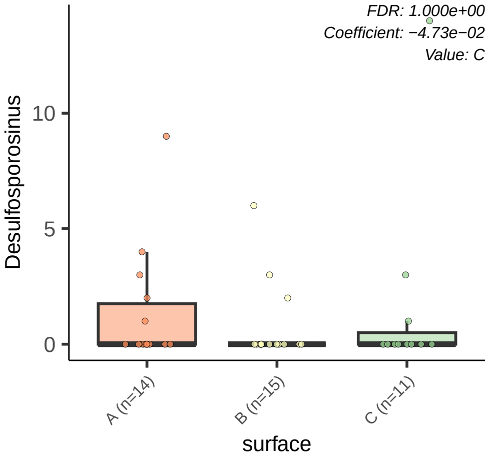

FDR: 1.000e+00
Coefficient: −4.73e−02
Value: C
Desulfosporosinus
10
5
0
A (n=14)
B (n=15)
C (n=11)
surface

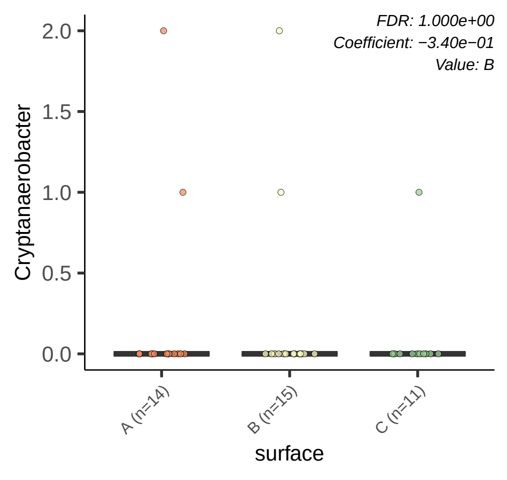
FDR: 1.000e+00
Coefficient: −3.40e−01
Value: B
Cryptanaerobacter
2.0
1.5
1.0
0.5
0.0
A (n=14)
B (n=15)
C (n=11)
surface

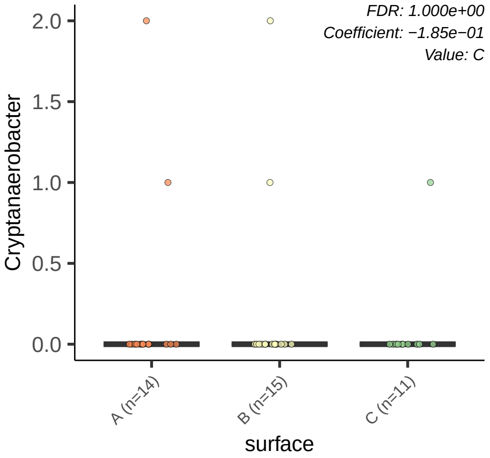

FDR: 1.000e+00
Coefficient: −1.85e−01
Value: C
Cryptanaerobacter
2.0
1.5
1.0
0.5
0.0
A (n=14)
B (n=15)
C (n=11)
surface

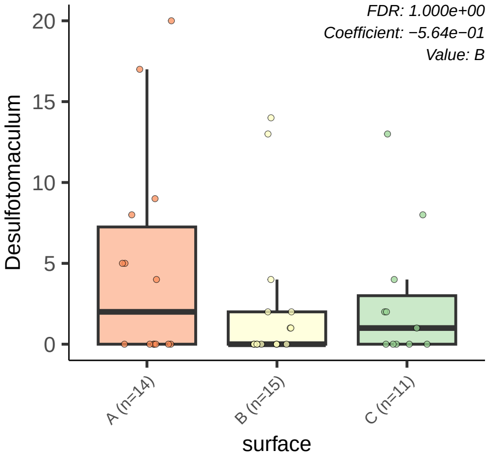

FDR: 1.000e+00
Coefficient: −5.64e−01
Value: B
Desulfotomaculum
20
15
10
5
0
A (n=14)
B (n=15)
C (n=11)
surface

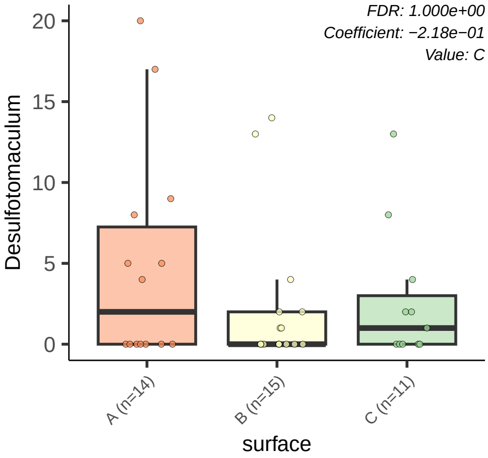

FDR: 1.000e+00
Coefficient: −2.18e−01
Value: C
Desulfotomaculum
20
15
10
5
0
A (n=14)
B (n=15)
C (n=11)
surface

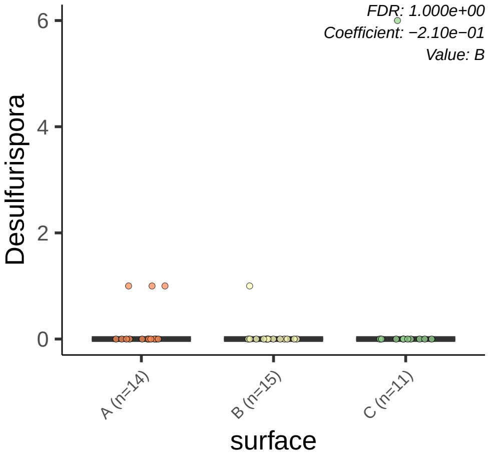

FDR: 1.000e+00
Coefficient: −2.10e−01
Value: B
Desulfurispora
6
4
2
0
A (n=14)
B (n=15)
C (n=11)
surface

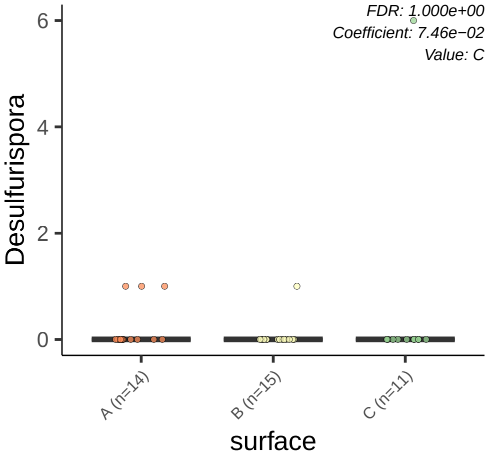

FDR: 1.000e+00
Coefficient: 7.46e−02
Value: C
Desulfurispora
6
4
2
0
A (n=14)
B (n=15)
C (n=11)
surface

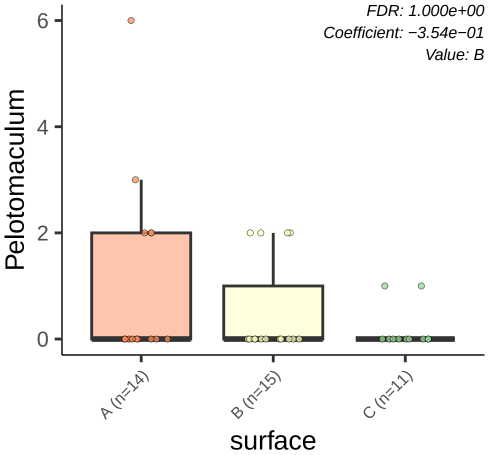
FDR: 1.000e+00
Coefficient: −3.54e−01
Value: B
Pelotomaculum
6
4
2
0
A (n=14)
B (n=15)
C (n=11)
surface

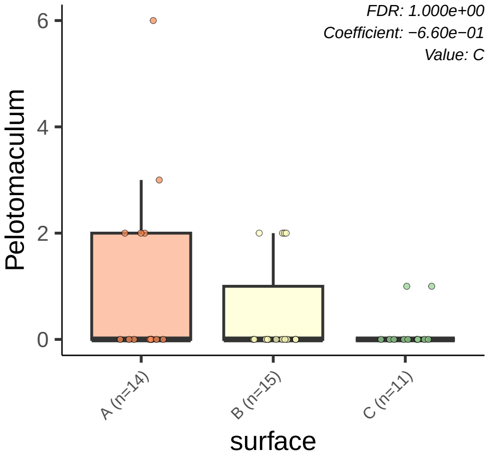
FDR: 1.000e+00
Coefficient: −6.60e−01
Value: C
Pelotomaculum
0
2
4
6
A (n=14)
B (n=15)
C (n=11)
surface

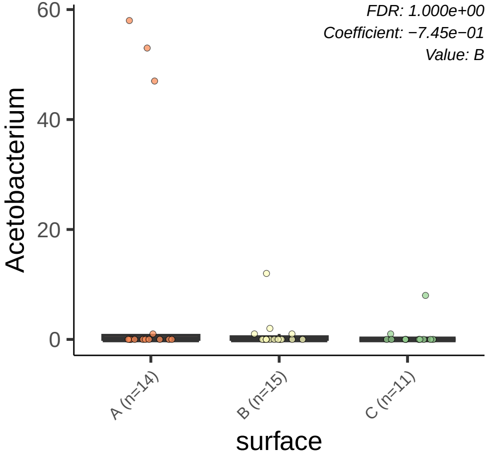
FDR: 1.000e+00
Coefficient: −7.45e−01
Value: B
Acetobacterium
60
40
20
0
A (n=14)
B (n=15)
C (n=11)
surface

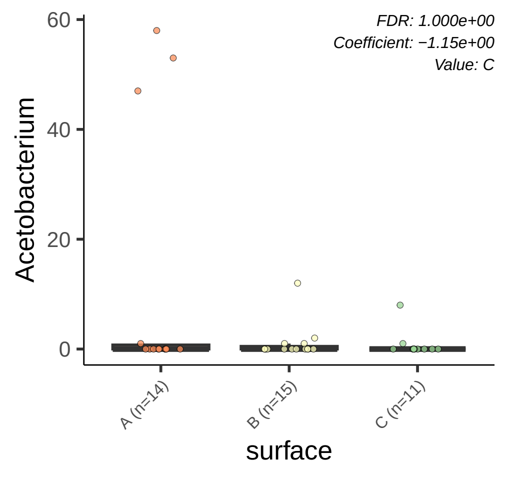
FDR: 1.000e+00
Coefficient: −1.15e+00
Value: C
60
40
20
0
Acetobacterium
A (n=14)
B (n=15)
C (n=11)
surface

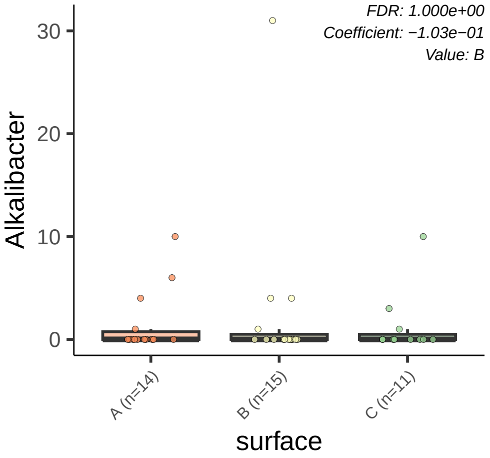

FDR: 1.000e+00
Coefficient: −1.03e−01
Value: B
Alkalibacter
0
10
20
30
A (n=14)
B (n=15)
C (n=11)
surface

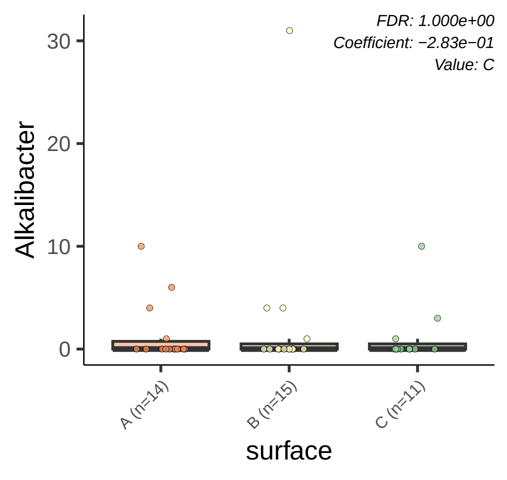

FDR: 1.000e+00
Coefficient: −2.83e−01
Value: C
Alkalibacter
30
20
10
0
A (n=14)
B (n=15)
C (n=11)
surface

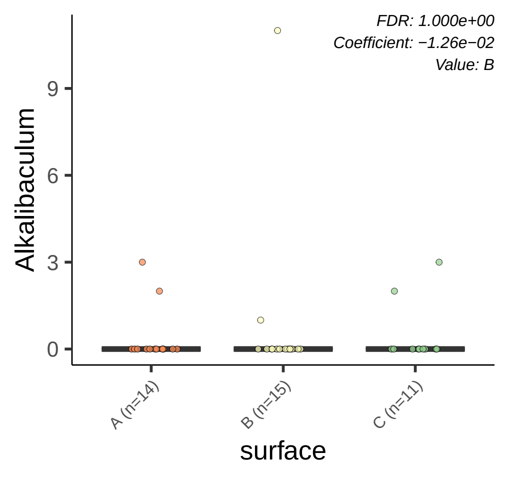
FDR: 1.000e+00
Coefficient: −1.26e−02
Value: B
Alkalibaculum
0
3
6
9
A (n=14)
B (n=15)
C (n=11)
surface

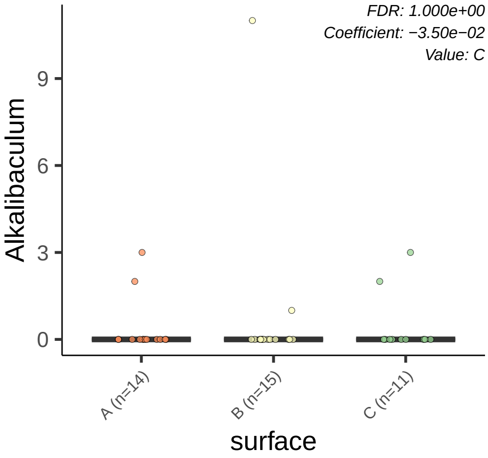
FDR: 1.000e+00
Coefficient: −3.50e−02
Value: C
Alkalibaculum
0
3
6
9
A (n=14)
B (n=15)
C (n=11)
surface

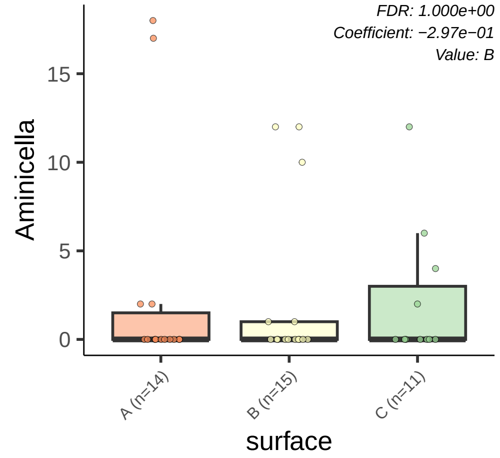
FDR: 1.000e+00
Coefficient: −2.97e−01
Value: B
Aminicella
15
10
5
0
A (n=14)
B (n=15)
C (n=11)
surface

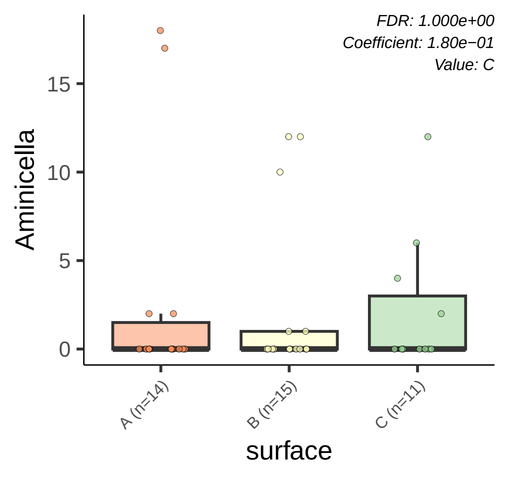
FDR: 1.000e+00
Coefficient: 1.80e−01
Value: C
Aminicella
0
5
10
15
A (n=14)
B (n=15)
C (n=11)
surface

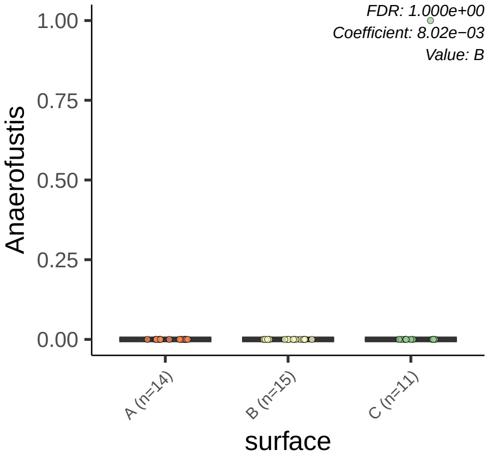
FDR: 1.000e+00
Coefficient: 8.02e−03
Value: B
Anaerofustis
1.00
0.75
0.50
0.25
0.00
A (n=14)
B (n=15)
C (n=11)
surface

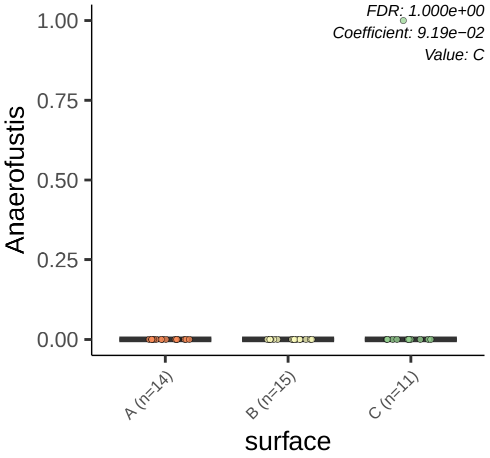

FDR: 1.000e+00
Coefficient: 9.19e−02
Value: C
Anaerofustis
1.00
0.75
0.50
0.25
0.00
A (n=14)
B (n=15)
C (n=11)
surface

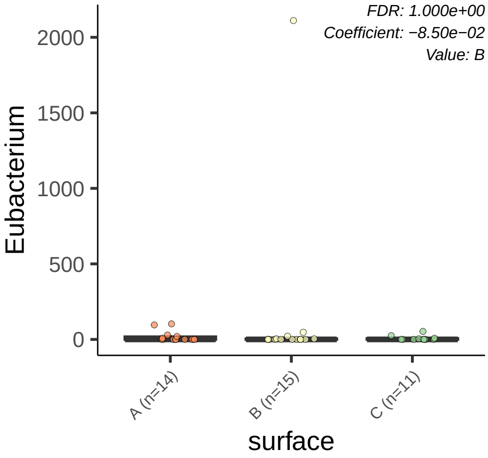
FDR: 1.000e+00
Coefficient: −8.50e−02
Value: B
Eubacterium
2000
1500
1000
500
0
A (n=14)
B (n=15)
C (n=11)
surface

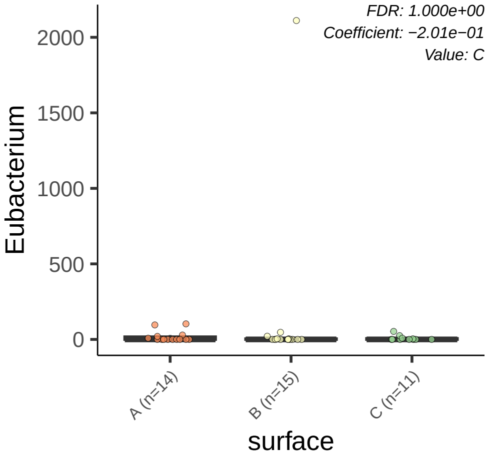

FDR: 1.000e+00
Coefficient: −2.01e−01
Value: C
Eubacterium
2000
1500
1000
500
0
A (n=14)
B (n=15)
C (n=11)
surface

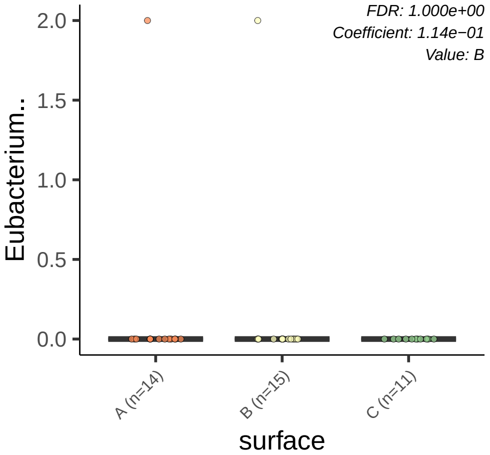

FDR: 1.000e+00
Coefficient: 1.14e−01
Value: B
Eubacterium..
2.0
1.5
1.0
0.5
0.0
A (n=14)
B (n=15)
C (n=11)
surface

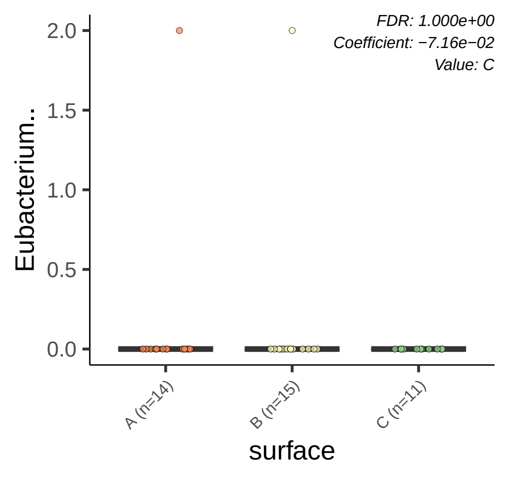
FDR: 1.000e+00
Coefficient: −7.16e−02
Value: C
Eubacterium..
2.0
1.5
1.0
0.5
0.0
A (n=14)
B (n=15)
C (n=11)
surface

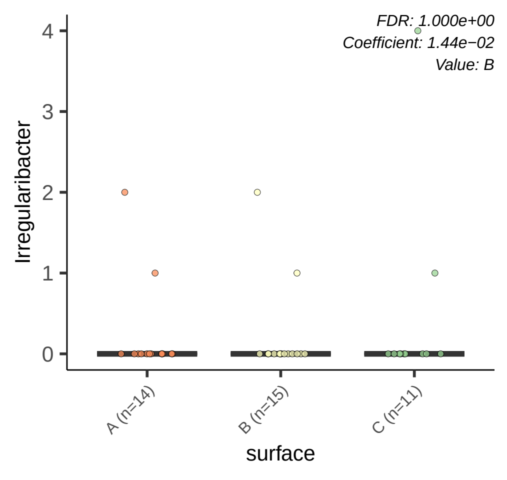

FDR: 1.000e+00
Coefficient: 1.44e−02
Value: B
Irregularibacter
0
1
2
3
4
A (n=14)
B (n=15)
C (n=11)
surface

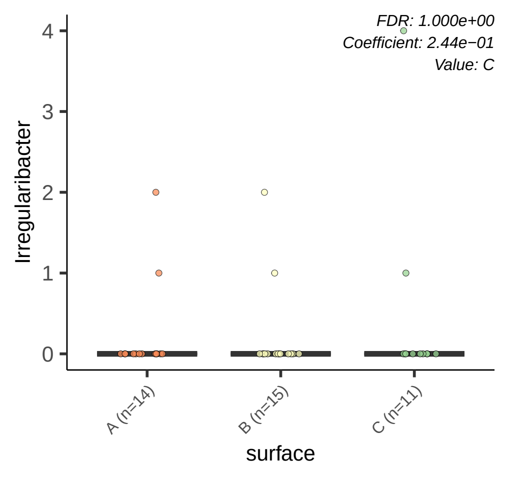
FDR: 1.000e+00
Coefficient: 2.44e−01
Value: C
Irregularibacter
0
1
2
3
4
A (n=14)
B (n=15)
C (n=11)
surface

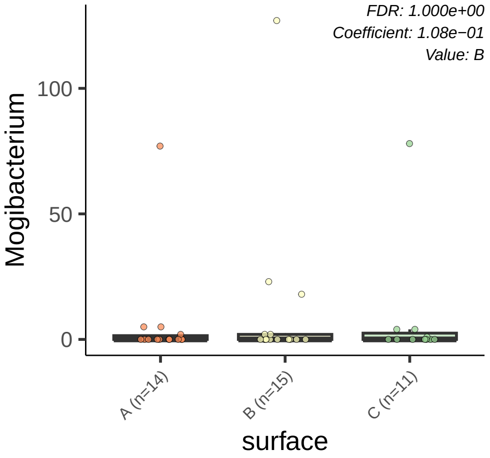
FDR: 1.000e+00
Coefficient: 1.08e−01
Value: B
Mogibacterium
100
50
0
A (n=14)
B (n=15)
C (n=11)
surface

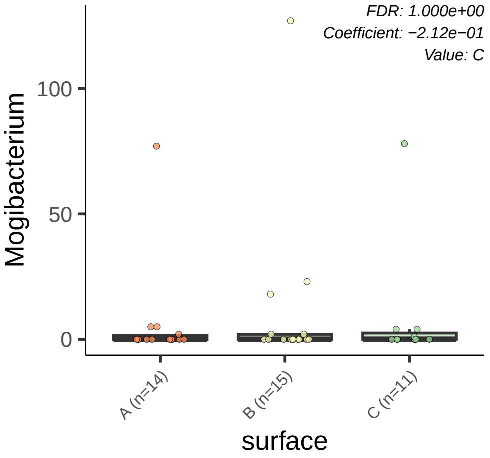
FDR: 1.000e+00
Coefficient: −2.12e−01
Value: C
Mogibacterium
100
50
0
A (n=14)
B (n=15)
C (n=11)
surface

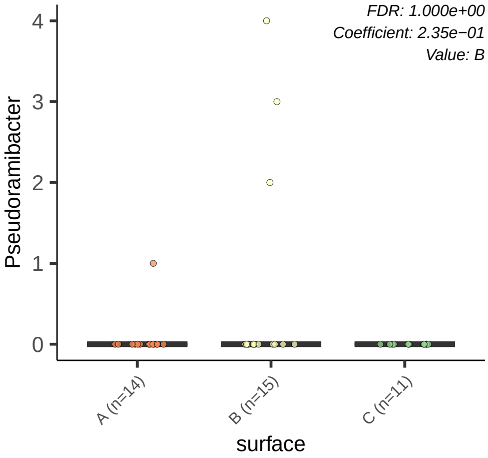
FDR: 1.000e+00
Coefficient: 2.35e−01
Value: B
Pseudoramibacter
4
3
2
1
0
A (n=14)
B (n=15)
C (n=11)
surface

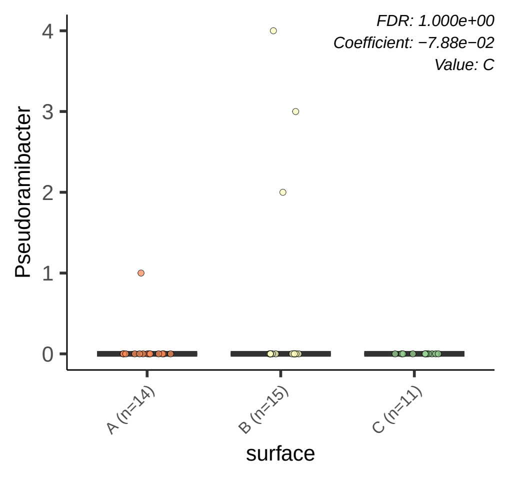

FDR: 1.000e+00
Coefficient: −7.88e−02
Value: C
Pseudoramibacter
0
1
2
3
4
A (n=14)
B (n=15)
C (n=11)
surface

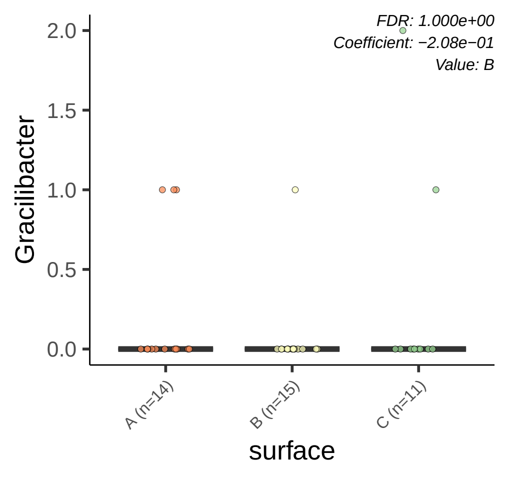

FDR: 1.000e+00
Coefficient: −2.08e−01
Value: B
Gracilibacter
2.0
1.5
1.0
0.5
0.0
A (n=14)
B (n=15)
C (n=11)
surface

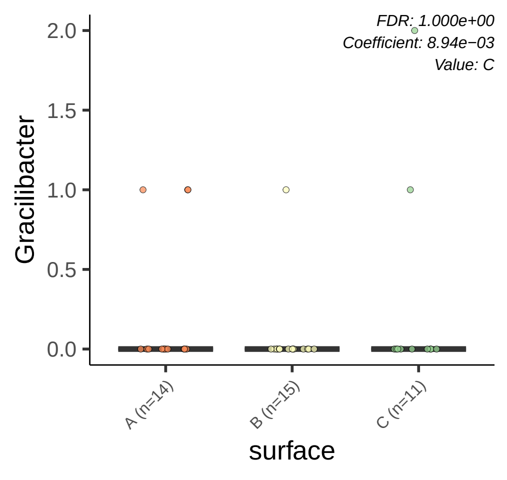

FDR: 1.000e+00
Coefficient: 8.94e−03
Value: C
Gracilibacter
2.0
1.5
1.0
0.5
0.0
A (n=14)
B (n=15)
C (n=11)
surface

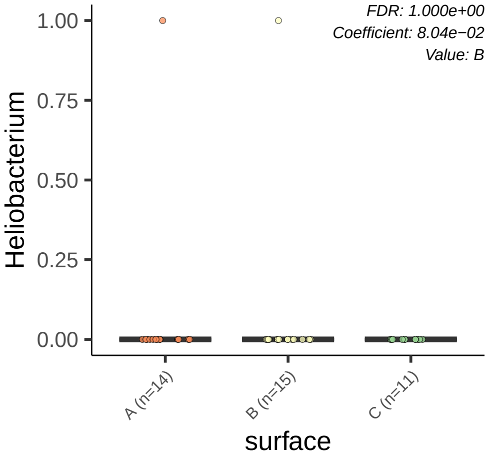
FDR: 1.000e+00
Coefficient: 8.04e−02
Value: B
Heliobacterium
1.00
0.75
0.50
0.25
0.00
A (n=14)
B (n=15)
C (n=11)
surface

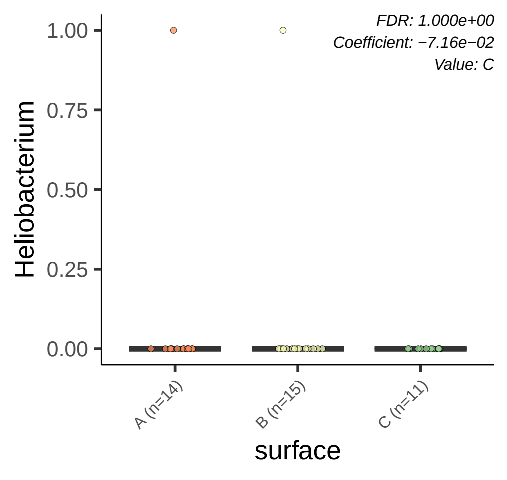

FDR: 1.000e+00
Coefficient: −7.16e−02
Value: C
Heliobacterium
1.00
0.75
0.50
0.25
0.00
A (n=14)
B (n=15)
C (n=11)
surface

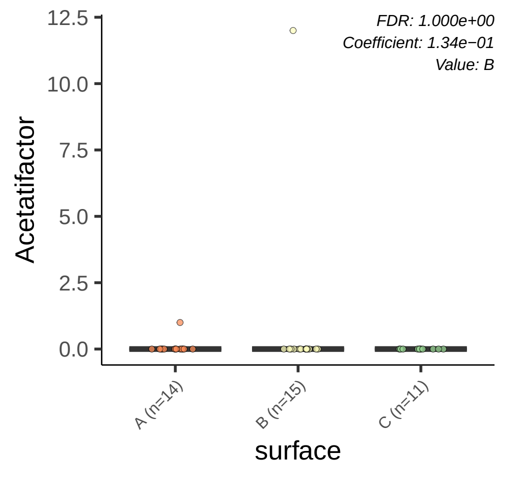
FDR: 1.000e+00
Coefficient: 1.34e−01
Value: B
Acetatifactor
12.5
10.0
7.5
5.0
2.5
0.0
A (n=14)
B (n=15)
C (n=11)
surface

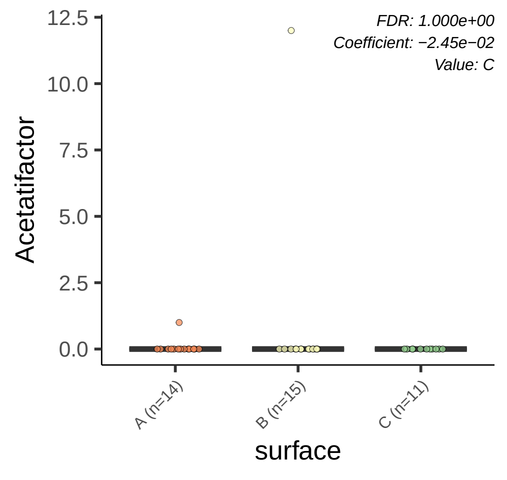
FDR: 1.000e+00
Coefficient: −2.45e−02
Value: C
Acetatifactor
12.5
10.0
7.5
5.0
2.5
0.0
A (n=14)
B (n=15)
C (n=11)
surface

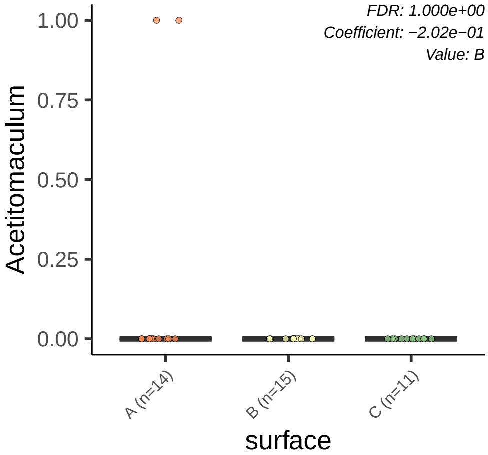
FDR: 1.000e+00
Coefficient: −2.02e−01
Value: B
Acetitomaculum
1.00
0.75
0.50
0.25
0.00
A (n=14)
B (n=15)
C (n=11)
surface

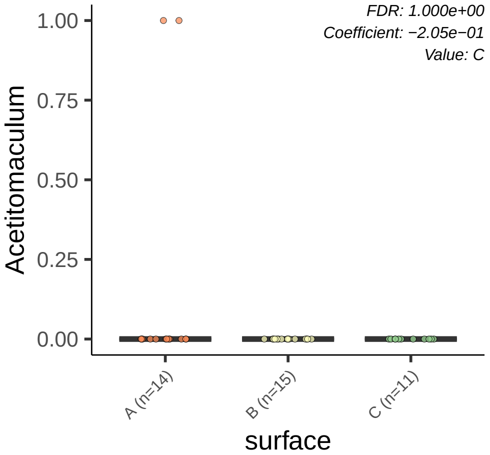
FDR: 1.000e+00
Coefficient: −2.05e−01
Value: C
Acetitomaculum
1.00
0.75
0.50
0.25
0.00
A (n=14)
B (n=15)
C (n=11)
surface

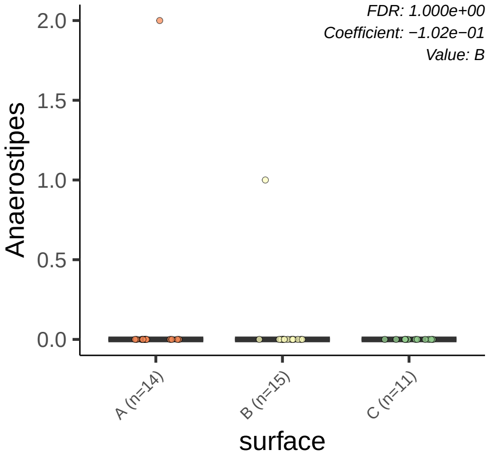

FDR: 1.000e+00
Coefficient: −1.02e−01
Value: B
Anaerostipes
2.0
1.5
1.0
0.5
0.0
A (n=14)
B (n=15)
C (n=11)
surface

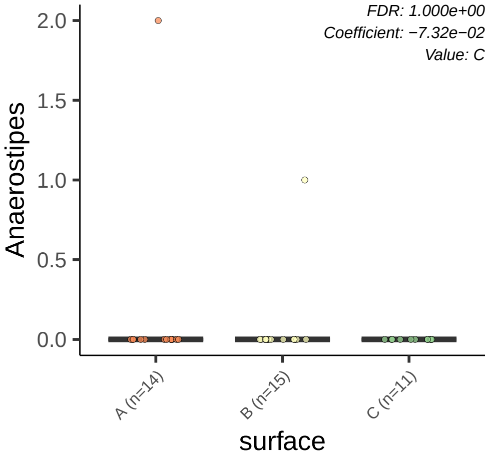

FDR: 1.000e+00
Coefficient: −7.32e−02
Value: C
Anaerostipes
2.0
1.5
1.0
0.5
0.0
A (n=14)
B (n=15)
C (n=11)
surface

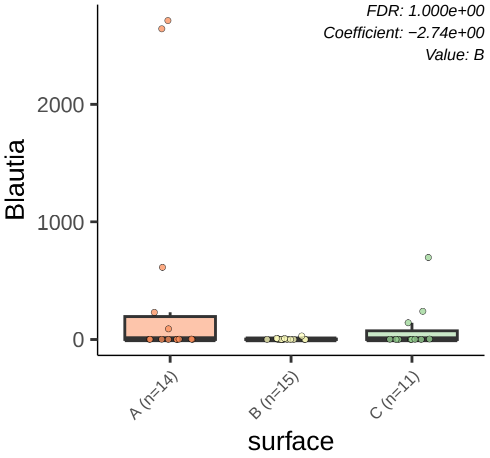

FDR: 1.000e+00
Coefficient: −2.74e+00
Value: B
Blautia
2000
1000
0
A (n=14)
B (n=15)
C (n=11)
surface

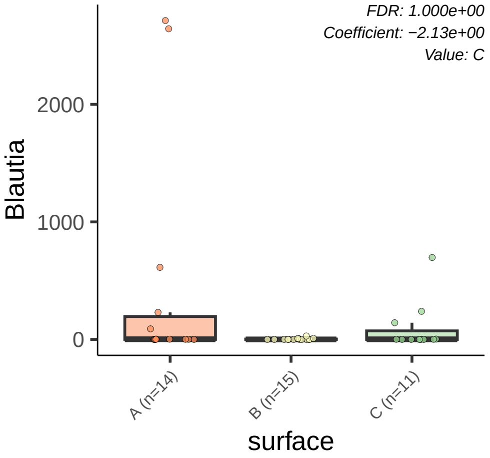
FDR: 1.000e+00
Coefficient: −2.13e+00
Value: C
Blautia
2000
1000
0
A (n=14)
B (n=15)
C (n=11)
surface

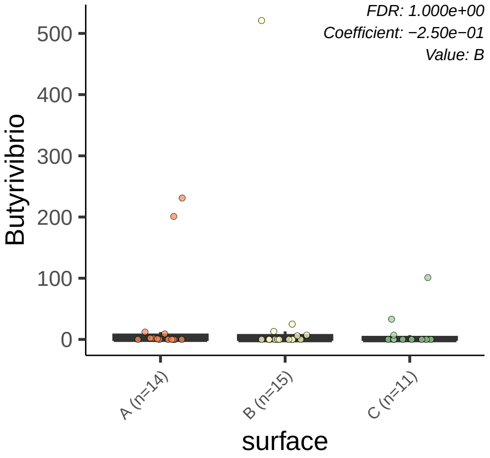

FDR: 1.000e+00
Coefficient: −2.50e−01
Value: B
Butyrivibrio
500
400
300
200
100
0
A (n=14)
B (n=15)
C (n=11)
surface

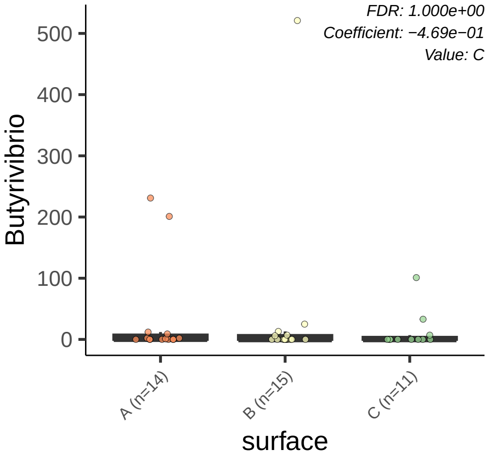
FDR: 1.000e+00
Coefficient: −4.69e−01
Value: C
Butyrivibrio
500
400
300
200
100
0
A (n=14)
B (n=15)
C (n=11)
surface

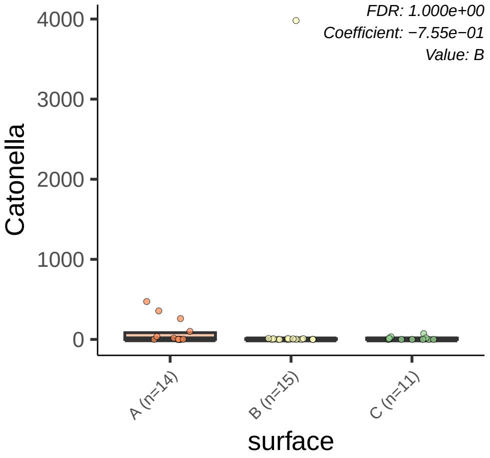

FDR: 1.000e+00
Coefficient: −7.55e−01
Value: B
4000
3000
2000
1000
0
Catonella
A (n=14)
B (n=15)
C (n=11)
surface

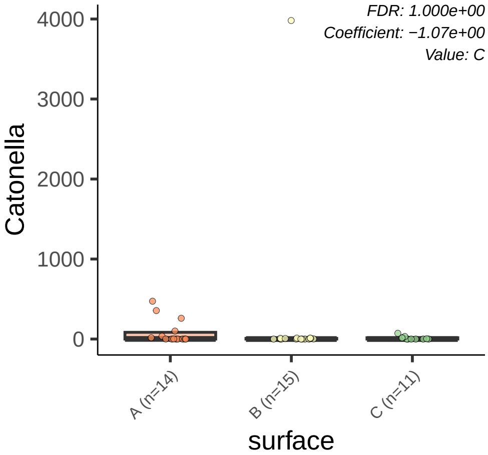
FDR: 1.000e+00
Coefficient: −1.07e+00
Value: C
4000
3000
2000
1000
0
Catonella
A (n=14)
B (n=15)
C (n=11)
surface

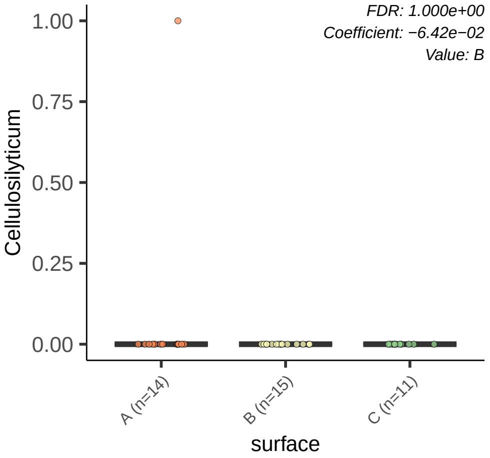
FDR: 1.000e+00
Coefficient: −6.42e−02
Value: B
Cellulosilyticum
1.00
0.75
0.50
0.25
0.00
A (n=14)
B (n=15)
C (n=11)
surface

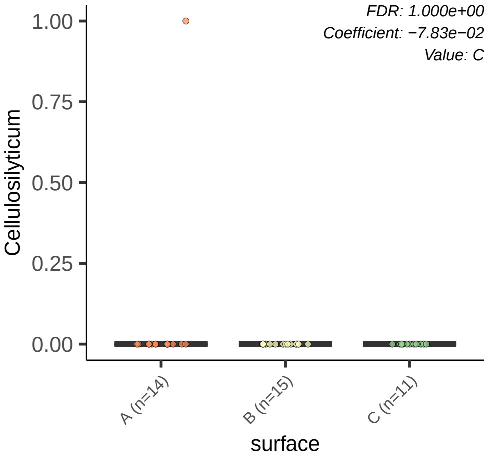

FDR: 1.000e+00
Coefficient: −7.83e−02
Value: C
Cellulosilyticum
1.00
0.75
0.50
0.25
0.00
A (n=14)
B (n=15)
C (n=11)
surface

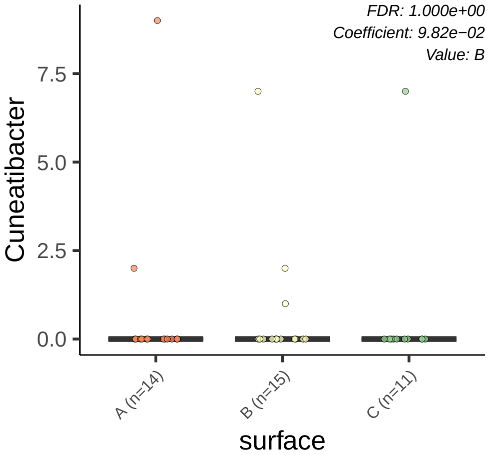
FDR: 1.000e+00
Coefficient: 9.82e−02
Value: B
Cuneatibacter
7.5
5.0
2.5
0.0
A (n=14)
B (n=15)
C (n=11)
surface

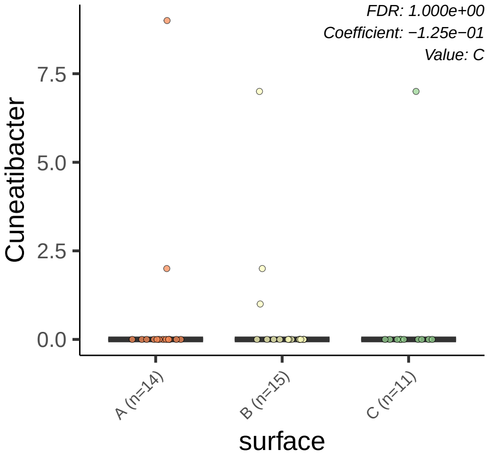

FDR: 1.000e+00
Coefficient: −1.25e−01
Value: C
Cuneatibacter
7.5
5.0
2.5
0.0
A (n=14)
B (n=15)
C (n=11)
surface

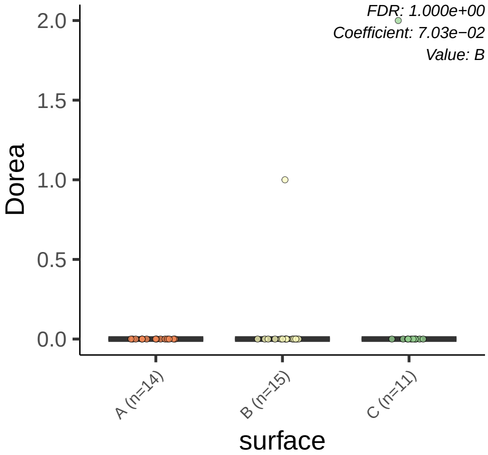
FDR: 1.000e+00
Coefficient: 7.03e−02
Value: B
Dorea
2.0
1.5
1.0
0.5
0.0
A (n=14)
B (n=15)
C (n=11)
surface

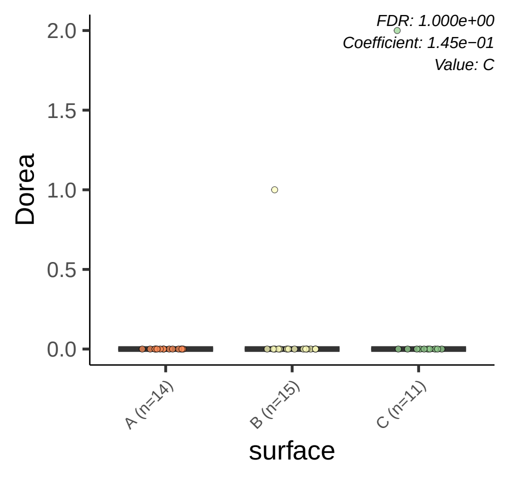
FDR: 1.000e+00
Coefficient: 1.45e−01
Value: C
Dorea
2.0
1.5
1.0
0.5
0.0
A (n=14)
B (n=15)
C (n=11)
surface

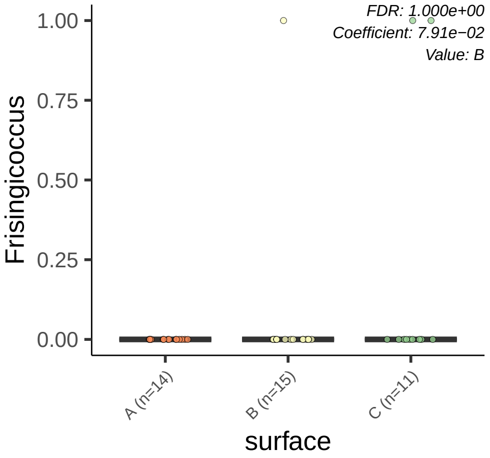

FDR: 1.000e+00
Coefficient: 7.91e−02
Value: B
Frisingicoccus
1.00
0.75
0.50
0.25
0.00
A (n=14)
B (n=15)
C (n=11)
surface

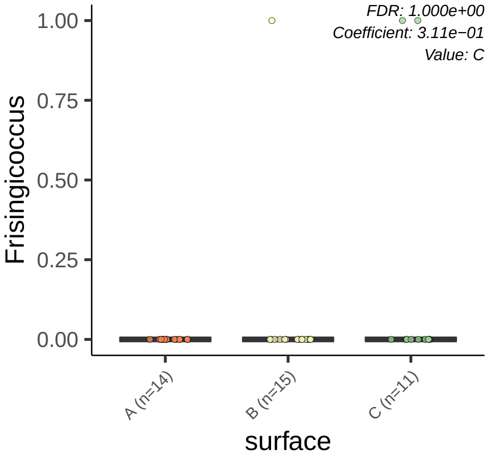
FDR: 1.000e+00
Coefficient: 3.11e−01
Value: C
1.00
0.75
0.50
0.25
0.00
Frisingicoccus
A (n=14)
B (n=15)
C (n=11)
surface

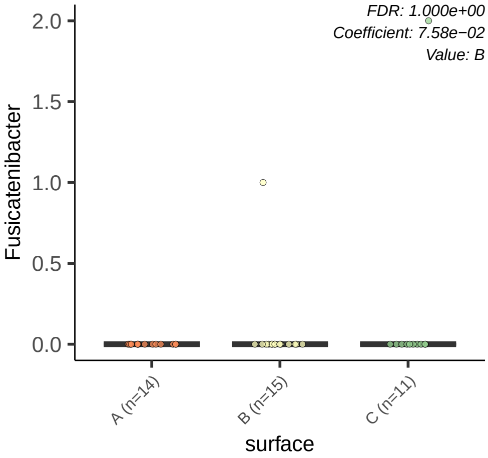
FDR: 1.000e+00
Coefficient: 7.58e−02
Value: B
Fusicatenibacter
2.0
1.5
1.0
0.5
0.0
A (n=14)
B (n=15)
C (n=11)
surface

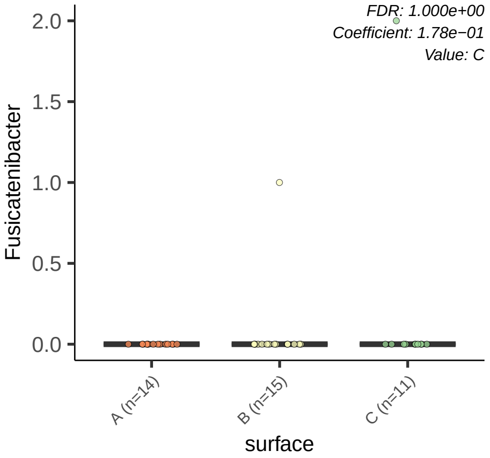
FDR: 1.000e+00
Coefficient: 1.78e−01
Value: C
Fusicatenibacter
2.0
1.5
1.0
0.5
0.0
A (n=14)
B (n=15)
C (n=11)
surface

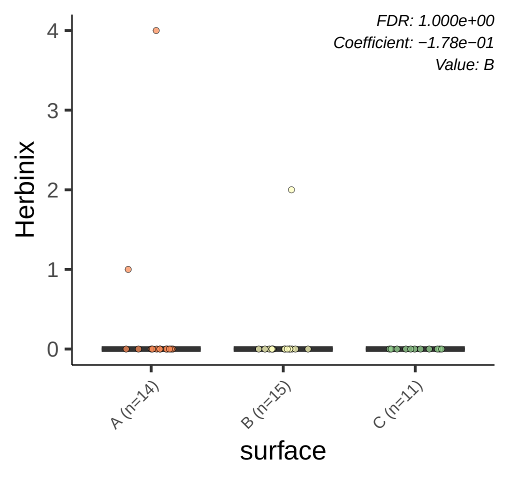
FDR: 1.000e+00
Coefficient: −1.78e−01
Value: B
Herbinix
0
1
2
3
4
A (n=14)
B (n=15)
C (n=11)
surface

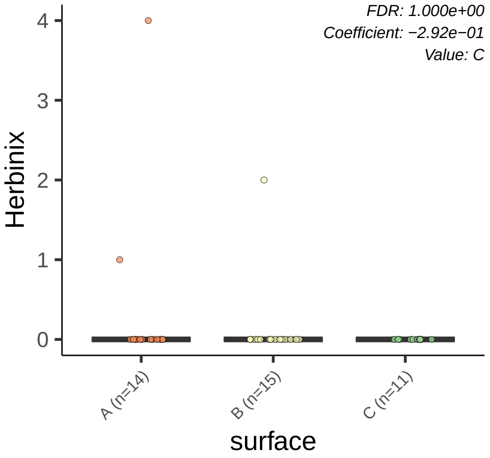
FDR: 1.000e+00
Coefficient: −2.92e−01
Value: C
Herbinix
4
3
2
1
0
A (n=14)
B (n=15)
C (n=11)
surface

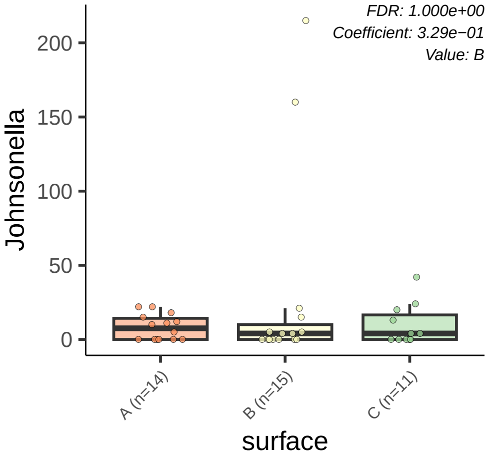
FDR: 1.000e+00
Coefficient: 3.29e−01
Value: B
Johnsonella
200
150
100
50
0
A (n=14)
B (n=15)
C (n=11)
surface

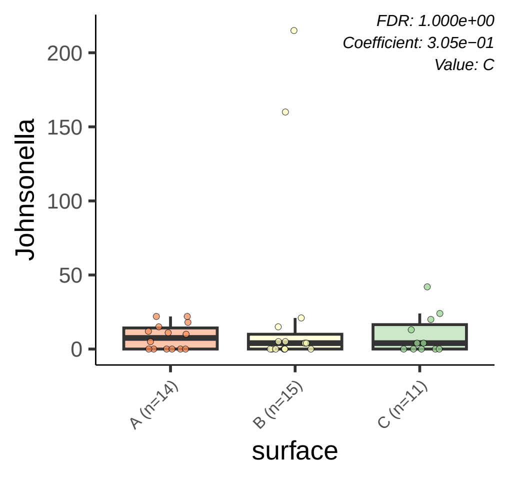

FDR: 1.000e+00
Coefficient: 3.05e−01
Value: C
Johnsonella
0
50
100
150
200
A (n=14)
B (n=15)
C (n=11)
surface

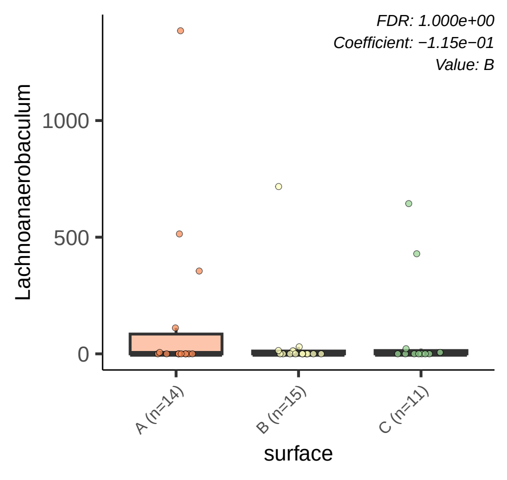
FDR: 1.000e+00
Coefficient: −1.15e−01
Value: B
Lachnoanaerobaculum
1000
500
0
A (n=14)
B (n=15)
C (n=11)
surface

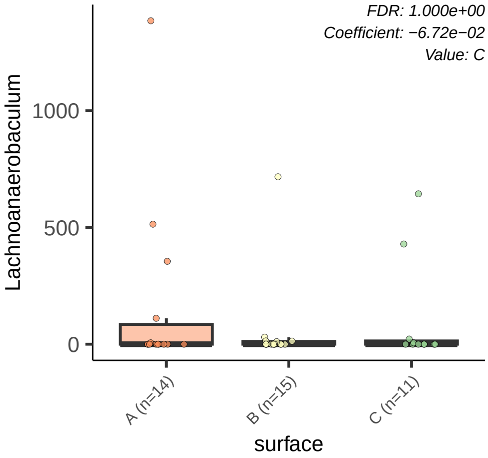
FDR: 1.000e+00
Coefficient: −6.72e−02
Value: C
Lachnoanaerobaculum
1000
500
0
A (n=14)
B (n=15)
C (n=11)
surface

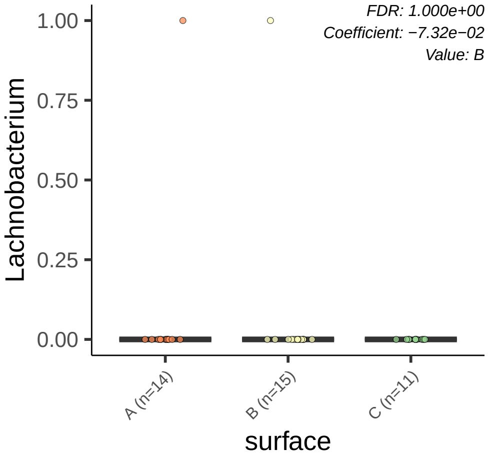
FDR: 1.000e+00
Coefficient: −7.32e−02
Value: B
Lachnobacterium
1.00
0.75
0.50
0.25
0.00
A (n=14)
B (n=15)
C (n=11)
surface

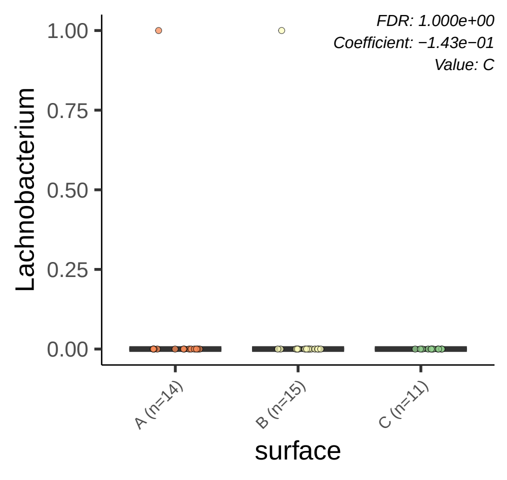

FDR: 1.000e+00
Coefficient: −1.43e−01
Value: C
Lachnobacterium
1.00
0.75
0.50
0.25
0.00
A (n=14)
B (n=15)
C (n=11)
surface

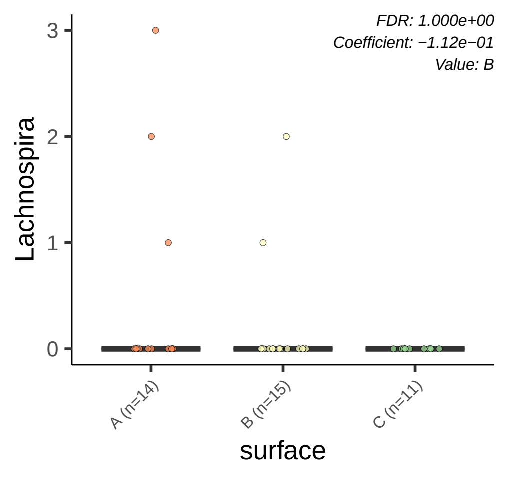
FDR: 1.000e+00
Coefficient: −1.12e−01
Value: B
Lachnospira
3
2
1
0
A (n=14)
B (n=15)
C (n=11)
surface

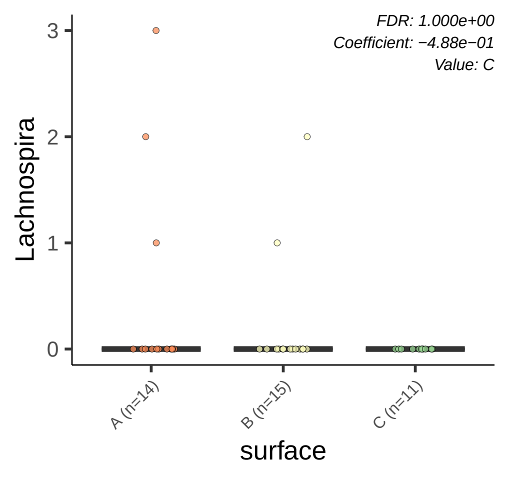

FDR: 1.000e+00
Coefficient: −4.88e−01
Value: C
3
2
1
0
Lachnospira
A (n=14)
B (n=15)
C (n=11)
surface

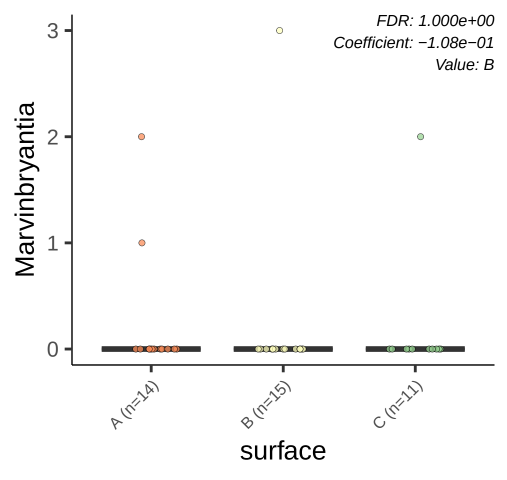
FDR: 1.000e+00
Coefficient: −1.08e−01
Value: B
Marvinbryantia
3
2
1
0
A (n=14)
B (n=15)
C (n=11)
surface

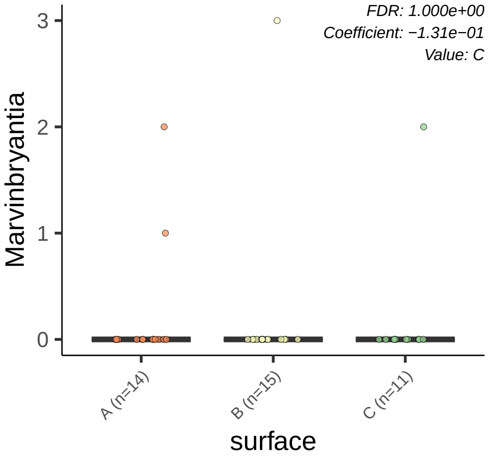

FDR: 1.000e+00
Coefficient: −1.31e−01
Value: C
Marvinbryantia
0
1
2
3
A (n=14)
B (n=15)
C (n=11)
surface

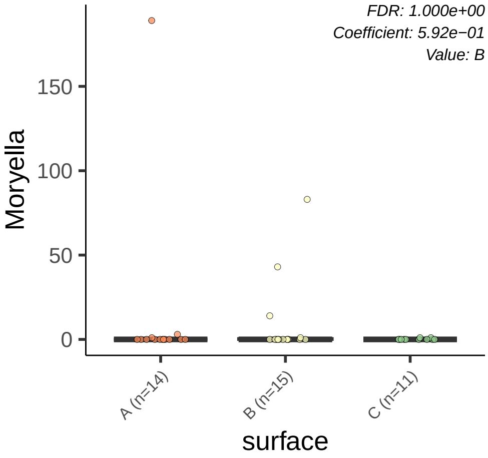
FDR: 1.000e+00
Coefficient: 5.92e−01
Value: B
Moryella
150
100
50
0
A (n=14)
B (n=15)
C (n=11)
surface

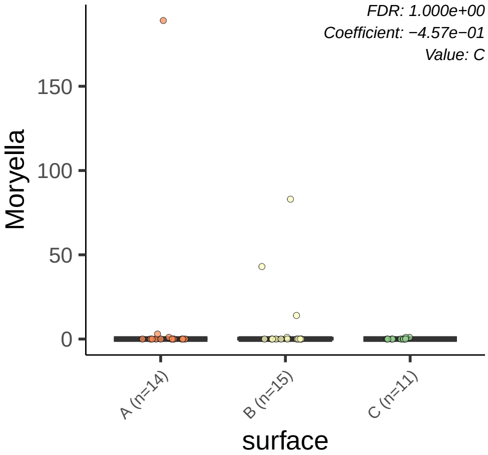

FDR: 1.000e+00
Coefficient: −4.57e−01
Value: C
Moryella
150
100
50
0
A (n=14)
B (n=15)
C (n=11)
surface

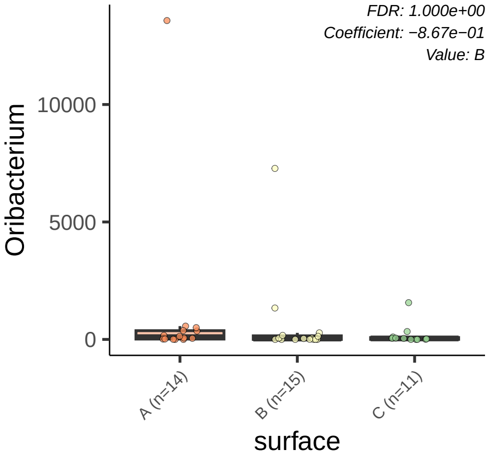
FDR: 1.000e+00
Coefficient: −8.67e−01
Value: B
Oribacterium
10000
5000
0
A (n=14)
B (n=15)
C (n=11)
surface

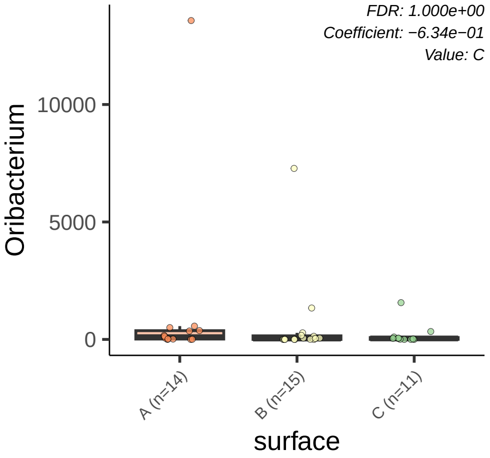

FDR: 1.000e+00
Coefficient: −6.34e−01
Value: C
Oribacterium
10000
5000
0
A (n=14)
B (n=15)
C (n=11)
surface

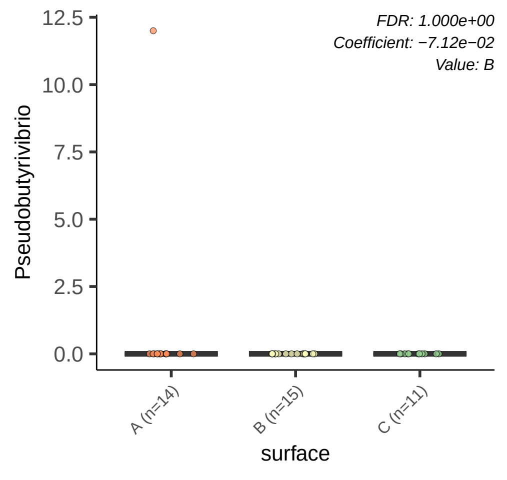
FDR: 1.000e+00
Coefficient: −7.12e−02
Value: B
Pseudobutyrivibrio
12.5
10.0
7.5
5.0
2.5
0.0
A (n=14)
B (n=15)
C (n=11)
surface

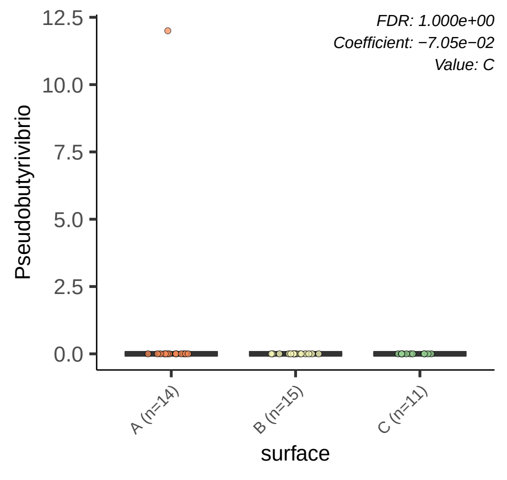

FDR: 1.000e+00
Coefficient: −7.05e−02
Value: C
Pseudobutyrivibrio
12.5
10.0
7.5
5.0
2.5
0.0
A (n=14)
B (n=15)
C (n=11)
surface

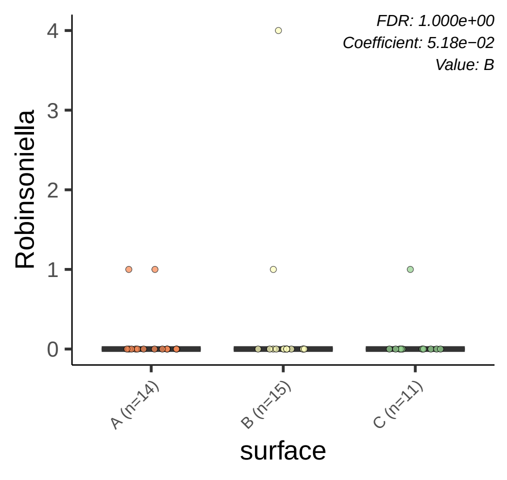
FDR: 1.000e+00
Coefficient: 5.18e−02
Value: B
Robinsoniella
4
3
2
1
0
A (n=14)
B (n=15)
C (n=11)
surface

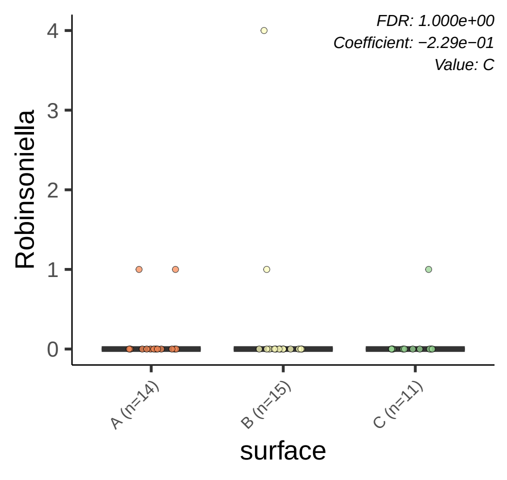
FDR: 1.000e+00
Coefficient: −2.29e−01
Value: C
Robinsoniella
4
3
2
1
0
A (n=14)
B (n=15)
C (n=11)
surface

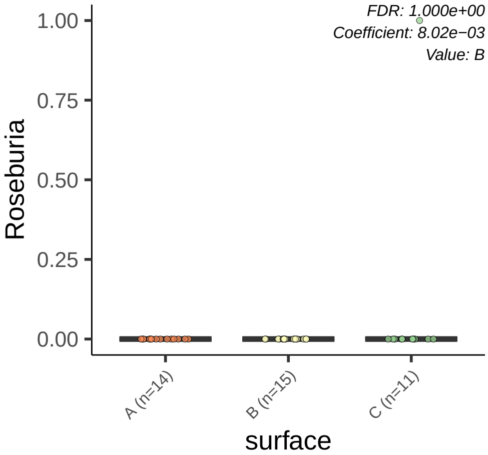
FDR: 1.000e+00
Coefficient: 8.02e−03
Value: B
Roseburia
1.00
0.75
0.50
0.25
0.00
A (n=14)
B (n=15)
C (n=11)
surface

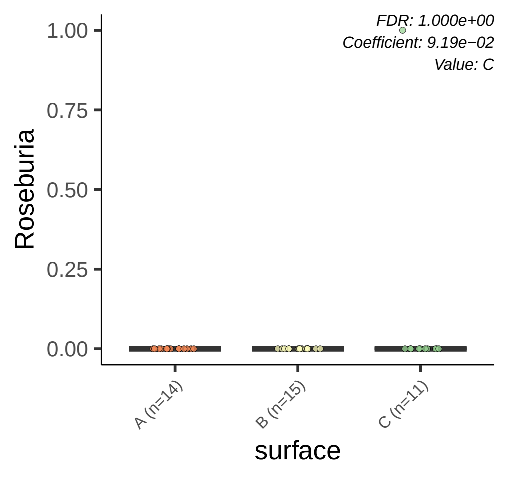
FDR: 1.000e+00
Coefficient: 9.19e−02
Value: C
Roseburia
1.00
0.75
0.50
0.25
0.00
A (n=14)
B (n=15)
C (n=11)
surface

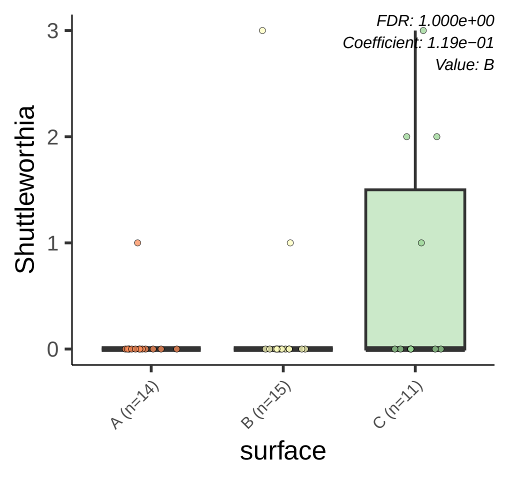
FDR: 1.000e+00
Coefficient: 1.19e−01
Value: B
Shuttleworthia
3
2
1
0
A (n=14)
B (n=15)
C (n=11)
surface

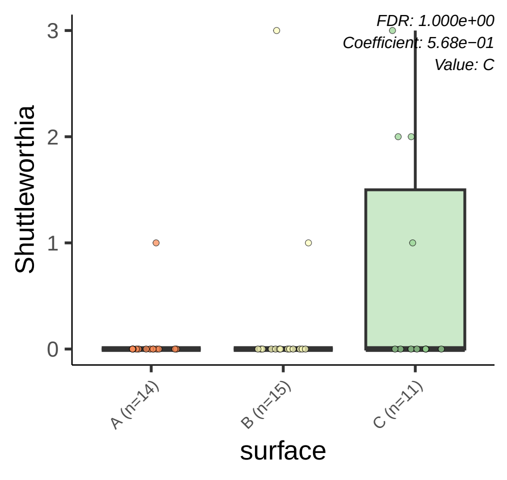

FDR: 1.000e+00
Coefficient: 5.68e−01
Value: C
Shuttleworthia
3
2
1
0
A (n=14)
B (n=15)
C (n=11)
surface

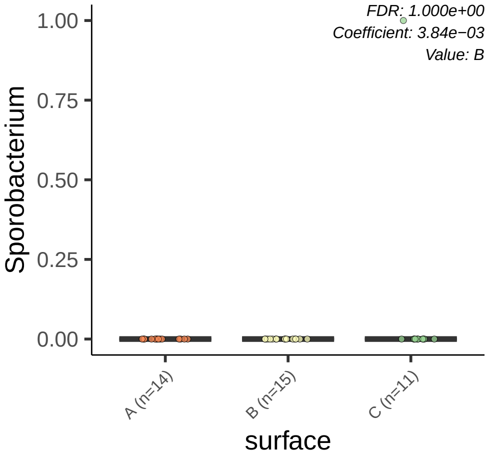

FDR: 1.000e+00
Coefficient: 3.84e−03
Value: B
Sporobacterium
1.00
0.75
0.50
0.25
0.00
A (n=14)
B (n=15)
C (n=11)
surface

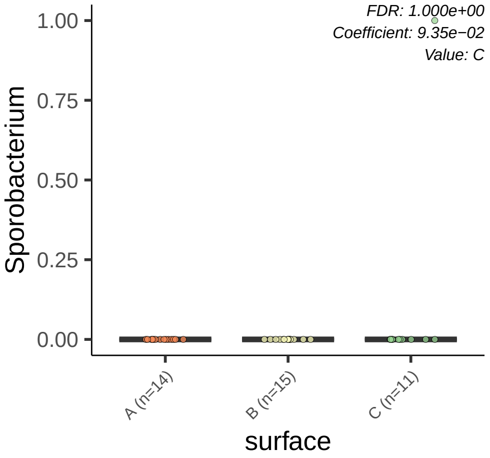

FDR: 1.000e+00
Coefficient: 9.35e−02
Value: C
Sporobacterium
1.00
0.75
0.50
0.25
0.00
A (n=14)
B (n=15)
C (n=11)
surface

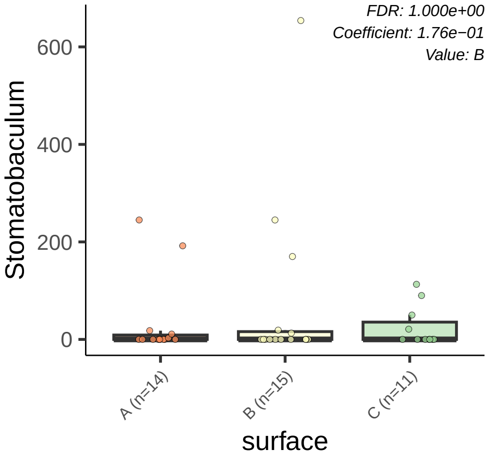

FDR: 1.000e+00
Coefficient: 1.76e−01
Value: B
Stomatobaculum
600
400
200
0
A (n=14)
B (n=15)
C (n=11)
surface

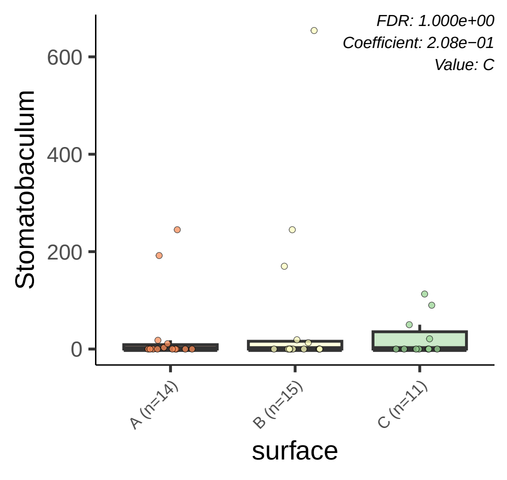
FDR: 1.000e+00
Coefficient: 2.08e−01
Value: C
Stomatobaculum
600
400
200
0
A (n=14)
B (n=15)
C (n=11)
surface

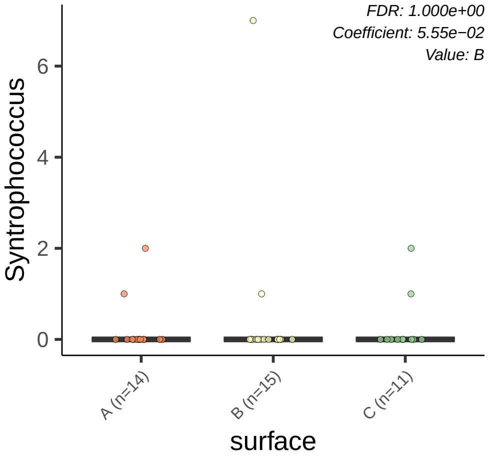
FDR: 1.000e+00
Coefficient: 5.55e−02
Value: B
Syntrophococcus
0
2
4
6
A (n=14)
B (n=15)
C (n=11)
surface

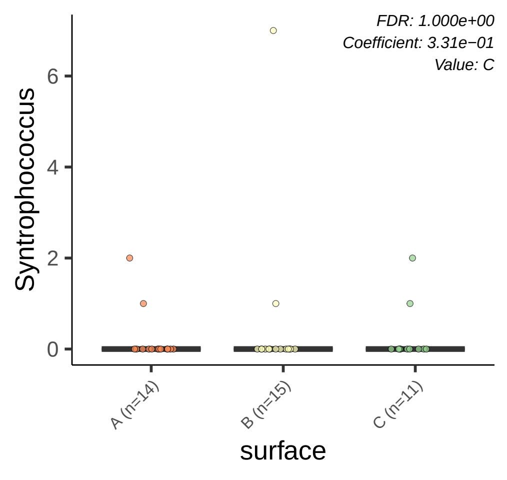

FDR: 1.000e+00
Coefficient: 3.31e−01
Value: C
Syntrophococcus
0
2
4
6
A (n=14)
B (n=15)
C (n=11)
surface

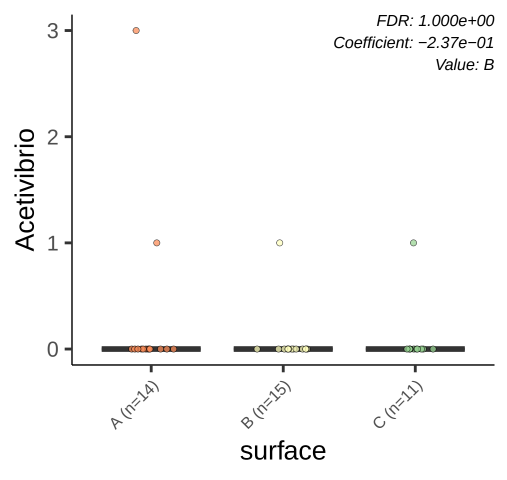

FDR: 1.000e+00
Coefficient: −2.37e−01
Value: B
Acetivibrio
3
2
1
0
A (n=14)
B (n=15)
C (n=11)
surface

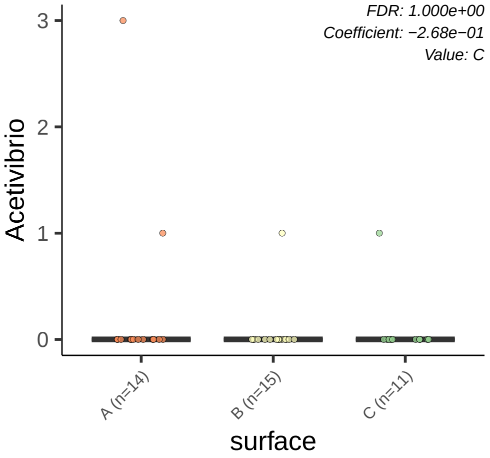

FDR: 1.000e+00
Coefficient: −2.68e−01
Value: C
Acetivibrio
3
2
1
0
A (n=14)
B (n=15)
C (n=11)
surface

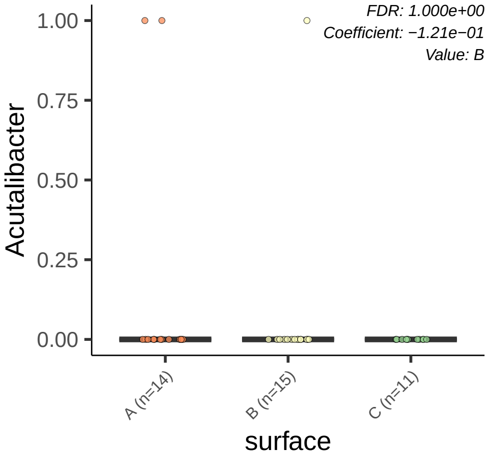

FDR: 1.000e+00
Coefficient: −1.21e−01
Value: B
Acutalibacter
1.00
0.75
0.50
0.25
0.00
A (n=14)
B (n=15)
C (n=11)
surface

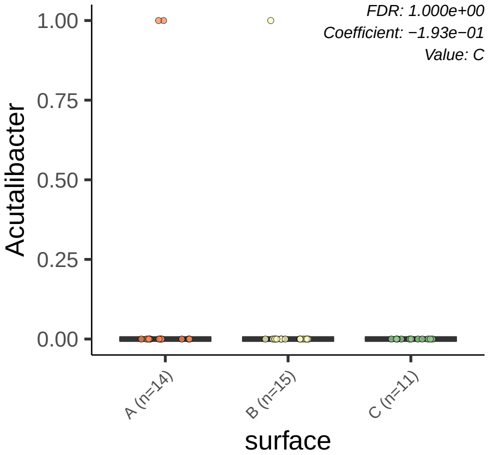

FDR: 1.000e+00
Coefficient: −1.93e−01
Value: C
Acutalibacter
1.00
0.75
0.50
0.25
0.00
A (n=14)
B (n=15)
C (n=11)
surface

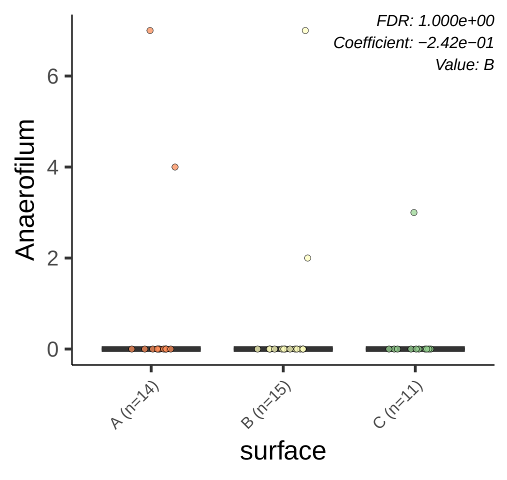
FDR: 1.000e+00
Coefficient: −2.42e−01
Value: B
Anaerofilum
0
2
4
6
A (n=14)
B (n=15)
C (n=11)
surface

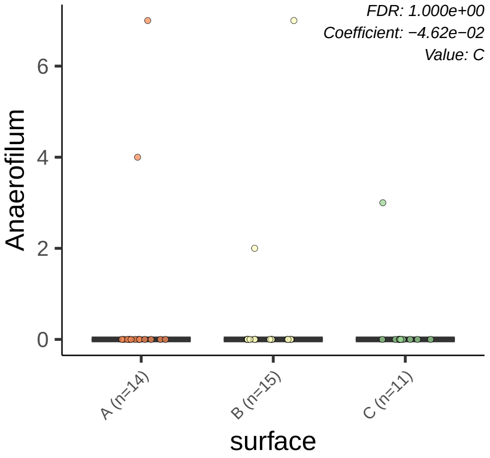
FDR: 1.000e+00
Coefficient: −4.62e−02
Value: C
Anaerofilum
0
2
4
6
A (n=14)
B (n=15)
C (n=11)
surface

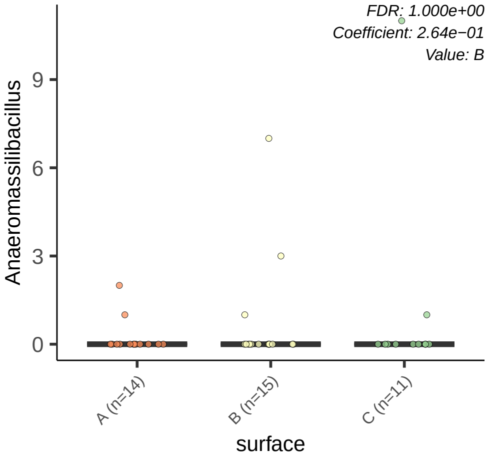

FDR: 1.000e+00
Coefficient: 2.64e−01
Value: B
Anaeromassilibacillus
9
6
3
0
A (n=14)
B (n=15)
C (n=11)
surface

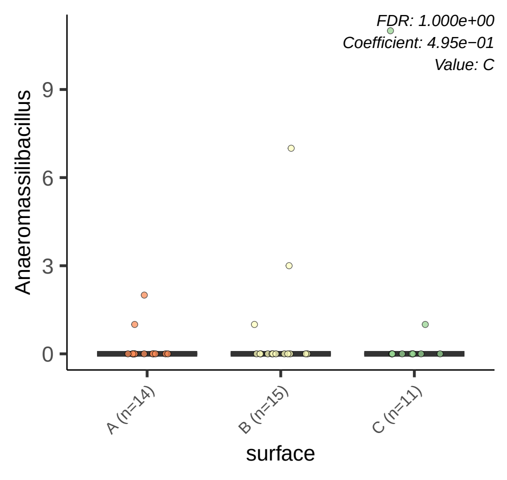

FDR: 1.000e+00
Coefficient: 4.95e−01
Value: C
Anaeromassilibacillus
0
3
6
9
A (n=14)
B (n=15)
C (n=11)
surface

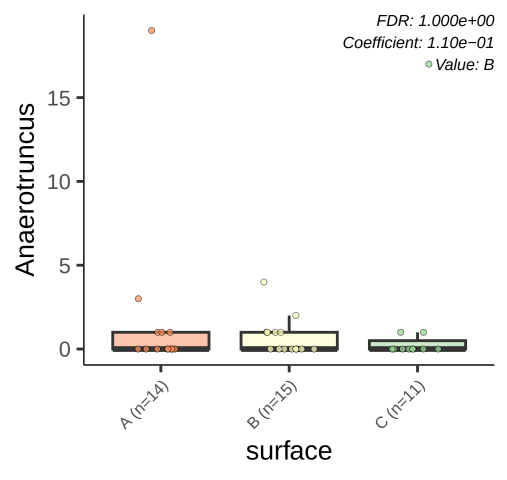
FDR: 1.000e+00
Coefficient: 1.10e−01
Value: B
Anaerotruncus
15
10
5
0
A (n=14)
B (n=15)
C (n=11)
surface

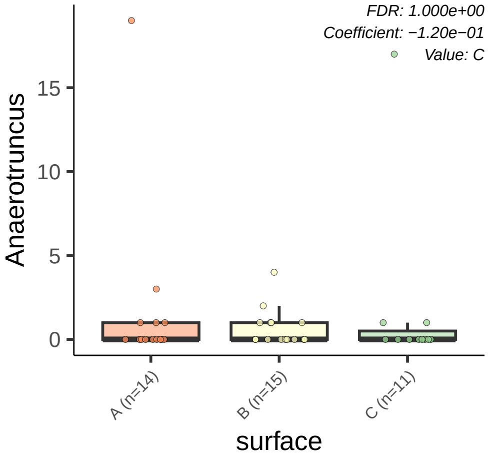
FDR: 1.000e+00
Coefficient: −1.20e−01
Value: C
Anaerotruncus
15
10
5
0
A (n=14)
B (n=15)
C (n=11)
surface

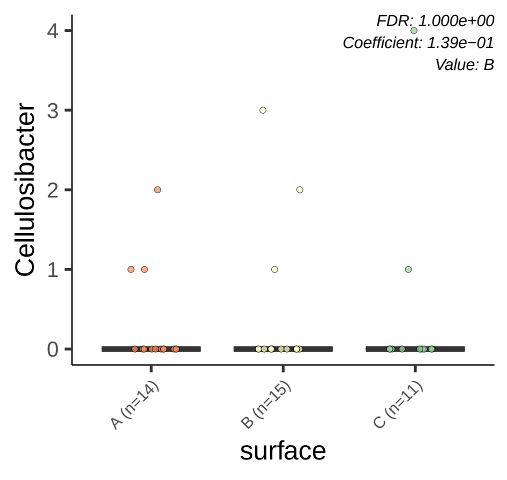
FDR: 1.000e+00
Coefficient: 1.39e−01
Value: B
Cellulosibacter
0
1
2
3
4
A (n=14)
B (n=15)
C (n=11)
surface

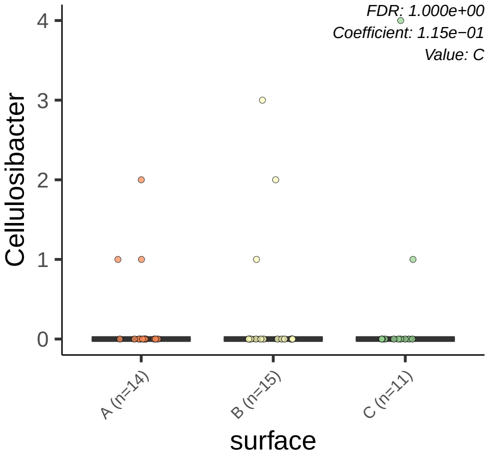
FDR: 1.000e+00
Coefficient: 1.15e−01
Value: C
Cellulosibacter
0
1
2
3
4
A (n=14)
B (n=15)
C (n=11)
surface

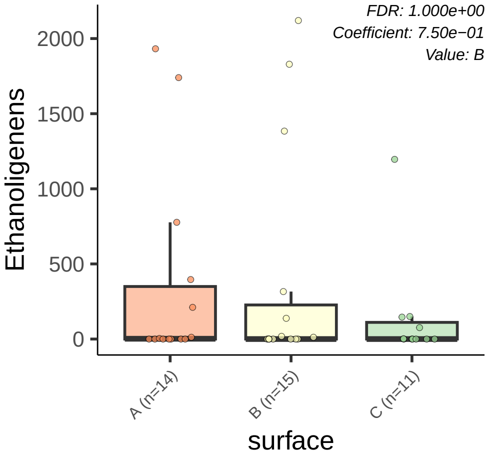

FDR: 1.000e+00
Coefficient: 7.50e−01
Value: B
Ethanoligenens
2000
1500
1000
500
0
A (n=14)
B (n=15)
C (n=11)
surface

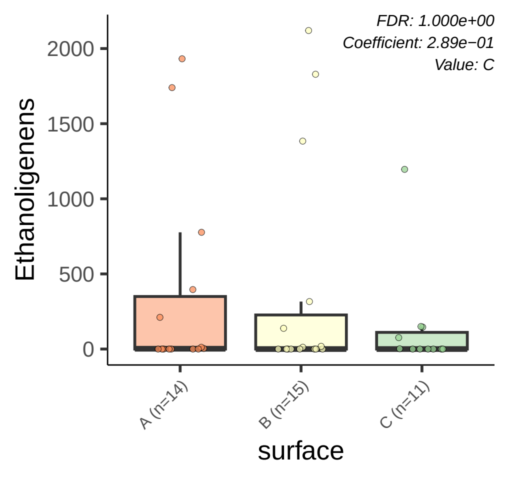

FDR: 1.000e+00
Coefficient: 2.89e−01
Value: C
Ethanoligenens
2000
1500
1000
500
0
A (n=14)
B (n=15)
C (n=11)
surface

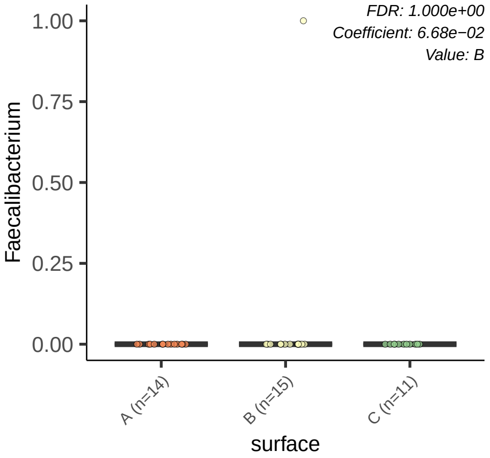
FDR: 1.000e+00
Coefficient: 6.68e−02
Value: B
Faecalibacterium
1.00
0.75
0.50
0.25
0.00
A (n=14)
B (n=15)
C (n=11)
surface

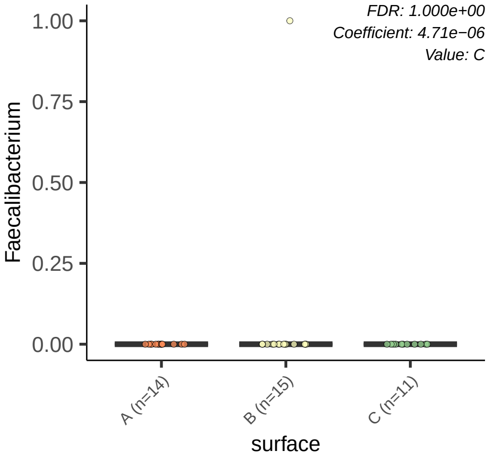

FDR: 1.000e+00
Coefficient: 4.71e−06
Value: C
Faecalibacterium
1.00
0.75
0.50
0.25
0.00
A (n=14)
B (n=15)
C (n=11)
surface

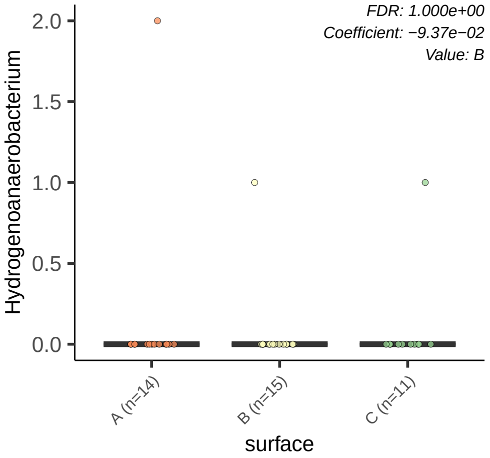

FDR: 1.000e+00
Coefficient: −9.37e−02
Value: B
Hydrogenoanaerobacterium
2.0
1.5
1.0
0.5
0.0
A (n=14)
B (n=15)
C (n=11)
surface

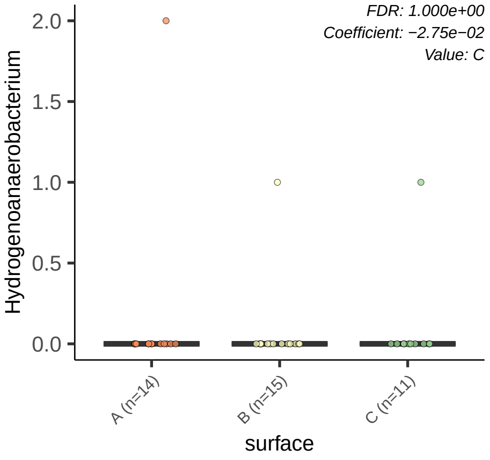
FDR: 1.000e+00
Coefficient: −2.75e−02
Value: C
Hydrogenoanaerobacterium
2.0
1.5
1.0
0.5
0.0
A (n=14)
B (n=15)
C (n=11)
surface

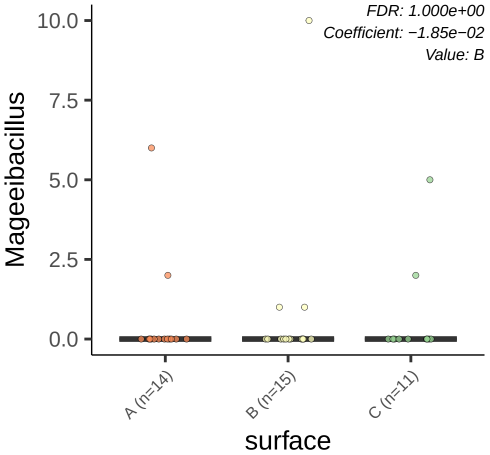
FDR: 1.000e+00
Coefficient: −1.85e−02
Value: B
Mageeibacillus
10.0
7.5
5.0
2.5
0.0
A (n=14)
B (n=15)
C (n=11)
surface

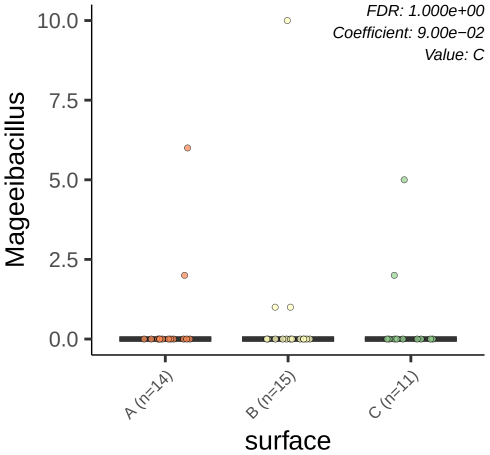
FDR: 1.000e+00
Coefficient: 9.00e−02
Value: C
Mageeibacillus
10.0
7.5
5.0
2.5
0.0
A (n=14)
B (n=15)
C (n=11)
surface

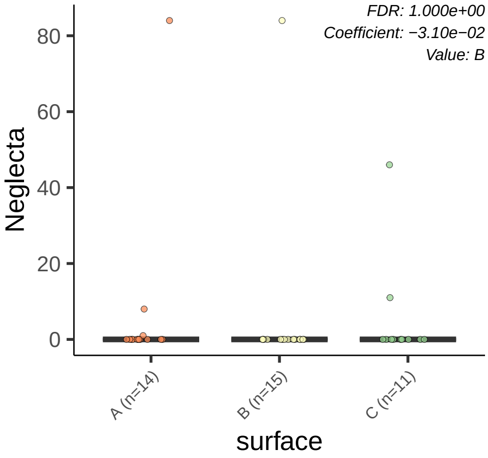

FDR: 1.000e+00
Coefficient: −3.10e−02
Value: B
Neglecta
0
20
40
60
80
A (n=14)
B (n=15)
C (n=11)
surface

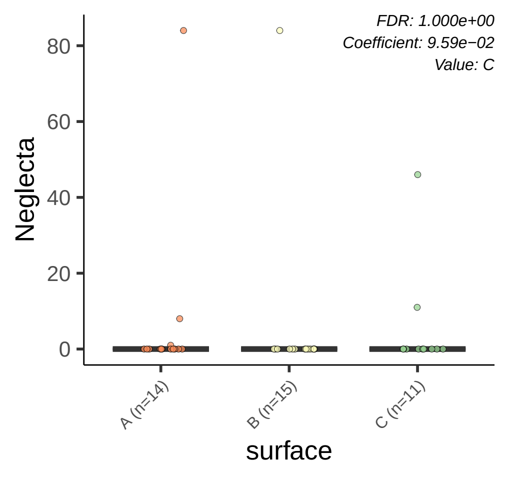

FDR: 1.000e+00
Coefficient: 9.59e−02
Value: C
Neglecta
0
20
40
60
80
A (n=14)
B (n=15)
C (n=11)
surface

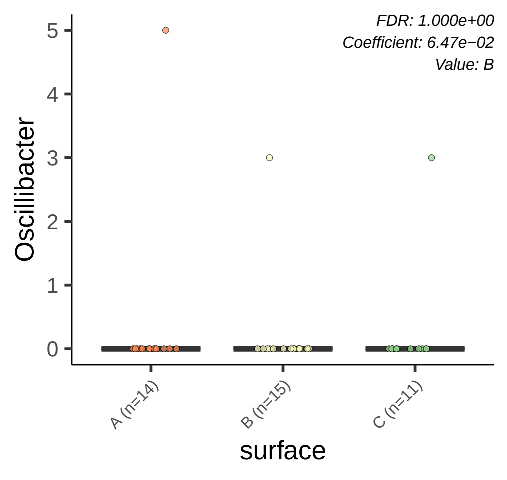
FDR: 1.000e+00
Coefficient: 6.47e−02
Value: B
Oscillibacter
5
4
3
2
1
0
A (n=14)
B (n=15)
C (n=11)
surface

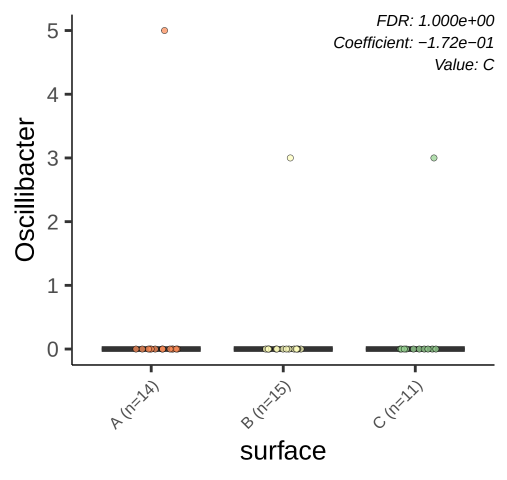

FDR: 1.000e+00
Coefficient: −1.72e−01
Value: C
Oscillibacter
5
4
3
2
1
0
A (n=14)
B (n=15)
C (n=11)
surface

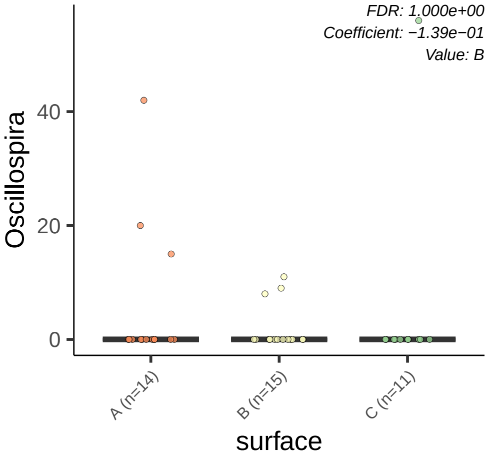

FDR: 1.000e+00
Coefficient: −1.39e−01
Value: B
Oscillospira
0
20
40
A (n=14)
B (n=15)
C (n=11)
surface

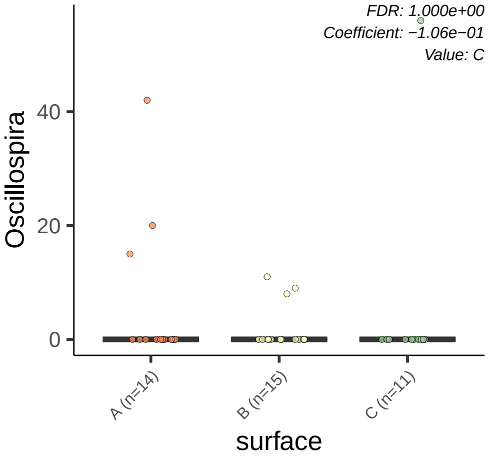

FDR: 1.000e+00
Coefficient: −1.06e−01
Value: C
Oscillospira
40
20
0
A (n=14)
B (n=15)
C (n=11)
surface

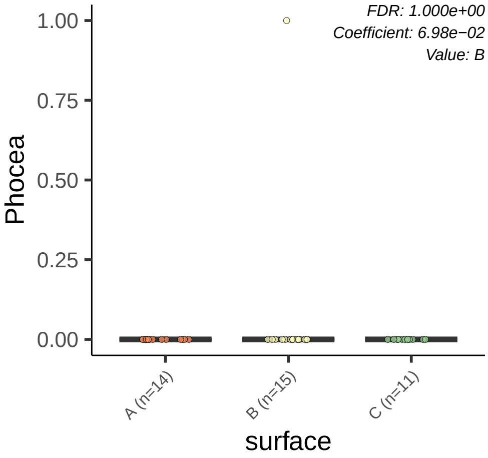

FDR: 1.000e+00
Coefficient: 6.98e−02
Value: B
Phocea
1.00
0.75
0.50
0.25
0.00
A (n=14)
B (n=15)
C (n=11)
surface

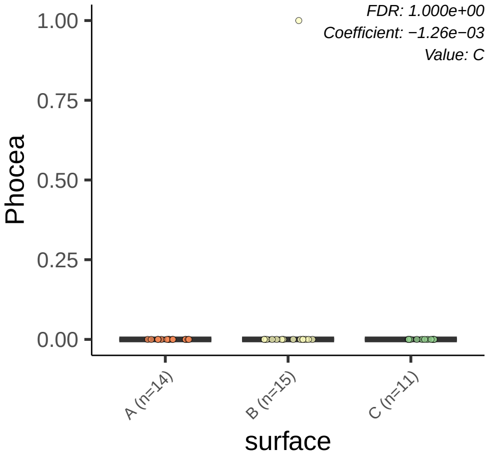
FDR: 1.000e+00
Coefficient: −1.26e−03
Value: C
Phocea
1.00
0.75
0.50
0.25
0.00
A (n=14)
B (n=15)
C (n=11)
surface

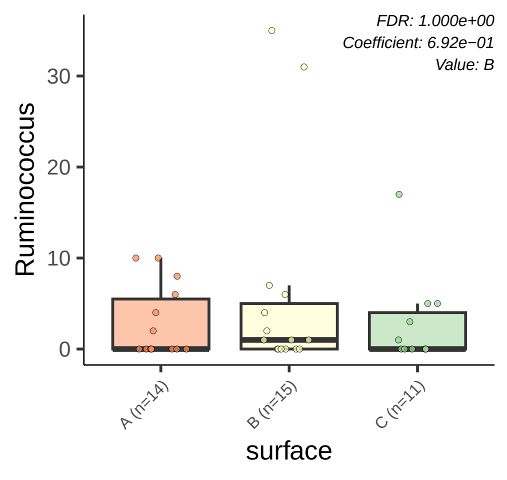

FDR: 1.000e+00
Coefficient: 6.92e−01
Value: B
Ruminococcus
0
10
20
30
A (n=14)
B (n=15)
C (n=11)
surface

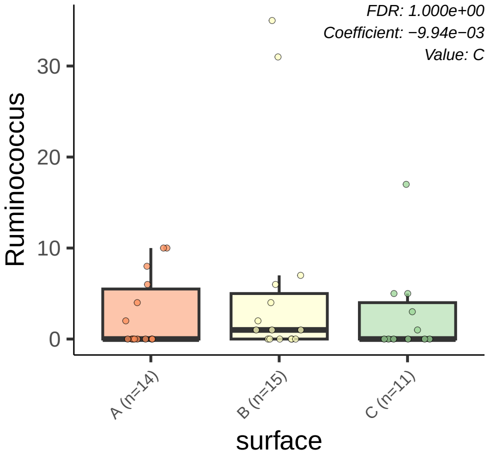
FDR: 1.000e+00
Coefficient: −9.94e−03
Value: C
Ruminococcus
0
10
20
30
A (n=14)
B (n=15)
C (n=11)
surface

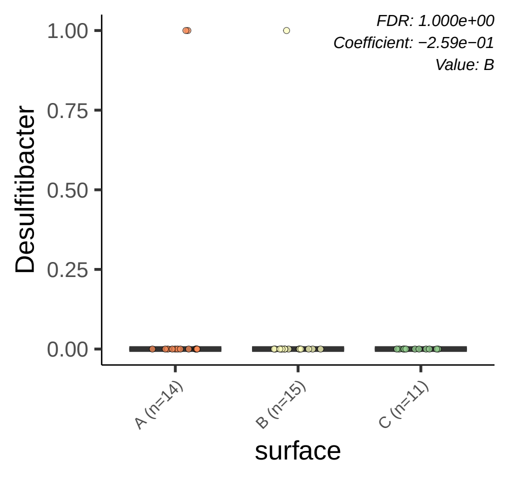
FDR: 1.000e+00
Coefficient: −2.59e−01
Value: B
Desulfitibacter
1.00
0.75
0.50
0.25
0.00
A (n=14)
B (n=15)
C (n=11)
surface

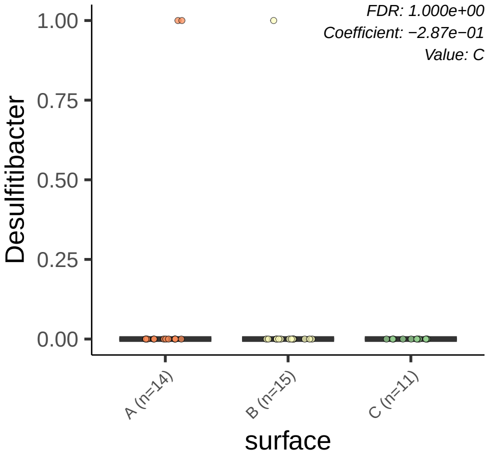

FDR: 1.000e+00
Coefficient: −2.87e−01
Value: C
Desulfitibacter
1.00
0.75
0.50
0.25
0.00
A (n=14)
B (n=15)
C (n=11)
surface

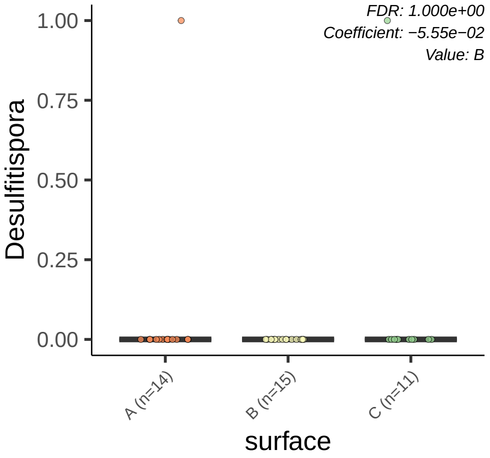

FDR: 1.000e+00
Coefficient: −5.55e−02
Value: B
Desulfitispora
1.00
0.75
0.50
0.25
0.00
A (n=14)
B (n=15)
C (n=11)
surface

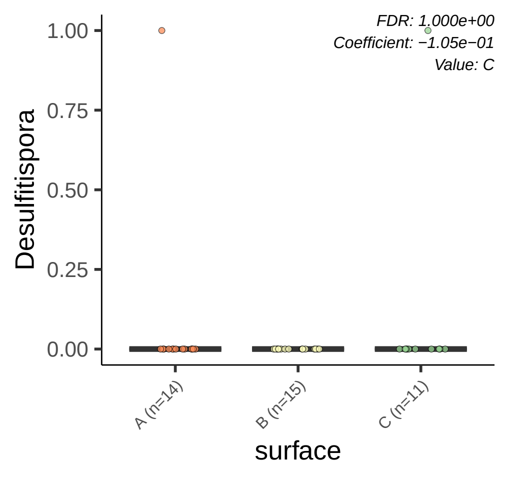
FDR: 1.000e+00
Coefficient: −1.05e−01
Value: C
Desulfitispora
1.00
0.75
0.50
0.25
0.00
A (n=14)
B (n=15)
C (n=11)
surface

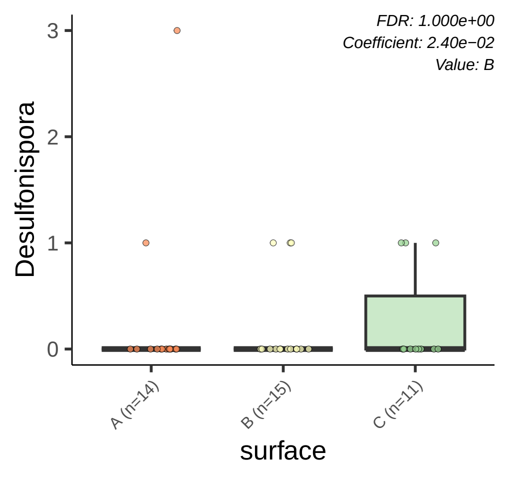
FDR: 1.000e+00
Coefficient: 2.40e−02
Value: B
Desulfonispora
3
2
1
0
A (n=14)
B (n=15)
C (n=11)
surface

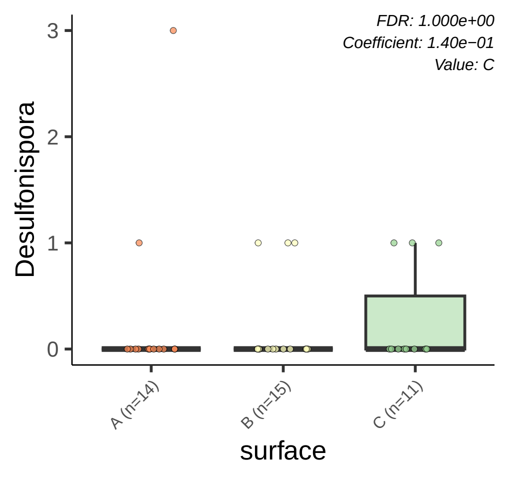
FDR: 1.000e+00
Coefficient: 1.40e−01
Value: C
Desulfonispora
3
2
1
0
A (n=14)
B (n=15)
C (n=11)
surface

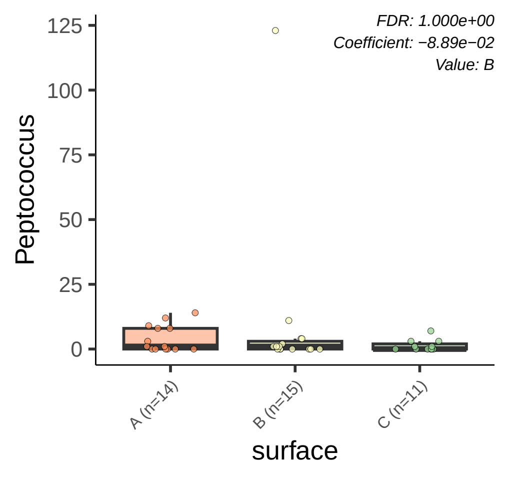
FDR: 1.000e+00
Coefficient: −8.89e−02
Value: B
Peptococcus
125
100
75
50
25
0
A (n=14)
B (n=15)
C (n=11)
surface

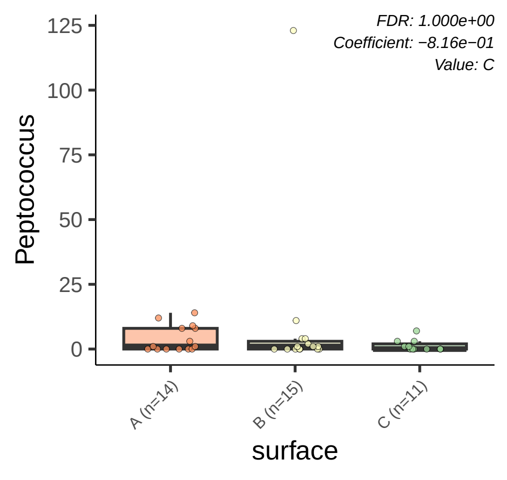

FDR: 1.000e+00
Coefficient: −8.16e−01
Value: C
Peptococcus
125
100
75
50
25
0
A (n=14)
B (n=15)
C (n=11)
surface

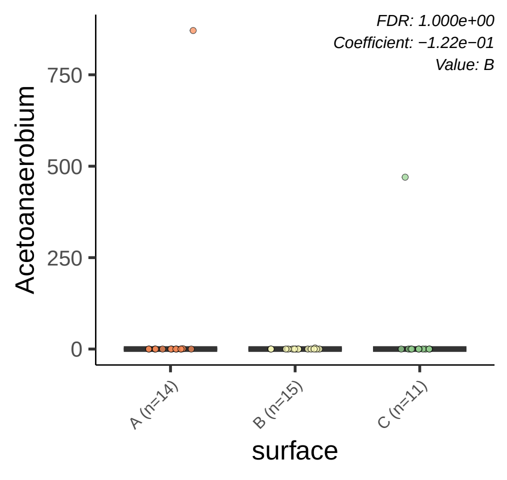
FDR: 1.000e+00
Coefficient: −1.22e−01
Value: B
Acetoanaerobium
750
500
250
0
A (n=14)
B (n=15)
C (n=11)
surface

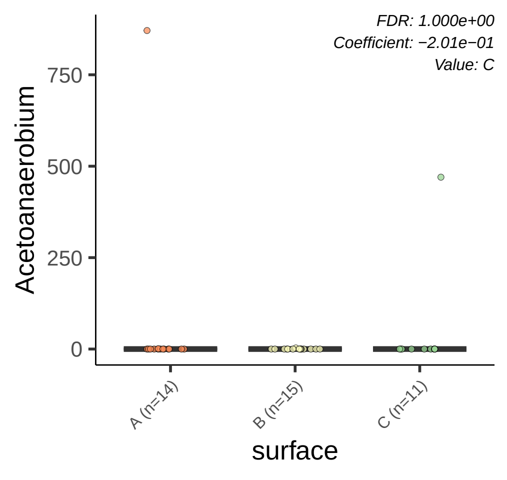
FDR: 1.000e+00
Coefficient: −2.01e−01
Value: C
Acetoanaerobium
750
500
250
0
A (n=14)
B (n=15)
C (n=11)
surface

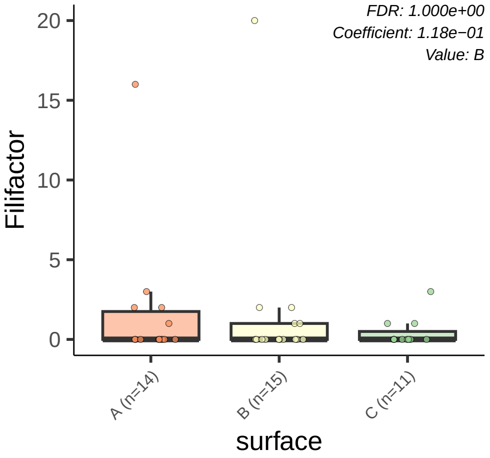
FDR: 1.000e+00
Coefficient: 1.18e−01
Value: B
Filifactor
20
15
10
5
0
A (n=14)
B (n=15)
C (n=11)
surface

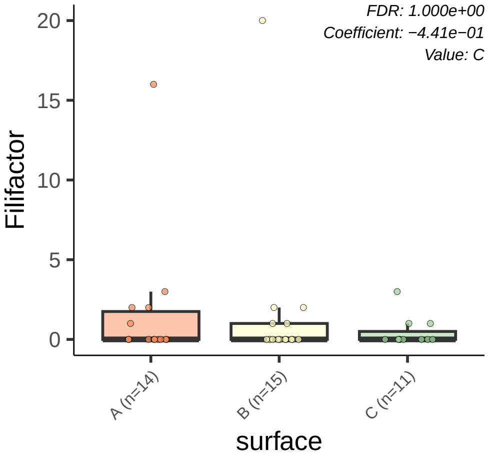

FDR: 1.000e+00
Coefficient: −4.41e−01
Value: C
Filifactor
20
15
10
5
0
A (n=14)
B (n=15)
C (n=11)
surface

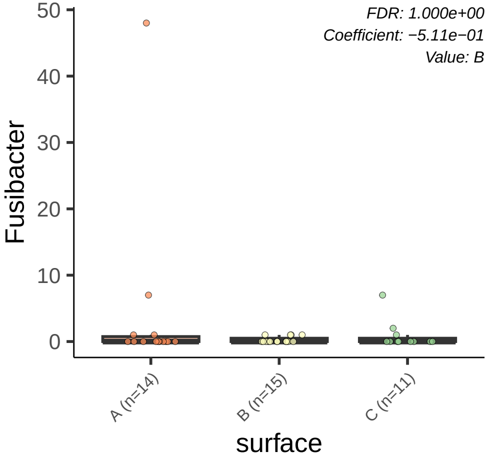

FDR: 1.000e+00
Coefficient: −5.11e−01
Value: B
Fusibacter
50
40
30
20
10
0
A (n=14)
B (n=15)
C (n=11)
surface

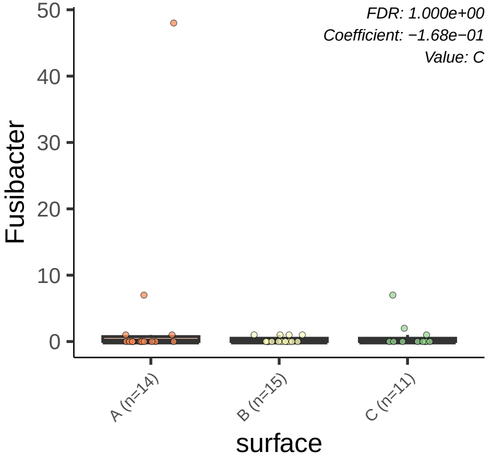
FDR: 1.000e+00
Coefficient: −1.68e−01
Value: C
Fusibacter
0
10
20
30
40
50
A (n=14)
B (n=15)
C (n=11)
surface

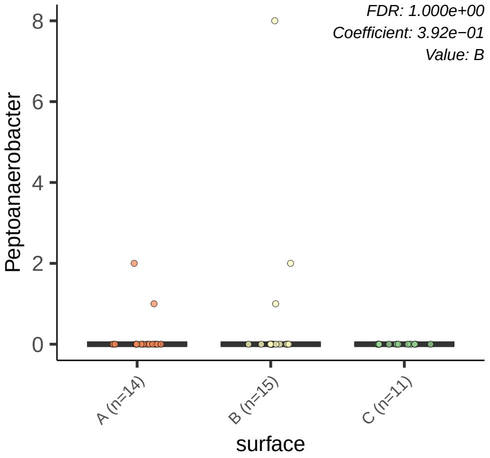

FDR: 1.000e+00
Coefficient: 3.92e−01
Value: B
Peptoanaerobacter
8
6
4
2
0
A (n=14)
B (n=15)
C (n=11)
surface

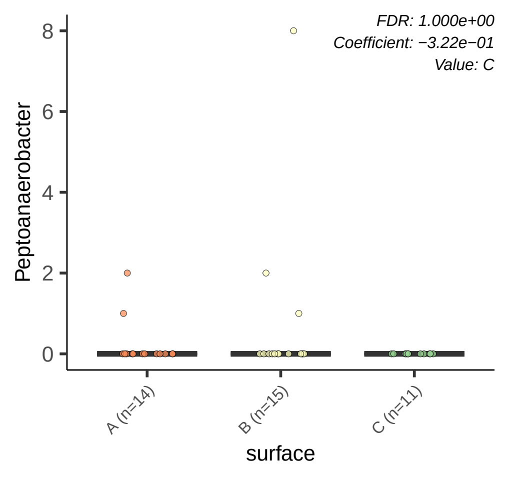

FDR: 1.000e+00
Coefficient: −3.22e−01
Value: C
Peptoanaerobacter
0
2
4
6
8
A (n=14)
B (n=15)
C (n=11)
surface

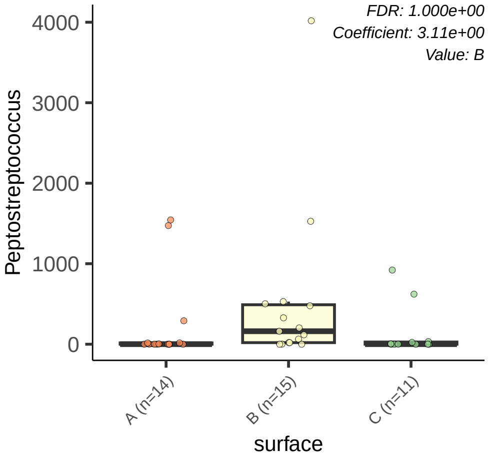
FDR: 1.000e+00
Coefficient: 3.11e+00
Value: B
Peptostreptococcus
4000
3000
2000
1000
0
A (n=14)
B (n=15)
C (n=11)
surface

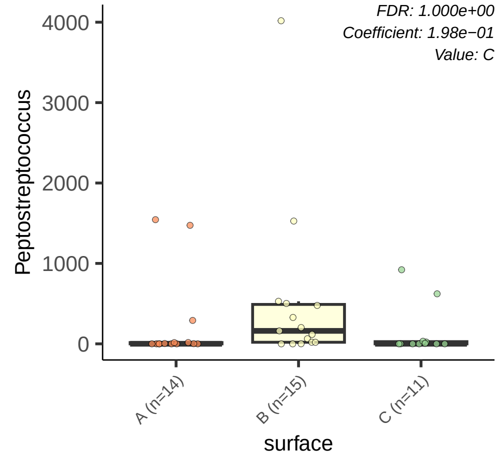

FDR: 1.000e+00
Coefficient: 1.98e−01
Value: C
Peptostreptococcus
4000
3000
2000
1000
0
A (n=14)
B (n=15)
C (n=11)
surface

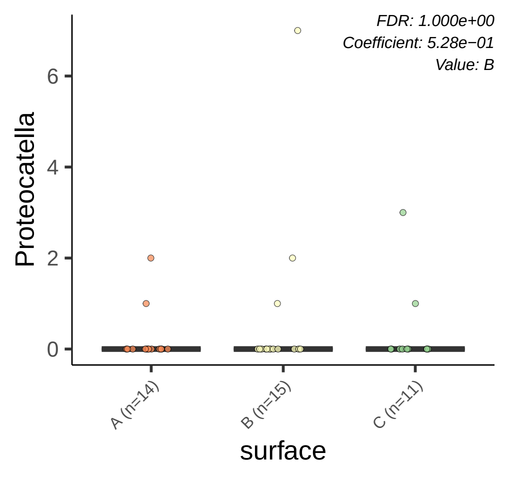
FDR: 1.000e+00
Coefficient: 5.28e−01
Value: B
Proteocatella
0
2
4
6
A (n=14)
B (n=15)
C (n=11)
surface

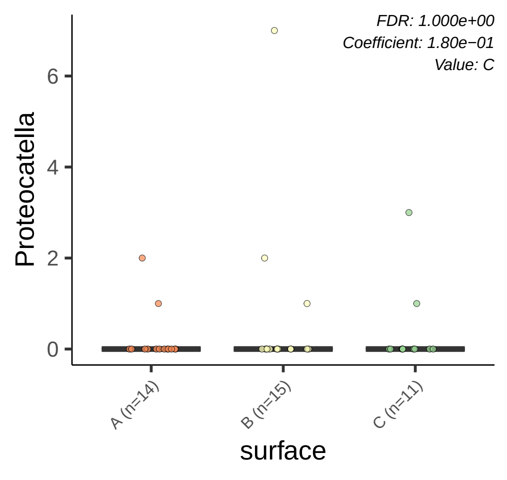

FDR: 1.000e+00
Coefficient: 1.80e−01
Value: C
Proteocatella
0
2
4
6
A (n=14)
B (n=15)
C (n=11)
surface

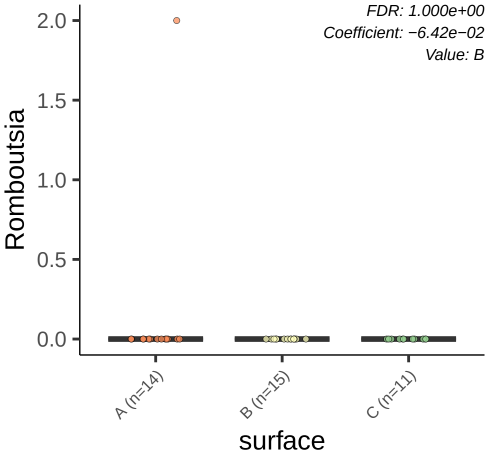
FDR: 1.000e+00
Coefficient: −6.42e−02
Value: B
Romboutsia
2.0
1.5
1.0
0.5
0.0
A (n=14)
B (n=15)
C (n=11)
surface

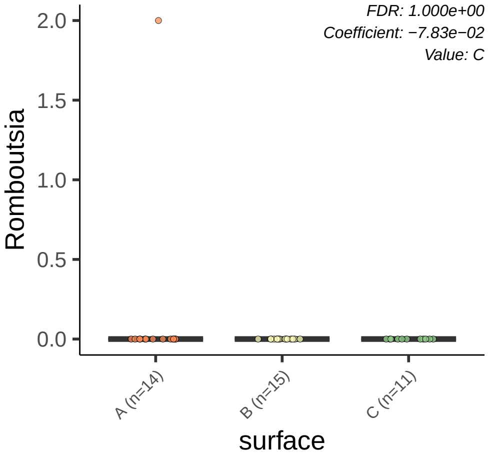
FDR: 1.000e+00
Coefficient: −7.83e−02
Value: C
Romboutsia
2.0
1.5
1.0
0.5
0.0
A (n=14)
B (n=15)
C (n=11)
surface

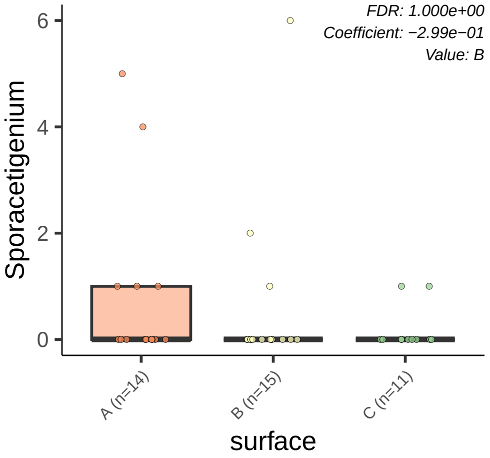

FDR: 1.000e+00
Coefficient: −2.99e−01
Value: B
Sporacetigenium
0
2
4
6
A (n=14)
B (n=15)
C (n=11)
surface

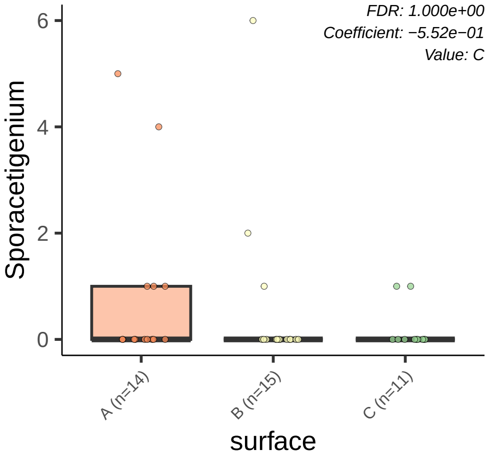

FDR: 1.000e+00
Coefficient: −5.52e−01
Value: C
Sporacetigenium
0
2
4
6
A (n=14)
B (n=15)
C (n=11)
surface

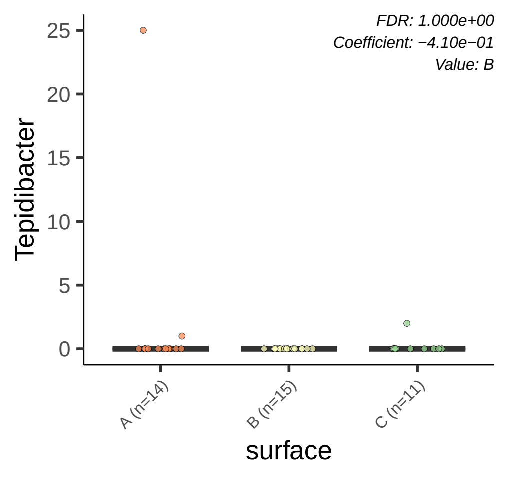

FDR: 1.000e+00
Coefficient: −4.10e−01
Value: B
Tepidibacter
25
20
15
10
5
0
A (n=14)
B (n=15)
C (n=11)
surface

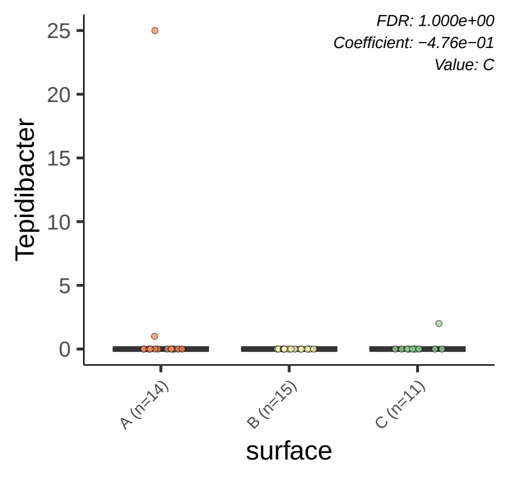

FDR: 1.000e+00
Coefficient: −4.76e−01
Value: C
Tepidibacter
25
20
15
10
5
0
A (n=14)
B (n=15)
C (n=11)
surface

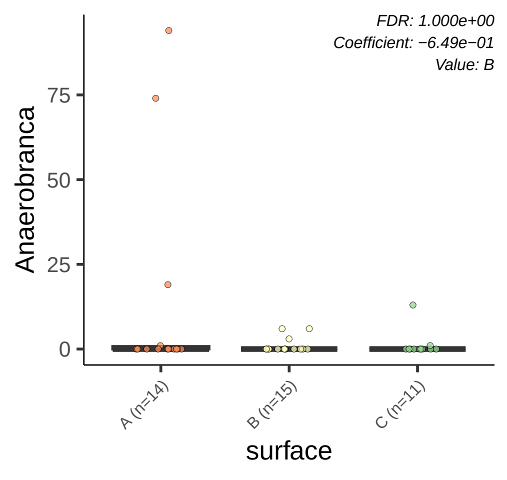
FDR: 1.000e+00
Coefficient: −6.49e−01
Value: B
Anaerobranca
75
50
25
0
A (n=14)
B (n=15)
C (n=11)
surface

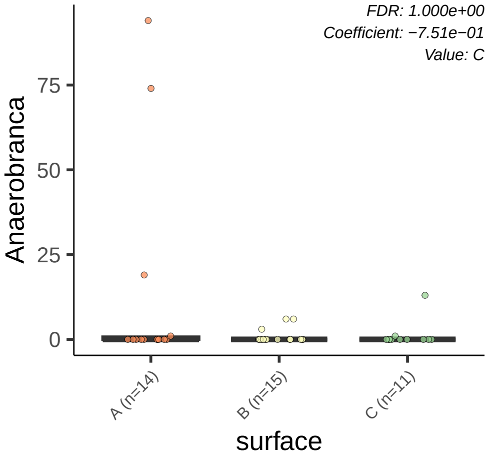
FDR: 1.000e+00
Coefficient: −7.51e−01
Value: C
Anaerobranca
0
25
50
75
A (n=14)
B (n=15)
C (n=11)
surface

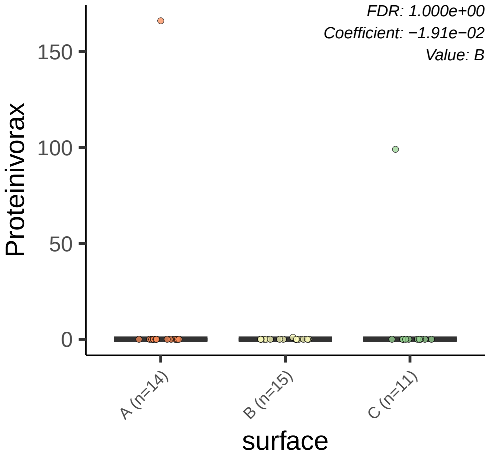
FDR: 1.000e+00
Coefficient: −1.91e−02
Value: B
Proteinivorax
150
100
50
0
A (n=14)
B (n=15)
C (n=11)
surface

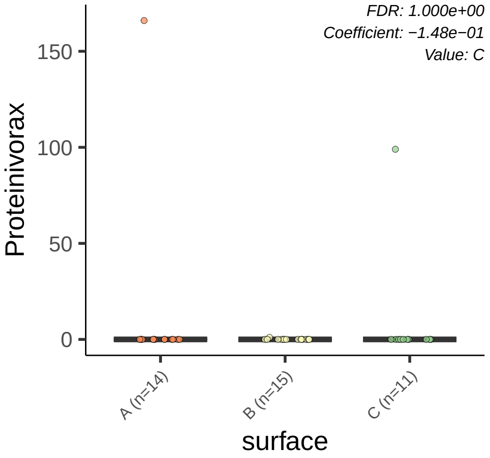
FDR: 1.000e+00
Coefficient: −1.48e−01
Value: C
Proteinivorax
150
100
50
0
A (n=14)
B (n=15)
C (n=11)
surface

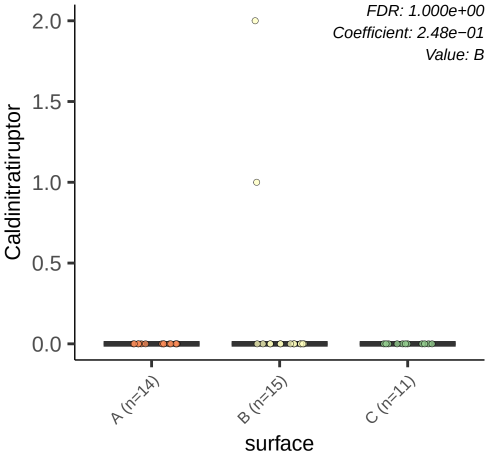
FDR: 1.000e+00
Coefficient: 2.48e−01
Value: B
2.0
1.5
1.0
0.5
0.0
Caldinitratiruptor
A (n=14)
B (n=15)
C (n=11)
surface

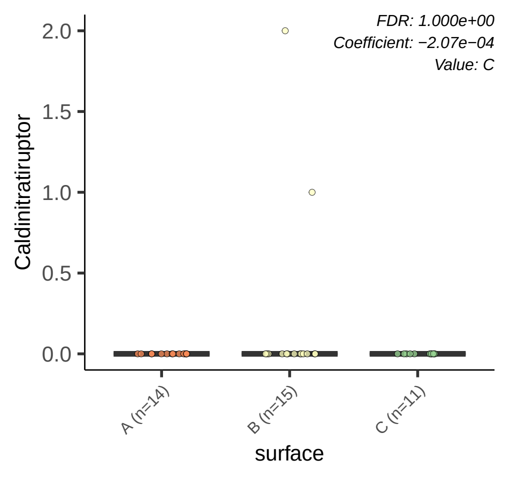
FDR: 1.000e+00
Coefficient: −2.07e−04
Value: C
Caldinitratiruptor
2.0
1.5
1.0
0.5
0.0
A (n=14)
B (n=15)
C (n=11)
surface

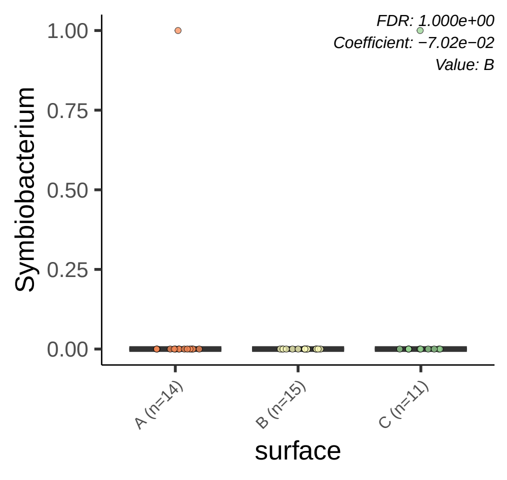
FDR: 1.000e+00
Coefficient: −7.02e−02
Value: B
Symbiobacterium
1.00
0.75
0.50
0.25
0.00
A (n=14)
B (n=15)
C (n=11)
surface

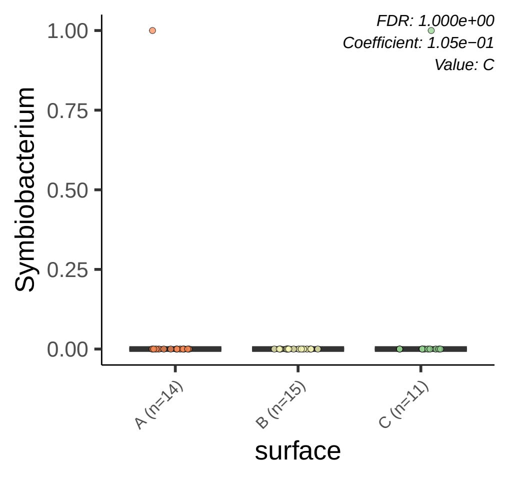

FDR: 1.000e+00
Coefficient: 1.05e−01
Value: C
1.00
0.75
0.50
0.25
0.00
Symbiobacterium
A (n=14)
B (n=15)
C (n=11)
surface

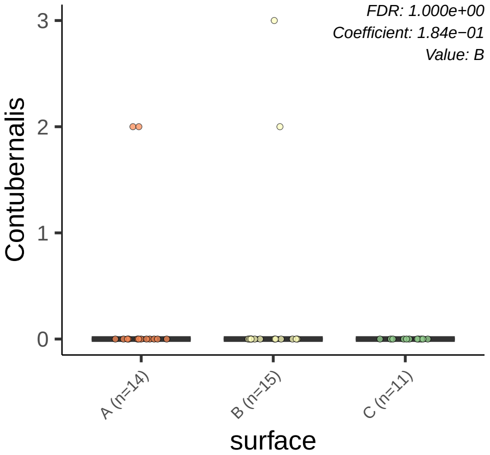
FDR: 1.000e+00
Coefficient: 1.84e−01
Value: B
Contubernalis
3
2
1
0
A (n=14)
B (n=15)
C (n=11)
surface

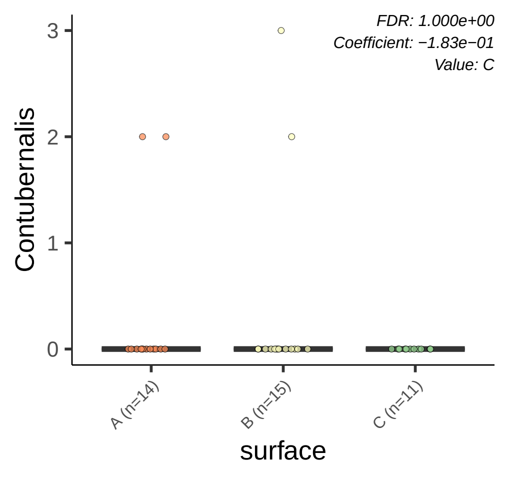
FDR: 1.000e+00
Coefficient: −1.83e−01
Value: C
Contubernalis
3
2
1
0
A (n=14)
B (n=15)
C (n=11)
surface

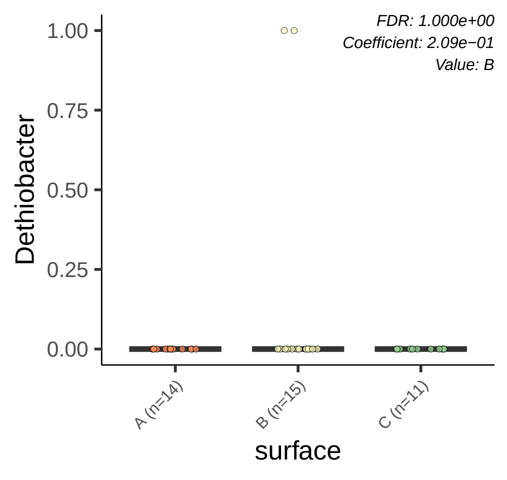
FDR: 1.000e+00
Coefficient: 2.09e−01
Value: B
Dethiobacter
1.00
0.75
0.50
0.25
0.00
A (n=14)
B (n=15)
C (n=11)
surface

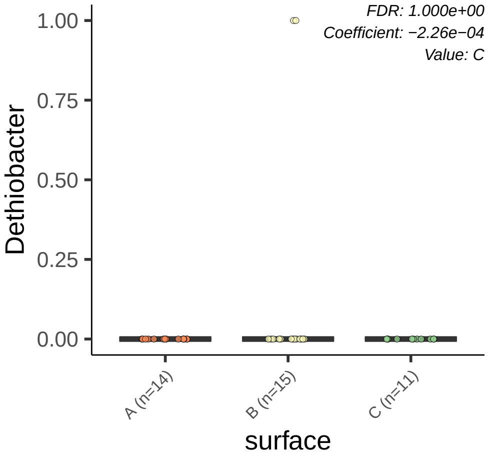

FDR: 1.000e+00
Coefficient: −2.26e−04
Value: C
1.00
0.75
0.50
0.25
0.00
Dethiobacter
A (n=14)
B (n=15)
C (n=11)
surface

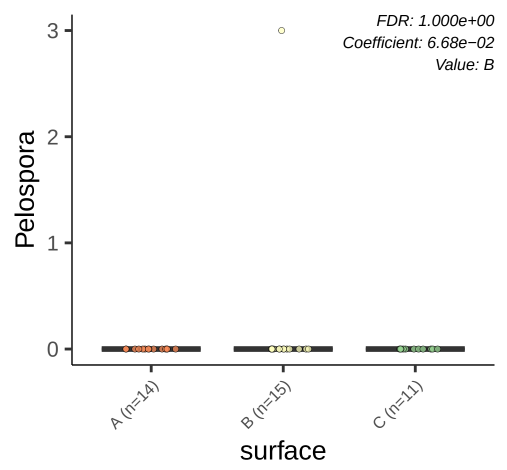

FDR: 1.000e+00
Coefficient: 6.68e−02
Value: B
Pelospora
3
2
1
0
A (n=14)
B (n=15)
C (n=11)
surface

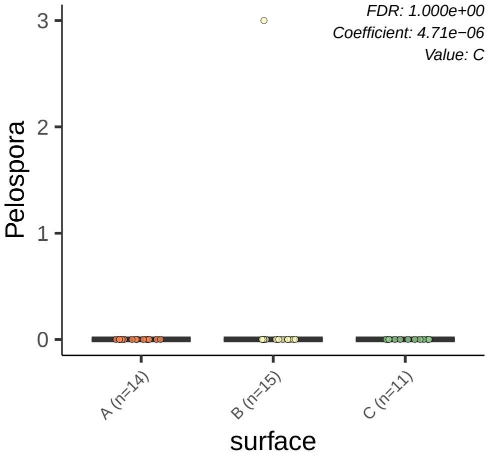

FDR: 1.000e+00
Coefficient: 4.71e−06
Value: C
Pelospora
3
2
1
0
A (n=14)
B (n=15)
C (n=11)
surface

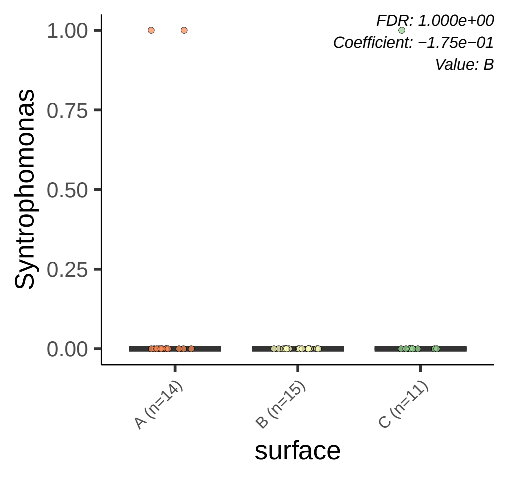
FDR: 1.000e+00
Coefficient: −1.75e−01
Value: B
Syntrophomonas
1.00
0.75
0.50
0.25
0.00
A (n=14)
B (n=15)
C (n=11)
surface

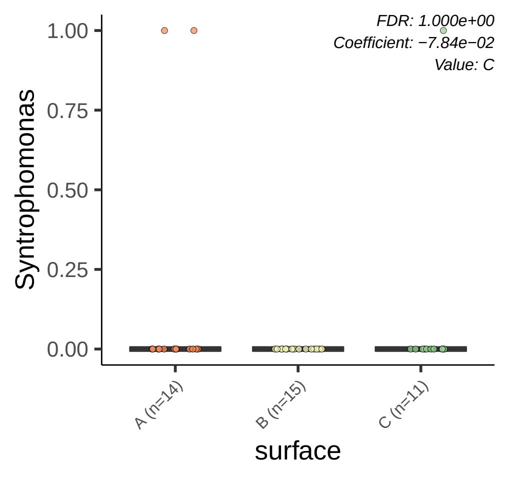

FDR: 1.000e+00
Coefficient: −7.84e−02
Value: C
Syntrophomonas
1.00
0.75
0.50
0.25
0.00
A (n=14)
B (n=15)
C (n=11)
surface

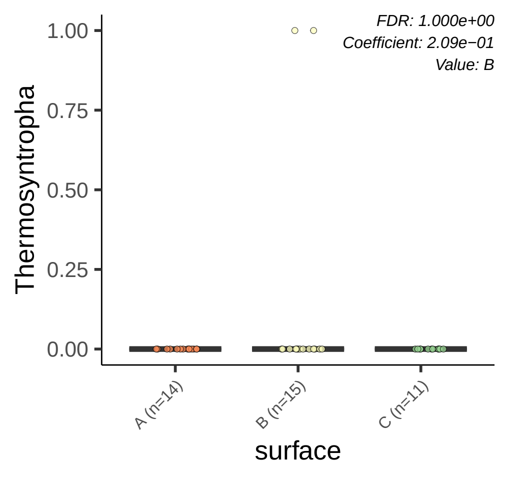

FDR: 1.000e+00
Coefficient: 2.09e−01
Value: B
Thermosyntropha
1.00
0.75
0.50
0.25
0.00
A (n=14)
B (n=15)
C (n=11)
surface

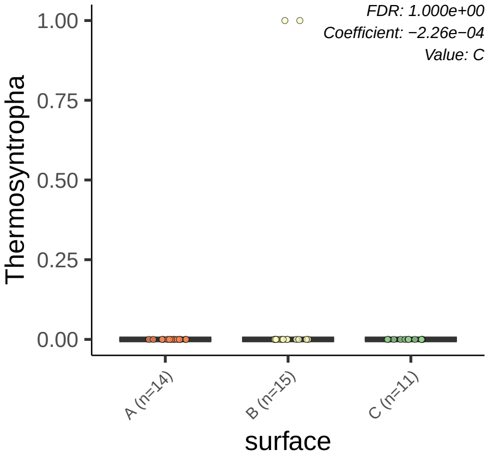
FDR: 1.000e+00
Coefficient: −2.26e−04
Value: C
Thermosyntropha
1.00
0.75
0.50
0.25
0.00
A (n=14)
B (n=15)
C (n=11)
surface

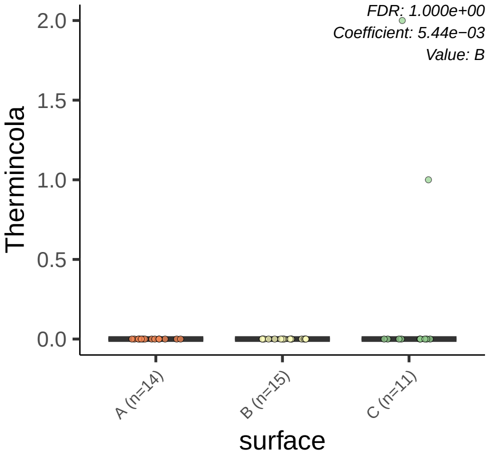

FDR: 1.000e+00
Coefficient: 5.44e−03
Value: B
Thermincola
2.0
1.5
1.0
0.5
0.0
A (n=14)
B (n=15)
C (n=11)
surface

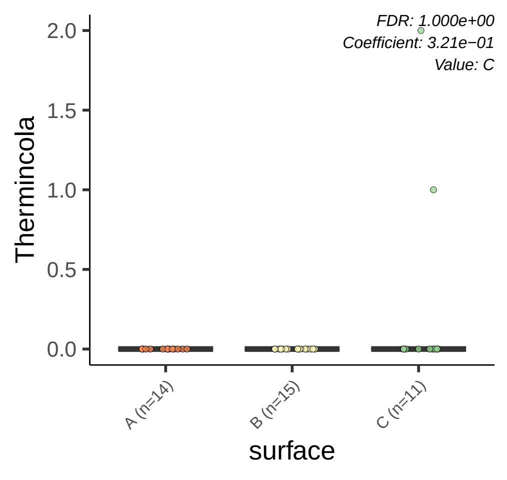
FDR: 1.000e+00
Coefficient: 3.21e−01
Value: C
Thermincola
2.0
1.5
1.0
0.5
0.0
A (n=14)
B (n=15)
C (n=11)
surface

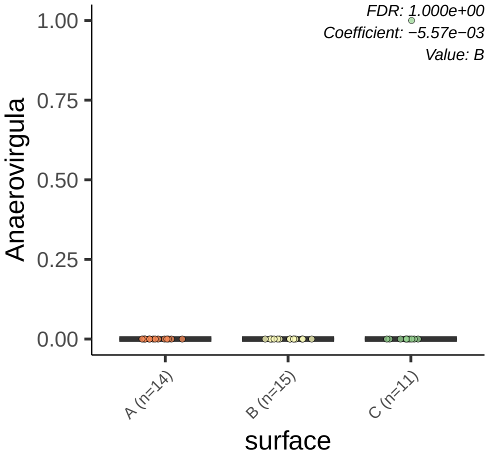
FDR: 1.000e+00
Coefficient: −5.57e−03
Value: B
Anaerovirgula
1.00
0.75
0.50
0.25
0.00
A (n=14)
B (n=15)
C (n=11)
surface

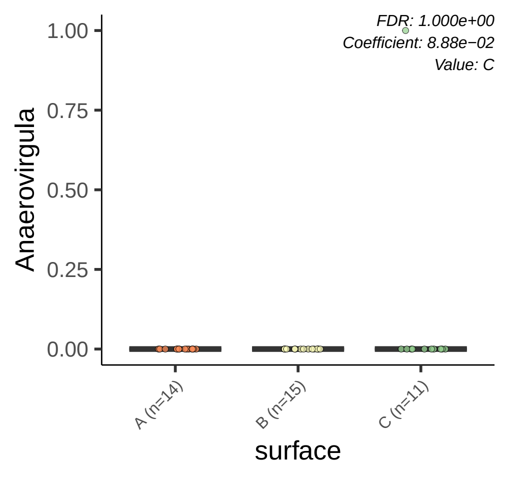
FDR: 1.000e+00
Coefficient: 8.88e−02
Value: C
Anaerovirgula
1.00
0.75
0.50
0.25
0.00
A (n=14)
B (n=15)
C (n=11)
surface

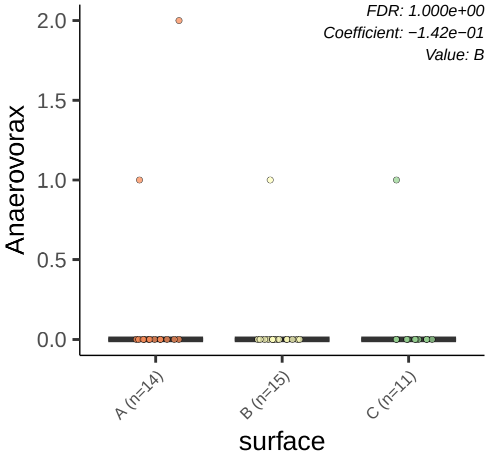
FDR: 1.000e+00
Coefficient: −1.42e−01
Value: B
Anaerovorax
2.0
1.5
1.0
0.5
0.0
A (n=14)
B (n=15)
C (n=11)
surface

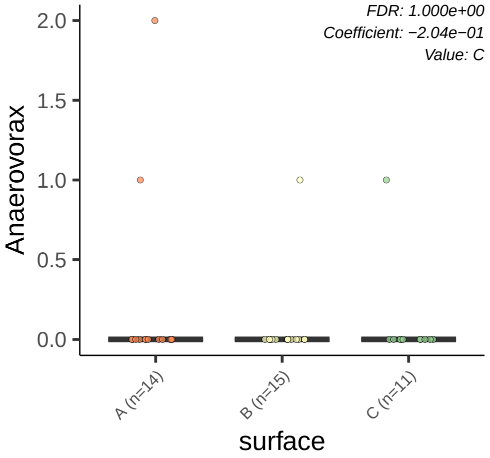

FDR: 1.000e+00
Coefficient: −2.04e−01
Value: C
Anaerovorax
2.0
1.5
1.0
0.5
0.0
A (n=14)
B (n=15)
C (n=11)
surface

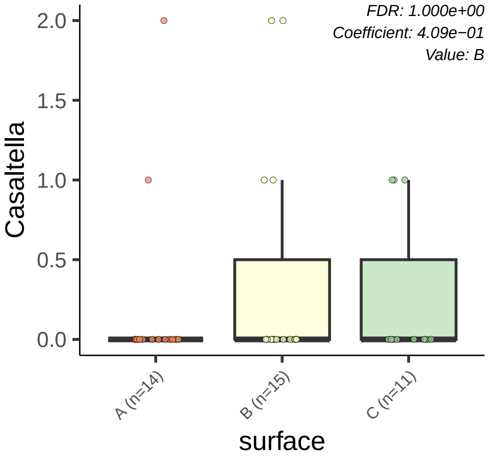
FDR: 1.000e+00
Coefficient: 4.09e−01
Value: B
Casaltella
2.0
1.5
1.0
0.5
0.0
A (n=14)
B (n=15)
C (n=11)
surface

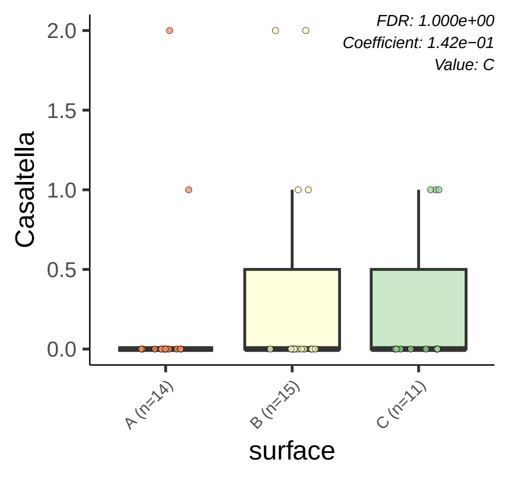
FDR: 1.000e+00
Coefficient: 1.42e−01
Value: C
Casaltella
2.0
1.5
1.0
0.5
0.0
A (n=14)
B (n=15)
C (n=11)
surface

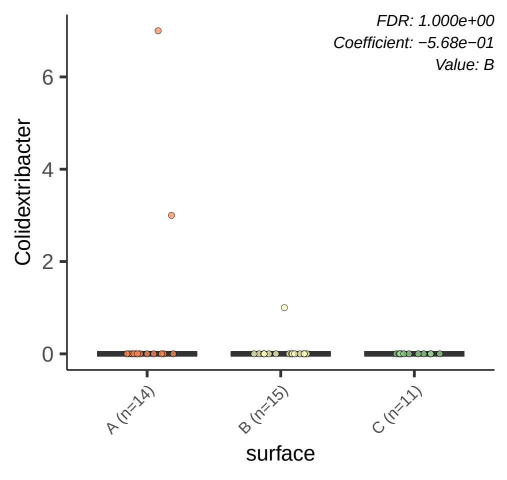
FDR: 1.000e+00
Coefficient: −5.68e−01
Value: B
Colidextribacter
0
2
4
6
A (n=14)
B (n=15)
C (n=11)
surface

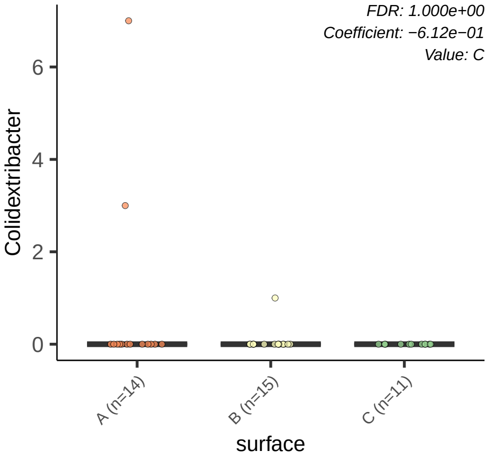
FDR: 1.000e+00
Coefficient: −6.12e−01
Value: C
Colidextribacter
0
2
4
6
A (n=14)
B (n=15)
C (n=11)
surface

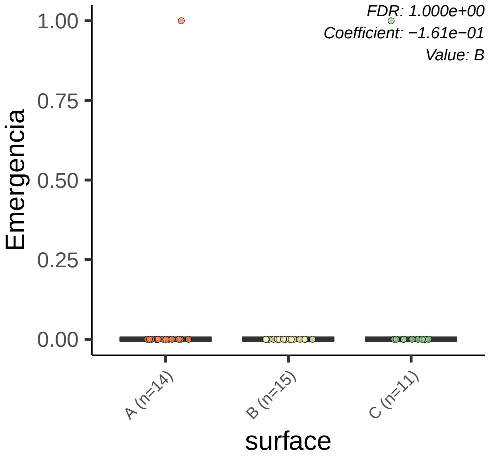
FDR: 1.000e+00
Coefficient: −1.61e−01
Value: B
Emergencia
1.00
0.75
0.50
0.25
0.00
A (n=14)
B (n=15)
C (n=11)
surface

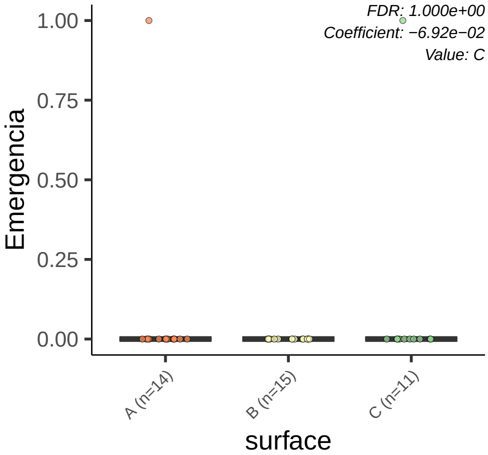
FDR: 1.000e+00
Coefficient: −6.92e−02
Value: C
Emergencia
1.00
0.75
0.50
0.25
0.00
A (n=14)
B (n=15)
C (n=11)
surface

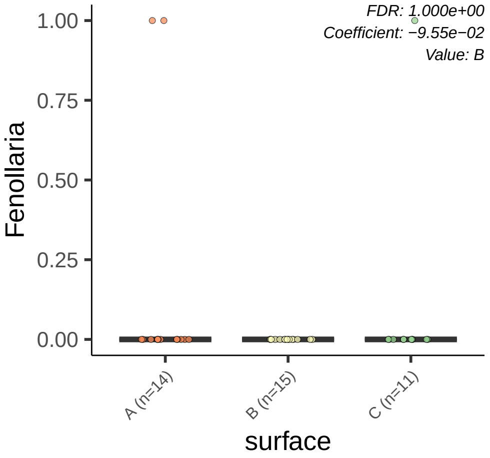
FDR: 1.000e+00
Coefficient: −9.55e−02
Value: B
Fenollaria
1.00
0.75
0.50
0.25
0.00
A (n=14)
B (n=15)
C (n=11)
surface

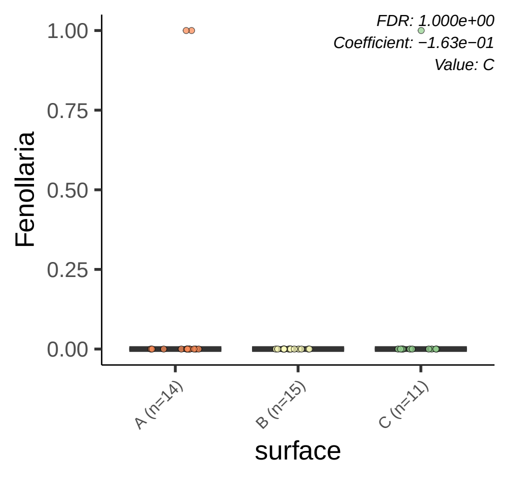
FDR: 1.000e+00
Coefficient: −1.63e−01
Value: C
Fenollaria
1.00
0.75
0.50
0.25
0.00
A (n=14)
B (n=15)
C (n=11)
surface

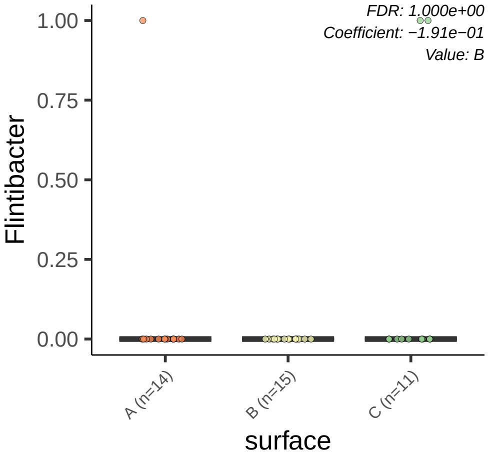

FDR: 1.000e+00
Coefficient: −1.91e−01
Value: B
Flintibacter
1.00
0.75
0.50
0.25
0.00
A (n=14)
B (n=15)
C (n=11)
surface

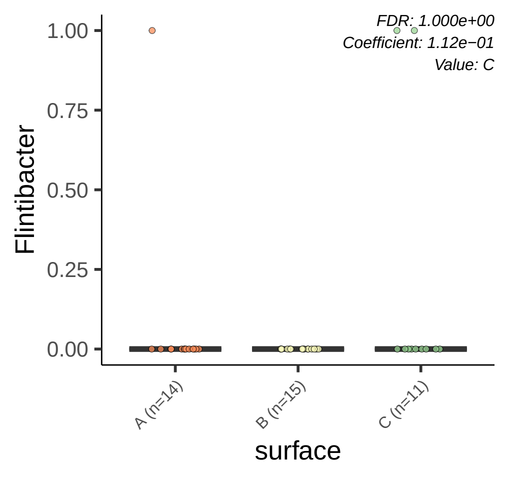

FDR: 1.000e+00
Coefficient: 1.12e−01
Value: C
Flintibacter
1.00
0.75
0.50
0.25
0.00
A (n=14)
B (n=15)
C (n=11)
surface

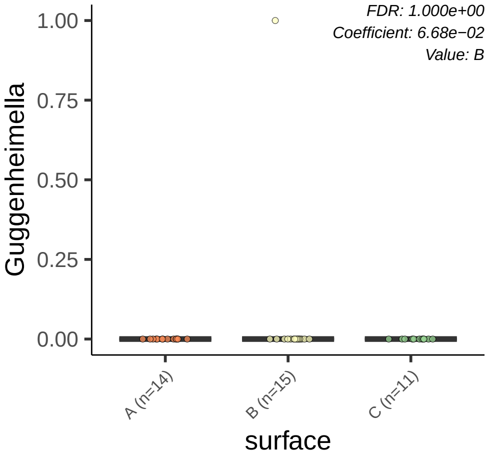
FDR: 1.000e+00
Coefficient: 6.68e−02
Value: B
Guggenheimella
1.00
0.75
0.50
0.25
0.00
A (n=14)
B (n=15)
C (n=11)
surface

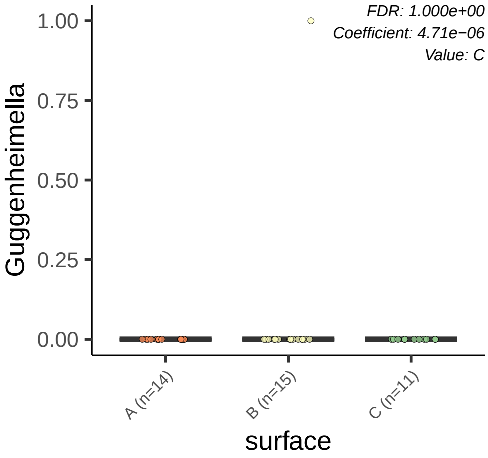
FDR: 1.000e+00
Coefficient: 4.71e−06
Value: C
Guggenheimella
1.00
0.75
0.50
0.25
0.00
A (n=14)
B (n=15)
C (n=11)
surface

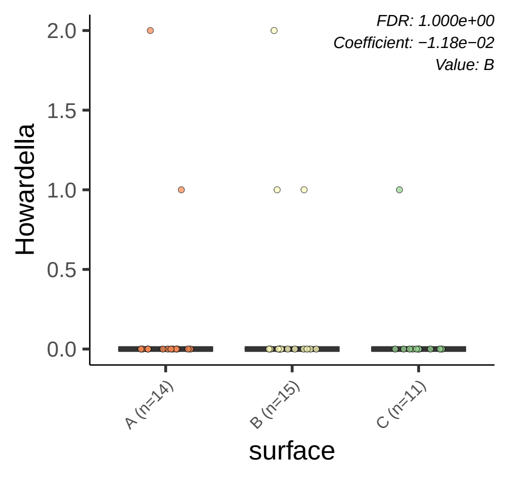
FDR: 1.000e+00
Coefficient: −1.18e−02
Value: B
Howardella
2.0
1.5
1.0
0.5
0.0
A (n=14)
B (n=15)
C (n=11)
surface

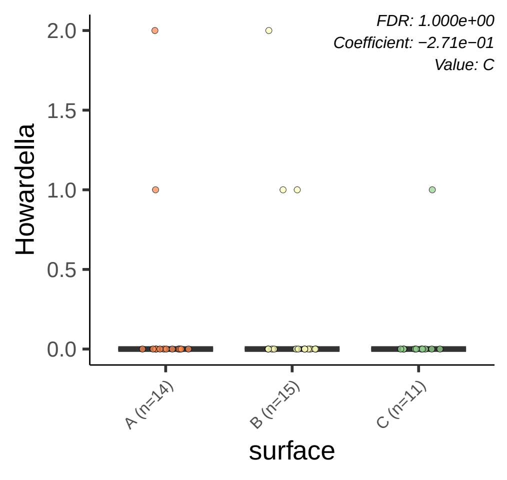
FDR: 1.000e+00
Coefficient: −2.71e−01
Value: C
Howardella
2.0
1.5
1.0
0.5
0.0
A (n=14)
B (n=15)
C (n=11)
surface

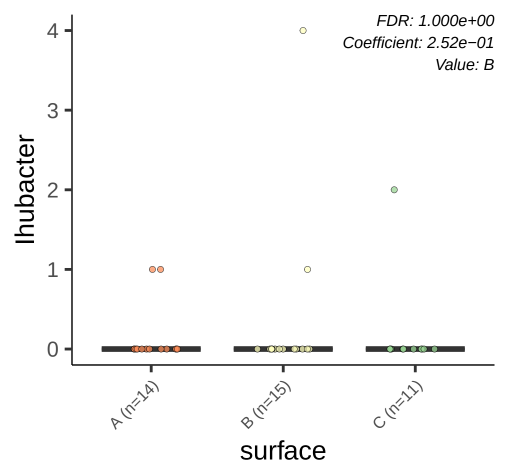

FDR: 1.000e+00
Coefficient: 2.52e−01
Value: B
lhubacter
4
3
2
1
0
A (n=14)
B (n=15)
C (n=11)
surface

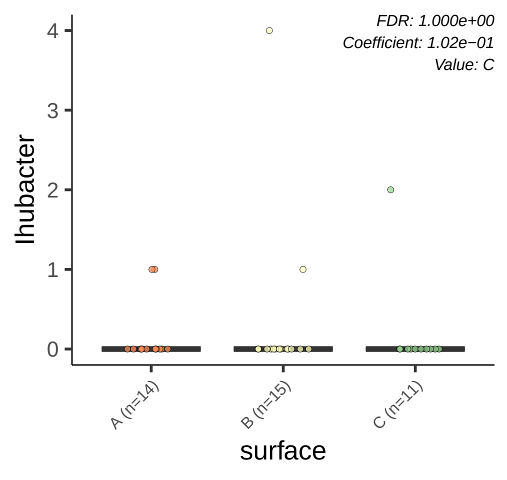
FDR: 1.000e+00
Coefficient: 1.02e−01
Value: C
Ihubacter
0
1
2
3
4
A (n=14)
B (n=15)
C (n=11)
surface

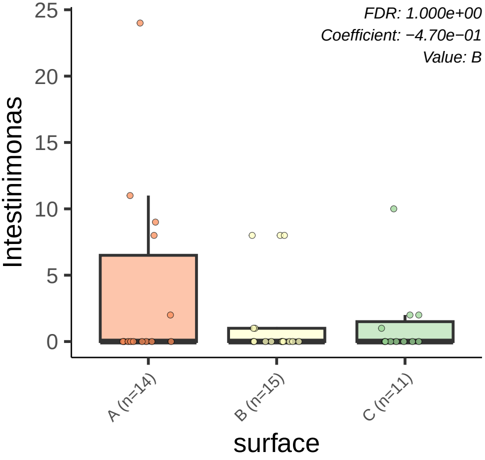
FDR: 1.000e+00
Coefficient: −4.70e−01
Value: B
Intestinimonas
0
5
10
15
20
25
A (n=14)
B (n=15)
C (n=11)
surface

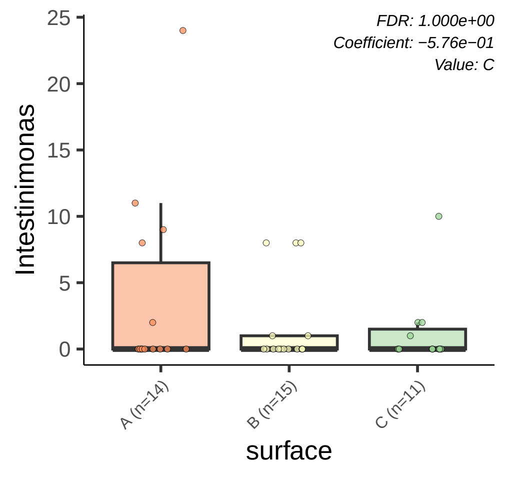

FDR: 1.000e+00
Coefficient: −5.76e−01
Value: C
Intestinimonas
surface
A (n=14)
B (n=15)
C (n=11)
0
5
10
15
20
25

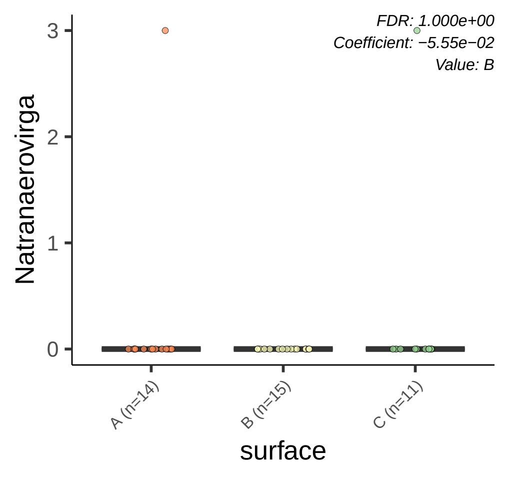
FDR: 1.000e+00
Coefficient: −5.55e−02
Value: B
Natranaerovirga
3
2
1
0
A (n=14)
B (n=15)
C (n=11)
surface

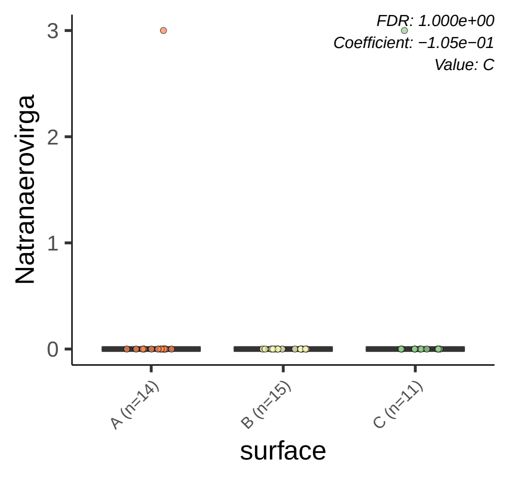
FDR: 1.000e+00
Coefficient: −1.05e−01
Value: C
Natranaerovirga
3
2
1
0
A (n=14)
B (n=15)
C (n=11)
surface

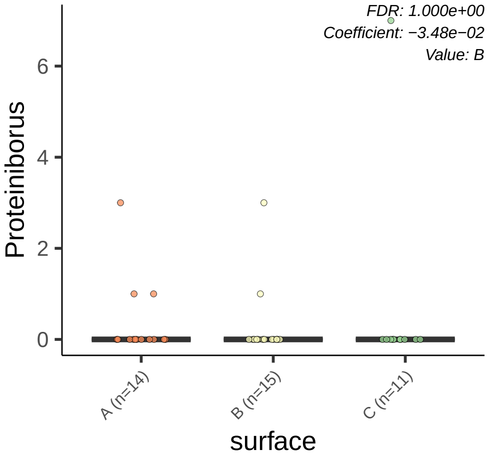
FDR: 1.000e+00
Coefficient: −3.48e−02
Value: B
Proteiniborus
0
2
4
6
A (n=14)
B (n=15)
C (n=11)
surface

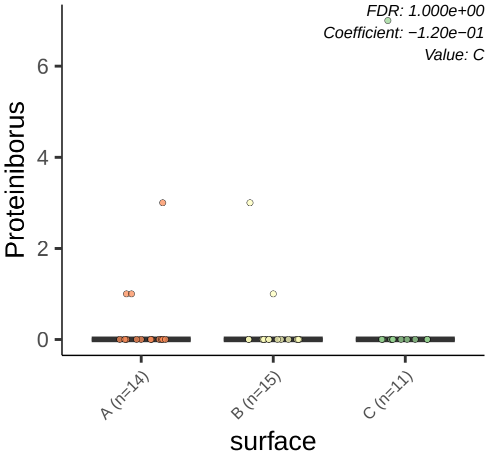
FDR: 1.000e+00
Coefficient: −1.20e−01
Value: C
Proteiniborus
0
2
4
6
A (n=14)
B (n=15)
C (n=11)
surface

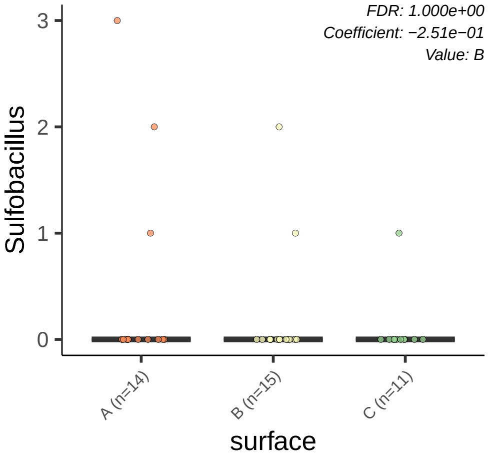
FDR: 1.000e+00
Coefficient: −2.51e−01
Value: B
Sulfobacillus
3
2
1
0
A (n=14)
B (n=15)
C (n=11)
surface

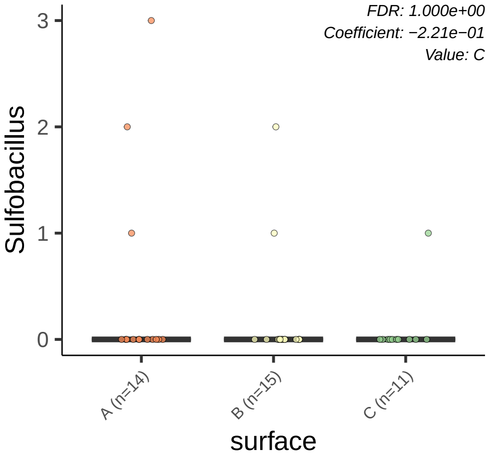

FDR: 1.000e+00
Coefficient: −2.21e−01
Value: C
Sulfobacillus
3
2
1
0
A (n=14)
B (n=15)
C (n=11)
surface

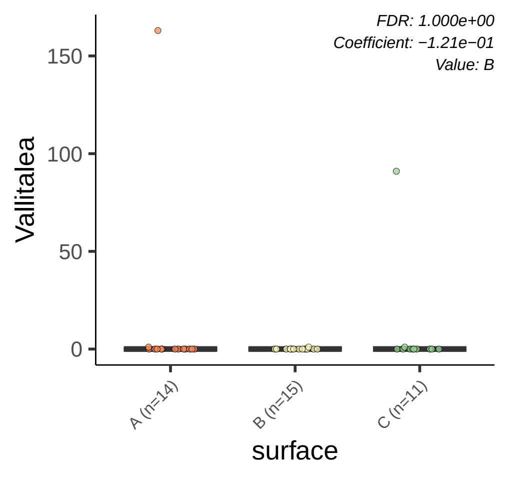

FDR: 1.000e+00
Coefficient: −1.21e−01
Value: B
Vallitalea
150
100
50
0
A (n=14)
B (n=15)
C (n=11)
surface

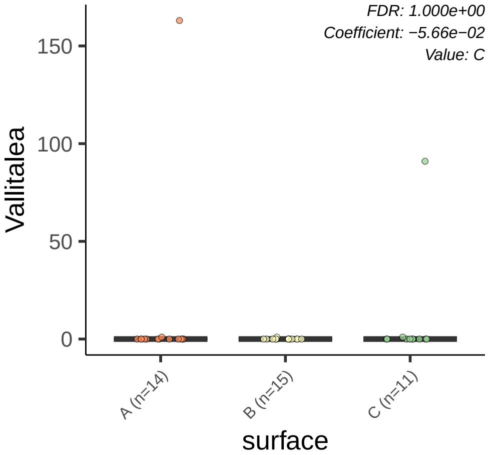
FDR: 1.000e+00
Coefficient: −5.66e−02
Value: C
Vallitalea
150
100
50
0
A (n=14)
B (n=15)
C (n=11)
surface

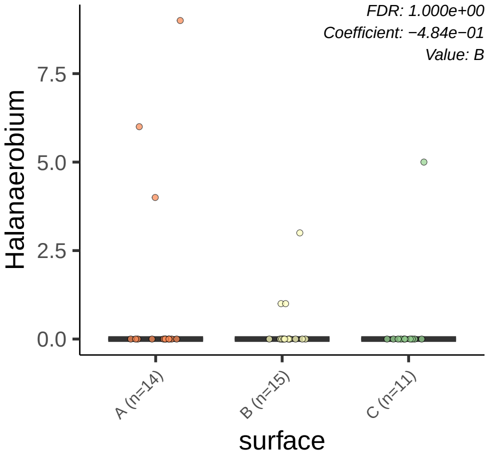

FDR: 1.000e+00
Coefficient: −4.84e−01
Value: B
Halanaerobium
7.5
5.0
2.5
0.0
A (n=14)
B (n=15)
C (n=11)
surface

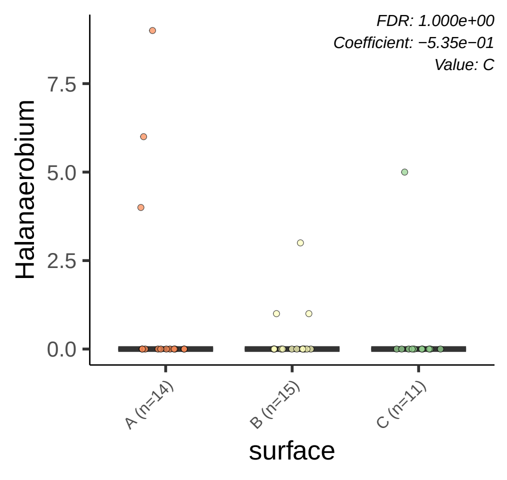
FDR: 1.000e+00
Coefficient: −5.35e−01
Value: C
Halanaerobium
7.5
5.0
2.5
0.0
A (n=14)
B (n=15)
C (n=11)
surface

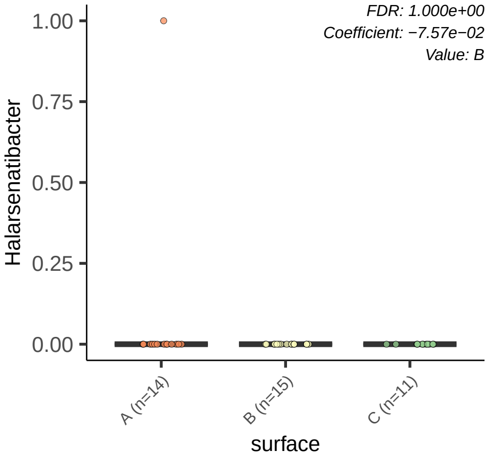
FDR: 1.000e+00
Coefficient: −7.57e−02
Value: B
Halarsenatibacter
1.00
0.75
0.50
0.25
0.00
A (n=14)
B (n=15)
C (n=11)
surface

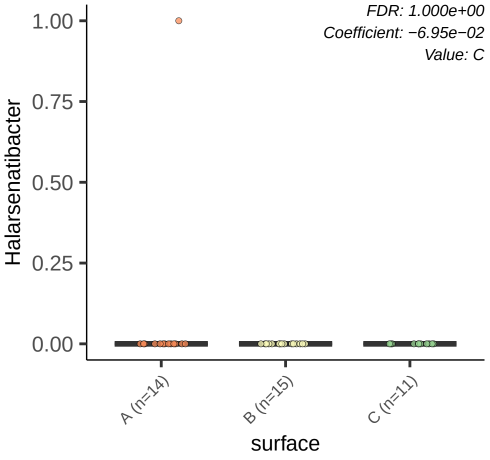

FDR: 1.000e+00
Coefficient: −6.95e−02
Value: C
Halarsenatibacter
1.00
0.75
0.50
0.25
0.00
A (n=14)
B (n=15)
C (n=11)
surface

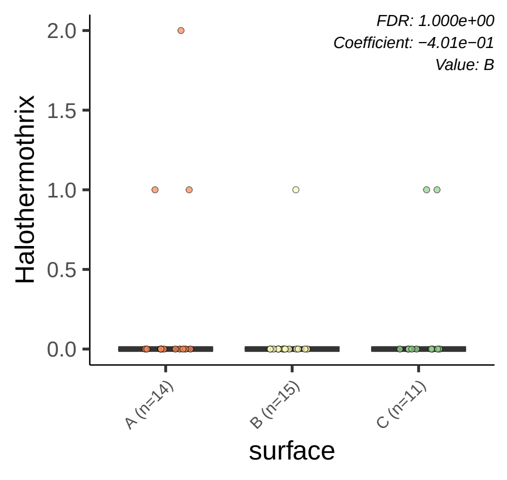

FDR: 1.000e+00
Coefficient: −4.01e−01
Value: B
Halothermothrix
2.0
1.5
1.0
0.5
0.0
A (n=14)
B (n=15)
C (n=11)
surface

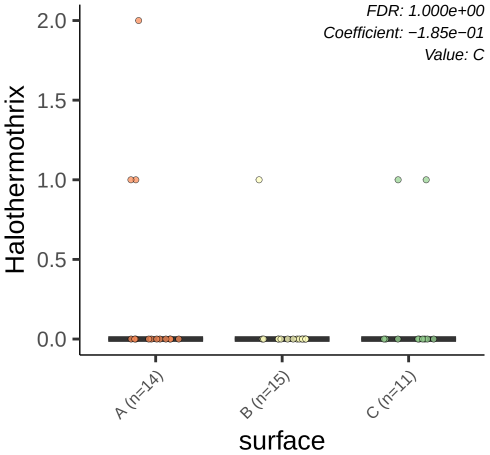

FDR: 1.000e+00
Coefficient: −1.85e−01
Value: C
Halothermothrix
2.0
1.5
1.0
0.5
0.0
A (n=14)
B (n=15)
C (n=11)
surface

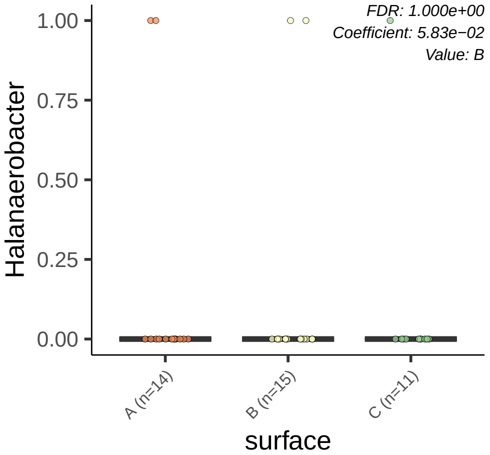
FDR: 1.000e+00
Coefficient: 5.83e−02
Value: B
Halanaerobacter
1.00
0.75
0.50
0.25
0.00
A (n=14)
B (n=15)
C (n=11)
surface

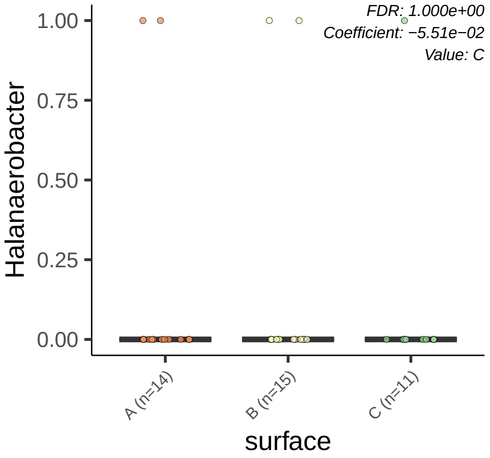

FDR: 1.000e+00
Coefficient: −5.51e−02
Value: C
Halanaerobacter
1.00
0.75
0.50
0.25
0.00
A (n=14)
B (n=15)
C (n=11)
surface

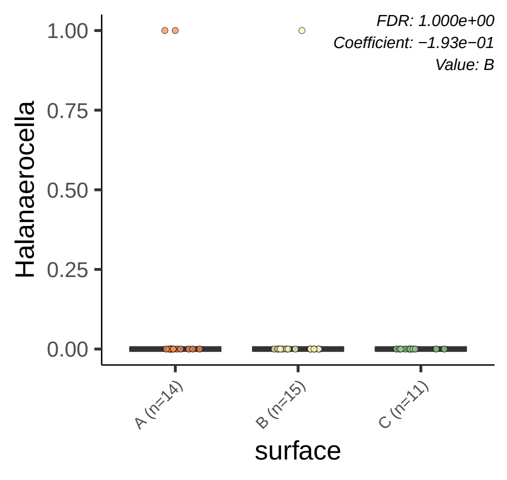

FDR: 1.000e+00
Coefficient: −1.93e−01
Value: B
Halanaerocella
1.00
0.75
0.50
0.25
0.00
A (n=14)
B (n=15)
C (n=11)
surface

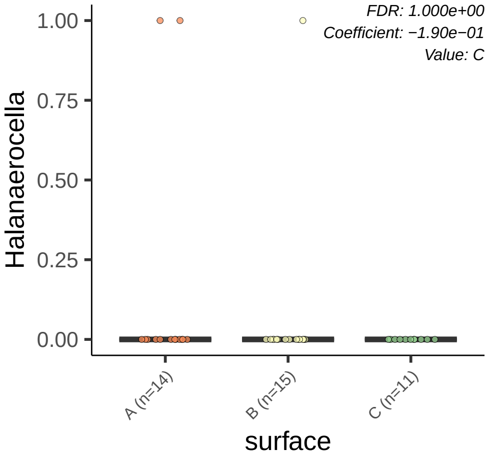

FDR: 1.000e+00
Coefficient: −1.90e−01
Value: C
Halanaerocella
1.00
0.75
0.50
0.25
0.00
A (n=14)
B (n=15)
C (n=11)
surface

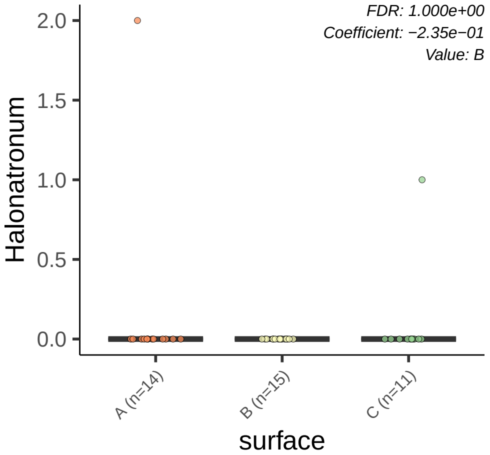

FDR: 1.000e+00
Coefficient: −2.35e−01
Value: B
Halonatronum
2.0
1.5
1.0
0.5
0.0
A (n=14)
B (n=15)
C (n=11)
surface

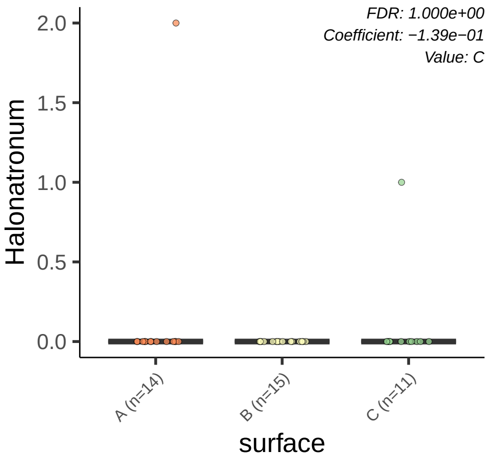

FDR: 1.000e+00
Coefficient: −1.39e−01
Value: C
Halonatronum
2.0
1.5
1.0
0.5
0.0
A (n=14)
B (n=15)
C (n=11)
surface

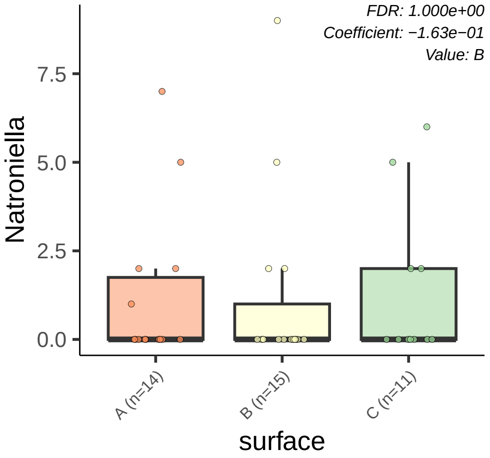
FDR: 1.000e+00
Coefficient: −1.63e−01
Value: B
Natroniella
7.5
5.0
2.5
0.0
A (n=14)
B (n=15)
C (n=11)
surface

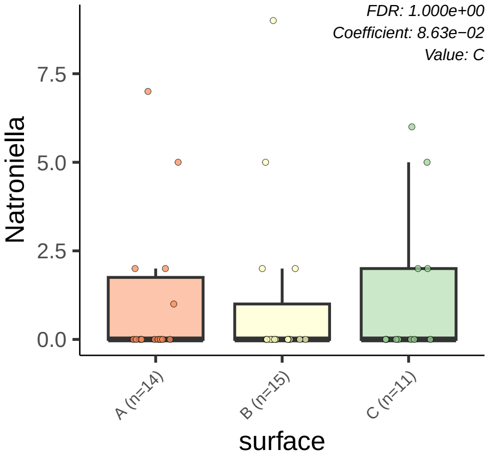
FDR: 1.000e+00
Coefficient: 8.63e−02
Value: C
Natroniella
7.5
5.0
2.5
0.0
A (n=14)
B (n=15)
C (n=11)
surface

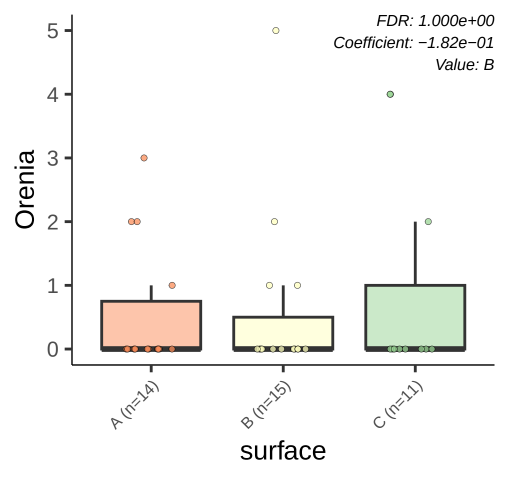
FDR: 1.000e+00
Coefficient: −1.82e−01
Value: B
Orenia
5
4
3
2
1
0
A (n=14)
B (n=15)
C (n=11)
surface

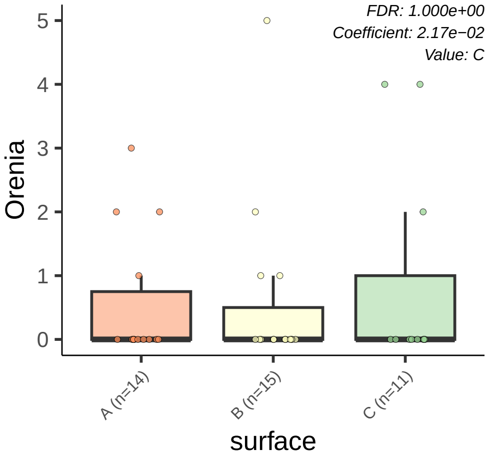

FDR: 1.000e+00
Coefficient: 2.17e−02
Value: C
Orenia
0
1
2
3
4
5
A (n=14)
B (n=15)
C (n=11)
surface

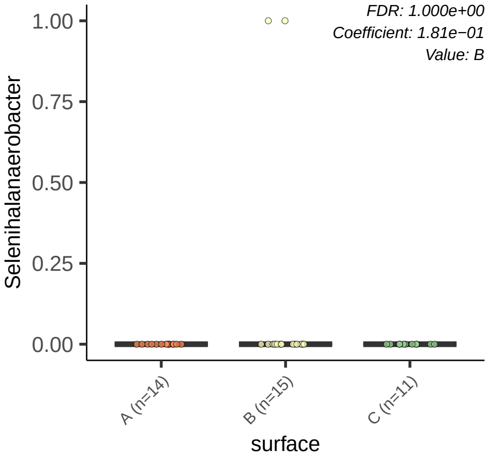
FDR: 1.000e+00
Coefficient: 1.81e−01
Value: B
Selenihalanaerobacter
1.00
0.75
0.50
0.25
0.00
A (n=14)
B (n=15)
C (n=11)
surface

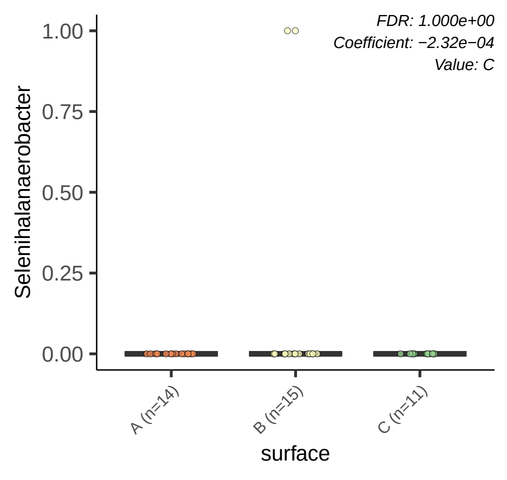
FDR: 1.000e+00
Coefficient: −2.32e−04
Value: C
Selenihalanaerobacter
1.00
0.75
0.50
0.25
0.00
A (n=14)
B (n=15)
C (n=11)
surface

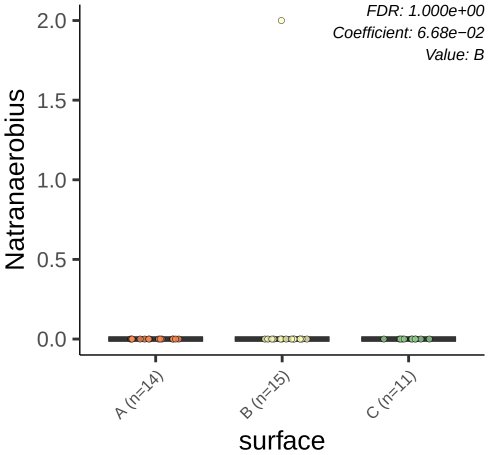
FDR: 1.000e+00
Coefficient: 6.68e−02
Value: B
Natranaerobius
2.0
1.5
1.0
0.5
0.0
A (n=14)
B (n=15)
C (n=11)
surface

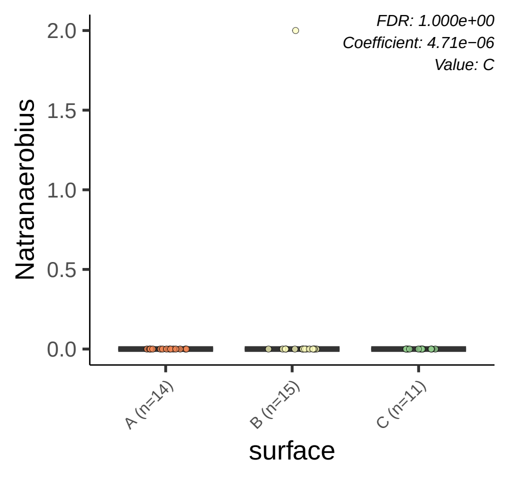
FDR: 1.000e+00
Coefficient: 4.71e−06
Value: C
Natranaerobius
2.0
1.5
1.0
0.5
0.0
A (n=14)
B (n=15)
C (n=11)
surface

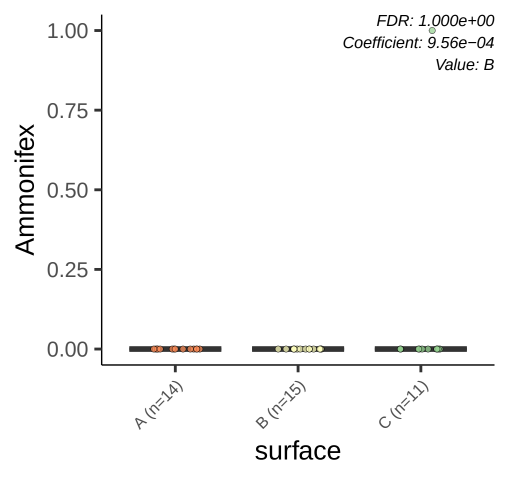

FDR: 1.000e+00
Coefficient: 9.56e−04
Value: B
Ammonifex
1.00
0.75
0.50
0.25
0.00
A (n=14)
B (n=15)
C (n=11)
surface

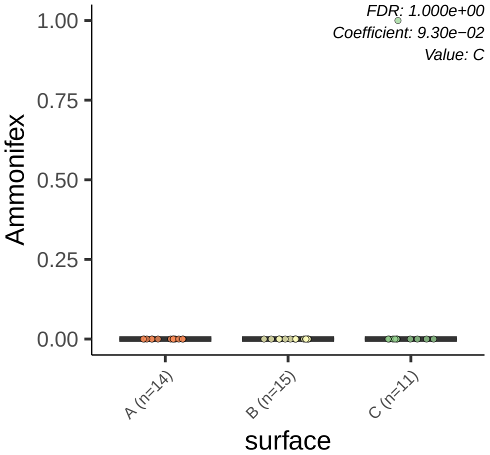
FDR: 1.000e+00
Coefficient: 9.30e−02
Value: C
Ammonifex
1.00
0.75
0.50
0.25
0.00
A (n=14)
B (n=15)
C (n=11)
surface

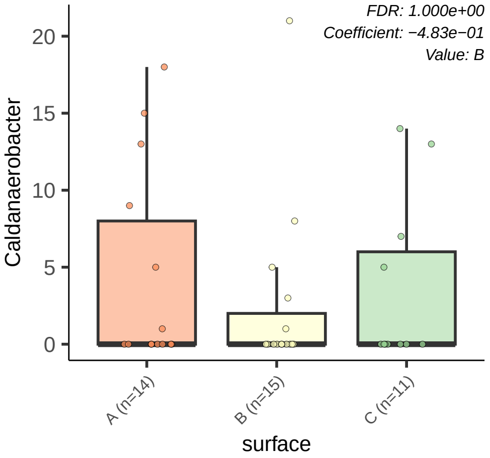
FDR: 1.000e+00
Coefficient: −4.83e−01
Value: B
Caldanaerobacter
0
5
10
15
20
A (n=14)
B (n=15)
C (n=11)
surface

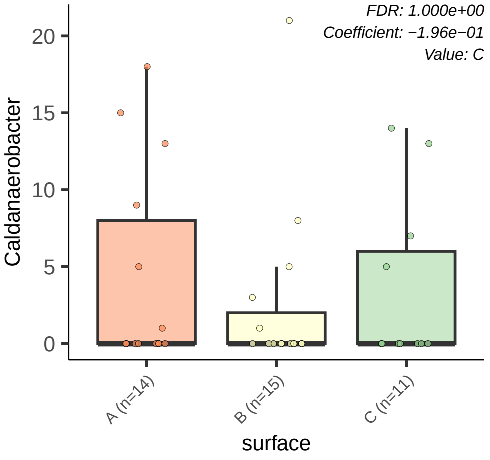
FDR: 1.000e+00
Coefficient: −1.96e−01
Value: C
Caldanaerobacter
0
5
10
15
20
A (n=14)
B (n=15)
C (n=11)
surface

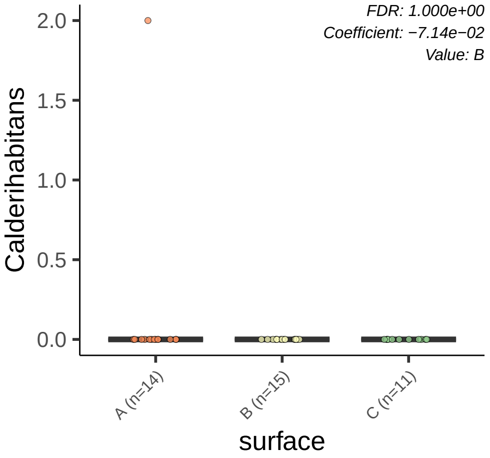

FDR: 1.000e+00
Coefficient: −7.14e−02
Value: B
Calderihabitans
2.0
1.5
1.0
0.5
0.0
A (n=14)
B (n=15)
C (n=11)
surface

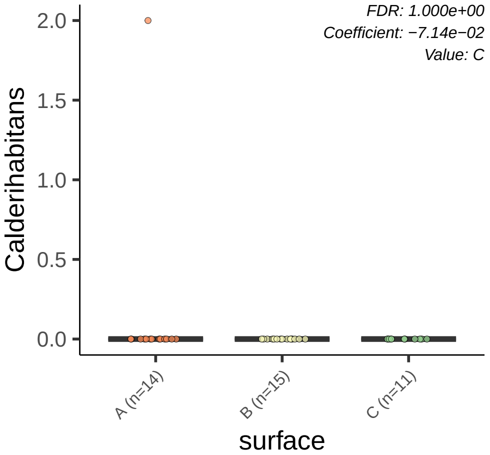

FDR: 1.000e+00
Coefficient: −7.14e−02
Value: C
Calderihabitans
2.0
1.5
1.0
0.5
0.0
A (n=14)
B (n=15)
C (n=11)
surface

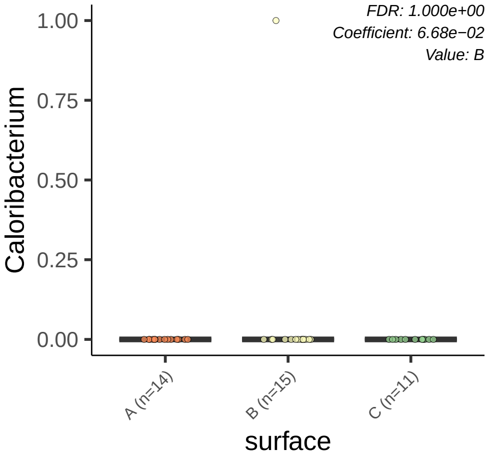
FDR: 1.000e+00
Coefficient: 6.68e−02
Value: B
Caloribacterium
1.00
0.75
0.50
0.25
0.00
A (n=14)
B (n=15)
C (n=11)
surface

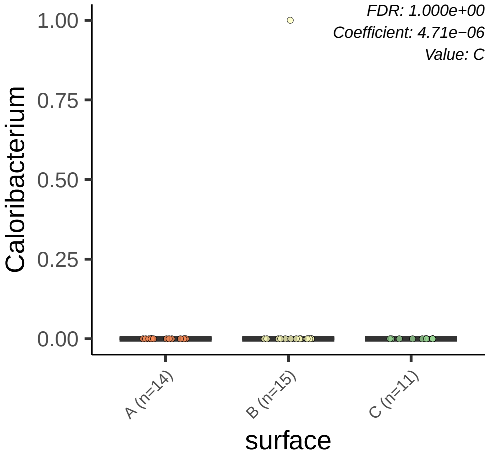
FDR: 1.000e+00
Coefficient: 4.71e−06
Value: C
Caloribacterium
1.00
0.75
0.50
0.25
0.00
A (n=14)
B (n=15)
C (n=11)
surface

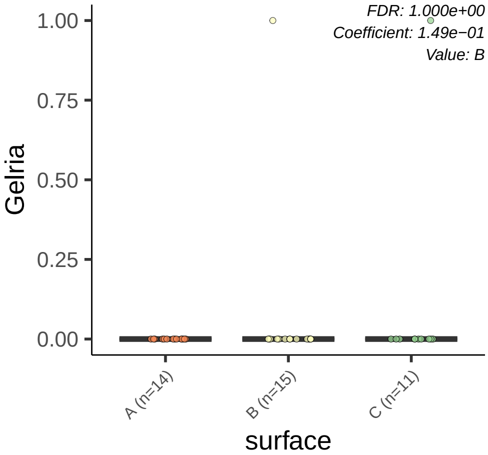

FDR: 1.000e+00
Coefficient: 1.49e−01
Value: B
Gelria
1.00
0.75
0.50
0.25
0.00
A (n=14)
B (n=15)
C (n=11)
surface

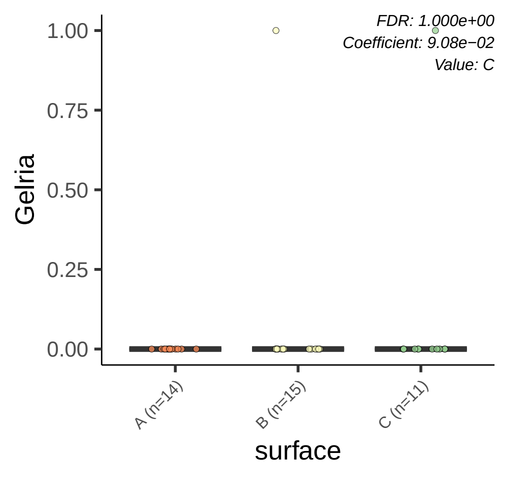

FDR: 1.000e+00
Coefficient: 9.08e−02
Value: C
Gelria
1.00
0.75
0.50
0.25
0.00
A (n=14)
B (n=15)
C (n=11)
surface

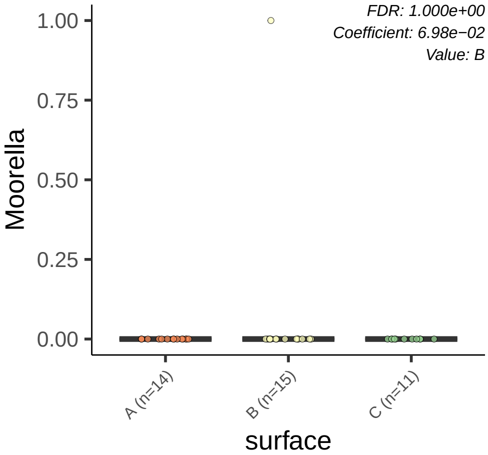

FDR: 1.000e+00
Coefficient: 6.98e−02
Value: B
Moorella
1.00
0.75
0.50
0.25
0.00
A (n=14)
B (n=15)
C (n=11)
surface

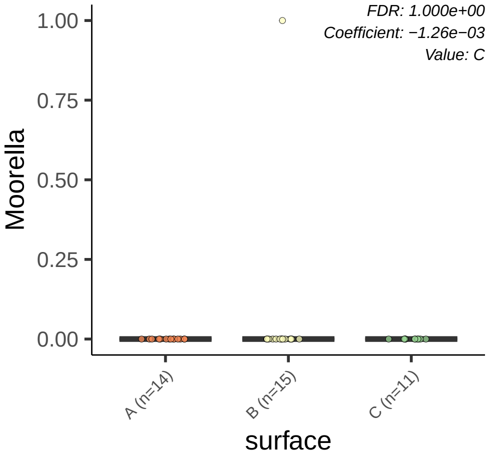

FDR: 1.000e+00
Coefficient: −1.26e−03
Value: C
Moorella
1.00
0.75
0.50
0.25
0.00
A (n=14)
B (n=15)
C (n=11)
surface

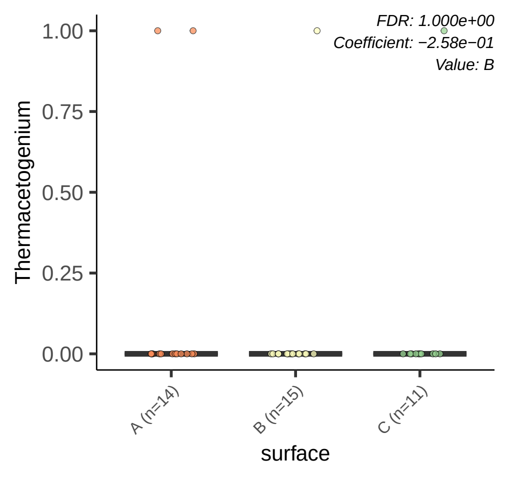
FDR: 1.000e+00
Coefficient: −2.58e−01
Value: B
Thermacetogenium
1.00
0.75
0.50
0.25
0.00
A (n=14)
B (n=15)
C (n=11)
surface

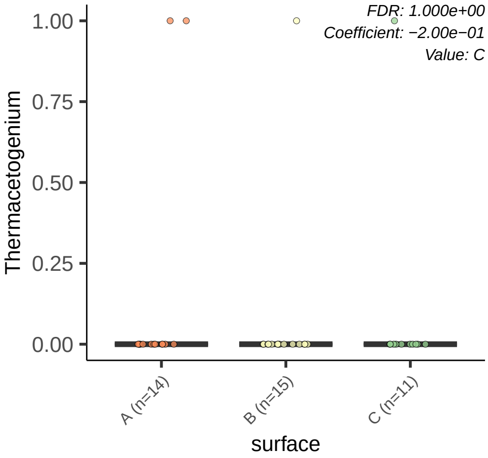
FDR: 1.000e+00
Coefficient: −2.00e−01
Value: C
Thermacetogenium
1.00
0.75
0.50
0.25
0.00
A (n=14)
B (n=15)
C (n=11)
surface

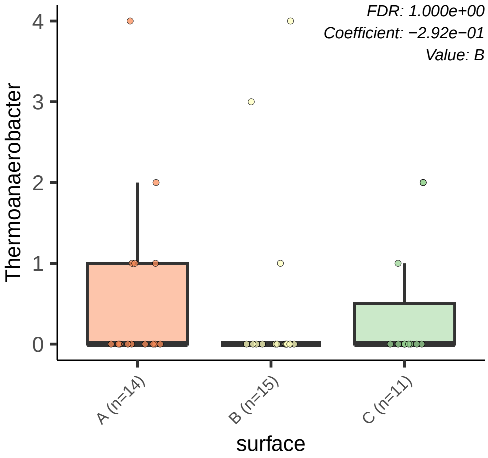

FDR: 1.000e+00
Coefficient: −2.92e−01
Value: B
Thermoanaerobacter
4
3
2
1
0
A (n=14)
B (n=15)
C (n=11)
surface

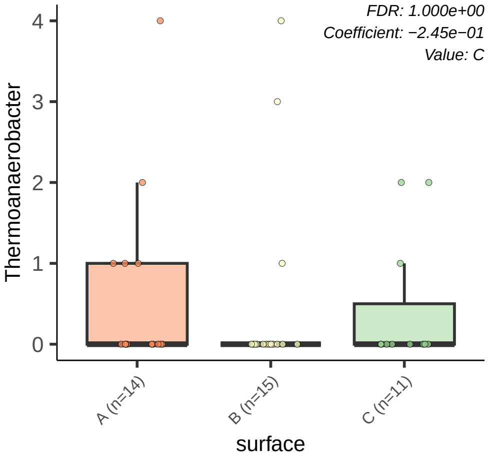

FDR: 1.000e+00
Coefficient: −2.45e−01
Value: C
Thermoanaerobacter
0
1
2
3
4
A (n=14)
B (n=15)
C (n=11)
surface

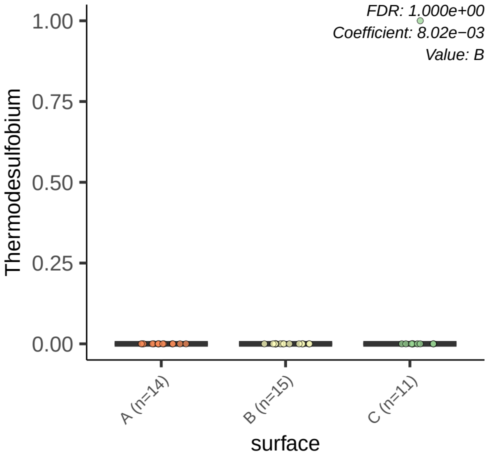
FDR: 1.000e+00
Coefficient: 8.02e−03
Value: B
Thermodesulfobium
1.00
0.75
0.50
0.25
0.00
A (n=14)
B (n=15)
C (n=11)
surface

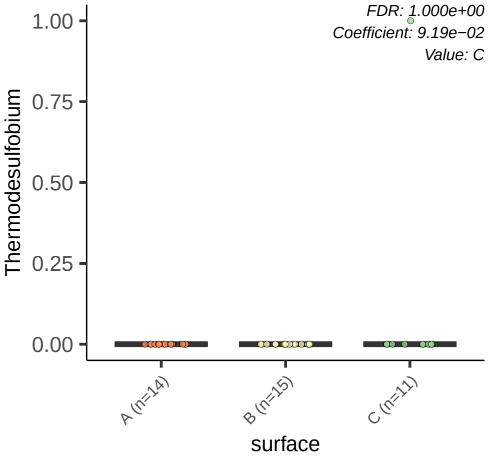

FDR: 1.000e+00
Coefficient: 9.19e−02
Value: C
Thermodesulfobium
1.00
0.75
0.50
0.25
0.00
A (n=14)
B (n=15)
C (n=11)
surface

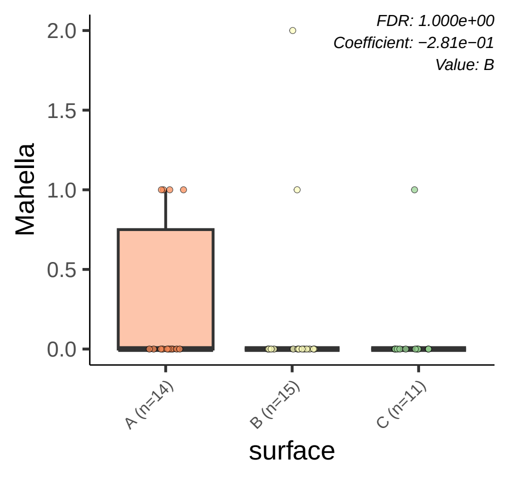
FDR: 1.000e+00
Coefficient: −2.81e−01
Value: B
Mahella
2.0
1.5
1.0
0.5
0.0
A (n=14)
B (n=15)
C (n=11)
surface

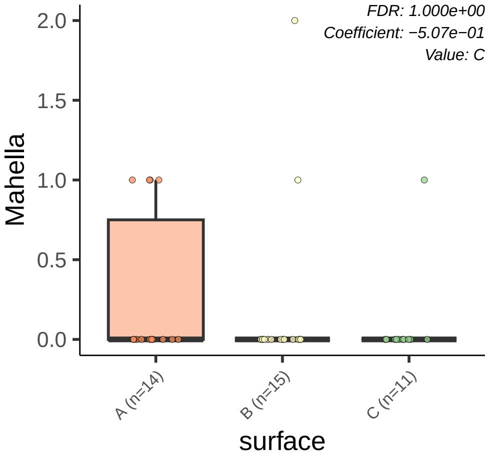

FDR: 1.000e+00
Coefficient: −5.07e−01
Value: C
Mahella
2.0
1.5
1.0
0.5
0.0
A (n=14)
B (n=15)
C (n=11)
surface

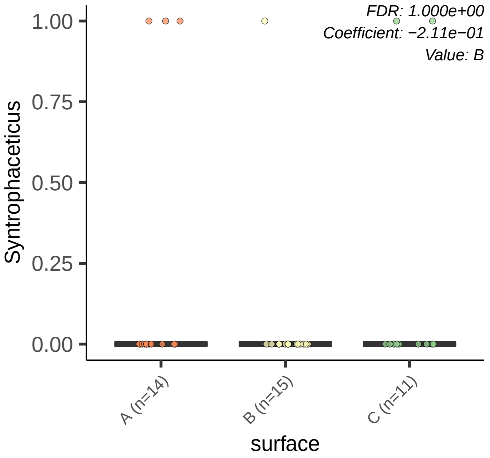

FDR: 1.000e+00
Coefficient: −2.11e−01
Value: B
Syntrophaceticus
1.00
0.75
0.50
0.25
0.00
A (n=14)
B (n=15)
C (n=11)
surface

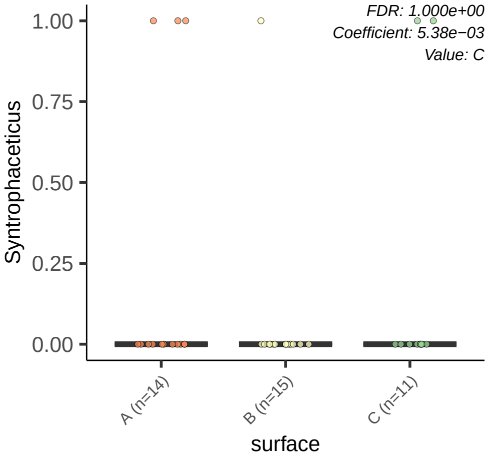

FDR: 1.000e+00
Coefficient: 5.38e−03
Value: C
Syntrophaceticus
1.00
0.75
0.50
0.25
0.00
A (n=14)
B (n=15)
C (n=11)
surface

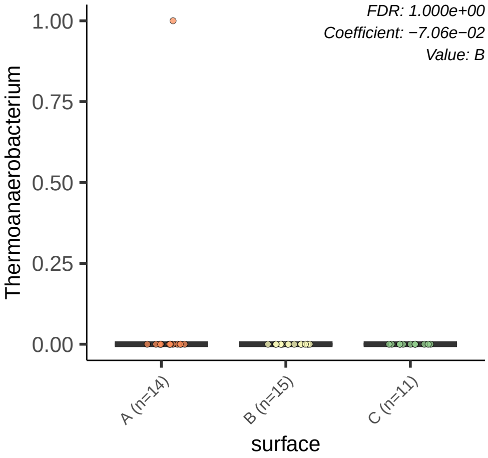
FDR: 1.000e+00
Coefficient: −7.06e−02
Value: B
Thermoanaerobacterium
1.00
0.75
0.50
0.25
0.00
A (n=14)
B (n=15)
C (n=11)
surface

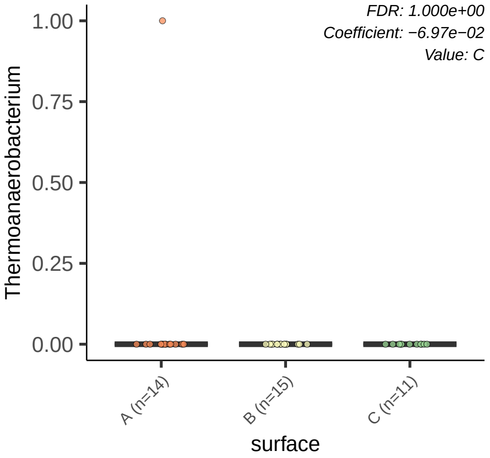

FDR: 1.000e+00
Coefficient: −6.97e−02
Value: C
Thermoanaerobacterium
1.00
0.75
0.50
0.25
0.00
A (n=14)
B (n=15)
C (n=11)
surface

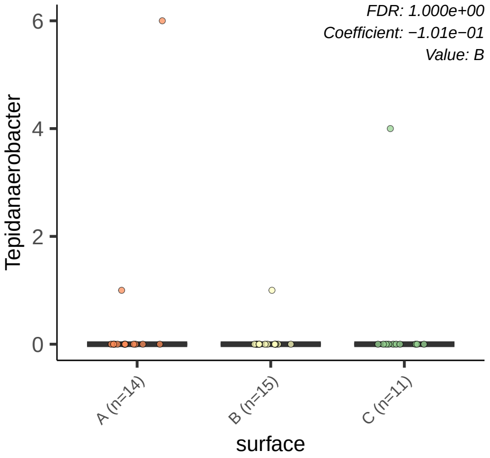

FDR: 1.000e+00
Coefficient: −1.01e−01
Value: B
Tepidanaerobacter
0
2
4
6
A (n=14)
B (n=15)
C (n=11)
surface

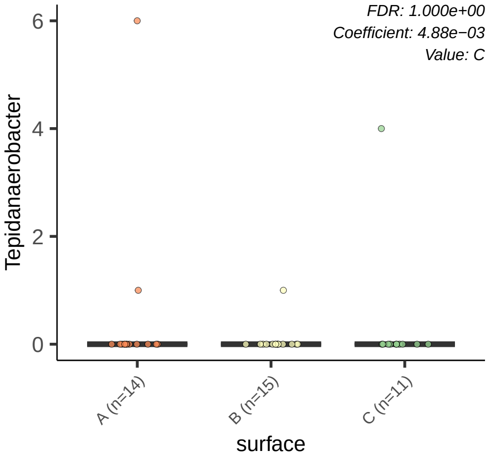
FDR: 1.000e+00
Coefficient: 4.88e−03
Value: C
Tepidanaerobacter
0
2
4
6
A (n=14)
B (n=15)
C (n=11)
surface

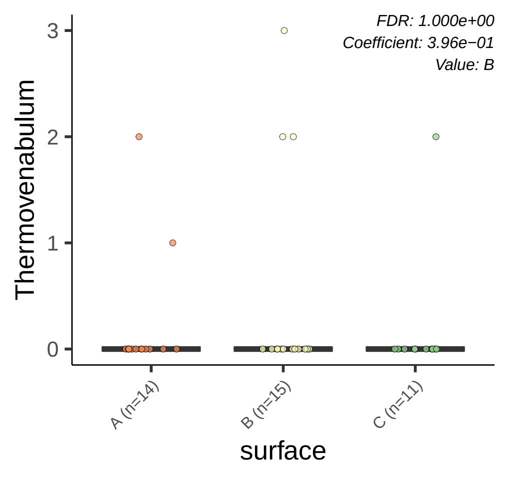

FDR: 1.000e+00
Coefficient: 3.96e−01
Value: B
Thermovenabulum
0
1
2
3
A (n=14)
B (n=15)
C (n=11)
surface

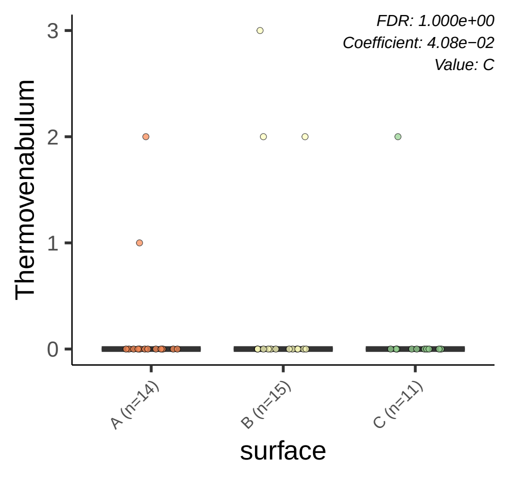
FDR: 1.000e+00
Coefficient: 4.08e−02
Value: C
Thermovenabulum
0
1
2
3
A (n=14)
B (n=15)
C (n=11)
surface

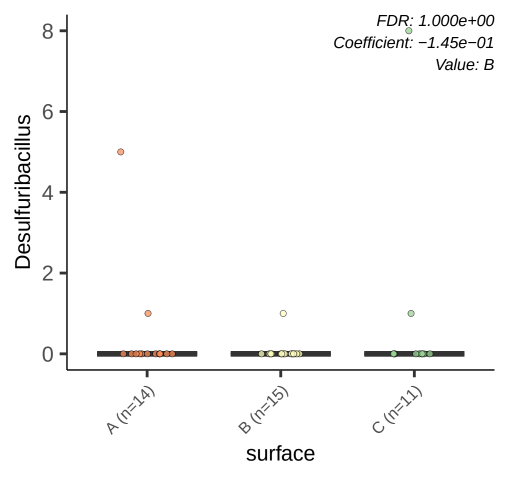
FDR: 1.000e+00
Coefficient: −1.45e−01
Value: B
Desulfuribacillus
8
6
4
2
0
A (n=14)
B (n=15)
C (n=11)
surface

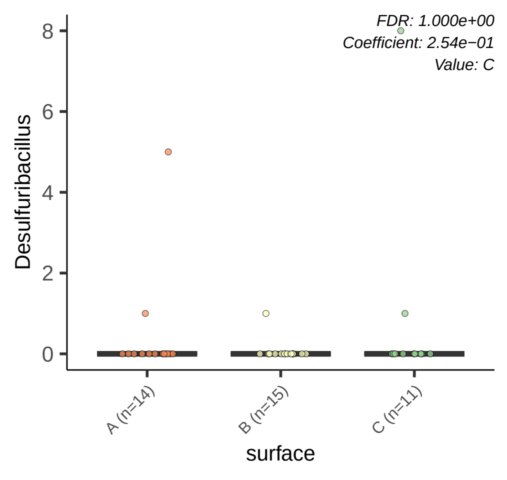
FDR: 1.000e+00
Coefficient: 2.54e−01
Value: C
Desulfuribacillus
0
2
4
6
8
A (n=14)
B (n=15)
C (n=11)
surface

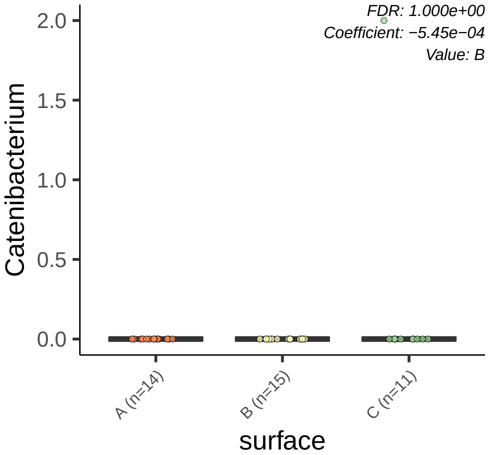
FDR: 1.000e+00
Coefficient: −5.45e−04
Value: B
Catenibacterium
2.0
1.5
1.0
0.5
0.0
A (n=14)
B (n=15)
C (n=11)
surface

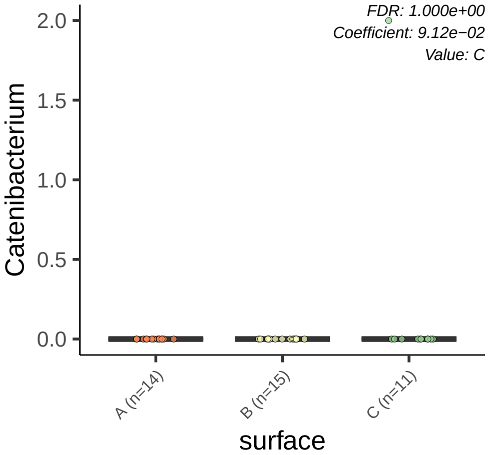
FDR: 1.000e+00
Coefficient: 9.12e−02
Value: C
Catenibacterium
2.0
1.5
1.0
0.5
0.0
A (n=14)
B (n=15)
C (n=11)
surface

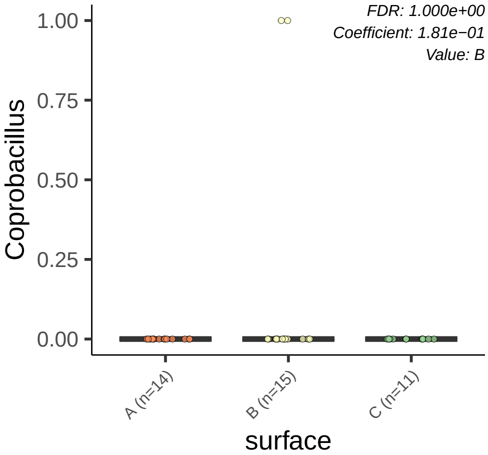

FDR: 1.000e+00
Coefficient: 1.81e−01
Value: B
1.00
0.75
0.50
0.25
0.00
Coprobacillus
A (n=14)
B (n=15)
C (n=11)
surface

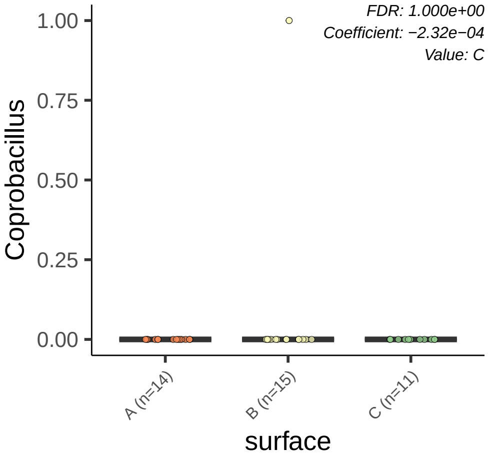
FDR: 1.000e+00
Coefficient: −2.32e−04
Value: C
Coprobacillus
1.00
0.75
0.50
0.25
0.00
A (n=14)
B (n=15)
C (n=11)
surface

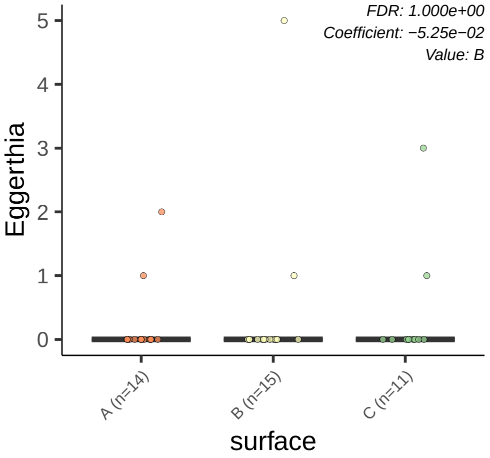
FDR: 1.000e+00
Coefficient: −5.25e−02
Value: B
Eggerthia
0
1
2
3
4
5
A (n=14)
B (n=15)
C (n=11)
surface

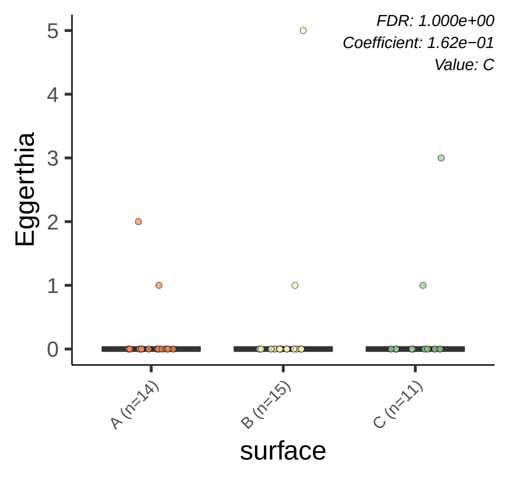
FDR: 1.000e+00
Coefficient: 1.62e−01
Value: C
Eggerthia
0
1
2
3
4
5
A (n=14)
B (n=15)
C (n=11)
surface

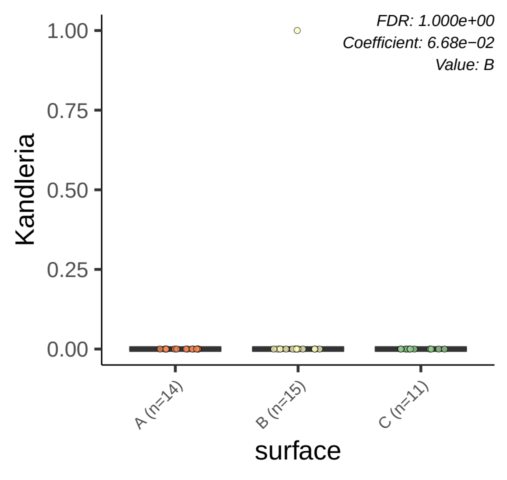
FDR: 1.000e+00
Coefficient: 6.68e−02
Value: B
1.00
0.75
0.50
0.25
0.00
Kandleria
A (n=14)
B (n=15)
C (n=11)
surface

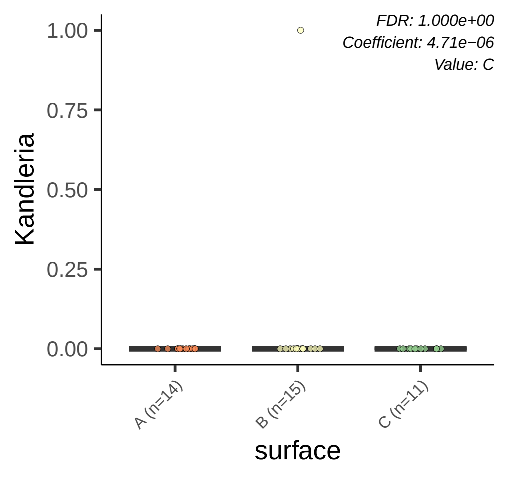

FDR: 1.000e+00
Coefficient: 4.71e−06
Value: C
Kandleria
1.00
0.75
0.50
0.25
0.00
A (n=14)
B (n=15)
C (n=11)
surface

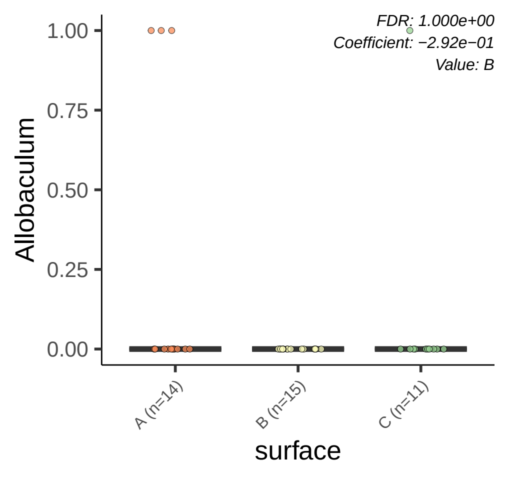
FDR: 1.000e+00
Coefficient: −2.92e−01
Value: B
Allobaculum
1.00
0.75
0.50
0.25
0.00
A (n=14)
B (n=15)
C (n=11)
surface

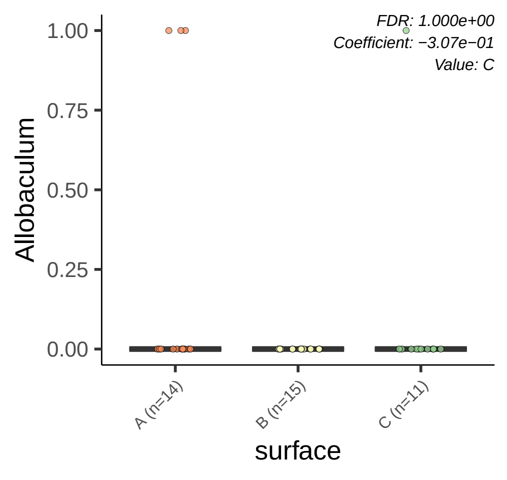

FDR: 1.000e+00
Coefficient: −3.07e−01
Value: C
Allobaculum
1.00
0.75
0.50
0.25
0.00
A (n=14)
B (n=15)
C (n=11)
surface

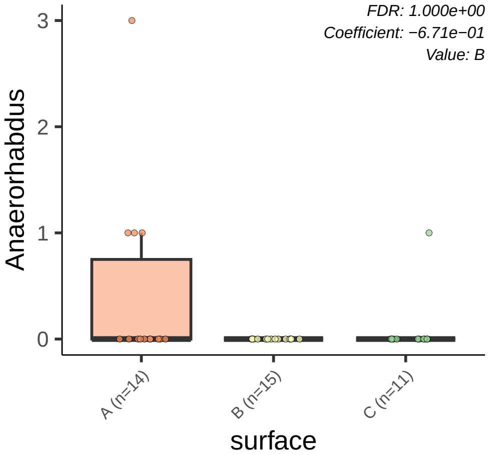
FDR: 1.000e+00
Coefficient: −6.71e−01
Value: B
Anaerorhabdus
3
2
1
0
A (n=14)
B (n=15)
C (n=11)
surface

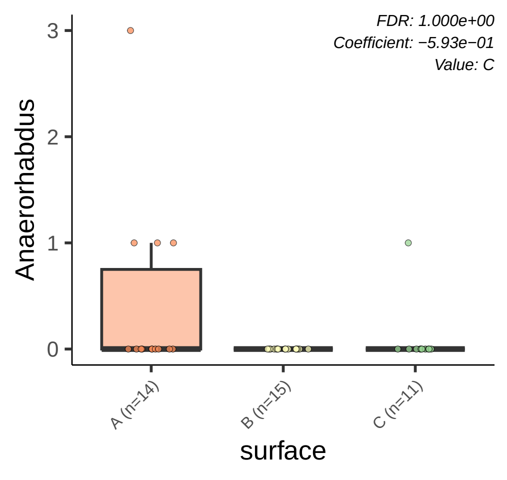
FDR: 1.000e+00
Coefficient: −5.93e−01
Value: C
Anaerorhabdus
3
2
1
0
A (n=14)
B (n=15)
C (n=11)
surface

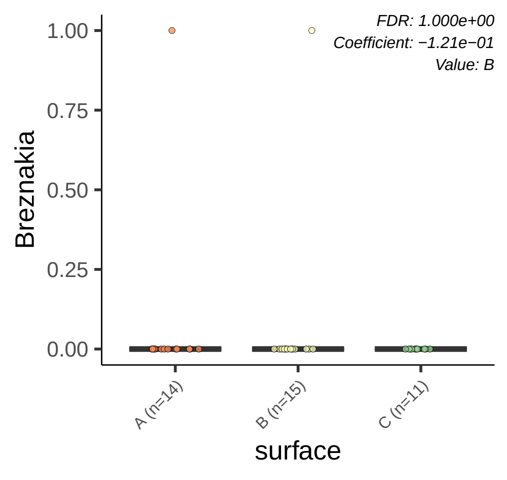
FDR: 1.000e+00
Coefficient: −1.21e−01
Value: B
Breznakia
1.00
0.75
0.50
0.25
0.00
A (n=14)
B (n=15)
C (n=11)
surface

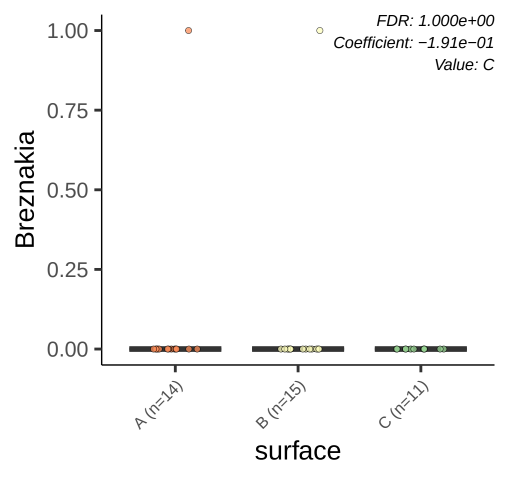

FDR: 1.000e+00
Coefficient: −1.91e−01
Value: C
Breznakia
1.00
0.75
0.50
0.25
0.00
A (n=14)
B (n=15)
C (n=11)
surface

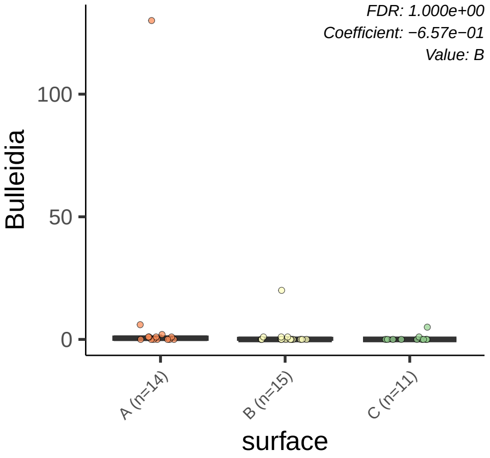
FDR: 1.000e+00
Coefficient: −6.57e−01
Value: B
Bulleidia
100
50
0
A (n=14)
B (n=15)
C (n=11)
surface

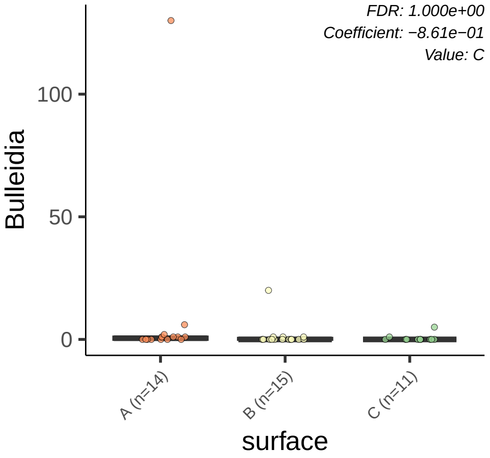
FDR: 1.000e+00
Coefficient: −8.61e−01
Value: C
Bulleidia
100
50
0
A (n=14)
B (n=15)
C (n=11)
surface

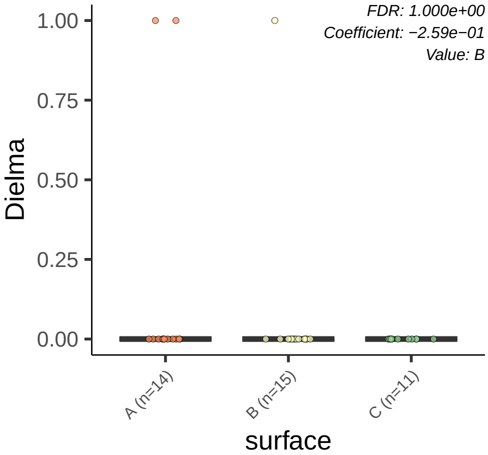

FDR: 1.000e+00
Coefficient: −2.59e−01
Value: B
Dielma
1.00
0.75
0.50
0.25
0.00
A (n=14)
B (n=15)
C (n=11)
surface

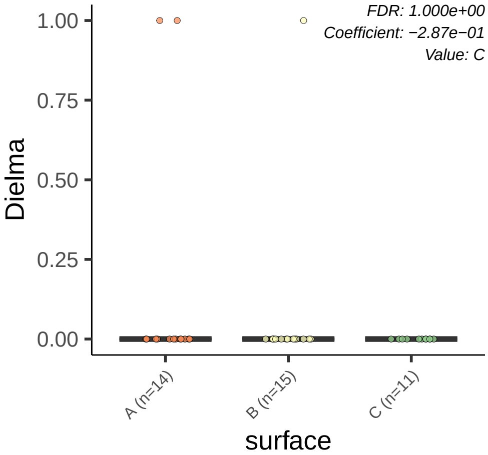

FDR: 1.000e+00
Coefficient: −2.87e−01
Value: C
Dielma
1.00
0.75
0.50
0.25
0.00
A (n=14)
B (n=15)
C (n=11)
surface

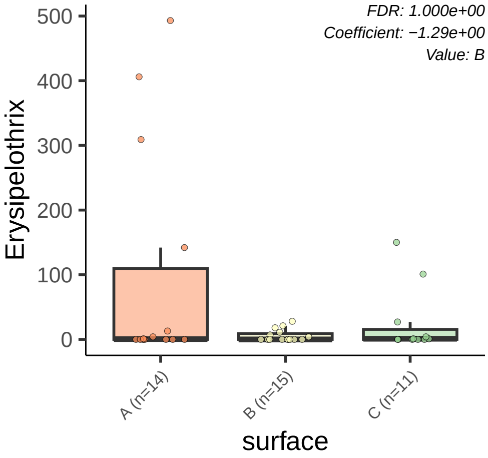

FDR: 1.000e+00
Coefficient: −1.29e+00
Value: B
Erysipelothrix
500
400
300
200
100
0
A (n=14)
B (n=15)
C (n=11)
surface

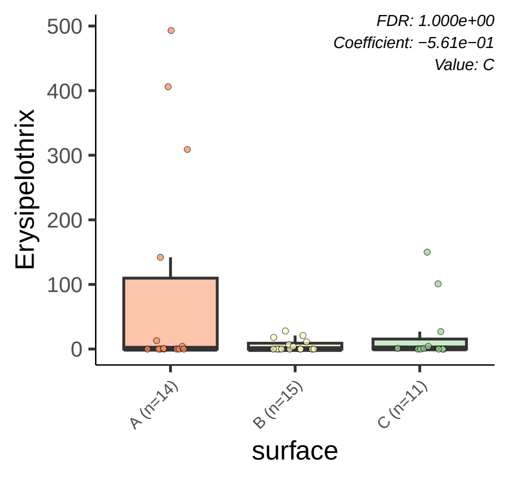

FDR: 1.000e+00
Coefficient: −5.61e−01
Value: C
Erysipelothrix
500
400
300
200
100
0
A (n=14)
B (n=15)
C (n=11)
surface

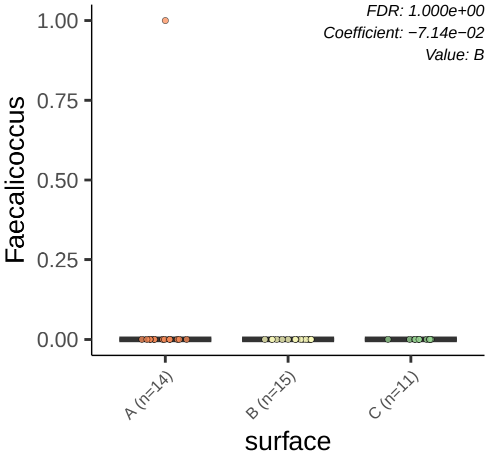
FDR: 1.000e+00
Coefficient: −7.14e−02
Value: B
Faecalicoccus
1.00
0.75
0.50
0.25
0.00
A (n=14)
B (n=15)
C (n=11)
surface

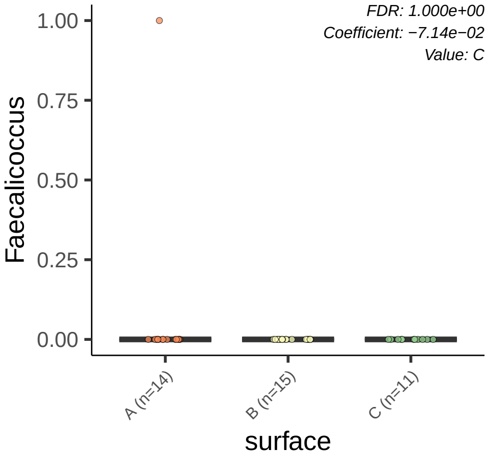

FDR: 1.000e+00
Coefficient: −7.14e−02
Value: C
Faecalicoccus
1.00
0.75
0.50
0.25
0.00
A (n=14)
B (n=15)
C (n=11)
surface

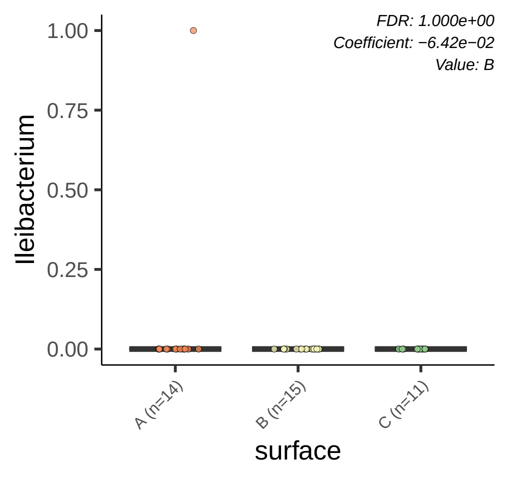

FDR: 1.000e+00
Coefficient: −6.42e−02
Value: B
Ileibacterium
1.00
0.75
0.50
0.25
0.00
A (n=14)
B (n=15)
C (n=11)
surface

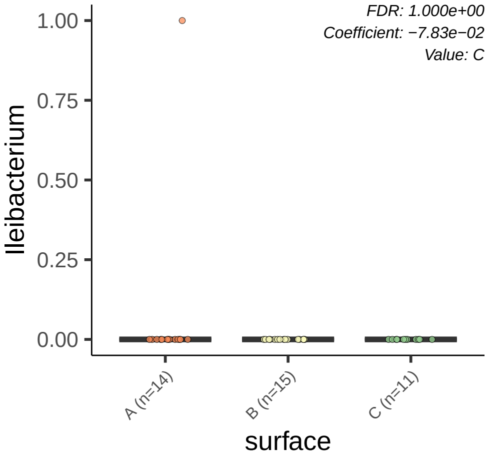

FDR: 1.000e+00
Coefficient: −7.83e−02
Value: C
Ileibacterium
1.00
0.75
0.50
0.25
0.00
A (n=14)
B (n=15)
C (n=11)
surface

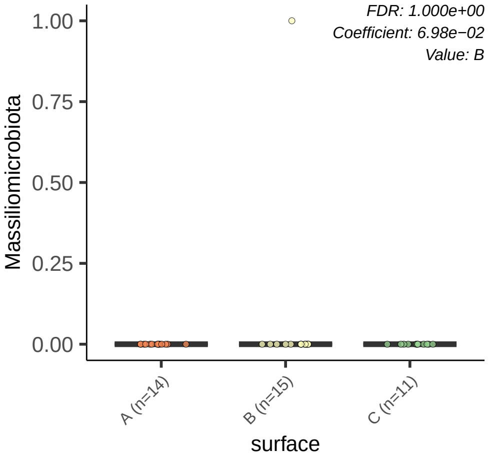

FDR: 1.000e+00
Coefficient: 6.98e−02
Value: B
Massiliomicrobiota
1.00
0.75
0.50
0.25
0.00
A (n=14)
B (n=15)
C (n=11)
surface

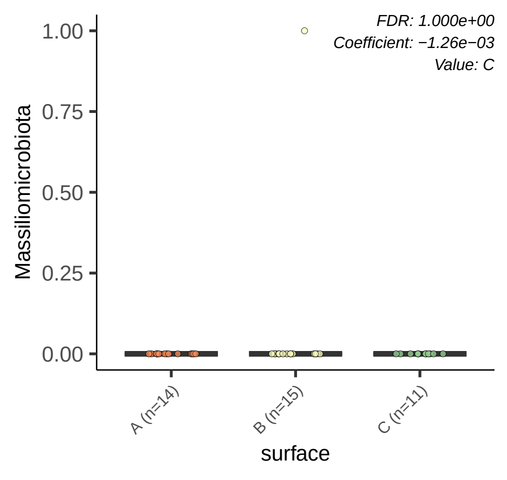
FDR: 1.000e+00
Coefficient: −1.26e−03
Value: C
Massiliomicrobiota
1.00
0.75
0.50
0.25
0.00
A (n=14)
B (n=15)
C (n=11)
surface

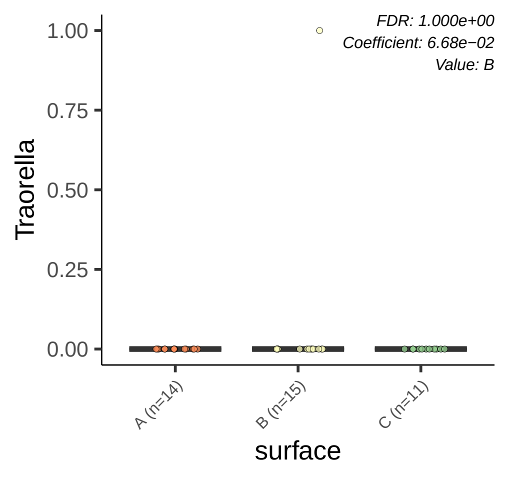
FDR: 1.000e+00
Coefficient: 6.68e−02
Value: B
Traorella
1.00
0.75
0.50
0.25
0.00
A (n=14)
B (n=15)
C (n=11)
surface

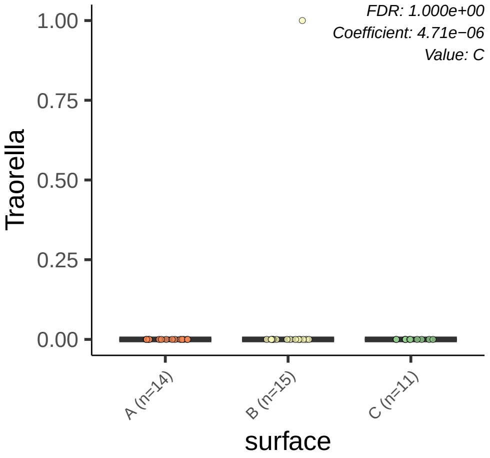
FDR: 1.000e+00
Coefficient: 4.71e−06
Value: C
Traorella
1.00
0.75
0.50
0.25
0.00
A (n=14)
B (n=15)
C (n=11)
surface

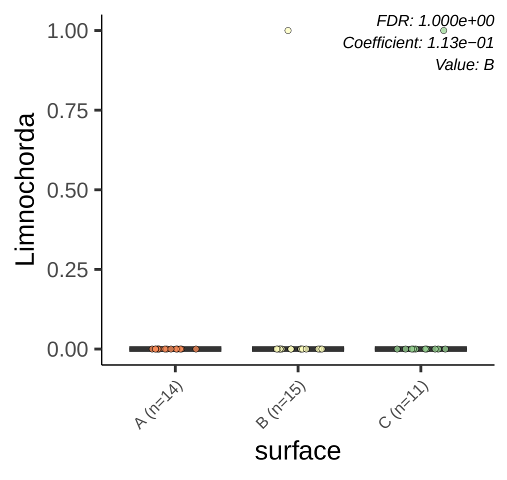

FDR: 1.000e+00
Coefficient: 1.13e−01
Value: B
Limnochorda
1.00
0.75
0.50
0.25
0.00
A (n=14)
B (n=15)
C (n=11)
surface

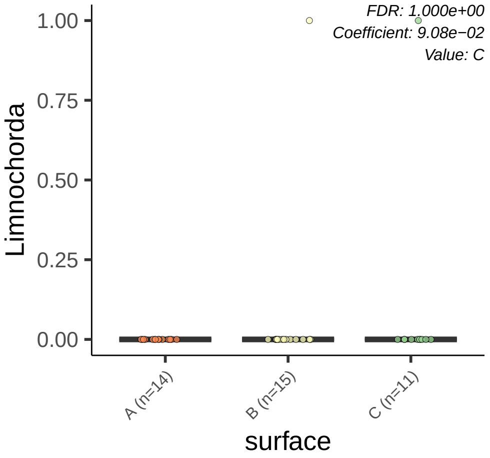
FDR: 1.000e+00
Coefficient: 9.08e−02
Value: C
Limnochorda
1.00
0.75
0.50
0.25
0.00
A (n=14)
B (n=15)
C (n=11)
surface

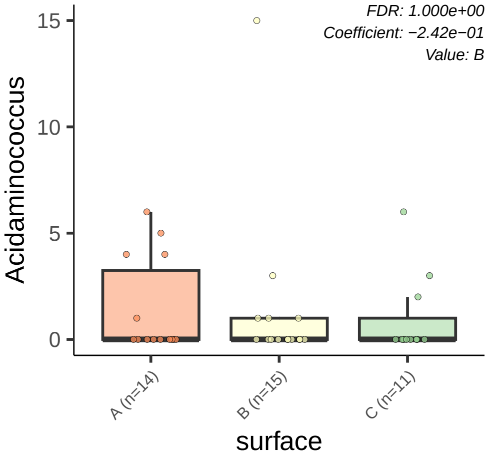
FDR: 1.000e+00
Coefficient: −2.42e−01
Value: B
Acidaminococcus
15
10
5
0
A (n=14)
B (n=15)
C (n=11)
surface

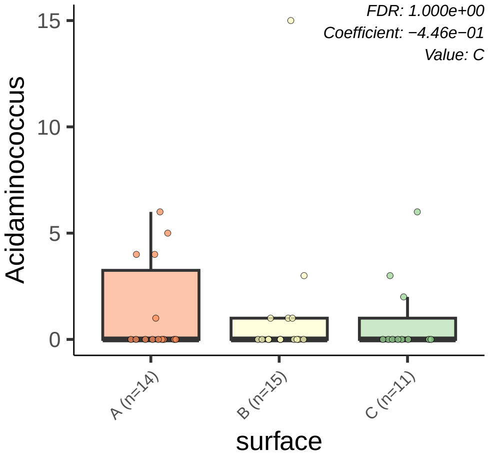
FDR: 1.000e+00
Coefficient: −4.46e−01
Value: C
Acidaminococcus
15
10
5
0
A (n=14)
B (n=15)
C (n=11)
surface

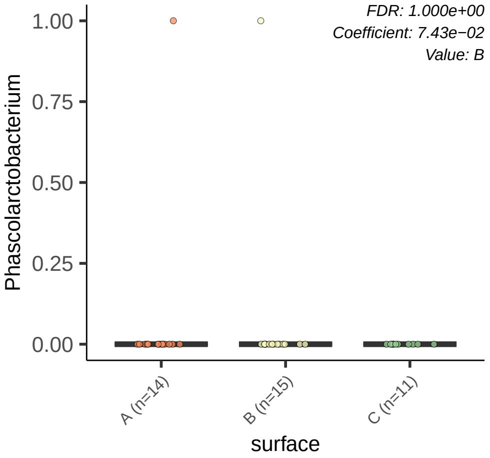
FDR: 1.000e+00
Coefficient: 7.43e−02
Value: B
Phascolarctobacterium
1.00
0.75
0.50
0.25
0.00
A (n=14)
B (n=15)
C (n=11)
surface

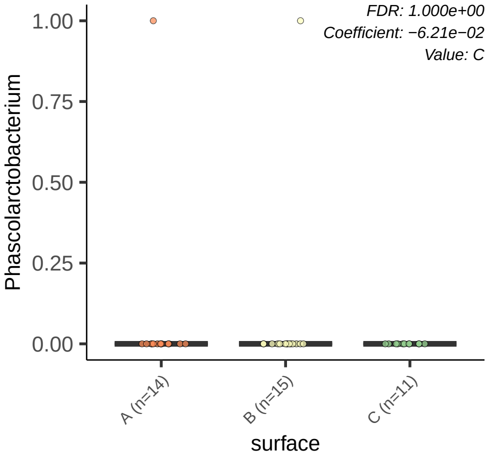

FDR: 1.000e+00
Coefficient: −6.21e−02
Value: C
Phascolarctobacterium
1.00
0.75
0.50
0.25
0.00
A (n=14)
B (n=15)
C (n=11)
surface

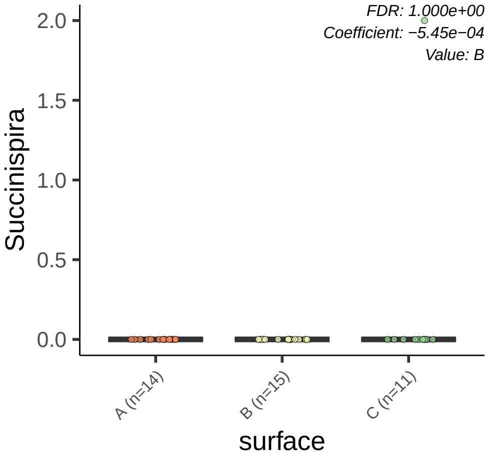
FDR: 1.000e+00
Coefficient: −5.45e−04
Value: B
Succinispira
2.0
1.5
1.0
0.5
0.0
A (n=14)
B (n=15)
C (n=11)
surface

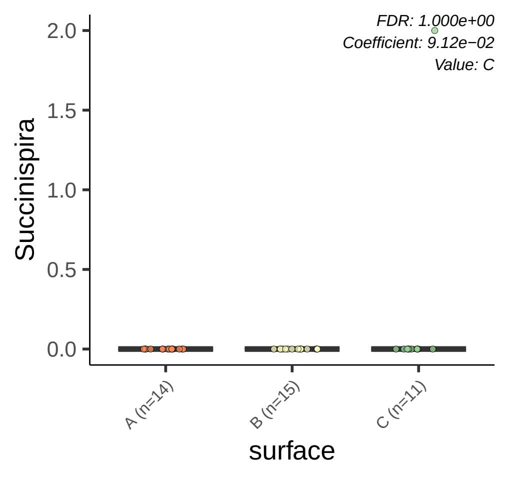

FDR: 1.000e+00
Coefficient: 9.12e−02
Value: C
Succinispira
2.0
1.5
1.0
0.5
0.0
A (n=14)
B (n=15)
C (n=11)
surface

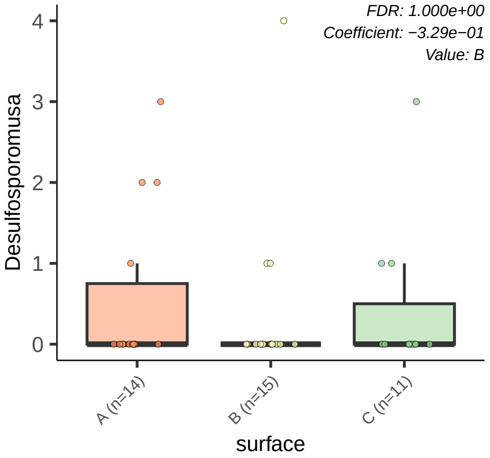
FDR: 1.000e+00
Coefficient: −3.29e−01
Value: B
Desulfosporomusa
0
1
2
3
4
A (n=14)
B (n=15)
C (n=11)
surface

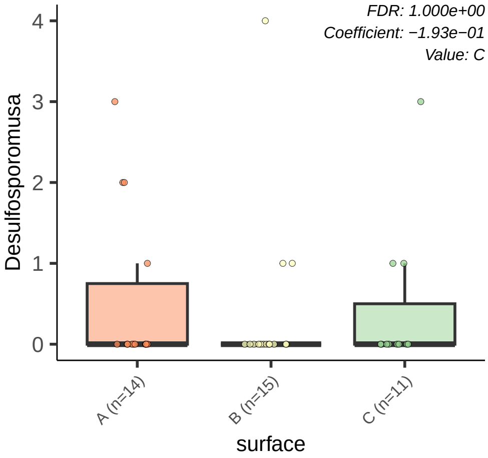

FDR: 1.000e+00
Coefficient: −1.93e−01
Value: C
Desulfosporomusa
4
3
2
1
0
A (n=14)
B (n=15)
C (n=11)
surface

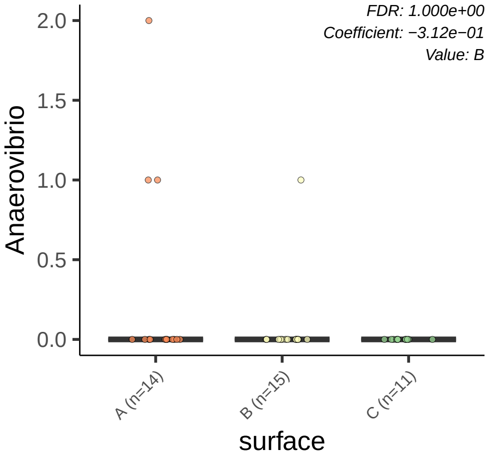
FDR: 1.000e+00
Coefficient: −3.12e−01
Value: B
Anaerovibrio
2.0
1.5
1.0
0.5
0.0
A (n=14)
B (n=15)
C (n=11)
surface

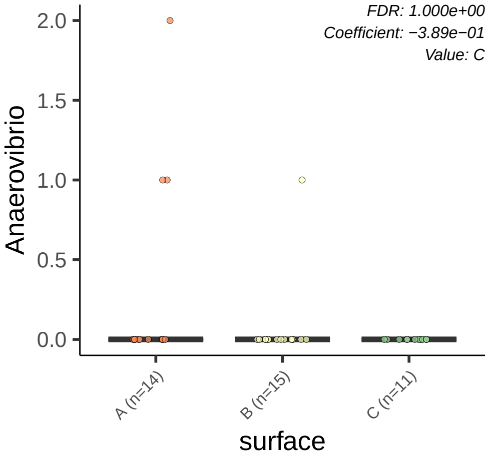
FDR: 1.000e+00
Coefficient: −3.89e−01
Value: C
Anaerovibrio
2.0
1.5
1.0
0.5
0.0
A (n=14)
B (n=15)
C (n=11)
surface

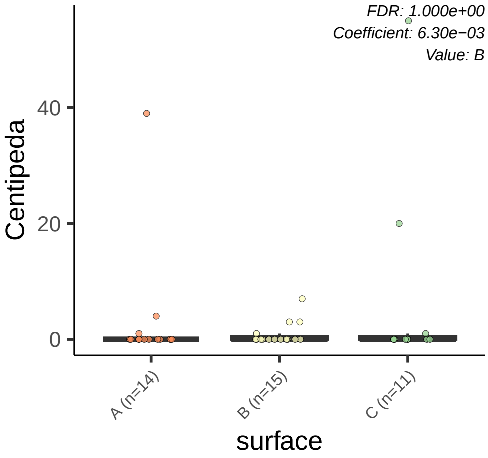
FDR: 1.000e+00
Coefficient: 6.30e−03
Value: B
Centipeda
40
20
0
A (n=14)
B (n=15)
C (n=11)
surface

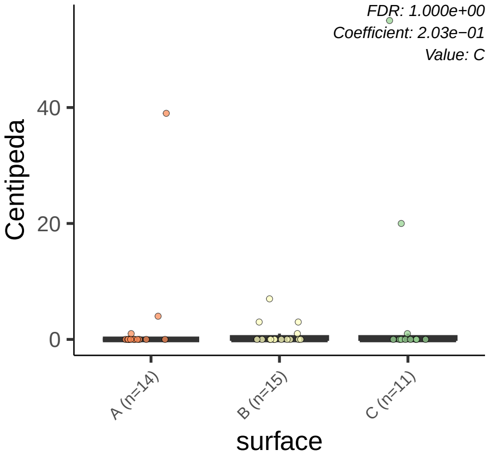

FDR: 1.000e+00
Coefficient: 2.03e−01
Value: C
Centipeda
40
20
0
A (n=14)
B (n=15)
C (n=11)
surface

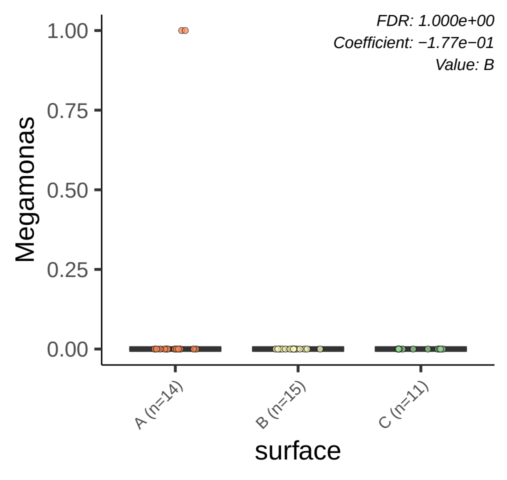
FDR: 1.000e+00
Coefficient: −1.77e−01
Value: B
Megamonas
1.00
0.75
0.50
0.25
0.00
A (n=14)
B (n=15)
C (n=11)
surface

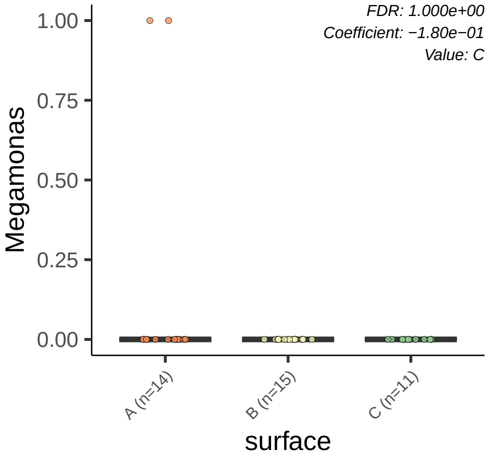
FDR: 1.000e+00
Coefficient: −1.80e−01
Value: C
Megamonas
1.00
0.75
0.50
0.25
0.00
A (n=14)
B (n=15)
C (n=11)
surface

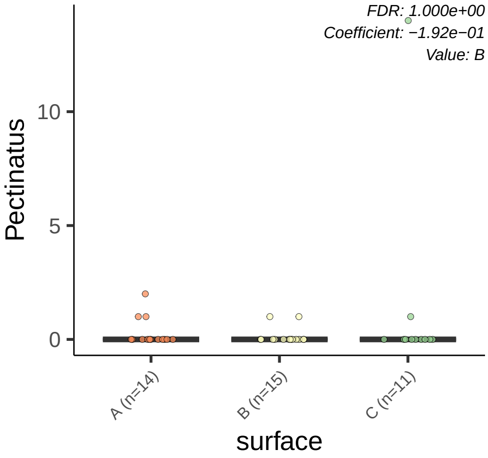

FDR: 1.000e+00
Coefficient: −1.92e−01
Value: B
Pectinatus
10
5
0
A (n=14)
B (n=15)
C (n=11)
surface

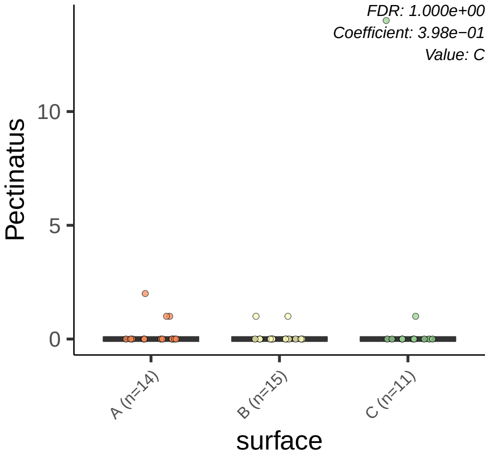

FDR: 1.000e+00
Coefficient: 3.98e−01
Value: C
Pectinatus
10
5
0
A (n=14)
B (n=15)
C (n=11)
surface

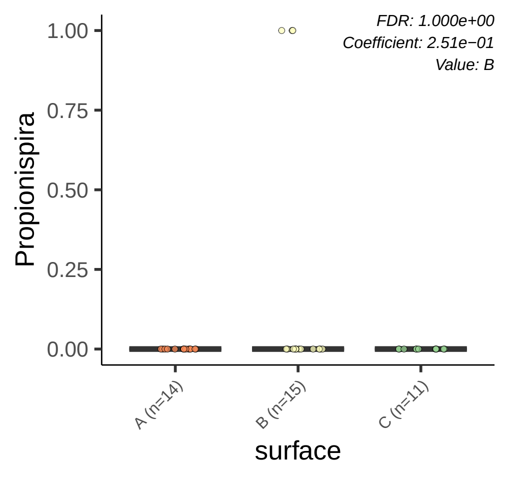

FDR: 1.000e+00
Coefficient: 2.51e−01
Value: B
Propionispira
1.00
0.75
0.50
0.25
0.00
A (n=14)
B (n=15)
C (n=11)
surface

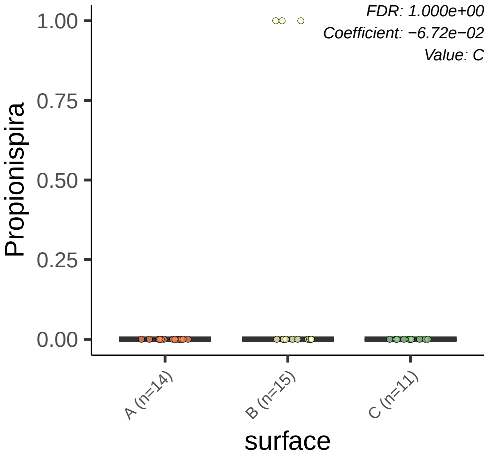

FDR: 1.000e+00
Coefficient: −6.72e−02
Value: C
Propionispira
1.00
0.75
0.50
0.25
0.00
A (n=14)
B (n=15)
C (n=11)
surface

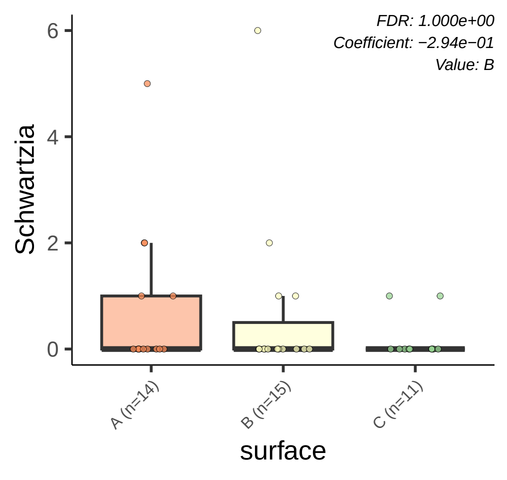
FDR: 1.000e+00
Coefficient: −2.94e−01
Value: B
Schwartzia
0
2
4
6
A (n=14)
B (n=15)
C (n=11)
surface

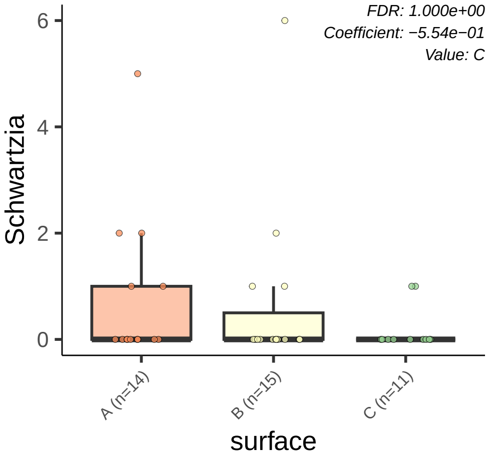

FDR: 1.000e+00
Coefficient: −5.54e−01
Value: C
Schwartzia
0
2
4
6
A (n=14)
B (n=15)
C (n=11)
surface

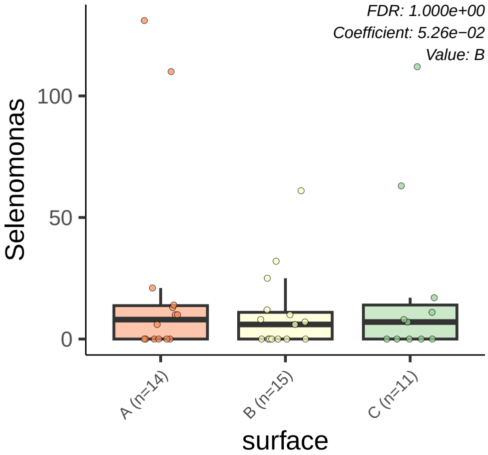
FDR: 1.000e+00
Coefficient: 5.26e−02
Value: B
Selenomonas
100
50
0
A (n=14)
B (n=15)
C (n=11)
surface

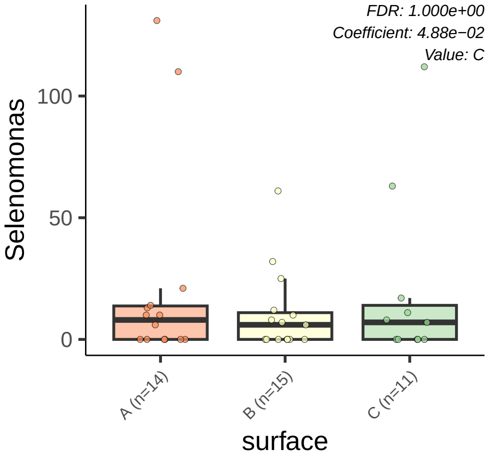
FDR: 1.000e+00
Coefficient: 4.88e−02
Value: C
Selenomonas
100
50
0
A (n=14)
B (n=15)
C (n=11)
surface

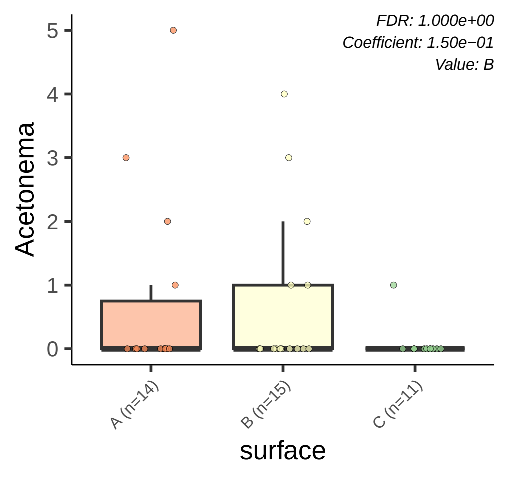

FDR: 1.000e+00
Coefficient: 1.50e−01
Value: B
Acetonema
0
1
2
3
4
5
A (n=14)
B (n=15)
C (n=11)
surface

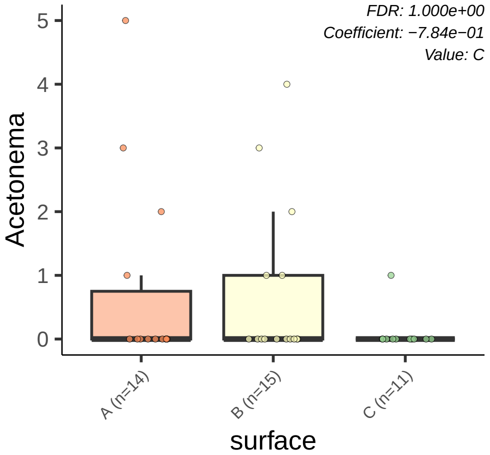

FDR: 1.000e+00
Coefficient: −7.84e−01
Value: C
Acetonema
0
1
2
3
4
5
A (n=14)
B (n=15)
C (n=11)
surface

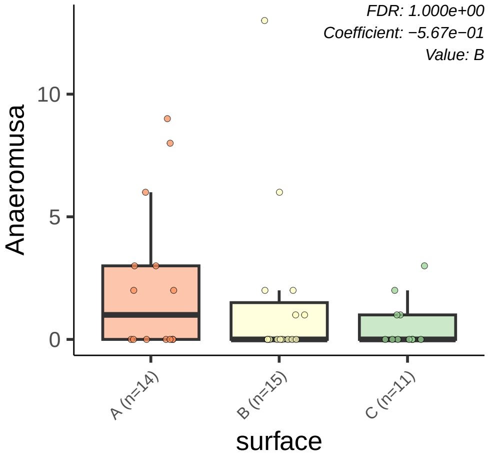
FDR: 1.000e+00
Coefficient: −5.67e−01
Value: B
Anaeromusa
10
5
0
A (n=14)
B (n=15)
C (n=11)
surface

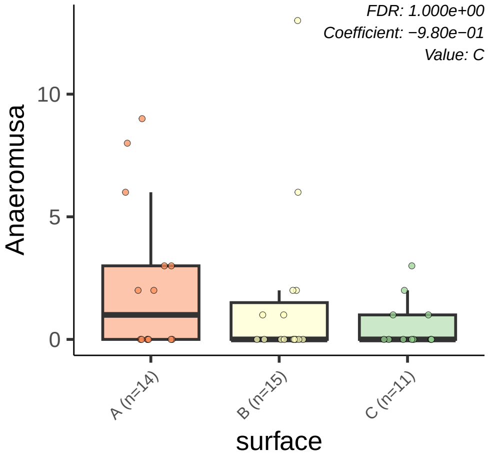

FDR: 1.000e+00
Coefficient: −9.80e−01
Value: C
Anaeromusa
10
5
0
A (n=14)
B (n=15)
C (n=11)
surface

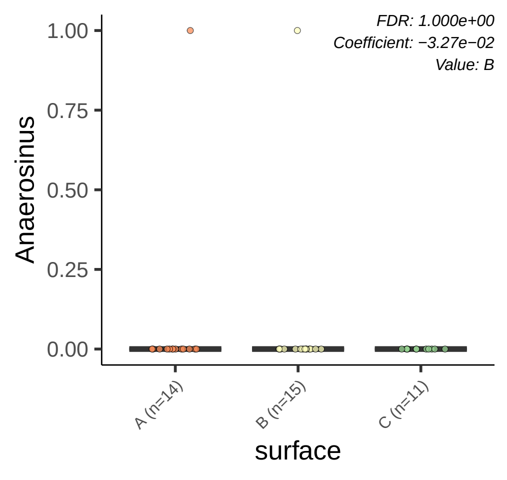

FDR: 1.000e+00
Coefficient: −3.27e−02
Value: B
Anaerosinus
1.00
0.75
0.50
0.25
0.00
A (n=14)
B (n=15)
C (n=11)
surface

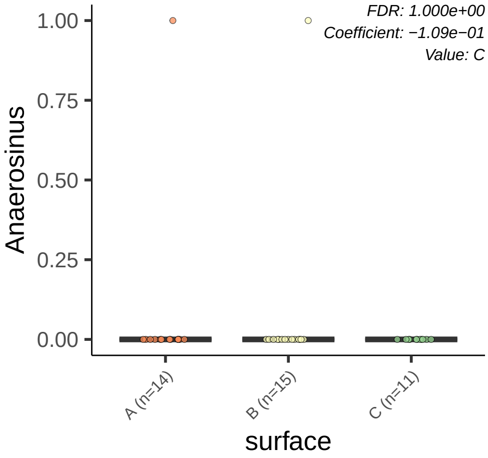

FDR: 1.000e+00
Coefficient: −1.09e−01
Value: C
Anaerosinus
1.00
0.75
0.50
0.25
0.00
A (n=14)
B (n=15)
C (n=11)
surface

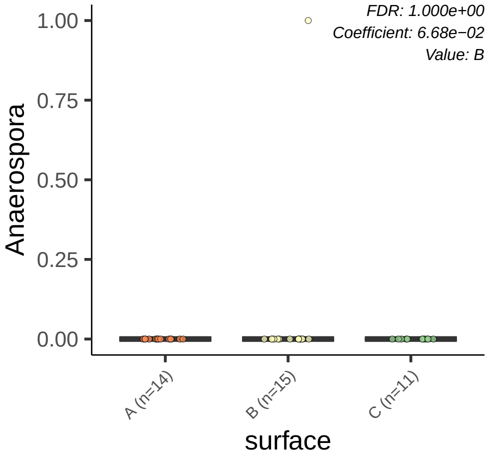
FDR: 1.000e+00
Coefficient: 6.68e−02
Value: B
1.00
0.75
0.50
0.25
0.00
Anaerospora
A (n=14)
B (n=15)
C (n=11)
surface

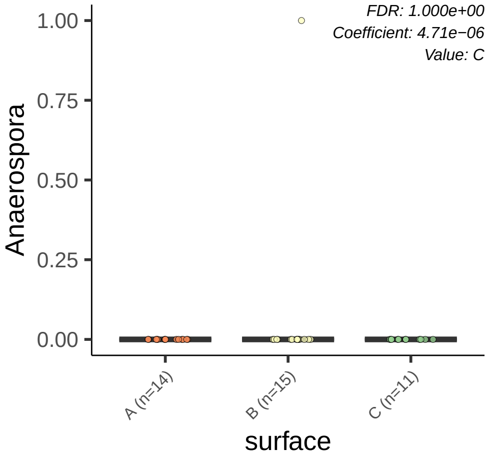
FDR: 1.000e+00
Coefficient: 4.71e−06
Value: C
Anaerospora
1.00
0.75
0.50
0.25
0.00
A (n=14)
B (n=15)
C (n=11)
surface

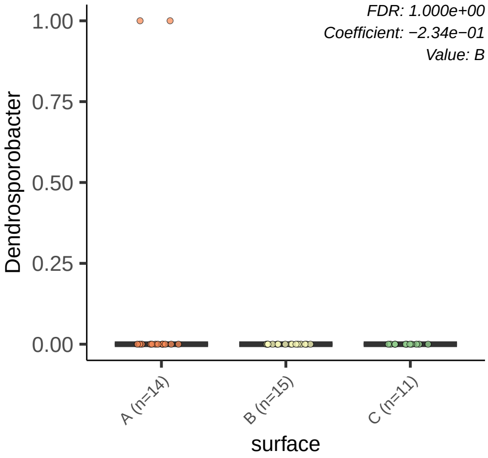
FDR: 1.000e+00
Coefficient: −2.34e−01
Value: B
Dendrosporobacter
1.00
0.75
0.50
0.25
0.00
A (n=14)
B (n=15)
C (n=11)
surface

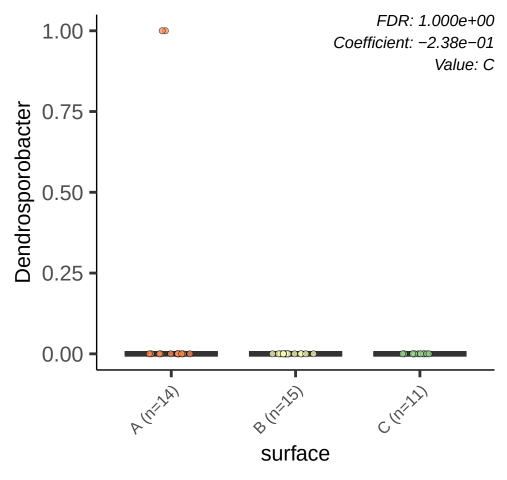
FDR: 1.000e+00
Coefficient: −2.38e−01
Value: C
Dendrosporobacter
1.00
0.75
0.50
0.25
0.00
A (n=14)
B (n=15)
C (n=11)
surface

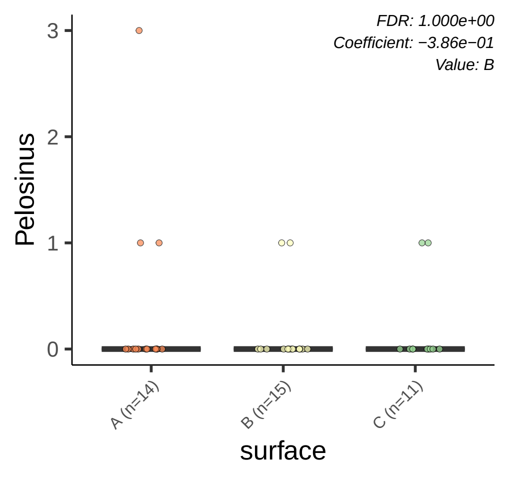
FDR: 1.000e+00
Coefficient: −3.86e−01
Value: B
Pelosinus
3
2
1
0
A (n=14)
B (n=15)
C (n=11)
surface

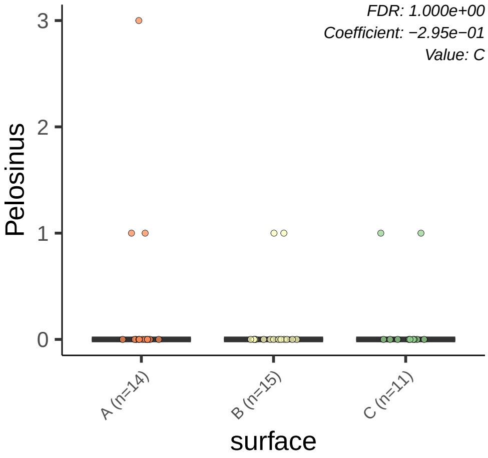

FDR: 1.000e+00
Coefficient: −2.95e−01
Value: C
Pelosinus
3
2
1
0
A (n=14)
B (n=15)
C (n=11)
surface

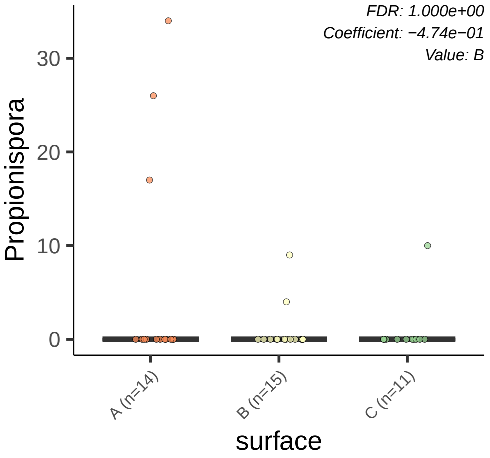
FDR: 1.000e+00
Coefficient: −4.74e−01
Value: B
Propionispora
30
20
10
0
A (n=14)
B (n=15)
C (n=11)
surface

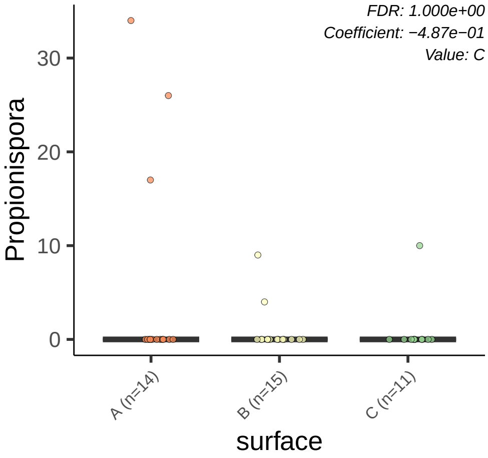
FDR: 1.000e+00
Coefficient: −4.87e−01
Value: C
Propionispora
0
10
20
30
A (n=14)
B (n=15)
C (n=11)
surface

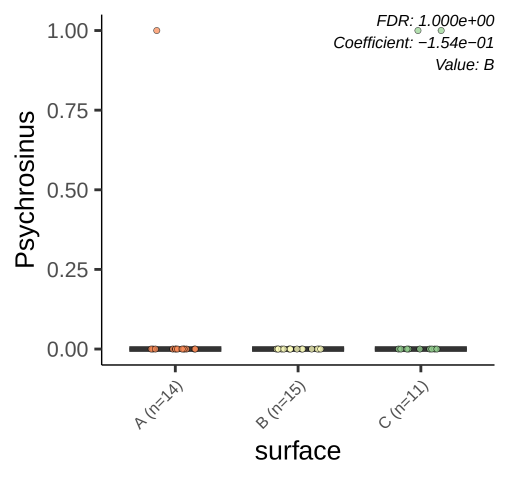
FDR: 1.000e+00
Coefficient: −1.54e−01
Value: B
Psychrosinus
1.00
0.75
0.50
0.25
0.00
A (n=14)
B (n=15)
C (n=11)
surface

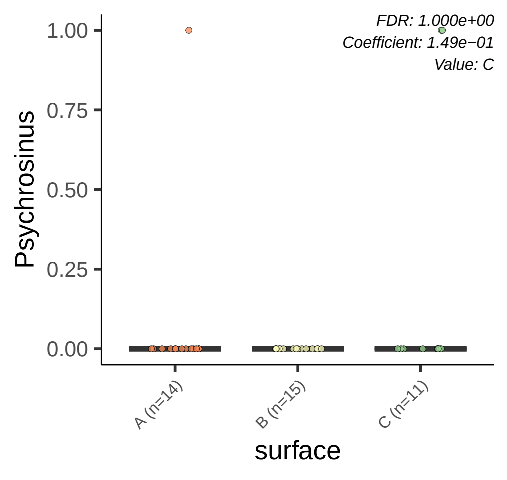
FDR: 1.000e+00
Coefficient: 1.49e−01
Value: C
Psychrosinus
1.00
0.75
0.50
0.25
0.00
A (n=14)
B (n=15)
C (n=11)
surface

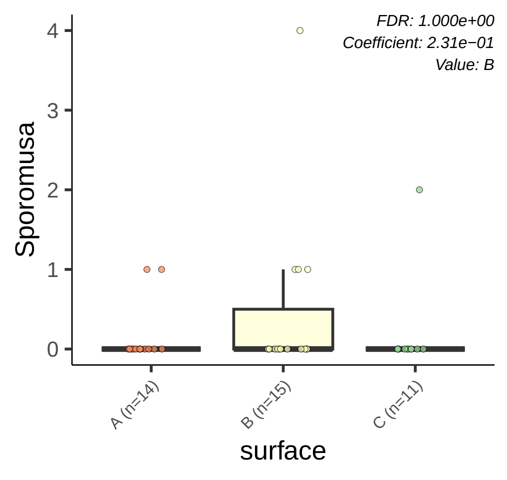

FDR: 1.000e+00
Coefficient: 2.31e−01
Value: B
Sporomusa
0
1
2
3
4
A (n=14)
B (n=15)
C (n=11)
surface

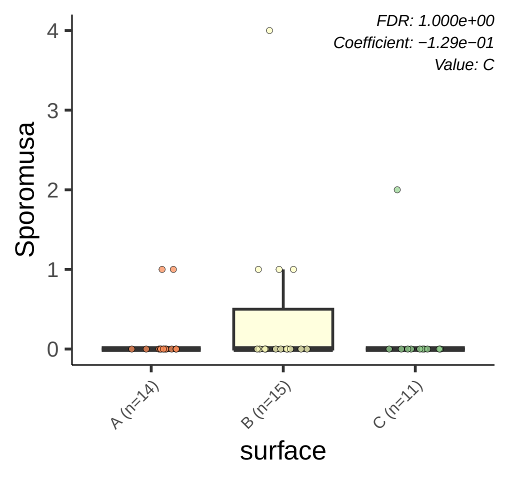

FDR: 1.000e+00
Coefficient: −1.29e−01
Value: C
Sporomusa
0
1
2
3
4
A (n=14)
B (n=15)
C (n=11)
surface

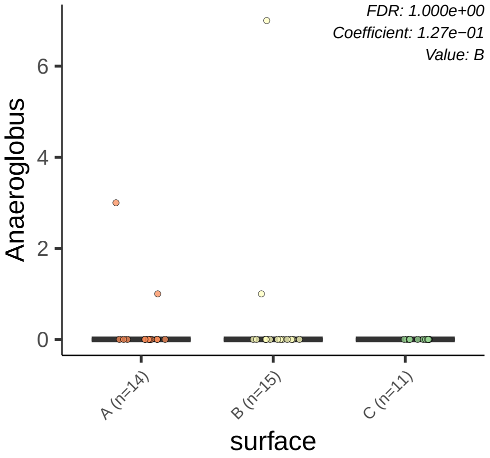
FDR: 1.000e+00
Coefficient: 1.27e−01
Value: B
Anaeroglobus
6
4
2
0
A (n=14)
B (n=15)
C (n=11)
surface

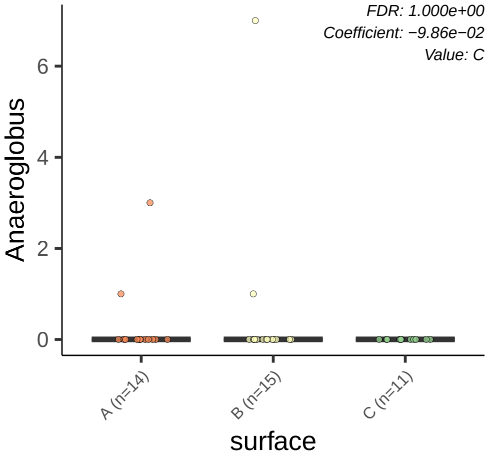
FDR: 1.000e+00
Coefficient: −9.86e−02
Value: C
Anaeroglobus
0
2
4
6
A (n=14)
B (n=15)
C (n=11)
surface

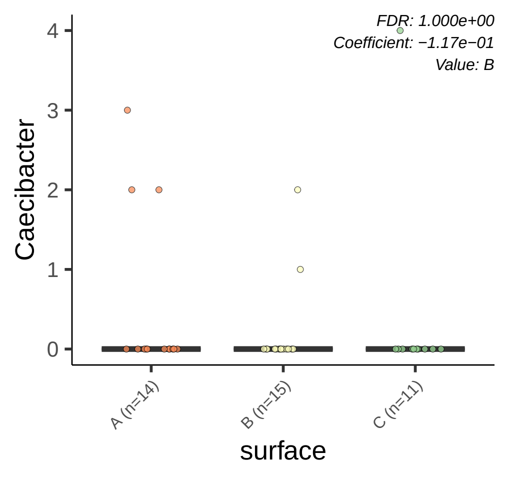
FDR: 1.000e+00
Coefficient: −1.17e−01
Value: B
Caecibacter
4
3
2
1
0
A (n=14)
B (n=15)
C (n=11)
surface

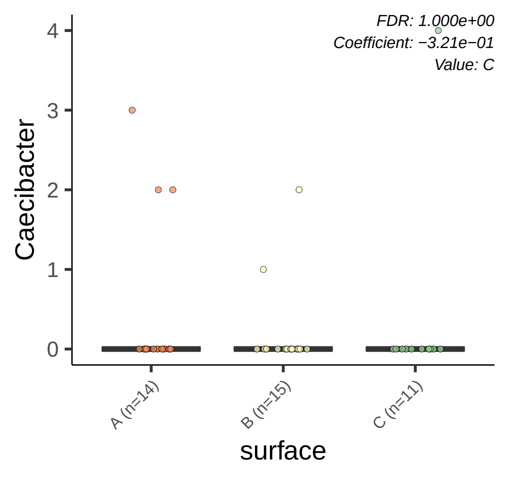
FDR: 1.000e+00
Coefficient: −3.21e−01
Value: C
Caecibacter
0
1
2
3
4
A (n=14)
B (n=15)
C (n=11)
surface

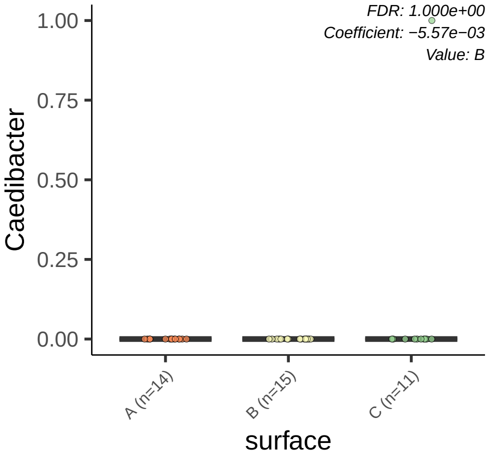

FDR: 1.000e+00
Coefficient: −5.57e−03
Value: B
Caedibacter
1.00
0.75
0.50
0.25
0.00
A (n=14)
B (n=15)
C (n=11)
surface

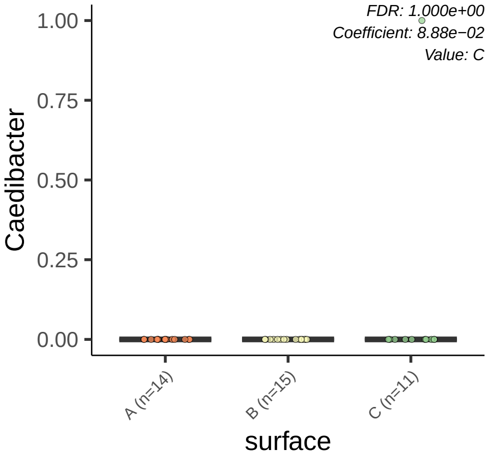

FDR: 1.000e+00
Coefficient: 8.88e−02
Value: C
Caedibacter
1.00
0.75
0.50
0.25
0.00
A (n=14)
B (n=15)
C (n=11)
surface

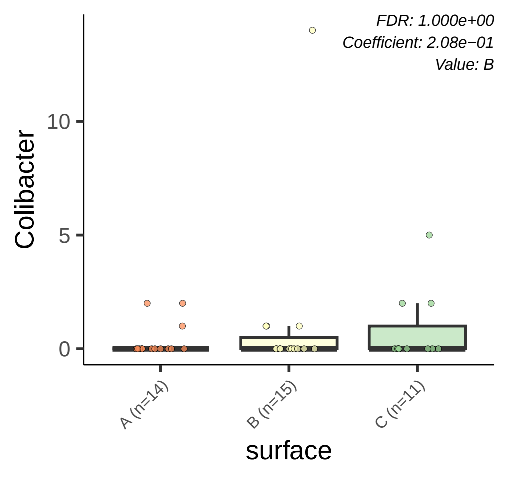
FDR: 1.000e+00
Coefficient: 2.08e−01
Value: B
Colibacter
10
5
0
A (n=14)
B (n=15)
C (n=11)
surface

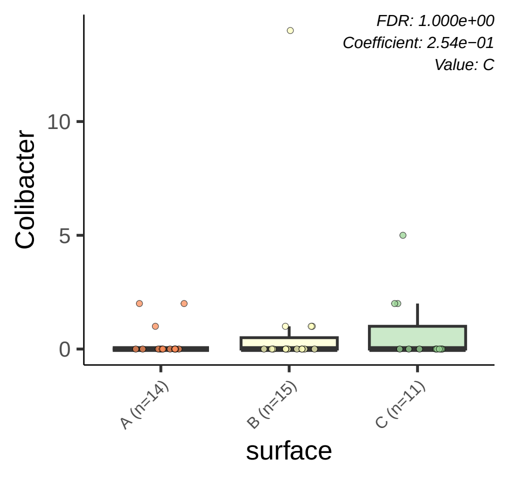
FDR: 1.000e+00
Coefficient: 2.54e−01
Value: C
Colibacter
10
5
0
A (n=14)
B (n=15)
C (n=11)
surface

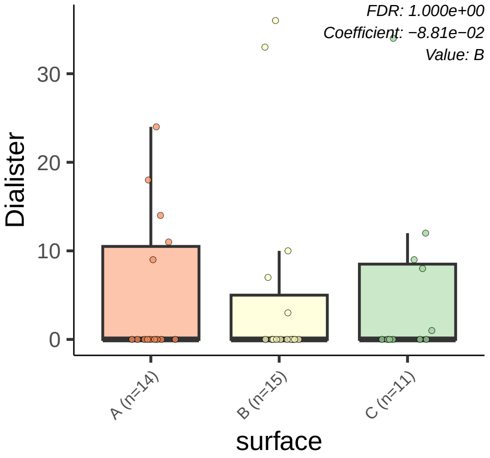

FDR: 1.000e+00
Coefficient: −8.81e−02
Value: B
Dialister
0
10
20
30
A (n=14)
B (n=15)
C (n=11)
surface

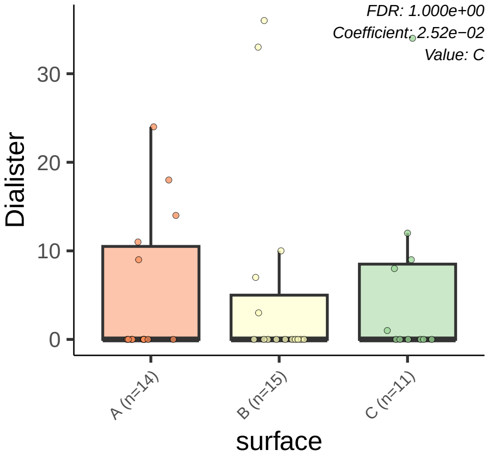
FDR: 1.000e+00
Coefficient: 2.52e−02
Value: C
Dialister
0
10
20
30
A (n=14)
B (n=15)
C (n=11)
surface

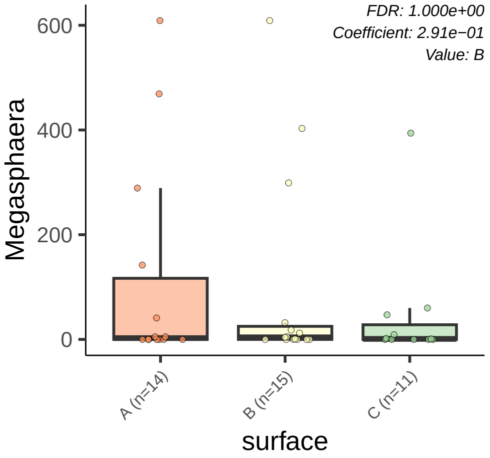

FDR: 1.000e+00
Coefficient: 2.91e−01
Value: B
Megasphaera
600
400
200
0
A (n=14)
B (n=15)
C (n=11)
surface

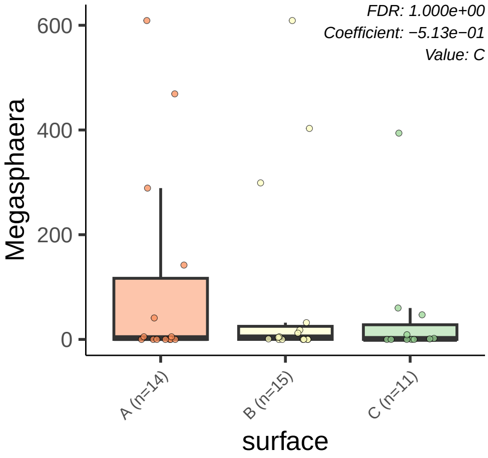
FDR: 1.000e+00
Coefficient: −5.13e−01
Value: C
Megasphaera
600
400
200
0
A (n=14)
B (n=15)
C (n=11)
surface

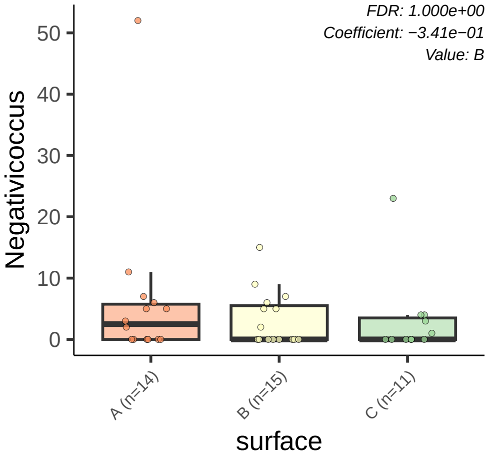

FDR: 1.000e+00
Coefficient: −3.41e−01
Value: B
Negativicoccus
50
40
30
20
10
0
A (n=14)
B (n=15)
C (n=11)
surface

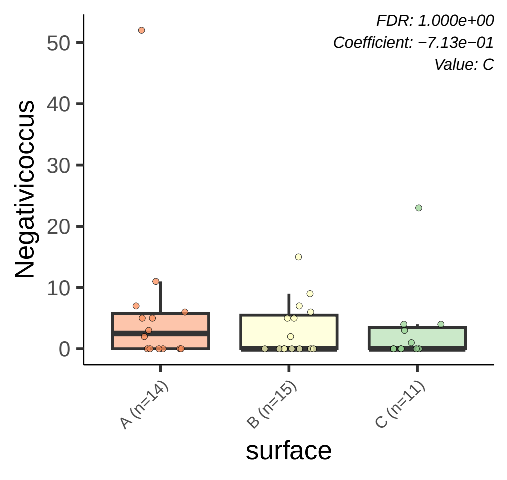

FDR: 1.000e+00
Coefficient: −7.13e−01
Value: C
Negativicoccus
0
10
20
30
40
50
A (n=14)
B (n=15)
C (n=11)
surface

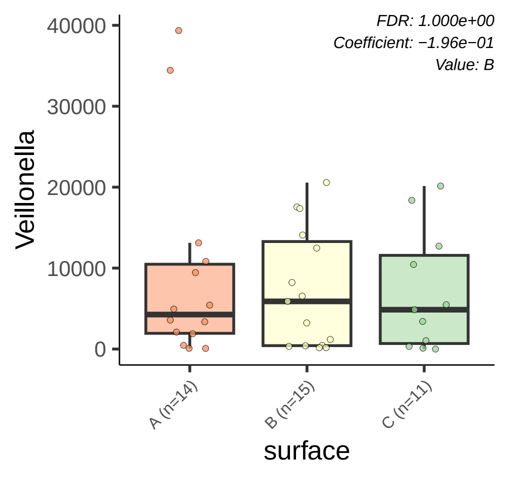

FDR: 1.000e+00
Coefficient: −1.96e−01
Value: B
Veillonella
40000
30000
20000
10000
0
A (n=14)
B (n=15)
C (n=11)
surface

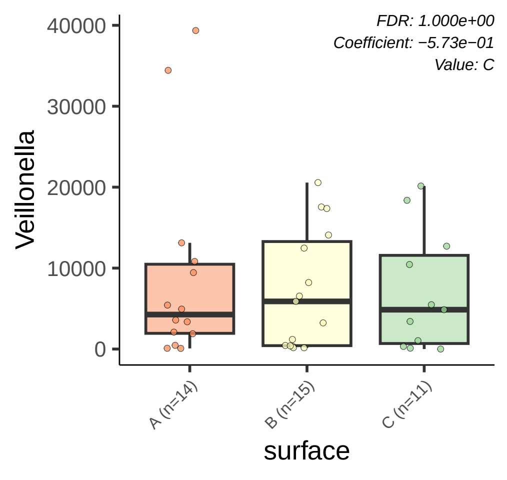

FDR: 1.000e+00
Coefficient: −5.73e−01
Value: C
Veillonella
40000
30000
20000
10000
0
A (n=14)
B (n=15)
C (n=11)
surface

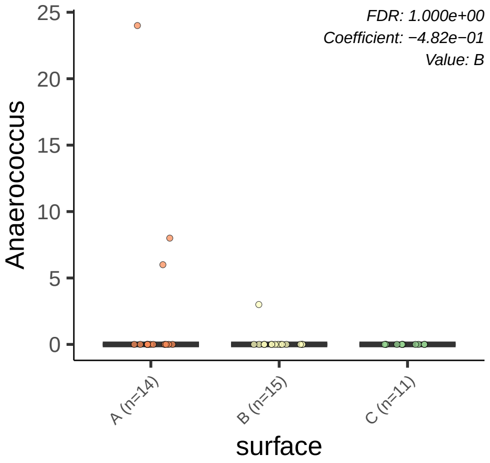

FDR: 1.000e+00
Coefficient: −4.82e−01
Value: B
Anaerococcus
25
20
15
10
5
0
A (n=14)
B (n=15)
C (n=11)
surface

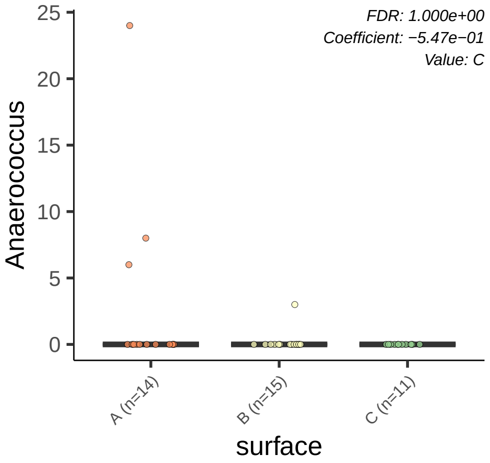
FDR: 1.000e+00
Coefficient: −5.47e−01
Value: C
Anaerococcus
25
20
15
10
5
0
A (n=14)
B (n=15)
C (n=11)
surface

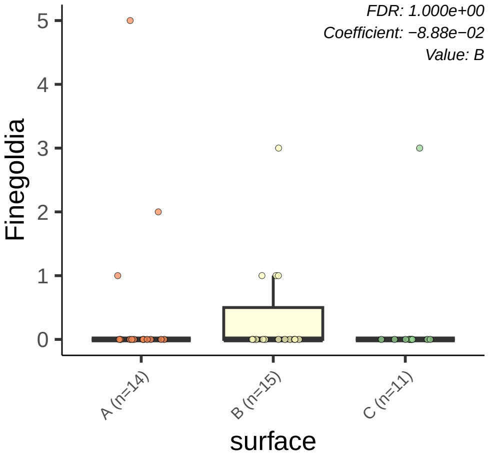
FDR: 1.000e+00
Coefficient: −8.88e−02
Value: B
Finegoldia
0
1
2
3
4
5
A (n=14)
B (n=15)
C (n=11)
surface

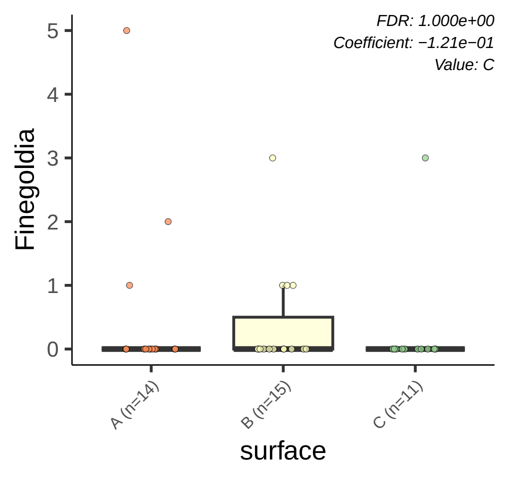
FDR: 1.000e+00
Coefficient: −1.21e−01
Value: C
Finegoldia
5
4
3
2
1
0
A (n=14)
B (n=15)
C (n=11)
surface

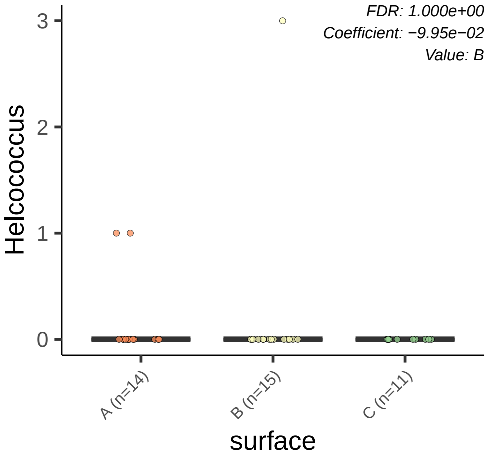

FDR: 1.000e+00
Coefficient: −9.95e−02
Value: B
Helcococcus
3
2
1
0
A (n=14)
B (n=15)
C (n=11)
surface

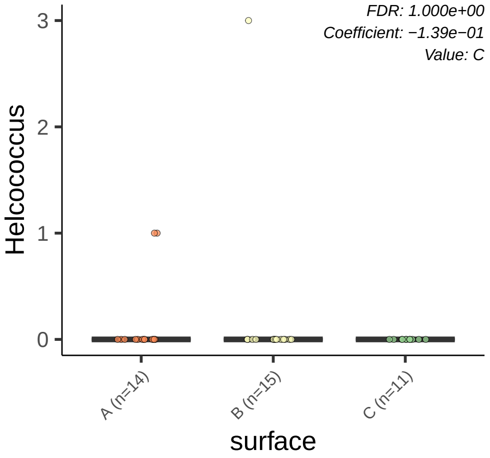
FDR: 1.000e+00
Coefficient: −1.39e−01
Value: C
Helcococcus
0
1
2
3
A (n=14)
B (n=15)
C (n=11)
surface

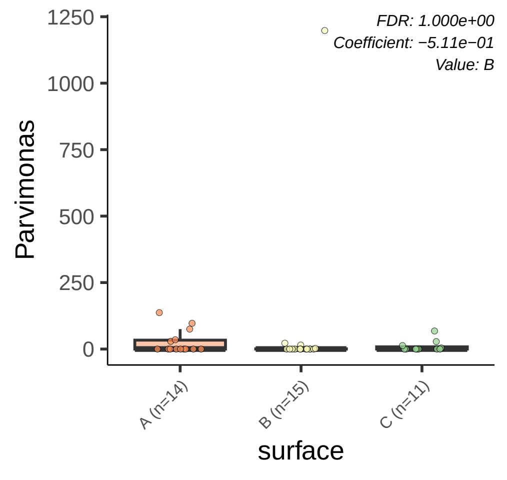
FDR: 1.000e+00
Coefficient: −5.11e−01
Value: B
Parvimonas
1250
1000
750
500
250
0
A (n=14)
B (n=15)
C (n=11)
surface

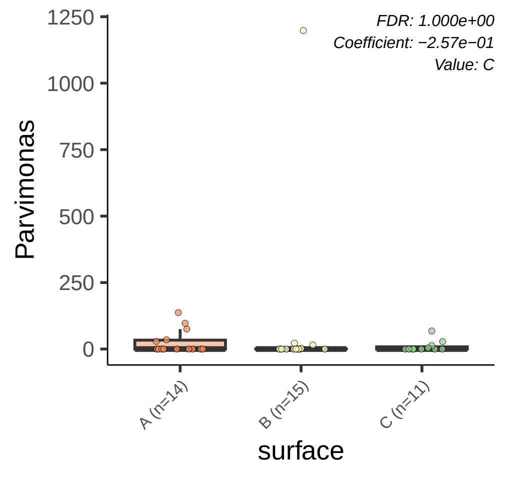

FDR: 1.000e+00
Coefficient: −2.57e−01
Value: C
Parvimonas
1250
1000
750
500
250
0
A (n=14)
B (n=15)
C (n=11)
surface

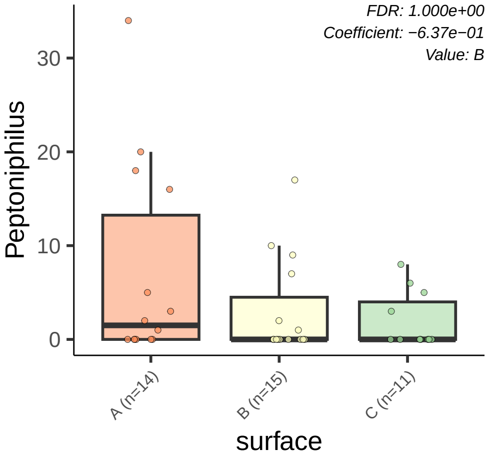
FDR: 1.000e+00
Coefficient: −6.37e−01
Value: B
Peptoniphilus
0
10
20
30
A (n=14)
B (n=15)
C (n=11)
surface

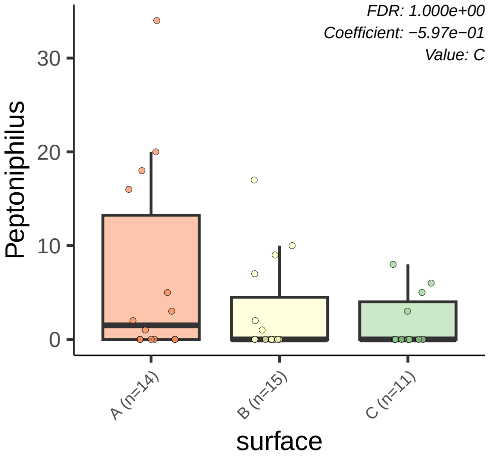

FDR: 1.000e+00
Coefficient: −5.97e−01
Value: C
Peptoniphilus
0
10
20
30
A (n=14)
B (n=15)
C (n=11)
surface

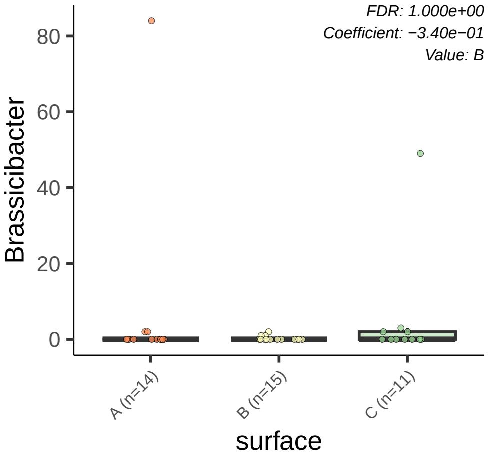

FDR: 1.000e+00
Coefficient: −3.40e−01
Value: B
Brassicibacter
80
60
40
20
0
A (n=14)
B (n=15)
C (n=11)
surface

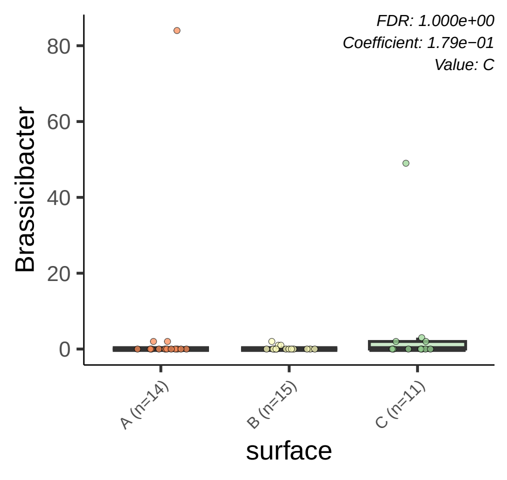
FDR: 1.000e+00
Coefficient: 1.79e−01
Value: C
Brassicibacter
80
60
40
20
0
A (n=14)
B (n=15)
C (n=11)
surface

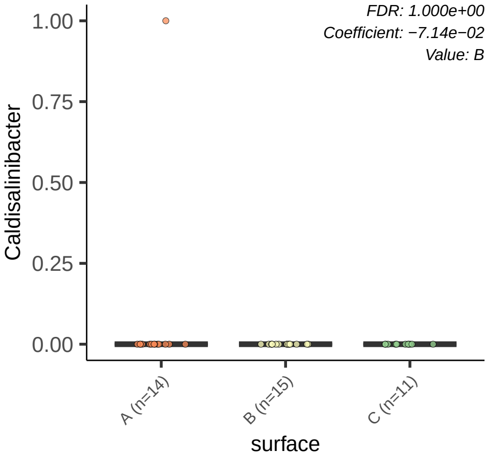

FDR: 1.000e+00
Coefficient: −7.14e−02
Value: B
Caldisalinibacter
1.00
0.75
0.50
0.25
0.00
A (n=14)
B (n=15)
C (n=11)
surface

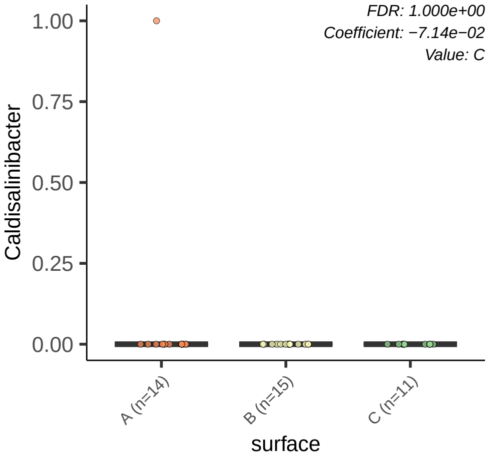
FDR: 1.000e+00
Coefficient: −7.14e−02
Value: C
Caldisalinibacter
1.00
0.75
0.50
0.25
0.00
A (n=14)
B (n=15)
C (n=11)
surface

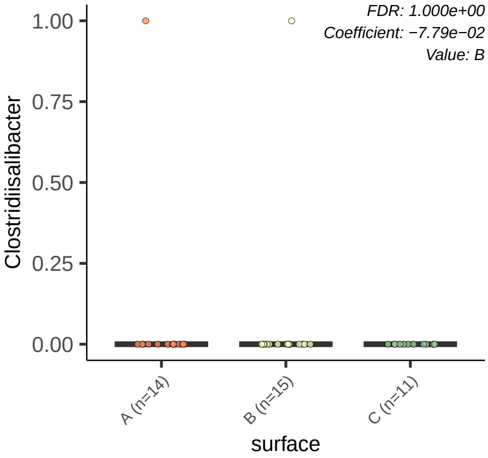

FDR: 1.000e+00
Coefficient: −7.79e−02
Value: B
Clostridiisalibacter
1.00
0.75
0.50
0.25
0.00
A (n=14)
B (n=15)
C (n=11)
surface

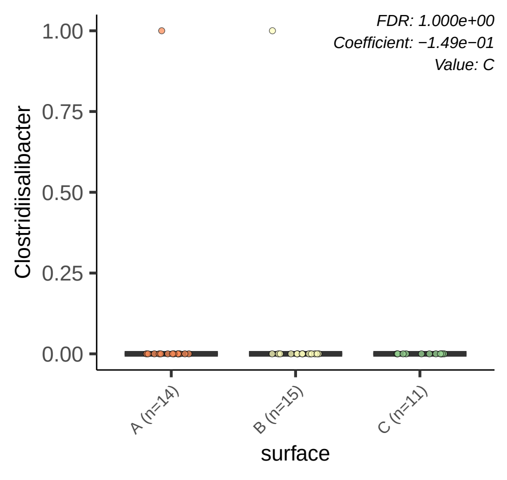

FDR: 1.000e+00
Coefficient: −1.49e−01
Value: C
Clostridiisalibacter
1.00
0.75
0.50
0.25
0.00
A (n=14)
B (n=15)
C (n=11)
surface

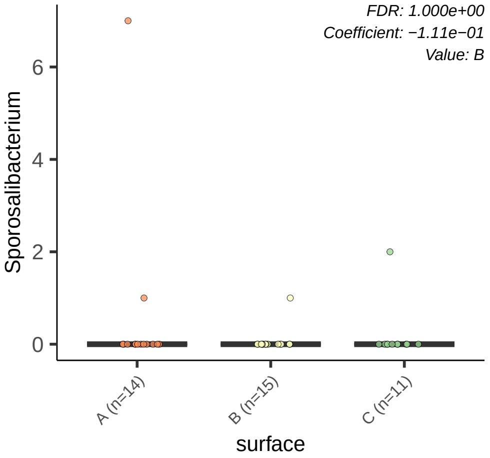
FDR: 1.000e+00
Coefficient: −1.11e−01
Value: B
Sporosalibacterium
0
2
4
6
A (n=14)
B (n=15)
C (n=11)
surface

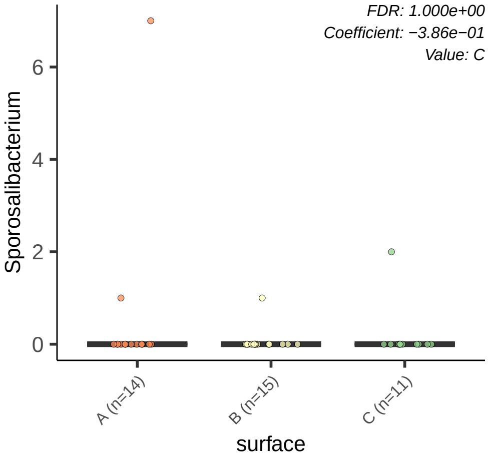

FDR: 1.000e+00
Coefficient: −3.86e−01
Value: C
Sporosalibacterium
0
2
4
6
A (n=14)
B (n=15)
C (n=11)
surface

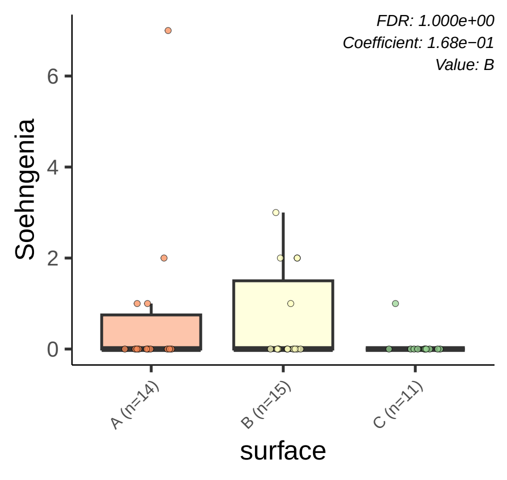
FDR: 1.000e+00
Coefficient: 1.68e−01
Value: B
Soehngenia
0
2
4
6
A (n=14)
B (n=15)
C (n=11)
surface

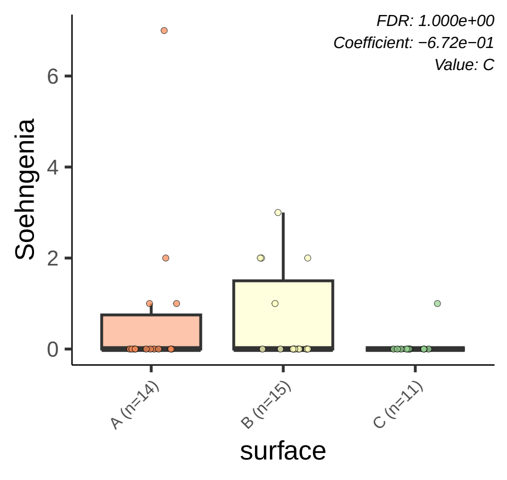
FDR: 1.000e+00
Coefficient: −6.72e−01
Value: C
Soehngenia
0
2
4
6
A (n=14)
B (n=15)
C (n=11)
surface

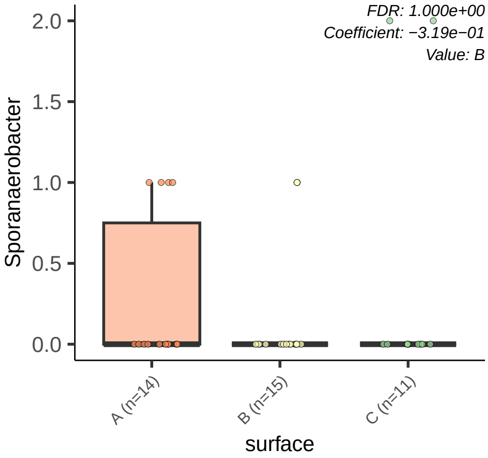
FDR: 1.000e+00
Coefficient: −3.19e−01
Value: B
Sporanaerobacter
2.0
1.5
1.0
0.5
0.0
A (n=14)
B (n=15)
C (n=11)
surface

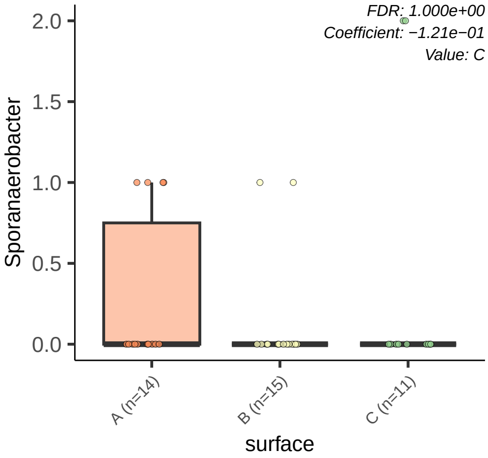

FDR: 1.000e+00
Coefficient: −1.21e−01
Value: C
Sporanaerobacter
2.0
1.5
1.0
0.5
0.0
A (n=14)
B (n=15)
C (n=11)
surface

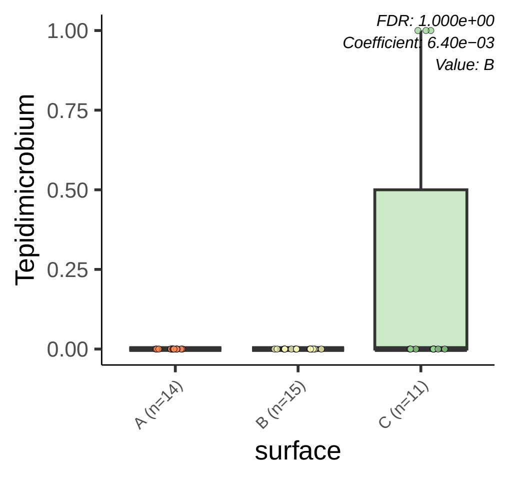
FDR: 1.000e+00
Coefficient: 6.40e−03
Value: B
Tepidimicrobium
1.00
0.75
0.50
0.25
0.00
A (n=14)
B (n=15)
C (n=11)
surface

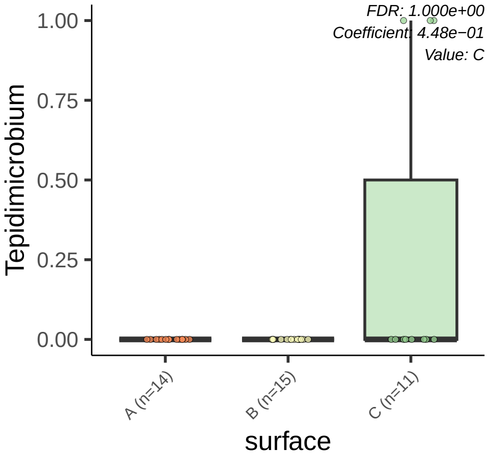

FDR: 1.000e+00
Coefficient: 4.48e−01
Value: C
Tepidimicrobium
1.00
0.75
0.50
0.25
0.00
A (n=14)
B (n=15)
C (n=11)
surface

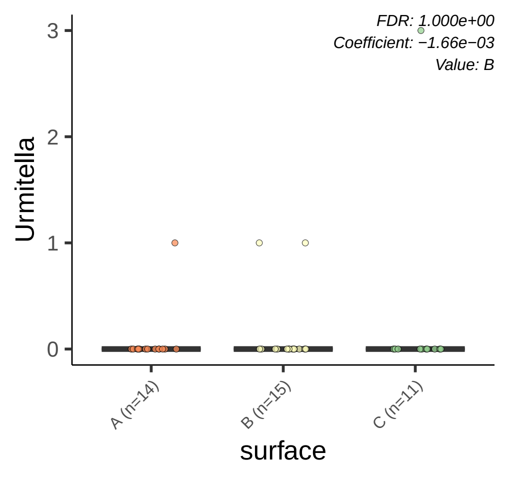
FDR: 1.000e+00
Coefficient: −1.66e−03
Value: B
Urmitella
3
2
1
0
A (n=14)
B (n=15)
C (n=11)
surface

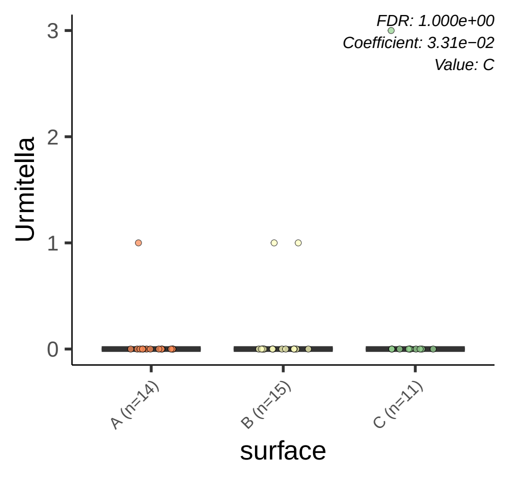

FDR: 1.000e+00
Coefficient: 3.31e−02
Value: C
Urmitella
3
2
1
0
A (n=14)
B (n=15)
C (n=11)
surface

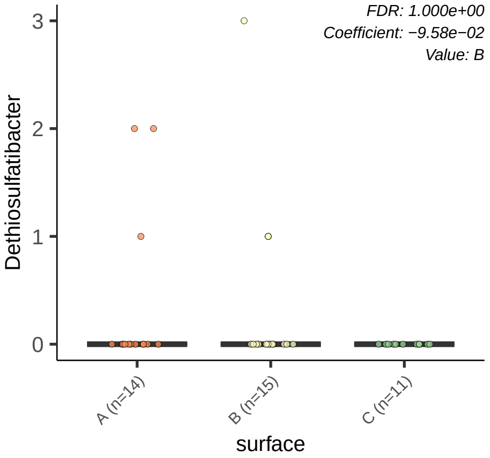
FDR: 1.000e+00
Coefficient: −9.58e−02
Value: B
Dethiosulfatibacter
0
1
2
3
A (n=14)
B (n=15)
C (n=11)
surface

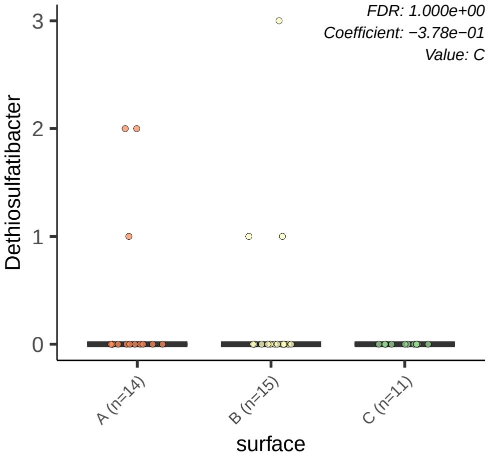

FDR: 1.000e+00
Coefficient: −3.78e−01
Value: C
Dethiosulfatibacter
3
2
1
0
A (n=14)
B (n=15)
C (n=11)
surface

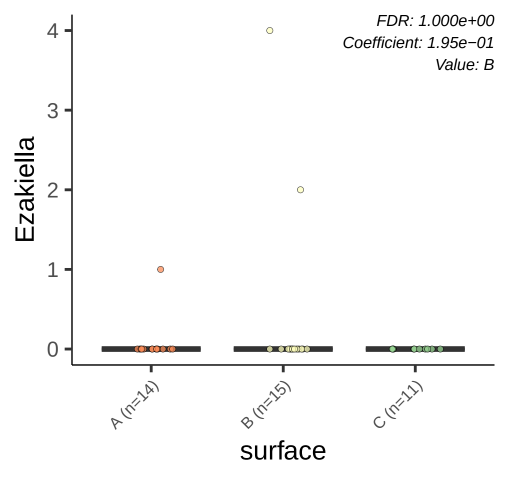
FDR: 1.000e+00
Coefficient: 1.95e−01
Value: B
Ezakiella
0
1
2
3
4
A (n=14)
B (n=15)
C (n=11)
surface

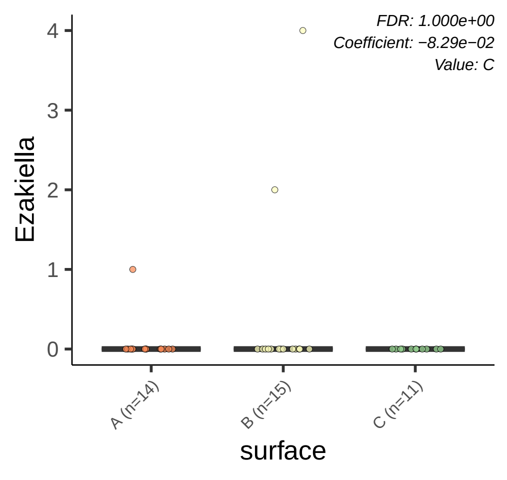
FDR: 1.000e+00
Coefficient: −8.29e−02
Value: C
Ezakiella
4
3
2
1
0
A (n=14)
B (n=15)
C (n=11)
surface

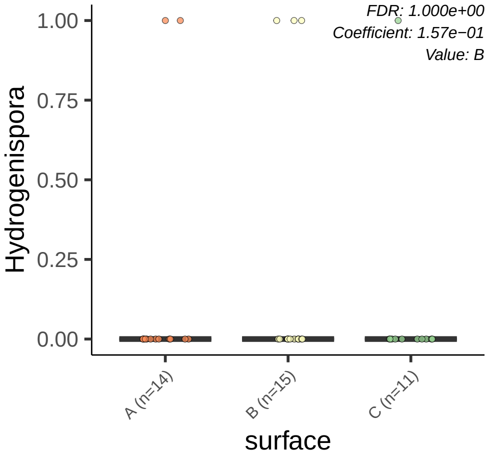

FDR: 1.000e+00
Coefficient: 1.57e−01
Value: B
Hydrogenispora
1.00
0.75
0.50
0.25
0.00
A (n=14)
B (n=15)
C (n=11)
surface

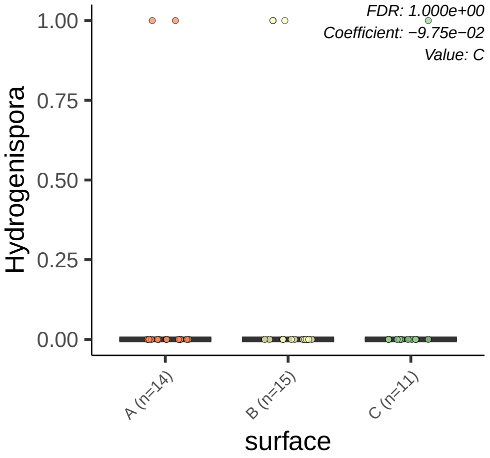
FDR: 1.000e+00
Coefficient: −9.75e−02
Value: C
Hydrogenispora
1.00
0.75
0.50
0.25
0.00
A (n=14)
B (n=15)
C (n=11)
surface

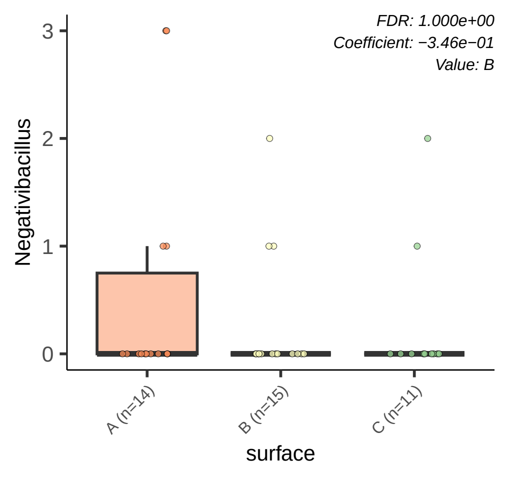

FDR: 1.000e+00
Coefficient: −3.46e−01
Value: B
Negativibacillus
3
2
1
0
A (n=14)
B (n=15)
C (n=11)
surface

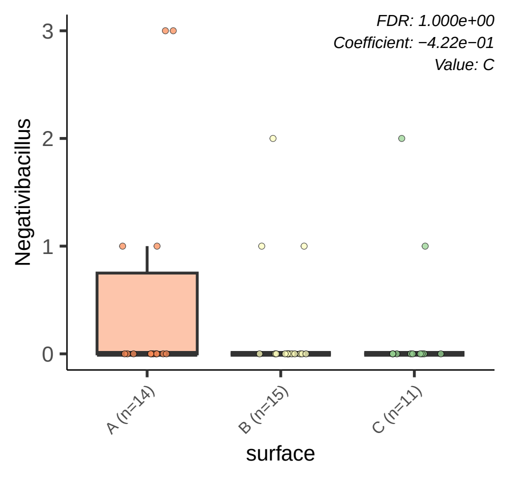

FDR: 1.000e+00
Coefficient: −4.22e−01
Value: C
Negativibacillus
3
2
1
0
A (n=14)
B (n=15)
C (n=11)
surface

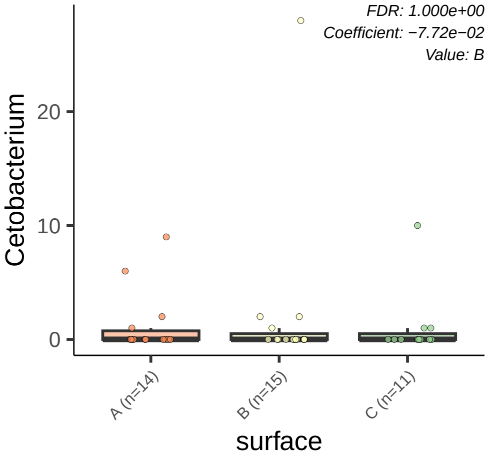
FDR: 1.000e+00
Coefficient: −7.72e−02
Value: B
Cetobacterium
0
10
20
A (n=14)
B (n=15)
C (n=11)
surface

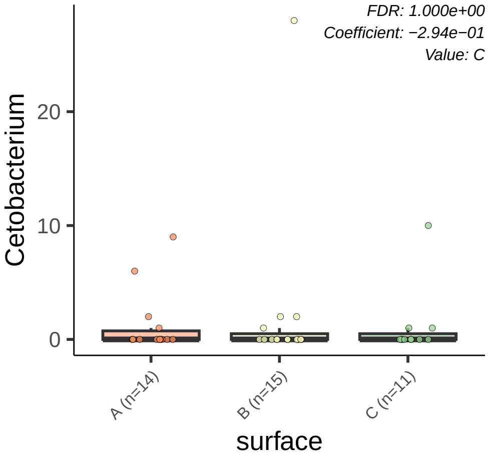

FDR: 1.000e+00
Coefficient: −2.94e−01
Value: C
Cetobacterium
20
10
0
A (n=14)
B (n=15)
C (n=11)
surface

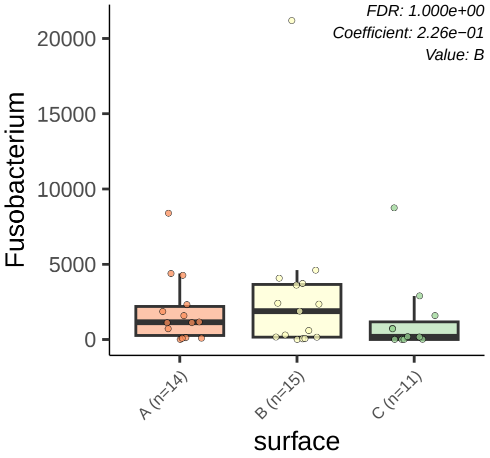
FDR: 1.000e+00
Coefficient: 2.26e−01
Value: B
Fusobacterium
20000
15000
10000
5000
0
A (n=14)
B (n=15)
C (n=11)
surface

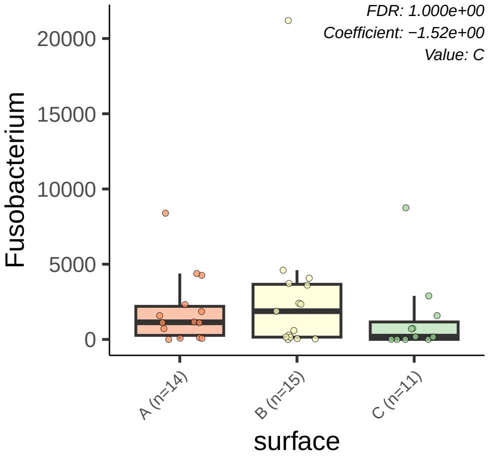

FDR: 1.000e+00
Coefficient: −1.52e+00
Value: C
Fusobacterium
20000
15000
10000
5000
0
A (n=14)
B (n=15)
C (n=11)
surface

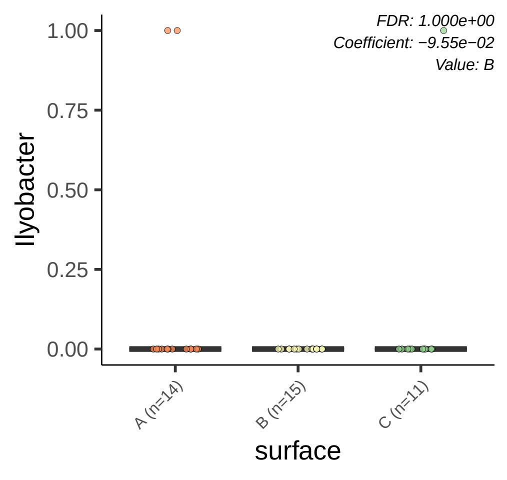
FDR: 1.000e+00
Coefficient: −9.55e−02
Value: B
Ilyobacter
1.00
0.75
0.50
0.25
0.00
A (n=14)
B (n=15)
C (n=11)
surface

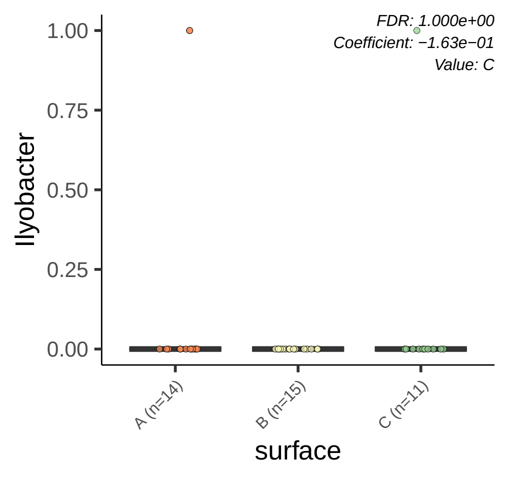
FDR: 1.000e+00
Coefficient: −1.63e−01
Value: C
Ilyobacter
1.00
0.75
0.50
0.25
0.00
A (n=14)
B (n=15)
C (n=11)
surface

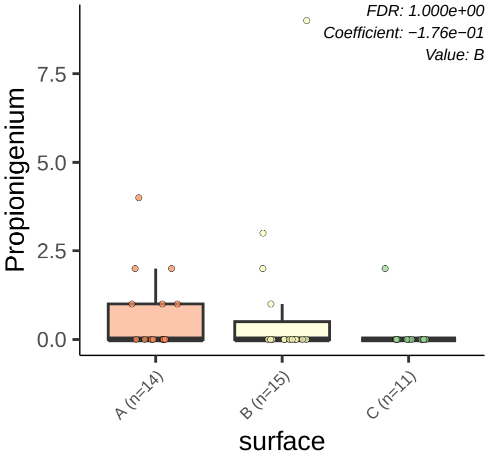
FDR: 1.000e+00
Coefficient: −1.76e−01
Value: B
Propionigenium
7.5
5.0
2.5
0.0
A (n=14)
B (n=15)
C (n=11)
surface

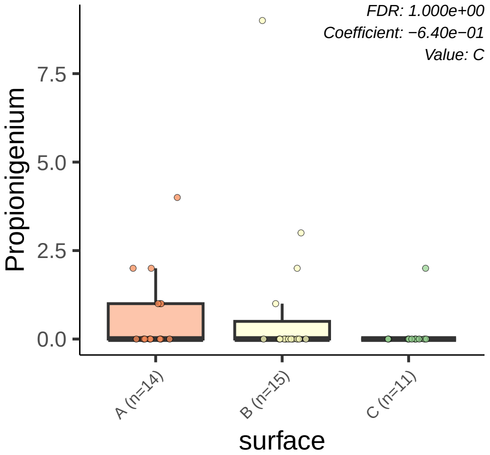
FDR: 1.000e+00
Coefficient: −6.40e−01
Value: C
Propionigenium
7.5
5.0
2.5
0.0
A (n=14)
B (n=15)
C (n=11)
surface

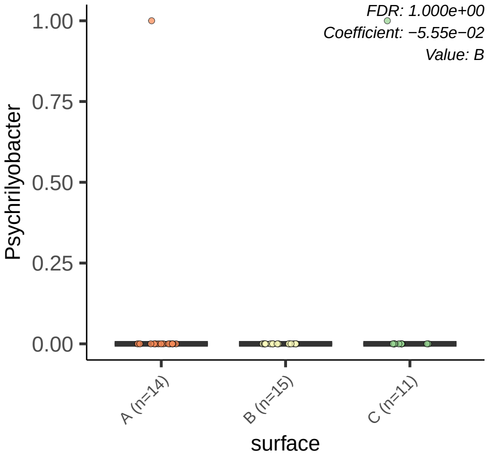
FDR: 1.000e+00
Coefficient: −5.55e−02
Value: B
Psychrilyobacter
1.00
0.75
0.50
0.25
0.00
A (n=14)
B (n=15)
C (n=11)
surface

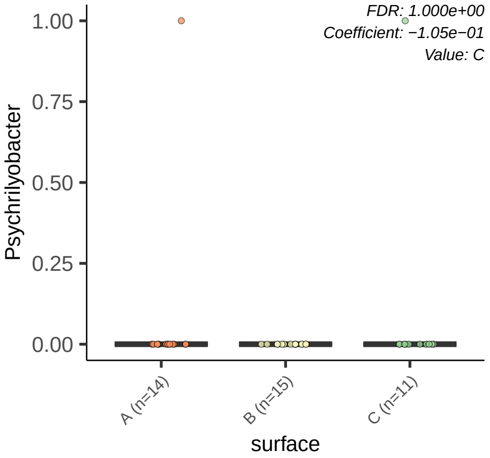

FDR: 1.000e+00
Coefficient: −1.05e−01
Value: C
Psychrilyobacter
1.00
0.75
0.50
0.25
0.00
A (n=14)
B (n=15)
C (n=11)
surface

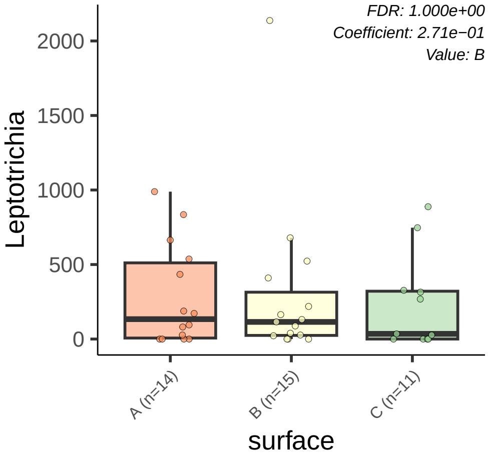
FDR: 1.000e+00
Coefficient: 2.71e−01
Value: B
Leptotrichia
2000
1500
1000
500
0
A (n=14)
B (n=15)
C (n=11)
surface

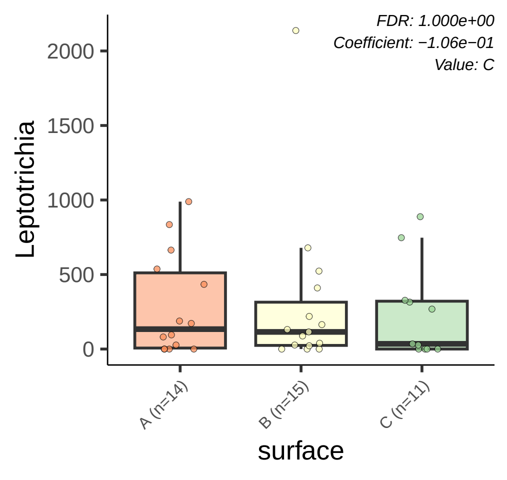
FDR: 1.000e+00
Coefficient: −1.06e−01
Value: C
Leptotrichia
2000
1500
1000
500
0
A (n=14)
B (n=15)
C (n=11)
surface

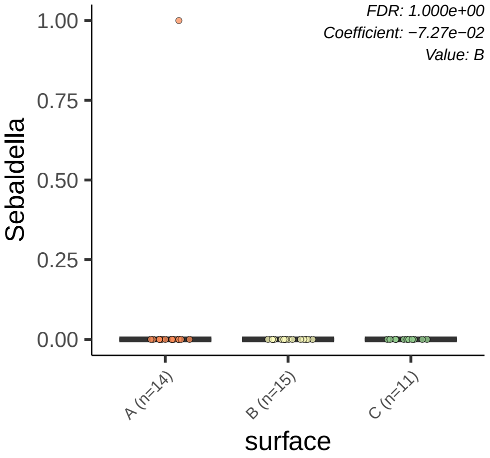

FDR: 1.000e+00
Coefficient: −7.27e−02
Value: B
1.00
0.75
0.50
0.25
0.00
Sebaldella
A (n=14)
B (n=15)
C (n=11)
surface

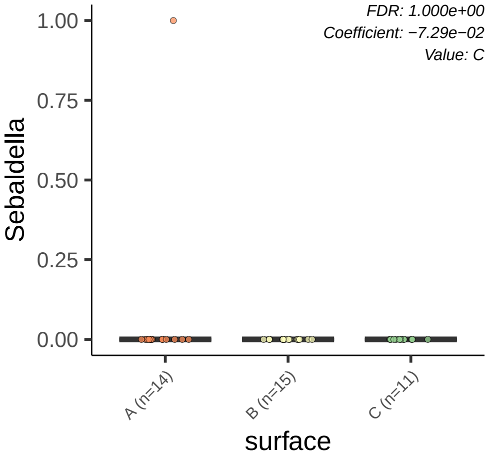
FDR: 1.000e+00
Coefficient: −7.29e−02
Value: C
Sebaldella
1.00
0.75
0.50
0.25
0.00
A (n=14)
B (n=15)
C (n=11)
surface

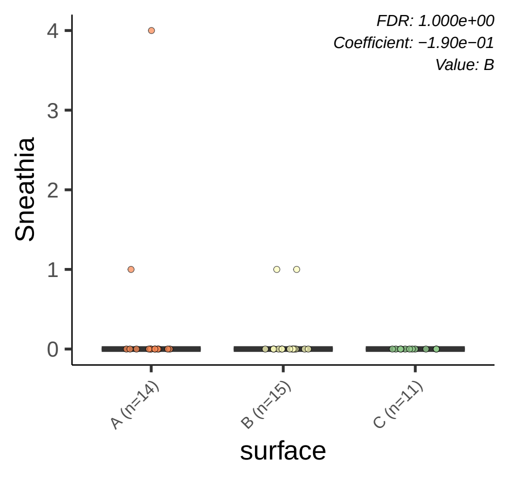
FDR: 1.000e+00
Coefficient: −1.90e−01
Value: B
Sneathia
0
1
2
3
4
A (n=14)
B (n=15)
C (n=11)
surface

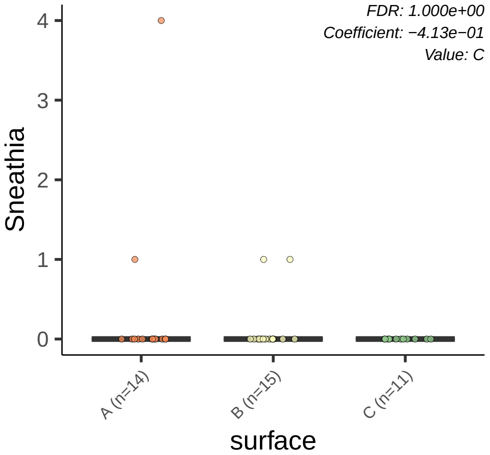
FDR: 1.000e+00
Coefficient: −4.13e−01
Value: C
Sneathia
0
1
2
3
4
A (n=14)
B (n=15)
C (n=11)
surface

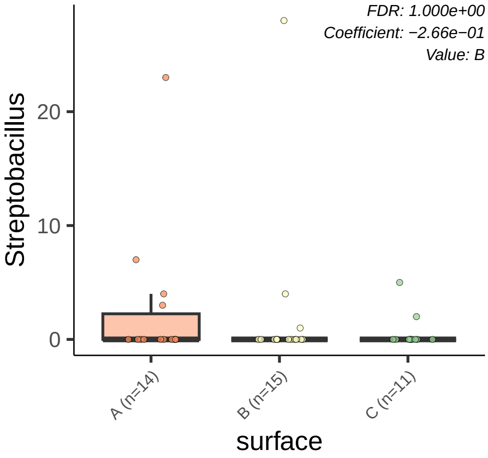
FDR: 1.000e+00
Coefficient: −2.66e−01
Value: B
Streptobacillus
0
10
20
A (n=14)
B (n=15)
C (n=11)
surface

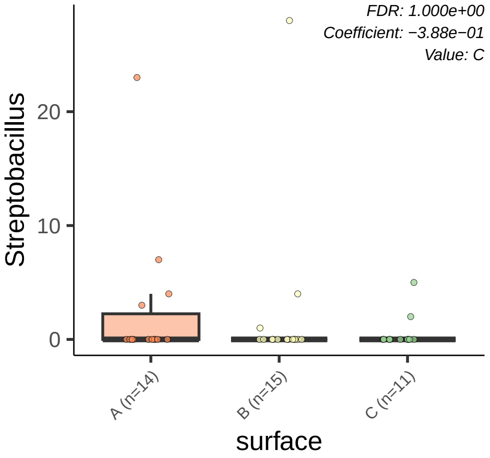
FDR: 1.000e+00
Coefficient: −3.88e−01
Value: C
Streptobacillus
20
10
0
A (n=14)
B (n=15)
C (n=11)
surface

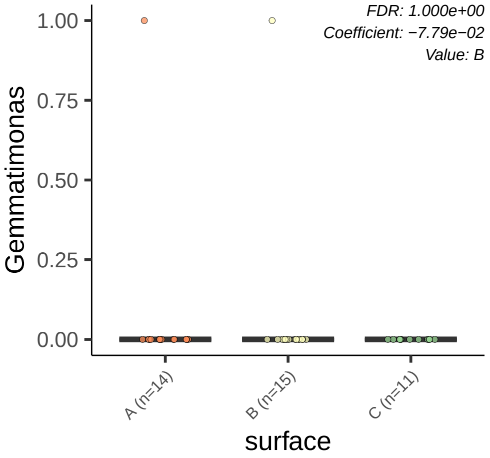
FDR: 1.000e+00
Coefficient: −7.79e−02
Value: B
Gemmatimonas
1.00
0.75
0.50
0.25
0.00
A (n=14)
B (n=15)
C (n=11)
surface

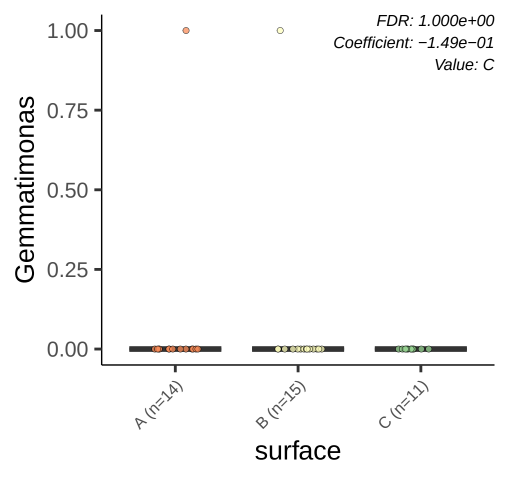

FDR: 1.000e+00
Coefficient: −1.49e−01
Value: C
Gemmatimonas
1.00
0.75
0.50
0.25
0.00
A (n=14)
B (n=15)
C (n=11)
surface

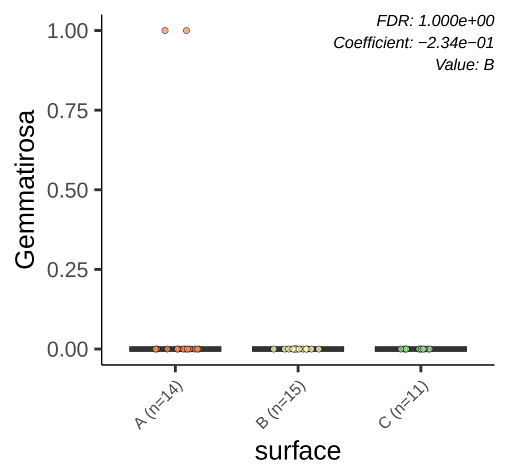
FDR: 1.000e+00
Coefficient: −2.34e−01
Value: B
Gemmatirosa
1.00
0.75
0.50
0.25
0.00
A (n=14)
B (n=15)
C (n=11)
surface

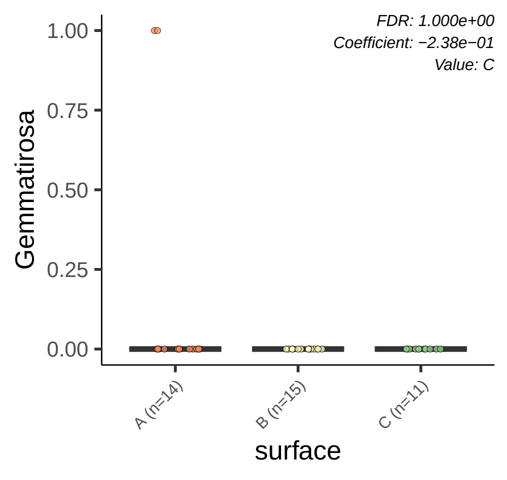
FDR: 1.000e+00
Coefficient: −2.38e−01
Value: C
Gemmatirosa
1.00
0.75
0.50
0.25
0.00
A (n=14)
B (n=15)
C (n=11)
surface

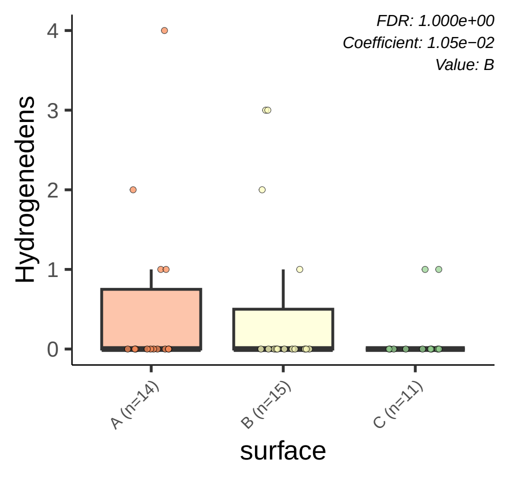

FDR: 1.000e+00
Coefficient: 1.05e−02
Value: B
Hydrogenedens
4
3
2
1
0
A (n=14)
B (n=15)
C (n=11)
surface

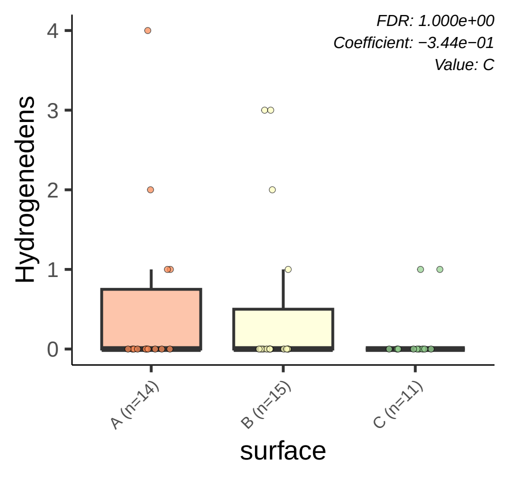

FDR: 1.000e+00
Coefficient: −3.44e−01
Value: C
Hydrogenedens
0
1
2
3
4
A (n=14)
B (n=15)
C (n=11)
surface

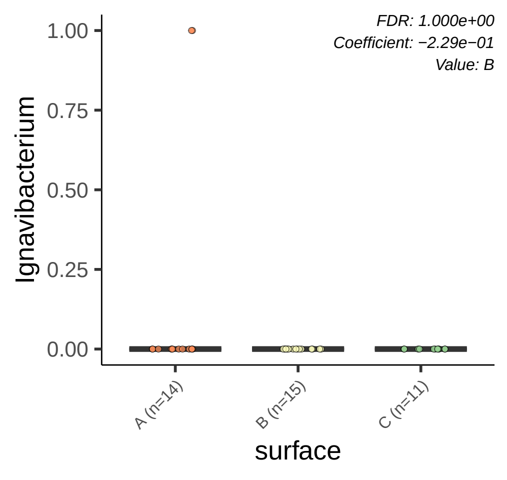

FDR: 1.000e+00
Coefficient: −2.29e−01
Value: B
Ignavibacterium
1.00
0.75
0.50
0.25
0.00
A (n=14)
B (n=15)
C (n=11)
surface

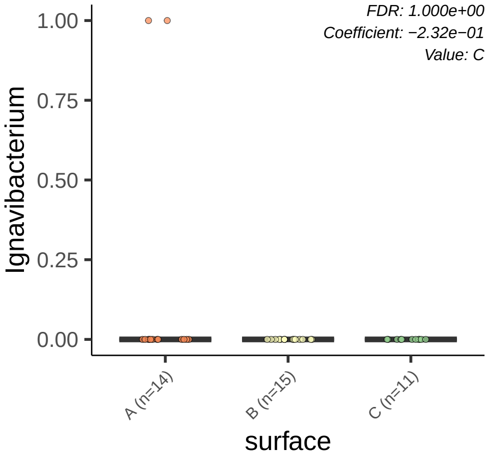
FDR: 1.000e+00
Coefficient: −2.32e−01
Value: C
Ignavibacterium
1.00
0.75
0.50
0.25
0.00
A (n=14)
B (n=15)
C (n=11)
surface

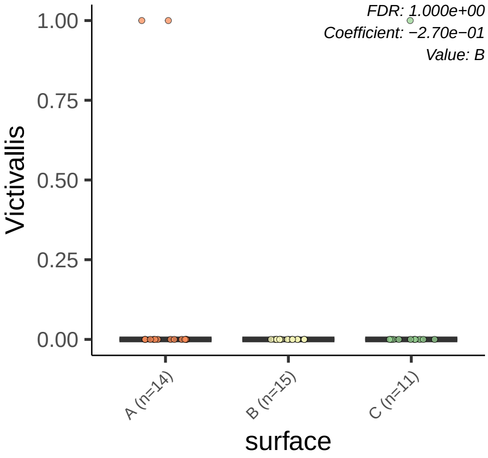
FDR: 1.000e+00
Coefficient: −2.70e−01
Value: B
Victivallis
1.00
0.75
0.50
0.25
0.00
A (n=14)
B (n=15)
C (n=11)
surface

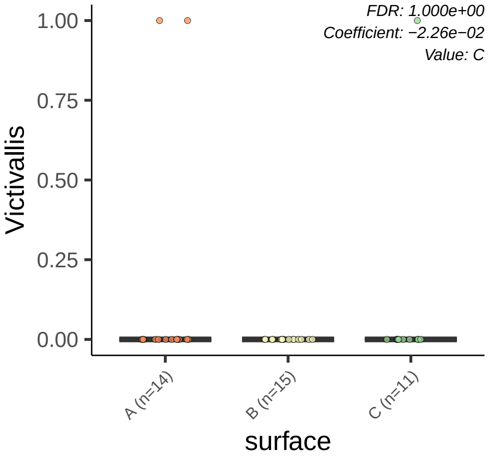
FDR: 1.000e+00
Coefficient: −2.26e−02
Value: C
Victivallis
1.00
0.75
0.50
0.25
0.00
A (n=14)
B (n=15)
C (n=11)
surface

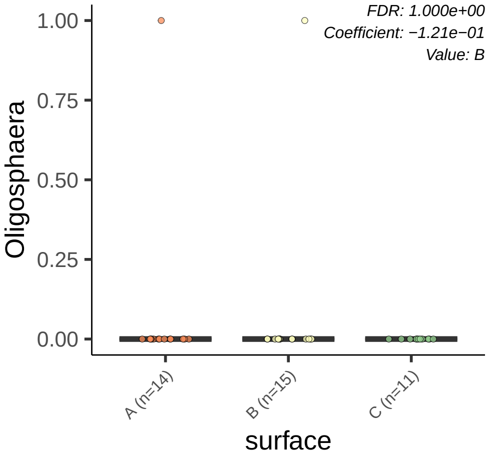
FDR: 1.000e+00
Coefficient: −1.21e−01
Value: B
Oligosphaera
1.00
0.75
0.50
0.25
0.00
A (n=14)
B (n=15)
C (n=11)
surface

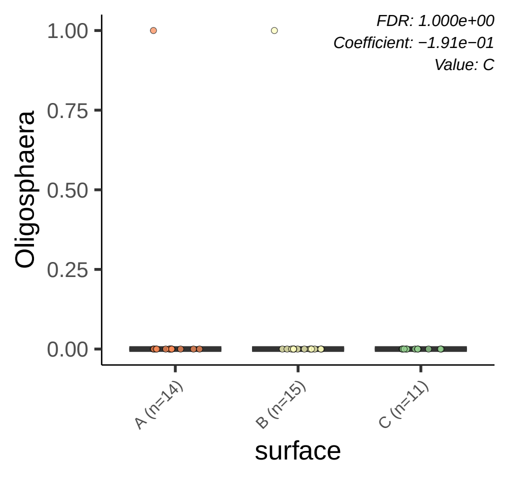

FDR: 1.000e+00
Coefficient: −1.91e−01
Value: C
Oligosphaera
1.00
0.75
0.50
0.25
0.00
A (n=14)
B (n=15)
C (n=11)
surface

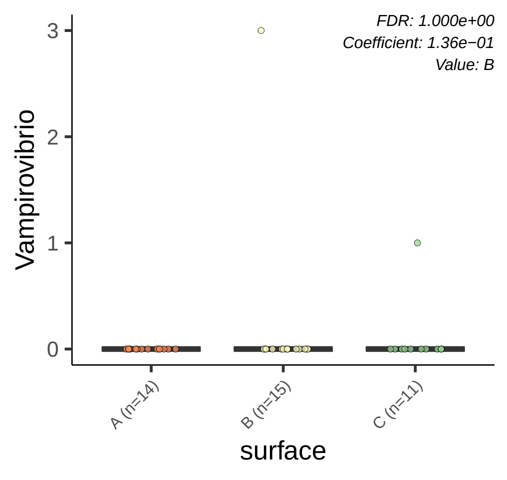

FDR: 1.000e+00
Coefficient: 1.36e−01
Value: B
Vampirovibrio
3
2
1
0
A (n=14)
B (n=15)
C (n=11)
surface

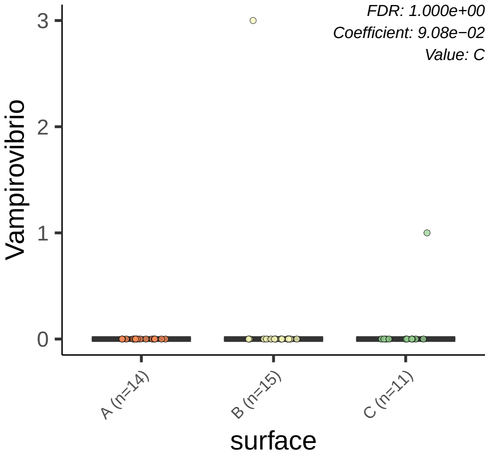

FDR: 1.000e+00
Coefficient: 9.08e−02
Value: C
Vampirovibrio
3
2
1
0
A (n=14)
B (n=15)
C (n=11)
surface

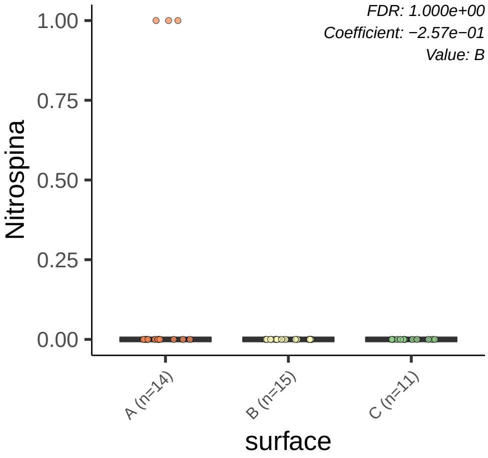

FDR: 1.000e+00
Coefficient: −2.57e−01
Value: B
Nitrospina
1.00
0.75
0.50
0.25
0.00
A (n=14)
B (n=15)
C (n=11)
surface

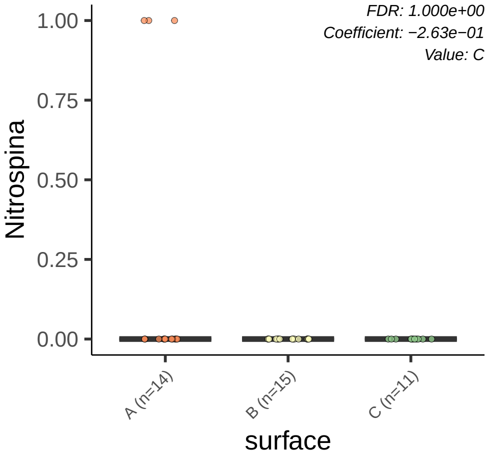

FDR: 1.000e+00
Coefficient: −2.63e−01
Value: C
Nitrospina
1.00
0.75
0.50
0.25
0.00
A (n=14)
B (n=15)
C (n=11)
surface

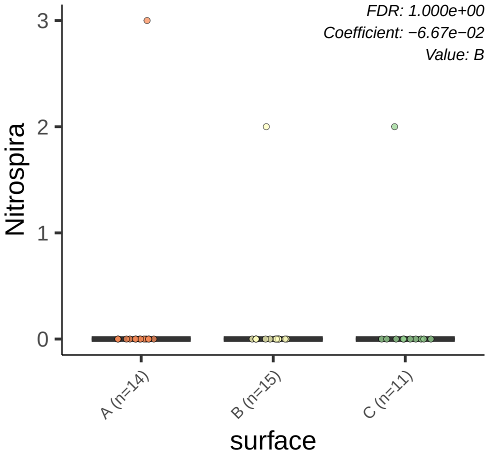

FDR: 1.000e+00
Coefficient: −6.67e−02
Value: B
Nitrospira
0
1
2
3
A (n=14)
B (n=15)
C (n=11)
surface

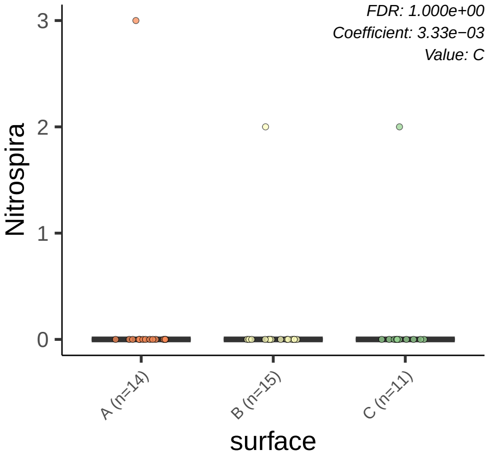
FDR: 1.000e+00
Coefficient: 3.33e−03
Value: C
Nitrospira
3
2
1
0
A (n=14)
B (n=15)
C (n=11)
surface

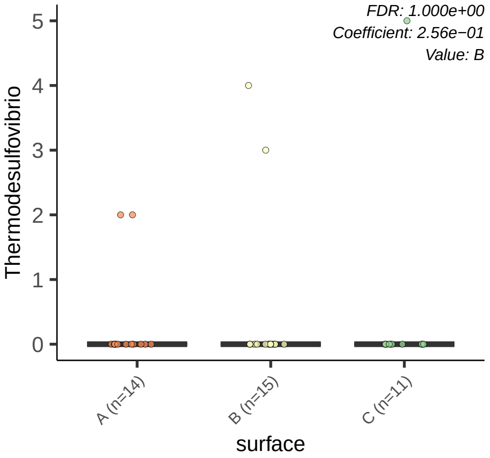

FDR: 1.000e+00
Coefficient: 2.56e−01
Value: B
Thermodesulfovibrio
0
1
2
3
4
5
A (n=14)
B (n=15)
C (n=11)
surface

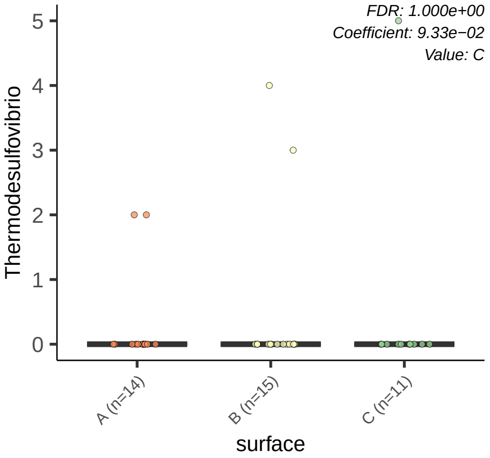
FDR: 1.000e+00
Coefficient: 9.33e−02
Value: C
Thermodesulfovibrio
5
4
3
2
1
0
A (n=14)
B (n=15)
C (n=11)
surface

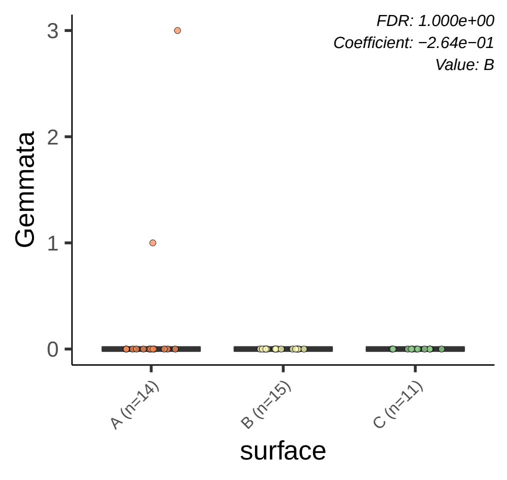

FDR: 1.000e+00
Coefficient: −2.64e−01
Value: B
Gemmata
0
1
2
3
A (n=14)
B (n=15)
C (n=11)
surface

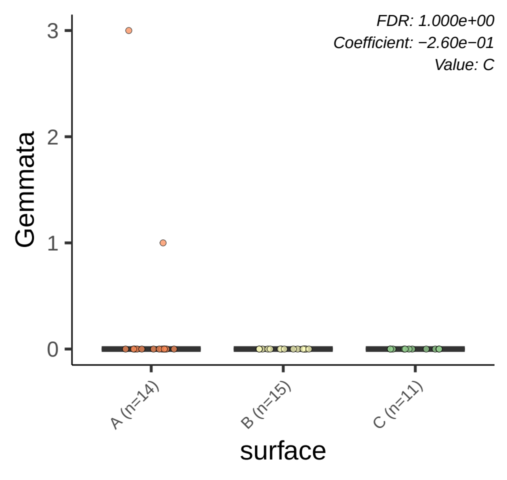
FDR: 1.000e+00
Coefficient: −2.60e−01
Value: C
Gemmata
3
2
1
0
A (n=14)
B (n=15)
C (n=11)
surface

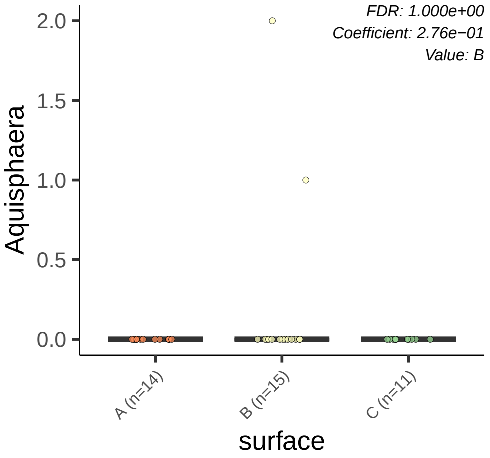
FDR: 1.000e+00
Coefficient: 2.76e−01
Value: B
Aquisphaera
2.0
1.5
1.0
0.5
0.0
A (n=14)
B (n=15)
C (n=11)
surface

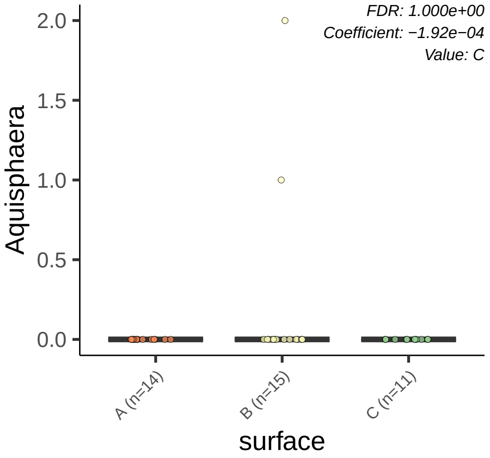

FDR: 1.000e+00
Coefficient: −1.92e−04
Value: C
Aquisphaera
2.0
1.5
1.0
0.5
0.0
A (n=14)
B (n=15)
C (n=11)
surface

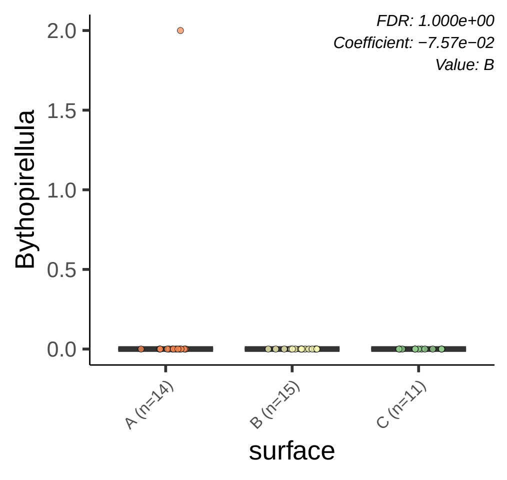
FDR: 1.000e+00
Coefficient: −7.57e−02
Value: B
Bythopirellula
2.0
1.5
1.0
0.5
0.0
A (n=14)
B (n=15)
C (n=11)
surface

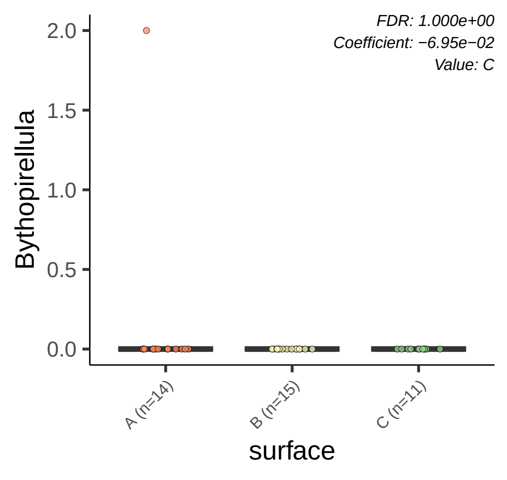
FDR: 1.000e+00
Coefficient: −6.95e−02
Value: C
Bythopirellula
2.0
1.5
1.0
0.5
0.0
A (n=14)
B (n=15)
C (n=11)
surface

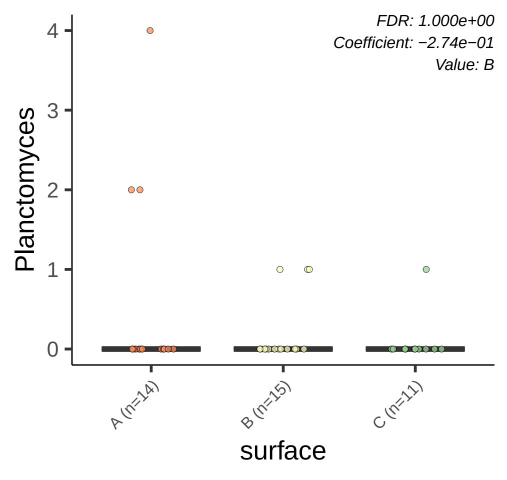
FDR: 1.000e+00
Coefficient: −2.74e−01
Value: B
Planctomyces
4
3
2
1
0
A (n=14)
B (n=15)
C (n=11)
surface

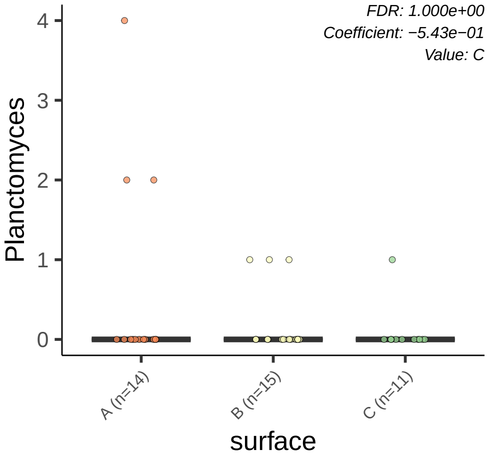
FDR: 1.000e+00
Coefficient: −5.43e−01
Value: C
Planctomyces
4
3
2
1
0
A (n=14)
B (n=15)
C (n=11)
surface

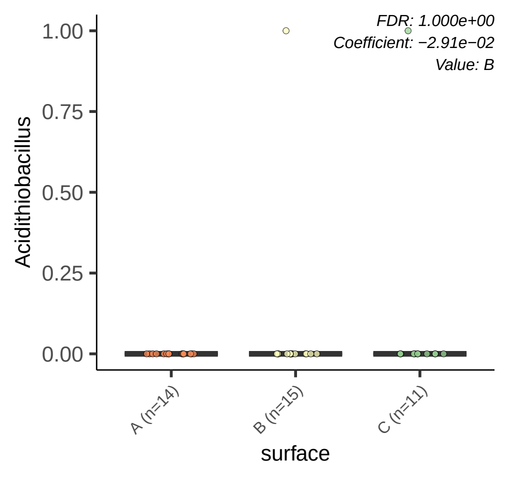
FDR: 1.000e+00
Coefficient: −2.91e−02
Value: B
Acidithiobacillus
1.00
0.75
0.50
0.25
0.00
A (n=14)
B (n=15)
C (n=11)
surface

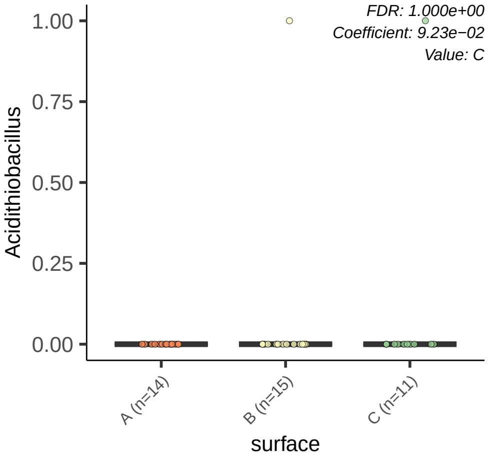

FDR: 1.000e+00
Coefficient: 9.23e−02
Value: C
Acidithiobacillus
1.00
0.75
0.50
0.25
0.00
A (n=14)
B (n=15)
C (n=11)
surface

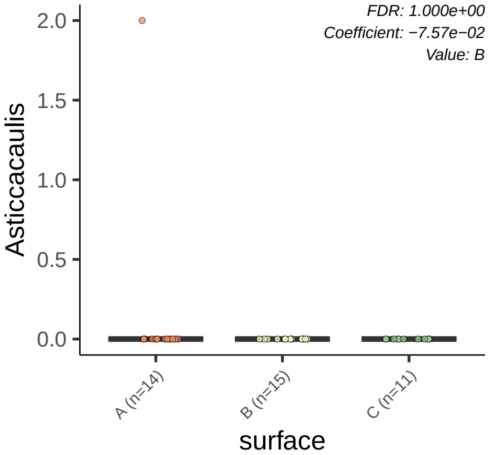
FDR: 1.000e+00
Coefficient: −7.57e−02
Value: B
2.0
1.5
1.0
0.5
0.0
Asticcacaulis
A (n=14)
B (n=15)
C (n=11)
surface

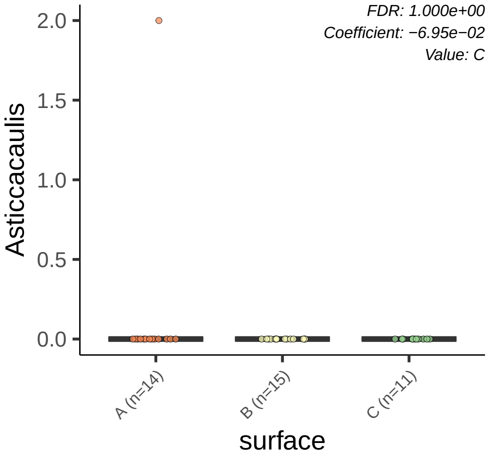
FDR: 1.000e+00
Coefficient: −6.95e−02
Value: C
Asticcacaulis
2.0
1.5
1.0
0.5
0.0
A (n=14)
B (n=15)
C (n=11)
surface

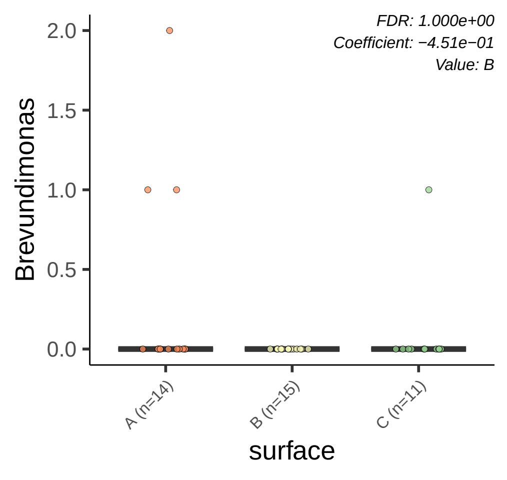
FDR: 1.000e+00
Coefficient: −4.51e−01
Value: B
Brevundimonas
2.0
1.5
1.0
0.5
0.0
A (n=14)
B (n=15)
C (n=11)
surface

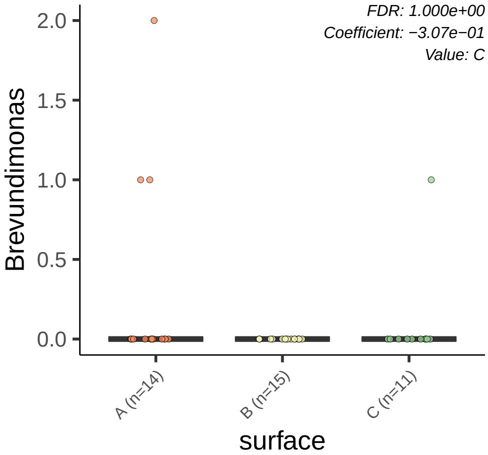

FDR: 1.000e+00
Coefficient: −3.07e−01
Value: C
Brevundimonas
2.0
1.5
1.0
0.5
0.0
A (n=14)
B (n=15)
C (n=11)
surface

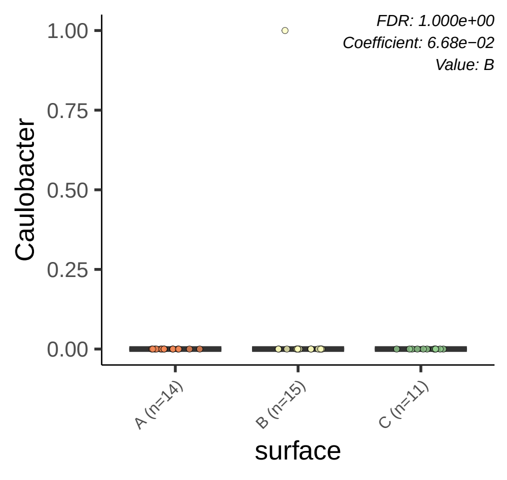

FDR: 1.000e+00
Coefficient: 6.68e−02
Value: B
Caulobacter
1.00
0.75
0.50
0.25
0.00
A (n=14)
B (n=15)
C (n=11)
surface

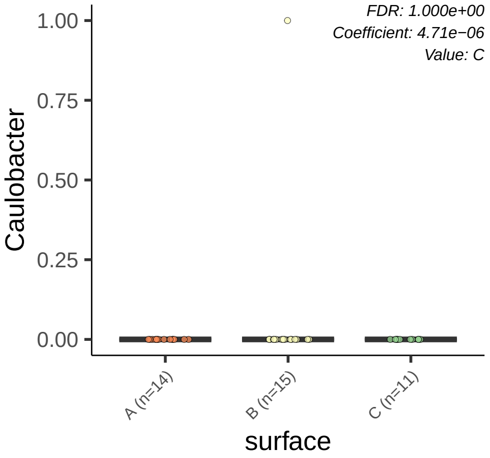

FDR: 1.000e+00
Coefficient: 4.71e−06
Value: C
Caulobacter
1.00
0.75
0.50
0.25
0.00
A (n=14)
B (n=15)
C (n=11)
surface

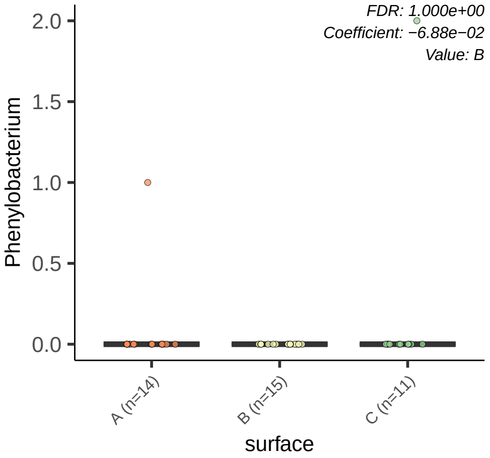

FDR: 1.000e+00
Coefficient: −6.88e−02
Value: B
Phenylobacterium
2.0
1.5
1.0
0.5
0.0
A (n=14)
B (n=15)
C (n=11)
surface

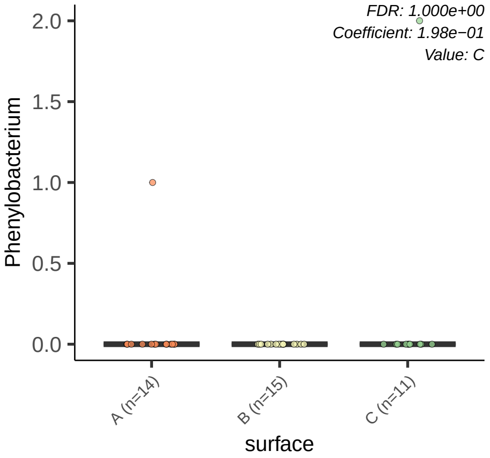
FDR: 1.000e+00
Coefficient: 1.98e−01
Value: C
Phenylobacterium
2.0
1.5
1.0
0.5
0.0
A (n=14)
B (n=15)
C (n=11)
surface

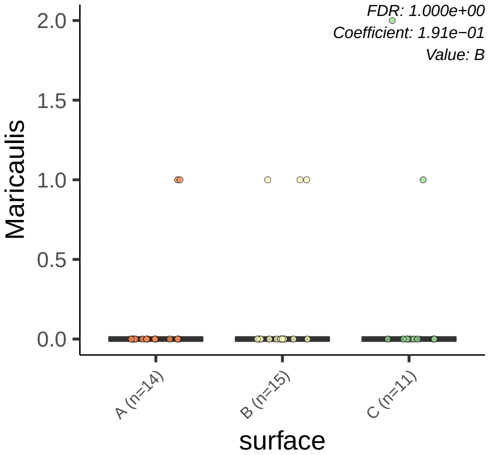
FDR: 1.000e+00
Coefficient: 1.91e−01
Value: B
Maricaulis
2.0
1.5
1.0
0.5
0.0
A (n=14)
B (n=15)
C (n=11)
surface

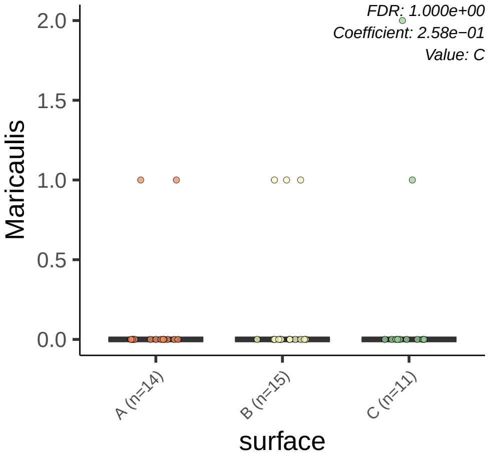

FDR: 1.000e+00
Coefficient: 2.58e−01
Value: C
Maricaulis
2.0
1.5
1.0
0.5
0.0
A (n=14)
B (n=15)
C (n=11)
surface

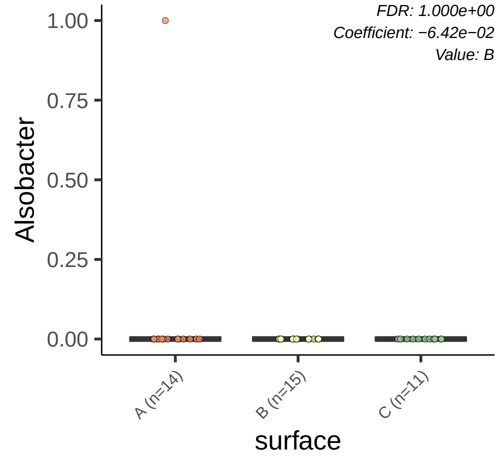
FDR: 1.000e+00
Coefficient: −6.42e−02
Value: B
Alsobacter
1.00
0.75
0.50
0.25
0.00
A (n=14)
B (n=15)
C (n=11)
surface

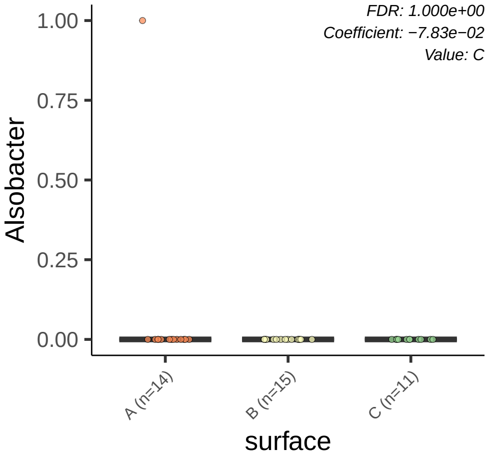

FDR: 1.000e+00
Coefficient: −7.83e−02
Value: C
Alsobacter
1.00
0.75
0.50
0.25
0.00
A (n=14)
B (n=15)
C (n=11)
surface

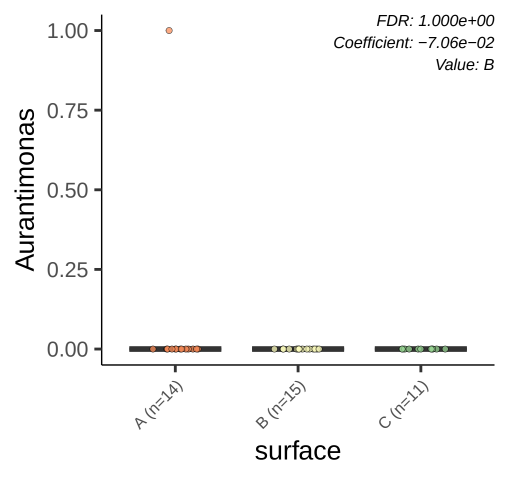
FDR: 1.000e+00
Coefficient: −7.06e−02
Value: B
Aurantimonas
1.00
0.75
0.50
0.25
0.00
A (n=14)
B (n=15)
C (n=11)
surface

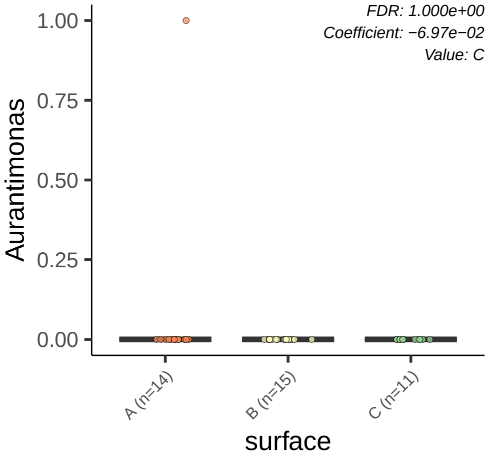
FDR: 1.000e+00
Coefficient: −6.97e−02
Value: C
Aurantimonas
1.00
0.75
0.50
0.25
0.00
A (n=14)
B (n=15)
C (n=11)
surface

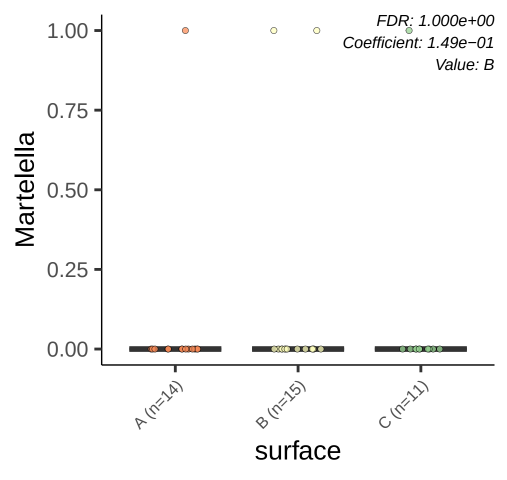
FDR: 1.000e+00
Coefficient: 1.49e−01
Value: B
Martelella
1.00
0.75
0.50
0.25
0.00
A (n=14)
B (n=15)
C (n=11)
surface

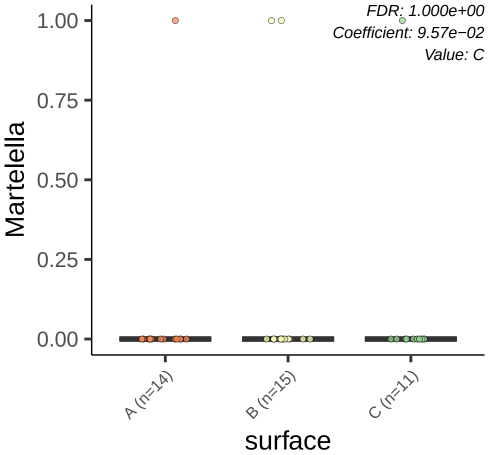
FDR: 1.000e+00
Coefficient: 9.57e−02
Value: C
Martelella
1.00
0.75
0.50
0.25
0.00
A (n=14)
B (n=15)
C (n=11)
surface

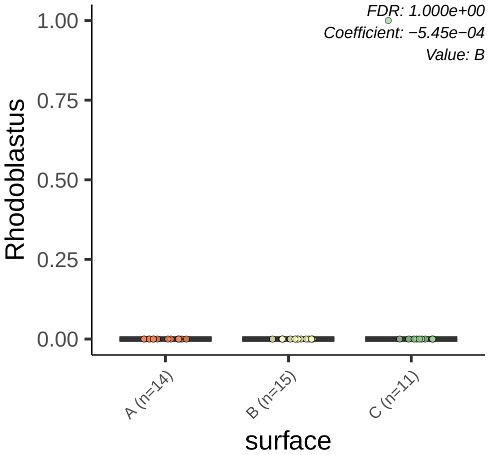

FDR: 1.000e+00
Coefficient: −5.45e−04
Value: B
Rhodoblastus
1.00
0.75
0.50
0.25
0.00
A (n=14)
B (n=15)
C (n=11)
surface

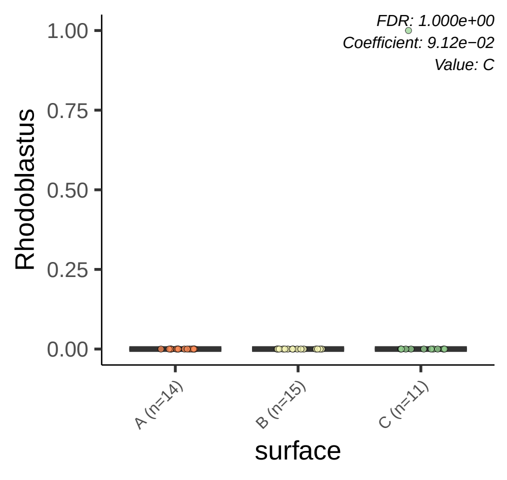

FDR: 1.000e+00
Coefficient: 9.12e−02
Value: C
Rhodoblastus
1.00
0.75
0.50
0.25
0.00
A (n=14)
B (n=15)
C (n=11)
surface

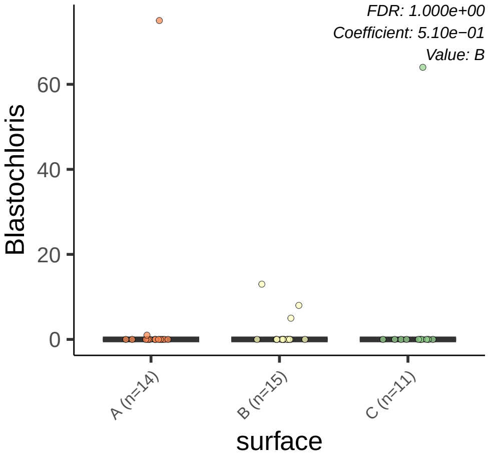
FDR: 1.000e+00
Coefficient: 5.10e−01
Value: B
Blastochloris
0
20
40
60
A (n=14)
B (n=15)
C (n=11)
surface

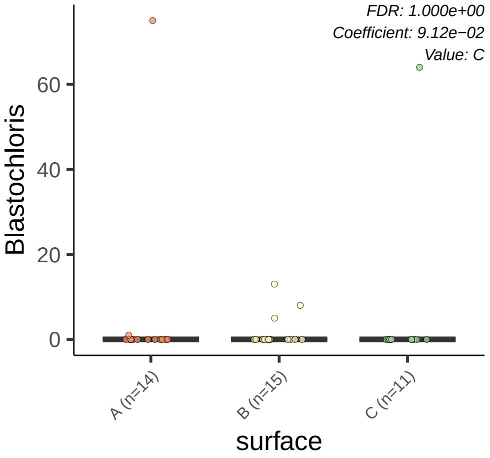
FDR: 1.000e+00
Coefficient: 9.12e−02
Value: C
Blastochloris
0
20
40
60
A (n=14)
B (n=15)
C (n=11)
surface

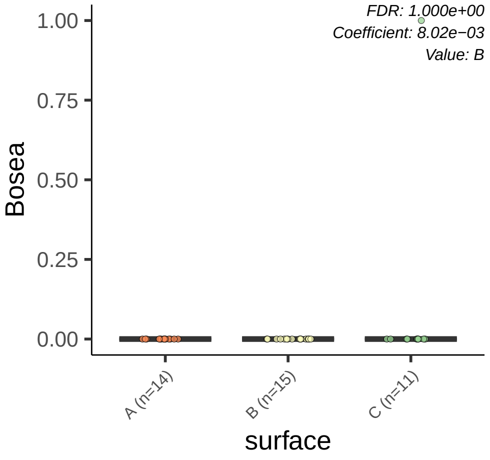
FDR: 1.000e+00
Coefficient: 8.02e−03
Value: B
Bosea
1.00
0.75
0.50
0.25
0.00
A (n=14)
B (n=15)
C (n=11)
surface

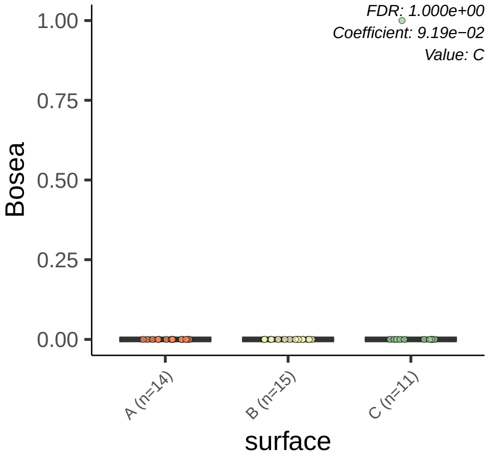
FDR: 1.000e+00
Coefficient: 9.19e−02
Value: C
Bosea
1.00
0.75
0.50
0.25
0.00
A (n=14)
B (n=15)
C (n=11)
surface

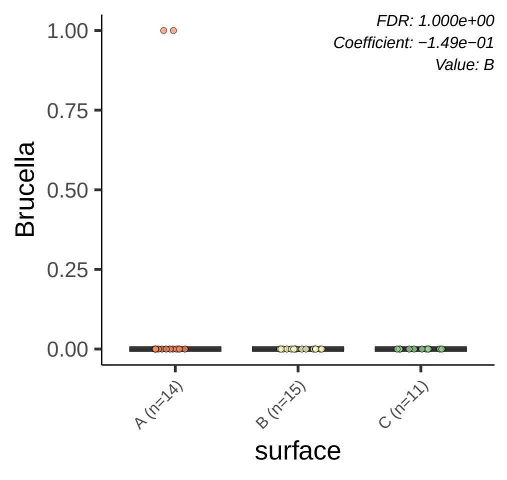
FDR: 1.000e+00
Coefficient: −1.49e−01
Value: B
Brucella
1.00
0.75
0.50
0.25
0.00
A (n=14)
B (n=15)
C (n=11)
surface

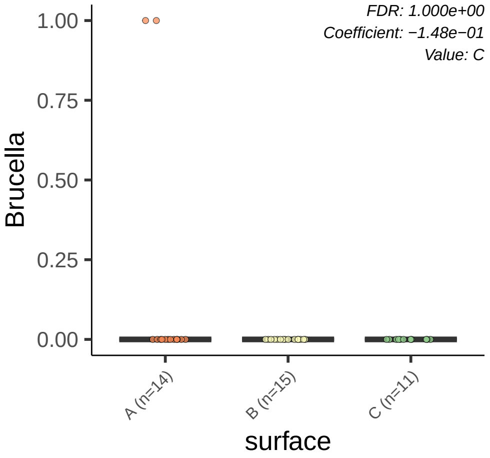

FDR: 1.000e+00
Coefficient: −1.48e−01
Value: C
Brucella
1.00
0.75
0.50
0.25
0.00
A (n=14)
B (n=15)
C (n=11)
surface

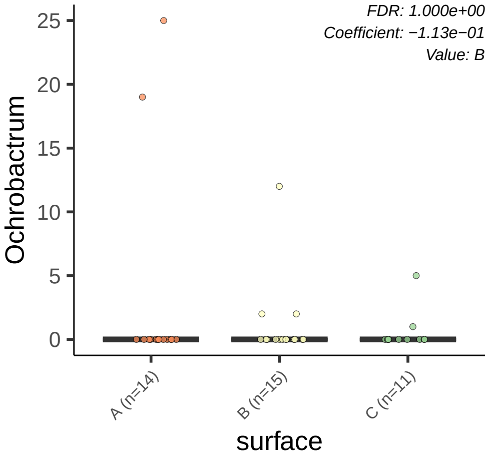
FDR: 1.000e+00
Coefficient: −1.13e−01
Value: B
Ochrobactrum
25
20
15
10
5
0
A (n=14)
B (n=15)
C (n=11)
surface

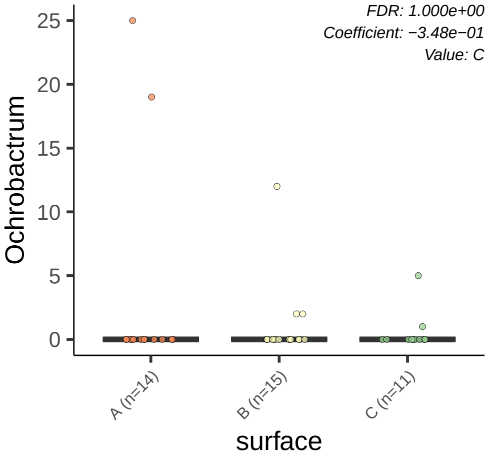
FDR: 1.000e+00
Coefficient: −3.48e−01
Value: C
Ochrobactrum
25
20
15
10
5
0
A (n=14)
B (n=15)
C (n=11)
surface

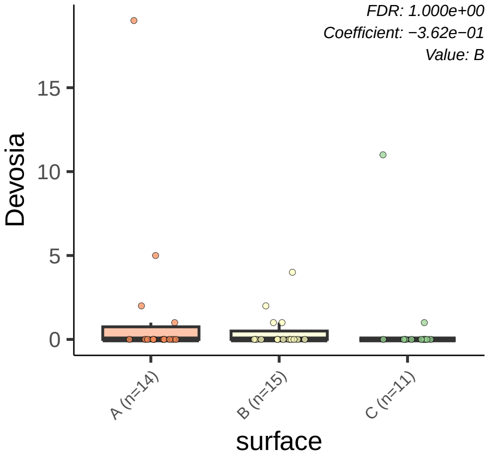

FDR: 1.000e+00
Coefficient: −3.62e−01
Value: B
Devosia
0
5
10
15
A (n=14)
B (n=15)
C (n=11)
surface

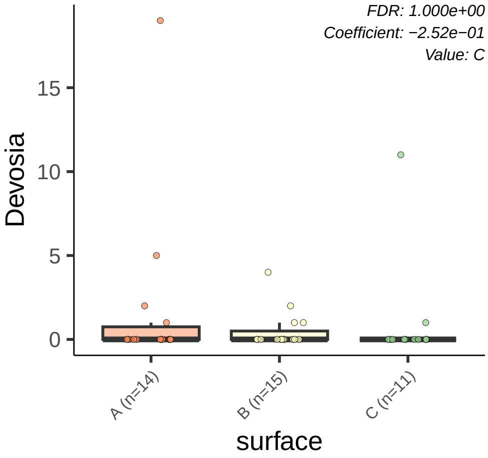
FDR: 1.000e+00
Coefficient: −2.52e−01
Value: C
Devosia
15
10
5
0
A (n=14)
B (n=15)
C (n=11)
surface

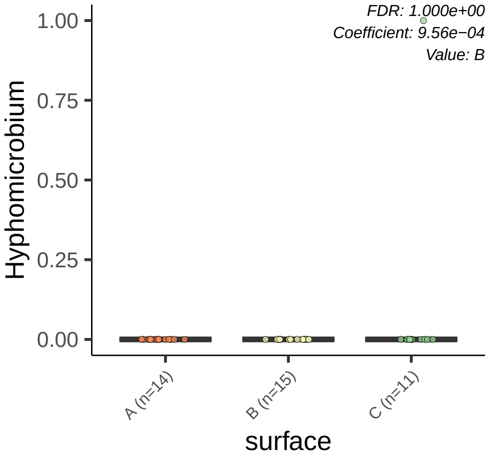
FDR: 1.000e+00
Coefficient: 9.56e−04
Value: B
Hyphomicrobium
1.00
0.75
0.50
0.25
0.00
A (n=14)
B (n=15)
C (n=11)
surface

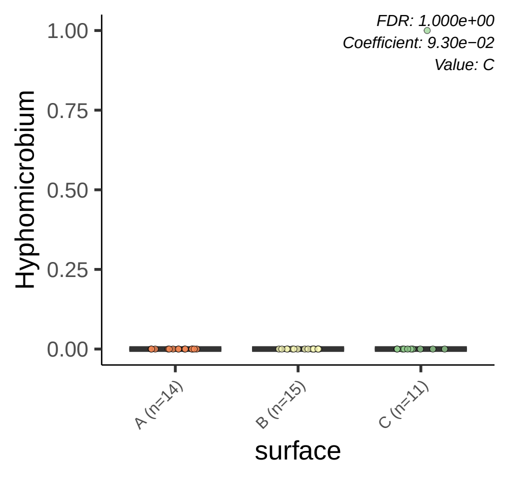
FDR: 1.000e+00
Coefficient: 9.30e−02
Value: C
Hyphomicrobium
1.00
0.75
0.50
0.25
0.00
A (n=14)
B (n=15)
C (n=11)
surface

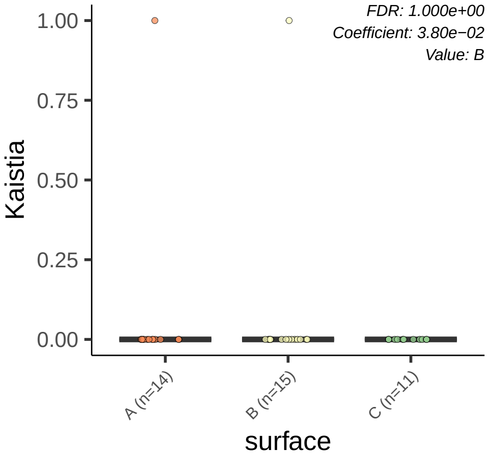
FDR: 1.000e+00
Coefficient: 3.80e−02
Value: B
Kaistia
1.00
0.75
0.50
0.25
0.00
A (n=14)
B (n=15)
C (n=11)
surface

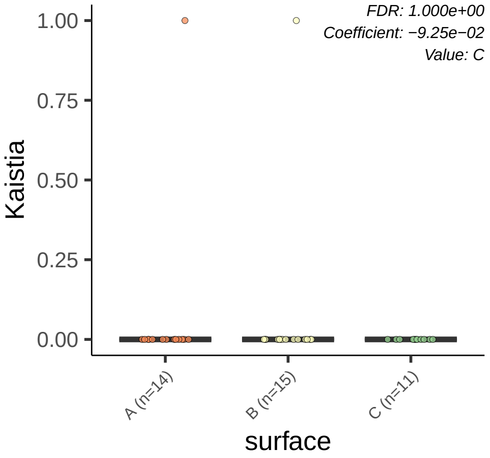
FDR: 1.000e+00
Coefficient: −9.25e−02
Value: C
Kaistia
1.00
0.75
0.50
0.25
0.00
A (n=14)
B (n=15)
C (n=11)
surface

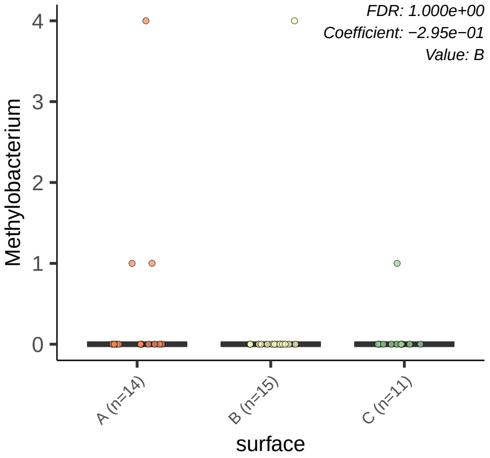

FDR: 1.000e+00
Coefficient: −2.95e−01
Value: B
Methylobacterium
4
3
2
1
0
A (n=14)
B (n=15)
C (n=11)
surface

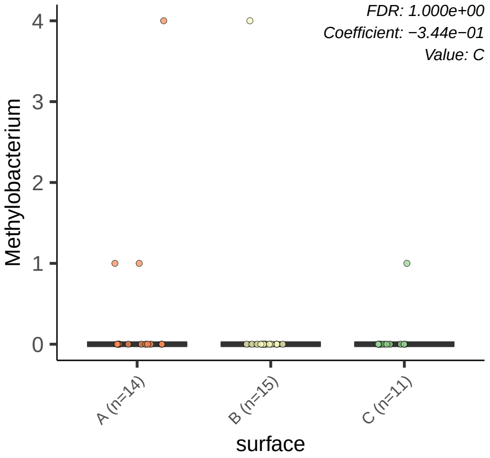

FDR: 1.000e+00
Coefficient: −3.44e−01
Value: C
Methylobacterium
4
3
2
1
0
A (n=14)
B (n=15)
C (n=11)
surface

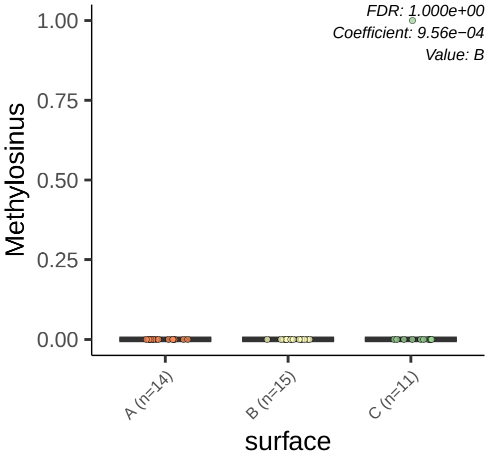

FDR: 1.000e+00
Coefficient: 9.56e−04
Value: B
Methylosinus
1.00
0.75
0.50
0.25
0.00
A (n=14)
B (n=15)
C (n=11)
surface

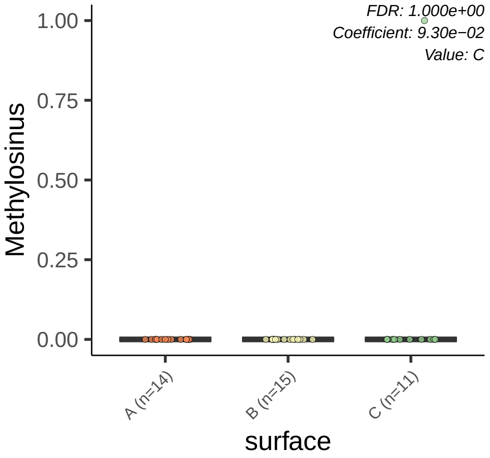
FDR: 1.000e+00
Coefficient: 9.30e−02
Value: C
Methylosinus
1.00
0.75
0.50
0.25
0.00
A (n=14)
B (n=15)
C (n=11)
surface

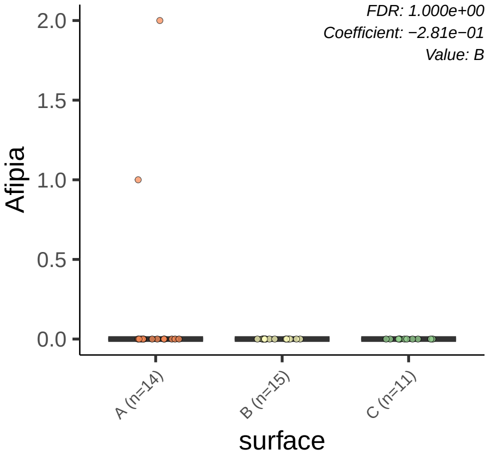
FDR: 1.000e+00
Coefficient: −2.81e−01
Value: B
Afipia
2.0
1.5
1.0
0.5
0.0
A (n=14)
B (n=15)
C (n=11)
surface

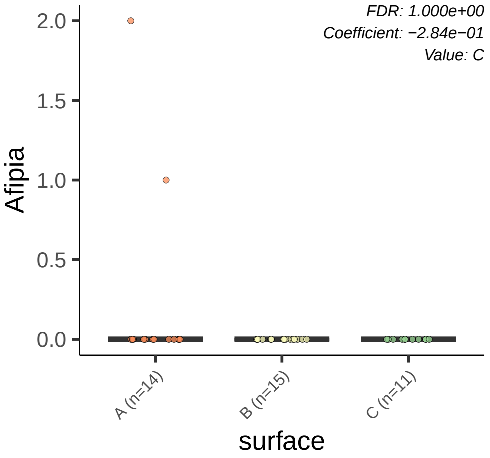
FDR: 1.000e+00
Coefficient: −2.84e−01
Value: C
Afipia
2.0
1.5
1.0
0.5
0.0
A (n=14)
B (n=15)
C (n=11)
surface

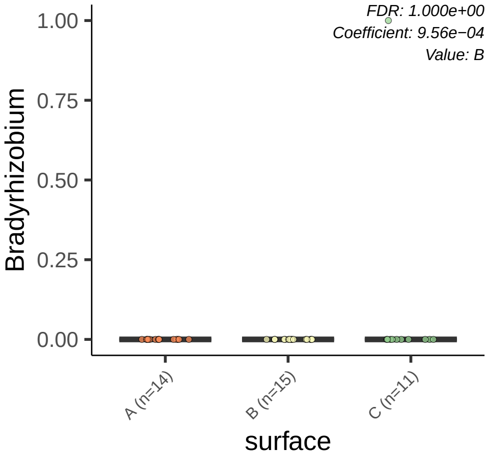

FDR: 1.000e+00
Coefficient: 9.56e−04
Value: B
Bradyrhizobium
1.00
0.75
0.50
0.25
0.00
A (n=14)
B (n=15)
C (n=11)
surface

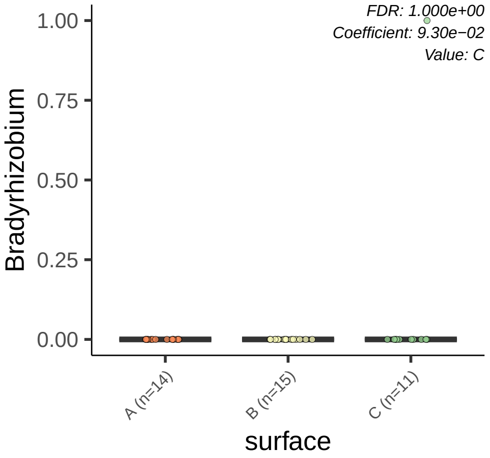

FDR: 1.000e+00
Coefficient: 9.30e−02
Value: C
Bradyrhizobium
1.00
0.75
0.50
0.25
0.00
A (n=14)
B (n=15)
C (n=11)
surface

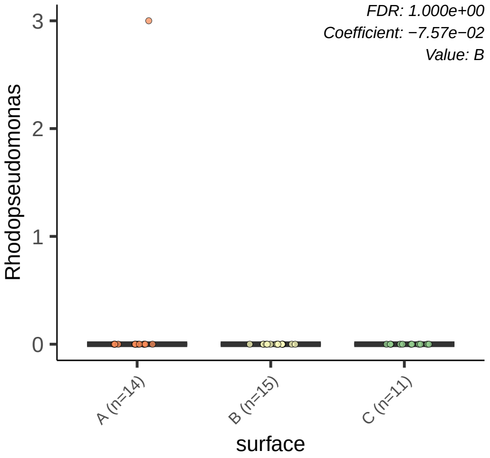
FDR: 1.000e+00
Coefficient: −7.57e−02
Value: B
Rhodopseudomonas
3
2
1
0
A (n=14)
B (n=15)
C (n=11)
surface

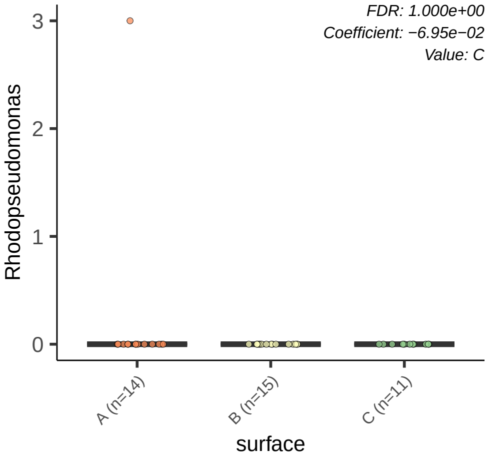

FDR: 1.000e+00
Coefficient: −6.95e−02
Value: C
Rhodopseudomonas
3
2
1
0
A (n=14)
B (n=15)
C (n=11)
surface

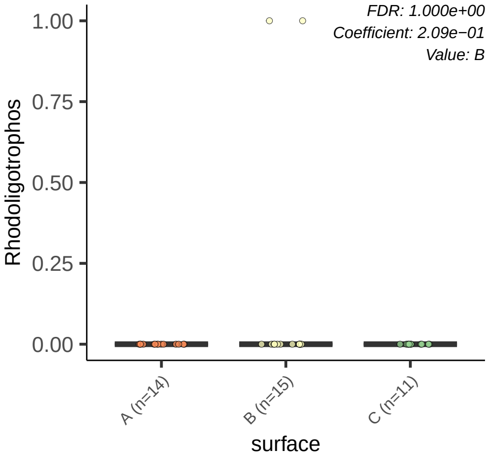

FDR: 1.000e+00
Coefficient: 2.09e−01
Value: B
Rhodoligotrophos
1.00
0.75
0.50
0.25
0.00
A (n=14)
B (n=15)
C (n=11)
surface

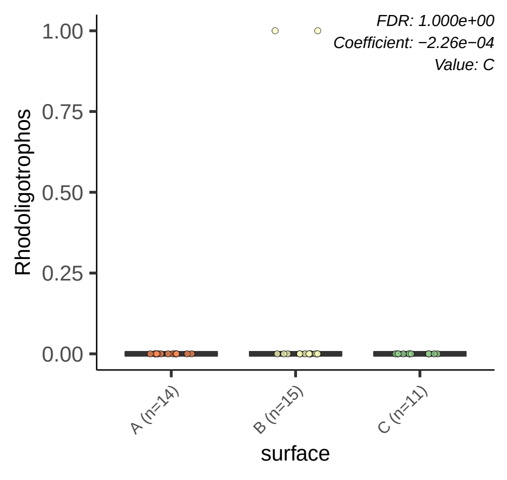

FDR: 1.000e+00
Coefficient: −2.26e−04
Value: C
Rhodoligotrophos
1.00
0.75
0.50
0.25
0.00
A (n=14)
B (n=15)
C (n=11)
surface

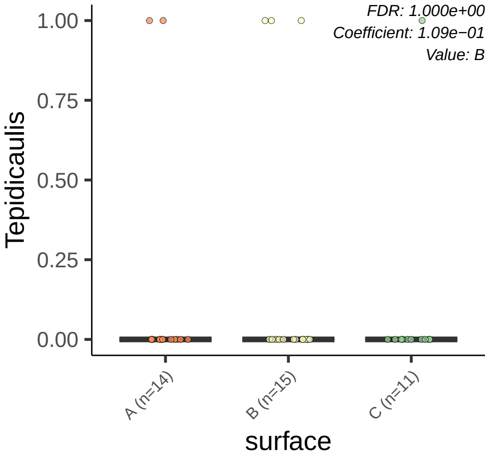

FDR: 1.000e+00
Coefficient: 1.09e−01
Value: B
1.00
0.75
0.50
0.25
0.00
Tepidicaulis
A (n=14)
B (n=15)
C (n=11)
surface

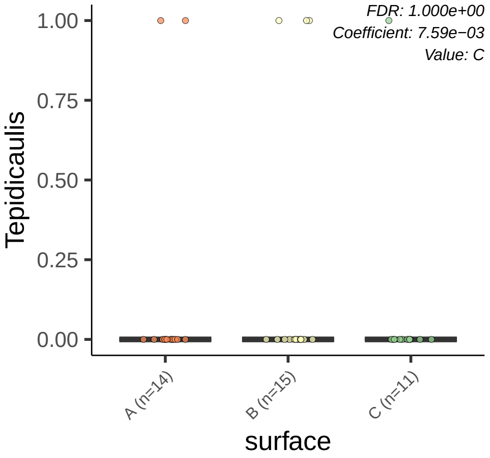

FDR: 1.000e+00
Coefficient: 7.59e−03
Value: C
Tepidicaulis
1.00
0.75
0.50
0.25
0.00
A (n=14)
B (n=15)
C (n=11)
surface

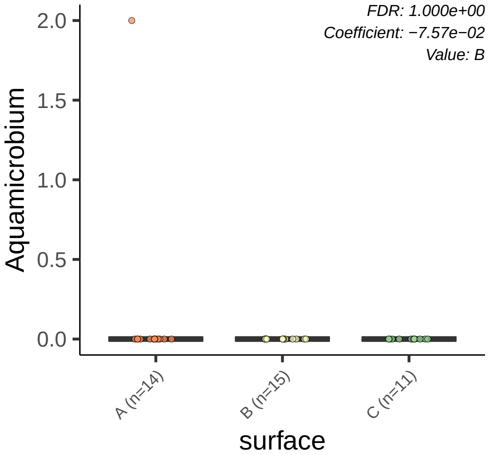
FDR: 1.000e+00
Coefficient: −7.57e−02
Value: B
Aquamicrobium
2.0
1.5
1.0
0.5
0.0
A (n=14)
B (n=15)
C (n=11)
surface

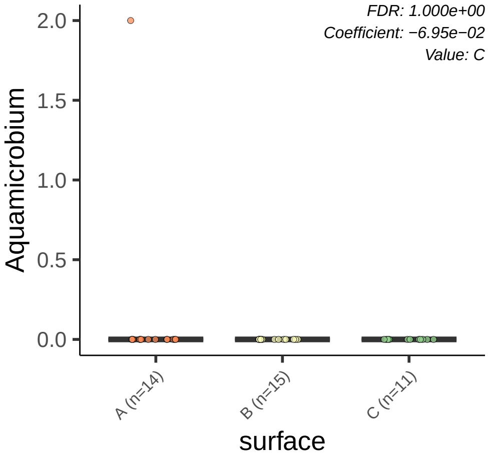

FDR: 1.000e+00
Coefficient: −6.95e−02
Value: C
Aquamicrobium
2.0
1.5
1.0
0.5
0.0
A (n=14)
B (n=15)
C (n=11)
surface

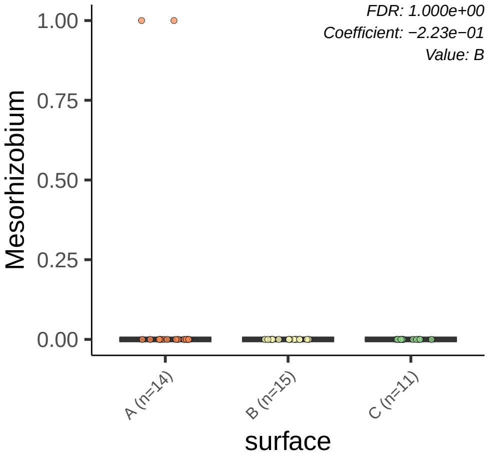
FDR: 1.000e+00
Coefficient: −2.23e−01
Value: B
Mesorhizobium
1.00
0.75
0.50
0.25
0.00
A (n=14)
B (n=15)
C (n=11)
surface

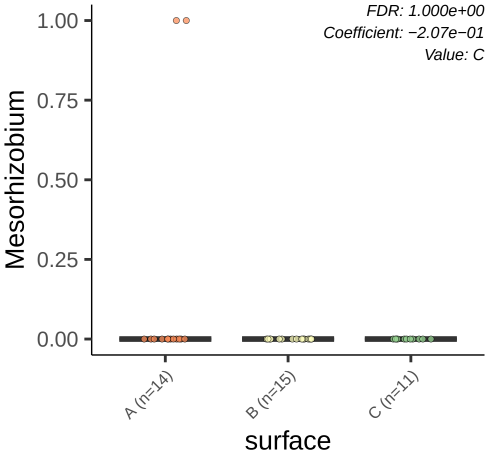

FDR: 1.000e+00
Coefficient: −2.07e−01
Value: C
Mesorhizobium
1.00
0.75
0.50
0.25
0.00
A (n=14)
B (n=15)
C (n=11)
surface

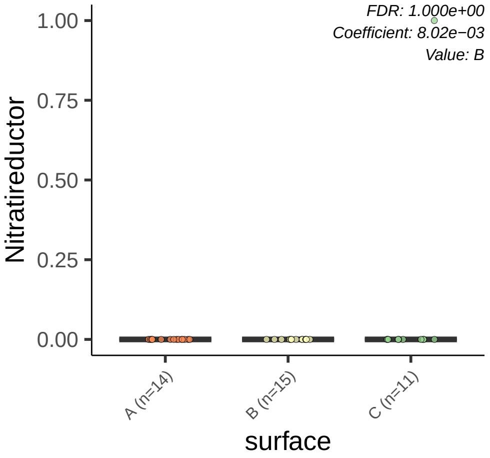

FDR: 1.000e+00
Coefficient: 8.02e−03
Value: B
Nitratireductor
1.00
0.75
0.50
0.25
0.00
A (n=14)
B (n=15)
C (n=11)
surface

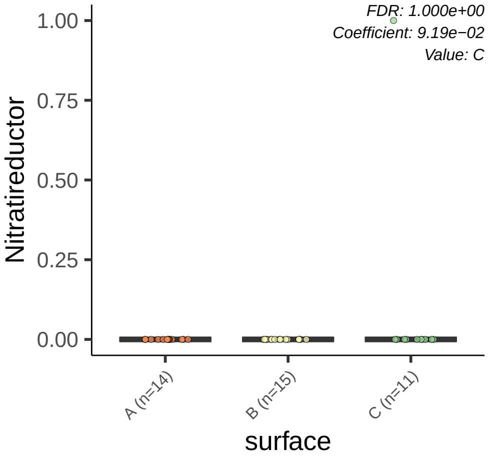

FDR: 1.000e+00
Coefficient: 9.19e−02
Value: C
Nitratireductor
1.00
0.75
0.50
0.25
0.00
A (n=14)
B (n=15)
C (n=11)
surface

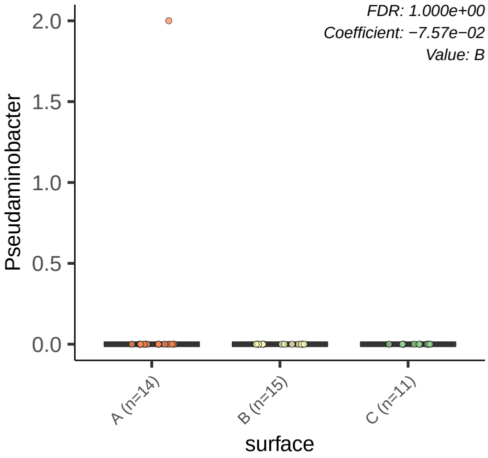
FDR: 1.000e+00
Coefficient: −7.57e−02
Value: B
Pseudaminobacter
2.0
1.5
1.0
0.5
0.0
A (n=14)
B (n=15)
C (n=11)
surface

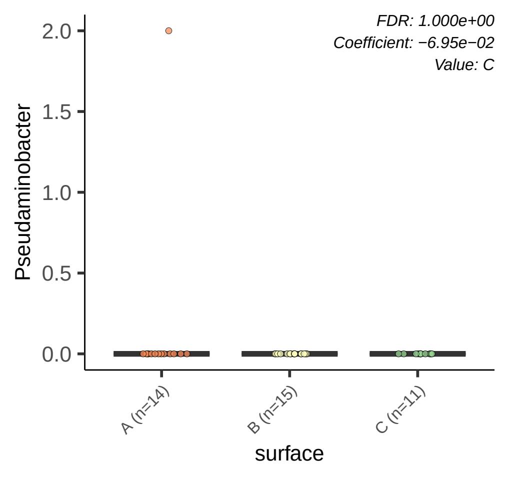

FDR: 1.000e+00
Coefficient: −6.95e−02
Value: C
Pseudaminobacter
2.0
1.5
1.0
0.5
0.0
A (n=14)
B (n=15)
C (n=11)
surface

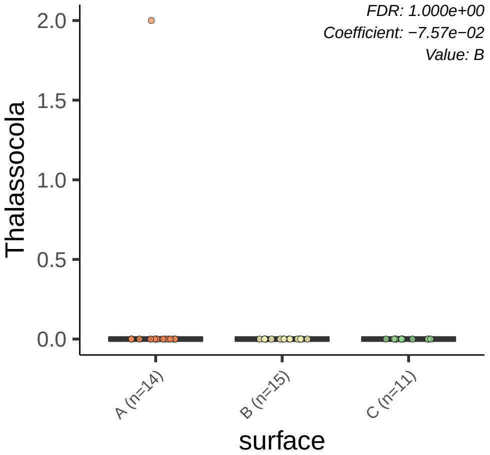

FDR: 1.000e+00
Coefficient: −7.57e−02
Value: B
Thalassocola
2.0
1.5
1.0
0.5
0.0
A (n=14)
B (n=15)
C (n=11)
surface

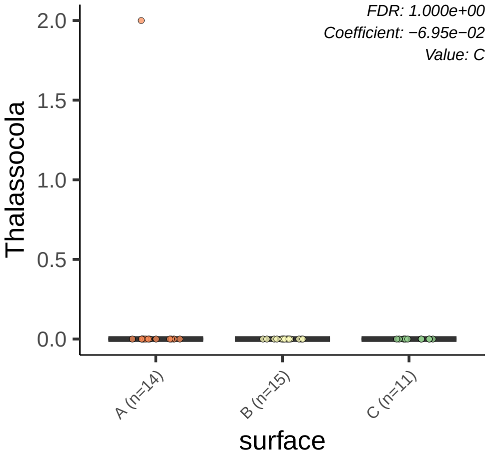

FDR: 1.000e+00
Coefficient: −6.95e−02
Value: C
Thalassocola
2.0
1.5
1.0
0.5
0.0
A (n=14)
B (n=15)
C (n=11)
surface

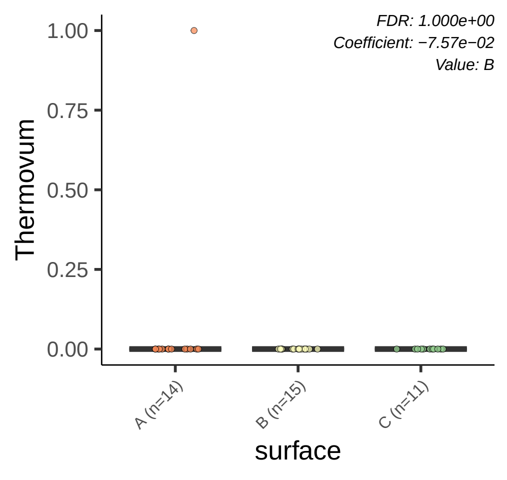

FDR: 1.000e+00
Coefficient: −7.57e−02
Value: B
Thermovum
1.00
0.75
0.50
0.25
0.00
A (n=14)
B (n=15)
C (n=11)
surface

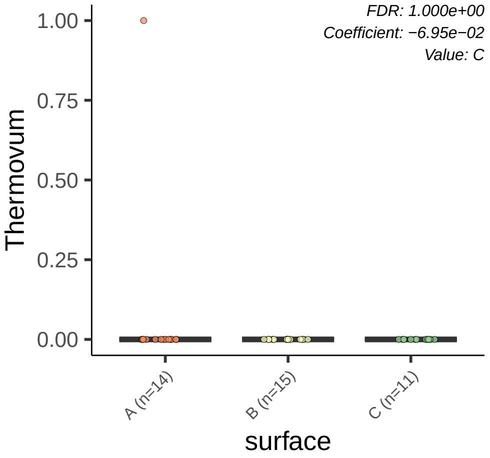

FDR: 1.000e+00
Coefficient: −6.95e−02
Value: C
1.00
0.75
0.50
0.25
0.00
Thermovum
A (n=14)
B (n=15)
C (n=11)
surface

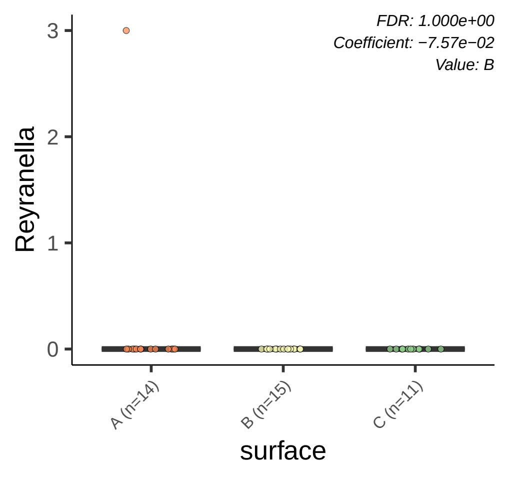

FDR: 1.000e+00
Coefficient: −7.57e−02
Value: B
Reyranella
3
2
1
0
A (n=14)
B (n=15)
C (n=11)
surface

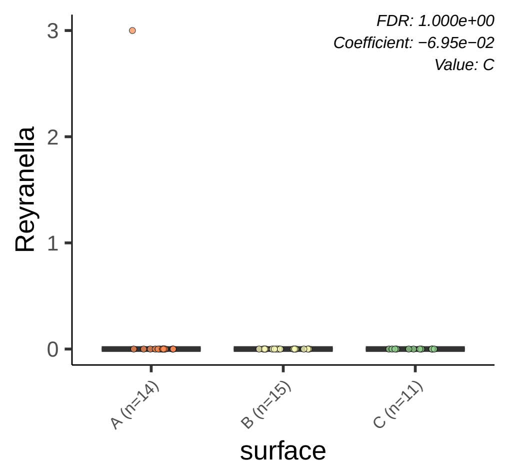

FDR: 1.000e+00
Coefficient: −6.95e−02
Value: C
Reyranella
3
2
1
0
A (n=14)
B (n=15)
C (n=11)
surface

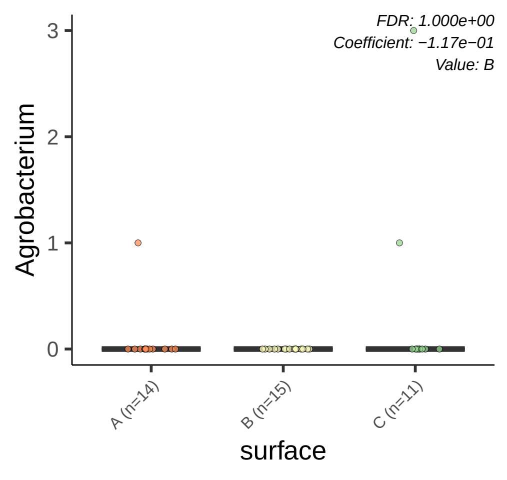
FDR: 1.000e+00
Coefficient: −1.17e−01
Value: B
Agrobacterium
3
2
1
0
A (n=14)
B (n=15)
C (n=11)
surface

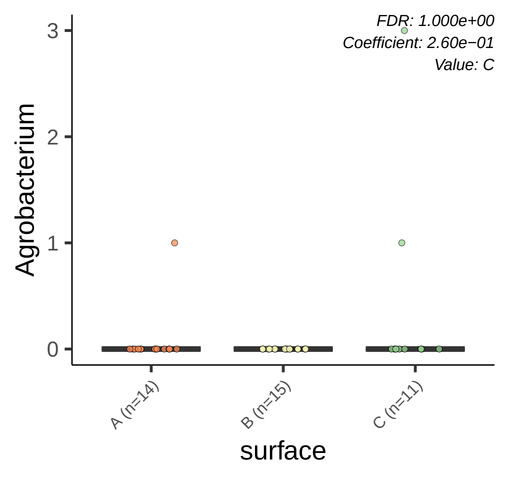
FDR: 1.000e+00
Coefficient: 2.60e−01
Value: C
Agrobacterium
0
1
2
3
A (n=14)
B (n=15)
C (n=11)
surface

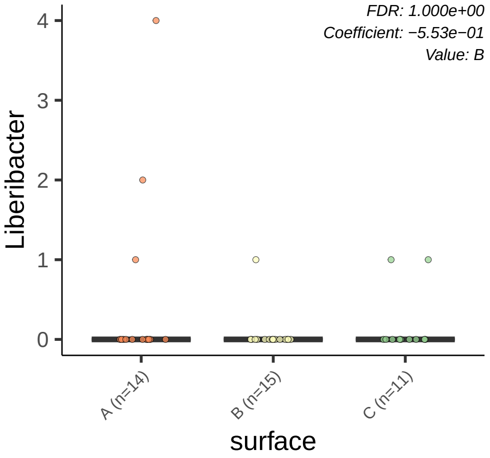
FDR: 1.000e+00
Coefficient: −5.53e−01
Value: B
Liberibacter
4
3
2
1
0
A (n=14)
B (n=15)
C (n=11)
surface

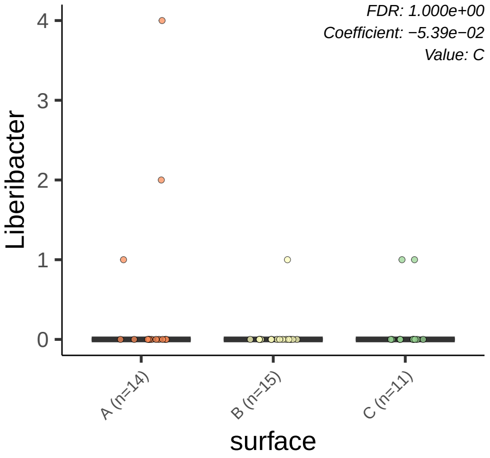
FDR: 1.000e+00
Coefficient: −5.39e−02
Value: C
Liberibacter
4
3
2
1
0
A (n=14)
B (n=15)
C (n=11)
surface

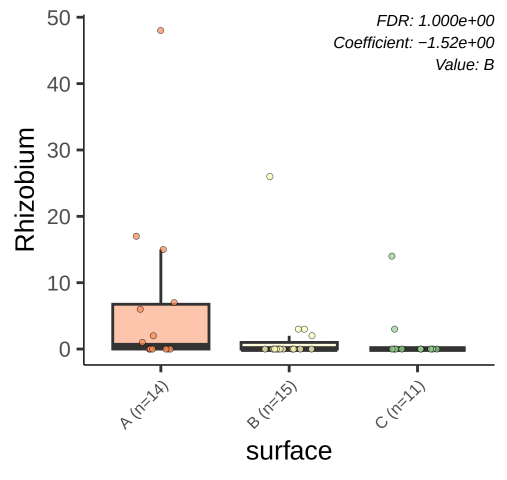
FDR: 1.000e+00
Coefficient: −1.52e+00
Value: B
Rhizobium
0
10
20
30
40
50
A (n=14)
B (n=15)
C (n=11)
surface

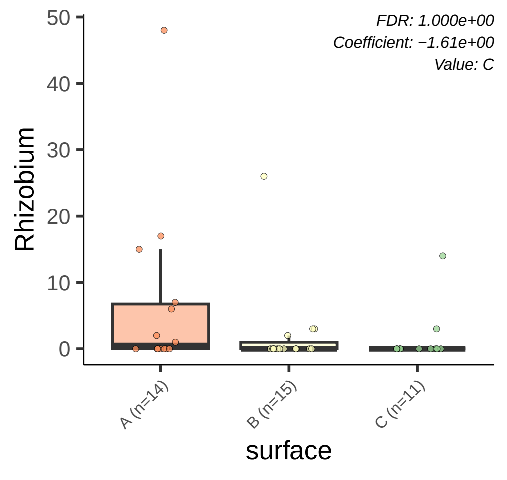
FDR: 1.000e+00
Coefficient: −1.61e+00
Value: C
Rhizobium
50
40
30
20
10
0
A (n=14)
B (n=15)
C (n=11)
surface

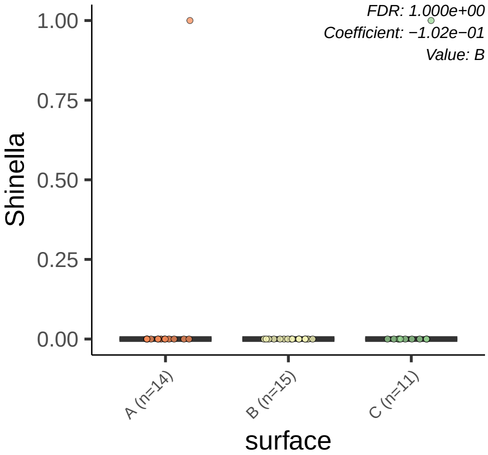
FDR: 1.000e+00
Coefficient: −1.02e−01
Value: B
Shinella
1.00
0.75
0.50
0.25
0.00
A (n=14)
B (n=15)
C (n=11)
surface

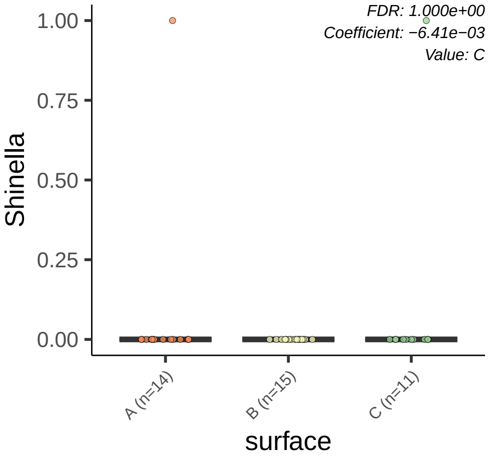

FDR: 1.000e+00
Coefficient: −6.41e−03
Value: C
Shinella
1.00
0.75
0.50
0.25
0.00
A (n=14)
B (n=15)
C (n=11)
surface

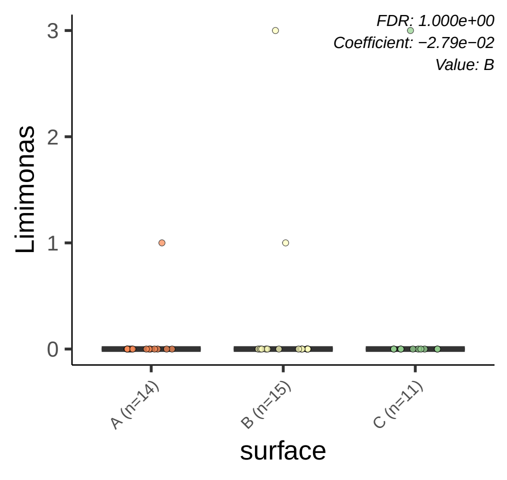
FDR: 1.000e+00
Coefficient: −2.79e−02
Value: B
Limimonas
3
2
1
0
A (n=14)
B (n=15)
C (n=11)
surface

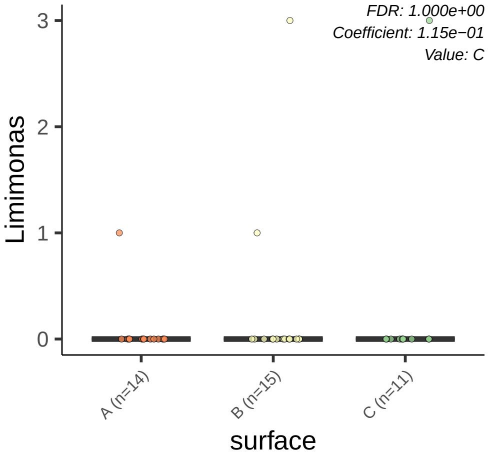

FDR: 1.000e+00
Coefficient: 1.15e−01
Value: C
Limimonas
0
1
2
3
A (n=14)
B (n=15)
C (n=11)
surface

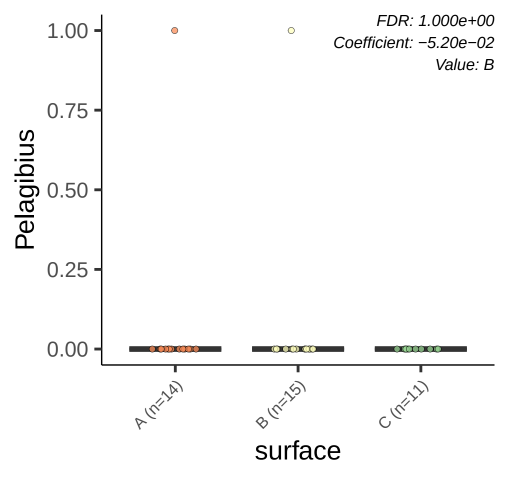
FDR: 1.000e+00
Coefficient: −5.20e−02
Value: B
Pelagibius
1.00
0.75
0.50
0.25
0.00
A (n=14)
B (n=15)
C (n=11)
surface

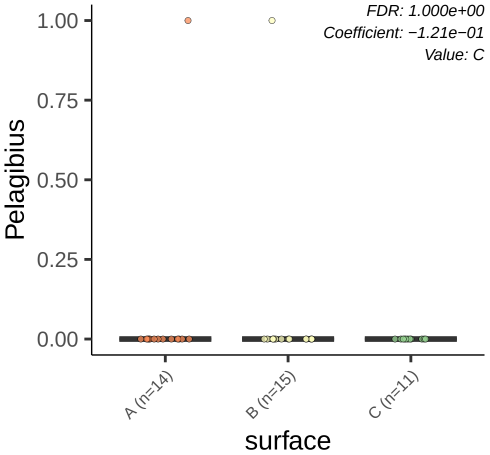
FDR: 1.000e+00
Coefficient: −1.21e−01
Value: C
Pelagibius
1.00
0.75
0.50
0.25
0.00
A (n=14)
B (n=15)
C (n=11)
surface

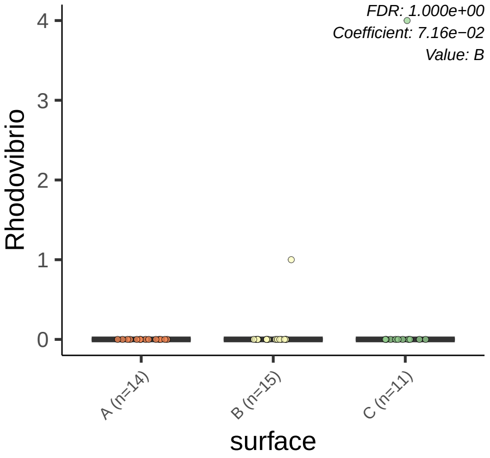

FDR: 1.000e+00
Coefficient: 7.16e−02
Value: B
Rhodovibrio
0
1
2
3
4
A (n=14)
B (n=15)
C (n=11)
surface

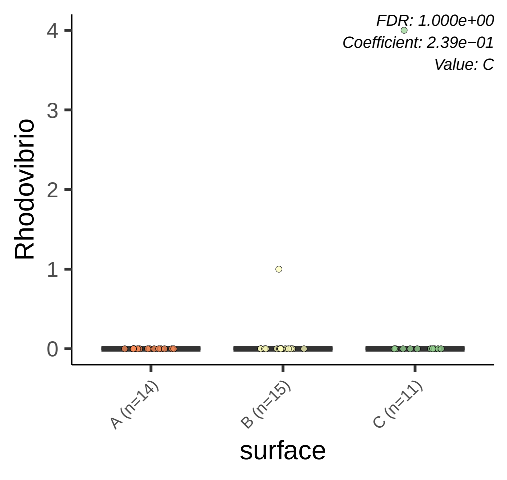
FDR: 1.000e+00
Coefficient: 2.39e−01
Value: C
Rhodovibrio
4
3
2
1
0
A (n=14)
B (n=15)
C (n=11)
surface

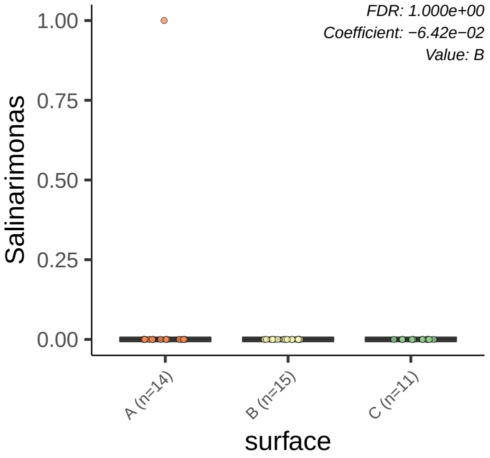
FDR: 1.000e+00
Coefficient: −6.42e−02
Value: B
Salinarimonas
1.00
0.75
0.50
0.25
0.00
A (n=14)
B (n=15)
C (n=11)
surface

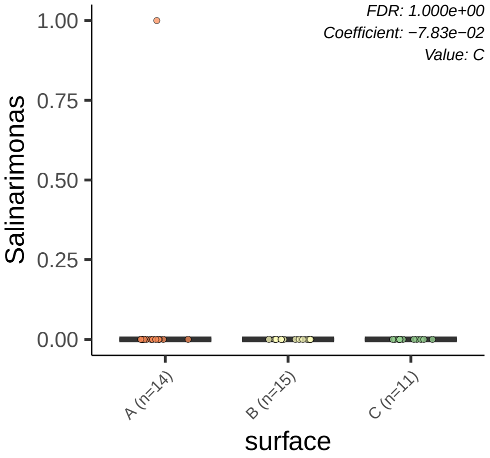
FDR: 1.000e+00
Coefficient: −7.83e−02
Value: C
Salinarimonas
1.00
0.75
0.50
0.25
0.00
A (n=14)
B (n=15)
C (n=11)
surface

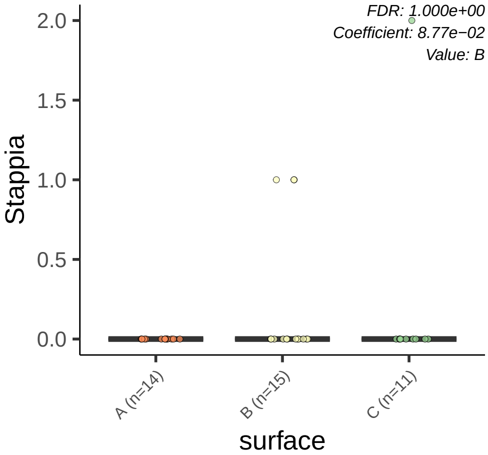
FDR: 1.000e+00
Coefficient: 8.77e−02
Value: B
Stappia
2.0
1.5
1.0
0.5
0.0
A (n=14)
B (n=15)
C (n=11)
surface

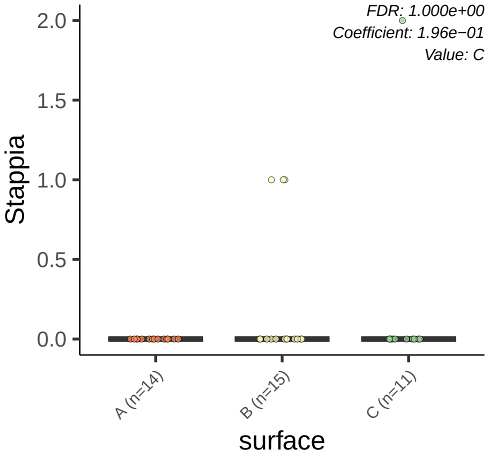
FDR: 1.000e+00
Coefficient: 1.96e−01
Value: C
Stappia
2.0
1.5
1.0
0.5
0.0
A (n=14)
B (n=15)
C (n=11)
surface

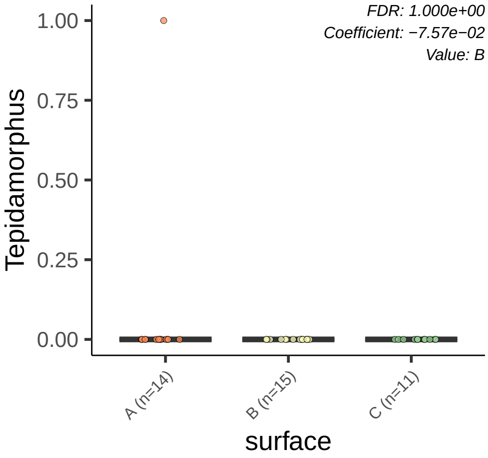
FDR: 1.000e+00
Coefficient: −7.57e−02
Value: B
Tepidamorphus
1.00
0.75
0.50
0.25
0.00
A (n=14)
B (n=15)
C (n=11)
surface

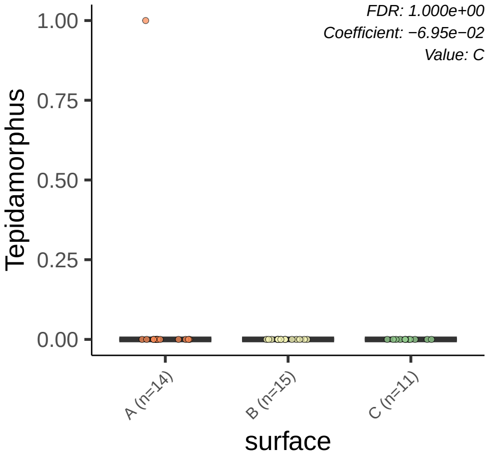
FDR: 1.000e+00
Coefficient: −6.95e−02
Value: C
Tepidamorphus
1.00
0.75
0.50
0.25
0.00
A (n=14)
B (n=15)
C (n=11)
surface

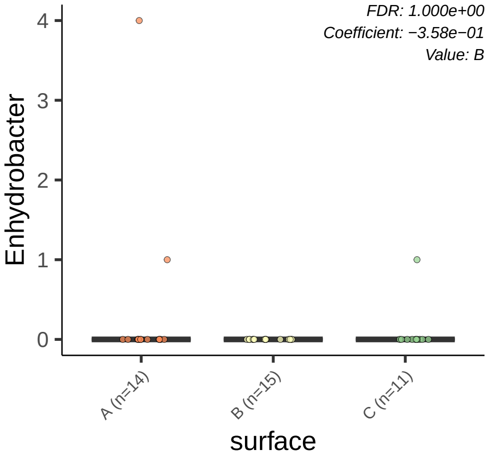

FDR: 1.000e+00
Coefficient: −3.58e−01
Value: B
Enhydrobacter
4
3
2
1
0
A (n=14)
B (n=15)
C (n=11)
surface

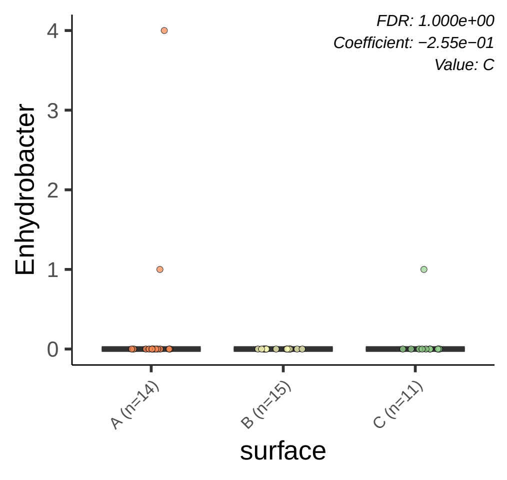
FDR: 1.000e+00
Coefficient: −2.55e−01
Value: C
Enhydrobacter
4
3
2
1
0
A (n=14)
B (n=15)
C (n=11)
surface

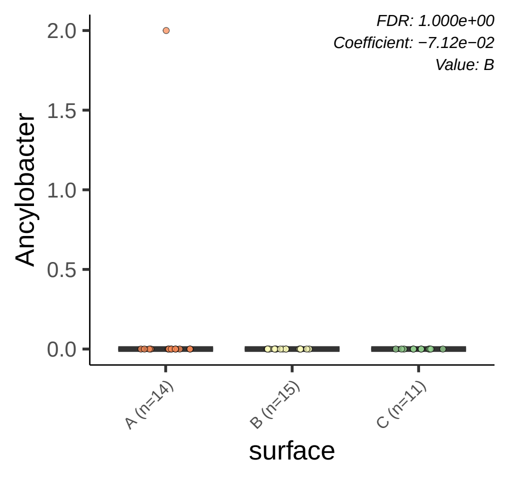
FDR: 1.000e+00
Coefficient: −7.12e−02
Value: B
Ancylobacter
2.0
1.5
1.0
0.5
0.0
A (n=14)
B (n=15)
C (n=11)
surface

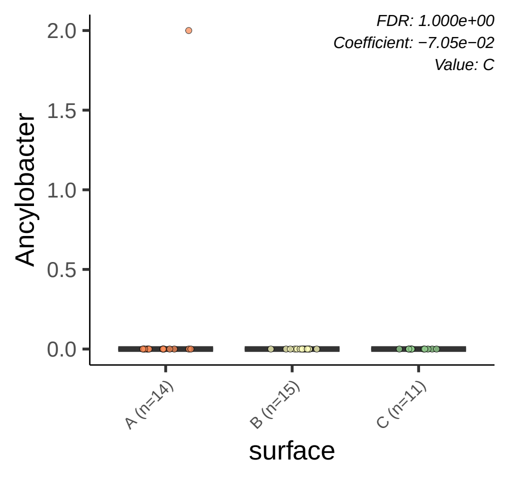
FDR: 1.000e+00
Coefficient: −7.05e−02
Value: C
Ancylobacter
2.0
1.5
1.0
0.5
0.0
A (n=14)
B (n=15)
C (n=11)
surface

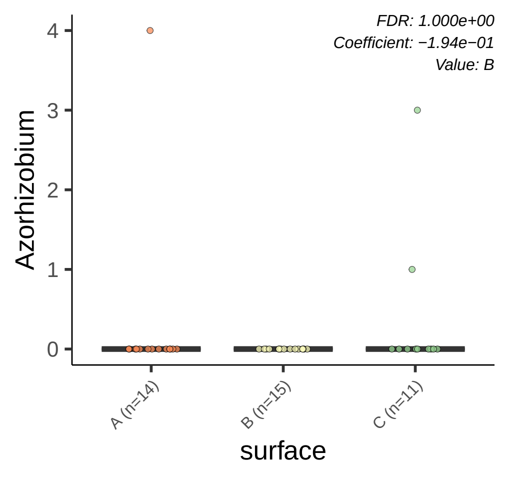

FDR: 1.000e+00
Coefficient: −1.94e−01
Value: B
Azorhizobium
4
3
2
1
0
A (n=14)
B (n=15)
C (n=11)
surface

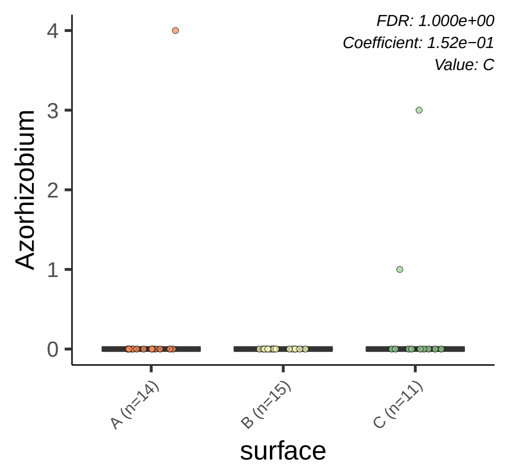

FDR: 1.000e+00
Coefficient: 1.52e−01
Value: C
Azorhizobium
0
1
2
3
4
A (n=14)
B (n=15)
C (n=11)
surface

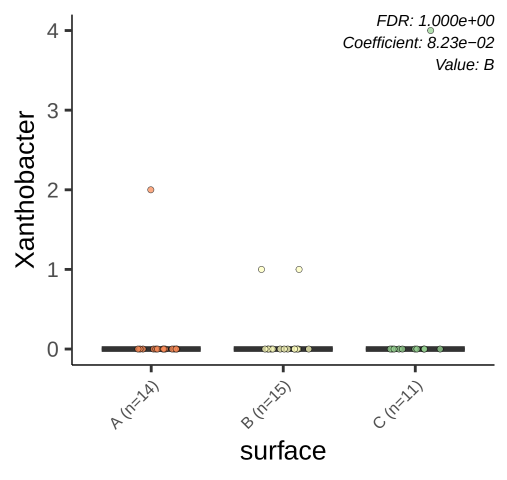

FDR: 1.000e+00
Coefficient: 8.23e−02
Value: B
Xanthobacter
4
3
2
1
0
A (n=14)
B (n=15)
C (n=11)
surface

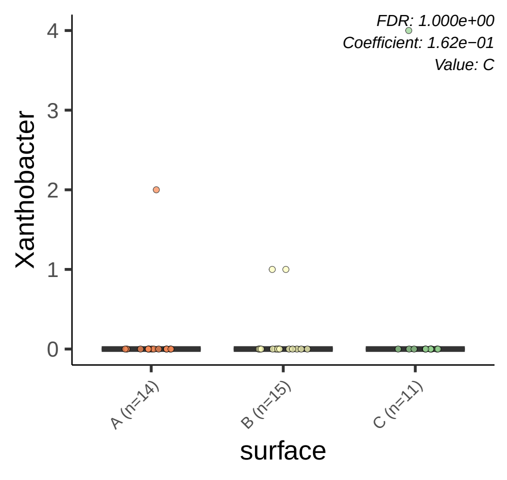

FDR: 1.000e+00
Coefficient: 1.62e−01
Value: C
Xanthobacter
0
1
2
3
4
A (n=14)
B (n=15)
C (n=11)
surface

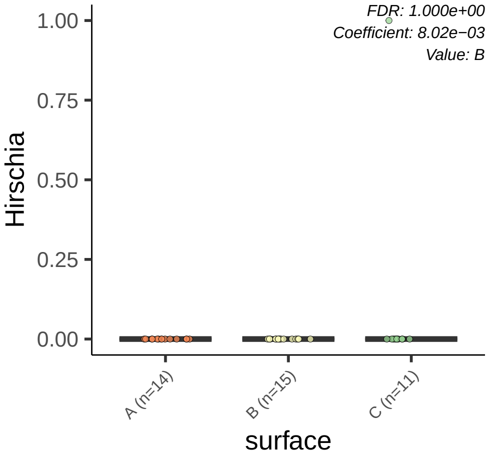

FDR: 1.000e+00
Coefficient: 8.02e−03
Value: B
Hirschia
1.00
0.75
0.50
0.25
0.00
A (n=14)
B (n=15)
C (n=11)
surface

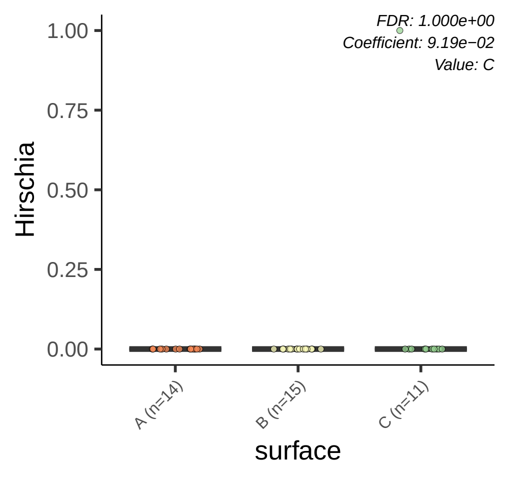

FDR: 1.000e+00
Coefficient: 9.19e−02
Value: C
Hirschia
1.00
0.75
0.50
0.25
0.00
A (n=14)
B (n=15)
C (n=11)
surface

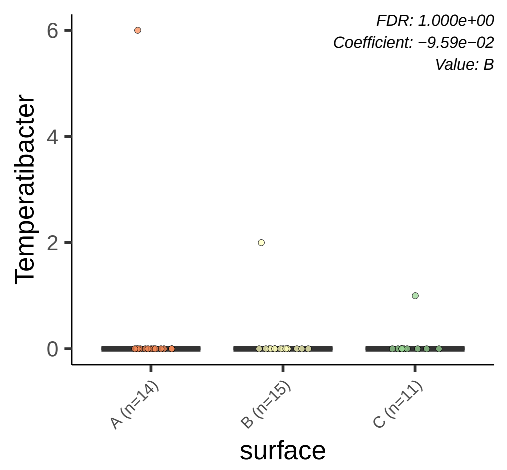
FDR: 1.000e+00
Coefficient: −9.59e−02
Value: B
Temperatibacter
6
4
2
0
A (n=14)
B (n=15)
C (n=11)
surface

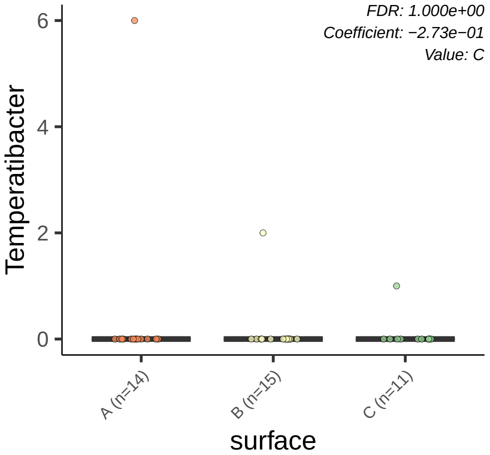
FDR: 1.000e+00
Coefficient: −2.73e−01
Value: C
Temperatibacter
6
4
2
0
A (n=14)
B (n=15)
C (n=11)
surface

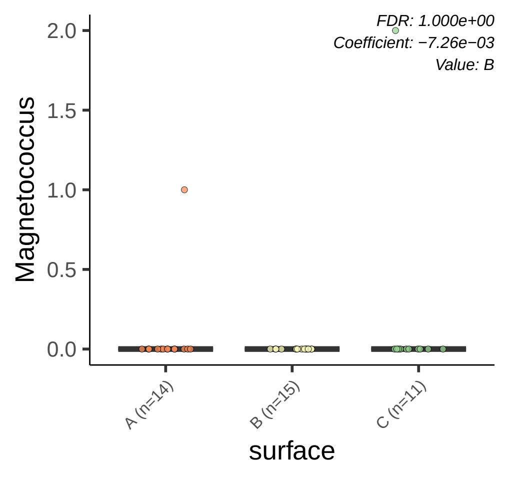

FDR: 1.000e+00
Coefficient: −7.26e−03
Value: B
Magnetococcus
2.0
1.5
1.0
0.5
0.0
A (n=14)
B (n=15)
C (n=11)
surface

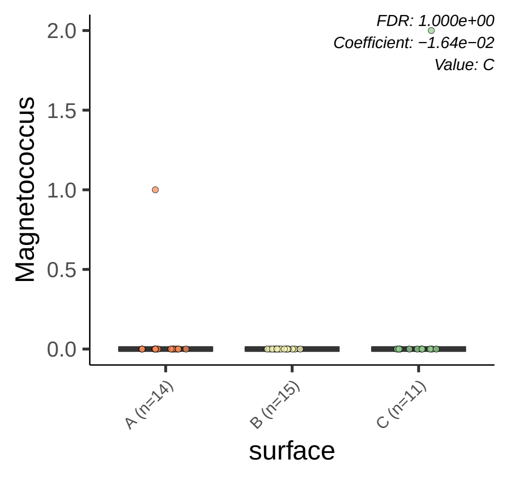

FDR: 1.000e+00
Coefficient: −1.64e−02
Value: C
Magnetococcus
2.0
1.5
1.0
0.5
0.0
A (n=14)
B (n=15)
C (n=11)
surface

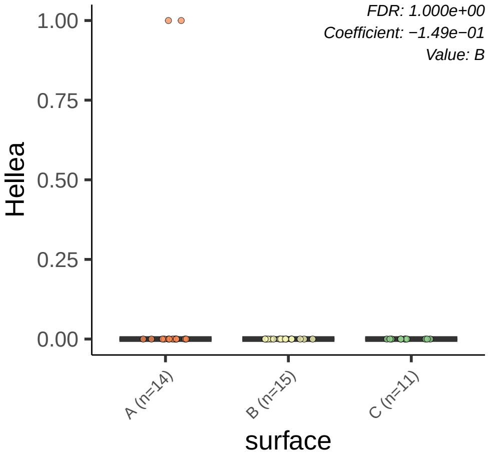
FDR: 1.000e+00
Coefficient: −1.49e−01
Value: B
Hellea
1.00
0.75
0.50
0.25
0.00
A (n=14)
B (n=15)
C (n=11)
surface

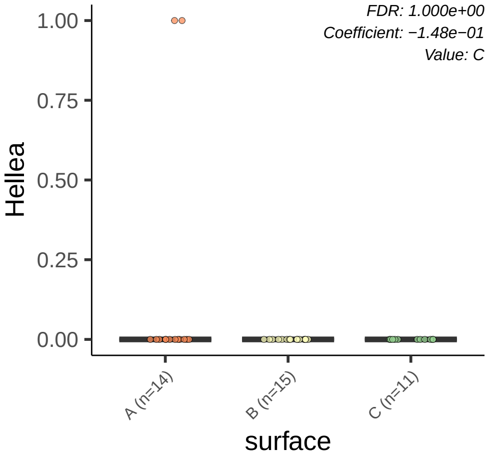
FDR: 1.000e+00
Coefficient: −1.48e−01
Value: C
Hellea
1.00
0.75
0.50
0.25
0.00
A (n=14)
B (n=15)
C (n=11)
surface

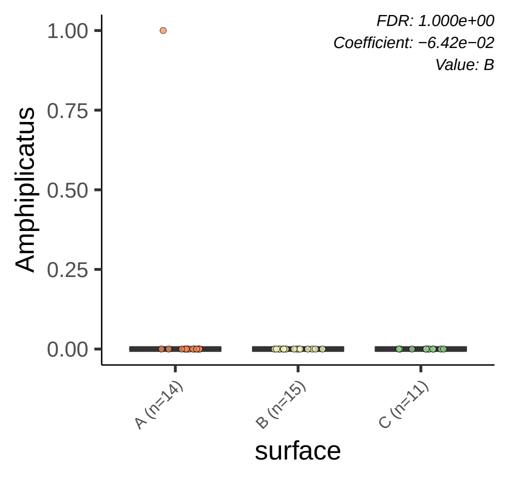

FDR: 1.000e+00
Coefficient: −6.42e−02
Value: B
Amphiplicatus
1.00
0.75
0.50
0.25
0.00
A (n=14)
B (n=15)
C (n=11)
surface

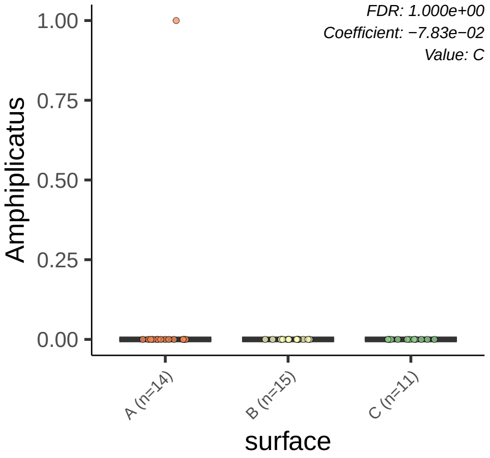
FDR: 1.000e+00
Coefficient: −7.83e−02
Value: C
Amphiplicatus
1.00
0.75
0.50
0.25
0.00
A (n=14)
B (n=15)
C (n=11)
surface

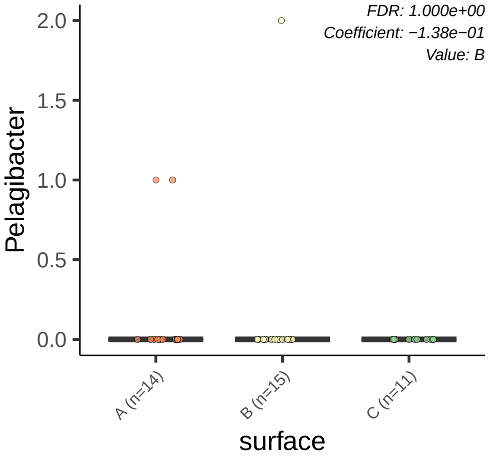

FDR: 1.000e+00
Coefficient: −1.38e−01
Value: B
Pelagibacter
2.0
1.5
1.0
0.5
0.0
A (n=14)
B (n=15)
C (n=11)
surface

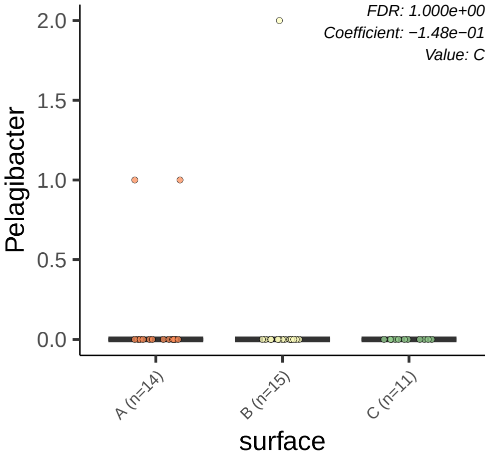

FDR: 1.000e+00
Coefficient: −1.48e−01
Value: C
Pelagibacter
2.0
1.5
1.0
0.5
0.0
A (n=14)
B (n=15)
C (n=11)
surface

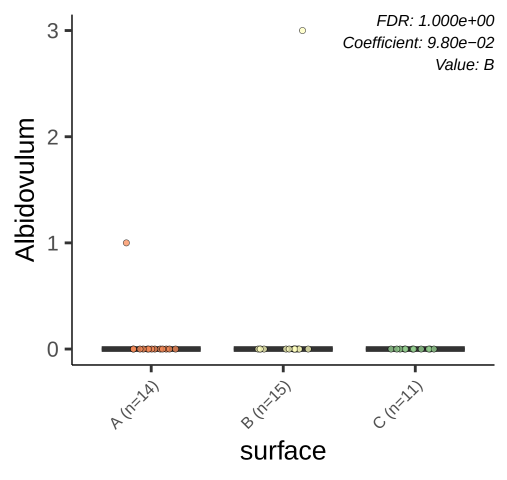
FDR: 1.000e+00
Coefficient: 9.80e−02
Value: B
Albidovulum
3
2
1
0
A (n=14)
B (n=15)
C (n=11)
surface

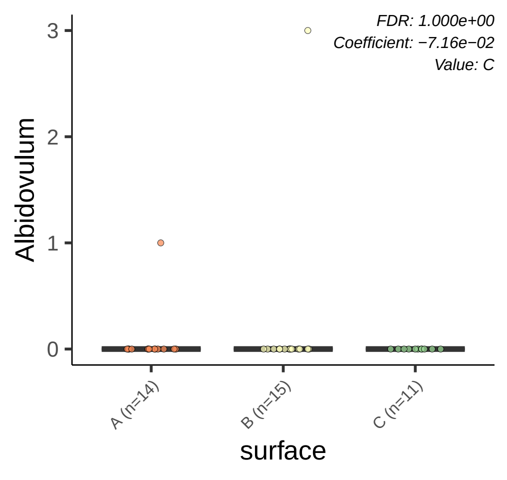
FDR: 1.000e+00
Coefficient: −7.16e−02
Value: C
Albidovulum
3
2
1
0
A (n=14)
B (n=15)
C (n=11)
surface

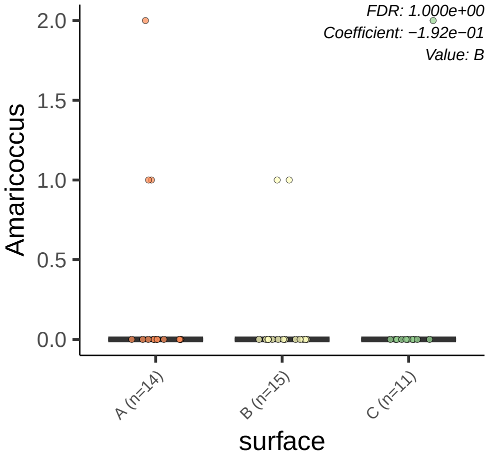
FDR: 1.000e+00
Coefficient: −1.92e−01
Value: B
Amaricoccus
2.0
1.5
1.0
0.5
0.0
A (n=14)
B (n=15)
C (n=11)
surface

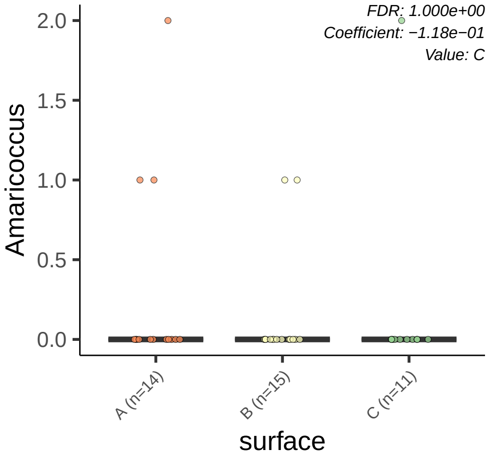

FDR: 1.000e+00
Coefficient: −1.18e−01
Value: C
Amaricoccus
2.0
1.5
1.0
0.5
0.0
A (n=14)
B (n=15)
C (n=11)
surface

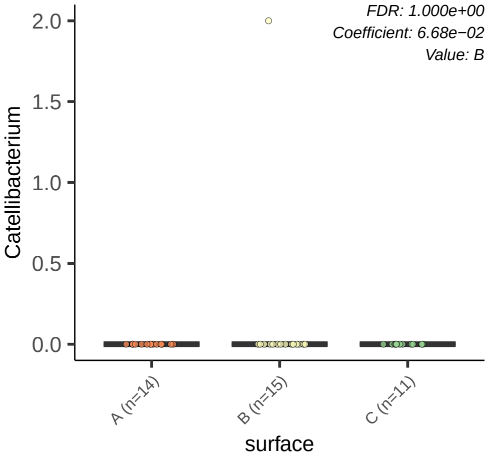
FDR: 1.000e+00
Coefficient: 6.68e−02
Value: B
2.0
1.5
1.0
0.5
0.0
Catellibacterium
A (n=14)
B (n=15)
C (n=11)
surface

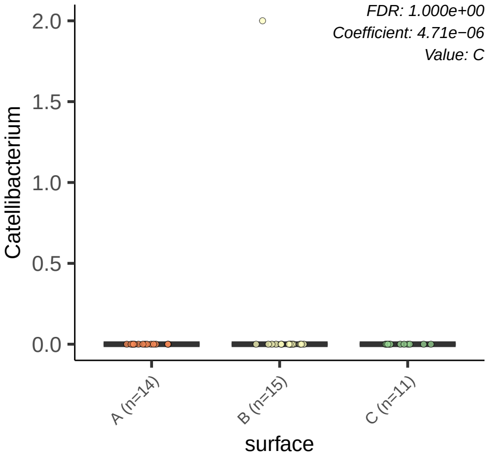
FDR: 1.000e+00
Coefficient: 4.71e−06
Value: C
Catellibacterium
2.0
1.5
1.0
0.5
0.0
A (n=14)
B (n=15)
C (n=11)
surface

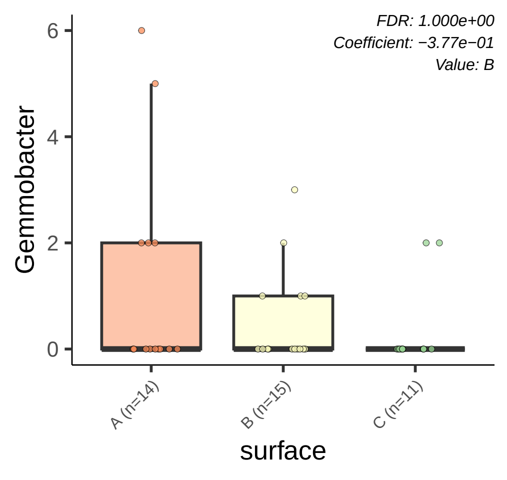
FDR: 1.000e+00
Coefficient: −3.77e−01
Value: B
Gemmobacter
0
2
4
6
A (n=14)
B (n=15)
C (n=11)
surface

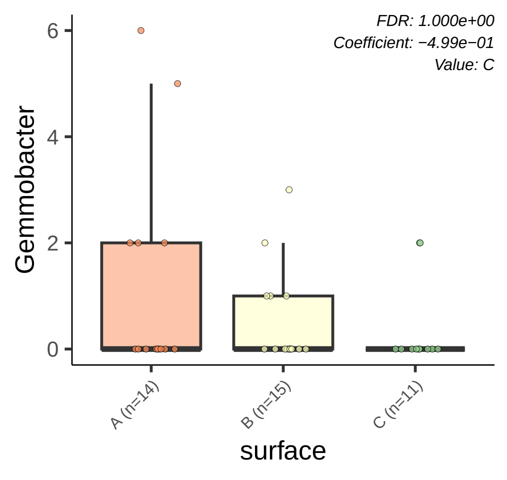
FDR: 1.000e+00
Coefficient: −4.99e−01
Value: C
Gemmobacter
0
2
4
6
A (n=14)
B (n=15)
C (n=11)
surface

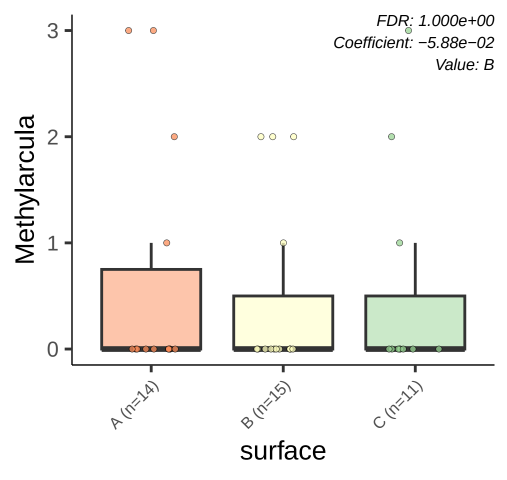
FDR: 1.000e+00
Coefficient: −5.88e−02
Value: B
Methylarcula
3
2
1
0
A (n=14)
B (n=15)
C (n=11)
surface

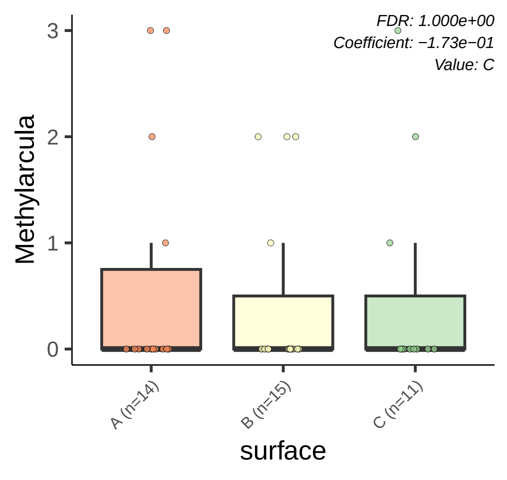
FDR: 1.000e+00
Coefficient: −1.73e−01
Value: C
Methylarcula
3
2
1
0
A (n=14)
B (n=15)
C (n=11)
surface

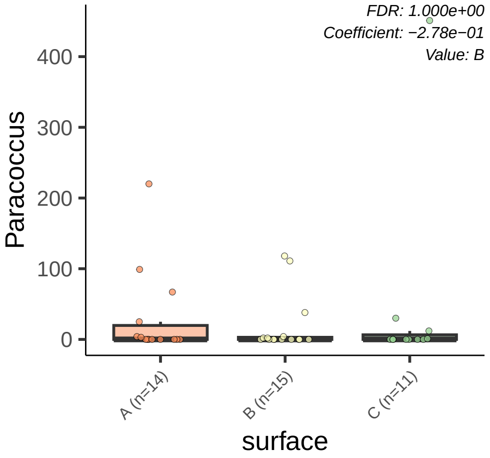
FDR: 1.000e+00
Coefficient: −2.78e−01
Value: B
Paracoccus
400
300
200
100
0
A (n=14)
B (n=15)
C (n=11)
surface

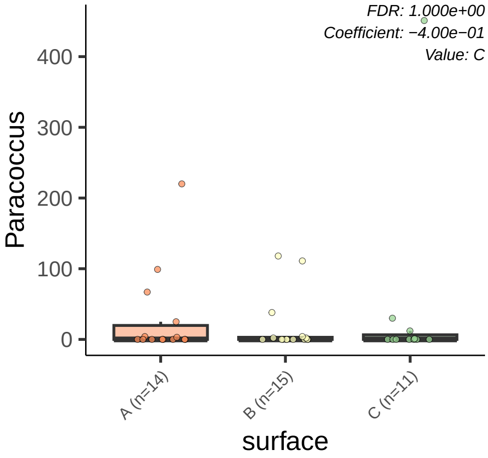

FDR: 1.000e+00
Coefficient: −4.00e−01
Value: C
Paracoccus
400
300
200
100
0
A (n=14)
B (n=15)
C (n=11)
surface

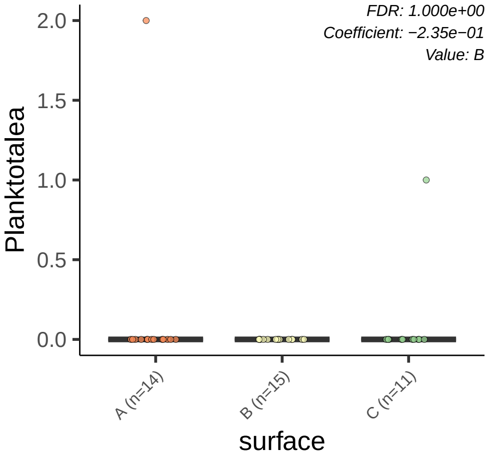
FDR: 1.000e+00
Coefficient: −2.35e−01
Value: B
Planktotalea
2.0
1.5
1.0
0.5
0.0
A (n=14)
B (n=15)
C (n=11)
surface

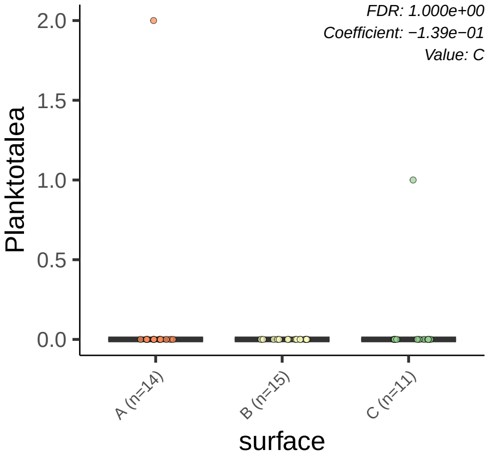
FDR: 1.000e+00
Coefficient: −1.39e−01
Value: C
Planktotalea
2.0
1.5
1.0
0.5
0.0
A (n=14)
B (n=15)
C (n=11)
surface

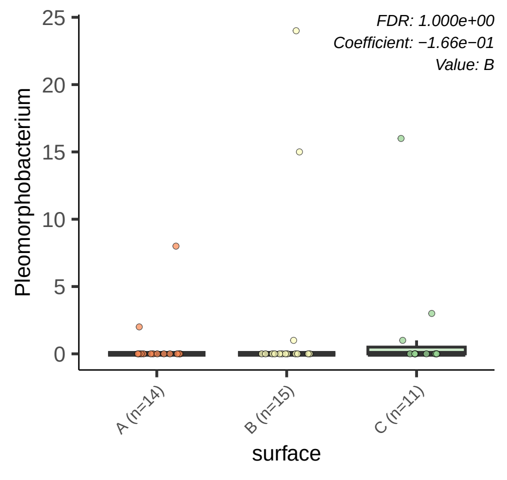
FDR: 1.000e+00
Coefficient: −1.66e−01
Value: B
Pleomorphobacterium
25
20
15
10
5
0
A (n=14)
B (n=15)
C (n=11)
surface

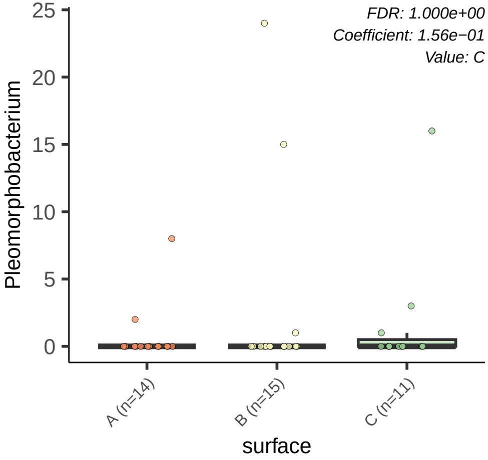

FDR: 1.000e+00
Coefficient: 1.56e−01
Value: C
Pleomorphobacterium
0
5
10
15
20
25
A (n=14)
B (n=15)
C (n=11)
surface

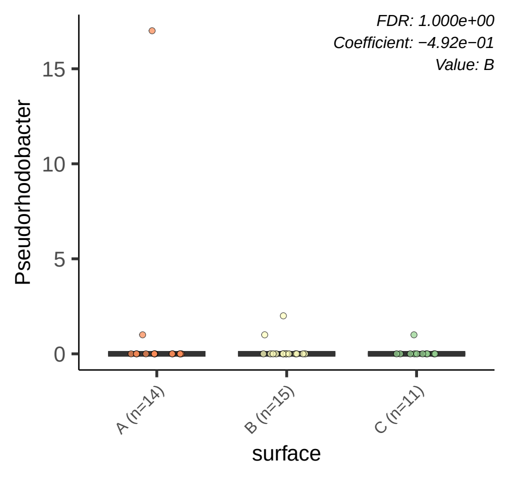
FDR: 1.000e+00
Coefficient: −4.92e−01
Value: B
Pseudorhodobacter
0
5
10
15
A (n=14)
B (n=15)
C (n=11)
surface

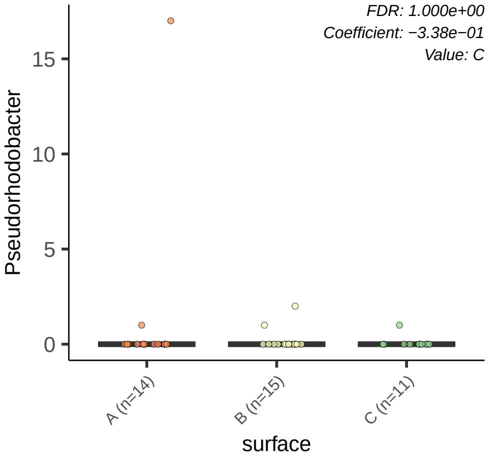
FDR: 1.000e+00
Coefficient: −3.38e−01
Value: C
Pseudorhodobacter
0
5
10
15
A (n=14)
B (n=15)
C (n=11)
surface

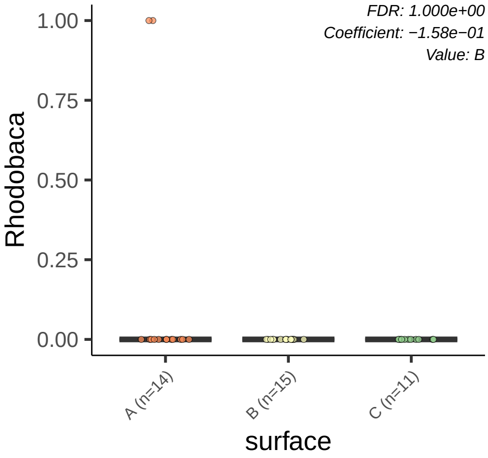

FDR: 1.000e+00
Coefficient: −1.58e−01
Value: B
Rhodobaca
1.00
0.75
0.50
0.25
0.00
A (n=14)
B (n=15)
C (n=11)
surface

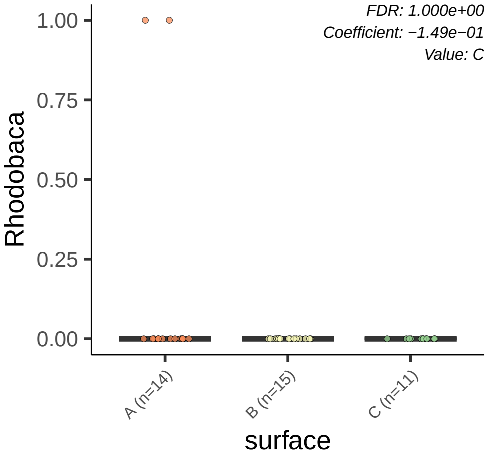

FDR: 1.000e+00
Coefficient: −1.49e−01
Value: C
Rhodobaca
1.00
0.75
0.50
0.25
0.00
A (n=14)
B (n=15)
C (n=11)
surface

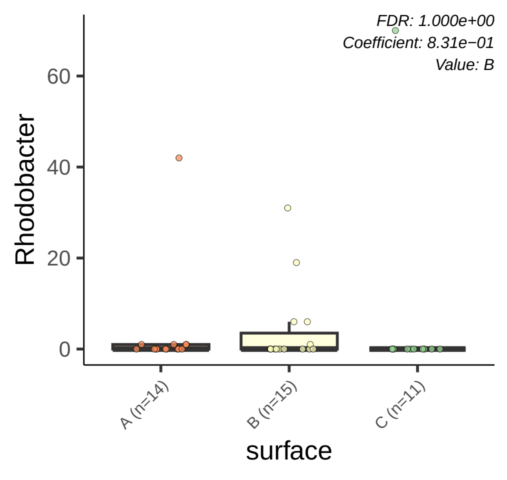

FDR: 1.000e+00
Coefficient: 8.31e−01
Value: B
Rhodobacter
60
40
20
0
A (n=14)
B (n=15)
C (n=11)
surface

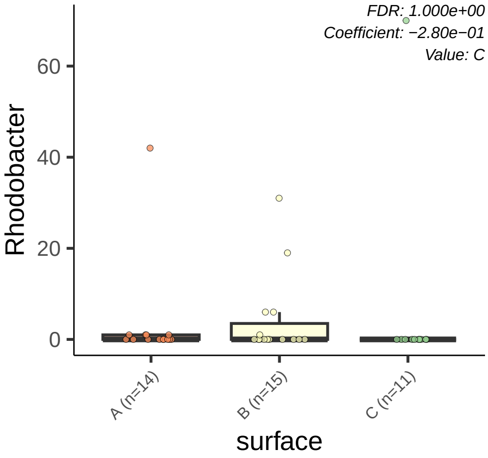

FDR: 1.000e+00
Coefficient: −2.80e−01
Value: C
Rhodobacter
60
40
20
0
A (n=14)
B (n=15)
C (n=11)
surface

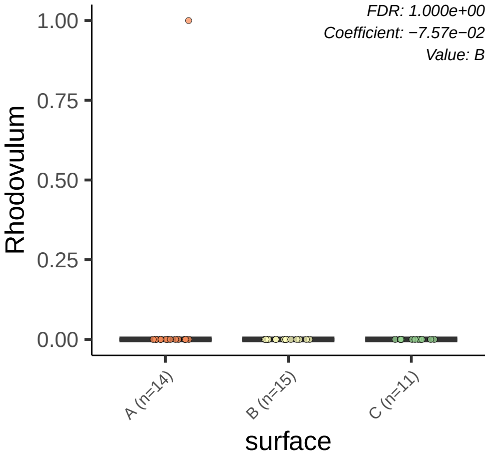

FDR: 1.000e+00
Coefficient: −7.57e−02
Value: B
Rhodovulum
1.00
0.75
0.50
0.25
0.00
A (n=14)
B (n=15)
C (n=11)
surface

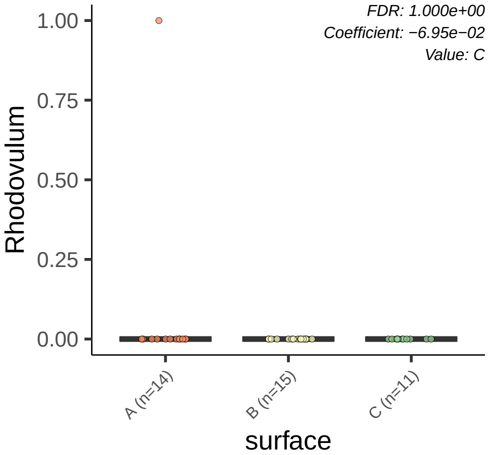
FDR: 1.000e+00
Coefficient: −6.95e−02
Value: C
Rhodovulum
1.00
0.75
0.50
0.25
0.00
A (n=14)
B (n=15)
C (n=11)
surface

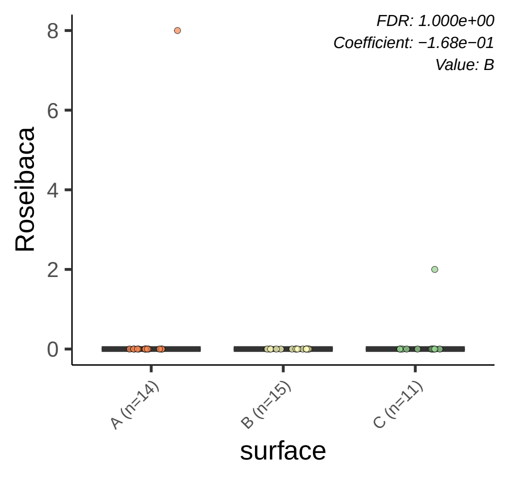
FDR: 1.000e+00
Coefficient: −1.68e−01
Value: B
Roseibaca
0
2
4
6
8
A (n=14)
B (n=15)
C (n=11)
surface

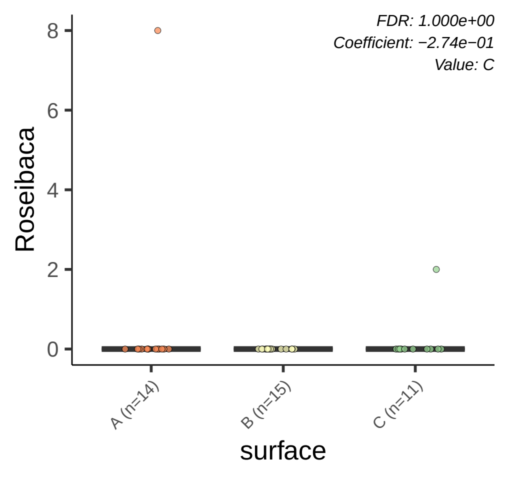

FDR: 1.000e+00
Coefficient: −2.74e−01
Value: C
Roseibaca
8
6
4
2
0
A (n=14)
B (n=15)
C (n=11)
surface

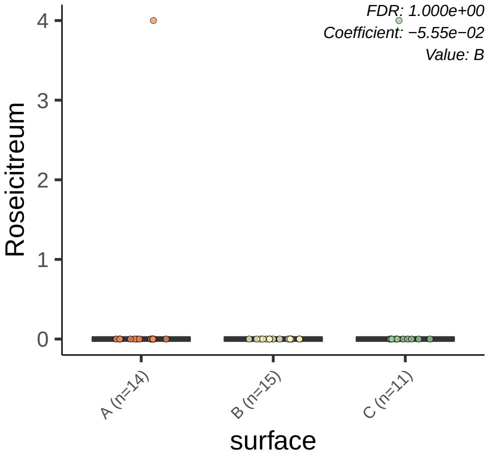
FDR: 1.000e+00
Coefficient: −5.55e−02
Value: B
Roseicitreum
0
1
2
3
4
A (n=14)
B (n=15)
C (n=11)
surface

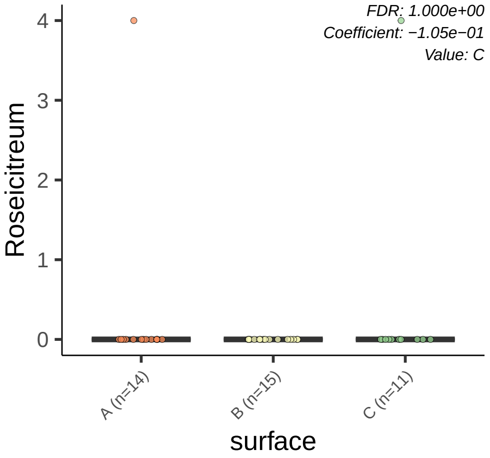

FDR: 1.000e+00
Coefficient: −1.05e−01
Value: C
Roseicitreum
0
1
2
3
4
A (n=14)
B (n=15)
C (n=11)
surface

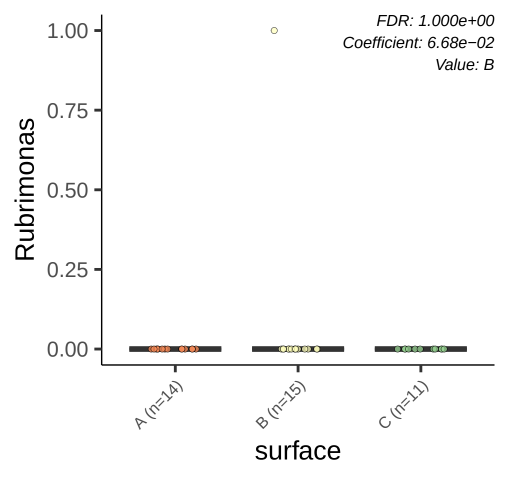

FDR: 1.000e+00
Coefficient: 6.68e−02
Value: B
1.00
0.75
0.50
0.25
0.00
Rubrimonas
A (n=14)
B (n=15)
C (n=11)
surface

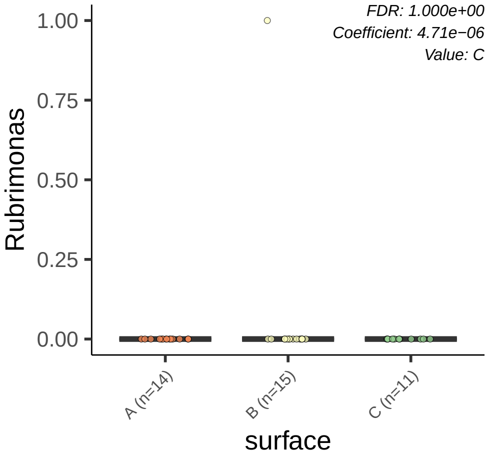

FDR: 1.000e+00
Coefficient: 4.71e−06
Value: C
Rubrimonas
1.00
0.75
0.50
0.25
0.00
A (n=14)
B (n=15)
C (n=11)
surface

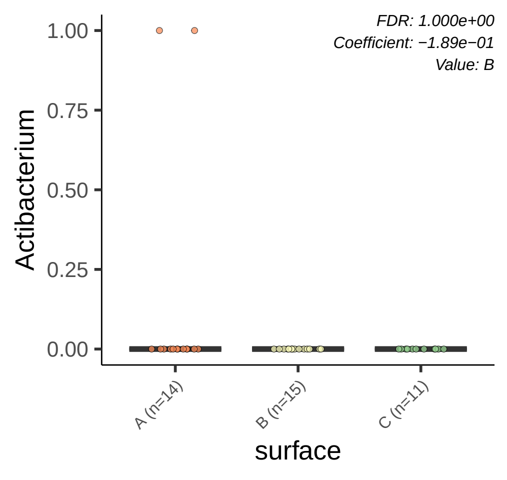
FDR: 1.000e+00
Coefficient: −1.89e−01
Value: B
Actibacterium
1.00
0.75
0.50
0.25
0.00
A (n=14)
B (n=15)
C (n=11)
surface

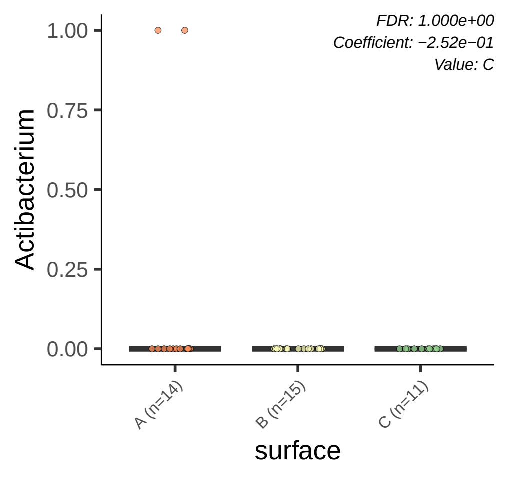
FDR: 1.000e+00
Coefficient: −2.52e−01
Value: C
Actibacterium
1.00
0.75
0.50
0.25
0.00
A (n=14)
B (n=15)
C (n=11)
surface

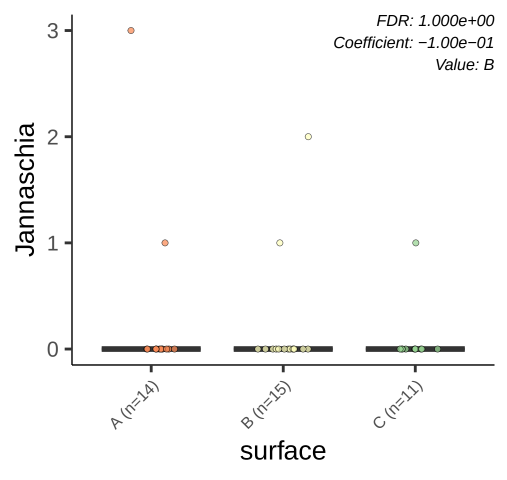
FDR: 1.000e+00
Coefficient: −1.00e−01
Value: B
Jannaschia
3
2
1
0
A (n=14)
B (n=15)
C (n=11)
surface

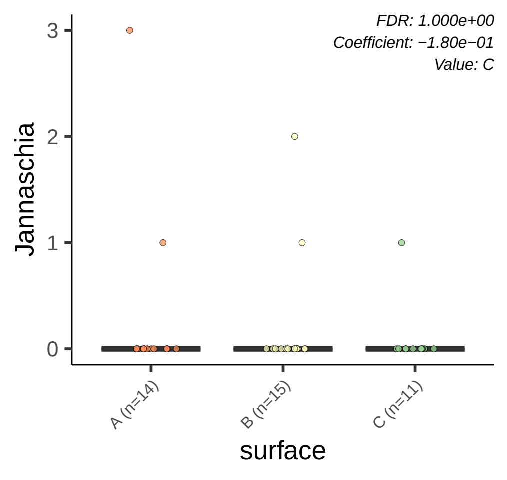

FDR: 1.000e+00
Coefficient: −1.80e−01
Value: C
Jannaschia
3
2
1
0
A (n=14)
B (n=15)
C (n=11)
surface

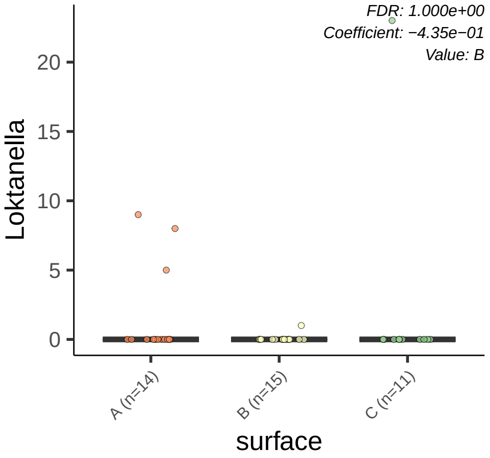

FDR: 1.000e+00
Coefficient: −4.35e−01
Value: B
Loktanella
20
15
10
5
0
A (n=14)
B (n=15)
C (n=11)
surface

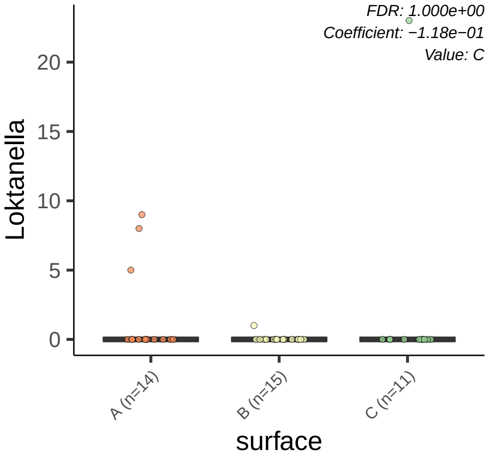
FDR: 1.000e+00
Coefficient: −1.18e−01
Value: C
Loktanella
0
5
10
15
20
A (n=14)
B (n=15)
C (n=11)
surface

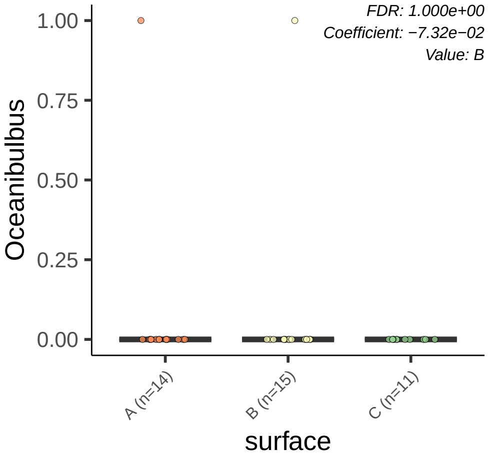
FDR: 1.000e+00
Coefficient: −7.32e−02
Value: B
Oceanibulbus
1.00
0.75
0.50
0.25
0.00
A (n=14)
B (n=15)
C (n=11)
surface

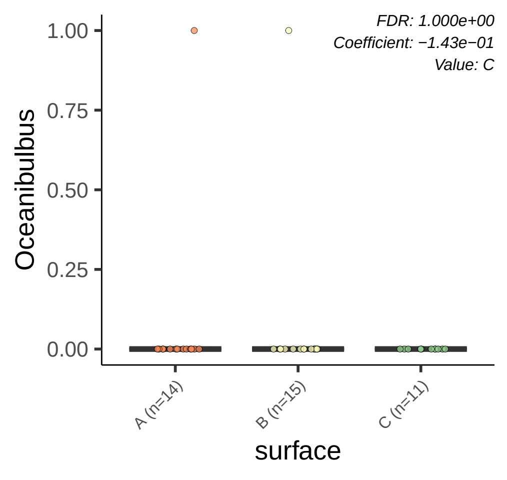
FDR: 1.000e+00
Coefficient: −1.43e−01
Value: C
Oceanibulbus
1.00
0.75
0.50
0.25
0.00
A (n=14)
B (n=15)
C (n=11)
surface

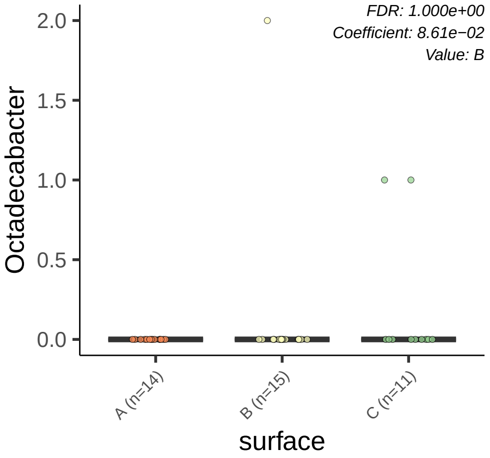
FDR: 1.000e+00
Coefficient: 8.61e−02
Value: B
Octadecabacter
2.0
1.5
1.0
0.5
0.0
A (n=14)
B (n=15)
C (n=11)
surface

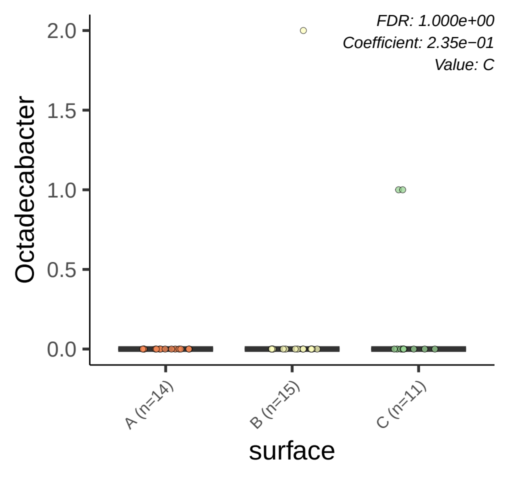

FDR: 1.000e+00
Coefficient: 2.35e−01
Value: C
Octadecabacter
2.0
1.5
1.0
0.5
0.0
A (n=14)
B (n=15)
C (n=11)
surface

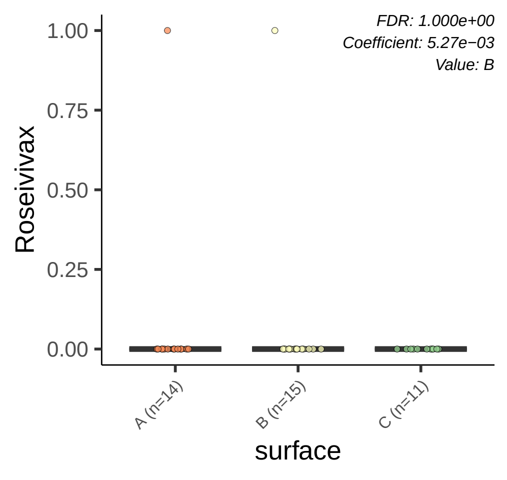
FDR: 1.000e+00
Coefficient: 5.27e−03
Value: B
Roseivivax
1.00
0.75
0.50
0.25
0.00
A (n=14)
B (n=15)
C (n=11)
surface

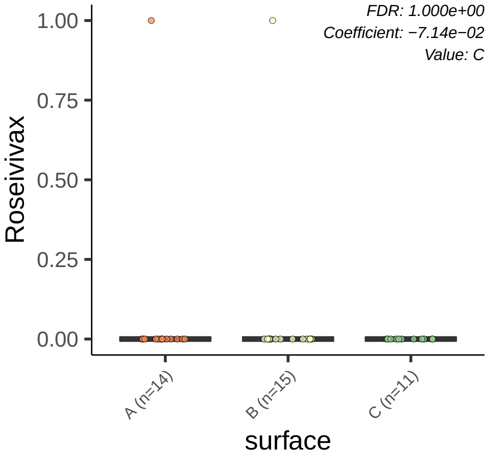
FDR: 1.000e+00
Coefficient: −7.14e−02
Value: C
Roseivivax
1.00
0.75
0.50
0.25
0.00
A (n=14)
B (n=15)
C (n=11)
surface

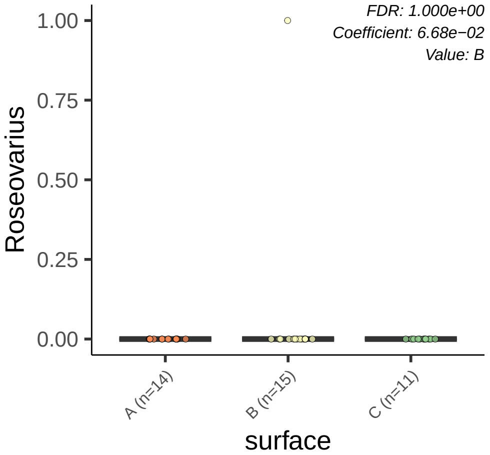
FDR: 1.000e+00
Coefficient: 6.68e−02
Value: B
Roseovarius
1.00
0.75
0.50
0.25
0.00
A (n=14)
B (n=15)
C (n=11)
surface

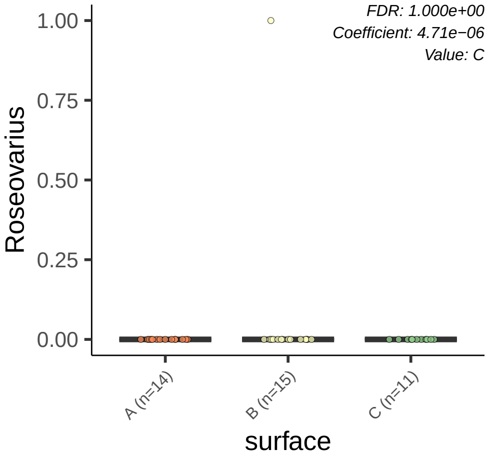
FDR: 1.000e+00
Coefficient: 4.71e−06
Value: C
Roseovarius
1.00
0.75
0.50
0.25
0.00
A (n=14)
B (n=15)
C (n=11)
surface

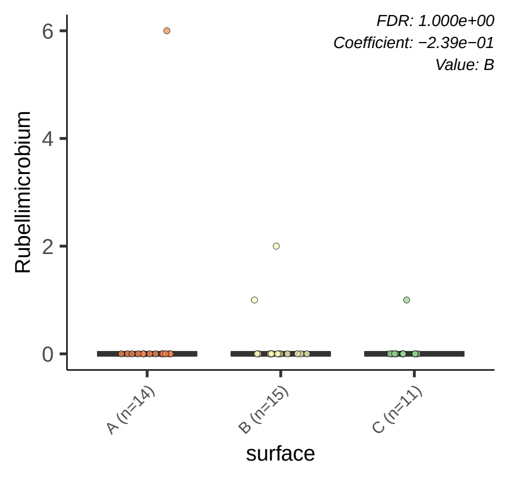
FDR: 1.000e+00
Coefficient: −2.39e−01
Value: B
Rubellimicrobium
6
4
2
0
A (n=14)
B (n=15)
C (n=11)
surface

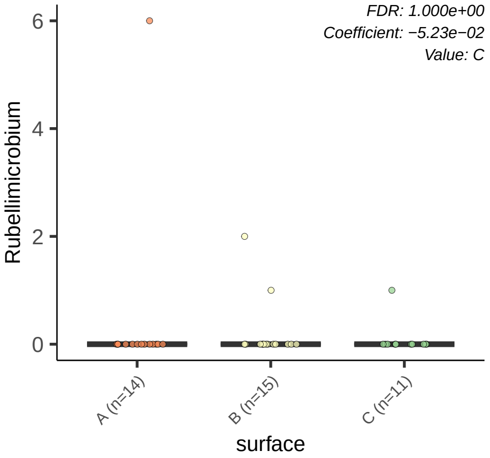
FDR: 1.000e+00
Coefficient: −5.23e−02
Value: C
Rubellimicrobium
6
4
2
0
A (n=14)
B (n=15)
C (n=11)
surface

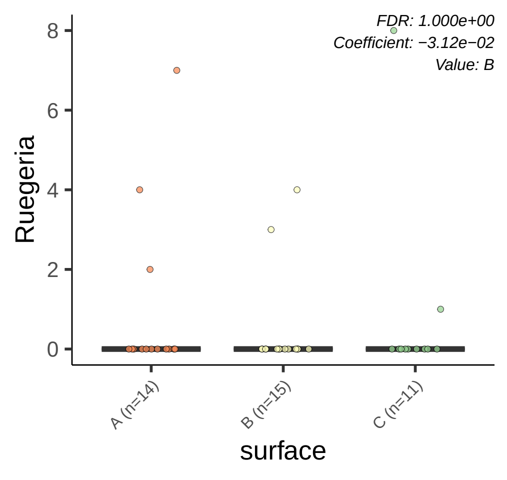
FDR: 1.000e+00
Coefficient: −3.12e−02
Value: B
Ruegeria
0
2
4
6
8
A (n=14)
B (n=15)
C (n=11)
surface

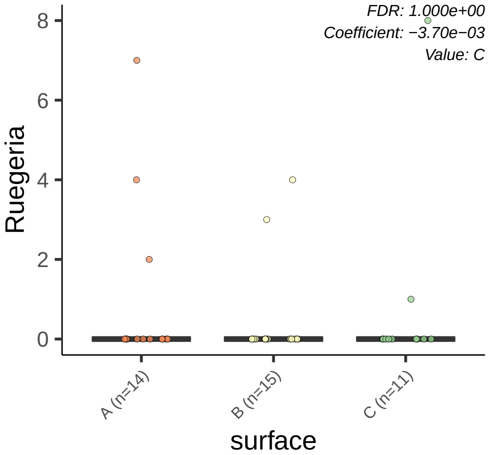
FDR: 1.000e+00
Coefficient: −3.70e−03
Value: C
Ruegeria
8
6
4
2
0
A (n=14)
B (n=15)
C (n=11)
surface

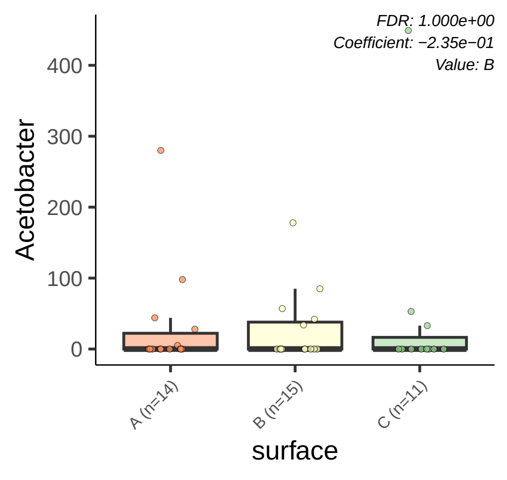
FDR: 1.000e+00
Coefficient: −2.35e−01
Value: B
Acetobacter
400
300
200
100
0
A (n=14)
B (n=15)
C (n=11)
surface

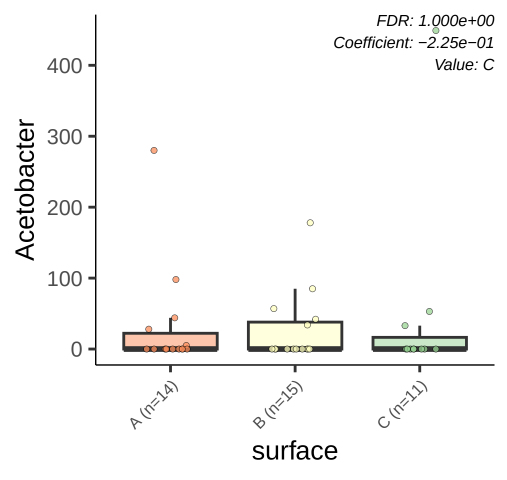
FDR: 1.000e+00
Coefficient: −2.25e−01
Value: C
Acetobacter
400
300
200
100
0
A (n=14)
B (n=15)
C (n=11)
surface

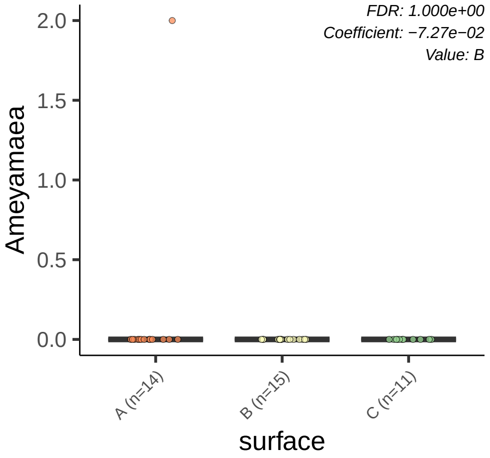

FDR: 1.000e+00
Coefficient: −7.27e−02
Value: B
Ameyamaea
2.0
1.5
1.0
0.5
0.0
A (n=14)
B (n=15)
C (n=11)
surface

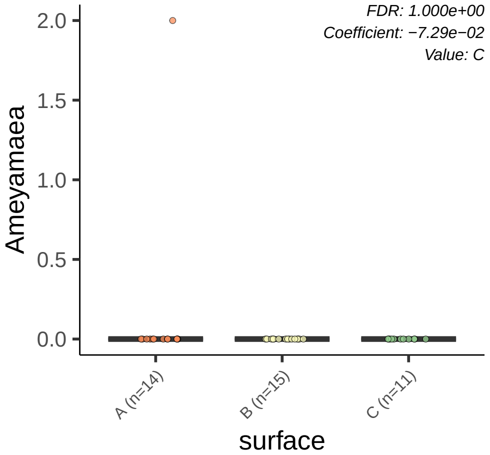
FDR: 1.000e+00
Coefficient: −7.29e−02
Value: C
Ameyamaea
2.0
1.5
1.0
0.5
0.0
A (n=14)
B (n=15)
C (n=11)
surface

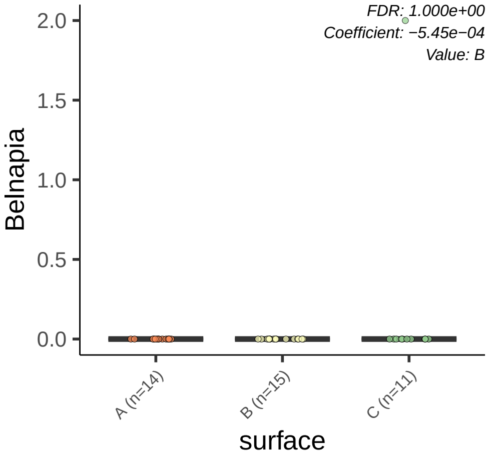
FDR: 1.000e+00
Coefficient: −5.45e−04
Value: B
Belnapia
2.0
1.5
1.0
0.5
0.0
A (n=14)
B (n=15)
C (n=11)
surface

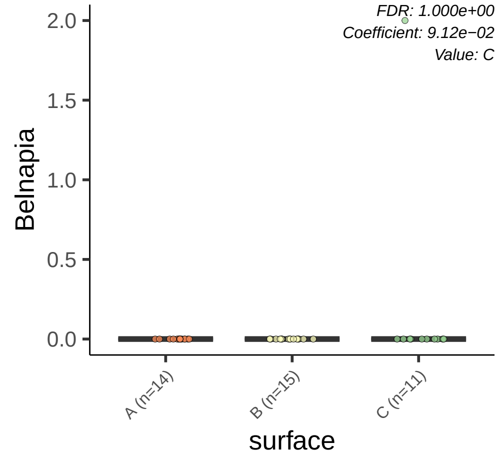
FDR: 1.000e+00
Coefficient: 9.12e−02
Value: C
Belnapia
2.0
1.5
1.0
0.5
0.0
A (n=14)
B (n=15)
C (n=11)
surface

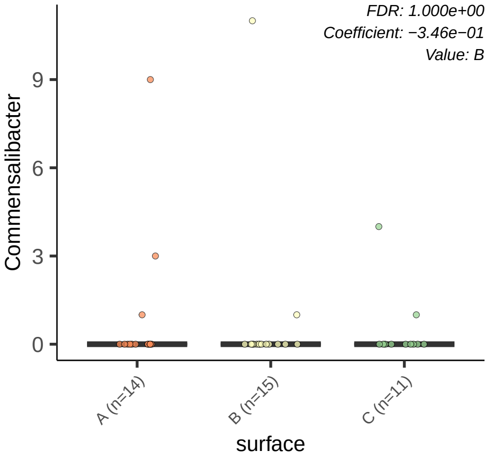

FDR: 1.000e+00
Coefficient: −3.46e−01
Value: B
Commensalibacter
0
3
6
9
A (n=14)
B (n=15)
C (n=11)
surface

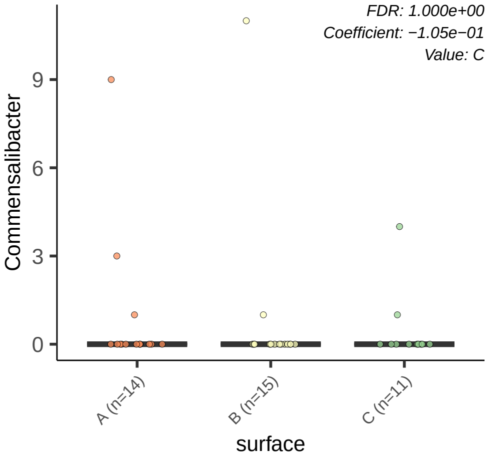
FDR: 1.000e+00
Coefficient: −1.05e−01
Value: C
Commensalibacter
0
3
6
9
A (n=14)
B (n=15)
C (n=11)
surface

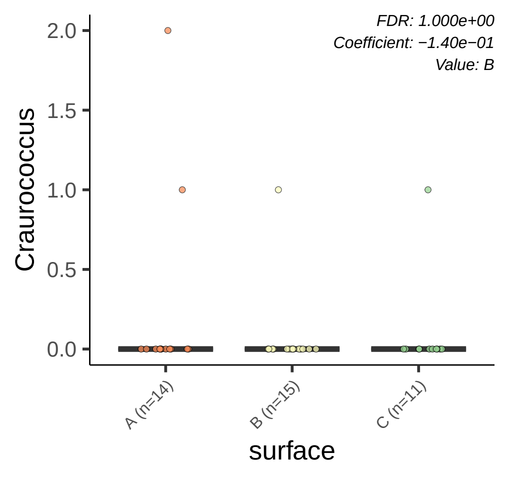

FDR: 1.000e+00
Coefficient: −1.40e−01
Value: B
Craurococcus
2.0
1.5
1.0
0.5
0.0
A (n=14)
B (n=15)
C (n=11)
surface

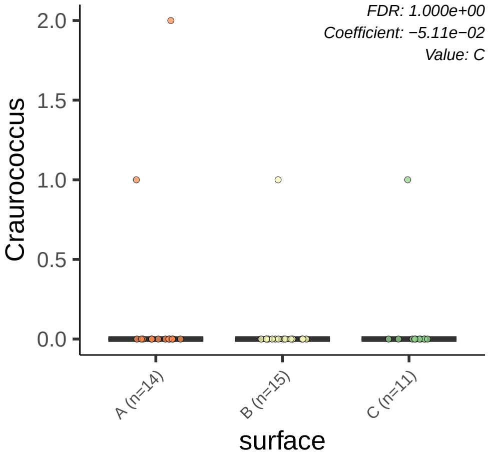

FDR: 1.000e+00
Coefficient: −5.11e−02
Value: C
Craurococcus
2.0
1.5
1.0
0.5
0.0
A (n=14)
B (n=15)
C (n=11)
surface

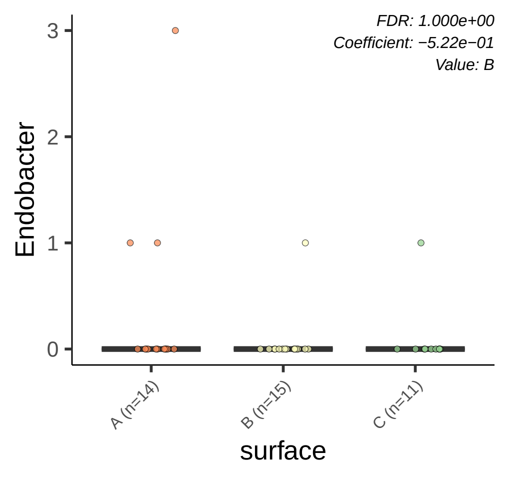

FDR: 1.000e+00
Coefficient: −5.22e−01
Value: B
Endobacter
3
2
1
0
A (n=14)
B (n=15)
C (n=11)
surface

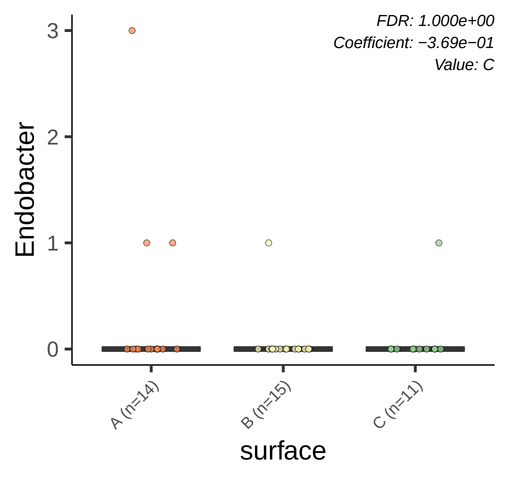
FDR: 1.000e+00
Coefficient: −3.69e−01
Value: C
Endobacter
3
2
1
0
A (n=14)
B (n=15)
C (n=11)
surface

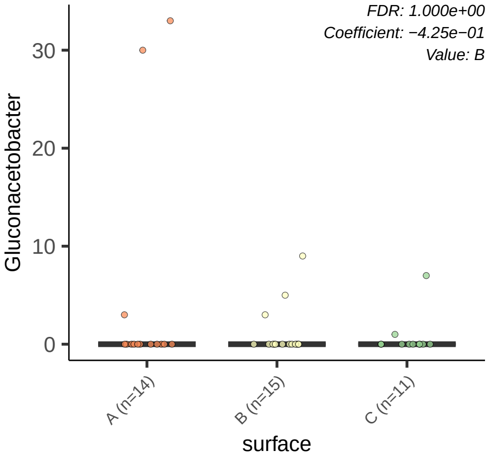
FDR: 1.000e+00
Coefficient: −4.25e−01
Value: B
Gluconacetobacter
30
20
10
0
A (n=14)
B (n=15)
C (n=11)
surface

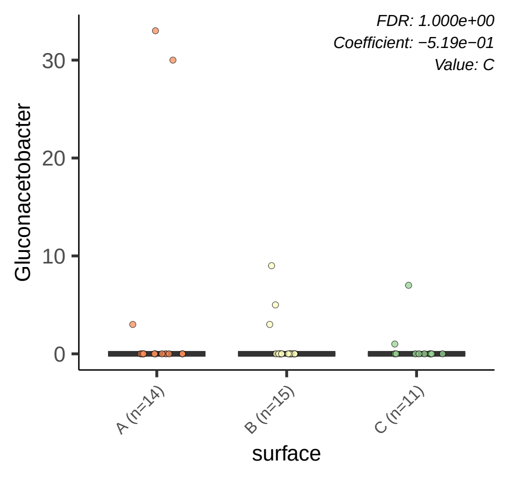
FDR: 1.000e+00
Coefficient: −5.19e−01
Value: C
Gluconacetobacter
30
20
10
0
A (n=14)
B (n=15)
C (n=11)
surface

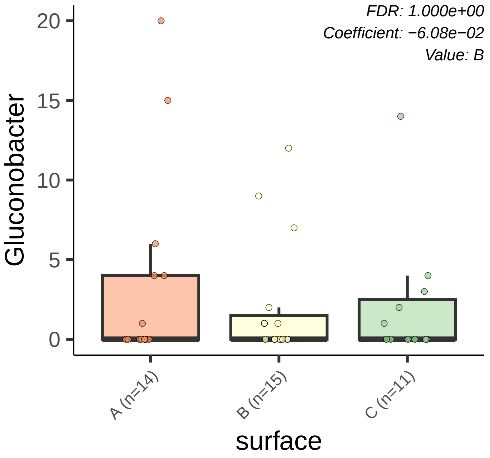
FDR: 1.000e+00
Coefficient: −6.08e−02
Value: B
Gluconobacter
20
15
10
5
0
A (n=14)
B (n=15)
C (n=11)
surface

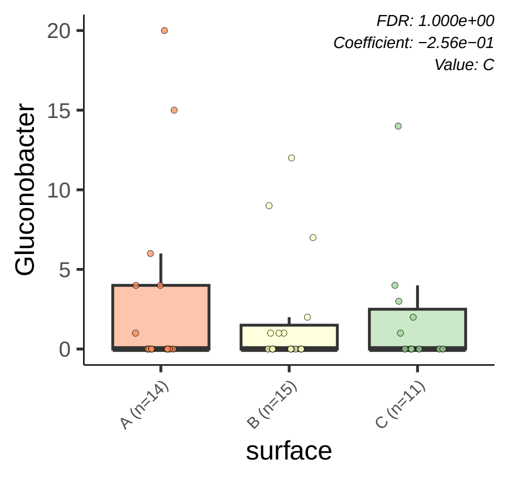
FDR: 1.000e+00
Coefficient: −2.56e−01
Value: C
Gluconobacter
20
15
10
5
0
A (n=14)
B (n=15)
C (n=11)
surface

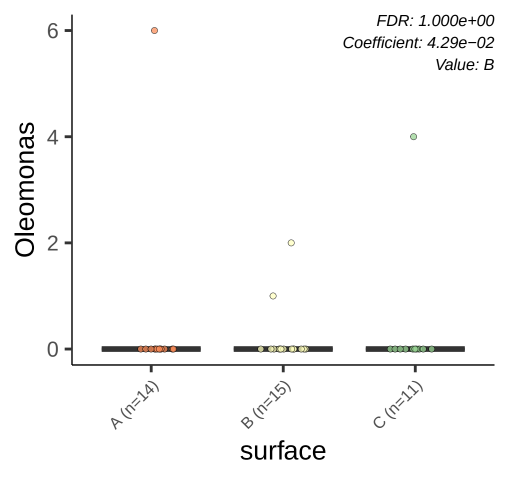

FDR: 1.000e+00
Coefficient: 4.29e−02
Value: B
Oleomonas
6
4
2
0
A (n=14)
B (n=15)
C (n=11)
surface

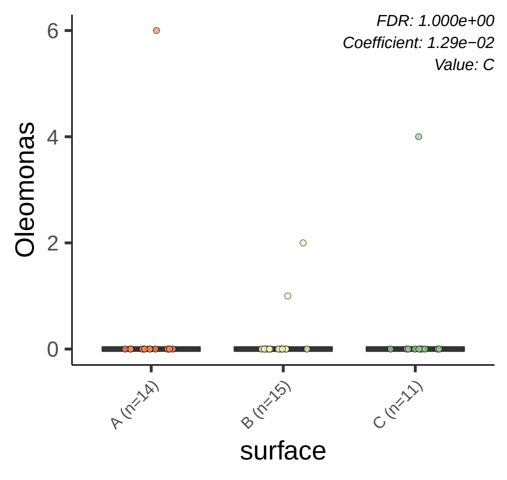
FDR: 1.000e+00
Coefficient: 1.29e−02
Value: C
Oleomonas
6
4
2
0
A (n=14)
B (n=15)
C (n=11)
surface

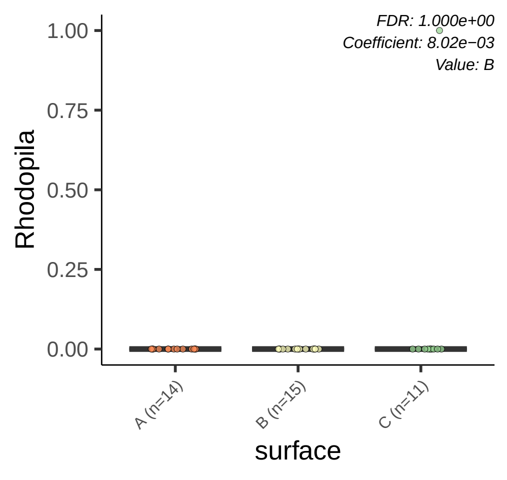
FDR: 1.000e+00
Coefficient: 8.02e−03
Value: B
Rhodopila
1.00
0.75
0.50
0.25
0.00
A (n=14)
B (n=15)
C (n=11)
surface

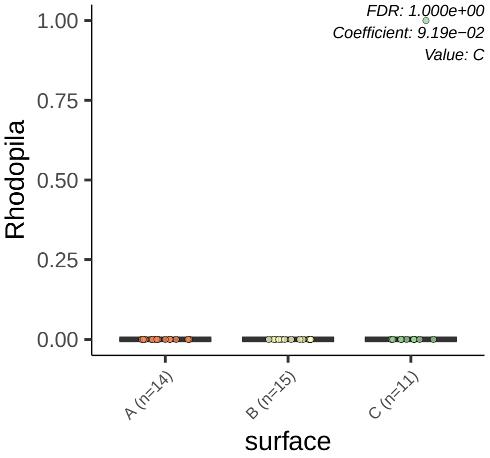
FDR: 1.000e+00
Coefficient: 9.19e−02
Value: C
Rhodopila
1.00
0.75
0.50
0.25
0.00
A (n=14)
B (n=15)
C (n=11)
surface

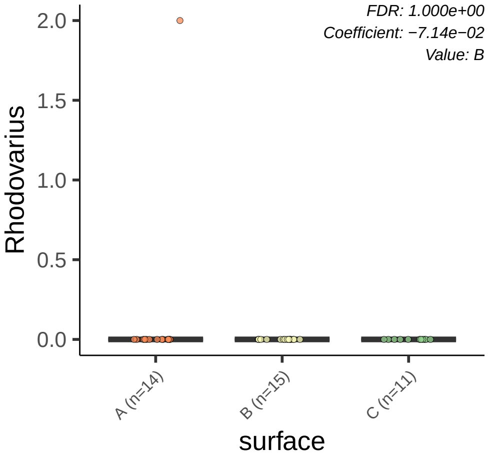

FDR: 1.000e+00
Coefficient: −7.14e−02
Value: B
Rhodovarius
2.0
1.5
1.0
0.5
0.0
A (n=14)
B (n=15)
C (n=11)
surface

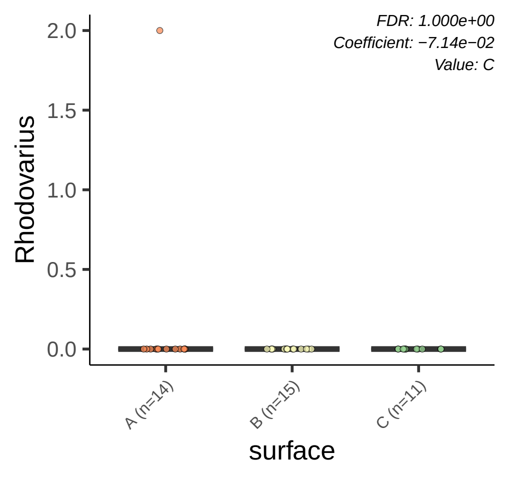

FDR: 1.000e+00
Coefficient: −7.14e−02
Value: C
Rhodovarius
2.0
1.5
1.0
0.5
0.0
A (n=14)
B (n=15)
C (n=11)
surface

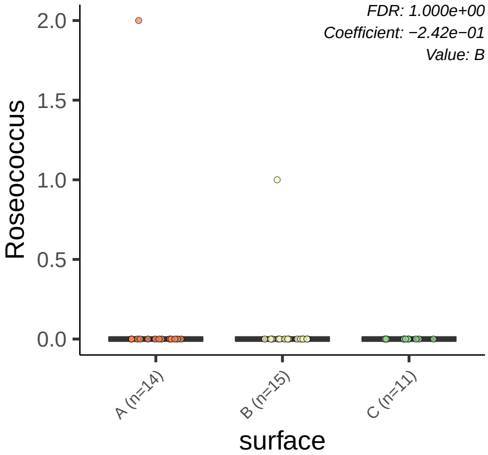

FDR: 1.000e+00
Coefficient: −2.42e−01
Value: B
Roseococcus
2.0
1.5
1.0
0.5
0.0
A (n=14)
B (n=15)
C (n=11)
surface

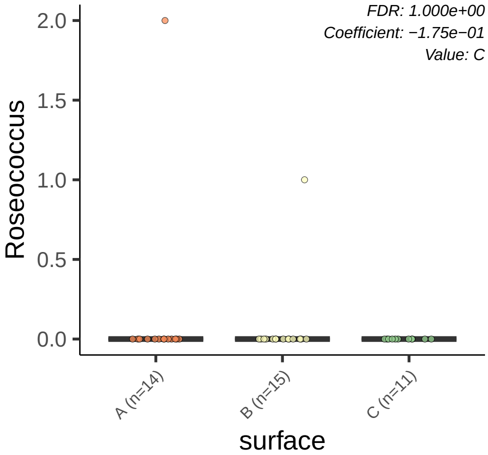
FDR: 1.000e+00
Coefficient: −1.75e−01
Value: C
Roseococcus
2.0
1.5
1.0
0.5
0.0
A (n=14)
B (n=15)
C (n=11)
surface

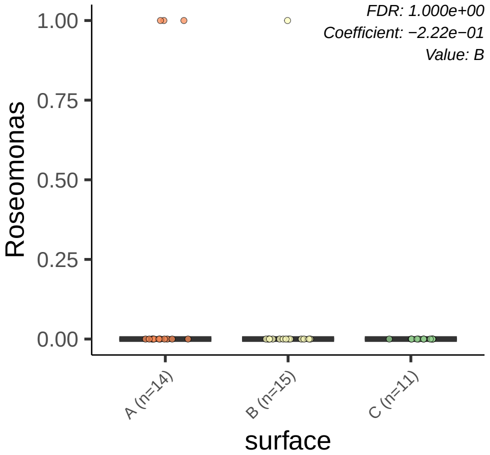

FDR: 1.000e+00
Coefficient: −2.22e−01
Value: B
Roseomonas
1.00
0.75
0.50
0.25
0.00
A (n=14)
B (n=15)
C (n=11)
surface

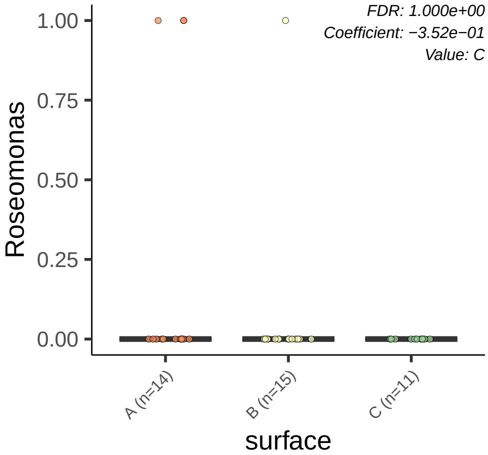
FDR: 1.000e+00
Coefficient: −3.52e−01
Value: C
1.00
0.75
0.50
0.25
0.00
Roseomonas
A (n=14)
B (n=15)
C (n=11)
surface

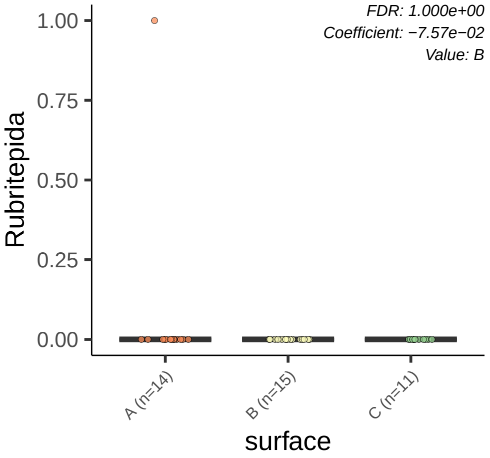

FDR: 1.000e+00
Coefficient: −7.57e−02
Value: B
Rubritepida
1.00
0.75
0.50
0.25
0.00
A (n=14)
B (n=15)
C (n=11)
surface

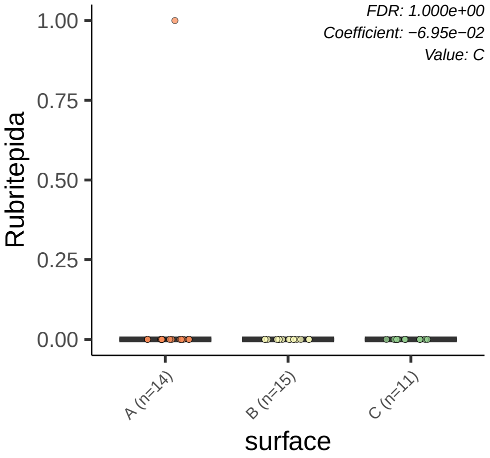
FDR: 1.000e+00
Coefficient: −6.95e−02
Value: C
Rubritepida
1.00
0.75
0.50
0.25
0.00
A (n=14)
B (n=15)
C (n=11)
surface

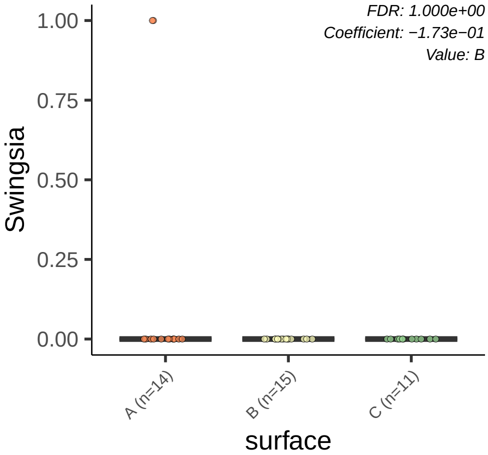
FDR: 1.000e+00
Coefficient: −1.73e−01
Value: B
Swingsia
1.00
0.75
0.50
0.25
0.00
A (n=14)
B (n=15)
C (n=11)
surface

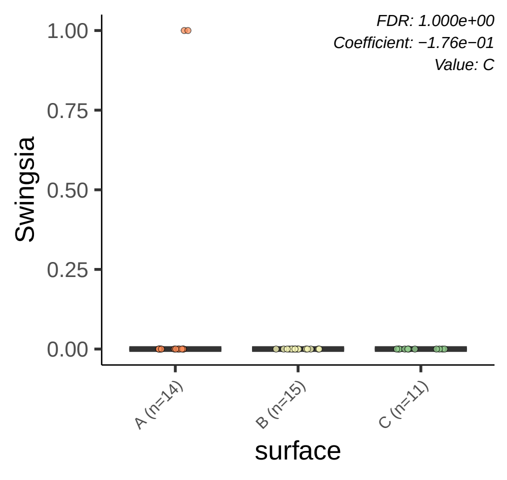

FDR: 1.000e+00
Coefficient: −1.76e−01
Value: C
Swingsia
1.00
0.75
0.50
0.25
0.00
A (n=14)
B (n=15)
C (n=11)
surface

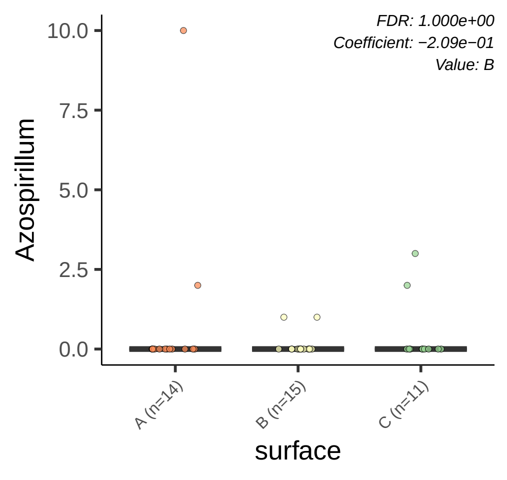
FDR: 1.000e+00
Coefficient: −2.09e−01
Value: B
Azospirillum
10.0
7.5
5.0
2.5
0.0
A (n=14)
B (n=15)
C (n=11)
surface

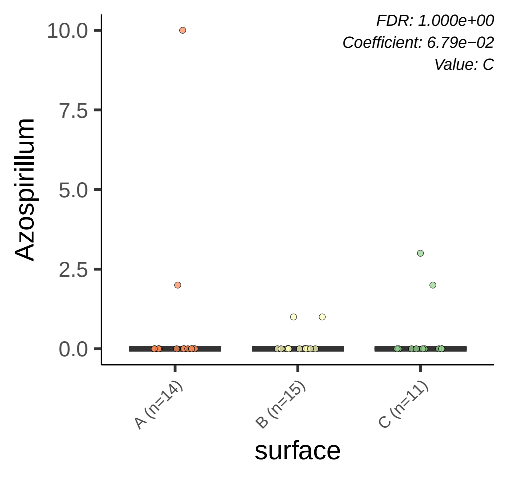
FDR: 1.000e+00
Coefficient: 6.79e−02
Value: C
Azospirillum
10.0
7.5
5.0
2.5
0.0
A (n=14)
B (n=15)
C (n=11)
surface

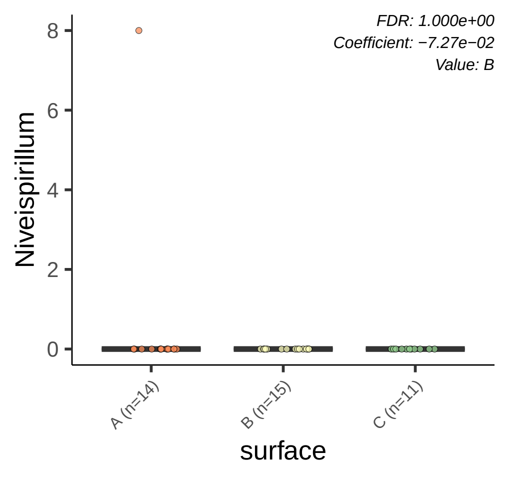
FDR: 1.000e+00
Coefficient: −7.27e−02
Value: B
Niveispirillum
8
6
4
2
0
A (n=14)
B (n=15)
C (n=11)
surface

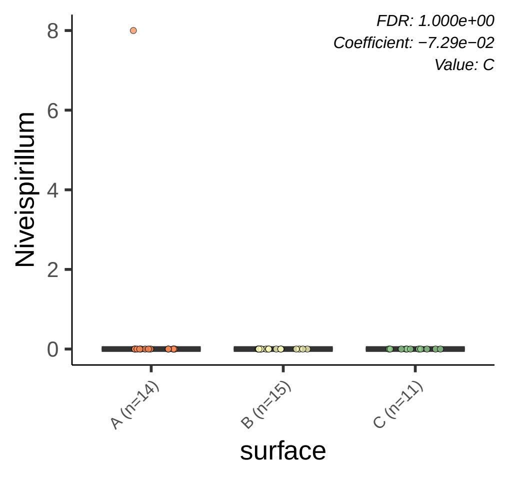

FDR: 1.000e+00
Coefficient: −7.29e−02
Value: C
Niveispirillum
8
6
4
2
0
A (n=14)
B (n=15)
C (n=11)
surface

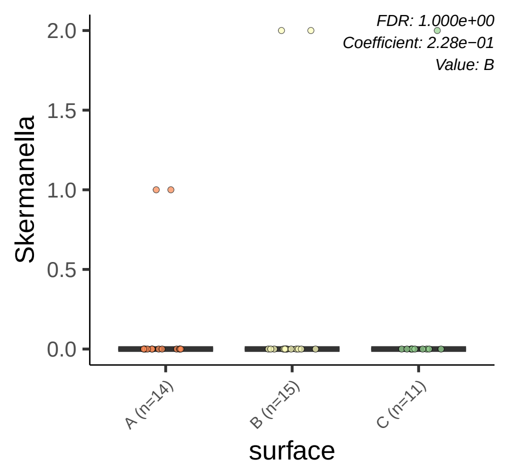
FDR: 1.000e+00
Coefficient: 2.28e−01
Value: B
Skermanella
2.0
1.5
1.0
0.5
0.0
A (n=14)
B (n=15)
C (n=11)
surface

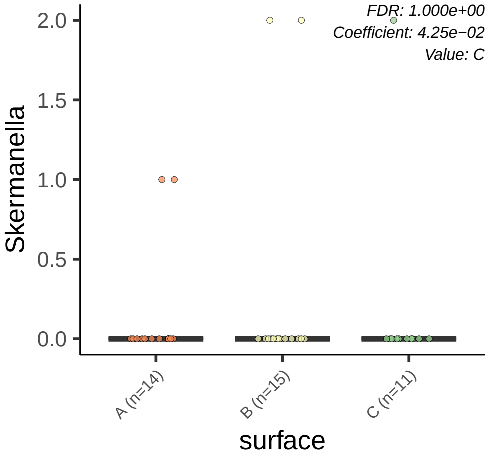
FDR: 1.000e+00
Coefficient: 4.25e−02
Value: C
Skermanella
2.0
1.5
1.0
0.5
0.0
A (n=14)
B (n=15)
C (n=11)
surface

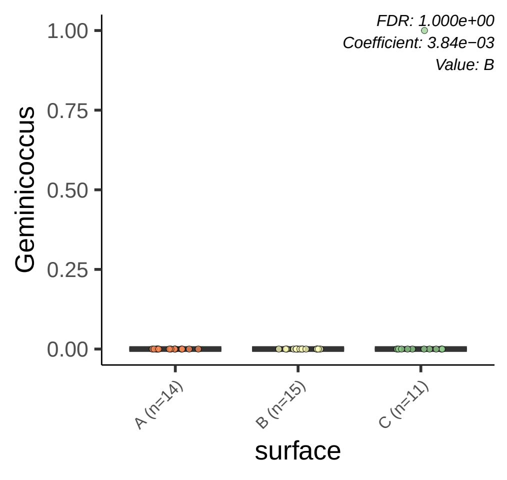
FDR: 1.000e+00
Coefficient: 3.84e−03
Value: B
Geminicoccus
1.00
0.75
0.50
0.25
0.00
A (n=14)
B (n=15)
C (n=11)
surface

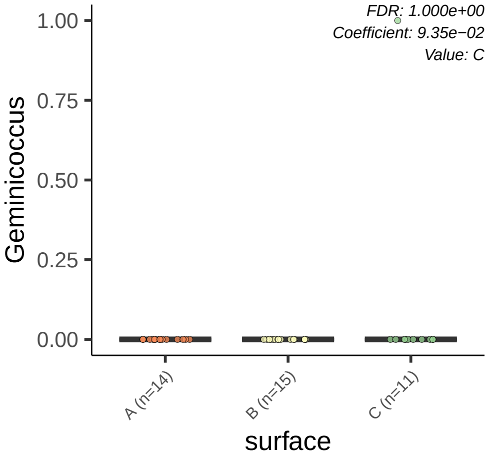
FDR: 1.000e+00
Coefficient: 9.35e−02
Value: C
Geminicoccus
1.00
0.75
0.50
0.25
0.00
A (n=14)
B (n=15)
C (n=11)
surface

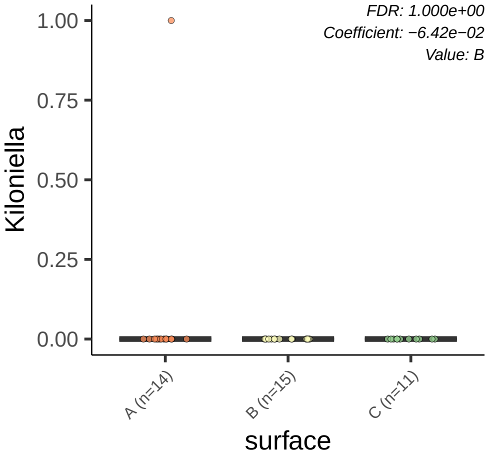

FDR: 1.000e+00
Coefficient: −6.42e−02
Value: B
Kiloniella
1.00
0.75
0.50
0.25
0.00
A (n=14)
B (n=15)
C (n=11)
surface

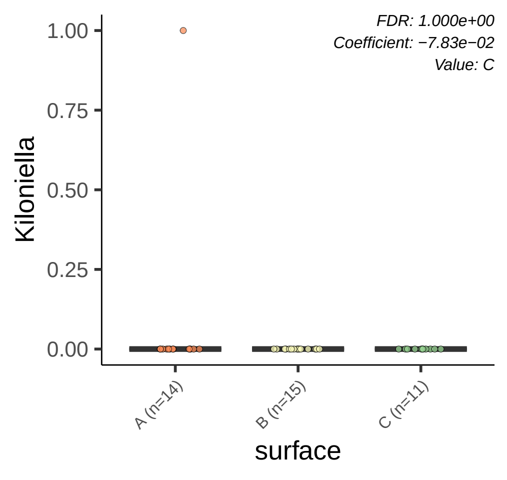
FDR: 1.000e+00
Coefficient: −7.83e−02
Value: C
Kiloniella
1.00
0.75
0.50
0.25
0.00
A (n=14)
B (n=15)
C (n=11)
surface

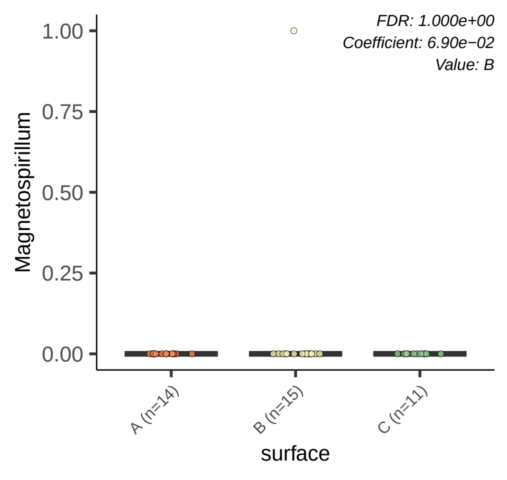
FDR: 1.000e+00
Coefficient: 6.90e−02
Value: B
Magnetospirillum
1.00
0.75
0.50
0.25
0.00
A (n=14)
B (n=15)
C (n=11)
surface

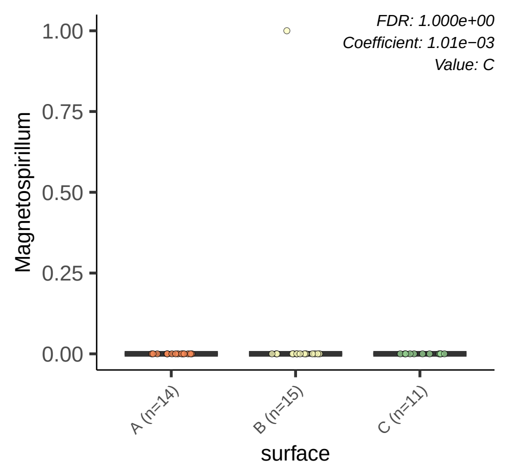
FDR: 1.000e+00
Coefficient: 1.01e−03
Value: C
Magnetospirillum
1.00
0.75
0.50
0.25
0.00
A (n=14)
B (n=15)
C (n=11)
surface

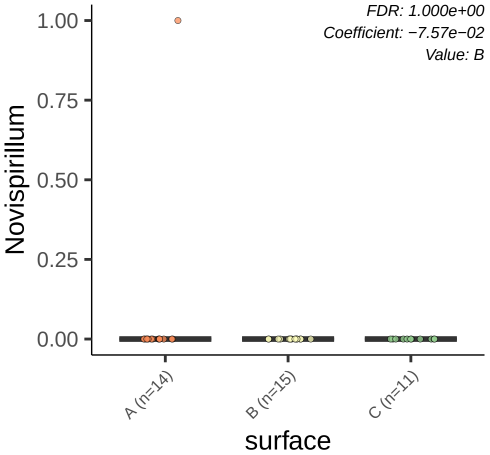

FDR: 1.000e+00
Coefficient: −7.57e−02
Value: B
Novispirillum
1.00
0.75
0.50
0.25
0.00
A (n=14)
B (n=15)
C (n=11)
surface

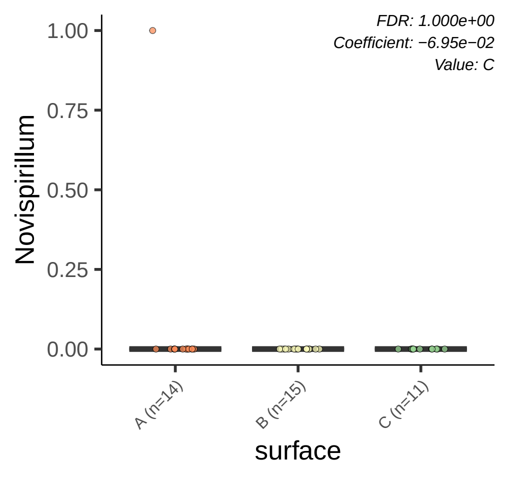
FDR: 1.000e+00
Coefficient: −6.95e−02
Value: C
Novispirillum
1.00
0.75
0.50
0.25
0.00
A (n=14)
B (n=15)
C (n=11)
surface

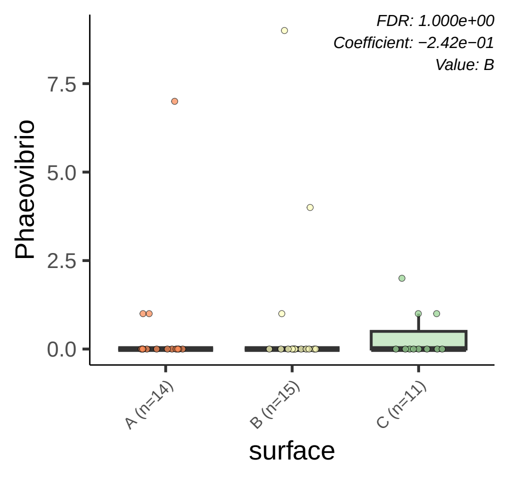

FDR: 1.000e+00
Coefficient: −2.42e−01
Value: B
Phaeovibrio
7.5
5.0
2.5
0.0
A (n=14)
B (n=15)
C (n=11)
surface

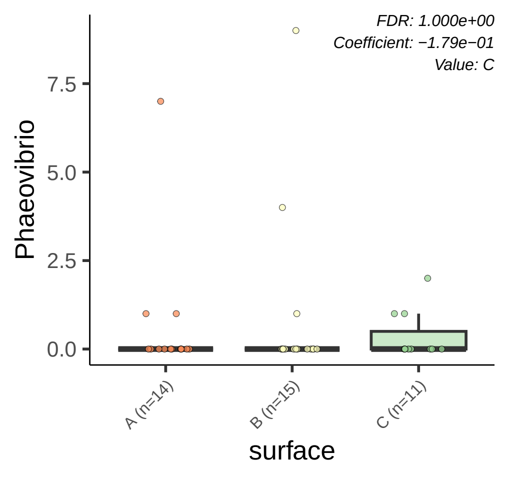
FDR: 1.000e+00
Coefficient: −1.79e−01
Value: C
Phaeovibrio
7.5
5.0
2.5
0.0
A (n=14)
B (n=15)
C (n=11)
surface

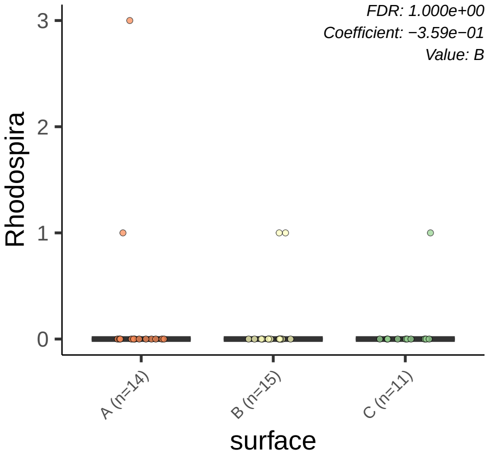

FDR: 1.000e+00
Coefficient: −3.59e−01
Value: B
Rhodospira
3
2
1
0
A (n=14)
B (n=15)
C (n=11)
surface

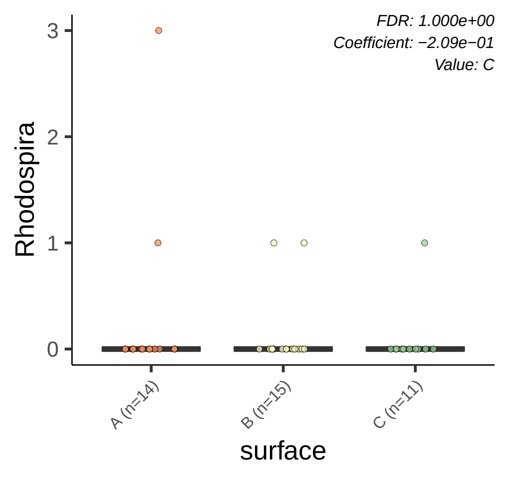
FDR: 1.000e+00
Coefficient: −2.09e−01
Value: C
Rhodospira
3
2
1
0
A (n=14)
B (n=15)
C (n=11)
surface

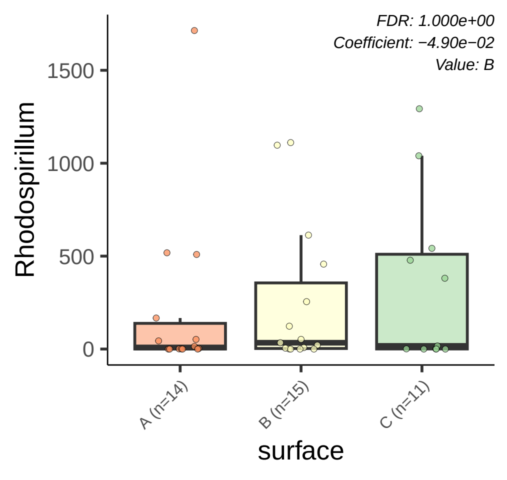
FDR: 1.000e+00
Coefficient: −4.90e−02
Value: B
Rhodospirillum
1500
1000
500
0
A (n=14)
B (n=15)
C (n=11)
surface

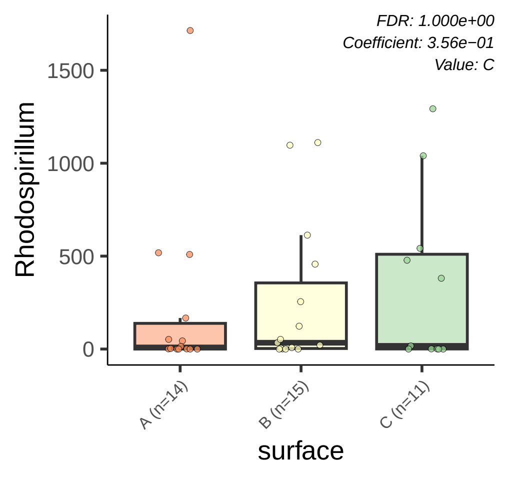
FDR: 1.000e+00
Coefficient: 3.56e−01
Value: C
Rhodospirillum
1500
1000
500
0
A (n=14)
B (n=15)
C (n=11)
surface

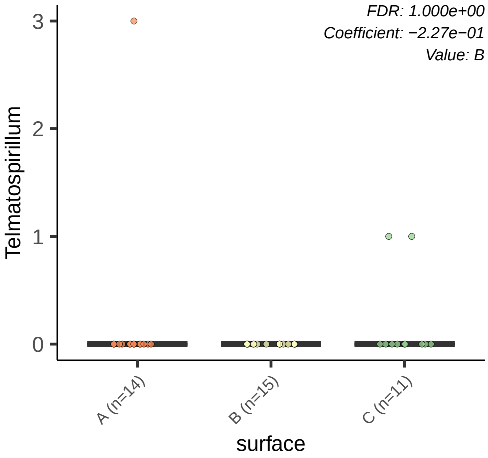
FDR: 1.000e+00
Coefficient: −2.27e−01
Value: B
Telmatospirillum
0
1
2
3
A (n=14)
B (n=15)
C (n=11)
surface

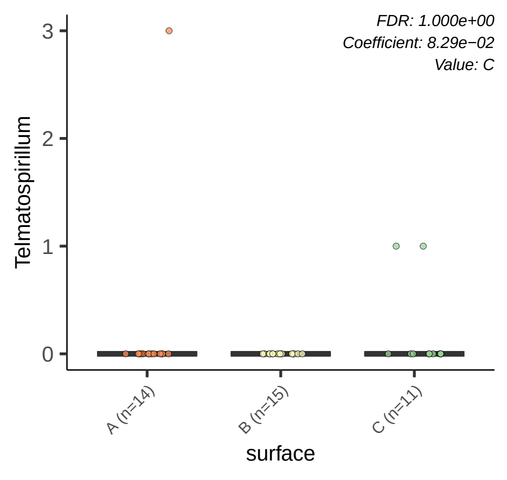
FDR: 1.000e+00
Coefficient: 8.29e−02
Value: C
Telmatospirillum
3
2
1
0
A (n=14)
B (n=15)
C (n=11)
surface

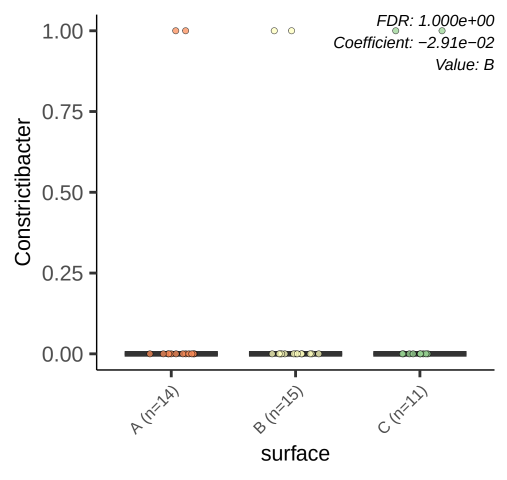

FDR: 1.000e+00
Coefficient: −2.91e−02
Value: B
Constrictibacter
1.00
0.75
0.50
0.25
0.00
A (n=14)
B (n=15)
C (n=11)
surface

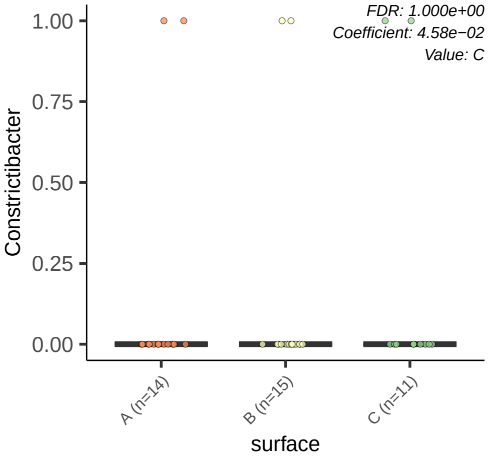
FDR: 1.000e+00
Coefficient: 4.58e−02
Value: C
Constrictibacter
1.00
0.75
0.50
0.25
0.00
A (n=14)
B (n=15)
C (n=11)
surface

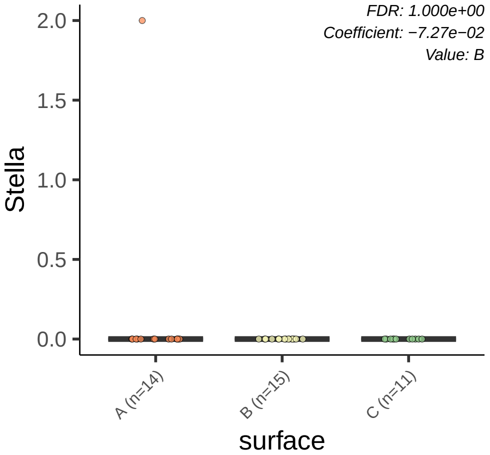
FDR: 1.000e+00
Coefficient: −7.27e−02
Value: B
Stella
2.0
1.5
1.0
0.5
0.0
A (n=14)
B (n=15)
C (n=11)
surface

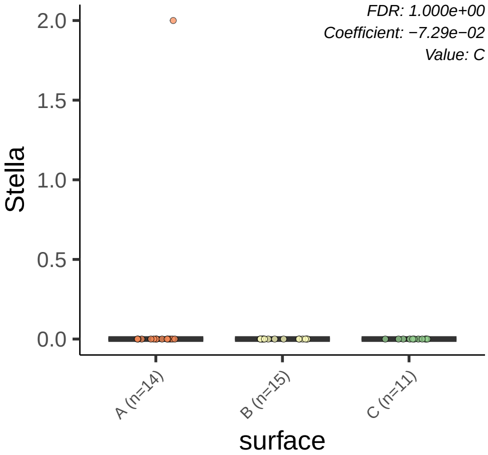
FDR: 1.000e+00
Coefficient: −7.29e−02
Value: C
Stella
2.0
1.5
1.0
0.5
0.0
A (n=14)
B (n=15)
C (n=11)
surface

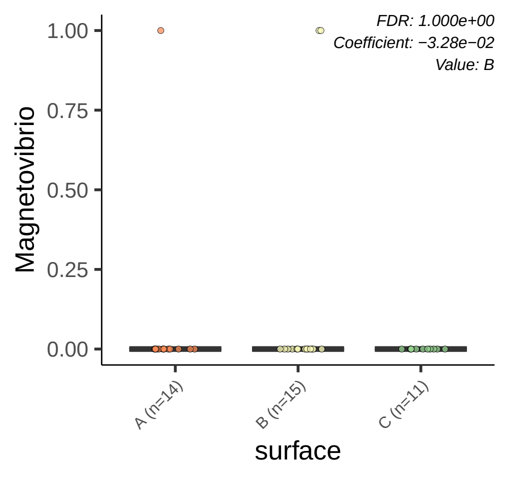

FDR: 1.000e+00
Coefficient: −3.28e−02
Value: B
Magnetovibrio
1.00
0.75
0.50
0.25
0.00
A (n=14)
B (n=15)
C (n=11)
surface

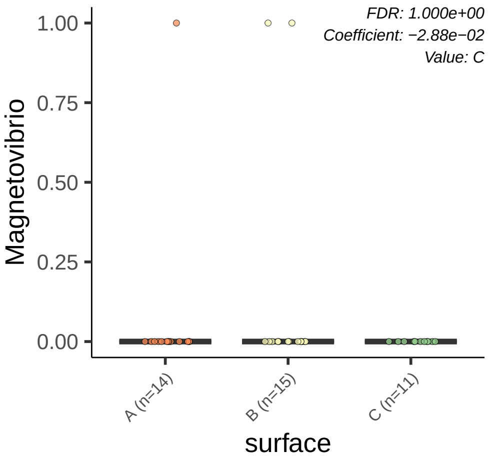
FDR: 1.000e+00
Coefficient: −2.88e−02
Value: C
Magnetovibrio
1.00
0.75
0.50
0.25
0.00
A (n=14)
B (n=15)
C (n=11)
surface

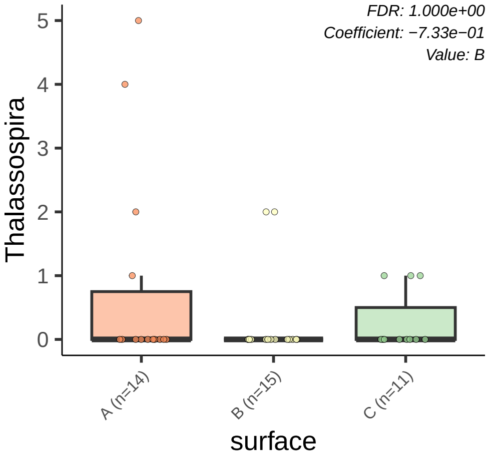
FDR: 1.000e+00
Coefficient: −7.33e−01
Value: B
Thalassospira
0
1
2
3
4
5
A (n=14)
B (n=15)
C (n=11)
surface

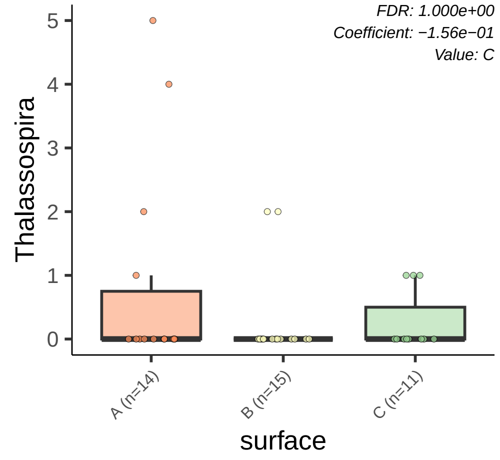

FDR: 1.000e+00
Coefficient: −1.56e−01
Value: C
Thalassospira
0
1
2
3
4
5
A (n=14)
B (n=15)
C (n=11)
surface

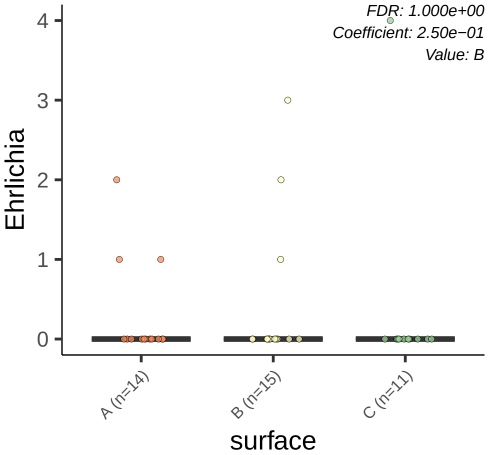
FDR: 1.000e+00
Coefficient: 2.50e−01
Value: B
Ehrlichia
0
1
2
3
4
A (n=14)
B (n=15)
C (n=11)
surface

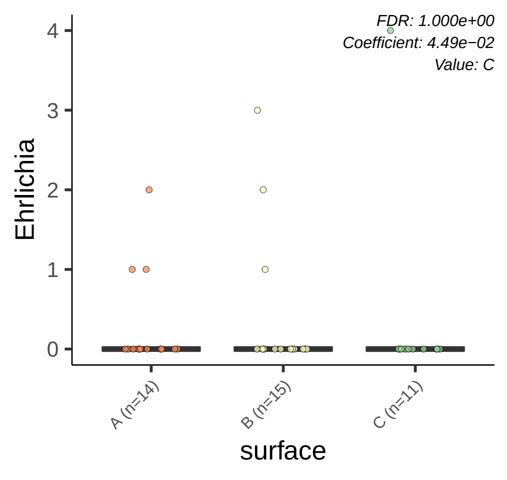

FDR: 1.000e+00
Coefficient: 4.49e−02
Value: C
Ehrlichia
0
1
2
3
4
A (n=14)
B (n=15)
C (n=11)
surface

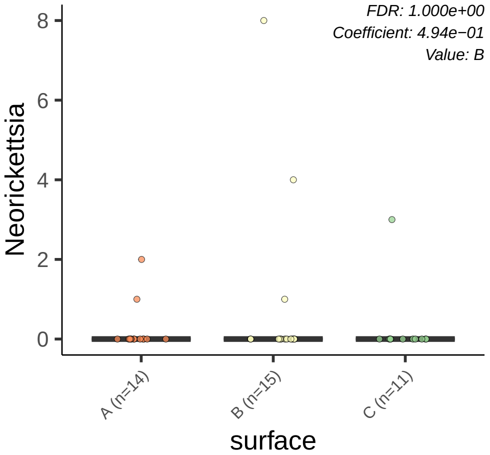
FDR: 1.000e+00
Coefficient: 4.94e−01
Value: B
Neorickettsia
0
2
4
6
8
A (n=14)
B (n=15)
C (n=11)
surface

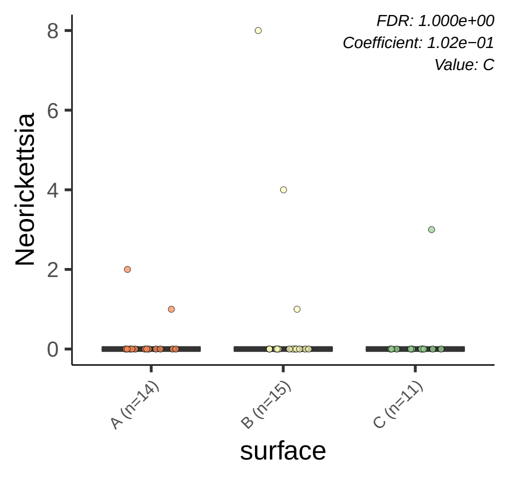
FDR: 1.000e+00
Coefficient: 1.02e−01
Value: C
Neorickettsia
8
6
4
2
0
A (n=14)
B (n=15)
C (n=11)
surface

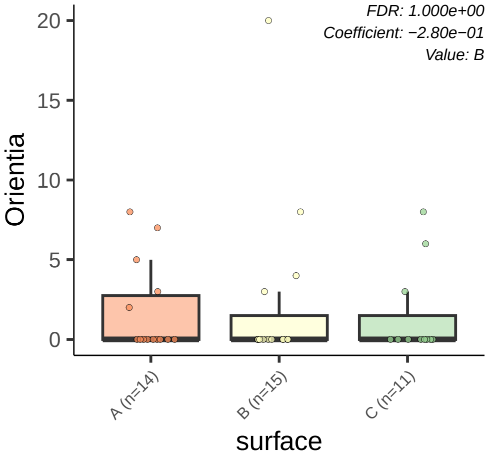

FDR: 1.000e+00
Coefficient: −2.80e−01
Value: B
Orientia
20
15
10
5
0
A (n=14)
B (n=15)
C (n=11)
surface

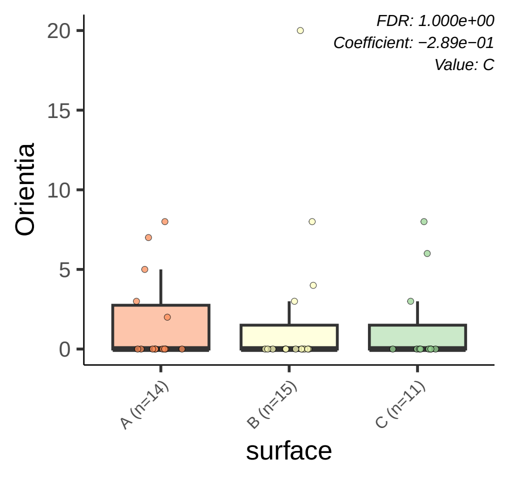

FDR: 1.000e+00
Coefficient: −2.89e−01
Value: C
Orientia
20
15
10
5
0
A (n=14)
B (n=15)
C (n=11)
surface

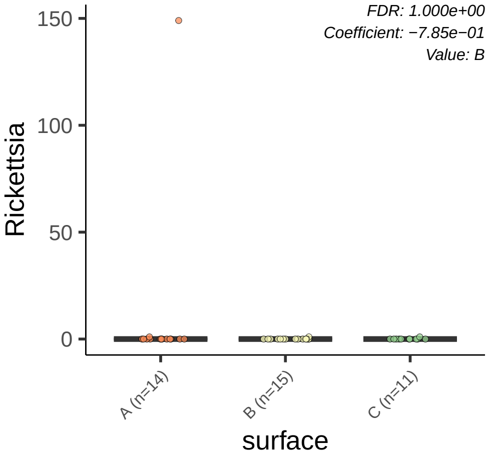

FDR: 1.000e+00
Coefficient: −7.85e−01
Value: B
Rickettsia
150
100
50
0
A (n=14)
B (n=15)
C (n=11)
surface

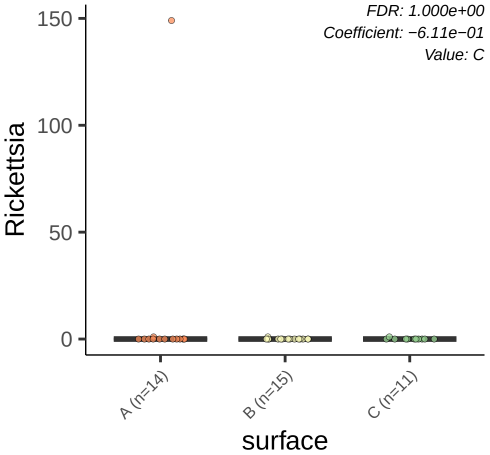
FDR: 1.000e+00
Coefficient: −6.11e−01
Value: C
Rickettsia
150
100
50
0
A (n=14)
B (n=15)
C (n=11)
surface

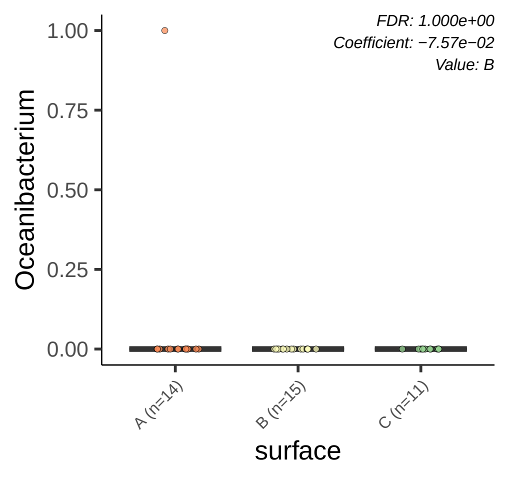
FDR: 1.000e+00
Coefficient: −7.57e−02
Value: B
Oceanibacterium
1.00
0.75
0.50
0.25
0.00
A (n=14)
B (n=15)
C (n=11)
surface

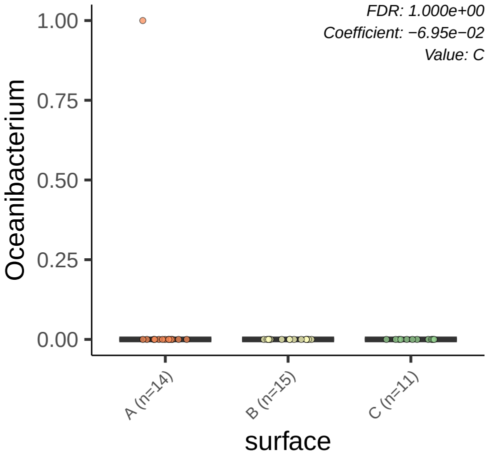
FDR: 1.000e+00
Coefficient: −6.95e−02
Value: C
Oceanibacterium
1.00
0.75
0.50
0.25
0.00
A (n=14)
B (n=15)
C (n=11)
surface

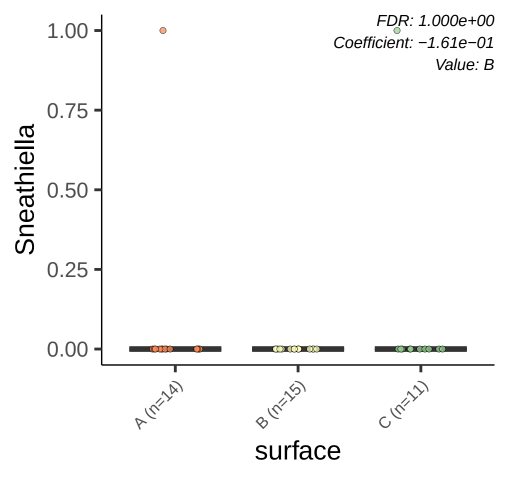

FDR: 1.000e+00
Coefficient: −1.61e−01
Value: B
Sneathiella
1.00
0.75
0.50
0.25
0.00
A (n=14)
B (n=15)
C (n=11)
surface

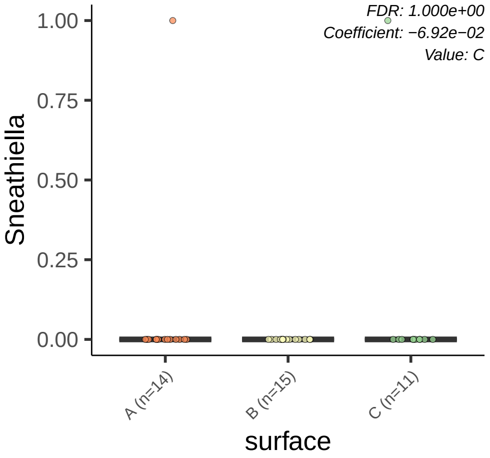

FDR: 1.000e+00
Coefficient: −6.92e−02
Value: C
1.00
0.75
0.50
0.25
0.00
Sneathiella
A (n=14)
B (n=15)
C (n=11)
surface

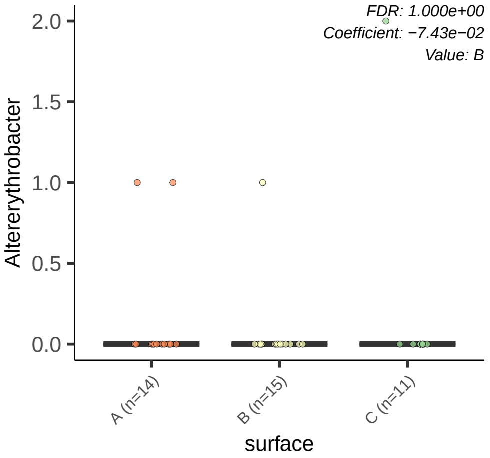
FDR: 1.000e+00
Coefficient: −7.43e−02
Value: B
Altererythrobacter
2.0
1.5
1.0
0.5
0.0
A (n=14)
B (n=15)
C (n=11)
surface

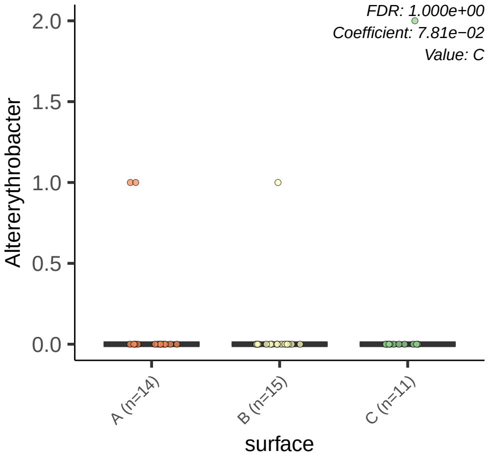

FDR: 1.000e+00
Coefficient: 7.81e−02
Value: C
Altererythrobacter
2.0
1.5
1.0
0.5
0.0
A (n=14)
B (n=15)
C (n=11)
surface

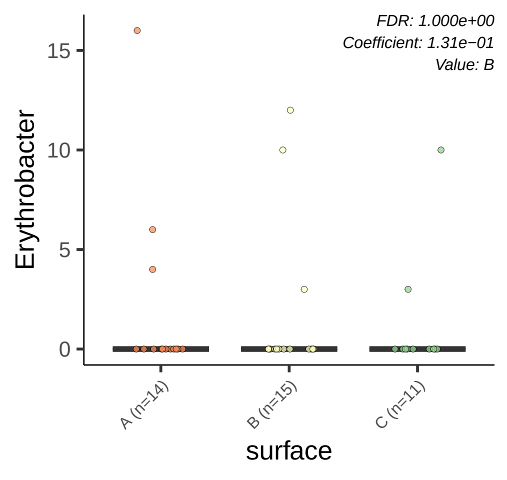
FDR: 1.000e+00
Coefficient: 1.31e−01
Value: B
Erythrobacter
15
10
5
0
A (n=14)
B (n=15)
C (n=11)
surface

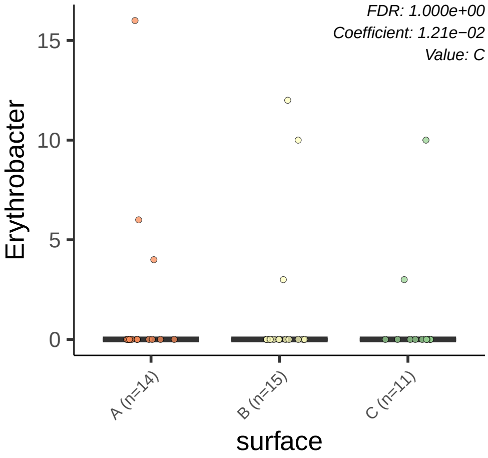

FDR: 1.000e+00
Coefficient: 1.21e−02
Value: C
Erythrobacter
15
10
5
0
A (n=14)
B (n=15)
C (n=11)
surface

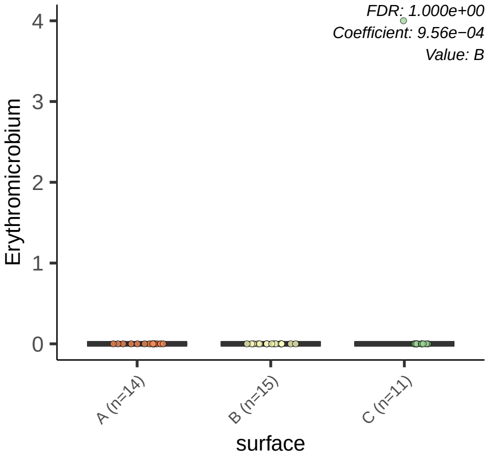
FDR: 1.000e+00
Coefficient: 9.56e−04
Value: B
Erythromicrobium
4
3
2
1
0
A (n=14)
B (n=15)
C (n=11)
surface

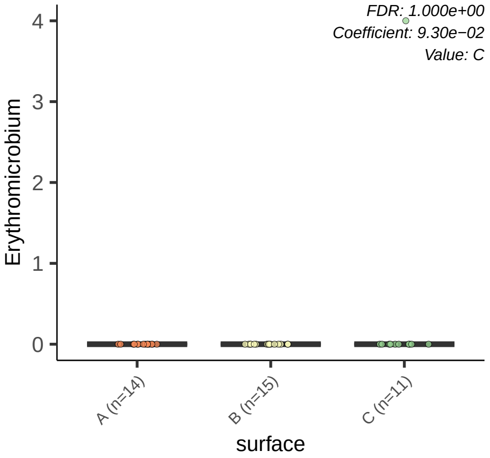
FDR: 1.000e+00
Coefficient: 9.30e−02
Value: C
Erythromicrobium
4
3
2
1
0
A (n=14)
B (n=15)
C (n=11)
surface

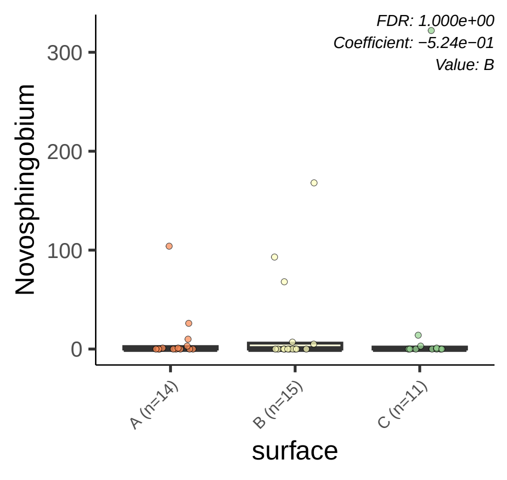
FDR: 1.000e+00
Coefficient: −5.24e−01
Value: B
Novosphingobium
300
200
100
0
A (n=14)
B (n=15)
C (n=11)
surface

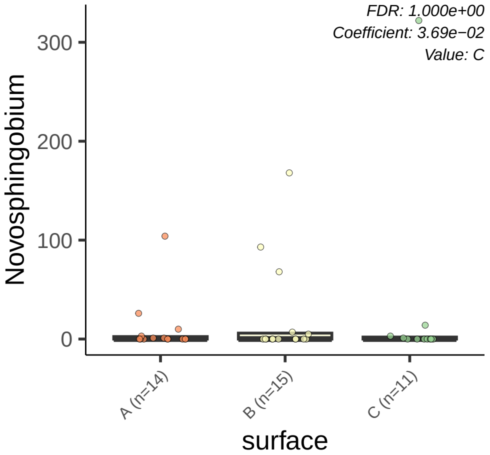
FDR: 1.000e+00
Coefficient: 3.69e−02
Value: C
Novosphingobium
300
200
100
0
A (n=14)
B (n=15)
C (n=11)
surface

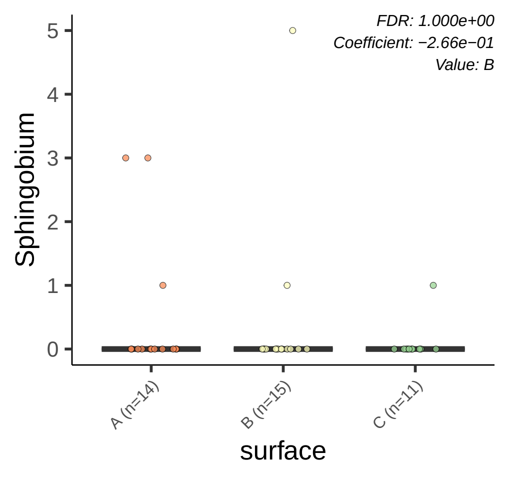
FDR: 1.000e+00
Coefficient: −2.66e−01
Value: B
Sphingobium
0
1
2
3
4
5
A (n=14)
B (n=15)
C (n=11)
surface

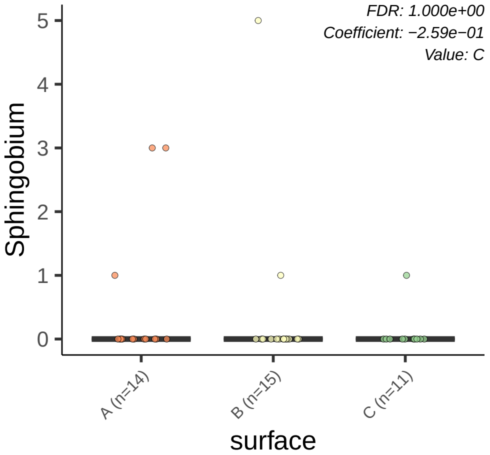
FDR: 1.000e+00
Coefficient: −2.59e−01
Value: C
Sphingobium
0
1
2
3
4
5
A (n=14)
B (n=15)
C (n=11)
surface

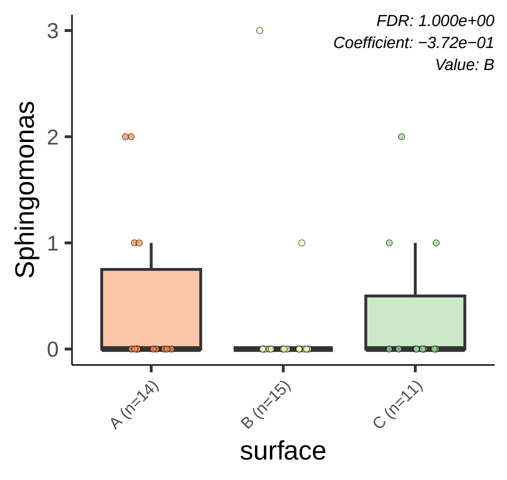
FDR: 1.000e+00
Coefficient: −3.72e−01
Value: B
Sphingomonas
3
2
1
0
A (n=14)
B (n=15)
C (n=11)
surface

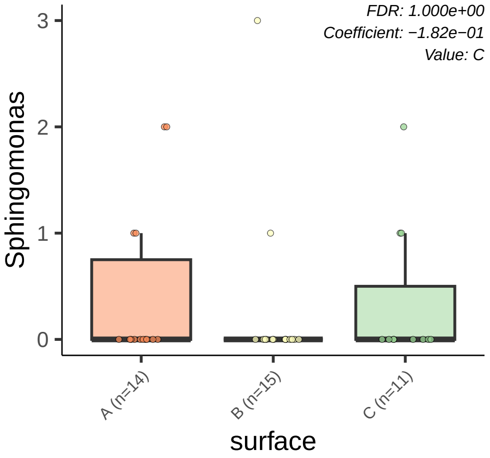
FDR: 1.000e+00
Coefficient: −1.82e−01
Value: C
Sphingomonas
3
2
1
0
A (n=14)
B (n=15)
C (n=11)
surface

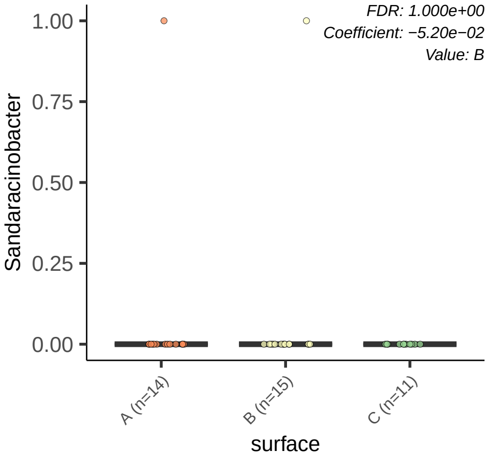
FDR: 1.000e+00
Coefficient: −5.20e−02
Value: B
Sandaracinobacter
1.00
0.75
0.50
0.25
0.00
A (n=14)
B (n=15)
C (n=11)
surface

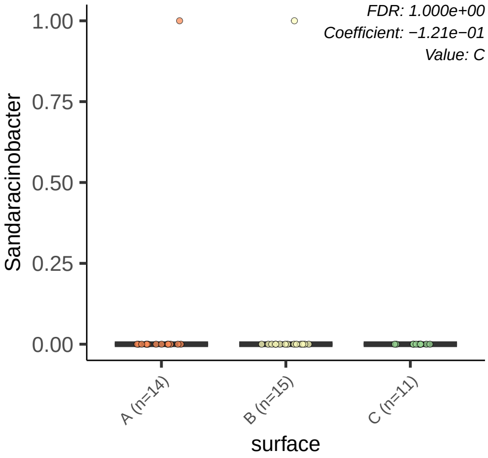
FDR: 1.000e+00
Coefficient: −1.21e−01
Value: C
Sandaracinobacter
1.00
0.75
0.50
0.25
0.00
A (n=14)
B (n=15)
C (n=11)
surface

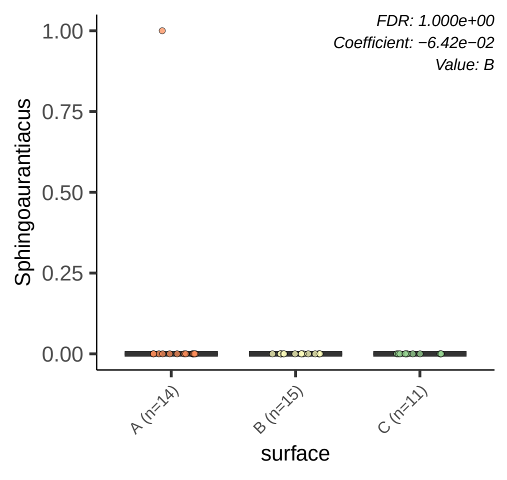

FDR: 1.000e+00
Coefficient: −6.42e−02
Value: B
Sphingoaurantiacus
1.00
0.75
0.50
0.25
0.00
A (n=14)
B (n=15)
C (n=11)
surface

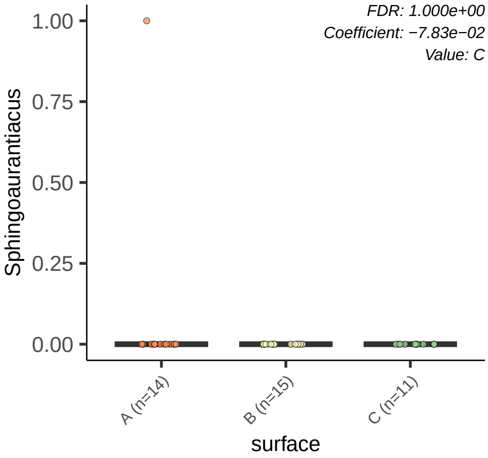
FDR: 1.000e+00
Coefficient: −7.83e−02
Value: C
Sphingoaurantiacus
1.00
0.75
0.50
0.25
0.00
A (n=14)
B (n=15)
C (n=11)
surface

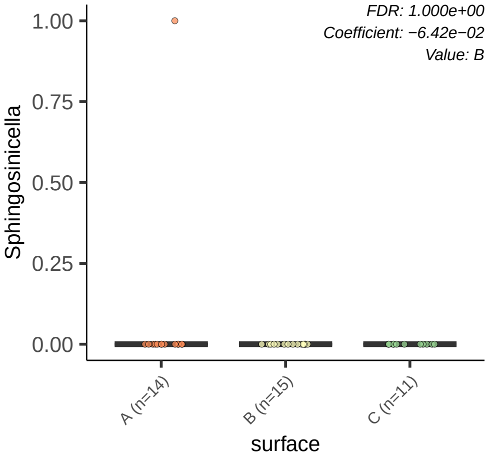
FDR: 1.000e+00
Coefficient: −6.42e−02
Value: B
Sphingosinicella
1.00
0.75
0.50
0.25
0.00
A (n=14)
B (n=15)
C (n=11)
surface

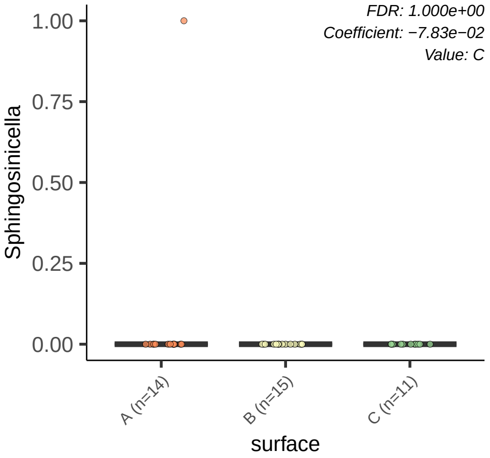
FDR: 1.000e+00
Coefficient: −7.83e−02
Value: C
Sphingosinicella
1.00
0.75
0.50
0.25
0.00
A (n=14)
B (n=15)
C (n=11)
surface

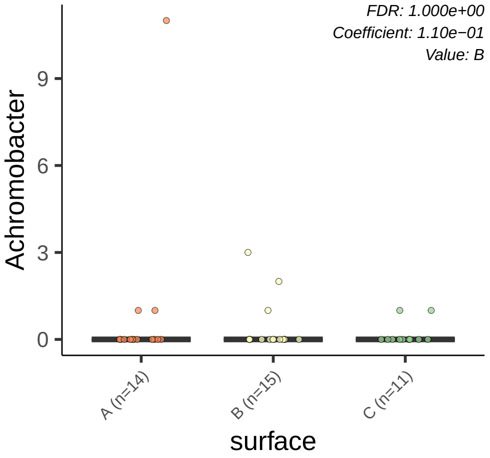
FDR: 1.000e+00
Coefficient: 1.10e−01
Value: B
Achromobacter
0
3
6
9
A (n=14)
B (n=15)
C (n=11)
surface

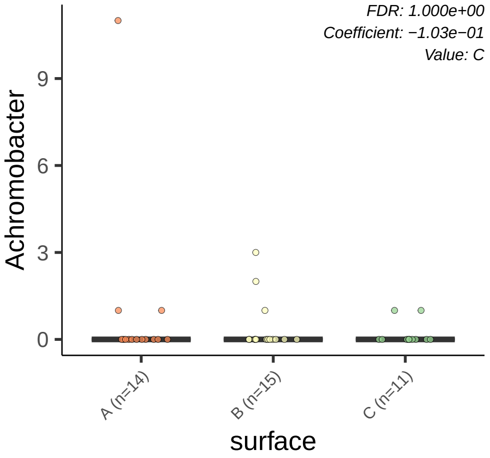

FDR: 1.000e+00
Coefficient: −1.03e−01
Value: C
Achromobacter
9
6
3
0
A (n=14)
B (n=15)
C (n=11)
surface

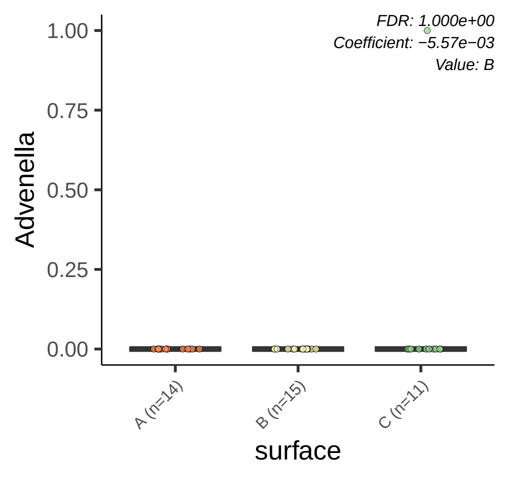

FDR: 1.000e+00
Coefficient: −5.57e−03
Value: B
1.00
0.75
0.50
0.25
0.00
Advenella
A (n=14)
B (n=15)
C (n=11)
surface

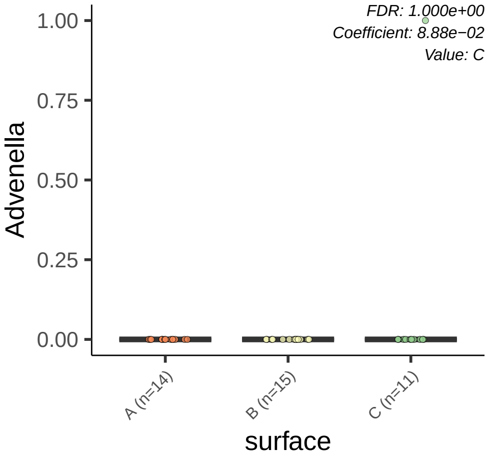
FDR: 1.000e+00
Coefficient: 8.88e−02
Value: C
Advenella
1.00
0.75
0.50
0.25
0.00
A (n=14)
B (n=15)
C (n=11)
surface

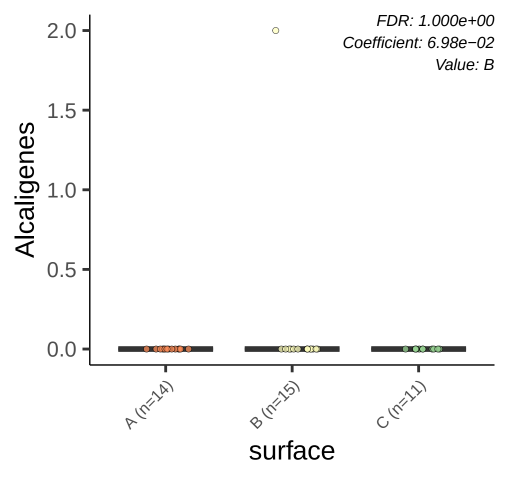

FDR: 1.000e+00
Coefficient: 6.98e−02
Value: B
Alcaligenes
2.0
1.5
1.0
0.5
0.0
A (n=14)
B (n=15)
C (n=11)
surface

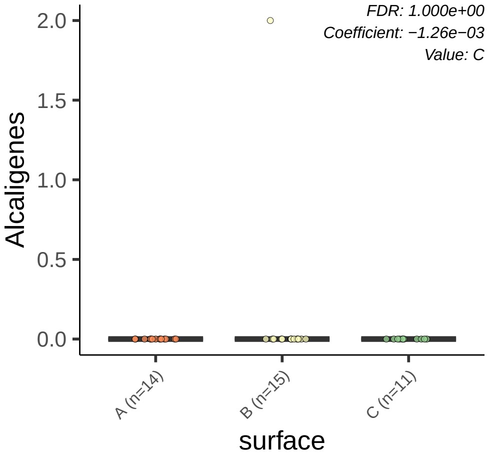
FDR: 1.000e+00
Coefficient: −1.26e−03
Value: C
Alcaligenes
2.0
1.5
1.0
0.5
0.0
A (n=14)
B (n=15)
C (n=11)
surface

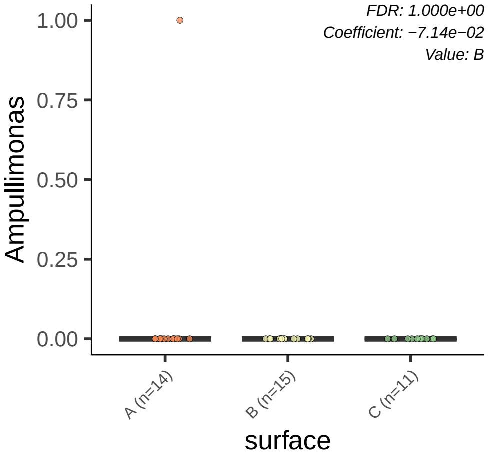
FDR: 1.000e+00
Coefficient: −7.14e−02
Value: B
Ampullimonas
1.00
0.75
0.50
0.25
0.00
A (n=14)
B (n=15)
C (n=11)
surface

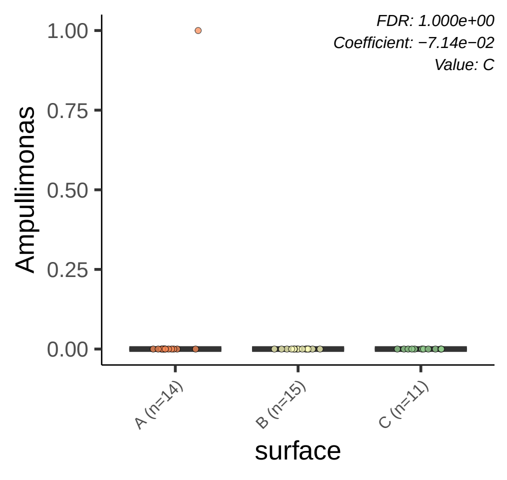
FDR: 1.000e+00
Coefficient: −7.14e−02
Value: C
Ampullimonas
1.00
0.75
0.50
0.25
0.00
A (n=14)
B (n=15)
C (n=11)
surface

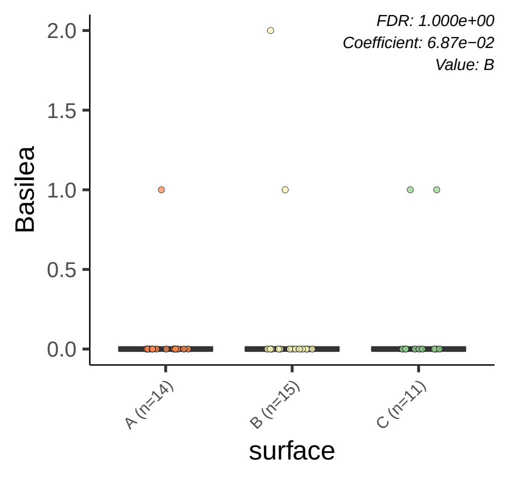
FDR: 1.000e+00
Coefficient: 6.87e−02
Value: B
Basilea
2.0
1.5
1.0
0.5
0.0
A (n=14)
B (n=15)
C (n=11)
surface

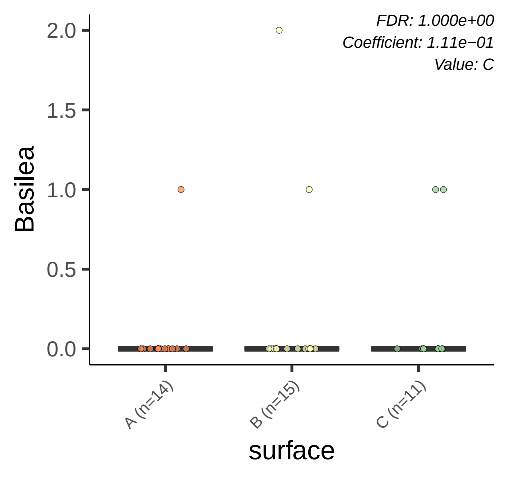

FDR: 1.000e+00
Coefficient: 1.11e−01
Value: C
2.0
1.5
1.0
0.5
0.0
Basilea
A (n=14)
B (n=15)
C (n=11)
surface

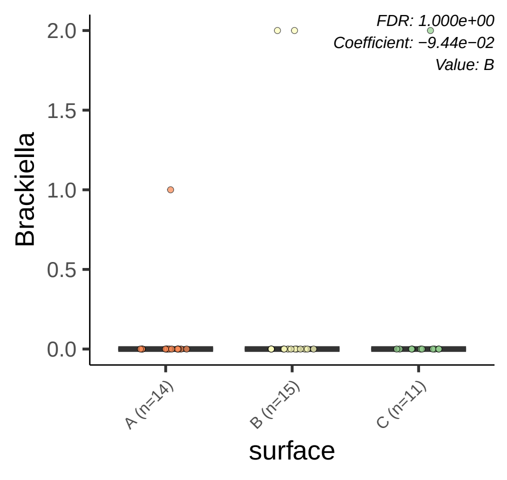
FDR: 1.000e+00
Coefficient: −9.44e−02
Value: B
Brackiella
2.0
1.5
1.0
0.5
0.0
A (n=14)
B (n=15)
C (n=11)
surface

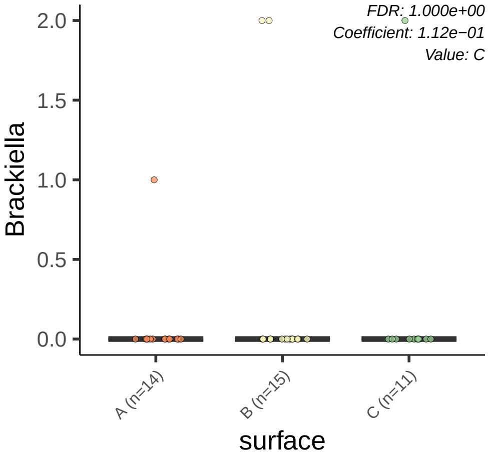

FDR: 1.000e+00
Coefficient: 1.12e−01
Value: C
Brackiella
2.0
1.5
1.0
0.5
0.0
A (n=14)
B (n=15)
C (n=11)
surface

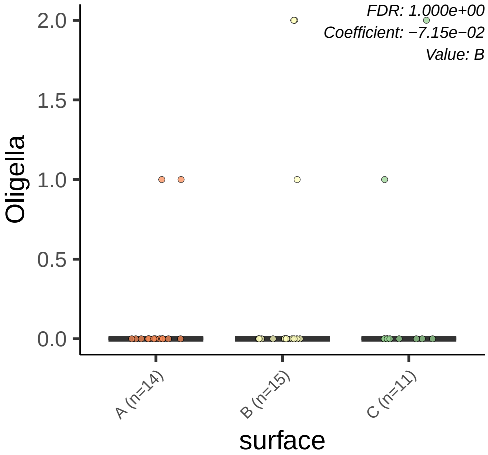

FDR: 1.000e+00
Coefficient: −7.15e−02
Value: B
Oligella
2.0
1.5
1.0
0.5
0.0
A (n=14)
B (n=15)
C (n=11)
surface

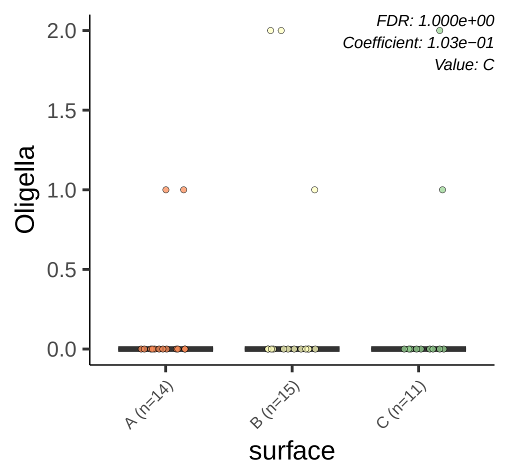
FDR: 1.000e+00
Coefficient: 1.03e−01
Value: C
Oligella
2.0
1.5
1.0
0.5
0.0
A (n=14)
B (n=15)
C (n=11)
surface

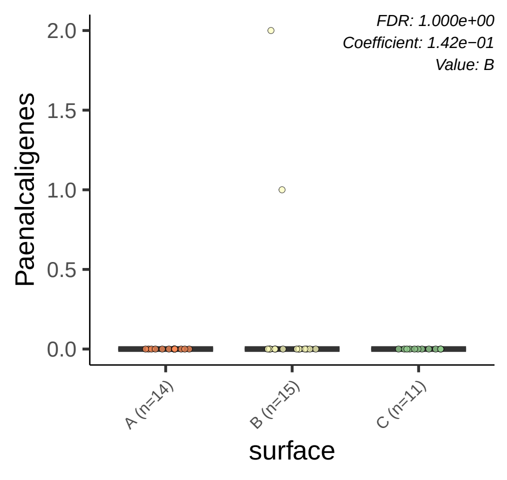
FDR: 1.000e+00
Coefficient: 1.42e−01
Value: B
Paenalcaligenes
2.0
1.5
1.0
0.5
0.0
A (n=14)
B (n=15)
C (n=11)
surface

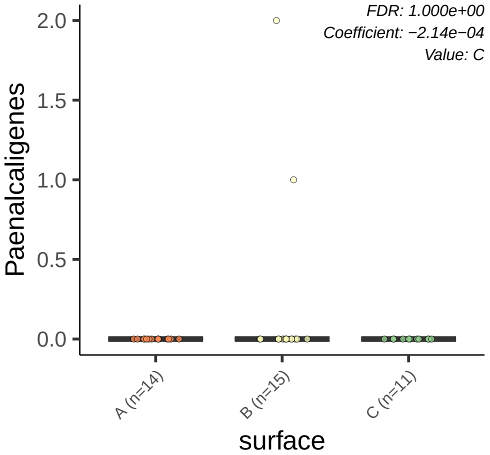

FDR: 1.000e+00
Coefficient: −2.14e−04
Value: C
Paenalcaligenes
2.0
1.5
1.0
0.5
0.0
A (n=14)
B (n=15)
C (n=11)
surface

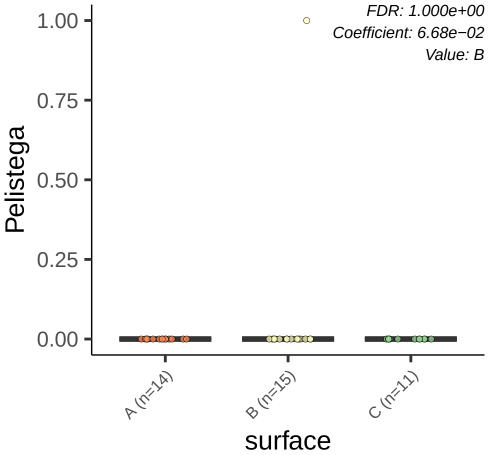
FDR: 1.000e+00
Coefficient: 6.68e−02
Value: B
Pelistega
1.00
0.75
0.50
0.25
0.00
A (n=14)
B (n=15)
C (n=11)
surface

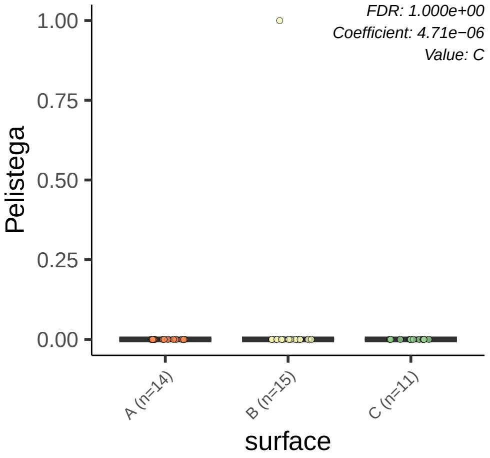
FDR: 1.000e+00
Coefficient: 4.71e−06
Value: C
Pelistega
1.00
0.75
0.50
0.25
0.00
A (n=14)
B (n=15)
C (n=11)
surface

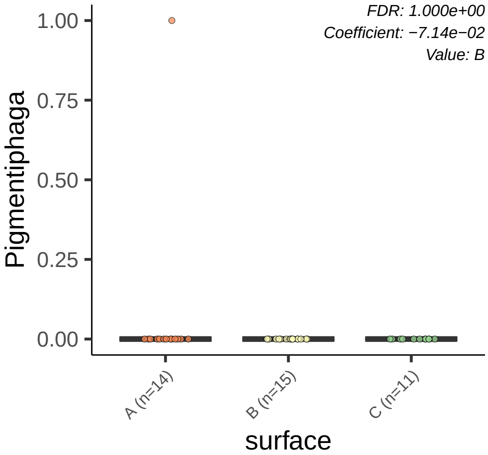

FDR: 1.000e+00
Coefficient: −7.14e−02
Value: B
Pigmentiphaga
1.00
0.75
0.50
0.25
0.00
A (n=14)
B (n=15)
C (n=11)
surface

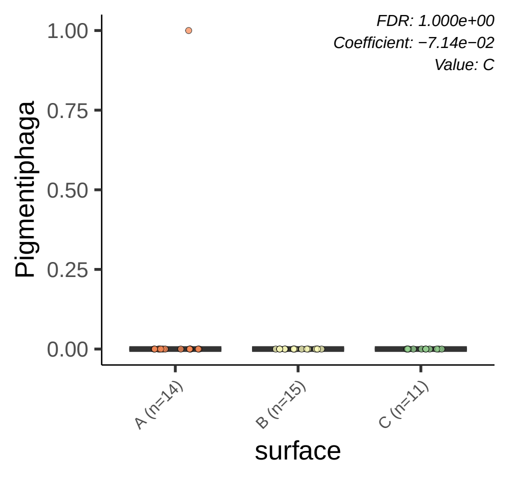
FDR: 1.000e+00
Coefficient: −7.14e−02
Value: C
1.00
0.75
0.50
0.25
0.00
Pigmentiphaga
A (n=14)
B (n=15)
C (n=11)
surface

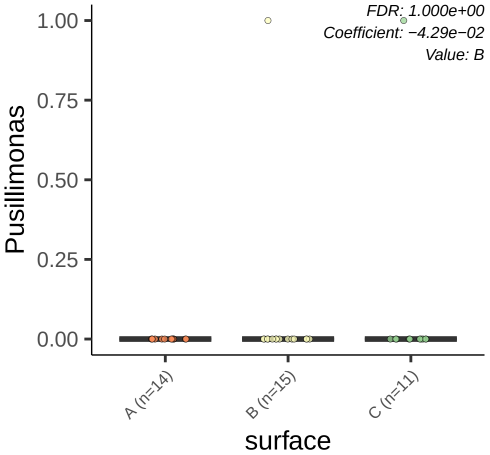
FDR: 1.000e+00
Coefficient: −4.29e−02
Value: B
Pusillimonas
1.00
0.75
0.50
0.25
0.00
A (n=14)
B (n=15)
C (n=11)
surface

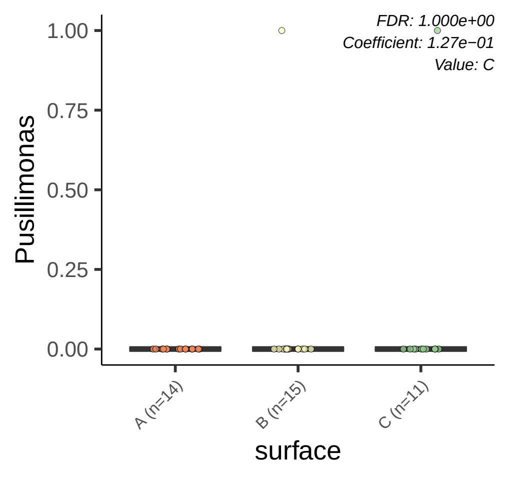
FDR: 1.000e+00
Coefficient: 1.27e−01
Value: C
Pusillimonas
1.00
0.75
0.50
0.25
0.00
A (n=14)
B (n=15)
C (n=11)
surface

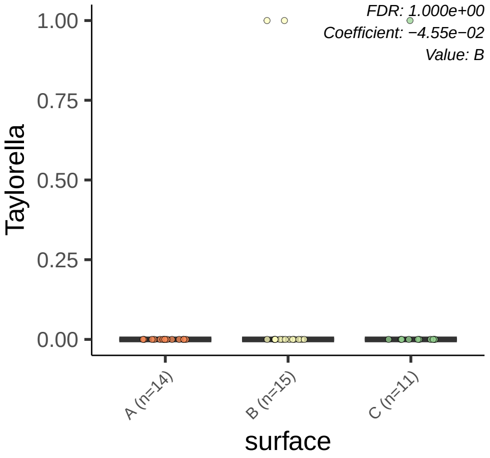
FDR: 1.000e+00
Coefficient: −4.55e−02
Value: B
Taylorella
1.00
0.75
0.50
0.25
0.00
A (n=14)
B (n=15)
C (n=11)
surface

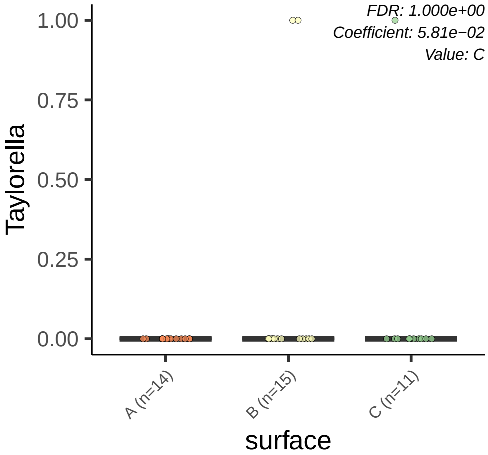
FDR: 1.000e+00
Coefficient: 5.81e−02
Value: C
Taylorella
1.00
0.75
0.50
0.25
0.00
A (n=14)
B (n=15)
C (n=11)
surface

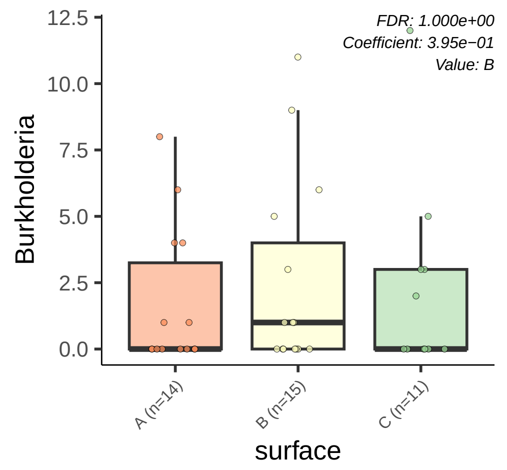
FDR: 1.000e+00
Coefficient: 3.95e−01
Value: B
12.5
10.0
7.5
5.0
2.5
0.0
Burkholderia
A (n=14)
B (n=15)
C (n=11)
surface

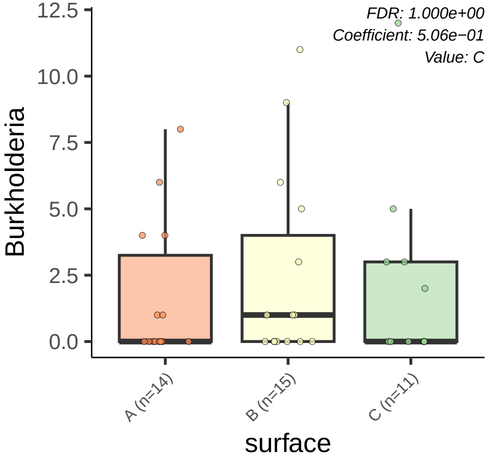

FDR: 1.000e+00
Coefficient: 5.06e−01
Value: C
Burkholderia
12.5
10.0
7.5
5.0
2.5
0.0
A (n=14)
B (n=15)
C (n=11)
surface

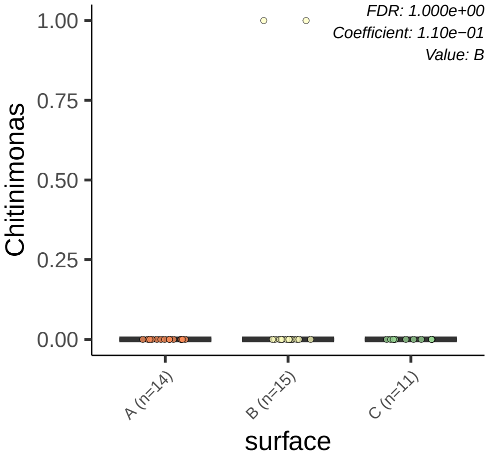

FDR: 1.000e+00
Coefficient: 1.10e−01
Value: B
1.00
0.75
0.50
0.25
0.00
Chitinimonas
A (n=14)
B (n=15)
C (n=11)
surface

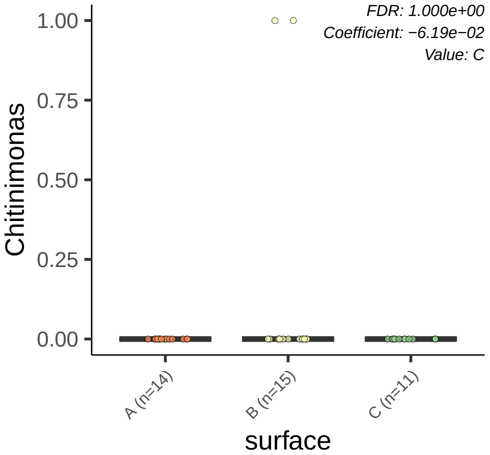

FDR: 1.000e+00
Coefficient: −6.19e−02
Value: C
Chitinimonas
1.00
0.75
0.50
0.25
0.00
A (n=14)
B (n=15)
C (n=11)
surface

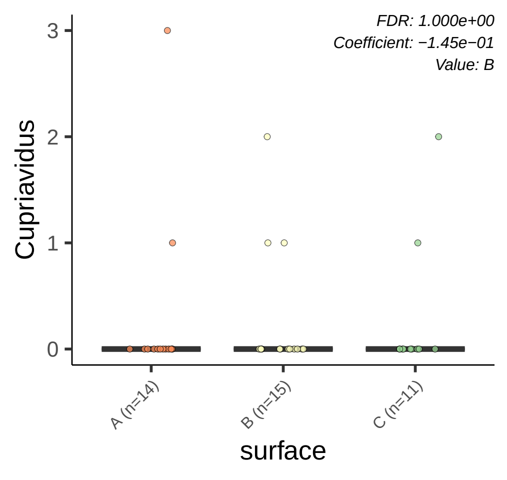
FDR: 1.000e+00
Coefficient: −1.45e−01
Value: B
Cupriavidus
3
2
1
0
A (n=14)
B (n=15)
C (n=11)
surface

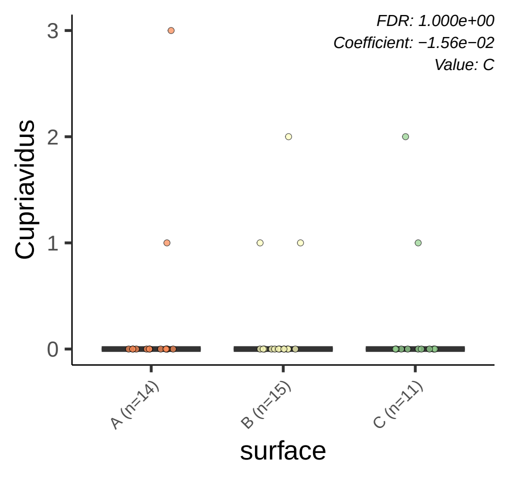
FDR: 1.000e+00
Coefficient: −1.56e−02
Value: C
Cupriavidus
3
2
1
0
A (n=14)
B (n=15)
C (n=11)
surface

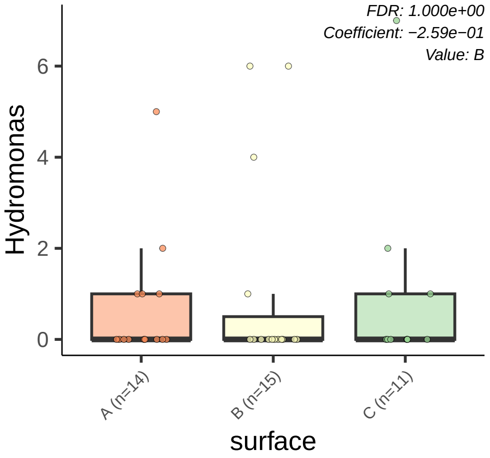

FDR: 1.000e+00
Coefficient: −2.59e−01
Value: B
Hydromonas
0
2
4
6
A (n=14)
B (n=15)
C (n=11)
surface

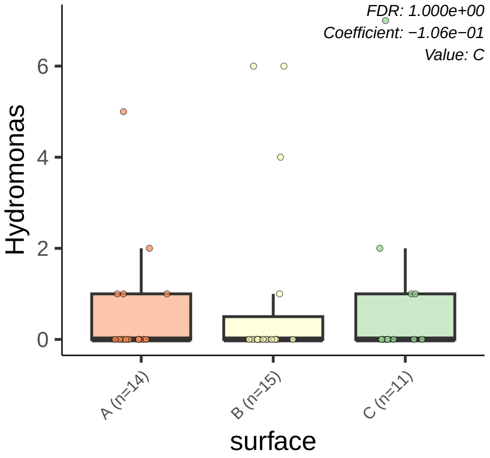

FDR: 1.000e+00
Coefficient: −1.06e−01
Value: C
Hydromonas
0
2
4
6
A (n=14)
B (n=15)
C (n=11)
surface

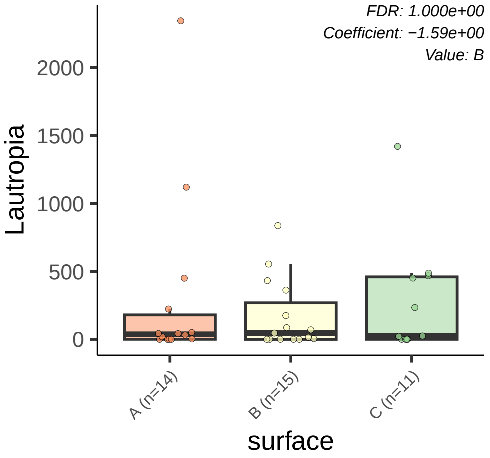
FDR: 1.000e+00
Coefficient: −1.59e+00
Value: B
Lautropia
2000
1500
1000
500
0
A (n=14)
B (n=15)
C (n=11)
surface

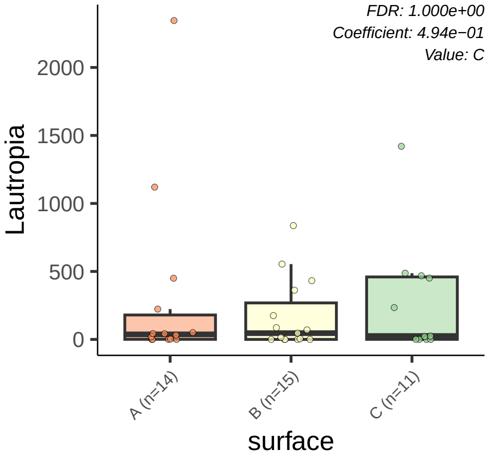

FDR: 1.000e+00
Coefficient: 4.94e−01
Value: C
Lautropia
2000
1500
1000
500
0
A (n=14)
B (n=15)
C (n=11)
surface

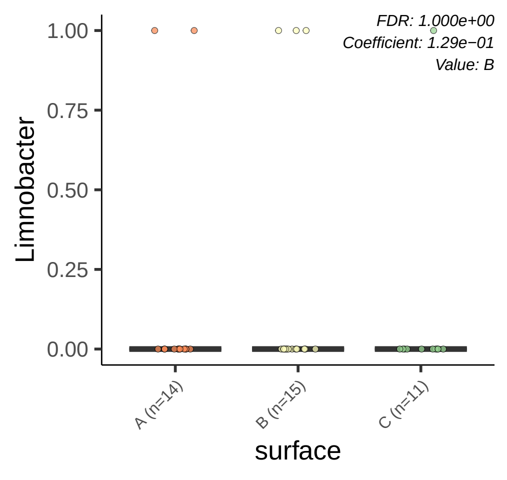
FDR: 1.000e+00
Coefficient: 1.29e−01
Value: B
Limnobacter
1.00
0.75
0.50
0.25
0.00
A (n=14)
B (n=15)
C (n=11)
surface

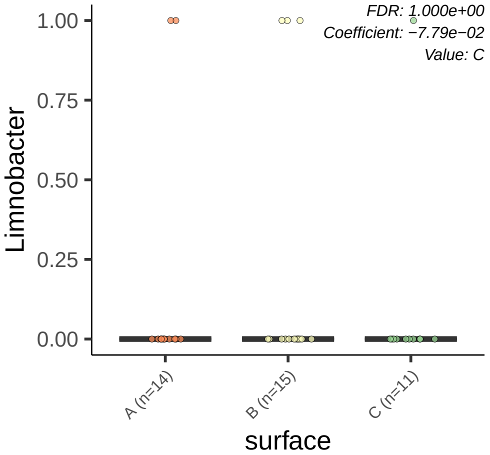

FDR: 1.000e+00
Coefficient: −7.79e−02
Value: C
Limnobacter
1.00
0.75
0.50
0.25
0.00
A (n=14)
B (n=15)
C (n=11)
surface

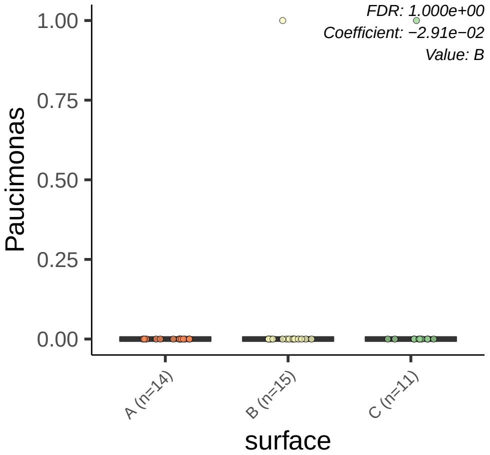
FDR: 1.000e+00
Coefficient: −2.91e−02
Value: B
Paucimonas
1.00
0.75
0.50
0.25
0.00
A (n=14)
B (n=15)
C (n=11)
surface

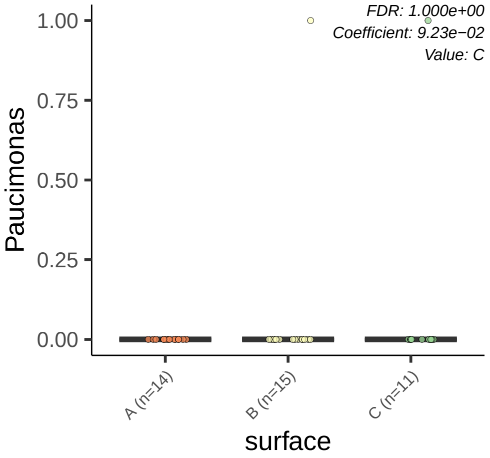
FDR: 1.000e+00
Coefficient: 9.23e−02
Value: C
Paucimonas
1.00
0.75
0.50
0.25
0.00
A (n=14)
B (n=15)
C (n=11)
surface

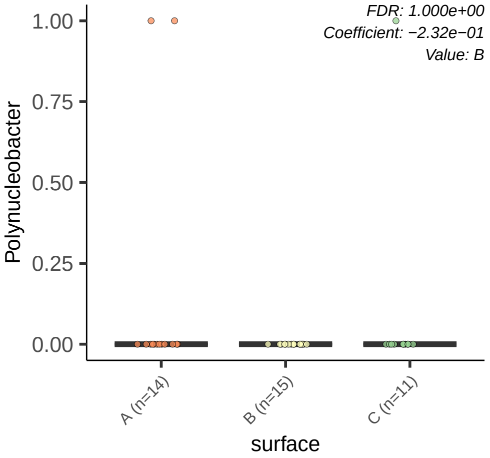

FDR: 1.000e+00
Coefficient: −2.32e−01
Value: B
Polynucleobacter
1.00
0.75
0.50
0.25
0.00
A (n=14)
B (n=15)
C (n=11)
surface

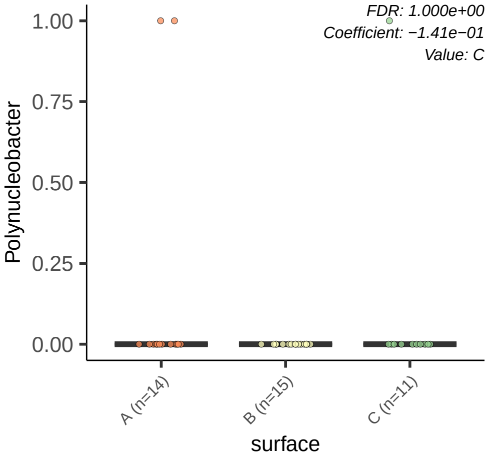

FDR: 1.000e+00
Coefficient: −1.41e−01
Value: C
Polynucleobacter
1.00
0.75
0.50
0.25
0.00
A (n=14)
B (n=15)
C (n=11)
surface

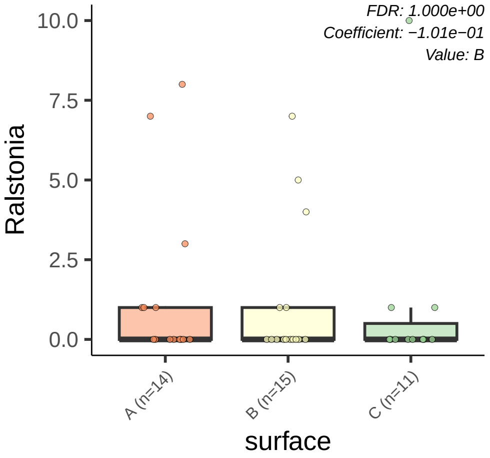

FDR: 1.000e+00
Coefficient: −1.01e−01
Value: B
Ralstonia
10.0
7.5
5.0
2.5
0.0
A (n=14)
B (n=15)
C (n=11)
surface

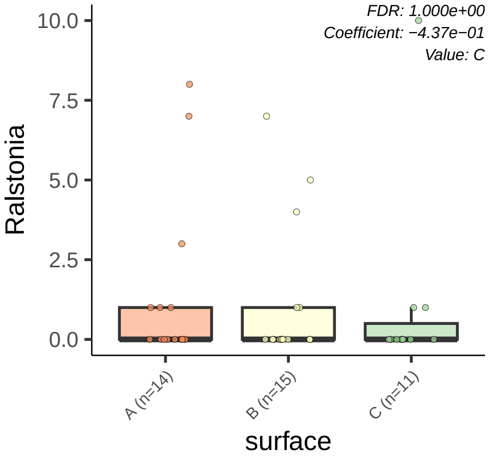
FDR: 1.000e+00
Coefficient: −4.37e−01
Value: C
Ralstonia
10.0
7.5
5.0
2.5
0.0
A (n=14)
B (n=15)
C (n=11)
surface

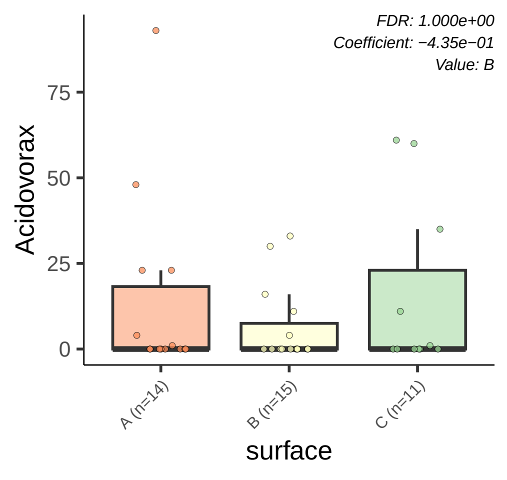
FDR: 1.000e+00
Coefficient: −4.35e−01
Value: B
Acidovorax
0
25
50
75
A (n=14)
B (n=15)
C (n=11)
surface

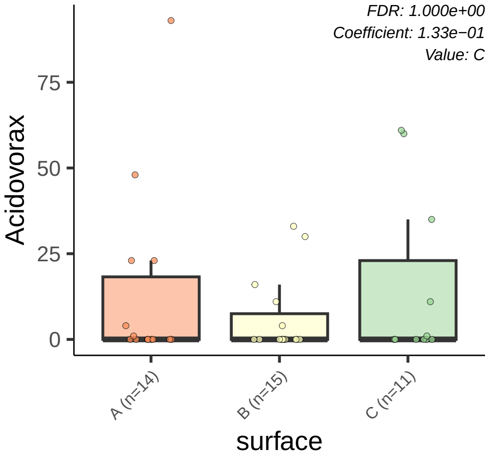
FDR: 1.000e+00
Coefficient: 1.33e−01
Value: C
Acidovorax
75
50
25
0
A (n=14)
B (n=15)
C (n=11)
surface

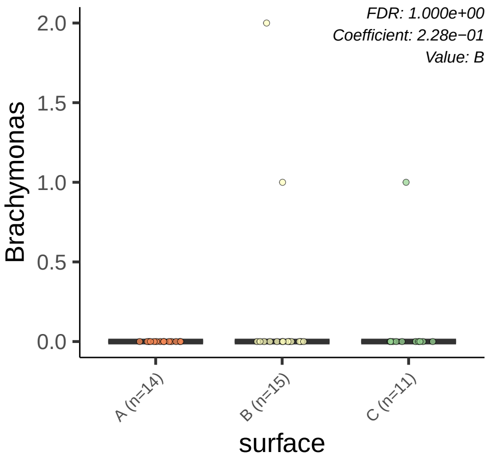
FDR: 1.000e+00
Coefficient: 2.28e−01
Value: B
Brachymonas
2.0
1.5
1.0
0.5
0.0
A (n=14)
B (n=15)
C (n=11)
surface

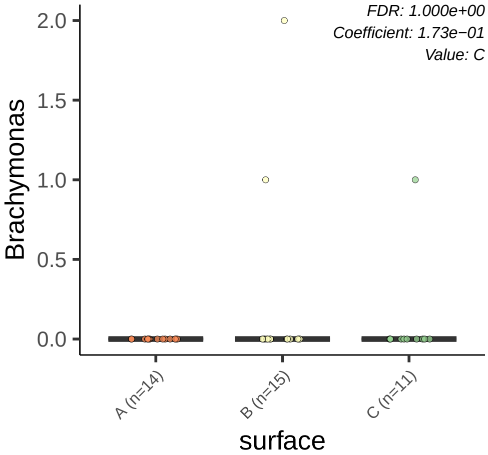
FDR: 1.000e+00
Coefficient: 1.73e−01
Value: C
Brachymonas
2.0
1.5
1.0
0.5
0.0
A (n=14)
B (n=15)
C (n=11)
surface

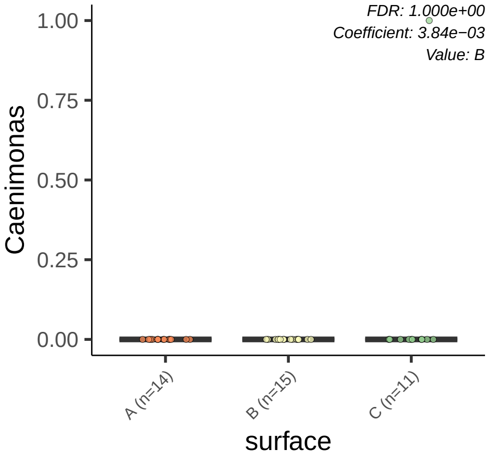
FDR: 1.000e+00
Coefficient: 3.84e−03
Value: B
Caenimonas
1.00
0.75
0.50
0.25
0.00
A (n=14)
B (n=15)
C (n=11)
surface

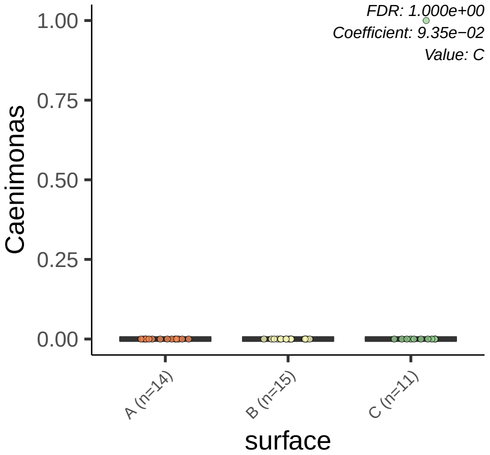

FDR: 1.000e+00
Coefficient: 9.35e−02
Value: C
Caenimonas
1.00
0.75
0.50
0.25
0.00
A (n=14)
B (n=15)
C (n=11)
surface

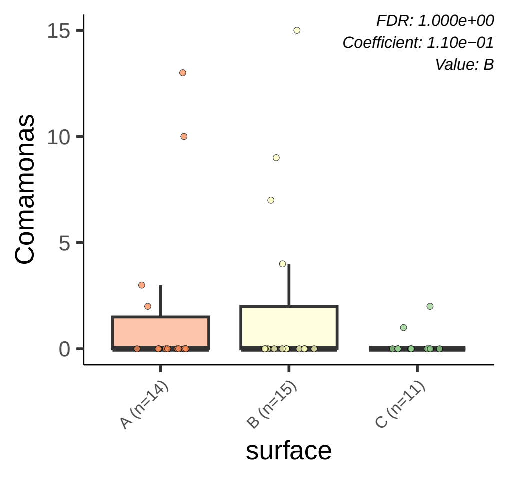
FDR: 1.000e+00
Coefficient: 1.10e−01
Value: B
Comamonas
15
10
5
0
A (n=14)
B (n=15)
C (n=11)
surface

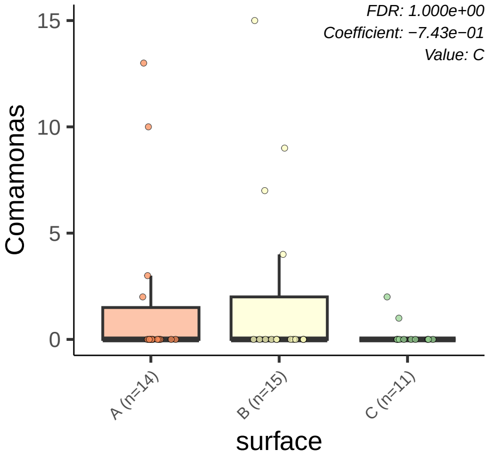

FDR: 1.000e+00
Coefficient: −7.43e−01
Value: C
Comamonas
15
10
5
0
A (n=14)
B (n=15)
C (n=11)
surface

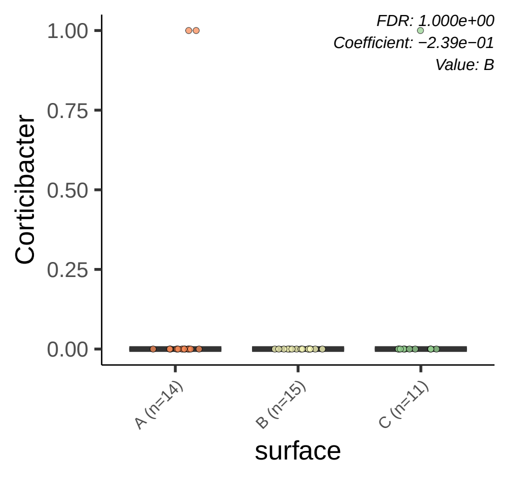

FDR: 1.000e+00
Coefficient: −2.39e−01
Value: B
Corticibacter
1.00
0.75
0.50
0.25
0.00
A (n=14)
B (n=15)
C (n=11)
surface

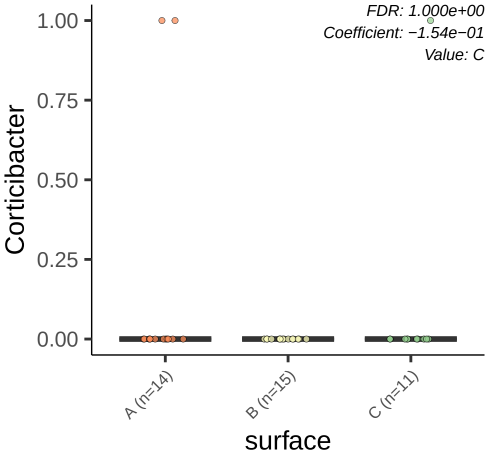
FDR: 1.000e+00
Coefficient: −1.54e−01
Value: C
Corticibacter
1.00
0.75
0.50
0.25
0.00
A (n=14)
B (n=15)
C (n=11)
surface

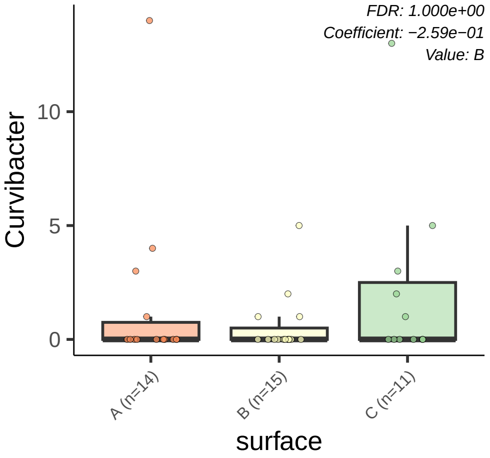
FDR: 1.000e+00
Coefficient: −2.59e−01
Value: B
Curvibacter
0
5
10
A (n=14)
B (n=15)
C (n=11)
surface

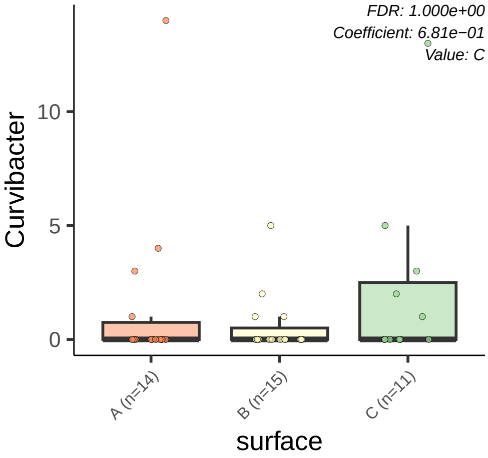

FDR: 1.000e+00
Coefficient: 6.81e−01
Value: C
Curvibacter
10
5
0
A (n=14)
B (n=15)
C (n=11)
surface

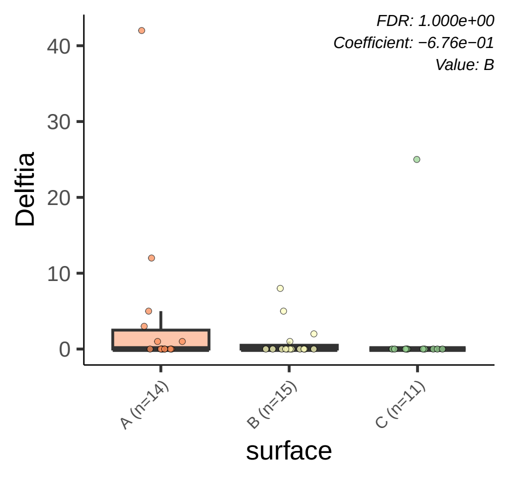
FDR: 1.000e+00
Coefficient: −6.76e−01
Value: B
Delftia
40
30
20
10
0
A (n=14)
B (n=15)
C (n=11)
surface

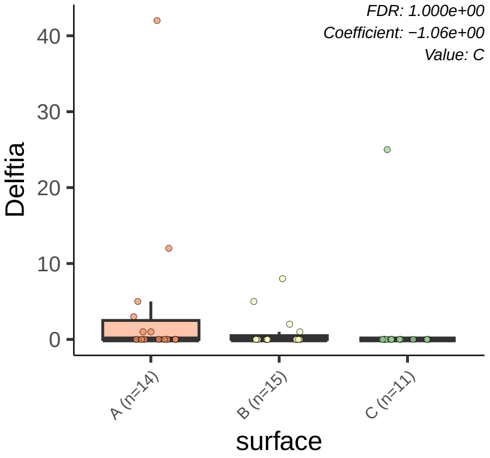

FDR: 1.000e+00
Coefficient: −1.06e+00
Value: C
Delftia
0
10
20
30
40
A (n=14)
B (n=15)
C (n=11)
surface

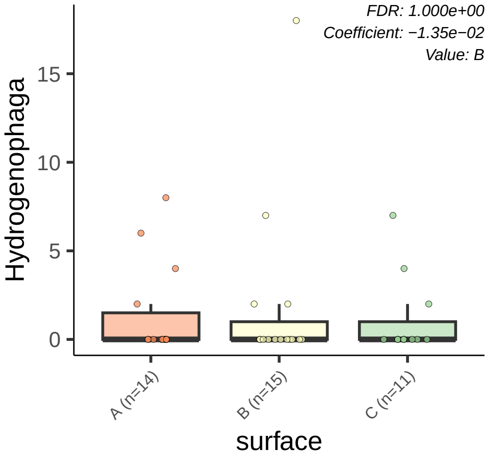
FDR: 1.000e+00
Coefficient: −1.35e−02
Value: B
Hydrogenophaga
15
10
5
0
A (n=14)
B (n=15)
C (n=11)
surface

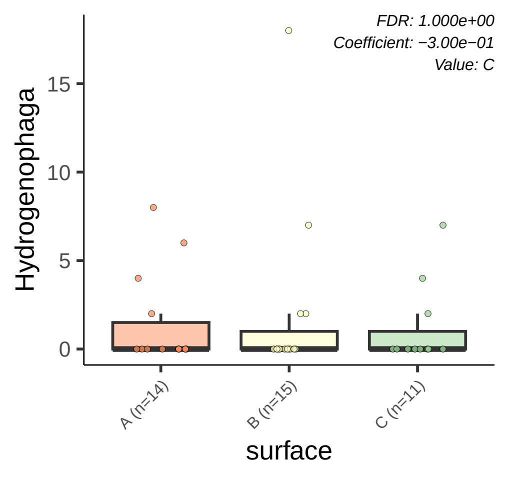

FDR: 1.000e+00
Coefficient: −3.00e−01
Value: C
Hydrogenophaga
15
10
5
0
A (n=14)
B (n=15)
C (n=11)
surface

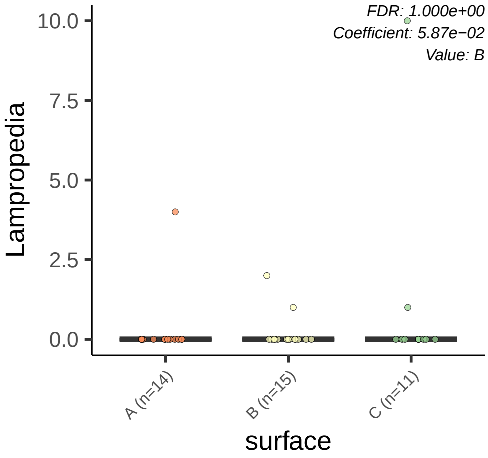

FDR: 1.000e+00
Coefficient: 5.87e−02
Value: B
Lampropedia
10.0
7.5
5.0
2.5
0.0
A (n=14)
B (n=15)
C (n=11)
surface

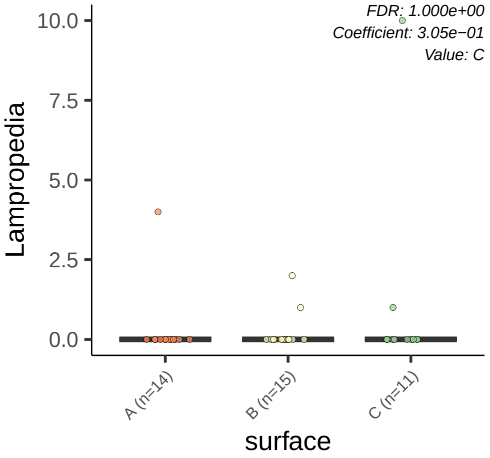

FDR: 1.000e+00
Coefficient: 3.05e−01
Value: C
Lampropedia
10.0
7.5
5.0
2.5
0.0
A (n=14)
B (n=15)
C (n=11)
surface

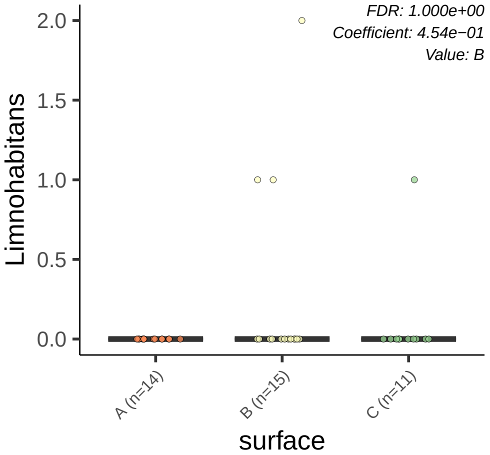
FDR: 1.000e+00
Coefficient: 4.54e−01
Value: B
Limnohabitans
2.0
1.5
1.0
0.5
0.0
A (n=14)
B (n=15)
C (n=11)
surface

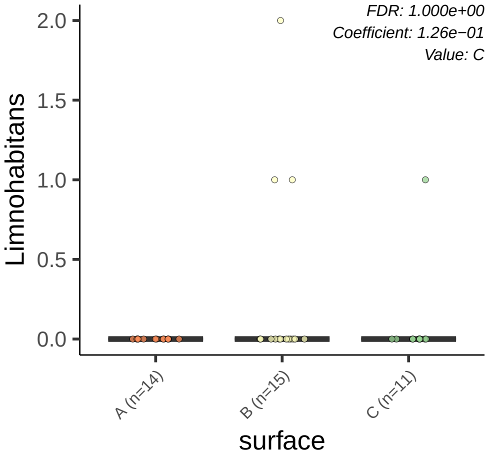

FDR: 1.000e+00
Coefficient: 1.26e−01
Value: C
Limnohabitans
2.0
1.5
1.0
0.5
0.0
A (n=14)
B (n=15)
C (n=11)
surface

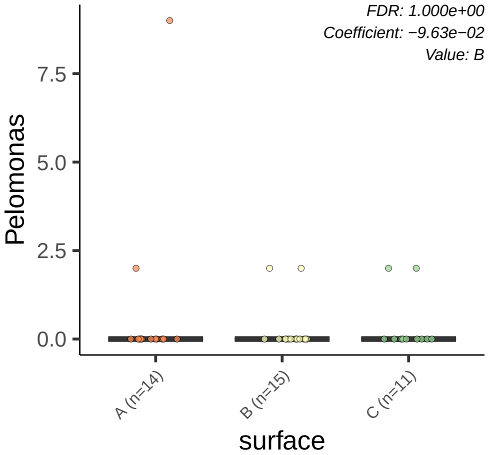

FDR: 1.000e+00
Coefficient: −9.63e−02
Value: B
Pelomonas
7.5
5.0
2.5
0.0
A (n=14)
B (n=15)
C (n=11)
surface

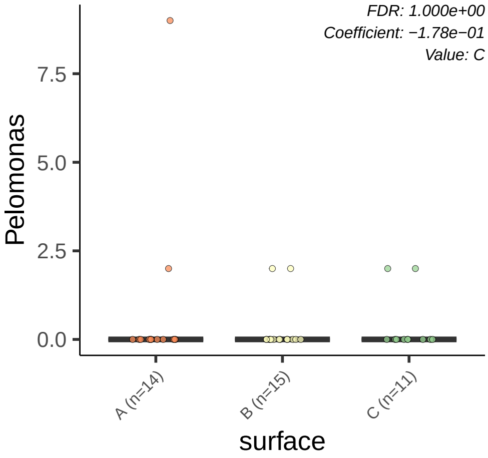

FDR: 1.000e+00
Coefficient: −1.78e−01
Value: C
Pelomonas
7.5
5.0
2.5
0.0
A (n=14)
B (n=15)
C (n=11)
surface

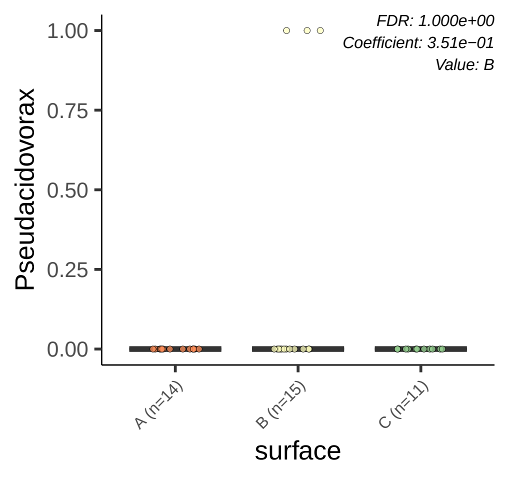
FDR: 1.000e+00
Coefficient: 3.51e−01
Value: B
Pseudacidovorax
1.00
0.75
0.50
0.25
0.00
A (n=14)
B (n=15)
C (n=11)
surface

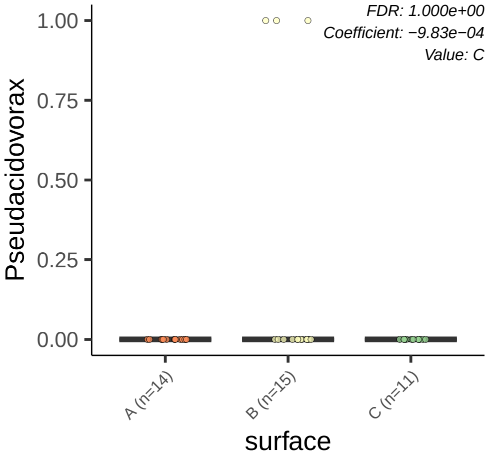
FDR: 1.000e+00
Coefficient: −9.83e−04
Value: C
Pseudacidovorax
1.00
0.75
0.50
0.25
0.00
A (n=14)
B (n=15)
C (n=11)
surface

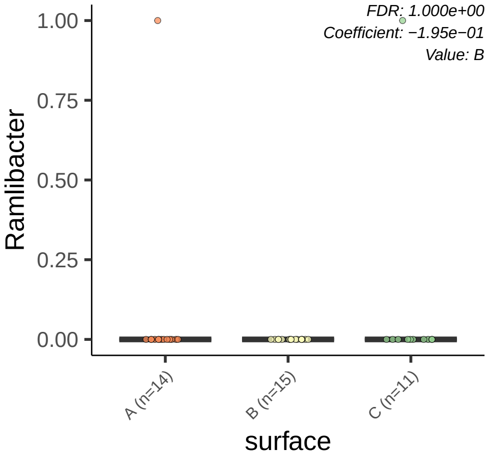
FDR: 1.000e+00
Coefficient: −1.95e−01
Value: B
Ramlibacter
1.00
0.75
0.50
0.25
0.00
A (n=14)
B (n=15)
C (n=11)
surface

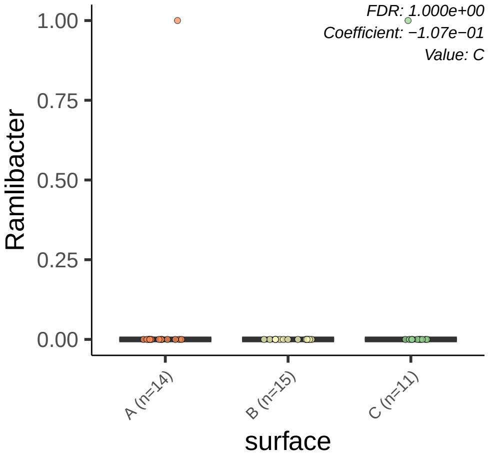
FDR: 1.000e+00
Coefficient: −1.07e−01
Value: C
Ramlibacter
1.00
0.75
0.50
0.25
0.00
A (n=14)
B (n=15)
C (n=11)
surface

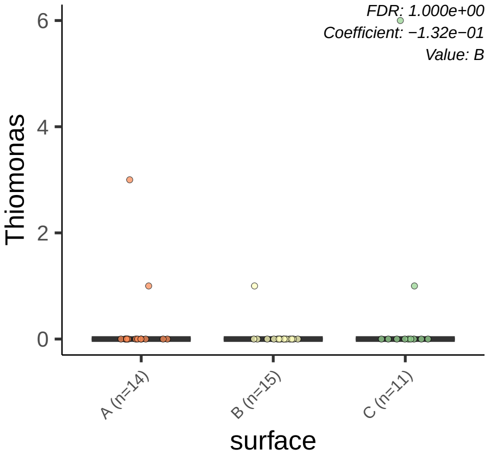
FDR: 1.000e+00
Coefficient: −1.32e−01
Value: B
Thiomonas
0
2
4
6
A (n=14)
B (n=15)
C (n=11)
surface

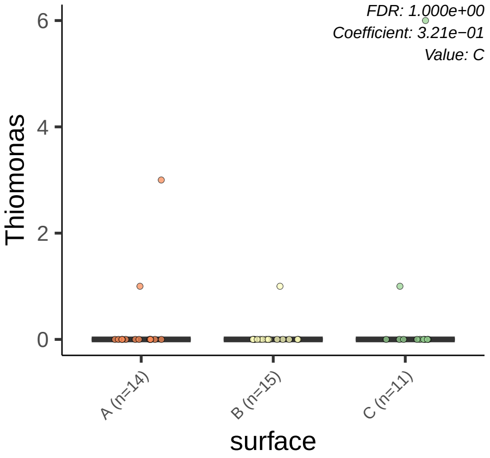

FDR: 1.000e+00
Coefficient: 3.21e−01
Value: C
Thiomonas
0
2
4
6
A (n=14)
B (n=15)
C (n=11)
surface

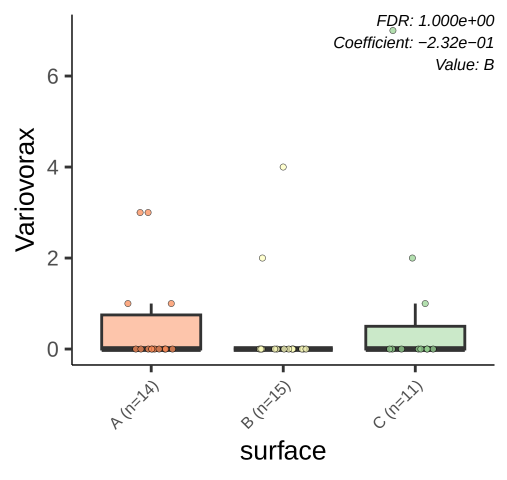

FDR: 1.000e+00
Coefficient: −2.32e−01
Value: B
Variovorax
0
2
4
6
A (n=14)
B (n=15)
C (n=11)
surface

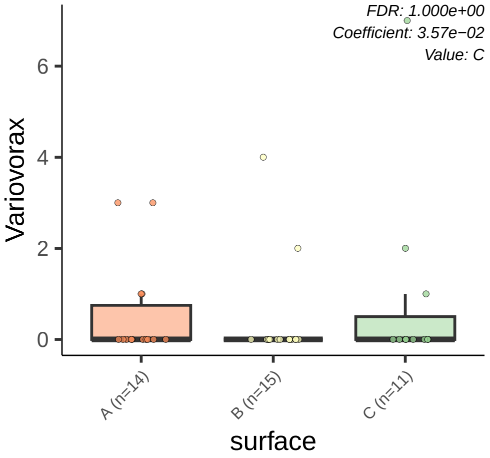

FDR: 1.000e+00
Coefficient: 3.57e−02
Value: C
Variovorax
0
2
4
6
A (n=14)
B (n=15)
C (n=11)
surface

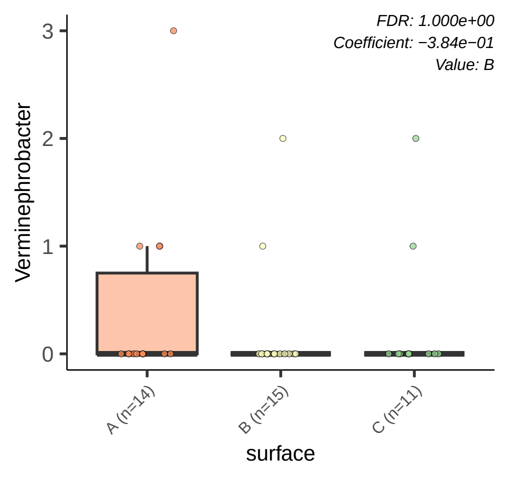
FDR: 1.000e+00
Coefficient: −3.84e−01
Value: B
Verminephrobacter
3
2
1
0
A (n=14)
B (n=15)
C (n=11)
surface

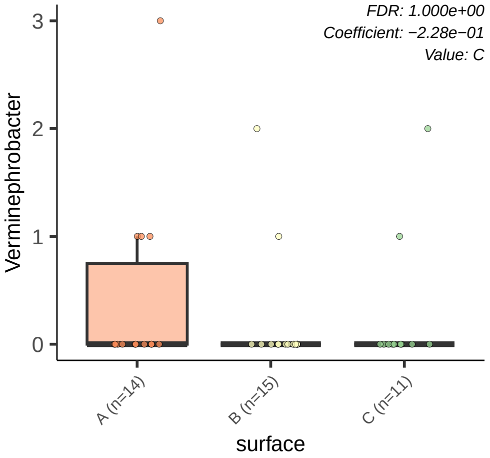

FDR: 1.000e+00
Coefficient: −2.28e−01
Value: C
Verminephrobacter
3
2
1
0
A (n=14)
B (n=15)
C (n=11)
surface

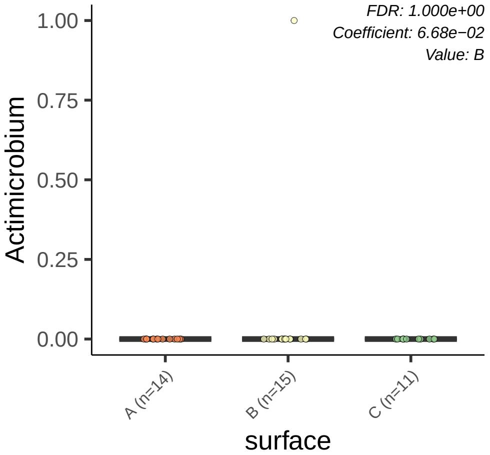

FDR: 1.000e+00
Coefficient: 6.68e−02
Value: B
Actimicrobium
1.00
0.75
0.50
0.25
0.00
A (n=14)
B (n=15)
C (n=11)
surface

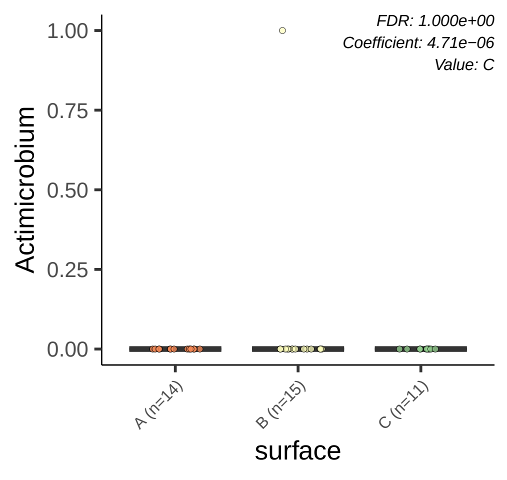
FDR: 1.000e+00
Coefficient: 4.71e−06
Value: C
Actimicrobium
1.00
0.75
0.50
0.25
0.00
A (n=14)
B (n=15)
C (n=11)
surface

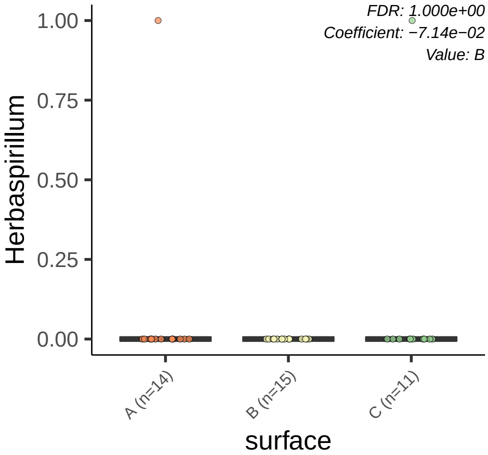
FDR: 1.000e+00
Coefficient: −7.14e−02
Value: B
Herbaspirillum
1.00
0.75
0.50
0.25
0.00
A (n=14)
B (n=15)
C (n=11)
surface

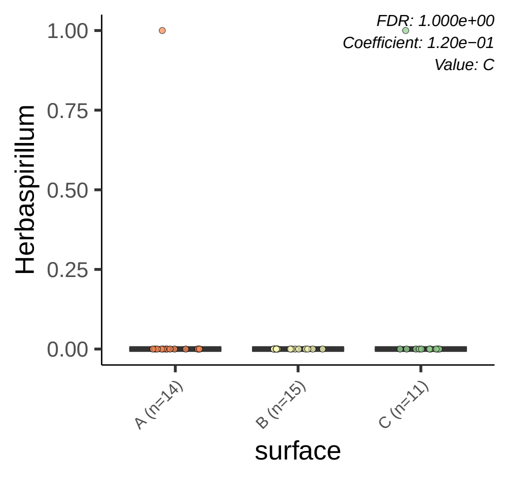

FDR: 1.000e+00
Coefficient: 1.20e−01
Value: C
1.00
0.75
0.50
0.25
0.00
Herbaspirillum
A (n=14)
B (n=15)
C (n=11)
surface

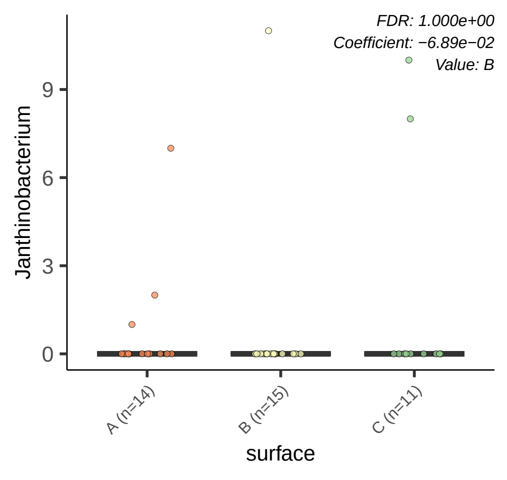
FDR: 1.000e+00
Coefficient: −6.89e−02
Value: B
Janthinobacterium
0
3
6
9
A (n=14)
B (n=15)
C (n=11)
surface

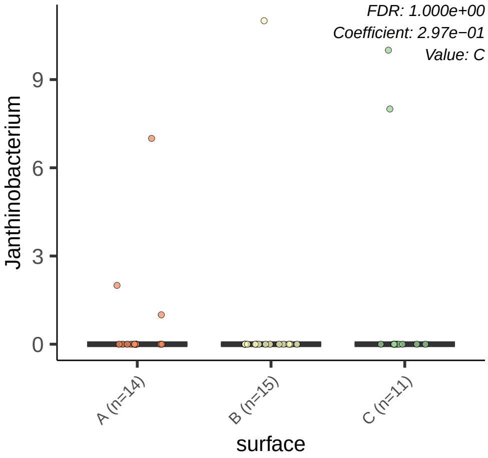
FDR: 1.000e+00
Coefficient: 2.97e−01
Value: C
Janthinobacterium
0
3
6
9
A (n=14)
B (n=15)
C (n=11)
surface

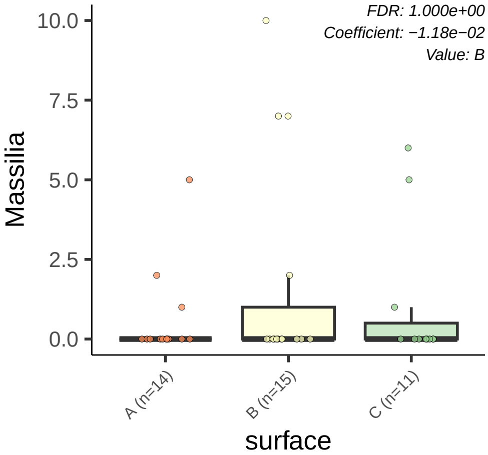
FDR: 1.000e+00
Coefficient: −1.18e−02
Value: B
Massilia
10.0
7.5
5.0
2.5
0.0
A (n=14)
B (n=15)
C (n=11)
surface

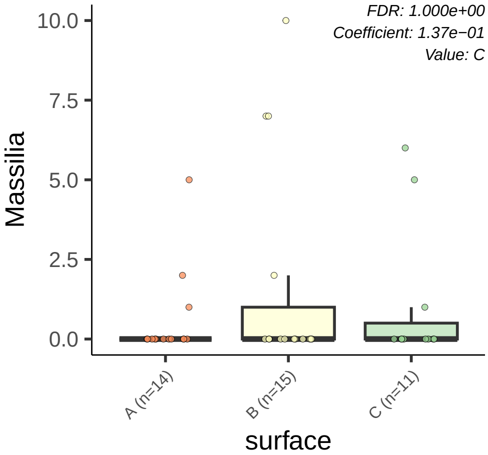
FDR: 1.000e+00
Coefficient: 1.37e−01
Value: C
Massilia
10.0
7.5
5.0
2.5
0.0
A (n=14)
B (n=15)
C (n=11)
surface

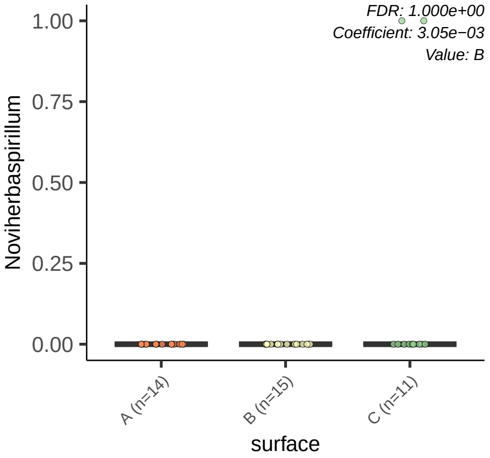
FDR: 1.000e+00
Coefficient: 3.05e−03
Value: B
Noviherbaspirillum
1.00
0.75
0.50
0.25
0.00
A (n=14)
B (n=15)
C (n=11)
surface

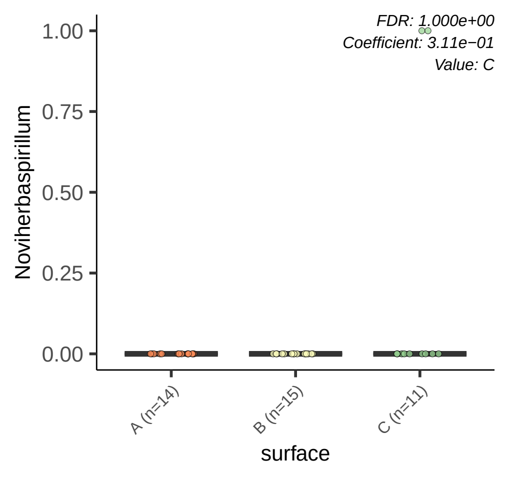

FDR: 1.000e+00
Coefficient: 3.11e−01
Value: C
Noviherbaspirillum
1.00
0.75
0.50
0.25
0.00
A (n=14)
B (n=15)
C (n=11)
surface

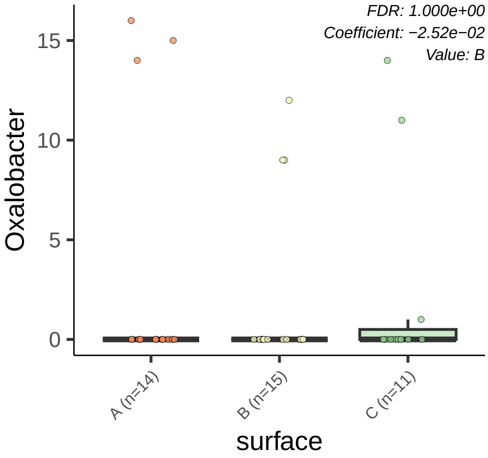

FDR: 1.000e+00
Coefficient: −2.52e−02
Value: B
Oxalobacter
15
10
5
0
A (n=14)
B (n=15)
C (n=11)
surface

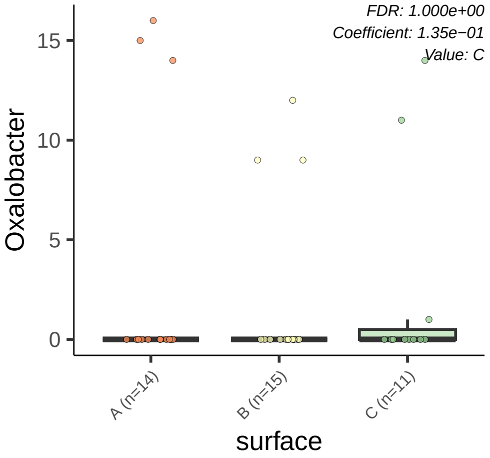

FDR: 1.000e+00
Coefficient: 1.35e−01
Value: C
Oxalobacter
15
10
5
0
A (n=14)
B (n=15)
C (n=11)
surface

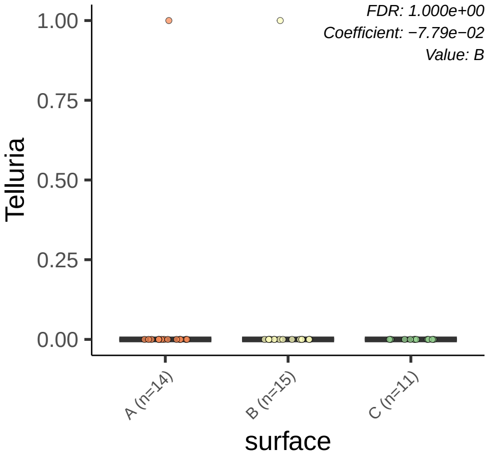
FDR: 1.000e+00
Coefficient: −7.79e−02
Value: B
Telluria
1.00
0.75
0.50
0.25
0.00
A (n=14)
B (n=15)
C (n=11)
surface

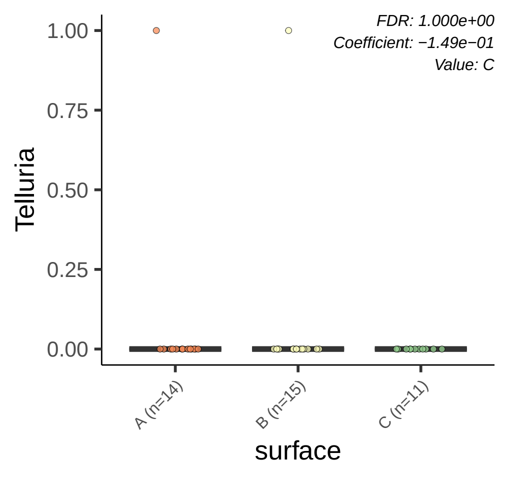
FDR: 1.000e+00
Coefficient: −1.49e−01
Value: C
Telluria
1.00
0.75
0.50
0.25
0.00
A (n=14)
B (n=15)
C (n=11)
surface

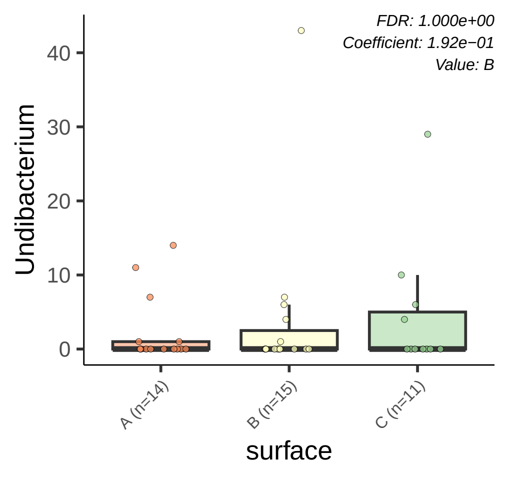
FDR: 1.000e+00
Coefficient: 1.92e−01
Value: B
Undibacterium
40
30
20
10
0
A (n=14)
B (n=15)
C (n=11)
surface

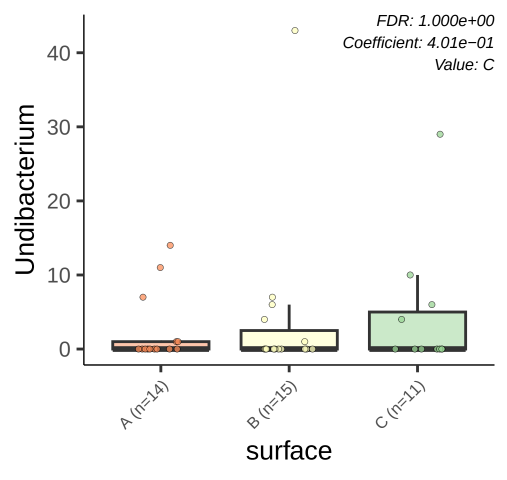

FDR: 1.000e+00
Coefficient: 4.01e−01
Value: C
Undibacterium
0
10
20
30
40
A (n=14)
B (n=15)
C (n=11)
surface

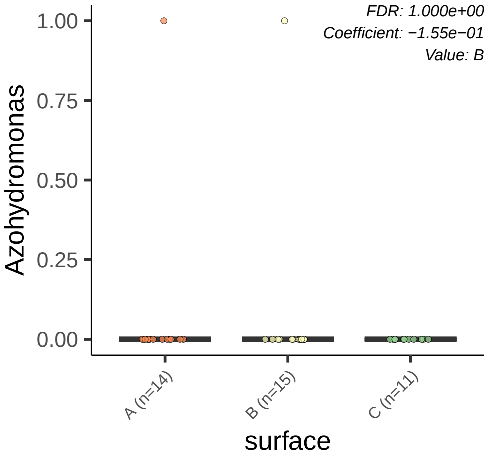

FDR: 1.000e+00
Coefficient: −1.55e−01
Value: B
Azohydromonas
1.00
0.75
0.50
0.25
0.00
A (n=14)
B (n=15)
C (n=11)
surface

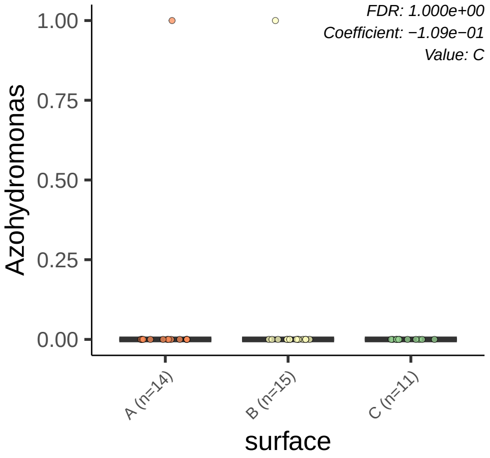

FDR: 1.000e+00
Coefficient: −1.09e−01
Value: C
Azohydromonas
1.00
0.75
0.50
0.25
0.00
A (n=14)
B (n=15)
C (n=11)
surface

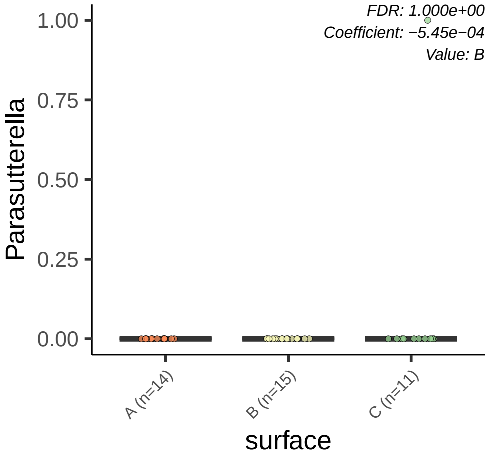
FDR: 1.000e+00
Coefficient: −5.45e−04
Value: B
Parasutterella
1.00
0.75
0.50
0.25
0.00
A (n=14)
B (n=15)
C (n=11)
surface

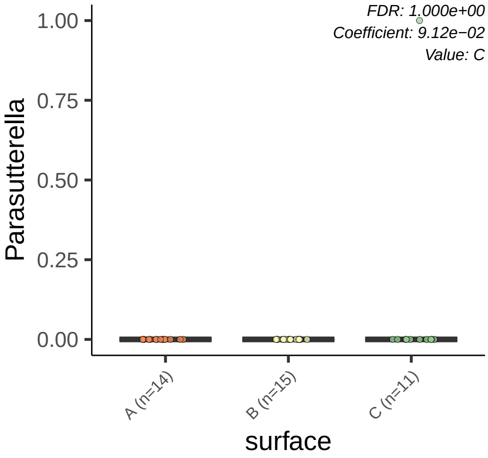

FDR: 1.000e+00
Coefficient: 9.12e−02
Value: C
Parasutterella
1.00
0.75
0.50
0.25
0.00
A (n=14)
B (n=15)
C (n=11)
surface

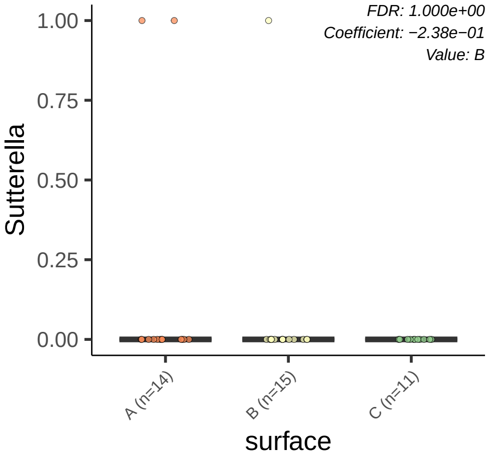
FDR: 1.000e+00
Coefficient: −2.38e−01
Value: B
Sutterella
1.00
0.75
0.50
0.25
0.00
A (n=14)
B (n=15)
C (n=11)
surface

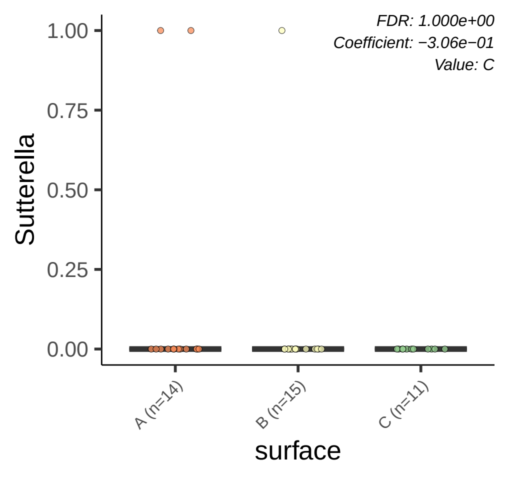
FDR: 1.000e+00
Coefficient: −3.06e−01
Value: C
Sutterella
1.00
0.75
0.50
0.25
0.00
A (n=14)
B (n=15)
C (n=11)
surface

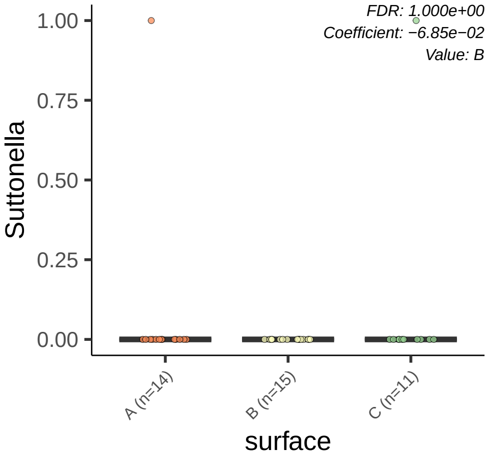

FDR: 1.000e+00
Coefficient: −6.85e−02
Value: B
Suttonella
1.00
0.75
0.50
0.25
0.00
A (n=14)
B (n=15)
C (n=11)
surface

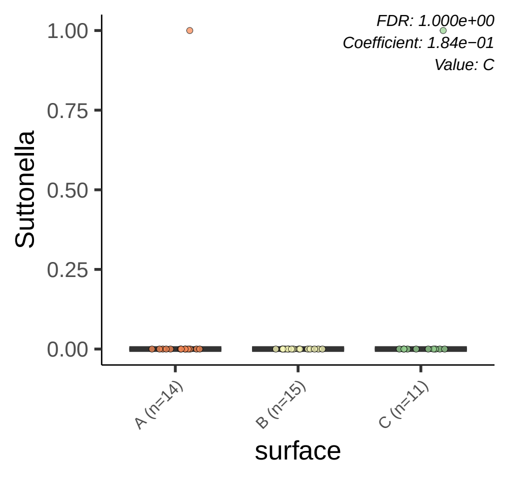
FDR: 1.000e+00
Coefficient: 1.84e−01
Value: C
Suttonella
1.00
0.75
0.50
0.25
0.00
A (n=14)
B (n=15)
C (n=11)
surface

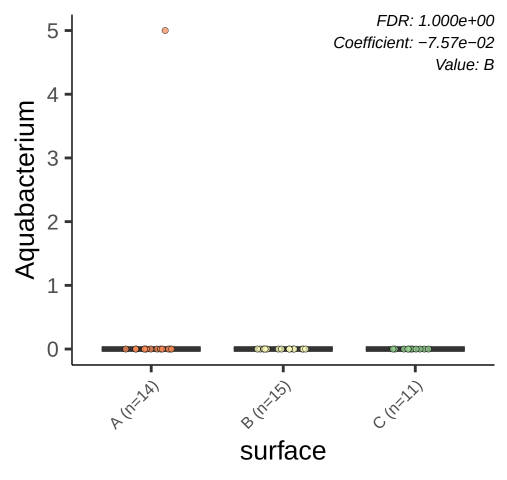

FDR: 1.000e+00
Coefficient: −7.57e−02
Value: B
Aquabacterium
5
4
3
2
1
0
A (n=14)
B (n=15)
C (n=11)
surface

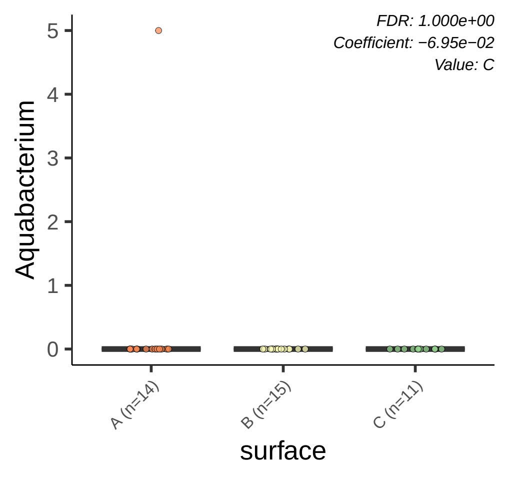

FDR: 1.000e+00
Coefficient: −6.95e−02
Value: C
Aquabacterium
5
4
3
2
1
0
A (n=14)
B (n=15)
C (n=11)
surface

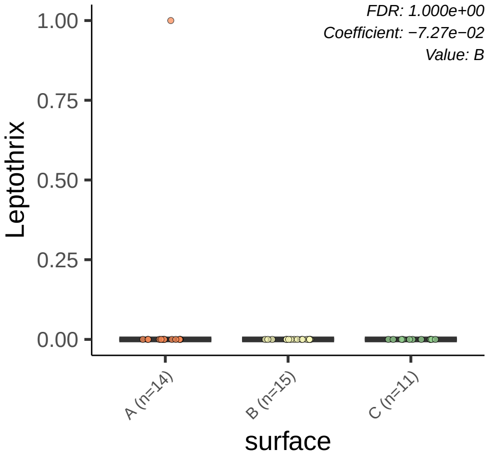
FDR: 1.000e+00
Coefficient: −7.27e−02
Value: B
Leptothrix
1.00
0.75
0.50
0.25
0.00
A (n=14)
B (n=15)
C (n=11)
surface

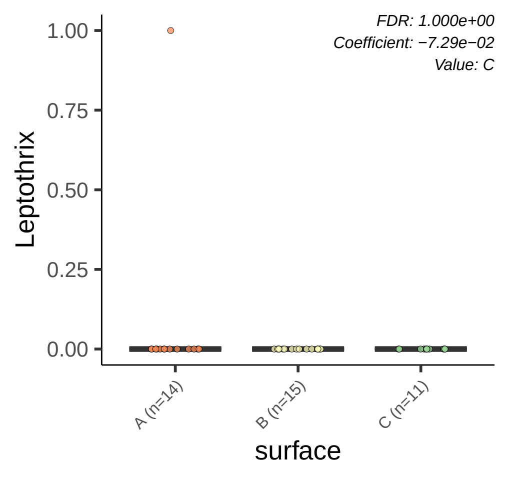
FDR: 1.000e+00
Coefficient: −7.29e−02
Value: C
Leptothrix
1.00
0.75
0.50
0.25
0.00
A (n=14)
B (n=15)
C (n=11)
surface

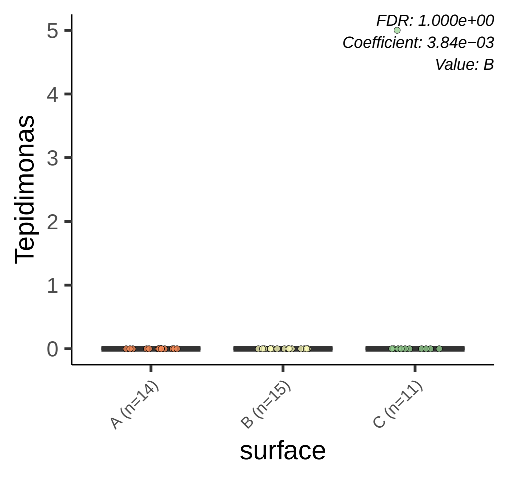
FDR: 1.000e+00
Coefficient: 3.84e−03
Value: B
Tepidimonas
0
1
2
3
4
5
A (n=14)
B (n=15)
C (n=11)
surface

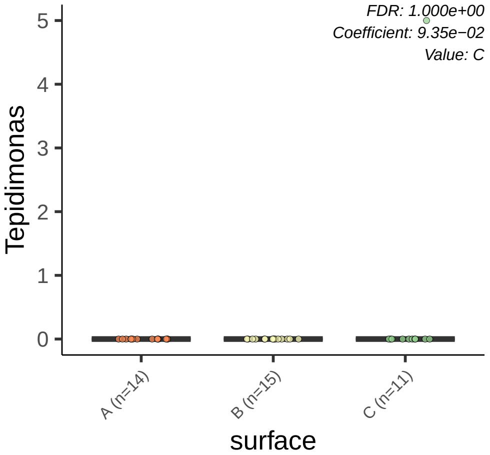

FDR: 1.000e+00
Coefficient: 9.35e−02
Value: C
Tepidimonas
0
1
2
3
4
5
A (n=14)
B (n=15)
C (n=11)
surface

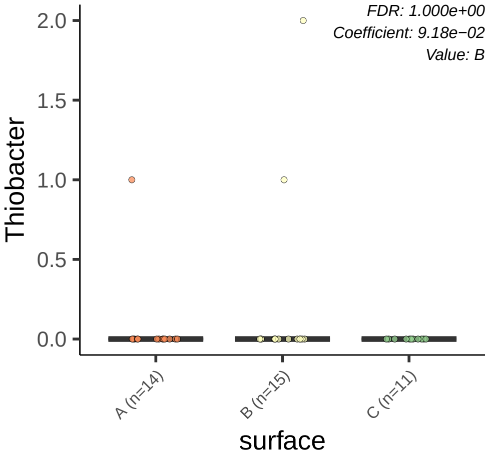
FDR: 1.000e+00
Coefficient: 9.18e−02
Value: B
Thiobacter
2.0
1.5
1.0
0.5
0.0
A (n=14)
B (n=15)
C (n=11)
surface

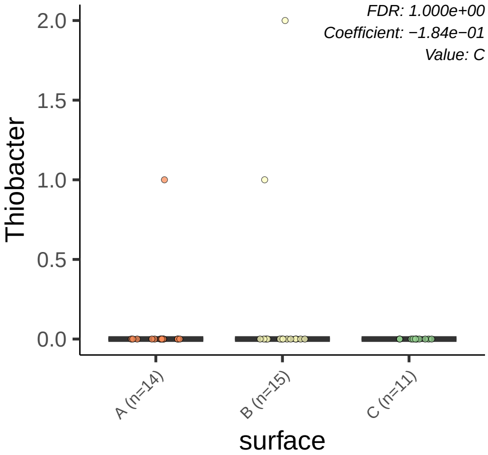
FDR: 1.000e+00
Coefficient: −1.84e−01
Value: C
Thiobacter
2.0
1.5
1.0
0.5
0.0
A (n=14)
B (n=15)
C (n=11)
surface

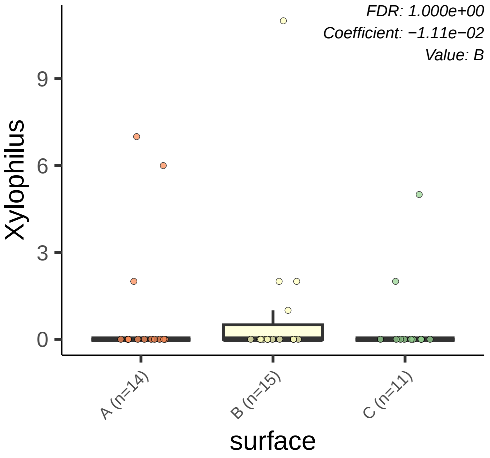

FDR: 1.000e+00
Coefficient: −1.11e−02
Value: B
Xylophilus
9
6
3
0
A (n=14)
B (n=15)
C (n=11)
surface

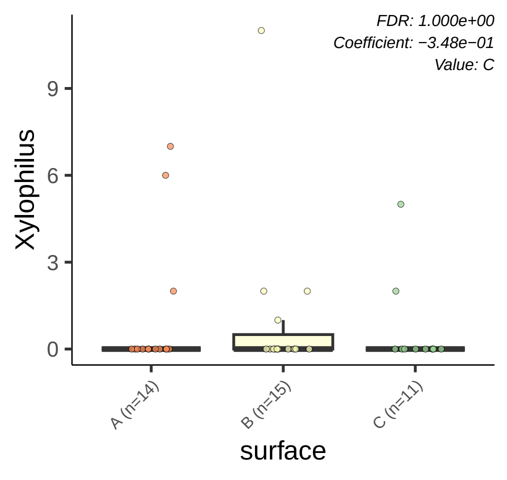
FDR: 1.000e+00
Coefficient: −3.48e−01
Value: C
Xylophilus
0
3
6
9
A (n=14)
B (n=15)
C (n=11)
surface

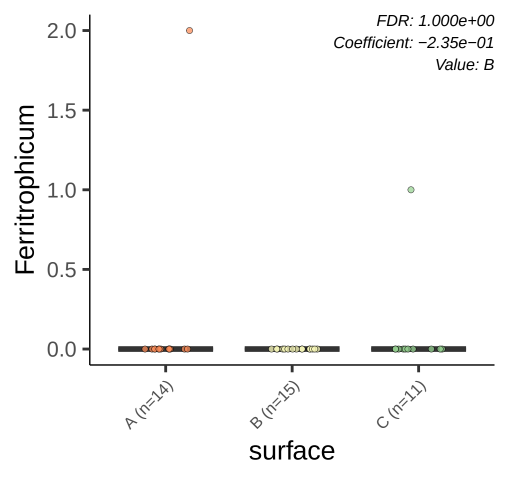
FDR: 1.000e+00
Coefficient: −2.35e−01
Value: B
Ferritrophicum
2.0
1.5
1.0
0.5
0.0
A (n=14)
B (n=15)
C (n=11)
surface

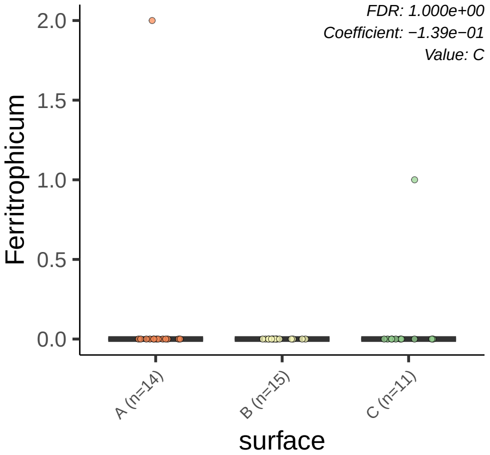
FDR: 1.000e+00
Coefficient: −1.39e−01
Value: C
Ferritrophicum
2.0
1.5
1.0
0.5
0.0
A (n=14)
B (n=15)
C (n=11)
surface

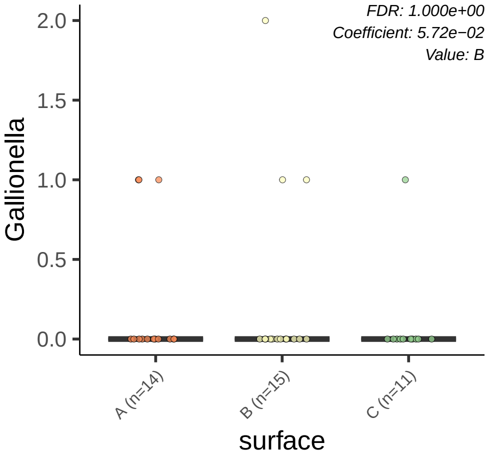
FDR: 1.000e+00
Coefficient: 5.72e−02
Value: B
Gallionella
2.0
1.5
1.0
0.5
0.0
A (n=14)
B (n=15)
C (n=11)
surface

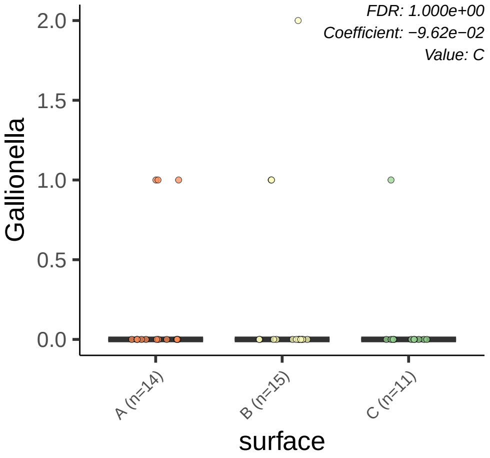
FDR: 1.000e+00
Coefficient: −9.62e−02
Value: C
Gallionella
2.0
1.5
1.0
0.5
0.0
A (n=14)
B (n=15)
C (n=11)
surface

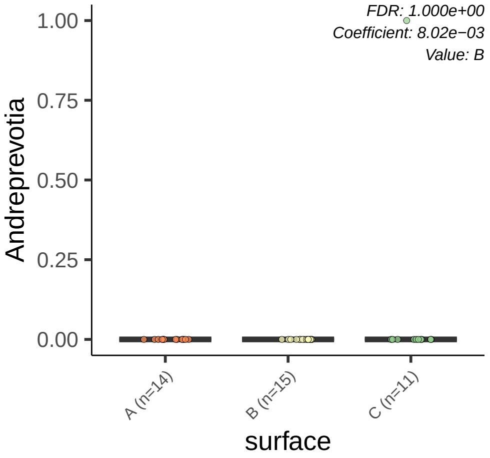

FDR: 1.000e+00
Coefficient: 8.02e−03
Value: B
Andreprevotia
1.00
0.75
0.50
0.25
0.00
A (n=14)
B (n=15)
C (n=11)
surface

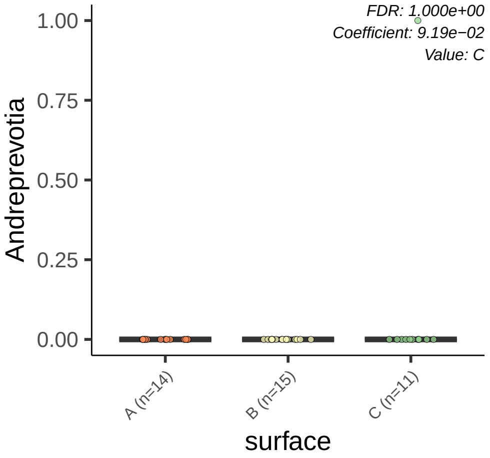

FDR: 1.000e+00
Coefficient: 9.19e−02
Value: C
Andreprevotia
1.00
0.75
0.50
0.25
0.00
A (n=14)
B (n=15)
C (n=11)
surface

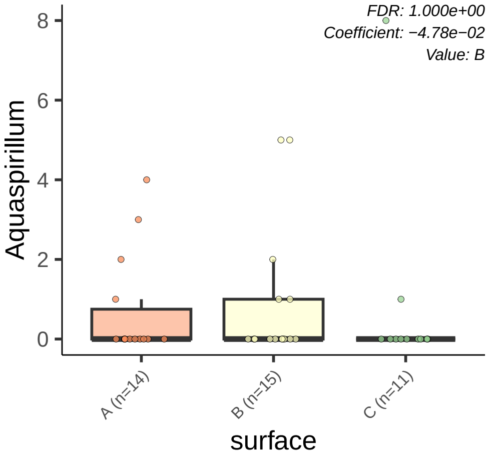
FDR: 1.000e+00
Coefficient: −4.78e−02
Value: B
Aquaspirillum
0
2
4
6
8
A (n=14)
B (n=15)
C (n=11)
surface

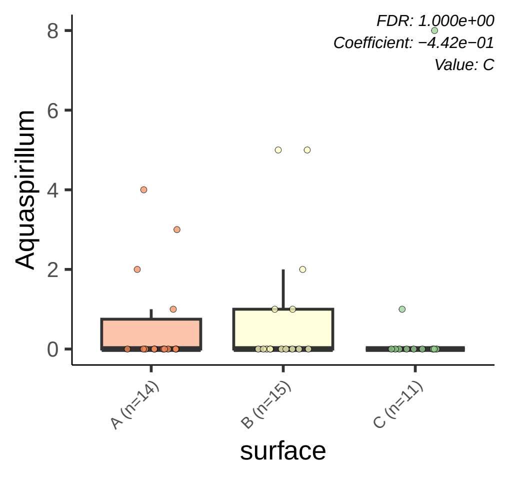

FDR: 1.000e+00
Coefficient: −4.42e−01
Value: C
Aquaspirillum
0
2
4
6
8
A (n=14)
B (n=15)
C (n=11)
surface

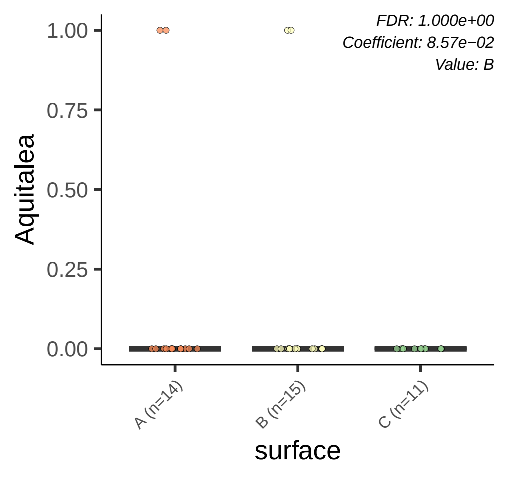
FDR: 1.000e+00
Coefficient: 8.57e−02
Value: B
Aquitalea
1.00
0.75
0.50
0.25
0.00
A (n=14)
B (n=15)
C (n=11)
surface

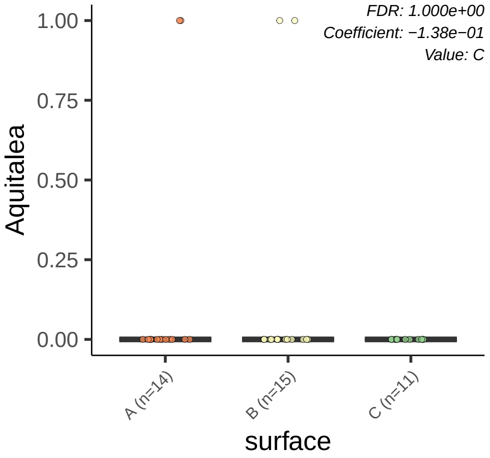
FDR: 1.000e+00
Coefficient: −1.38e−01
Value: C
Aquitalea
1.00
0.75
0.50
0.25
0.00
A (n=14)
B (n=15)
C (n=11)
surface

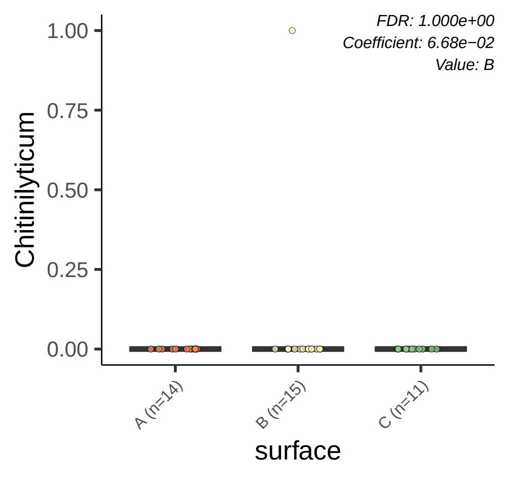
FDR: 1.000e+00
Coefficient: 6.68e−02
Value: B
1.00
0.75
0.50
0.25
0.00
Chitinilyticum
A (n=14)
B (n=15)
C (n=11)
surface

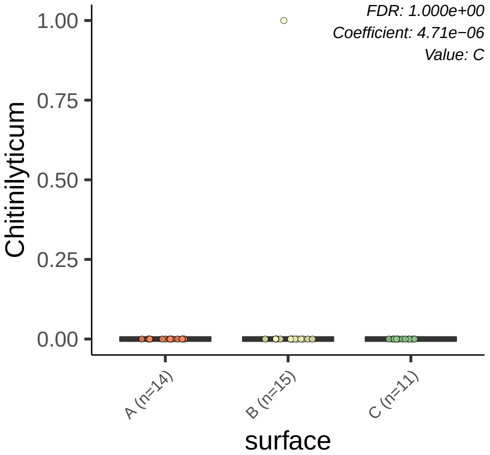
FDR: 1.000e+00
Coefficient: 4.71e−06
Value: C
Chitinilyticum
1.00
0.75
0.50
0.25
0.00
A (n=14)
B (n=15)
C (n=11)
surface

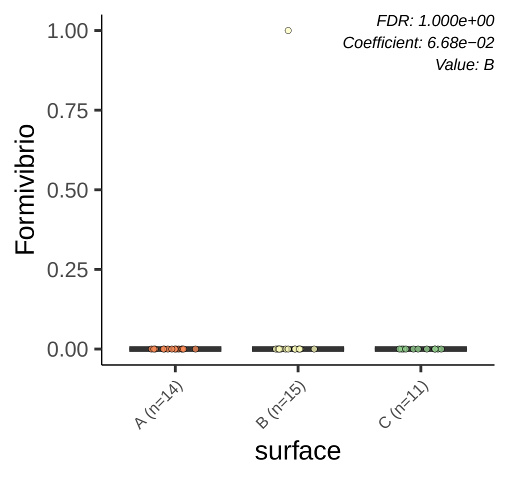
FDR: 1.000e+00
Coefficient: 6.68e−02
Value: B
Formivibrio
1.00
0.75
0.50
0.25
0.00
A (n=14)
B (n=15)
C (n=11)
surface

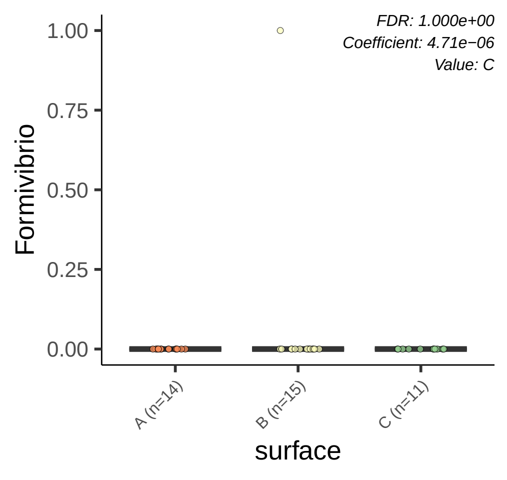
FDR: 1.000e+00
Coefficient: 4.71e−06
Value: C
Formivibrio
1.00
0.75
0.50
0.25
0.00
A (n=14)
B (n=15)
C (n=11)
surface

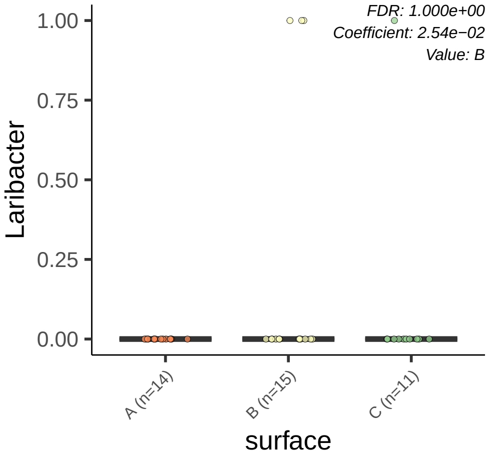
FDR: 1.000e+00
Coefficient: 2.54e−02
Value: B
Laribacter
1.00
0.75
0.50
0.25
0.00
A (n=14)
B (n=15)
C (n=11)
surface

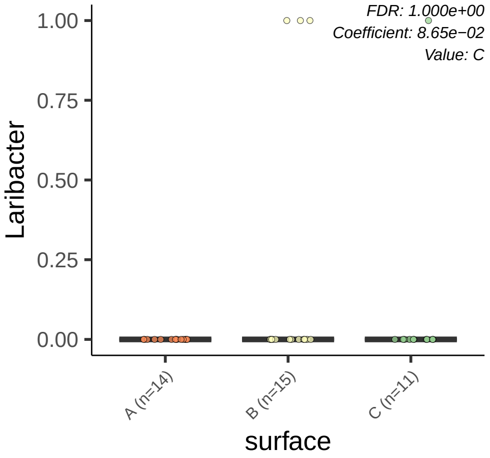

FDR: 1.000e+00
Coefficient: 8.65e−02
Value: C
Laribacter
1.00
0.75
0.50
0.25
0.00
A (n=14)
B (n=15)
C (n=11)
surface

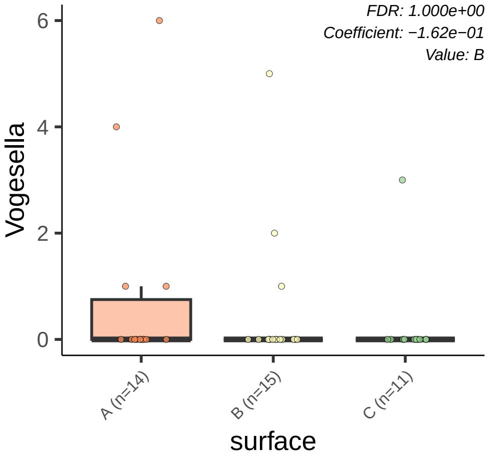

FDR: 1.000e+00
Coefficient: −1.62e−01
Value: B
Vogesella
6
4
2
0
A (n=14)
B (n=15)
C (n=11)
surface

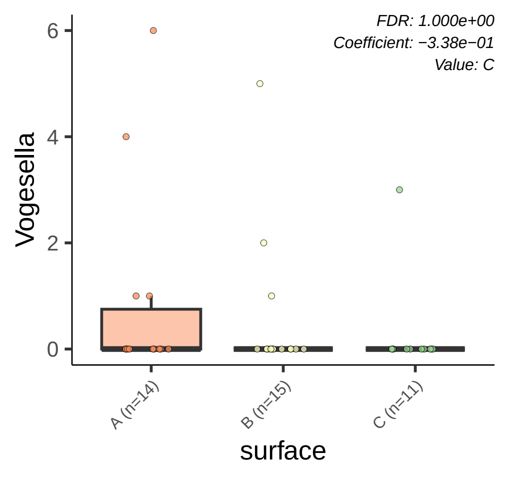
FDR: 1.000e+00
Coefficient: −3.38e−01
Value: C
Vogesella
0
2
4
6
A (n=14)
B (n=15)
C (n=11)
surface

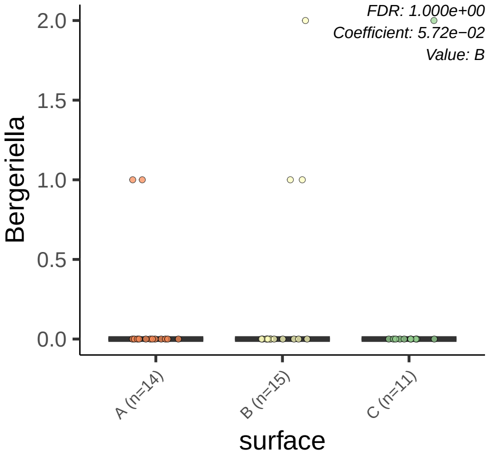

FDR: 1.000e+00
Coefficient: 5.72e−02
Value: B
Bergeriella
2.0
1.5
1.0
0.5
0.0
A (n=14)
B (n=15)
C (n=11)
surface

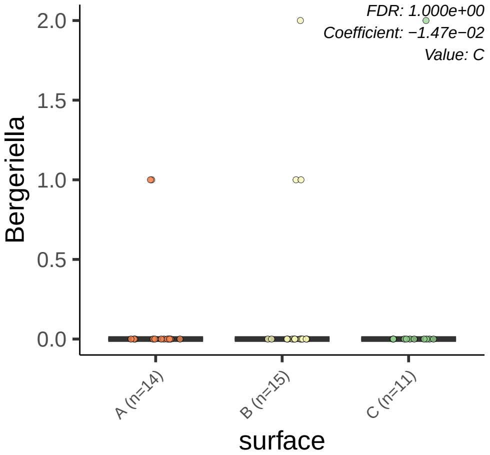
FDR: 1.000e+00
Coefficient: −1.47e−02
Value: C
Bergeriella
2.0
1.5
1.0
0.5
0.0
A (n=14)
B (n=15)
C (n=11)
surface

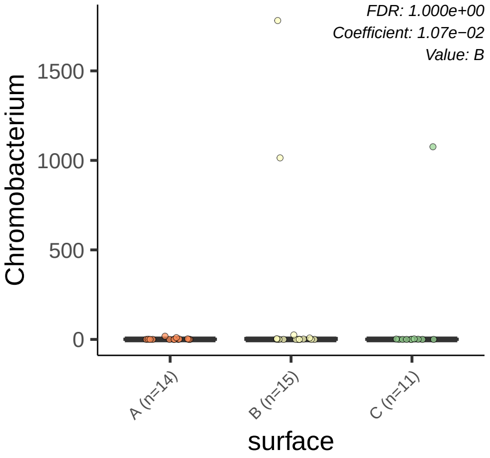
FDR: 1.000e+00
Coefficient: 1.07e−02
Value: B
Chromobacterium
1500
1000
500
0
A (n=14)
B (n=15)
C (n=11)
surface

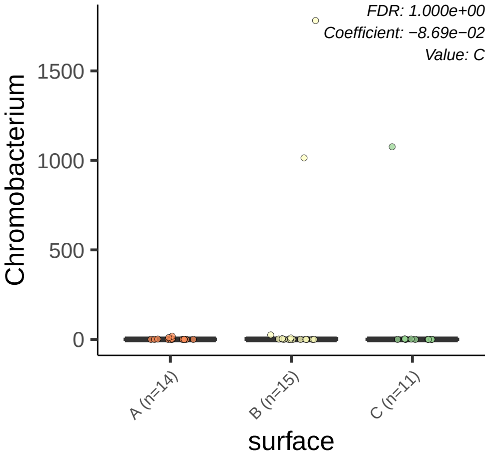

FDR: 1.000e+00
Coefficient: −8.69e−02
Value: C
Chromobacterium
1500
1000
500
0
A (n=14)
B (n=15)
C (n=11)
surface

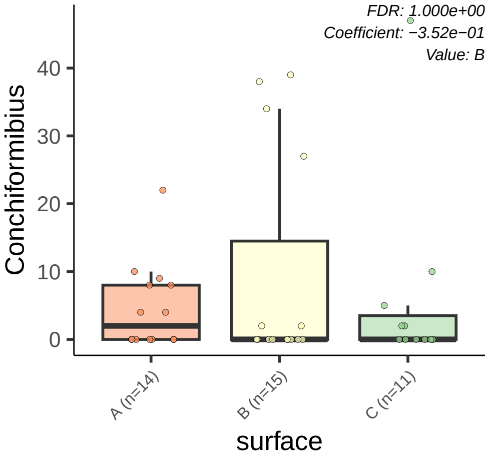
FDR: 1.000e+00
Coefficient: −3.52e−01
Value: B
Conchiformibius
0
10
20
30
40
A (n=14)
B (n=15)
C (n=11)
surface

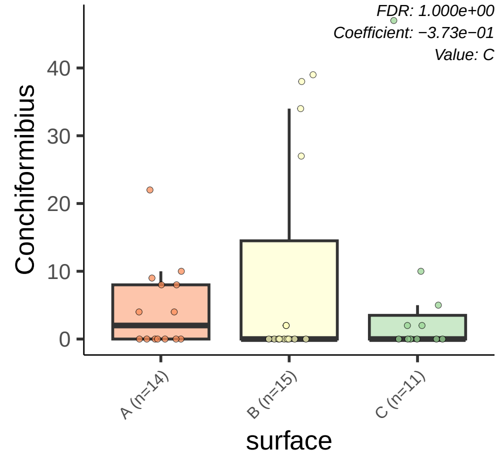
FDR: 1.000e+00
Coefficient: −3.73e−01
Value: C
Conchiformibius
0
10
20
30
40
A (n=14)
B (n=15)
C (n=11)
surface

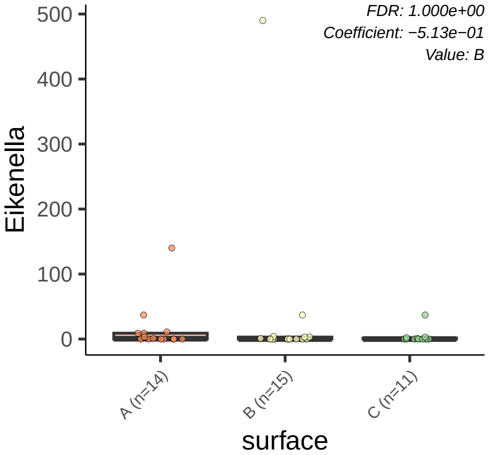
FDR: 1.000e+00
Coefficient: −5.13e−01
Value: B
Eikenella
500
400
300
200
100
0
A (n=14)
B (n=15)
C (n=11)
surface

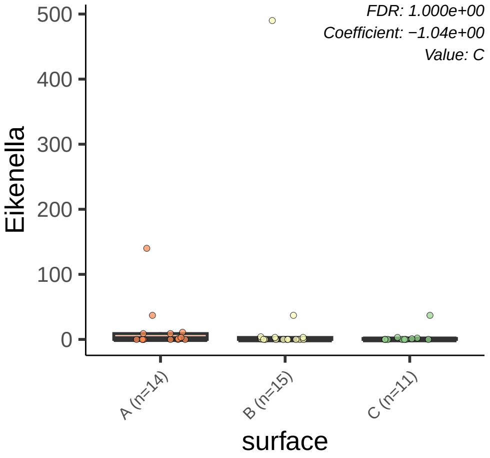
FDR: 1.000e+00
Coefficient: −1.04e+00
Value: C
Eikenella
500
400
300
200
100
0
A (n=14)
B (n=15)
C (n=11)
surface

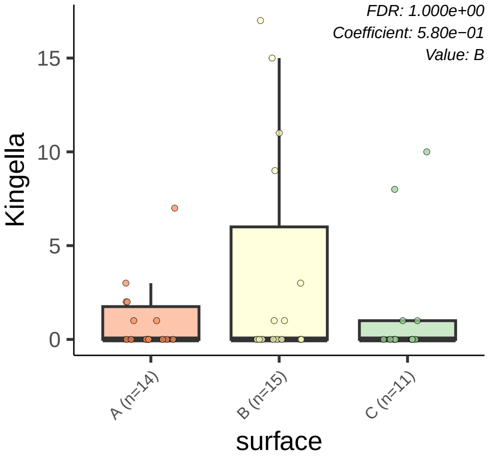
FDR: 1.000e+00
Coefficient: 5.80e−01
Value: B
Kingella
0
5
10
15
A (n=14)
B (n=15)
C (n=11)
surface

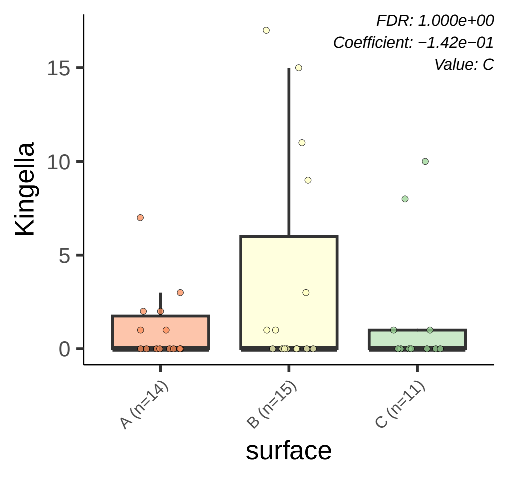
FDR: 1.000e+00
Coefficient: −1.42e−01
Value: C
Kingella
0
5
10
15
A (n=14)
B (n=15)
C (n=11)
surface

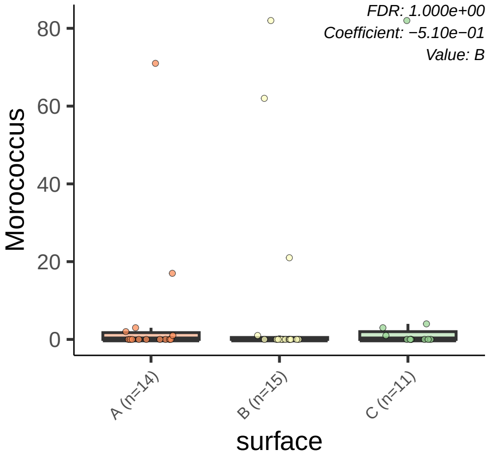
FDR: 1.000e+00
Coefficient: −5.10e−01
Value: B
Morococcus
80
60
40
20
0
A (n=14)
B (n=15)
C (n=11)
surface

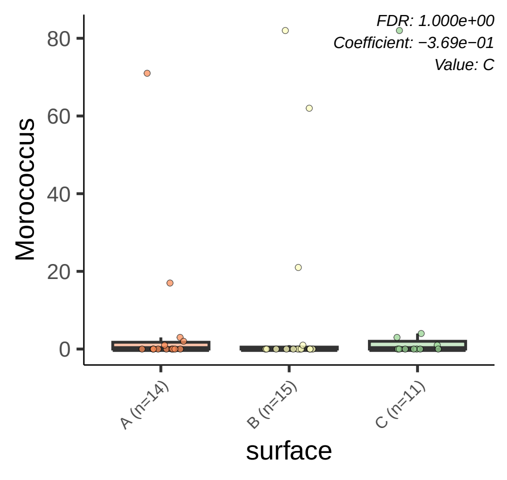
FDR: 1.000e+00
Coefficient: −3.69e−01
Value: C
Morococcus
0
20
40
60
80
A (n=14)
B (n=15)
C (n=11)
surface

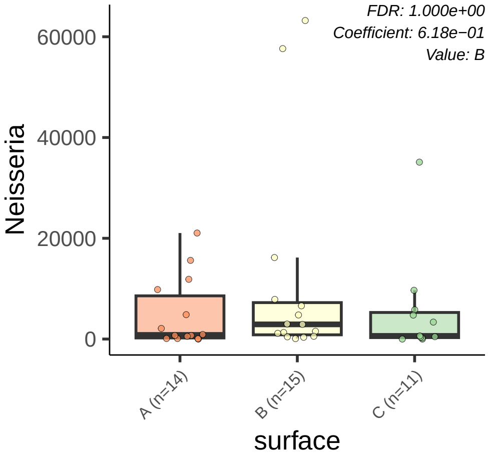

FDR: 1.000e+00
Coefficient: 6.18e−01
Value: B
Neisseria
60000
40000
20000
0
A (n=14)
B (n=15)
C (n=11)
surface

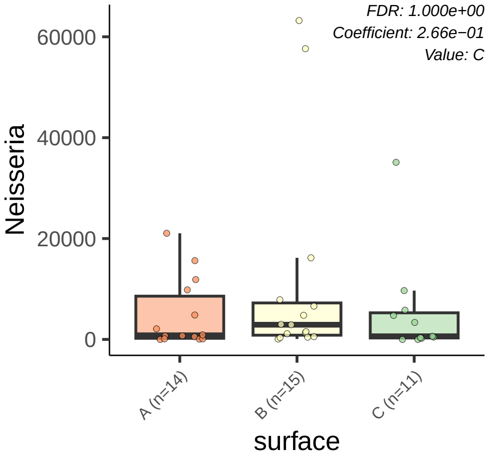
FDR: 1.000e+00
Coefficient: 2.66e−01
Value: C
Neisseria
60000
40000
20000
0
A (n=14)
B (n=15)
C (n=11)
surface

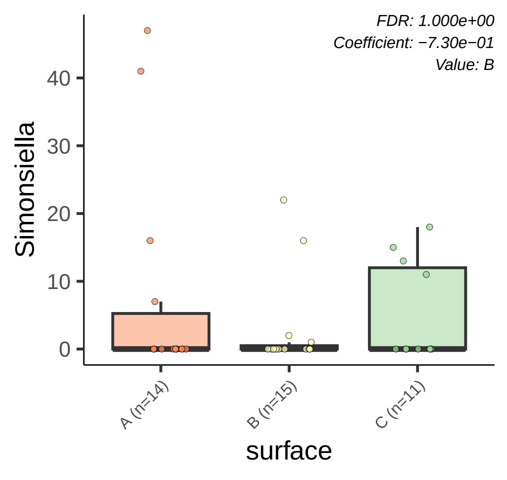

FDR: 1.000e+00
Coefficient: −7.30e−01
Value: B
Simonsiella
0
10
20
30
40
A (n=14)
B (n=15)
C (n=11)
surface

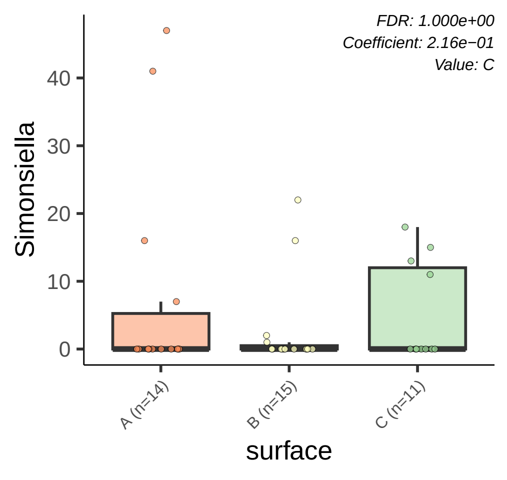
FDR: 1.000e+00
Coefficient: 2.16e−01
Value: C
Simonsiella
40
30
20
10
0
A (n=14)
B (n=15)
C (n=11)
surface

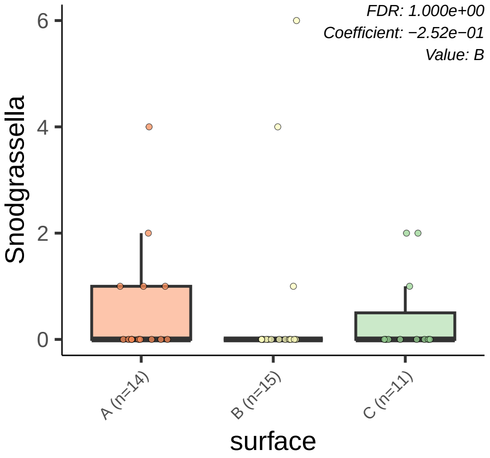

FDR: 1.000e+00
Coefficient: −2.52e−01
Value: B
Snodgrassella
0
2
4
6
A (n=14)
B (n=15)
C (n=11)
surface

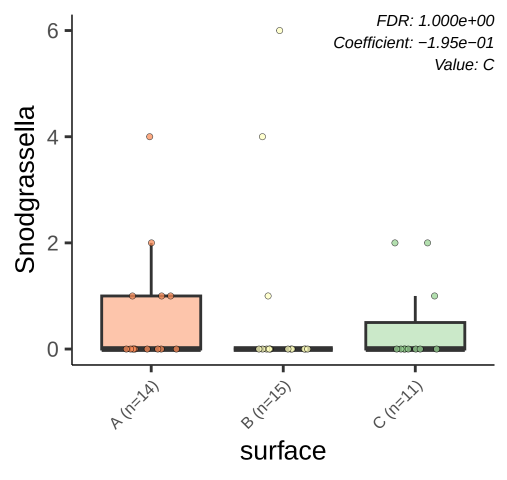

FDR: 1.000e+00
Coefficient: −1.95e−01
Value: C
Snodgrassella
0
2
4
6
A (n=14)
B (n=15)
C (n=11)
surface

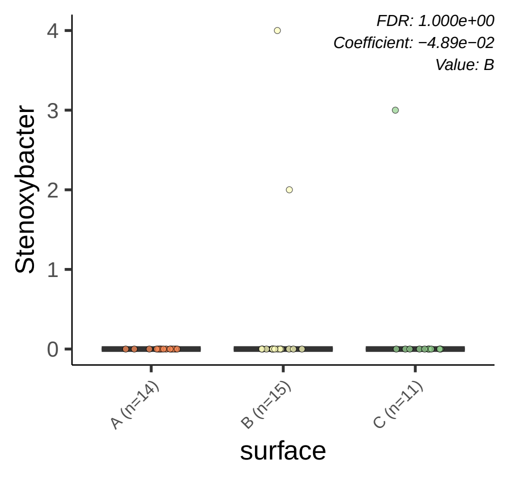

FDR: 1.000e+00
Coefficient: −4.89e−02
Value: B
Stenoxybacter
4
3
2
1
0
A (n=14)
B (n=15)
C (n=11)
surface

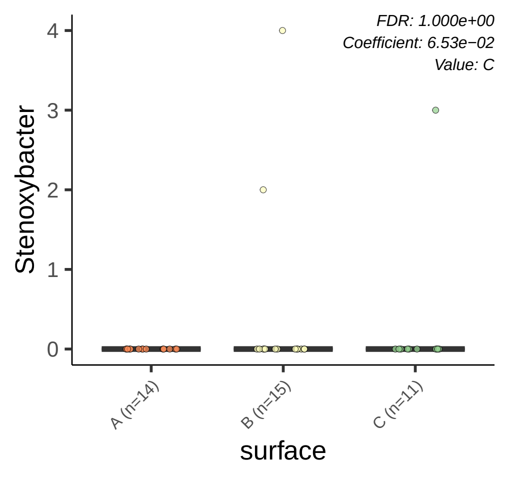
FDR: 1.000e+00
Coefficient: 6.53e−02
Value: C
Stenoxybacter
0
1
2
3
4
A (n=14)
B (n=15)
C (n=11)
surface

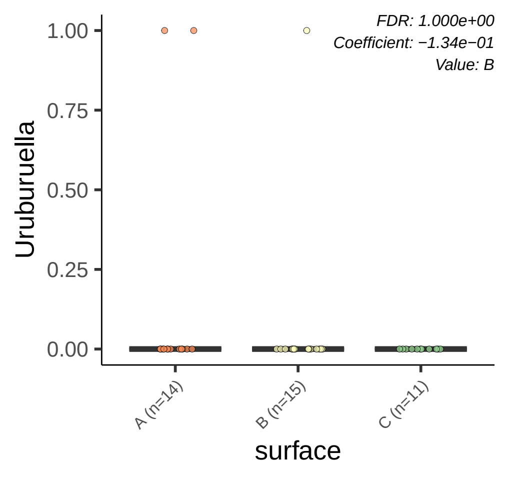
FDR: 1.000e+00
Coefficient: −1.34e−01
Value: B
Uruburuella
1.00
0.75
0.50
0.25
0.00
A (n=14)
B (n=15)
C (n=11)
surface

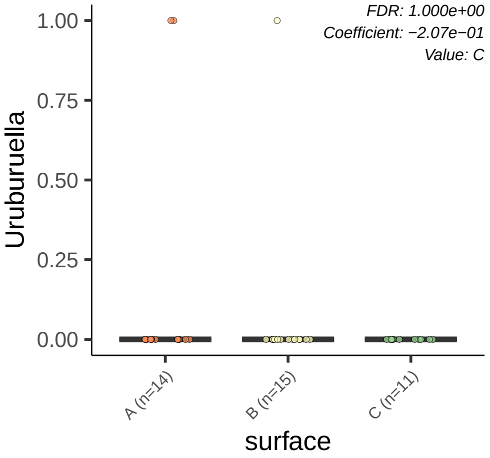

FDR: 1.000e+00
Coefficient: −2.07e−01
Value: C
1.00
0.75
0.50
0.25
0.00
Uruburuella
A (n=14)
B (n=15)
C (n=11)
surface

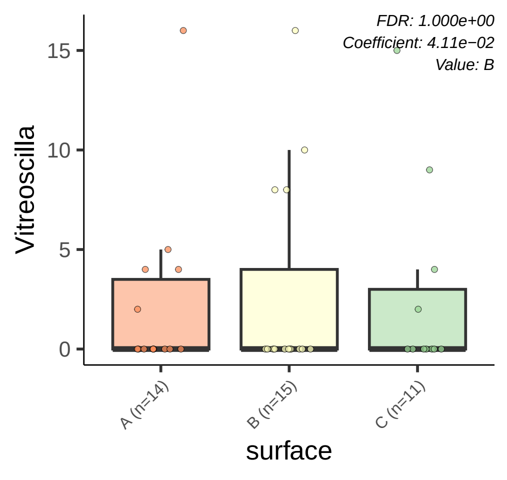
FDR: 1.000e+00
Coefficient: 4.11e−02
Value: B
Vitreoscilla
15
10
5
0
A (n=14)
B (n=15)
C (n=11)
surface

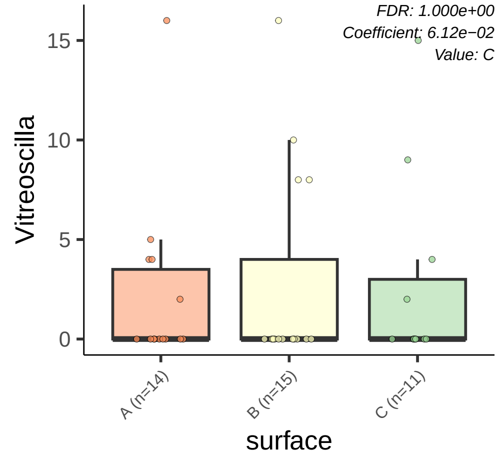
FDR: 1.000e+00
Coefficient: 6.12e−02
Value: C
Vitreoscilla
15
10
5
0
A (n=14)
B (n=15)
C (n=11)
surface

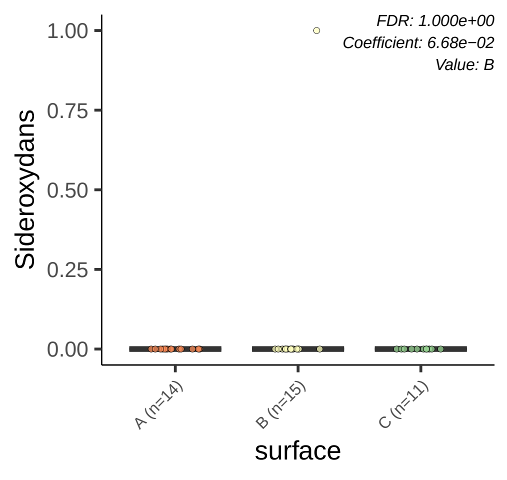
FDR: 1.000e+00
Coefficient: 6.68e−02
Value: B
Sideroxydans
1.00
0.75
0.50
0.25
0.00
A (n=14)
B (n=15)
C (n=11)
surface

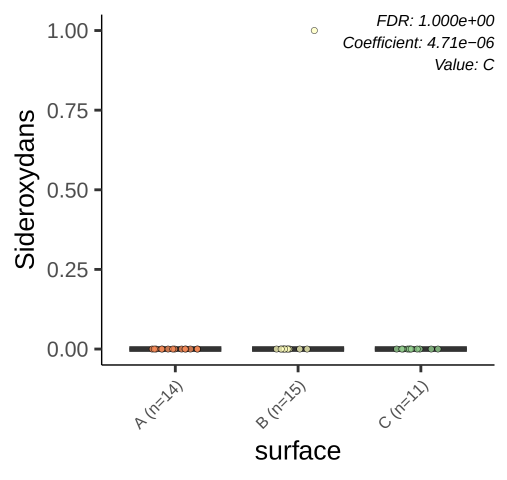

FDR: 1.000e+00
Coefficient: 4.71e−06
Value: C
Sideroxydans
1.00
0.75
0.50
0.25
0.00
A (n=14)
B (n=15)
C (n=11)
surface

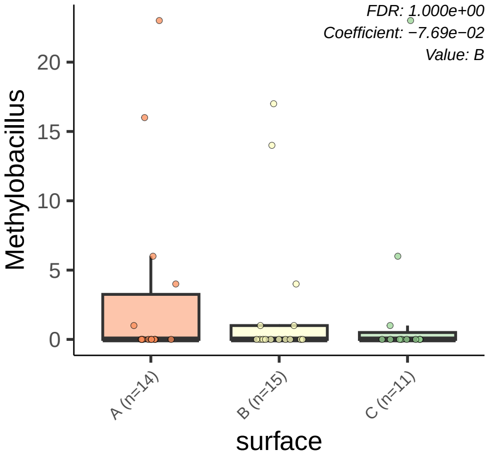

FDR: 1.000e+00
Coefficient: −7.69e−02
Value: B
Methylobacillus
20
15
10
5
0
A (n=14)
B (n=15)
C (n=11)
surface

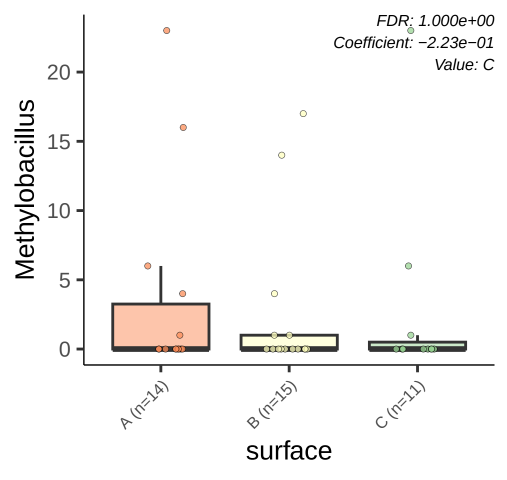
FDR: 1.000e+00
Coefficient: −2.23e−01
Value: C
Methylobacillus
0
5
10
15
20
A (n=14)
B (n=15)
C (n=11)
surface

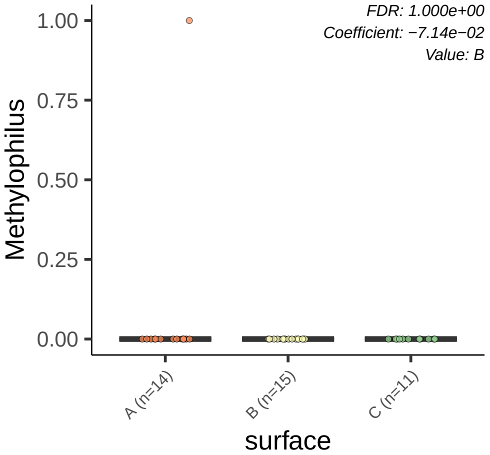
FDR: 1.000e+00
Coefficient: −7.14e−02
Value: B
Methylophilus
1.00
0.75
0.50
0.25
0.00
A (n=14)
B (n=15)
C (n=11)
surface

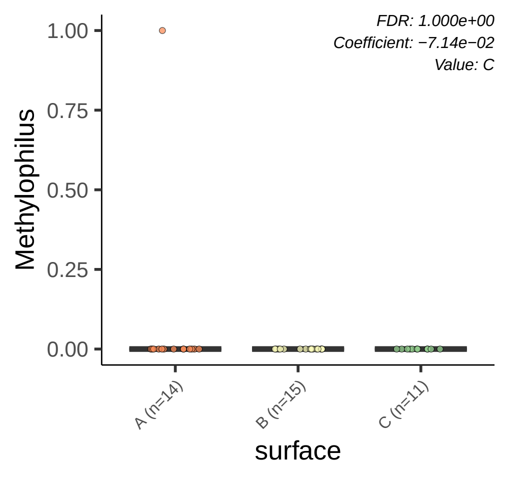
FDR: 1.000e+00
Coefficient: −7.14e−02
Value: C
Methylophilus
1.00
0.75
0.50
0.25
0.00
A (n=14)
B (n=15)
C (n=11)
surface

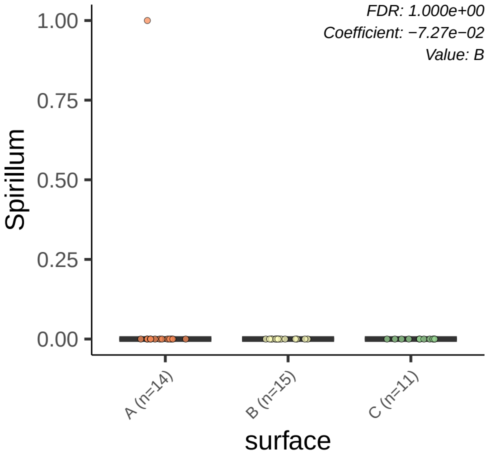
FDR: 1.000e+00
Coefficient: −7.27e−02
Value: B
Spirillum
1.00
0.75
0.50
0.25
0.00
A (n=14)
B (n=15)
C (n=11)
surface

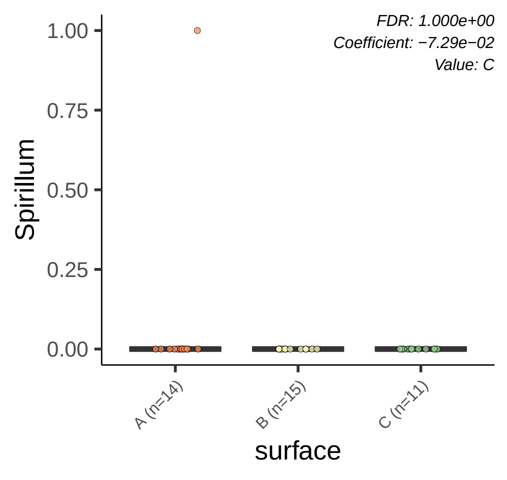
FDR: 1.000e+00
Coefficient: −7.29e−02
Value: C
Spirillum
1.00
0.75
0.50
0.25
0.00
A (n=14)
B (n=15)
C (n=11)
surface

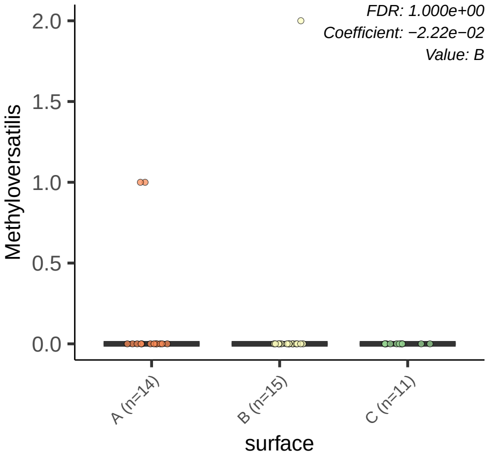

FDR: 1.000e+00
Coefficient: −2.22e−02
Value: B
Methyloversatilis
2.0
1.5
1.0
0.5
0.0
A (n=14)
B (n=15)
C (n=11)
surface

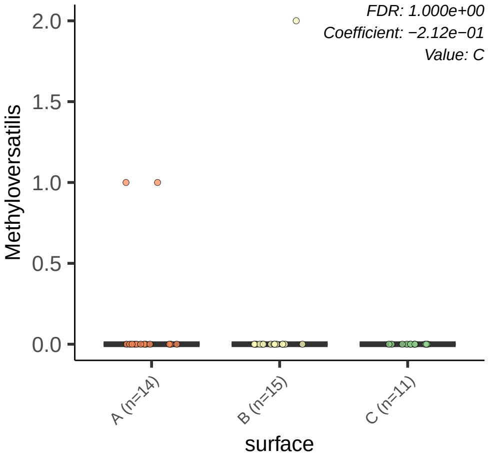

FDR: 1.000e+00
Coefficient: −2.12e−01
Value: C
Methyloversatilis
2.0
1.5
1.0
0.5
0.0
A (n=14)
B (n=15)
C (n=11)
surface

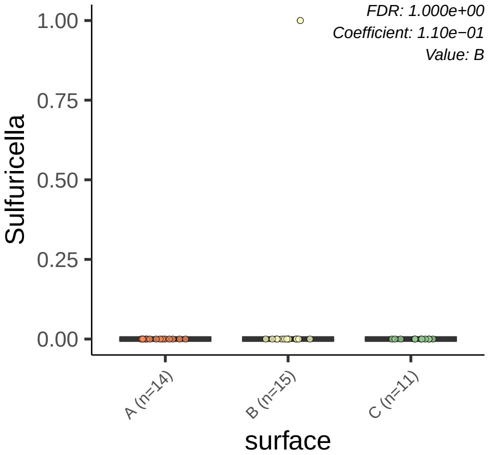
FDR: 1.000e+00
Coefficient: 1.10e−01
Value: B
Sulfuricella
1.00
0.75
0.50
0.25
0.00
A (n=14)
B (n=15)
C (n=11)
surface

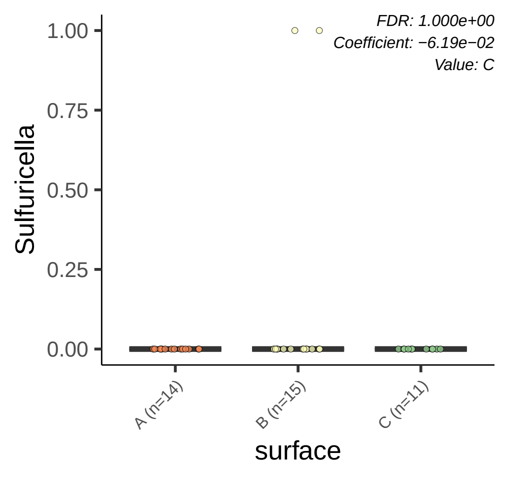

FDR: 1.000e+00
Coefficient: −6.19e−02
Value: C
Sulfuricella
1.00
0.75
0.50
0.25
0.00
A (n=14)
B (n=15)
C (n=11)
surface

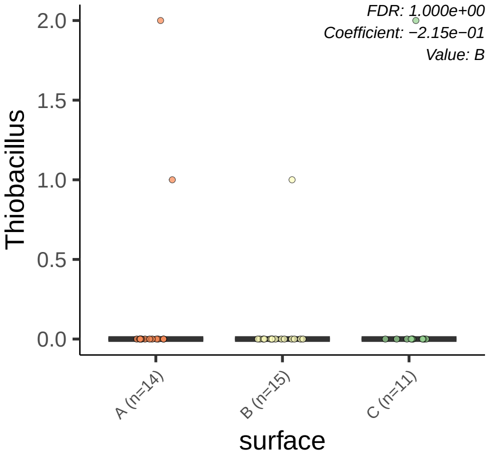
FDR: 1.000e+00
Coefficient: −2.15e−01
Value: B
Thiobacillus
2.0
1.5
1.0
0.5
0.0
A (n=14)
B (n=15)
C (n=11)
surface

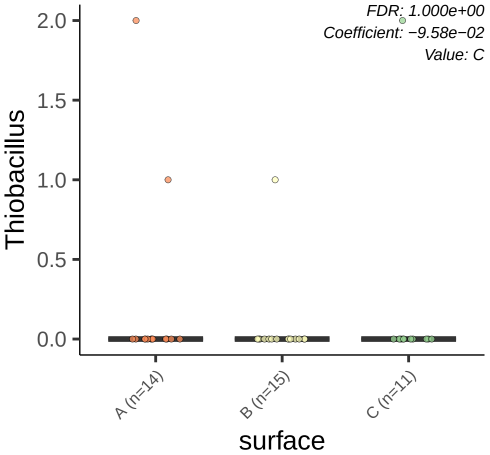
FDR: 1.000e+00
Coefficient: −9.58e−02
Value: C
Thiobacillus
2.0
1.5
1.0
0.5
0.0
A (n=14)
B (n=15)
C (n=11)
surface

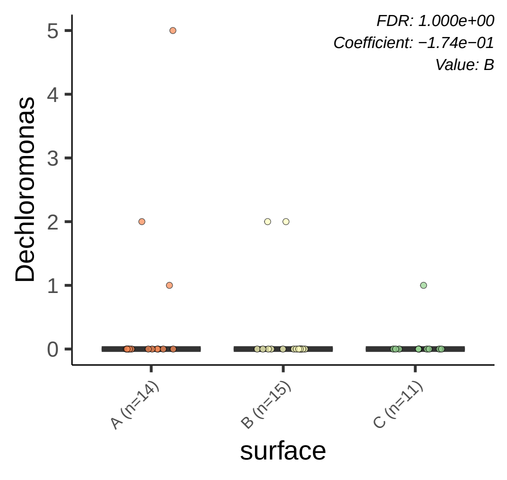
FDR: 1.000e+00
Coefficient: −1.74e−01
Value: B
Dechloromonas
5
4
3
2
1
0
A (n=14)
B (n=15)
C (n=11)
surface

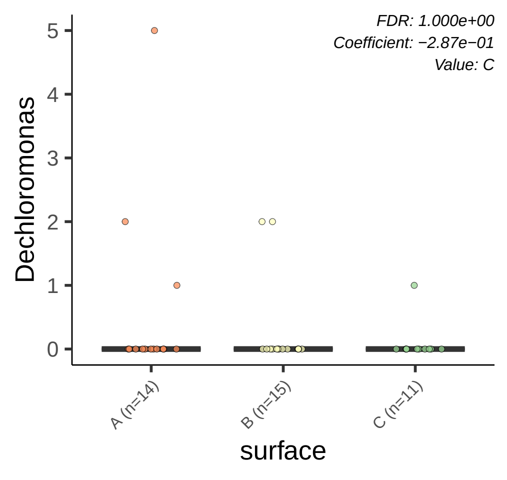
FDR: 1.000e+00
Coefficient: −2.87e−01
Value: C
Dechloromonas
5
4
3
2
1
0
A (n=14)
B (n=15)
C (n=11)
surface

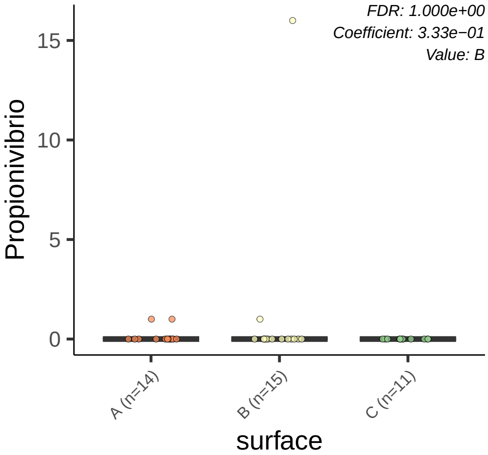
FDR: 1.000e+00
Coefficient: 3.33e−01
Value: B
Propionivibrio
15
10
5
0
A (n=14)
B (n=15)
C (n=11)
surface

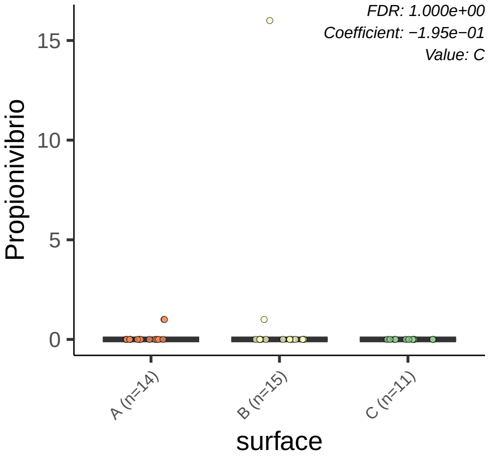
FDR: 1.000e+00
Coefficient: −1.95e−01
Value: C
Propionivibrio
15
10
5
0
A (n=14)
B (n=15)
C (n=11)
surface

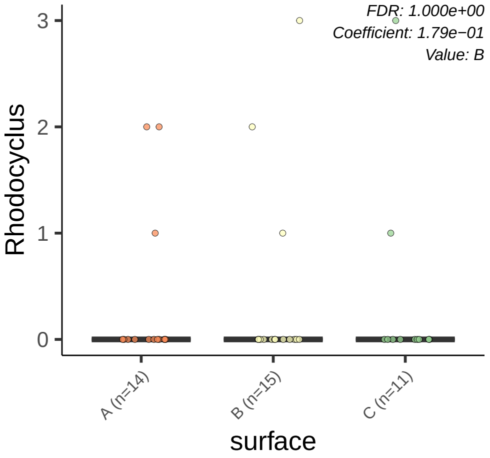
FDR: 1.000e+00
Coefficient: 1.79e−01
Value: B
Rhodocyclus
3
2
1
0
A (n=14)
B (n=15)
C (n=11)
surface

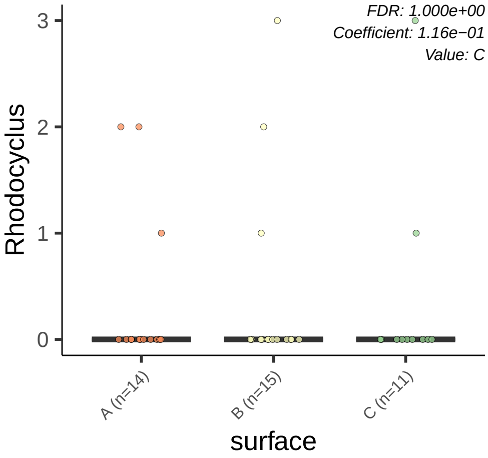

FDR: 1.000e+00
Coefficient: 1.16e−01
Value: C
Rhodocyclus
3
2
1
0
A (n=14)
B (n=15)
C (n=11)
surface

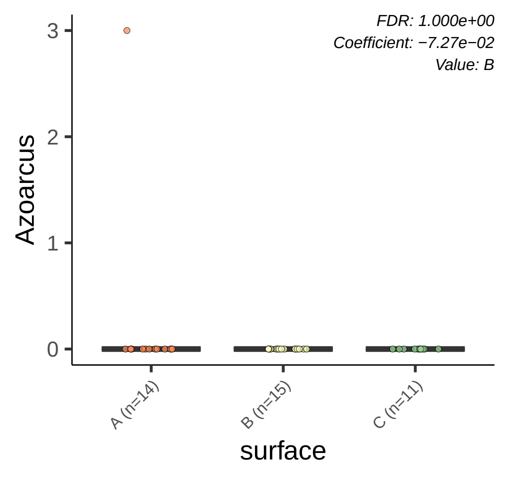
FDR: 1.000e+00
Coefficient: −7.27e−02
Value: B
Azoarcus
3
2
1
0
A (n=14)
B (n=15)
C (n=11)
surface

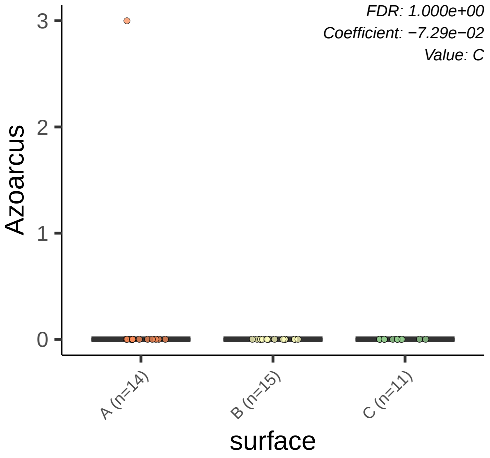

FDR: 1.000e+00
Coefficient: −7.29e−02
Value: C
Azoarcus
3
2
1
0
A (n=14)
B (n=15)
C (n=11)
surface

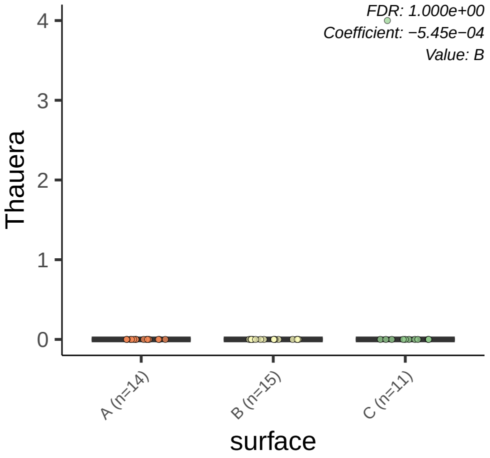
FDR: 1.000e+00
Coefficient: −5.45e−04
Value: B
Thauera
4
3
2
1
0
A (n=14)
B (n=15)
C (n=11)
surface

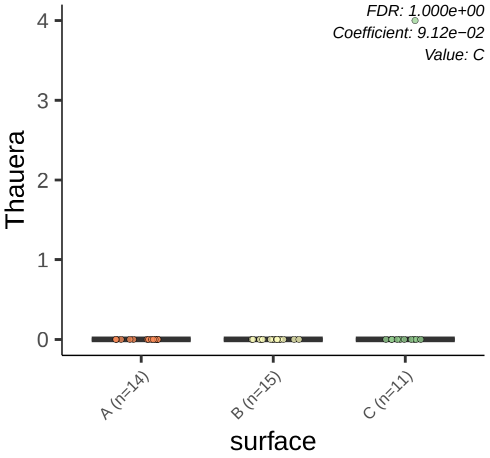
FDR: 1.000e+00
Coefficient: 9.12e−02
Value: C
Thauera
4
3
2
1
0
A (n=14)
B (n=15)
C (n=11)
surface

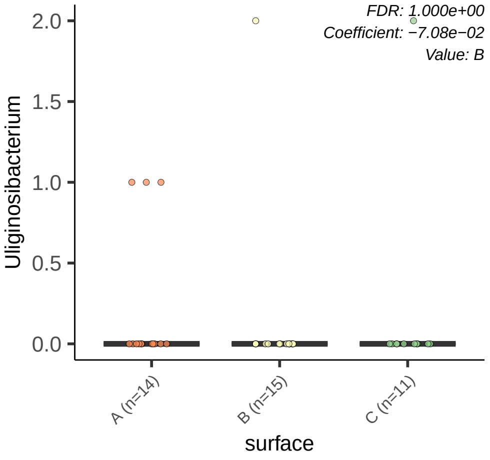

FDR: 1.000e+00
Coefficient: −7.08e−02
Value: B
Uliginosibacterium
2.0
1.5
1.0
0.5
0.0
A (n=14)
B (n=15)
C (n=11)
surface

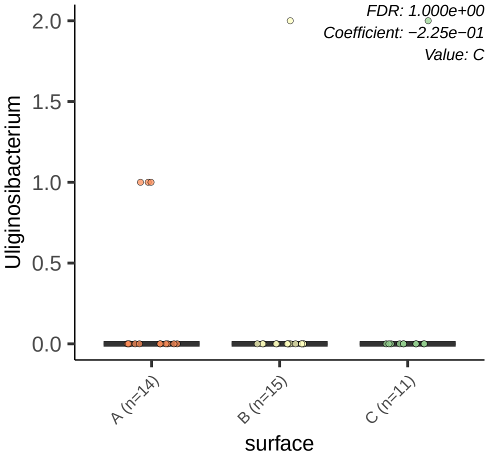

FDR: 1.000e+00
Coefficient: −2.25e−01
Value: C
Uliginosibacterium
2.0
1.5
1.0
0.5
0.0
A (n=14)
B (n=15)
C (n=11)
surface

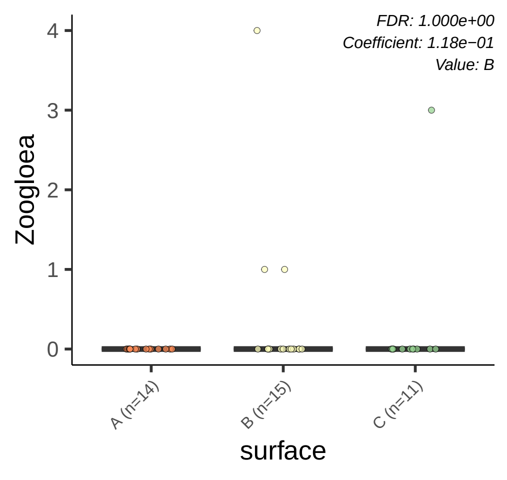

FDR: 1.000e+00
Coefficient: 1.18e−01
Value: B
Zoogloea
0
1
2
3
4
A (n=14)
B (n=15)
C (n=11)
surface

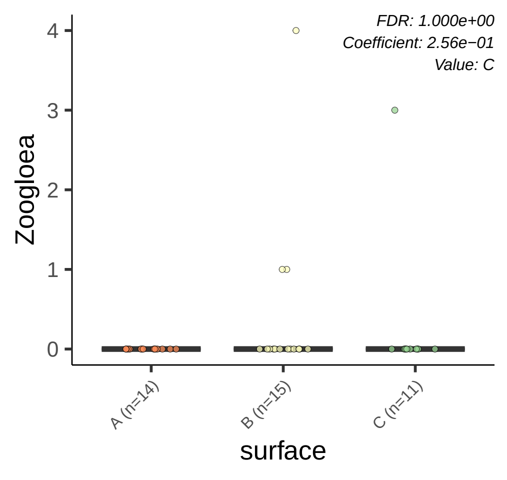

FDR: 1.000e+00
Coefficient: 2.56e−01
Value: C
Zoogloea
4
3
2
1
0
A (n=14)
B (n=15)
C (n=11)
surface

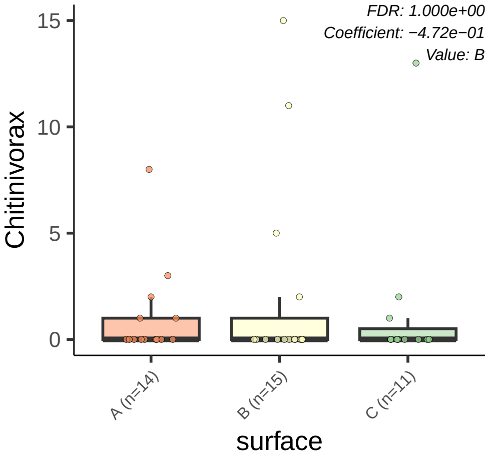
FDR: 1.000e+00
Coefficient: −4.72e−01
Value: B
Chitinivorax
15
10
5
0
A (n=14)
B (n=15)
C (n=11)
surface

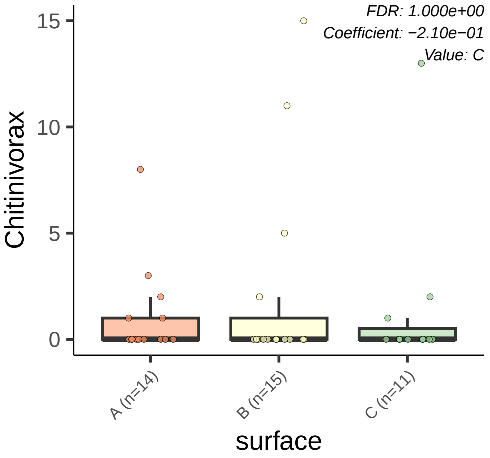
FDR: 1.000e+00
Coefficient: −2.10e−01
Value: C
Chitinivorax
15
10
5
0
A (n=14)
B (n=15)
C (n=11)
surface

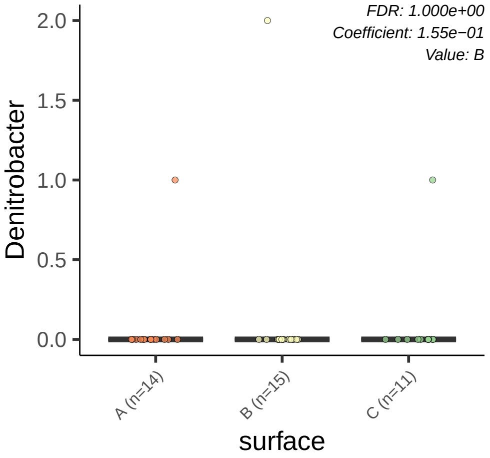

FDR: 1.000e+00
Coefficient: 1.55e−01
Value: B
Denitrobacter
2.0
1.5
1.0
0.5
0.0
A (n=14)
B (n=15)
C (n=11)
surface

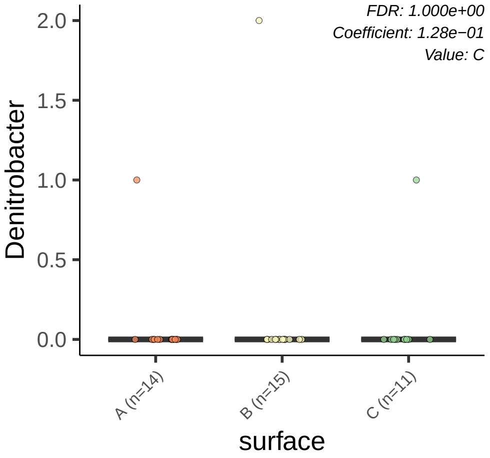
FDR: 1.000e+00
Coefficient: 1.28e−01
Value: C
Denitrobacter
2.0
1.5
1.0
0.5
0.0
A (n=14)
B (n=15)
C (n=11)
surface

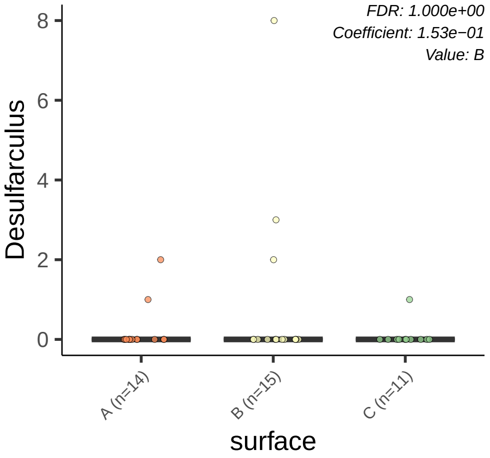

FDR: 1.000e+00
Coefficient: 1.53e−01
Value: B
Desulfarculus
0
2
4
6
8
A (n=14)
B (n=15)
C (n=11)
surface

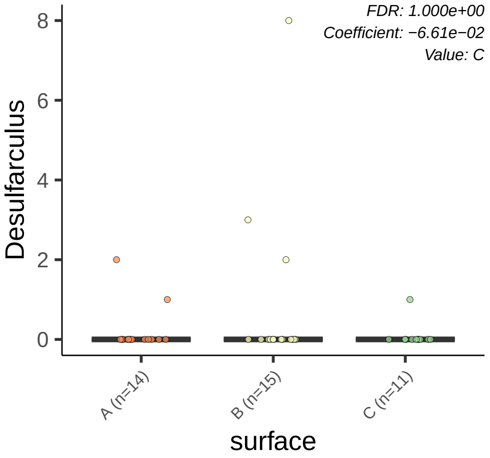
FDR: 1.000e+00
Coefficient: −6.61e−02
Value: C
Desulfarculus
8
6
4
2
0
A (n=14)
B (n=15)
C (n=11)
surface

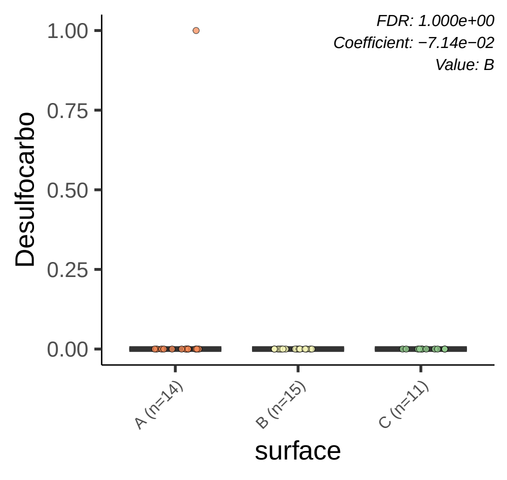

FDR: 1.000e+00
Coefficient: −7.14e−02
Value: B
Desulfocarbo
1.00
0.75
0.50
0.25
0.00
A (n=14)
B (n=15)
C (n=11)
surface

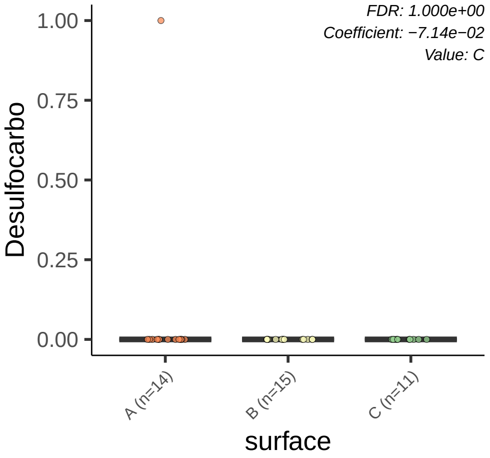

FDR: 1.000e+00
Coefficient: −7.14e−02
Value: C
Desulfocarbo
1.00
0.75
0.50
0.25
0.00
A (n=14)
B (n=15)
C (n=11)
surface

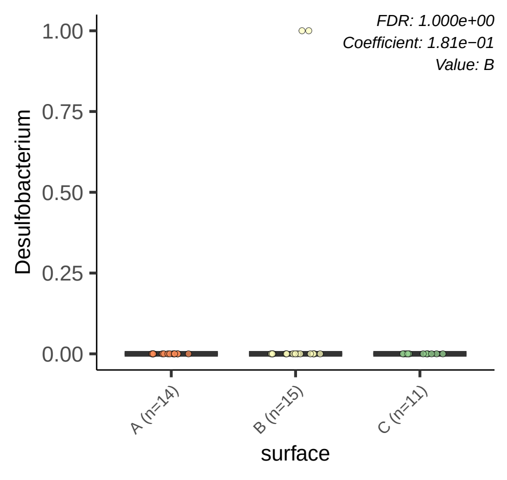

FDR: 1.000e+00
Coefficient: 1.81e−01
Value: B
Desulfobacterium
1.00
0.75
0.50
0.25
0.00
A (n=14)
B (n=15)
C (n=11)
surface

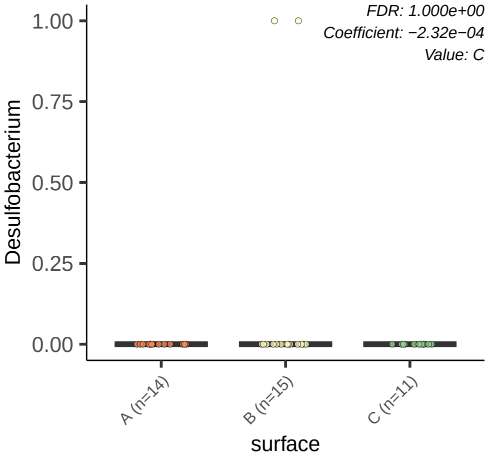
FDR: 1.000e+00
Coefficient: −2.32e−04
Value: C
Desulfobacterium
1.00
0.75
0.50
0.25
0.00
A (n=14)
B (n=15)
C (n=11)
surface

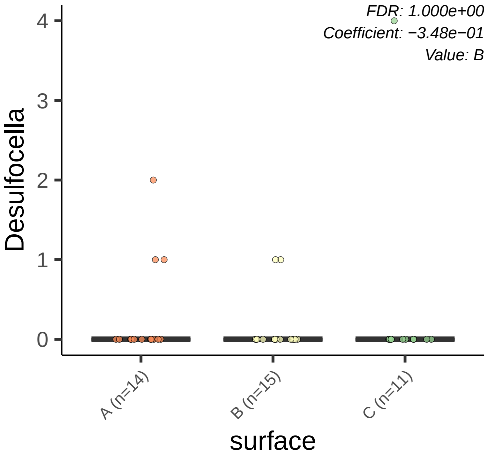
FDR: 1.000e+00
Coefficient: −3.48e−01
Value: B
Desulfocella
4
3
2
1
0
A (n=14)
B (n=15)
C (n=11)
surface

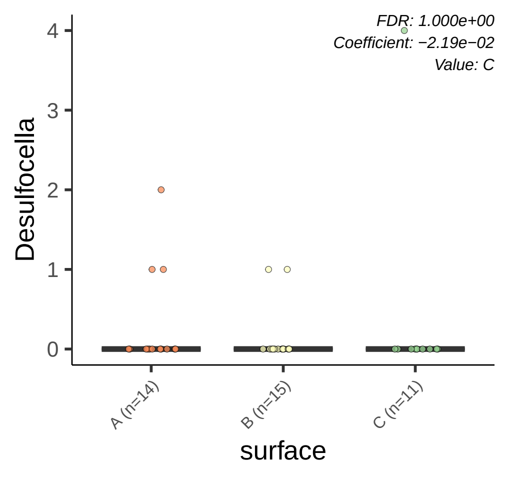

FDR: 1.000e+00
Coefficient: −2.19e−02
Value: C
Desulfocella
0
1
2
3
4
A (n=14)
B (n=15)
C (n=11)
surface

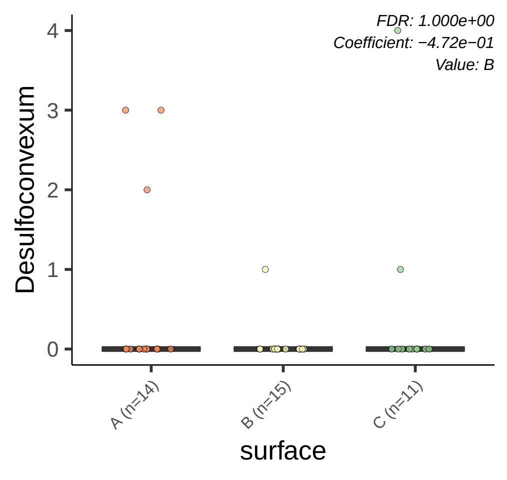
FDR: 1.000e+00
Coefficient: −4.72e−01
Value: B
Desulfoconvexum
4
3
2
1
0
A (n=14)
B (n=15)
C (n=11)
surface

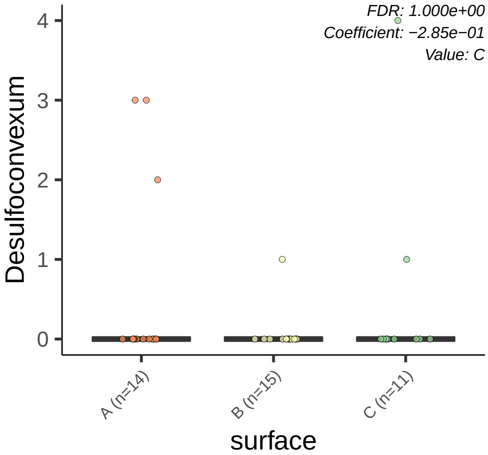

FDR: 1.000e+00
Coefficient: −2.85e−01
Value: C
Desulfoconvexum
0
1
2
3
4
A (n=14)
B (n=15)
C (n=11)
surface

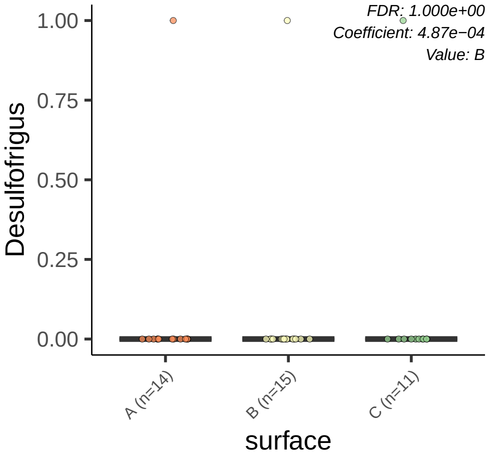

FDR: 1.000e+00
Coefficient: 4.87e−04
Value: B
Desulfofrigus
1.00
0.75
0.50
0.25
0.00
A (n=14)
B (n=15)
C (n=11)
surface

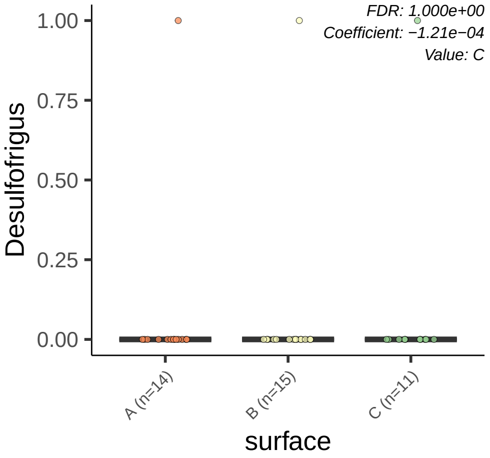
FDR: 1.000e+00
Coefficient: −1.21e−04
Value: C
Desulfofrigus
1.00
0.75
0.50
0.25
0.00
A (n=14)
B (n=15)
C (n=11)
surface

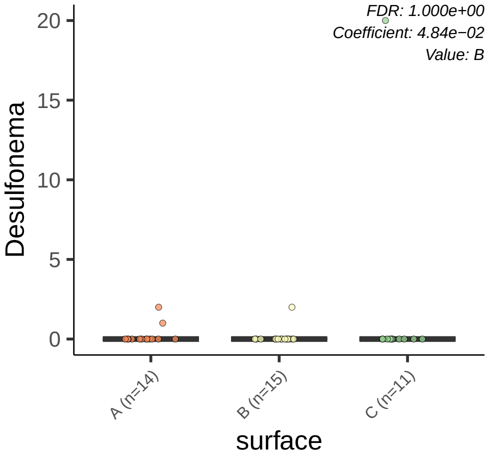
FDR: 1.000e+00
Coefficient: 4.84e−02
Value: B
Desulfonema
20
15
10
5
0
A (n=14)
B (n=15)
C (n=11)
surface

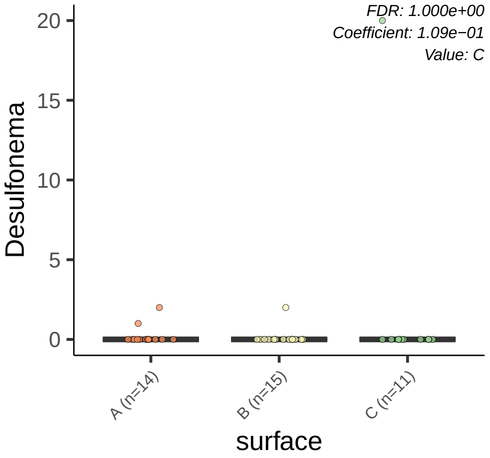

FDR: 1.000e+00
Coefficient: 1.09e−01
Value: C
Desulfonema
20
15
10
5
0
A (n=14)
B (n=15)
C (n=11)
surface

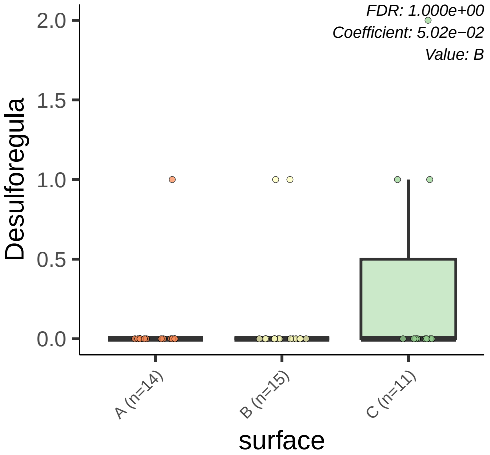

FDR: 1.000e+00
Coefficient: 5.02e−02
Value: B
Desulforegula
2.0
1.5
1.0
0.5
0.0
A (n=14)
B (n=15)
C (n=11)
surface

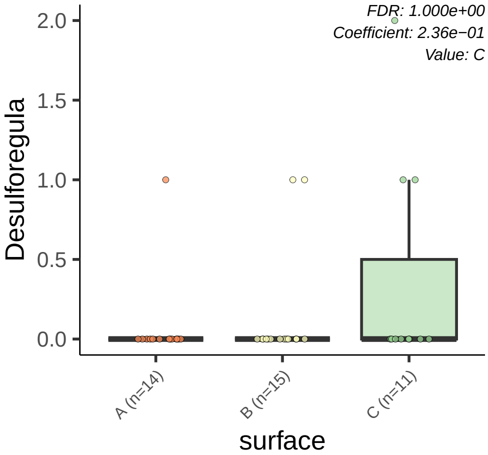
FDR: 1.000e+00
Coefficient: 2.36e−01
Value: C
Desulforegula
2.0
1.5
1.0
0.5
0.0
A (n=14)
B (n=15)
C (n=11)
surface

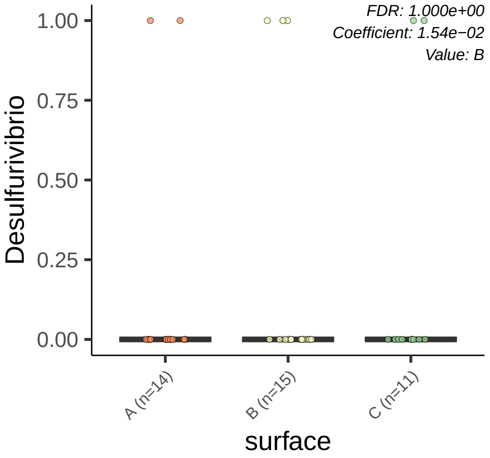

FDR: 1.000e+00
Coefficient: 1.54e−02
Value: B
Desulfurivibrio
1.00
0.75
0.50
0.25
0.00
A (n=14)
B (n=15)
C (n=11)
surface

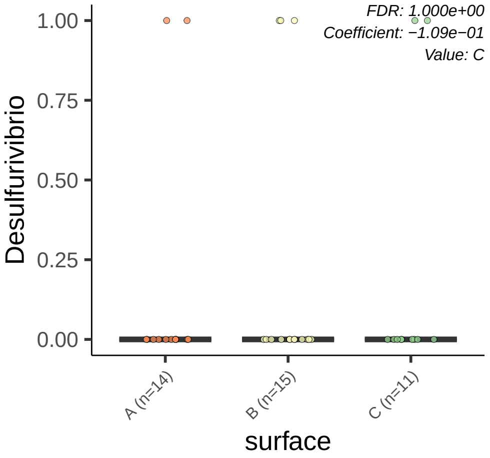
FDR: 1.000e+00
Coefficient: −1.09e−01
Value: C
Desulfurivibrio
1.00
0.75
0.50
0.25
0.00
A (n=14)
B (n=15)
C (n=11)
surface

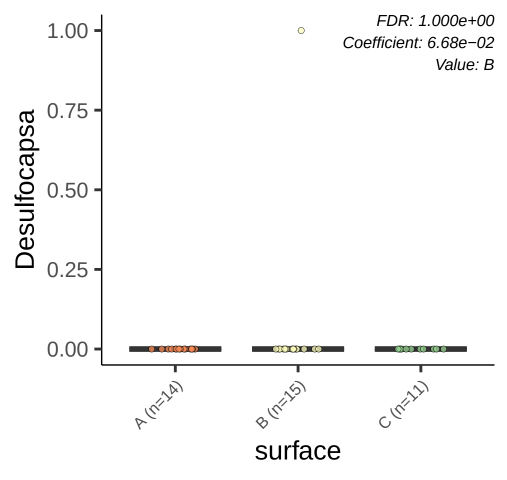

FDR: 1.000e+00
Coefficient: 6.68e−02
Value: B
Desulfocapsa
1.00
0.75
0.50
0.25
0.00
A (n=14)
B (n=15)
C (n=11)
surface

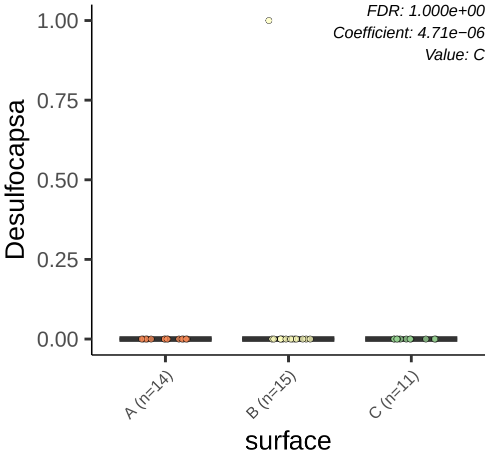
FDR: 1.000e+00
Coefficient: 4.71e−06
Value: C
1.00
0.75
0.50
0.25
0.00
Desulfocapsa
A (n=14)
B (n=15)
C (n=11)
surface

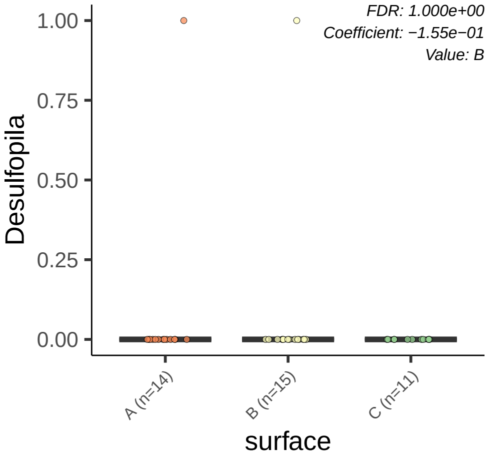
FDR: 1.000e+00
Coefficient: −1.55e−01
Value: B
Desulfopila
1.00
0.75
0.50
0.25
0.00
A (n=14)
B (n=15)
C (n=11)
surface

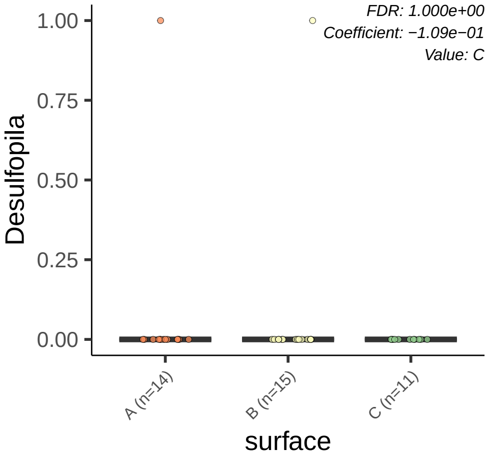

FDR: 1.000e+00
Coefficient: −1.09e−01
Value: C
Desulfopila
1.00
0.75
0.50
0.25
0.00
A (n=14)
B (n=15)
C (n=11)
surface

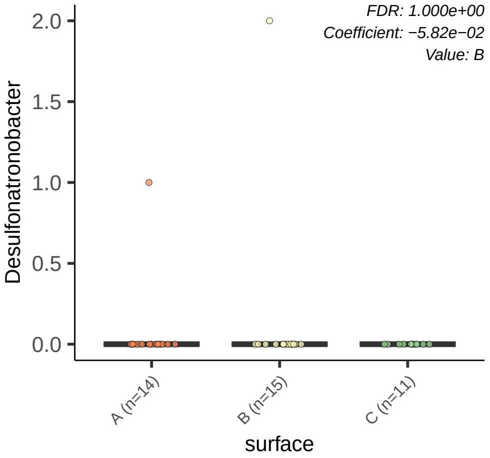

FDR: 1.000e+00
Coefficient: −5.82e−02
Value: B
Desulfonatronobacter
2.0
1.5
1.0
0.5
0.0
A (n=14)
B (n=15)
C (n=11)
surface

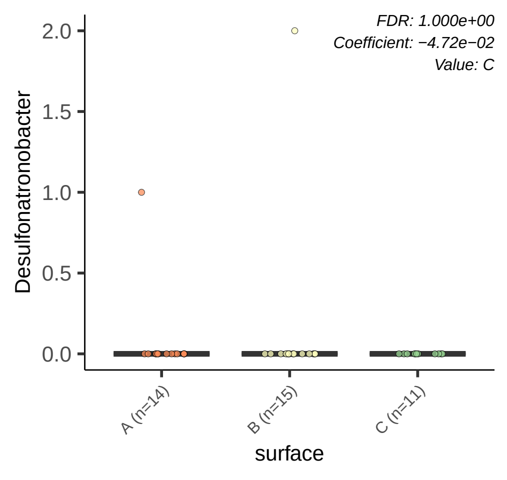
FDR: 1.000e+00
Coefficient: −4.72e−02
Value: C
Desulfonatronobacter
2.0
1.5
1.0
0.5
0.0
A (n=14)
B (n=15)
C (n=11)
surface

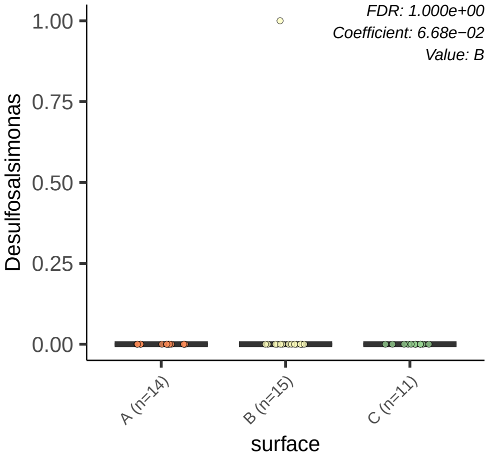

FDR: 1.000e+00
Coefficient: 6.68e−02
Value: B
Desulfosalsimonas
1.00
0.75
0.50
0.25
0.00
A (n=14)
B (n=15)
C (n=11)
surface

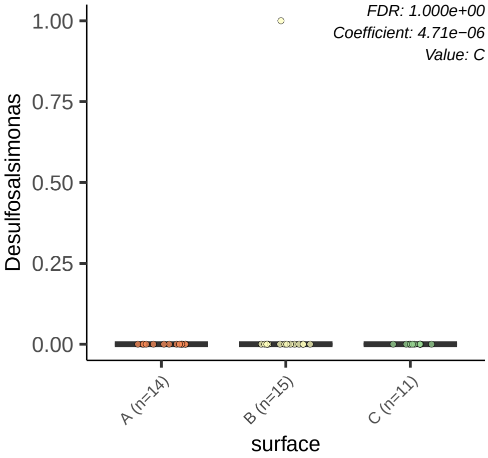

FDR: 1.000e+00
Coefficient: 4.71e−06
Value: C
Desulfosalsimonas
1.00
0.75
0.50
0.25
0.00
A (n=14)
B (n=15)
C (n=11)
surface

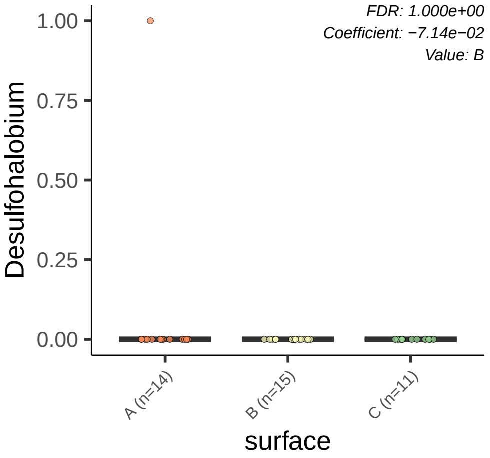
FDR: 1.000e+00
Coefficient: −7.14e−02
Value: B
Desulfohalobium
1.00
0.75
0.50
0.25
0.00
A (n=14)
B (n=15)
C (n=11)
surface

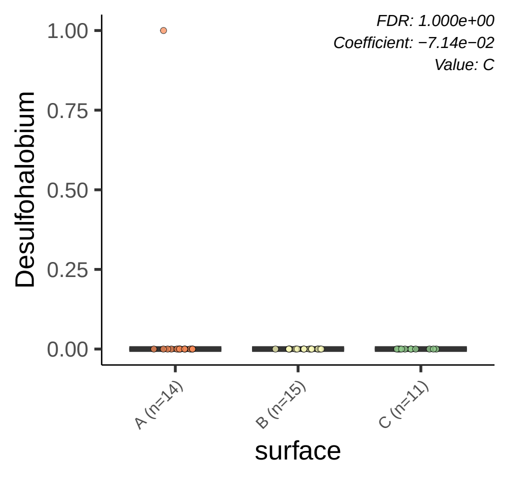

FDR: 1.000e+00
Coefficient: −7.14e−02
Value: C
Desulfohalobium
1.00
0.75
0.50
0.25
0.00
A (n=14)
B (n=15)
C (n=11)
surface

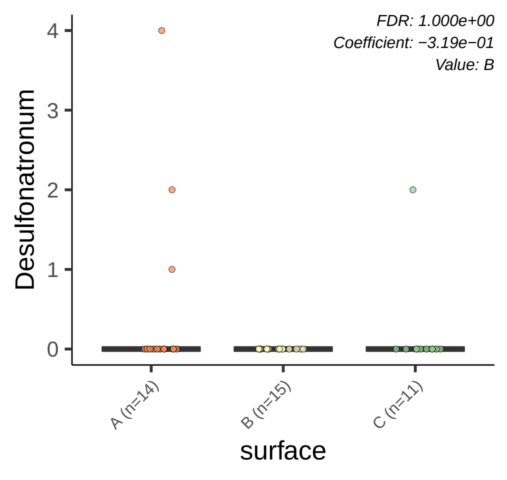
FDR: 1.000e+00
Coefficient: −3.19e−01
Value: B
Desulfonatronum
4
3
2
1
0
A (n=14)
B (n=15)
C (n=11)
surface

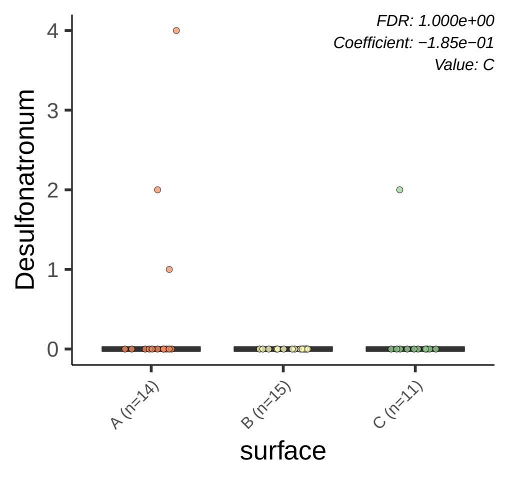
FDR: 1.000e+00
Coefficient: −1.85e−01
Value: C
Desulfonatronum
0
1
2
3
4
A (n=14)
B (n=15)
C (n=11)
surface

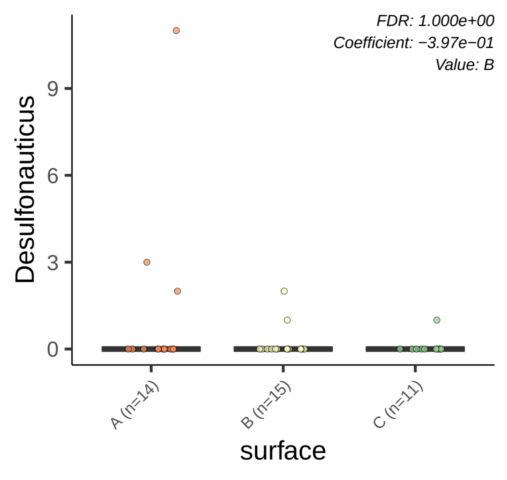

FDR: 1.000e+00
Coefficient: −3.97e−01
Value: B
Desulfonauticus
9
6
3
0
A (n=14)
B (n=15)
C (n=11)
surface

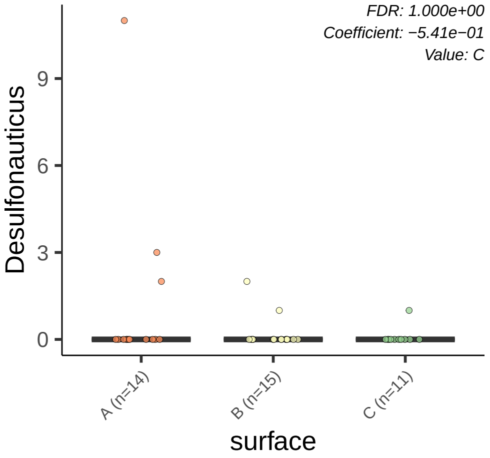
FDR: 1.000e+00
Coefficient: −5.41e−01
Value: C
Desulfonauticus
0
3
6
9
A (n=14)
B (n=15)
C (n=11)
surface

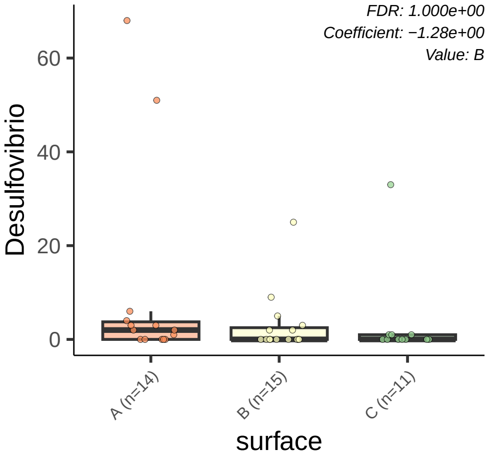
FDR: 1.000e+00
Coefficient: −1.28e+00
Value: B
Desulfovibrio
60
40
20
0
A (n=14)
B (n=15)
C (n=11)
surface

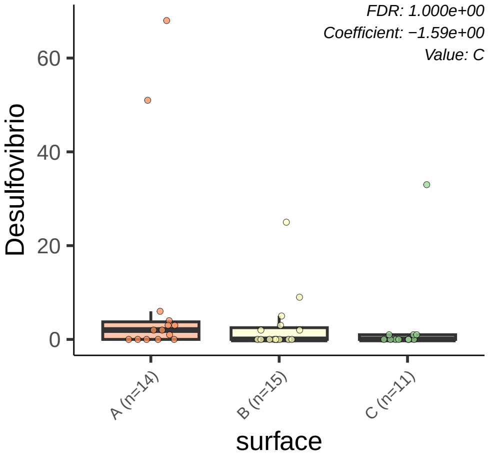

FDR: 1.000e+00
Coefficient: −1.59e+00
Value: C
Desulfovibrio
0
20
40
60
A (n=14)
B (n=15)
C (n=11)
surface

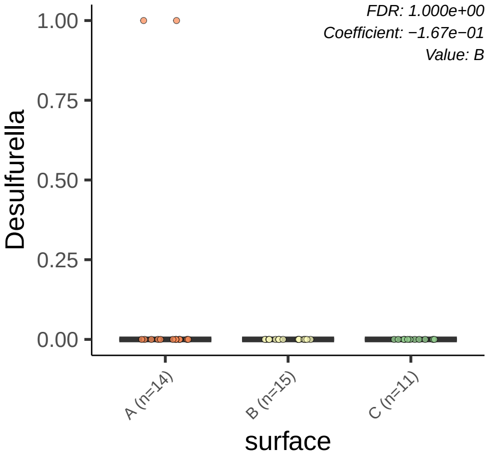
FDR: 1.000e+00
Coefficient: −1.67e−01
Value: B
1.00
0.75
0.50
0.25
0.00
Desulfurella
A (n=14)
B (n=15)
C (n=11)
surface

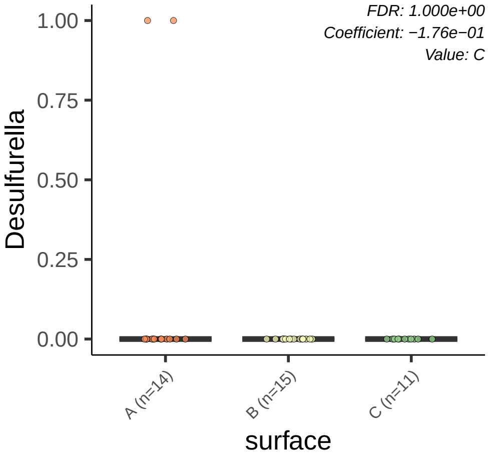
FDR: 1.000e+00
Coefficient: −1.76e−01
Value: C
Desulfurella
1.00
0.75
0.50
0.25
0.00
A (n=14)
B (n=15)
C (n=11)
surface

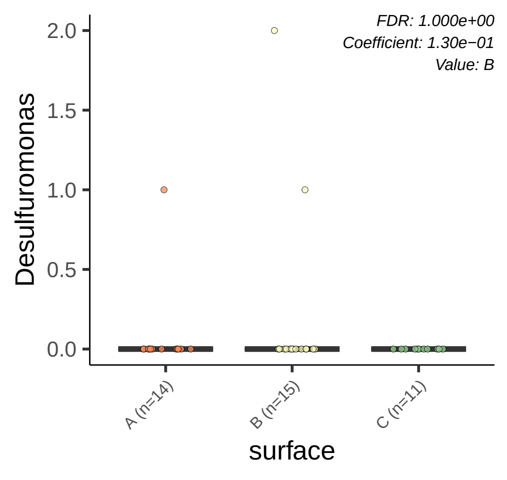

FDR: 1.000e+00
Coefficient: 1.30e−01
Value: B
Desulfuromonas
2.0
1.5
1.0
0.5
0.0
A (n=14)
B (n=15)
C (n=11)
surface

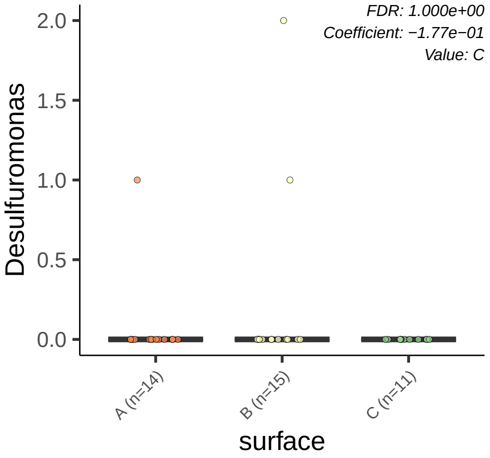
FDR: 1.000e+00
Coefficient: −1.77e−01
Value: C
Desulfuromonas
2.0
1.5
1.0
0.5
0.0
A (n=14)
B (n=15)
C (n=11)
surface

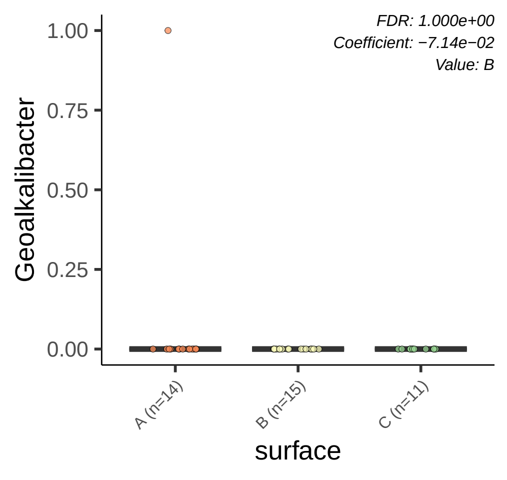
FDR: 1.000e+00
Coefficient: −7.14e−02
Value: B
Geoalkalibacter
1.00
0.75
0.50
0.25
0.00
A (n=14)
B (n=15)
C (n=11)
surface

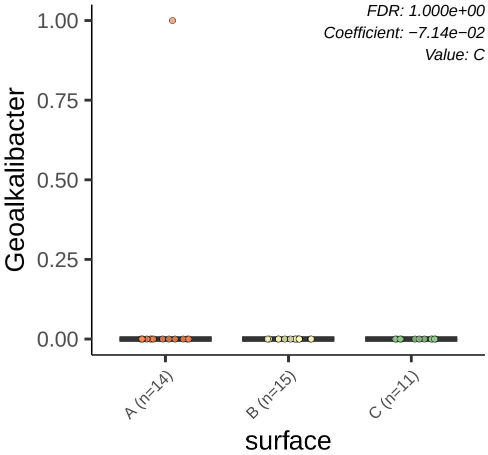

FDR: 1.000e+00
Coefficient: −7.14e−02
Value: C
Geoalkalibacter
1.00
0.75
0.50
0.25
0.00
A (n=14)
B (n=15)
C (n=11)
surface

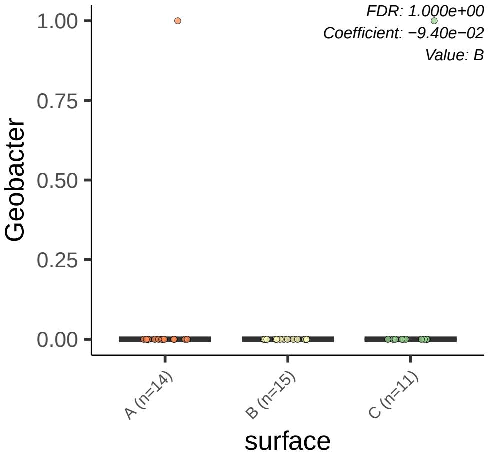
FDR: 1.000e+00
Coefficient: −9.40e−02
Value: B
Geobacter
1.00
0.75
0.50
0.25
0.00
A (n=14)
B (n=15)
C (n=11)
surface

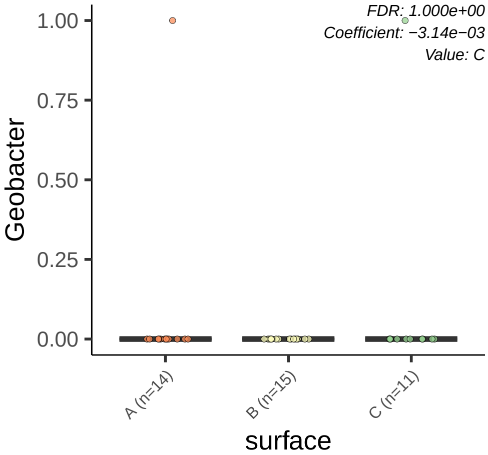
FDR: 1.000e+00
Coefficient: −3.14e−03
Value: C
Geobacter
1.00
0.75
0.50
0.25
0.00
A (n=14)
B (n=15)
C (n=11)
surface

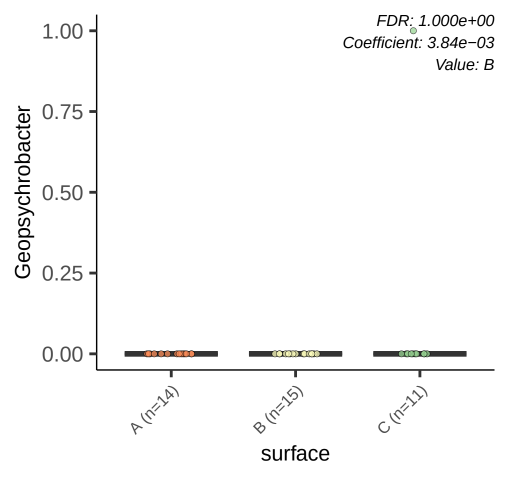
FDR: 1.000e+00
Coefficient: 3.84e−03
Value: B
Geopsychrobacter
1.00
0.75
0.50
0.25
0.00
A (n=14)
B (n=15)
C (n=11)
surface

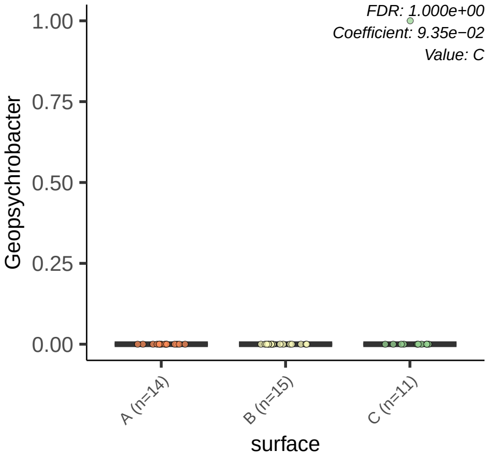

FDR: 1.000e+00
Coefficient: 9.35e−02
Value: C
Geopsychrobacter
1.00
0.75
0.50
0.25
0.00
A (n=14)
B (n=15)
C (n=11)
surface

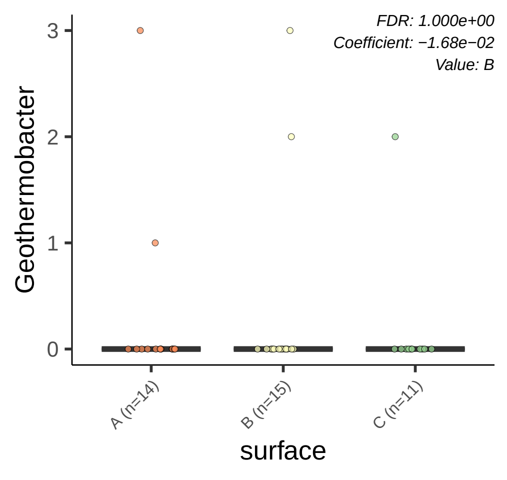
FDR: 1.000e+00
Coefficient: −1.68e−02
Value: B
Geothermobacter
3
2
1
0
A (n=14)
B (n=15)
C (n=11)
surface

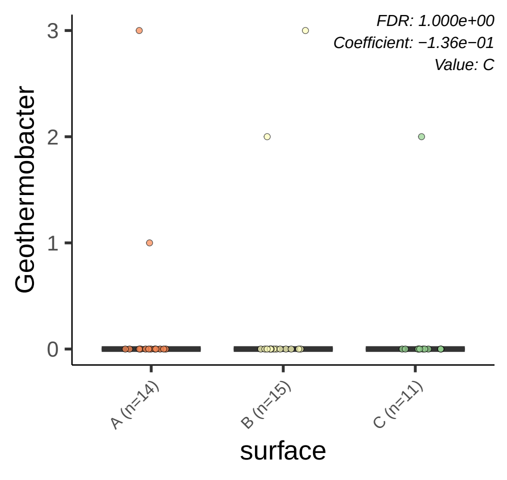

FDR: 1.000e+00
Coefficient: −1.36e−01
Value: C
Geothermobacter
3
2
1
0
A (n=14)
B (n=15)
C (n=11)
surface

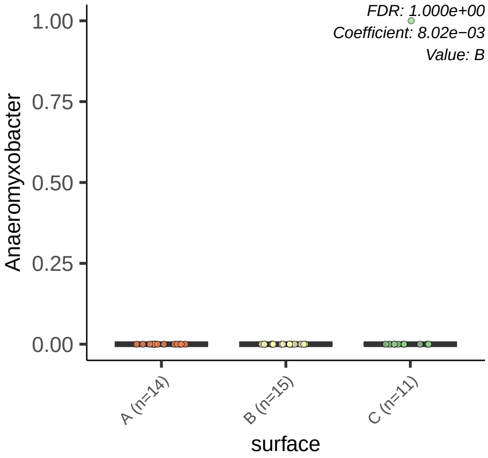

FDR: 1.000e+00
Coefficient: 8.02e−03
Value: B
Anaeromyxobacter
1.00
0.75
0.50
0.25
0.00
A (n=14)
B (n=15)
C (n=11)
surface

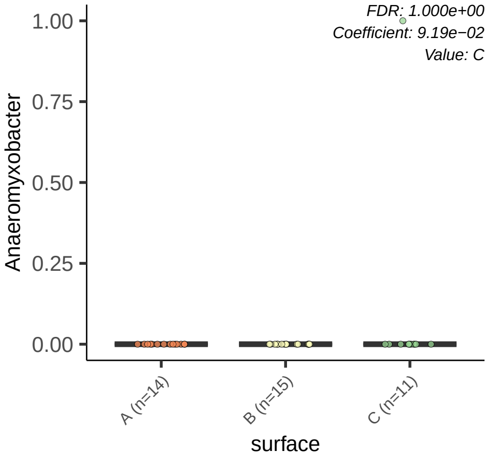
FDR: 1.000e+00
Coefficient: 9.19e−02
Value: C
Anaeromyxobacter
1.00
0.75
0.50
0.25
0.00
A (n=14)
B (n=15)
C (n=11)
surface

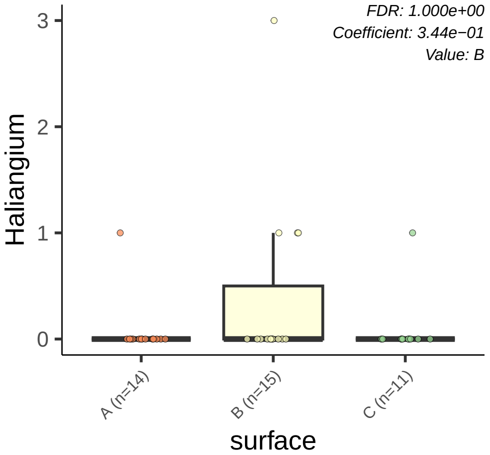
FDR: 1.000e+00
Coefficient: 3.44e−01
Value: B
Haliangium
3
2
1
0
A (n=14)
B (n=15)
C (n=11)
surface

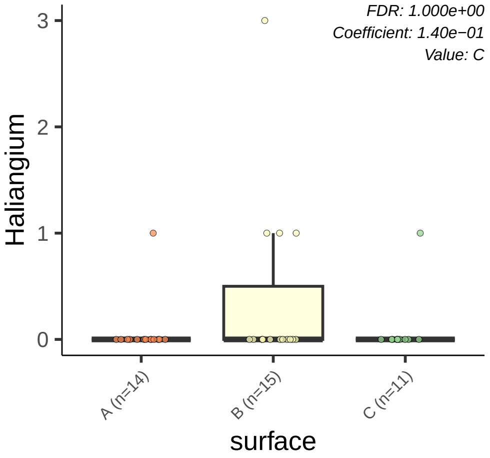
FDR: 1.000e+00
Coefficient: 1.40e−01
Value: C
Haliangium
0
1
2
3
A (n=14)
B (n=15)
C (n=11)
surface

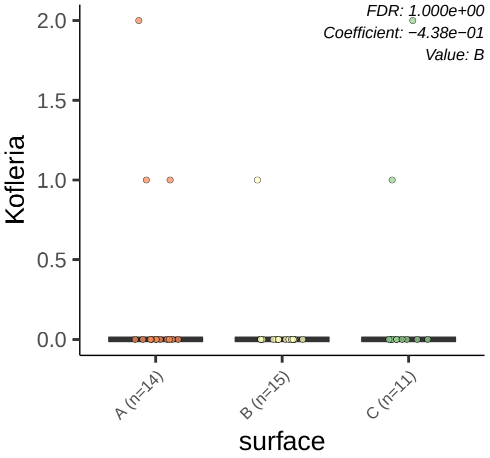

FDR: 1.000e+00
Coefficient: −4.38e−01
Value: B
Kofleria
2.0
1.5
1.0
0.5
0.0
A (n=14)
B (n=15)
C (n=11)
surface

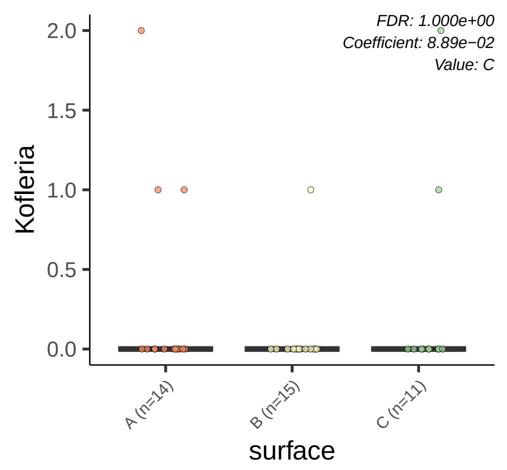
FDR: 1.000e+00
Coefficient: 8.89e−02
Value: C
Kofleria
2.0
1.5
1.0
0.5
0.0
A (n=14)
B (n=15)
C (n=11)
surface

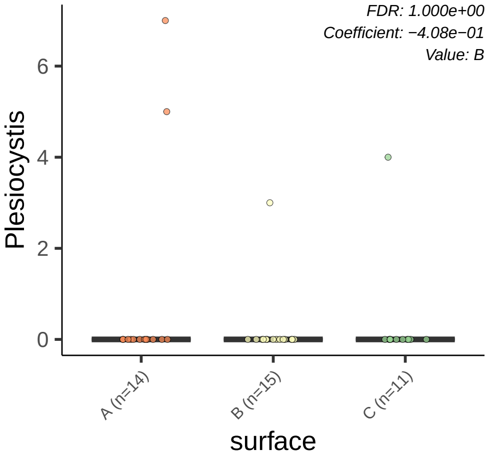
FDR: 1.000e+00
Coefficient: −4.08e−01
Value: B
Plesiocystis
6
4
2
0
A (n=14)
B (n=15)
C (n=11)
surface

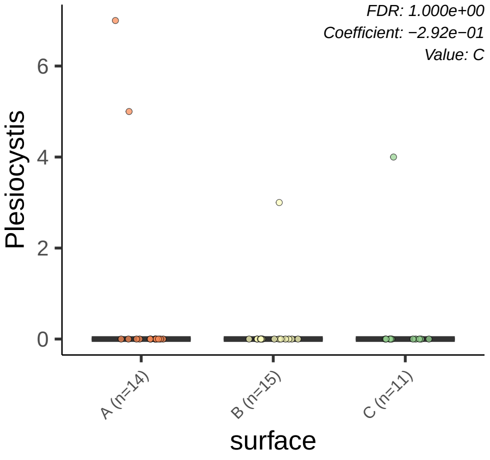

FDR: 1.000e+00
Coefficient: −2.92e−01
Value: C
Plesiocystis
6
4
2
0
A (n=14)
B (n=15)
C (n=11)
surface

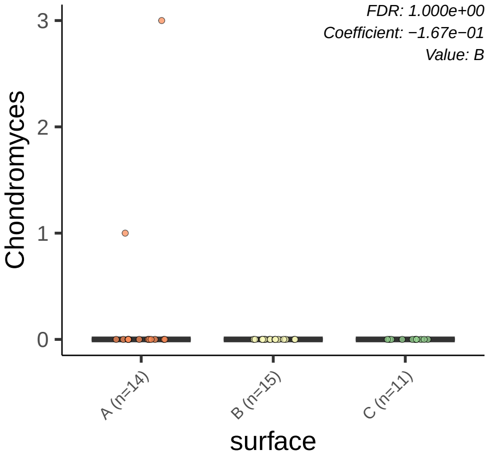

FDR: 1.000e+00
Coefficient: −1.67e−01
Value: B
Chondromyces
3
2
1
0
A (n=14)
B (n=15)
C (n=11)
surface

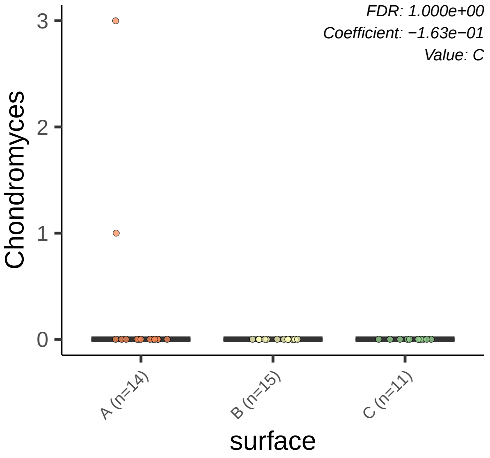
FDR: 1.000e+00
Coefficient: −1.63e−01
Value: C
Chondromyces
0
1
2
3
A (n=14)
B (n=15)
C (n=11)
surface

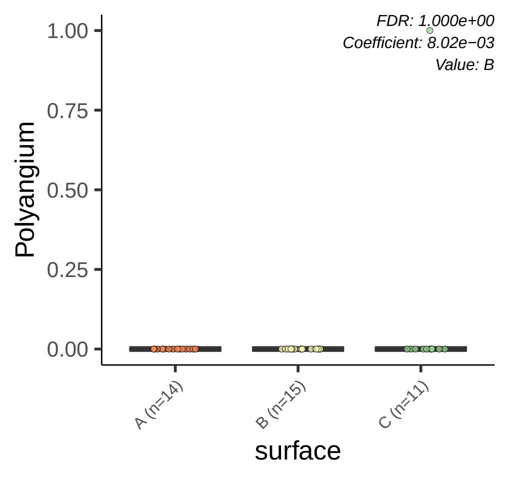
FDR: 1.000e+00
Coefficient: 8.02e−03
Value: B
Polyangium
1.00
0.75
0.50
0.25
0.00
A (n=14)
B (n=15)
C (n=11)
surface

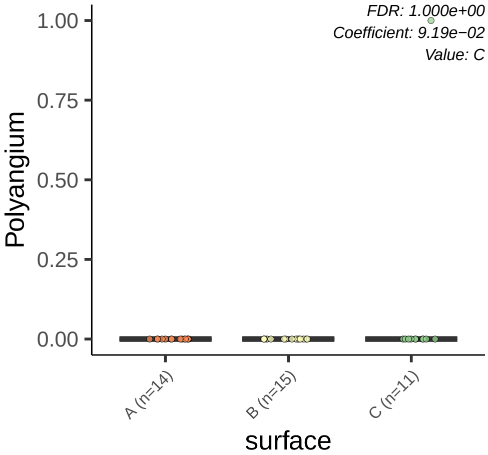

FDR: 1.000e+00
Coefficient: 9.19e−02
Value: C
1.00
0.75
0.50
0.25
0.00
Polyangium
A (n=14)
B (n=15)
C (n=11)
surface

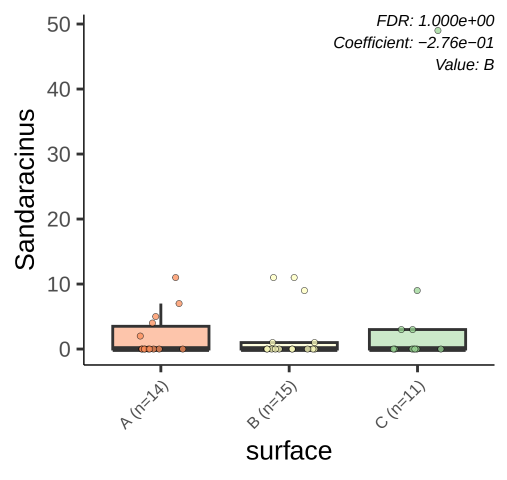
FDR: 1.000e+00
Coefficient: −2.76e−01
Value: B
Sandaracinus
0
10
20
30
40
50
A (n=14)
B (n=15)
C (n=11)
surface

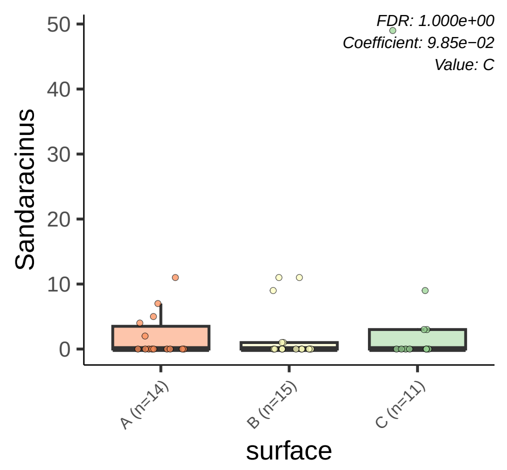

FDR: 1.000e+00
Coefficient: 9.85e−02
Value: C
Sandaracinus
0
10
20
30
40
50
A (n=14)
B (n=15)
C (n=11)
surface

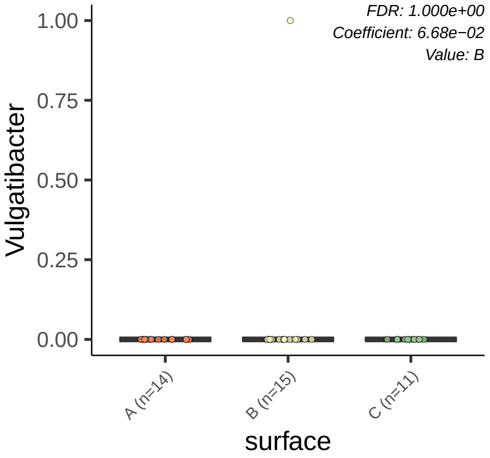

FDR: 1.000e+00
Coefficient: 6.68e−02
Value: B
Vulgatibacter
1.00
0.75
0.50
0.25
0.00
A (n=14)
B (n=15)
C (n=11)
surface

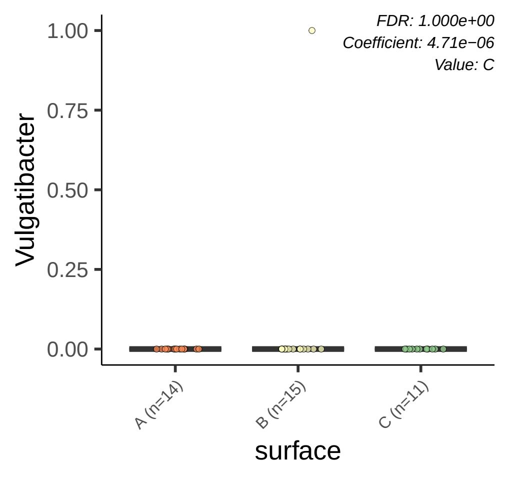

FDR: 1.000e+00
Coefficient: 4.71e−06
Value: C
Vulgatibacter
1.00
0.75
0.50
0.25
0.00
A (n=14)
B (n=15)
C (n=11)
surface

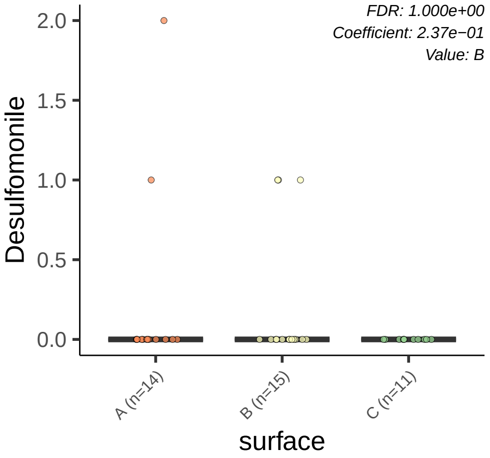

FDR: 1.000e+00
Coefficient: 2.37e−01
Value: B
Desulfomonile
2.0
1.5
1.0
0.5
0.0
A (n=14)
B (n=15)
C (n=11)
surface

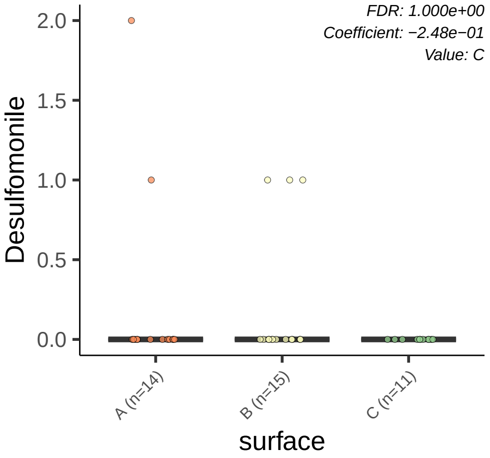
FDR: 1.000e+00
Coefficient: −2.48e−01
Value: C
Desulfomonile
2.0
1.5
1.0
0.5
0.0
A (n=14)
B (n=15)
C (n=11)
surface

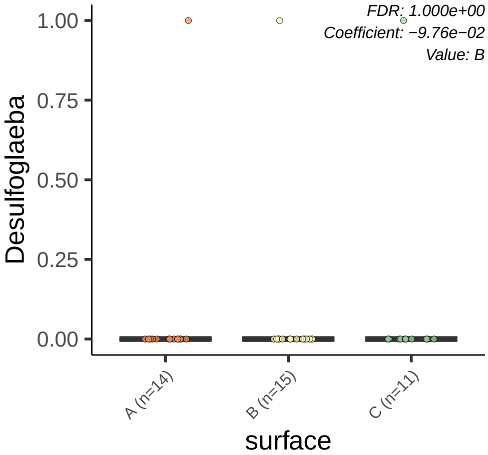
FDR: 1.000e+00
Coefficient: −9.76e−02
Value: B
Desulfoglaeba
1.00
0.75
0.50
0.25
0.00
A (n=14)
B (n=15)
C (n=11)
surface

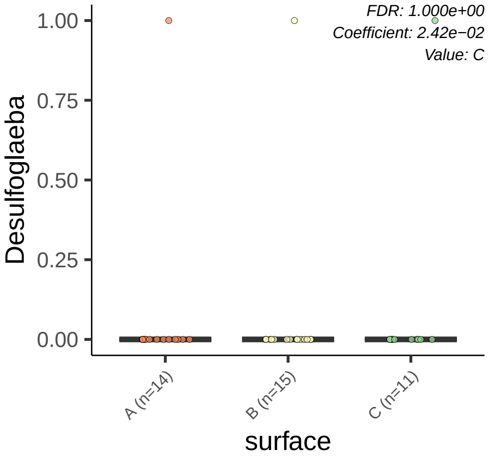

FDR: 1.000e+00
Coefficient: 2.42e−02
Value: C
Desulfoglaeba
1.00
0.75
0.50
0.25
0.00
A (n=14)
B (n=15)
C (n=11)
surface

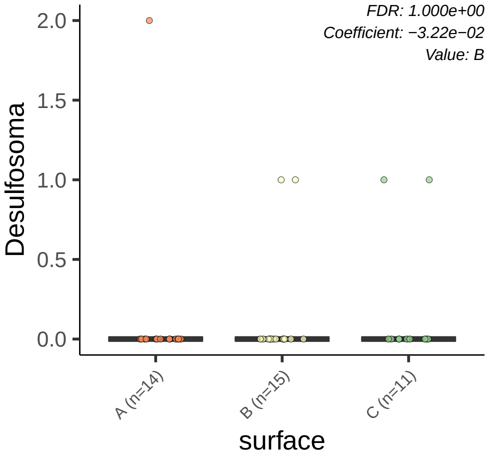

FDR: 1.000e+00
Coefficient: −3.22e−02
Value: B
Desulfosoma
2.0
1.5
1.0
0.5
0.0
A (n=14)
B (n=15)
C (n=11)
surface

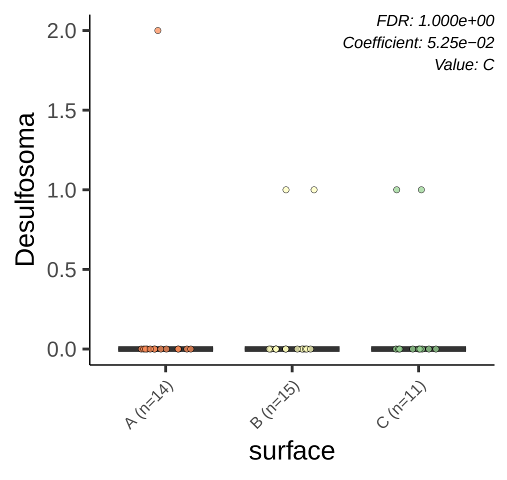
FDR: 1.000e+00
Coefficient: 5.25e−02
Value: C
Desulfosoma
2.0
1.5
1.0
0.5
0.0
A (n=14)
B (n=15)
C (n=11)
surface

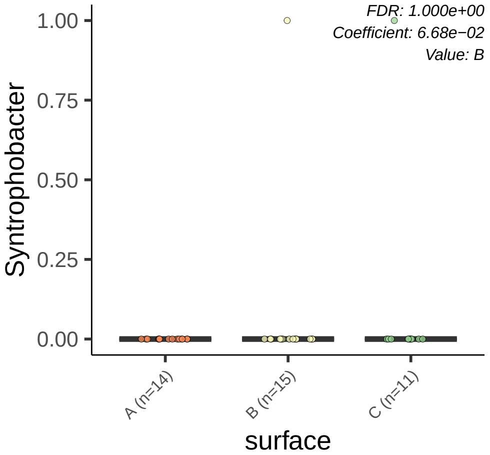

FDR: 1.000e+00
Coefficient: 6.68e−02
Value: B
Syntrophobacter
1.00
0.75
0.50
0.25
0.00
A (n=14)
B (n=15)
C (n=11)
surface

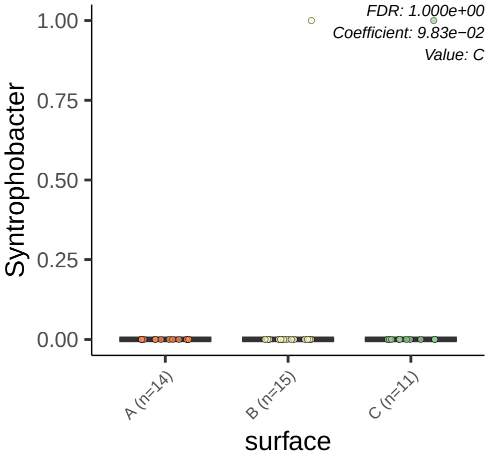
FDR: 1.000e+00
Coefficient: 9.83e−02
Value: C
Syntrophobacter
1.00
0.75
0.50
0.25
0.00
A (n=14)
B (n=15)
C (n=11)
surface

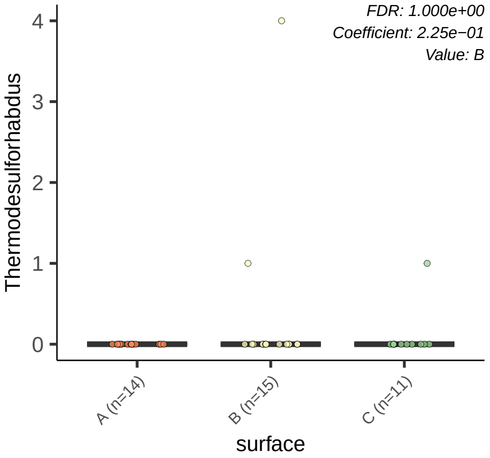

FDR: 1.000e+00
Coefficient: 2.25e−01
Value: B
Thermodesulforhabdus
4
3
2
1
0
A (n=14)
B (n=15)
C (n=11)
surface

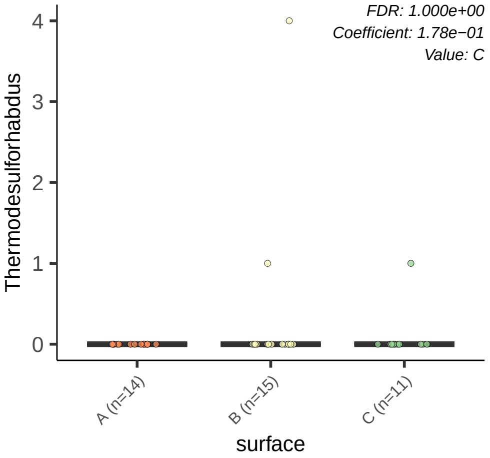

FDR: 1.000e+00
Coefficient: 1.78e−01
Value: C
Thermodesulforhabdus
4
3
2
1
0
A (n=14)
B (n=15)
C (n=11)
surface

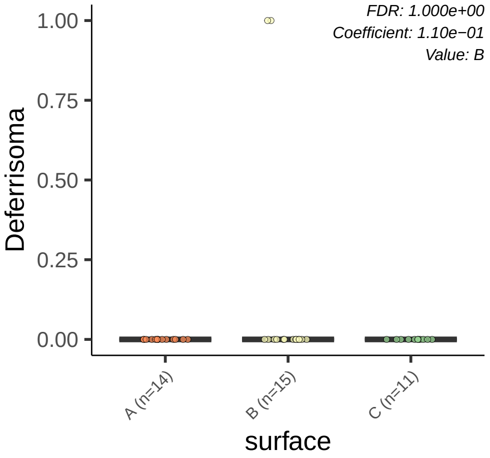
FDR: 1.000e+00
Coefficient: 1.10e−01
Value: B
Deferrisoma
1.00
0.75
0.50
0.25
0.00
A (n=14)
B (n=15)
C (n=11)
surface

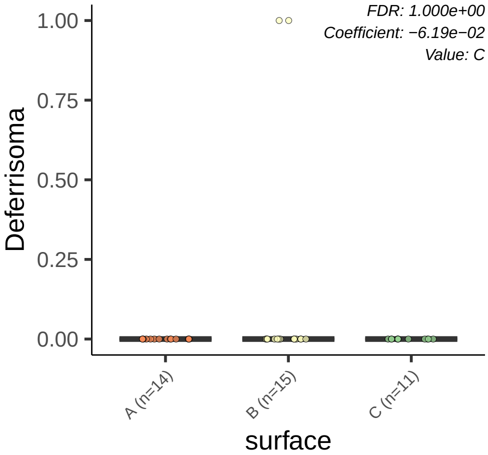
FDR: 1.000e+00
Coefficient: −6.19e−02
Value: C
Deferrisoma
1.00
0.75
0.50
0.25
0.00
A (n=14)
B (n=15)
C (n=11)
surface

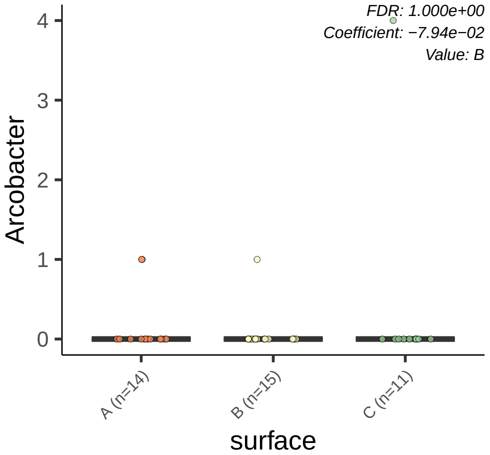
FDR: 1.000e+00
Coefficient: −7.94e−02
Value: B
Arcobacter
4
3
2
1
0
A (n=14)
B (n=15)
C (n=11)
surface

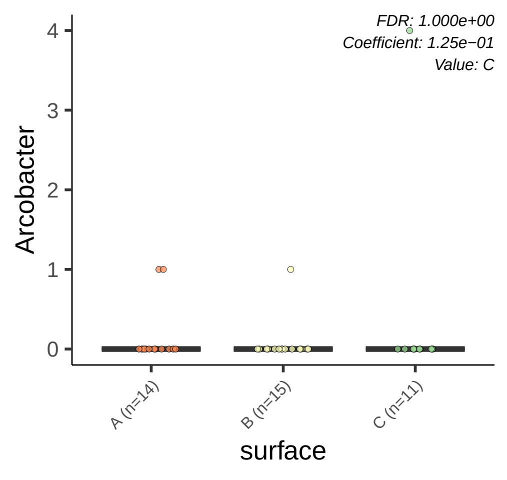

FDR: 1.000e+00
Coefficient: 1.25e−01
Value: C
Arcobacter
0
1
2
3
4
A (n=14)
B (n=15)
C (n=11)
surface

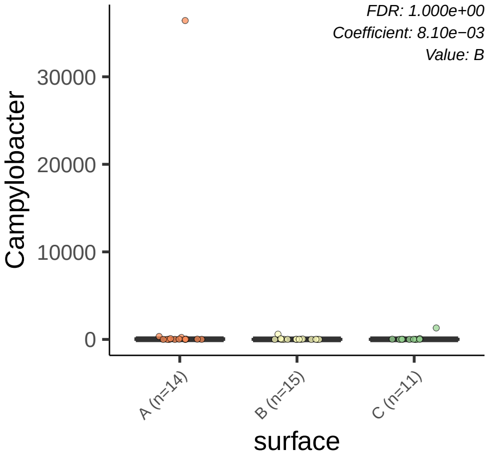
FDR: 1.000e+00
Coefficient: 8.10e−03
Value: B
Campylobacter
30000
20000
10000
0
A (n=14)
B (n=15)
C (n=11)
surface

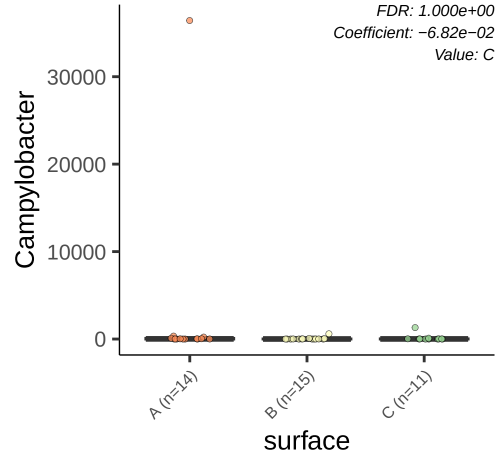
FDR: 1.000e+00
Coefficient: −6.82e−02
Value: C
Campylobacter
30000
20000
10000
0
A (n=14)
B (n=15)
C (n=11)
surface

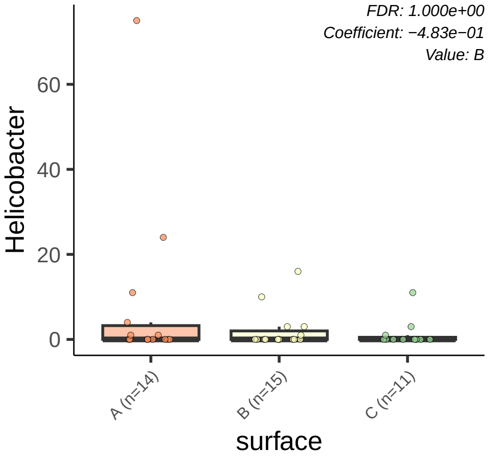
FDR: 1.000e+00
Coefficient: −4.83e−01
Value: B
Helicobacter
0
20
40
60
A (n=14)
B (n=15)
C (n=11)
surface

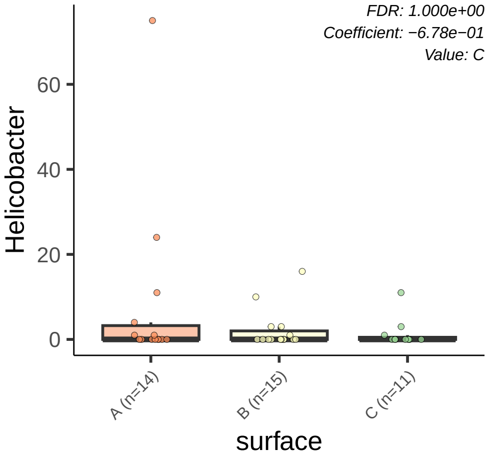
FDR: 1.000e+00
Coefficient: −6.78e−01
Value: C
Helicobacter
0
20
40
60
A (n=14)
B (n=15)
C (n=11)
surface

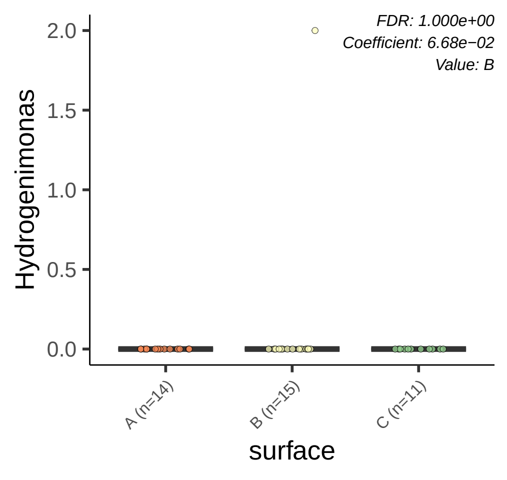
FDR: 1.000e+00
Coefficient: 6.68e−02
Value: B
Hydrogenimonas
2.0
1.5
1.0
0.5
0.0
A (n=14)
B (n=15)
C (n=11)
surface

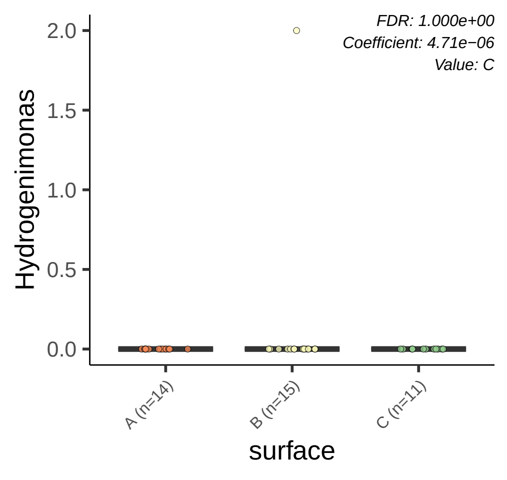
FDR: 1.000e+00
Coefficient: 4.71e−06
Value: C
Hydrogenimonas
2.0
1.5
1.0
0.5
0.0
A (n=14)
B (n=15)
C (n=11)
surface

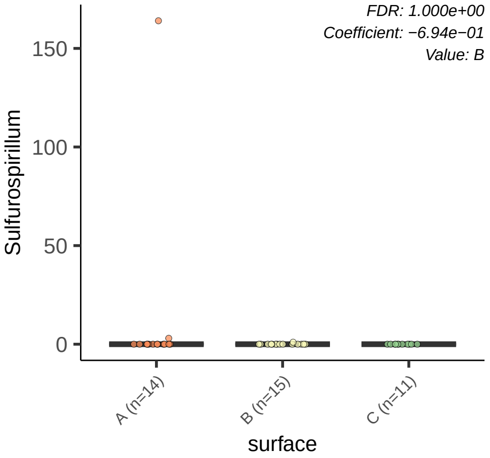
FDR: 1.000e+00
Coefficient: −6.94e−01
Value: B
Sulfurospirillum
150
100
50
0
A (n=14)
B (n=15)
C (n=11)
surface

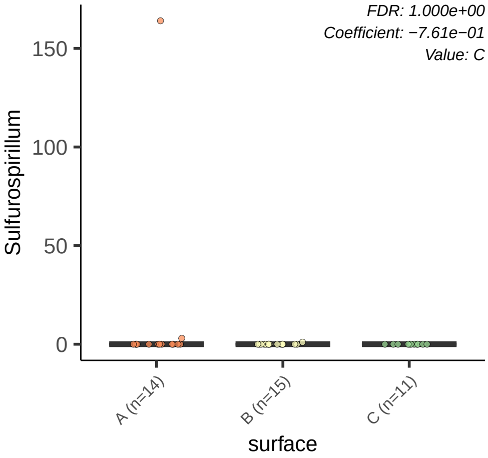
FDR: 1.000e+00
Coefficient: −7.61e−01
Value: C
Sulfurospirillum
150
100
50
0
A (n=14)
B (n=15)
C (n=11)
surface

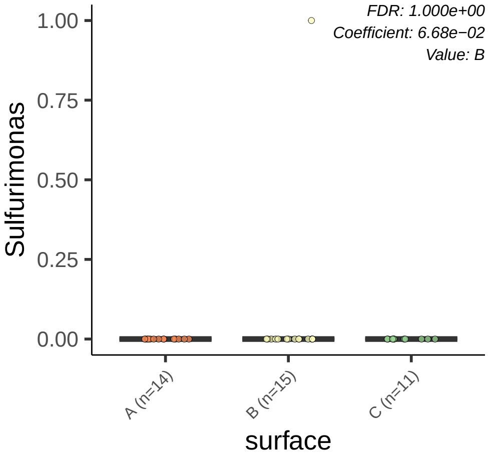
FDR: 1.000e+00
Coefficient: 6.68e−02
Value: B
Sulfurimonas
1.00
0.75
0.50
0.25
0.00
A (n=14)
B (n=15)
C (n=11)
surface

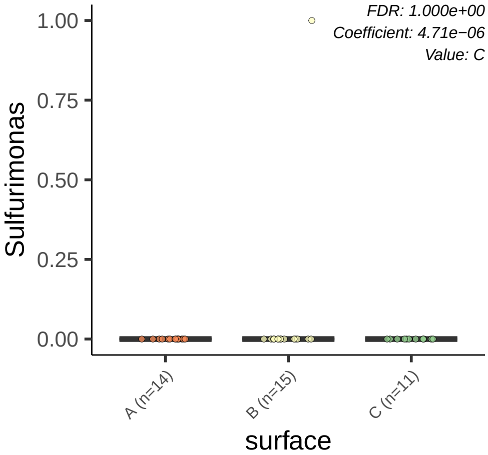

FDR: 1.000e+00
Coefficient: 4.71e−06
Value: C
Sulfurimonas
1.00
0.75
0.50
0.25
0.00
A (n=14)
B (n=15)
C (n=11)
surface

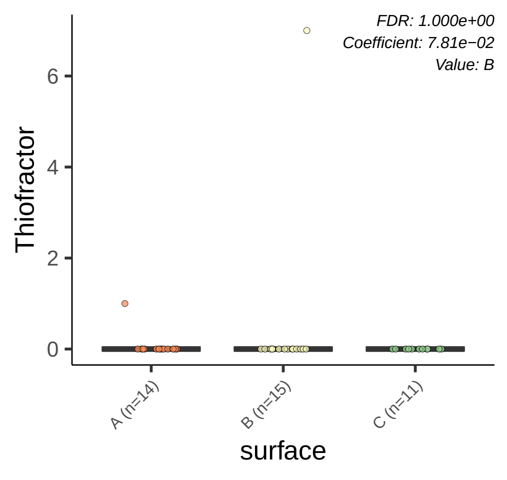

FDR: 1.000e+00
Coefficient: 7.81e−02
Value: B
Thiofractor
0
2
4
6
A (n=14)
B (n=15)
C (n=11)
surface

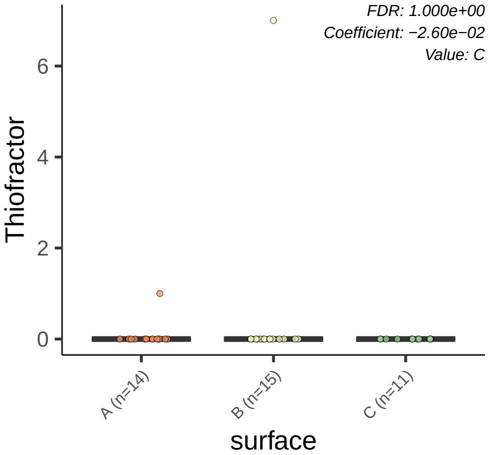

FDR: 1.000e+00
Coefficient: −2.60e−02
Value: C
Thiofractor
0
2
4
6
A (n=14)
B (n=15)
C (n=11)
surface

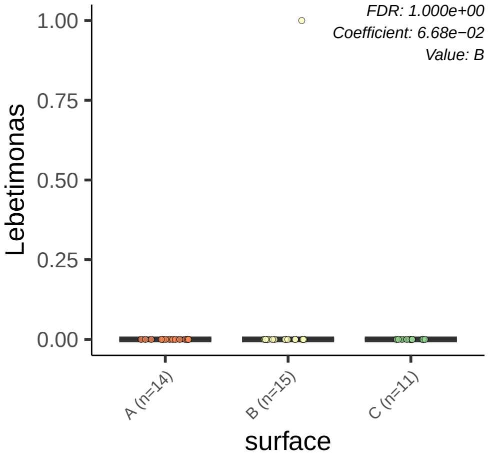
FDR: 1.000e+00
Coefficient: 6.68e−02
Value: B
Lebetimonas
1.00
0.75
0.50
0.25
0.00
A (n=14)
B (n=15)
C (n=11)
surface

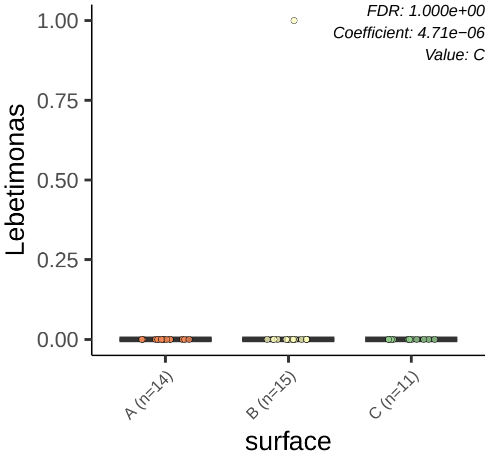
FDR: 1.000e+00
Coefficient: 4.71e−06
Value: C
Lebetimonas
1.00
0.75
0.50
0.25
0.00
A (n=14)
B (n=15)
C (n=11)
surface

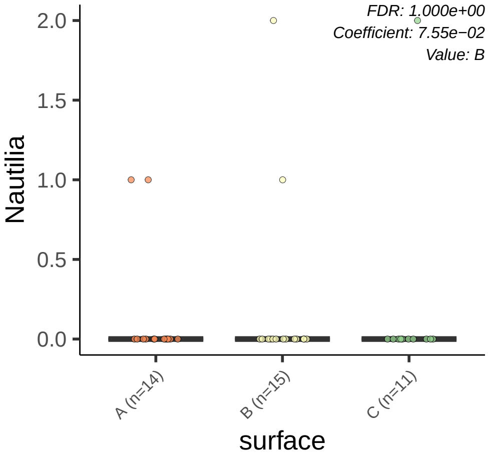
FDR: 1.000e+00
Coefficient: 7.55e−02
Value: B
Nautilia
2.0
1.5
1.0
0.5
0.0
A (n=14)
B (n=15)
C (n=11)
surface

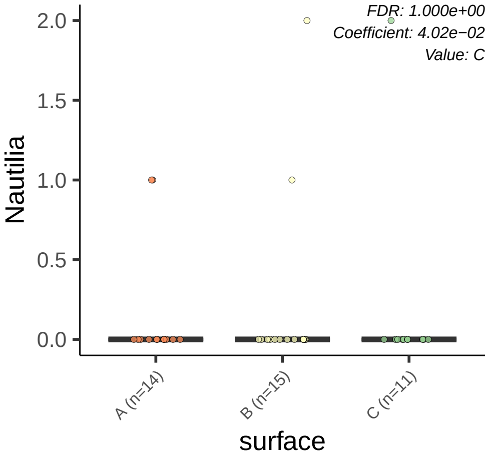
FDR: 1.000e+00
Coefficient: 4.02e−02
Value: C
Nautilia
2.0
1.5
1.0
0.5
0.0
A (n=14)
B (n=15)
C (n=11)
surface

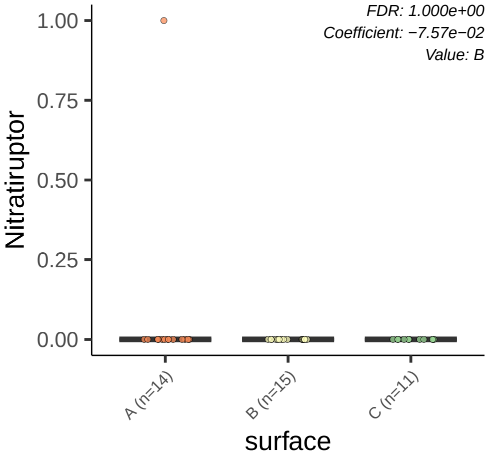

FDR: 1.000e+00
Coefficient: −7.57e−02
Value: B
Nitratiruptor
1.00
0.75
0.50
0.25
0.00
A (n=14)
B (n=15)
C (n=11)
surface

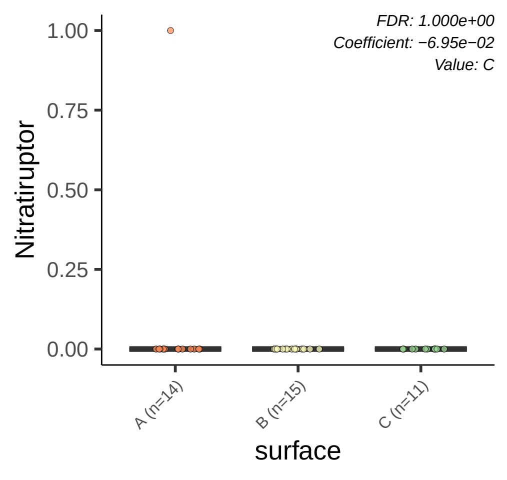
FDR: 1.000e+00
Coefficient: −6.95e−02
Value: C
Nitratiruptor
1.00
0.75
0.50
0.25
0.00
A (n=14)
B (n=15)
C (n=11)
surface

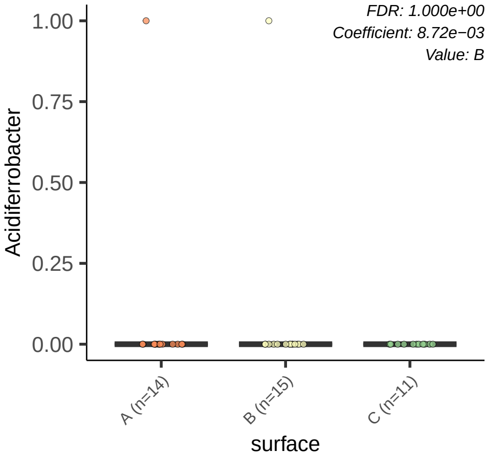

FDR: 1.000e+00
Coefficient: 8.72e−03
Value: B
Acidiferrobacter
1.00
0.75
0.50
0.25
0.00
A (n=14)
B (n=15)
C (n=11)
surface

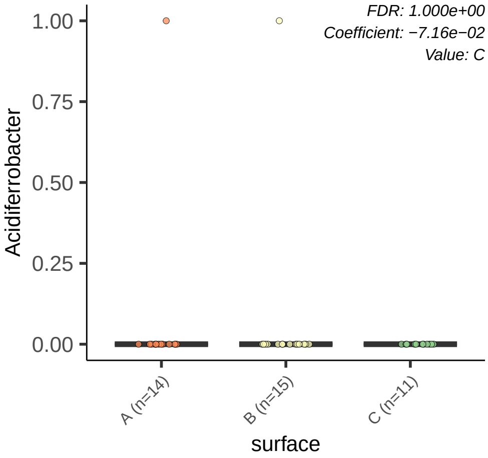

FDR: 1.000e+00
Coefficient: −7.16e−02
Value: C
Acidiferrobacter
1.00
0.75
0.50
0.25
0.00
A (n=14)
B (n=15)
C (n=11)
surface

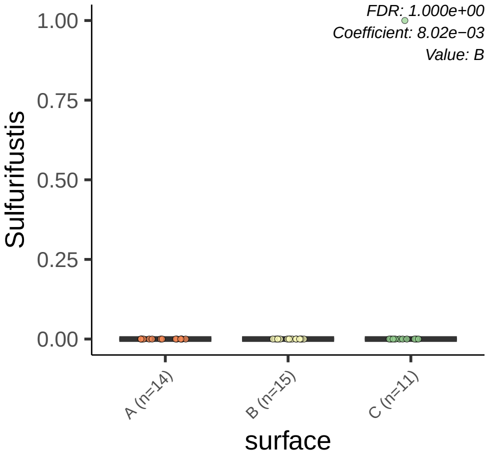
FDR: 1.000e+00
Coefficient: 8.02e−03
Value: B
Sulfurifustis
1.00
0.75
0.50
0.25
0.00
A (n=14)
B (n=15)
C (n=11)
surface

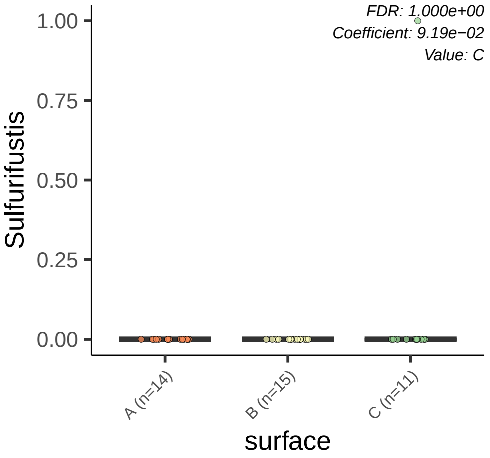
FDR: 1.000e+00
Coefficient: 9.19e−02
Value: C
1.00
0.75
0.50
0.25
0.00
Sulfurifustis
A (n=14)
B (n=15)
C (n=11)
surface

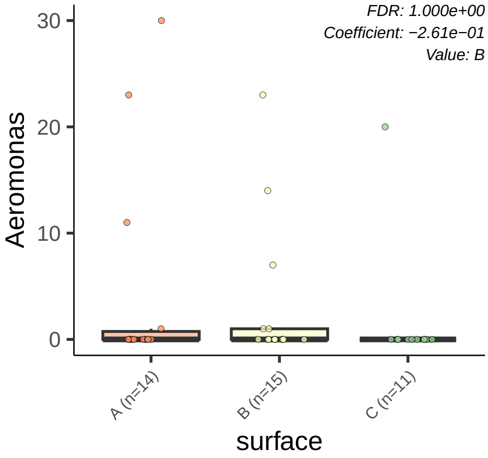

FDR: 1.000e+00
Coefficient: −2.61e−01
Value: B
Aeromonas
30
20
10
0
A (n=14)
B (n=15)
C (n=11)
surface

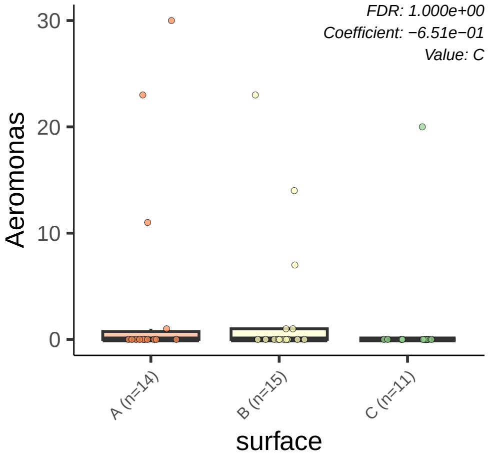

FDR: 1.000e+00
Coefficient: −6.51e−01
Value: C
Aeromonas
30
20
10
0
A (n=14)
B (n=15)
C (n=11)
surface

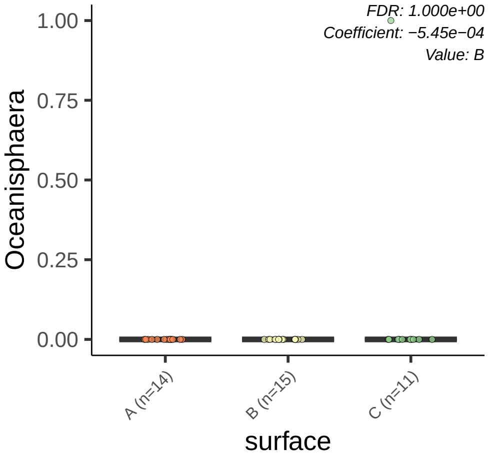
FDR: 1.000e+00
Coefficient: −5.45e−04
Value: B
Oceanisphaera
1.00
0.75
0.50
0.25
0.00
A (n=14)
B (n=15)
C (n=11)
surface

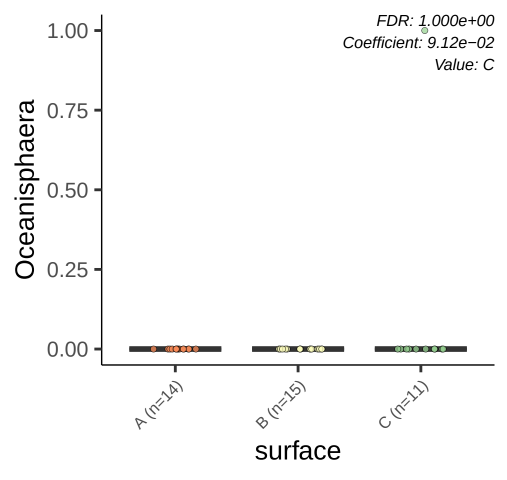

FDR: 1.000e+00
Coefficient: 9.12e−02
Value: C
Oceanisphaera
1.00
0.75
0.50
0.25
0.00
A (n=14)
B (n=15)
C (n=11)
surface

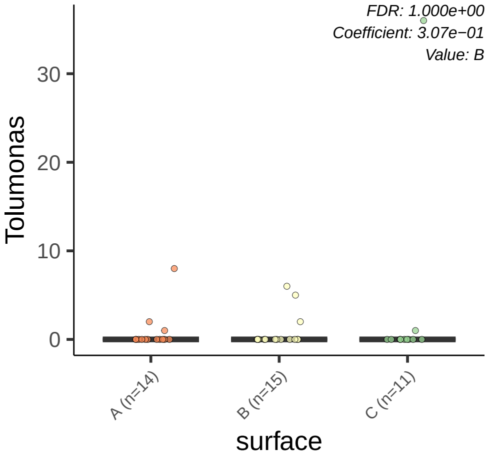

FDR: 1.000e+00
Coefficient: 3.07e−01
Value: B
Tolumonas
30
20
10
0
A (n=14)
B (n=15)
C (n=11)
surface

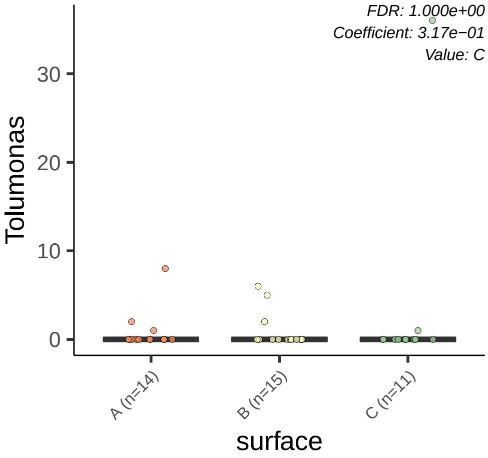

FDR: 1.000e+00
Coefficient: 3.17e−01
Value: C
Tolumonas
30
20
10
0
A (n=14)
B (n=15)
C (n=11)
surface

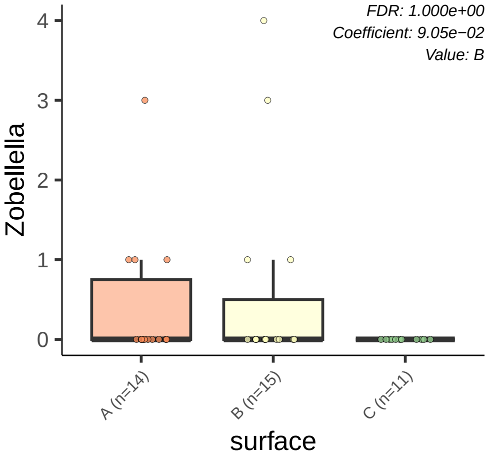
FDR: 1.000e+00
Coefficient: 9.05e−02
Value: B
Zobellella
0
1
2
3
4
A (n=14)
B (n=15)
C (n=11)
surface

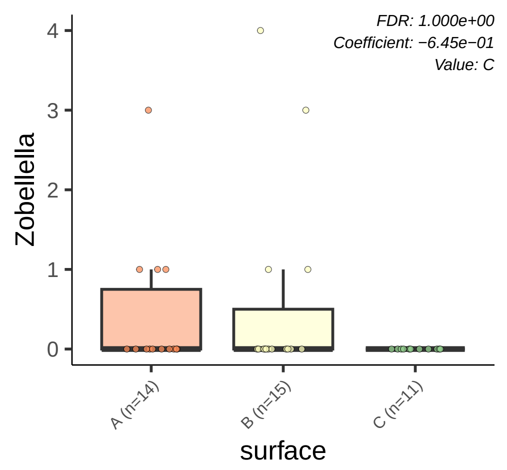

FDR: 1.000e+00
Coefficient: −6.45e−01
Value: C
Zobellella
4
3
2
1
0
A (n=14)
B (n=15)
C (n=11)
surface

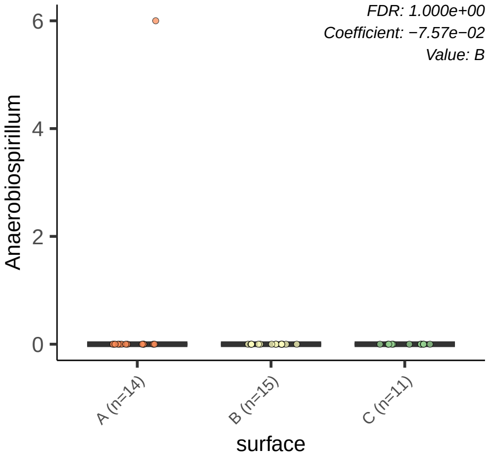

FDR: 1.000e+00
Coefficient: −7.57e−02
Value: B
Anaerobiospirillum
6
4
2
0
A (n=14)
B (n=15)
C (n=11)
surface

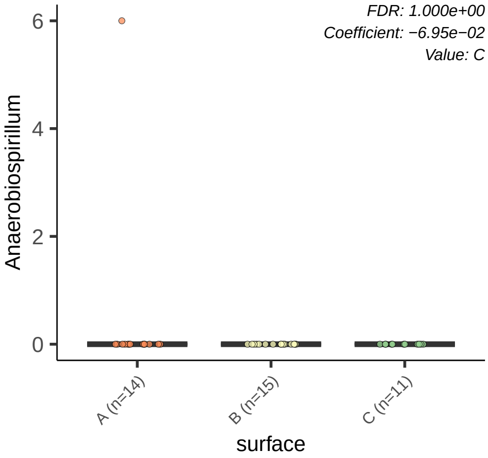

FDR: 1.000e+00
Coefficient: −6.95e−02
Value: C
Anaerobiospirillum
0
2
4
6
A (n=14)
B (n=15)
C (n=11)
surface

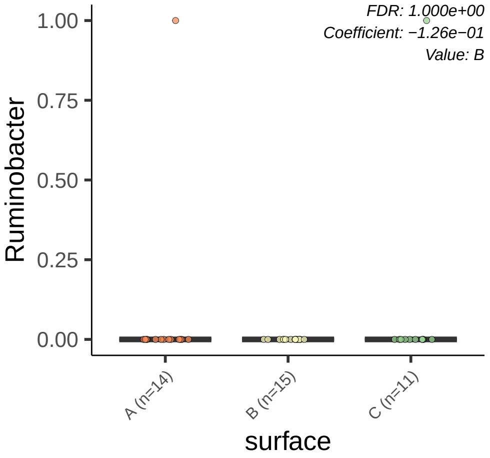
FDR: 1.000e+00
Coefficient: −1.26e−01
Value: B
Ruminobacter
1.00
0.75
0.50
0.25
0.00
A (n=14)
B (n=15)
C (n=11)
surface

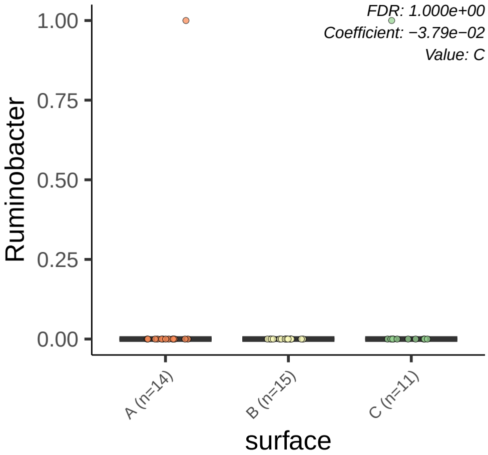
FDR: 1.000e+00
Coefficient: −3.79e−02
Value: C
Ruminobacter
1.00
0.75
0.50
0.25
0.00
A (n=14)
B (n=15)
C (n=11)
surface

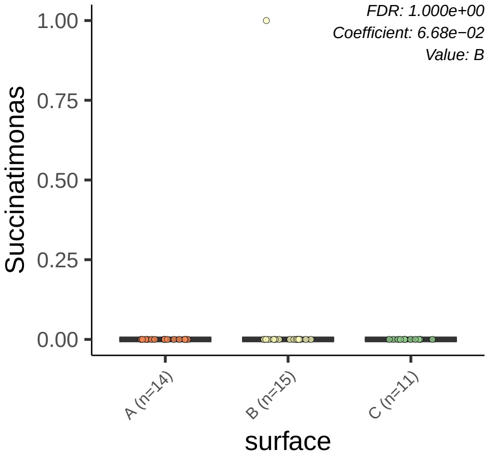
FDR: 1.000e+00
Coefficient: 6.68e−02
Value: B
Succinatimonas
1.00
0.75
0.50
0.25
0.00
A (n=14)
B (n=15)
C (n=11)
surface

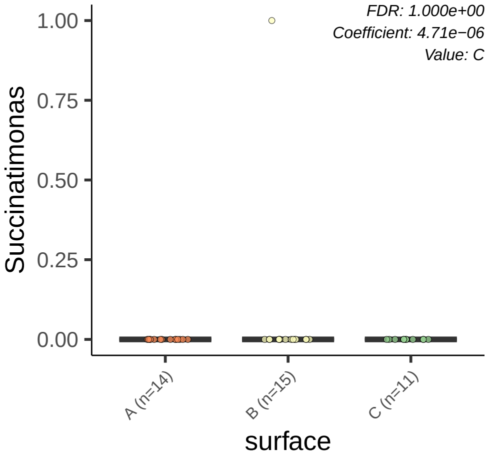
FDR: 1.000e+00
Coefficient: 4.71e−06
Value: C
Succinatimonas
1.00
0.75
0.50
0.25
0.00
A (n=14)
B (n=15)
C (n=11)
surface

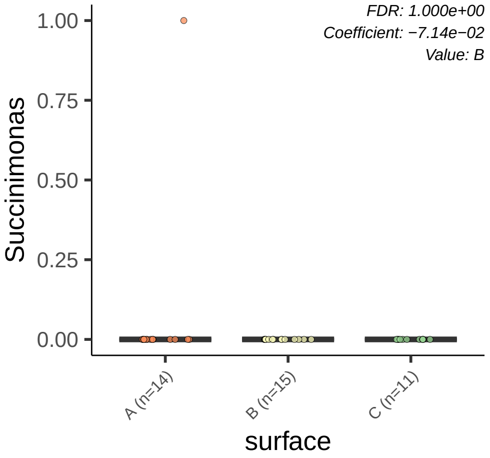
FDR: 1.000e+00
Coefficient: −7.14e−02
Value: B
Succinimonas
1.00
0.75
0.50
0.25
0.00
A (n=14)
B (n=15)
C (n=11)
surface

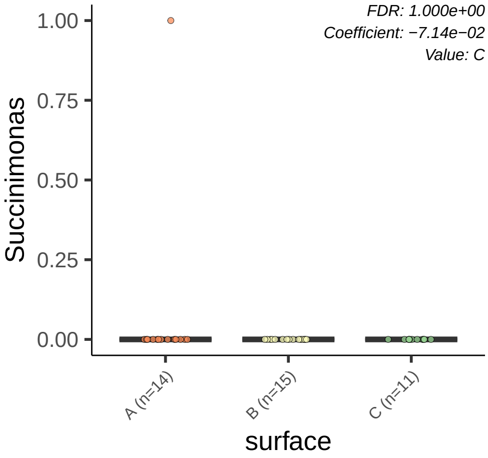
FDR: 1.000e+00
Coefficient: −7.14e−02
Value: C
Succinimonas
1.00
0.75
0.50
0.25
0.00
A (n=14)
B (n=15)
C (n=11)
surface

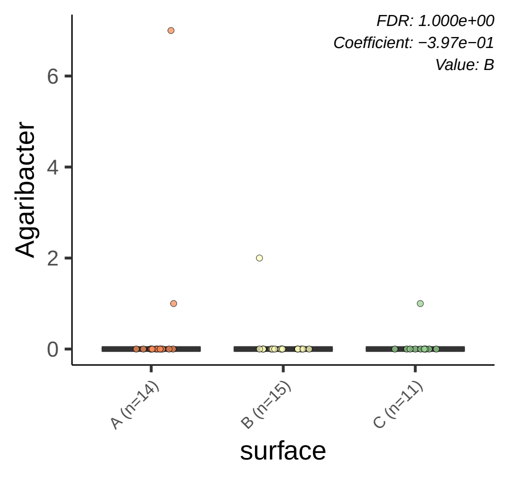

FDR: 1.000e+00
Coefficient: −3.97e−01
Value: B
Agaribacter
0
2
4
6
A (n=14)
B (n=15)
C (n=11)
surface

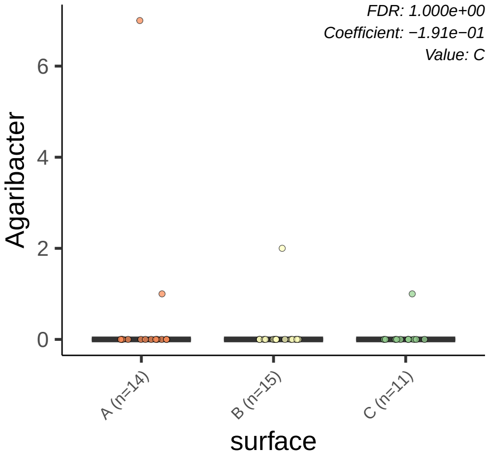
FDR: 1.000e+00
Coefficient: −1.91e−01
Value: C
Agaribacter
0
2
4
6
A (n=14)
B (n=15)
C (n=11)
surface

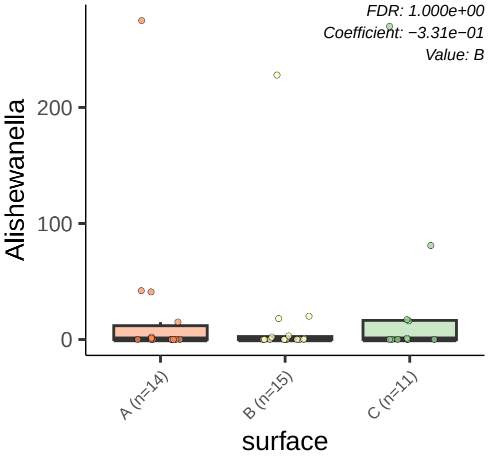
FDR: 1.000e+00
Coefficient: −3.31e−01
Value: B
Alishewanella
200
100
0
A (n=14)
B (n=15)
C (n=11)
surface

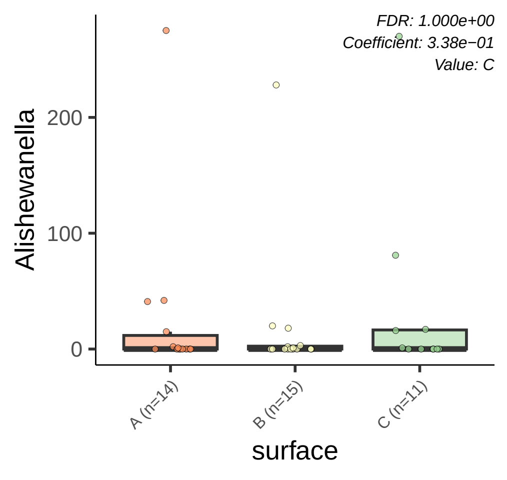
FDR: 1.000e+00
Coefficient: 3.38e−01
Value: C
Alishewanella
200
100
0
A (n=14)
B (n=15)
C (n=11)
surface

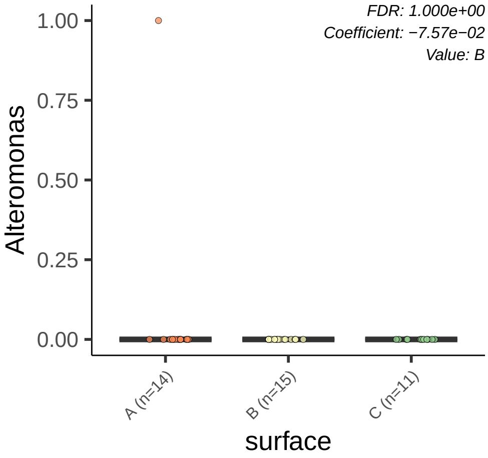
FDR: 1.000e+00
Coefficient: −7.57e−02
Value: B
1.00
0.75
0.50
0.25
0.00
Alteromonas
A (n=14)
B (n=15)
C (n=11)
surface

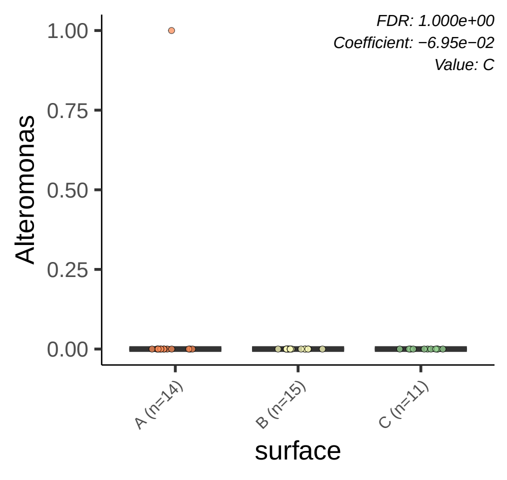
FDR: 1.000e+00
Coefficient: −6.95e−02
Value: C
1.00
0.75
0.50
0.25
0.00
Alteromonas
A (n=14)
B (n=15)
C (n=11)
surface

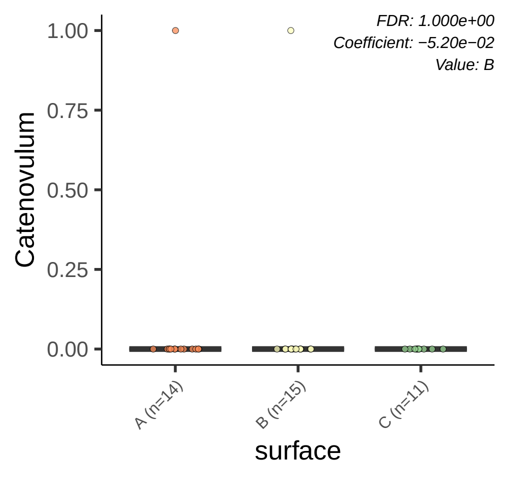

FDR: 1.000e+00
Coefficient: −5.20e−02
Value: B
Catenovulum
1.00
0.75
0.50
0.25
0.00
A (n=14)
B (n=15)
C (n=11)
surface

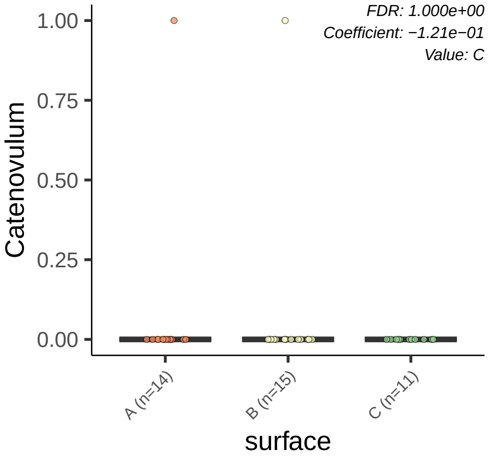
FDR: 1.000e+00
Coefficient: −1.21e−01
Value: C
Catenovulum
1.00
0.75
0.50
0.25
0.00
A (n=14)
B (n=15)
C (n=11)
surface

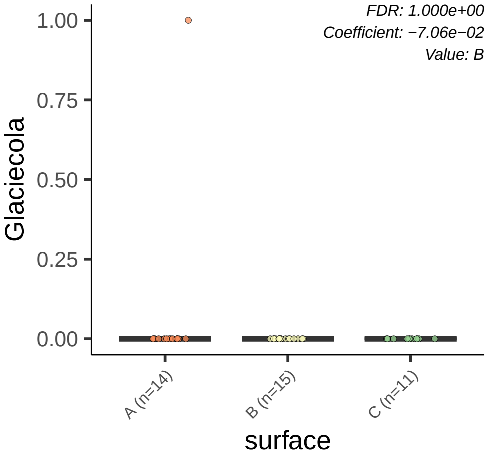

FDR: 1.000e+00
Coefficient: −7.06e−02
Value: B
Glaciecola
1.00
0.75
0.50
0.25
0.00
A (n=14)
B (n=15)
C (n=11)
surface

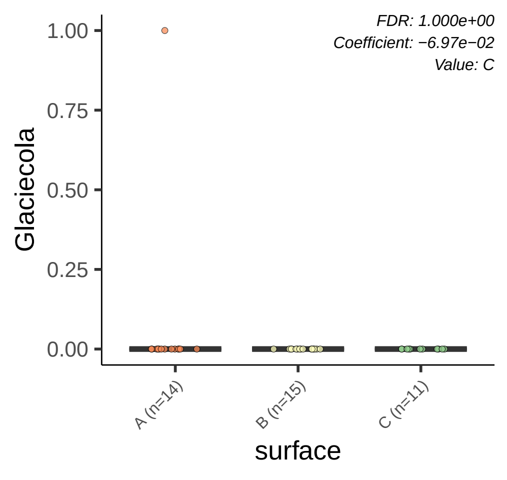
FDR: 1.000e+00
Coefficient: −6.97e−02
Value: C
Glaciecola
1.00
0.75
0.50
0.25
0.00
A (n=14)
B (n=15)
C (n=11)
surface

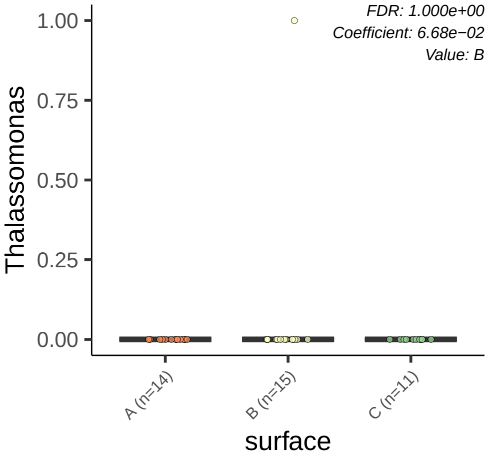

FDR: 1.000e+00
Coefficient: 6.68e−02
Value: B
Thalassomonas
1.00
0.75
0.50
0.25
0.00
A (n=14)
B (n=15)
C (n=11)
surface

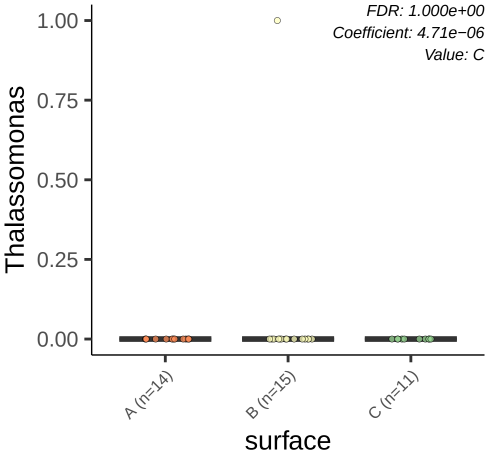

FDR: 1.000e+00
Coefficient: 4.71e−06
Value: C
Thalassomonas
1.00
0.75
0.50
0.25
0.00
A (n=14)
B (n=15)
C (n=11)
surface

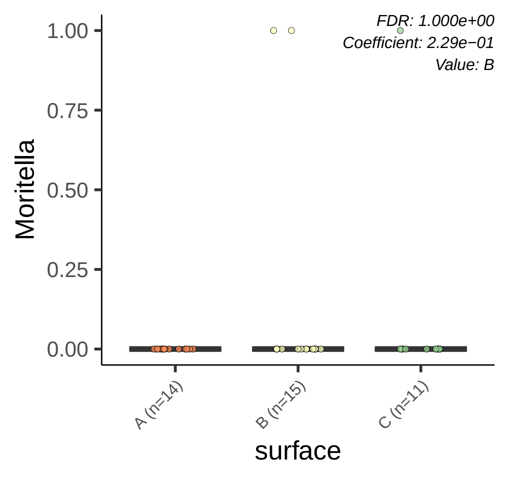
FDR: 1.000e+00
Coefficient: 2.29e−01
Value: B
Moritella
1.00
0.75
0.50
0.25
0.00
A (n=14)
B (n=15)
C (n=11)
surface

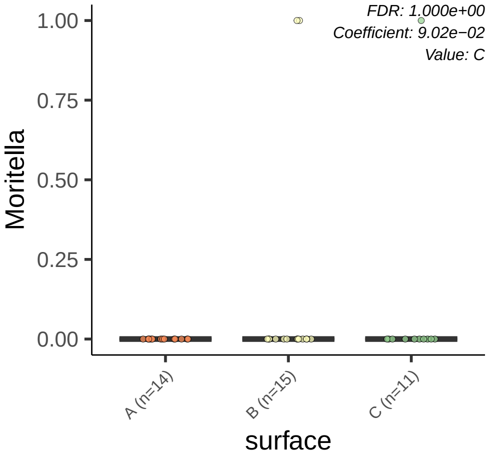
FDR: 1.000e+00
Coefficient: 9.02e−02
Value: C
Moritella
1.00
0.75
0.50
0.25
0.00
A (n=14)
B (n=15)
C (n=11)
surface

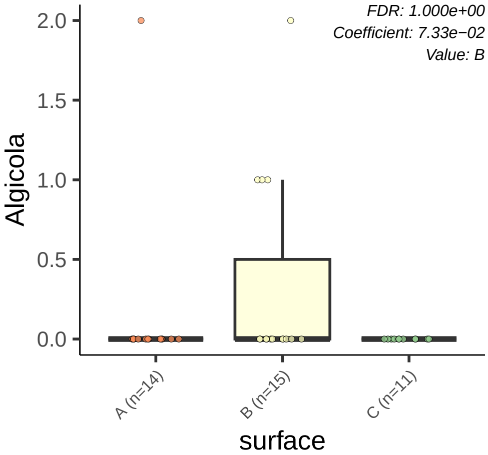

FDR: 1.000e+00
Coefficient: 7.33e−02
Value: B
Algicola
2.0
1.5
1.0
0.5
0.0
A (n=14)
B (n=15)
C (n=11)
surface

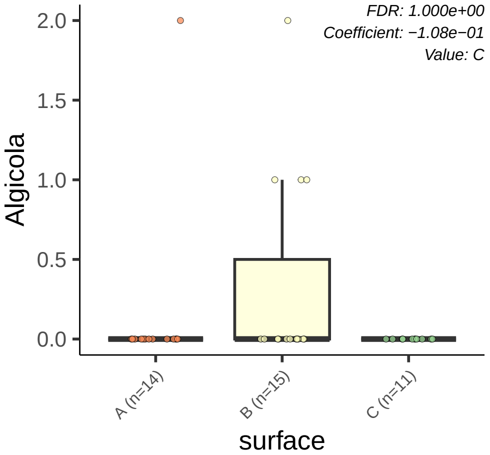
FDR: 1.000e+00
Coefficient: −1.08e−01
Value: C
Algicola
2.0
1.5
1.0
0.5
0.0
A (n=14)
B (n=15)
C (n=11)
surface

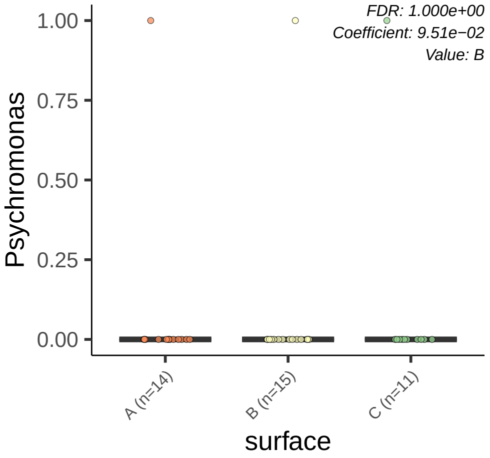
FDR: 1.000e+00
Coefficient: 9.51e−02
Value: B
Psychromonas
1.00
0.75
0.50
0.25
0.00
A (n=14)
B (n=15)
C (n=11)
surface

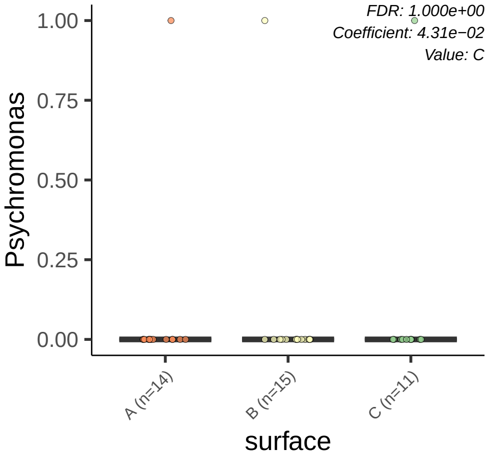

FDR: 1.000e+00
Coefficient: 4.31e−02
Value: C
Psychromonas
1.00
0.75
0.50
0.25
0.00
A (n=14)
B (n=15)
C (n=11)
surface

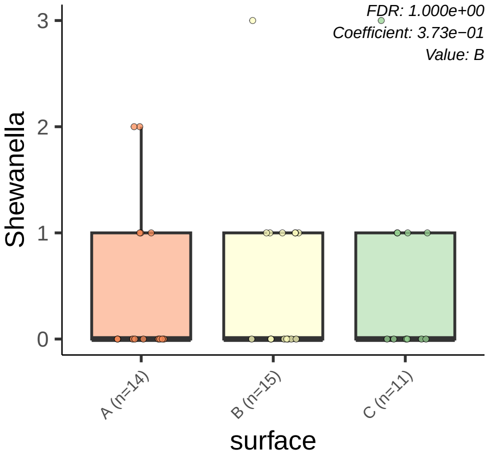
FDR: 1.000e+00
Coefficient: 3.73e−01
Value: B
Shewanella
0
1
2
3
A (n=14)
B (n=15)
C (n=11)
surface

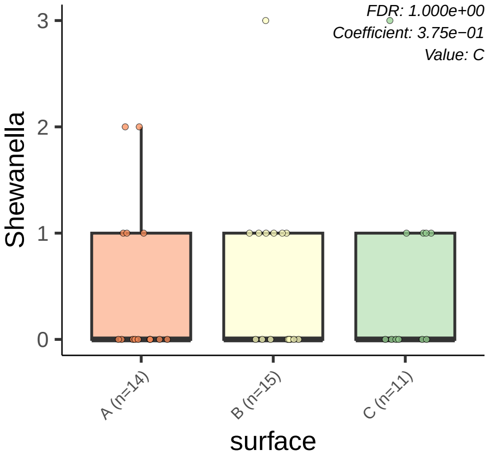
FDR: 1.000e+00
Coefficient: 3.75e−01
Value: C
Shewanella
3
2
1
0
A (n=14)
B (n=15)
C (n=11)
surface

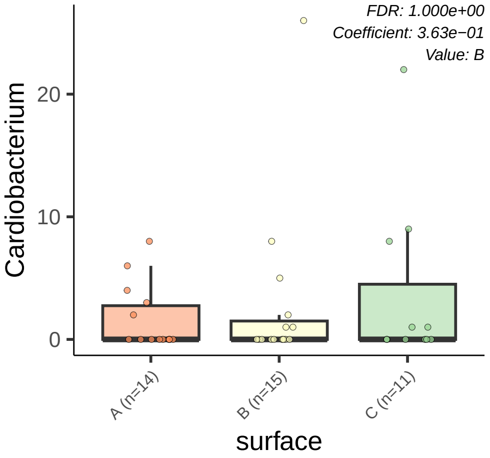
FDR: 1.000e+00
Coefficient: 3.63e−01
Value: B
Cardiobacterium
20
10
0
A (n=14)
B (n=15)
C (n=11)
surface

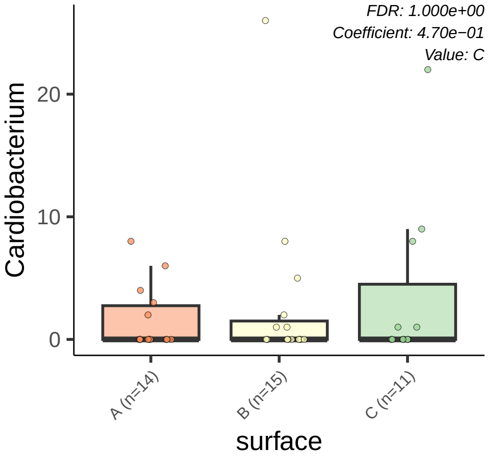
FDR: 1.000e+00
Coefficient: 4.70e−01
Value: C
Cardiobacterium
20
10
0
A (n=14)
B (n=15)
C (n=11)
surface

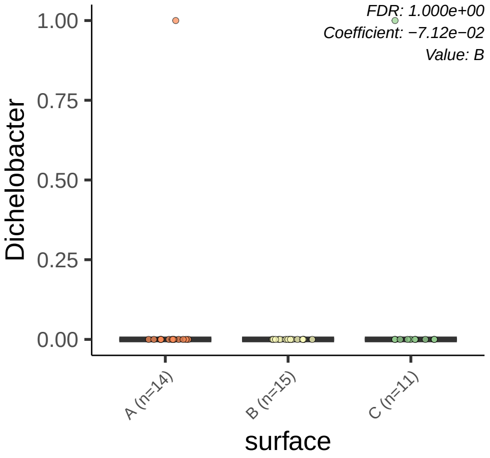
FDR: 1.000e+00
Coefficient: −7.12e−02
Value: B
Dichelobacter
1.00
0.75
0.50
0.25
0.00
A (n=14)
B (n=15)
C (n=11)
surface

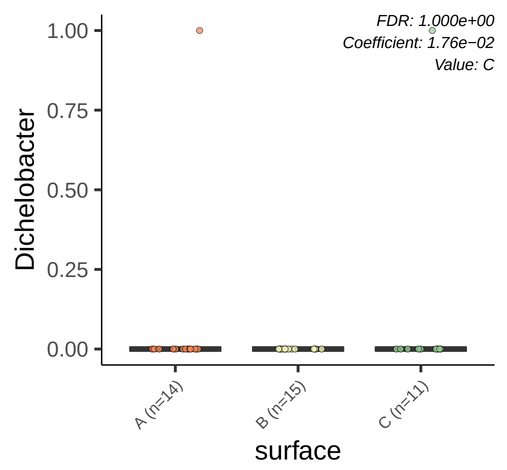
FDR: 1.000e+00
Coefficient: 1.76e−02
Value: C
Dichelobacter
1.00
0.75
0.50
0.25
0.00
A (n=14)
B (n=15)
C (n=11)
surface

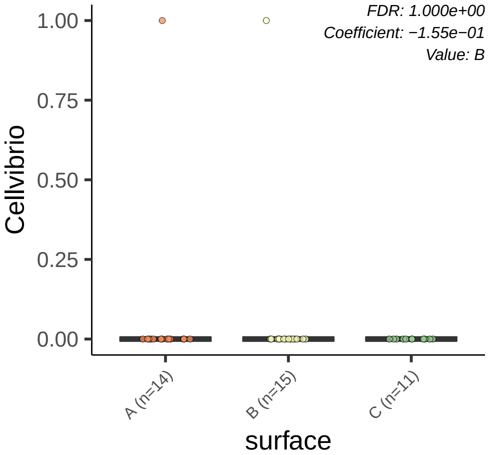

FDR: 1.000e+00
Coefficient: −1.55e−01
Value: B
Cellvibrio
1.00
0.75
0.50
0.25
0.00
A (n=14)
B (n=15)
C (n=11)
surface

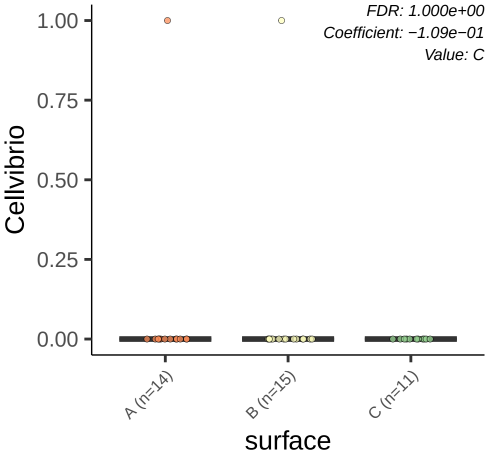
FDR: 1.000e+00
Coefficient: −1.09e−01
Value: C
Cellvibrio
1.00
0.75
0.50
0.25
0.00
A (n=14)
B (n=15)
C (n=11)
surface

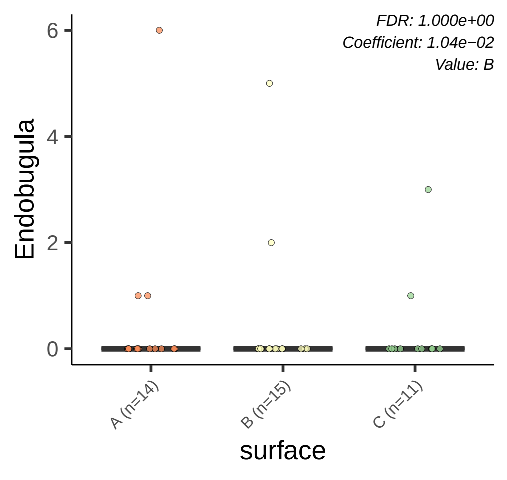
FDR: 1.000e+00
Coefficient: 1.04e−02
Value: B
Endobugula
0
2
4
6
A (n=14)
B (n=15)
C (n=11)
surface

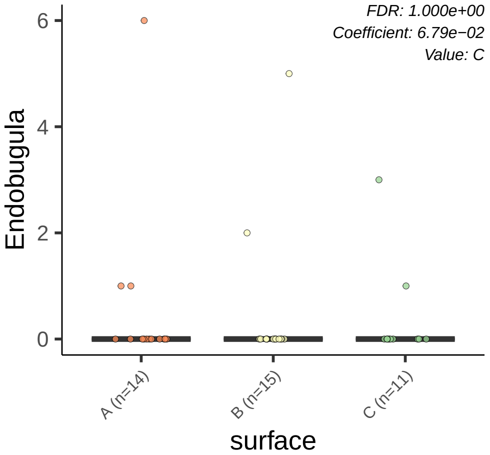
FDR: 1.000e+00
Coefficient: 6.79e−02
Value: C
Endobugula
0
2
4
6
A (n=14)
B (n=15)
C (n=11)
surface

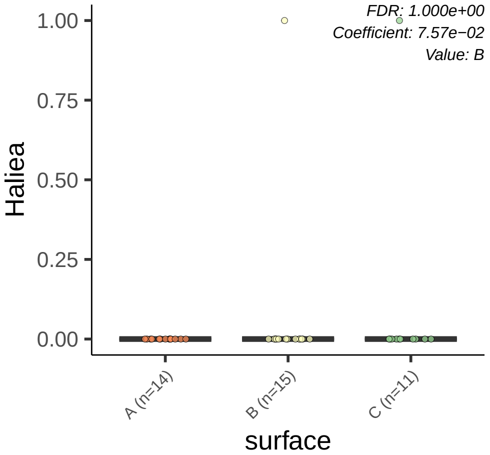
FDR: 1.000e+00
Coefficient: 7.57e−02
Value: B
Haliea
1.00
0.75
0.50
0.25
0.00
A (n=14)
B (n=15)
C (n=11)
surface

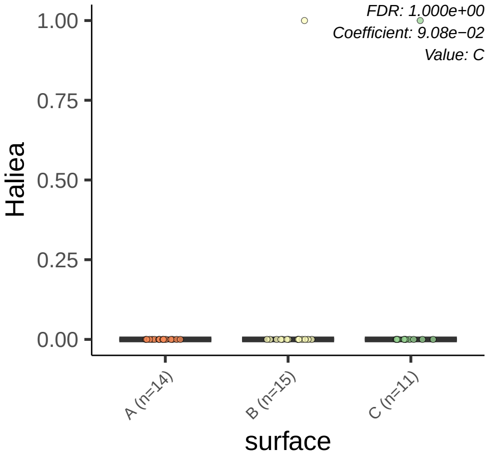

FDR: 1.000e+00
Coefficient: 9.08e−02
Value: C
1.00
0.75
0.50
0.25
0.00
Haliea
A (n=14)
B (n=15)
C (n=11)
surface

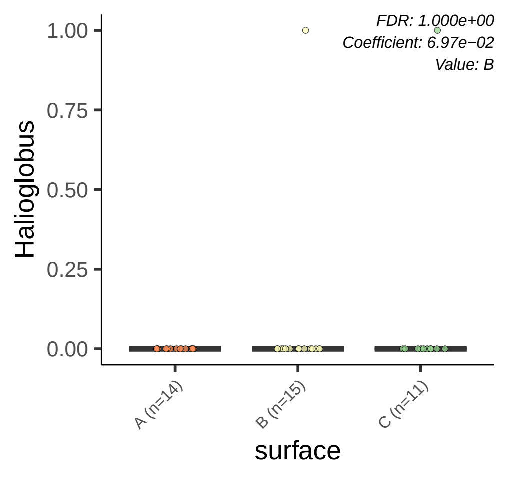
FDR: 1.000e+00
Coefficient: 6.97e−02
Value: B
Halioglobus
1.00
0.75
0.50
0.25
0.00
A (n=14)
B (n=15)
C (n=11)
surface

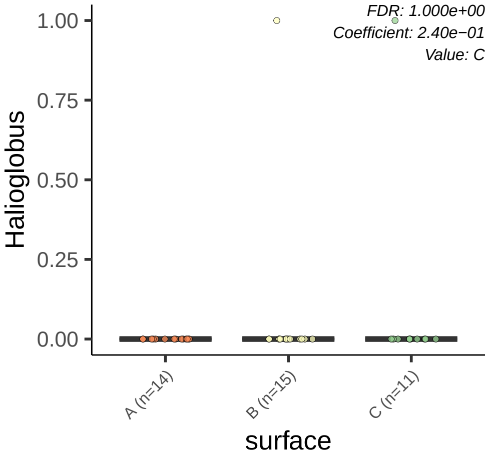

FDR: 1.000e+00
Coefficient: 2.40e−01
Value: C
Halioglobus
1.00
0.75
0.50
0.25
0.00
A (n=14)
B (n=15)
C (n=11)
surface

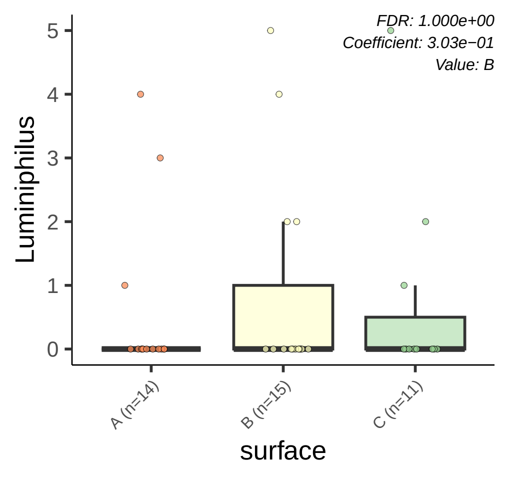

FDR: 1.000e+00
Coefficient: 3.03e−01
Value: B
Luminiphilus
0
1
2
3
4
5
A (n=14)
B (n=15)
C (n=11)
surface

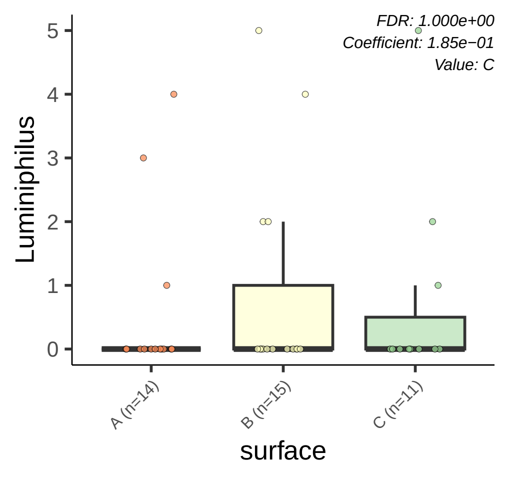
FDR: 1.000e+00
Coefficient: 1.85e−01
Value: C
Luminiphilus
0
1
2
3
4
5
A (n=14)
B (n=15)
C (n=11)
surface

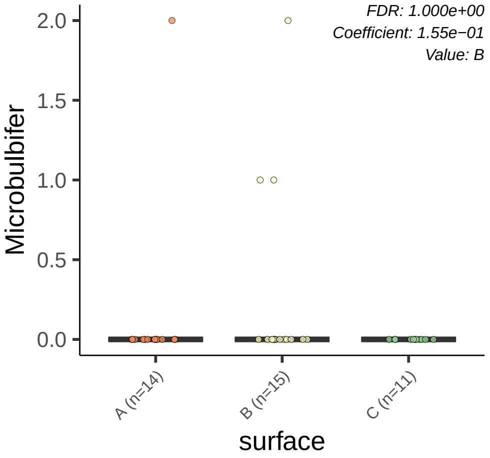

FDR: 1.000e+00
Coefficient: 1.55e−01
Value: B
Microbulbifer
2.0
1.5
1.0
0.5
0.0
A (n=14)
B (n=15)
C (n=11)
surface

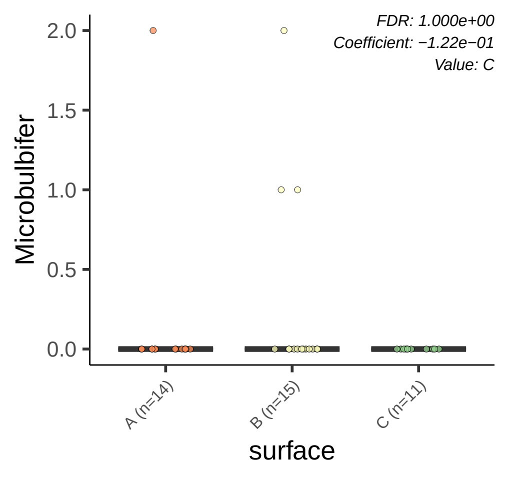
FDR: 1.000e+00
Coefficient: −1.22e−01
Value: C
Microbulbifer
2.0
1.5
1.0
0.5
0.0
A (n=14)
B (n=15)
C (n=11)
surface

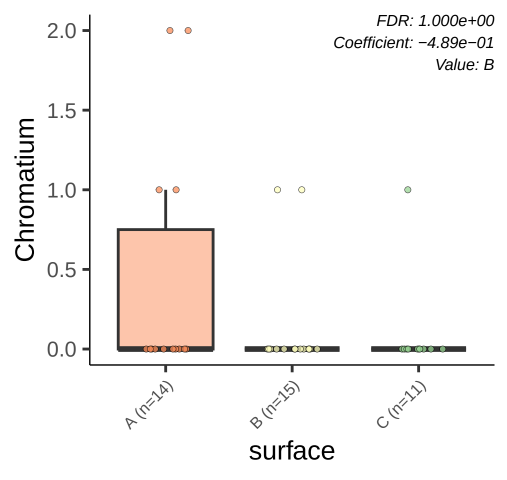
FDR: 1.000e+00
Coefficient: −4.89e−01
Value: B
Chromatium
2.0
1.5
1.0
0.5
0.0
A (n=14)
B (n=15)
C (n=11)
surface

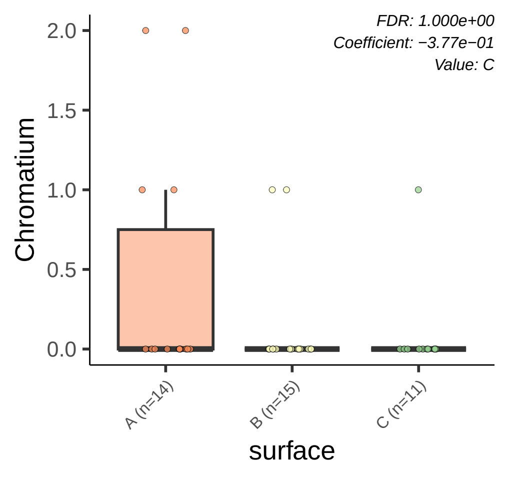
FDR: 1.000e+00
Coefficient: −3.77e−01
Value: C
Chromatium
2.0
1.5
1.0
0.5
0.0
A (n=14)
B (n=15)
C (n=11)
surface

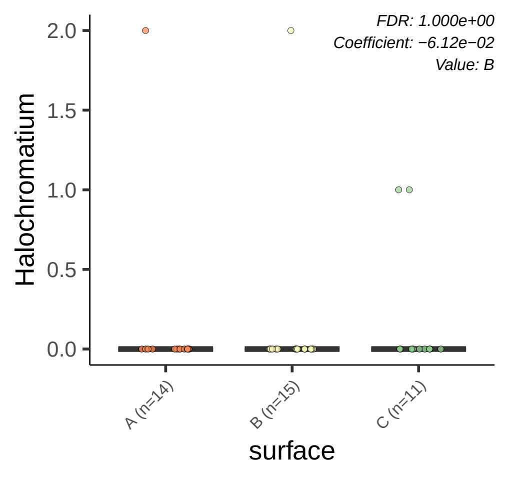
FDR: 1.000e+00
Coefficient: −6.12e−02
Value: B
Halochromatium
2.0
1.5
1.0
0.5
0.0
A (n=14)
B (n=15)
C (n=11)
surface

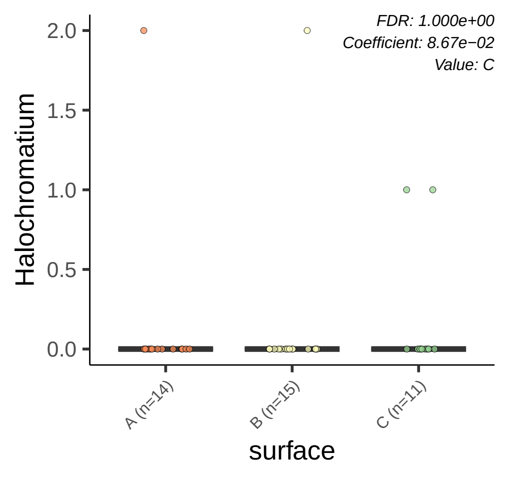

FDR: 1.000e+00
Coefficient: 8.67e−02
Value: C
Halochromatium
2.0
1.5
1.0
0.5
0.0
A (n=14)
B (n=15)
C (n=11)
surface

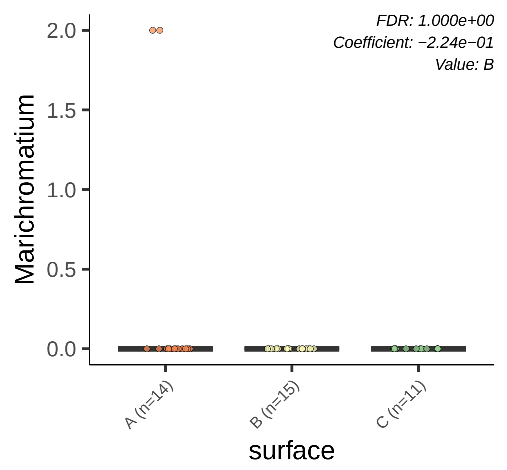
FDR: 1.000e+00
Coefficient: −2.24e−01
Value: B
Marichromatium
2.0
1.5
1.0
0.5
0.0
A (n=14)
B (n=15)
C (n=11)
surface

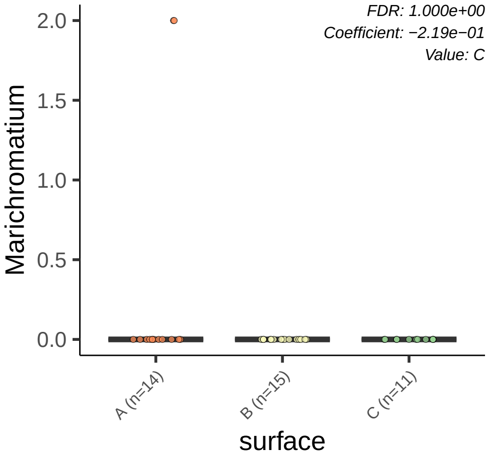

FDR: 1.000e+00
Coefficient: −2.19e−01
Value: C
Marichromatium
2.0
1.5
1.0
0.5
0.0
A (n=14)
B (n=15)
C (n=11)
surface

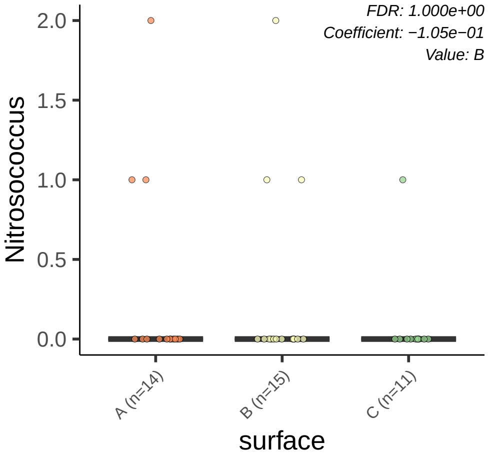
FDR: 1.000e+00
Coefficient: −1.05e−01
Value: B
Nitrosococcus
2.0
1.5
1.0
0.5
0.0
A (n=14)
B (n=15)
C (n=11)
surface

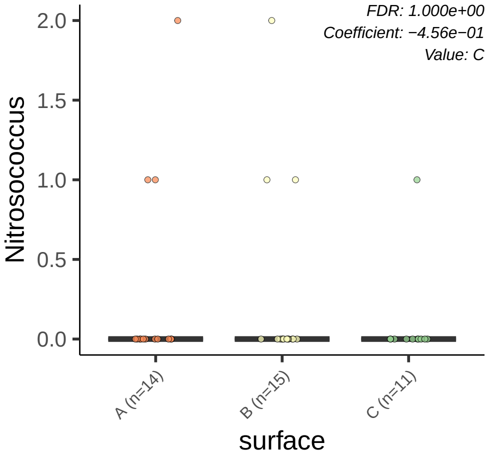
FDR: 1.000e+00
Coefficient: −4.56e−01
Value: C
Nitrosococcus
2.0
1.5
1.0
0.5
0.0
A (n=14)
B (n=15)
C (n=11)
surface

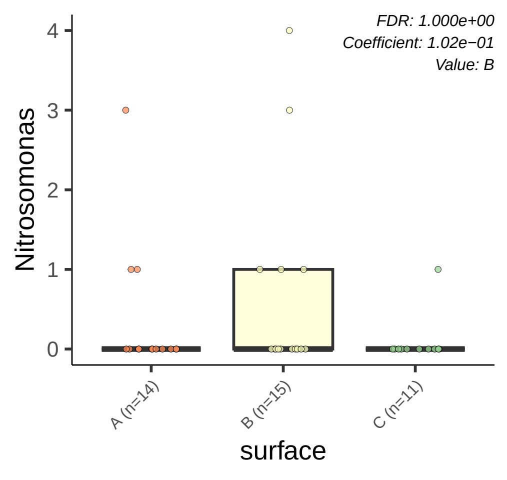
FDR: 1.000e+00
Coefficient: 1.02e−01
Value: B
Nitrosomonas
4
3
2
1
0
A (n=14)
B (n=15)
C (n=11)
surface

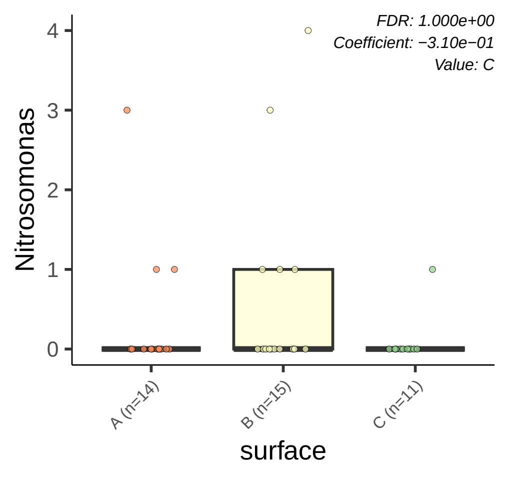

FDR: 1.000e+00
Coefficient: −3.10e−01
Value: C
Nitrosomonas
4
3
2
1
0
A (n=14)
B (n=15)
C (n=11)
surface

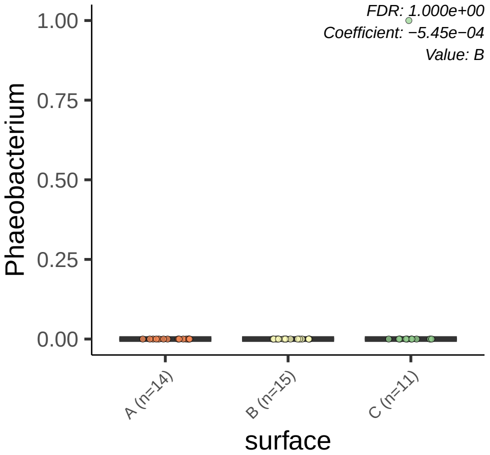
FDR: 1.000e+00
Coefficient: −5.45e−04
Value: B
Phaeobacterium
1.00
0.75
0.50
0.25
0.00
A (n=14)
B (n=15)
C (n=11)
surface

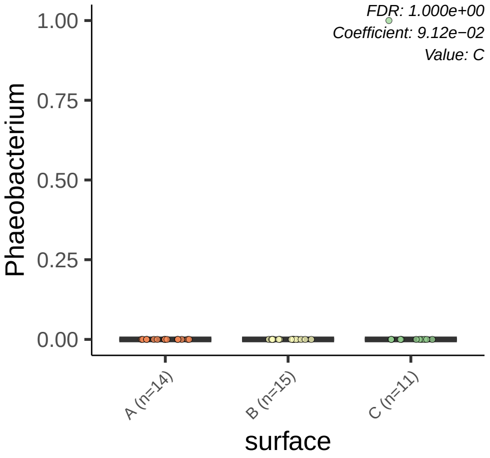
FDR: 1.000e+00
Coefficient: 9.12e−02
Value: C
Phaeobacterium
1.00
0.75
0.50
0.25
0.00
A (n=14)
B (n=15)
C (n=11)
surface

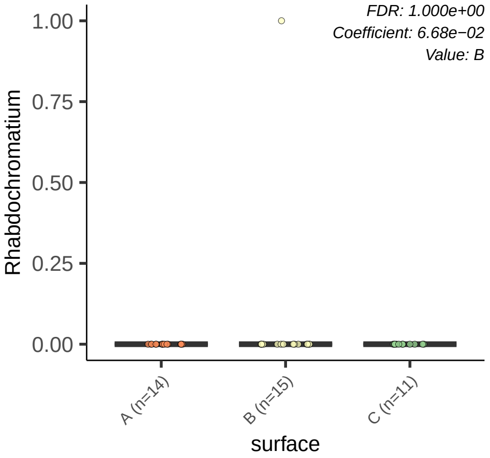
FDR: 1.000e+00
Coefficient: 6.68e−02
Value: B
Rhabdochromatium
1.00
0.75
0.50
0.25
0.00
A (n=14)
B (n=15)
C (n=11)
surface

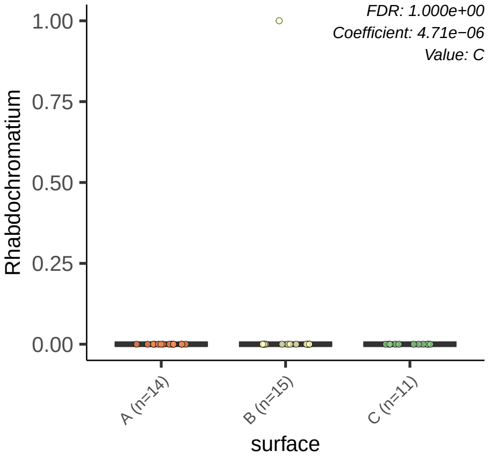

FDR: 1.000e+00
Coefficient: 4.71e−06
Value: C
Rhabdochromatium
1.00
0.75
0.50
0.25
0.00
A (n=14)
B (n=15)
C (n=11)
surface

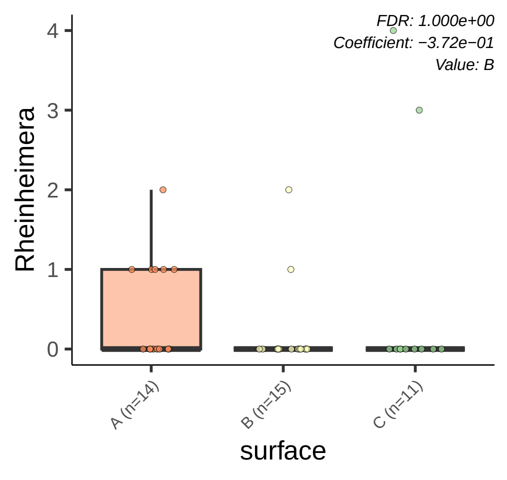
FDR: 1.000e+00
Coefficient: −3.72e−01
Value: B
Rheinheimera
0
1
2
3
4
A (n=14)
B (n=15)
C (n=11)
surface

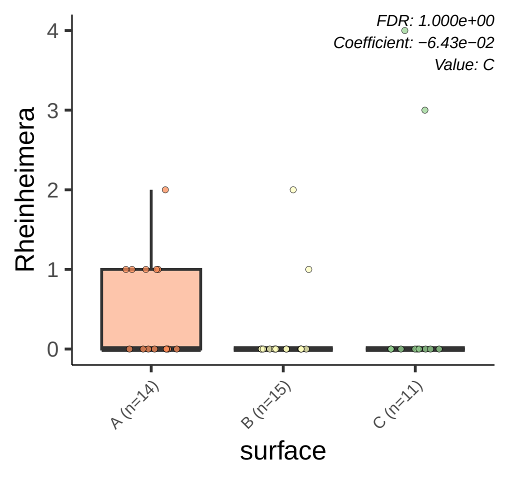

FDR: 1.000e+00
Coefficient: −6.43e−02
Value: C
Rheinheimera
0
1
2
3
4
A (n=14)
B (n=15)
C (n=11)
surface

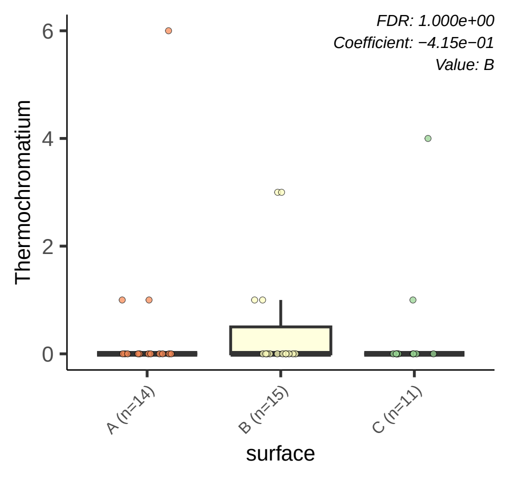

FDR: 1.000e+00
Coefficient: −4.15e−01
Value: B
Thermochromatium
6
4
2
0
A (n=14)
B (n=15)
C (n=11)
surface

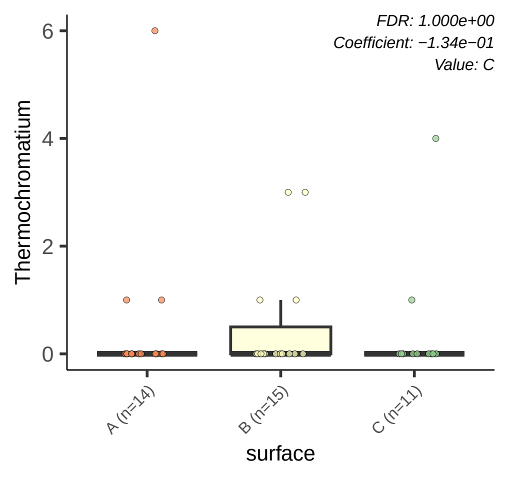

FDR: 1.000e+00
Coefficient: −1.34e−01
Value: C
Thermochromatium
6
4
2
0
A (n=14)
B (n=15)
C (n=11)
surface

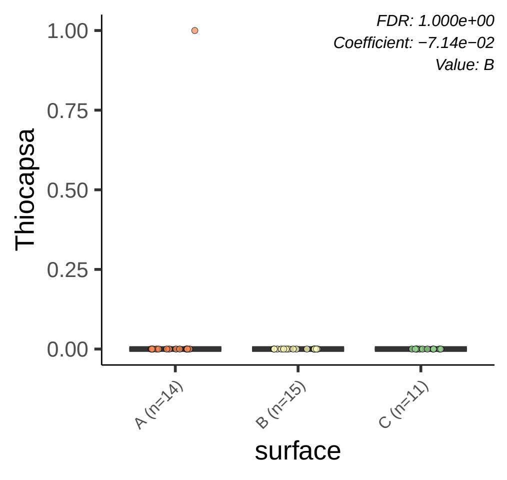
FDR: 1.000e+00
Coefficient: −7.14e−02
Value: B
Thiocapsa
1.00
0.75
0.50
0.25
0.00
A (n=14)
B (n=15)
C (n=11)
surface

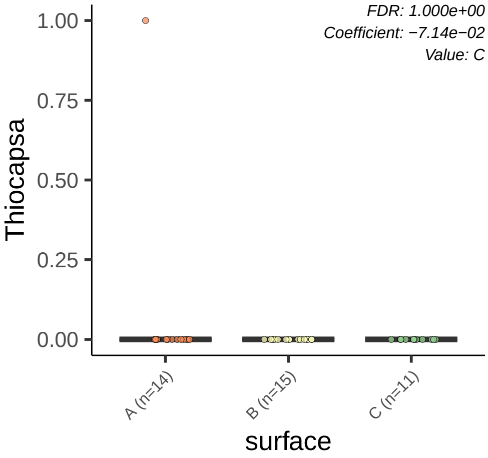
FDR: 1.000e+00
Coefficient: −7.14e−02
Value: C
Thiocapsa
1.00
0.75
0.50
0.25
0.00
A (n=14)
B (n=15)
C (n=11)
surface

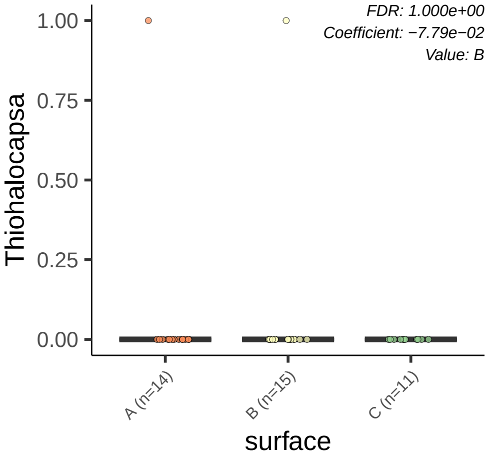

FDR: 1.000e+00
Coefficient: −7.79e−02
Value: B
Thiohalocapsa
1.00
0.75
0.50
0.25
0.00
A (n=14)
B (n=15)
C (n=11)
surface

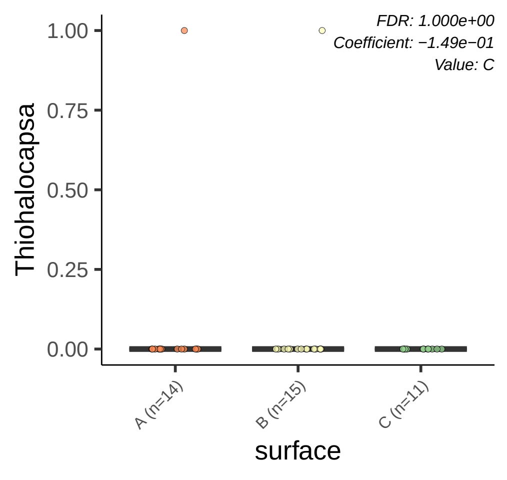
FDR: 1.000e+00
Coefficient: −1.49e−01
Value: C
Thiohalocapsa
1.00
0.75
0.50
0.25
0.00
A (n=14)
B (n=15)
C (n=11)
surface

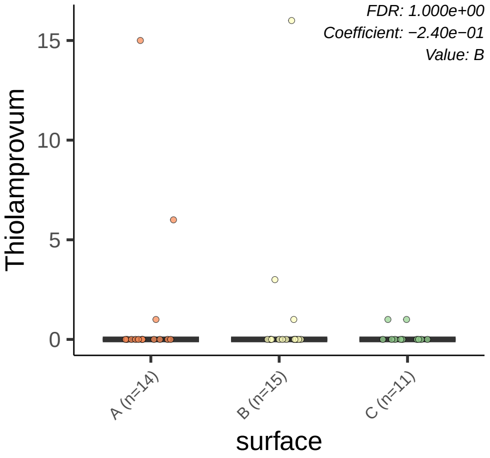
FDR: 1.000e+00
Coefficient: −2.40e−01
Value: B
Thiolamprovum
0
5
10
15
A (n=14)
B (n=15)
C (n=11)
surface

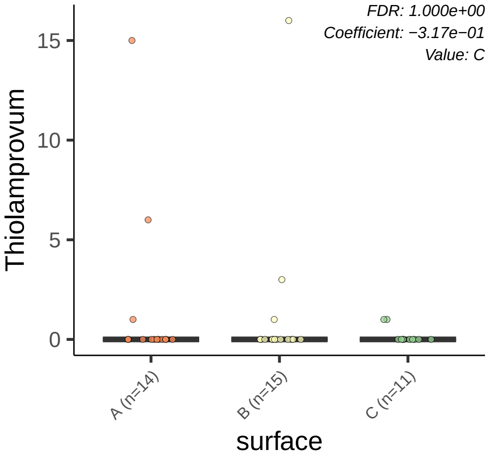

FDR: 1.000e+00
Coefficient: −3.17e−01
Value: C
Thiolamprovum
15
10
5
0
A (n=14)
B (n=15)
C (n=11)
surface

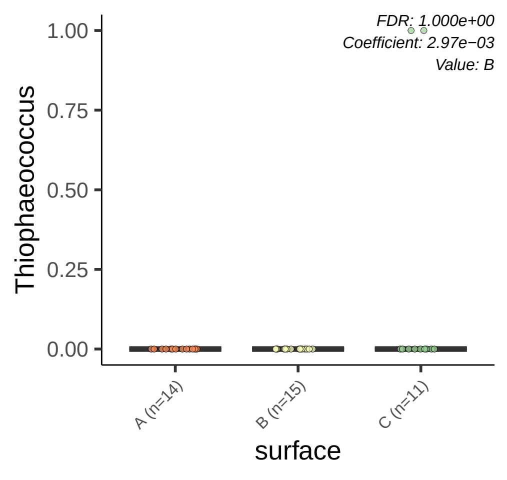

FDR: 1.000e+00
Coefficient: 2.97e−03
Value: B
Thiophaeococcus
1.00
0.75
0.50
0.25
0.00
A (n=14)
B (n=15)
C (n=11)
surface

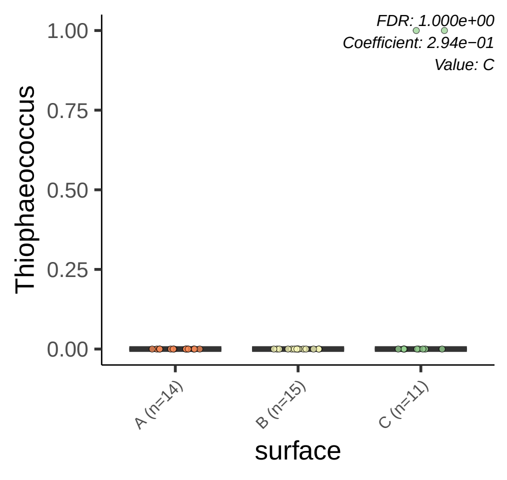

FDR: 1.000e+00
Coefficient: 2.94e−01
Value: C
Thiophaeococcus
1.00
0.75
0.50
0.25
0.00
A (n=14)
B (n=15)
C (n=11)
surface

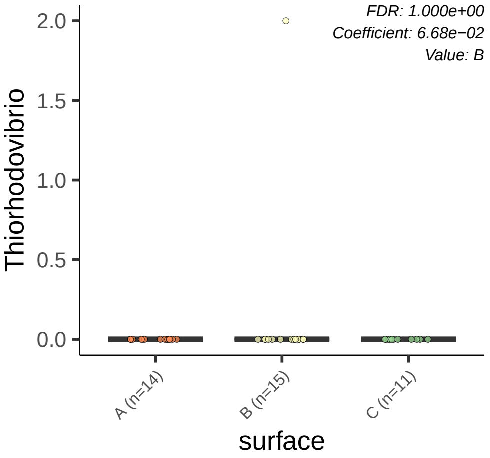
FDR: 1.000e+00
Coefficient: 6.68e−02
Value: B
Thiorhodovibrio
2.0
1.5
1.0
0.5
0.0
A (n=14)
B (n=15)
C (n=11)
surface

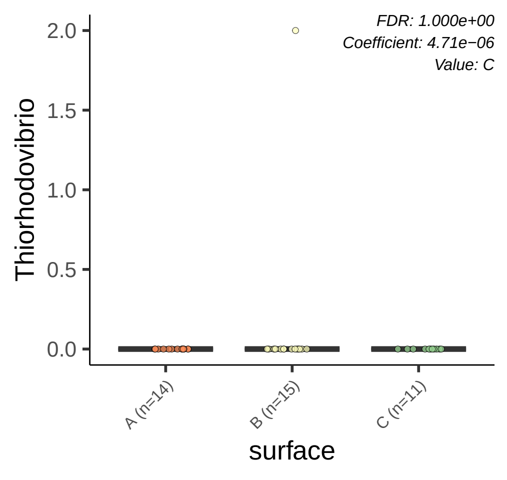
FDR: 1.000e+00
Coefficient: 4.71e−06
Value: C
Thiorhodovibrio
2.0
1.5
1.0
0.5
0.0
A (n=14)
B (n=15)
C (n=11)
surface

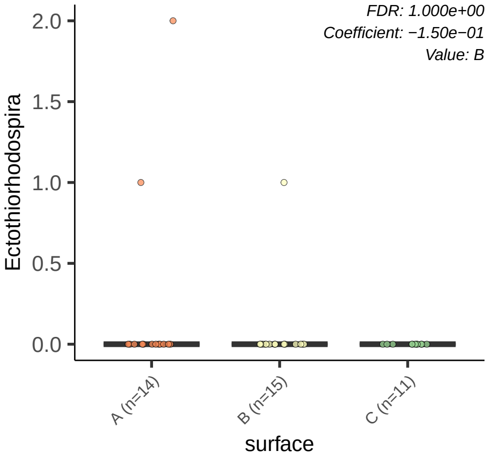
FDR: 1.000e+00
Coefficient: −1.50e−01
Value: B
Ectothiorhodospira
2.0
1.5
1.0
0.5
0.0
A (n=14)
B (n=15)
C (n=11)
surface

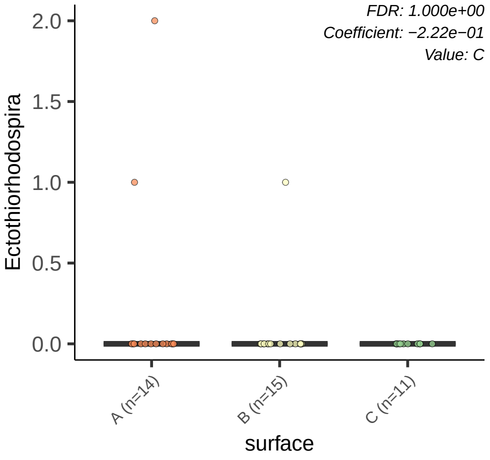
FDR: 1.000e+00
Coefficient: −2.22e−01
Value: C
Ectothiorhodospira
2.0
1.5
1.0
0.5
0.0
A (n=14)
B (n=15)
C (n=11)
surface

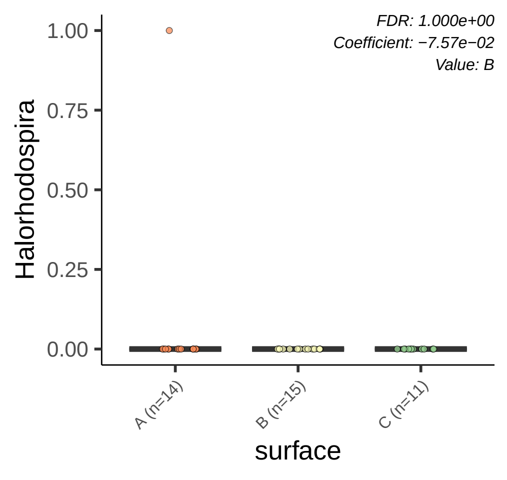

FDR: 1.000e+00
Coefficient: −7.57e−02
Value: B
Halorhodospira
1.00
0.75
0.50
0.25
0.00
A (n=14)
B (n=15)
C (n=11)
surface

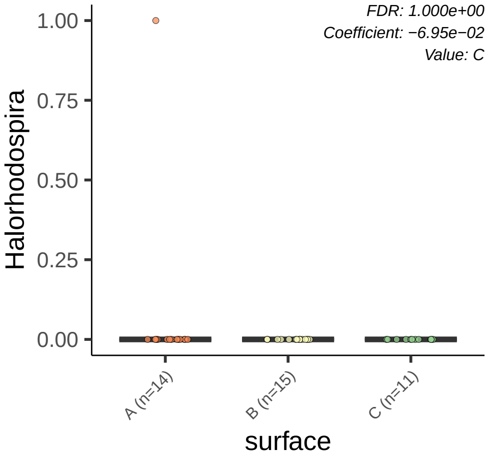
FDR: 1.000e+00
Coefficient: −6.95e−02
Value: C
Halorhodospira
1.00
0.75
0.50
0.25
0.00
A (n=14)
B (n=15)
C (n=11)
surface

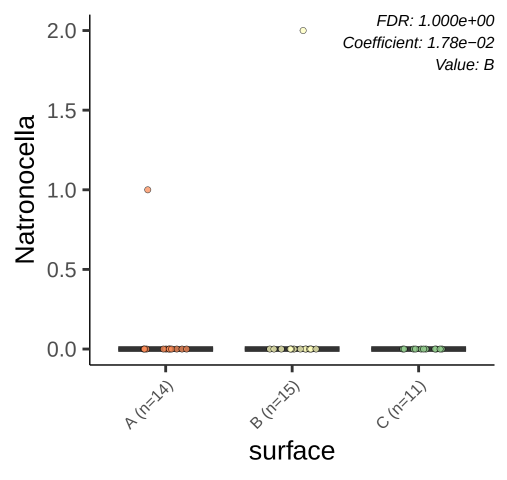
FDR: 1.000e+00
Coefficient: 1.78e−02
Value: B
2.0
1.5
1.0
0.5
0.0
Natronocella
A (n=14)
B (n=15)
C (n=11)
surface

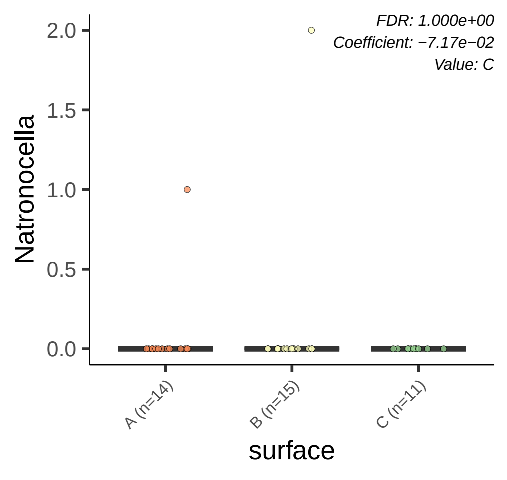
FDR: 1.000e+00
Coefficient: −7.17e−02
Value: C
Natronocella
2.0
1.5
1.0
0.5
0.0
A (n=14)
B (n=15)
C (n=11)
surface

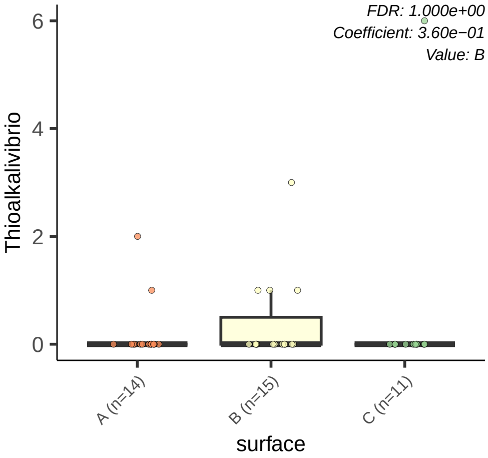
FDR: 1.000e+00
Coefficient: 3.60e−01
Value: B
Thioalkalivibrio
6
4
2
0
A (n=14)
B (n=15)
C (n=11)
surface

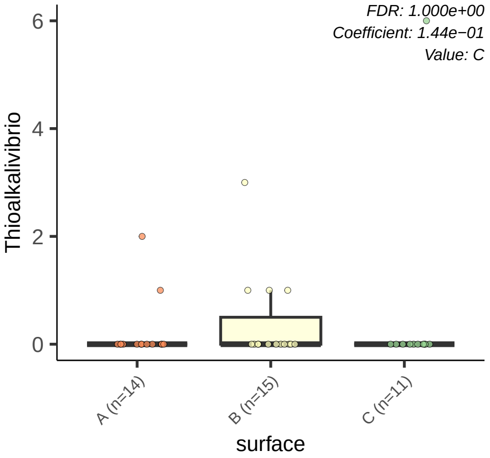

FDR: 1.000e+00
Coefficient: 1.44e−01
Value: C
Thioalkalivibrio
0
2
4
6
A (n=14)
B (n=15)
C (n=11)
surface

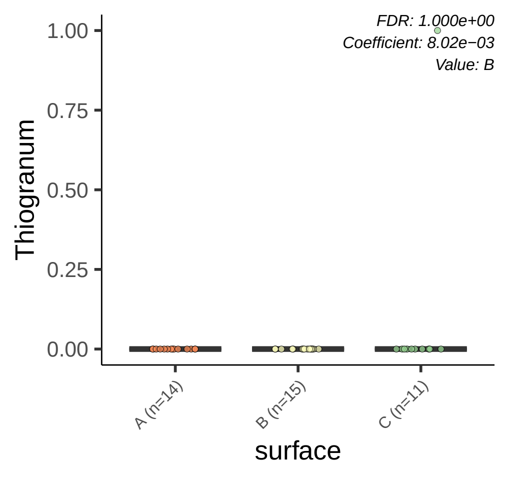
FDR: 1.000e+00
Coefficient: 8.02e−03
Value: B
1.00
0.75
0.50
0.25
0.00
Thiogranum
A (n=14)
B (n=15)
C (n=11)
surface

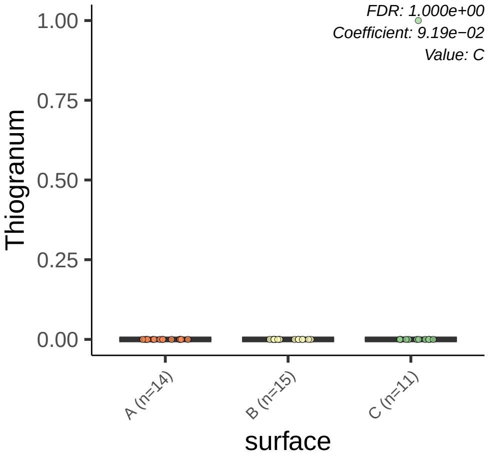
FDR: 1.000e+00
Coefficient: 9.19e−02
Value: C
Thiogranum
1.00
0.75
0.50
0.25
0.00
A (n=14)
B (n=15)
C (n=11)
surface

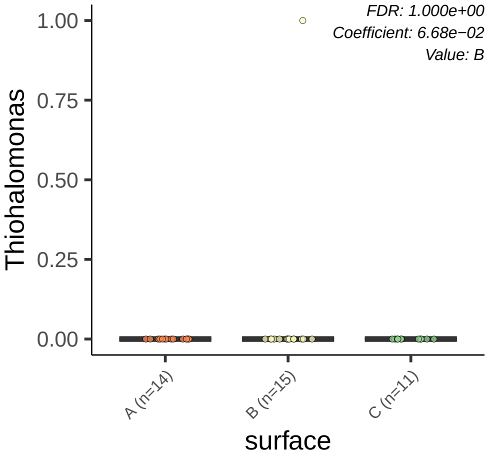
FDR: 1.000e+00
Coefficient: 6.68e−02
Value: B
Thiohalomonas
1.00
0.75
0.50
0.25
0.00
A (n=14)
B (n=15)
C (n=11)
surface

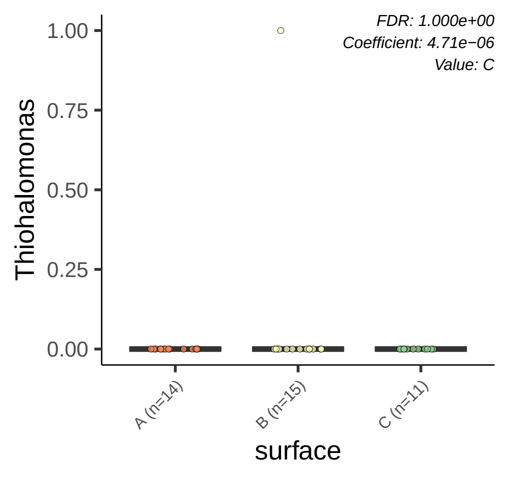
FDR: 1.000e+00
Coefficient: 4.71e−06
Value: C
Thiohalomonas
1.00
0.75
0.50
0.25
0.00
A (n=14)
B (n=15)
C (n=11)
surface

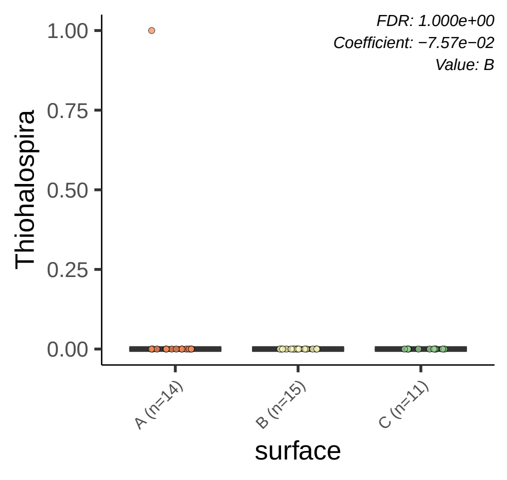

FDR: 1.000e+00
Coefficient: −7.57e−02
Value: B
Thiohalospira
1.00
0.75
0.50
0.25
0.00
A (n=14)
B (n=15)
C (n=11)
surface

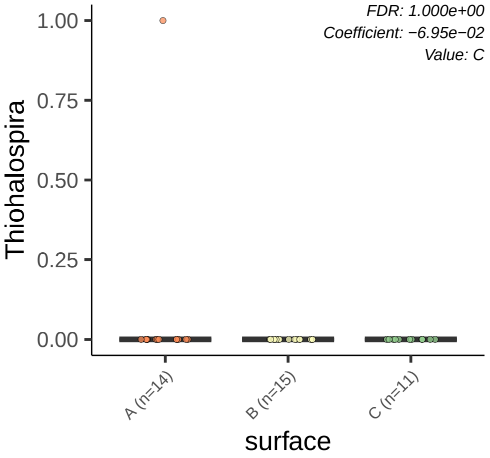
FDR: 1.000e+00
Coefficient: −6.95e−02
Value: C
1.00
0.75
0.50
0.25
0.00
Thiohalospira
A (n=14)
B (n=15)
C (n=11)
surface

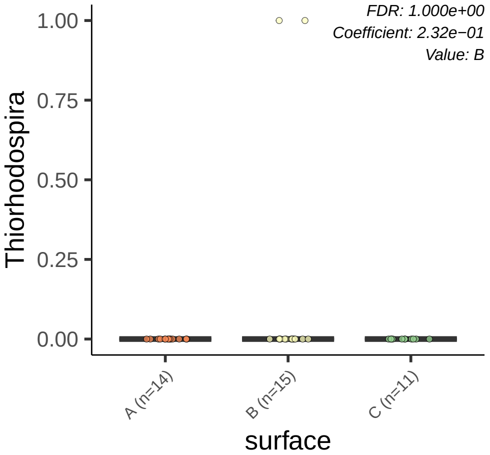

FDR: 1.000e+00
Coefficient: 2.32e−01
Value: B
Thiorhodospira
1.00
0.75
0.50
0.25
0.00
A (n=14)
B (n=15)
C (n=11)
surface

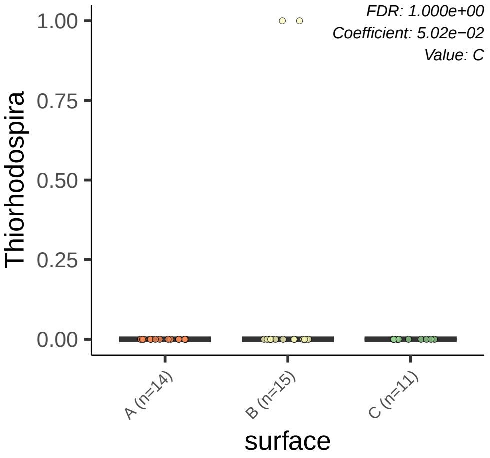

FDR: 1.000e+00
Coefficient: 5.02e−02
Value: C
Thiorhodospira
1.00
0.75
0.50
0.25
0.00
A (n=14)
B (n=15)
C (n=11)
surface

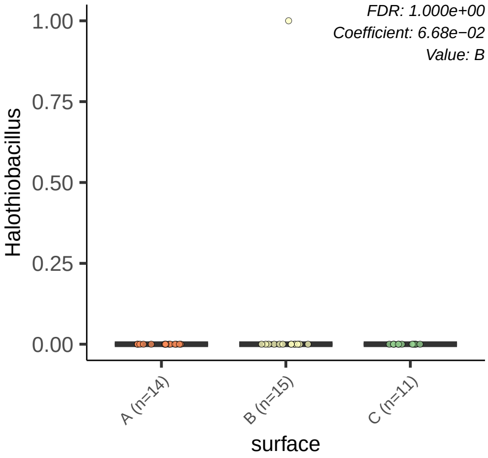
FDR: 1.000e+00
Coefficient: 6.68e−02
Value: B
Halothiobacillus
1.00
0.75
0.50
0.25
0.00
A (n=14)
B (n=15)
C (n=11)
surface

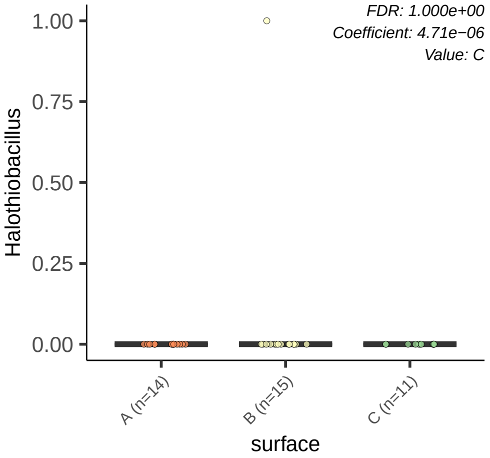

FDR: 1.000e+00
Coefficient: 4.71e−06
Value: C
Halothiobacillus
1.00
0.75
0.50
0.25
0.00
A (n=14)
B (n=15)
C (n=11)
surface

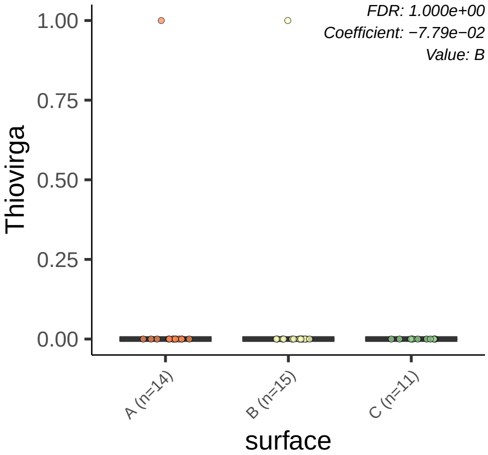
FDR: 1.000e+00
Coefficient: −7.79e−02
Value: B
Thiovirga
1.00
0.75
0.50
0.25
0.00
A (n=14)
B (n=15)
C (n=11)
surface

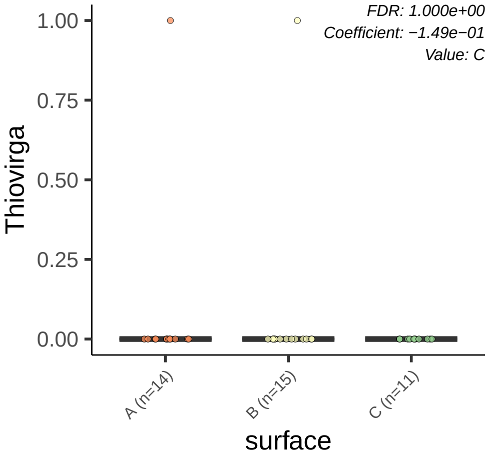

FDR: 1.000e+00
Coefficient: −1.49e−01
Value: C
Thiovirga
1.00
0.75
0.50
0.25
0.00
A (n=14)
B (n=15)
C (n=11)
surface

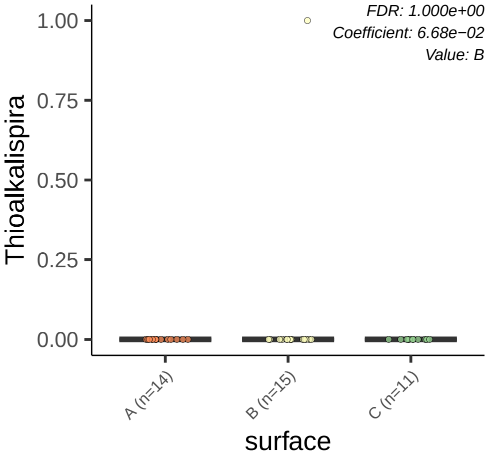
FDR: 1.000e+00
Coefficient: 6.68e−02
Value: B
Thioalkalispira
1.00
0.75
0.50
0.25
0.00
A (n=14)
B (n=15)
C (n=11)
surface

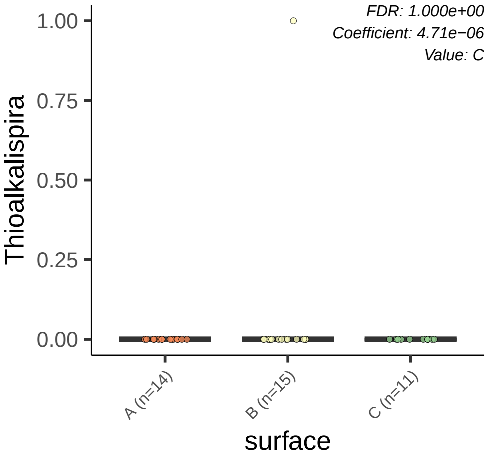
FDR: 1.000e+00
Coefficient: 4.71e−06
Value: C
Thioalkalispira
1.00
0.75
0.50
0.25
0.00
A (n=14)
B (n=15)
C (n=11)
surface

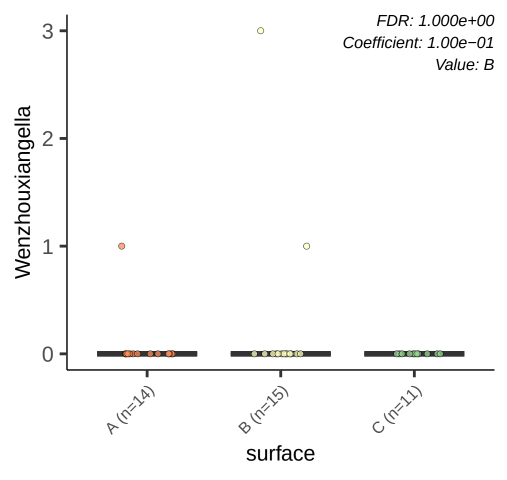

FDR: 1.000e+00
Coefficient: 1.00e−01
Value: B
Wenzhouxiangella
0
1
2
3
A (n=14)
B (n=15)
C (n=11)
surface

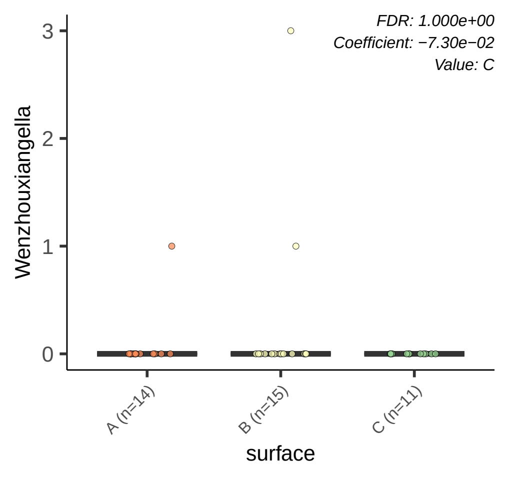
FDR: 1.000e+00
Coefficient: −7.30e−02
Value: C
Wenzhouxiangella
3
2
1
0
A (n=14)
B (n=15)
C (n=11)
surface

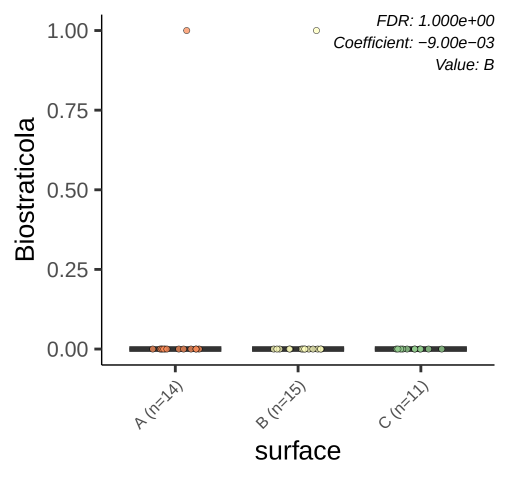
FDR: 1.000e+00
Coefficient: −9.00e−03
Value: B
Biostraticola
1.00
0.75
0.50
0.25
0.00
A (n=14)
B (n=15)
C (n=11)
surface

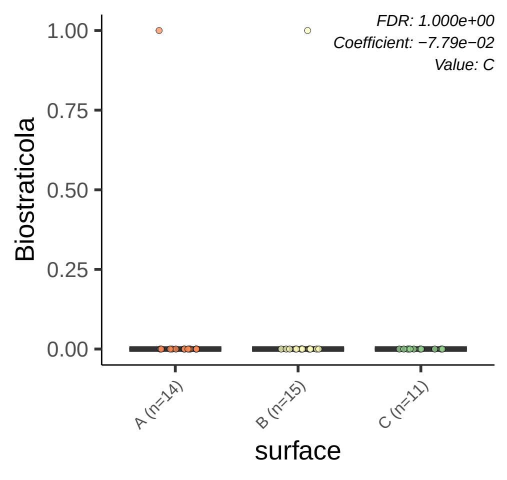
FDR: 1.000e+00
Coefficient: −7.79e−02
Value: C
Biostraticola
1.00
0.75
0.50
0.25
0.00
A (n=14)
B (n=15)
C (n=11)
surface

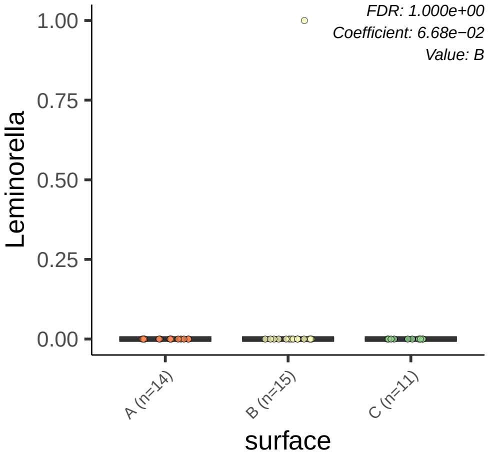
FDR: 1.000e+00
Coefficient: 6.68e−02
Value: B
Leminorella
1.00
0.75
0.50
0.25
0.00
A (n=14)
B (n=15)
C (n=11)
surface

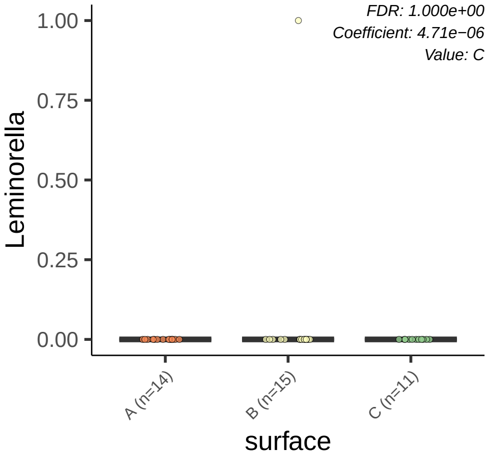
FDR: 1.000e+00
Coefficient: 4.71e−06
Value: C
Leminorella
1.00
0.75
0.50
0.25
0.00
A (n=14)
B (n=15)
C (n=11)
surface

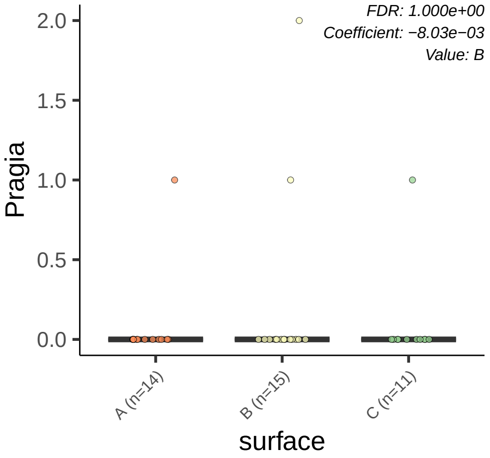

FDR: 1.000e+00
Coefficient: −8.03e−03
Value: B
Pragia
2.0
1.5
1.0
0.5
0.0
A (n=14)
B (n=15)
C (n=11)
surface

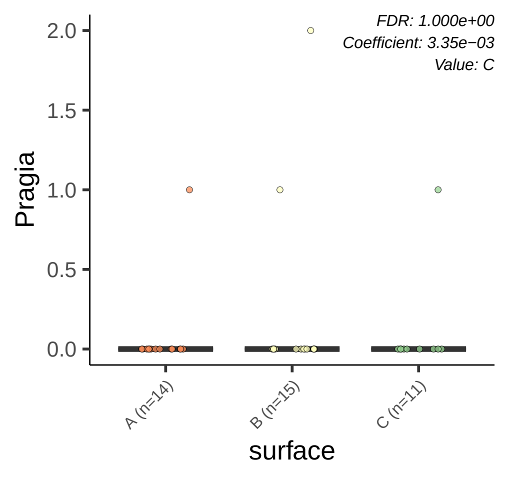
FDR: 1.000e+00
Coefficient: 3.35e−03
Value: C
Pragia
2.0
1.5
1.0
0.5
0.0
A (n=14)
B (n=15)
C (n=11)
surface

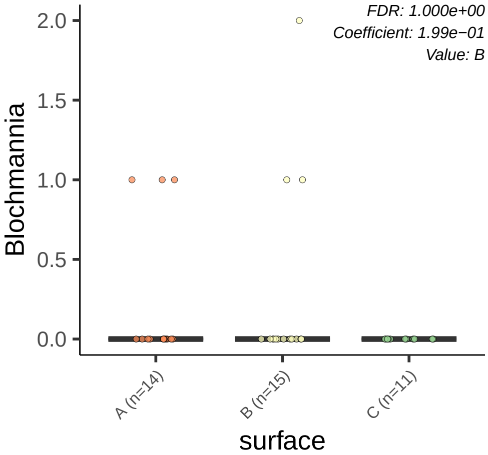
FDR: 1.000e+00
Coefficient: 1.99e−01
Value: B
Blochmannia
2.0
1.5
1.0
0.5
0.0
A (n=14)
B (n=15)
C (n=11)
surface

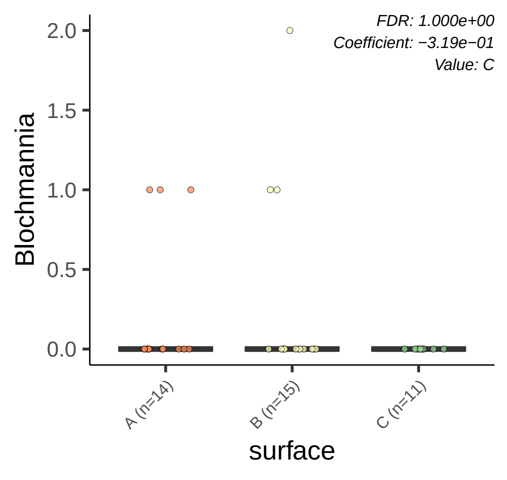

FDR: 1.000e+00
Coefficient: −3.19e−01
Value: C
Blochmannia
2.0
1.5
1.0
0.5
0.0
A (n=14)
B (n=15)
C (n=11)
surface

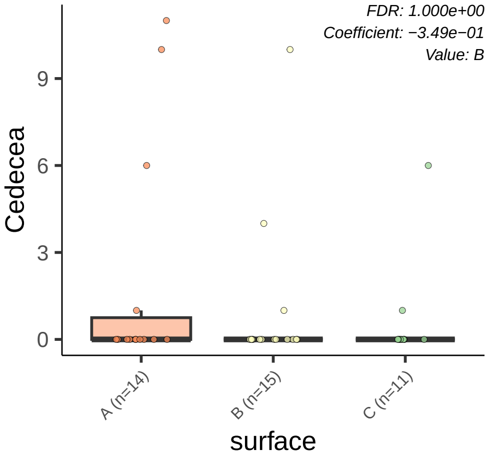

FDR: 1.000e+00
Coefficient: −3.49e−01
Value: B
Cedecea
0
3
6
9
A (n=14)
B (n=15)
C (n=11)
surface

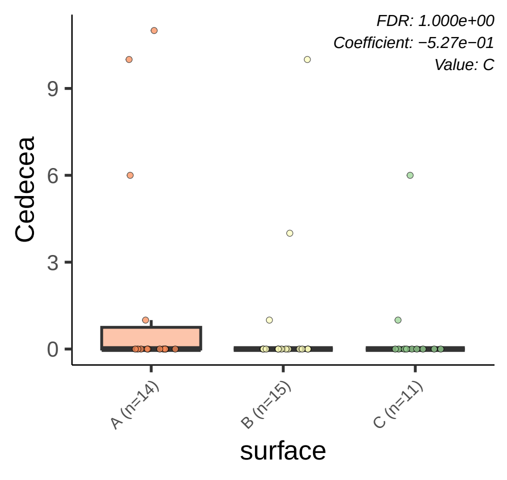

FDR: 1.000e+00
Coefficient: −5.27e−01
Value: C
Cedecea
9
6
3
0
A (n=14)
B (n=15)
C (n=11)
surface

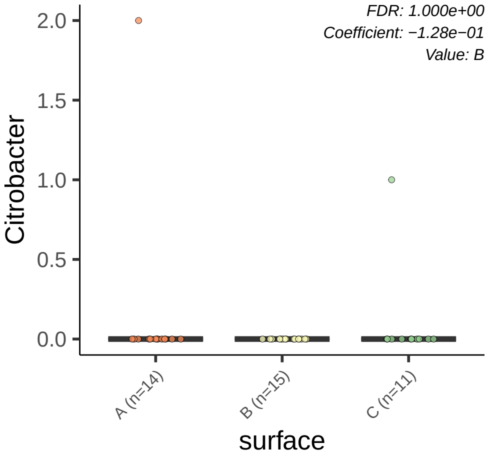
FDR: 1.000e+00
Coefficient: −1.28e−01
Value: B
Citrobacter
2.0
1.5
1.0
0.5
0.0
A (n=14)
B (n=15)
C (n=11)
surface

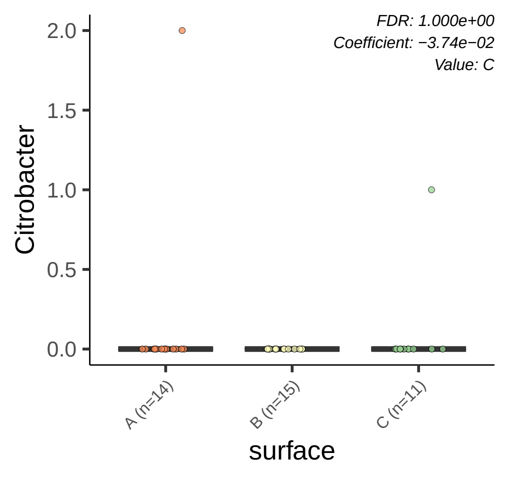
FDR: 1.000e+00
Coefficient: −3.74e−02
Value: C
Citrobacter
2.0
1.5
1.0
0.5
0.0
A (n=14)
B (n=15)
C (n=11)
surface

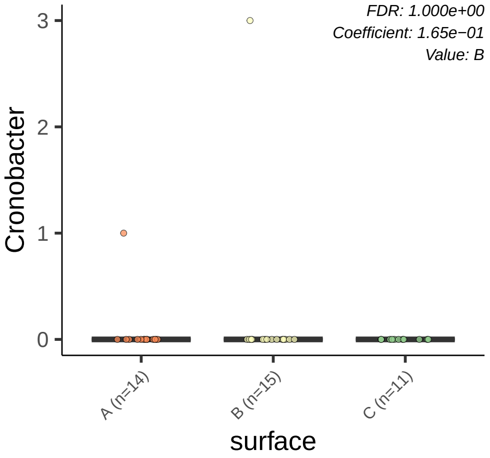
FDR: 1.000e+00
Coefficient: 1.65e−01
Value: B
Cronobacter
0
1
2
3
A (n=14)
B (n=15)
C (n=11)
surface

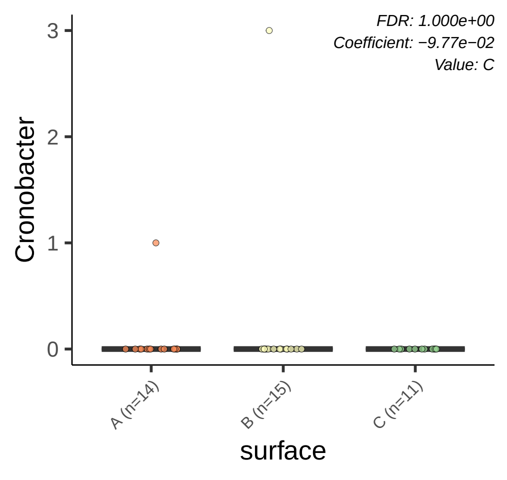
FDR: 1.000e+00
Coefficient: −9.77e−02
Value: C
Cronobacter
3
2
1
0
A (n=14)
B (n=15)
C (n=11)
surface

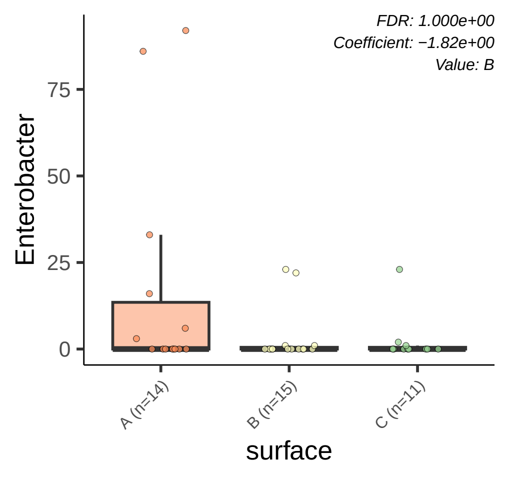

FDR: 1.000e+00
Coefficient: −1.82e+00
Value: B
Enterobacter
0
25
50
75
A (n=14)
B (n=15)
C (n=11)
surface

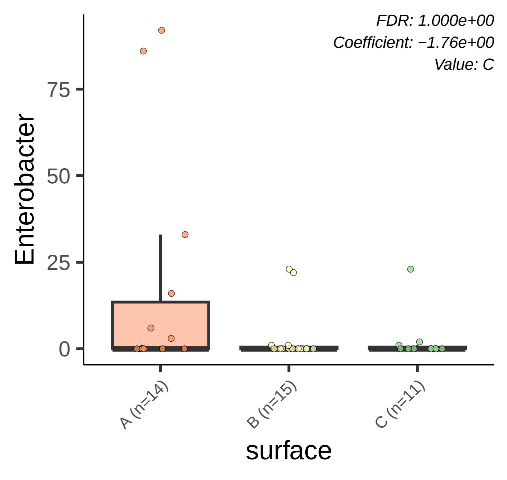
FDR: 1.000e+00
Coefficient: −1.76e+00
Value: C
Enterobacter
0
25
50
75
A (n=14)
B (n=15)
C (n=11)
surface

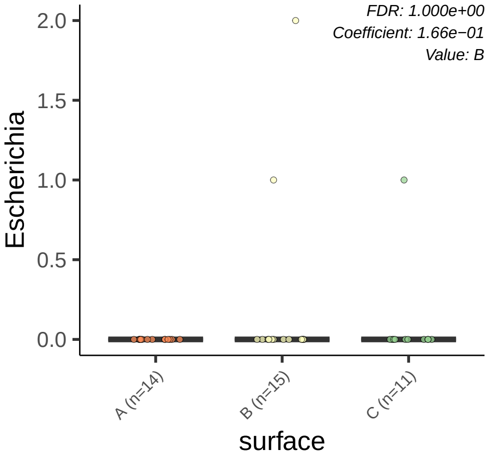
FDR: 1.000e+00
Coefficient: 1.66e−01
Value: B
Escherichia
2.0
1.5
1.0
0.5
0.0
A (n=14)
B (n=15)
C (n=11)
surface

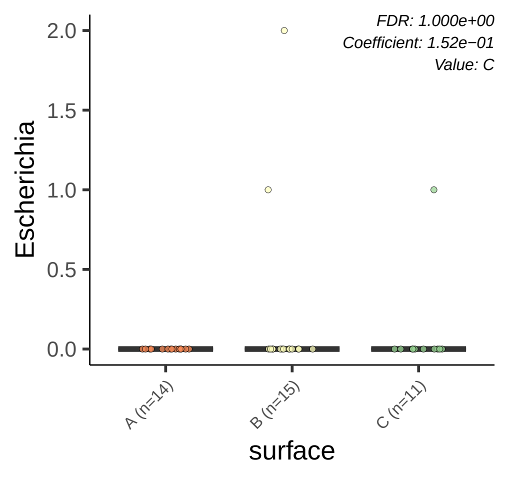
FDR: 1.000e+00
Coefficient: 1.52e−01
Value: C
Escherichia
2.0
1.5
1.0
0.5
0.0
A (n=14)
B (n=15)
C (n=11)
surface

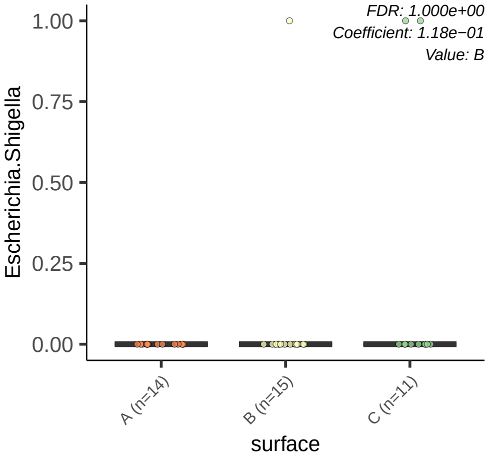

FDR: 1.000e+00
Coefficient: 1.18e−01
Value: B
Escherichia.Shigella
1.00
0.75
0.50
0.25
0.00
A (n=14)
B (n=15)
C (n=11)
surface

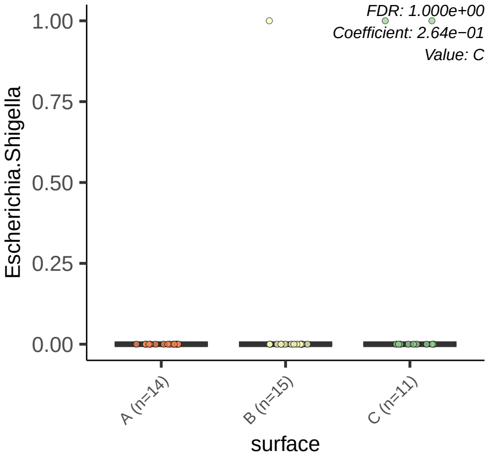

FDR: 1.000e+00
Coefficient: 2.64e−01
Value: C
Escherichia.Shigella
1.00
0.75
0.50
0.25
0.00
A (n=14)
B (n=15)
C (n=11)
surface

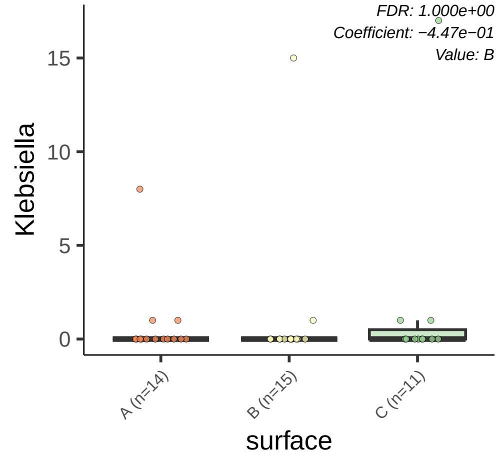
FDR: 1.000e+00
Coefficient: −4.47e−01
Value: B
Klebsiella
15
10
5
0
A (n=14)
B (n=15)
C (n=11)
surface

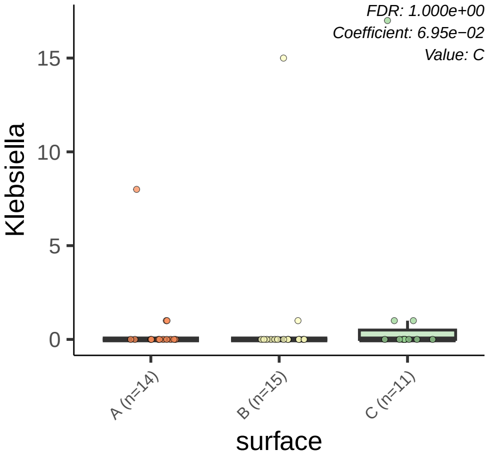

FDR: 1.000e+00
Coefficient: 6.95e−02
Value: C
Klebsiella
0
5
10
15
A (n=14)
B (n=15)
C (n=11)
surface

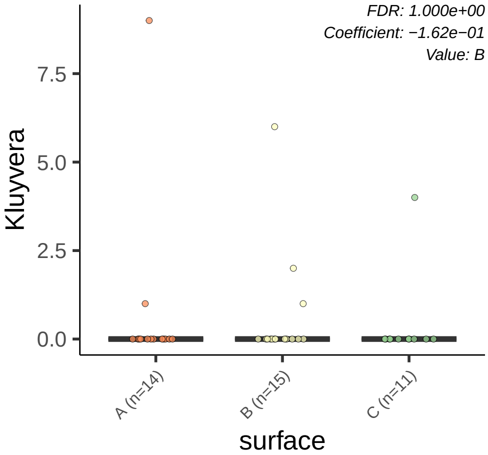
FDR: 1.000e+00
Coefficient: −1.62e−01
Value: B
Kluyvera
7.5
5.0
2.5
0.0
A (n=14)
B (n=15)
C (n=11)
surface

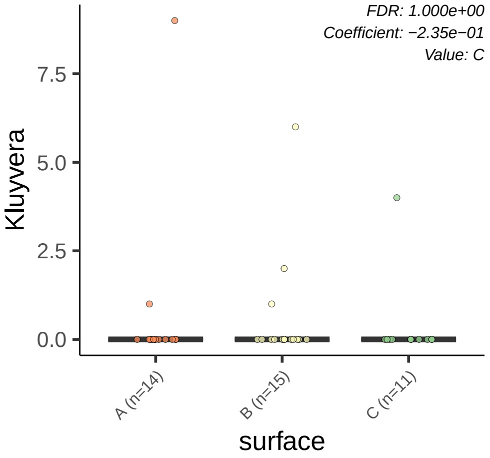

FDR: 1.000e+00
Coefficient: −2.35e−01
Value: C
Kluyvera
7.5
5.0
2.5
0.0
A (n=14)
B (n=15)
C (n=11)
surface

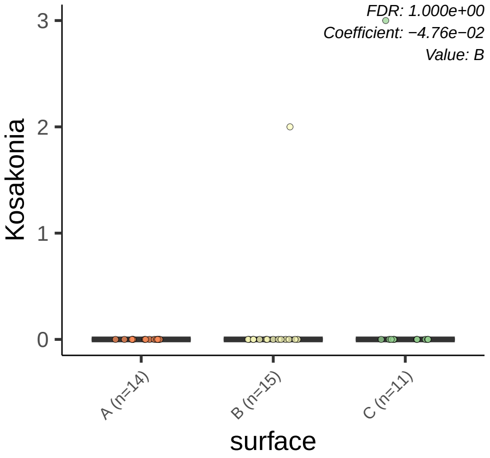
FDR: 1.000e+00
Coefficient: −4.76e−02
Value: B
Kosakonia
0
1
2
3
A (n=14)
B (n=15)
C (n=11)
surface

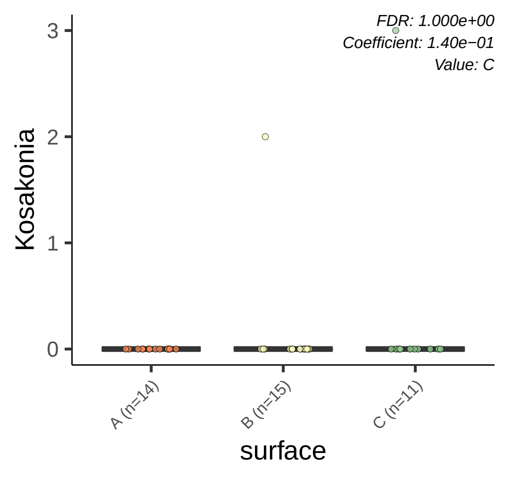

FDR: 1.000e+00
Coefficient: 1.40e−01
Value: C
Kosakonia
3
2
1
0
A (n=14)
B (n=15)
C (n=11)
surface

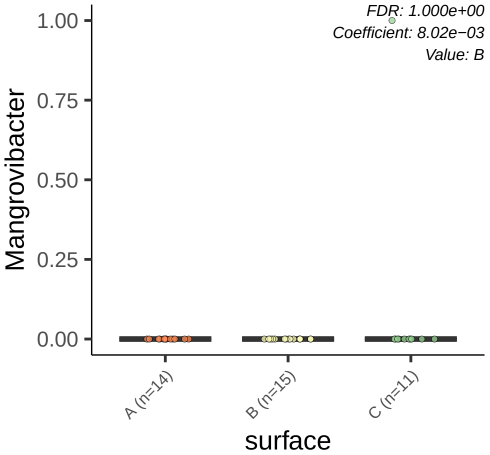
FDR: 1.000e+00
Coefficient: 8.02e−03
Value: B
Mangrovibacter
1.00
0.75
0.50
0.25
0.00
A (n=14)
B (n=15)
C (n=11)
surface

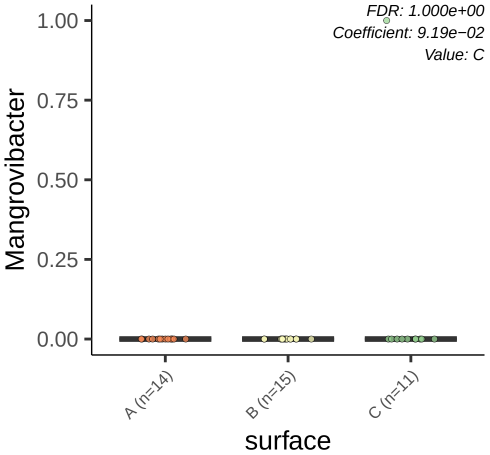
FDR: 1.000e+00
Coefficient: 9.19e−02
Value: C
1.00
0.75
0.50
0.25
0.00
Mangrovibacter
A (n=14)
B (n=15)
C (n=11)
surface

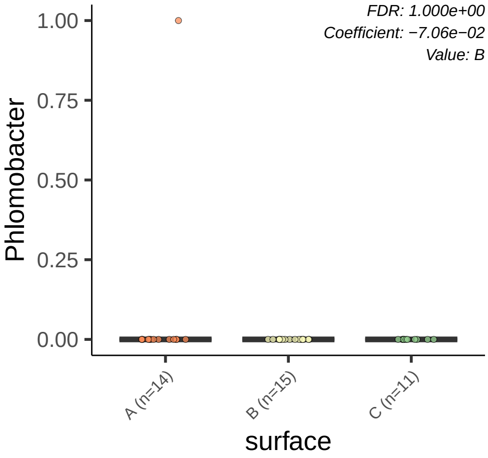
FDR: 1.000e+00
Coefficient: −7.06e−02
Value: B
Phlomobacter
1.00
0.75
0.50
0.25
0.00
A (n=14)
B (n=15)
C (n=11)
surface

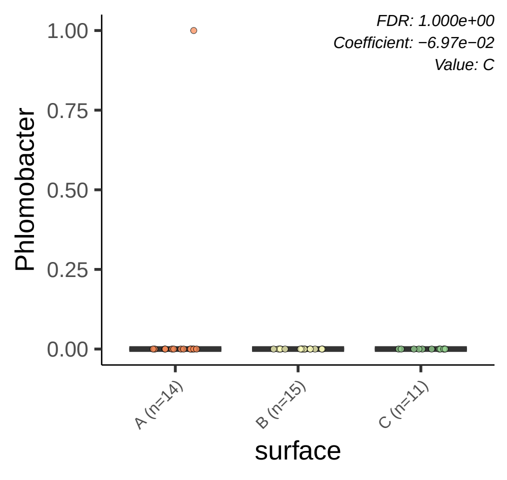

FDR: 1.000e+00
Coefficient: −6.97e−02
Value: C
Phlomobacter
1.00
0.75
0.50
0.25
0.00
A (n=14)
B (n=15)
C (n=11)
surface

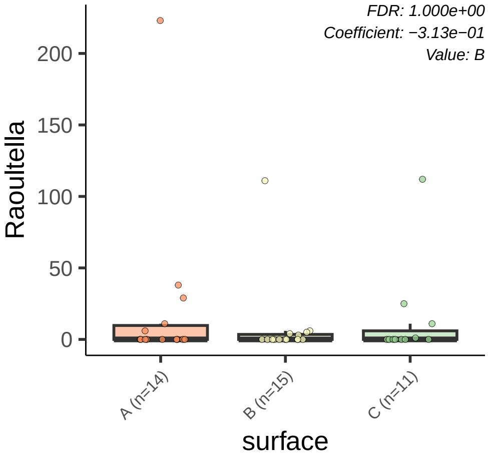
FDR: 1.000e+00
Coefficient: −3.13e−01
Value: B
Raoultella
200
150
100
50
0
A (n=14)
B (n=15)
C (n=11)
surface

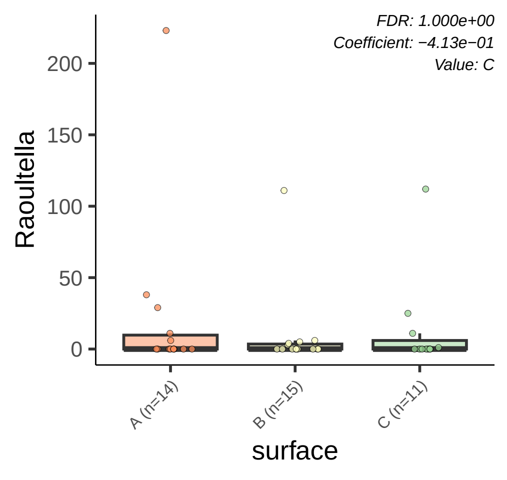
FDR: 1.000e+00
Coefficient: −4.13e−01
Value: C
Raoultella
200
150
100
50
0
A (n=14)
B (n=15)
C (n=11)
surface

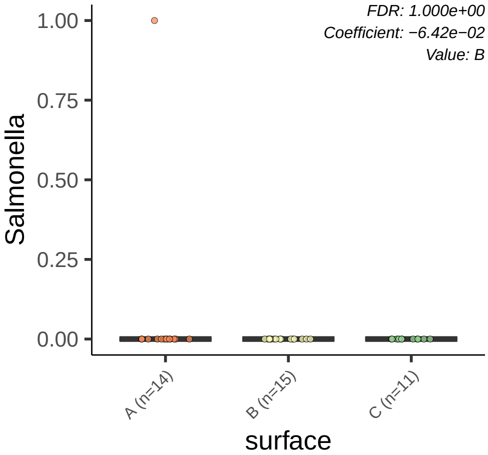
FDR: 1.000e+00
Coefficient: −6.42e−02
Value: B
Salmonella
1.00
0.75
0.50
0.25
0.00
A (n=14)
B (n=15)
C (n=11)
surface

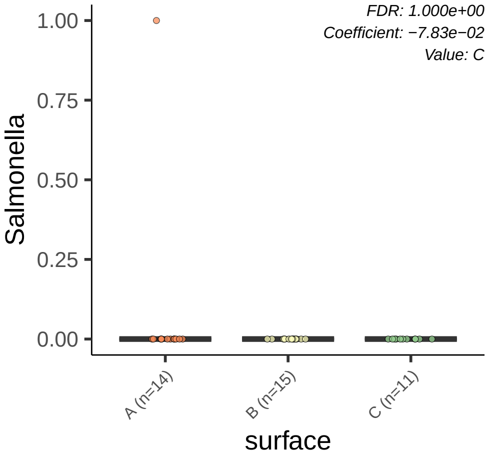
FDR: 1.000e+00
Coefficient: −7.83e−02
Value: C
Salmonella
1.00
0.75
0.50
0.25
0.00
A (n=14)
B (n=15)
C (n=11)
surface

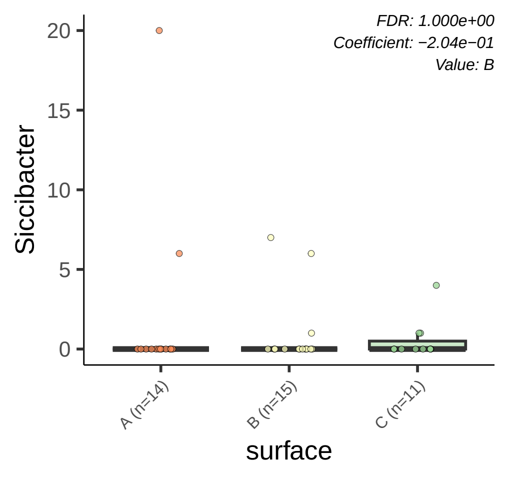
FDR: 1.000e+00
Coefficient: −2.04e−01
Value: B
Siccibacter
20
15
10
5
0
A (n=14)
B (n=15)
C (n=11)
surface

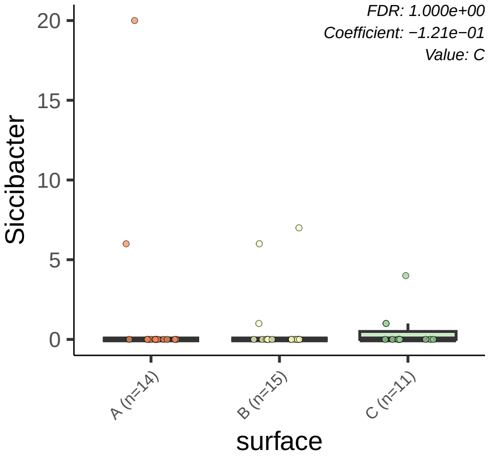
FDR: 1.000e+00
Coefficient: −1.21e−01
Value: C
Siccibacter
20
15
10
5
0
A (n=14)
B (n=15)
C (n=11)
surface

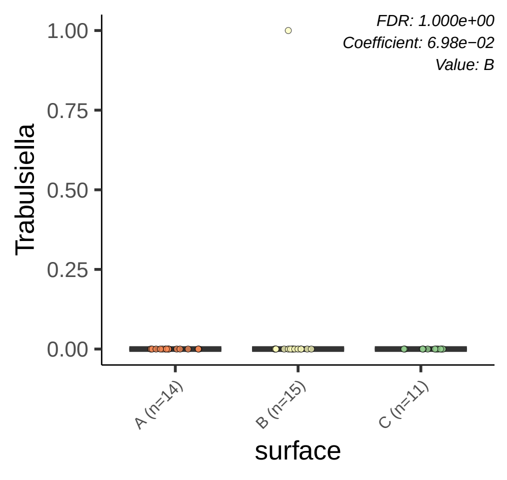
FDR: 1.000e+00
Coefficient: 6.98e−02
Value: B
Trabulsiella
1.00
0.75
0.50
0.25
0.00
A (n=14)
B (n=15)
C (n=11)
surface

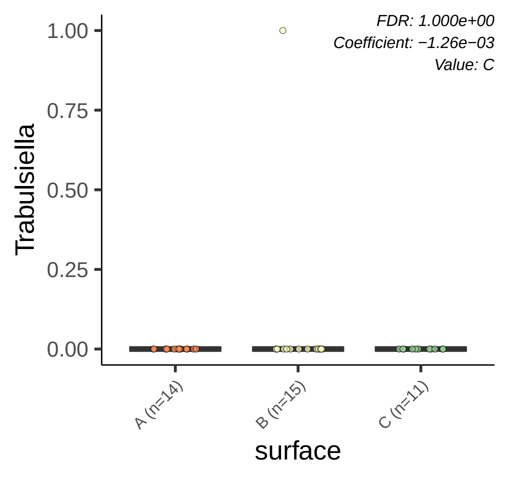

FDR: 1.000e+00
Coefficient: −1.26e−03
Value: C
Trabulsiella
1.00
0.75
0.50
0.25
0.00
A (n=14)
B (n=15)
C (n=11)
surface

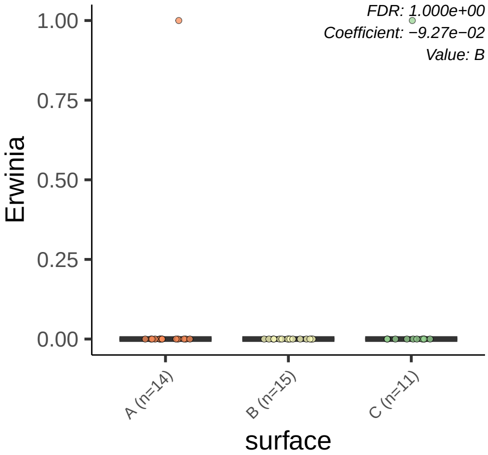
FDR: 1.000e+00
Coefficient: −9.27e−02
Value: B
Erwinia
1.00
0.75
0.50
0.25
0.00
A (n=14)
B (n=15)
C (n=11)
surface

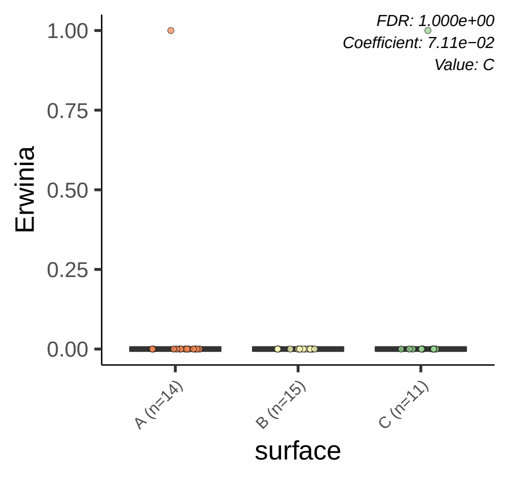

FDR: 1.000e+00
Coefficient: 7.11e−02
Value: C
Erwinia
1.00
0.75
0.50
0.25
0.00
A (n=14)
B (n=15)
C (n=11)
surface

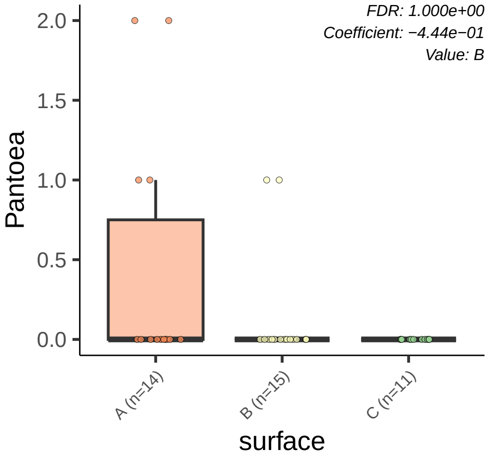
FDR: 1.000e+00
Coefficient: −4.44e−01
Value: B
Pantoea
2.0
1.5
1.0
0.5
0.0
A (n=14)
B (n=15)
C (n=11)
surface

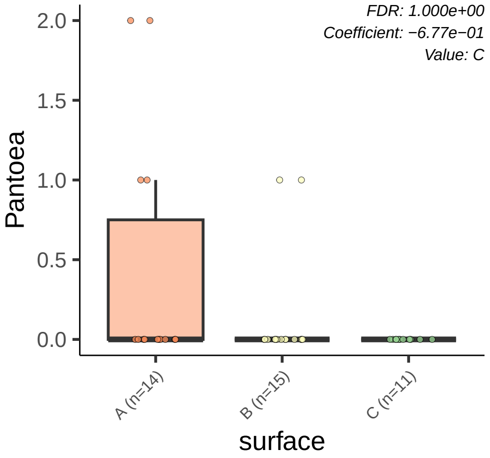

FDR: 1.000e+00
Coefficient: −6.77e−01
Value: C
Pantoea
2.0
1.5
1.0
0.5
0.0
A (n=14)
B (n=15)
C (n=11)
surface

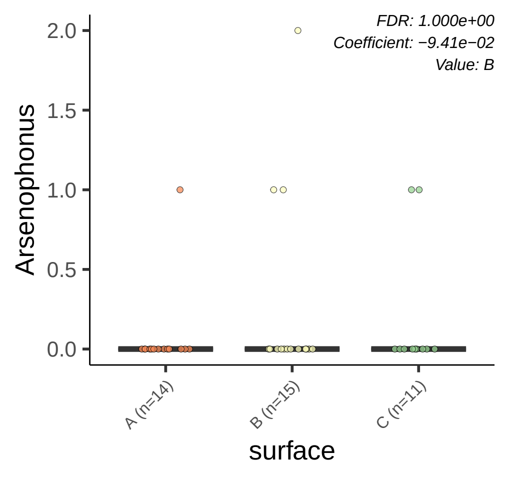

FDR: 1.000e+00
Coefficient: −9.41e−02
Value: B
Arsenophonus
2.0
1.5
1.0
0.5
0.0
A (n=14)
B (n=15)
C (n=11)
surface

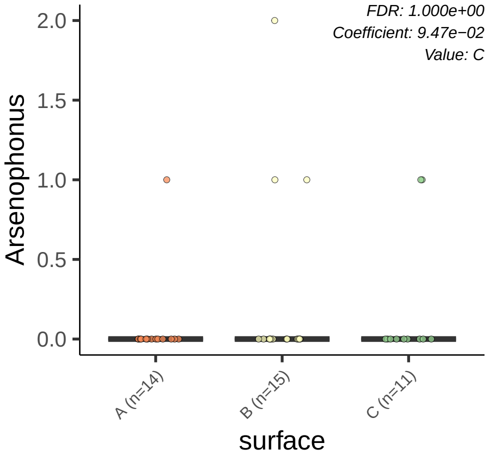
FDR: 1.000e+00
Coefficient: 9.47e−02
Value: C
Arsenophonus
2.0
1.5
1.0
0.5
0.0
A (n=14)
B (n=15)
C (n=11)
surface

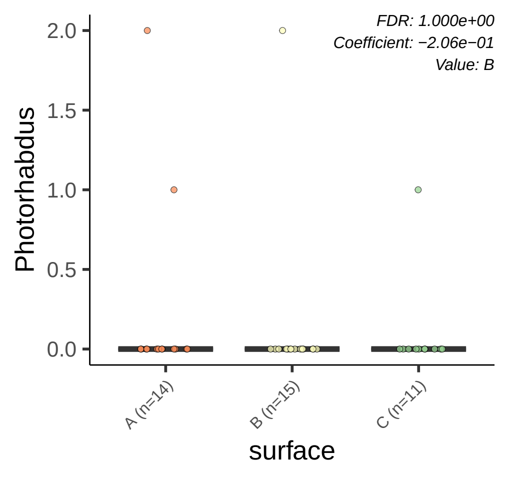

FDR: 1.000e+00
Coefficient: −2.06e−01
Value: B
Photorhabdus
2.0
1.5
1.0
0.5
0.0
A (n=14)
B (n=15)
C (n=11)
surface

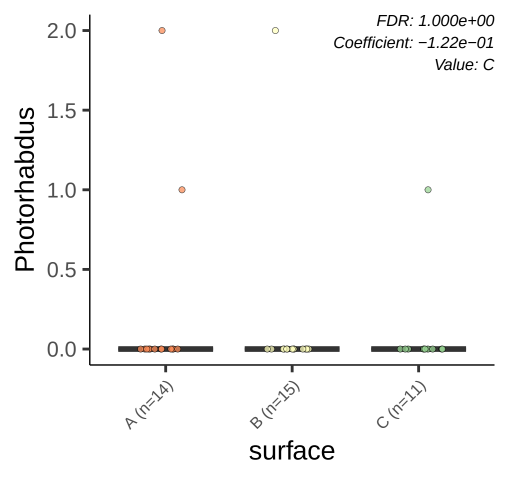

FDR: 1.000e+00
Coefficient: −1.22e−01
Value: C
Photorhabdus
2.0
1.5
1.0
0.5
0.0
A (n=14)
B (n=15)
C (n=11)
surface

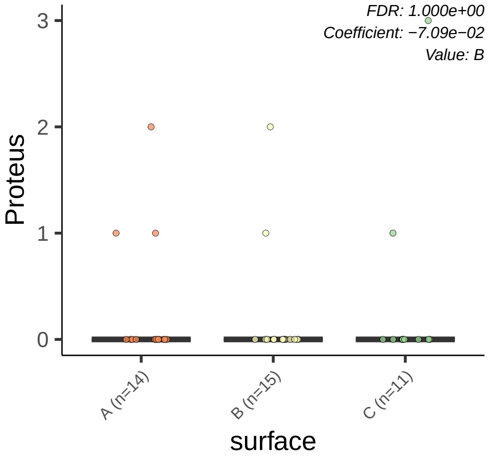
FDR: 1.000e+00
Coefficient: −7.09e−02
Value: B
Proteus
3
2
1
0
A (n=14)
B (n=15)
C (n=11)
surface

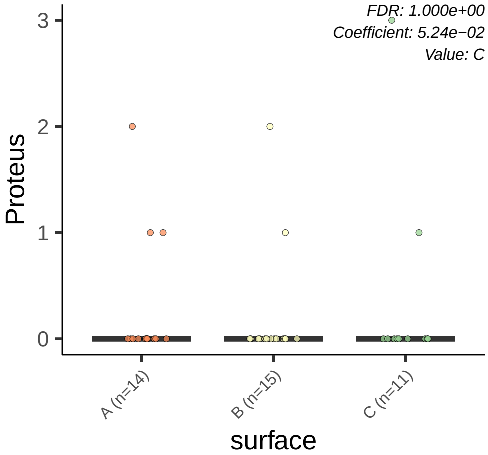

FDR: 1.000e+00
Coefficient: 5.24e−02
Value: C
Proteus
0
1
2
3
A (n=14)
B (n=15)
C (n=11)
surface

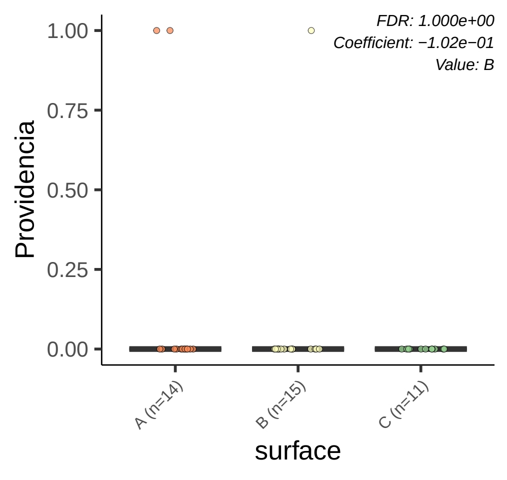

FDR: 1.000e+00
Coefficient: −1.02e−01
Value: B
Providencia
1.00
0.75
0.50
0.25
0.00
A (n=14)
B (n=15)
C (n=11)
surface

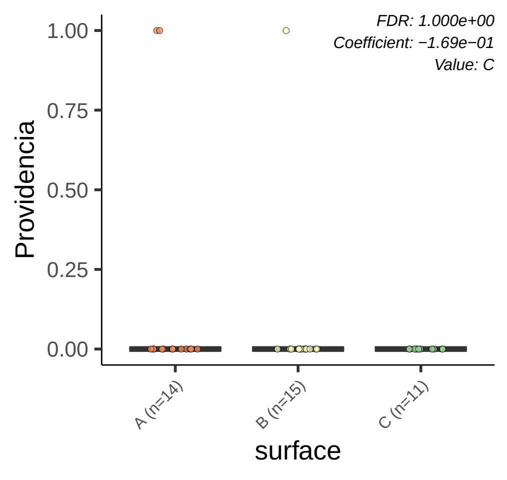

FDR: 1.000e+00
Coefficient: −1.69e−01
Value: C
Providencia
1.00
0.75
0.50
0.25
0.00
A (n=14)
B (n=15)
C (n=11)
surface

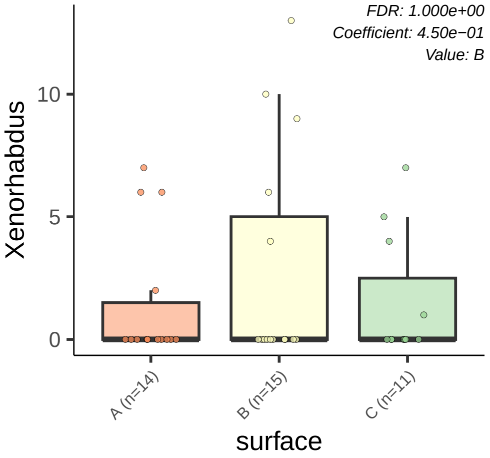

FDR: 1.000e+00
Coefficient: 4.50e−01
Value: B
Xenorhabdus
0
5
10
A (n=14)
B (n=15)
C (n=11)
surface

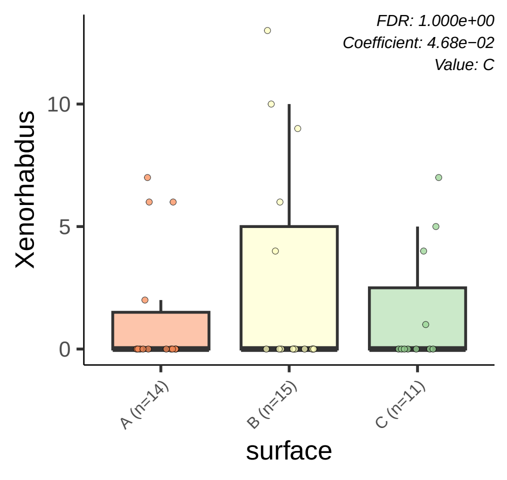

FDR: 1.000e+00
Coefficient: 4.68e−02
Value: C
Xenorhabdus
0
5
10
A (n=14)
B (n=15)
C (n=11)
surface

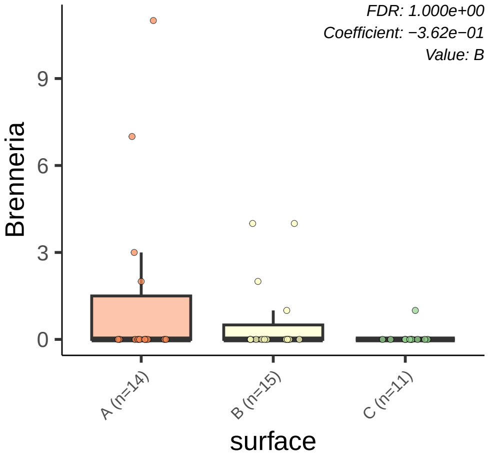

FDR: 1.000e+00
Coefficient: −3.62e−01
Value: B
Brenneria
0
3
6
9
A (n=14)
B (n=15)
C (n=11)
surface

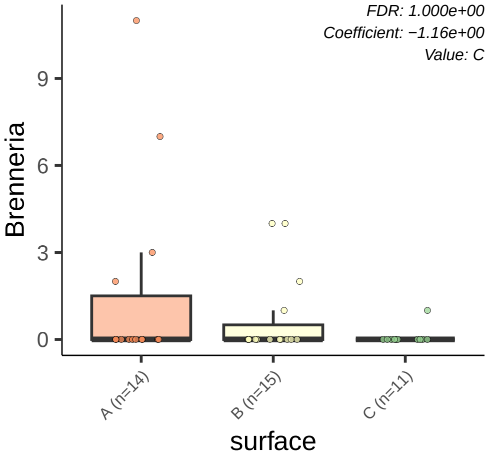

FDR: 1.000e+00
Coefficient: −1.16e+00
Value: C
Brenneria
0
3
6
9
A (n=14)
B (n=15)
C (n=11)
surface

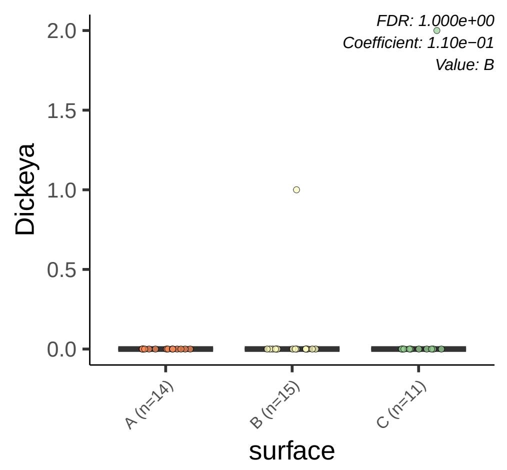

FDR: 1.000e+00
Coefficient: 1.10e−01
Value: B
Dickeya
2.0
1.5
1.0
0.5
0.0
A (n=14)
B (n=15)
C (n=11)
surface

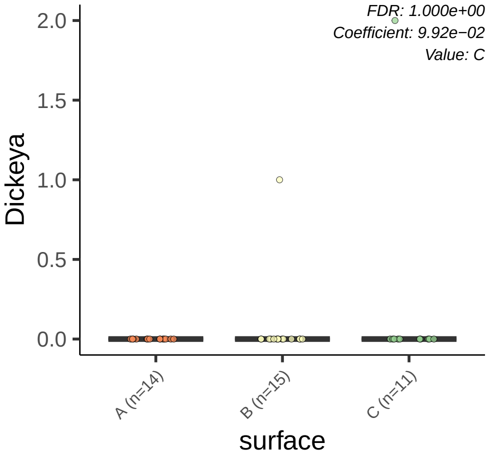
FDR: 1.000e+00
Coefficient: 9.92e−02
Value: C
Dickeya
2.0
1.5
1.0
0.5
0.0
A (n=14)
B (n=15)
C (n=11)
surface

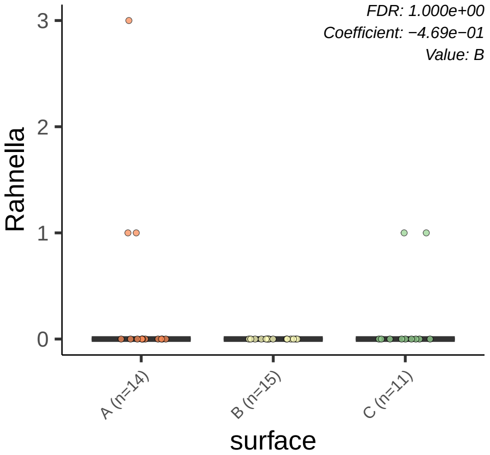
FDR: 1.000e+00
Coefficient: −4.69e−01
Value: B
Rahnella
3
2
1
0
A (n=14)
B (n=15)
C (n=11)
surface

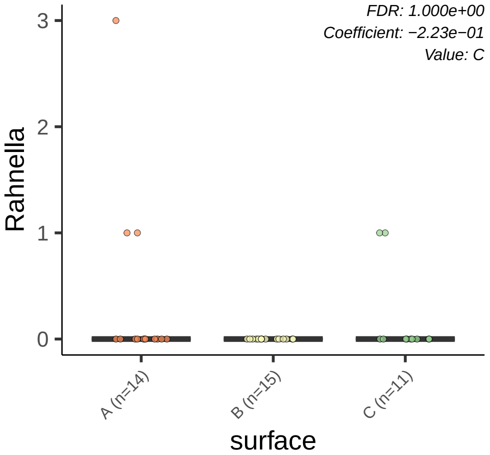
FDR: 1.000e+00
Coefficient: −2.23e−01
Value: C
Rahnella
0
1
2
3
A (n=14)
B (n=15)
C (n=11)
surface

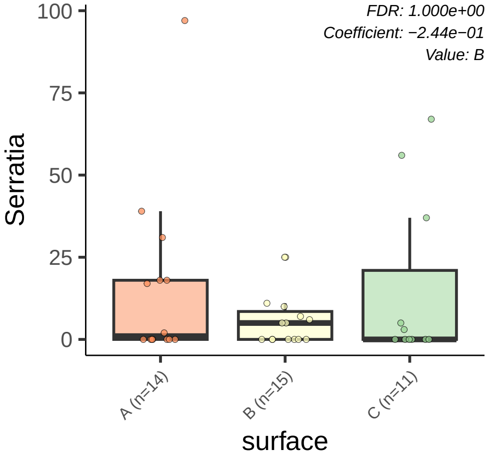
FDR: 1.000e+00
Coefficient: −2.44e−01
Value: B
Serratia
100
75
50
25
0
A (n=14)
B (n=15)
C (n=11)
surface

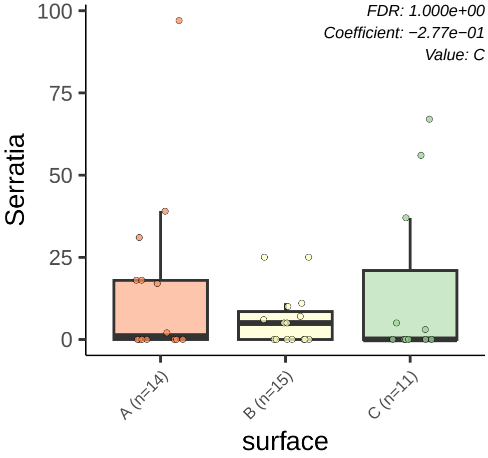
FDR: 1.000e+00
Coefficient: −2.77e−01
Value: C
Serratia
100
75
50
25
0
A (n=14)
B (n=15)
C (n=11)
surface

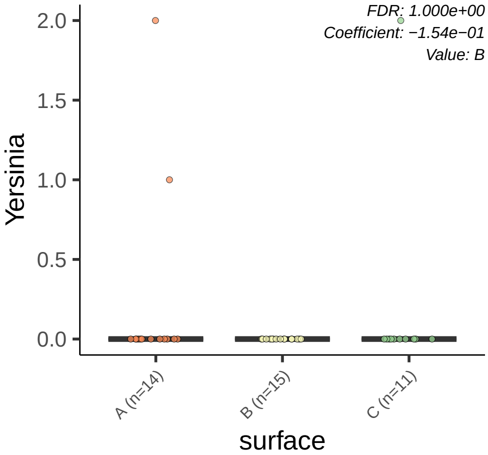

FDR: 1.000e+00
Coefficient: −1.54e−01
Value: B
Yersinia
2.0
1.5
1.0
0.5
0.0
A (n=14)
B (n=15)
C (n=11)
surface

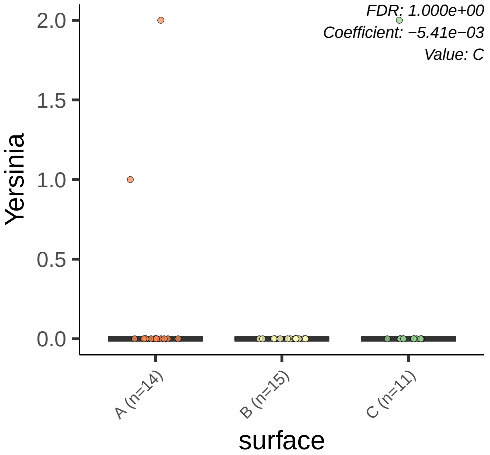
FDR: 1.000e+00
Coefficient: −5.41e−03
Value: C
Yersinia
2.0
1.5
1.0
0.5
0.0
A (n=14)
B (n=15)
C (n=11)
surface

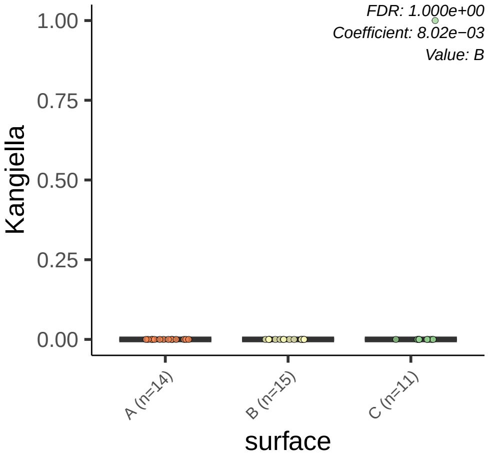
FDR: 1.000e+00
Coefficient: 8.02e−03
Value: B
Kangiella
1.00
0.75
0.50
0.25
0.00
A (n=14)
B (n=15)
C (n=11)
surface

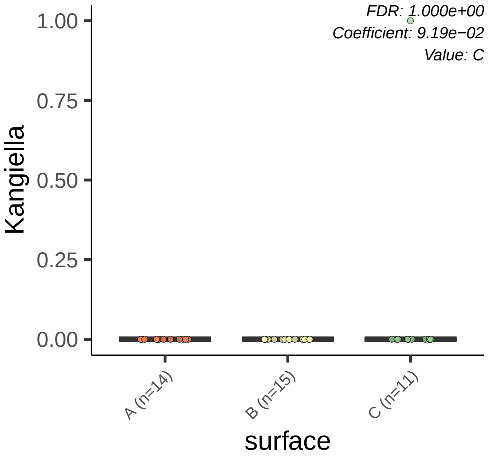
FDR: 1.000e+00
Coefficient: 9.19e−02
Value: C
Kangiella
1.00
0.75
0.50
0.25
0.00
A (n=14)
B (n=15)
C (n=11)
surface

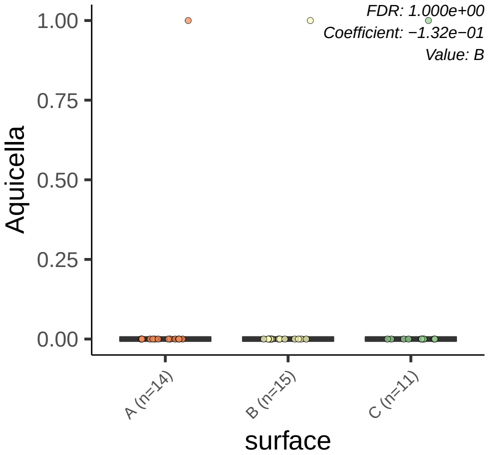

FDR: 1.000e+00
Coefficient: −1.32e−01
Value: B
Aquicella
1.00
0.75
0.50
0.25
0.00
A (n=14)
B (n=15)
C (n=11)
surface

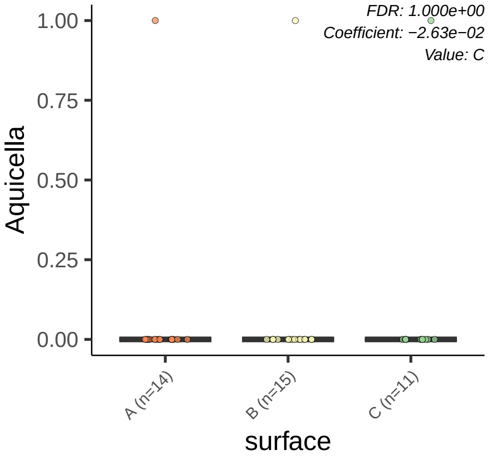
FDR: 1.000e+00
Coefficient: −2.63e−02
Value: C
Aquicella
1.00
0.75
0.50
0.25
0.00
A (n=14)
B (n=15)
C (n=11)
surface

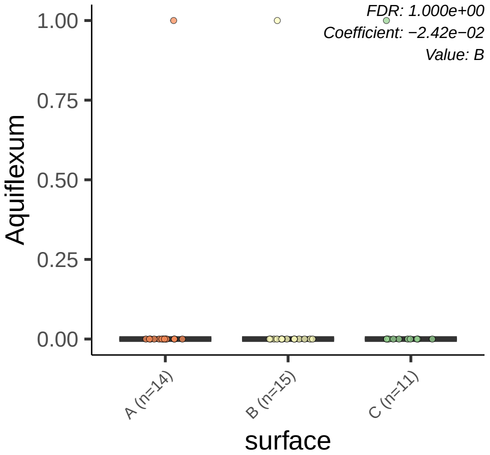

FDR: 1.000e+00
Coefficient: −2.42e−02
Value: B
1.00
0.75
0.50
0.25
0.00
Aquiflexum
A (n=14)
B (n=15)
C (n=11)
surface

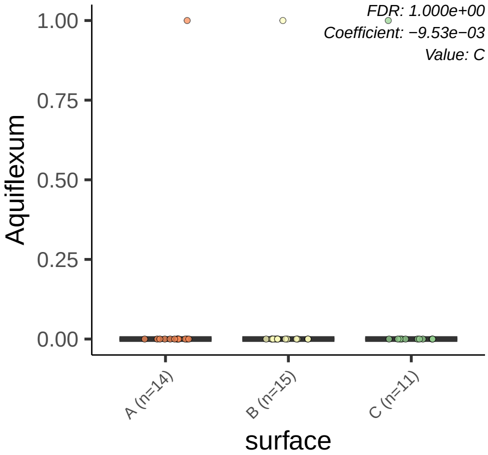
FDR: 1.000e+00
Coefficient: −9.53e−03
Value: C
Aquiflexum
1.00
0.75
0.50
0.25
0.00
A (n=14)
B (n=15)
C (n=11)
surface

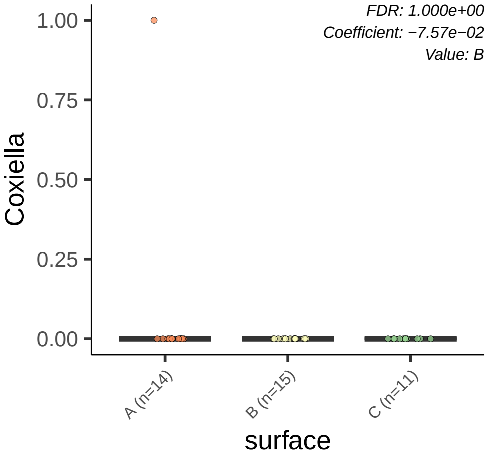

FDR: 1.000e+00
Coefficient: −7.57e−02
Value: B
Coxiella
1.00
0.75
0.50
0.25
0.00
A (n=14)
B (n=15)
C (n=11)
surface

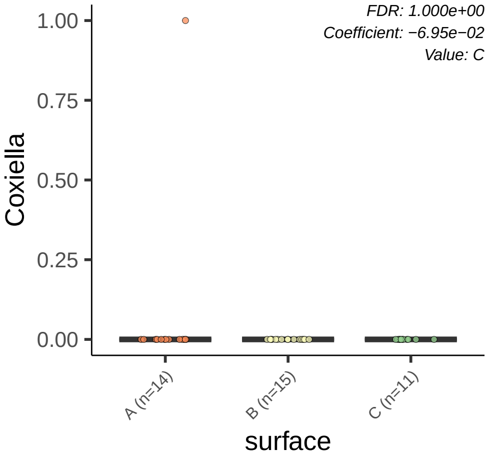

FDR: 1.000e+00
Coefficient: −6.95e−02
Value: C
Coxiella
1.00
0.75
0.50
0.25
0.00
A (n=14)
B (n=15)
C (n=11)
surface

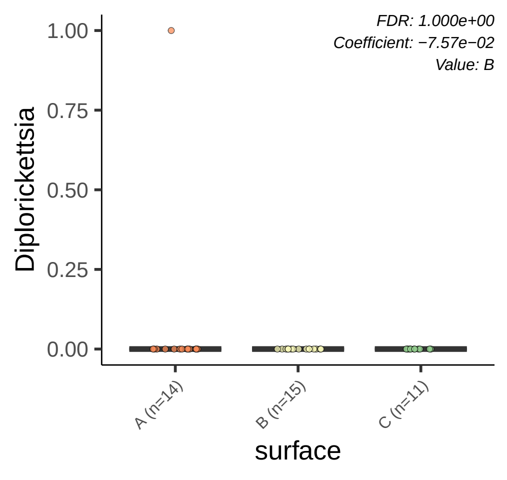
FDR: 1.000e+00
Coefficient: −7.57e−02
Value: B
Diplorickettsia
1.00
0.75
0.50
0.25
0.00
A (n=14)
B (n=15)
C (n=11)
surface

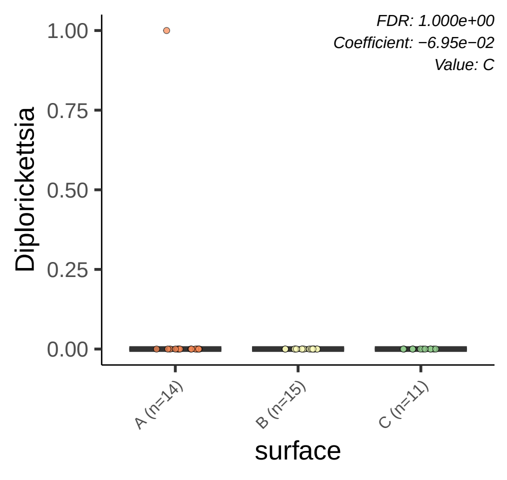
FDR: 1.000e+00
Coefficient: −6.95e−02
Value: C
Diploricketttsia
1.00
0.75
0.50
0.25
0.00
A (n=14)
B (n=15)
C (n=11)
surface

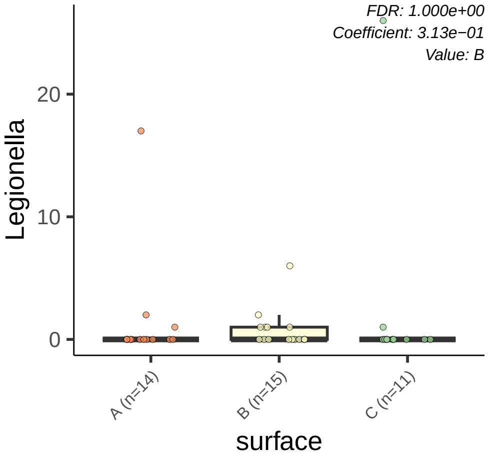

FDR: 1.000e+00
Coefficient: 3.13e−01
Value: B
Legionella
20
10
0
A (n=14)
B (n=15)
C (n=11)
surface

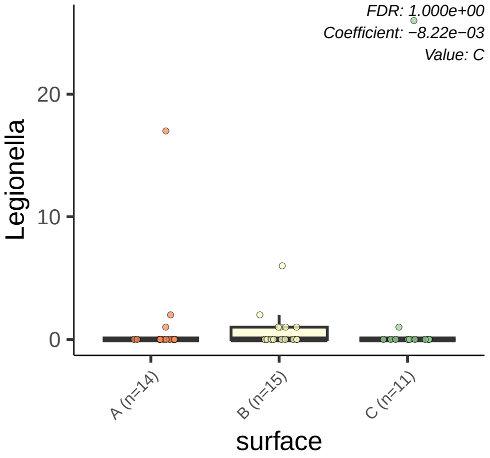
FDR: 1.000e+00
Coefficient: −8.22e−03
Value: C
Legionella
20
10
0
A (n=14)
B (n=15)
C (n=11)
surface

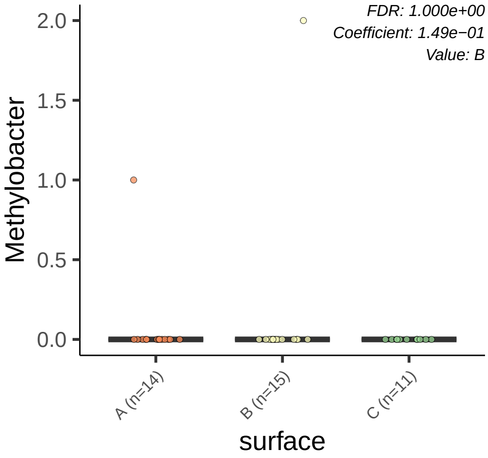
FDR: 1.000e+00
Coefficient: 1.49e−01
Value: B
Methylobacter
2.0
1.5
1.0
0.5
0.0
A (n=14)
B (n=15)
C (n=11)
surface

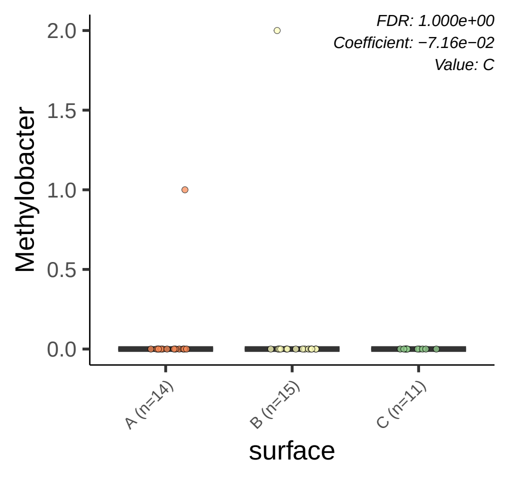
FDR: 1.000e+00
Coefficient: −7.16e−02
Value: C
Methylobacter
2.0
1.5
1.0
0.5
0.0
A (n=14)
B (n=15)
C (n=11)
surface

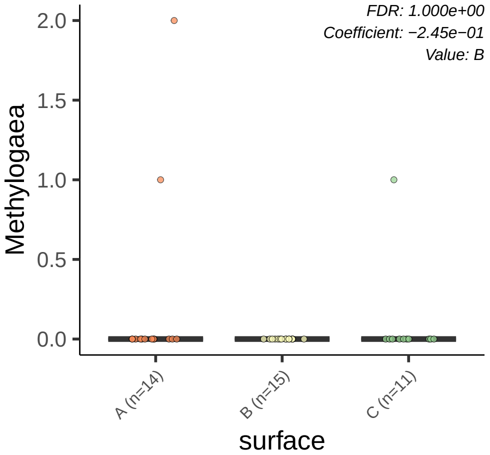
FDR: 1.000e+00
Coefficient: −2.45e−01
Value: B
Methylogaea
2.0
1.5
1.0
0.5
0.0
A (n=14)
B (n=15)
C (n=11)
surface

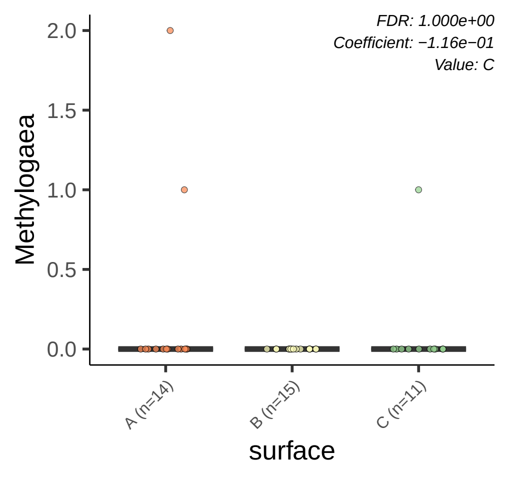

FDR: 1.000e+00
Coefficient: −1.16e−01
Value: C
Methylogaea
2.0
1.5
1.0
0.5
0.0
A (n=14)
B (n=15)
C (n=11)
surface

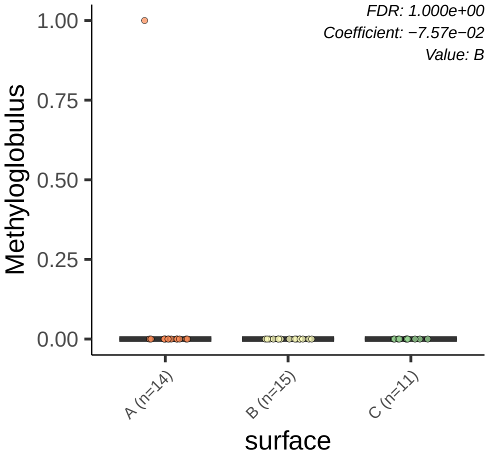
FDR: 1.000e+00
Coefficient: −7.57e−02
Value: B
Methyloglobulus
1.00
0.75
0.50
0.25
0.00
A (n=14)
B (n=15)
C (n=11)
surface

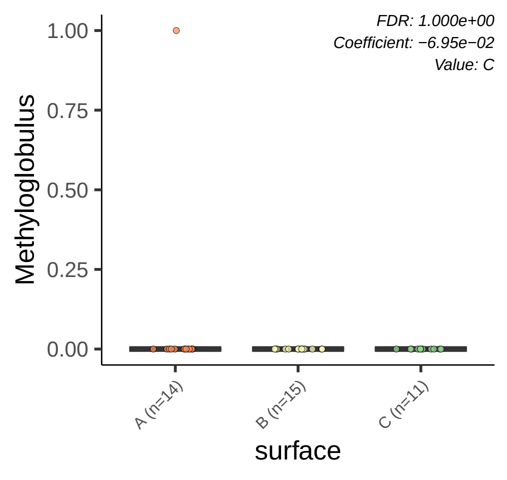
FDR: 1.000e+00
Coefficient: −6.95e−02
Value: C
Methyloglobulus
1.00
0.75
0.50
0.25
0.00
A (n=14)
B (n=15)
C (n=11)
surface

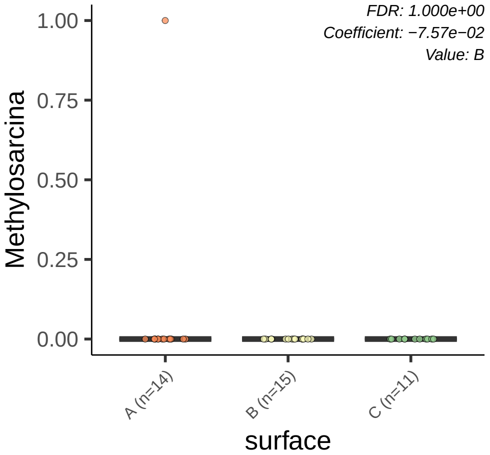
FDR: 1.000e+00
Coefficient: −7.57e−02
Value: B
Methylosarcina
1.00
0.75
0.50
0.25
0.00
A (n=14)
B (n=15)
C (n=11)
surface

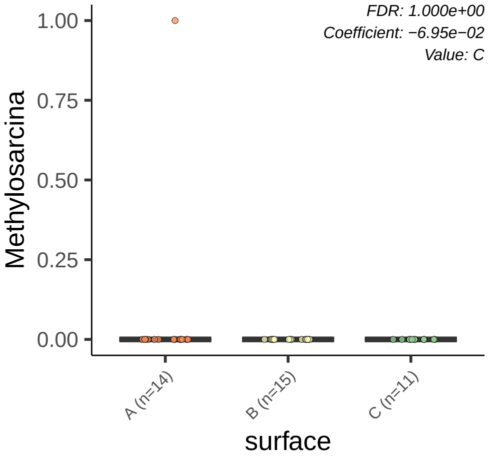
FDR: 1.000e+00
Coefficient: −6.95e−02
Value: C
Methylosarcina
1.00
0.75
0.50
0.25
0.00
A (n=14)
B (n=15)
C (n=11)
surface

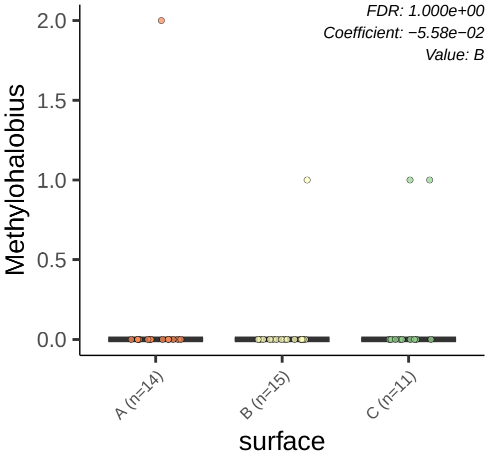
FDR: 1.000e+00
Coefficient: −5.58e−02
Value: B
Methylohalobius
2.0
1.5
1.0
0.5
0.0
A (n=14)
B (n=15)
C (n=11)
surface

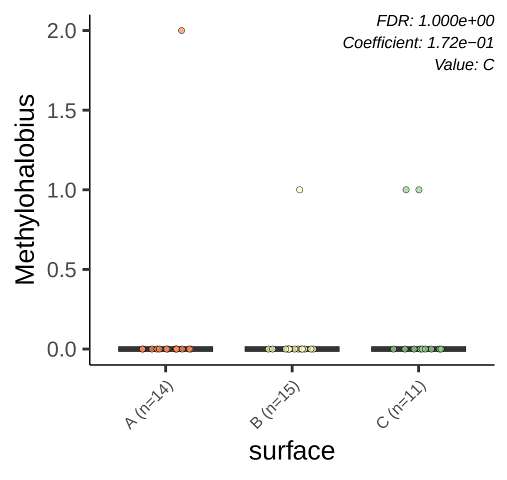

FDR: 1.000e+00
Coefficient: 1.72e−01
Value: C
Methylohalobius
2.0
1.5
1.0
0.5
0.0
A (n=14)
B (n=15)
C (n=11)
surface

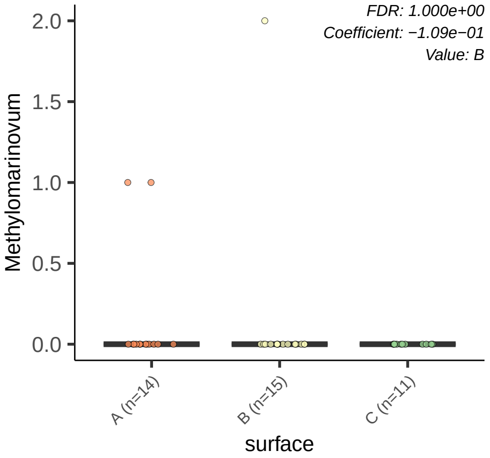

FDR: 1.000e+00
Coefficient: −1.09e−01
Value: B
Methylomarinovum
2.0
1.5
1.0
0.5
0.0
A (n=14)
B (n=15)
C (n=11)
surface

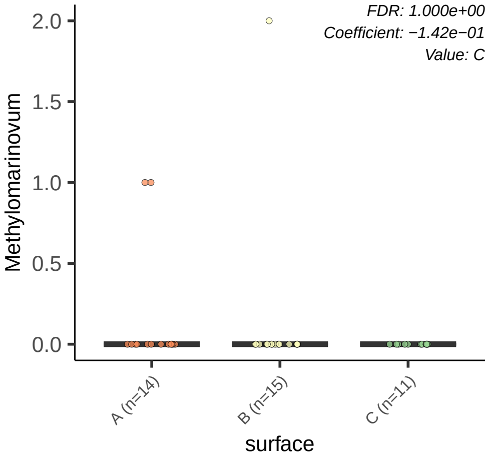
FDR: 1.000e+00
Coefficient: −1.42e−01
Value: C
Methylomarinovum
2.0
1.5
1.0
0.5
0.0
A (n=14)
B (n=15)
C (n=11)
surface

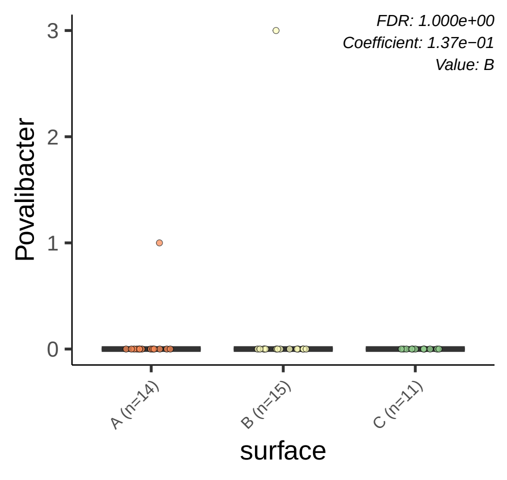

FDR: 1.000e+00
Coefficient: 1.37e−01
Value: B
Povalibacter
3
2
1
0
A (n=14)
B (n=15)
C (n=11)
surface

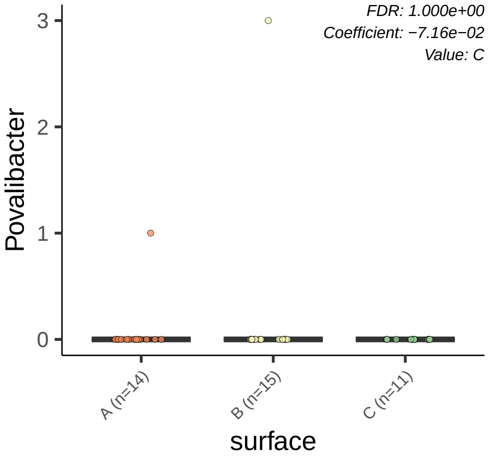

FDR: 1.000e+00
Coefficient: −7.16e−02
Value: C
Povalibacter
3
2
1
0
A (n=14)
B (n=15)
C (n=11)
surface

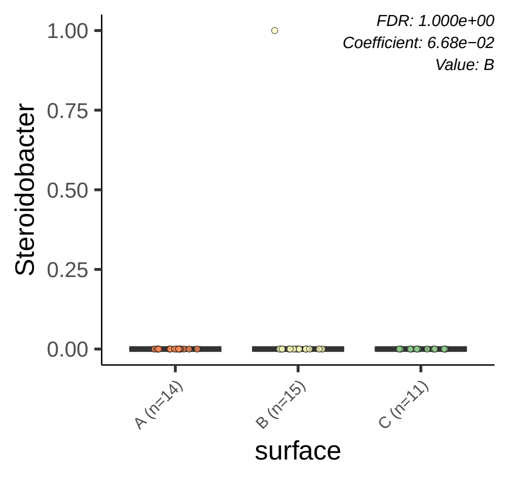
FDR: 1.000e+00
Coefficient: 6.68e−02
Value: B
Steroidobacter
1.00
0.75
0.50
0.25
0.00
A (n=14)
B (n=15)
C (n=11)
surface

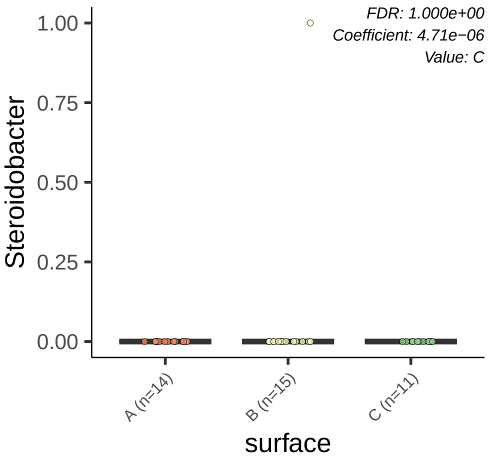
FDR: 1.000e+00
Coefficient: 4.71e−06
Value: C
Steroidobacter
1.00
0.75
0.50
0.25
0.00
A (n=14)
B (n=15)
C (n=11)
surface

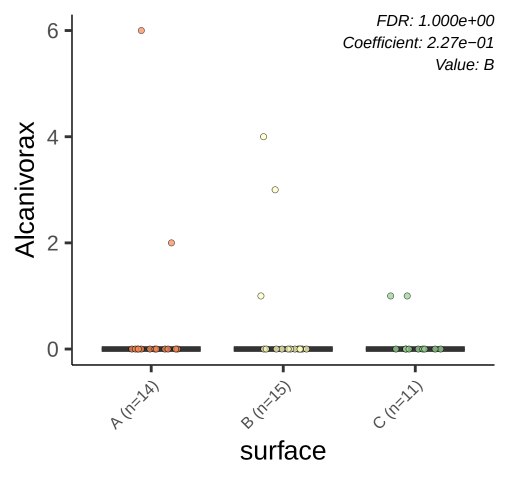
FDR: 1.000e+00
Coefficient: 2.27e−01
Value: B
Alcanivorax
0
2
4
6
A (n=14)
B (n=15)
C (n=11)
surface

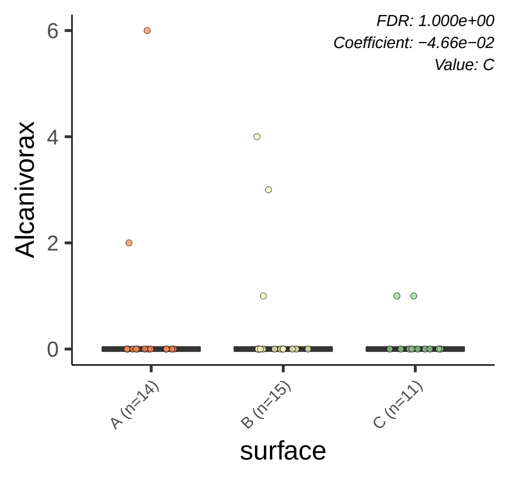
FDR: 1.000e+00
Coefficient: −4.66e−02
Value: C
Alcanivorax
6
4
2
0
A (n=14)
B (n=15)
C (n=11)
surface

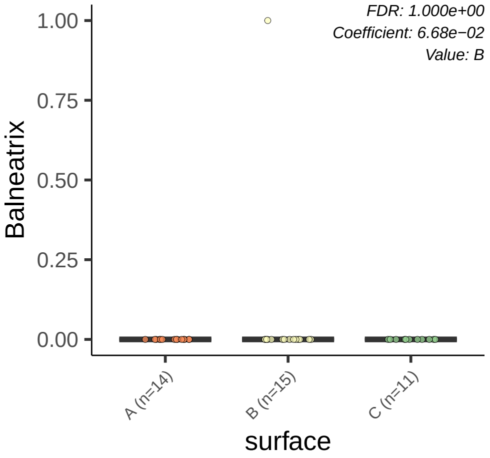
FDR: 1.000e+00
Coefficient: 6.68e−02
Value: B
Balneatrix
1.00
0.75
0.50
0.25
0.00
A (n=14)
B (n=15)
C (n=11)
surface

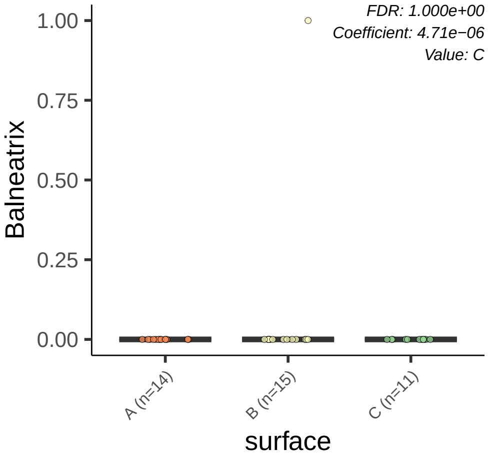

FDR: 1.000e+00
Coefficient: 4.71e−06
Value: C
Balneatrix
1.00
0.75
0.50
0.25
0.00
A (n=14)
B (n=15)
C (n=11)
surface

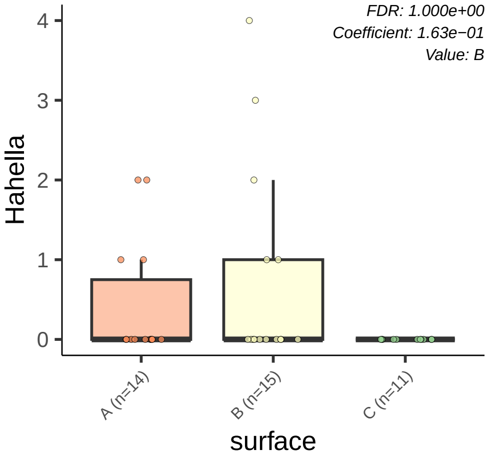

FDR: 1.000e+00
Coefficient: 1.63e−01
Value: B
Hahella
0
1
2
3
4
A (n=14)
B (n=15)
C (n=11)
surface

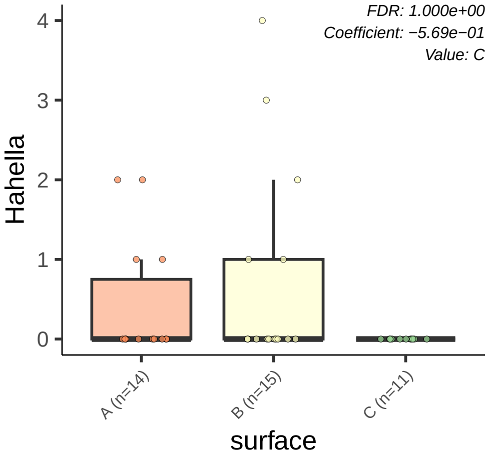

FDR: 1.000e+00
Coefficient: −5.69e−01
Value: C
Hahella
4
3
2
1
0
A (n=14)
B (n=15)
C (n=11)
surface

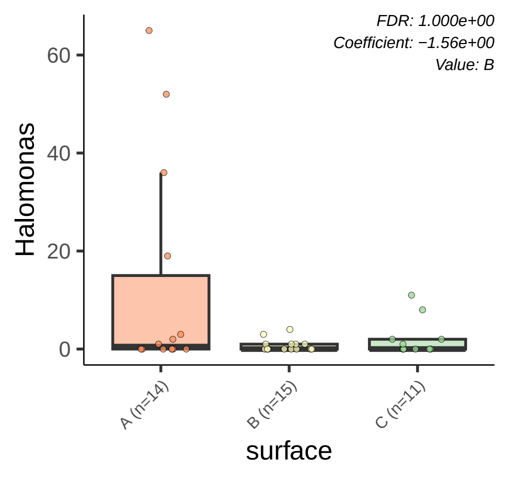
FDR: 1.000e+00
Coefficient: −1.56e+00
Value: B
Halomonas
0
20
40
60
A (n=14)
B (n=15)
C (n=11)
surface

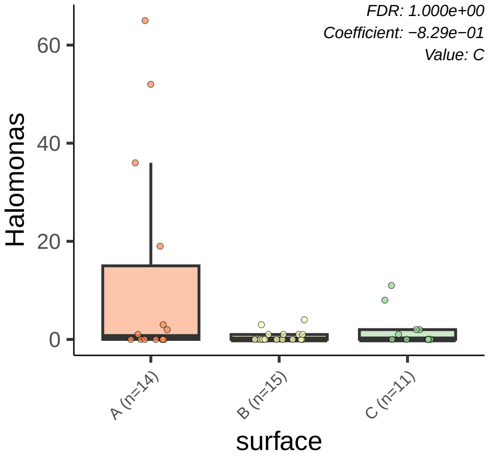

FDR: 1.000e+00
Coefficient: −8.29e−01
Value: C
Halomonas
60
40
20
0
A (n=14)
B (n=15)
C (n=11)
surface

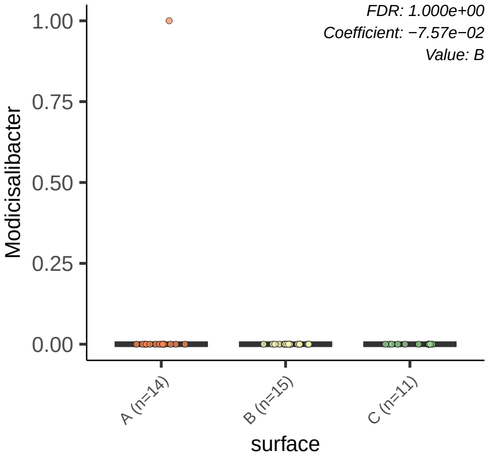

FDR: 1.000e+00
Coefficient: −7.57e−02
Value: B
Modicisalibacter
1.00
0.75
0.50
0.25
0.00
A (n=14)
B (n=15)
C (n=11)
surface

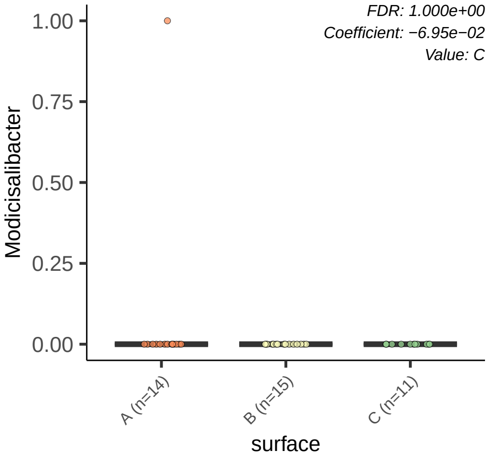
FDR: 1.000e+00
Coefficient: −6.95e−02
Value: C
Modicisalibacter
1.00
0.75
0.50
0.25
0.00
A (n=14)
B (n=15)
C (n=11)
surface

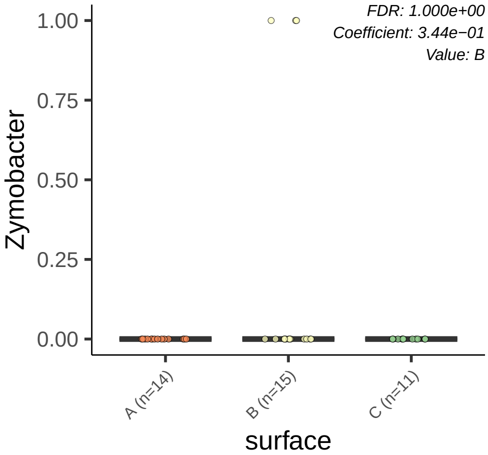
FDR: 1.000e+00
Coefficient: 3.44e−01
Value: B
1.00
0.75
0.50
0.25
0.00
Zymobacter
A (n=14)
B (n=15)
C (n=11)
surface

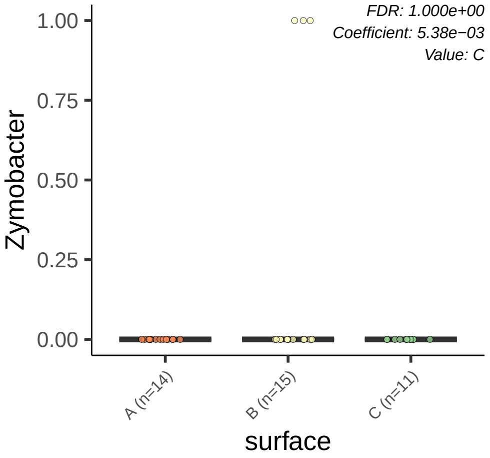

FDR: 1.000e+00
Coefficient: 5.38e−03
Value: C
Zymobacter
1.00
0.75
0.50
0.25
0.00
A (n=14)
B (n=15)
C (n=11)
surface

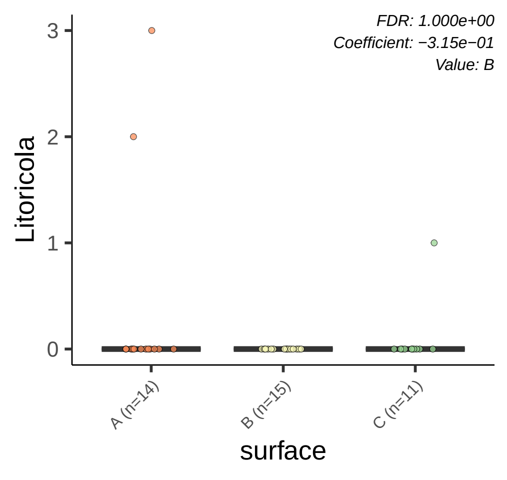

FDR: 1.000e+00
Coefficient: −3.15e−01
Value: B
Litoricola
3
2
1
0
A (n=14)
B (n=15)
C (n=11)
surface

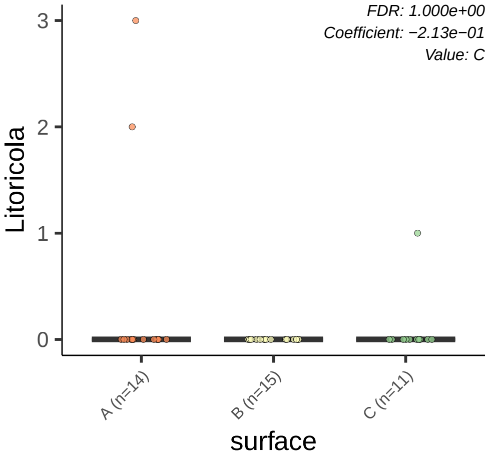
FDR: 1.000e+00
Coefficient: −2.13e−01
Value: C
Litoricola
3
2
1
0
A (n=14)
B (n=15)
C (n=11)
surface

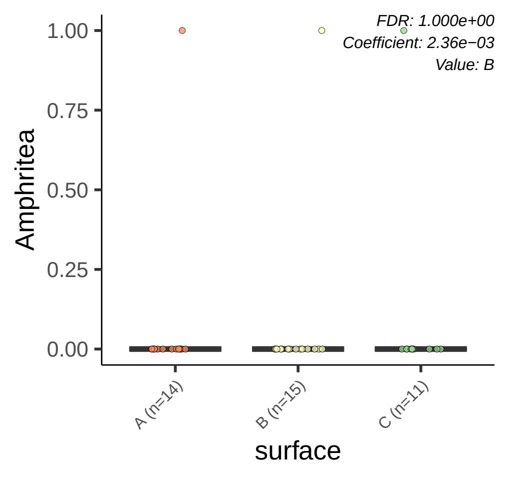

FDR: 1.000e+00
Coefficient: 2.36e−03
Value: B
Amphritea
1.00
0.75
0.50
0.25
0.00
A (n=14)
B (n=15)
C (n=11)
surface

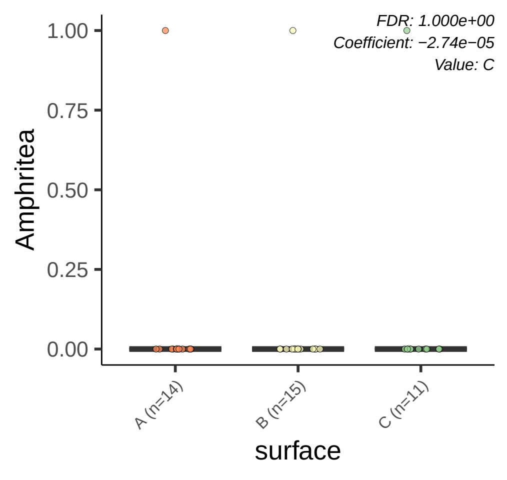

FDR: 1.000e+00
Coefficient: −2.74e−05
Value: C
Amphritea
1.00
0.75
0.50
0.25
0.00
A (n=14)
B (n=15)
C (n=11)
surface

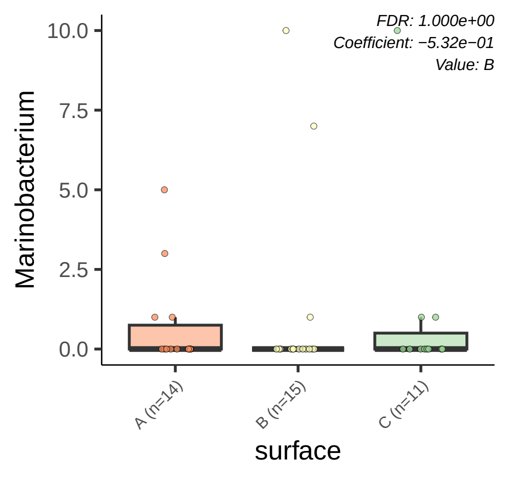
FDR: 1.000e+00
Coefficient: −5.32e−01
Value: B
Marinobacterium
10.0
7.5
5.0
2.5
0.0
A (n=14)
B (n=15)
C (n=11)
surface

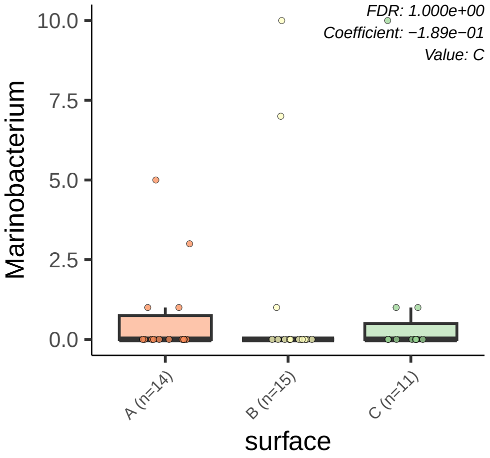
FDR: 1.000e+00
Coefficient: −1.89e−01
Value: C
Marinobacterium
10.0
7.5
5.0
2.5
0.0
A (n=14)
B (n=15)
C (n=11)
surface

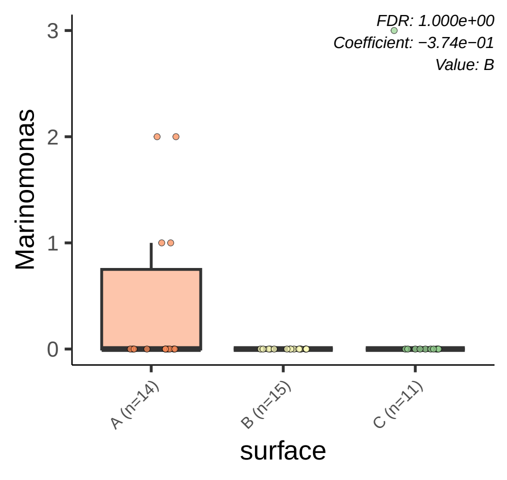
FDR: 1.000e+00
Coefficient: −3.74e−01
Value: B
Marinomonas
0
1
2
3
A (n=14)
B (n=15)
C (n=11)
surface

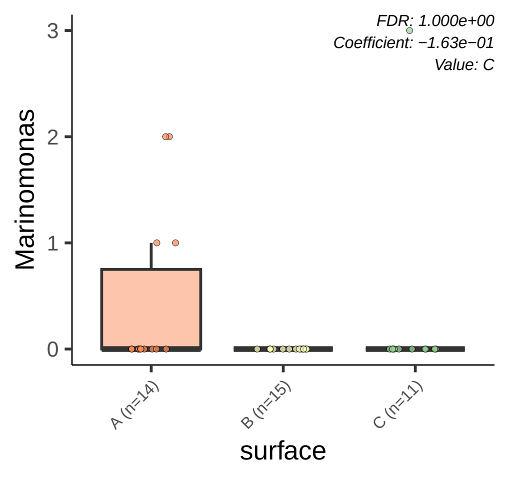
FDR: 1.000e+00
Coefficient: −1.63e−01
Value: C
Marinomonas
3
2
1
0
A (n=14)
B (n=15)
C (n=11)
surface

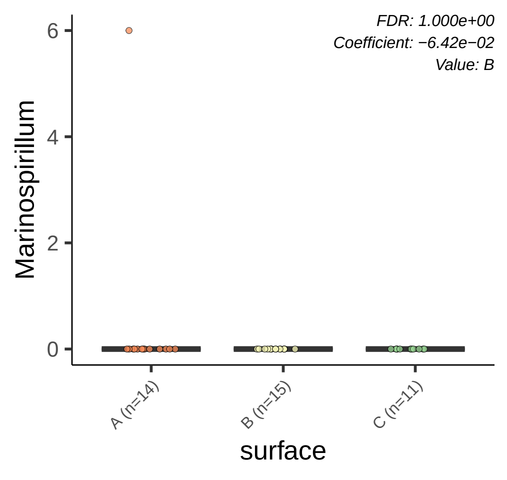
FDR: 1.000e+00
Coefficient: −6.42e−02
Value: B
Marinospirillum
6
4
2
0
A (n=14)
B (n=15)
C (n=11)
surface

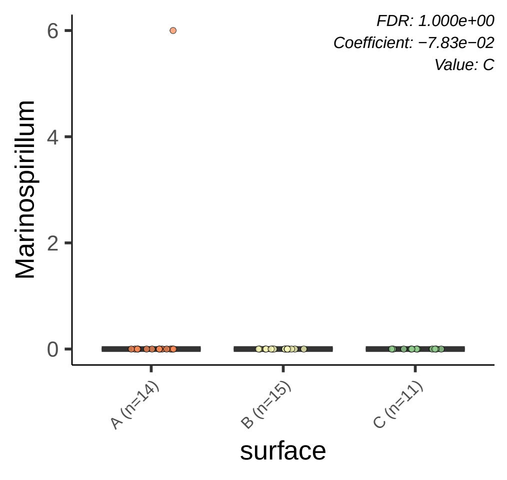
FDR: 1.000e+00
Coefficient: −7.83e−02
Value: C
Marinospirillum
6
4
2
0
A (n=14)
B (n=15)
C (n=11)
surface

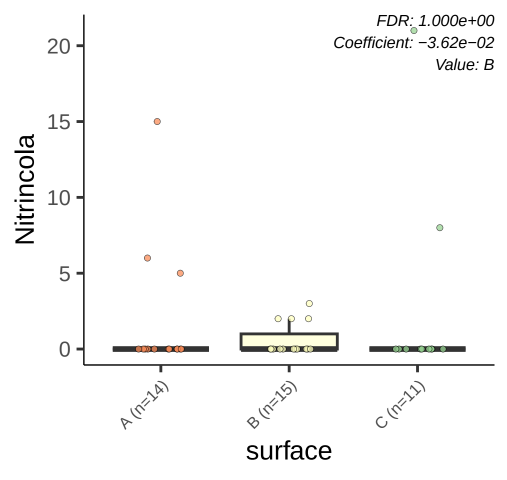
FDR: 1.000e+00
Coefficient: −3.62e−02
Value: B
Nitrincola
20
15
10
5
0
A (n=14)
B (n=15)
C (n=11)
surface

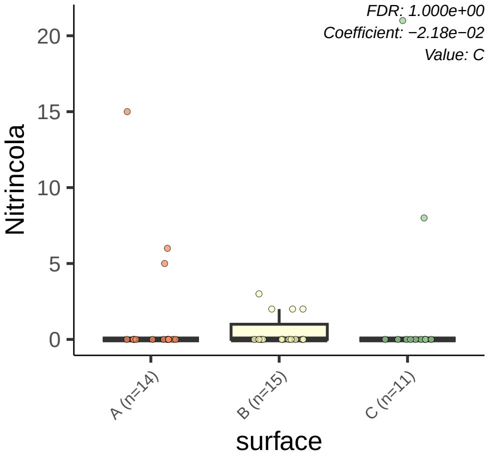
FDR: 1.000e+00
Coefficient: −2.18e−02
Value: C
Nitrincola
20
15
10
5
0
A (n=14)
B (n=15)
C (n=11)
surface

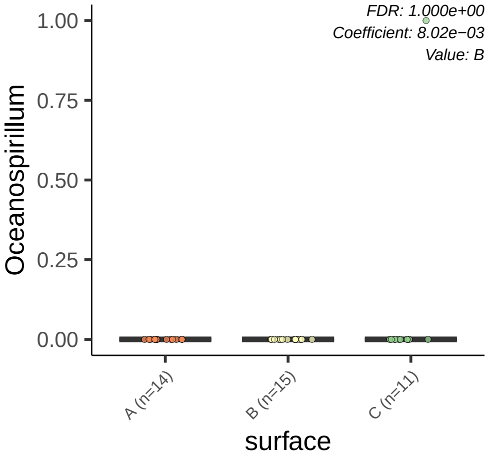
FDR: 1.000e+00
Coefficient: 8.02e−03
Value: B
Oceanospirillum
1.00
0.75
0.50
0.25
0.00
A (n=14)
B (n=15)
C (n=11)
surface

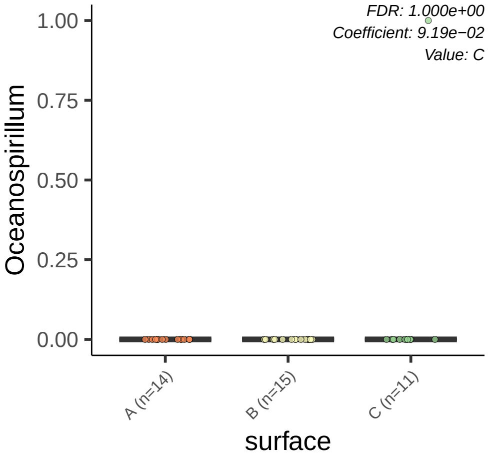

FDR: 1.000e+00
Coefficient: 9.19e−02
Value: C
Oceanospirillum
1.00
0.75
0.50
0.25
0.00
A (n=14)
B (n=15)
C (n=11)
surface

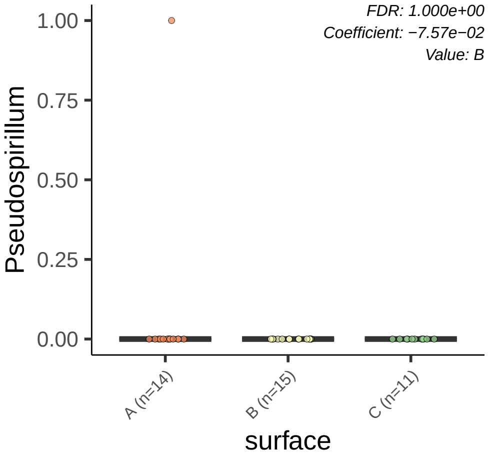
FDR: 1.000e+00
Coefficient: −7.57e−02
Value: B
Pseudospirillum
1.00
0.75
0.50
0.25
0.00
A (n=14)
B (n=15)
C (n=11)
surface

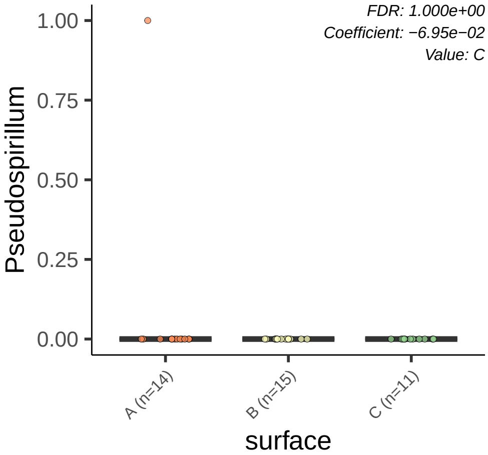

FDR: 1.000e+00
Coefficient: −6.95e−02
Value: C
Pseudospirillum
1.00
0.75
0.50
0.25
0.00
A (n=14)
B (n=15)
C (n=11)
surface

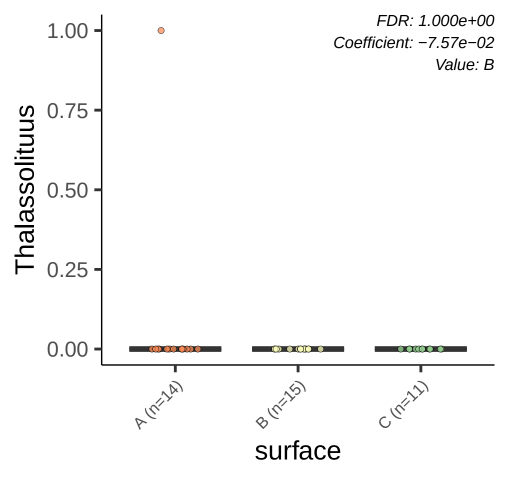
FDR: 1.000e+00
Coefficient: −7.57e−02
Value: B
Thalassolituus
1.00
0.75
0.50
0.25
0.00
A (n=14)
B (n=15)
C (n=11)
surface

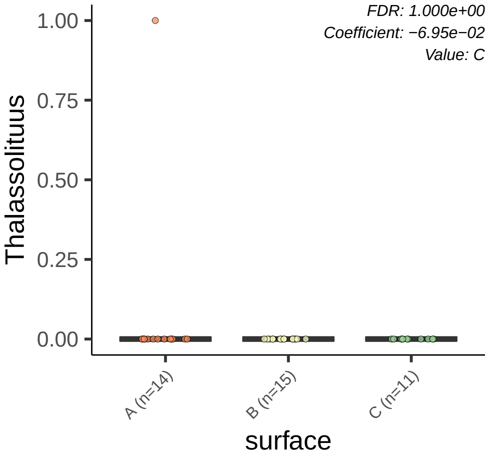

FDR: 1.000e+00
Coefficient: −6.95e−02
Value: C
Thalassolituus
1.00
0.75
0.50
0.25
0.00
A (n=14)
B (n=15)
C (n=11)
surface

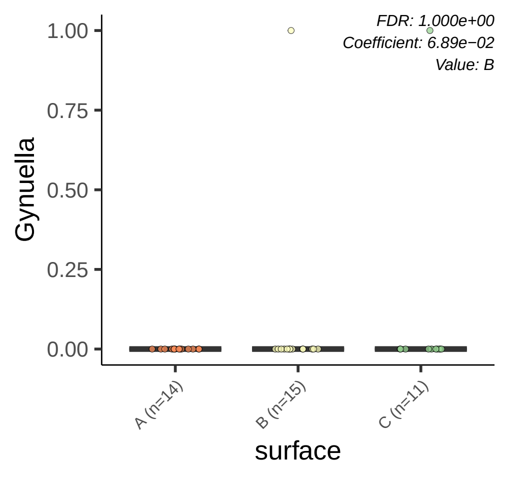
FDR: 1.000e+00
Coefficient: 6.89e−02
Value: B
Gynuella
1.00
0.75
0.50
0.25
0.00
A (n=14)
B (n=15)
C (n=11)
surface

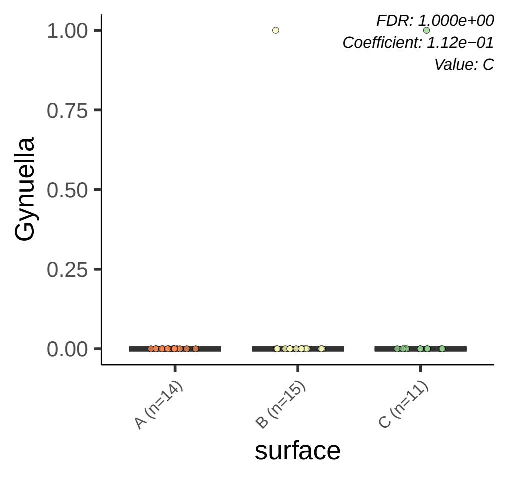

FDR: 1.000e+00
Coefficient: 1.12e−01
Value: C
1.00
0.75
0.50
0.25
0.00
Gynuella
A (n=14)
B (n=15)
C (n=11)
surface

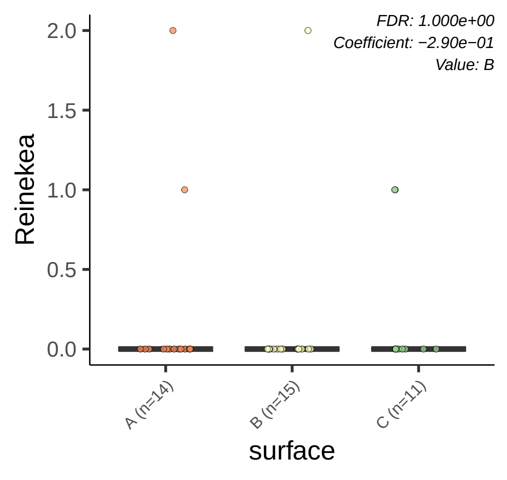

FDR: 1.000e+00
Coefficient: −2.90e−01
Value: B
Reinekea
2.0
1.5
1.0
0.5
0.0
A (n=14)
B (n=15)
C (n=11)
surface

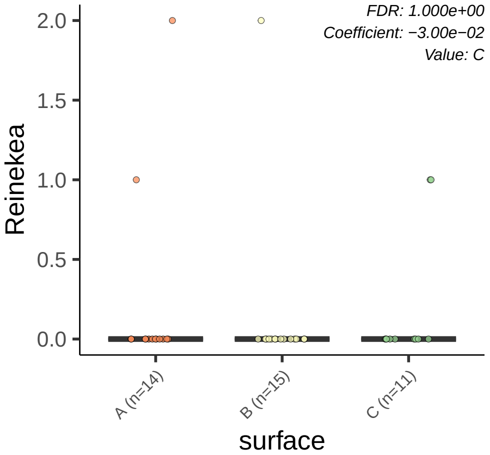

FDR: 1.000e+00
Coefficient: −3.00e−02
Value: C
Reinekea
2.0
1.5
1.0
0.5
0.0
A (n=14)
B (n=15)
C (n=11)
surface

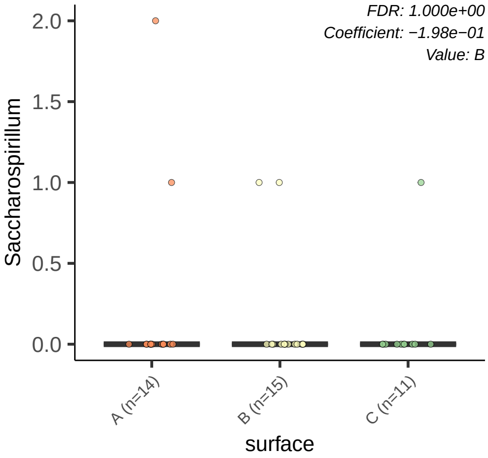

FDR: 1.000e+00
Coefficient: −1.98e−01
Value: B
Saccharospirillum
2.0
1.5
1.0
0.5
0.0
A (n=14)
B (n=15)
C (n=11)
surface

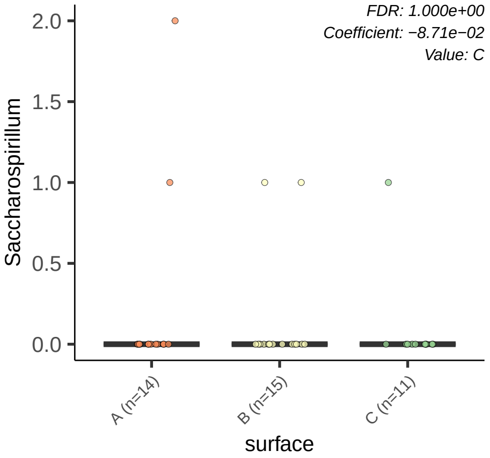

FDR: 1.000e+00
Coefficient: −8.71e−02
Value: C
Saccharospirillum
2.0
1.5
1.0
0.5
0.0
A (n=14)
B (n=15)
C (n=11)
surface

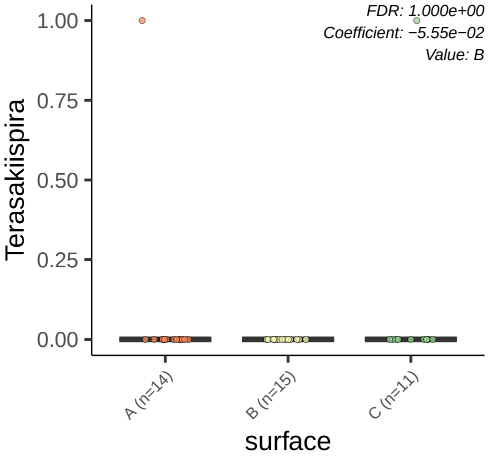

FDR: 1.000e+00
Coefficient: −5.55e−02
Value: B
Terasakiispira
1.00
0.75
0.50
0.25
0.00
A (n=14)
B (n=15)
C (n=11)
surface

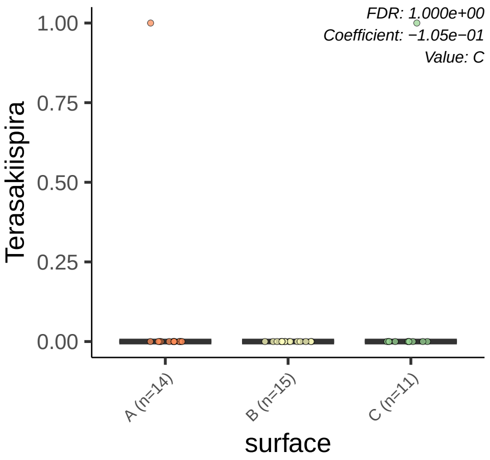

FDR: 1.000e+00
Coefficient: −1.05e−01
Value: C
Terasakiispira
1.00
0.75
0.50
0.25
0.00
A (n=14)
B (n=15)
C (n=11)
surface

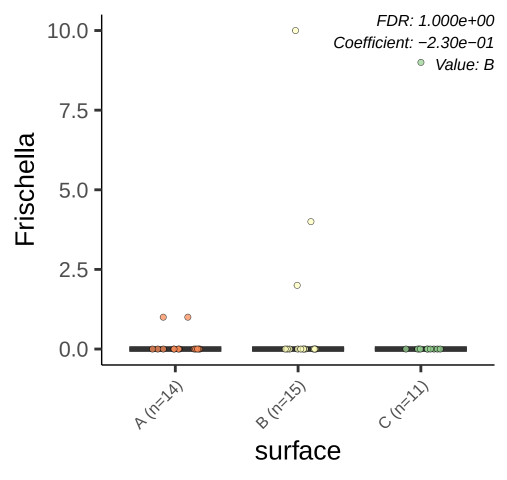

FDR: 1.000e+00
Coefficient: −2.30e−01
Value: B
Frischella
10.0
7.5
5.0
2.5
0.0
A (n=14)
B (n=15)
C (n=11)
surface

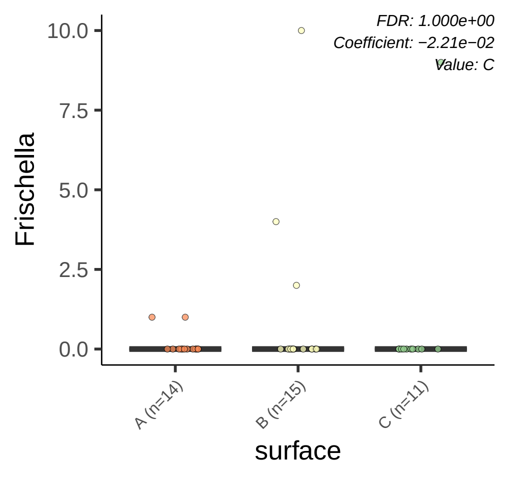

FDR: 1.000e+00
Coefficient: −2.21e−02
Value: C
Frischella
10.0
7.5
5.0
2.5
0.0
A (n=14)
B (n=15)
C (n=11)
surface

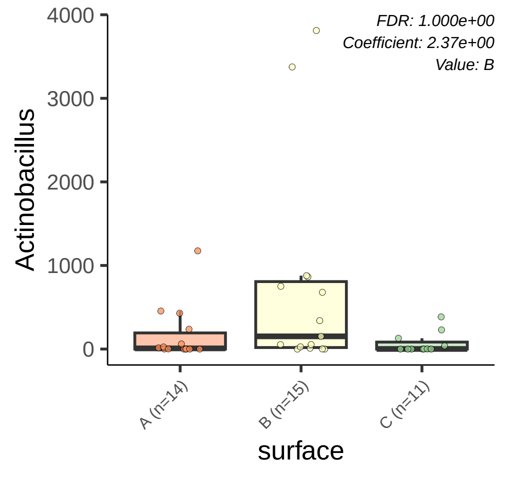
FDR: 1.000e+00
Coefficient: 2.37e+00
Value: B
Actinobacillus
4000
3000
2000
1000
0
A (n=14)
B (n=15)
C (n=11)
surface

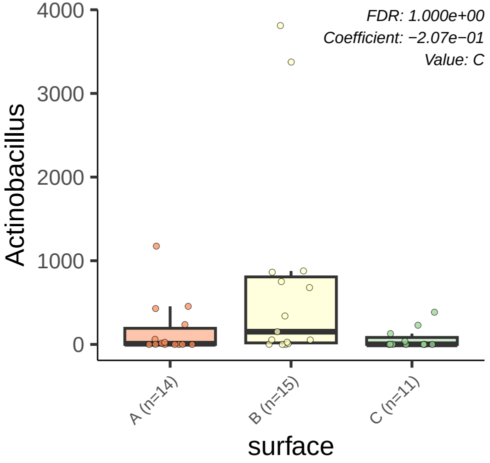
FDR: 1.000e+00
Coefficient: −2.07e−01
Value: C
Actinobacillus
4000
3000
2000
1000
0
A (n=14)
B (n=15)
C (n=11)
surface

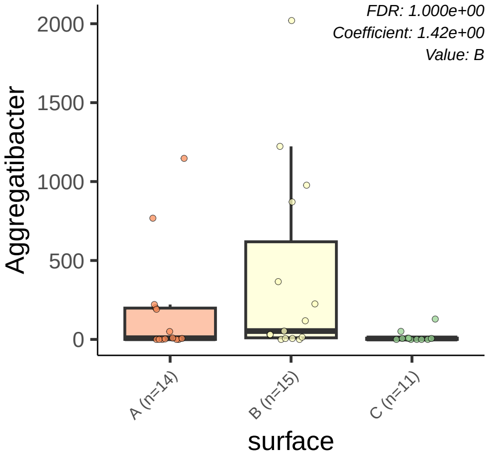
FDR: 1.000e+00
Coefficient: 1.42e+00
Value: B
Aggregatibacter
2000
1500
1000
500
0
A (n=14)
B (n=15)
C (n=11)
surface

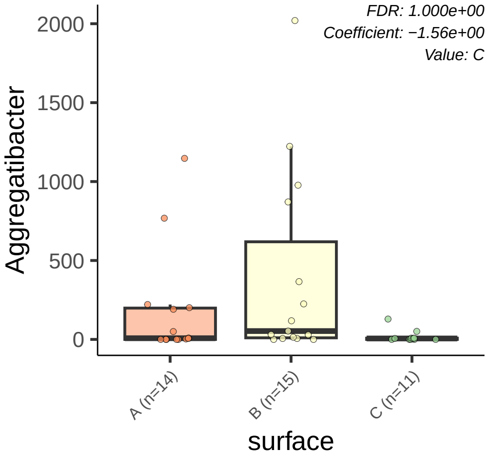

FDR: 1.000e+00
Coefficient: −1.56e+00
Value: C
Aggregatibacter
2000
1500
1000
500
0
A (n=14)
B (n=15)
C (n=11)
surface

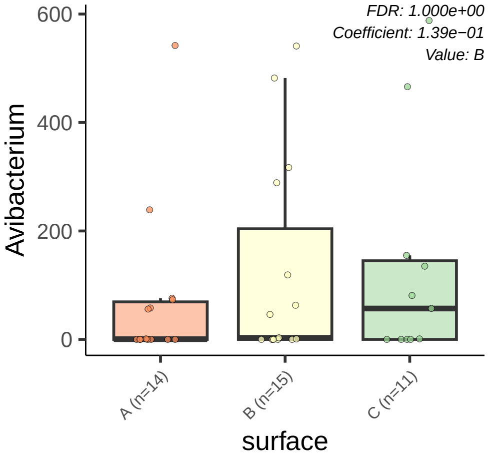
FDR: 1.000e+00
Coefficient: 1.39e−01
Value: B
Avibacterium
600
400
200
0
A (n=14)
B (n=15)
C (n=11)
surface

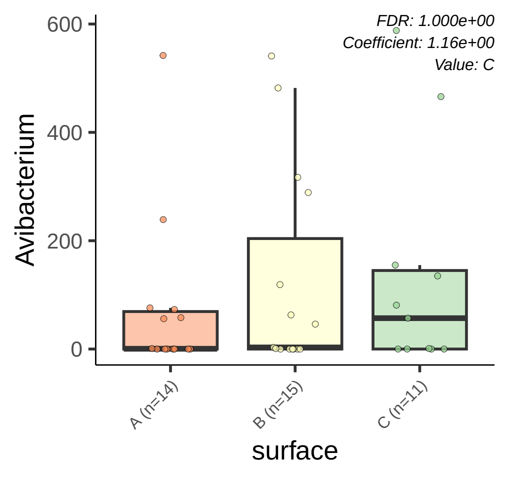

FDR: 1.000e+00
Coefficient: 1.16e+00
Value: C
600
400
200
0
Avibacterium
A (n=14)
B (n=15)
C (n=11)
surface

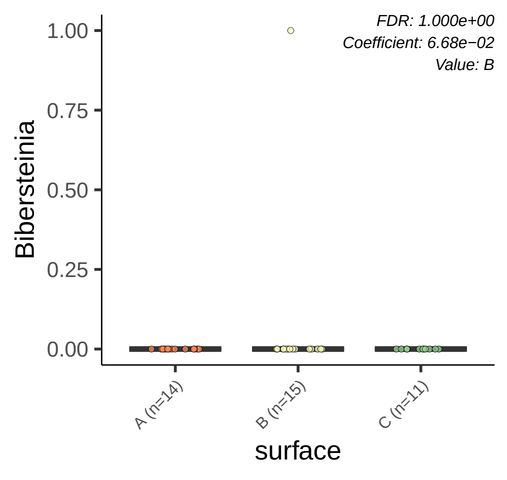
FDR: 1.000e+00
Coefficient: 6.68e−02
Value: B
Bibersteinia
1.00
0.75
0.50
0.25
0.00
A (n=14)
B (n=15)
C (n=11)
surface

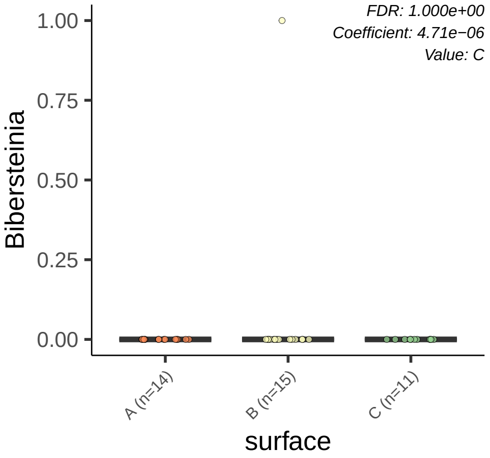

FDR: 1.000e+00
Coefficient: 4.71e−06
Value: C
Bibersteinia
1.00
0.75
0.50
0.25
0.00
A (n=14)
B (n=15)
C (n=11)
surface

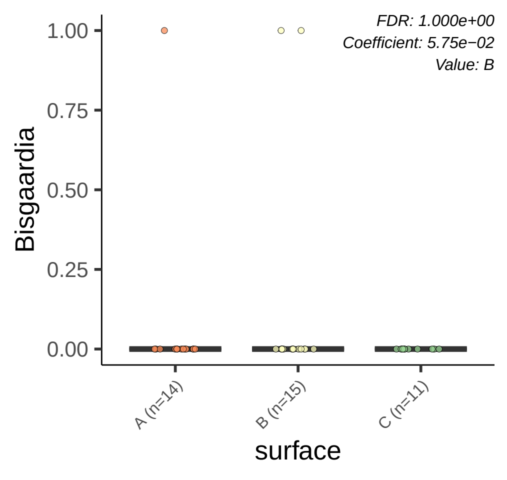

FDR: 1.000e+00
Coefficient: 5.75e−02
Value: B
Bisgaardia
1.00
0.75
0.50
0.25
0.00
A (n=14)
B (n=15)
C (n=11)
surface

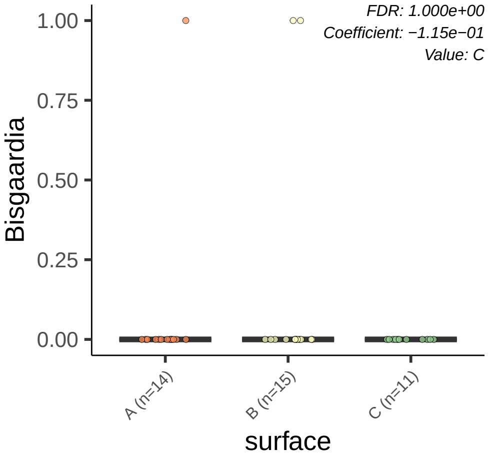
FDR: 1.000e+00
Coefficient: −1.15e−01
Value: C
Bisgaardia
1.00
0.75
0.50
0.25
0.00
A (n=14)
B (n=15)
C (n=11)
surface

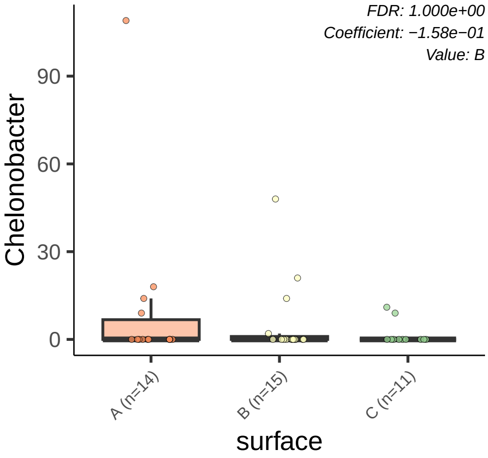
FDR: 1.000e+00
Coefficient: −1.58e−01
Value: B
Chelonobacter
90
60
30
0
A (n=14)
B (n=15)
C (n=11)
surface

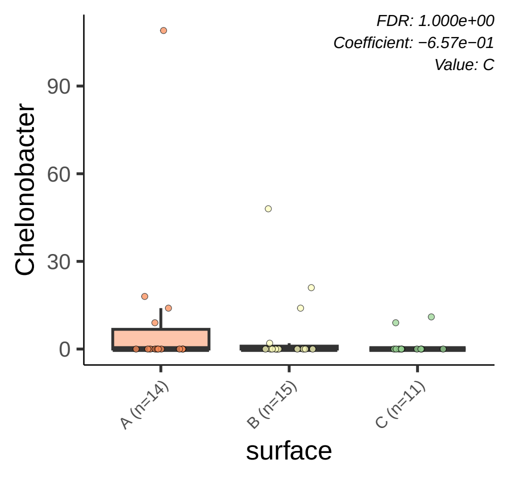

FDR: 1.000e+00
Coefficient: −6.57e−01
Value: C
Chelonobacter
90
60
30
0
A (n=14)
B (n=15)
C (n=11)
surface

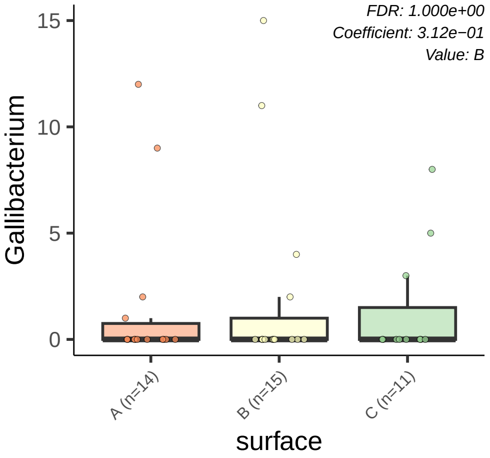
FDR: 1.000e+00
Coefficient: 3.12e−01
Value: B
Gallibacterium
15
10
5
0
A (n=14)
B (n=15)
C (n=11)
surface

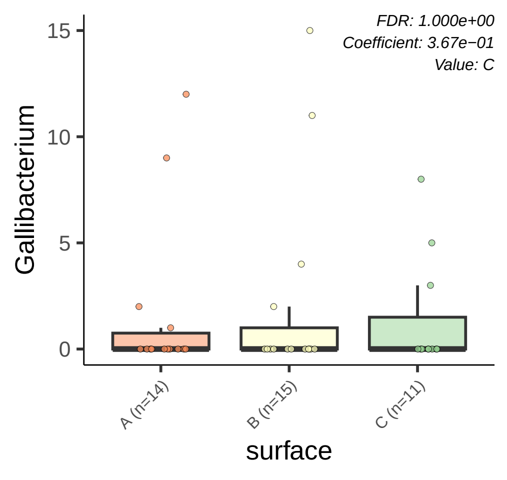
FDR: 1.000e+00
Coefficient: 3.67e−01
Value: C
Gallibacterium
15
10
5
0
A (n=14)
B (n=15)
C (n=11)
surface

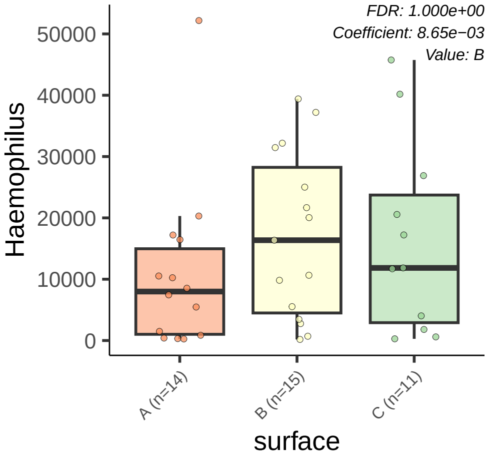

FDR: 1.000e+00
Coefficient: 8.65e−03
Value: B
Haemophilus
50000
40000
30000
20000
10000
0
A (n=14)
B (n=15)
C (n=11)
surface

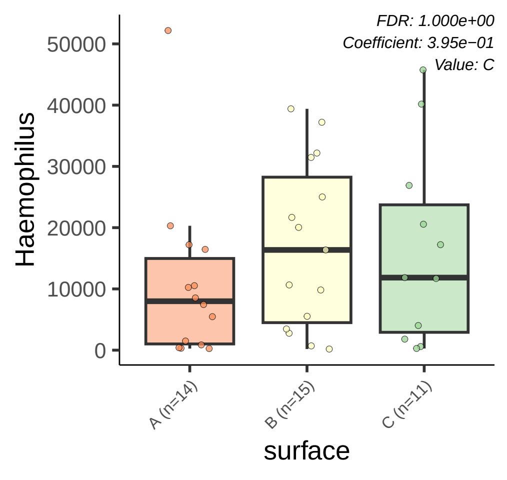
FDR: 1.000e+00
Coefficient: 3.95e−01
Value: C
Haemophilus
50000
40000
30000
20000
10000
0
A (n=14)
B (n=15)
C (n=11)
surface

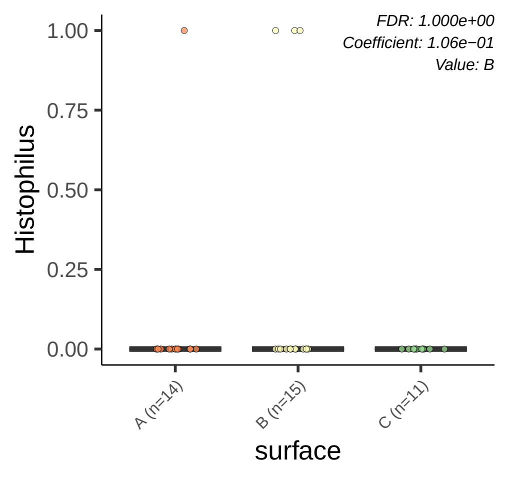
FDR: 1.000e+00
Coefficient: 1.06e−01
Value: B
Histophilus
1.00
0.75
0.50
0.25
0.00
A (n=14)
B (n=15)
C (n=11)
surface

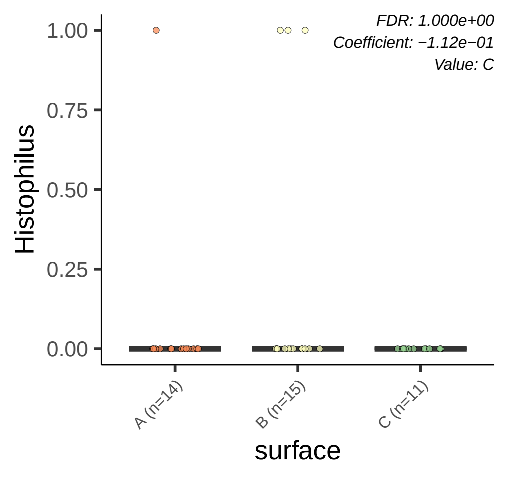

FDR: 1.000e+00
Coefficient: −1.12e−01
Value: C
Histophilus
1.00
0.75
0.50
0.25
0.00
A (n=14)
B (n=15)
C (n=11)
surface

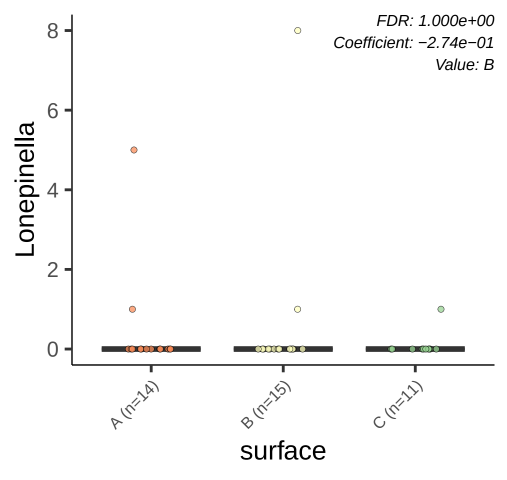
FDR: 1.000e+00
Coefficient: −2.74e−01
Value: B
Lonepinella
8
6
4
2
0
A (n=14)
B (n=15)
C (n=11)
surface

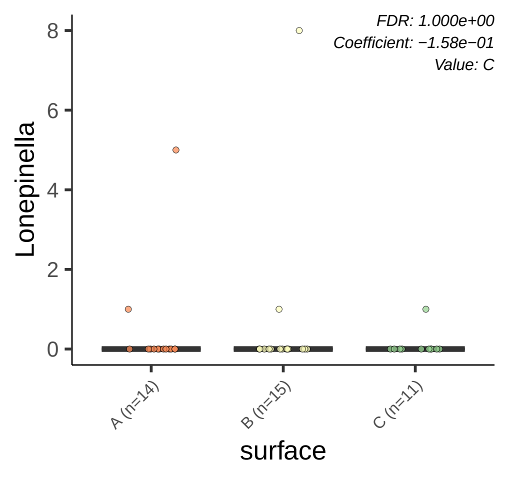

FDR: 1.000e+00
Coefficient: −1.58e−01
Value: C
Lonepinella
0
2
4
6
8
A (n=14)
B (n=15)
C (n=11)
surface

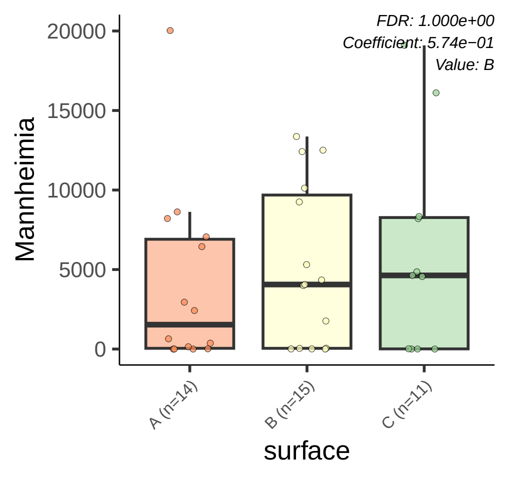
FDR: 1.000e+00
Coefficient: 5.74e−01
Value: B
Mannheimia
20000
15000
10000
5000
0
A (n=14)
B (n=15)
C (n=11)
surface

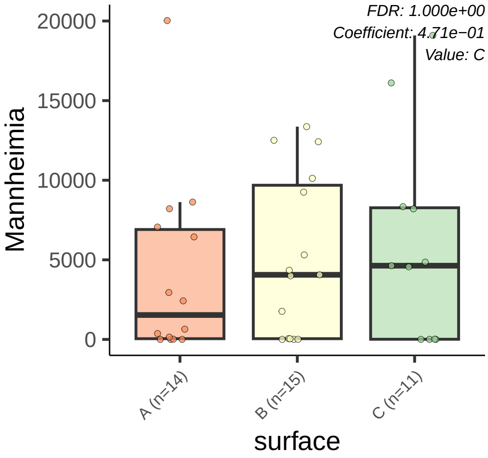
FDR: 1.000e+00
Coefficient: 4.71e−01
Value: C
Mannheimia
20000
15000
10000
5000
0
A (n=14)
B (n=15)
C (n=11)
surface

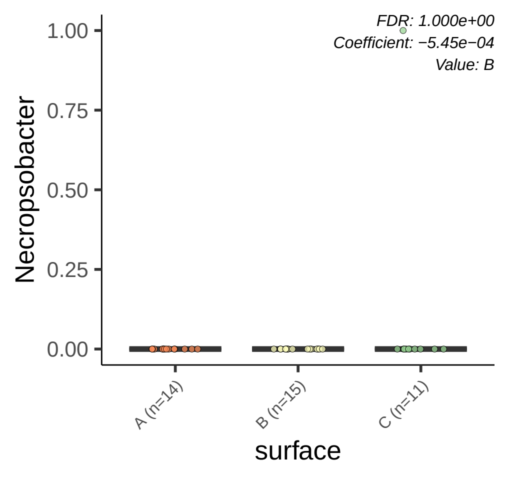
FDR: 1.000e+00
Coefficient: −5.45e−04
Value: B
Necropsobacter
1.00
0.75
0.50
0.25
0.00
A (n=14)
B (n=15)
C (n=11)
surface

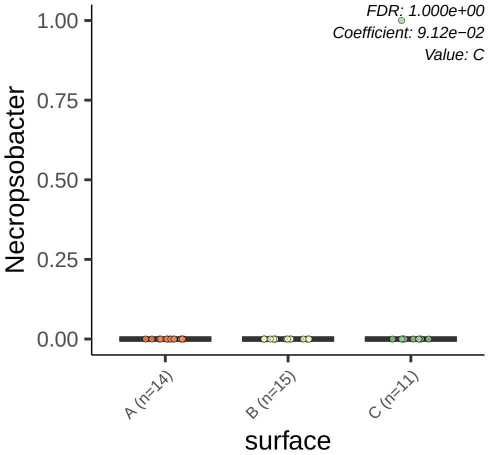

FDR: 1.000e+00
Coefficient: 9.12e−02
Value: C
Necropsobacter
1.00
0.75
0.50
0.25
0.00
A (n=14)
B (n=15)
C (n=11)
surface

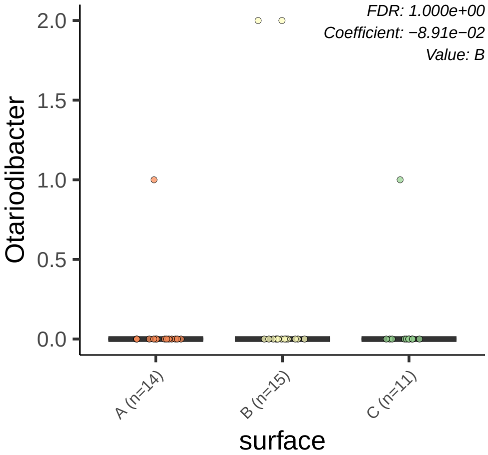
FDR: 1.000e+00
Coefficient: −8.91e−02
Value: B
Otariodibacter
2.0
1.5
1.0
0.5
0.0
A (n=14)
B (n=15)
C (n=11)
surface

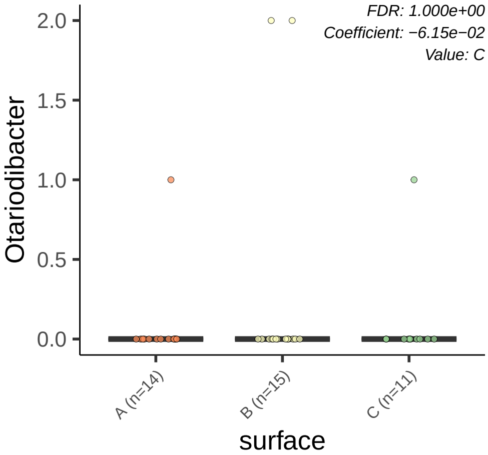

FDR: 1.000e+00
Coefficient: −6.15e−02
Value: C
Otariodibacter
2.0
1.5
1.0
0.5
0.0
A (n=14)
B (n=15)
C (n=11)
surface

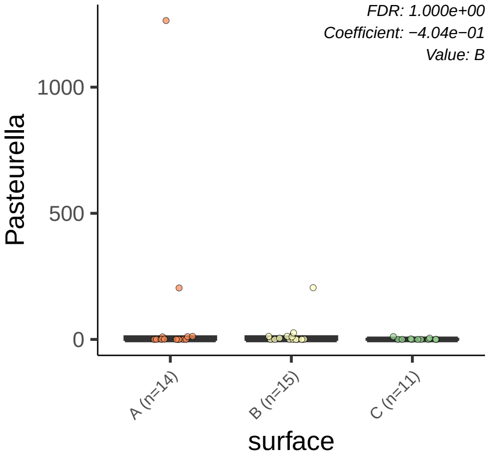
FDR: 1.000e+00
Coefficient: −4.04e−01
Value: B
Pasteurella
1000
500
0
A (n=14)
B (n=15)
C (n=11)
surface

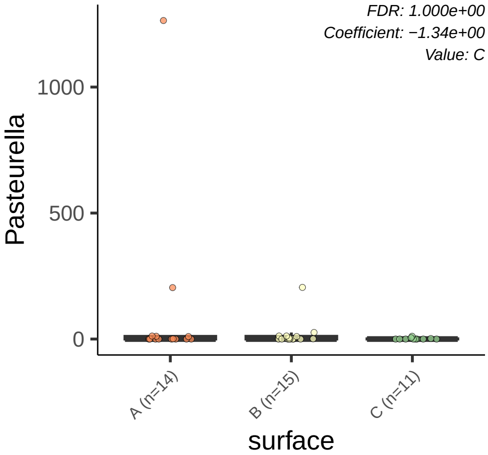
FDR: 1.000e+00
Coefficient: −1.34e+00
Value: C
Pasteurella
1000
500
0
A (n=14)
B (n=15)
C (n=11)
surface

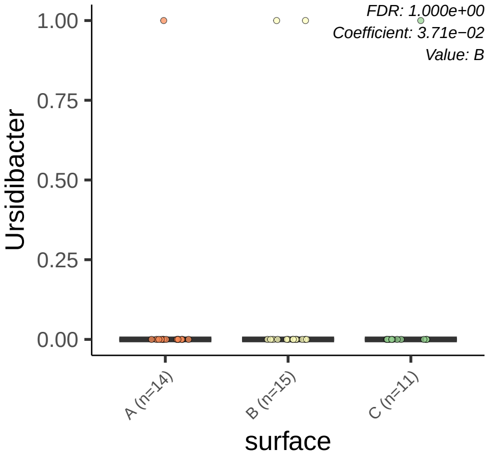

FDR: 1.000e+00
Coefficient: 3.71e−02
Value: B
Ursidibacter
1.00
0.75
0.50
0.25
0.00
A (n=14)
B (n=15)
C (n=11)
surface

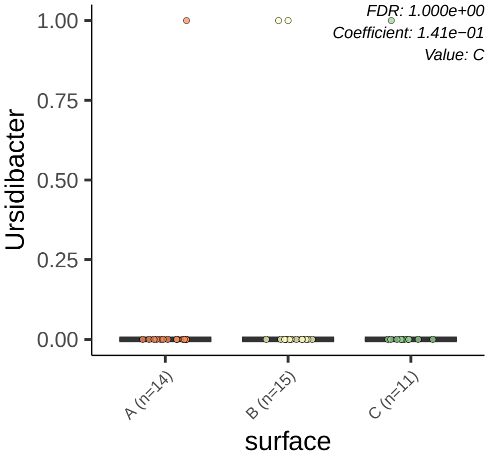

FDR: 1.000e+00
Coefficient: 1.41e−01
Value: C
Ursidibacter
1.00
0.75
0.50
0.25
0.00
A (n=14)
B (n=15)
C (n=11)
surface

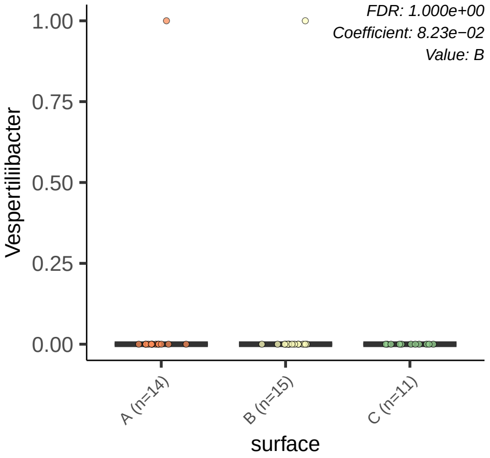
FDR: 1.000e+00
Coefficient: 8.23e−02
Value: B
Vespertiliibacter
1.00
0.75
0.50
0.25
0.00
A (n=14)
B (n=15)
C (n=11)
surface

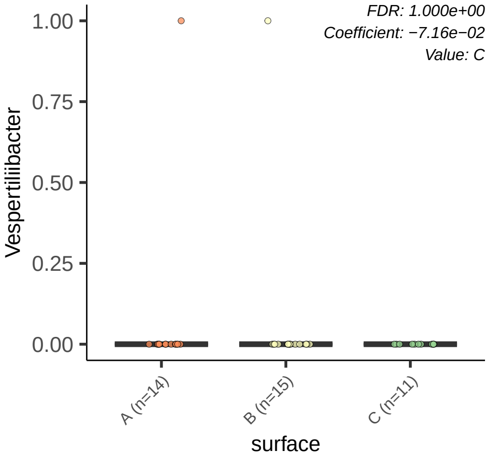

FDR: 1.000e+00
Coefficient: −7.16e−02
Value: C
Vespertiliibacter
1.00
0.75
0.50
0.25
0.00
A (n=14)
B (n=15)
C (n=11)
surface

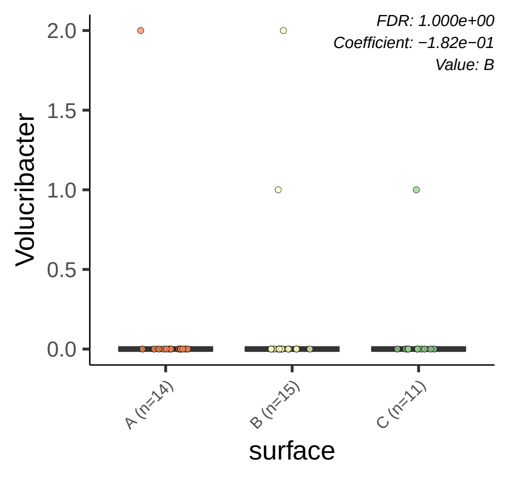

FDR: 1.000e+00
Coefficient: −1.82e−01
Value: B
Volucribacter
2.0
1.5
1.0
0.5
0.0
A (n=14)
B (n=15)
C (n=11)
surface

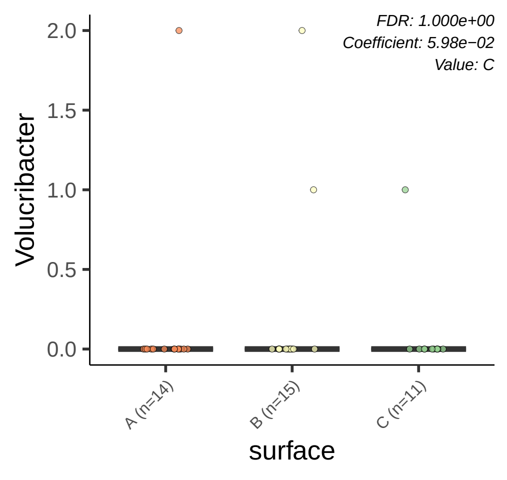

FDR: 1.000e+00
Coefficient: 5.98e−02
Value: C
Volucribacter
2.0
1.5
1.0
0.5
0.0
A (n=14)
B (n=15)
C (n=11)
surface

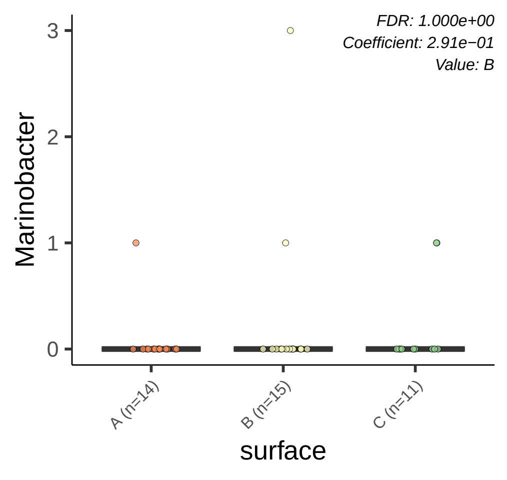

FDR: 1.000e+00
Coefficient: 2.91e−01
Value: B
Marinobacter
0
1
2
3
A (n=14)
B (n=15)
C (n=11)
surface

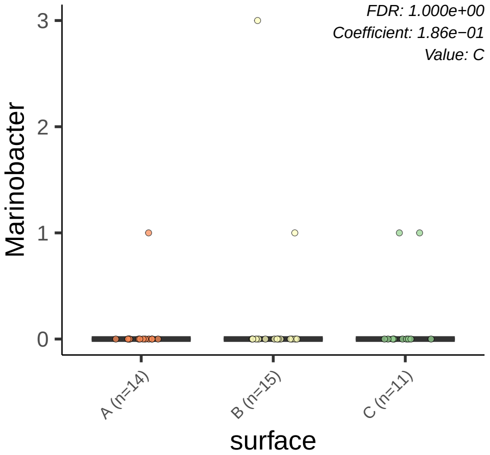
FDR: 1.000e+00
Coefficient: 1.86e−01
Value: C
Marinobacter
3
2
1
0
A (n=14)
B (n=15)
C (n=11)
surface

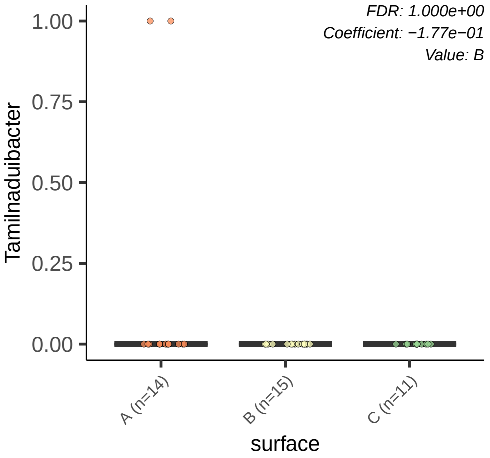

FDR: 1.000e+00
Coefficient: −1.77e−01
Value: B
Tamilnaduibacter
1.00
0.75
0.50
0.25
0.00
A (n=14)
B (n=15)
C (n=11)
surface

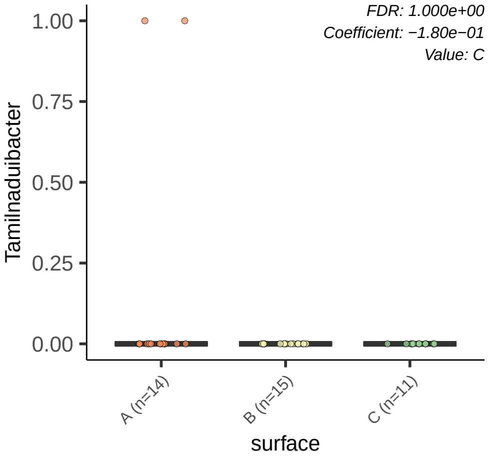

FDR: 1.000e+00
Coefficient: −1.80e−01
Value: C
Tamilnaduibacter
1.00
0.75
0.50
0.25
0.00
A (n=14)
B (n=15)
C (n=11)
surface

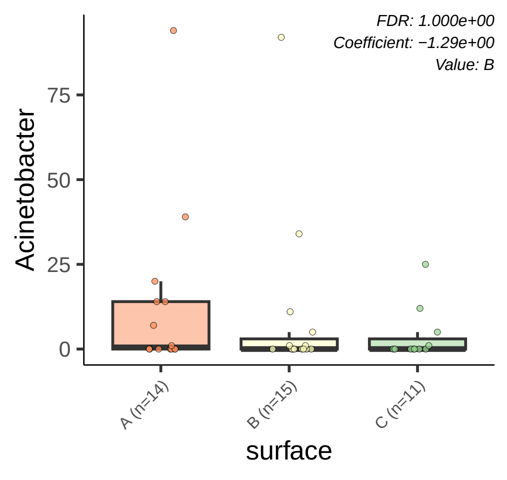
FDR: 1.000e+00
Coefficient: −1.29e+00
Value: B
Acinetobacter
75
50
25
0
A (n=14)
B (n=15)
C (n=11)
surface

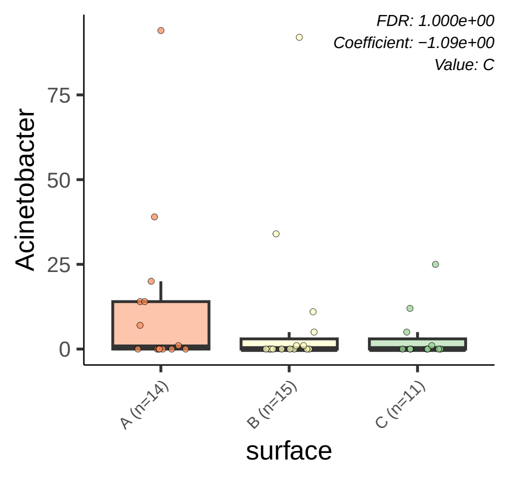

FDR: 1.000e+00
Coefficient: −1.09e+00
Value: C
Acinetobacter
75
50
25
0
A (n=14)
B (n=15)
C (n=11)
surface

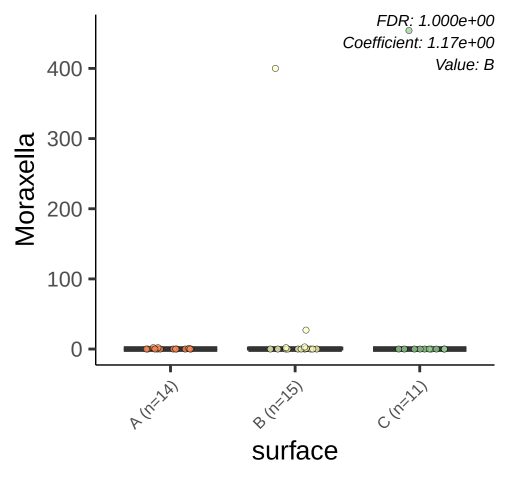

FDR: 1.000e+00
Coefficient: 1.17e+00
Value: B
Moraxella
400
300
200
100
0
A (n=14)
B (n=15)
C (n=11)
surface

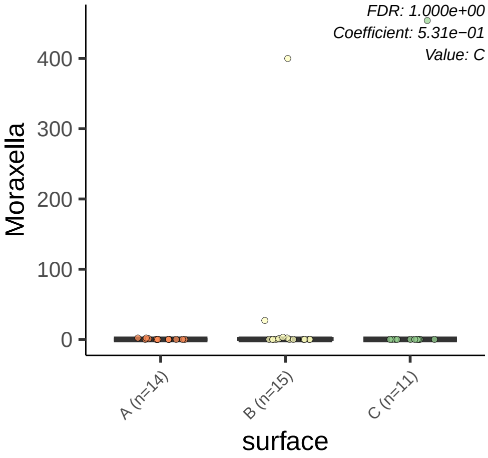
FDR: 1.000e+00
Coefficient: 5.31e−01
Value: C
Moraxella
400
300
200
100
0
A (n=14)
B (n=15)
C (n=11)
surface

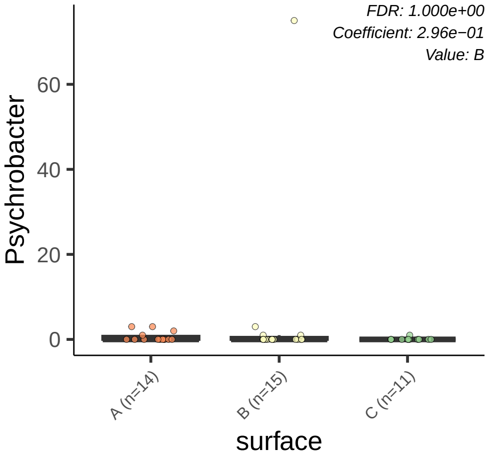

FDR: 1.000e+00
Coefficient: 2.96e−01
Value: B
Psychrobacter
0
20
40
60
A (n=14)
B (n=15)
C (n=11)
surface

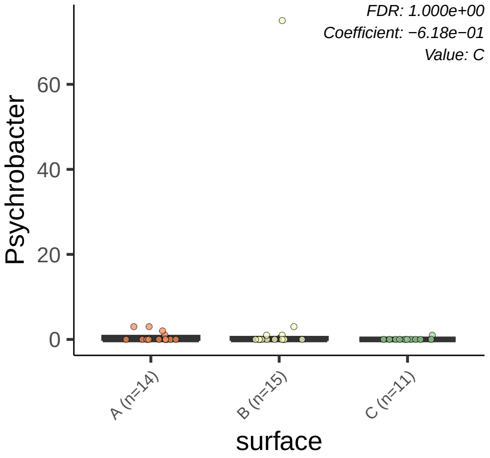
FDR: 1.000e+00
Coefficient: −6.18e−01
Value: C
Psychrobacter
60
40
20
0
A (n=14)
B (n=15)
C (n=11)
surface

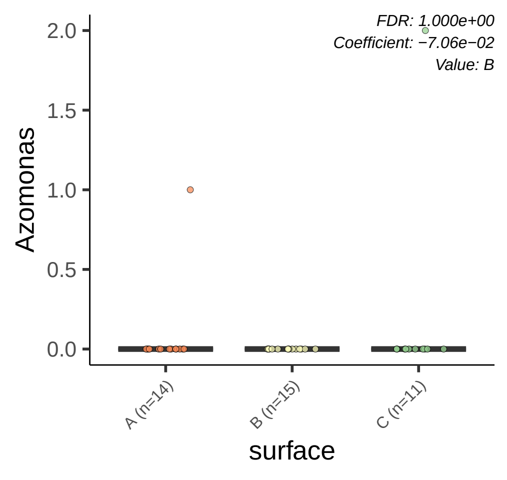
FDR: 1.000e+00
Coefficient: −7.06e−02
Value: B
Azomonas
2.0
1.5
1.0
0.5
0.0
A (n=14)
B (n=15)
C (n=11)
surface

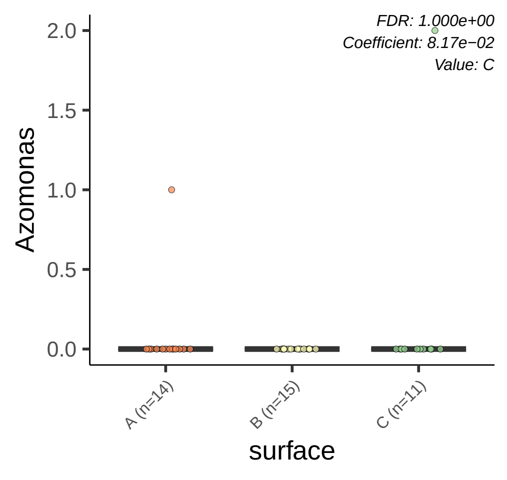
FDR: 1.000e+00
Coefficient: 8.17e−02
Value: C
Azomonas
2.0
1.5
1.0
0.5
0.0
A (n=14)
B (n=15)
C (n=11)
surface

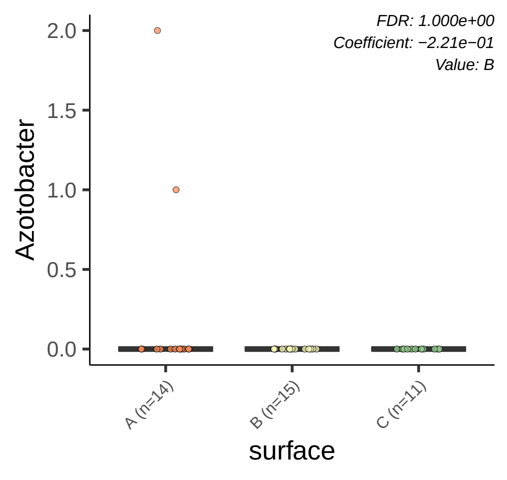
FDR: 1.000e+00
Coefficient: −2.21e−01
Value: B
Azotobacter
2.0
1.5
1.0
0.5
0.0
A (n=14)
B (n=15)
C (n=11)
surface

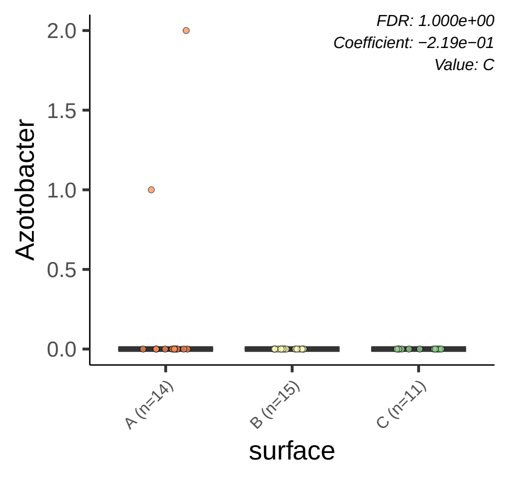
FDR: 1.000e+00
Coefficient: −2.19e−01
Value: C
Azotobacter
2.0
1.5
1.0
0.5
0.0
A (n=14)
B (n=15)
C (n=11)
surface

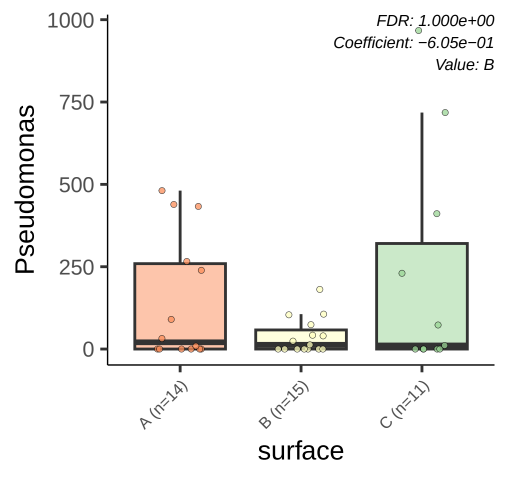
FDR: 1.000e+00
Coefficient: −6.05e−01
Value: B
Pseudomonas
1000
750
500
250
0
A (n=14)
B (n=15)
C (n=11)
surface

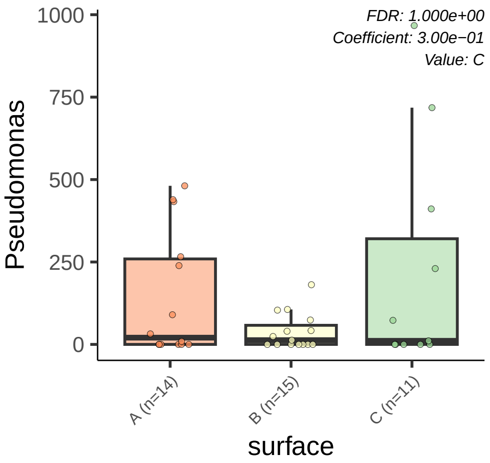

FDR: 1.000e+00
Coefficient: 3.00e−01
Value: C
Pseudomonas
1000
750
500
250
0
A (n=14)
B (n=15)
C (n=11)
surface

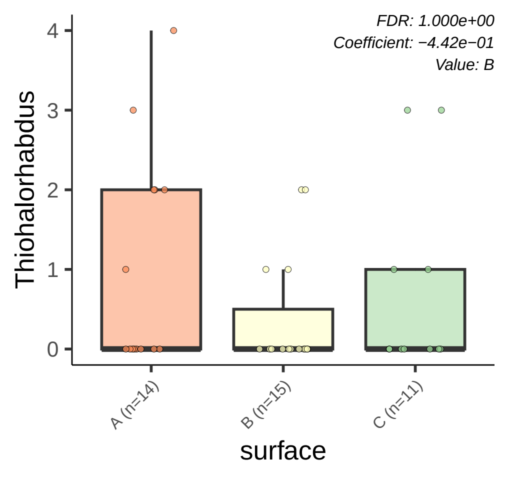

FDR: 1.000e+00
Coefficient: −4.42e−01
Value: B
Thiohalorhabdus
4
3
2
1
0
A (n=14)
B (n=15)
C (n=11)
surface

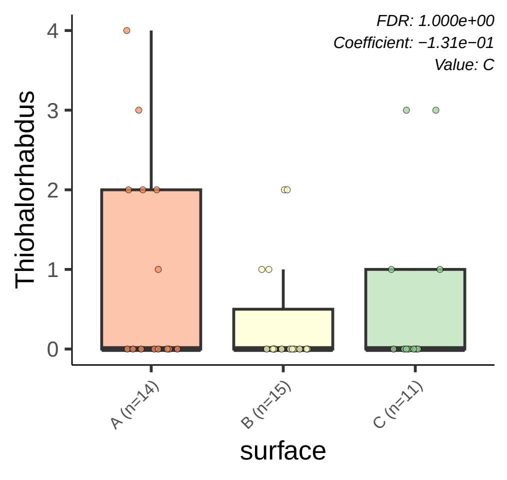

FDR: 1.000e+00
Coefficient: −1.31e−01
Value: C
Thiohalorhabdus
0
1
2
3
4
A (n=14)
B (n=15)
C (n=11)
surface

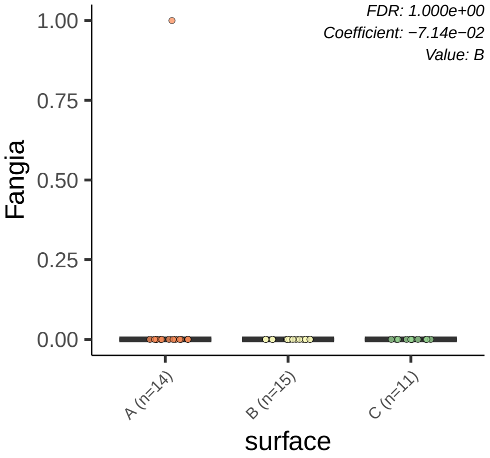
FDR: 1.000e+00
Coefficient: −7.14e−02
Value: B
Fangia
1.00
0.75
0.50
0.25
0.00
A (n=14)
B (n=15)
C (n=11)
surface

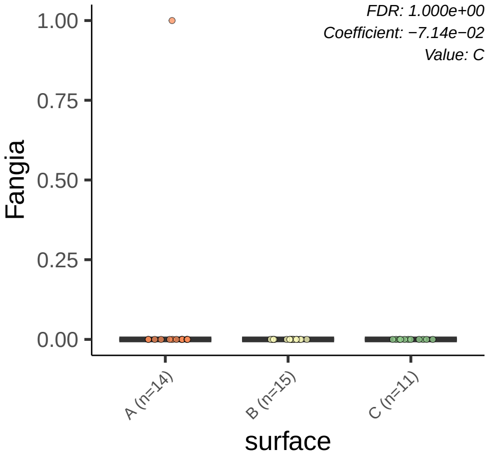

FDR: 1.000e+00
Coefficient: −7.14e−02
Value: C
Fangia
1.00
0.75
0.50
0.25
0.00
A (n=14)
B (n=15)
C (n=11)
surface

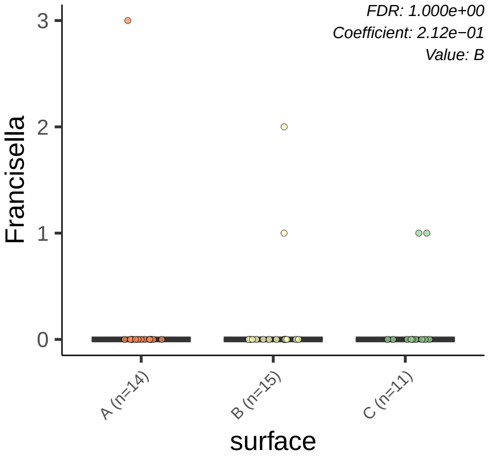

FDR: 1.000e+00
Coefficient: 2.12e−01
Value: B
Francisella
3
2
1
0
A (n=14)
B (n=15)
C (n=11)
surface

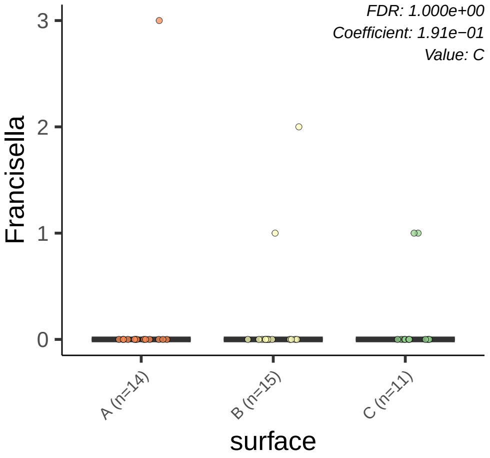
FDR: 1.000e+00
Coefficient: 1.91e−01
Value: C
Francisella
3
2
1
0
A (n=14)
B (n=15)
C (n=11)
surface

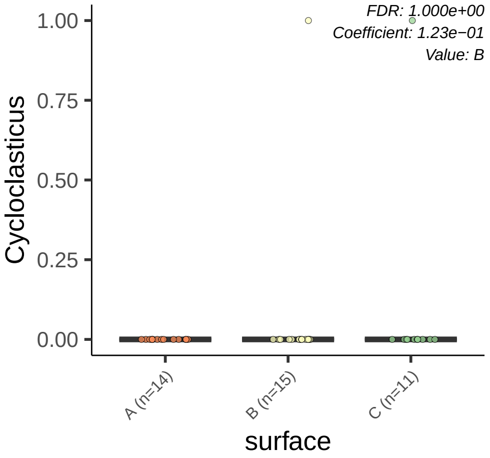
FDR: 1.000e+00
Coefficient: 1.23e−01
Value: B
Cycloclasticus
1.00
0.75
0.50
0.25
0.00
A (n=14)
B (n=15)
C (n=11)
surface

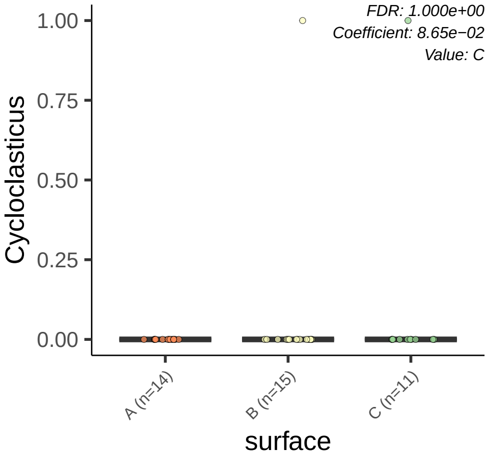
FDR: 1.000e+00
Coefficient: 8.65e−02
Value: C
Cycloclasticus
1.00
0.75
0.50
0.25
0.00
A (n=14)
B (n=15)
C (n=11)
surface

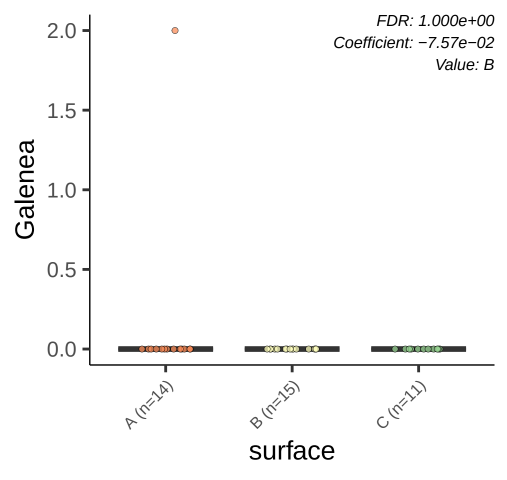

FDR: 1.000e+00
Coefficient: −7.57e−02
Value: B
Galenea
2.0
1.5
1.0
0.5
0.0
A (n=14)
B (n=15)
C (n=11)
surface

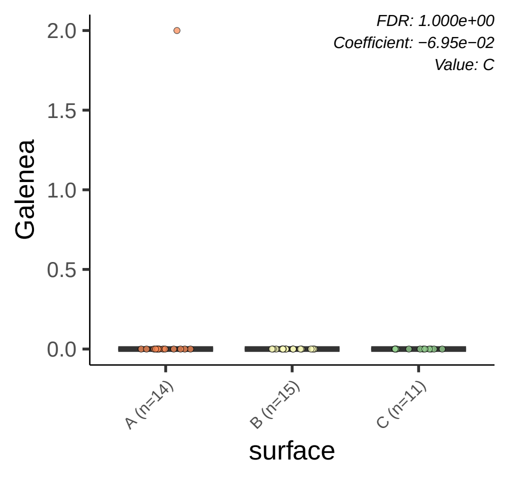
FDR: 1.000e+00
Coefficient: −6.95e−02
Value: C
Galenea
2.0
1.5
1.0
0.5
0.0
A (n=14)
B (n=15)
C (n=11)
surface

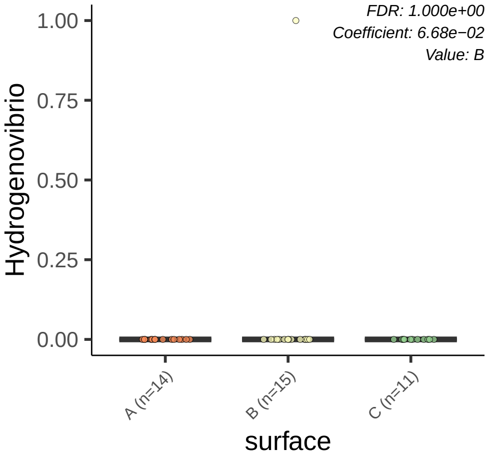
FDR: 1.000e+00
Coefficient: 6.68e−02
Value: B
Hydrogenovibrio
1.00
0.75
0.50
0.25
0.00
A (n=14)
B (n=15)
C (n=11)
surface

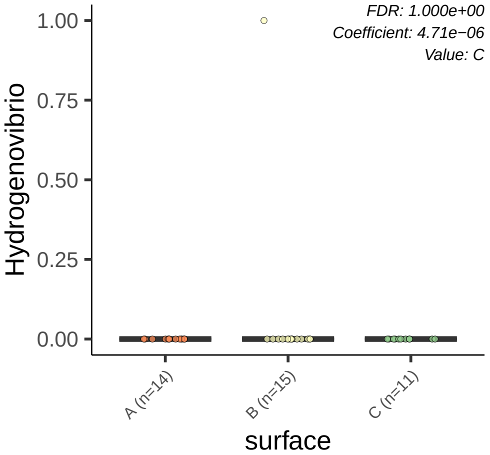
FDR: 1.000e+00
Coefficient: 4.71e−06
Value: C
Hydrogenovibrio
1.00
0.75
0.50
0.25
0.00
A (n=14)
B (n=15)
C (n=11)
surface

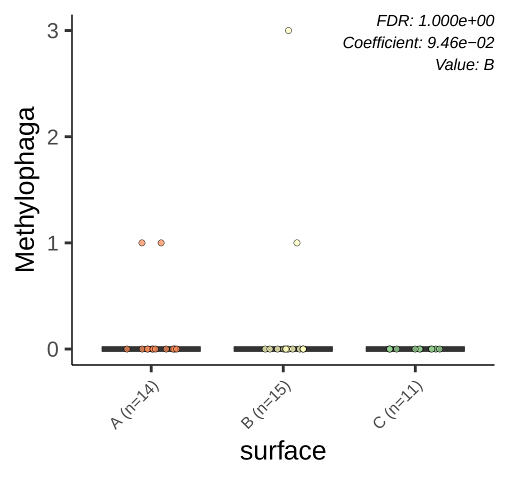

FDR: 1.000e+00
Coefficient: 9.46e−02
Value: B
Methylophaga
3
2
1
0
A (n=14)
B (n=15)
C (n=11)
surface

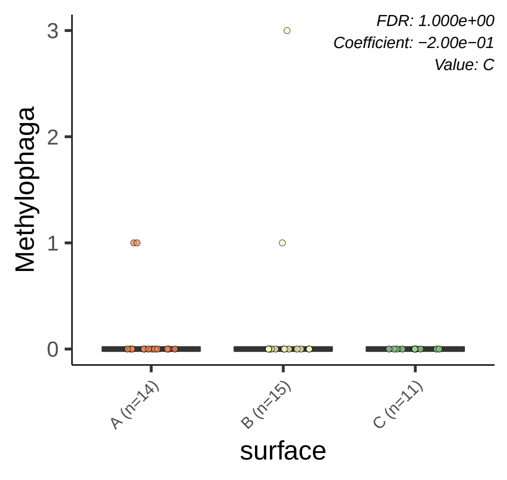

FDR: 1.000e+00
Coefficient: −2.00e−01
Value: C
Methylophaga
3
2
1
0
A (n=14)
B (n=15)
C (n=11)
surface

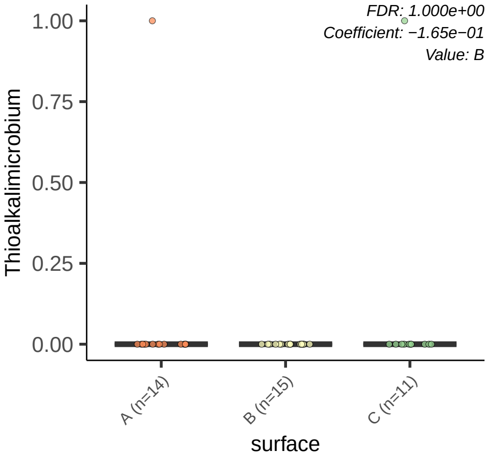
FDR: 1.000e+00
Coefficient: −1.65e−01
Value: B
1.00
0.75
0.50
0.25
0.00
Thioalkalimicrobium
A (n=14)
B (n=15)
C (n=11)
surface

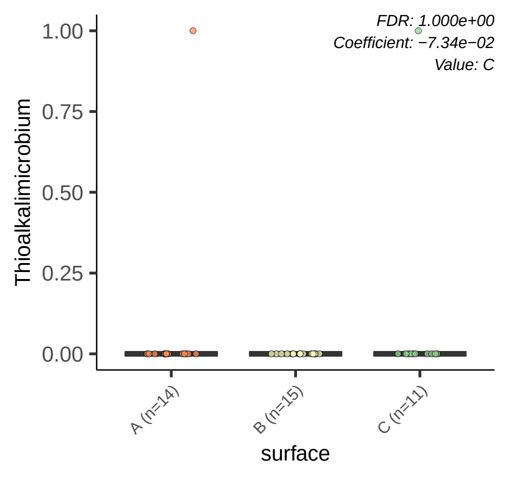
FDR: 1.000e+00
Coefficient: −7.34e−02
Value: C
Thioalkalimicrobium
1.00
0.75
0.50
0.25
0.00
A (n=14)
B (n=15)
C (n=11)
surface

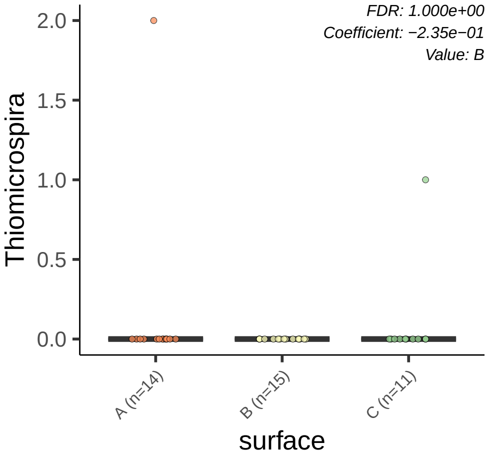
FDR: 1.000e+00
Coefficient: −2.35e−01
Value: B
Thiomicrospira
2.0
1.5
1.0
0.5
0.0
A (n=14)
B (n=15)
C (n=11)
surface

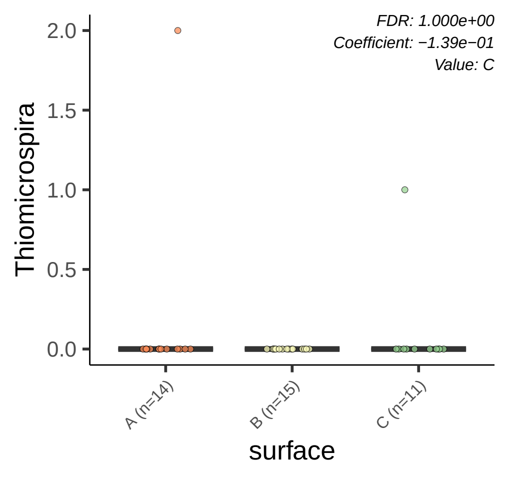
FDR: 1.000e+00
Coefficient: −1.39e−01
Value: C
Thiomicrospira
2.0
1.5
1.0
0.5
0.0
A (n=14)
B (n=15)
C (n=11)
surface

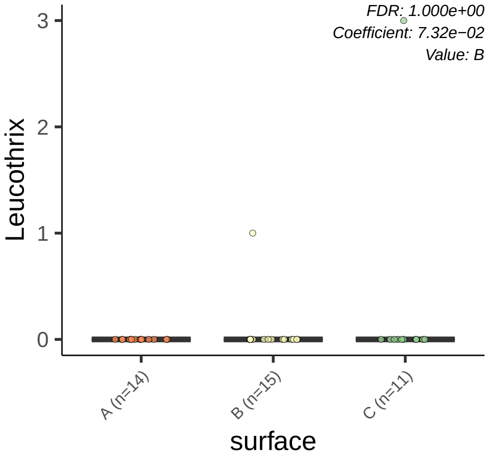
FDR: 1.000e+00
Coefficient: 7.32e−02
Value: B
Leucothrix
3
2
1
0
A (n=14)
B (n=15)
C (n=11)
surface

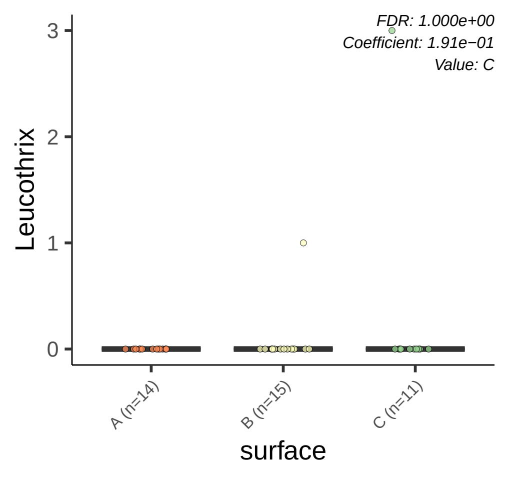

FDR: 1.000e+00
Coefficient: 1.91e−01
Value: C
Leucothrix
3
2
1
0
A (n=14)
B (n=15)
C (n=11)
surface

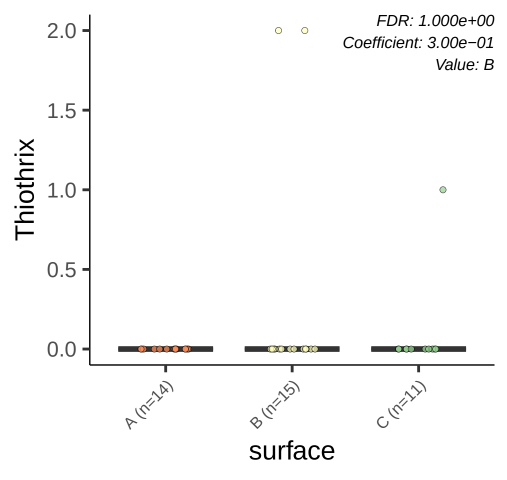
FDR: 1.000e+00
Coefficient: 3.00e−01
Value: B
Thiothrix
2.0
1.5
1.0
0.5
0.0
A (n=14)
B (n=15)
C (n=11)
surface

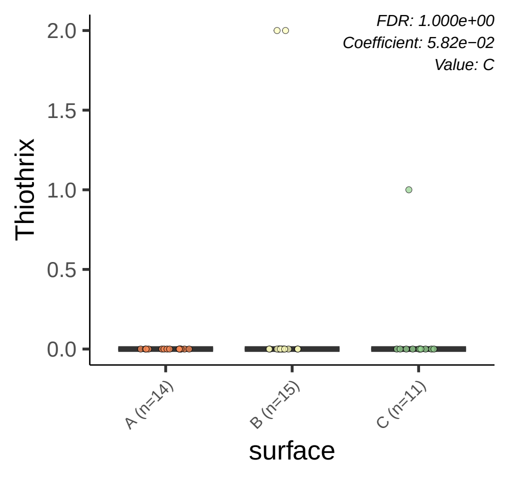
FDR: 1.000e+00
Coefficient: 5.82e−02
Value: C
Thiothrix
2.0
1.5
1.0
0.5
0.0
A (n=14)
B (n=15)
C (n=11)
surface

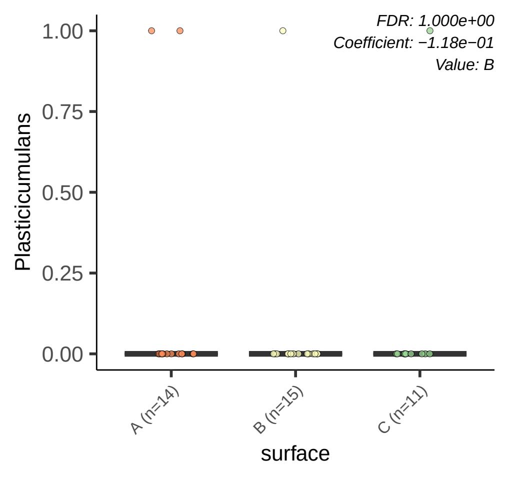
FDR: 1.000e+00
Coefficient: −1.18e−01
Value: B
Plasticicumulans
1.00
0.75
0.50
0.25
0.00
A (n=14)
B (n=15)
C (n=11)
surface

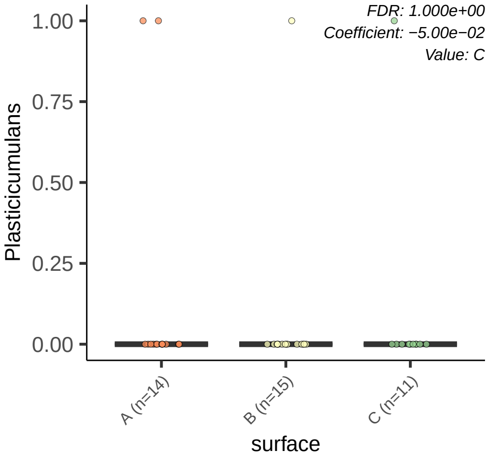

FDR: 1.000e+00
Coefficient: −5.00e−02
Value: C
Plasticicumulans
1.00
0.75
0.50
0.25
0.00
A (n=14)
B (n=15)
C (n=11)
surface

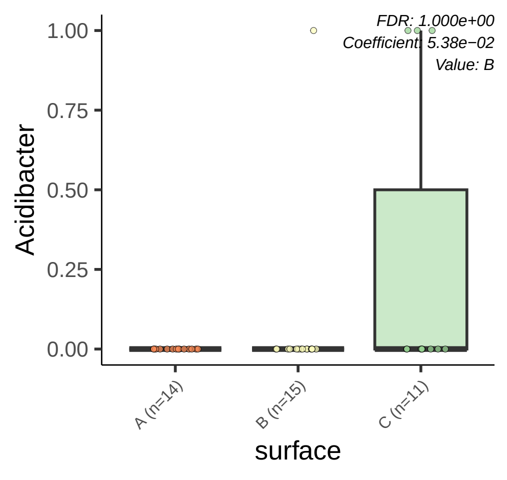
FDR: 1.000e+00
Coefficient: 5.38e−02
Value: B
Acidibacter
1.00
0.75
0.50
0.25
0.00
A (n=14)
B (n=15)
C (n=11)
surface

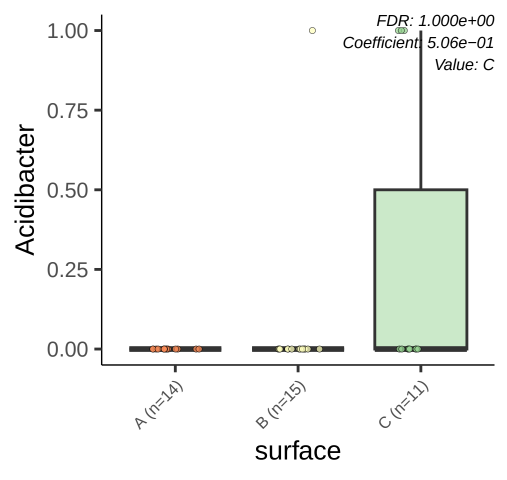
FDR: 1.000e+00
Coefficient: 5.06e−01
Value: C
1.00
0.75
0.50
0.25
0.00
Acidibacter
A (n=14)
B (n=15)
C (n=11)
surface

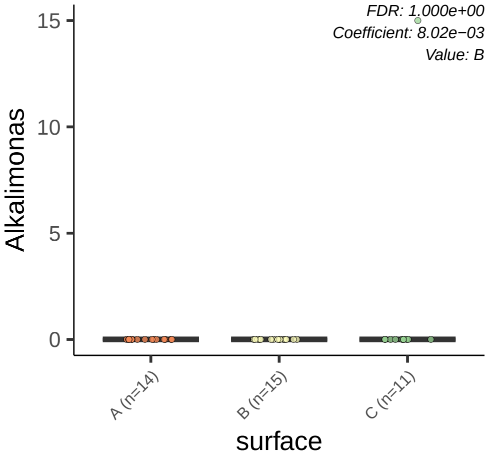
FDR: 1.000e+00
Coefficient: 8.02e−03
Value: B
Alkalimonas
15
10
5
0
A (n=14)
B (n=15)
C (n=11)
surface

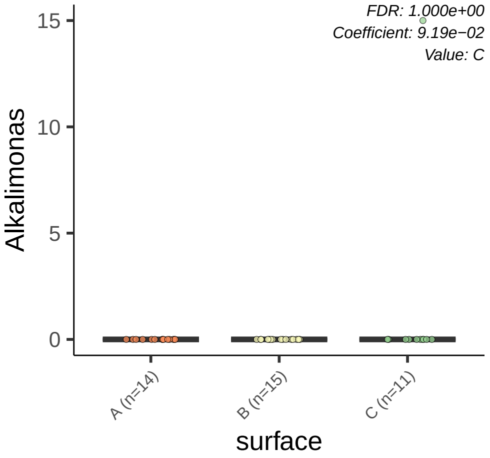
FDR: 1.000e+00
Coefficient: 9.19e−02
Value: C
Alkalimonas
15
10
5
0
A (n=14)
B (n=15)
C (n=11)
surface

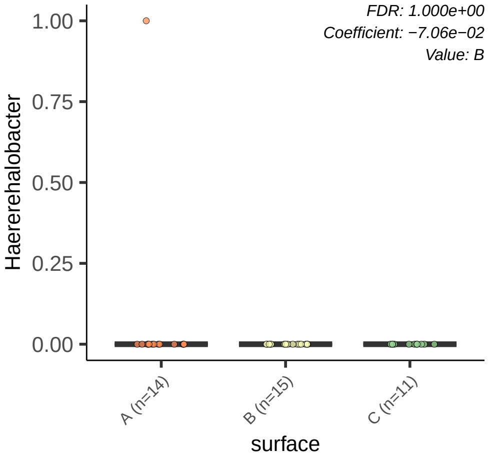
FDR: 1.000e+00
Coefficient: −7.06e−02
Value: B
Haererehalobacter
1.00
0.75
0.50
0.25
0.00
A (n=14)
B (n=15)
C (n=11)
surface

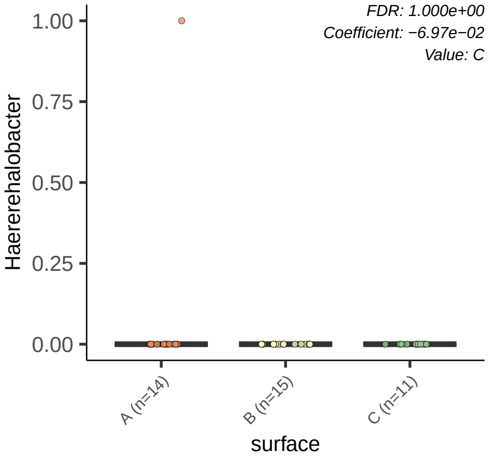
FDR: 1.000e+00
Coefficient: −6.97e−02
Value: C
Haererehalobacter
1.00
0.75
0.50
0.25
0.00
A (n=14)
B (n=15)
C (n=11)
surface

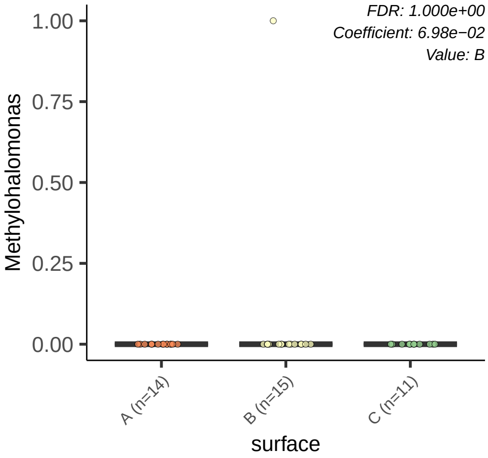

FDR: 1.000e+00
Coefficient: 6.98e−02
Value: B
Methylohalomonas
1.00
0.75
0.50
0.25
0.00
A (n=14)
B (n=15)
C (n=11)
surface

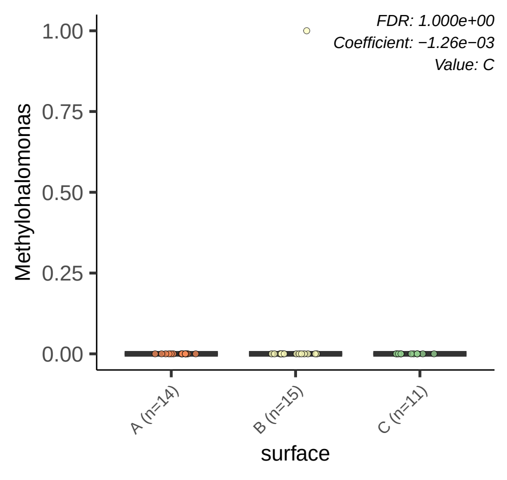

FDR: 1.000e+00
Coefficient: −1.26e−03
Value: C
Methylohalomonas
1.00
0.75
0.50
0.25
0.00
A (n=14)
B (n=15)
C (n=11)
surface

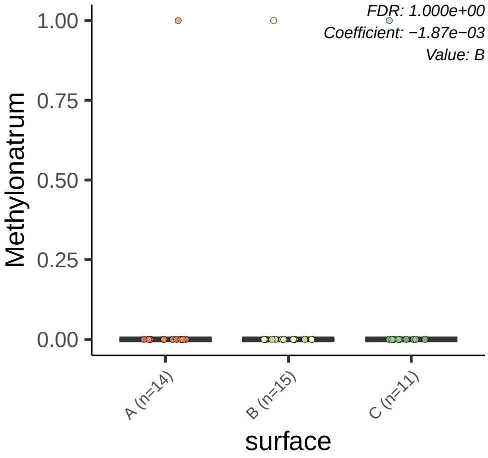
FDR: 1.000e+00
Coefficient: −1.87e−03
Value: B
Methylonatrum
1.00
0.75
0.50
0.25
0.00
A (n=14)
B (n=15)
C (n=11)
surface

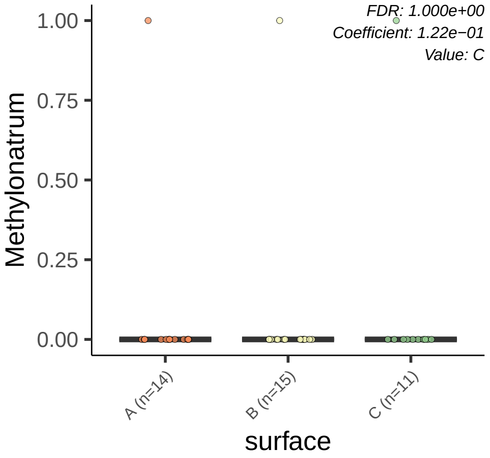

FDR: 1.000e+00
Coefficient: 1.22e−01
Value: C
Methylonatrum
1.00
0.75
0.50
0.25
0.00
A (n=14)
B (n=15)
C (n=11)
surface

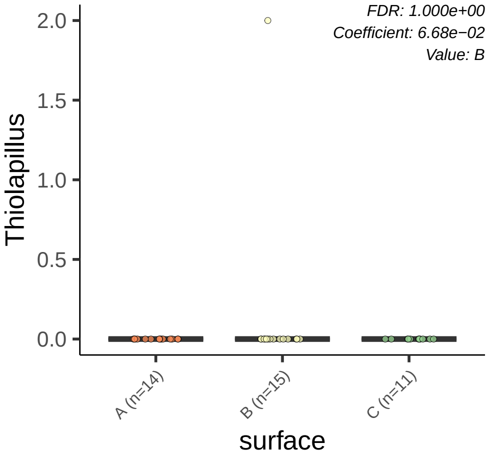
FDR: 1.000e+00
Coefficient: 6.68e−02
Value: B
Thiolapillus
2.0
1.5
1.0
0.5
0.0
A (n=14)
B (n=15)
C (n=11)
surface

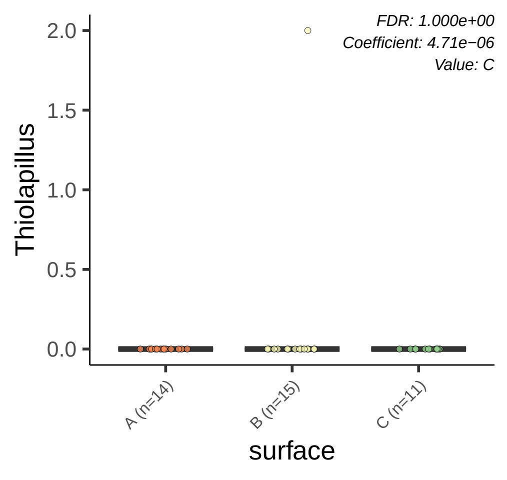

FDR: 1.000e+00
Coefficient: 4.71e−06
Value: C
Thiolapillus
2.0
1.5
1.0
0.5
0.0
A (n=14)
B (n=15)
C (n=11)
surface

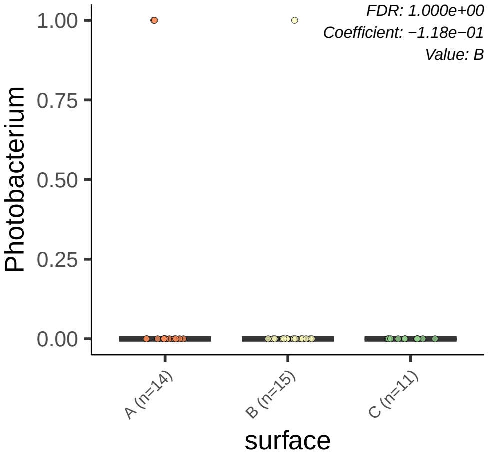

FDR: 1.000e+00
Coefficient: −1.18e−01
Value: B
Photobacterium
1.00
0.75
0.50
0.25
0.00
A (n=14)
B (n=15)
C (n=11)
surface

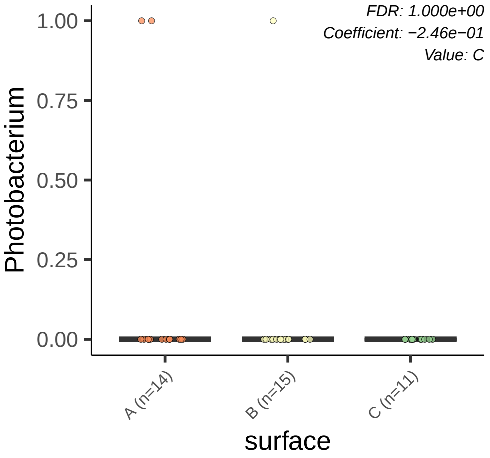
FDR: 1.000e+00
Coefficient: −2.46e−01
Value: C
Photobacterium
1.00
0.75
0.50
0.25
0.00
A (n=14)
B (n=15)
C (n=11)
surface

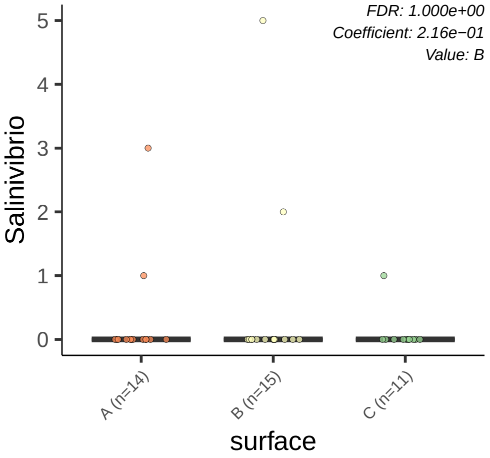
FDR: 1.000e+00
Coefficient: 2.16e−01
Value: B
Salinivibrio
0
1
2
3
4
5
A (n=14)
B (n=15)
C (n=11)
surface

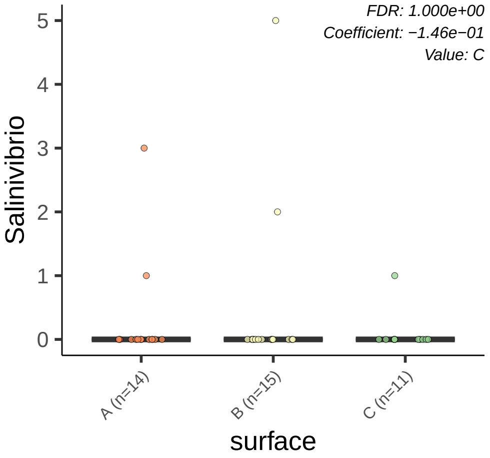
FDR: 1.000e+00
Coefficient: −1.46e−01
Value: C
Salinivibrio
5
4
3
2
1
0
A (n=14)
B (n=15)
C (n=11)
surface

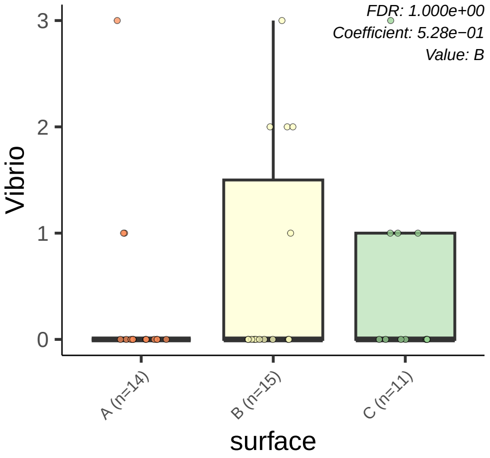

FDR: 1.000e+00
Coefficient: 5.28e−01
Value: B
Vibrio
0
1
2
3
A (n=14)
B (n=15)
C (n=11)
surface

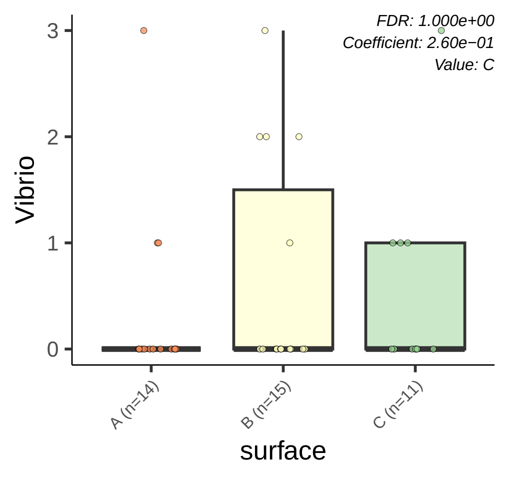
FDR: 1.000e+00
Coefficient: 2.60e−01
Value: C
Vibrio
0
1
2
3
A (n=14)
B (n=15)
C (n=11)
surface

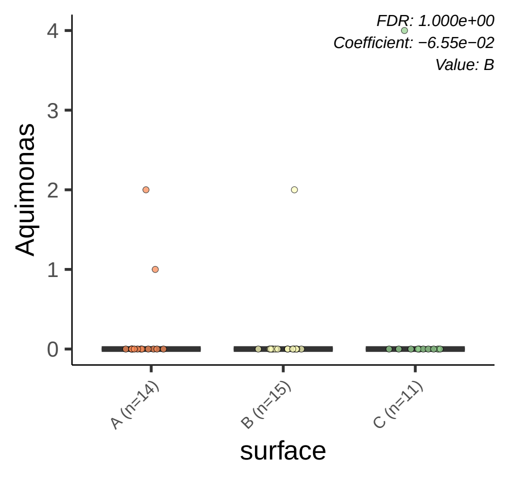

FDR: 1.000e+00
Coefficient: −6.55e−02
Value: B
Aquimonas
0
1
2
3
4
A (n=14)
B (n=15)
C (n=11)
surface

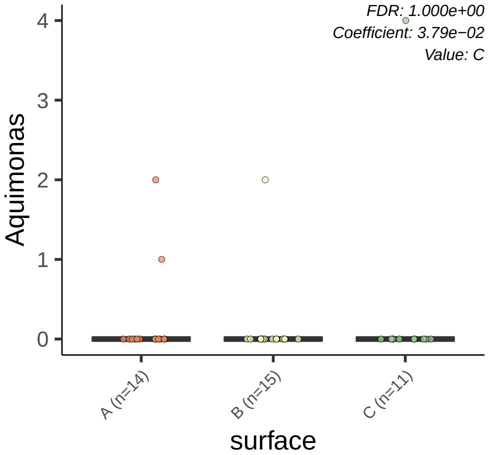
FDR: 1.000e+00
Coefficient: 3.79e−02
Value: C
Aquimonas
0
1
2
3
4
A (n=14)
B (n=15)
C (n=11)
surface

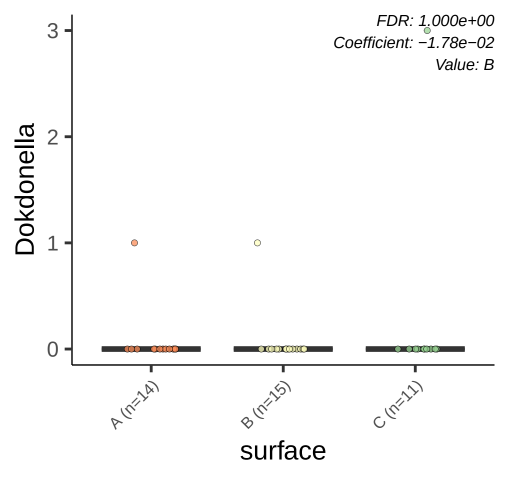
FDR: 1.000e+00
Coefficient: −1.78e−02
Value: B
Dokdonella
3
2
1
0
A (n=14)
B (n=15)
C (n=11)
surface

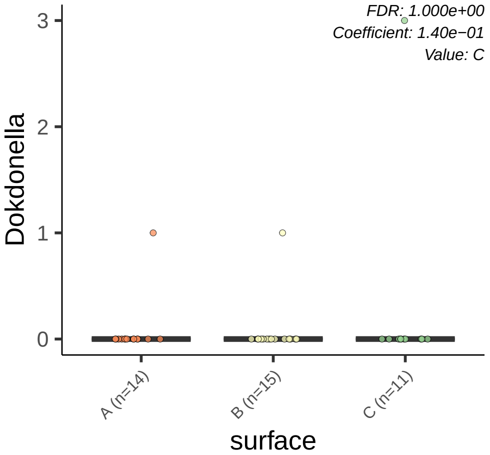

FDR: 1.000e+00
Coefficient: 1.40e−01
Value: C
Dokdonella
3
2
1
0
A (n=14)
B (n=15)
C (n=11)
surface

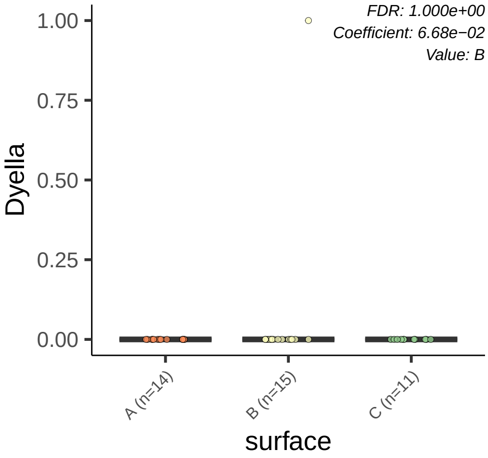

FDR: 1.000e+00
Coefficient: 6.68e−02
Value: B
Dyella
1.00
0.75
0.50
0.25
0.00
A (n=14)
B (n=15)
C (n=11)
surface

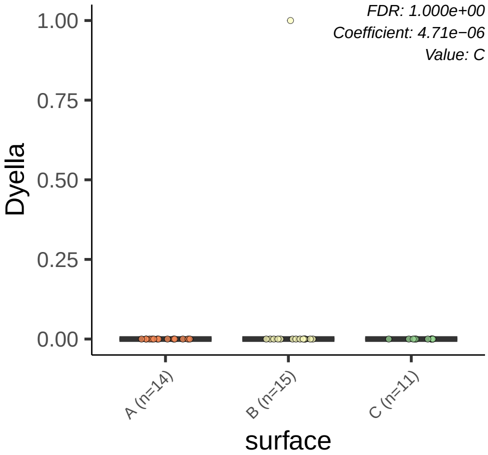
FDR: 1.000e+00
Coefficient: 4.71e−06
Value: C
Dyella
1.00
0.75
0.50
0.25
0.00
A (n=14)
B (n=15)
C (n=11)
surface

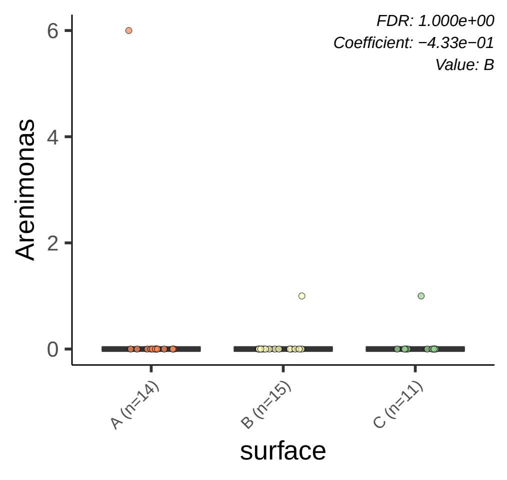
FDR: 1.000e+00
Coefficient: −4.33e−01
Value: B
Arenimonas
6
4
2
0
A (n=14)
B (n=15)
C (n=11)
surface

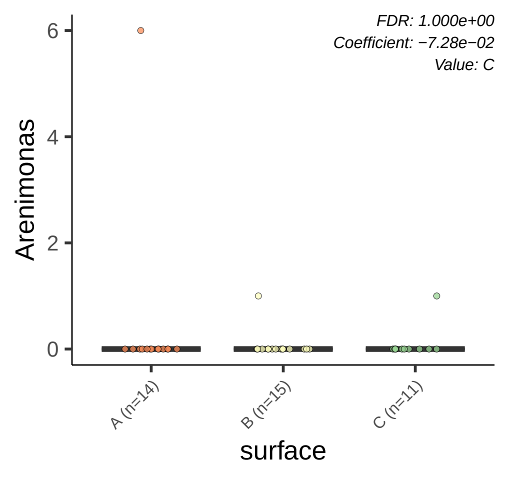
FDR: 1.000e+00
Coefficient: −7.28e−02
Value: C
Arenimonas
6
4
2
0
A (n=14)
B (n=15)
C (n=11)
surface

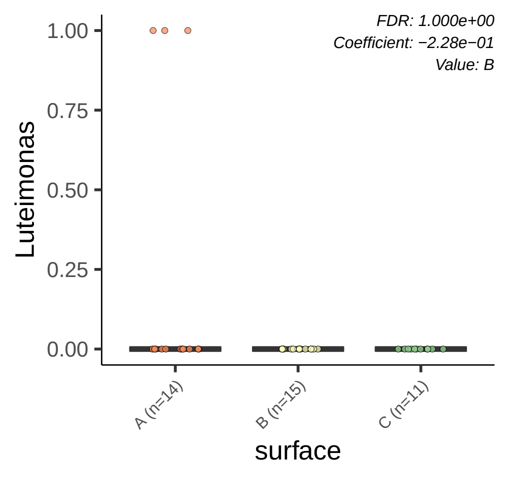
FDR: 1.000e+00
Coefficient: −2.28e−01
Value: B
Luteimonas
1.00
0.75
0.50
0.25
0.00
A (n=14)
B (n=15)
C (n=11)
surface

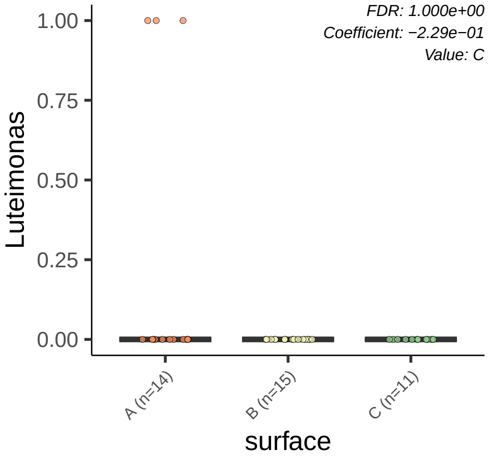

FDR: 1.000e+00
Coefficient: −2.29e−01
Value: C
Luteimonas
1.00
0.75
0.50
0.25
0.00
A (n=14)
B (n=15)
C (n=11)
surface

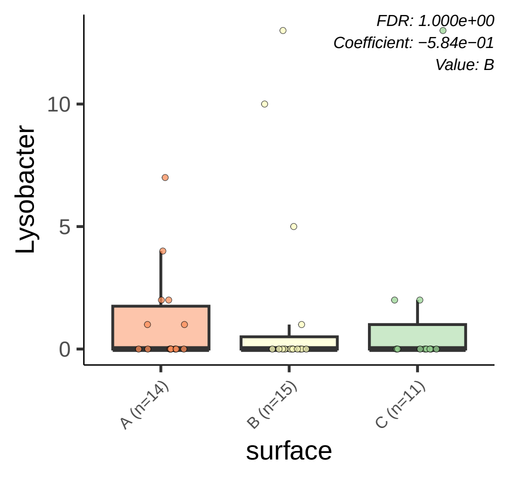
FDR: 1.000e+00
Coefficient: −5.84e−01
Value: B
Lysobacter
0
5
10
A (n=14)
B (n=15)
C (n=11)
surface

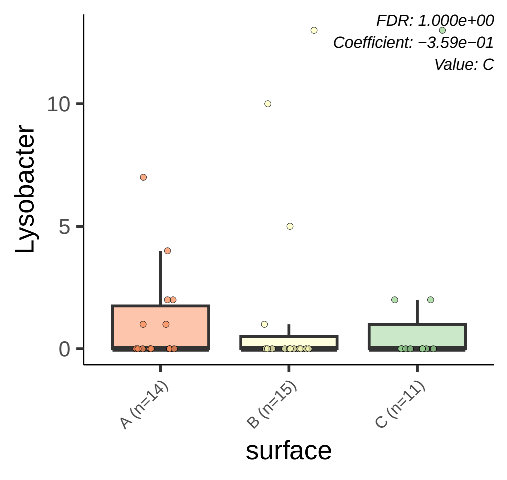

FDR: 1.000e+00
Coefficient: −3.59e−01
Value: C
Lysobacter
10
5
0
A (n=14)
B (n=15)
C (n=11)
surface

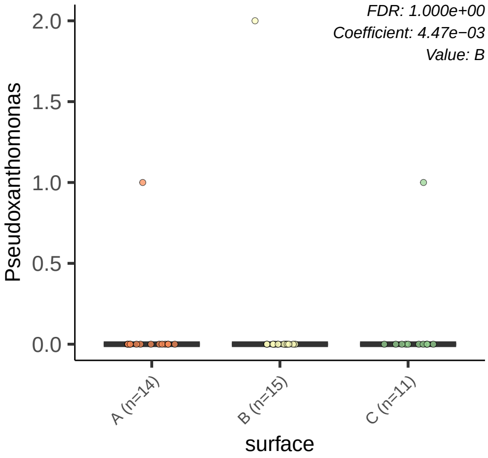

FDR: 1.000e+00
Coefficient: 4.47e−03
Value: B
Pseudoxanthomonas
2.0
1.5
1.0
0.5
0.0
A (n=14)
B (n=15)
C (n=11)
surface

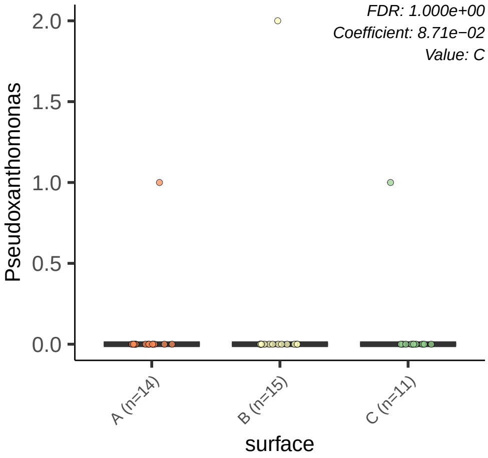
FDR: 1.000e+00
Coefficient: 8.71e−02
Value: C
Pseudoxanthomonas
2.0
1.5
1.0
0.5
0.0
A (n=14)
B (n=15)
C (n=11)
surface

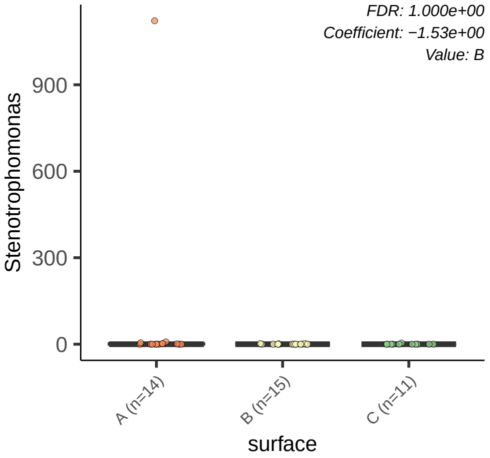

FDR: 1.000e+00
Coefficient: −1.53e+00
Value: B
Stenotrophomonas
900
600
300
0
A (n=14)
B (n=15)
C (n=11)
surface

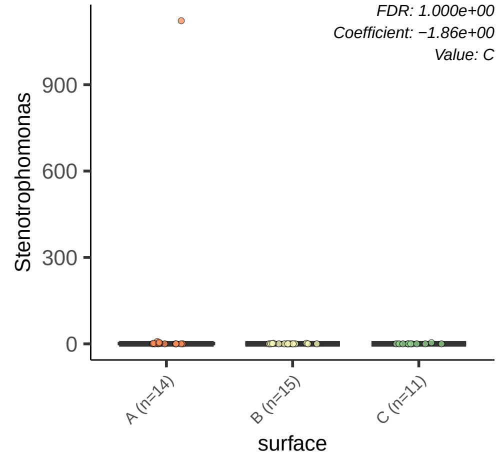
FDR: 1.000e+00
Coefficient: −1.86e+00
Value: C
Stenotrophomonas
900
600
300
0
A (n=14)
B (n=15)
C (n=11)
surface

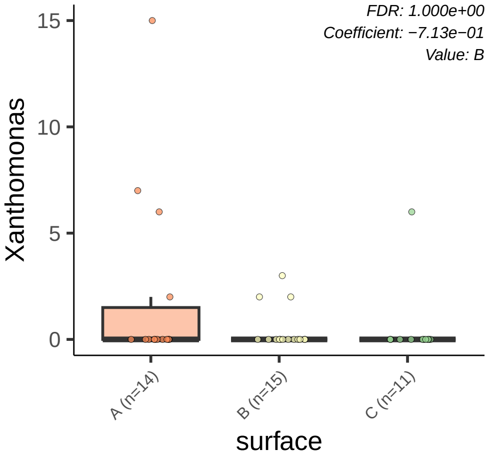

FDR: 1.000e+00
Coefficient: −7.13e−01
Value: B
Xanthomonas
15
10
5
0
A (n=14)
B (n=15)
C (n=11)
surface

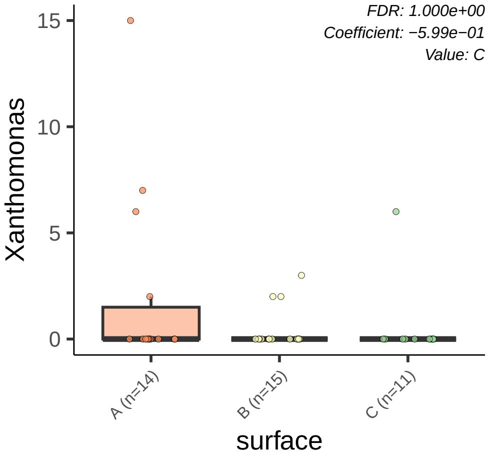

FDR: 1.000e+00
Coefficient: −5.99e−01
Value: C
Xanthomonas
15
10
5
0
A (n=14)
B (n=15)
C (n=11)
surface

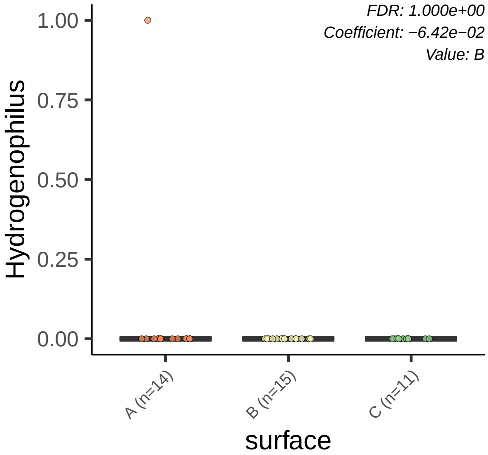
FDR: 1.000e+00
Coefficient: −6.42e−02
Value: B
Hydrogenophilus
1.00
0.75
0.50
0.25
0.00
A (n=14)
B (n=15)
C (n=11)
surface

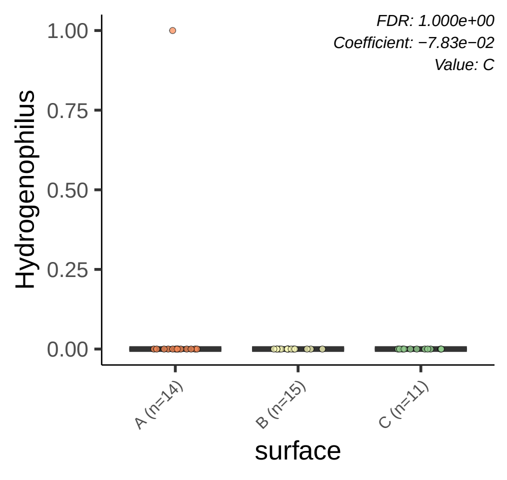
FDR: 1.000e+00
Coefficient: −7.83e−02
Value: C
Hydrogenophilus
1.00
0.75
0.50
0.25
0.00
A (n=14)
B (n=15)
C (n=11)
surface

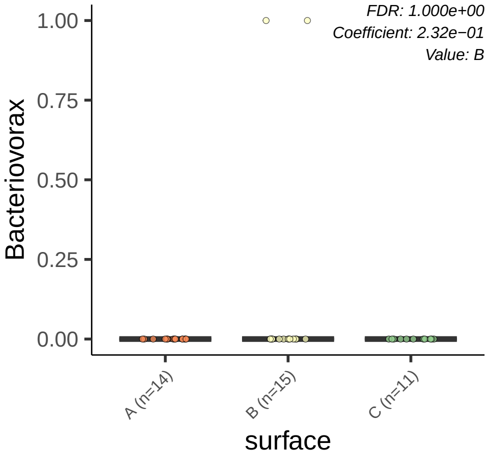

FDR: 1.000e+00
Coefficient: 2.32e−01
Value: B
Bacteriovorax
1.00
0.75
0.50
0.25
0.00
A (n=14)
B (n=15)
C (n=11)
surface

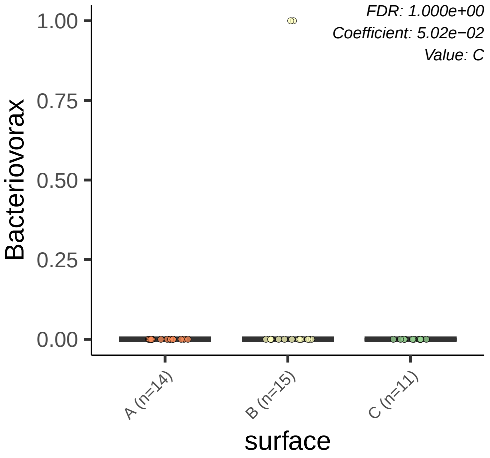
FDR: 1.000e+00
Coefficient: 5.02e−02
Value: C
Bacteriovorax
1.00
0.75
0.50
0.25
0.00
A (n=14)
B (n=15)
C (n=11)
surface

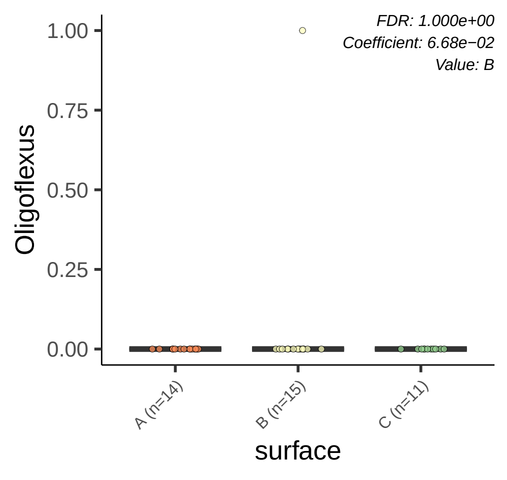
FDR: 1.000e+00
Coefficient: 6.68e−02
Value: B
Oligoflexus
1.00
0.75
0.50
0.25
0.00
A (n=14)
B (n=15)
C (n=11)
surface

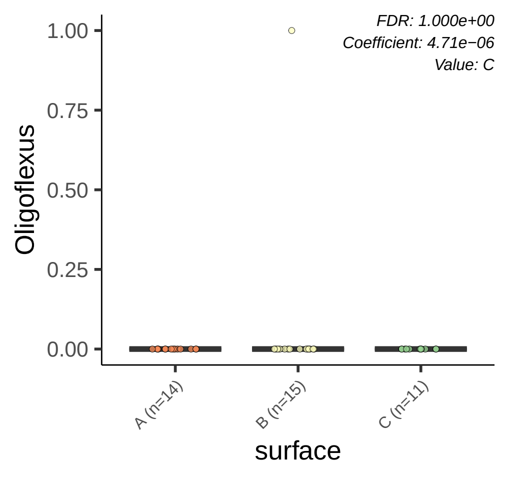

FDR: 1.000e+00
Coefficient: 4.71e−06
Value: C
Oligoflexus
1.00
0.75
0.50
0.25
0.00
A (n=14)
B (n=15)
C (n=11)
surface

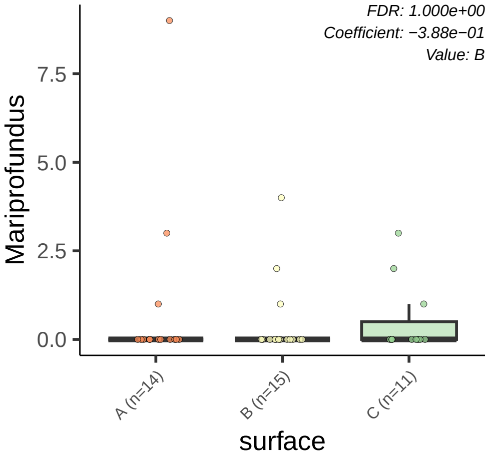
FDR: 1.000e+00
Coefficient: −3.88e−01
Value: B
Mariprofundus
7.5
5.0
2.5
0.0
A (n=14)
B (n=15)
C (n=11)
surface

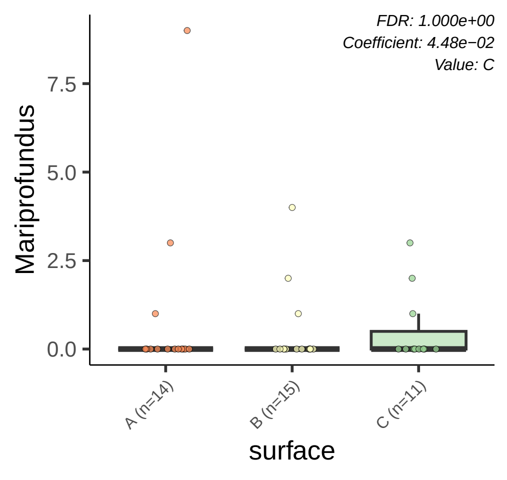
FDR: 1.000e+00
Coefficient: 4.48e−02
Value: C
Mariprofundus
7.5
5.0
2.5
0.0
A (n=14)
B (n=15)
C (n=11)
surface

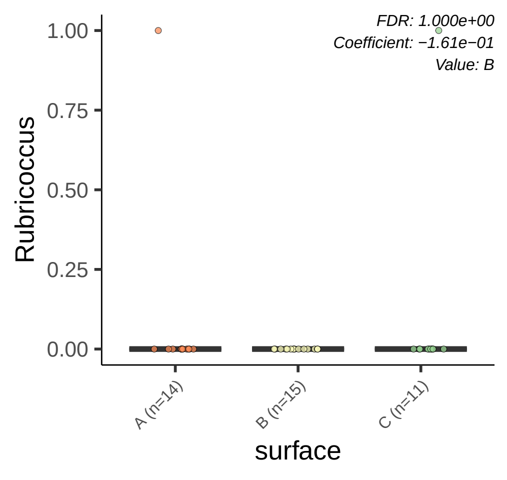
FDR: 1.000e+00
Coefficient: −1.61e−01
Value: B
Rubricoccus
1.00
0.75
0.50
0.25
0.00
A (n=14)
B (n=15)
C (n=11)
surface

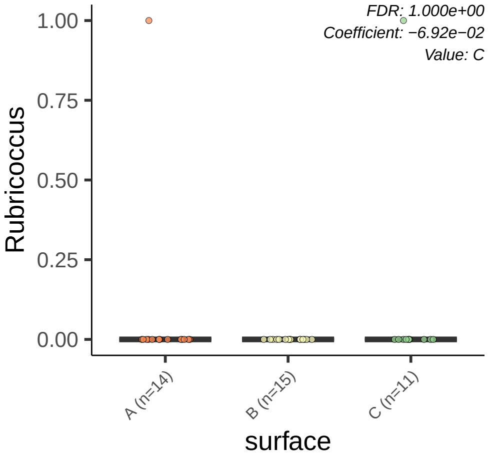
FDR: 1.000e+00
Coefficient: −6.92e−02
Value: C
1.00
0.75
0.50
0.25
0.00
Rubricoccus
A (n=14)
B (n=15)
C (n=11)
surface

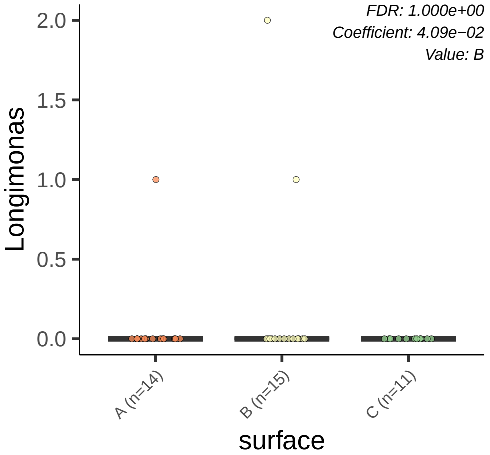
FDR: 1.000e+00
Coefficient: 4.09e−02
Value: B
Longimonas
2.0
1.5
1.0
0.5
0.0
A (n=14)
B (n=15)
C (n=11)
surface

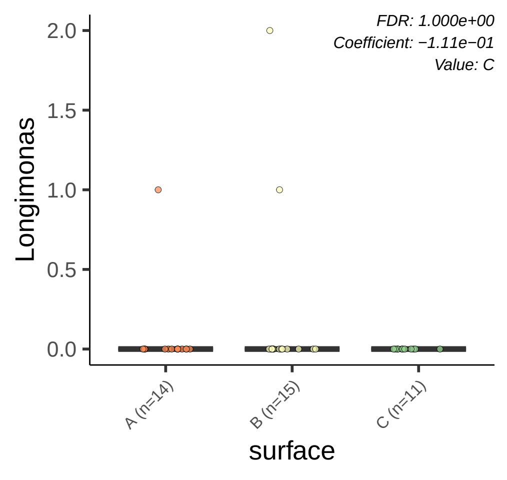
FDR: 1.000e+00
Coefficient: −1.11e−01
Value: C
Longimonas
2.0
1.5
1.0
0.5
0.0
A (n=14)
B (n=15)
C (n=11)
surface

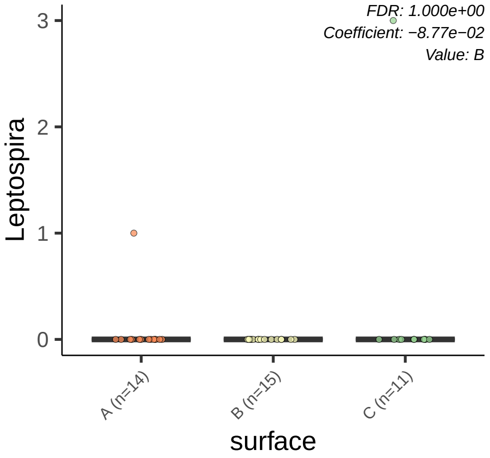
FDR: 1.000e+00
Coefficient: −8.77e−02
Value: B
Leptospira
3
2
1
0
A (n=14)
B (n=15)
C (n=11)
surface

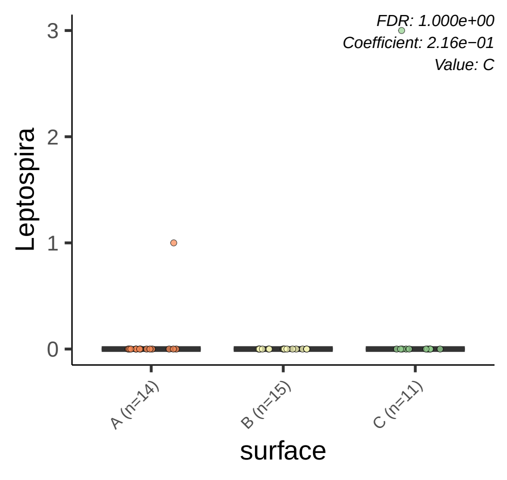

FDR: 1.000e+00
Coefficient: 2.16e−01
Value: C
Leptospira
0
1
2
3
A (n=14)
B (n=15)
C (n=11)
surface

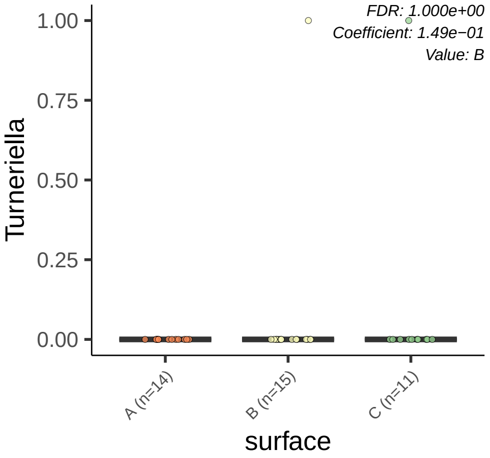

FDR: 1.000e+00
Coefficient: 1.49e−01
Value: B
Turneriella
1.00
0.75
0.50
0.25
0.00
A (n=14)
B (n=15)
C (n=11)
surface

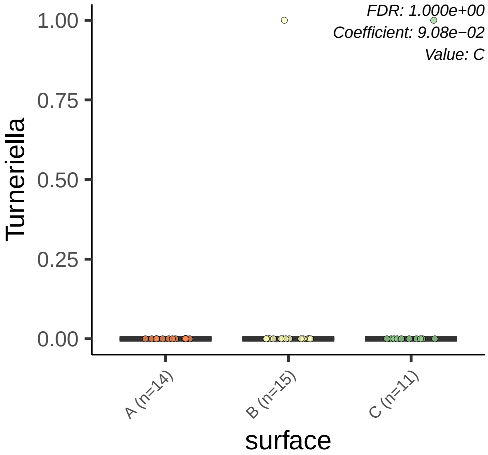

FDR: 1.000e+00
Coefficient: 9.08e−02
Value: C
Turneriella
1.00
0.75
0.50
0.25
0.00
A (n=14)
B (n=15)
C (n=11)
surface

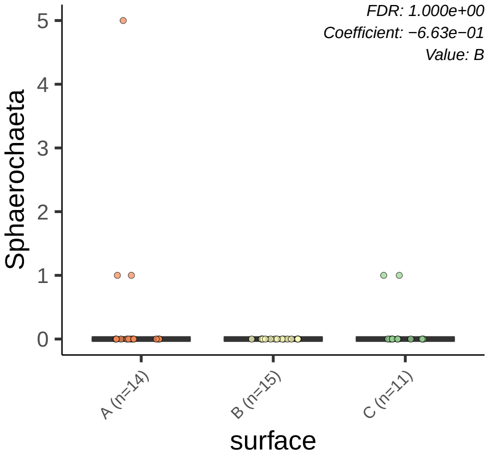
FDR: 1.000e+00
Coefficient: −6.63e−01
Value: B
Sphaerochaeta
5
4
3
2
1
0
A (n=14)
B (n=15)
C (n=11)
surface

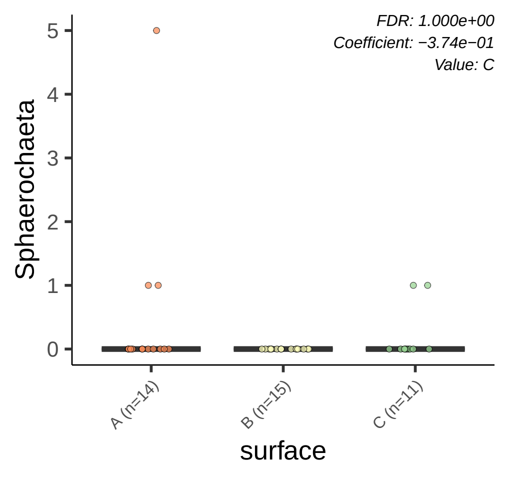
FDR: 1.000e+00
Coefficient: −3.74e−01
Value: C
Sphaerochaeta
5
4
3
2
1
0
A (n=14)
B (n=15)
C (n=11)
surface

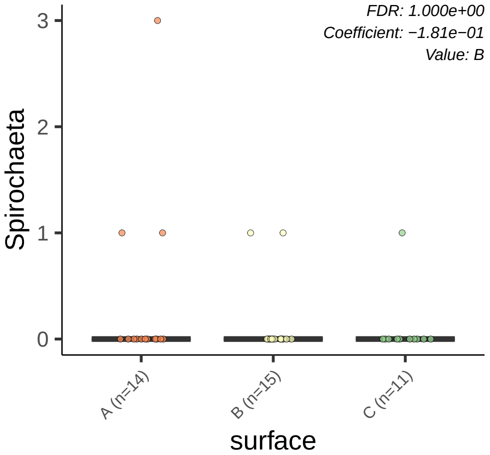

FDR: 1.000e+00
Coefficient: −1.81e−01
Value: B
Spirochaeta
3
2
1
0
A (n=14)
B (n=15)
C (n=11)
surface

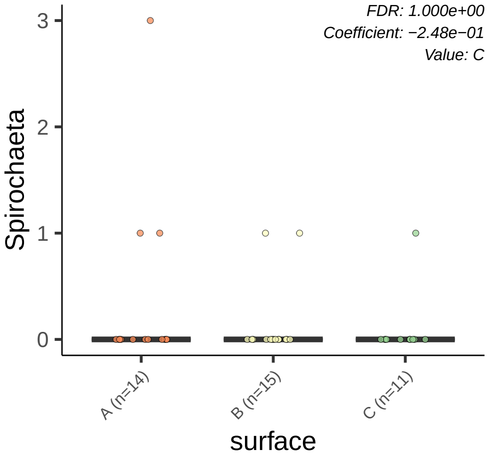
FDR: 1.000e+00
Coefficient: −2.48e−01
Value: C
Spirochaeta
0
1
2
3
A (n=14)
B (n=15)
C (n=11)
surface

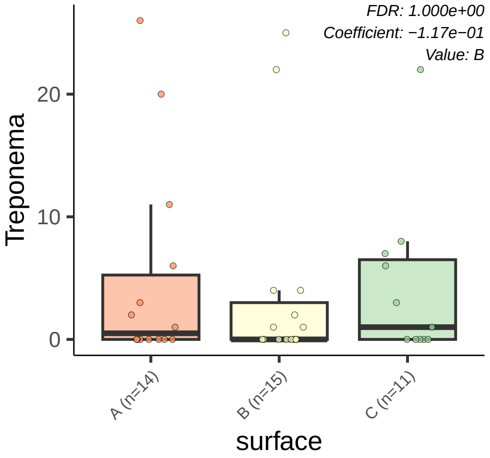

FDR: 1.000e+00
Coefficient: −1.17e−01
Value: B
Treponema
0
10
20
A (n=14)
B (n=15)
C (n=11)
surface

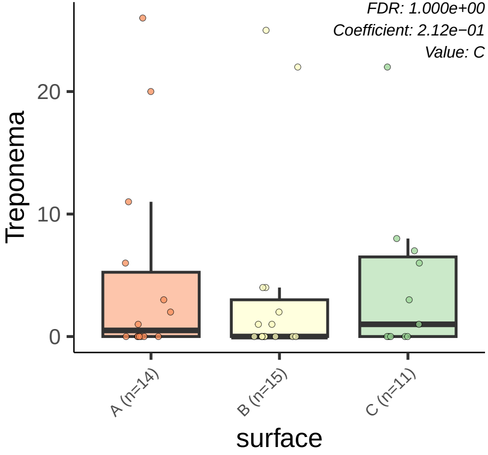

FDR: 1.000e+00
Coefficient: 2.12e−01
Value: C
Treponema
20
10
0
A (n=14)
B (n=15)
C (n=11)
surface

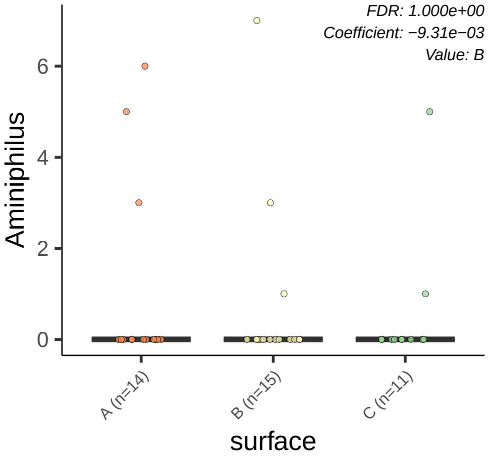

FDR: 1.000e+00
Coefficient: −9.31e−03
Value: B
Aminiphilus
0
2
4
6
A (n=14)
B (n=15)
C (n=11)
surface

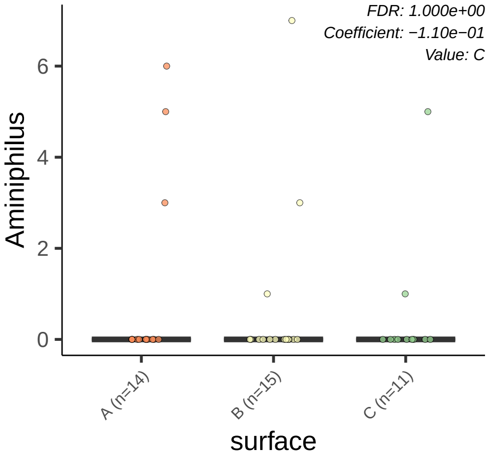

FDR: 1.000e+00
Coefficient: −1.10e−01
Value: C
Aminiphilus
0
2
4
6
A (n=14)
B (n=15)
C (n=11)
surface

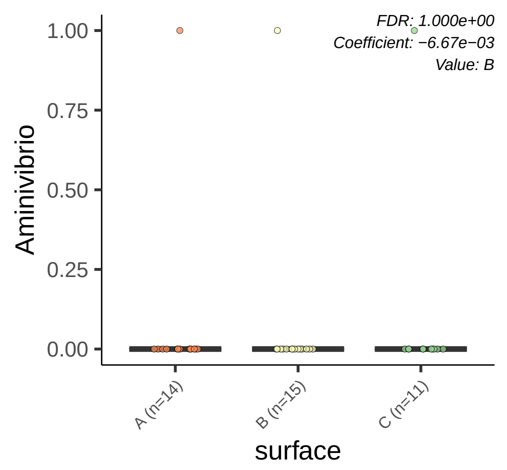

FDR: 1.000e+00
Coefficient: −6.67e−03
Value: B
1.00
0.75
0.50
0.25
0.00
Aminivibrio
A (n=14)
B (n=15)
C (n=11)
surface

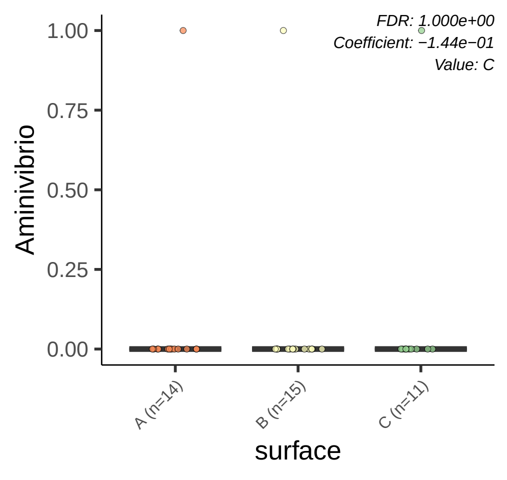
FDR: 1.000e+00
Coefficient: −1.44e−01
Value: C
Aminivibrio
1.00
0.75
0.50
0.25
0.00
A (n=14)
B (n=15)
C (n=11)
surface

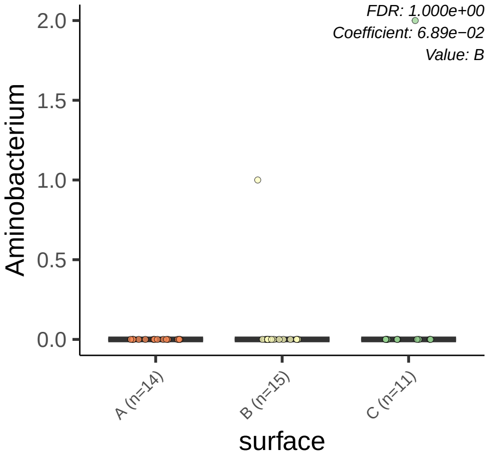
FDR: 1.000e+00
Coefficient: 6.89e−02
Value: B
Aminobacterium
2.0
1.5
1.0
0.5
0.0
A (n=14)
B (n=15)
C (n=11)
surface

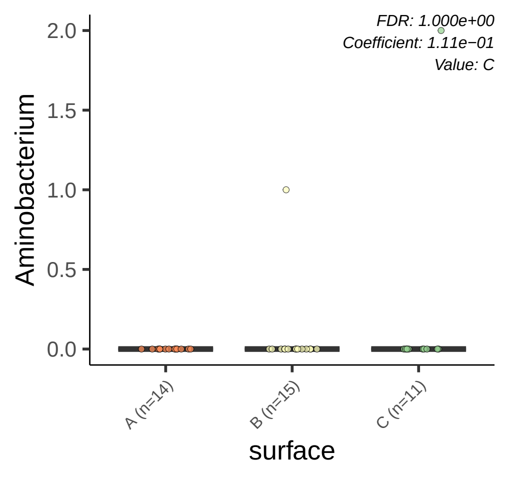

FDR: 1.000e+00
Coefficient: 1.11e−01
Value: C
Aminobacterium
2.0
1.5
1.0
0.5
0.0
A (n=14)
B (n=15)
C (n=11)
surface

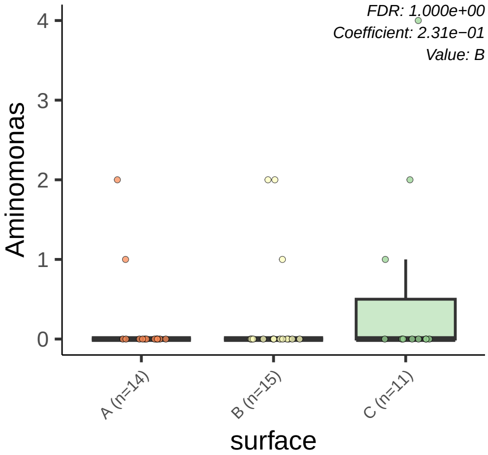
FDR: 1.000e+00
Coefficient: 2.31e−01
Value: B
Aminomonas
0
1
2
3
4
A (n=14)
B (n=15)
C (n=11)
surface

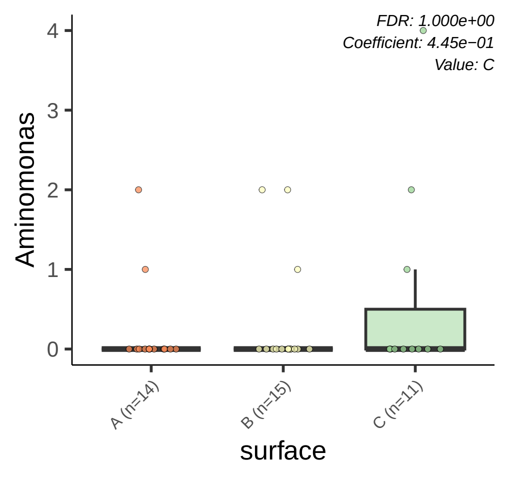
FDR: 1.000e+00
Coefficient: 4.45e−01
Value: C
Aminomonas
0
1
2
3
4
A (n=14)
B (n=15)
C (n=11)
surface

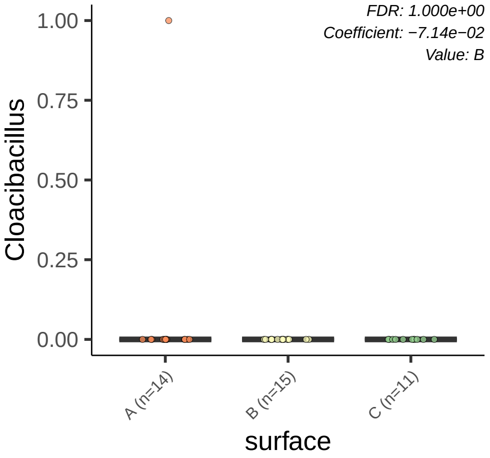
FDR: 1.000e+00
Coefficient: −7.14e−02
Value: B
Cloacibacillus
1.00
0.75
0.50
0.25
0.00
A (n=14)
B (n=15)
C (n=11)
surface

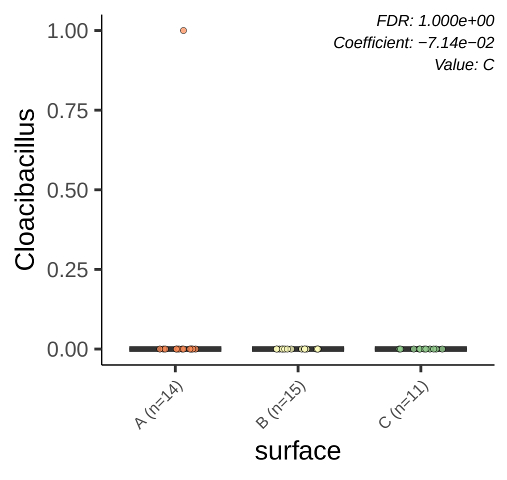
FDR: 1.000e+00
Coefficient: −7.14e−02
Value: C
Cloacibacillus
1.00
0.75
0.50
0.25
0.00
A (n=14)
B (n=15)
C (n=11)
surface

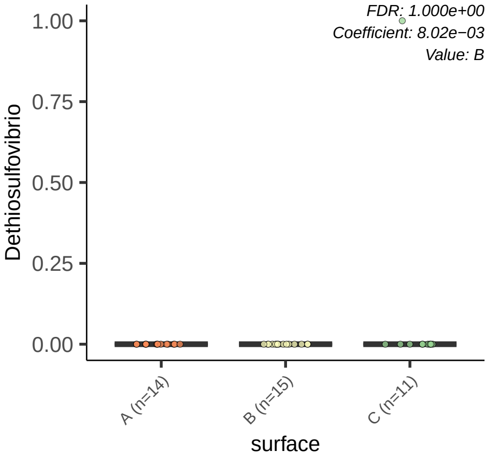
FDR: 1.000e+00
Coefficient: 8.02e−03
Value: B
Dethiosulfovibrio
1.00
0.75
0.50
0.25
0.00
A (n=14)
B (n=15)
C (n=11)
surface

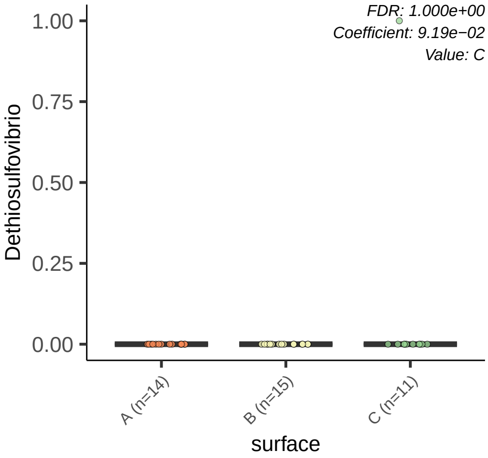

FDR: 1.000e+00
Coefficient: 9.19e−02
Value: C
1.00
0.75
0.50
0.25
0.00
Dethiosulfovibrio
A (n=14)
B (n=15)
C (n=11)
surface

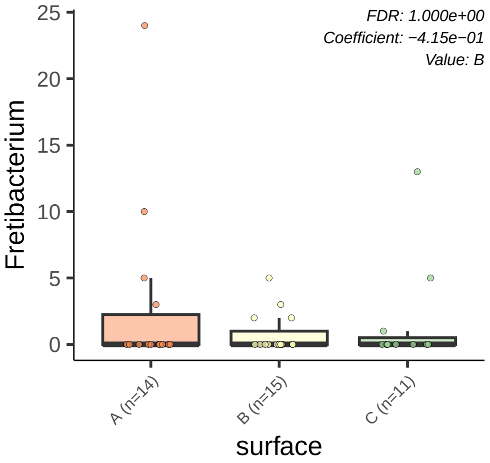

FDR: 1.000e+00
Coefficient: −4.15e−01
Value: B
Fretibacterium
25
20
15
10
5
0
A (n=14)
B (n=15)
C (n=11)
surface

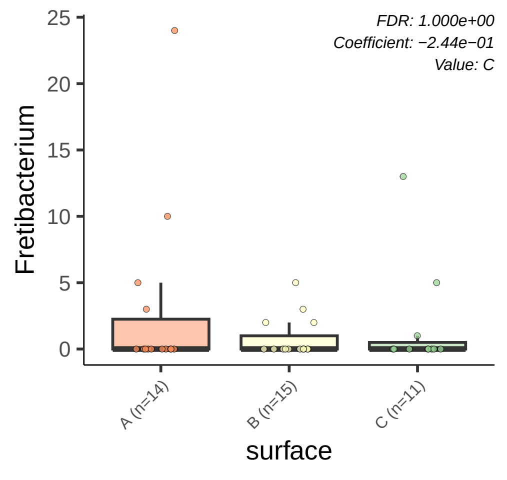
FDR: 1.000e+00
Coefficient: −2.44e−01
Value: C
Fretibacterium
25
20
15
10
5
0
A (n=14)
B (n=15)
C (n=11)
surface

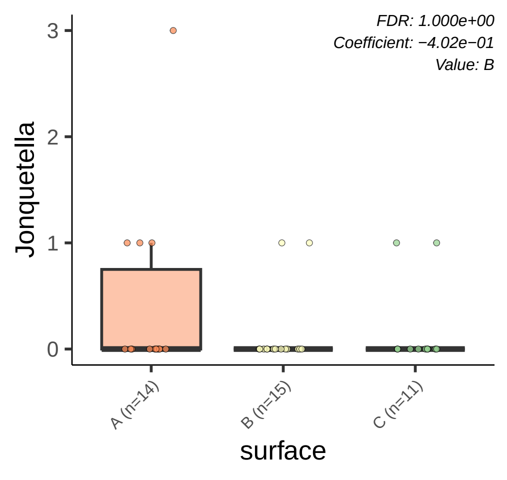
FDR: 1.000e+00
Coefficient: −4.02e−01
Value: B
Jonquetella
3
2
1
0
A (n=14)
B (n=15)
C (n=11)
surface

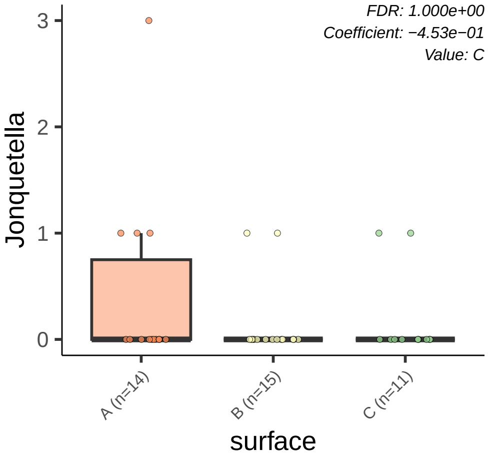

FDR: 1.000e+00
Coefficient: −4.53e−01
Value: C
Jonquetella
3
2
1
0
A (n=14)
B (n=15)
C (n=11)
surface

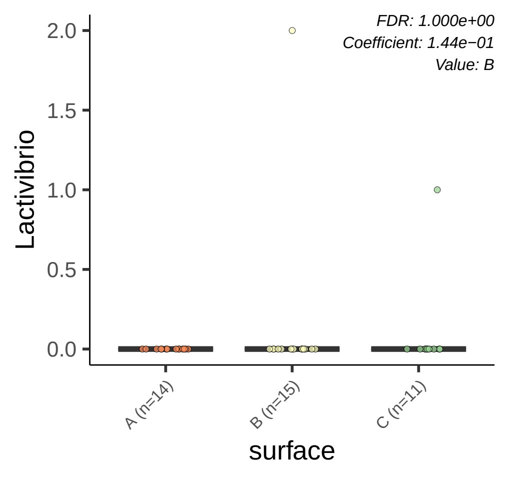

FDR: 1.000e+00
Coefficient: 1.44e−01
Value: B
Lactivibrio
2.0
1.5
1.0
0.5
0.0
A (n=14)
B (n=15)
C (n=11)
surface

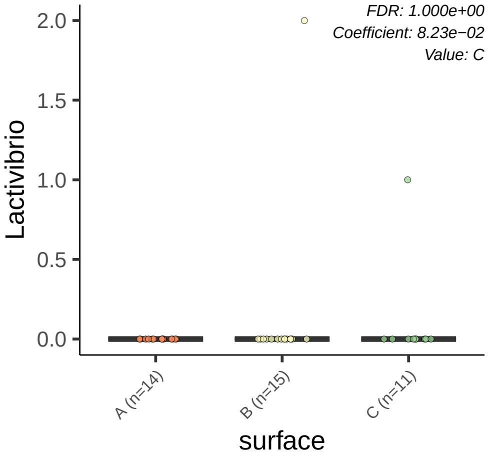
FDR: 1.000e+00
Coefficient: 8.23e−02
Value: C
Lactivibrio
2.0
1.5
1.0
0.5
0.0
A (n=14)
B (n=15)
C (n=11)
surface

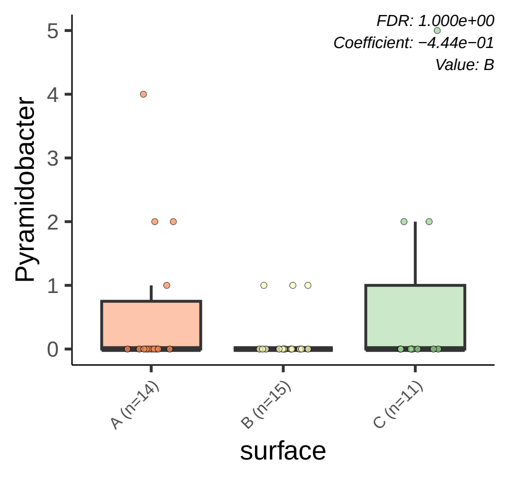

FDR: 1.000e+00
Coefficient: −4.44e−01
Value: B
Pyramidobacter
0
1
2
3
4
5
A (n=14)
B (n=15)
C (n=11)
surface

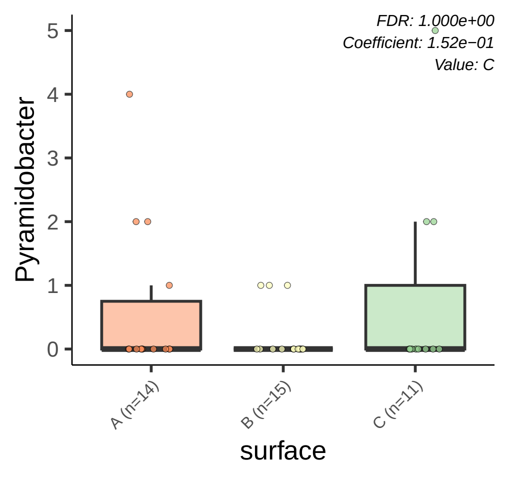

FDR: 1.000e+00
Coefficient: 1.52e−01
Value: C
Pyramidobacter
0
1
2
3
4
5
A (n=14)
B (n=15)
C (n=11)
surface

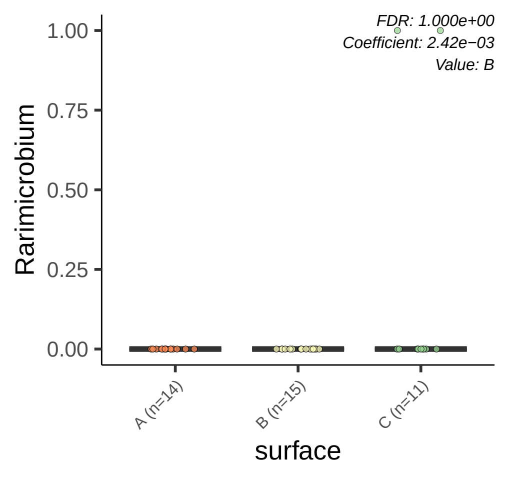
FDR: 1.000e+00
Coefficient: 2.42e−03
Value: B
Rarimicrobium
1.00
0.75
0.50
0.25
0.00
A (n=14)
B (n=15)
C (n=11)
surface

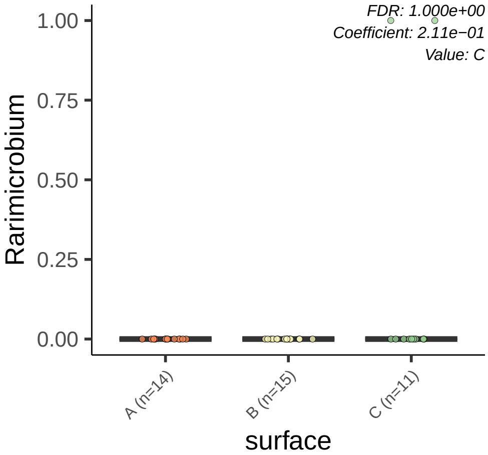
FDR: 1.000e+00
Coefficient: 2.11e−01
Value: C
Rarimicrobium
1.00
0.75
0.50
0.25
0.00
A (n=14)
B (n=15)
C (n=11)
surface

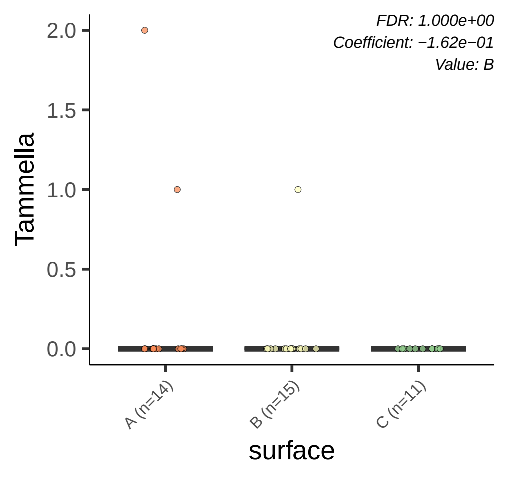
FDR: 1.000e+00
Coefficient: −1.62e−01
Value: B
Tammella
2.0
1.5
1.0
0.5
0.0
A (n=14)
B (n=15)
C (n=11)
surface

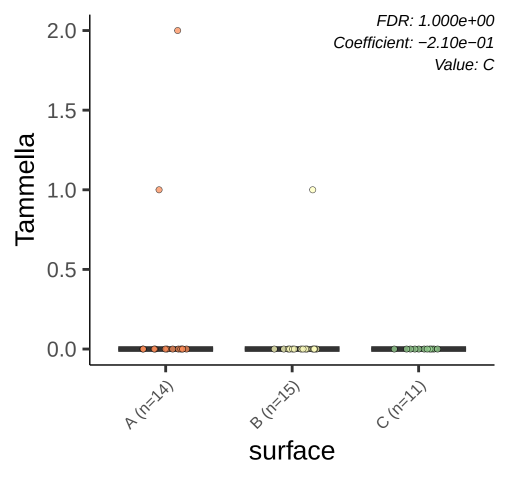
FDR: 1.000e+00
Coefficient: −2.10e−01
Value: C
Tammella
2.0
1.5
1.0
0.5
0.0
A (n=14)
B (n=15)
C (n=11)
surface

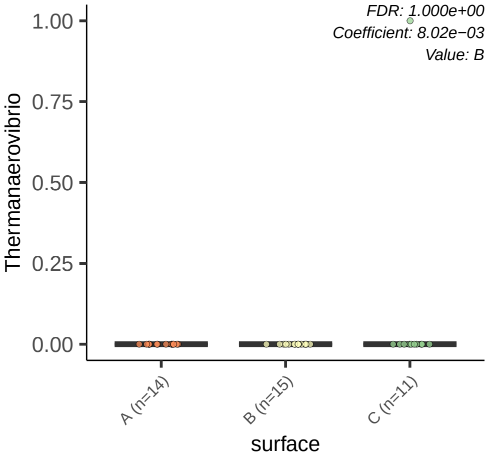
FDR: 1.000e+00
Coefficient: 8.02e−03
Value: B
Thermanaerovibrio
1.00
0.75
0.50
0.25
0.00
A (n=14)
B (n=15)
C (n=11)
surface

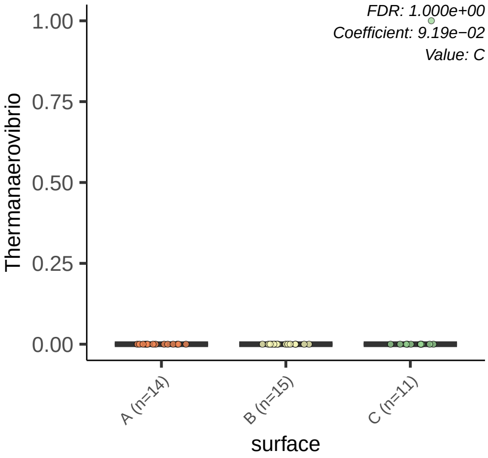

FDR: 1.000e+00
Coefficient: 9.19e−02
Value: C
Thermanaerovibrio
1.00
0.75
0.50
0.25
0.00
A (n=14)
B (n=15)
C (n=11)
surface

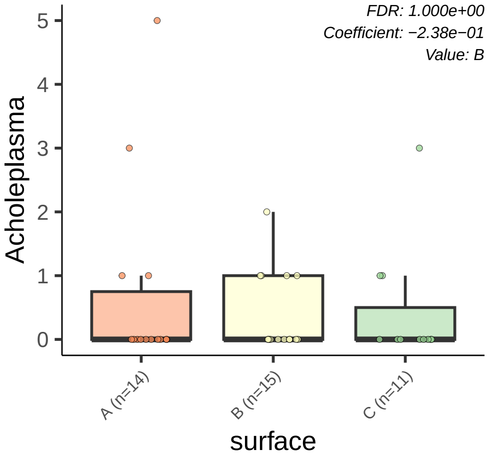
FDR: 1.000e+00
Coefficient: −2.38e−01
Value: B
Acholeplasma
0
1
2
3
4
5
A (n=14)
B (n=15)
C (n=11)
surface

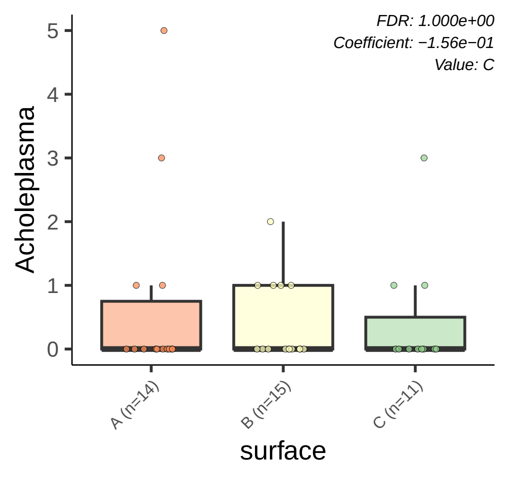

FDR: 1.000e+00
Coefficient: −1.56e−01
Value: C
Acholeplasma
0
1
2
3
4
5
A (n=14)
B (n=15)
C (n=11)
surface

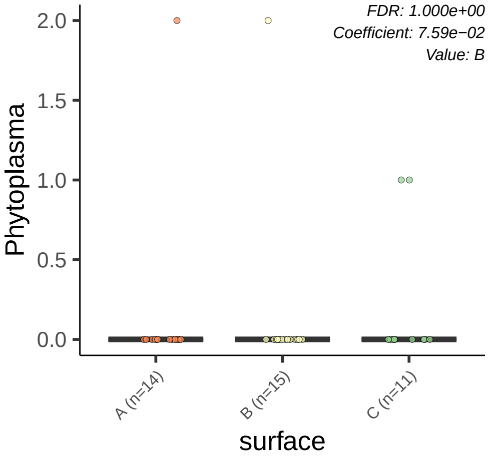
FDR: 1.000e+00
Coefficient: 7.59e−02
Value: B
Phytoplasma
2.0
1.5
1.0
0.5
0.0
A (n=14)
B (n=15)
C (n=11)
surface

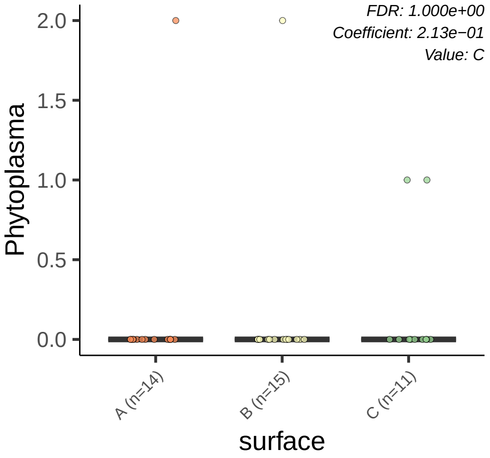
FDR: 1.000e+00
Coefficient: 2.13e−01
Value: C
Phytoplasma
2.0
1.5
1.0
0.5
0.0
A (n=14)
B (n=15)
C (n=11)
surface

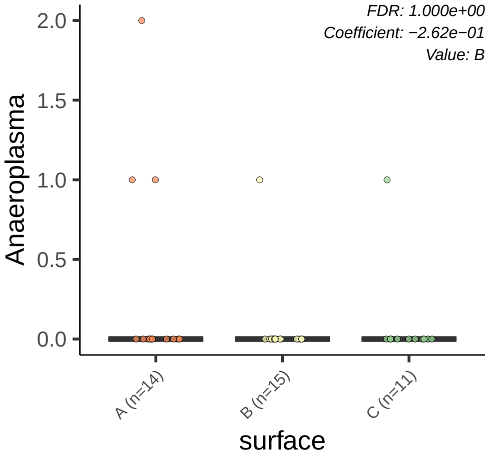
FDR: 1.000e+00
Coefficient: −2.62e−01
Value: B
Anaeroplasma
2.0
1.5
1.0
0.5
0.0
A (n=14)
B (n=15)
C (n=11)
surface

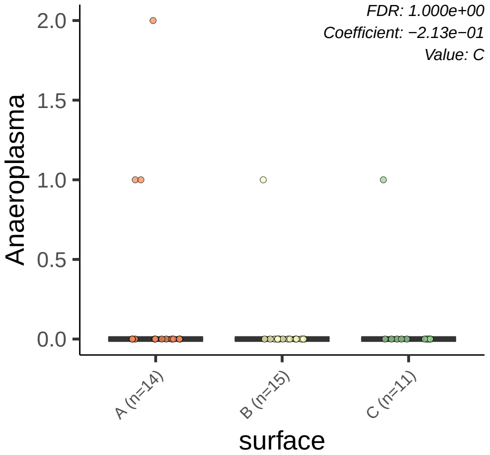
FDR: 1.000e+00
Coefficient: −2.13e−01
Value: C
Anaeroplasma
2.0
1.5
1.0
0.5
0.0
A (n=14)
B (n=15)
C (n=11)
surface

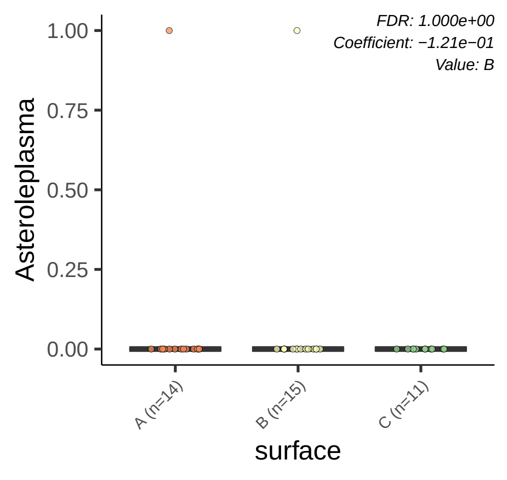
FDR: 1.000e+00
Coefficient: −1.21e−01
Value: B
Asteroleplasma
1.00
0.75
0.50
0.25
0.00
A (n=14)
B (n=15)
C (n=11)
surface

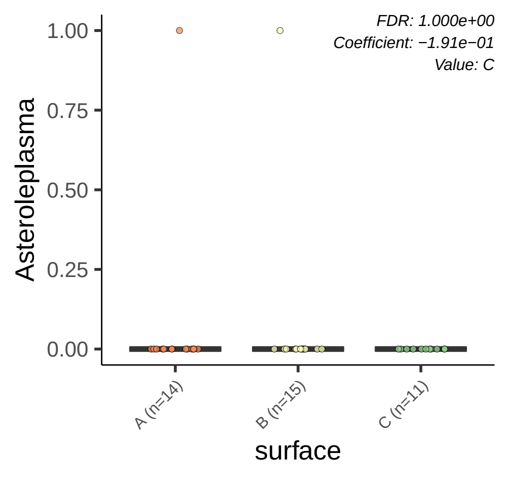

FDR: 1.000e+00
Coefficient: −1.91e−01
Value: C
Asteroleplasma
1.00
0.75
0.50
0.25
0.00
A (n=14)
B (n=15)
C (n=11)
surface

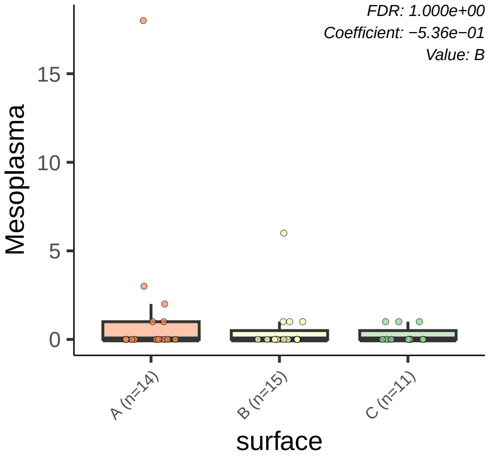
FDR: 1.000e+00
Coefficient: −5.36e−01
Value: B
Mesoplasma
0
5
10
15
A (n=14)
B (n=15)
C (n=11)
surface

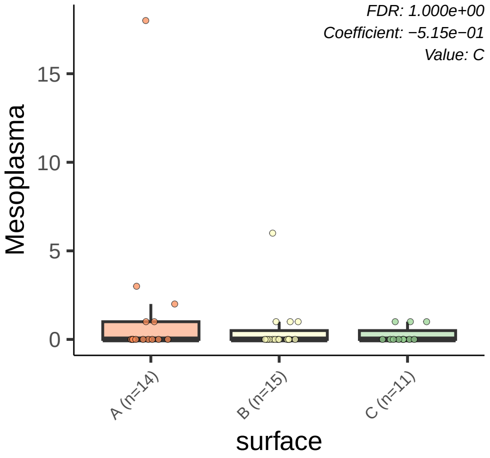
FDR: 1.000e+00
Coefficient: −5.15e−01
Value: C
Mesoplasma
15
10
5
0
A (n=14)
B (n=15)
C (n=11)
surface

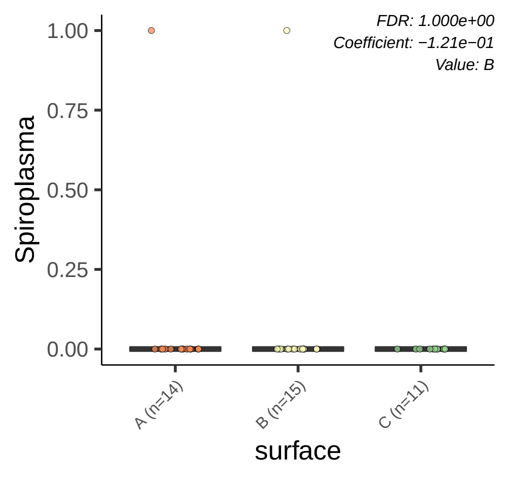
FDR: 1.000e+00
Coefficient: −1.21e−01
Value: B
Spiroplasma
1.00
0.75
0.50
0.25
0.00
A (n=14)
B (n=15)
C (n=11)
surface

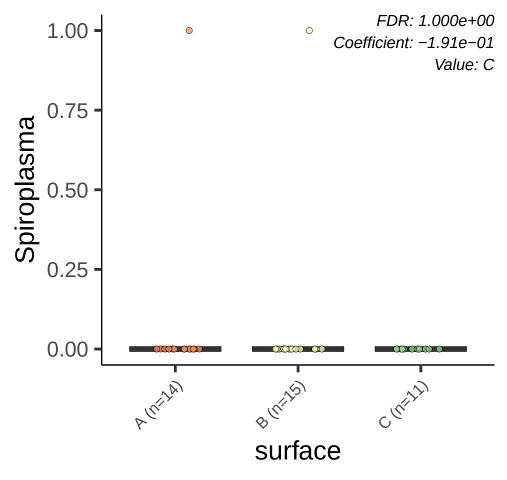
FDR: 1.000e+00
Coefficient: −1.91e−01
Value: C
Spiroplasma
1.00
0.75
0.50
0.25
0.00
A (n=14)
B (n=15)
C (n=11)
surface

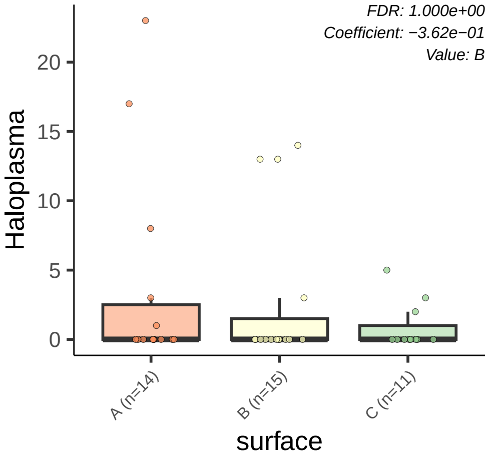

FDR: 1.000e+00
Coefficient: −3.62e−01
Value: B
Haloplasma
20
15
10
5
0
A (n=14)
B (n=15)
C (n=11)
surface

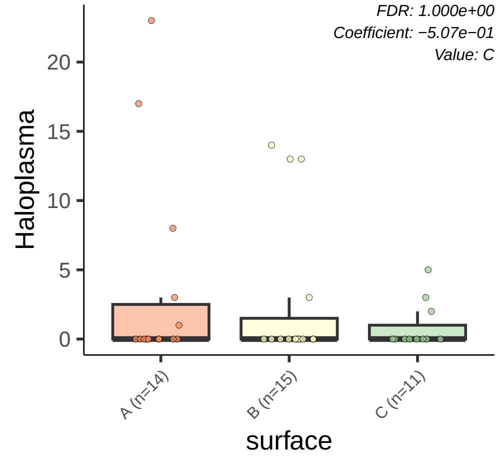
FDR: 1.000e+00
Coefficient: −5.07e−01
Value: C
Haloplasma
0
5
10
15
20
A (n=14)
B (n=15)
C (n=11)
surface

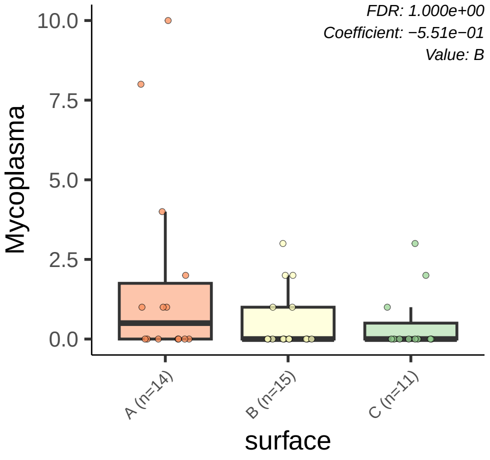
FDR: 1.000e+00
Coefficient: −5.51e−01
Value: B
Mycoplasma
10.0
7.5
5.0
2.5
0.0
A (n=14)
B (n=15)
C (n=11)
surface

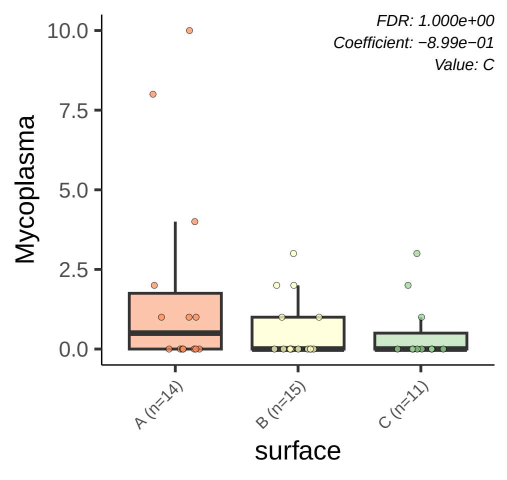

FDR: 1.000e+00
Coefficient: −8.99e−01
Value: C
Mycoplasma
10.0
7.5
5.0
2.5
0.0
A (n=14)
B (n=15)
C (n=11)
surface

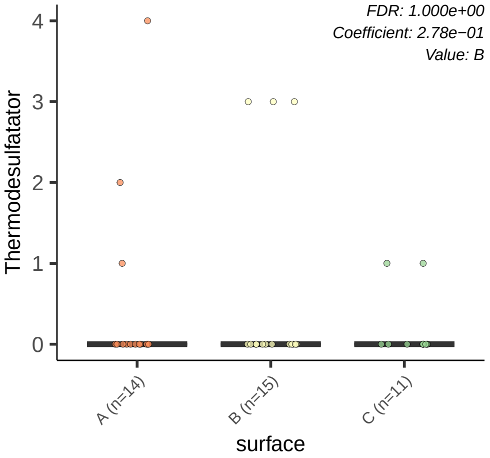

FDR: 1.000e+00
Coefficient: 2.78e−01
Value: B
Thermodesulfatator
0
1
2
3
4
A (n=14)
B (n=15)
C (n=11)
surface

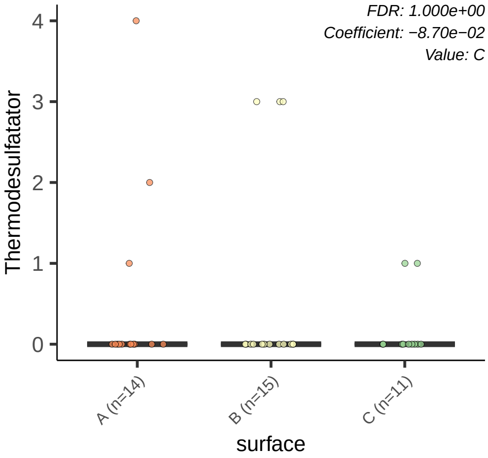

FDR: 1.000e+00
Coefficient: −8.70e−02
Value: C
Thermodesulfatator
0
1
2
3
4
A (n=14)
B (n=15)
C (n=11)
surface

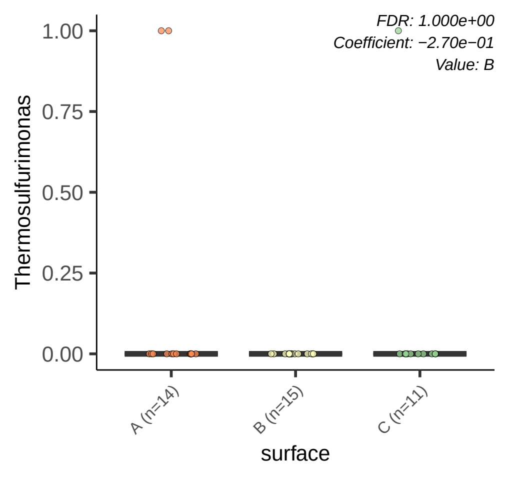

FDR: 1.000e+00
Coefficient: −2.70e−01
Value: B
Thermosulfurimonas
1.00
0.75
0.50
0.25
0.00
A (n=14)
B (n=15)
C (n=11)
surface

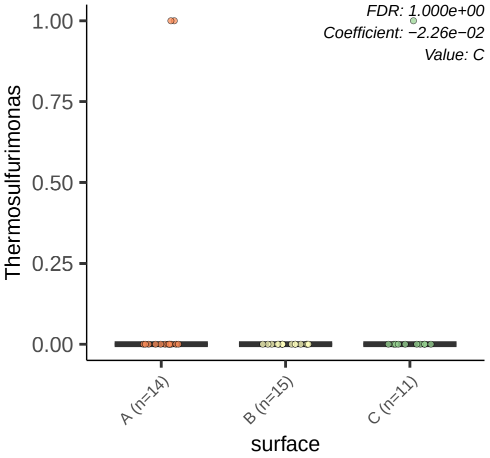
FDR: 1.000e+00
Coefficient: −2.26e−02
Value: C
Thermosulfurimonas
1.00
0.75
0.50
0.25
0.00
A (n=14)
B (n=15)
C (n=11)
surface

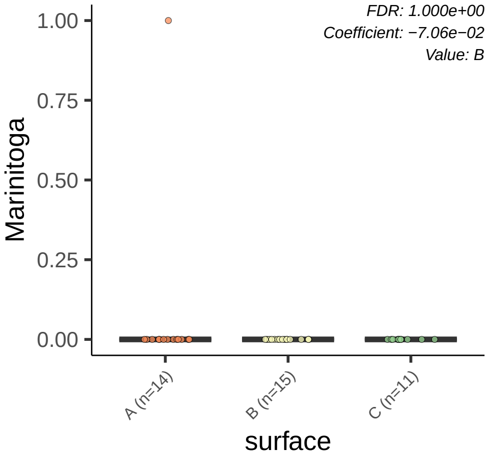

FDR: 1.000e+00
Coefficient: −7.06e−02
Value: B
Marinitoga
1.00
0.75
0.50
0.25
0.00
A (n=14)
B (n=15)
C (n=11)
surface

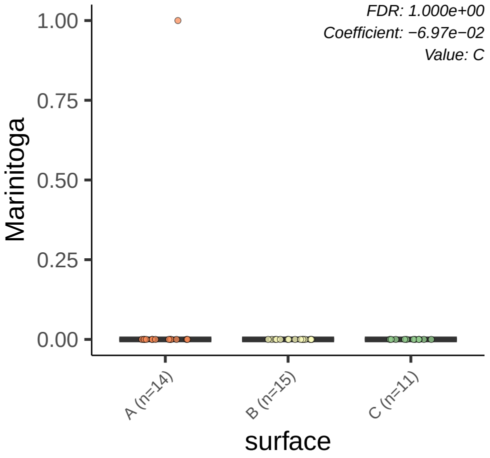
FDR: 1.000e+00
Coefficient: −6.97e−02
Value: C
Marinitoga
1.00
0.75
0.50
0.25
0.00
A (n=14)
B (n=15)
C (n=11)
surface

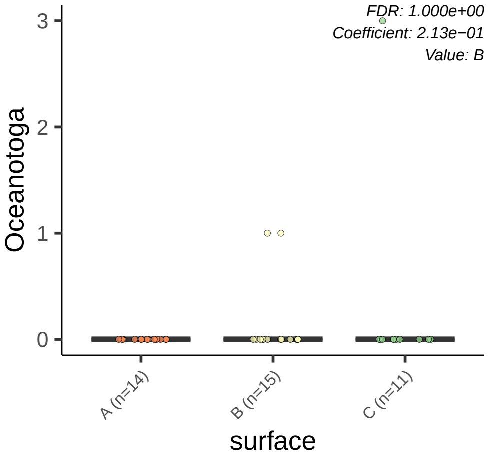

FDR: 1.000e+00
Coefficient: 2.13e−01
Value: B
Oceanotoga
3
2
1
0
A (n=14)
B (n=15)
C (n=11)
surface

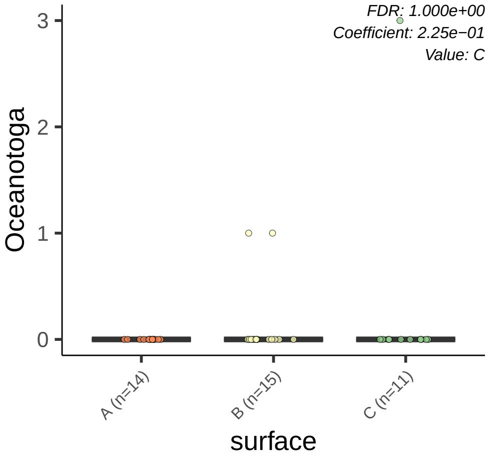
FDR: 1.000e+00
Coefficient: 2.25e−01
Value: C
Oceanotoga
3
2
1
0
A (n=14)
B (n=15)
C (n=11)
surface

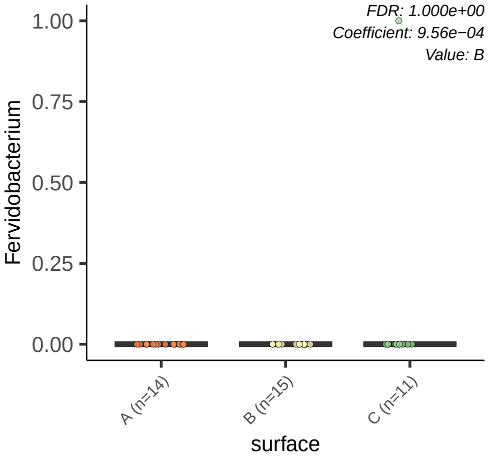

FDR: 1.000e+00
Coefficient: 9.56e−04
Value: B
1.00
0.75
0.50
0.25
0.00
Fervidobacterium
A (n=14)
B (n=15)
C (n=11)
surface

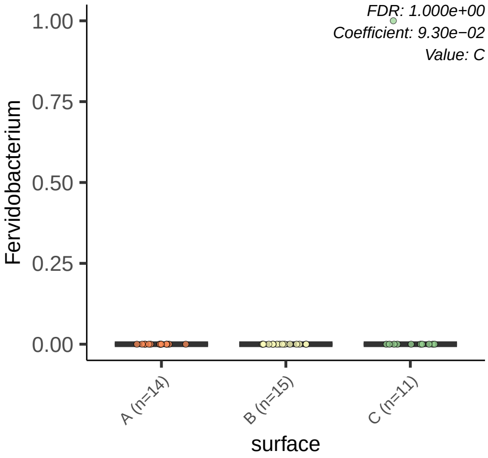
FDR: 1.000e+00
Coefficient: 9.30e−02
Value: C
Fervidobacterium
1.00
0.75
0.50
0.25
0.00
A (n=14)
B (n=15)
C (n=11)
surface

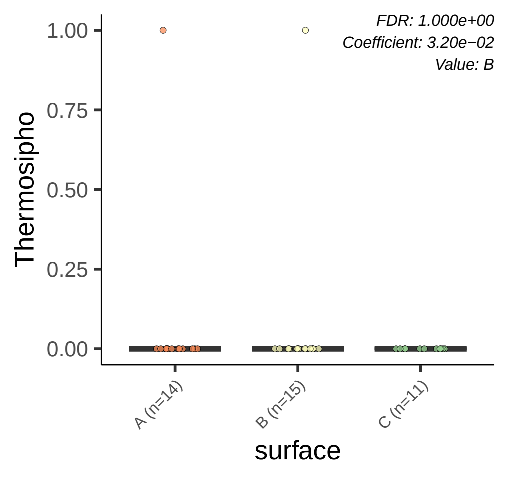

FDR: 1.000e+00
Coefficient: 3.20e−02
Value: B
Thermosipho
1.00
0.75
0.50
0.25
0.00
A (n=14)
B (n=15)
C (n=11)
surface

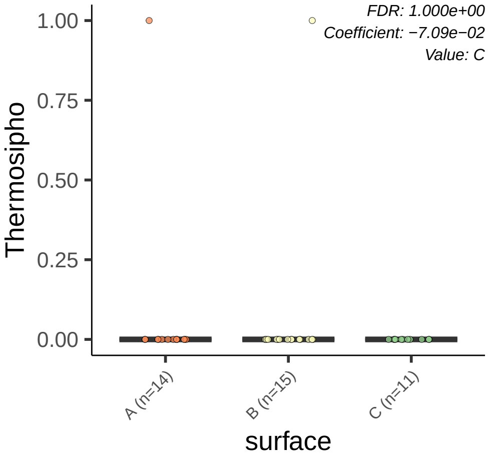
FDR: 1.000e+00
Coefficient: −7.09e−02
Value: C
Thermosipho
1.00
0.75
0.50
0.25
0.00
A (n=14)
B (n=15)
C (n=11)
surface

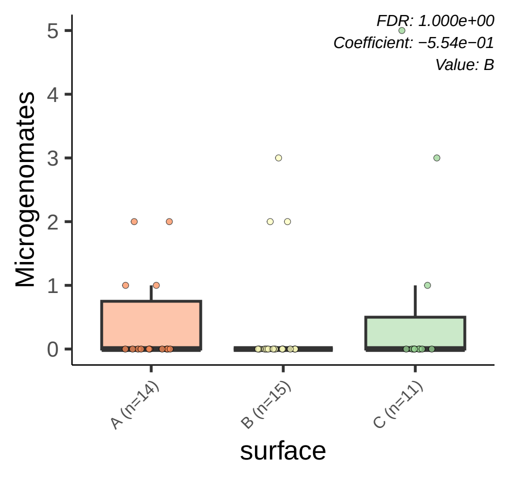

FDR: 1.000e+00
Coefficient: −5.54e−01
Value: B
Microgenomates
0
1
2
3
4
5
A (n=14)
B (n=15)
C (n=11)
surface

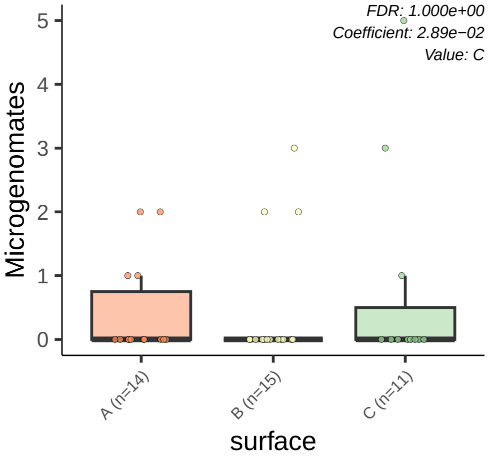

FDR: 1.000e+00
Coefficient: 2.89e−02
Value: C
Microgenomates
0
1
2
3
4
5
A (n=14)
B (n=15)
C (n=11)
surface

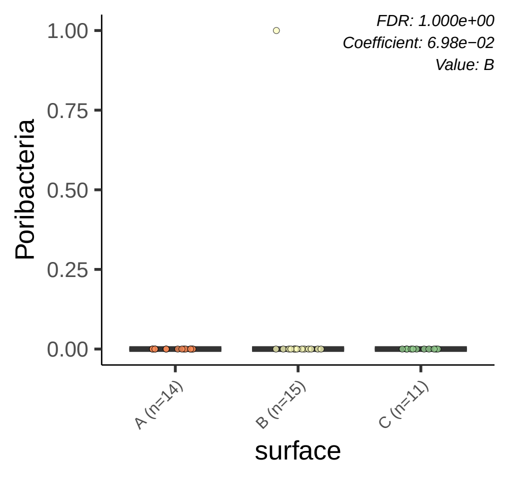

FDR: 1.000e+00
Coefficient: 6.98e−02
Value: B
Poribacteria
1.00
0.75
0.50
0.25
0.00
A (n=14)
B (n=15)
C (n=11)
surface

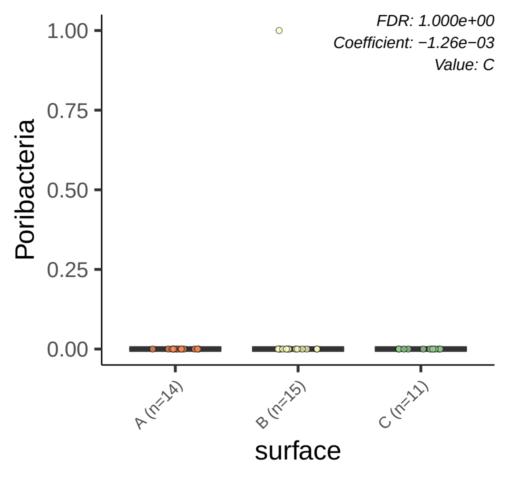
FDR: 1.000e+00
Coefficient: −1.26e−03
Value: C
Poribacteria
1.00
0.75
0.50
0.25
0.00
A (n=14)
B (n=15)
C (n=11)
surface

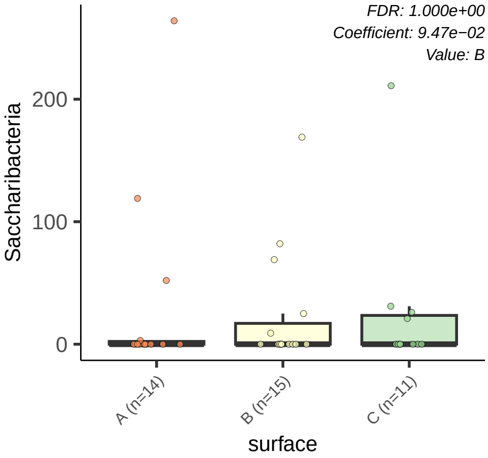

FDR: 1.000e+00
Coefficient: 9.47e−02
Value: B
Saccharibacteria
200
100
0
A (n=14)
B (n=15)
C (n=11)
surface

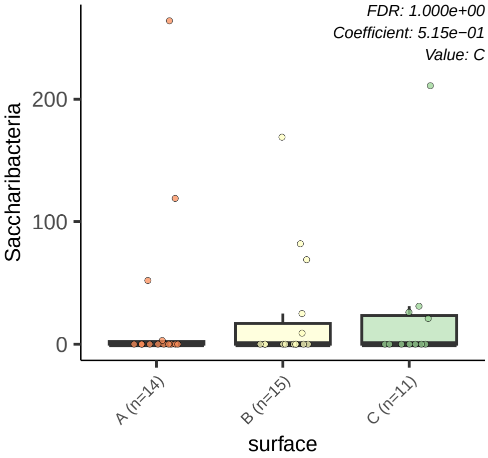

FDR: 1.000e+00
Coefficient: 5.15e−01
Value: C
Saccharibacteria
200
100
0
A (n=14)
B (n=15)
C (n=11)
surface

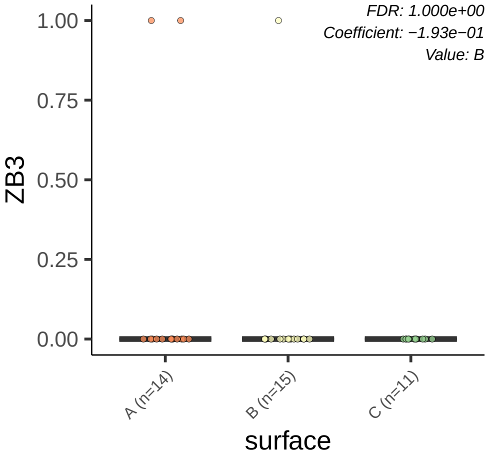
FDR: 1.000e+00
Coefficient: −1.93e−01
Value: B
ZB3
1.00
0.75
0.50
0.25
0.00
A (n=14)
B (n=15)
C (n=11)
surface

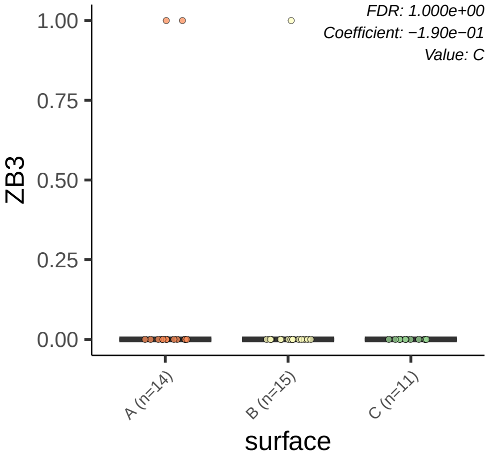

FDR: 1.000e+00
Coefficient: −1.90e−01
Value: C
ZB3
1.00
0.75
0.50
0.25
0.00
A (n=14)
B (n=15)
C (n=11)
surface

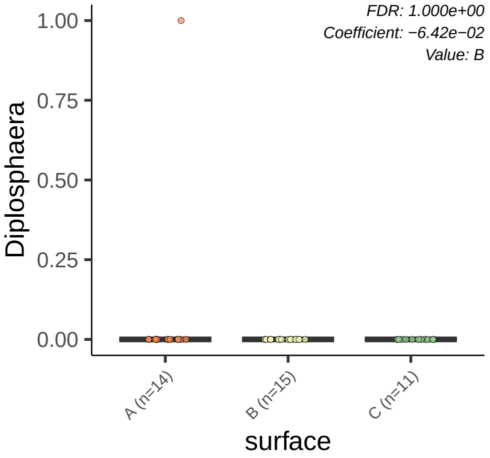

FDR: 1.000e+00
Coefficient: −6.42e−02
Value: B
Diplosphaera
1.00
0.75
0.50
0.25
0.00
A (n=14)
B (n=15)
C (n=11)
surface

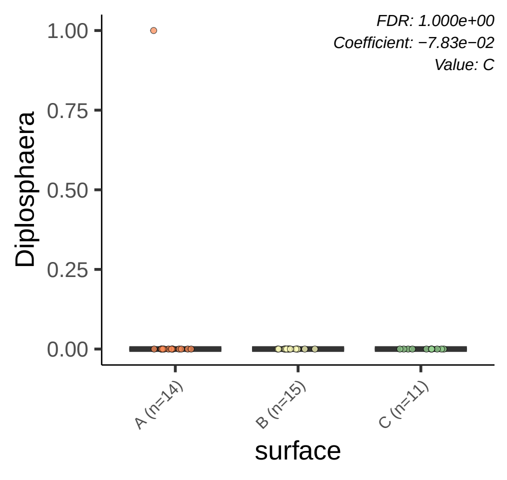
FDR: 1.000e+00
Coefficient: −7.83e−02
Value: C
Diplosphaera
1.00
0.75
0.50
0.25
0.00
A (n=14)
B (n=15)
C (n=11)
surface

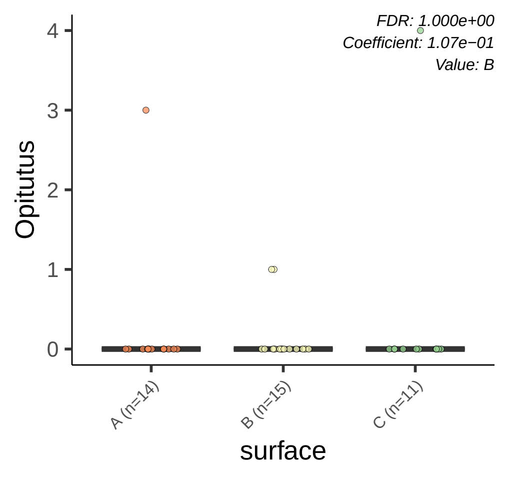
FDR: 1.000e+00
Coefficient: 1.07e−01
Value: B
Opitutus
4
3
2
1
0
A (n=14)
B (n=15)
C (n=11)
surface

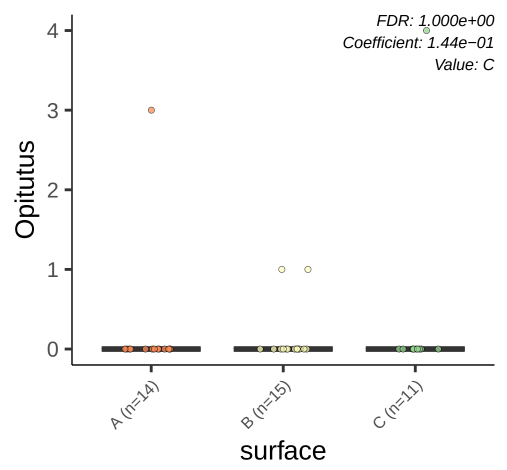
FDR: 1.000e+00
Coefficient: 1.44e−01
Value: C
Opitutus
4
3
2
1
0
A (n=14)
B (n=15)
C (n=11)
surface

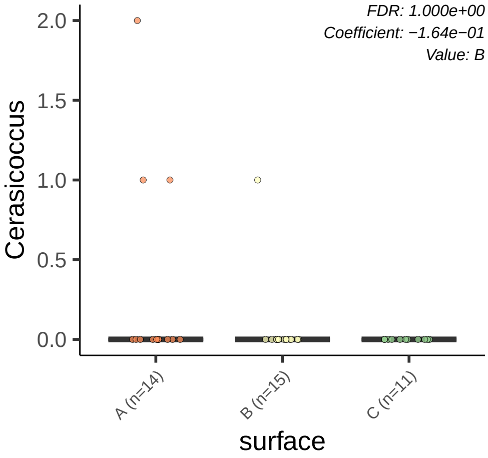
FDR: 1.000e+00
Coefficient: −1.64e−01
Value: B
Cerasicoccus
2.0
1.5
1.0
0.5
0.0
A (n=14)
B (n=15)
C (n=11)
surface

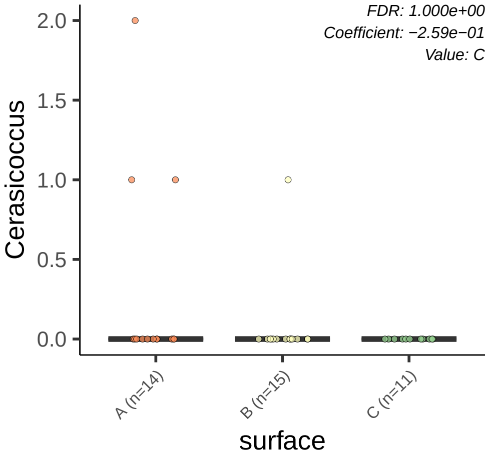
FDR: 1.000e+00
Coefficient: −2.59e−01
Value: C
Cerasicoccus
2.0
1.5
1.0
0.5
0.0
A (n=14)
B (n=15)
C (n=11)
surface

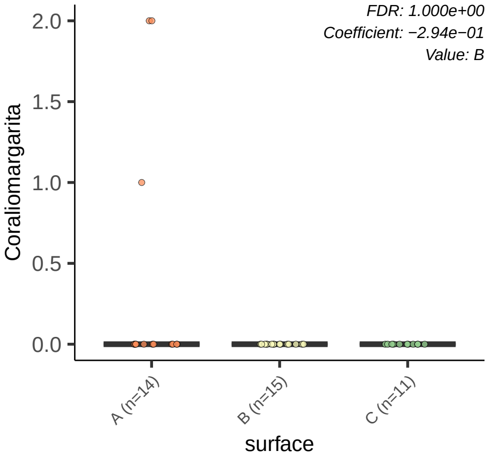
FDR: 1.000e+00
Coefficient: −2.94e−01
Value: B
Coraliomargarita
2.0
1.5
1.0
0.5
0.0
A (n=14)
B (n=15)
C (n=11)
surface

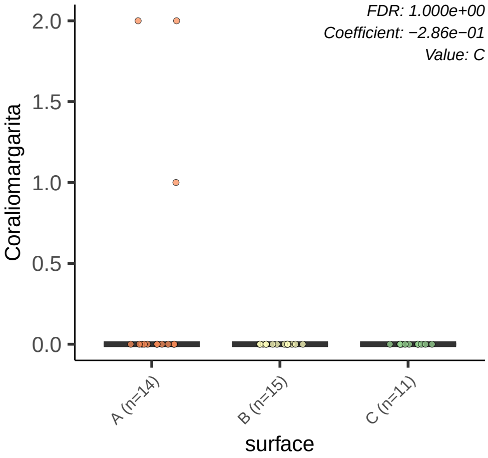
FDR: 1.000e+00
Coefficient: −2.86e−01
Value: C
Coraliomargarita
2.0
1.5
1.0
0.5
0.0
A (n=14)
B (n=15)
C (n=11)
surface

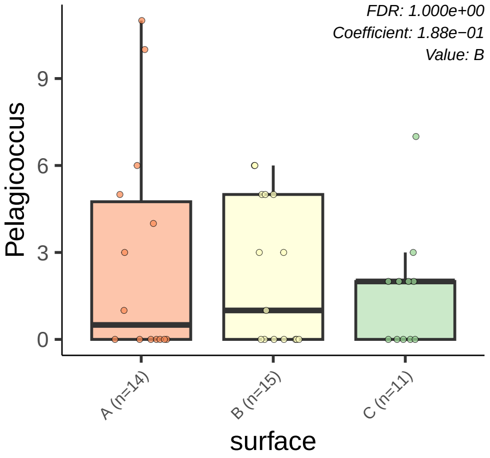
FDR: 1.000e+00
Coefficient: 1.88e−01
Value: B
Pelagicoccus
9
6
3
0
A (n=14)
B (n=15)
C (n=11)
surface

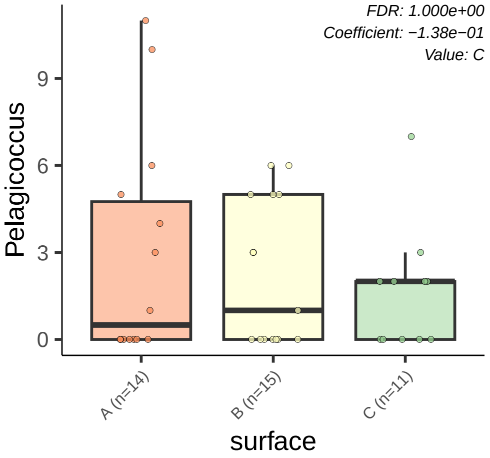
FDR: 1.000e+00
Coefficient: −1.38e−01
Value: C
Pelagicoccus
0
3
6
9
A (n=14)
B (n=15)
C (n=11)
surface

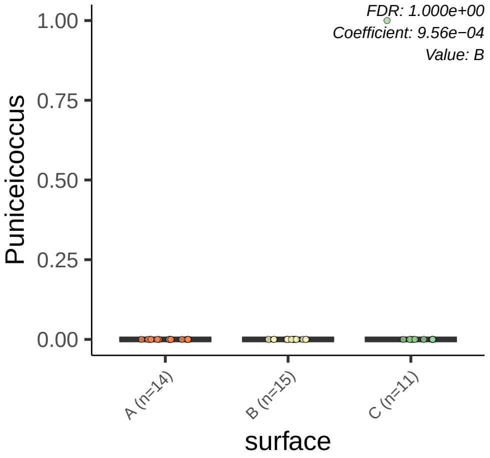

FDR: 1.000e+00
Coefficient: 9.56e−04
Value: B
Puniceicoccus
1.00
0.75
0.50
0.25
0.00
A (n=14)
B (n=15)
C (n=11)
surface

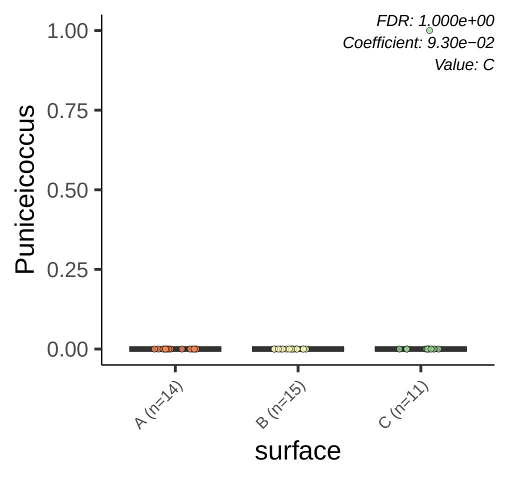
FDR: 1.000e+00
Coefficient: 9.30e−02
Value: C
Puniceicoccus
1.00
0.75
0.50
0.25
0.00
A (n=14)
B (n=15)
C (n=11)
surface

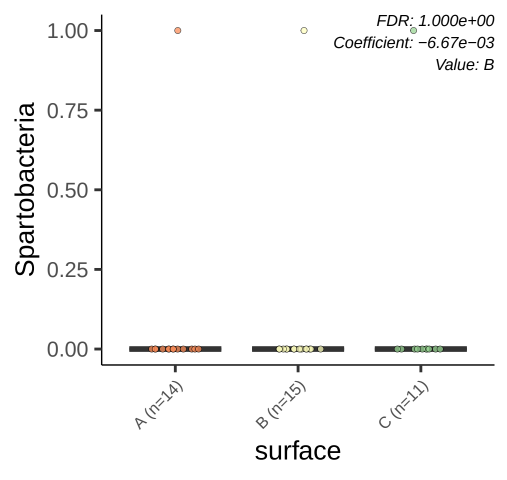

FDR: 1.000e+00
Coefficient: −6.67e−03
Value: B
Spartobacteria
1.00
0.75
0.50
0.25
0.00
A (n=14)
B (n=15)
C (n=11)
surface

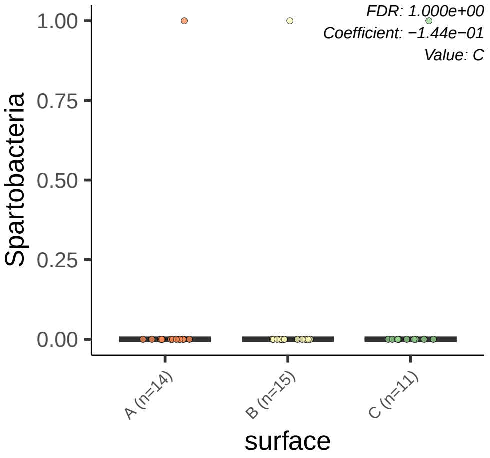

FDR: 1.000e+00
Coefficient: −1.44e−01
Value: C
Spartobacteria
1.00
0.75
0.50
0.25
0.00
A (n=14)
B (n=15)
C (n=11)
surface

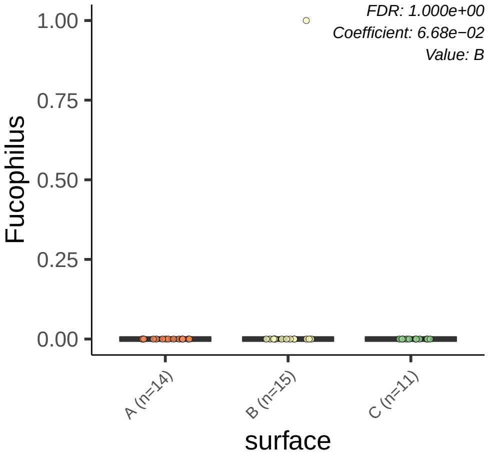
FDR: 1.000e+00
Coefficient: 6.68e−02
Value: B
Fucophilus
1.00
0.75
0.50
0.25
0.00
A (n=14)
B (n=15)
C (n=11)
surface

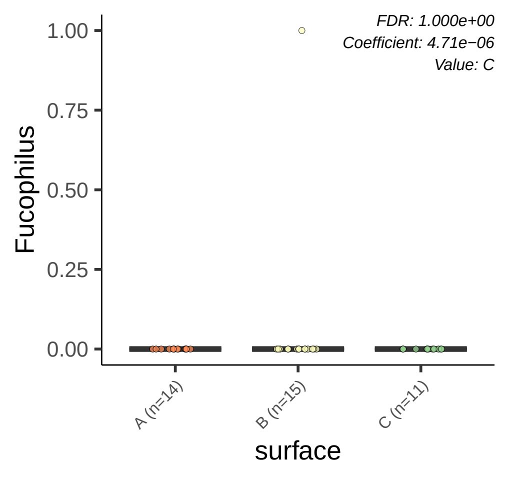
FDR: 1.000e+00
Coefficient: 4.71e−06
Value: C
Fucophilus
1.00
0.75
0.50
0.25
0.00
A (n=14)
B (n=15)
C (n=11)
surface

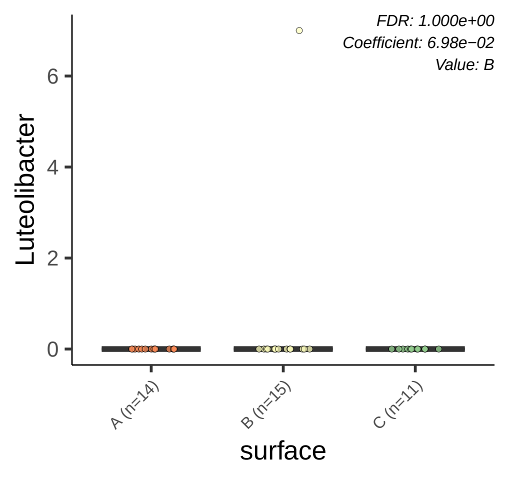

FDR: 1.000e+00
Coefficient: 6.98e−02
Value: B
Luteolibacter
0
2
4
6
A (n=14)
B (n=15)
C (n=11)
surface

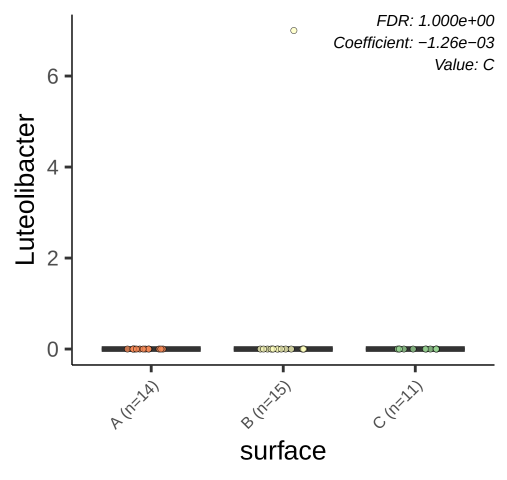
FDR: 1.000e+00
Coefficient: −1.26e−03
Value: C
Luteolibacter
6
4
2
0
A (n=14)
B (n=15)
C (n=11)
surface

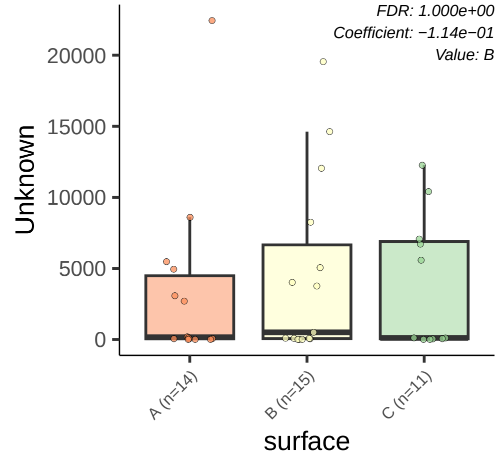
FDR: 1.000e+00
Coefficient: −1.14e−01
Value: B
Unknown
20000
15000
10000
5000
0
A (n=14)
B (n=15)
C (n=11)
surface

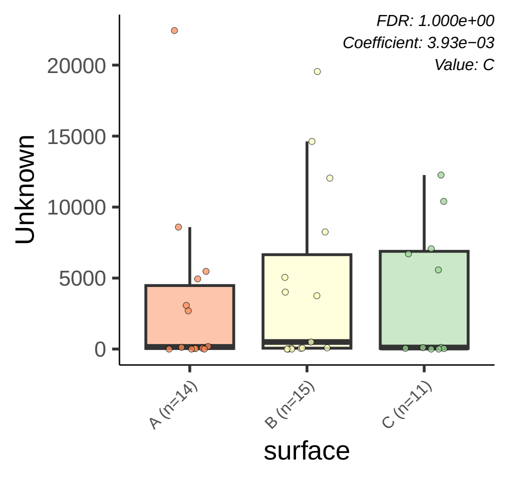

FDR: 1.000e+00
Coefficient: 3.93e−03
Value: C
Unknown
20000
15000
10000
5000
0
A (n=14)
B (n=15)
C (n=11)
surface

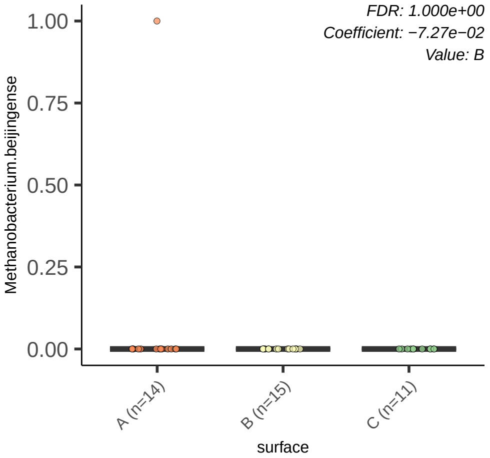

FDR: 1.000e+00
Coefficient: −7.27e−02
Value: B
Methanobacterium.beijingense
1.00
0.75
0.50
0.25
0.00
A (n=14)
B (n=15)
C (n=11)
surface

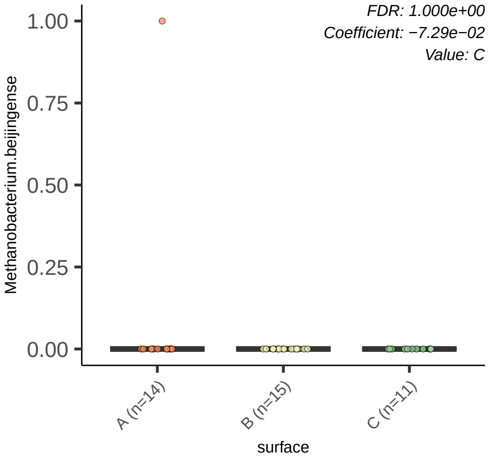
FDR: 1.000e+00
Coefficient: −7.29e−02
Value: C
Methanobacterium.beijingense
1.00
0.75
0.50
0.25
0.00
A (n=14)
B (n=15)
C (n=11)
surface

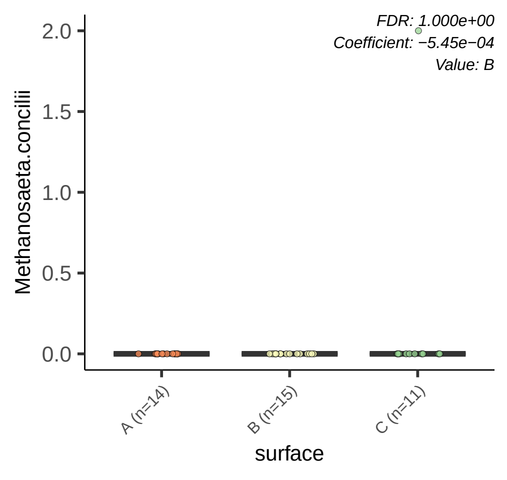
FDR: 1.000e+00
Coefficient: −5.45e−04
Value: B
Methanosaeta.concilii
2.0
1.5
1.0
0.5
0.0
A (n=14)
B (n=15)
C (n=11)
surface

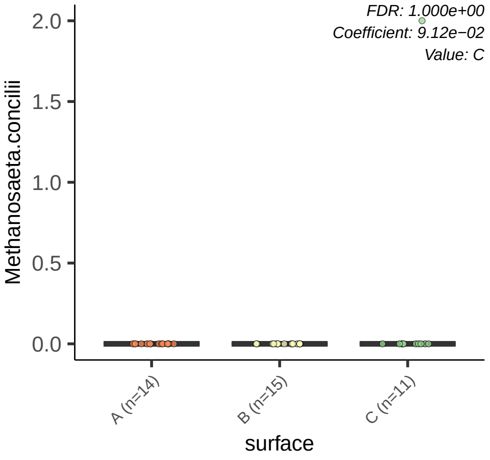
FDR: 1.000e+00
Coefficient: 9.12e−02
Value: C
Methanosaeta.concilii
2.0
1.5
1.0
0.5
0.0
A (n=14)
B (n=15)
C (n=11)
surface

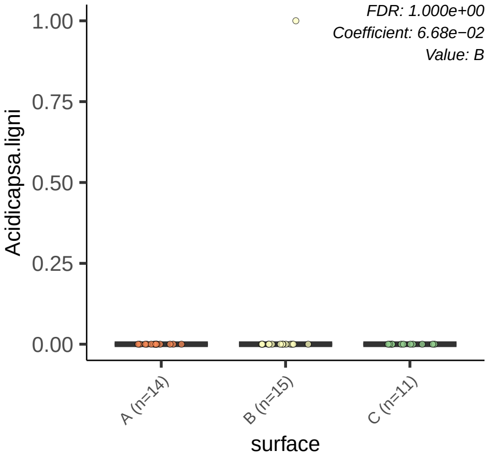
FDR: 1.000e+00
Coefficient: 6.68e−02
Value: B
Acidicapsa.ligni
1.00
0.75
0.50
0.25
0.00
A (n=14)
B (n=15)
C (n=11)
surface

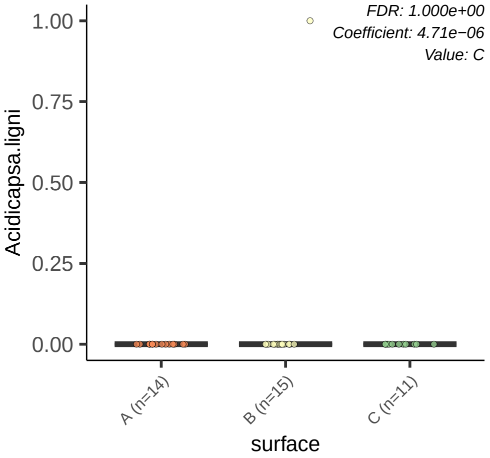
FDR: 1.000e+00
Coefficient: 4.71e−06
Value: C
Acidicapsa.ligni
1.00
0.75
0.50
0.25
0.00
A (n=14)
B (n=15)
C (n=11)
surface

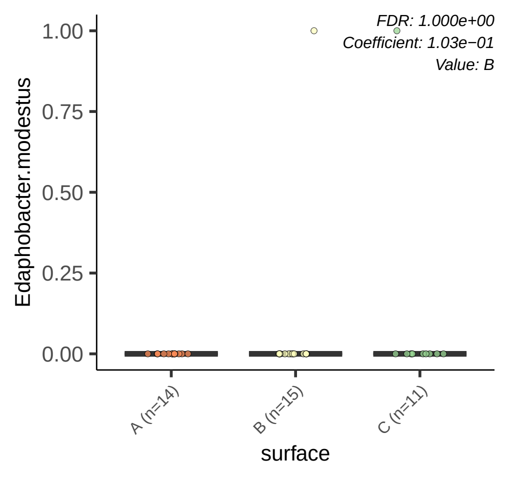
FDR: 1.000e+00
Coefficient: 1.03e−01
Value: B
Edaphobacter.modestus
1.00
0.75
0.50
0.25
0.00
A (n=14)
B (n=15)
C (n=11)
surface

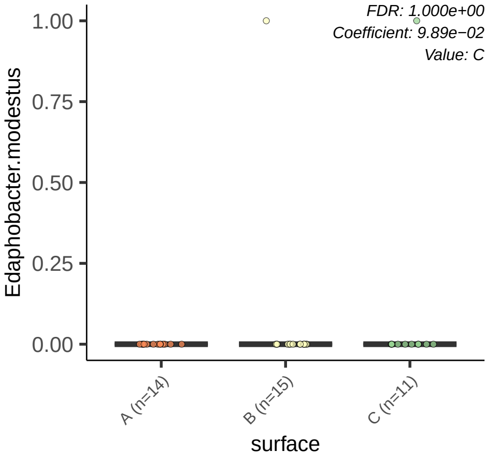
FDR: 1.000e+00
Coefficient: 9.89e−02
Value: C
Edaphobacter.modestus
1.00
0.75
0.50
0.25
0.00
A (n=14)
B (n=15)
C (n=11)
surface

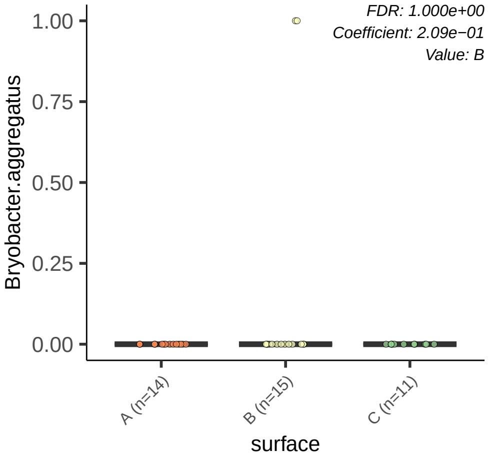
FDR: 1.000e+00
Coefficient: 2.09e−01
Value: B
Bryobacter.aggregatus
1.00
0.75
0.50
0.25
0.00
A (n=14)
B (n=15)
C (n=11)
surface

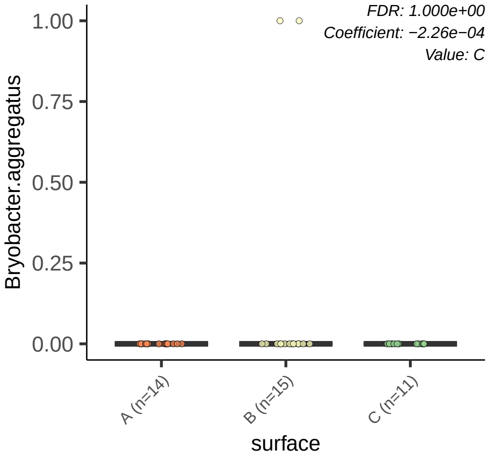
FDR: 1.000e+00
Coefficient: −2.26e−04
Value: C
Bryobacter.aggregatus
1.00
0.75
0.50
0.25
0.00
A (n=14)
B (n=15)
C (n=11)
surface

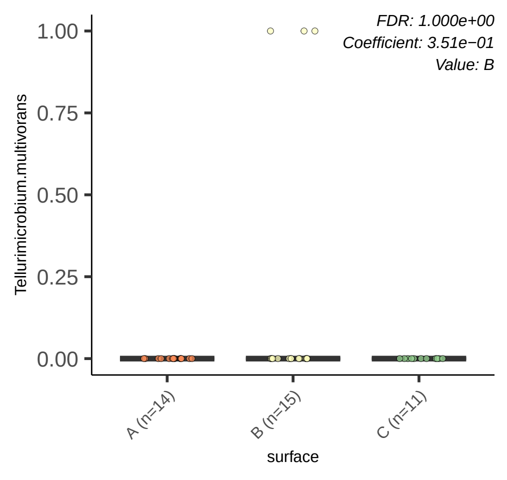
FDR: 1.000e+00
Coefficient: 3.51e−01
Value: B
Tellurimicrobium.multivorans
1.00
0.75
0.50
0.25
0.00
A (n=14)
B (n=15)
C (n=11)
surface

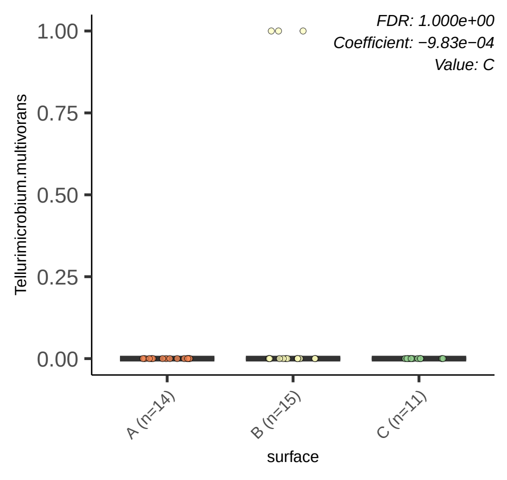
FDR: 1.000e+00
Coefficient: −9.83e−04
Value: C
Tellurimicrobium.multivorans
1.00
0.75
0.50
0.25
0.00
A (n=14)
B (n=15)
C (n=11)
surface

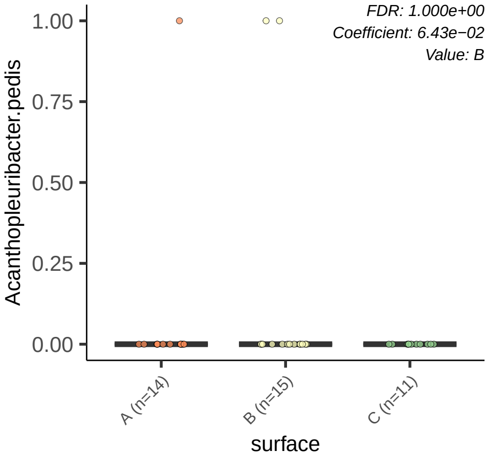

FDR: 1.000e+00
Coefficient: 6.43e−02
Value: B
Acanthopleuribacter.pedis
1.00
0.75
0.50
0.25
0.00
A (n=14)
B (n=15)
C (n=11)
surface

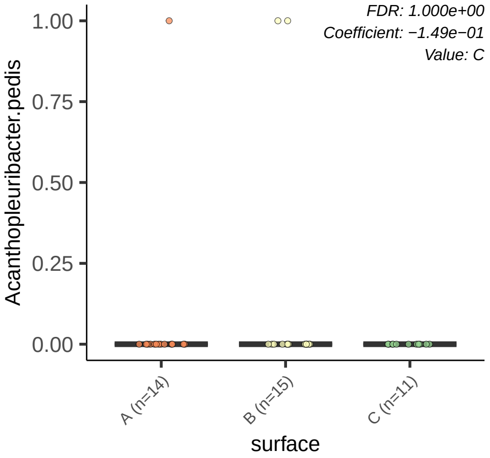
FDR: 1.000e+00
Coefficient: −1.49e−01
Value: C
Acanthopleuribacter.pedis
1.00
0.75
0.50
0.25
0.00
A (n=14)
B (n=15)
C (n=11)
surface

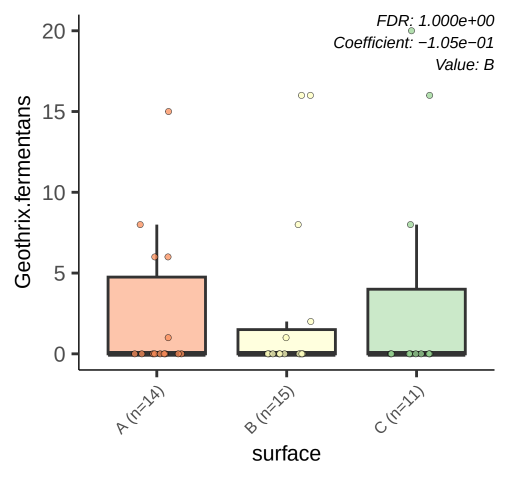

FDR: 1.000e+00
Coefficient: −1.05e−01
Value: B
Geothrix.fermentans
0
5
10
15
20
A (n=14)
B (n=15)
C (n=11)
surface

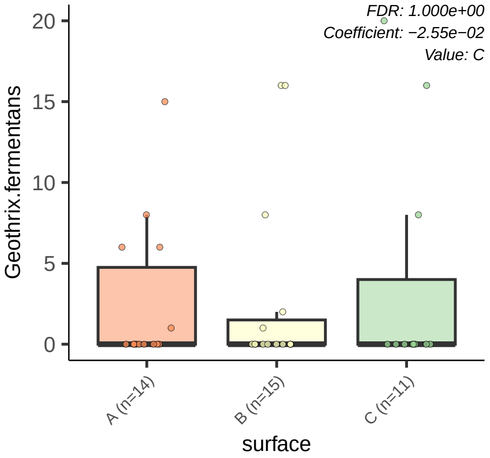
FDR: 1.000e+00
Coefficient: −2.55e−02
Value: C
Geothrix.fermentans
20
15
10
5
0
A (n=14)
B (n=15)
C (n=11)
surface

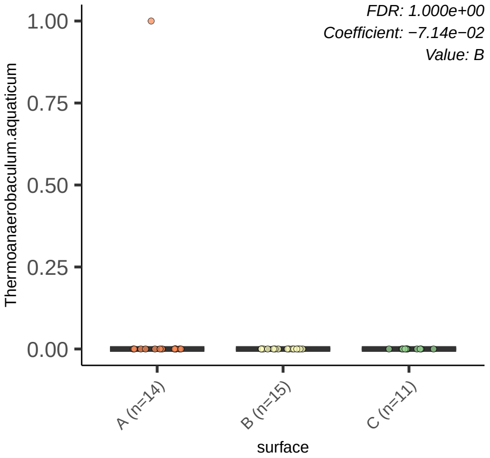

FDR: 1.000e+00
Coefficient: −7.14e−02
Value: B
Thermoanaerobaculum.aquaticum
1.00
0.75
0.50
0.25
0.00
A (n=14)
B (n=15)
C (n=11)
surface

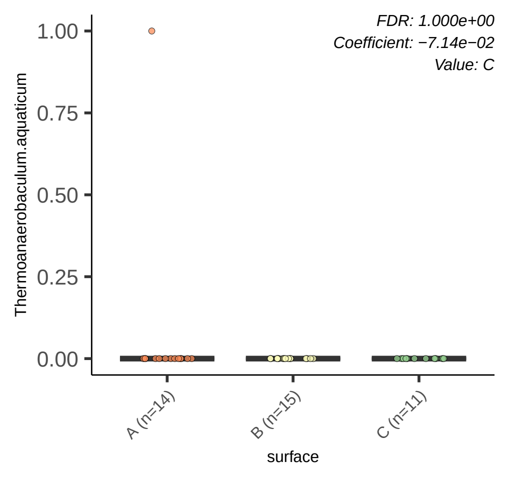

FDR: 1.000e+00
Coefficient: −7.14e−02
Value: C
Thermoanaerobaculum.aquaticum
1.00
0.75
0.50
0.25
0.00
A (n=14)
B (n=15)
C (n=11)
surface

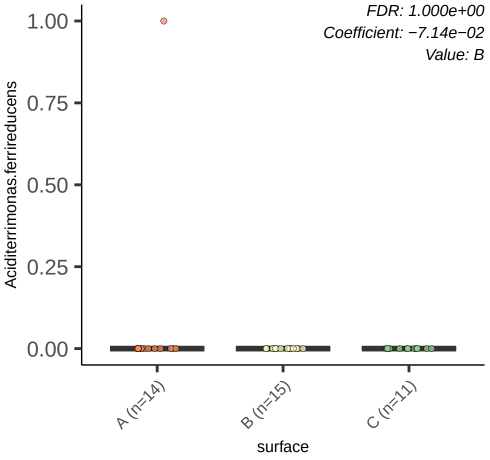

FDR: 1.000e+00
Coefficient: −7.14e−02
Value: B
Aciditerrimonas.ferrireducens
1.00
0.75
0.50
0.25
0.00
A (n=14)
B (n=15)
C (n=11)
surface

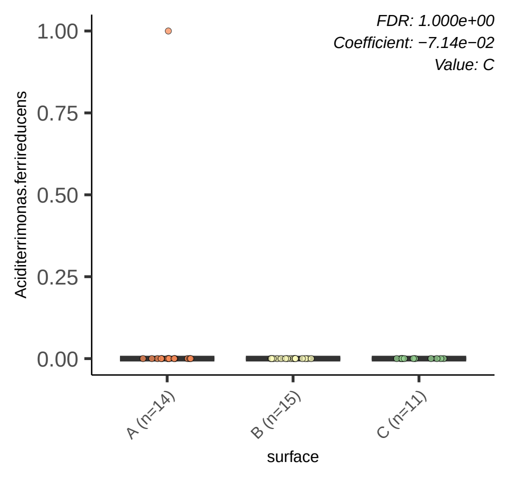
FDR: 1.000e+00
Coefficient: −7.14e−02
Value: C
Aciditerrimonas.ferrireducens
1.00
0.75
0.50
0.25
0.00
A (n=14)
B (n=15)
C (n=11)
surface

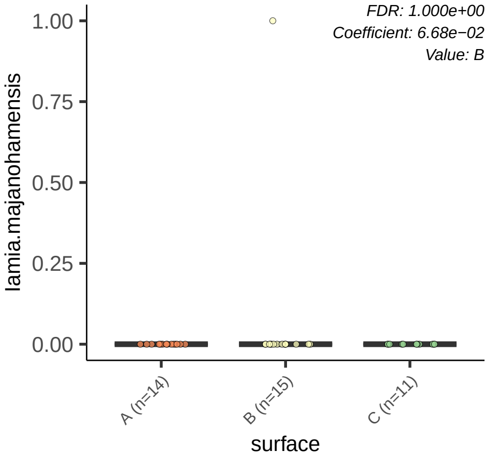

FDR: 1.000e+00
Coefficient: 6.68e−02
Value: B
lamia.majanohamensis
1.00
0.75
0.50
0.25
0.00
A (n=14)
B (n=15)
C (n=11)
surface

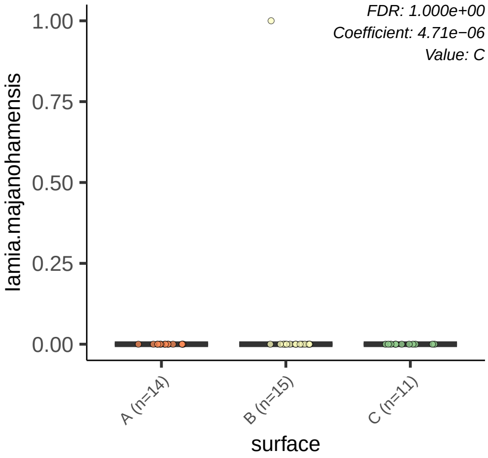
FDR: 1.000e+00
Coefficient: 4.71e−06
Value: C
lamia.majanohamensis
1.00
0.75
0.50
0.25
0.00
A (n=14)
B (n=15)
C (n=11)
surface

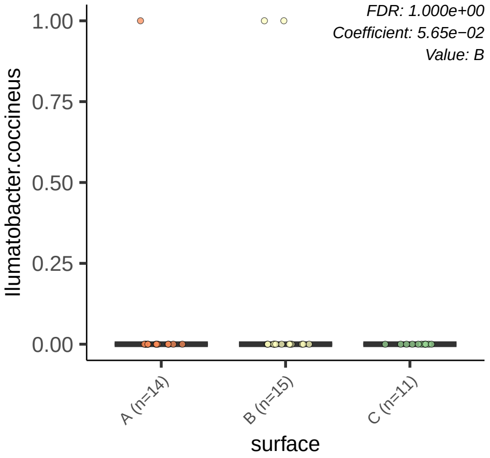
FDR: 1.000e+00
Coefficient: 5.65e−02
Value: B
Ilumatobacter.coccineus
1.00
0.75
0.50
0.25
0.00
A (n=14)
B (n=15)
C (n=11)
surface

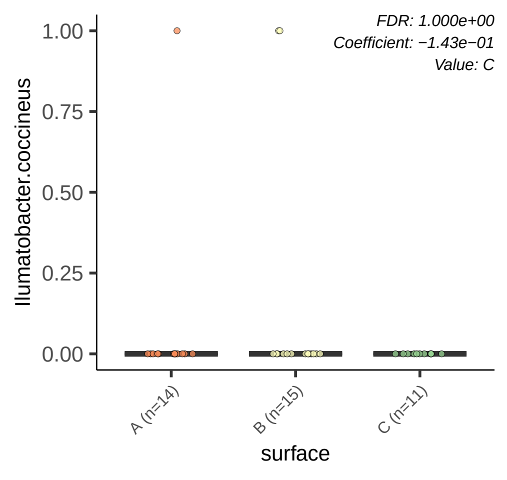
FDR: 1.000e+00
Coefficient: −1.43e−01
Value: C
Ilumatobacter.coccineus
1.00
0.75
0.50
0.25
0.00
A (n=14)
B (n=15)
C (n=11)
surface

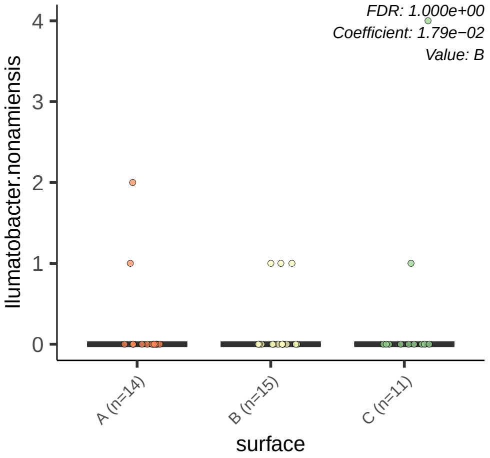
FDR: 1.000e+00
Coefficient: 1.79e−02
Value: B
Ilumatobacter.nonamiensis
0
1
2
3
4
A (n=14)
B (n=15)
C (n=11)
surface

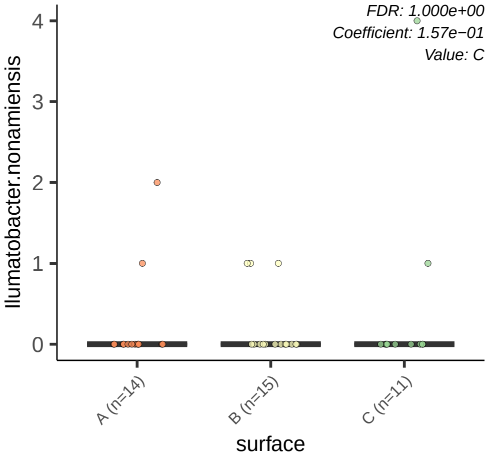

FDR: 1.000e+00
Coefficient: 1.57e−01
Value: C
Ilumatobacter.nonamiensis
0
1
2
3
4
A (n=14)
B (n=15)
C (n=11)
surface

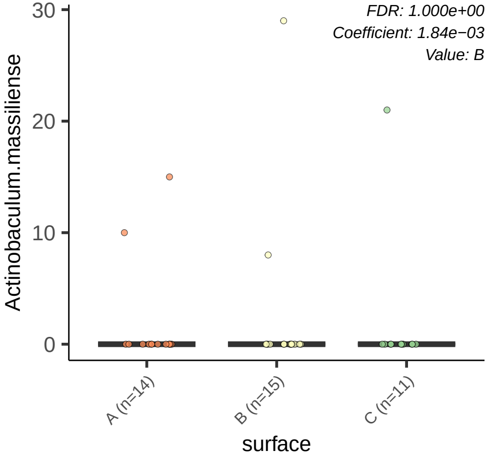
FDR: 1.000e+00
Coefficient: 1.84e−03
Value: B
Actinobaculum.massiliense
0
10
20
30
A (n=14)
B (n=15)
C (n=11)
surface

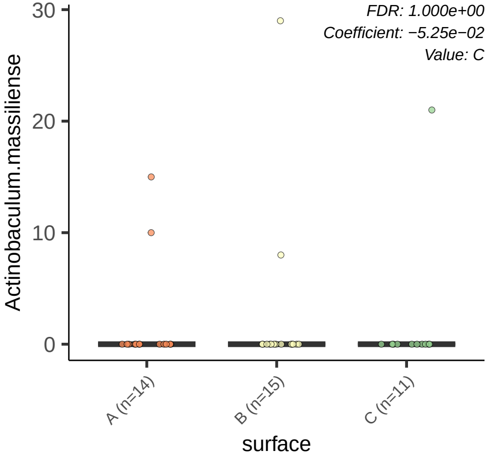

FDR: 1.000e+00
Coefficient: −5.25e−02
Value: C
Actinobaculum.massiliense
0
10
20
30
A (n=14)
B (n=15)
C (n=11)
surface

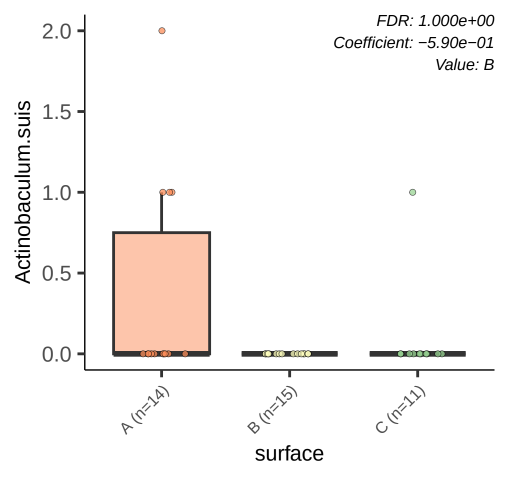
FDR: 1.000e+00
Coefficient: −5.90e−01
Value: B
Actinobaculum.suis
2.0
1.5
1.0
0.5
0.0
A (n=14)
B (n=15)
C (n=11)
surface

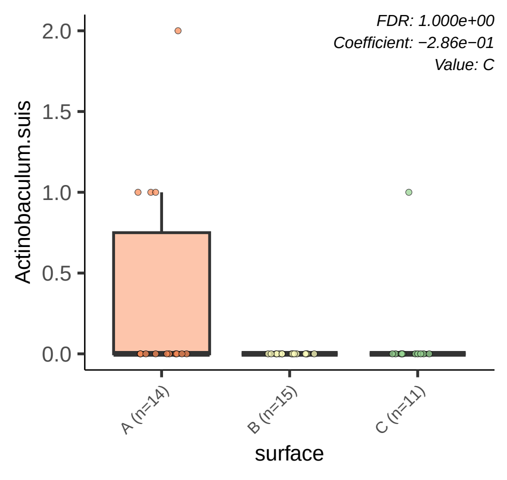
FDR: 1.000e+00
Coefficient: −2.86e−01
Value: C
Actinobaculum.suis
2.0
1.5
1.0
0.5
0.0
A (n=14)
B (n=15)
C (n=11)
surface

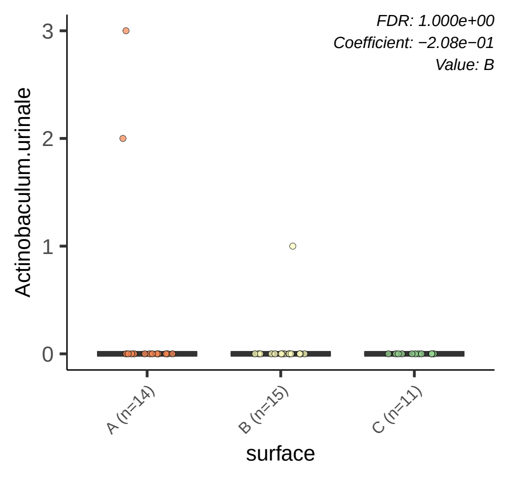

FDR: 1.000e+00
Coefficient: −2.08e−01
Value: B
3
2
1
0
Actinobaculum.urinale
A (n=14)
B (n=15)
C (n=11)
surface

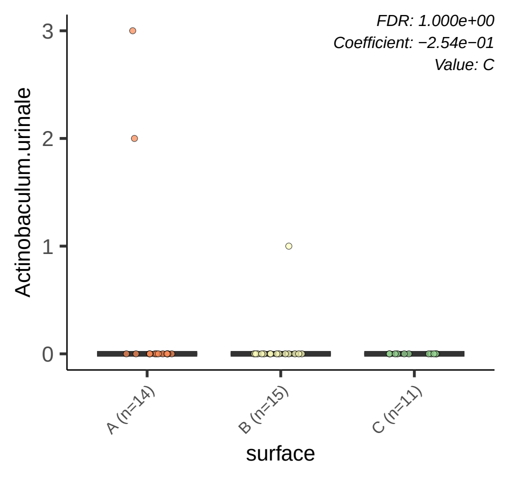
FDR: 1.000e+00
Coefficient: −2.54e−01
Value: C
Actinobaculum.urinale
3
2
1
0
A (n=14)
B (n=15)
C (n=11)
surface

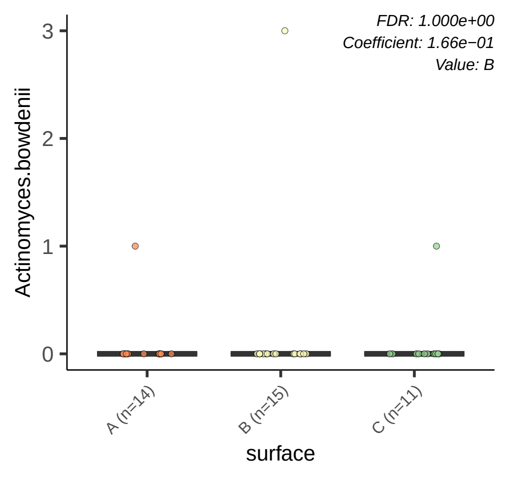
FDR: 1.000e+00
Coefficient: 1.66e−01
Value: B
Actinomyces.bowdenii
0
1
2
3
A (n=14)
B (n=15)
C (n=11)
surface

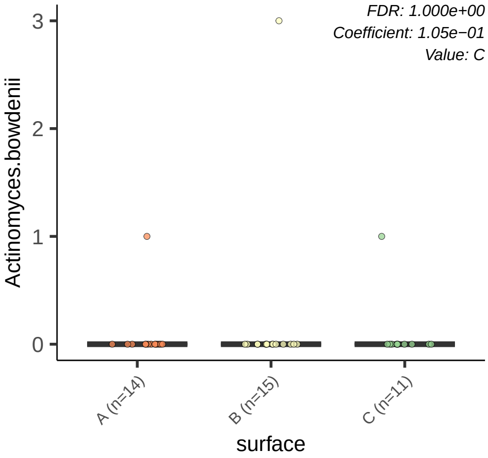
FDR: 1.000e+00
Coefficient: 1.05e−01
Value: C
Actinomyces.bowdenii
3
2
1
0
A (n=14)
B (n=15)
C (n=11)
surface

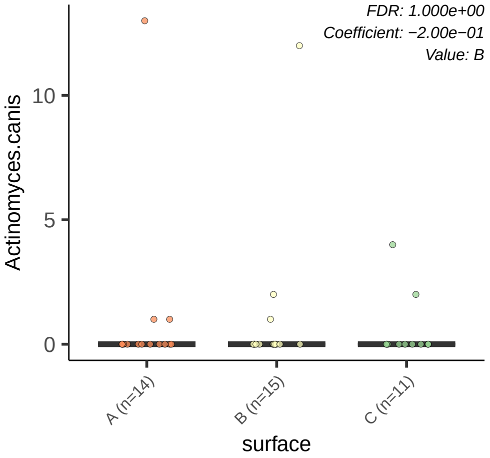

FDR: 1.000e+00
Coefficient: −2.00e−01
Value: B
Actinomyces.canis
0
5
10
A (n=14)
B (n=15)
C (n=11)
surface

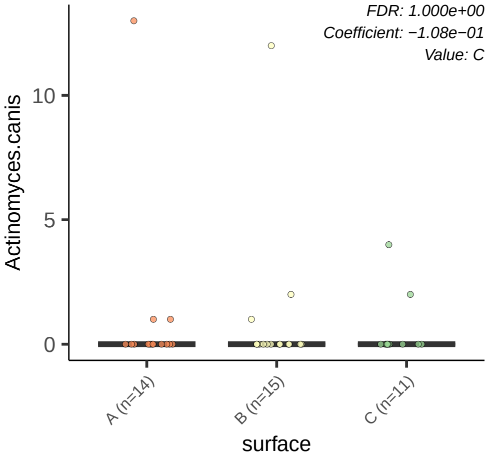

FDR: 1.000e+00
Coefficient: −1.08e−01
Value: C
Actinomyces.canis
10
5
0
A (n=14)
B (n=15)
C (n=11)
surface

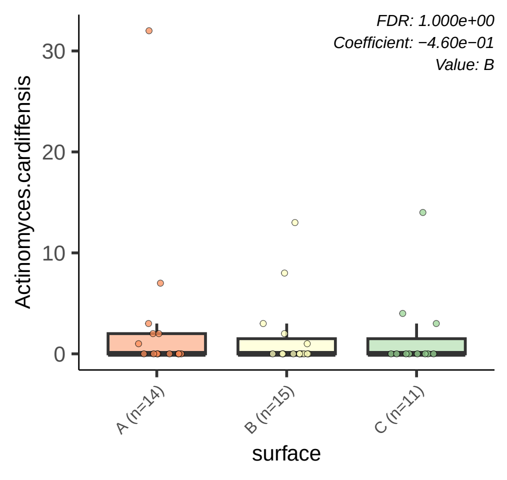

FDR: 1.000e+00
Coefficient: −4.60e−01
Value: B
Actinomyces.cardiffensis
30
20
10
0
A (n=14)
B (n=15)
C (n=11)
surface

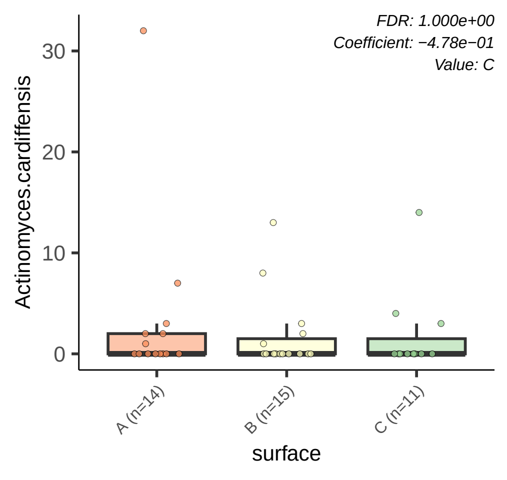

FDR: 1.000e+00
Coefficient: −4.78e−01
Value: C
Actinomyces.cardiffensis
0
10
20
30
A (n=14)
B (n=15)
C (n=11)
surface

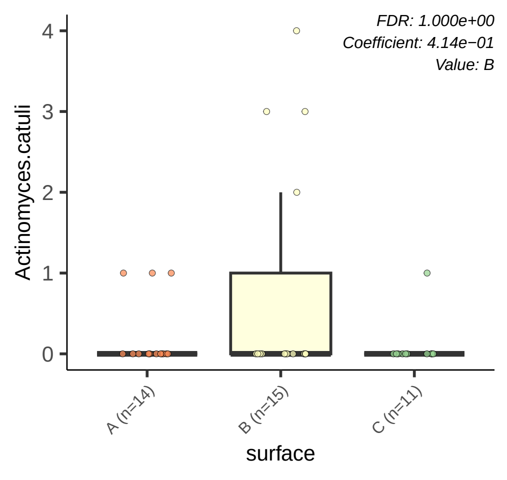

FDR: 1.000e+00
Coefficient: 4.14e−01
Value: B
Actinomyces.catuli
0
1
2
3
4
A (n=14)
B (n=15)
C (n=11)
surface

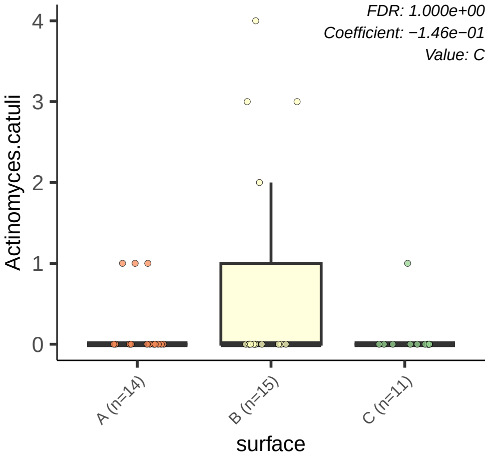

FDR: 1.000e+00
Coefficient: −1.46e−01
Value: C
Actinomyces.catuli
0
1
2
3
4
A (n=14)
B (n=15)
C (n=11)
surface

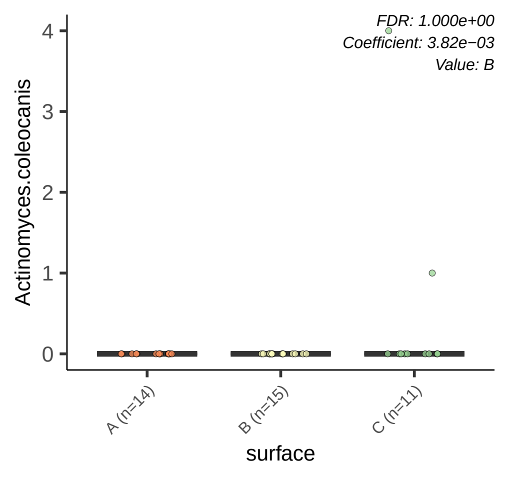

FDR: 1.000e+00
Coefficient: 3.82e−03
Value: B
Actinomyces.coleocanis
4
3
2
1
0
A (n=14)
B (n=15)
C (n=11)
surface

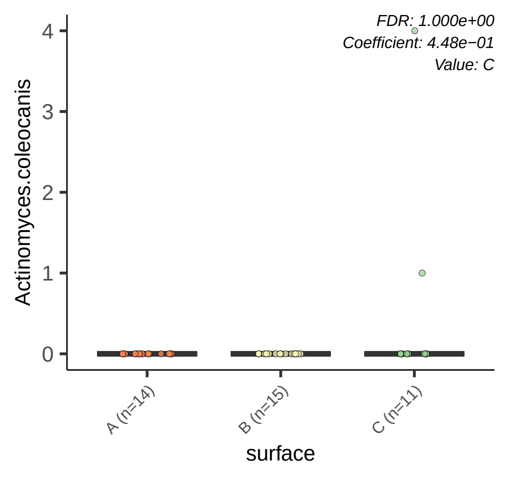
FDR: 1.000e+00
Coefficient: 4.48e−01
Value: C
Actinomyces.coleocanis
4
3
2
1
0
A (n=14)
B (n=15)
C (n=11)
surface

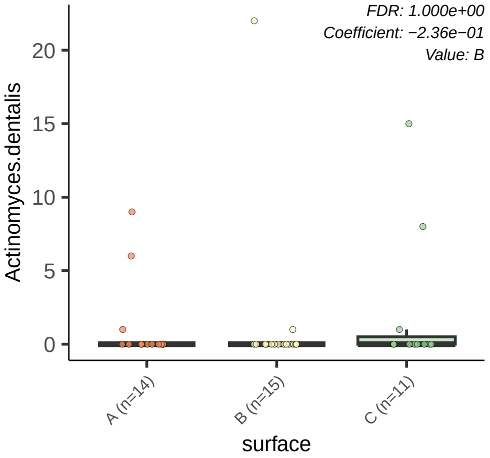
FDR: 1.000e+00
Coefficient: −2.36e−01
Value: B
Actinomyces.dentalis
0
5
10
15
20
A (n=14)
B (n=15)
C (n=11)
surface

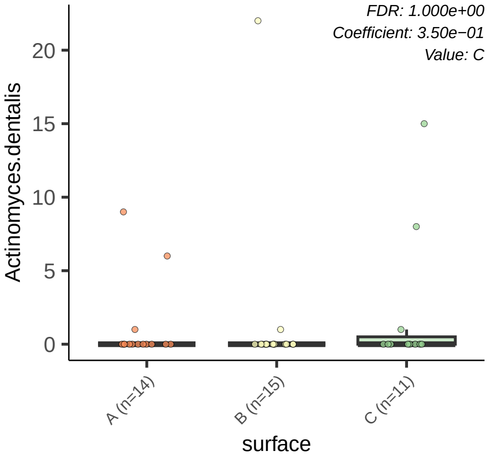
FDR: 1.000e+00
Coefficient: 3.50e−01
Value: C
Actinomyces.dentalis
0
5
10
15
20
A (n=14)
B (n=15)
C (n=11)
surface

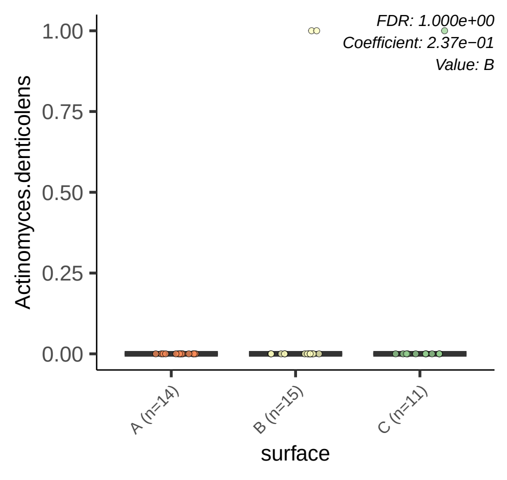

FDR: 1.000e+00
Coefficient: 2.37e−01
Value: B
Actinomyces.denticolens
1.00
0.75
0.50
0.25
0.00
A (n=14)
B (n=15)
C (n=11)
surface

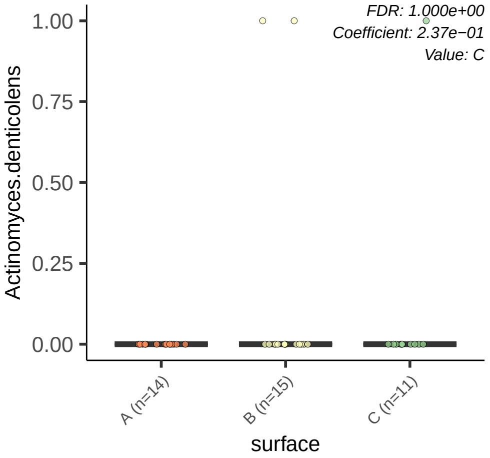

FDR: 1.000e+00
Coefficient: 2.37e−01
Value: C
Actinomyces.denticolens
1.00
0.75
0.50
0.25
0.00
A (n=14)
B (n=15)
C (n=11)
surface

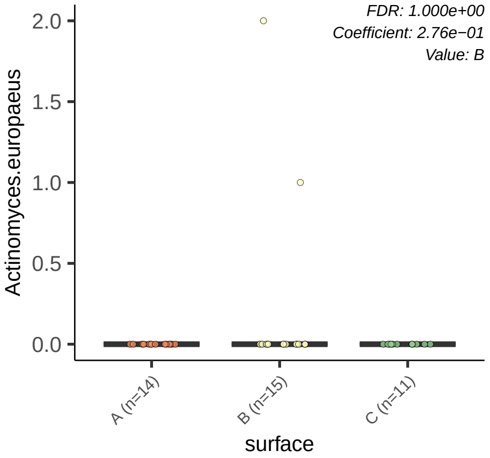
FDR: 1.000e+00
Coefficient: 2.76e−01
Value: B
Actinomyces.europaeus
2.0
1.5
1.0
0.5
0.0
A (n=14)
B (n=15)
C (n=11)
surface

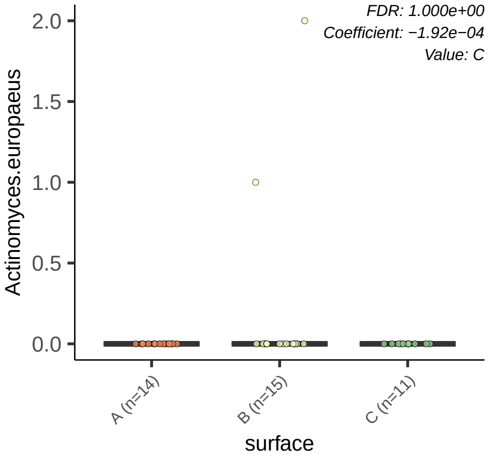
FDR: 1.000e+00
Coefficient: −1.92e−04
Value: C
Actinomyces.europaeus
2.0
1.5
1.0
0.5
0.0
A (n=14)
B (n=15)
C (n=11)
surface

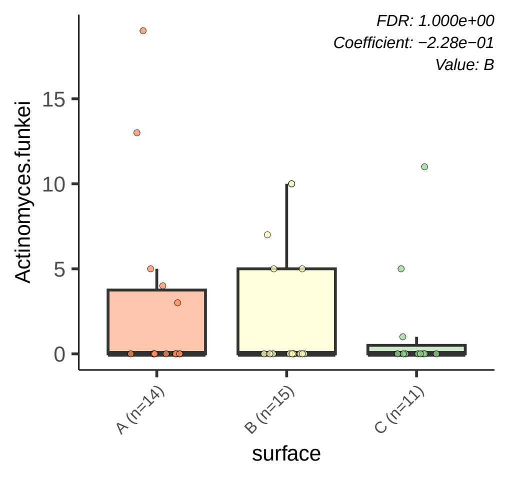

FDR: 1.000e+00
Coefficient: −2.28e−01
Value: B
Actinomyces.funkei
0
5
10
15
A (n=14)
B (n=15)
C (n=11)
surface

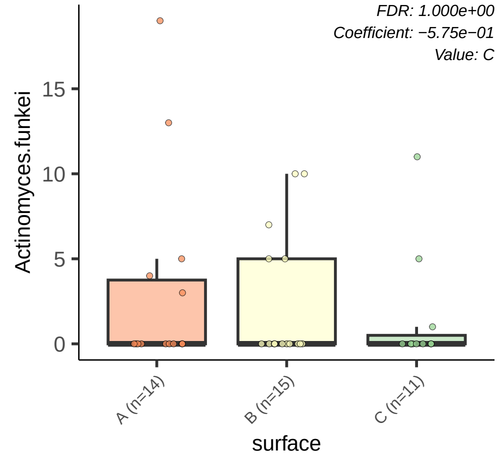
FDR: 1.000e+00
Coefficient: −5.75e−01
Value: C
Actinomyces.funkei
0
5
10
15
A (n=14)
B (n=15)
C (n=11)
surface

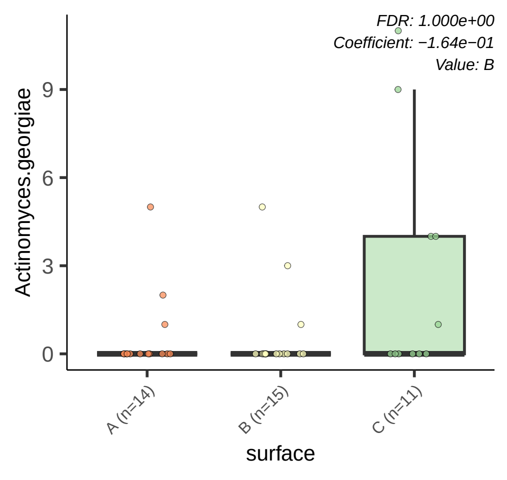
FDR: 1.000e+00
Coefficient: −1.64e−01
Value: B
Actinomyces.georgiae
0
3
6
9
A (n=14)
B (n=15)
C (n=11)
surface

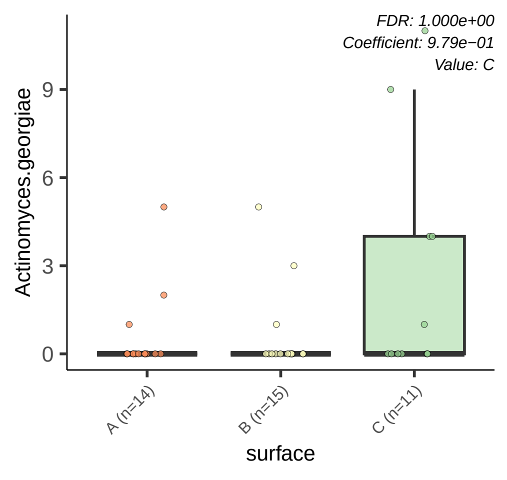

FDR: 1.000e+00
Coefficient: 9.79e−01
Value: C
Actinomyces.georgiae
0
3
6
9
A (n=14)
B (n=15)
C (n=11)
surface

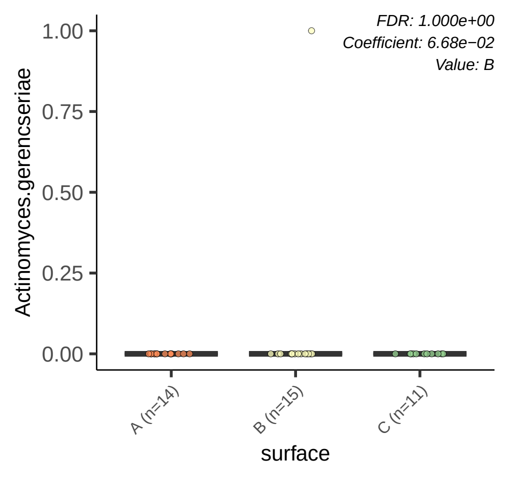

FDR: 1.000e+00
Coefficient: 6.68e−02
Value: B
Actinomyces.gerencseriae
1.00
0.75
0.50
0.25
0.00
A (n=14)
B (n=15)
C (n=11)
surface

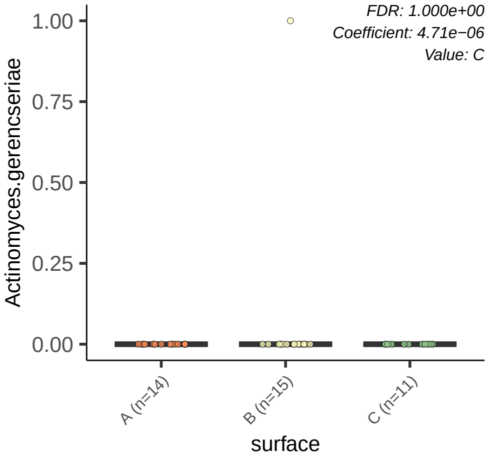

FDR: 1.000e+00
Coefficient: 4.71e−06
Value: C
Actinomyces.gerencseriae
1.00
0.75
0.50
0.25
0.00
A (n=14)
B (n=15)
C (n=11)
surface

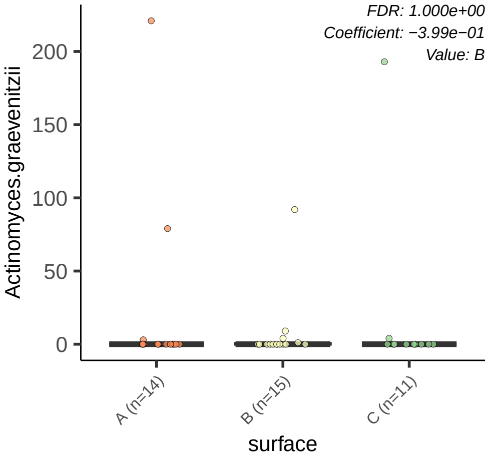
FDR: 1.000e+00
Coefficient: −3.99e−01
Value: B
Actinomyces.graevenitzii
200
150
100
50
0
A (n=14)
B (n=15)
C (n=11)
surface

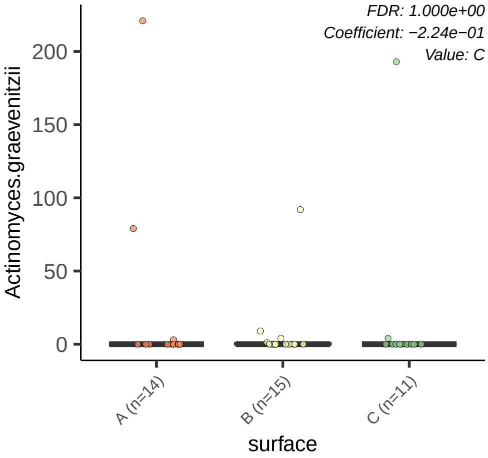

FDR: 1.000e+00
Coefficient: −2.24e−01
Value: C
Actinomyces.graevenitzii
200
150
100
50
0
A (n=14)
B (n=15)
C (n=11)
surface

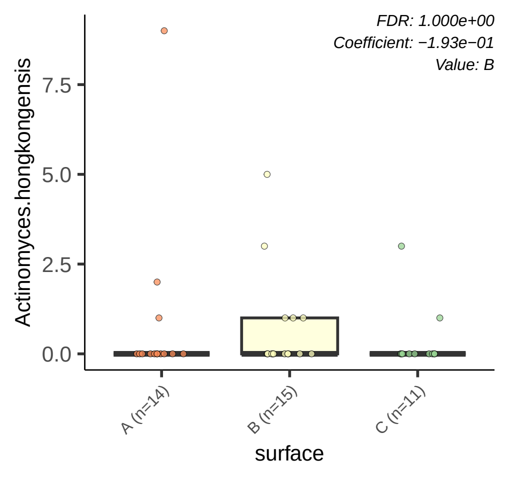
FDR: 1.000e+00
Coefficient: −1.93e−01
Value: B
Actinomyces.hongkongensis
7.5
5.0
2.5
0.0
A (n=14)
B (n=15)
C (n=11)
surface

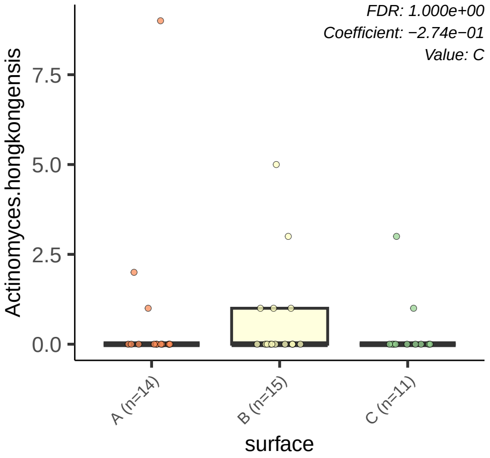

FDR: 1.000e+00
Coefficient: −2.74e−01
Value: C
Actinomyces.hongkongensis
7.5
5.0
2.5
0.0
A (n=14)
B (n=15)
C (n=11)
surface

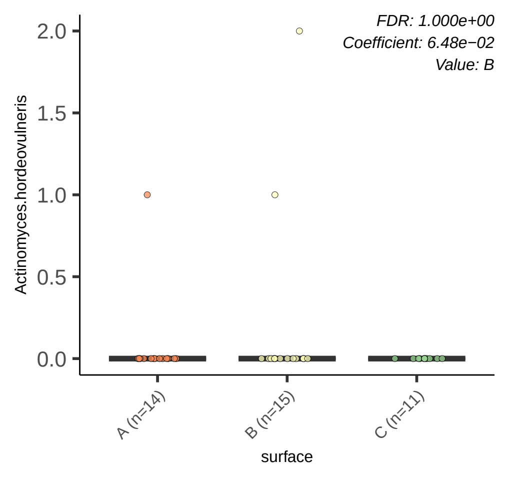
FDR: 1.000e+00
Coefficient: 6.48e−02
Value: B
Actinomyces.hordeovulneris
2.0
1.5
1.0
0.5
0.0
A (n=14)
B (n=15)
C (n=11)
surface

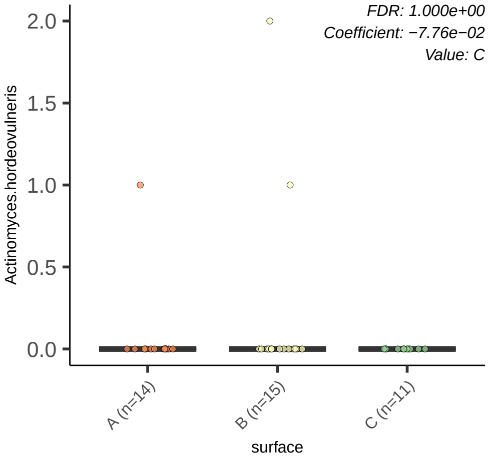

FDR: 1.000e+00
Coefficient: −7.76e−02
Value: C
Actinomyces.hordeovulneris
2.0
1.5
1.0
0.5
0.0
A (n=14)
B (n=15)
C (n=11)
surface

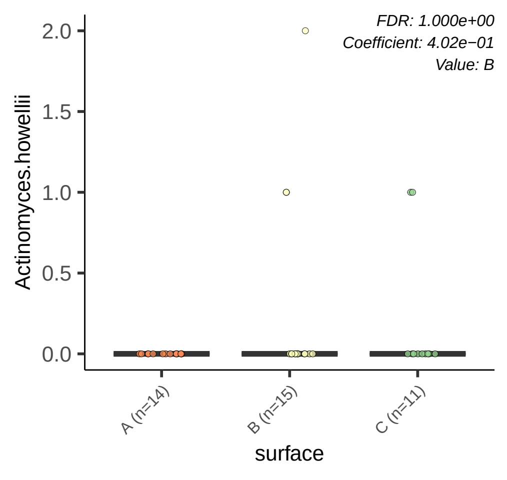
FDR: 1.000e+00
Coefficient: 4.02e−01
Value: B
Actinomyces.howellii
2.0
1.5
1.0
0.5
0.0
A (n=14)
B (n=15)
C (n=11)
surface

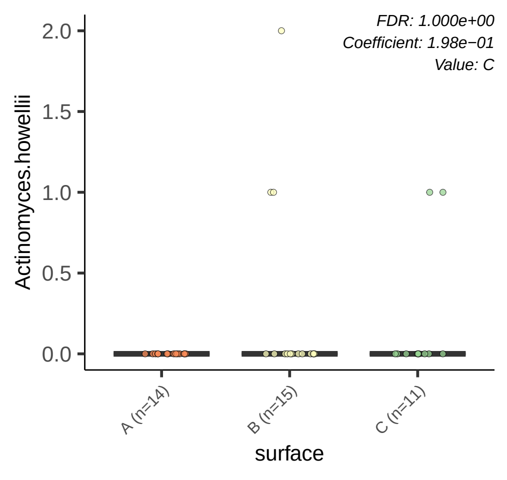
FDR: 1.000e+00
Coefficient: 1.98e−01
Value: C
Actinomyces.howellii
2.0
1.5
1.0
0.5
0.0
A (n=14)
B (n=15)
C (n=11)
surface

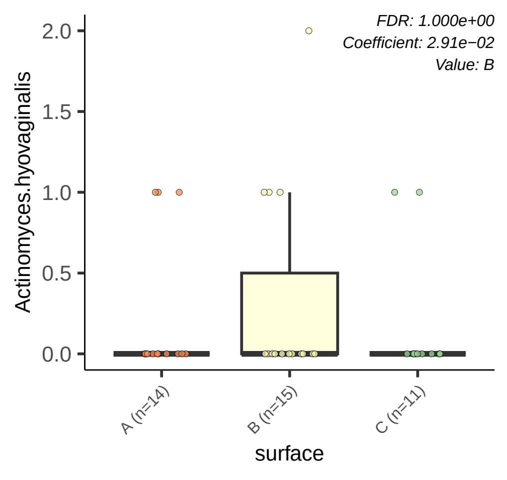
FDR: 1.000e+00
Coefficient: 2.91e−02
Value: B
Actinomyces.hyovaginalis
2.0
1.5
1.0
0.5
0.0
A (n=14)
B (n=15)
C (n=11)
surface

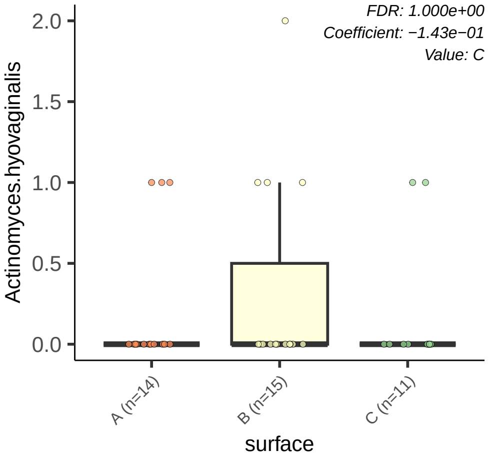

FDR: 1.000e+00
Coefficient: −1.43e−01
Value: C
Actinomyces.hyovaginalis
2.0
1.5
1.0
0.5
0.0
A (n=14)
B (n=15)
C (n=11)
surface

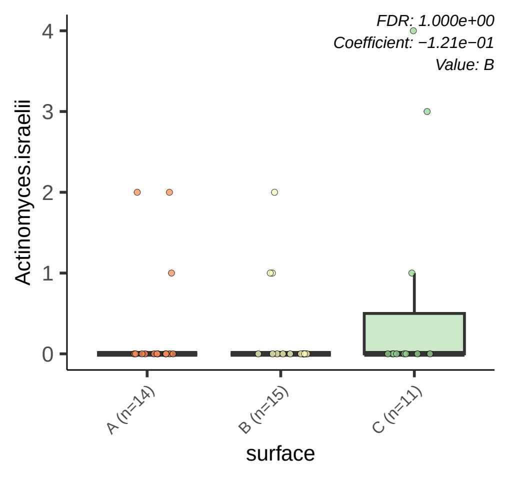
FDR: 1.000e+00
Coefficient: −1.21e−01
Value: B
Actinomyces.israelii
0
1
2
3
4
A (n=14)
B (n=15)
C (n=11)
surface

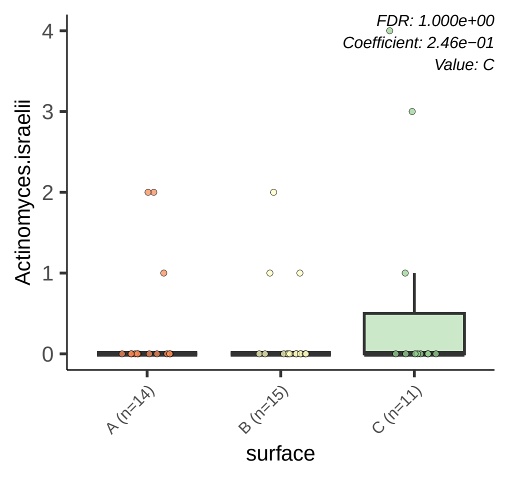
FDR: 1.000e+00
Coefficient: 2.46e−01
Value: C
Actinomyces.israelii
0
1
2
3
4
A (n=14)
B (n=15)
C (n=11)
surface

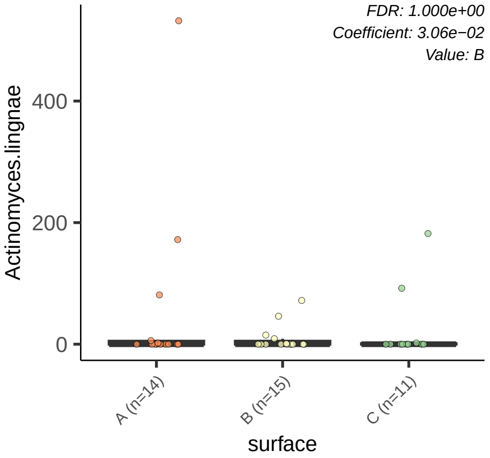
FDR: 1.000e+00
Coefficient: 3.06e−02
Value: B
Actinomyces.lingnae
400
200
0
A (n=14)
B (n=15)
C (n=11)
surface

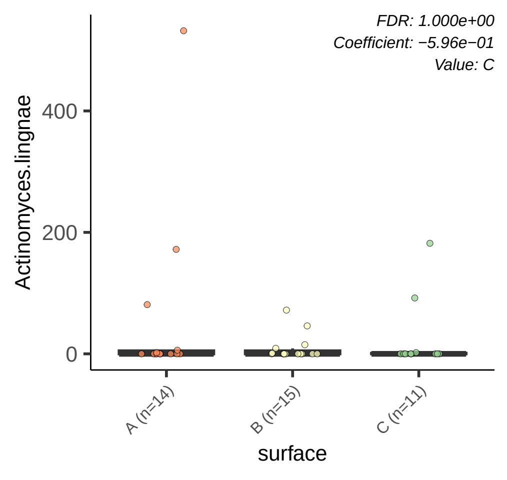

FDR: 1.000e+00
Coefficient: −5.96e−01
Value: C
Actinomyces.lingnae
400
200
0
A (n=14)
B (n=15)
C (n=11)
surface

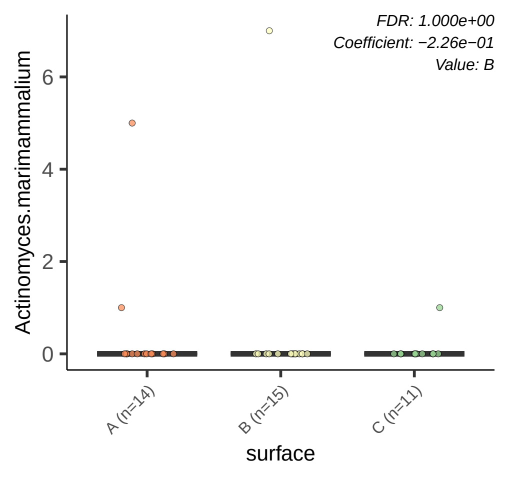

FDR: 1.000e+00
Coefficient: −2.26e−01
Value: B
Actinomyces.marimammalium
0
2
4
6
A (n=14)
B (n=15)
C (n=11)
surface

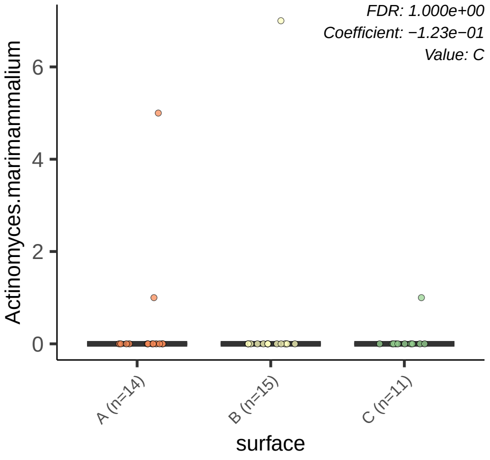

FDR: 1.000e+00
Coefficient: −1.23e−01
Value: C
Actinomyces.marimammalium
0
2
4
6
A (n=14)
B (n=15)
C (n=11)
surface

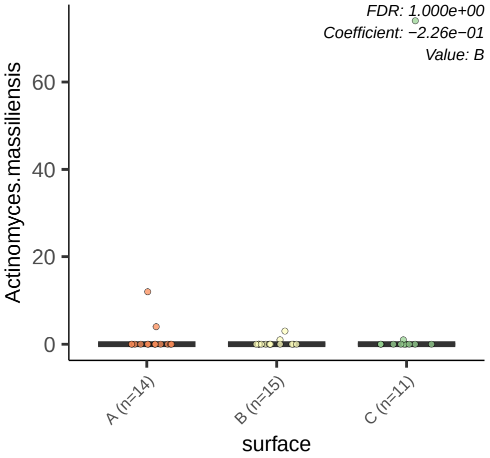
FDR: 1.000e+00
Coefficient: −2.26e−01
Value: B
Actinomyces.massiliensis
60
40
20
0
A (n=14)
B (n=15)
C (n=11)
surface

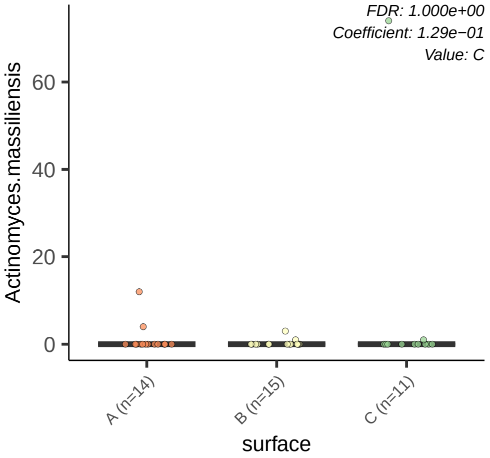
FDR: 1.000e+00
Coefficient: 1.29e−01
Value: C
Actinomyces.massiliensis
0
20
40
60
A (n=14)
B (n=15)
C (n=11)
surface

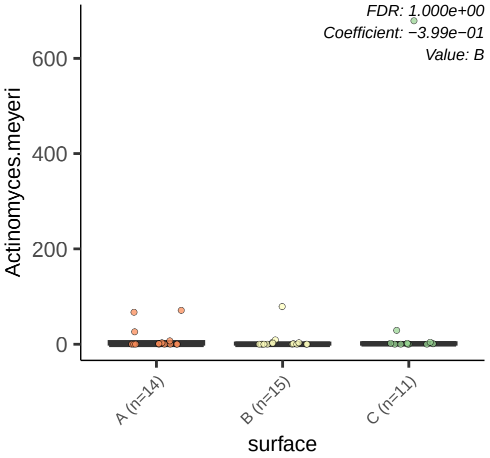
FDR: 1.000e+00
Coefficient: −3.99e−01
Value: B
600
400
200
0
Actinomyces.meyeri
A (n=14)
B (n=15)
C (n=11)
surface

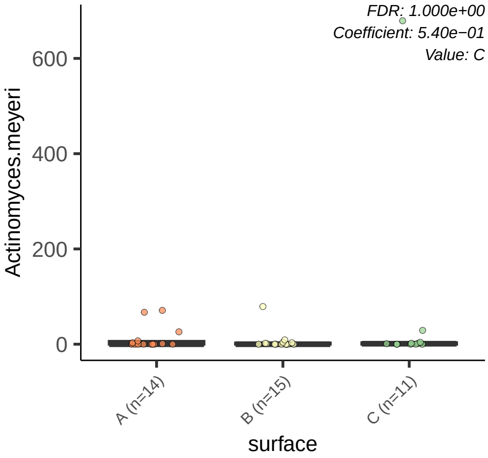
FDR: 1.000e+00
Coefficient: 5.40e−01
Value: C
Actinomyces.meyeri
600
400
200
0
A (n=14)
B (n=15)
C (n=11)
surface

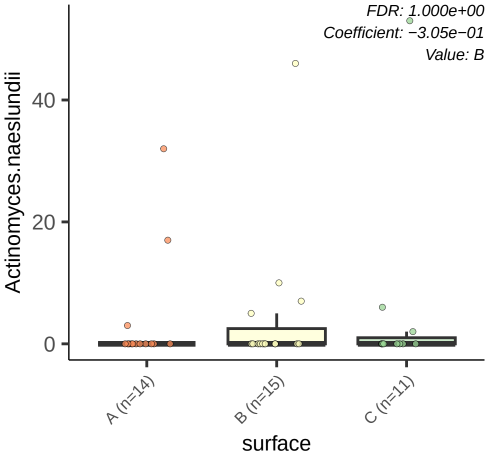

FDR: 1.000e+00
Coefficient: −3.05e−01
Value: B
Actinomyces.naeslundii
0
20
40
A (n=14)
B (n=15)
C (n=11)
surface

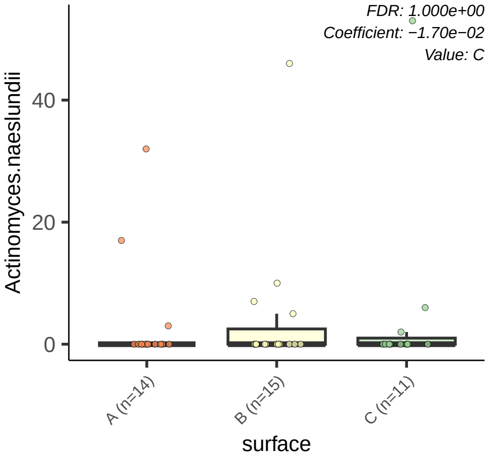
FDR: 1.000e+00
Coefficient: −1.70e−02
Value: C
Actinomyces.naeslundii
40
20
0
A (n=14)
B (n=15)
C (n=11)
surface

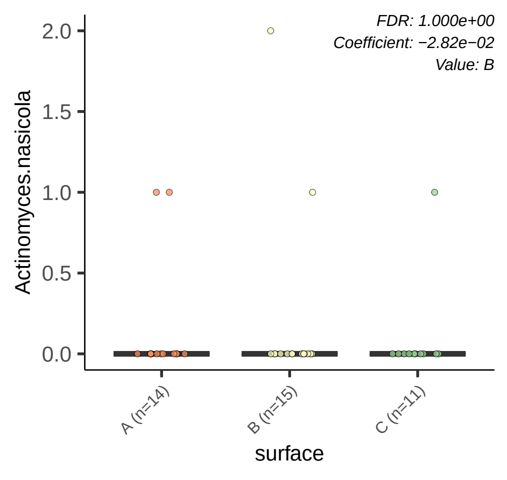
FDR: 1.000e+00
Coefficient: −2.82e−02
Value: B
Actinomyces.nasicola
2.0
1.5
1.0
0.5
0.0
A (n=14)
B (n=15)
C (n=11)
surface

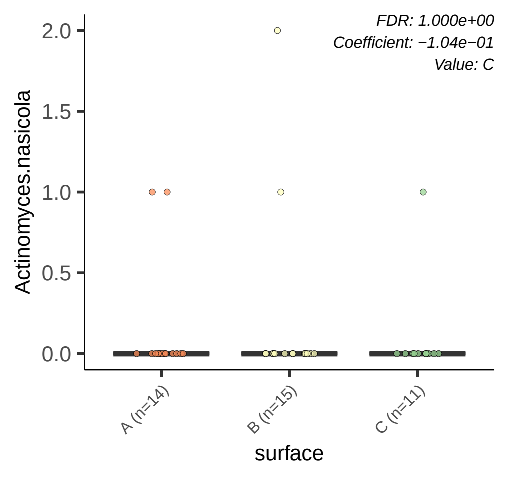
FDR: 1.000e+00
Coefficient: −1.04e−01
Value: C
Actinomyces.nasicola
2.0
1.5
1.0
0.5
0.0
A (n=14)
B (n=15)
C (n=11)
surface

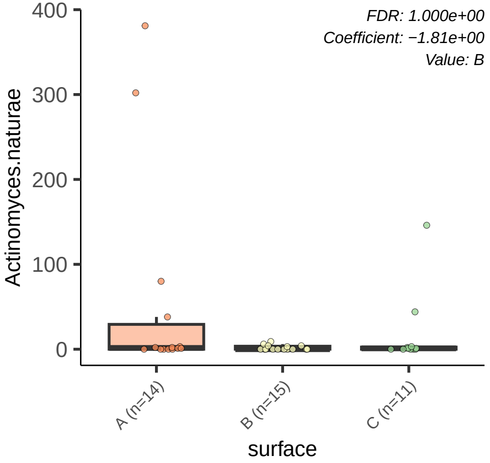
FDR: 1.000e+00
Coefficient: −1.81e+00
Value: B
Actinomyces.naturae
400
300
200
100
0
A (n=14)
B (n=15)
C (n=11)
surface

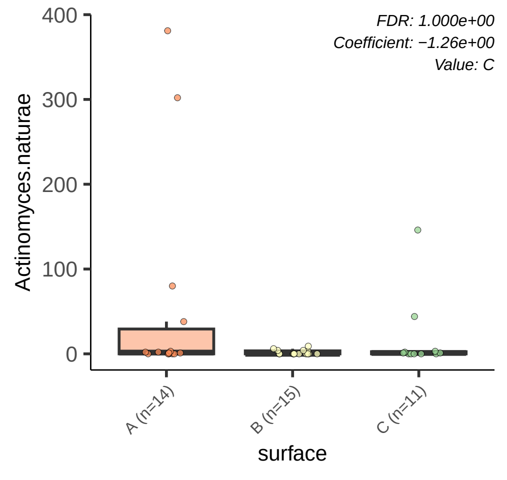
FDR: 1.000e+00
Coefficient: −1.26e+00
Value: C
Actinomyces.naturae
400
300
200
100
0
A (n=14)
B (n=15)
C (n=11)
surface

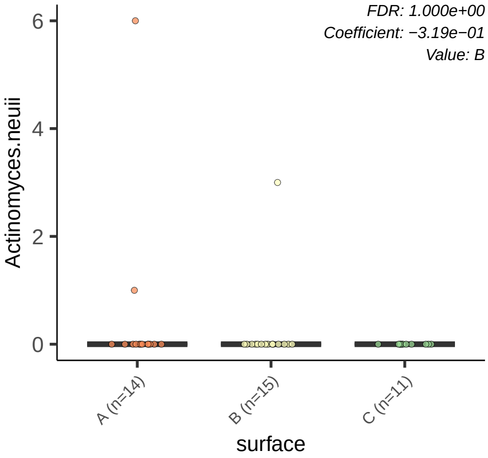

FDR: 1.000e+00
Coefficient: −3.19e−01
Value: B
Actinomyces.neuii
6
4
2
0
A (n=14)
B (n=15)
C (n=11)
surface

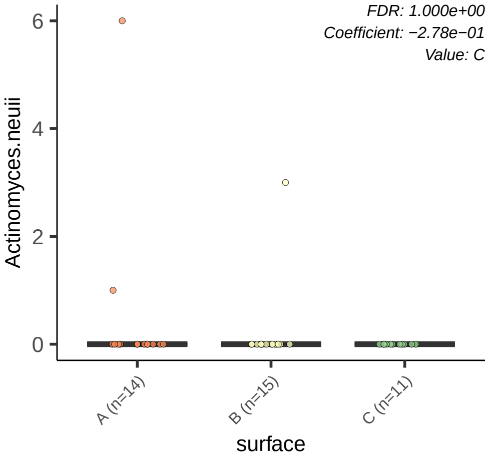
FDR: 1.000e+00
Coefficient: −2.78e−01
Value: C
Actinomyces.neuii
0
2
4
6
A (n=14)
B (n=15)
C (n=11)
surface

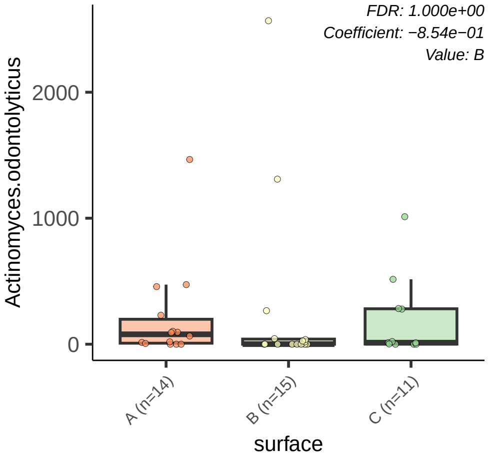

FDR: 1.000e+00
Coefficient: −8.54e−01
Value: B
Actinomyces.odontolyticus
2000
1000
0
A (n=14)
B (n=15)
C (n=11)
surface

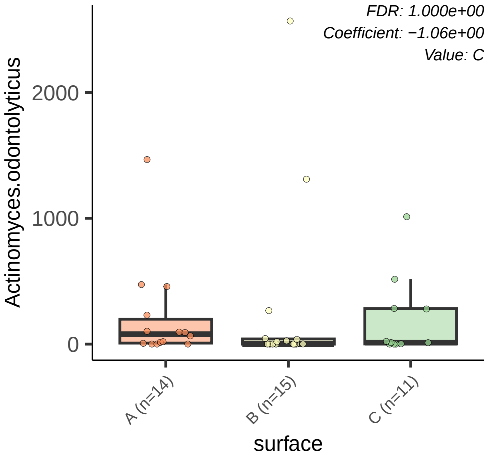

FDR: 1.000e+00
Coefficient: −1.06e+00
Value: C
Actinomyces.odontolyticus
2000
1000
0
A (n=14)
B (n=15)
C (n=11)
surface

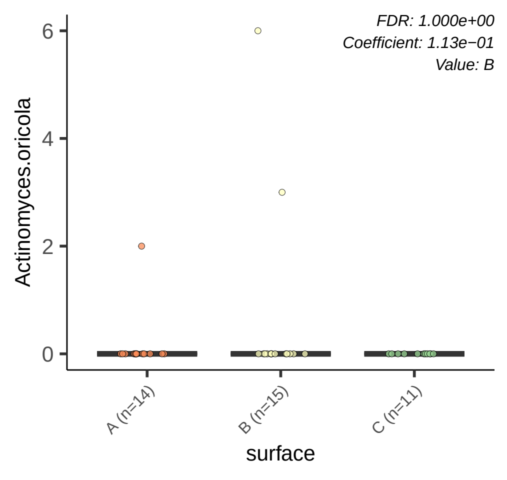

FDR: 1.000e+00
Coefficient: 1.13e−01
Value: B
Actinomyces.oricola
0
2
4
6
A (n=14)
B (n=15)
C (n=11)
surface

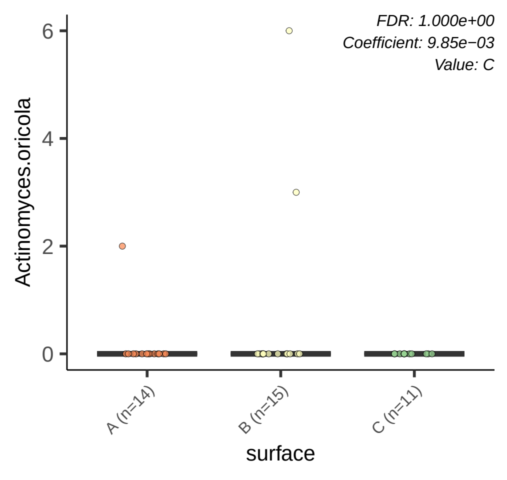

FDR: 1.000e+00
Coefficient: 9.85e−03
Value: C
Actinomyces.oricola
6
4
2
0
A (n=14)
B (n=15)
C (n=11)
surface

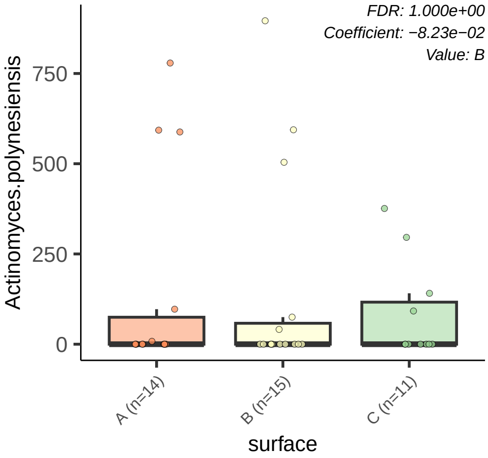
FDR: 1.000e+00
Coefficient: −8.23e−02
Value: B
Actinomyces.polynesiensis
750
500
250
0
A (n=14)
B (n=15)
C (n=11)
surface

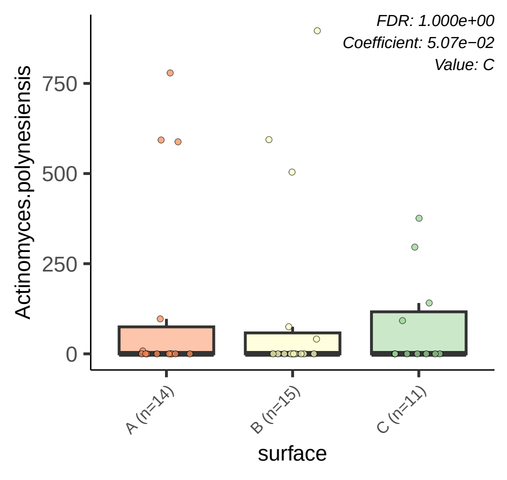

FDR: 1.000e+00
Coefficient: 5.07e−02
Value: C
Actinomyces.polynesiensis
750
500
250
0
A (n=14)
B (n=15)
C (n=11)
surface

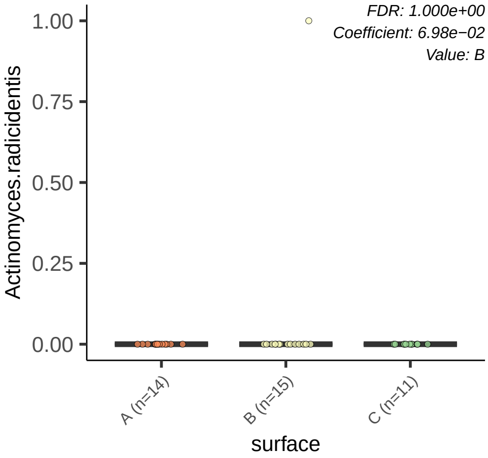
FDR: 1.000e+00
Coefficient: 6.98e−02
Value: B
Actinomyces.radicidentis
1.00
0.75
0.50
0.25
0.00
A (n=14)
B (n=15)
C (n=11)
surface

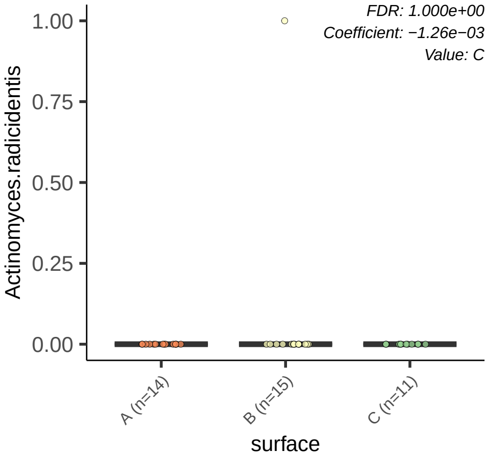

FDR: 1.000e+00
Coefficient: −1.26e−03
Value: C
Actinomyces.radicidentis
1.00
0.75
0.50
0.25
0.00
A (n=14)
B (n=15)
C (n=11)
surface

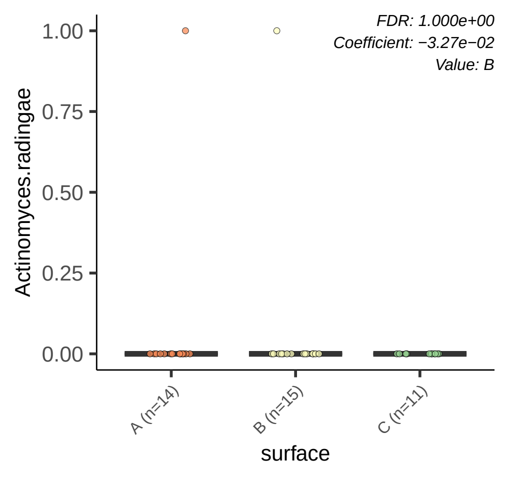
FDR: 1.000e+00
Coefficient: −3.27e−02
Value: B
Actinomyces.radingae
1.00
0.75
0.50
0.25
0.00
A (n=14)
B (n=15)
C (n=11)
surface

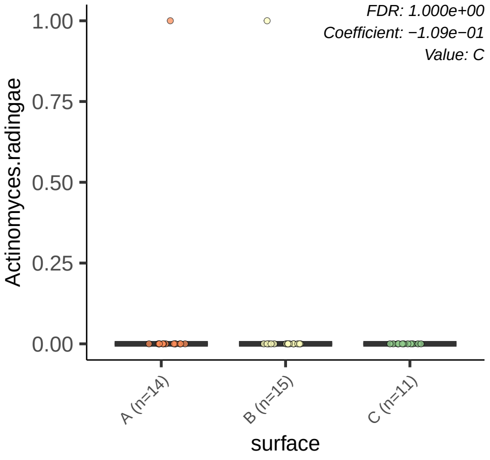
FDR: 1.000e+00
Coefficient: −1.09e−01
Value: C
Actinomyces.radingae
1.00
0.75
0.50
0.25
0.00
A (n=14)
B (n=15)
C (n=11)
surface

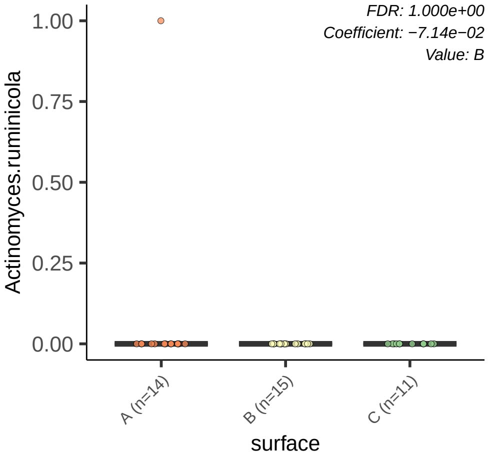

FDR: 1.000e+00
Coefficient: −7.14e−02
Value: B
Actinomyces.ruminicola
1.00
0.75
0.50
0.25
0.00
A (n=14)
B (n=15)
C (n=11)
surface

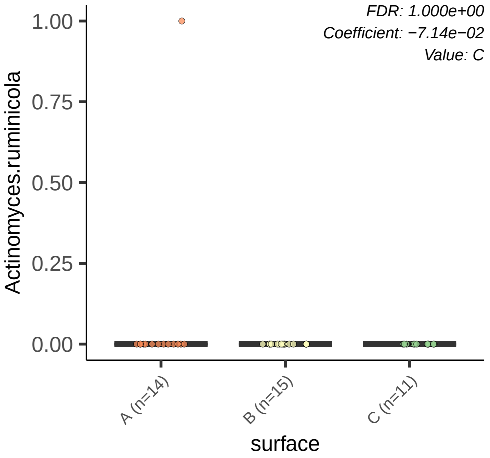
FDR: 1.000e+00
Coefficient: −7.14e−02
Value: C
Actinomyces.ruminicola
1.00
0.75
0.50
0.25
0.00
A (n=14)
B (n=15)
C (n=11)
surface

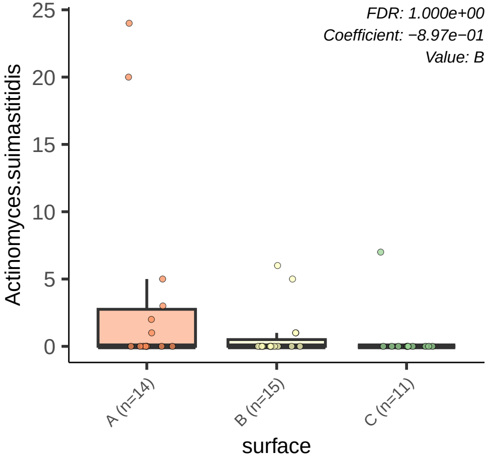

FDR: 1.000e+00
Coefficient: −8.97e−01
Value: B
Actinomyces.suimastitidis
25
20
15
10
5
0
A (n=14)
B (n=15)
C (n=11)
surface

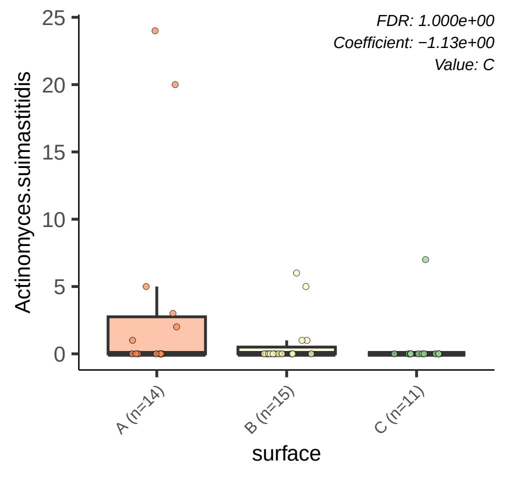
FDR: 1.000e+00
Coefficient: −1.13e+00
Value: C
Actinomyces.suimastitidis
0
5
10
15
20
25
A (n=14)
B (n=15)
C (n=11)
surface

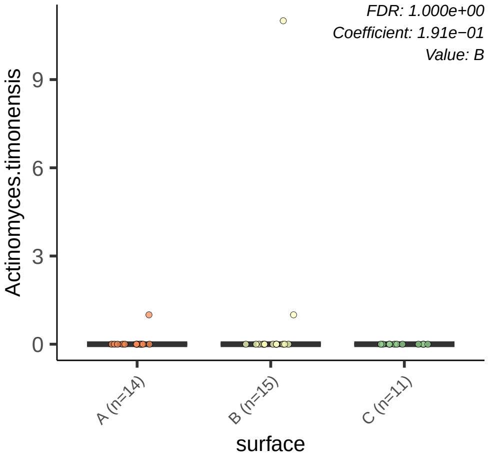

FDR: 1.000e+00
Coefficient: 1.91e−01
Value: B
Actinomyces.timonensis
0
3
6
9
A (n=14)
B (n=15)
C (n=11)
surface

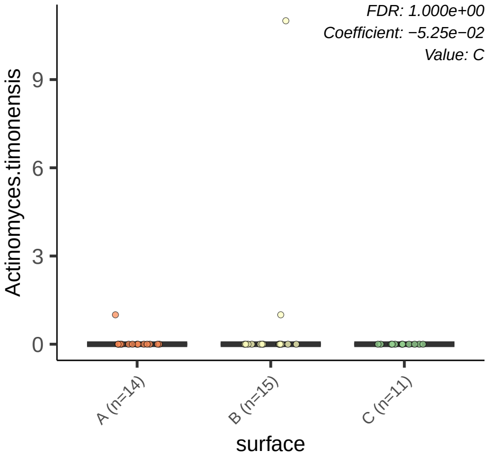
FDR: 1.000e+00
Coefficient: −5.25e−02
Value: C
Actinomyces.timonensis
0
3
6
9
A (n=14)
B (n=15)
C (n=11)
surface

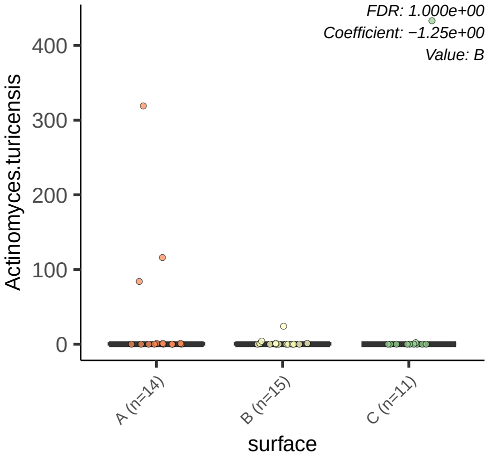
FDR: 1.000e+00
Coefficient: −1.25e+00
Value: B
Actinomyces.turicensis
400
300
200
100
0
A (n=14)
B (n=15)
C (n=11)
surface

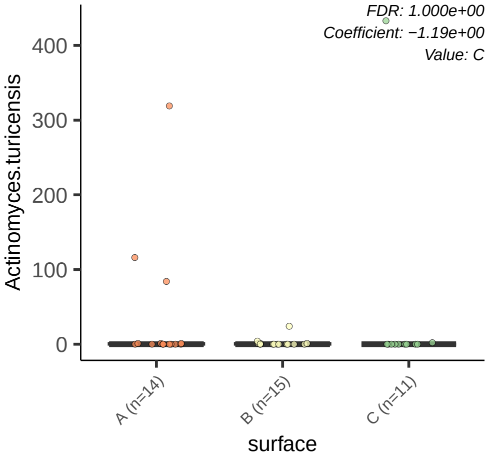

FDR: 1.000e+00
Coefficient: −1.19e+00
Value: C
Actinomyces.turicensis
0
100
200
300
400
A (n=14)
B (n=15)
C (n=11)
surface

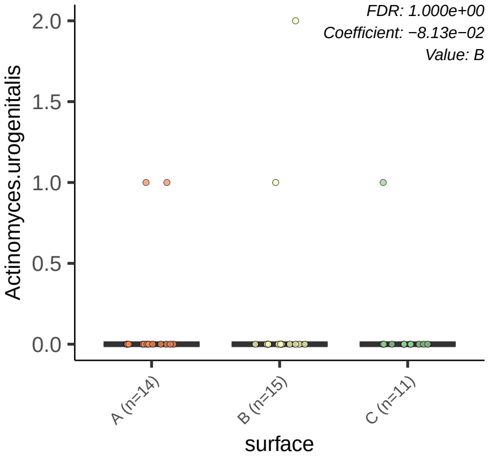

FDR: 1.000e+00
Coefficient: −8.13e−02
Value: B
Actinomyces.urogenitalis
2.0
1.5
1.0
0.5
0.0
A (n=14)
B (n=15)
C (n=11)
surface

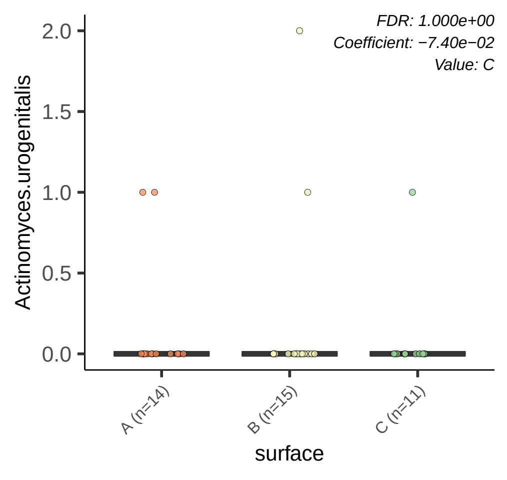
FDR: 1.000e+00
Coefficient: −7.40e−02
Value: C
Actinomyces.urogenitalis
2.0
1.5
1.0
0.5
0.0
A (n=14)
B (n=15)
C (n=11)
surface

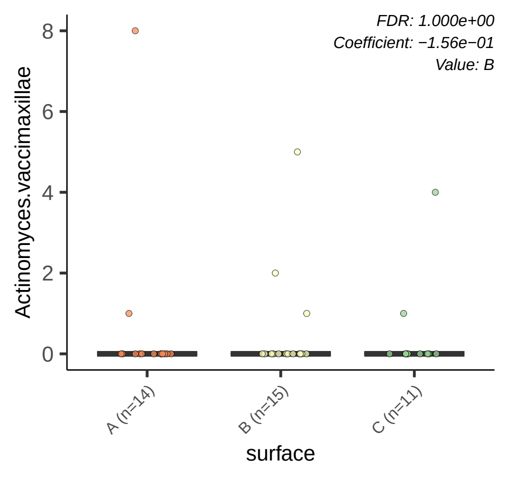
FDR: 1.000e+00
Coefficient: −1.56e−01
Value: B
Actinomyces.vaccimaxillae
0
2
4
6
8
A (n=14)
B (n=15)
C (n=11)
surface

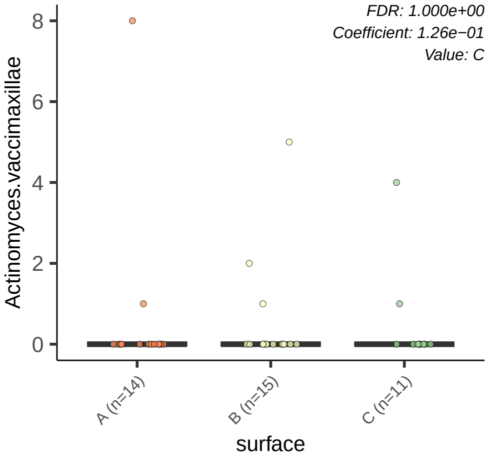
FDR: 1.000e+00
Coefficient: 1.26e−01
Value: C
Actinomyces.vaccimaxillae
8
6
4
2
0
A (n=14)
B (n=15)
C (n=11)
surface

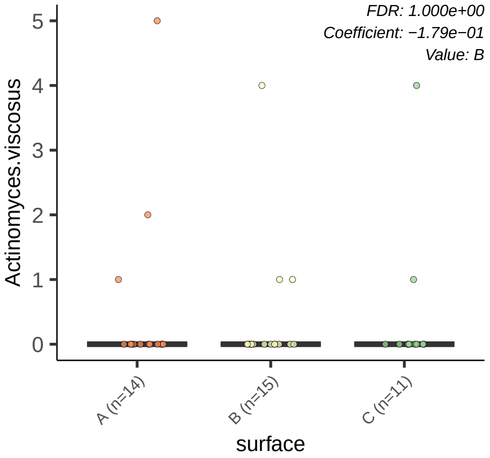
FDR: 1.000e+00
Coefficient: −1.79e−01
Value: B
Actinomyces.viscosus
0
1
2
3
4
5
A (n=14)
B (n=15)
C (n=11)
surface

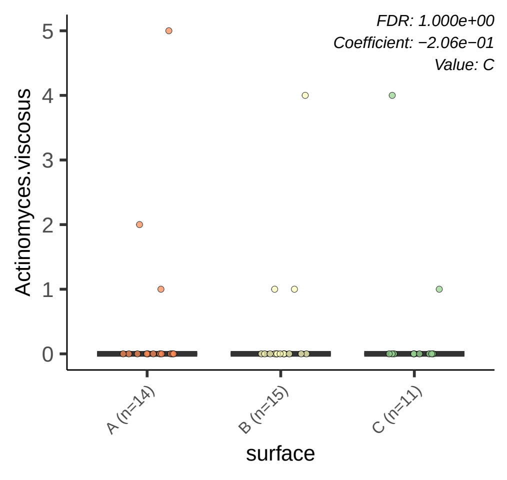

FDR: 1.000e+00
Coefficient: −2.06e−01
Value: C
Actinomyces.viscosus
5
4
3
2
1
0
A (n=14)
B (n=15)
C (n=11)
surface

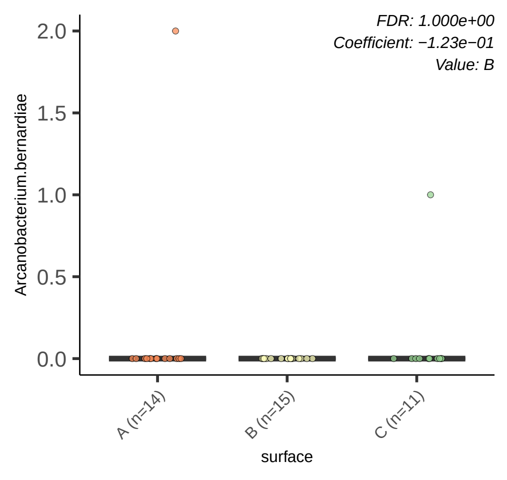
FDR: 1.000e+00
Coefficient: −1.23e−01
Value: B
2.0
1.5
1.0
0.5
0.0
Arcanobacterium.bernardiae
A (n=14)
B (n=15)
C (n=11)
surface

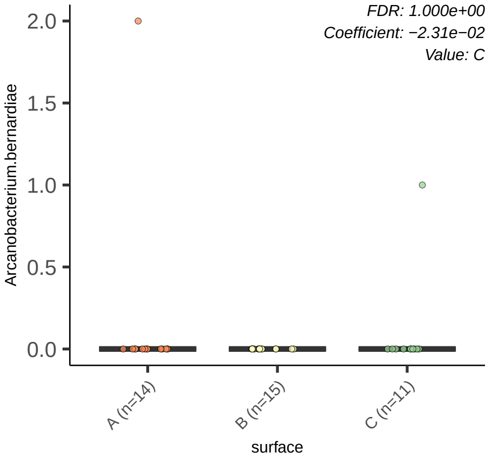
FDR: 1.000e+00
Coefficient: −2.31e−02
Value: C
Arcanobacterium.bernardiae
2.0
1.5
1.0
0.5
0.0
A (n=14)
B (n=15)
C (n=11)
surface

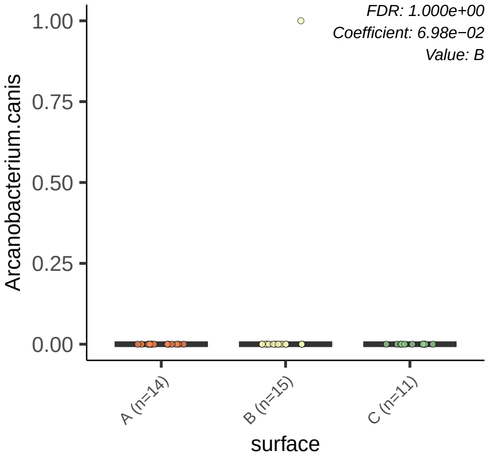
FDR: 1.000e+00
Coefficient: 6.98e−02
Value: B
Arcanobacterium.canis
1.00
0.75
0.50
0.25
0.00
A (n=14)
B (n=15)
C (n=11)
surface

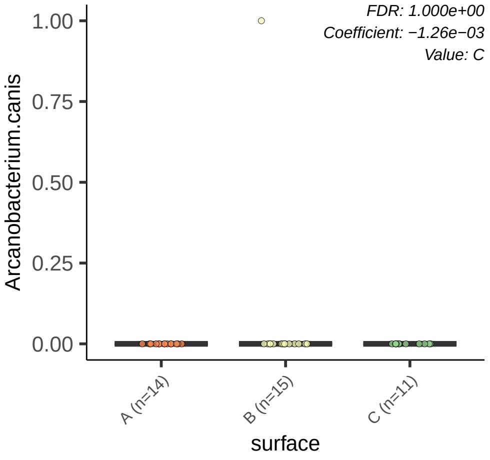
FDR: 1.000e+00
Coefficient: −1.26e−03
Value: C
Arcanobacterium.canis
1.00
0.75
0.50
0.25
0.00
A (n=14)
B (n=15)
C (n=11)
surface

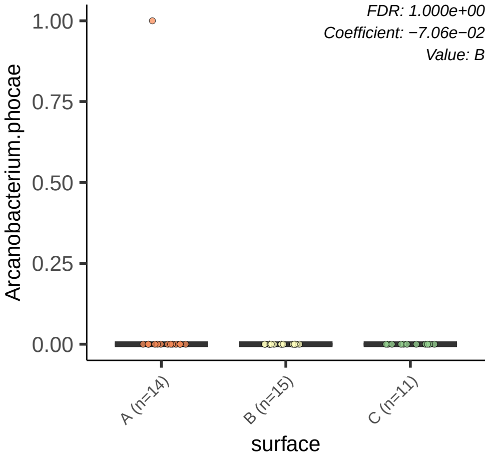

FDR: 1.000e+00
Coefficient: −7.06e−02
Value: B
Arcanobacterium.phocae
1.00
0.75
0.50
0.25
0.00
A (n=14)
B (n=15)
C (n=11)
surface

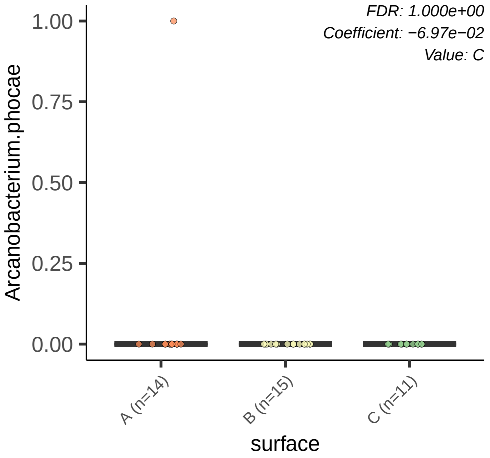
FDR: 1.000e+00
Coefficient: −6.97e−02
Value: C
Arcanobacterium.phocae
1.00
0.75
0.50
0.25
0.00
A (n=14)
B (n=15)
C (n=11)
surface

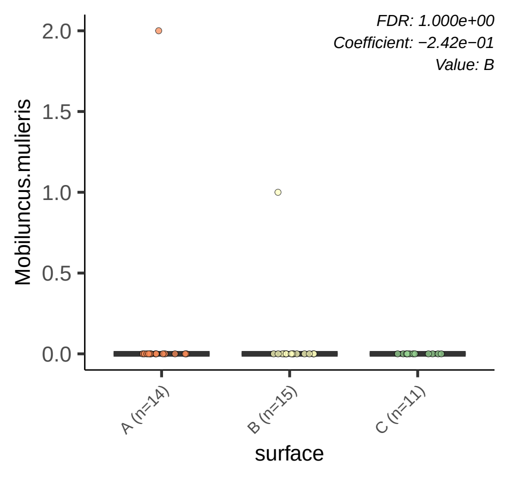

FDR: 1.000e+00
Coefficient: −2.42e−01
Value: B
Mobiluncus.mulieris
2.0
1.5
1.0
0.5
0.0
A (n=14)
B (n=15)
C (n=11)
surface

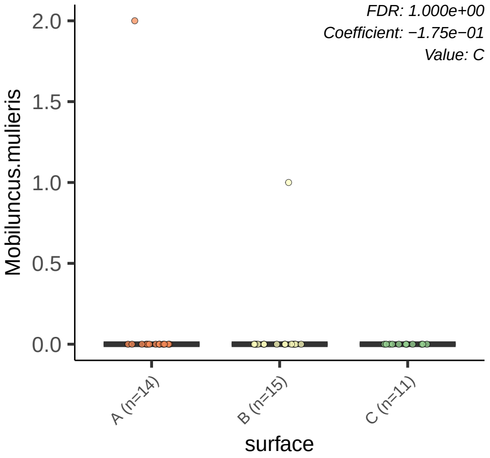
FDR: 1.000e+00
Coefficient: −1.75e−01
Value: C
Mobiluncus.mulieris
2.0
1.5
1.0
0.5
0.0
A (n=14)
B (n=15)
C (n=11)
surface

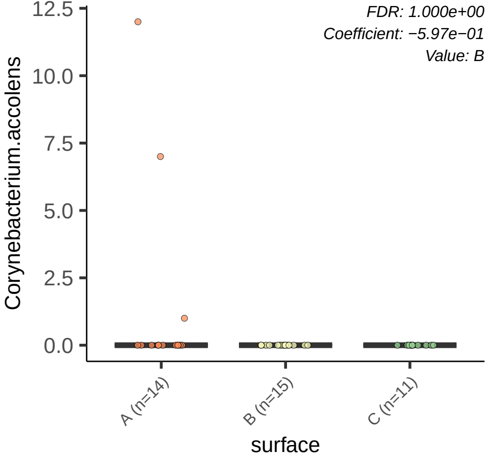
FDR: 1.000e+00
Coefficient: −5.97e−01
Value: B
Corynebacterium.accolens
12.5
10.0
7.5
5.0
2.5
0.0
A (n=14)
B (n=15)
C (n=11)
surface

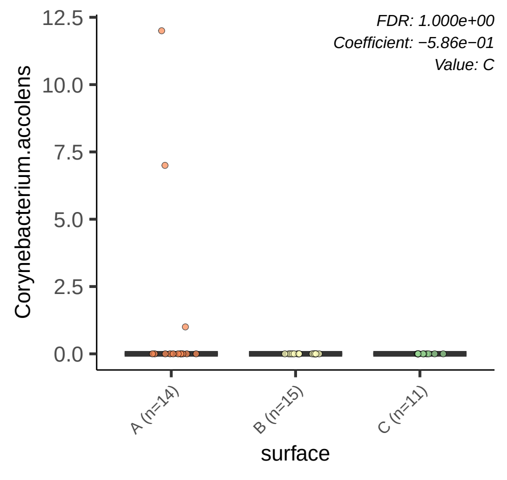

FDR: 1.000e+00
Coefficient: −5.86e−01
Value: C
Corynebacterium.accolens
12.5
10.0
7.5
5.0
2.5
0.0
A (n=14)
B (n=15)
C (n=11)
surface

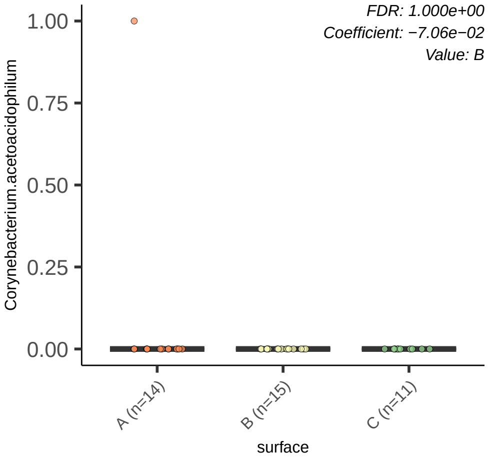

FDR: 1.000e+00
Coefficient: −7.06e−02
Value: B
Corynebacterium.acetoacidophilum
1.00
0.75
0.50
0.25
0.00
A (n=14)
B (n=15)
C (n=11)
surface

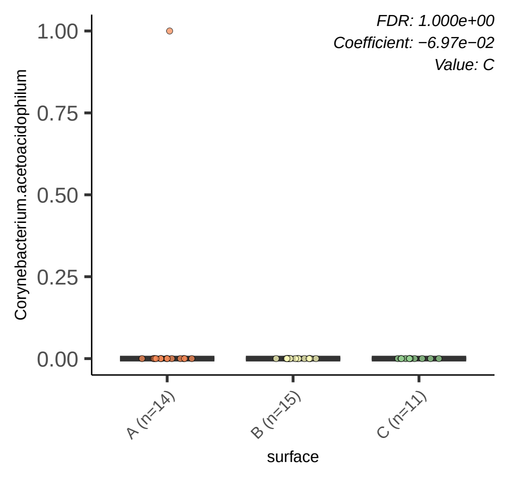

FDR: 1.000e+00
Coefficient: −6.97e−02
Value: C
Corynebacterium.acetoacidophilum
1.00
0.75
0.50
0.25
0.00
A (n=14)
B (n=15)
C (n=11)
surface

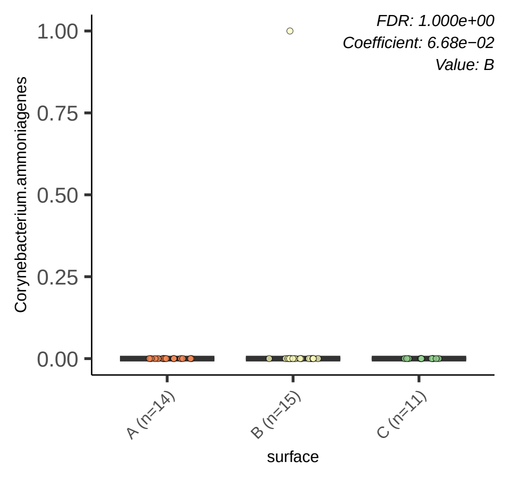
FDR: 1.000e+00
Coefficient: 6.68e−02
Value: B
1.00
0.75
0.50
0.25
0.00
Corynebacterium.ammoniagenes
A (n=14)
B (n=15)
C (n=11)
surface

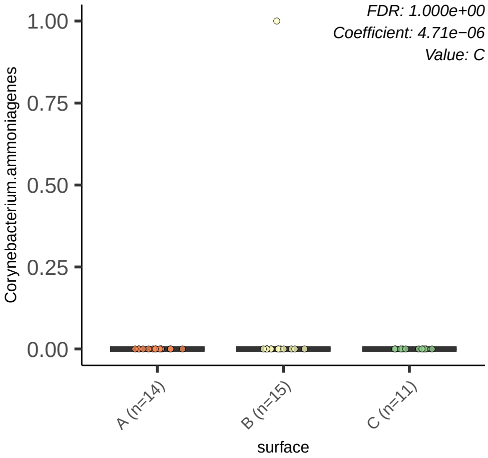
FDR: 1.000e+00
Coefficient: 4.71e−06
Value: C
Corynebacterium.ammoniagenes
1.00
0.75
0.50
0.25
0.00
A (n=14)
B (n=15)
C (n=11)
surface

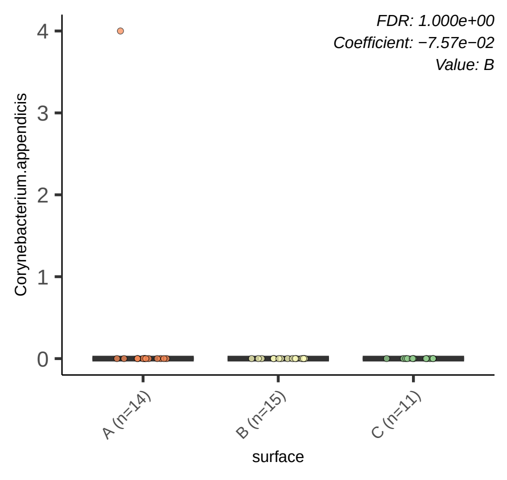
FDR: 1.000e+00
Coefficient: −7.57e−02
Value: B
Corynebacterium.appendicis
4
3
2
1
0
A (n=14)
B (n=15)
C (n=11)
surface

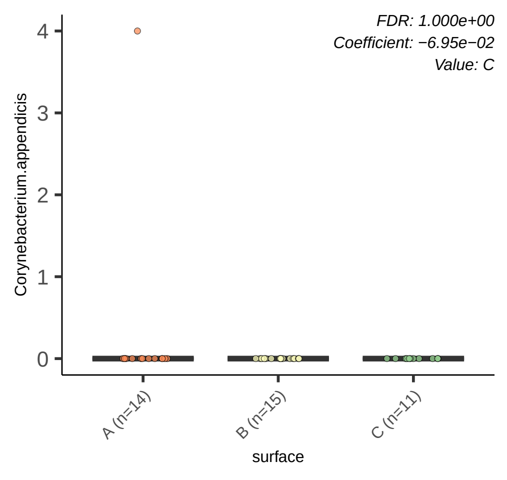

FDR: 1.000e+00
Coefficient: −6.95e−02
Value: C
Corynebacterium.appendicis
4
3
2
1
0
A (n=14)
B (n=15)
C (n=11)
surface

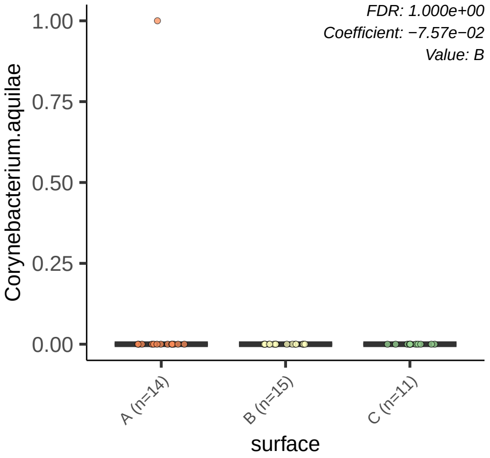

FDR: 1.000e+00
Coefficient: −7.57e−02
Value: B
1.00
0.75
0.50
0.25
0.00
Corynebacterium.aquilae
A (n=14)
B (n=15)
C (n=11)
surface

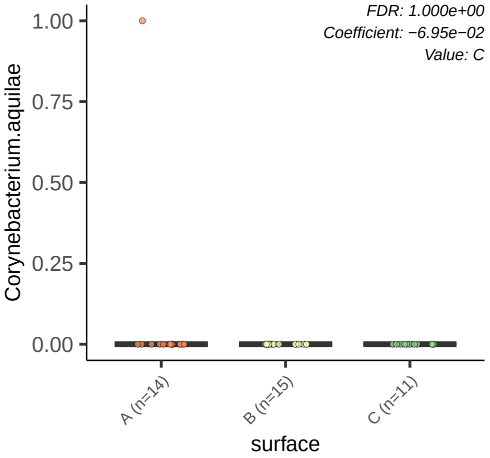

FDR: 1.000e+00
Coefficient: −6.95e−02
Value: C
Corynebacterium.aquilae
1.00
0.75
0.50
0.25
0.00
A (n=14)
B (n=15)
C (n=11)
surface

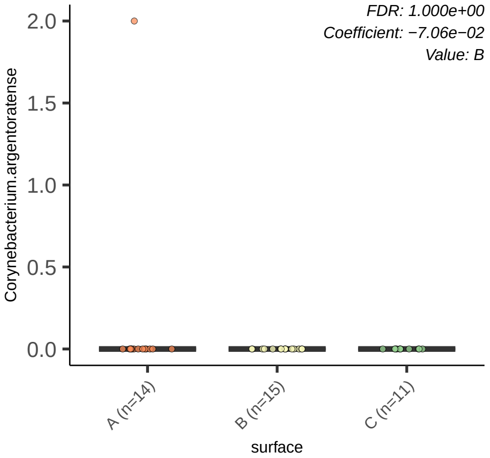
FDR: 1.000e+00
Coefficient: −7.06e−02
Value: B
Corynebacterium.argentoratense
2.0
1.5
1.0
0.5
0.0
A (n=14)
B (n=15)
C (n=11)
surface

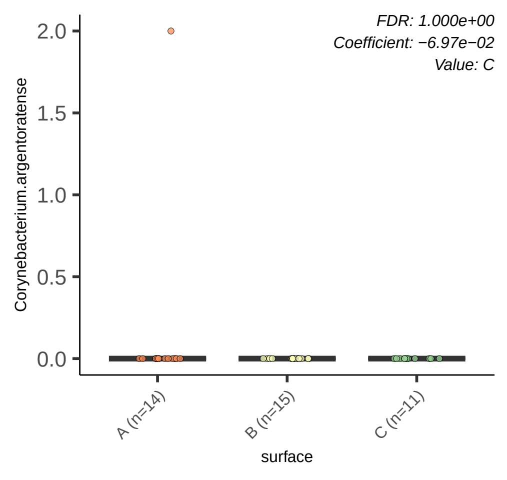
FDR: 1.000e+00
Coefficient: −6.97e−02
Value: C
Corynebacterium.argentoratense
2.0
1.5
1.0
0.5
0.0
A (n=14)
B (n=15)
C (n=11)
surface

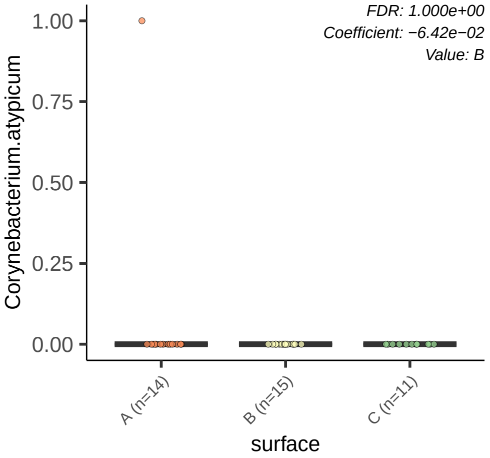
FDR: 1.000e+00
Coefficient: −6.42e−02
Value: B
Corynebacterium.atypicum
1.00
0.75
0.50
0.25
0.00
A (n=14)
B (n=15)
C (n=11)
surface

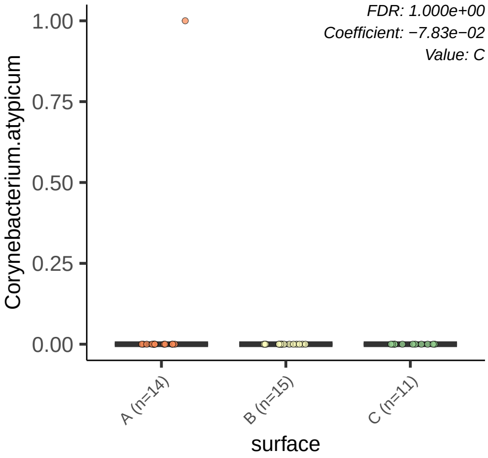

FDR: 1.000e+00
Coefficient: −7.83e−02
Value: C
Corynebacterium.atypicum
1.00
0.75
0.50
0.25
0.00
A (n=14)
B (n=15)
C (n=11)
surface

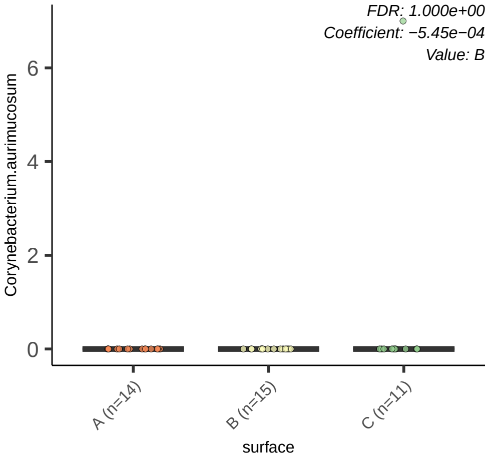
FDR: 1.000e+00
Coefficient: −5.45e−04
Value: B
Corynebacterium.aurimucosum
0
2
4
6
A (n=14)
B (n=15)
C (n=11)
surface

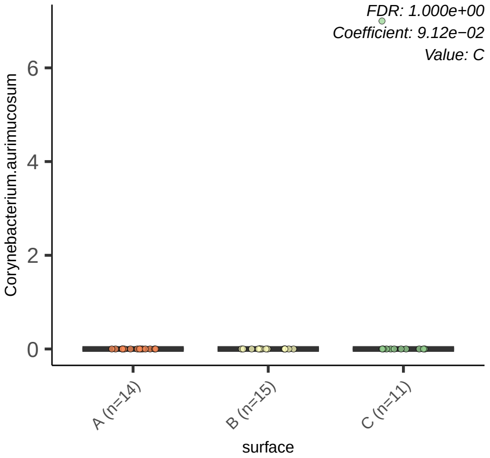

FDR: 1.000e+00
Coefficient: 9.12e−02
Value: C
Corynebacterium.aurimucosum
0
2
4
6
A (n=14)
B (n=15)
C (n=11)
surface

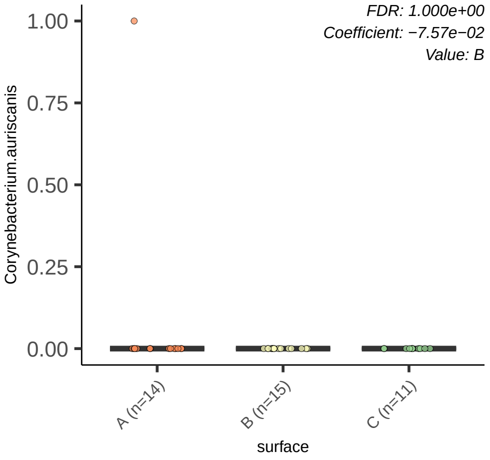
FDR: 1.000e+00
Coefficient: −7.57e−02
Value: B
1.00
0.75
0.50
0.25
0.00
Corynebacterium.auriscanis
A (n=14)
B (n=15)
C (n=11)
surface

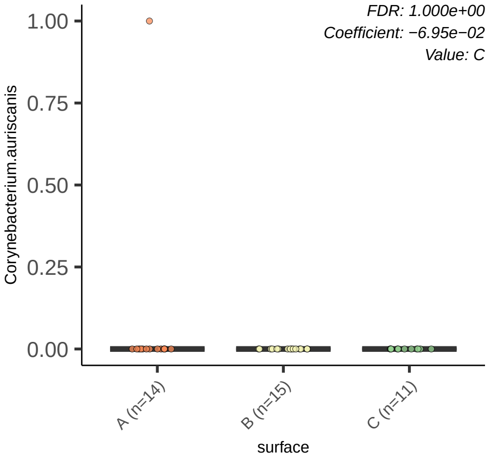

FDR: 1.000e+00
Coefficient: −6.95e−02
Value: C
Corynebacterium.auriscanis
1.00
0.75
0.50
0.25
0.00
A (n=14)
B (n=15)
C (n=11)
surface

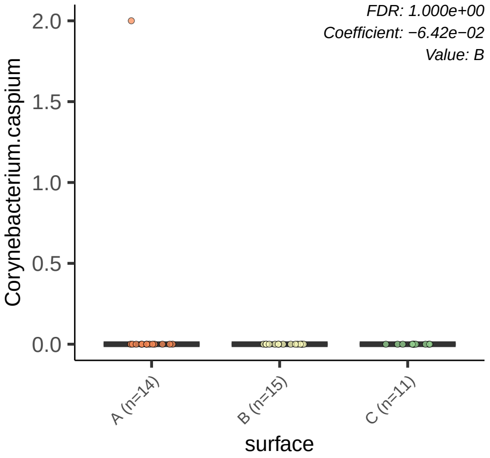
FDR: 1.000e+00
Coefficient: −6.42e−02
Value: B
Corynebacterium.caspium
2.0
1.5
1.0
0.5
0.0
A (n=14)
B (n=15)
C (n=11)
surface

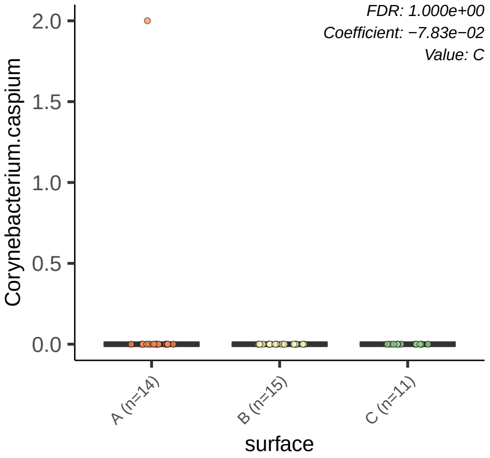

FDR: 1.000e+00
Coefficient: −7.83e−02
Value: C
Corynebacterium.caspium
2.0
1.5
1.0
0.5
0.0
A (n=14)
B (n=15)
C (n=11)
surface

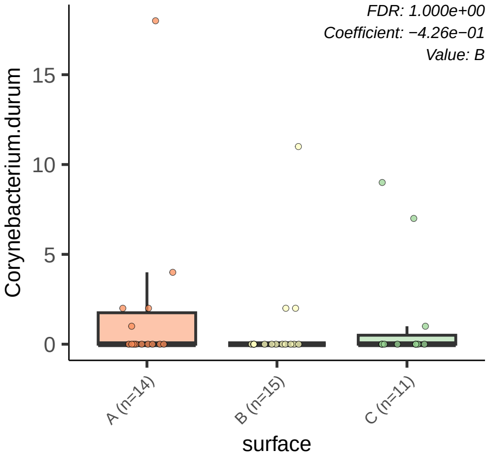
FDR: 1.000e+00
Coefficient: −4.26e−01
Value: B
Corynebacterium.durum
0
5
10
15
A (n=14)
B (n=15)
C (n=11)
surface

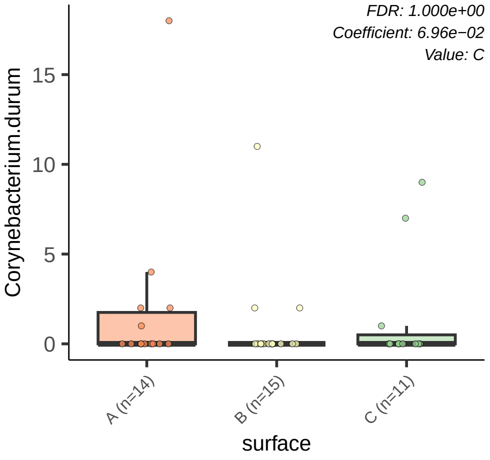
FDR: 1.000e+00
Coefficient: 6.96e−02
Value: C
Corynebacterium.durum
0
5
10
15
A (n=14)
B (n=15)
C (n=11)
surface

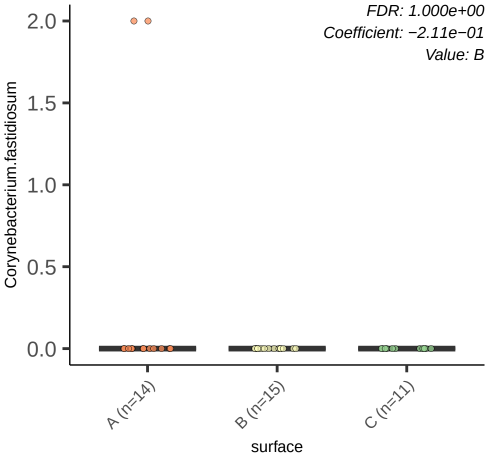
FDR: 1.000e+00
Coefficient: −2.11e−01
Value: B
Corynebacterium.fastidiosum
2.0
1.5
1.0
0.5
0.0
A (n=14)
B (n=15)
C (n=11)
surface

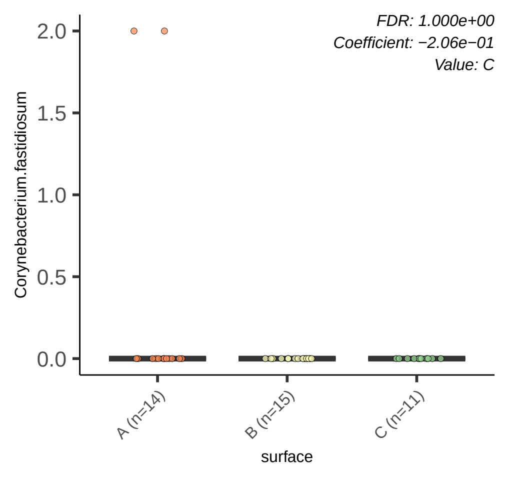

FDR: 1.000e+00
Coefficient: −2.06e−01
Value: C
Corynebacterium.fastidiosum
2.0
1.5
1.0
0.5
0.0
A (n=14)
B (n=15)
C (n=11)
surface

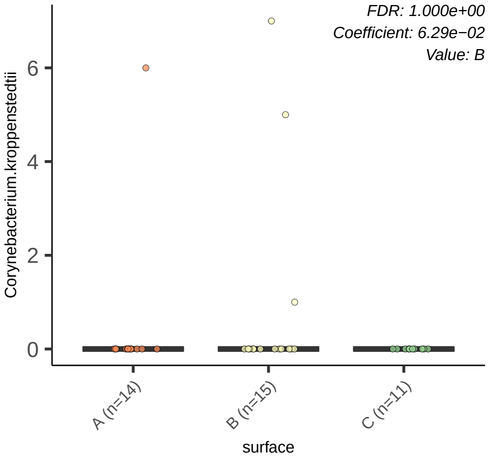

FDR: 1.000e+00
Coefficient: 6.29e−02
Value: B
Corynebacterium.kroppenstedtii
0
2
4
6
A (n=14)
B (n=15)
C (n=11)
surface

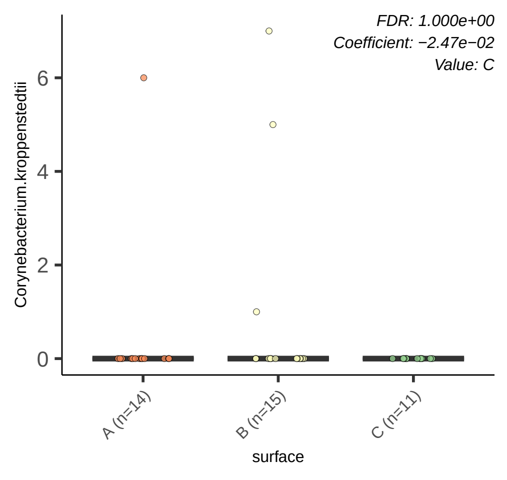

FDR: 1.000e+00
Coefficient: −2.47e−02
Value: C
Corynebacterium.kroppenstedtii
0
2
4
6
A (n=14)
B (n=15)
C (n=11)
surface

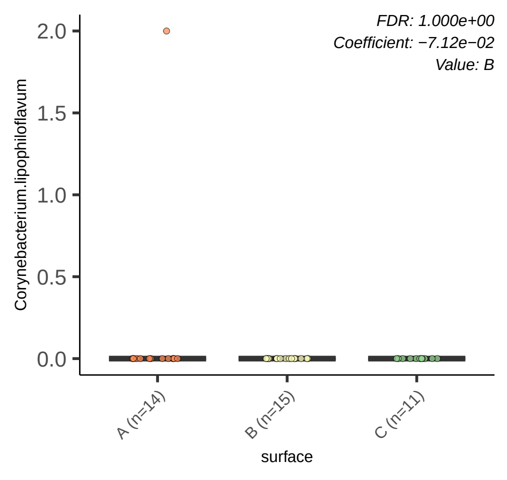
FDR: 1.000e+00
Coefficient: −7.12e−02
Value: B
Corynebacterium.lipophiloflavum
2.0
1.5
1.0
0.5
0.0
A (n=14)
B (n=15)
C (n=11)
surface

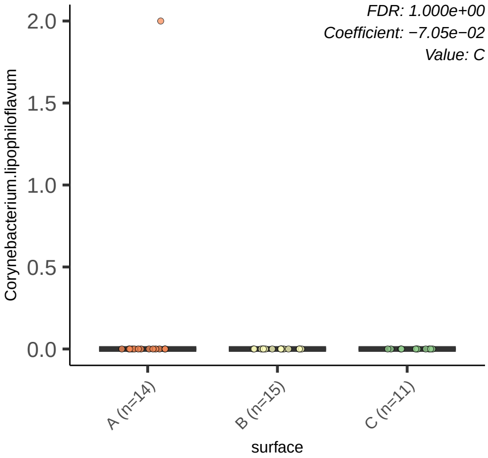
FDR: 1.000e+00
Coefficient: −7.05e−02
Value: C
Corynebacterium.lipophiloflavum
2.0
1.5
1.0
0.5
0.0
A (n=14)
B (n=15)
C (n=11)
surface

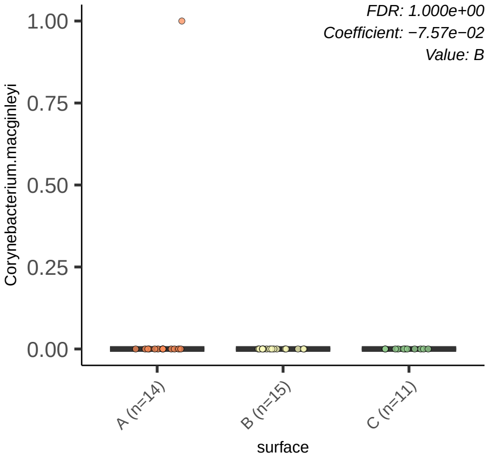
FDR: 1.000e+00
Coefficient: −7.57e−02
Value: B
Corynebacterium.macginleyi
1.00
0.75
0.50
0.25
0.00
A (n=14)
B (n=15)
C (n=11)
surface

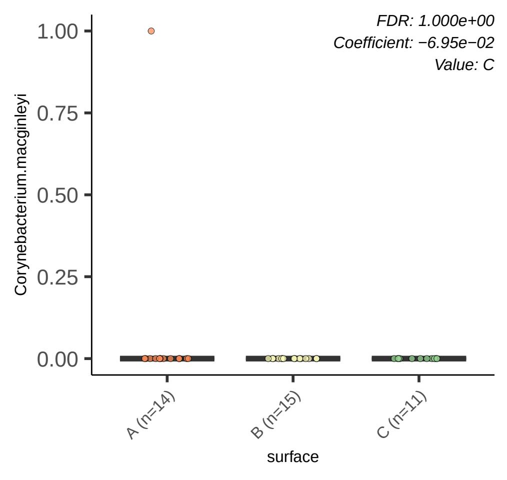

FDR: 1.000e+00
Coefficient: −6.95e−02
Value: C
Corynebacterium.macginleyi
1.00
0.75
0.50
0.25
0.00
A (n=14)
B (n=15)
C (n=11)
surface

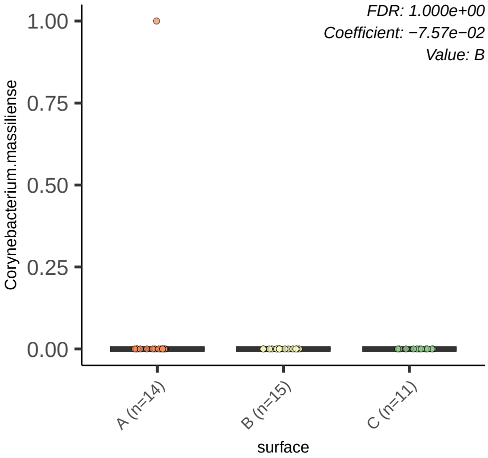
FDR: 1.000e+00
Coefficient: −7.57e−02
Value: B
Corynebacterium.massiliense
1.00
0.75
0.50
0.25
0.00
A (n=14)
B (n=15)
C (n=11)
surface

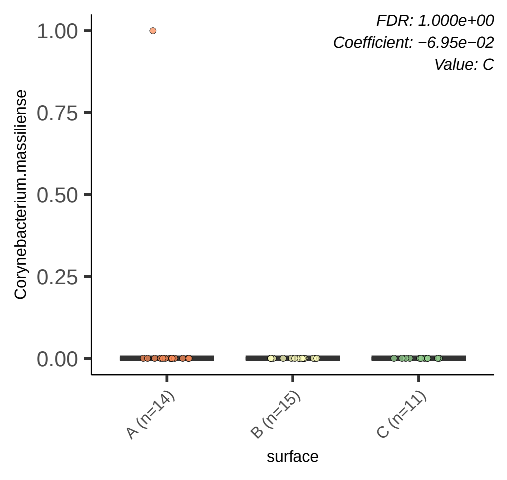
FDR: 1.000e+00
Coefficient: −6.95e−02
Value: C
Corynebacterium.massiliense
1.00
0.75
0.50
0.25
0.00
A (n=14)
B (n=15)
C (n=11)
surface

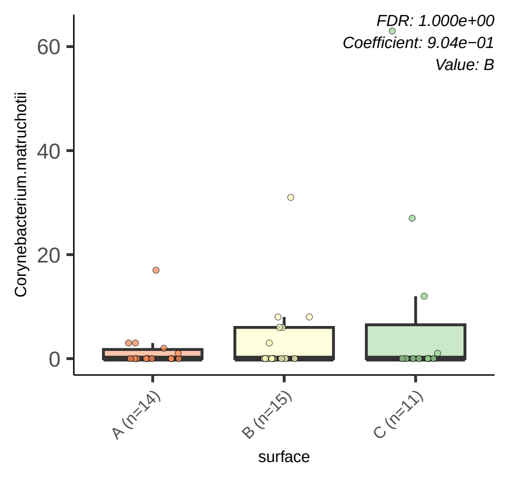

FDR: 1.000e+00
Coefficient: 9.04e−01
Value: B
Corynebacterium.matruchotii
60
40
20
0
A (n=14)
B (n=15)
C (n=11)
surface

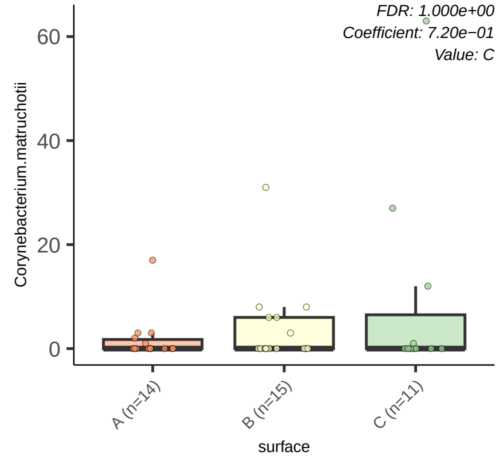
FDR: 1.000e+00
Coefficient: 7.20e−01
Value: C
Corynebacterium.matruchotii
60
40
20
0
A (n=14)
B (n=15)
C (n=11)
surface

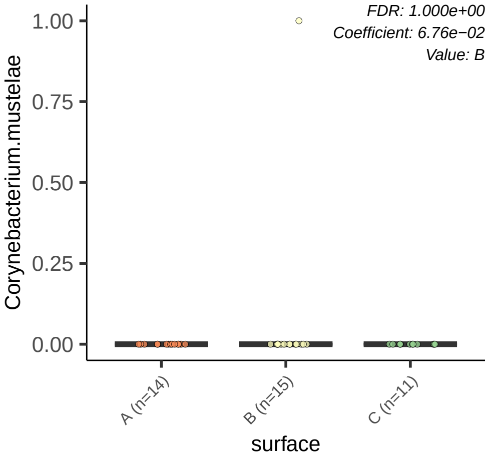
FDR: 1.000e+00
Coefficient: 6.76e−02
Value: B
Corynebacterium.mustelae
1.00
0.75
0.50
0.25
0.00
A (n=14)
B (n=15)
C (n=11)
surface

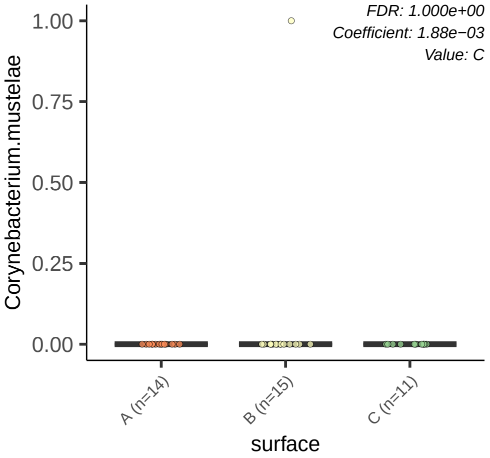
FDR: 1.000e+00
Coefficient: 1.88e−03
Value: C
Corynebacterium.mustelae
1.00
0.75
0.50
0.25
0.00
A (n=14)
B (n=15)
C (n=11)
surface

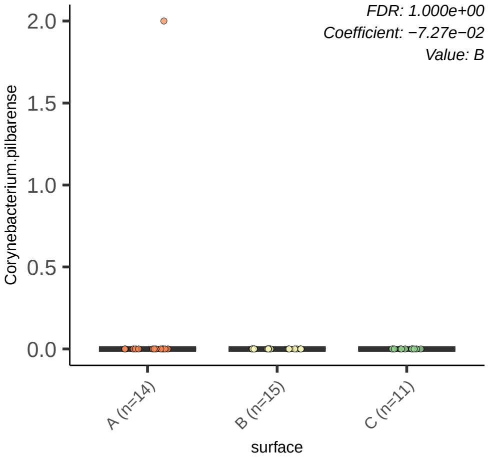
FDR: 1.000e+00
Coefficient: −7.27e−02
Value: B
Corynebacterium.pilbarense
2.0
1.5
1.0
0.5
0.0
A (n=14)
B (n=15)
C (n=11)
surface

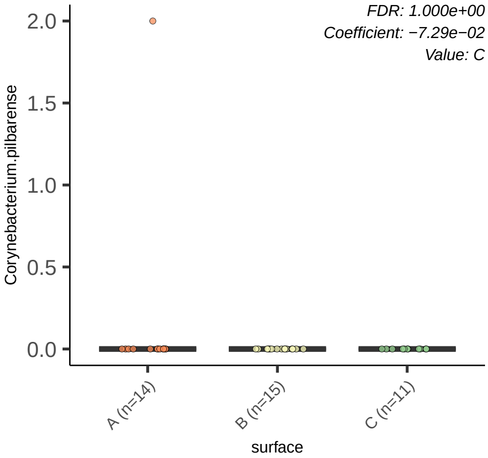

FDR: 1.000e+00
Coefficient: −7.29e−02
Value: C
2.0
1.5
1.0
0.5
0.0
Corynebacterium.pilbarense
A (n=14)
B (n=15)
C (n=11)
surface

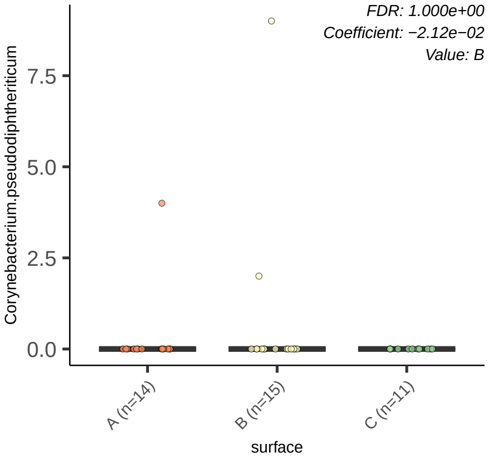

FDR: 1.000e+00
Coefficient: −2.12e−02
Value: B
Corynebacterium.pseudodiphtheriticum
7.5
5.0
2.5
0.0
A (n=14)
B (n=15)
C (n=11)
surface

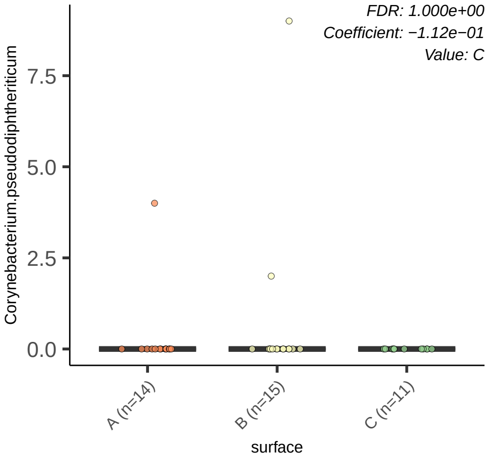
FDR: 1.000e+00
Coefficient: −1.12e−01
Value: C
Corynebacterium.pseudodiphtheriticum
7.5
5.0
2.5
0.0
A (n=14)
B (n=15)
C (n=11)
surface

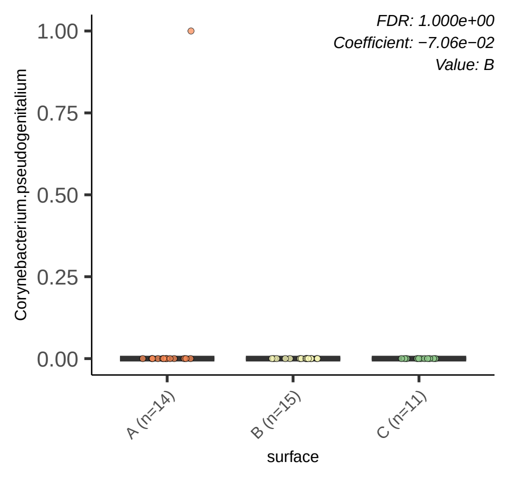
FDR: 1.000e+00
Coefficient: −7.06e−02
Value: B
Corynebacterium.pseudogenitalium
1.00
0.75
0.50
0.25
0.00
A (n=14)
B (n=15)
C (n=11)
surface

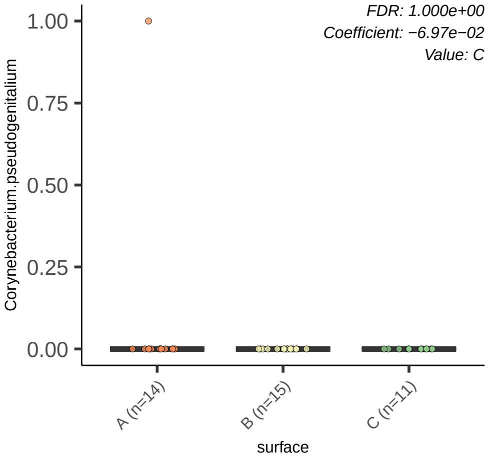
FDR: 1.000e+00
Coefficient: −6.97e−02
Value: C
Corynebacterium.pseudogenitalium
1.00
0.75
0.50
0.25
0.00
A (n=14)
B (n=15)
C (n=11)
surface

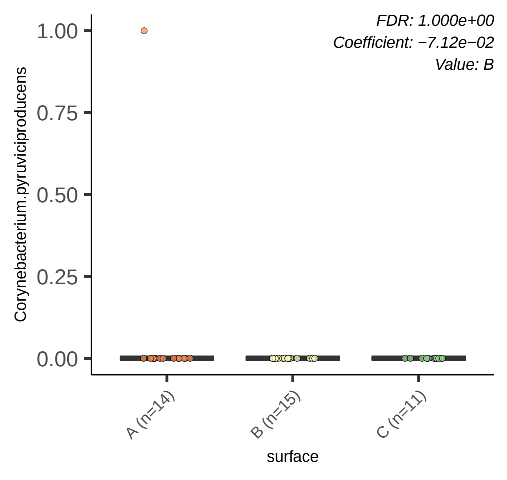

FDR: 1.000e+00
Coefficient: −7.12e−02
Value: B
1.00
0.75
0.50
0.25
0.00
Corynebacterium.pyruviciproducens
A (n=14)
B (n=15)
C (n=11)
surface

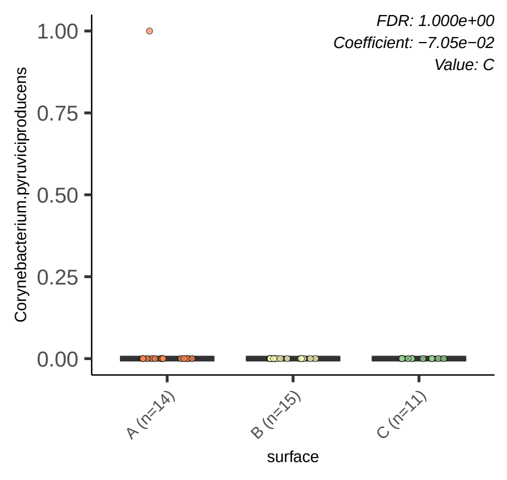

FDR: 1.000e+00
Coefficient: −7.05e−02
Value: C
1.00
0.75
0.50
0.25
0.00
Corynebacterium.pyruviciproducens
A (n=14)
B (n=15)
C (n=11)
surface

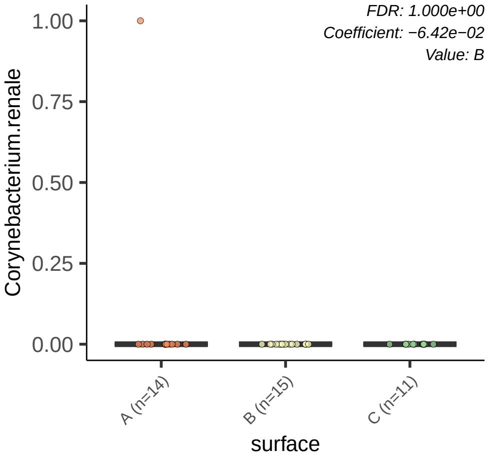

FDR: 1.000e+00
Coefficient: −6.42e−02
Value: B
Corynebacterium.renale
1.00
0.75
0.50
0.25
0.00
A (n=14)
B (n=15)
C (n=11)
surface

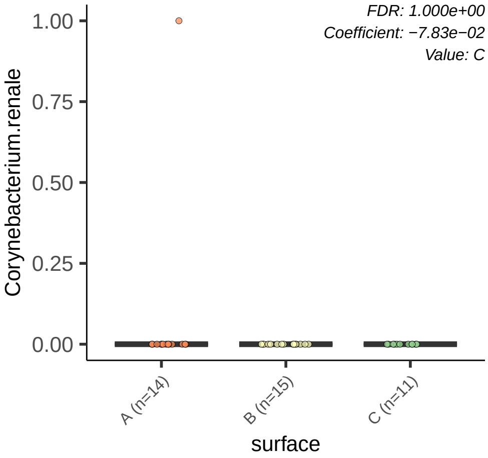

FDR: 1.000e+00
Coefficient: −7.83e−02
Value: C
Corynebacterium.renale
1.00
0.75
0.50
0.25
0.00
A (n=14)
B (n=15)
C (n=11)
surface

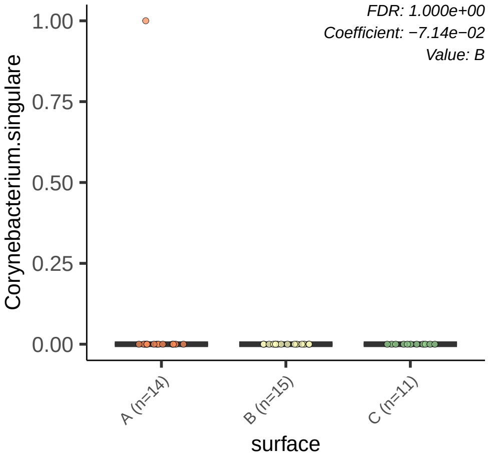

FDR: 1.000e+00
Coefficient: −7.14e−02
Value: B
Corynebacterium.singulare
1.00
0.75
0.50
0.25
0.00
A (n=14)
B (n=15)
C (n=11)
surface

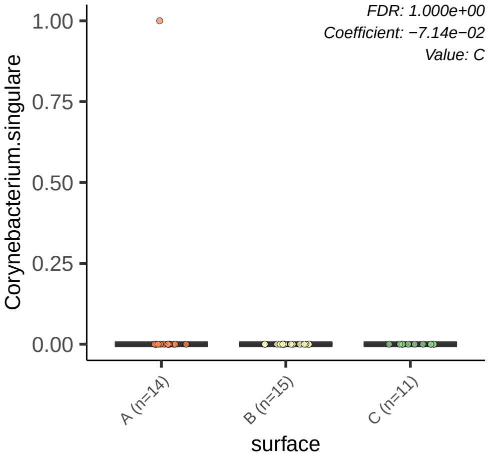
FDR: 1.000e+00
Coefficient: −7.14e−02
Value: C
Corynebacterium.singulare
1.00
0.75
0.50
0.25
0.00
A (n=14)
B (n=15)
C (n=11)
surface

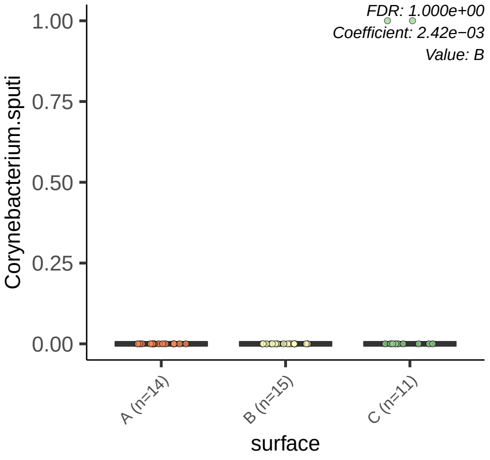
FDR: 1.000e+00
Coefficient: 2.42e−03
Value: B
Corynebacterium.sputi
1.00
0.75
0.50
0.25
0.00
A (n=14)
B (n=15)
C (n=11)
surface

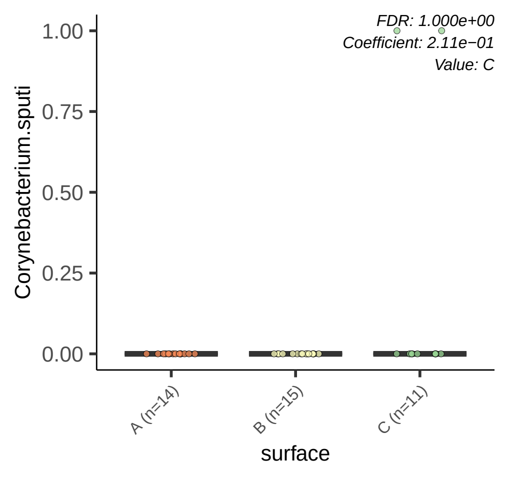
FDR: 1.000e+00
Coefficient: 2.11e−01
Value: C
Corynebacterium.sputi
1.00
0.75
0.50
0.25
0.00
A (n=14)
B (n=15)
C (n=11)
surface

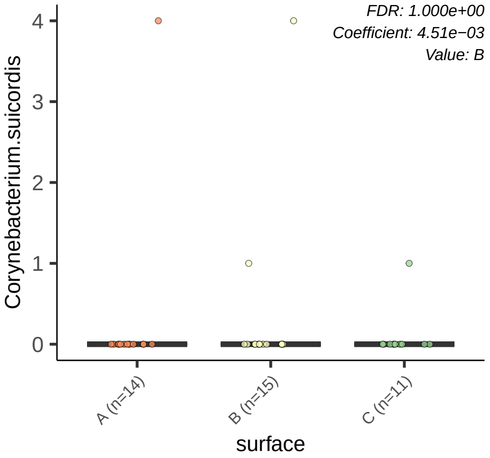
FDR: 1.000e+00
Coefficient: 4.51e−03
Value: B
Corynebacterium.suicordis
0
1
2
3
4
A (n=14)
B (n=15)
C (n=11)
surface

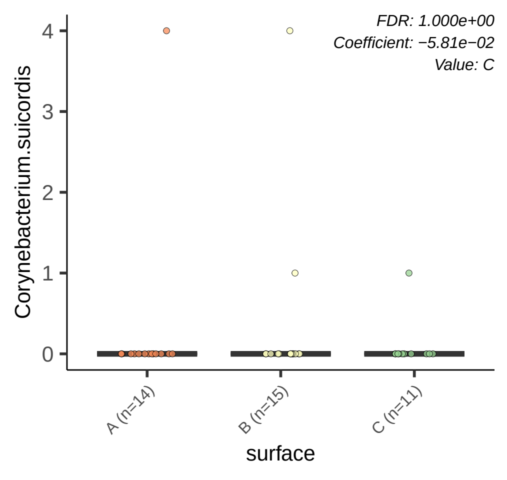

FDR: 1.000e+00
Coefficient: −5.81e−02
Value: C
Corynebacterium.suicordis
0
1
2
3
4
A (n=14)
B (n=15)
C (n=11)
surface

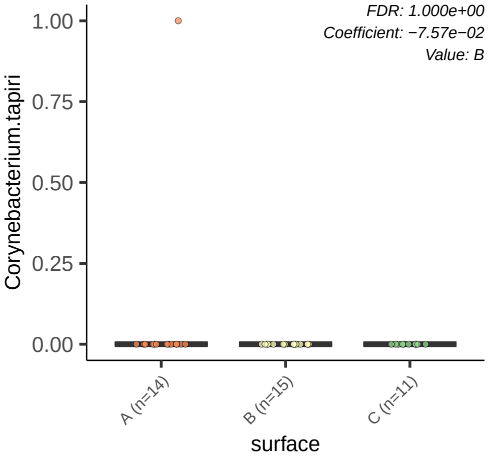

FDR: 1.000e+00
Coefficient: −7.57e−02
Value: B
Corynebacterium.tapiri
1.00
0.75
0.50
0.25
0.00
A (n=14)
B (n=15)
C (n=11)
surface

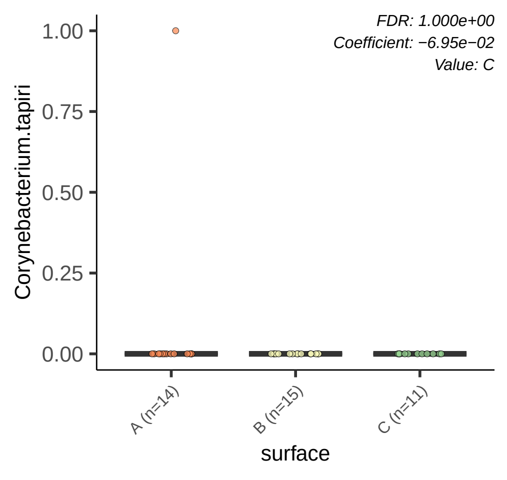

FDR: 1.000e+00
Coefficient: −6.95e−02
Value: C
Corynebacterium.tapiri
1.00
0.75
0.50
0.25
0.00
A (n=14)
B (n=15)
C (n=11)
surface

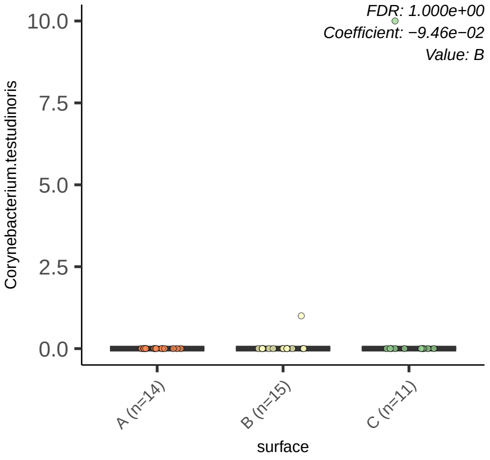
FDR: 1.000e+00
Coefficient: −9.46e−02
Value: B
10.0
7.5
5.0
2.5
0.0
Corynebacterium.testudinoris
A (n=14)
B (n=15)
C (n=11)
surface

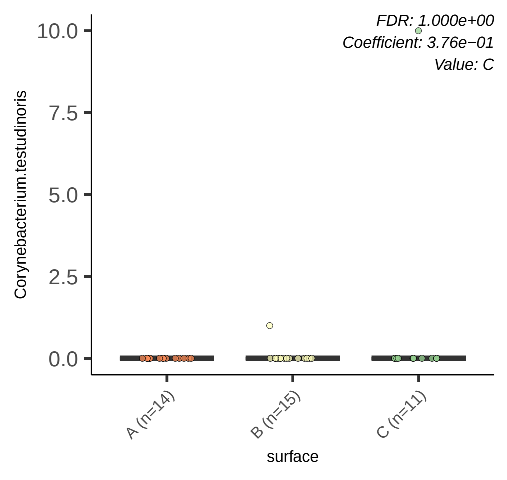
FDR: 1.000e+00
Coefficient: 3.76e−01
Value: C
Corynebacterium.testudinoris
10.0
7.5
5.0
2.5
0.0
A (n=14)
B (n=15)
C (n=11)
surface

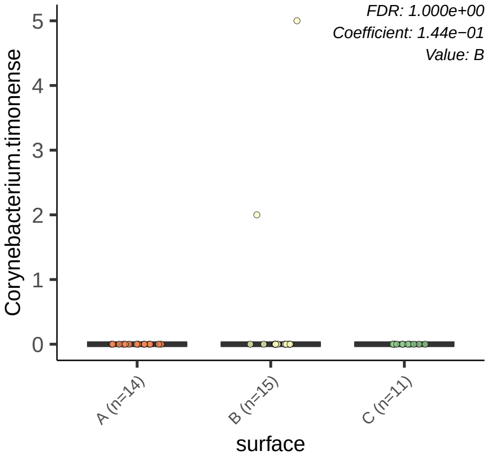
FDR: 1.000e+00
Coefficient: 1.44e−01
Value: B
Corynebacterium.timonense
5
4
3
2
1
0
A (n=14)
B (n=15)
C (n=11)
surface

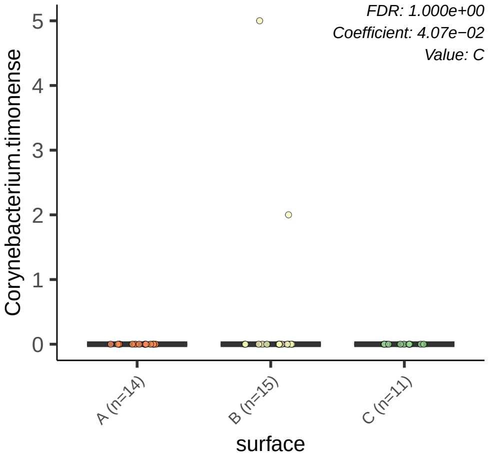

FDR: 1.000e+00
Coefficient: 4.07e−02
Value: C
Corynebacterium.timonense
0
1
2
3
4
5
A (n=14)
B (n=15)
C (n=11)
surface

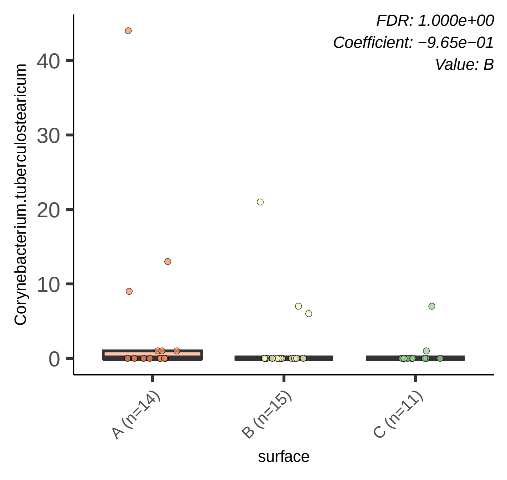

FDR: 1.000e+00
Coefficient: −9.65e−01
Value: B
Corynebacterium.tuberculostearicum
40
30
20
10
0
A (n=14)
B (n=15)
C (n=11)
surface

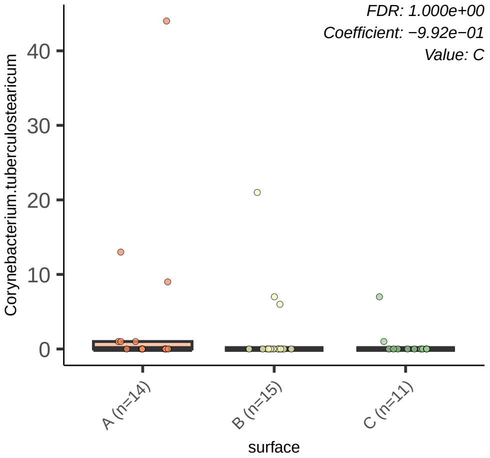
FDR: 1.000e+00
Coefficient: −9.92e−01
Value: C
Corynebacterium.tuberculostearicum
0
10
20
30
40
A (n=14)
B (n=15)
C (n=11)
surface

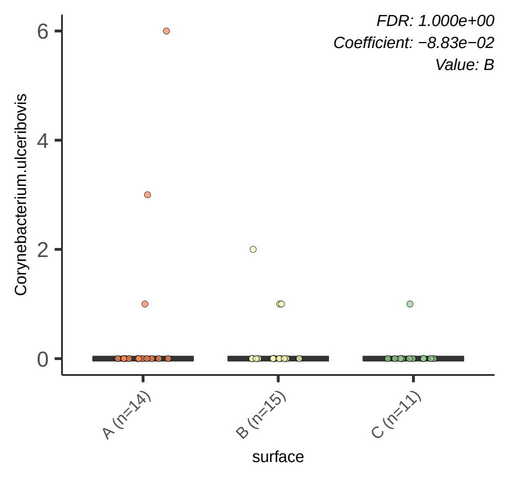
FDR: 1.000e+00
Coefficient: −8.83e−02
Value: B
Corynebacterium.ulceribovis
0
2
4
6
A (n=14)
B (n=15)
C (n=11)
surface

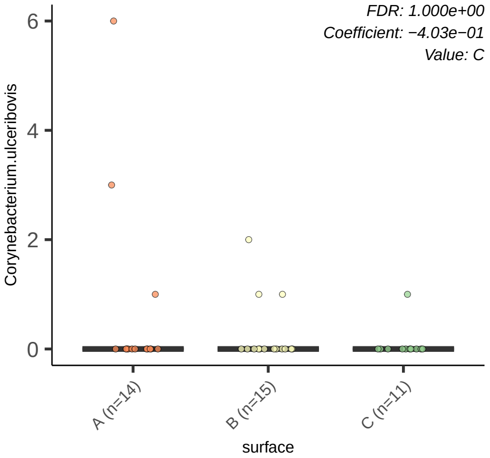

FDR: 1.000e+00
Coefficient: −4.03e−01
Value: C
Corynebacterium.ulceribovis
0
2
4
6
A (n=14)
B (n=15)
C (n=11)
surface

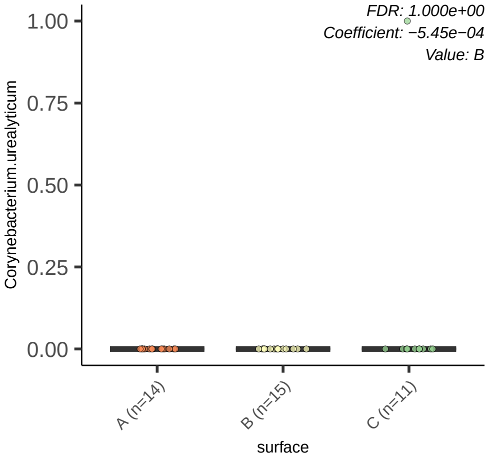
FDR: 1.000e+00
Coefficient: −5.45e−04
Value: B
Corynebacterium.urealyticum
1.00
0.75
0.50
0.25
0.00
A (n=14)
B (n=15)
C (n=11)
surface

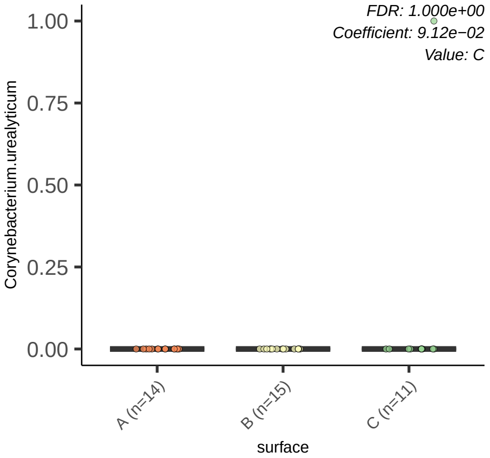

FDR: 1.000e+00
Coefficient: 9.12e−02
Value: C
Corynebacterium.urealyticum
1.00
0.75
0.50
0.25
0.00
A (n=14)
B (n=15)
C (n=11)
surface

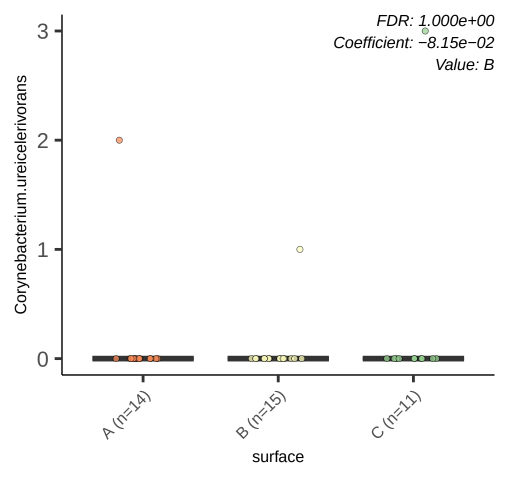
FDR: 1.000e+00
Coefficient: −8.15e−02
Value: B
Corynebacterium.ureicelerivorans
3
2
1
0
A (n=14)
B (n=15)
C (n=11)
surface

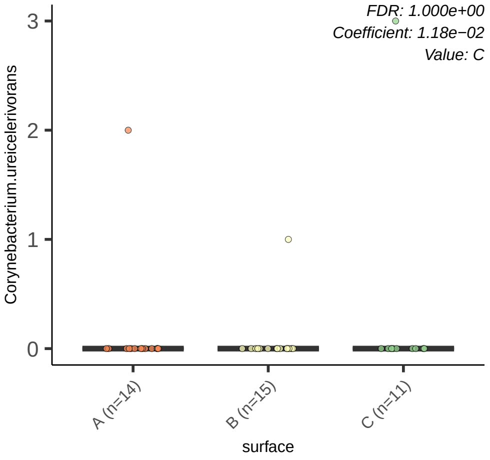

FDR: 1.000e+00
Coefficient: 1.18e−02
Value: C
Corynebacterium.ureicelerivorans
3
2
1
0
A (n=14)
B (n=15)
C (n=11)
surface

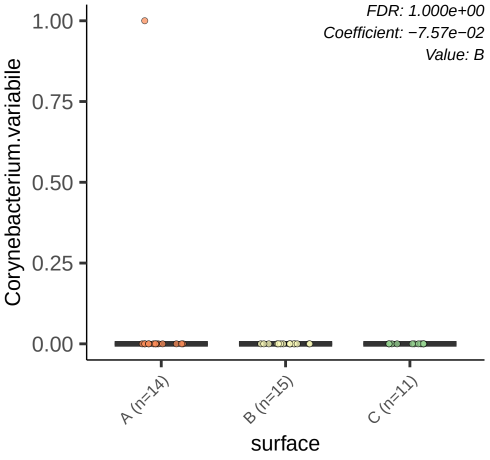
FDR: 1.000e+00
Coefficient: −7.57e−02
Value: B
Corynebacterium.variabile
1.00
0.75
0.50
0.25
0.00
A (n=14)
B (n=15)
C (n=11)
surface

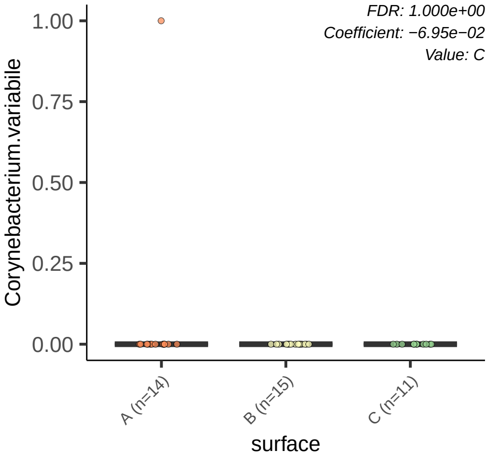
FDR: 1.000e+00
Coefficient: −6.95e−02
Value: C
Corynebacterium.variabile
1.00
0.75
0.50
0.25
0.00
A (n=14)
B (n=15)
C (n=11)
surface

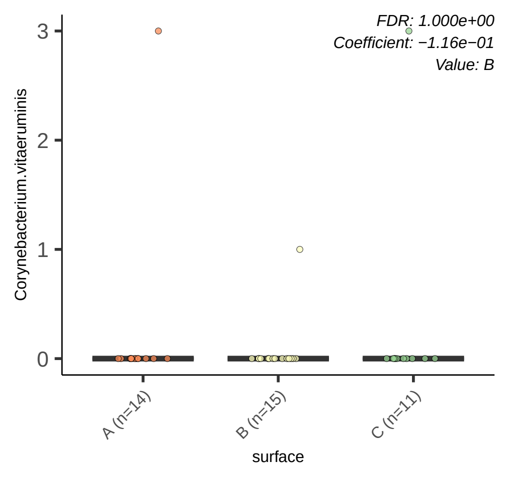

FDR: 1.000e+00
Coefficient: −1.16e−01
Value: B
Corynebacterium.vitaeruminis
3
2
1
0
A (n=14)
B (n=15)
C (n=11)
surface

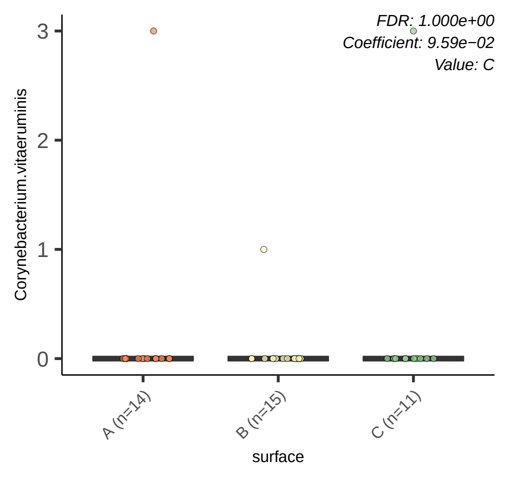
FDR: 1.000e+00
Coefficient: 9.59e−02
Value: C
Corynebacterium.vitaeruminis
3
2
1
0
A (n=14)
B (n=15)
C (n=11)
surface

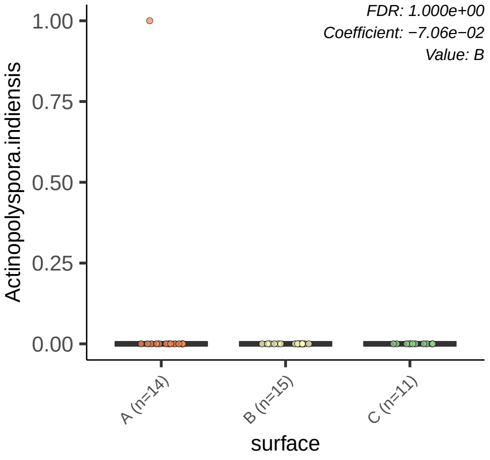
FDR: 1.000e+00
Coefficient: −7.06e−02
Value: B
Actinopolyspora.indiensis
1.00
0.75
0.50
0.25
0.00
A (n=14)
B (n=15)
C (n=11)
surface

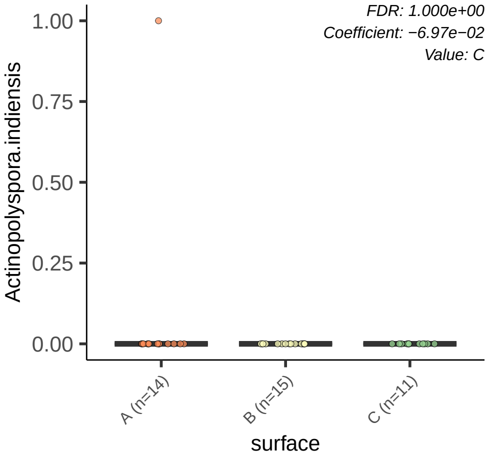
FDR: 1.000e+00
Coefficient: −6.97e−02
Value: C
Actinopolyspora.indiensis
1.00
0.75
0.50
0.25
0.00
A (n=14)
B (n=15)
C (n=11)
surface

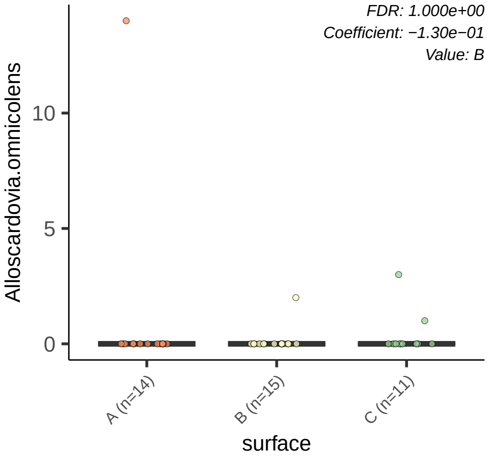

FDR: 1.000e+00
Coefficient: −1.30e−01
Value: B
Alloscardovia.omnicolens
10
5
0
A (n=14)
B (n=15)
C (n=11)
surface

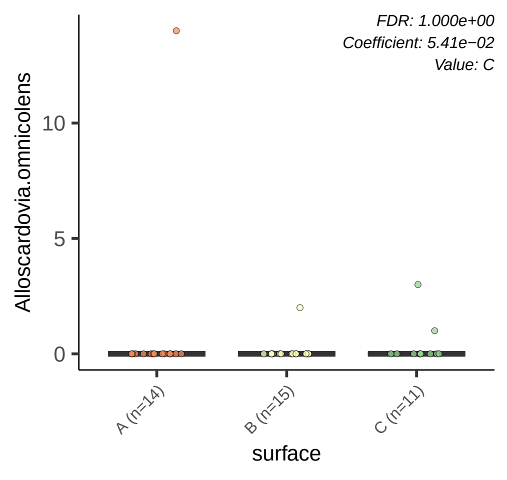
FDR: 1.000e+00
Coefficient: 5.41e−02
Value: C
Alloscardovia.omnicolens
0
5
10
A (n=14)
B (n=15)
C (n=11)
surface

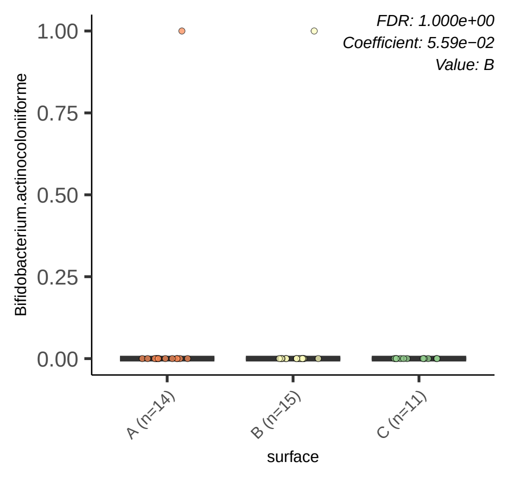

FDR: 1.000e+00
Coefficient: 5.59e−02
Value: B
Bifidobacterium.actinocoloniiforme
1.00
0.75
0.50
0.25
0.00
A (n=14)
B (n=15)
C (n=11)
surface

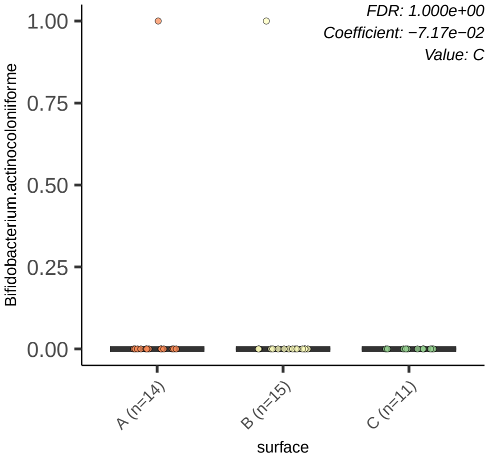
FDR: 1.000e+00
Coefficient: −7.17e−02
Value: C
Bifidobacterium.actinocoloniiforme
1.00
0.75
0.50
0.25
0.00
A (n=14)
B (n=15)
C (n=11)
surface

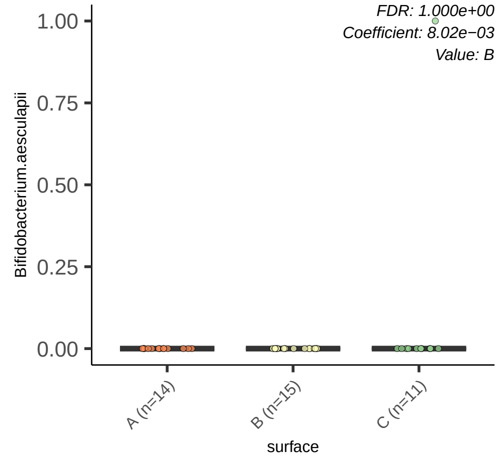
FDR: 1.000e+00
Coefficient: 8.02e−03
Value: B
1.00
0.75
0.50
0.25
0.00
Bifidobacterium.aesculapii
A (n=14)
B (n=15)
C (n=11)
surface

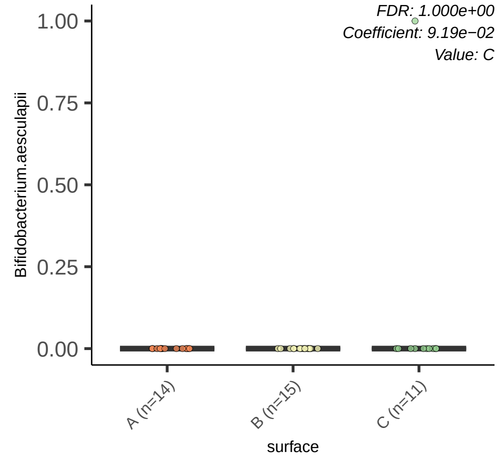
FDR: 1.000e+00
Coefficient: 9.19e−02
Value: C
Bifidobacterium.aesculapii
1.00
0.75
0.50
0.25
0.00
A (n=14)
B (n=15)
C (n=11)
surface

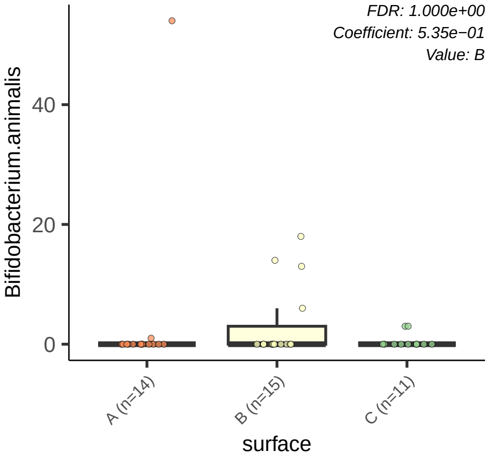

FDR: 1.000e+00
Coefficient: 5.35e−01
Value: B
Bifidobacterium.animalis
40
20
0
A (n=14)
B (n=15)
C (n=11)
surface

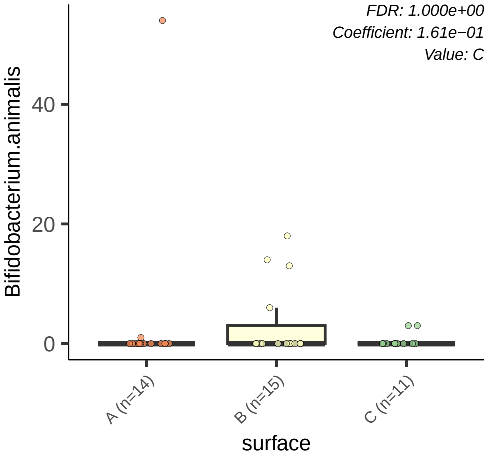
FDR: 1.000e+00
Coefficient: 1.61e−01
Value: C
Bifidobacterium.animalis
0
20
40
A (n=14)
B (n=15)
C (n=11)
surface

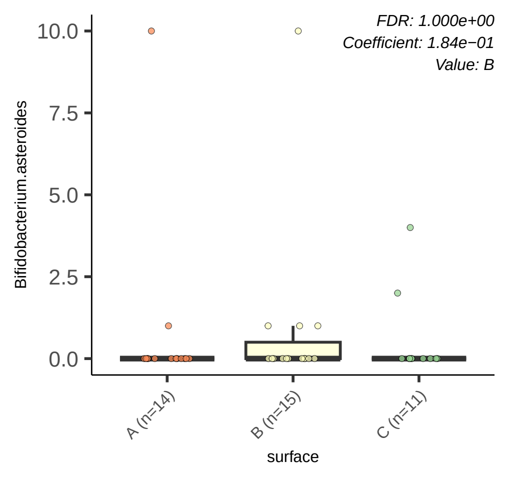

FDR: 1.000e+00
Coefficient: 1.84e−01
Value: B
Bifidobacterium.asteroides
10.0
7.5
5.0
2.5
0.0
A (n=14)
B (n=15)
C (n=11)
surface

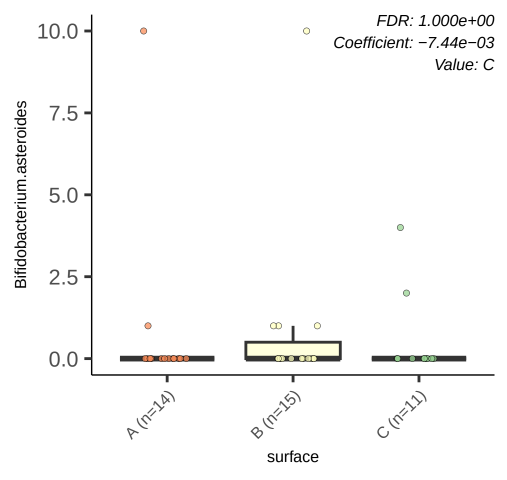

FDR: 1.000e+00
Coefficient: −7.44e−03
Value: C
Bifidobacterium.asteroides
10.0
7.5
5.0
2.5
0.0
A (n=14)
B (n=15)
C (n=11)
surface

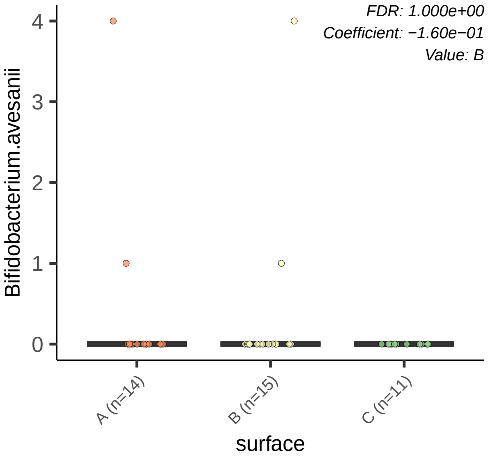
FDR: 1.000e+00
Coefficient: −1.60e−01
Value: B
Bifidobacterium.avesanii
4
3
2
1
0
A (n=14)
B (n=15)
C (n=11)
surface

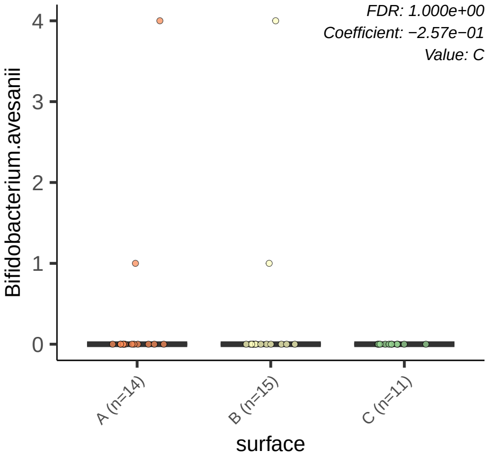
FDR: 1.000e+00
Coefficient: −2.57e−01
Value: C
Bifidobacterium.avesanii
0
1
2
3
4
A (n=14)
B (n=15)
C (n=11)
surface

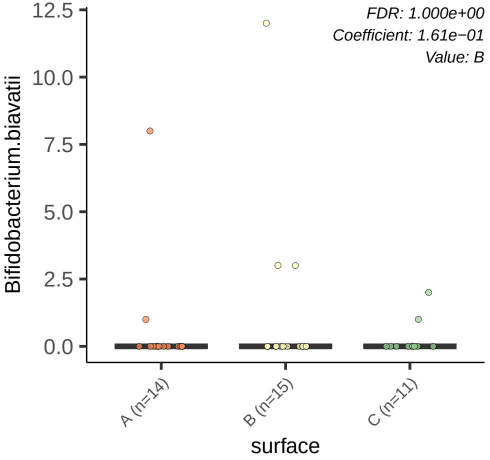
FDR: 1.000e+00
Coefficient: 1.61e−01
Value: B
Bifidobacterium.biavatii
12.5
10.0
7.5
5.0
2.5
0.0
A (n=14)
B (n=15)
C (n=11)
surface

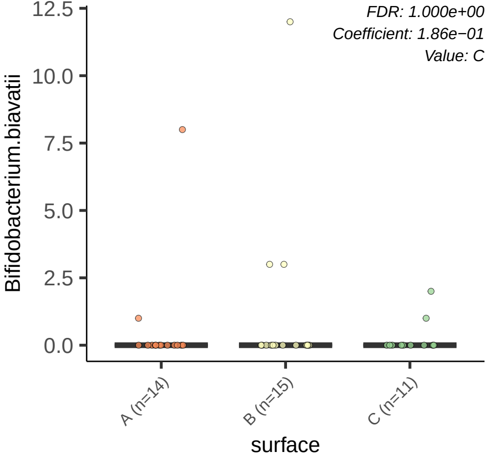
FDR: 1.000e+00
Coefficient: 1.86e−01
Value: C
Bifidobacterium.biavatii
12.5
10.0
7.5
5.0
2.5
0.0
A (n=14)
B (n=15)
C (n=11)
surface

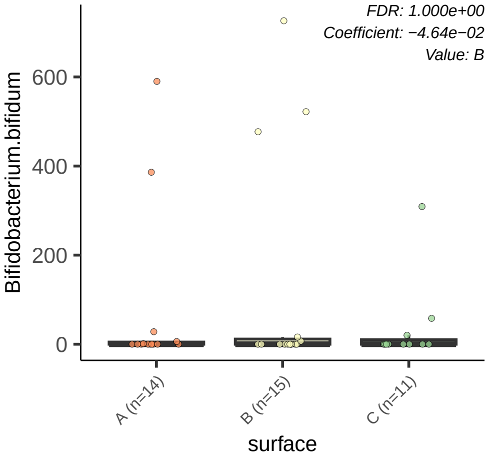

FDR: 1.000e+00
Coefficient: −4.64e−02
Value: B
Bifidobacterium.bifidum
0
200
400
600
A (n=14)
B (n=15)
C (n=11)
surface

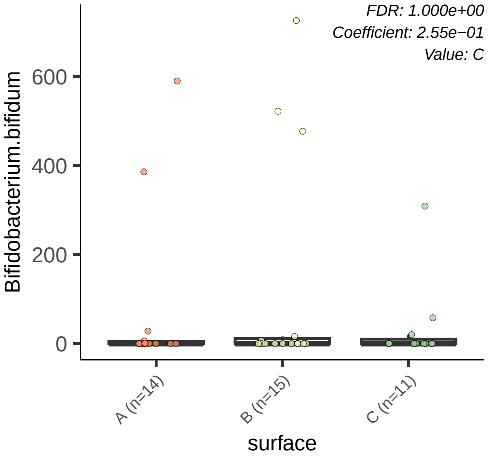

FDR: 1.000e+00
Coefficient: 2.55e−01
Value: C
Bifidobacterium.bifidum
600
400
200
0
A (n=14)
B (n=15)
C (n=11)
surface

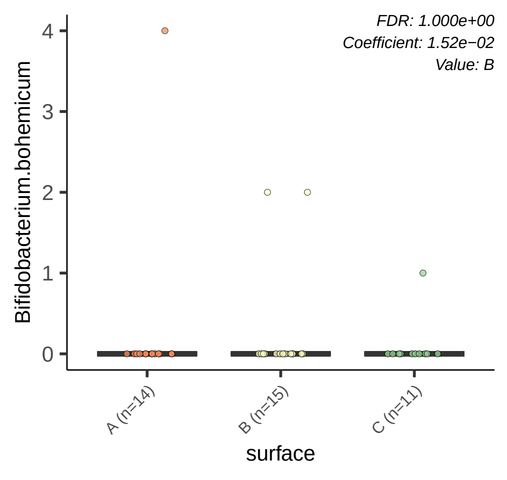

FDR: 1.000e+00
Coefficient: 1.52e−02
Value: B
Bifidobacterium.bohemicum
4
3
2
1
0
A (n=14)
B (n=15)
C (n=11)
surface

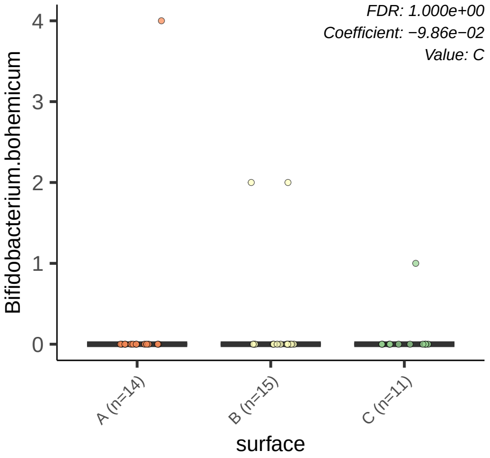
FDR: 1.000e+00
Coefficient: −9.86e−02
Value: C
Bifidobacterium.bohemicum
4
3
2
1
0
A (n=14)
B (n=15)
C (n=11)
surface

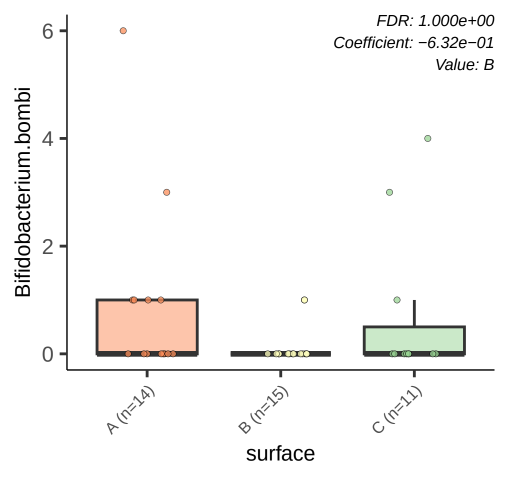
FDR: 1.000e+00
Coefficient: −6.32e−01
Value: B
Bifidobacterium.bombi
6
4
2
0
A (n=14)
B (n=15)
C (n=11)
surface

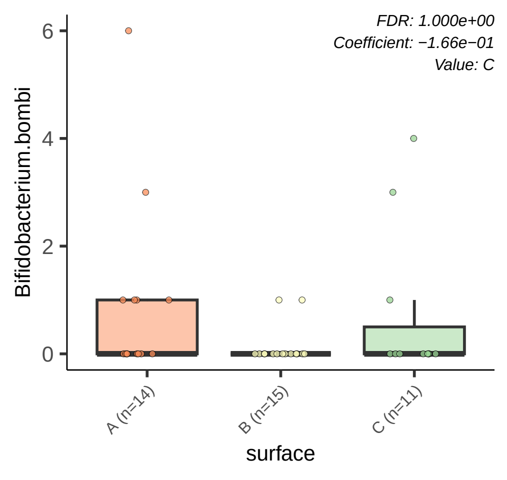

FDR: 1.000e+00
Coefficient: −1.66e−01
Value: C
Bifidobacterium.bombi
6
4
2
0
A (n=14)
B (n=15)
C (n=11)
surface

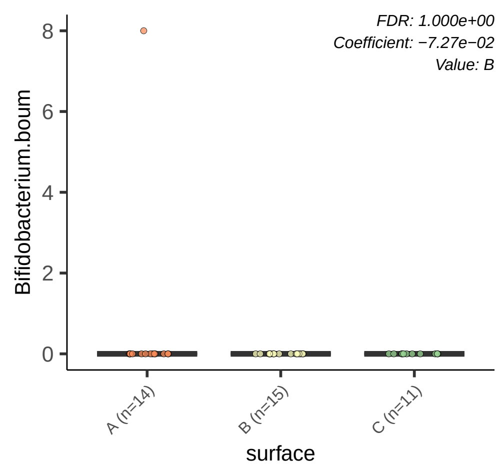

FDR: 1.000e+00
Coefficient: −7.27e−02
Value: B
Bifidobacterium.boum
8
6
4
2
0
A (n=14)
B (n=15)
C (n=11)
surface

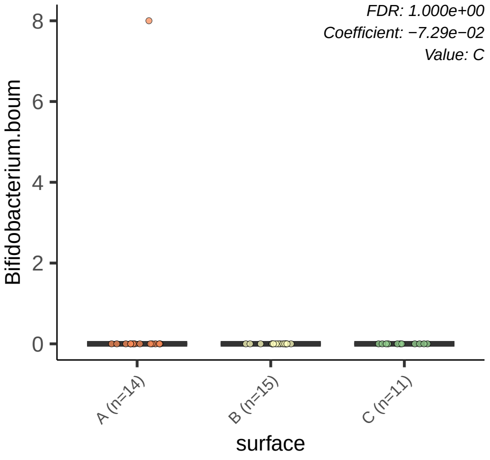
FDR: 1.000e+00
Coefficient: −7.29e−02
Value: C
Bifidobacterium.boum
8
6
4
2
0
A (n=14)
B (n=15)
C (n=11)
surface

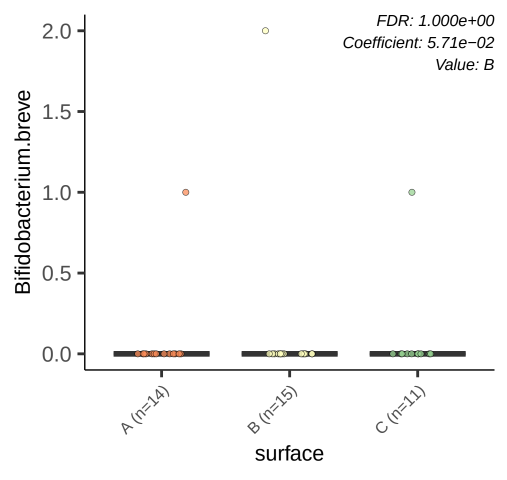

FDR: 1.000e+00
Coefficient: 5.71e−02
Value: B
Bifidobacterium.breve
2.0
1.5
1.0
0.5
0.0
A (n=14)
B (n=15)
C (n=11)
surface

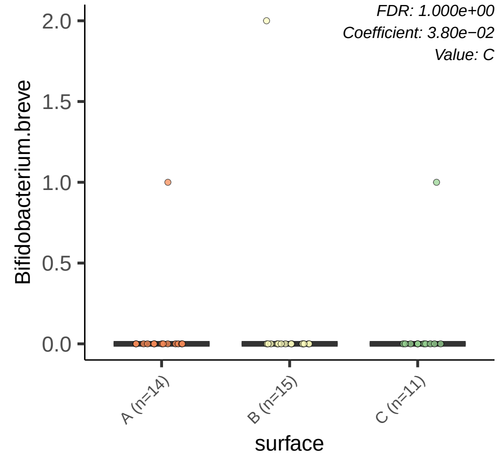
FDR: 1.000e+00
Coefficient: 3.80e−02
Value: C
Bifidobacterium.breve
2.0
1.5
1.0
0.5
0.0
A (n=14)
B (n=15)
C (n=11)
surface

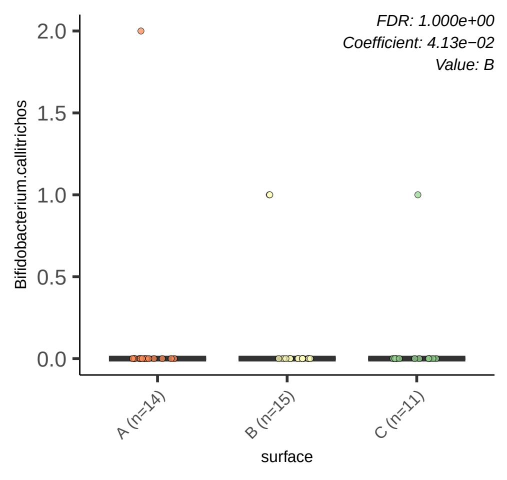
FDR: 1.000e+00
Coefficient: 4.13e−02
Value: B
Bifidobacterium.callitrichos
2.0
1.5
1.0
0.5
0.0
A (n=14)
B (n=15)
C (n=11)
surface

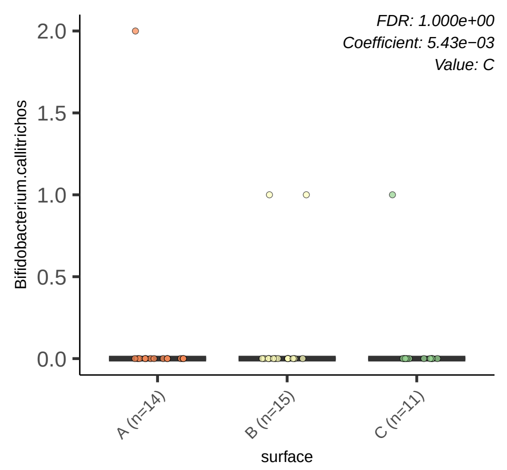
FDR: 1.000e+00
Coefficient: 5.43e−03
Value: C
Bifidobacterium.callitrichos
2.0
1.5
1.0
0.5
0.0
A (n=14)
B (n=15)
C (n=11)
surface

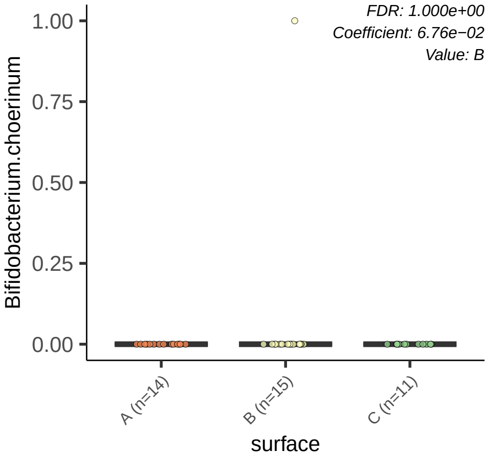

FDR: 1.000e+00
Coefficient: 6.76e−02
Value: B
Bifidobacterium.choerinum
1.00
0.75
0.50
0.25
0.00
A (n=14)
B (n=15)
C (n=11)
surface

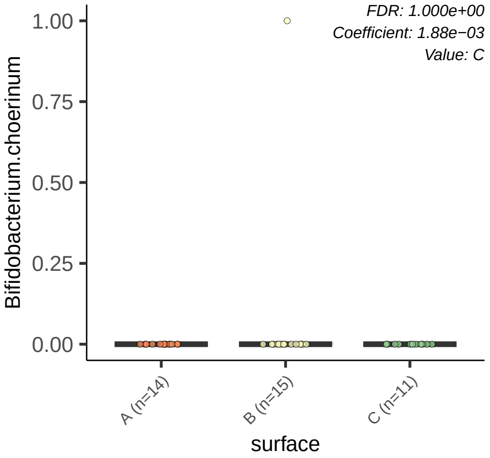

FDR: 1.000e+00
Coefficient: 1.88e−03
Value: C
Bifidobacterium.choerinum
1.00
0.75
0.50
0.25
0.00
A (n=14)
B (n=15)
C (n=11)
surface

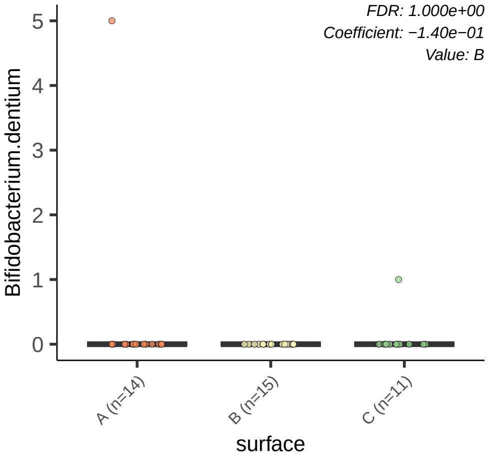
FDR: 1.000e+00
Coefficient: −1.40e−01
Value: B
Bifidobacterium.dentium
5
4
3
2
1
0
A (n=14)
B (n=15)
C (n=11)
surface

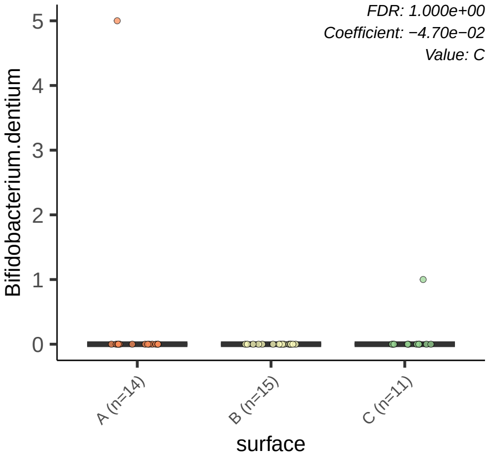
FDR: 1.000e+00
Coefficient: −4.70e−02
Value: C
Bifidobacterium.dentium
0
1
2
3
4
5
A (n=14)
B (n=15)
C (n=11)
surface

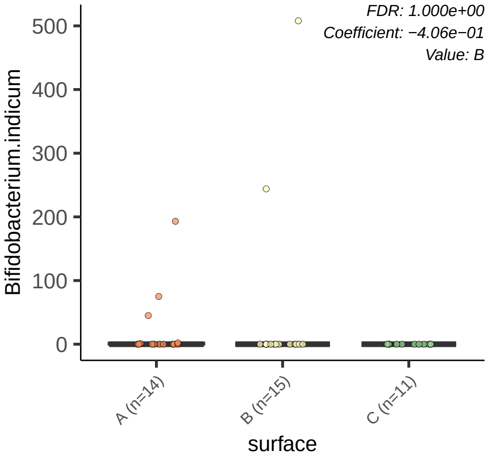
FDR: 1.000e+00
Coefficient: −4.06e−01
Value: B
Bifidobacterium.indicum
500
400
300
200
100
0
A (n=14)
B (n=15)
C (n=11)
surface

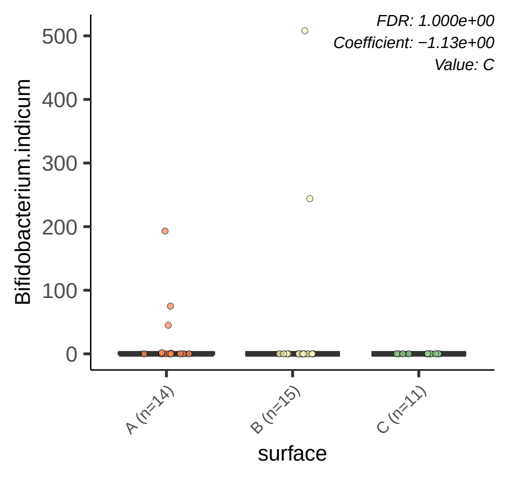
FDR: 1.000e+00
Coefficient: −1.13e+00
Value: C
Bifidobacterium.indicum
500
400
300
200
100
0
A (n=14)
B (n=15)
C (n=11)
surface

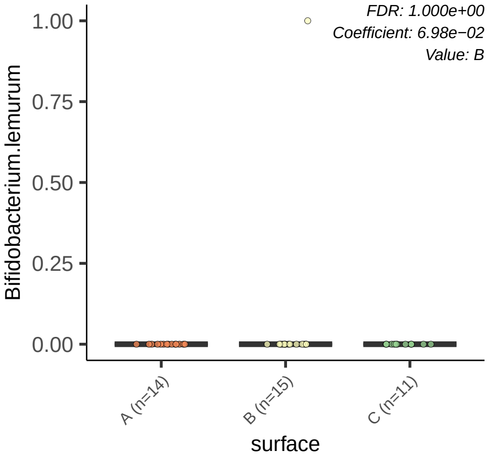

FDR: 1.000e+00
Coefficient: 6.98e−02
Value: B
Bifidobacterium.lemurum
1.00
0.75
0.50
0.25
0.00
A (n=14)
B (n=15)
C (n=11)
surface

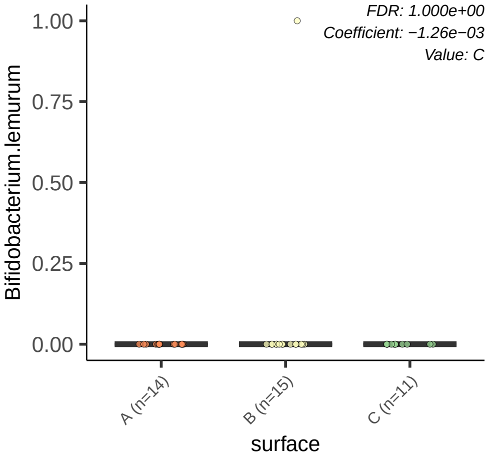
FDR: 1.000e+00
Coefficient: −1.26e−03
Value: C
Bifidobacterium.lemurum
1.00
0.75
0.50
0.25
0.00
A (n=14)
B (n=15)
C (n=11)
surface

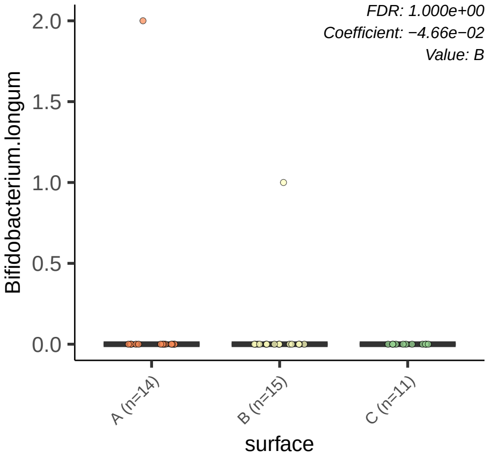
FDR: 1.000e+00
Coefficient: −4.66e−02
Value: B
Bifidobacterium.longum
2.0
1.5
1.0
0.5
0.0
A (n=14)
B (n=15)
C (n=11)
surface

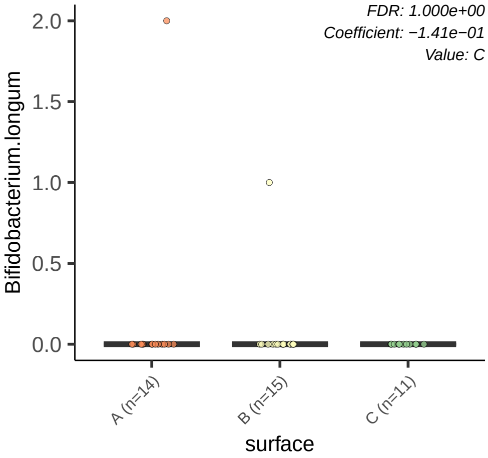
FDR: 1.000e+00
Coefficient: −1.41e−01
Value: C
Bifidobacterium.longum
2.0
1.5
1.0
0.5
0.0
A (n=14)
B (n=15)
C (n=11)
surface

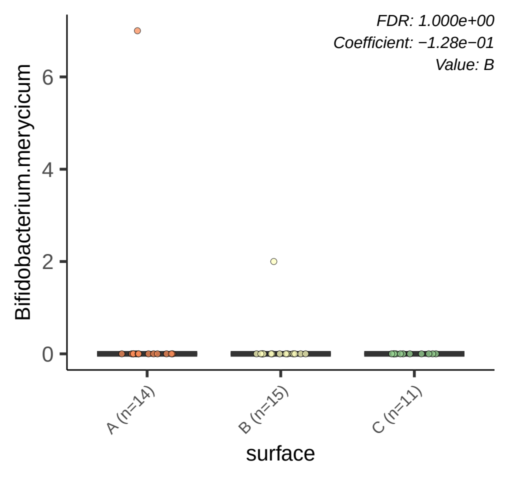

FDR: 1.000e+00
Coefficient: −1.28e−01
Value: B
Bifidobacterium.merycicum
0
2
4
6
A (n=14)
B (n=15)
C (n=11)
surface

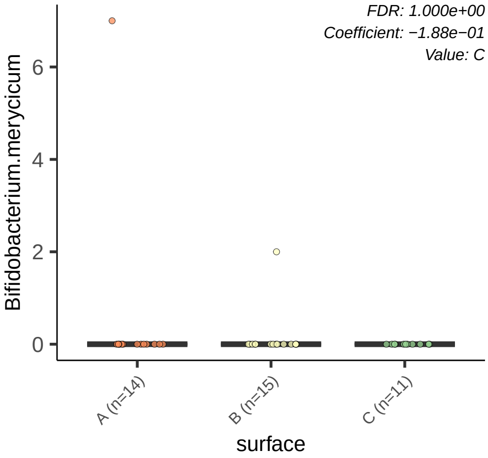
FDR: 1.000e+00
Coefficient: −1.88e−01
Value: C
Bifidobacterium.merycicum
0
2
4
6
A (n=14)
B (n=15)
C (n=11)
surface

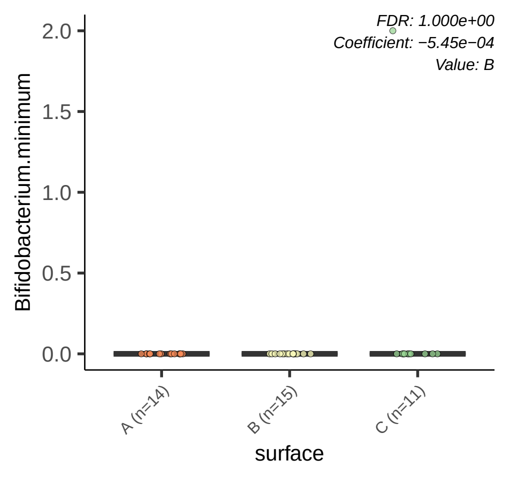
FDR: 1.000e+00
Coefficient: −5.45e−04
Value: B
Bifidobacterium.minimum
2.0
1.5
1.0
0.5
0.0
A (n=14)
B (n=15)
C (n=11)
surface

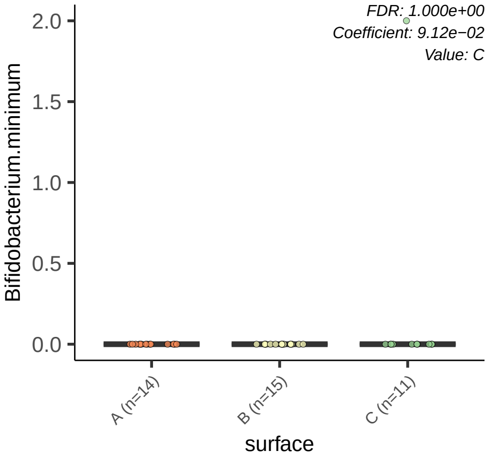

FDR: 1.000e+00
Coefficient: 9.12e−02
Value: C
Bifidobacterium.minimum
2.0
1.5
1.0
0.5
0.0
A (n=14)
B (n=15)
C (n=11)
surface

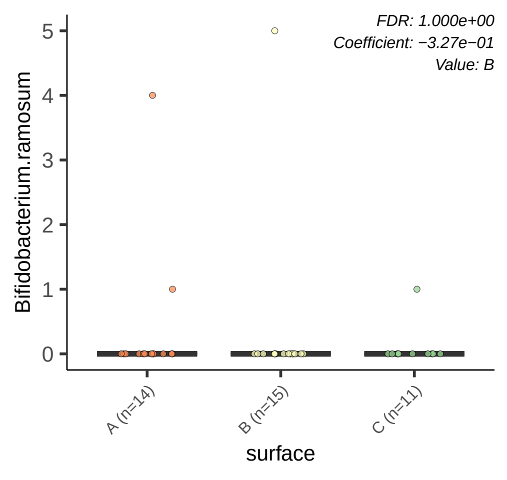
FDR: 1.000e+00
Coefficient: −3.27e−01
Value: B
Bifidobacterium.ramosum
0
1
2
3
4
5
A (n=14)
B (n=15)
C (n=11)
surface

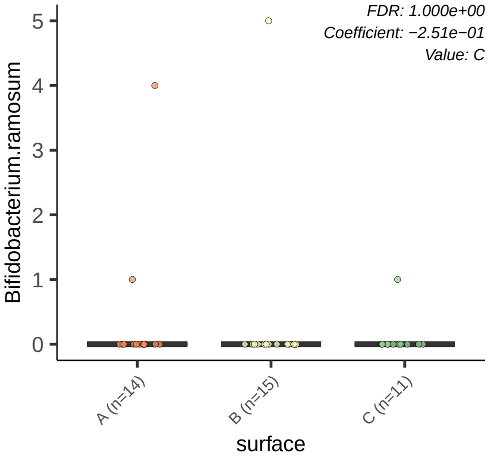

FDR: 1.000e+00
Coefficient: −2.51e−01
Value: C
Bifidobacterium.ramosum
0
1
2
3
4
5
A (n=14)
B (n=15)
C (n=11)
surface

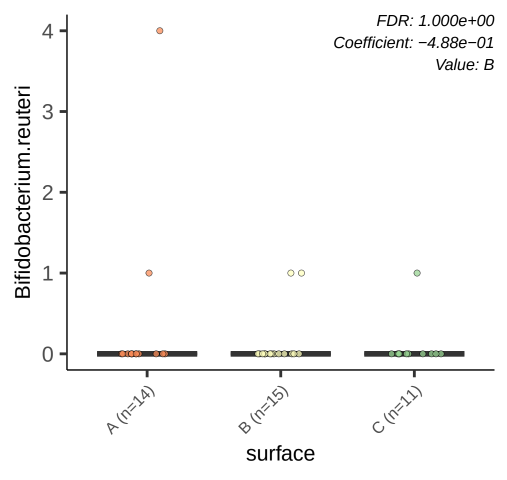
FDR: 1.000e+00
Coefficient: −4.88e−01
Value: B
Bifidobacterium.reuteri
0
1
2
3
4
A (n=14)
B (n=15)
C (n=11)
surface

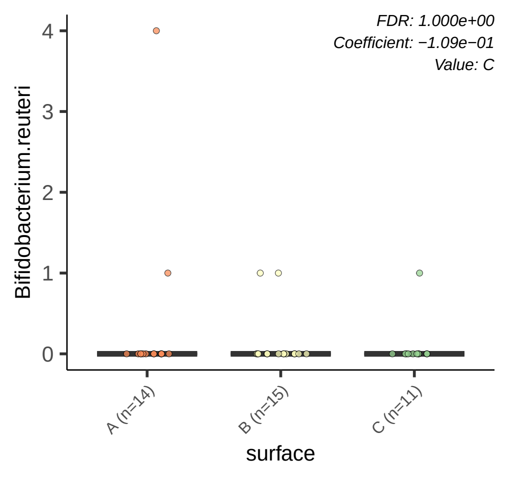

FDR: 1.000e+00
Coefficient: −1.09e−01
Value: C
Bifidobacterium.reuteri
0
1
2
3
4
A (n=14)
B (n=15)
C (n=11)
surface

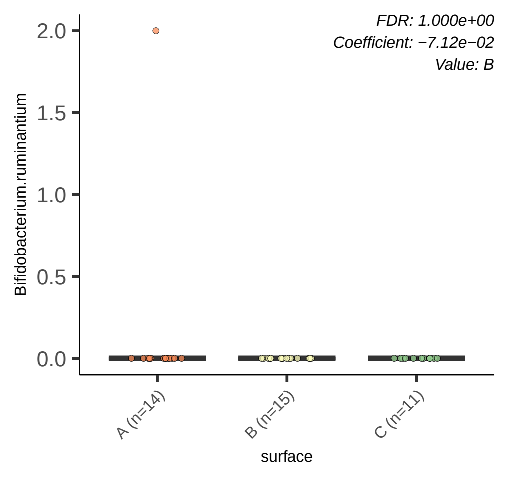

FDR: 1.000e+00
Coefficient: −7.12e−02
Value: B
Bifidobacterium.ruminantium
2.0
1.5
1.0
0.5
0.0
A (n=14)
B (n=15)
C (n=11)
surface

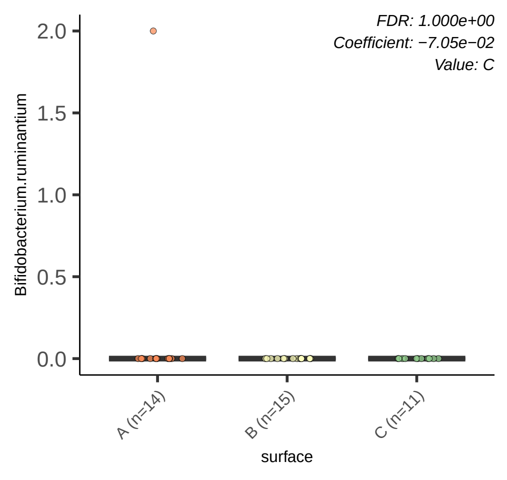

FDR: 1.000e+00
Coefficient: −7.05e−02
Value: C
Bifidobacterium.ruminantium
2.0
1.5
1.0
0.5
0.0
A (n=14)
B (n=15)
C (n=11)
surface

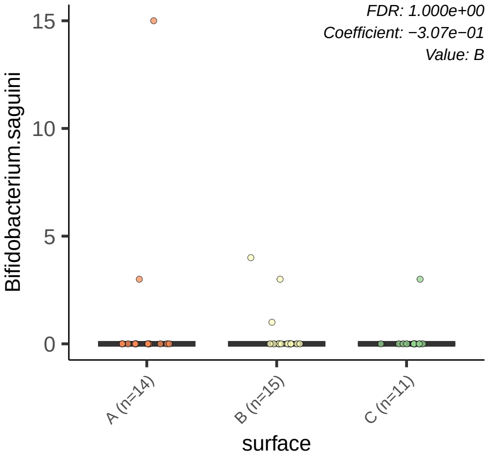
FDR: 1.000e+00
Coefficient: −3.07e−01
Value: B
Bifidobacterium.saguini
15
10
5
0
A (n=14)
B (n=15)
C (n=11)
surface

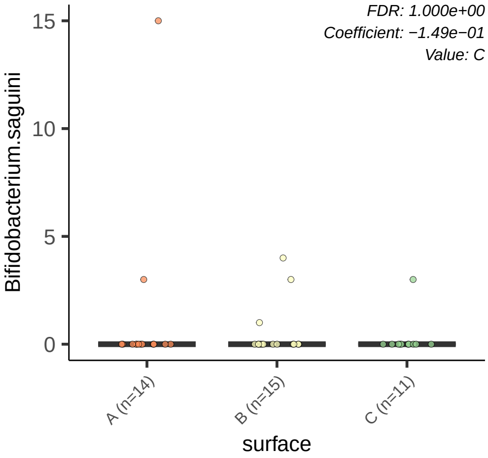
FDR: 1.000e+00
Coefficient: −1.49e−01
Value: C
Bifidobacterium.saguini
15
10
5
0
A (n=14)
B (n=15)
C (n=11)
surface

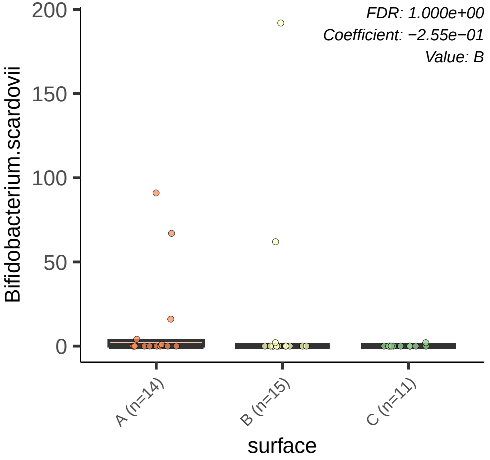

FDR: 1.000e+00
Coefficient: −2.55e−01
Value: B
Bifidobacterium.scardovii
200
150
100
50
0
A (n=14)
B (n=15)
C (n=11)
surface

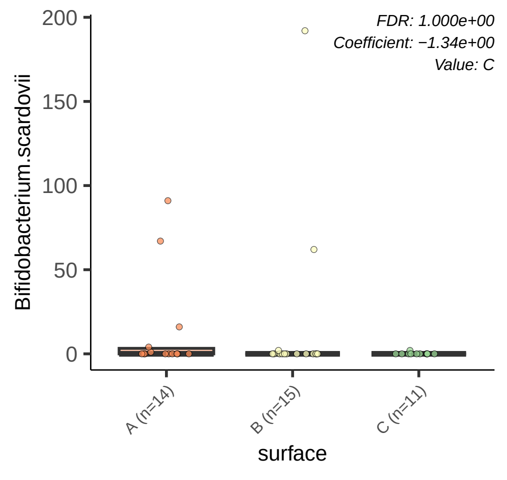
FDR: 1.000e+00
Coefficient: −1.34e+00
Value: C
Bifidobacterium.scardovii
200
150
100
50
0
A (n=14)
B (n=15)
C (n=11)
surface

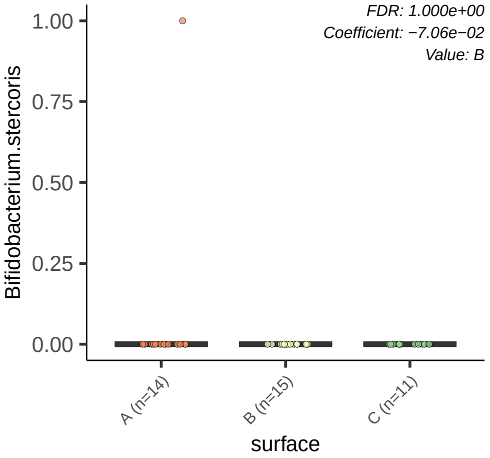
FDR: 1.000e+00
Coefficient: −7.06e−02
Value: B
Bifidobacterium.stercoris
1.00
0.75
0.50
0.25
0.00
A (n=14)
B (n=15)
C (n=11)
surface

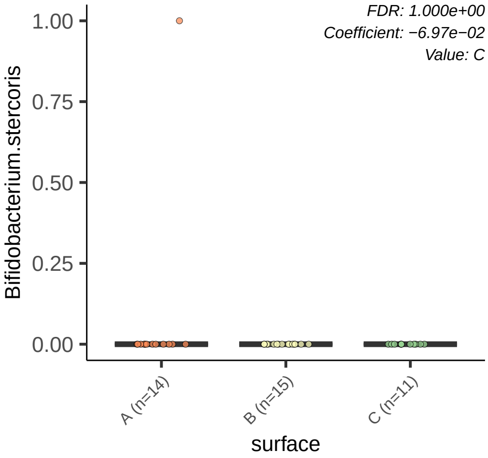
FDR: 1.000e+00
Coefficient: −6.97e−02
Value: C
Bifidobacterium.stercoris
1.00
0.75
0.50
0.25
0.00
A (n=14)
B (n=15)
C (n=11)
surface

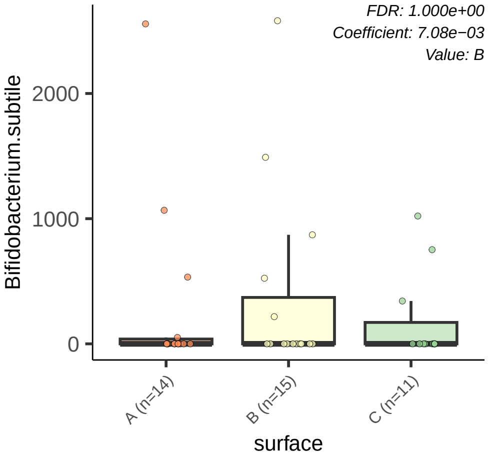

FDR: 1.000e+00
Coefficient: 7.08e−03
Value: B
Bifidobacterium.subtile
2000
1000
0
A (n=14)
B (n=15)
C (n=11)
surface

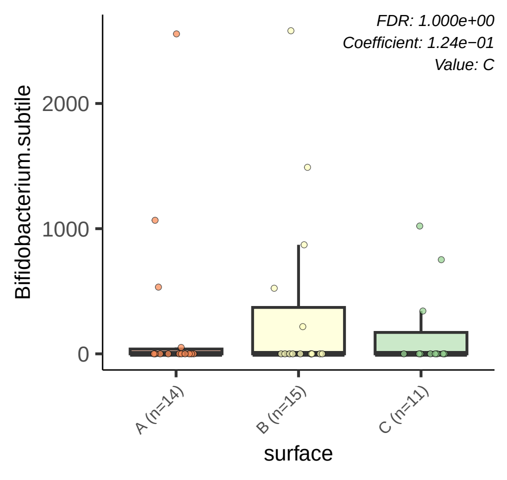

FDR: 1.000e+00
Coefficient: 1.24e−01
Value: C
Bifidobacterium.subtile
2000
1000
0
A (n=14)
B (n=15)
C (n=11)
surface

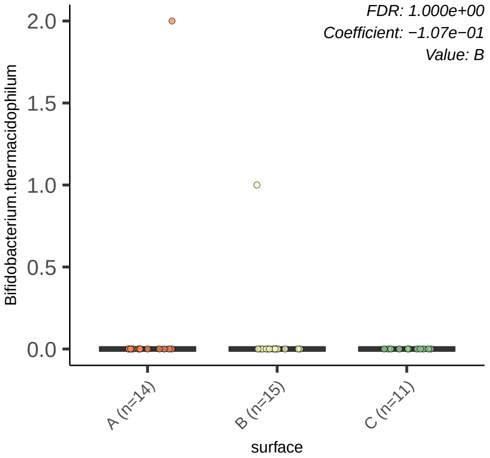
FDR: 1.000e+00
Coefficient: −1.07e−01
Value: B
Bifidobacterium.thermacidophilum
2.0
1.5
1.0
0.5
0.0
A (n=14)
B (n=15)
C (n=11)
surface

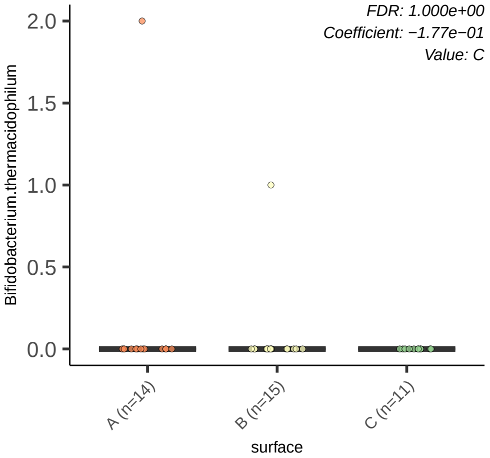
FDR: 1.000e+00
Coefficient: −1.77e−01
Value: C
Bifidobacterium.thermacidophilum
2.0
1.5
1.0
0.5
0.0
A (n=14)
B (n=15)
C (n=11)
surface

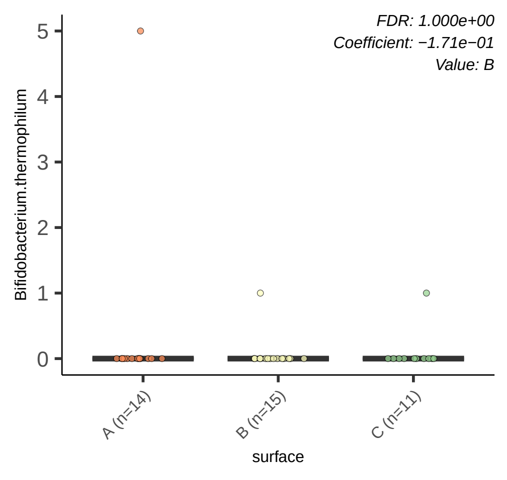

FDR: 1.000e+00
Coefficient: −1.71e−01
Value: B
Bifidobacterium.thermophilum
0
1
2
3
4
5
A (n=14)
B (n=15)
C (n=11)
surface

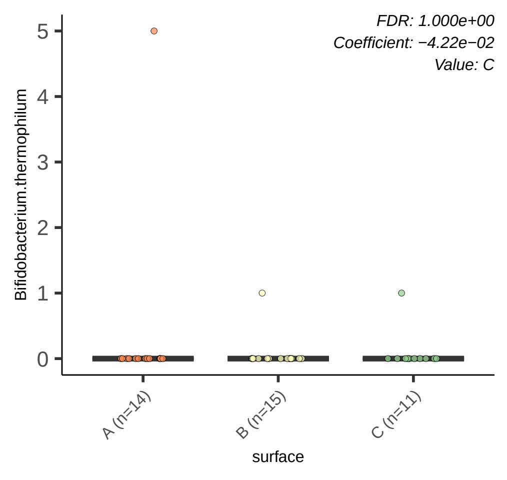
FDR: 1.000e+00
Coefficient: −4.22e−02
Value: C
Bifidobacterium.thermophilum
0
1
2
3
4
5
A (n=14)
B (n=15)
C (n=11)
surface

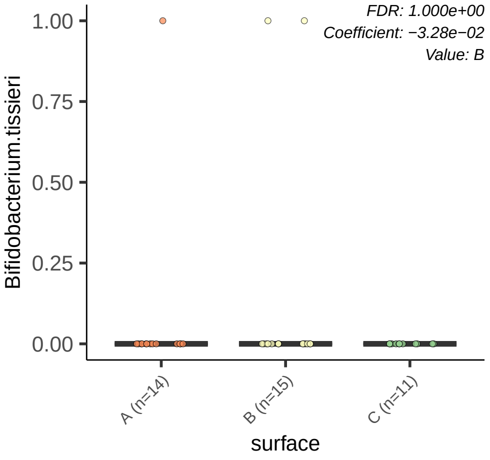
FDR: 1.000e+00
Coefficient: −3.28e−02
Value: B
Bifidobacterium.tissieri
1.00
0.75
0.50
0.25
0.00
A (n=14)
B (n=15)
C (n=11)
surface

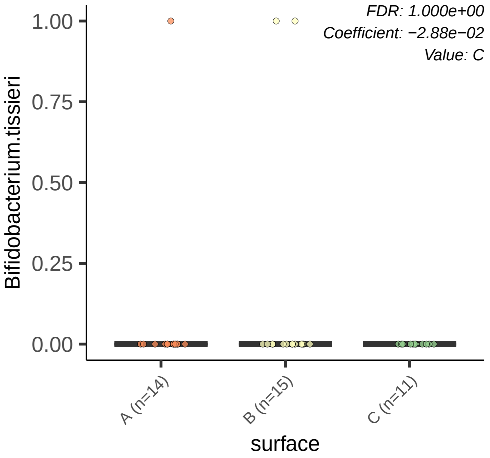

FDR: 1.000e+00
Coefficient: −2.88e−02
Value: C
Bifidobacterium.tissieri
1.00
0.75
0.50
0.25
0.00
A (n=14)
B (n=15)
C (n=11)
surface

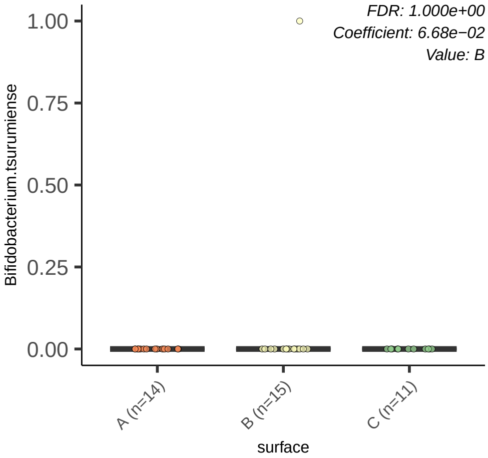
FDR: 1.000e+00
Coefficient: 6.68e−02
Value: B
Bifidobacterium.tsurumiense
1.00
0.75
0.50
0.25
0.00
A (n=14)
B (n=15)
C (n=11)
surface

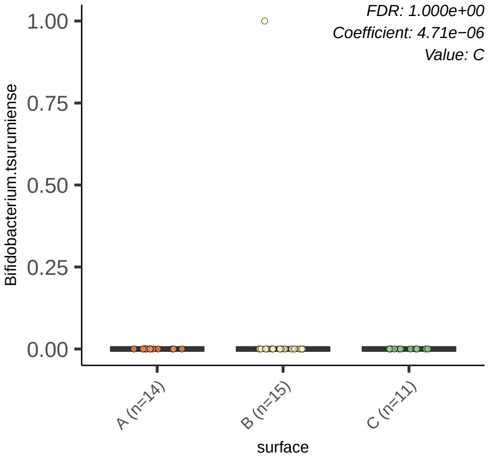
FDR: 1.000e+00
Coefficient: 4.71e−06
Value: C
1.00
0.75
0.50
0.25
0.00
Bifidobacterium.tsurumiense
A (n=14)
B (n=15)
C (n=11)
surface

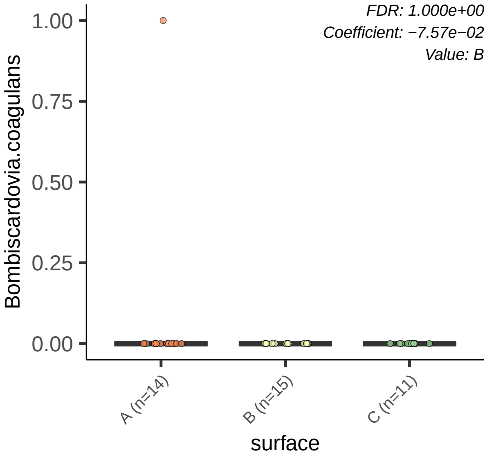

FDR: 1.000e+00
Coefficient: −7.57e−02
Value: B
Bombiscardovia.coagulans
1.00
0.75
0.50
0.25
0.00
A (n=14)
B (n=15)
C (n=11)
surface

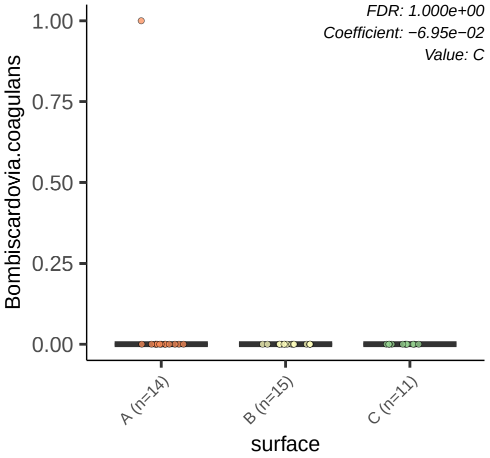

FDR: 1.000e+00
Coefficient: −6.95e−02
Value: C
Bombiscardovia.coagulans
1.00
0.75
0.50
0.25
0.00
A (n=14)
B (n=15)
C (n=11)
surface

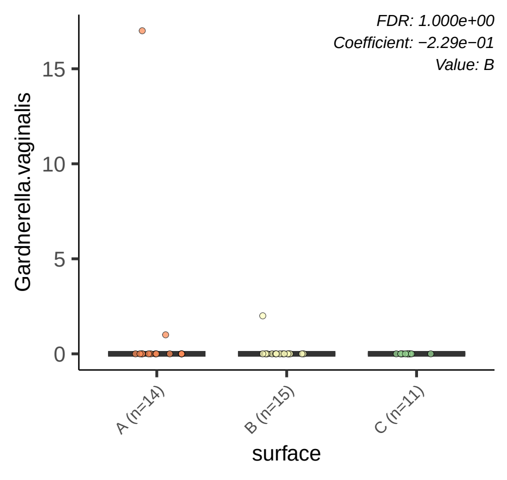

FDR: 1.000e+00
Coefficient: −2.29e−01
Value: B
Gardnerella.vaginalis
15
10
5
0
A (n=14)
B (n=15)
C (n=11)
surface

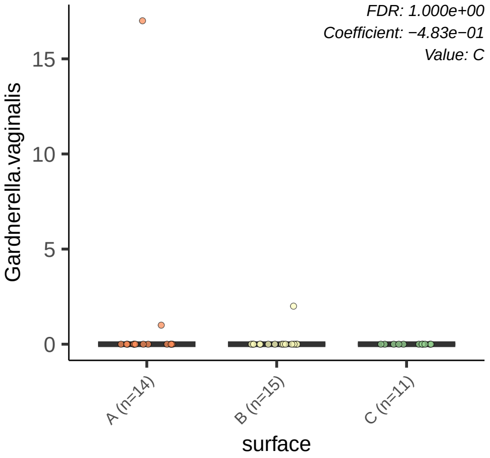

FDR: 1.000e+00
Coefficient: −4.83e−01
Value: C
Gardnerella.vaginalis
15
10
5
0
A (n=14)
B (n=15)
C (n=11)
surface

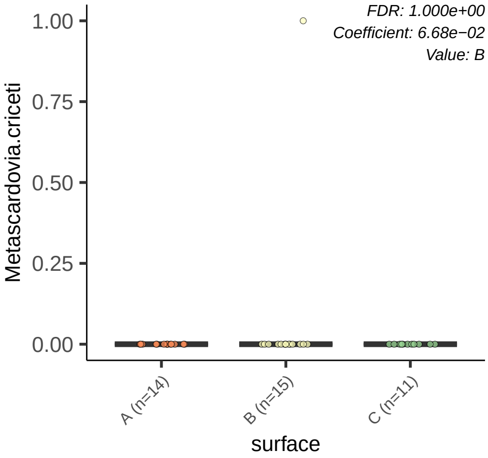

FDR: 1.000e+00
Coefficient: 6.68e−02
Value: B
Metascardovia.criceti
1.00
0.75
0.50
0.25
0.00
A (n=14)
B (n=15)
C (n=11)
surface

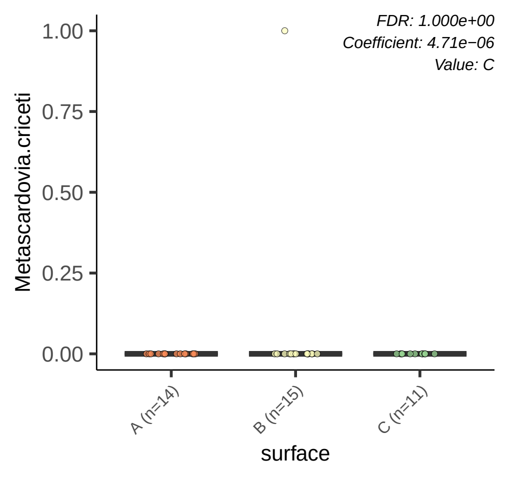
FDR: 1.000e+00
Coefficient: 4.71e−06
Value: C
Metascardovia.criceti
1.00
0.75
0.50
0.25
0.00
A (n=14)
B (n=15)
C (n=11)
surface

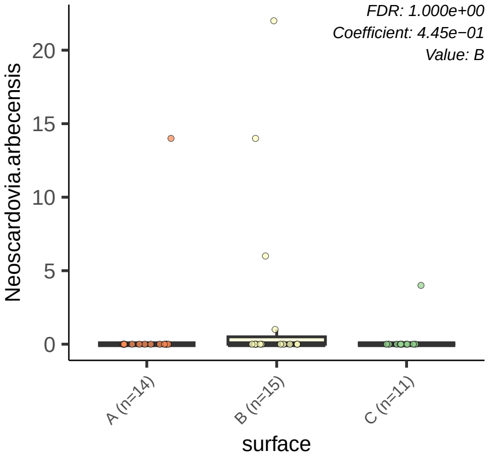

FDR: 1.000e+00
Coefficient: 4.45e−01
Value: B
Neoscardovia.arbecensis
20
15
10
5
0
A (n=14)
B (n=15)
C (n=11)
surface

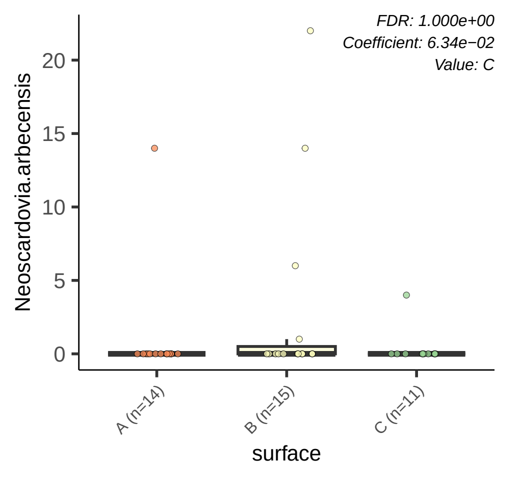
FDR: 1.000e+00
Coefficient: 6.34e−02
Value: C
Neoscardovia.arbecensis
20
15
10
5
0
A (n=14)
B (n=15)
C (n=11)
surface

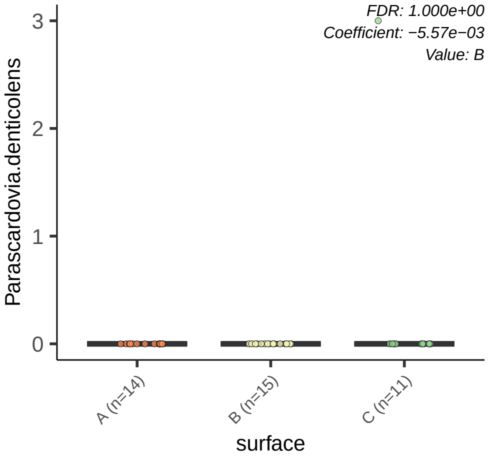
FDR: 1.000e+00
Coefficient: −5.57e−03
Value: B
Parascardovia.denticolens
3
2
1
0
A (n=14)
B (n=15)
C (n=11)
surface

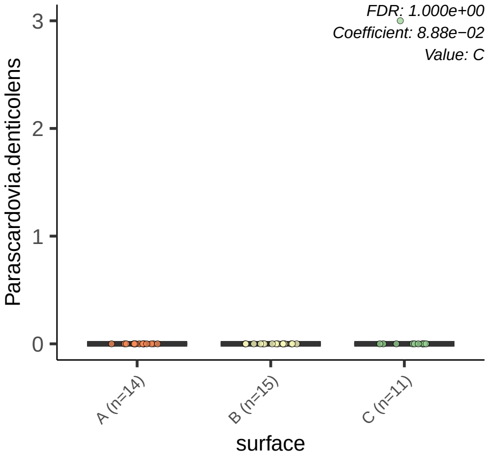

FDR: 1.000e+00
Coefficient: 8.88e−02
Value: C
Parascardovia.denticolens
3
2
1
0
A (n=14)
B (n=15)
C (n=11)
surface

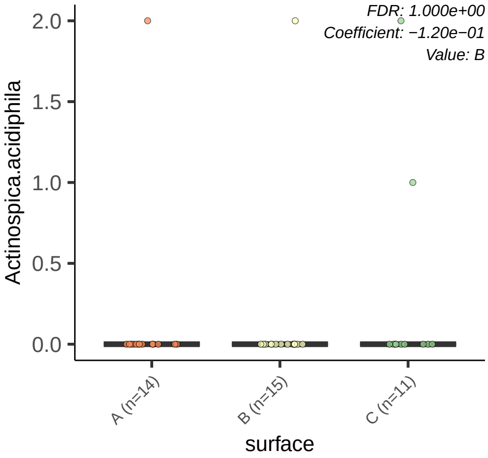

FDR: 1.000e+00
Coefficient: −1.20e−01
Value: B
Actinospica.acidiphila
2.0
1.5
1.0
0.5
0.0
A (n=14)
B (n=15)
C (n=11)
surface

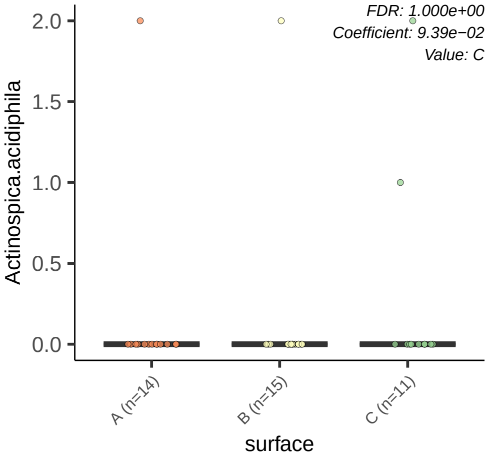

FDR: 1.000e+00
Coefficient: 9.39e−02
Value: C
Actinospica.acidiphila
2.0
1.5
1.0
0.5
0.0
A (n=14)
B (n=15)
C (n=11)
surface

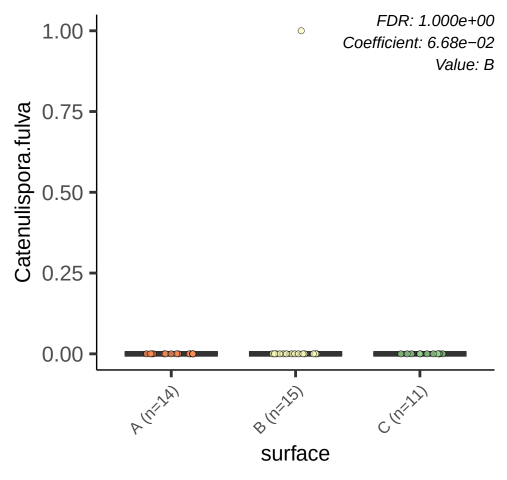
FDR: 1.000e+00
Coefficient: 6.68e−02
Value: B
Catenulispora.fulva
1.00
0.75
0.50
0.25
0.00
A (n=14)
B (n=15)
C (n=11)
surface

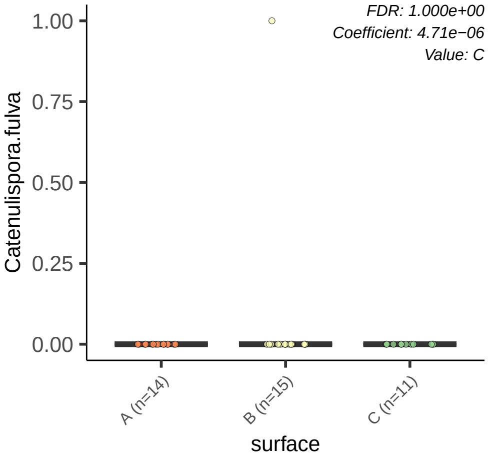
FDR: 1.000e+00
Coefficient: 4.71e−06
Value: C
Catenulispora.fulva
1.00
0.75
0.50
0.25
0.00
A (n=14)
B (n=15)
C (n=11)
surface

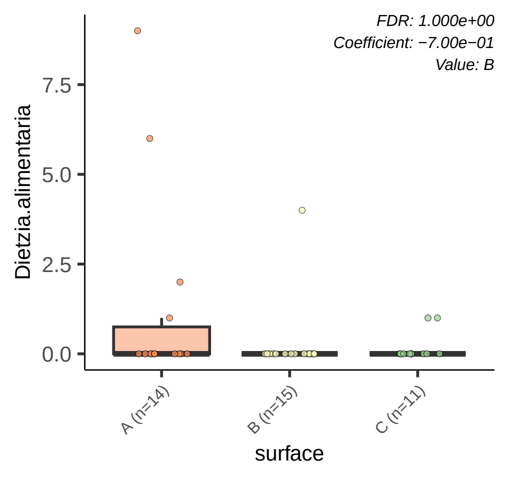

FDR: 1.000e+00
Coefficient: −7.00e−01
Value: B
Dietzia.alimentaria
7.5
5.0
2.5
0.0
A (n=14)
B (n=15)
C (n=11)
surface

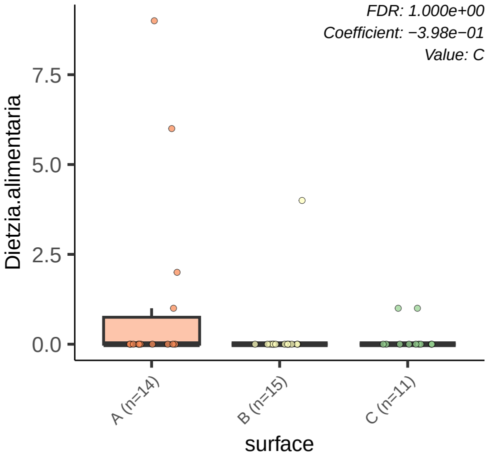

FDR: 1.000e+00
Coefficient: −3.98e−01
Value: C
Dietzia.alimentaria
7.5
5.0
2.5
0.0
A (n=14)
B (n=15)
C (n=11)
surface

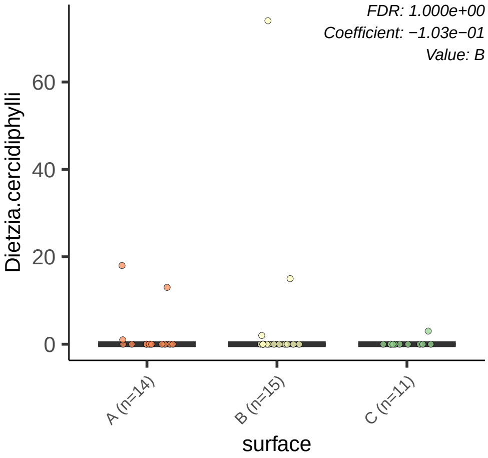

FDR: 1.000e+00
Coefficient: −1.03e−01
Value: B
Dietzia.cercidiphylli
0
20
40
60
A (n=14)
B (n=15)
C (n=11)
surface

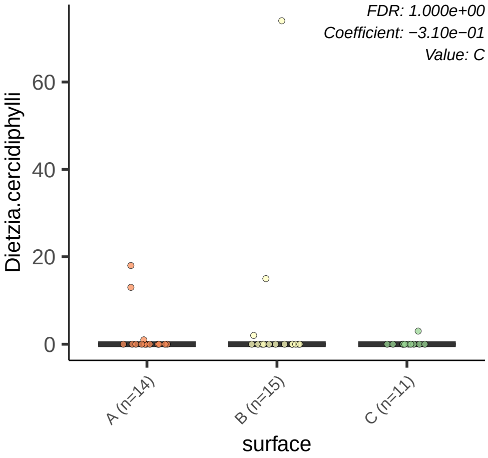

FDR: 1.000e+00
Coefficient: −3.10e−01
Value: C
Dietzia.cercidiphylli
0
20
40
60
A (n=14)
B (n=15)
C (n=11)
surface

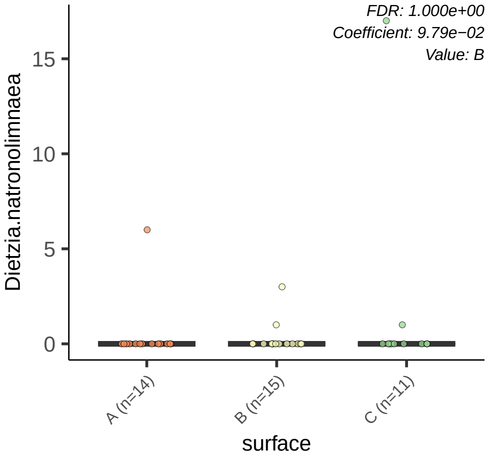

FDR: 1.000e+00
Coefficient: 9.79e−02
Value: B
Dietzia.natronolimnaea
15
10
5
0
A (n=14)
B (n=15)
C (n=11)
surface

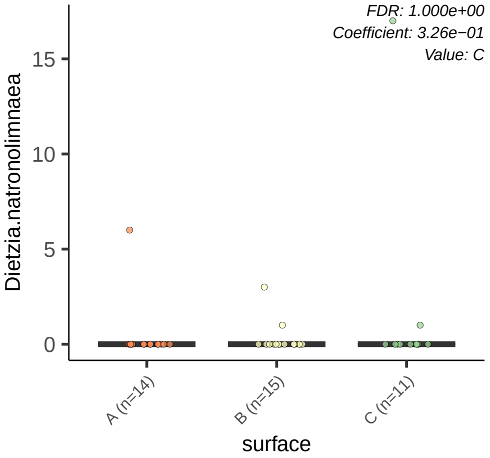

FDR: 1.000e+00
Coefficient: 3.26e−01
Value: C
Dietzia.natronolimnaea
15
10
5
0
A (n=14)
B (n=15)
C (n=11)
surface

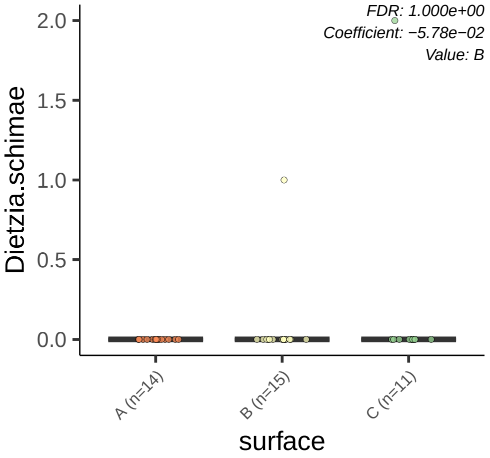

FDR: 1.000e+00
Coefficient: −5.78e−02
Value: B
Dietzia.schimae
2.0
1.5
1.0
0.5
0.0
A (n=14)
B (n=15)
C (n=11)
surface

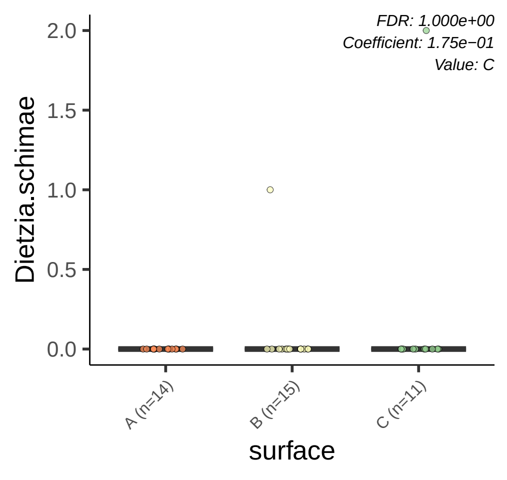
FDR: 1.000e+00
Coefficient: 1.75e−01
Value: C
Dietzia.schimae
2.0
1.5
1.0
0.5
0.0
A (n=14)
B (n=15)
C (n=11)
surface

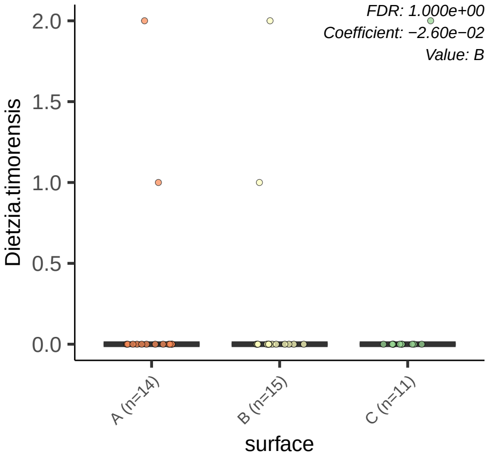
FDR: 1.000e+00
Coefficient: −2.60e−02
Value: B
Dietzia.timorensis
2.0
1.5
1.0
0.5
0.0
A (n=14)
B (n=15)
C (n=11)
surface

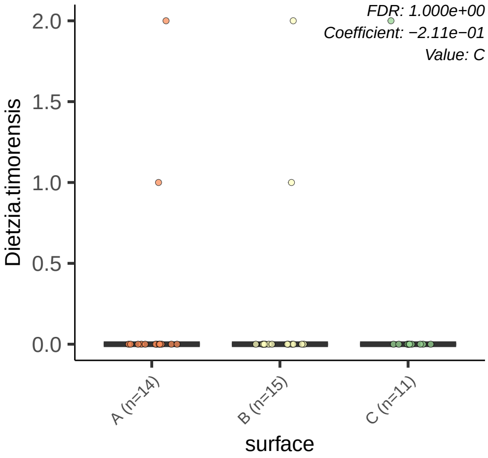

FDR: 1.000e+00
Coefficient: −2.11e−01
Value: C
Dietzia.timorensis
2.0
1.5
1.0
0.5
0.0
A (n=14)
B (n=15)
C (n=11)
surface

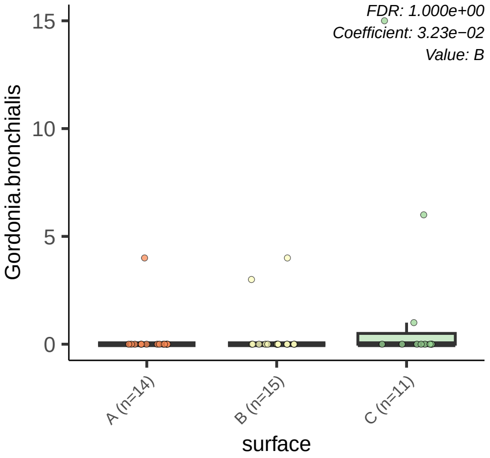

FDR: 1.000e+00
Coefficient: 3.23e−02
Value: B
Gordonia.bronchialis
15
10
5
0
A (n=14)
B (n=15)
C (n=11)
surface

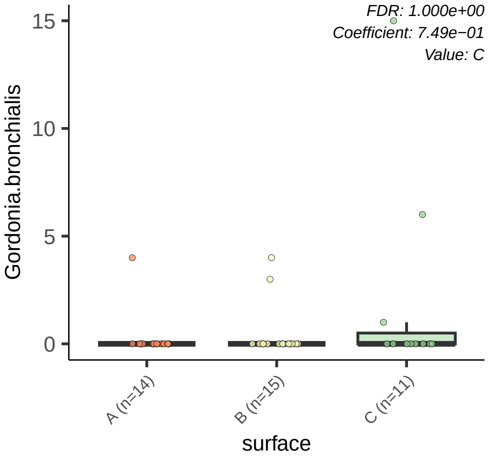
FDR: 1.000e+00
Coefficient: 7.49e−01
Value: C
Gordonia.bronchialis
15
10
5
0
A (n=14)
B (n=15)
C (n=11)
surface

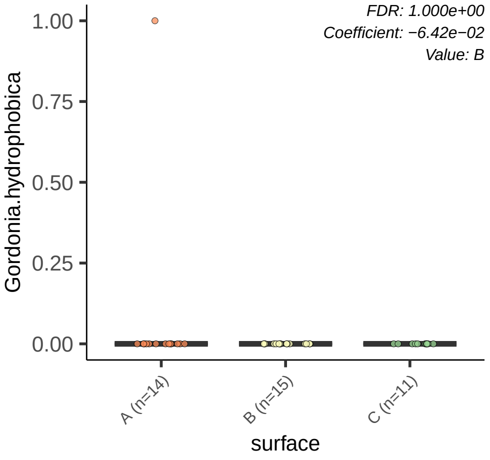
FDR: 1.000e+00
Coefficient: −6.42e−02
Value: B
Gordonia.hydrophobica
1.00
0.75
0.50
0.25
0.00
A (n=14)
B (n=15)
C (n=11)
surface

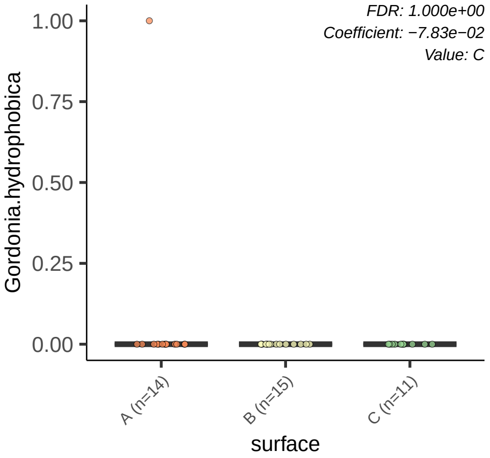

FDR: 1.000e+00
Coefficient: −7.83e−02
Value: C
Gordonia.hydrophobica
1.00
0.75
0.50
0.25
0.00
A (n=14)
B (n=15)
C (n=11)
surface

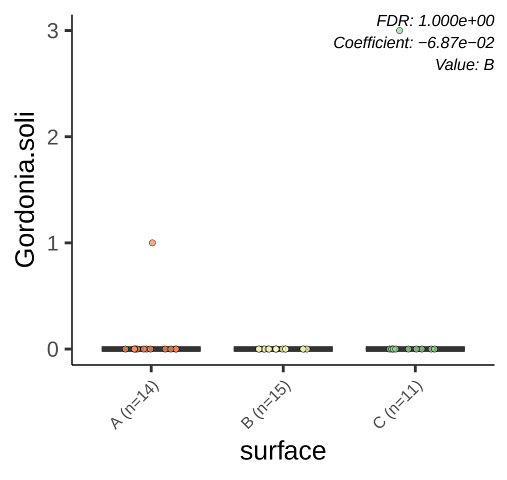
FDR: 1.000e+00
Coefficient: −6.87e−02
Value: B
Gordonia.soli
0
1
2
3
A (n=14)
B (n=15)
C (n=11)
surface

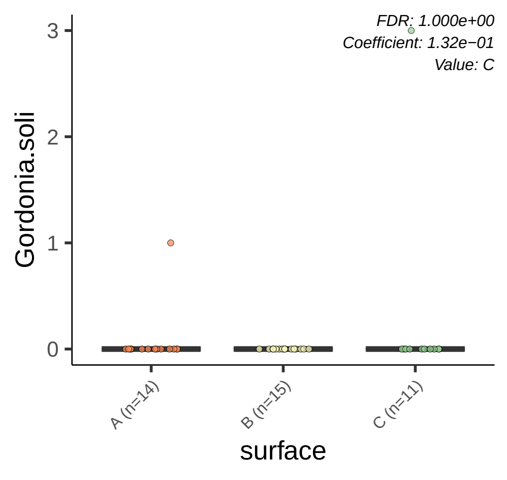
FDR: 1.000e+00
Coefficient: 1.32e−01
Value: C
Gordonia.soli
3
2
1
0
A (n=14)
B (n=15)
C (n=11)
surface

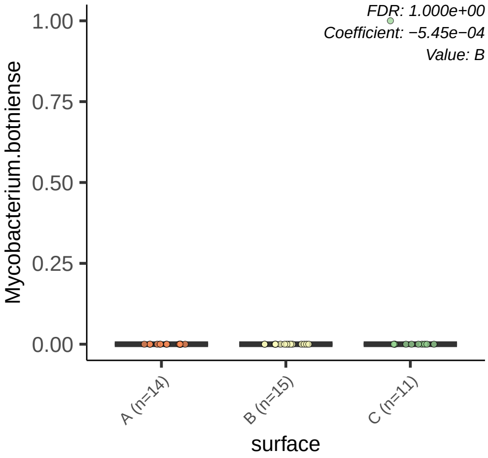
FDR: 1.000e+00
Coefficient: −5.45e−04
Value: B
Mycobacterium.botniense
1.00
0.75
0.50
0.25
0.00
A (n=14)
B (n=15)
C (n=11)
surface

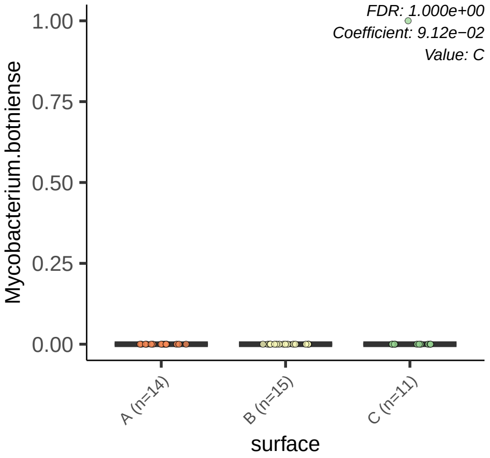

FDR: 1.000e+00
Coefficient: 9.12e−02
Value: C
1.00
0.75
0.50
0.25
0.00
Mycobacterium.botniense
A (n=14)
B (n=15)
C (n=11)
surface

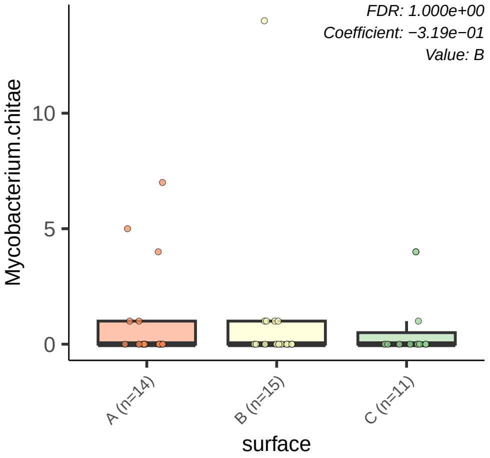
FDR: 1.000e+00
Coefficient: −3.19e−01
Value: B
Mycobacterium.chitae
0
5
10
A (n=14)
B (n=15)
C (n=11)
surface

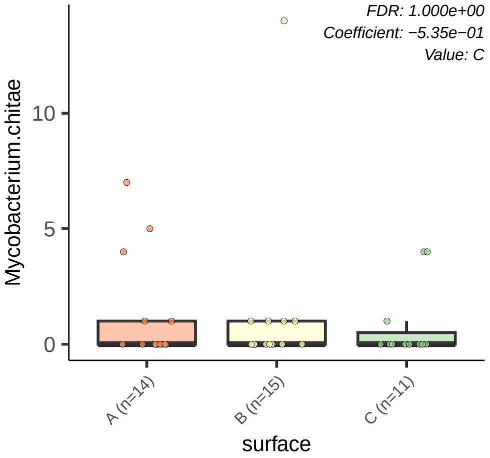
FDR: 1.000e+00
Coefficient: −5.35e−01
Value: C
Mycobacterium.chitae
10
5
0
A (n=14)
B (n=15)
C (n=11)
surface

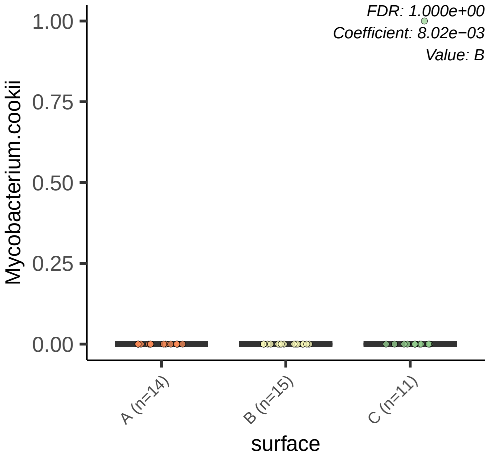

FDR: 1.000e+00
Coefficient: 8.02e−03
Value: B
1.00
0.75
0.50
0.25
0.00
Mycobacterium.cookii
A (n=14)
B (n=15)
C (n=11)
surface

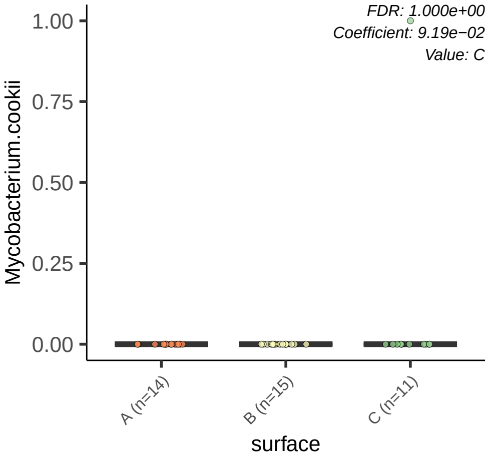

FDR: 1.000e+00
Coefficient: 9.19e−02
Value: C
1.00
0.75
0.50
0.25
0.00
Mycobacterium.cookii
A (n=14)
B (n=15)
C (n=11)
surface

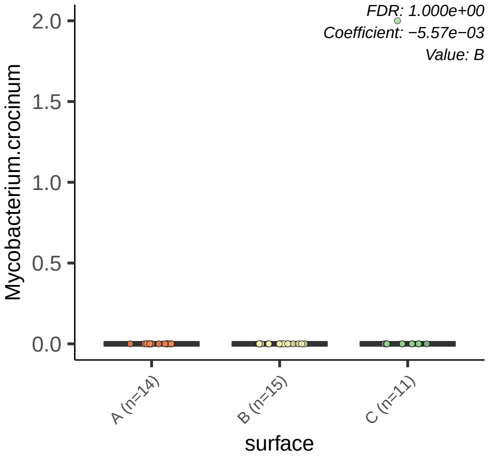

FDR: 1.000e+00
Coefficient: −5.57e−03
Value: B
2.0
1.5
1.0
0.5
0.0
Mycobacterium.crocinum
A (n=14)
B (n=15)
C (n=11)
surface

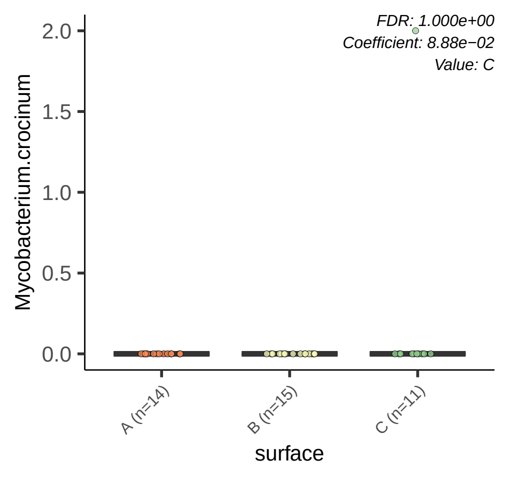

FDR: 1.000e+00
Coefficient: 8.88e−02
Value: C
Mycobacterium.crocinum
2.0
1.5
1.0
0.5
0.0
A (n=14)
B (n=15)
C (n=11)
surface

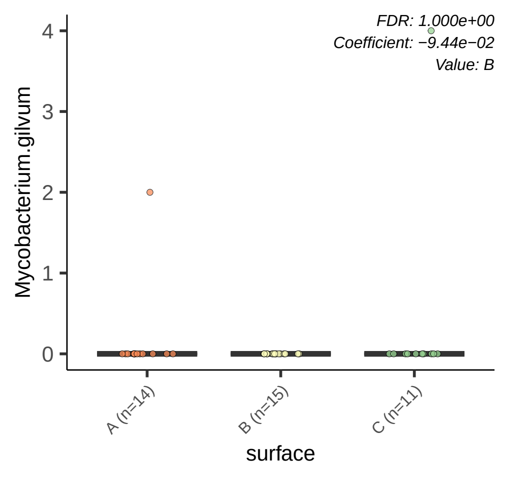
FDR: 1.000e+00
Coefficient: −9.44e−02
Value: B
Mycobacterium.gilvum
0
1
2
3
4
A (n=14)
B (n=15)
C (n=11)
surface

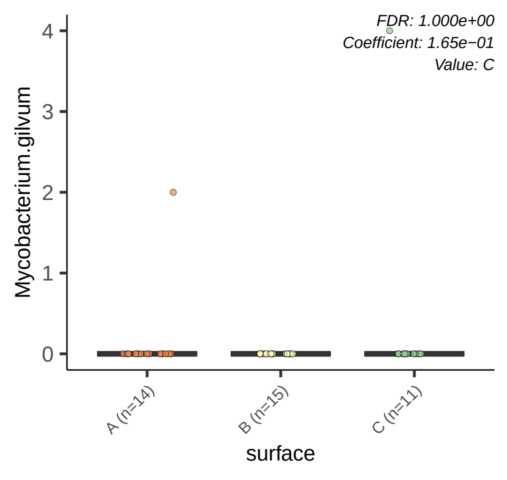
FDR: 1.000e+00
Coefficient: 1.65e−01
Value: C
Mycobacterium.gilvum
0
1
2
3
4
A (n=14)
B (n=15)
C (n=11)
surface

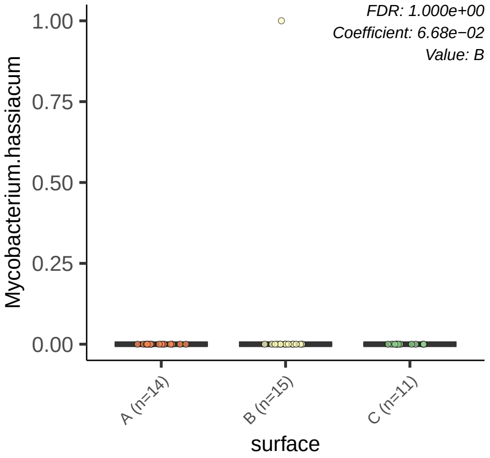

FDR: 1.000e+00
Coefficient: 6.68e−02
Value: B
Mycobacterium.hassiacum
1.00
0.75
0.50
0.25
0.00
A (n=14)
B (n=15)
C (n=11)
surface

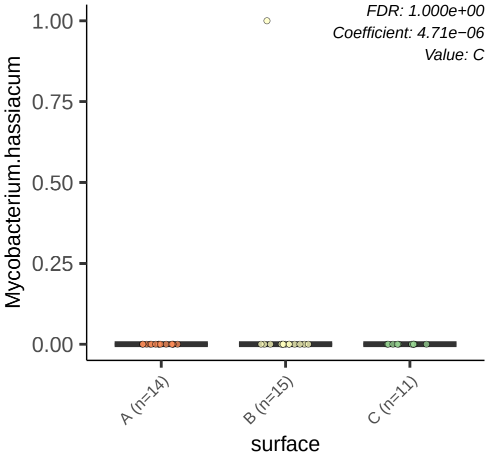
FDR: 1.000e+00
Coefficient: 4.71e−06
Value: C
Mycobacterium.hassiacum
1.00
0.75
0.50
0.25
0.00
A (n=14)
B (n=15)
C (n=11)
surface

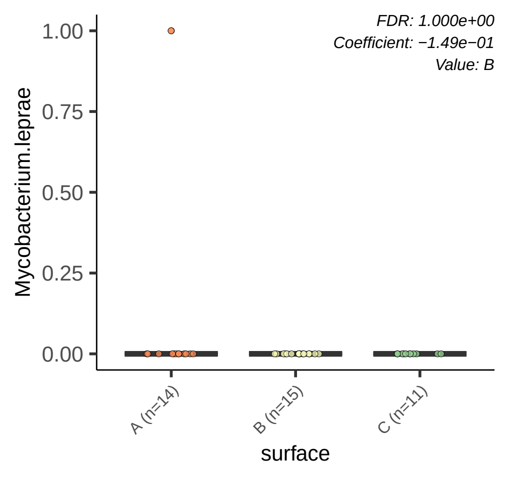

FDR: 1.000e+00
Coefficient: −1.49e−01
Value: B
Mycobacterium.leprae
1.00
0.75
0.50
0.25
0.00
A (n=14)
B (n=15)
C (n=11)
surface

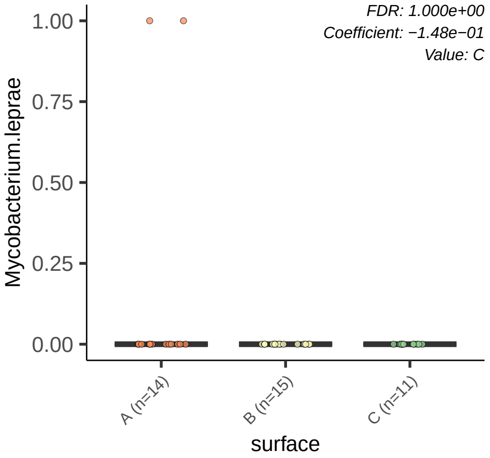
FDR: 1.000e+00
Coefficient: −1.48e−01
Value: C
Mycobacterium.leprae
1.00
0.75
0.50
0.25
0.00
A (n=14)
B (n=15)
C (n=11)
surface

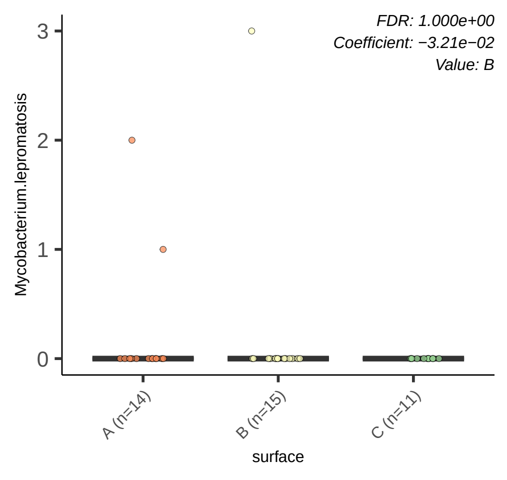

FDR: 1.000e+00
Coefficient: −3.21e−02
Value: B
Mycobacterium.lepromatosis
3
2
1
0
A (n=14)
B (n=15)
C (n=11)
surface

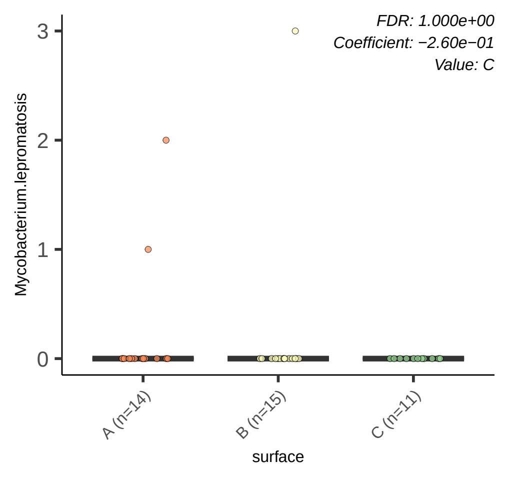

FDR: 1.000e+00
Coefficient: −2.60e−01
Value: C
Mycobacterium.lepromatosis
3
2
1
0
A (n=14)
B (n=15)
C (n=11)
surface

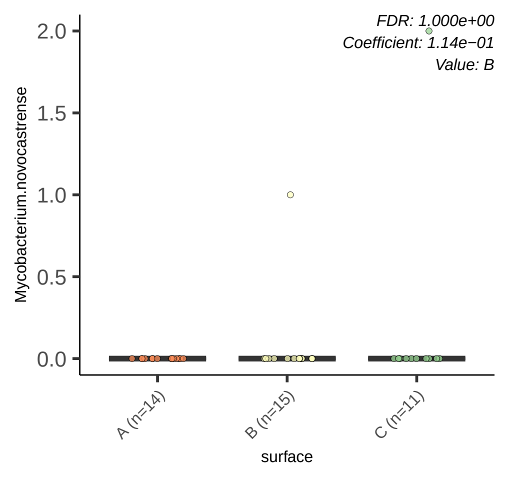
FDR: 1.000e+00
Coefficient: 1.14e−01
Value: B
Mycobacterium.novocastrense
2.0
1.5
1.0
0.5
0.0
A (n=14)
B (n=15)
C (n=11)
surface

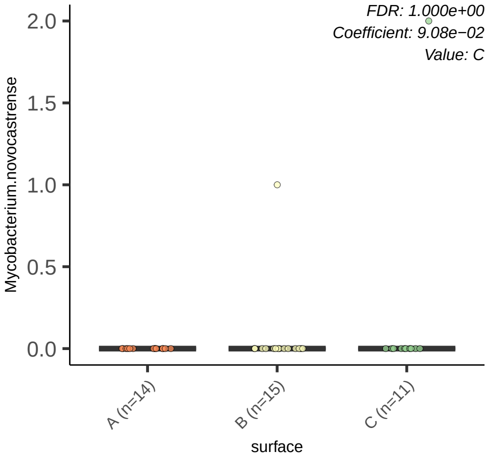
FDR: 1.000e+00
Coefficient: 9.08e−02
Value: C
Mycobacterium.novocastrense
2.0
1.5
1.0
0.5
0.0
A (n=14)
B (n=15)
C (n=11)
surface

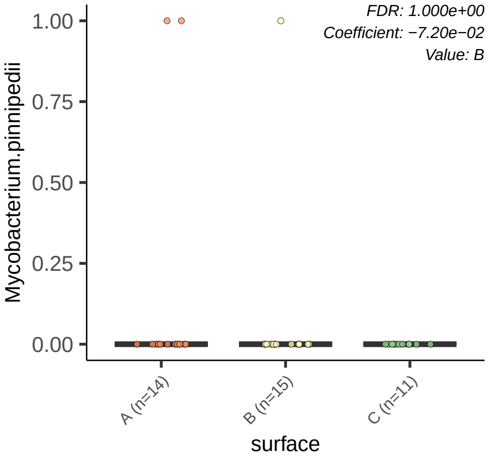
FDR: 1.000e+00
Coefficient: −7.20e−02
Value: B
Mycobacterium.pinnipedii
1.00
0.75
0.50
0.25
0.00
A (n=14)
B (n=15)
C (n=11)
surface

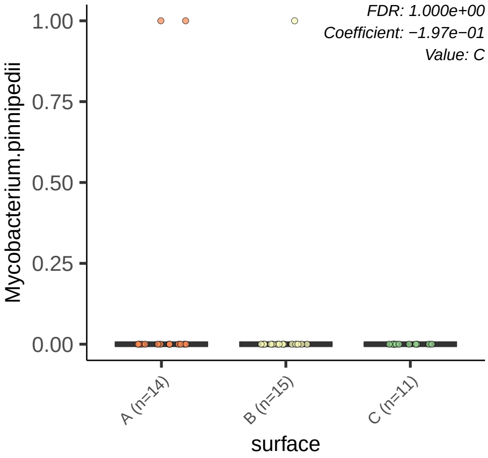
FDR: 1.000e+00
Coefficient: −1.97e−01
Value: C
Mycobacterium.pinnipedii
1.00
0.75
0.50
0.25
0.00
A (n=14)
B (n=15)
C (n=11)
surface

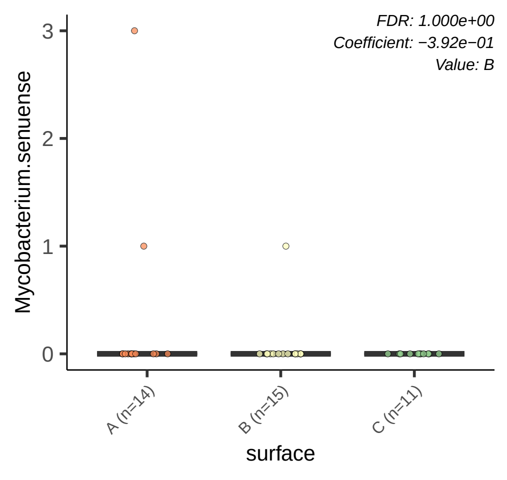
FDR: 1.000e+00
Coefficient: −3.92e−01
Value: B
Mycobacterium.senuense
3
2
1
0
A (n=14)
B (n=15)
C (n=11)
surface

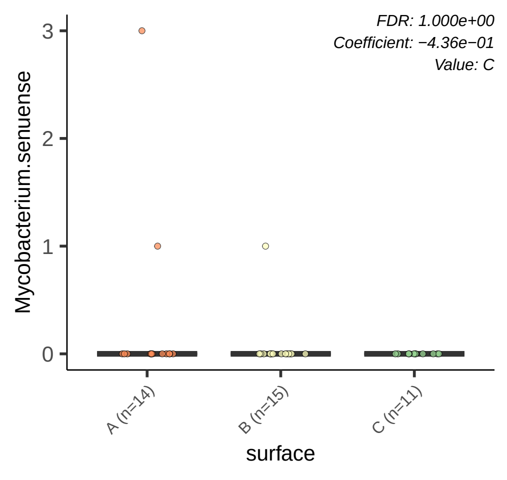

FDR: 1.000e+00
Coefficient: −4.36e−01
Value: C
Mycobacterium.senuense
3
2
1
0
A (n=14)
B (n=15)
C (n=11)
surface

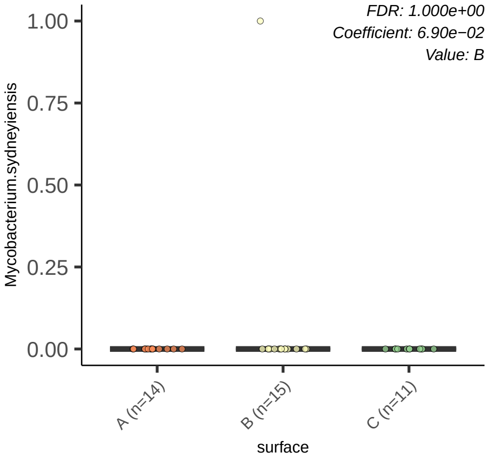

FDR: 1.000e+00
Coefficient: 6.90e−02
Value: B
1.00
0.75
0.50
0.25
0.00
Mycobacterium.sydneyiensis
A (n=14)
B (n=15)
C (n=11)
surface

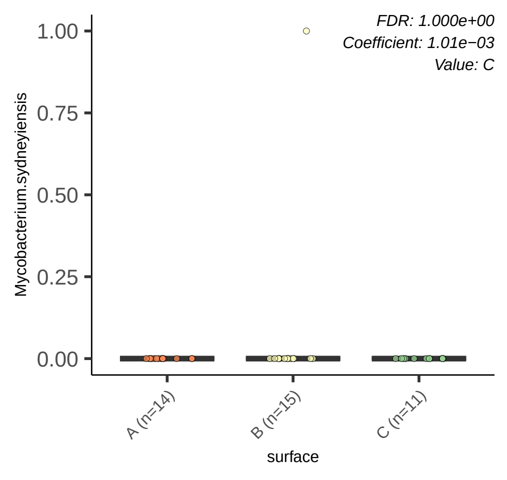

FDR: 1.000e+00
Coefficient: 1.01e−03
Value: C
1.00
0.75
0.50
0.25
0.00
Mycobacterium.sydneyiensis
A (n=14)
B (n=15)
C (n=11)
surface

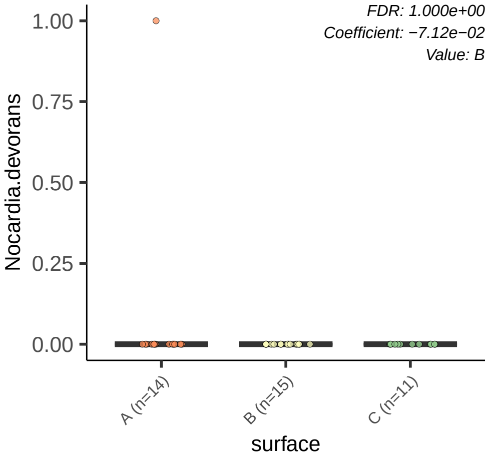
FDR: 1.000e+00
Coefficient: −7.12e−02
Value: B
Nocardia.devorans
1.00
0.75
0.50
0.25
0.00
A (n=14)
B (n=15)
C (n=11)
surface

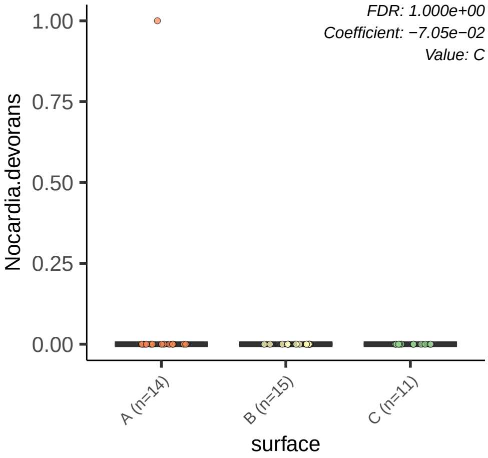

FDR: 1.000e+00
Coefficient: −7.05e−02
Value: C
Nocardia.devorans
1.00
0.75
0.50
0.25
0.00
A (n=14)
B (n=15)
C (n=11)
surface

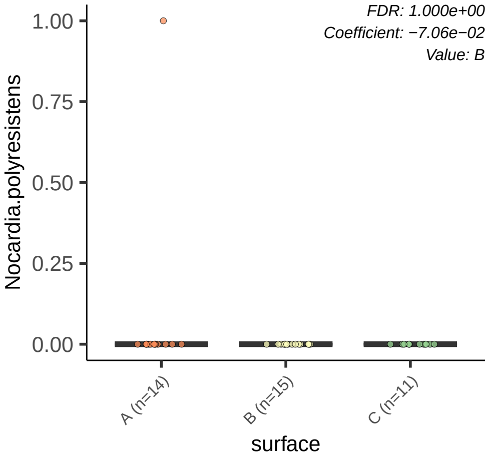
FDR: 1.000e+00
Coefficient: −7.06e−02
Value: B
Nocardia.polyresistens
1.00
0.75
0.50
0.25
0.00
A (n=14)
B (n=15)
C (n=11)
surface

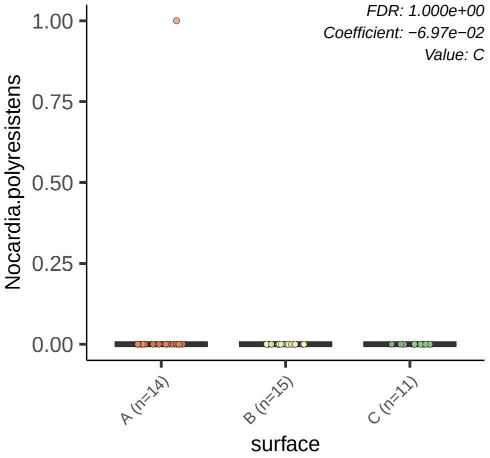
FDR: 1.000e+00
Coefficient: −6.97e−02
Value: C
Nocardia.polyresistens
1.00
0.75
0.50
0.25
0.00
A (n=14)
B (n=15)
C (n=11)
surface

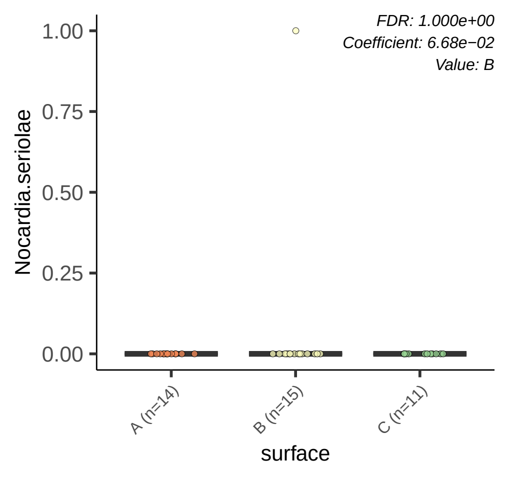
FDR: 1.000e+00
Coefficient: 6.68e−02
Value: B
Nocardia.seriolae
1.00
0.75
0.50
0.25
0.00
A (n=14)
B (n=15)
C (n=11)
surface

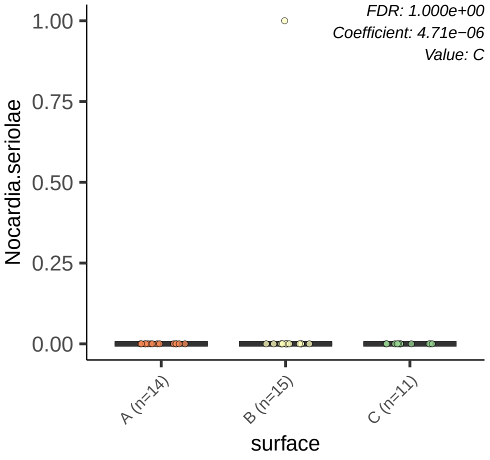
FDR: 1.000e+00
Coefficient: 4.71e−06
Value: C
Nocardia.seriolae
1.00
0.75
0.50
0.25
0.00
A (n=14)
B (n=15)
C (n=11)
surface

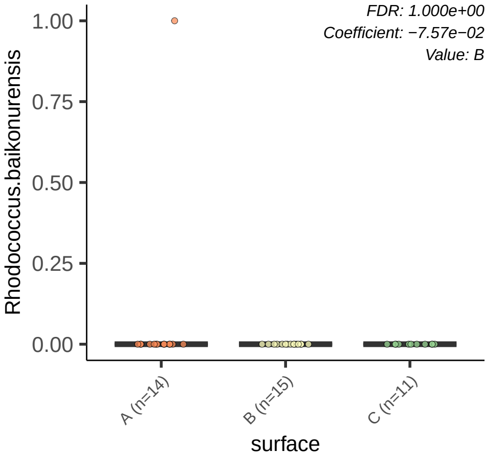
FDR: 1.000e+00
Coefficient: −7.57e−02
Value: B
Rhodococcus.baikonurensis
1.00
0.75
0.50
0.25
0.00
A (n=14)
B (n=15)
C (n=11)
surface

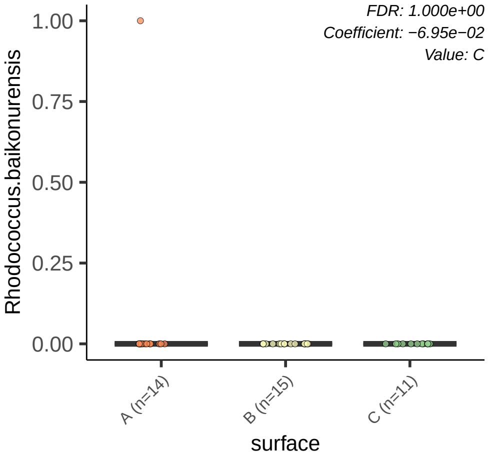

FDR: 1.000e+00
Coefficient: −6.95e−02
Value: C
Rhodococcus.baikonurensis
1.00
0.75
0.50
0.25
0.00
A (n=14)
B (n=15)
C (n=11)
surface

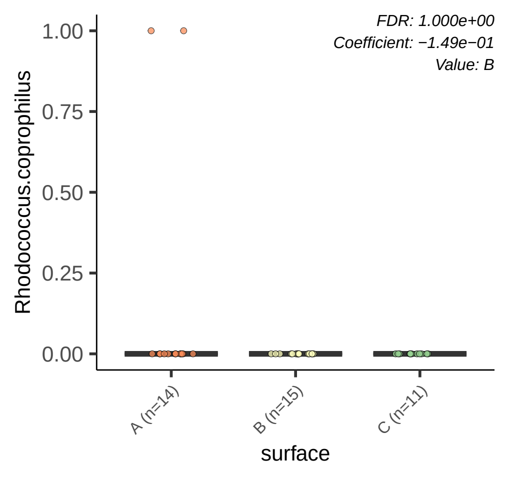
FDR: 1.000e+00
Coefficient: −1.49e−01
Value: B
Rhodococcus.coprophilus
1.00
0.75
0.50
0.25
0.00
A (n=14)
B (n=15)
C (n=11)
surface

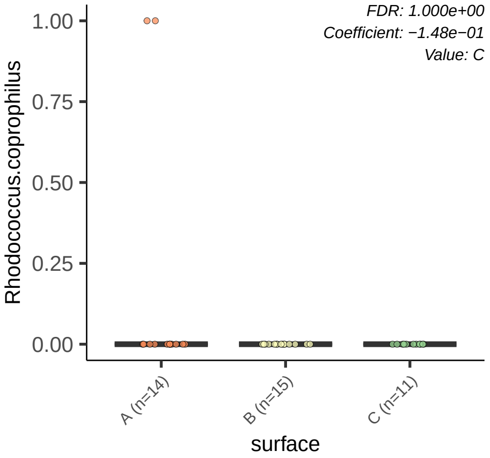
FDR: 1.000e+00
Coefficient: −1.48e−01
Value: C
Rhodococcus.coprophilus
1.00
0.75
0.50
0.25
0.00
A (n=14)
B (n=15)
C (n=11)
surface

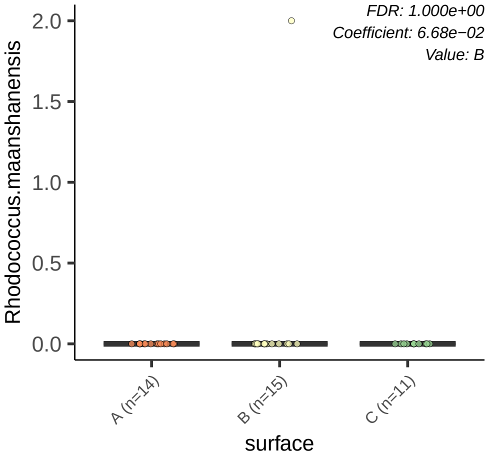
FDR: 1.000e+00
Coefficient: 6.68e−02
Value: B
Rhodococcus.maanshanensis
2.0
1.5
1.0
0.5
0.0
A (n=14)
B (n=15)
C (n=11)
surface

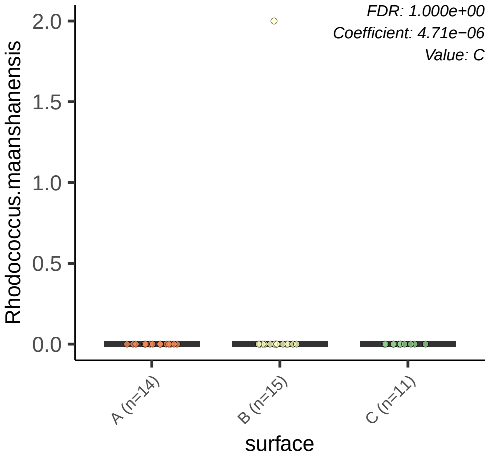
FDR: 1.000e+00
Coefficient: 4.71e−06
Value: C
Rhodococcus.maanshanensis
2.0
1.5
1.0
0.5
0.0
A (n=14)
B (n=15)
C (n=11)
surface

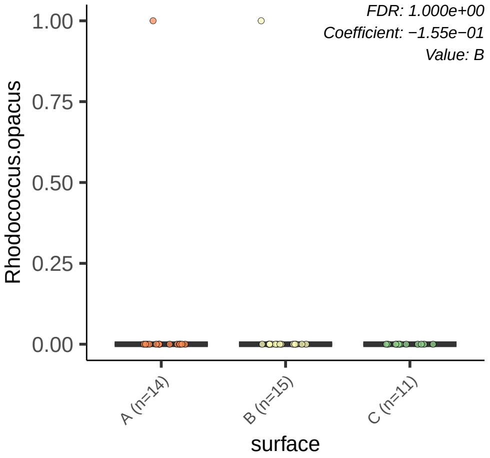
FDR: 1.000e+00
Coefficient: −1.55e−01
Value: B
Rhodococcus.opacus
1.00
0.75
0.50
0.25
0.00
A (n=14)
B (n=15)
C (n=11)
surface

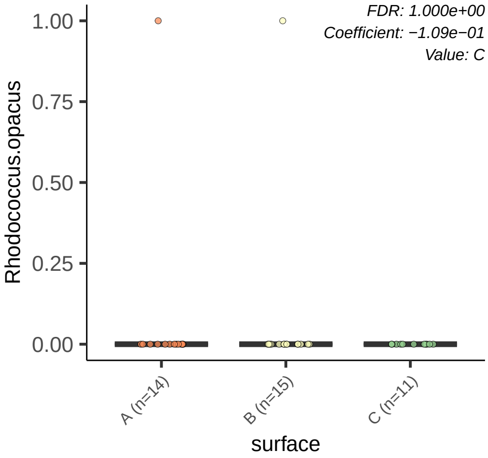
FDR: 1.000e+00
Coefficient: −1.09e−01
Value: C
Rhodococcus.opacus
1.00
0.75
0.50
0.25
0.00
A (n=14)
B (n=15)
C (n=11)
surface

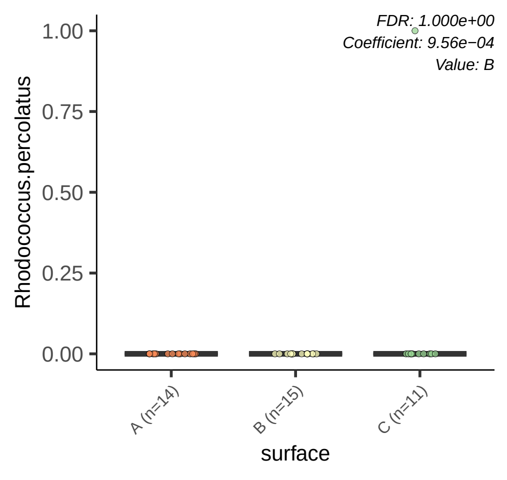

FDR: 1.000e+00
Coefficient: 9.56e−04
Value: B
Rhodococcus.percolatus
1.00
0.75
0.50
0.25
0.00
A (n=14)
B (n=15)
C (n=11)
surface

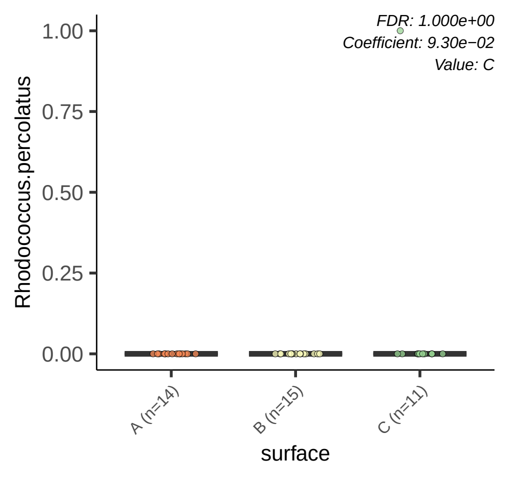

FDR: 1.000e+00
Coefficient: 9.30e−02
Value: C
Rhodococcus.percolatus
1.00
0.75
0.50
0.25
0.00
A (n=14)
B (n=15)
C (n=11)
surface

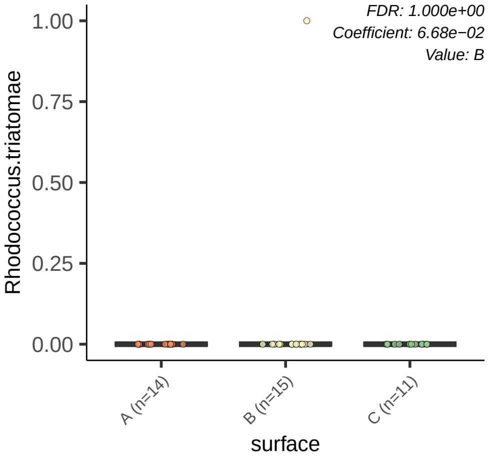

FDR: 1.000e+00
Coefficient: 6.68e−02
Value: B
Rhodococcus.triatomae
1.00
0.75
0.50
0.25
0.00
A (n=14)
B (n=15)
C (n=11)
surface

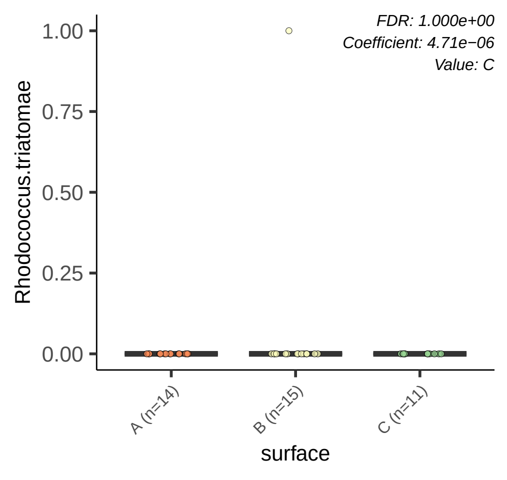
FDR: 1.000e+00
Coefficient: 4.71e−06
Value: C
1.00
0.75
0.50
0.25
0.00
Rhodococcus.triatomae
A (n=14)
B (n=15)
C (n=11)
surface

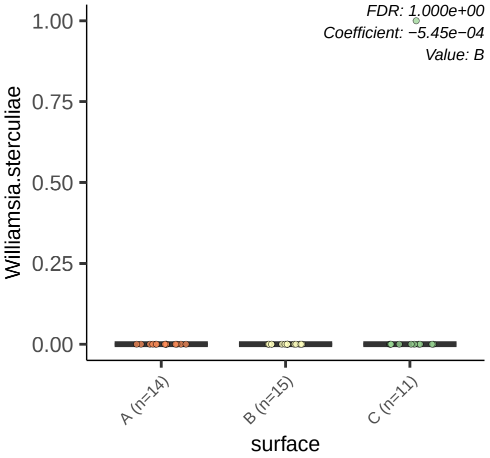
FDR: 1.000e+00
Coefficient: −5.45e−04
Value: B
Williamsia.sterculiae
1.00
0.75
0.50
0.25
0.00
A (n=14)
B (n=15)
C (n=11)
surface

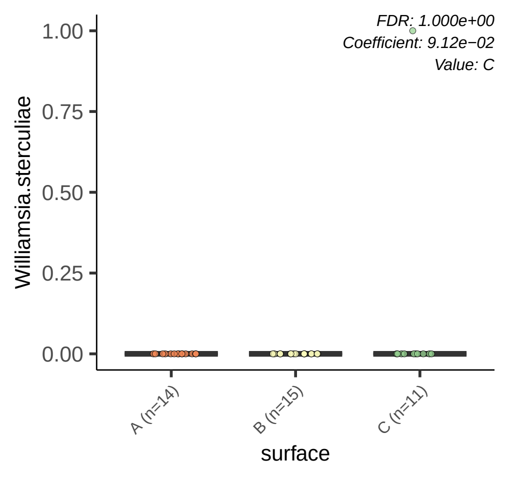
FDR: 1.000e+00
Coefficient: 9.12e−02
Value: C
Williamsia.sterculiae
1.00
0.75
0.50
0.25
0.00
A (n=14)
B (n=15)
C (n=11)
surface

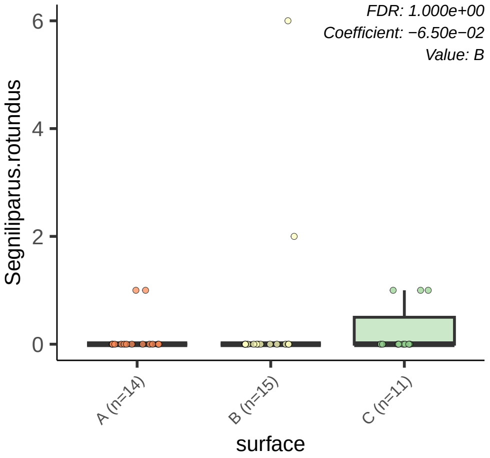

FDR: 1.000e+00
Coefficient: −6.50e−02
Value: B
Segniliparus.rotundus
0
2
4
6
A (n=14)
B (n=15)
C (n=11)
surface

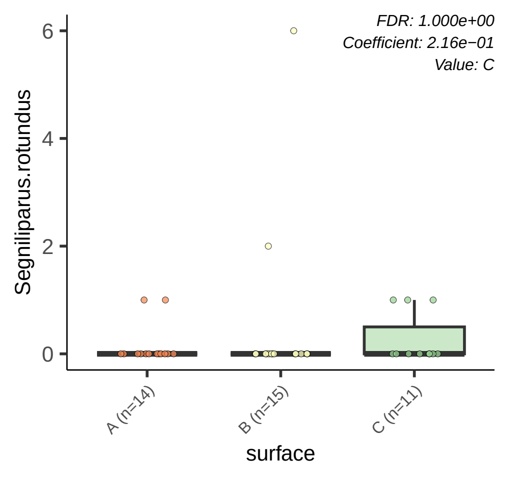
FDR: 1.000e+00
Coefficient: 2.16e−01
Value: C
Segniliparus.rotundus
6
4
2
0
A (n=14)
B (n=15)
C (n=11)
surface

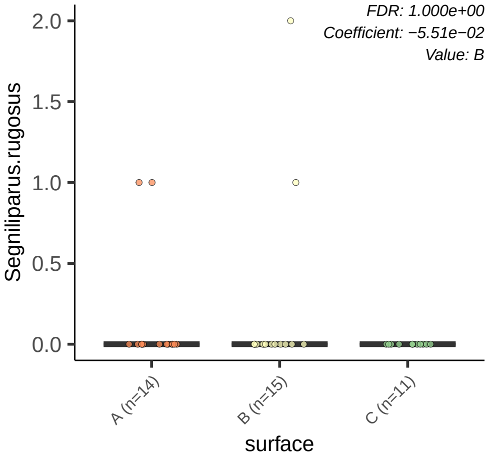

FDR: 1.000e+00
Coefficient: −5.51e−02
Value: B
Segniliparus.rugosus
2.0
1.5
1.0
0.5
0.0
A (n=14)
B (n=15)
C (n=11)
surface

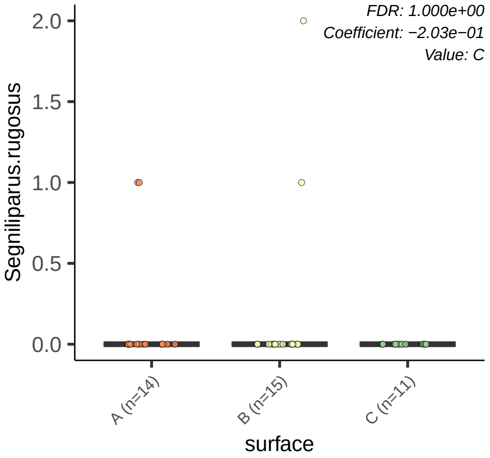

FDR: 1.000e+00
Coefficient: −2.03e−01
Value: C
Segniliparus.rugosus
2.0
1.5
1.0
0.5
0.0
A (n=14)
B (n=15)
C (n=11)
surface

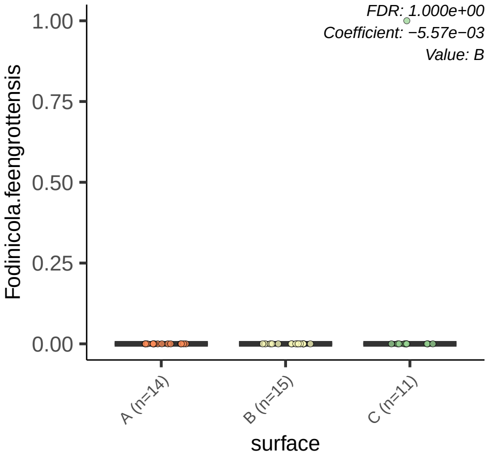
FDR: 1.000e+00
Coefficient: −5.57e−03
Value: B
Fodinicola.feengrottensis
1.00
0.75
0.50
0.25
0.00
A (n=14)
B (n=15)
C (n=11)
surface

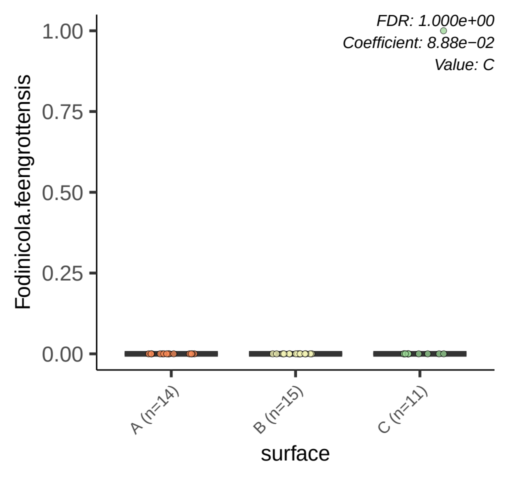
FDR: 1.000e+00
Coefficient: 8.88e−02
Value: C
Fodinicola.feengrottensis
1.00
0.75
0.50
0.25
0.00
A (n=14)
B (n=15)
C (n=11)
surface

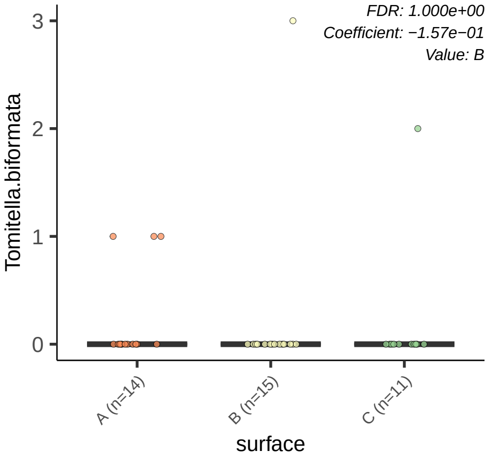
FDR: 1.000e+00
Coefficient: −1.57e−01
Value: B
Tomitella.biformata
0
1
2
3
A (n=14)
B (n=15)
C (n=11)
surface

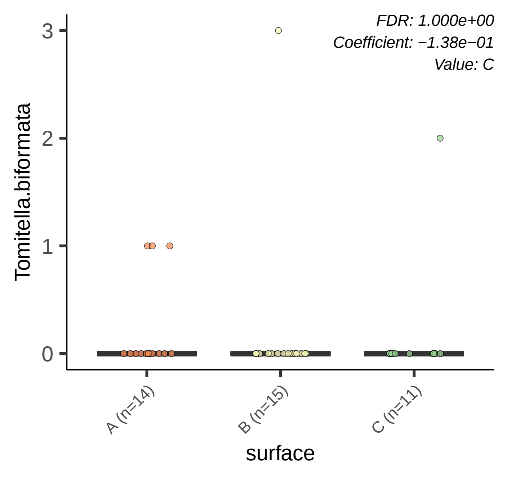
FDR: 1.000e+00
Coefficient: −1.38e−01
Value: C
Tomitella.biformata
3
2
1
0
A (n=14)
B (n=15)
C (n=11)
surface

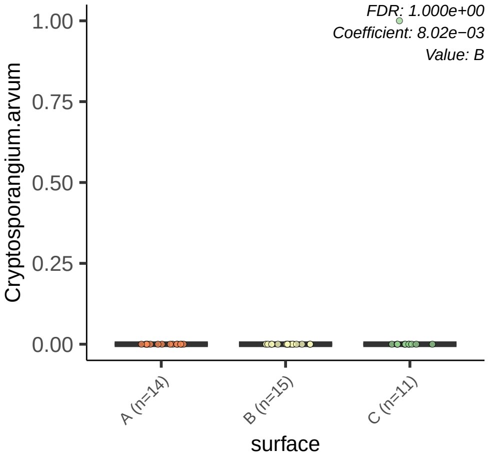
FDR: 1.000e+00
Coefficient: 8.02e−03
Value: B
Cryptosporangium.arvum
1.00
0.75
0.50
0.25
0.00
A (n=14)
B (n=15)
C (n=11)
surface

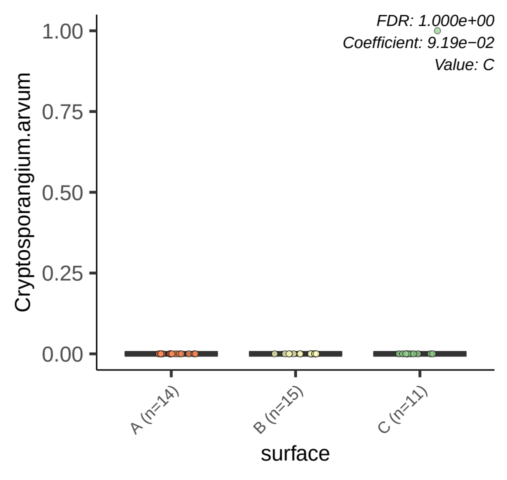
FDR: 1.000e+00
Coefficient: 9.19e−02
Value: C
1.00
0.75
0.50
0.25
0.00
Cryptosporangium.arvum
A (n=14)
B (n=15)
C (n=11)
surface

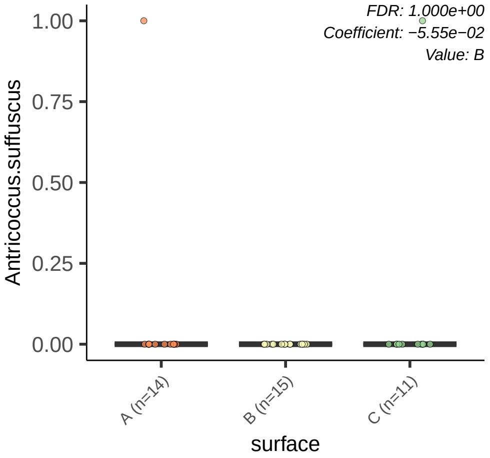

FDR: 1.000e+00
Coefficient: −5.55e−02
Value: B
Antricoccus.suffuscus
1.00
0.75
0.50
0.25
0.00
A (n=14)
B (n=15)
C (n=11)
surface

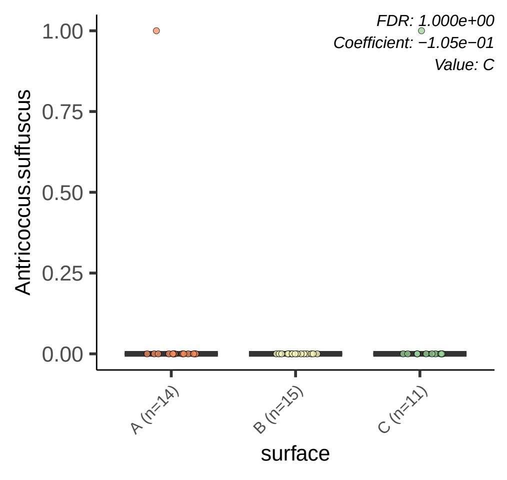

FDR: 1.000e+00
Coefficient: −1.05e−01
Value: C
Antricoccus.suffuscus
1.00
0.75
0.50
0.25
0.00
A (n=14)
B (n=15)
C (n=11)
surface

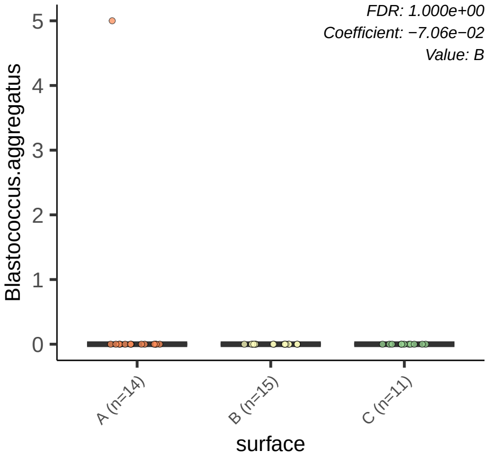

FDR: 1.000e+00
Coefficient: −7.06e−02
Value: B
Blastococcus.aggregatus
5
4
3
2
1
0
A (n=14)
B (n=15)
C (n=11)
surface

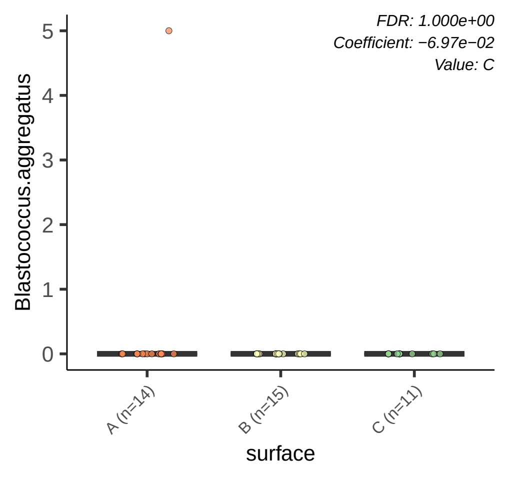

FDR: 1.000e+00
Coefficient: −6.97e−02
Value: C
Blastococcus.aggregatus
0
1
2
3
4
5
A (n=14)
B (n=15)
C (n=11)
surface

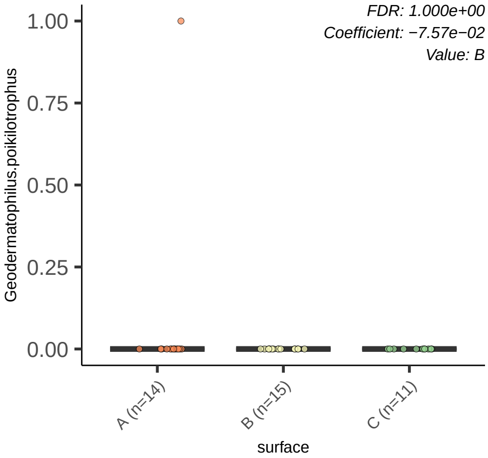

FDR: 1.000e+00
Coefficient: −7.57e−02
Value: B
Geodermatophilus.poikilotrophus
1.00
0.75
0.50
0.25
0.00
A (n=14)
B (n=15)
C (n=11)
surface

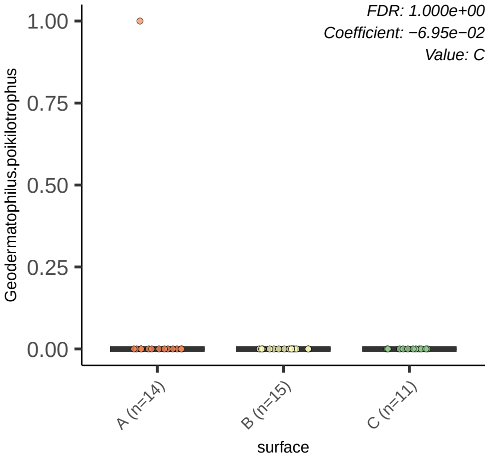

FDR: 1.000e+00
Coefficient: −6.95e−02
Value: C
Geodermatophilus.poikilotrophus
1.00
0.75
0.50
0.25
0.00
A (n=14)
B (n=15)
C (n=11)
surface

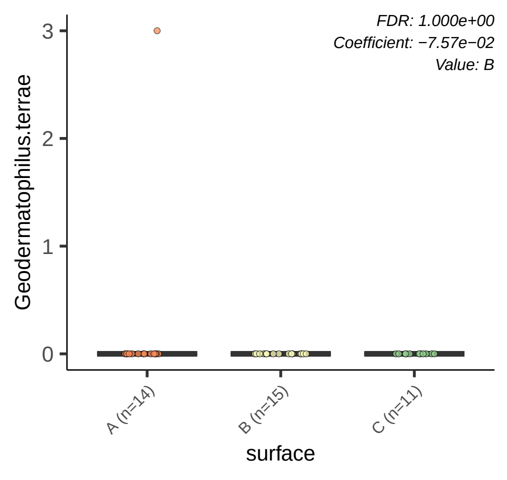

FDR: 1.000e+00
Coefficient: −7.57e−02
Value: B
Geodermatophilus.terrae
3
2
1
0
A (n=14)
B (n=15)
C (n=11)
surface

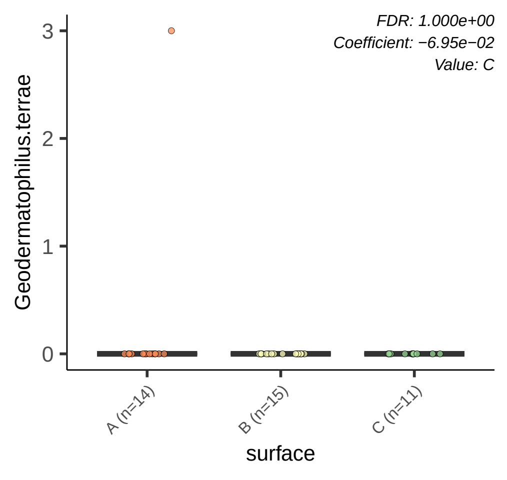

FDR: 1.000e+00
Coefficient: −6.95e−02
Value: C
Geodermatophilus.terrae
3
2
1
0
A (n=14)
B (n=15)
C (n=11)
surface

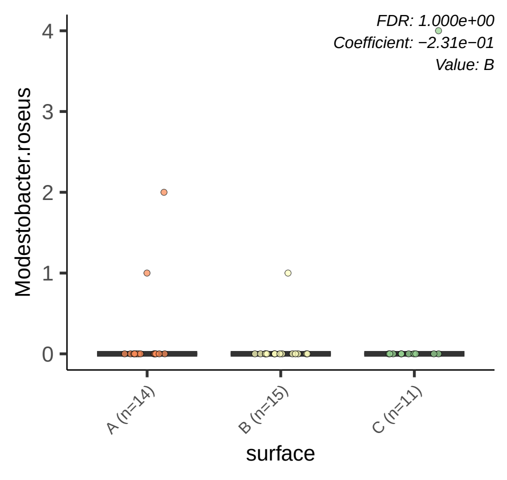

FDR: 1.000e+00
Coefficient: −2.31e−01
Value: B
Modestobacter.roseus
0
1
2
3
4
A (n=14)
B (n=15)
C (n=11)
surface

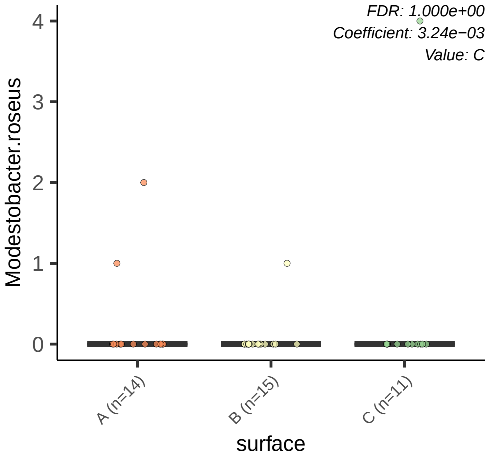
FDR: 1.000e+00
Coefficient: 3.24e−03
Value: C
Modestobacter.roseus
0
1
2
3
4
A (n=14)
B (n=15)
C (n=11)
surface

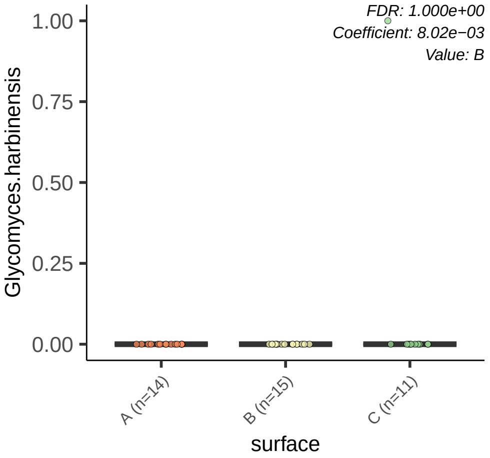

FDR: 1.000e+00
Coefficient: 8.02e−03
Value: B
Glycomyces.harbinensis
1.00
0.75
0.50
0.25
0.00
A (n=14)
B (n=15)
C (n=11)
surface

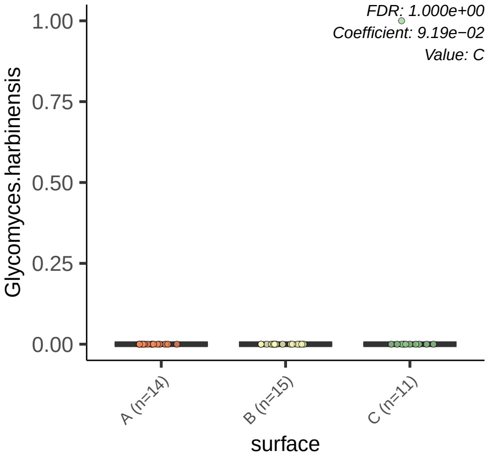
FDR: 1.000e+00
Coefficient: 9.19e−02
Value: C
Glycomyces.harbinensis
1.00
0.75
0.50
0.25
0.00
A (n=14)
B (n=15)
C (n=11)
surface

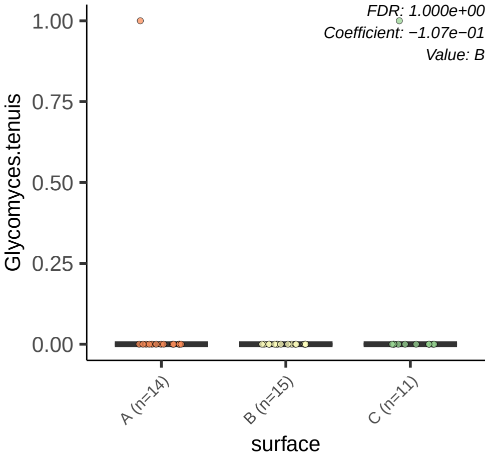
FDR: 1.000e+00
Coefficient: −1.07e−01
Value: B
Glycomyces.tenuis
1.00
0.75
0.50
0.25
0.00
A (n=14)
B (n=15)
C (n=11)
surface

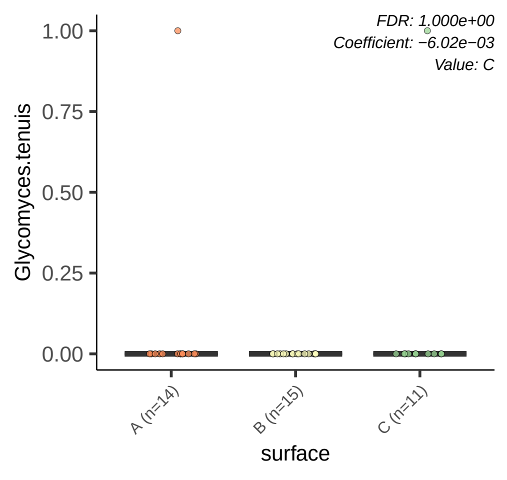

FDR: 1.000e+00
Coefficient: −6.02e−03
Value: C
Glycomyces.tenuis
1.00
0.75
0.50
0.25
0.00
A (n=14)
B (n=15)
C (n=11)
surface

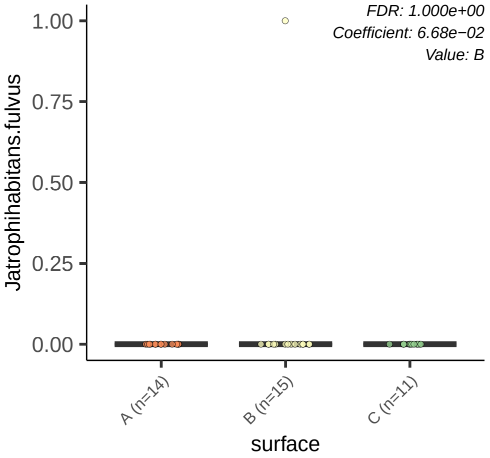
FDR: 1.000e+00
Coefficient: 6.68e−02
Value: B
Jatrophihabitans.fulvus
1.00
0.75
0.50
0.25
0.00
A (n=14)
B (n=15)
C (n=11)
surface

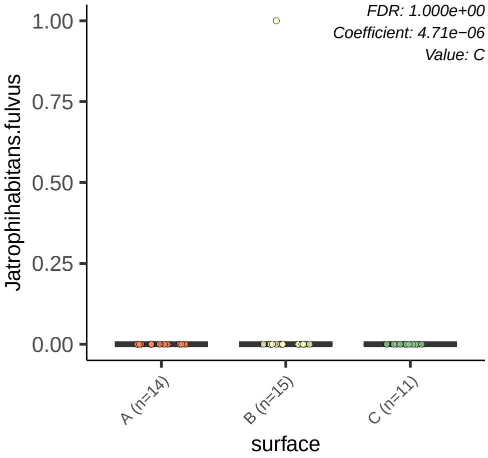
FDR: 1.000e+00
Coefficient: 4.71e−06
Value: C
Jatrophihabitans.fulvus
1.00
0.75
0.50
0.25
0.00
A (n=14)
B (n=15)
C (n=11)
surface

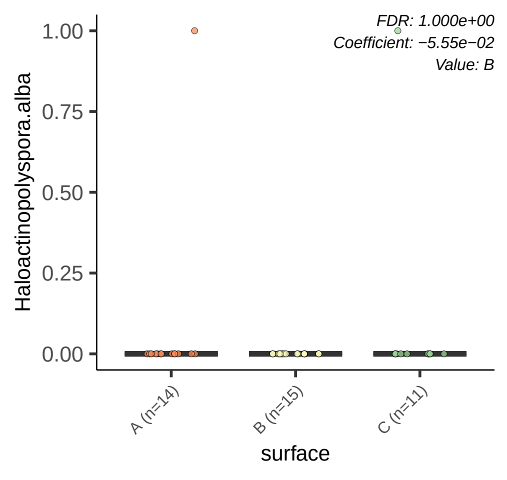

FDR: 1.000e+00
Coefficient: −5.55e−02
Value: B
Haloactinopolyspora.alba
1.00
0.75
0.50
0.25
0.00
A (n=14)
B (n=15)
C (n=11)
surface

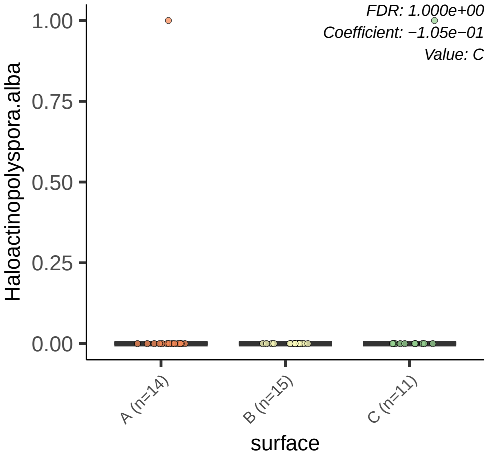
FDR: 1.000e+00
Coefficient: −1.05e−01
Value: C
Haloactinopolyspora.alba
1.00
0.75
0.50
0.25
0.00
A (n=14)
B (n=15)
C (n=11)
surface

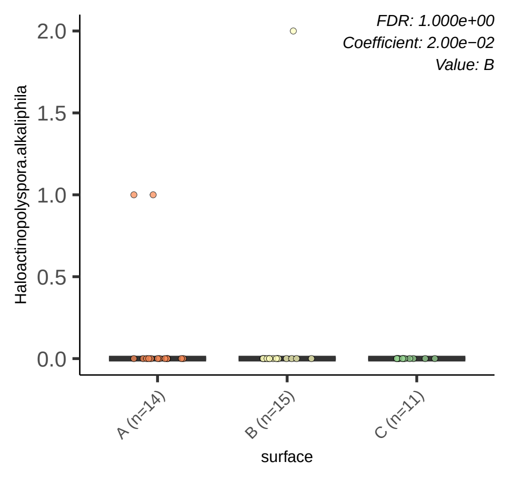
FDR: 1.000e+00
Coefficient: 2.00e−02
Value: B
Haloactinopolyspora.alkaliphila
2.0
1.5
1.0
0.5
0.0
A (n=14)
B (n=15)
C (n=11)
surface

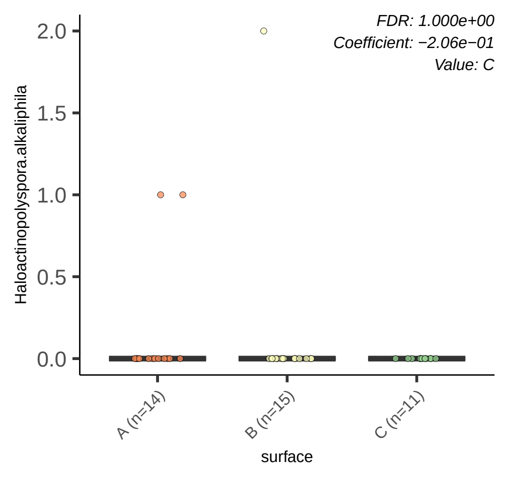

FDR: 1.000e+00
Coefficient: −2.06e−01
Value: C
Haloactinopolyspora.alkaliphila
2.0
1.5
1.0
0.5
0.0
A (n=14)
B (n=15)
C (n=11)
surface

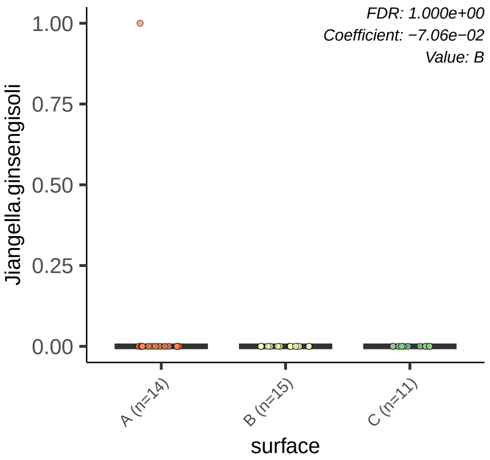

FDR: 1.000e+00
Coefficient: −7.06e−02
Value: B
Jiangella.ginsengisoli
1.00
0.75
0.50
0.25
0.00
A (n=14)
B (n=15)
C (n=11)
surface

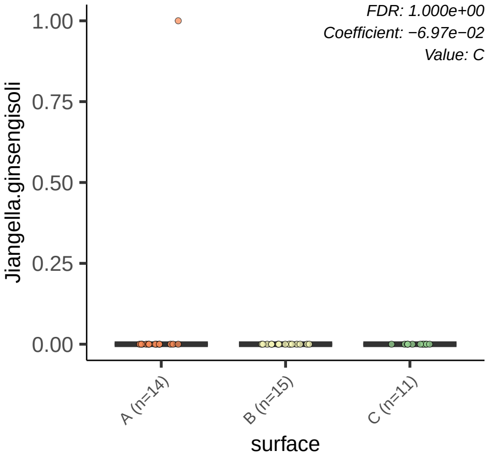
FDR: 1.000e+00
Coefficient: −6.97e−02
Value: C
Jiangella.ginsengisoli
1.00
0.75
0.50
0.25
0.00
A (n=14)
B (n=15)
C (n=11)
surface

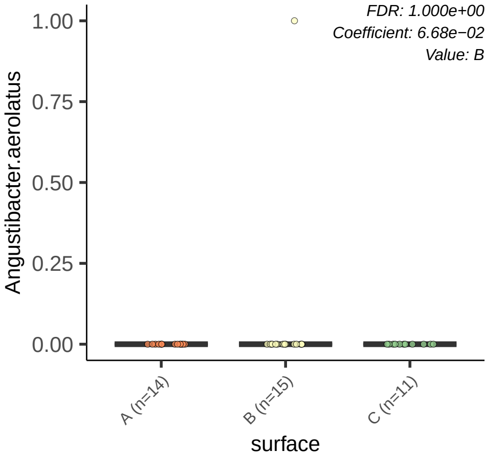

FDR: 1.000e+00
Coefficient: 6.68e−02
Value: B
Angustibacter.aerolatus
1.00
0.75
0.50
0.25
0.00
A (n=14)
B (n=15)
C (n=11)
surface

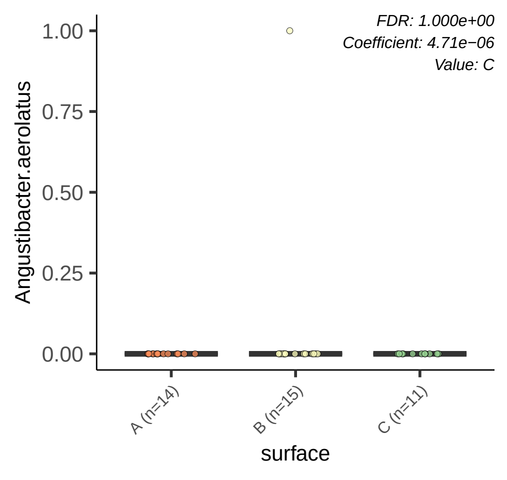
FDR: 1.000e+00
Coefficient: 4.71e−06
Value: C
Angustibacter.aerolatus
1.00
0.75
0.50
0.25
0.00
A (n=14)
B (n=15)
C (n=11)
surface

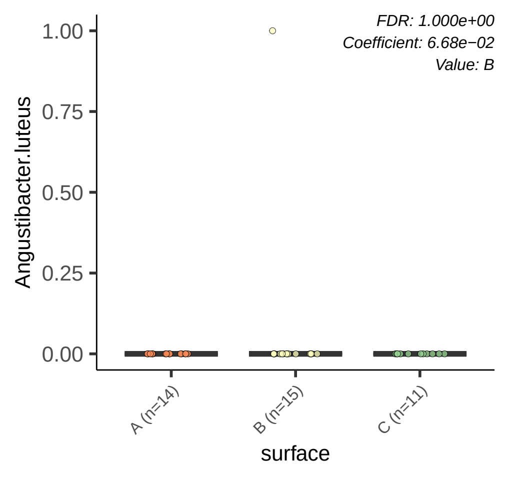
FDR: 1.000e+00
Coefficient: 6.68e−02
Value: B
Angustibacter.luteus
1.00
0.75
0.50
0.25
0.00
A (n=14)
B (n=15)
C (n=11)
surface

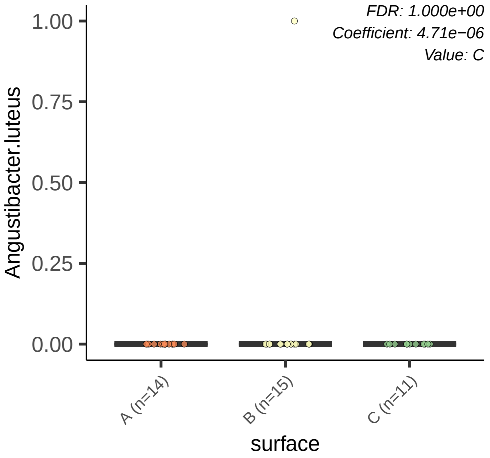
FDR: 1.000e+00
Coefficient: 4.71e−06
Value: C
Angustibacter.luteus
1.00
0.75
0.50
0.25
0.00
A (n=14)
B (n=15)
C (n=11)
surface

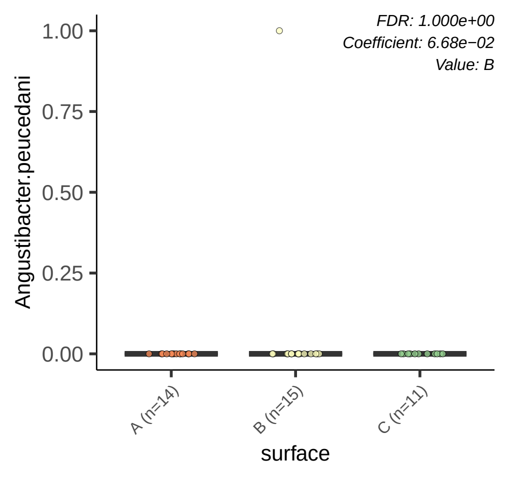
FDR: 1.000e+00
Coefficient: 6.68e−02
Value: B
Angustibacter.peucedani
1.00
0.75
0.50
0.25
0.00
A (n=14)
B (n=15)
C (n=11)
surface

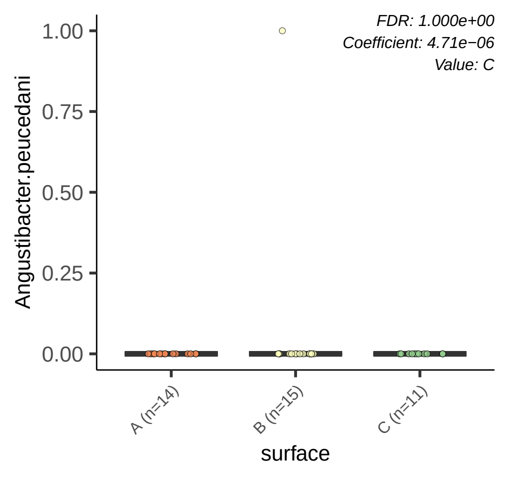
FDR: 1.000e+00
Coefficient: 4.71e−06
Value: C
Angustibacter.peucedani
1.00
0.75
0.50
0.25
0.00
A (n=14)
B (n=15)
C (n=11)
surface

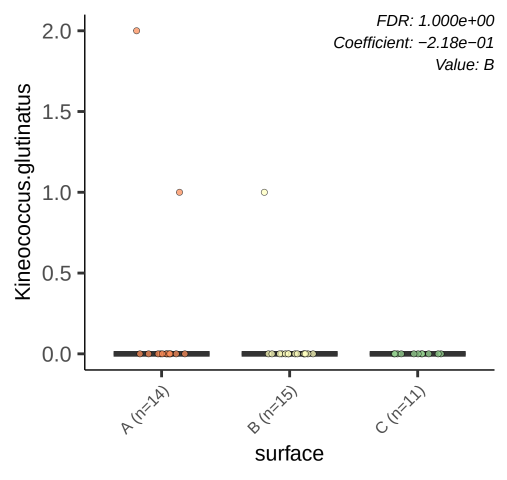
FDR: 1.000e+00
Coefficient: −2.18e−01
Value: B
Kineococcus.glutinatus
2.0
1.5
1.0
0.5
0.0
A (n=14)
B (n=15)
C (n=11)
surface

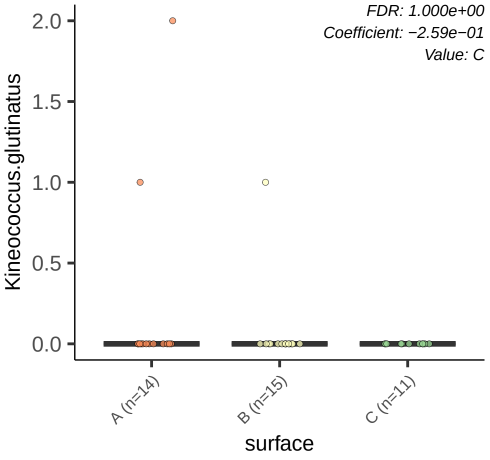
FDR: 1.000e+00
Coefficient: −2.59e−01
Value: C
Kineococcus.glutinatus
2.0
1.5
1.0
0.5
0.0
A (n=14)
B (n=15)
C (n=11)
surface

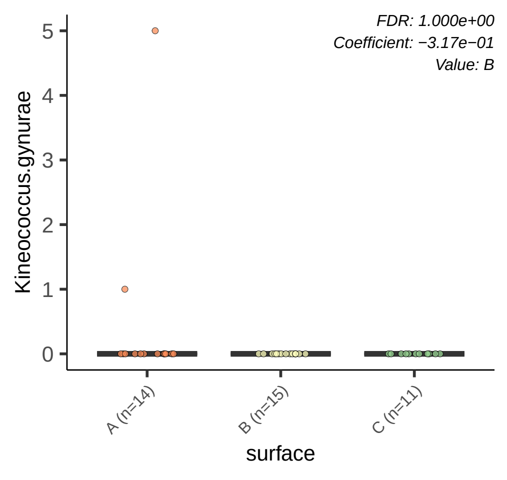

FDR: 1.000e+00
Coefficient: −3.17e−01
Value: B
Kineococcus.gynurae
0
1
2
3
4
5
A (n=14)
B (n=15)
C (n=11)
surface

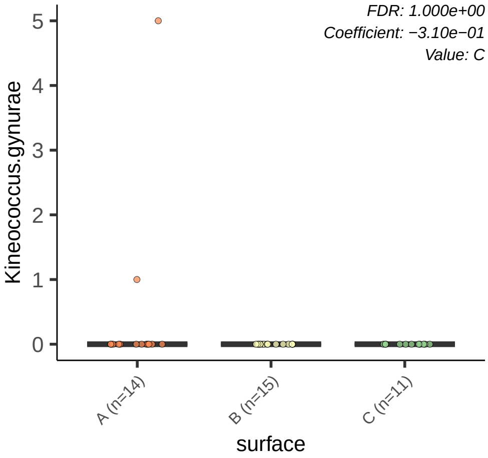

FDR: 1.000e+00
Coefficient: −3.10e−01
Value: C
Kineococcus.gynurae
0
1
2
3
4
5
A (n=14)
B (n=15)
C (n=11)
surface

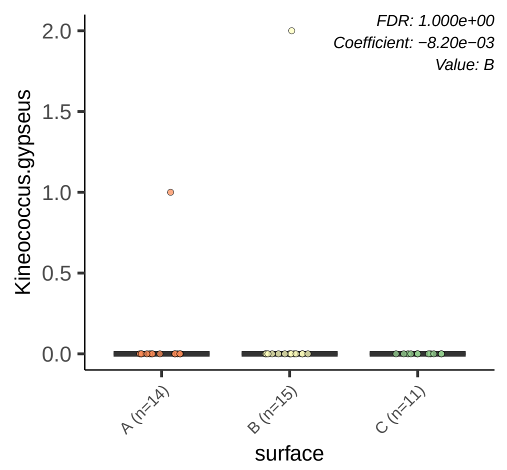
FDR: 1.000e+00
Coefficient: −8.20e−03
Value: B
Kineococcus.gypseus
2.0
1.5
1.0
0.5
0.0
A (n=14)
B (n=15)
C (n=11)
surface

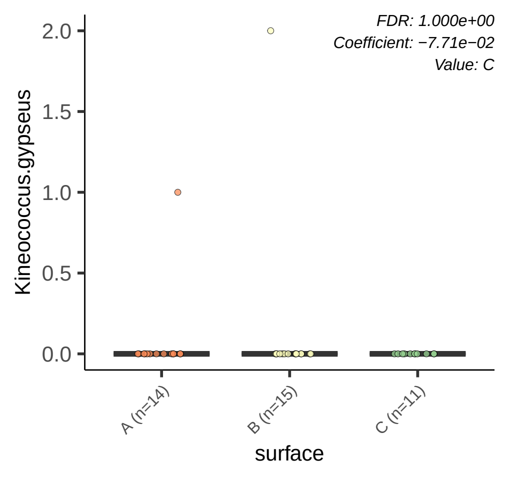
FDR: 1.000e+00
Coefficient: −7.71e−02
Value: C
Kineococcus.gypseus
2.0
1.5
1.0
0.5
0.0
A (n=14)
B (n=15)
C (n=11)
surface

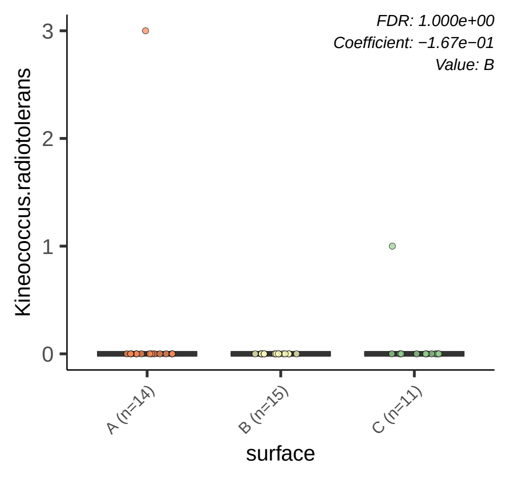

FDR: 1.000e+00
Coefficient: −1.67e−01
Value: B
Kineococcus.radiotolerans
3
2
1
0
A (n=14)
B (n=15)
C (n=11)
surface

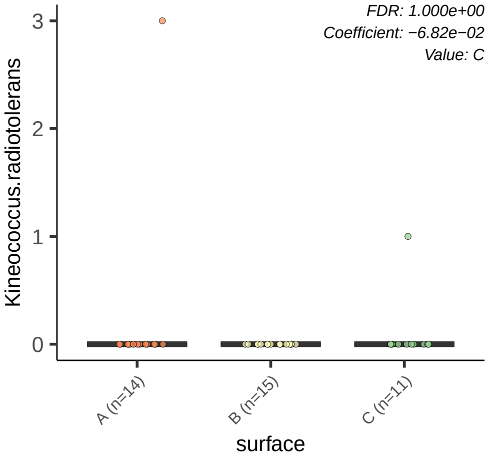
FDR: 1.000e+00
Coefficient: −6.82e−02
Value: C
Kineococcus.radiotolerans
3
2
1
0
A (n=14)
B (n=15)
C (n=11)
surface

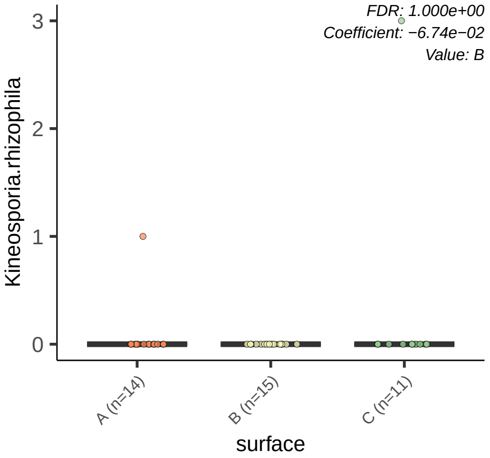
FDR: 1.000e+00
Coefficient: −6.74e−02
Value: B
Kineosporia.rhizophila
3
2
1
0
A (n=14)
B (n=15)
C (n=11)
surface

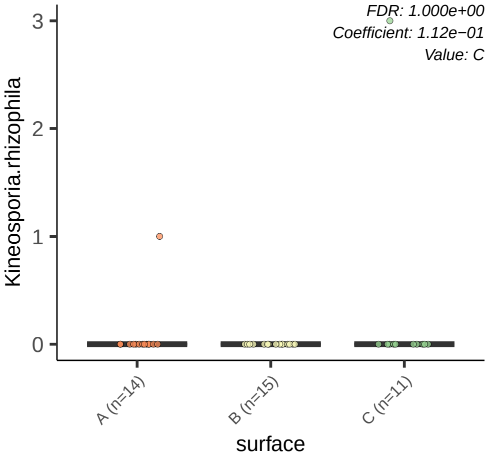
FDR: 1.000e+00
Coefficient: 1.12e−01
Value: C
Kineosporia.rhizophila
0
1
2
3
A (n=14)
B (n=15)
C (n=11)
surface

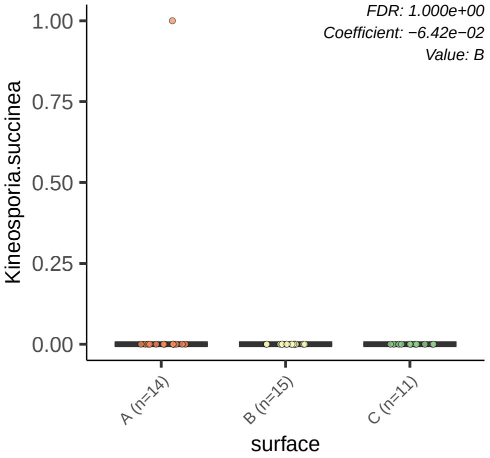
FDR: 1.000e+00
Coefficient: −6.42e−02
Value: B
Kineosporia.succinea
1.00
0.75
0.50
0.25
0.00
A (n=14)
B (n=15)
C (n=11)
surface

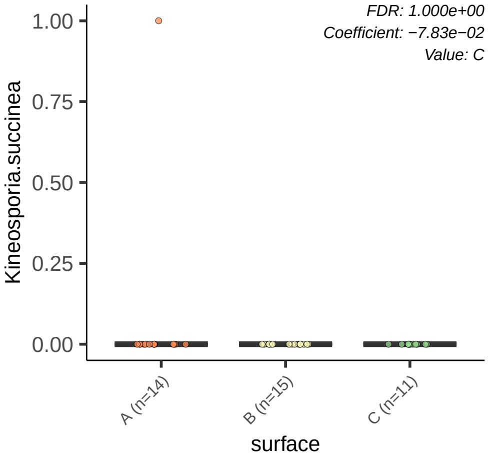

FDR: 1.000e+00
Coefficient: −7.83e−02
Value: C
Kineosporia.succinea
1.00
0.75
0.50
0.25
0.00
A (n=14)
B (n=15)
C (n=11)
surface

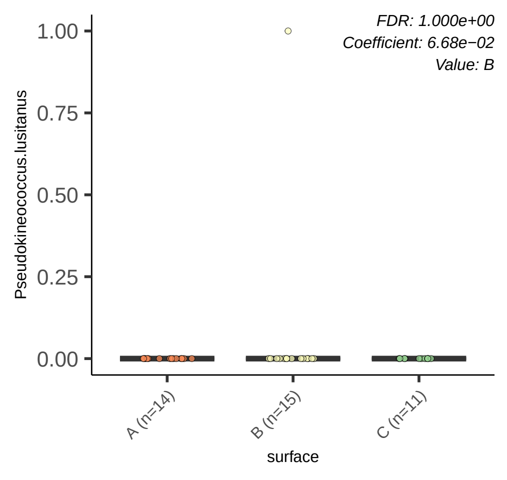
FDR: 1.000e+00
Coefficient: 6.68e−02
Value: B
Pseudokineococcus.lusitanus
1.00
0.75
0.50
0.25
0.00
A (n=14)
B (n=15)
C (n=11)
surface

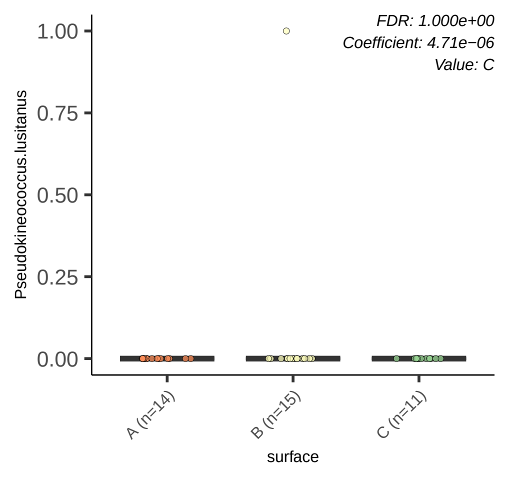
FDR: 1.000e+00
Coefficient: 4.71e−06
Value: C
Pseudokineococcus.lusitanus
1.00
0.75
0.50
0.25
0.00
A (n=14)
B (n=15)
C (n=11)
surface

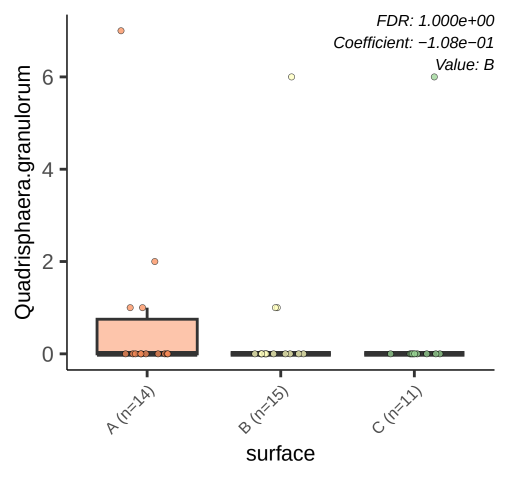

FDR: 1.000e+00
Coefficient: −1.08e−01
Value: B
Quadrisphaera.granulorum
0
2
4
6
A (n=14)
B (n=15)
C (n=11)
surface

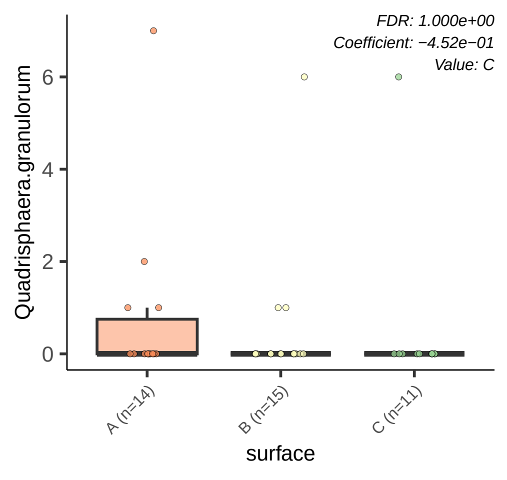
FDR: 1.000e+00
Coefficient: −4.52e−01
Value: C
Quadrisphaera.granulorum
0
2
4
6
A (n=14)
B (n=15)
C (n=11)
surface

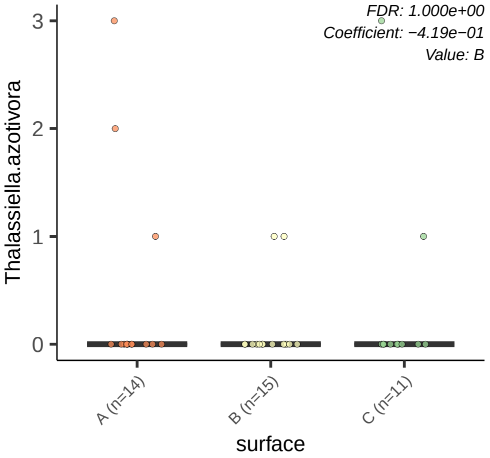

FDR: 1.000e+00
Coefficient: −4.19e−01
Value: B
Thalassiella.azotivora
0
1
2
3
A (n=14)
B (n=15)
C (n=11)
surface

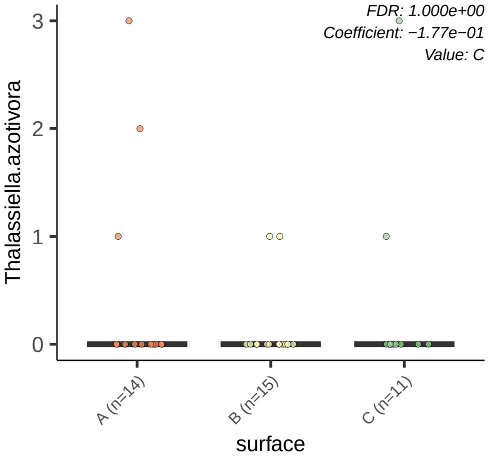

FDR: 1.000e+00
Coefficient: −1.77e−01
Value: C
Thalassiella.azotivora
0
1
2
3
A (n=14)
B (n=15)
C (n=11)
surface

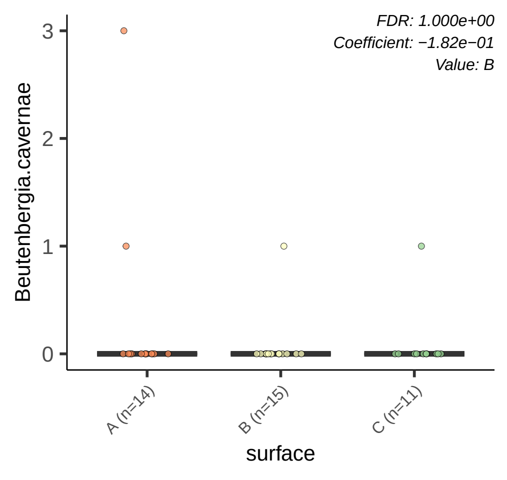
FDR: 1.000e+00
Coefficient: −1.82e−01
Value: B
Beutenbergia.cavernae
3
2
1
0
A (n=14)
B (n=15)
C (n=11)
surface

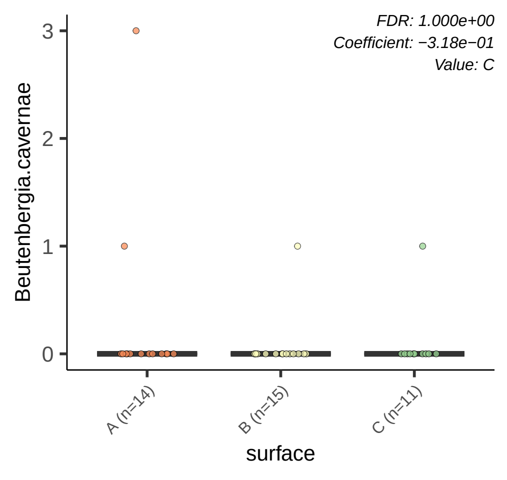

FDR: 1.000e+00
Coefficient: −3.18e−01
Value: C
Beutenbergia.cavernae
3
2
1
0
A (n=14)
B (n=15)
C (n=11)
surface

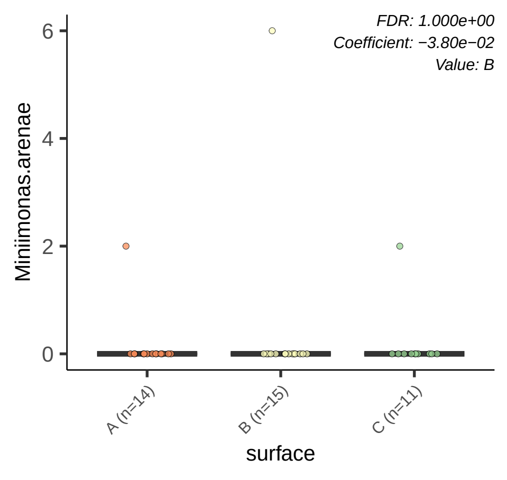
FDR: 1.000e+00
Coefficient: −3.80e−02
Value: B
Miniimonas.arenae
0
2
4
6
A (n=14)
B (n=15)
C (n=11)
surface

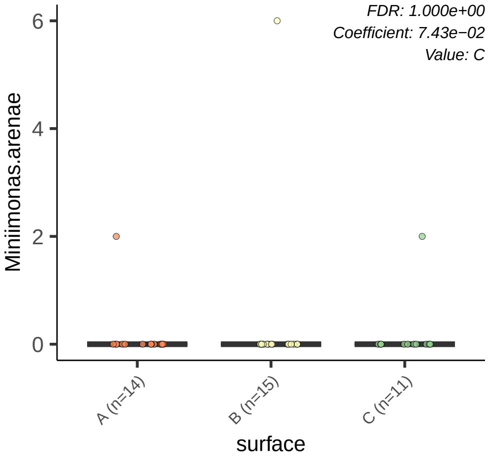
FDR: 1.000e+00
Coefficient: 7.43e−02
Value: C
Miniimonas.arenae
0
2
4
6
A (n=14)
B (n=15)
C (n=11)
surface

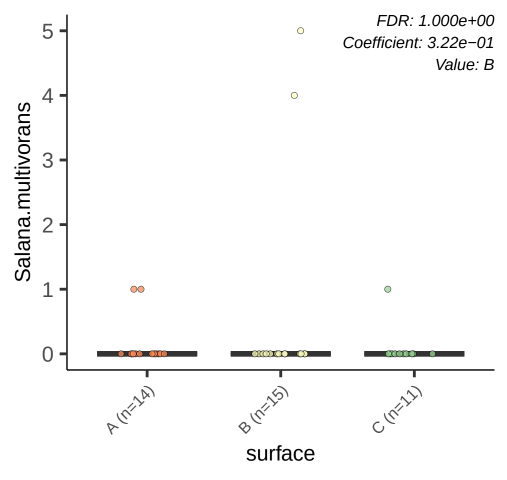
FDR: 1.000e+00
Coefficient: 3.22e−01
Value: B
Salana.multivorans
5
4
3
2
1
0
A (n=14)
B (n=15)
C (n=11)
surface

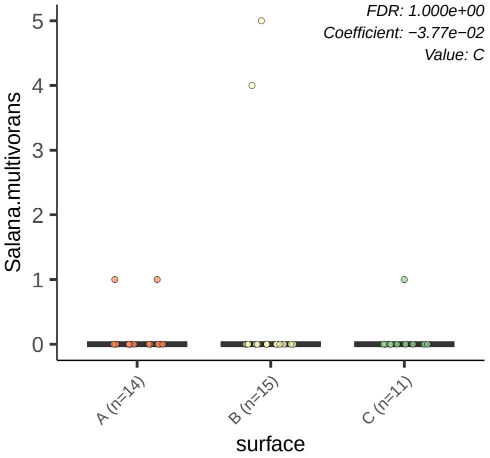

FDR: 1.000e+00
Coefficient: −3.77e−02
Value: C
Salana.multivorans
0
1
2
3
4
5
A (n=14)
B (n=15)
C (n=11)
surface

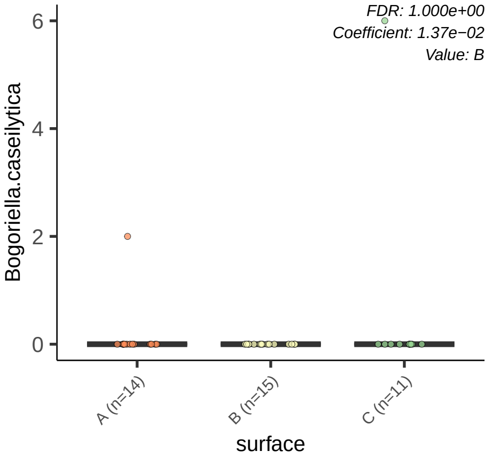

FDR: 1.000e+00
Coefficient: 1.37e−02
Value: B
Bogoriella.caseilytica
0
2
4
6
A (n=14)
B (n=15)
C (n=11)
surface

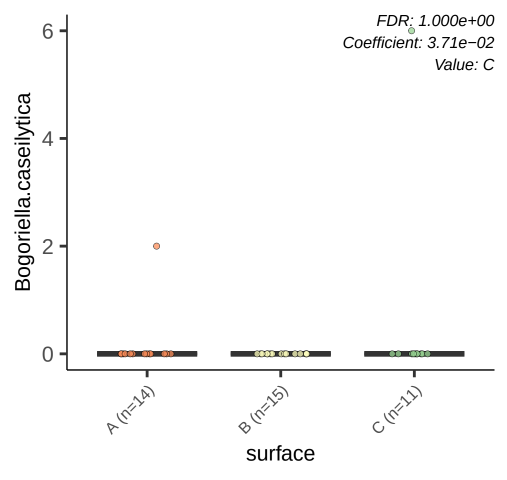
FDR: 1.000e+00
Coefficient: 3.71e−02
Value: C
Bogoriella.caseilytica
6
4
2
0
A (n=14)
B (n=15)
C (n=11)
surface

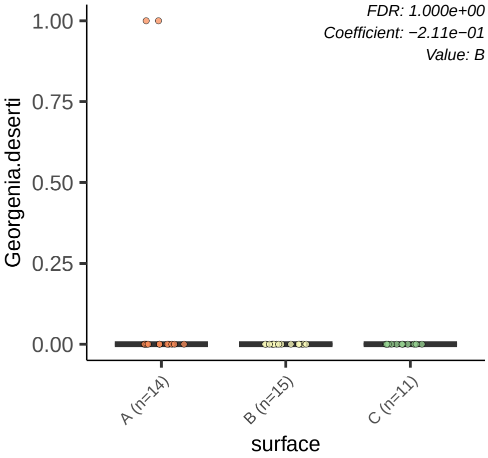

FDR: 1.000e+00
Coefficient: −2.11e−01
Value: B
Georgenia.deserti
1.00
0.75
0.50
0.25
0.00
A (n=14)
B (n=15)
C (n=11)
surface

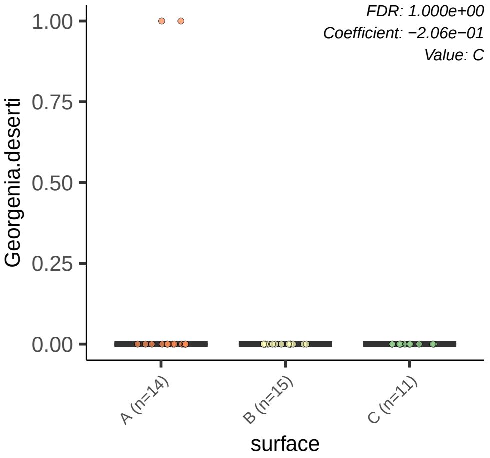
FDR: 1.000e+00
Coefficient: −2.06e−01
Value: C
Georgenia.deserti
1.00
0.75
0.50
0.25
0.00
A (n=14)
B (n=15)
C (n=11)
surface

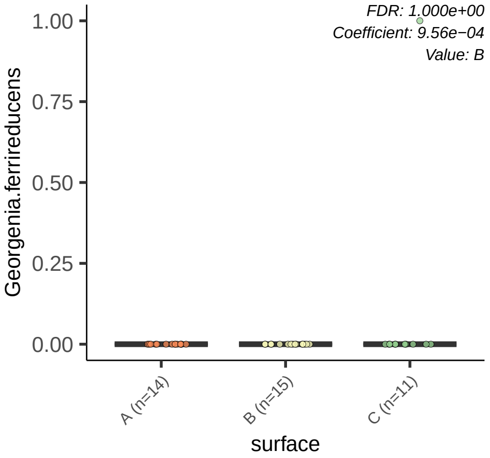

FDR: 1.000e+00
Coefficient: 9.56e−04
Value: B
Georgenia.ferrireducens
1.00
0.75
0.50
0.25
0.00
A (n=14)
B (n=15)
C (n=11)
surface

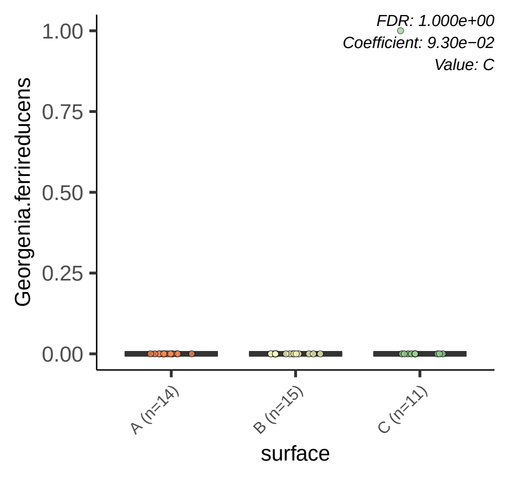

FDR: 1.000e+00
Coefficient: 9.30e−02
Value: C
Georgenia.ferrireducens
1.00
0.75
0.50
0.25
0.00
A (n=14)
B (n=15)
C (n=11)
surface

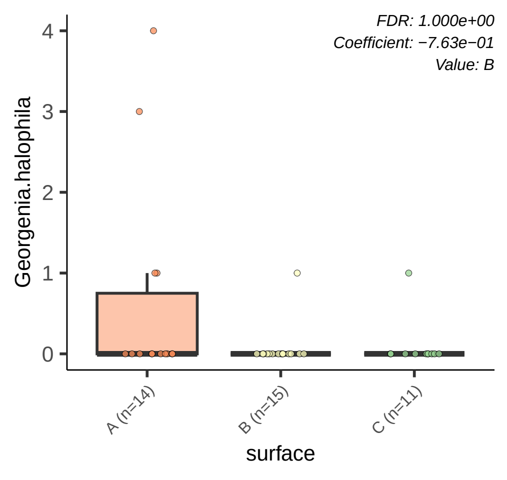

FDR: 1.000e+00
Coefficient: −7.63e−01
Value: B
Georgenia.halophila
0
1
2
3
4
A (n=14)
B (n=15)
C (n=11)
surface

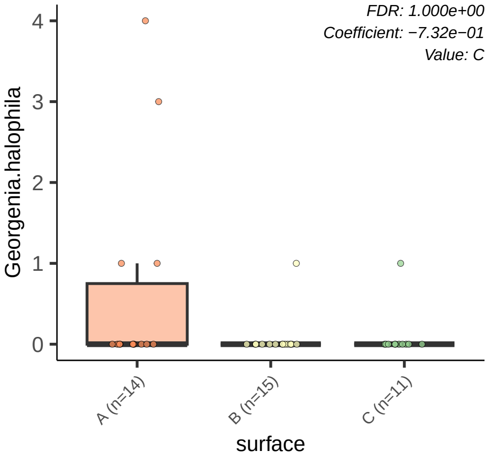
FDR: 1.000e+00
Coefficient: −7.32e−01
Value: C
Georgenia.halophila
0
1
2
3
4
A (n=14)
B (n=15)
C (n=11)
surface

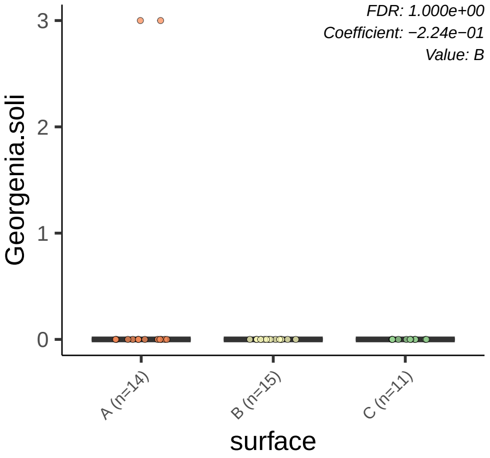
FDR: 1.000e+00
Coefficient: −2.24e−01
Value: B
Georgenia.soli
3
2
1
0
A (n=14)
B (n=15)
C (n=11)
surface

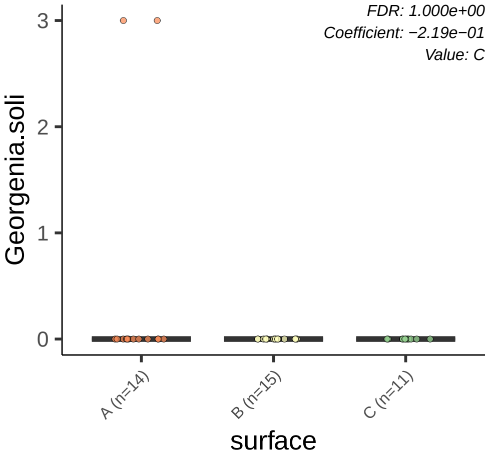

FDR: 1.000e+00
Coefficient: −2.19e−01
Value: C
Georgenia.soli
3
2
1
0
A (n=14)
B (n=15)
C (n=11)
surface

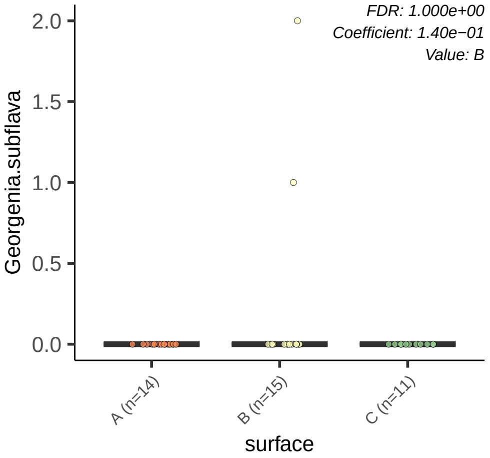
FDR: 1.000e+00
Coefficient: 1.40e−01
Value: B
Georgenia.subflava
2.0
1.5
1.0
0.5
0.0
A (n=14)
B (n=15)
C (n=11)
surface

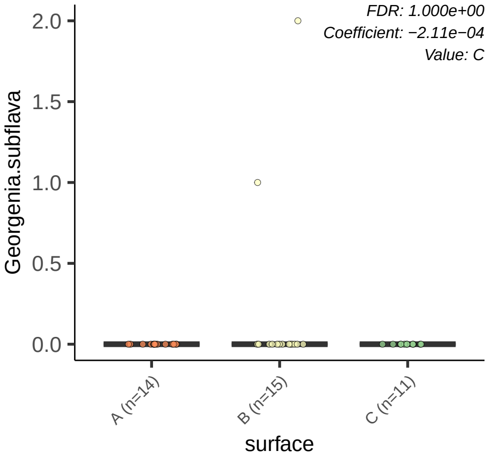
FDR: 1.000e+00
Coefficient: −2.11e−04
Value: C
Georgenia.subflava
2.0
1.5
1.0
0.5
0.0
A (n=14)
B (n=15)
C (n=11)
surface

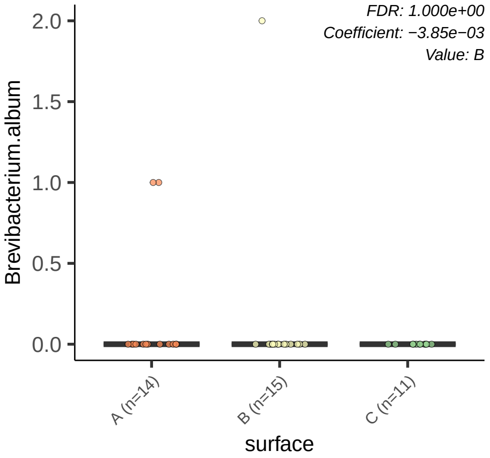
FDR: 1.000e+00
Coefficient: −3.85e−03
Value: B
Brevibacterium.album
2.0
1.5
1.0
0.5
0.0
A (n=14)
B (n=15)
C (n=11)
surface

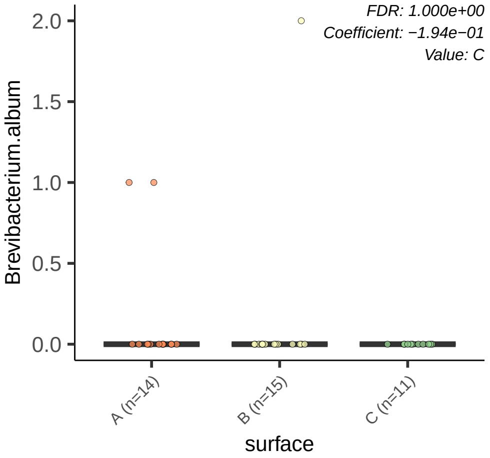

FDR: 1.000e+00
Coefficient: −1.94e−01
Value: C
Brevibacterium.album
2.0
1.5
1.0
0.5
0.0
A (n=14)
B (n=15)
C (n=11)
surface

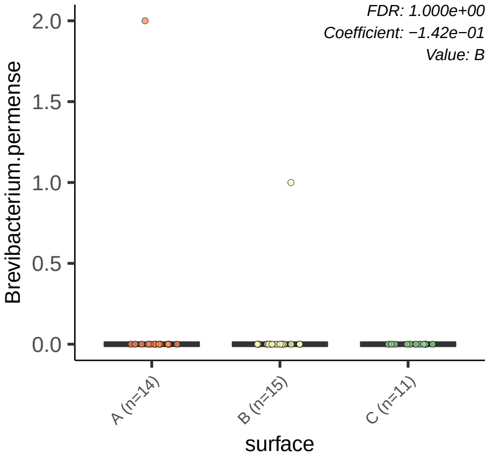
FDR: 1.000e+00
Coefficient: −1.42e−01
Value: B
Brevibacterium.permense
2.0
1.5
1.0
0.5
0.0
A (n=14)
B (n=15)
C (n=11)
surface

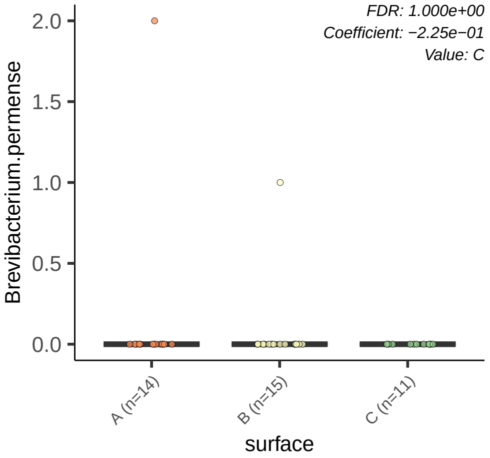
FDR: 1.000e+00
Coefficient: −2.25e−01
Value: C
Brevibacterium.permense
2.0
1.5
1.0
0.5
0.0
A (n=14)
B (n=15)
C (n=11)
surface

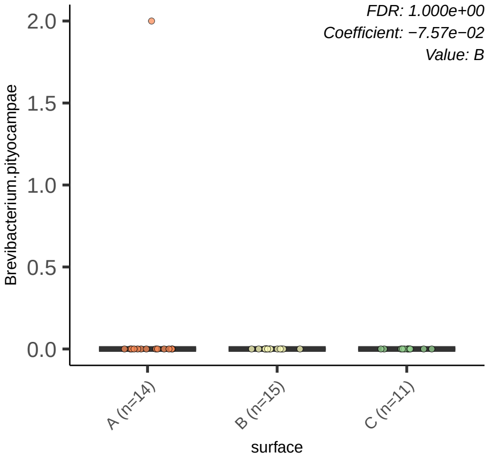

FDR: 1.000e+00
Coefficient: −7.57e−02
Value: B
Brevibacterium.pityocampae
2.0
1.5
1.0
0.5
0.0
A (n=14)
B (n=15)
C (n=11)
surface

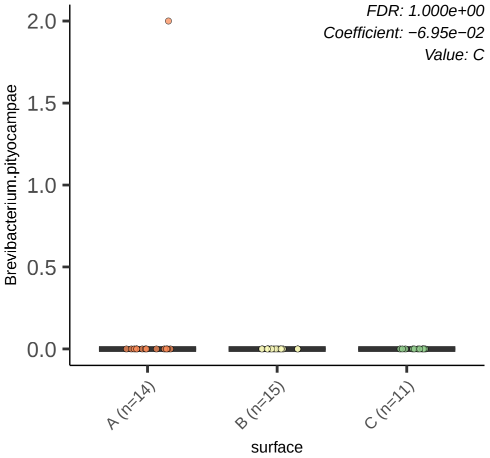

FDR: 1.000e+00
Coefficient: −6.95e−02
Value: C
Brevibacterium.pityocampae
2.0
1.5
1.0
0.5
0.0
A (n=14)
B (n=15)
C (n=11)
surface

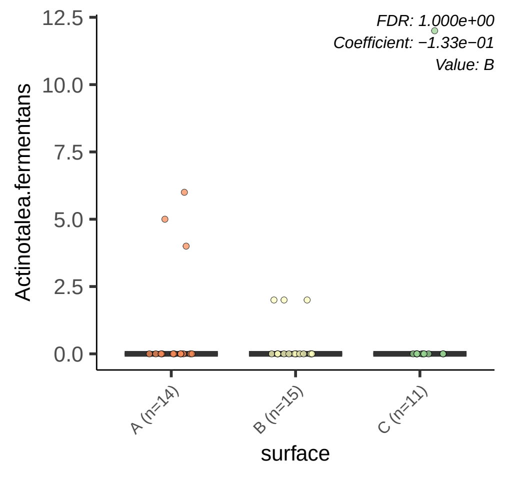

FDR: 1.000e+00
Coefficient: −1.33e−01
Value: B
Actinotalea.fermentans
12.5
10.0
7.5
5.0
2.5
0.0
A (n=14)
B (n=15)
C (n=11)
surface

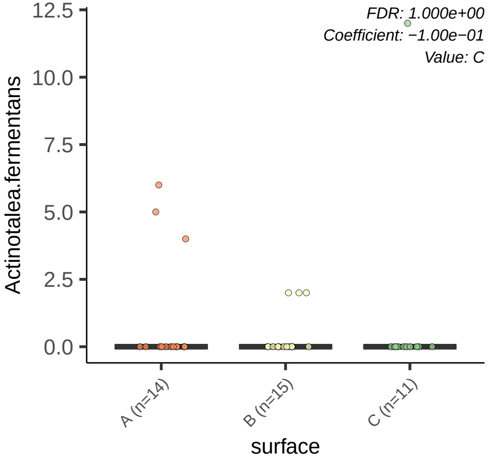
FDR: 1.000e+00
Coefficient: −1.00e−01
Value: C
Actinotalea.fermentans
12.5
10.0
7.5
5.0
2.5
0.0
A (n=14)
B (n=15)
C (n=11)
surface

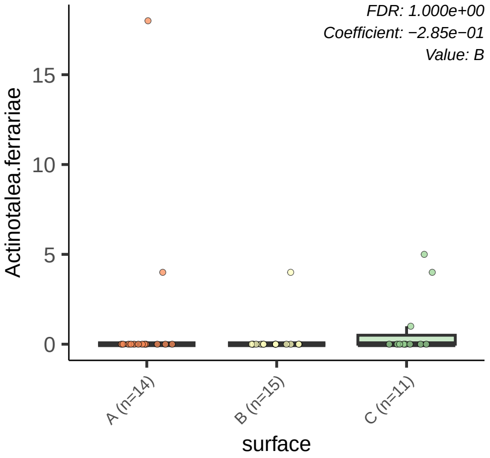

FDR: 1.000e+00
Coefficient: −2.85e−01
Value: B
Actinotalea.ferrariae
15
10
5
0
A (n=14)
B (n=15)
C (n=11)
surface

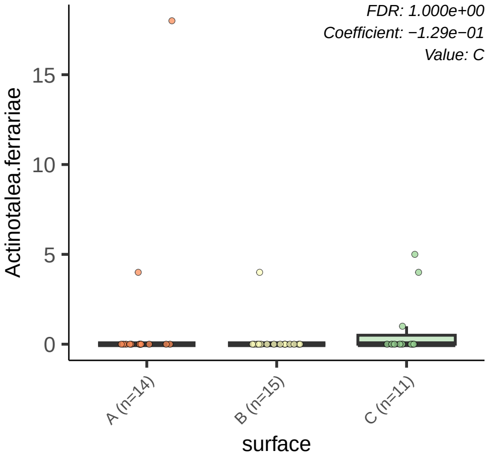
FDR: 1.000e+00
Coefficient: −1.29e−01
Value: C
Actinotalea.ferrariae
15
10
5
0
A (n=14)
B (n=15)
C (n=11)
surface

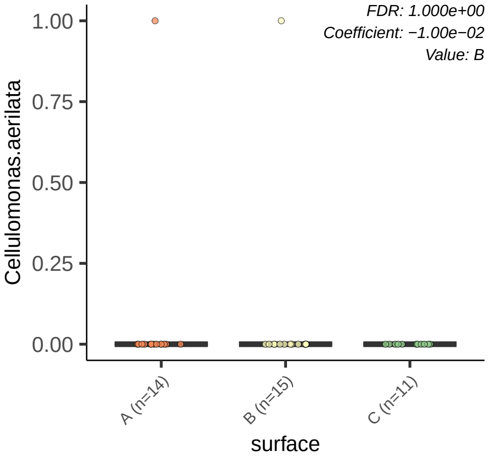

FDR: 1.000e+00
Coefficient: −1.00e−02
Value: B
Cellulomonas.aerilata
1.00
0.75
0.50
0.25
0.00
A (n=14)
B (n=15)
C (n=11)
surface

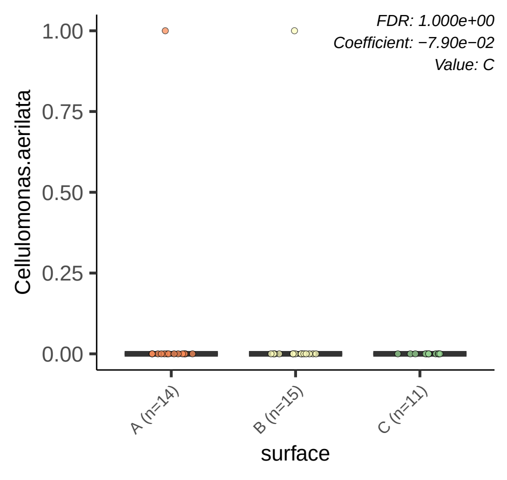
FDR: 1.000e+00
Coefficient: −7.90e−02
Value: C
Cellulomonas.aerilata
1.00
0.75
0.50
0.25
0.00
A (n=14)
B (n=15)
C (n=11)
surface

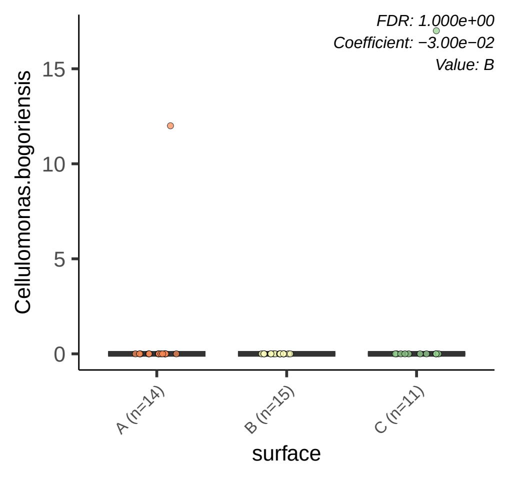
FDR: 1.000e+00
Coefficient: −3.00e−02
Value: B
Cellulomonas.bogoriensis
15
10
5
0
A (n=14)
B (n=15)
C (n=11)
surface

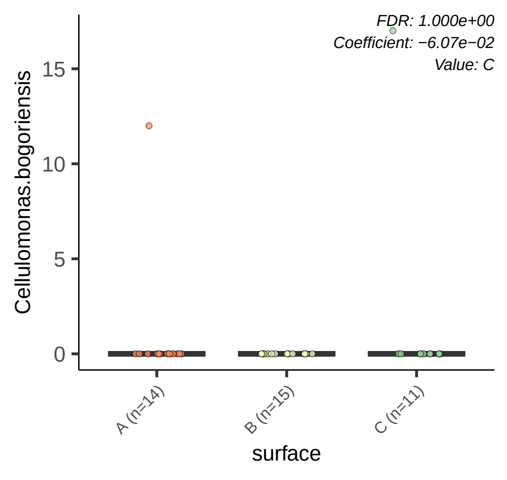
FDR: 1.000e+00
Coefficient: −6.07e−02
Value: C
Cellulomonas.bogoriensis
15
10
5
0
A (n=14)
B (n=15)
C (n=11)
surface

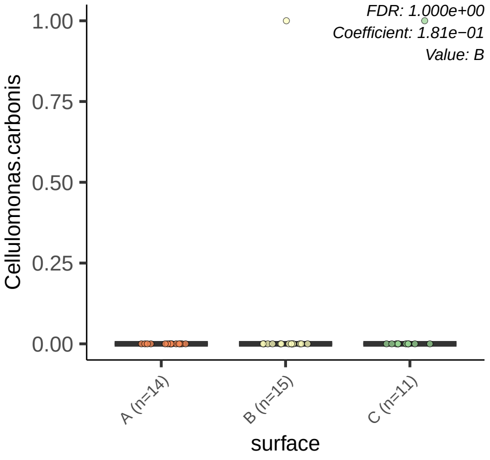
FDR: 1.000e+00
Coefficient: 1.81e−01
Value: B
Cellulomonas.carbonis
1.00
0.75
0.50
0.25
0.00
A (n=14)
B (n=15)
C (n=11)
surface

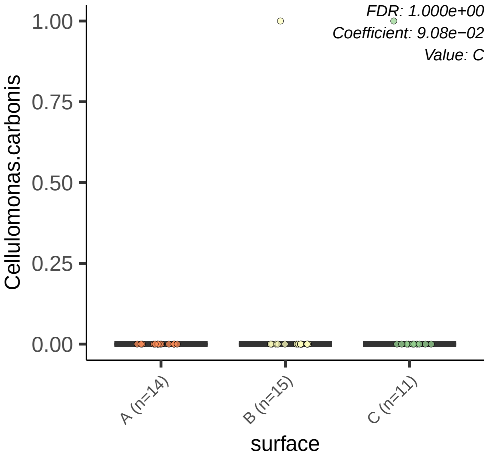
FDR: 1.000e+00
Coefficient: 9.08e−02
Value: C
Cellulomonas.carbonis
1.00
0.75
0.50
0.25
0.00
A (n=14)
B (n=15)
C (n=11)
surface

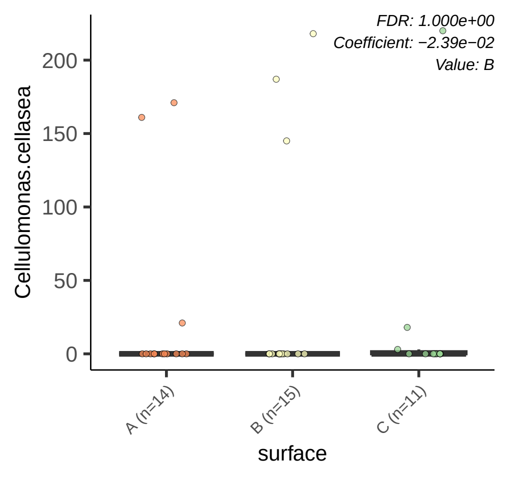

FDR: 1.000e+00
Coefficient: −2.39e−02
Value: B
Cellulomonas.cellasea
200
150
100
50
0
A (n=14)
B (n=15)
C (n=11)
surface

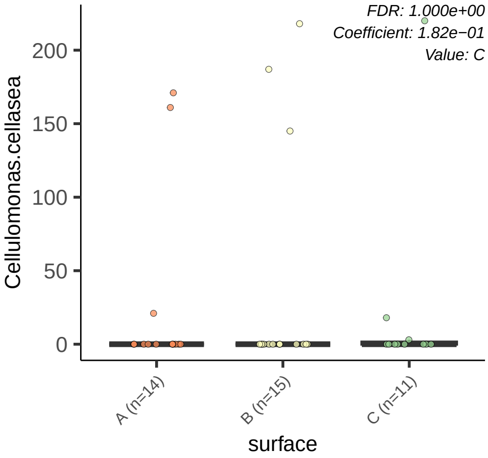

FDR: 1.000e+00
Coefficient: 1.82e−01
Value: C
Cellulomonas.cellasea
200
150
100
50
0
A (n=14)
B (n=15)
C (n=11)
surface

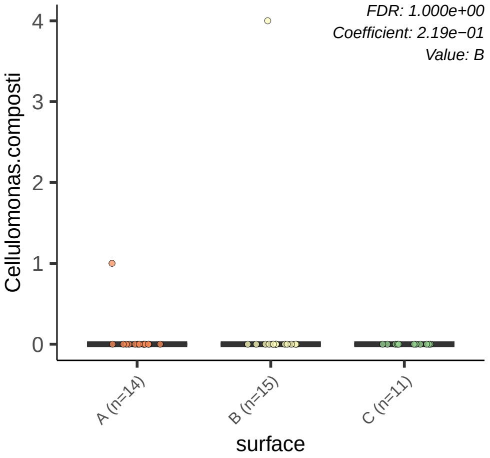
FDR: 1.000e+00
Coefficient: 2.19e−01
Value: B
Cellulomonas.composti
4
3
2
1
0
A (n=14)
B (n=15)
C (n=11)
surface

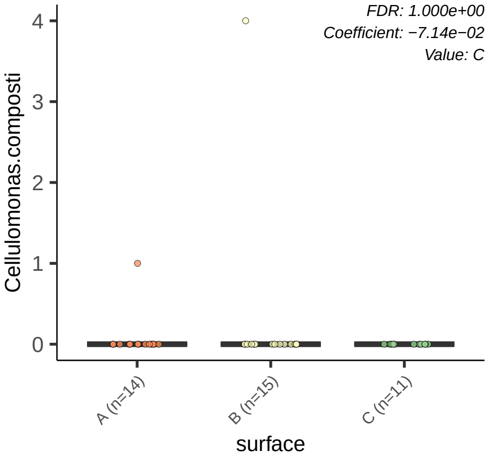
FDR: 1.000e+00
Coefficient: −7.14e−02
Value: C
Cellulomonas.composti
0
1
2
3
4
A (n=14)
B (n=15)
C (n=11)
surface

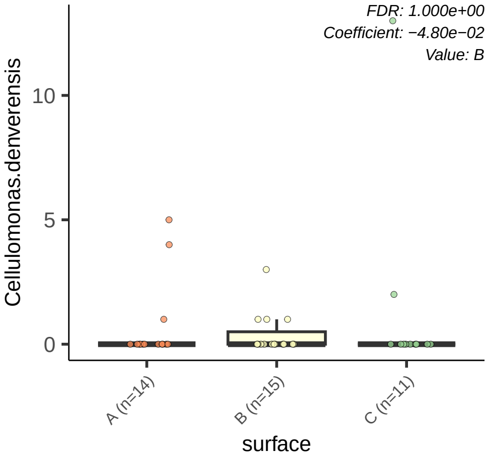

FDR: 1.000e+00
Coefficient: −4.80e−02
Value: B
Cellulomonas.denverensis
10
5
0
A (n=14)
B (n=15)
C (n=11)
surface

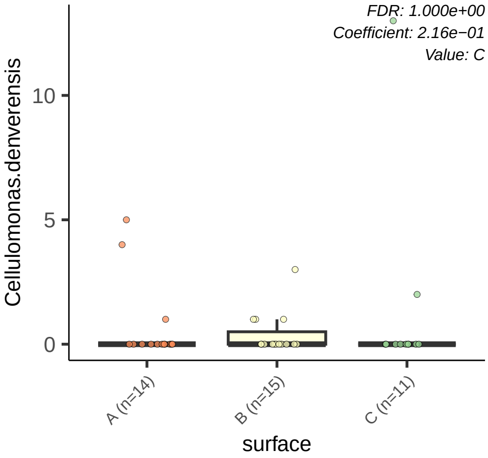
FDR: 1.000e+00
Coefficient: 2.16e−01
Value: C
Cellulomonas.denverensis
0
5
10
A (n=14)
B (n=15)
C (n=11)
surface

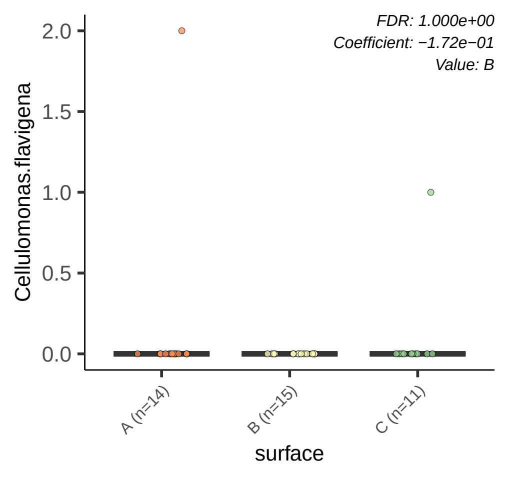
FDR: 1.000e+00
Coefficient: −1.72e−01
Value: B
Cellulomonas.flavigena
2.0
1.5
1.0
0.5
0.0
A (n=14)
B (n=15)
C (n=11)
surface

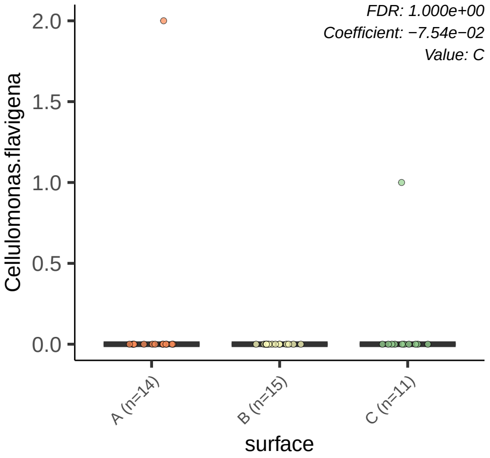
FDR: 1.000e+00
Coefficient: −7.54e−02
Value: C
Cellulomonas.flavigena
2.0
1.5
1.0
0.5
0.0
A (n=14)
B (n=15)
C (n=11)
surface

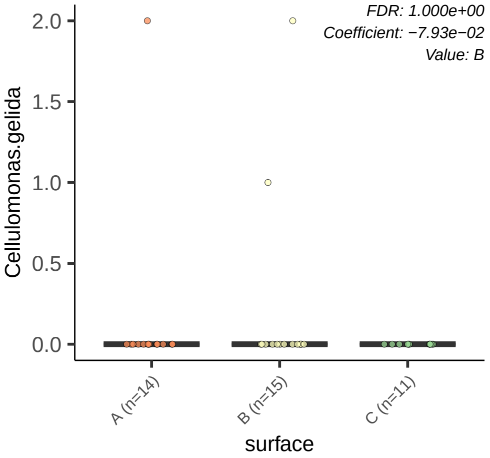

FDR: 1.000e+00
Coefficient: −7.93e−02
Value: B
Cellulomonas.gelida
2.0
1.5
1.0
0.5
0.0
A (n=14)
B (n=15)
C (n=11)
surface

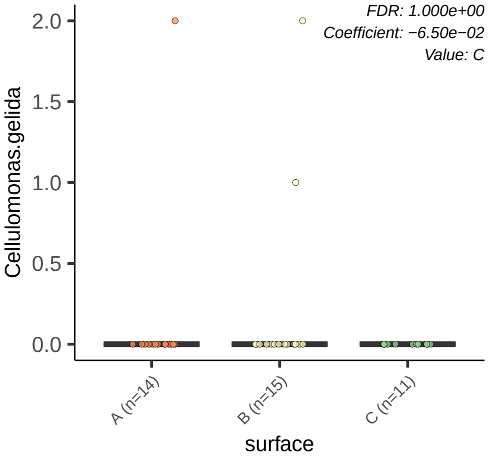
FDR: 1.000e+00
Coefficient: −6.50e−02
Value: C
Cellulomonas.gelida
2.0
1.5
1.0
0.5
0.0
A (n=14)
B (n=15)
C (n=11)
surface

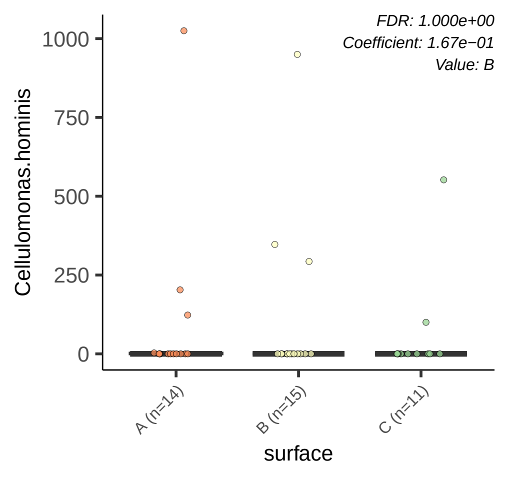

FDR: 1.000e+00
Coefficient: 1.67e−01
Value: B
Cellulomonas.hominis
1000
750
500
250
0
A (n=14)
B (n=15)
C (n=11)
surface

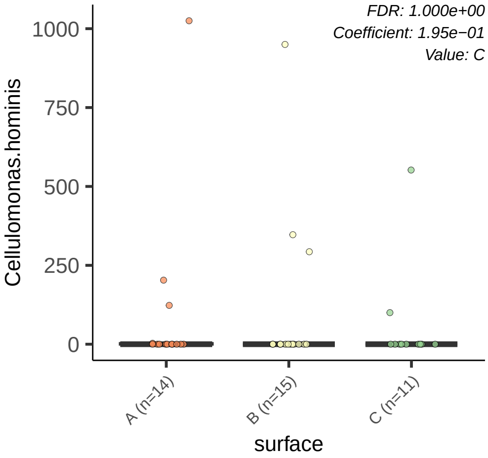
FDR: 1.000e+00
Coefficient: 1.95e−01
Value: C
1000
750
500
250
0
Cellulomonas.hominis
A (n=14)
B (n=15)
C (n=11)
surface

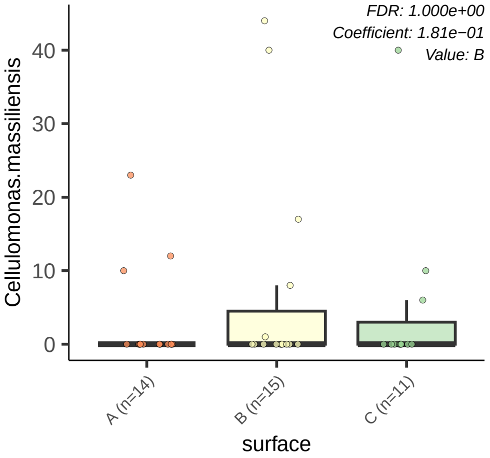

FDR: 1.000e+00
Coefficient: 1.81e−01
Value: B
Cellulomonas.massiliensis
0
10
20
30
40
A (n=14)
B (n=15)
C (n=11)
surface

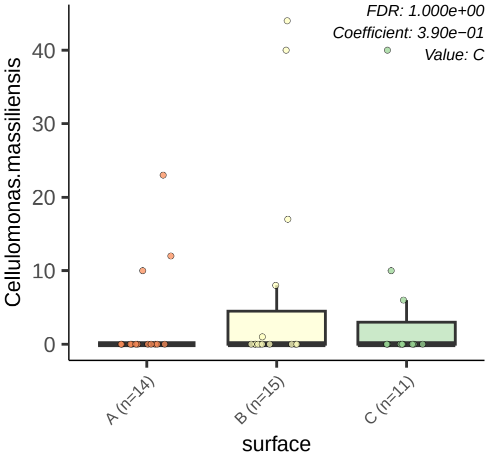

FDR: 1.000e+00
Coefficient: 3.90e−01
Value: C
Cellulomonas.massiliensis
0
10
20
30
40
A (n=14)
B (n=15)
C (n=11)
surface

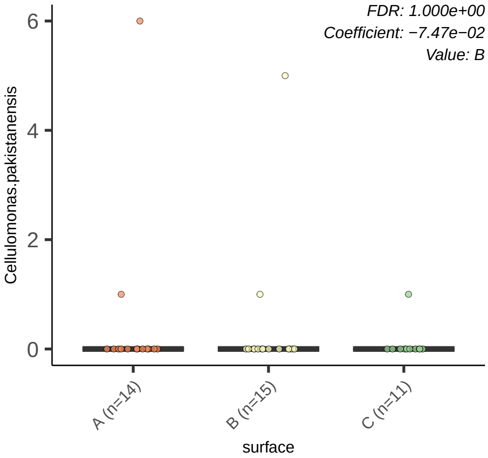

FDR: 1.000e+00
Coefficient: −7.47e−02
Value: B
Cellulomonas.pakistanensis
0
2
4
6
A (n=14)
B (n=15)
C (n=11)
surface

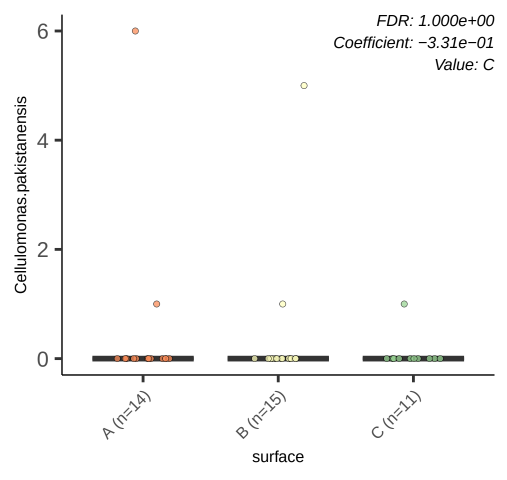

FDR: 1.000e+00
Coefficient: −3.31e−01
Value: C
Cellulomonas.pakistanensis
0
2
4
6
A (n=14)
B (n=15)
C (n=11)
surface

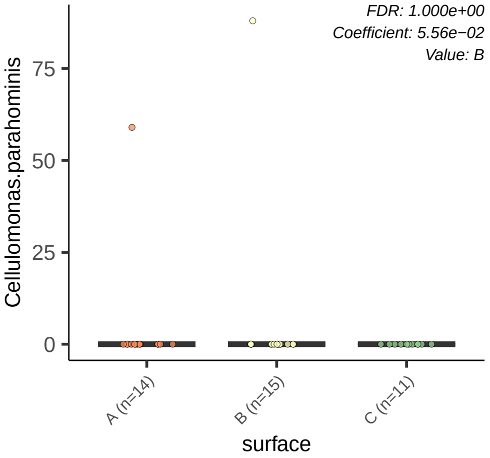

FDR: 1.000e+00
Coefficient: 5.56e−02
Value: B
Cellulomonas.parahominis
75
50
25
0
A (n=14)
B (n=15)
C (n=11)
surface

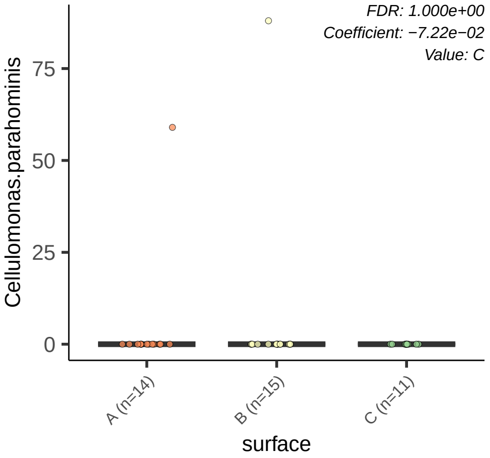
FDR: 1.000e+00
Coefficient: −7.22e−02
Value: C
Cellulomonas.parahominis
75
50
25
0
A (n=14)
B (n=15)
C (n=11)
surface

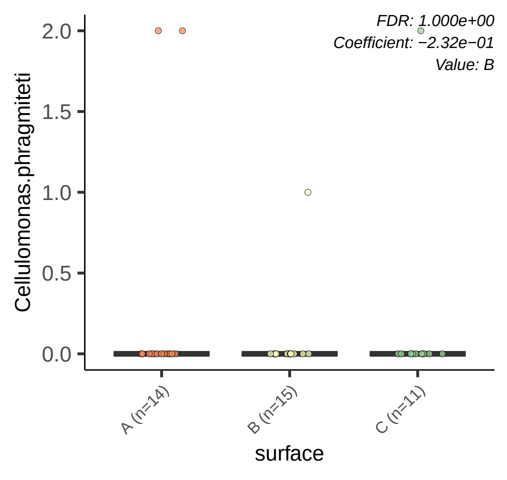
FDR: 1.000e+00
Coefficient: −2.32e−01
Value: B
Cellulomonas.phragmiteti
2.0
1.5
1.0
0.5
0.0
A (n=14)
B (n=15)
C (n=11)
surface

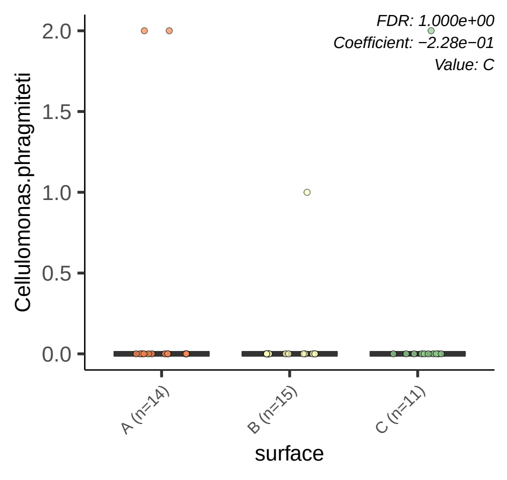

FDR: 1.000e+00
Coefficient: −2.28e−01
Value: C
Cellulomonas.phragmiteti
2.0
1.5
1.0
0.5
0.0
A (n=14)
B (n=15)
C (n=11)
surface

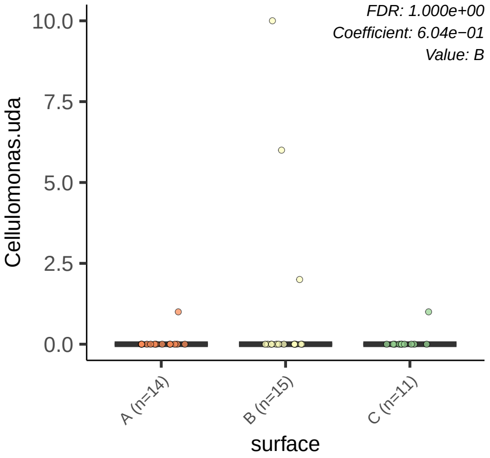
FDR: 1.000e+00
Coefficient: 6.04e−01
Value: B
Cellulomonas.uda
10.0
7.5
5.0
2.5
0.0
A (n=14)
B (n=15)
C (n=11)
surface

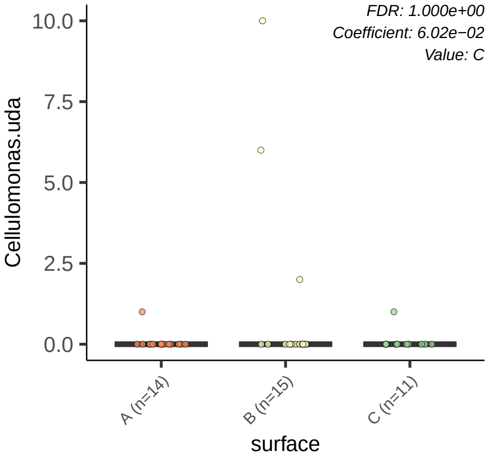

FDR: 1.000e+00
Coefficient: 6.02e−02
Value: C
Cellulomonas.uda
10.0
7.5
5.0
2.5
0.0
A (n=14)
B (n=15)
C (n=11)
surface

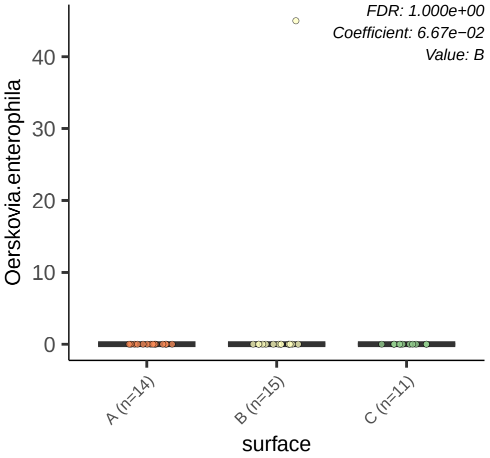
FDR: 1.000e+00
Coefficient: 6.67e−02
Value: B
Oerskovia.enterophila
0
10
20
30
40
A (n=14)
B (n=15)
C (n=11)
surface

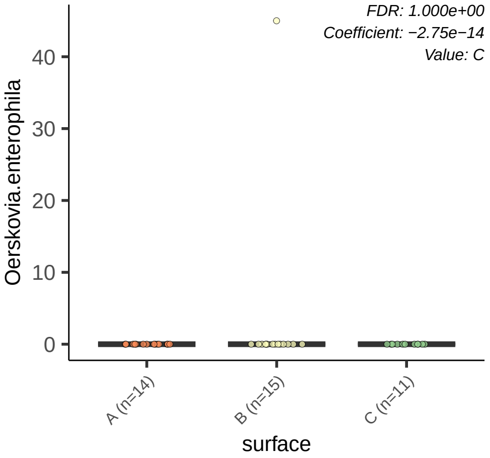
FDR: 1.000e+00
Coefficient: −2.75e−14
Value: C
Oerskovia.enterophila
40
30
20
10
0
A (n=14)
B (n=15)
C (n=11)
surface

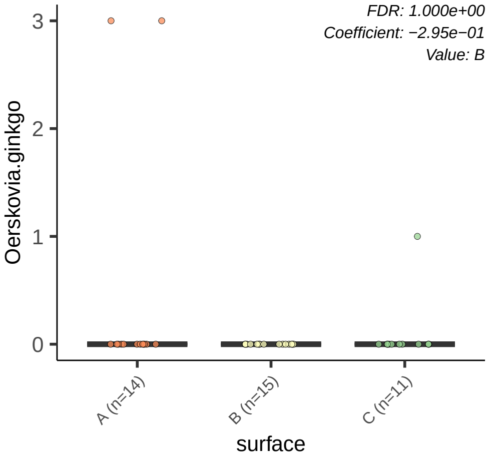

FDR: 1.000e+00
Coefficient: −2.95e−01
Value: B
Oerskovia.ginkgo
3
2
1
0
A (n=14)
B (n=15)
C (n=11)
surface

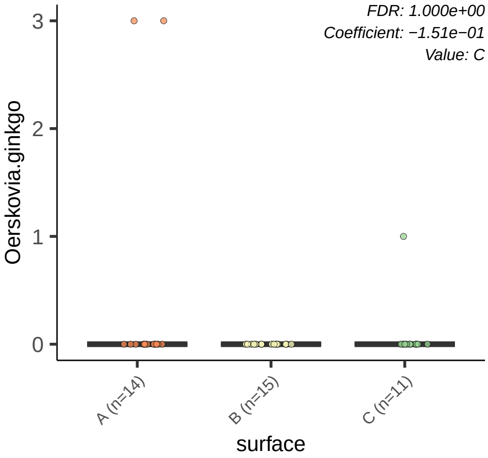
FDR: 1.000e+00
Coefficient: −1.51e−01
Value: C
Oerskovia.ginkgo
0
1
2
3
A (n=14)
B (n=15)
C (n=11)
surface

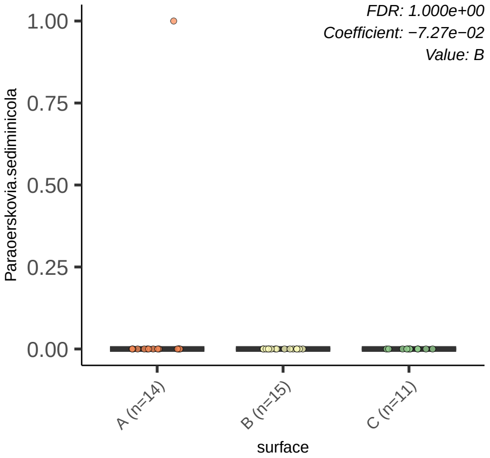
FDR: 1.000e+00
Coefficient: −7.27e−02
Value: B
Paraoerskovia.sedimincola
1.00
0.75
0.50
0.25
0.00
A (n=14)
B (n=15)
C (n=11)
surface

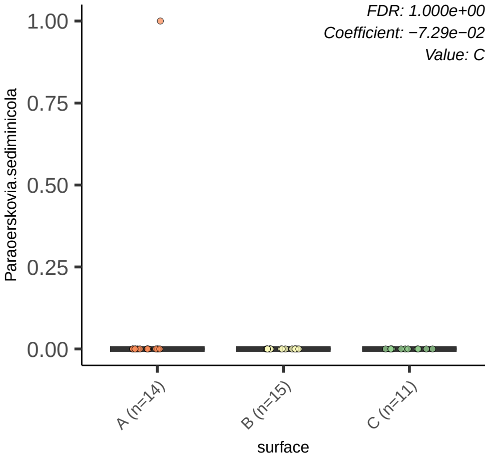
FDR: 1.000e+00
Coefficient: −7.29e−02
Value: C
Paraoerskovia.sediminicola
1.00
0.75
0.50
0.25
0.00
A (n=14)
B (n=15)
C (n=11)
surface

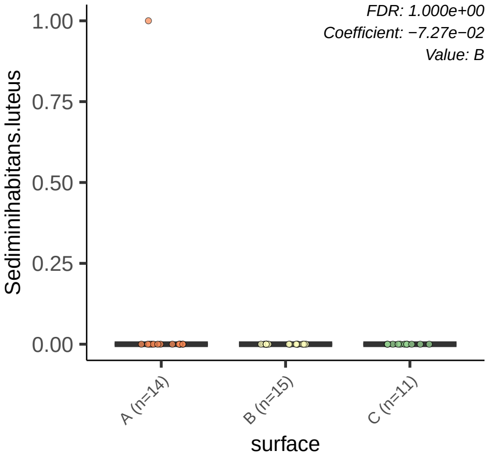
FDR: 1.000e+00
Coefficient: −7.27e−02
Value: B
Sediminihabitans.luteus
1.00
0.75
0.50
0.25
0.00
A (n=14)
B (n=15)
C (n=11)
surface

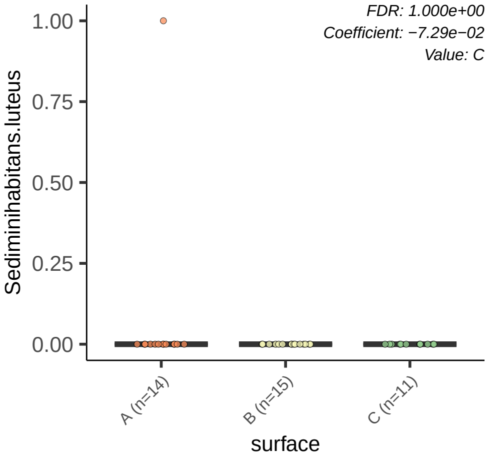
FDR: 1.000e+00
Coefficient: −7.29e−02
Value: C
Sediminihabitans.luteus
1.00
0.75
0.50
0.25
0.00
A (n=14)
B (n=15)
C (n=11)
surface

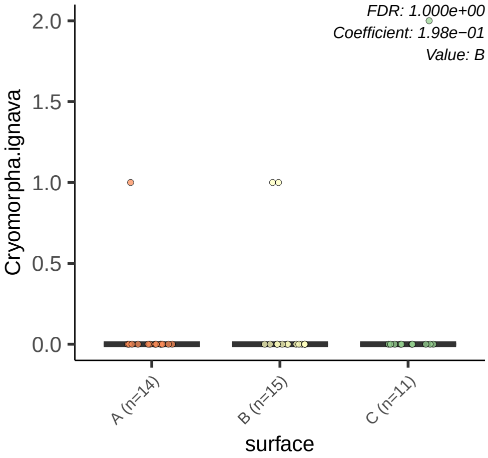
FDR: 1.000e+00
Coefficient: 1.98e−01
Value: B
Cryomorpha.ignava
2.0
1.5
1.0
0.5
0.0
A (n=14)
B (n=15)
C (n=11)
surface

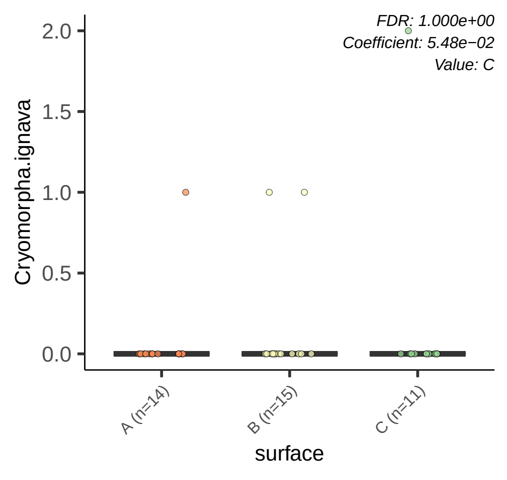
FDR: 1.000e+00
Coefficient: 5.48e−02
Value: C
Cryomorpha.ignava
2.0
1.5
1.0
0.5
0.0
A (n=14)
B (n=15)
C (n=11)
surface

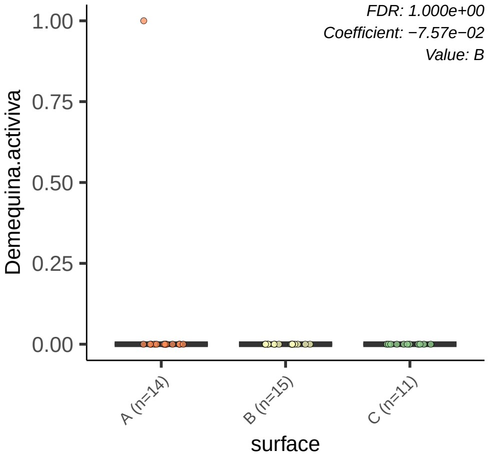
FDR: 1.000e+00
Coefficient: −7.57e−02
Value: B
Demequina.activiva
1.00
0.75
0.50
0.25
0.00
A (n=14)
B (n=15)
C (n=11)
surface

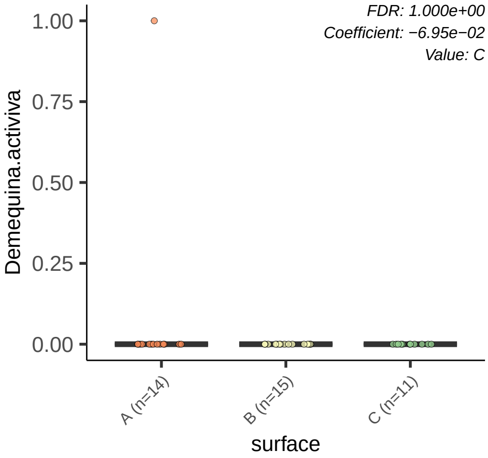

FDR: 1.000e+00
Coefficient: −6.95e−02
Value: C
Demequina.activiva
1.00
0.75
0.50
0.25
0.00
A (n=14)
B (n=15)
C (n=11)
surface

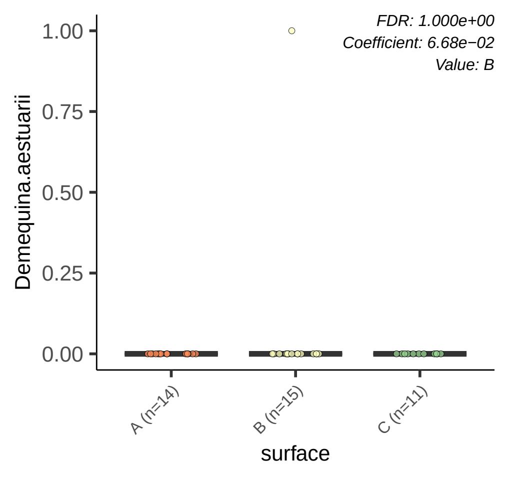

FDR: 1.000e+00
Coefficient: 6.68e−02
Value: B
Demequina.aestuarii
1.00
0.75
0.50
0.25
0.00
A (n=14)
B (n=15)
C (n=11)
surface

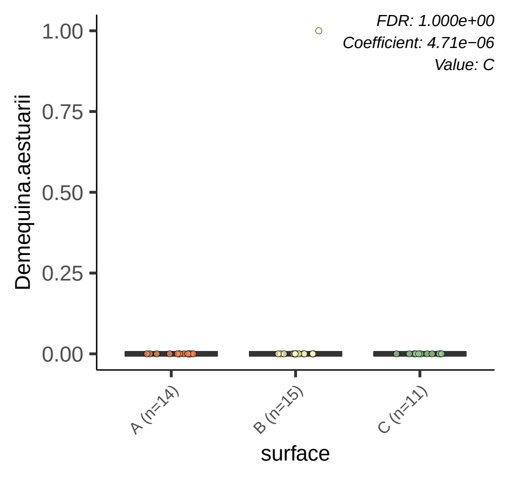
FDR: 1.000e+00
Coefficient: 4.71e−06
Value: C
Demequina.aestuarii
1.00
0.75
0.50
0.25
0.00
A (n=14)
B (n=15)
C (n=11)
surface

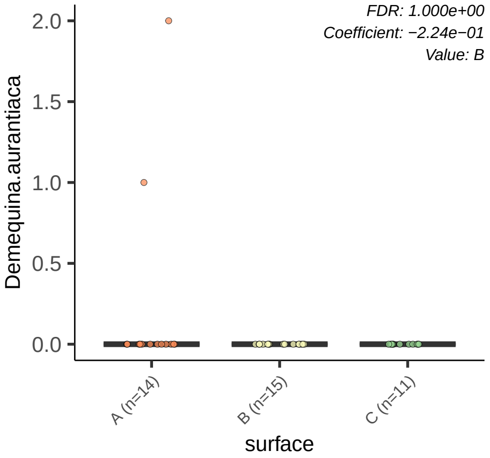

FDR: 1.000e+00
Coefficient: −2.24e−01
Value: B
Demequina.aurantiaca
2.0
1.5
1.0
0.5
0.0
A (n=14)
B (n=15)
C (n=11)
surface

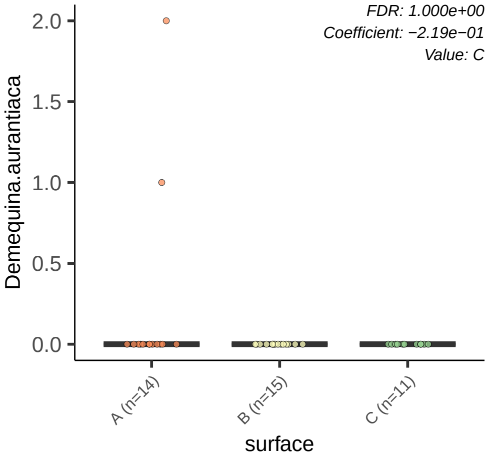
FDR: 1.000e+00
Coefficient: −2.19e−01
Value: C
Demequina.aurantiaca
2.0
1.5
1.0
0.5
0.0
A (n=14)
B (n=15)
C (n=11)
surface

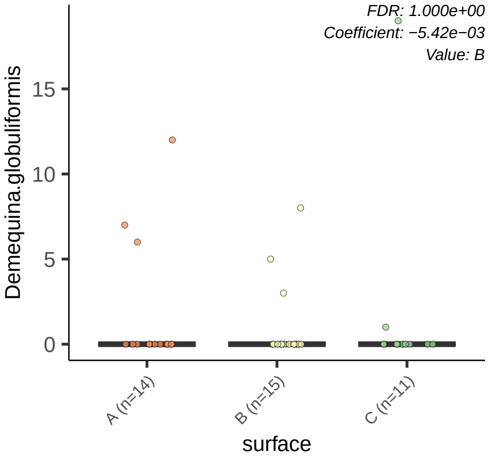

FDR: 1.000e+00
Coefficient: −5.42e−03
Value: B
Demequina.globuliformis
0
5
10
15
A (n=14)
B (n=15)
C (n=11)
surface

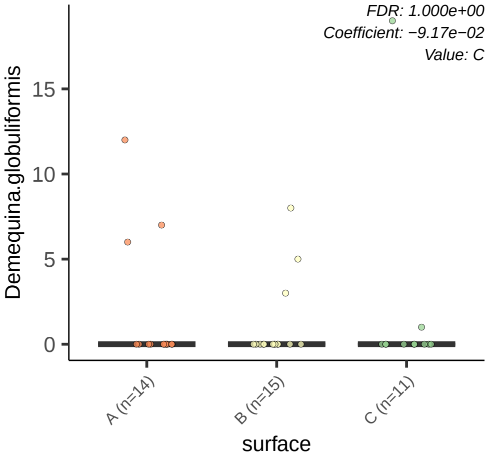
FDR: 1.000e+00
Coefficient: −9.17e−02
Value: C
Demequina.globuliformis
15
10
5
0
A (n=14)
B (n=15)
C (n=11)
surface

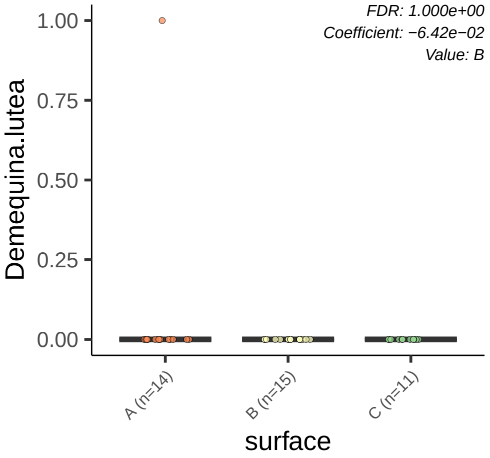
FDR: 1.000e+00
Coefficient: −6.42e−02
Value: B
Demequina.lutea
1.00
0.75
0.50
0.25
0.00
A (n=14)
B (n=15)
C (n=11)
surface

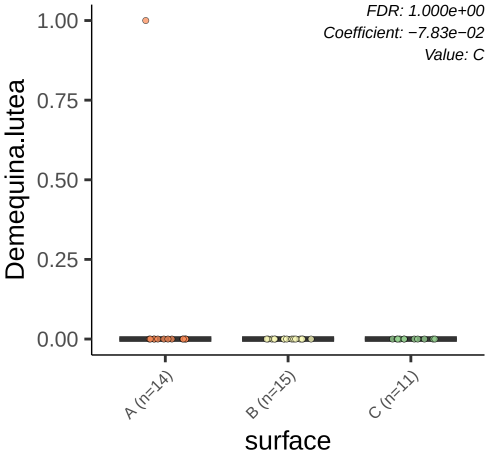

FDR: 1.000e+00
Coefficient: −7.83e−02
Value: C
Demequina.lutea
1.00
0.75
0.50
0.25
0.00
A (n=14)
B (n=15)
C (n=11)
surface

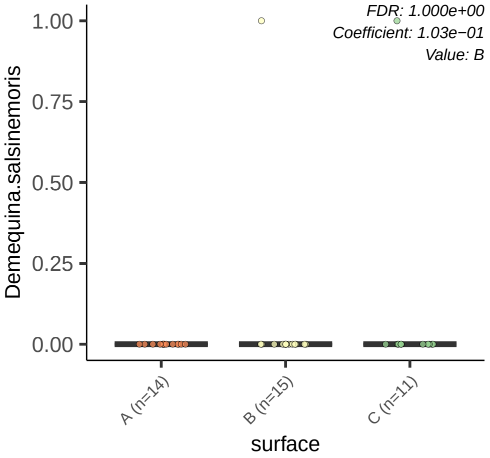

FDR: 1.000e+00
Coefficient: 1.03e−01
Value: B
Demequina.salsinemoris
1.00
0.75
0.50
0.25
0.00
A (n=14)
B (n=15)
C (n=11)
surface

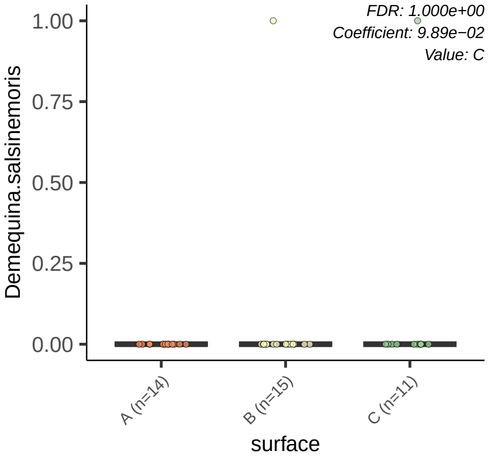

FDR: 1.000e+00
Coefficient: 9.89e−02
Value: C
Demequina.salsinemoris
1.00
0.75
0.50
0.25
0.00
A (n=14)
B (n=15)
C (n=11)
surface

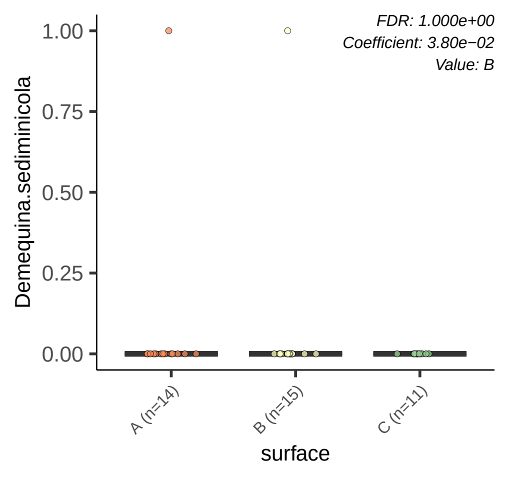

FDR: 1.000e+00
Coefficient: 3.80e−02
Value: B
Demequina.sediminicola
1.00
0.75
0.50
0.25
0.00
A (n=14)
B (n=15)
C (n=11)
surface

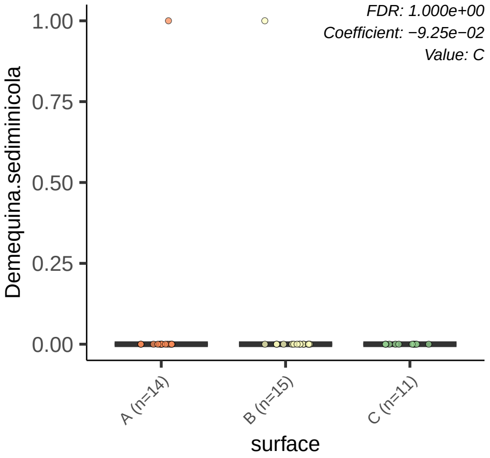

FDR: 1.000e+00
Coefficient: −9.25e−02
Value: C
Demequina.sedimenicola
1.00
0.75
0.50
0.25
0.00
A (n=14)
B (n=15)
C (n=11)
surface

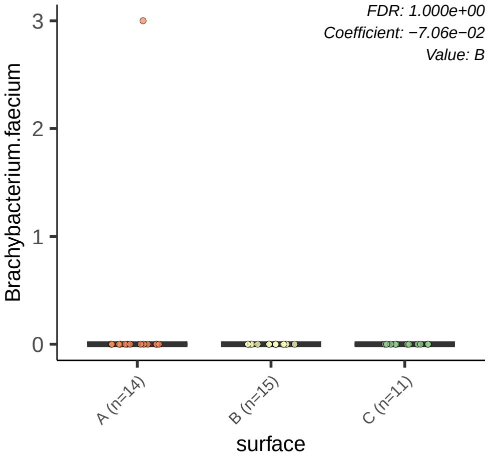

FDR: 1.000e+00
Coefficient: −7.06e−02
Value: B
Brachybacterium.faecium
3
2
1
0
A (n=14)
B (n=15)
C (n=11)
surface

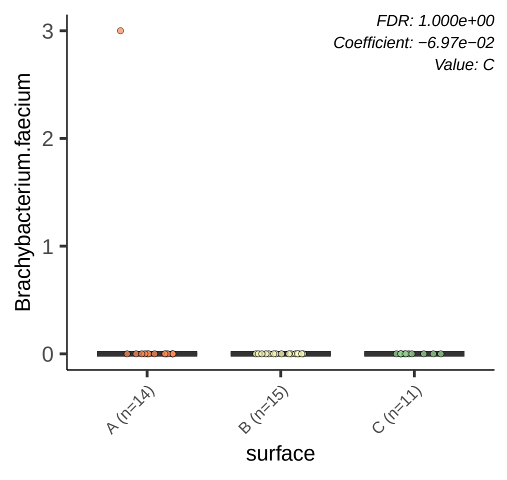
FDR: 1.000e+00
Coefficient: −6.97e−02
Value: C
Brachybacterium.faecium
3
2
1
0
A (n=14)
B (n=15)
C (n=11)
surface

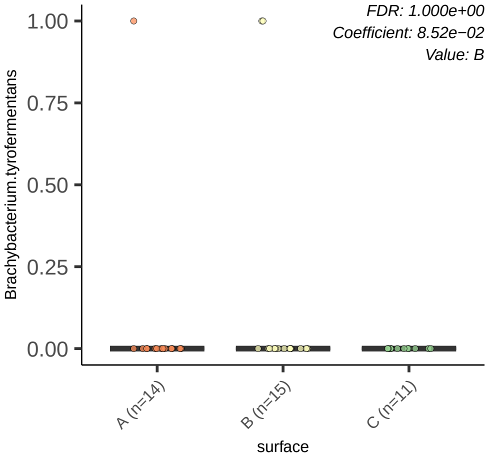

FDR: 1.000e+00
Coefficient: 8.52e−02
Value: B
Brachybacterium.tyrofermentans
1.00
0.75
0.50
0.25
0.00
A (n=14)
B (n=15)
C (n=11)
surface

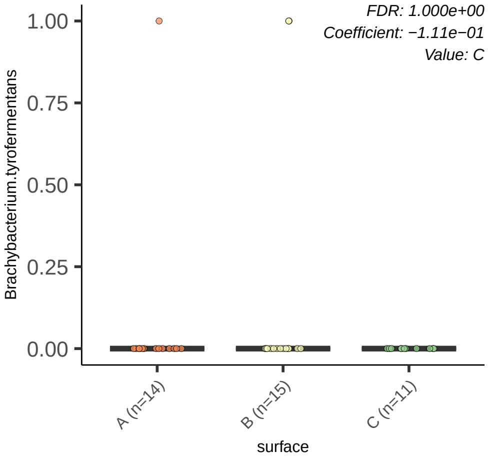
FDR: 1.000e+00
Coefficient: −1.11e−01
Value: C
Brachybacterium.tyrofermentans
1.00
0.75
0.50
0.25
0.00
A (n=14)
B (n=15)
C (n=11)
surface

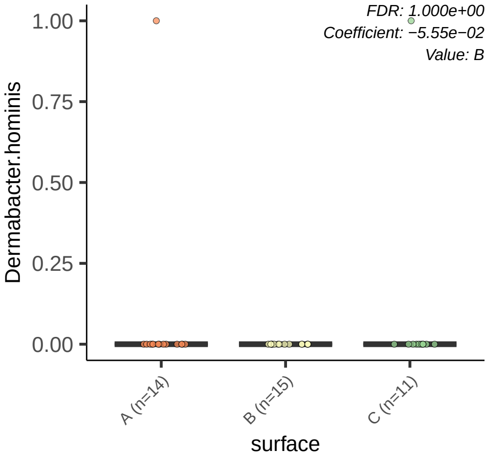
FDR: 1.000e+00
Coefficient: −5.55e−02
Value: B
Dermabacter.hominis
1.00
0.75
0.50
0.25
0.00
A (n=14)
B (n=15)
C (n=11)
surface

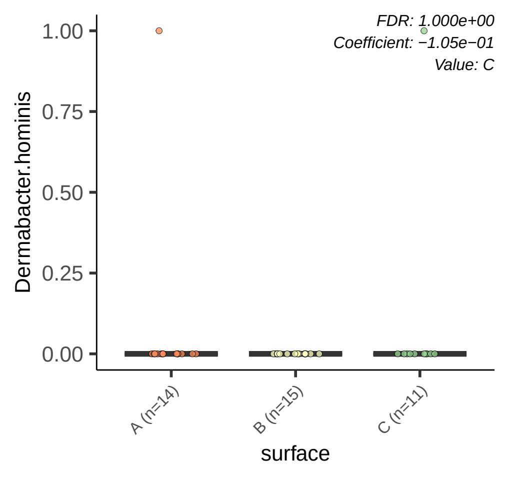
FDR: 1.000e+00
Coefficient: −1.05e−01
Value: C
Dermabacter.hominis
1.00
0.75
0.50
0.25
0.00
A (n=14)
B (n=15)
C (n=11)
surface

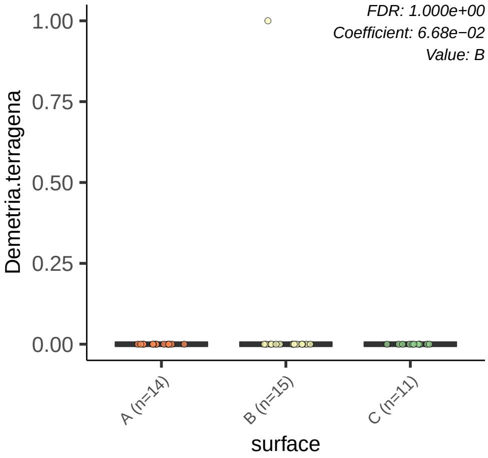

FDR: 1.000e+00
Coefficient: 6.68e−02
Value: B
Demetria.terragena
1.00
0.75
0.50
0.25
0.00
A (n=14)
B (n=15)
C (n=11)
surface

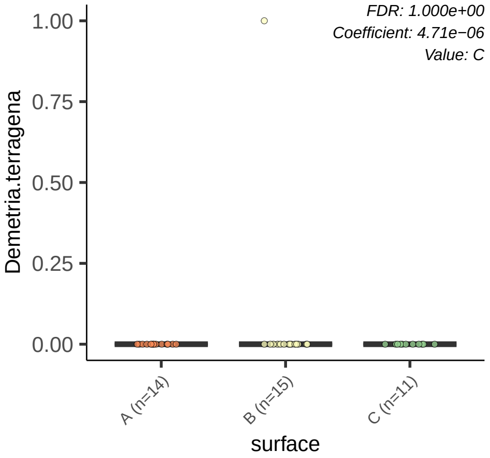
FDR: 1.000e+00
Coefficient: 4.71e−06
Value: C
Demetria.terragena
1.00
0.75
0.50
0.25
0.00
A (n=14)
B (n=15)
C (n=11)
surface

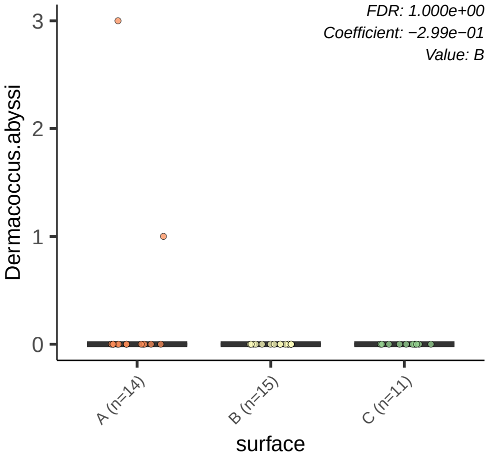
FDR: 1.000e+00
Coefficient: −2.99e−01
Value: B
Dermacoccus.abyssi
3
2
1
0
A (n=14)
B (n=15)
C (n=11)
surface

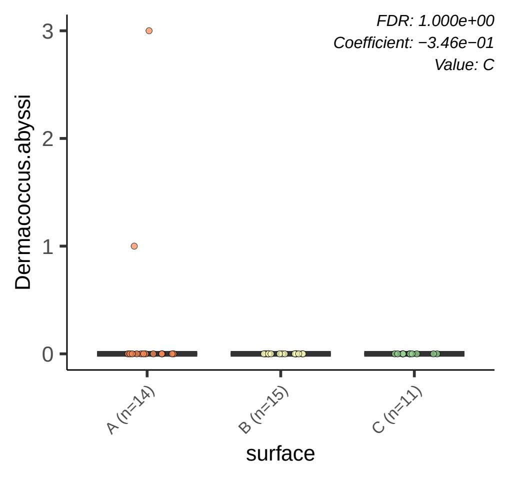
FDR: 1.000e+00
Coefficient: −3.46e−01
Value: C
Dermacoccus.abyssi
3
2
1
0
A (n=14)
B (n=15)
C (n=11)
surface

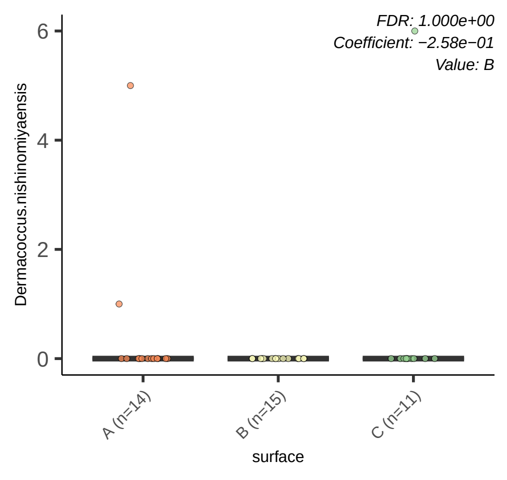
FDR: 1.000e+00
Coefficient: −2.58e−01
Value: B
Dermacoccus.nishinomiyaensis
6
4
2
0
A (n=14)
B (n=15)
C (n=11)
surface

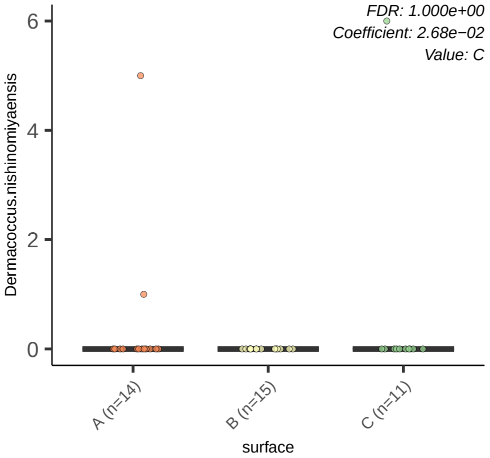

FDR: 1.000e+00
Coefficient: 2.68e−02
Value: C
Dermacoccus.nishinomiyaensis
6
4
2
0
A (n=14)
B (n=15)
C (n=11)
surface

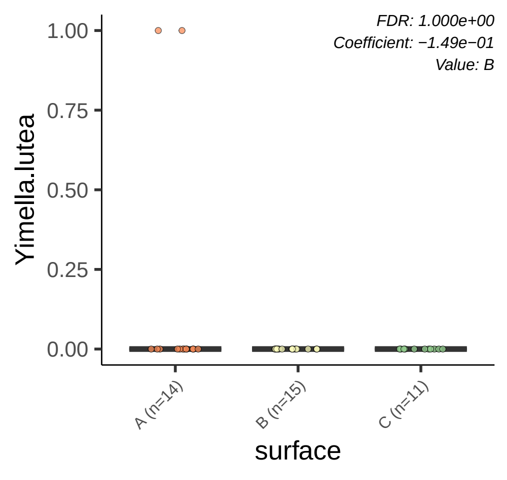

FDR: 1.000e+00
Coefficient: −1.49e−01
Value: B
Yimella.lutea
1.00
0.75
0.50
0.25
0.00
A (n=14)
B (n=15)
C (n=11)
surface

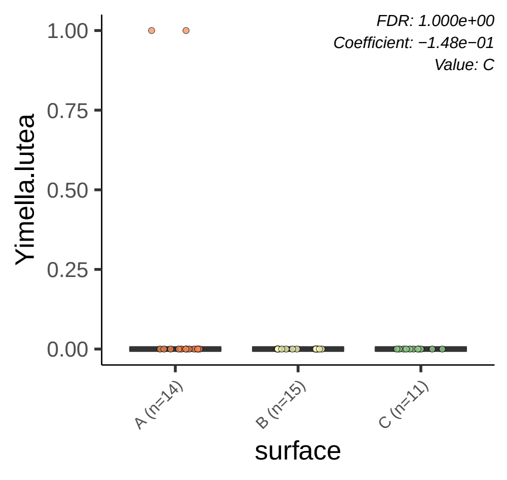

FDR: 1.000e+00
Coefficient: −1.48e−01
Value: C
Yimella.lutea
1.00
0.75
0.50
0.25
0.00
A (n=14)
B (n=15)
C (n=11)
surface

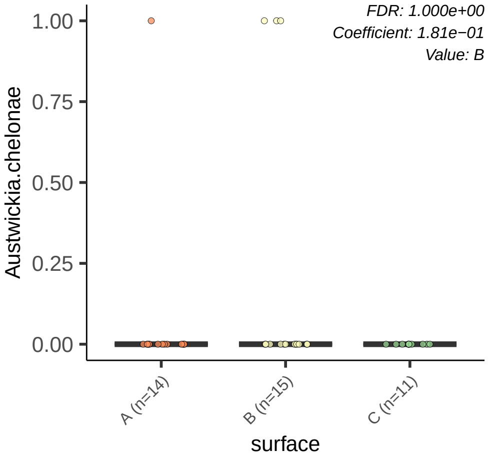
FDR: 1.000e+00
Coefficient: 1.81e−01
Value: B
Austwickia.chelonae
1.00
0.75
0.50
0.25
0.00
A (n=14)
B (n=15)
C (n=11)
surface

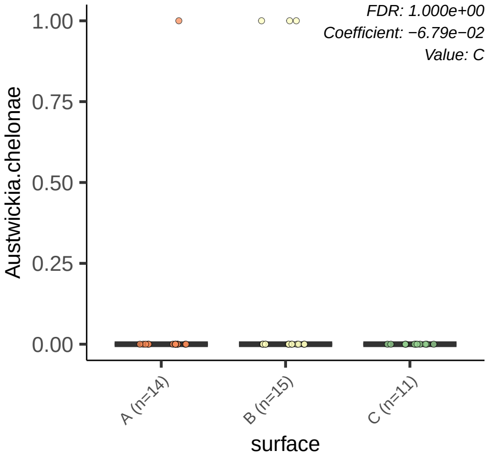
FDR: 1.000e+00
Coefficient: −6.79e−02
Value: C
Austwickia.chelonae
1.00
0.75
0.50
0.25
0.00
A (n=14)
B (n=15)
C (n=11)
surface

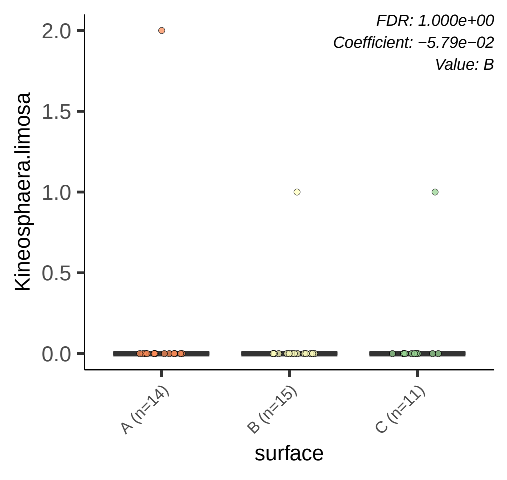

FDR: 1.000e+00
Coefficient: −5.79e−02
Value: B
Kineosphaera.limosa
2.0
1.5
1.0
0.5
0.0
A (n=14)
B (n=15)
C (n=11)
surface

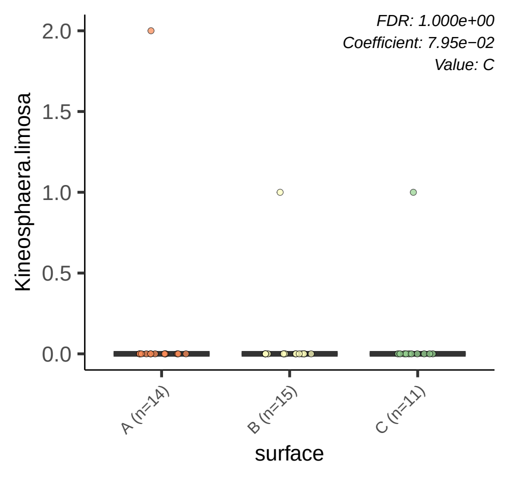

FDR: 1.000e+00
Coefficient: 7.95e−02
Value: C
Kineosphaera.limosa
2.0
1.5
1.0
0.5
0.0
A (n=14)
B (n=15)
C (n=11)
surface

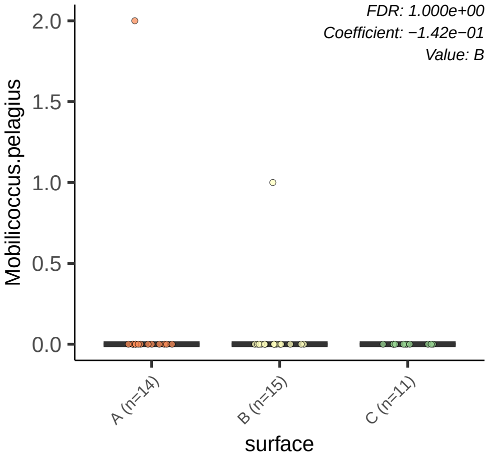
FDR: 1.000e+00
Coefficient: −1.42e−01
Value: B
Mobilicoccus.pelagius
2.0
1.5
1.0
0.5
0.0
A (n=14)
B (n=15)
C (n=11)
surface

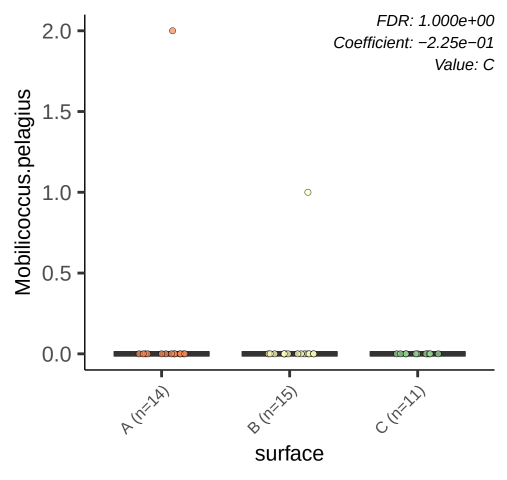

FDR: 1.000e+00
Coefficient: −2.25e−01
Value: C
Mobilicoccus.pelagius
2.0
1.5
1.0
0.5
0.0
A (n=14)
B (n=15)
C (n=11)
surface

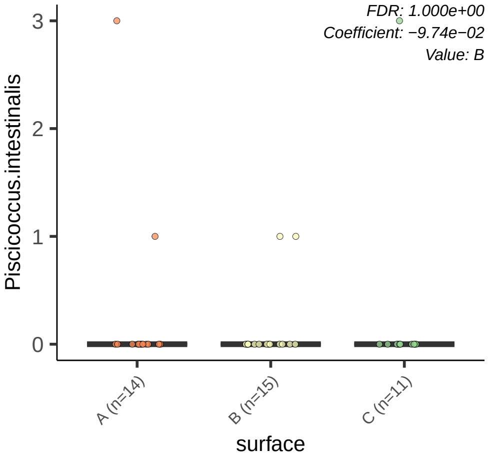
FDR: 1.000e+00
Coefficient: −9.74e−02
Value: B
Piscicoccus.intestinalis
3
2
1
0
A (n=14)
B (n=15)
C (n=11)
surface

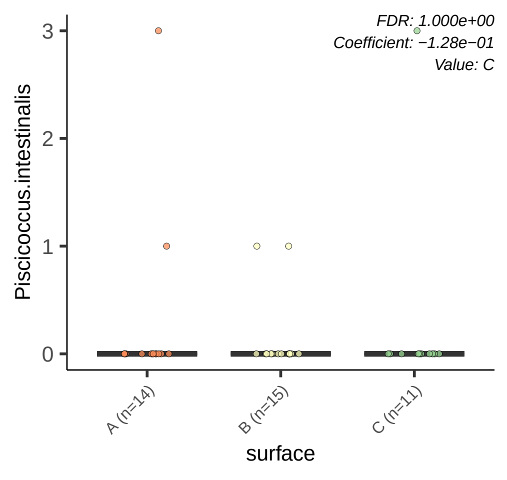

FDR: 1.000e+00
Coefficient: −1.28e−01
Value: C
Piscicoccus.intestinalis
3
2
1
0
A (n=14)
B (n=15)
C (n=11)
surface

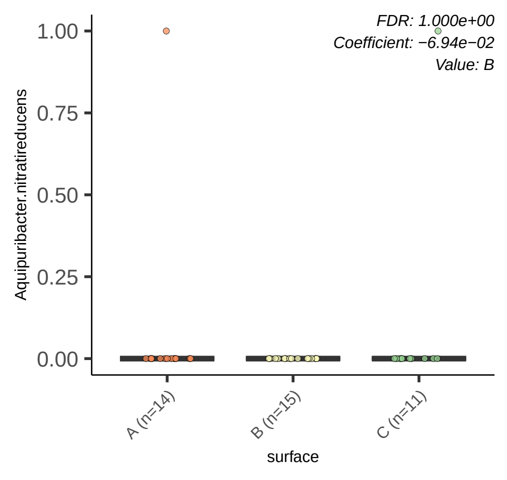
FDR: 1.000e+00
Coefficient: −6.94e−02
Value: B
Aquipuribacter.nitratireducens
1.00
0.75
0.50
0.25
0.00
A (n=14)
B (n=15)
C (n=11)
surface

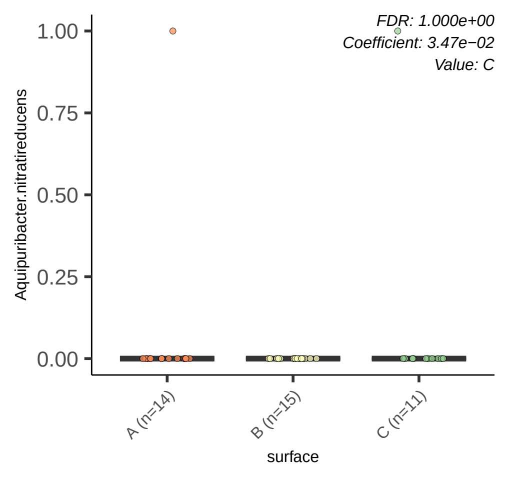

FDR: 1.000e+00
Coefficient: 3.47e−02
Value: C
Aquipuribacter.nitratireducens
1.00
0.75
0.50
0.25
0.00
A (n=14)
B (n=15)
C (n=11)
surface

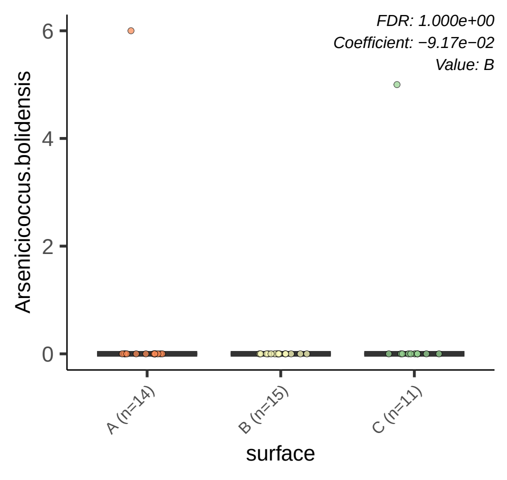

FDR: 1.000e+00
Coefficient: −9.17e−02
Value: B
Arsenicicoccus.bolidensis
6
4
2
0
A (n=14)
B (n=15)
C (n=11)
surface

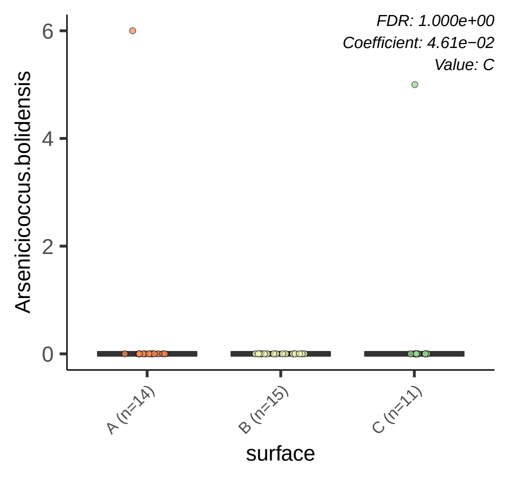

FDR: 1.000e+00
Coefficient: 4.61e−02
Value: C
Arsenicicoccus.bolidensis
6
4
2
0
A (n=14)
B (n=15)
C (n=11)
surface

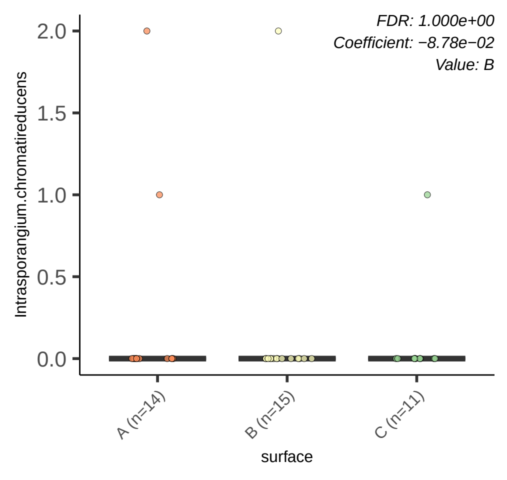

FDR: 1.000e+00
Coefficient: −8.78e−02
Value: B
2.0
1.5
1.0
0.5
0.0
Intrasporangium.chromatireducens
A (n=14)
B (n=15)
C (n=11)
surface

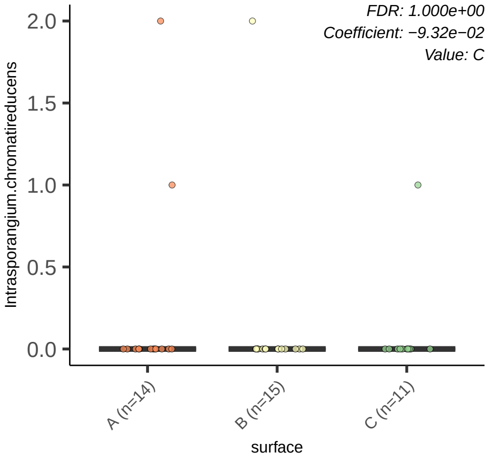

FDR: 1.000e+00
Coefficient: −9.32e−02
Value: C
Intrasporangium.chromatireducens
2.0
1.5
1.0
0.5
0.0
A (n=14)
B (n=15)
C (n=11)
surface

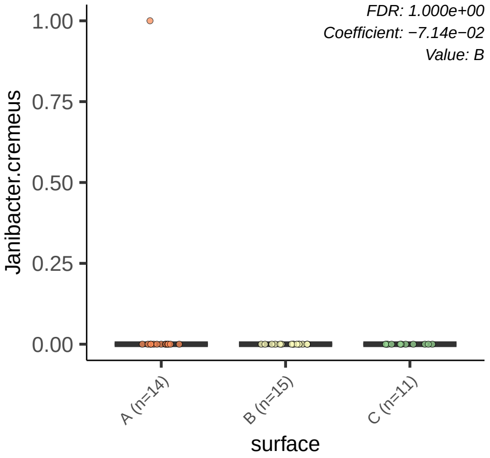

FDR: 1.000e+00
Coefficient: −7.14e−02
Value: B
Janibacter.cremeus
1.00
0.75
0.50
0.25
0.00
A (n=14)
B (n=15)
C (n=11)
surface

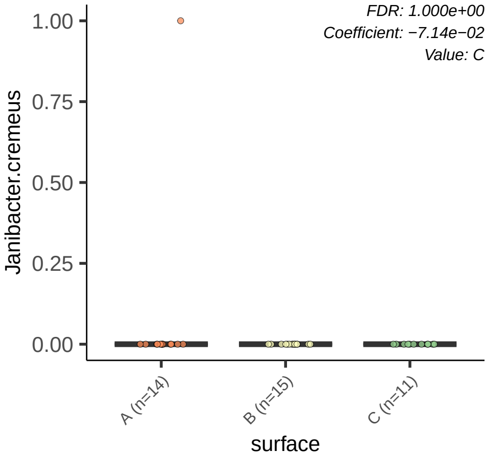

FDR: 1.000e+00
Coefficient: −7.14e−02
Value: C
Janibacter.cremeus
1.00
0.75
0.50
0.25
0.00
A (n=14)
B (n=15)
C (n=11)
surface

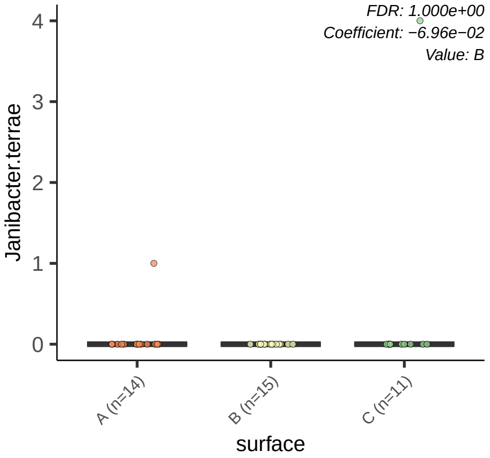
FDR: 1.000e+00
Coefficient: −6.96e−02
Value: B
Janibacter.terrae
4
3
2
1
0
A (n=14)
B (n=15)
C (n=11)
surface

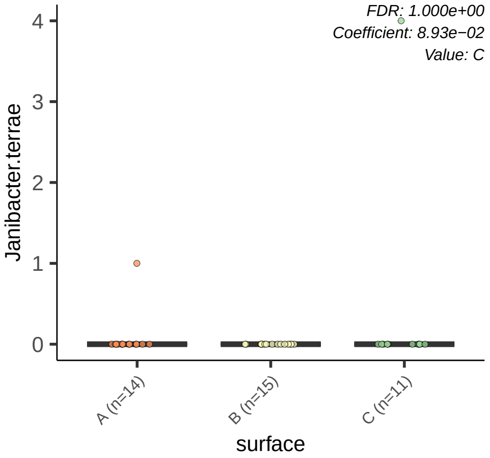
FDR: 1.000e+00
Coefficient: 8.93e−02
Value: C
Janibacter.terrae
4
3
2
1
0
A (n=14)
B (n=15)
C (n=11)
surface

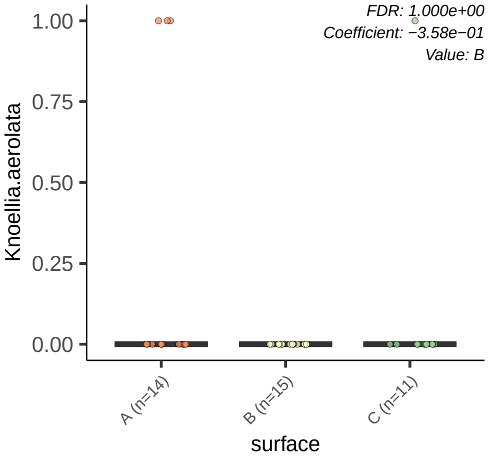

FDR: 1.000e+00
Coefficient: −3.58e−01
Value: B
Knoellia.aerolata
1.00
0.75
0.50
0.25
0.00
A (n=14)
B (n=15)
C (n=11)
surface

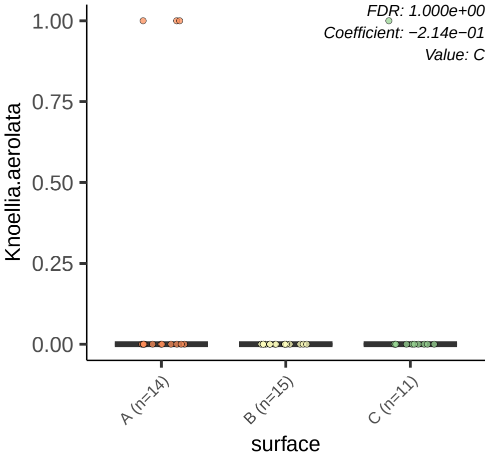

FDR: 1.000e+00
Coefficient: −2.14e−01
Value: C
Knoellia.aerolata
1.00
0.75
0.50
0.25
0.00
A (n=14)
B (n=15)
C (n=11)
surface

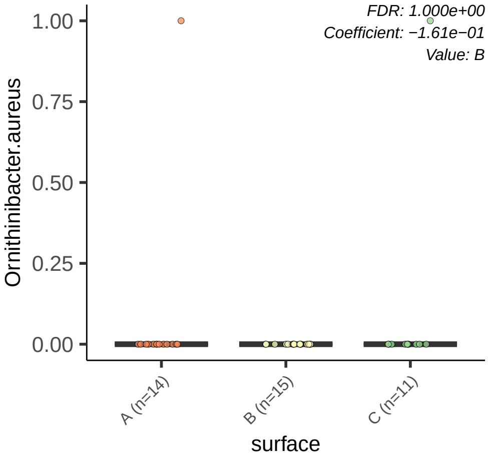

FDR: 1.000e+00
Coefficient: −1.61e−01
Value: B
Ornithinibacter.aureus
1.00
0.75
0.50
0.25
0.00
A (n=14)
B (n=15)
C (n=11)
surface

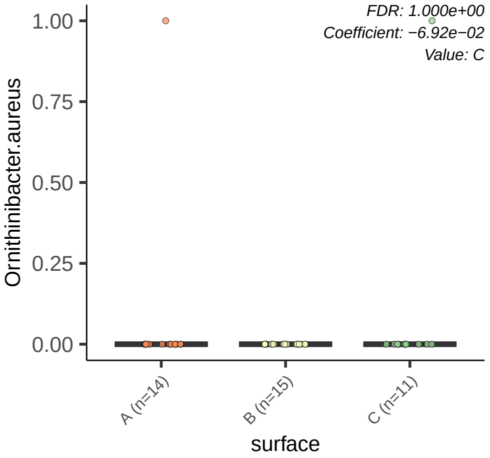
FDR: 1.000e+00
Coefficient: −6.92e−02
Value: C
Ornithinibacter.aureus
1.00
0.75
0.50
0.25
0.00
A (n=14)
B (n=15)
C (n=11)
surface

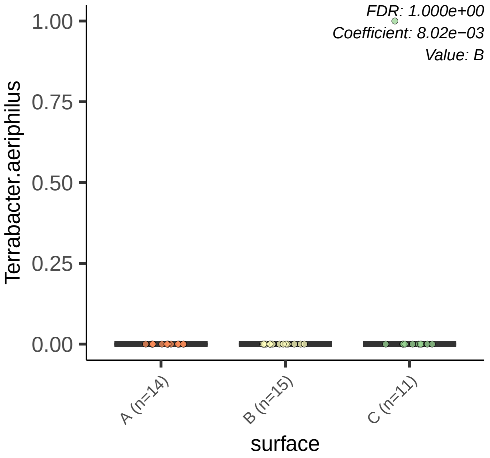
FDR: 1.000e+00
Coefficient: 8.02e−03
Value: B
Terrabacter.aeriphilus
1.00
0.75
0.50
0.25
0.00
A (n=14)
B (n=15)
C (n=11)
surface

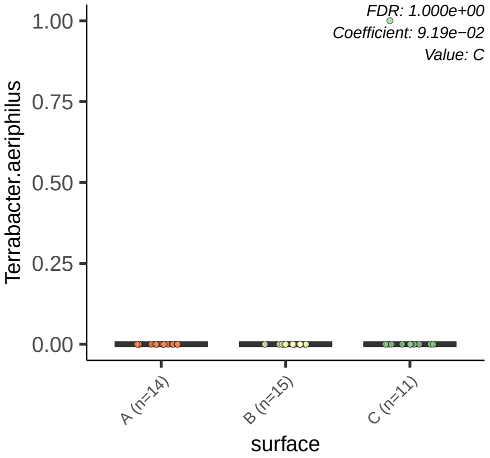
FDR: 1.000e+00
Coefficient: 9.19e−02
Value: C
Terrabacter.aeriphilus
1.00
0.75
0.50
0.25
0.00
A (n=14)
B (n=15)
C (n=11)
surface

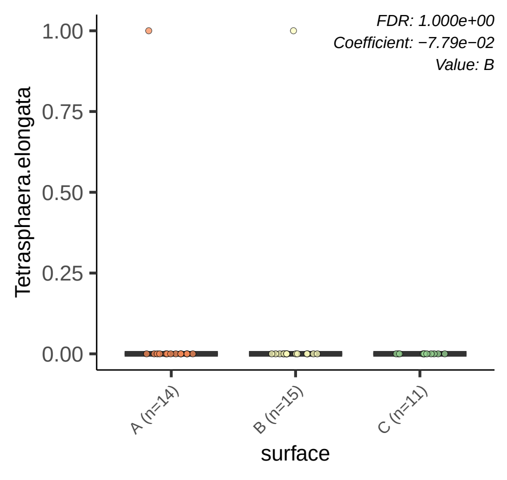
FDR: 1.000e+00
Coefficient: −7.79e−02
Value: B
Tetrasphaera.elongata
1.00
0.75
0.50
0.25
0.00
A (n=14)
B (n=15)
C (n=11)
surface

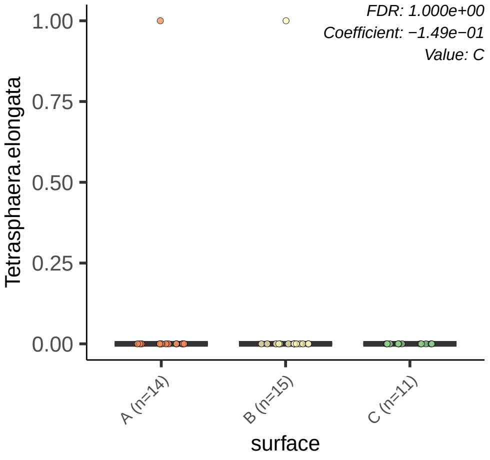

FDR: 1.000e+00
Coefficient: −1.49e−01
Value: C
Tetrasphaera.elongata
1.00
0.75
0.50
0.25
0.00
A (n=14)
B (n=15)
C (n=11)
surface

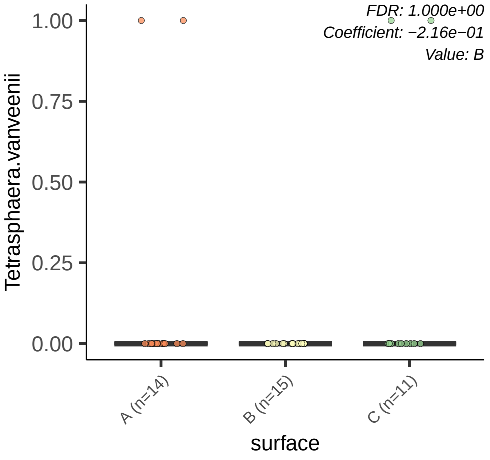
FDR: 1.000e+00
Coefficient: −2.16e−01
Value: B
Tetrasphaera.vanveenii
1.00
0.75
0.50
0.25
0.00
A (n=14)
B (n=15)
C (n=11)
surface

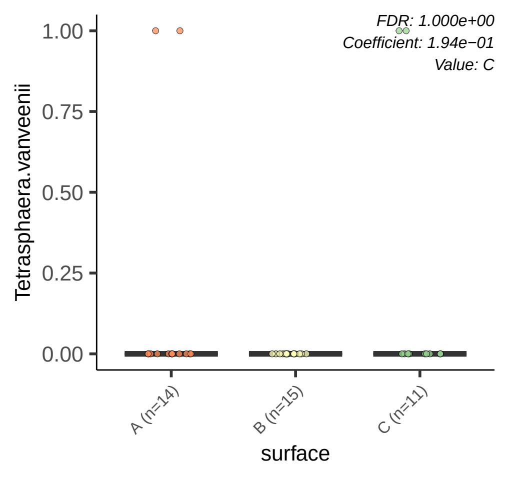

FDR: 1.000e+00
Coefficient: 1.94e−01
Value: C
Tetrasphaera.vanveenii
1.00
0.75
0.50
0.25
0.00
A (n=14)
B (n=15)
C (n=11)
surface

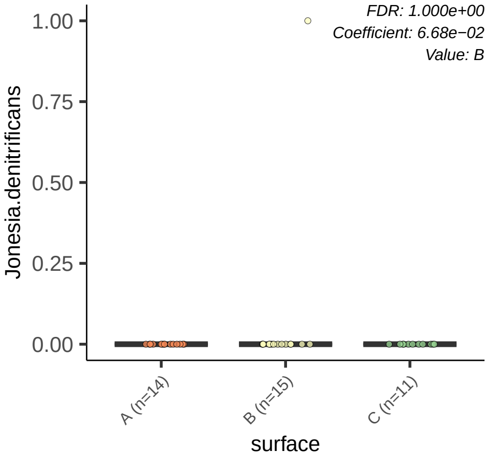
FDR: 1.000e+00
Coefficient: 6.68e−02
Value: B
Jonesia.denitrificans
1.00
0.75
0.50
0.25
0.00
A (n=14)
B (n=15)
C (n=11)
surface

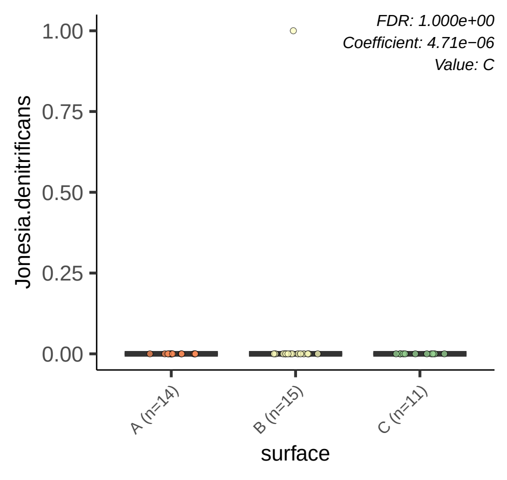

FDR: 1.000e+00
Coefficient: 4.71e−06
Value: C
Jonesia.denitrificans
1.00
0.75
0.50
0.25
0.00
A (n=14)
B (n=15)
C (n=11)
surface

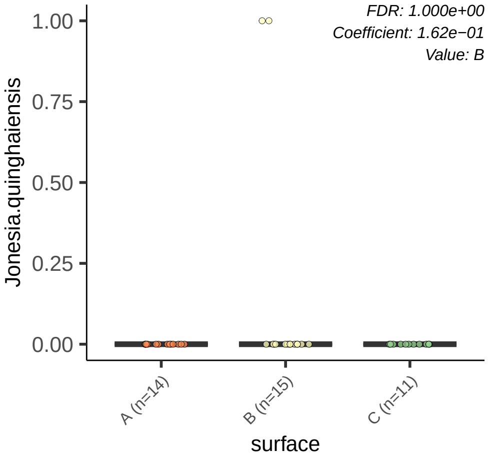
FDR: 1.000e+00
Coefficient: 1.62e−01
Value: B
Jonesia.quinghaiensis
1.00
0.75
0.50
0.25
0.00
A (n=14)
B (n=15)
C (n=11)
surface

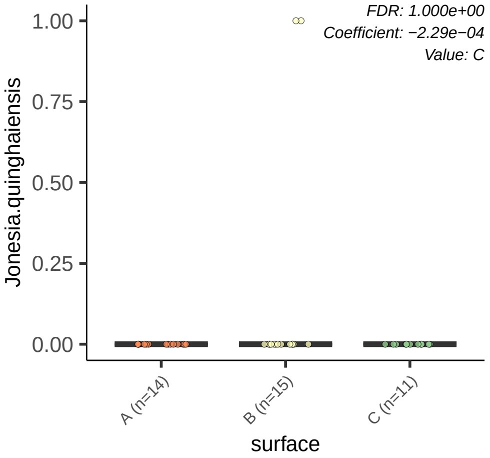
FDR: 1.000e+00
Coefficient: −2.29e−04
Value: C
Jonesia.quinghaiensis
1.00
0.75
0.50
0.25
0.00
A (n=14)
B (n=15)
C (n=11)
surface

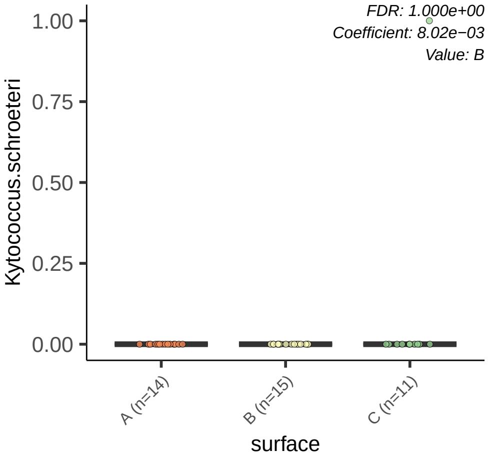

FDR: 1.000e+00
Coefficient: 8.02e−03
Value: B
Kytococcus.schroeteri
1.00
0.75
0.50
0.25
0.00
A (n=14)
B (n=15)
C (n=11)
surface

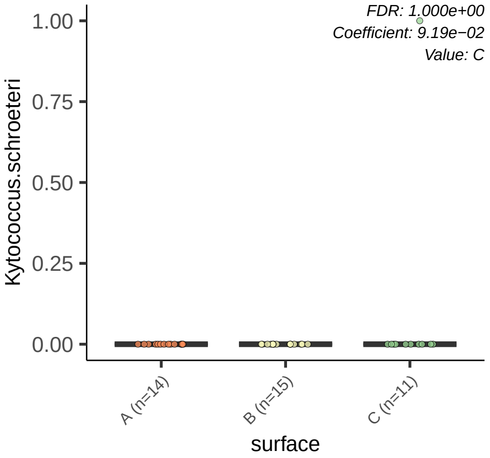
FDR: 1.000e+00
Coefficient: 9.19e−02
Value: C
Kytococcus.schroeteri
1.00
0.75
0.50
0.25
0.00
A (n=14)
B (n=15)
C (n=11)
surface

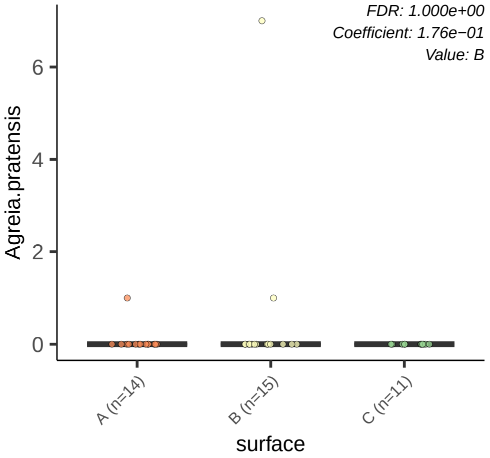
FDR: 1.000e+00
Coefficient: 1.76e−01
Value: B
Agreia.pratensis
0
2
4
6
A (n=14)
B (n=15)
C (n=11)
surface

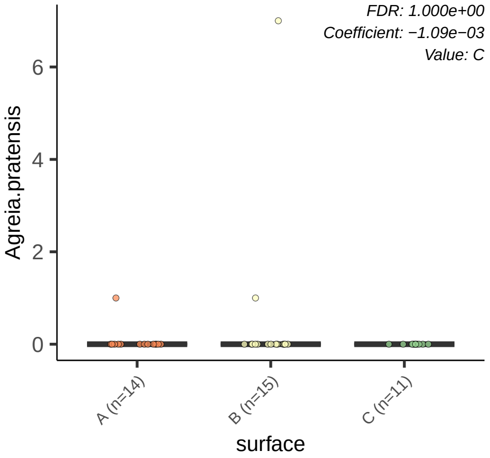

FDR: 1.000e+00
Coefficient: −1.09e−03
Value: C
Agreia.pratensis
0
2
4
6
A (n=14)
B (n=15)
C (n=11)
surface

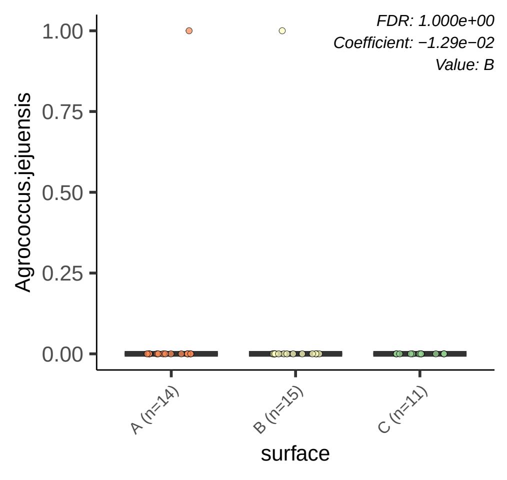
FDR: 1.000e+00
Coefficient: −1.29e−02
Value: B
Agrococcus.jejuensis
1.00
0.75
0.50
0.25
0.00
A (n=14)
B (n=15)
C (n=11)
surface

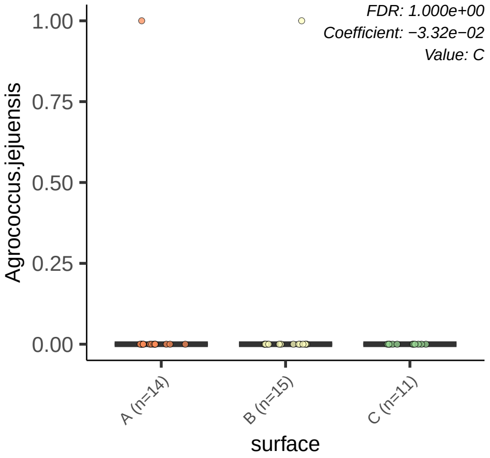
FDR: 1.000e+00
Coefficient: −3.32e−02
Value: C
Agrococcus.jejuensis
1.00
0.75
0.50
0.25
0.00
A (n=14)
B (n=15)
C (n=11)
surface

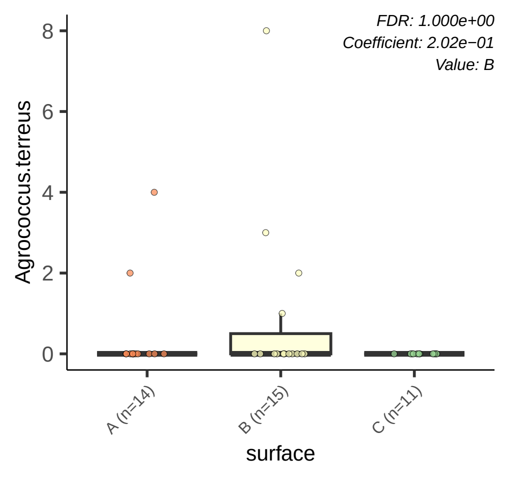

FDR: 1.000e+00
Coefficient: 2.02e−01
Value: B
Agrococcus.terreus
8
6
4
2
0
A (n=14)
B (n=15)
C (n=11)
surface

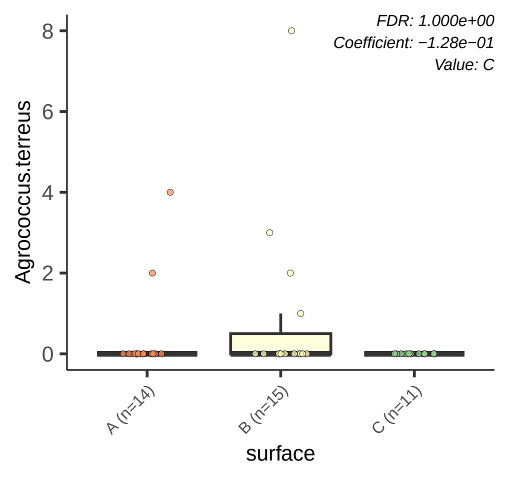
FDR: 1.000e+00
Coefficient: −1.28e−01
Value: C
Agrococcus.terreus
0
2
4
6
8
A (n=14)
B (n=15)
C (n=11)
surface

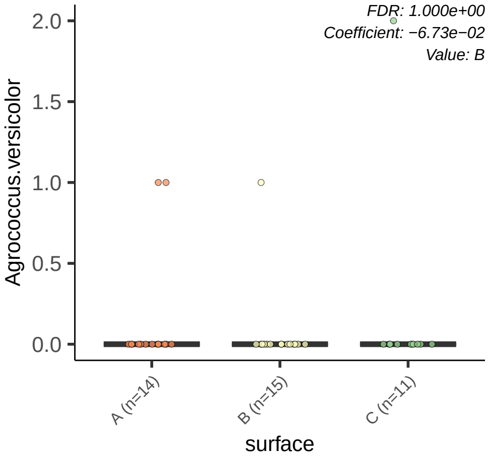
FDR: 1.000e+00
Coefficient: −6.73e−02
Value: B
Agrococcus.versicolor
2.0
1.5
1.0
0.5
0.0
A (n=14)
B (n=15)
C (n=11)
surface

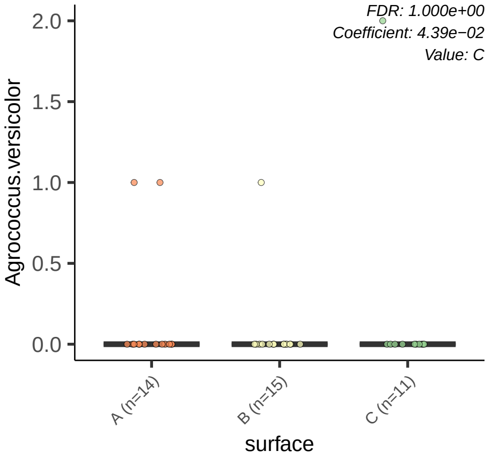
FDR: 1.000e+00
Coefficient: 4.39e−02
Value: C
Agrococcus.versicolor
2.0
1.5
1.0
0.5
0.0
A (n=14)
B (n=15)
C (n=11)
surface

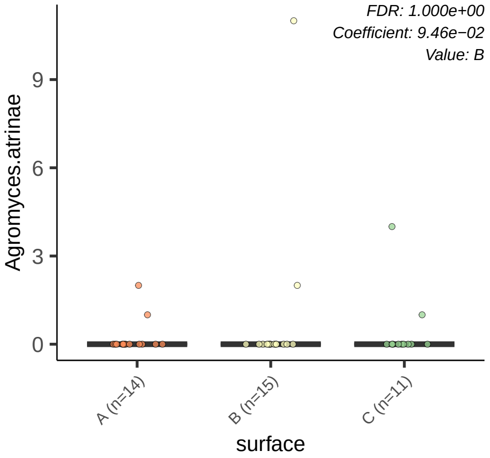

FDR: 1.000e+00
Coefficient: 9.46e−02
Value: B
Agromyces.atrinae
0
3
6
9
A (n=14)
B (n=15)
C (n=11)
surface

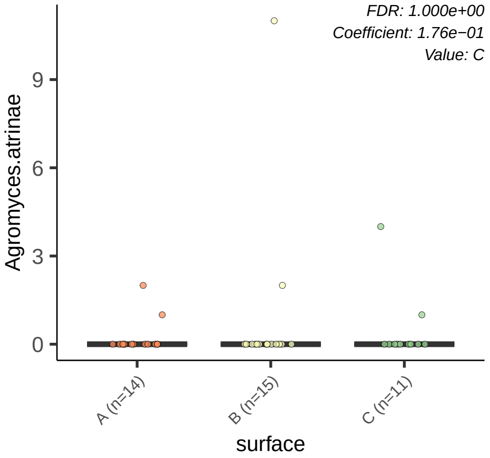
FDR: 1.000e+00
Coefficient: 1.76e−01
Value: C
Agromyces.atrinae
0
3
6
9
A (n=14)
B (n=15)
C (n=11)
surface

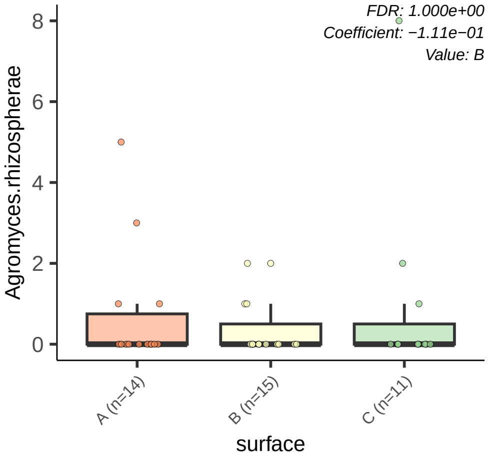

FDR: 1.000e+00
Coefficient: −1.11e−01
Value: B
Agromyces.rhizospherae
0
2
4
6
8
A (n=14)
B (n=15)
C (n=11)
surface

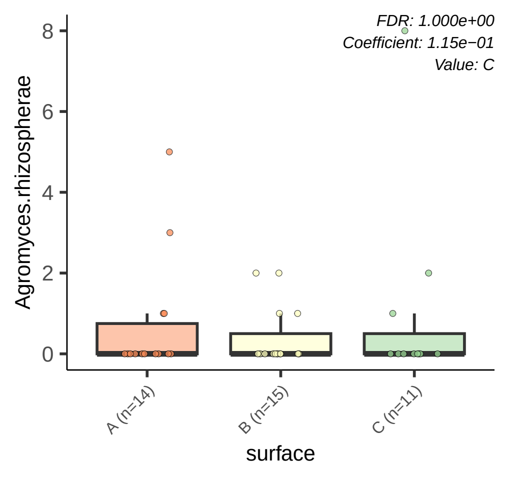
FDR: 1.000e+00
Coefficient: 1.15e−01
Value: C
Agromyces.rhizospherae
0
2
4
6
8
A (n=14)
B (n=15)
C (n=11)
surface

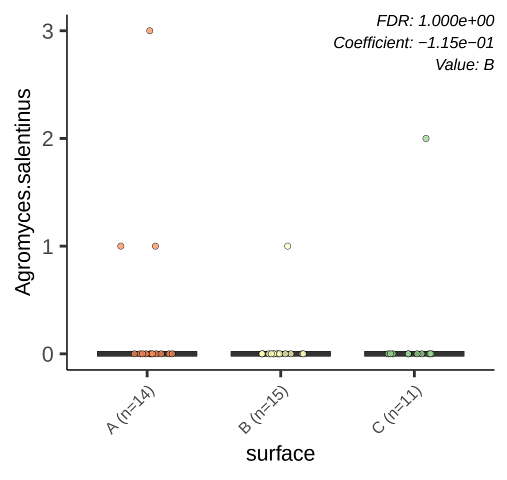
FDR: 1.000e+00
Coefficient: −1.15e−01
Value: B
Agromyces.salentinus
3
2
1
0
A (n=14)
B (n=15)
C (n=11)
surface

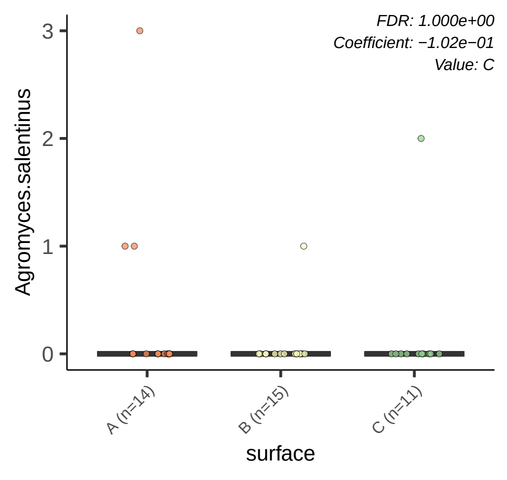
FDR: 1.000e+00
Coefficient: −1.02e−01
Value: C
Agromyces.salentinus
0
1
2
3
A (n=14)
B (n=15)
C (n=11)
surface

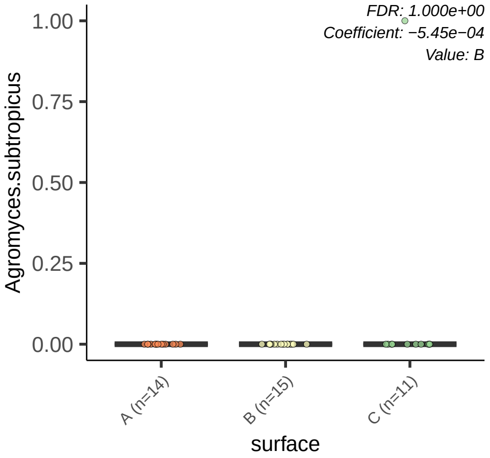
FDR: 1.000e+00
Coefficient: −5.45e−04
Value: B
Agromyces.subtropicus
1.00
0.75
0.50
0.25
0.00
A (n=14)
B (n=15)
C (n=11)
surface

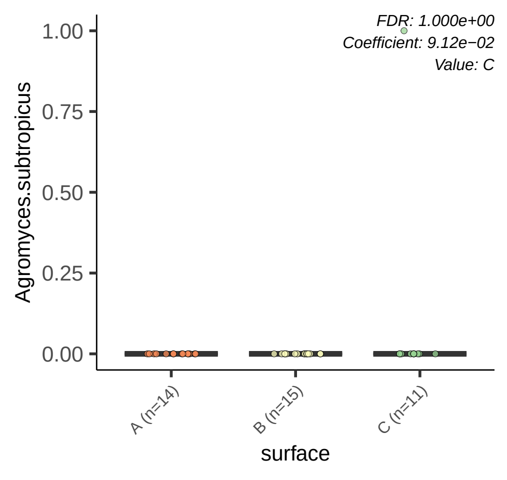
FDR: 1.000e+00
Coefficient: 9.12e−02
Value: C
Agromyces.subtropicus
1.00
0.75
0.50
0.25
0.00
A (n=14)
B (n=15)
C (n=11)
surface

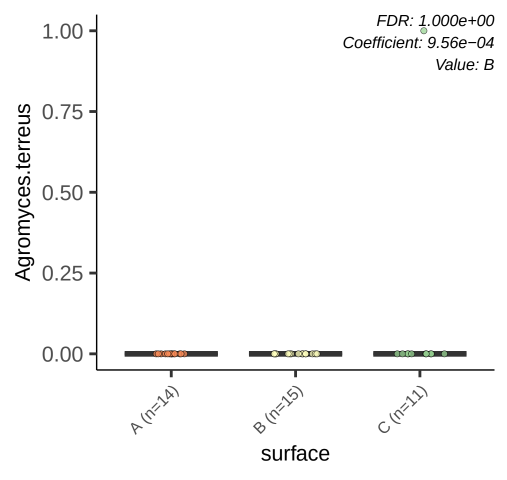
FDR: 1.000e+00
Coefficient: 9.56e−04
Value: B
Agromyces.terreus
1.00
0.75
0.50
0.25
0.00
A (n=14)
B (n=15)
C (n=11)
surface

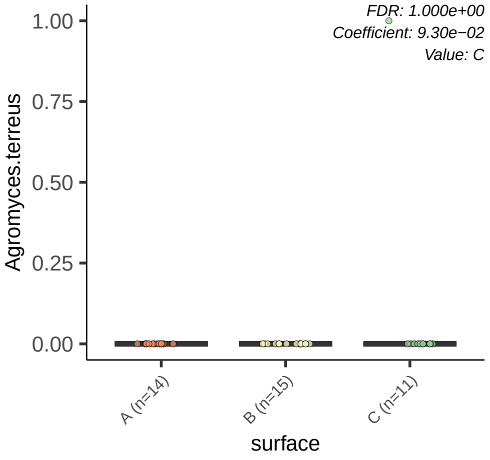
FDR: 1.000e+00
Coefficient: 9.30e−02
Value: C
Agromyces.terreus
1.00
0.75
0.50
0.25
0.00
A (n=14)
B (n=15)
C (n=11)
surface

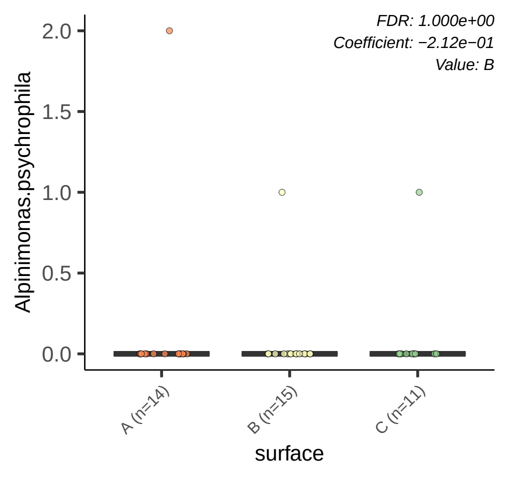

FDR: 1.000e+00
Coefficient: −2.12e−01
Value: B
Alpinimonas.psychrophila
2.0
1.5
1.0
0.5
0.0
A (n=14)
B (n=15)
C (n=11)
surface

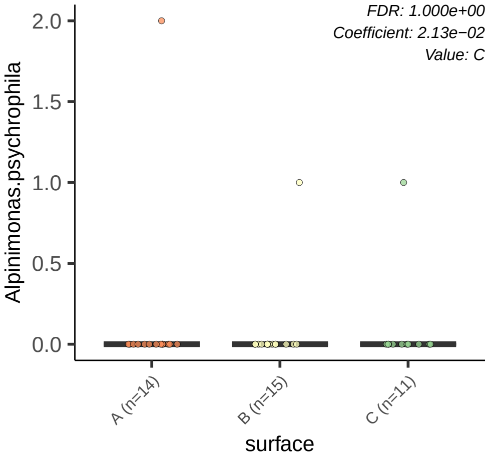

FDR: 1.000e+00
Coefficient: 2.13e−02
Value: C
Alpinimonas.psychrophila
2.0
1.5
1.0
0.5
0.0
A (n=14)
B (n=15)
C (n=11)
surface

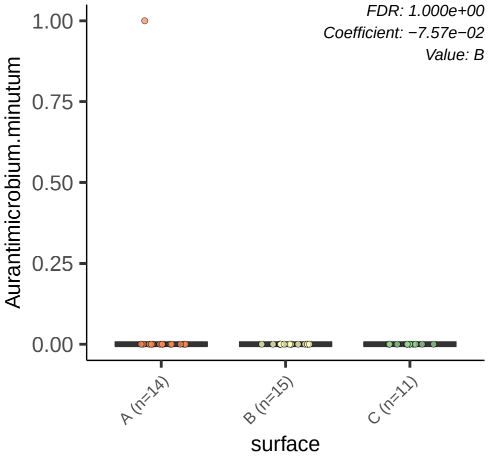

FDR: 1.000e+00
Coefficient: −7.57e−02
Value: B
Aurantimicrobium.minutum
1.00
0.75
0.50
0.25
0.00
A (n=14)
B (n=15)
C (n=11)
surface

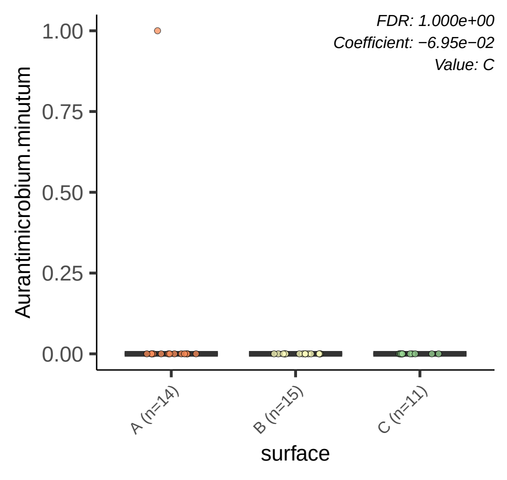
FDR: 1.000e+00
Coefficient: −6.95e−02
Value: C
Aurantimicrobium.minutum
1.00
0.75
0.50
0.25
0.00
A (n=14)
B (n=15)
C (n=11)
surface

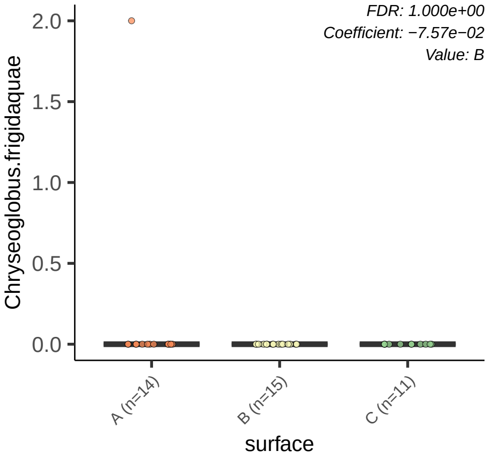
FDR: 1.000e+00
Coefficient: −7.57e−02
Value: B
Chryseoglobus.frigidaquae
2.0
1.5
1.0
0.5
0.0
A (n=14)
B (n=15)
C (n=11)
surface

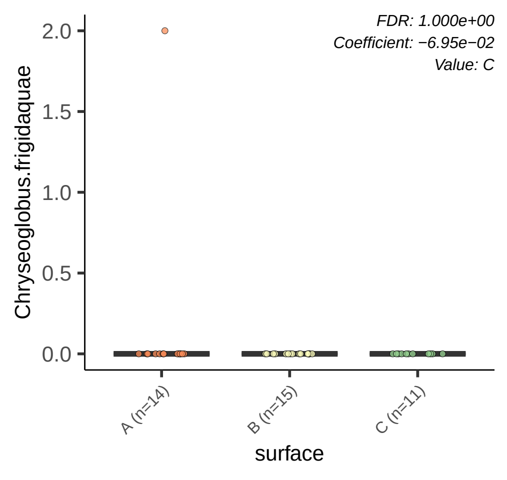
FDR: 1.000e+00
Coefficient: −6.95e−02
Value: C
Chryseoglobus.frigidaquae
2.0
1.5
1.0
0.5
0.0
A (n=14)
B (n=15)
C (n=11)
surface

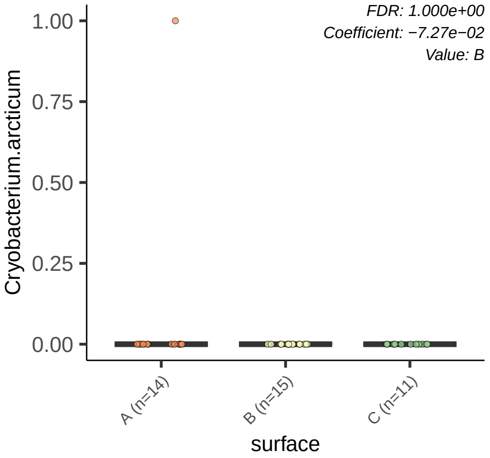
FDR: 1.000e+00
Coefficient: −7.27e−02
Value: B
Cryobacterium.arcticum
1.00
0.75
0.50
0.25
0.00
A (n=14)
B (n=15)
C (n=11)
surface

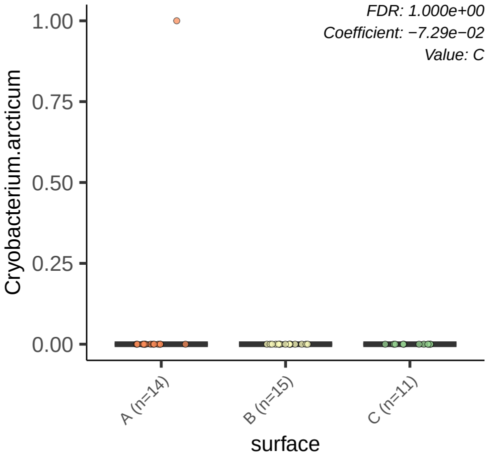
FDR: 1.000e+00
Coefficient: −7.29e−02
Value: C
Cryobacterium.arcticum
1.00
0.75
0.50
0.25
0.00
A (n=14)
B (n=15)
C (n=11)
surface

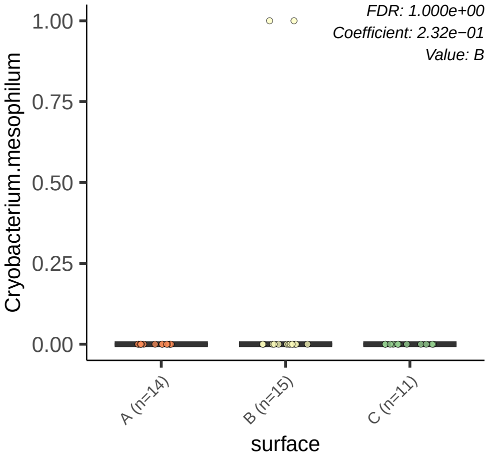
FDR: 1.000e+00
Coefficient: 2.32e−01
Value: B
Cryobacterium.mesophilum
1.00
0.75
0.50
0.25
0.00
A (n=14)
B (n=15)
C (n=11)
surface

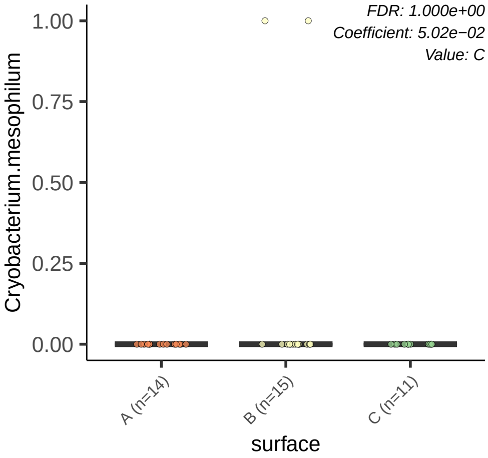

FDR: 1.000e+00
Coefficient: 5.02e−02
Value: C
Cryobacterium.mesophilum
1.00
0.75
0.50
0.25
0.00
A (n=14)
B (n=15)
C (n=11)
surface

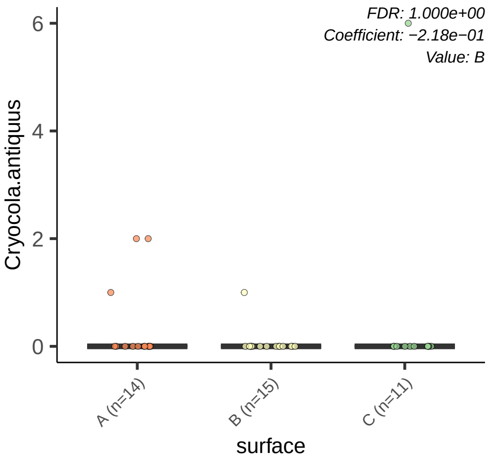
FDR: 1.000e+00
Coefficient: −2.18e−01
Value: B
Cryocola.antiquus
0
2
4
6
A (n=14)
B (n=15)
C (n=11)
surface

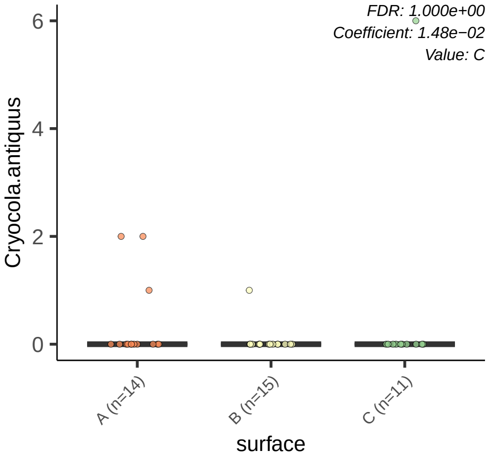
FDR: 1.000e+00
Coefficient: 1.48e−02
Value: C
Cryocola.antiquus
6
4
2
0
A (n=14)
B (n=15)
C (n=11)
surface

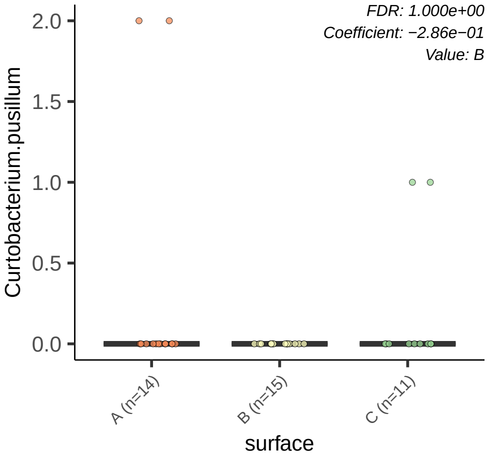
FDR: 1.000e+00
Coefficient: −2.86e−01
Value: B
Curtobacterium.pusillum
2.0
1.5
1.0
0.5
0.0
A (n=14)
B (n=15)
C (n=11)
surface

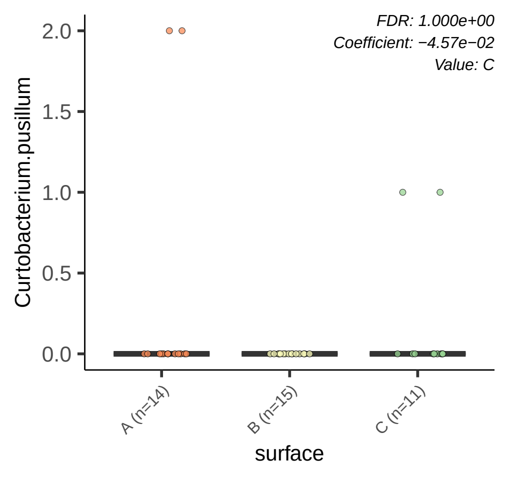

FDR: 1.000e+00
Coefficient: −4.57e−02
Value: C
Curtobacterium.pusillum
2.0
1.5
1.0
0.5
0.0
A (n=14)
B (n=15)
C (n=11)
surface

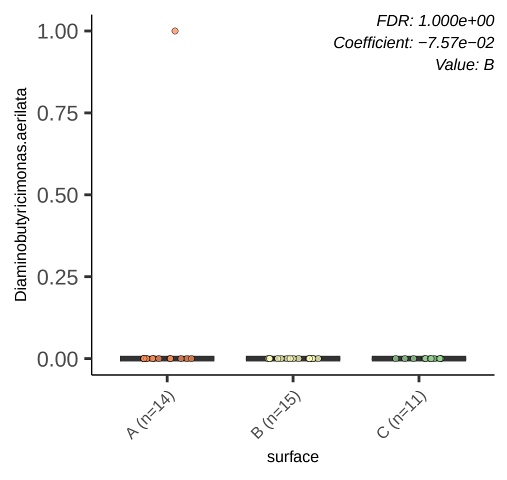

FDR: 1.000e+00
Coefficient: −7.57e−02
Value: B
Diaminobutyricimonas.aerilata
1.00
0.75
0.50
0.25
0.00
A (n=14)
B (n=15)
C (n=11)
surface

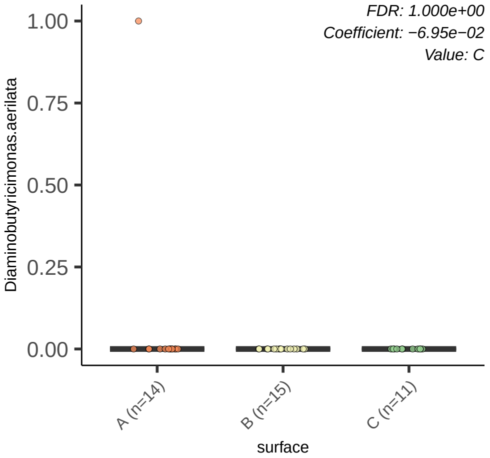

FDR: 1.000e+00
Coefficient: −6.95e−02
Value: C
Diaminobutyricimonas.aerilata
1.00
0.75
0.50
0.25
0.00
A (n=14)
B (n=15)
C (n=11)
surface

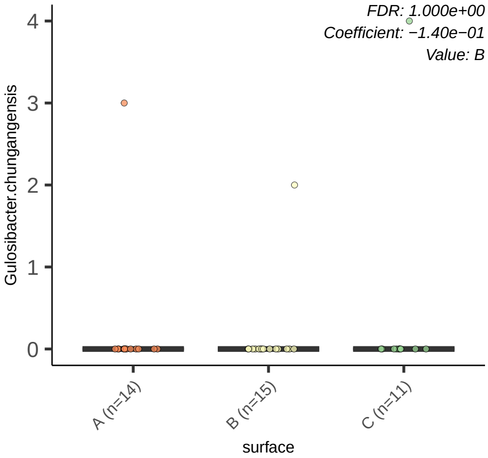
FDR: 1.000e+00
Coefficient: −1.40e−01
Value: B
Gulosibacter.chungangensis
0
1
2
3
4
A (n=14)
B (n=15)
C (n=11)
surface

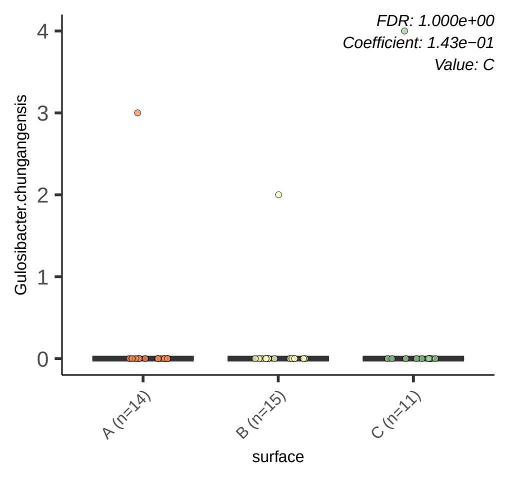
FDR: 1.000e+00
Coefficient: 1.43e−01
Value: C
Gulosibacter.chungangensis
0
1
2
3
4
A (n=14)
B (n=15)
C (n=11)
surface

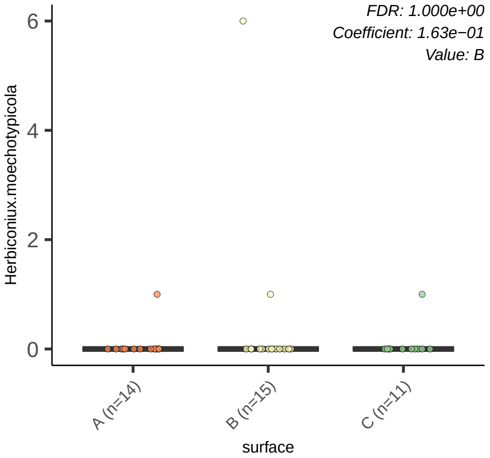
FDR: 1.000e+00
Coefficient: 1.63e−01
Value: B
Herbiconiux.moechotypicola
0
2
4
6
A (n=14)
B (n=15)
C (n=11)
surface

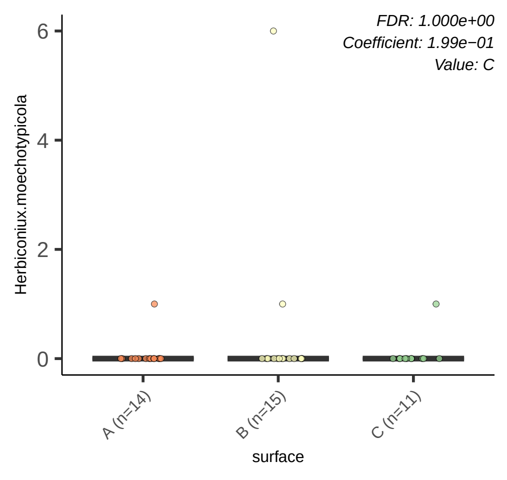
FDR: 1.000e+00
Coefficient: 1.99e−01
Value: C
Herbiconiux.moechotypicola
0
2
4
6
A (n=14)
B (n=15)
C (n=11)
surface

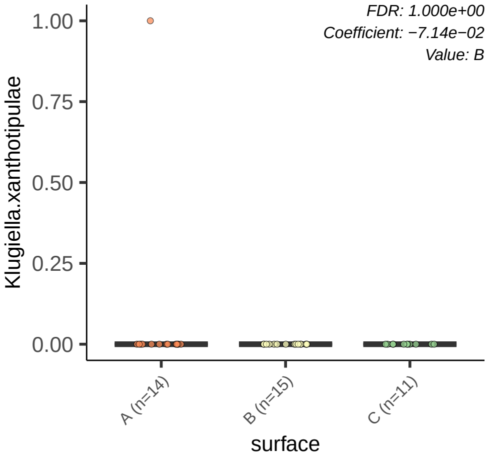
FDR: 1.000e+00
Coefficient: −7.14e−02
Value: B
Klugiella.xanthotipulae
1.00
0.75
0.50
0.25
0.00
A (n=14)
B (n=15)
C (n=11)
surface

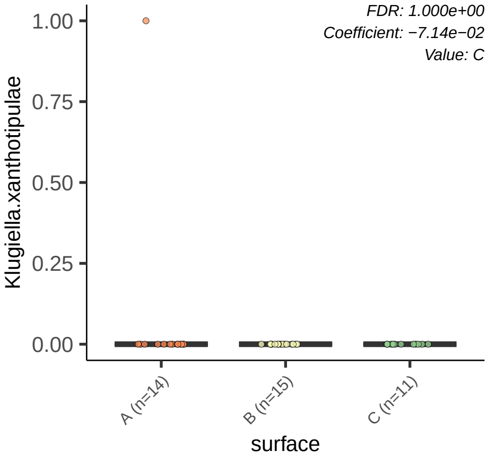
FDR: 1.000e+00
Coefficient: −7.14e−02
Value: C
Klugiella.xanthotipulae
1.00
0.75
0.50
0.25
0.00
A (n=14)
B (n=15)
C (n=11)
surface

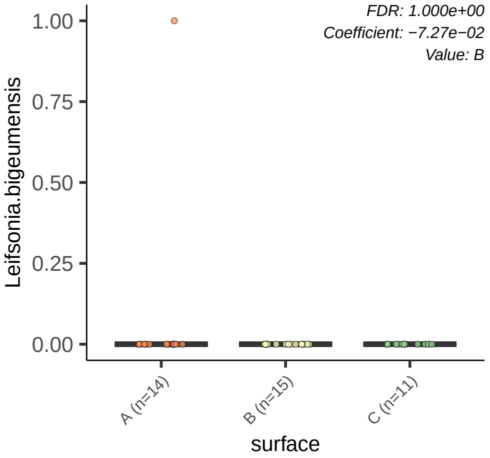

FDR: 1.000e+00
Coefficient: −7.27e−02
Value: B
Leifsonia.bigeumensis
1.00
0.75
0.50
0.25
0.00
A (n=14)
B (n=15)
C (n=11)
surface

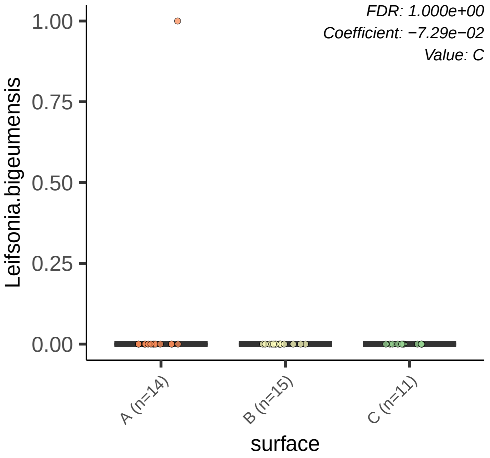

FDR: 1.000e+00
Coefficient: −7.29e−02
Value: C
Leifsonia.bigeumensis
1.00
0.75
0.50
0.25
0.00
A (n=14)
B (n=15)
C (n=11)
surface

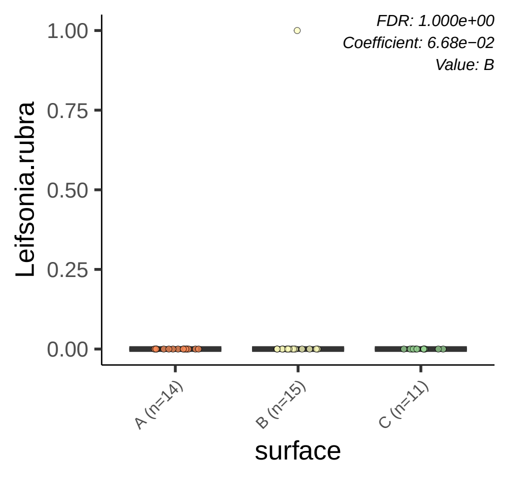
FDR: 1.000e+00
Coefficient: 6.68e−02
Value: B
Leifsonia.rubra
1.00
0.75
0.50
0.25
0.00
A (n=14)
B (n=15)
C (n=11)
surface

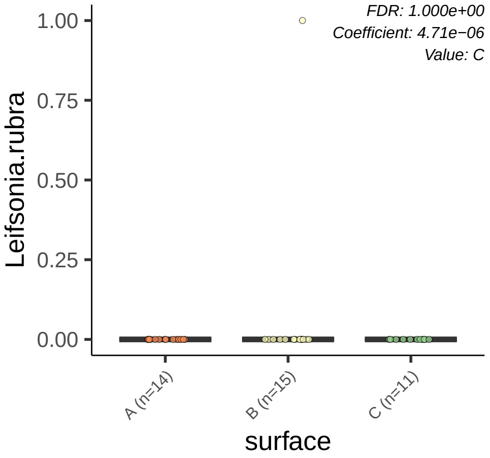
FDR: 1.000e+00
Coefficient: 4.71e−06
Value: C
1.00
0.75
0.50
0.25
0.00
Leifsonia.rubra
A (n=14)
B (n=15)
C (n=11)
surface

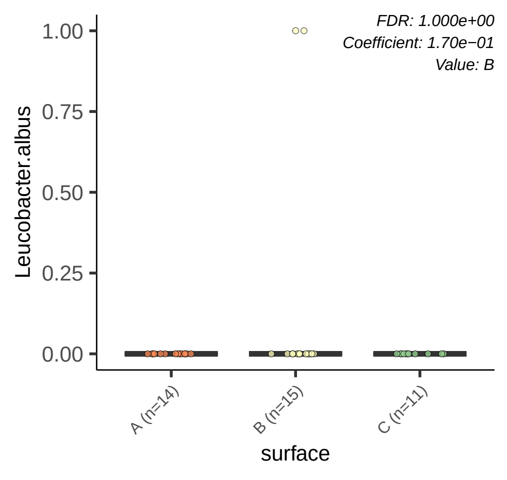

FDR: 1.000e+00
Coefficient: 1.70e−01
Value: B
Leucobacter.albus
1.00
0.75
0.50
0.25
0.00
A (n=14)
B (n=15)
C (n=11)
surface

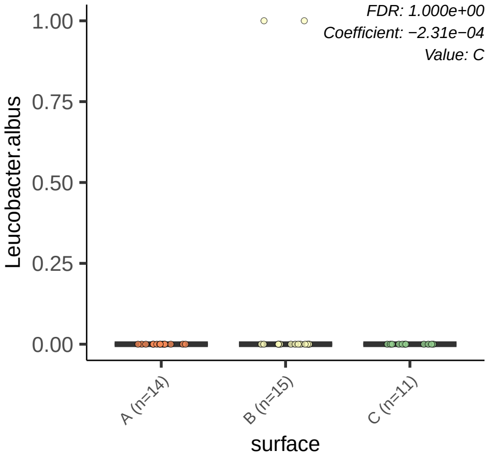
FDR: 1.000e+00
Coefficient: −2.31e−04
Value: C
1.00
0.75
0.50
0.25
0.00
Leucobacter.albus
A (n=14)
B (n=15)
C (n=11)
surface

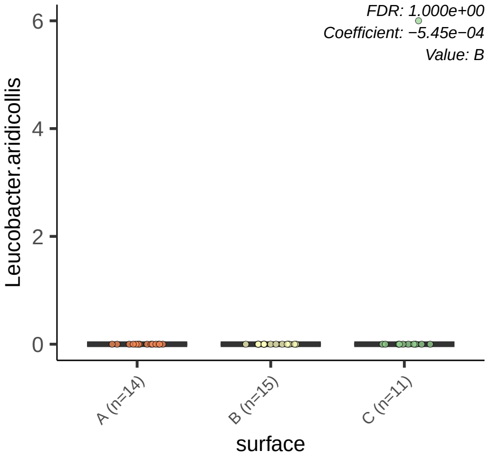

FDR: 1.000e+00
Coefficient: −5.45e−04
Value: B
Leucobacter.aridicollis
6
4
2
0
A (n=14)
B (n=15)
C (n=11)
surface

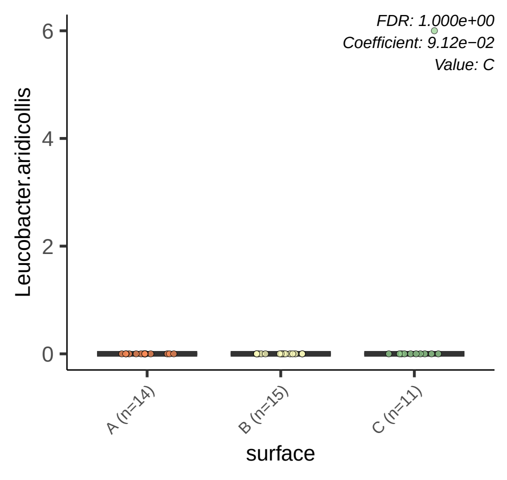
FDR: 1.000e+00
Coefficient: 9.12e−02
Value: C
Leucobacter.aridicollis
0
2
4
6
A (n=14)
B (n=15)
C (n=11)
surface

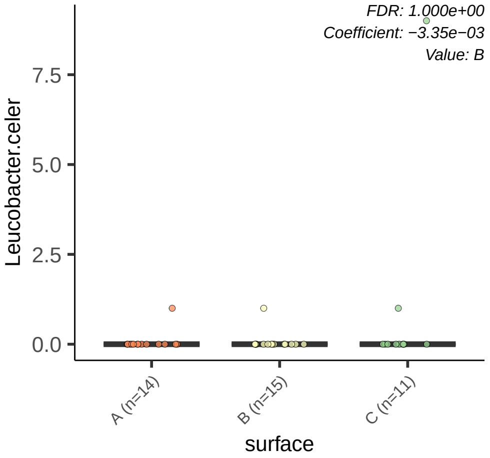
FDR: 1.000e+00
Coefficient: −3.35e−03
Value: B
Leucobacter.celer
7.5
5.0
2.5
0.0
A (n=14)
B (n=15)
C (n=11)
surface

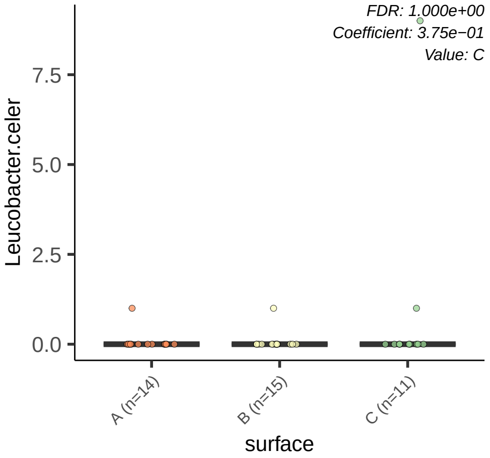
FDR: 1.000e+00
Coefficient: 3.75e−01
Value: C
Leucobacter.celer
7.5
5.0
2.5
0.0
A (n=14)
B (n=15)
C (n=11)
surface

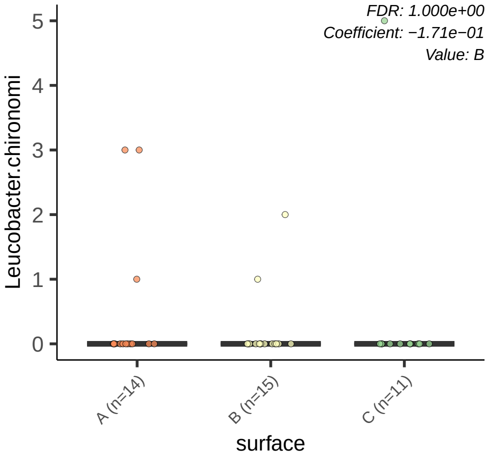

FDR: 1.000e+00
Coefficient: −1.71e−01
Value: B
Leucobacter.chironomi
0
1
2
3
4
5
A (n=14)
B (n=15)
C (n=11)
surface

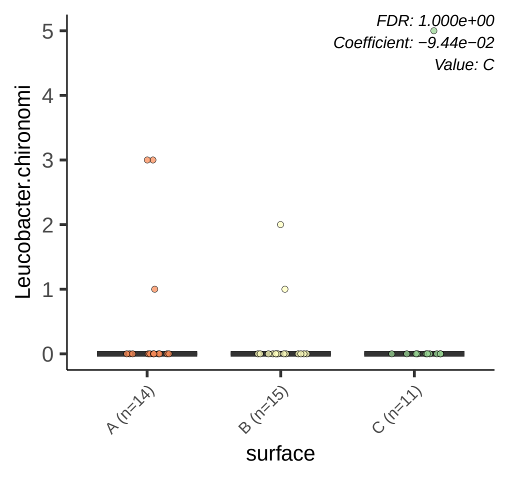
FDR: 1.000e+00
Coefficient: −9.44e−02
Value: C
Leucobacter.chironomi
0
1
2
3
4
5
A (n=14)
B (n=15)
C (n=11)
surface

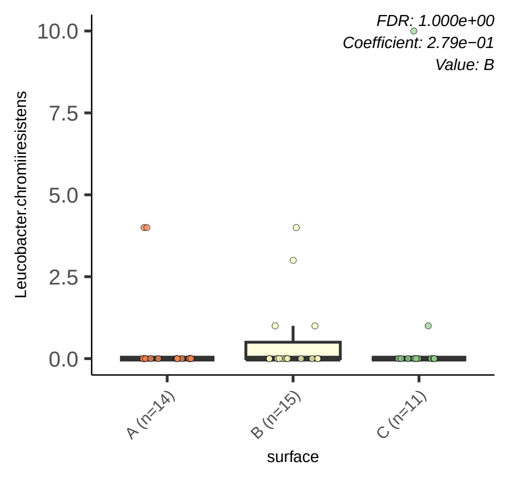

FDR: 1.000e+00
Coefficient: 2.79e−01
Value: B
Leucobacter.chromiiresistens
10.0
7.5
5.0
2.5
0.0
A (n=14)
B (n=15)
C (n=11)
surface

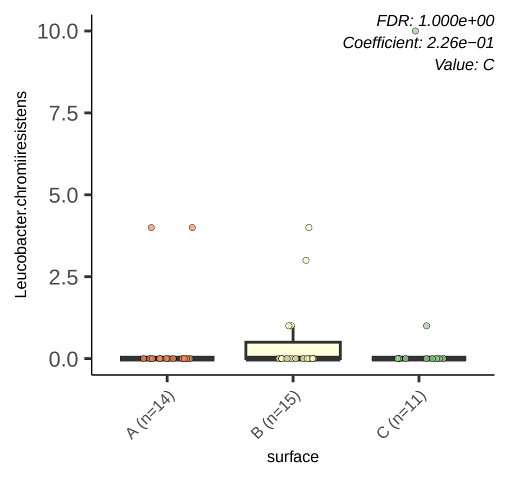
FDR: 1.000e+00
Coefficient: 2.26e−01
Value: C
Leucobacter.chromiiresistens
10.0
7.5
5.0
2.5
0.0
A (n=14)
B (n=15)
C (n=11)
surface

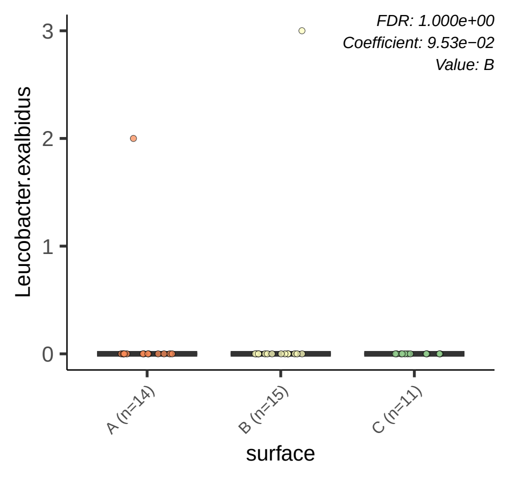

FDR: 1.000e+00
Coefficient: 9.53e−02
Value: B
Leucobacter.exalbidus
3
2
1
0
A (n=14)
B (n=15)
C (n=11)
surface

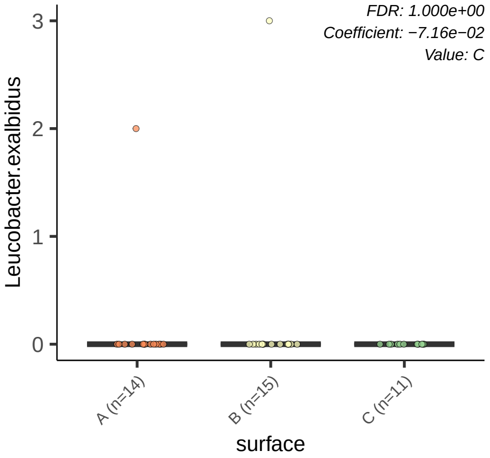

FDR: 1.000e+00
Coefficient: −7.16e−02
Value: C
Leucobacter.exalbidus
0
1
2
3
A (n=14)
B (n=15)
C (n=11)
surface

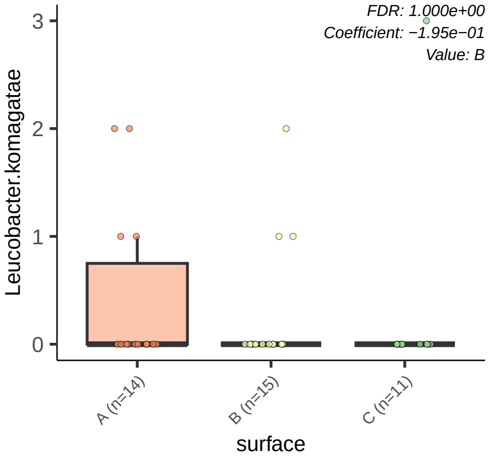

FDR: 1.000e+00
Coefficient: −1.95e−01
Value: B
Leucobacter.komagatae
3
2
1
0
A (n=14)
B (n=15)
C (n=11)
surface

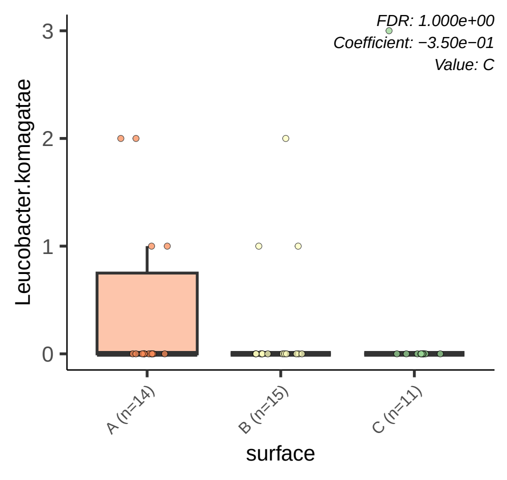
FDR: 1.000e+00
Coefficient: −3.50e−01
Value: C
Leucobacter.komagatae
0
1
2
3
A (n=14)
B (n=15)
C (n=11)
surface

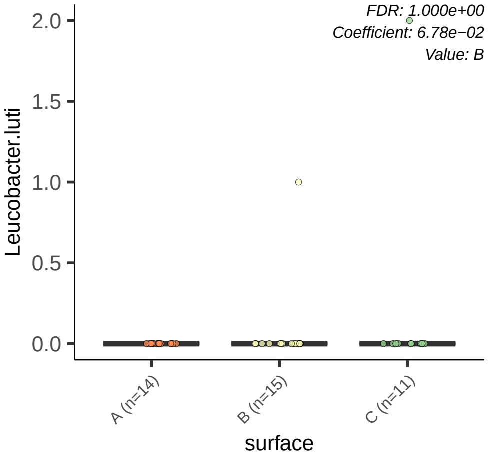
FDR: 1.000e+00
Coefficient: 6.78e−02
Value: B
Leucobacter.luti
2.0
1.5
1.0
0.5
0.0
A (n=14)
B (n=15)
C (n=11)
surface

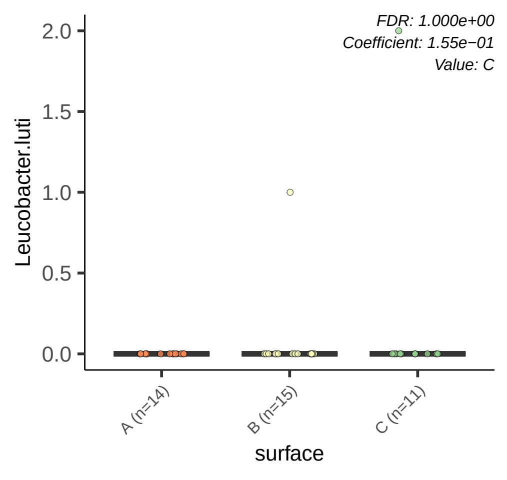
FDR: 1.000e+00
Coefficient: 1.55e−01
Value: C
Leucobacter.luti
2.0
1.5
1.0
0.5
0.0
A (n=14)
B (n=15)
C (n=11)
surface

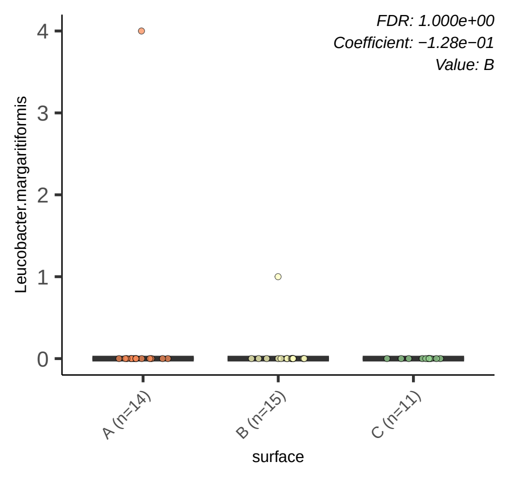
FDR: 1.000e+00
Coefficient: −1.28e−01
Value: B
Leucobacter.margaritiformis
0
1
2
3
4
A (n=14)
B (n=15)
C (n=11)
surface

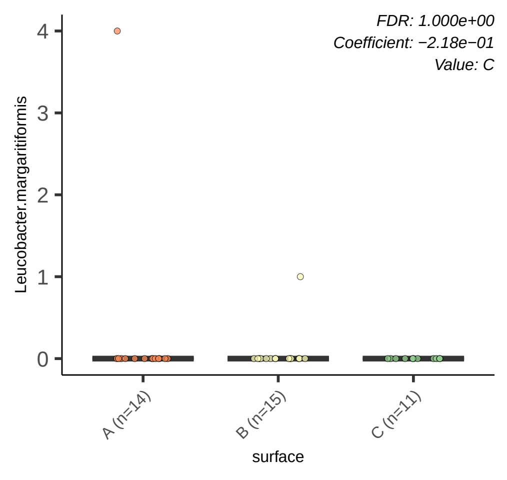

FDR: 1.000e+00
Coefficient: −2.18e−01
Value: C
4
3
2
1
0
Leucobacter.margaritiformis
A (n=14)
B (n=15)
C (n=11)
surface

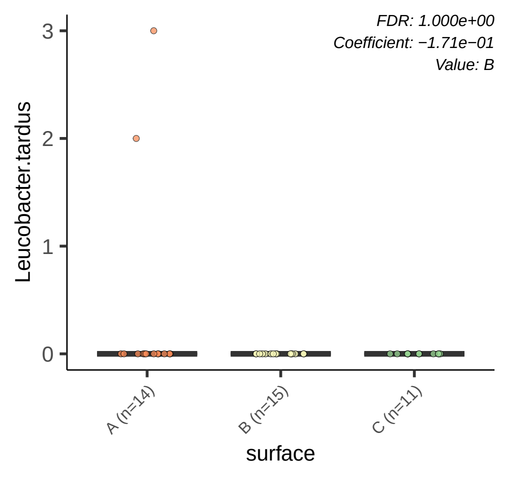

FDR: 1.000e+00
Coefficient: −1.71e−01
Value: B
Leucobacter.tardus
3
2
1
0
A (n=14)
B (n=15)
C (n=11)
surface

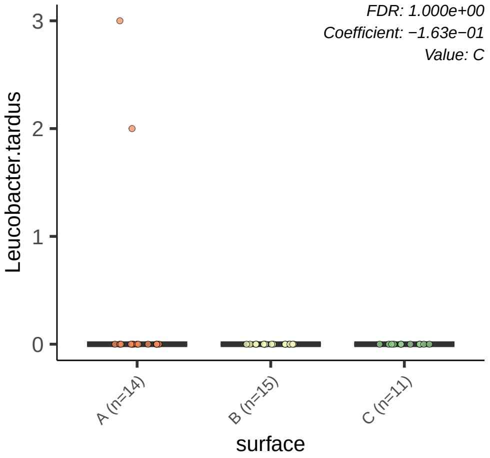
FDR: 1.000e+00
Coefficient: −1.63e−01
Value: C
Leucobacter.tardus
3
2
1
0
A (n=14)
B (n=15)
C (n=11)
surface

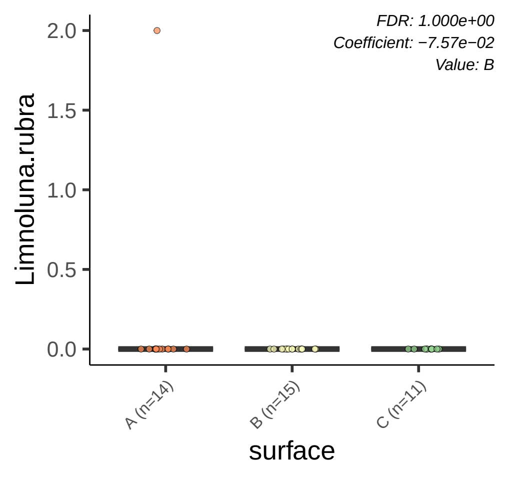
FDR: 1.000e+00
Coefficient: −7.57e−02
Value: B
Limnoluna.rubra
2.0
1.5
1.0
0.5
0.0
A (n=14)
B (n=15)
C (n=11)
surface

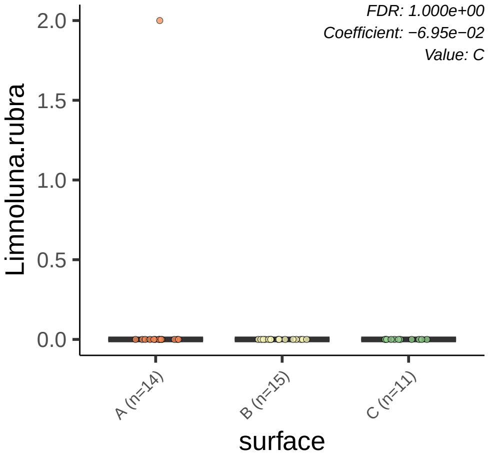
FDR: 1.000e+00
Coefficient: −6.95e−02
Value: C
Limnoluna.rubra
2.0
1.5
1.0
0.5
0.0
A (n=14)
B (n=15)
C (n=11)
surface

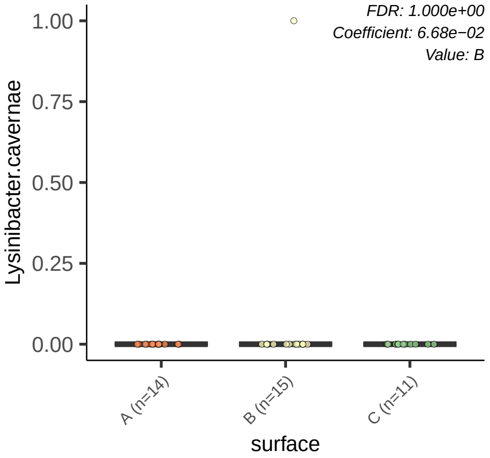

FDR: 1.000e+00
Coefficient: 6.68e−02
Value: B
Lysinibacter.cavernae
1.00
0.75
0.50
0.25
0.00
A (n=14)
B (n=15)
C (n=11)
surface

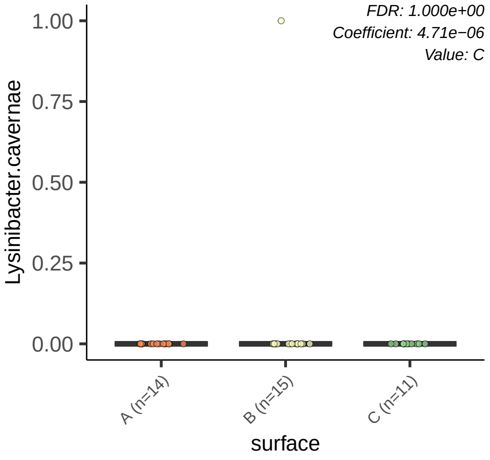
FDR: 1.000e+00
Coefficient: 4.71e−06
Value: C
Lysinibacter.cavernae
1.00
0.75
0.50
0.25
0.00
A (n=14)
B (n=15)
C (n=11)
surface

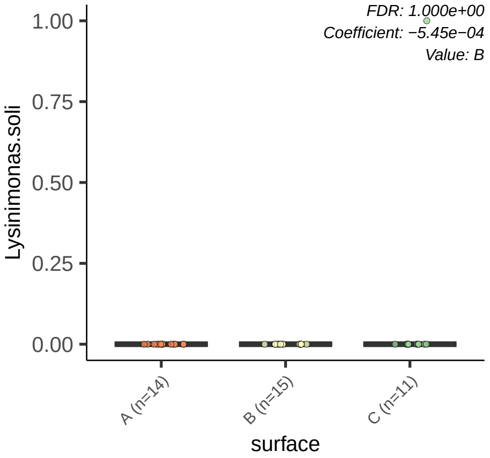
FDR: 1.000e+00
Coefficient: −5.45e−04
Value: B
Lysinimonas.soli
1.00
0.75
0.50
0.25
0.00
A (n=14)
B (n=15)
C (n=11)
surface

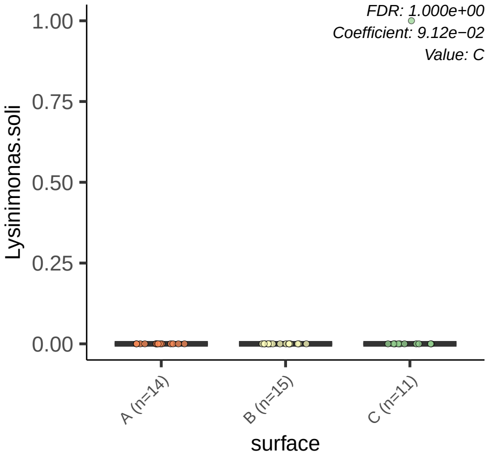
FDR: 1.000e+00
Coefficient: 9.12e−02
Value: C
Lysinimonas.soli
1.00
0.75
0.50
0.25
0.00
A (n=14)
B (n=15)
C (n=11)
surface

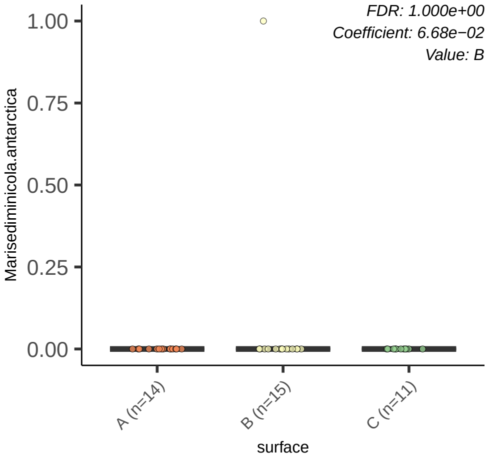
FDR: 1.000e+00
Coefficient: 6.68e−02
Value: B
1.00
0.75
0.50
0.25
0.00
Marisediminicola.antarctica
A (n=14)
B (n=15)
C (n=11)
surface

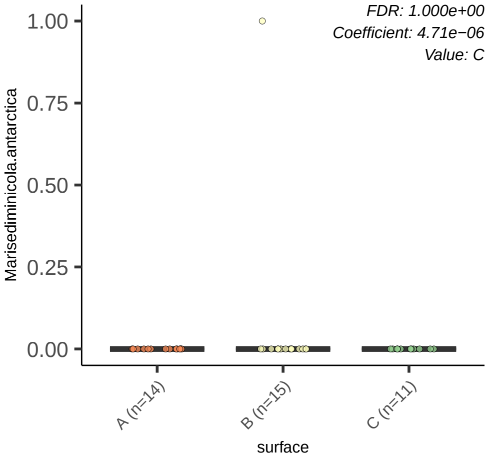
FDR: 1.000e+00
Coefficient: 4.71e−06
Value: C
1.00
0.75
0.50
0.25
0.00
Marisediminicola.antarctica
A (n=14)
B (n=15)
C (n=11)
surface

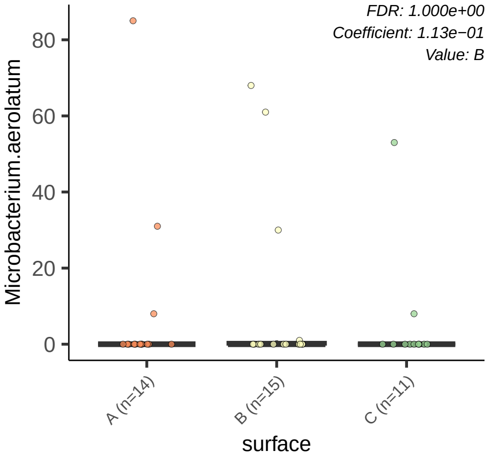

FDR: 1.000e+00
Coefficient: 1.13e−01
Value: B
Microbacterium.aerolatum
80
60
40
20
0
A (n=14)
B (n=15)
C (n=11)
surface

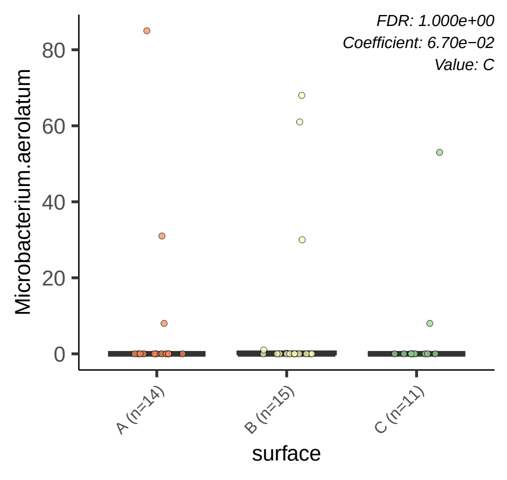

FDR: 1.000e+00
Coefficient: 6.70e−02
Value: C
Microbacterium.aerolatum
0
20
40
60
80
A (n=14)
B (n=15)
C (n=11)
surface

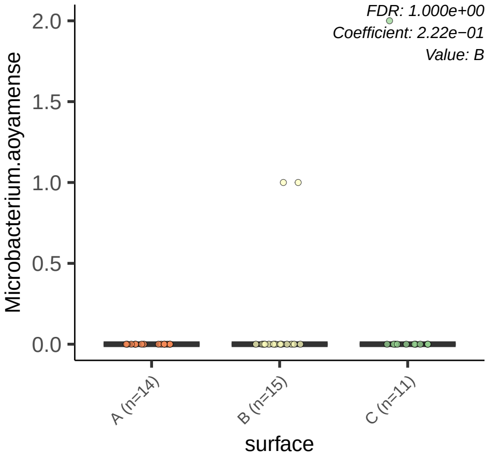
FDR: 1.000e+00
Coefficient: 2.22e−01
Value: B
Microbacterium.aoyamense
2.0
1.5
1.0
0.5
0.0
A (n=14)
B (n=15)
C (n=11)
surface

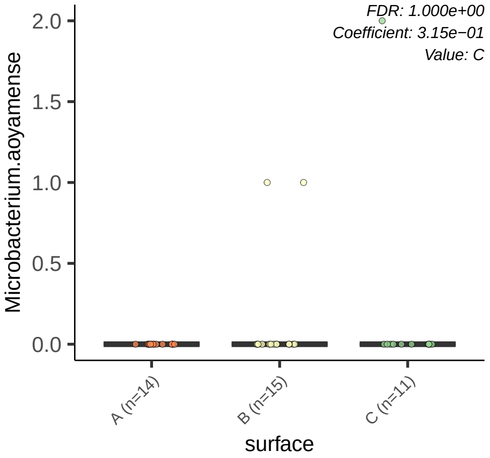

FDR: 1.000e+00
Coefficient: 3.15e−01
Value: C
Microbacterium.aoyamense
2.0
1.5
1.0
0.5
0.0
A (n=14)
B (n=15)
C (n=11)
surface

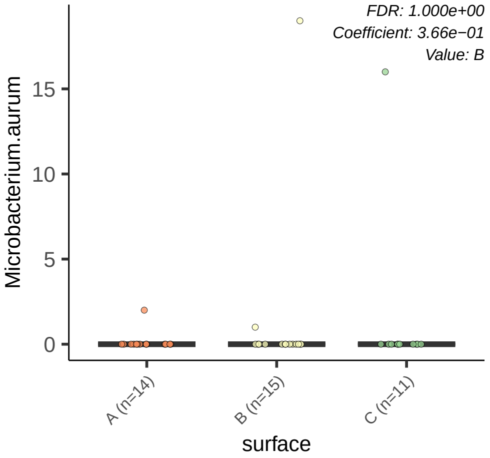

FDR: 1.000e+00
Coefficient: 3.66e−01
Value: B
Microbacterium.aurum
0
5
10
15
A (n=14)
B (n=15)
C (n=11)
surface

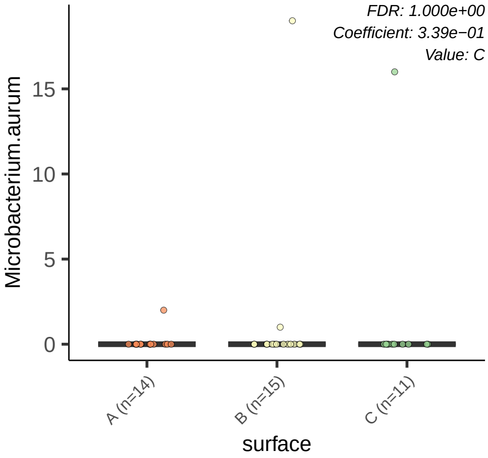

FDR: 1.000e+00
Coefficient: 3.39e−01
Value: C
Microbacterium.aurum
0
5
10
15
A (n=14)
B (n=15)
C (n=11)
surface

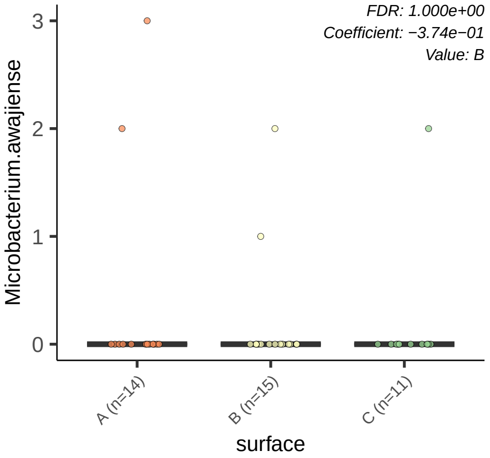
FDR: 1.000e+00
Coefficient: −3.74e−01
Value: B
Microbacterium.awajiense
0
1
2
3
A (n=14)
B (n=15)
C (n=11)
surface

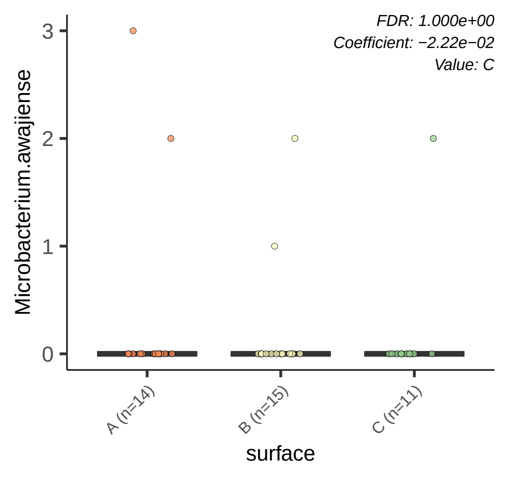

FDR: 1.000e+00
Coefficient: −2.22e−02
Value: C
Microbacterium.awajiense
3
2
1
0
A (n=14)
B (n=15)
C (n=11)
surface

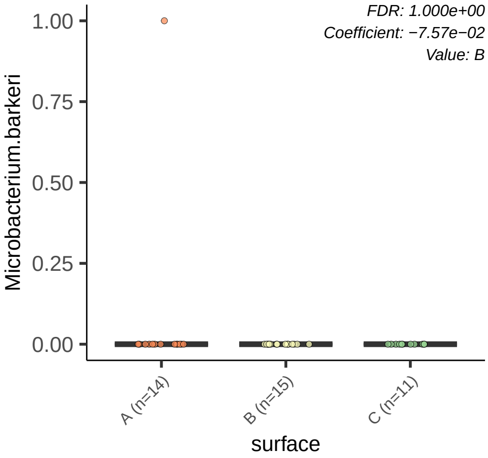
FDR: 1.000e+00
Coefficient: −7.57e−02
Value: B
Microbacterium.barkeri
1.00
0.75
0.50
0.25
0.00
A (n=14)
B (n=15)
C (n=11)
surface

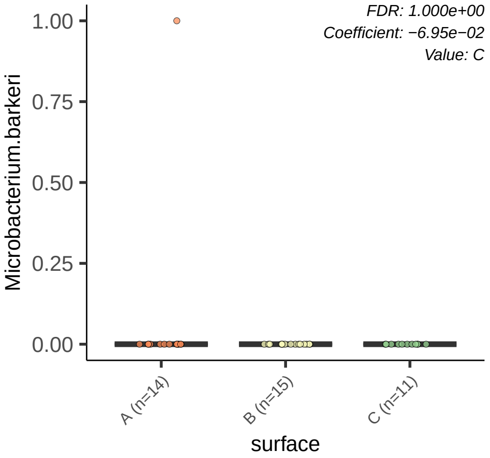

FDR: 1.000e+00
Coefficient: −6.95e−02
Value: C
Microbacterium.barkeri
1.00
0.75
0.50
0.25
0.00
A (n=14)
B (n=15)
C (n=11)
surface

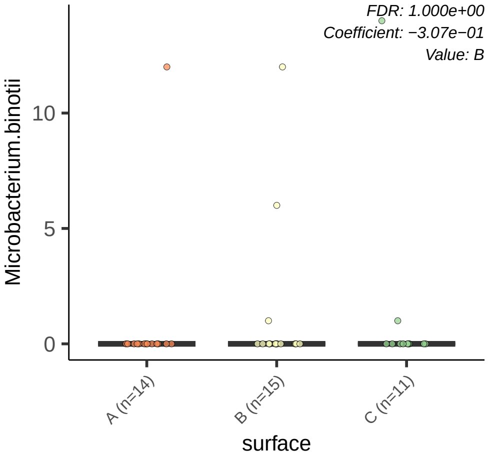

FDR: 1.000e+00
Coefficient: −3.07e−01
Value: B
Microbacterium.binotii
0
5
10
A (n=14)
B (n=15)
C (n=11)
surface

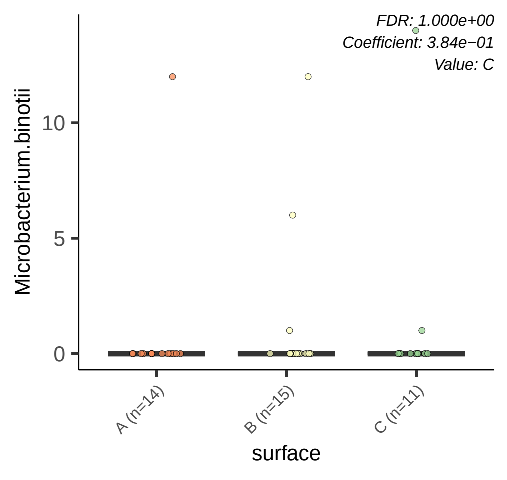

FDR: 1.000e+00
Coefficient: 3.84e−01
Value: C
Microbacterium.binotii
10
5
0
A (n=14)
B (n=15)
C (n=11)
surface

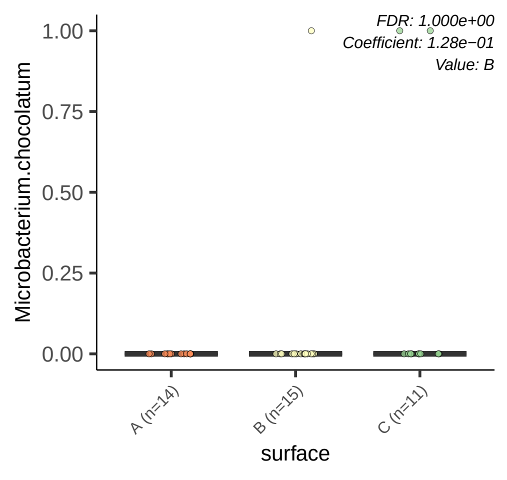
FDR: 1.000e+00
Coefficient: 1.28e−01
Value: B
Microbacterium.chocolatum
1.00
0.75
0.50
0.25
0.00
A (n=14)
B (n=15)
C (n=11)
surface

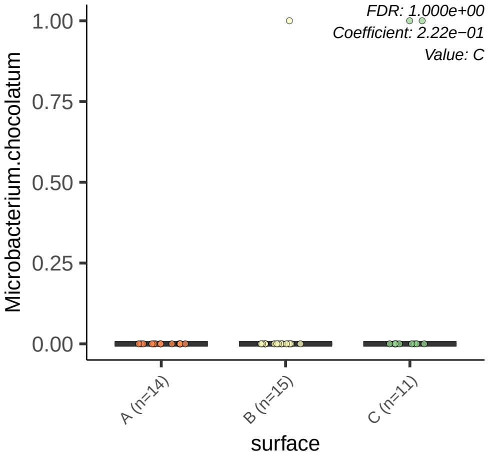

FDR: 1.000e+00
Coefficient: 2.22e−01
Value: C
Microbacterium.chocolatum
1.00
0.75
0.50
0.25
0.00
A (n=14)
B (n=15)
C (n=11)
surface

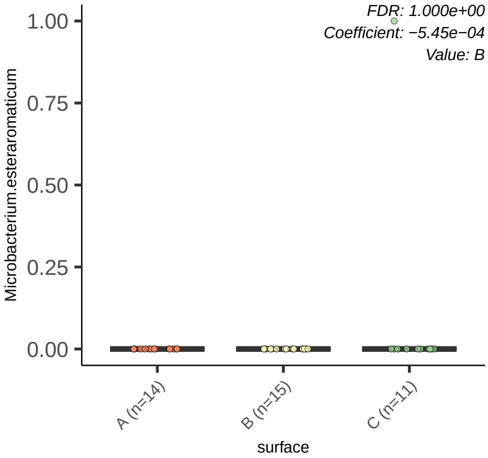
FDR: 1.000e+00
Coefficient: −5.45e−04
Value: B
Microbacterium.esteraromaticum
1.00
0.75
0.50
0.25
0.00
A (n=14)
B (n=15)
C (n=11)
surface

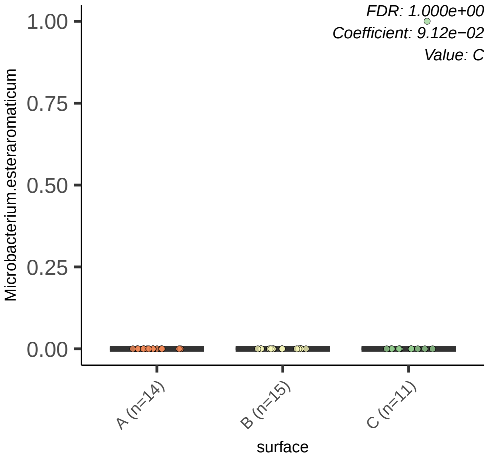

FDR: 1.000e+00
Coefficient: 9.12e−02
Value: C
Microbacterium.esteraromaticum
1.00
0.75
0.50
0.25
0.00
A (n=14)
B (n=15)
C (n=11)
surface

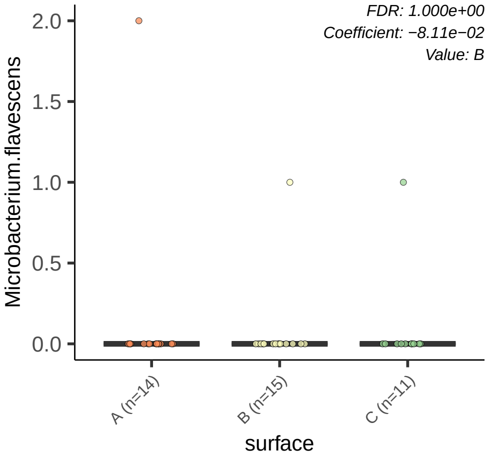

FDR: 1.000e+00
Coefficient: −8.11e−02
Value: B
Microbacterium.flavescens
2.0
1.5
1.0
0.5
0.0
A (n=14)
B (n=15)
C (n=11)
surface

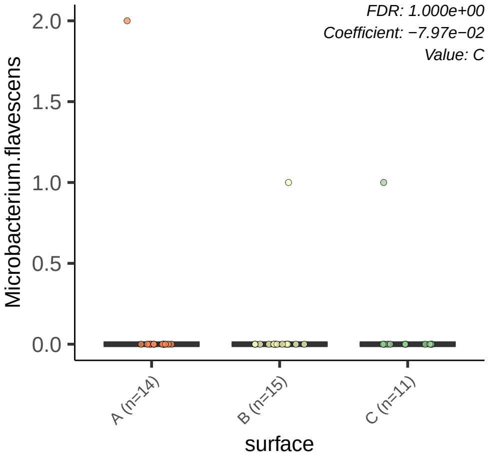
FDR: 1.000e+00
Coefficient: −7.97e−02
Value: C
Microbacterium.flavescens
2.0
1.5
1.0
0.5
0.0
A (n=14)
B (n=15)
C (n=11)
surface

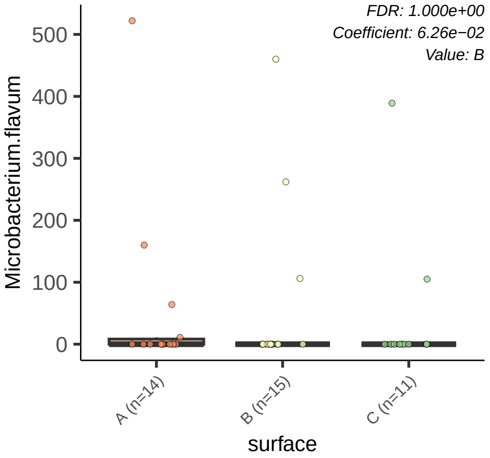
FDR: 1.000e+00
Coefficient: 6.26e−02
Value: B
Microbacterium.flavum
0
100
200
300
400
500
A (n=14)
B (n=15)
C (n=11)
surface

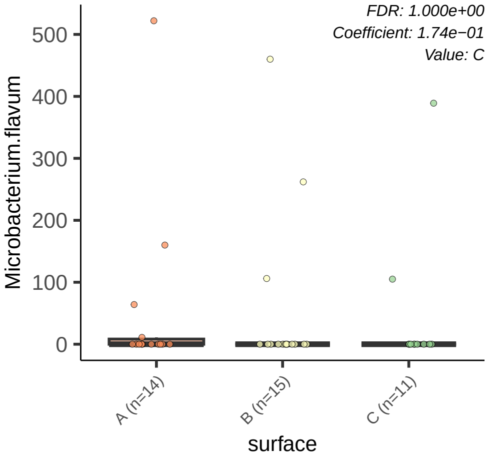
FDR: 1.000e+00
Coefficient: 1.74e−01
Value: C
Microbacterium.flavum
500
400
300
200
100
0
A (n=14)
B (n=15)
C (n=11)
surface

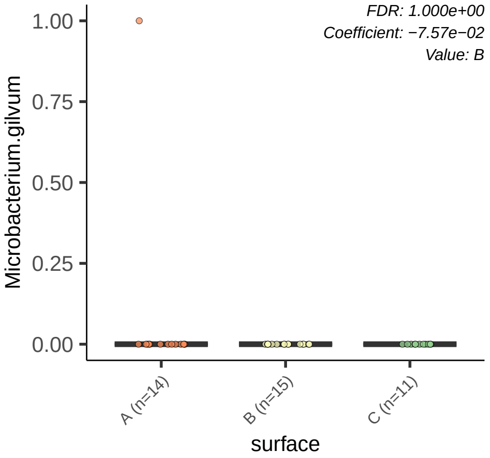

FDR: 1.000e+00
Coefficient: −7.57e−02
Value: B
Microbacterium.gilvum
1.00
0.75
0.50
0.25
0.00
A (n=14)
B (n=15)
C (n=11)
surface

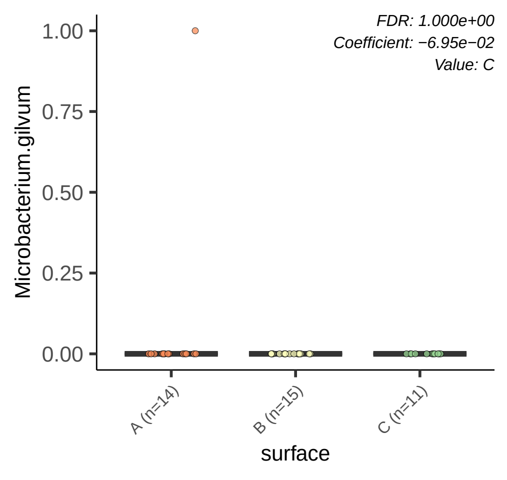

FDR: 1.000e+00
Coefficient: −6.95e−02
Value: C
Microbacterium.gilvum
1.00
0.75
0.50
0.25
0.00
A (n=14)
B (n=15)
C (n=11)
surface

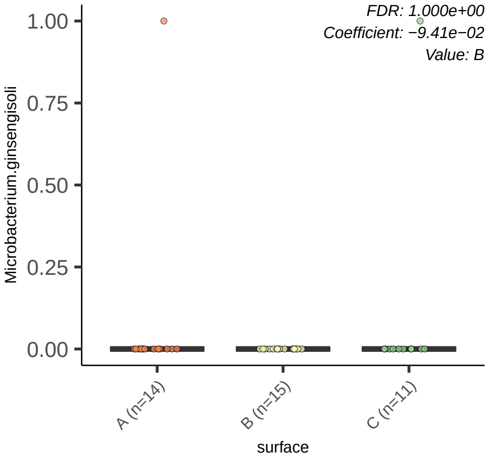

FDR: 1.000e+00
Coefficient: −9.41e−02
Value: B
Microbacterium.ginsengisoli
1.00
0.75
0.50
0.25
0.00
A (n=14)
B (n=15)
C (n=11)
surface

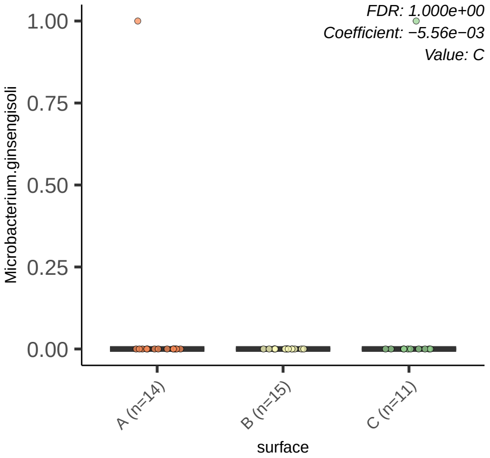
FDR: 1.000e+00
Coefficient: −5.56e−03
Value: C
Microbacterium.ginsengisoli
1.00
0.75
0.50
0.25
0.00
A (n=14)
B (n=15)
C (n=11)
surface

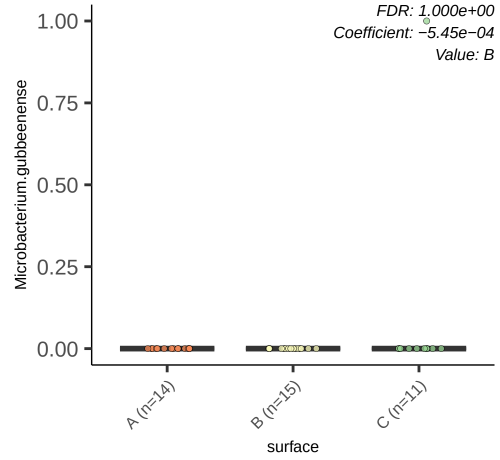

FDR: 1.000e+00
Coefficient: −5.45e−04
Value: B
Microbacterium.gubbeenense
1.00
0.75
0.50
0.25
0.00
A (n=14)
B (n=15)
C (n=11)
surface

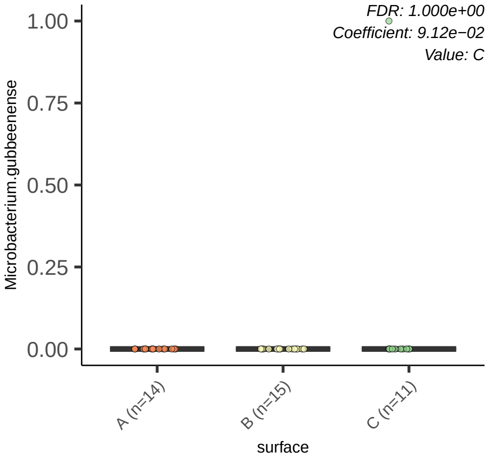

FDR: 1.000e+00
Coefficient: 9.12e−02
Value: C
Microbacterium.gubbeenense
1.00
0.75
0.50
0.25
0.00
A (n=14)
B (n=15)
C (n=11)
surface

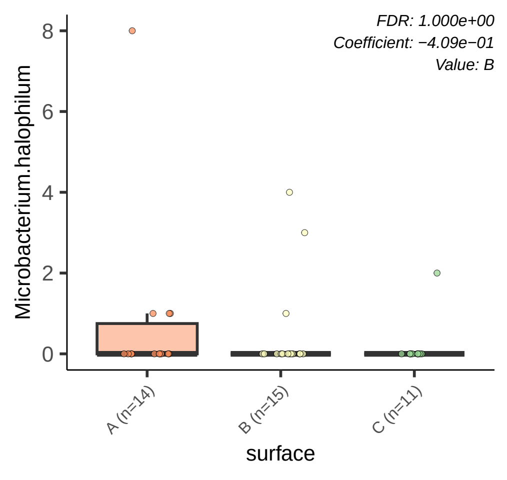
FDR: 1.000e+00
Coefficient: −4.09e−01
Value: B
Microbacterium.halophilum
8
6
4
2
0
A (n=14)
B (n=15)
C (n=11)
surface

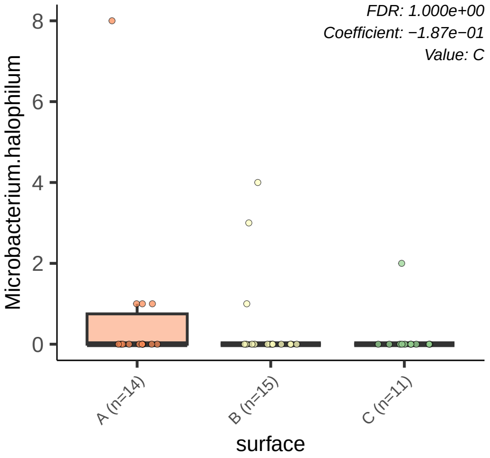
FDR: 1.000e+00
Coefficient: −1.87e−01
Value: C
Microbacterium.halophilum
8
6
4
2
0
A (n=14)
B (n=15)
C (n=11)
surface

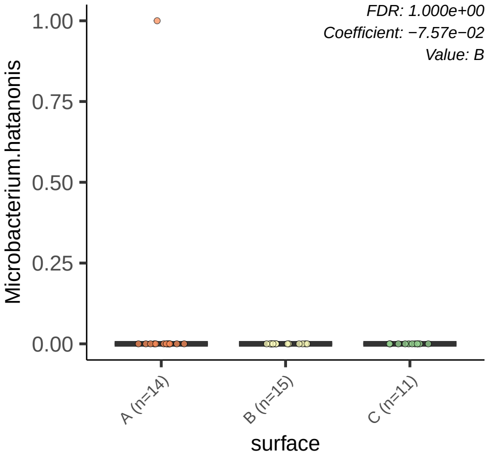

FDR: 1.000e+00
Coefficient: −7.57e−02
Value: B
Microbacterium.hatanonis
1.00
0.75
0.50
0.25
0.00
A (n=14)
B (n=15)
C (n=11)
surface

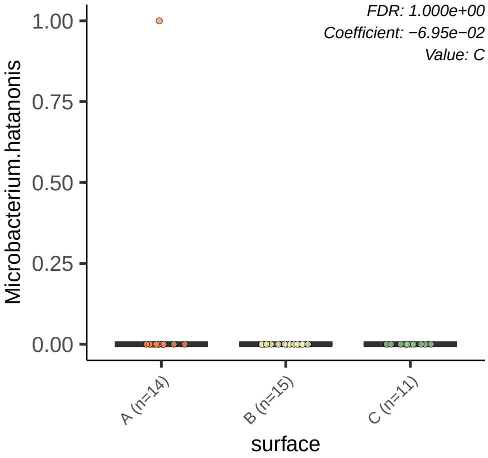
FDR: 1.000e+00
Coefficient: −6.95e−02
Value: C
Microbacterium.hatanonis
1.00
0.75
0.50
0.25
0.00
A (n=14)
B (n=15)
C (n=11)
surface

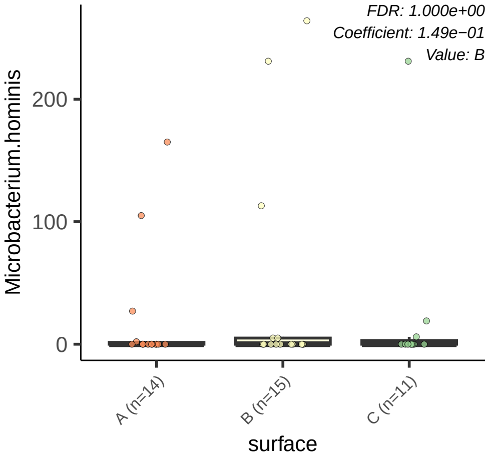

FDR: 1.000e+00
Coefficient: 1.49e−01
Value: B
Microbacterium.hominis
200
100
0
A (n=14)
B (n=15)
C (n=11)
surface

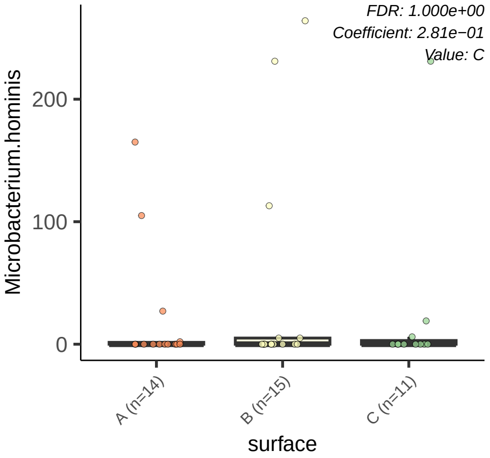
FDR: 1.000e+00
Coefficient: 2.81e−01
Value: C
Microbacterium.hominis
200
100
0
A (n=14)
B (n=15)
C (n=11)
surface

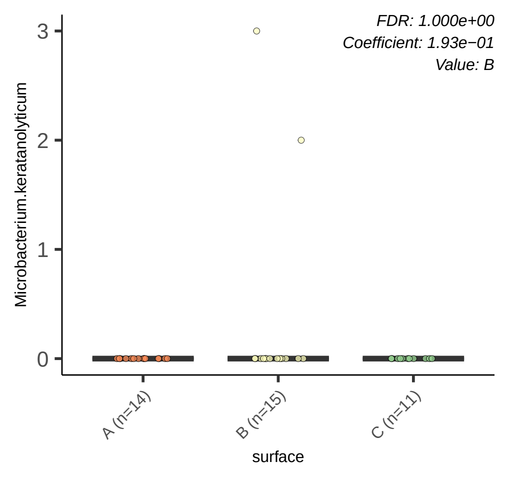

FDR: 1.000e+00
Coefficient: 1.93e−01
Value: B
Microbacterium.keratanolyticum
3
2
1
0
A (n=14)
B (n=15)
C (n=11)
surface

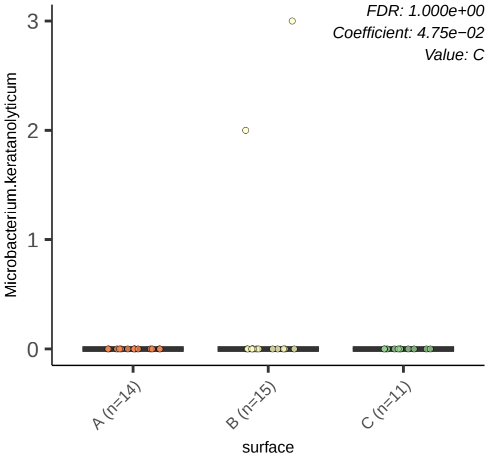

FDR: 1.000e+00
Coefficient: 4.75e−02
Value: C
Microbacterium.keratanolyticum
3
2
1
0
A (n=14)
B (n=15)
C (n=11)
surface

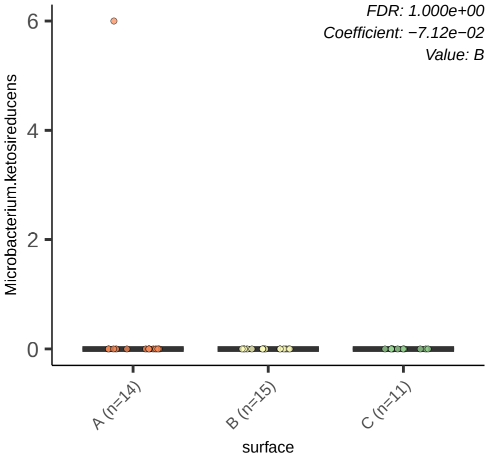
FDR: 1.000e+00
Coefficient: −7.12e−02
Value: B
Microbacterium.ketosireducens
6
4
2
0
A (n=14)
B (n=15)
C (n=11)
surface

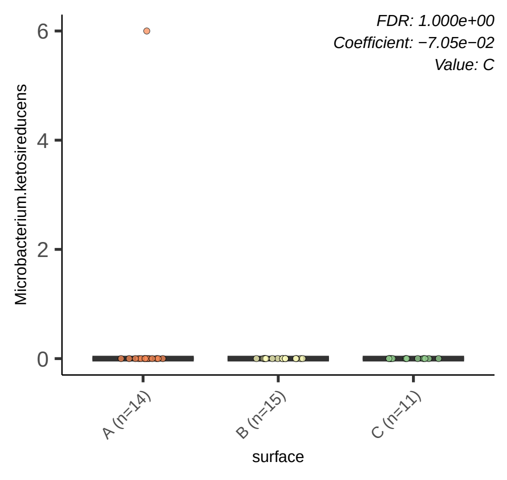

FDR: 1.000e+00
Coefficient: −7.05e−02
Value: C
Microbacterium.ketosireducens
6
4
2
0
A (n=14)
B (n=15)
C (n=11)
surface

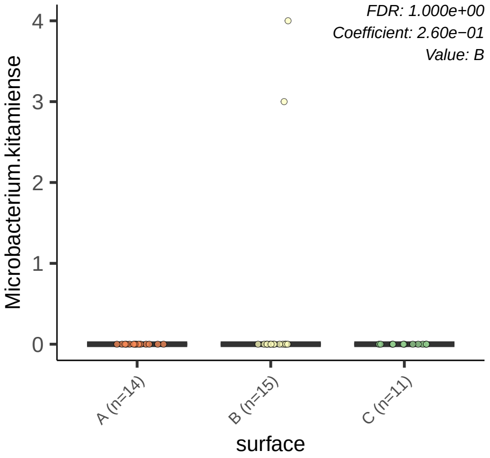
FDR: 1.000e+00
Coefficient: 2.60e−01
Value: B
Microbacterium.kitamiense
0
1
2
3
4
A (n=14)
B (n=15)
C (n=11)
surface

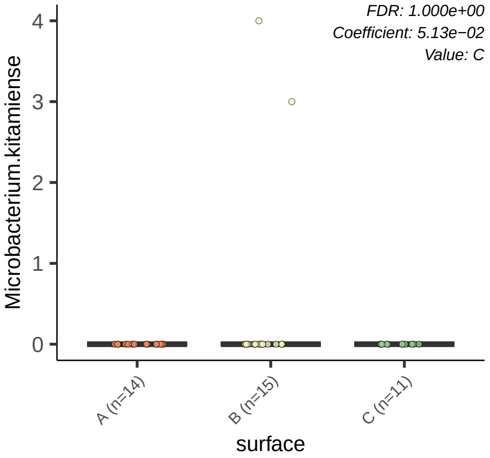
FDR: 1.000e+00
Coefficient: 5.13e−02
Value: C
Microbacterium.kitamiense
4
3
2
1
0
A (n=14)
B (n=15)
C (n=11)
surface

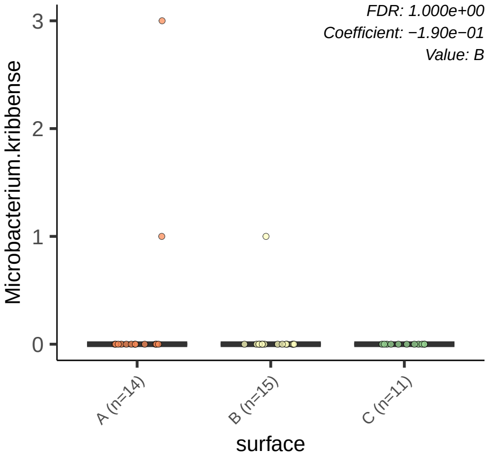
FDR: 1.000e+00
Coefficient: −1.90e−01
Value: B
Microbacterium.kribbense
3
2
1
0
A (n=14)
B (n=15)
C (n=11)
surface

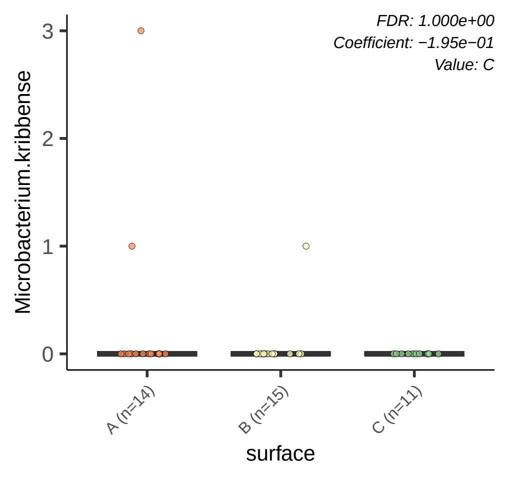
FDR: 1.000e+00
Coefficient: −1.95e−01
Value: C
Microbacterium.kribbense
3
2
1
0
A (n=14)
B (n=15)
C (n=11)
surface

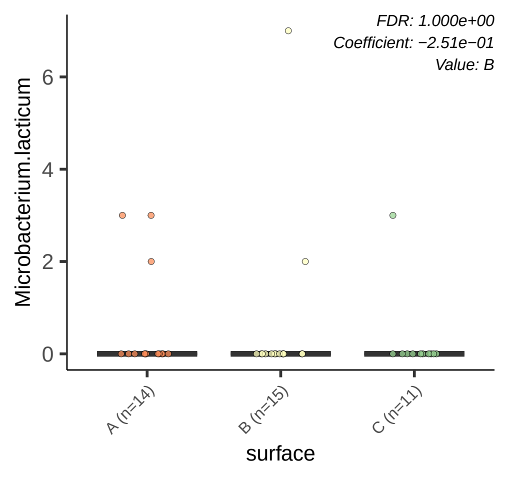
FDR: 1.000e+00
Coefficient: −2.51e−01
Value: B
Microbacterium.lacticum
0
2
4
6
A (n=14)
B (n=15)
C (n=11)
surface

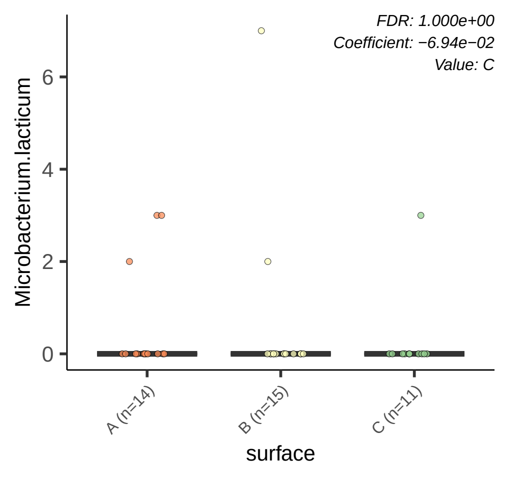
FDR: 1.000e+00
Coefficient: −6.94e−02
Value: C
Microbacterium.lacticum
0
2
4
6
A (n=14)
B (n=15)
C (n=11)
surface

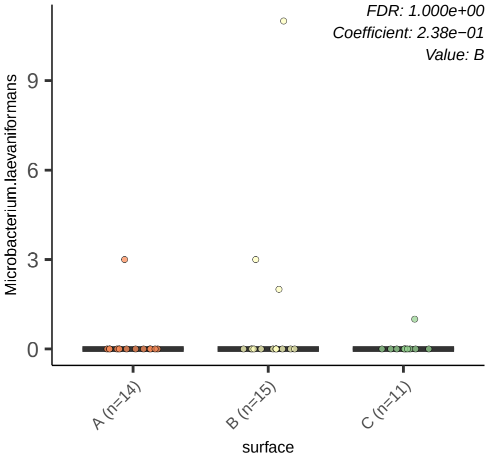
FDR: 1.000e+00
Coefficient: 2.38e−01
Value: B
Microbacterium.laevaniformans
0
3
6
9
A (n=14)
B (n=15)
C (n=11)
surface

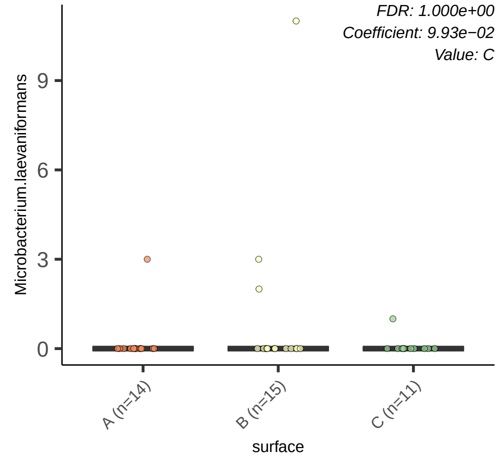
FDR: 1.000e+00
Coefficient: 9.93e−02
Value: C
Microbacterium.laevaniformans
0
3
6
9
A (n=14)
B (n=15)
C (n=11)
surface

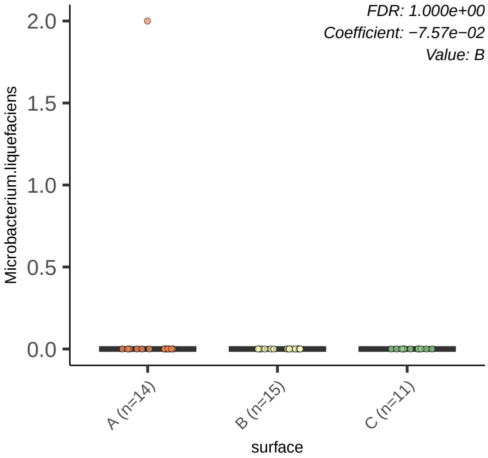

FDR: 1.000e+00
Coefficient: −7.57e−02
Value: B
Microbacterium.liquefaciens
2.0
1.5
1.0
0.5
0.0
A (n=14)
B (n=15)
C (n=11)
surface

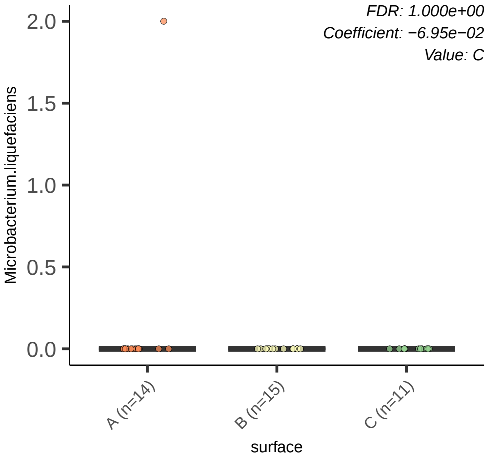
FDR: 1.000e+00
Coefficient: −6.95e−02
Value: C
Microbacterium.liquefaciens
2.0
1.5
1.0
0.5
0.0
A (n=14)
B (n=15)
C (n=11)
surface

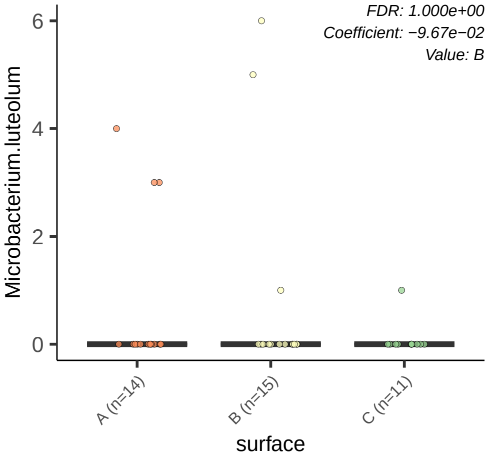
FDR: 1.000e+00
Coefficient: −9.67e−02
Value: B
Microbacterium.luteolum
0
2
4
6
A (n=14)
B (n=15)
C (n=11)
surface

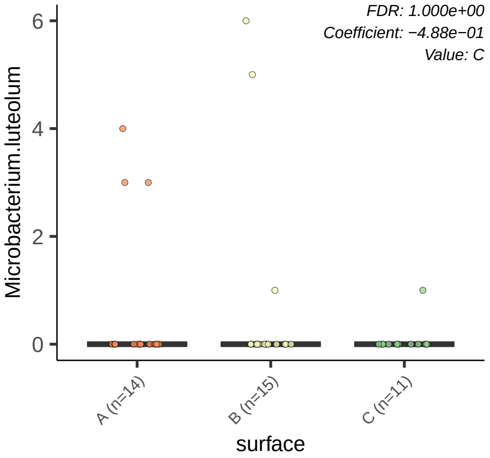
FDR: 1.000e+00
Coefficient: −4.88e−01
Value: C
Microbacterium.luteolum
6
4
2
0
A (n=14)
B (n=15)
C (n=11)
surface

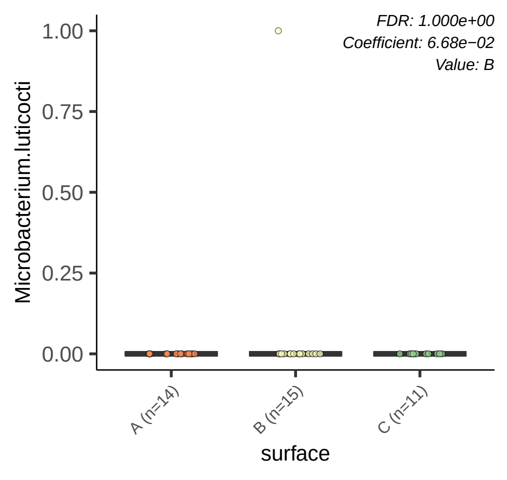
FDR: 1.000e+00
Coefficient: 6.68e−02
Value: B
Microbacterium.luticocti
1.00
0.75
0.50
0.25
0.00
A (n=14)
B (n=15)
C (n=11)
surface

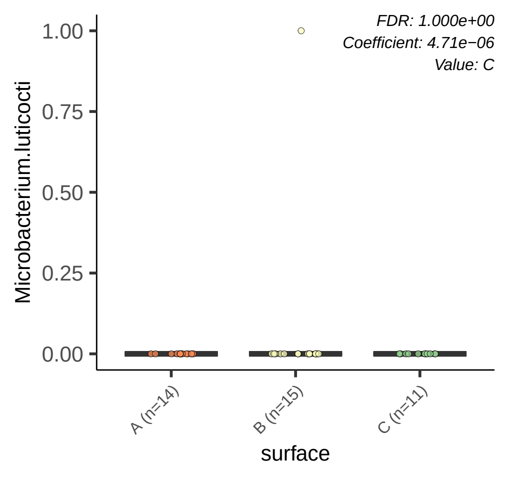
FDR: 1.000e+00
Coefficient: 4.71e−06
Value: C
Microbacterium.luticocti
1.00
0.75
0.50
0.25
0.00
A (n=14)
B (n=15)
C (n=11)
surface

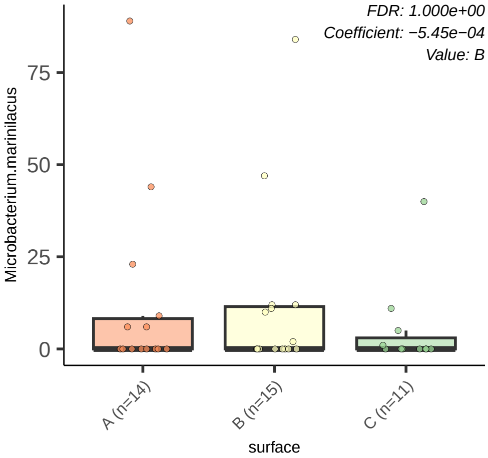

FDR: 1.000e+00
Coefficient: −5.45e−04
Value: B
Microbacterium.marinilacus
0
25
50
75
A (n=14)
B (n=15)
C (n=11)
surface

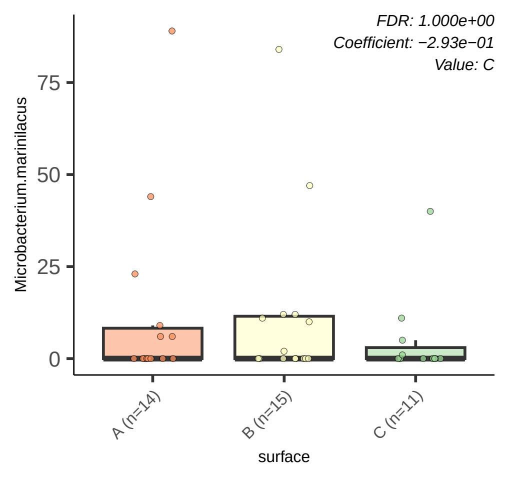
FDR: 1.000e+00
Coefficient: −2.93e−01
Value: C
Microbacterium.marinilacus
0
25
50
75
A (n=14)
B (n=15)
C (n=11)
surface

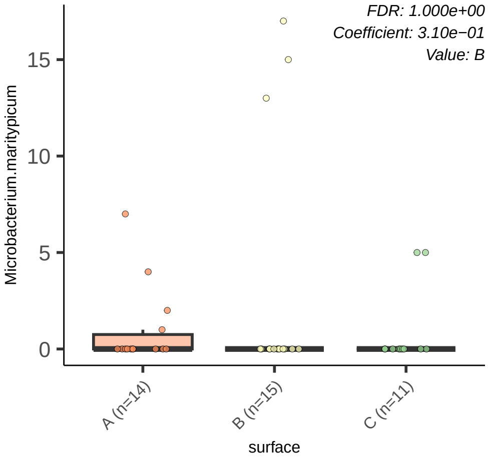
FDR: 1.000e+00
Coefficient: 3.10e−01
Value: B
Microbacterium.maritypicum
0
5
10
15
A (n=14)
B (n=15)
C (n=11)
surface

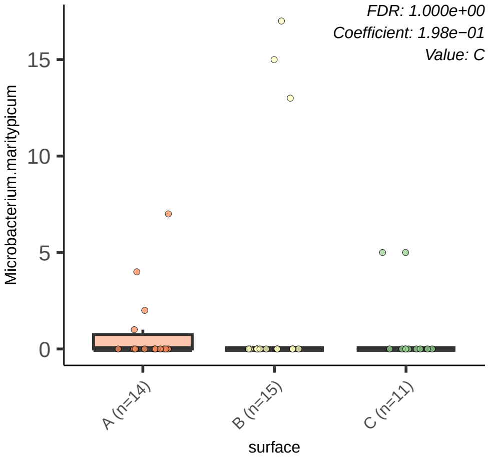
FDR: 1.000e+00
Coefficient: 1.98e−01
Value: C
Microbacterium.maritypicum
15
10
5
0
A (n=14)
B (n=15)
C (n=11)
surface

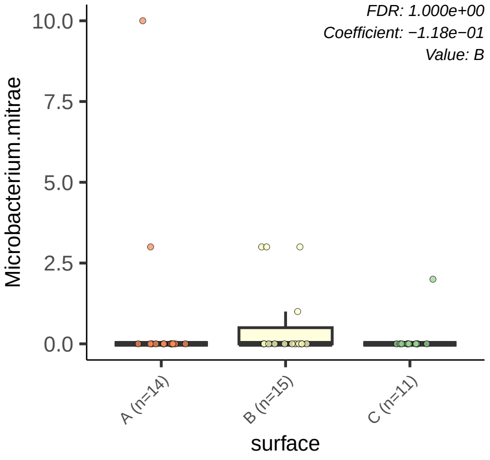
FDR: 1.000e+00
Coefficient: −1.18e−01
Value: B
Microbacterium.mitrae
10.0
7.5
5.0
2.5
0.0
A (n=14)
B (n=15)
C (n=11)
surface

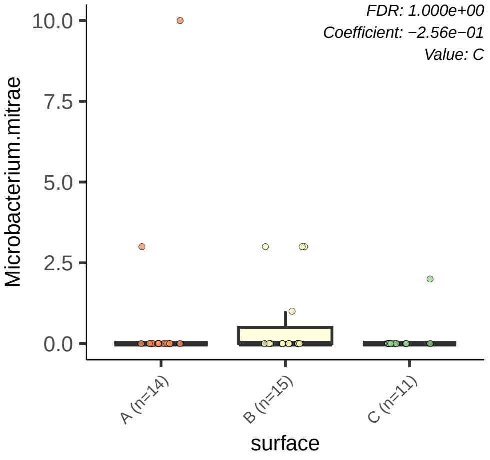
FDR: 1.000e+00
Coefficient: −2.56e−01
Value: C
Microbacterium.mitrae
10.0
7.5
5.0
2.5
0.0
A (n=14)
B (n=15)
C (n=11)
surface

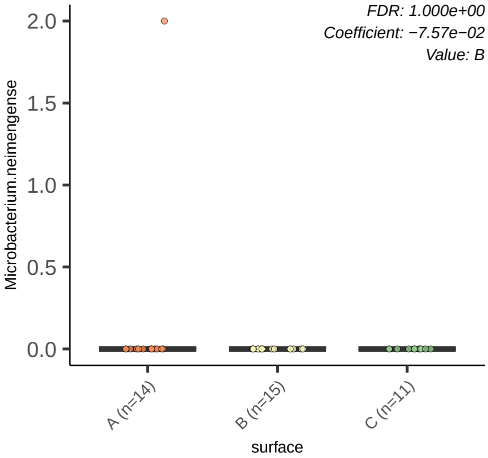
FDR: 1.000e+00
Coefficient: −7.57e−02
Value: B
Microbacterium.neimengense
2.0
1.5
1.0
0.5
0.0
A (n=14)
B (n=15)
C (n=11)
surface

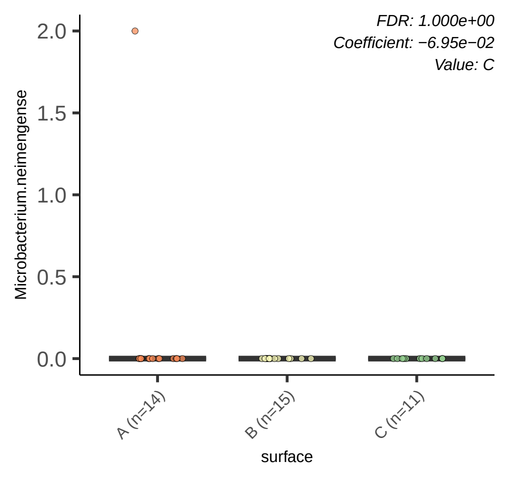

FDR: 1.000e+00
Coefficient: −6.95e−02
Value: C
Microbacterium.neimengense
2.0
1.5
1.0
0.5
0.0
A (n=14)
B (n=15)
C (n=11)
surface

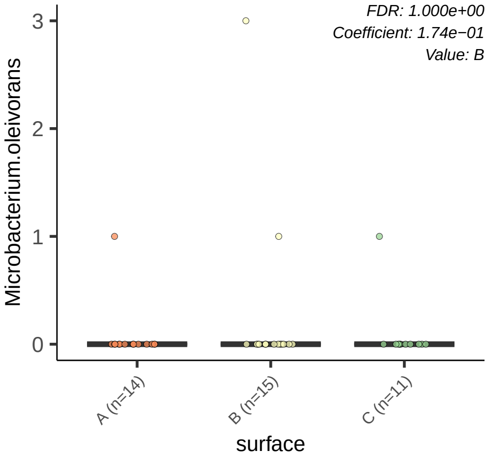

FDR: 1.000e+00
Coefficient: 1.74e−01
Value: B
Microbacterium.oleivorans
3
2
1
0
A (n=14)
B (n=15)
C (n=11)
surface

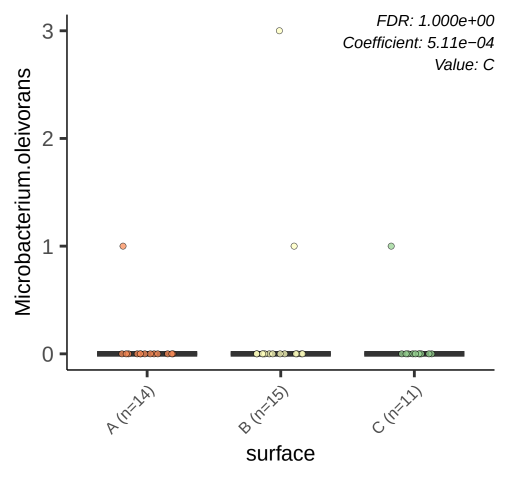

FDR: 1.000e+00
Coefficient: 5.11e−04
Value: C
Microbacterium.oleivorans
3
2
1
0
A (n=14)
B (n=15)
C (n=11)
surface

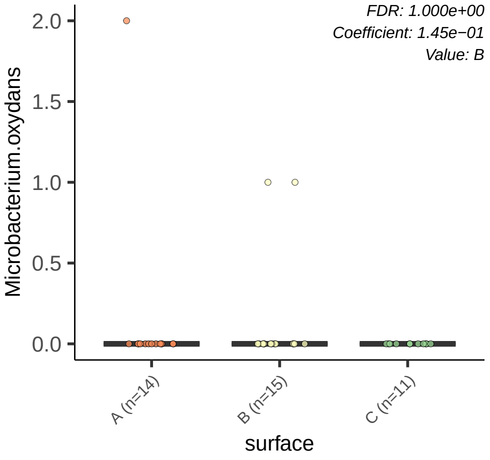

FDR: 1.000e+00
Coefficient: 1.45e−01
Value: B
Microbacterium.oxydans
2.0
1.5
1.0
0.5
0.0
A (n=14)
B (n=15)
C (n=11)
surface

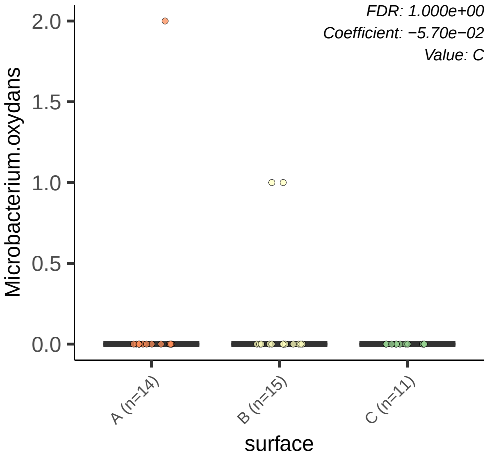
FDR: 1.000e+00
Coefficient: −5.70e−02
Value: C
Microbacterium.oxydans
2.0
1.5
1.0
0.5
0.0
A (n=14)
B (n=15)
C (n=11)
surface

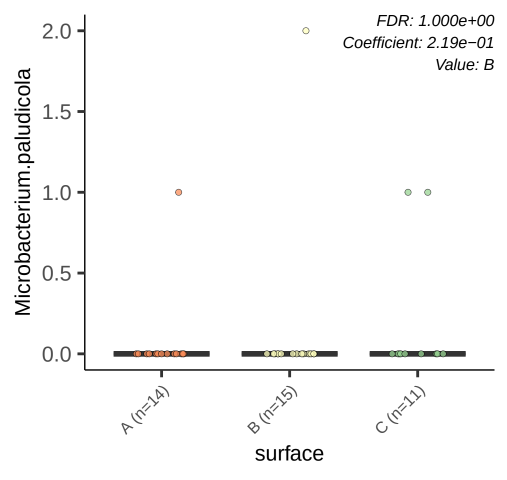

FDR: 1.000e+00
Coefficient: 2.19e−01
Value: B
Microbacterium.paludicola
2.0
1.5
1.0
0.5
0.0
A (n=14)
B (n=15)
C (n=11)
surface

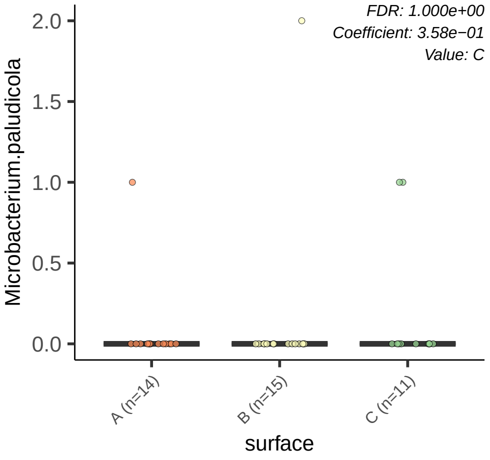
FDR: 1.000e+00
Coefficient: 3.58e−01
Value: C
Microbacterium.paludicola
2.0
1.5
1.0
0.5
0.0
A (n=14)
B (n=15)
C (n=11)
surface

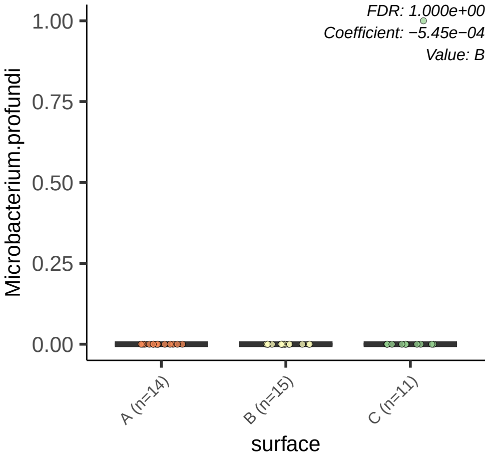
FDR: 1.000e+00
Coefficient: −5.45e−04
Value: B
Microbacterium.profundi
1.00
0.75
0.50
0.25
0.00
A (n=14)
B (n=15)
C (n=11)
surface

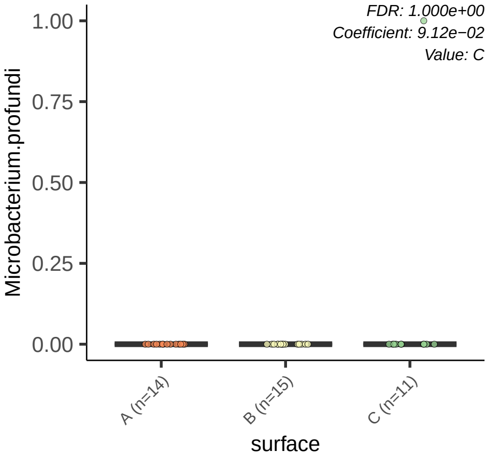

FDR: 1.000e+00
Coefficient: 9.12e−02
Value: C
Microbacterium.profundi
1.00
0.75
0.50
0.25
0.00
A (n=14)
B (n=15)
C (n=11)
surface

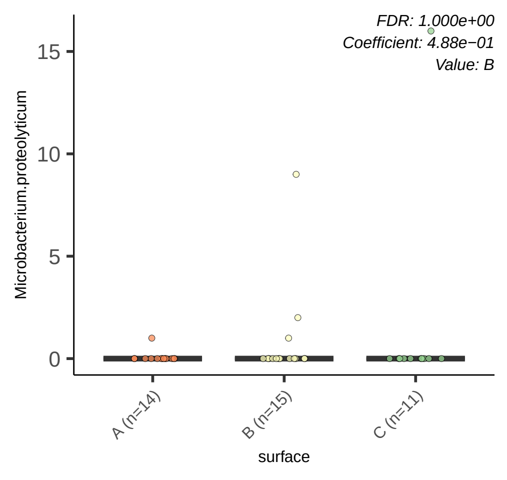
FDR: 1.000e+00
Coefficient: 4.88e−01
Value: B
Microbacterium.proteolyticum
15
10
5
0
A (n=14)
B (n=15)
C (n=11)
surface

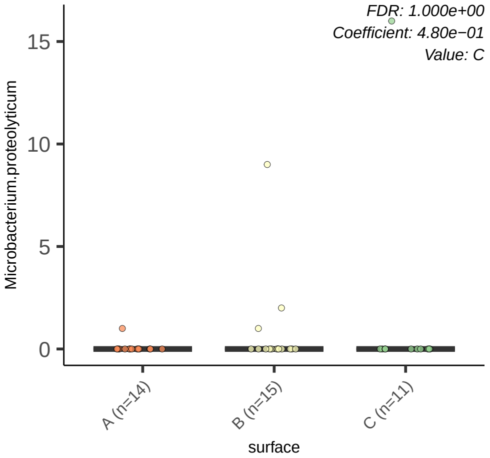

FDR: 1.000e+00
Coefficient: 4.80e−01
Value: C
Microbacterium.proteolyticum
15
10
5
0
A (n=14)
B (n=15)
C (n=11)
surface

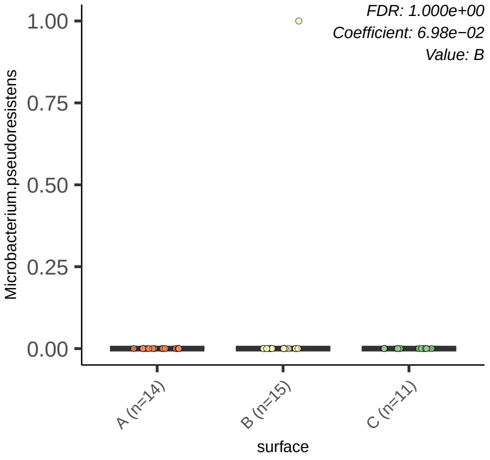

FDR: 1.000e+00
Coefficient: 6.98e−02
Value: B
Microbacterium.pseudoresistens
1.00
0.75
0.50
0.25
0.00
A (n=14)
B (n=15)
C (n=11)
surface

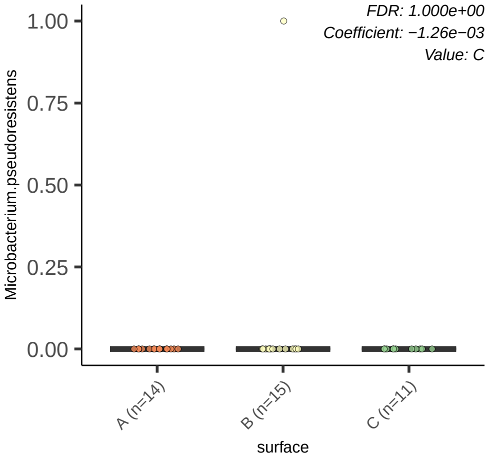
FDR: 1.000e+00
Coefficient: −1.26e−03
Value: C
Microbacterium.pseudoresistens
1.00
0.75
0.50
0.25
0.00
A (n=14)
B (n=15)
C (n=11)
surface

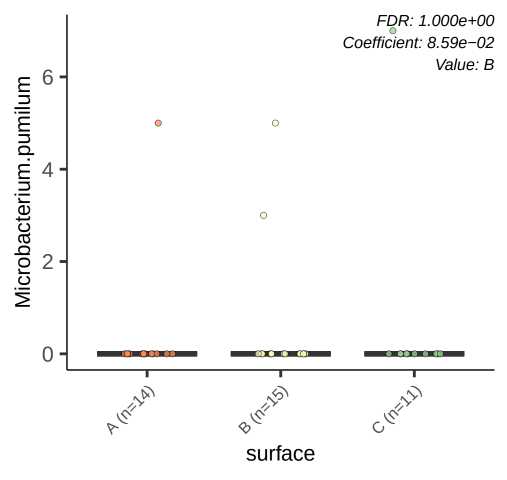

FDR: 1.000e+00
Coefficient: 8.59e−02
Value: B
Microbacterium.pumilum
0
2
4
6
A (n=14)
B (n=15)
C (n=11)
surface

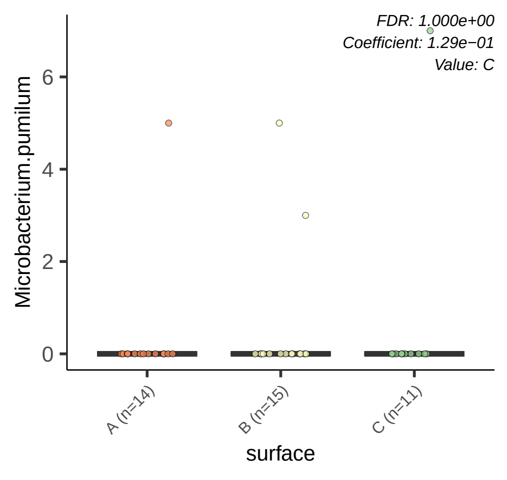
FDR: 1.000e+00
Coefficient: 1.29e−01
Value: C
Microbacterium.pumilum
0
2
4
6
A (n=14)
B (n=15)
C (n=11)
surface

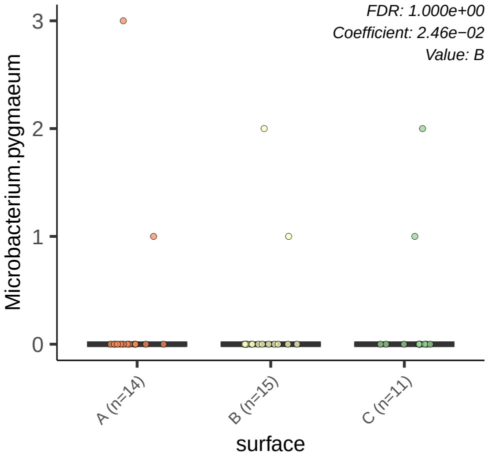
FDR: 1.000e+00
Coefficient: 2.46e−02
Value: B
Microbacterium.pygmaeum
0
1
2
3
A (n=14)
B (n=15)
C (n=11)
surface

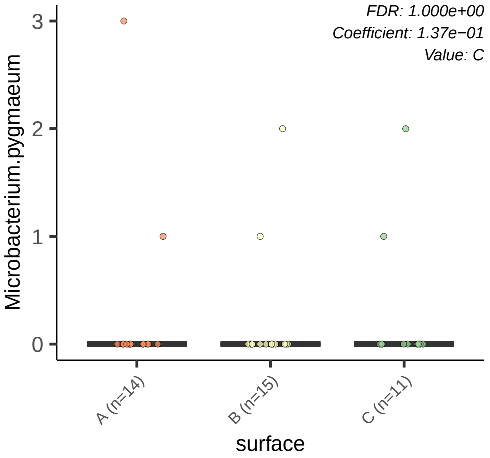

FDR: 1.000e+00
Coefficient: 1.37e−01
Value: C
Microbacterium.pygmaeum
3
2
1
0
A (n=14)
B (n=15)
C (n=11)
surface

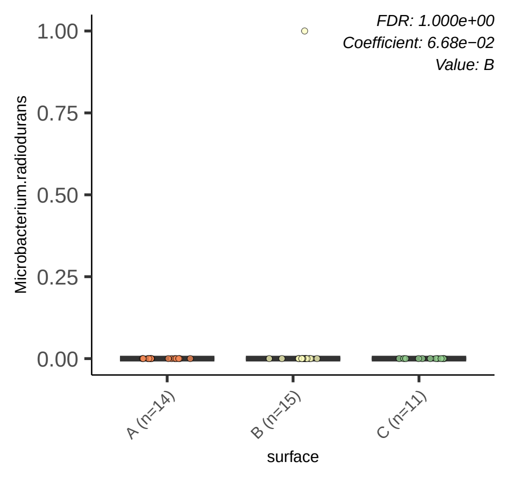

FDR: 1.000e+00
Coefficient: 6.68e−02
Value: B
1.00
0.75
0.50
0.25
0.00
Microbacterium.radiodurans
A (n=14)
B (n=15)
C (n=11)
surface

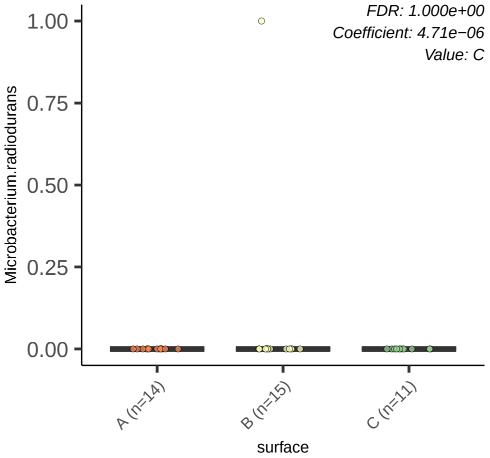
FDR: 1.000e+00
Coefficient: 4.71e−06
Value: C
Microbacterium.radiodurans
1.00
0.75
0.50
0.25
0.00
A (n=14)
B (n=15)
C (n=11)
surface

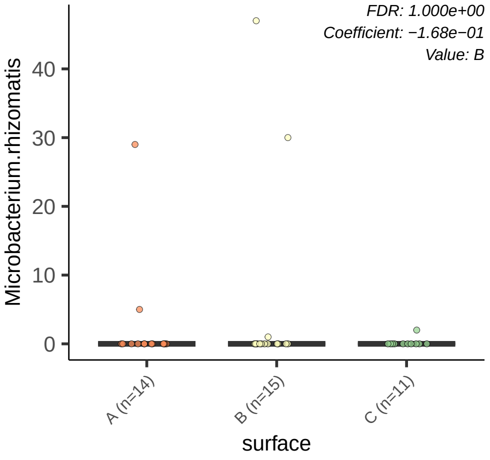
FDR: 1.000e+00
Coefficient: −1.68e−01
Value: B
Microbacterium.rhizomatis
40
30
20
10
0
A (n=14)
B (n=15)
C (n=11)
surface

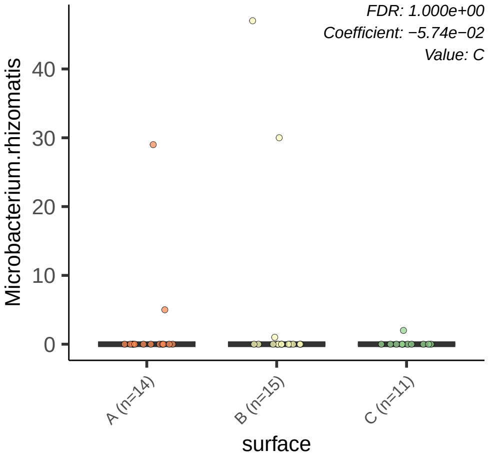

FDR: 1.000e+00
Coefficient: −5.74e−02
Value: C
Microbacterium.rhizomatis
0
10
20
30
40
A (n=14)
B (n=15)
C (n=11)
surface

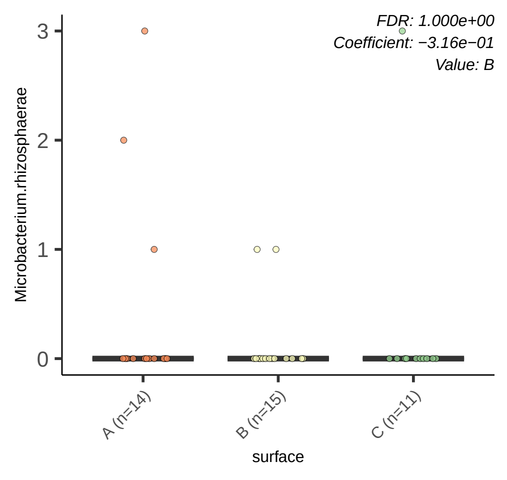
FDR: 1.000e+00
Coefficient: −3.16e−01
Value: B
Microbacterium.rhizosphaerae
0
1
2
3
A (n=14)
B (n=15)
C (n=11)
surface

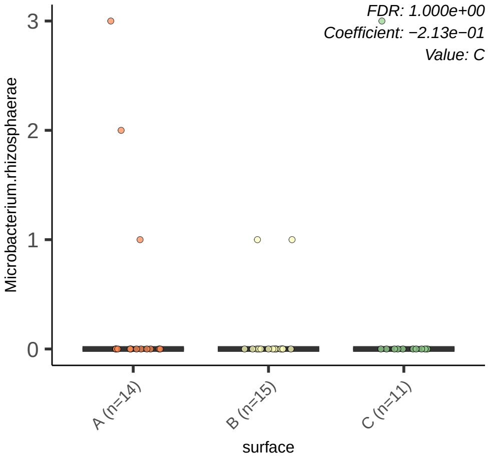

FDR: 1.000e+00
Coefficient: −2.13e−01
Value: C
Microbacterium.rhizosphaerae
3
2
1
0
A (n=14)
B (n=15)
C (n=11)
surface

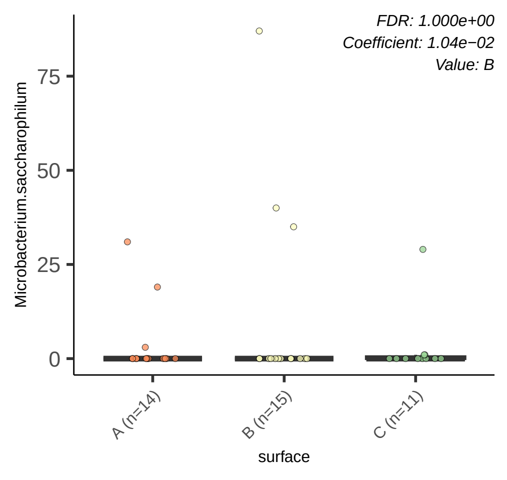
FDR: 1.000e+00
Coefficient: 1.04e−02
Value: B
Microbacterium.saccharophilum
75
50
25
0
A (n=14)
B (n=15)
C (n=11)
surface

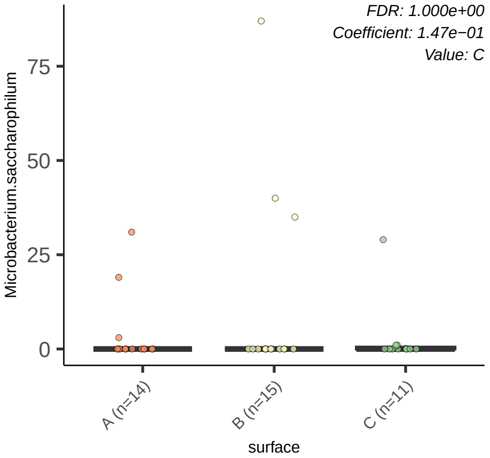
FDR: 1.000e+00
Coefficient: 1.47e−01
Value: C
Microbacterium.saccharophilum
75
50
25
0
A (n=14)
B (n=15)
C (n=11)
surface

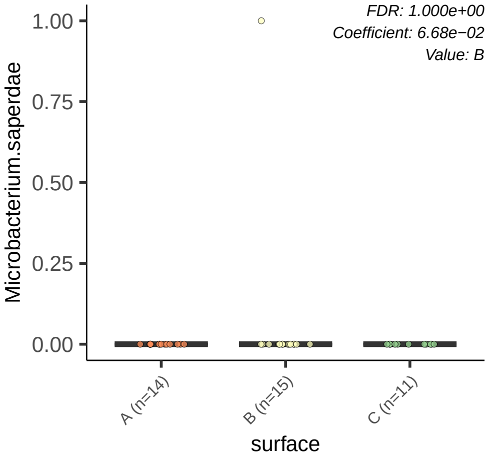
FDR: 1.000e+00
Coefficient: 6.68e−02
Value: B
Microbacterium.saperdae
1.00
0.75
0.50
0.25
0.00
A (n=14)
B (n=15)
C (n=11)
surface

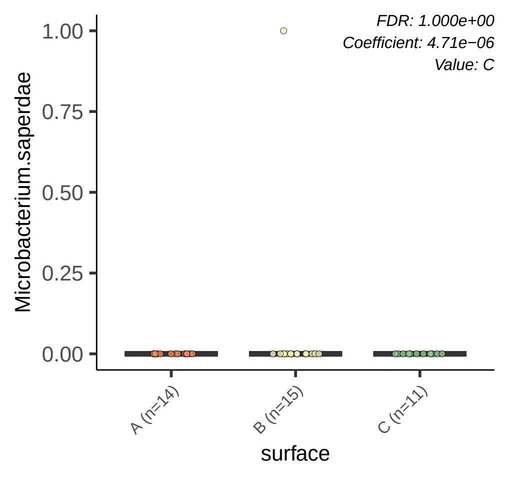
FDR: 1.000e+00
Coefficient: 4.71e−06
Value: C
Microbacterium.saperdae
1.00
0.75
0.50
0.25
0.00
A (n=14)
B (n=15)
C (n=11)
surface

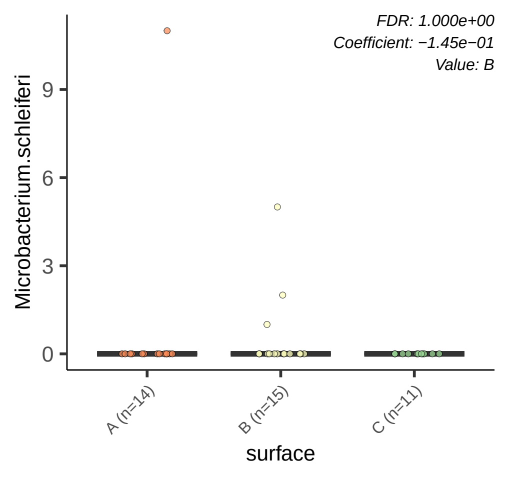

FDR: 1.000e+00
Coefficient: −1.45e−01
Value: B
Microbacterium.schleiferi
0
3
6
9
A (n=14)
B (n=15)
C (n=11)
surface

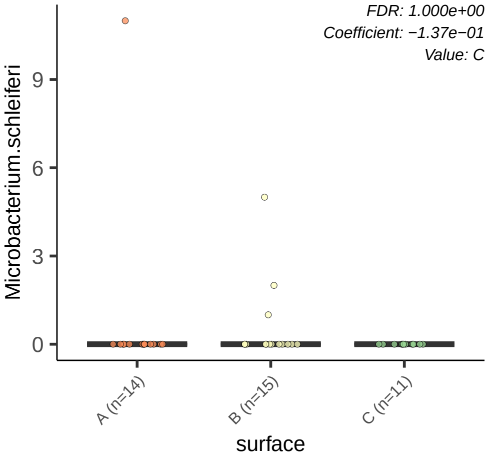
FDR: 1.000e+00
Coefficient: −1.37e−01
Value: C
Microbacterium.schleiferi
0
3
6
9
A (n=14)
B (n=15)
C (n=11)
surface

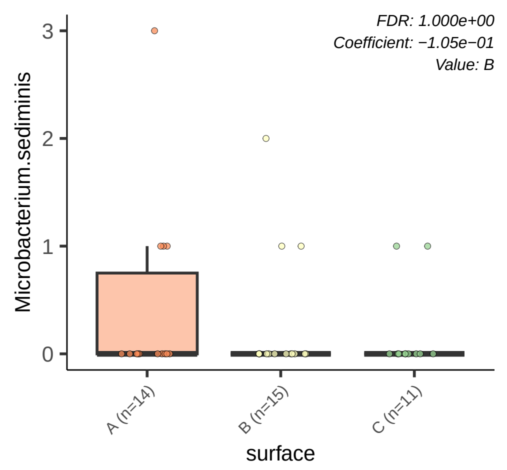
FDR: 1.000e+00
Coefficient: −1.05e−01
Value: B
Microbacterium.sedimінis
3
2
1
0
A (n=14)
B (n=15)
C (n=11)
surface

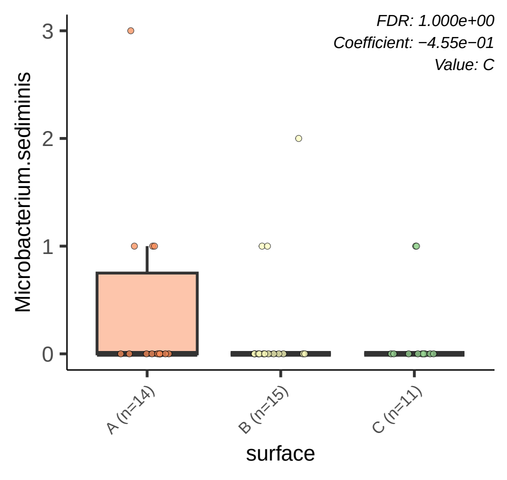

FDR: 1.000e+00
Coefficient: −4.55e−01
Value: C
Microbacterium.sediminis
3
2
1
0
A (n=14)
B (n=15)
C (n=11)
surface

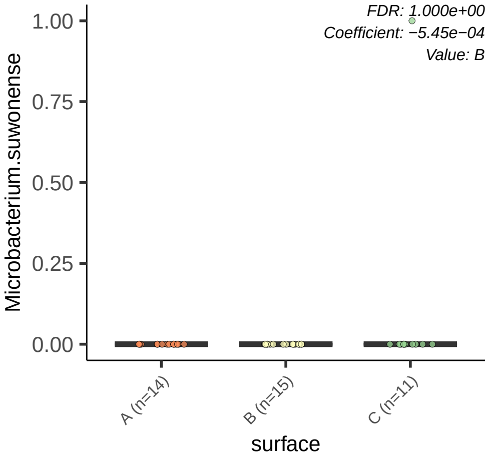
FDR: 1.000e+00
Coefficient: −5.45e−04
Value: B
Microbacterium.suwonense
1.00
0.75
0.50
0.25
0.00
A (n=14)
B (n=15)
C (n=11)
surface

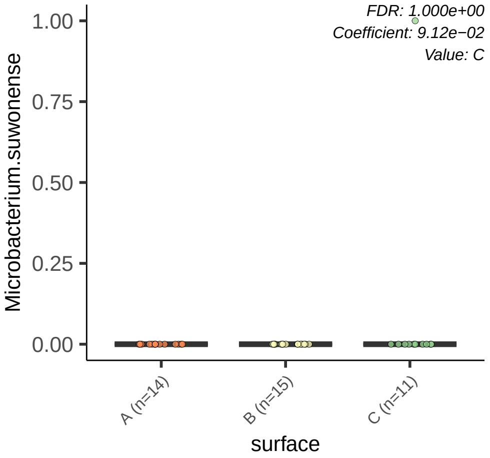
FDR: 1.000e+00
Coefficient: 9.12e−02
Value: C
Microbacterium.suwonense
1.00
0.75
0.50
0.25
0.00
A (n=14)
B (n=15)
C (n=11)
surface

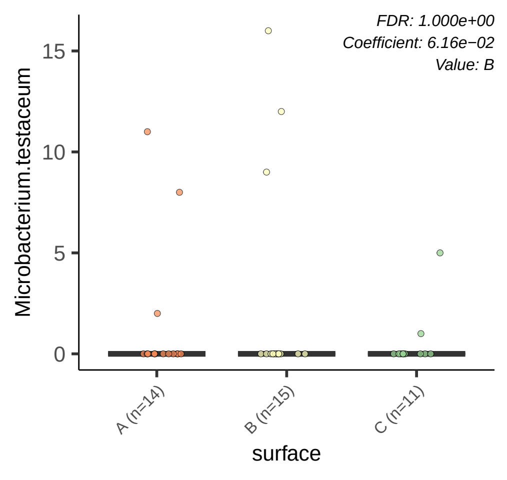
FDR: 1.000e+00
Coefficient: 6.16e−02
Value: B
Microbacterium.testaceum
0
5
10
15
A (n=14)
B (n=15)
C (n=11)
surface

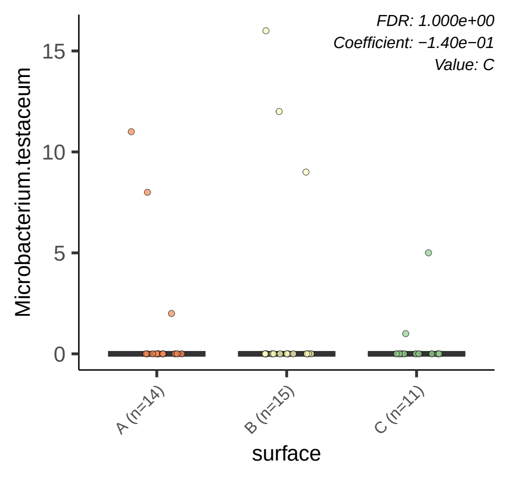
FDR: 1.000e+00
Coefficient: −1.40e−01
Value: C
Microbacterium.testaceum
15
10
5
0
A (n=14)
B (n=15)
C (n=11)
surface

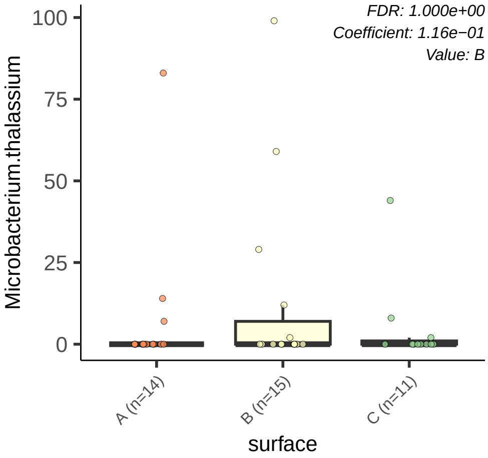
FDR: 1.000e+00
Coefficient: 1.16e−01
Value: B
Microbacterium.thalassium
100
75
50
25
0
A (n=14)
B (n=15)
C (n=11)
surface

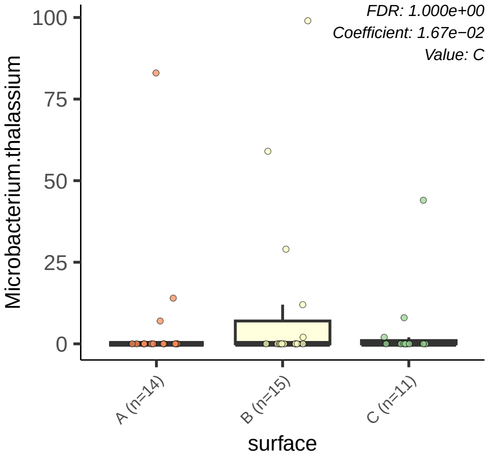

FDR: 1.000e+00
Coefficient: 1.67e−02
Value: C
Microbacterium.thalassium
100
75
50
25
0
A (n=14)
B (n=15)
C (n=11)
surface

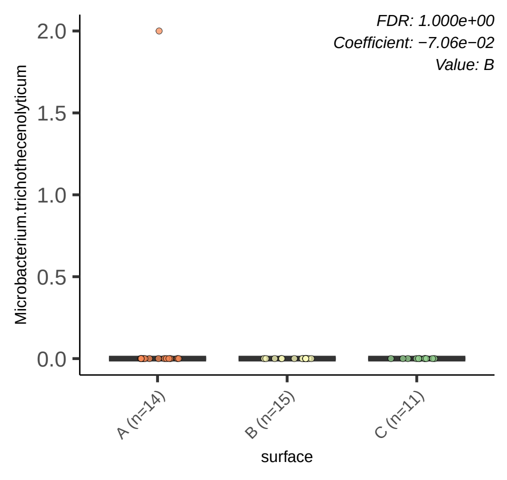
FDR: 1.000e+00
Coefficient: −7.06e−02
Value: B
Microbacterium.trichothecenolyticum
2.0
1.5
1.0
0.5
0.0
A (n=14)
B (n=15)
C (n=11)
surface

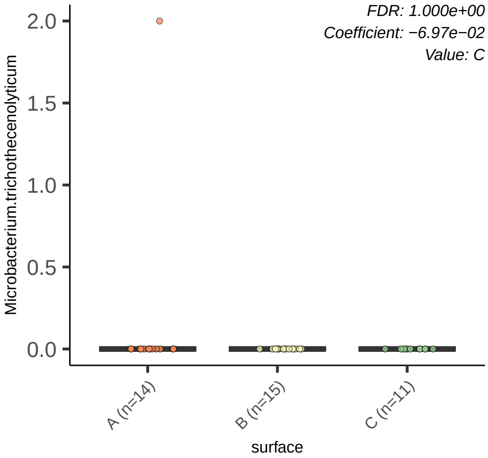

FDR: 1.000e+00
Coefficient: −6.97e−02
Value: C
Microbacterium.trichothecenolyticum
2.0
1.5
1.0
0.5
0.0
A (n=14)
B (n=15)
C (n=11)
surface

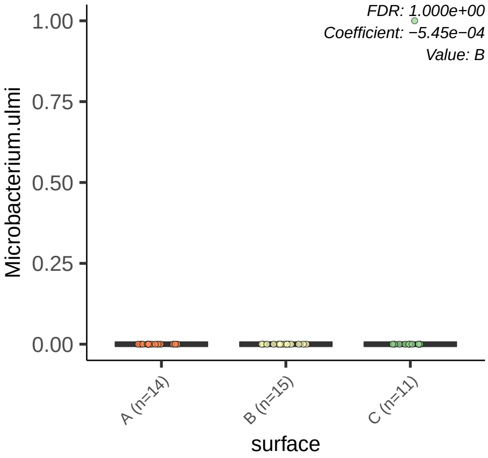

FDR: 1.000e+00
Coefficient: −5.45e−04
Value: B
Microbacterium.ulmi
1.00
0.75
0.50
0.25
0.00
A (n=14)
B (n=15)
C (n=11)
surface

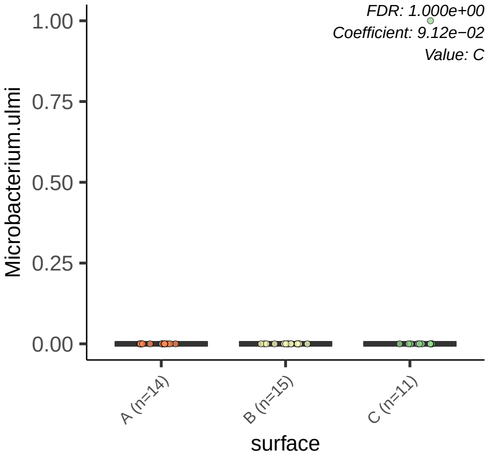

FDR: 1.000e+00
Coefficient: 9.12e−02
Value: C
Microbacterium.ulmi
1.00
0.75
0.50
0.25
0.00
A (n=14)
B (n=15)
C (n=11)
surface

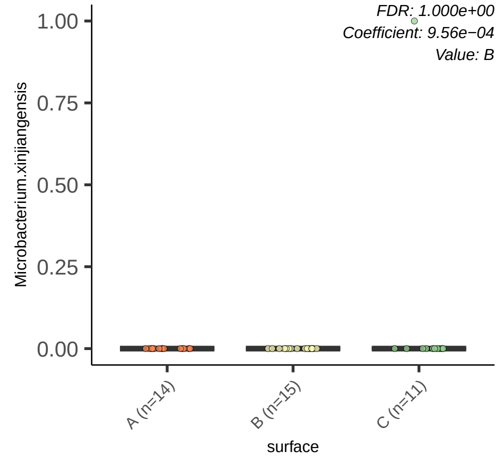

FDR: 1.000e+00
Coefficient: 9.56e−04
Value: B
1.00
0.75
0.50
0.25
0.00
Microbacterium.xinjiangensis
A (n=14)
B (n=15)
C (n=11)
surface

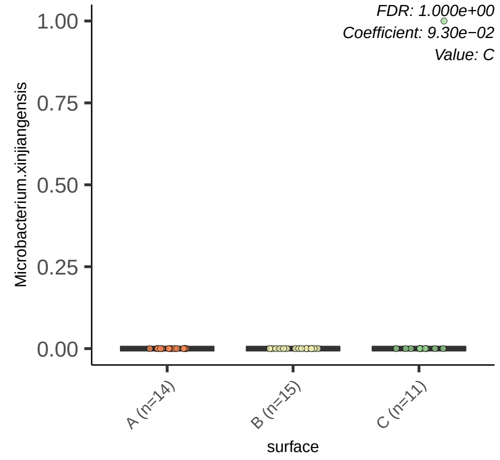
FDR: 1.000e+00
Coefficient: 9.30e−02
Value: C
Microbacterium.xinjiangensis
1.00
0.75
0.50
0.25
0.00
A (n=14)
B (n=15)
C (n=11)
surface

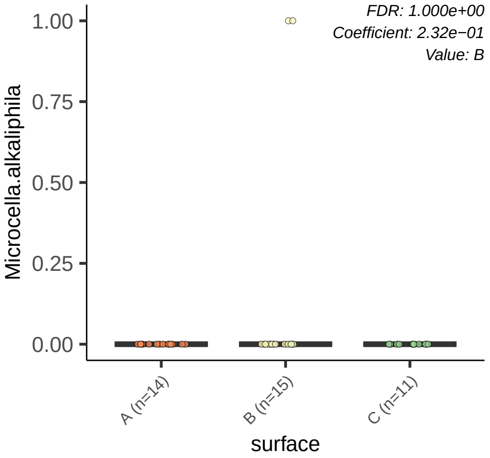

FDR: 1.000e+00
Coefficient: 2.32e−01
Value: B
Microcella.alkaliphila
1.00
0.75
0.50
0.25
0.00
A (n=14)
B (n=15)
C (n=11)
surface

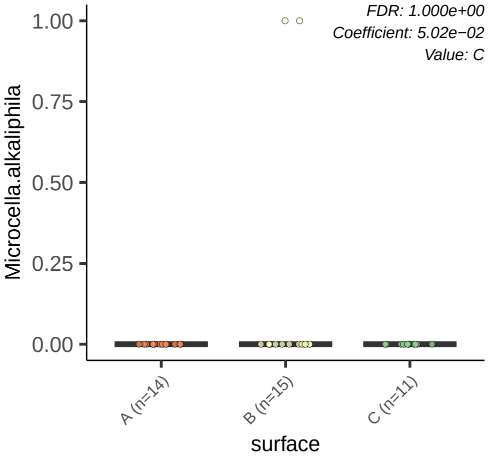
FDR: 1.000e+00
Coefficient: 5.02e−02
Value: C
Microcella.alkaliphila
1.00
0.75
0.50
0.25
0.00
A (n=14)
B (n=15)
C (n=11)
surface

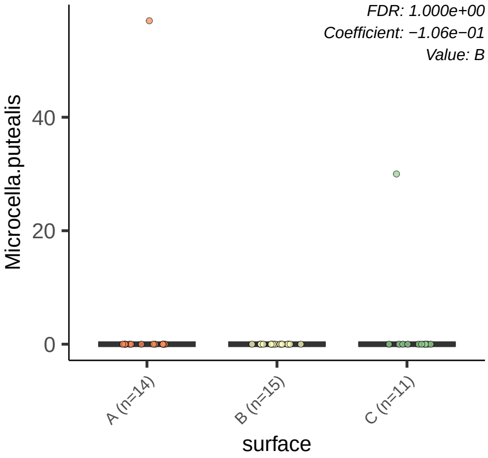
FDR: 1.000e+00
Coefficient: −1.06e−01
Value: B
Microcella.putealis
40
20
0
A (n=14)
B (n=15)
C (n=11)
surface

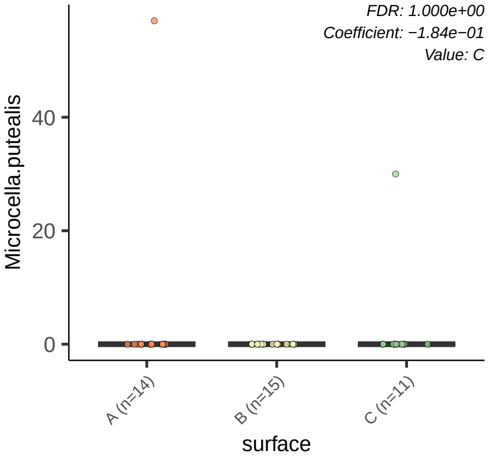
FDR: 1.000e+00
Coefficient: −1.84e−01
Value: C
Microcella.putealis
40
20
0
A (n=14)
B (n=15)
C (n=11)
surface

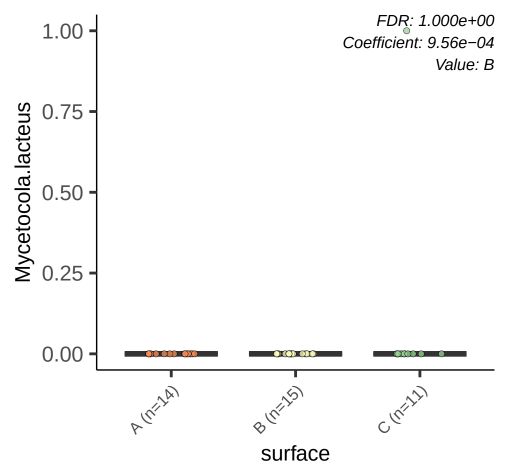
FDR: 1.000e+00
Coefficient: 9.56e−04
Value: B
Mycetocola.lacteus
1.00
0.75
0.50
0.25
0.00
A (n=14)
B (n=15)
C (n=11)
surface

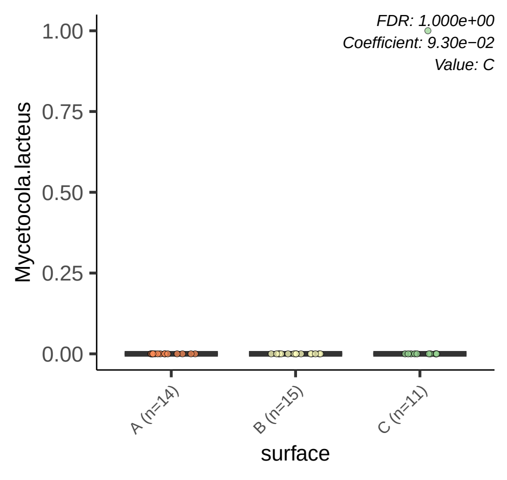
FDR: 1.000e+00
Coefficient: 9.30e−02
Value: C
Mycetocola.lacteus
1.00
0.75
0.50
0.25
0.00
A (n=14)
B (n=15)
C (n=11)
surface

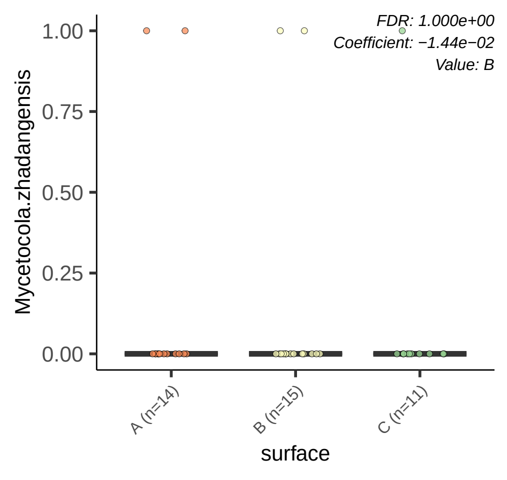

FDR: 1.000e+00
Coefficient: −1.44e−02
Value: B
Mycetocola.zhadangensis
1.00
0.75
0.50
0.25
0.00
A (n=14)
B (n=15)
C (n=11)
surface

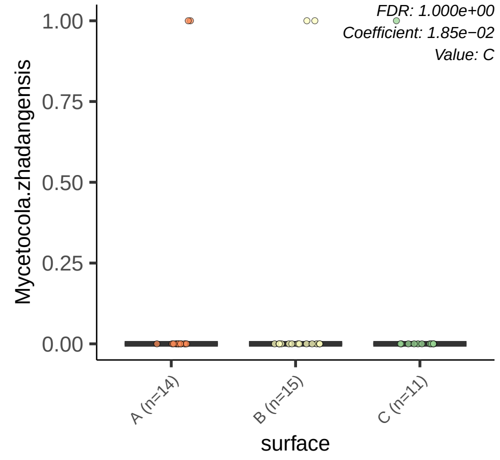

FDR: 1.000e+00
Coefficient: 1.85e−02
Value: C
Mycetocola.zhadangensis
1.00
0.75
0.50
0.25
0.00
A (n=14)
B (n=15)
C (n=11)
surface

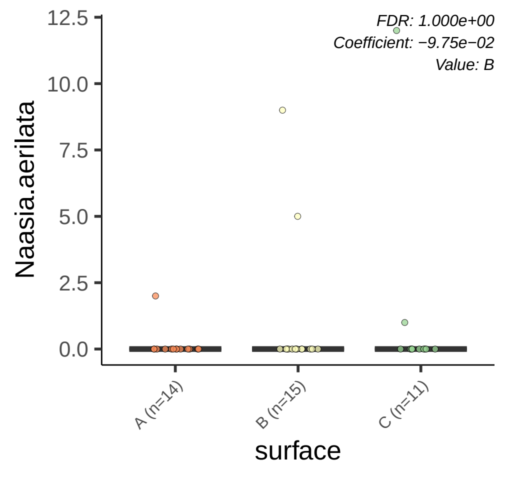
FDR: 1.000e+00
Coefficient: −9.75e−02
Value: B
Naasia.aerilata
12.5
10.0
7.5
5.0
2.5
0.0
A (n=14)
B (n=15)
C (n=11)
surface

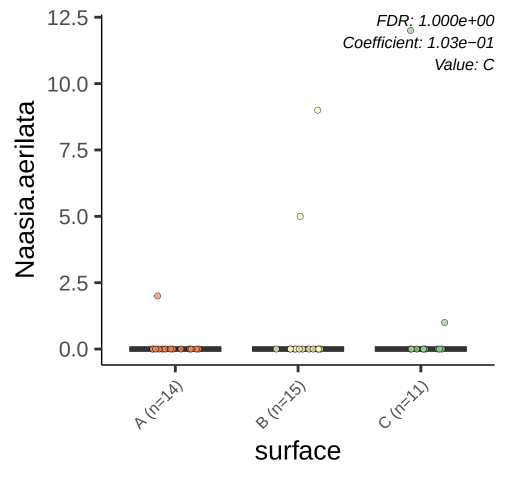

FDR: 1.000e+00
Coefficient: 1.03e−01
Value: C
Naasia.aerilata
12.5
10.0
7.5
5.0
2.5
0.0
A (n=14)
B (n=15)
C (n=11)
surface

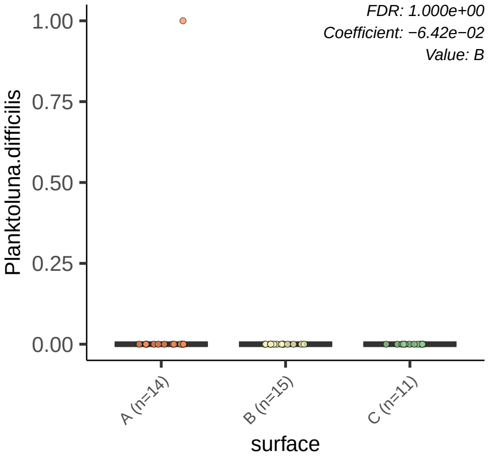
FDR: 1.000e+00
Coefficient: −6.42e−02
Value: B
Planktoluna.difficilis
1.00
0.75
0.50
0.25
0.00
A (n=14)
B (n=15)
C (n=11)
surface

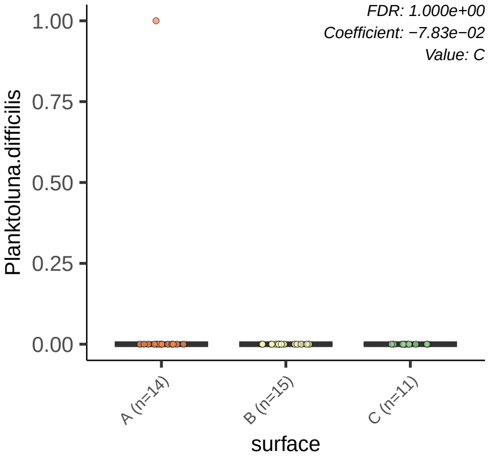
FDR: 1.000e+00
Coefficient: −7.83e−02
Value: C
Planktoluna.difficilis
1.00
0.75
0.50
0.25
0.00
A (n=14)
B (n=15)
C (n=11)
surface

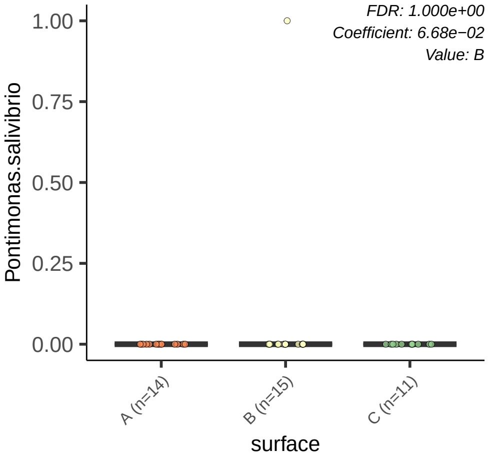

FDR: 1.000e+00
Coefficient: 6.68e−02
Value: B
Pontimonas.salivibrio
1.00
0.75
0.50
0.25
0.00
A (n=14)
B (n=15)
C (n=11)
surface

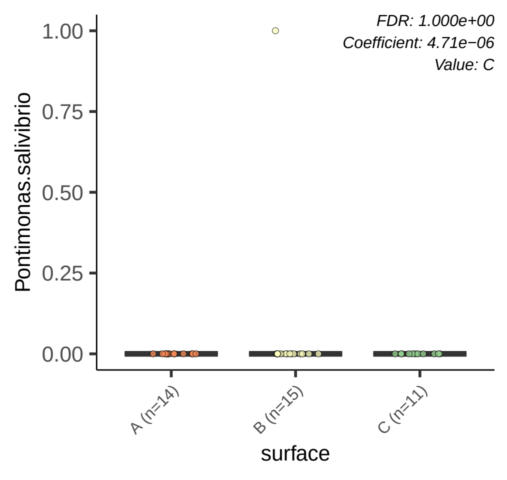
FDR: 1.000e+00
Coefficient: 4.71e−06
Value: C
Pontimonas.salivibrio
1.00
0.75
0.50
0.25
0.00
A (n=14)
B (n=15)
C (n=11)
surface

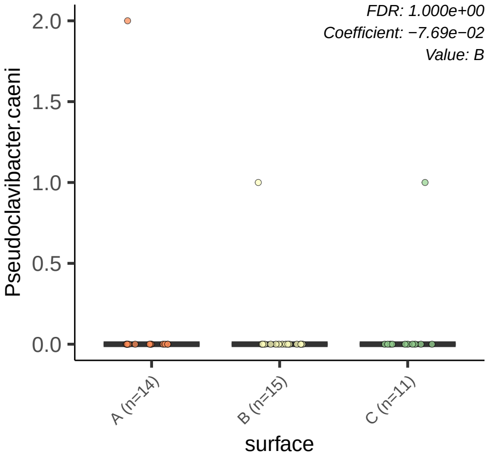
FDR: 1.000e+00
Coefficient: −7.69e−02
Value: B
Pseudoclavibacter.caeni
2.0
1.5
1.0
0.5
0.0
A (n=14)
B (n=15)
C (n=11)
surface

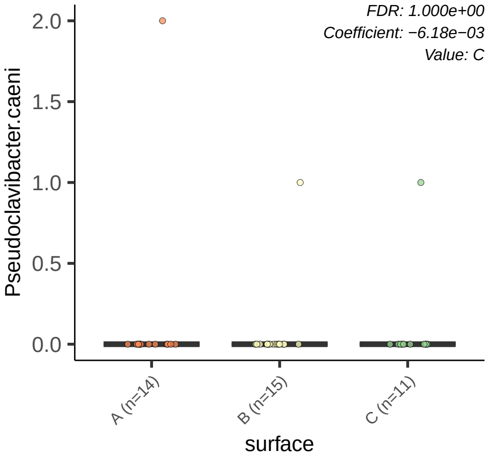

FDR: 1.000e+00
Coefficient: −6.18e−03
Value: C
Pseudoclavibacter.caeni
2.0
1.5
1.0
0.5
0.0
A (n=14)
B (n=15)
C (n=11)
surface

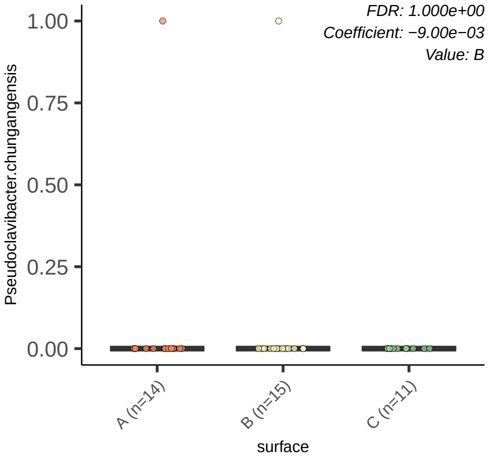
FDR: 1.000e+00
Coefficient: −9.00e−03
Value: B
Pseudoclavibacter.chungangensis
1.00
0.75
0.50
0.25
0.00
A (n=14)
B (n=15)
C (n=11)
surface

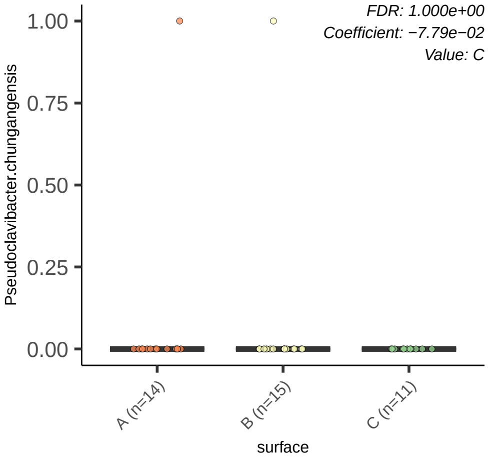
FDR: 1.000e+00
Coefficient: −7.79e−02
Value: C
Pseudoclavibacter.chungangensis
1.00
0.75
0.50
0.25
0.00
A (n=14)
B (n=15)
C (n=11)
surface

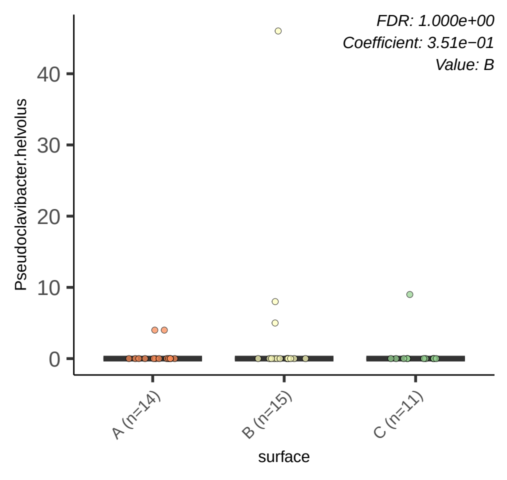

FDR: 1.000e+00
Coefficient: 3.51e−01
Value: B
Pseudoclavibacter.helvolus
40
30
20
10
0
A (n=14)
B (n=15)
C (n=11)
surface

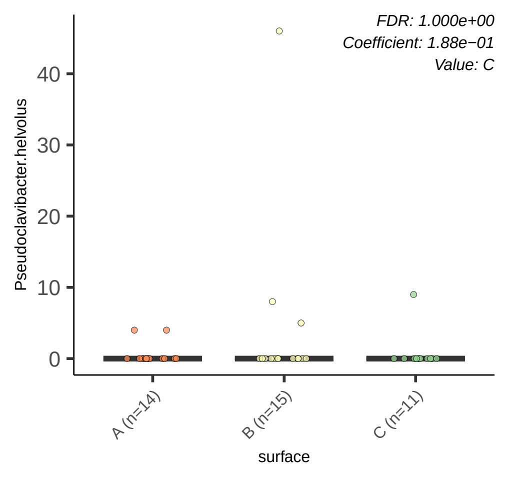

FDR: 1.000e+00
Coefficient: 1.88e−01
Value: C
Pseudoclavibacter.helvolus
40
30
20
10
0
A (n=14)
B (n=15)
C (n=11)
surface

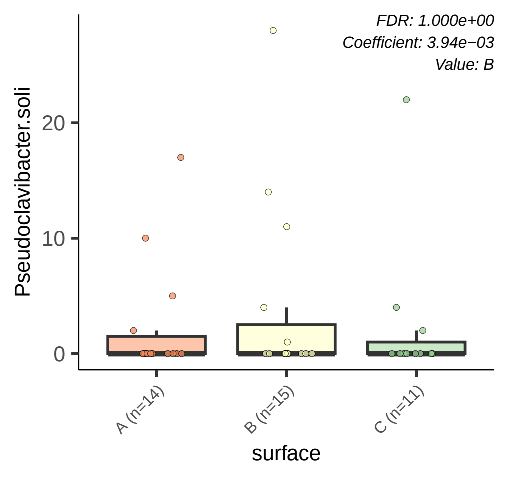

FDR: 1.000e+00
Coefficient: 3.94e−03
Value: B
Pseudoclavibacter.soli
0
10
20
A (n=14)
B (n=15)
C (n=11)
surface

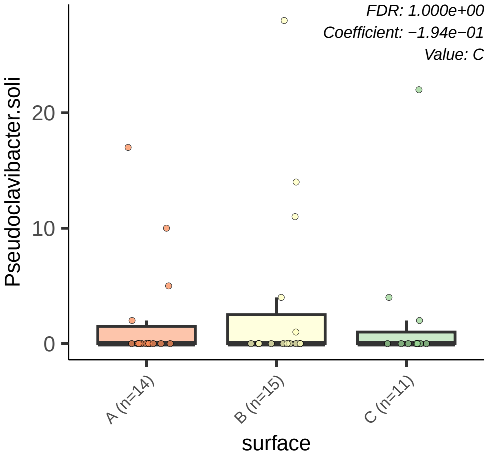

FDR: 1.000e+00
Coefficient: −1.94e−01
Value: C
Pseudoclavibacter.soli
20
10
0
A (n=14)
B (n=15)
C (n=11)
surface

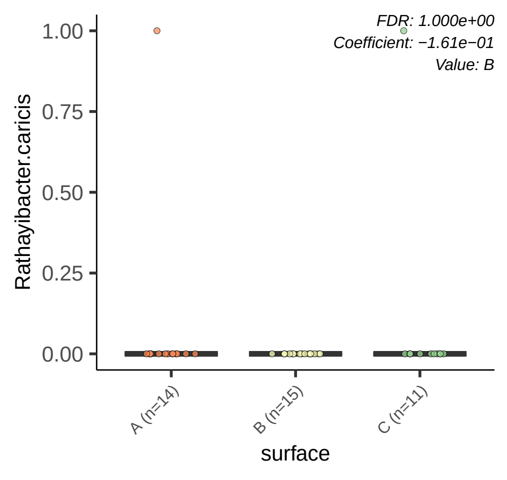
FDR: 1.000e+00
Coefficient: −1.61e−01
Value: B
Rathayibacter.caricis
1.00
0.75
0.50
0.25
0.00
A (n=14)
B (n=15)
C (n=11)
surface

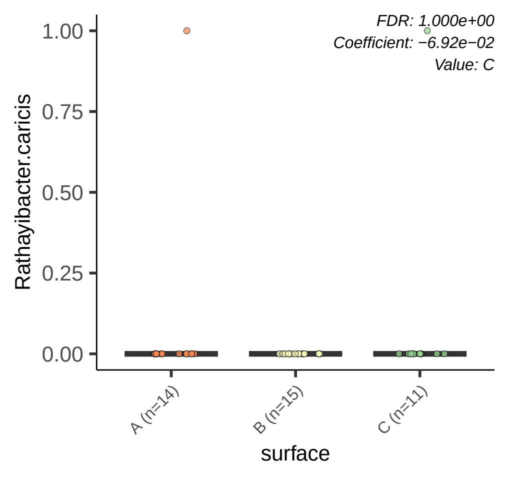

FDR: 1.000e+00
Coefficient: −6.92e−02
Value: C
Rathayibacter.caricis
1.00
0.75
0.50
0.25
0.00
A (n=14)
B (n=15)
C (n=11)
surface

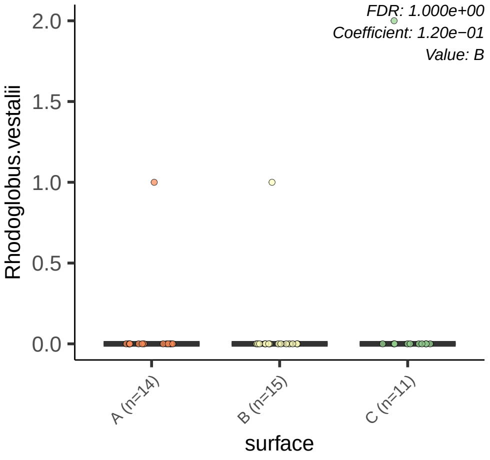
FDR: 1.000e+00
Coefficient: 1.20e−01
Value: B
Rhodoglobus.vestalii
2.0
1.5
1.0
0.5
0.0
A (n=14)
B (n=15)
C (n=11)
surface

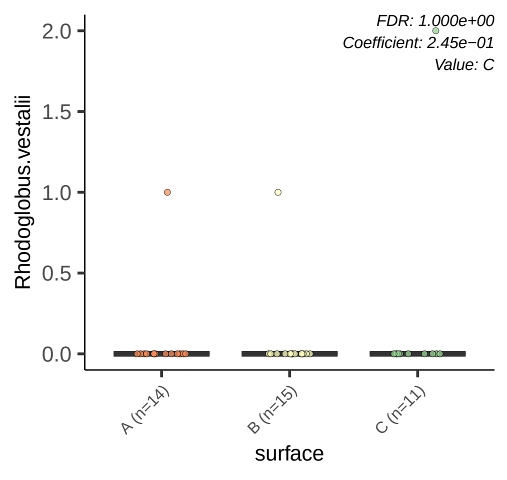
FDR: 1.000e+00
Coefficient: 2.45e−01
Value: C
Rhodoglobus.vestalii
2.0
1.5
1.0
0.5
0.0
A (n=14)
B (n=15)
C (n=11)
surface

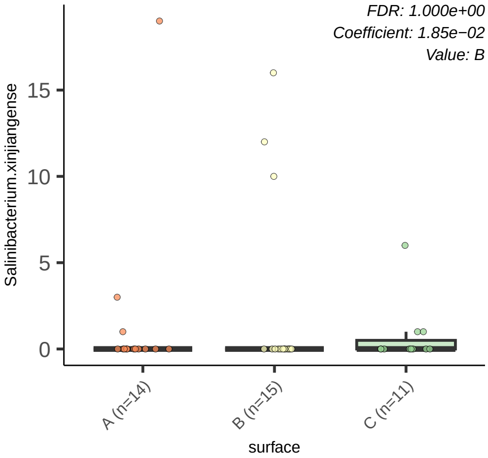
FDR: 1.000e+00
Coefficient: 1.85e−02
Value: B
Salinibacterium.xinjiangense
0
5
10
15
A (n=14)
B (n=15)
C (n=11)
surface

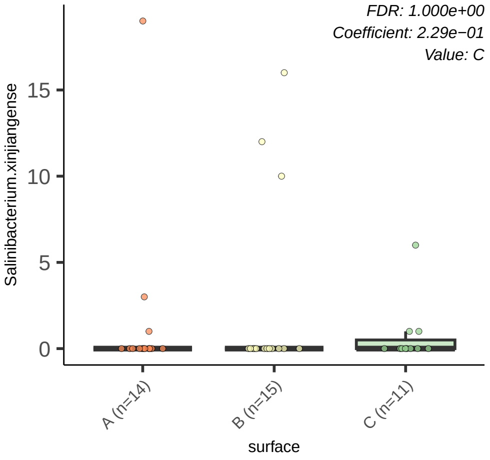

FDR: 1.000e+00
Coefficient: 2.29e−01
Value: C
Salinibacterium.xinjiangense
0
5
10
15
A (n=14)
B (n=15)
C (n=11)
surface

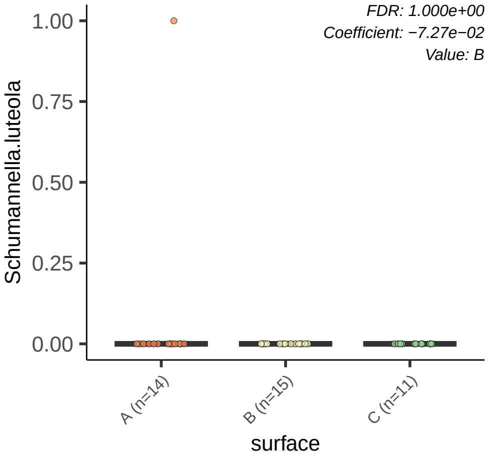

FDR: 1.000e+00
Coefficient: −7.27e−02
Value: B
Schumannella.luteola
1.00
0.75
0.50
0.25
0.00
A (n=14)
B (n=15)
C (n=11)
surface

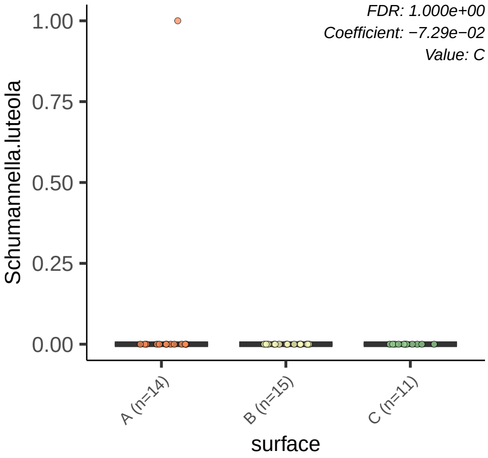
FDR: 1.000e+00
Coefficient: −7.29e−02
Value: C
Schumannella.luteola
1.00
0.75
0.50
0.25
0.00
A (n=14)
B (n=15)
C (n=11)
surface

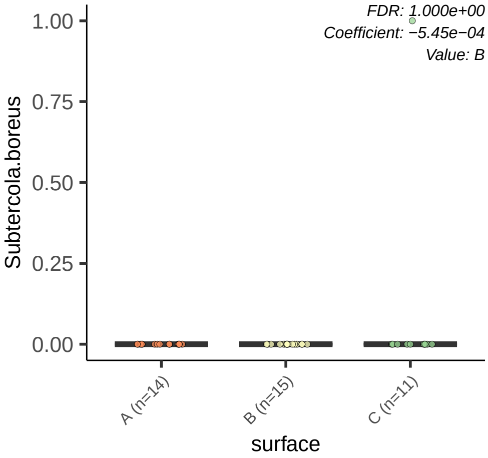

FDR: 1.000e+00
Coefficient: −5.45e−04
Value: B
Subtercola.boreus
1.00
0.75
0.50
0.25
0.00
A (n=14)
B (n=15)
C (n=11)
surface

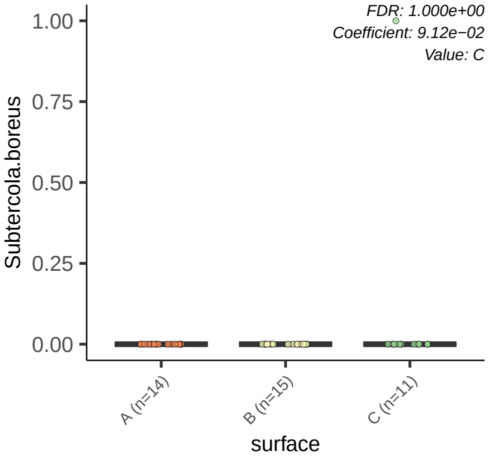
FDR: 1.000e+00
Coefficient: 9.12e−02
Value: C
Subtercola.boreus
1.00
0.75
0.50
0.25
0.00
A (n=14)
B (n=15)
C (n=11)
surface

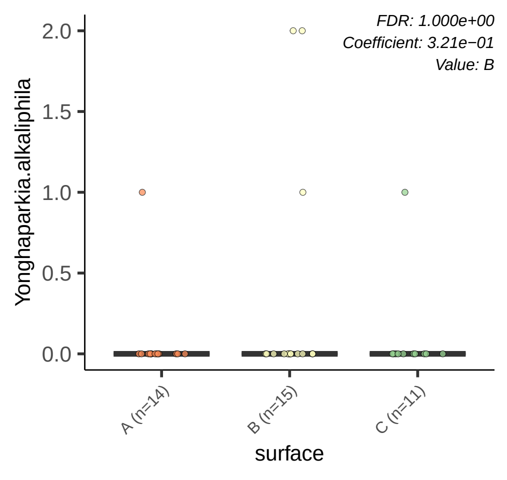
FDR: 1.000e+00
Coefficient: 3.21e−01
Value: B
Yonghaparkia.alkaliphila
2.0
1.5
1.0
0.5
0.0
A (n=14)
B (n=15)
C (n=11)
surface

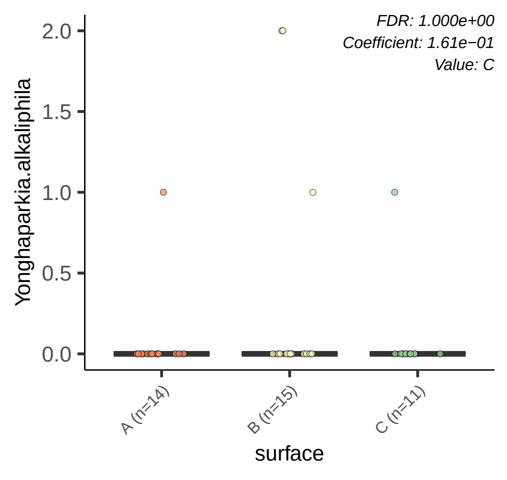
FDR: 1.000e+00
Coefficient: 1.61e−01
Value: C
Yonghaparkia.alkaliphila
2.0
1.5
1.0
0.5
0.0
A (n=14)
B (n=15)
C (n=11)
surface

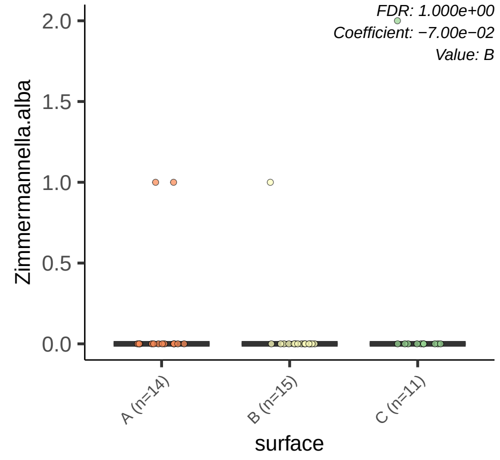
FDR: 1.000e+00
Coefficient: −7.00e−02
Value: B
Zimmermannella.alba
2.0
1.5
1.0
0.5
0.0
A (n=14)
B (n=15)
C (n=11)
surface

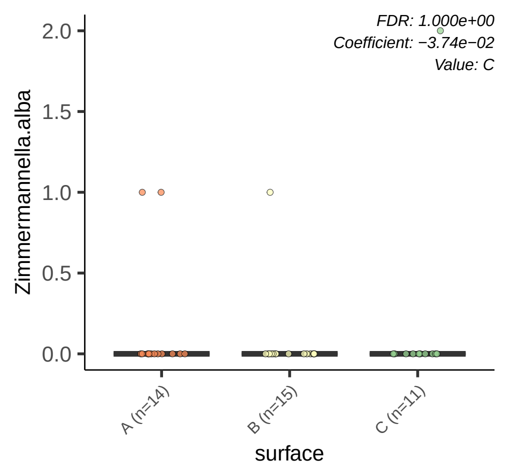

FDR: 1.000e+00
Coefficient: −3.74e−02
Value: C
Zimmermannella.alba
2.0
1.5
1.0
0.5
0.0
A (n=14)
B (n=15)
C (n=11)
surface

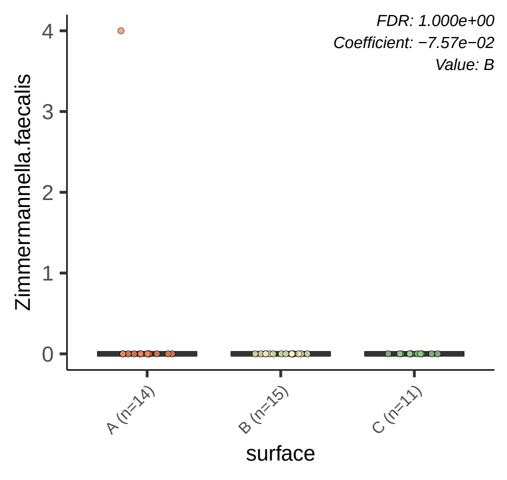

FDR: 1.000e+00
Coefficient: −7.57e−02
Value: B
Zimmermannella.faecalis
0
1
2
3
4
A (n=14)
B (n=15)
C (n=11)
surface

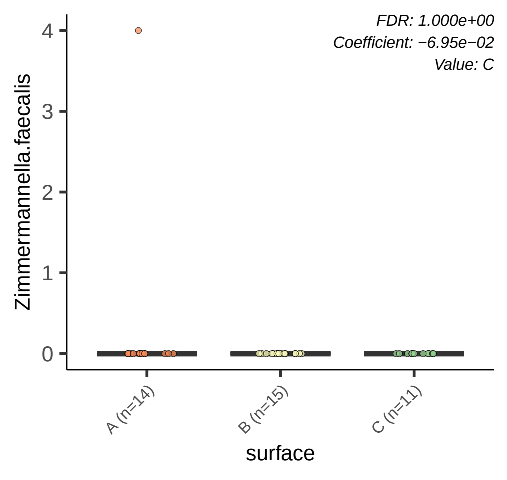
FDR: 1.000e+00
Coefficient: −6.95e−02
Value: C
Zimmermannella.faecalis
4
3
2
1
0
A (n=14)
B (n=15)
C (n=11)
surface

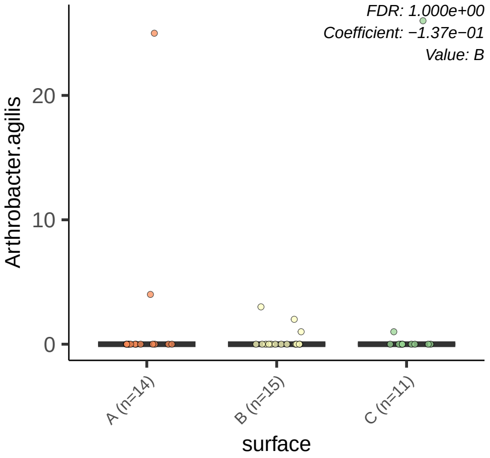

FDR: 1.000e+00
Coefficient: −1.37e−01
Value: B
Arthrobacter.agilis
20
10
0
A (n=14)
B (n=15)
C (n=11)
surface

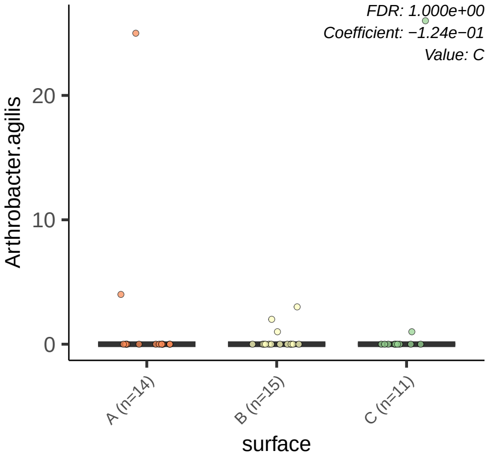

FDR: 1.000e+00
Coefficient: −1.24e−01
Value: C
Arthrobacter.agilis
20
10
0
A (n=14)
B (n=15)
C (n=11)
surface

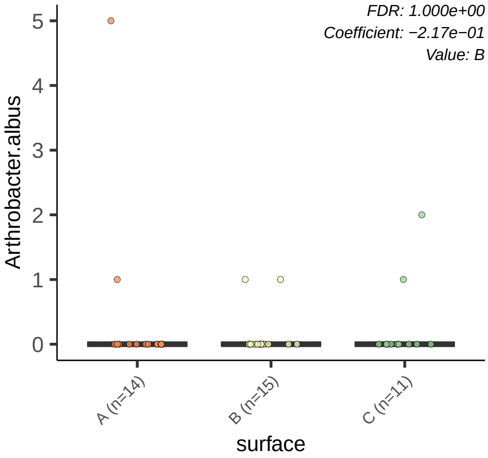

FDR: 1.000e+00
Coefficient: −2.17e−01
Value: B
Arthrobacter.albus
5
4
3
2
1
0
A (n=14)
B (n=15)
C (n=11)
surface

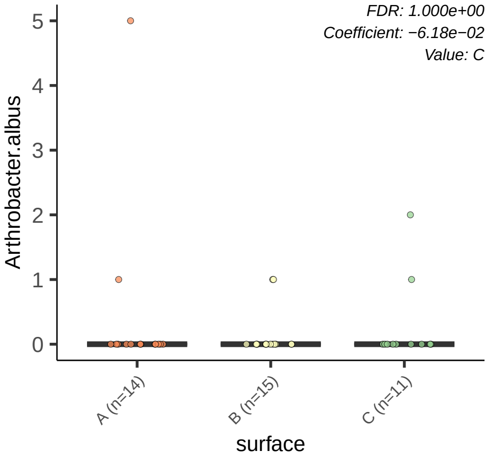
FDR: 1.000e+00
Coefficient: −6.18e−02
Value: C
Arthrobacter.albus
0
1
2
3
4
5
A (n=14)
B (n=15)
C (n=11)
surface

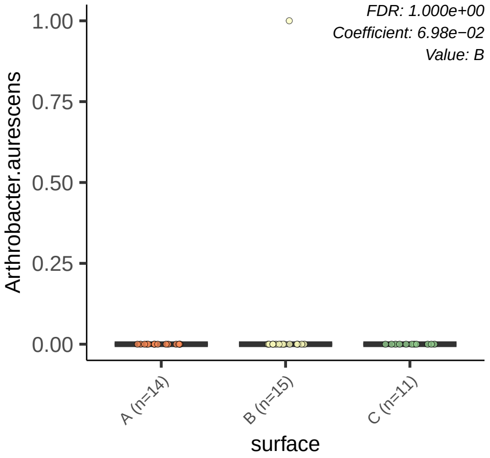
FDR: 1.000e+00
Coefficient: 6.98e−02
Value: B
Arthrobacter.aurescens
1.00
0.75
0.50
0.25
0.00
A (n=14)
B (n=15)
C (n=11)
surface

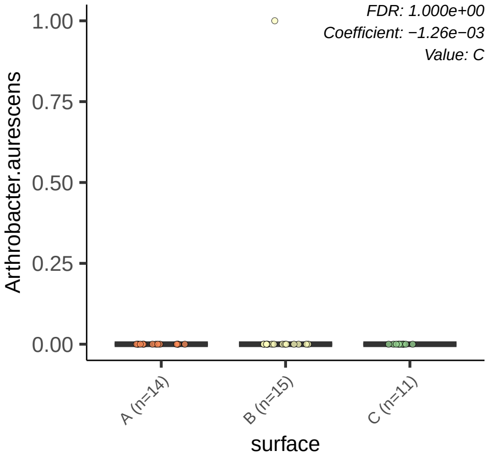
FDR: 1.000e+00
Coefficient: −1.26e−03
Value: C
Arthrobacter.aurescens
1.00
0.75
0.50
0.25
0.00
A (n=14)
B (n=15)
C (n=11)
surface

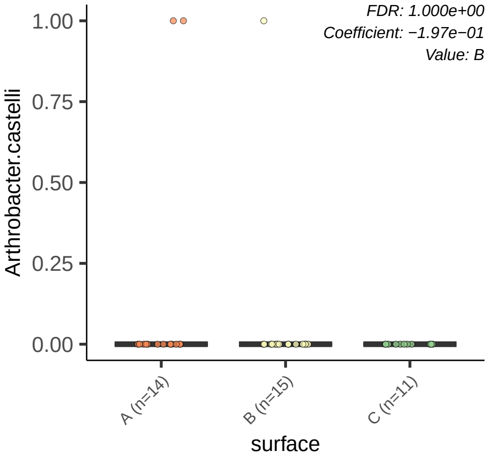

FDR: 1.000e+00
Coefficient: −1.97e−01
Value: B
Arthrobacter.castelli
1.00
0.75
0.50
0.25
0.00
A (n=14)
B (n=15)
C (n=11)
surface

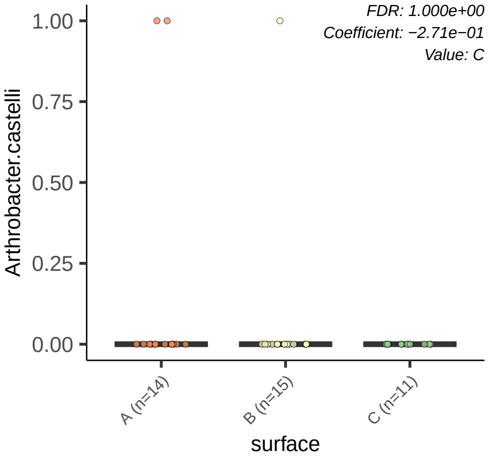

FDR: 1.000e+00
Coefficient: −2.71e−01
Value: C
Arthrobacter.castelli
1.00
0.75
0.50
0.25
0.00
A (n=14)
B (n=15)
C (n=11)
surface

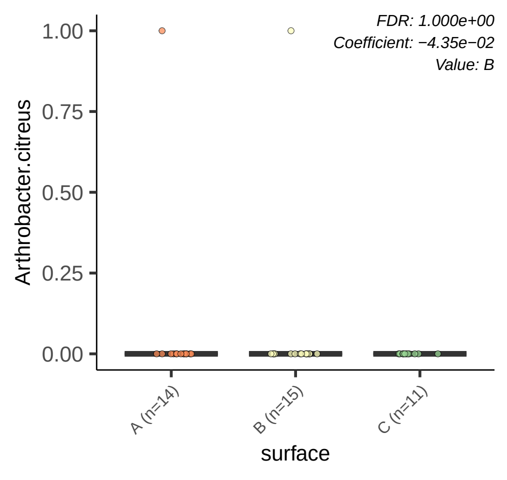

FDR: 1.000e+00
Coefficient: −4.35e−02
Value: B
Arthrobacter.citreus
1.00
0.75
0.50
0.25
0.00
A (n=14)
B (n=15)
C (n=11)
surface

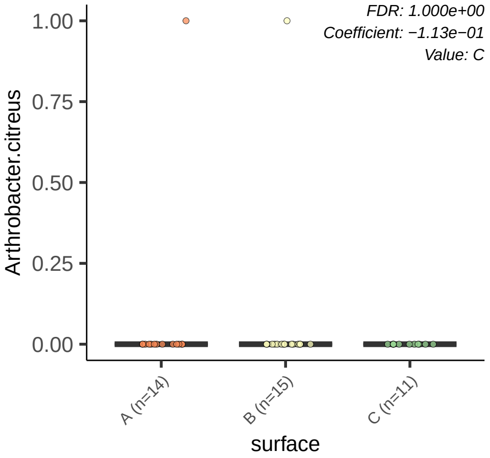
FDR: 1.000e+00
Coefficient: −1.13e−01
Value: C
Arthrobacter.citreus
1.00
0.75
0.50
0.25
0.00
A (n=14)
B (n=15)
C (n=11)
surface

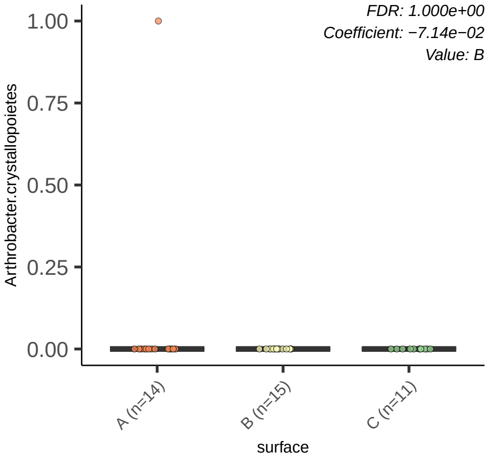
FDR: 1.000e+00
Coefficient: −7.14e−02
Value: B
1.00
0.75
0.50
0.25
0.00
Arthrobacter.crystallopoietes
A (n=14)
B (n=15)
C (n=11)
surface

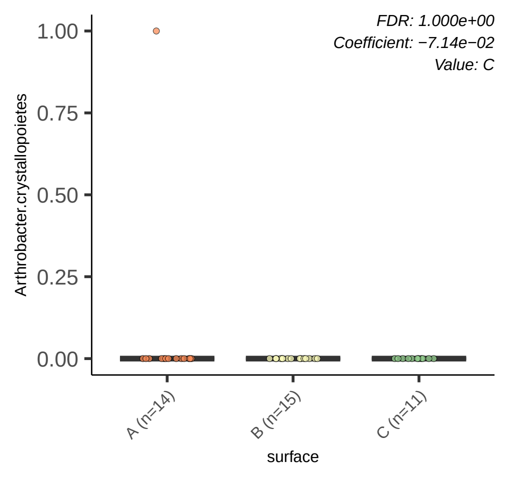

FDR: 1.000e+00
Coefficient: −7.14e−02
Value: C
Arthrobacter.crystallopoietes
1.00
0.75
0.50
0.25
0.00
A (n=14)
B (n=15)
C (n=11)
surface

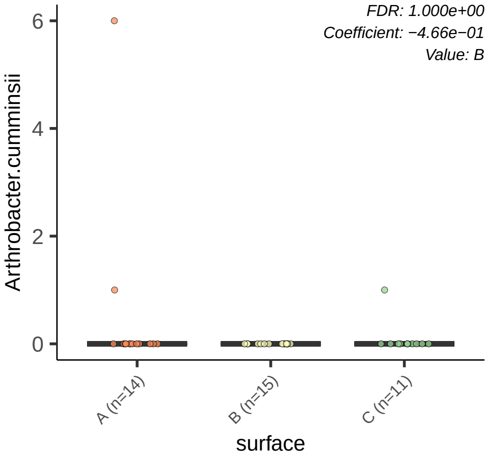
FDR: 1.000e+00
Coefficient: −4.66e−01
Value: B
Arthrobacter.cumminsii
0
2
4
6
A (n=14)
B (n=15)
C (n=11)
surface

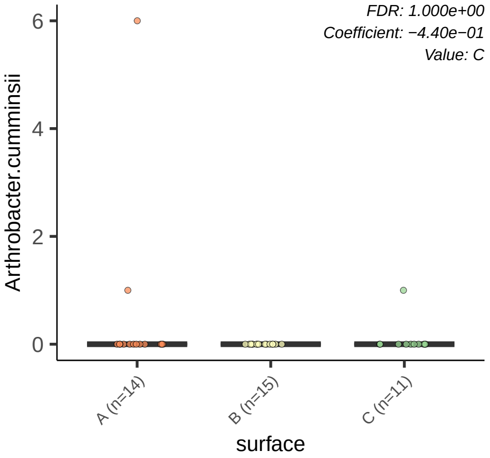

FDR: 1.000e+00
Coefficient: −4.40e−01
Value: C
Arthrobacter.cumminsii
0
2
4
6
A (n=14)
B (n=15)
C (n=11)
surface

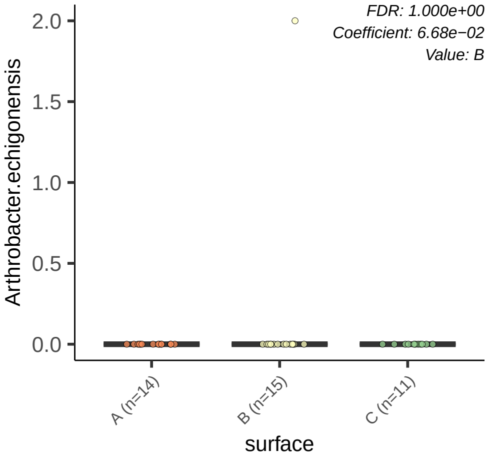

FDR: 1.000e+00
Coefficient: 6.68e−02
Value: B
Arthrobacter.echigonensis
2.0
1.5
1.0
0.5
0.0
A (n=14)
B (n=15)
C (n=11)
surface

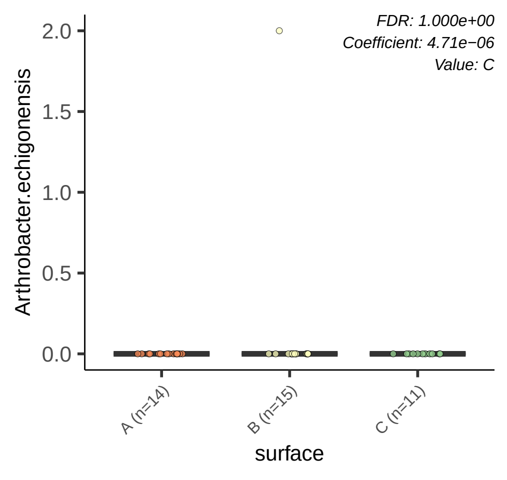
FDR: 1.000e+00
Coefficient: 4.71e−06
Value: C
Arthrobacter.echigonensis
2.0
1.5
1.0
0.5
0.0
A (n=14)
B (n=15)
C (n=11)
surface

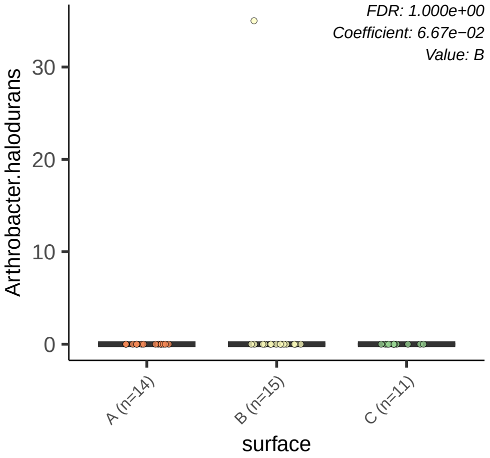

FDR: 1.000e+00
Coefficient: 6.67e−02
Value: B
Arthrobacter.halodurans
30
20
10
0
A (n=14)
B (n=15)
C (n=11)
surface

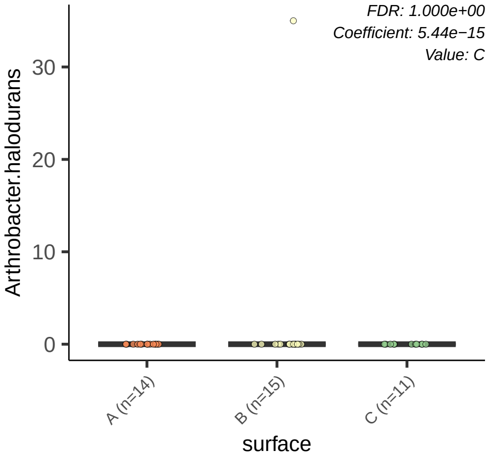
FDR: 1.000e+00
Coefficient: 5.44e−15
Value: C
Arthrobacter.halodurans
30
20
10
0
A (n=14)
B (n=15)
C (n=11)
surface

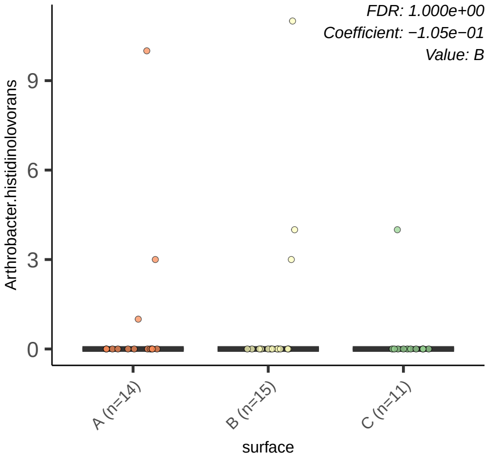

FDR: 1.000e+00
Coefficient: −1.05e−01
Value: B
Arthrobacter.histidinolovorans
0
3
6
9
A (n=14)
B (n=15)
C (n=11)
surface

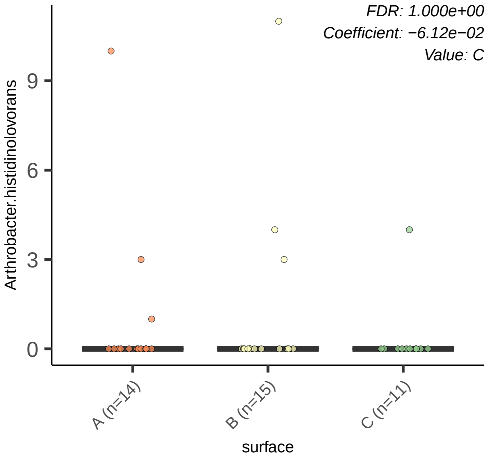
FDR: 1.000e+00
Coefficient: −6.12e−02
Value: C
Arthrobacter.histidinolovorans
9
6
3
0
A (n=14)
B (n=15)
C (n=11)
surface

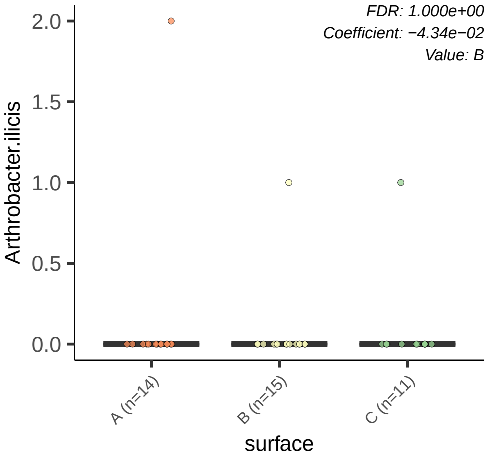

FDR: 1.000e+00
Coefficient: −4.34e−02
Value: B
Arthrobacter.ilicis
2.0
1.5
1.0
0.5
0.0
A (n=14)
B (n=15)
C (n=11)
surface

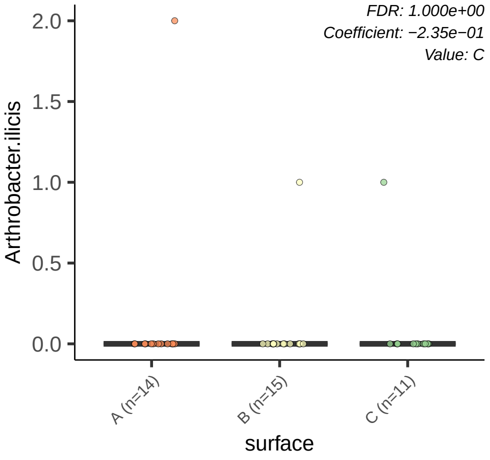
FDR: 1.000e+00
Coefficient: −2.35e−01
Value: C
Arthrobacter.ilicis
2.0
1.5
1.0
0.5
0.0
A (n=14)
B (n=15)
C (n=11)
surface

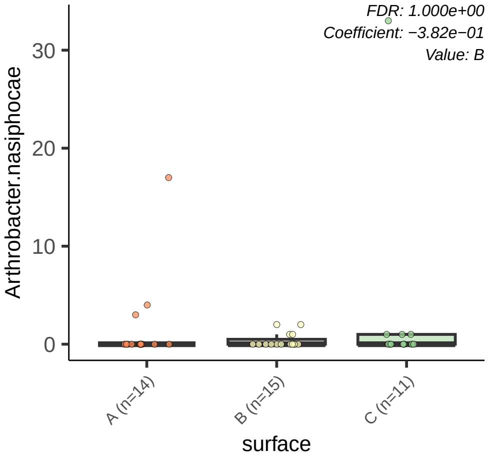

FDR: 1.000e+00
Coefficient: −3.82e−01
Value: B
Arthrobacter.nasiphocae
0
10
20
30
A (n=14)
B (n=15)
C (n=11)
surface

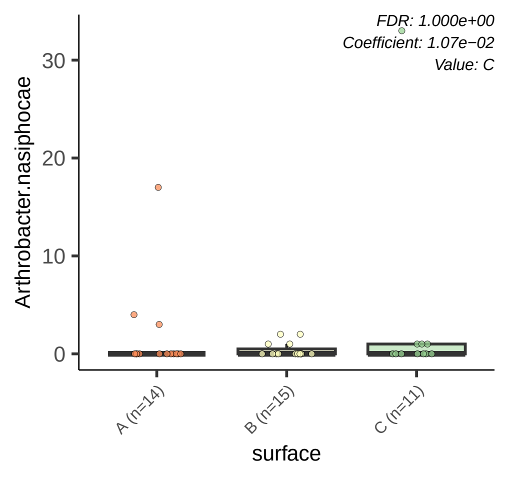

FDR: 1.000e+00
Coefficient: 1.07e−02
Value: C
Arthrobacter.nasiphocae
30
20
10
0
A (n=14)
B (n=15)
C (n=11)
surface

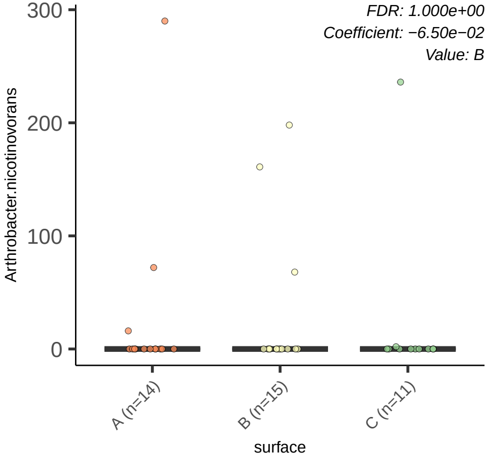

FDR: 1.000e+00
Coefficient: −6.50e−02
Value: B
Arthrobacter.nicotinovorans
300
200
100
0
A (n=14)
B (n=15)
C (n=11)
surface

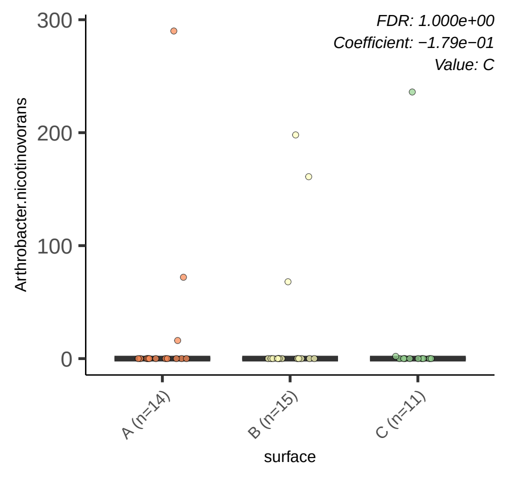

FDR: 1.000e+00
Coefficient: −1.79e−01
Value: C
Arthrobacter.nicotinovorans
300
200
100
0
A (n=14)
B (n=15)
C (n=11)
surface

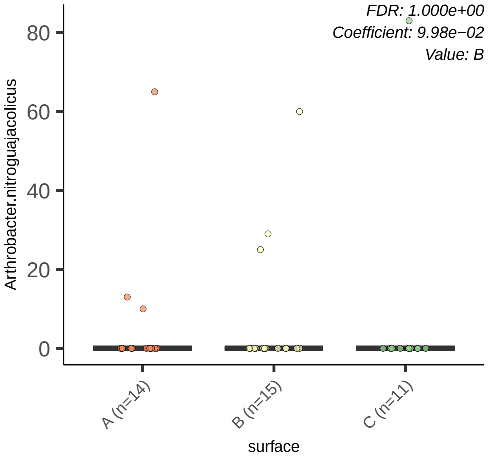
FDR: 1.000e+00
Coefficient: 9.98e−02
Value: B
Arthrobacter.nitroguajacolicus
0
20
40
60
80
A (n=14)
B (n=15)
C (n=11)
surface

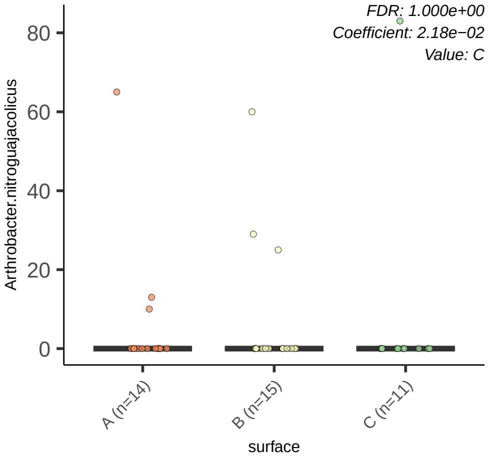
FDR: 1.000e+00
Coefficient: 2.18e−02
Value: C
Arthrobacter.nitroguajacolicus
80
60
40
20
0
A (n=14)
B (n=15)
C (n=11)
surface

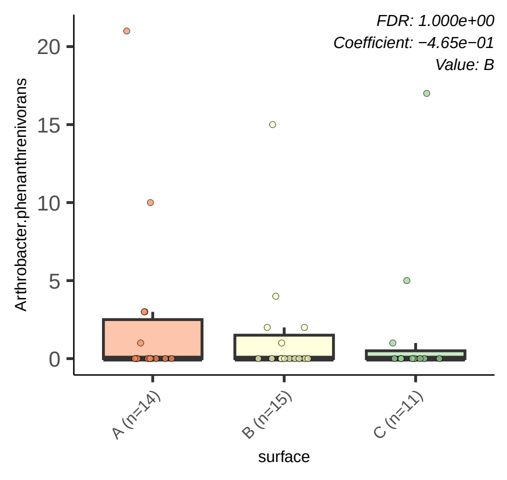

FDR: 1.000e+00
Coefficient: −4.65e−01
Value: B
Arthrobacter.phenanthrenivorans
20
15
10
5
0
A (n=14)
B (n=15)
C (n=11)
surface

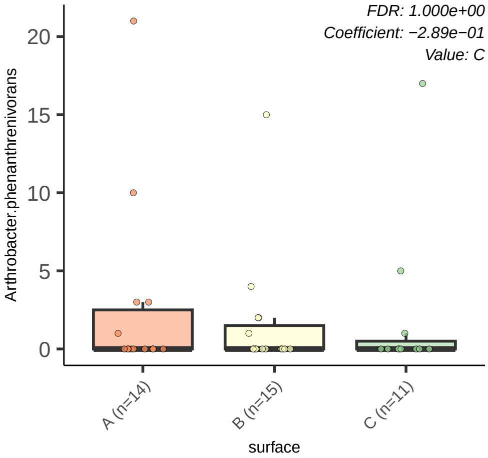
FDR: 1.000e+00
Coefficient: −2.89e−01
Value: C
Arthrobacter.phenanthrenivorans
20
15
10
5
0
A (n=14)
B (n=15)
C (n=11)
surface

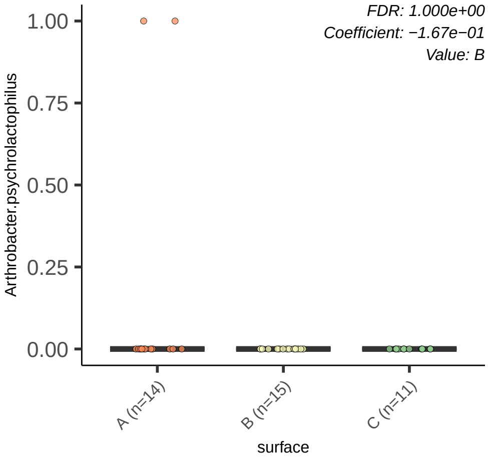
FDR: 1.000e+00
Coefficient: −1.67e−01
Value: B
Arthrobacter.psychrolactophilus
1.00
0.75
0.50
0.25
0.00
A (n=14)
B (n=15)
C (n=11)
surface

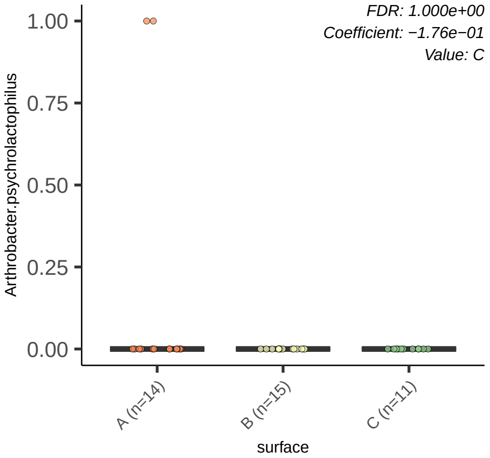
FDR: 1.000e+00
Coefficient: −1.76e−01
Value: C
Arthrobacter.psychrolactophilus
1.00
0.75
0.50
0.25
0.00
A (n=14)
B (n=15)
C (n=11)
surface

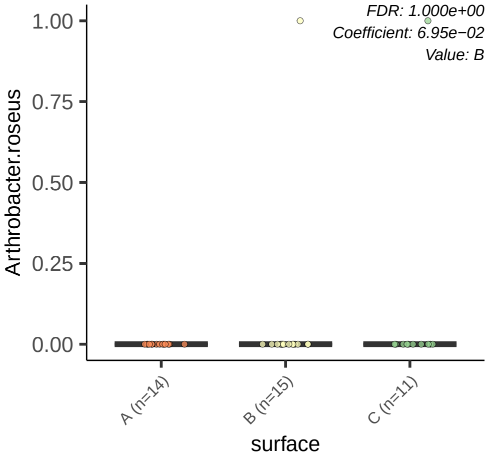

FDR: 1.000e+00
Coefficient: 6.95e−02
Value: B
1.00
0.75
0.50
0.25
0.00
Arthrobacter.roseus
A (n=14)
B (n=15)
C (n=11)
surface

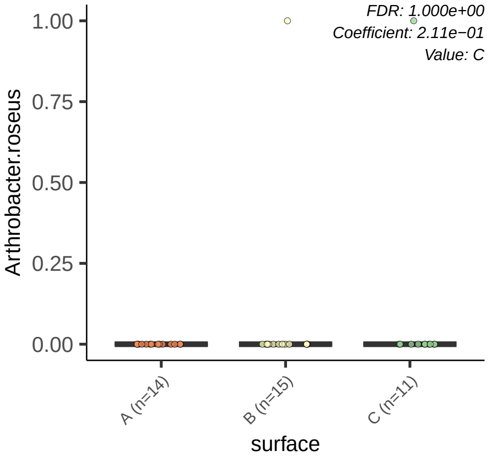
FDR: 1.000e+00
Coefficient: 2.11e−01
Value: C
Arthrobacter.roseus
1.00
0.75
0.50
0.25
0.00
A (n=14)
B (n=15)
C (n=11)
surface

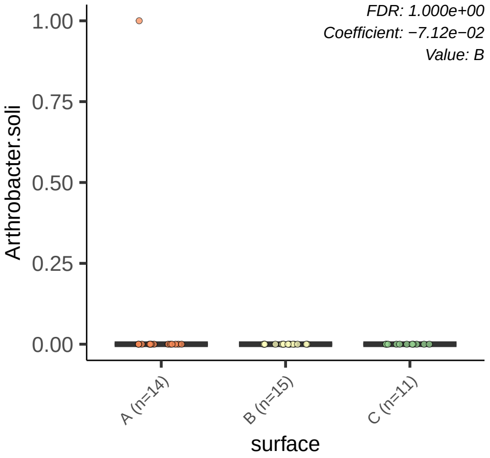
FDR: 1.000e+00
Coefficient: −7.12e−02
Value: B
Arthrobacter.soli
1.00
0.75
0.50
0.25
0.00
A (n=14)
B (n=15)
C (n=11)
surface

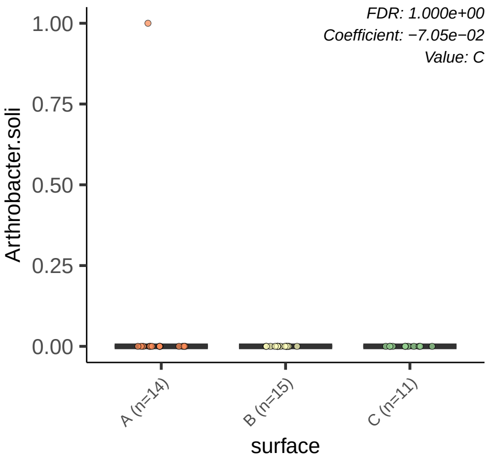

FDR: 1.000e+00
Coefficient: −7.05e−02
Value: C
Arthrobacter.soli
1.00
0.75
0.50
0.25
0.00
A (n=14)
B (n=15)
C (n=11)
surface

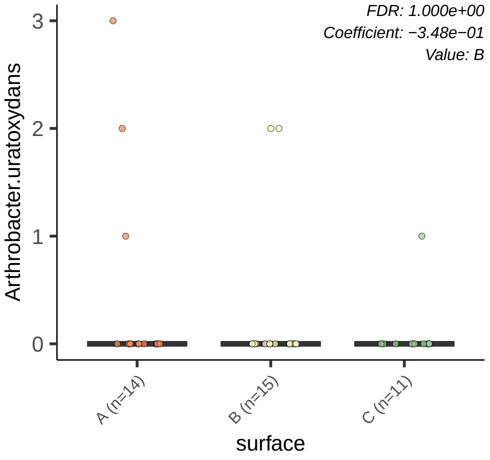

FDR: 1.000e+00
Coefficient: −3.48e−01
Value: B
Arthrobacter.uratoxydans
3
2
1
0
A (n=14)
B (n=15)
C (n=11)
surface

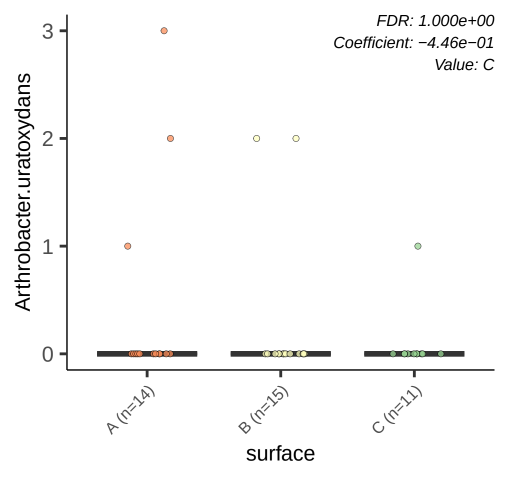
FDR: 1.000e+00
Coefficient: −4.46e−01
Value: C
Arthrobacter.uratoxydans
3
2
1
0
A (n=14)
B (n=15)
C (n=11)
surface

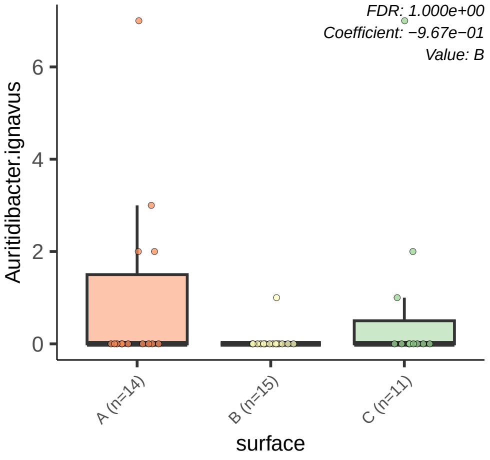

FDR: 1.000e+00
Coefficient: −9.67e−01
Value: B
Auritidibacter.ignavus
0
2
4
6
A (n=14)
B (n=15)
C (n=11)
surface

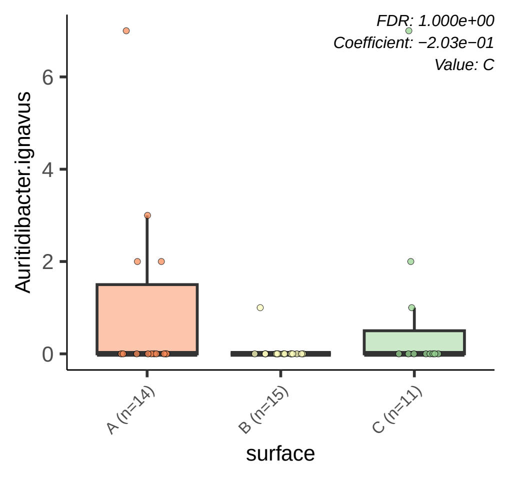
FDR: 1.000e+00
Coefficient: −2.03e−01
Value: C
Auritidibacter.ignavus
0
2
4
6
A (n=14)
B (n=15)
C (n=11)
surface

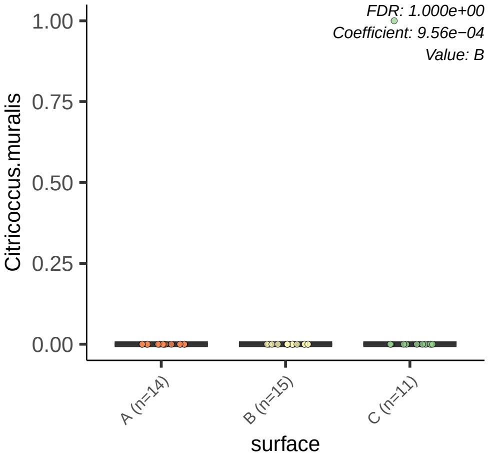
FDR: 1.000e+00
Coefficient: 9.56e−04
Value: B
Citricoccus.muralis
1.00
0.75
0.50
0.25
0.00
A (n=14)
B (n=15)
C (n=11)
surface

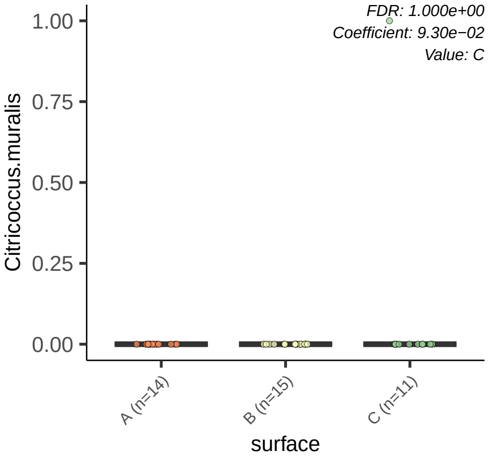
FDR: 1.000e+00
Coefficient: 9.30e−02
Value: C
Citricoccus.muralis
1.00
0.75
0.50
0.25
0.00
A (n=14)
B (n=15)
C (n=11)
surface

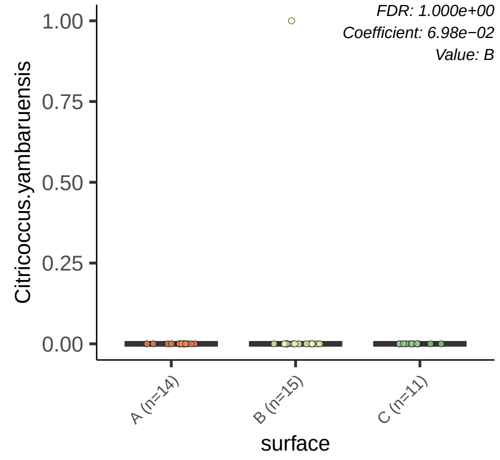
FDR: 1.000e+00
Coefficient: 6.98e−02
Value: B
Citricoccus.yambaruensis
1.00
0.75
0.50
0.25
0.00
A (n=14)
B (n=15)
C (n=11)
surface

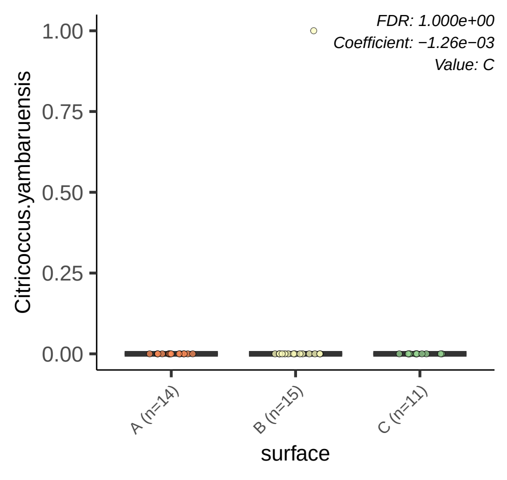
FDR: 1.000e+00
Coefficient: −1.26e−03
Value: C
Citricoccus.yambaruensis
1.00
0.75
0.50
0.25
0.00
A (n=14)
B (n=15)
C (n=11)
surface

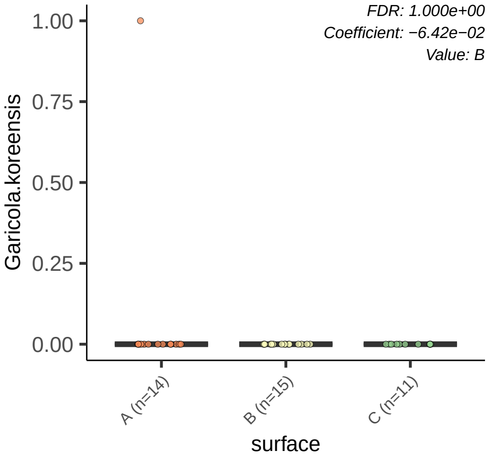
FDR: 1.000e+00
Coefficient: −6.42e−02
Value: B
Garicola.koreensis
1.00
0.75
0.50
0.25
0.00
A (n=14)
B (n=15)
C (n=11)
surface

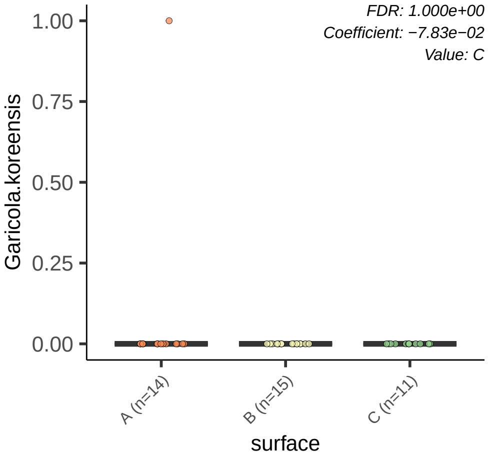

FDR: 1.000e+00
Coefficient: −7.83e−02
Value: C
Garicola.koreensis
1.00
0.75
0.50
0.25
0.00
A (n=14)
B (n=15)
C (n=11)
surface

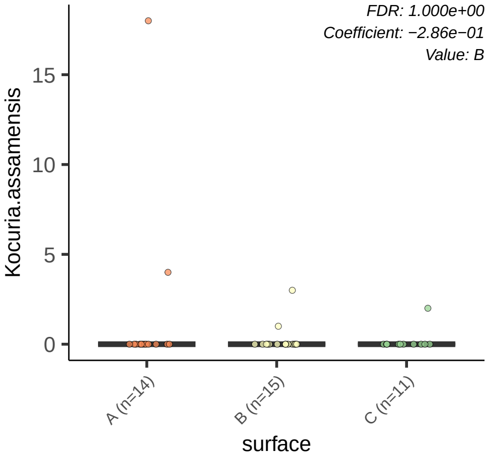
FDR: 1.000e+00
Coefficient: −2.86e−01
Value: B
Kocuria.assamensis
15
10
5
0
A (n=14)
B (n=15)
C (n=11)
surface

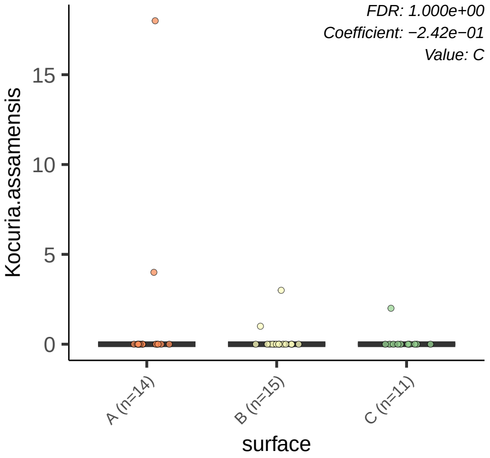

FDR: 1.000e+00
Coefficient: −2.42e−01
Value: C
Kocuria.assamensis
0
5
10
15
A (n=14)
B (n=15)
C (n=11)
surface

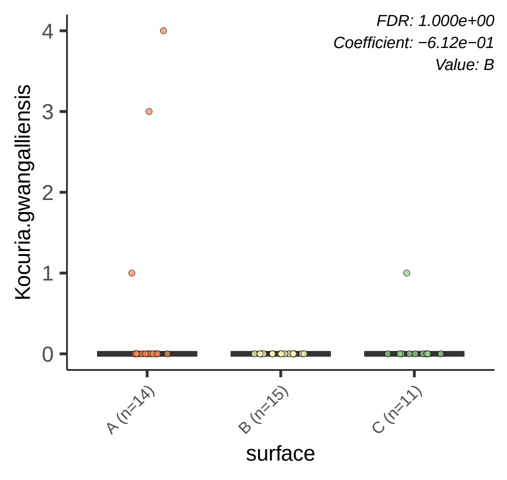

FDR: 1.000e+00
Coefficient: −6.12e−01
Value: B
Kocuria.gwangalliensis
4
3
2
1
0
A (n=14)
B (n=15)
C (n=11)
surface

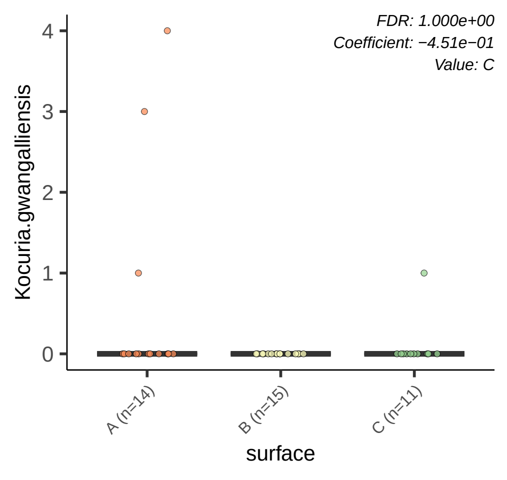

FDR: 1.000e+00
Coefficient: −4.51e−01
Value: C
Kocuria.gwangalliensis
0
1
2
3
4
A (n=14)
B (n=15)
C (n=11)
surface

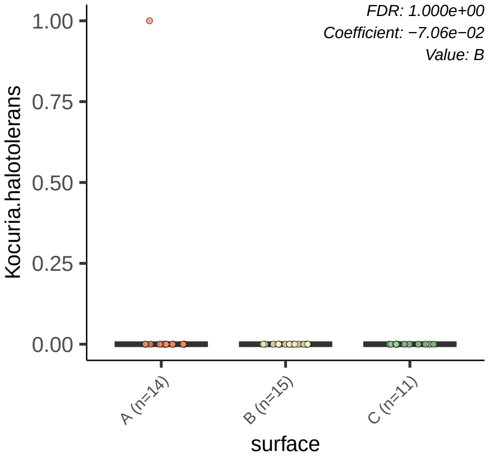
FDR: 1.000e+00
Coefficient: −7.06e−02
Value: B
Kocuria.halotolerans
1.00
0.75
0.50
0.25
0.00
A (n=14)
B (n=15)
C (n=11)
surface

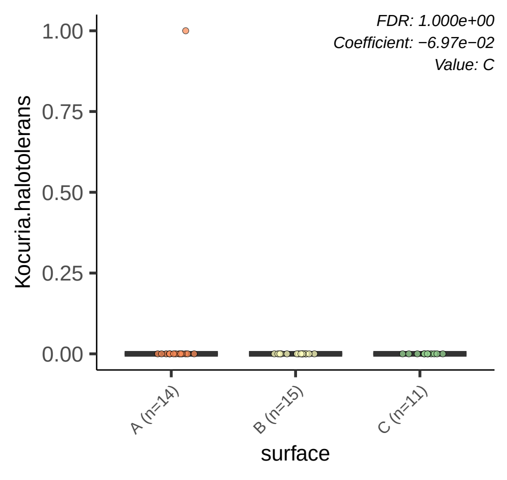

FDR: 1.000e+00
Coefficient: −6.97e−02
Value: C
Kocuria.halotolerans
1.00
0.75
0.50
0.25
0.00
A (n=14)
B (n=15)
C (n=11)
surface

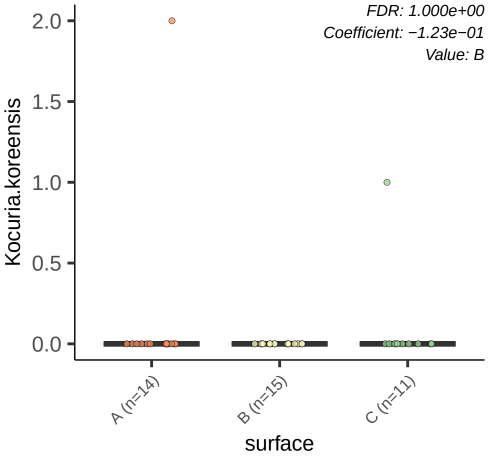

FDR: 1.000e+00
Coefficient: −1.23e−01
Value: B
Kocuria.koreensis
2.0
1.5
1.0
0.5
0.0
A (n=14)
B (n=15)
C (n=11)
surface

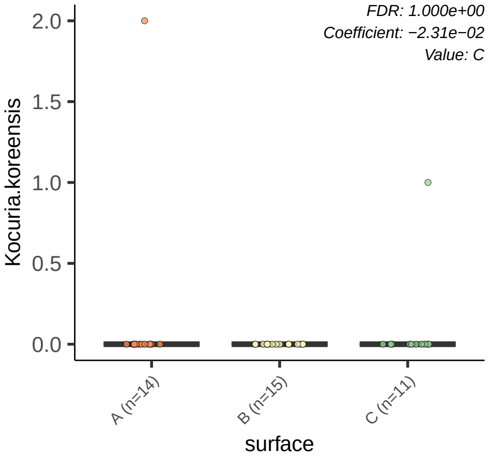
FDR: 1.000e+00
Coefficient: −2.31e−02
Value: C
Kocuria.koreensis
2.0
1.5
1.0
0.5
0.0
A (n=14)
B (n=15)
C (n=11)
surface

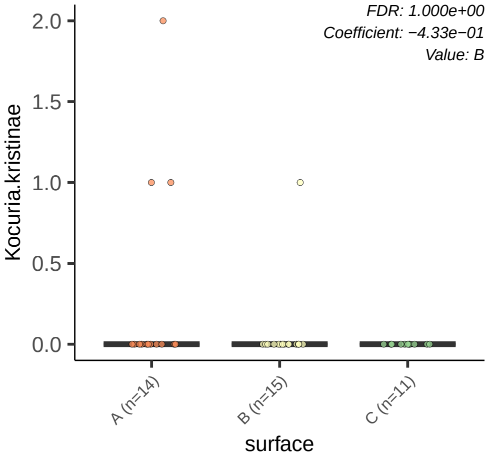
FDR: 1.000e+00
Coefficient: −4.33e−01
Value: B
Kocuria.kristinae
2.0
1.5
1.0
0.5
0.0
A (n=14)
B (n=15)
C (n=11)
surface

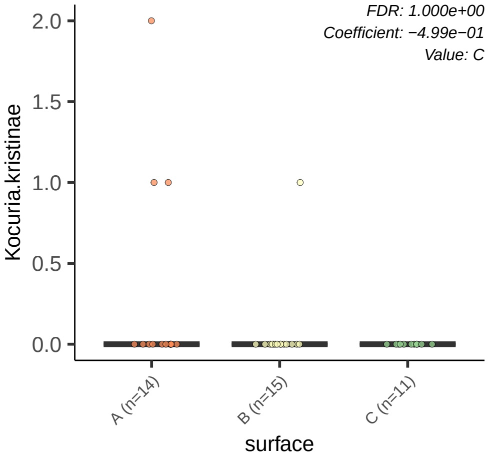
FDR: 1.000e+00
Coefficient: −4.99e−01
Value: C
Kocuria.kristinae
2.0
1.5
1.0
0.5
0.0
A (n=14)
B (n=15)
C (n=11)
surface

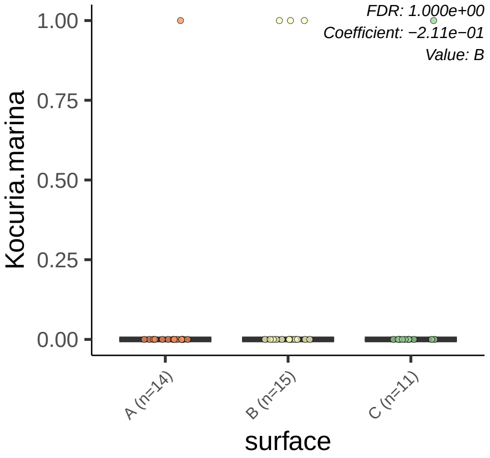
FDR: 1.000e+00
Coefficient: −2.11e−01
Value: B
Kocuria.marina
1.00
0.75
0.50
0.25
0.00
A (n=14)
B (n=15)
C (n=11)
surface

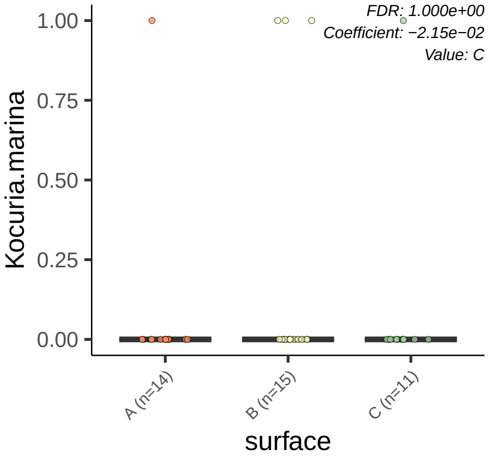
FDR: 1.000e+00
Coefficient: −2.15e−02
Value: C
Kocuria.marina
1.00
0.75
0.50
0.25
0.00
A (n=14)
B (n=15)
C (n=11)
surface

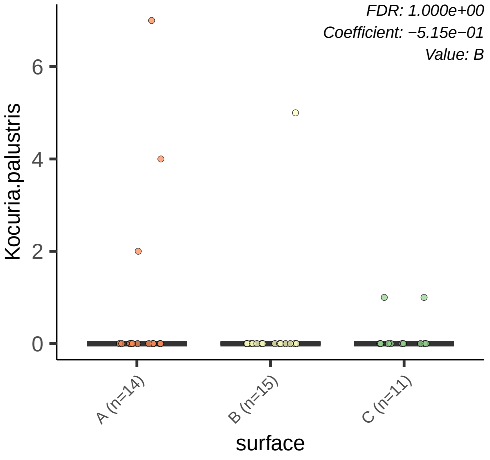

FDR: 1.000e+00
Coefficient: −5.15e−01
Value: B
Kocuria.palustris
0
2
4
6
A (n=14)
B (n=15)
C (n=11)
surface

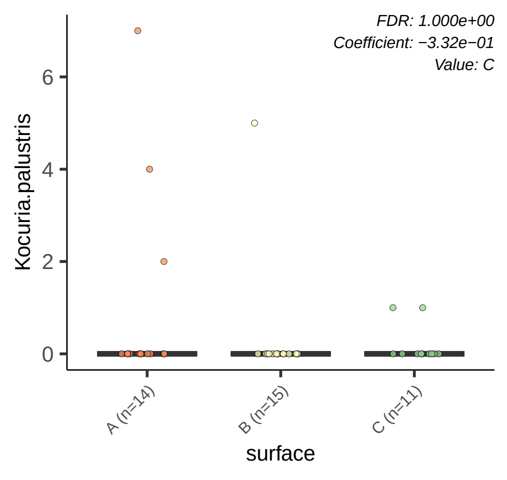
FDR: 1.000e+00
Coefficient: −3.32e−01
Value: C
Kocuria.palustris
0
2
4
6
A (n=14)
B (n=15)
C (n=11)
surface

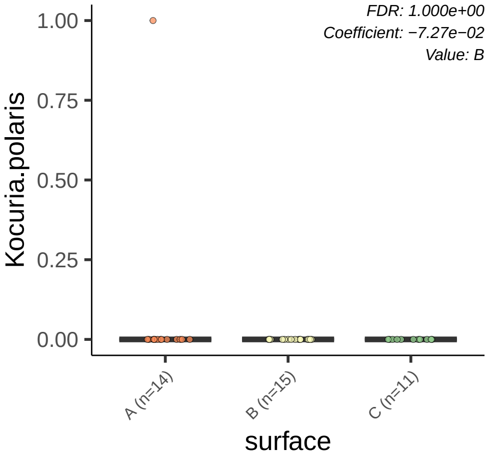
FDR: 1.000e+00
Coefficient: −7.27e−02
Value: B
Kocuria.polaris
1.00
0.75
0.50
0.25
0.00
A (n=14)
B (n=15)
C (n=11)
surface

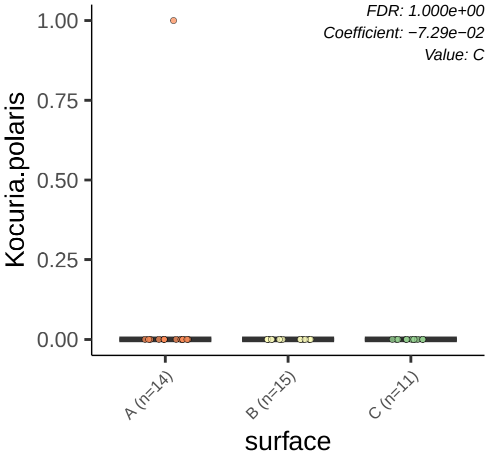
FDR: 1.000e+00
Coefficient: −7.29e−02
Value: C
Kocuria.polaris
1.00
0.75
0.50
0.25
0.00
A (n=14)
B (n=15)
C (n=11)
surface

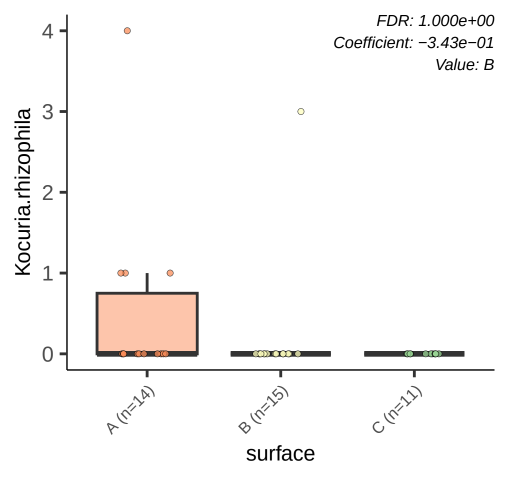

FDR: 1.000e+00
Coefficient: −3.43e−01
Value: B
Kocuria.rhizophila
4
3
2
1
0
A (n=14)
B (n=15)
C (n=11)
surface

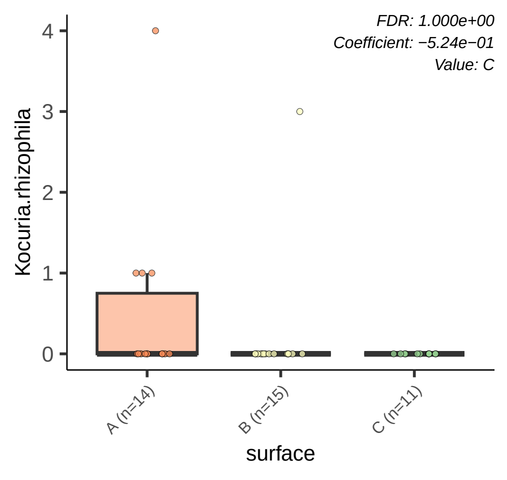

FDR: 1.000e+00
Coefficient: −5.24e−01
Value: C
Kocuria.rhizophila
4
3
2
1
0
A (n=14)
B (n=15)
C (n=11)
surface

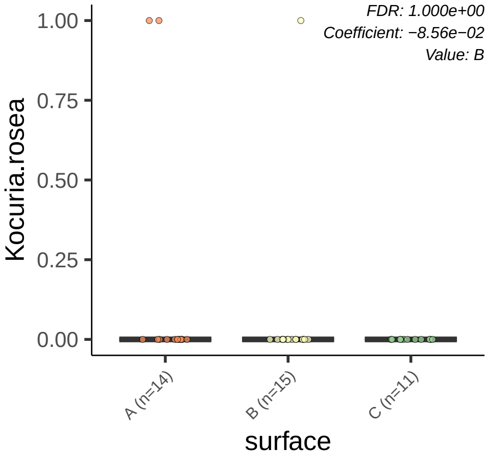
FDR: 1.000e+00
Coefficient: −8.56e−02
Value: B
Kocuria.rosea
1.00
0.75
0.50
0.25
0.00
A (n=14)
B (n=15)
C (n=11)
surface

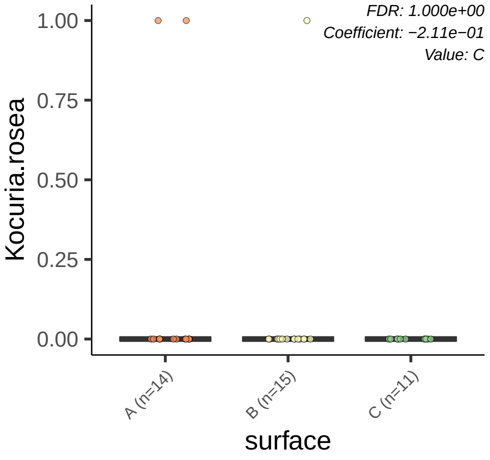
FDR: 1.000e+00
Coefficient: −2.11e−01
Value: C
Kocuria.rosea
1.00
0.75
0.50
0.25
0.00
A (n=14)
B (n=15)
C (n=11)
surface

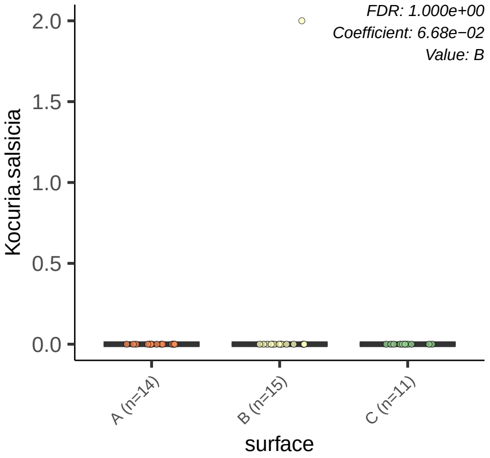
FDR: 1.000e+00
Coefficient: 6.68e−02
Value: B
Kocuria.salsicia
2.0
1.5
1.0
0.5
0.0
A (n=14)
B (n=15)
C (n=11)
surface

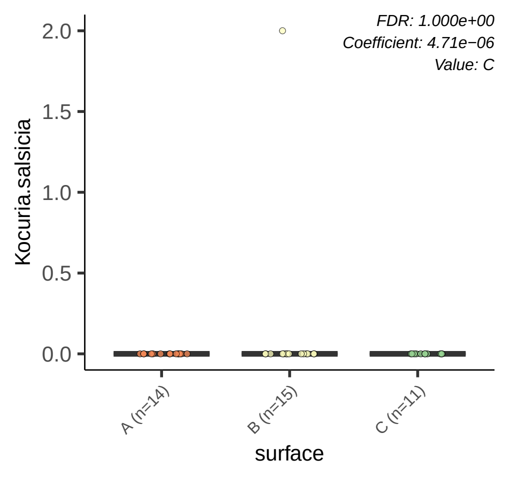
FDR: 1.000e+00
Coefficient: 4.71e−06
Value: C
Kocuria.salsicia
2.0
1.5
1.0
0.5
0.0
A (n=14)
B (n=15)
C (n=11)
surface

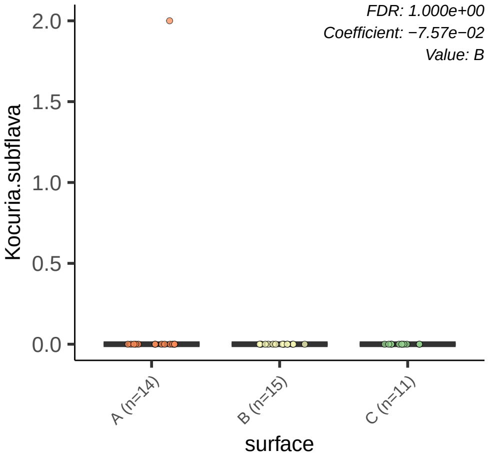

FDR: 1.000e+00
Coefficient: −7.57e−02
Value: B
Kocuria.subflava
2.0
1.5
1.0
0.5
0.0
A (n=14)
B (n=15)
C (n=11)
surface

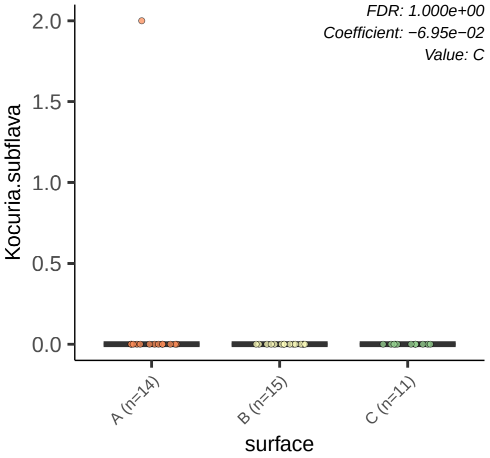
FDR: 1.000e+00
Coefficient: −6.95e−02
Value: C
Kocuria.subflava
2.0
1.5
1.0
0.5
0.0
A (n=14)
B (n=15)
C (n=11)
surface

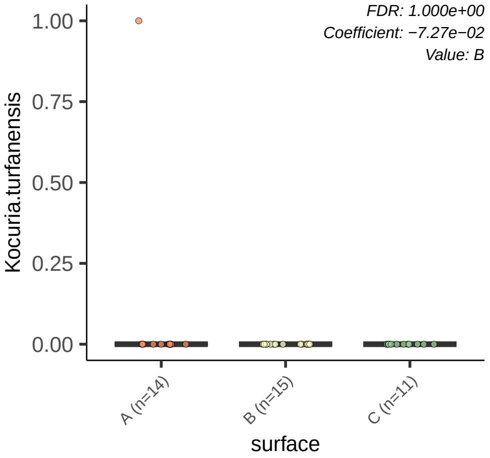

FDR: 1.000e+00
Coefficient: −7.27e−02
Value: B
Kocuria.turfanensis
1.00
0.75
0.50
0.25
0.00
A (n=14)
B (n=15)
C (n=11)
surface

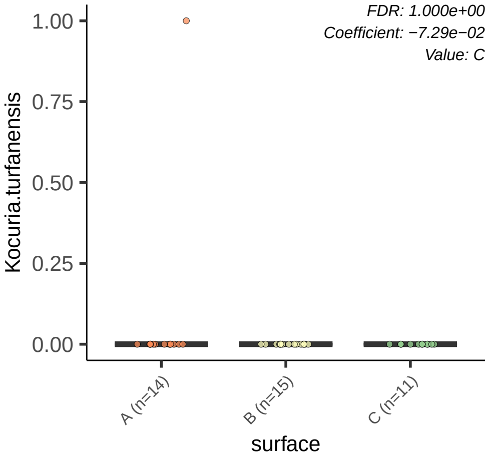
FDR: 1.000e+00
Coefficient: −7.29e−02
Value: C
Kocuria.turfanensis
1.00
0.75
0.50
0.25
0.00
A (n=14)
B (n=15)
C (n=11)
surface

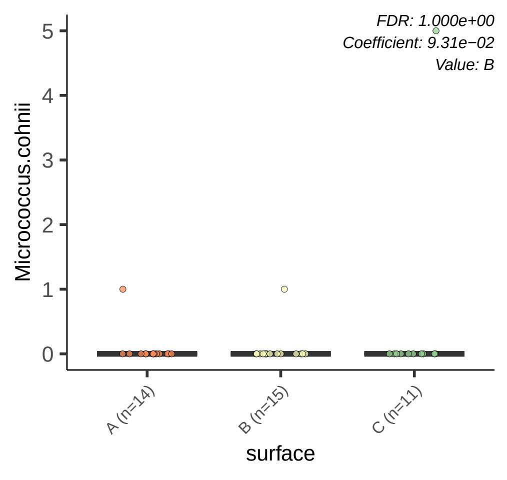

FDR: 1.000e+00
Coefficient: 9.31e−02
Value: B
Micrococcus.cohnii
0
1
2
3
4
5
A (n=14)
B (n=15)
C (n=11)
surface

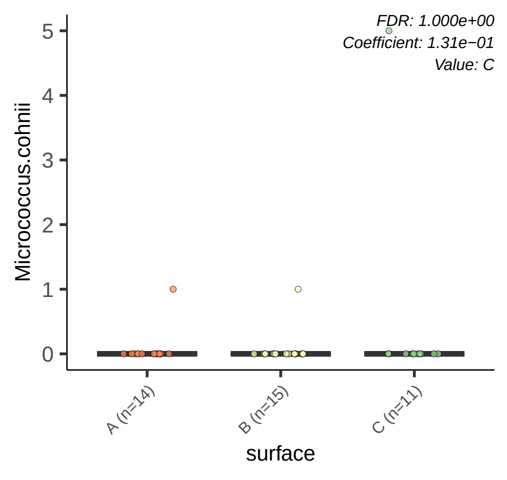
FDR: 1.000e+00
Coefficient: 1.31e−01
Value: C
Micrococcus.cohnii
0
1
2
3
4
5
A (n=14)
B (n=15)
C (n=11)
surface

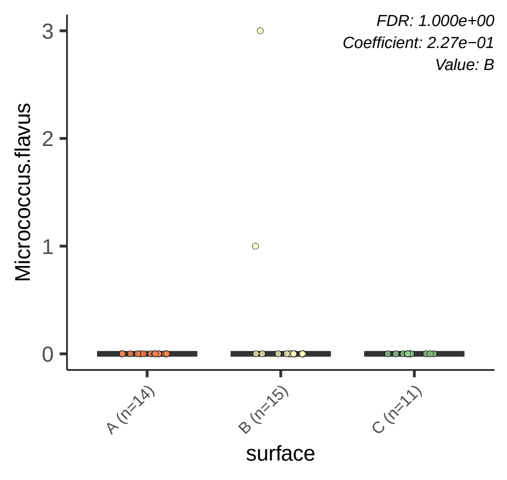
FDR: 1.000e+00
Coefficient: 2.27e−01
Value: B
Micrococcus.flavus
3
2
1
0
A (n=14)
B (n=15)
C (n=11)
surface

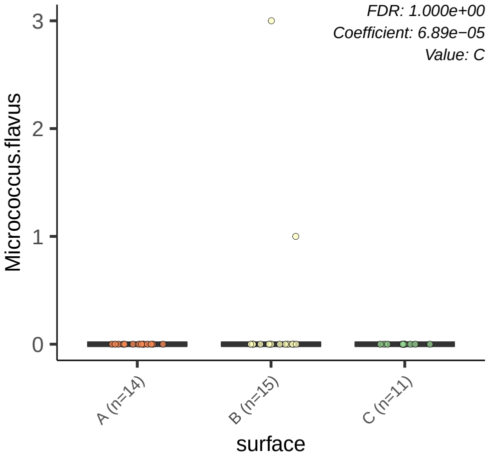

FDR: 1.000e+00
Coefficient: 6.89e−05
Value: C
Micrococcus.flavus
3
2
1
0
A (n=14)
B (n=15)
C (n=11)
surface

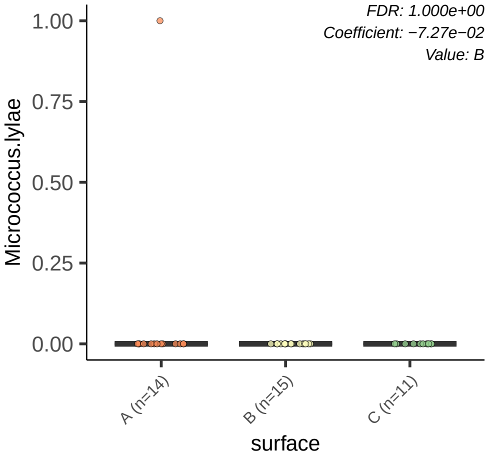
FDR: 1.000e+00
Coefficient: −7.27e−02
Value: B
Micrococcus.lylae
1.00
0.75
0.50
0.25
0.00
A (n=14)
B (n=15)
C (n=11)
surface

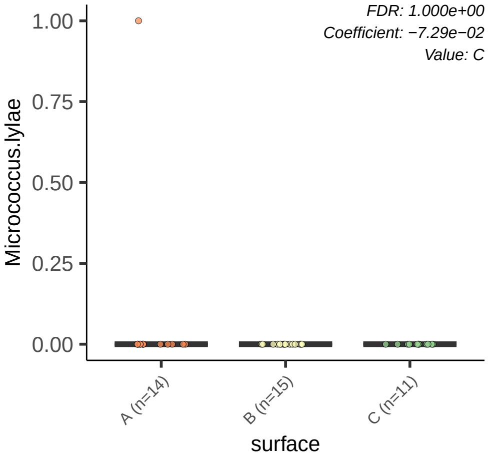

FDR: 1.000e+00
Coefficient: −7.29e−02
Value: C
Micrococcus.lylae
1.00
0.75
0.50
0.25
0.00
A (n=14)
B (n=15)
C (n=11)
surface

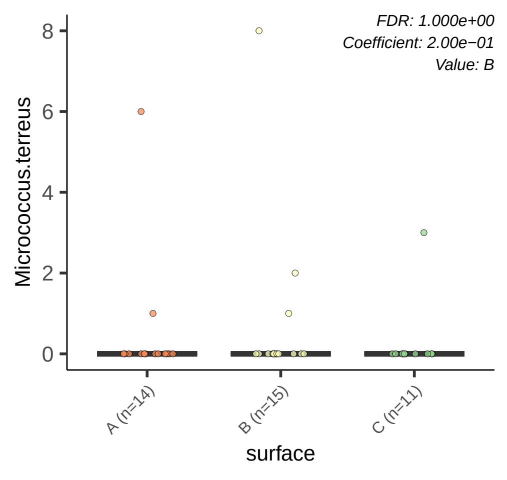

FDR: 1.000e+00
Coefficient: 2.00e−01
Value: B
Micrococcus.terreus
8
6
4
2
0
A (n=14)
B (n=15)
C (n=11)
surface

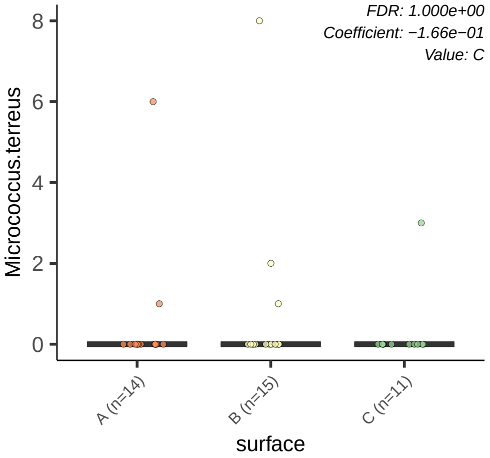

FDR: 1.000e+00
Coefficient: −1.66e−01
Value: C
Micrococcus.terreus
0
2
4
6
8
A (n=14)
B (n=15)
C (n=11)
surface

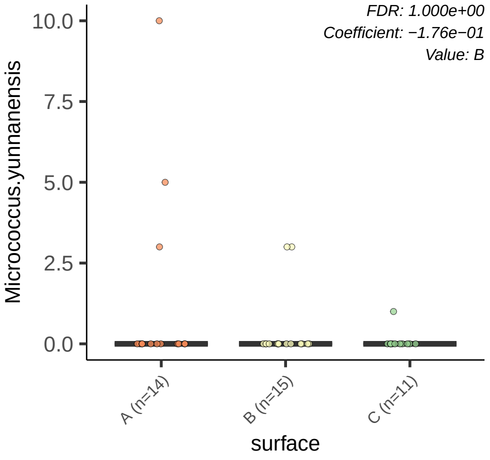
FDR: 1.000e+00
Coefficient: −1.76e−01
Value: B
Micrococcus.yunnanensis
10.0
7.5
5.0
2.5
0.0
A (n=14)
B (n=15)
C (n=11)
surface

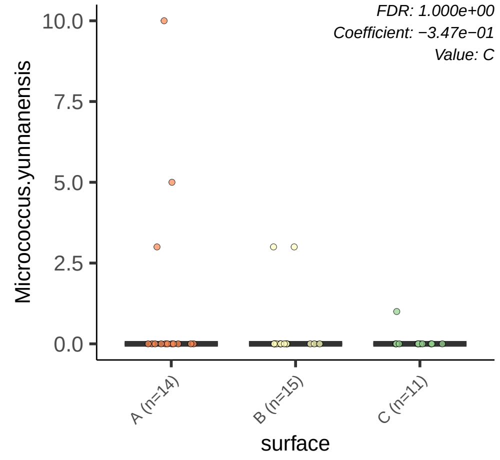

FDR: 1.000e+00
Coefficient: −3.47e−01
Value: C
Micrococcus.yunnanensis
10.0
7.5
5.0
2.5
0.0
A (n=14)
B (n=15)
C (n=11)
surface

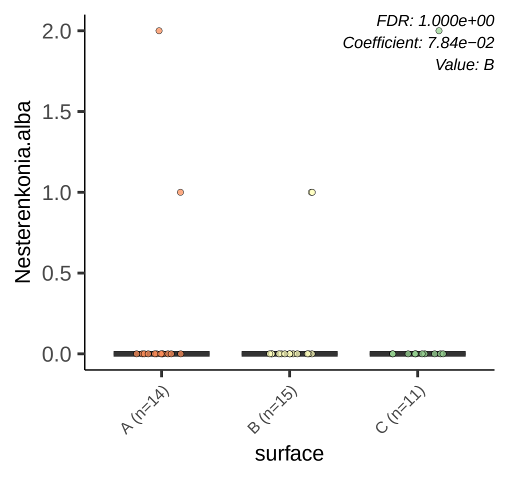
FDR: 1.000e+00
Coefficient: 7.84e−02
Value: B
Nesterenkonia.alba
2.0
1.5
1.0
0.5
0.0
A (n=14)
B (n=15)
C (n=11)
surface

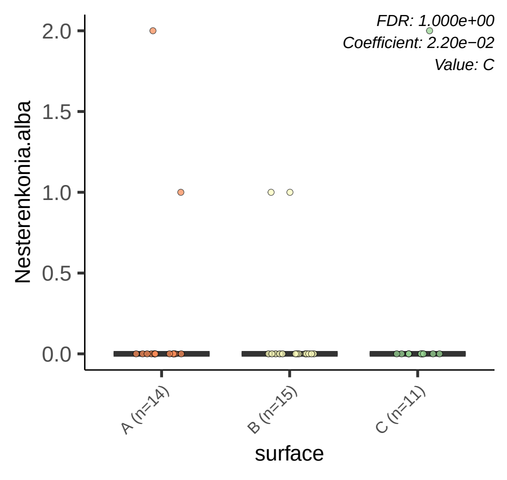
FDR: 1.000e+00
Coefficient: 2.20e−02
Value: C
Nesterenkonia.alba
2.0
1.5
1.0
0.5
0.0
A (n=14)
B (n=15)
C (n=11)
surface

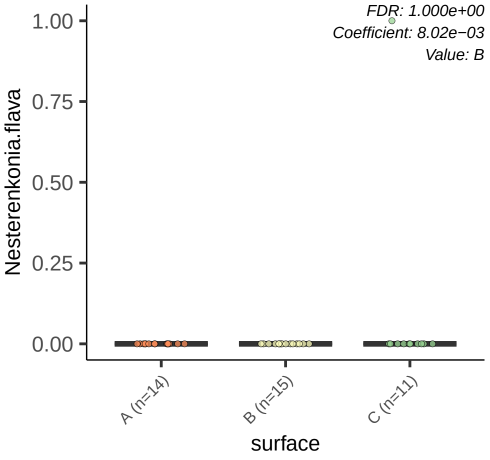
FDR: 1.000e+00
Coefficient: 8.02e−03
Value: B
1.00
0.75
0.50
0.25
0.00
Nesterenkonia.flava
A (n=14)
B (n=15)
C (n=11)
surface

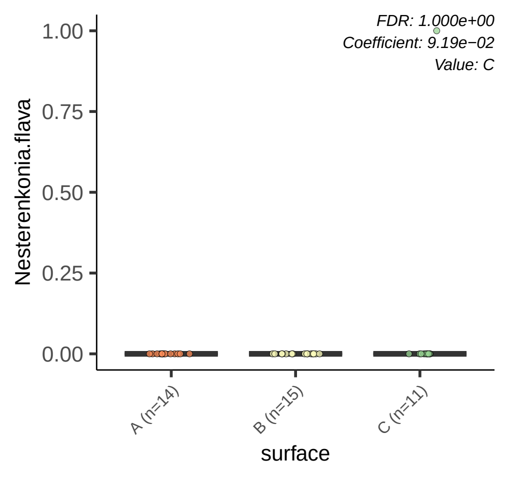
FDR: 1.000e+00
Coefficient: 9.19e−02
Value: C
Nesterenkonia.flava
1.00
0.75
0.50
0.25
0.00
A (n=14)
B (n=15)
C (n=11)
surface

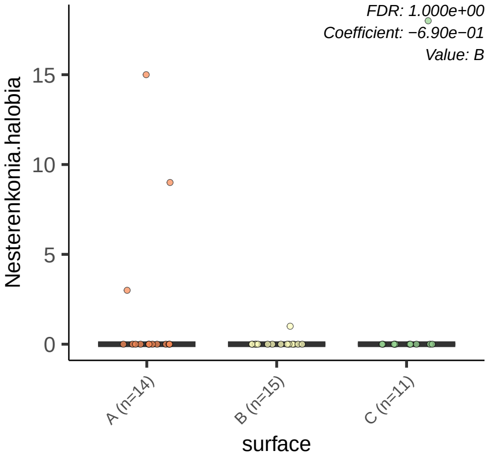
FDR: 1.000e+00
Coefficient: −6.90e−01
Value: B
Nesterenkonia.halobia
0
5
10
15
A (n=14)
B (n=15)
C (n=11)
surface

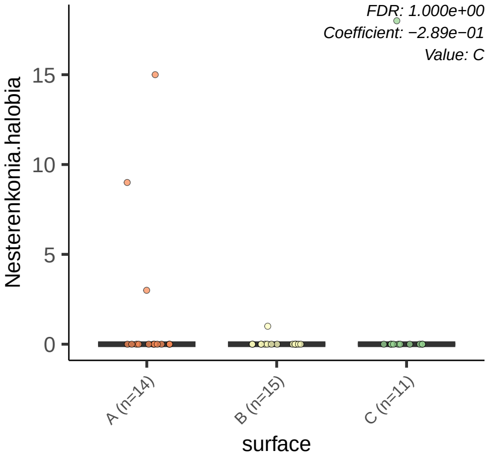

FDR: 1.000e+00
Coefficient: −2.89e−01
Value: C
Nesterenkonia.halobia
15
10
5
0
A (n=14)
B (n=15)
C (n=11)
surface

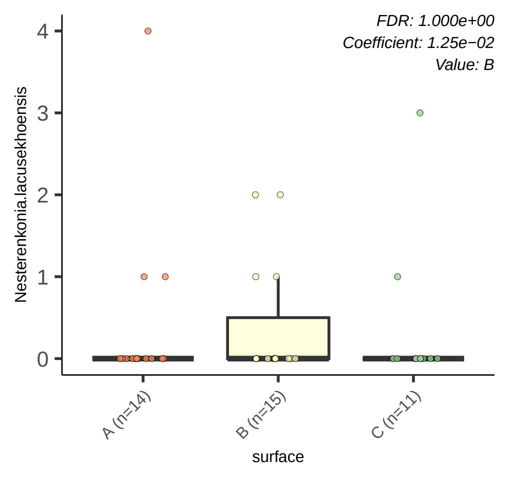
FDR: 1.000e+00
Coefficient: 1.25e−02
Value: B
Nesterenkonia.lacusekhoensis
0
1
2
3
4
A (n=14)
B (n=15)
C (n=11)
surface

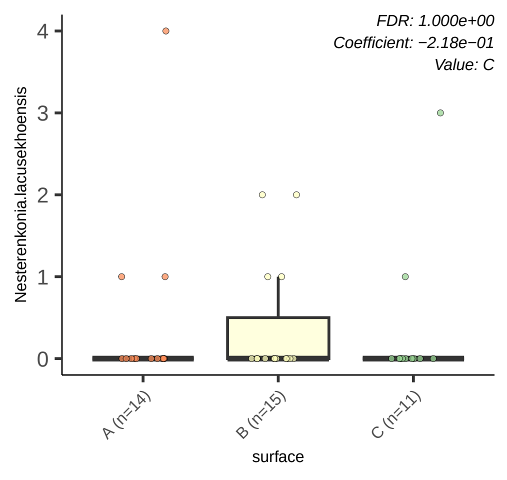

FDR: 1.000e+00
Coefficient: −2.18e−01
Value: C
Nesterenkonia.lacusekhoensis
0
1
2
3
4
A (n=14)
B (n=15)
C (n=11)
surface

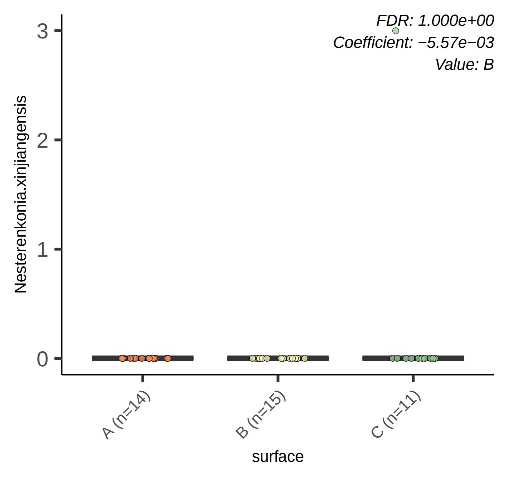
FDR: 1.000e+00
Coefficient: −5.57e−03
Value: B
Nesterenkonia.xinjiangensis
3
2
1
0
A (n=14)
B (n=15)
C (n=11)
surface

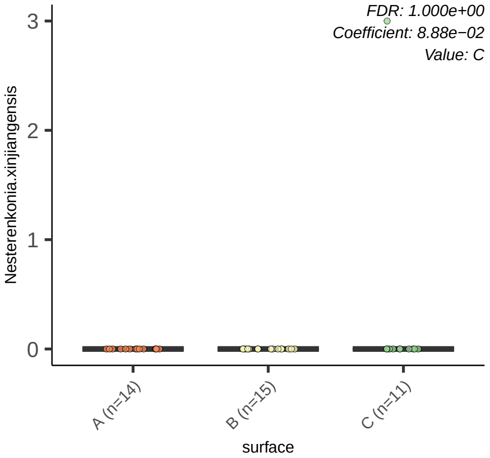

FDR: 1.000e+00
Coefficient: 8.88e−02
Value: C
Nesterenkonia.xinjiangensis
0
1
2
3
A (n=14)
B (n=15)
C (n=11)
surface

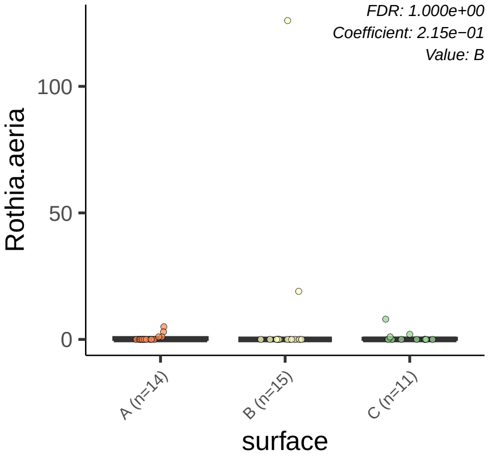
FDR: 1.000e+00
Coefficient: 2.15e−01
Value: B
Rothia.aeria
100
50
0
A (n=14)
B (n=15)
C (n=11)
surface

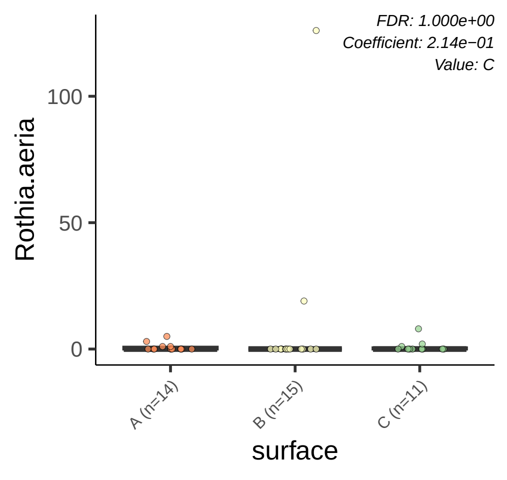

FDR: 1.000e+00
Coefficient: 2.14e−01
Value: C
Rothia.aeria
100
50
0
A (n=14)
B (n=15)
C (n=11)
surface

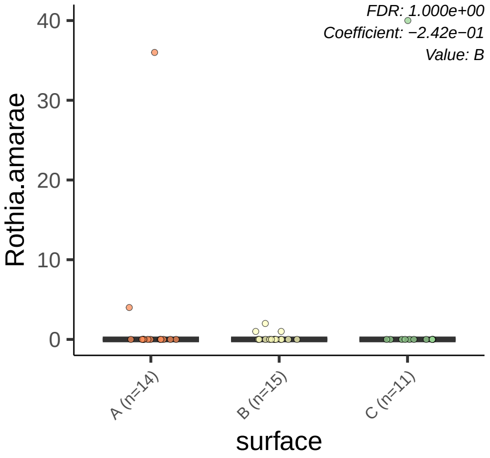
FDR: 1.000e+00
Coefficient: −2.42e−01
Value: B
Rothia.amarae
40
30
20
10
0
A (n=14)
B (n=15)
C (n=11)
surface

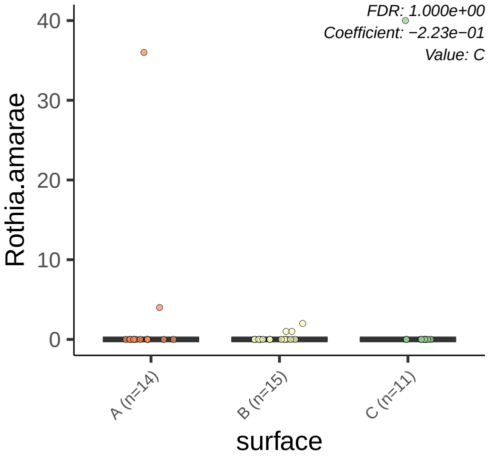

FDR: 1.000e+00
Coefficient: −2.23e−01
Value: C
Rothia.amarae
40
30
20
10
0
A (n=14)
B (n=15)
C (n=11)
surface

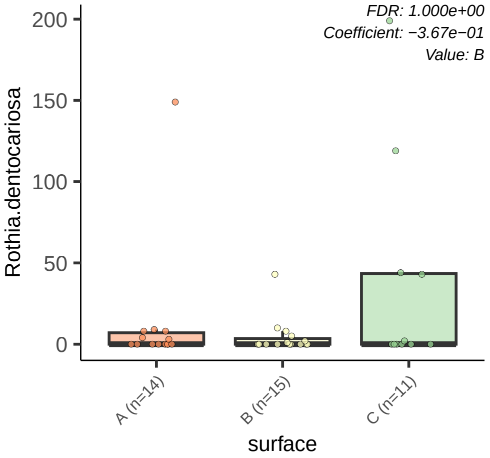

FDR: 1.000e+00
Coefficient: −3.67e−01
Value: B
Rothia.dentocariosa
200
150
100
50
0
A (n=14)
B (n=15)
C (n=11)
surface

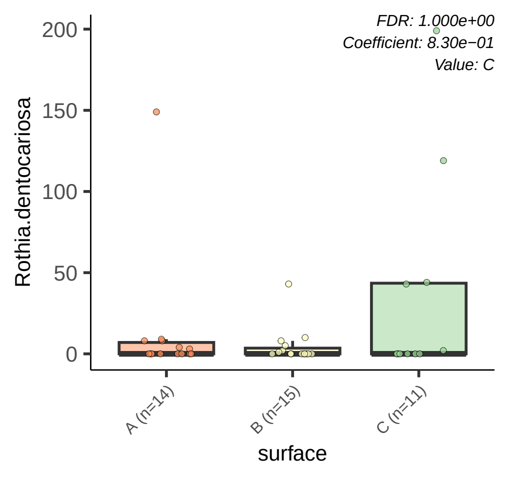
FDR: 1.000e+00
Coefficient: 8.30e−01
Value: C
Rothia.dentocariosa
200
150
100
50
0
A (n=14)
B (n=15)
C (n=11)
surface

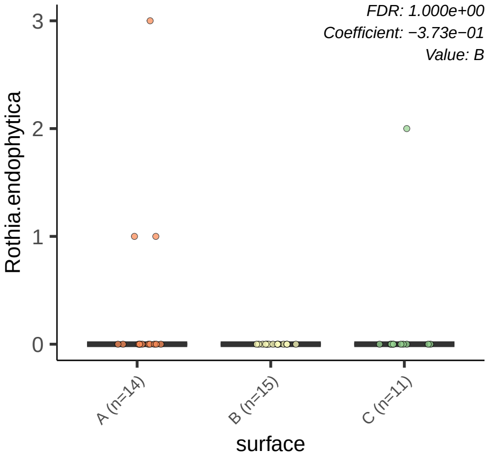

FDR: 1.000e+00
Coefficient: −3.73e−01
Value: B
Rothia.endophytica
3
2
1
0
A (n=14)
B (n=15)
C (n=11)
surface

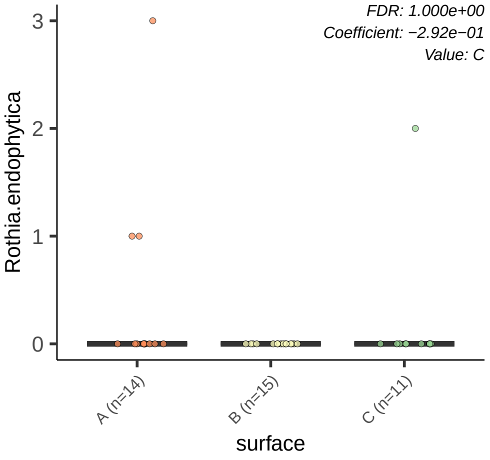

FDR: 1.000e+00
Coefficient: −2.92e−01
Value: C
Rothia.endophytica
3
2
1
0
A (n=14)
B (n=15)
C (n=11)
surface

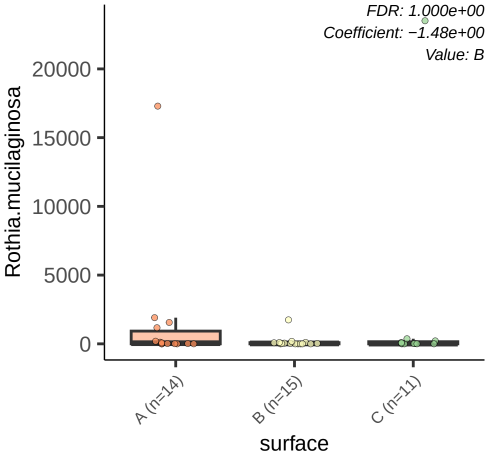

FDR: 1.000e+00
Coefficient: −1.48e+00
Value: B
Rothia.mucilaginosa
20000
15000
10000
5000
0
A (n=14)
B (n=15)
C (n=11)
surface

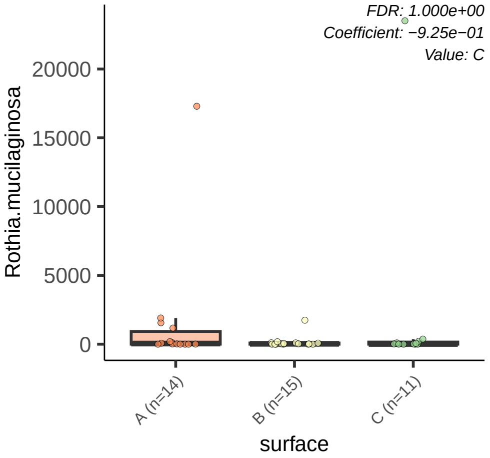
FDR: 1.000e+00
Coefficient: −9.25e−01
Value: C
Rothia.mucilaginosa
20000
15000
10000
5000
0
A (n=14)
B (n=15)
C (n=11)
surface

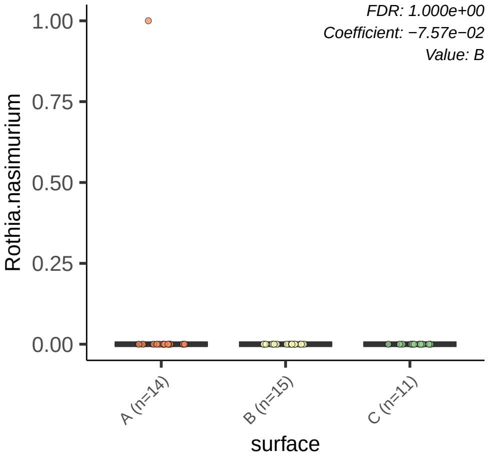
FDR: 1.000e+00
Coefficient: −7.57e−02
Value: B
Rothia.nasimurium
1.00
0.75
0.50
0.25
0.00
A (n=14)
B (n=15)
C (n=11)
surface

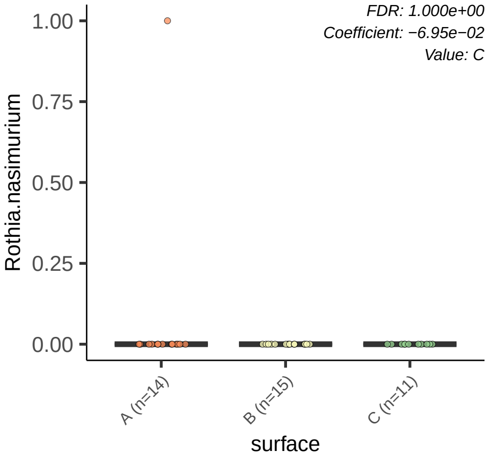

FDR: 1.000e+00
Coefficient: −6.95e−02
Value: C
Rothia.nasimurium
1.00
0.75
0.50
0.25
0.00
A (n=14)
B (n=15)
C (n=11)
surface

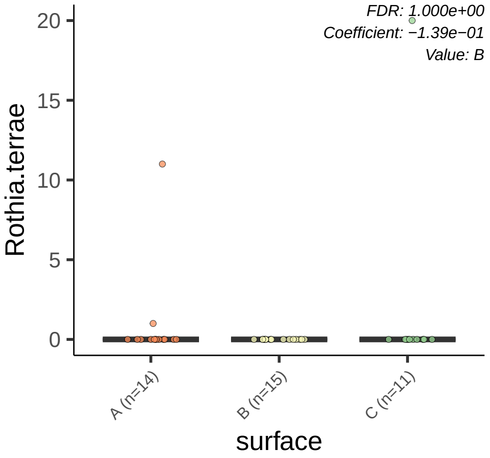
FDR: 1.000e+00
Coefficient: −1.39e−01
Value: B
Rothia.terrae
20
15
10
5
0
A (n=14)
B (n=15)
C (n=11)
surface

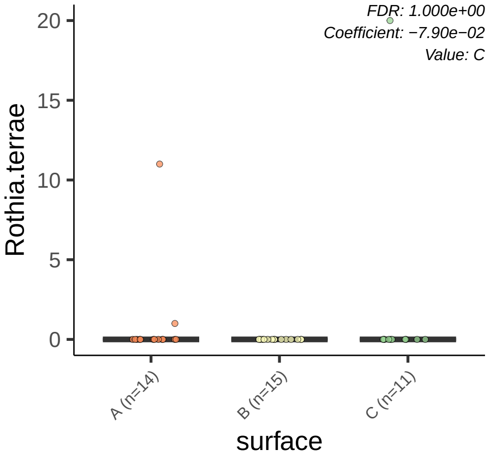

FDR: 1.000e+00
Coefficient: −7.90e−02
Value: C
Rothia.terrae
20
15
10
5
0
A (n=14)
B (n=15)
C (n=11)
surface

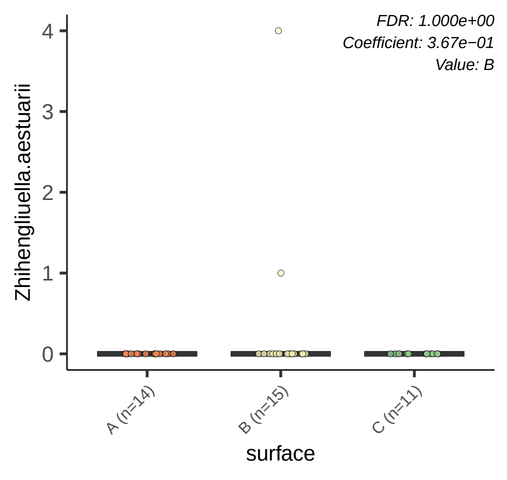
FDR: 1.000e+00
Coefficient: 3.67e−01
Value: B
Zhihengliuella.aestuarii
4
3
2
1
0
A (n=14)
B (n=15)
C (n=11)
surface

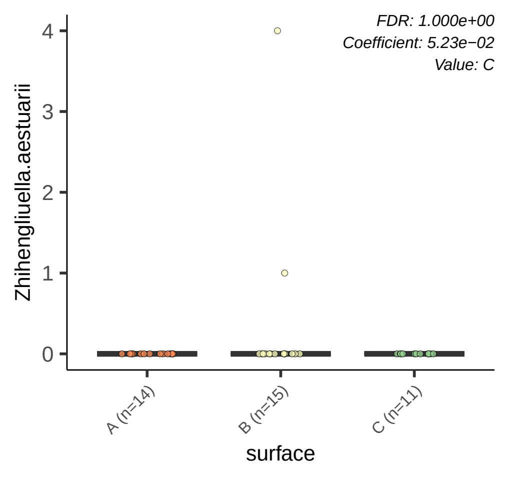

FDR: 1.000e+00
Coefficient: 5.23e−02
Value: C
Zhihengliuella.aestuarii
0
1
2
3
4
A (n=14)
B (n=15)
C (n=11)
surface

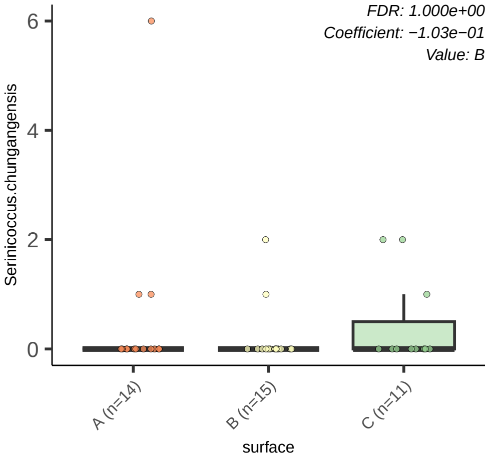
FDR: 1.000e+00
Coefficient: −1.03e−01
Value: B
Serinicoccus.chungangensis
6
4
2
0
A (n=14)
B (n=15)
C (n=11)
surface

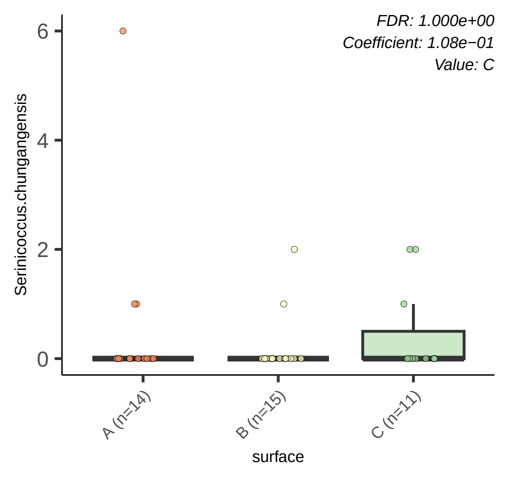

FDR: 1.000e+00
Coefficient: 1.08e−01
Value: C
Serinicoccus.chungangensis
0
2
4
6
A (n=14)
B (n=15)
C (n=11)
surface

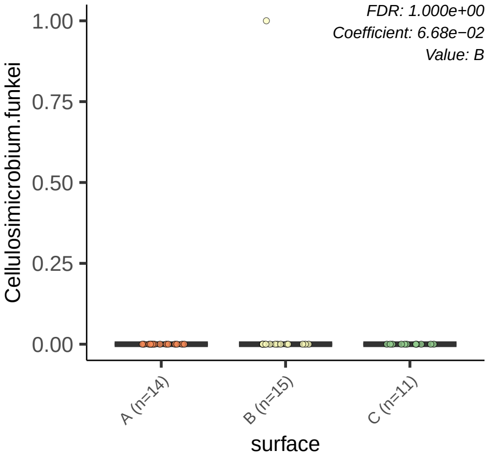
FDR: 1.000e+00
Coefficient: 6.68e−02
Value: B
Cellulosimicrobium.funkei
1.00
0.75
0.50
0.25
0.00
A (n=14)
B (n=15)
C (n=11)
surface

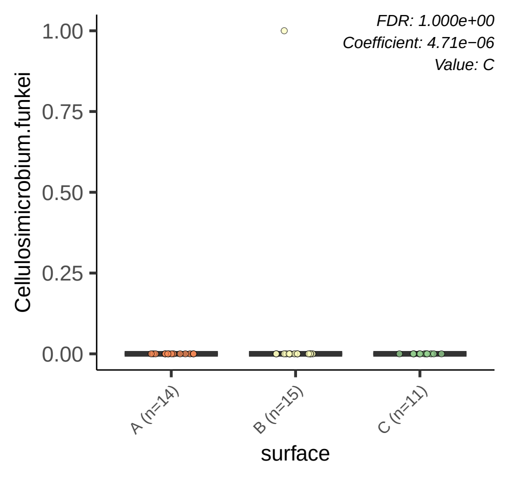
FDR: 1.000e+00
Coefficient: 4.71e−06
Value: C
Cellulosimicrobium.funkei
1.00
0.75
0.50
0.25
0.00
A (n=14)
B (n=15)
C (n=11)
surface

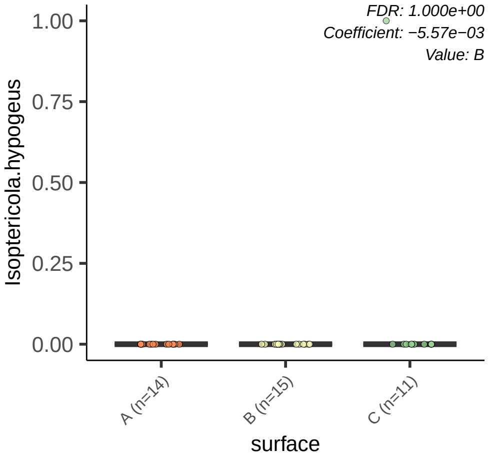

FDR: 1.000e+00
Coefficient: −5.57e−03
Value: B
1.00
0.75
0.50
0.25
0.00
Isoptericola.hypogeus
A (n=14)
B (n=15)
C (n=11)
surface

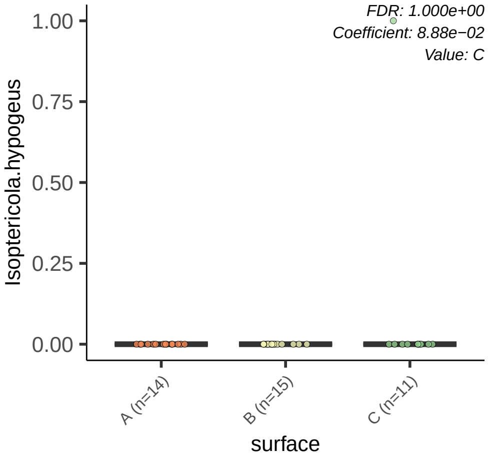

FDR: 1.000e+00
Coefficient: 8.88e−02
Value: C
Isoptericola.hypogeus
1.00
0.75
0.50
0.25
0.00
A (n=14)
B (n=15)
C (n=11)
surface

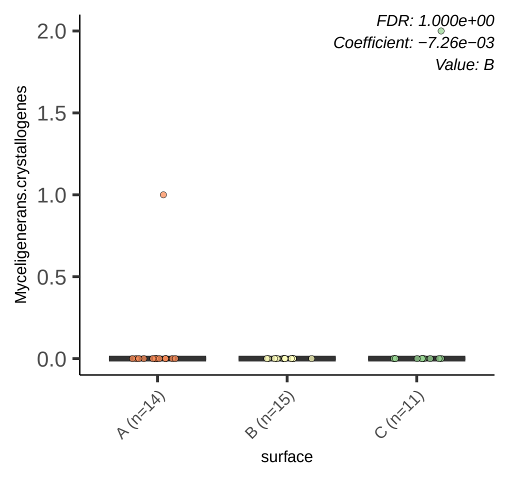

FDR: 1.000e+00
Coefficient: −7.26e−03
Value: B
Myceligenerans.crystallogenes
2.0
1.5
1.0
0.5
0.0
A (n=14)
B (n=15)
C (n=11)
surface

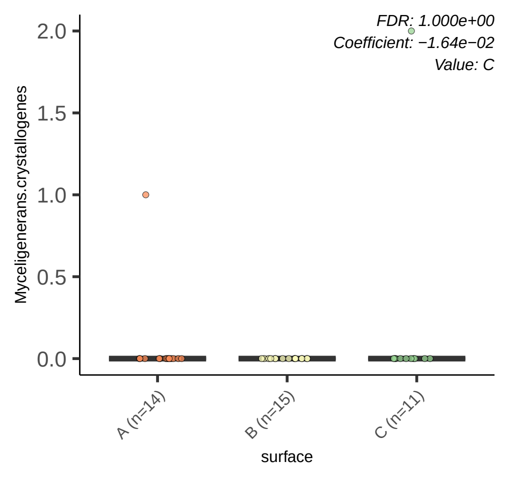

FDR: 1.000e+00
Coefficient: −1.64e−02
Value: C
2.0
1.5
1.0
0.5
0.0
Myceligenerans.crystallogenes
A (n=14)
B (n=15)
C (n=11)
surface

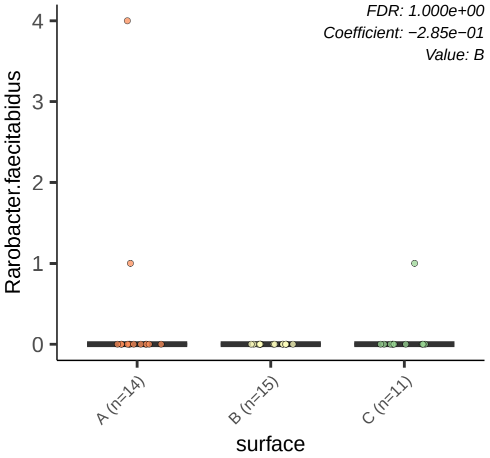
FDR: 1.000e+00
Coefficient: −2.85e−01
Value: B
Rarobacter.faecitabidus
4
3
2
1
0
A (n=14)
B (n=15)
C (n=11)
surface

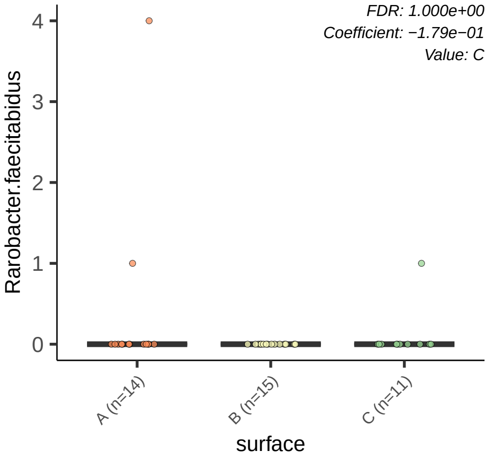

FDR: 1.000e+00
Coefficient: −1.79e−01
Value: C
Rarobacter.faecitabidus
0
1
2
3
4
A (n=14)
B (n=15)
C (n=11)
surface

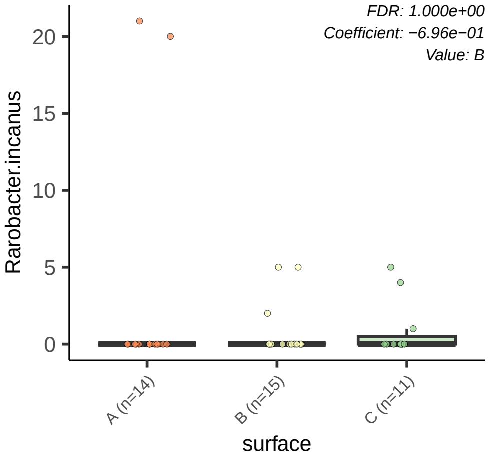
FDR: 1.000e+00
Coefficient: −6.96e−01
Value: B
Rarobacter.incanus
20
15
10
5
0
A (n=14)
B (n=15)
C (n=11)
surface

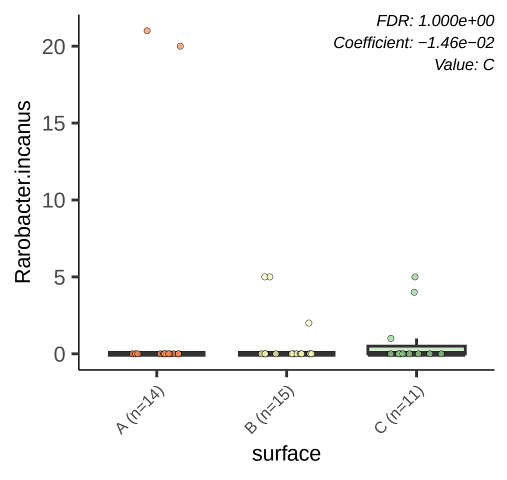

FDR: 1.000e+00
Coefficient: −1.46e−02
Value: C
Rarobacter.incanus
20
15
10
5
0
A (n=14)
B (n=15)
C (n=11)
surface

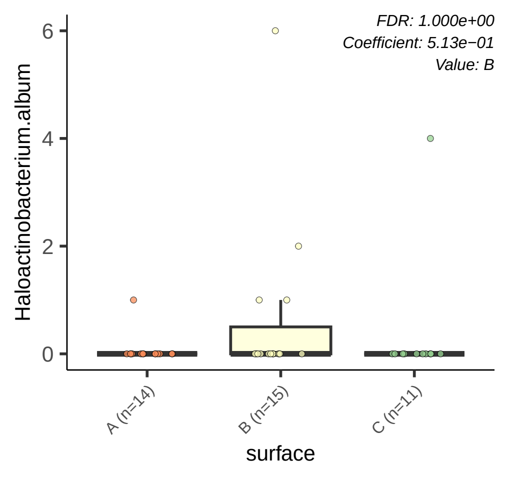
FDR: 1.000e+00
Coefficient: 5.13e−01
Value: B
Haloactinobacterium.album
0
2
4
6
A (n=14)
B (n=15)
C (n=11)
surface

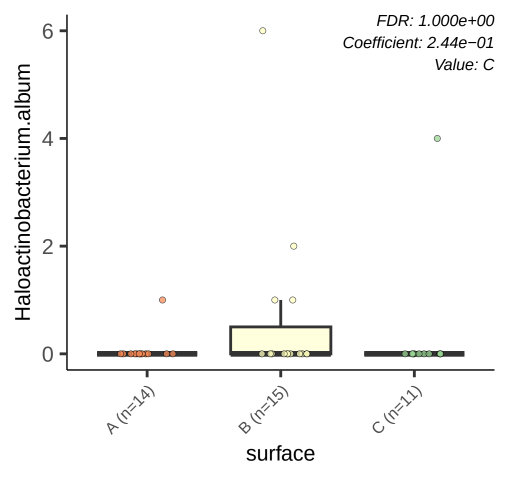

FDR: 1.000e+00
Coefficient: 2.44e−01
Value: C
Haloactinobacterium.album
0
2
4
6
A (n=14)
B (n=15)
C (n=11)
surface

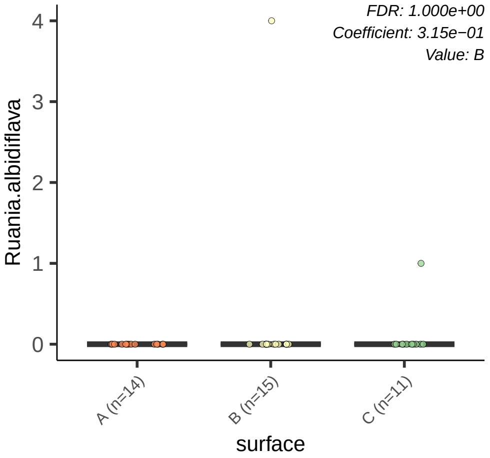

FDR: 1.000e+00
Coefficient: 3.15e−01
Value: B
Ruania.albidiflava
0
1
2
3
4
A (n=14)
B (n=15)
C (n=11)
surface

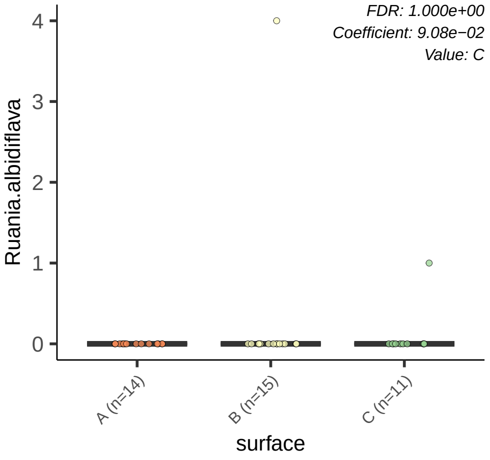

FDR: 1.000e+00
Coefficient: 9.08e−02
Value: C
Ruania.albidiflava
4
3
2
1
0
A (n=14)
B (n=15)
C (n=11)
surface

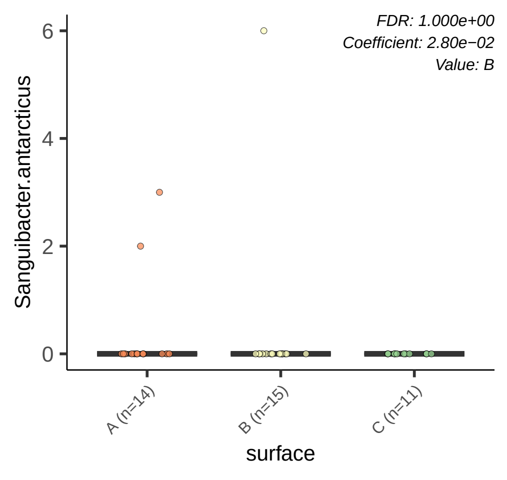
FDR: 1.000e+00
Coefficient: 2.80e−02
Value: B
Sanguibacter.antarcticus
0
2
4
6
A (n=14)
B (n=15)
C (n=11)
surface

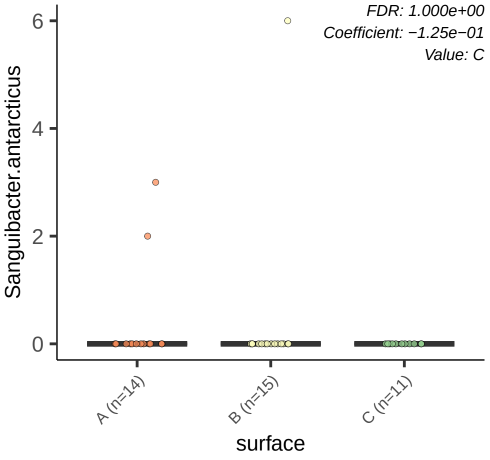

FDR: 1.000e+00
Coefficient: −1.25e−01
Value: C
Sanguibacter.antarcticus
0
2
4
6
A (n=14)
B (n=15)
C (n=11)
surface

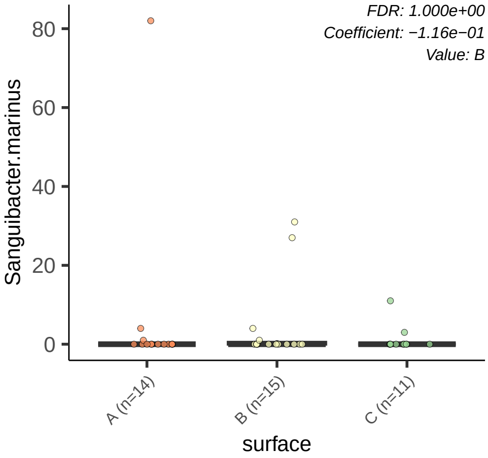
FDR: 1.000e+00
Coefficient: −1.16e−01
Value: B
Sanguibacter.marinus
0
20
40
60
80
A (n=14)
B (n=15)
C (n=11)
surface

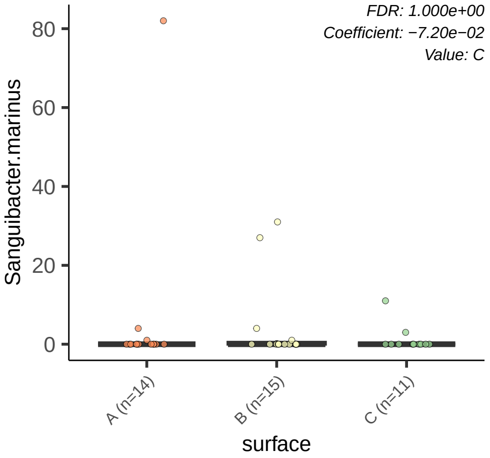
FDR: 1.000e+00
Coefficient: −7.20e−02
Value: C
Sanguibacter.marinus
0
20
40
60
80
A (n=14)
B (n=15)
C (n=11)
surface

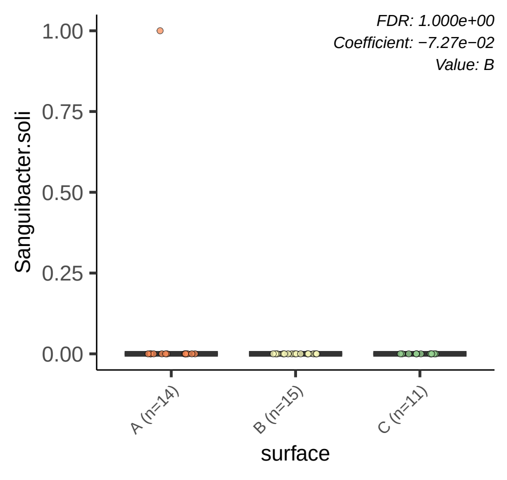

FDR: 1.000e+00
Coefficient: −7.27e−02
Value: B
Sanguibacter.soli
1.00
0.75
0.50
0.25
0.00
A (n=14)
B (n=15)
C (n=11)
surface

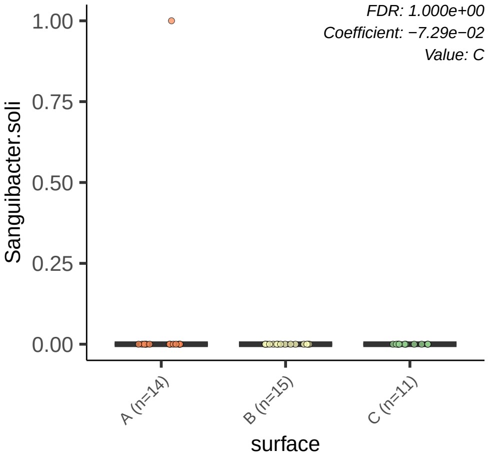
FDR: 1.000e+00
Coefficient: −7.29e−02
Value: C
Sanguibacter.soli
1.00
0.75
0.50
0.25
0.00
A (n=14)
B (n=15)
C (n=11)
surface

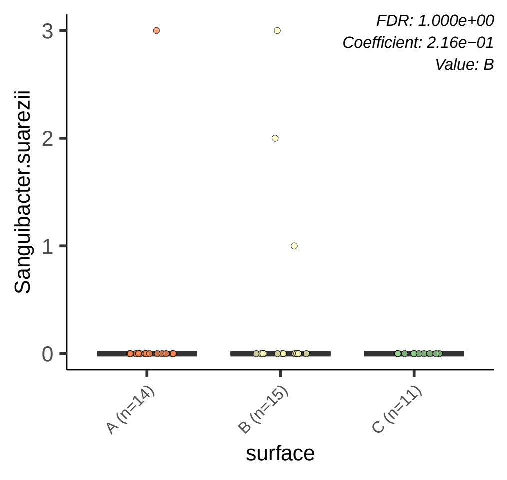
FDR: 1.000e+00
Coefficient: 2.16e−01
Value: B
Sanguibacter.suarezii
0
1
2
3
A (n=14)
B (n=15)
C (n=11)
surface

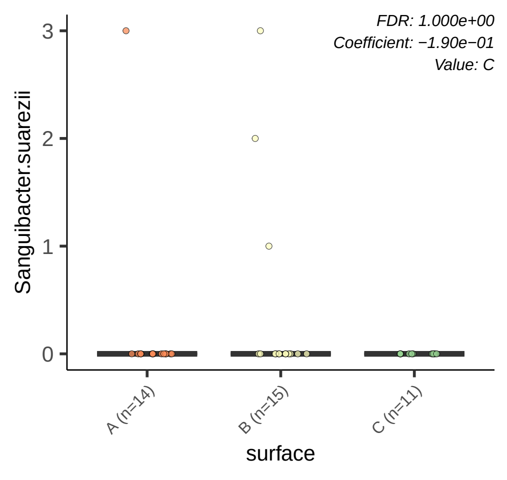

FDR: 1.000e+00
Coefficient: −1.90e−01
Value: C
Sanguibacter.suarezii
3
2
1
0
A (n=14)
B (n=15)
C (n=11)
surface

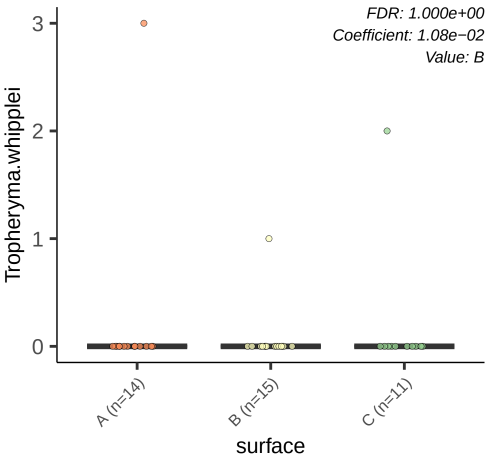
FDR: 1.000e+00
Coefficient: 1.08e−02
Value: B
Tropheryma.whipplei
3
2
1
0
A (n=14)
B (n=15)
C (n=11)
surface

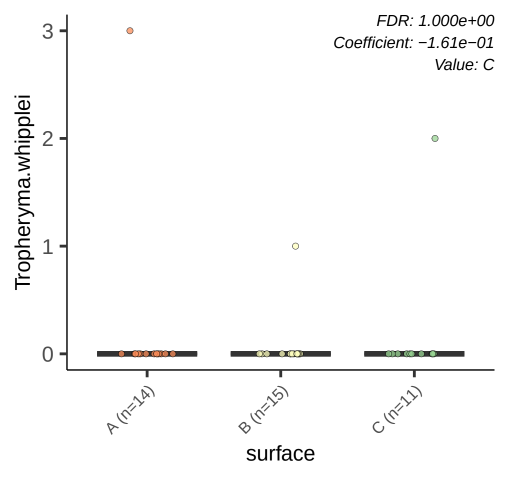
FDR: 1.000e+00
Coefficient: −1.61e−01
Value: C
Tropheryma.whipplei
3
2
1
0
A (n=14)
B (n=15)
C (n=11)
surface

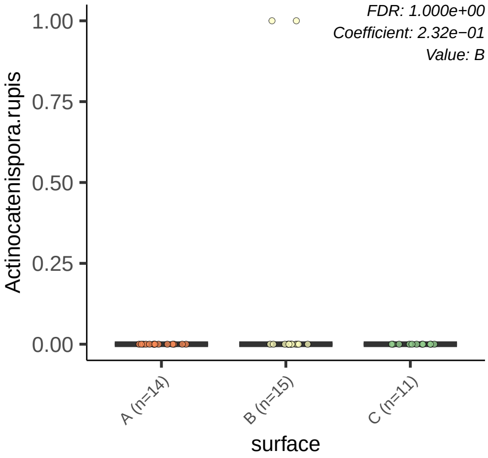
FDR: 1.000e+00
Coefficient: 2.32e−01
Value: B
Actinocatenispora.rupis
1.00
0.75
0.50
0.25
0.00
A (n=14)
B (n=15)
C (n=11)
surface

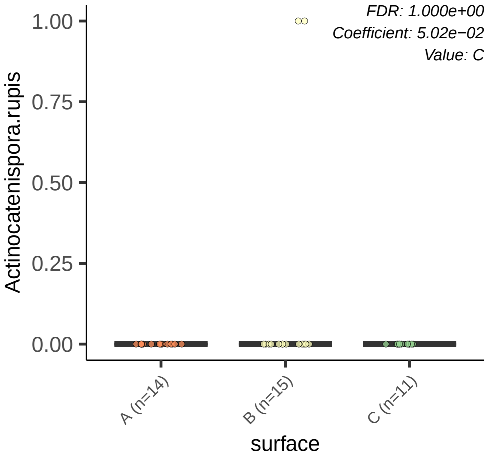
FDR: 1.000e+00
Coefficient: 5.02e−02
Value: C
Actinocatenispora.rupis
1.00
0.75
0.50
0.25
0.00
A (n=14)
B (n=15)
C (n=11)
surface

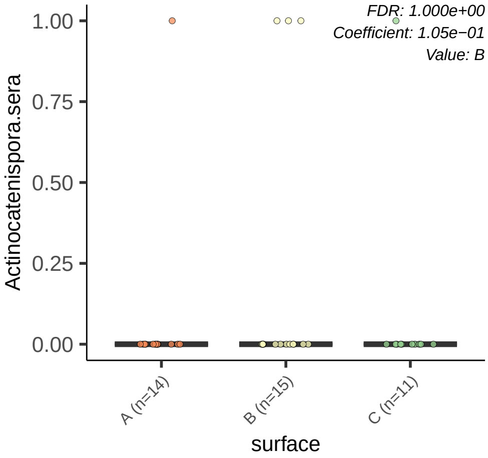
FDR: 1.000e+00
Coefficient: 1.05e−01
Value: B
Actinocatenispora.sera
1.00
0.75
0.50
0.25
0.00
A (n=14)
B (n=15)
C (n=11)
surface

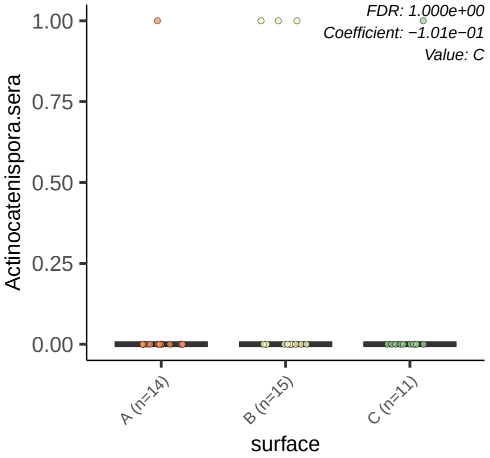
FDR: 1.000e+00
Coefficient: −1.01e−01
Value: C
Actinocatenispora.sera
1.00
0.75
0.50
0.25
0.00
A (n=14)
B (n=15)
C (n=11)
surface

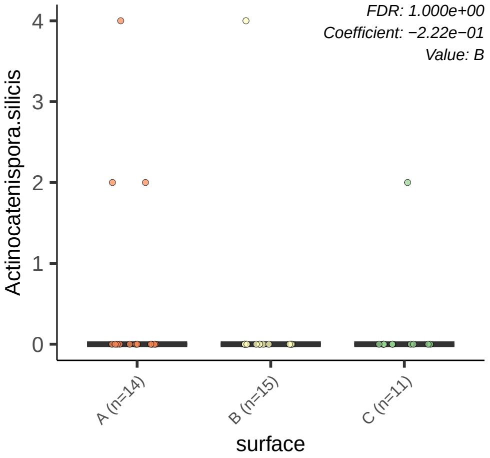

FDR: 1.000e+00
Coefficient: −2.22e−01
Value: B
Actinocatenispora.silicis
4
3
2
1
0
A (n=14)
B (n=15)
C (n=11)
surface

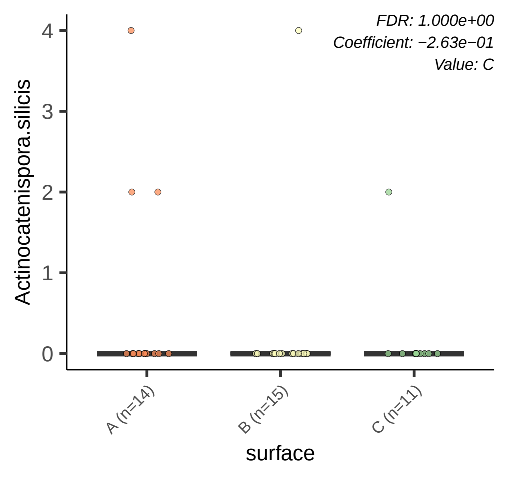

FDR: 1.000e+00
Coefficient: −2.63e−01
Value: C
Actinocatenispora.silicis
0
1
2
3
4
A (n=14)
B (n=15)
C (n=11)
surface

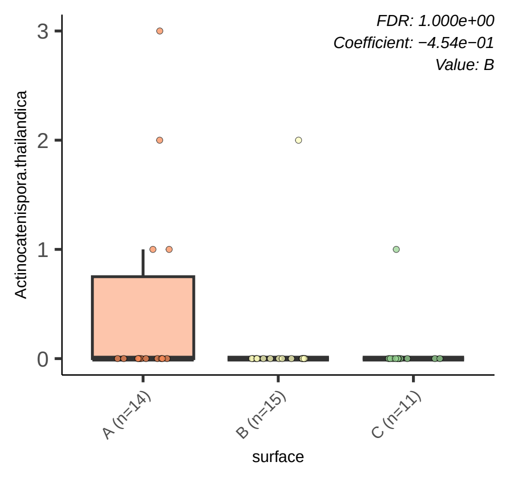
FDR: 1.000e+00
Coefficient: −4.54e−01
Value: B
Actinocatenispora.thailandica
0
1
2
3
A (n=14)
B (n=15)
C (n=11)
surface

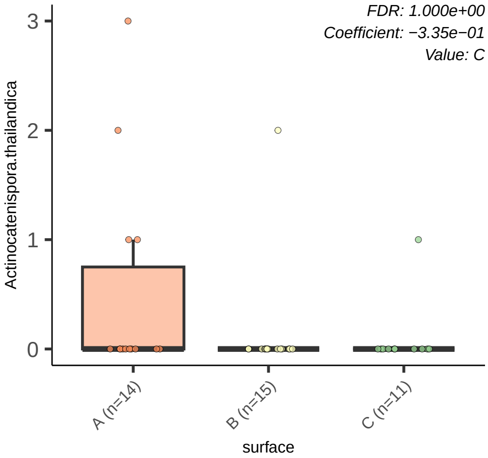

FDR: 1.000e+00
Coefficient: −3.35e−01
Value: C
Actinocatenispora.thailandica
0
1
2
3
A (n=14)
B (n=15)
C (n=11)
surface

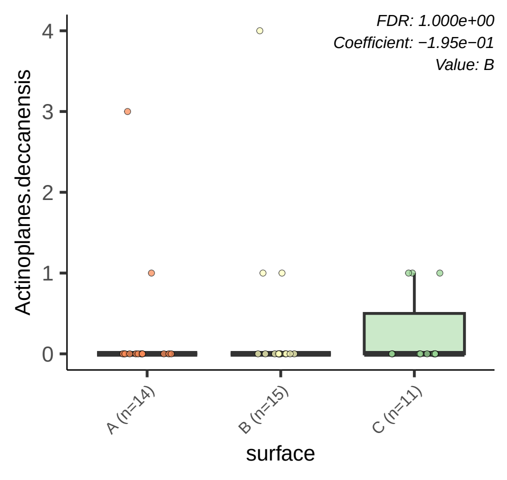

FDR: 1.000e+00
Coefficient: −1.95e−01
Value: B
Actinoplanes.deccanensis
4
3
2
1
0
A (n=14)
B (n=15)
C (n=11)
surface

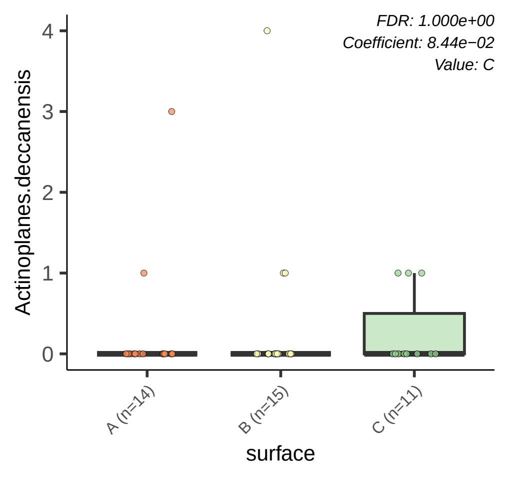

FDR: 1.000e+00
Coefficient: 8.44e−02
Value: C
Actinoplanes.deccanensis
0
1
2
3
4
A (n=14)
B (n=15)
C (n=11)
surface

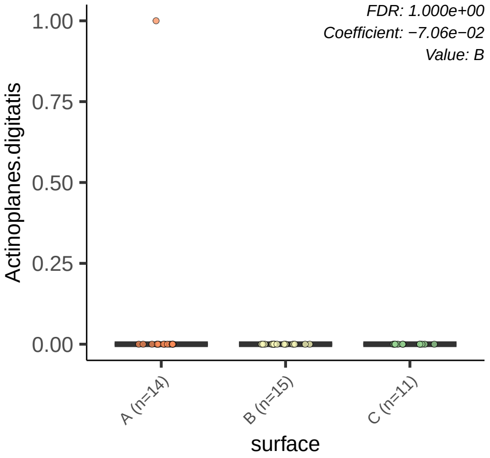
FDR: 1.000e+00
Coefficient: −7.06e−02
Value: B
Actinoplanes.digitatis
1.00
0.75
0.50
0.25
0.00
A (n=14)
B (n=15)
C (n=11)
surface

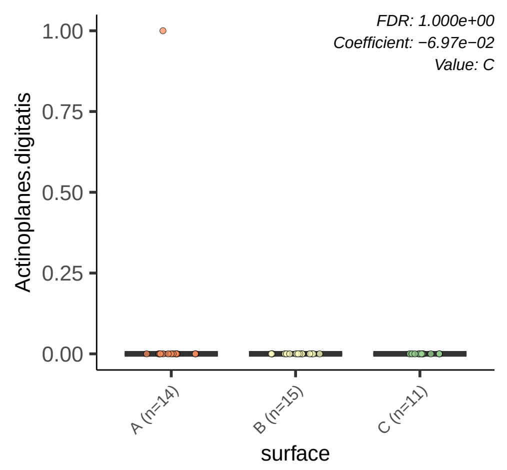
FDR: 1.000e+00
Coefficient: −6.97e−02
Value: C
Actinoplanes.digitatis
1.00
0.75
0.50
0.25
0.00
A (n=14)
B (n=15)
C (n=11)
surface

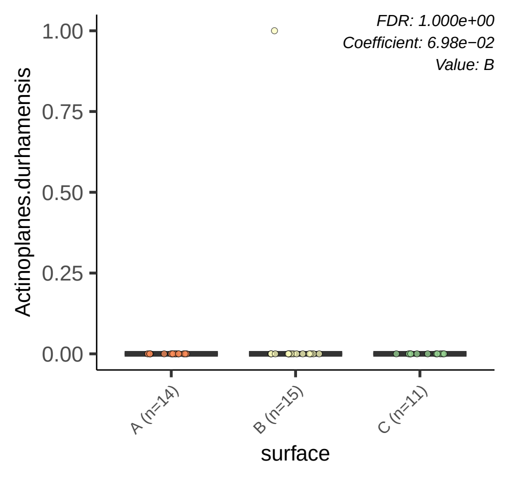
FDR: 1.000e+00
Coefficient: 6.98e−02
Value: B
Actinoplanes.durhamensis
1.00
0.75
0.50
0.25
0.00
A (n=14)
B (n=15)
C (n=11)
surface

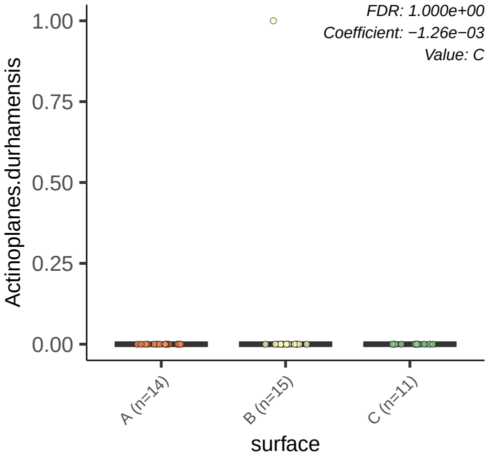
FDR: 1.000e+00
Coefficient: −1.26e−03
Value: C
Actinoplanes.durhamensis
1.00
0.75
0.50
0.25
0.00
A (n=14)
B (n=15)
C (n=11)
surface

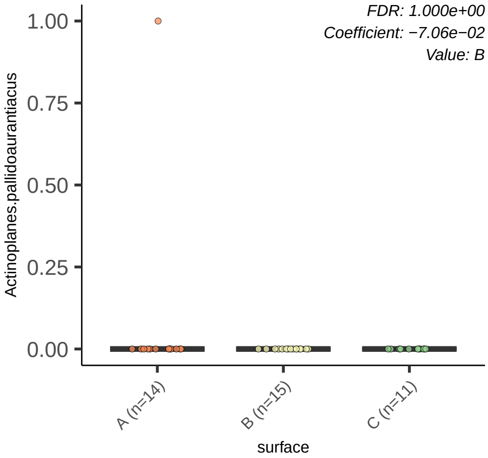

FDR: 1.000e+00
Coefficient: −7.06e−02
Value: B
Actinoplanes.pallidoaurantiacus
1.00
0.75
0.50
0.25
0.00
A (n=14)
B (n=15)
C (n=11)
surface

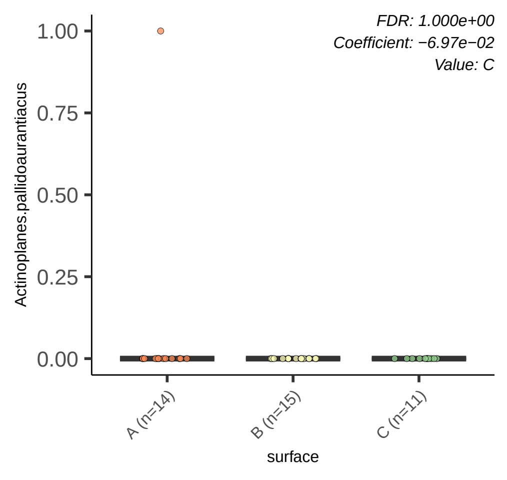

FDR: 1.000e+00
Coefficient: −6.97e−02
Value: C
1.00
0.75
0.50
0.25
0.00
Actinoplanes.pallidoaurantiacus
A (n=14)
B (n=15)
C (n=11)
surface

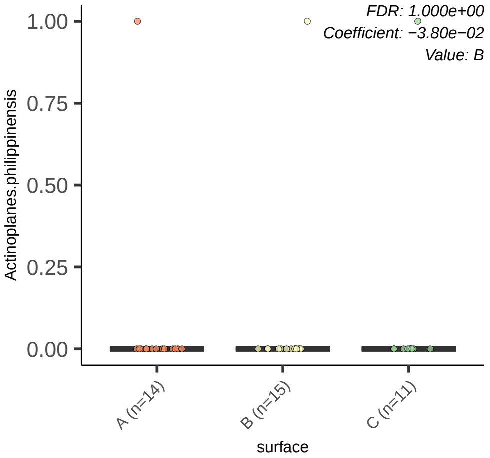
FDR: 1.000e+00
Coefficient: −3.80e−02
Value: B
Actinoplanes.philippinensis
1.00
0.75
0.50
0.25
0.00
A (n=14)
B (n=15)
C (n=11)
surface

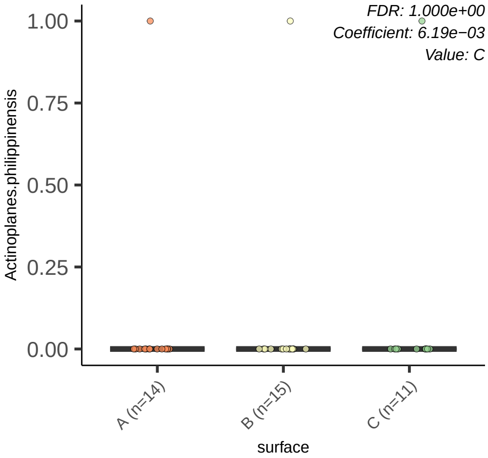
FDR: 1.000e+00
Coefficient: 6.19e−03
Value: C
1.00
0.75
0.50
0.25
0.00
Actinoplanes.philippinensis
A (n=14)
B (n=15)
C (n=11)
surface

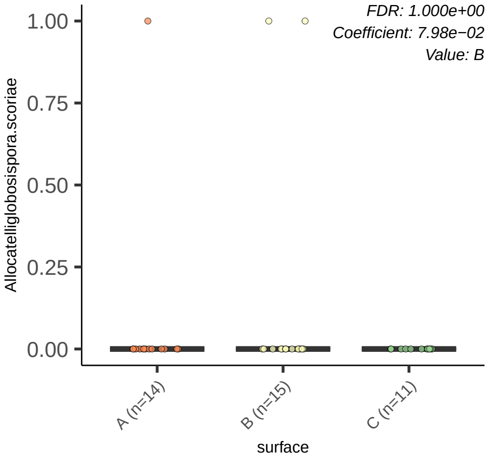
FDR: 1.000e+00
Coefficient: 7.98e−02
Value: B
Allocatelliglobosispora.scoriae
1.00
0.75
0.50
0.25
0.00
A (n=14)
B (n=15)
C (n=11)
surface

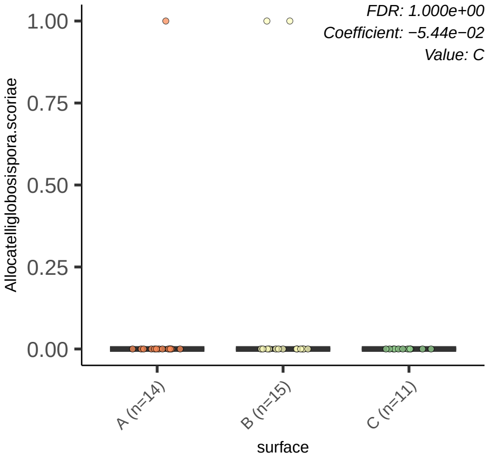
FDR: 1.000e+00
Coefficient: −5.44e−02
Value: C
1.00
0.75
0.50
0.25
0.00
Allocatelliglobosispora.scoriae
A (n=14)
B (n=15)
C (n=11)
surface

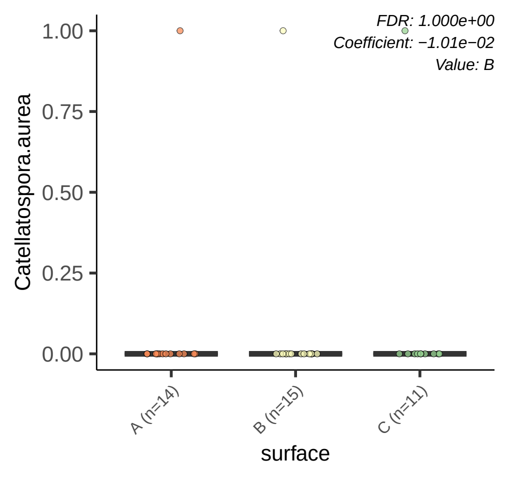

FDR: 1.000e+00
Coefficient: −1.01e−02
Value: B
Catellatospora.aurea
1.00
0.75
0.50
0.25
0.00
A (n=14)
B (n=15)
C (n=11)
surface

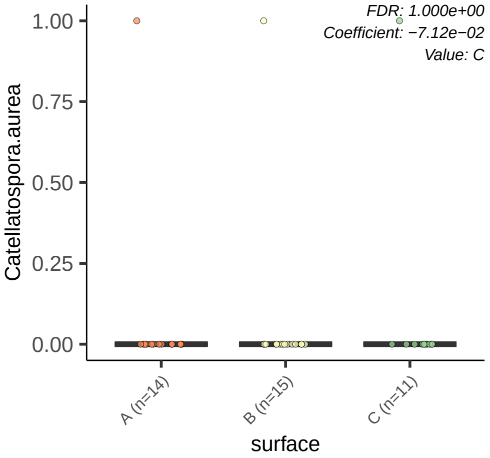
FDR: 1.000e+00
Coefficient: −7.12e−02
Value: C
Catellatospora.aurea
1.00
0.75
0.50
0.25
0.00
A (n=14)
B (n=15)
C (n=11)
surface

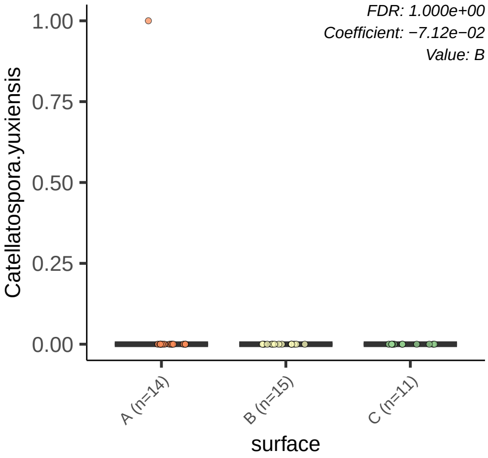

FDR: 1.000e+00
Coefficient: −7.12e−02
Value: B
Catellatospora.yuxiensis
1.00
0.75
0.50
0.25
0.00
A (n=14)
B (n=15)
C (n=11)
surface

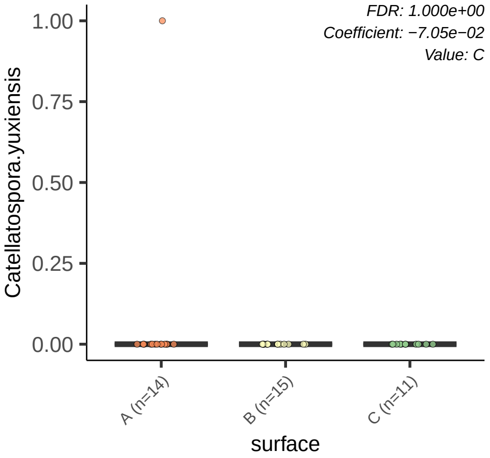
FDR: 1.000e+00
Coefficient: −7.05e−02
Value: C
Catellatospora.yuxiensis
1.00
0.75
0.50
0.25
0.00
A (n=14)
B (n=15)
C (n=11)
surface

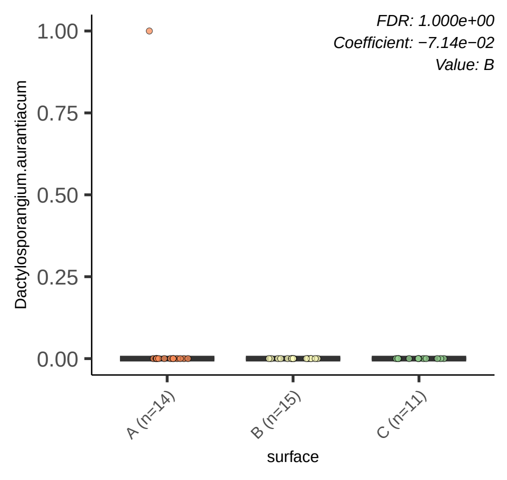
FDR: 1.000e+00
Coefficient: −7.14e−02
Value: B
1.00
0.75
0.50
0.25
0.00
Dactylosporangium.aurantiacum
A (n=14)
B (n=15)
C (n=11)
surface

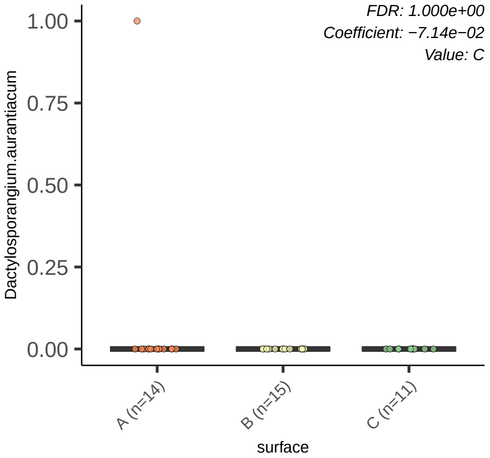
FDR: 1.000e+00
Coefficient: −7.14e−02
Value: C
1.00
0.75
0.50
0.25
0.00
Dactylosporangium.aurantiacum
A (n=14)
B (n=15)
C (n=11)
surface

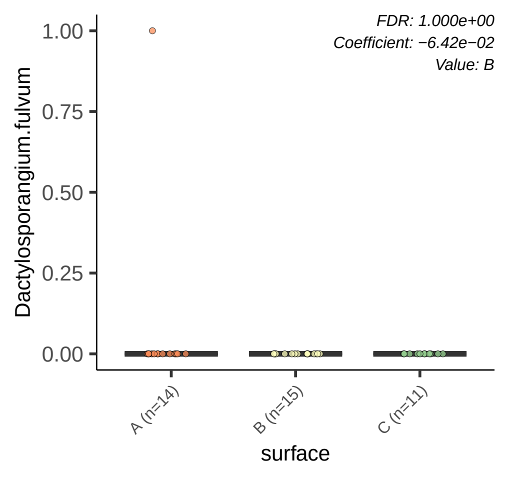

FDR: 1.000e+00
Coefficient: −6.42e−02
Value: B
Dactylosporangium.fulvum
1.00
0.75
0.50
0.25
0.00
A (n=14)
B (n=15)
C (n=11)
surface

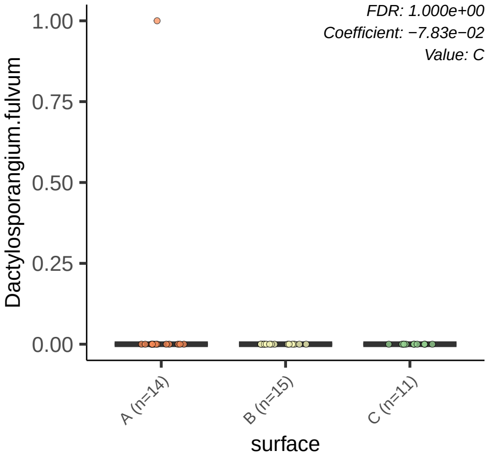
FDR: 1.000e+00
Coefficient: −7.83e−02
Value: C
Dactylosporangium.fulvum
1.00
0.75
0.50
0.25
0.00
A (n=14)
B (n=15)
C (n=11)
surface

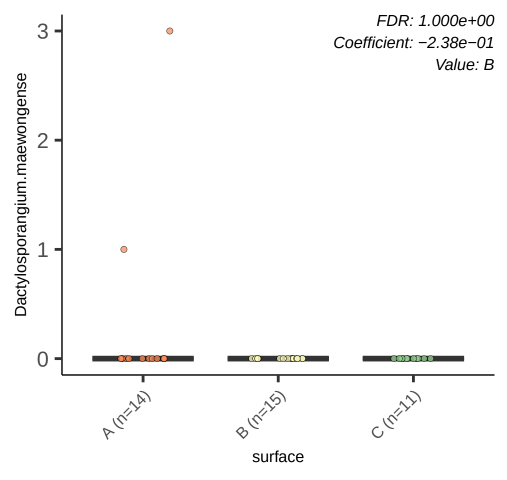
FDR: 1.000e+00
Coefficient: −2.38e−01
Value: B
Dactylosporangium.maewongense
3
2
1
0
A (n=14)
B (n=15)
C (n=11)
surface

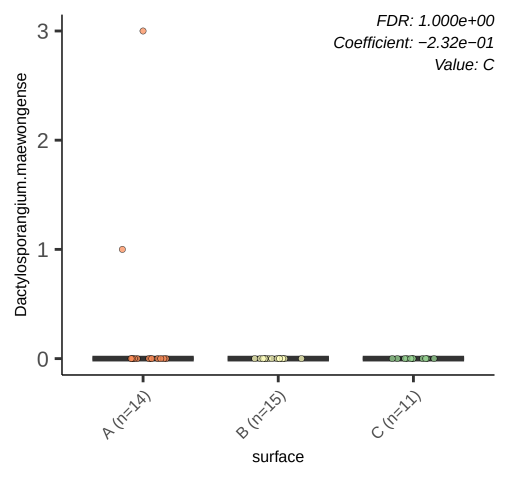

FDR: 1.000e+00
Coefficient: −2.32e−01
Value: C
Dactylosporangium.maewongense
0
1
2
3
A (n=14)
B (n=15)
C (n=11)
surface

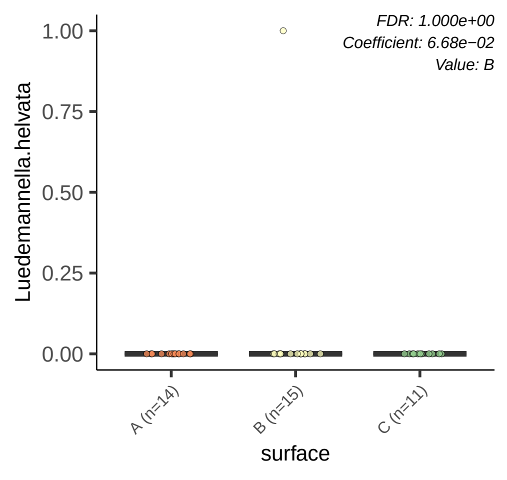
FDR: 1.000e+00
Coefficient: 6.68e−02
Value: B
Luedemannella.helvata
1.00
0.75
0.50
0.25
0.00
A (n=14)
B (n=15)
C (n=11)
surface

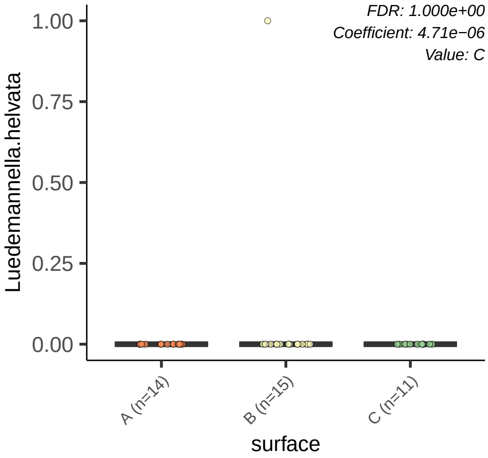
FDR: 1.000e+00
Coefficient: 4.71e−06
Value: C
Luedemannella.helvata
1.00
0.75
0.50
0.25
0.00
A (n=14)
B (n=15)
C (n=11)
surface

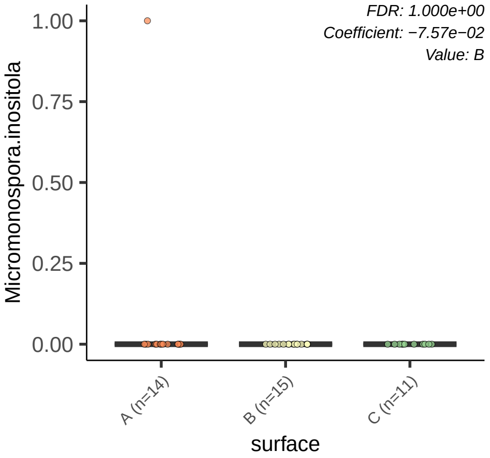
FDR: 1.000e+00
Coefficient: −7.57e−02
Value: B
Micromonospora.inositola
1.00
0.75
0.50
0.25
0.00
A (n=14)
B (n=15)
C (n=11)
surface

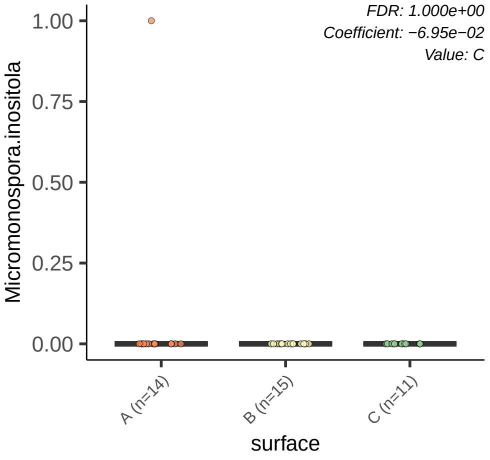

FDR: 1.000e+00
Coefficient: −6.95e−02
Value: C
Micromonospora.inositola
1.00
0.75
0.50
0.25
0.00
A (n=14)
B (n=15)
C (n=11)
surface

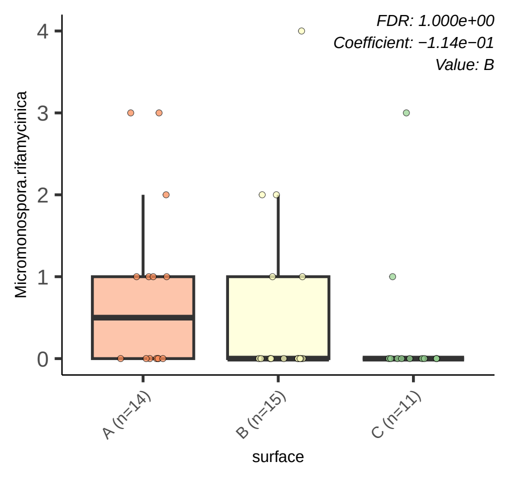
FDR: 1.000e+00
Coefficient: −1.14e−01
Value: B
Micromonospora.rifamycinica
0
1
2
3
4
A (n=14)
B (n=15)
C (n=11)
surface

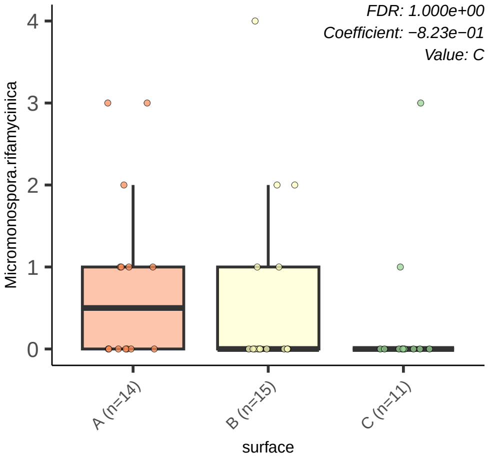

FDR: 1.000e+00
Coefficient: −8.23e−01
Value: C
Micromonospora.rifamycinica
0
1
2
3
4
A (n=14)
B (n=15)
C (n=11)
surface

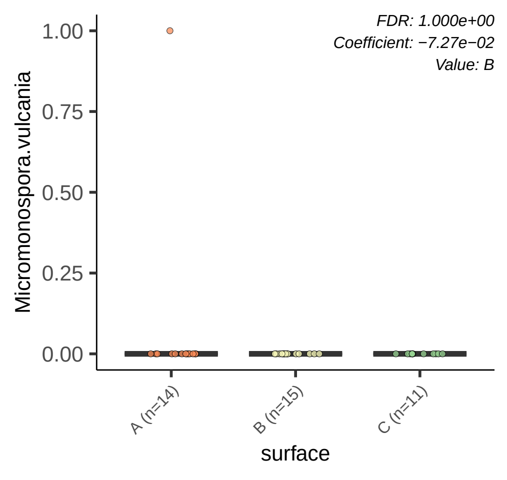

FDR: 1.000e+00
Coefficient: −7.27e−02
Value: B
Micromonospora.vulcania
1.00
0.75
0.50
0.25
0.00
A (n=14)
B (n=15)
C (n=11)
surface

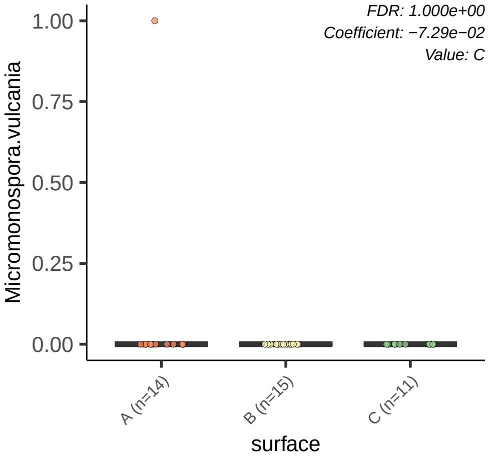

FDR: 1.000e+00
Coefficient: −7.29e−02
Value: C
Micromonospora.vulcania
1.00
0.75
0.50
0.25
0.00
A (n=14)
B (n=15)
C (n=11)
surface

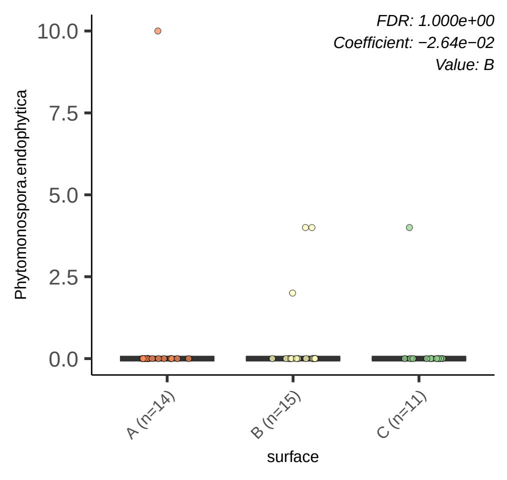
FDR: 1.000e+00
Coefficient: −2.64e−02
Value: B
Phytomonospora.endophytica
10.0
7.5
5.0
2.5
0.0
A (n=14)
B (n=15)
C (n=11)
surface

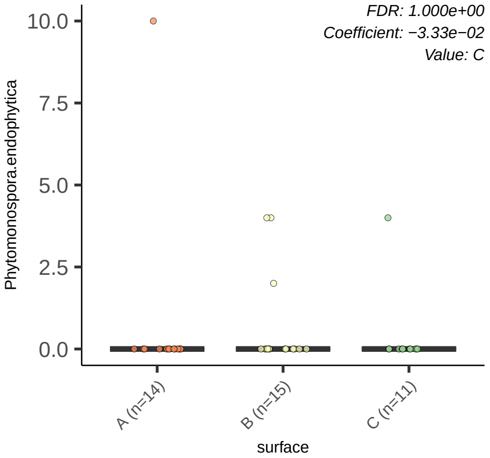

FDR: 1.000e+00
Coefficient: −3.33e−02
Value: C
Phytomonospora.endophytica
10.0
7.5
5.0
2.5
0.0
A (n=14)
B (n=15)
C (n=11)
surface

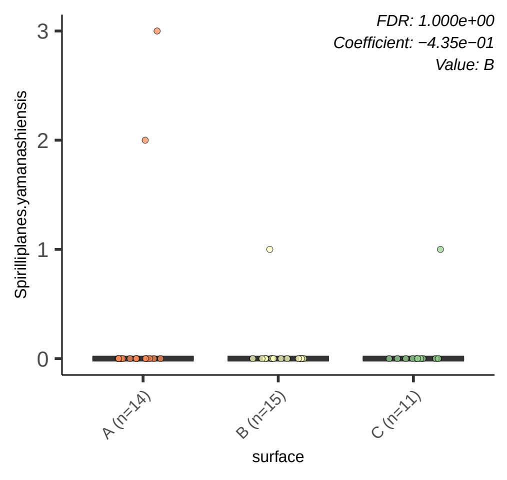
FDR: 1.000e+00
Coefficient: −4.35e−01
Value: B
Spirilliplanes.yamanashiensis
3
2
1
0
A (n=14)
B (n=15)
C (n=11)
surface

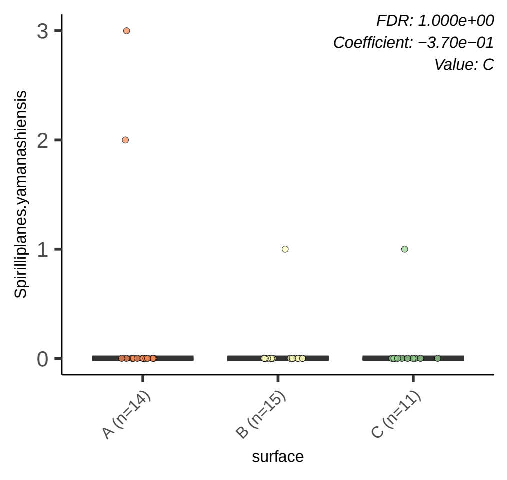
FDR: 1.000e+00
Coefficient: −3.70e−01
Value: C
Spirilliplanes.yamanashiensis
3
2
1
0
A (n=14)
B (n=15)
C (n=11)
surface

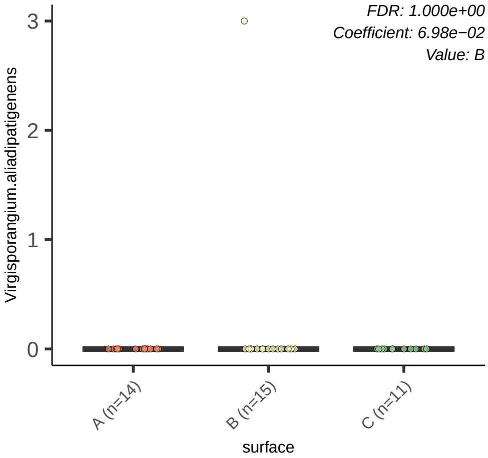

FDR: 1.000e+00
Coefficient: 6.98e−02
Value: B
Virgisporangium.aliadipatigenens
3
2
1
0
A (n=14)
B (n=15)
C (n=11)
surface

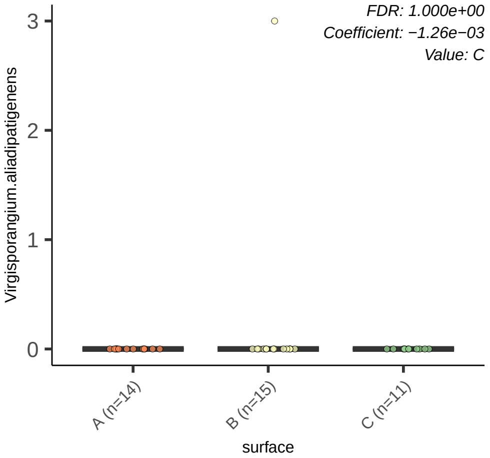
FDR: 1.000e+00
Coefficient: −1.26e−03
Value: C
Virgisporangium.aliadipatigenens
3
2
1
0
A (n=14)
B (n=15)
C (n=11)
surface

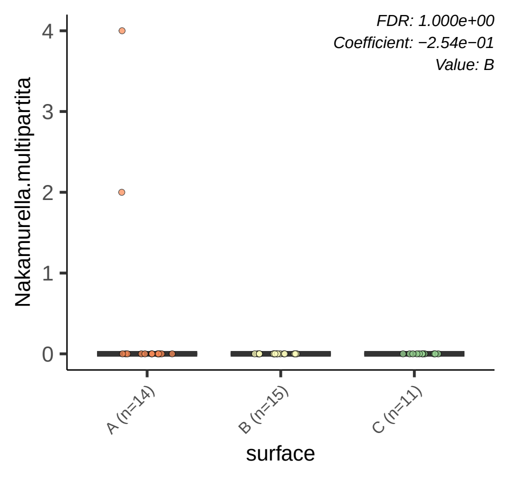
FDR: 1.000e+00
Coefficient: −2.54e−01
Value: B
Nakamurella.multipartita
0
1
2
3
4
A (n=14)
B (n=15)
C (n=11)
surface

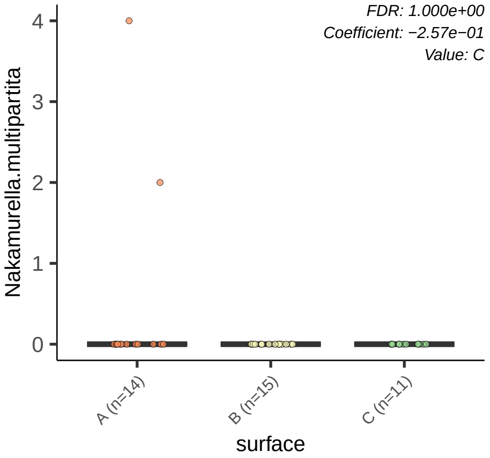
FDR: 1.000e+00
Coefficient: −2.57e−01
Value: C
Nakamurella.multipartita
0
1
2
3
4
A (n=14)
B (n=15)
C (n=11)
surface

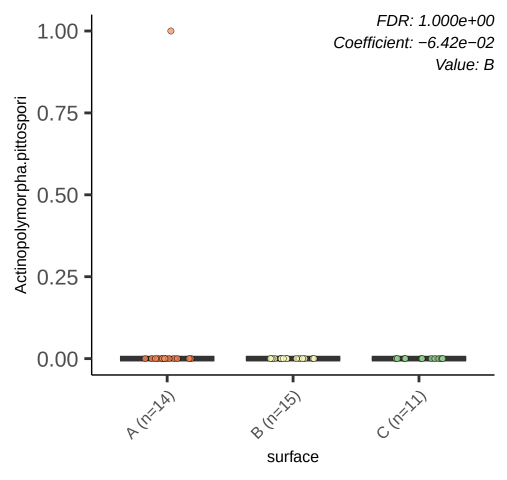
FDR: 1.000e+00
Coefficient: −6.42e−02
Value: B
Actinopolymorpha.pittospori
1.00
0.75
0.50
0.25
0.00
A (n=14)
B (n=15)
C (n=11)
surface

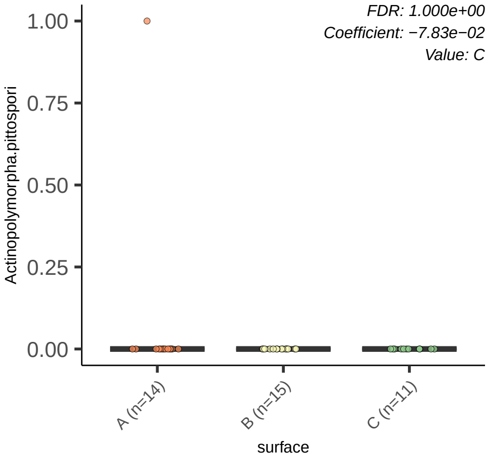

FDR: 1.000e+00
Coefficient: −7.83e−02
Value: C
Actinopolymorpha.pittospori
1.00
0.75
0.50
0.25
0.00
A (n=14)
B (n=15)
C (n=11)
surface

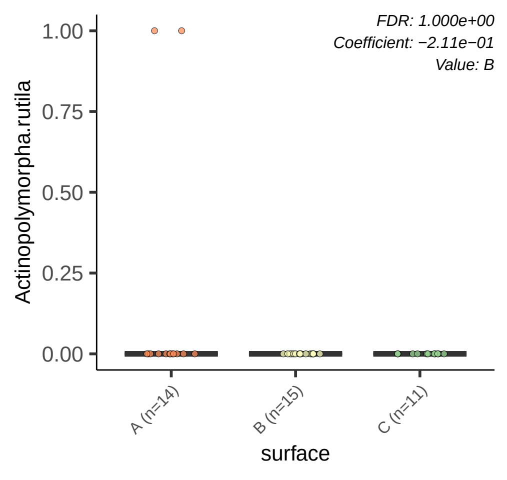

FDR: 1.000e+00
Coefficient: −2.11e−01
Value: B
Actinopolymorpha.rutila
1.00
0.75
0.50
0.25
0.00
A (n=14)
B (n=15)
C (n=11)
surface

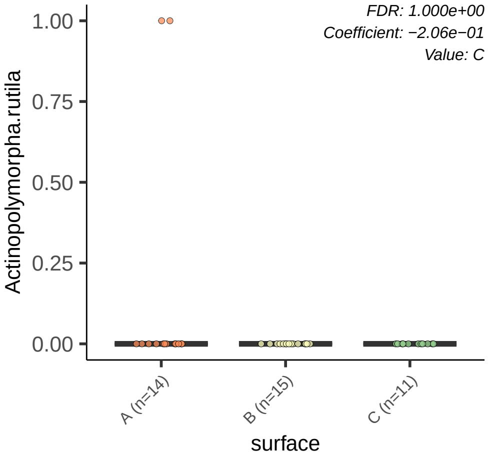
FDR: 1.000e+00
Coefficient: −2.06e−01
Value: C
Actinopolymorpha.rutila
1.00
0.75
0.50
0.25
0.00
A (n=14)
B (n=15)
C (n=11)
surface

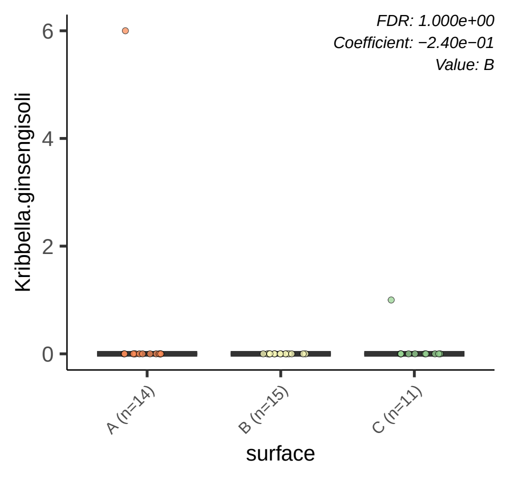

FDR: 1.000e+00
Coefficient: −2.40e−01
Value: B
Kribbella.ginsengisoli
6
4
2
0
A (n=14)
B (n=15)
C (n=11)
surface

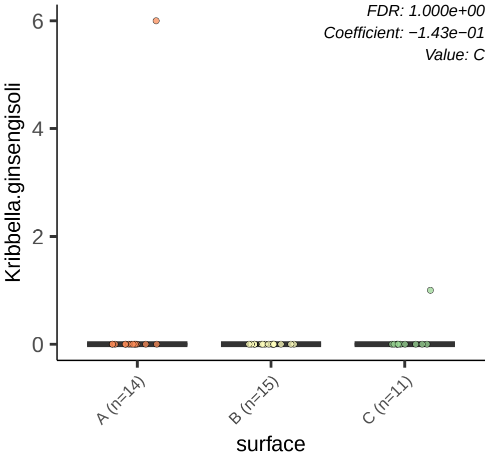
FDR: 1.000e+00
Coefficient: −1.43e−01
Value: C
Kribbella.ginsengisoli
0
2
4
6
A (n=14)
B (n=15)
C (n=11)
surface

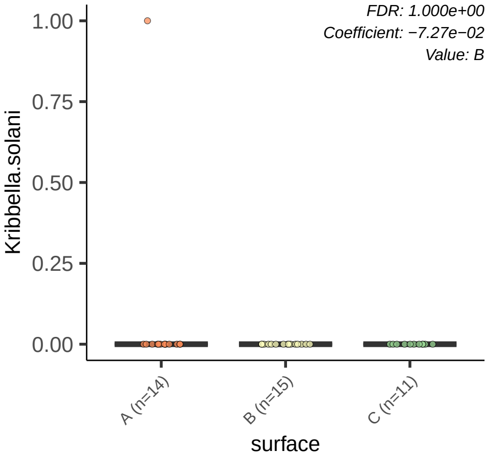
FDR: 1.000e+00
Coefficient: −7.27e−02
Value: B
Kribbella.solani
1.00
0.75
0.50
0.25
0.00
A (n=14)
B (n=15)
C (n=11)
surface

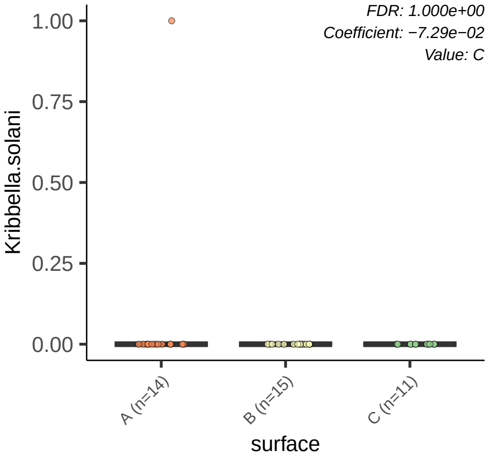

FDR: 1.000e+00
Coefficient: −7.29e−02
Value: C
Kribbella.solani
1.00
0.75
0.50
0.25
0.00
A (n=14)
B (n=15)
C (n=11)
surface

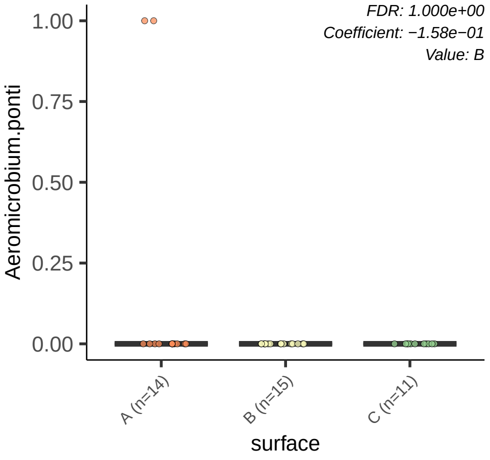
FDR: 1.000e+00
Coefficient: −1.58e−01
Value: B
Aeromicrobium.ponti
1.00
0.75
0.50
0.25
0.00
A (n=14)
B (n=15)
C (n=11)
surface

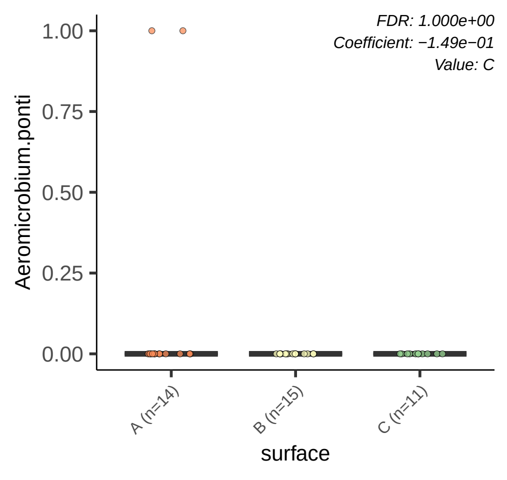
FDR: 1.000e+00
Coefficient: −1.49e−01
Value: C
Aeromicrobium.ponti
1.00
0.75
0.50
0.25
0.00
A (n=14)
B (n=15)
C (n=11)
surface

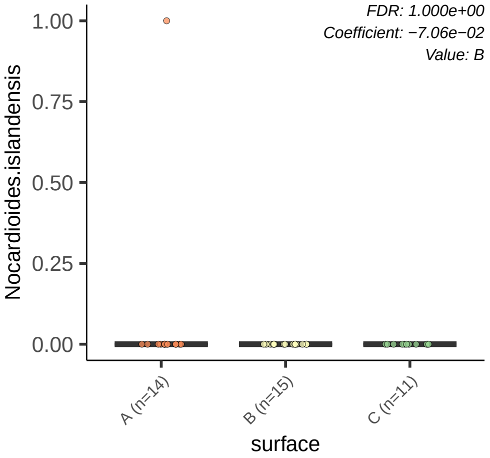
FDR: 1.000e+00
Coefficient: −7.06e−02
Value: B
Nocardioides.islandensis
1.00
0.75
0.50
0.25
0.00
A (n=14)
B (n=15)
C (n=11)
surface

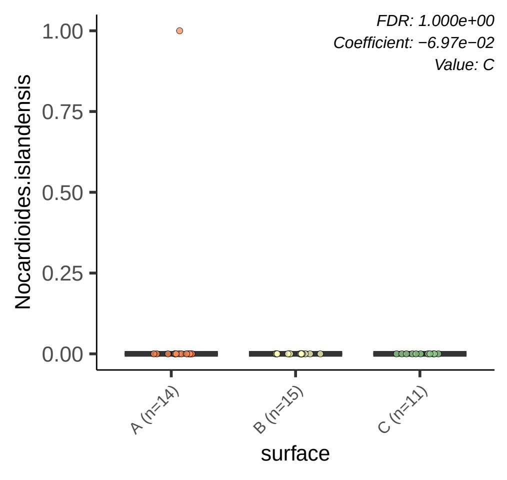
FDR: 1.000e+00
Coefficient: −6.97e−02
Value: C
Nocardioides.islandensis
1.00
0.75
0.50
0.25
0.00
A (n=14)
B (n=15)
C (n=11)
surface

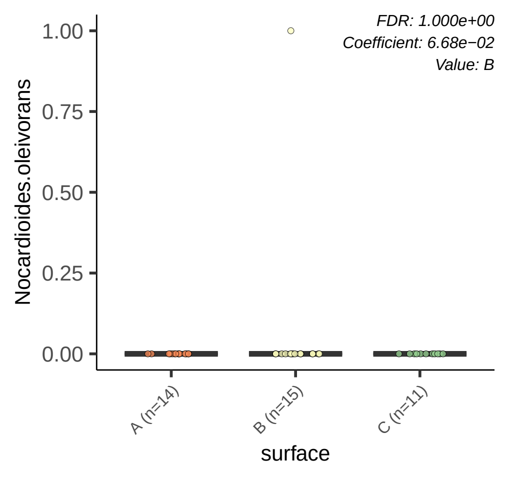
FDR: 1.000e+00
Coefficient: 6.68e−02
Value: B
Nocardioides.oleivorans
1.00
0.75
0.50
0.25
0.00
A (n=14)
B (n=15)
C (n=11)
surface

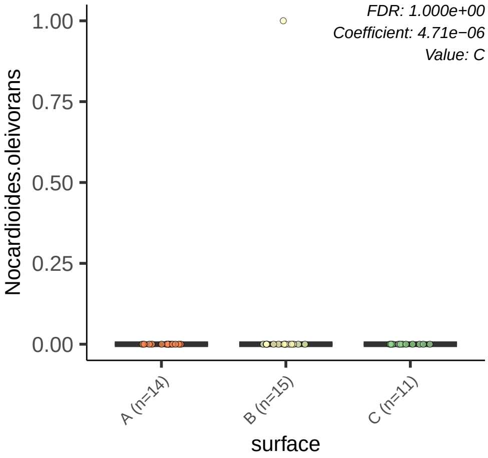
FDR: 1.000e+00
Coefficient: 4.71e−06
Value: C
Nocardioides.oleivorans
1.00
0.75
0.50
0.25
0.00
A (n=14)
B (n=15)
C (n=11)
surface

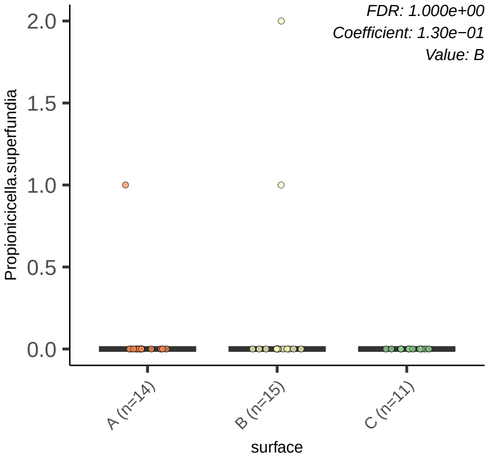

FDR: 1.000e+00
Coefficient: 1.30e−01
Value: B
Propionicicella.superfundia
2.0
1.5
1.0
0.5
0.0
A (n=14)
B (n=15)
C (n=11)
surface

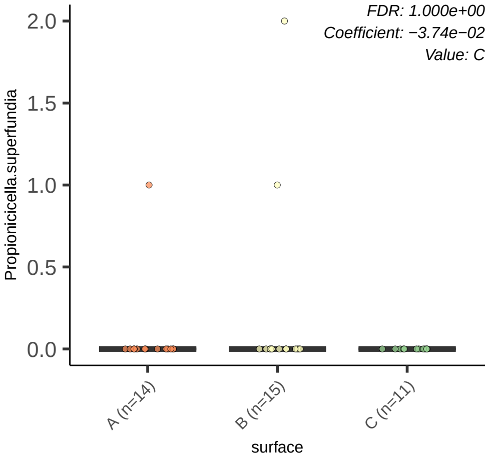
FDR: 1.000e+00
Coefficient: −3.74e−02
Value: C
Propionicicella.superfundia
2.0
1.5
1.0
0.5
0.0
A (n=14)
B (n=15)
C (n=11)
surface

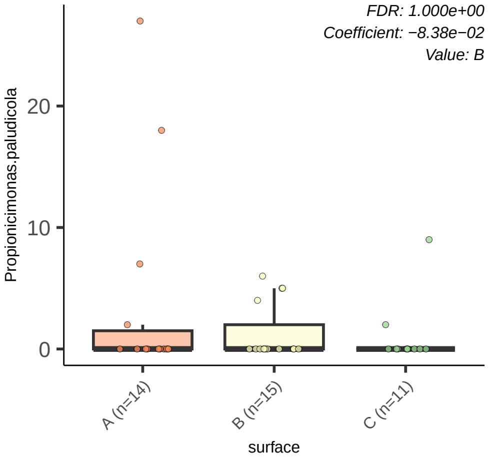

FDR: 1.000e+00
Coefficient: −8.38e−02
Value: B
Propionicimonas.paludicola
20
10
0
A (n=14)
B (n=15)
C (n=11)
surface

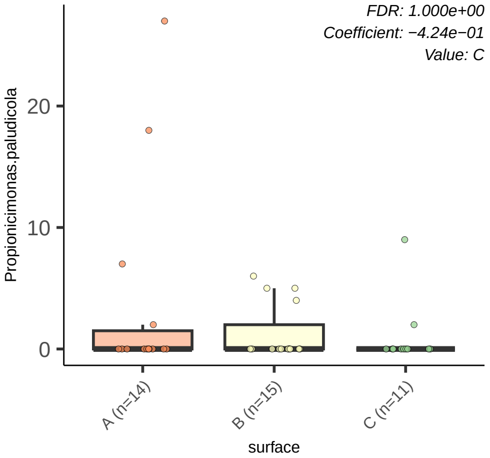
FDR: 1.000e+00
Coefficient: −4.24e−01
Value: C
Propionicimonas.paludicola
0
10
20
A (n=14)
B (n=15)
C (n=11)
surface

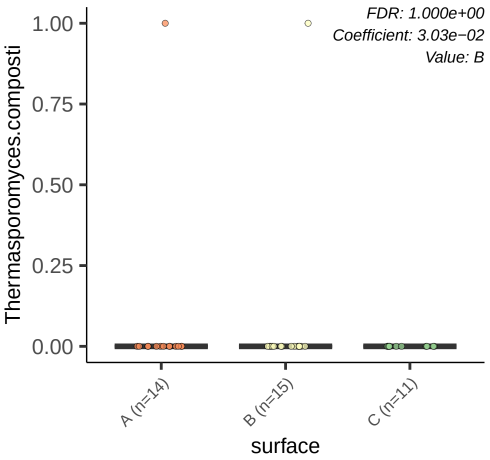
FDR: 1.000e+00
Coefficient: 3.03e−02
Value: B
Thermasporomyces.composti
1.00
0.75
0.50
0.25
0.00
A (n=14)
B (n=15)
C (n=11)
surface

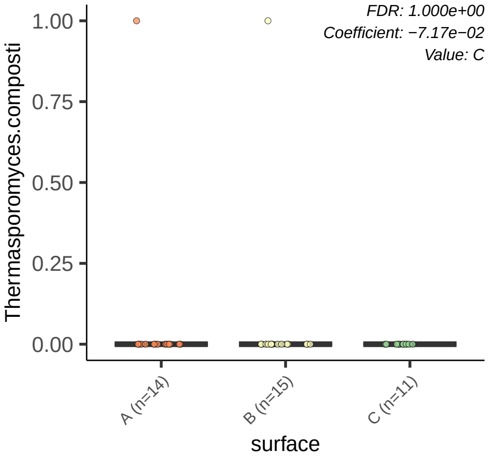

FDR: 1.000e+00
Coefficient: −7.17e−02
Value: C
Thermasporomyces.composti
1.00
0.75
0.50
0.25
0.00
A (n=14)
B (n=15)
C (n=11)
surface

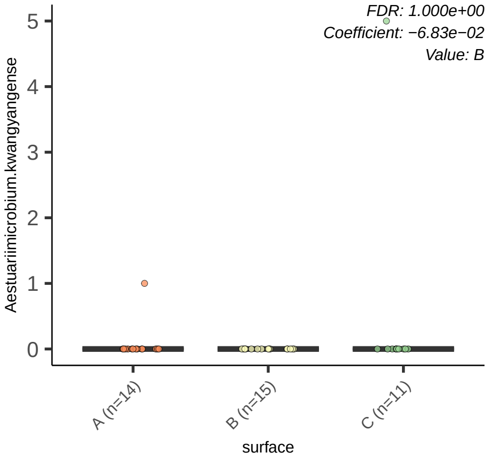

FDR: 1.000e+00
Coefficient: −6.83e−02
Value: B
Aestuariimicrobium.kwangyangense
0
1
2
3
4
5
A (n=14)
B (n=15)
C (n=11)
surface

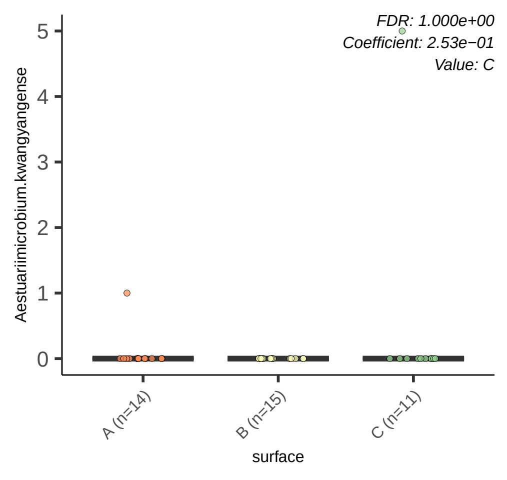

FDR: 1.000e+00
Coefficient: 2.53e−01
Value: C
Aestuariimicrobium.kwangyangense
0
1
2
3
4
5
A (n=14)
B (n=15)
C (n=11)
surface

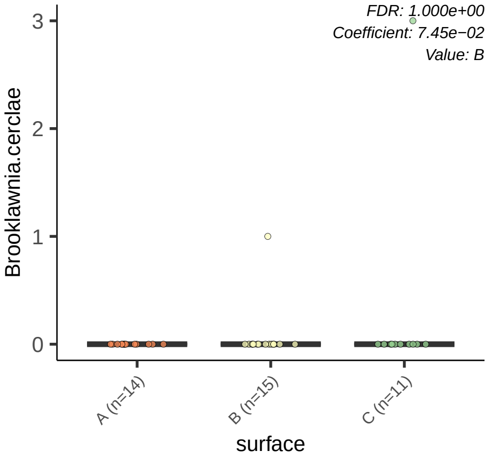

FDR: 1.000e+00
Coefficient: 7.45e−02
Value: B
Brooklawnia.cerclae
0
1
2
3
A (n=14)
B (n=15)
C (n=11)
surface

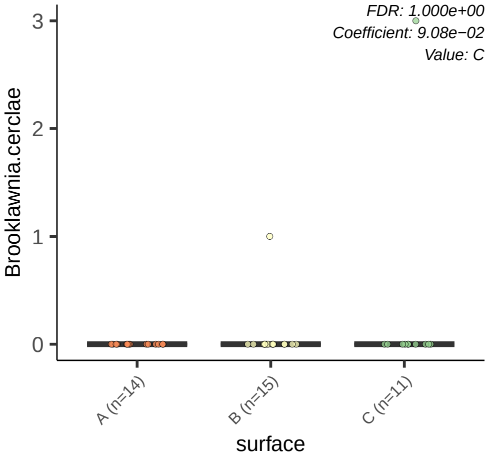
FDR: 1.000e+00
Coefficient: 9.08e−02
Value: C
Brooklawnia.cerclae
3
2
1
0
A (n=14)
B (n=15)
C (n=11)
surface

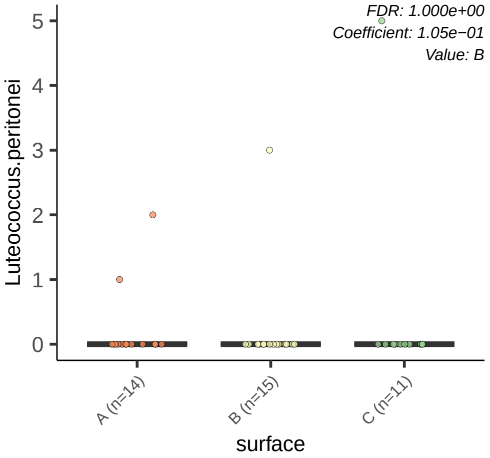

FDR: 1.000e+00
Coefficient: 1.05e−01
Value: B
Luteococcus.peritonei
0
1
2
3
4
5
A (n=14)
B (n=15)
C (n=11)
surface

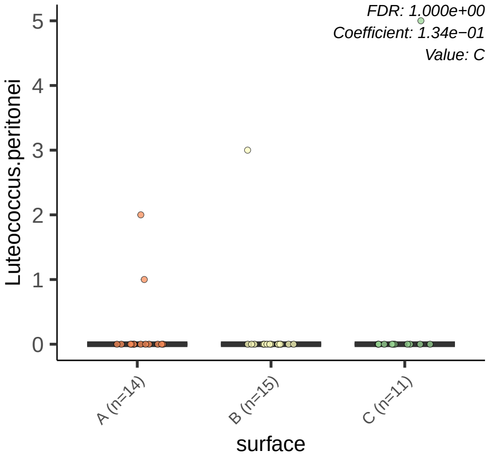

FDR: 1.000e+00
Coefficient: 1.34e−01
Value: C
Luteococcus.peritonei
0
1
2
3
4
5
A (n=14)
B (n=15)
C (n=11)
surface

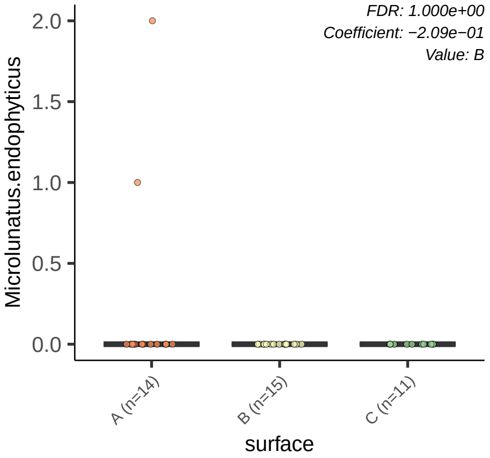
FDR: 1.000e+00
Coefficient: −2.09e−01
Value: B
Microlunatus.endophyticus
2.0
1.5
1.0
0.5
0.0
A (n=14)
B (n=15)
C (n=11)
surface

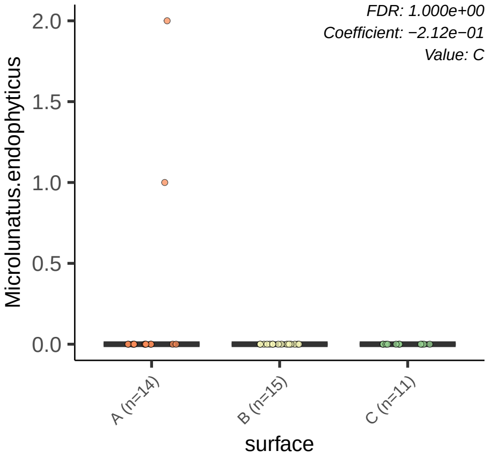
FDR: 1.000e+00
Coefficient: −2.12e−01
Value: C
Microlunatus.endophyticus
2.0
1.5
1.0
0.5
0.0
A (n=14)
B (n=15)
C (n=11)
surface

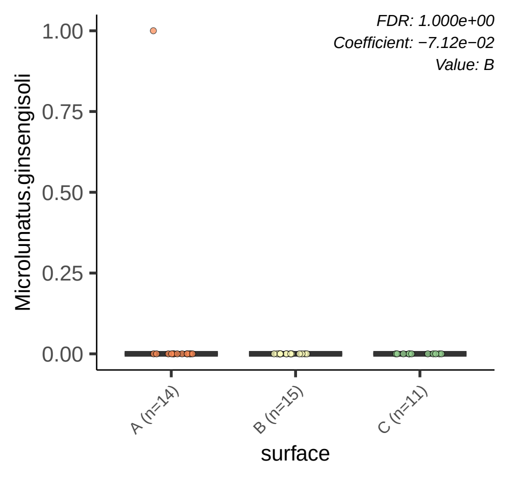
FDR: 1.000e+00
Coefficient: −7.12e−02
Value: B
Microlunatus.ginsengisoli
1.00
0.75
0.50
0.25
0.00
A (n=14)
B (n=15)
C (n=11)
surface

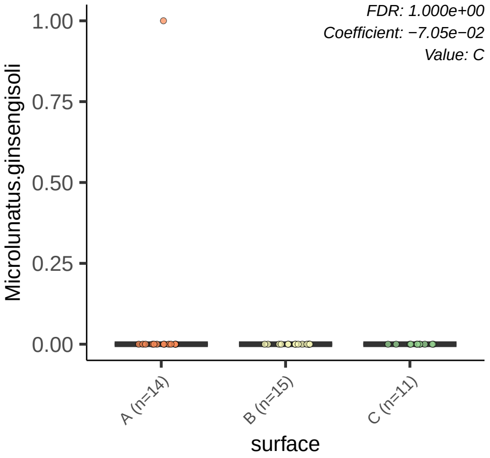
FDR: 1.000e+00
Coefficient: −7.05e−02
Value: C
Microlunatus.ginsengisoli
1.00
0.75
0.50
0.25
0.00
A (n=14)
B (n=15)
C (n=11)
surface

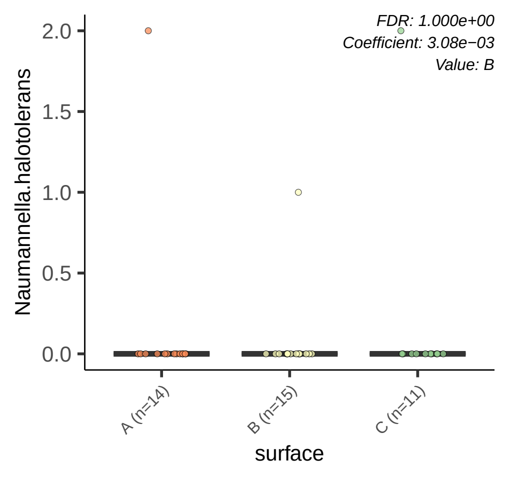
FDR: 1.000e+00
Coefficient: 3.08e−03
Value: B
Naumannella.halotolerans
2.0
1.5
1.0
0.5
0.0
A (n=14)
B (n=15)
C (n=11)
surface

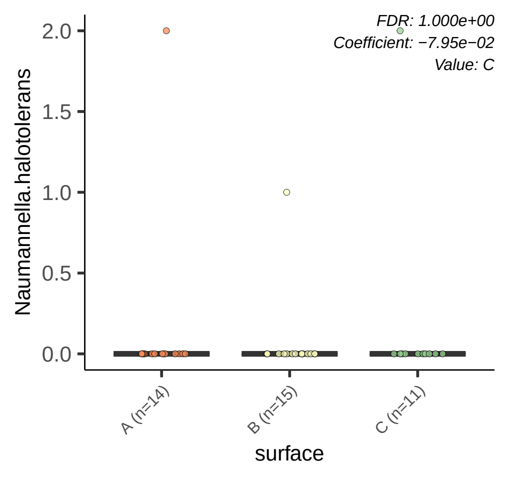
FDR: 1.000e+00
Coefficient: −7.95e−02
Value: C
Naumannella.halotolerans
2.0
1.5
1.0
0.5
0.0
A (n=14)
B (n=15)
C (n=11)
surface

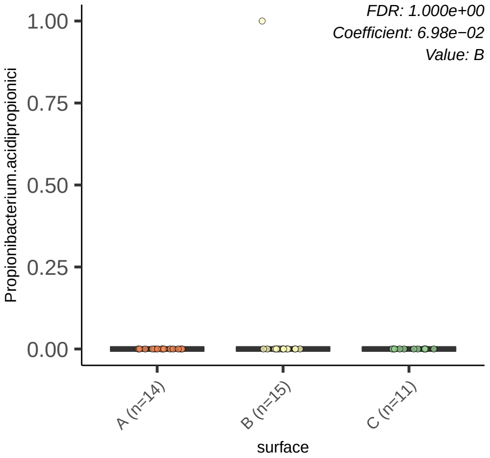
FDR: 1.000e+00
Coefficient: 6.98e−02
Value: B
1.00
0.75
0.50
0.25
0.00
Propionibacterium.acidipropionici
A (n=14)
B (n=15)
C (n=11)
surface

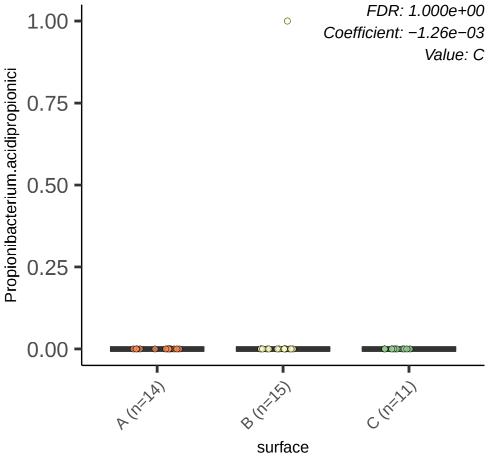

FDR: 1.000e+00
Coefficient: −1.26e−03
Value: C
Propionibacterium.acidipropionici
1.00
0.75
0.50
0.25
0.00
A (n=14)
B (n=15)
C (n=11)
surface

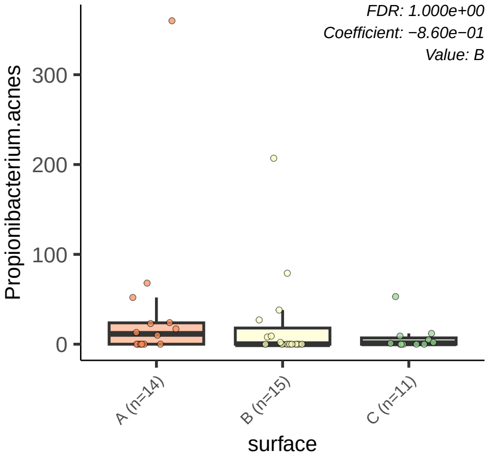

FDR: 1.000e+00
Coefficient: −8.60e−01
Value: B
Propionibacterium.acnes
300
200
100
0
A (n=14)
B (n=15)
C (n=11)
surface

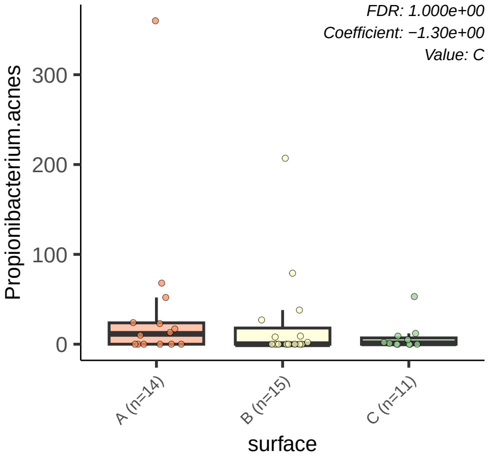

FDR: 1.000e+00
Coefficient: −1.30e+00
Value: C
Propionibacterium.acnes
300
200
100
0
A (n=14)
B (n=15)
C (n=11)
surface

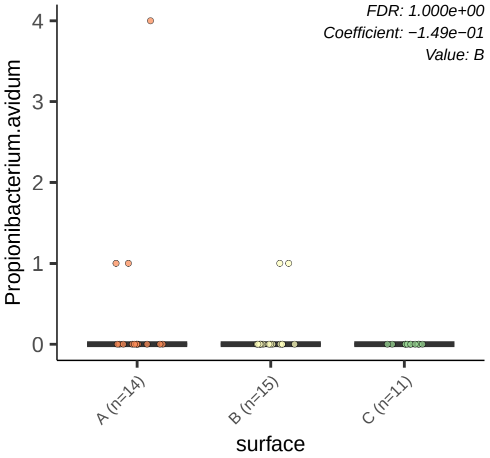
FDR: 1.000e+00
Coefficient: −1.49e−01
Value: B
Propionibacterium.avidum
4
3
2
1
0
A (n=14)
B (n=15)
C (n=11)
surface

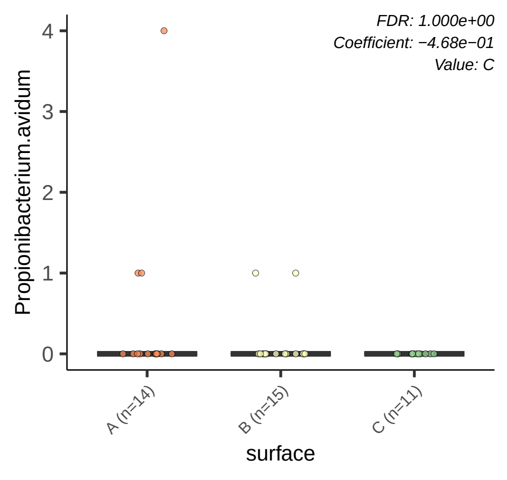
FDR: 1.000e+00
Coefficient: −4.68e−01
Value: C
Propionibacterium.avidum
0
1
2
3
4
A (n=14)
B (n=15)
C (n=11)
surface

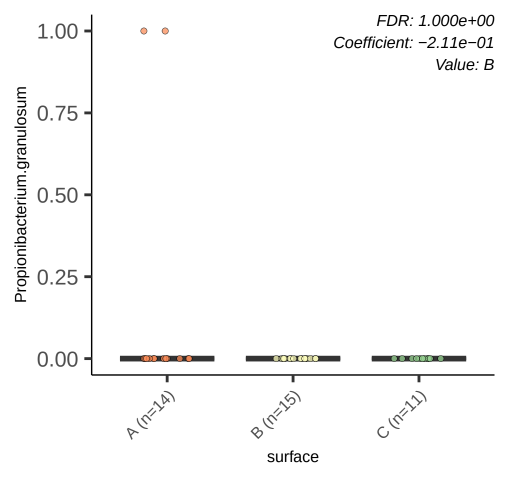
FDR: 1.000e+00
Coefficient: −2.11e−01
Value: B
Propionibacterium.granulosum
1.00
0.75
0.50
0.25
0.00
A (n=14)
B (n=15)
C (n=11)
surface

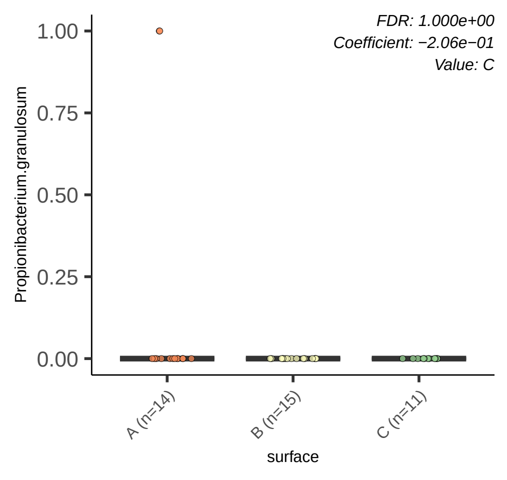
FDR: 1.000e+00
Coefficient: −2.06e−01
Value: C
Propionibacterium.granulosum
1.00
0.75
0.50
0.25
0.00
A (n=14)
B (n=15)
C (n=11)
surface

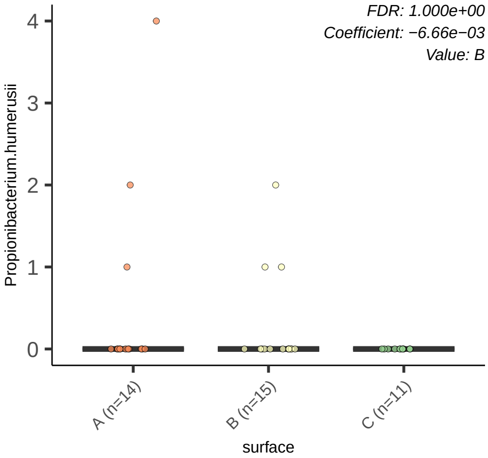

FDR: 1.000e+00
Coefficient: −6.66e−03
Value: B
Propionibacterium.humerusii
4
3
2
1
0
A (n=14)
B (n=15)
C (n=11)
surface

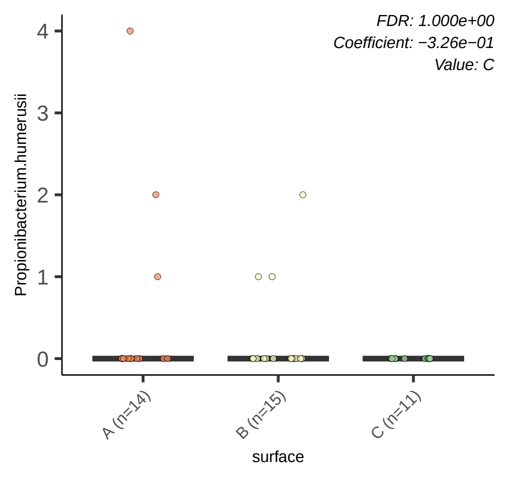

FDR: 1.000e+00
Coefficient: −3.26e−01
Value: C
Propionibacterium.humerusii
0
1
2
3
4
A (n=14)
B (n=15)
C (n=11)
surface

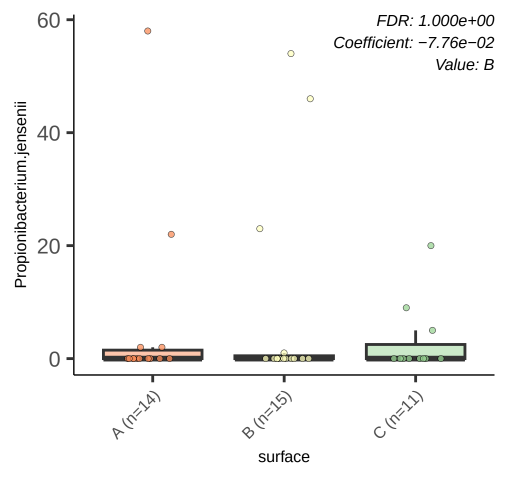

FDR: 1.000e+00
Coefficient: −7.76e−02
Value: B
Propionibacterium.jensenii
60
40
20
0
A (n=14)
B (n=15)
C (n=11)
surface

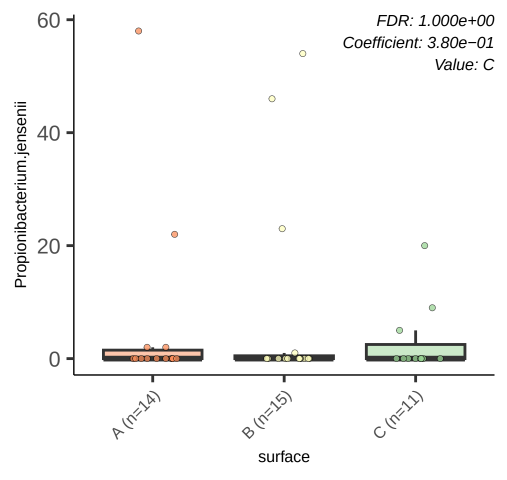

FDR: 1.000e+00
Coefficient: 3.80e−01
Value: C
Propionibacterium.jensenii
0
20
40
60
A (n=14)
B (n=15)
C (n=11)
surface

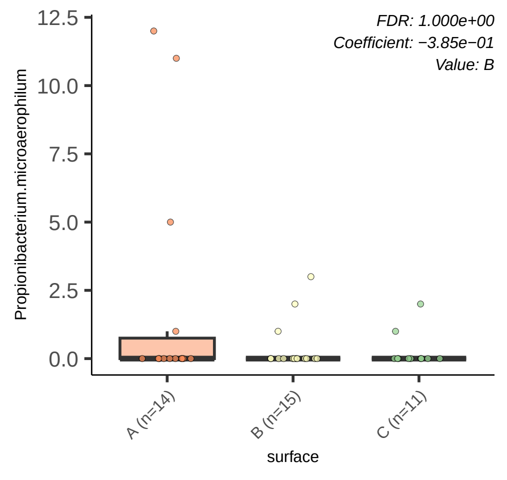

FDR: 1.000e+00
Coefficient: −3.85e−01
Value: B
12.5
10.0
7.5
5.0
2.5
0.0
Propionibacterium.microaerophilum
A (n=14)
B (n=15)
C (n=11)
surface

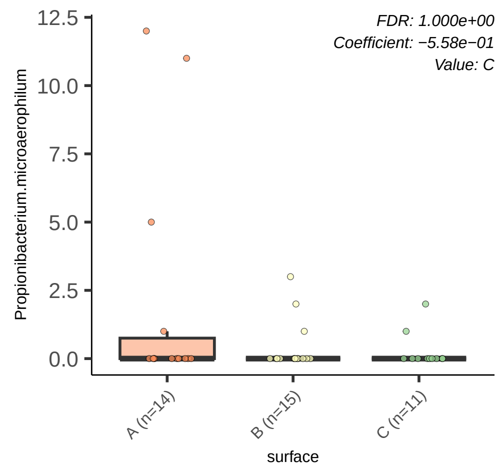

FDR: 1.000e+00
Coefficient: −5.58e−01
Value: C
12.5
10.0
7.5
5.0
2.5
0.0
Propionibacterium.microaerophilum
A (n=14)
B (n=15)
C (n=11)
surface

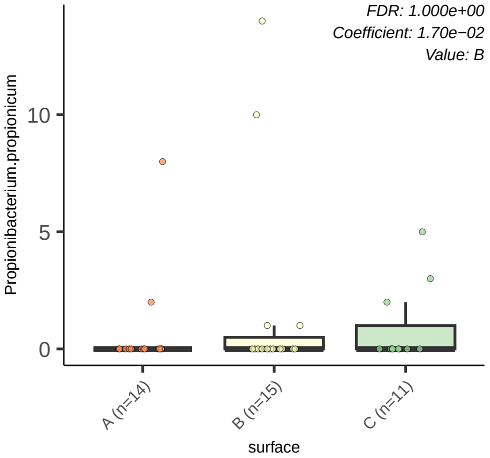
FDR: 1.000e+00
Coefficient: 1.70e−02
Value: B
Propionibacterium.propionicum
0
5
10
A (n=14)
B (n=15)
C (n=11)
surface

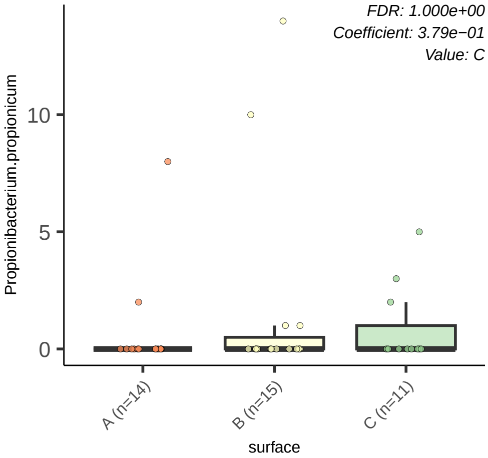

FDR: 1.000e+00
Coefficient: 3.79e−01
Value: C
Propionibacterium.propionicum
10
5
0
A (n=14)
B (n=15)
C (n=11)
surface

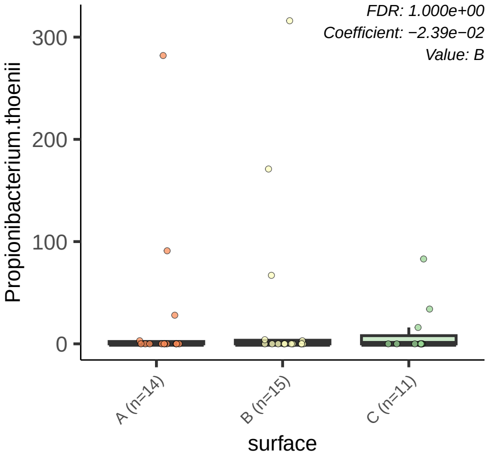

FDR: 1.000e+00
Coefficient: −2.39e−02
Value: B
Propionibacterium.thoenii
300
200
100
0
A (n=14)
B (n=15)
C (n=11)
surface

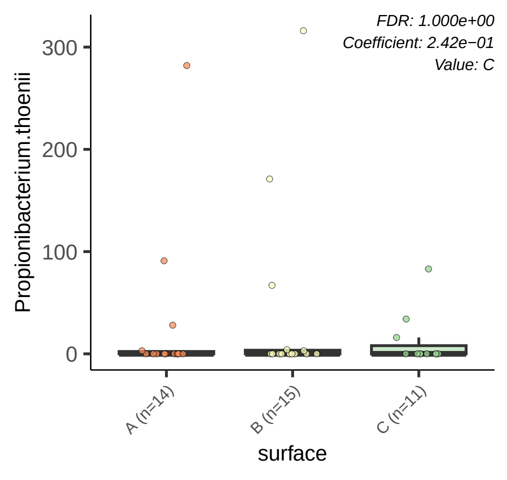

FDR: 1.000e+00
Coefficient: 2.42e−01
Value: C
Propionibacterium.thoenii
300
200
100
0
A (n=14)
B (n=15)
C (n=11)
surface

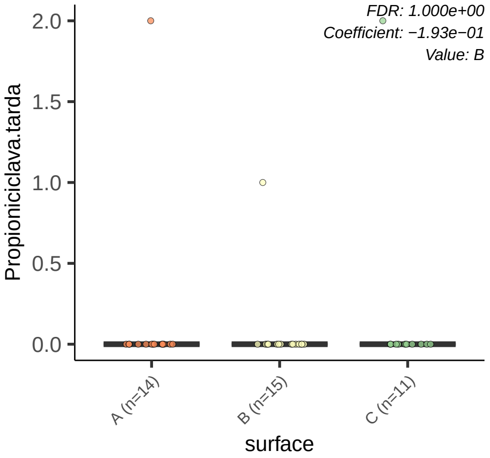
FDR: 1.000e+00
Coefficient: −1.93e−01
Value: B
Propioniciclava.tarda
2.0
1.5
1.0
0.5
0.0
A (n=14)
B (n=15)
C (n=11)
surface

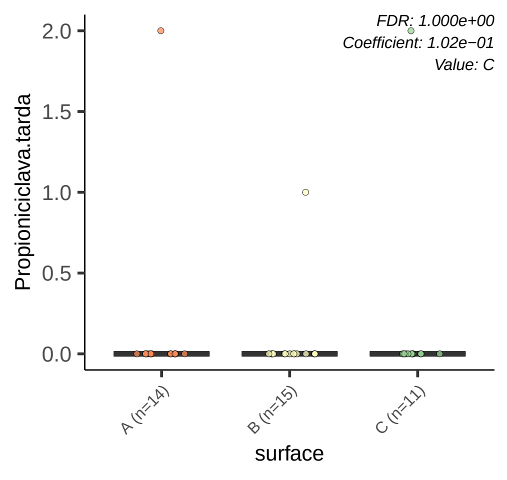

FDR: 1.000e+00
Coefficient: 1.02e−01
Value: C
Propioniciclava.tarda
2.0
1.5
1.0
0.5
0.0
A (n=14)
B (n=15)
C (n=11)
surface

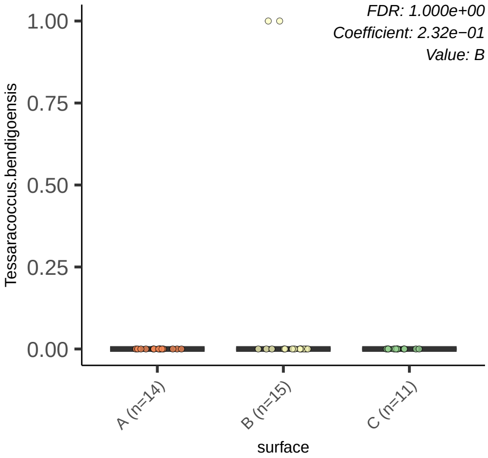

FDR: 1.000e+00
Coefficient: 2.32e−01
Value: B
Tessaracoccus.bendigoensis
1.00
0.75
0.50
0.25
0.00
A (n=14)
B (n=15)
C (n=11)
surface

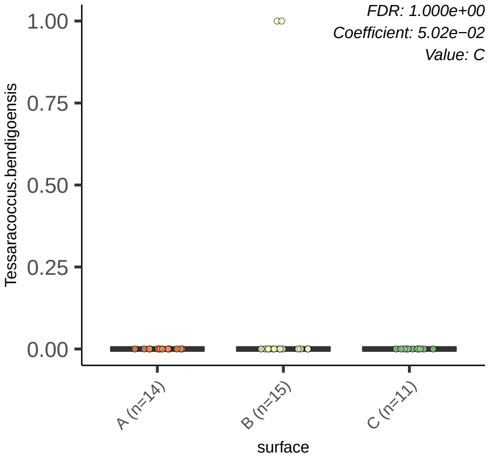

FDR: 1.000e+00
Coefficient: 5.02e−02
Value: C
Tessaracoccus.bendigoensis
1.00
0.75
0.50
0.25
0.00
A (n=14)
B (n=15)
C (n=11)
surface

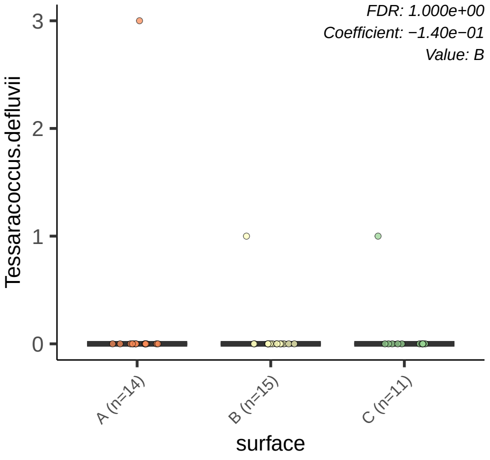
FDR: 1.000e+00
Coefficient: −1.40e−01
Value: B
Tessaracoccus.defluvii
3
2
1
0
A (n=14)
B (n=15)
C (n=11)
surface

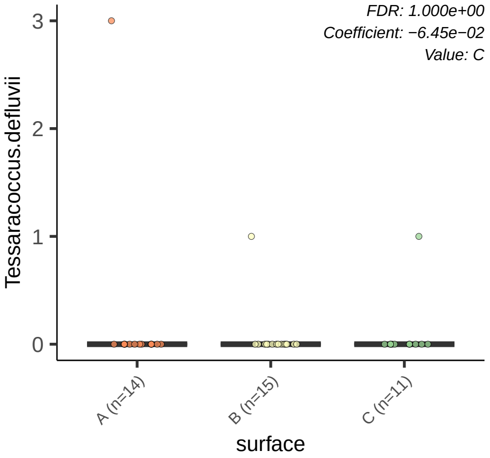
FDR: 1.000e+00
Coefficient: −6.45e−02
Value: C
Tessaracoccus.defluvii
3
2
1
0
A (n=14)
B (n=15)
C (n=11)
surface

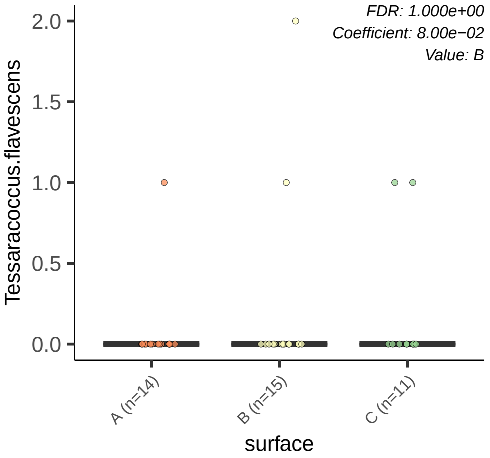

FDR: 1.000e+00
Coefficient: 8.00e−02
Value: B
Tessaracoccus.flavescens
2.0
1.5
1.0
0.5
0.0
A (n=14)
B (n=15)
C (n=11)
surface

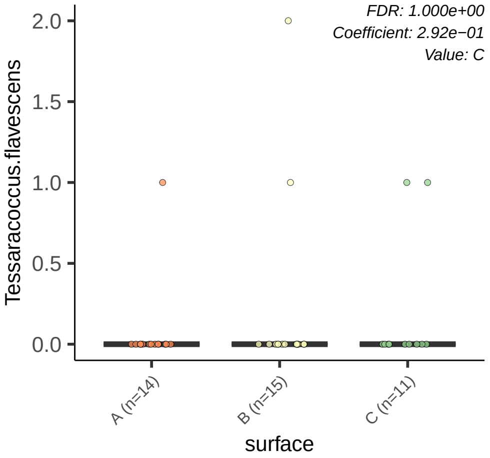
FDR: 1.000e+00
Coefficient: 2.92e−01
Value: C
Tessaracoccus.flavescens
2.0
1.5
1.0
0.5
0.0
A (n=14)
B (n=15)
C (n=11)
surface

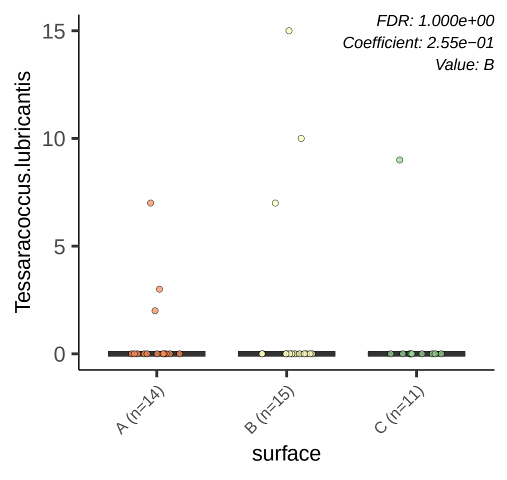
FDR: 1.000e+00
Coefficient: 2.55e−01
Value: B
Tessaracoccus.lubricantis
15
10
5
0
A (n=14)
B (n=15)
C (n=11)
surface

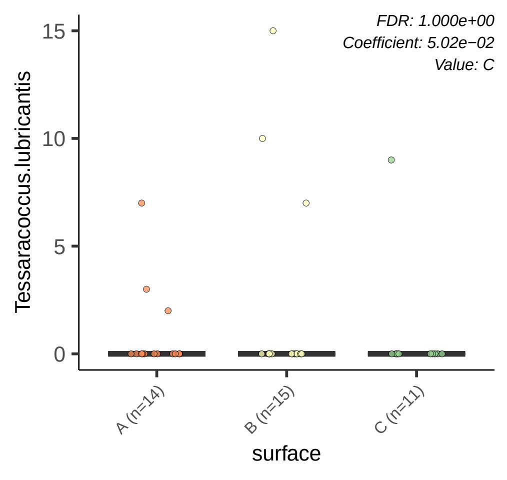
FDR: 1.000e+00
Coefficient: 5.02e−02
Value: C
Tessaracoccus.lubricantis
15
10
5
0
A (n=14)
B (n=15)
C (n=11)
surface

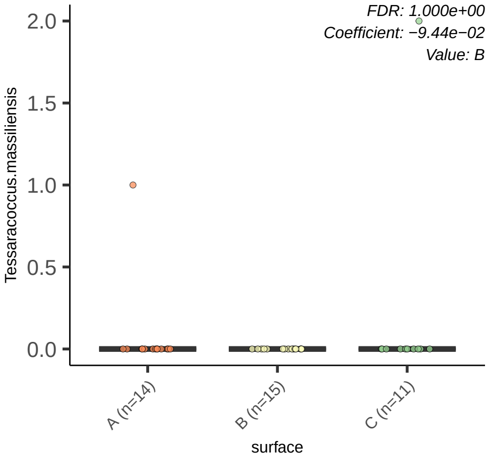

FDR: 1.000e+00
Coefficient: −9.44e−02
Value: B
Tessaracoccus.massiliensis
2.0
1.5
1.0
0.5
0.0
A (n=14)
B (n=15)
C (n=11)
surface

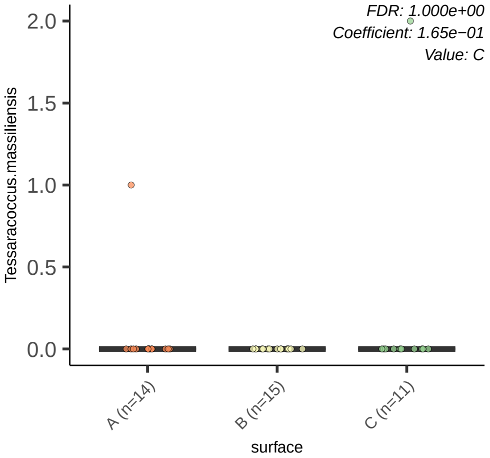
FDR: 1.000e+00
Coefficient: 1.65e−01
Value: C
2.0
1.5
1.0
0.5
0.0
Tessaracoccus.massiliensis
A (n=14)
B (n=15)
C (n=11)
surface

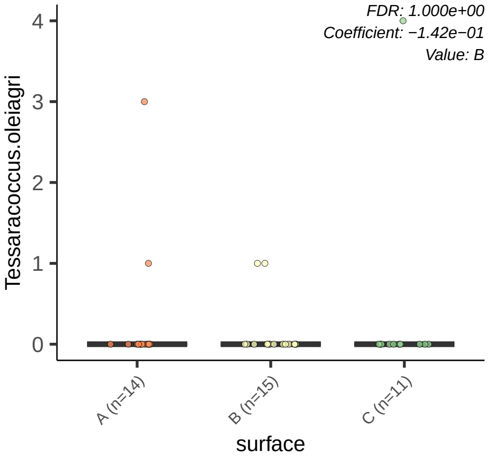

FDR: 1.000e+00
Coefficient: −1.42e−01
Value: B
Tessaracoccus.oleiagri
4
3
2
1
0
A (n=14)
B (n=15)
C (n=11)
surface

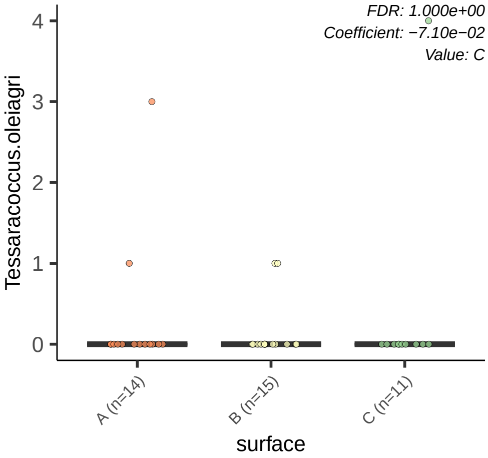
FDR: 1.000e+00
Coefficient: −7.10e−02
Value: C
Tessaracoccus.oleiagri
0
1
2
3
4
A (n=14)
B (n=15)
C (n=11)
surface

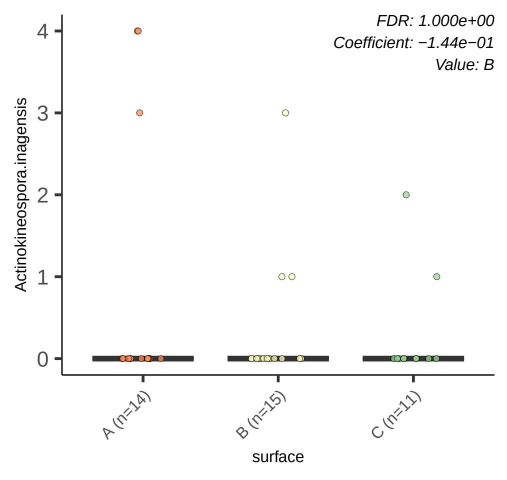
FDR: 1.000e+00
Coefficient: −1.44e−01
Value: B
Actinokineospora.inagensis
0
1
2
3
4
A (n=14)
B (n=15)
C (n=11)
surface

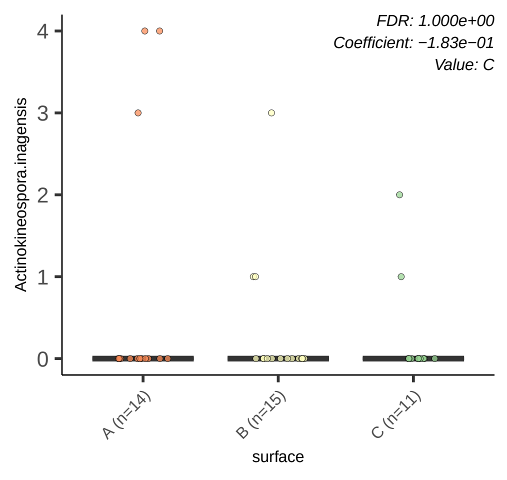
FDR: 1.000e+00
Coefficient: −1.83e−01
Value: C
Actinokineospora.inagensis
4
3
2
1
0
A (n=14)
B (n=15)
C (n=11)
surface

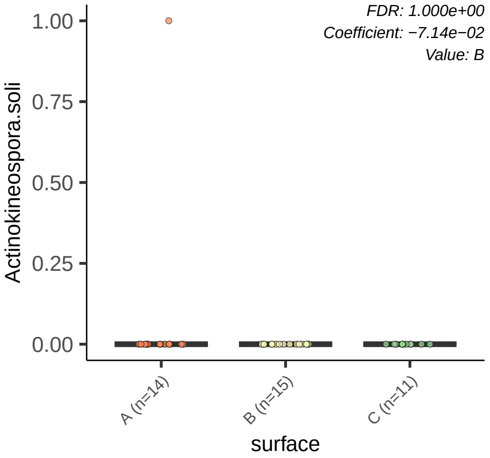
FDR: 1.000e+00
Coefficient: −7.14e−02
Value: B
Actinokineospora.soli
1.00
0.75
0.50
0.25
0.00
A (n=14)
B (n=15)
C (n=11)
surface

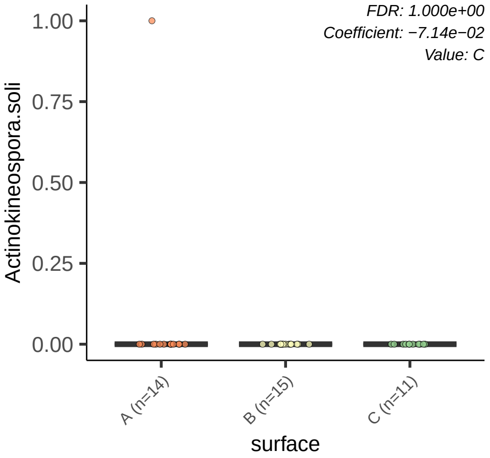
FDR: 1.000e+00
Coefficient: −7.14e−02
Value: C
Actinokineospora.soli
1.00
0.75
0.50
0.25
0.00
A (n=14)
B (n=15)
C (n=11)
surface

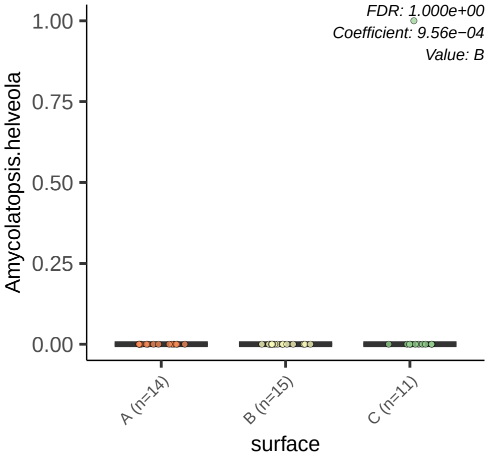
FDR: 1.000e+00
Coefficient: 9.56e−04
Value: B
Amycolatopsis.helveola
1.00
0.75
0.50
0.25
0.00
A (n=14)
B (n=15)
C (n=11)
surface

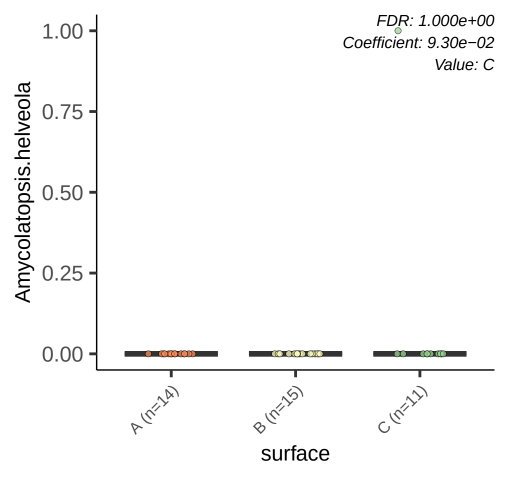
FDR: 1.000e+00
Coefficient: 9.30e−02
Value: C
Amycolatopsis.helveola
1.00
0.75
0.50
0.25
0.00
A (n=14)
B (n=15)
C (n=11)
surface

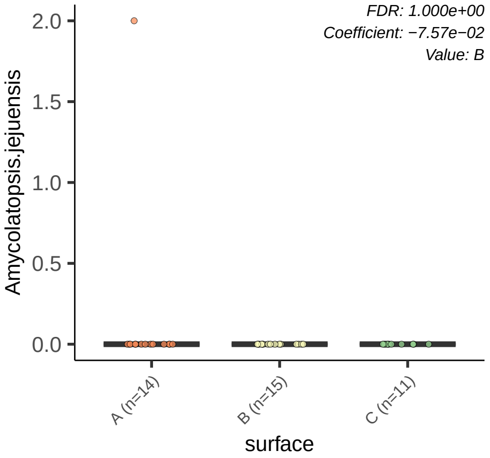

FDR: 1.000e+00
Coefficient: −7.57e−02
Value: B
Amycolatopsis.jejuensis
2.0
1.5
1.0
0.5
0.0
A (n=14)
B (n=15)
C (n=11)
surface

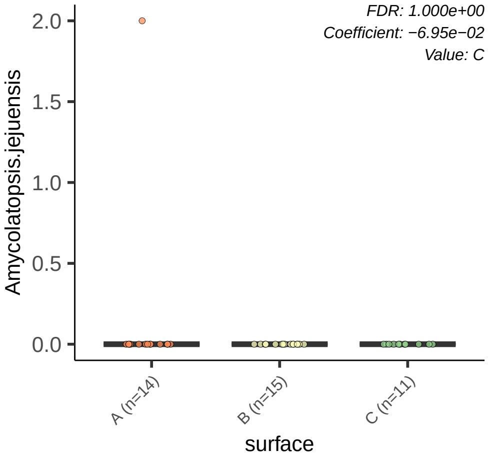

FDR: 1.000e+00
Coefficient: −6.95e−02
Value: C
Amycolatopsis.jejuensis
2.0
1.5
1.0
0.5
0.0
A (n=14)
B (n=15)
C (n=11)
surface

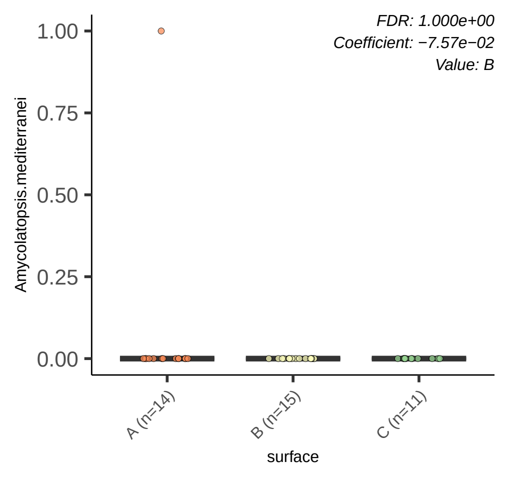

FDR: 1.000e+00
Coefficient: −7.57e−02
Value: B
Amycolatopsis.mediterranei
1.00
0.75
0.50
0.25
0.00
A (n=14)
B (n=15)
C (n=11)
surface

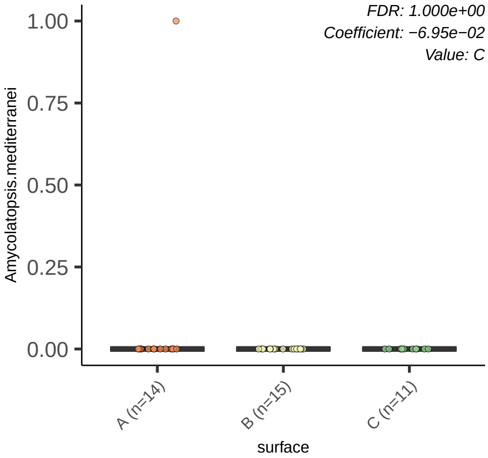

FDR: 1.000e+00
Coefficient: −6.95e−02
Value: C
1.00
0.75
0.50
0.25
0.00
Amycolatopsis.mediterranei
A (n=14)
B (n=15)
C (n=11)
surface

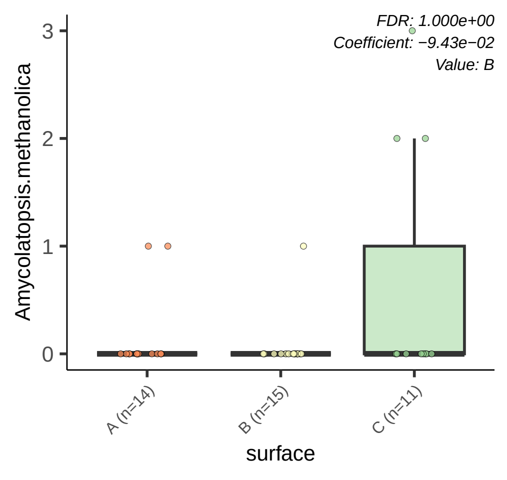

FDR: 1.000e+00
Coefficient: −9.43e−02
Value: B
Amycolatopsis.methanolica
3
2
1
0
A (n=14)
B (n=15)
C (n=11)
surface

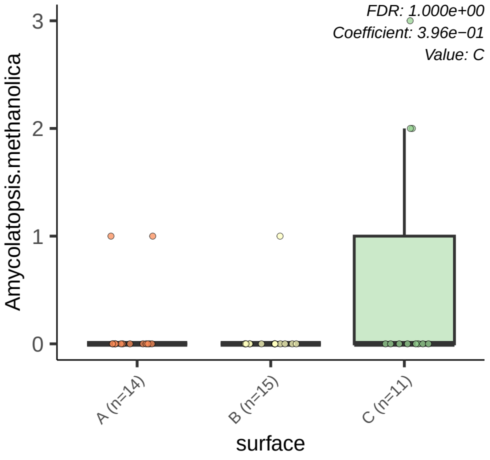
FDR: 1.000e+00
Coefficient: 3.96e−01
Value: C
Amycolatopsis.methanolica
3
2
1
0
A (n=14)
B (n=15)
C (n=11)
surface

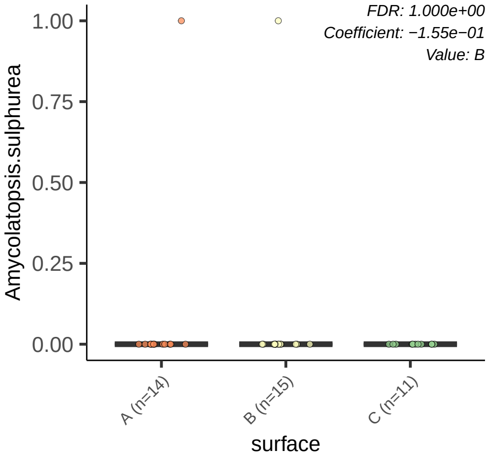

FDR: 1.000e+00
Coefficient: −1.55e−01
Value: B
Amycolatopsis.sulphurea
1.00
0.75
0.50
0.25
0.00
A (n=14)
B (n=15)
C (n=11)
surface

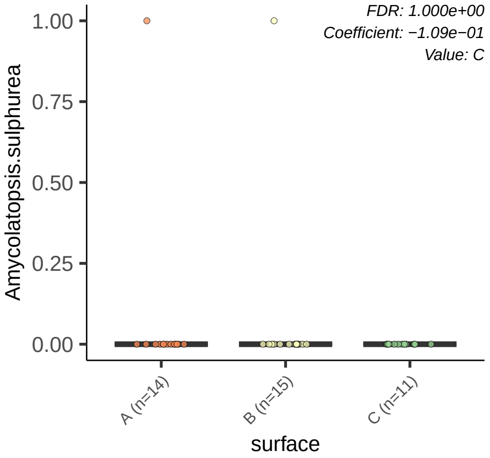

FDR: 1.000e+00
Coefficient: −1.09e−01
Value: C
Amycolatopsis.sulphurea
1.00
0.75
0.50
0.25
0.00
A (n=14)
B (n=15)
C (n=11)
surface

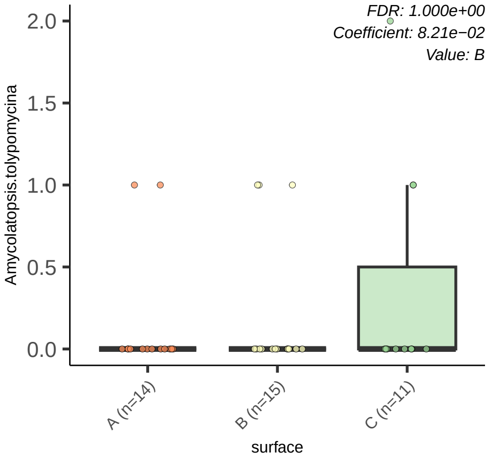

FDR: 1.000e+00
Coefficient: 8.21e−02
Value: B
Amycolatopsis.tolypomycina
2.0
1.5
1.0
0.5
0.0
A (n=14)
B (n=15)
C (n=11)
surface

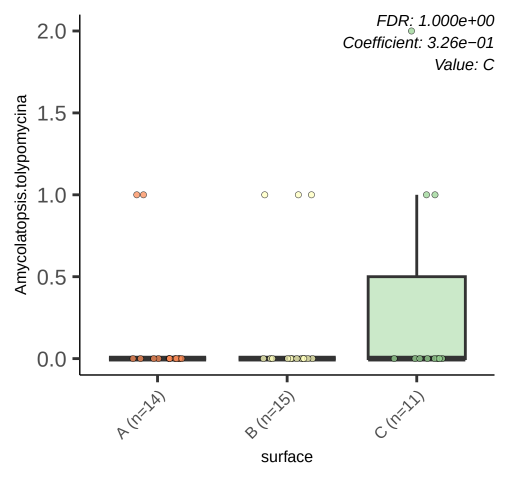

FDR: 1.000e+00
Coefficient: 3.26e−01
Value: C
Amycolatopsis.tolypomycina
2.0
1.5
1.0
0.5
0.0
A (n=14)
B (n=15)
C (n=11)
surface

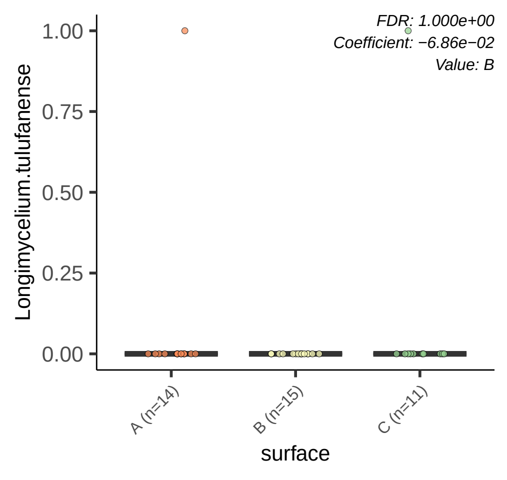

FDR: 1.000e+00
Coefficient: −6.86e−02
Value: B
Longimycelium.tulufanense
1.00
0.75
0.50
0.25
0.00
A (n=14)
B (n=15)
C (n=11)
surface

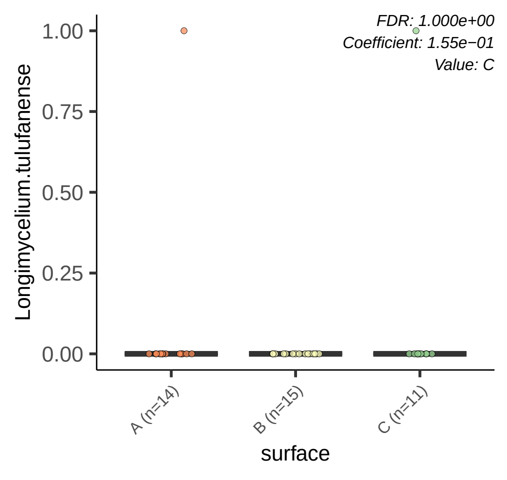
FDR: 1.000e+00
Coefficient: 1.55e−01
Value: C
Longimycelium.tulufanense
1.00
0.75
0.50
0.25
0.00
A (n=14)
B (n=15)
C (n=11)
surface

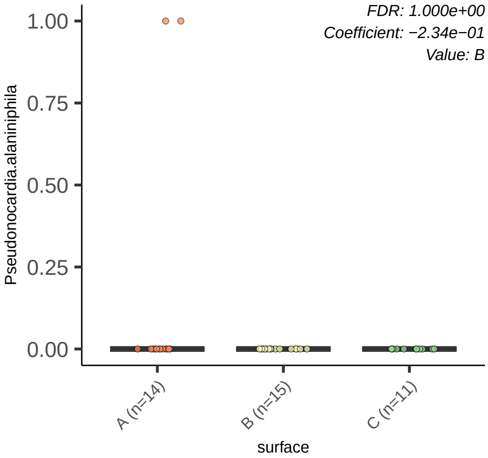
FDR: 1.000e+00
Coefficient: −2.34e−01
Value: B
1.00
0.75
0.50
0.25
0.00
Pseudonocardia.alaniniphila
A (n=14)
B (n=15)
C (n=11)
surface

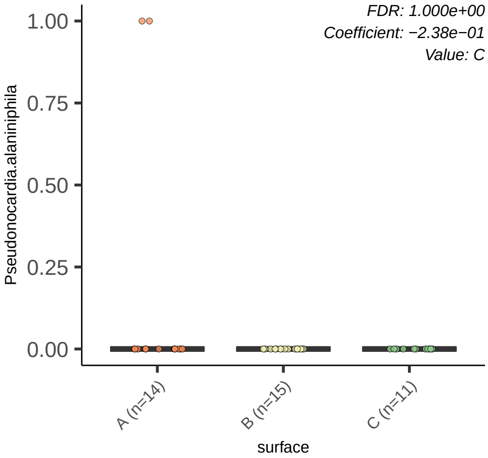
FDR: 1.000e+00
Coefficient: −2.38e−01
Value: C
Pseudonocardia.alaniniphila
1.00
0.75
0.50
0.25
0.00
A (n=14)
B (n=15)
C (n=11)
surface

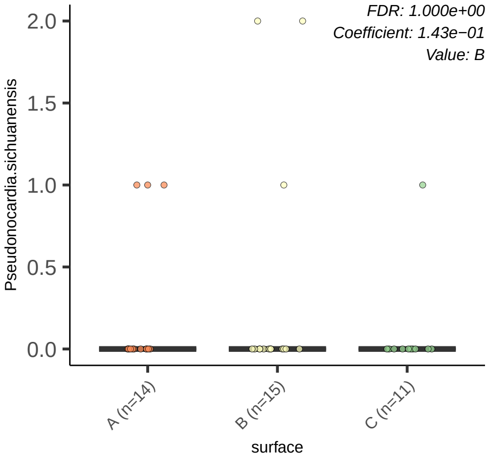

FDR: 1.000e+00
Coefficient: 1.43e−01
Value: B
Pseudonocardia.sichuanensis
2.0
1.5
1.0
0.5
0.0
A (n=14)
B (n=15)
C (n=11)
surface

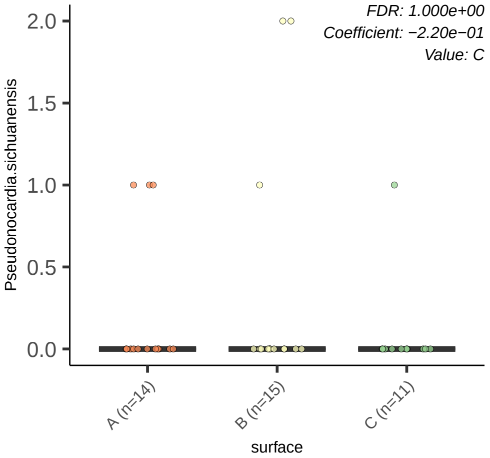

FDR: 1.000e+00
Coefficient: −2.20e−01
Value: C
Pseudonocardia.sichuanensis
2.0
1.5
1.0
0.5
0.0
A (n=14)
B (n=15)
C (n=11)
surface

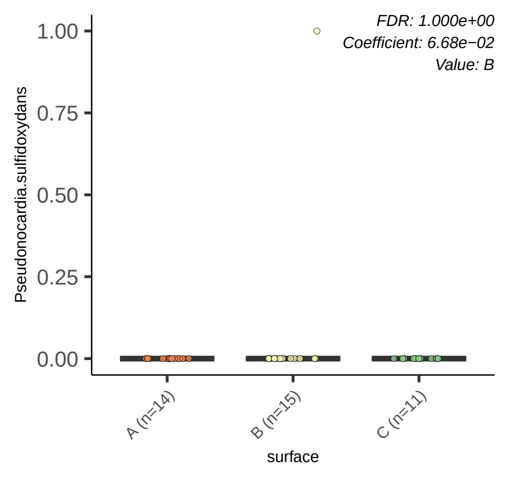

FDR: 1.000e+00
Coefficient: 6.68e−02
Value: B
Pseudonocardia.sulfidoxydans
1.00
0.75
0.50
0.25
0.00
A (n=14)
B (n=15)
C (n=11)
surface

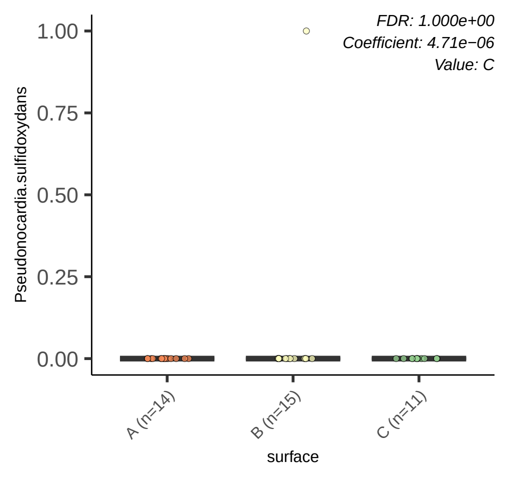
FDR: 1.000e+00
Coefficient: 4.71e−06
Value: C
1.00
0.75
0.50
0.25
0.00
Pseudonocardia.sulfidoxydans
A (n=14)
B (n=15)
C (n=11)
surface

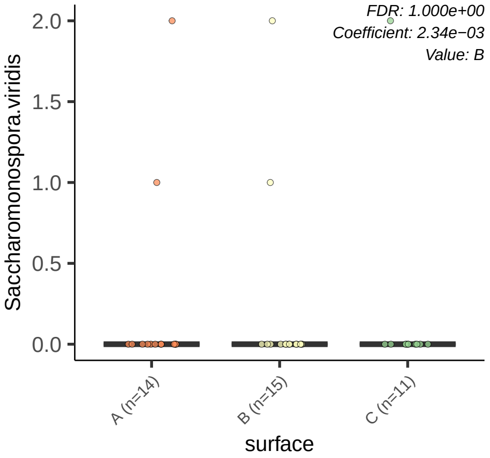
FDR: 1.000e+00
Coefficient: 2.34e−03
Value: B
Saccharomonospora.viridis
2.0
1.5
1.0
0.5
0.0
A (n=14)
B (n=15)
C (n=11)
surface

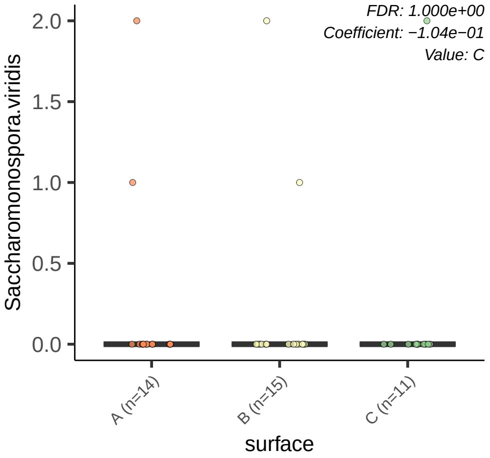

FDR: 1.000e+00
Coefficient: −1.04e−01
Value: C
Saccharomonospora.viridis
2.0
1.5
1.0
0.5
0.0
A (n=14)
B (n=15)
C (n=11)
surface

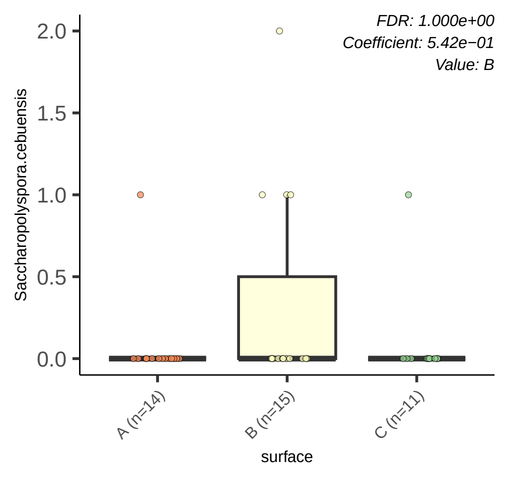

FDR: 1.000e+00
Coefficient: 5.42e−01
Value: B
Saccharopolyspora.cebuensis
2.0
1.5
1.0
0.5
0.0
A (n=14)
B (n=15)
C (n=11)
surface

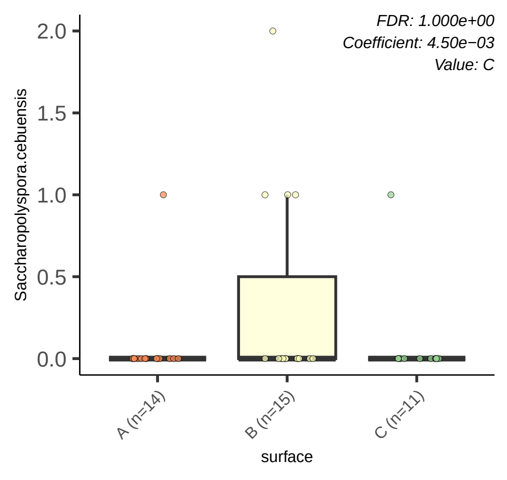
FDR: 1.000e+00
Coefficient: 4.50e−03
Value: C
Saccharopolyspora.cebuensis
2.0
1.5
1.0
0.5
0.0
A (n=14)
B (n=15)
C (n=11)
surface

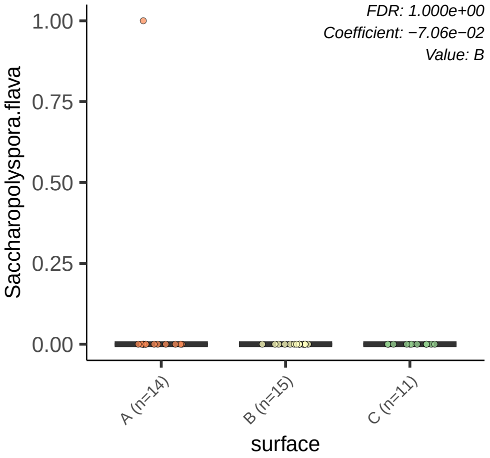

FDR: 1.000e+00
Coefficient: −7.06e−02
Value: B
Saccharopolyspora.flava
1.00
0.75
0.50
0.25
0.00
A (n=14)
B (n=15)
C (n=11)
surface

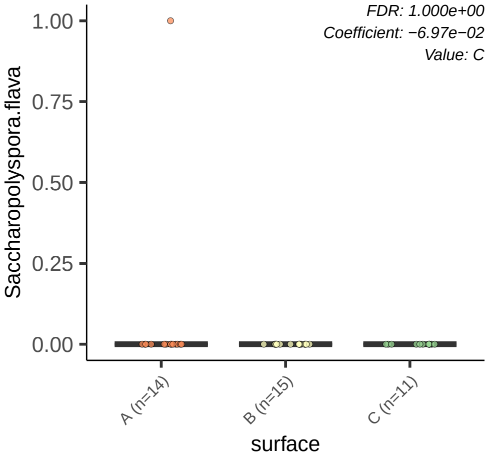

FDR: 1.000e+00
Coefficient: −6.97e−02
Value: C
Saccharopolyspora.flava
1.00
0.75
0.50
0.25
0.00
A (n=14)
B (n=15)
C (n=11)
surface

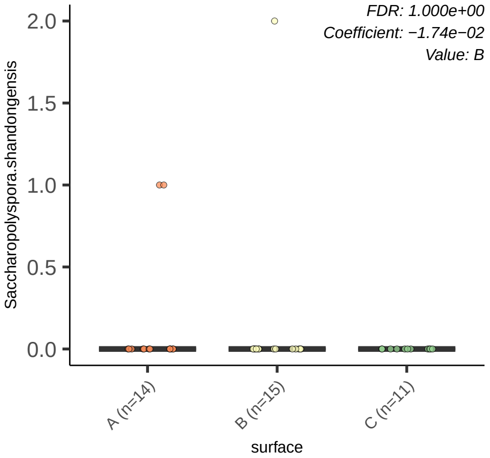
FDR: 1.000e+00
Coefficient: −1.74e−02
Value: B
Saccharopolyspora.shandongensis
2.0
1.5
1.0
0.5
0.0
A (n=14)
B (n=15)
C (n=11)
surface

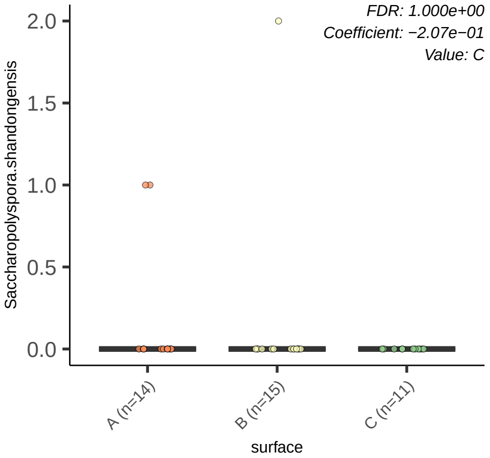
FDR: 1.000e+00
Coefficient: −2.07e−01
Value: C
Saccharopolyspora.shandongensis
2.0
1.5
1.0
0.5
0.0
A (n=14)
B (n=15)
C (n=11)
surface

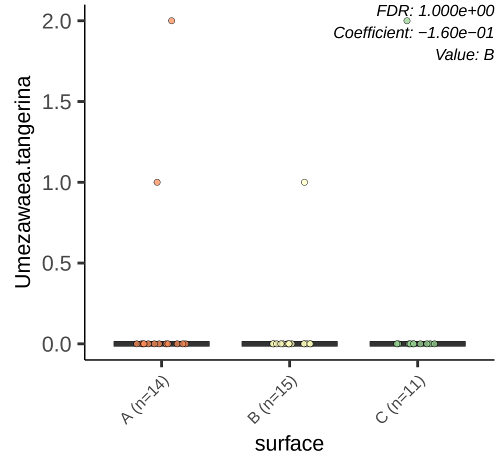
FDR: 1.000e+00
Coefficient: −1.60e−01
Value: B
Umezawaea.tangerina
2.0
1.5
1.0
0.5
0.0
A (n=14)
B (n=15)
C (n=11)
surface

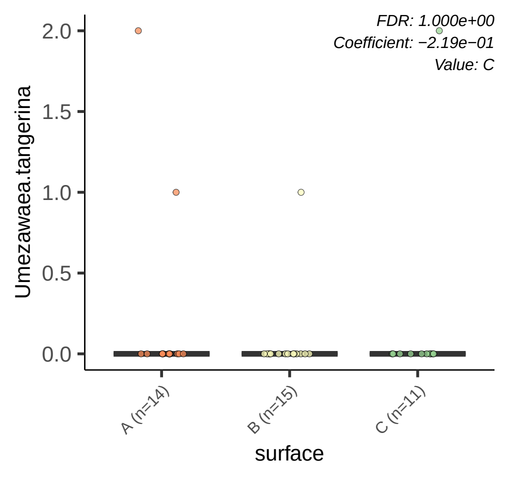
FDR: 1.000e+00
Coefficient: −2.19e−01
Value: C
Umezawaea.tangerina
2.0
1.5
1.0
0.5
0.0
A (n=14)
B (n=15)
C (n=11)
surface

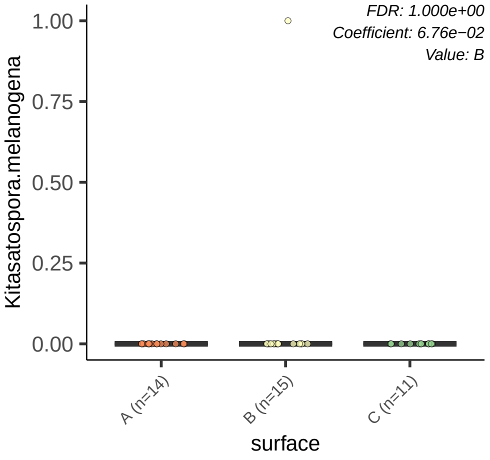
FDR: 1.000e+00
Coefficient: 6.76e−02
Value: B
Kitasatospora.melanogena
1.00
0.75
0.50
0.25
0.00
A (n=14)
B (n=15)
C (n=11)
surface

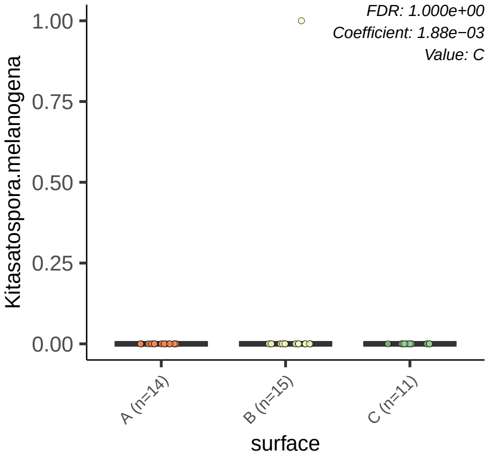
FDR: 1.000e+00
Coefficient: 1.88e−03
Value: C
Kitasatospora.melanogena
1.00
0.75
0.50
0.25
0.00
A (n=14)
B (n=15)
C (n=11)
surface

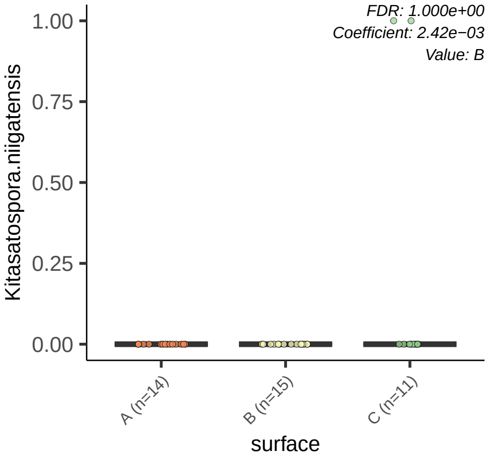

FDR: 1.000e+00
Coefficient: 2.42e−03
Value: B
Kitasatospora.niigatensis
1.00
0.75
0.50
0.25
0.00
A (n=14)
B (n=15)
C (n=11)
surface

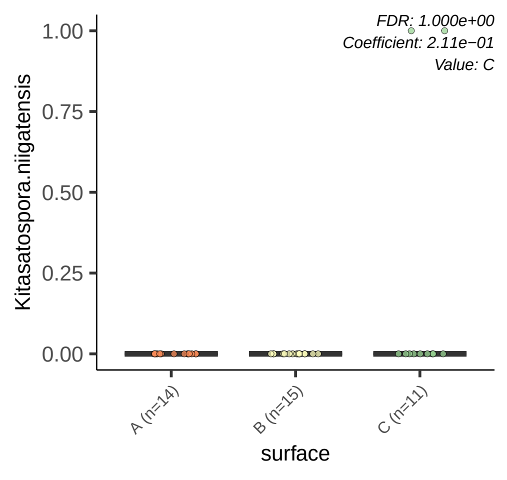

FDR: 1.000e+00
Coefficient: 2.11e−01
Value: C
Kitasatospora.niigatensis
1.00
0.75
0.50
0.25
0.00
A (n=14)
B (n=15)
C (n=11)
surface

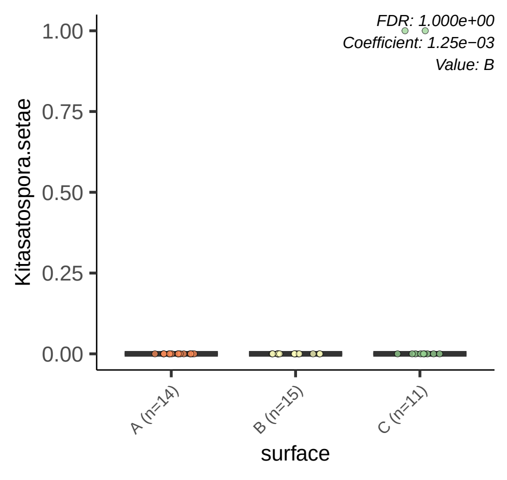
FDR: 1.000e+00
Coefficient: 1.25e−03
Value: B
Kitasatospora.setae
1.00
0.75
0.50
0.25
0.00
A (n=14)
B (n=15)
C (n=11)
surface

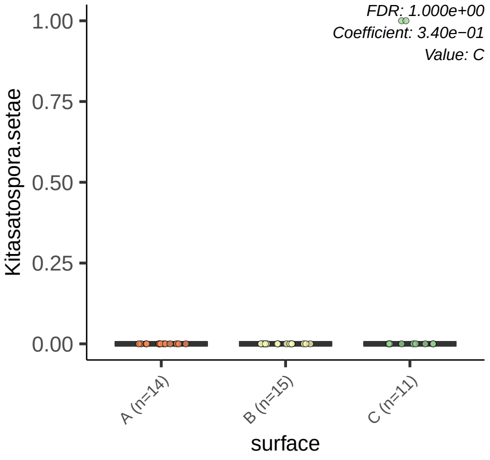

FDR: 1.000e+00
Coefficient: 3.40e−01
Value: C
Kitasatospora.setae
1.00
0.75
0.50
0.25
0.00
A (n=14)
B (n=15)
C (n=11)
surface

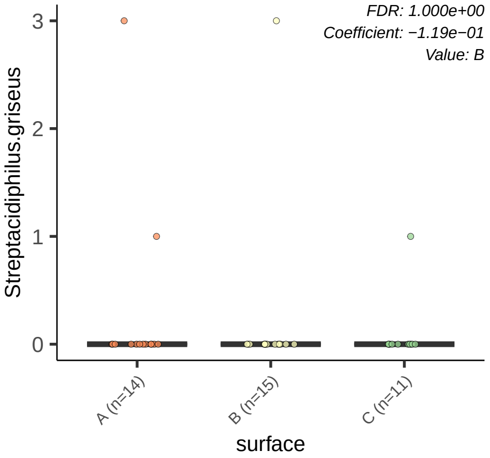

FDR: 1.000e+00
Coefficient: −1.19e−01
Value: B
Streptacidiphilus.griseus
3
2
1
0
A (n=14)
B (n=15)
C (n=11)
surface

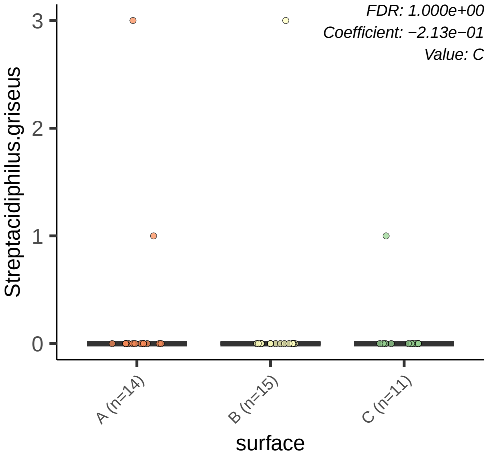
FDR: 1.000e+00
Coefficient: −2.13e−01
Value: C
Streptacidiphilus.griseus
3
2
1
0
A (n=14)
B (n=15)
C (n=11)
surface

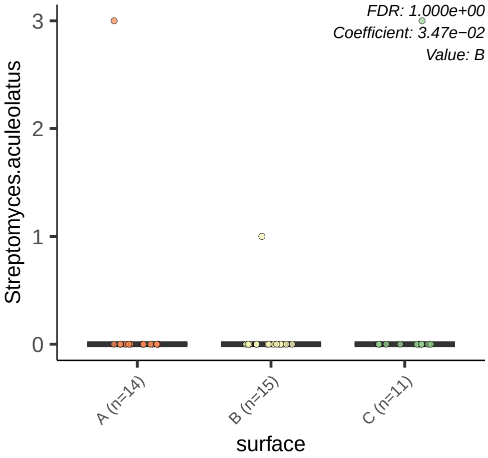
FDR: 1.000e+00
Coefficient: 3.47e−02
Value: B
Streptomyces.aculeolatus
3
2
1
0
A (n=14)
B (n=15)
C (n=11)
surface

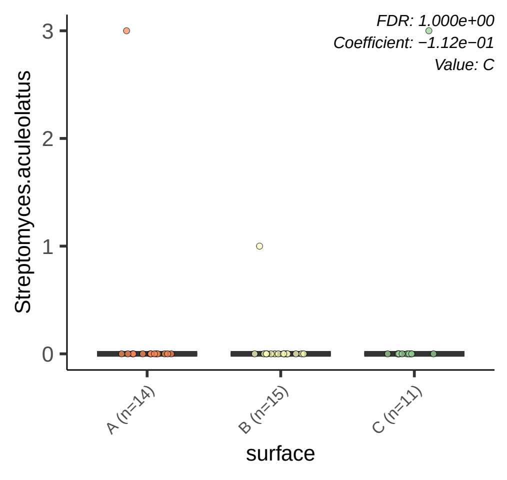

FDR: 1.000e+00
Coefficient: −1.12e−01
Value: C
Streptomyces.aculeolatus
3
2
1
0
A (n=14)
B (n=15)
C (n=11)
surface

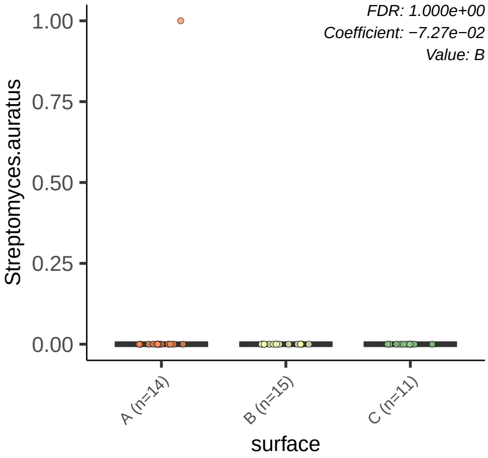

FDR: 1.000e+00
Coefficient: −7.27e−02
Value: B
Streptomyces.auratus
1.00
0.75
0.50
0.25
0.00
A (n=14)
B (n=15)
C (n=11)
surface

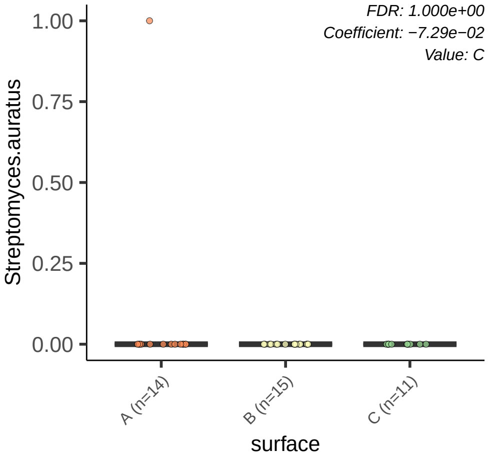

FDR: 1.000e+00
Coefficient: −7.29e−02
Value: C
1.00
0.75
0.50
0.25
0.00
Streptomyces.auratus
A (n=14)
B (n=15)
C (n=11)
surface

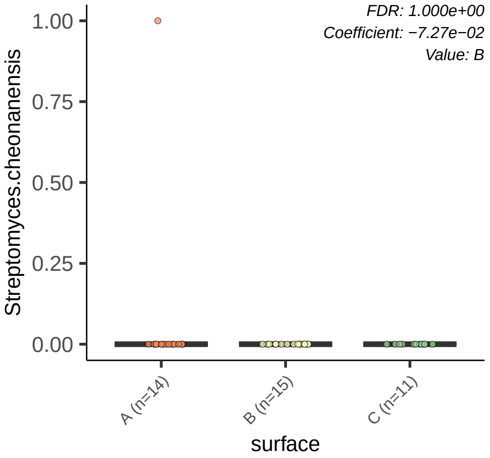

FDR: 1.000e+00
Coefficient: −7.27e−02
Value: B
Streptomyces.cheonanensis
1.00
0.75
0.50
0.25
0.00
A (n=14)
B (n=15)
C (n=11)
surface

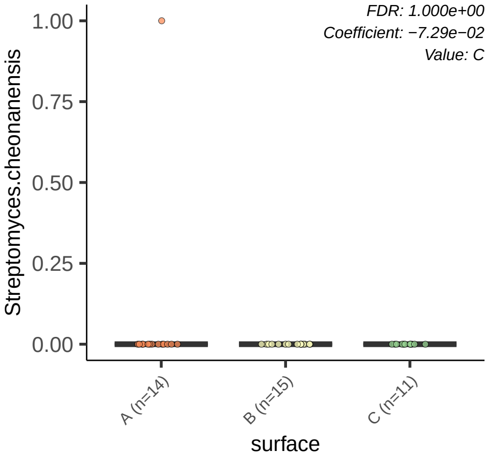

FDR: 1.000e+00
Coefficient: −7.29e−02
Value: C
Streptomyces.cheonanensis
1.00
0.75
0.50
0.25
0.00
A (n=14)
B (n=15)
C (n=11)
surface

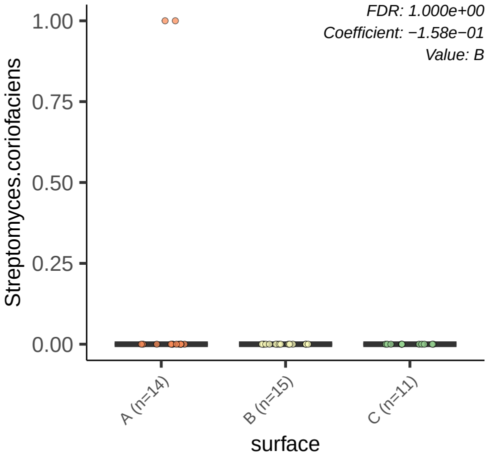
FDR: 1.000e+00
Coefficient: −1.58e−01
Value: B
Streptomyces.coriofaciens
1.00
0.75
0.50
0.25
0.00
A (n=14)
B (n=15)
C (n=11)
surface

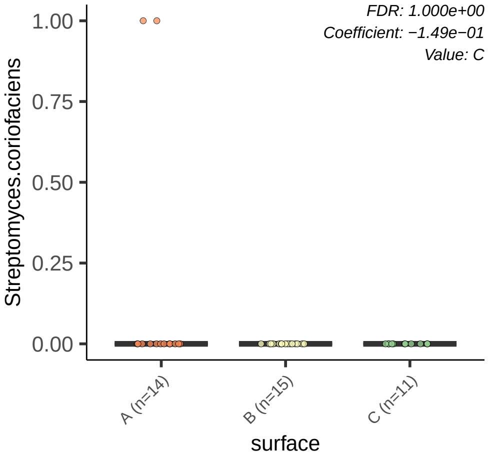
FDR: 1.000e+00
Coefficient: −1.49e−01
Value: C
Streptomyces.coriofaciens
1.00
0.75
0.50
0.25
0.00
A (n=14)
B (n=15)
C (n=11)
surface

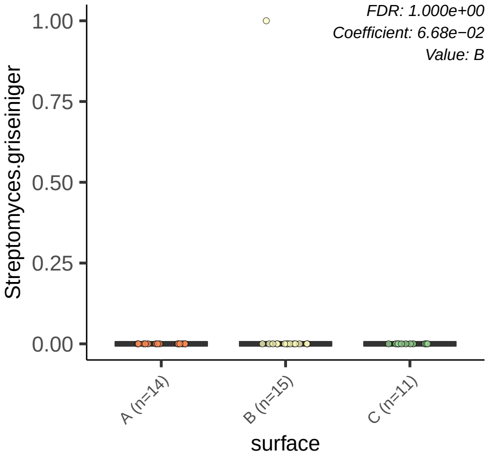

FDR: 1.000e+00
Coefficient: 6.68e−02
Value: B
Streptomyces.griseiniger
1.00
0.75
0.50
0.25
0.00
A (n=14)
B (n=15)
C (n=11)
surface

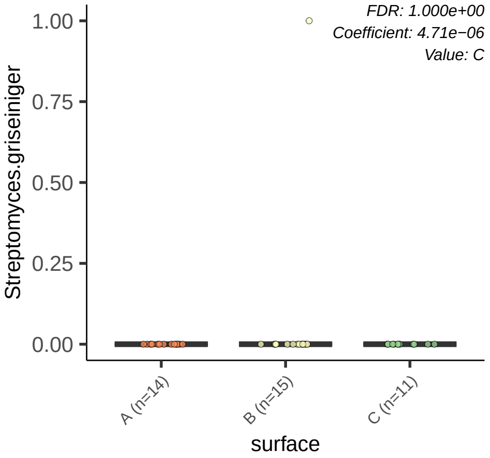
FDR: 1.000e+00
Coefficient: 4.71e−06
Value: C
Streptomyces.griseiniger
1.00
0.75
0.50
0.25
0.00
A (n=14)
B (n=15)
C (n=11)
surface

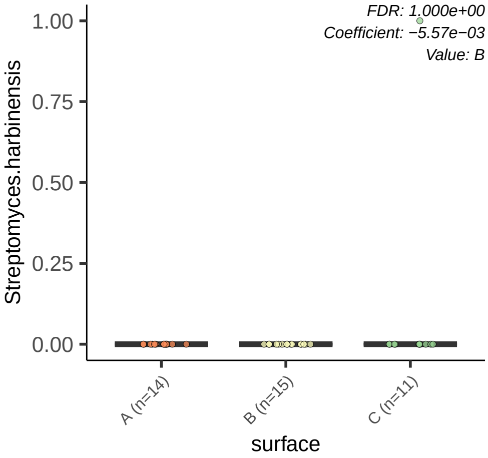
FDR: 1.000e+00
Coefficient: −5.57e−03
Value: B
Streptomyces.harbinensis
1.00
0.75
0.50
0.25
0.00
A (n=14)
B (n=15)
C (n=11)
surface

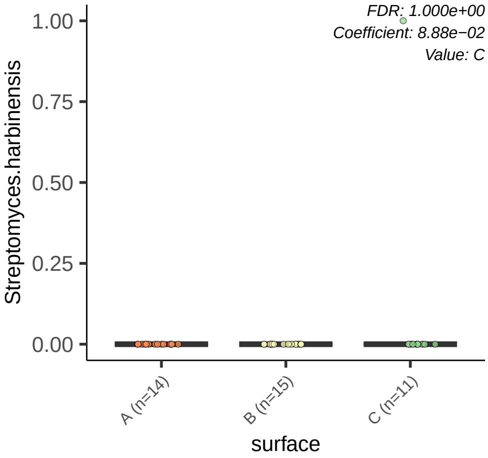

FDR: 1.000e+00
Coefficient: 8.88e−02
Value: C
Streptomyces.harbinensis
1.00
0.75
0.50
0.25
0.00
A (n=14)
B (n=15)
C (n=11)
surface

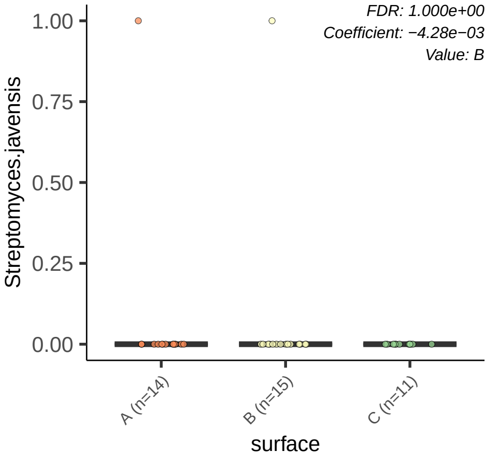
FDR: 1.000e+00
Coefficient: −4.28e−03
Value: B
Streptomyces.javensis
1.00
0.75
0.50
0.25
0.00
A (n=14)
B (n=15)
C (n=11)
surface

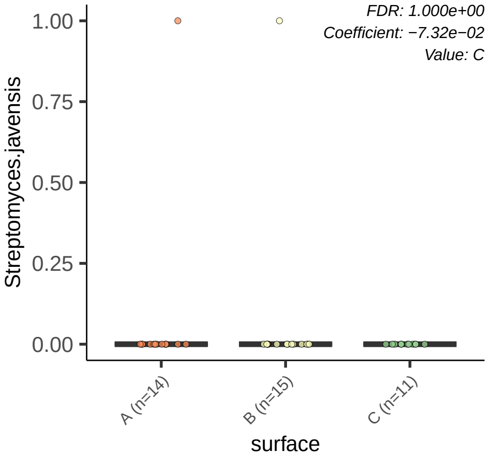

FDR: 1.000e+00
Coefficient: −7.32e−02
Value: C
Streptomyces.javensis
1.00
0.75
0.50
0.25
0.00
A (n=14)
B (n=15)
C (n=11)
surface

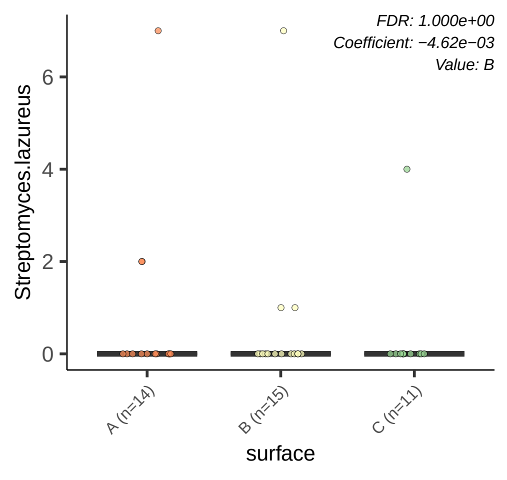
FDR: 1.000e+00
Coefficient: −4.62e−03
Value: B
Streptomyces.lazureus
0
2
4
6
A (n=14)
B (n=15)
C (n=11)
surface

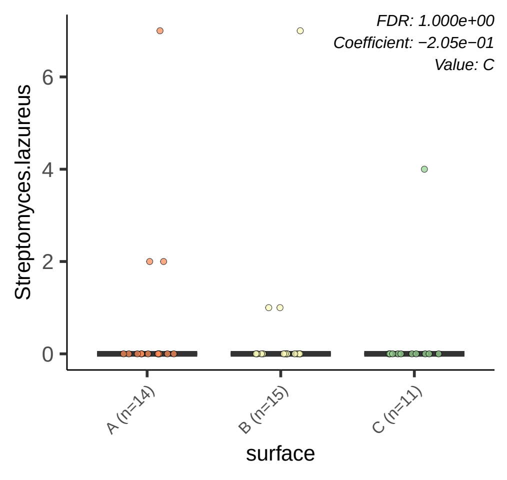
FDR: 1.000e+00
Coefficient: −2.05e−01
Value: C
Streptomyces.lazureus
0
2
4
6
A (n=14)
B (n=15)
C (n=11)
surface

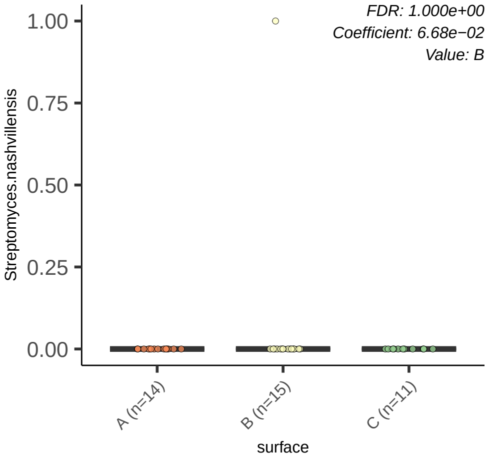

FDR: 1.000e+00
Coefficient: 6.68e−02
Value: B
Streptomyces.nashvillensis
1.00
0.75
0.50
0.25
0.00
A (n=14)
B (n=15)
C (n=11)
surface

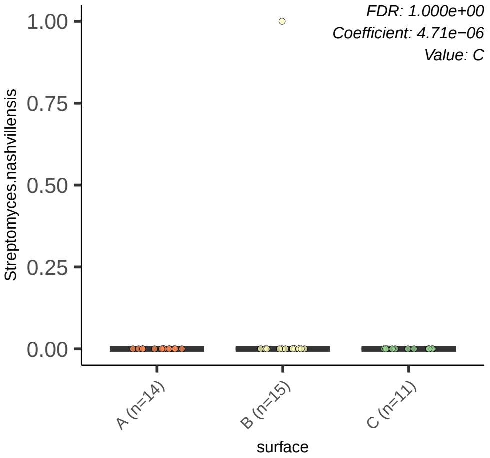
FDR: 1.000e+00
Coefficient: 4.71e−06
Value: C
Streptomyces.nashvillensis
1.00
0.75
0.50
0.25
0.00
A (n=14)
B (n=15)
C (n=11)
surface

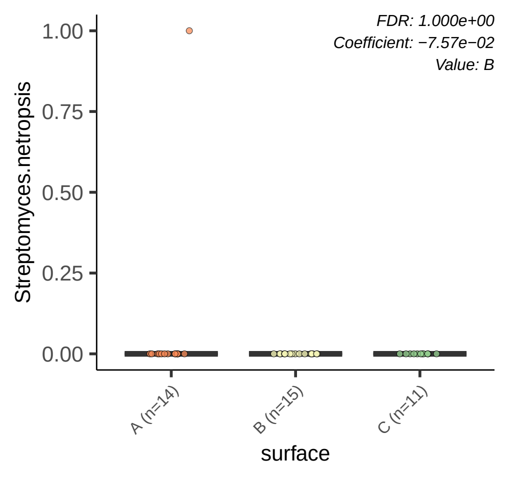
FDR: 1.000e+00
Coefficient: −7.57e−02
Value: B
Streptomyces.netropsis
1.00
0.75
0.50
0.25
0.00
A (n=14)
B (n=15)
C (n=11)
surface

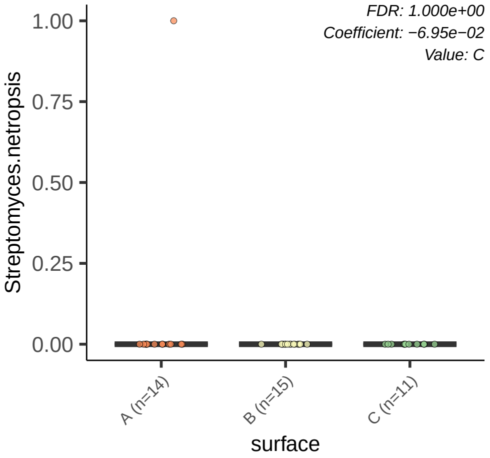
FDR: 1.000e+00
Coefficient: −6.95e−02
Value: C
Streptomyces.netropsis
1.00
0.75
0.50
0.25
0.00
A (n=14)
B (n=15)
C (n=11)
surface

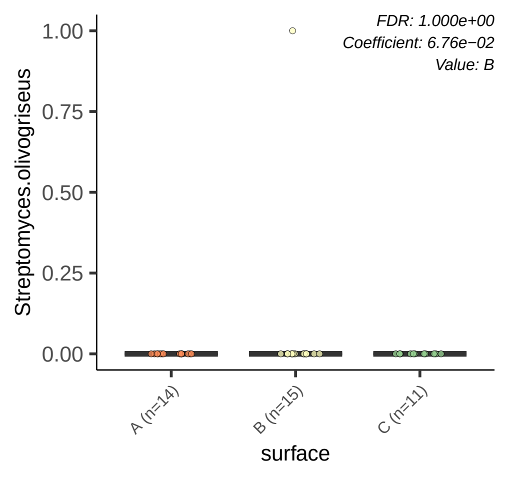

FDR: 1.000e+00
Coefficient: 6.76e−02
Value: B
Streptomyces.olivogriseus
1.00
0.75
0.50
0.25
0.00
A (n=14)
B (n=15)
C (n=11)
surface

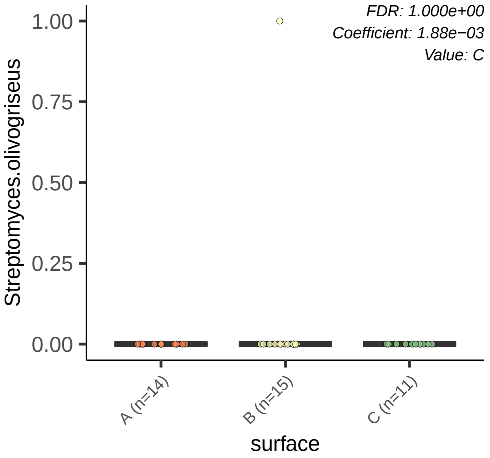
FDR: 1.000e+00
Coefficient: 1.88e−03
Value: C
Streptomyces.olivogriseus
1.00
0.75
0.50
0.25
0.00
A (n=14)
B (n=15)
C (n=11)
surface

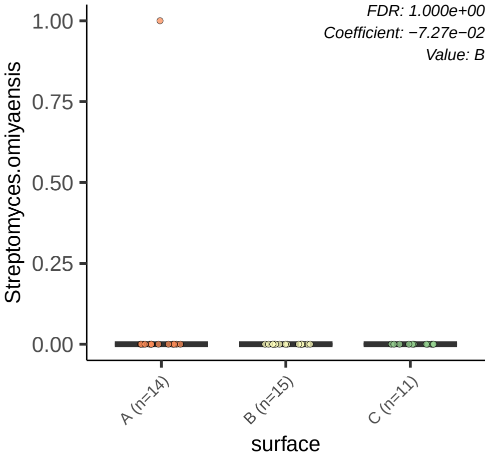

FDR: 1.000e+00
Coefficient: −7.27e−02
Value: B
1.00
0.75
0.50
0.25
0.00
Streptomyces.omiyaensis
A (n=14)
B (n=15)
C (n=11)
surface

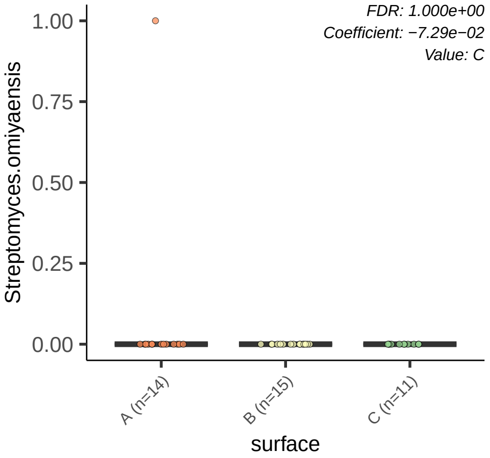
FDR: 1.000e+00
Coefficient: −7.29e−02
Value: C
Streptomyces.omiyaensis
1.00
0.75
0.50
0.25
0.00
A (n=14)
B (n=15)
C (n=11)
surface

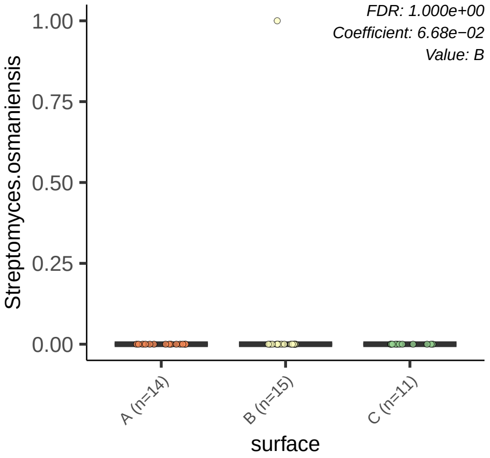

FDR: 1.000e+00
Coefficient: 6.68e−02
Value: B
Streptomyces.osmaniensis
1.00
0.75
0.50
0.25
0.00
A (n=14)
B (n=15)
C (n=11)
surface

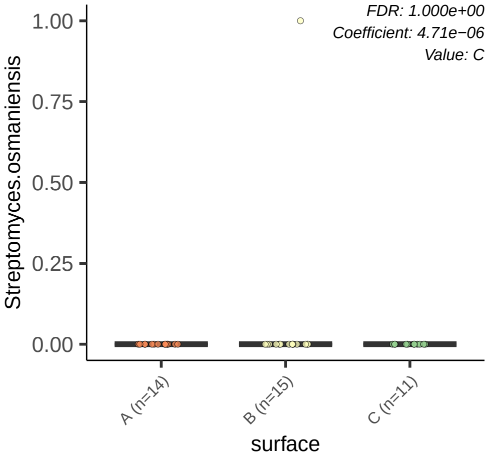

FDR: 1.000e+00
Coefficient: 4.71e−06
Value: C
Streptomyces.osmaniensis
1.00
0.75
0.50
0.25
0.00
A (n=14)
B (n=15)
C (n=11)
surface

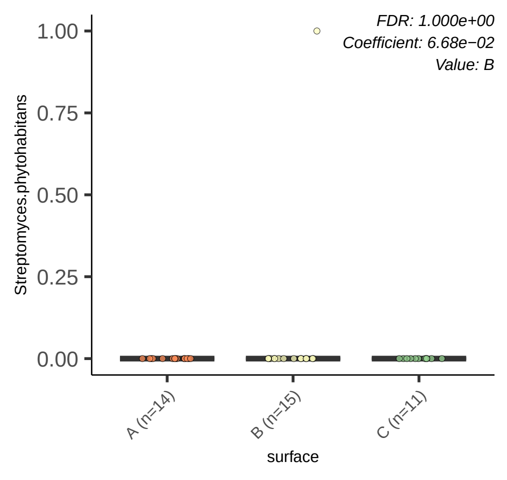
FDR: 1.000e+00
Coefficient: 6.68e−02
Value: B
1.00
0.75
0.50
0.25
0.00
Streptomyces.phytohabitans
A (n=14)
B (n=15)
C (n=11)
surface

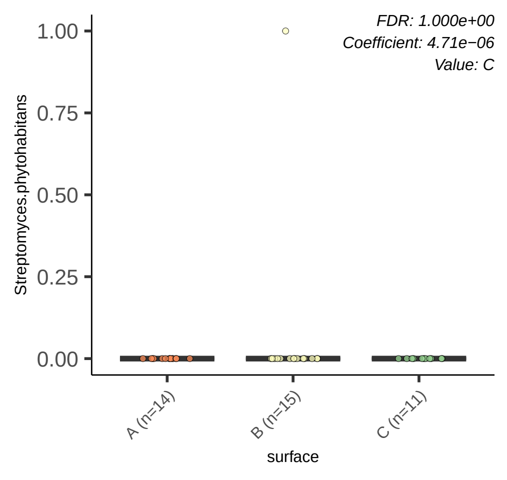
FDR: 1.000e+00
Coefficient: 4.71e−06
Value: C
Streptomyces.phytohabitans
1.00
0.75
0.50
0.25
0.00
A (n=14)
B (n=15)
C (n=11)
surface

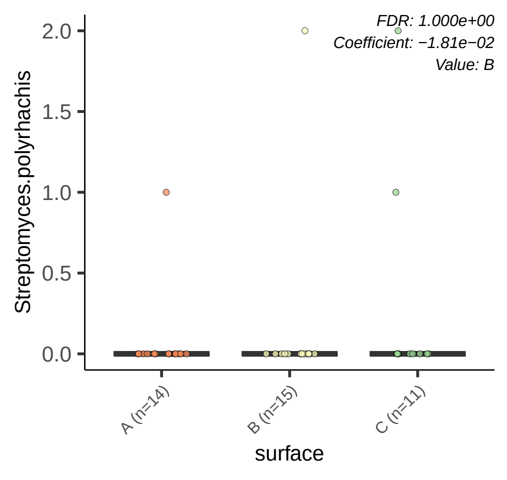
FDR: 1.000e+00
Coefficient: −1.81e−02
Value: B
Streptomyces.polyrhachis
2.0
1.5
1.0
0.5
0.0
A (n=14)
B (n=15)
C (n=11)
surface

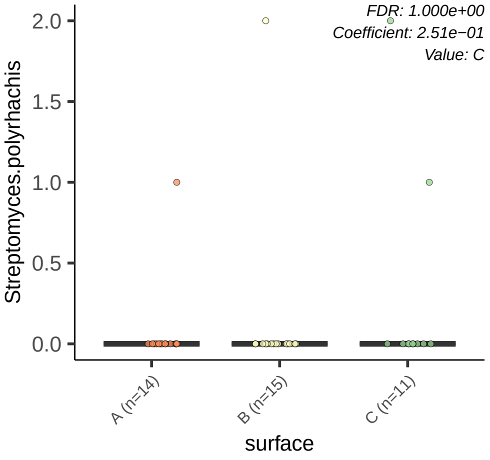
FDR: 1.000e+00
Coefficient: 2.51e−01
Value: C
Streptomyces.polyrhachis
2.0
1.5
1.0
0.5
0.0
A (n=14)
B (n=15)
C (n=11)
surface

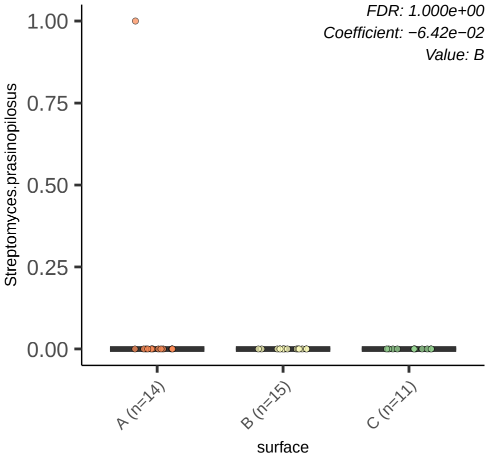
FDR: 1.000e+00
Coefficient: −6.42e−02
Value: B
Streptomyces.prasinopilosus
1.00
0.75
0.50
0.25
0.00
A (n=14)
B (n=15)
C (n=11)
surface

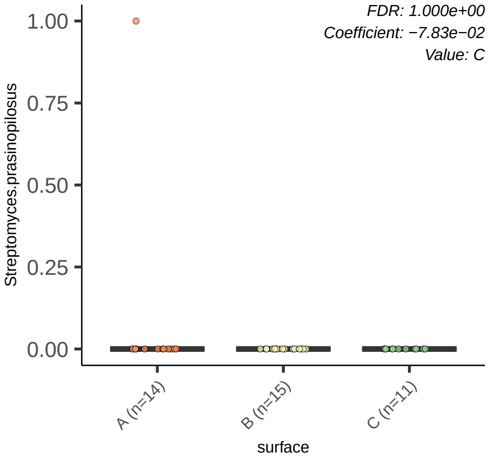
FDR: 1.000e+00
Coefficient: −7.83e−02
Value: C
Streptomyces.prasinopilosus
1.00
0.75
0.50
0.25
0.00
A (n=14)
B (n=15)
C (n=11)
surface

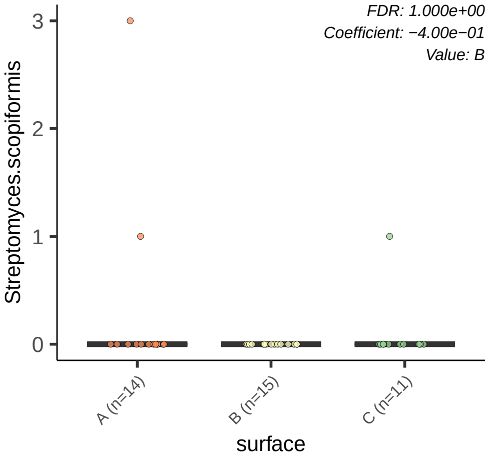
FDR: 1.000e+00
Coefficient: −4.00e−01
Value: B
Streptomyces.scopiformis
0
1
2
3
A (n=14)
B (n=15)
C (n=11)
surface

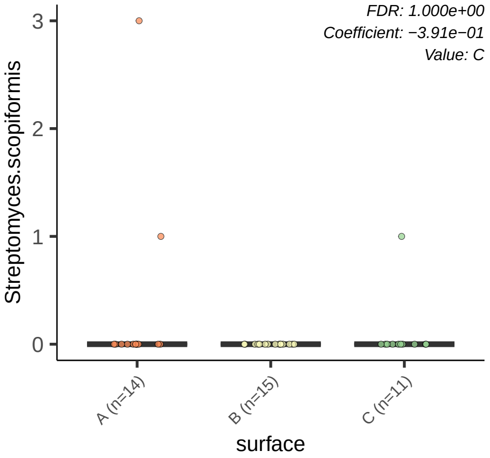

FDR: 1.000e+00
Coefficient: −3.91e−01
Value: C
Streptomyces.scopiformis
3
2
1
0
A (n=14)
B (n=15)
C (n=11)
surface

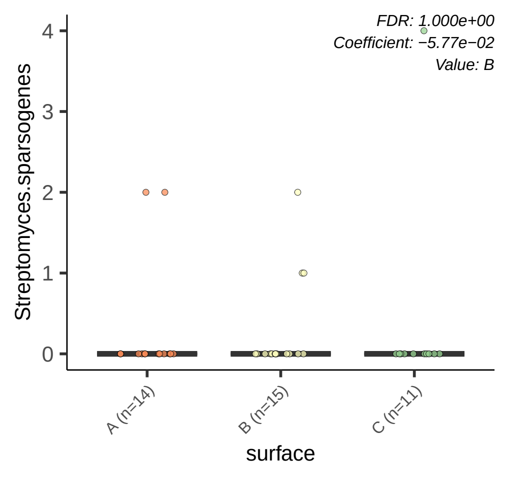
FDR: 1.000e+00
Coefficient: −5.77e−02
Value: B
Streptomyces.sparsogenes
0
1
2
3
4
A (n=14)
B (n=15)
C (n=11)
surface

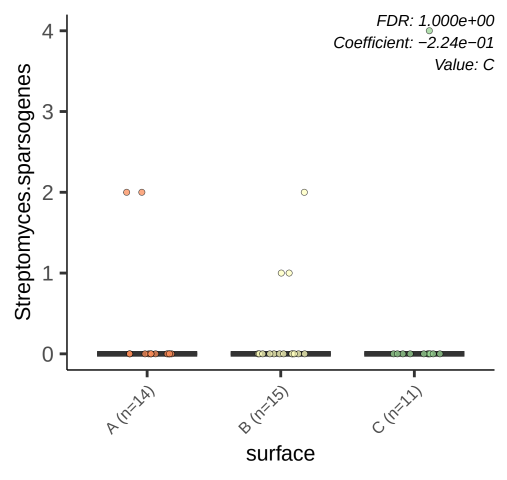

FDR: 1.000e+00
Coefficient: −2.24e−01
Value: C
Streptomyces.sparsogenes
0
1
2
3
4
A (n=14)
B (n=15)
C (n=11)
surface

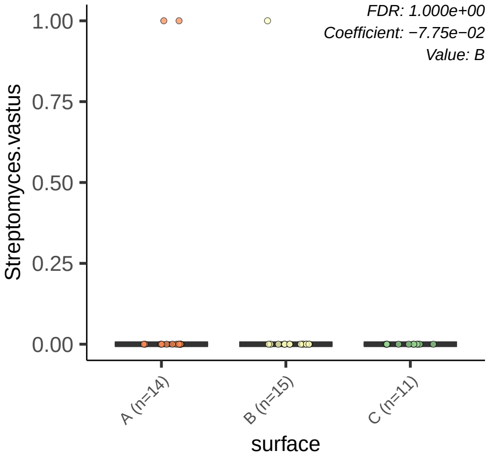
FDR: 1.000e+00
Coefficient: −7.75e−02
Value: B
Streptomyces.vastus
1.00
0.75
0.50
0.25
0.00
A (n=14)
B (n=15)
C (n=11)
surface

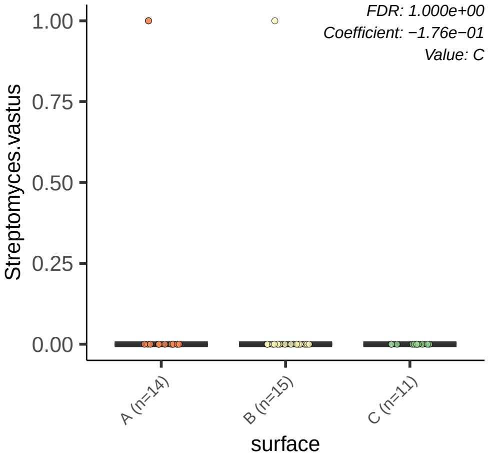
FDR: 1.000e+00
Coefficient: −1.76e−01
Value: C
Streptomyces.vastus
1.00
0.75
0.50
0.25
0.00
A (n=14)
B (n=15)
C (n=11)
surface

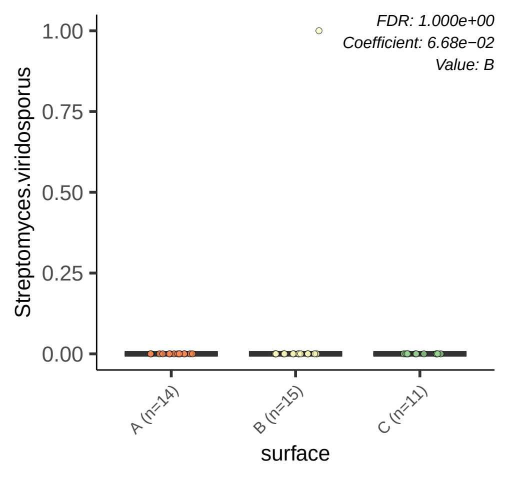
FDR: 1.000e+00
Coefficient: 6.68e−02
Value: B
Streptomyces.viridosporus
1.00
0.75
0.50
0.25
0.00
A (n=14)
B (n=15)
C (n=11)
surface

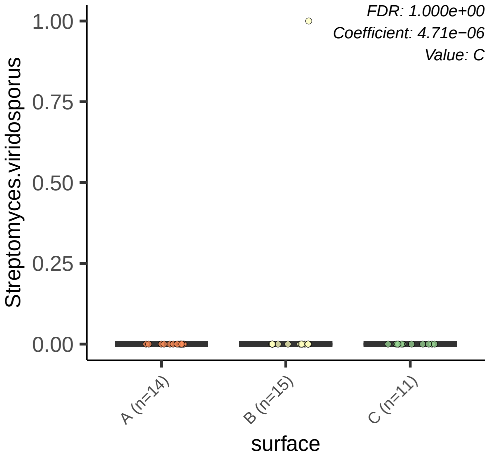
FDR: 1.000e+00
Coefficient: 4.71e−06
Value: C
Streptomyces.viridosporus
1.00
0.75
0.50
0.25
0.00
A (n=14)
B (n=15)
C (n=11)
surface

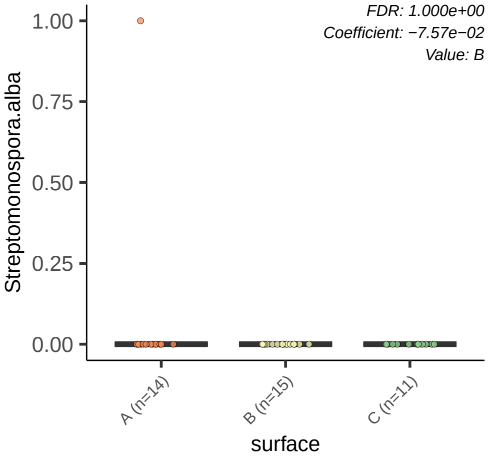
FDR: 1.000e+00
Coefficient: −7.57e−02
Value: B
Streptomonospora.alba
1.00
0.75
0.50
0.25
0.00
A (n=14)
B (n=15)
C (n=11)
surface

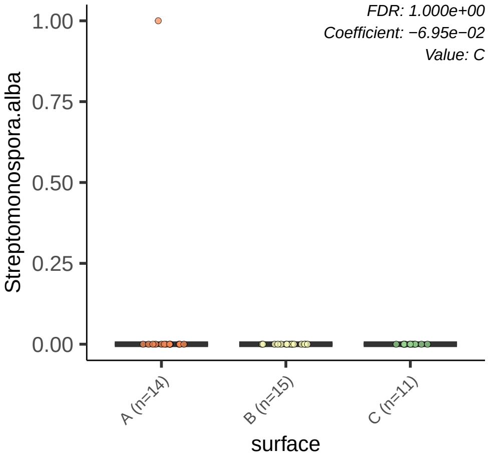

FDR: 1.000e+00
Coefficient: −6.95e−02
Value: C
Streptomonospora.alba
1.00
0.75
0.50
0.25
0.00
A (n=14)
B (n=15)
C (n=11)
surface

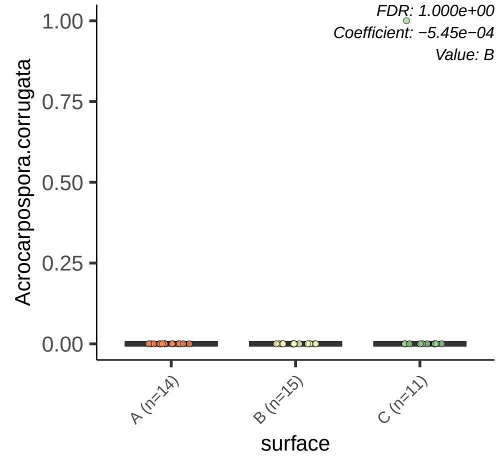

FDR: 1.000e+00
Coefficient: −5.45e−04
Value: B
Acrocarpospora.corrugata
1.00
0.75
0.50
0.25
0.00
A (n=14)
B (n=15)
C (n=11)
surface

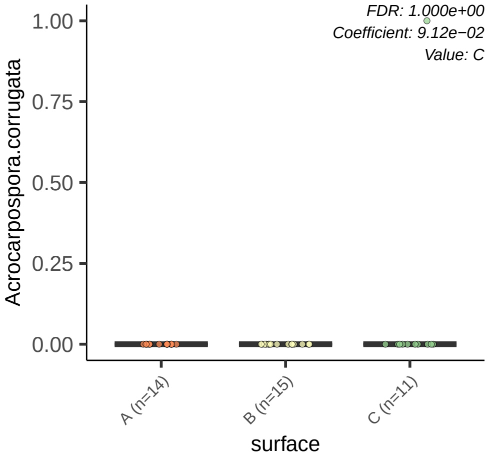

FDR: 1.000e+00
Coefficient: 9.12e−02
Value: C
Acrocarpospora.corrugata
1.00
0.75
0.50
0.25
0.00
A (n=14)
B (n=15)
C (n=11)
surface

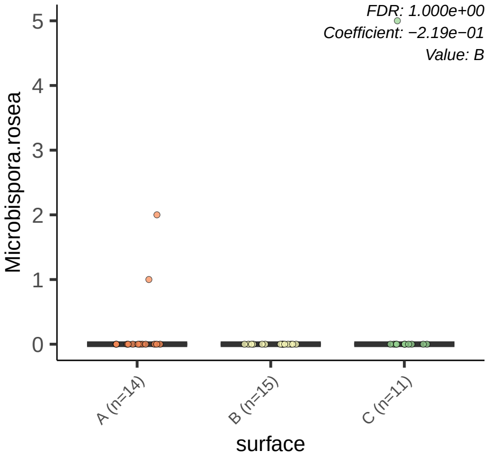
FDR: 1.000e+00
Coefficient: −2.19e−01
Value: B
Microbispora.rosea
0
1
2
3
4
5
A (n=14)
B (n=15)
C (n=11)
surface

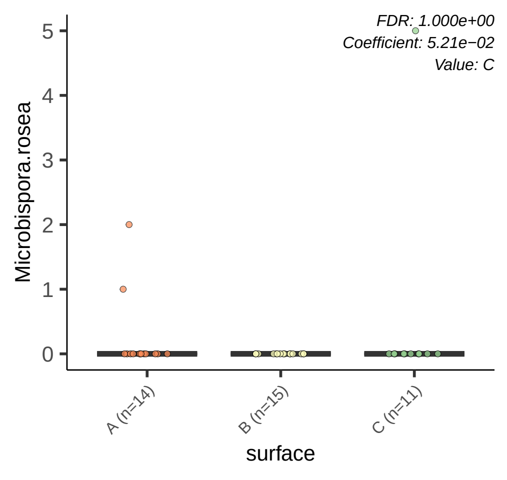

FDR: 1.000e+00
Coefficient: 5.21e−02
Value: C
Microbispora.rosea
5
4
3
2
1
0
A (n=14)
B (n=15)
C (n=11)
surface

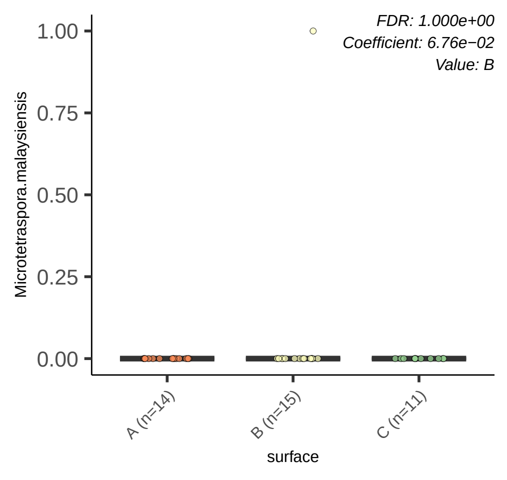

FDR: 1.000e+00
Coefficient: 6.76e−02
Value: B
Microtetraspora.malaysiensis
1.00
0.75
0.50
0.25
0.00
A (n=14)
B (n=15)
C (n=11)
surface

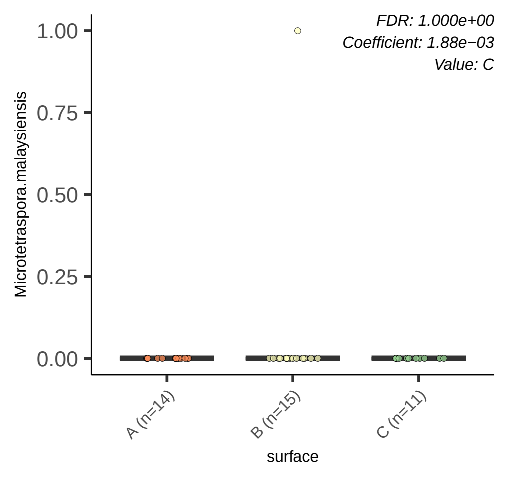
FDR: 1.000e+00
Coefficient: 1.88e−03
Value: C
Microtetraspora.malaysiensis
1.00
0.75
0.50
0.25
0.00
A (n=14)
B (n=15)
C (n=11)
surface

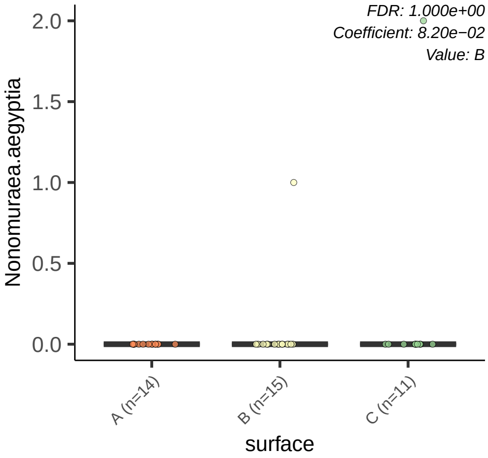
FDR: 1.000e+00
Coefficient: 8.20e−02
Value: B
Nonomuraea.aegyptia
2.0
1.5
1.0
0.5
0.0
A (n=14)
B (n=15)
C (n=11)
surface

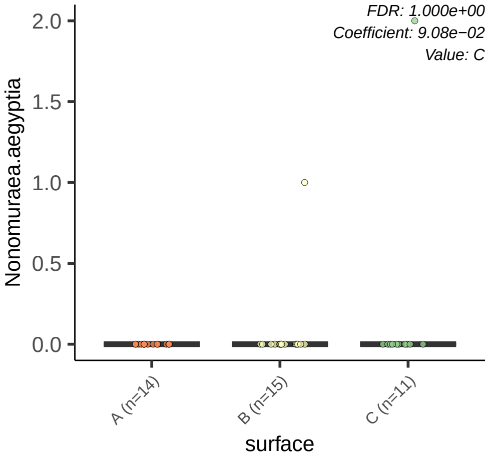

FDR: 1.000e+00
Coefficient: 9.08e−02
Value: C
Nonomuraea.aegyptia
2.0
1.5
1.0
0.5
0.0
A (n=14)
B (n=15)
C (n=11)
surface

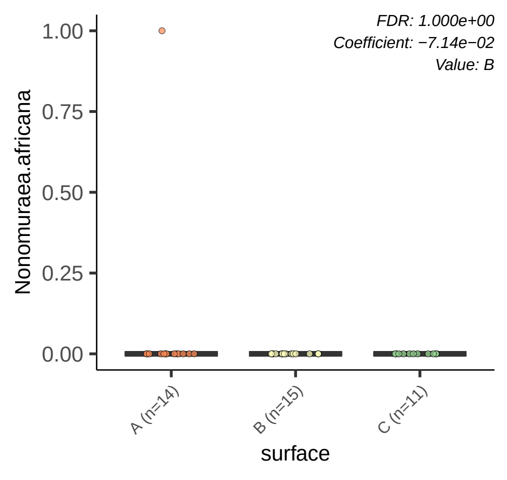

FDR: 1.000e+00
Coefficient: −7.14e−02
Value: B
1.00
0.75
0.50
0.25
0.00
Nonomuraea.africana
A (n=14)
B (n=15)
C (n=11)
surface

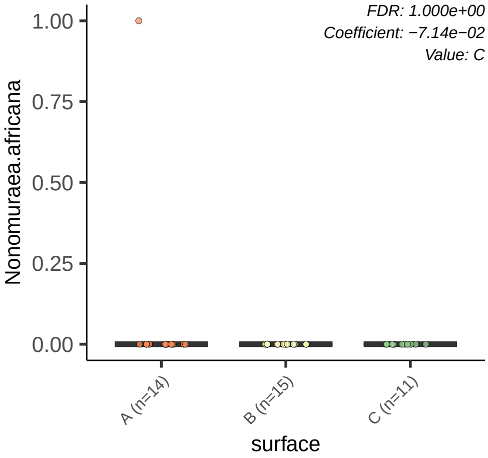

FDR: 1.000e+00
Coefficient: −7.14e−02
Value: C
Nonomuraea.africana
1.00
0.75
0.50
0.25
0.00
A (n=14)
B (n=15)
C (n=11)
surface

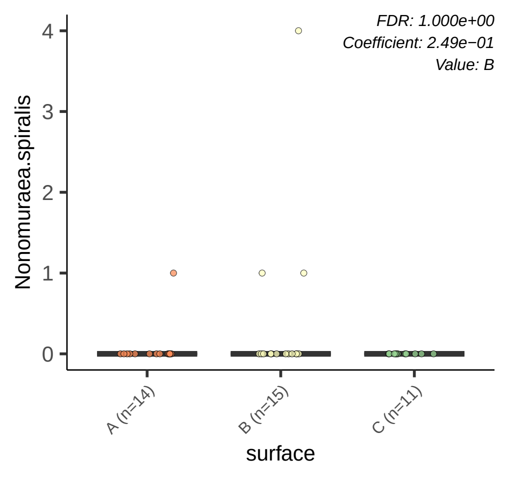
FDR: 1.000e+00
Coefficient: 2.49e−01
Value: B
Nonomuraea.spiralis
4
3
2
1
0
A (n=14)
B (n=15)
C (n=11)
surface

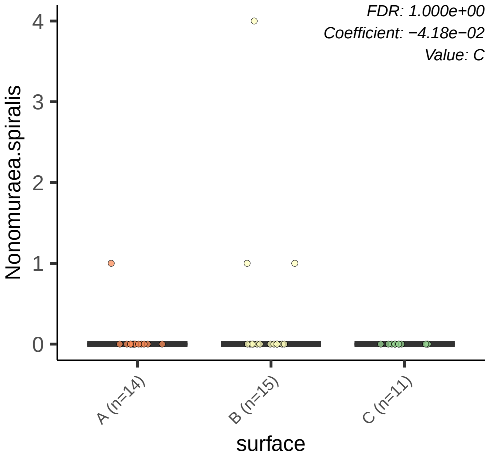
FDR: 1.000e+00
Coefficient: −4.18e−02
Value: C
Nonomuraea.spiralis
0
1
2
3
4
A (n=14)
B (n=15)
C (n=11)
surface

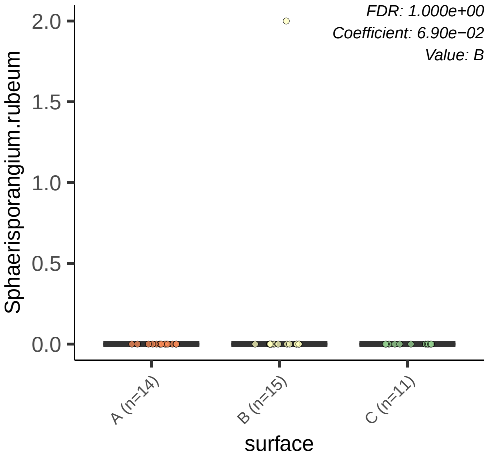
FDR: 1.000e+00
Coefficient: 6.90e−02
Value: B
Sphaerisporangium.rubeum
2.0
1.5
1.0
0.5
0.0
A (n=14)
B (n=15)
C (n=11)
surface

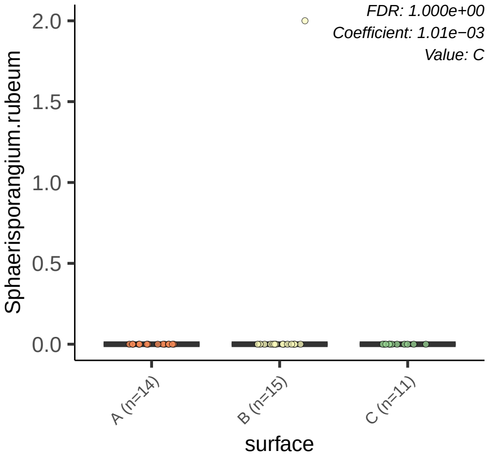

FDR: 1.000e+00
Coefficient: 1.01e−03
Value: C
Sphaerisporangium.rubeum
2.0
1.5
1.0
0.5
0.0
A (n=14)
B (n=15)
C (n=11)
surface

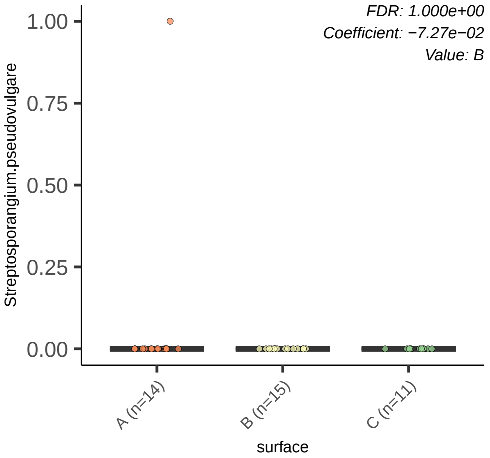
FDR: 1.000e+00
Coefficient: −7.27e−02
Value: B
Streptosporangium.pseudovulgare
1.00
0.75
0.50
0.25
0.00
A (n=14)
B (n=15)
C (n=11)
surface

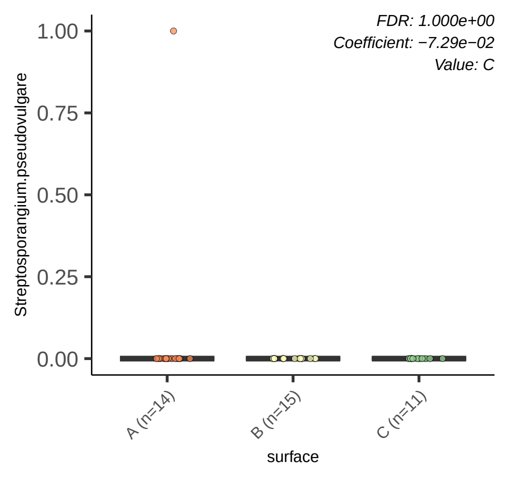

FDR: 1.000e+00
Coefficient: −7.29e−02
Value: C
Streptosporangium.pseudovulgare
1.00
0.75
0.50
0.25
0.00
A (n=14)
B (n=15)
C (n=11)
surface

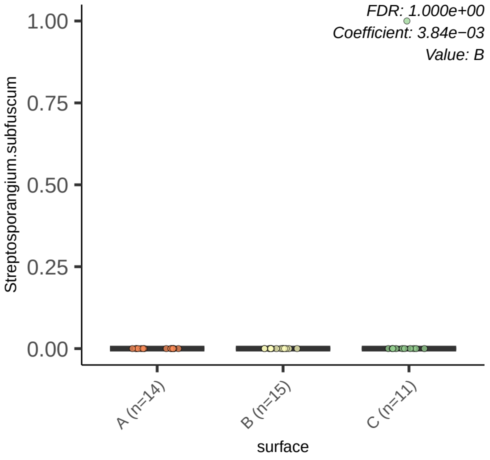
FDR: 1.000e+00
Coefficient: 3.84e−03
Value: B
1.00
0.75
0.50
0.25
0.00
Streptosporangium.subfuscum
A (n=14)
B (n=15)
C (n=11)
surface

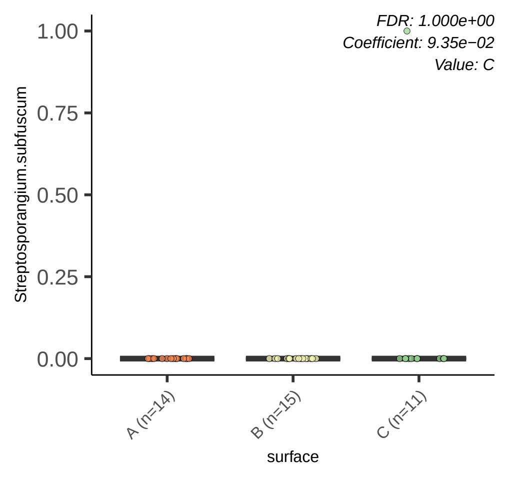
FDR: 1.000e+00
Coefficient: 9.35e−02
Value: C
Streptosporangium.subfuscum
1.00
0.75
0.50
0.25
0.00
A (n=14)
B (n=15)
C (n=11)
surface

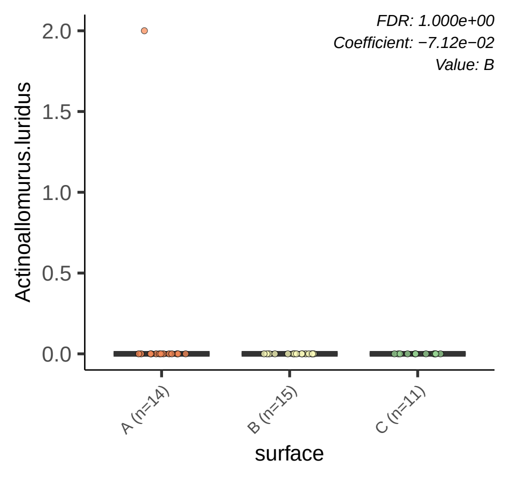
FDR: 1.000e+00
Coefficient: −7.12e−02
Value: B
Actinoallomurus.luridus
2.0
1.5
1.0
0.5
0.0
A (n=14)
B (n=15)
C (n=11)
surface

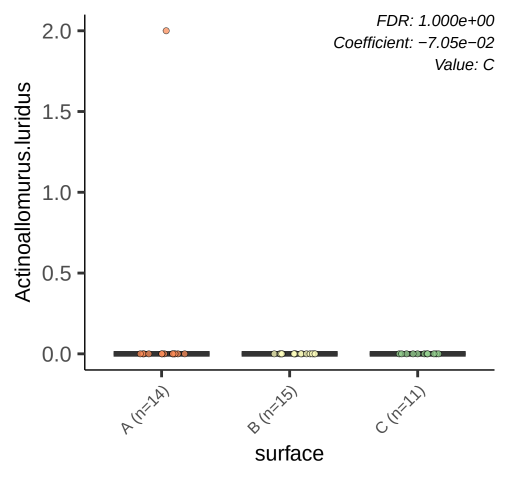
FDR: 1.000e+00
Coefficient: −7.05e−02
Value: C
Actinoallomurus.luridus
2.0
1.5
1.0
0.5
0.0
A (n=14)
B (n=15)
C (n=11)
surface

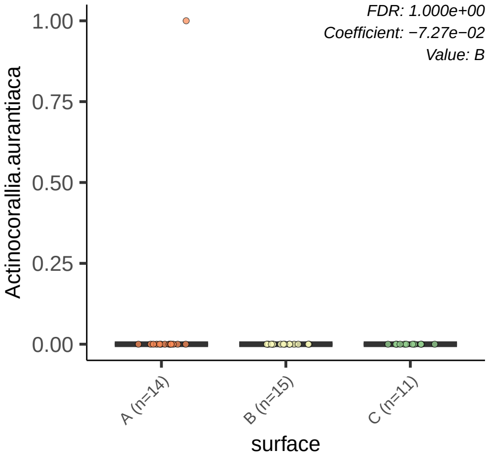
FDR: 1.000e+00
Coefficient: −7.27e−02
Value: B
Actinocorallia.aurantiaca
1.00
0.75
0.50
0.25
0.00
A (n=14)
B (n=15)
C (n=11)
surface

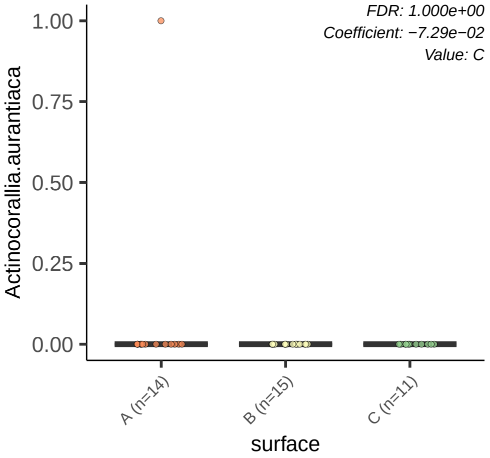

FDR: 1.000e+00
Coefficient: −7.29e−02
Value: C
Actinocorallia.aurantiaca
1.00
0.75
0.50
0.25
0.00
A (n=14)
B (n=15)
C (n=11)
surface

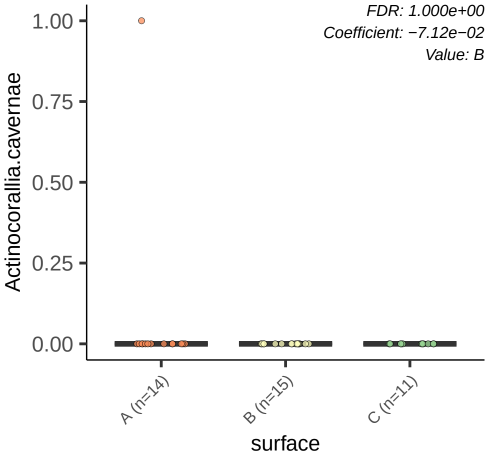
FDR: 1.000e+00
Coefficient: −7.12e−02
Value: B
Actinocorallia.cavernae
1.00
0.75
0.50
0.25
0.00
A (n=14)
B (n=15)
C (n=11)
surface

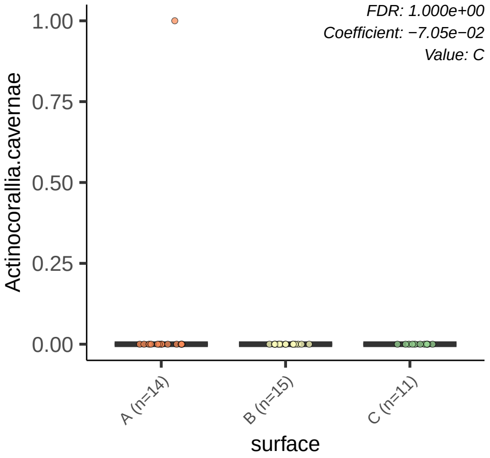
FDR: 1.000e+00
Coefficient: −7.05e−02
Value: C
Actinocorallia.cavernae
1.00
0.75
0.50
0.25
0.00
A (n=14)
B (n=15)
C (n=11)
surface

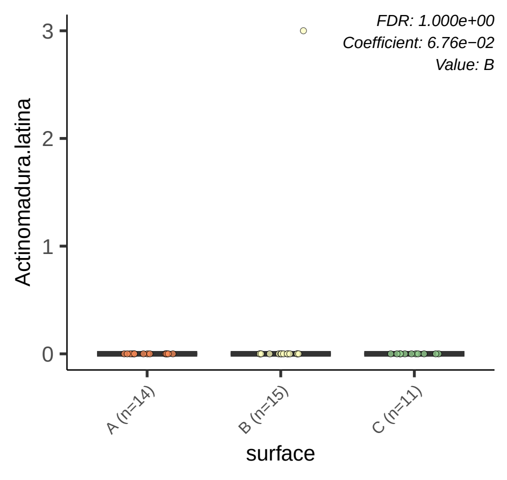

FDR: 1.000e+00
Coefficient: 6.76e−02
Value: B
Actinomadura.latina
3
2
1
0
A (n=14)
B (n=15)
C (n=11)
surface

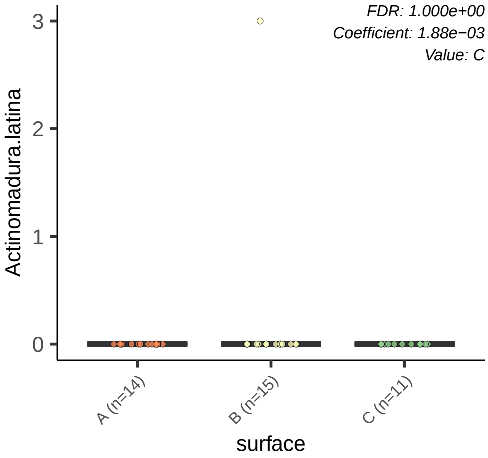
FDR: 1.000e+00
Coefficient: 1.88e−03
Value: C
Actinomadura.latina
3
2
1
0
A (n=14)
B (n=15)
C (n=11)
surface

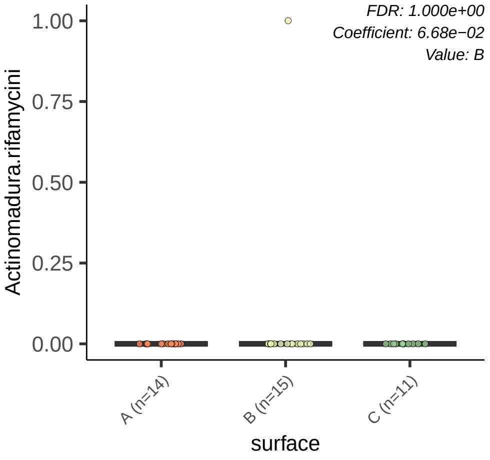
FDR: 1.000e+00
Coefficient: 6.68e−02
Value: B
Actinomadura.rifamycini
1.00
0.75
0.50
0.25
0.00
A (n=14)
B (n=15)
C (n=11)
surface

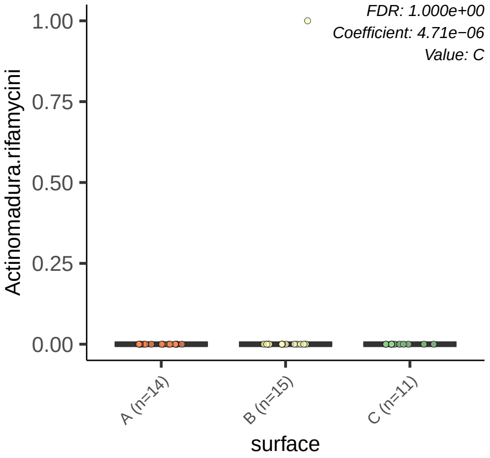
FDR: 1.000e+00
Coefficient: 4.71e−06
Value: C
Actinomadura.rifamycini
1.00
0.75
0.50
0.25
0.00
A (n=14)
B (n=15)
C (n=11)
surface

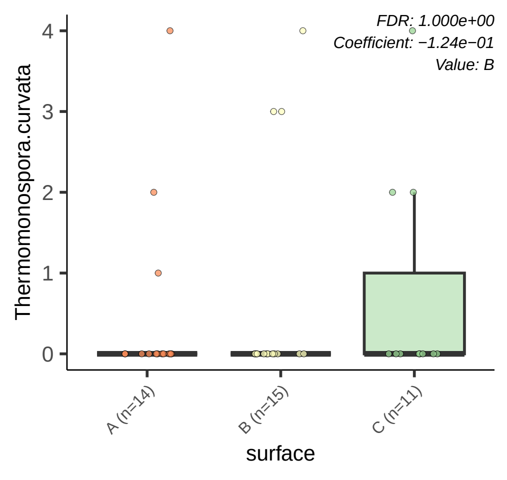

FDR: 1.000e+00
Coefficient: −1.24e−01
Value: B
Thermomonospora.curvata
0
1
2
3
4
A (n=14)
B (n=15)
C (n=11)
surface

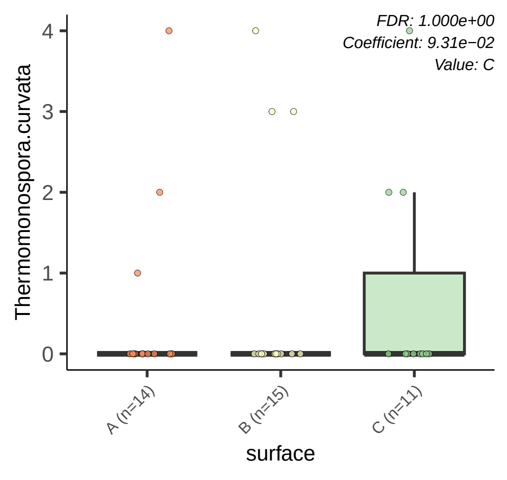

FDR: 1.000e+00
Coefficient: 9.31e−02
Value: C
Thermomonospora.curvata
0
1
2
3
4
A (n=14)
B (n=15)
C (n=11)
surface

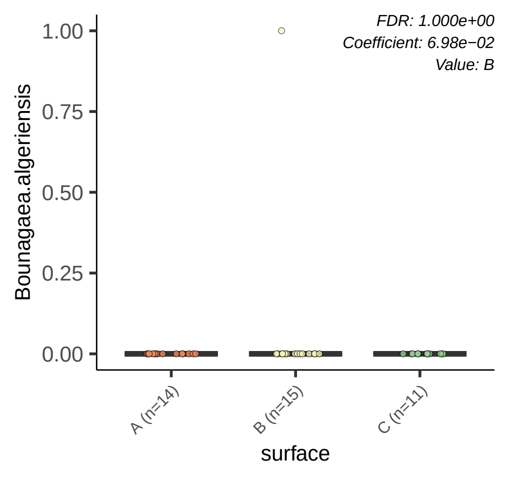
FDR: 1.000e+00
Coefficient: 6.98e−02
Value: B
Bounagaea.algeriensis
1.00
0.75
0.50
0.25
0.00
A (n=14)
B (n=15)
C (n=11)
surface

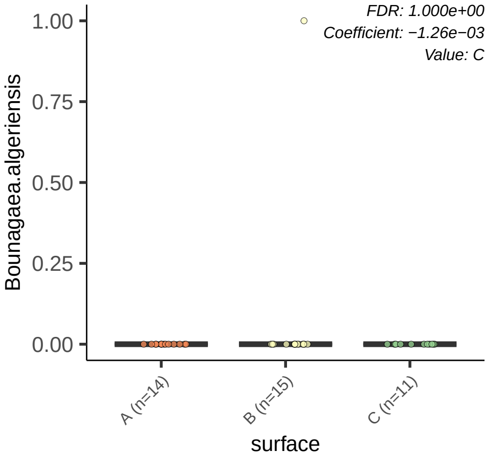
FDR: 1.000e+00
Coefficient: −1.26e−03
Value: C
Bounagaea.algeriensis
1.00
0.75
0.50
0.25
0.00
A (n=14)
B (n=15)
C (n=11)
surface

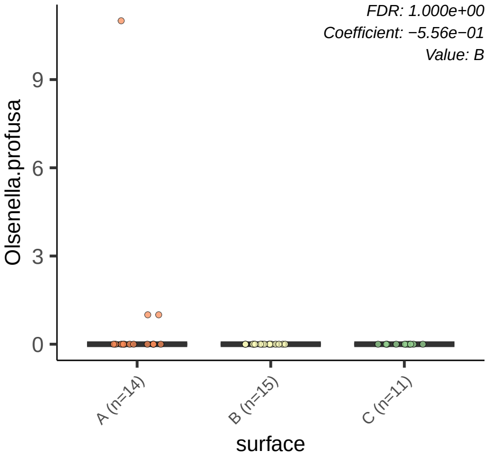
FDR: 1.000e+00
Coefficient: −5.56e−01
Value: B
Olsenella.profusa
0
3
6
9
A (n=14)
B (n=15)
C (n=11)
surface

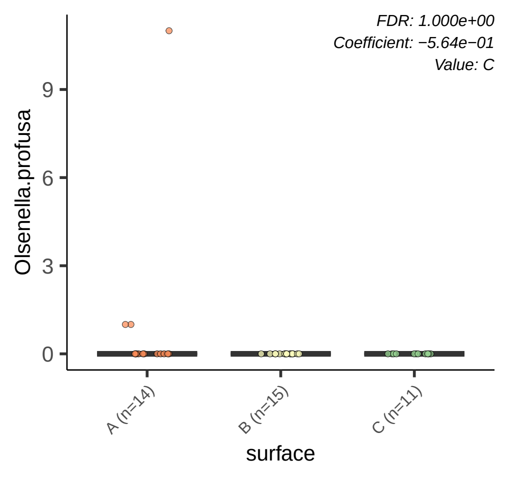

FDR: 1.000e+00
Coefficient: −5.64e−01
Value: C
Olsenella.profusa
0
3
6
9
A (n=14)
B (n=15)
C (n=11)
surface

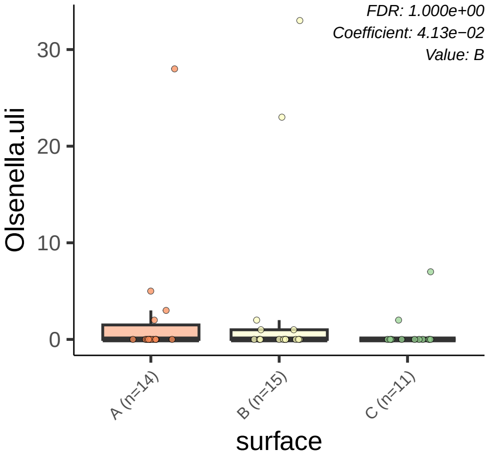
FDR: 1.000e+00
Coefficient: 4.13e−02
Value: B
Olsenella.uli
30
20
10
0
A (n=14)
B (n=15)
C (n=11)
surface

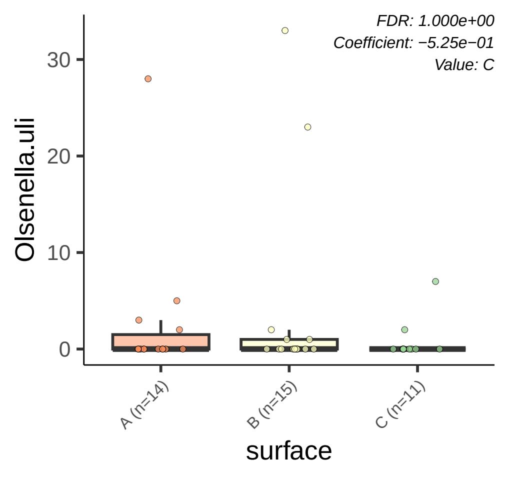

FDR: 1.000e+00
Coefficient: −5.25e−01
Value: C
Olsenella.uli
30
20
10
0
A (n=14)
B (n=15)
C (n=11)
surface

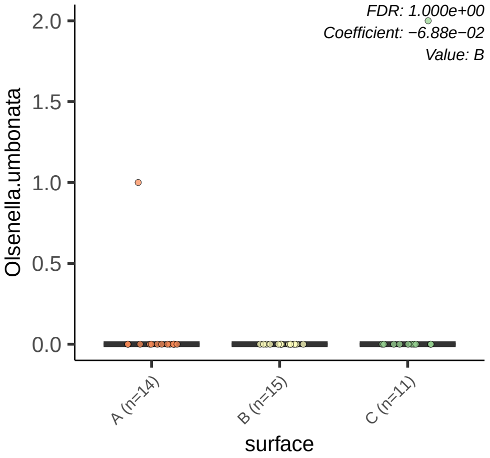
FDR: 1.000e+00
Coefficient: −6.88e−02
Value: B
Olsenella.umbonata
2.0
1.5
1.0
0.5
0.0
A (n=14)
B (n=15)
C (n=11)
surface

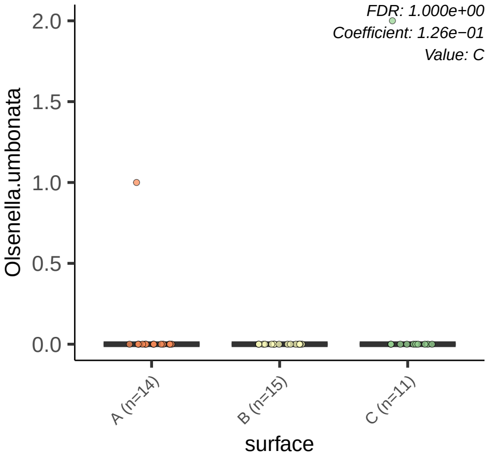
FDR: 1.000e+00
Coefficient: 1.26e−01
Value: C
Olsenella.umbonata
2.0
1.5
1.0
0.5
0.0
A (n=14)
B (n=15)
C (n=11)
surface

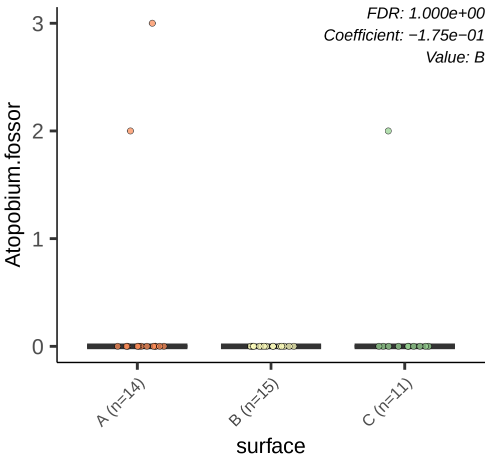
FDR: 1.000e+00
Coefficient: −1.75e−01
Value: B
Atopobium.fossor
3
2
1
0
A (n=14)
B (n=15)
C (n=11)
surface

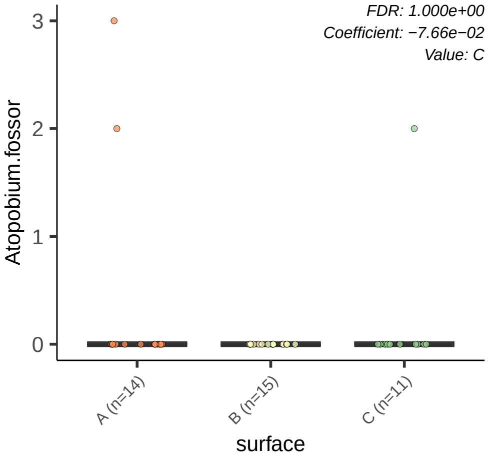

FDR: 1.000e+00
Coefficient: −7.66e−02
Value: C
Atopobium.fossor
3
2
1
0
A (n=14)
B (n=15)
C (n=11)
surface

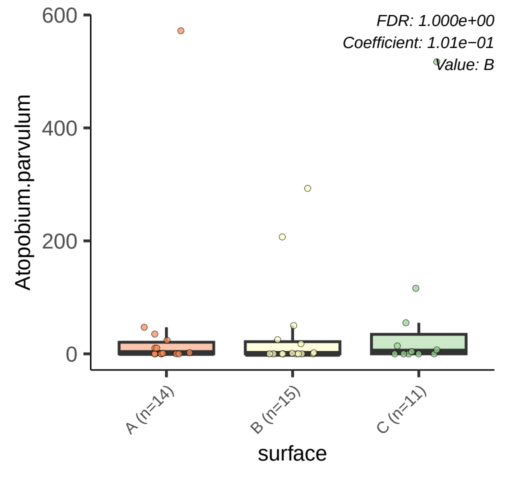

FDR: 1.000e+00
Coefficient: 1.01e−01
Value: B
Atopobium.parvulum
600
400
200
0
A (n=14)
B (n=15)
C (n=11)
surface

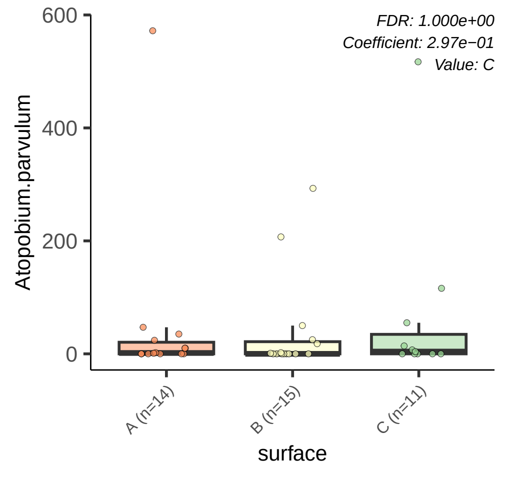
FDR: 1.000e+00
Coefficient: 2.97e−01
Value: C
600
400
200
0
Atopobium.parvulum
A (n=14)
B (n=15)
C (n=11)
surface

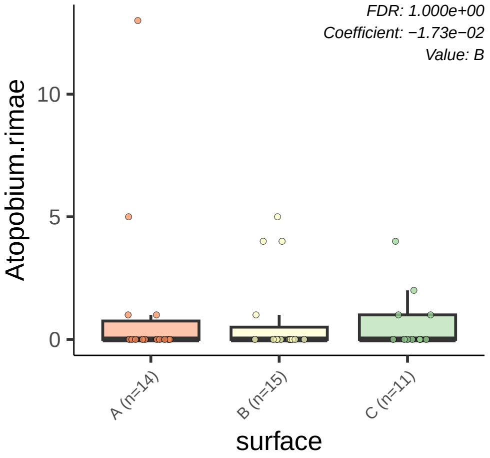

FDR: 1.000e+00
Coefficient: −1.73e−02
Value: B
Atopobium.rimae
10
5
0
A (n=14)
B (n=15)
C (n=11)
surface

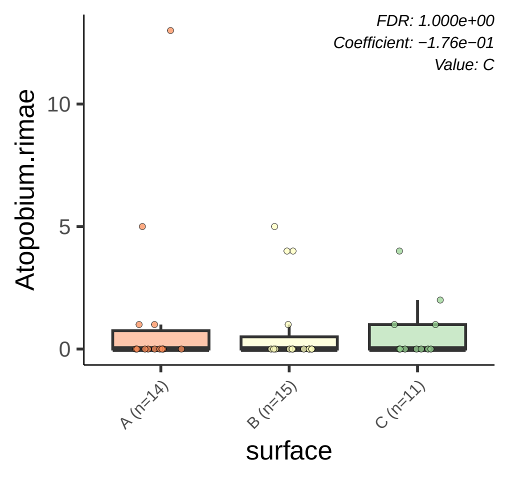
FDR: 1.000e+00
Coefficient: −1.76e−01
Value: C
Atopobium.rimae
10
5
0
A (n=14)
B (n=15)
C (n=11)
surface

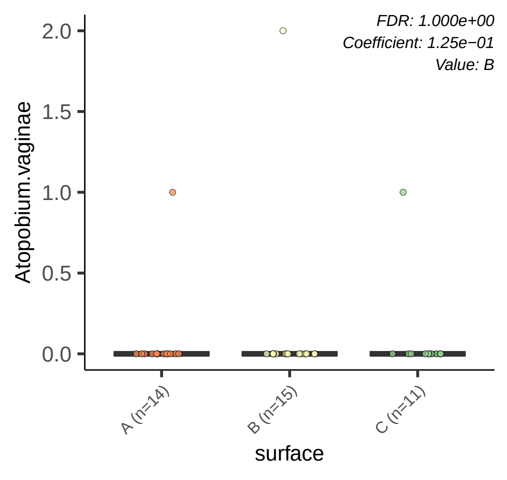
FDR: 1.000e+00
Coefficient: 1.25e−01
Value: B
Atopobium.vaginae
2.0
1.5
1.0
0.5
0.0
A (n=14)
B (n=15)
C (n=11)
surface

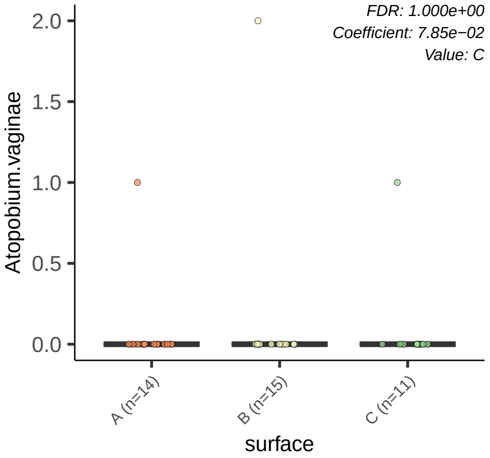

FDR: 1.000e+00
Coefficient: 7.85e−02
Value: C
Atopobium.vaginae
2.0
1.5
1.0
0.5
0.0
A (n=14)
B (n=15)
C (n=11)
surface

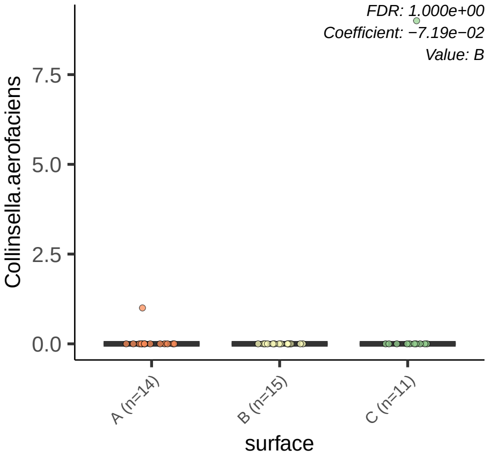
FDR: 1.000e+00
Coefficient: −7.19e−02
Value: B
Collinsella.aerofaciens
7.5
5.0
2.5
0.0
A (n=14)
B (n=15)
C (n=11)
surface

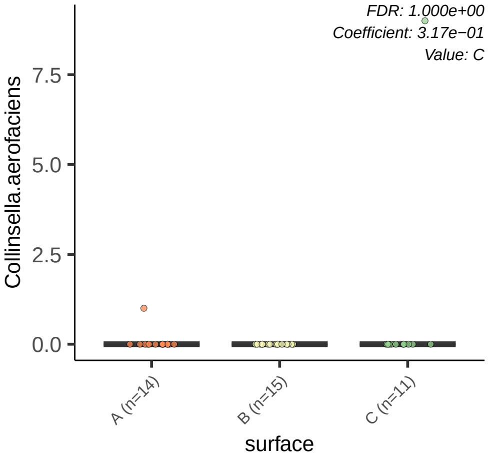
FDR: 1.000e+00
Coefficient: 3.17e−01
Value: C
Collinsella.aerofaciens
7.5
5.0
2.5
0.0
A (n=14)
B (n=15)
C (n=11)
surface

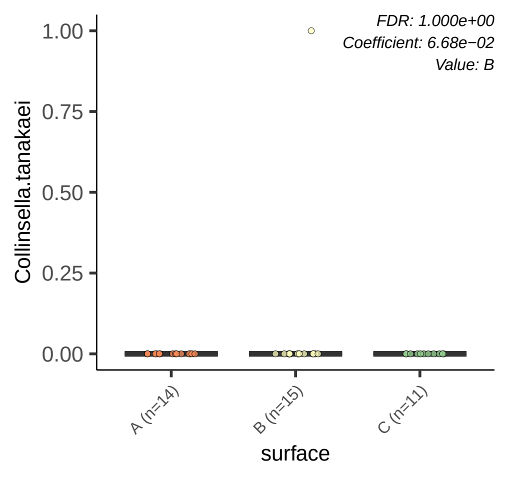

FDR: 1.000e+00
Coefficient: 6.68e−02
Value: B
Collinsella.tanakaei
1.00
0.75
0.50
0.25
0.00
A (n=14)
B (n=15)
C (n=11)
surface

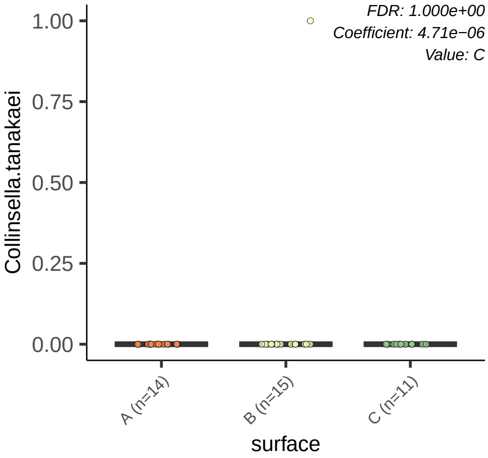

FDR: 1.000e+00
Coefficient: 4.71e−06
Value: C
Collinsella.tanakaei
1.00
0.75
0.50
0.25
0.00
A (n=14)
B (n=15)
C (n=11)
surface

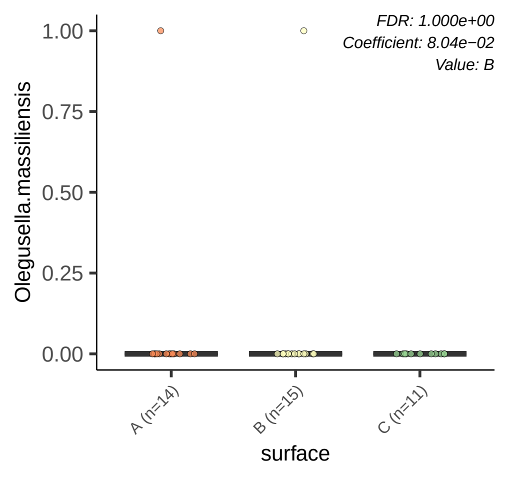

FDR: 1.000e+00
Coefficient: 8.04e−02
Value: B
Olegusella.massiliensis
1.00
0.75
0.50
0.25
0.00
A (n=14)
B (n=15)
C (n=11)
surface

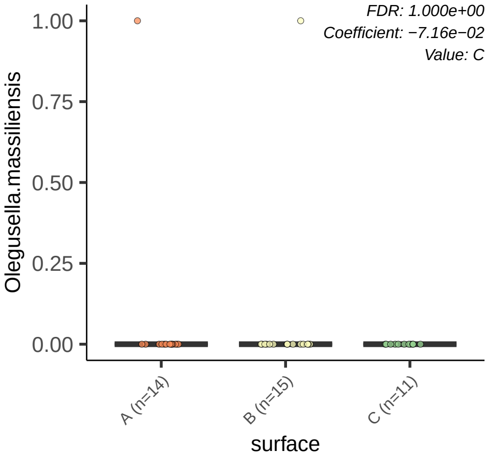
FDR: 1.000e+00
Coefficient: −7.16e−02
Value: C
1.00
0.75
0.50
0.25
0.00
Olegusella.massiliensis
A (n=14)
B (n=15)
C (n=11)
surface

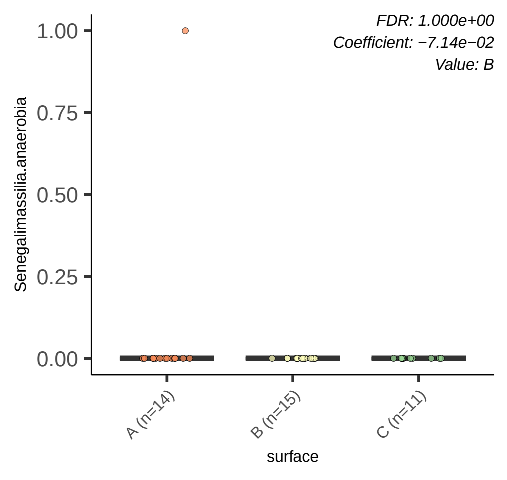
FDR: 1.000e+00
Coefficient: −7.14e−02
Value: B
1.00
0.75
0.50
0.25
0.00
Senegalimassilia.anaerobia
A (n=14)
B (n=15)
C (n=11)
surface

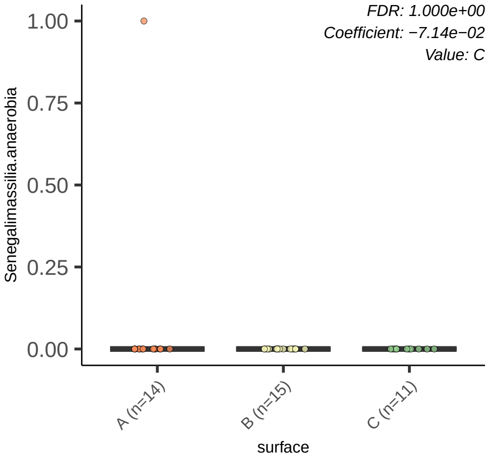

FDR: 1.000e+00
Coefficient: −7.14e−02
Value: C
1.00
0.75
0.50
0.25
0.00
Senegalimassilia.anaerobia
A (n=14)
B (n=15)
C (n=11)
surface

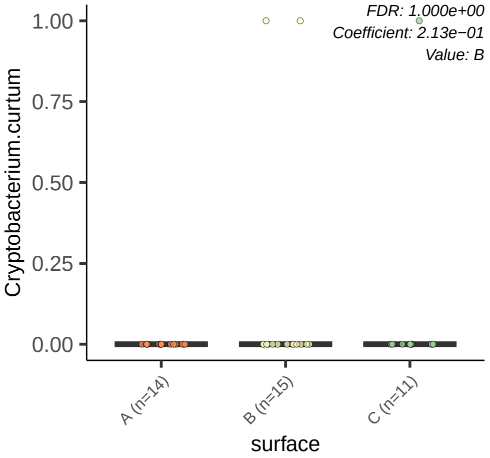
FDR: 1.000e+00
Coefficient: 2.13e−01
Value: B
Cryptobacterium.curtum
1.00
0.75
0.50
0.25
0.00
A (n=14)
B (n=15)
C (n=11)
surface

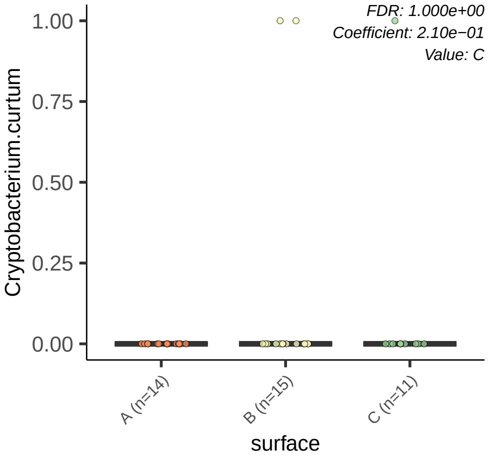
FDR: 1.000e+00
Coefficient: 2.10e−01
Value: C
Cryptobacterium.curtum
1.00
0.75
0.50
0.25
0.00
A (n=14)
B (n=15)
C (n=11)
surface

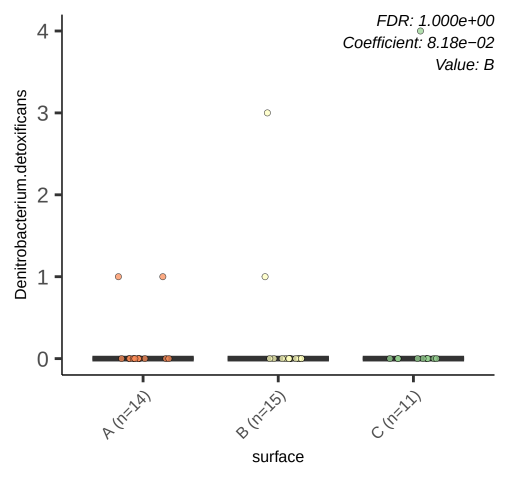

FDR: 1.000e+00
Coefficient: 8.18e−02
Value: B
Denitrobacterium.detoxificans
0
1
2
3
4
A (n=14)
B (n=15)
C (n=11)
surface

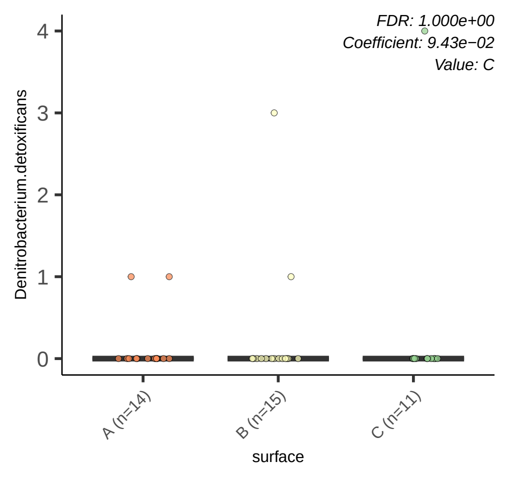

FDR: 1.000e+00
Coefficient: 9.43e−02
Value: C
Denitrobacterium.detoxificans
4
3
2
1
0
A (n=14)
B (n=15)
C (n=11)
surface

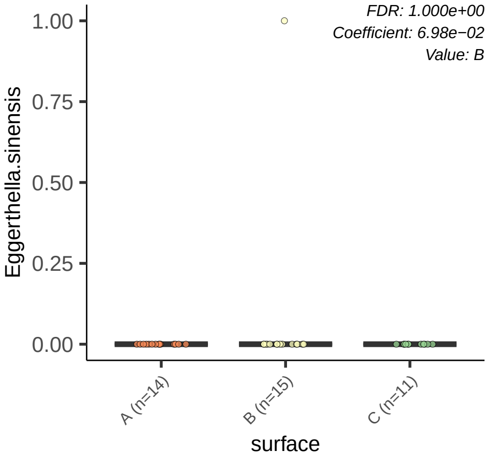
FDR: 1.000e+00
Coefficient: 6.98e−02
Value: B
Eggerthella.sinensis
1.00
0.75
0.50
0.25
0.00
A (n=14)
B (n=15)
C (n=11)
surface

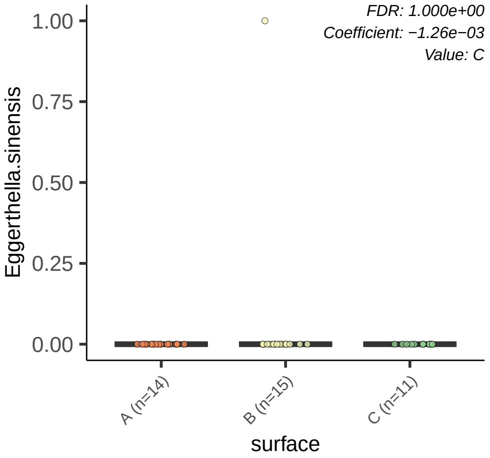
FDR: 1.000e+00
Coefficient: −1.26e−03
Value: C
Eggerthella.sinensis
1.00
0.75
0.50
0.25
0.00
A (n=14)
B (n=15)
C (n=11)
surface

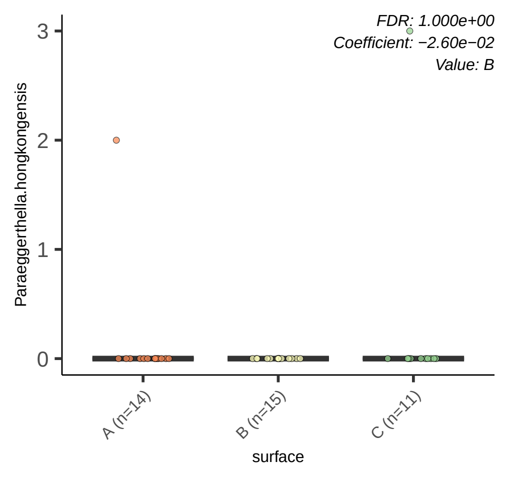
FDR: 1.000e+00
Coefficient: −2.60e−02
Value: B
Paraeggerthella.hongkongensis
3
2
1
0
A (n=14)
B (n=15)
C (n=11)
surface

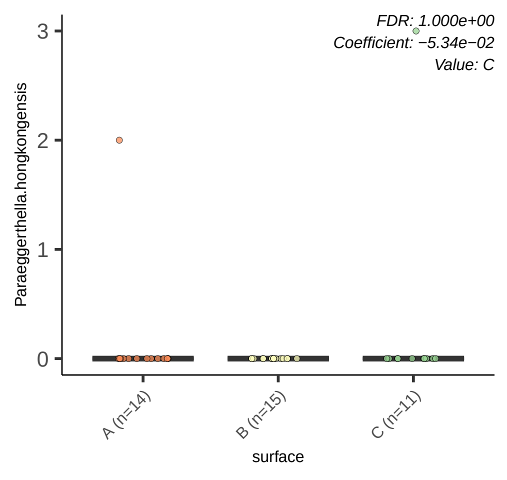

FDR: 1.000e+00
Coefficient: −5.34e−02
Value: C
Paraeggerthella.hongkongensis
3
2
1
0
A (n=14)
B (n=15)
C (n=11)
surface

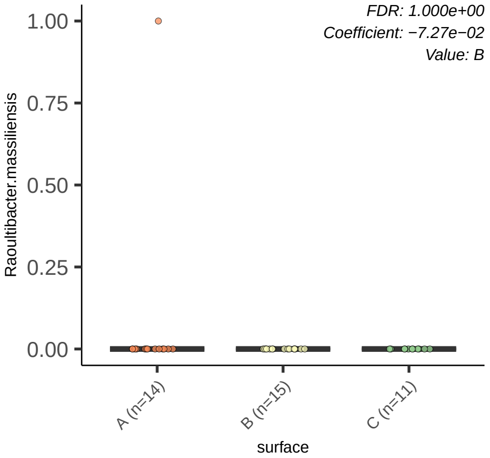
FDR: 1.000e+00
Coefficient: −7.27e−02
Value: B
Raoultibacter.massiliensis
1.00
0.75
0.50
0.25
0.00
A (n=14)
B (n=15)
C (n=11)
surface

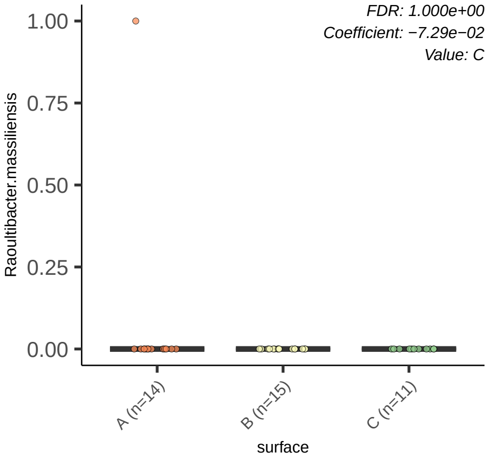
FDR: 1.000e+00
Coefficient: −7.29e−02
Value: C
1.00
0.75
0.50
0.25
0.00
Raoultibacter.massiliensis
A (n=14)
B (n=15)
C (n=11)
surface

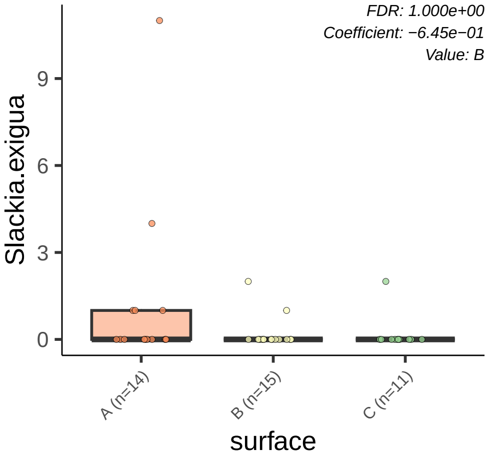

FDR: 1.000e+00
Coefficient: −6.45e−01
Value: B
Slackia.exigua
9
6
3
0
A (n=14)
B (n=15)
C (n=11)
surface

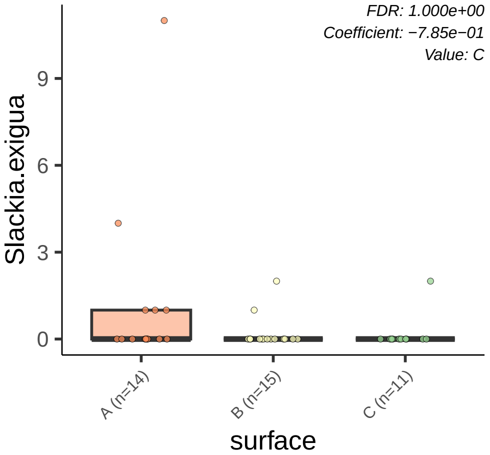

FDR: 1.000e+00
Coefficient: −7.85e−01
Value: C
Slackia.exigua
9
6
3
0
A (n=14)
B (n=15)
C (n=11)
surface

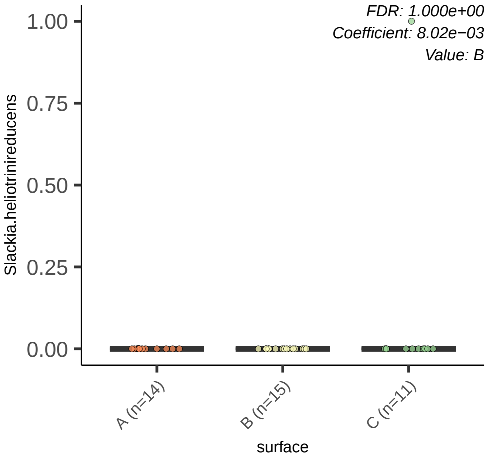
FDR: 1.000e+00
Coefficient: 8.02e−03
Value: B
1.00
0.75
0.50
0.25
0.00
Slackia.heliotrinireducens
A (n=14)
B (n=15)
C (n=11)
surface

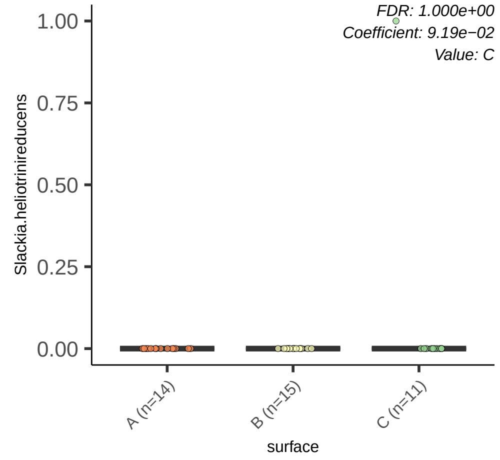

FDR: 1.000e+00
Coefficient: 9.19e−02
Value: C
1.00
0.75
0.50
0.25
0.00
Slackia.heliotrinireducens
A (n=14)
B (n=15)
C (n=11)
surface

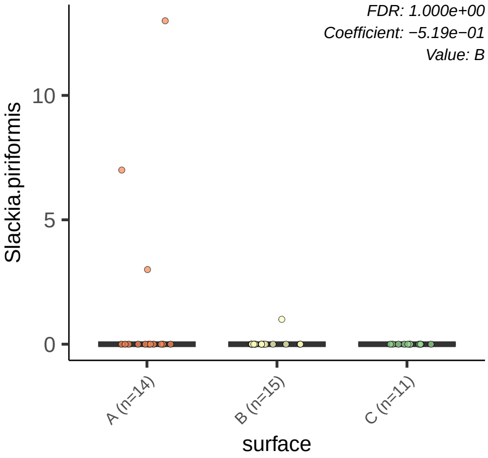

FDR: 1.000e+00
Coefficient: −5.19e−01
Value: B
Slackia.piriformis
0
5
10
A (n=14)
B (n=15)
C (n=11)
surface

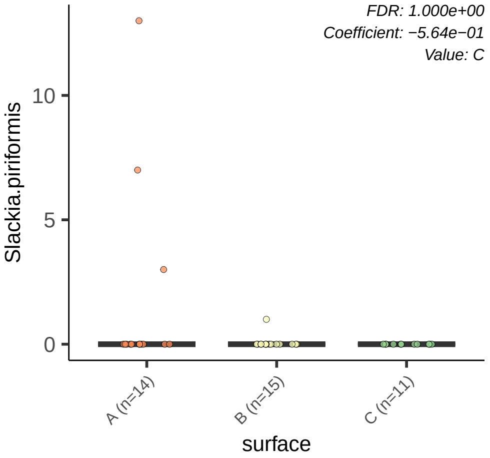

FDR: 1.000e+00
Coefficient: −5.64e−01
Value: C
Slackia.piriformis
10
5
0
A (n=14)
B (n=15)
C (n=11)
surface

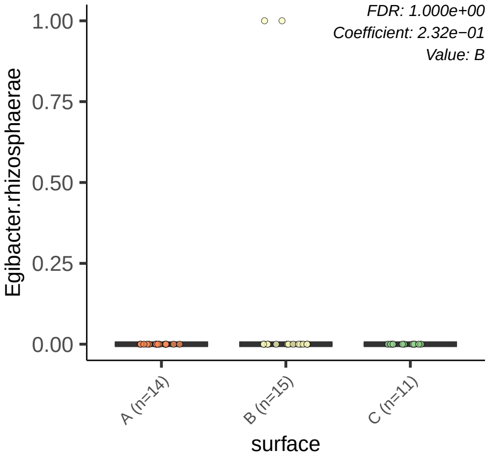
FDR: 1.000e+00
Coefficient: 2.32e−01
Value: B
Egibacter.rhizosphaerae
1.00
0.75
0.50
0.25
0.00
A (n=14)
B (n=15)
C (n=11)
surface

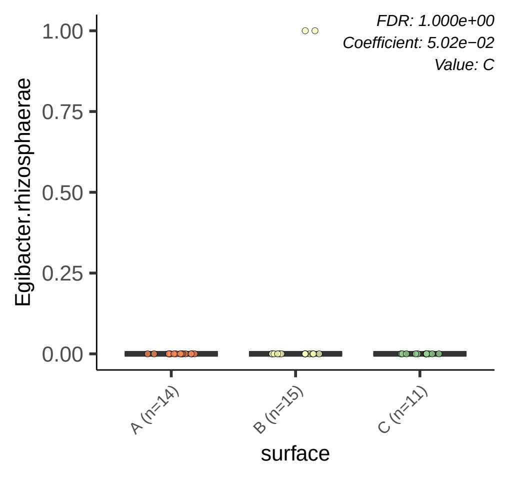

FDR: 1.000e+00
Coefficient: 5.02e−02
Value: C
1.00
0.75
0.50
0.25
0.00
Egibacter.rhizosphaerae
A (n=14)
B (n=15)
C (n=11)
surface

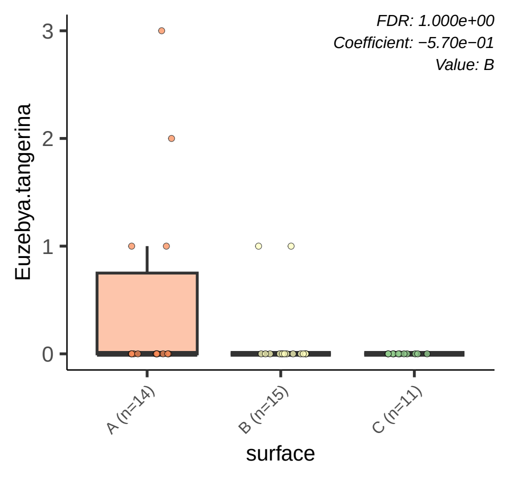
FDR: 1.000e+00
Coefficient: −5.70e−01
Value: B
Euzebya.tangerina
3
2
1
0
A (n=14)
B (n=15)
C (n=11)
surface

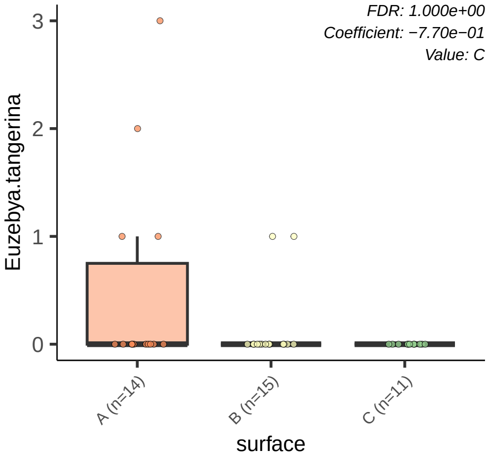

FDR: 1.000e+00
Coefficient: −7.70e−01
Value: C
Euzebya.tangerina
3
2
1
0
A (n=14)
B (n=15)
C (n=11)
surface

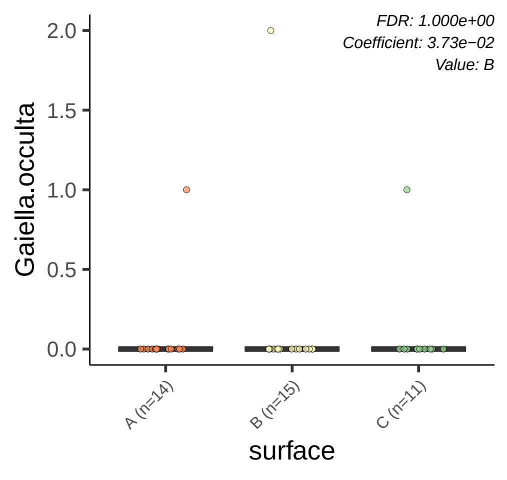
FDR: 1.000e+00
Coefficient: 3.73e−02
Value: B
Gaiella.occulta
2.0
1.5
1.0
0.5
0.0
A (n=14)
B (n=15)
C (n=11)
surface

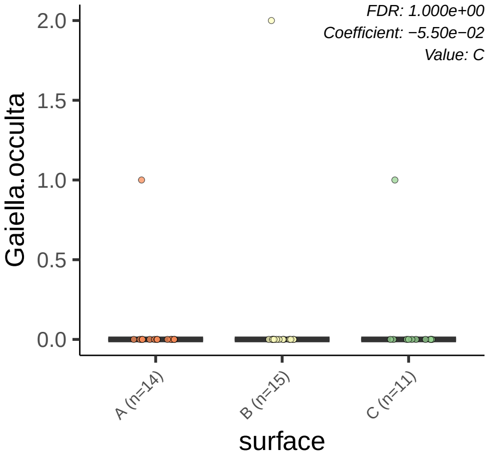

FDR: 1.000e+00
Coefficient: −5.50e−02
Value: C
2.0
1.5
1.0
0.5
0.0
Gaiella.occulta
A (n=14)
B (n=15)
C (n=11)
surface

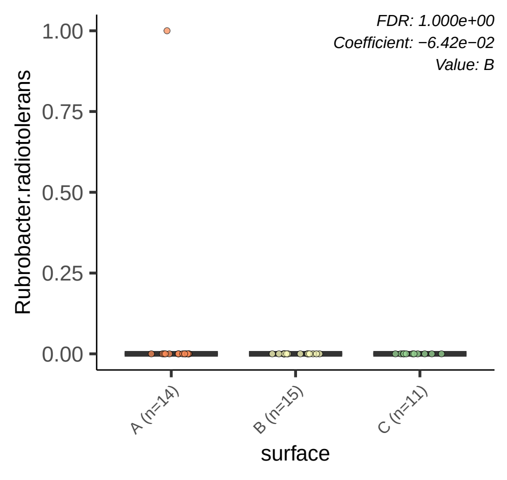
FDR: 1.000e+00
Coefficient: −6.42e−02
Value: B
Rubrobacter.radiotolerans
1.00
0.75
0.50
0.25
0.00
A (n=14)
B (n=15)
C (n=11)
surface

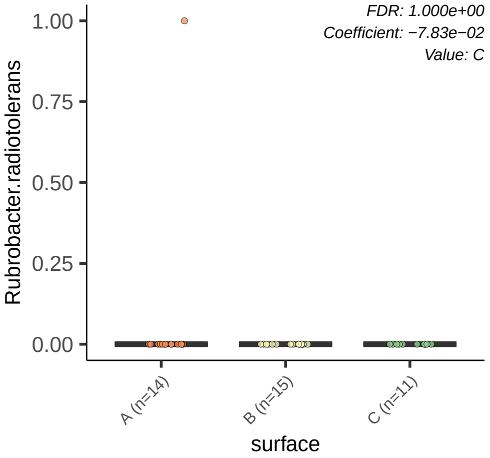

FDR: 1.000e+00
Coefficient: −7.83e−02
Value: C
Rubrobacter.radiotolerans
1.00
0.75
0.50
0.25
0.00
A (n=14)
B (n=15)
C (n=11)
surface

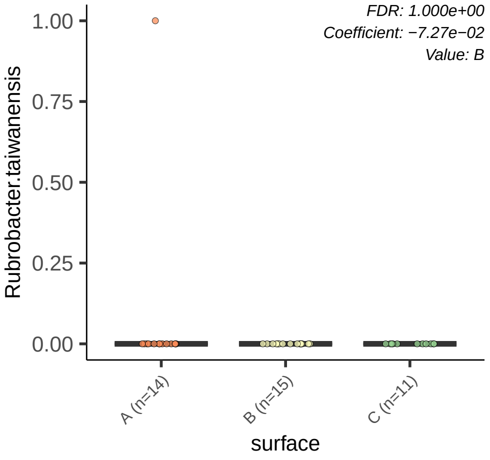
FDR: 1.000e+00
Coefficient: −7.27e−02
Value: B
Rubrobacter.taiwanensis
1.00
0.75
0.50
0.25
0.00
A (n=14)
B (n=15)
C (n=11)
surface

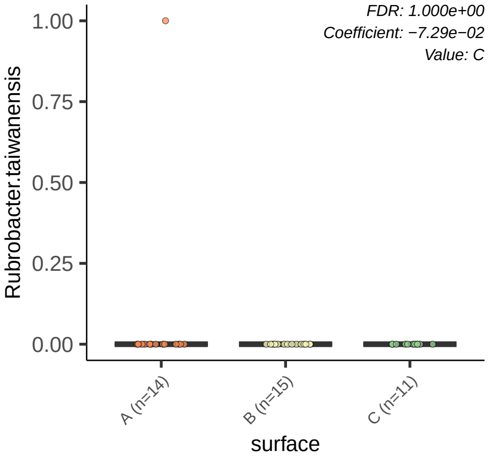

FDR: 1.000e+00
Coefficient: −7.29e−02
Value: C
Rubrobacter.taiwanensis
1.00
0.75
0.50
0.25
0.00
A (n=14)
B (n=15)
C (n=11)
surface

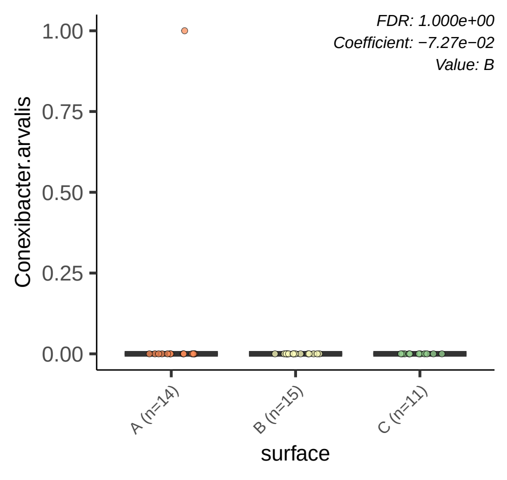
FDR: 1.000e+00
Coefficient: −7.27e−02
Value: B
Conexibacter.arvalis
1.00
0.75
0.50
0.25
0.00
A (n=14)
B (n=15)
C (n=11)
surface

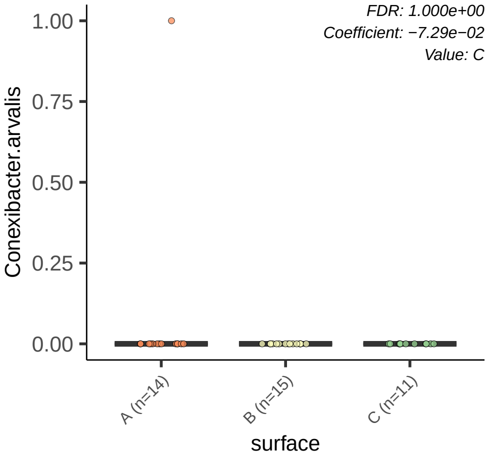
FDR: 1.000e+00
Coefficient: −7.29e−02
Value: C
Conexibacter.arvalis
1.00
0.75
0.50
0.25
0.00
A (n=14)
B (n=15)
C (n=11)
surface

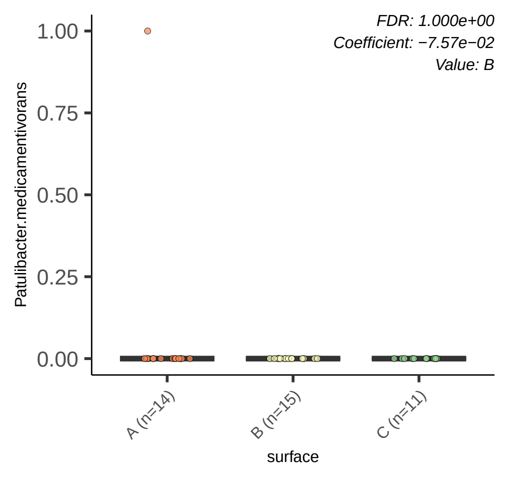
FDR: 1.000e+00
Coefficient: −7.57e−02
Value: B
Patulibacter.medicamentivorans
1.00
0.75
0.50
0.25
0.00
A (n=14)
B (n=15)
C (n=11)
surface

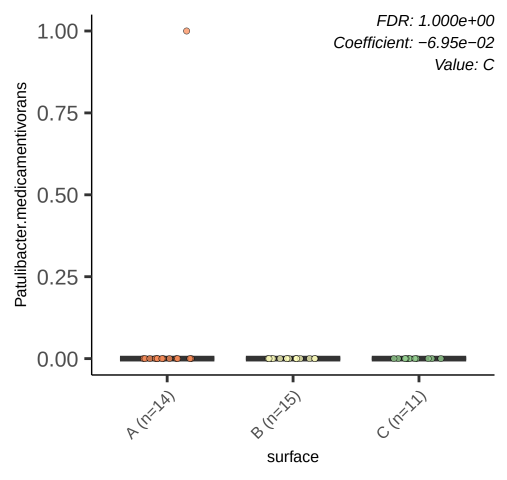
FDR: 1.000e+00
Coefficient: −6.95e−02
Value: C
Patulibacter.medicamentivorans
1.00
0.75
0.50
0.25
0.00
A (n=14)
B (n=15)
C (n=11)
surface

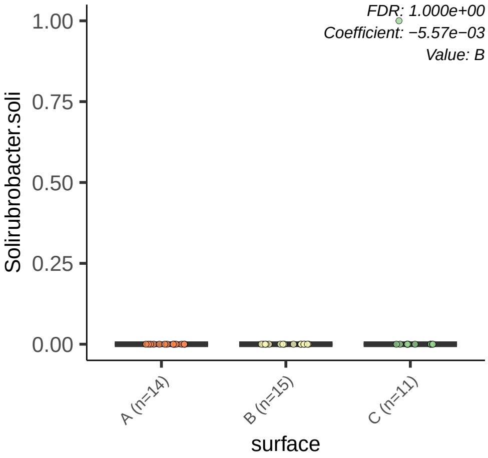
FDR: 1.000e+00
Coefficient: −5.57e−03
Value: B
Solirubrobacter.soli
1.00
0.75
0.50
0.25
0.00
A (n=14)
B (n=15)
C (n=11)
surface

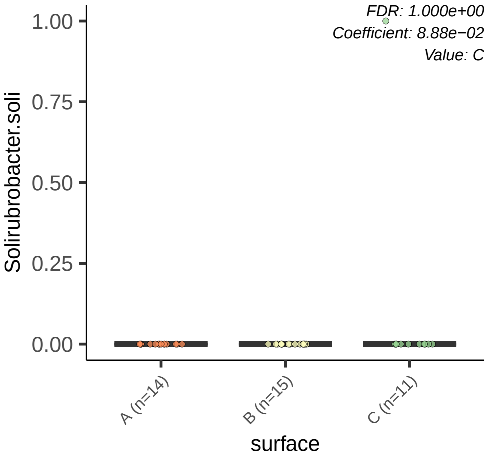
FDR: 1.000e+00
Coefficient: 8.88e−02
Value: C
Solirubrobacter.soli
1.00
0.75
0.50
0.25
0.00
A (n=14)
B (n=15)
C (n=11)
surface

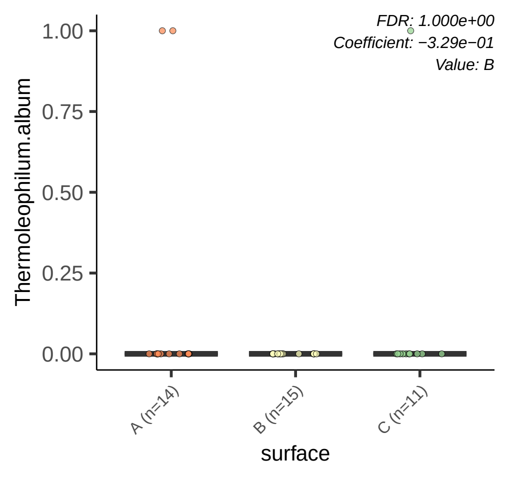
FDR: 1.000e+00
Coefficient: −3.29e−01
Value: B
Thermoleophilum.album
1.00
0.75
0.50
0.25
0.00
A (n=14)
B (n=15)
C (n=11)
surface

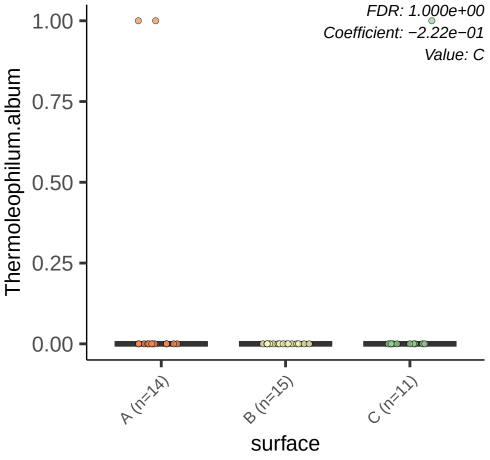

FDR: 1.000e+00
Coefficient: −2.22e−01
Value: C
Thermoleophilum.album
1.00
0.75
0.50
0.25
0.00
A (n=14)
B (n=15)
C (n=11)
surface

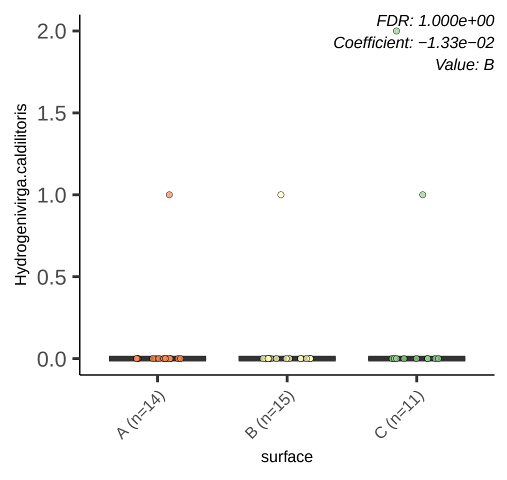
FDR: 1.000e+00
Coefficient: −1.33e−02
Value: B
Hydrogenivirga.caldilitoris
2.0
1.5
1.0
0.5
0.0
A (n=14)
B (n=15)
C (n=11)
surface

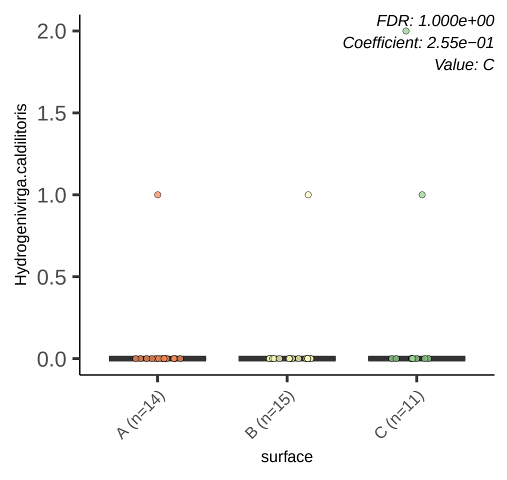
FDR: 1.000e+00
Coefficient: 2.55e−01
Value: C
Hydrogenivirga.caldilitoris
2.0
1.5
1.0
0.5
0.0
A (n=14)
B (n=15)
C (n=11)
surface

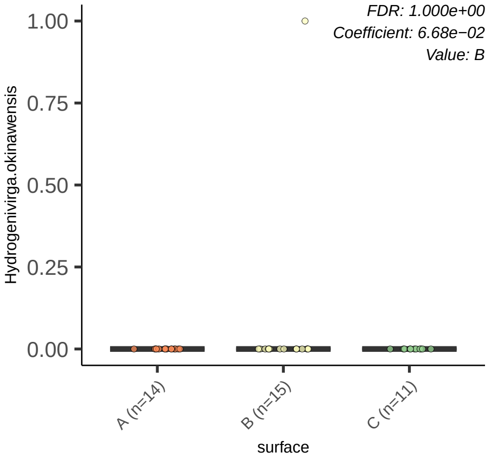
FDR: 1.000e+00
Coefficient: 6.68e−02
Value: B
Hydrogenivirga.okinawensis
1.00
0.75
0.50
0.25
0.00
A (n=14)
B (n=15)
C (n=11)
surface

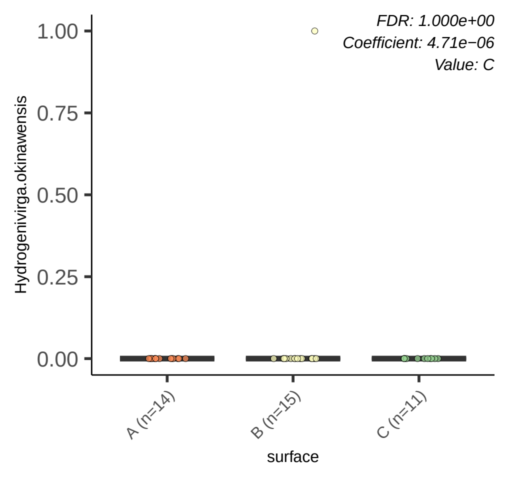
FDR: 1.000e+00
Coefficient: 4.71e−06
Value: C
Hydrogenivirga.okinawensis
1.00
0.75
0.50
0.25
0.00
A (n=14)
B (n=15)
C (n=11)
surface

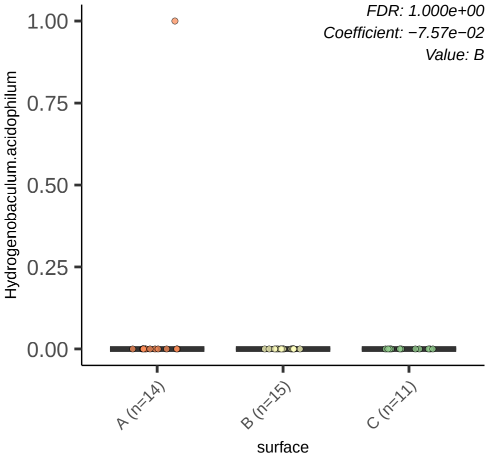

FDR: 1.000e+00
Coefficient: −7.57e−02
Value: B
Hydrogenobaculum.acidophilum
1.00
0.75
0.50
0.25
0.00
A (n=14)
B (n=15)
C (n=11)
surface

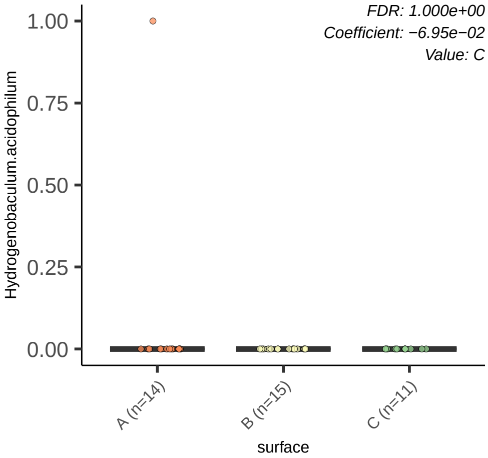
FDR: 1.000e+00
Coefficient: −6.95e−02
Value: C
Hydrogenobaculum.acidophilum
1.00
0.75
0.50
0.25
0.00
A (n=14)
B (n=15)
C (n=11)
surface

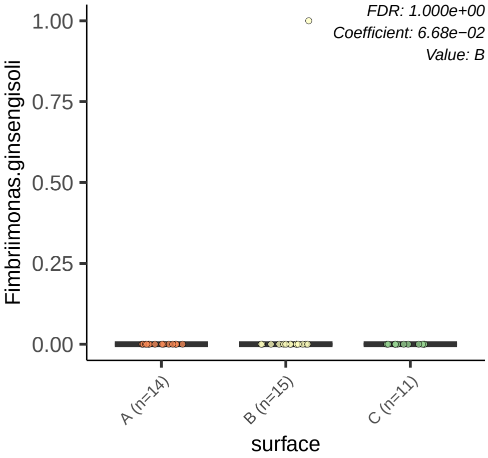

FDR: 1.000e+00
Coefficient: 6.68e−02
Value: B
1.00
0.75
0.50
0.25
0.00
Fimbriimonas.ginsengisoli
A (n=14)
B (n=15)
C (n=11)
surface

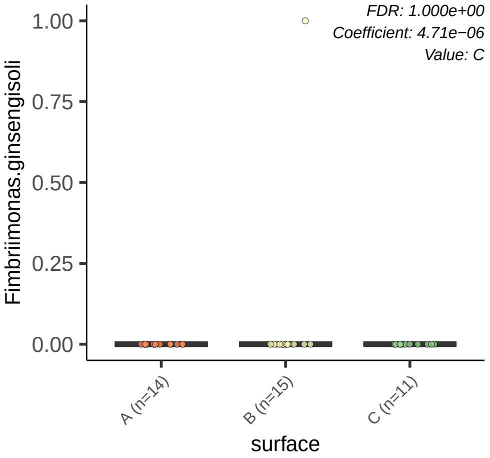
FDR: 1.000e+00
Coefficient: 4.71e−06
Value: C
Fimbriimonas.ginsengisoli
1.00
0.75
0.50
0.25
0.00
A (n=14)
B (n=15)
C (n=11)
surface

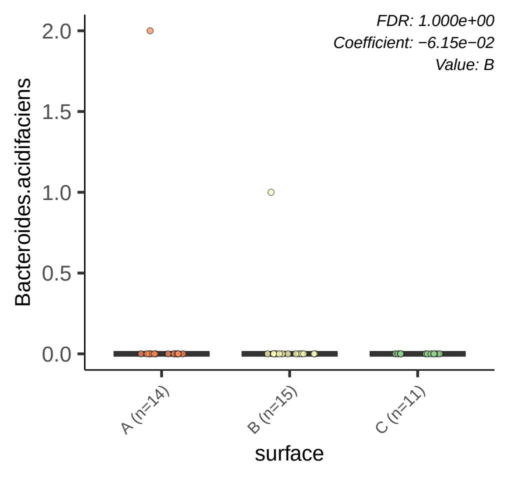
FDR: 1.000e+00
Coefficient: −6.15e−02
Value: B
Bacteroides.acidifaciens
2.0
1.5
1.0
0.5
0.0
A (n=14)
B (n=15)
C (n=11)
surface

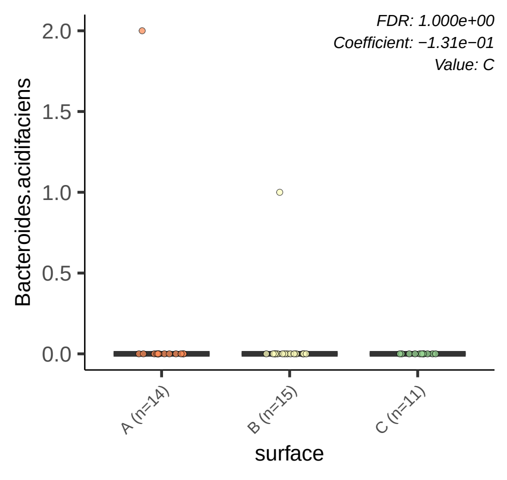

FDR: 1.000e+00
Coefficient: −1.31e−01
Value: C
Bacteroides.acidifaciens
2.0
1.5
1.0
0.5
0.0
A (n=14)
B (n=15)
C (n=11)
surface

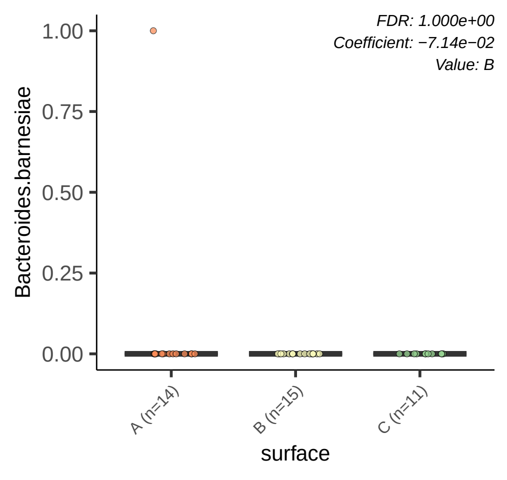
FDR: 1.000e+00
Coefficient: −7.14e−02
Value: B
Bacteroides.barnesiae
1.00
0.75
0.50
0.25
0.00
A (n=14)
B (n=15)
C (n=11)
surface

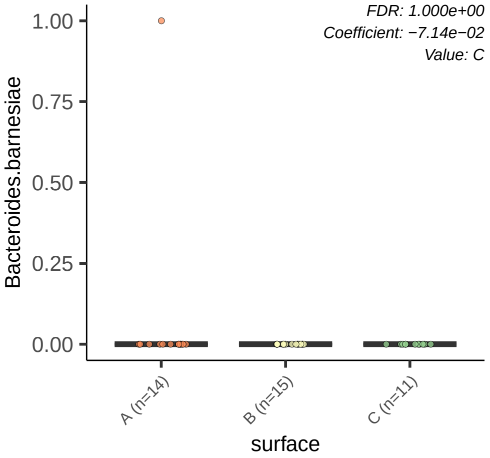

FDR: 1.000e+00
Coefficient: −7.14e−02
Value: C
Bacteroides.barnesiae
1.00
0.75
0.50
0.25
0.00
A (n=14)
B (n=15)
C (n=11)
surface

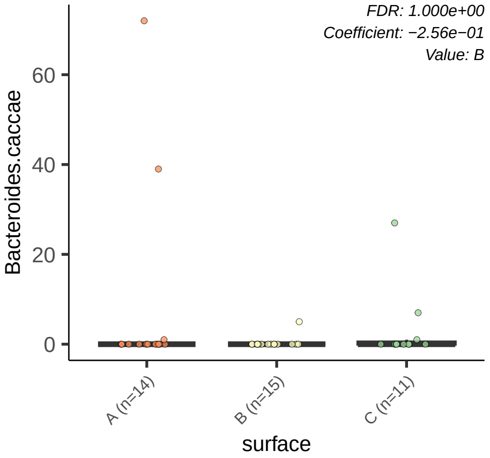
FDR: 1.000e+00
Coefficient: −2.56e−01
Value: B
Bacteroides.caccae
60
40
20
0
A (n=14)
B (n=15)
C (n=11)
surface

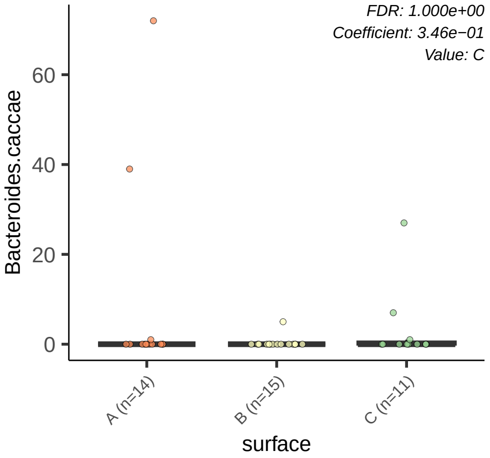

FDR: 1.000e+00
Coefficient: 3.46e−01
Value: C
Bacteroides.caccae
0
20
40
60
A (n=14)
B (n=15)
C (n=11)
surface

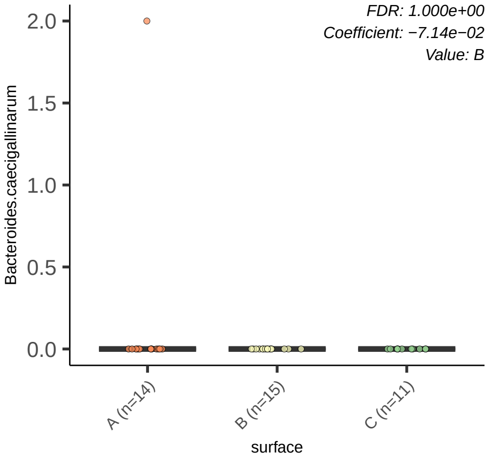

FDR: 1.000e+00
Coefficient: −7.14e−02
Value: B
Bacteroides.caecigallinarum
2.0
1.5
1.0
0.5
0.0
A (n=14)
B (n=15)
C (n=11)
surface

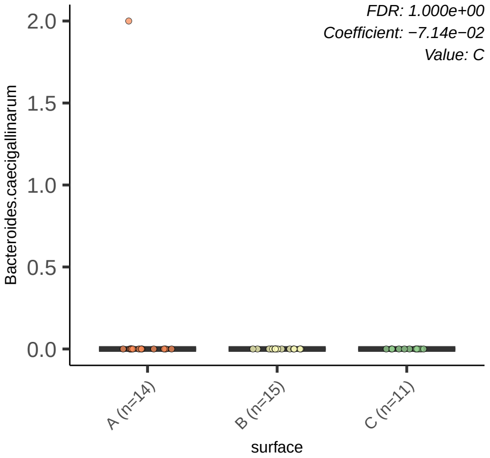
FDR: 1.000e+00
Coefficient: −7.14e−02
Value: C
Bacteroides.caecigallinarum
2.0
1.5
1.0
0.5
0.0
A (n=14)
B (n=15)
C (n=11)
surface

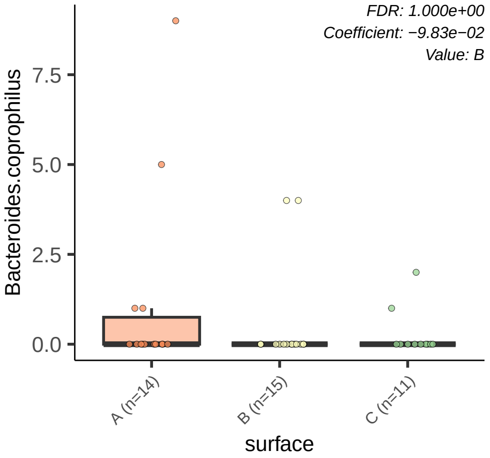
FDR: 1.000e+00
Coefficient: −9.83e−02
Value: B
Bacteroides.coprophilus
7.5
5.0
2.5
0.0
A (n=14)
B (n=15)
C (n=11)
surface

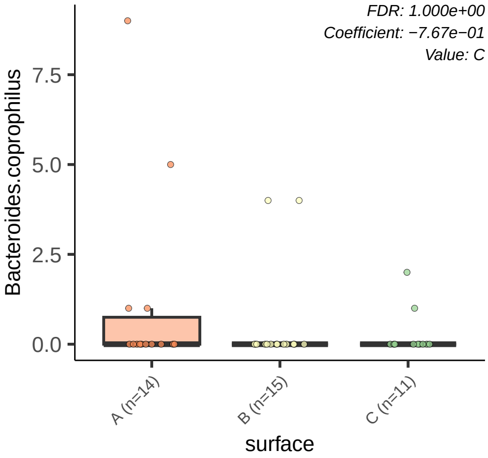

FDR: 1.000e+00
Coefficient: −7.67e−01
Value: C
Bacteroides.coprophilus
7.5
5.0
2.5
0.0
A (n=14)
B (n=15)
C (n=11)
surface

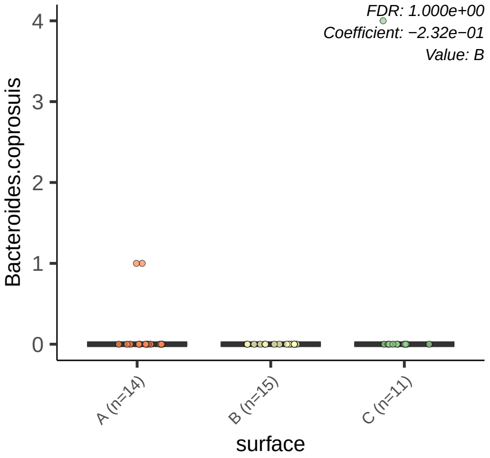
FDR: 1.000e+00
Coefficient: −2.32e−01
Value: B
Bacteroides.coprosuis
4
3
2
1
0
A (n=14)
B (n=15)
C (n=11)
surface

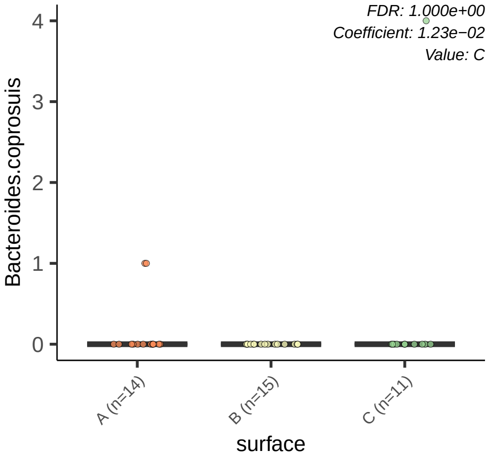

FDR: 1.000e+00
Coefficient: 1.23e−02
Value: C
Bacteroides.coprosuis
0
1
2
3
4
A (n=14)
B (n=15)
C (n=11)
surface

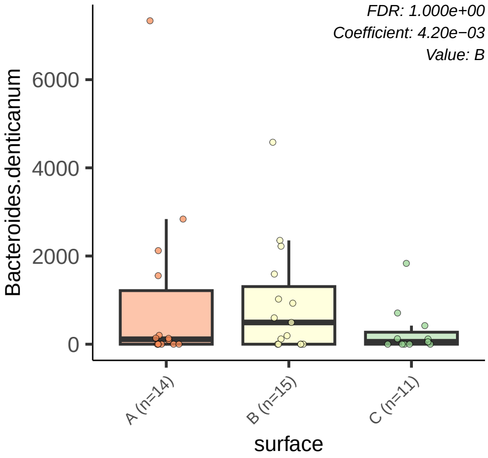
FDR: 1.000e+00
Coefficient: 4.20e−03
Value: B
Bacteroides.denticanum
6000
4000
2000
0
A (n=14)
B (n=15)
C (n=11)
surface

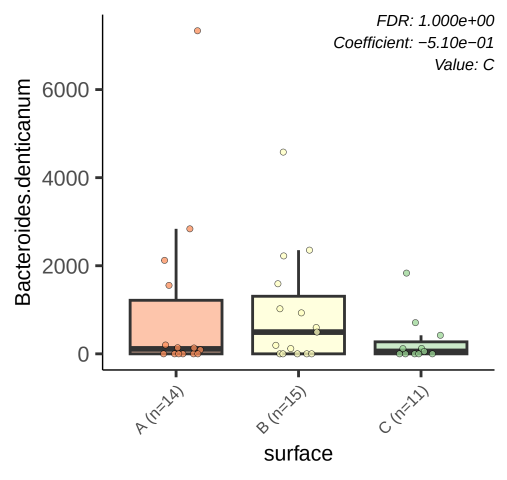
FDR: 1.000e+00
Coefficient: −5.10e−01
Value: C
Bacteroides.denticanum
0
2000
4000
6000
A (n=14)
B (n=15)
C (n=11)
surface

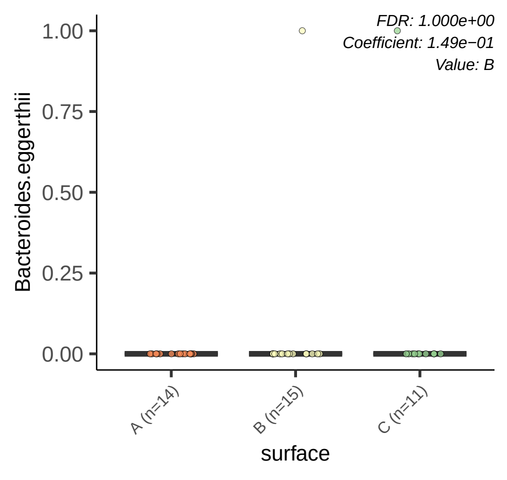
FDR: 1.000e+00
Coefficient: 1.49e−01
Value: B
Bacteroides.eggerthii
1.00
0.75
0.50
0.25
0.00
A (n=14)
B (n=15)
C (n=11)
surface

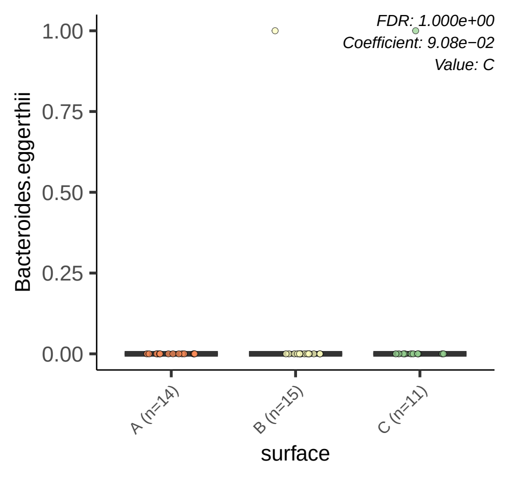
FDR: 1.000e+00
Coefficient: 9.08e−02
Value: C
Bacteroides.eggerthii
1.00
0.75
0.50
0.25
0.00
A (n=14)
B (n=15)
C (n=11)
surface

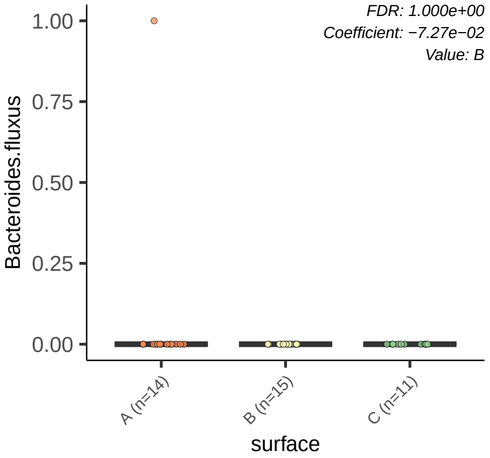
FDR: 1.000e+00
Coefficient: −7.27e−02
Value: B
Bacteroides.fluxus
1.00
0.75
0.50
0.25
0.00
A (n=14)
B (n=15)
C (n=11)
surface

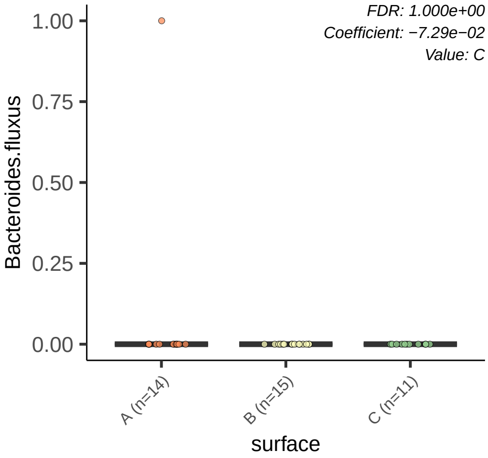
FDR: 1.000e+00
Coefficient: −7.29e−02
Value: C
1.00
0.75
0.50
0.25
0.00
Bacteroides.fluxus
A (n=14)
B (n=15)
C (n=11)
surface

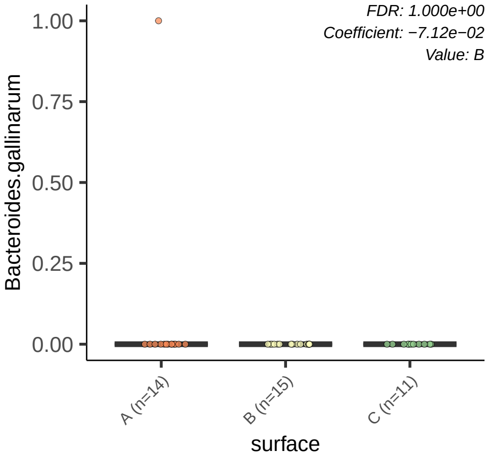
FDR: 1.000e+00
Coefficient: −7.12e−02
Value: B
Bacteroides.gallinarum
1.00
0.75
0.50
0.25
0.00
A (n=14)
B (n=15)
C (n=11)
surface

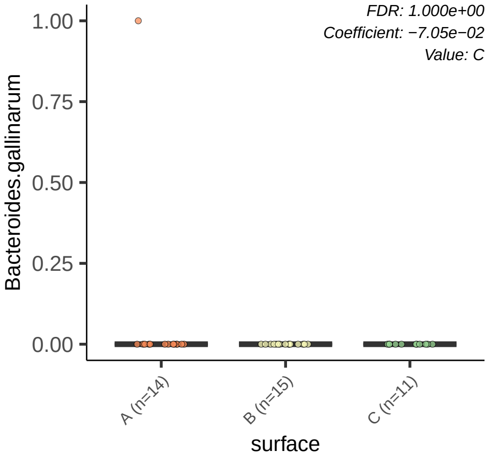
FDR: 1.000e+00
Coefficient: −7.05e−02
Value: C
Bacteroides.gallinarum
1.00
0.75
0.50
0.25
0.00
A (n=14)
B (n=15)
C (n=11)
surface

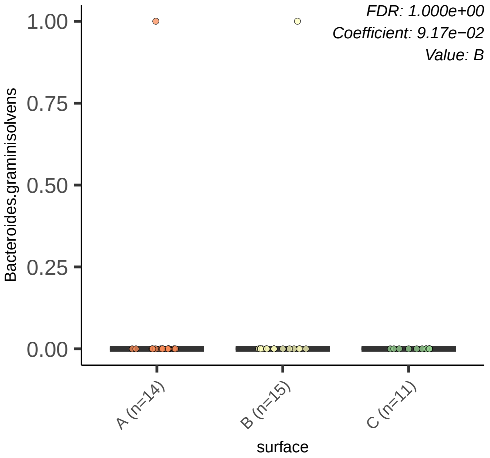

FDR: 1.000e+00
Coefficient: 9.17e−02
Value: B
Bacteroides.graminisolvens
1.00
0.75
0.50
0.25
0.00
A (n=14)
B (n=15)
C (n=11)
surface

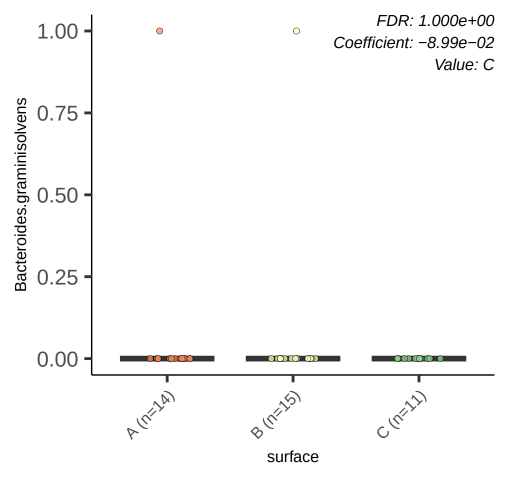

FDR: 1.000e+00
Coefficient: −8.99e−02
Value: C
1.00
0.75
0.50
0.25
0.00
Bacteroides.graminisolvens
A (n=14)
B (n=15)
C (n=11)
surface

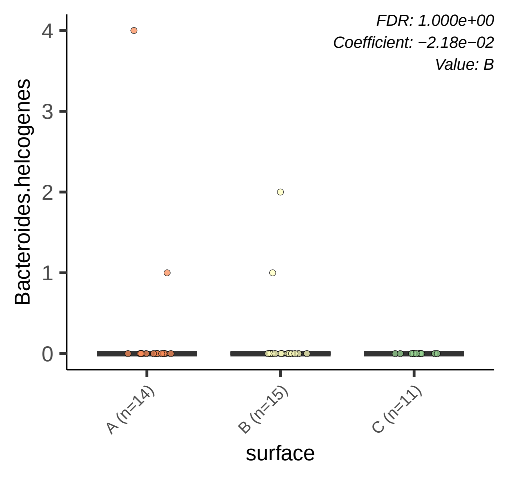
FDR: 1.000e+00
Coefficient: −2.18e−02
Value: B
Bacteroides.helcogenes
0
1
2
3
4
A (n=14)
B (n=15)
C (n=11)
surface

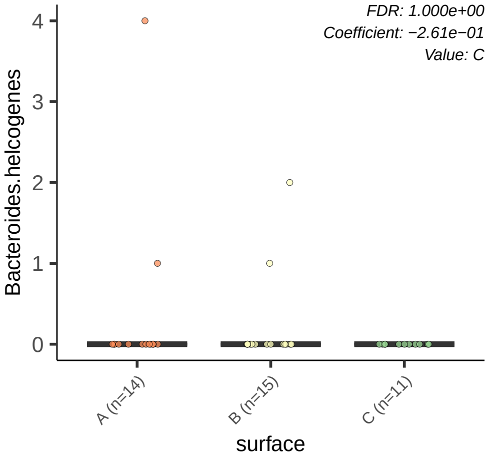
FDR: 1.000e+00
Coefficient: −2.61e−01
Value: C
Bacteroides.helcogenes
0
1
2
3
4
A (n=14)
B (n=15)
C (n=11)
surface

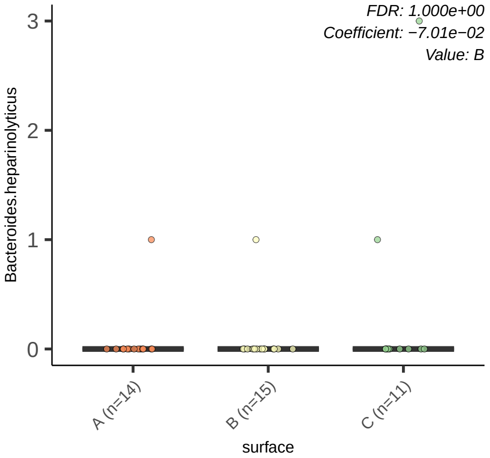
FDR: 1.000e+00
Coefficient: −7.01e−02
Value: B
Bacteroides.heparinolyticus
0
1
2
3
A (n=14)
B (n=15)
C (n=11)
surface

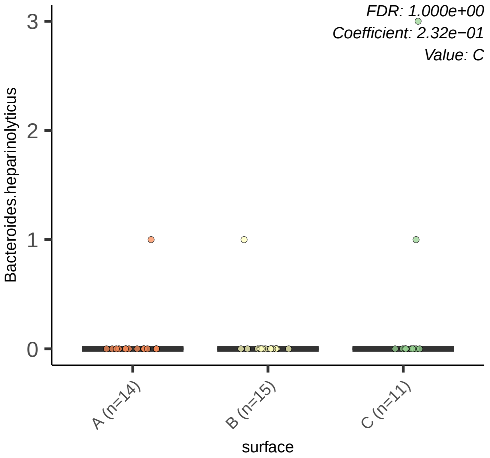
FDR: 1.000e+00
Coefficient: 2.32e−01
Value: C
Bacteroides.heparinolyticus
3
2
1
0
A (n=14)
B (n=15)
C (n=11)
surface

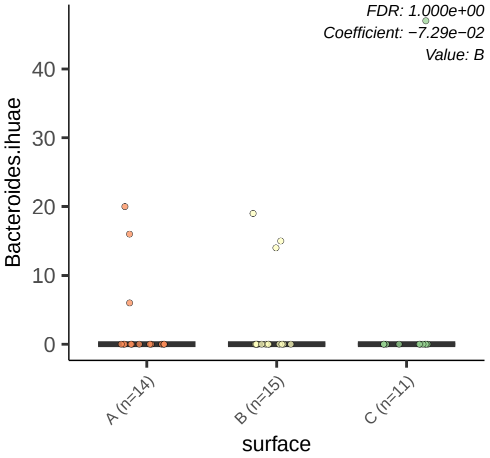
FDR: 1.000e+00
Coefficient: −7.29e−02
Value: B
Bacteroides.ihuae
40
30
20
10
0
A (n=14)
B (n=15)
C (n=11)
surface

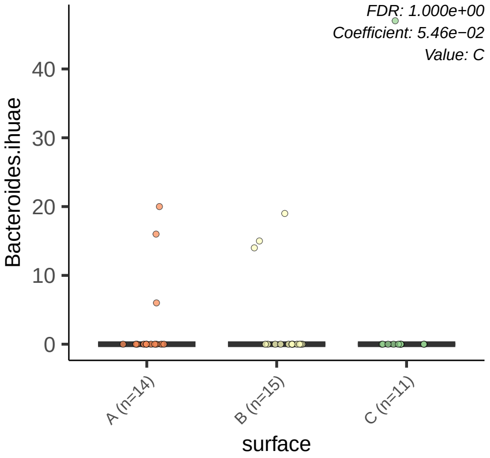
FDR: 1.000e+00
Coefficient: 5.46e−02
Value: C
Bacteroides.ihuae
40
30
20
10
0
A (n=14)
B (n=15)
C (n=11)
surface

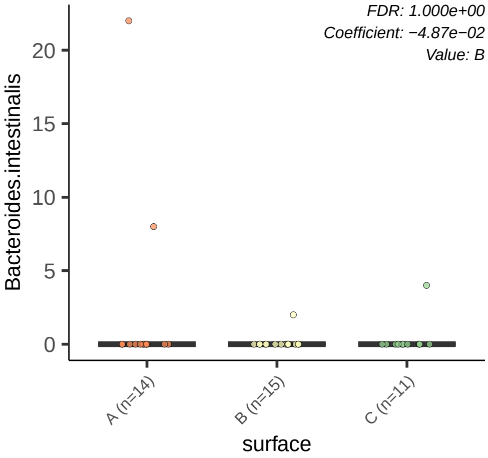
FDR: 1.000e+00
Coefficient: −4.87e−02
Value: B
Bacteroides.intestinalis
0
5
10
15
20
A (n=14)
B (n=15)
C (n=11)
surface

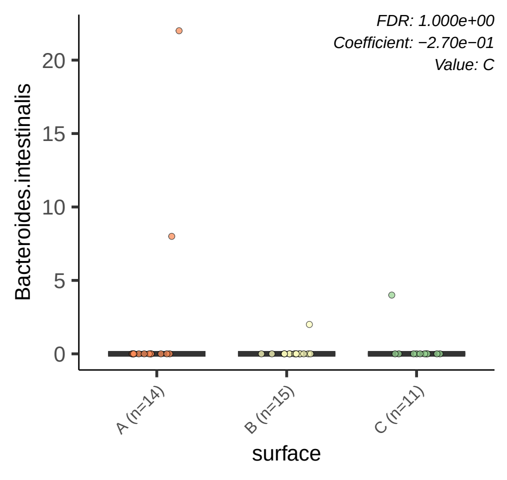

FDR: 1.000e+00
Coefficient: −2.70e−01
Value: C
Bacteroides.intestinalis
20
15
10
5
0
A (n=14)
B (n=15)
C (n=11)
surface

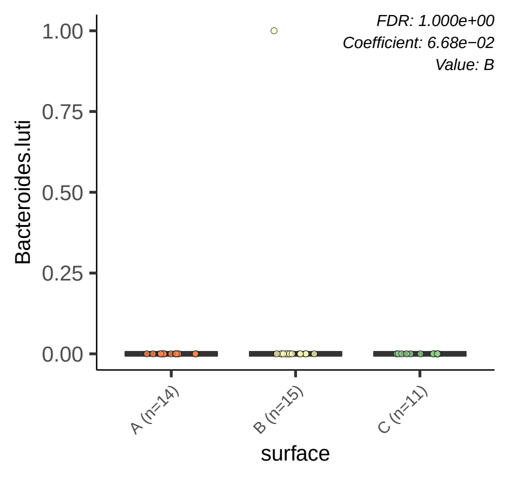

FDR: 1.000e+00
Coefficient: 6.68e−02
Value: B
Bacteroides.luti
1.00
0.75
0.50
0.25
0.00
A (n=14)
B (n=15)
C (n=11)
surface

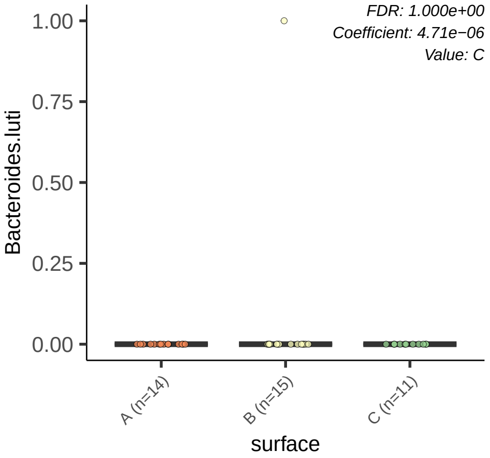

FDR: 1.000e+00
Coefficient: 4.71e−06
Value: C
1.00
0.75
0.50
0.25
0.00
Bacteroides.luti
A (n=14)
B (n=15)
C (n=11)
surface

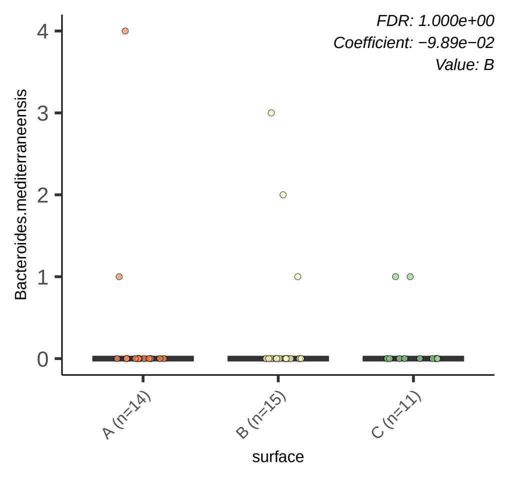
FDR: 1.000e+00
Coefficient: −9.89e−02
Value: B
Bacteroides.mediterraneensis
4
3
2
1
0
A (n=14)
B (n=15)
C (n=11)
surface

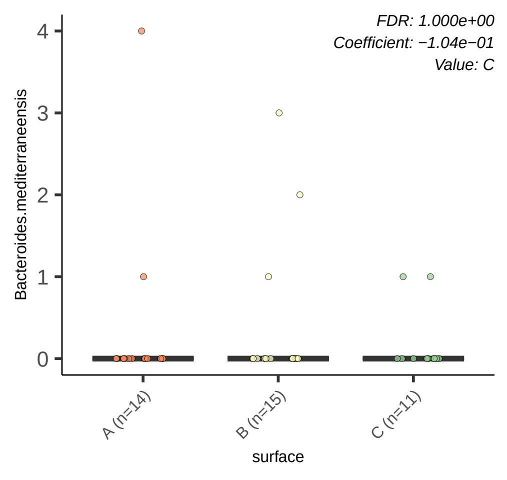

FDR: 1.000e+00
Coefficient: −1.04e−01
Value: C
Bacteroides.mediterraneensis
4
3
2
1
0
A (n=14)
B (n=15)
C (n=11)
surface

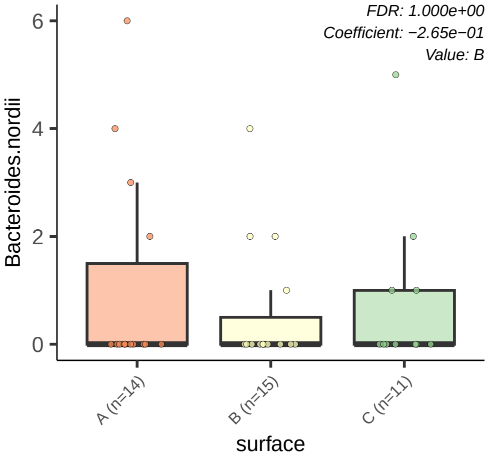

FDR: 1.000e+00
Coefficient: −2.65e−01
Value: B
Bacteroides.nordii
0
2
4
6
A (n=14)
B (n=15)
C (n=11)
surface

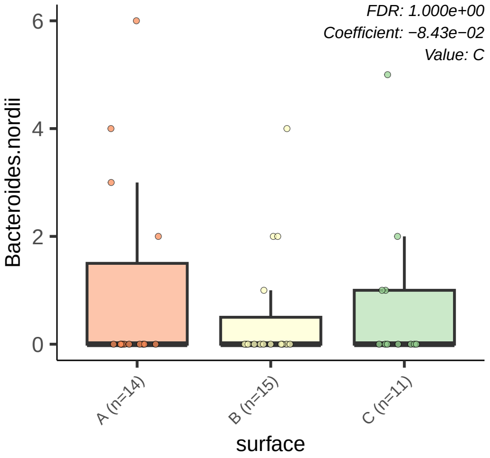
FDR: 1.000e+00
Coefficient: −8.43e−02
Value: C
Bacteroides.nordii
0
2
4
6
A (n=14)
B (n=15)
C (n=11)
surface

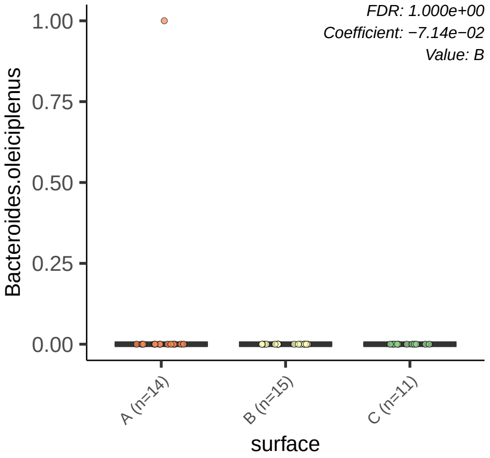
FDR: 1.000e+00
Coefficient: −7.14e−02
Value: B
Bacteroides.oleiciplenus
1.00
0.75
0.50
0.25
0.00
A (n=14)
B (n=15)
C (n=11)
surface

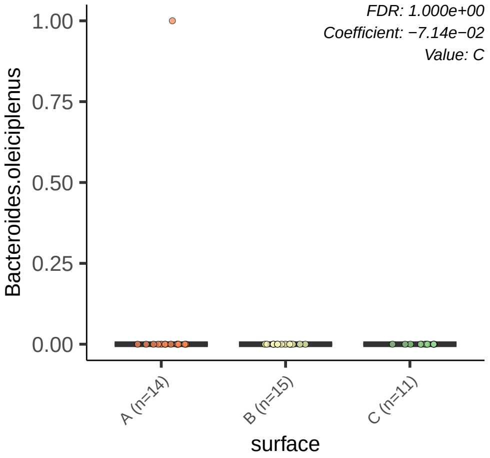
FDR: 1.000e+00
Coefficient: −7.14e−02
Value: C
Bacteroides.oleiciplenus
1.00
0.75
0.50
0.25
0.00
A (n=14)
B (n=15)
C (n=11)
surface

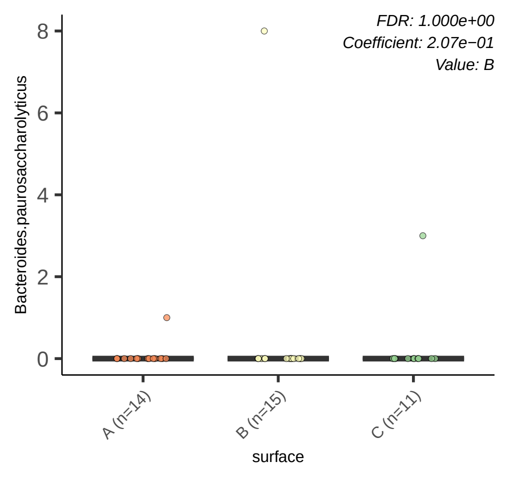
FDR: 1.000e+00
Coefficient: 2.07e−01
Value: B
Bacteroides.paurosaccharolyticus
8
6
4
2
0
A (n=14)
B (n=15)
C (n=11)
surface

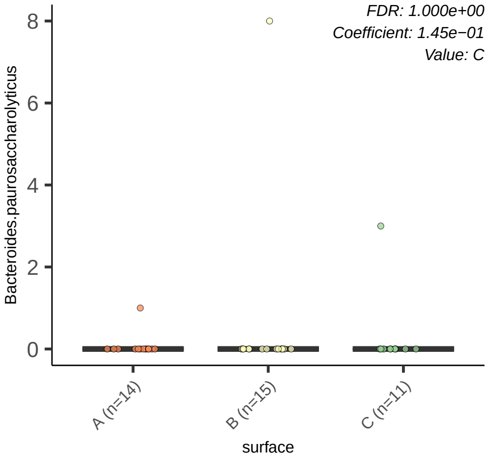
FDR: 1.000e+00
Coefficient: 1.45e−01
Value: C
Bacteroides.paurosaccharolyticus
8
6
4
2
0
A (n=14)
B (n=15)
C (n=11)
surface

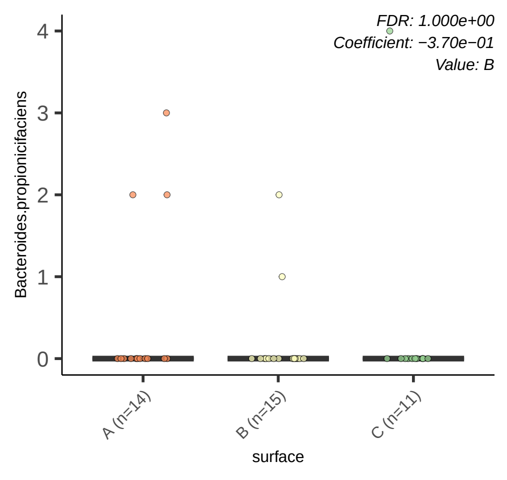
FDR: 1.000e+00
Coefficient: −3.70e−01
Value: B
Bacteroides.propionicifaciens
4
3
2
1
0
A (n=14)
B (n=15)
C (n=11)
surface

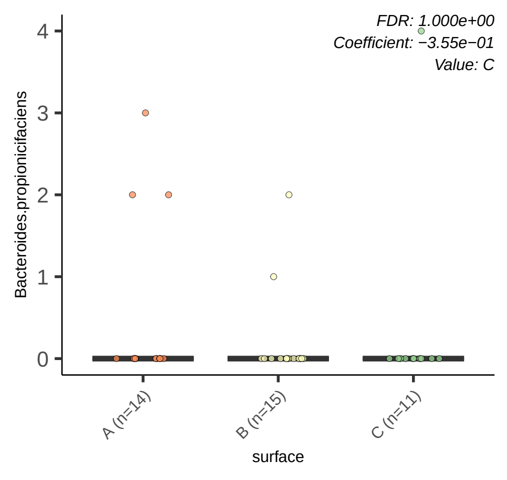

FDR: 1.000e+00
Coefficient: −3.55e−01
Value: C
Bacteroides.propionicifaciens
0
1
2
3
4
A (n=14)
B (n=15)
C (n=11)
surface

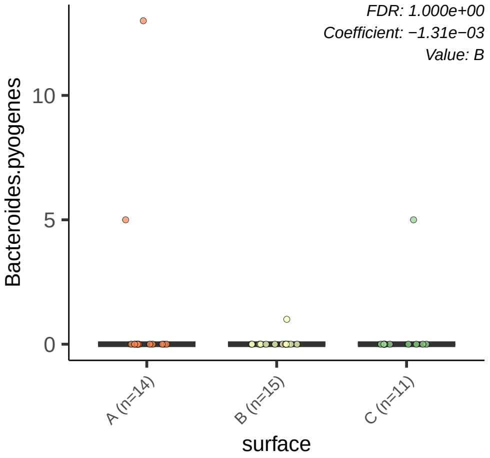

FDR: 1.000e+00
Coefficient: −1.31e−03
Value: B
Bacteroides.pyogenes
10
5
0
A (n=14)
B (n=15)
C (n=11)
surface

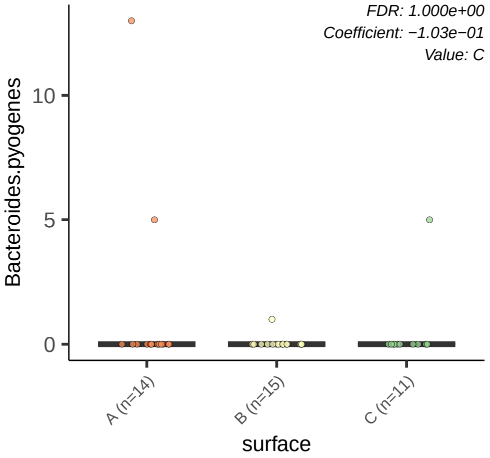
FDR: 1.000e+00
Coefficient: −1.03e−01
Value: C
Bacteroides.pyogenes
10
5
0
A (n=14)
B (n=15)
C (n=11)
surface

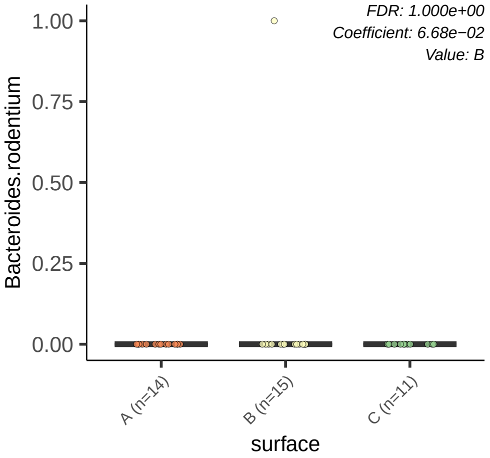
FDR: 1.000e+00
Coefficient: 6.68e−02
Value: B
Bacteroides.rodentium
1.00
0.75
0.50
0.25
0.00
A (n=14)
B (n=15)
C (n=11)
surface

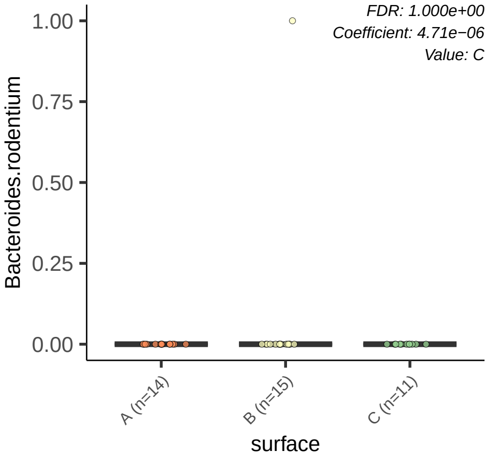

FDR: 1.000e+00
Coefficient: 4.71e−06
Value: C
Bacteroides.rodentium
1.00
0.75
0.50
0.25
0.00
A (n=14)
B (n=15)
C (n=11)
surface

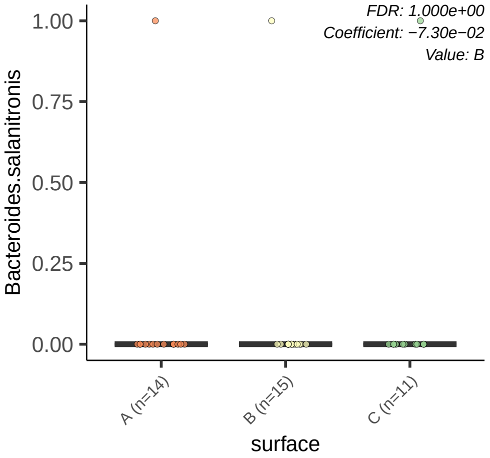

FDR: 1.000e+00
Coefficient: −7.30e−02
Value: B
Bacteroides.salanitronis
1.00
0.75
0.50
0.25
0.00
A (n=14)
B (n=15)
C (n=11)
surface

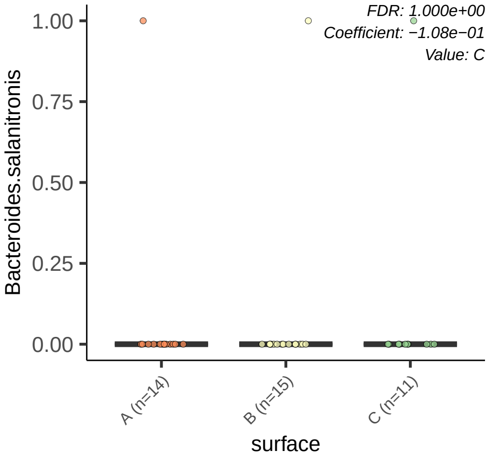

FDR: 1.000e+00
Coefficient: −1.08e−01
Value: C
Bacteroides.salanitronis
1.00
0.75
0.50
0.25
0.00
A (n=14)
B (n=15)
C (n=11)
surface

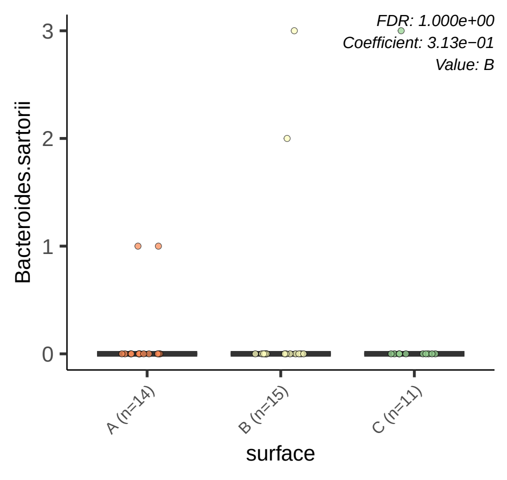
FDR: 1.000e+00
Coefficient: 3.13e−01
Value: B
Bacteroides.sartorii
3
2
1
0
A (n=14)
B (n=15)
C (n=11)
surface

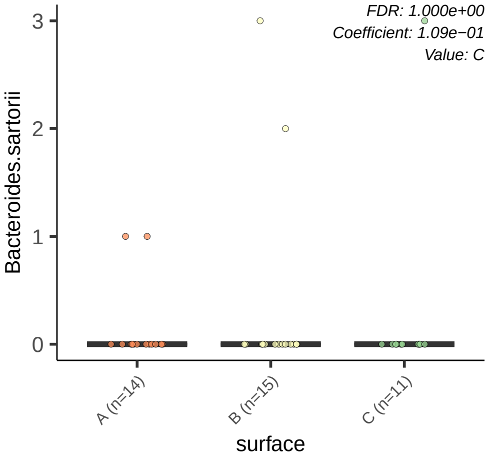
FDR: 1.000e+00
Coefficient: 1.09e−01
Value: C
Bacteroides.sartorii
3
2
1
0
A (n=14)
B (n=15)
C (n=11)
surface

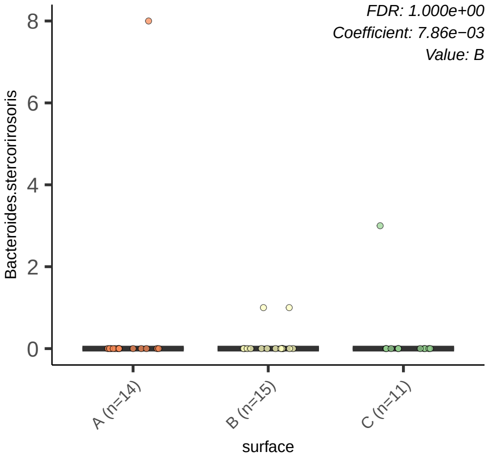

FDR: 1.000e+00
Coefficient: 7.86e−03
Value: B
Bacteroides.stercorirosoris
0
2
4
6
8
A (n=14)
B (n=15)
C (n=11)
surface

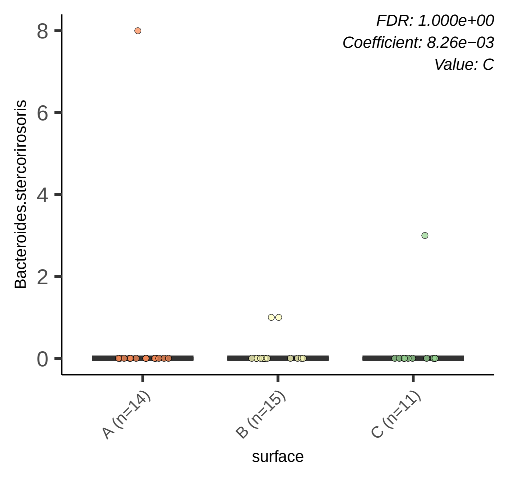

FDR: 1.000e+00
Coefficient: 8.26e−03
Value: C
Bacteroides.stercorirosoris
0
2
4
6
8
A (n=14)
B (n=15)
C (n=11)
surface

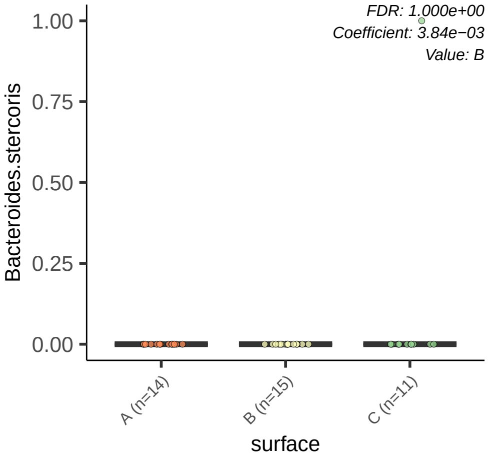
FDR: 1.000e+00
Coefficient: 3.84e−03
Value: B
Bacteroides.stercoris
1.00
0.75
0.50
0.25
0.00
A (n=14)
B (n=15)
C (n=11)
surface

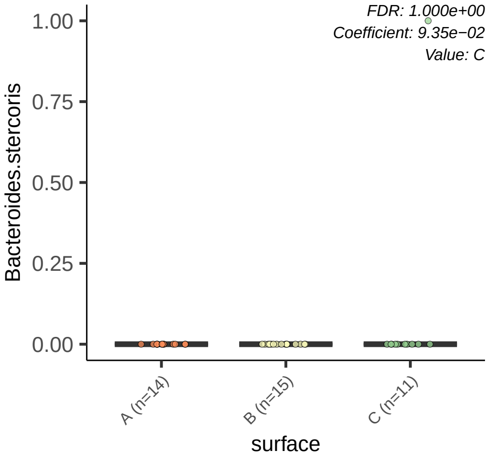
FDR: 1.000e+00
Coefficient: 9.35e−02
Value: C
Bacteroides.stercoris
1.00
0.75
0.50
0.25
0.00
A (n=14)
B (n=15)
C (n=11)
surface

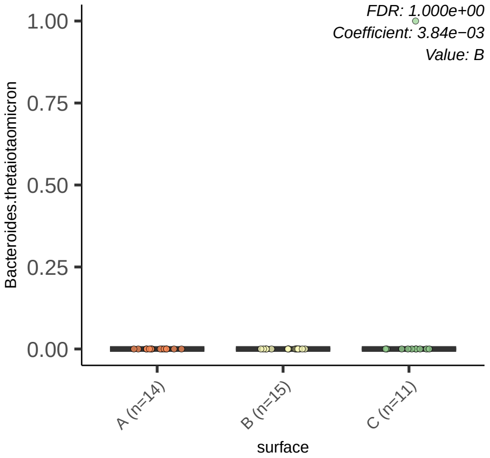
FDR: 1.000e+00
Coefficient: 3.84e−03
Value: B
1.00
0.75
0.50
0.25
0.00
Bacteroides.thetaiotaomicron
A (n=14)
B (n=15)
C (n=11)
surface

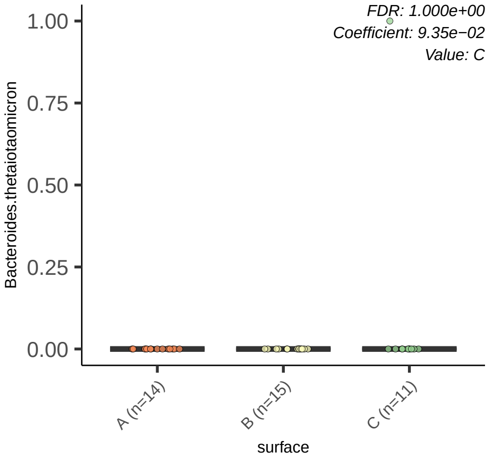

FDR: 1.000e+00
Coefficient: 9.35e−02
Value: C
1.00
0.75
0.50
0.25
0.00
Bacteroides.thetaiotaomicron
A (n=14)
B (n=15)
C (n=11)
surface

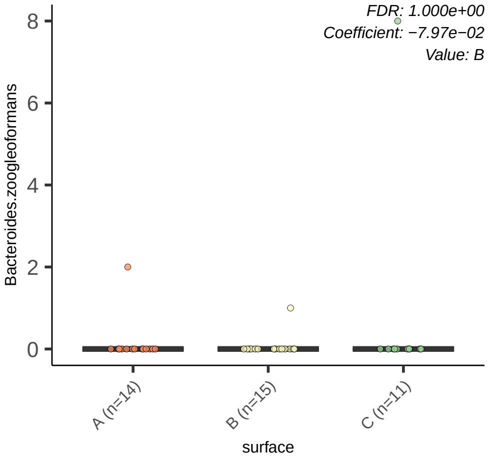

FDR: 1.000e+00
Coefficient: −7.97e−02
Value: B
Bacteroides.zoogleoformans
8
6
4
2
0
A (n=14)
B (n=15)
C (n=11)
surface

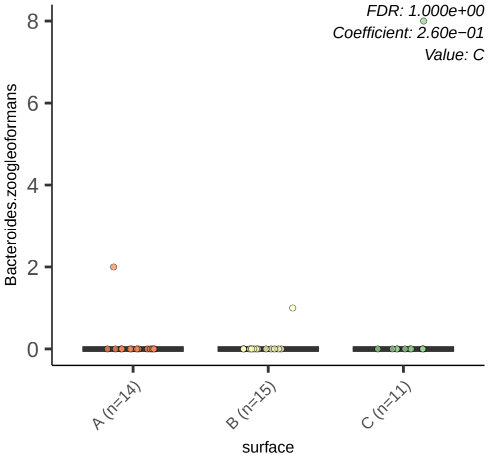
FDR: 1.000e+00
Coefficient: 2.60e−01
Value: C
Bacteroides.zoogleoformans
8
6
4
2
0
A (n=14)
B (n=15)
C (n=11)
surface

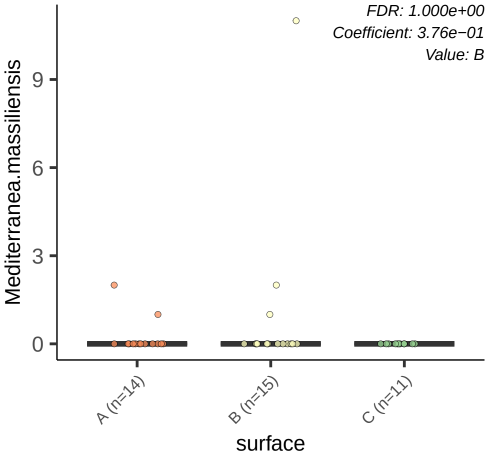
FDR: 1.000e+00
Coefficient: 3.76e−01
Value: B
Mediterranea.massiliensis
0
3
6
9
A (n=14)
B (n=15)
C (n=11)
surface

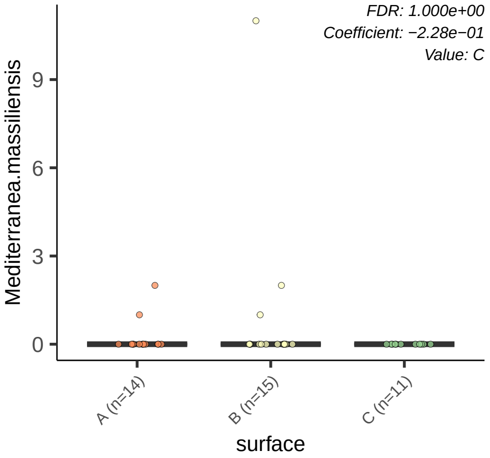
FDR: 1.000e+00
Coefficient: −2.28e−01
Value: C
Mediterranea.massiliensis
0
3
6
9
A (n=14)
B (n=15)
C (n=11)
surface

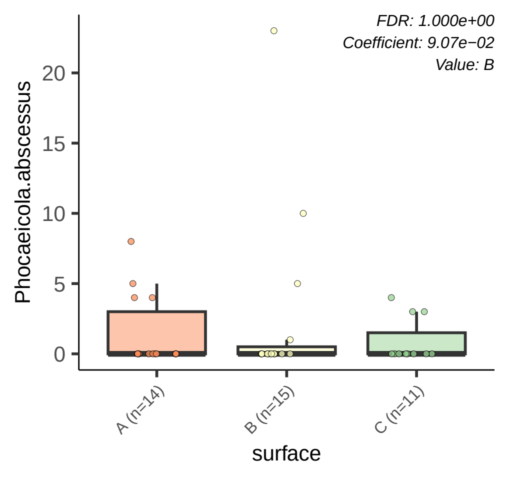

FDR: 1.000e+00
Coefficient: 9.07e−02
Value: B
Phocaeicola.abscessus
0
5
10
15
20
A (n=14)
B (n=15)
C (n=11)
surface

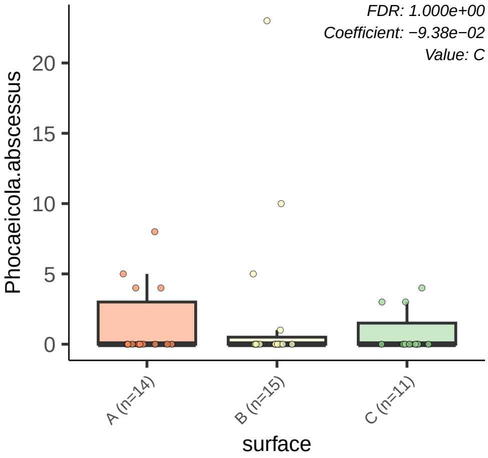

FDR: 1.000e+00
Coefficient: −9.38e−02
Value: C
Phocaeicola.abscessus
0
5
10
15
20
A (n=14)
B (n=15)
C (n=11)
surface

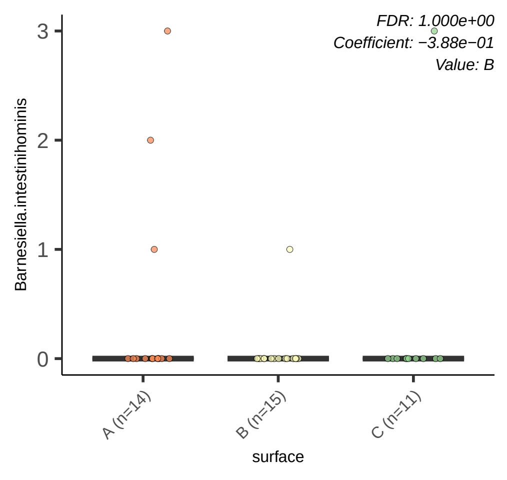
FDR: 1.000e+00
Coefficient: −3.88e−01
Value: B
Barnesiella.intestinihominis
0
1
2
3
A (n=14)
B (n=15)
C (n=11)
surface

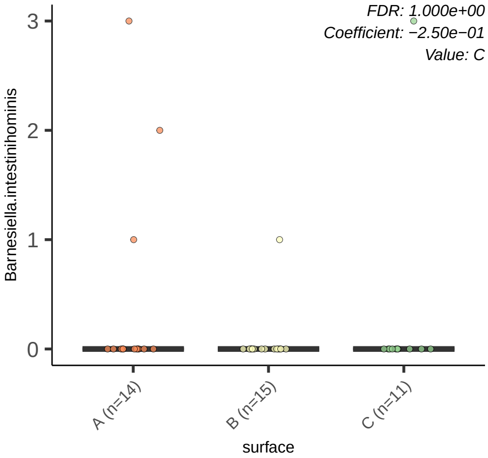
FDR: 1.000e+00
Coefficient: −2.50e−01
Value: C
Barnesiella.intestinihominis
3
2
1
0
A (n=14)
B (n=15)
C (n=11)
surface

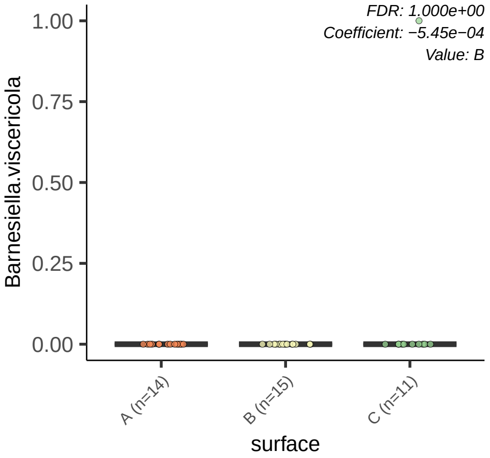
FDR: 1.000e+00
Coefficient: −5.45e−04
Value: B
Barnesiella.viscericola
1.00
0.75
0.50
0.25
0.00
A (n=14)
B (n=15)
C (n=11)
surface

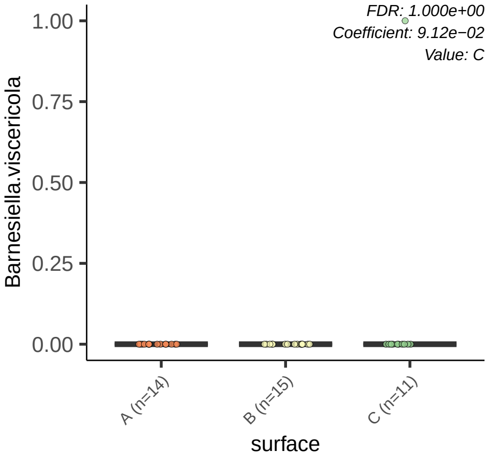

FDR: 1.000e+00
Coefficient: 9.12e−02
Value: C
1.00
0.75
0.50
0.25
0.00
Barnesiella.viscericola
A (n=14)
B (n=15)
C (n=11)
surface

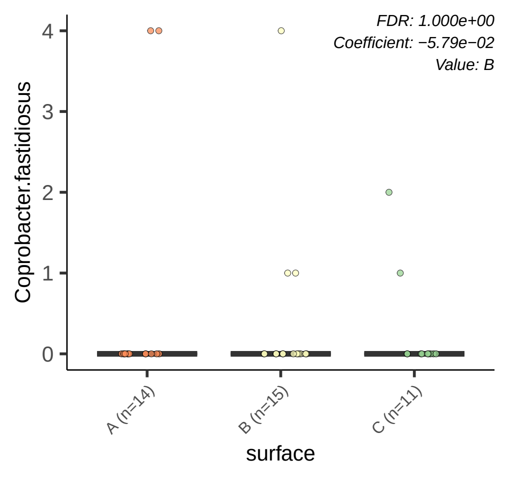
FDR: 1.000e+00
Coefficient: −5.79e−02
Value: B
Coprobacter.fastidiosus
4
3
2
1
0
A (n=14)
B (n=15)
C (n=11)
surface

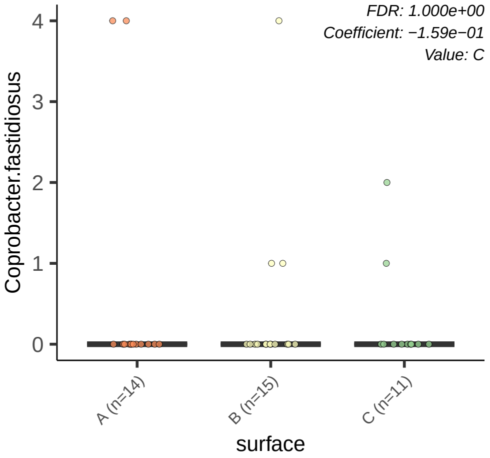
FDR: 1.000e+00
Coefficient: −1.59e−01
Value: C
Coprobacter.fastidiosus
4
3
2
1
0
A (n=14)
B (n=15)
C (n=11)
surface

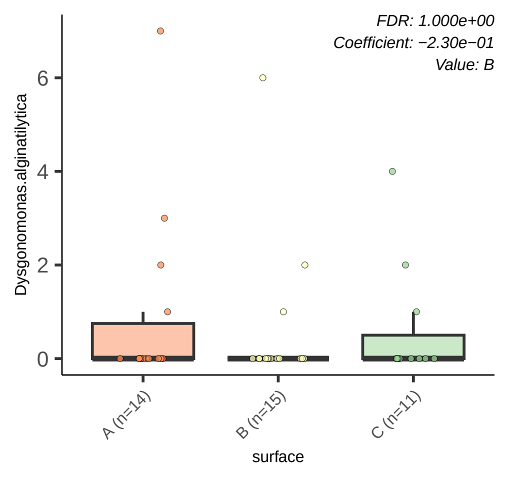
FDR: 1.000e+00
Coefficient: −2.30e−01
Value: B
Dysgonomonas.alginatilytica
0
2
4
6
A (n=14)
B (n=15)
C (n=11)
surface

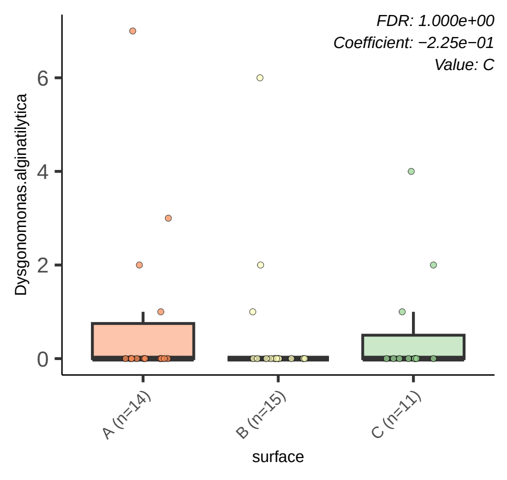

FDR: 1.000e+00
Coefficient: −2.25e−01
Value: C
Dysgonomonas.alginatilytica
0
2
4
6
A (n=14)
B (n=15)
C (n=11)
surface

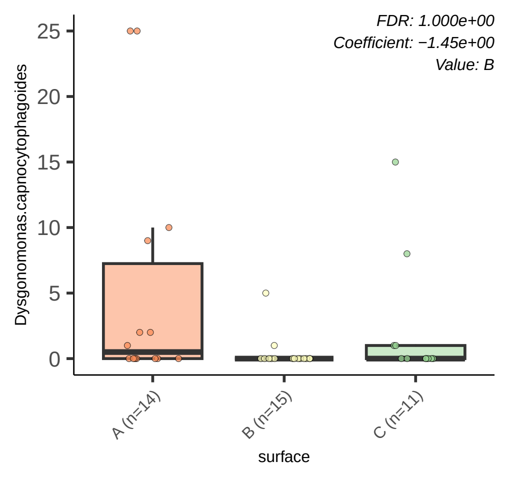

FDR: 1.000e+00
Coefficient: −1.45e+00
Value: B
Dysgonomonas.capnocytophagoides
0
5
10
15
20
25
A (n=14)
B (n=15)
C (n=11)
surface

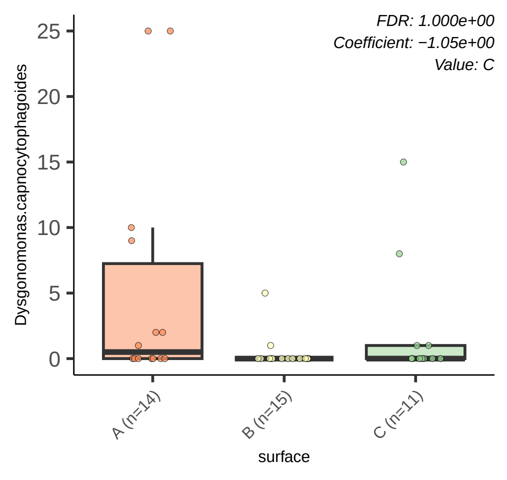
FDR: 1.000e+00
Coefficient: −1.05e+00
Value: C
Dysgonomonas.capnocytophagoides
0
5
10
15
20
25
A (n=14)
B (n=15)
C (n=11)
surface

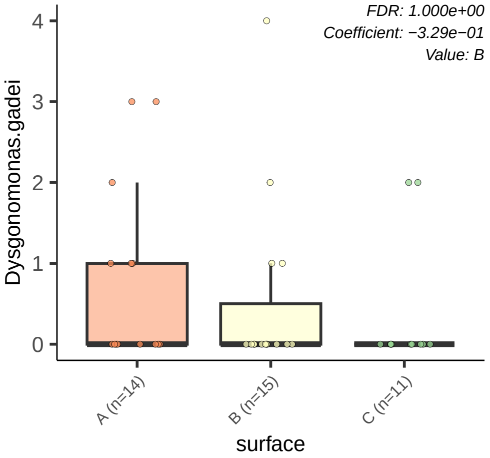
FDR: 1.000e+00
Coefficient: −3.29e−01
Value: B
Dysgonomonas.gadei
0
1
2
3
4
A (n=14)
B (n=15)
C (n=11)
surface

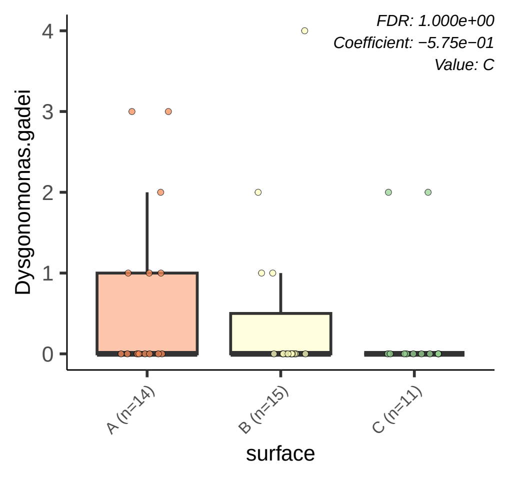

FDR: 1.000e+00
Coefficient: −5.75e−01
Value: C
Dysgonomonas.gadei
0
1
2
3
4
A (n=14)
B (n=15)
C (n=11)
surface

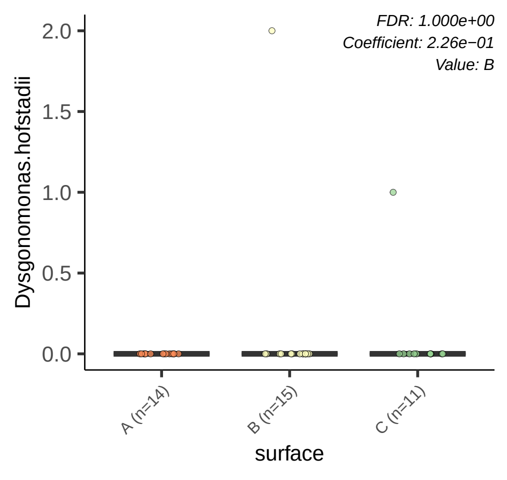
FDR: 1.000e+00
Coefficient: 2.26e−01
Value: B
Dysgonomonas.hofstadii
2.0
1.5
1.0
0.5
0.0
A (n=14)
B (n=15)
C (n=11)
surface

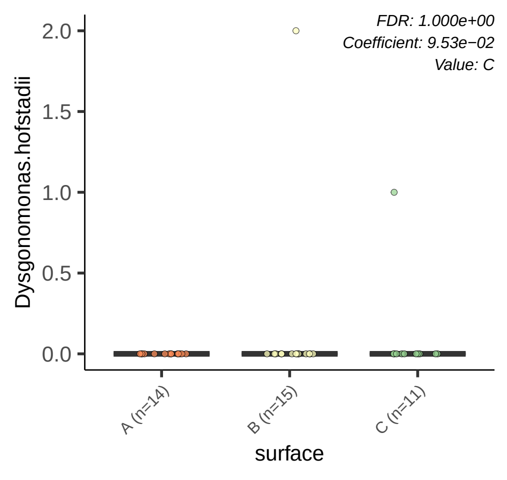
FDR: 1.000e+00
Coefficient: 9.53e−02
Value: C
Dysgonomonas.hofstadii
2.0
1.5
1.0
0.5
0.0
A (n=14)
B (n=15)
C (n=11)
surface

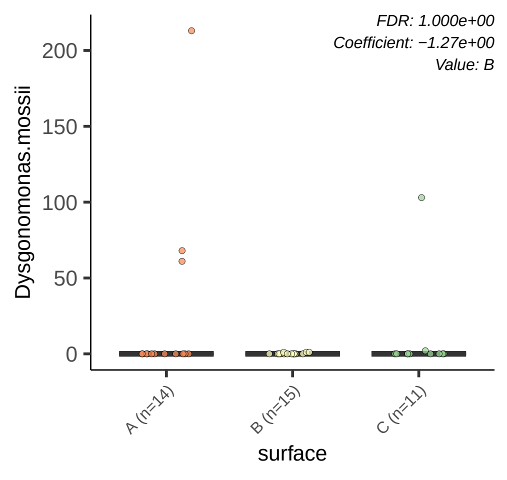
FDR: 1.000e+00
Coefficient: −1.27e+00
Value: B
Dysgonomonas.mossii
200
150
100
50
0
A (n=14)
B (n=15)
C (n=11)
surface

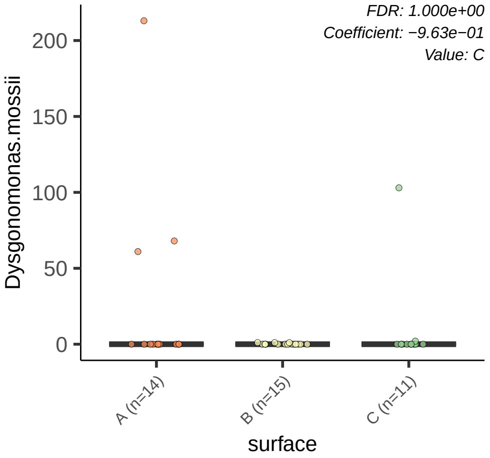

FDR: 1.000e+00
Coefficient: −9.63e−01
Value: C
Dysgonomonas.mossii
200
150
100
50
0
A (n=14)
B (n=15)
C (n=11)
surface

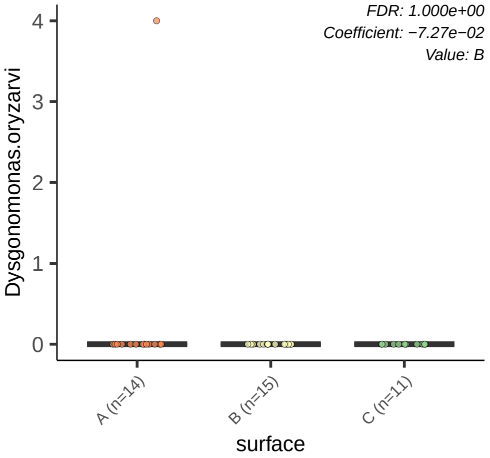

FDR: 1.000e+00
Coefficient: −7.27e−02
Value: B
Dysgonomonas.oryzarvi
4
3
2
1
0
A (n=14)
B (n=15)
C (n=11)
surface

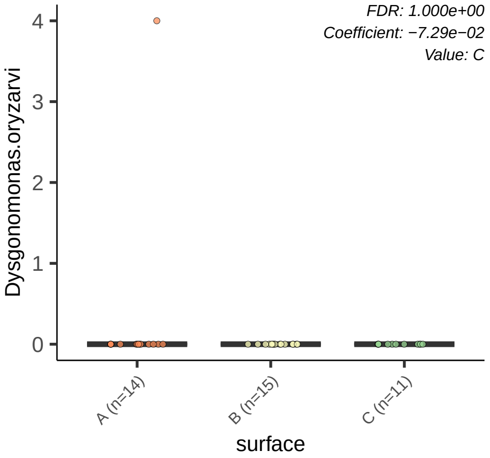

FDR: 1.000e+00
Coefficient: −7.29e−02
Value: C
Dysgonomonas.oryzarvi
4
3
2
1
0
A (n=14)
B (n=15)
C (n=11)
surface

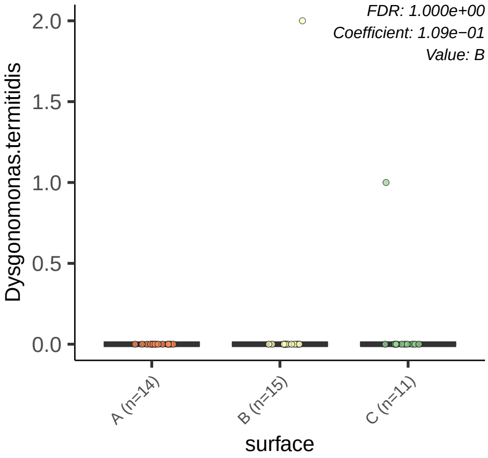
FDR: 1.000e+00
Coefficient: 1.09e−01
Value: B
Dysgonomonas.termitidis
2.0
1.5
1.0
0.5
0.0
A (n=14)
B (n=15)
C (n=11)
surface

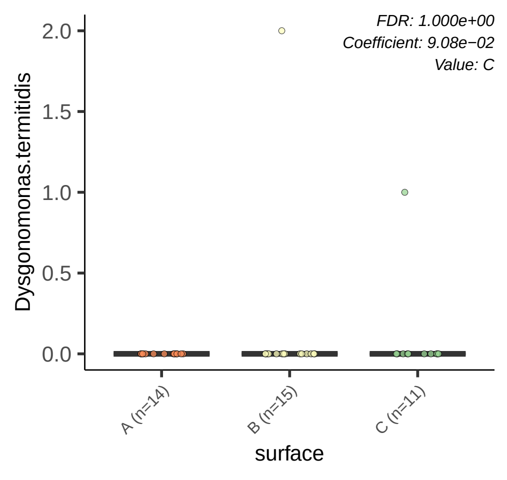
FDR: 1.000e+00
Coefficient: 9.08e−02
Value: C
Dysgonomonas.termitidis
2.0
1.5
1.0
0.5
0.0
A (n=14)
B (n=15)
C (n=11)
surface

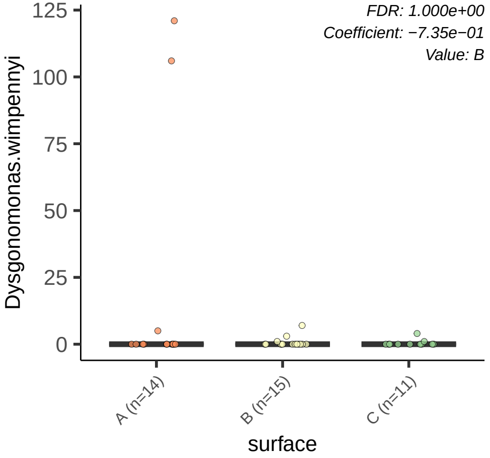

FDR: 1.000e+00
Coefficient: −7.35e−01
Value: B
Dysgonomonas.wimpennyi
125
100
75
50
25
0
A (n=14)
B (n=15)
C (n=11)
surface

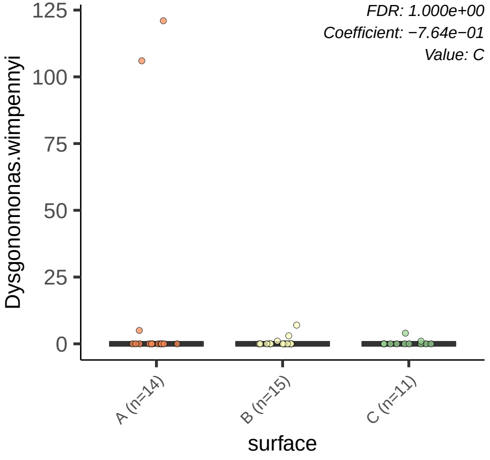
FDR: 1.000e+00
Coefficient: −7.64e−01
Value: C
Dysgonomonas.wimpennyi
125
100
75
50
25
0
A (n=14)
B (n=15)
C (n=11)
surface

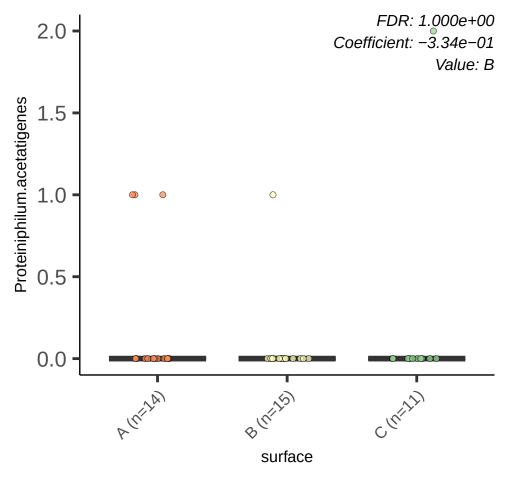

FDR: 1.000e+00
Coefficient: −3.34e−01
Value: B
Proteiniphilum.acetatigenes
2.0
1.5
1.0
0.5
0.0
A (n=14)
B (n=15)
C (n=11)
surface

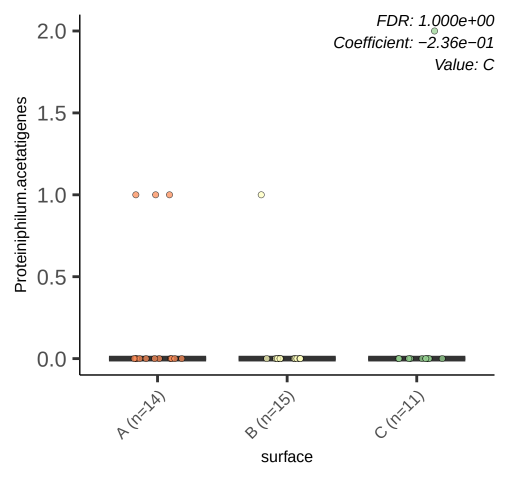

FDR: 1.000e+00
Coefficient: −2.36e−01
Value: C
Proteiniphilum.acetatigenes
2.0
1.5
1.0
0.5
0.0
A (n=14)
B (n=15)
C (n=11)
surface

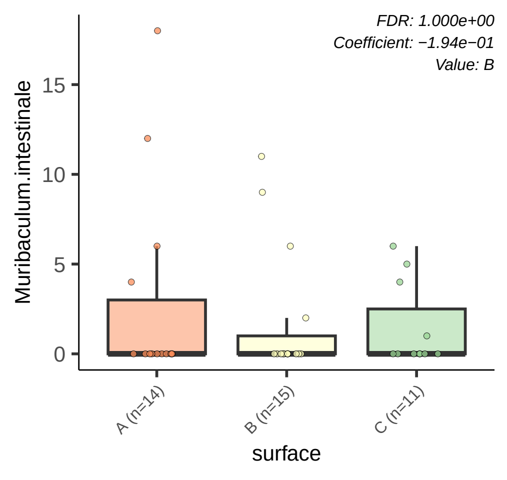

FDR: 1.000e+00
Coefficient: −1.94e−01
Value: B
Muribaculum.intestinale
0
5
10
15
A (n=14)
B (n=15)
C (n=11)
surface

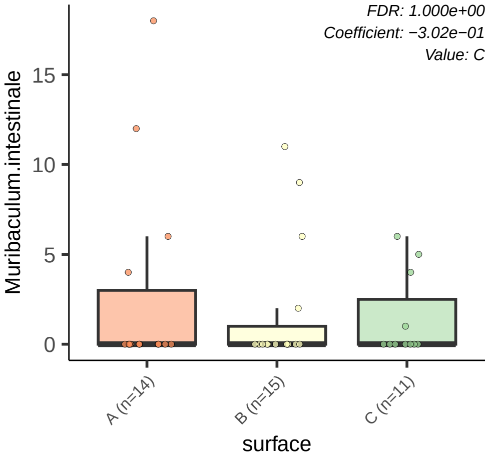
FDR: 1.000e+00
Coefficient: −3.02e−01
Value: C
Muribaculum.intestinale
0
5
10
15
A (n=14)
B (n=15)
C (n=11)
surface

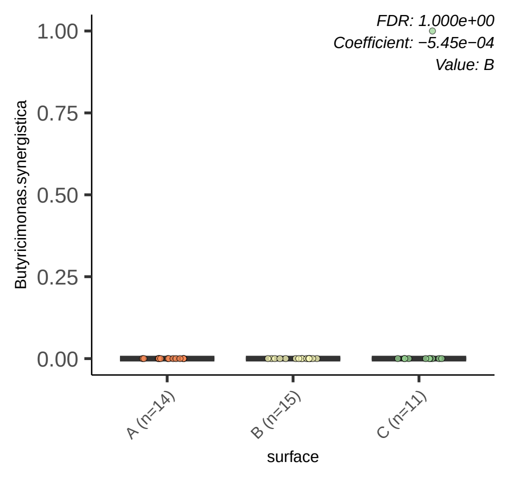
FDR: 1.000e+00
Coefficient: −5.45e−04
Value: B
Butyricimonas.synergistica
1.00
0.75
0.50
0.25
0.00
A (n=14)
B (n=15)
C (n=11)
surface

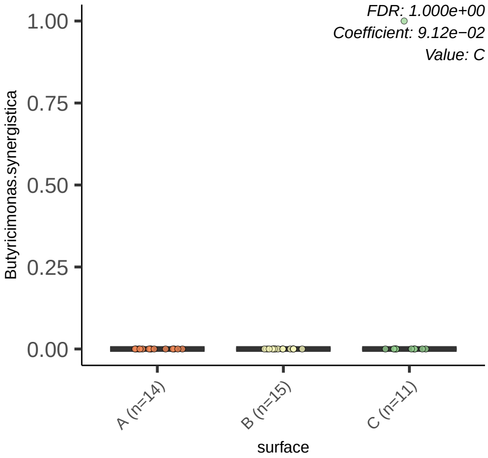
FDR: 1.000e+00
Coefficient: 9.12e−02
Value: C
1.00
0.75
0.50
0.25
0.00
Butyricimonas.synergistica
A (n=14)
B (n=15)
C (n=11)
surface

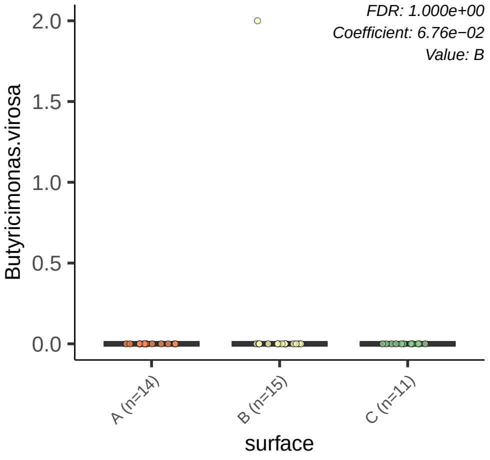
FDR: 1.000e+00
Coefficient: 6.76e−02
Value: B
Butyricimonas.virosa
2.0
1.5
1.0
0.5
0.0
A (n=14)
B (n=15)
C (n=11)
surface

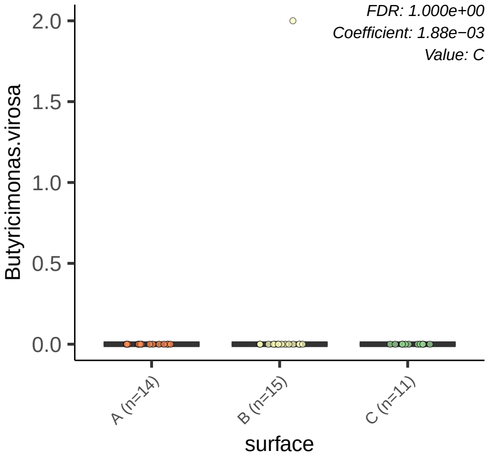
FDR: 1.000e+00
Coefficient: 1.88e−03
Value: C
Butyricimonas.virosa
2.0
1.5
1.0
0.5
0.0
A (n=14)
B (n=15)
C (n=11)
surface

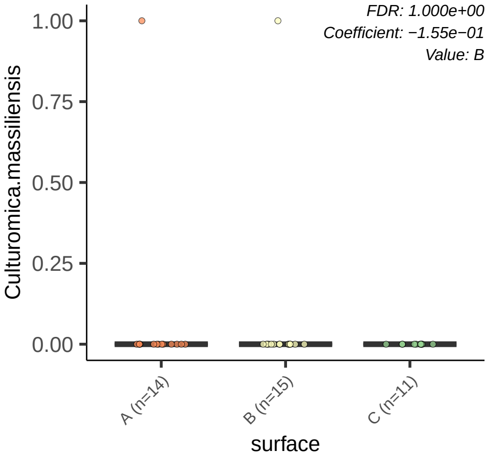
FDR: 1.000e+00
Coefficient: −1.55e−01
Value: B
Culturomica.massiliensis
1.00
0.75
0.50
0.25
0.00
A (n=14)
B (n=15)
C (n=11)
surface

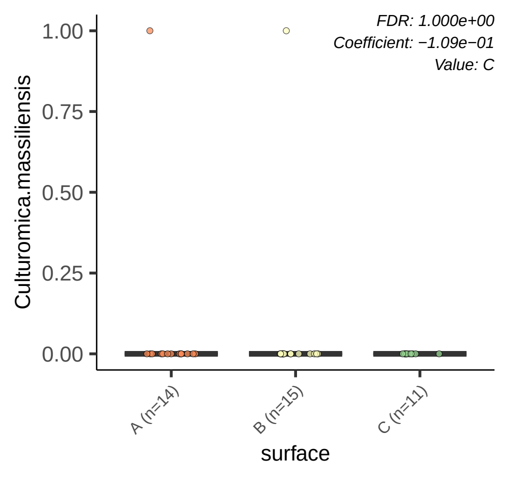
FDR: 1.000e+00
Coefficient: −1.09e−01
Value: C
1.00
0.75
0.50
0.25
0.00
Culturomica.massiliensis
A (n=14)
B (n=15)
C (n=11)
surface

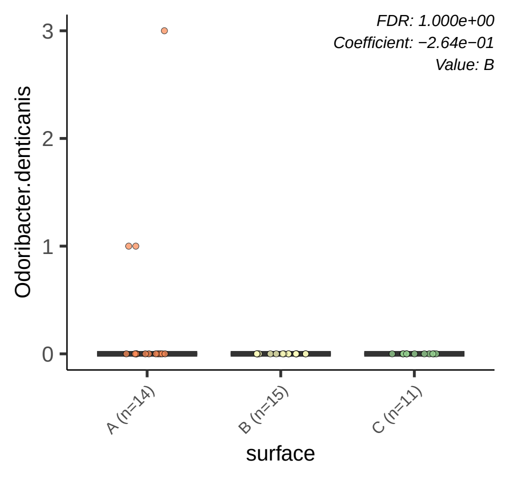

FDR: 1.000e+00
Coefficient: −2.64e−01
Value: B
Odoribacter.denticanis
3
2
1
0
A (n=14)
B (n=15)
C (n=11)
surface

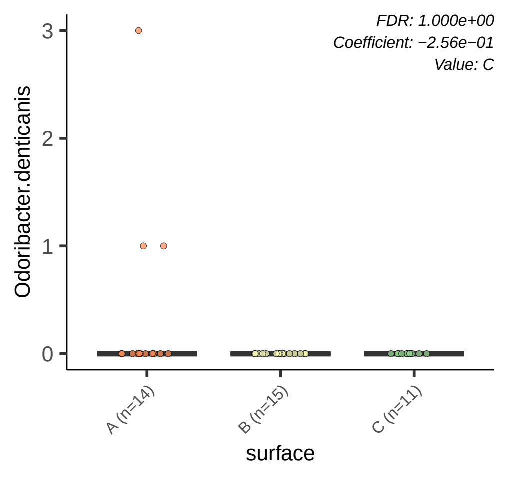

FDR: 1.000e+00
Coefficient: −2.56e−01
Value: C
Odoribacter.denticanis
3
2
1
0
A (n=14)
B (n=15)
C (n=11)
surface

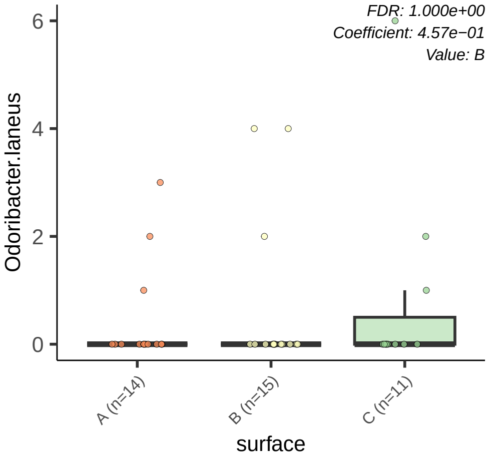
FDR: 1.000e+00
Coefficient: 4.57e−01
Value: B
Odoribacter.laneus
0
2
4
6
A (n=14)
B (n=15)
C (n=11)
surface

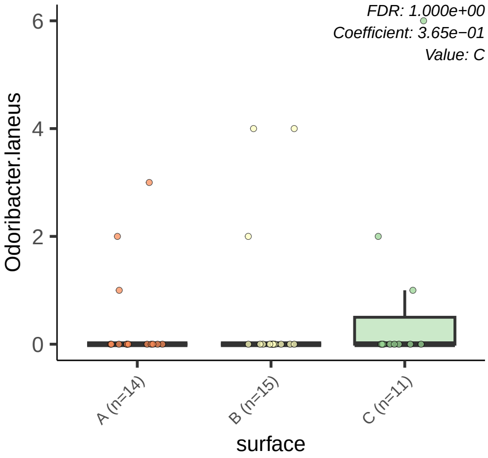
FDR: 1.000e+00
Coefficient: 3.65e−01
Value: C
Odoribacter.laneus
0
2
4
6
A (n=14)
B (n=15)
C (n=11)
surface

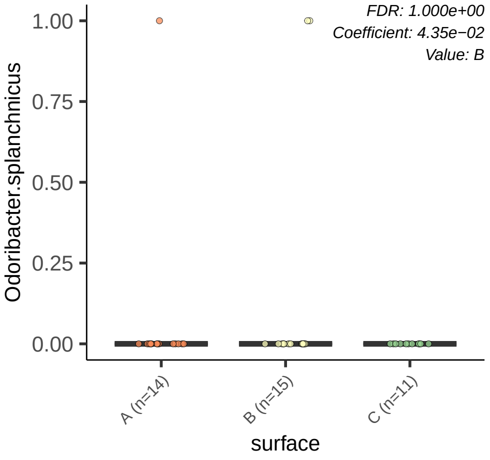

FDR: 1.000e+00
Coefficient: 4.35e−02
Value: B
Odoribacter.splanchnicus
1.00
0.75
0.50
0.25
0.00
A (n=14)
B (n=15)
C (n=11)
surface

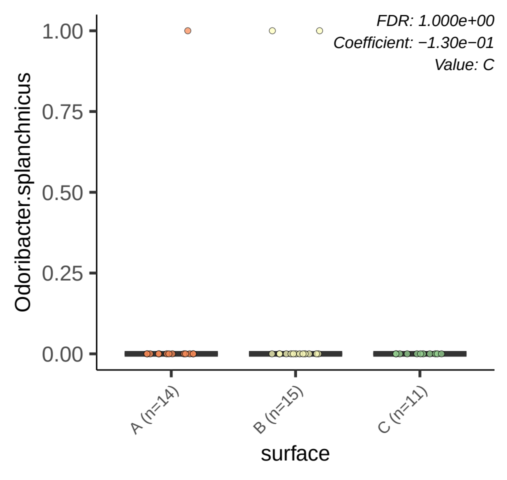

FDR: 1.000e+00
Coefficient: −1.30e−01
Value: C
Odoribacter.splanchnicus
1.00
0.75
0.50
0.25
0.00
A (n=14)
B (n=15)
C (n=11)
surface

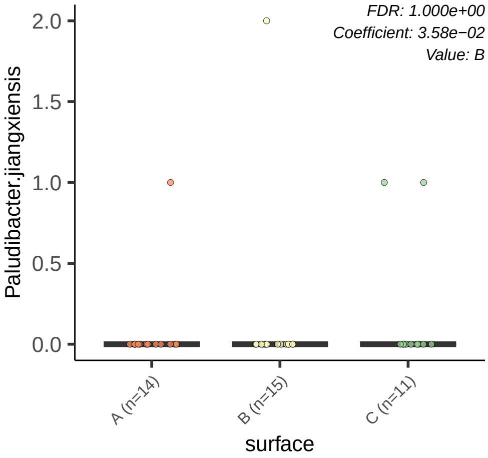
FDR: 1.000e+00
Coefficient: 3.58e−02
Value: B
Paludibacter.jiangxiensis
2.0
1.5
1.0
0.5
0.0
A (n=14)
B (n=15)
C (n=11)
surface

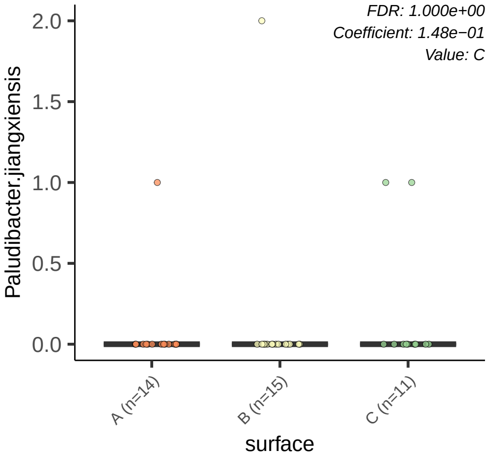
FDR: 1.000e+00
Coefficient: 1.48e−01
Value: C
Paludibacter.jiangxiensis
2.0
1.5
1.0
0.5
0.0
A (n=14)
B (n=15)
C (n=11)
surface

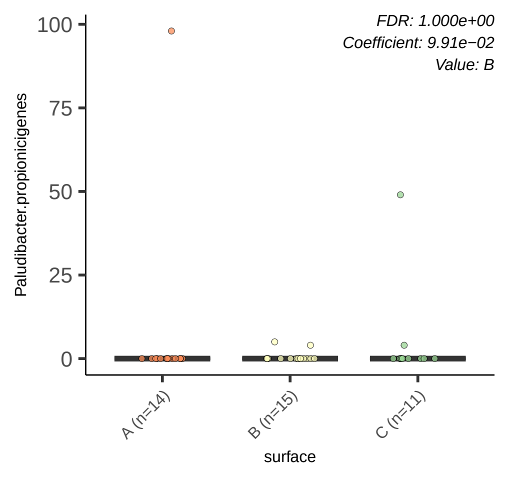
FDR: 1.000e+00
Coefficient: 9.91e−02
Value: B
Paludibacter.propionicigenes
100
75
50
25
0
A (n=14)
B (n=15)
C (n=11)
surface

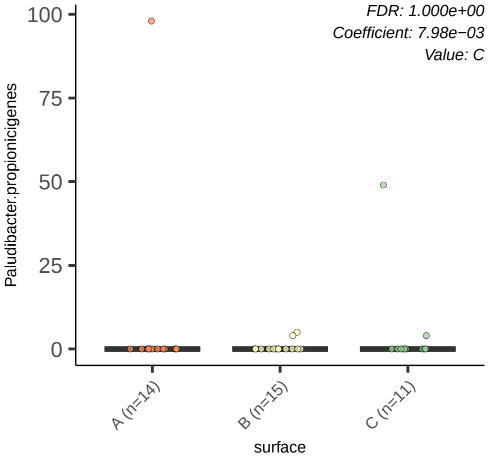
FDR: 1.000e+00
Coefficient: 7.98e−03
Value: C
Paludibacter.propionicigenes
100
75
50
25
0
A (n=14)
B (n=15)
C (n=11)
surface

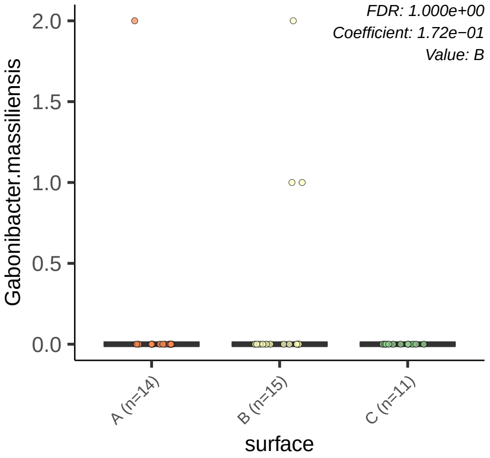
FDR: 1.000e+00
Coefficient: 1.72e−01
Value: B
Gabonibacter.massiliensis
2.0
1.5
1.0
0.5
0.0
A (n=14)
B (n=15)
C (n=11)
surface

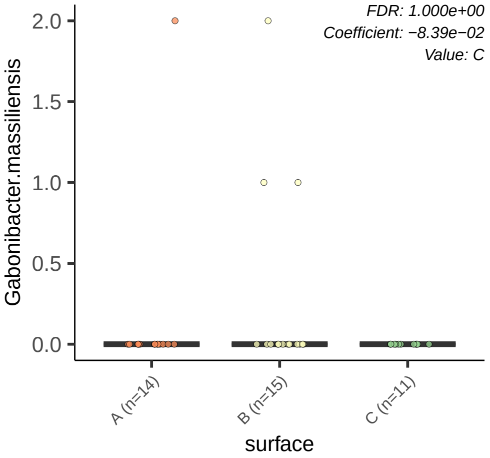
FDR: 1.000e+00
Coefficient: −8.39e−02
Value: C
Gabonibacter.massiliensis
2.0
1.5
1.0
0.5
0.0
A (n=14)
B (n=15)
C (n=11)
surface

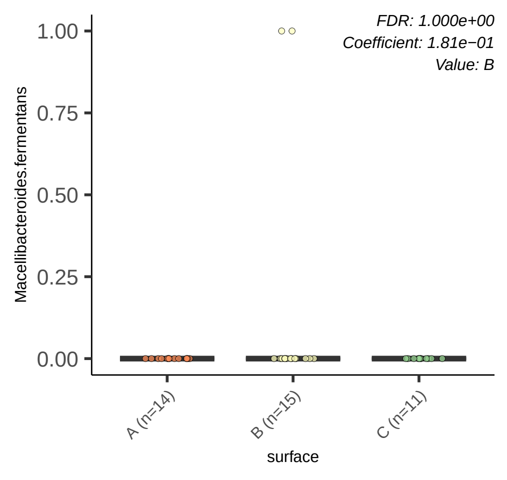
FDR: 1.000e+00
Coefficient: 1.81e−01
Value: B
Macellibacteroides.fermentans
1.00
0.75
0.50
0.25
0.00
A (n=14)
B (n=15)
C (n=11)
surface

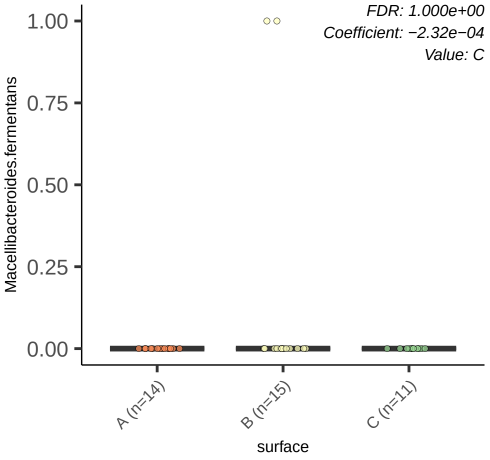

FDR: 1.000e+00
Coefficient: −2.32e−04
Value: C
Macellibacteroides.fermentans
1.00
0.75
0.50
0.25
0.00
A (n=14)
B (n=15)
C (n=11)
surface

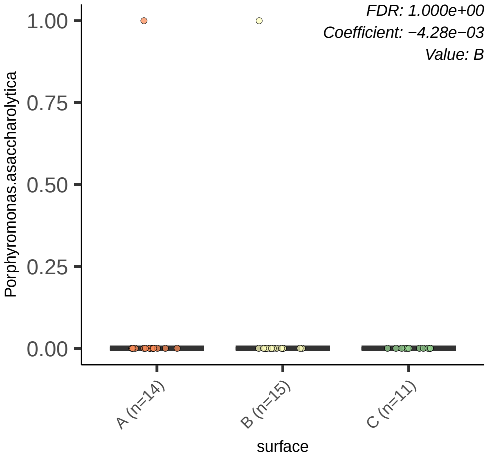
FDR: 1.000e+00
Coefficient: −4.28e−03
Value: B
Porphyromonas.asaccharolytica
1.00
0.75
0.50
0.25
0.00
A (n=14)
B (n=15)
C (n=11)
surface

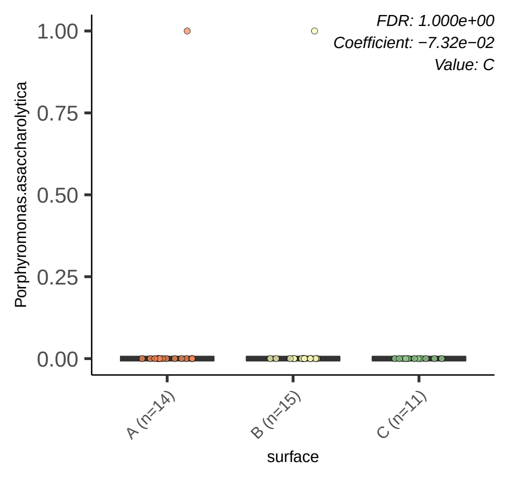
FDR: 1.000e+00
Coefficient: −7.32e−02
Value: C
Porphyromonas.asaccharolytica
1.00
0.75
0.50
0.25
0.00
A (n=14)
B (n=15)
C (n=11)
surface

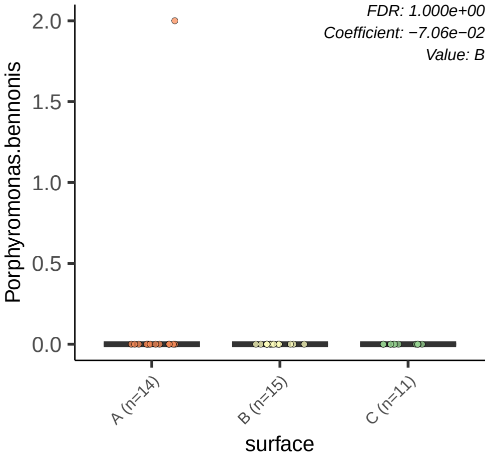

FDR: 1.000e+00
Coefficient: −7.06e−02
Value: B
Porphyromonas.bennonis
2.0
1.5
1.0
0.5
0.0
A (n=14)
B (n=15)
C (n=11)
surface

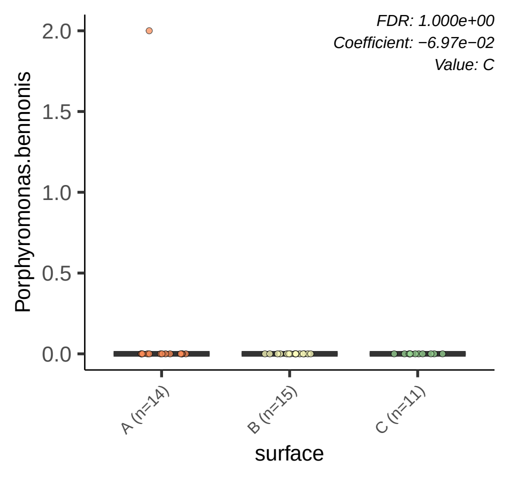
FDR: 1.000e+00
Coefficient: −6.97e−02
Value: C
Porphyromonas.bennonis
2.0
1.5
1.0
0.5
0.0
A (n=14)
B (n=15)
C (n=11)
surface

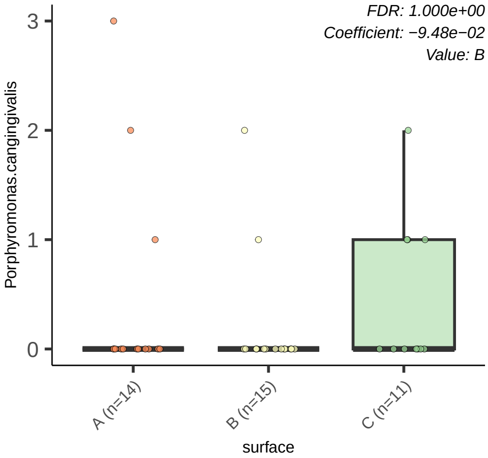
FDR: 1.000e+00
Coefficient: −9.48e−02
Value: B
Porphyromonas.cangingivalis
0
1
2
3
A (n=14)
B (n=15)
C (n=11)
surface

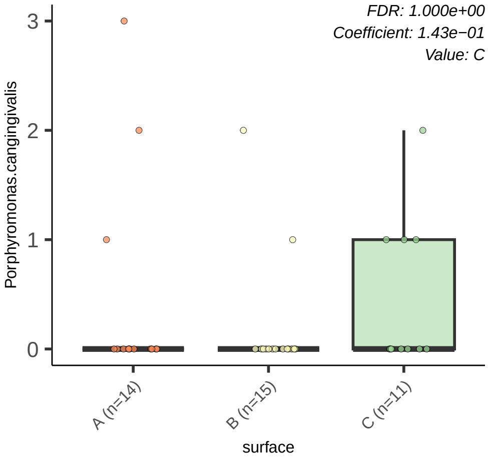

FDR: 1.000e+00
Coefficient: 1.43e−01
Value: C
Porphyromonas.cangingivalis
3
2
1
0
A (n=14)
B (n=15)
C (n=11)
surface

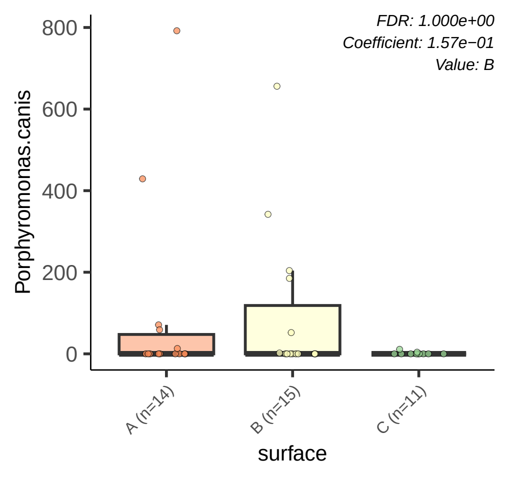
FDR: 1.000e+00
Coefficient: 1.57e−01
Value: B
Porphyromonas.canis
800
600
400
200
0
A (n=14)
B (n=15)
C (n=11)
surface

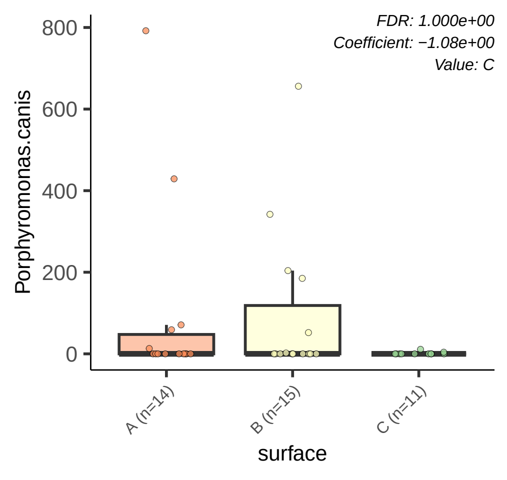
FDR: 1.000e+00
Coefficient: −1.08e+00
Value: C
Porphyromonas.canis
800
600
400
200
0
A (n=14)
B (n=15)
C (n=11)
surface

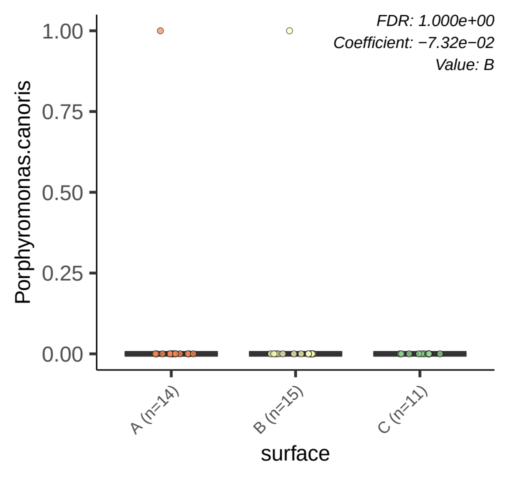

FDR: 1.000e+00
Coefficient: −7.32e−02
Value: B
Porphyromonas.canoris
1.00
0.75
0.50
0.25
0.00
A (n=14)
B (n=15)
C (n=11)
surface

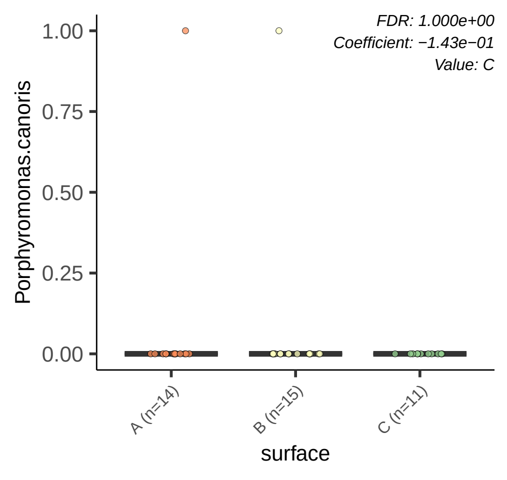
FDR: 1.000e+00
Coefficient: −1.43e−01
Value: C
Porphyromonas.canoris
1.00
0.75
0.50
0.25
0.00
A (n=14)
B (n=15)
C (n=11)
surface

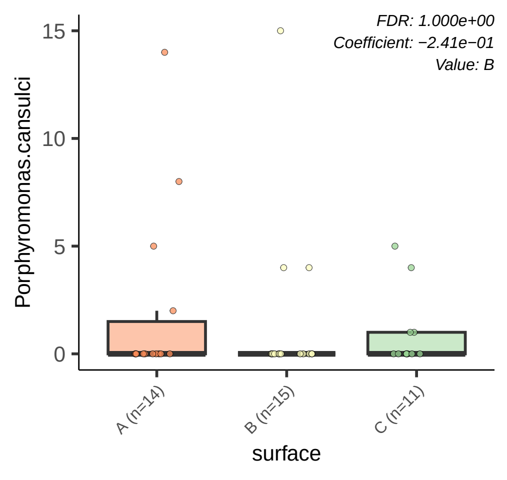

FDR: 1.000e+00
Coefficient: −2.41e−01
Value: B
Porphyromonas.cansulci
0
5
10
15
A (n=14)
B (n=15)
C (n=11)
surface

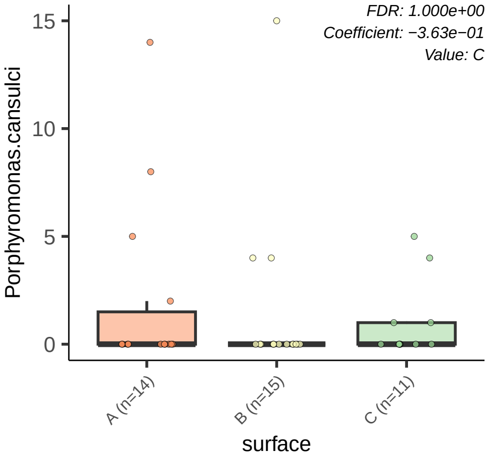
FDR: 1.000e+00
Coefficient: −3.63e−01
Value: C
Porphyromonas.cansulci
15
10
5
0
A (n=14)
B (n=15)
C (n=11)
surface

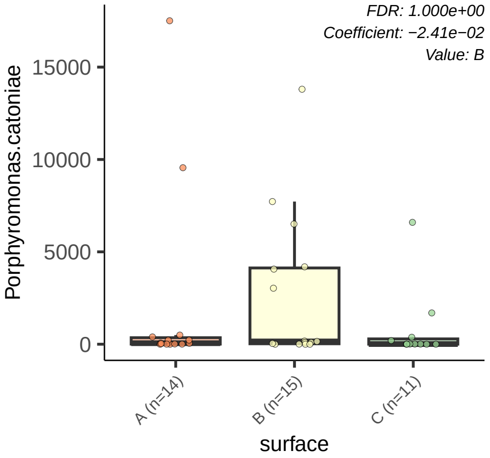

FDR: 1.000e+00
Coefficient: −2.41e−02
Value: B
Porphyromonas.catoniae
15000
10000
5000
0
A (n=14)
B (n=15)
C (n=11)
surface

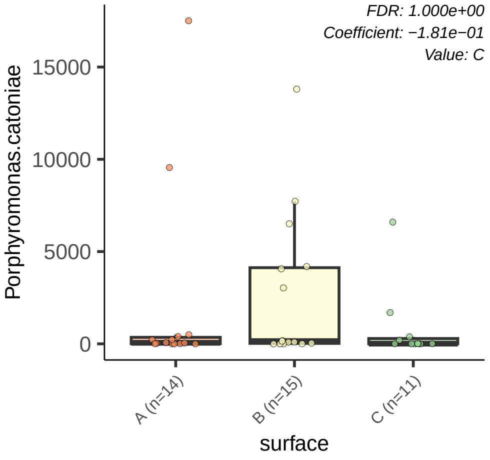
FDR: 1.000e+00
Coefficient: −1.81e−01
Value: C
Porphyromonas.catoniae
15000
10000
5000
0
A (n=14)
B (n=15)
C (n=11)
surface

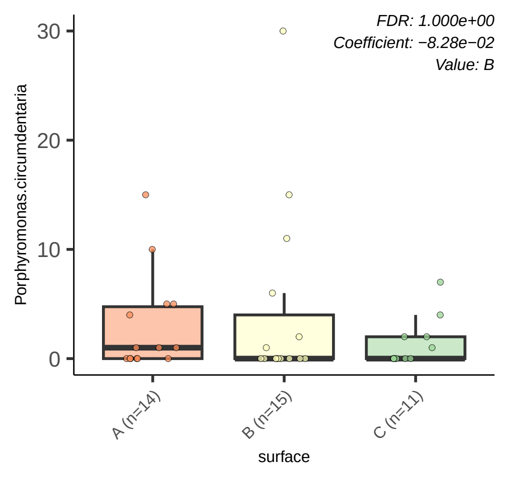
FDR: 1.000e+00
Coefficient: −8.28e−02
Value: B
Porphyromonas.circumdentaria
30
20
10
0
A (n=14)
B (n=15)
C (n=11)
surface

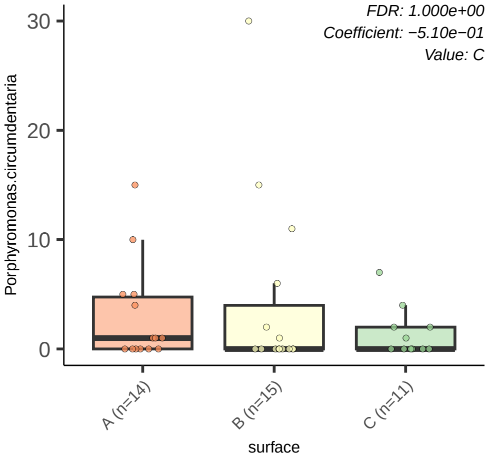
FDR: 1.000e+00
Coefficient: −5.10e−01
Value: C
Porphyromonas.circumdentaria
30
20
10
0
A (n=14)
B (n=15)
C (n=11)
surface

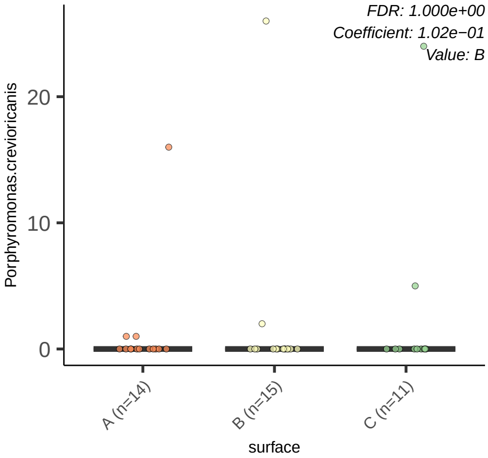
FDR: 1.000e+00
Coefficient: 1.02e−01
Value: B
Porphyromonas.crevioricanis
20
10
0
A (n=14)
B (n=15)
C (n=11)
surface

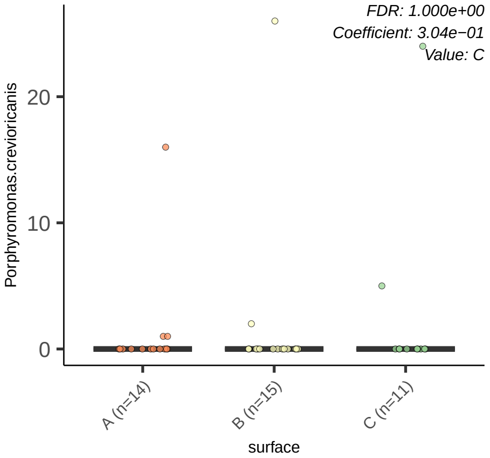
FDR: 1.000e+00
Coefficient: 3.04e−01
Value: C
Porphyromonas.crevioricanis
0
10
20
A (n=14)
B (n=15)
C (n=11)
surface

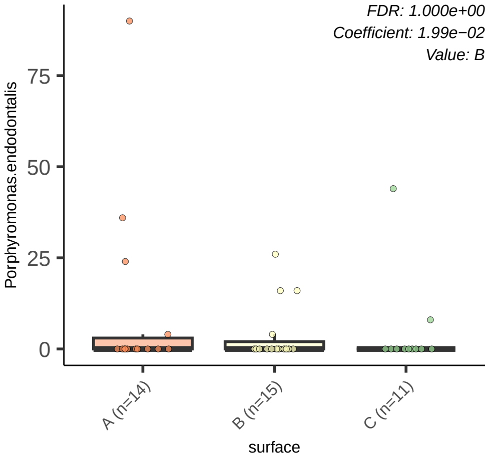
FDR: 1.000e+00
Coefficient: 1.99e−02
Value: B
Porphyromonas.endodontalis
0
25
50
75
A (n=14)
B (n=15)
C (n=11)
surface

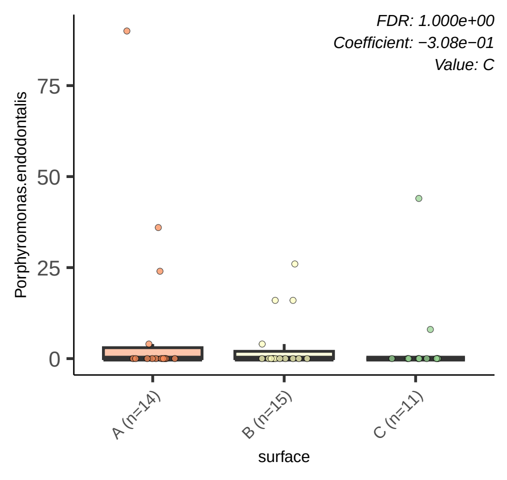

FDR: 1.000e+00
Coefficient: −3.08e−01
Value: C
Porphyromonas.endodontalis
75
50
25
0
A (n=14)
B (n=15)
C (n=11)
surface

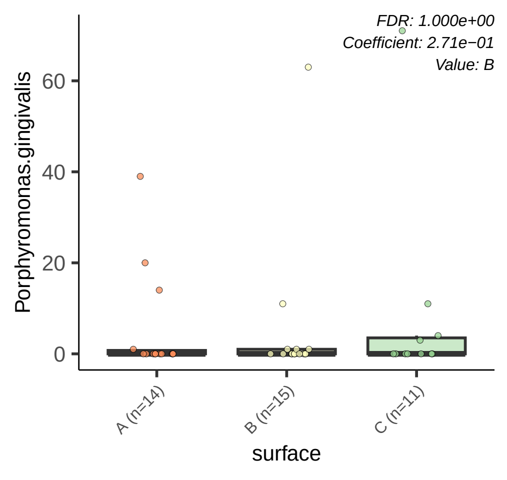
FDR: 1.000e+00
Coefficient: 2.71e−01
Value: B
Porphyromonas.gingivalis
60
40
20
0
A (n=14)
B (n=15)
C (n=11)
surface

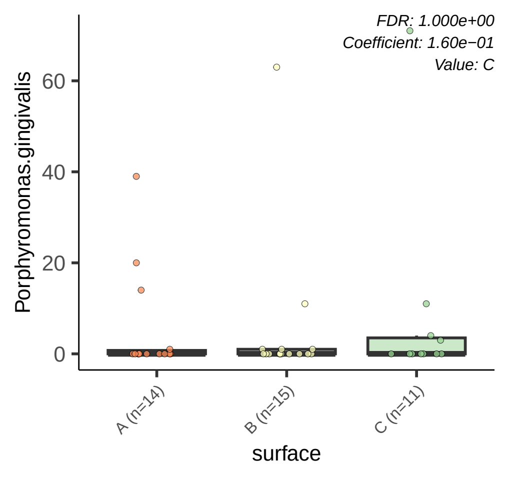
FDR: 1.000e+00
Coefficient: 1.60e−01
Value: C
Porphyromonas.gingivalis
0
20
40
60
A (n=14)
B (n=15)
C (n=11)
surface

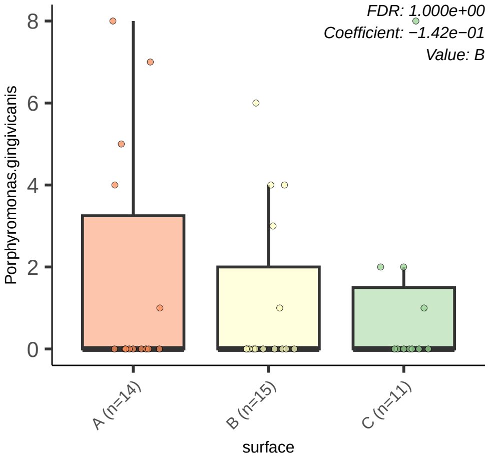
FDR: 1.000e+00
Coefficient: −1.42e−01
Value: B
Porphyromonas.gingivicanis
0
2
4
6
8
A (n=14)
B (n=15)
C (n=11)
surface

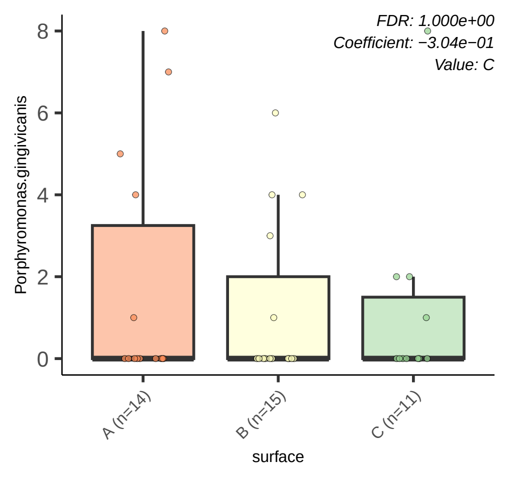

FDR: 1.000e+00
Coefficient: −3.04e−01
Value: C
Porphyromonas.gingivicanis
0
2
4
6
8
A (n=14)
B (n=15)
C (n=11)
surface

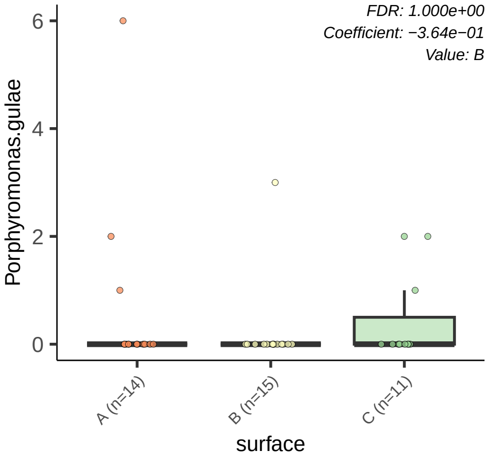
FDR: 1.000e+00
Coefficient: −3.64e−01
Value: B
Porphyromonas.gulae
0
2
4
6
A (n=14)
B (n=15)
C (n=11)
surface

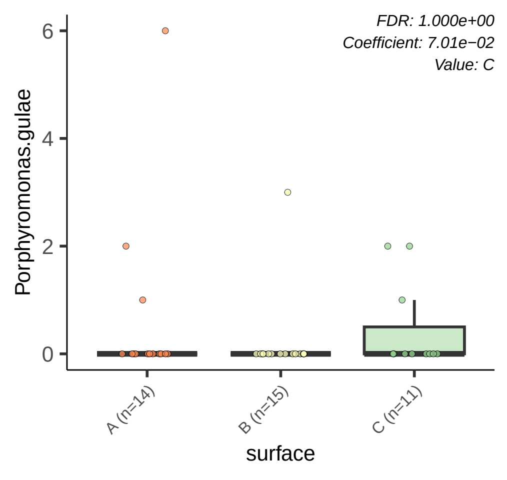
FDR: 1.000e+00
Coefficient: 7.01e−02
Value: C
Porphyromonas.gulae
6
4
2
0
A (n=14)
B (n=15)
C (n=11)
surface

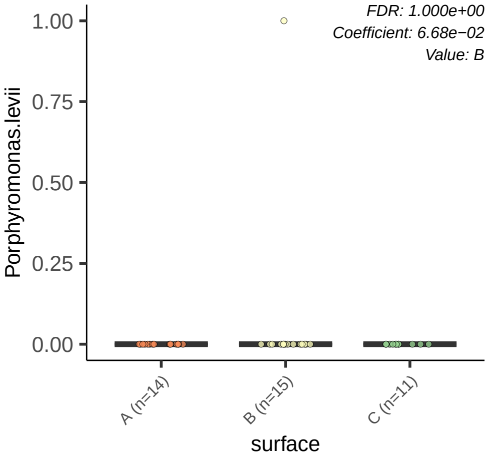
FDR: 1.000e+00
Coefficient: 6.68e−02
Value: B
Porphyromonas.levii
1.00
0.75
0.50
0.25
0.00
A (n=14)
B (n=15)
C (n=11)
surface

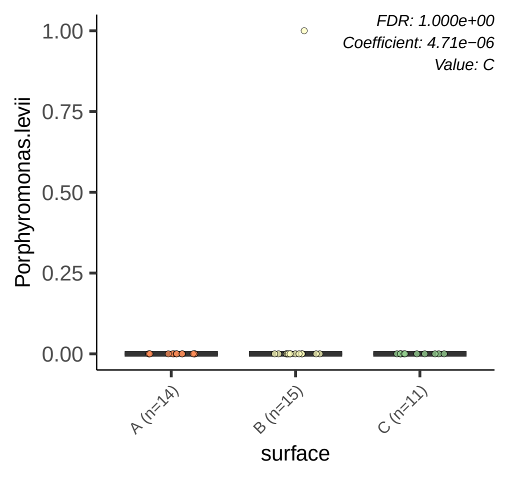
FDR: 1.000e+00
Coefficient: 4.71e−06
Value: C
1.00
0.75
0.50
0.25
0.00
Porphyromonas.levii
A (n=14)
B (n=15)
C (n=11)
surface

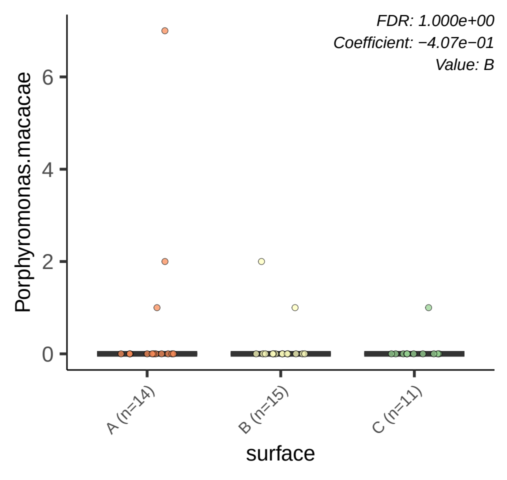
FDR: 1.000e+00
Coefficient: −4.07e−01
Value: B
Porphyromonas.macacae
0
2
4
6
A (n=14)
B (n=15)
C (n=11)
surface

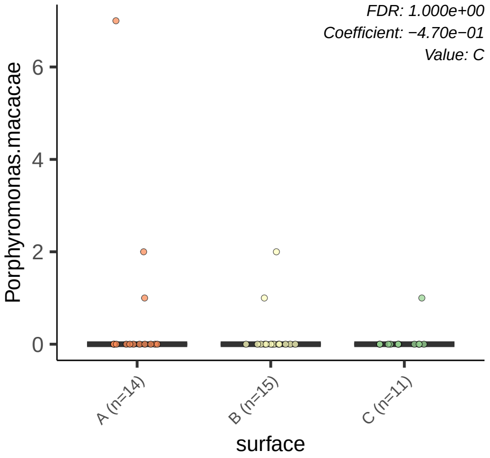

FDR: 1.000e+00
Coefficient: −4.70e−01
Value: C
Porphyromonas.macacae
0
2
4
6
A (n=14)
B (n=15)
C (n=11)
surface

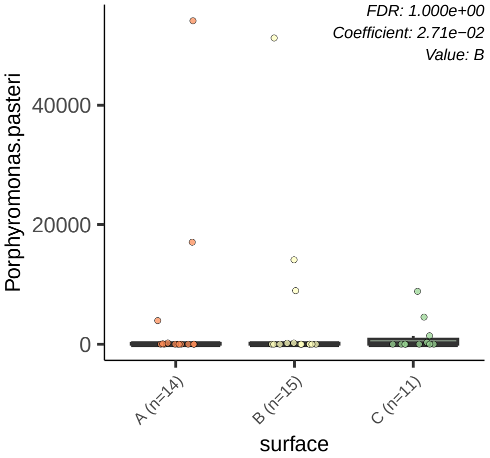
FDR: 1.000e+00
Coefficient: 2.71e−02
Value: B
Porphyromonas.pasteri
40000
20000
0
A (n=14)
B (n=15)
C (n=11)
surface

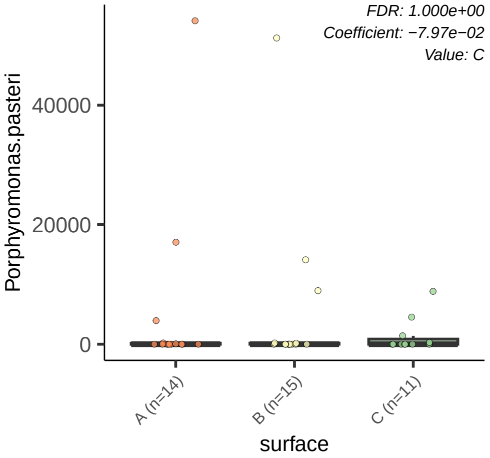

FDR: 1.000e+00
Coefficient: −7.97e−02
Value: C
Porphyromonas.pasteri
40000
20000
0
A (n=14)
B (n=15)
C (n=11)
surface

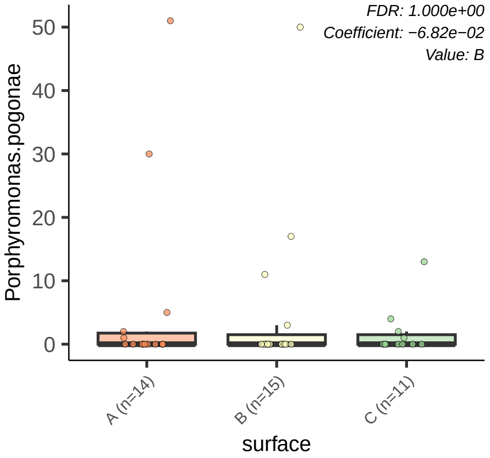
FDR: 1.000e+00
Coefficient: −6.82e−02
Value: B
Porphyromonas.pogonae
0
10
20
30
40
50
A (n=14)
B (n=15)
C (n=11)
surface

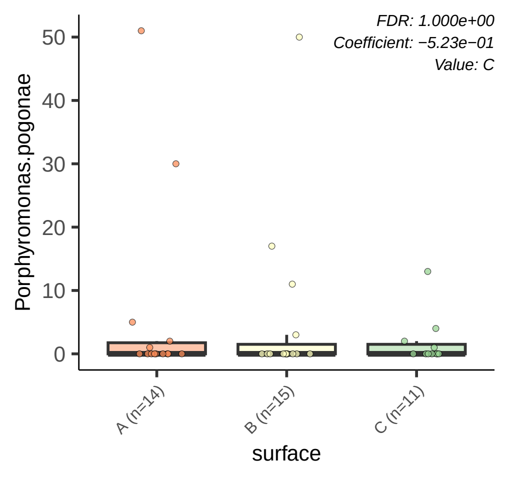
FDR: 1.000e+00
Coefficient: −5.23e−01
Value: C
Porphyromonas.pogonae
0
10
20
30
40
50
A (n=14)
B (n=15)
C (n=11)
surface

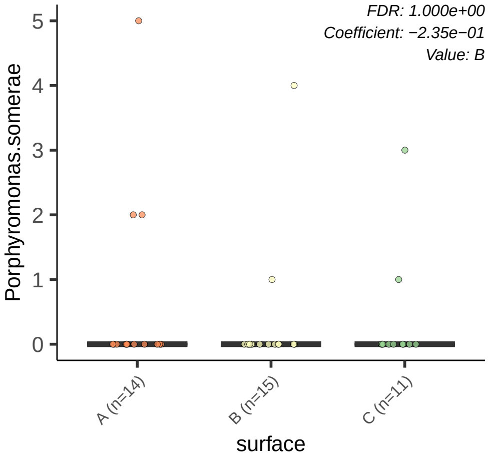
FDR: 1.000e+00
Coefficient: −2.35e−01
Value: B
Porphyromonas.somerae
0
1
2
3
4
5
A (n=14)
B (n=15)
C (n=11)
surface

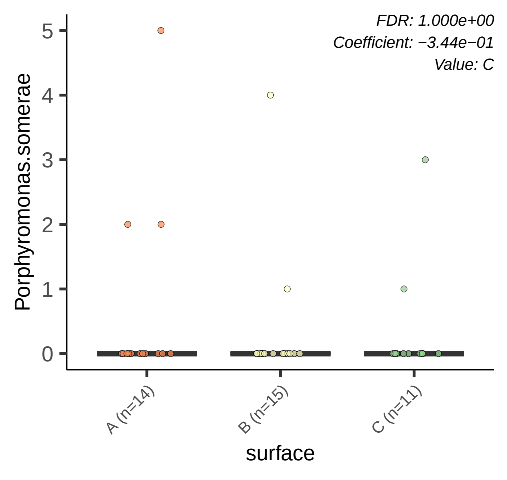

FDR: 1.000e+00
Coefficient: −3.44e−01
Value: C
Porphyromonas.somerae
5
4
3
2
1
0
A (n=14)
B (n=15)
C (n=11)
surface

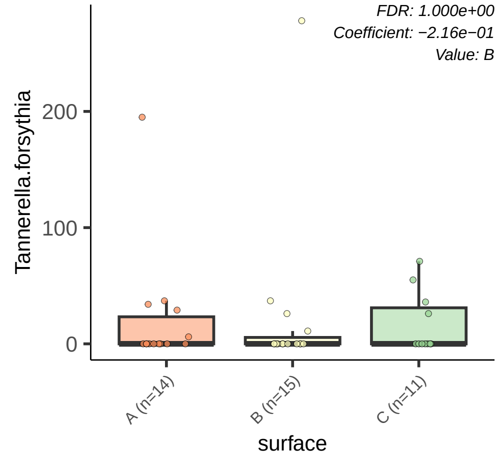

FDR: 1.000e+00
Coefficient: −2.16e−01
Value: B
Tannerella.forsythia
200
100
0
A (n=14)
B (n=15)
C (n=11)
surface

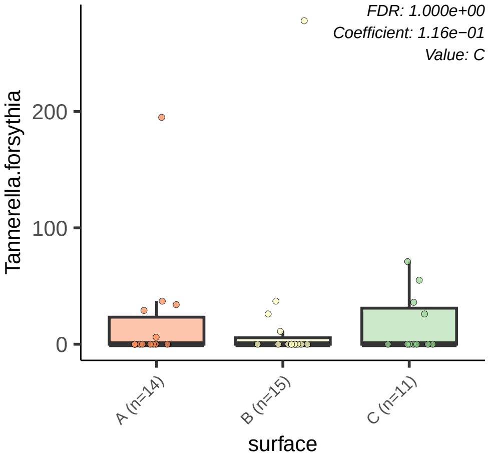
FDR: 1.000e+00
Coefficient: 1.16e−01
Value: C
Tannerella.forsythia
200
100
0
A (n=14)
B (n=15)
C (n=11)
surface

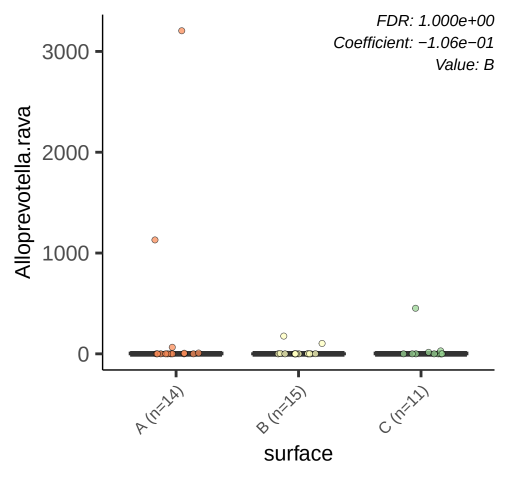

FDR: 1.000e+00
Coefficient: −1.06e−01
Value: B
Alloprevotella.rava
3000
2000
1000
0
A (n=14)
B (n=15)
C (n=11)
surface

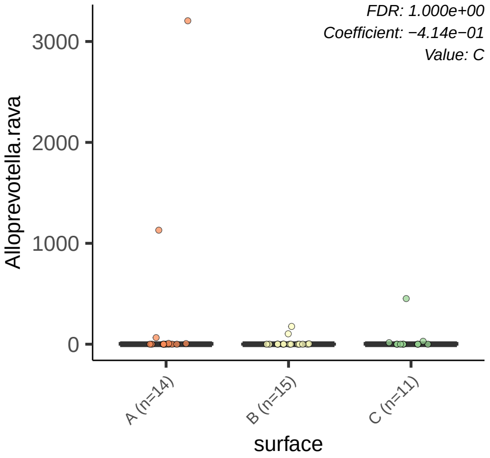

FDR: 1.000e+00
Coefficient: −4.14e−01
Value: C
Alloprevotella.rava
3000
2000
1000
0
A (n=14)
B (n=15)
C (n=11)
surface

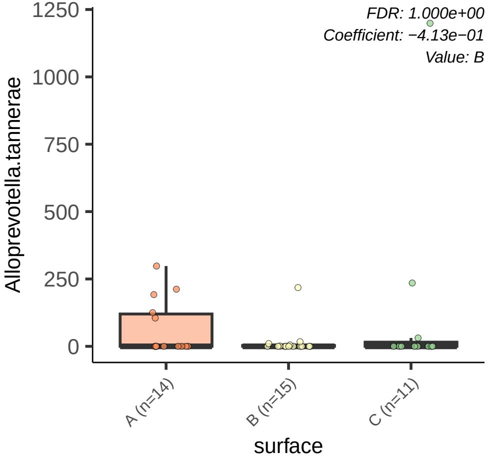
FDR: 1.000e+00
Coefficient: −4.13e−01
Value: B
Alloprevotella.tannerae
1250
1000
750
500
250
0
A (n=14)
B (n=15)
C (n=11)
surface

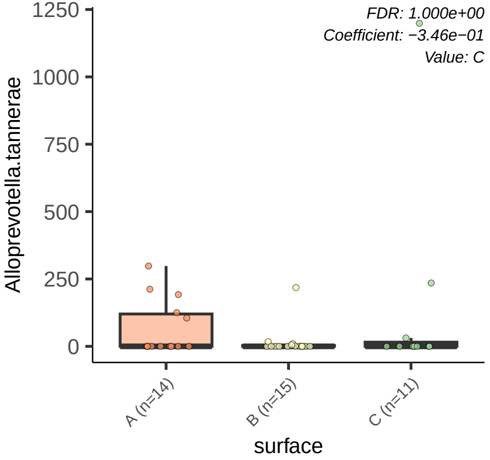

FDR: 1.000e+00
Coefficient: −3.46e−01
Value: C
Alloprevotella.tannerae
1250
1000
750
500
250
0
A (n=14)
B (n=15)
C (n=11)
surface

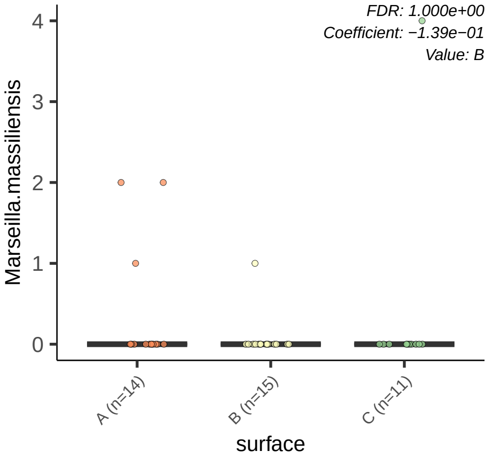
FDR: 1.000e+00
Coefficient: −1.39e−01
Value: B
Marseilla.massiliensis
0
1
2
3
4
A (n=14)
B (n=15)
C (n=11)
surface

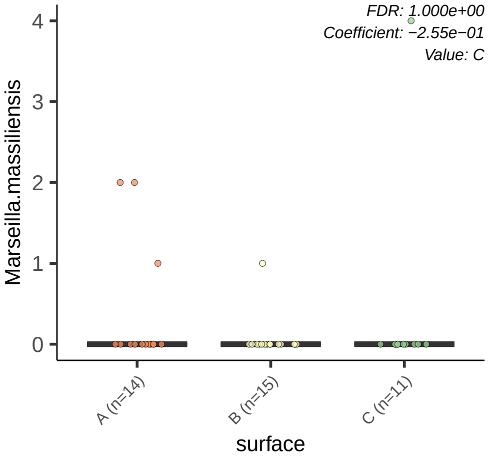

FDR: 1.000e+00
Coefficient: −2.55e−01
Value: C
Marseilla.massiliensis
0
1
2
3
4
A (n=14)
B (n=15)
C (n=11)
surface

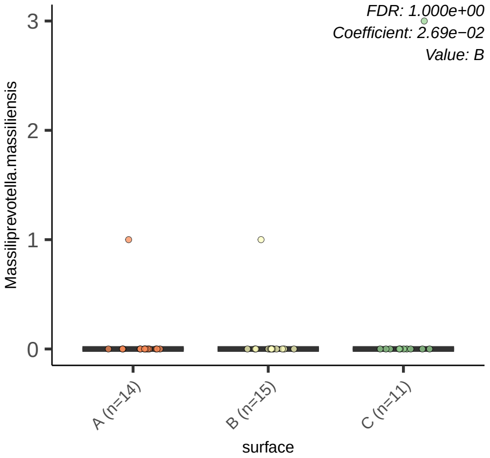

FDR: 1.000e+00
Coefficient: 2.69e−02
Value: B
Massiliprevotella.massiliensis
0
1
2
3
A (n=14)
B (n=15)
C (n=11)
surface

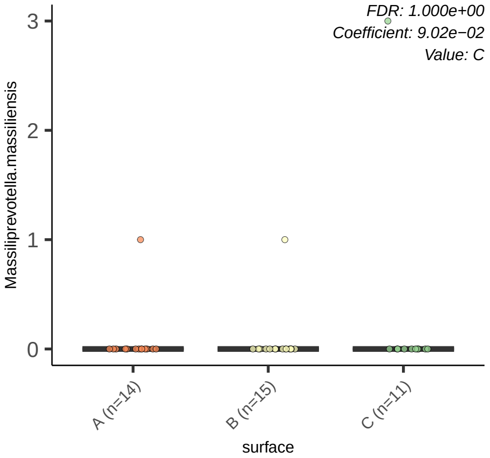

FDR: 1.000e+00
Coefficient: 9.02e−02
Value: C
Massiliprevotella.massiliensis
0
1
2
3
A (n=14)
B (n=15)
C (n=11)
surface

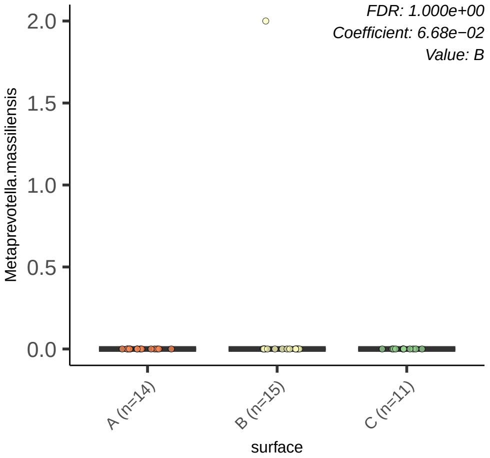
FDR: 1.000e+00
Coefficient: 6.68e−02
Value: B
2.0
1.5
1.0
0.5
0.0
Metaprevotella.massiliensis
A (n=14)
B (n=15)
C (n=11)
surface

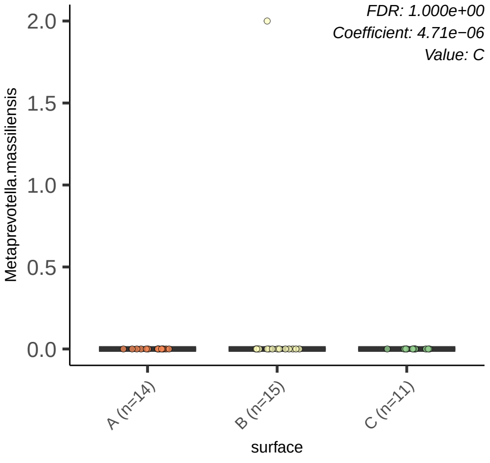

FDR: 1.000e+00
Coefficient: 4.71e−06
Value: C
Metaprevotella.massiliensis
2.0
1.5
1.0
0.5
0.0
A (n=14)
B (n=15)
C (n=11)
surface

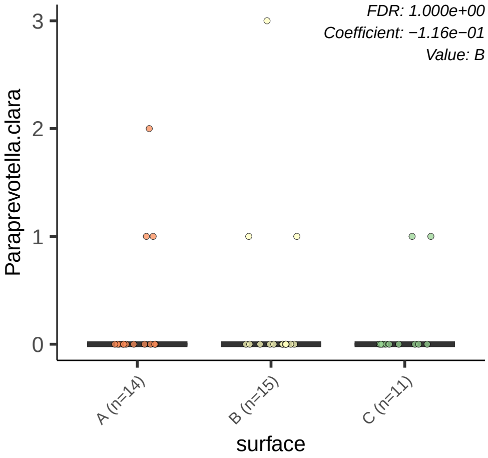

FDR: 1.000e+00
Coefficient: −1.16e−01
Value: B
Paraprevotella.clara
3
2
1
0
A (n=14)
B (n=15)
C (n=11)
surface

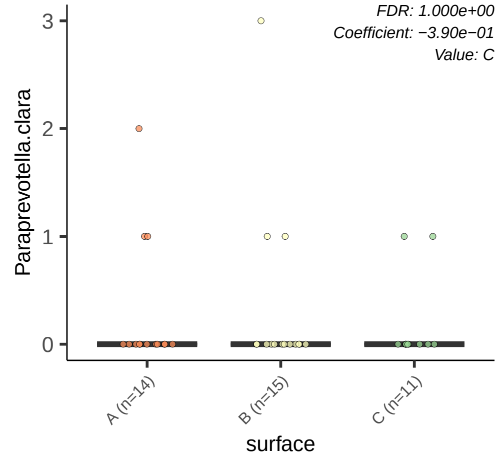

FDR: 1.000e+00
Coefficient: −3.90e−01
Value: C
Paraprevotella.clara
3
2
1
0
A (n=14)
B (n=15)
C (n=11)
surface

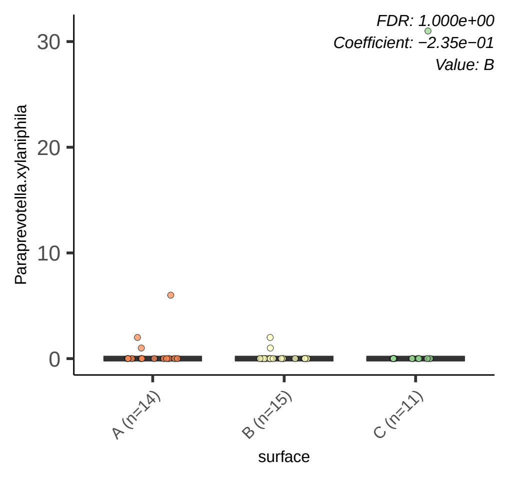
FDR: 1.000e+00
Coefficient: −2.35e−01
Value: B
Paraprevotella.xylaniphila
30
20
10
0
A (n=14)
B (n=15)
C (n=11)
surface

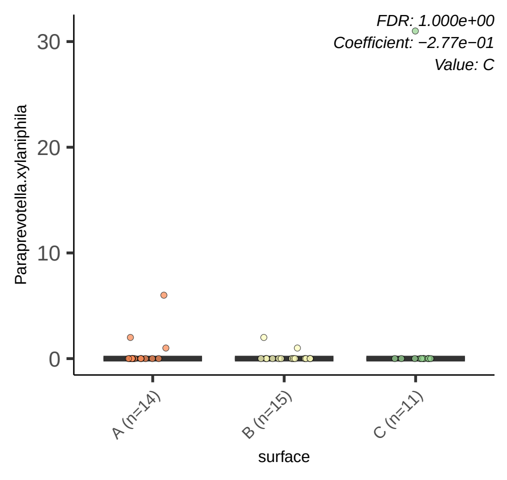
FDR: 1.000e+00
Coefficient: −2.77e−01
Value: C
Paraprevotella.xylaniphila
30
20
10
0
A (n=14)
B (n=15)
C (n=11)
surface

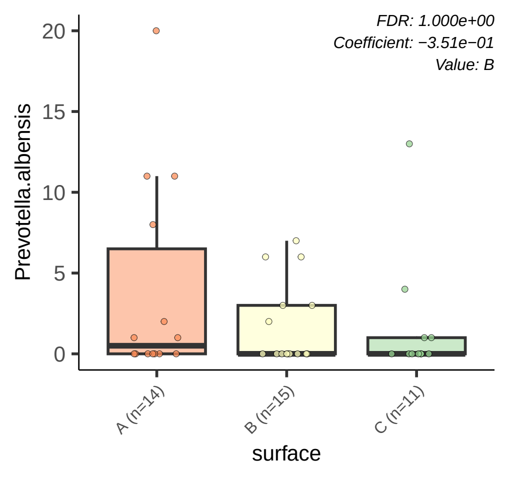
FDR: 1.000e+00
Coefficient: −3.51e−01
Value: B
Prevotella.albensis
20
15
10
5
0
A (n=14)
B (n=15)
C (n=11)
surface

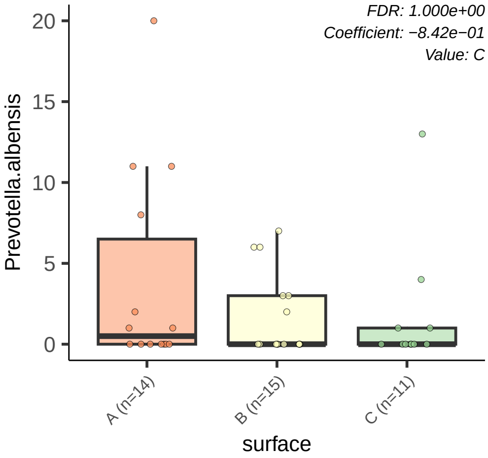
FDR: 1.000e+00
Coefficient: −8.42e−01
Value: C
Prevotella.albensis
20
15
10
5
0
A (n=14)
B (n=15)
C (n=11)
surface

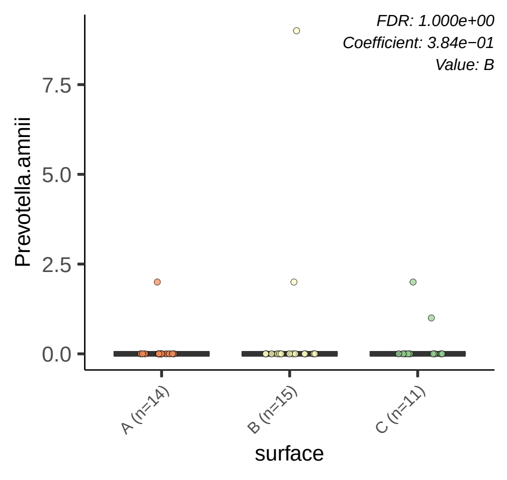

FDR: 1.000e+00
Coefficient: 3.84e−01
Value: B
Prevotella.amnii
7.5
5.0
2.5
0.0
A (n=14)
B (n=15)
C (n=11)
surface

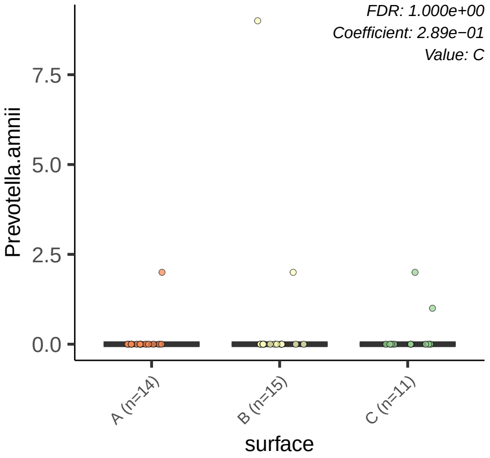

FDR: 1.000e+00
Coefficient: 2.89e−01
Value: C
Prevotella.amnii
7.5
5.0
2.5
0.0
A (n=14)
B (n=15)
C (n=11)
surface

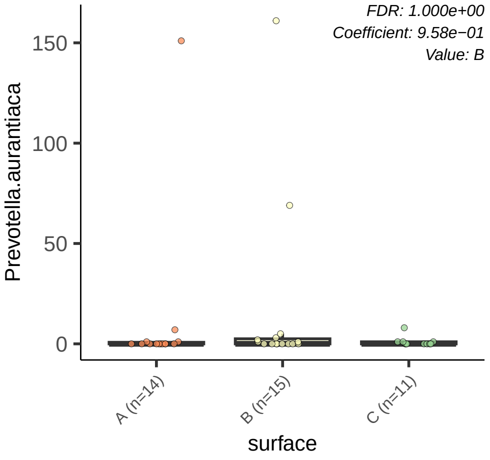
FDR: 1.000e+00
Coefficient: 9.58e−01
Value: B
Prevotella.aurantiaca
150
100
50
0
A (n=14)
B (n=15)
C (n=11)
surface

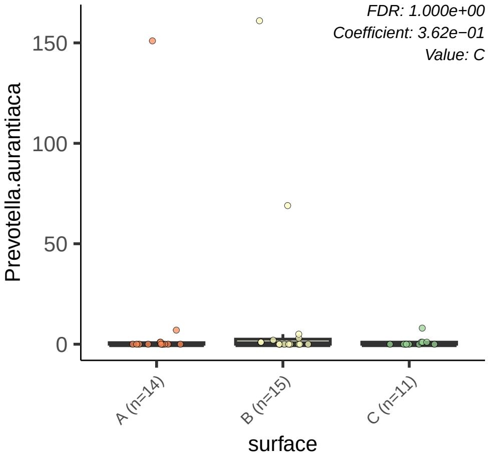

FDR: 1.000e+00
Coefficient: 3.62e−01
Value: C
Prevotella.aurantiaca
150
100
50
0
A (n=14)
B (n=15)
C (n=11)
surface

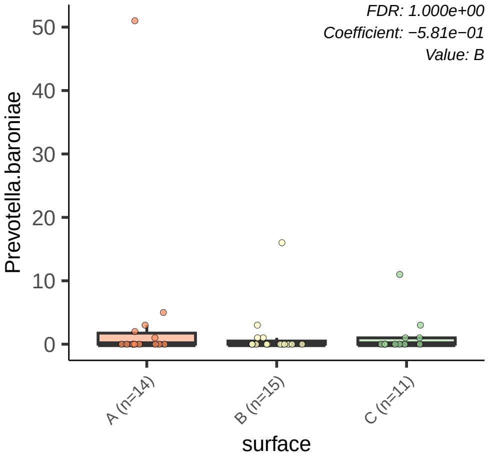
FDR: 1.000e+00
Coefficient: −5.81e−01
Value: B
Prevotella.baroniae
50
40
30
20
10
0
A (n=14)
B (n=15)
C (n=11)
surface

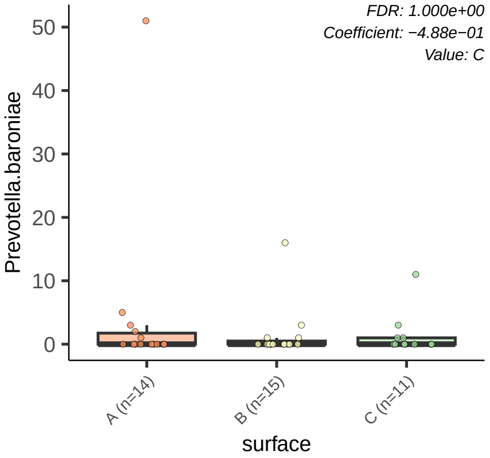
FDR: 1.000e+00
Coefficient: −4.88e−01
Value: C
Prevotella.baroniae
50
40
30
20
10
0
A (n=14)
B (n=15)
C (n=11)
surface

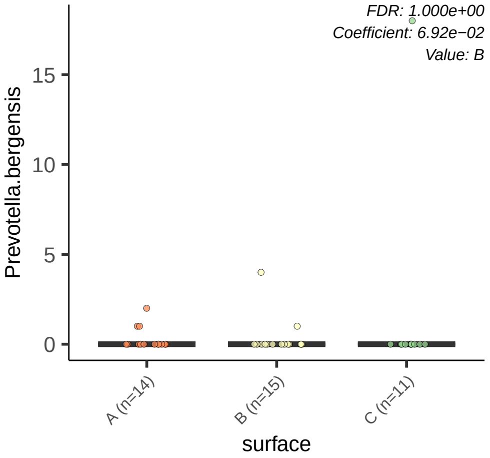

FDR: 1.000e+00
Coefficient: 6.92e−02
Value: B
Prevotella.bergensis
15
10
5
0
A (n=14)
B (n=15)
C (n=11)
surface

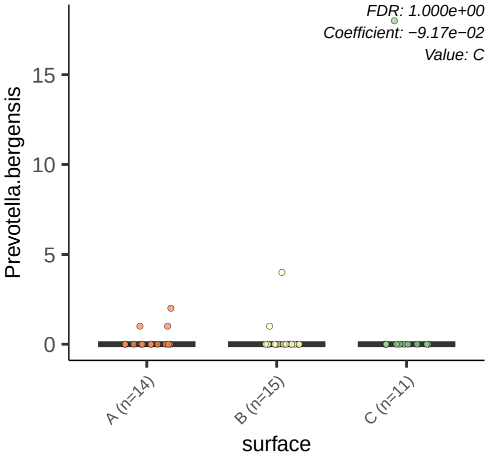

FDR: 1.000e+00
Coefficient: −9.17e−02
Value: C
Prevotella.bergensis
0
5
10
15
A (n=14)
B (n=15)
C (n=11)
surface

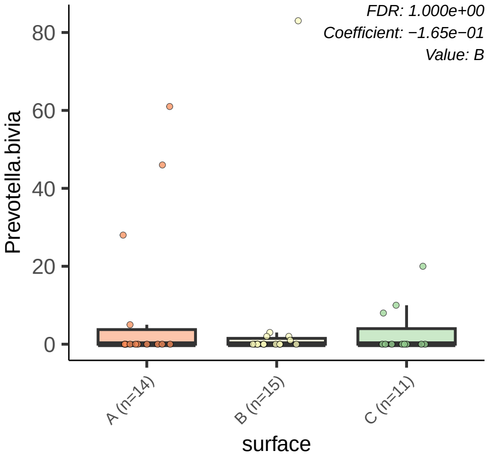
FDR: 1.000e+00
Coefficient: −1.65e−01
Value: B
Prevotella.bivia
80
60
40
20
0
A (n=14)
B (n=15)
C (n=11)
surface

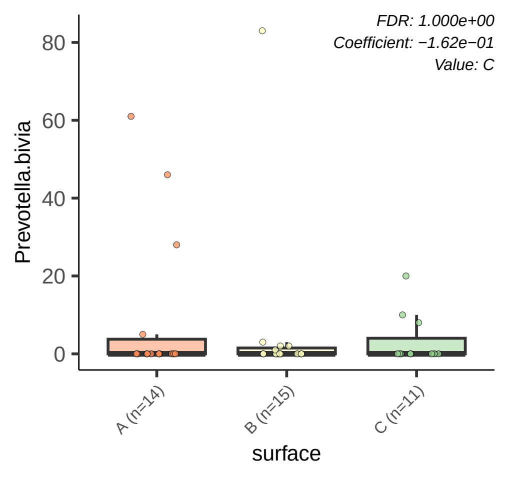
FDR: 1.000e+00
Coefficient: −1.62e−01
Value: C
Prevotella.bivia
0
20
40
60
80
A (n=14)
B (n=15)
C (n=11)
surface

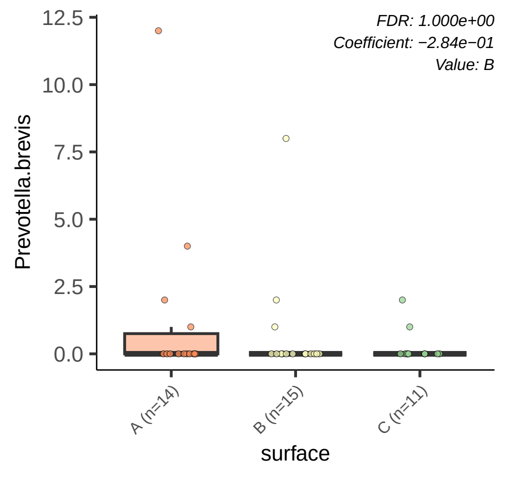
FDR: 1.000e+00
Coefficient: −2.84e−01
Value: B
Prevotella.brevis
12.5
10.0
7.5
5.0
2.5
0.0
A (n=14)
B (n=15)
C (n=11)
surface

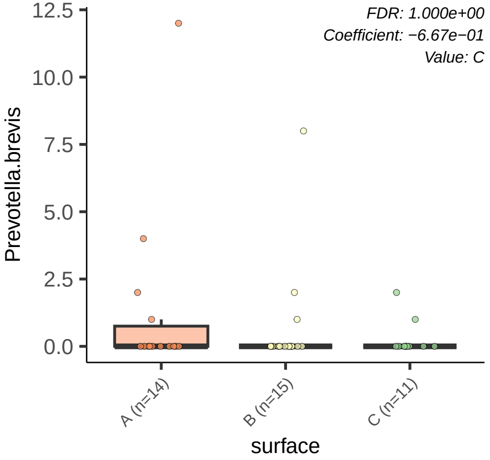
FDR: 1.000e+00
Coefficient: −6.67e−01
Value: C
Prevotella.brevis
12.5
10.0
7.5
5.0
2.5
0.0
A (n=14)
B (n=15)
C (n=11)
surface

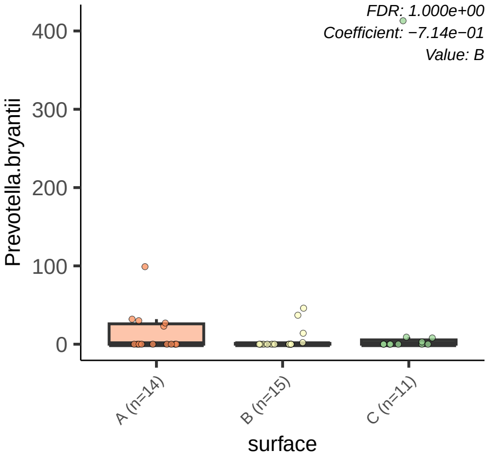

FDR: 1.000e+00
Coefficient: −7.14e−01
Value: B
Prevotella.bryantii
400
300
200
100
0
A (n=14)
B (n=15)
C (n=11)
surface

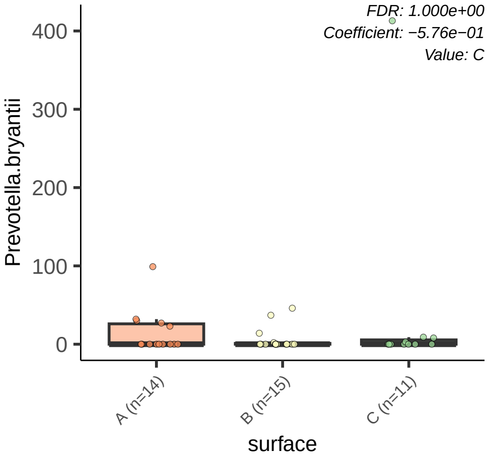
FDR: 1.000e+00
Coefficient: −5.76e−01
Value: C
Prevotella.bryantii
400
300
200
100
0
A (n=14)
B (n=15)
C (n=11)
surface

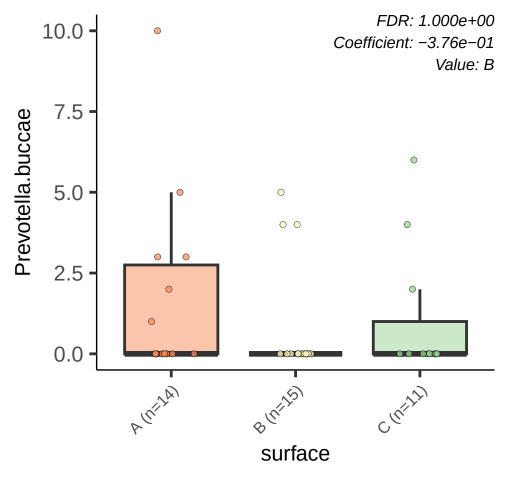
FDR: 1.000e+00
Coefficient: −3.76e−01
Value: B
Prevotella.buccae
10.0
7.5
5.0
2.5
0.0
A (n=14)
B (n=15)
C (n=11)
surface

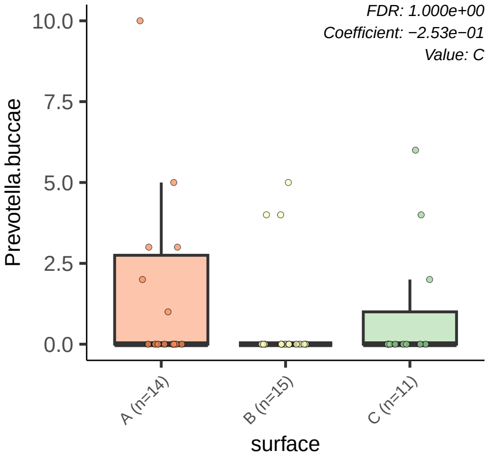

FDR: 1.000e+00
Coefficient: −2.53e−01
Value: C
Prevotella.buccae
10.0
7.5
5.0
2.5
0.0
A (n=14)
B (n=15)
C (n=11)
surface

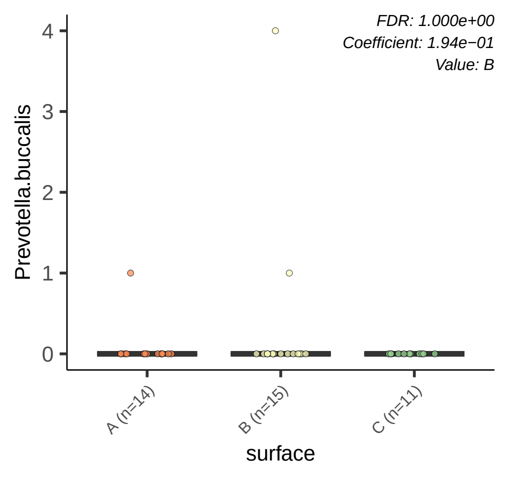
FDR: 1.000e+00
Coefficient: 1.94e−01
Value: B
Prevotella.buccalis
4
3
2
1
0
A (n=14)
B (n=15)
C (n=11)
surface

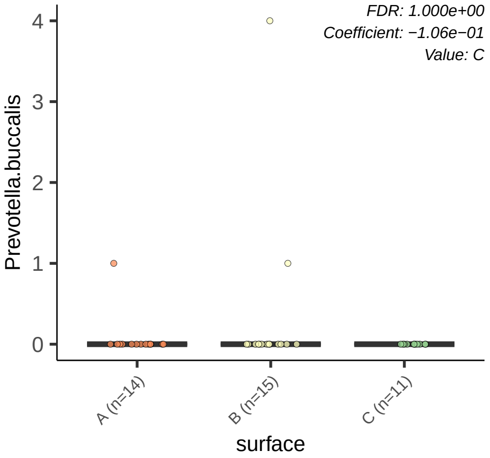
FDR: 1.000e+00
Coefficient: −1.06e−01
Value: C
Prevotella.buccalis
4
3
2
1
0
A (n=14)
B (n=15)
C (n=11)
surface

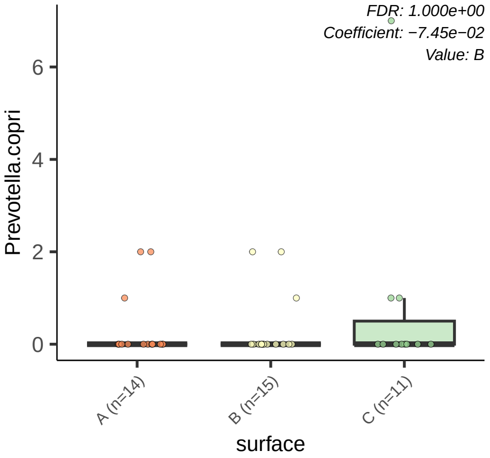

FDR: 1.000e+00
Coefficient: −7.45e−02
Value: B
Prevotella.copri
0
2
4
6
A (n=14)
B (n=15)
C (n=11)
surface

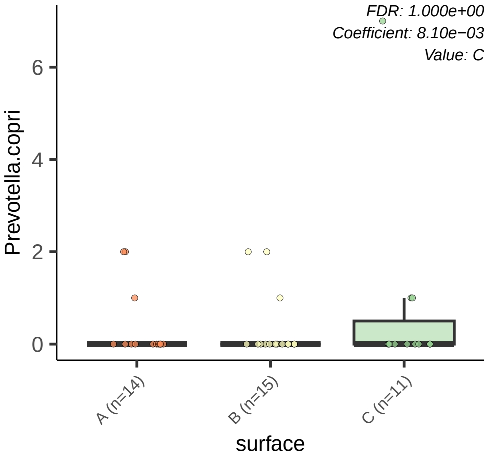
FDR: 1.000e+00
Coefficient: 8.10e−03
Value: C
Prevotella.copri
0
2
4
6
A (n=14)
B (n=15)
C (n=11)
surface

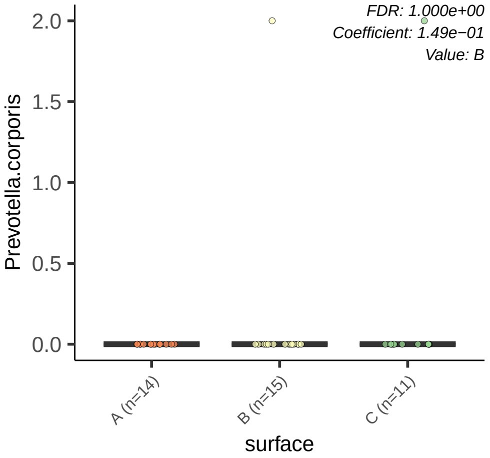

FDR: 1.000e+00
Coefficient: 1.49e−01
Value: B
Prevotella.corporis
2.0
1.5
1.0
0.5
0.0
A (n=14)
B (n=15)
C (n=11)
surface

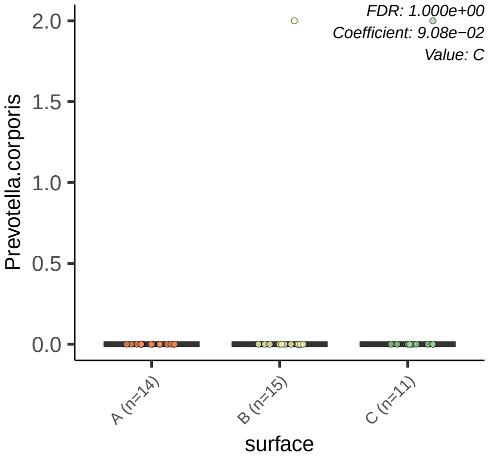
FDR: 1.000e+00
Coefficient: 9.08e−02
Value: C
Prevotella.corporis
2.0
1.5
1.0
0.5
0.0
A (n=14)
B (n=15)
C (n=11)
surface

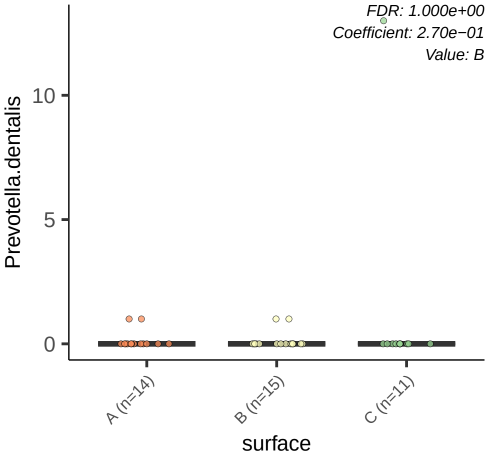

FDR: 1.000e+00
Coefficient: 2.70e−01
Value: B
Prevotella.dentalis
10
5
0
A (n=14)
B (n=15)
C (n=11)
surface

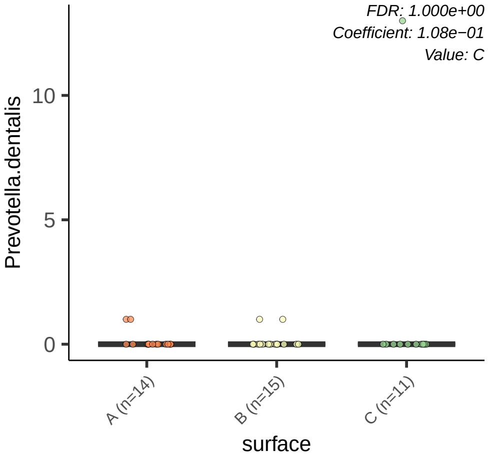
FDR: 1.000e+00
Coefficient: 1.08e−01
Value: C
Prevotella.dentalis
10
5
0
A (n=14)
B (n=15)
C (n=11)
surface

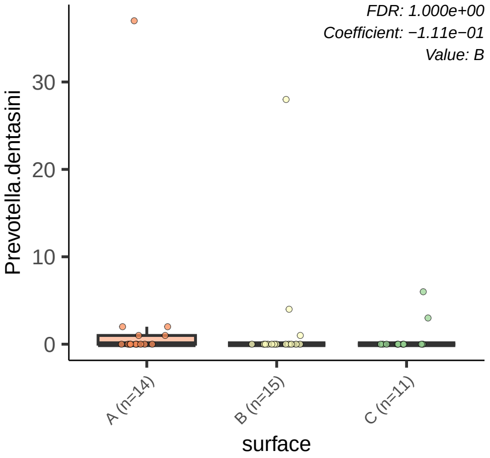

FDR: 1.000e+00
Coefficient: −1.11e−01
Value: B
Prevotella.dentasini
30
20
10
0
A (n=14)
B (n=15)
C (n=11)
surface

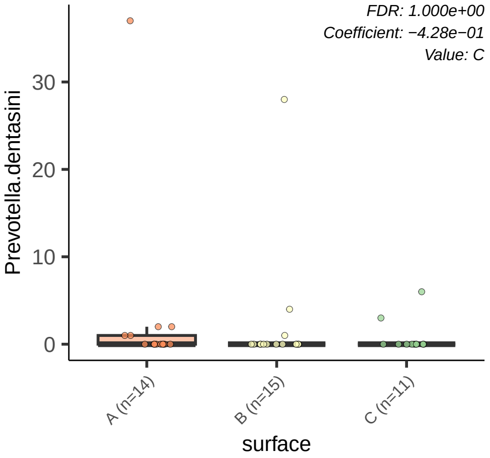
FDR: 1.000e+00
Coefficient: −4.28e−01
Value: C
Prevotella.dentasini
0
10
20
30
A (n=14)
B (n=15)
C (n=11)
surface

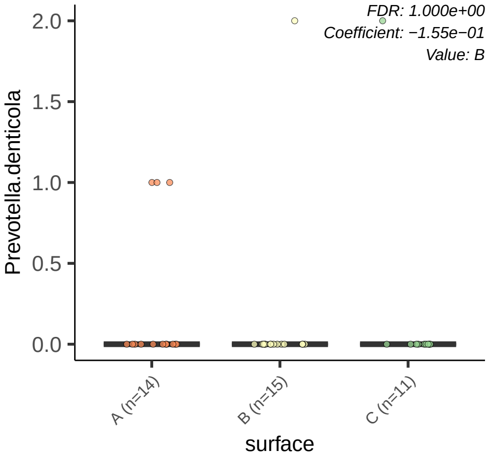

FDR: 1.000e+00
Coefficient: −1.55e−01
Value: B
Prevotella.denticola
2.0
1.5
1.0
0.5
0.0
A (n=14)
B (n=15)
C (n=11)
surface

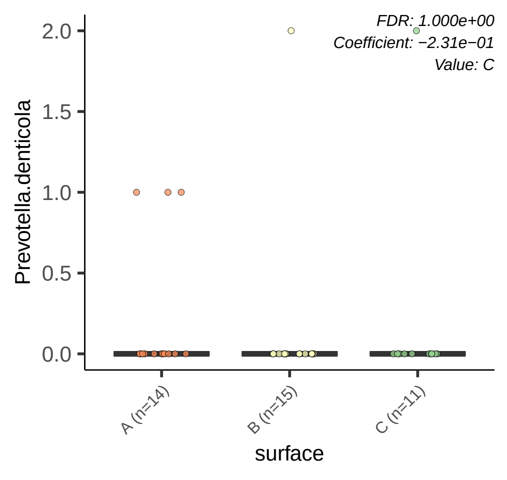
FDR: 1.000e+00
Coefficient: −2.31e−01
Value: C
Prevotella.denticola
2.0
1.5
1.0
0.5
0.0
A (n=14)
B (n=15)
C (n=11)
surface

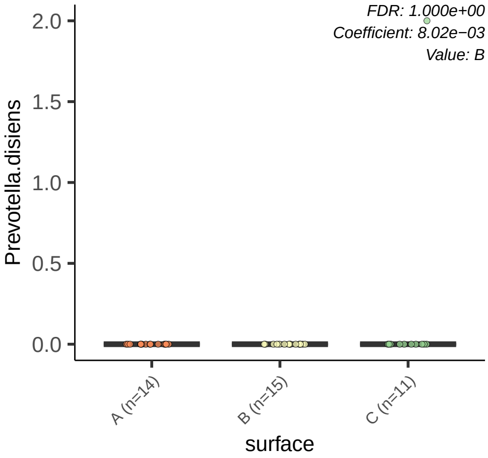
FDR: 1.000e+00
Coefficient: 8.02e−03
Value: B
Prevotella.disiens
2.0
1.5
1.0
0.5
0.0
A (n=14)
B (n=15)
C (n=11)
surface

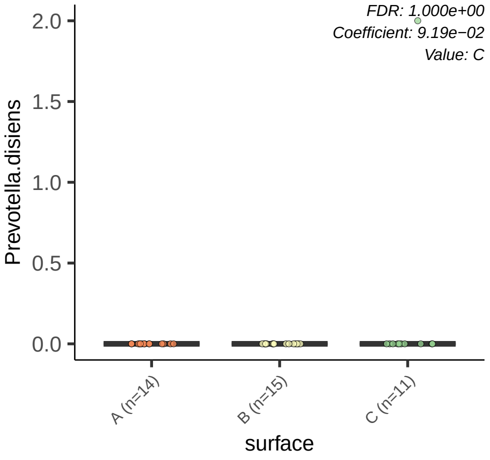
FDR: 1.000e+00
Coefficient: 9.19e−02
Value: C
Prevotella.disiens
2.0
1.5
1.0
0.5
0.0
A (n=14)
B (n=15)
C (n=11)
surface

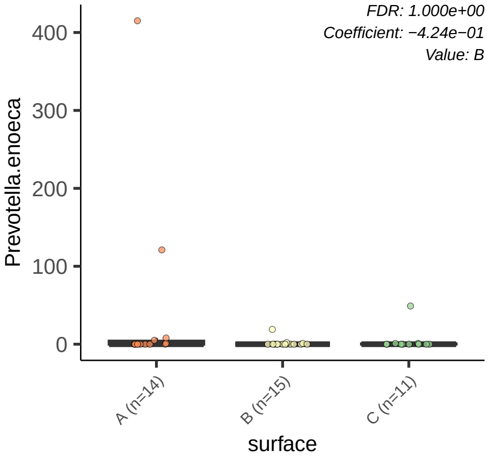
FDR: 1.000e+00
Coefficient: −4.24e−01
Value: B
Prevotella.enoeca
400
300
200
100
0
A (n=14)
B (n=15)
C (n=11)
surface

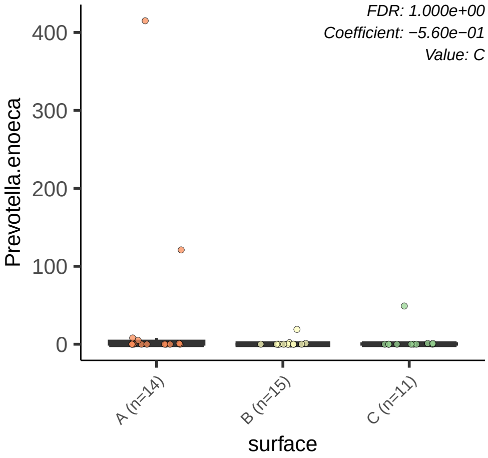

FDR: 1.000e+00
Coefficient: −5.60e−01
Value: C
Prevotella.enoeca
400
300
200
100
0
A (n=14)
B (n=15)
C (n=11)
surface

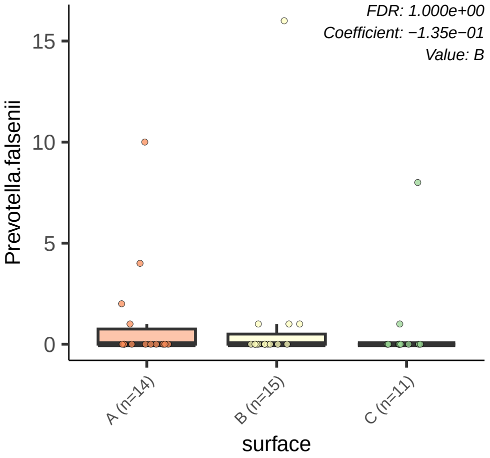

FDR: 1.000e+00
Coefficient: −1.35e−01
Value: B
Prevotella.falsenii
15
10
5
0
A (n=14)
B (n=15)
C (n=11)
surface

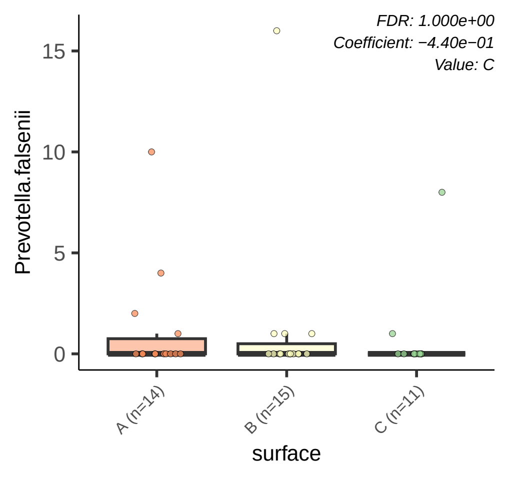

FDR: 1.000e+00
Coefficient: −4.40e−01
Value: C
Prevotella.falsenii
15
10
5
0
A (n=14)
B (n=15)
C (n=11)
surface

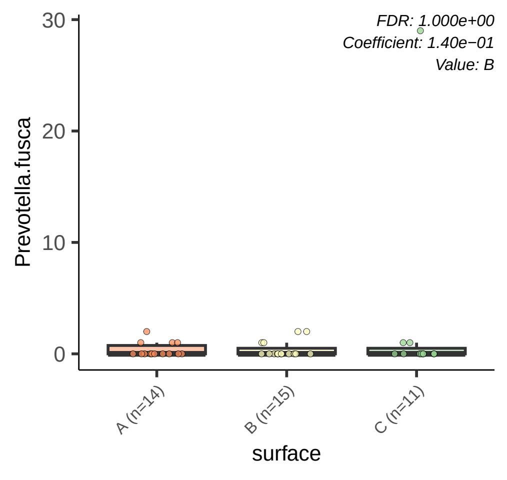
FDR: 1.000e+00
Coefficient: 1.40e−01
Value: B
Prevotella.fusca
30
20
10
0
A (n=14)
B (n=15)
C (n=11)
surface

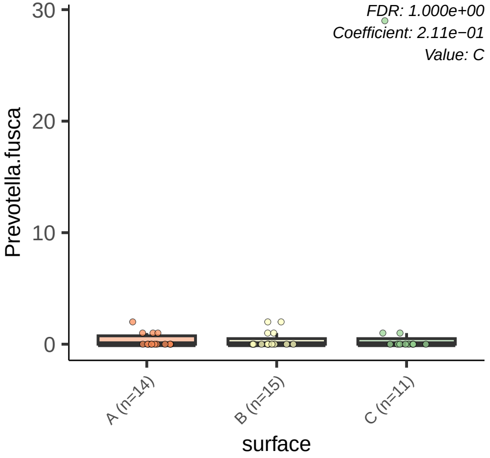
FDR: 1.000e+00
Coefficient: 2.11e−01
Value: C
Prevotella.fusca
30
20
10
0
A (n=14)
B (n=15)
C (n=11)
surface

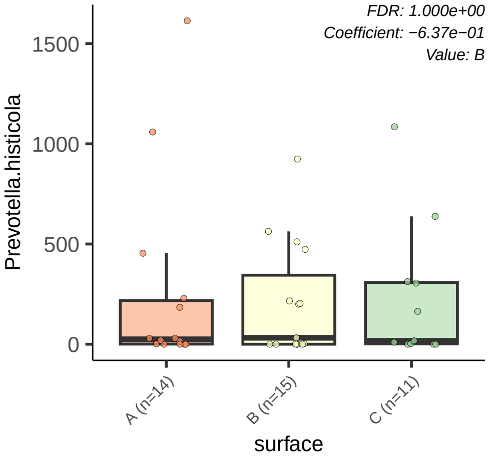

FDR: 1.000e+00
Coefficient: −6.37e−01
Value: B
Prevotella.histicola
1500
1000
500
0
A (n=14)
B (n=15)
C (n=11)
surface

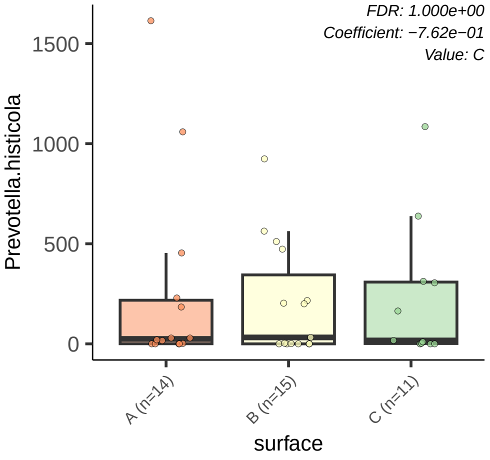
FDR: 1.000e+00
Coefficient: −7.62e−01
Value: C
Prevotella.histicola
1500
1000
500
0
A (n=14)
B (n=15)
C (n=11)
surface

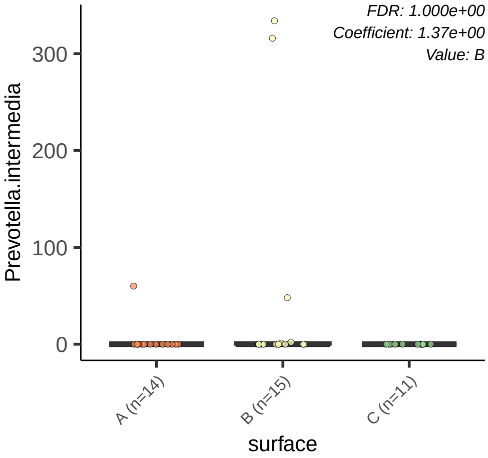
FDR: 1.000e+00
Coefficient: 1.37e+00
Value: B
Prevotella.intermedia
300
200
100
0
A (n=14)
B (n=15)
C (n=11)
surface

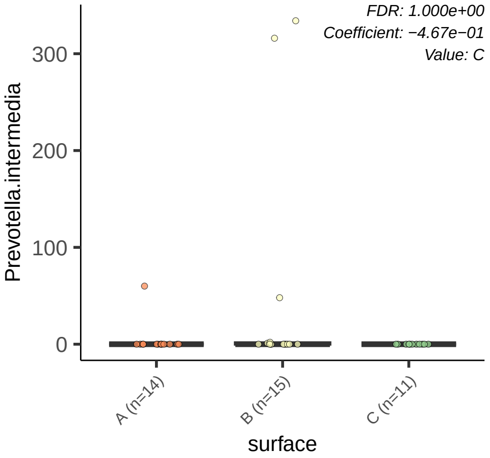

FDR: 1.000e+00
Coefficient: −4.67e−01
Value: C
Prevotella.intermedia
300
200
100
0
A (n=14)
B (n=15)
C (n=11)
surface

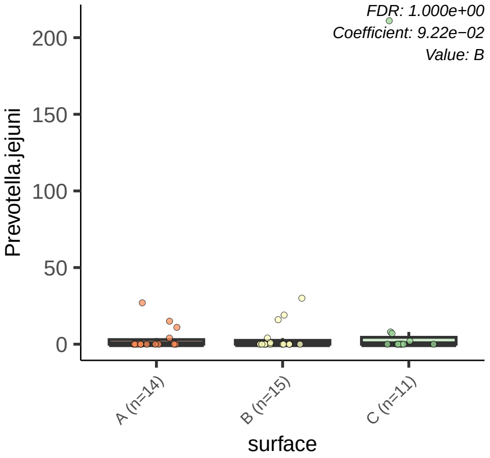
FDR: 1.000e+00
Coefficient: 9.22e−02
Value: B
Prevotella.jejuni
200
150
100
50
0
A (n=14)
B (n=15)
C (n=11)
surface

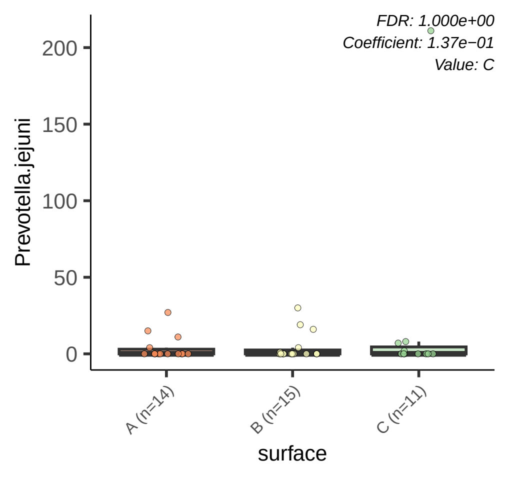
FDR: 1.000e+00
Coefficient: 1.37e−01
Value: C
Prevotella.jejuni
200
150
100
50
0
A (n=14)
B (n=15)
C (n=11)
surface

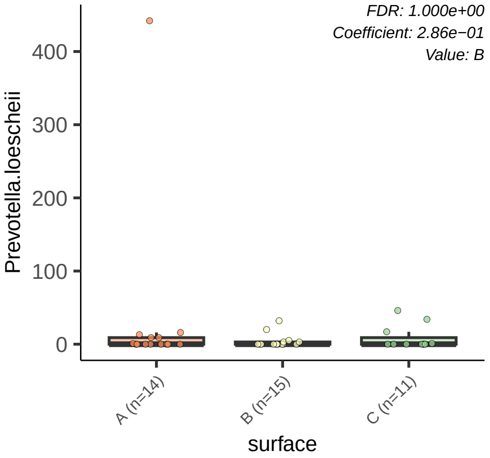
FDR: 1.000e+00
Coefficient: 2.86e−01
Value: B
Prevotella.loescheii
400
300
200
100
0
A (n=14)
B (n=15)
C (n=11)
surface

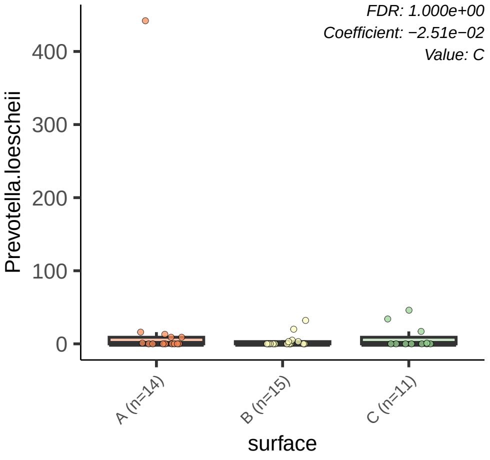

FDR: 1.000e+00
Coefficient: −2.51e−02
Value: C
Prevotella.loescheii
400
300
200
100
0
A (n=14)
B (n=15)
C (n=11)
surface

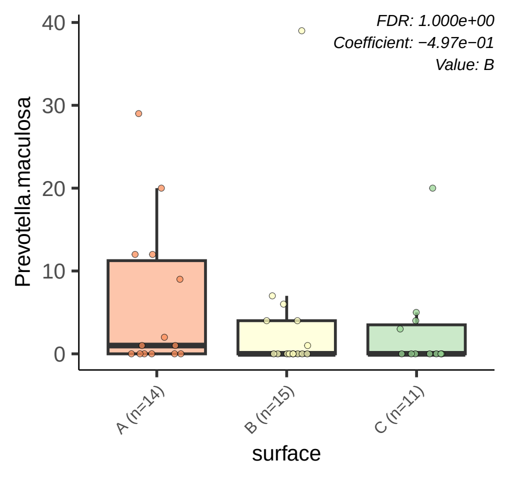

FDR: 1.000e+00
Coefficient: −4.97e−01
Value: B
Prevotella.maculosa
40
30
20
10
0
A (n=14)
B (n=15)
C (n=11)
surface

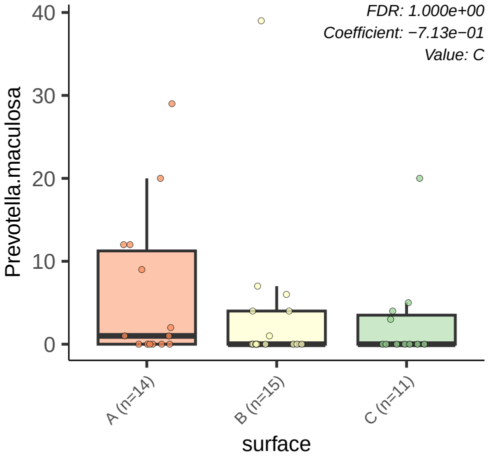

FDR: 1.000e+00
Coefficient: −7.13e−01
Value: C
Prevotella.maculosa
40
30
20
10
0
A (n=14)
B (n=15)
C (n=11)
surface

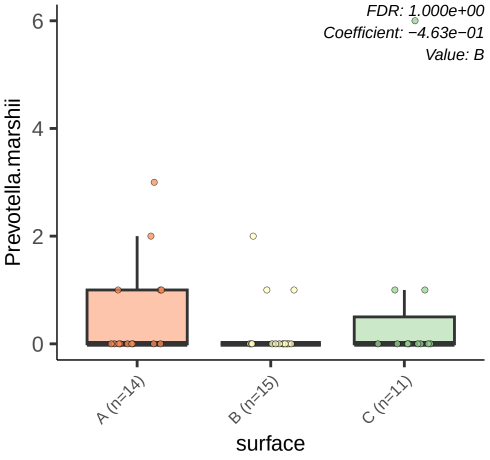

FDR: 1.000e+00
Coefficient: −4.63e−01
Value: B
Prevotella.marshii
6
4
2
0
A (n=14)
B (n=15)
C (n=11)
surface

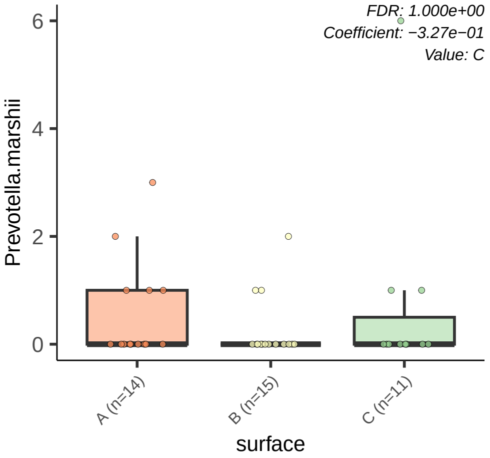

FDR: 1.000e+00
Coefficient: −3.27e−01
Value: C
Prevotella.marshii
6
4
2
0
A (n=14)
B (n=15)
C (n=11)
surface

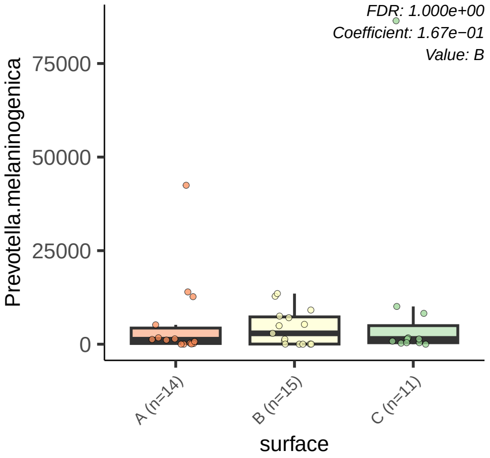
FDR: 1.000e+00
Coefficient: 1.67e−01
Value: B
Prevotella.melaninogenica
75000
50000
25000
0
A (n=14)
B (n=15)
C (n=11)
surface

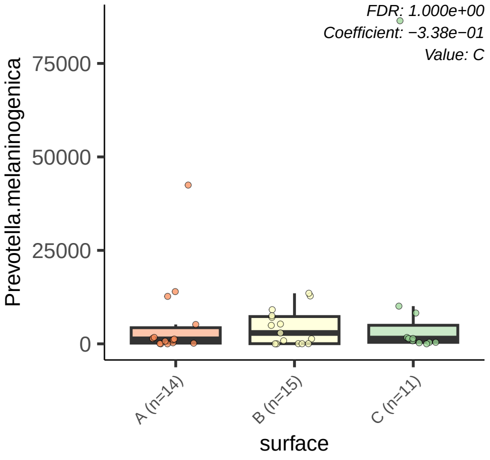

FDR: 1.000e+00
Coefficient: −3.38e−01
Value: C
Prevotella.melaninogenica
75000
50000
25000
0
A (n=14)
B (n=15)
C (n=11)
surface

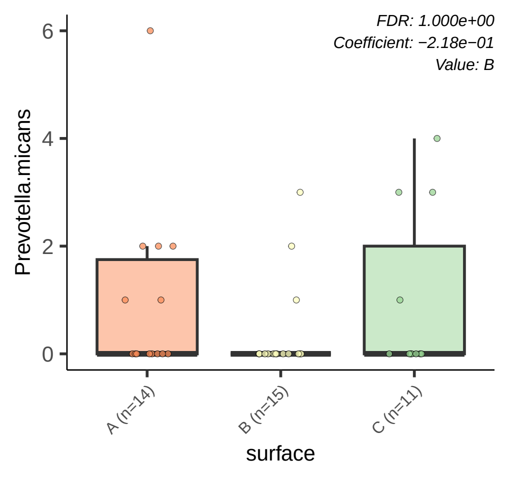

FDR: 1.000e+00
Coefficient: −2.18e−01
Value: B
Prevotella.micans
6
4
2
0
A (n=14)
B (n=15)
C (n=11)
surface

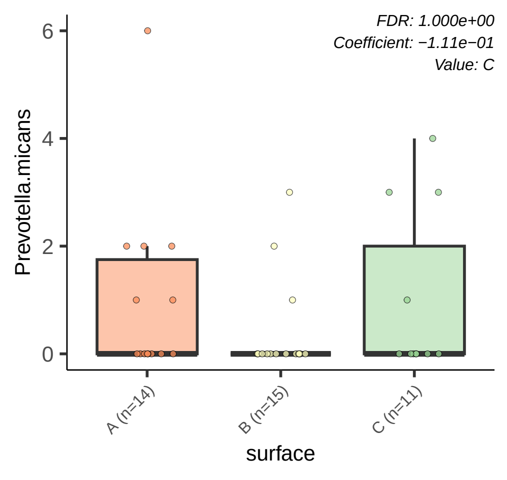

FDR: 1.000e+00
Coefficient: −1.11e−01
Value: C
Prevotella.micans
6
4
2
0
A (n=14)
B (n=15)
C (n=11)
surface

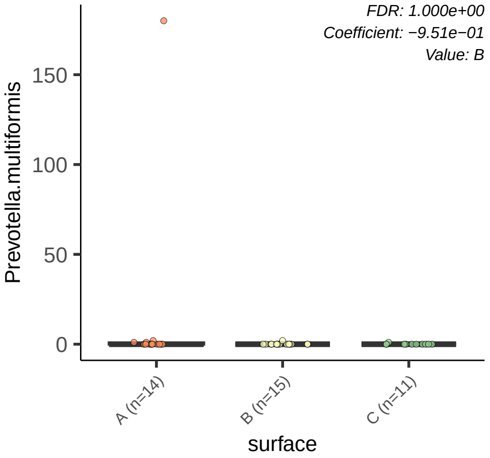

FDR: 1.000e+00
Coefficient: −9.51e−01
Value: B
Prevotella.multiformis
150
100
50
0
A (n=14)
B (n=15)
C (n=11)
surface

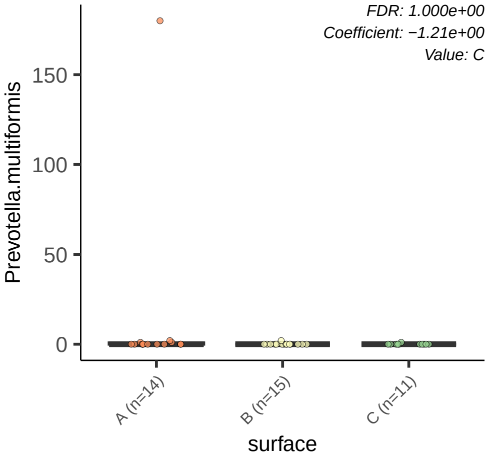

FDR: 1.000e+00
Coefficient: −1.21e+00
Value: C
Prevotella.multiformis
150
100
50
0
A (n=14)
B (n=15)
C (n=11)
surface

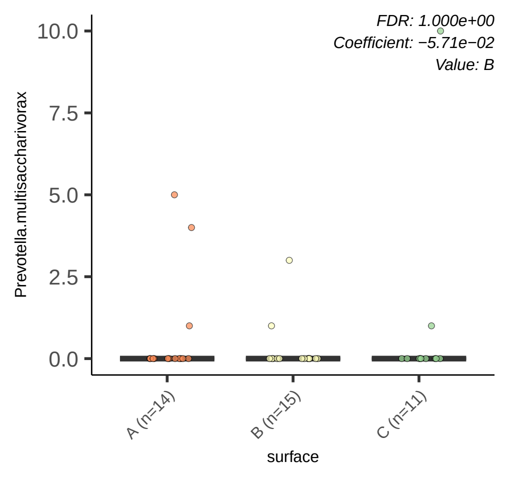
FDR: 1.000e+00
Coefficient: −5.71e−02
Value: B
Prevotella.multisaccharivorax
10.0
7.5
5.0
2.5
0.0
A (n=14)
B (n=15)
C (n=11)
surface

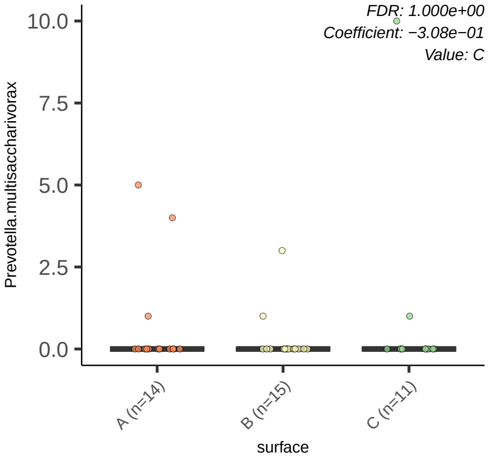
FDR: 1.000e+00
Coefficient: −3.08e−01
Value: C
10.0
7.5
5.0
2.5
0.0
Prevotella.multisaccharivorax
A (n=14)
B (n=15)
C (n=11)
surface

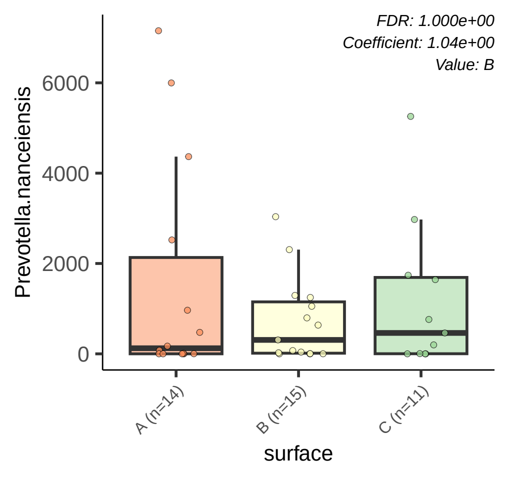
FDR: 1.000e+00
Coefficient: 1.04e+00
Value: B
Prevotella.nanceiensis
6000
4000
2000
0
A (n=14)
B (n=15)
C (n=11)
surface

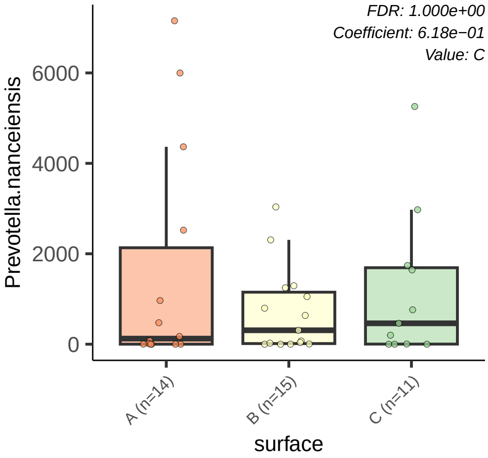

FDR: 1.000e+00
Coefficient: 6.18e−01
Value: C
Prevotella.nanceiensis
0
2000
4000
6000
A (n=14)
B (n=15)
C (n=11)
surface

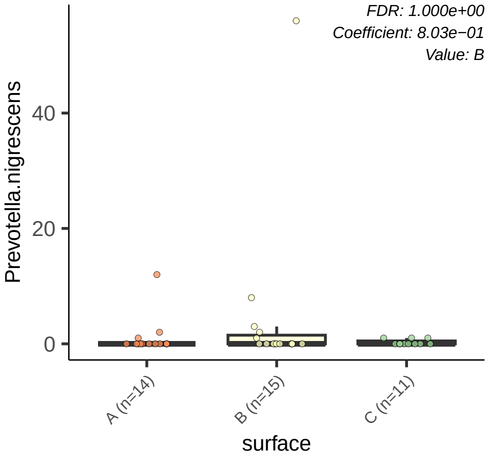
FDR: 1.000e+00
Coefficient: 8.03e−01
Value: B
Prevotella.nigrescens
40
20
0
A (n=14)
B (n=15)
C (n=11)
surface

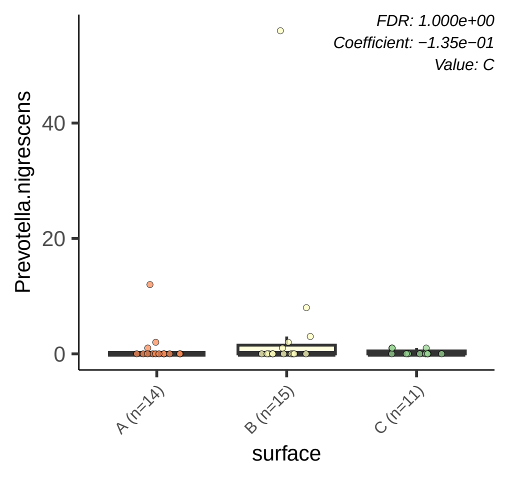

FDR: 1.000e+00
Coefficient: −1.35e−01
Value: C
Prevotella.nigrescens
0
20
40
A (n=14)
B (n=15)
C (n=11)
surface

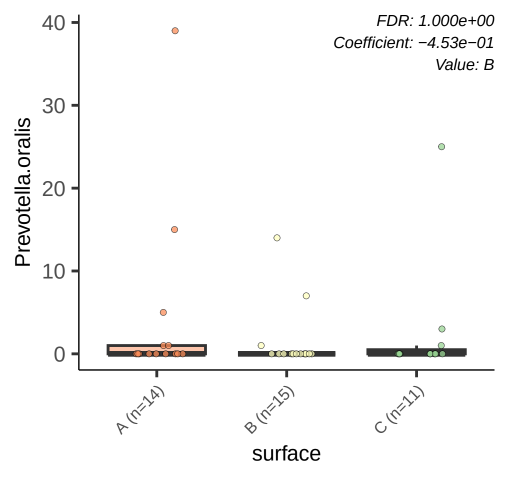

FDR: 1.000e+00
Coefficient: −4.53e−01
Value: B
Prevotella.oralis
40
30
20
10
0
A (n=14)
B (n=15)
C (n=11)
surface

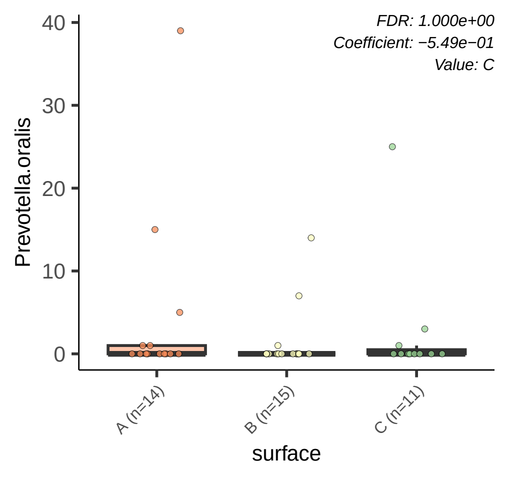
FDR: 1.000e+00
Coefficient: −5.49e−01
Value: C
Prevotella.oralis
0
10
20
30
40
A (n=14)
B (n=15)
C (n=11)
surface

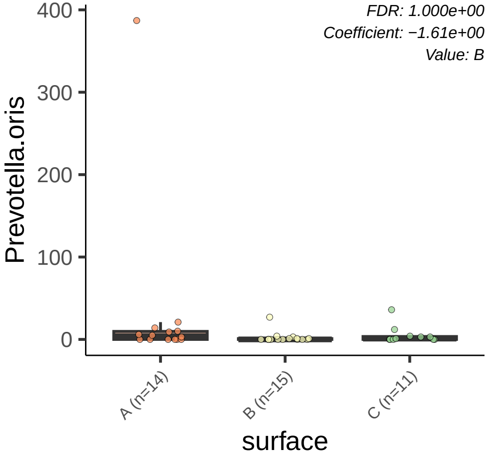
FDR: 1.000e+00
Coefficient: −1.61e+00
Value: B
Prevotella.oris
400
300
200
100
0
A (n=14)
B (n=15)
C (n=11)
surface

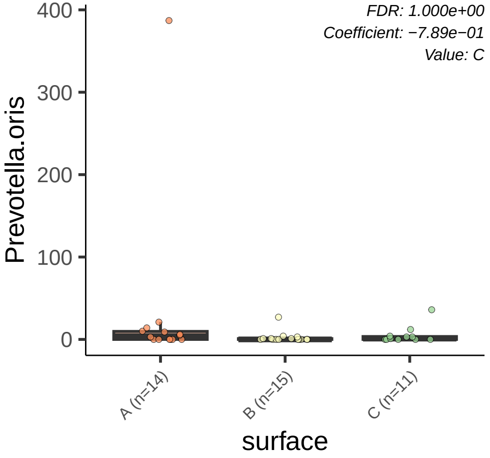

FDR: 1.000e+00
Coefficient: −7.89e−01
Value: C
400
300
200
100
0
Prevotella.oris
A (n=14)
B (n=15)
C (n=11)
surface

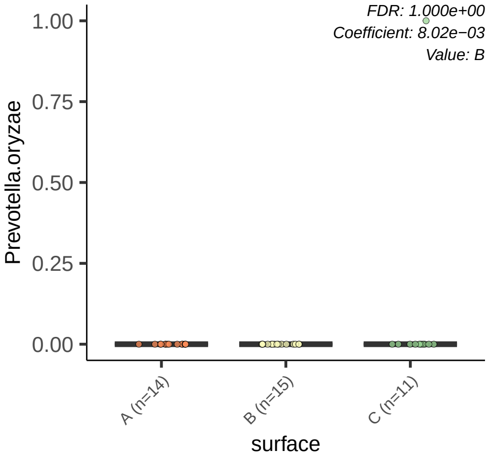

FDR: 1.000e+00
Coefficient: 8.02e−03
Value: B
Prevotella.oryzae
1.00
0.75
0.50
0.25
0.00
A (n=14)
B (n=15)
C (n=11)
surface

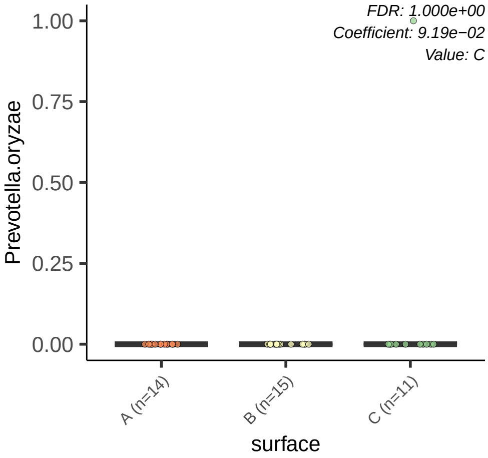
FDR: 1.000e+00
Coefficient: 9.19e−02
Value: C
Prevotella.oryzae
1.00
0.75
0.50
0.25
0.00
A (n=14)
B (n=15)
C (n=11)
surface

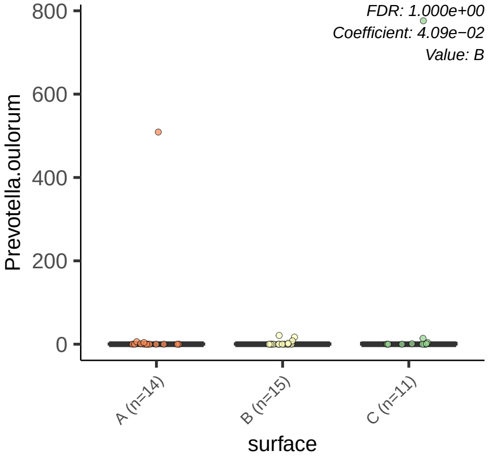
FDR: 1.000e+00
Coefficient: 4.09e−02
Value: B
800
600
400
200
0
Prevotella.oulorum
A (n=14)
B (n=15)
C (n=11)
surface

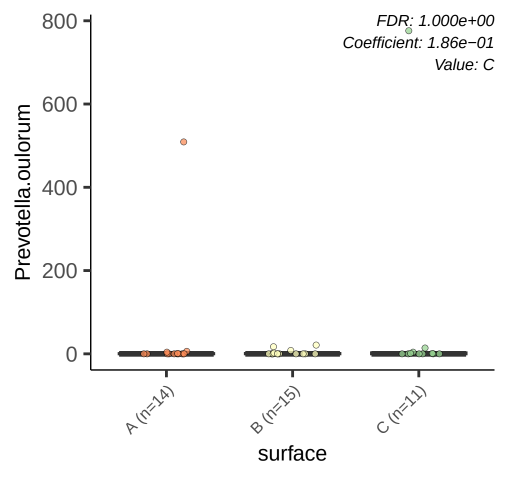
FDR: 1.000e+00
Coefficient: 1.86e−01
Value: C
Prevotella.oulorum
800
600
400
200
0
A (n=14)
B (n=15)
C (n=11)
surface

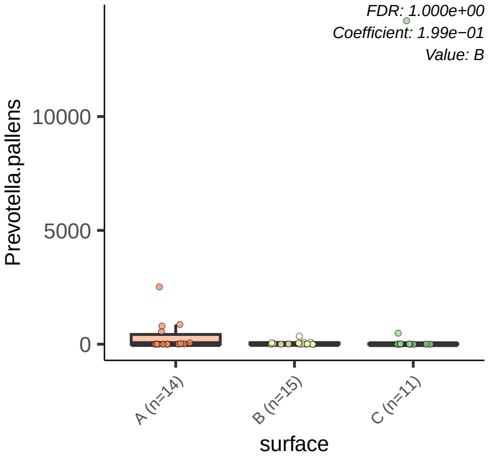

FDR: 1.000e+00
Coefficient: 1.99e−01
Value: B
Prevotella.pallens
10000
5000
0
A (n=14)
B (n=15)
C (n=11)
surface

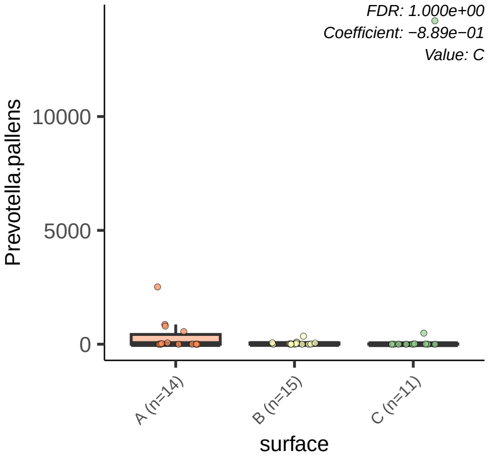

FDR: 1.000e+00
Coefficient: −8.89e−01
Value: C
Prevotella.pallens
10000
5000
0
A (n=14)
B (n=15)
C (n=11)
surface

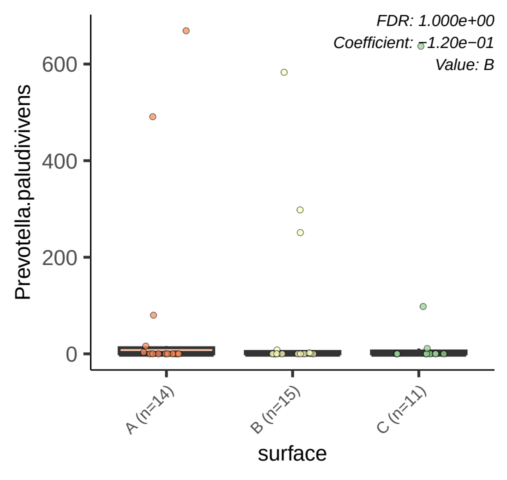
FDR: 1.000e+00
Coefficient: −1.20e−01
Value: B
Prevotella.paludivivens
600
400
200
0
A (n=14)
B (n=15)
C (n=11)
surface

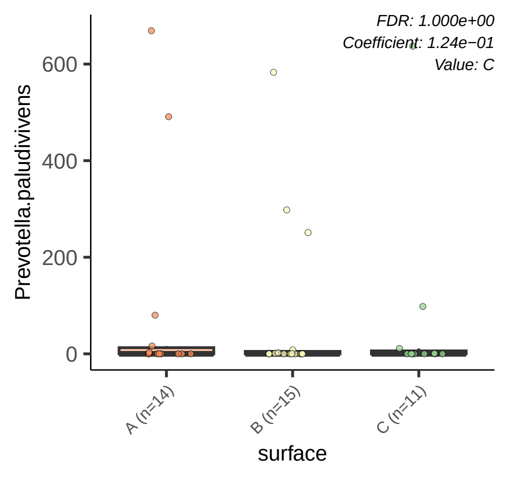

FDR: 1.000e+00
Coefficient: 1.24e−01
Value: C
Prevotella.paludivivens
600
400
200
0
A (n=14)
B (n=15)
C (n=11)
surface

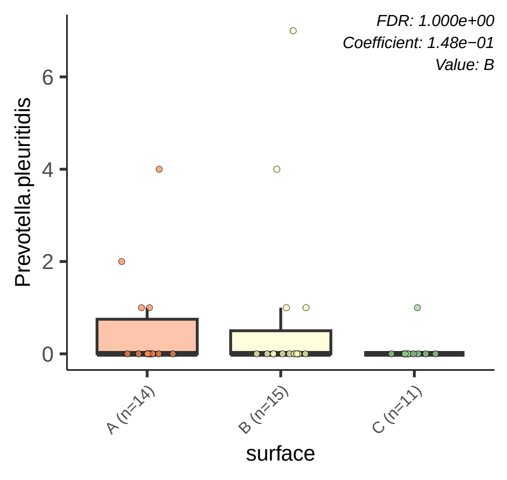
FDR: 1.000e+00
Coefficient: 1.48e−01
Value: B
Prevotella.pleuritidis
0
2
4
6
A (n=14)
B (n=15)
C (n=11)
surface

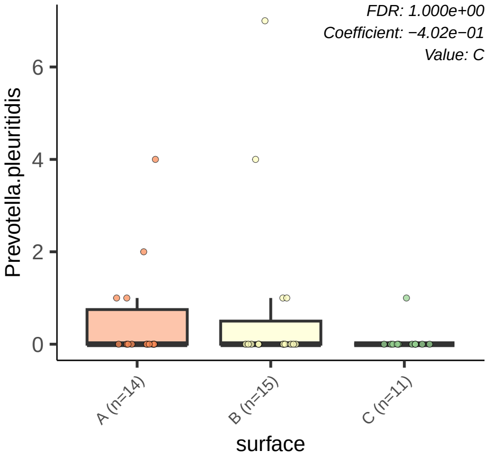
FDR: 1.000e+00
Coefficient: −4.02e−01
Value: C
Prevotella.pleuritidis
0
2
4
6
A (n=14)
B (n=15)
C (n=11)
surface

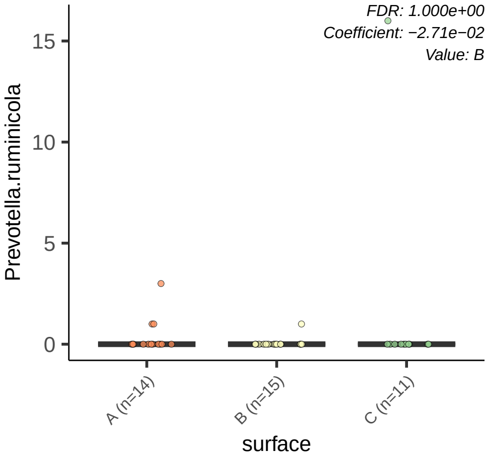

FDR: 1.000e+00
Coefficient: −2.71e−02
Value: B
Prevotella.ruminicola
15
10
5
0
A (n=14)
B (n=15)
C (n=11)
surface

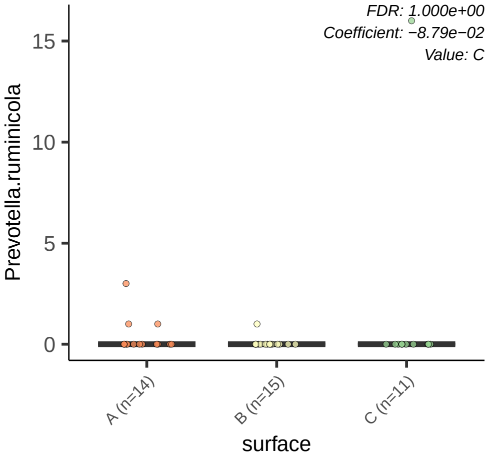
FDR: 1.000e+00
Coefficient: −8.79e−02
Value: C
Prevotella.ruminicola
15
10
5
0
A (n=14)
B (n=15)
C (n=11)
surface

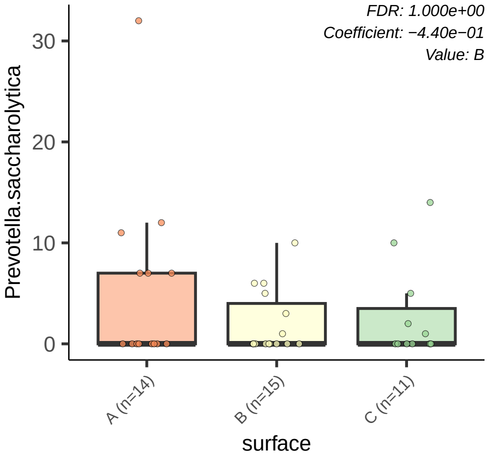

FDR: 1.000e+00
Coefficient: −4.40e−01
Value: B
Prevotella.saccharolytica
0
10
20
30
A (n=14)
B (n=15)
C (n=11)
surface

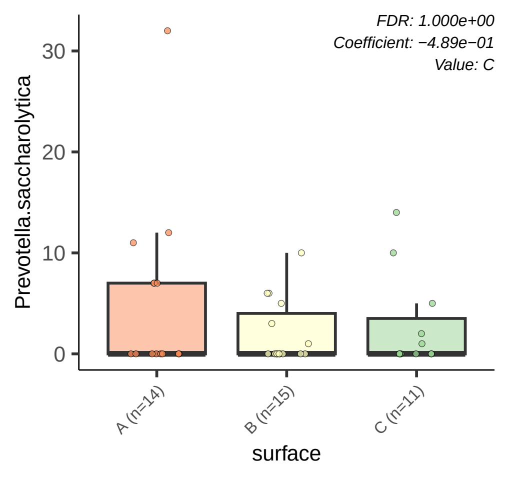
FDR: 1.000e+00
Coefficient: −4.89e−01
Value: C
Prevotella.saccharolytica
30
20
10
0
A (n=14)
B (n=15)
C (n=11)
surface

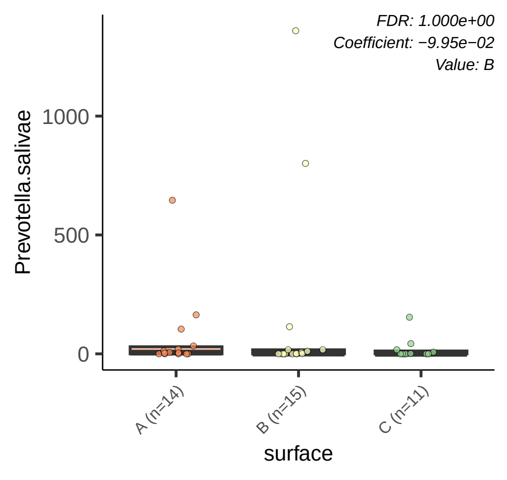

FDR: 1.000e+00
Coefficient: −9.95e−02
Value: B
Prevotella.salivae
1000
500
0
A (n=14)
B (n=15)
C (n=11)
surface

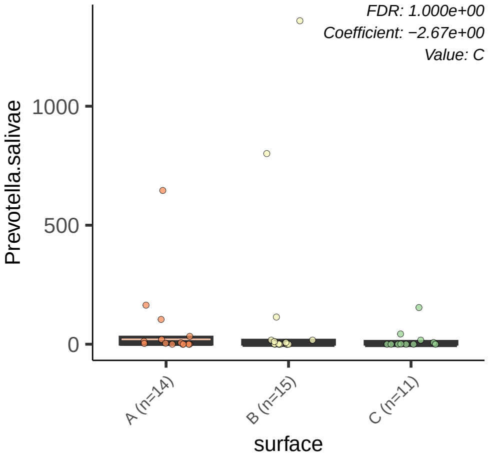

FDR: 1.000e+00
Coefficient: −2.67e+00
Value: C
Prevotella.salivae
1000
500
0
A (n=14)
B (n=15)
C (n=11)
surface

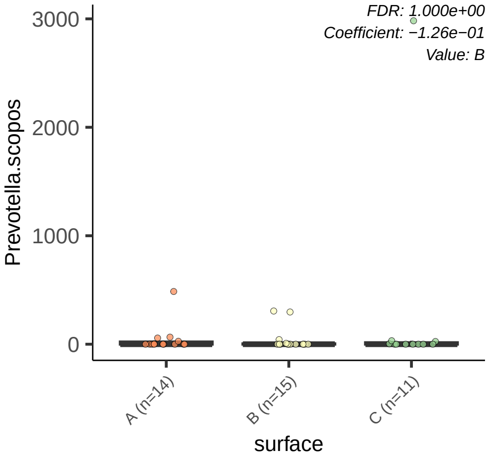

FDR: 1.000e+00
Coefficient: −1.26e−01
Value: B
Prevotella.scopos
3000
2000
1000
0
A (n=14)
B (n=15)
C (n=11)
surface

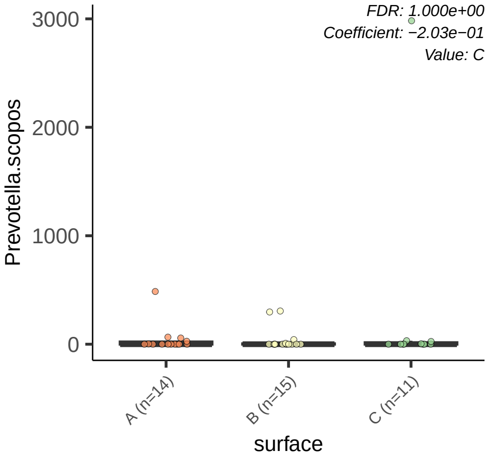

FDR: 1.000e+00
Coefficient: −2.03e−01
Value: C
Prevotella.scopos
3000
2000
1000
0
A (n=14)
B (n=15)
C (n=11)
surface

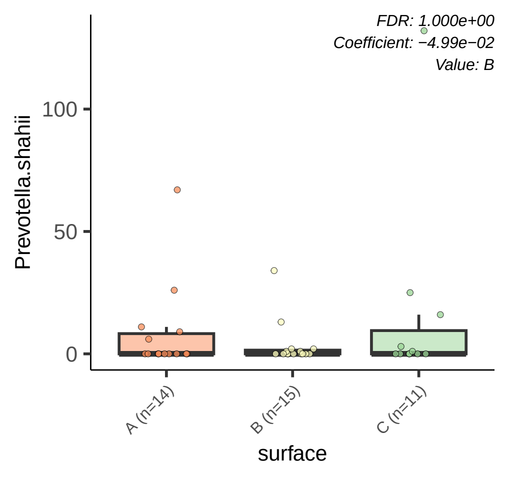

FDR: 1.000e+00
Coefficient: −4.99e−02
Value: B
Prevotella.shahii
100
50
0
A (n=14)
B (n=15)
C (n=11)
surface

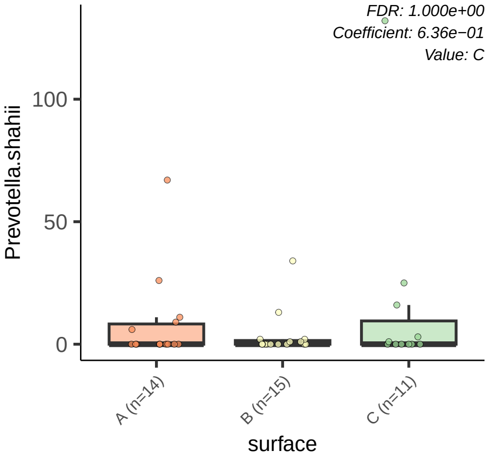

FDR: 1.000e+00
Coefficient: 6.36e−01
Value: C
Prevotella.shahii
100
50
0
A (n=14)
B (n=15)
C (n=11)
surface

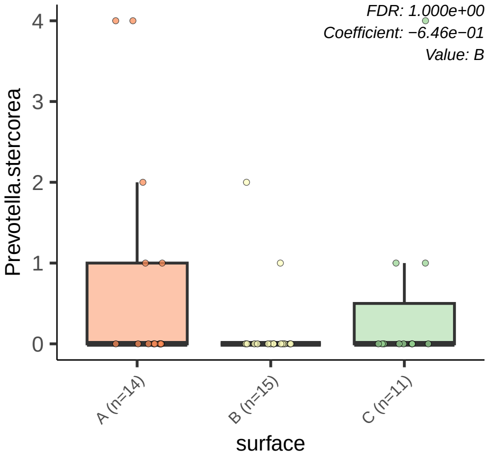
FDR: 1.000e+00
Coefficient: −6.46e−01
Value: B
Prevotella.stercorea
4
3
2
1
0
A (n=14)
B (n=15)
C (n=11)
surface

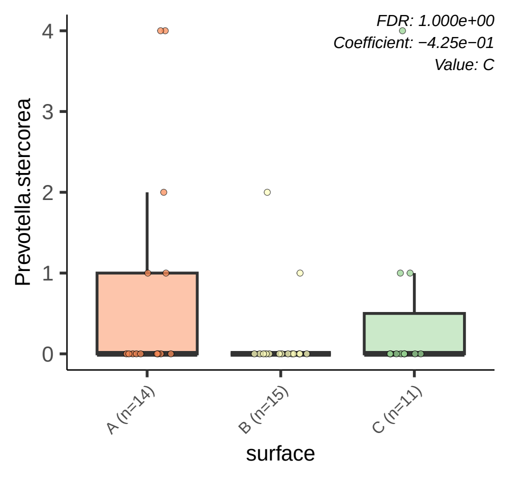
FDR: 1.000e+00
Coefficient: −4.25e−01
Value: C
Prevotella.stercorea
0
1
2
3
4
A (n=14)
B (n=15)
C (n=11)
surface

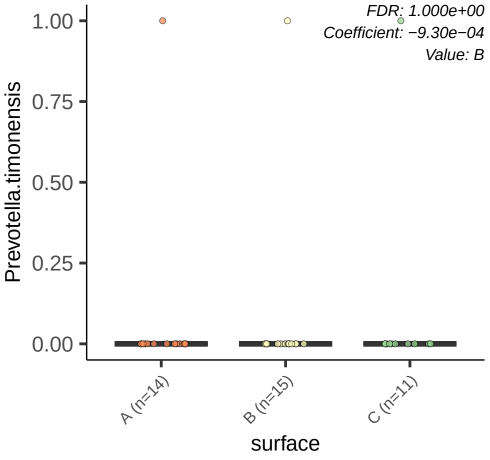
FDR: 1.000e+00
Coefficient: −9.30e−04
Value: B
1.00
0.75
0.50
0.25
0.00
Prevotella.timonensis
A (n=14)
B (n=15)
C (n=11)
surface

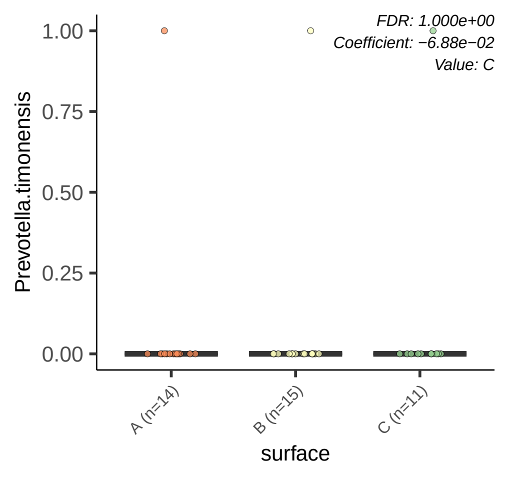
FDR: 1.000e+00
Coefficient: −6.88e−02
Value: C
Prevotella.timonensis
1.00
0.75
0.50
0.25
0.00
A (n=14)
B (n=15)
C (n=11)
surface

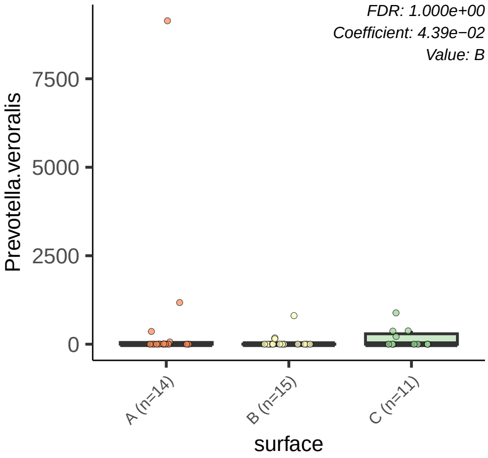

FDR: 1.000e+00
Coefficient: 4.39e−02
Value: B
Prevotella.veroralis
7500
5000
2500
0
A (n=14)
B (n=15)
C (n=11)
surface

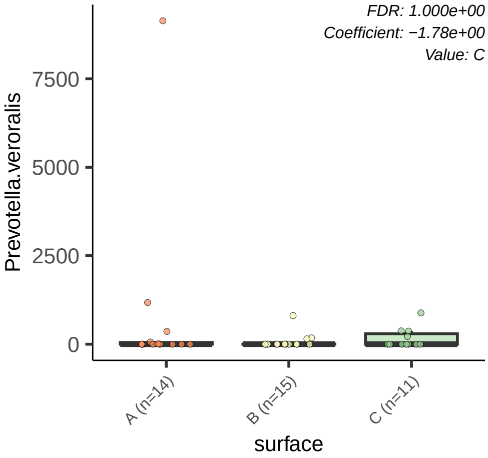

FDR: 1.000e+00
Coefficient: −1.78e+00
Value: C
7500
5000
2500
0
Prevotella.veroralis
A (n=14)
B (n=15)
C (n=11)
surface

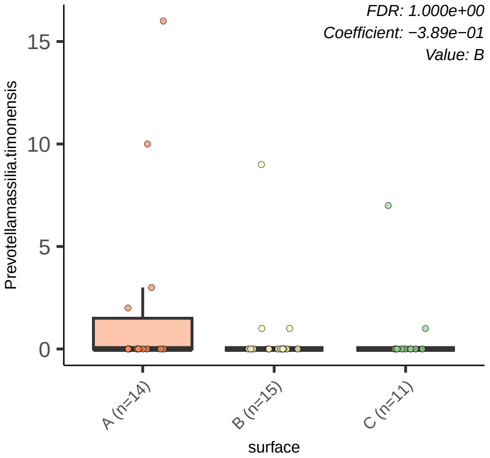

FDR: 1.000e+00
Coefficient: −3.89e−01
Value: B
Prevotellamassilia.timonensis
15
10
5
0
A (n=14)
B (n=15)
C (n=11)
surface

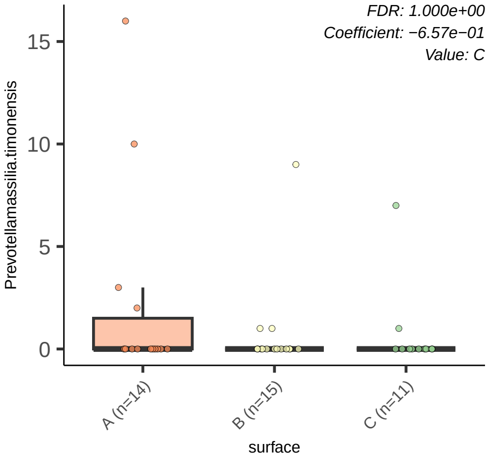

FDR: 1.000e+00
Coefficient: −6.57e−01
Value: C
Prevotellamassilia.timonensis
15
10
5
0
A (n=14)
B (n=15)
C (n=11)
surface

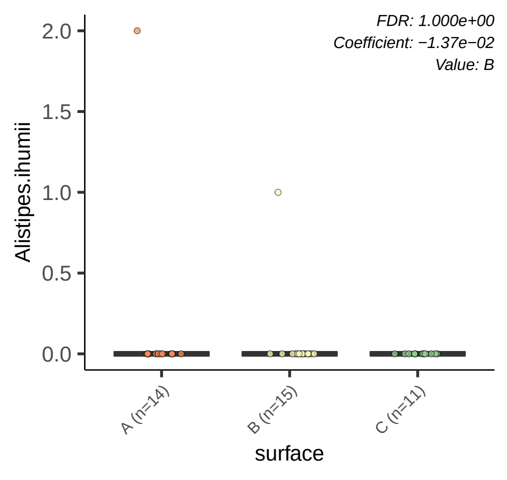

FDR: 1.000e+00
Coefficient: −1.37e−02
Value: B
Alistipes.ihumii
2.0
1.5
1.0
0.5
0.0
A (n=14)
B (n=15)
C (n=11)
surface

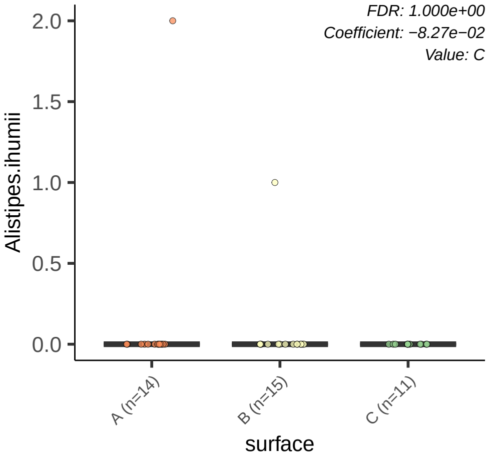
FDR: 1.000e+00
Coefficient: −8.27e−02
Value: C
Alistipes.ihumii
2.0
1.5
1.0
0.5
0.0
A (n=14)
B (n=15)
C (n=11)
surface

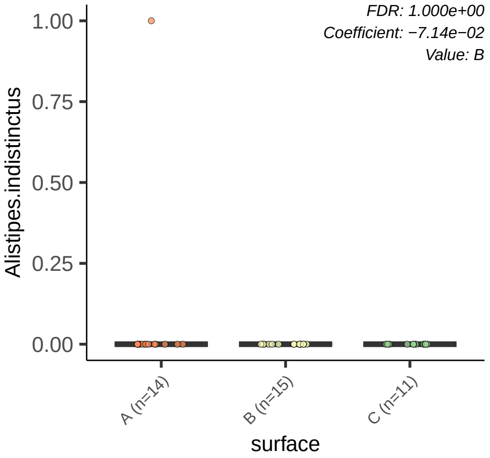

FDR: 1.000e+00
Coefficient: −7.14e−02
Value: B
Alistipes.indistinctus
1.00
0.75
0.50
0.25
0.00
A (n=14)
B (n=15)
C (n=11)
surface

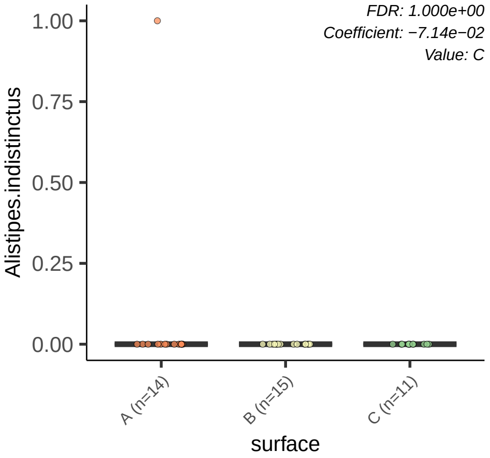
FDR: 1.000e+00
Coefficient: −7.14e−02
Value: C
Alistipes.indistinctus
1.00
0.75
0.50
0.25
0.00
A (n=14)
B (n=15)
C (n=11)
surface

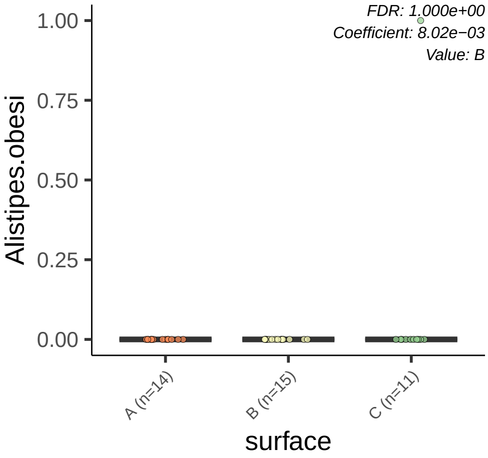
FDR: 1.000e+00
Coefficient: 8.02e−03
Value: B
1.00
0.75
0.50
0.25
0.00
Alistipes.obesi
A (n=14)
B (n=15)
C (n=11)
surface

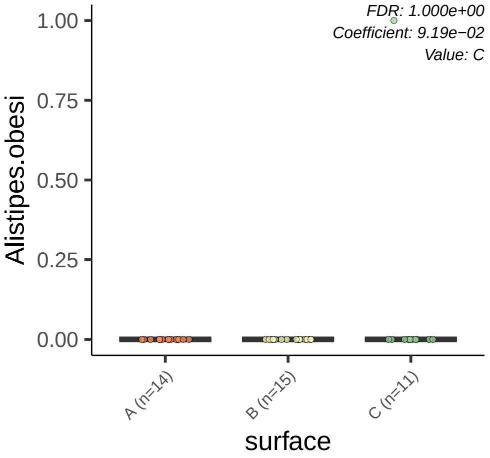
FDR: 1.000e+00
Coefficient: 9.19e−02
Value: C
Alistipes.obesi
1.00
0.75
0.50
0.25
0.00
A (n=14)
B (n=15)
C (n=11)
surface

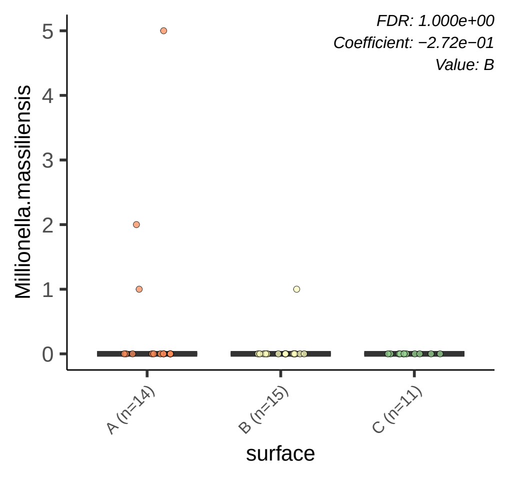

FDR: 1.000e+00
Coefficient: −2.72e−01
Value: B
Millionella.massiliensis
0
1
2
3
4
5
A (n=14)
B (n=15)
C (n=11)
surface

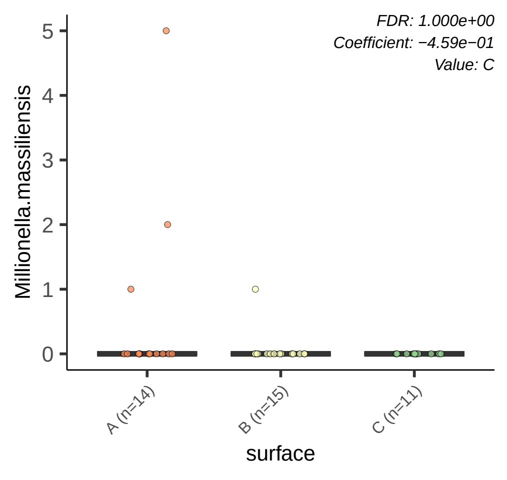

FDR: 1.000e+00
Coefficient: −4.59e−01
Value: C
Millionella.massiliensis
0
1
2
3
4
5
A (n=14)
B (n=15)
C (n=11)
surface

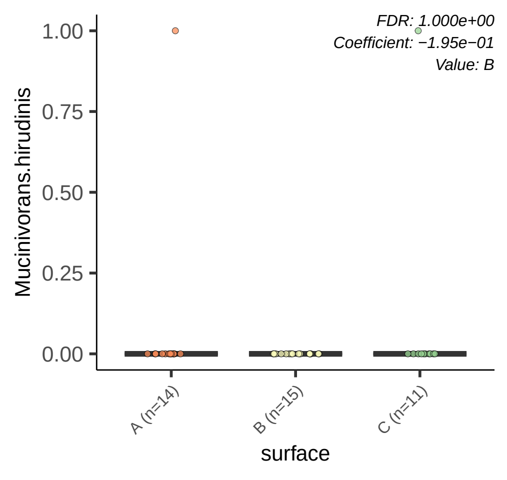
FDR: 1.000e+00
Coefficient: −1.95e−01
Value: B
Mucinivorans.hirudinis
1.00
0.75
0.50
0.25
0.00
A (n=14)
B (n=15)
C (n=11)
surface

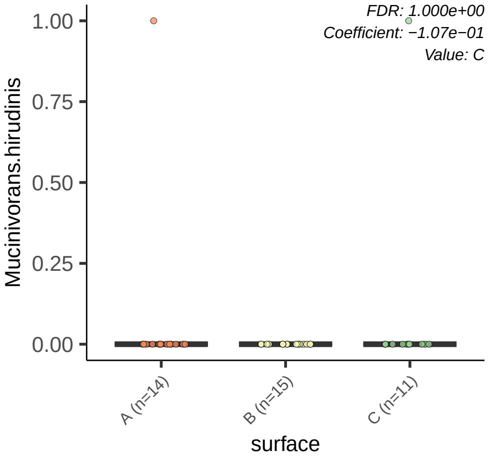
FDR: 1.000e+00
Coefficient: −1.07e−01
Value: C
Mucinivorans.hirudinis
1.00
0.75
0.50
0.25
0.00
A (n=14)
B (n=15)
C (n=11)
surface

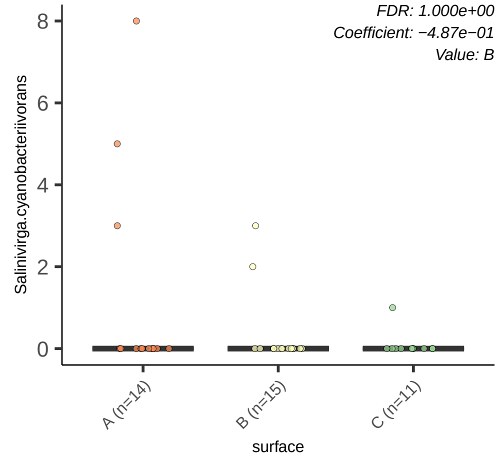

FDR: 1.000e+00
Coefficient: −4.87e−01
Value: B
Saliniviirga.cyanobacteriivorans
8
6
4
2
0
A (n=14)
B (n=15)
C (n=11)
surface

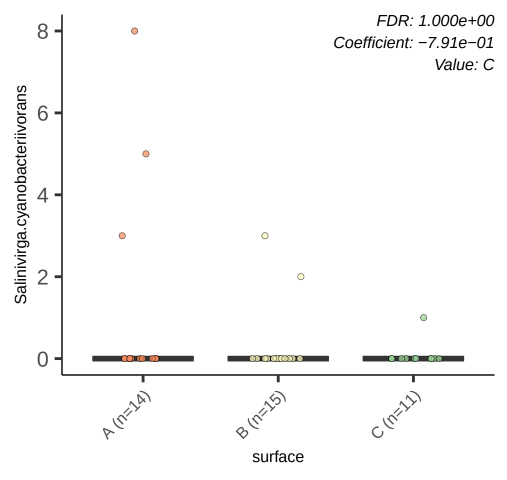

FDR: 1.000e+00
Coefficient: −7.91e−01
Value: C
Saliniviirga.cyanobacteriivorans
0
2
4
6
8
A (n=14)
B (n=15)
C (n=11)
surface

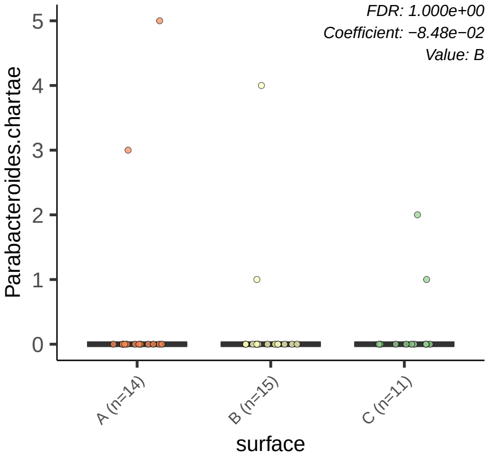
FDR: 1.000e+00
Coefficient: −8.48e−02
Value: B
Parabacteroides.chartae
0
1
2
3
4
5
A (n=14)
B (n=15)
C (n=11)
surface

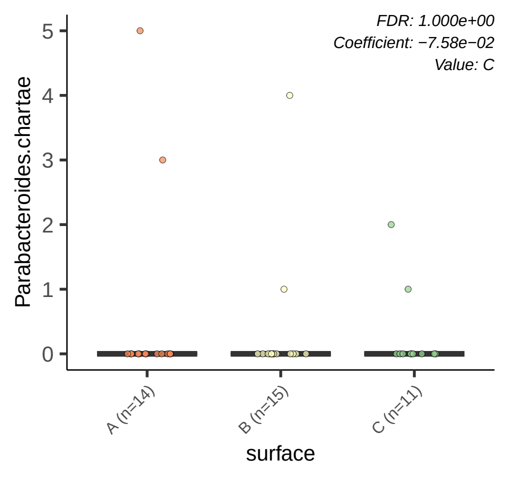

FDR: 1.000e+00
Coefficient: −7.58e−02
Value: C
Parabacteroides.chartae
0
1
2
3
4
5
A (n=14)
B (n=15)
C (n=11)
surface

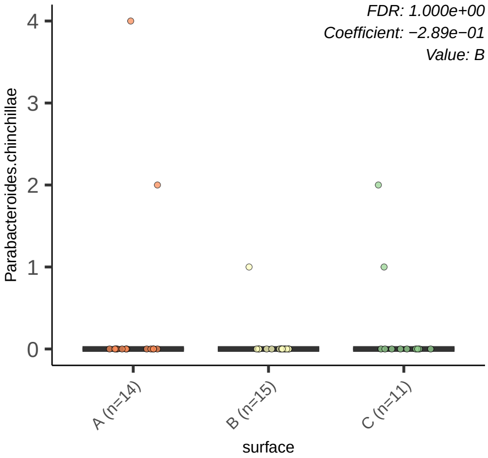

FDR: 1.000e+00
Coefficient: −2.89e−01
Value: B
Parabacteroides.chinchillae
0
1
2
3
4
A (n=14)
B (n=15)
C (n=11)
surface

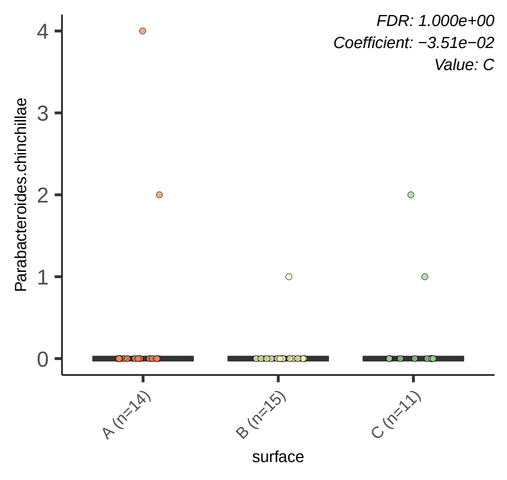
FDR: 1.000e+00
Coefficient: −3.51e−02
Value: C
Parabacteroides.chinchillae
0
1
2
3
4
A (n=14)
B (n=15)
C (n=11)
surface

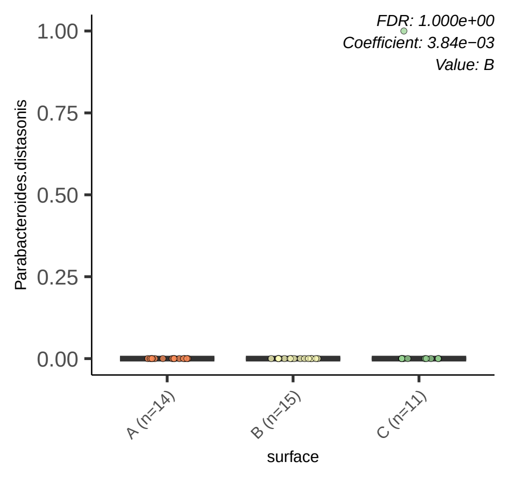
FDR: 1.000e+00
Coefficient: 3.84e−03
Value: B
1.00
0.75
0.50
0.25
0.00
Parabacteroides.distasonis
A (n=14)
B (n=15)
C (n=11)
surface

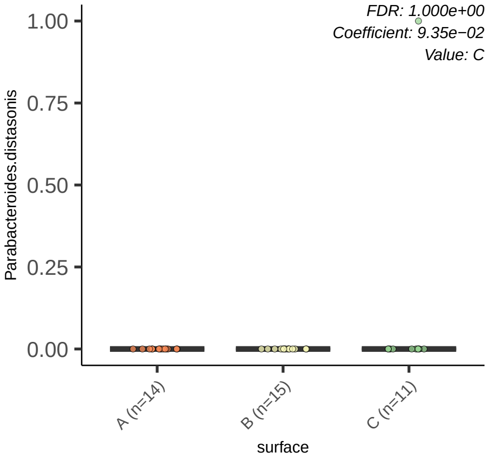
FDR: 1.000e+00
Coefficient: 9.35e−02
Value: C
Parabacteroides.distasonis
1.00
0.75
0.50
0.25
0.00
A (n=14)
B (n=15)
C (n=11)
surface

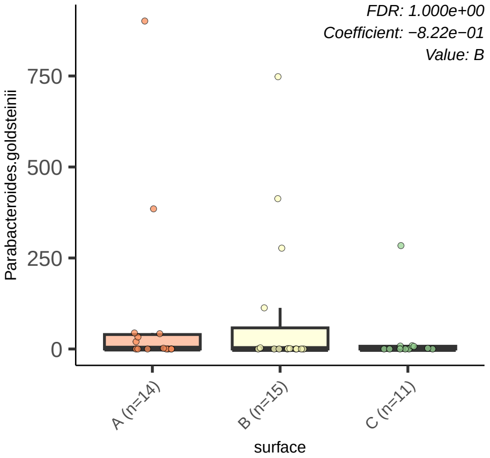
FDR: 1.000e+00
Coefficient: −8.22e−01
Value: B
750
500
250
0
Parabacteroides.goldsteinii
A (n=14)
B (n=15)
C (n=11)
surface

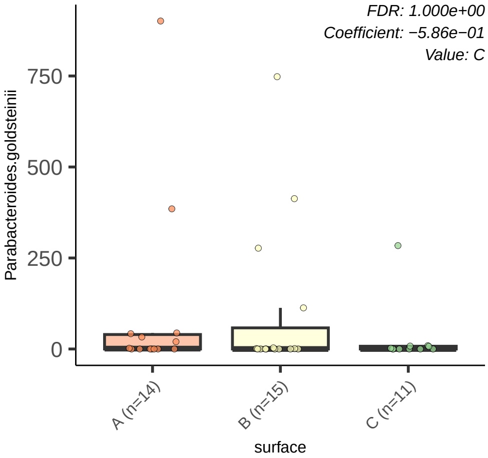
FDR: 1.000e+00
Coefficient: −5.86e−01
Value: C
Parabacteroides.goldsteinii
750
500
250
0
A (n=14)
B (n=15)
C (n=11)
surface

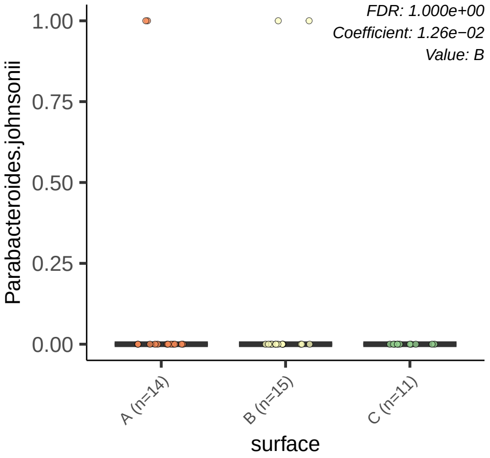

FDR: 1.000e+00
Coefficient: 1.26e−02
Value: B
Parabacteroides.johnsonii
1.00
0.75
0.50
0.25
0.00
A (n=14)
B (n=15)
C (n=11)
surface

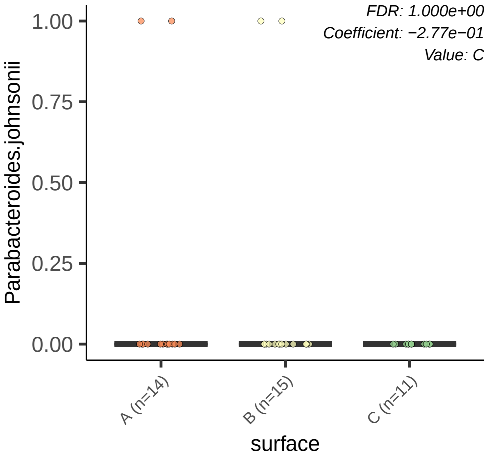
FDR: 1.000e+00
Coefficient: −2.77e−01
Value: C
Parabacteroides.johnsonii
1.00
0.75
0.50
0.25
0.00
A (n=14)
B (n=15)
C (n=11)
surface

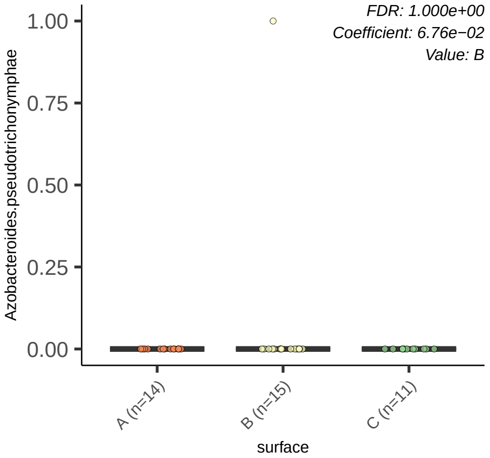
FDR: 1.000e+00
Coefficient: 6.76e−02
Value: B
Azobacteroides.pseudotrichonymphae
1.00
0.75
0.50
0.25
0.00
A (n=14)
B (n=15)
C (n=11)
surface

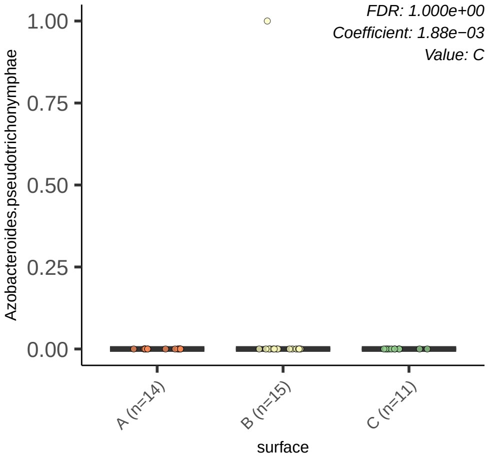

FDR: 1.000e+00
Coefficient: 1.88e−03
Value: C
Azobacteroides.pseudotrichonymphae
1.00
0.75
0.50
0.25
0.00
A (n=14)
B (n=15)
C (n=11)
surface

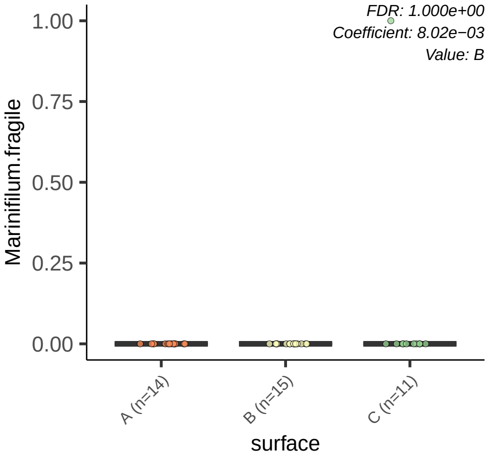
FDR: 1.000e+00
Coefficient: 8.02e−03
Value: B
Marinifilum.fragile
1.00
0.75
0.50
0.25
0.00
A (n=14)
B (n=15)
C (n=11)
surface

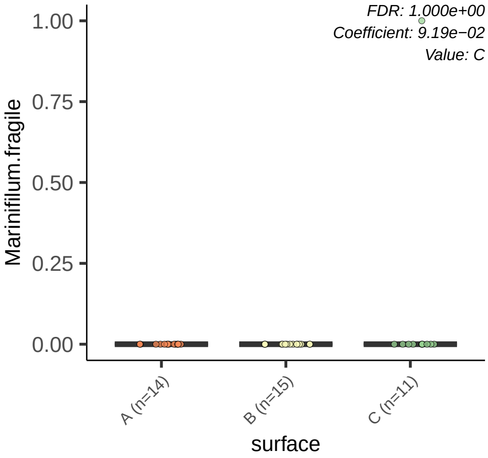
FDR: 1.000e+00
Coefficient: 9.19e−02
Value: C
Marinifilum.fragile
1.00
0.75
0.50
0.25
0.00
A (n=14)
B (n=15)
C (n=11)
surface

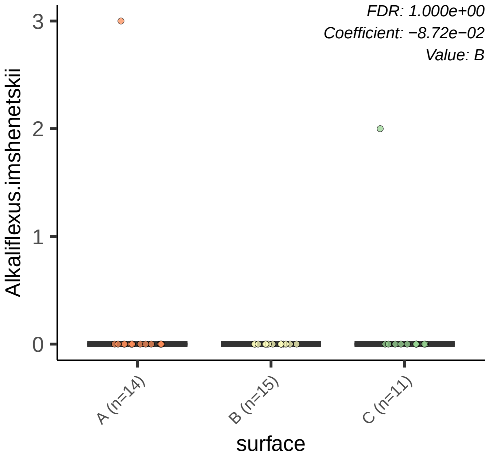
FDR: 1.000e+00
Coefficient: −8.72e−02
Value: B
Alkaliflexus.imshenetskii
3
2
1
0
A (n=14)
B (n=15)
C (n=11)
surface

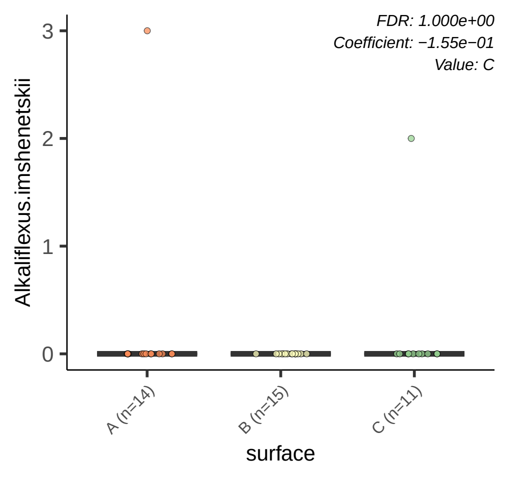
FDR: 1.000e+00
Coefficient: −1.55e−01
Value: C
Alkaliflexus.imshenetskii
3
2
1
0
A (n=14)
B (n=15)
C (n=11)
surface

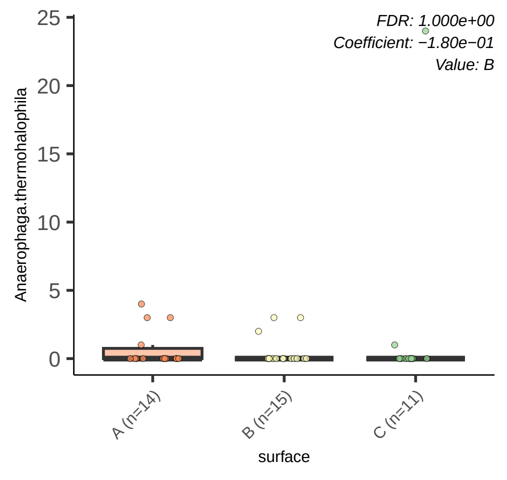

FDR: 1.000e+00
Coefficient: −1.80e−01
Value: B
Anaerophaga.thermohalophila
0
5
10
15
20
25
A (n=14)
B (n=15)
C (n=11)
surface

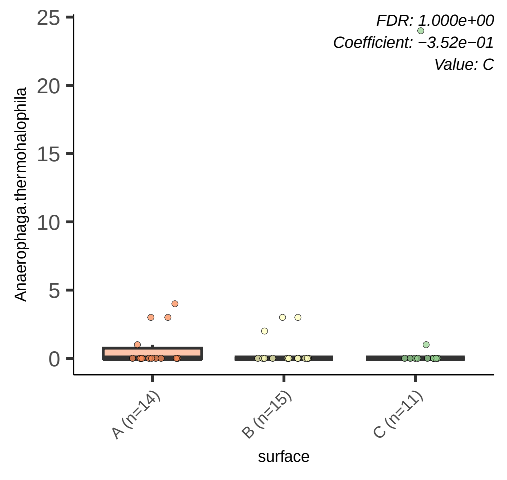

FDR: 1.000e+00
Coefficient: −3.52e−01
Value: C
Anaerophaga.thermohalophila
25
20
15
10
5
0
A (n=14)
B (n=15)
C (n=11)
surface

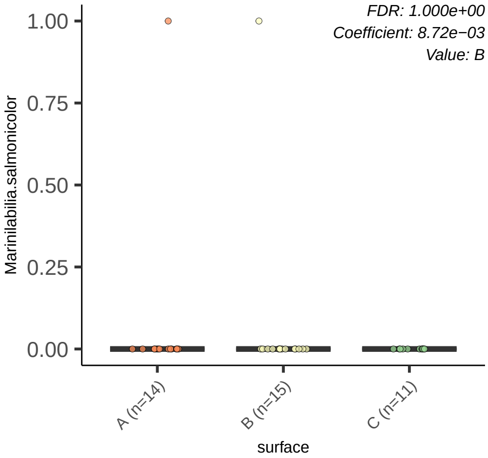
FDR: 1.000e+00
Coefficient: 8.72e−03
Value: B
Marinilabilia.salmonicolor
1.00
0.75
0.50
0.25
0.00
A (n=14)
B (n=15)
C (n=11)
surface

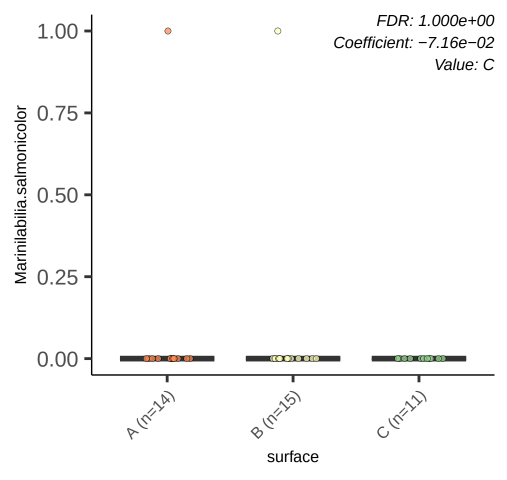
FDR: 1.000e+00
Coefficient: −7.16e−02
Value: C
Marinilabilia.salmonicolor
1.00
0.75
0.50
0.25
0.00
A (n=14)
B (n=15)
C (n=11)
surface

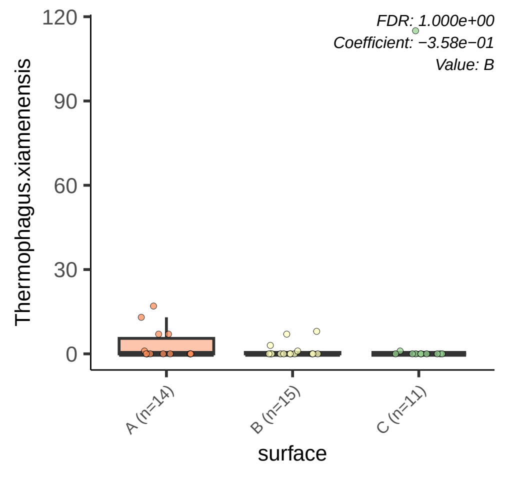

FDR: 1.000e+00
Coefficient: −3.58e−01
Value: B
Thermophagus.xiamenensis
120
90
60
30
0
A (n=14)
B (n=15)
C (n=11)
surface

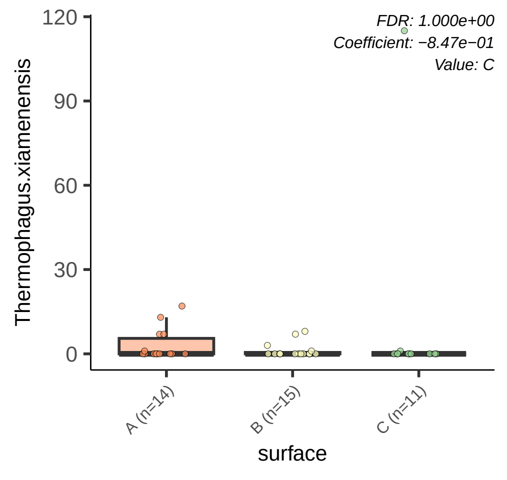
FDR: 1.000e+00
Coefficient: −8.47e−01
Value: C
Thermophagus.xiamenensis
120
90
60
30
0
A (n=14)
B (n=15)
C (n=11)
surface

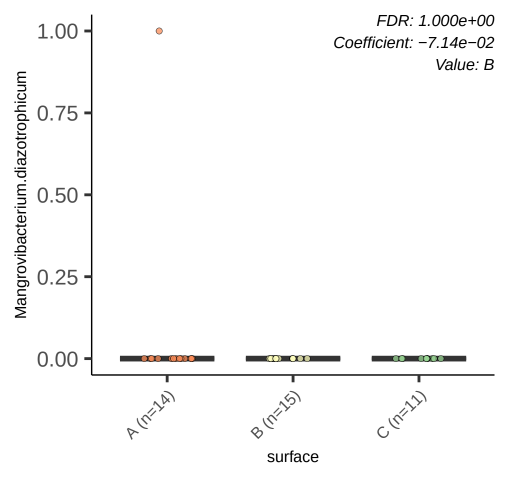
FDR: 1.000e+00
Coefficient: −7.14e−02
Value: B
Mangrovibacterium.diazotrophicum
1.00
0.75
0.50
0.25
0.00
A (n=14)
B (n=15)
C (n=11)
surface

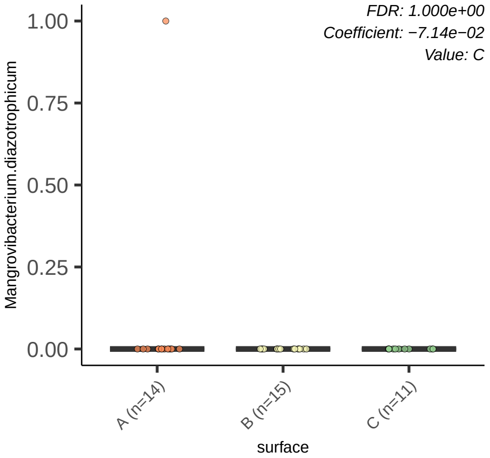

FDR: 1.000e+00
Coefficient: −7.14e−02
Value: C
1.00
0.75
0.50
0.25
0.00
Mangrovibacterium.diazotrophicum
A (n=14)
B (n=15)
C (n=11)
surface

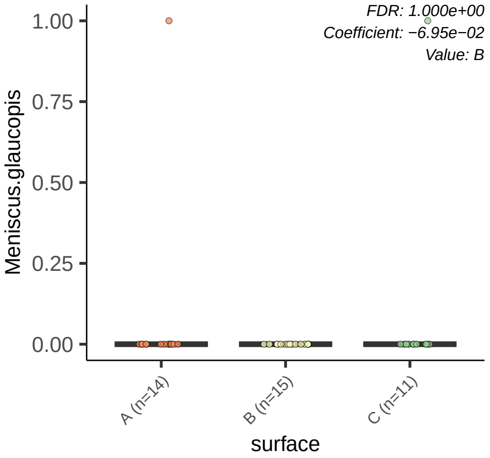

FDR: 1.000e+00
Coefficient: −6.95e−02
Value: B
Meniscus.glaucopis
1.00
0.75
0.50
0.25
0.00
A (n=14)
B (n=15)
C (n=11)
surface

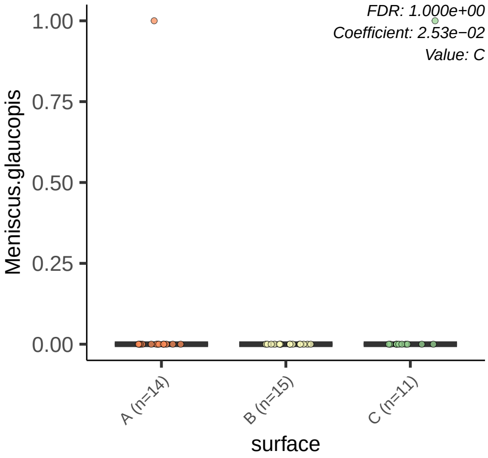

FDR: 1.000e+00
Coefficient: 2.53e−02
Value: C
Meniscus.glaucopis
1.00
0.75
0.50
0.25
0.00
A (n=14)
B (n=15)
C (n=11)
surface

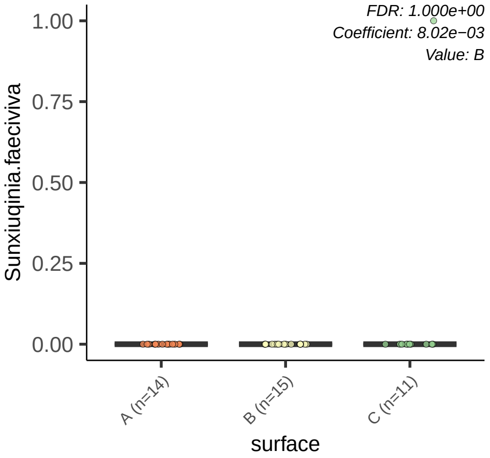
FDR: 1.000e+00
Coefficient: 8.02e−03
Value: B
Sunxiuqinia.faeciviva
1.00
0.75
0.50
0.25
0.00
A (n=14)
B (n=15)
C (n=11)
surface

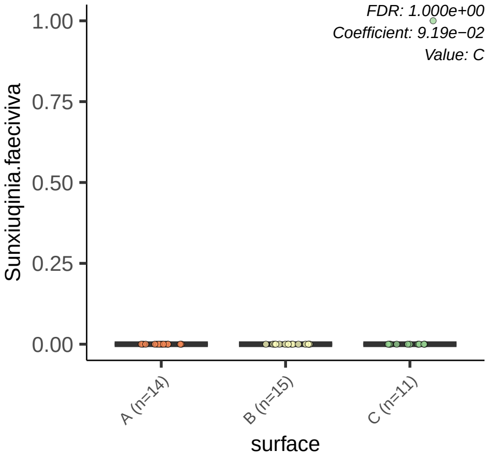
FDR: 1.000e+00
Coefficient: 9.19e−02
Value: C
Sunxiuqinia.faeciviva
1.00
0.75
0.50
0.25
0.00
A (n=14)
B (n=15)
C (n=11)
surface

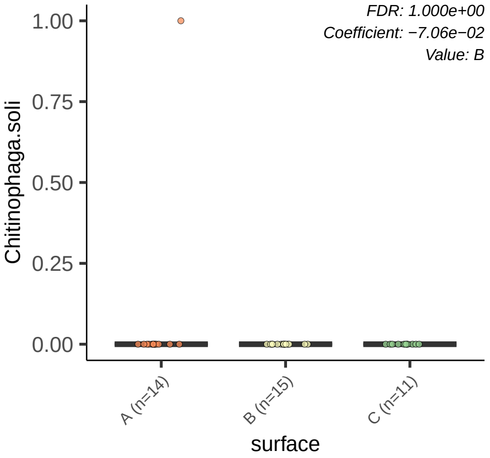
FDR: 1.000e+00
Coefficient: −7.06e−02
Value: B
Chitinophaga.soli
1.00
0.75
0.50
0.25
0.00
A (n=14)
B (n=15)
C (n=11)
surface

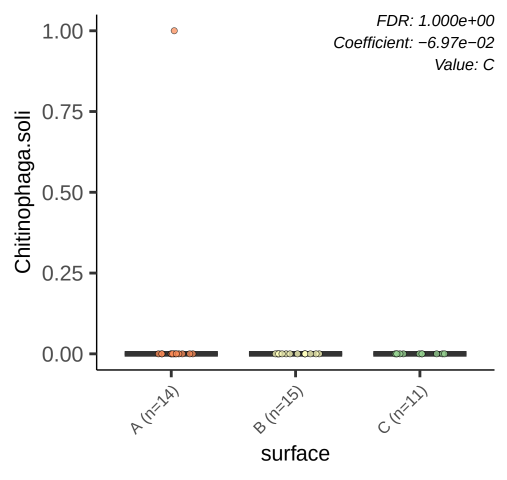

FDR: 1.000e+00
Coefficient: −6.97e−02
Value: C
Chitinophaga.soli
1.00
0.75
0.50
0.25
0.00
A (n=14)
B (n=15)
C (n=11)
surface

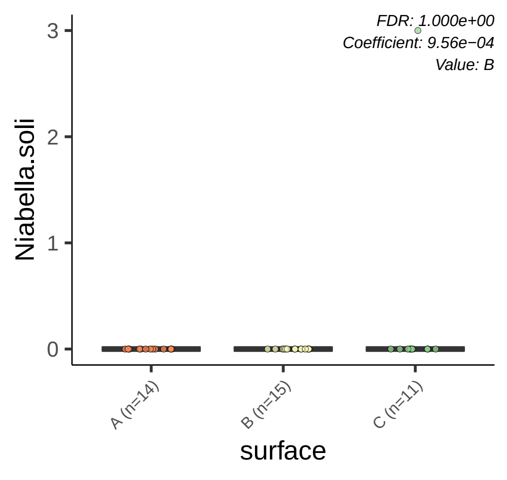

FDR: 1.000e+00
Coefficient: 9.56e−04
Value: B
Niabella.soli
3
2
1
0
A (n=14)
B (n=15)
C (n=11)
surface

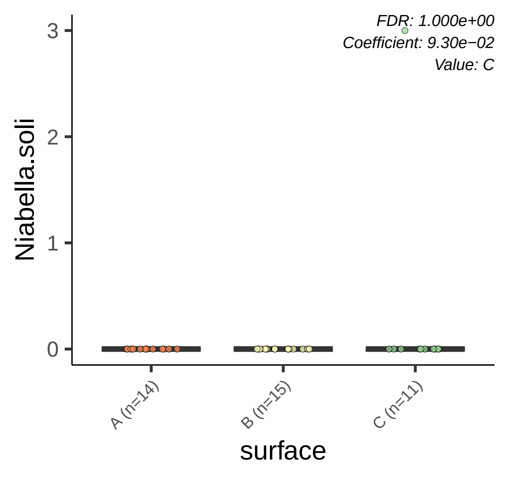

FDR: 1.000e+00
Coefficient: 9.30e−02
Value: C
Niabella.soli
3
2
1
0
A (n=14)
B (n=15)
C (n=11)
surface

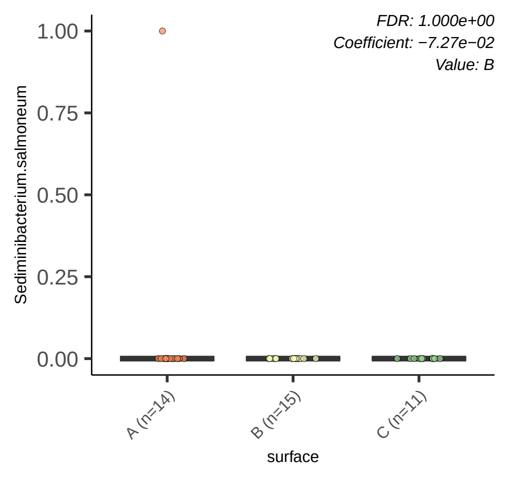

FDR: 1.000e+00
Coefficient: −7.27e−02
Value: B
Sediminibacterium.salmoneum
1.00
0.75
0.50
0.25
0.00
A (n=14)
B (n=15)
C (n=11)
surface

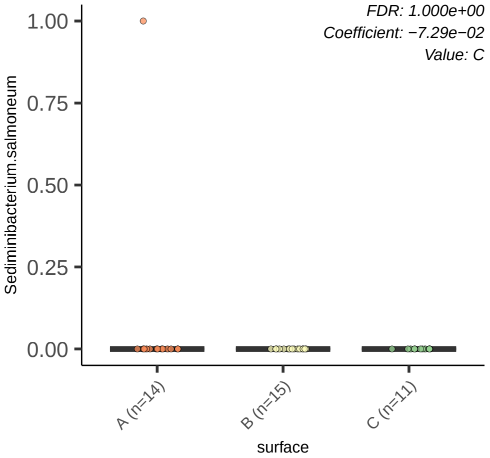

FDR: 1.000e+00
Coefficient: −7.29e−02
Value: C
Sediminibacterium.salmoneum
1.00
0.75
0.50
0.25
0.00
A (n=14)
B (n=15)
C (n=11)
surface

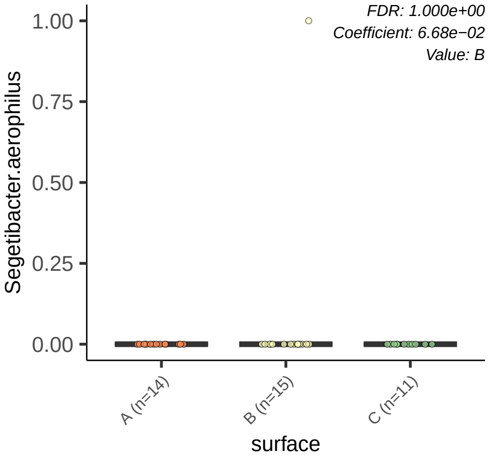
FDR: 1.000e+00
Coefficient: 6.68e−02
Value: B
Segetibacter.aerophilus
1.00
0.75
0.50
0.25
0.00
A (n=14)
B (n=15)
C (n=11)
surface

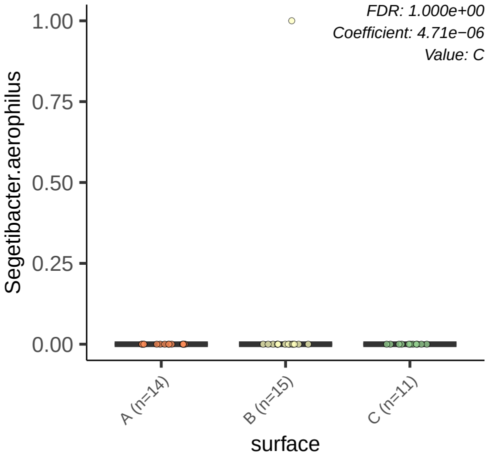

FDR: 1.000e+00
Coefficient: 4.71e−06
Value: C
Segetibacter.aerophilus
1.00
0.75
0.50
0.25
0.00
A (n=14)
B (n=15)
C (n=11)
surface

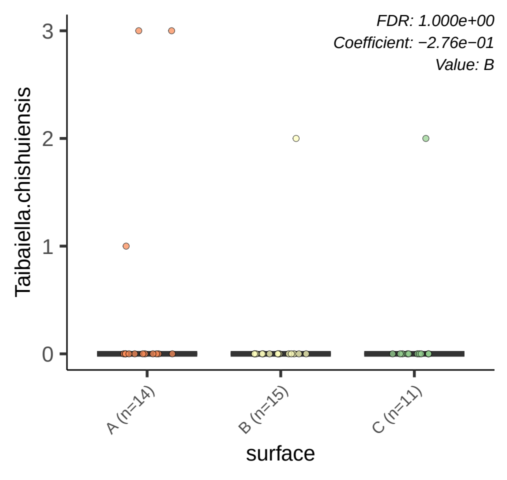

FDR: 1.000e+00
Coefficient: −2.76e−01
Value: B
Taibaiella.chishuiensis
3
2
1
0
A (n=14)
B (n=15)
C (n=11)
surface

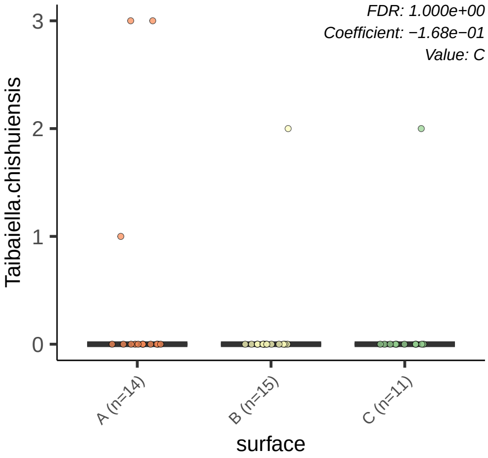

FDR: 1.000e+00
Coefficient: −1.68e−01
Value: C
Taibaiella.chishuiensis
3
2
1
0
A (n=14)
B (n=15)
C (n=11)
surface

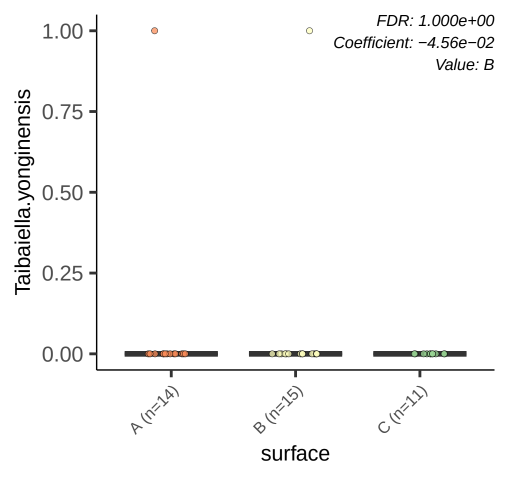

FDR: 1.000e+00
Coefficient: −4.56e−02
Value: B
Taibaiella.yonginensis
1.00
0.75
0.50
0.25
0.00
A (n=14)
B (n=15)
C (n=11)
surface

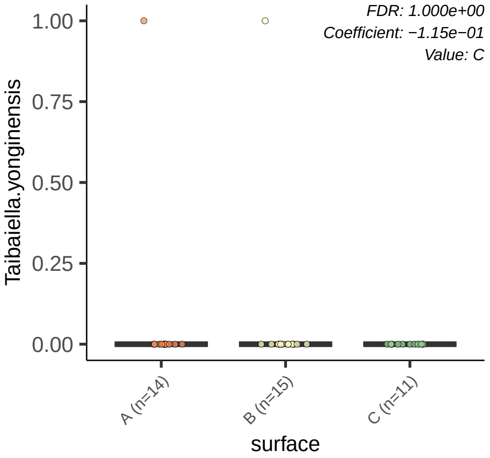

FDR: 1.000e+00
Coefficient: −1.15e−01
Value: C
Taibaiella.yonginensis
1.00
0.75
0.50
0.25
0.00
A (n=14)
B (n=15)
C (n=11)
surface

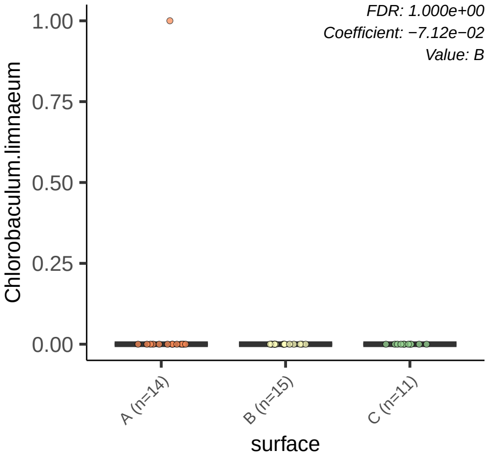
FDR: 1.000e+00
Coefficient: −7.12e−02
Value: B
Chlorobaculum.limnaeum
1.00
0.75
0.50
0.25
0.00
A (n=14)
B (n=15)
C (n=11)
surface

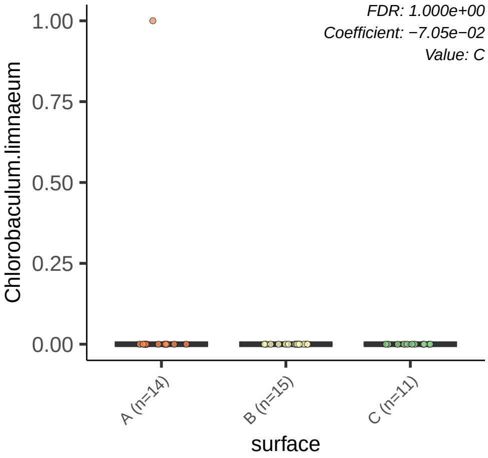
FDR: 1.000e+00
Coefficient: −7.05e−02
Value: C
Chlorobaculum.limnaeum
1.00
0.75
0.50
0.25
0.00
A (n=14)
B (n=15)
C (n=11)
surface

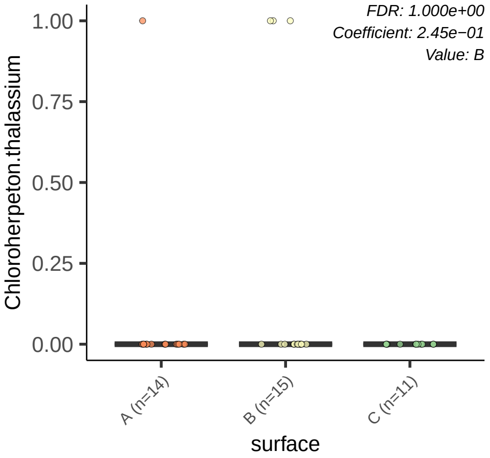
FDR: 1.000e+00
Coefficient: 2.45e−01
Value: B
Chloroherpeton.thalassium
1.00
0.75
0.50
0.25
0.00
A (n=14)
B (n=15)
C (n=11)
surface

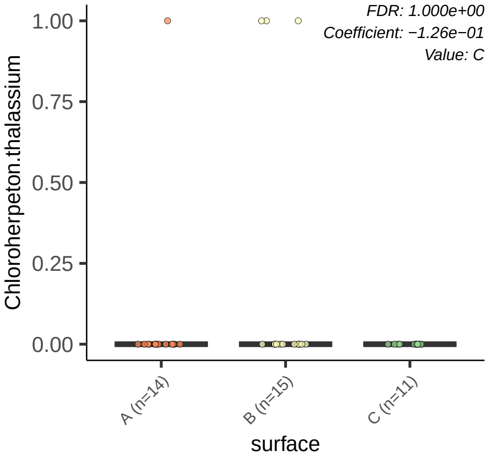

FDR: 1.000e+00
Coefficient: −1.26e−01
Value: C
Chloroherpeton.thalassium
1.00
0.75
0.50
0.25
0.00
A (n=14)
B (n=15)
C (n=11)
surface

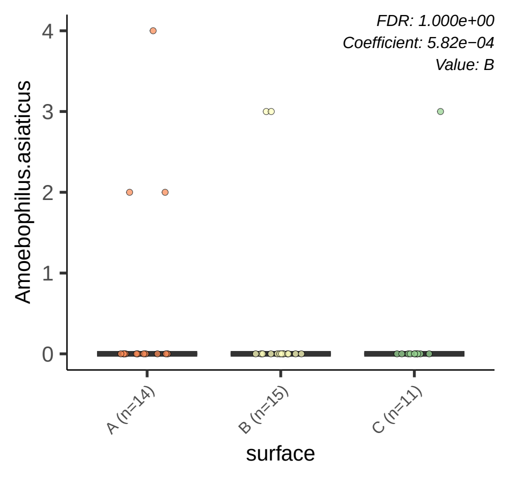

FDR: 1.000e+00
Coefficient: 5.82e−04
Value: B
Amoebophilus.asiaticus
0
1
2
3
4
A (n=14)
B (n=15)
C (n=11)
surface

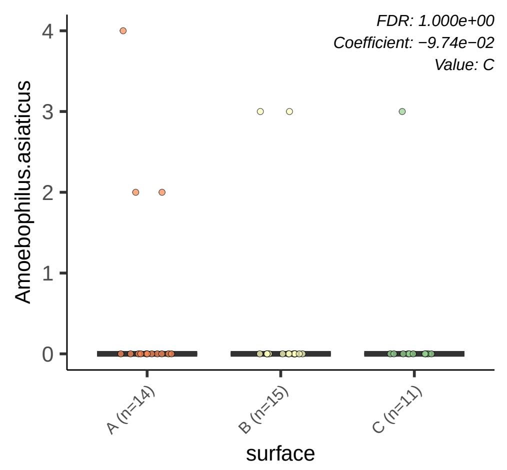

FDR: 1.000e+00
Coefficient: −9.74e−02
Value: C
Amoebophilus.asiaticus
4
3
2
1
0
A (n=14)
B (n=15)
C (n=11)
surface

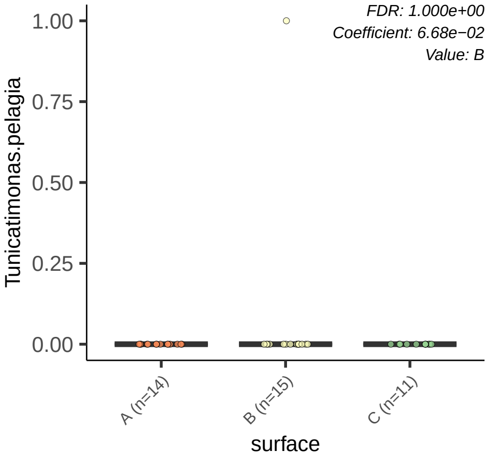
FDR: 1.000e+00
Coefficient: 6.68e−02
Value: B
Tunicatimonas.pelagia
1.00
0.75
0.50
0.25
0.00
A (n=14)
B (n=15)
C (n=11)
surface

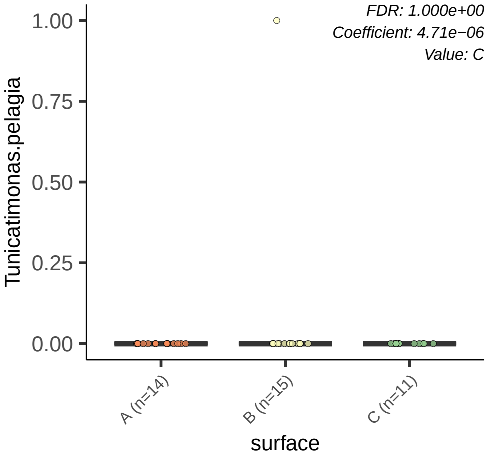
FDR: 1.000e+00
Coefficient: 4.71e−06
Value: C
Tunicatimonas.pelagia
1.00
0.75
0.50
0.25
0.00
A (n=14)
B (n=15)
C (n=11)
surface

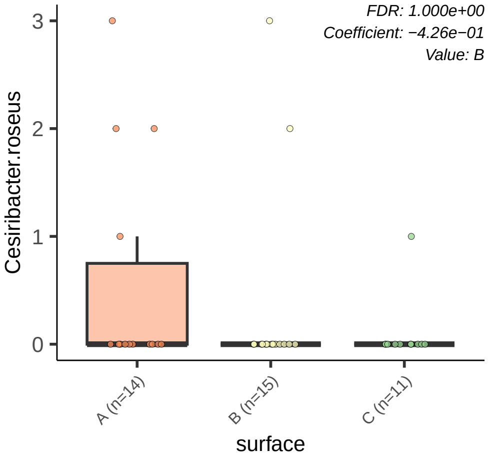
FDR: 1.000e+00
Coefficient: −4.26e−01
Value: B
Cesiribacter.roseus
3
2
1
0
A (n=14)
B (n=15)
C (n=11)
surface

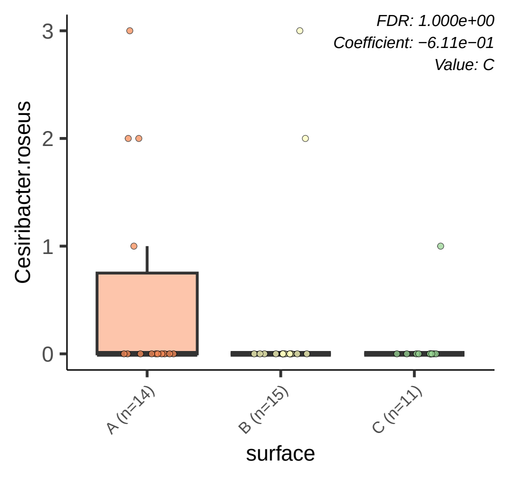
FDR: 1.000e+00
Coefficient: −6.11e−01
Value: C
Cesiribacter.roseus
3
2
1
0
A (n=14)
B (n=15)
C (n=11)
surface

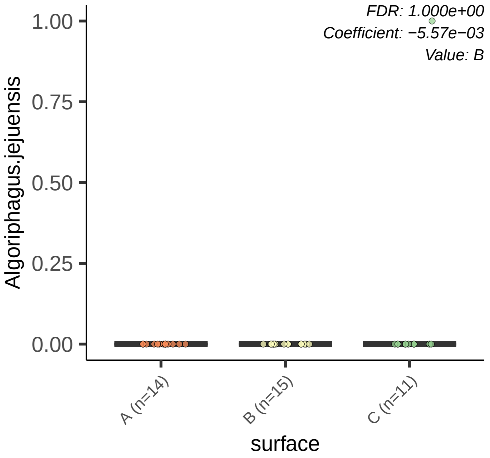
FDR: 1.000e+00
Coefficient: −5.57e−03
Value: B
Algoriphagus.jejuensis
1.00
0.75
0.50
0.25
0.00
A (n=14)
B (n=15)
C (n=11)
surface

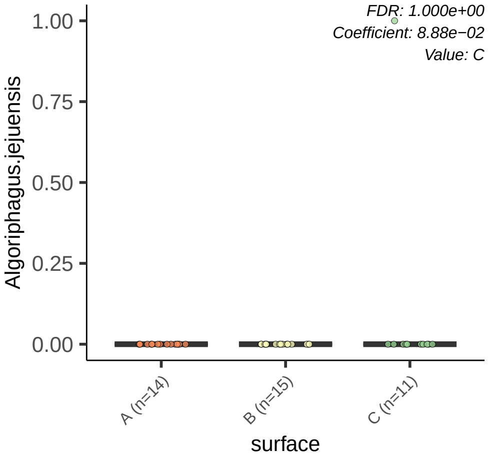
FDR: 1.000e+00
Coefficient: 8.88e−02
Value: C
1.00
0.75
0.50
0.25
0.00
Algoriphagus.jejuensis
A (n=14)
B (n=15)
C (n=11)
surface

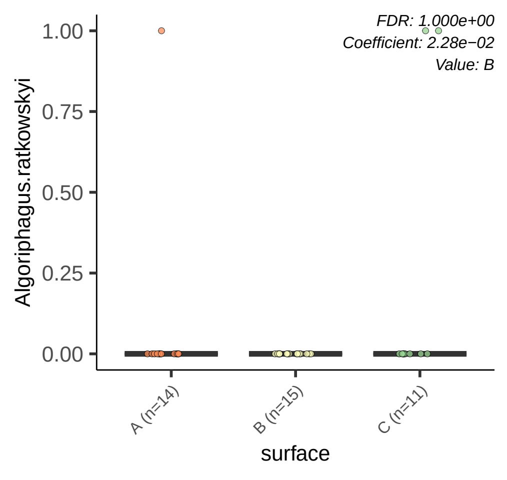

FDR: 1.000e+00
Coefficient: 2.28e−02
Value: B
Algoriphagus.ratkowskyi
1.00
0.75
0.50
0.25
0.00
A (n=14)
B (n=15)
C (n=11)
surface

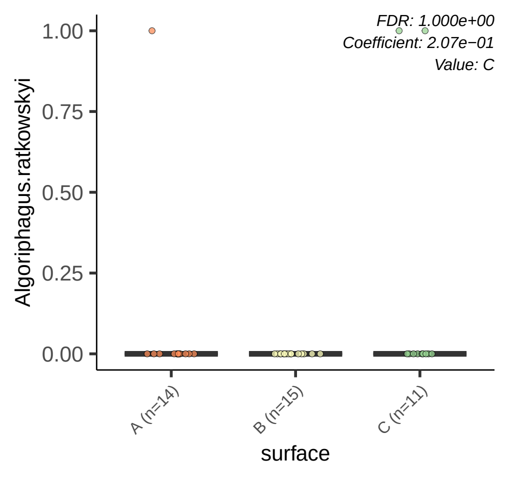
FDR: 1.000e+00
Coefficient: 2.07e−01
Value: C
Algoriphagus.ratkowskyi
1.00
0.75
0.50
0.25
0.00
A (n=14)
B (n=15)
C (n=11)
surface

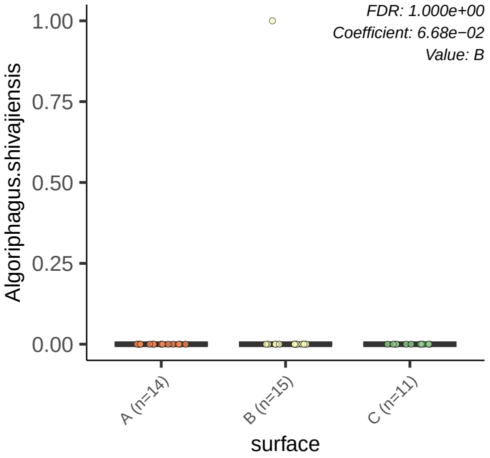
FDR: 1.000e+00
Coefficient: 6.68e−02
Value: B
Algoriphagus.shivajiensis
1.00
0.75
0.50
0.25
0.00
A (n=14)
B (n=15)
C (n=11)
surface

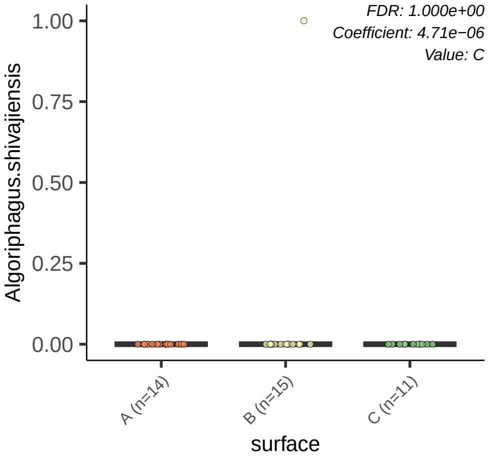
FDR: 1.000e+00
Coefficient: 4.71e−06
Value: C
Algoriphagus.shivajiensis
1.00
0.75
0.50
0.25
0.00
A (n=14)
B (n=15)
C (n=11)
surface

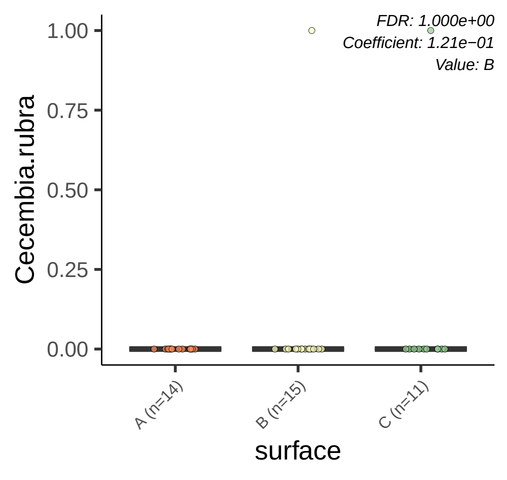
FDR: 1.000e+00
Coefficient: 1.21e−01
Value: B
Cecembia.rubra
1.00
0.75
0.50
0.25
0.00
A (n=14)
B (n=15)
C (n=11)
surface

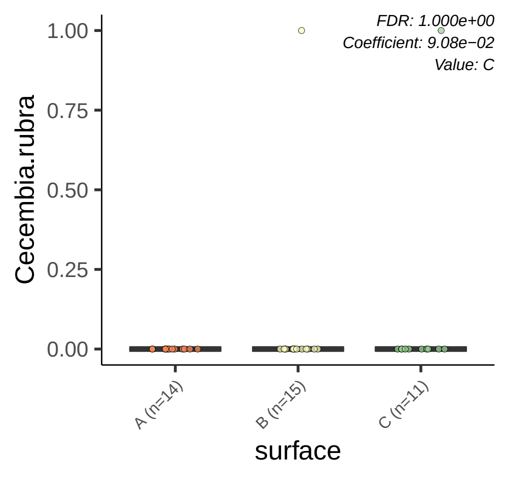

FDR: 1.000e+00
Coefficient: 9.08e−02
Value: C
Cecembia.rubra
1.00
0.75
0.50
0.25
0.00
A (n=14)
B (n=15)
C (n=11)
surface

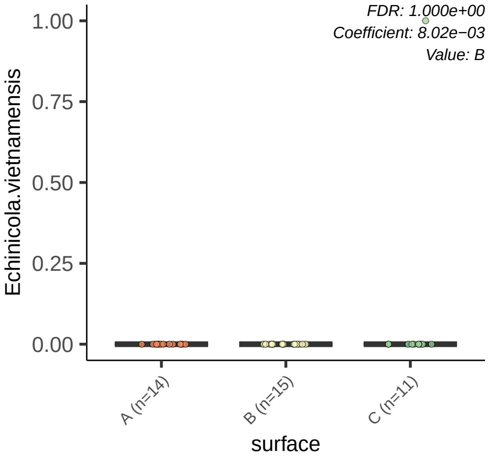
FDR: 1.000e+00
Coefficient: 8.02e−03
Value: B
Echinicola.vietnamensis
1.00
0.75
0.50
0.25
0.00
A (n=14)
B (n=15)
C (n=11)
surface

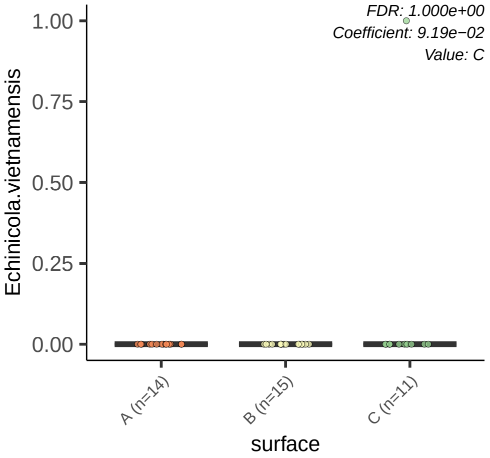

FDR: 1.000e+00
Coefficient: 9.19e−02
Value: C
Echinicola.vietnamensis
1.00
0.75
0.50
0.25
0.00
A (n=14)
B (n=15)
C (n=11)
surface

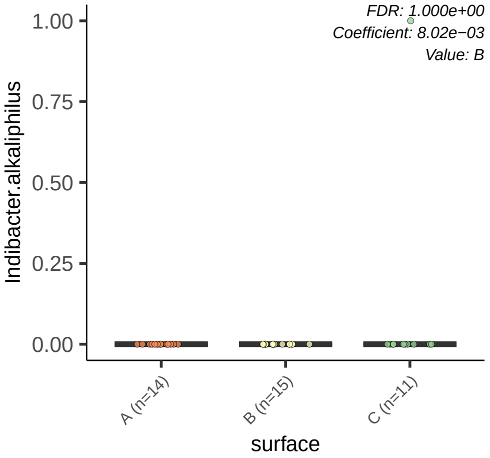

FDR: 1.000e+00
Coefficient: 8.02e−03
Value: B
1.00
0.75
0.50
0.25
0.00
Indibacter.alkaliphilus
A (n=14)
B (n=15)
C (n=11)
surface

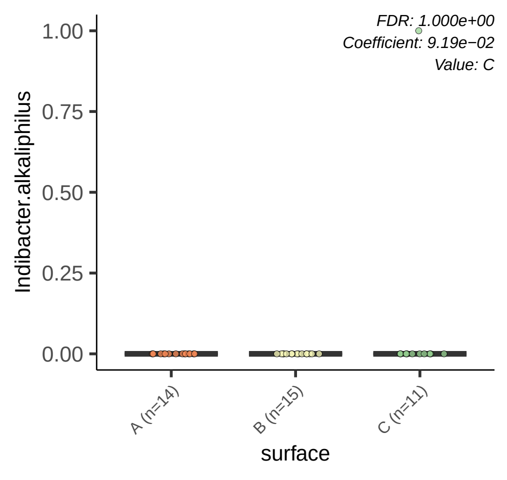

FDR: 1.000e+00
Coefficient: 9.19e−02
Value: C
Indibacter.alkaliphilus
1.00
0.75
0.50
0.25
0.00
A (n=14)
B (n=15)
C (n=11)
surface

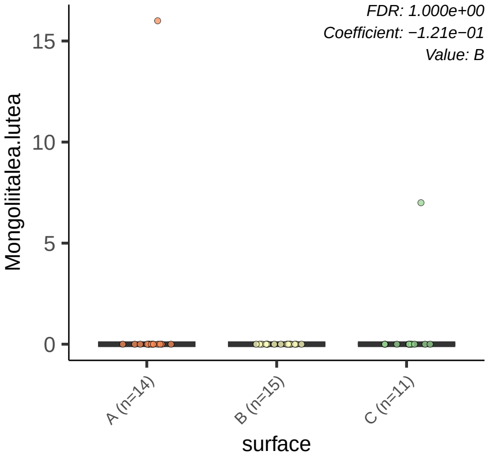
FDR: 1.000e+00
Coefficient: −1.21e−01
Value: B
Mongoliitalea.lutea
15
10
5
0
A (n=14)
B (n=15)
C (n=11)
surface

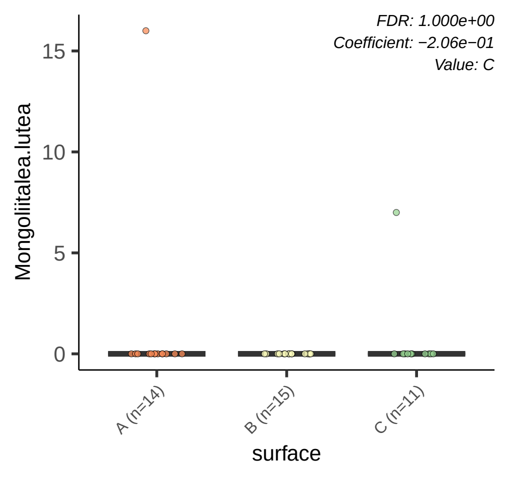

FDR: 1.000e+00
Coefficient: −2.06e−01
Value: C
Mongoliitalea.lutea
15
10
5
0
A (n=14)
B (n=15)
C (n=11)
surface

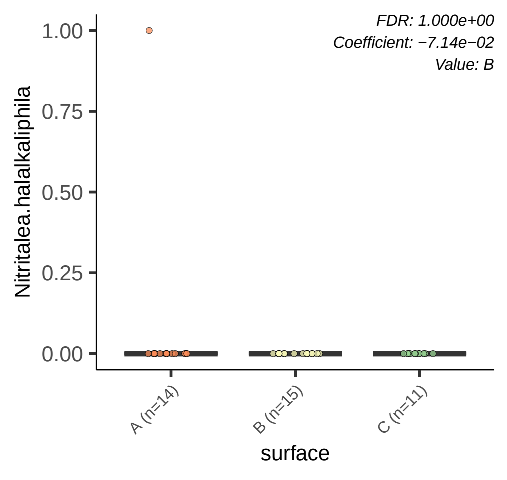

FDR: 1.000e+00
Coefficient: −7.14e−02
Value: B
Nitritalea.halalkaliphila
1.00
0.75
0.50
0.25
0.00
A (n=14)
B (n=15)
C (n=11)
surface

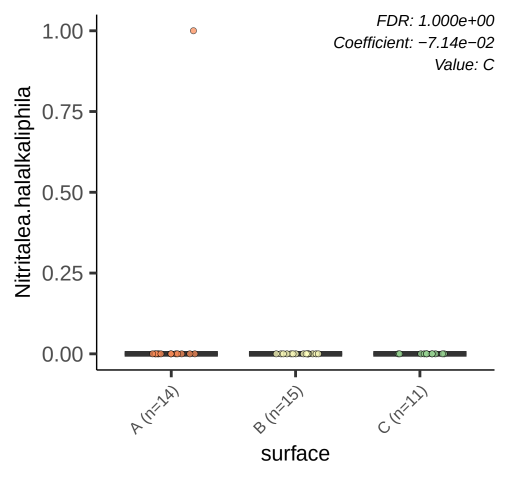
FDR: 1.000e+00
Coefficient: −7.14e−02
Value: C
Nitritalea.halalkaliphila
1.00
0.75
0.50
0.25
0.00
A (n=14)
B (n=15)
C (n=11)
surface

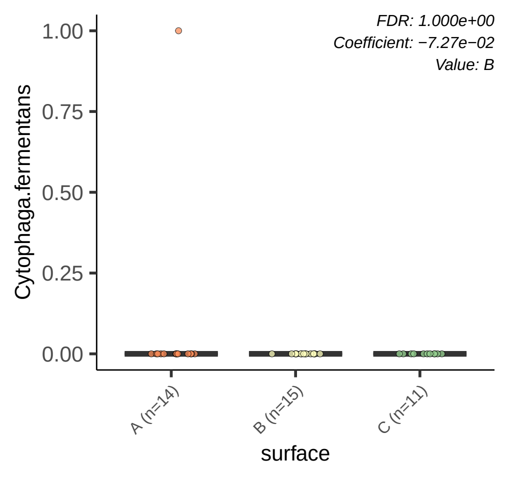
FDR: 1.000e+00
Coefficient: −7.27e−02
Value: B
Cytophaga.fermentans
1.00
0.75
0.50
0.25
0.00
A (n=14)
B (n=15)
C (n=11)
surface

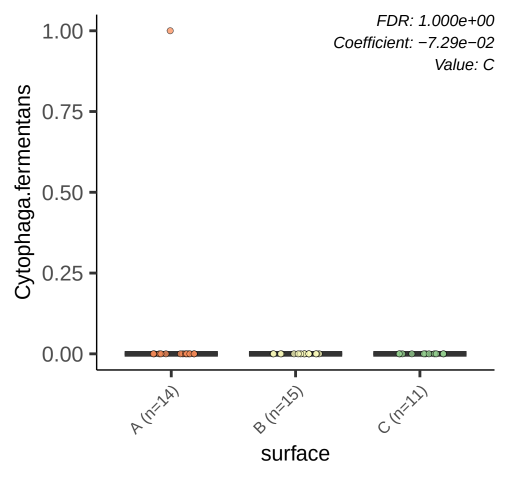
FDR: 1.000e+00
Coefficient: −7.29e−02
Value: C
Cytophaga.fermentans
1.00
0.75
0.50
0.25
0.00
A (n=14)
B (n=15)
C (n=11)
surface

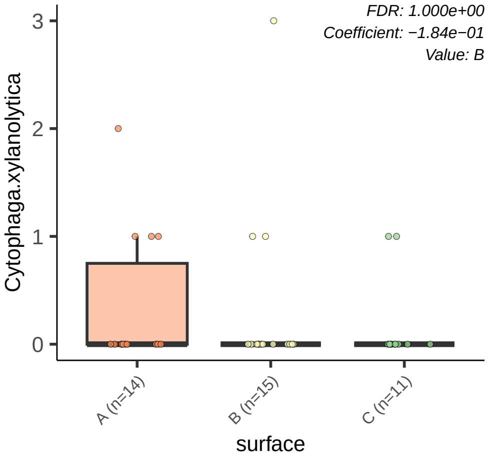

FDR: 1.000e+00
Coefficient: −1.84e−01
Value: B
Cytophaga.xylanolytica
3
2
1
0
A (n=14)
B (n=15)
C (n=11)
surface

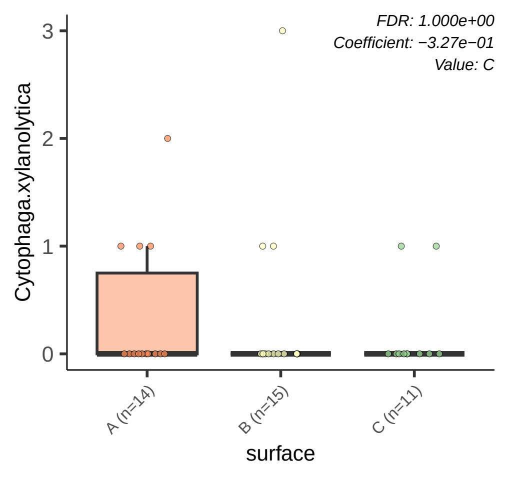

FDR: 1.000e+00
Coefficient: −3.27e−01
Value: C
Cytophaga.xylanolytica
3
2
1
0
A (n=14)
B (n=15)
C (n=11)
surface

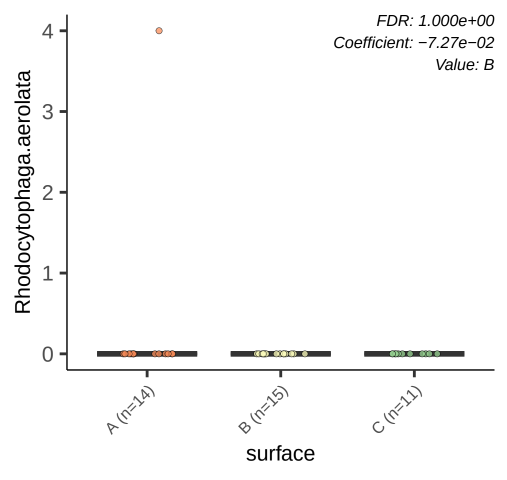
FDR: 1.000e+00
Coefficient: −7.27e−02
Value: B
Rhodocytophaga.aerolata
0
1
2
3
4
A (n=14)
B (n=15)
C (n=11)
surface

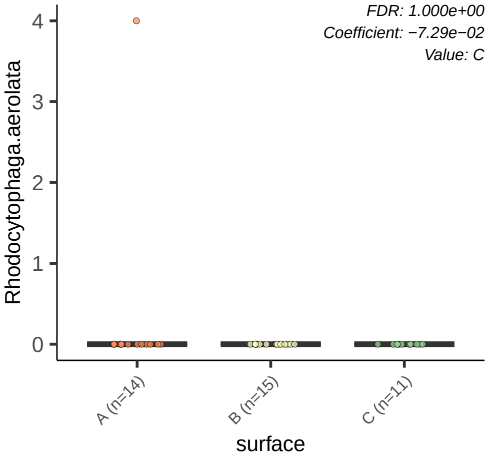

FDR: 1.000e+00
Coefficient: −7.29e−02
Value: C
Rhodocytophaga.aerolata
4
3
2
1
0
A (n=14)
B (n=15)
C (n=11)
surface

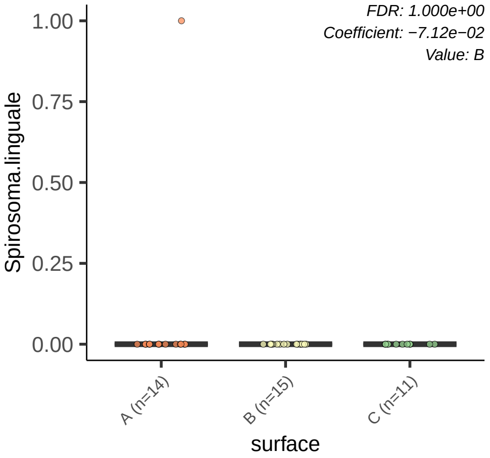
FDR: 1.000e+00
Coefficient: −7.12e−02
Value: B
Spirosoma.linguale
1.00
0.75
0.50
0.25
0.00
A (n=14)
B (n=15)
C (n=11)
surface

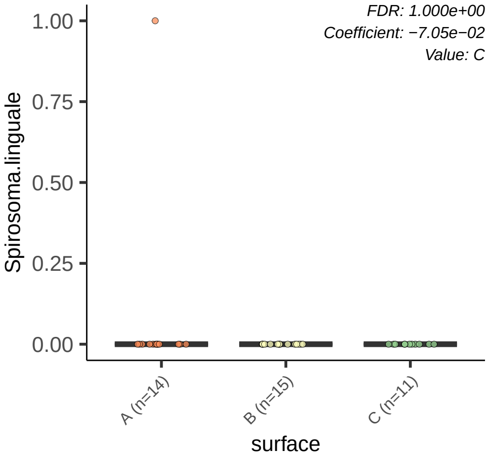
FDR: 1.000e+00
Coefficient: −7.05e−02
Value: C
Spirosoma.linguale
1.00
0.75
0.50
0.25
0.00
A (n=14)
B (n=15)
C (n=11)
surface

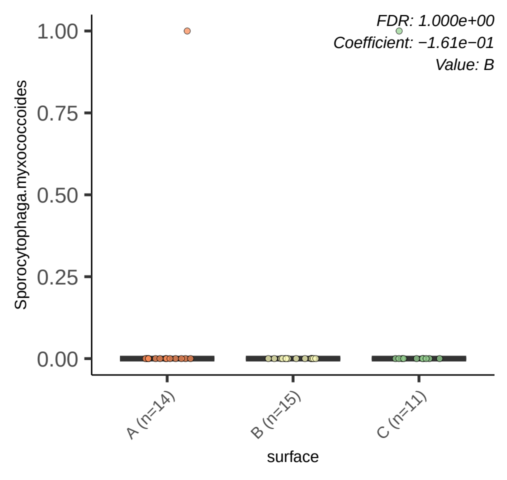

FDR: 1.000e+00
Coefficient: −1.61e−01
Value: B
Sporocytophaga.myxococcoides
1.00
0.75
0.50
0.25
0.00
A (n=14)
B (n=15)
C (n=11)
surface

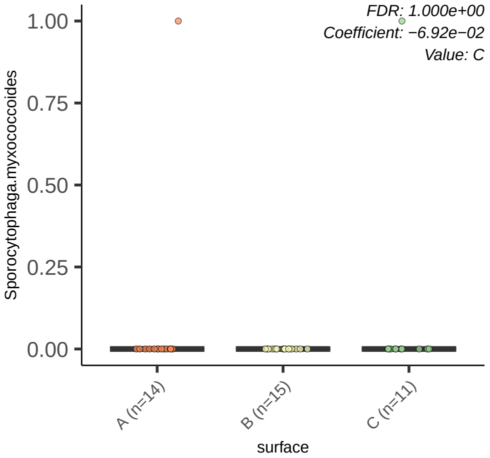

FDR: 1.000e+00
Coefficient: −6.92e−02
Value: C
Sporocytophaga.myxococcoides
1.00
0.75
0.50
0.25
0.00
A (n=14)
B (n=15)
C (n=11)
surface

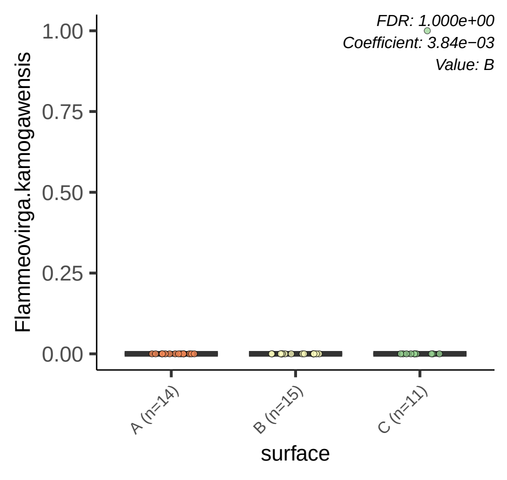

FDR: 1.000e+00
Coefficient: 3.84e−03
Value: B
Flammeovirga.kamogawensis
1.00
0.75
0.50
0.25
0.00
A (n=14)
B (n=15)
C (n=11)
surface

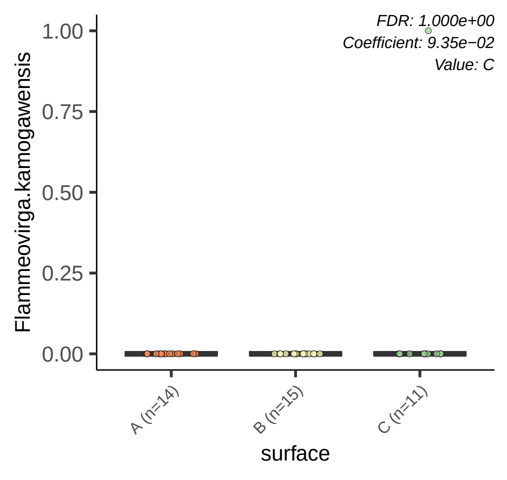
FDR: 1.000e+00
Coefficient: 9.35e−02
Value: C
Flammeovirga.kamogawensis
1.00
0.75
0.50
0.25
0.00
A (n=14)
B (n=15)
C (n=11)
surface

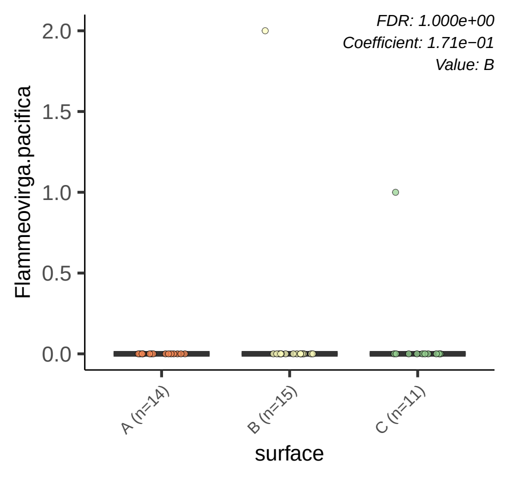
FDR: 1.000e+00
Coefficient: 1.71e−01
Value: B
Flammeovirga.pacifica
2.0
1.5
1.0
0.5
0.0
A (n=14)
B (n=15)
C (n=11)
surface

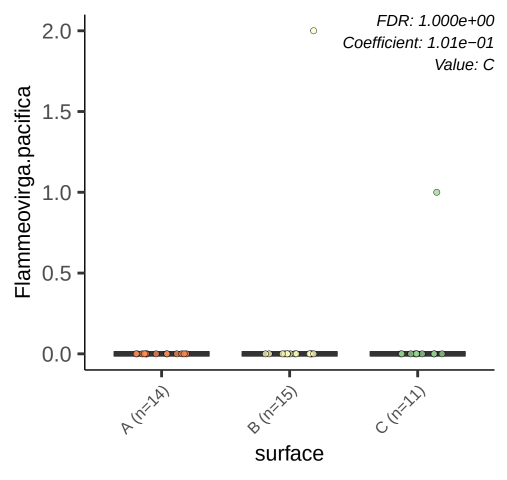
FDR: 1.000e+00
Coefficient: 1.01e−01
Value: C
Flammeovirga.pacifica
2.0
1.5
1.0
0.5
0.0
A (n=14)
B (n=15)
C (n=11)
surface

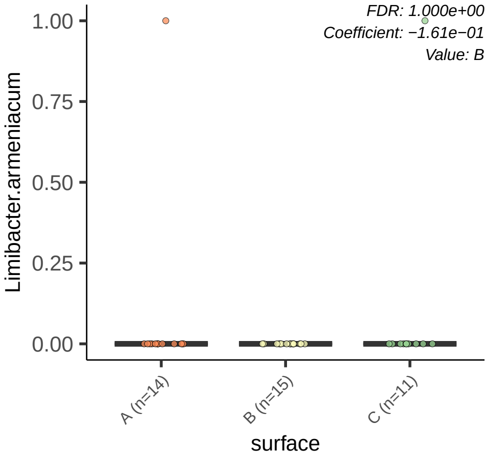
FDR: 1.000e+00
Coefficient: −1.61e−01
Value: B
Limibacter.armeniacum
1.00
0.75
0.50
0.25
0.00
A (n=14)
B (n=15)
C (n=11)
surface

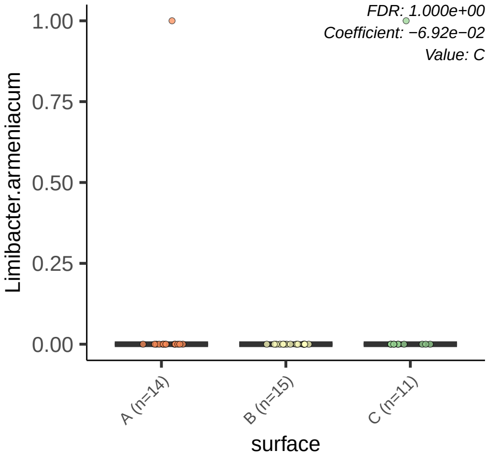

FDR: 1.000e+00
Coefficient: −6.92e−02
Value: C
Limibacter.armeniacum
1.00
0.75
0.50
0.25
0.00
A (n=14)
B (n=15)
C (n=11)
surface

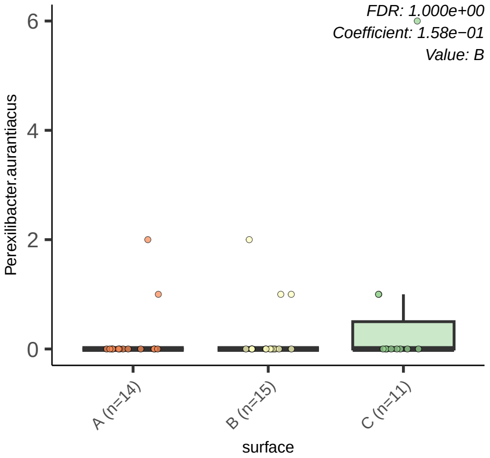

FDR: 1.000e+00
Coefficient: 1.58e−01
Value: B
Perexilibacter.aurantiacus
0
2
4
6
A (n=14)
B (n=15)
C (n=11)
surface

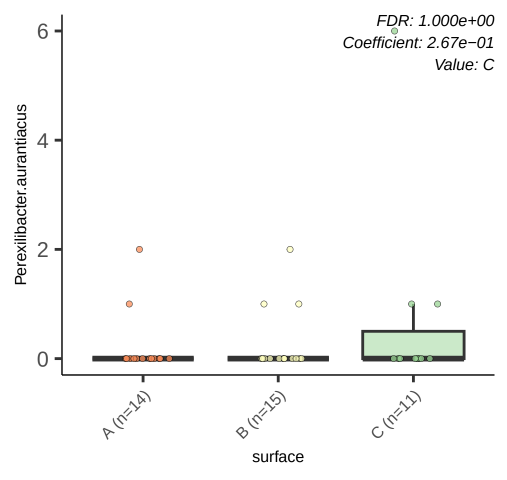
FDR: 1.000e+00
Coefficient: 2.67e−01
Value: C
Perexilibacter.aurantiacus
0
2
4
6
A (n=14)
B (n=15)
C (n=11)
surface

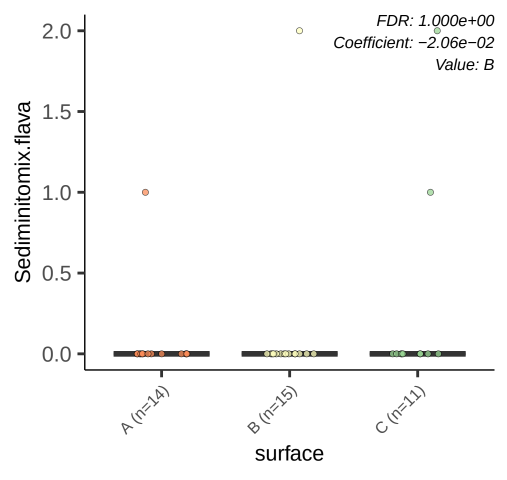

FDR: 1.000e+00
Coefficient: −2.06e−02
Value: B
Sediminitomix.flava
2.0
1.5
1.0
0.5
0.0
A (n=14)
B (n=15)
C (n=11)
surface

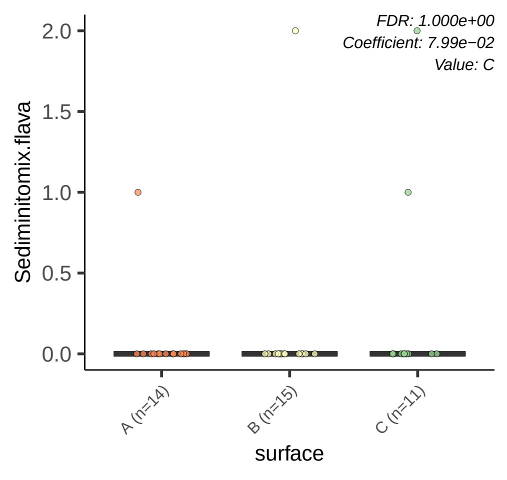
FDR: 1.000e+00
Coefficient: 7.99e−02
Value: C
Sediminitomix.flava
2.0
1.5
1.0
0.5
0.0
A (n=14)
B (n=15)
C (n=11)
surface

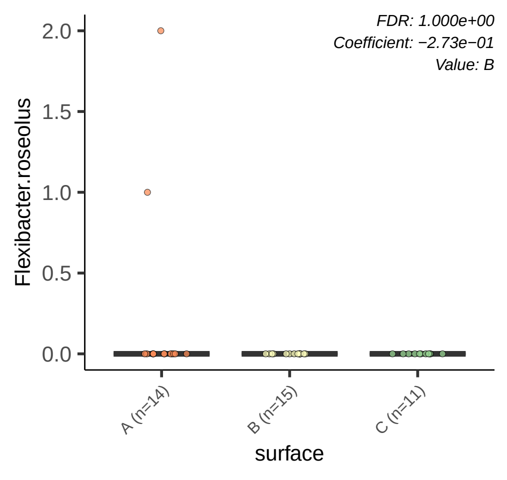
FDR: 1.000e+00
Coefficient: −2.73e−01
Value: B
Flexibacter.roseolus
2.0
1.5
1.0
0.5
0.0
A (n=14)
B (n=15)
C (n=11)
surface

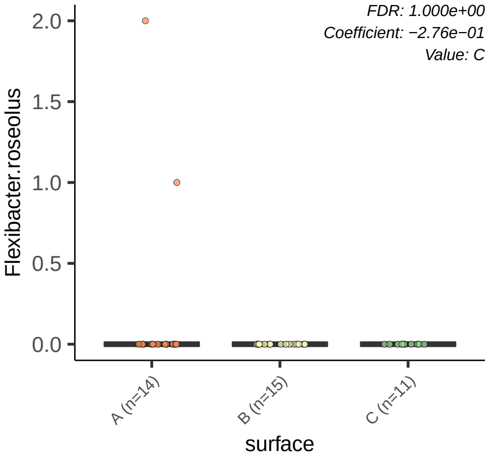

FDR: 1.000e+00
Coefficient: −2.76e−01
Value: C
Flexibacter.roseolus
2.0
1.5
1.0
0.5
0.0
A (n=14)
B (n=15)
C (n=11)
surface

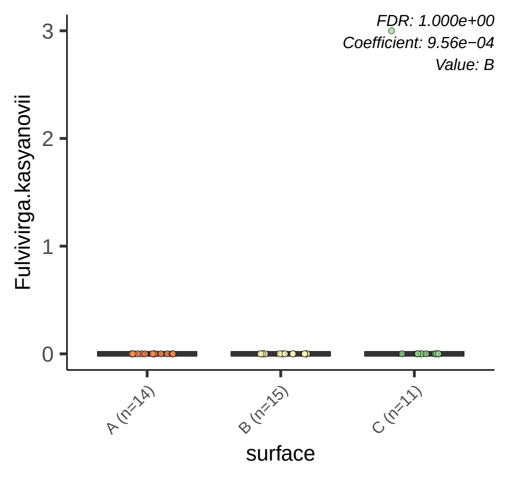
FDR: 1.000e+00
Coefficient: 9.56e−04
Value: B
Fulvivirga.kasyanovii
0
1
2
3
A (n=14)
B (n=15)
C (n=11)
surface

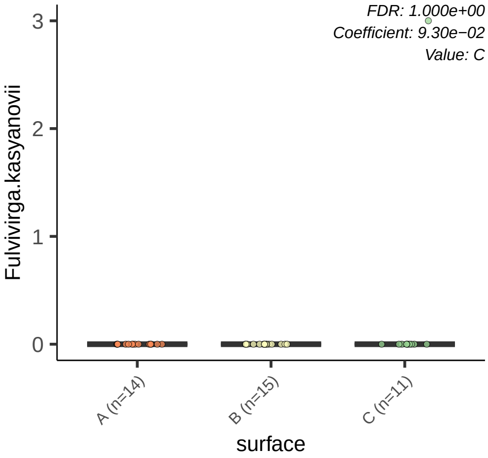
FDR: 1.000e+00
Coefficient: 9.30e−02
Value: C
3
2
1
0
Fulvivirga.kasyanovii
A (n=14)
B (n=15)
C (n=11)
surface

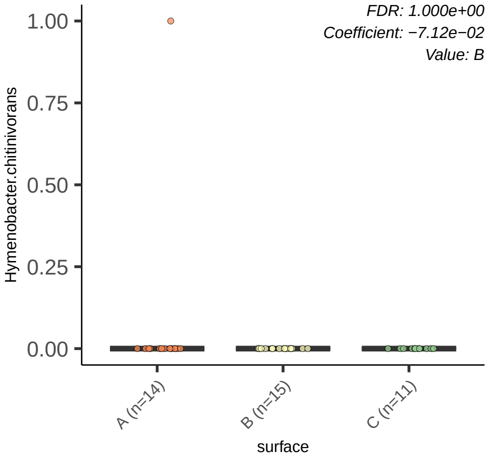
FDR: 1.000e+00
Coefficient: −7.12e−02
Value: B
Hymenobacter.chitinivorans
1.00
0.75
0.50
0.25
0.00
A (n=14)
B (n=15)
C (n=11)
surface

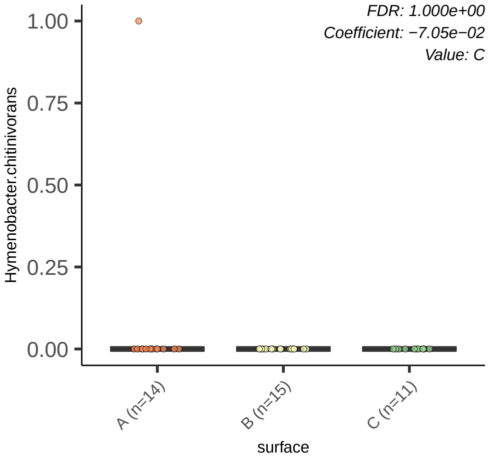

FDR: 1.000e+00
Coefficient: −7.05e−02
Value: C
Hymenobacter.chitinivorans
1.00
0.75
0.50
0.25
0.00
A (n=14)
B (n=15)
C (n=11)
surface

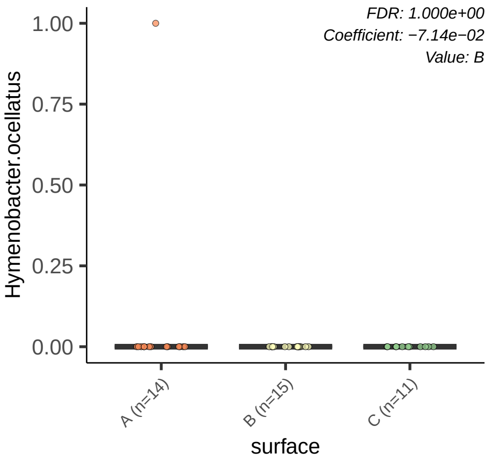
FDR: 1.000e+00
Coefficient: −7.14e−02
Value: B
Hymenobacter.ocellatus
1.00
0.75
0.50
0.25
0.00
A (n=14)
B (n=15)
C (n=11)
surface

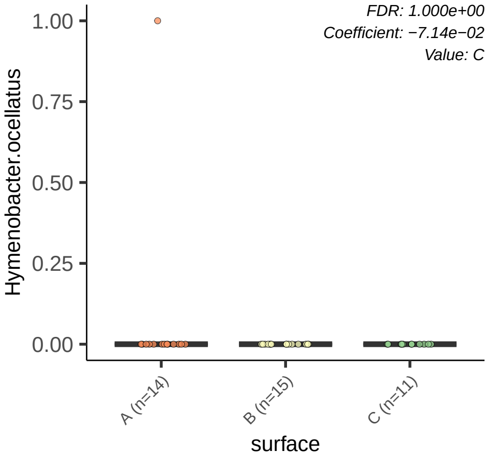
FDR: 1.000e+00
Coefficient: −7.14e−02
Value: C
Hymenobacter.ocellatus
1.00
0.75
0.50
0.25
0.00
A (n=14)
B (n=15)
C (n=11)
surface

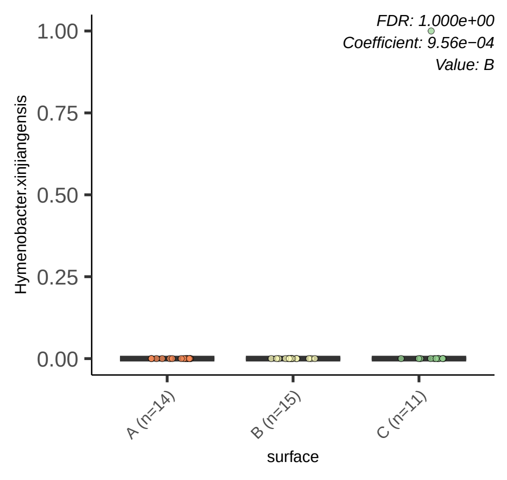

FDR: 1.000e+00
Coefficient: 9.56e−04
Value: B
Hymenobacter.xinjiangensis
1.00
0.75
0.50
0.25
0.00
A (n=14)
B (n=15)
C (n=11)
surface

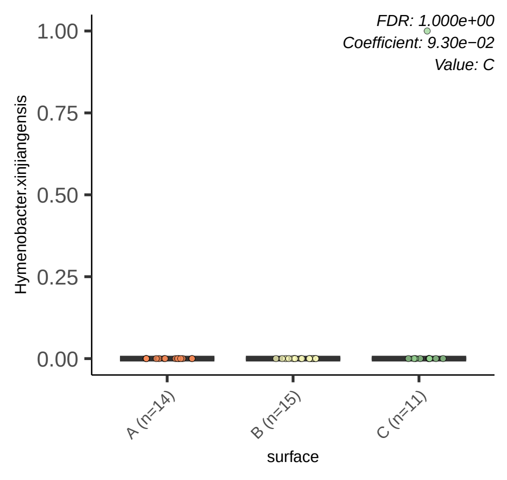

FDR: 1.000e+00
Coefficient: 9.30e−02
Value: C
1.00
0.75
0.50
0.25
0.00
Hymenobacter.xinjiangensis
A (n=14)
B (n=15)
C (n=11)
surface

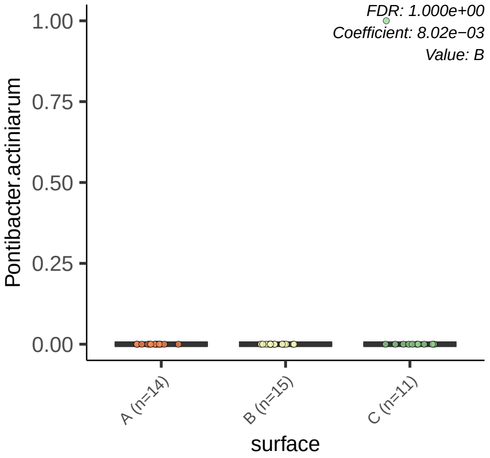

FDR: 1.000e+00
Coefficient: 8.02e−03
Value: B
Pontibacter.actiniarum
1.00
0.75
0.50
0.25
0.00
A (n=14)
B (n=15)
C (n=11)
surface

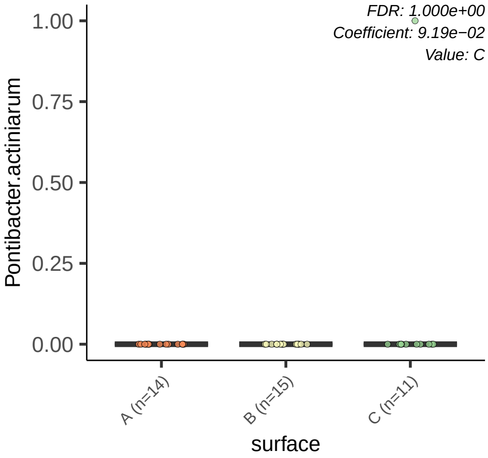
FDR: 1.000e+00
Coefficient: 9.19e−02
Value: C
Pontibacter.actiniarum
1.00
0.75
0.50
0.25
0.00
A (n=14)
B (n=15)
C (n=11)
surface

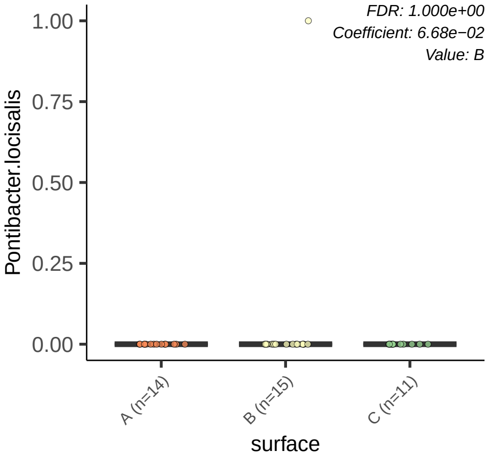

FDR: 1.000e+00
Coefficient: 6.68e−02
Value: B
Pontibacter.locisalis
1.00
0.75
0.50
0.25
0.00
A (n=14)
B (n=15)
C (n=11)
surface

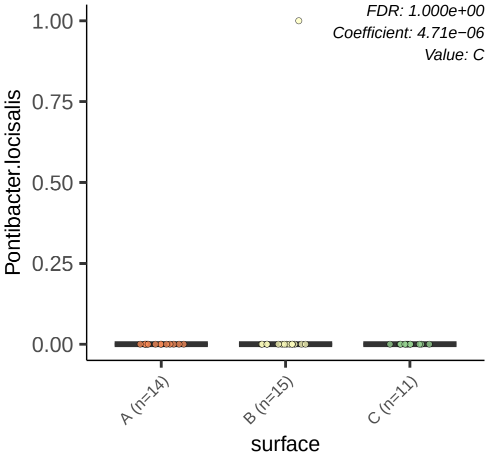

FDR: 1.000e+00
Coefficient: 4.71e−06
Value: C
Pontibacter.locisalis
1.00
0.75
0.50
0.25
0.00
A (n=14)
B (n=15)
C (n=11)
surface

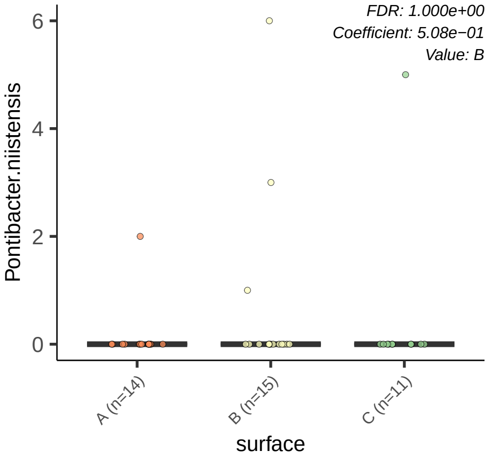

FDR: 1.000e+00
Coefficient: 5.08e−01
Value: B
Pontibacter.niistensis
6
4
2
0
A (n=14)
B (n=15)
C (n=11)
surface

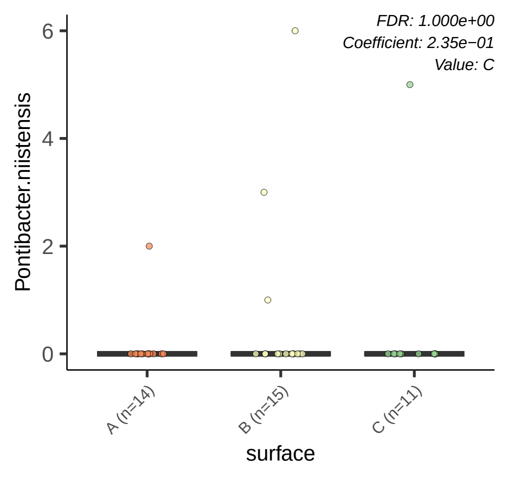
FDR: 1.000e+00
Coefficient: 2.35e−01
Value: C
Pontibacter.niistensis
6
4
2
0
A (n=14)
B (n=15)
C (n=11)
surface

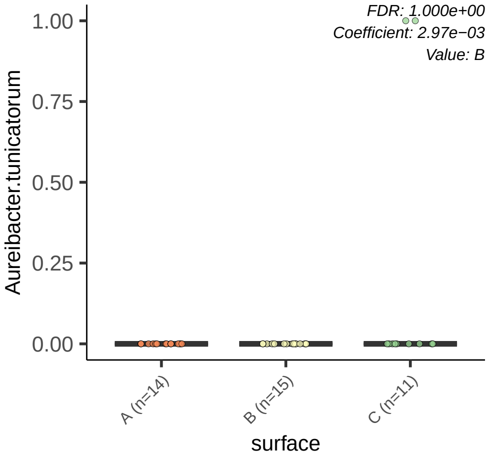
FDR: 1.000e+00
Coefficient: 2.97e−03
Value: B
Aureibacter.tunicatorum
1.00
0.75
0.50
0.25
0.00
A (n=14)
B (n=15)
C (n=11)
surface

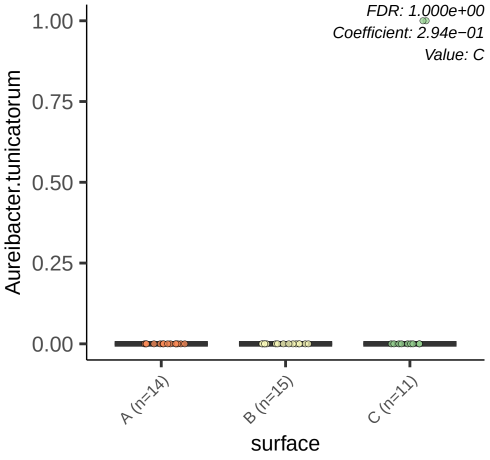
FDR: 1.000e+00
Coefficient: 2.94e−01
Value: C
1.00
0.75
0.50
0.25
0.00
Aureibacter.tunicatorum
A (n=14)
B (n=15)
C (n=11)
surface

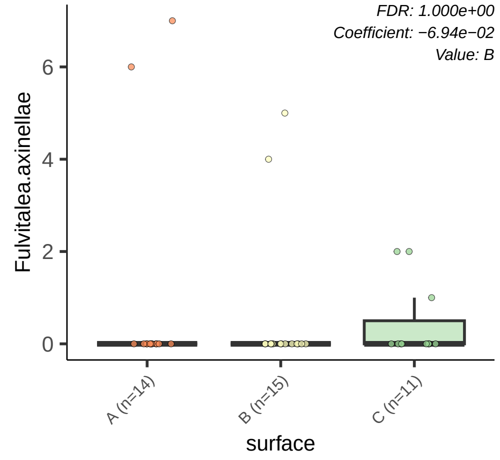
FDR: 1.000e+00
Coefficient: −6.94e−02
Value: B
Fulvitalea.axinellae
0
2
4
6
A (n=14)
B (n=15)
C (n=11)
surface

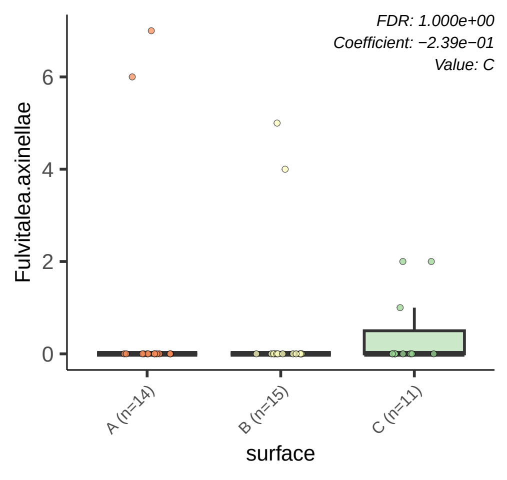
FDR: 1.000e+00
Coefficient: −2.39e−01
Value: C
Fulvitalea.axinellae
0
2
4
6
A (n=14)
B (n=15)
C (n=11)
surface

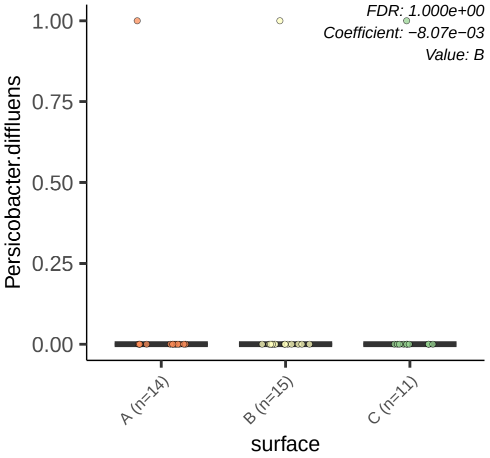

FDR: 1.000e+00
Coefficient: −8.07e−03
Value: B
Persicobacter.diffluens
1.00
0.75
0.50
0.25
0.00
A (n=14)
B (n=15)
C (n=11)
surface

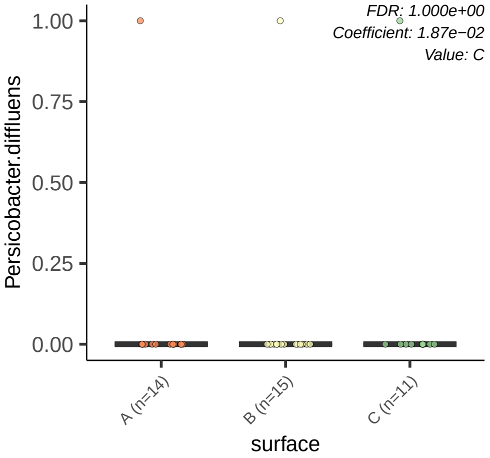
FDR: 1.000e+00
Coefficient: 1.87e−02
Value: C
Persicobacter.diffluens
1.00
0.75
0.50
0.25
0.00
A (n=14)
B (n=15)
C (n=11)
surface

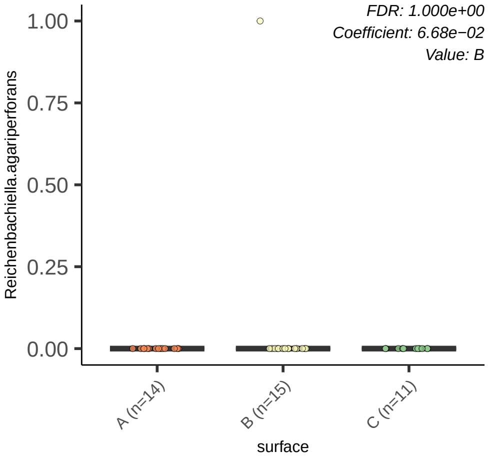
FDR: 1.000e+00
Coefficient: 6.68e−02
Value: B
Reichenbachiella.agariperforans
1.00
0.75
0.50
0.25
0.00
A (n=14)
B (n=15)
C (n=11)
surface

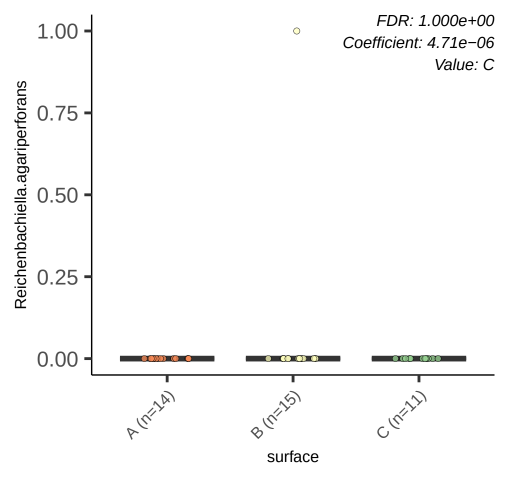

FDR: 1.000e+00
Coefficient: 4.71e−06
Value: C
1.00
0.75
0.50
0.25
0.00
Reichenbachiella.agariperforans
A (n=14)
B (n=15)
C (n=11)
surface

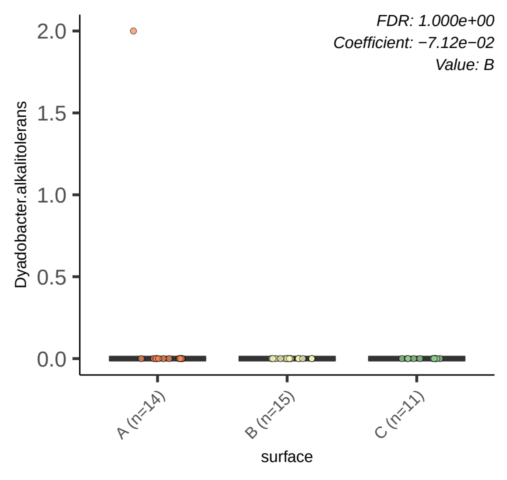
FDR: 1.000e+00
Coefficient: −7.12e−02
Value: B
Dyadobacter.alkalitolerans
2.0
1.5
1.0
0.5
0.0
A (n=14)
B (n=15)
C (n=11)
surface

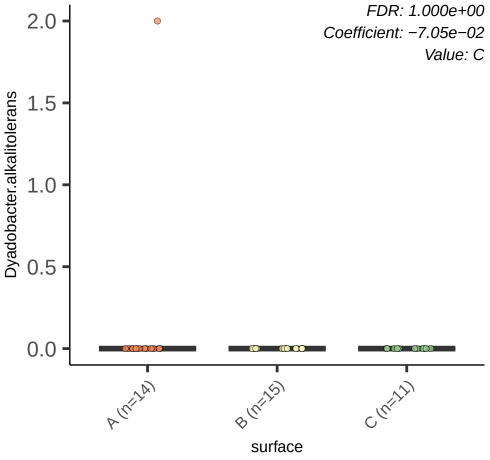

FDR: 1.000e+00
Coefficient: −7.05e−02
Value: C
Dyadobacter.alkalitolerans
2.0
1.5
1.0
0.5
0.0
A (n=14)
B (n=15)
C (n=11)
surface

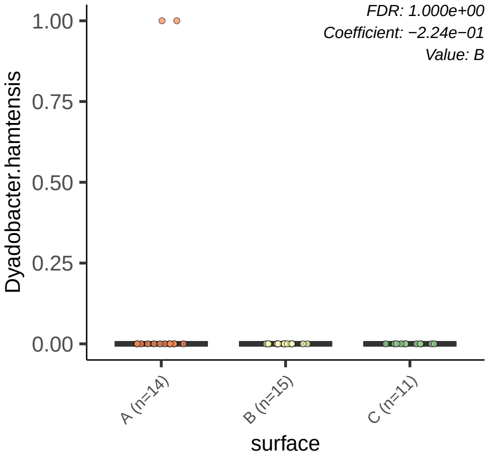

FDR: 1.000e+00
Coefficient: −2.24e−01
Value: B
Dyadobacter.hamtensis
1.00
0.75
0.50
0.25
0.00
A (n=14)
B (n=15)
C (n=11)
surface

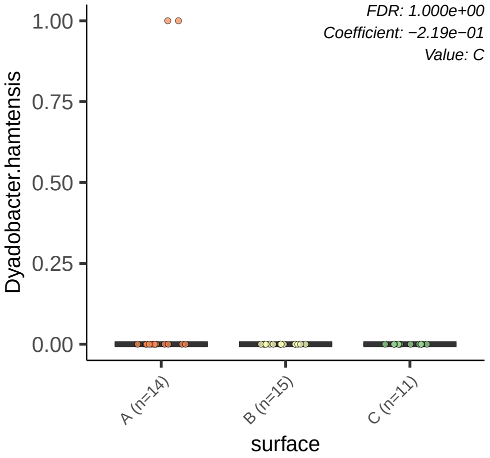
FDR: 1.000e+00
Coefficient: −2.19e−01
Value: C
Dyadobacter.hamtensis
1.00
0.75
0.50
0.25
0.00
A (n=14)
B (n=15)
C (n=11)
surface

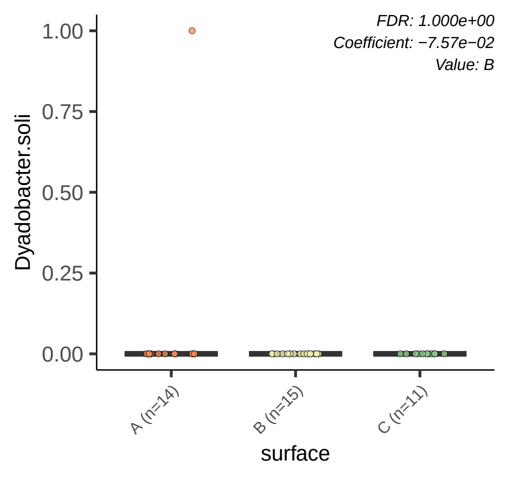

FDR: 1.000e+00
Coefficient: −7.57e−02
Value: B
Dyadobacter.soli
1.00
0.75
0.50
0.25
0.00
A (n=14)
B (n=15)
C (n=11)
surface

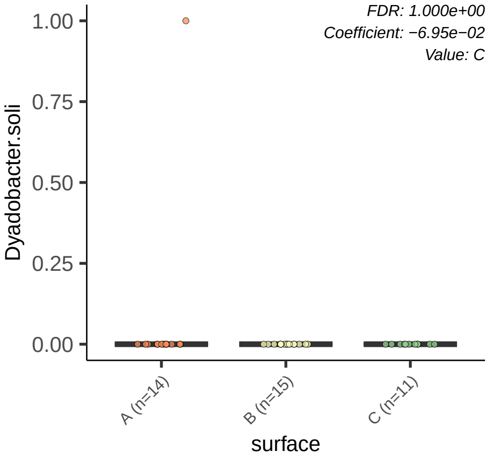

FDR: 1.000e+00
Coefficient: −6.95e−02
Value: C
Dyadobacter.soli
1.00
0.75
0.50
0.25
0.00
A (n=14)
B (n=15)
C (n=11)
surface

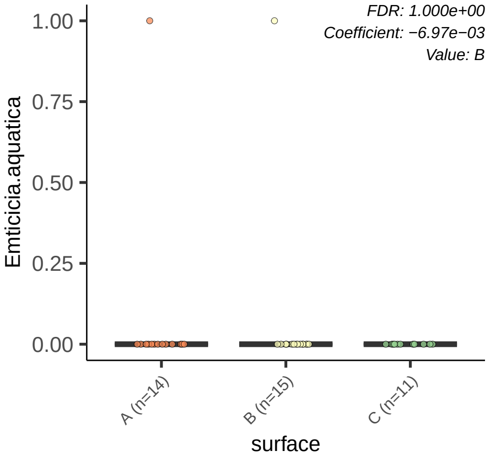
FDR: 1.000e+00
Coefficient: −6.97e−03
Value: B
Emticicia.aquatica
1.00
0.75
0.50
0.25
0.00
A (n=14)
B (n=15)
C (n=11)
surface

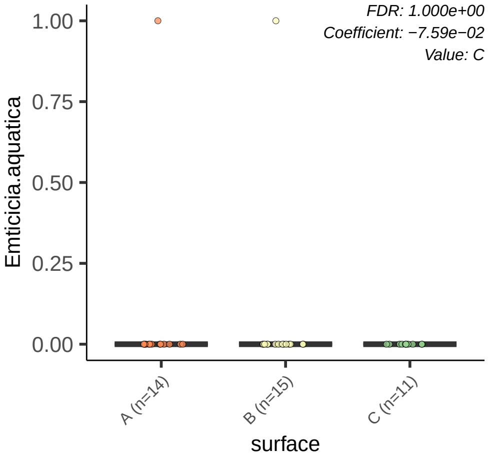

FDR: 1.000e+00
Coefficient: −7.59e−02
Value: C
Emticicia.aquatica
1.00
0.75
0.50
0.25
0.00
A (n=14)
B (n=15)
C (n=11)
surface

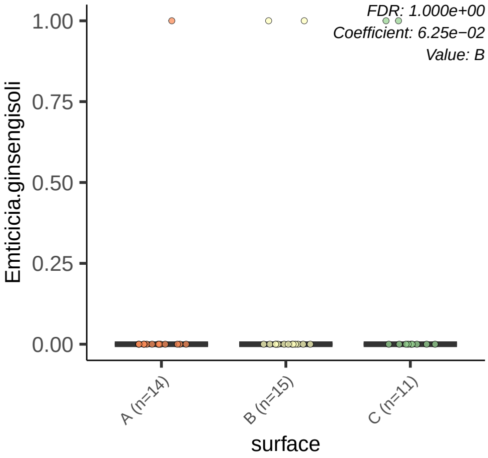

FDR: 1.000e+00
Coefficient: 6.25e−02
Value: B
Emticicia.ginsengisoli
1.00
0.75
0.50
0.25
0.00
A (n=14)
B (n=15)
C (n=11)
surface

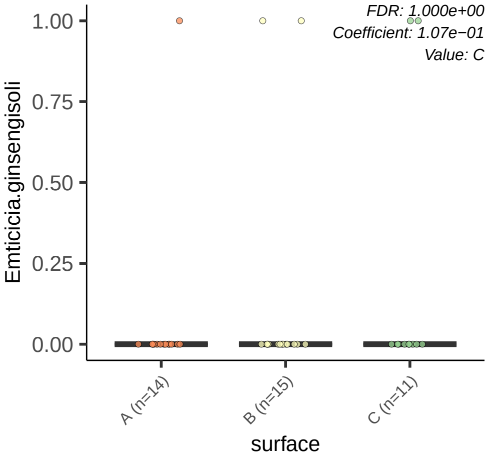

FDR: 1.000e+00
Coefficient: 1.07e−01
Value: C
Emticicia.ginsengisoli
1.00
0.75
0.50
0.25
0.00
A (n=14)
B (n=15)
C (n=11)
surface

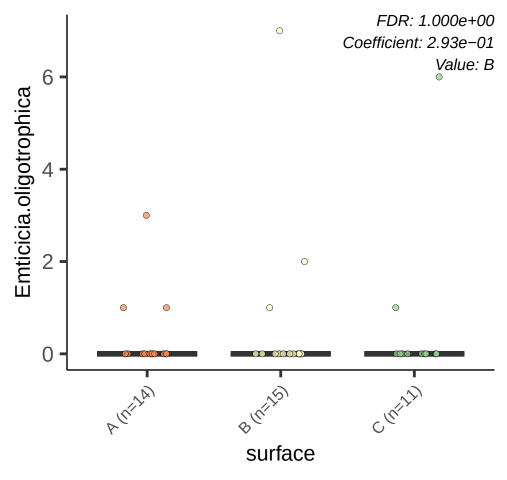
FDR: 1.000e+00
Coefficient: 2.93e−01
Value: B
Emticicia.oligotrophica
0
2
4
6
A (n=14)
B (n=15)
C (n=11)
surface

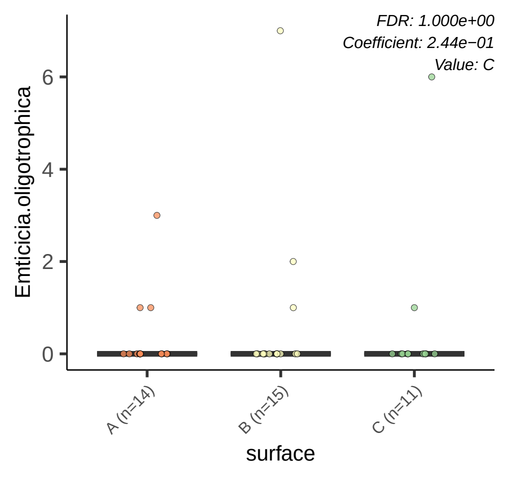
FDR: 1.000e+00
Coefficient: 2.44e−01
Value: C
Emticicia.oligotrophica
0
2
4
6
A (n=14)
B (n=15)
C (n=11)
surface

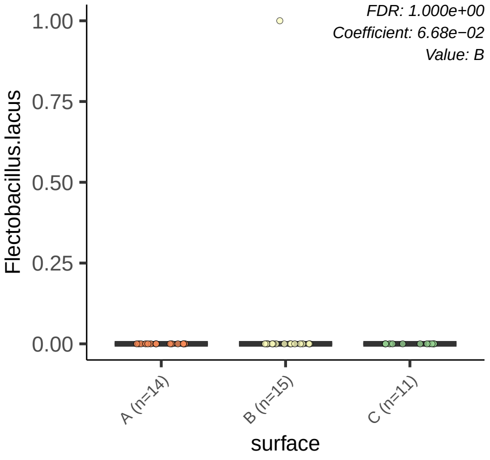
FDR: 1.000e+00
Coefficient: 6.68e−02
Value: B
Flectobacillus.lacus
1.00
0.75
0.50
0.25
0.00
A (n=14)
B (n=15)
C (n=11)
surface

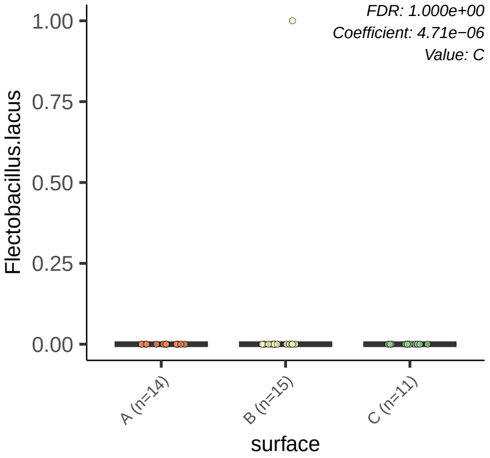
FDR: 1.000e+00
Coefficient: 4.71e−06
Value: C
Flectobacillus.lacus
1.00
0.75
0.50
0.25
0.00
A (n=14)
B (n=15)
C (n=11)
surface

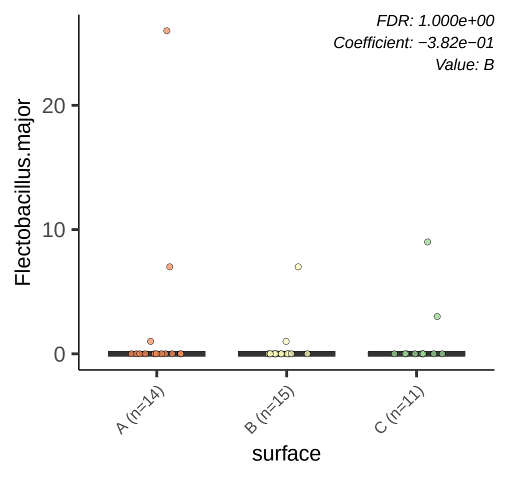
FDR: 1.000e+00
Coefficient: −3.82e−01
Value: B
Flectobacillus.major
20
10
0
A (n=14)
B (n=15)
C (n=11)
surface

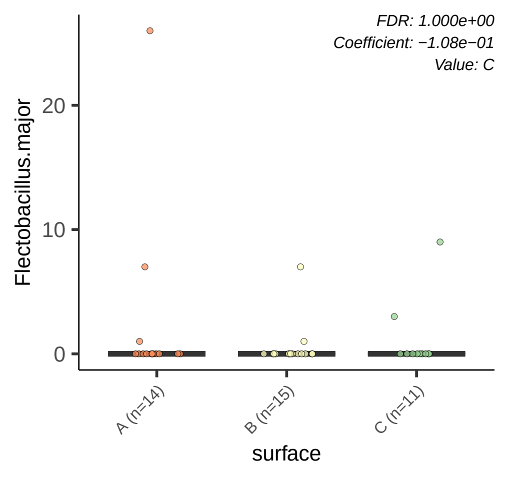

FDR: 1.000e+00
Coefficient: −1.08e−01
Value: C
Flectobacillus.major
20
10
0
A (n=14)
B (n=15)
C (n=11)
surface

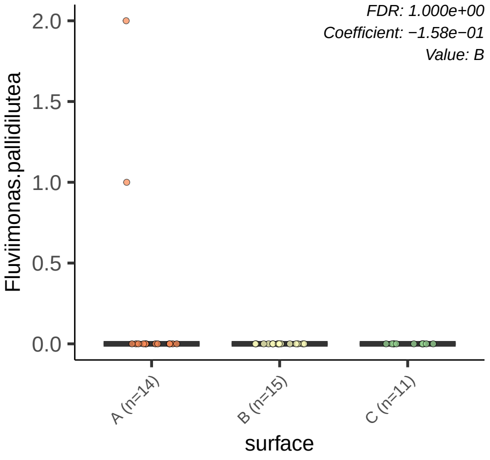

FDR: 1.000e+00
Coefficient: −1.58e−01
Value: B
Fluviimonas.pallidilutea
2.0
1.5
1.0
0.5
0.0
A (n=14)
B (n=15)
C (n=11)
surface

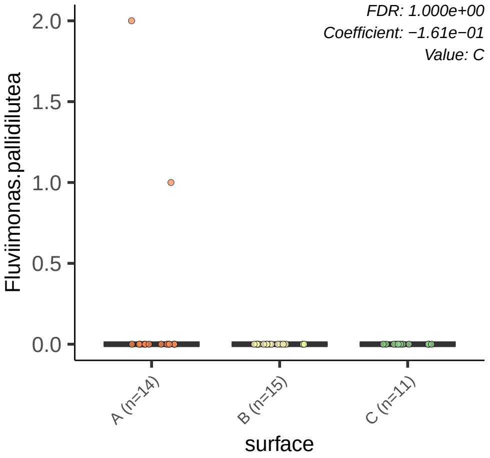
FDR: 1.000e+00
Coefficient: −1.61e−01
Value: C
Fluviimonas.pallidilutea
2.0
1.5
1.0
0.5
0.0
A (n=14)
B (n=15)
C (n=11)
surface

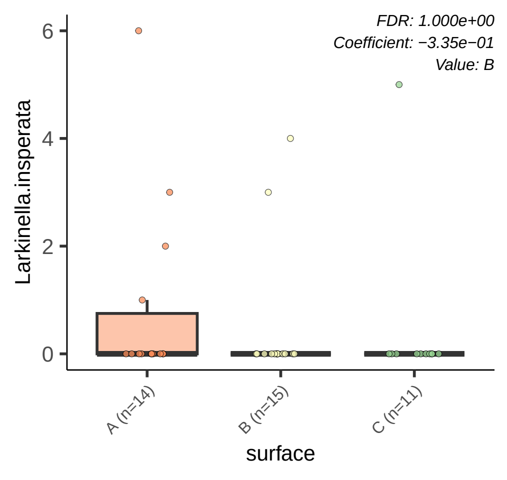
FDR: 1.000e+00
Coefficient: −3.35e−01
Value: B
Larkinella.insperata
6
4
2
0
A (n=14)
B (n=15)
C (n=11)
surface

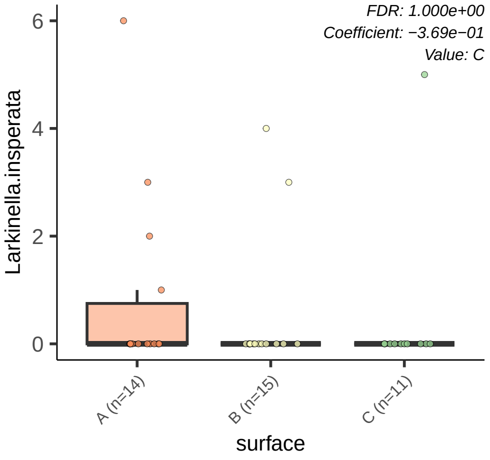

FDR: 1.000e+00
Coefficient: −3.69e−01
Value: C
Larkinella.insperata
0
2
4
6
A (n=14)
B (n=15)
C (n=11)
surface

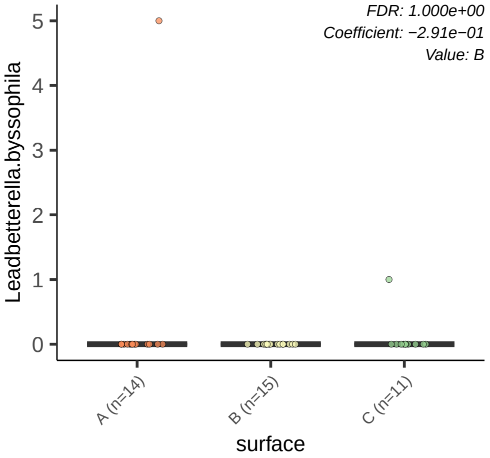

FDR: 1.000e+00
Coefficient: −2.91e−01
Value: B
Leadbetterella.byssophila
0
1
2
3
4
5
A (n=14)
B (n=15)
C (n=11)
surface

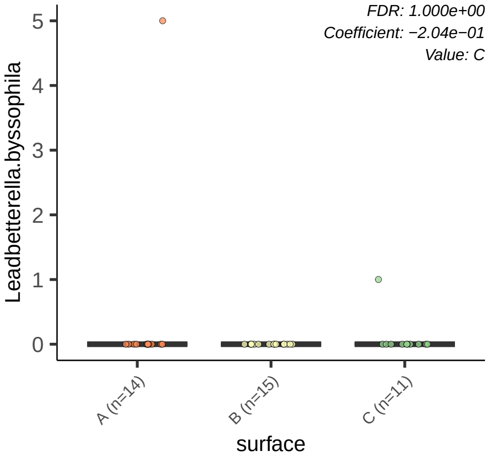

FDR: 1.000e+00
Coefficient: −2.04e−01
Value: C
Leadbetterella.byssophila
0
1
2
3
4
5
A (n=14)
B (n=15)
C (n=11)
surface

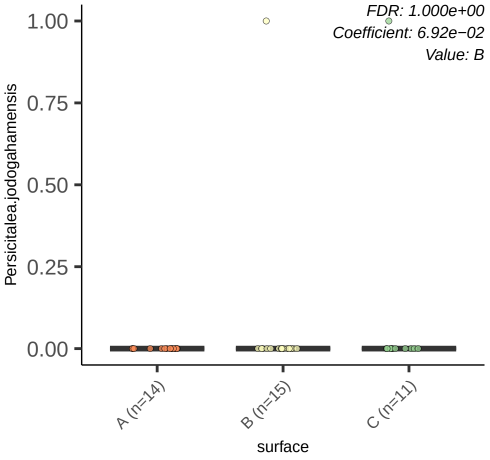

FDR: 1.000e+00
Coefficient: 6.92e−02
Value: B
1.00
0.75
0.50
0.25
0.00
Persicitalea.jodogahamensis
A (n=14)
B (n=15)
C (n=11)
surface

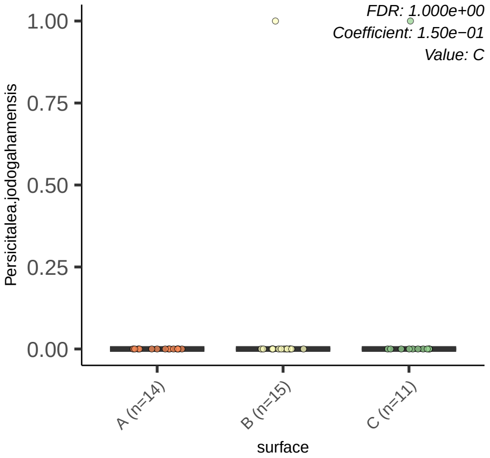

FDR: 1.000e+00
Coefficient: 1.50e−01
Value: C
1.00
0.75
0.50
0.25
0.00
Persicitalea.jodogahamensis
A (n=14)
B (n=15)
C (n=11)
surface

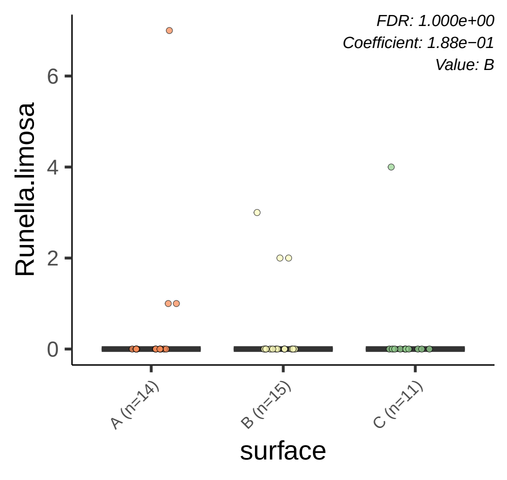

FDR: 1.000e+00
Coefficient: 1.88e−01
Value: B
Runella.limosa
0
2
4
6
A (n=14)
B (n=15)
C (n=11)
surface

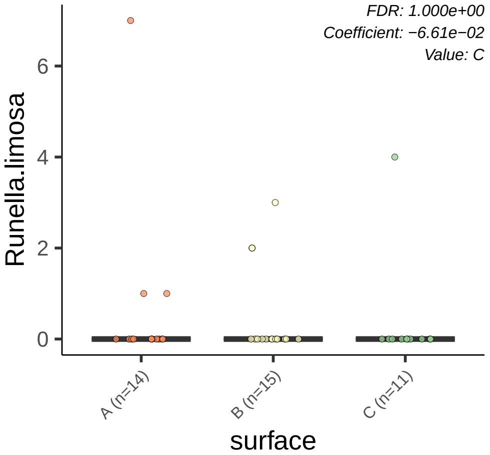
FDR: 1.000e+00
Coefficient: −6.61e−02
Value: C
Runella.limosa
0
2
4
6
A (n=14)
B (n=15)
C (n=11)
surface

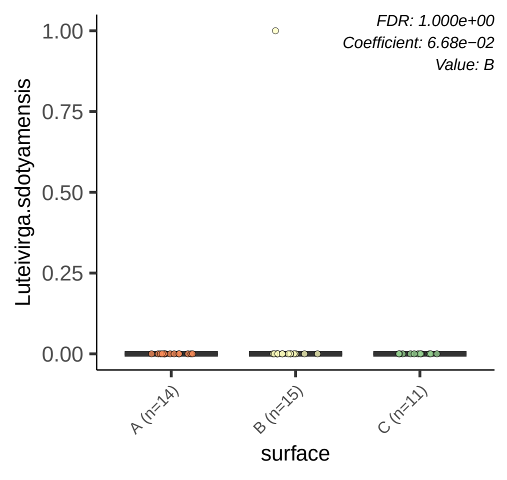

FDR: 1.000e+00
Coefficient: 6.68e−02
Value: B
Luteivirga.sdotyamensis
1.00
0.75
0.50
0.25
0.00
A (n=14)
B (n=15)
C (n=11)
surface

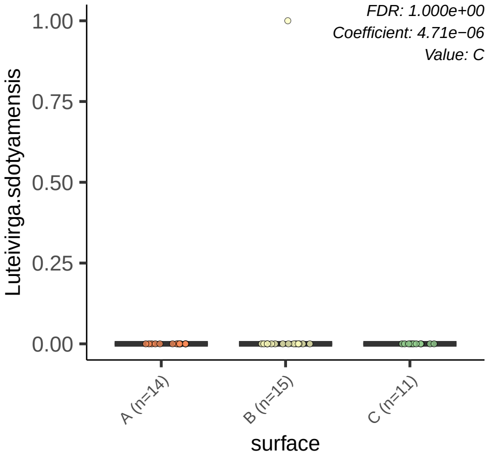

FDR: 1.000e+00
Coefficient: 4.71e−06
Value: C
Luteivirga.sdotyamensis
1.00
0.75
0.50
0.25
0.00
A (n=14)
B (n=15)
C (n=11)
surface

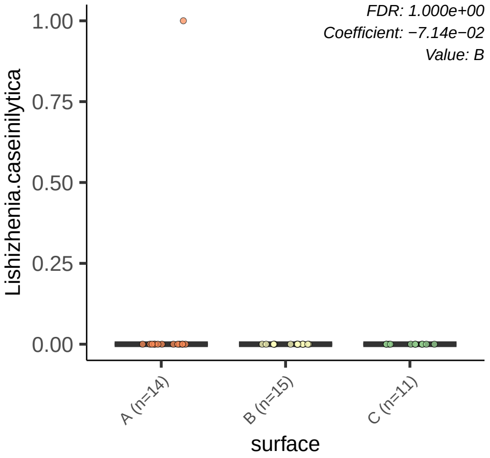

FDR: 1.000e+00
Coefficient: −7.14e−02
Value: B
Lishizhenia.caseinilytica
1.00
0.75
0.50
0.25
0.00
A (n=14)
B (n=15)
C (n=11)
surface

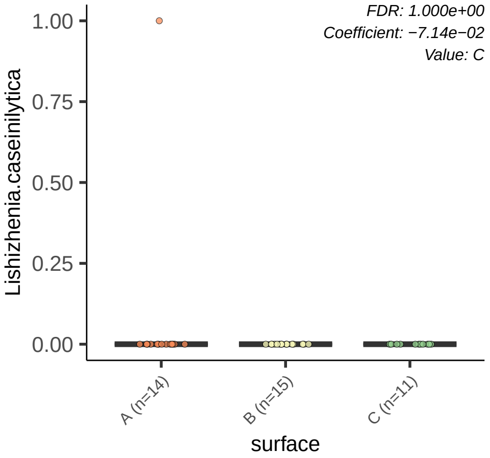

FDR: 1.000e+00
Coefficient: −7.14e−02
Value: C
Lishizhenia.caseinilytica
1.00
0.75
0.50
0.25
0.00
A (n=14)
B (n=15)
C (n=11)
surface

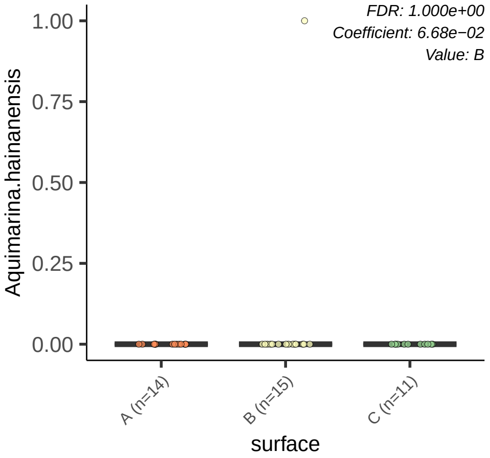
FDR: 1.000e+00
Coefficient: 6.68e−02
Value: B
Aquimarina.hainanensis
1.00
0.75
0.50
0.25
0.00
A (n=14)
B (n=15)
C (n=11)
surface

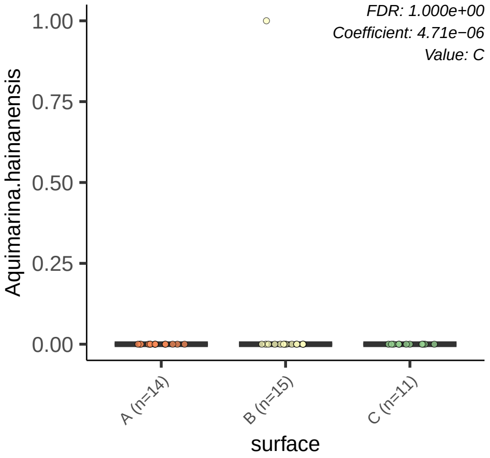
FDR: 1.000e+00
Coefficient: 4.71e−06
Value: C
Aquimarina.hainanensis
1.00
0.75
0.50
0.25
0.00
A (n=14)
B (n=15)
C (n=11)
surface

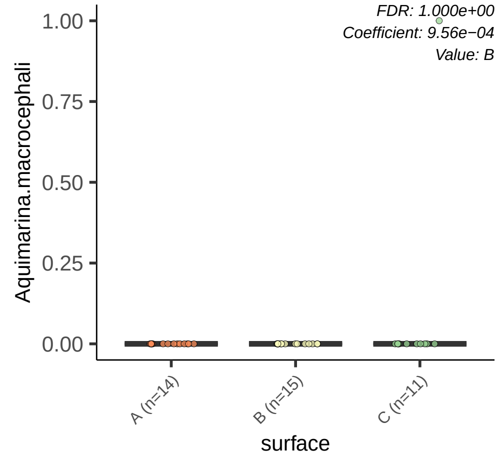

FDR: 1.000e+00
Coefficient: 9.56e−04
Value: B
Aquimarina.macrocephali
1.00
0.75
0.50
0.25
0.00
A (n=14)
B (n=15)
C (n=11)
surface

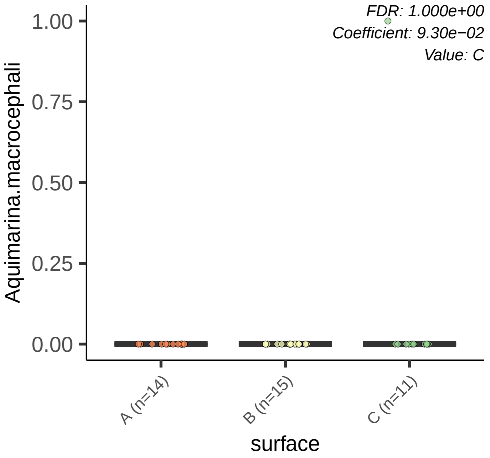
FDR: 1.000e+00
Coefficient: 9.30e−02
Value: C
Aquimarina.macrocephali
1.00
0.75
0.50
0.25
0.00
A (n=14)
B (n=15)
C (n=11)
surface

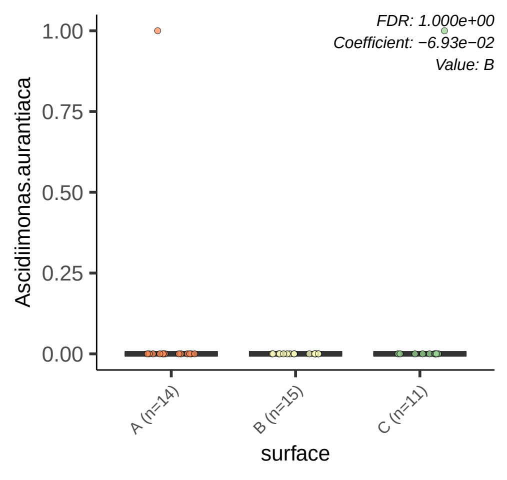
FDR: 1.000e+00
Coefficient: −6.93e−02
Value: B
Ascidiimonas.aurantiaca
1.00
0.75
0.50
0.25
0.00
A (n=14)
B (n=15)
C (n=11)
surface

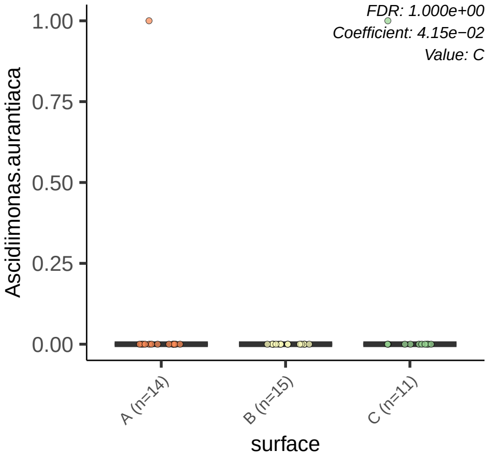
FDR: 1.000e+00
Coefficient: 4.15e−02
Value: C
Ascidiimonas.aurantiaca
1.00
0.75
0.50
0.25
0.00
A (n=14)
B (n=15)
C (n=11)
surface

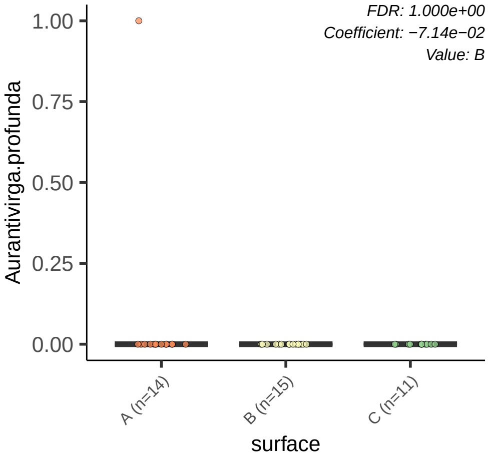
FDR: 1.000e+00
Coefficient: −7.14e−02
Value: B
Aurantivirga.profunda
1.00
0.75
0.50
0.25
0.00
A (n=14)
B (n=15)
C (n=11)
surface

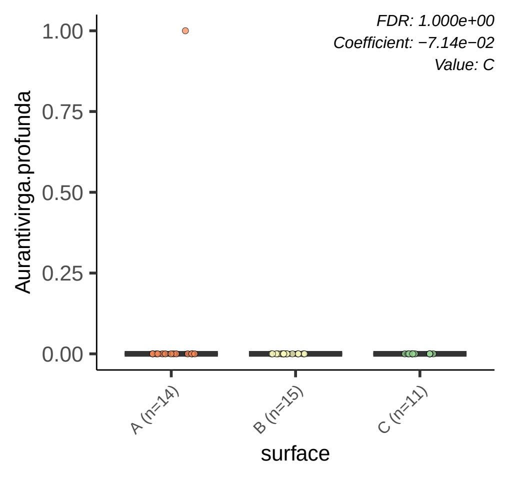

FDR: 1.000e+00
Coefficient: −7.14e−02
Value: C
Aurantivirga.profunda
1.00
0.75
0.50
0.25
0.00
A (n=14)
B (n=15)
C (n=11)
surface

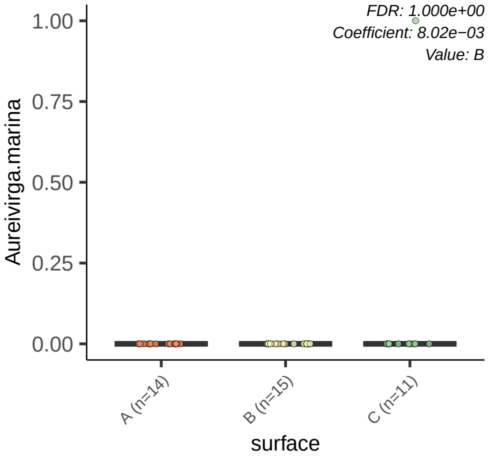
FDR: 1.000e+00
Coefficient: 8.02e−03
Value: B
Aureivirga.marina
1.00
0.75
0.50
0.25
0.00
A (n=14)
B (n=15)
C (n=11)
surface

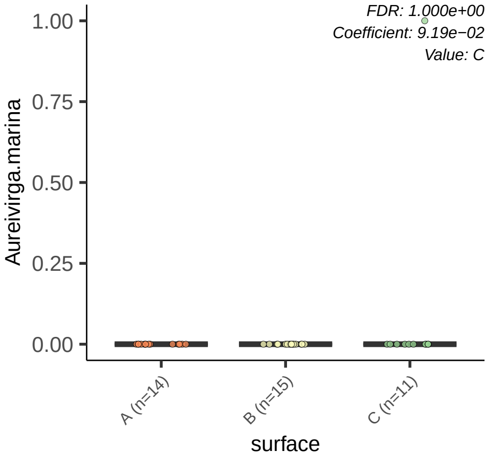
FDR: 1.000e+00
Coefficient: 9.19e−02
Value: C
Aureivirga.marina
1.00
0.75
0.50
0.25
0.00
A (n=14)
B (n=15)
C (n=11)
surface

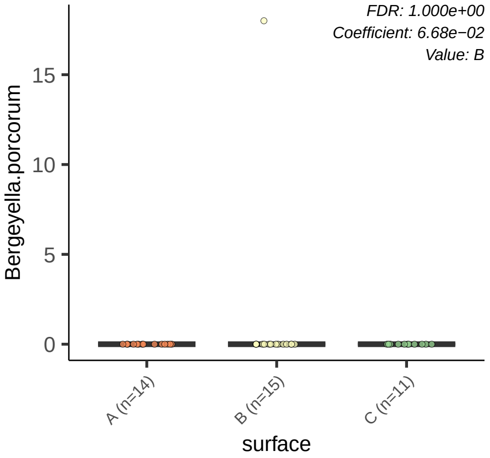
FDR: 1.000e+00
Coefficient: 6.68e−02
Value: B
Bergeyella.porcorum
0
5
10
15
A (n=14)
B (n=15)
C (n=11)
surface

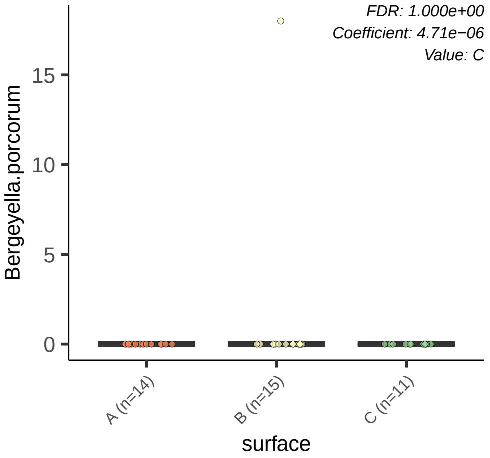

FDR: 1.000e+00
Coefficient: 4.71e−06
Value: C
Bergeyella.porcorum
0
5
10
15
A (n=14)
B (n=15)
C (n=11)
surface

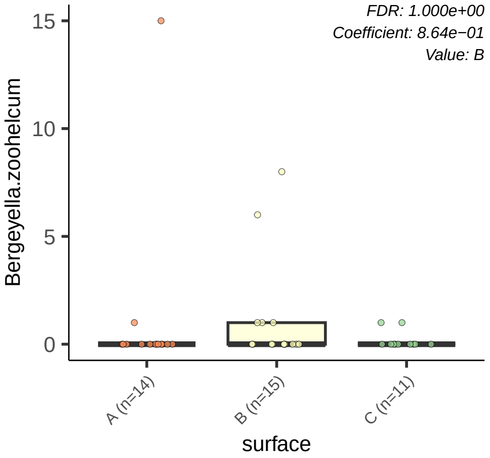

FDR: 1.000e+00
Coefficient: 8.64e−01
Value: B
15
10
5
0
Bergeyella.zoohelcum
A (n=14)
B (n=15)
C (n=11)
surface

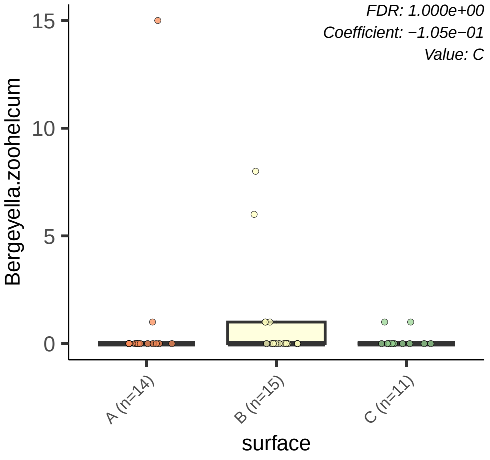
FDR: 1.000e+00
Coefficient: −1.05e−01
Value: C
Bergeyella.zoohelcum
15
10
5
0
A (n=14)
B (n=15)
C (n=11)
surface

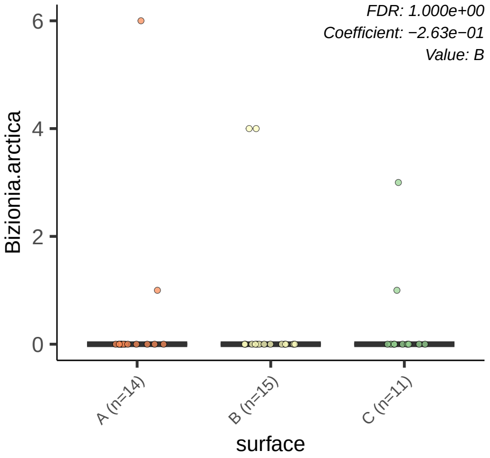
FDR: 1.000e+00
Coefficient: −2.63e−01
Value: B
Bizionia.arctica
0
2
4
6
A (n=14)
B (n=15)
C (n=11)
surface

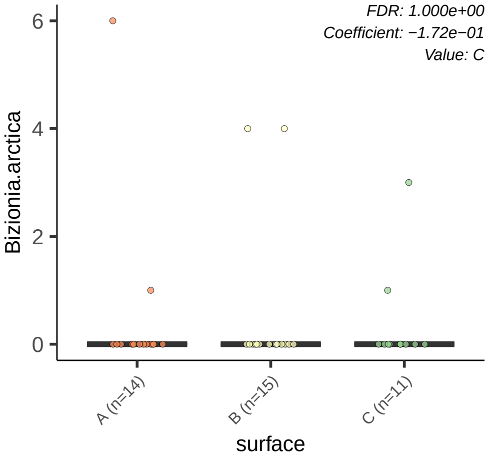

FDR: 1.000e+00
Coefficient: −1.72e−01
Value: C
Bizionia.arctica
6
4
2
0
A (n=14)
B (n=15)
C (n=11)
surface

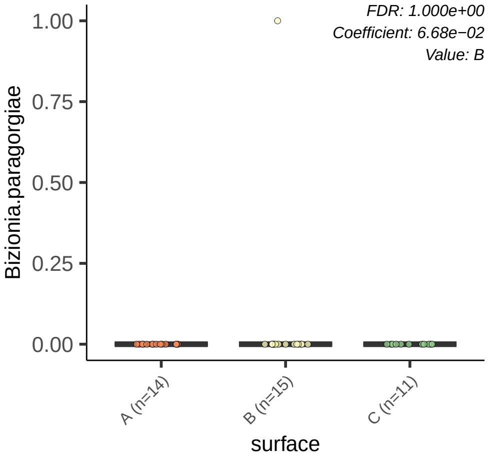
FDR: 1.000e+00
Coefficient: 6.68e−02
Value: B
Bizionia.paragorgiae
1.00
0.75
0.50
0.25
0.00
A (n=14)
B (n=15)
C (n=11)
surface

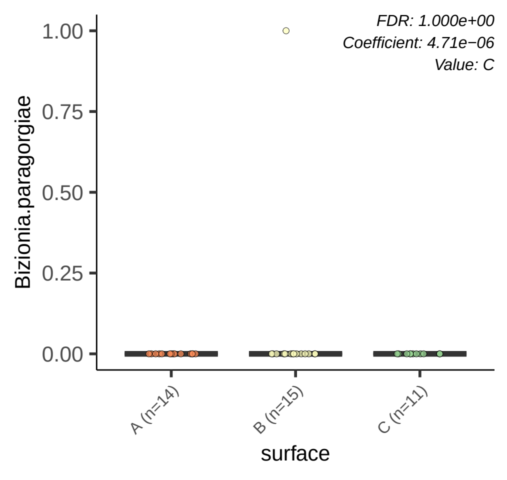
FDR: 1.000e+00
Coefficient: 4.71e−06
Value: C
Bizionia.paragorgiae
1.00
0.75
0.50
0.25
0.00
A (n=14)
B (n=15)
C (n=11)
surface

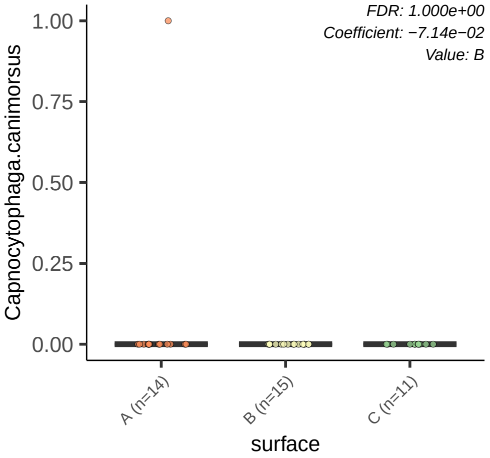

FDR: 1.000e+00
Coefficient: −7.14e−02
Value: B
Capnocytophaga.canimorsus
1.00
0.75
0.50
0.25
0.00
A (n=14)
B (n=15)
C (n=11)
surface

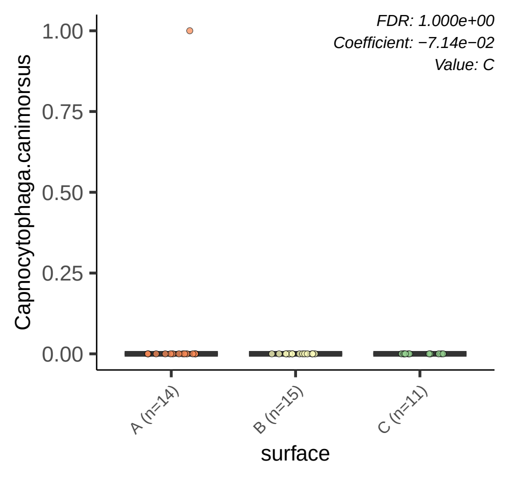
FDR: 1.000e+00
Coefficient: −7.14e−02
Value: C
Capnocytophaga.canimorsus
1.00
0.75
0.50
0.25
0.00
A (n=14)
B (n=15)
C (n=11)
surface

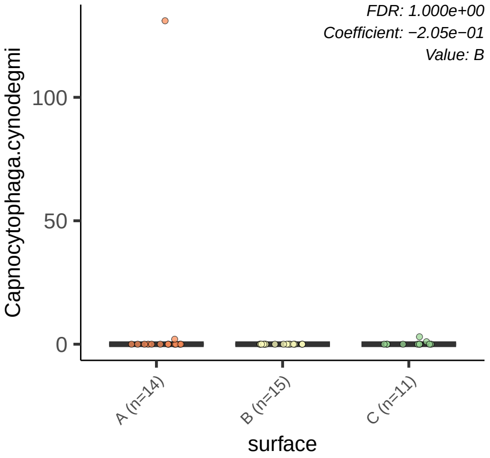
FDR: 1.000e+00
Coefficient: −2.05e−01
Value: B
Capnocytophaga.cynodegmi
100
50
0
A (n=14)
B (n=15)
C (n=11)
surface

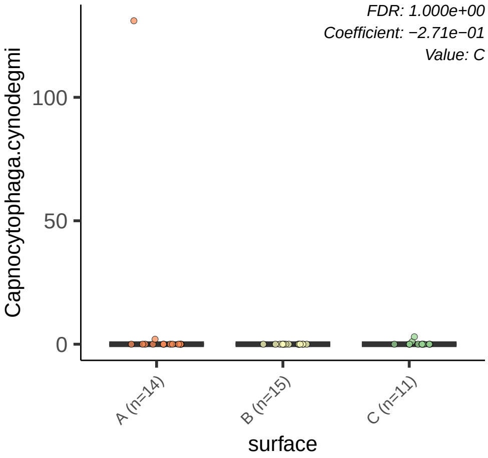
FDR: 1.000e+00
Coefficient: −2.71e−01
Value: C
Capnocytophaga.cynodegmi
100
50
0
A (n=14)
B (n=15)
C (n=11)
surface

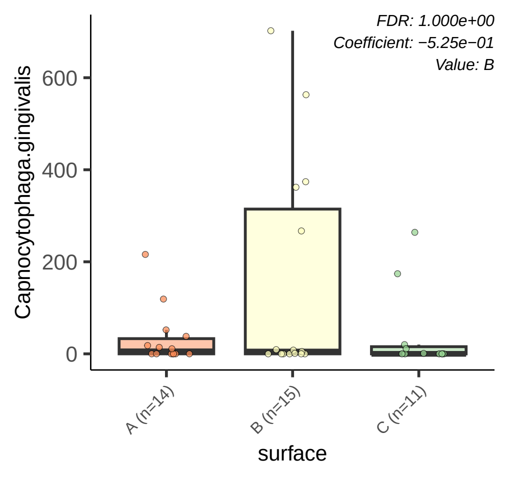

FDR: 1.000e+00
Coefficient: −5.25e−01
Value: B
Capnocytophaga.gingivalis
600
400
200
0
A (n=14)
B (n=15)
C (n=11)
surface

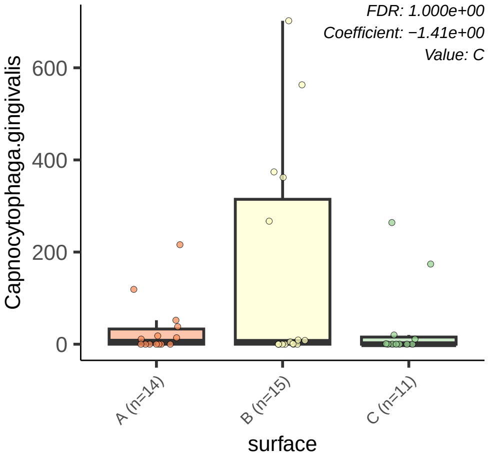
FDR: 1.000e+00
Coefficient: −1.41e+00
Value: C
Capnocytophaga.gingivalis
0
200
400
600
A (n=14)
B (n=15)
C (n=11)
surface

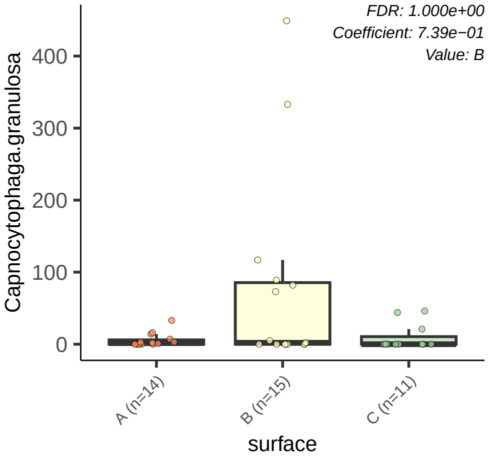
FDR: 1.000e+00
Coefficient: 7.39e−01
Value: B
Capnocytophaga.granulosa
400
300
200
100
0
A (n=14)
B (n=15)
C (n=11)
surface

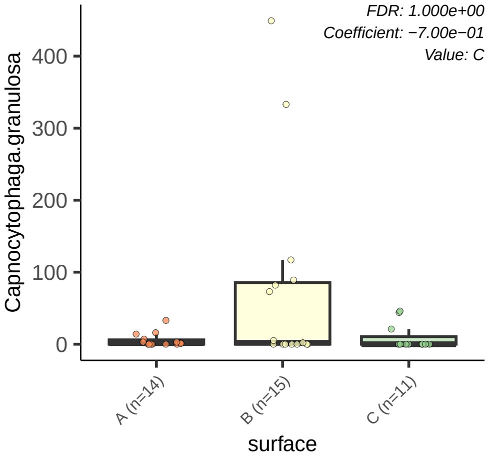
FDR: 1.000e+00
Coefficient: −7.00e−01
Value: C
Capnocytophaga.granulosa
0
100
200
300
400
A (n=14)
B (n=15)
C (n=11)
surface

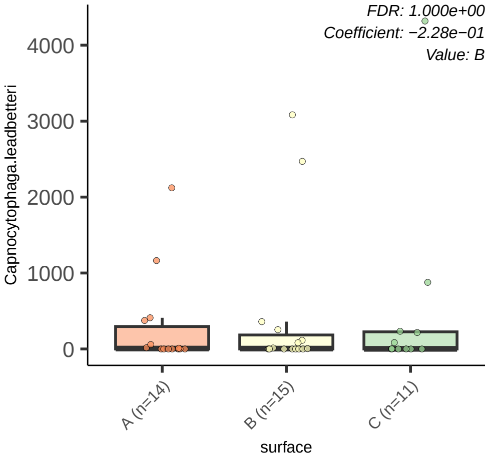

FDR: 1.000e+00
Coefficient: −2.28e−01
Value: B
4000
3000
2000
1000
0
Capnocytophaga.leadbetteri
A (n=14)
B (n=15)
C (n=11)
surface

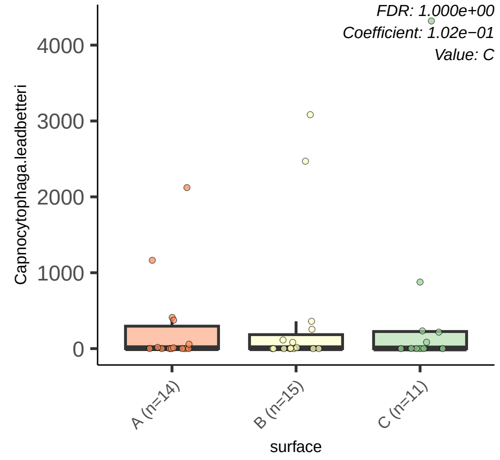

FDR: 1.000e+00
Coefficient: 1.02e−01
Value: C
Capnocytophaga.leadbetteri
4000
3000
2000
1000
0
A (n=14)
B (n=15)
C (n=11)
surface

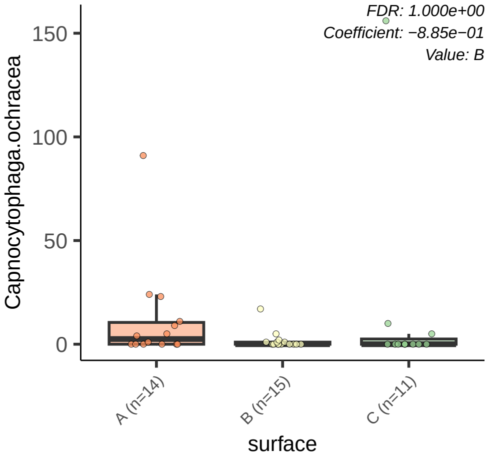
FDR: 1.000e+00
Coefficient: −8.85e−01
Value: B
Capnocytophaga.ochracea
150
100
50
0
A (n=14)
B (n=15)
C (n=11)
surface

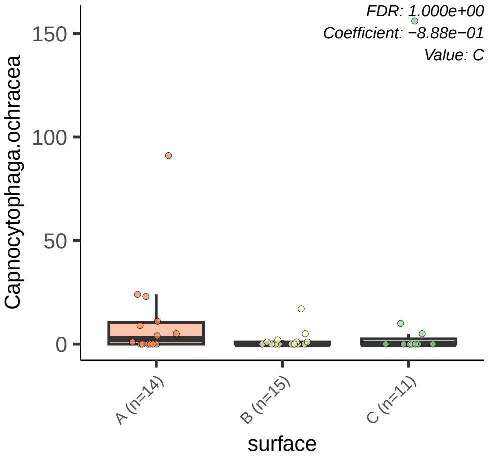
FDR: 1.000e+00
Coefficient: −8.88e−01
Value: C
Capnocytophaga.ochracea
150
100
50
0
A (n=14)
B (n=15)
C (n=11)
surface

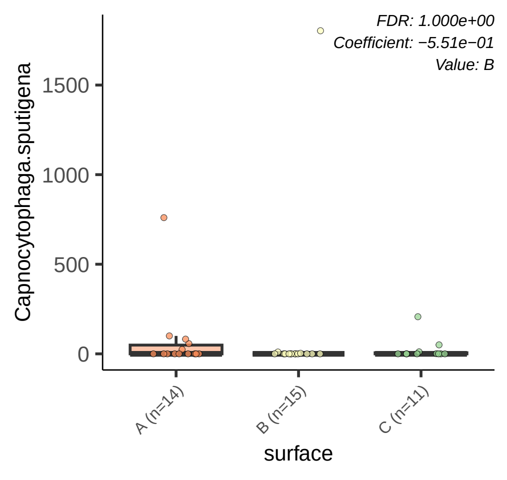
FDR: 1.000e+00
Coefficient: −5.51e−01
Value: B
Capnocytophaga.sputigena
0
500
1000
1500
A (n=14)
B (n=15)
C (n=11)
surface

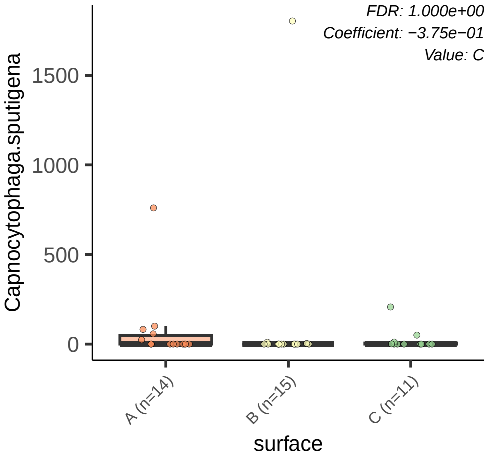

FDR: 1.000e+00
Coefficient: −3.75e−01
Value: C
Capnocytophaga.sputigena
1500
1000
500
0
A (n=14)
B (n=15)
C (n=11)
surface

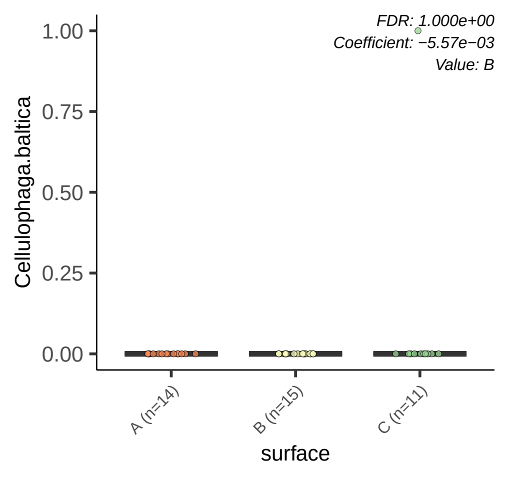
FDR: 1.000e+00
Coefficient: −5.57e−03
Value: B
Cellulophaga.baltica
1.00
0.75
0.50
0.25
0.00
A (n=14)
B (n=15)
C (n=11)
surface

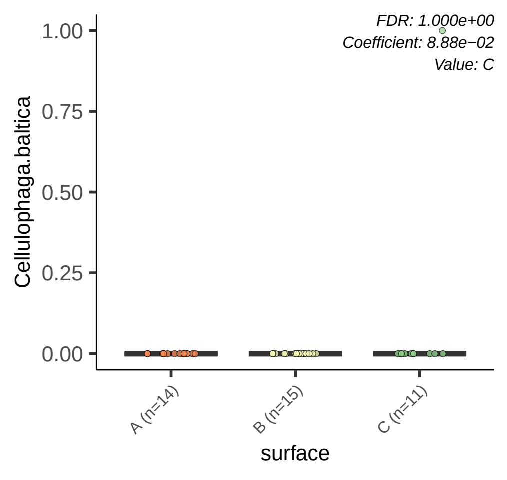
FDR: 1.000e+00
Coefficient: 8.88e−02
Value: C
Cellulophaga.baltica
1.00
0.75
0.50
0.25
0.00
A (n=14)
B (n=15)
C (n=11)
surface

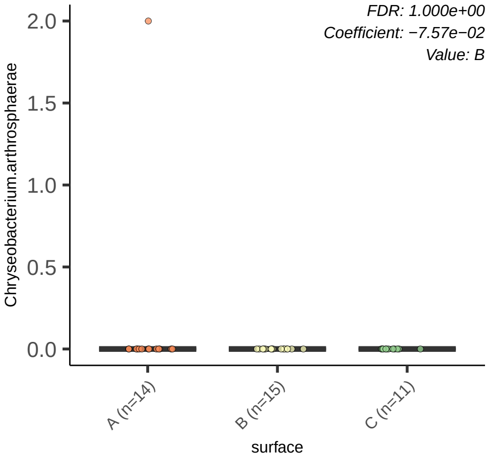
FDR: 1.000e+00
Coefficient: −7.57e−02
Value: B
Chryseobacterium.arthrosphaerae
2.0
1.5
1.0
0.5
0.0
A (n=14)
B (n=15)
C (n=11)
surface

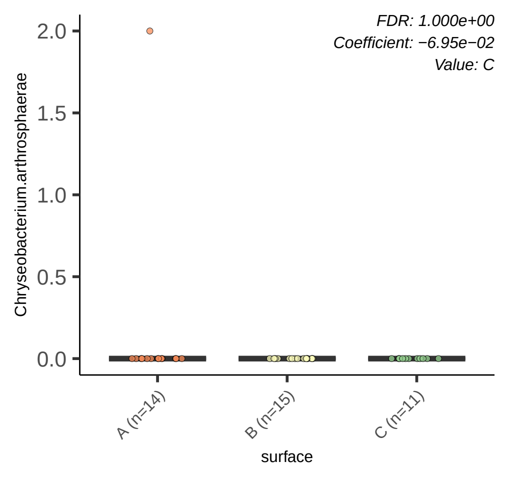
FDR: 1.000e+00
Coefficient: −6.95e−02
Value: C
Chryseobacterium.arthrosphaerae
2.0
1.5
1.0
0.5
0.0
A (n=14)
B (n=15)
C (n=11)
surface

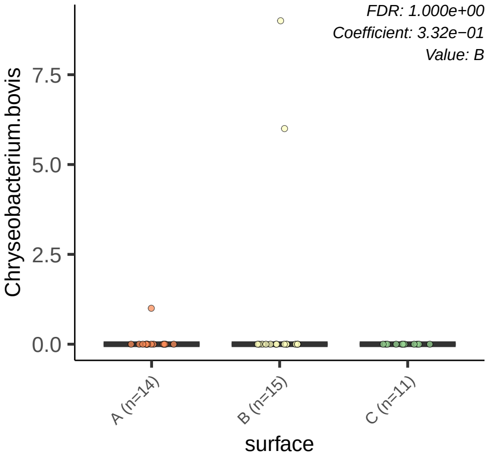
FDR: 1.000e+00
Coefficient: 3.32e−01
Value: B
Chryseobacterium.bovis
7.5
5.0
2.5
0.0
A (n=14)
B (n=15)
C (n=11)
surface

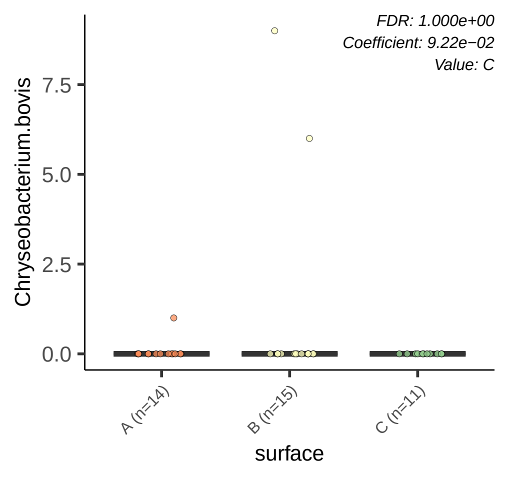
FDR: 1.000e+00
Coefficient: 9.22e−02
Value: C
Chryseobacterium.bovis
7.5
5.0
2.5
0.0
A (n=14)
B (n=15)
C (n=11)
surface

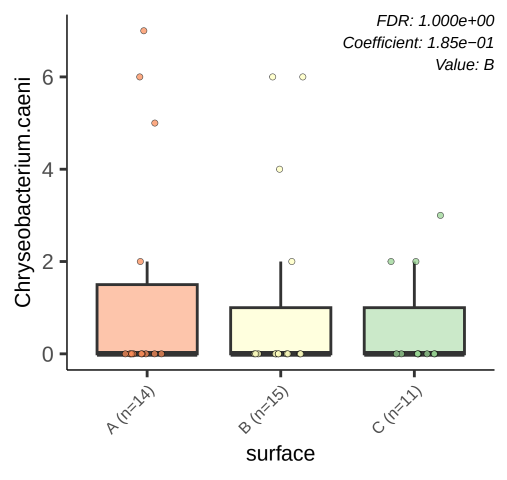

FDR: 1.000e+00
Coefficient: 1.85e−01
Value: B
Chryseobacterium.caeni
0
2
4
6
A (n=14)
B (n=15)
C (n=11)
surface

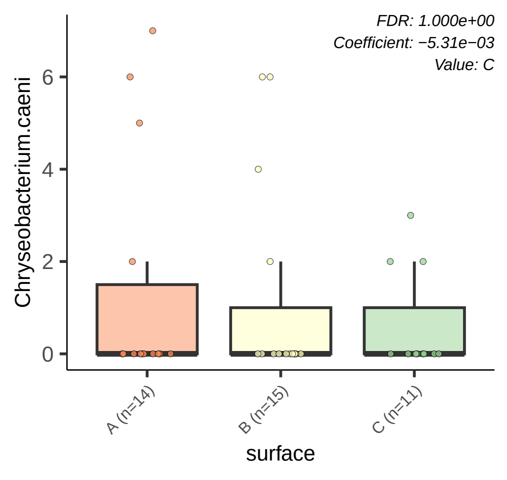
FDR: 1.000e+00
Coefficient: −5.31e−03
Value: C
Chryseobacterium.caeni
0
2
4
6
A (n=14)
B (n=15)
C (n=11)
surface

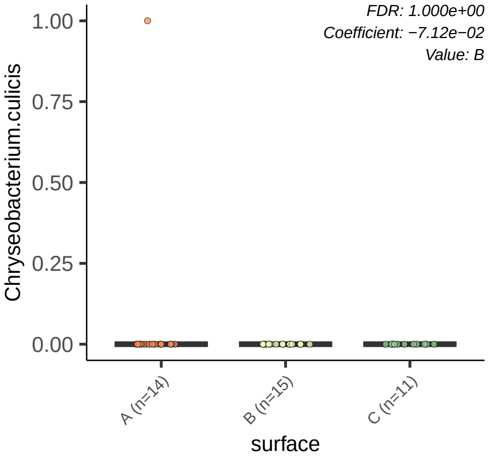
FDR: 1.000e+00
Coefficient: −7.12e−02
Value: B
Chryseobacterium.culicis
1.00
0.75
0.50
0.25
0.00
A (n=14)
B (n=15)
C (n=11)
surface

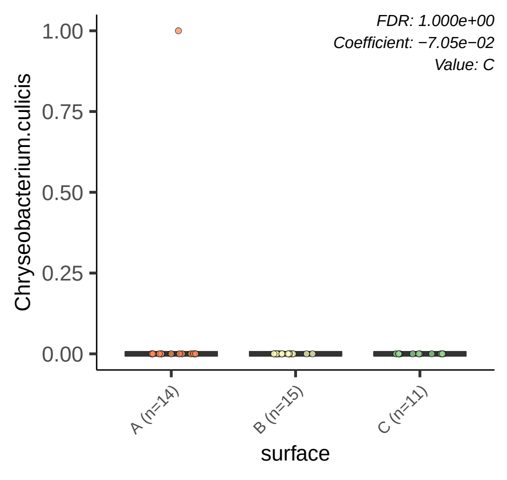

FDR: 1.000e+00
Coefficient: −7.05e−02
Value: C
Chryseobacterium.culicis
1.00
0.75
0.50
0.25
0.00
A (n=14)
B (n=15)
C (n=11)
surface

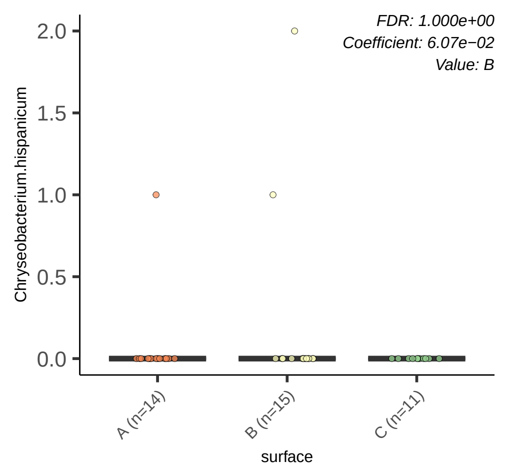
FDR: 1.000e+00
Coefficient: 6.07e−02
Value: B
Chryseobacterium.hispanicum
2.0
1.5
1.0
0.5
0.0
A (n=14)
B (n=15)
C (n=11)
surface

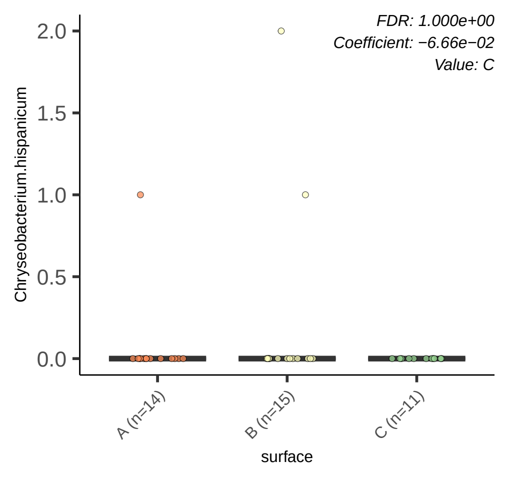
FDR: 1.000e+00
Coefficient: −6.66e−02
Value: C
Chryseobacterium.hispanicum
2.0
1.5
1.0
0.5
0.0
A (n=14)
B (n=15)
C (n=11)
surface

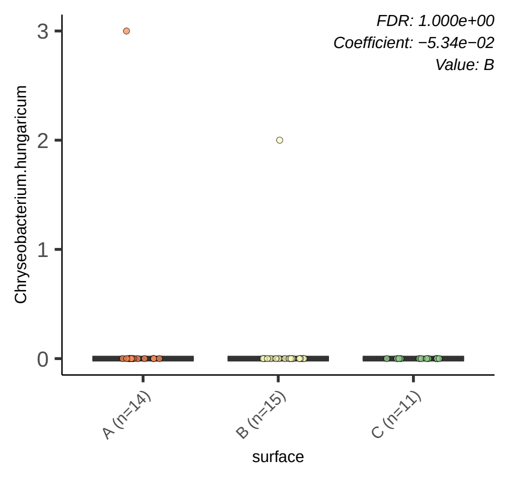

FDR: 1.000e+00
Coefficient: −5.34e−02
Value: B
Chryseobacterium.hungaricum
3
2
1
0
A (n=14)
B (n=15)
C (n=11)
surface

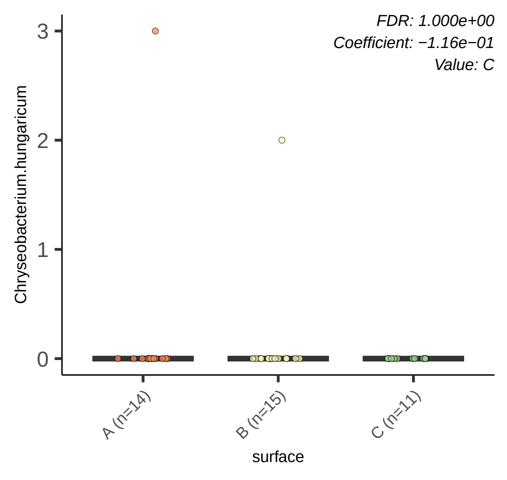
FDR: 1.000e+00
Coefficient: −1.16e−01
Value: C
Chryseobacterium.hungaricum
3
2
1
0
A (n=14)
B (n=15)
C (n=11)
surface

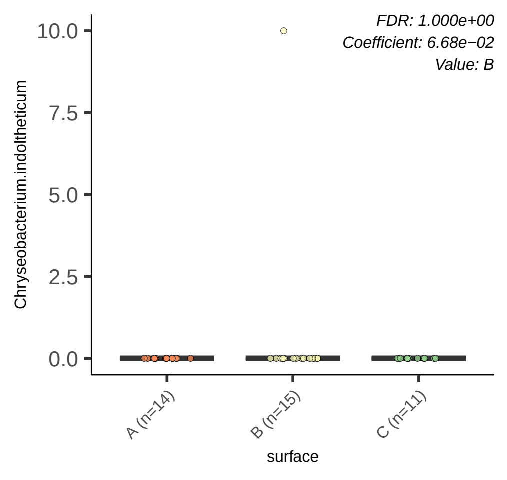

FDR: 1.000e+00
Coefficient: 6.68e−02
Value: B
10.0
7.5
5.0
2.5
0.0
Chryseobacterium.indoltheticum
A (n=14)
B (n=15)
C (n=11)
surface

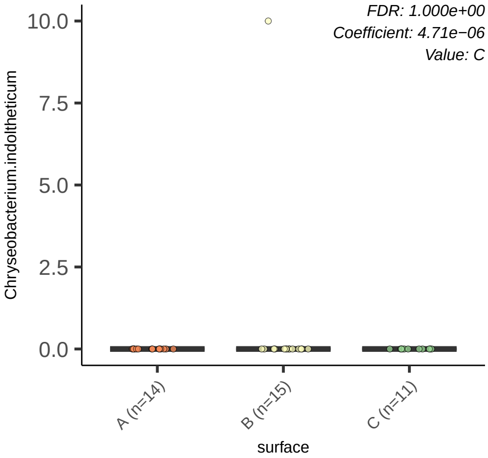
FDR: 1.000e+00
Coefficient: 4.71e−06
Value: C
10.0
7.5
5.0
2.5
0.0
Chryseobacterium.indoltheticum
A (n=14)
B (n=15)
C (n=11)
surface

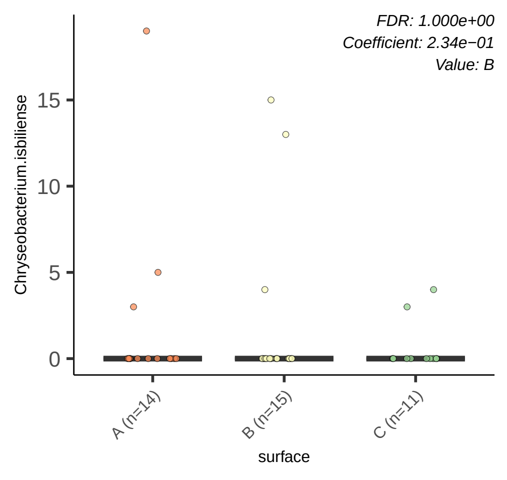
FDR: 1.000e+00
Coefficient: 2.34e−01
Value: B
Chryseobacterium.isbiliense
0
5
10
15
A (n=14)
B (n=15)
C (n=11)
surface

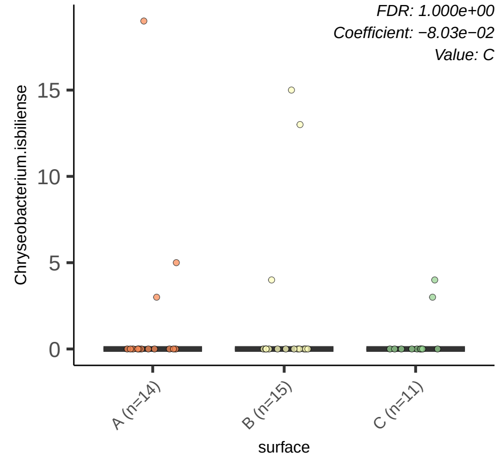

FDR: 1.000e+00
Coefficient: −8.03e−02
Value: C
Chryseobacterium.isbiliense
15
10
5
0
A (n=14)
B (n=15)
C (n=11)
surface

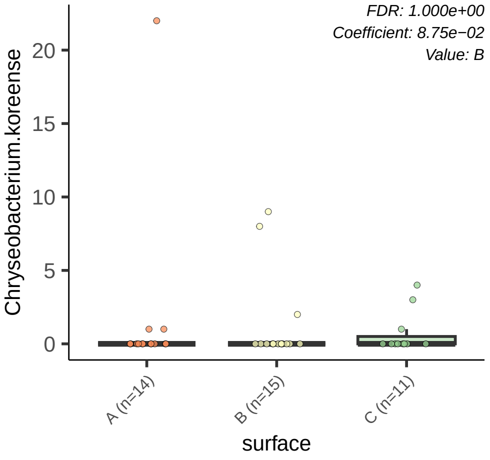
FDR: 1.000e+00
Coefficient: 8.75e−02
Value: B
Chryseobacterium.koreense
0
5
10
15
20
A (n=14)
B (n=15)
C (n=11)
surface

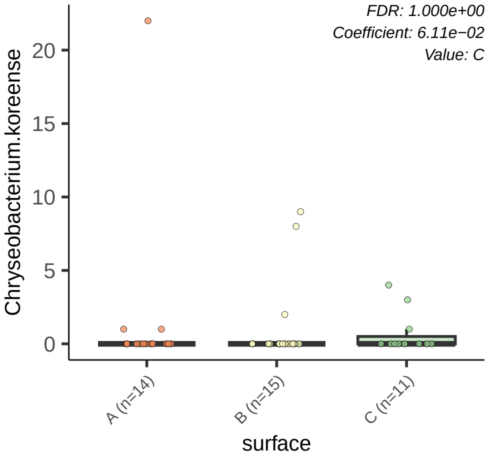

FDR: 1.000e+00
Coefficient: 6.11e−02
Value: C
Chryseobacterium.koreense
0
5
10
15
20
A (n=14)
B (n=15)
C (n=11)
surface

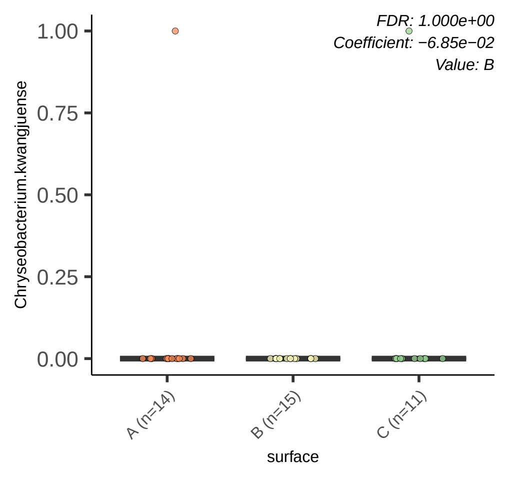
FDR: 1.000e+00
Coefficient: −6.85e−02
Value: B
Chryseobacterium.kwangjuense
1.00
0.75
0.50
0.25
0.00
A (n=14)
B (n=15)
C (n=11)
surface

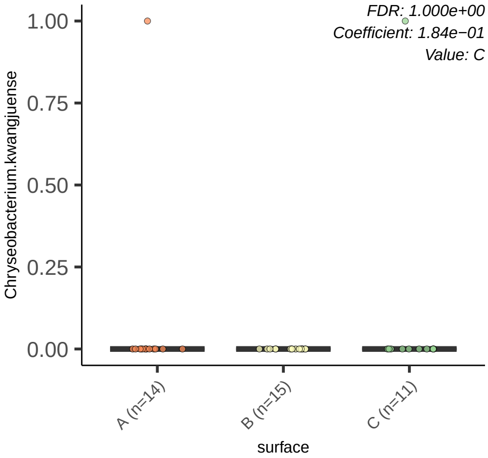
FDR: 1.000e+00
Coefficient: 1.84e−01
Value: C
Chryseobacterium.kwangjuense
1.00
0.75
0.50
0.25
0.00
A (n=14)
B (n=15)
C (n=11)
surface

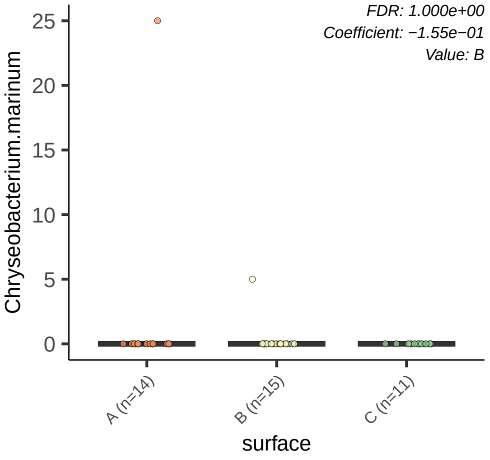
FDR: 1.000e+00
Coefficient: −1.55e−01
Value: B
Chryseobacterium.marinum
25
20
15
10
5
0
A (n=14)
B (n=15)
C (n=11)
surface

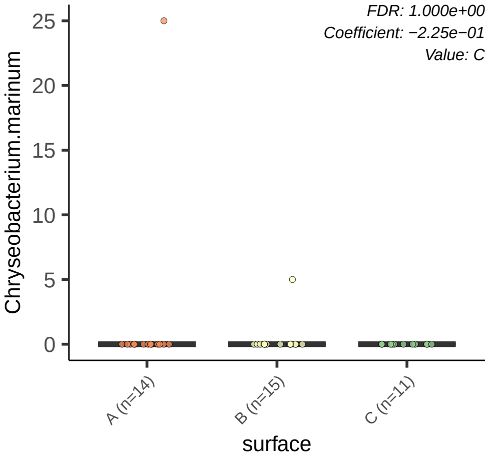

FDR: 1.000e+00
Coefficient: −2.25e−01
Value: C
Chryseobacterium.marinum
0
5
10
15
20
25
A (n=14)
B (n=15)
C (n=11)
surface

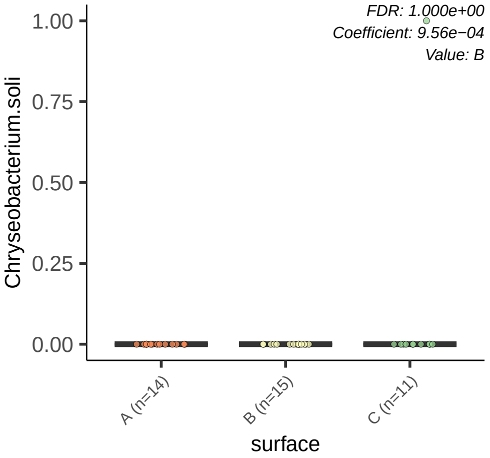

FDR: 1.000e+00
Coefficient: 9.56e−04
Value: B
Chryseobacterium.soli
1.00
0.75
0.50
0.25
0.00
A (n=14)
B (n=15)
C (n=11)
surface

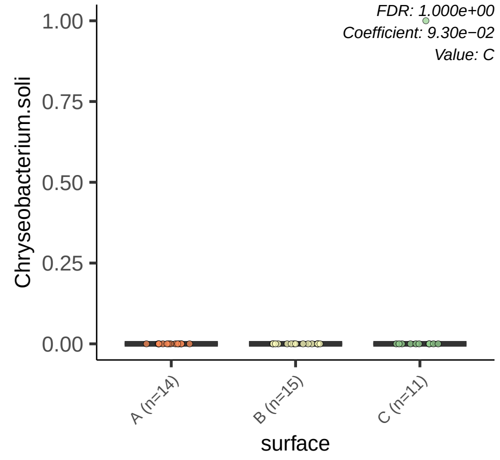
FDR: 1.000e+00
Coefficient: 9.30e−02
Value: C
1.00
0.75
0.50
0.25
0.00
Chryseobacterium.soli
A (n=14)
B (n=15)
C (n=11)
surface

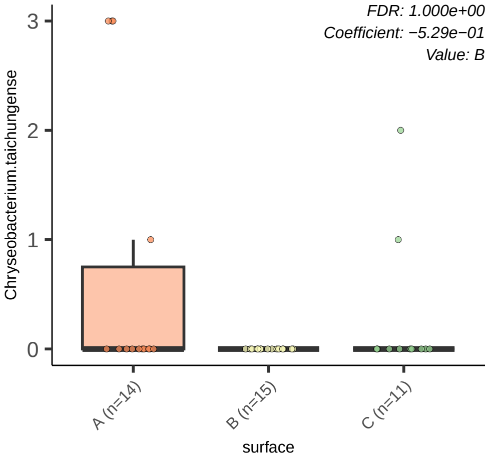
FDR: 1.000e+00
Coefficient: −5.29e−01
Value: B
Chryseobacterium.taichungense
3
2
1
0
A (n=14)
B (n=15)
C (n=11)
surface

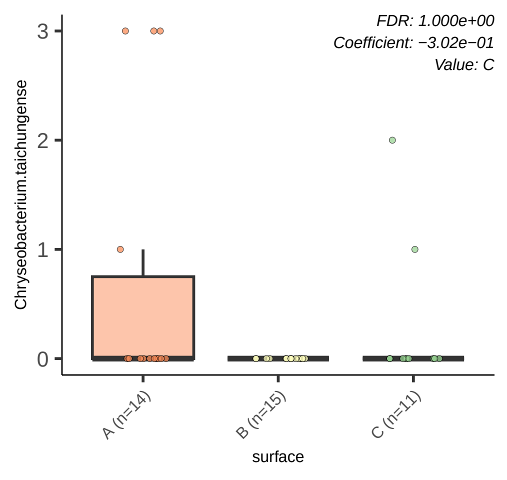
FDR: 1.000e+00
Coefficient: −3.02e−01
Value: C
Chryseobacterium.taichungense
3
2
1
0
A (n=14)
B (n=15)
C (n=11)
surface

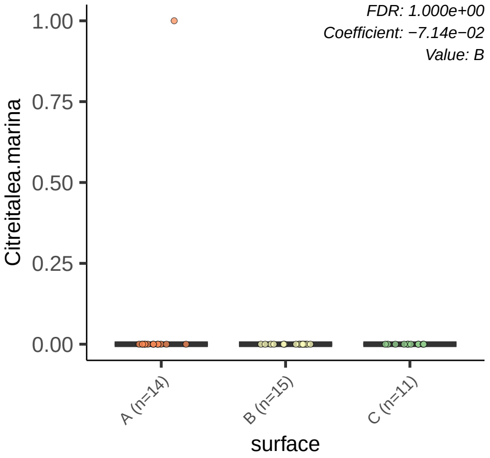
FDR: 1.000e+00
Coefficient: −7.14e−02
Value: B
Citreitalea.marina
1.00
0.75
0.50
0.25
0.00
A (n=14)
B (n=15)
C (n=11)
surface

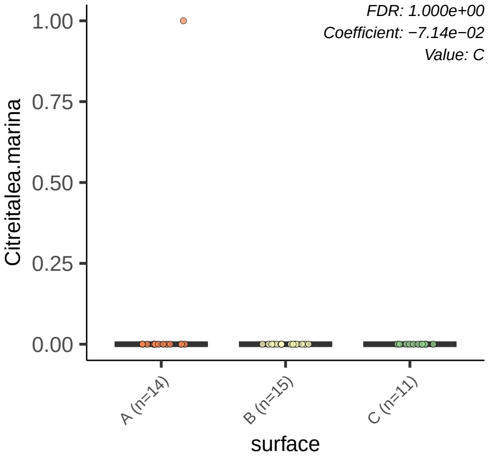
FDR: 1.000e+00
Coefficient: −7.14e−02
Value: C
Citreitalea.marina
1.00
0.75
0.50
0.25
0.00
A (n=14)
B (n=15)
C (n=11)
surface

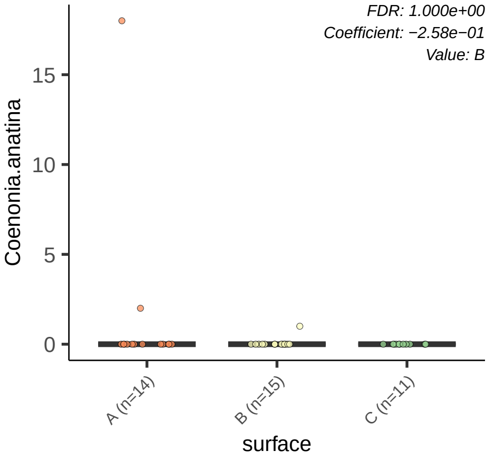

FDR: 1.000e+00
Coefficient: −2.58e−01
Value: B
Coenonia.anatina
15
10
5
0
A (n=14)
B (n=15)
C (n=11)
surface

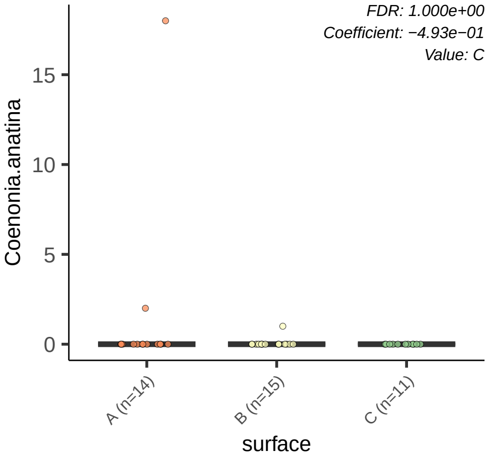

FDR: 1.000e+00
Coefficient: −4.93e−01
Value: C
Coenonia.anatina
0
5
10
15
A (n=14)
B (n=15)
C (n=11)
surface

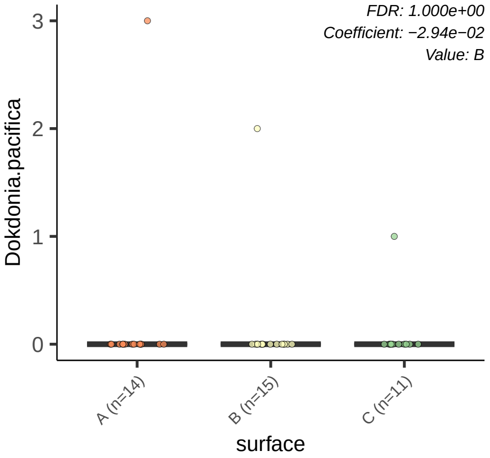

FDR: 1.000e+00
Coefficient: −2.94e−02
Value: B
Dokdonia.pacifica
3
2
1
0
A (n=14)
B (n=15)
C (n=11)
surface

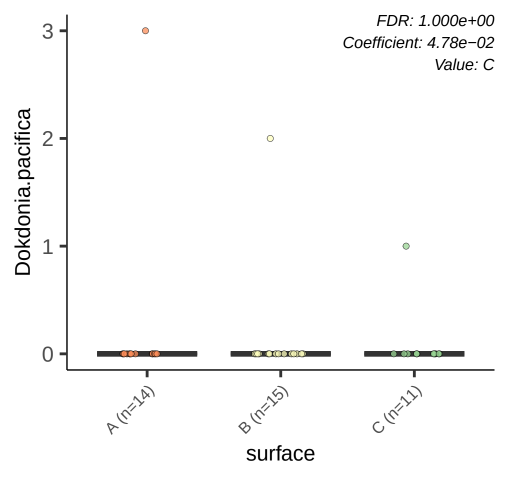
FDR: 1.000e+00
Coefficient: 4.78e−02
Value: C
Dokdonia.pacifica
0
1
2
3
A (n=14)
B (n=15)
C (n=11)
surface

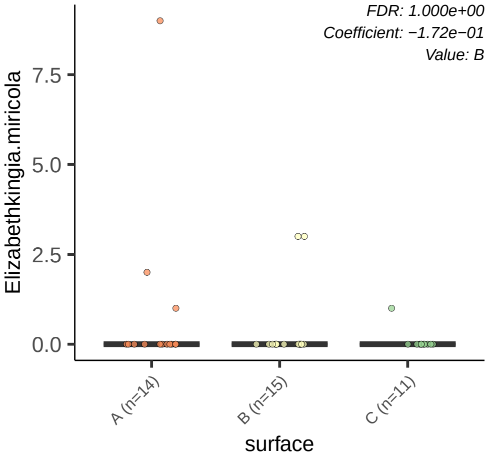
FDR: 1.000e+00
Coefficient: −1.72e−01
Value: B
Elizabethkingia.miricola
7.5
5.0
2.5
0.0
A (n=14)
B (n=15)
C (n=11)
surface

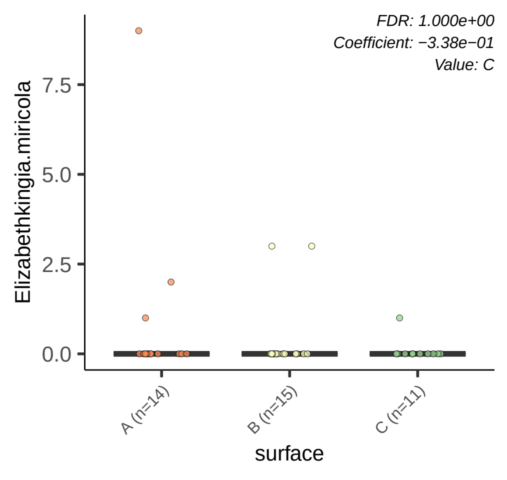
FDR: 1.000e+00
Coefficient: −3.38e−01
Value: C
Elizabethkingia.miricola
7.5
5.0
2.5
0.0
A (n=14)
B (n=15)
C (n=11)
surface

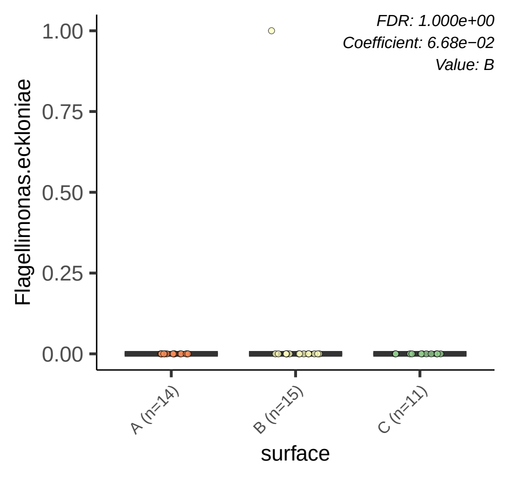

FDR: 1.000e+00
Coefficient: 6.68e−02
Value: B
Flagellimonas.eckloniae
1.00
0.75
0.50
0.25
0.00
A (n=14)
B (n=15)
C (n=11)
surface

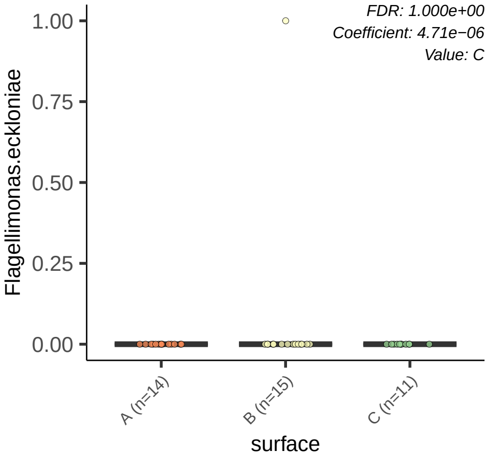

FDR: 1.000e+00
Coefficient: 4.71e−06
Value: C
Flagellimonas.ecklonia e
1.00
0.75
0.50
0.25
0.00
A (n=14)
B (n=15)
C (n=11)
surface

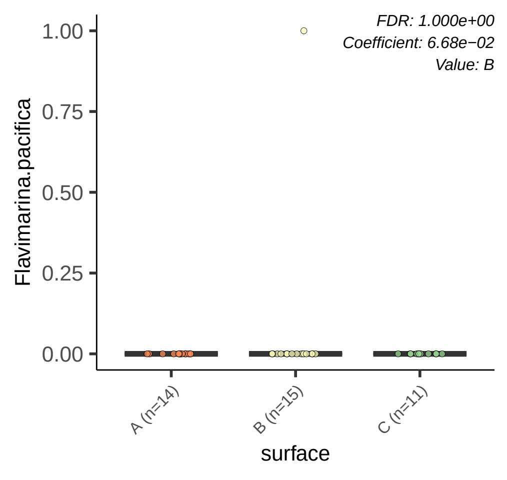
FDR: 1.000e+00
Coefficient: 6.68e−02
Value: B
Flavimarina.pacifica
1.00
0.75
0.50
0.25
0.00
A (n=14)
B (n=15)
C (n=11)
surface

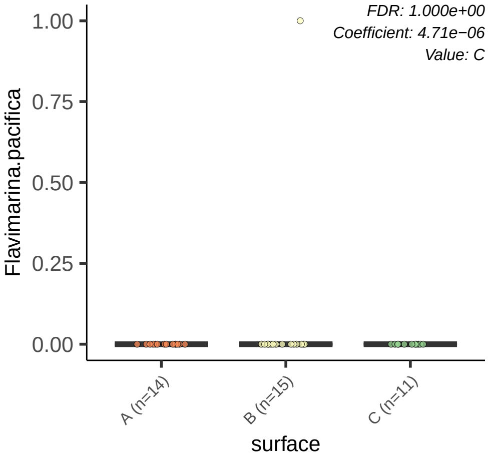

FDR: 1.000e+00
Coefficient: 4.71e−06
Value: C
Flavimarina.pacifica
1.00
0.75
0.50
0.25
0.00
A (n=14)
B (n=15)
C (n=11)
surface

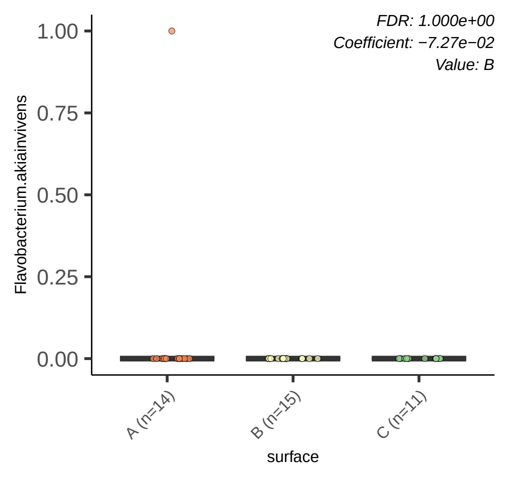
FDR: 1.000e+00
Coefficient: −7.27e−02
Value: B
Flavobacterium.akiainvivens
1.00
0.75
0.50
0.25
0.00
A (n=14)
B (n=15)
C (n=11)
surface

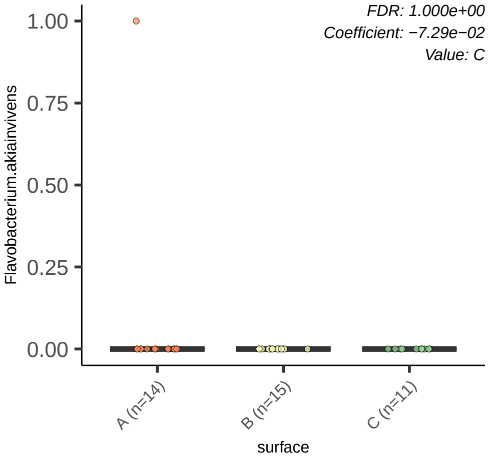
FDR: 1.000e+00
Coefficient: −7.29e−02
Value: C
Flavobacterium.akiainvivens
1.00
0.75
0.50
0.25
0.00
A (n=14)
B (n=15)
C (n=11)
surface

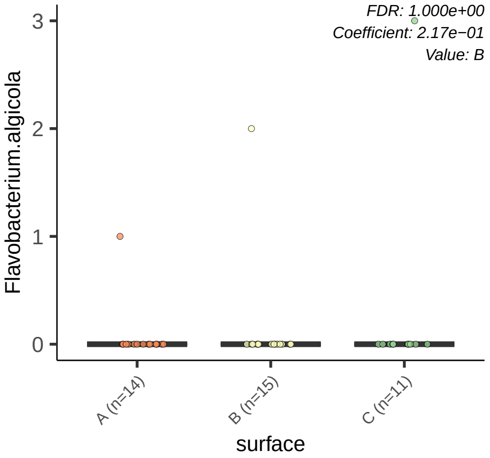
FDR: 1.000e+00
Coefficient: 2.17e−01
Value: B
Flavobacterium.algicola
3
2
1
0
A (n=14)
B (n=15)
C (n=11)
surface

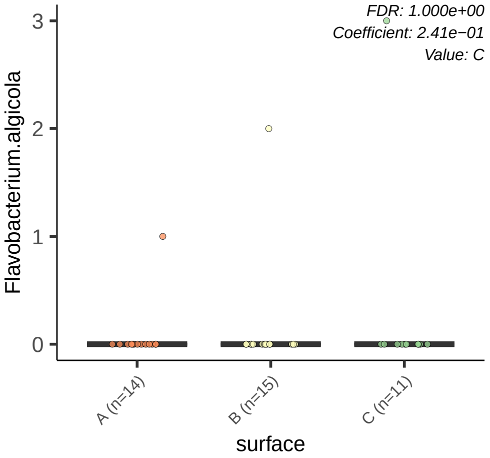
FDR: 1.000e+00
Coefficient: 2.41e−01
Value: C
Flavobacterium.algicola
0
1
2
3
A (n=14)
B (n=15)
C (n=11)
surface

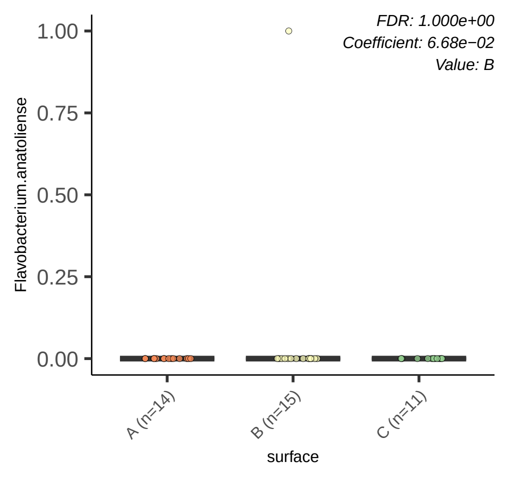
FDR: 1.000e+00
Coefficient: 6.68e−02
Value: B
1.00
0.75
0.50
0.25
0.00
Flavobacterium.anatoliense
A (n=14)
B (n=15)
C (n=11)
surface

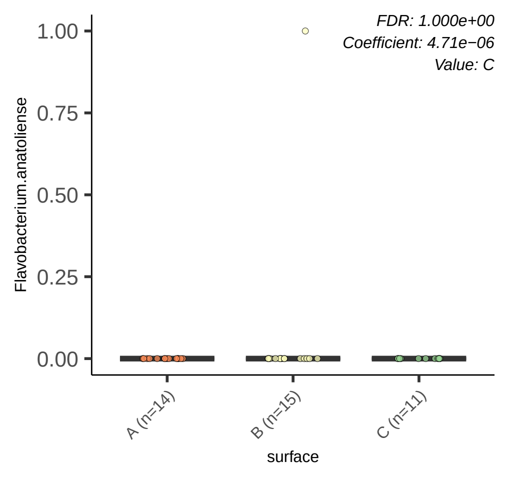
FDR: 1.000e+00
Coefficient: 4.71e−06
Value: C
Flavobacterium.anatoliense
1.00
0.75
0.50
0.25
0.00
A (n=14)
B (n=15)
C (n=11)
surface

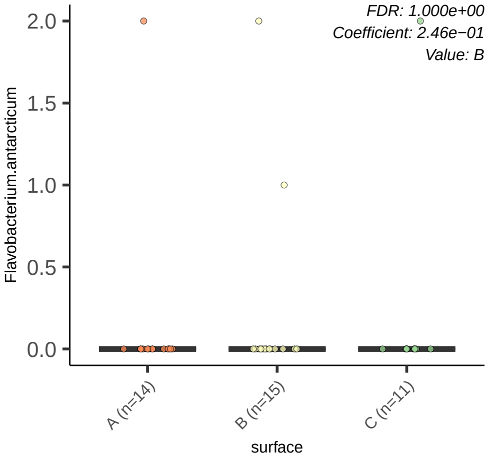

FDR: 1.000e+00
Coefficient: 2.46e−01
Value: B
Flavobacterium.antarcticum
2.0
1.5
1.0
0.5
0.0
A (n=14)
B (n=15)
C (n=11)
surface

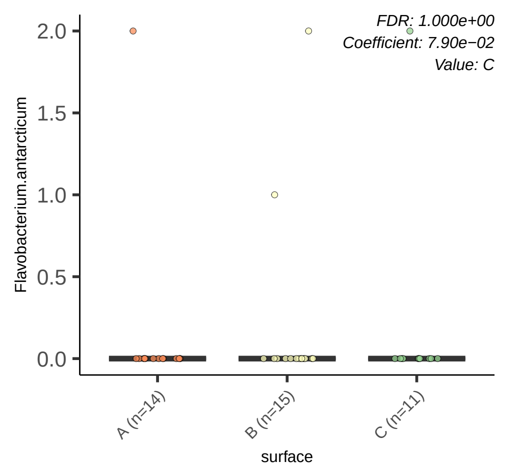

FDR: 1.000e+00
Coefficient: 7.90e−02
Value: C
Flavobacterium.antarcticum
2.0
1.5
1.0
0.5
0.0
A (n=14)
B (n=15)
C (n=11)
surface

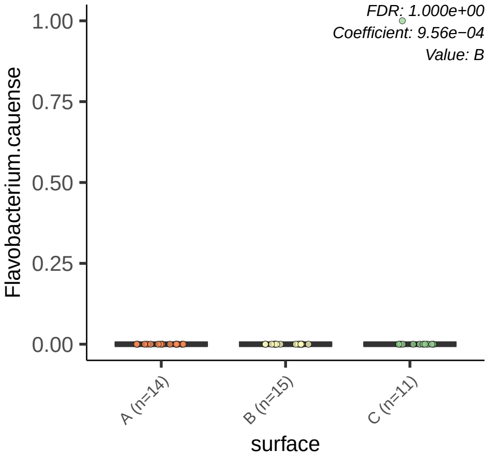

FDR: 1.000e+00
Coefficient: 9.56e−04
Value: B
1.00
0.75
0.50
0.25
0.00
Flavobacterium.cauense
A (n=14)
B (n=15)
C (n=11)
surface

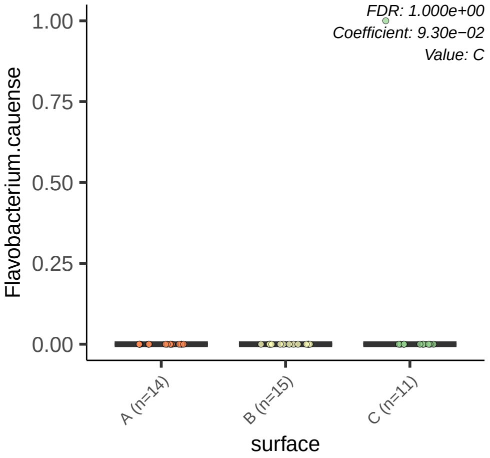
FDR: 1.000e+00
Coefficient: 9.30e−02
Value: C
Flavobacterium.cauense
1.00
0.75
0.50
0.25
0.00
A (n=14)
B (n=15)
C (n=11)
surface

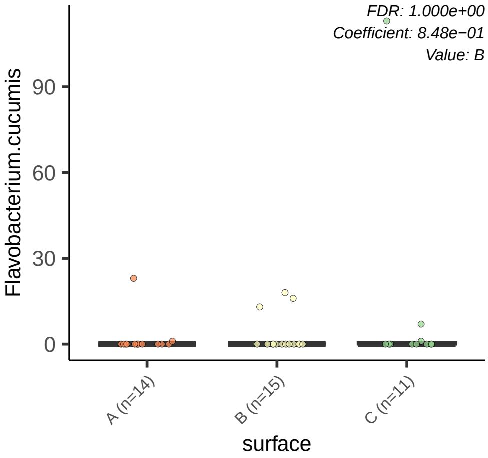
FDR: 1.000e+00
Coefficient: 8.48e−01
Value: B
Flavobacterium.cucumis
90
60
30
0
A (n=14)
B (n=15)
C (n=11)
surface

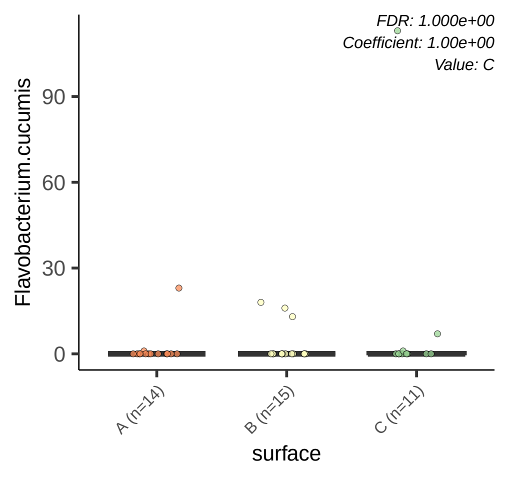

FDR: 1.000e+00
Coefficient: 1.00e+00
Value: C
Flavobacterium.cucumis
90
60
30
0
A (n=14)
B (n=15)
C (n=11)
surface

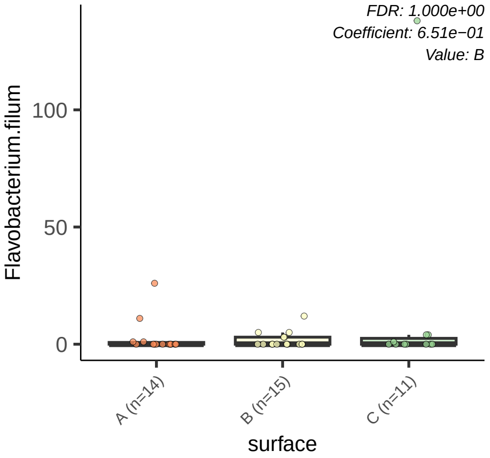
FDR: 1.000e+00
Coefficient: 6.51e−01
Value: B
Flavobacterium.filum
100
50
0
A (n=14)
B (n=15)
C (n=11)
surface

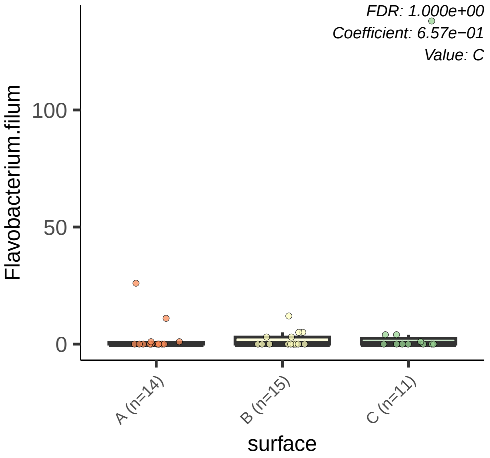
FDR: 1.000e+00
Coefficient: 6.57e−01
Value: C
Flavobacterium.filum
100
50
0
A (n=14)
B (n=15)
C (n=11)
surface

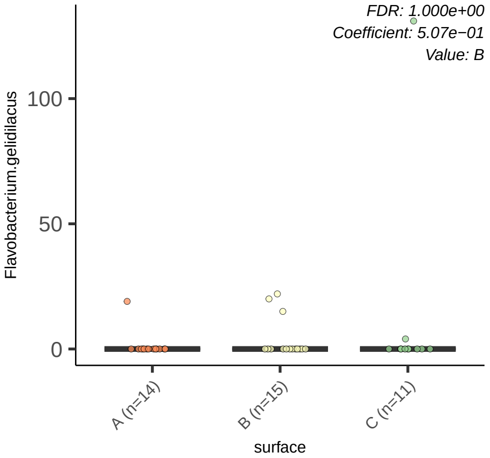

FDR: 1.000e+00
Coefficient: 5.07e−01
Value: B
Flavobacterium.gelidilacus
100
50
0
A (n=14)
B (n=15)
C (n=11)
surface

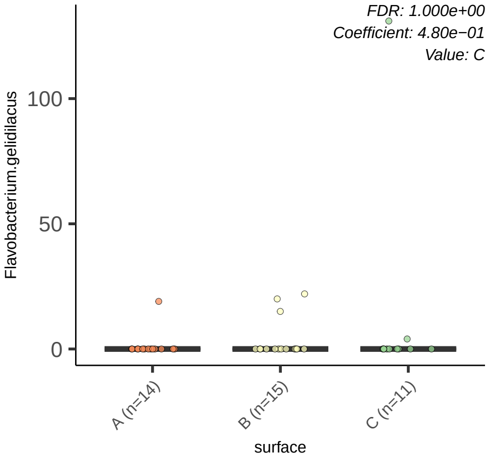
FDR: 1.000e+00
Coefficient: 4.80e−01
Value: C
Flavobacterium.gelidilacus
100
50
0
A (n=14)
B (n=15)
C (n=11)
surface

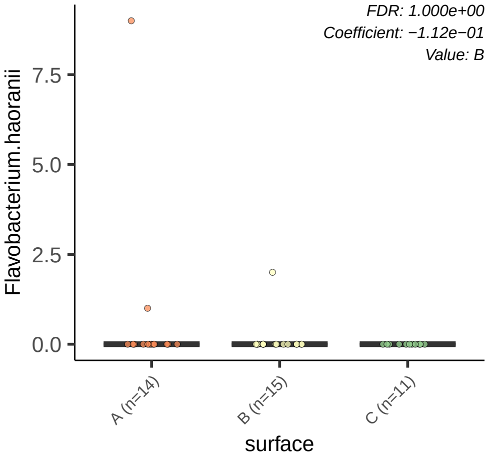

FDR: 1.000e+00
Coefficient: −1.12e−01
Value: B
Flavobacterium.haoranii
7.5
5.0
2.5
0.0
A (n=14)
B (n=15)
C (n=11)
surface

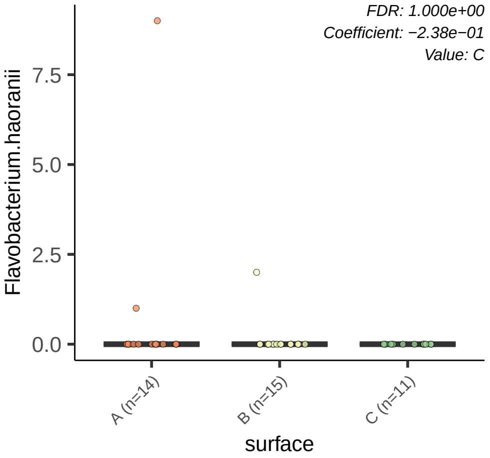
FDR: 1.000e+00
Coefficient: −2.38e−01
Value: C
Flavobacterium.haoranii
0.0
2.5
5.0
7.5
A (n=14)
B (n=15)
C (n=11)
surface

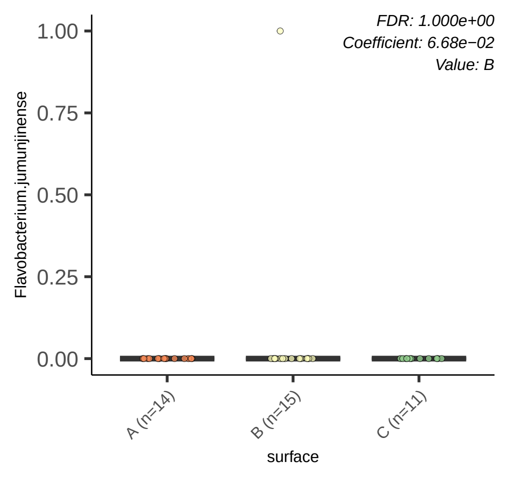

FDR: 1.000e+00
Coefficient: 6.68e−02
Value: B
Flavobacterium.jumunjinense
1.00
0.75
0.50
0.25
0.00
A (n=14)
B (n=15)
C (n=11)
surface

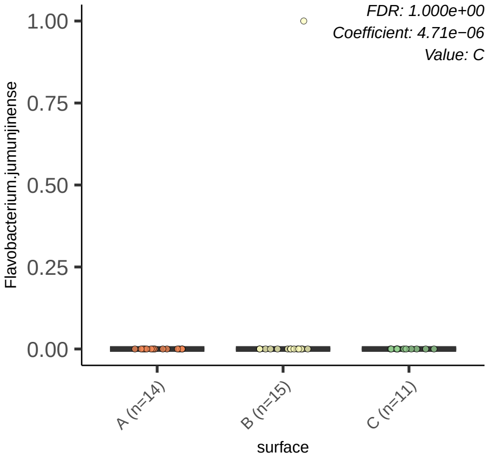

FDR: 1.000e+00
Coefficient: 4.71e−06
Value: C
Flavobacterium.jumunjinense
1.00
0.75
0.50
0.25
0.00
A (n=14)
B (n=15)
C (n=11)
surface

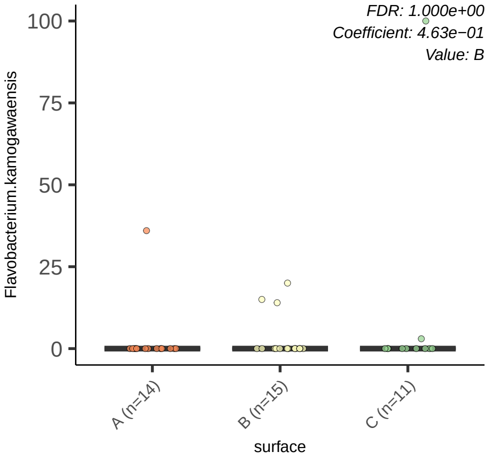
FDR: 1.000e+00
Coefficient: 4.63e−01
Value: B
Flavobacterium.kamogawaensis
100
75
50
25
0
A (n=14)
B (n=15)
C (n=11)
surface

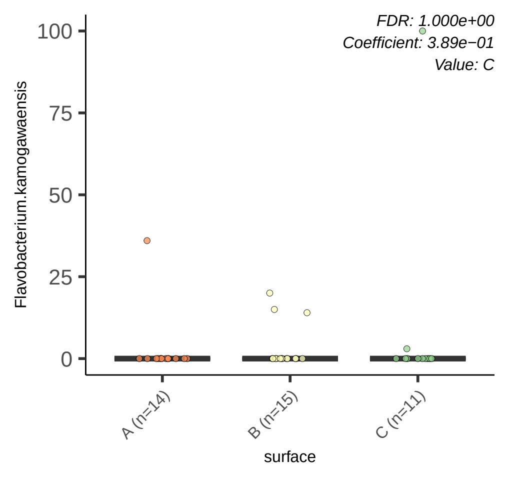
FDR: 1.000e+00
Coefficient: 3.89e−01
Value: C
100
75
50
25
0
Flavobacterium.kamogawaensis
A (n=14)
B (n=15)
C (n=11)
surface

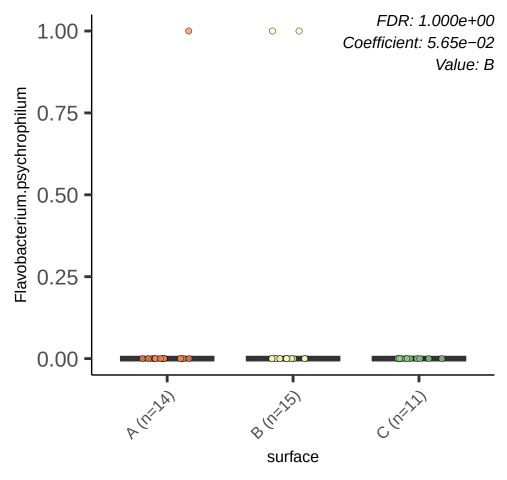
FDR: 1.000e+00
Coefficient: 5.65e−02
Value: B
Flavobacterium.psychrophilum
1.00
0.75
0.50
0.25
0.00
A (n=14)
B (n=15)
C (n=11)
surface

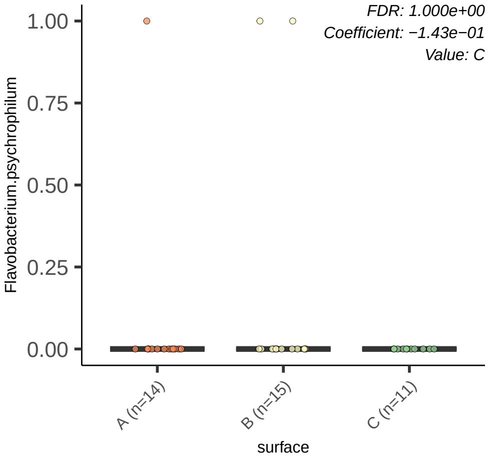

FDR: 1.000e+00
Coefficient: −1.43e−01
Value: C
Flavobacterium.psychrophilum
1.00
0.75
0.50
0.25
0.00
A (n=14)
B (n=15)
C (n=11)
surface

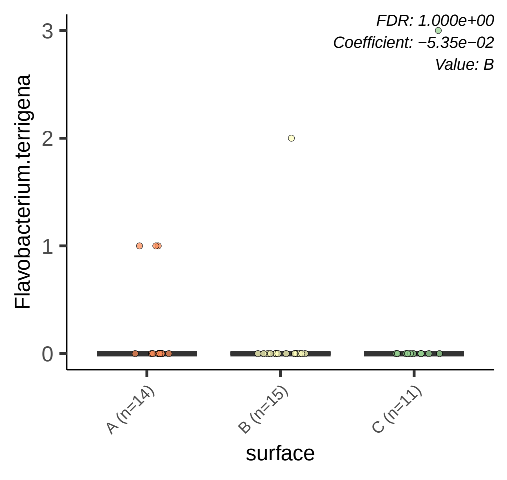

FDR: 1.000e+00
Coefficient: −5.35e−02
Value: B
Flavobacterium.terrigena
3
2
1
0
A (n=14)
B (n=15)
C (n=11)
surface

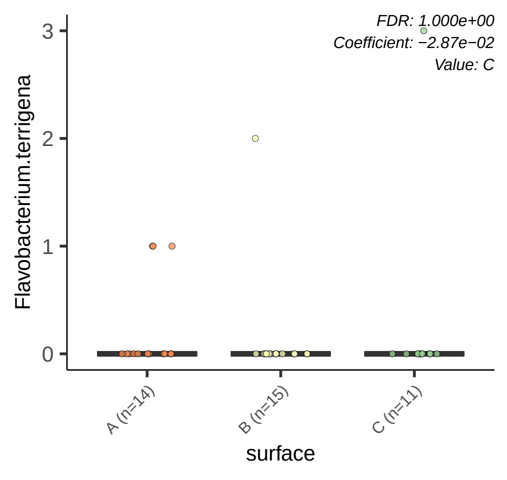

FDR: 1.000e+00
Coefficient: −2.87e−02
Value: C
Flavobacterium.terrigena
3
2
1
0
A (n=14)
B (n=15)
C (n=11)
surface

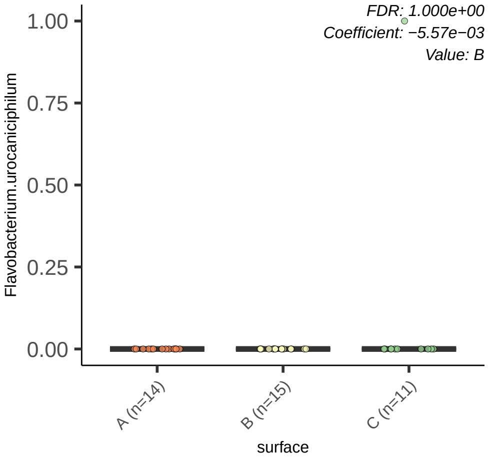

FDR: 1.000e+00
Coefficient: −5.57e−03
Value: B
Flavobacterium.urocaniciphilum
1.00
0.75
0.50
0.25
0.00
A (n=14)
B (n=15)
C (n=11)
surface

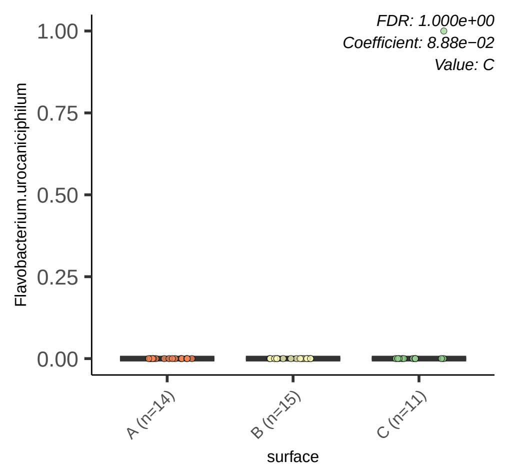
FDR: 1.000e+00
Coefficient: 8.88e−02
Value: C
Flavobacterium.urocaniciphilum
1.00
0.75
0.50
0.25
0.00
A (n=14)
B (n=15)
C (n=11)
surface

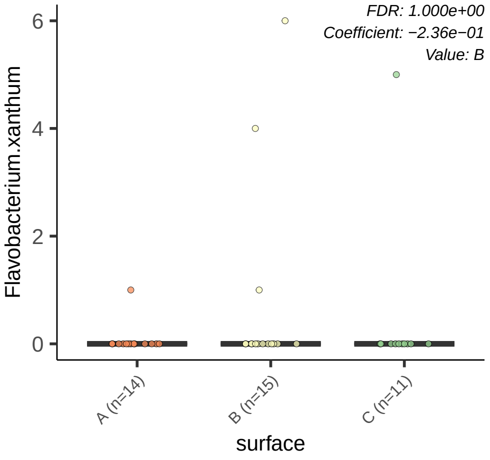
FDR: 1.000e+00
Coefficient: −2.36e−01
Value: B
Flavobacterium.xanthum
6
4
2
0
A (n=14)
B (n=15)
C (n=11)
surface

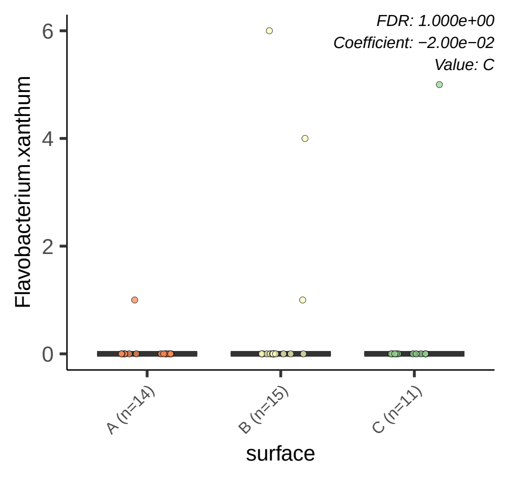
FDR: 1.000e+00
Coefficient: −2.00e−02
Value: C
Flavobacterium.xanthum
0
2
4
6
A (n=14)
B (n=15)
C (n=11)
surface

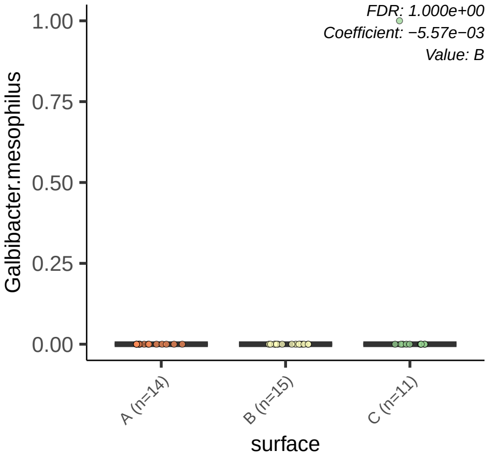

FDR: 1.000e+00
Coefficient: −5.57e−03
Value: B
Galbibacter.mesophilus
1.00
0.75
0.50
0.25
0.00
A (n=14)
B (n=15)
C (n=11)
surface

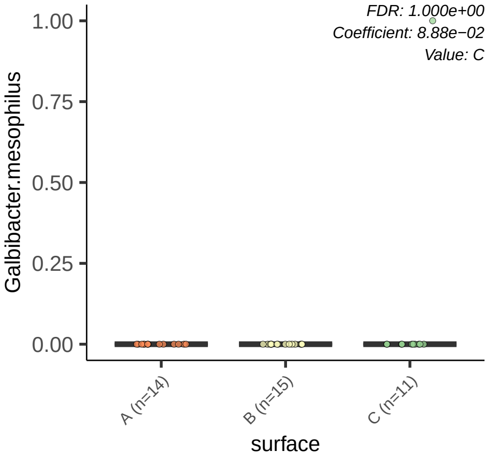
FDR: 1.000e+00
Coefficient: 8.88e−02
Value: C
Galbibacter.mesophilus
1.00
0.75
0.50
0.25
0.00
A (n=14)
B (n=15)
C (n=11)
surface

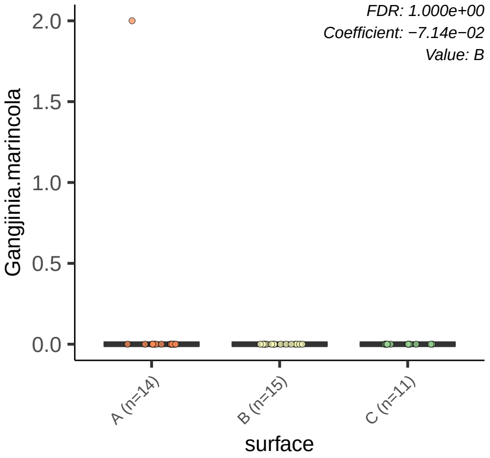
FDR: 1.000e+00
Coefficient: −7.14e−02
Value: B
Gangjinia.marincola
2.0
1.5
1.0
0.5
0.0
A (n=14)
B (n=15)
C (n=11)
surface

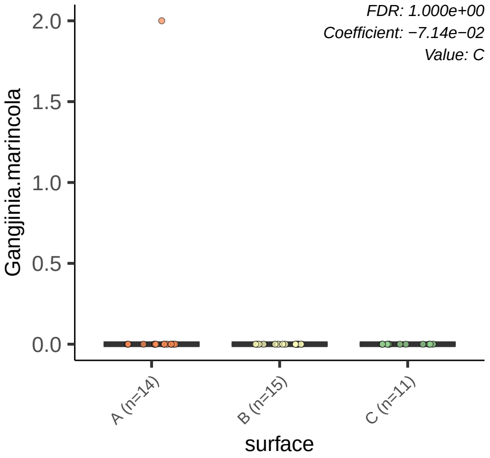
FDR: 1.000e+00
Coefficient: −7.14e−02
Value: C
Gangjinia.marincola
2.0
1.5
1.0
0.5
0.0
A (n=14)
B (n=15)
C (n=11)
surface

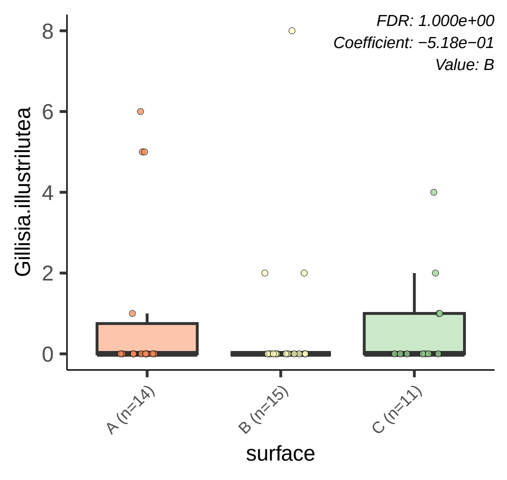
FDR: 1.000e+00
Coefficient: −5.18e−01
Value: B
Gillisia.illustrilutea
0
2
4
6
8
A (n=14)
B (n=15)
C (n=11)
surface

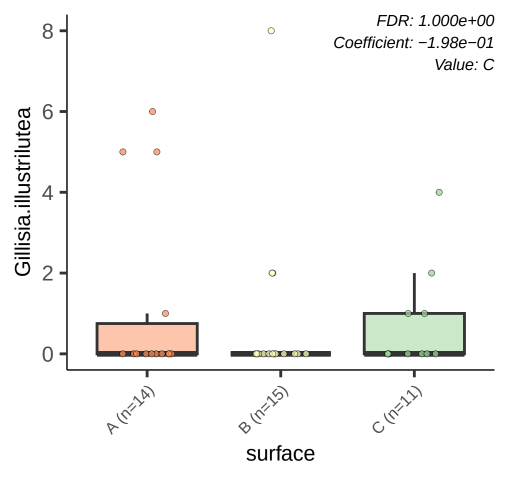

FDR: 1.000e+00
Coefficient: −1.98e−01
Value: C
Gillisia.illustrilutea
8
6
4
2
0
A (n=14)
B (n=15)
C (n=11)
surface

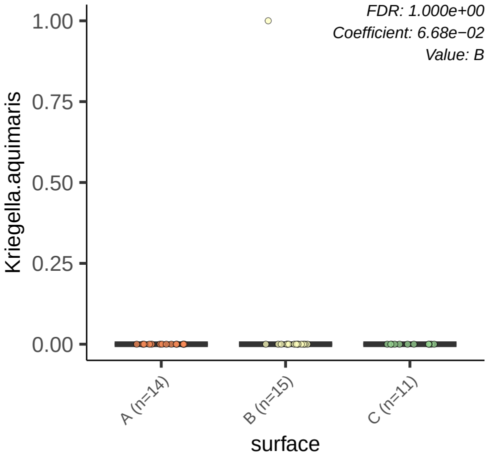
FDR: 1.000e+00
Coefficient: 6.68e−02
Value: B
Kriegella.aquimaris
1.00
0.75
0.50
0.25
0.00
A (n=14)
B (n=15)
C (n=11)
surface

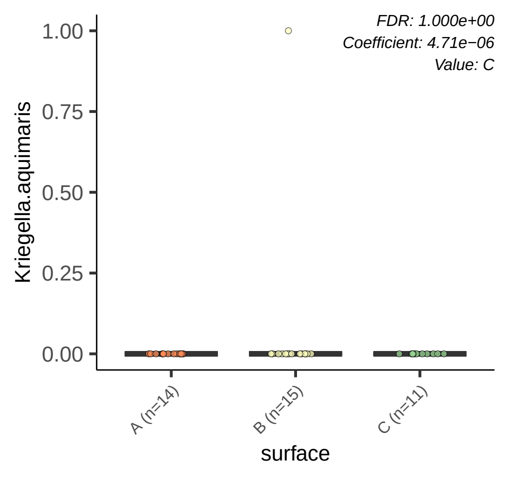
FDR: 1.000e+00
Coefficient: 4.71e−06
Value: C
Kriegella.aquimaris
1.00
0.75
0.50
0.25
0.00
A (n=14)
B (n=15)
C (n=11)
surface

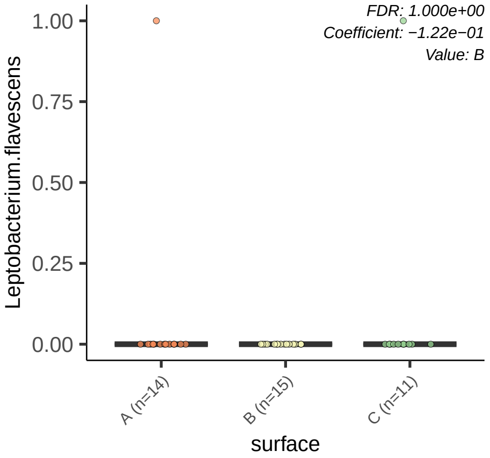

FDR: 1.000e+00
Coefficient: −1.22e−01
Value: B
Leptobacterium.flavescens
1.00
0.75
0.50
0.25
0.00
A (n=14)
B (n=15)
C (n=11)
surface

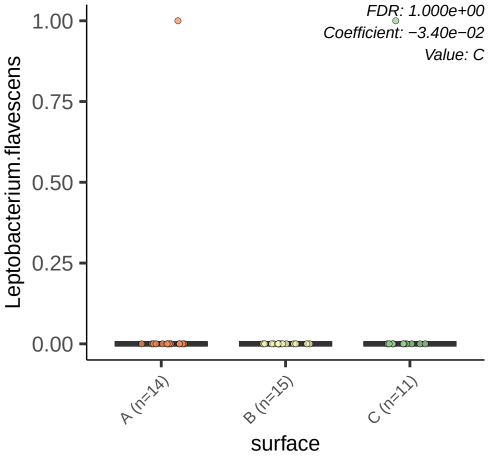

FDR: 1.000e+00
Coefficient: −3.40e−02
Value: C
Leptobacterium.flavescens
1.00
0.75
0.50
0.25
0.00
A (n=14)
B (n=15)
C (n=11)
surface

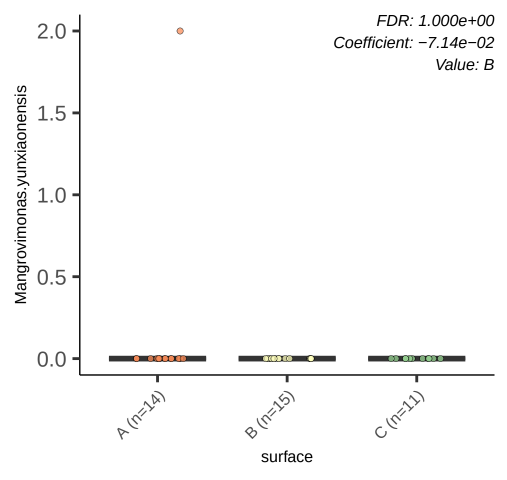
FDR: 1.000e+00
Coefficient: −7.14e−02
Value: B
Mangrovimonas.yunxiaonensis
2.0
1.5
1.0
0.5
0.0
A (n=14)
B (n=15)
C (n=11)
surface

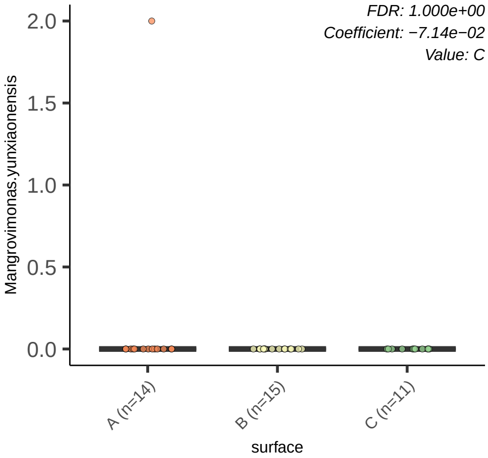

FDR: 1.000e+00
Coefficient: −7.14e−02
Value: C
Mangrovimonas.yunxiaonensis
2.0
1.5
1.0
0.5
0.0
A (n=14)
B (n=15)
C (n=11)
surface

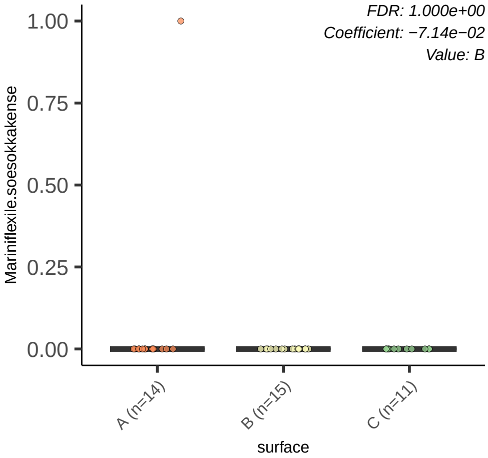

FDR: 1.000e+00
Coefficient: −7.14e−02
Value: B
Mariniflexile.soesokkakense
1.00
0.75
0.50
0.25
0.00
A (n=14)
B (n=15)
C (n=11)
surface

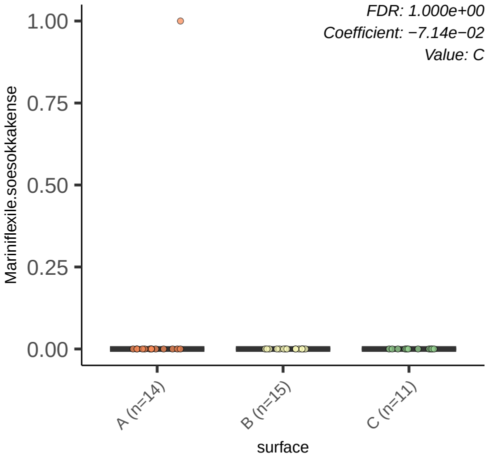
FDR: 1.000e+00
Coefficient: −7.14e−02
Value: C
Mariniflexile.soesokkakense
1.00
0.75
0.50
0.25
0.00
A (n=14)
B (n=15)
C (n=11)
surface

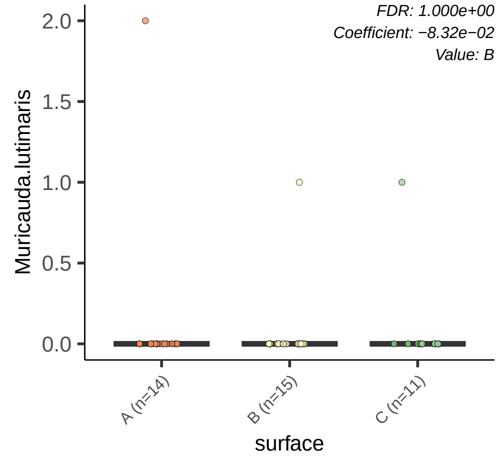

FDR: 1.000e+00
Coefficient: −8.32e−02
Value: B
Muricauda.lutimaris
2.0
1.5
1.0
0.5
0.0
A (n=14)
B (n=15)
C (n=11)
surface

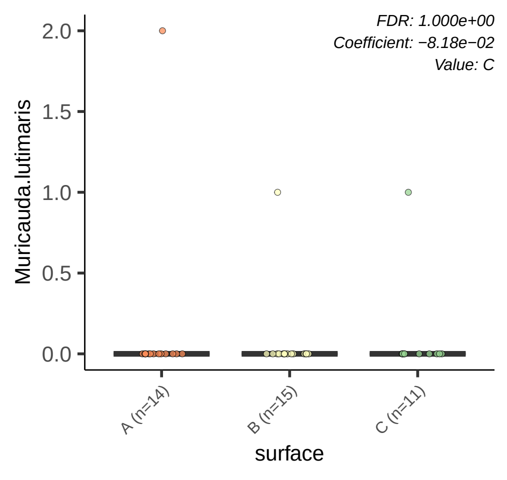

FDR: 1.000e+00
Coefficient: −8.18e−02
Value: C
Muricauda.lutimaris
2.0
1.5
1.0
0.5
0.0
A (n=14)
B (n=15)
C (n=11)
surface

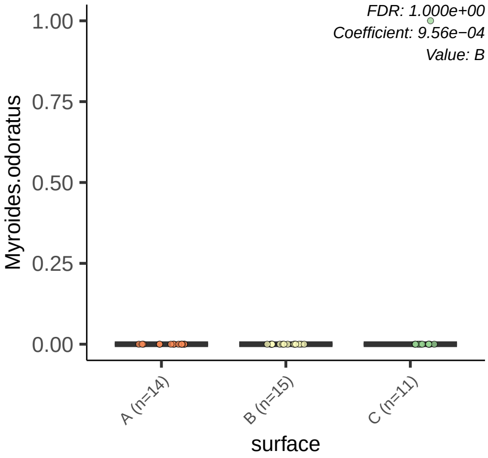
FDR: 1.000e+00
Coefficient: 9.56e−04
Value: B
Myroides.odoratus
1.00
0.75
0.50
0.25
0.00
A (n=14)
B (n=15)
C (n=11)
surface

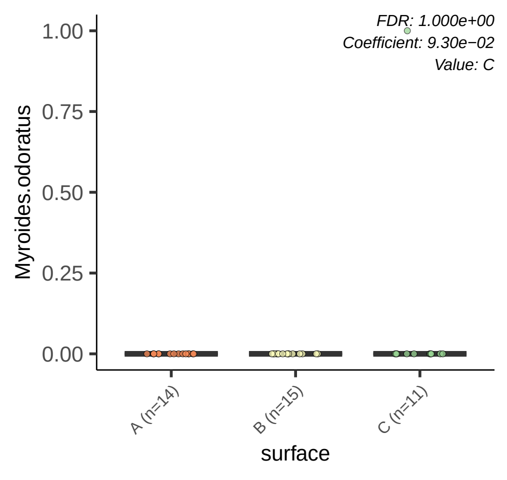
FDR: 1.000e+00
Coefficient: 9.30e−02
Value: C
1.00
0.75
0.50
0.25
0.00
Myroides.odoratus
A (n=14)
B (n=15)
C (n=11)
surface

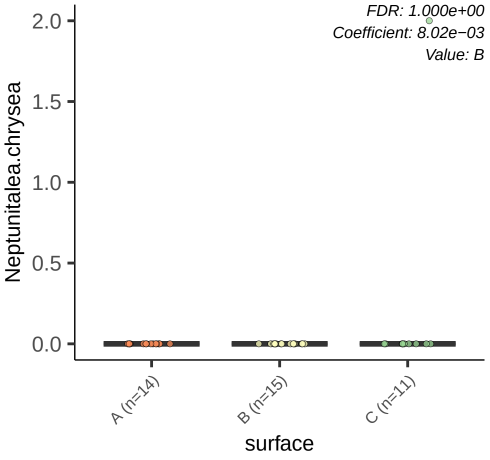
FDR: 1.000e+00
Coefficient: 8.02e−03
Value: B
Neptunitalea.chrysea
2.0
1.5
1.0
0.5
0.0
A (n=14)
B (n=15)
C (n=11)
surface

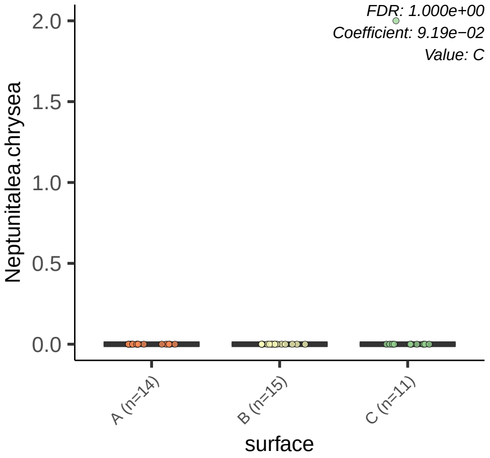

FDR: 1.000e+00
Coefficient: 9.19e−02
Value: C
Neptunitalea.chrysea
2.0
1.5
1.0
0.5
0.0
A (n=14)
B (n=15)
C (n=11)
surface

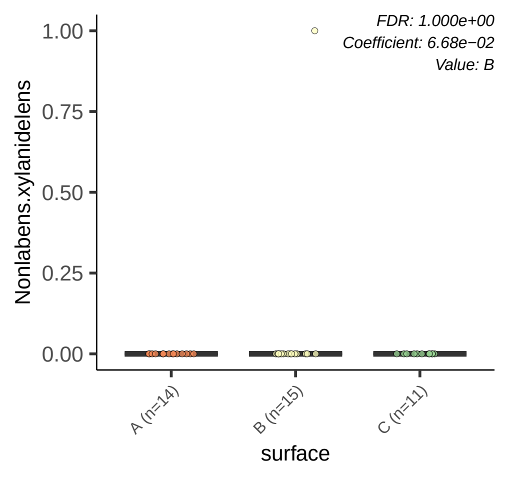
FDR: 1.000e+00
Coefficient: 6.68e−02
Value: B
Nonlabens.xylanidelens
1.00
0.75
0.50
0.25
0.00
A (n=14)
B (n=15)
C (n=11)
surface

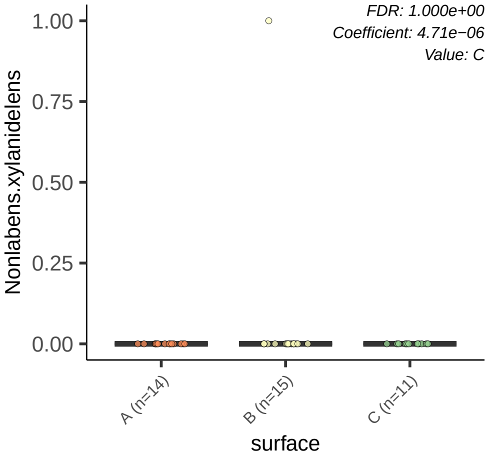
FDR: 1.000e+00
Coefficient: 4.71e−06
Value: C
Nonlabens.xylanidelens
1.00
0.75
0.50
0.25
0.00
A (n=14)
B (n=15)
C (n=11)
surface

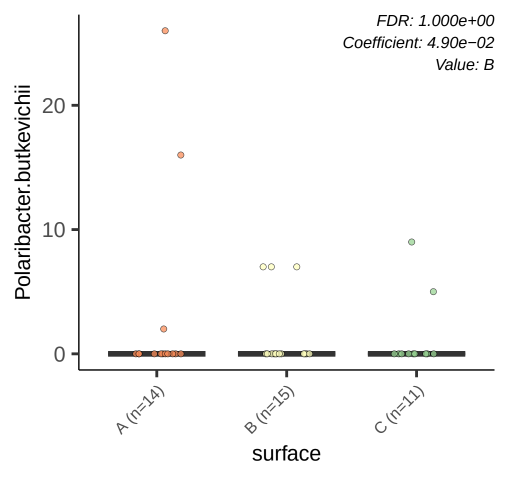
FDR: 1.000e+00
Coefficient: 4.90e−02
Value: B
Polaribacter.butkevichii
0
10
20
A (n=14)
B (n=15)
C (n=11)
surface

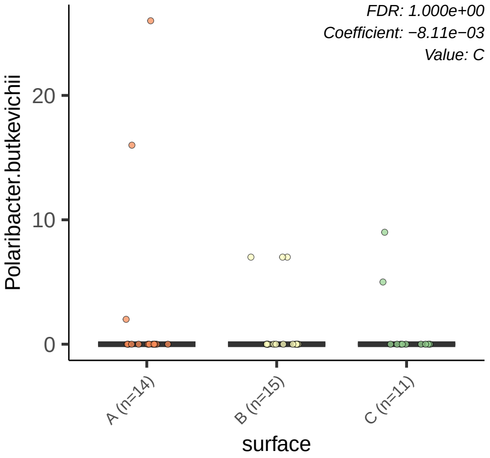
FDR: 1.000e+00
Coefficient: −8.11e−03
Value: C
Polaribacter.butkevichii
20
10
0
A (n=14)
B (n=15)
C (n=11)
surface

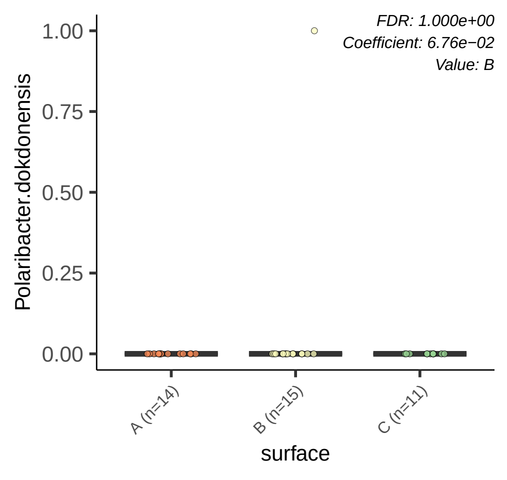
FDR: 1.000e+00
Coefficient: 6.76e−02
Value: B
Polaribacter.dokdonensis
1.00
0.75
0.50
0.25
0.00
A (n=14)
B (n=15)
C (n=11)
surface

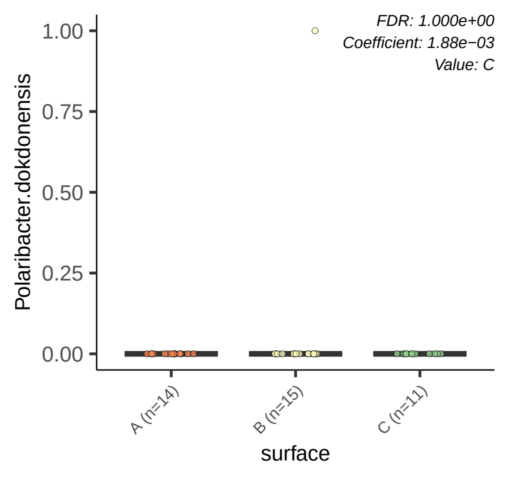

FDR: 1.000e+00
Coefficient: 1.88e−03
Value: C
Polaribacter.dokdonensis
1.00
0.75
0.50
0.25
0.00
A (n=14)
B (n=15)
C (n=11)
surface

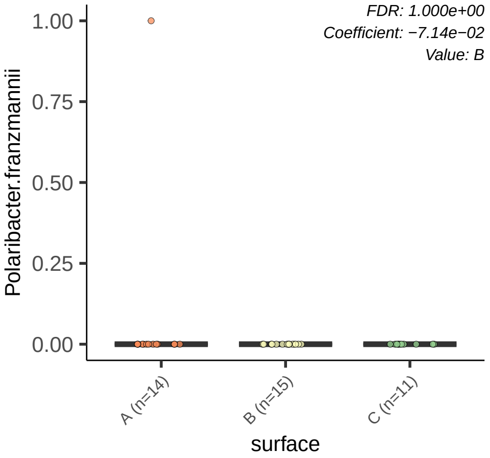
FDR: 1.000e+00
Coefficient: −7.14e−02
Value: B
1.00
0.75
0.50
0.25
0.00
Polaribacter.franzmannii
A (n=14)
B (n=15)
C (n=11)
surface

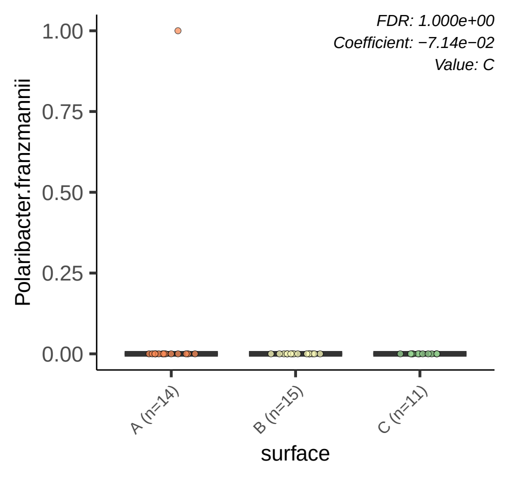
FDR: 1.000e+00
Coefficient: −7.14e−02
Value: C
1.00
0.75
0.50
0.25
0.00
Polaribacter.franzmannii
A (n=14)
B (n=15)
C (n=11)
surface

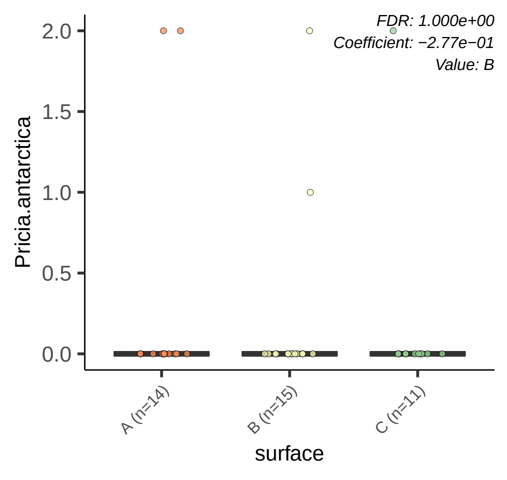

FDR: 1.000e+00
Coefficient: −2.77e−01
Value: B
Pricia.antarctica
2.0
1.5
1.0
0.5
0.0
A (n=14)
B (n=15)
C (n=11)
surface

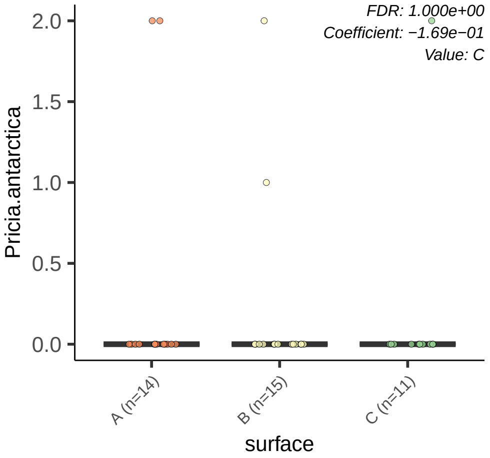

FDR: 1.000e+00
Coefficient: −1.69e−01
Value: C
Pricia.antarctica
2.0
1.5
1.0
0.5
0.0
A (n=14)
B (n=15)
C (n=11)
surface

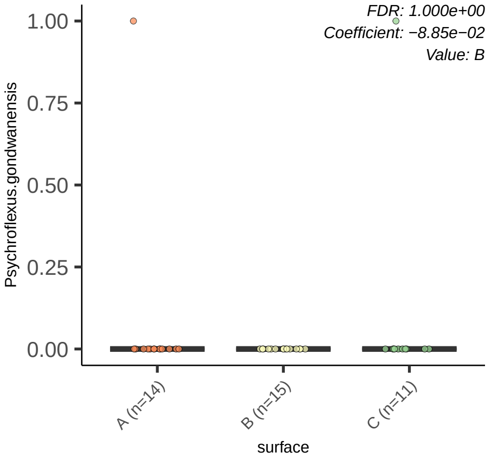

FDR: 1.000e+00
Coefficient: −8.85e−02
Value: B
1.00
0.75
0.50
0.25
0.00
Psychroflexus.gondwanensis
A (n=14)
B (n=15)
C (n=11)
surface

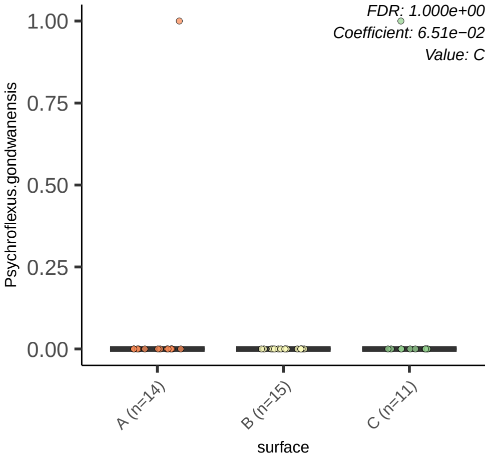

FDR: 1.000e+00
Coefficient: 6.51e−02
Value: C
Psychroflexus.gondwanensis
1.00
0.75
0.50
0.25
0.00
A (n=14)
B (n=15)
C (n=11)
surface

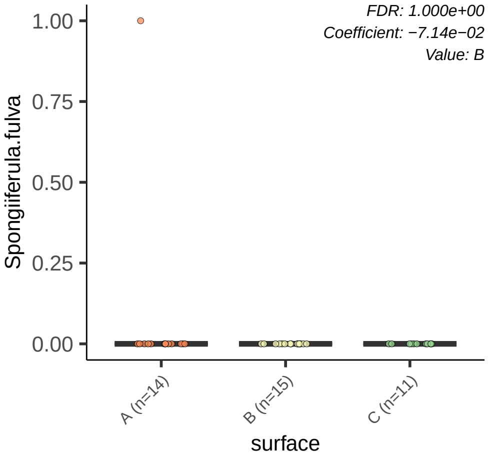
FDR: 1.000e+00
Coefficient: −7.14e−02
Value: B
Spongiiferula.fulva
1.00
0.75
0.50
0.25
0.00
A (n=14)
B (n=15)
C (n=11)
surface

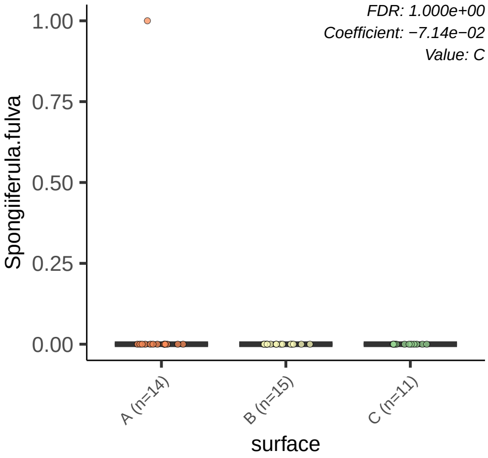

FDR: 1.000e+00
Coefficient: −7.14e−02
Value: C
Spongiiferula.fulva
1.00
0.75
0.50
0.25
0.00
A (n=14)
B (n=15)
C (n=11)
surface

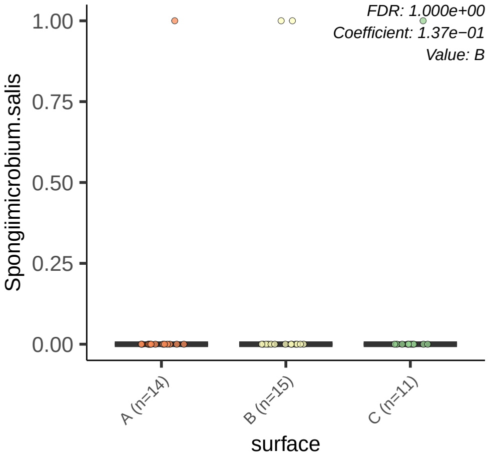
FDR: 1.000e+00
Coefficient: 1.37e−01
Value: B
Spongiimicrobium.salis
1.00
0.75
0.50
0.25
0.00
A (n=14)
B (n=15)
C (n=11)
surface

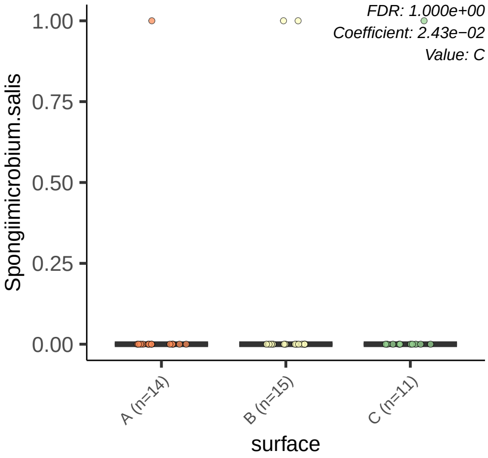
FDR: 1.000e+00
Coefficient: 2.43e−02
Value: C
Spongiimicrobium.salis
1.00
0.75
0.50
0.25
0.00
A (n=14)
B (n=15)
C (n=11)
surface

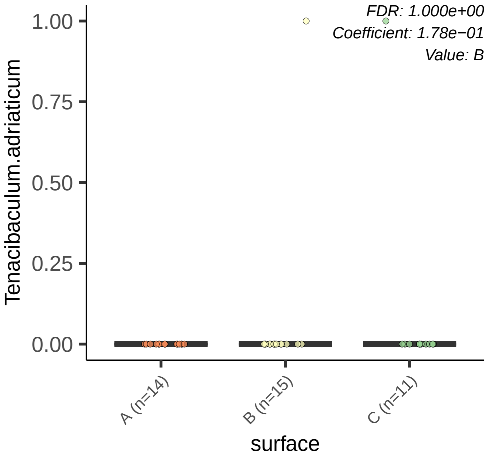
FDR: 1.000e+00
Coefficient: 1.78e−01
Value: B
Tenacibaculum.adriaticum
1.00
0.75
0.50
0.25
0.00
A (n=14)
B (n=15)
C (n=11)
surface

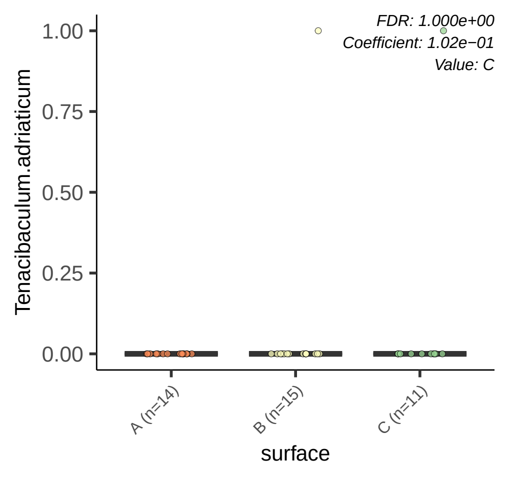

FDR: 1.000e+00
Coefficient: 1.02e−01
Value: C
Tenacibaculum.adriaticum
1.00
0.75
0.50
0.25
0.00
A (n=14)
B (n=15)
C (n=11)
surface

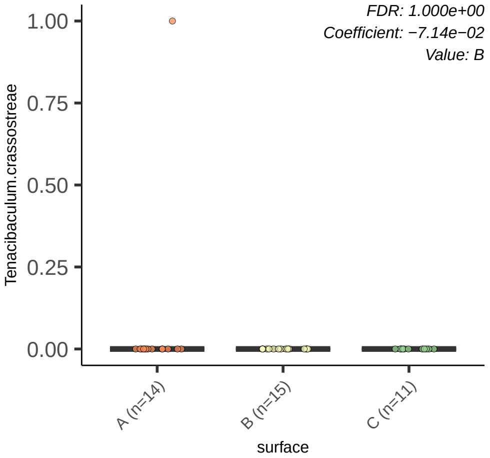

FDR: 1.000e+00
Coefficient: −7.14e−02
Value: B
Tenacibaculum.crassostreae
1.00
0.75
0.50
0.25
0.00
A (n=14)
B (n=15)
C (n=11)
surface

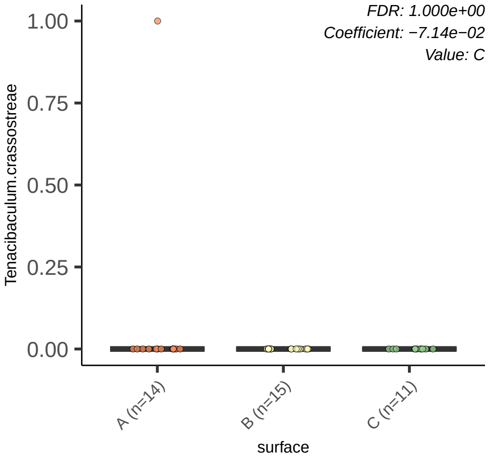

FDR: 1.000e+00
Coefficient: −7.14e−02
Value: C
Tenacibaculum.crassostreae
1.00
0.75
0.50
0.25
0.00
A (n=14)
B (n=15)
C (n=11)
surface

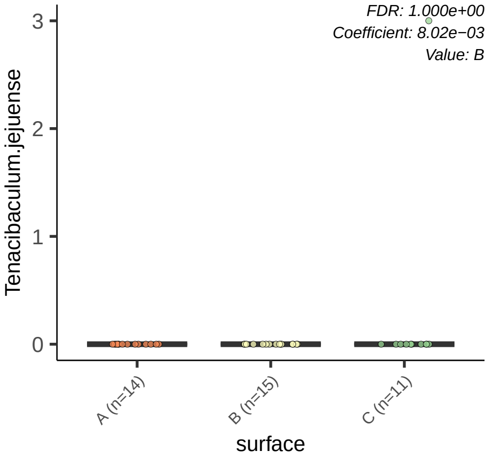
FDR: 1.000e+00
Coefficient: 8.02e−03
Value: B
Tenacibaculum.jejuense
3
2
1
0
A (n=14)
B (n=15)
C (n=11)
surface

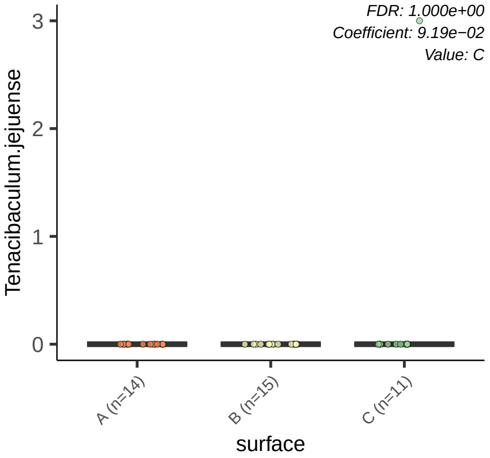
FDR: 1.000e+00
Coefficient: 9.19e−02
Value: C
Tenacibaculum.jejuense
3
2
1
0
A (n=14)
B (n=15)
C (n=11)
surface

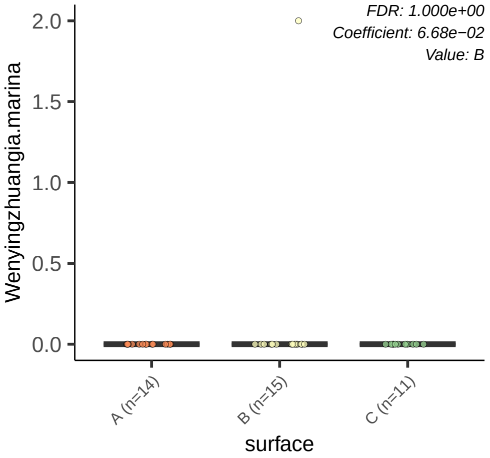

FDR: 1.000e+00
Coefficient: 6.68e−02
Value: B
Wenyingzhuangia.marina
2.0
1.5
1.0
0.5
0.0
A (n=14)
B (n=15)
C (n=11)
surface

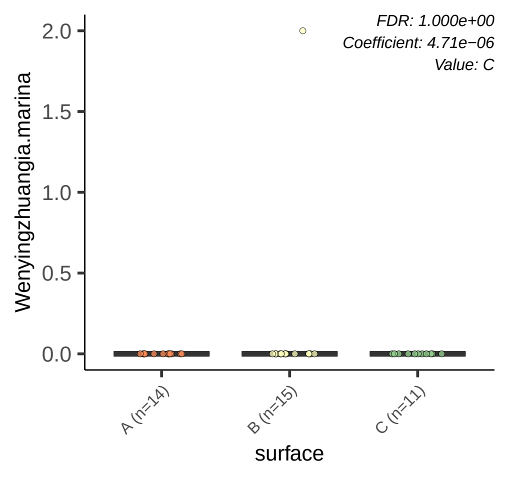

FDR: 1.000e+00
Coefficient: 4.71e−06
Value: C
Wenyingzhuangia.marina
2.0
1.5
1.0
0.5
0.0
A (n=14)
B (n=15)
C (n=11)
surface

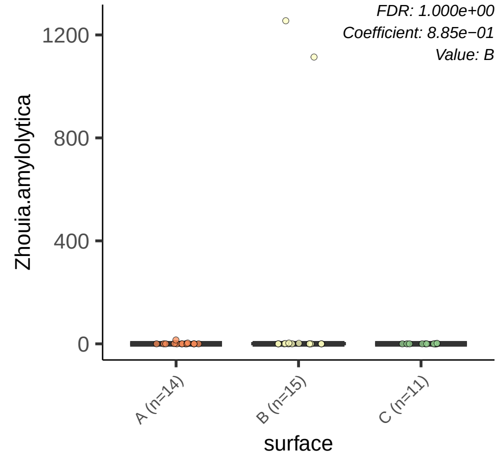

FDR: 1.000e+00
Coefficient: 8.85e−01
Value: B
1200
800
400
0
Zhouia.amylolytica
A (n=14)
B (n=15)
C (n=11)
surface

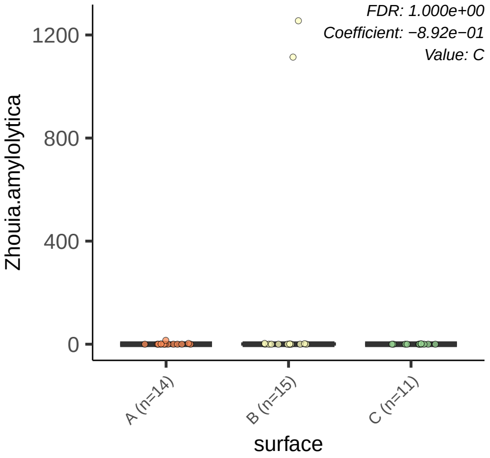

FDR: 1.000e+00
Coefficient: −8.92e−01
Value: C
1200
800
400
0
Zhouia.amylolytica
A (n=14)
B (n=15)
C (n=11)
surface

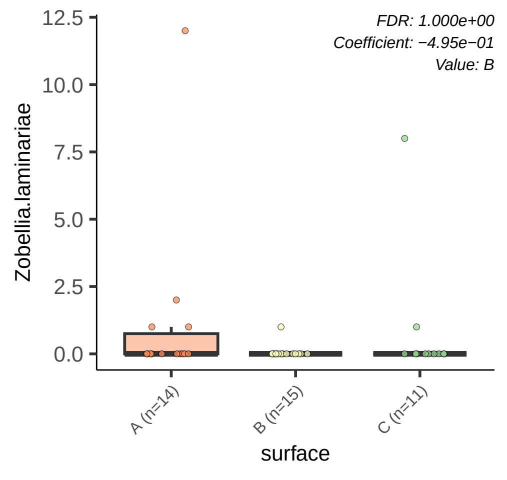

FDR: 1.000e+00
Coefficient: −4.95e−01
Value: B
Zobellia.laminariae
12.5
10.0
7.5
5.0
2.5
0.0
A (n=14)
B (n=15)
C (n=11)
surface

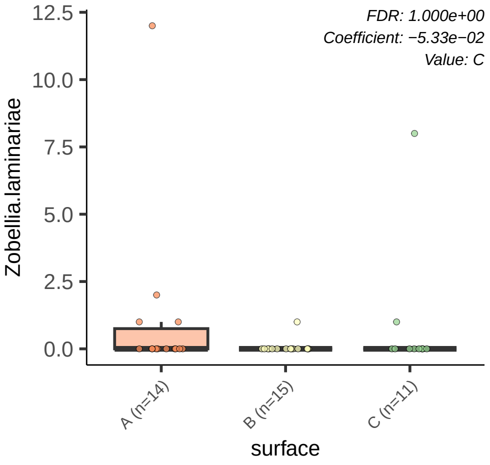

FDR: 1.000e+00
Coefficient: −5.33e−02
Value: C
Zobellia.laminariae
12.5
10.0
7.5
5.0
2.5
0.0
A (n=14)
B (n=15)
C (n=11)
surface

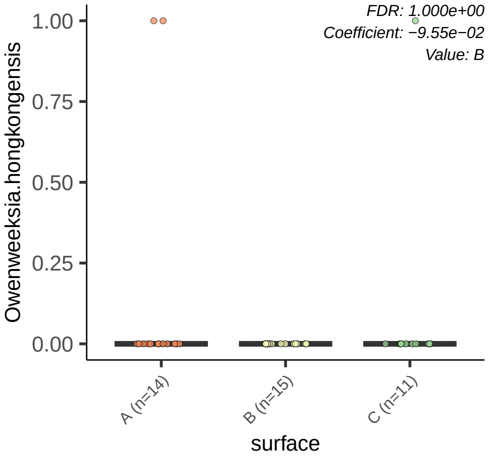
FDR: 1.000e+00
Coefficient: −9.55e−02
Value: B
Owenweeksia.hongkongensis
1.00
0.75
0.50
0.25
0.00
A (n=14)
B (n=15)
C (n=11)
surface

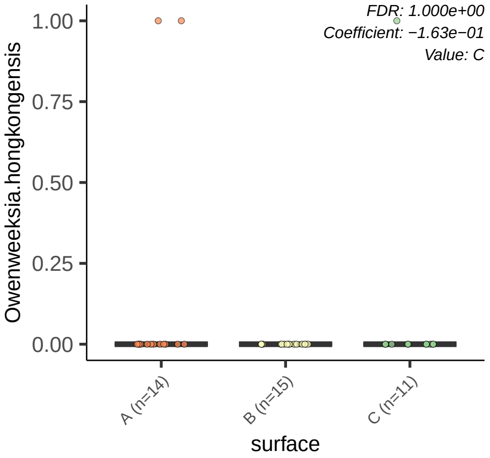
FDR: 1.000e+00
Coefficient: −1.63e−01
Value: C
Owenweeksia.hongkongensis
1.00
0.75
0.50
0.25
0.00
A (n=14)
B (n=15)
C (n=11)
surface

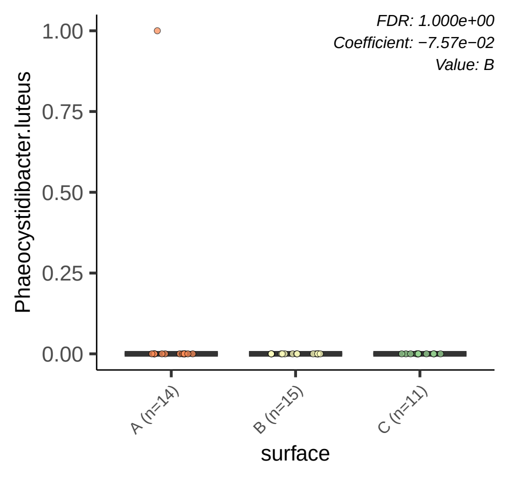
FDR: 1.000e+00
Coefficient: −7.57e−02
Value: B
Phaeocystidibacter.luteus
1.00
0.75
0.50
0.25
0.00
A (n=14)
B (n=15)
C (n=11)
surface

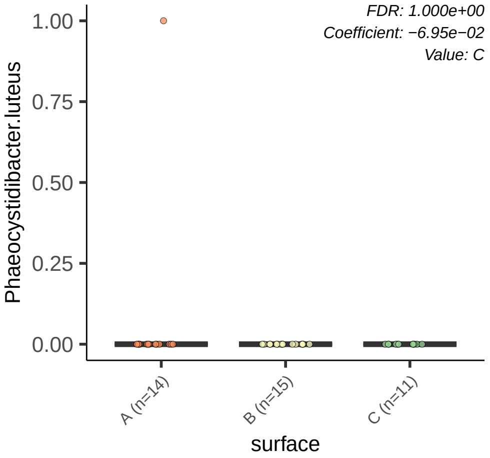
FDR: 1.000e+00
Coefficient: −6.95e−02
Value: C
Phaeocystidibacter.luteus
1.00
0.75
0.50
0.25
0.00
A (n=14)
B (n=15)
C (n=11)
surface

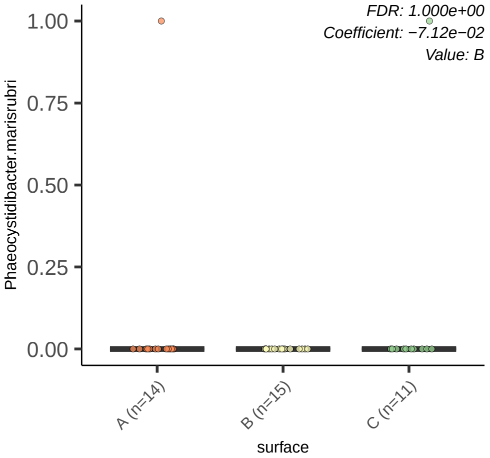
FDR: 1.000e+00
Coefficient: −7.12e−02
Value: B
Phaeocystidibacter.marisrubri
1.00
0.75
0.50
0.25
0.00
A (n=14)
B (n=15)
C (n=11)
surface

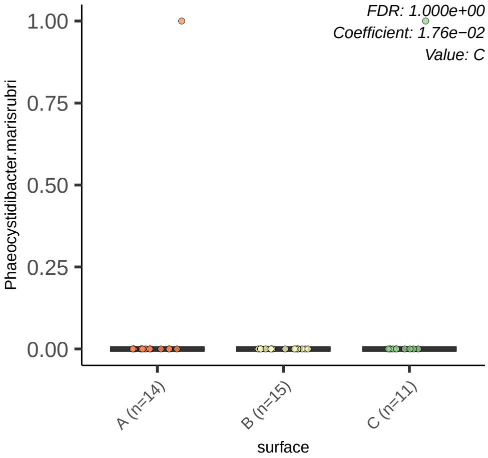
FDR: 1.000e+00
Coefficient: 1.76e−02
Value: C
Phaeocystidibacter.marisrubri
1.00
0.75
0.50
0.25
0.00
A (n=14)
B (n=15)
C (n=11)
surface

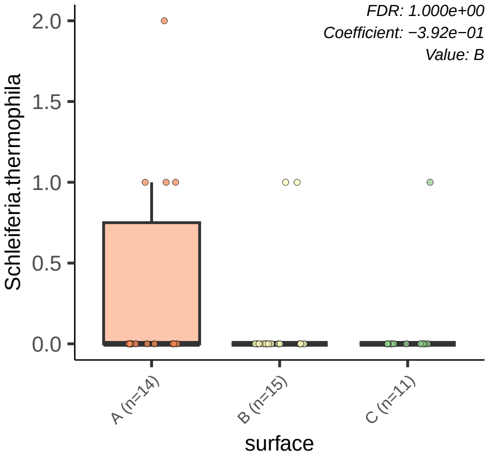
FDR: 1.000e+00
Coefficient: −3.92e−01
Value: B
Schleiferia.thermophila
2.0
1.5
1.0
0.5
0.0
A (n=14)
B (n=15)
C (n=11)
surface

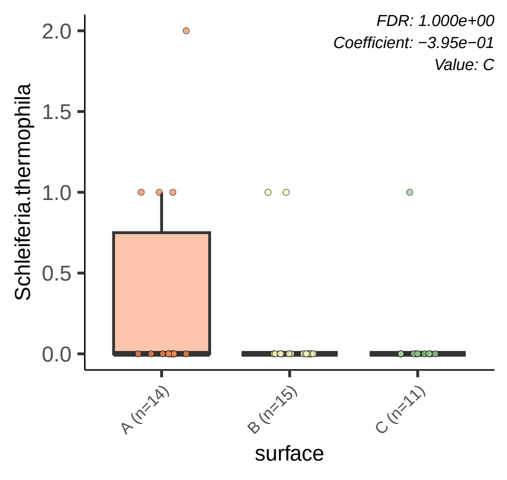
FDR: 1.000e+00
Coefficient: −3.95e−01
Value: C
Schleiferia.thermophila
2.0
1.5
1.0
0.5
0.0
A (n=14)
B (n=15)
C (n=11)
surface

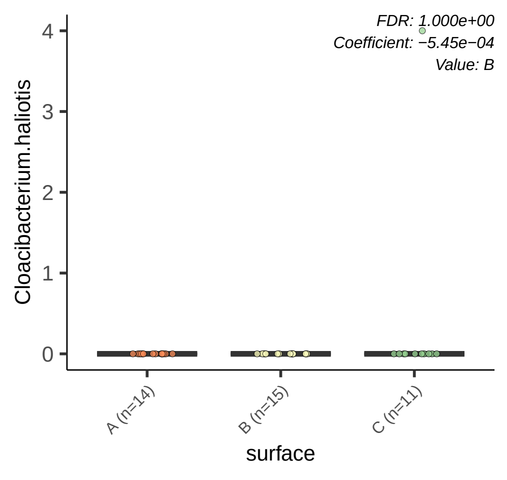
FDR: 1.000e+00
Coefficient: −5.45e−04
Value: B
Cloacibacterium.haliotis
0
1
2
3
4
A (n=14)
B (n=15)
C (n=11)
surface

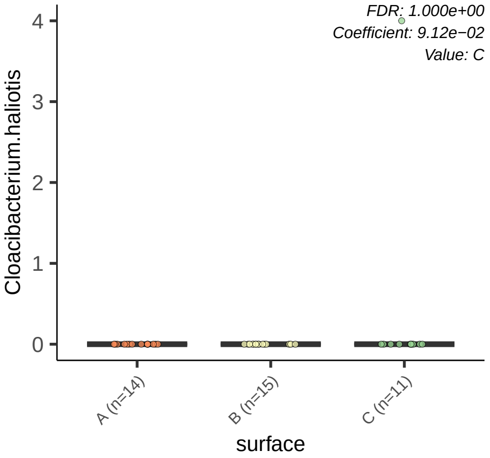
FDR: 1.000e+00
Coefficient: 9.12e−02
Value: C
Cloacibacterium.haliotis
4
3
2
1
0
A (n=14)
B (n=15)
C (n=11)
surface

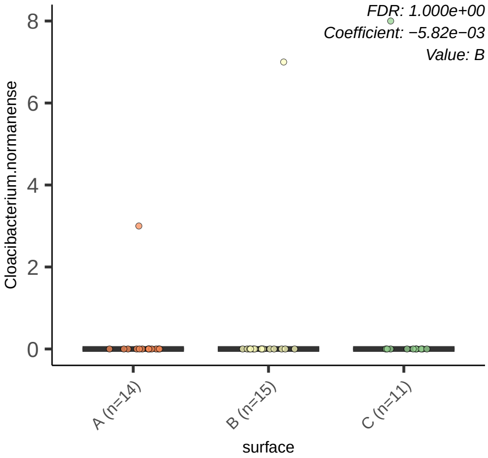

FDR: 1.000e+00
Coefficient: −5.82e−03
Value: B
Cloacibacterium.normanense
0
2
4
6
8
A (n=14)
B (n=15)
C (n=11)
surface

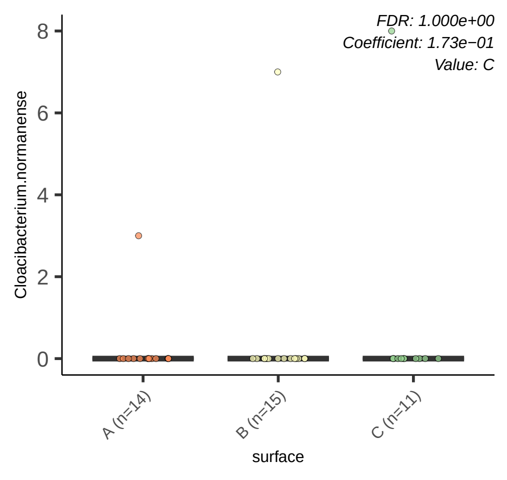
FDR: 1.000e+00
Coefficient: 1.73e−01
Value: C
Cloacibacterium.normanense
8
6
4
2
0
A (n=14)
B (n=15)
C (n=11)
surface

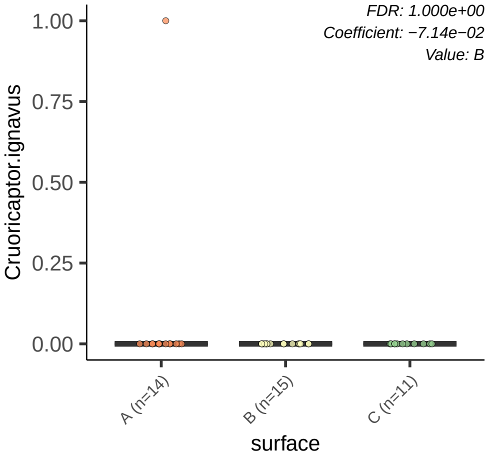

FDR: 1.000e+00
Coefficient: −7.14e−02
Value: B
Cruoricaptor.ignavus
1.00
0.75
0.50
0.25
0.00
A (n=14)
B (n=15)
C (n=11)
surface

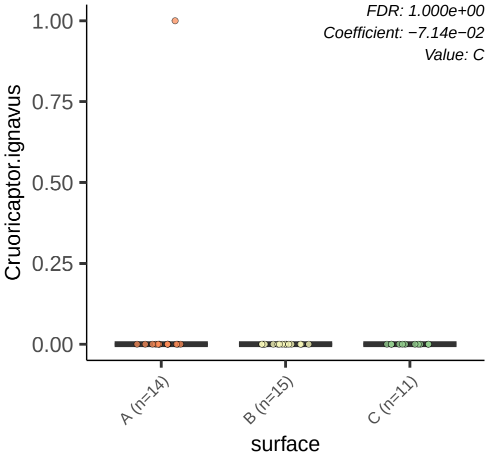
FDR: 1.000e+00
Coefficient: −7.14e−02
Value: C
Cruoricaptor.ignavus
1.00
0.75
0.50
0.25
0.00
A (n=14)
B (n=15)
C (n=11)
surface

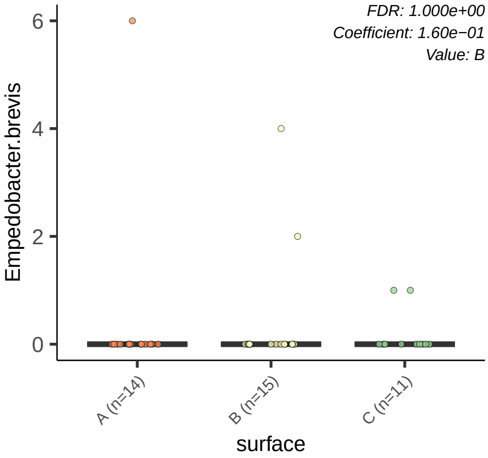

FDR: 1.000e+00
Coefficient: 1.60e−01
Value: B
Empedobacter.brevis
0
2
4
6
A (n=14)
B (n=15)
C (n=11)
surface

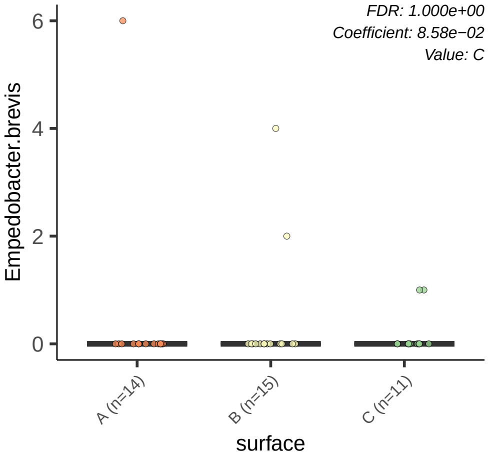

FDR: 1.000e+00
Coefficient: 8.58e−02
Value: C
Empedobacter.brevis
6
4
2
0
A (n=14)
B (n=15)
C (n=11)
surface

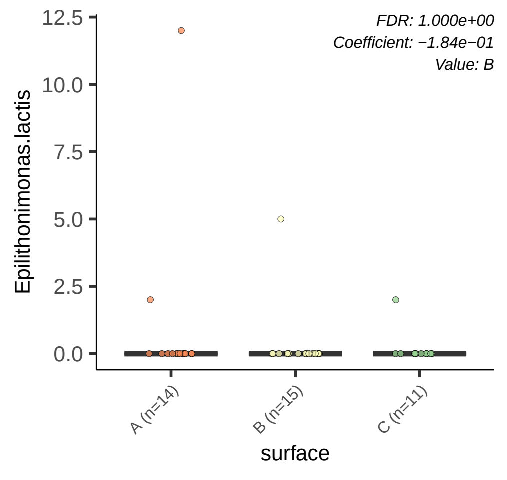
FDR: 1.000e+00
Coefficient: −1.84e−01
Value: B
Epilithonimonas.lactis
12.5
10.0
7.5
5.0
2.5
0.0
A (n=14)
B (n=15)
C (n=11)
surface

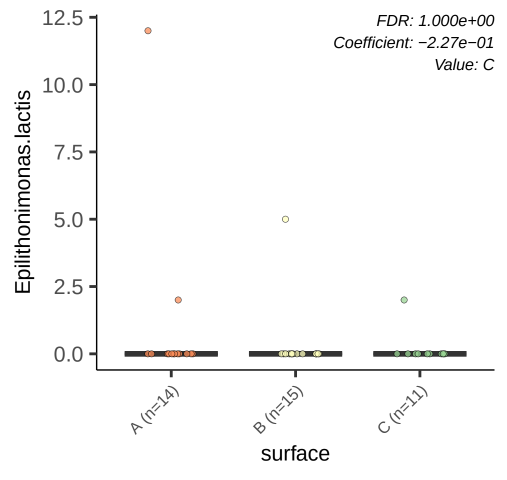
FDR: 1.000e+00
Coefficient: −2.27e−01
Value: C
Epilithonimonas.lactis
12.5
10.0
7.5
5.0
2.5
0.0
A (n=14)
B (n=15)
C (n=11)
surface

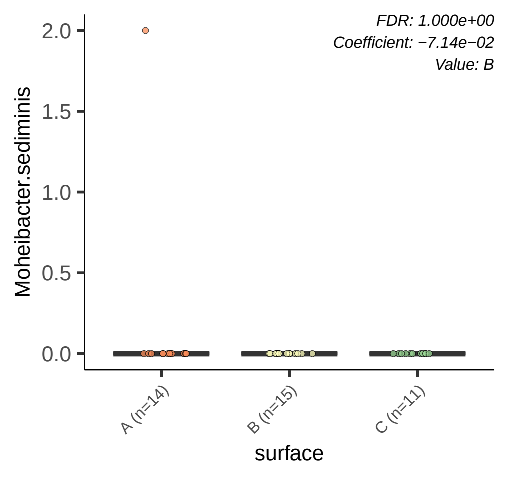
FDR: 1.000e+00
Coefficient: −7.14e−02
Value: B
Moheibacter.sedimini
2.0
1.5
1.0
0.5
0.0
A (n=14)
B (n=15)
C (n=11)
surface

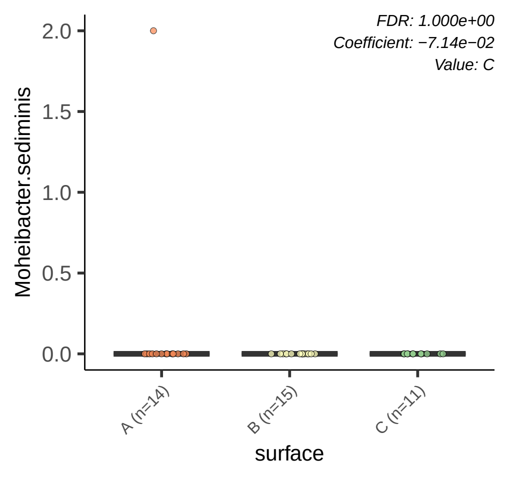

FDR: 1.000e+00
Coefficient: −7.14e−02
Value: C
Moheibacter.sedimminis
2.0
1.5
1.0
0.5
0.0
A (n=14)
B (n=15)
C (n=11)
surface

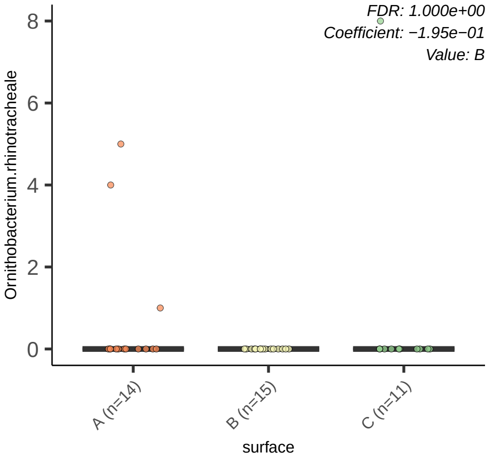
FDR: 1.000e+00
Coefficient: −1.95e−01
Value: B
Ornithobacterium.rhinotracheale
0
2
4
6
8
A (n=14)
B (n=15)
C (n=11)
surface

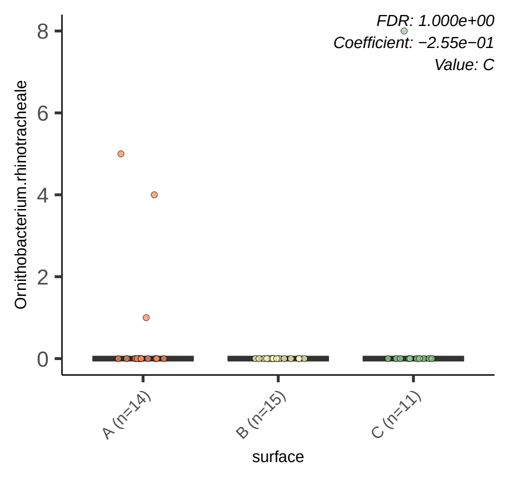
FDR: 1.000e+00
Coefficient: −2.55e−01
Value: C
Ornithobacterium.rhinotracheale
0
2
4
6
8
A (n=14)
B (n=15)
C (n=11)
surface

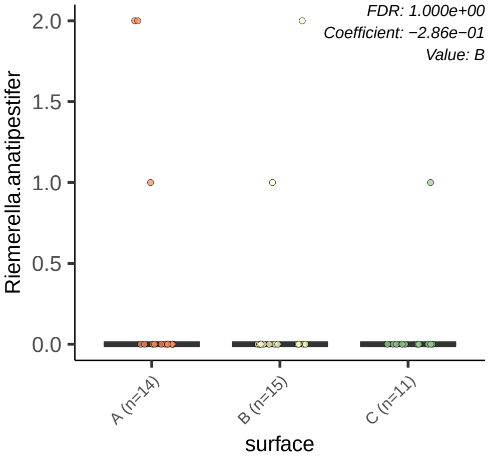

FDR: 1.000e+00
Coefficient: −2.86e−01
Value: B
Riemerella.anatipestifer
2.0
1.5
1.0
0.5
0.0
A (n=14)
B (n=15)
C (n=11)
surface

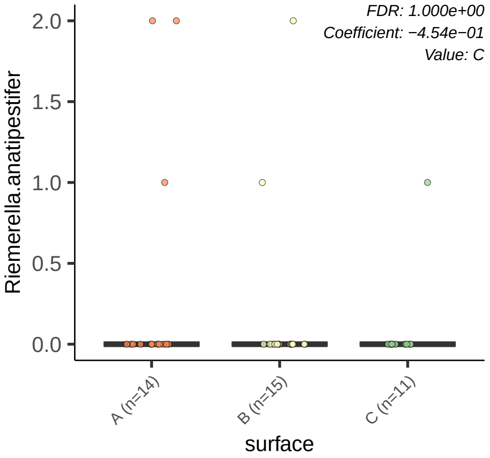
FDR: 1.000e+00
Coefficient: −4.54e−01
Value: C
2.0
1.5
1.0
0.5
0.0
Riemerella.anatipestifer
A (n=14)
B (n=15)
C (n=11)
surface

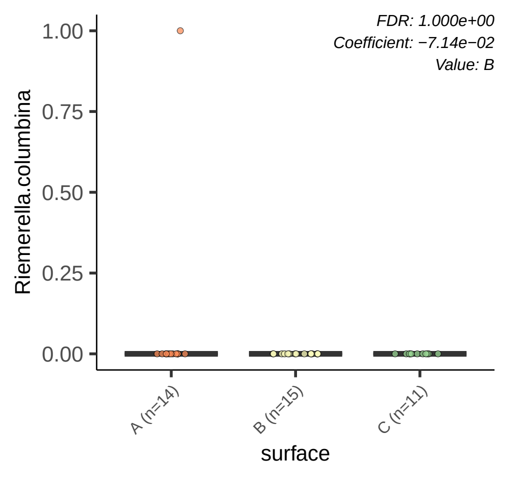

FDR: 1.000e+00
Coefficient: −7.14e−02
Value: B
Riemerella.columbina
1.00
0.75
0.50
0.25
0.00
A (n=14)
B (n=15)
C (n=11)
surface

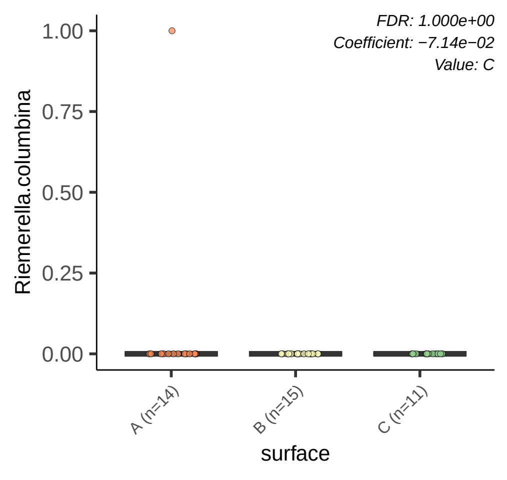

FDR: 1.000e+00
Coefficient: −7.14e−02
Value: C
Riemerella.columbina
1.00
0.75
0.50
0.25
0.00
A (n=14)
B (n=15)
C (n=11)
surface

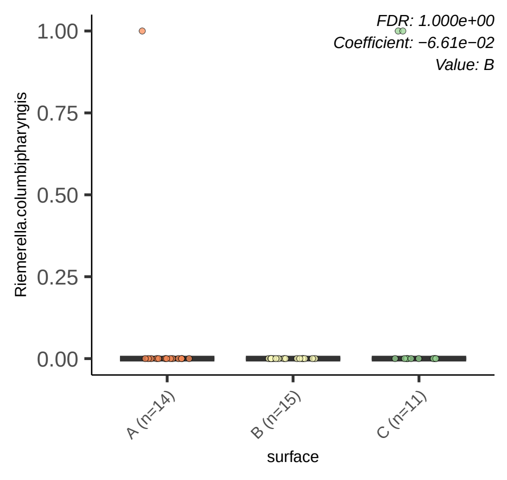
FDR: 1.000e+00
Coefficient: −6.61e−02
Value: B
Riemerella.columbipharyngis
1.00
0.75
0.50
0.25
0.00
A (n=14)
B (n=15)
C (n=11)
surface

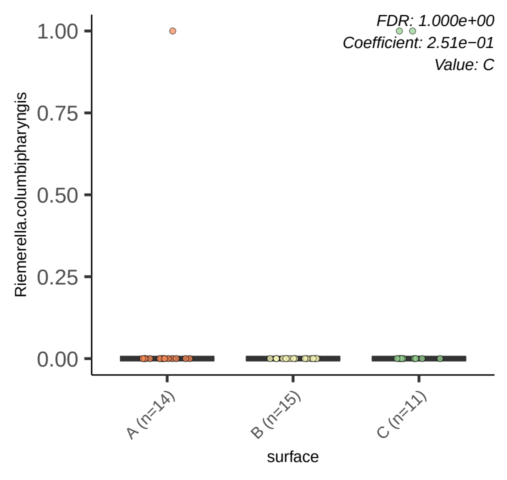

FDR: 1.000e+00
Coefficient: 2.51e−01
Value: C
Riemerella.columbipharyngis
1.00
0.75
0.50
0.25
0.00
A (n=14)
B (n=15)
C (n=11)
surface

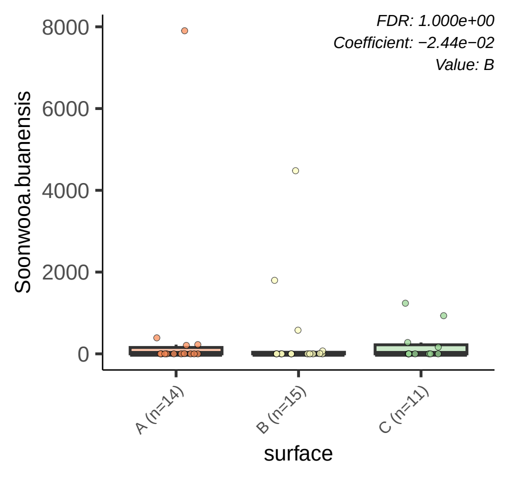

FDR: 1.000e+00
Coefficient: −2.44e−02
Value: B
Soonwooa.buanensis
8000
6000
4000
2000
0
A (n=14)
B (n=15)
C (n=11)
surface

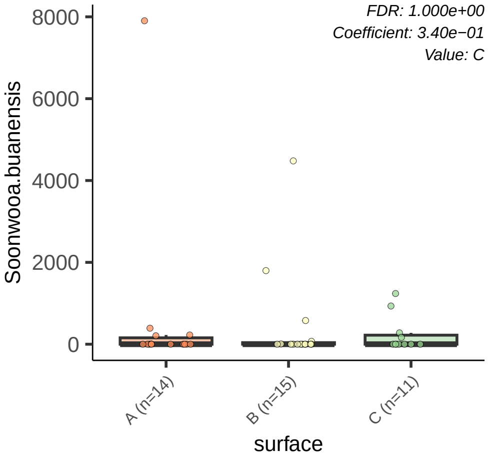

FDR: 1.000e+00
Coefficient: 3.40e−01
Value: C
Soonwooa.buanensis
8000
6000
4000
2000
0
A (n=14)
B (n=15)
C (n=11)
surface

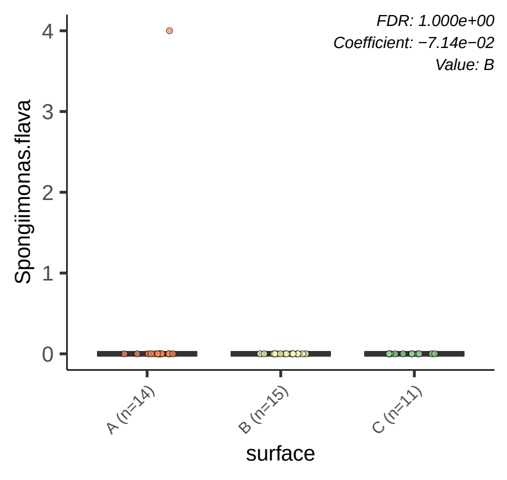

FDR: 1.000e+00
Coefficient: −7.14e−02
Value: B
Spongiimonas.flava
4
3
2
1
0
A (n=14)
B (n=15)
C (n=11)
surface

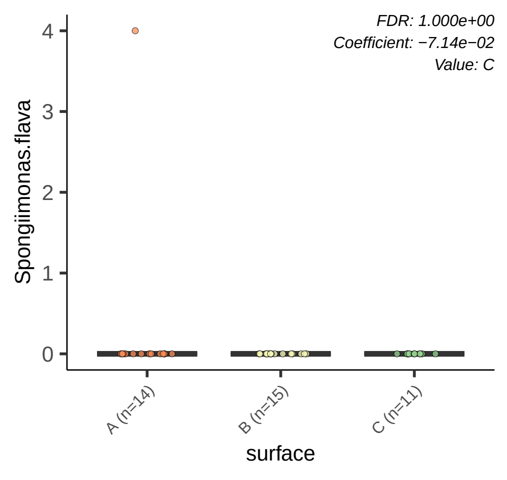
FDR: 1.000e+00
Coefficient: −7.14e−02
Value: C
Spongiimonas.flava
4
3
2
1
0
A (n=14)
B (n=15)
C (n=11)
surface

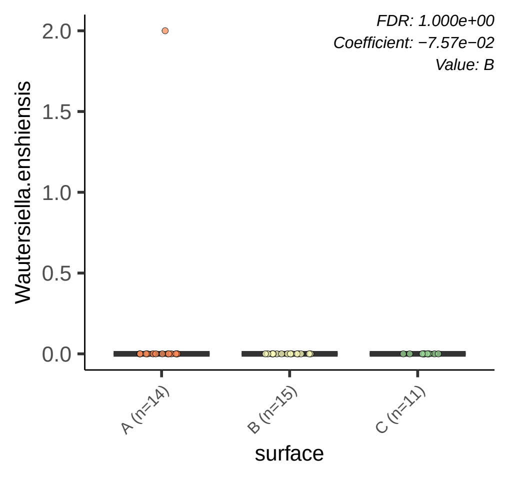
FDR: 1.000e+00
Coefficient: −7.57e−02
Value: B
Wautersiella.enshiensis
2.0
1.5
1.0
0.5
0.0
A (n=14)
B (n=15)
C (n=11)
surface

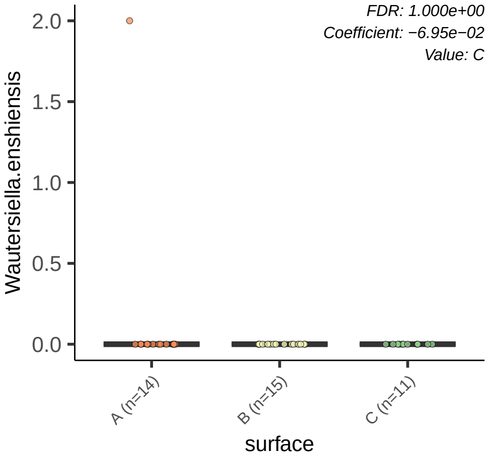

FDR: 1.000e+00
Coefficient: −6.95e−02
Value: C
Wautersiella.enshiensis
2.0
1.5
1.0
0.5
0.0
A (n=14)
B (n=15)
C (n=11)
surface

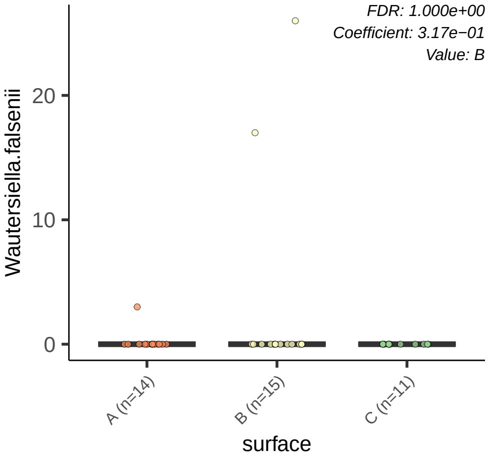

FDR: 1.000e+00
Coefficient: 3.17e−01
Value: B
Wautersiella.falsenii
20
10
0
A (n=14)
B (n=15)
C (n=11)
surface

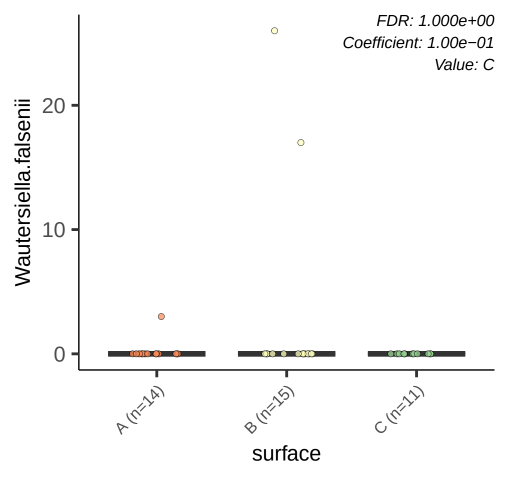
FDR: 1.000e+00
Coefficient: 1.00e−01
Value: C
Wautersiella.falsenii
20
10
0
A (n=14)
B (n=15)
C (n=11)
surface

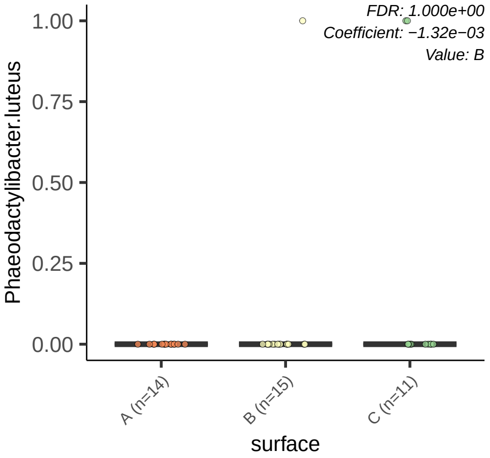

FDR: 1.000e+00
Coefficient: −1.32e−03
Value: B
Phaeodactylibacter.luteus
1.00
0.75
0.50
0.25
0.00
A (n=14)
B (n=15)
C (n=11)
surface

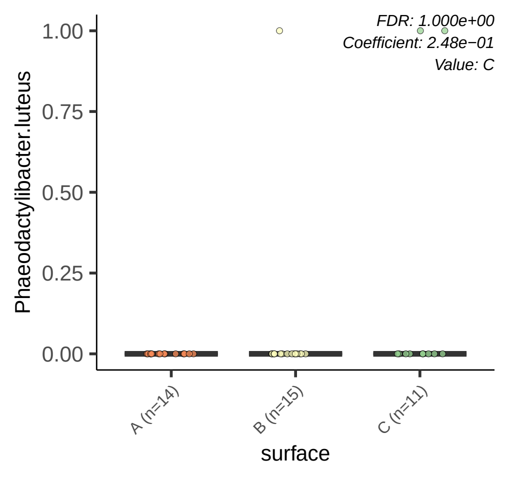
FDR: 1.000e+00
Coefficient: 2.48e−01
Value: C
Phaeodactylibacter.luteus
1.00
0.75
0.50
0.25
0.00
A (n=14)
B (n=15)
C (n=11)
surface

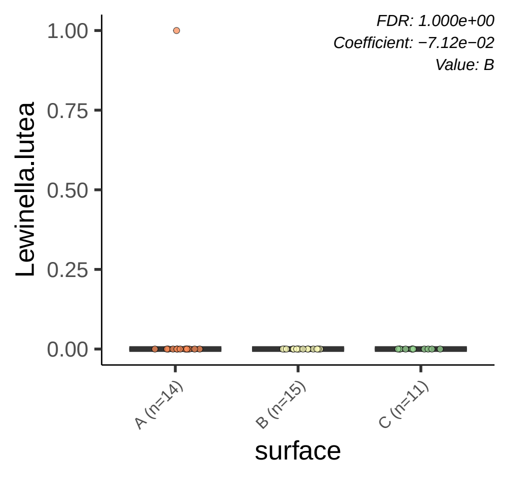
FDR: 1.000e+00
Coefficient: −7.12e−02
Value: B
Lewinella.lutea
1.00
0.75
0.50
0.25
0.00
A (n=14)
B (n=15)
C (n=11)
surface

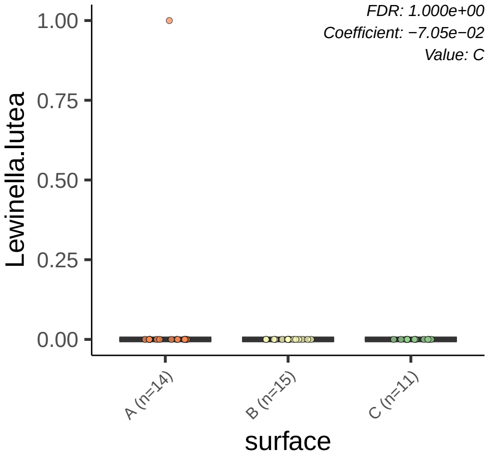

FDR: 1.000e+00
Coefficient: −7.05e−02
Value: C
Lewinella.lutea
1.00
0.75
0.50
0.25
0.00
A (n=14)
B (n=15)
C (n=11)
surface

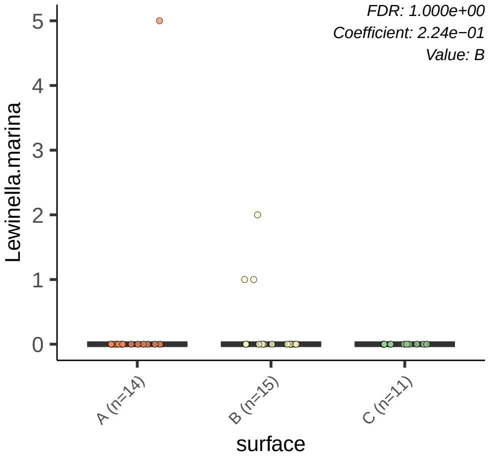

FDR: 1.000e+00
Coefficient: 2.24e−01
Value: B
Lewinella.marina
5
4
3
2
1
0
A (n=14)
B (n=15)
C (n=11)
surface

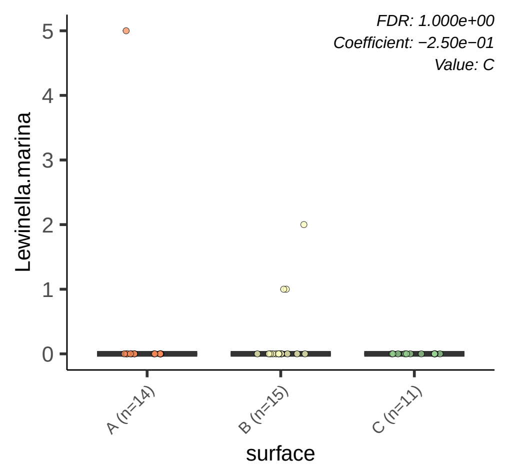
FDR: 1.000e+00
Coefficient: −2.50e−01
Value: C
Lewinella.marina
5
4
3
2
1
0
A (n=14)
B (n=15)
C (n=11)
surface

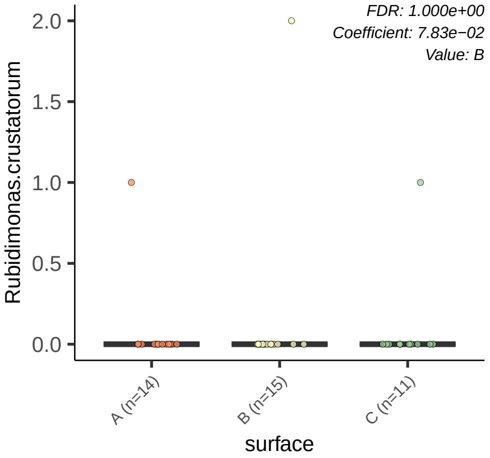
FDR: 1.000e+00
Coefficient: 7.83e−02
Value: B
Rubidimonas.crustatorum
2.0
1.5
1.0
0.5
0.0
A (n=14)
B (n=15)
C (n=11)
surface

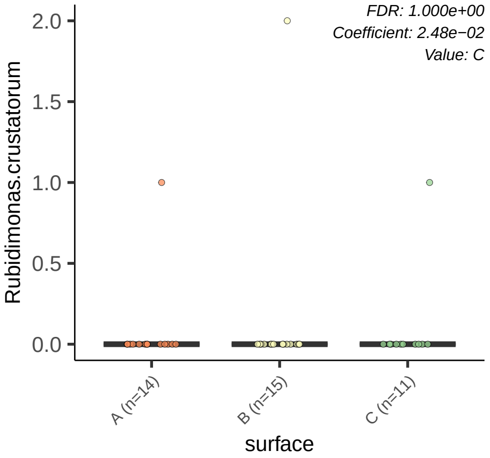
FDR: 1.000e+00
Coefficient: 2.48e−02
Value: C
Rubidimonas.crustatorum
2.0
1.5
1.0
0.5
0.0
A (n=14)
B (n=15)
C (n=11)
surface

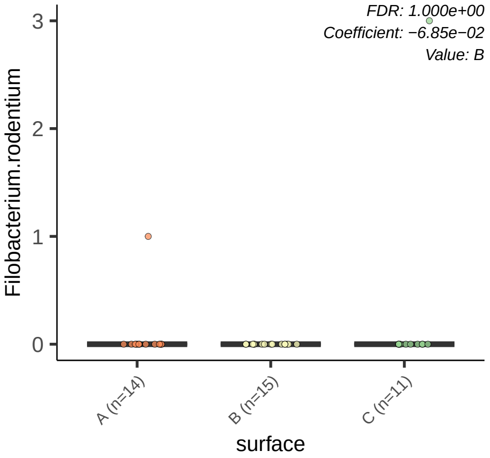
FDR: 1.000e+00
Coefficient: −6.85e−02
Value: B
Filobacterium.rodentium
0
1
2
3
A (n=14)
B (n=15)
C (n=11)
surface

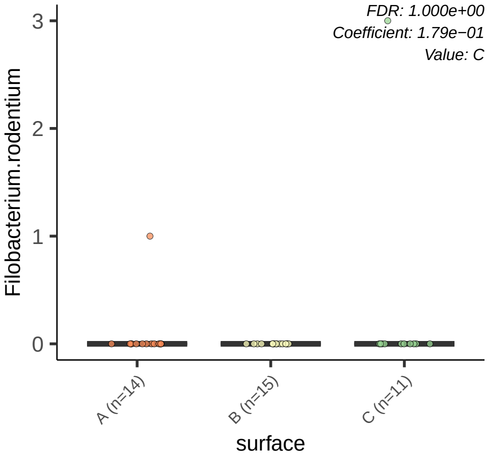
FDR: 1.000e+00
Coefficient: 1.79e−01
Value: C
Filobacterium.rodentium
3
2
1
0
A (n=14)
B (n=15)
C (n=11)
surface

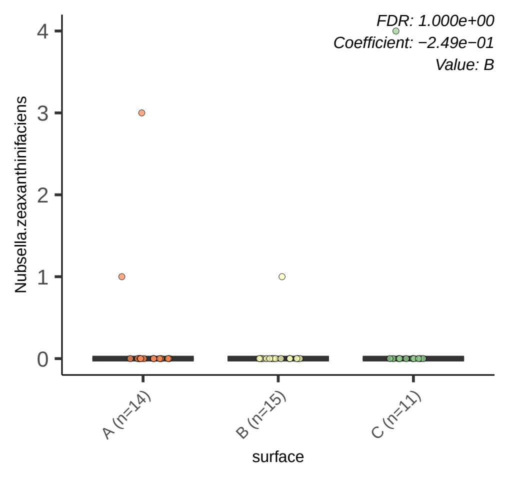
FDR: 1.000e+00
Coefficient: −2.49e−01
Value: B
Nubsella.zeaxanthinifaciens
0
1
2
3
4
A (n=14)
B (n=15)
C (n=11)
surface

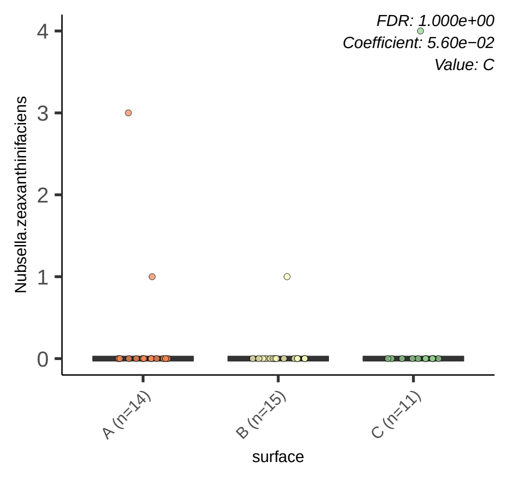

FDR: 1.000e+00
Coefficient: 5.60e−02
Value: C
Nubsella.zeaxanthinifaciens
0
1
2
3
4
A (n=14)
B (n=15)
C (n=11)
surface

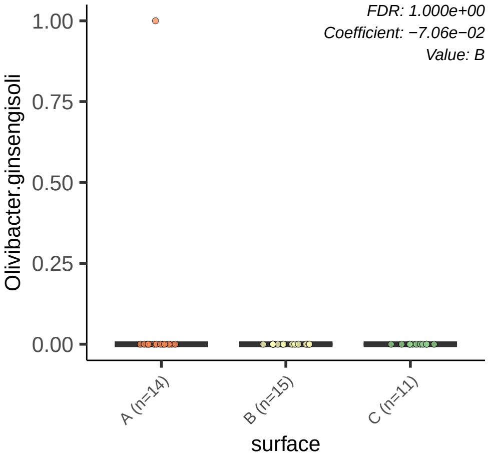
FDR: 1.000e+00
Coefficient: −7.06e−02
Value: B
Olivibacter.ginsengisoli
1.00
0.75
0.50
0.25
0.00
A (n=14)
B (n=15)
C (n=11)
surface

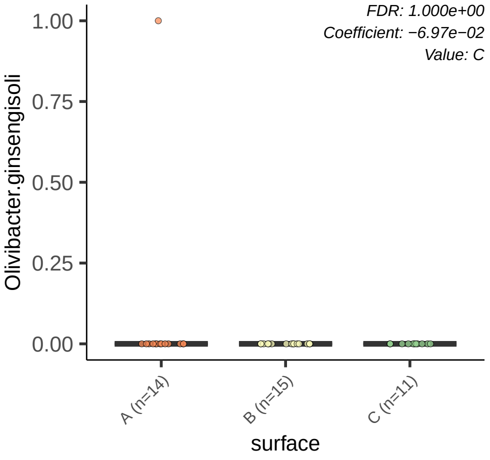

FDR: 1.000e+00
Coefficient: −6.97e−02
Value: C
Olivibacter.ginsengisoli
1.00
0.75
0.50
0.25
0.00
A (n=14)
B (n=15)
C (n=11)
surface

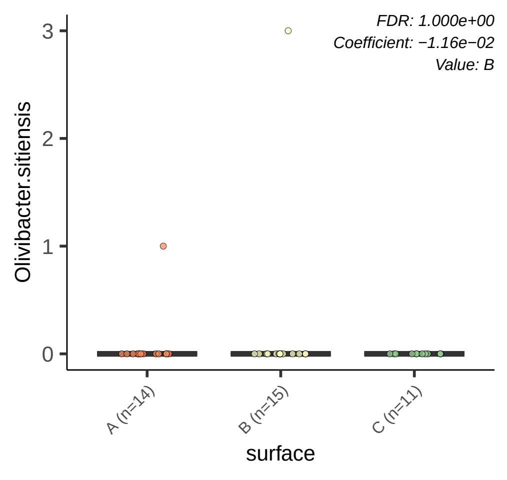

FDR: 1.000e+00
Coefficient: −1.16e−02
Value: B
Olivibacter.sitiensis
0
1
2
3
A (n=14)
B (n=15)
C (n=11)
surface

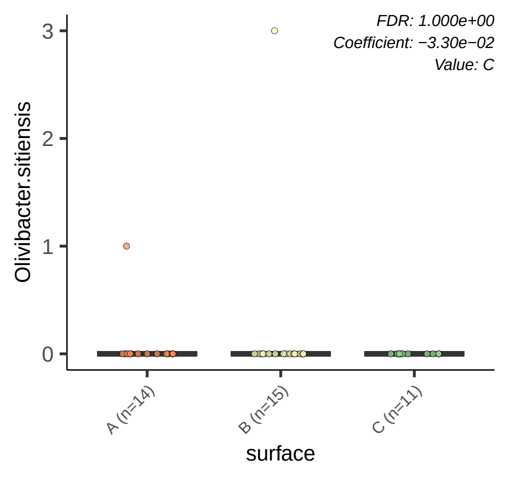

FDR: 1.000e+00
Coefficient: −3.30e−02
Value: C
Olivibacter.sitiensis
3
2
1
0
A (n=14)
B (n=15)
C (n=11)
surface

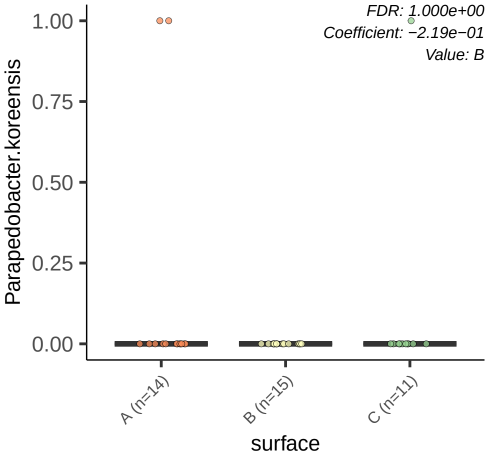
FDR: 1.000e+00
Coefficient: −2.19e−01
Value: B
Parapedobacter.koreensis
1.00
0.75
0.50
0.25
0.00
A (n=14)
B (n=15)
C (n=11)
surface

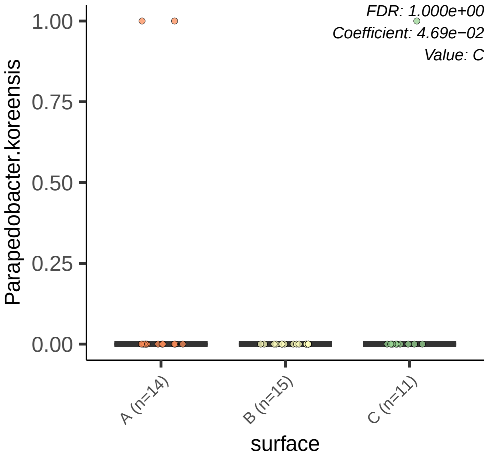
FDR: 1.000e+00
Coefficient: 4.69e−02
Value: C
Parapedobacter.koreensis
1.00
0.75
0.50
0.25
0.00
A (n=14)
B (n=15)
C (n=11)
surface

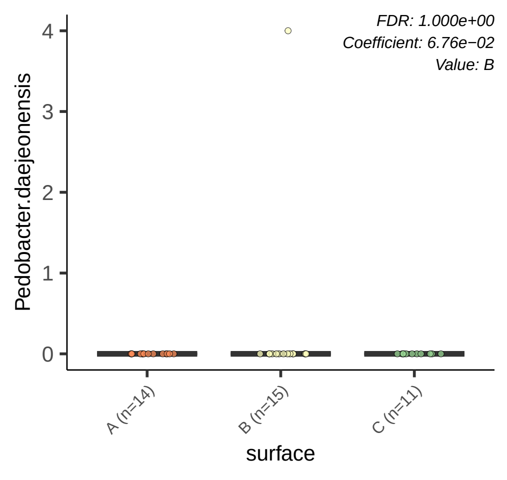
FDR: 1.000e+00
Coefficient: 6.76e−02
Value: B
Pedobacter.daejeonensis
4
3
2
1
0
A (n=14)
B (n=15)
C (n=11)
surface

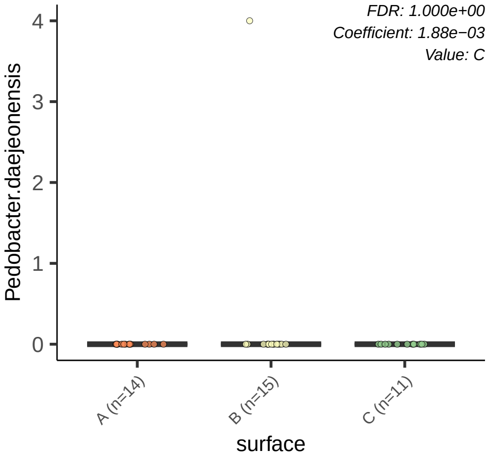
FDR: 1.000e+00
Coefficient: 1.88e−03
Value: C
Pedobacter.daejeonensis
0
1
2
3
4
A (n=14)
B (n=15)
C (n=11)
surface

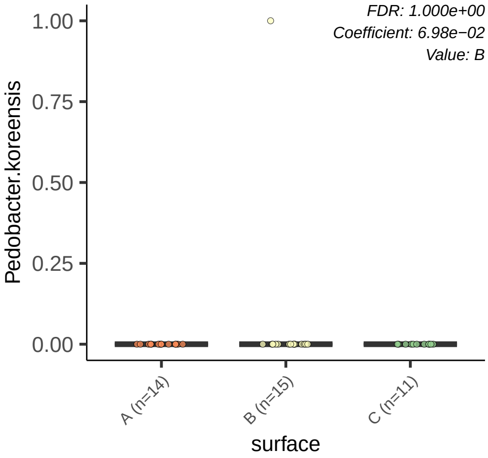

FDR: 1.000e+00
Coefficient: 6.98e−02
Value: B
Pedobacter.koreensis
1.00
0.75
0.50
0.25
0.00
A (n=14)
B (n=15)
C (n=11)
surface

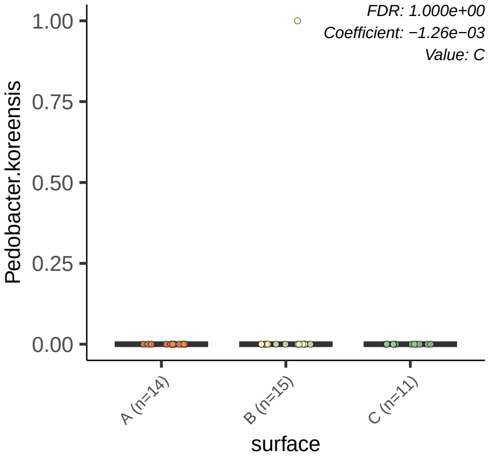
FDR: 1.000e+00
Coefficient: −1.26e−03
Value: C
Pedobacter.koreensis
1.00
0.75
0.50
0.25
0.00
A (n=14)
B (n=15)
C (n=11)
surface

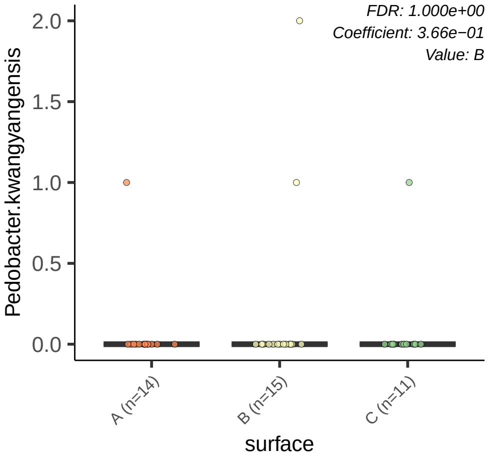
FDR: 1.000e+00
Coefficient: 3.66e−01
Value: B
Pedobacter.kwangyangensis
2.0
1.5
1.0
0.5
0.0
A (n=14)
B (n=15)
C (n=11)
surface

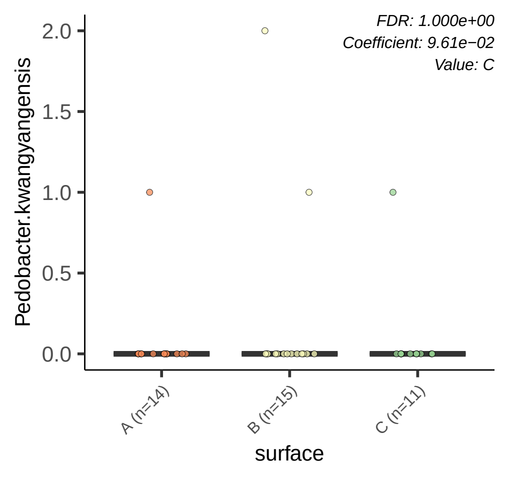
FDR: 1.000e+00
Coefficient: 9.61e−02
Value: C
Pedobacter.kwangyangensis
2.0
1.5
1.0
0.5
0.0
A (n=14)
B (n=15)
C (n=11)
surface

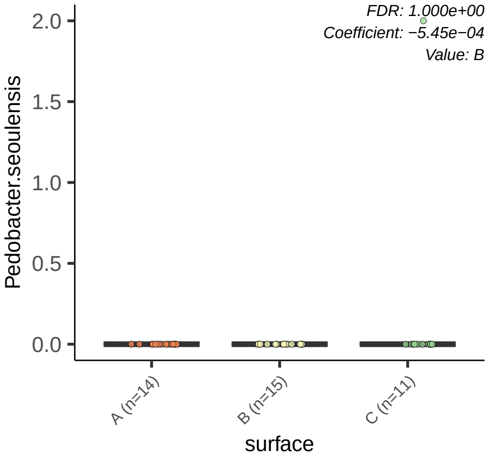
FDR: 1.000e+00
Coefficient: −5.45e−04
Value: B
Pedobacter.seoulensis
2.0
1.5
1.0
0.5
0.0
A (n=14)
B (n=15)
C (n=11)
surface

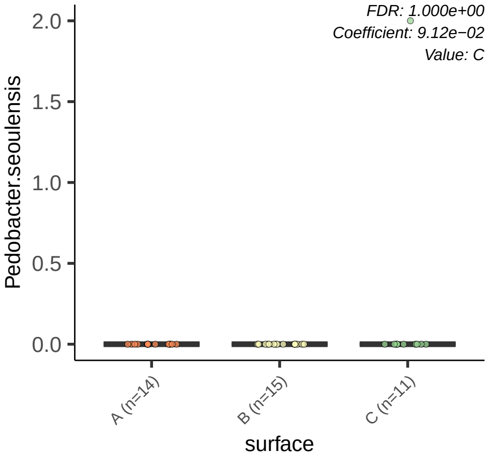

FDR: 1.000e+00
Coefficient: 9.12e−02
Value: C
Pedobacter.seoulensis
2.0
1.5
1.0
0.5
0.0
A (n=14)
B (n=15)
C (n=11)
surface

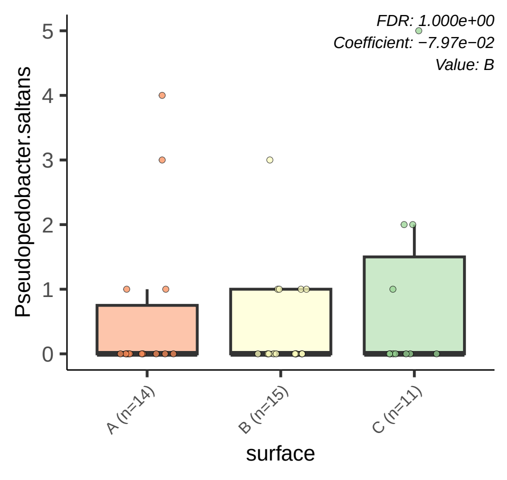

FDR: 1.000e+00
Coefficient: −7.97e−02
Value: B
Pseudopedobacter.saltans
0
1
2
3
4
5
A (n=14)
B (n=15)
C (n=11)
surface

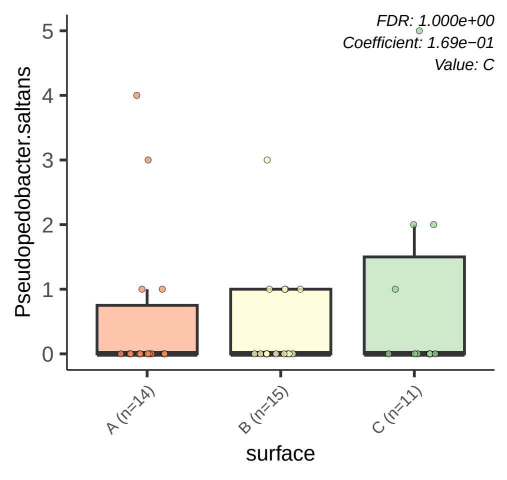

FDR: 1.000e+00
Coefficient: 1.69e−01
Value: C
Pseudopedobacter.saltans
0
1
2
3
4
5
A (n=14)
B (n=15)
C (n=11)
surface

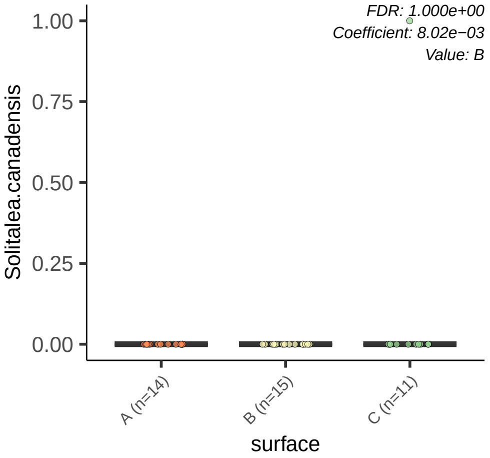
FDR: 1.000e+00
Coefficient: 8.02e−03
Value: B
Solitalea.canadensis
1.00
0.75
0.50
0.25
0.00
A (n=14)
B (n=15)
C (n=11)
surface

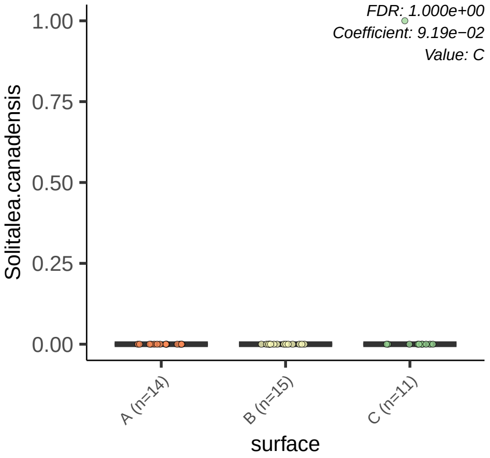
FDR: 1.000e+00
Coefficient: 9.19e−02
Value: C
1.00
0.75
0.50
0.25
0.00
Solitalea.canadensis
A (n=14)
B (n=15)
C (n=11)
surface

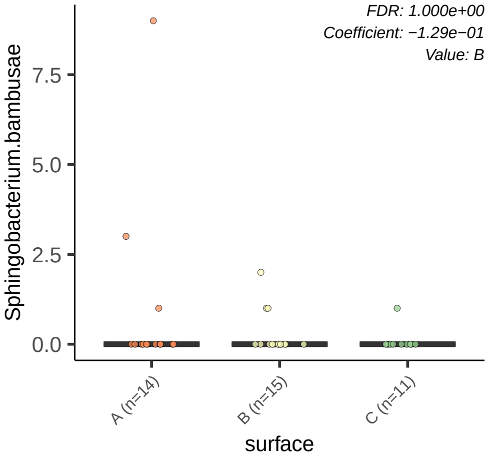

FDR: 1.000e+00
Coefficient: −1.29e−01
Value: B
Sphingobacterium.bambusae
7.5
5.0
2.5
0.0
A (n=14)
B (n=15)
C (n=11)
surface

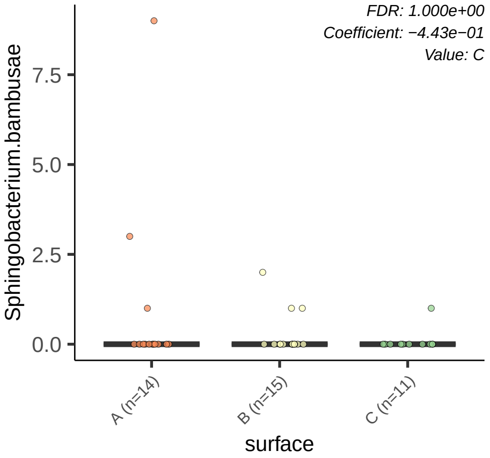

FDR: 1.000e+00
Coefficient: −4.43e−01
Value: C
Sphingobacterium.bambusae
7.5
5.0
2.5
0.0
A (n=14)
B (n=15)
C (n=11)
surface

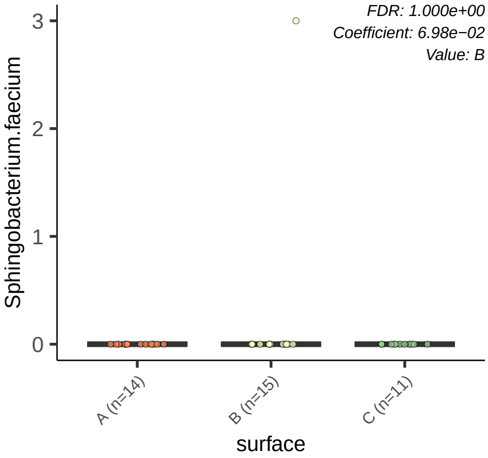
FDR: 1.000e+00
Coefficient: 6.98e−02
Value: B
Sphingobacterium.faecium
3
2
1
0
A (n=14)
B (n=15)
C (n=11)
surface

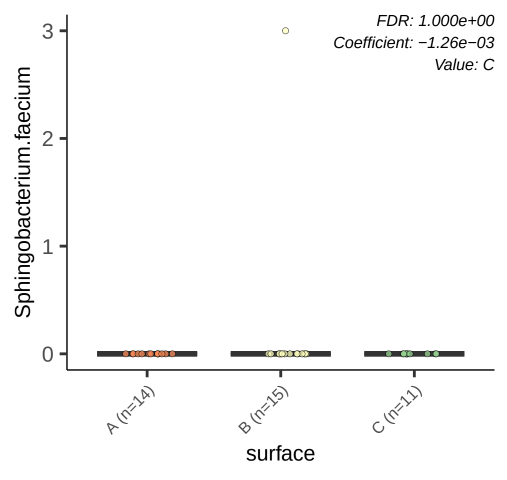
FDR: 1.000e+00
Coefficient: −1.26e−03
Value: C
Sphingobacterium.faecium
3
2
1
0
A (n=14)
B (n=15)
C (n=11)
surface

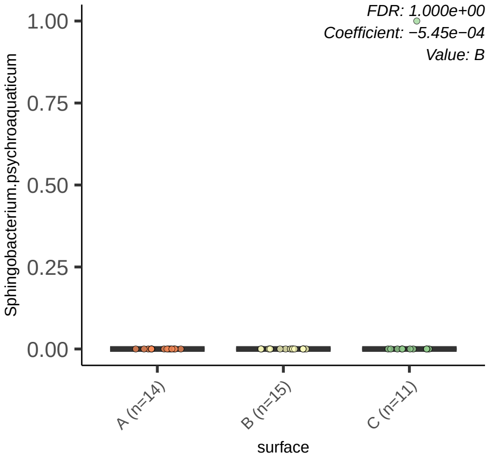
FDR: 1.000e+00
Coefficient: −5.45e−04
Value: B
Sphingobacterium.psychroaquaticum
1.00
0.75
0.50
0.25
0.00
A (n=14)
B (n=15)
C (n=11)
surface

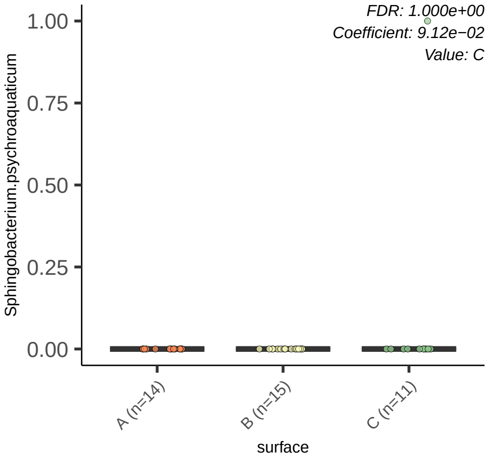
FDR: 1.000e+00
Coefficient: 9.12e−02
Value: C
Sphingobacterium.psychroaquaticum
1.00
0.75
0.50
0.25
0.00
A (n=14)
B (n=15)
C (n=11)
surface

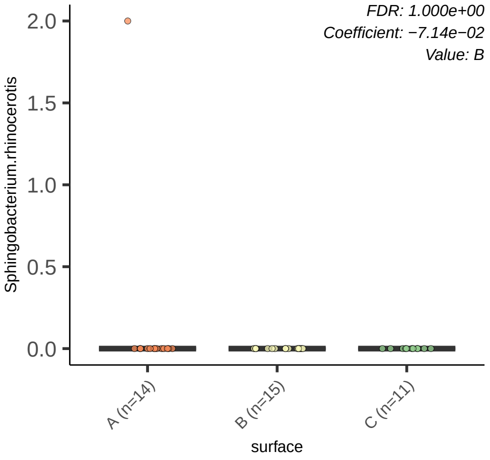

FDR: 1.000e+00
Coefficient: −7.14e−02
Value: B
Sphingobacterium.rhinocerotis
2.0
1.5
1.0
0.5
0.0
A (n=14)
B (n=15)
C (n=11)
surface

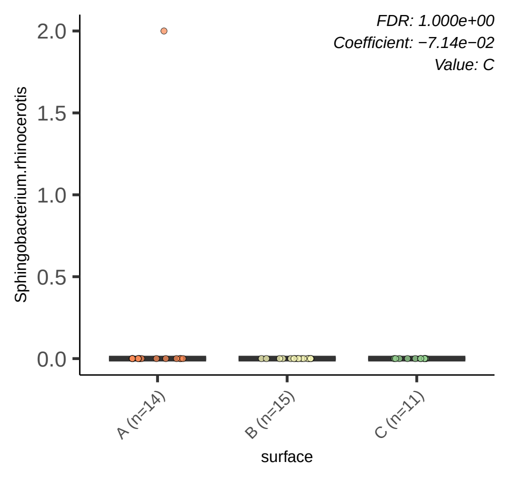

FDR: 1.000e+00
Coefficient: −7.14e−02
Value: C
Sphingobacterium.rhinocerotis
2.0
1.5
1.0
0.5
0.0
A (n=14)
B (n=15)
C (n=11)
surface

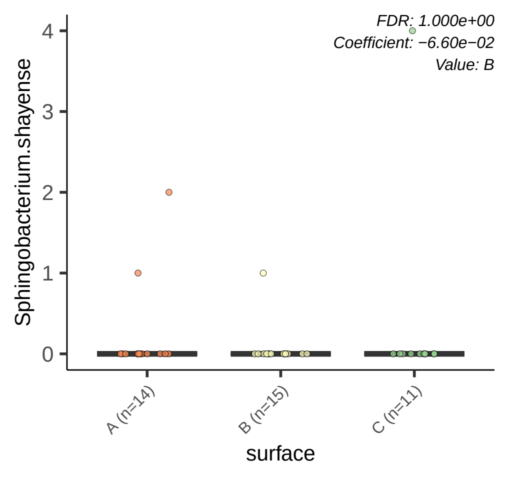

FDR: 1.000e+00
Coefficient: −6.60e−02
Value: B
Sphingobacterium.shayense
4
3
2
1
0
A (n=14)
B (n=15)
C (n=11)
surface

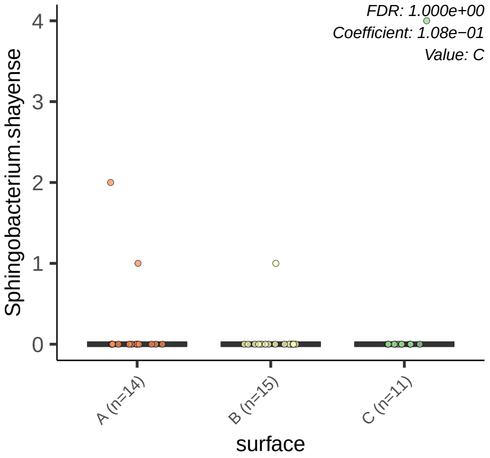

FDR: 1.000e+00
Coefficient: 1.08e−01
Value: C
Sphingobacterium.shayense
0
1
2
3
4
A (n=14)
B (n=15)
C (n=11)
surface

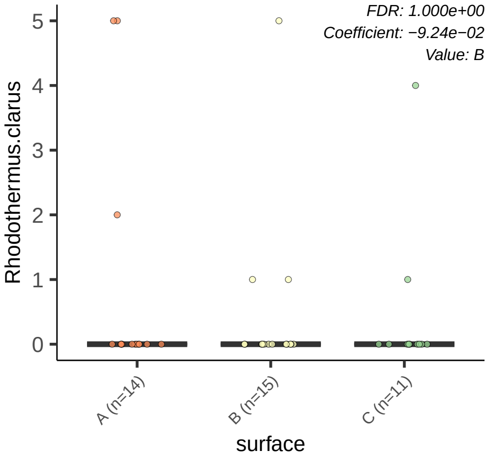

FDR: 1.000e+00
Coefficient: −9.24e−02
Value: B
Rhodothermus.clarus
0
1
2
3
4
5
A (n=14)
B (n=15)
C (n=11)
surface

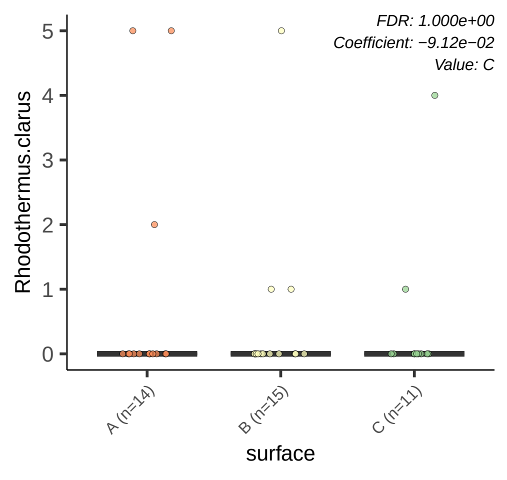

FDR: 1.000e+00
Coefficient: −9.12e−02
Value: C
Rhodothermus.clarus
0
1
2
3
4
5
A (n=14)
B (n=15)
C (n=11)
surface

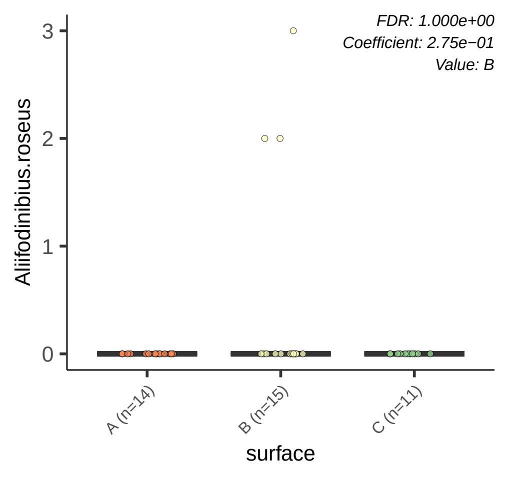

FDR: 1.000e+00
Coefficient: 2.75e−01
Value: B
Aliifodinibius.roseus
3
2
1
0
A (n=14)
B (n=15)
C (n=11)
surface

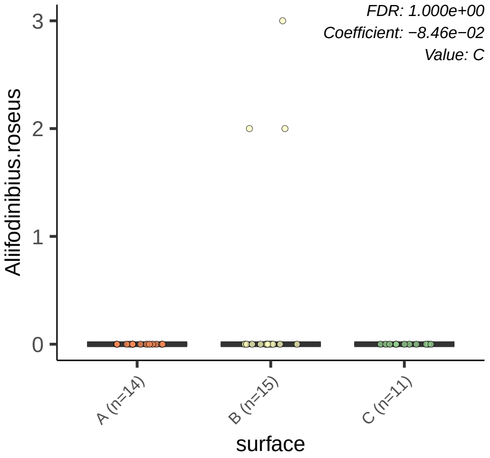

FDR: 1.000e+00
Coefficient: −8.46e−02
Value: C
Aliifodinibius.roseus
3
2
1
0
A (n=14)
B (n=15)
C (n=11)
surface

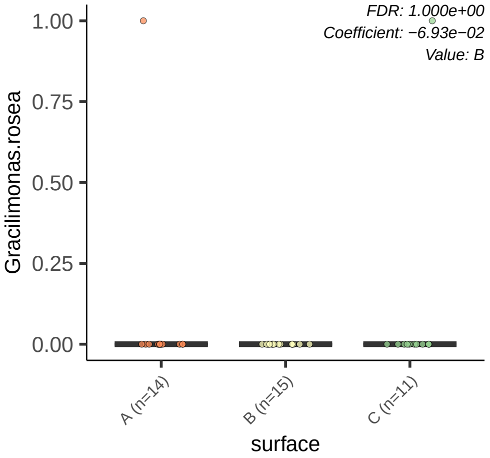

FDR: 1.000e+00
Coefficient: −6.93e−02
Value: B
Gracilimonas.rosea
1.00
0.75
0.50
0.25
0.00
A (n=14)
B (n=15)
C (n=11)
surface

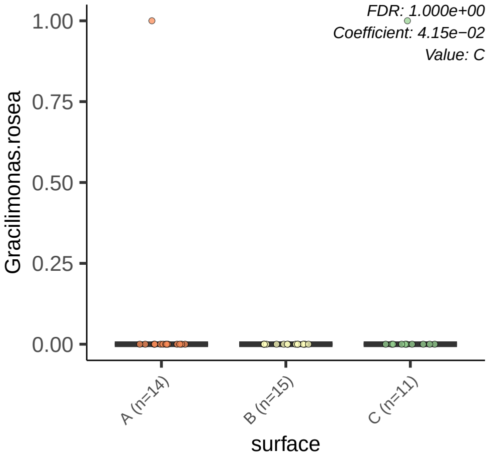
FDR: 1.000e+00
Coefficient: 4.15e−02
Value: C
Gracilimonas.rosea
1.00
0.75
0.50
0.25
0.00
A (n=14)
B (n=15)
C (n=11)
surface

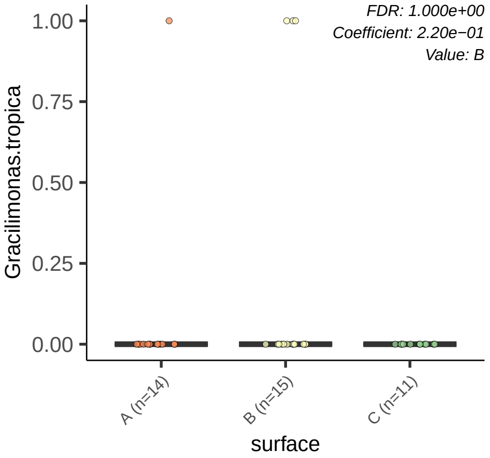
FDR: 1.000e+00
Coefficient: 2.20e−01
Value: B
Gracilimonas.tropica
1.00
0.75
0.50
0.25
0.00
A (n=14)
B (n=15)
C (n=11)
surface

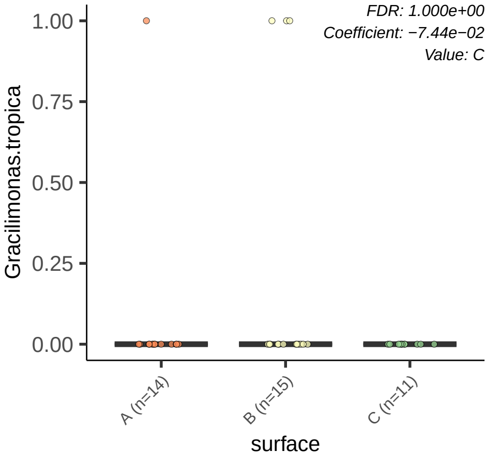
FDR: 1.000e+00
Coefficient: −7.44e−02
Value: C
Gracilimonas.tropica
1.00
0.75
0.50
0.25
0.00
A (n=14)
B (n=15)
C (n=11)
surface

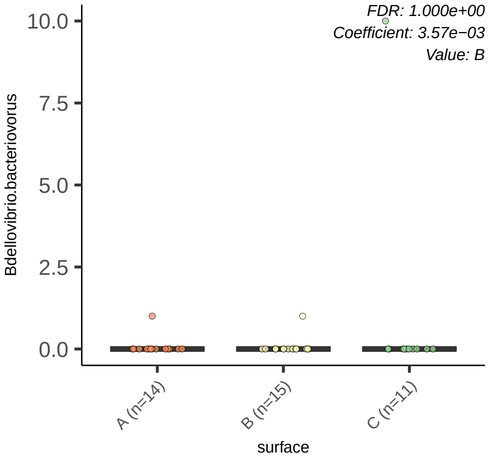
FDR: 1.000e+00
Coefficient: 3.57e−03
Value: B
10.0
7.5
5.0
2.5
0.0
Bdellovibrio.bacteriovorus
A (n=14)
B (n=15)
C (n=11)
surface

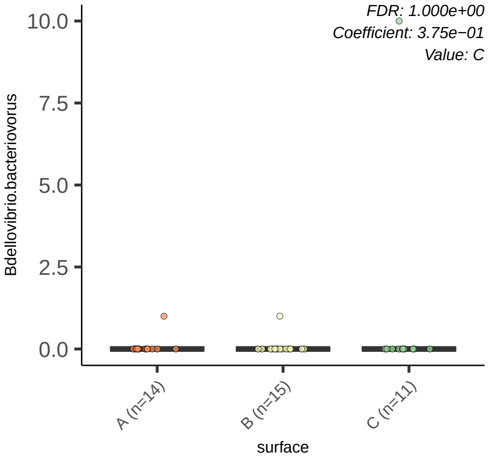
FDR: 1.000e+00
Coefficient: 3.75e−01
Value: C
10.0
7.5
5.0
2.5
0.0
Bdellovibrio.bacteriovorus
A (n=14)
B (n=15)
C (n=11)
surface

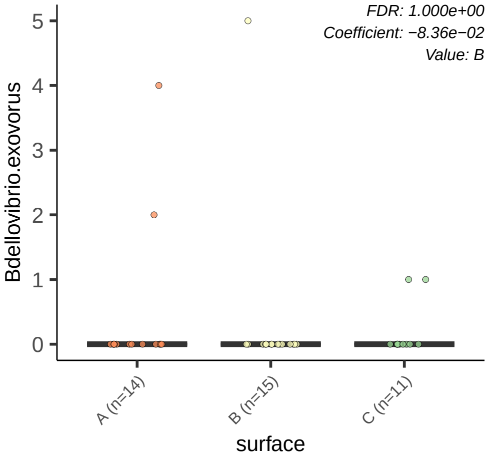

FDR: 1.000e+00
Coefficient: −8.36e−02
Value: B
Bdellovibrio.exovorus
0
1
2
3
4
5
A (n=14)
B (n=15)
C (n=11)
surface

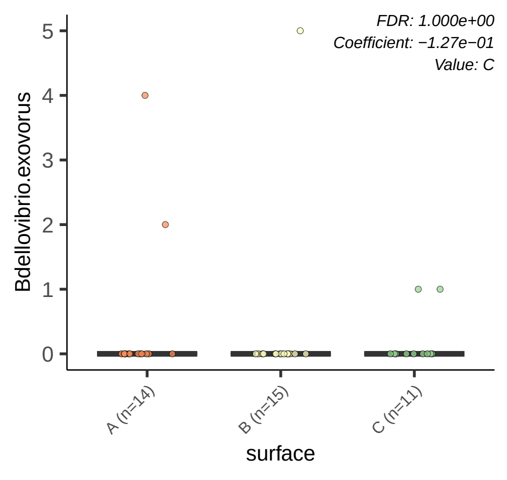

FDR: 1.000e+00
Coefficient: −1.27e−01
Value: C
Bdellovibrio.exovorus
0
1
2
3
4
5
A (n=14)
B (n=15)
C (n=11)
surface

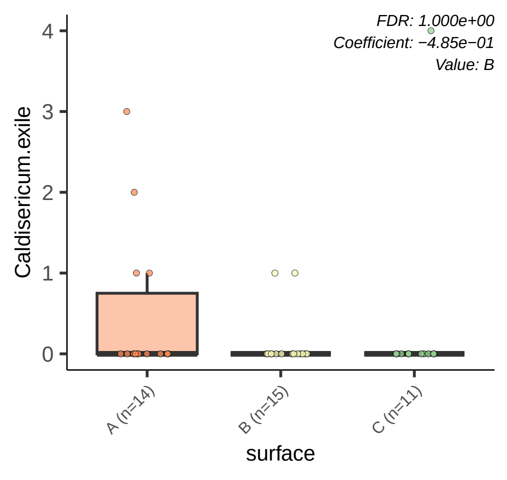
FDR: 1.000e+00
Coefficient: −4.85e−01
Value: B
Caldisericum.exile
4
3
2
1
0
A (n=14)
B (n=15)
C (n=11)
surface

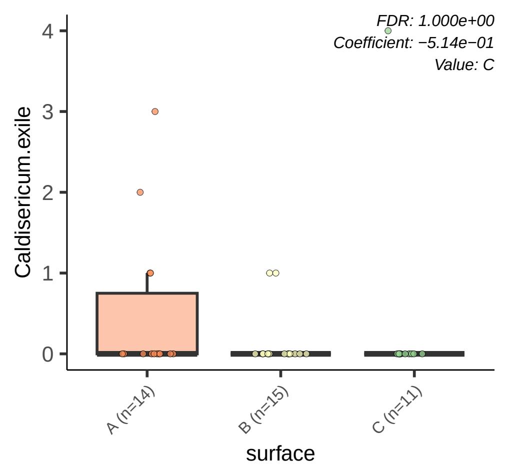
FDR: 1.000e+00
Coefficient: −5.14e−01
Value: C
Caldisericum.exile
0
1
2
3
4
A (n=14)
B (n=15)
C (n=11)
surface

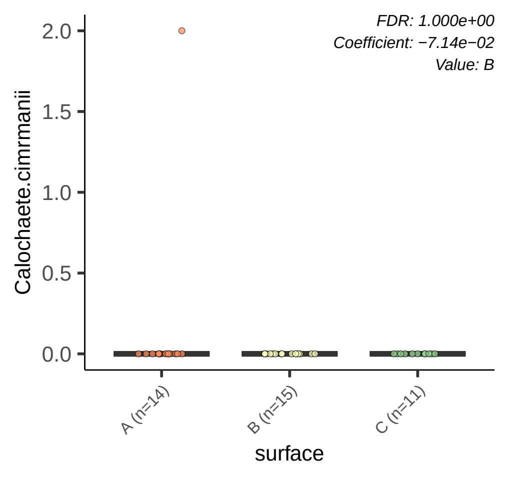

FDR: 1.000e+00
Coefficient: −7.14e−02
Value: B
Calochaete.cimrmanii
2.0
1.5
1.0
0.5
0.0
A (n=14)
B (n=15)
C (n=11)
surface

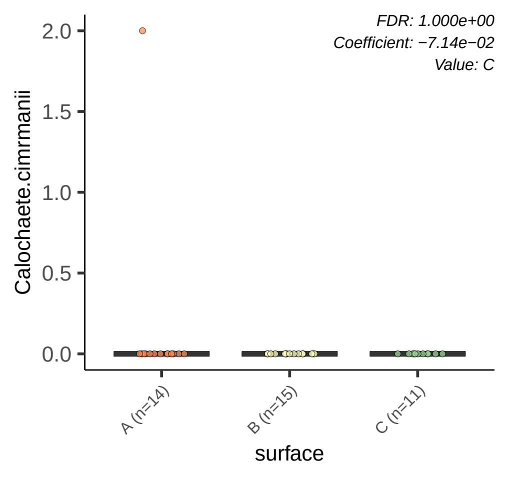
FDR: 1.000e+00
Coefficient: −7.14e−02
Value: C
Calochaete.cimrmanii
2.0
1.5
1.0
0.5
0.0
A (n=14)
B (n=15)
C (n=11)
surface

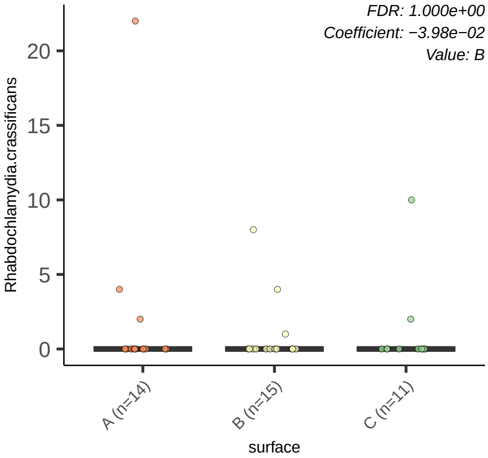
FDR: 1.000e+00
Coefficient: −3.98e−02
Value: B
Rhabdochlamydia.crassificans
0
5
10
15
20
A (n=14)
B (n=15)
C (n=11)
surface

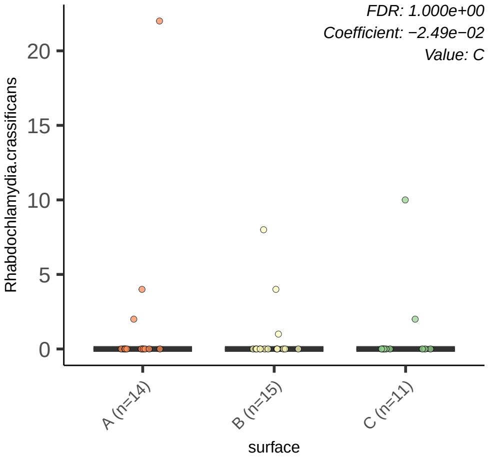
FDR: 1.000e+00
Coefficient: −2.49e−02
Value: C
Rhabdochlamydia.crassificans
0
5
10
15
20
A (n=14)
B (n=15)
C (n=11)
surface

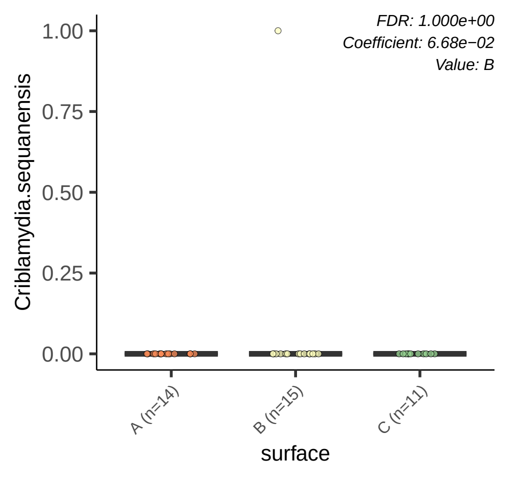
FDR: 1.000e+00
Coefficient: 6.68e−02
Value: B
Criblamydia.sequanensis
1.00
0.75
0.50
0.25
0.00
A (n=14)
B (n=15)
C (n=11)
surface

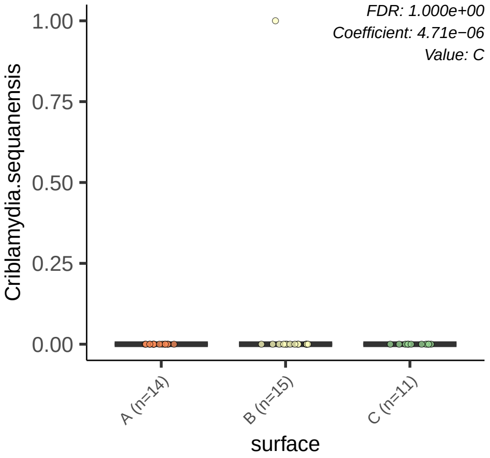
FDR: 1.000e+00
Coefficient: 4.71e−06
Value: C
Criblamydia.sequanensis
1.00
0.75
0.50
0.25
0.00
A (n=14)
B (n=15)
C (n=11)
surface

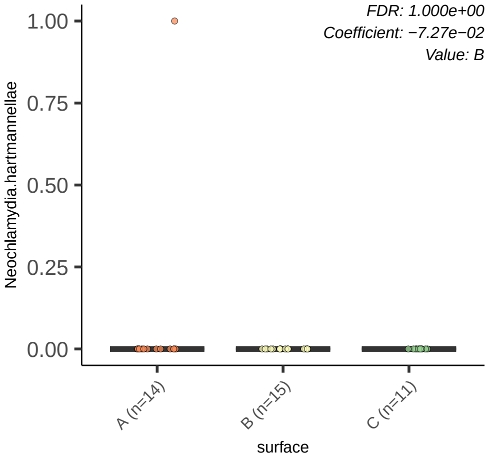
FDR: 1.000e+00
Coefficient: −7.27e−02
Value: B
Neochlamydia.hartmannellae
1.00
0.75
0.50
0.25
0.00
A (n=14)
B (n=15)
C (n=11)
surface

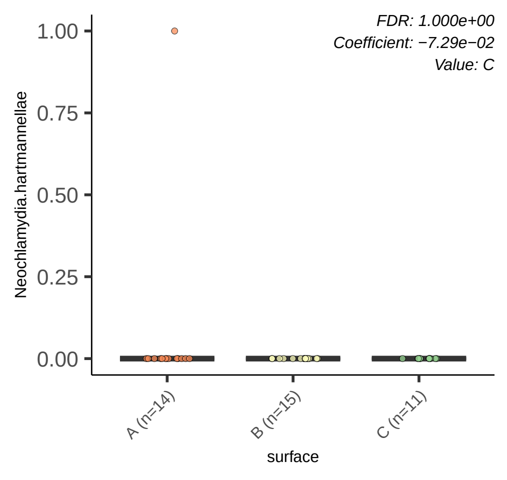

FDR: 1.000e+00
Coefficient: −7.29e−02
Value: C
Neochlamydia.hartmannellae
1.00
0.75
0.50
0.25
0.00
A (n=14)
B (n=15)
C (n=11)
surface

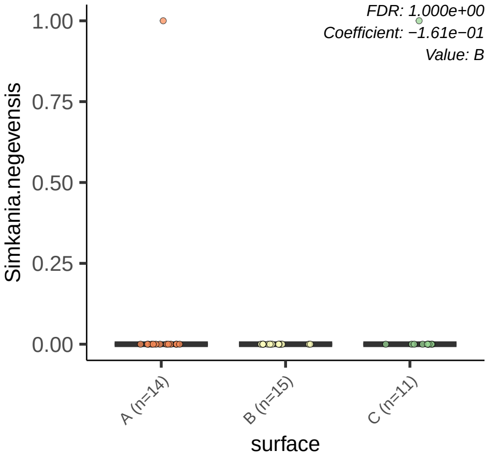

FDR: 1.000e+00
Coefficient: −1.61e−01
Value: B
Simkania.negevensis
1.00
0.75
0.50
0.25
0.00
A (n=14)
B (n=15)
C (n=11)
surface

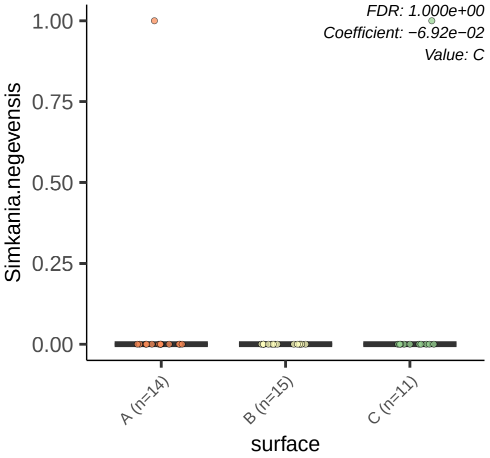

FDR: 1.000e+00
Coefficient: −6.92e−02
Value: C
1.00
0.75
0.50
0.25
0.00
Simkania.negevensis
A (n=14)
B (n=15)
C (n=11)
surface

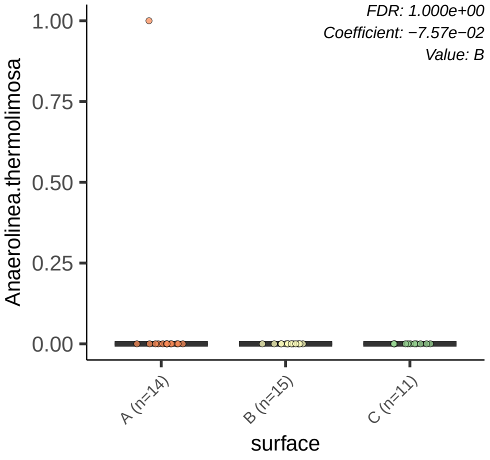

FDR: 1.000e+00
Coefficient: −7.57e−02
Value: B
Anaerolinea.thermolimosa
1.00
0.75
0.50
0.25
0.00
A (n=14)
B (n=15)
C (n=11)
surface

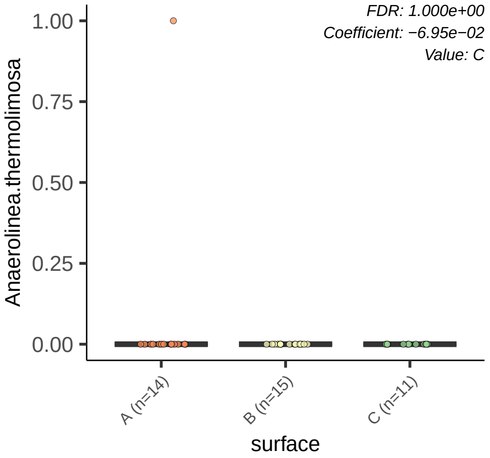

FDR: 1.000e+00
Coefficient: −6.95e−02
Value: C
Anaerolinea.thermolimosa
1.00
0.75
0.50
0.25
0.00
A (n=14)
B (n=15)
C (n=11)
surface

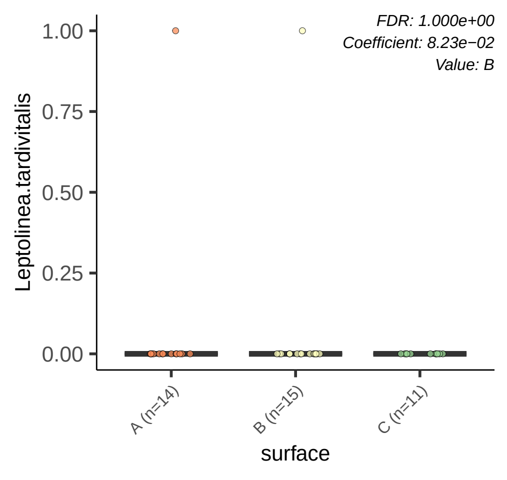
FDR: 1.000e+00
Coefficient: 8.23e−02
Value: B
Leptolinea.tardivitalis
1.00
0.75
0.50
0.25
0.00
A (n=14)
B (n=15)
C (n=11)
surface

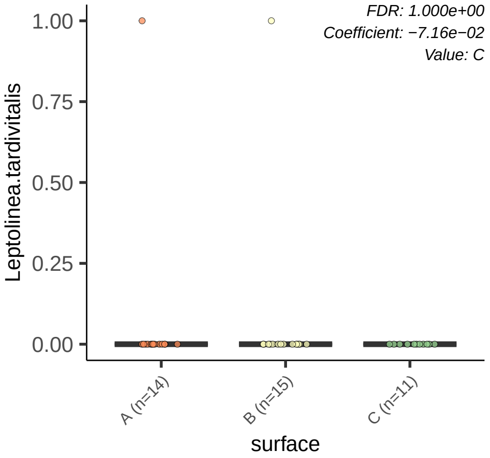
FDR: 1.000e+00
Coefficient: −7.16e−02
Value: C
Leptolinea.tardivitalis
1.00
0.75
0.50
0.25
0.00
A (n=14)
B (n=15)
C (n=11)
surface

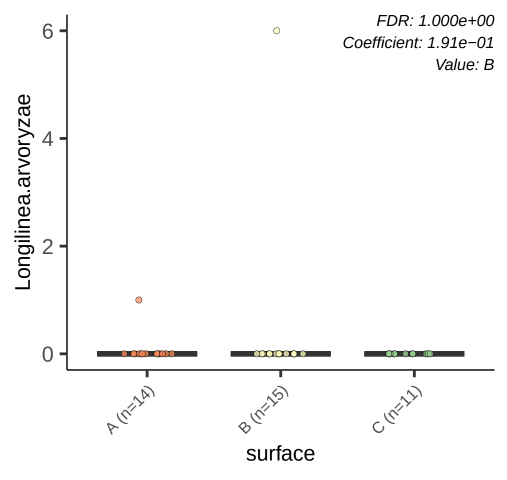
FDR: 1.000e+00
Coefficient: 1.91e−01
Value: B
Longilinea.arvoryzae
0
2
4
6
A (n=14)
B (n=15)
C (n=11)
surface

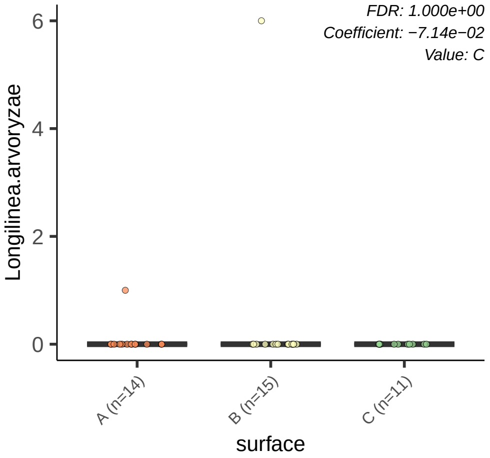
FDR: 1.000e+00
Coefficient: −7.14e−02
Value: C
Longilinea.arvoryzae
0
2
4
6
A (n=14)
B (n=15)
C (n=11)
surface

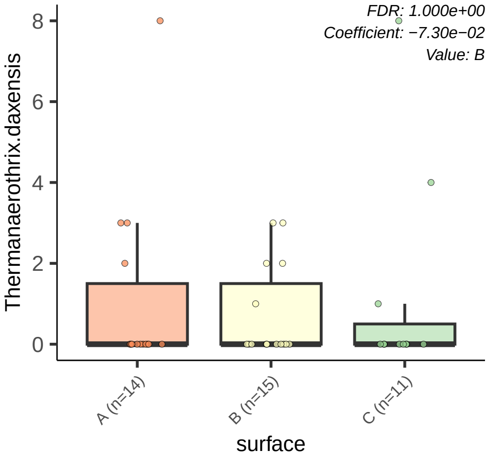

FDR: 1.000e+00
Coefficient: −7.30e−02
Value: B
Thermanaerothrix.daxensis
0
2
4
6
8
A (n=14)
B (n=15)
C (n=11)
surface

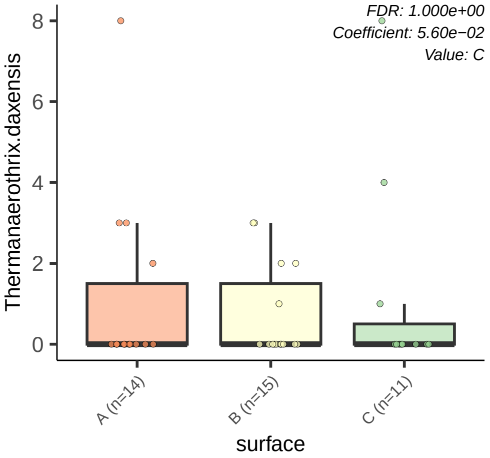
FDR: 1.000e+00
Coefficient: 5.60e−02
Value: C
Thermanaerothrix.daxensis
0
2
4
6
8
A (n=14)
B (n=15)
C (n=11)
surface

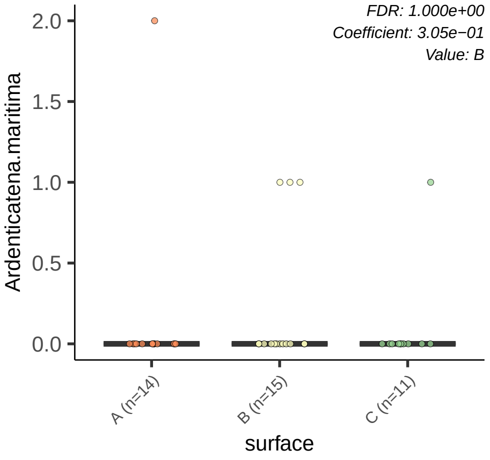
FDR: 1.000e+00
Coefficient: 3.05e−01
Value: B
Ardenticatena.maritima
2.0
1.5
1.0
0.5
0.0
A (n=14)
B (n=15)
C (n=11)
surface

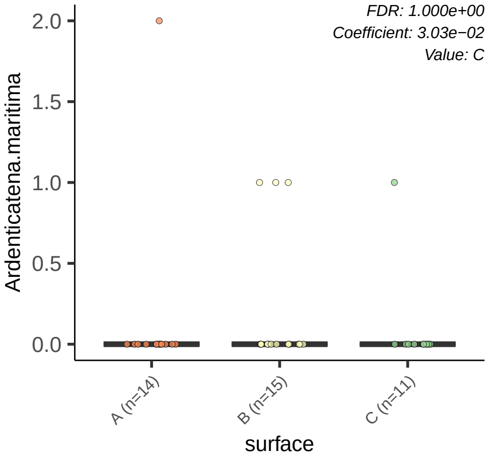
FDR: 1.000e+00
Coefficient: 3.03e−02
Value: C
Ardenticatena.maritima
2.0
1.5
1.0
0.5
0.0
A (n=14)
B (n=15)
C (n=11)
surface

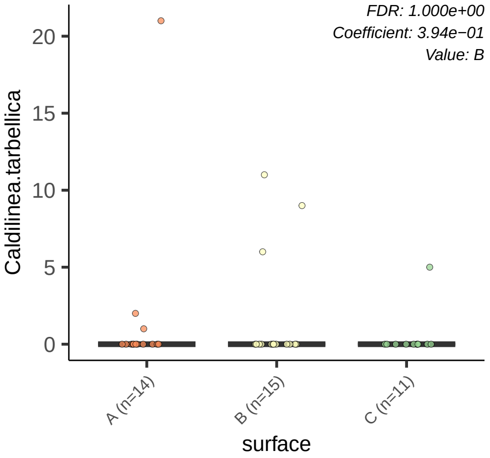

FDR: 1.000e+00
Coefficient: 3.94e−01
Value: B
Caldilinea.tarbellica
20
15
10
5
0
A (n=14)
B (n=15)
C (n=11)
surface

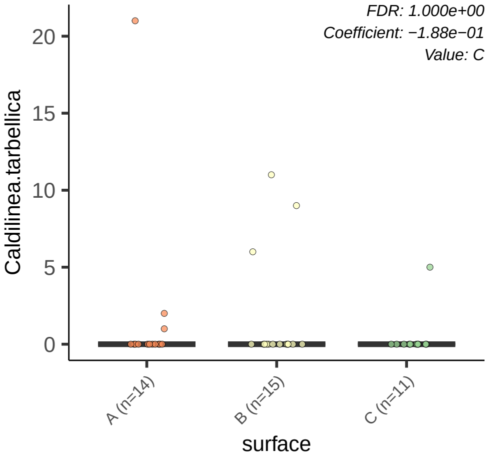
FDR: 1.000e+00
Coefficient: −1.88e−01
Value: C
Caldilinea.tarbellica
0
5
10
15
20
A (n=14)
B (n=15)
C (n=11)
surface

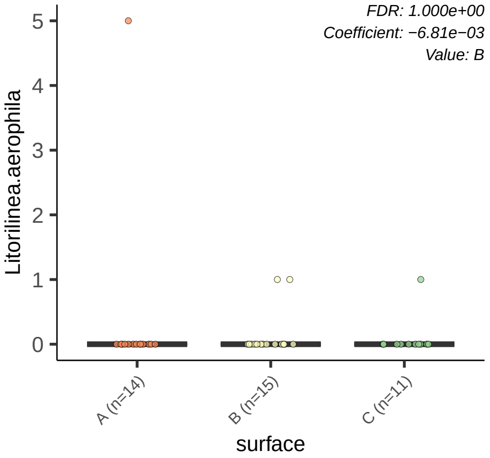
FDR: 1.000e+00
Coefficient: −6.81e−03
Value: B
Litorilinea.aerophila
5
4
3
2
1
0
A (n=14)
B (n=15)
C (n=11)
surface

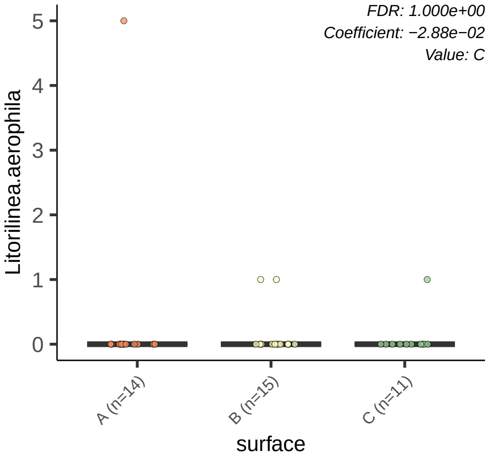

FDR: 1.000e+00
Coefficient: −2.88e−02
Value: C
Litorilinea.aerophila
0
1
2
3
4
5
A (n=14)
B (n=15)
C (n=11)
surface

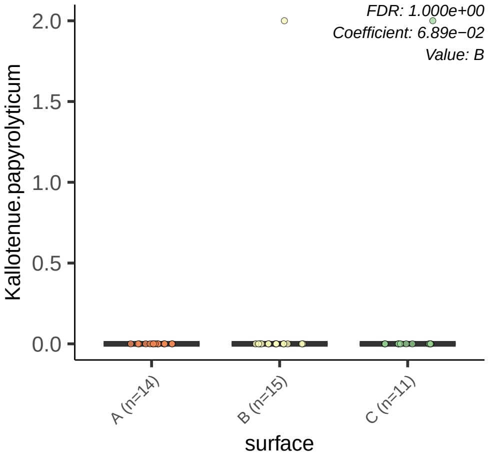

FDR: 1.000e+00
Coefficient: 6.89e−02
Value: B
Kallotenue.papyrolyticum
2.0
1.5
1.0
0.5
0.0
A (n=14)
B (n=15)
C (n=11)
surface

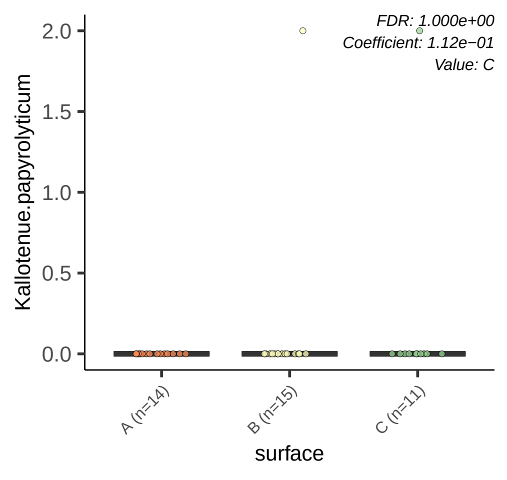
FDR: 1.000e+00
Coefficient: 1.12e−01
Value: C
Kallotenue.papyrolyticum
2.0
1.5
1.0
0.5
0.0
A (n=14)
B (n=15)
C (n=11)
surface

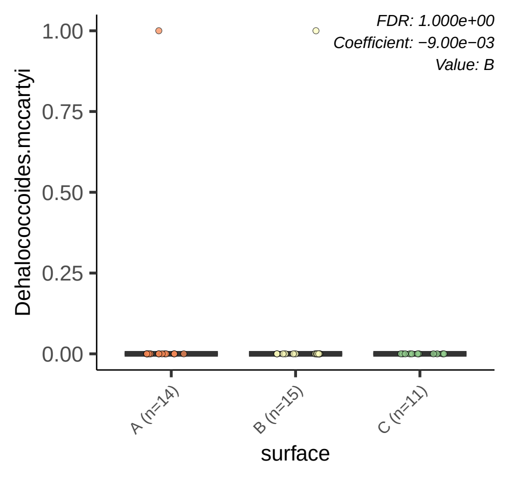
FDR: 1.000e+00
Coefficient: −9.00e−03
Value: B
Dehalococcoides.mccartyi
1.00
0.75
0.50
0.25
0.00
A (n=14)
B (n=15)
C (n=11)
surface

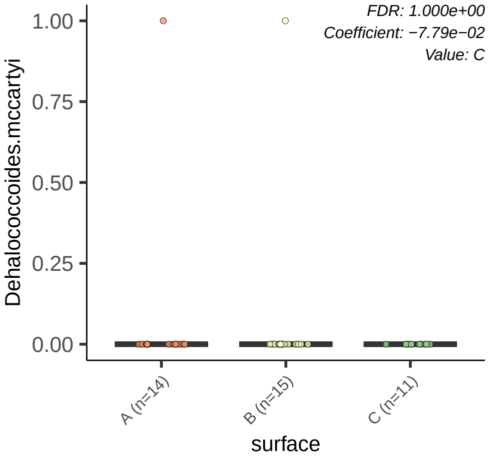

FDR: 1.000e+00
Coefficient: −7.79e−02
Value: C
Dehalococcoides.mccartyi
1.00
0.75
0.50
0.25
0.00
A (n=14)
B (n=15)
C (n=11)
surface

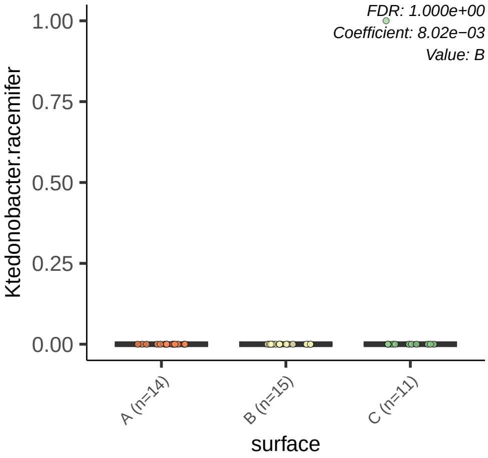
FDR: 1.000e+00
Coefficient: 8.02e−03
Value: B
Ktedonobacter.racemifer
1.00
0.75
0.50
0.25
0.00
A (n=14)
B (n=15)
C (n=11)
surface

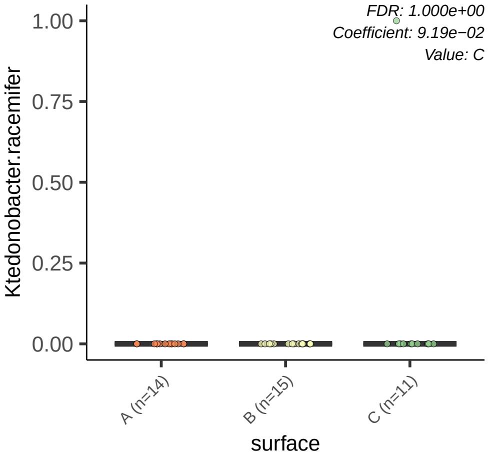
FDR: 1.000e+00
Coefficient: 9.19e−02
Value: C
Ktedonobacter.racemifer
1.00
0.75
0.50
0.25
0.00
A (n=14)
B (n=15)
C (n=11)
surface

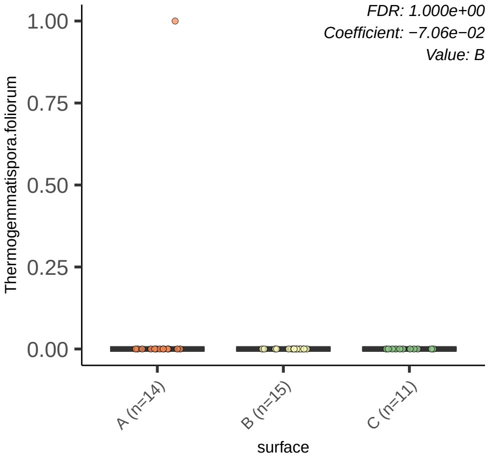
FDR: 1.000e+00
Coefficient: −7.06e−02
Value: B
Thermogemmatispora.foliorum
1.00
0.75
0.50
0.25
0.00
A (n=14)
B (n=15)
C (n=11)
surface

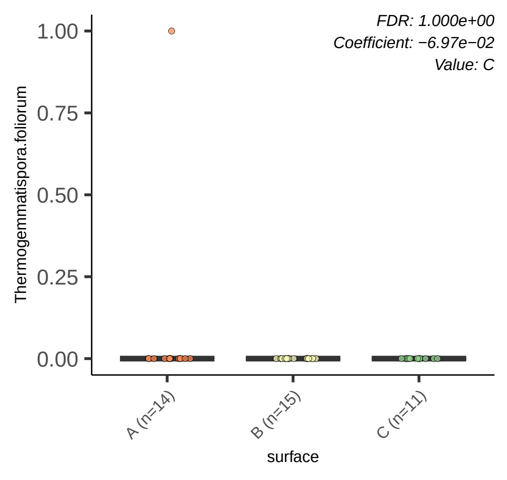

FDR: 1.000e+00
Coefficient: −6.97e−02
Value: C
Thermogemmatispora.foliorum
1.00
0.75
0.50
0.25
0.00
A (n=14)
B (n=15)
C (n=11)
surface

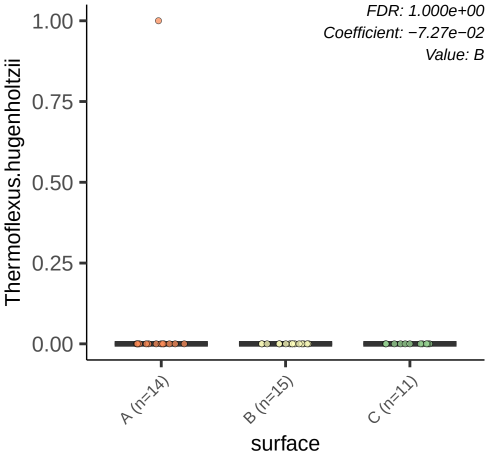

FDR: 1.000e+00
Coefficient: −7.27e−02
Value: B
Thermoflexus.hugenholtzii
1.00
0.75
0.50
0.25
0.00
A (n=14)
B (n=15)
C (n=11)
surface

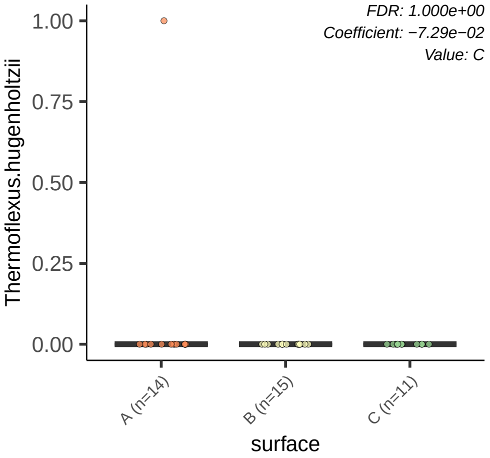
FDR: 1.000e+00
Coefficient: −7.29e−02
Value: C
Thermoflexus.hugenholtzii
1.00
0.75
0.50
0.25
0.00
A (n=14)
B (n=15)
C (n=11)
surface

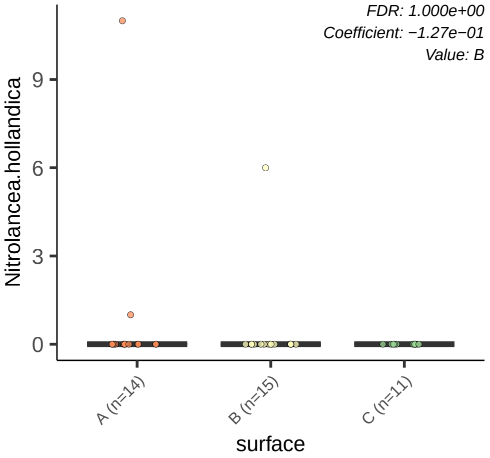
FDR: 1.000e+00
Coefficient: −1.27e−01
Value: B
Nitrolancea.hollandica
0
3
6
9
A (n=14)
B (n=15)
C (n=11)
surface

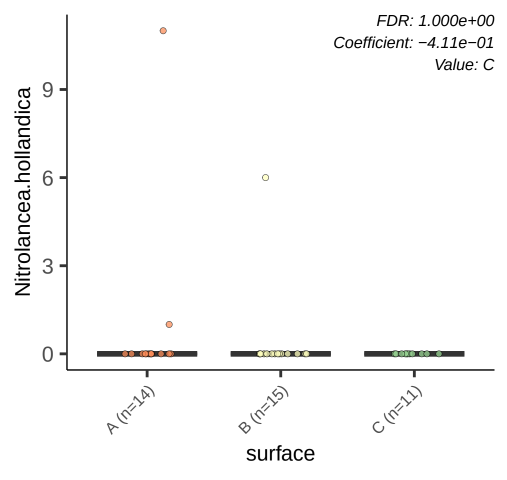

FDR: 1.000e+00
Coefficient: −4.11e−01
Value: C
Nitrolancea.hollandica
9
6
3
0
A (n=14)
B (n=15)
C (n=11)
surface

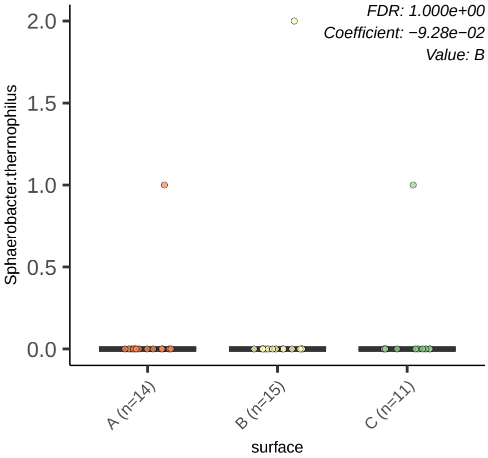

FDR: 1.000e+00
Coefficient: −9.28e−02
Value: B
Sphaerobacter.thermophilus
2.0
1.5
1.0
0.5
0.0
A (n=14)
B (n=15)
C (n=11)
surface

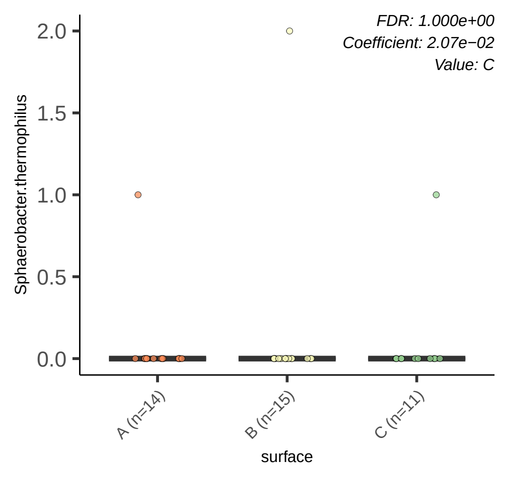
FDR: 1.000e+00
Coefficient: 2.07e−02
Value: C
Sphaerobacter.thermophilus
2.0
1.5
1.0
0.5
0.0
A (n=14)
B (n=15)
C (n=11)
surface

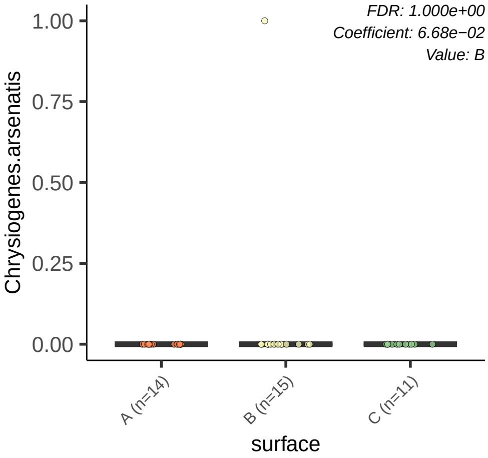
FDR: 1.000e+00
Coefficient: 6.68e−02
Value: B
Chrysiogenes.arsenatis
1.00
0.75
0.50
0.25
0.00
A (n=14)
B (n=15)
C (n=11)
surface

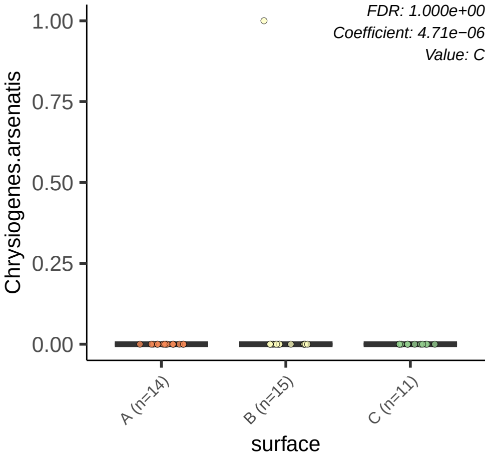

FDR: 1.000e+00
Coefficient: 4.71e−06
Value: C
Chrysiogenes.arsenatis
1.00
0.75
0.50
0.25
0.00
A (n=14)
B (n=15)
C (n=11)
surface

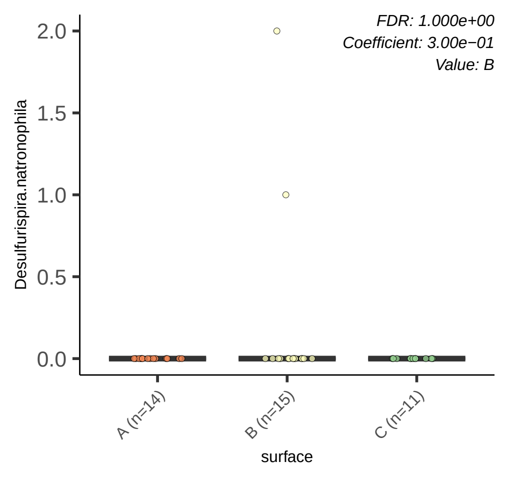
FDR: 1.000e+00
Coefficient: 3.00e−01
Value: B
Desulfurispira.natronophila
2.0
1.5
1.0
0.5
0.0
A (n=14)
B (n=15)
C (n=11)
surface

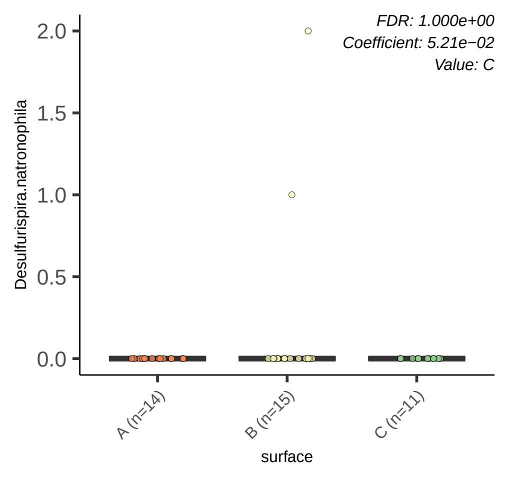
FDR: 1.000e+00
Coefficient: 5.21e−02
Value: C
Desulfurispira.natronophila
2.0
1.5
1.0
0.5
0.0
A (n=14)
B (n=15)
C (n=11)
surface

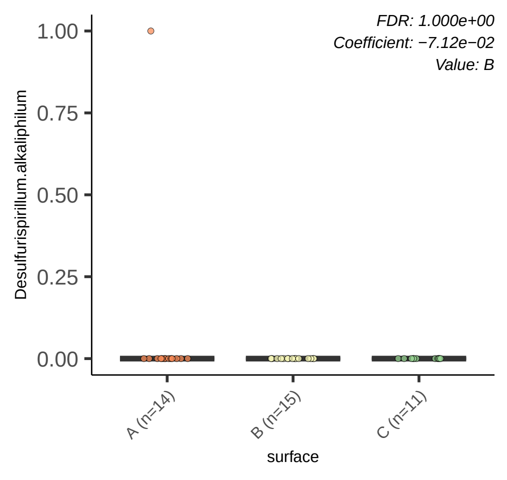
FDR: 1.000e+00
Coefficient: −7.12e−02
Value: B
1.00
0.75
0.50
0.25
0.00
Desulfurispirillum.alkaliphilum
A (n=14)
B (n=15)
C (n=11)
surface

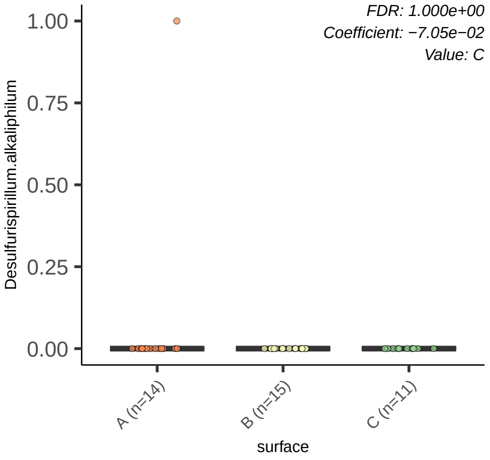
FDR: 1.000e+00
Coefficient: −7.05e−02
Value: C
Desulfurispirillum.alkaliphilum
1.00
0.75
0.50
0.25
0.00
A (n=14)
B (n=15)
C (n=11)
surface

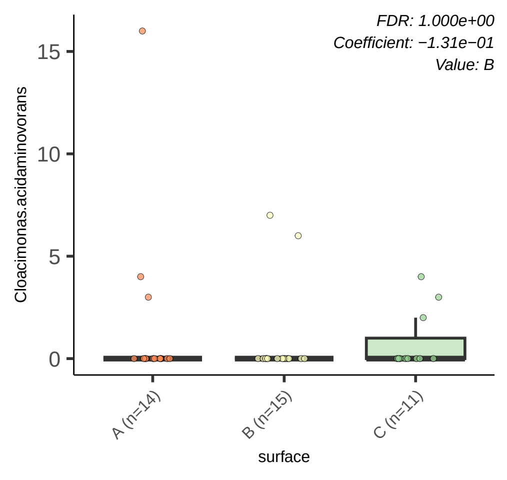
FDR: 1.000e+00
Coefficient: −1.31e−01
Value: B
Cloacimonas.acidaminovorans
15
10
5
0
A (n=14)
B (n=15)
C (n=11)
surface

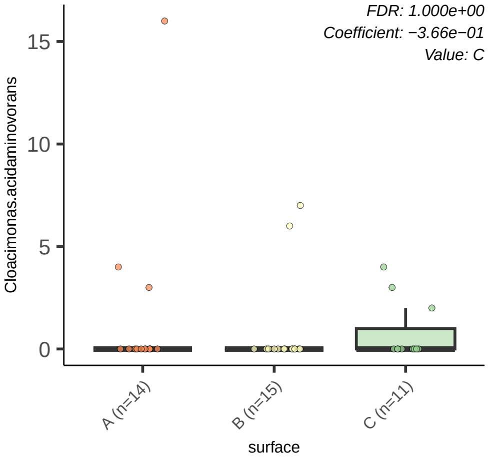

FDR: 1.000e+00
Coefficient: −3.66e−01
Value: C
Cloacimonas.acidaminovorans
15
10
5
0
A (n=14)
B (n=15)
C (n=11)
surface

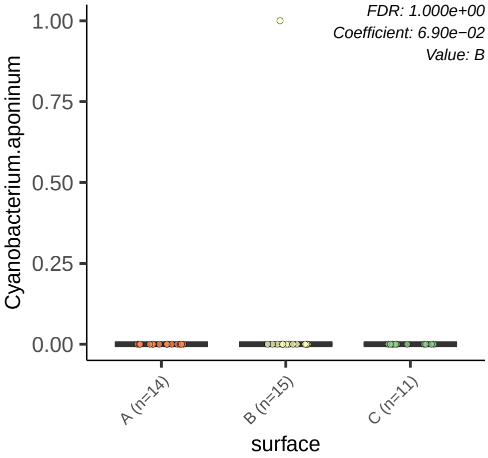

FDR: 1.000e+00
Coefficient: 6.90e−02
Value: B
Cyanobacterium.aponinum
1.00
0.75
0.50
0.25
0.00
A (n=14)
B (n=15)
C (n=11)
surface

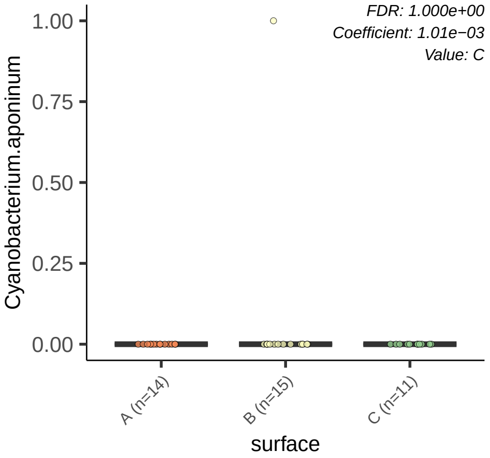

FDR: 1.000e+00
Coefficient: 1.01e−03
Value: C
Cyanobacterium.aponinum
1.00
0.75
0.50
0.25
0.00
A (n=14)
B (n=15)
C (n=11)
surface

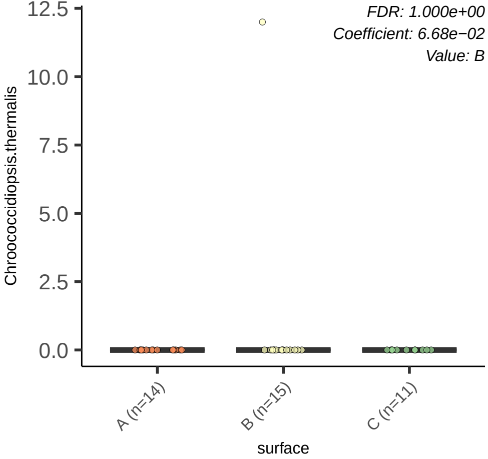
FDR: 1.000e+00
Coefficient: 6.68e−02
Value: B
Chroococcidiopsis.thermalis
12.5
10.0
7.5
5.0
2.5
0.0
A (n=14)
B (n=15)
C (n=11)
surface

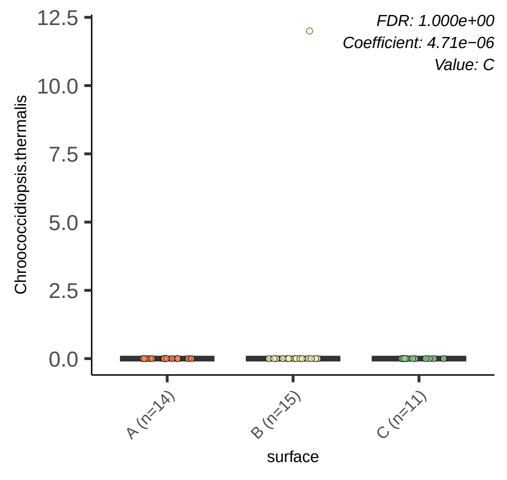

FDR: 1.000e+00
Coefficient: 4.71e−06
Value: C
12.5
10.0
7.5
5.0
2.5
0.0
Chroococcidiopsis.thermalis
A (n=14)
B (n=15)
C (n=11)
surface

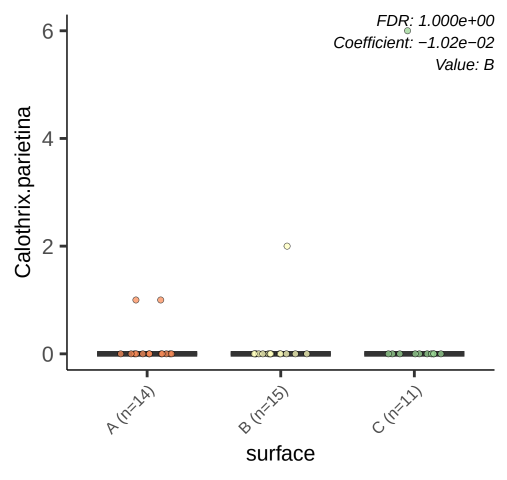
FDR: 1.000e+00
Coefficient: −1.02e−02
Value: B
Calothrix.parietina
6
4
2
0
A (n=14)
B (n=15)
C (n=11)
surface

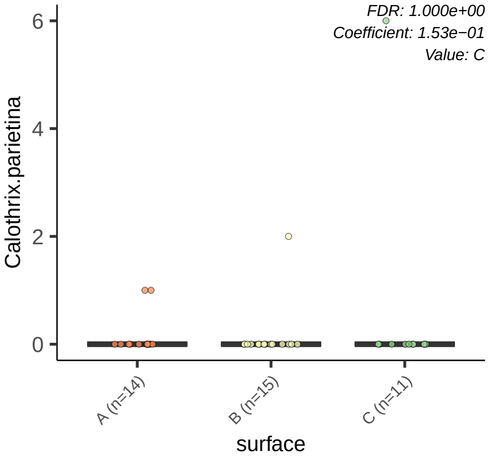

FDR: 1.000e+00
Coefficient: 1.53e−01
Value: C
Calothrix.parietina
0
2
4
6
A (n=14)
B (n=15)
C (n=11)
surface

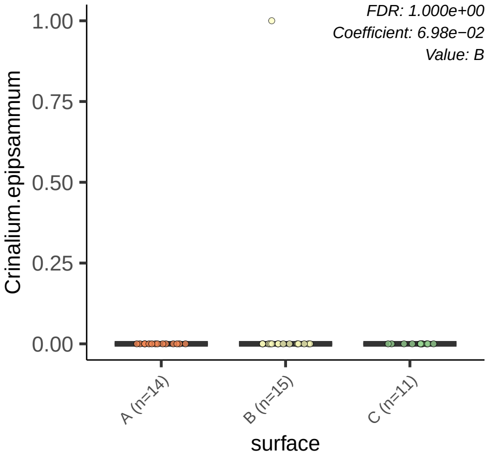
FDR: 1.000e+00
Coefficient: 6.98e−02
Value: B
Crinalium.epipsammum
1.00
0.75
0.50
0.25
0.00
A (n=14)
B (n=15)
C (n=11)
surface

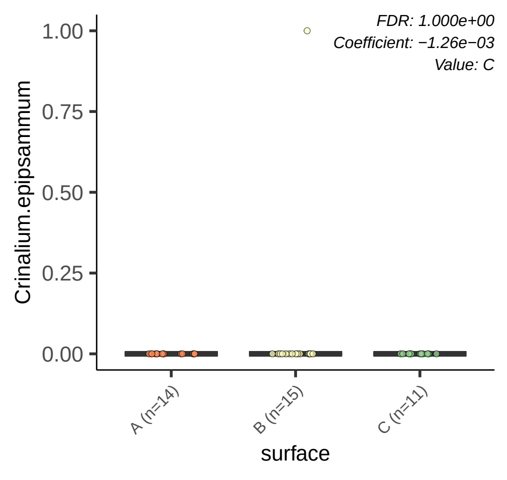
FDR: 1.000e+00
Coefficient: −1.26e−03
Value: C
Crinalium.epipsammum
1.00
0.75
0.50
0.25
0.00
A (n=14)
B (n=15)
C (n=11)
surface

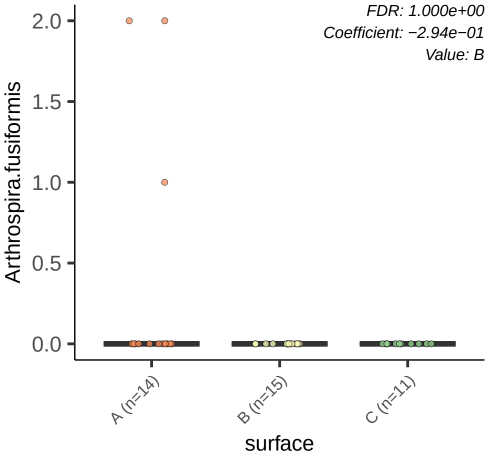
FDR: 1.000e+00
Coefficient: −2.94e−01
Value: B
Arthrospira.fusiformis
2.0
1.5
1.0
0.5
0.0
A (n=14)
B (n=15)
C (n=11)
surface

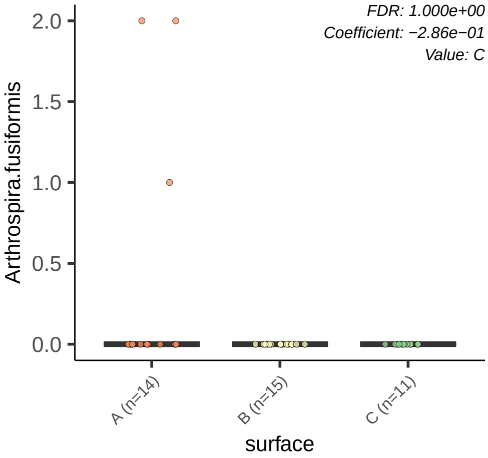

FDR: 1.000e+00
Coefficient: −2.86e−01
Value: C
Arthrospira.fusiformis
2.0
1.5
1.0
0.5
0.0
A (n=14)
B (n=15)
C (n=11)
surface

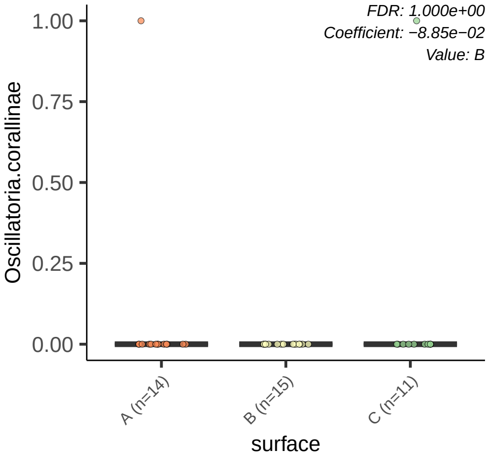

FDR: 1.000e+00
Coefficient: −8.85e−02
Value: B
Oscillatoria.coralline
1.00
0.75
0.50
0.25
0.00
A (n=14)
B (n=15)
C (n=11)
surface

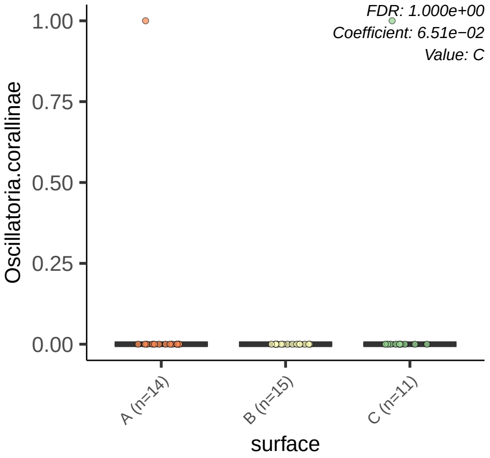

FDR: 1.000e+00
Coefficient: 6.51e−02
Value: C
Oscillatoria.corallinae
1.00
0.75
0.50
0.25
0.00
A (n=14)
B (n=15)
C (n=11)
surface

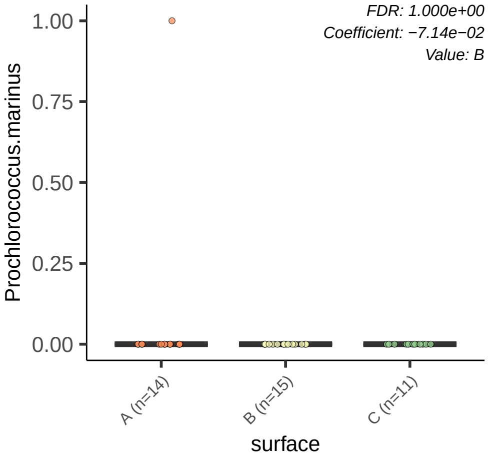

FDR: 1.000e+00
Coefficient: −7.14e−02
Value: B
Prochlorococcus.marinus
1.00
0.75
0.50
0.25
0.00
A (n=14)
B (n=15)
C (n=11)
surface

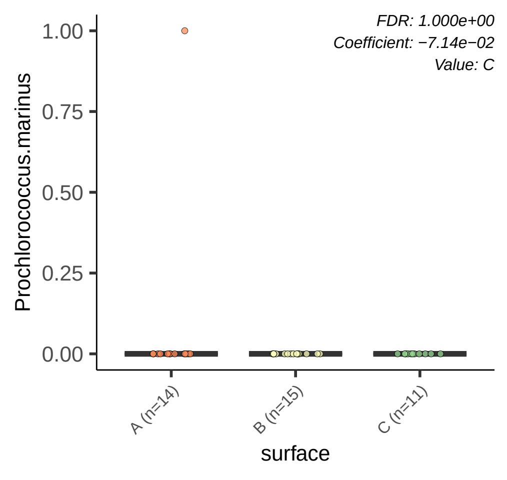
FDR: 1.000e+00
Coefficient: −7.14e−02
Value: C
Prochlorococcus.marinus
1.00
0.75
0.50
0.25
0.00
A (n=14)
B (n=15)
C (n=11)
surface

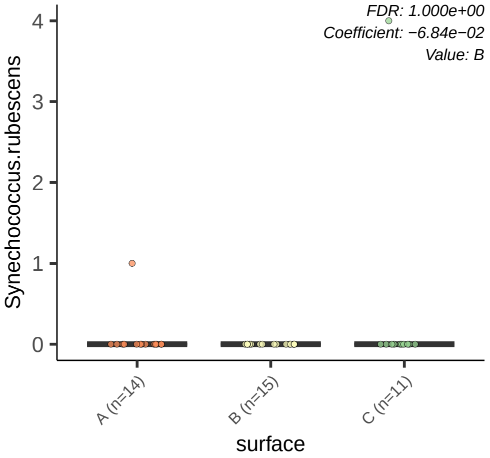
FDR: 1.000e+00
Coefficient: −6.84e−02
Value: B
Synechococcus.rubescens
4
3
2
1
0
A (n=14)
B (n=15)
C (n=11)
surface

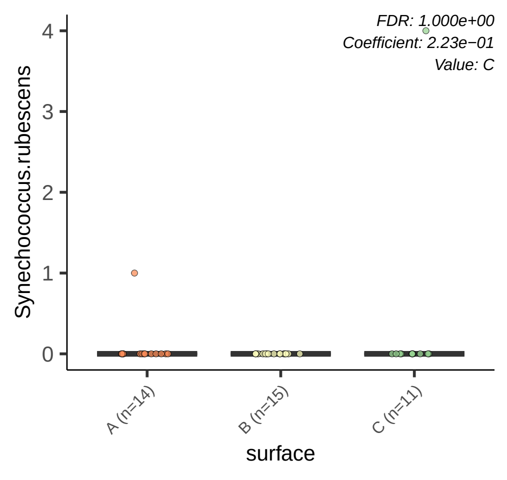
FDR: 1.000e+00
Coefficient: 2.23e−01
Value: C
Synechococcus.rubescens
4
3
2
1
0
A (n=14)
B (n=15)
C (n=11)
surface

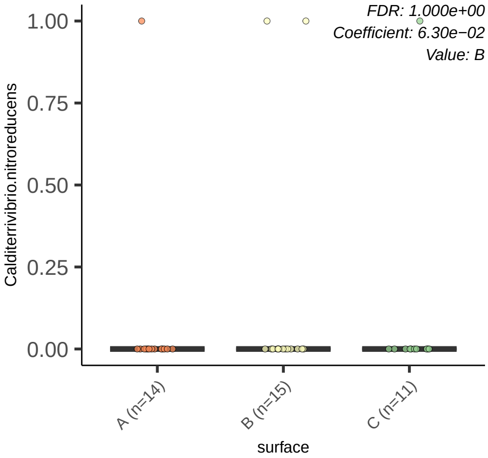
FDR: 1.000e+00
Coefficient: 6.30e−02
Value: B
Calditerrivibrio.nitroreducens
1.00
0.75
0.50
0.25
0.00
A (n=14)
B (n=15)
C (n=11)
surface

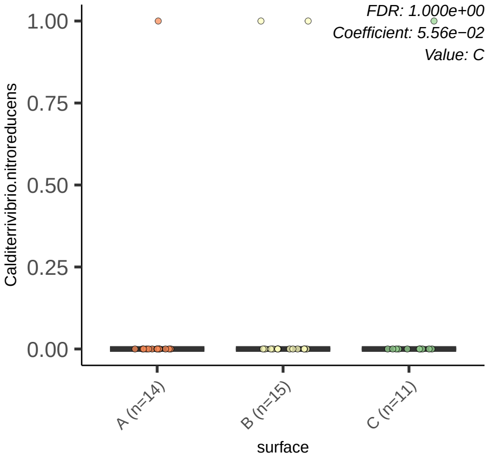

FDR: 1.000e+00
Coefficient: 5.56e−02
Value: C
Calditerrivibrio.nitroreducens
1.00
0.75
0.50
0.25
0.00
A (n=14)
B (n=15)
C (n=11)
surface

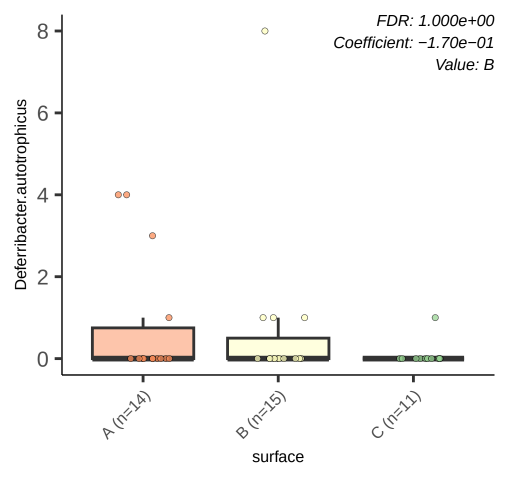

FDR: 1.000e+00
Coefficient: −1.70e−01
Value: B
Deferribacter.autotrophicus
0
2
4
6
8
A (n=14)
B (n=15)
C (n=11)
surface

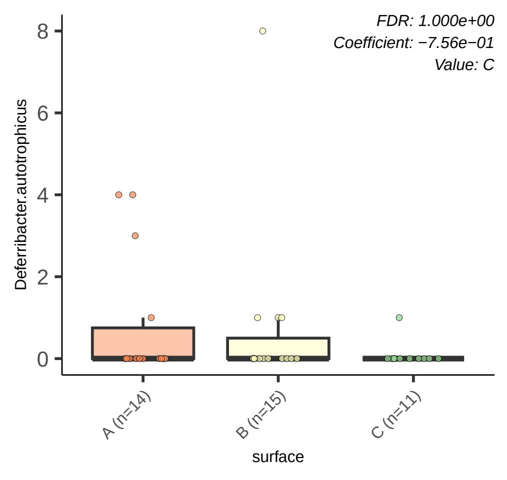
FDR: 1.000e+00
Coefficient: −7.56e−01
Value: C
Deferribacter.autotrophicus
0
2
4
6
8
A (n=14)
B (n=15)
C (n=11)
surface

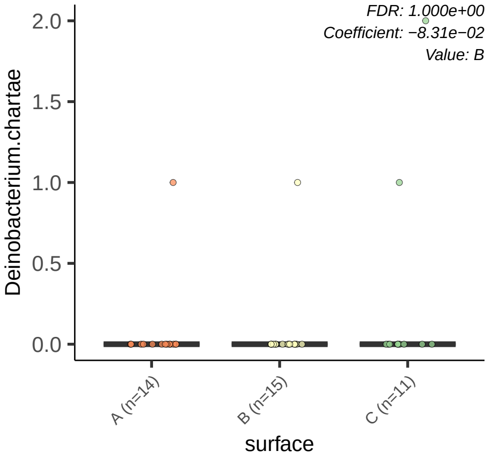

FDR: 1.000e+00
Coefficient: −8.31e−02
Value: B
Deinobacterium.chartae
2.0
1.5
1.0
0.5
0.0
A (n=14)
B (n=15)
C (n=11)
surface

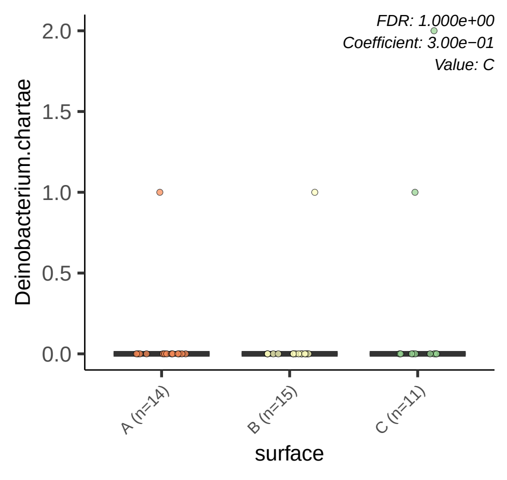
FDR: 1.000e+00
Coefficient: 3.00e−01
Value: C
Deinobacterium.chartae
2.0
1.5
1.0
0.5
0.0
A (n=14)
B (n=15)
C (n=11)
surface

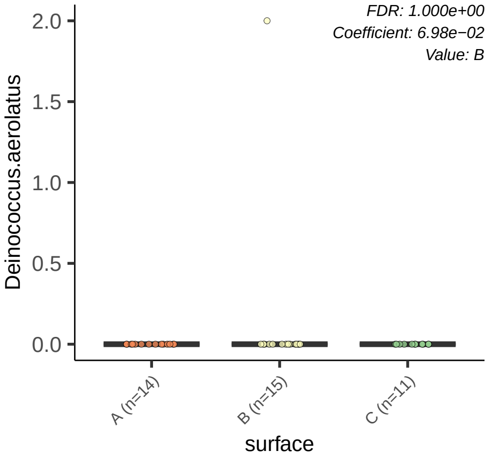

FDR: 1.000e+00
Coefficient: 6.98e−02
Value: B
Deinococcus.aerolatus
2.0
1.5
1.0
0.5
0.0
A (n=14)
B (n=15)
C (n=11)
surface

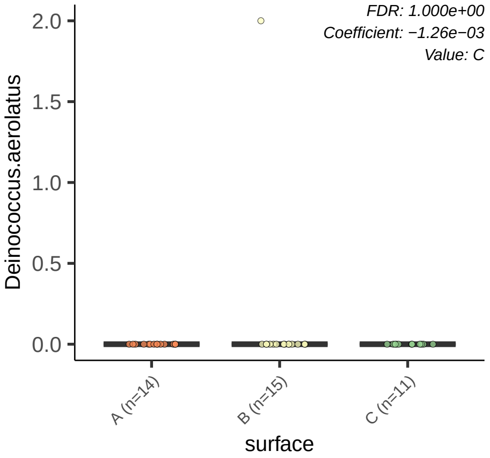
FDR: 1.000e+00
Coefficient: −1.26e−03
Value: C
Deinococcus.aerolatus
2.0
1.5
1.0
0.5
0.0
A (n=14)
B (n=15)
C (n=11)
surface

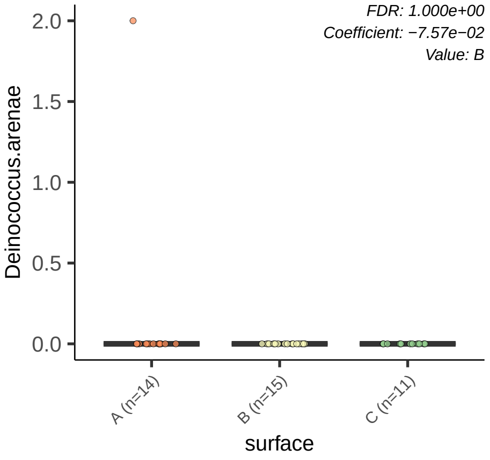

FDR: 1.000e+00
Coefficient: −7.57e−02
Value: B
Deinococcus.arenae
2.0
1.5
1.0
0.5
0.0
A (n=14)
B (n=15)
C (n=11)
surface

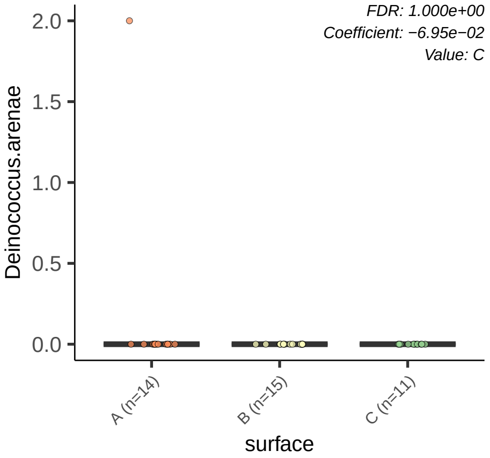
FDR: 1.000e+00
Coefficient: −6.95e−02
Value: C
Deinococcus.arenae
2.0
1.5
1.0
0.5
0.0
A (n=14)
B (n=15)
C (n=11)
surface

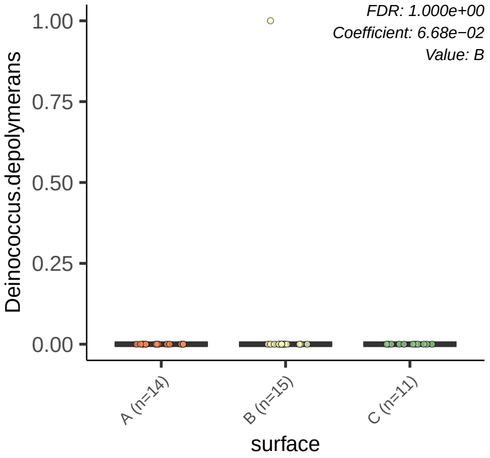
FDR: 1.000e+00
Coefficient: 6.68e−02
Value: B
Deinococcus.depolymerans
1.00
0.75
0.50
0.25
0.00
A (n=14)
B (n=15)
C (n=11)
surface

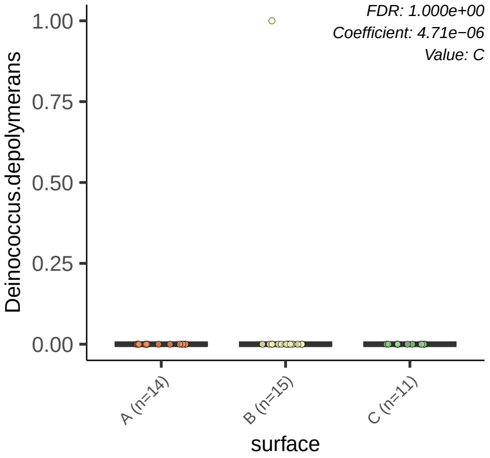
FDR: 1.000e+00
Coefficient: 4.71e−06
Value: C
Deinococcus.depolymerans
1.00
0.75
0.50
0.25
0.00
A (n=14)
B (n=15)
C (n=11)
surface

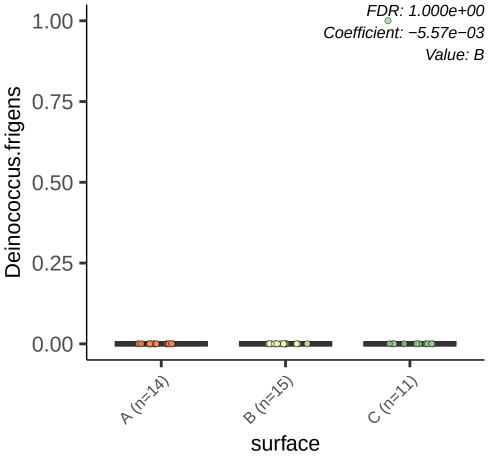
FDR: 1.000e+00
Coefficient: −5.57e−03
Value: B
Deinococcus.frigens
1.00
0.75
0.50
0.25
0.00
A (n=14)
B (n=15)
C (n=11)
surface

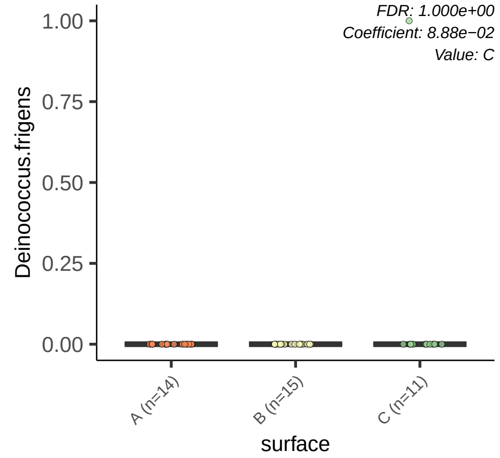
FDR: 1.000e+00
Coefficient: 8.88e−02
Value: C
Deinococcus.frigens
1.00
0.75
0.50
0.25
0.00
A (n=14)
B (n=15)
C (n=11)
surface

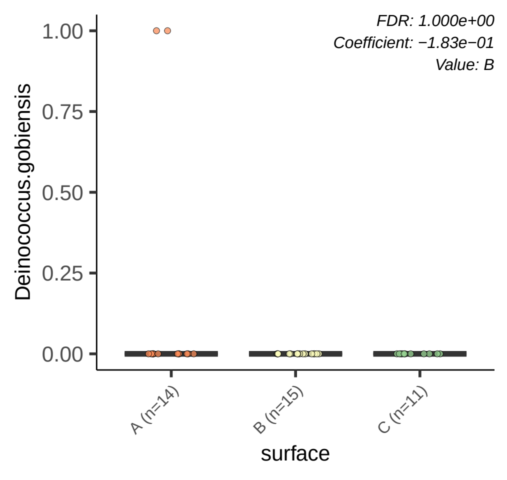

FDR: 1.000e+00
Coefficient: −1.83e−01
Value: B
Deinococcus.gobiensis
1.00
0.75
0.50
0.25
0.00
A (n=14)
B (n=15)
C (n=11)
surface

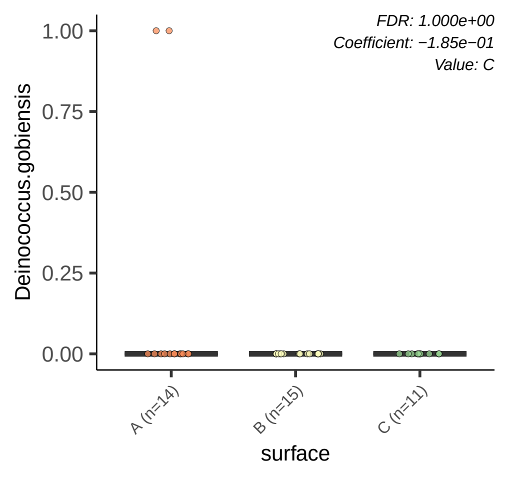
FDR: 1.000e+00
Coefficient: −1.85e−01
Value: C
Deinococcus.gobiensis
1.00
0.75
0.50
0.25
0.00
A (n=14)
B (n=15)
C (n=11)
surface

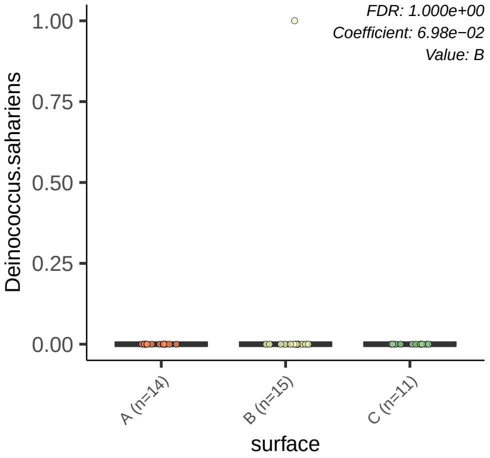

FDR: 1.000e+00
Coefficient: 6.98e−02
Value: B
Deinococcus.sahariens
1.00
0.75
0.50
0.25
0.00
A (n=14)
B (n=15)
C (n=11)
surface

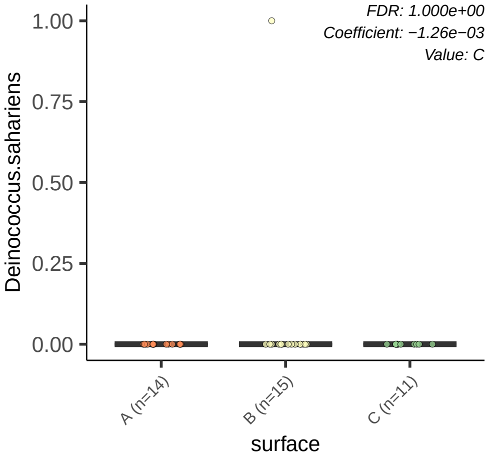
FDR: 1.000e+00
Coefficient: −1.26e−03
Value: C
Deinococcus.sahariens
1.00
0.75
0.50
0.25
0.00
A (n=14)
B (n=15)
C (n=11)
surface

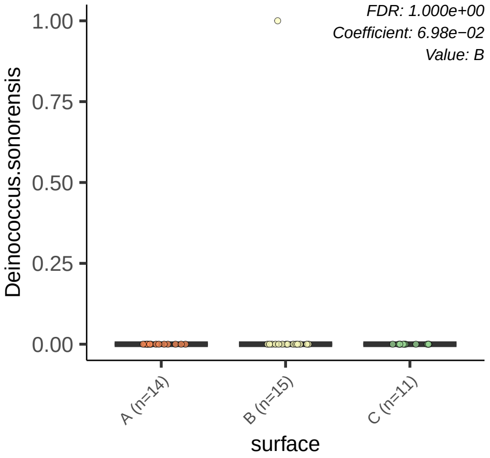
FDR: 1.000e+00
Coefficient: 6.98e−02
Value: B
Deinococcus.sonorensis
1.00
0.75
0.50
0.25
0.00
A (n=14)
B (n=15)
C (n=11)
surface

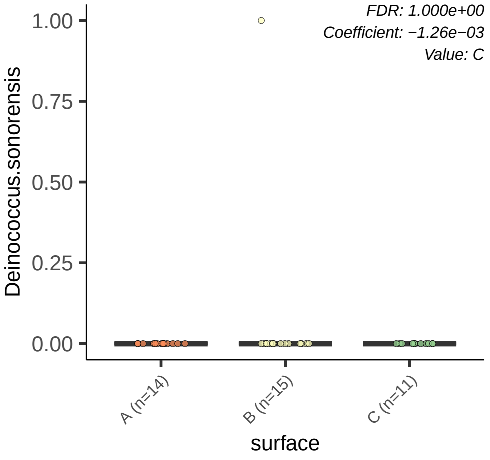

FDR: 1.000e+00
Coefficient: −1.26e−03
Value: C
Deinococcus.sonorensis
1.00
0.75
0.50
0.25
0.00
A (n=14)
B (n=15)
C (n=11)
surface

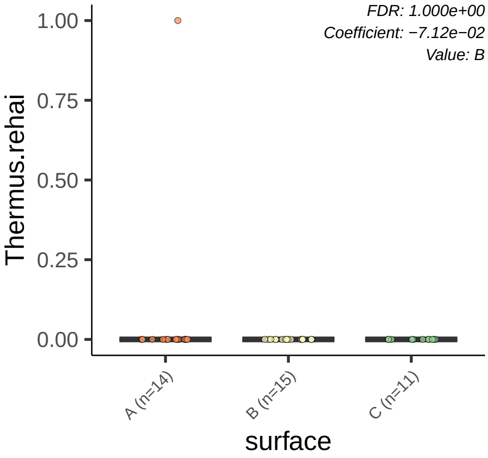
FDR: 1.000e+00
Coefficient: −7.12e−02
Value: B
Thermus.rehai
1.00
0.75
0.50
0.25
0.00
A (n=14)
B (n=15)
C (n=11)
surface

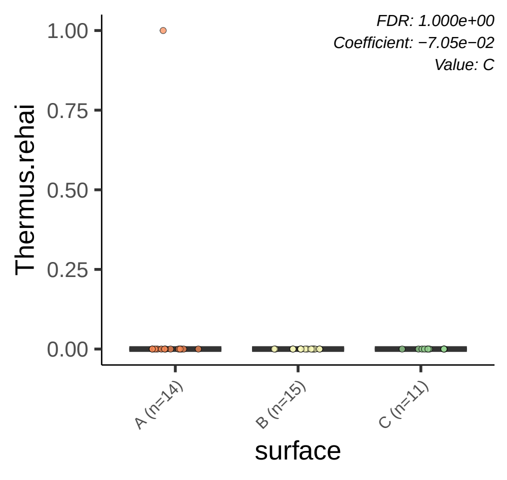
FDR: 1.000e+00
Coefficient: −7.05e−02
Value: C
Thermus.rehai
1.00
0.75
0.50
0.25
0.00
A (n=14)
B (n=15)
C (n=11)
surface

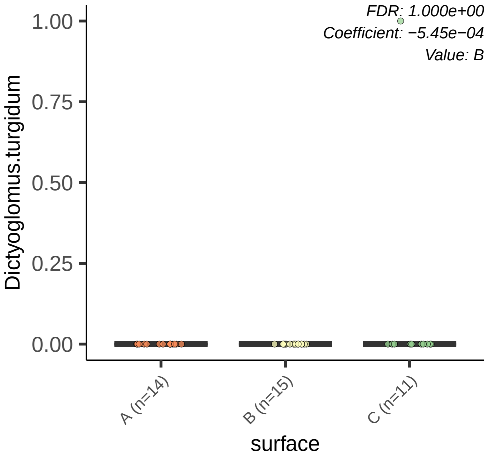

FDR: 1.000e+00
Coefficient: −5.45e−04
Value: B
Dictyoglomus.turgidum
1.00
0.75
0.50
0.25
0.00
A (n=14)
B (n=15)
C (n=11)
surface

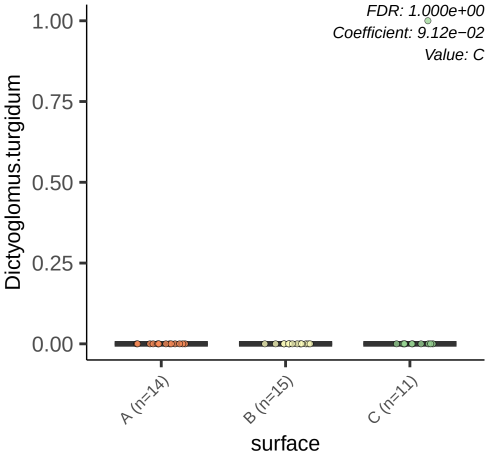

FDR: 1.000e+00
Coefficient: 9.12e−02
Value: C
Dictyoglomus.turgidum
1.00
0.75
0.50
0.25
0.00
A (n=14)
B (n=15)
C (n=11)
surface

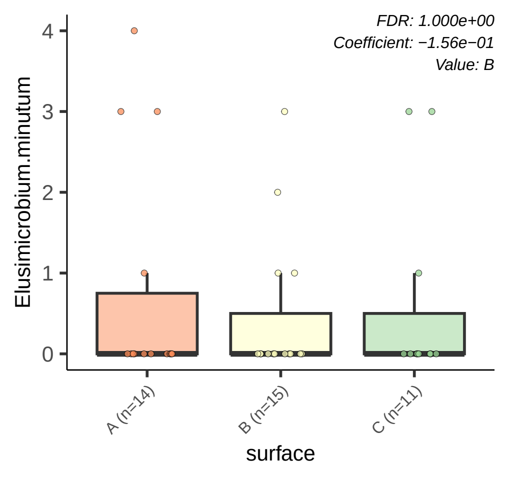

FDR: 1.000e+00
Coefficient: −1.56e−01
Value: B
Elusimicrobium.minutum
4
3
2
1
0
A (n=14)
B (n=15)
C (n=11)
surface

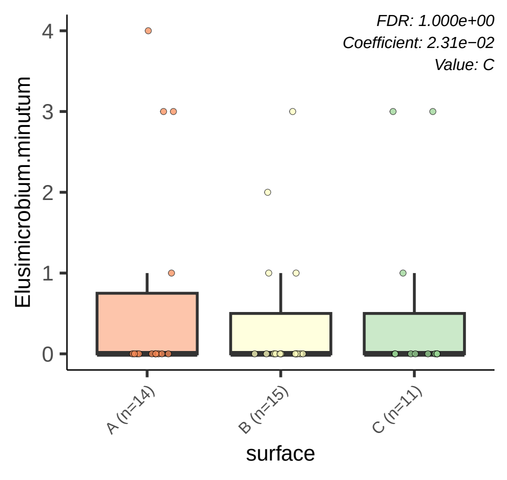
FDR: 1.000e+00
Coefficient: 2.31e−02
Value: C
Elusimicrobium.minutum
0
1
2
3
4
A (n=14)
B (n=15)
C (n=11)
surface

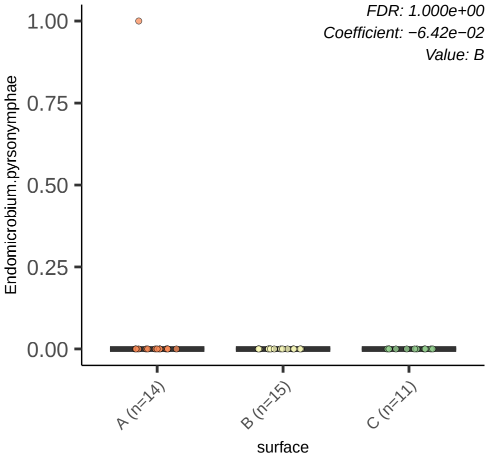
FDR: 1.000e+00
Coefficient: −6.42e−02
Value: B
Endomicrobium.pyrsonymphae
1.00
0.75
0.50
0.25
0.00
A (n=14)
B (n=15)
C (n=11)
surface

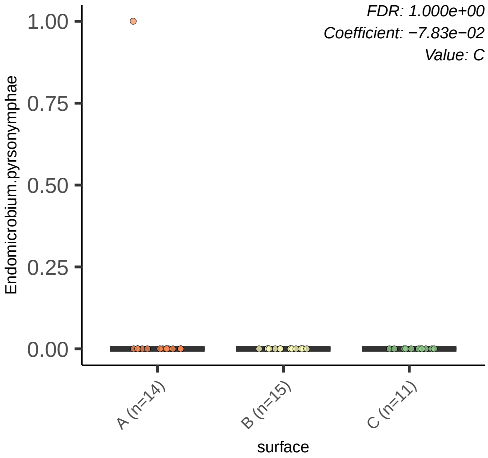
FDR: 1.000e+00
Coefficient: −7.83e−02
Value: C
1.00
0.75
0.50
0.25
0.00
Endomicrobium.pyrsonymphae
A (n=14)
B (n=15)
C (n=11)
surface

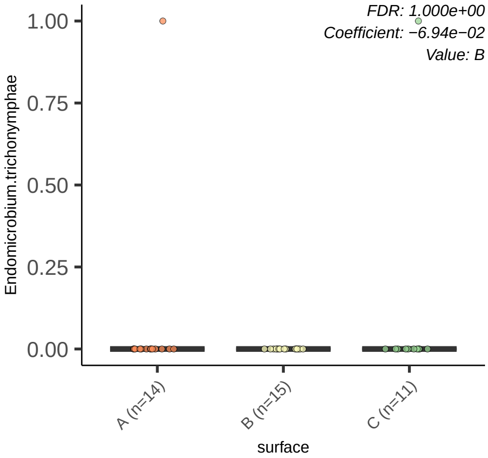

FDR: 1.000e+00
Coefficient: −6.94e−02
Value: B
Endomicrobium.trichonymphae
1.00
0.75
0.50
0.25
0.00
A (n=14)
B (n=15)
C (n=11)
surface

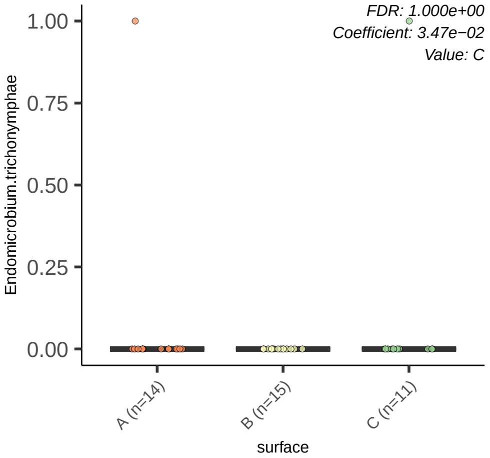

FDR: 1.000e+00
Coefficient: 3.47e−02
Value: C
Endomicrobium.trichonymphae
1.00
0.75
0.50
0.25
0.00
A (n=14)
B (n=15)
C (n=11)
surface

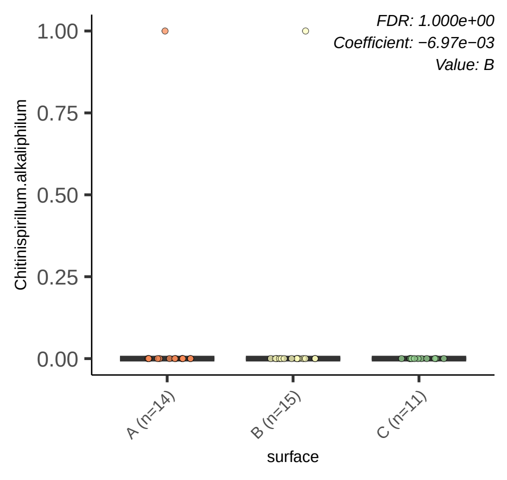
FDR: 1.000e+00
Coefficient: −6.97e−03
Value: B
Chitinispirillum.alkaliphilum
1.00
0.75
0.50
0.25
0.00
A (n=14)
B (n=15)
C (n=11)
surface

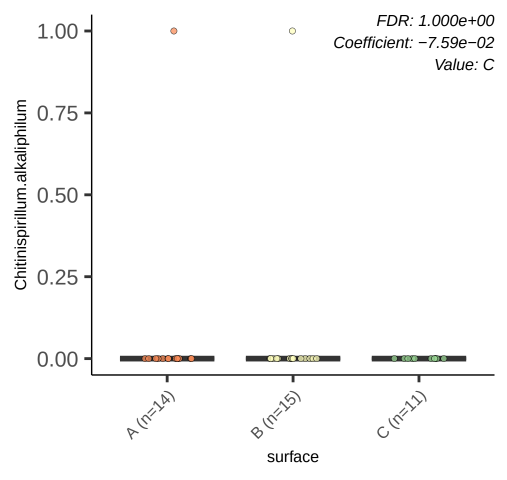

FDR: 1.000e+00
Coefficient: −7.59e−02
Value: C
1.00
0.75
0.50
0.25
0.00
Chitinispirillum.alkaliphilum
A (n=14)
B (n=15)
C (n=11)
surface

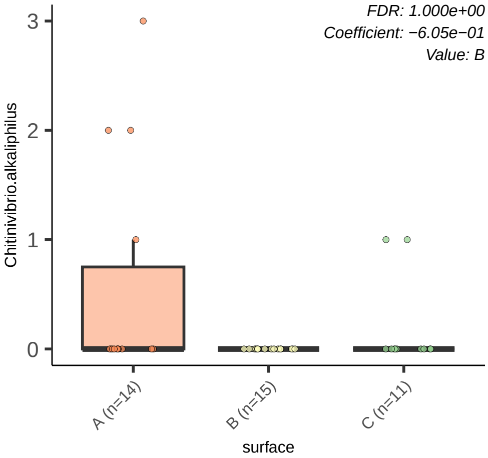

FDR: 1.000e+00
Coefficient: −6.05e−01
Value: B
Chitinivibrio.alkaliphilus
3
2
1
0
A (n=14)
B (n=15)
C (n=11)
surface

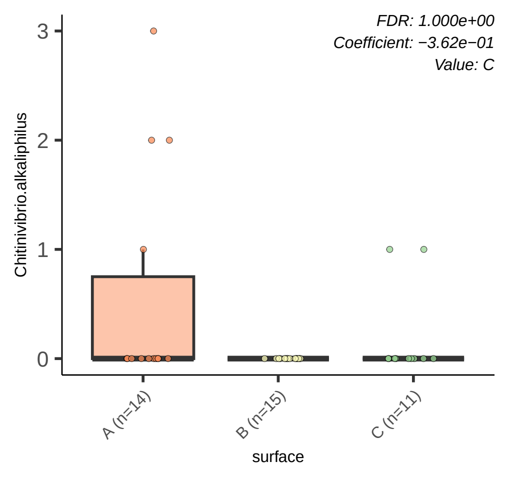

FDR: 1.000e+00
Coefficient: −3.62e−01
Value: C
Chitinivibrio.alkaliphilus
3
2
1
0
A (n=14)
B (n=15)
C (n=11)
surface

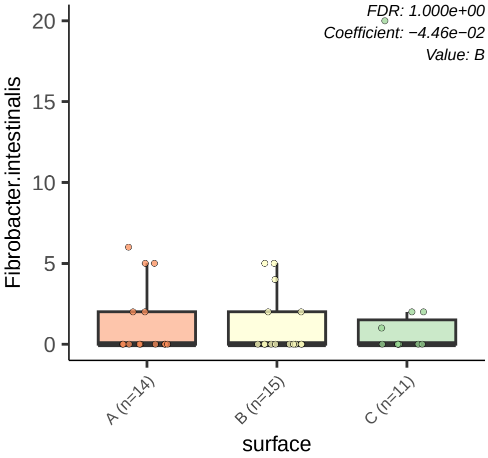

FDR: 1.000e+00
Coefficient: −4.46e−02
Value: B
Fibrobacter.intestinalis
20
15
10
5
0
A (n=14)
B (n=15)
C (n=11)
surface

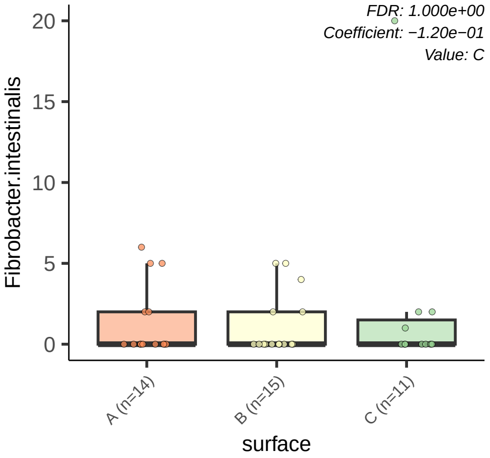
FDR: 1.000e+00
Coefficient: −1.20e−01
Value: C
Fibrobacter.intestinalis
20
15
10
5
0
A (n=14)
B (n=15)
C (n=11)
surface

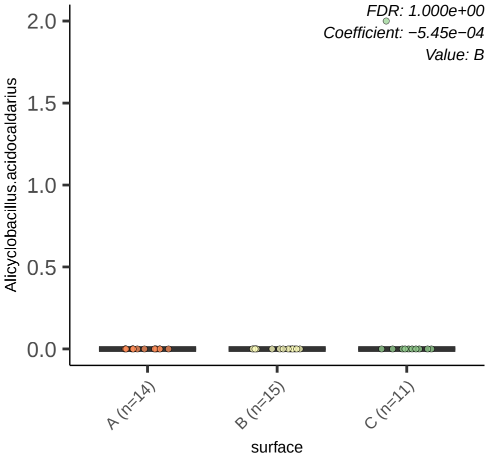
FDR: 1.000e+00
Coefficient: −5.45e−04
Value: B
Alicyclobacillus.acidocaldarius
2.0
1.5
1.0
0.5
0.0
A (n=14)
B (n=15)
C (n=11)
surface

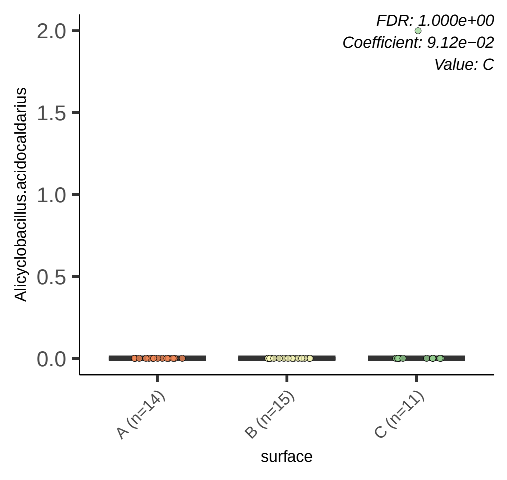

FDR: 1.000e+00
Coefficient: 9.12e−02
Value: C
Alicyclobacillus.acidocaldarius
2.0
1.5
1.0
0.5
0.0
A (n=14)
B (n=15)
C (n=11)
surface

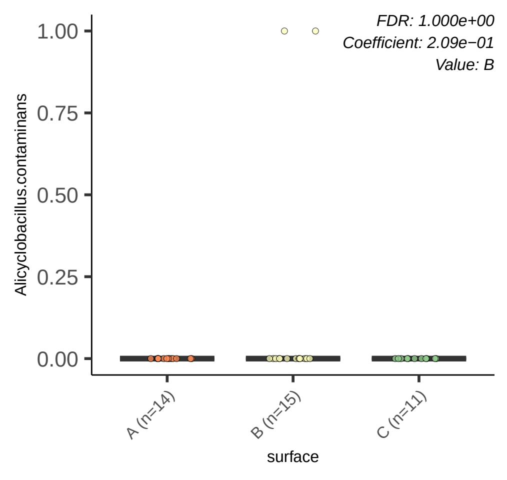

FDR: 1.000e+00
Coefficient: 2.09e−01
Value: B
Alicyclobacillus.contaminans
1.00
0.75
0.50
0.25
0.00
A (n=14)
B (n=15)
C (n=11)
surface

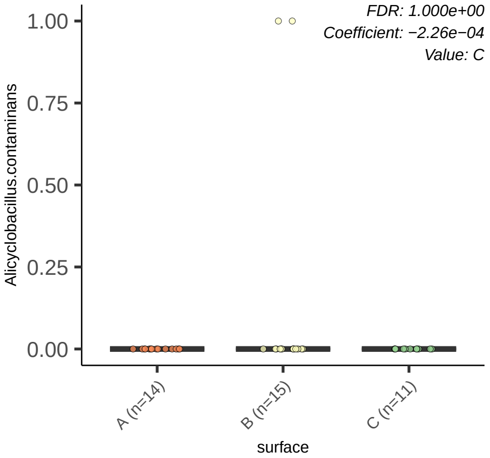
FDR: 1.000e+00
Coefficient: −2.26e−04
Value: C
Alicyclobacillus.contaminans
1.00
0.75
0.50
0.25
0.00
A (n=14)
B (n=15)
C (n=11)
surface

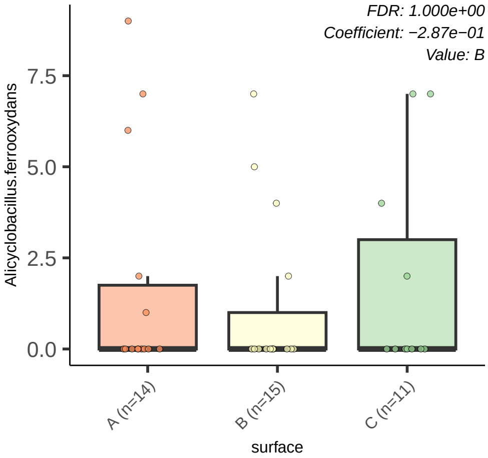

FDR: 1.000e+00
Coefficient: −2.87e−01
Value: B
Alicyclobacillus.ferrooxydans
7.5
5.0
2.5
0.0
A (n=14)
B (n=15)
C (n=11)
surface

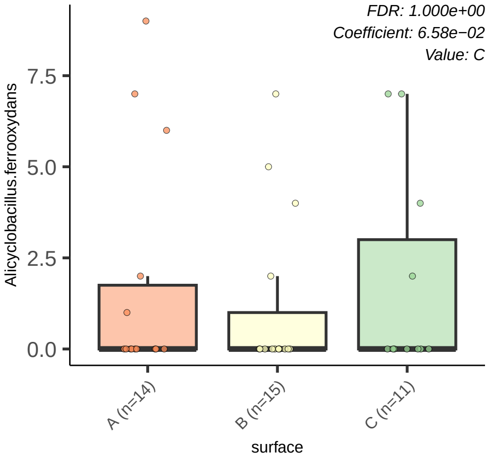

FDR: 1.000e+00
Coefficient: 6.58e−02
Value: C
Alicyclobacillus.ferrooxydans
7.5
5.0
2.5
0.0
A (n=14)
B (n=15)
C (n=11)
surface

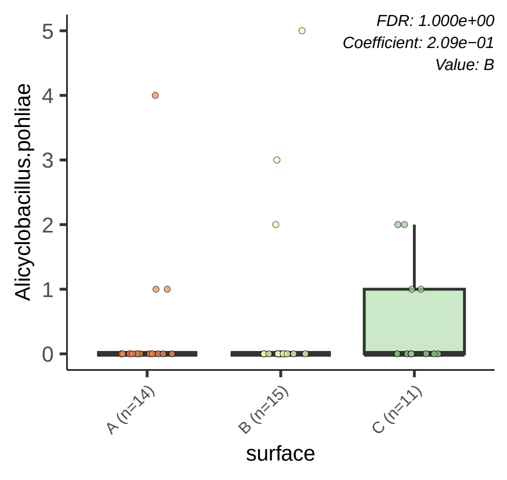
FDR: 1.000e+00
Coefficient: 2.09e−01
Value: B
Alicyclobacillus.pohliae
0
1
2
3
4
5
A (n=14)
B (n=15)
C (n=11)
surface

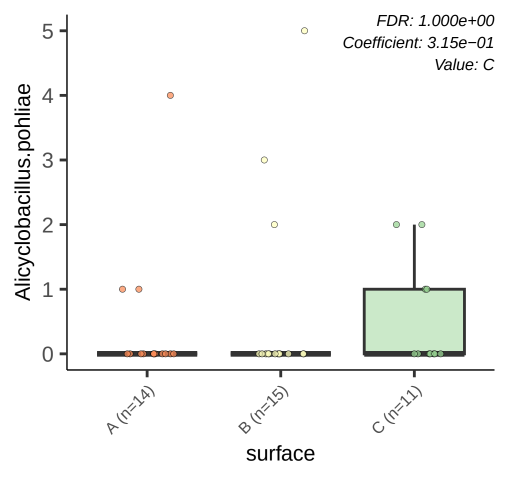
FDR: 1.000e+00
Coefficient: 3.15e−01
Value: C
Alicyclobacillus.pohliae
0
1
2
3
4
5
A (n=14)
B (n=15)
C (n=11)
surface

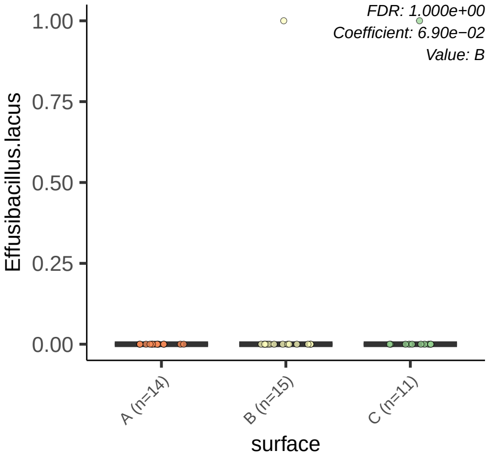

FDR: 1.000e+00
Coefficient: 6.90e−02
Value: B
Effusibacillus.lacus
1.00
0.75
0.50
0.25
0.00
A (n=14)
B (n=15)
C (n=11)
surface

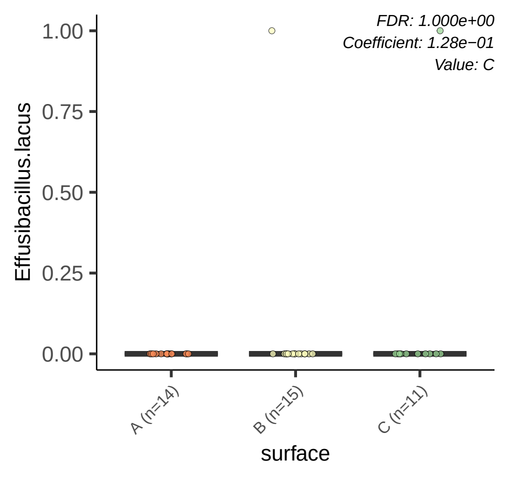
FDR: 1.000e+00
Coefficient: 1.28e−01
Value: C
Effusibacillus.lacus
1.00
0.75
0.50
0.25
0.00
A (n=14)
B (n=15)
C (n=11)
surface

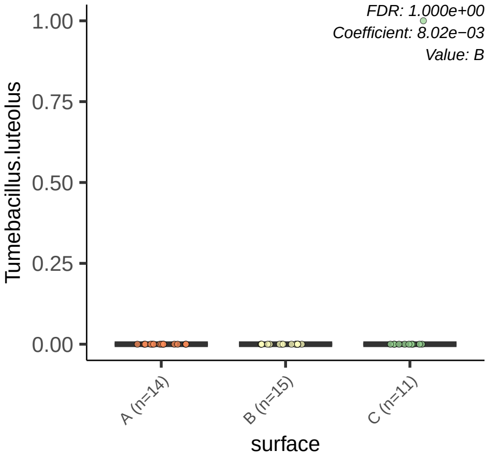

FDR: 1.000e+00
Coefficient: 8.02e−03
Value: B
Tumebacillus.luteolus
1.00
0.75
0.50
0.25
0.00
A (n=14)
B (n=15)
C (n=11)
surface

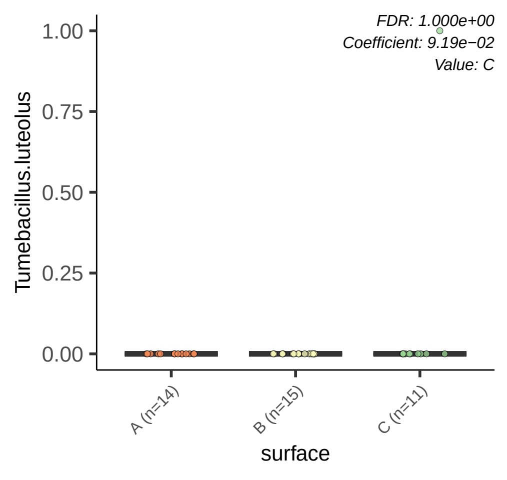

FDR: 1.000e+00
Coefficient: 9.19e−02
Value: C
Tumebacillus.luteolus
1.00
0.75
0.50
0.25
0.00
A (n=14)
B (n=15)
C (n=11)
surface

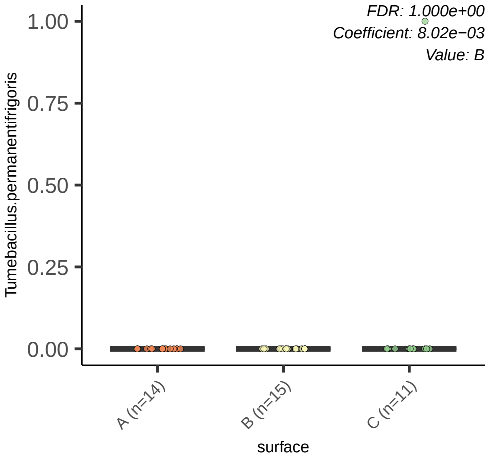

FDR: 1.000e+00
Coefficient: 8.02e−03
Value: B
Tumebacillus.permanentifrigoris
1.00
0.75
0.50
0.25
0.00
A (n=14)
B (n=15)
C (n=11)
surface

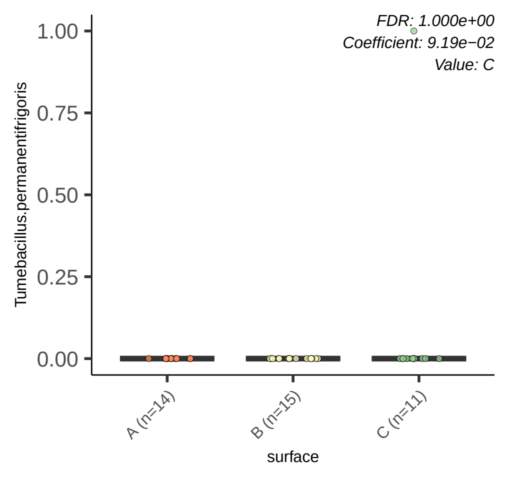

FDR: 1.000e+00
Coefficient: 9.19e−02
Value: C
Tumebacillus.permanentifrigoris
1.00
0.75
0.50
0.25
0.00
A (n=14)
B (n=15)
C (n=11)
surface

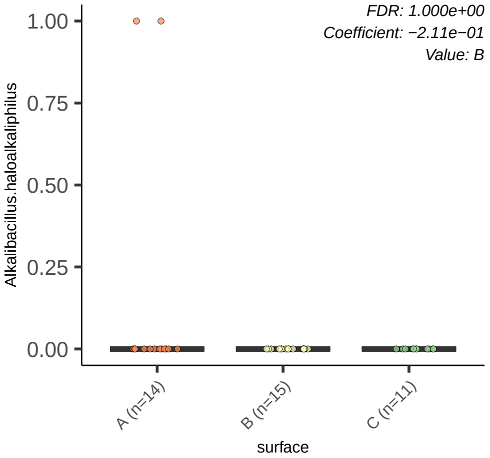

FDR: 1.000e+00
Coefficient: −2.11e−01
Value: B
Alkalibacillus.haloalkaliphilus
1.00
0.75
0.50
0.25
0.00
A (n=14)
B (n=15)
C (n=11)
surface

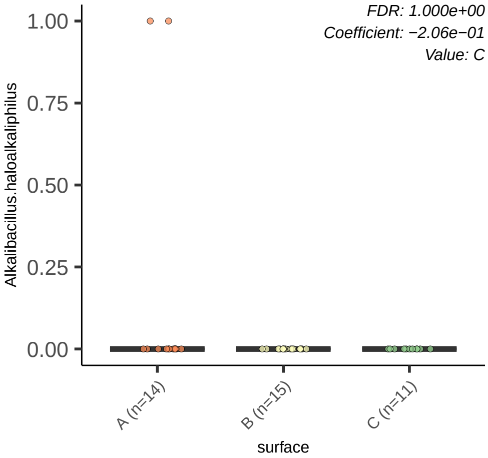
FDR: 1.000e+00
Coefficient: −2.06e−01
Value: C
Alkalibacillus.haloalkaliphilus
1.00
0.75
0.50
0.25
0.00
A (n=14)
B (n=15)
C (n=11)
surface

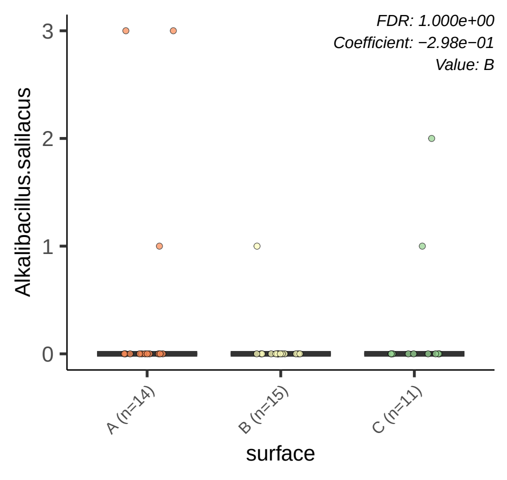

FDR: 1.000e+00
Coefficient: −2.98e−01
Value: B
Alkalibacillus.salilacus
3
2
1
0
A (n=14)
B (n=15)
C (n=11)
surface

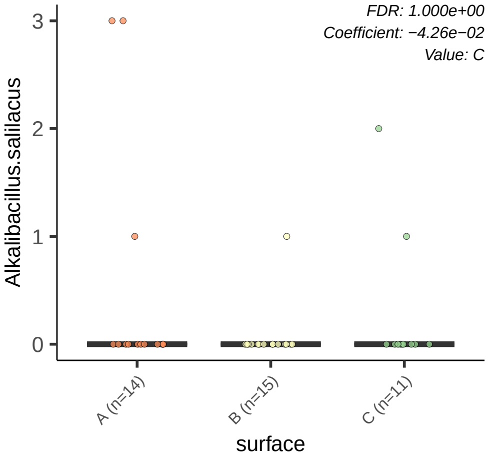

FDR: 1.000e+00
Coefficient: −4.26e−02
Value: C
Alkalibacillus.salilacus
3
2
1
0
A (n=14)
B (n=15)
C (n=11)
surface

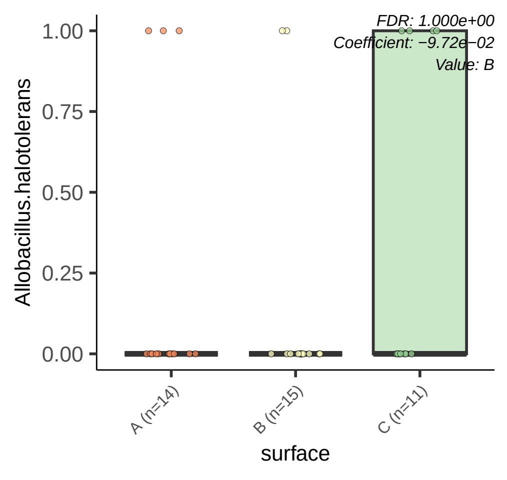
FDR: 1.000e+00
Coefficient: −9.72e−02
Value: B
Allobacillus.halotolerans
1.00
0.75
0.50
0.25
0.00
A (n=14)
B (n=15)
C (n=11)
surface

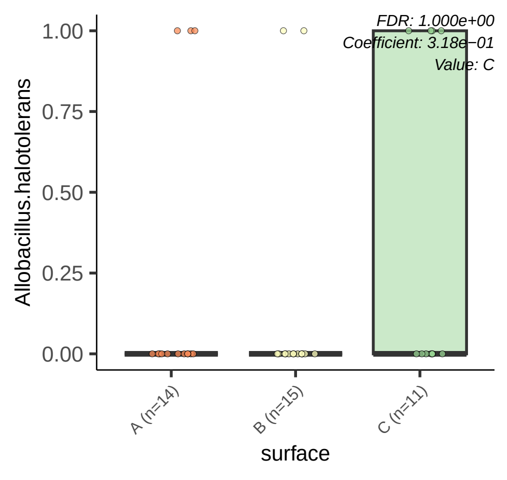

FDR: 1.000e+00
Coefficient: 3.18e−01
Value: C
Allobacillus.halotolerans
1.00
0.75
0.50
0.25
0.00
A (n=14)
B (n=15)
C (n=11)
surface

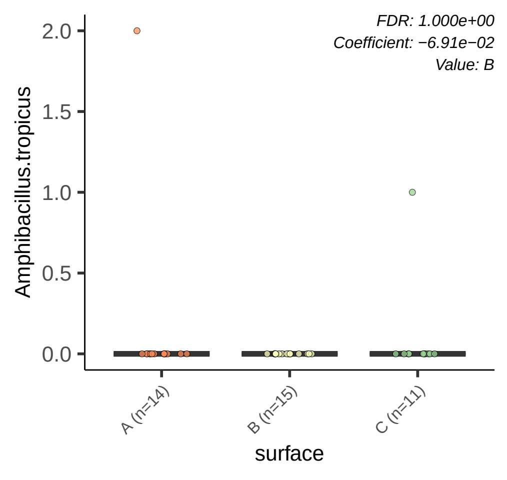
FDR: 1.000e+00
Coefficient: −6.91e−02
Value: B
Amphibacillus.tropicus
2.0
1.5
1.0
0.5
0.0
A (n=14)
B (n=15)
C (n=11)
surface

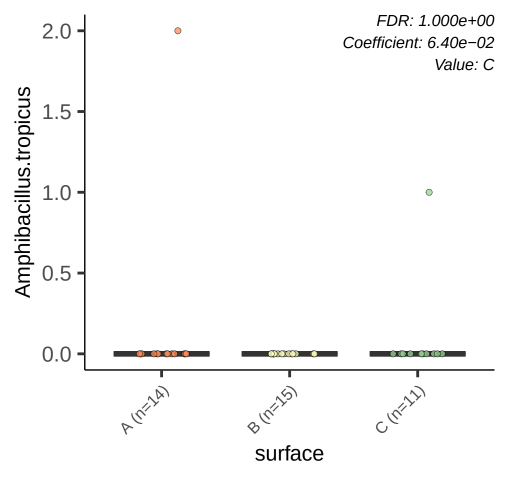
FDR: 1.000e+00
Coefficient: 6.40e−02
Value: C
Amphibacillus.tropicus
2.0
1.5
1.0
0.5
0.0
A (n=14)
B (n=15)
C (n=11)
surface

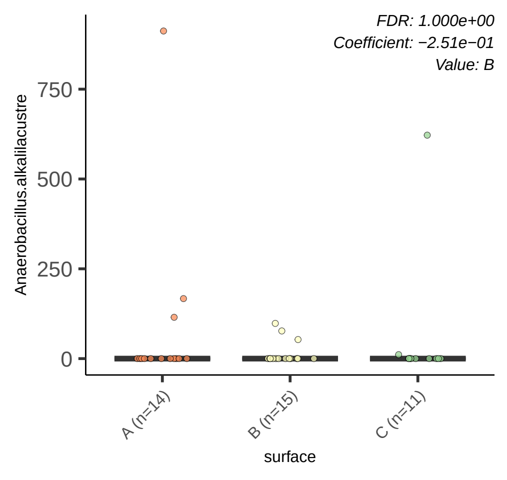

FDR: 1.000e+00
Coefficient: −2.51e−01
Value: B
Anaerobacillus.alkaliacustre
750
500
250
0
A (n=14)
B (n=15)
C (n=11)
surface

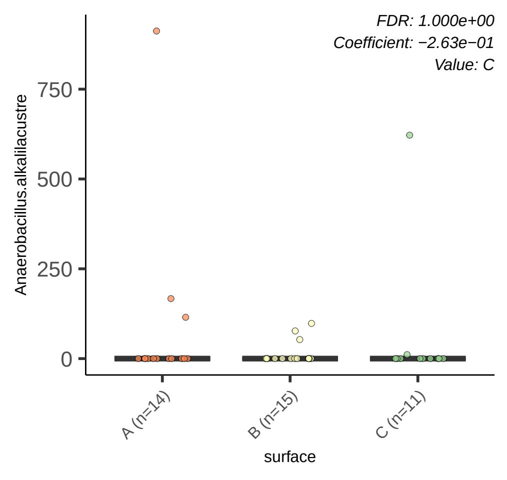

FDR: 1.000e+00
Coefficient: −2.63e−01
Value: C
Anaerobacillus.alkaliacustre
750
500
250
0
A (n=14)
B (n=15)
C (n=11)
surface

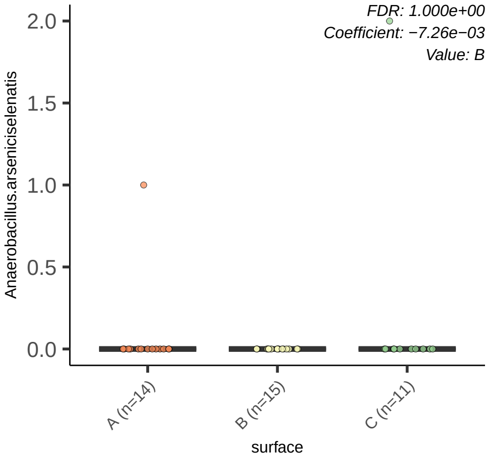
FDR: 1.000e+00
Coefficient: −7.26e−03
Value: B
Anaerobacillus.arseniciselenatis
2.0
1.5
1.0
0.5
0.0
A (n=14)
B (n=15)
C (n=11)
surface

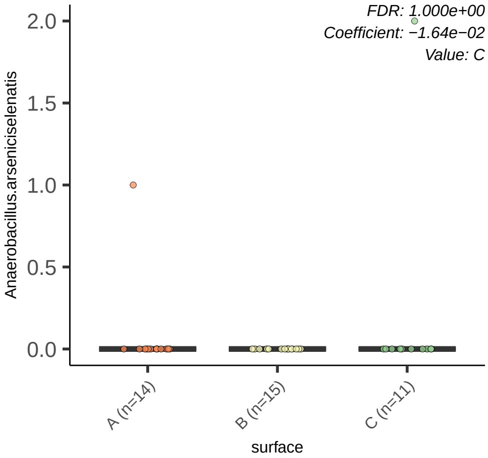

FDR: 1.000e+00
Coefficient: −1.64e−02
Value: C
Anaerobacillus.arseniciselenatis
2.0
1.5
1.0
0.5
0.0
A (n=14)
B (n=15)
C (n=11)
surface

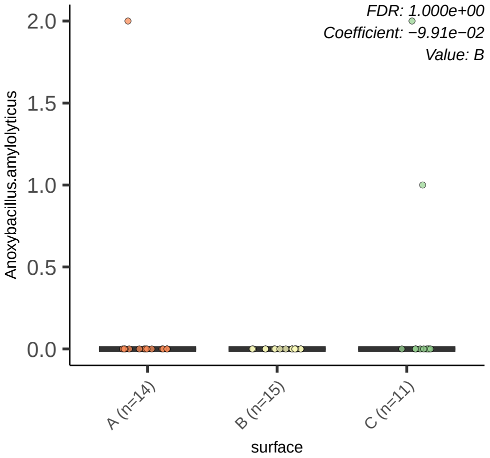
FDR: 1.000e+00
Coefficient: −9.91e−02
Value: B
Anoxybacillus.amylolyticus
2.0
1.5
1.0
0.5
0.0
A (n=14)
B (n=15)
C (n=11)
surface

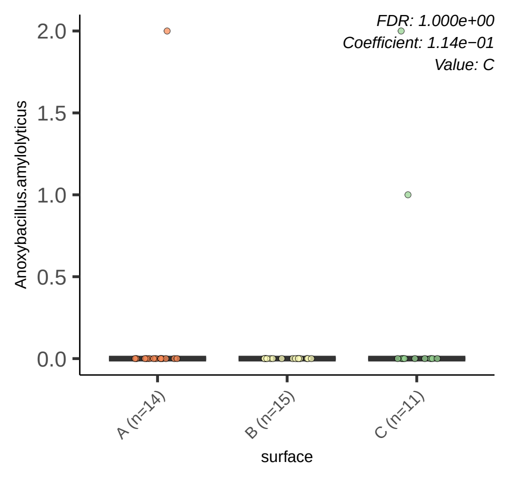
FDR: 1.000e+00
Coefficient: 1.14e−01
Value: C
Anoxybacillus.amylolyticus
2.0
1.5
1.0
0.5
0.0
A (n=14)
B (n=15)
C (n=11)
surface

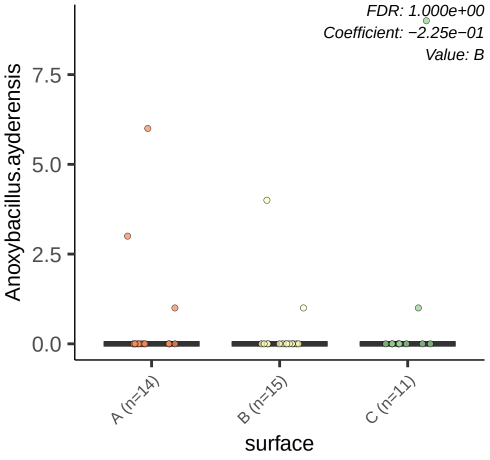

FDR: 1.000e+00
Coefficient: −2.25e−01
Value: B
Anoxybacillus.ayderensis
7.5
5.0
2.5
0.0
A (n=14)
B (n=15)
C (n=11)
surface

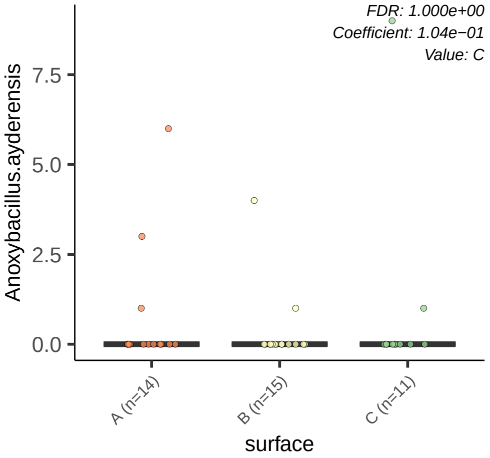

FDR: 1.000e+00
Coefficient: 1.04e−01
Value: C
Anoxybacillus.ayderensis
7.5
5.0
2.5
0.0
A (n=14)
B (n=15)
C (n=11)
surface

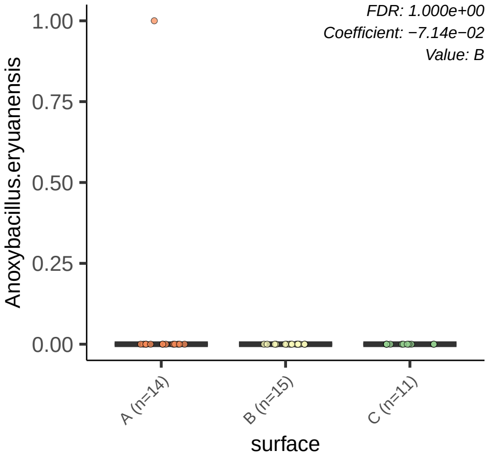
FDR: 1.000e+00
Coefficient: −7.14e−02
Value: B
Anoxybacillus.eryuanensis
1.00
0.75
0.50
0.25
0.00
A (n=14)
B (n=15)
C (n=11)
surface

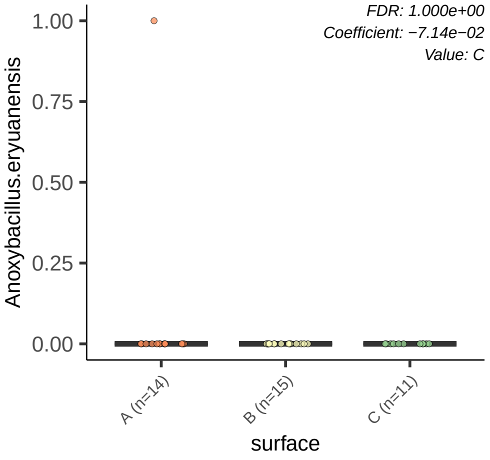

FDR: 1.000e+00
Coefficient: −7.14e−02
Value: C
Anoxybacillus.eryuanensis
1.00
0.75
0.50
0.25
0.00
A (n=14)
B (n=15)
C (n=11)
surface

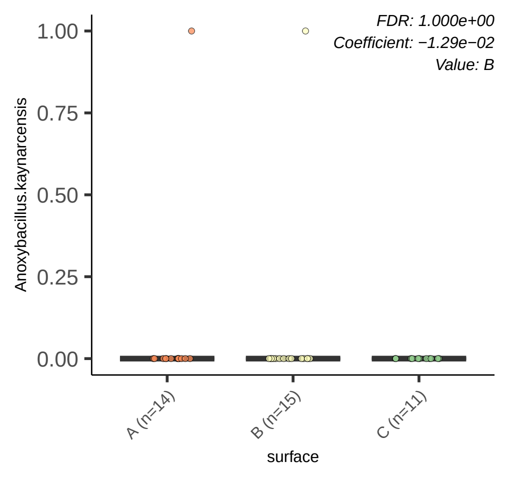

FDR: 1.000e+00
Coefficient: −1.29e−02
Value: B
Anoxybacillus.kaynarcensis
1.00
0.75
0.50
0.25
0.00
A (n=14)
B (n=15)
C (n=11)
surface

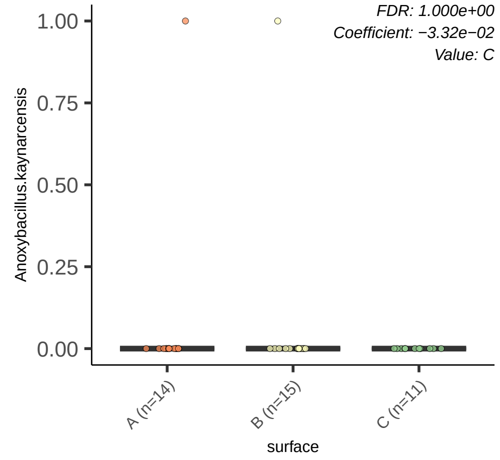

FDR: 1.000e+00
Coefficient: −3.32e−02
Value: C
Anoxybacillus.kaynarcensis
1.00
0.75
0.50
0.25
0.00
A (n=14)
B (n=15)
C (n=11)
surface

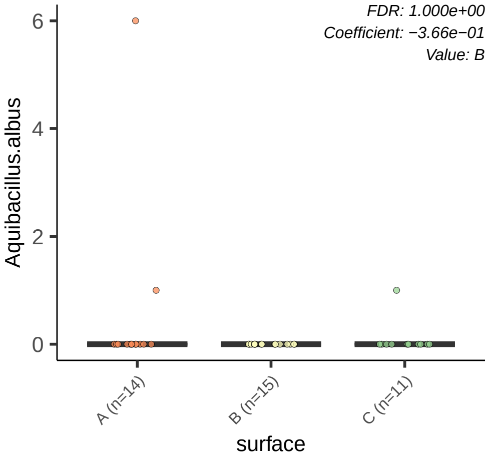
FDR: 1.000e+00
Coefficient: −3.66e−01
Value: B
Aquibacillus.albus
6
4
2
0
A (n=14)
B (n=15)
C (n=11)
surface

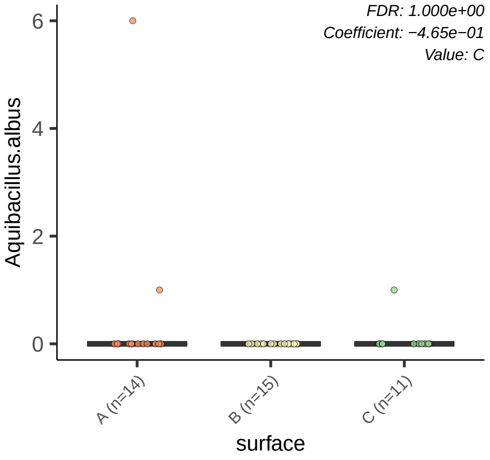
FDR: 1.000e+00
Coefficient: −4.65e−01
Value: C
Aquibacillus.albus
6
4
2
0
A (n=14)
B (n=15)
C (n=11)
surface

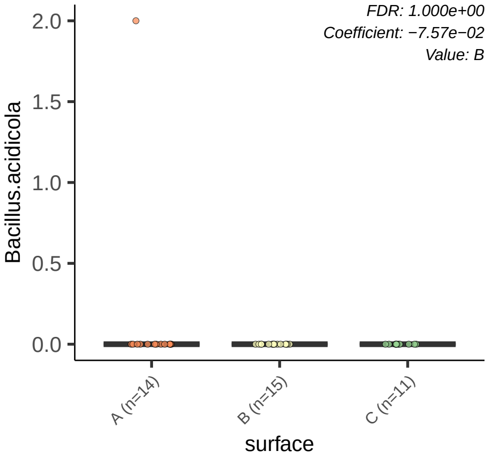

FDR: 1.000e+00
Coefficient: −7.57e−02
Value: B
Bacillus.acidicola
2.0
1.5
1.0
0.5
0.0
A (n=14)
B (n=15)
C (n=11)
surface

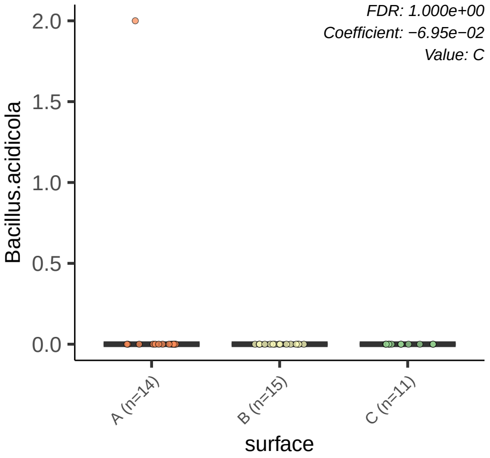
FDR: 1.000e+00
Coefficient: −6.95e−02
Value: C
Bacillus.acidicola
2.0
1.5
1.0
0.5
0.0
A (n=14)
B (n=15)
C (n=11)
surface

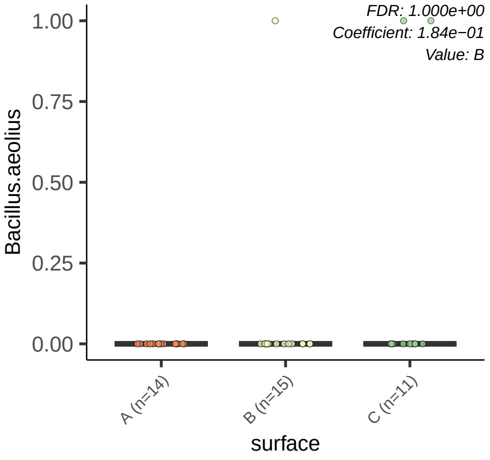
FDR: 1.000e+00
Coefficient: 1.84e−01
Value: B
1.00
0.75
0.50
0.25
0.00
Bacillus.aeolius
A (n=14)
B (n=15)
C (n=11)
surface

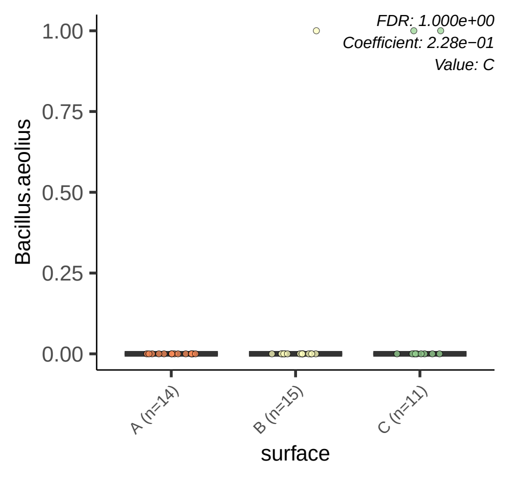
FDR: 1.000e+00
Coefficient: 2.28e−01
Value: C
Bacillus.aeolius
1.00
0.75
0.50
0.25
0.00
A (n=14)
B (n=15)
C (n=11)
surface

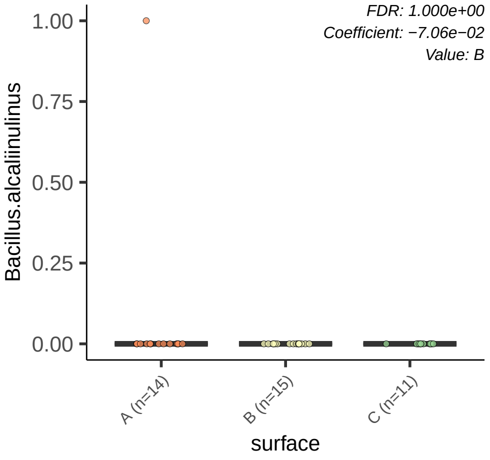

FDR: 1.000e+00
Coefficient: −7.06e−02
Value: B
Bacillus.alcaliinulinus
1.00
0.75
0.50
0.25
0.00
A (n=14)
B (n=15)
C (n=11)
surface

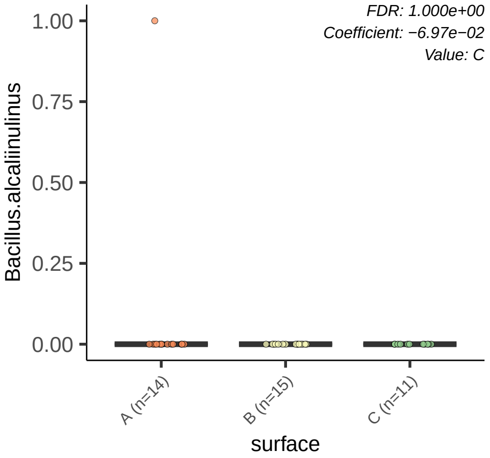

FDR: 1.000e+00
Coefficient: −6.97e−02
Value: C
Bacillus.alcalinulinus
1.00
0.75
0.50
0.25
0.00
A (n=14)
B (n=15)
C (n=11)
surface

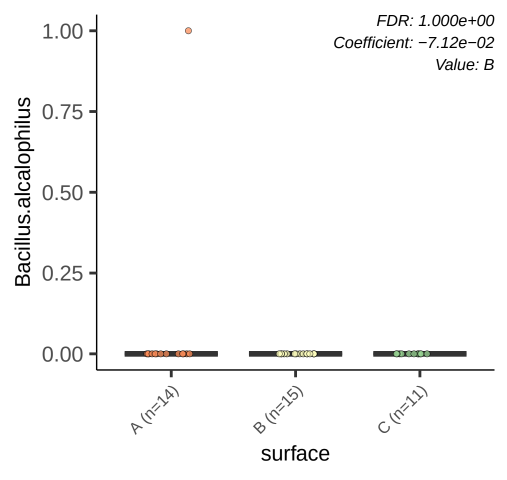
FDR: 1.000e+00
Coefficient: −7.12e−02
Value: B
Bacillus.alcalophilus
1.00
0.75
0.50
0.25
0.00
A (n=14)
B (n=15)
C (n=11)
surface

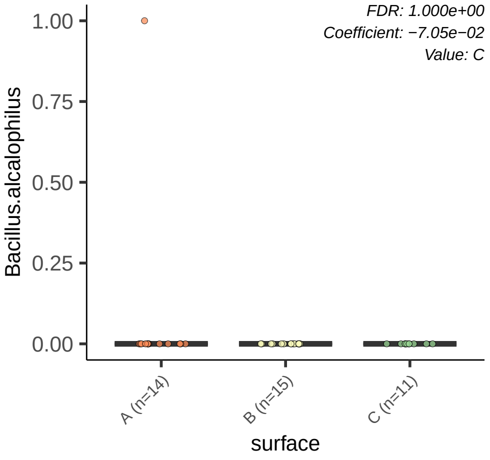
FDR: 1.000e+00
Coefficient: −7.05e−02
Value: C
Bacillus.alcalophilus
1.00
0.75
0.50
0.25
0.00
A (n=14)
B (n=15)
C (n=11)
surface

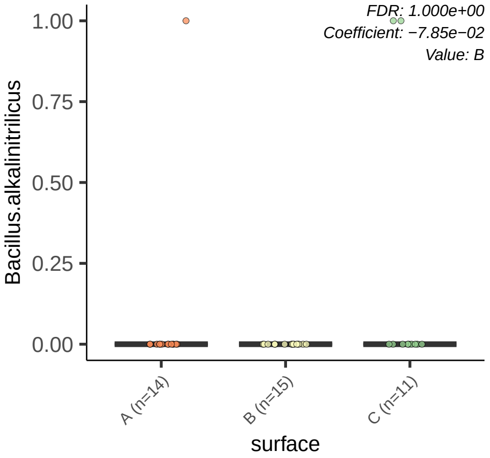

FDR: 1.000e+00
Coefficient: −7.85e−02
Value: B
Bacillus.alkalinitrilicus
1.00
0.75
0.50
0.25
0.00
A (n=14)
B (n=15)
C (n=11)
surface

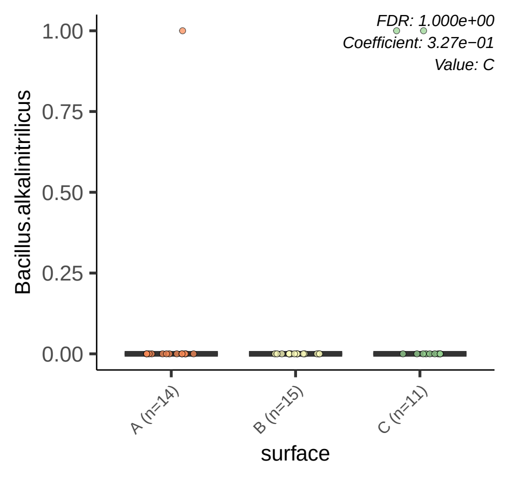
FDR: 1.000e+00
Coefficient: 3.27e−01
Value: C
Bacillus.alkalinitrilicus
1.00
0.75
0.50
0.25
0.00
A (n=14)
B (n=15)
C (n=11)
surface

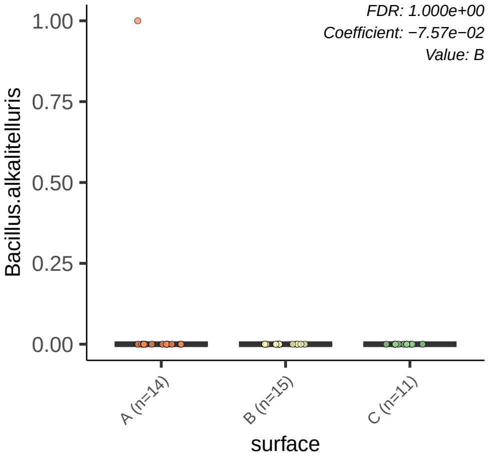
FDR: 1.000e+00
Coefficient: −7.57e−02
Value: B
Bacillus.alkalitelluris
1.00
0.75
0.50
0.25
0.00
A (n=14)
B (n=15)
C (n=11)
surface

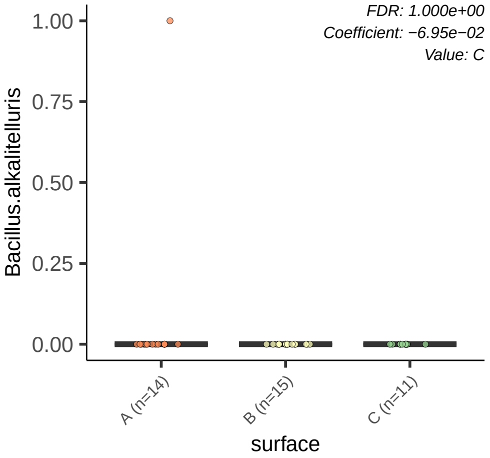

FDR: 1.000e+00
Coefficient: −6.95e−02
Value: C
Bacillus.alkalitelluris
1.00
0.75
0.50
0.25
0.00
A (n=14)
B (n=15)
C (n=11)
surface

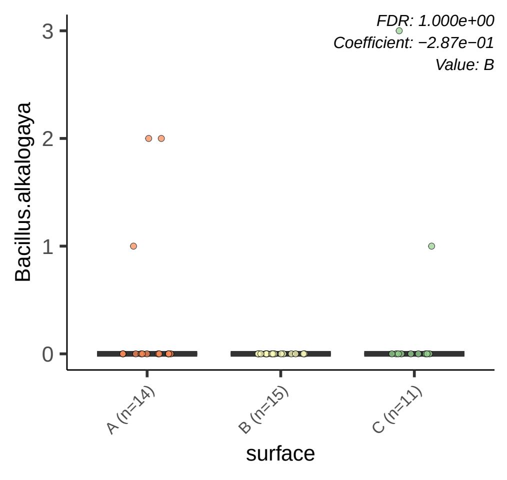

FDR: 1.000e+00
Coefficient: −2.87e−01
Value: B
Bacillus.alkalogaya
3
2
1
0
A (n=14)
B (n=15)
C (n=11)
surface

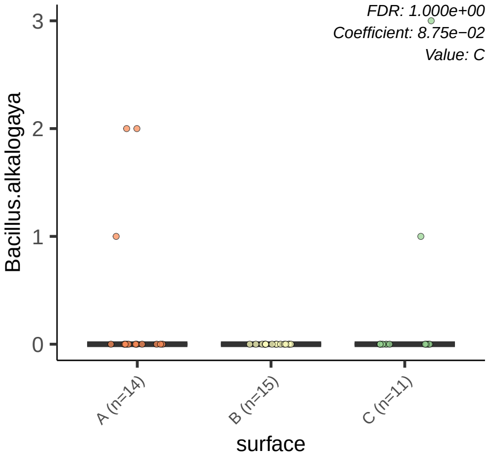
FDR: 1.000e+00
Coefficient: 8.75e−02
Value: C
Bacillus.alkalogaya
0
1
2
3
A (n=14)
B (n=15)
C (n=11)
surface

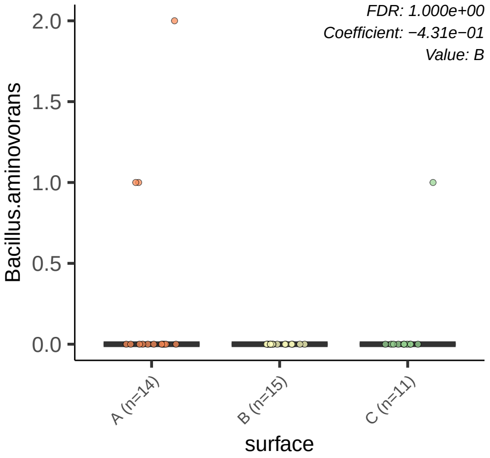
FDR: 1.000e+00
Coefficient: −4.31e−01
Value: B
Bacillus.aminovorans
2.0
1.5
1.0
0.5
0.0
A (n=14)
B (n=15)
C (n=11)
surface

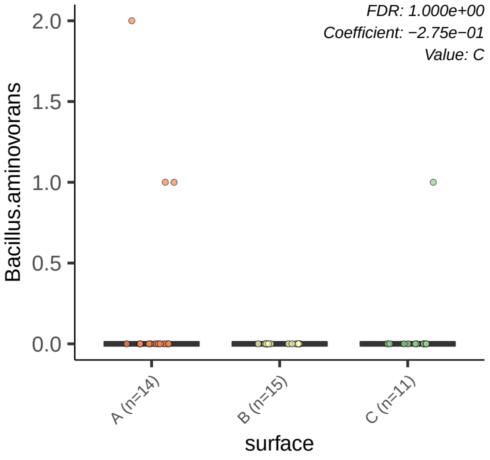
FDR: 1.000e+00
Coefficient: −2.75e−01
Value: C
Bacillus.aminovorans
2.0
1.5
1.0
0.5
0.0
A (n=14)
B (n=15)
C (n=11)
surface

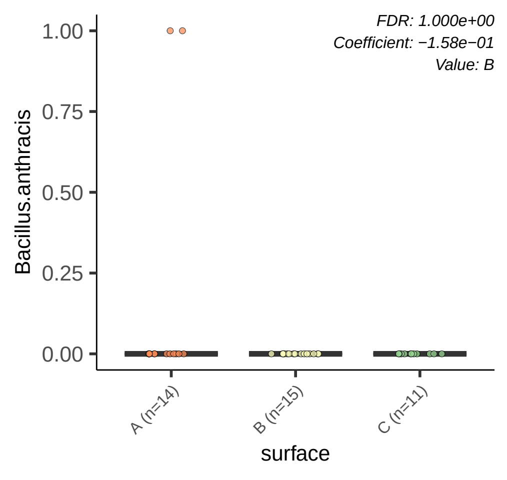
FDR: 1.000e+00
Coefficient: −1.58e−01
Value: B
Bacillus.anthracis
1.00
0.75
0.50
0.25
0.00
A (n=14)
B (n=15)
C (n=11)
surface

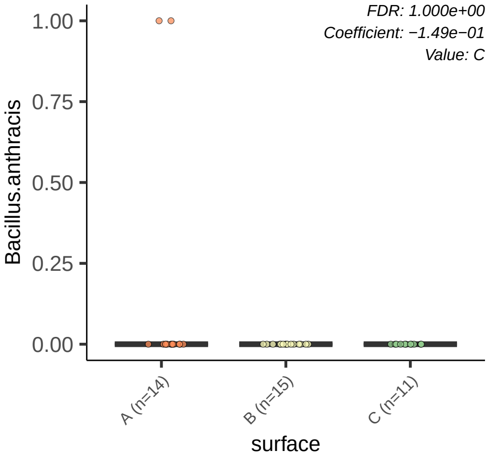

FDR: 1.000e+00
Coefficient: −1.49e−01
Value: C
Bacillus.anthracis
1.00
0.75
0.50
0.25
0.00
A (n=14)
B (n=15)
C (n=11)
surface

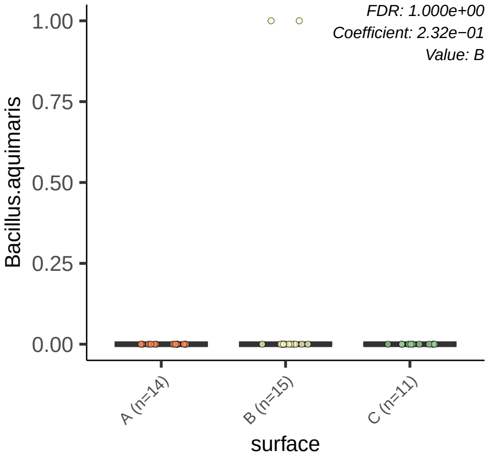
FDR: 1.000e+00
Coefficient: 2.32e−01
Value: B
Bacillus.aquimaris
1.00
0.75
0.50
0.25
0.00
A (n=14)
B (n=15)
C (n=11)
surface

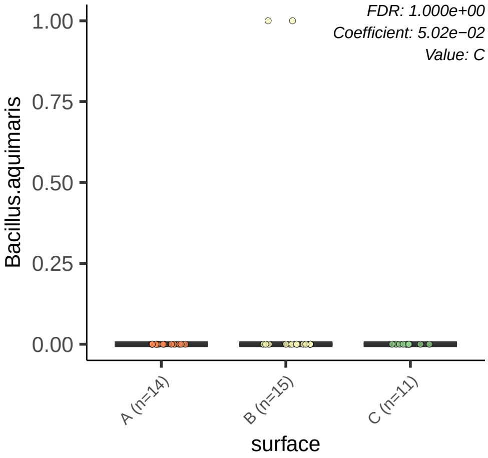
FDR: 1.000e+00
Coefficient: 5.02e−02
Value: C
Bacillus.aquimaris
1.00
0.75
0.50
0.25
0.00
A (n=14)
B (n=15)
C (n=11)
surface

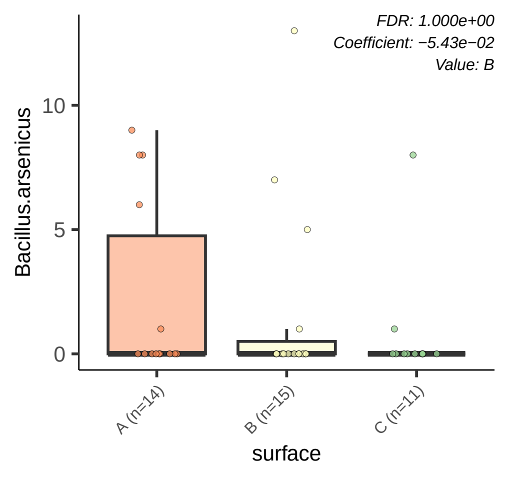

FDR: 1.000e+00
Coefficient: −5.43e−02
Value: B
Bacillus.arsenicus
10
5
0
A (n=14)
B (n=15)
C (n=11)
surface

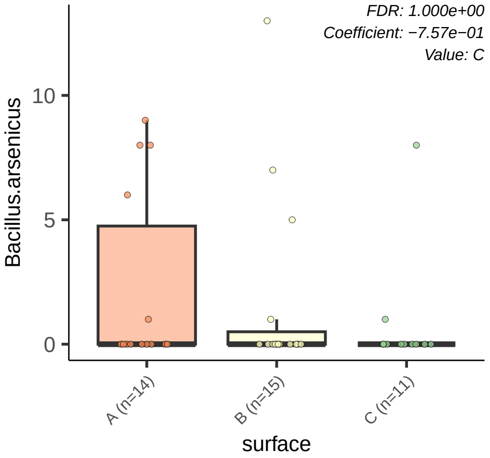

FDR: 1.000e+00
Coefficient: −7.57e−01
Value: C
Bacillus.arsenicus
10
5
0
A (n=14)
B (n=15)
C (n=11)
surface

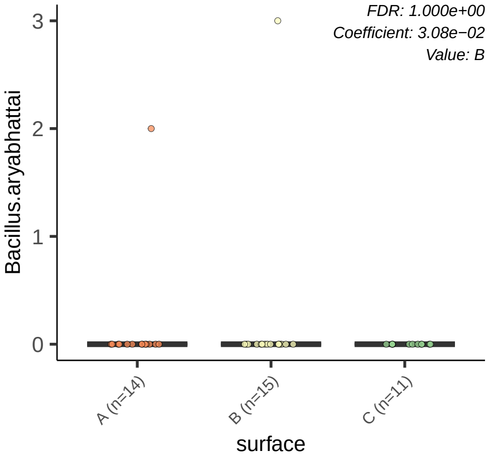

FDR: 1.000e+00
Coefficient: 3.08e−02
Value: B
Bacillus.aryabhattai
0
1
2
3
A (n=14)
B (n=15)
C (n=11)
surface

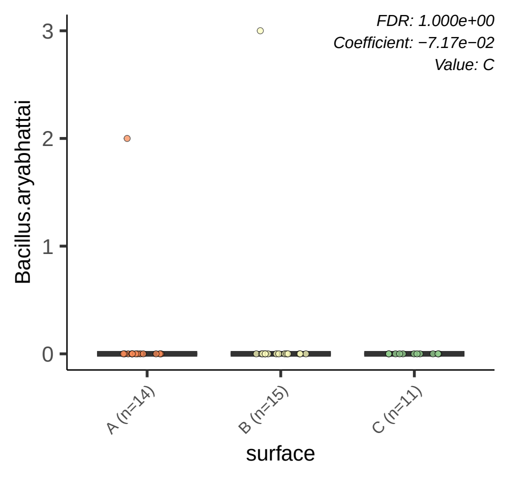

FDR: 1.000e+00
Coefficient: −7.17e−02
Value: C
Bacillus.aryabhattai
0
1
2
3
A (n=14)
B (n=15)
C (n=11)
surface

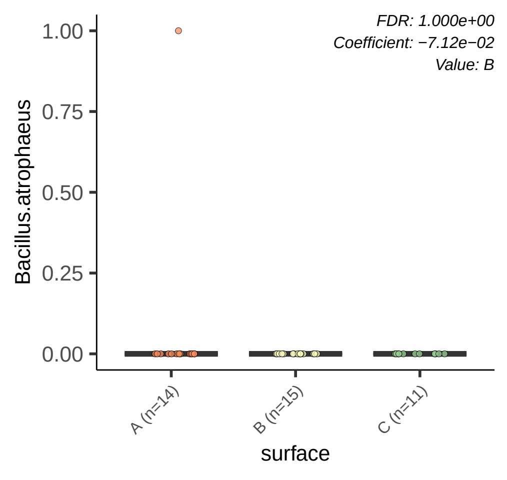
FDR: 1.000e+00
Coefficient: −7.12e−02
Value: B
Bacillus.atrophaeus
1.00
0.75
0.50
0.25
0.00
A (n=14)
B (n=15)
C (n=11)
surface

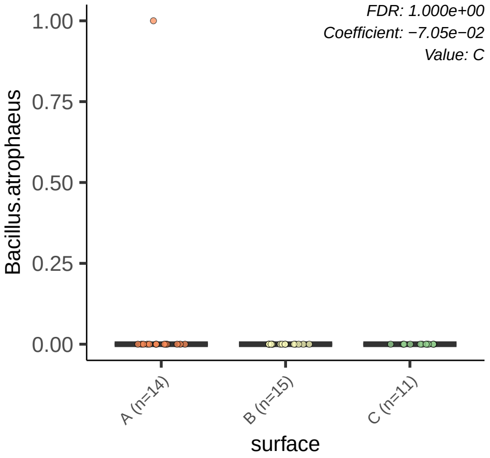
FDR: 1.000e+00
Coefficient: −7.05e−02
Value: C
Bacillus.atrophaeus
1.00
0.75
0.50
0.25
0.00
A (n=14)
B (n=15)
C (n=11)
surface

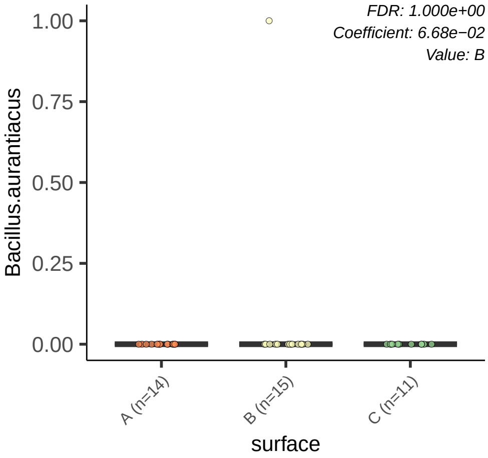
FDR: 1.000e+00
Coefficient: 6.68e−02
Value: B
Bacillus.aurantiacus
1.00
0.75
0.50
0.25
0.00
A (n=14)
B (n=15)
C (n=11)
surface

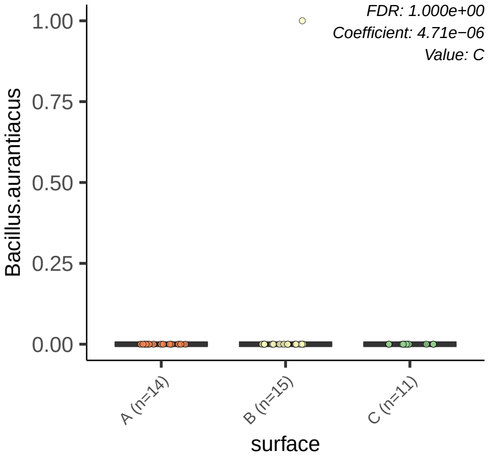
FDR: 1.000e+00
Coefficient: 4.71e−06
Value: C
Bacillus.aurantiacus
1.00
0.75
0.50
0.25
0.00
A (n=14)
B (n=15)
C (n=11)
surface

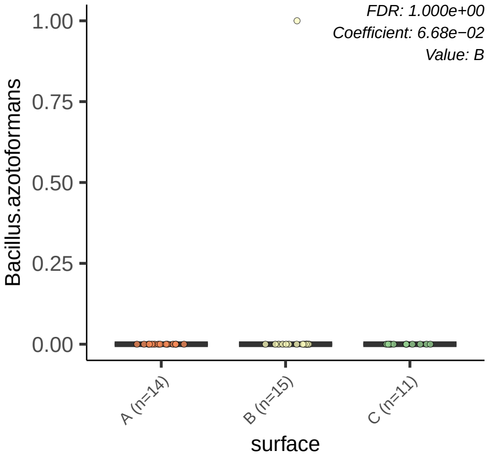

FDR: 1.000e+00
Coefficient: 6.68e−02
Value: B
Bacillus.azotoformans
1.00
0.75
0.50
0.25
0.00
A (n=14)
B (n=15)
C (n=11)
surface

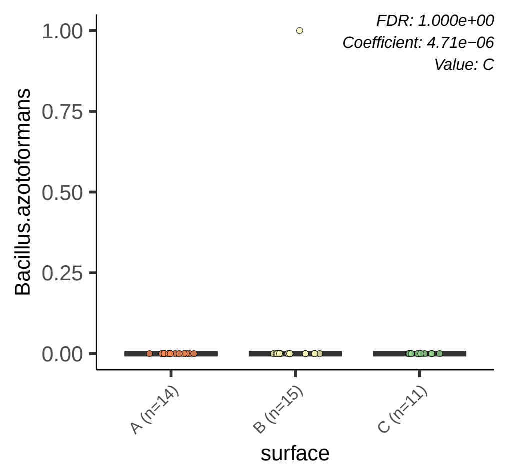

FDR: 1.000e+00
Coefficient: 4.71e−06
Value: C
Bacillus.azotoformans
1.00
0.75
0.50
0.25
0.00
A (n=14)
B (n=15)
C (n=11)
surface

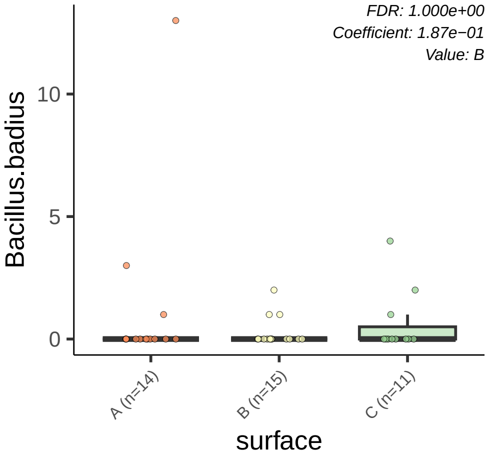
FDR: 1.000e+00
Coefficient: 1.87e−01
Value: B
Bacillus.badius
10
5
0
A (n=14)
B (n=15)
C (n=11)
surface

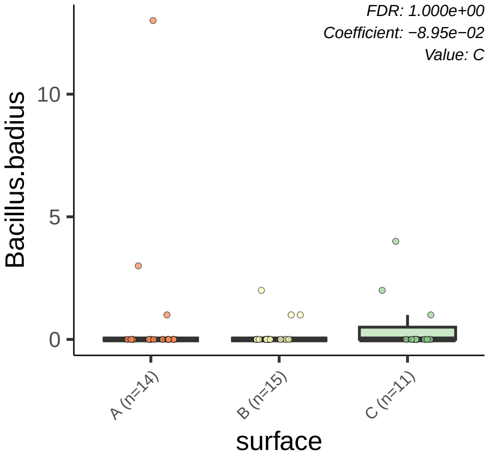
FDR: 1.000e+00
Coefficient: −8.95e−02
Value: C
Bacillus.badius
10
5
0
A (n=14)
B (n=15)
C (n=11)
surface

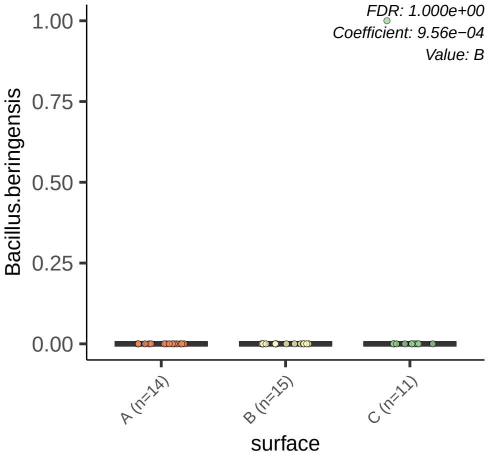
FDR: 1.000e+00
Coefficient: 9.56e−04
Value: B
Bacillus.beringensis
1.00
0.75
0.50
0.25
0.00
A (n=14)
B (n=15)
C (n=11)
surface

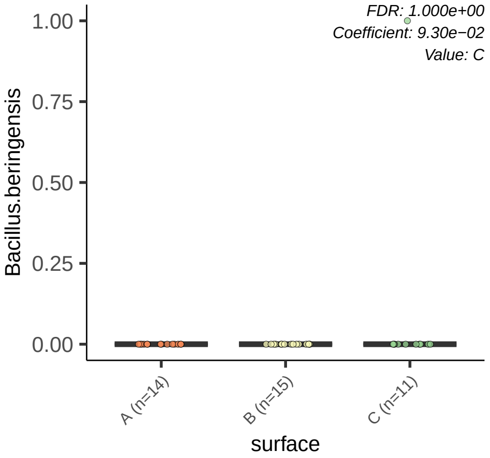
FDR: 1.000e+00
Coefficient: 9.30e−02
Value: C
Bacillus.beringensis
1.00
0.75
0.50
0.25
0.00
A (n=14)
B (n=15)
C (n=11)
surface

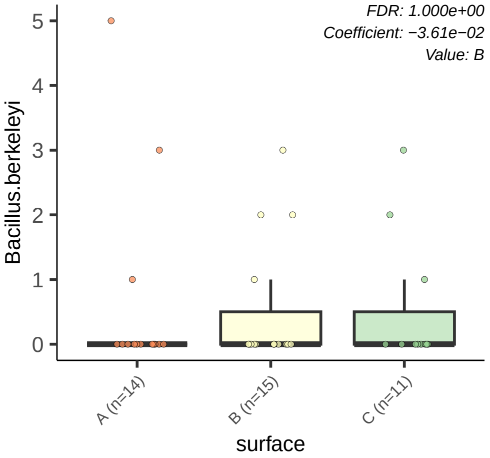

FDR: 1.000e+00
Coefficient: −3.61e−02
Value: B
Bacillus.berkeleyi
0
1
2
3
4
5
A (n=14)
B (n=15)
C (n=11)
surface

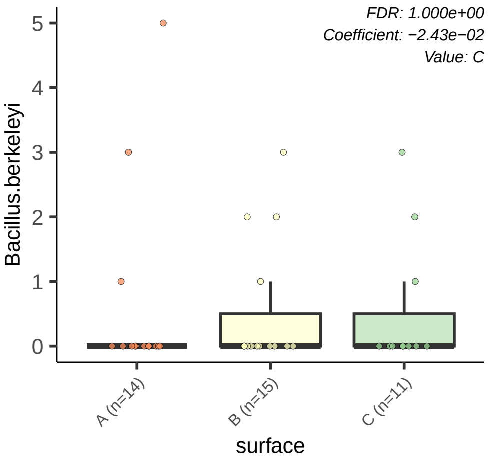
FDR: 1.000e+00
Coefficient: −2.43e−02
Value: C
Bacillus.berkeleyi
0
1
2
3
4
5
A (n=14)
B (n=15)
C (n=11)
surface

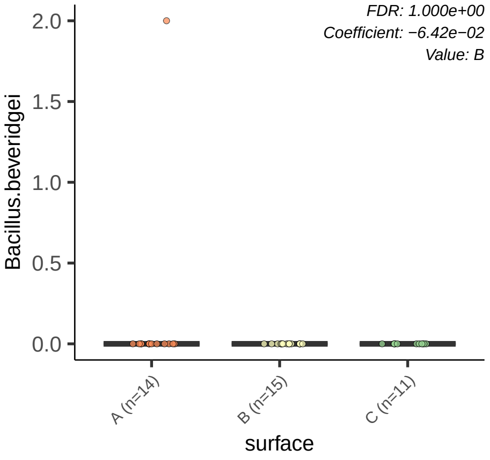
FDR: 1.000e+00
Coefficient: −6.42e−02
Value: B
Bacillus.beveridgei
2.0
1.5
1.0
0.5
0.0
A (n=14)
B (n=15)
C (n=11)
surface

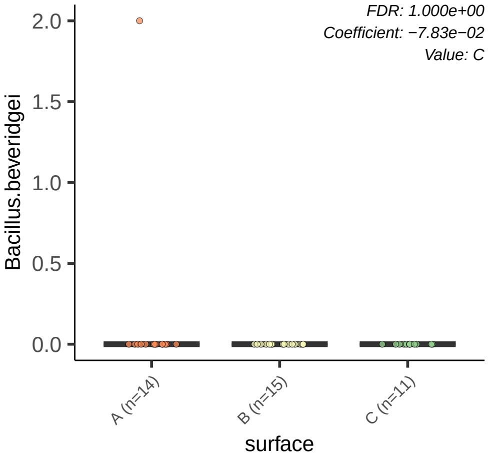
FDR: 1.000e+00
Coefficient: −7.83e−02
Value: C
Bacillus.beveridgei
2.0
1.5
1.0
0.5
0.0
A (n=14)
B (n=15)
C (n=11)
surface

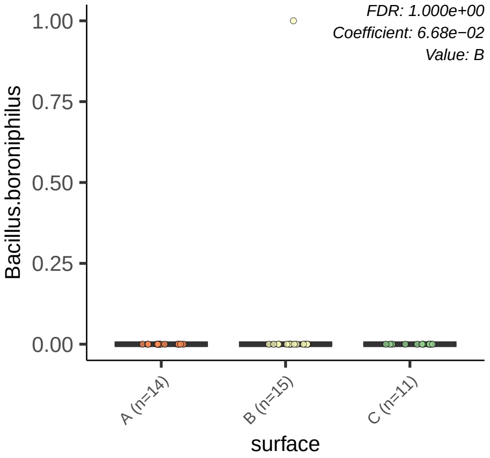

FDR: 1.000e+00
Coefficient: 6.68e−02
Value: B
Bacillus.boroniphilus
1.00
0.75
0.50
0.25
0.00
A (n=14)
B (n=15)
C (n=11)
surface

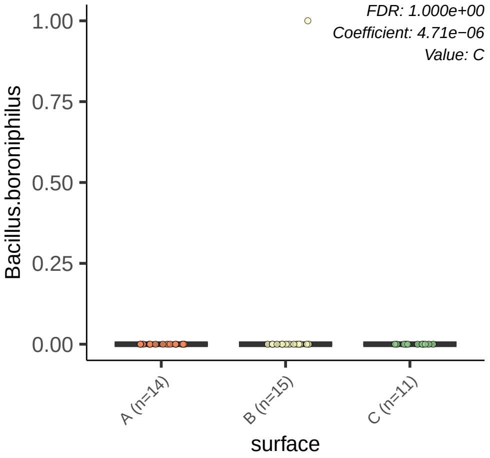
FDR: 1.000e+00
Coefficient: 4.71e−06
Value: C
Bacillus.boroniphilus
1.00
0.75
0.50
0.25
0.00
A (n=14)
B (n=15)
C (n=11)
surface

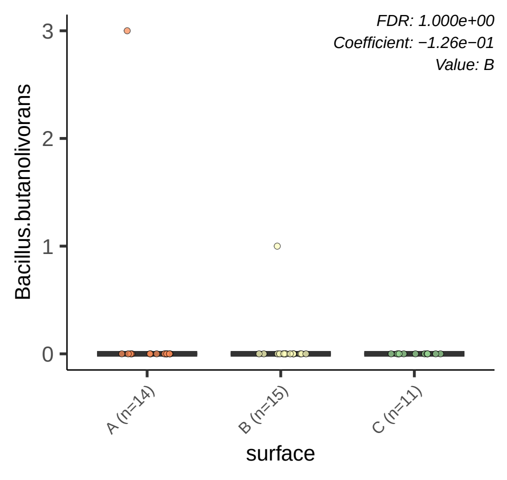
FDR: 1.000e+00
Coefficient: −1.26e−01
Value: B
Bacillus.butanolivorans
3
2
1
0
A (n=14)
B (n=15)
C (n=11)
surface

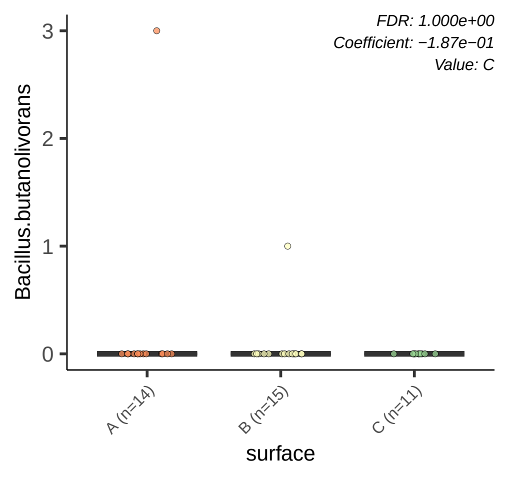
FDR: 1.000e+00
Coefficient: −1.87e−01
Value: C
Bacillus.butanolivorans
3
2
1
0
A (n=14)
B (n=15)
C (n=11)
surface

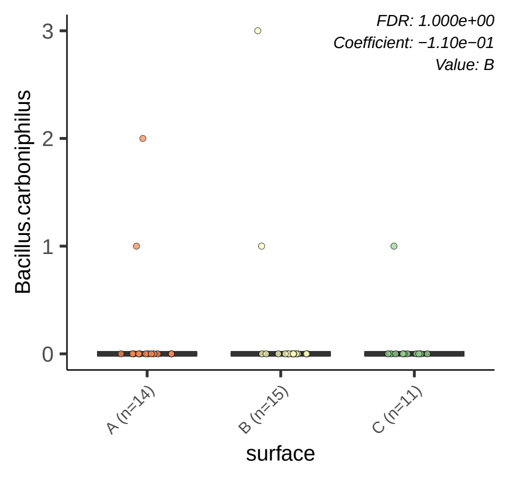
FDR: 1.000e+00
Coefficient: −1.10e−01
Value: B
Bacillus.carboniphilus
3
2
1
0
A (n=14)
B (n=15)
C (n=11)
surface

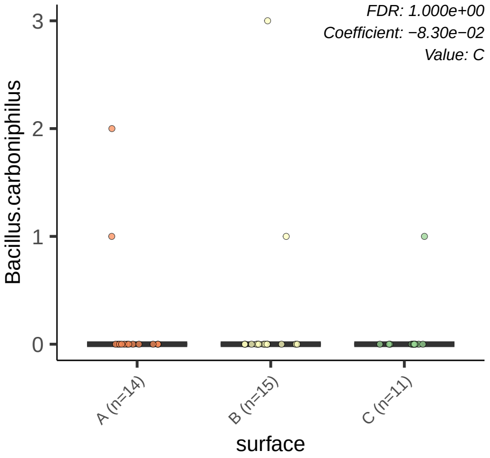
FDR: 1.000e+00
Coefficient: −8.30e−02
Value: C
Bacillus.carbonphilus
3
2
1
0
A (n=14)
B (n=15)
C (n=11)
surface

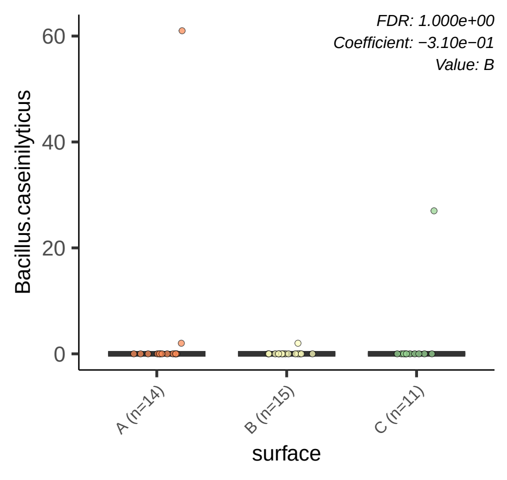

FDR: 1.000e+00
Coefficient: −3.10e−01
Value: B
Bacillus.caseinilyticus
0
20
40
60
A (n=14)
B (n=15)
C (n=11)
surface

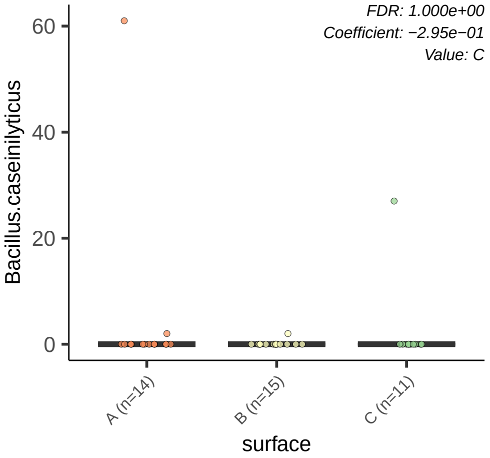

FDR: 1.000e+00
Coefficient: −2.95e−01
Value: C
60
40
20
0
Bacillus.caseinilyticus
A (n=14)
B (n=15)
C (n=11)
surface

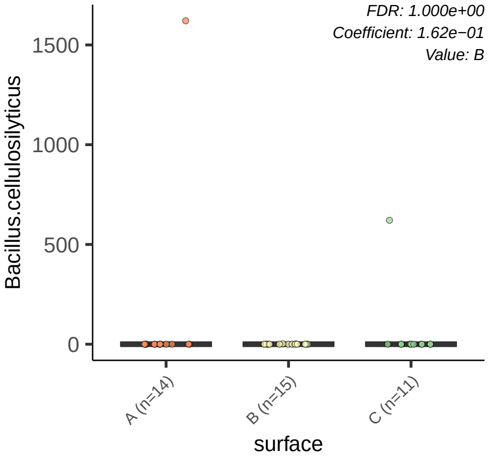
FDR: 1.000e+00
Coefficient: 1.62e−01
Value: B
Bacillus.cellulosilyticus
1500
1000
500
0
A (n=14)
B (n=15)
C (n=11)
surface

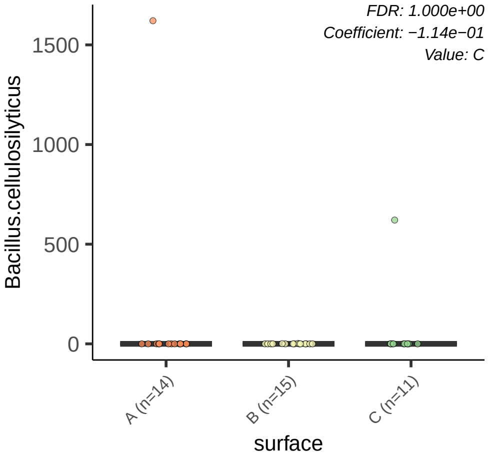

FDR: 1.000e+00
Coefficient: −1.14e−01
Value: C
Bacillus.cellulosilyticus
1500
1000
500
0
A (n=14)
B (n=15)
C (n=11)
surface

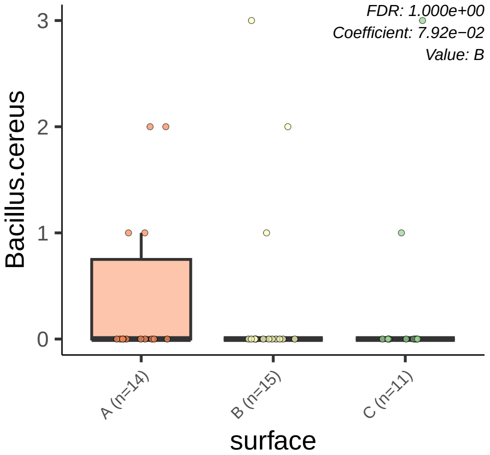

FDR: 1.000e+00
Coefficient: 7.92e−02
Value: B
3
2
1
0
Bacillus.cereus
A (n=14)
B (n=15)
C (n=11)
surface

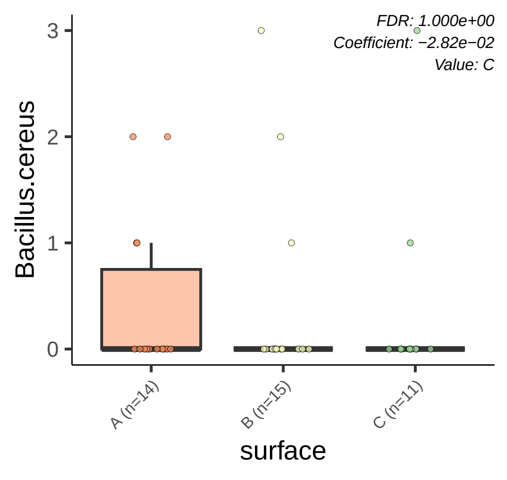

FDR: 1.000e+00
Coefficient: −2.82e−02
Value: C
Bacillus.cereus
3
2
1
0
A (n=14)
B (n=15)
C (n=11)
surface

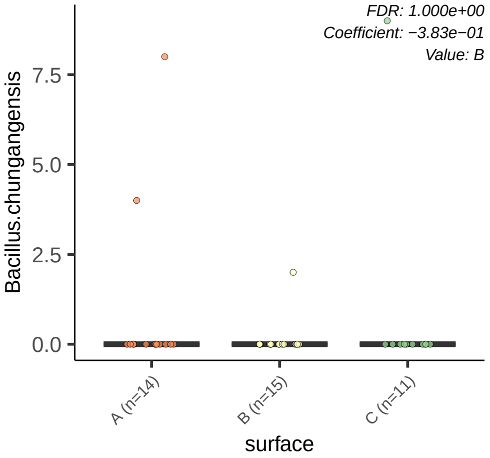

FDR: 1.000e+00
Coefficient: −3.83e−01
Value: B
Bacillus.chungangensis
7.5
5.0
2.5
0.0
A (n=14)
B (n=15)
C (n=11)
surface

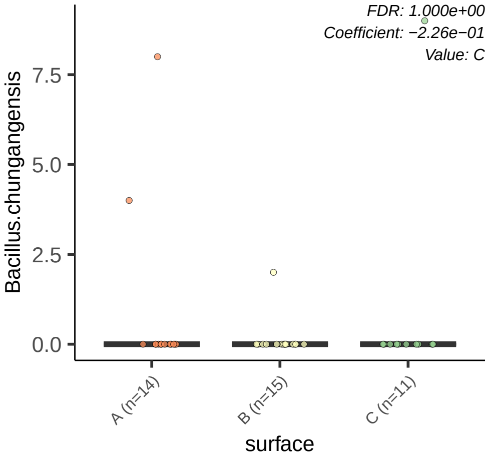

FDR: 1.000e+00
Coefficient: −2.26e−01
Value: C
Bacillus.chungangensis
7.5
5.0
2.5
0.0
A (n=14)
B (n=15)
C (n=11)
surface

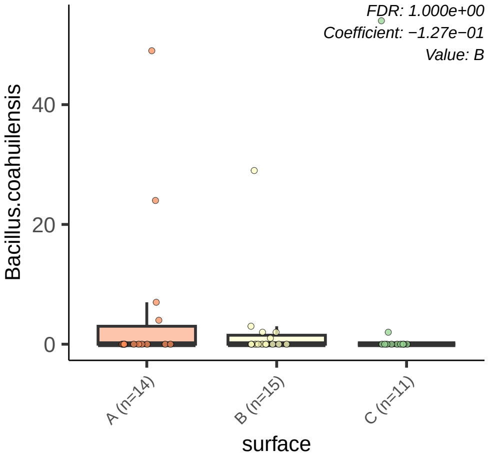
FDR: 1.000e+00
Coefficient: −1.27e−01
Value: B
Bacillus.coahuilensis
40
20
0
A (n=14)
B (n=15)
C (n=11)
surface

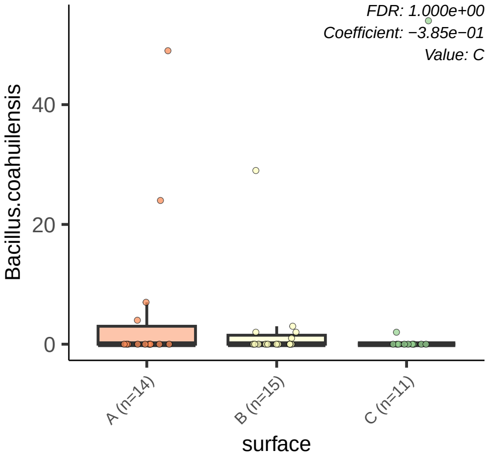

FDR: 1.000e+00
Coefficient: −3.85e−01
Value: C
Bacillus.coahuilensis
40
20
0
A (n=14)
B (n=15)
C (n=11)
surface

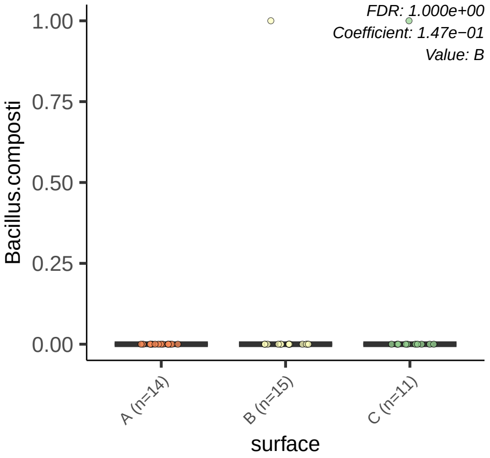

FDR: 1.000e+00
Coefficient: 1.47e−01
Value: B
Bacillus.composti
1.00
0.75
0.50
0.25
0.00
A (n=14)
B (n=15)
C (n=11)
surface

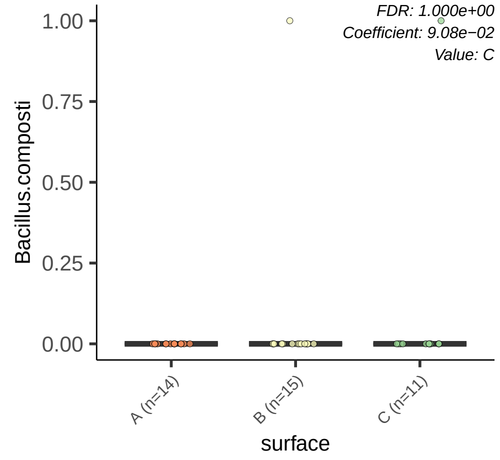

FDR: 1.000e+00
Coefficient: 9.08e−02
Value: C
Bacillus.composti
1.00
0.75
0.50
0.25
0.00
A (n=14)
B (n=15)
C (n=11)
surface

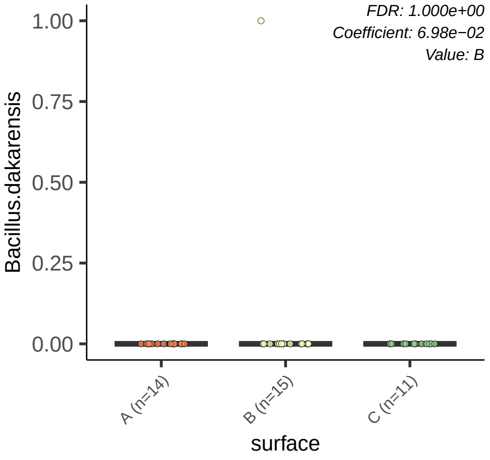
FDR: 1.000e+00
Coefficient: 6.98e−02
Value: B
Bacillus.dakarensis
1.00
0.75
0.50
0.25
0.00
A (n=14)
B (n=15)
C (n=11)
surface

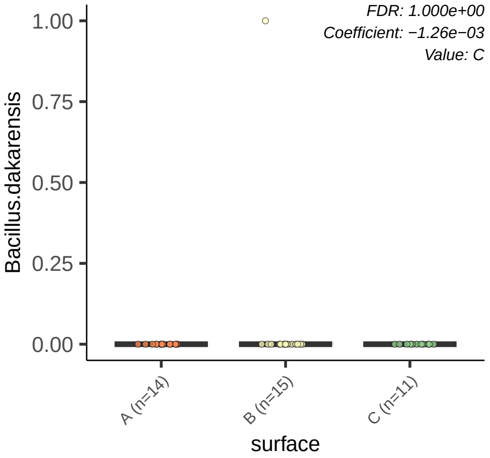
FDR: 1.000e+00
Coefficient: −1.26e−03
Value: C
Bacillus.dakarensis
1.00
0.75
0.50
0.25
0.00
A (n=14)
B (n=15)
C (n=11)
surface

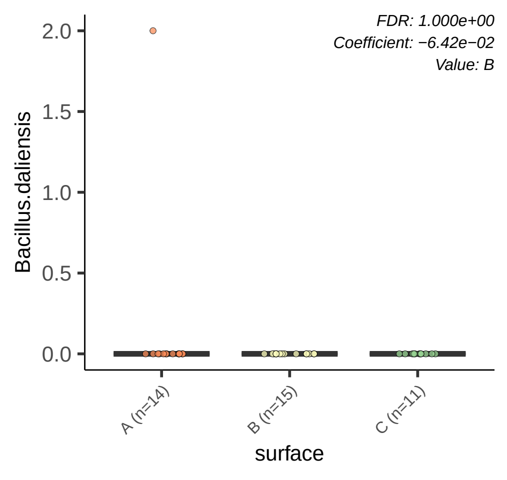
FDR: 1.000e+00
Coefficient: −6.42e−02
Value: B
Bacillus.daliensis
2.0
1.5
1.0
0.5
0.0
A (n=14)
B (n=15)
C (n=11)
surface

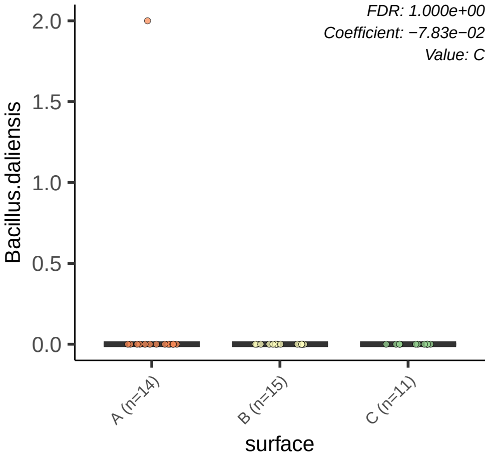
FDR: 1.000e+00
Coefficient: −7.83e−02
Value: C
Bacillus.daliensis
2.0
1.5
1.0
0.5
0.0
A (n=14)
B (n=15)
C (n=11)
surface

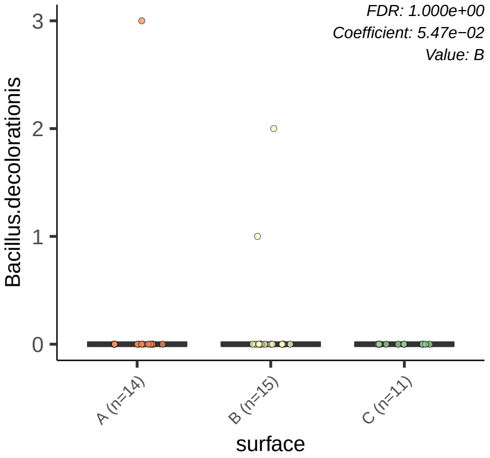
FDR: 1.000e+00
Coefficient: 5.47e−02
Value: B
Bacillus.decolorationis
0
1
2
3
A (n=14)
B (n=15)
C (n=11)
surface

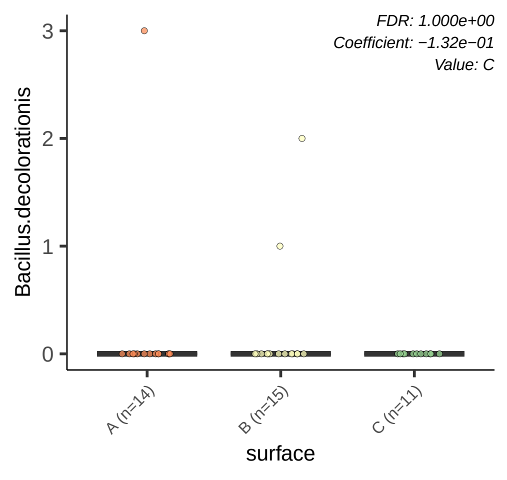

FDR: 1.000e+00
Coefficient: −1.32e−01
Value: C
Bacillus.decolorationis
0
1
2
3
A (n=14)
B (n=15)
C (n=11)
surface

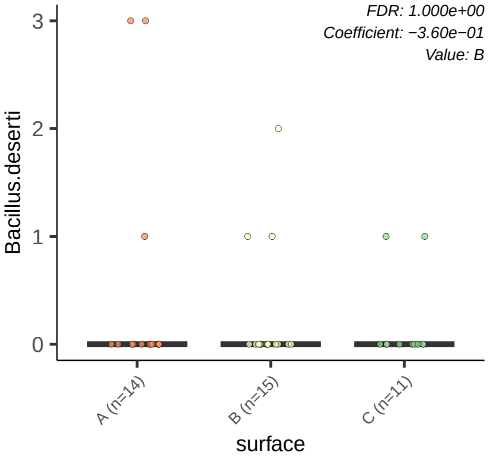

FDR: 1.000e+00
Coefficient: −3.60e−01
Value: B
Bacillus.deserti
3
2
1
0
A (n=14)
B (n=15)
C (n=11)
surface

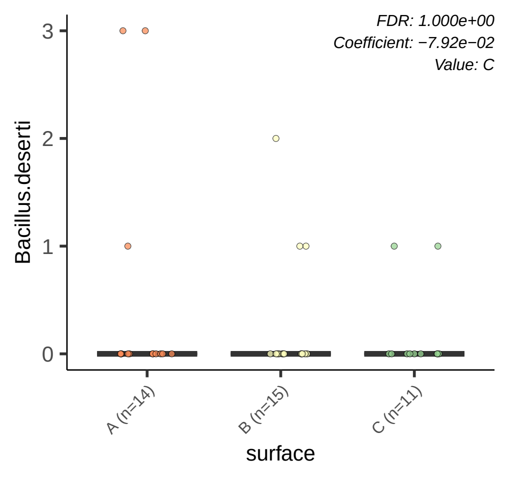

FDR: 1.000e+00
Coefficient: −7.92e−02
Value: C
Bacillus.deserti
3
2
1
0
A (n=14)
B (n=15)
C (n=11)
surface

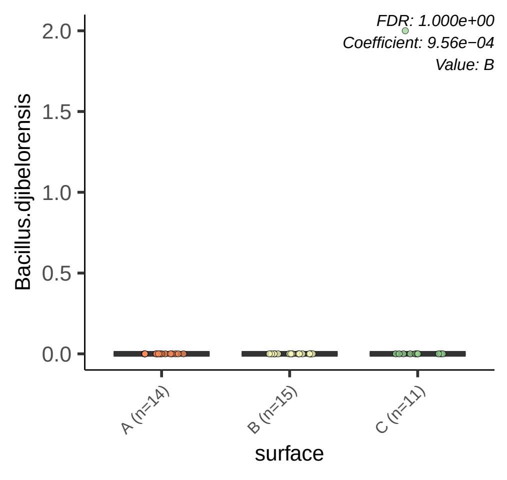

FDR: 1.000e+00
Coefficient: 9.56e−04
Value: B
2.0
1.5
1.0
0.5
0.0
Bacillus.djibelorensis
A (n=14)
B (n=15)
C (n=11)
surface

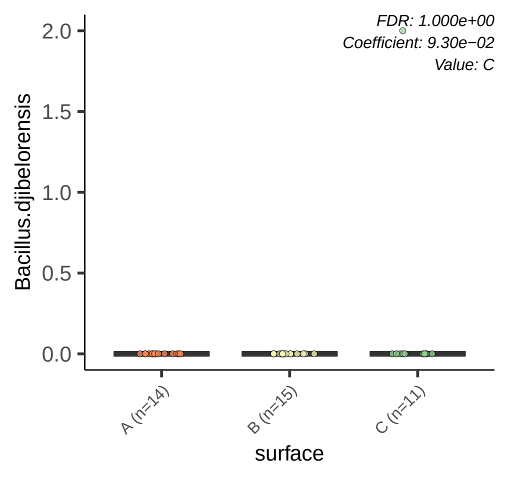
FDR: 1.000e+00
Coefficient: 9.30e−02
Value: C
Bacillus.djibelorensis
2.0
1.5
1.0
0.5
0.0
A (n=14)
B (n=15)
C (n=11)
surface

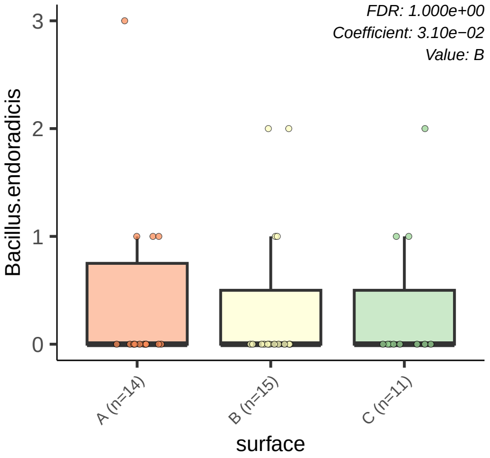
FDR: 1.000e+00
Coefficient: 3.10e−02
Value: B
Bacillus.endoradicis
3
2
1
0
A (n=14)
B (n=15)
C (n=11)
surface

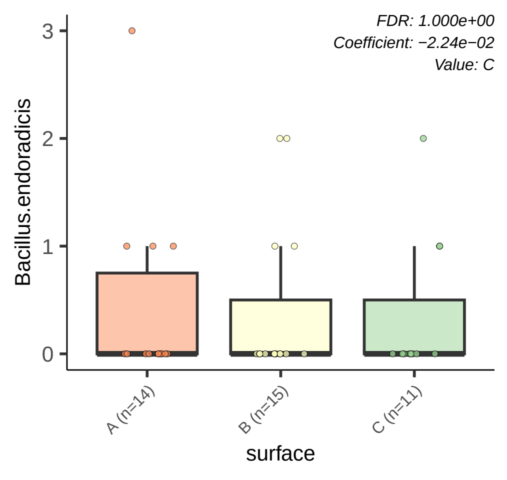

FDR: 1.000e+00
Coefficient: −2.24e−02
Value: C
Bacillus.endoradicis
3
2
1
0
A (n=14)
B (n=15)
C (n=11)
surface

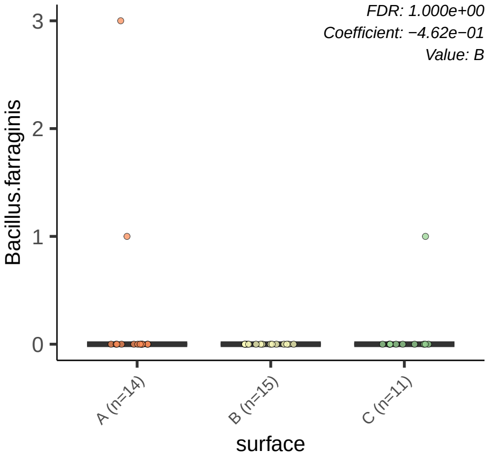

FDR: 1.000e+00
Coefficient: −4.62e−01
Value: B
Bacillus.farraginis
3
2
1
0
A (n=14)
B (n=15)
C (n=11)
surface

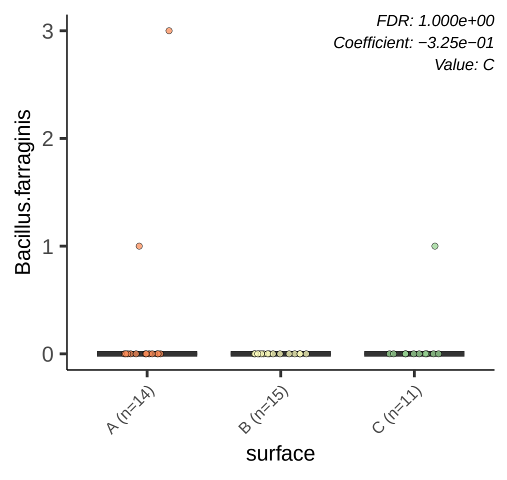
FDR: 1.000e+00
Coefficient: −3.25e−01
Value: C
Bacillus.farraginis
3
2
1
0
A (n=14)
B (n=15)
C (n=11)
surface

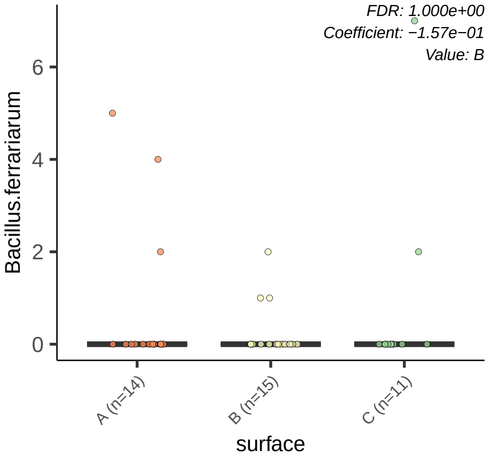
FDR: 1.000e+00
Coefficient: −1.57e−01
Value: B
Bacillus.ferrariarum
0
2
4
6
A (n=14)
B (n=15)
C (n=11)
surface

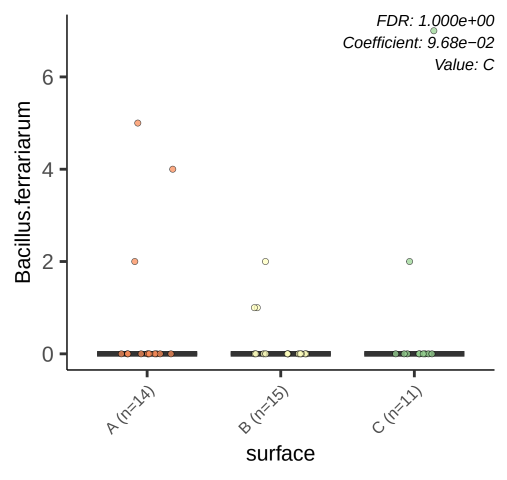

FDR: 1.000e+00
Coefficient: 9.68e−02
Value: C
Bacillus.ferrariarum
0
2
4
6
A (n=14)
B (n=15)
C (n=11)
surface

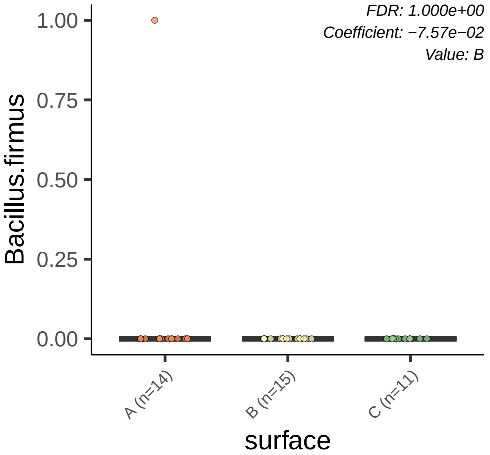

FDR: 1.000e+00
Coefficient: −7.57e−02
Value: B
Bacillus.firmus
1.00
0.75
0.50
0.25
0.00
A (n=14)
B (n=15)
C (n=11)
surface

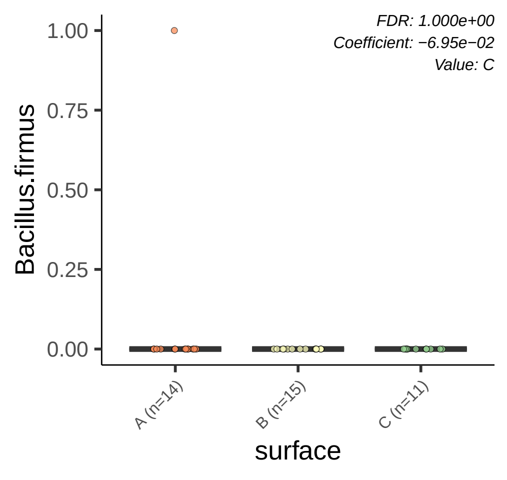

FDR: 1.000e+00
Coefficient: −6.95e−02
Value: C
Bacillus.firmus
1.00
0.75
0.50
0.25
0.00
A (n=14)
B (n=15)
C (n=11)
surface

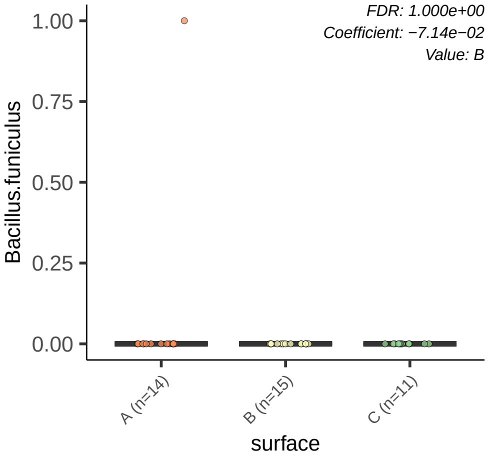
FDR: 1.000e+00
Coefficient: −7.14e−02
Value: B
Bacillus.funiculus
1.00
0.75
0.50
0.25
0.00
A (n=14)
B (n=15)
C (n=11)
surface

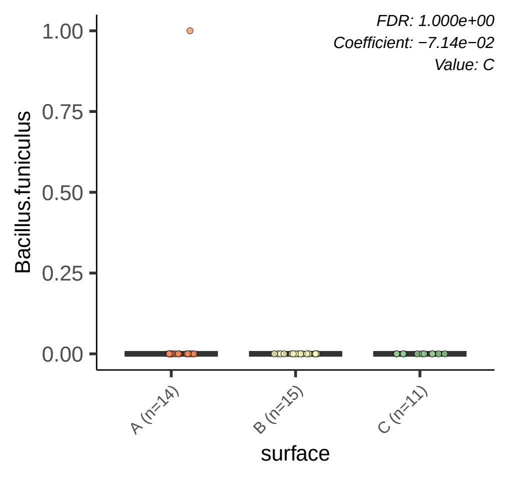

FDR: 1.000e+00
Coefficient: −7.14e−02
Value: C
Bacillus.funiculus
1.00
0.75
0.50
0.25
0.00
A (n=14)
B (n=15)
C (n=11)
surface

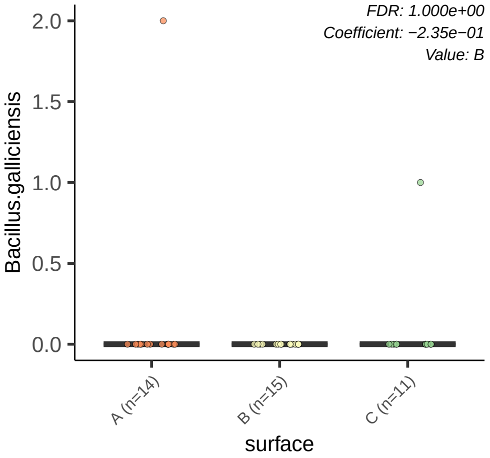
FDR: 1.000e+00
Coefficient: −2.35e−01
Value: B
Bacillus.galliciensis
2.0
1.5
1.0
0.5
0.0
A (n=14)
B (n=15)
C (n=11)
surface

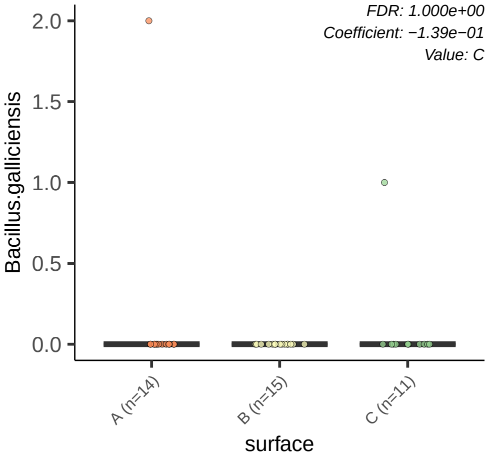

FDR: 1.000e+00
Coefficient: −1.39e−01
Value: C
Bacillus.galliciensis
2.0
1.5
1.0
0.5
0.0
A (n=14)
B (n=15)
C (n=11)
surface

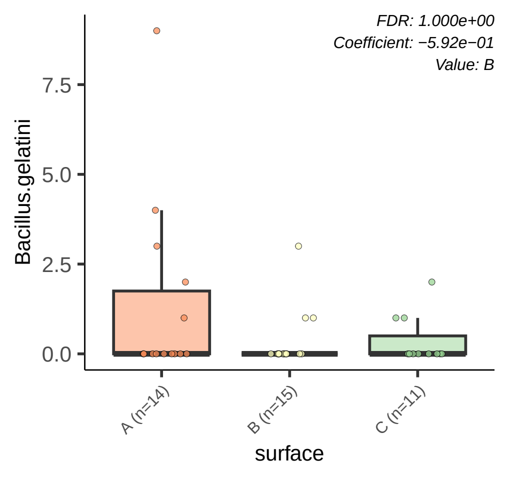
FDR: 1.000e+00
Coefficient: −5.92e−01
Value: B
Bacillus.gelatini
7.5
5.0
2.5
0.0
A (n=14)
B (n=15)
C (n=11)
surface

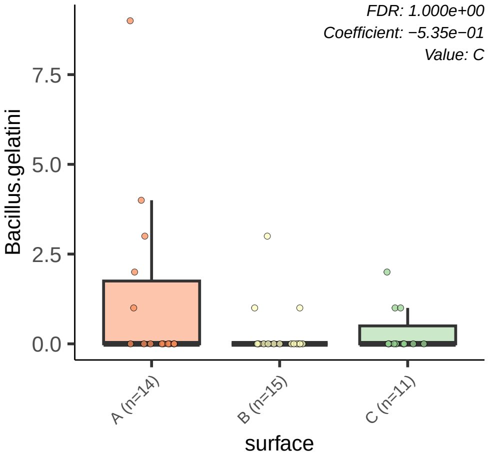
FDR: 1.000e+00
Coefficient: −5.35e−01
Value: C
Bacillus.gelatini
7.5
5.0
2.5
0.0
A (n=14)
B (n=15)
C (n=11)
surface

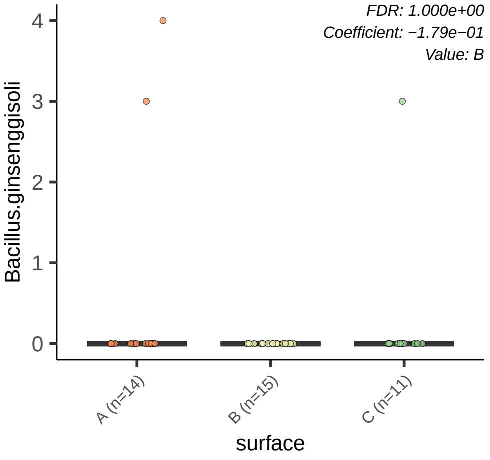

FDR: 1.000e+00
Coefficient: −1.79e−01
Value: B
Bacillus.ginsenggisoli
4
3
2
1
0
A (n=14)
B (n=15)
C (n=11)
surface

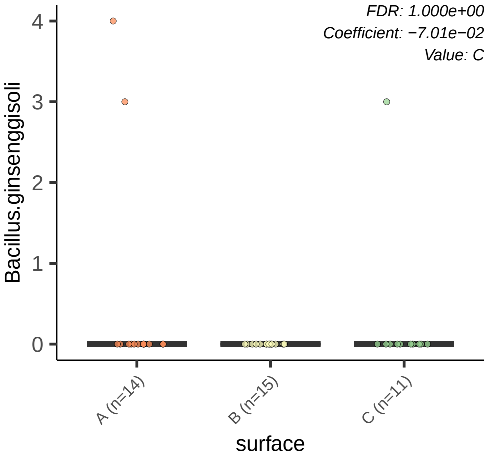

FDR: 1.000e+00
Coefficient: −7.01e−02
Value: C
Bacillus.ginsenggisoli
4
3
2
1
0
A (n=14)
B (n=15)
C (n=11)
surface

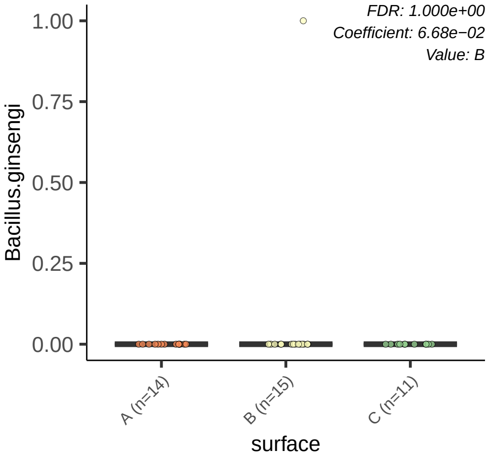

FDR: 1.000e+00
Coefficient: 6.68e−02
Value: B
Bacillus.ginsengi
1.00
0.75
0.50
0.25
0.00
A (n=14)
B (n=15)
C (n=11)
surface

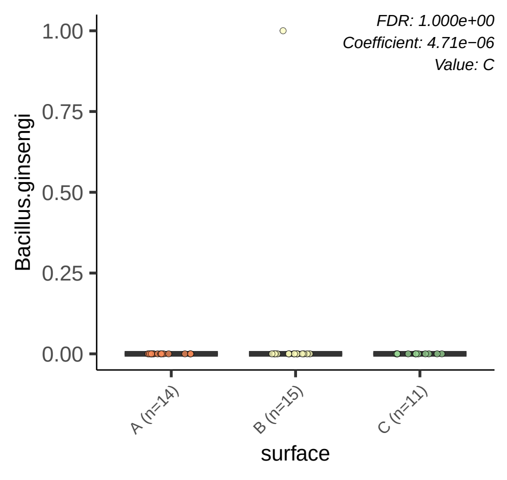
FDR: 1.000e+00
Coefficient: 4.71e−06
Value: C
Bacillus.ginsengi
1.00
0.75
0.50
0.25
0.00
A (n=14)
B (n=15)
C (n=11)
surface

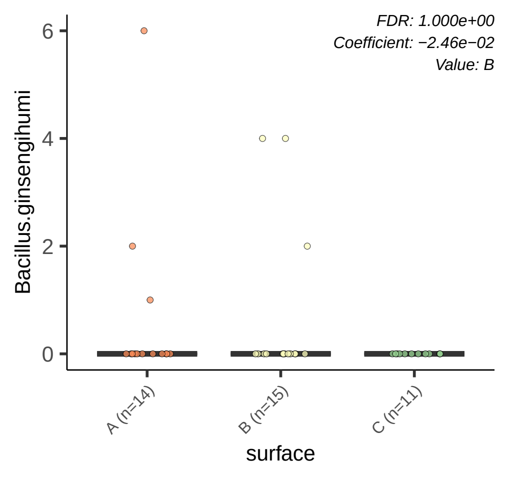
FDR: 1.000e+00
Coefficient: −2.46e−02
Value: B
Bacillus.ginsengihumi
6
4
2
0
A (n=14)
B (n=15)
C (n=11)
surface

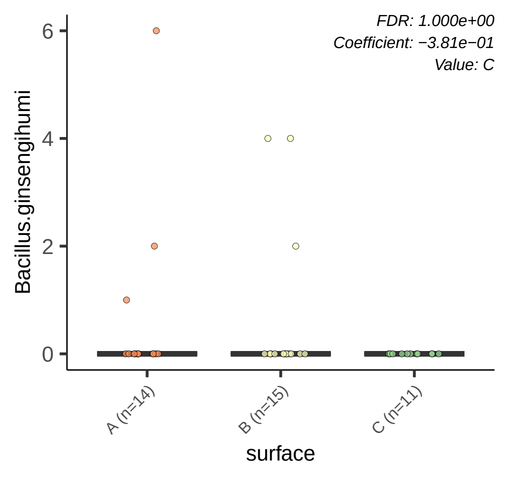
FDR: 1.000e+00
Coefficient: −3.81e−01
Value: C
Bacillus.ginsenghumi
6
4
2
0
A (n=14)
B (n=15)
C (n=11)
surface

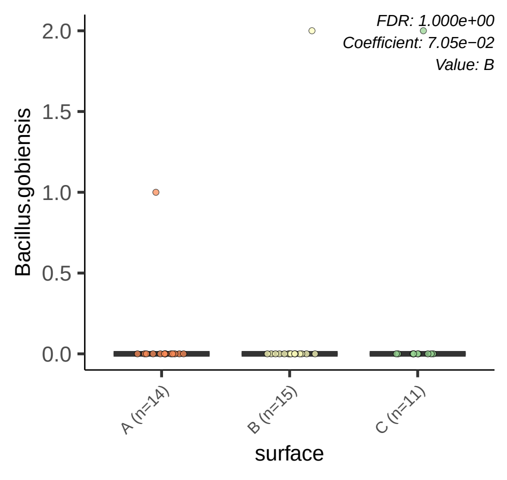

FDR: 1.000e+00
Coefficient: 7.05e−02
Value: B
Bacillus.gobiensis
2.0
1.5
1.0
0.5
0.0
A (n=14)
B (n=15)
C (n=11)
surface

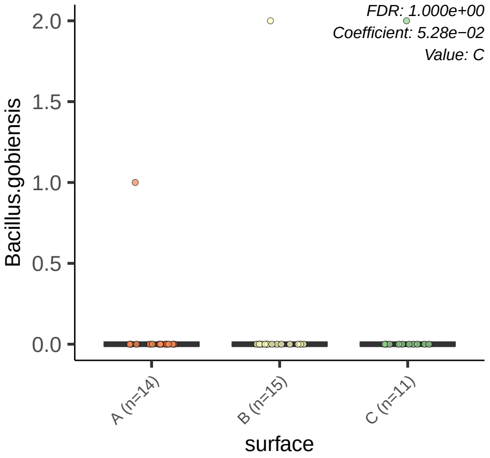
FDR: 1.000e+00
Coefficient: 5.28e−02
Value: C
Bacillus.gobiensis
2.0
1.5
1.0
0.5
0.0
A (n=14)
B (n=15)
C (n=11)
surface

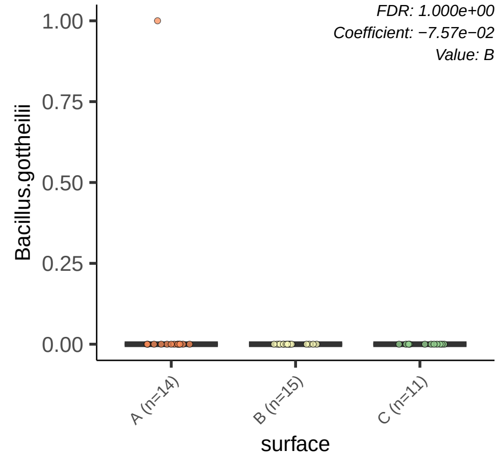
FDR: 1.000e+00
Coefficient: −7.57e−02
Value: B
1.00
0.75
0.50
0.25
0.00
Bacillus.gottheilii
A (n=14)
B (n=15)
C (n=11)
surface

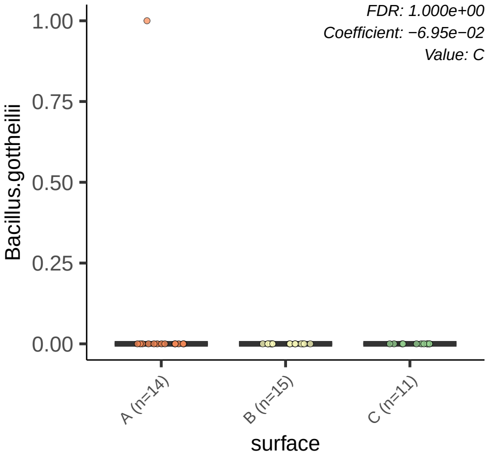
FDR: 1.000e+00
Coefficient: −6.95e−02
Value: C
Bacillus.gottheilii
1.00
0.75
0.50
0.25
0.00
A (n=14)
B (n=15)
C (n=11)
surface

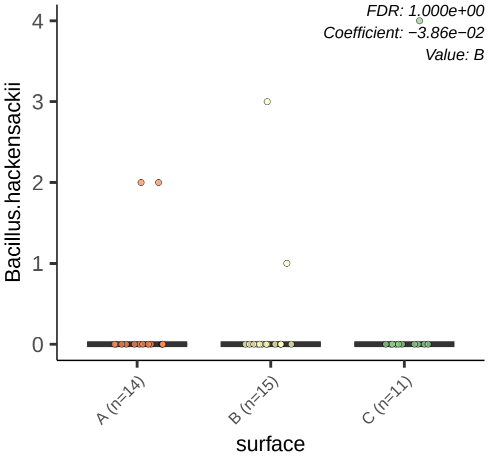
FDR: 1.000e+00
Coefficient: −3.86e−02
Value: B
Bacillus.hackensackii
4
3
2
1
0
A (n=14)
B (n=15)
C (n=11)
surface

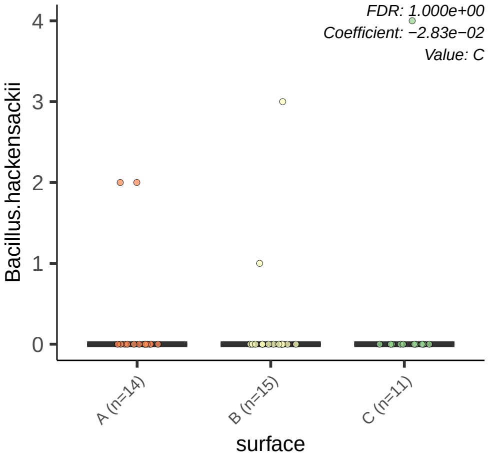

FDR: 1.000e+00
Coefficient: −2.83e−02
Value: C
Bacillus.hackensackii
0
1
2
3
4
A (n=14)
B (n=15)
C (n=11)
surface

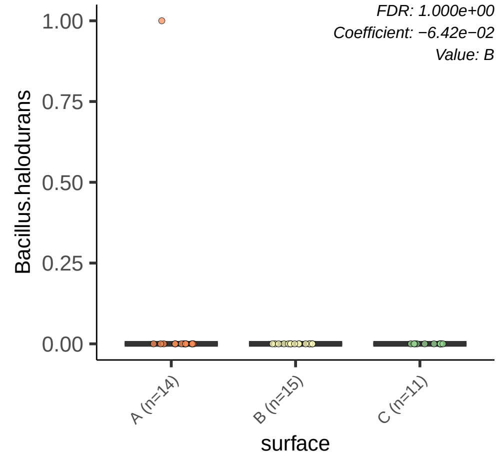
FDR: 1.000e+00
Coefficient: −6.42e−02
Value: B
Bacillus.halodurans
1.00
0.75
0.50
0.25
0.00
A (n=14)
B (n=15)
C (n=11)
surface

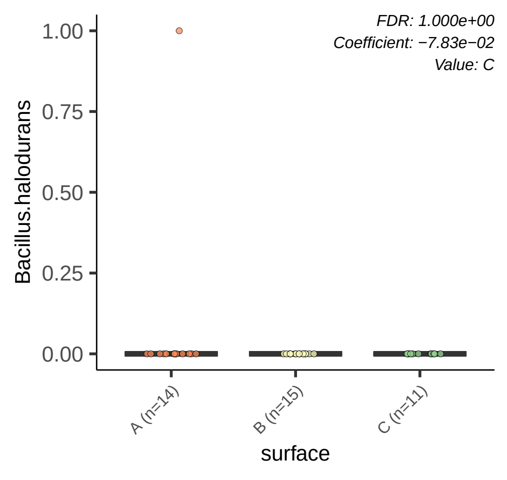
FDR: 1.000e+00
Coefficient: −7.83e−02
Value: C
Bacillus.halodurans
1.00
0.75
0.50
0.25
0.00
A (n=14)
B (n=15)
C (n=11)
surface

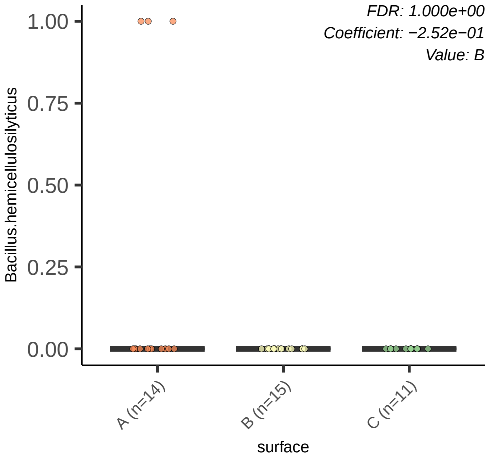
FDR: 1.000e+00
Coefficient: −2.52e−01
Value: B
1.00
0.75
0.50
0.25
0.00
Bacillus.hemicellulosilyticus
A (n=14)
B (n=15)
C (n=11)
surface

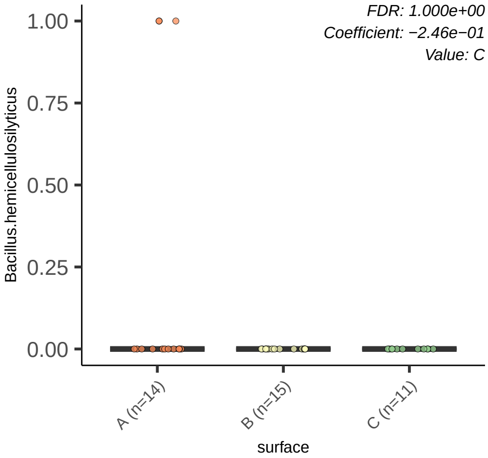
FDR: 1.000e+00
Coefficient: −2.46e−01
Value: C
Bacillus.hemicellulosilyticus
1.00
0.75
0.50
0.25
0.00
A (n=14)
B (n=15)
C (n=11)
surface

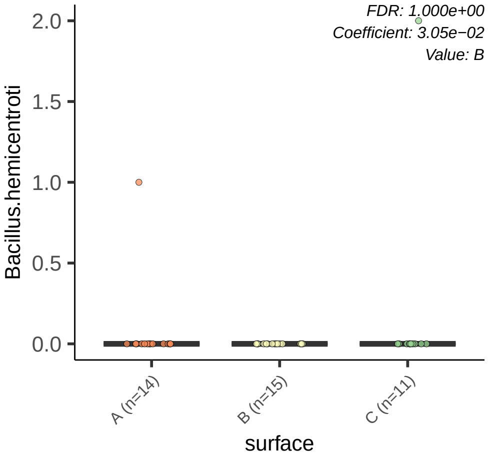
FDR: 1.000e+00
Coefficient: 3.05e−02
Value: B
Bacillus.hemicentroti
2.0
1.5
1.0
0.5
0.0
A (n=14)
B (n=15)
C (n=11)
surface

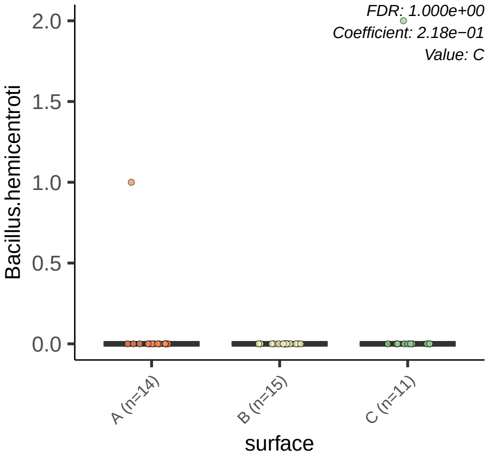

FDR: 1.000e+00
Coefficient: 2.18e−01
Value: C
Bacillus.hemicentroti
2.0
1.5
1.0
0.5
0.0
A (n=14)
B (n=15)
C (n=11)
surface

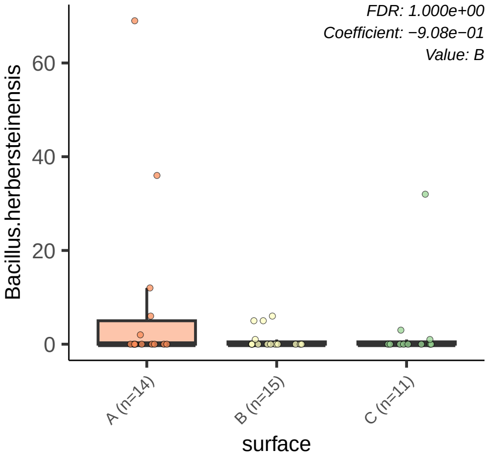
FDR: 1.000e+00
Coefficient: −9.08e−01
Value: B
Bacillus.herbersteinensis
60
40
20
0
A (n=14)
B (n=15)
C (n=11)
surface

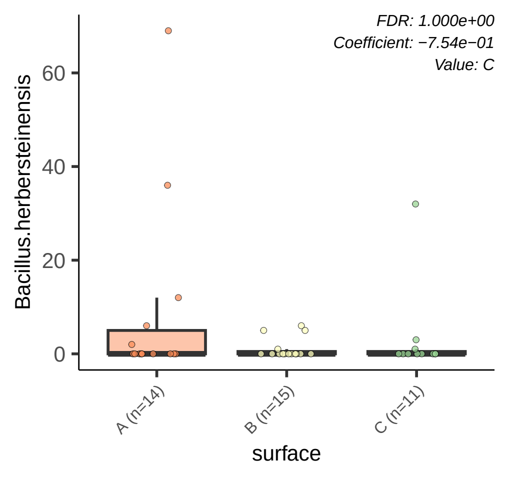
FDR: 1.000e+00
Coefficient: −7.54e−01
Value: C
Bacillus.herbersteinensis
0
20
40
60
A (n=14)
B (n=15)
C (n=11)
surface

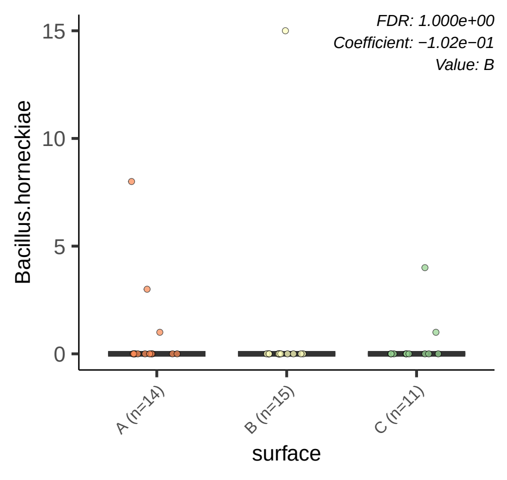

FDR: 1.000e+00
Coefficient: −1.02e−01
Value: B
Bacillus.horneckiae
15
10
5
0
A (n=14)
B (n=15)
C (n=11)
surface

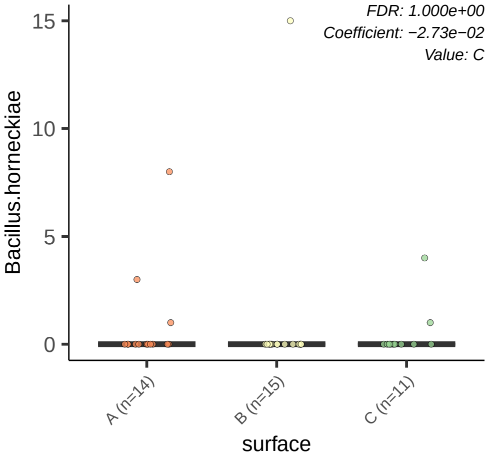
FDR: 1.000e+00
Coefficient: −2.73e−02
Value: C
Bacillus.horneckiae
15
10
5
0
A (n=14)
B (n=15)
C (n=11)
surface

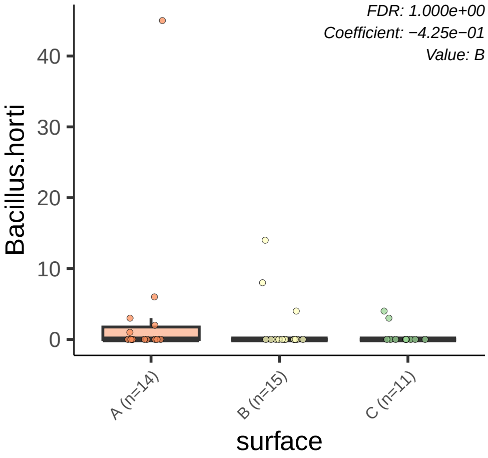
FDR: 1.000e+00
Coefficient: −4.25e−01
Value: B
Bacillus.horti
40
30
20
10
0
A (n=14)
B (n=15)
C (n=11)
surface

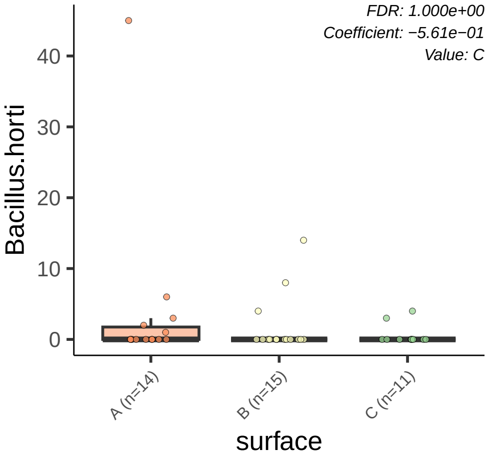

FDR: 1.000e+00
Coefficient: −5.61e−01
Value: C
Bacillus.horti
0
10
20
30
40
A (n=14)
B (n=15)
C (n=11)
surface

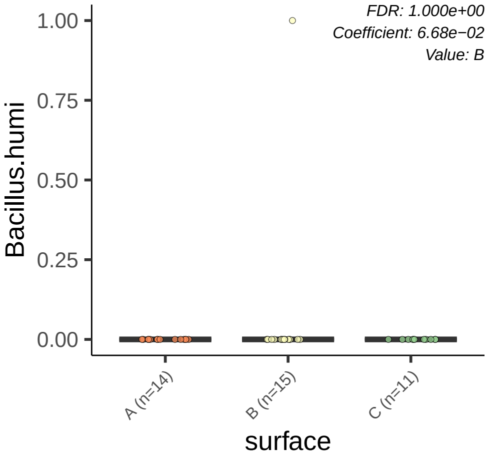
FDR: 1.000e+00
Coefficient: 6.68e−02
Value: B
Bacillus.humi
1.00
0.75
0.50
0.25
0.00
A (n=14)
B (n=15)
C (n=11)
surface

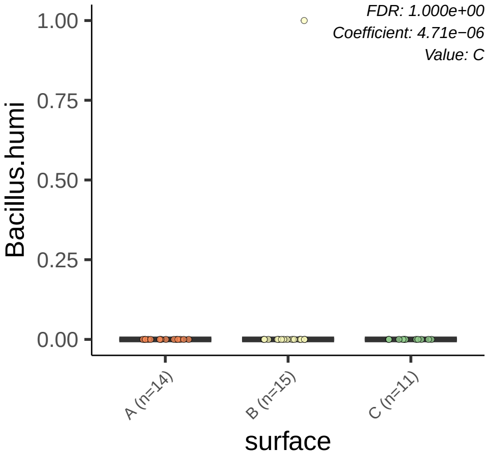

FDR: 1.000e+00
Coefficient: 4.71e−06
Value: C
Bacillus.humi
1.00
0.75
0.50
0.25
0.00
A (n=14)
B (n=15)
C (n=11)
surface

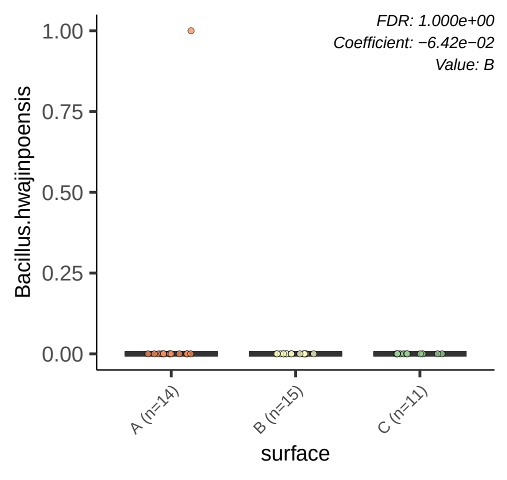
FDR: 1.000e+00
Coefficient: −6.42e−02
Value: B
Bacillus.hwajinpoensis
1.00
0.75
0.50
0.25
0.00
A (n=14)
B (n=15)
C (n=11)
surface

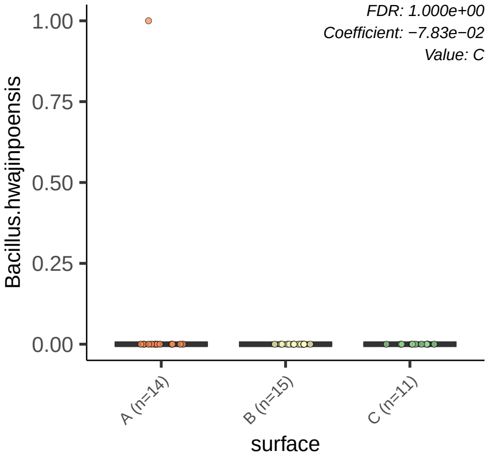

FDR: 1.000e+00
Coefficient: −7.83e−02
Value: C
Bacillus.hwajinpoensis
1.00
0.75
0.50
0.25
0.00
A (n=14)
B (n=15)
C (n=11)
surface

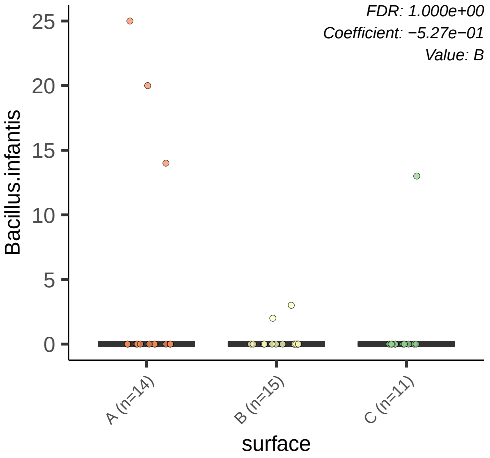
FDR: 1.000e+00
Coefficient: −5.27e−01
Value: B
Bacillus.infantis
25
20
15
10
5
0
A (n=14)
B (n=15)
C (n=11)
surface

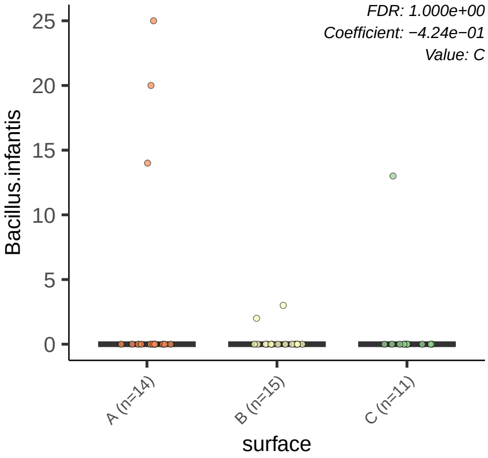
FDR: 1.000e+00
Coefficient: −4.24e−01
Value: C
Bacillus.infantis
25
20
15
10
5
0
A (n=14)
B (n=15)
C (n=11)
surface

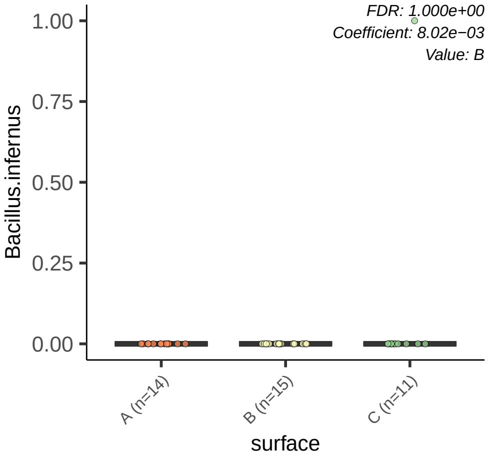
FDR: 1.000e+00
Coefficient: 8.02e−03
Value: B
Bacillus.infernus
1.00
0.75
0.50
0.25
0.00
A (n=14)
B (n=15)
C (n=11)
surface

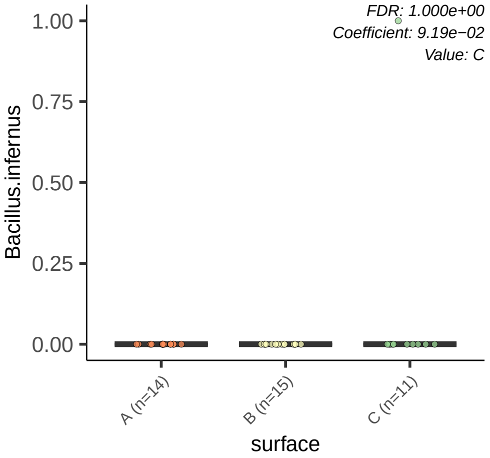
FDR: 1.000e+00
Coefficient: 9.19e−02
Value: C
Bacillus.infernus
1.00
0.75
0.50
0.25
0.00
A (n=14)
B (n=15)
C (n=11)
surface

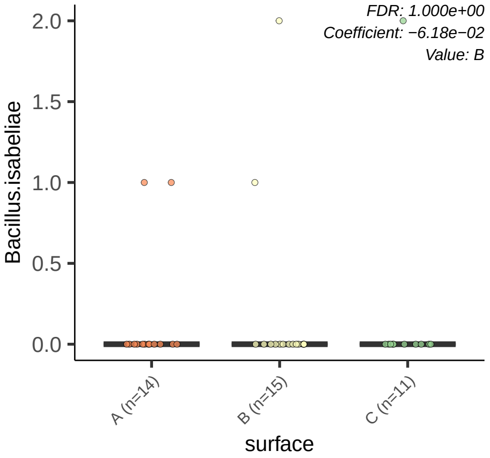

FDR: 1.000e+00
Coefficient: −6.18e−02
Value: B
Bacillus.isabeliae
2.0
1.5
1.0
0.5
0.0
A (n=14)
B (n=15)
C (n=11)
surface

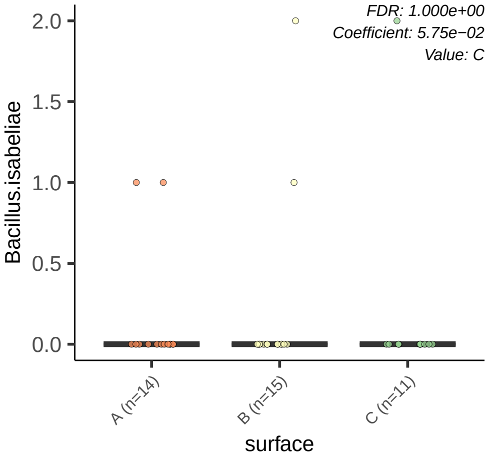
FDR: 1.000e+00
Coefficient: 5.75e−02
Value: C
Bacillus.isabeliae
2.0
1.5
1.0
0.5
0.0
A (n=14)
B (n=15)
C (n=11)
surface

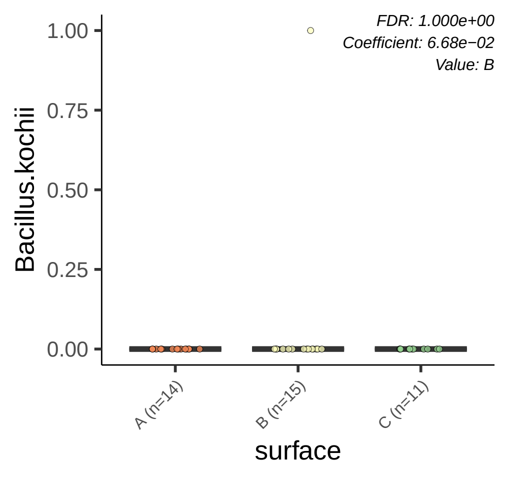
FDR: 1.000e+00
Coefficient: 6.68e−02
Value: B
Bacillus.kochii
1.00
0.75
0.50
0.25
0.00
A (n=14)
B (n=15)
C (n=11)
surface

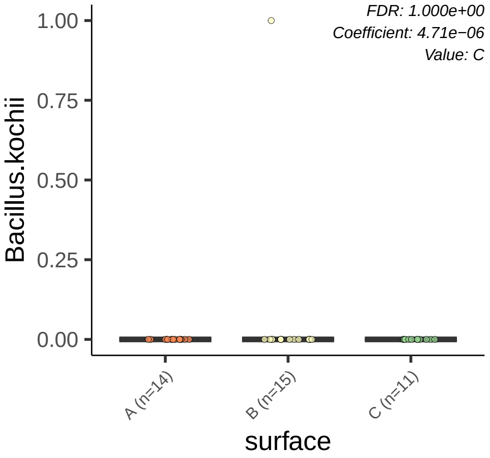

FDR: 1.000e+00
Coefficient: 4.71e−06
Value: C
Bacillus.kochii
1.00
0.75
0.50
0.25
0.00
A (n=14)
B (n=15)
C (n=11)
surface

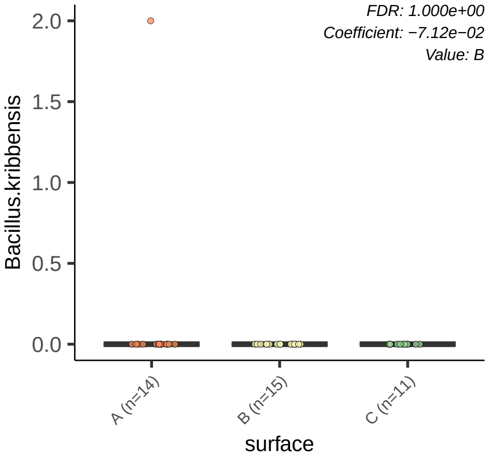

FDR: 1.000e+00
Coefficient: −7.12e−02
Value: B
Bacillus.kribbensis
2.0
1.5
1.0
0.5
0.0
A (n=14)
B (n=15)
C (n=11)
surface

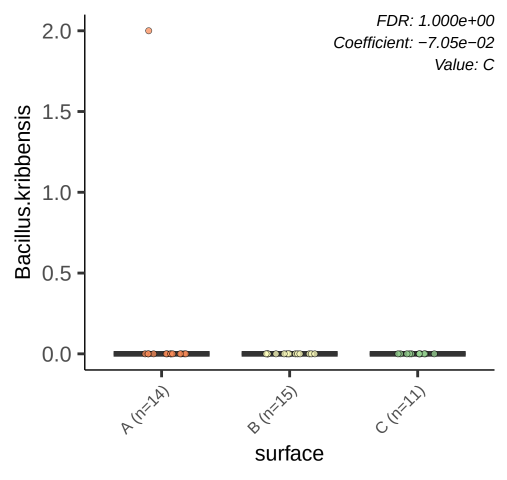

FDR: 1.000e+00
Coefficient: −7.05e−02
Value: C
Bacillus.kribbensis
2.0
1.5
1.0
0.5
0.0
A (n=14)
B (n=15)
C (n=11)
surface

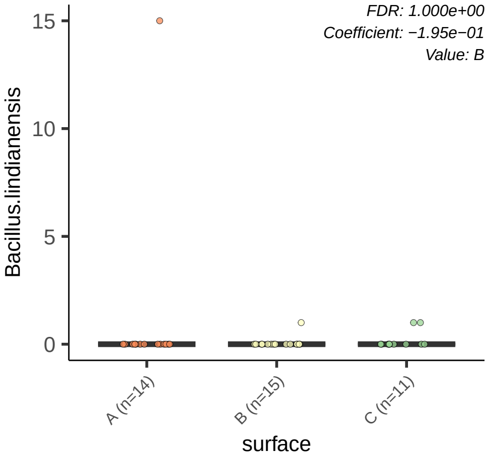

FDR: 1.000e+00
Coefficient: −1.95e−01
Value: B
Bacillus.lindianensis
15
10
5
0
A (n=14)
B (n=15)
C (n=11)
surface

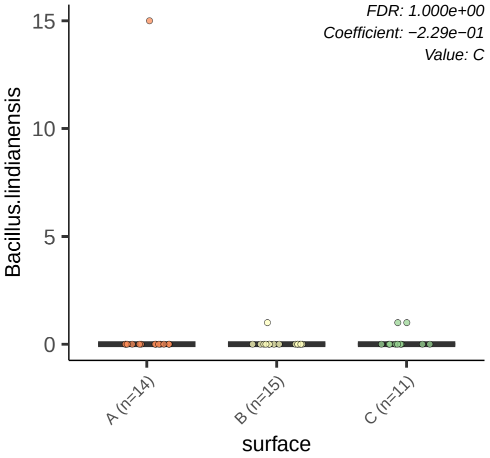

FDR: 1.000e+00
Coefficient: −2.29e−01
Value: C
Bacillus.lindianensis
15
10
5
0
A (n=14)
B (n=15)
C (n=11)
surface

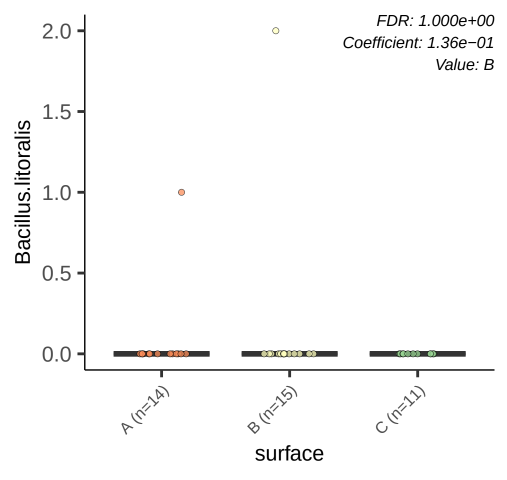
FDR: 1.000e+00
Coefficient: 1.36e−01
Value: B
2.0
1.5
1.0
0.5
0.0
Bacillus.litoralis
A (n=14)
B (n=15)
C (n=11)
surface

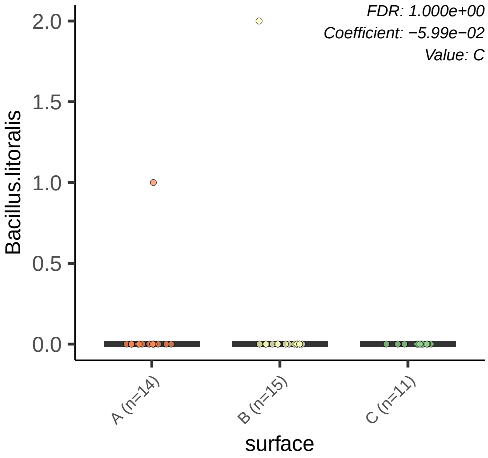

FDR: 1.000e+00
Coefficient: −5.99e−02
Value: C
2.0
1.5
1.0
0.5
0.0
Bacillus.litoralis
A (n=14)
B (n=15)
C (n=11)
surface

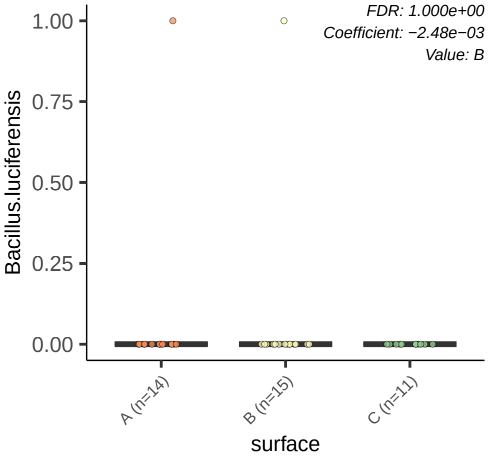
FDR: 1.000e+00
Coefficient: −2.48e−03
Value: B
Bacillus.luciferensis
1.00
0.75
0.50
0.25
0.00
A (n=14)
B (n=15)
C (n=11)
surface

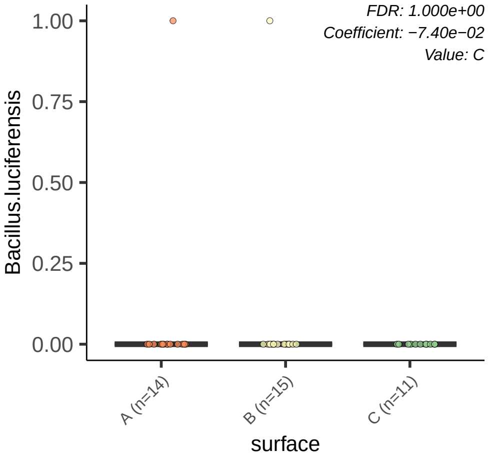
FDR: 1.000e+00
Coefficient: −7.40e−02
Value: C
Bacillus.luciferensis
1.00
0.75
0.50
0.25
0.00
A (n=14)
B (n=15)
C (n=11)
surface

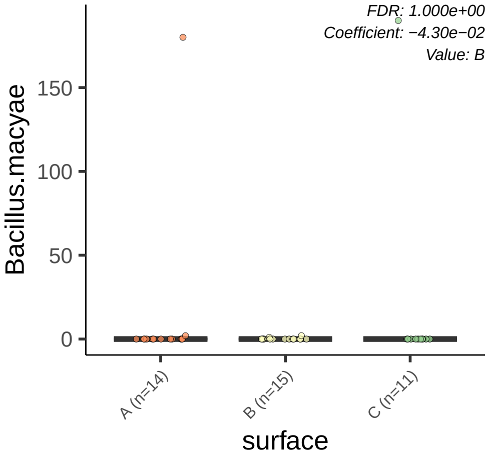

FDR: 1.000e+00
Coefficient: −4.30e−02
Value: B
Bacillus.macyae
150
100
50
0
A (n=14)
B (n=15)
C (n=11)
surface

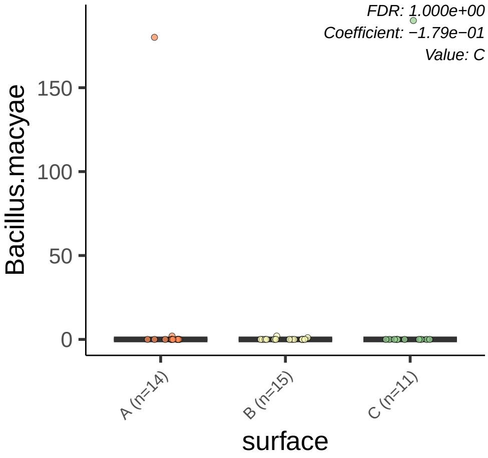
FDR: 1.000e+00
Coefficient: −1.79e−01
Value: C
Bacillus.macyae
150
100
50
0
A (n=14)
B (n=15)
C (n=11)
surface

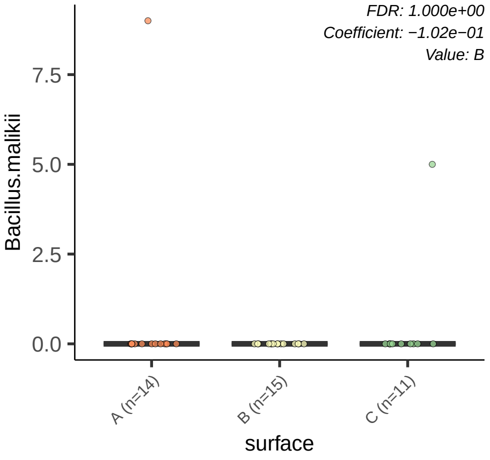

FDR: 1.000e+00
Coefficient: −1.02e−01
Value: B
Bacillus.malikii
7.5
5.0
2.5
0.0
A (n=14)
B (n=15)
C (n=11)
surface

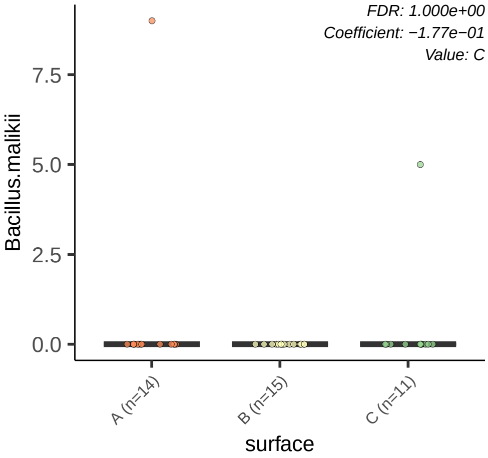
FDR: 1.000e+00
Coefficient: −1.77e−01
Value: C
Bacillus.malikii
7.5
5.0
2.5
0.0
A (n=14)
B (n=15)
C (n=11)
surface

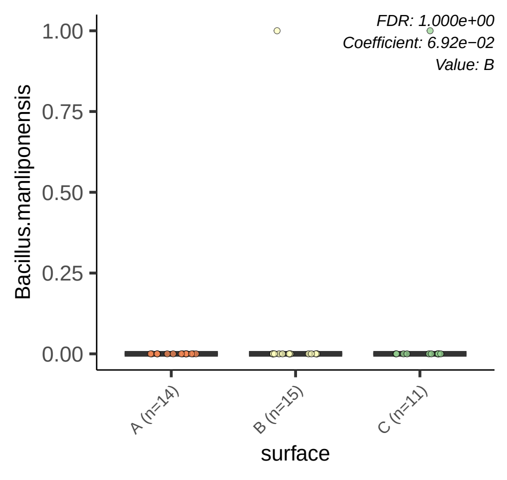
FDR: 1.000e+00
Coefficient: 6.92e−02
Value: B
Bacillus.manliponensis
1.00
0.75
0.50
0.25
0.00
A (n=14)
B (n=15)
C (n=11)
surface

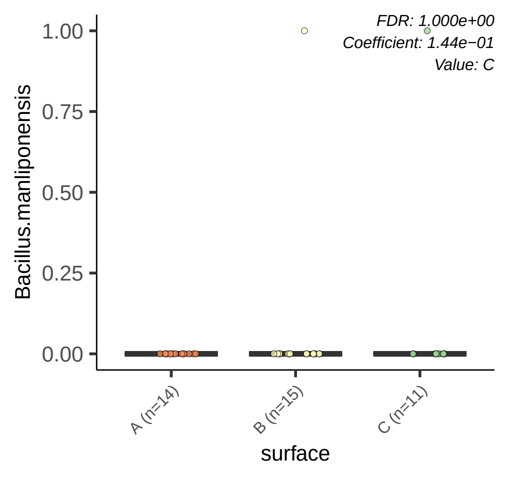
FDR: 1.000e+00
Coefficient: 1.44e−01
Value: C
1.00
0.75
0.50
0.25
0.00
Bacillus.manliponensis
A (n=14)
B (n=15)
C (n=11)
surface

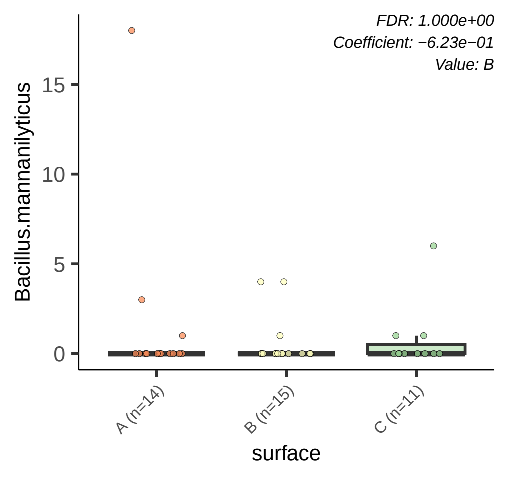
FDR: 1.000e+00
Coefficient: −6.23e−01
Value: B
Bacillus.mannanilyticus
15
10
5
0
A (n=14)
B (n=15)
C (n=11)
surface

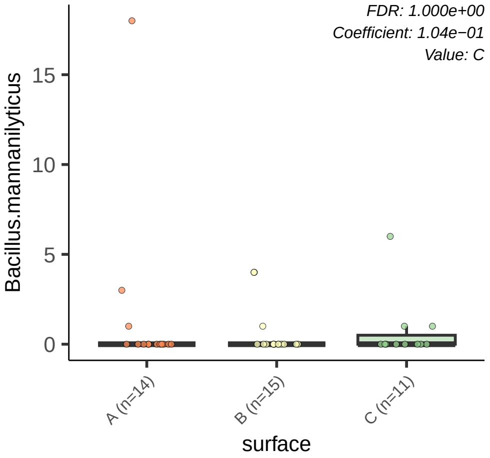
FDR: 1.000e+00
Coefficient: 1.04e−01
Value: C
Bacillus.mannanilyticus
15
10
5
0
A (n=14)
B (n=15)
C (n=11)
surface

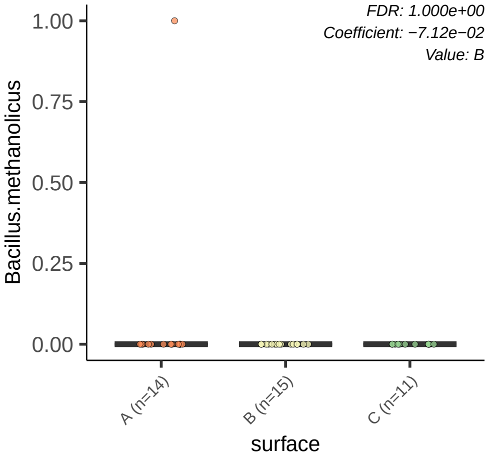
FDR: 1.000e+00
Coefficient: −7.12e−02
Value: B
Bacillus.methanolicus
1.00
0.75
0.50
0.25
0.00
A (n=14)
B (n=15)
C (n=11)
surface

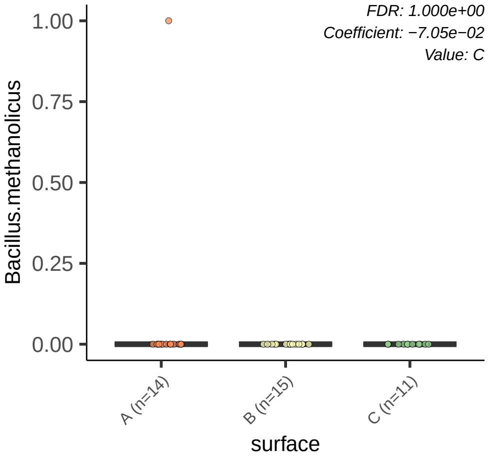
FDR: 1.000e+00
Coefficient: −7.05e−02
Value: C
Bacillus.methanolicus
1.00
0.75
0.50
0.25
0.00
A (n=14)
B (n=15)
C (n=11)
surface

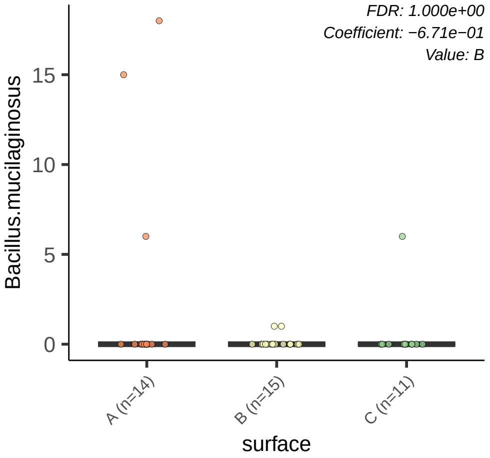

FDR: 1.000e+00
Coefficient: −6.71e−01
Value: B
Bacillus.mucilaginosus
0
5
10
15
A (n=14)
B (n=15)
C (n=11)
surface

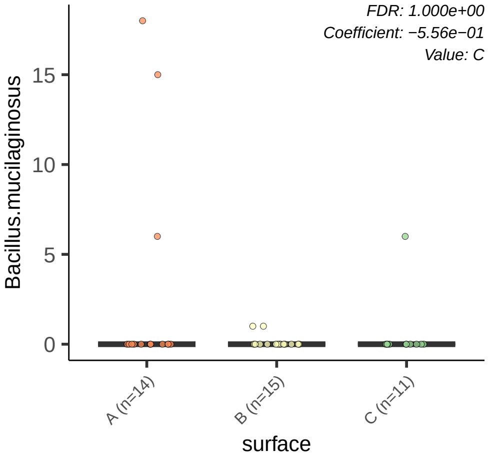
FDR: 1.000e+00
Coefficient: −5.56e−01
Value: C
Bacillus.mucilaginosus
0
5
10
15
A (n=14)
B (n=15)
C (n=11)
surface

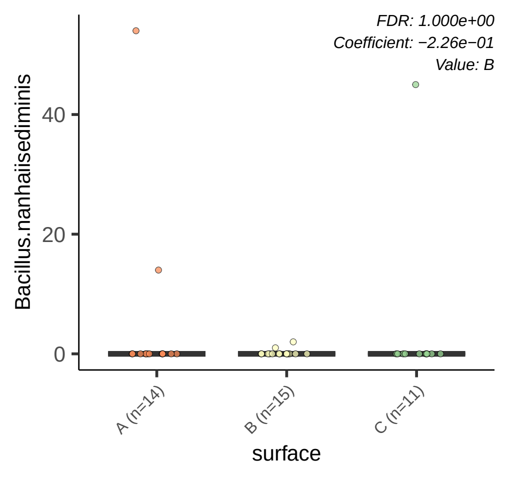

FDR: 1.000e+00
Coefficient: −2.26e−01
Value: B
Bacillus.nanhaisediminis
40
20
0
A (n=14)
B (n=15)
C (n=11)
surface

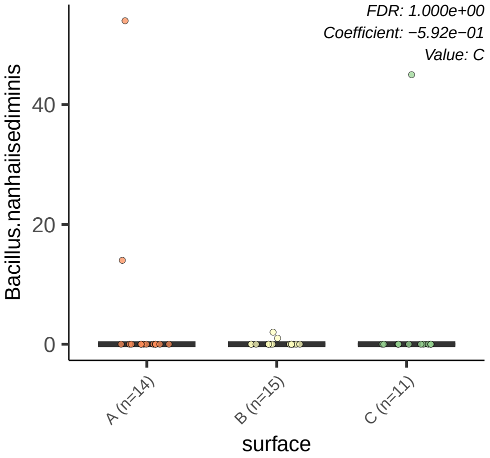
FDR: 1.000e+00
Coefficient: −5.92e−01
Value: C
Bacillus.nanhaisediminis
40
20
0
A (n=14)
B (n=15)
C (n=11)
surface

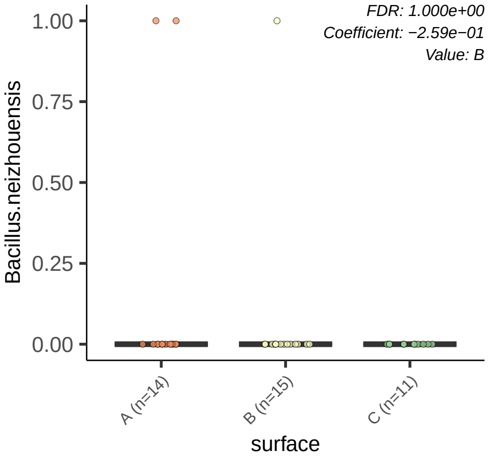
FDR: 1.000e+00
Coefficient: −2.59e−01
Value: B
Bacillus.neizhouensis
1.00
0.75
0.50
0.25
0.00
A (n=14)
B (n=15)
C (n=11)
surface

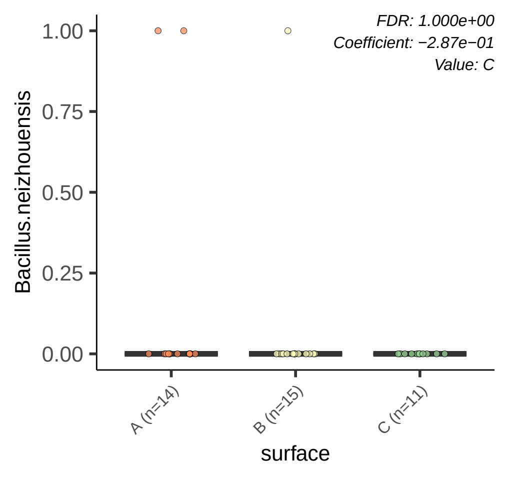

FDR: 1.000e+00
Coefficient: −2.87e−01
Value: C
Bacillus.neizhouensis
1.00
0.75
0.50
0.25
0.00
A (n=14)
B (n=15)
C (n=11)
surface

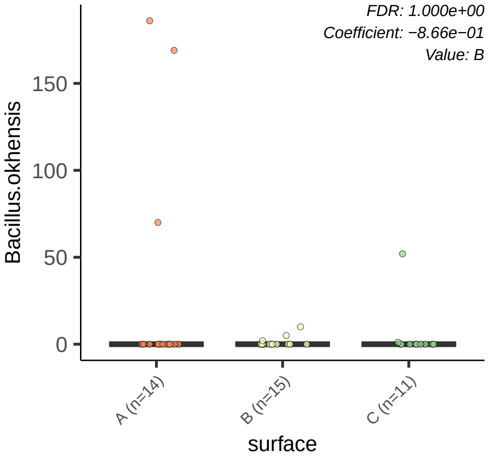
FDR: 1.000e+00
Coefficient: −8.66e−01
Value: B
Bacillus.okhensis
150
100
50
0
A (n=14)
B (n=15)
C (n=11)
surface

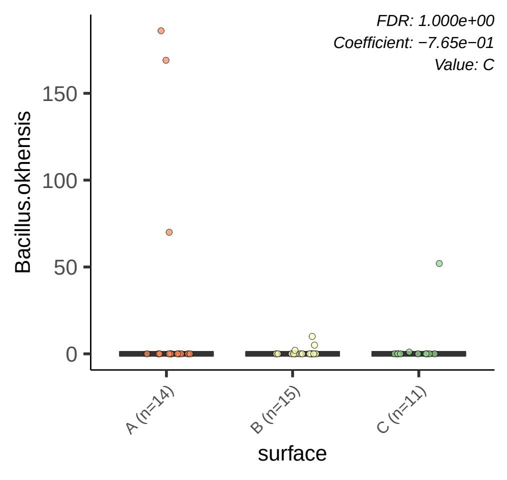
FDR: 1.000e+00
Coefficient: −7.65e−01
Value: C
Bacillus.okhensis
150
100
50
0
A (n=14)
B (n=15)
C (n=11)
surface

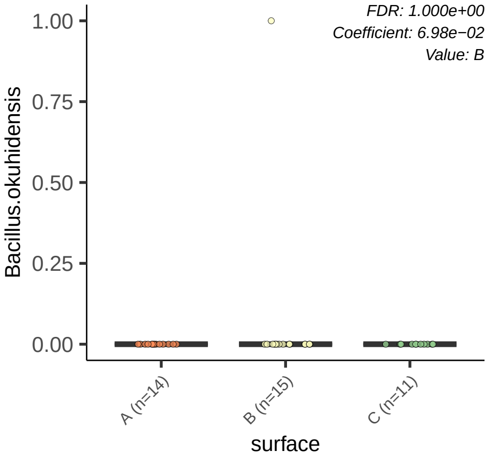
FDR: 1.000e+00
Coefficient: 6.98e−02
Value: B
Bacillus.okuhidensis
1.00
0.75
0.50
0.25
0.00
A (n=14)
B (n=15)
C (n=11)
surface

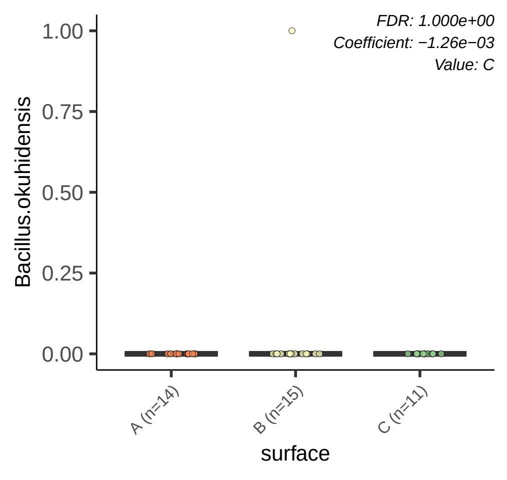

FDR: 1.000e+00
Coefficient: −1.26e−03
Value: C
Bacillus.okuhidensis
1.00
0.75
0.50
0.25
0.00
A (n=14)
B (n=15)
C (n=11)
surface

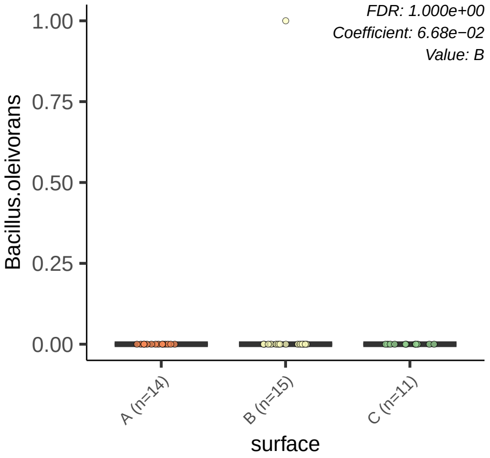
FDR: 1.000e+00
Coefficient: 6.68e−02
Value: B
Bacillus.oleivorans
1.00
0.75
0.50
0.25
0.00
A (n=14)
B (n=15)
C (n=11)
surface

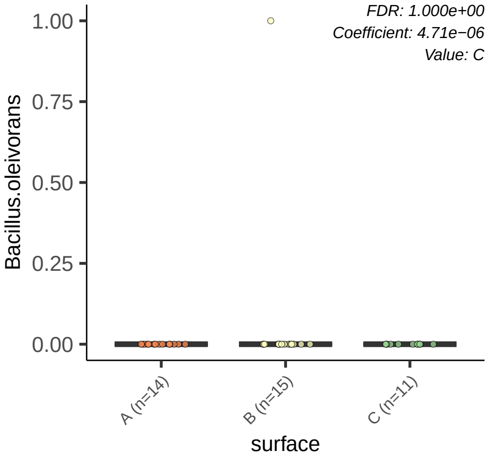

FDR: 1.000e+00
Coefficient: 4.71e−06
Value: C
1.00
0.75
0.50
0.25
0.00
Bacillus.oleivorans
A (n=14)
B (n=15)
C (n=11)
surface

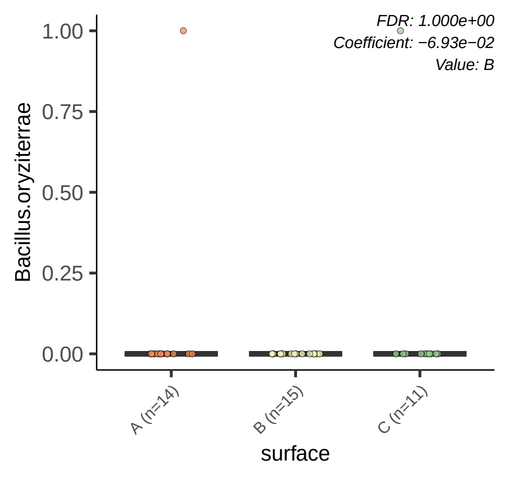
FDR: 1.000e+00
Coefficient: −6.93e−02
Value: B
Bacillus.oryziterrae
1.00
0.75
0.50
0.25
0.00
A (n=14)
B (n=15)
C (n=11)
surface

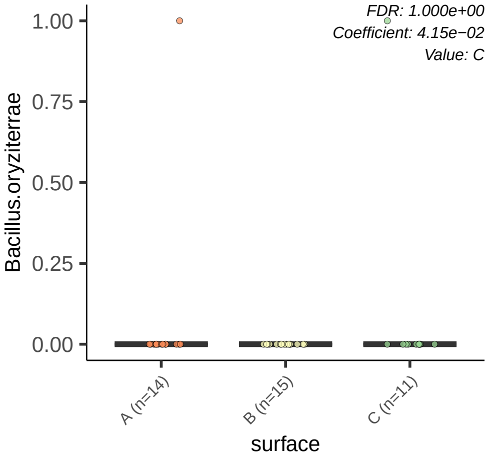
FDR: 1.000e+00
Coefficient: 4.15e−02
Value: C
Bacillus.oryziterrae
1.00
0.75
0.50
0.25
0.00
A (n=14)
B (n=15)
C (n=11)
surface

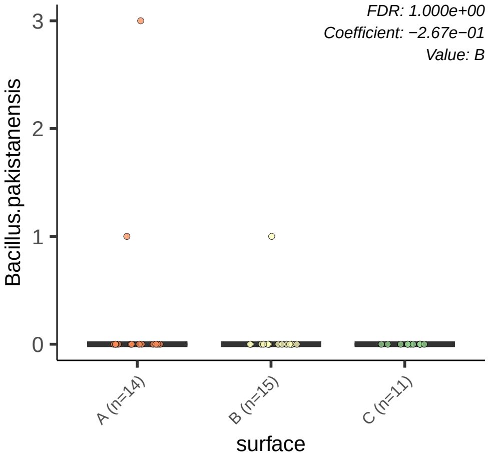

FDR: 1.000e+00
Coefficient: −2.67e−01
Value: B
Bacillus.pakistanensis
3
2
1
0
A (n=14)
B (n=15)
C (n=11)
surface

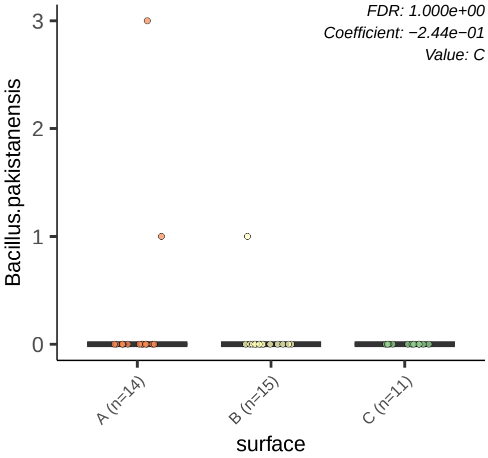
FDR: 1.000e+00
Coefficient: −2.44e−01
Value: C
Bacillus.pakistanensis
3
2
1
0
A (n=14)
B (n=15)
C (n=11)
surface

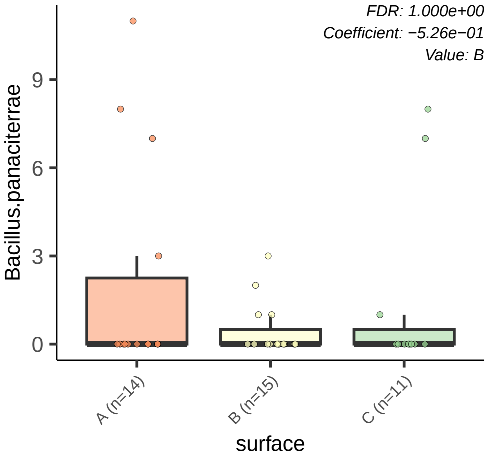

FDR: 1.000e+00
Coefficient: −5.26e−01
Value: B
Bacillus.panaciterrae
0
3
6
9
A (n=14)
B (n=15)
C (n=11)
surface

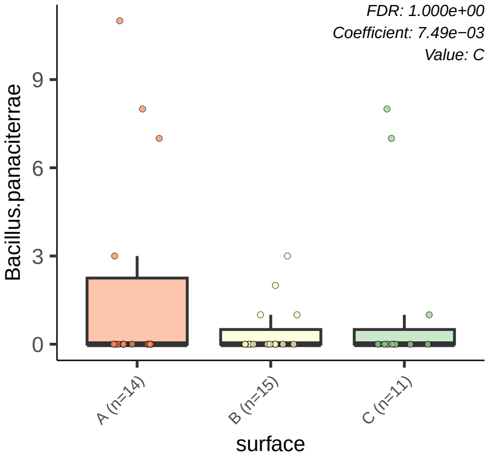
FDR: 1.000e+00
Coefficient: 7.49e−03
Value: C
Bacillus.panaciterrae
0
3
6
9
A (n=14)
B (n=15)
C (n=11)
surface

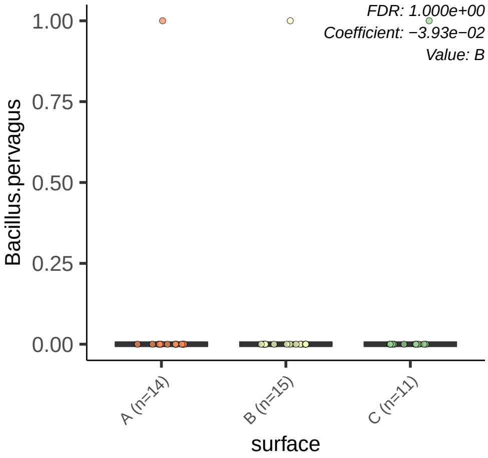
FDR: 1.000e+00
Coefficient: −3.93e−02
Value: B
Bacillus.pervagus
1.00
0.75
0.50
0.25
0.00
A (n=14)
B (n=15)
C (n=11)
surface

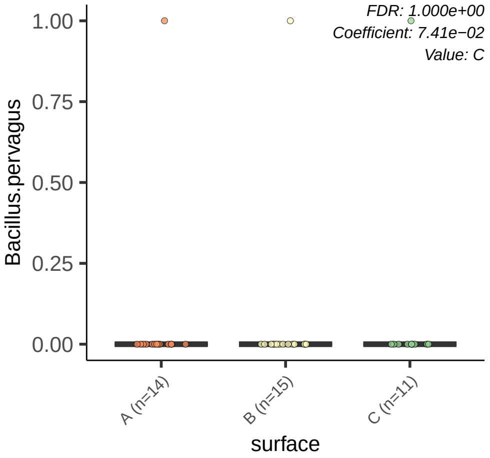
FDR: 1.000e+00
Coefficient: 7.41e−02
Value: C
Bacillus.pervagus
1.00
0.75
0.50
0.25
0.00
A (n=14)
B (n=15)
C (n=11)
surface

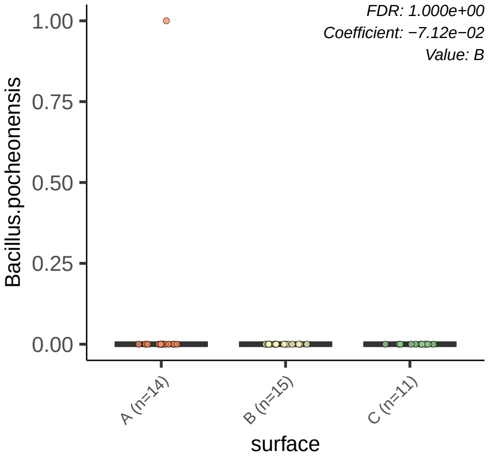
FDR: 1.000e+00
Coefficient: −7.12e−02
Value: B
Bacillus.pocheonensis
1.00
0.75
0.50
0.25
0.00
A (n=14)
B (n=15)
C (n=11)
surface

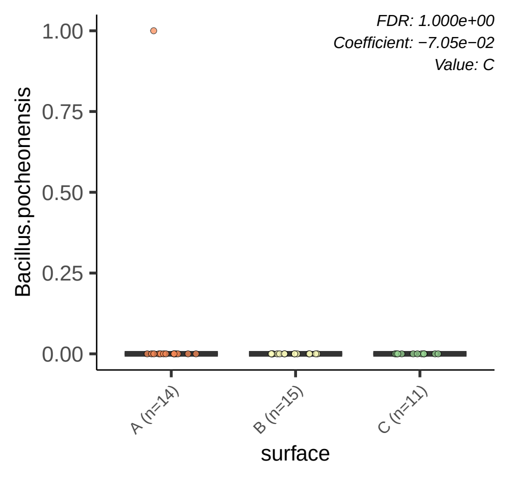
FDR: 1.000e+00
Coefficient: −7.05e−02
Value: C
Bacillus.pocheonensis
1.00
0.75
0.50
0.25
0.00
A (n=14)
B (n=15)
C (n=11)
surface

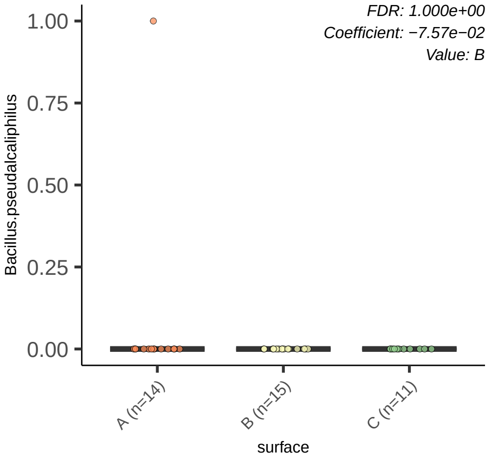
FDR: 1.000e+00
Coefficient: −7.57e−02
Value: B
Bacillus.pseudalcaliphilus
1.00
0.75
0.50
0.25
0.00
A (n=14)
B (n=15)
C (n=11)
surface

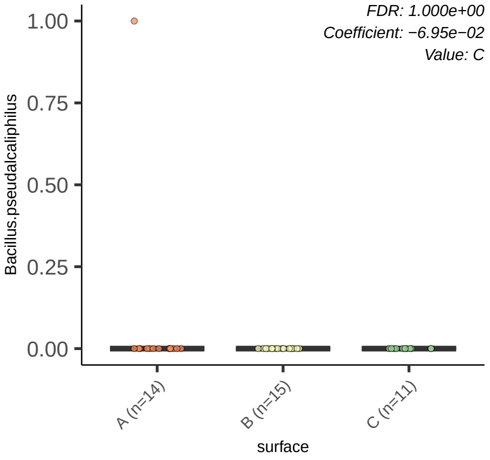
FDR: 1.000e+00
Coefficient: −6.95e−02
Value: C
Bacillus.pseudalcaliphilus
1.00
0.75
0.50
0.25
0.00
A (n=14)
B (n=15)
C (n=11)
surface

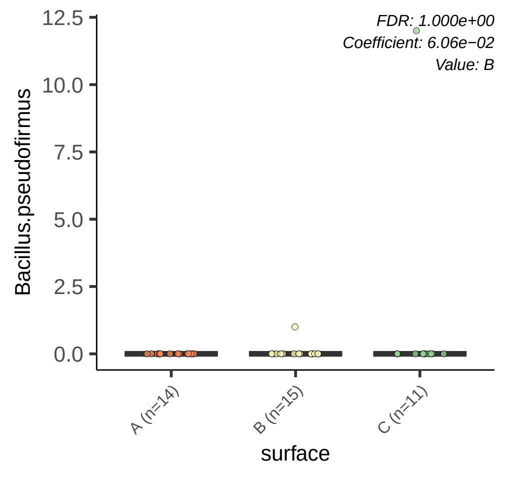
FDR: 1.000e+00
Coefficient: 6.06e−02
Value: B
Bacillus.pseudofirmus
12.5
10.0
7.5
5.0
2.5
0.0
A (n=14)
B (n=15)
C (n=11)
surface

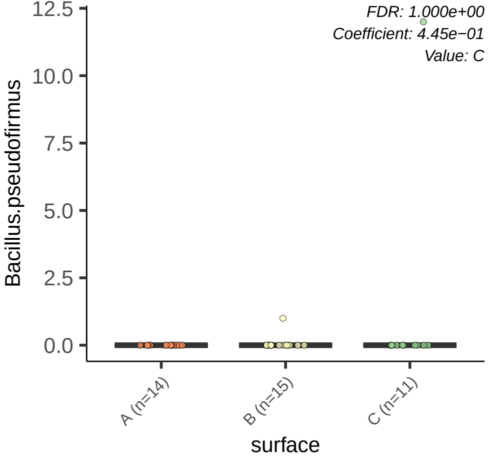
FDR: 1.000e+00
Coefficient: 4.45e−01
Value: C
Bacillus.pseudofirmus
12.5
10.0
7.5
5.0
2.5
0.0
A (n=14)
B (n=15)
C (n=11)
surface

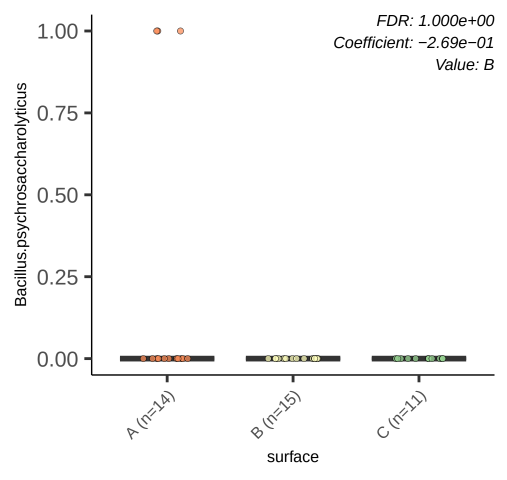
FDR: 1.000e+00
Coefficient: −2.69e−01
Value: B
1.00
0.75
0.50
0.25
0.00
Bacillus.psychrosaccharolyticus
A (n=14)
B (n=15)
C (n=11)
surface

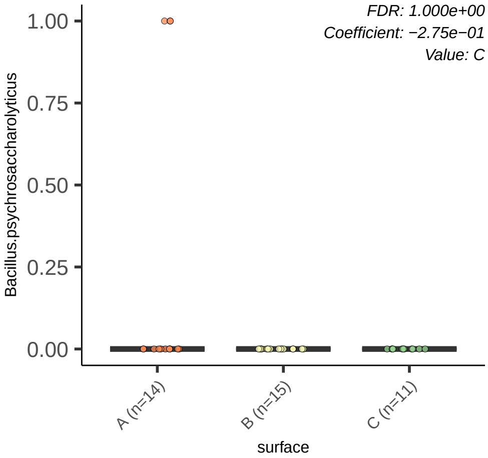
FDR: 1.000e+00
Coefficient: −2.75e−01
Value: C
1.00
0.75
0.50
0.25
0.00
Bacillus.psychrosaccharolyticus
A (n=14)
B (n=15)
C (n=11)
surface

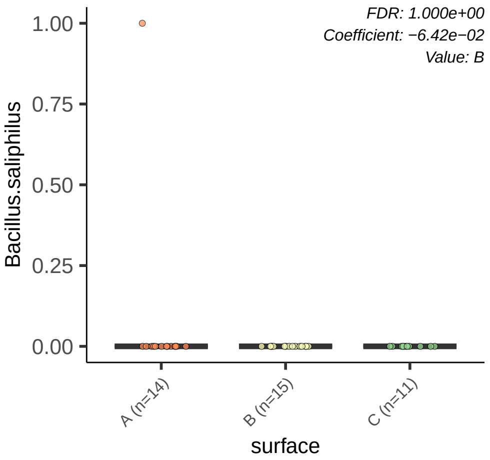

FDR: 1.000e+00
Coefficient: −6.42e−02
Value: B
Bacillus.saliphilus
1.00
0.75
0.50
0.25
0.00
A (n=14)
B (n=15)
C (n=11)
surface

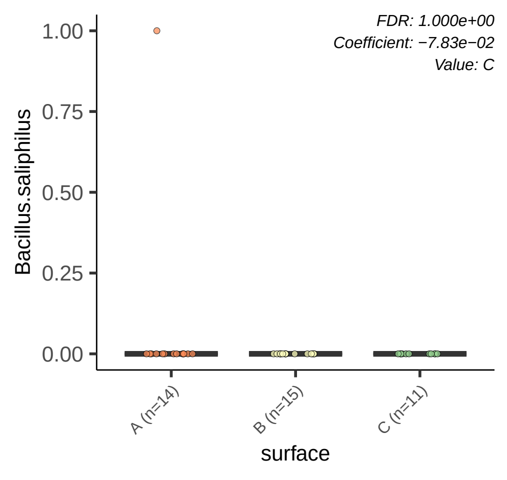
FDR: 1.000e+00
Coefficient: −7.83e−02
Value: C
Bacillus.saliphilus
1.00
0.75
0.50
0.25
0.00
A (n=14)
B (n=15)
C (n=11)
surface

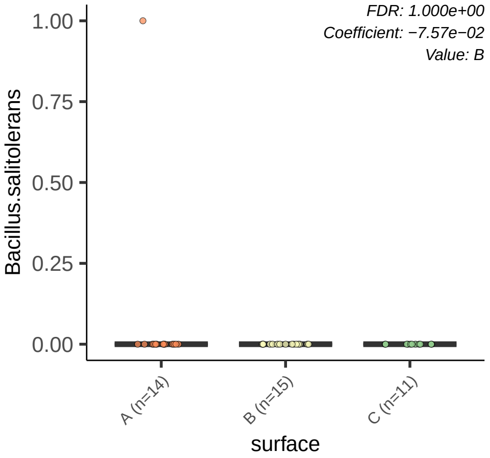

FDR: 1.000e+00
Coefficient: −7.57e−02
Value: B
Bacillus.salitolerans
1.00
0.75
0.50
0.25
0.00
A (n=14)
B (n=15)
C (n=11)
surface

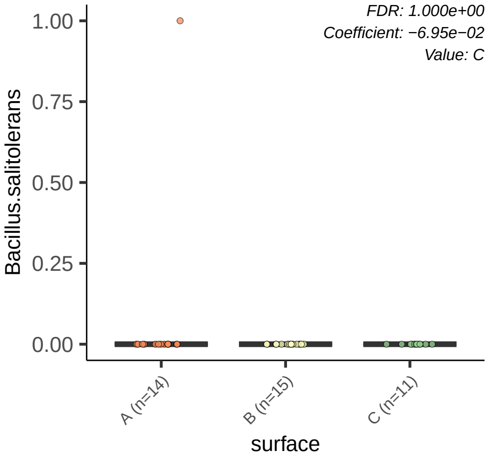
FDR: 1.000e+00
Coefficient: −6.95e−02
Value: C
Bacillus.salitolerans
1.00
0.75
0.50
0.25
0.00
A (n=14)
B (n=15)
C (n=11)
surface

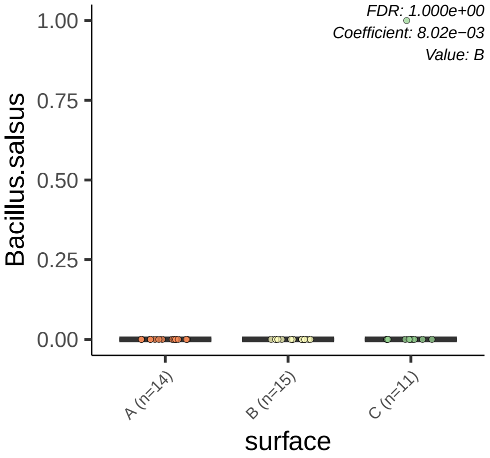
FDR: 1.000e+00
Coefficient: 8.02e−03
Value: B
Bacillus.salsus
1.00
0.75
0.50
0.25
0.00
A (n=14)
B (n=15)
C (n=11)
surface

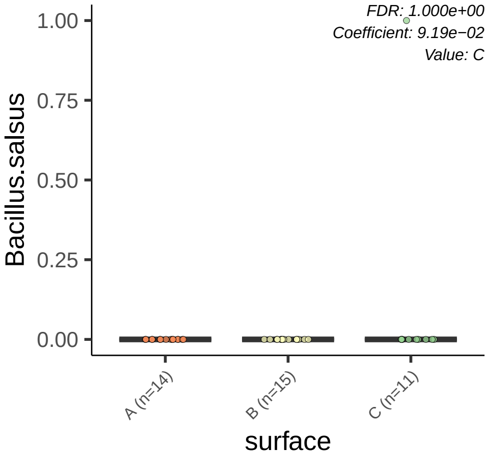
FDR: 1.000e+00
Coefficient: 9.19e−02
Value: C
Bacillus.salsus
1.00
0.75
0.50
0.25
0.00
A (n=14)
B (n=15)
C (n=11)
surface

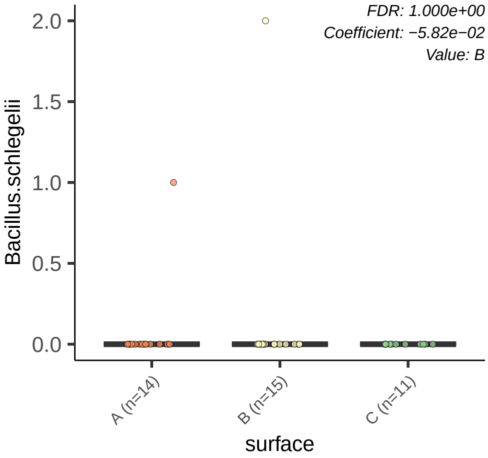
FDR: 1.000e+00
Coefficient: −5.82e−02
Value: B
Bacillus.schlegelii
2.0
1.5
1.0
0.5
0.0
A (n=14)
B (n=15)
C (n=11)
surface

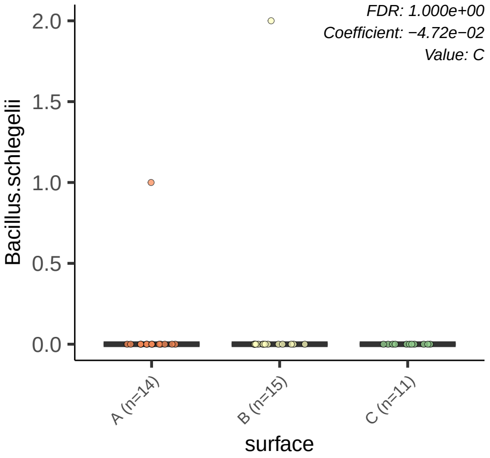

FDR: 1.000e+00
Coefficient: −4.72e−02
Value: C
Bacillus.schlegelii
2.0
1.5
1.0
0.5
0.0
A (n=14)
B (n=15)
C (n=11)
surface

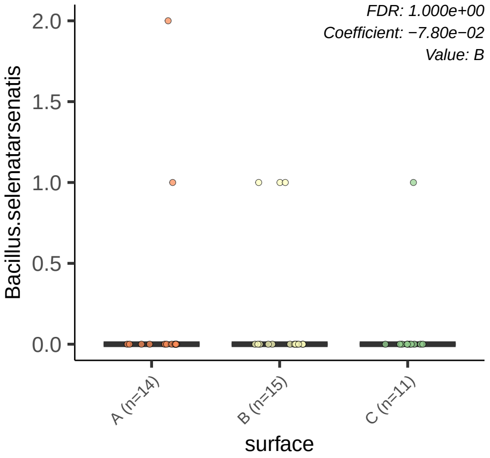
FDR: 1.000e+00
Coefficient: −7.80e−02
Value: B
Bacillus.selenatarsenatis
2.0
1.5
1.0
0.5
0.0
A (n=14)
B (n=15)
C (n=11)
surface

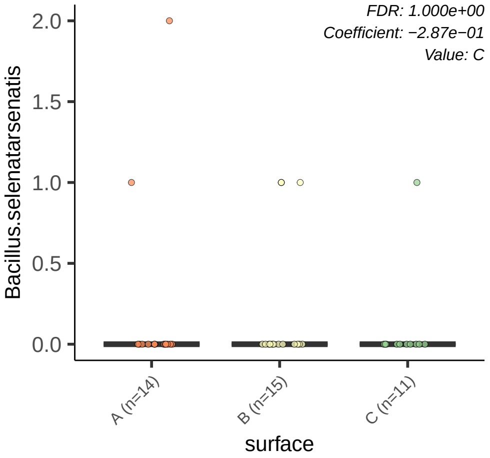
FDR: 1.000e+00
Coefficient: −2.87e−01
Value: C
Bacillus.selenatarsenatis
2.0
1.5
1.0
0.5
0.0
A (n=14)
B (n=15)
C (n=11)
surface

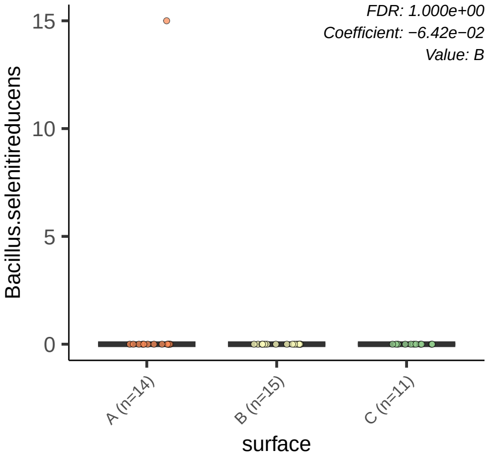

FDR: 1.000e+00
Coefficient: −6.42e−02
Value: B
Bacillus.selenitireducens
15
10
5
0
A (n=14)
B (n=15)
C (n=11)
surface

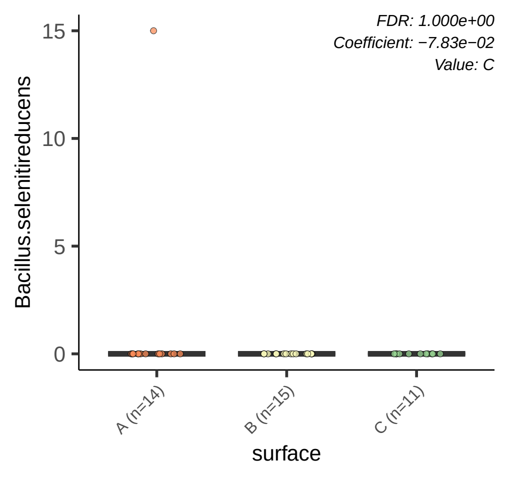

FDR: 1.000e+00
Coefficient: −7.83e−02
Value: C
Bacillus.selenitireducens
15
10
5
0
A (n=14)
B (n=15)
C (n=11)
surface

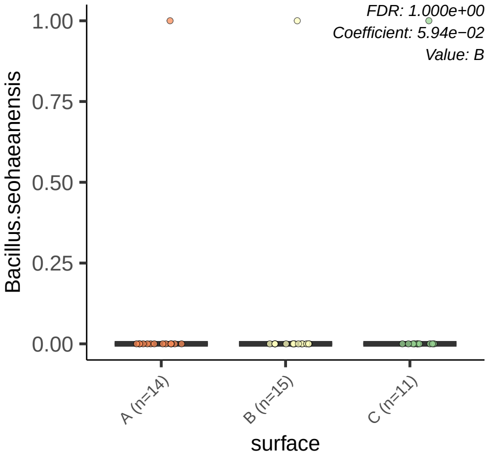

FDR: 1.000e+00
Coefficient: 5.94e−02
Value: B
Bacillus.seohaeanensis
1.00
0.75
0.50
0.25
0.00
A (n=14)
B (n=15)
C (n=11)
surface

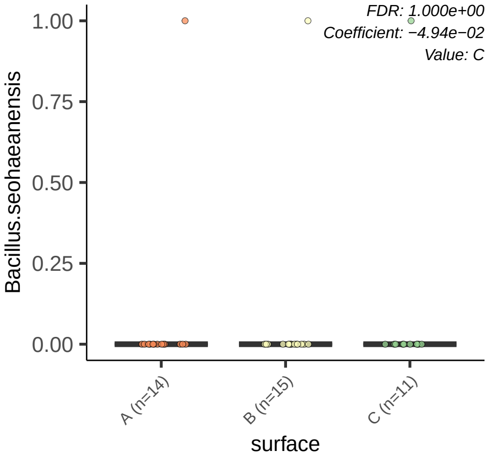
FDR: 1.000e+00
Coefficient: −4.94e−02
Value: C
1.00
0.75
0.50
0.25
0.00
Bacillus.seohaeanensis
A (n=14)
B (n=15)
C (n=11)
surface

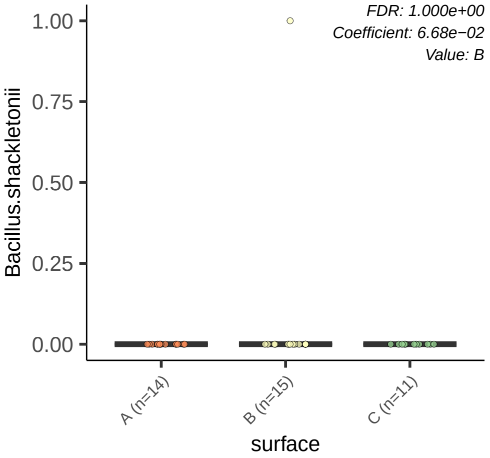
FDR: 1.000e+00
Coefficient: 6.68e−02
Value: B
Bacillus.shackletonii
1.00
0.75
0.50
0.25
0.00
A (n=14)
B (n=15)
C (n=11)
surface

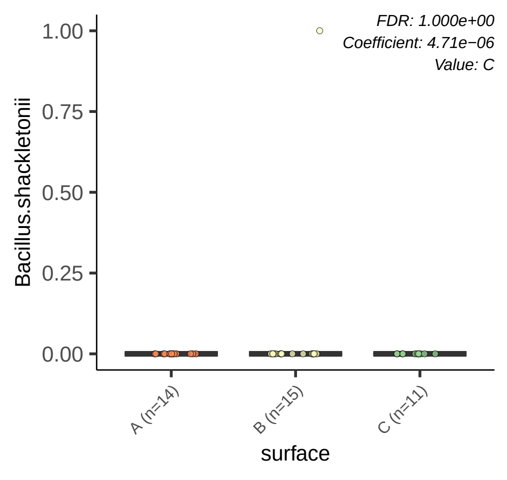
FDR: 1.000e+00
Coefficient: 4.71e−06
Value: C
Bacillus.shackletonii
1.00
0.75
0.50
0.25
0.00
A (n=14)
B (n=15)
C (n=11)
surface

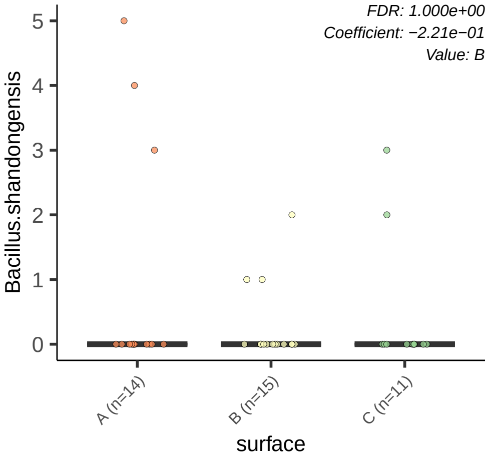

FDR: 1.000e+00
Coefficient: −2.21e−01
Value: B
Bacillus.shandongensis
0
1
2
3
4
5
A (n=14)
B (n=15)
C (n=11)
surface

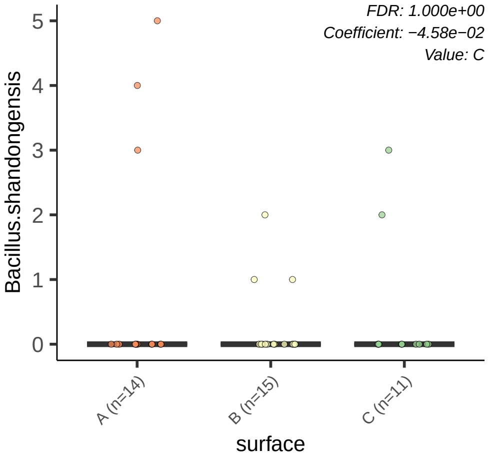

FDR: 1.000e+00
Coefficient: −4.58e−02
Value: C
Bacillus.shandongensis
0
1
2
3
4
5
A (n=14)
B (n=15)
C (n=11)
surface

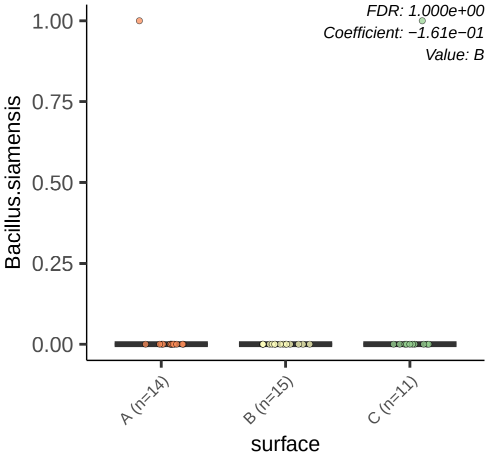
FDR: 1.000e+00
Coefficient: −1.61e−01
Value: B
1.00
0.75
0.50
0.25
0.00
Bacillus.siamensis
A (n=14)
B (n=15)
C (n=11)
surface

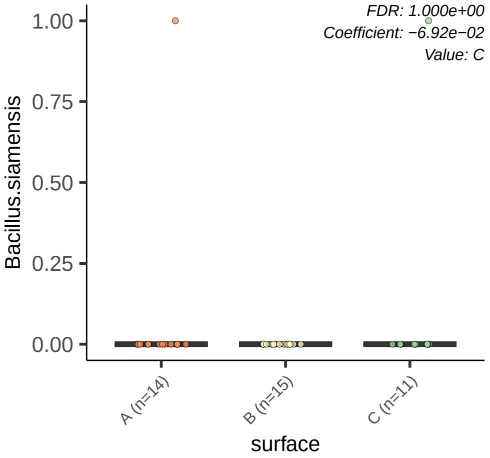
FDR: 1.000e+00
Coefficient: −6.92e−02
Value: C
Bacillus.siamensis
1.00
0.75
0.50
0.25
0.00
A (n=14)
B (n=15)
C (n=11)
surface

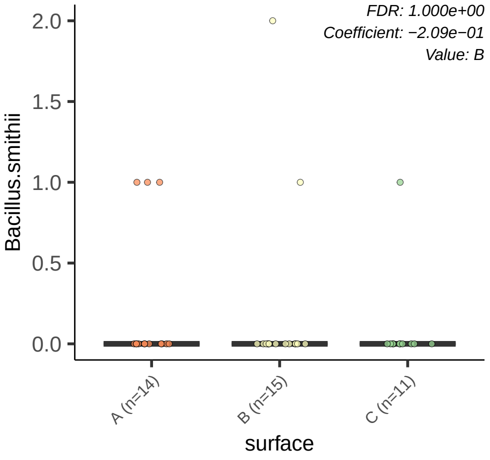

FDR: 1.000e+00
Coefficient: −2.09e−01
Value: B
Bacillus.smithii
2.0
1.5
1.0
0.5
0.0
A (n=14)
B (n=15)
C (n=11)
surface

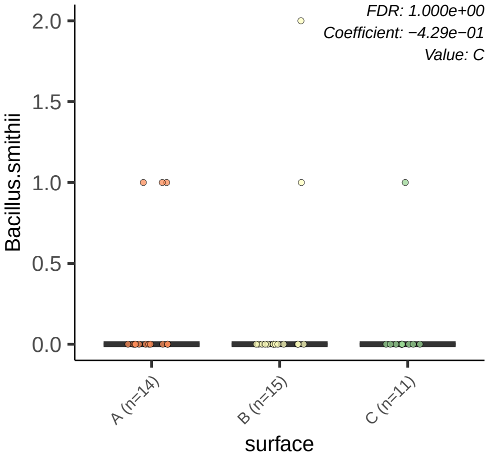

FDR: 1.000e+00
Coefficient: −4.29e−01
Value: C
Bacillus.smithii
2.0
1.5
1.0
0.5
0.0
A (n=14)
B (n=15)
C (n=11)
surface

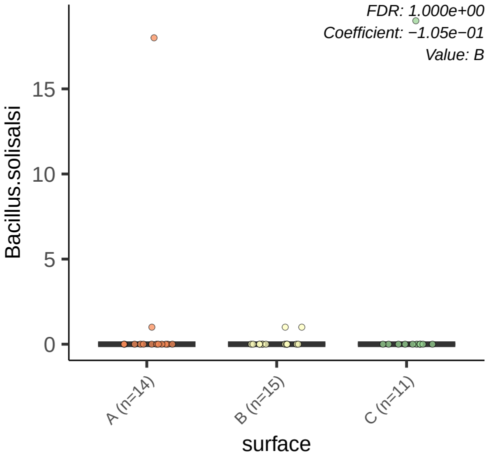
FDR: 1.000e+00
Coefficient: −1.05e−01
Value: B
Bacillus.solisalsi
15
10
5
0
A (n=14)
B (n=15)
C (n=11)
surface

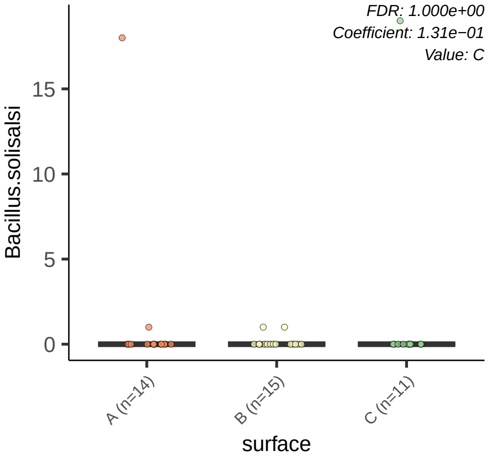

FDR: 1.000e+00
Coefficient: 1.31e−01
Value: C
Bacillus.solisalsi
0
5
10
15
A (n=14)
B (n=15)
C (n=11)
surface

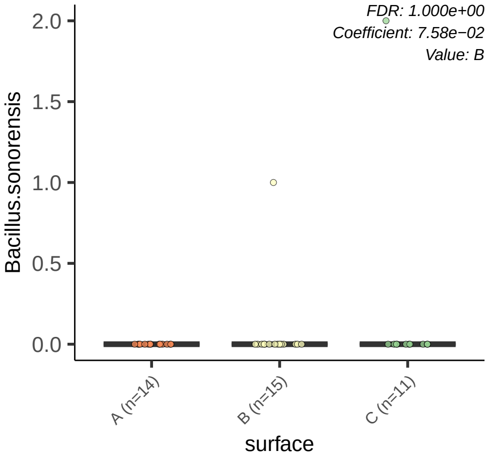
FDR: 1.000e+00
Coefficient: 7.58e−02
Value: B
Bacillus.sonorensis
2.0
1.5
1.0
0.5
0.0
A (n=14)
B (n=15)
C (n=11)
surface

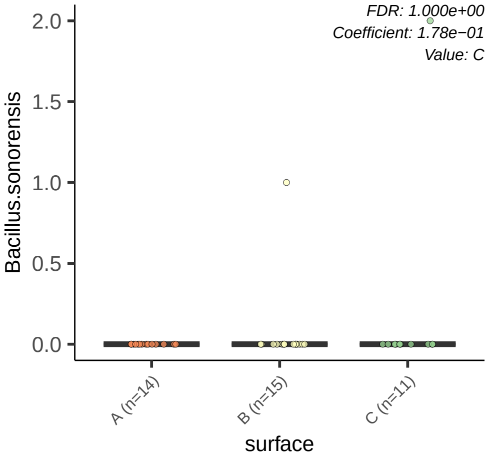
FDR: 1.000e+00
Coefficient: 1.78e−01
Value: C
Bacillus.sonorensis
2.0
1.5
1.0
0.5
0.0
A (n=14)
B (n=15)
C (n=11)
surface

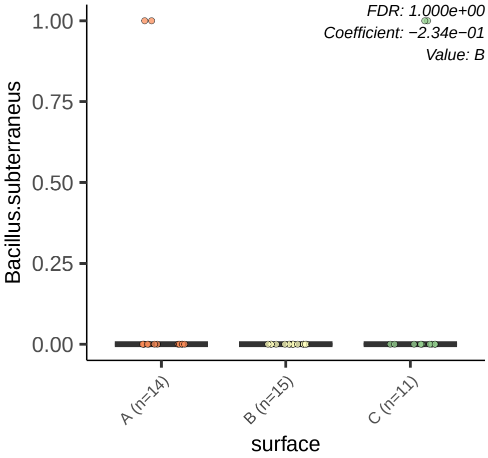

FDR: 1.000e+00
Coefficient: −2.34e−01
Value: B
Bacillus.subterraneus
1.00
0.75
0.50
0.25
0.00
A (n=14)
B (n=15)
C (n=11)
surface

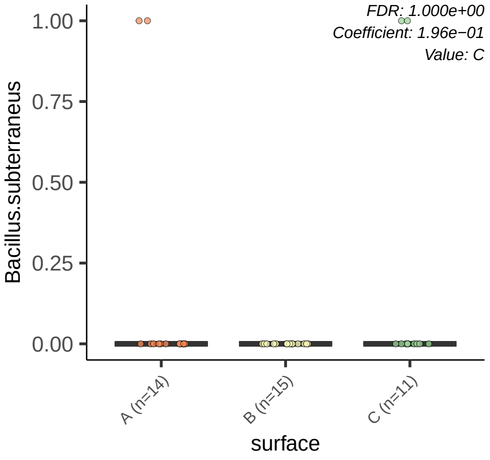

FDR: 1.000e+00
Coefficient: 1.96e−01
Value: C
1.00
0.75
0.50
0.25
0.00
Bacillus.subterraneus
A (n=14)
B (n=15)
C (n=11)
surface

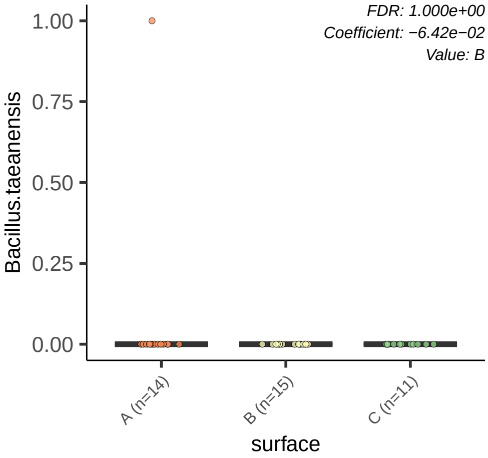

FDR: 1.000e+00
Coefficient: −6.42e−02
Value: B
Bacillus.taeanensis
1.00
0.75
0.50
0.25
0.00
A (n=14)
B (n=15)
C (n=11)
surface

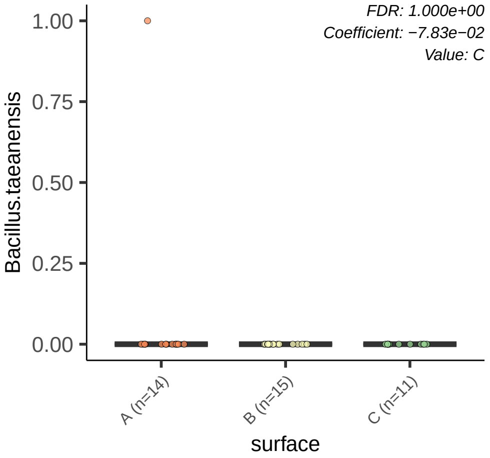
FDR: 1.000e+00
Coefficient: −7.83e−02
Value: C
Bacillus.taeanensis
1.00
0.75
0.50
0.25
0.00
A (n=14)
B (n=15)
C (n=11)
surface

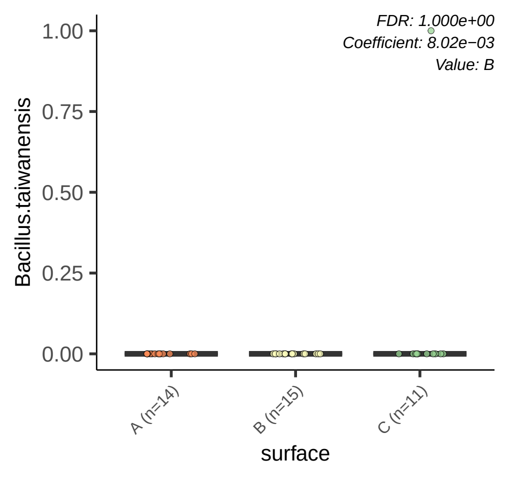
FDR: 1.000e+00
Coefficient: 8.02e−03
Value: B
Bacillus.taiwanensis
1.00
0.75
0.50
0.25
0.00
A (n=14)
B (n=15)
C (n=11)
surface

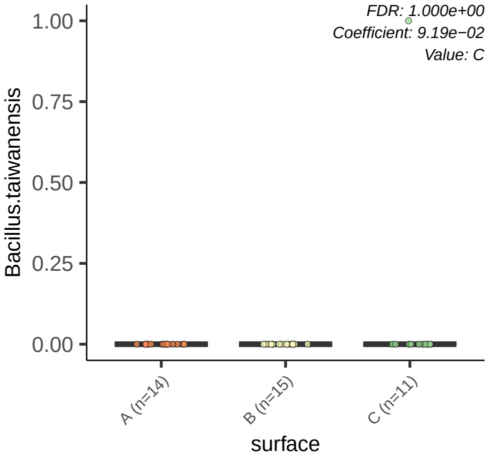
FDR: 1.000e+00
Coefficient: 9.19e−02
Value: C
Bacillus.taiwanensis
1.00
0.75
0.50
0.25
0.00
A (n=14)
B (n=15)
C (n=11)
surface

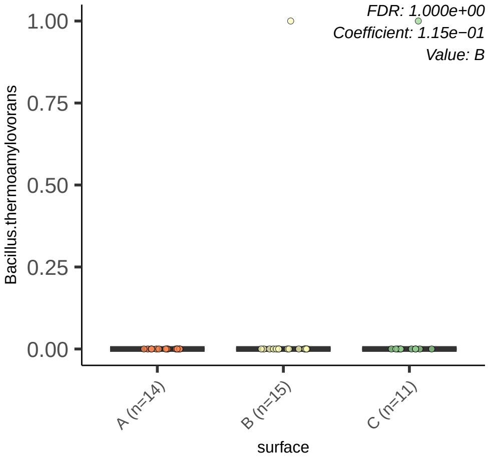
FDR: 1.000e+00
Coefficient: 1.15e−01
Value: B
Bacillus.thermoamylovorans
1.00
0.75
0.50
0.25
0.00
A (n=14)
B (n=15)
C (n=11)
surface

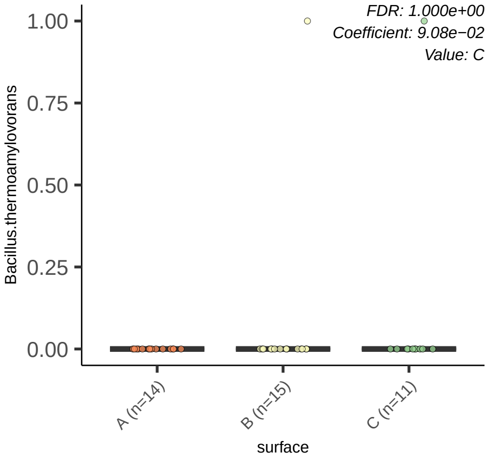
FDR: 1.000e+00
Coefficient: 9.08e−02
Value: C
1.00
0.75
0.50
0.25
0.00
Bacillus.thermoamylovorans
A (n=14)
B (n=15)
C (n=11)
surface

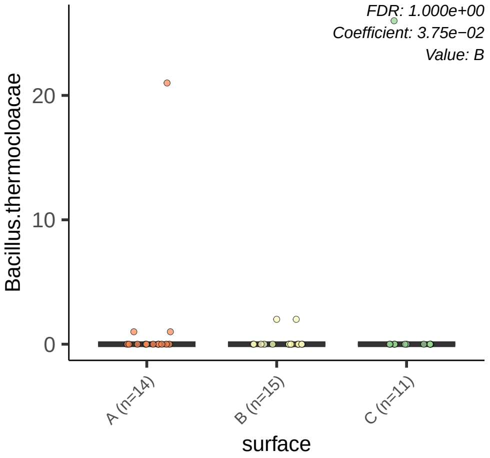
FDR: 1.000e+00
Coefficient: 3.75e−02
Value: B
Bacillus.thermocloacae
20
10
0
A (n=14)
B (n=15)
C (n=11)
surface

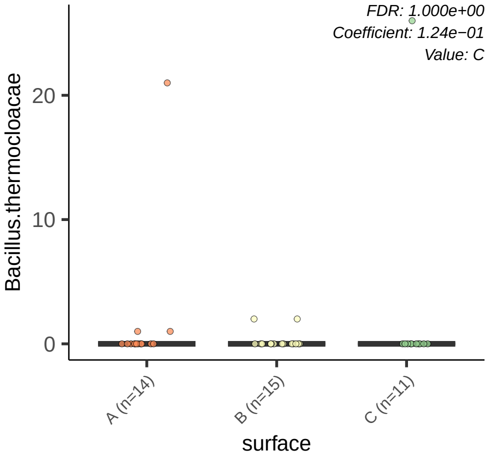
FDR: 1.000e+00
Coefficient: 1.24e−01
Value: C
Bacillus.thermocloacae
20
10
0
A (n=14)
B (n=15)
C (n=11)
surface

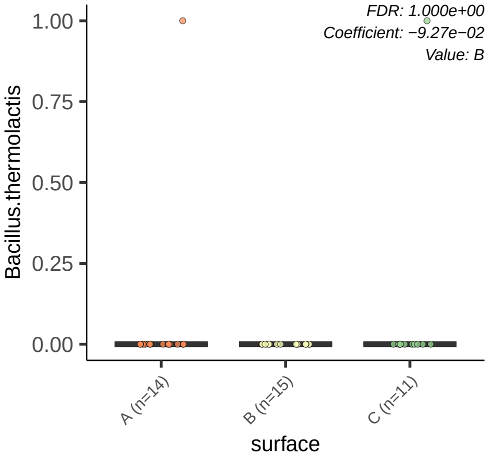
FDR: 1.000e+00
Coefficient: −9.27e−02
Value: B
Bacillus.thermolactis
1.00
0.75
0.50
0.25
0.00
A (n=14)
B (n=15)
C (n=11)
surface

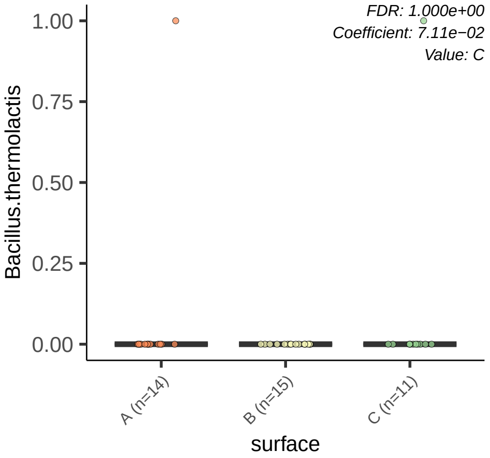
FDR: 1.000e+00
Coefficient: 7.11e−02
Value: C
Bacillus.thermolactis
1.00
0.75
0.50
0.25
0.00
A (n=14)
B (n=15)
C (n=11)
surface

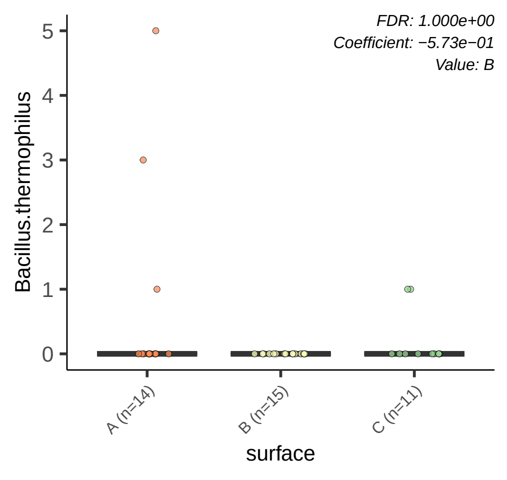

FDR: 1.000e+00
Coefficient: −5.73e−01
Value: B
Bacillus.thermophilus
0
1
2
3
4
5
A (n=14)
B (n=15)
C (n=11)
surface

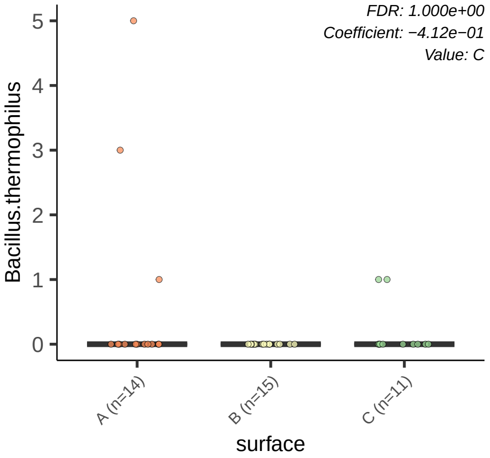

FDR: 1.000e+00
Coefficient: −4.12e−01
Value: C
Bacillus.thermophilus
0
1
2
3
4
5
A (n=14)
B (n=15)
C (n=11)
surface

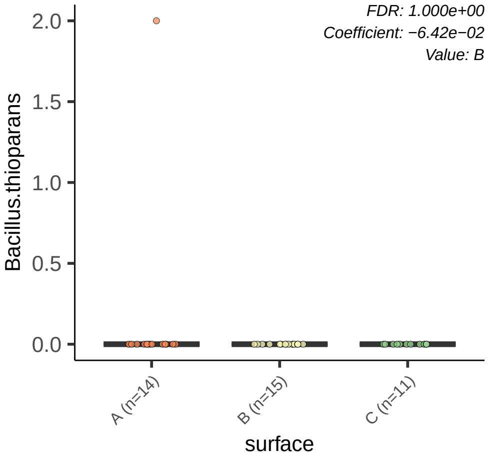
FDR: 1.000e+00
Coefficient: −6.42e−02
Value: B
Bacillus.thioparans
2.0
1.5
1.0
0.5
0.0
A (n=14)
B (n=15)
C (n=11)
surface

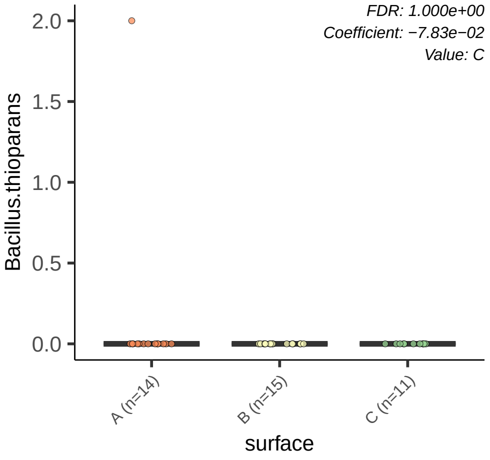
FDR: 1.000e+00
Coefficient: −7.83e−02
Value: C
Bacillus.thioparans
2.0
1.5
1.0
0.5
0.0
A (n=14)
B (n=15)
C (n=11)
surface

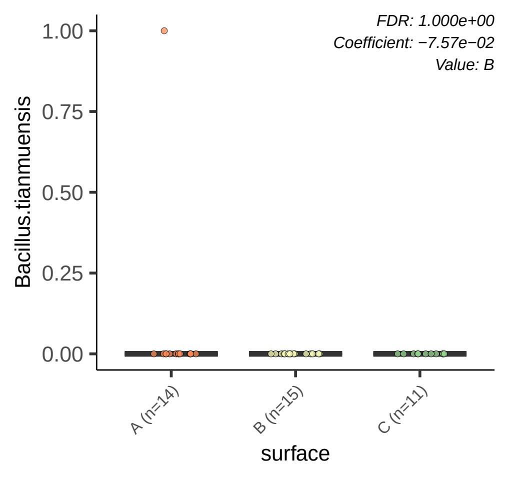
FDR: 1.000e+00
Coefficient: −7.57e−02
Value: B
Bacillus.tianmuensis
1.00
0.75
0.50
0.25
0.00
A (n=14)
B (n=15)
C (n=11)
surface

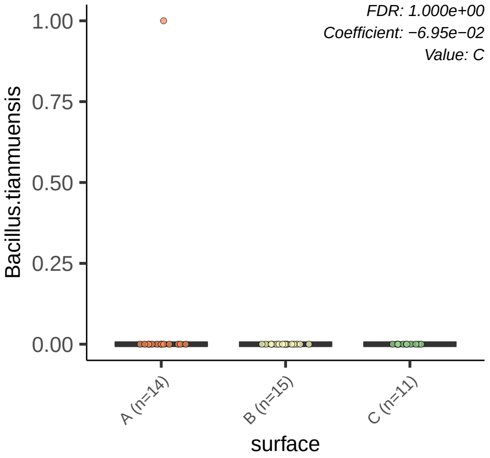
FDR: 1.000e+00
Coefficient: −6.95e−02
Value: C
Bacillus.tianmuensis
1.00
0.75
0.50
0.25
0.00
A (n=14)
B (n=15)
C (n=11)
surface

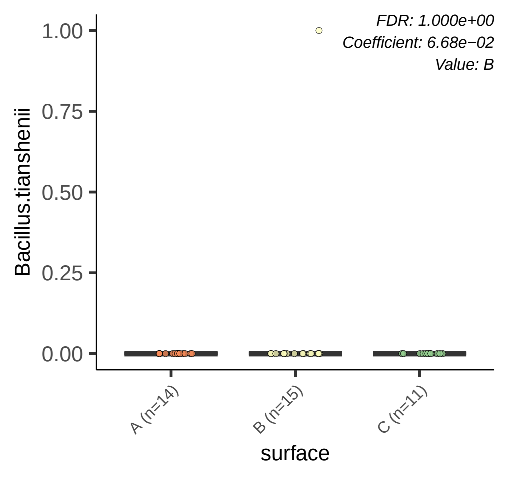

FDR: 1.000e+00
Coefficient: 6.68e−02
Value: B
Bacillus.tianshenii
1.00
0.75
0.50
0.25
0.00
A (n=14)
B (n=15)
C (n=11)
surface

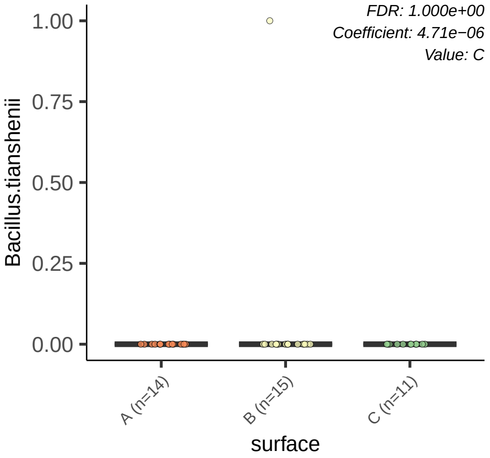

FDR: 1.000e+00
Coefficient: 4.71e−06
Value: C
Bacillus.tianshenii
1.00
0.75
0.50
0.25
0.00
A (n=14)
B (n=15)
C (n=11)
surface

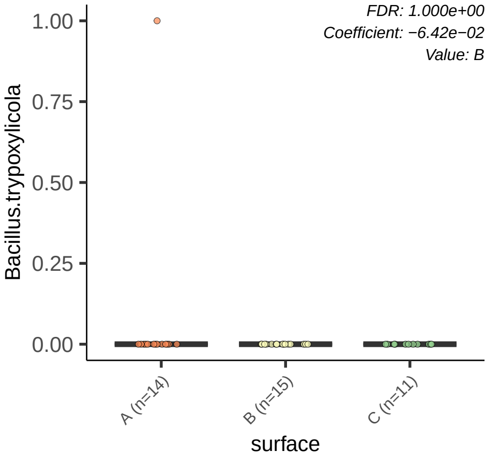
FDR: 1.000e+00
Coefficient: −6.42e−02
Value: B
Bacillus.trypoxylicola
1.00
0.75
0.50
0.25
0.00
A (n=14)
B (n=15)
C (n=11)
surface

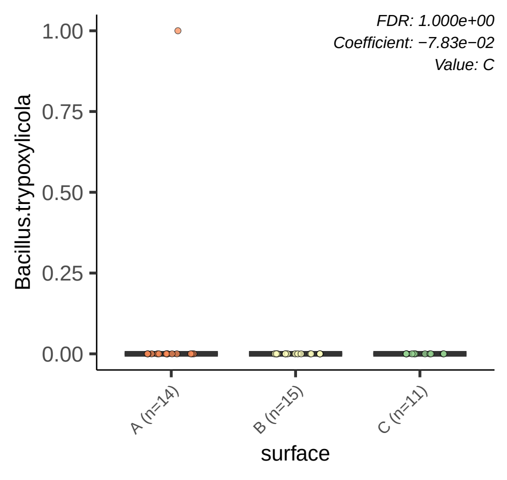

FDR: 1.000e+00
Coefficient: −7.83e−02
Value: C
Bacillus.trypoxylicola
1.00
0.75
0.50
0.25
0.00
A (n=14)
B (n=15)
C (n=11)
surface

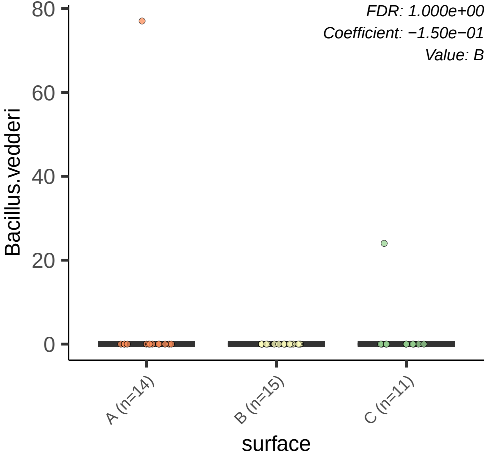

FDR: 1.000e+00
Coefficient: −1.50e−01
Value: B
Bacillus.vedderi
80
60
40
20
0
A (n=14)
B (n=15)
C (n=11)
surface

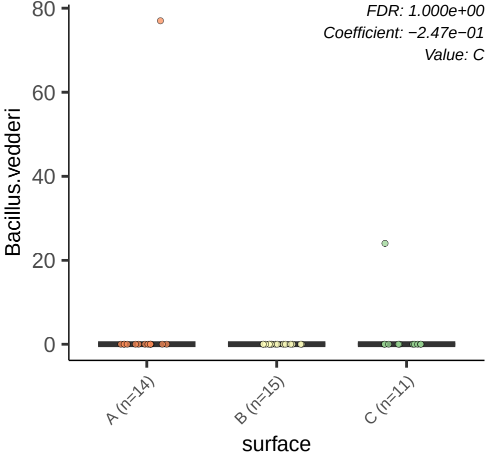
FDR: 1.000e+00
Coefficient: −2.47e−01
Value: C
Bacillus.vedderi
80
60
40
20
0
A (n=14)
B (n=15)
C (n=11)
surface

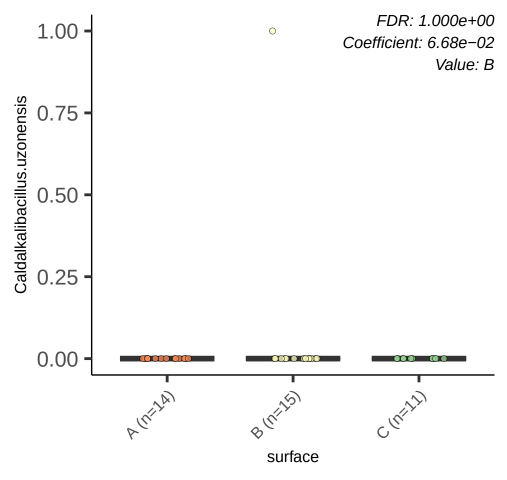

FDR: 1.000e+00
Coefficient: 6.68e−02
Value: B
Caldalkalibacillus.uzonensis
1.00
0.75
0.50
0.25
0.00
A (n=14)
B (n=15)
C (n=11)
surface

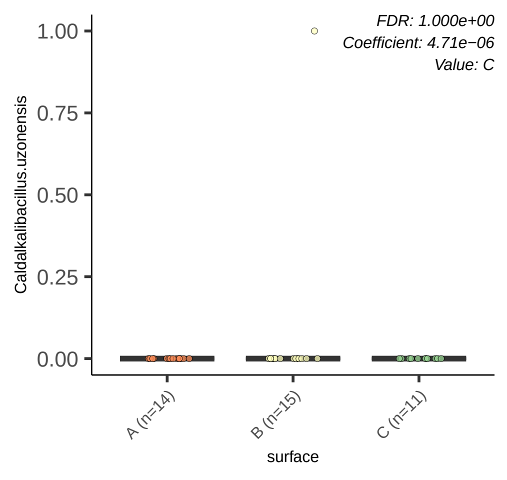

FDR: 1.000e+00
Coefficient: 4.71e−06
Value: C
1.00
0.75
0.50
0.25
0.00
Caldalkalibacillus.uzonensis
A (n=14)
B (n=15)
C (n=11)
surface

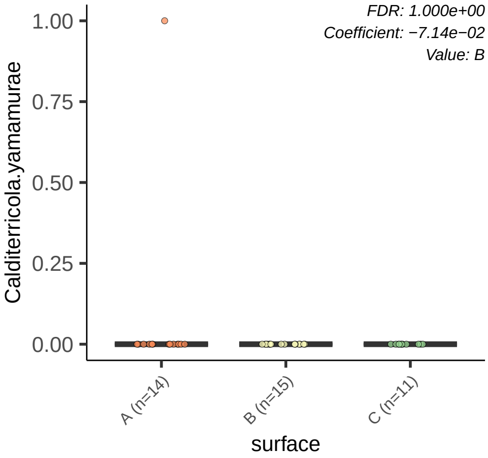

FDR: 1.000e+00
Coefficient: −7.14e−02
Value: B
Calditerricola.yamamurae
1.00
0.75
0.50
0.25
0.00
A (n=14)
B (n=15)
C (n=11)
surface

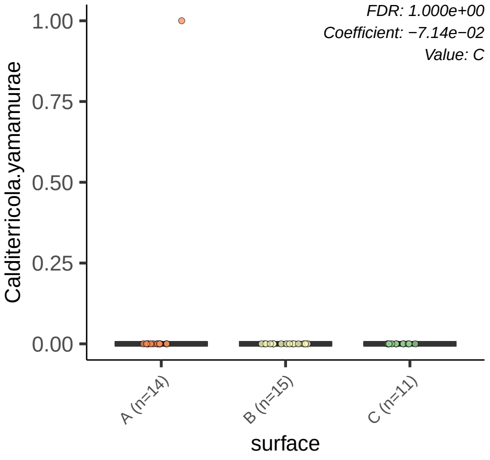

FDR: 1.000e+00
Coefficient: −7.14e−02
Value: C
Calditerricola.yamamurae
1.00
0.75
0.50
0.25
0.00
A (n=14)
B (n=15)
C (n=11)
surface

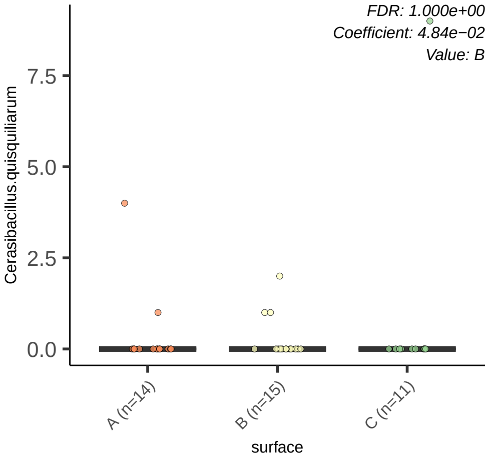

FDR: 1.000e+00
Coefficient: 4.84e−02
Value: B
Cerasibacillus.quisquiliarum
7.5
5.0
2.5
0.0
A (n=14)
B (n=15)
C (n=11)
surface

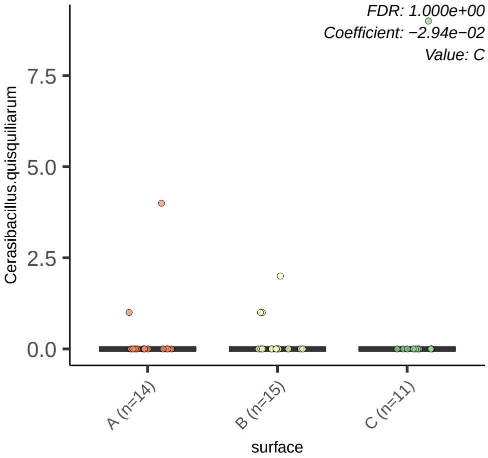
FDR: 1.000e+00
Coefficient: −2.94e−02
Value: C
Cerasibacillus.quisquiliarum
7.5
5.0
2.5
0.0
A (n=14)
B (n=15)
C (n=11)
surface

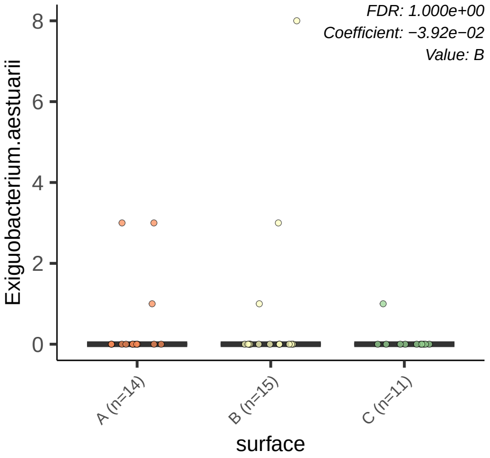

FDR: 1.000e+00
Coefficient: −3.92e−02
Value: B
Exiguobacterium.aestuarii
0
2
4
6
8
A (n=14)
B (n=15)
C (n=11)
surface

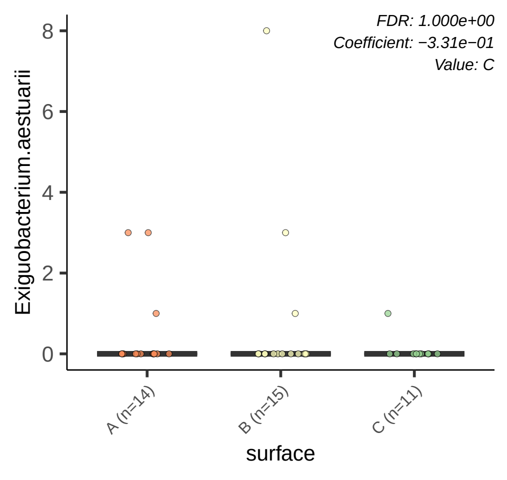

FDR: 1.000e+00
Coefficient: −3.31e−01
Value: C
Exiguobacterium.aestuarii
0
2
4
6
8
A (n=14)
B (n=15)
C (n=11)
surface

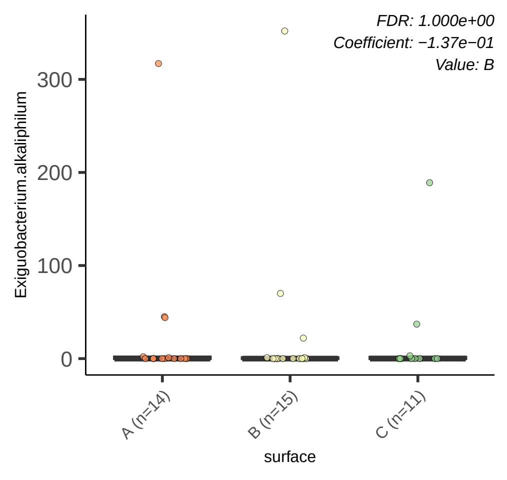
FDR: 1.000e+00
Coefficient: −1.37e−01
Value: B
Exiguobacterium.alkaliphilum
300
200
100
0
A (n=14)
B (n=15)
C (n=11)
surface

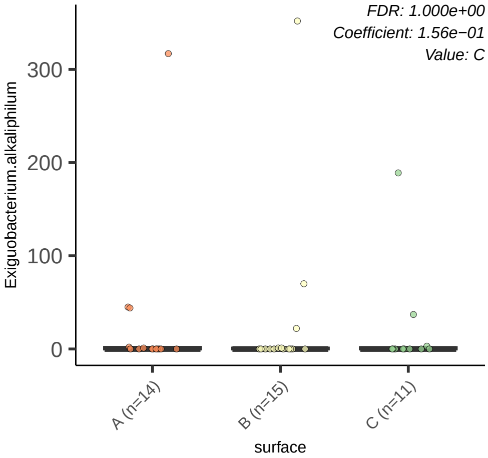
FDR: 1.000e+00
Coefficient: 1.56e−01
Value: C
Exiguobacterium.alkaliphilum
300
200
100
0
A (n=14)
B (n=15)
C (n=11)
surface

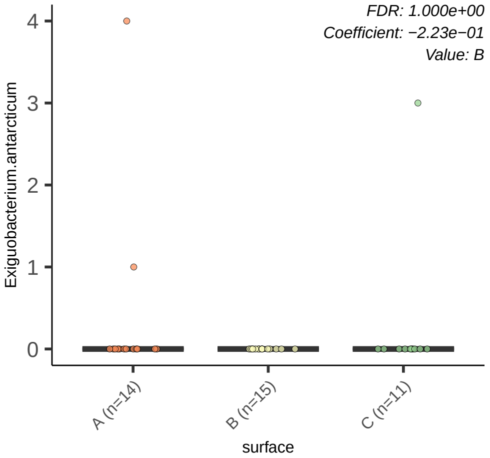

FDR: 1.000e+00
Coefficient: −2.23e−01
Value: B
Exiguobacterium.antarcticum
0
1
2
3
4
A (n=14)
B (n=15)
C (n=11)
surface

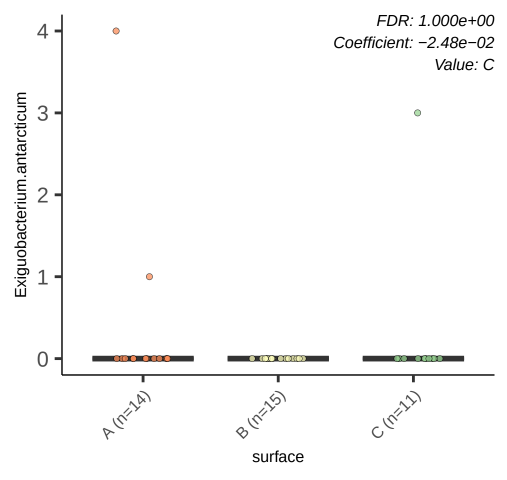

FDR: 1.000e+00
Coefficient: −2.48e−02
Value: C
Exiguobacterium.antarcticum
4
3
2
1
0
A (n=14)
B (n=15)
C (n=11)
surface

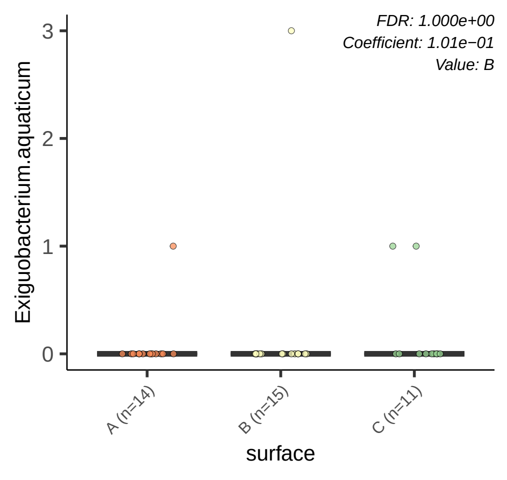
FDR: 1.000e+00
Coefficient: 1.01e−01
Value: B
Exiguobacterium.aquaticum
3
2
1
0
A (n=14)
B (n=15)
C (n=11)
surface

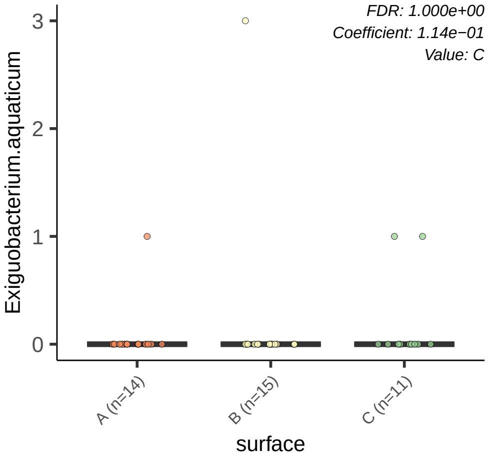
FDR: 1.000e+00
Coefficient: 1.14e−01
Value: C
3
2
1
0
Exiguobacterium.aquaticum
A (n=14)
B (n=15)
C (n=11)
surface

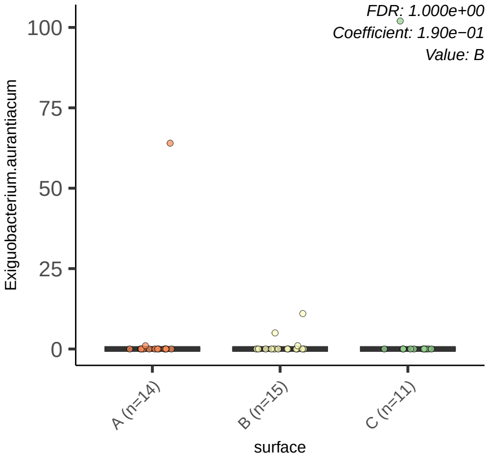

FDR: 1.000e+00
Coefficient: 1.90e−01
Value: B
Exiguobacterium.aurantiacum
100
75
50
25
0
A (n=14)
B (n=15)
C (n=11)
surface

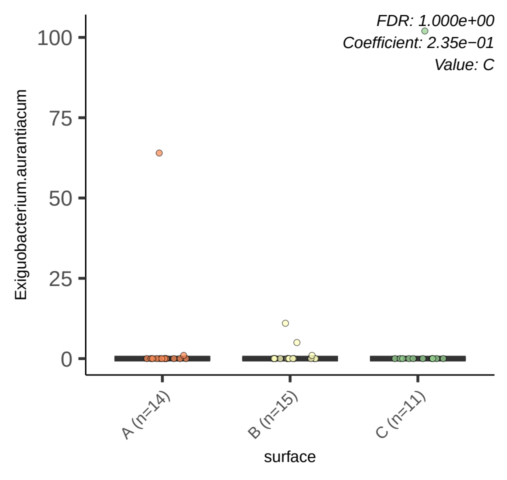
FDR: 1.000e+00
Coefficient: 2.35e−01
Value: C
Exiguobacterium.aurantiacum
100
75
50
25
0
A (n=14)
B (n=15)
C (n=11)
surface

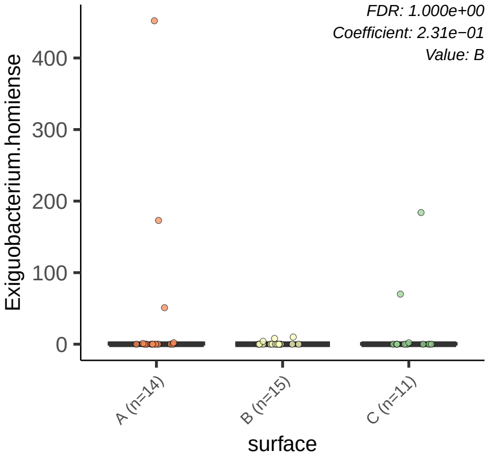

FDR: 1.000e+00
Coefficient: 2.31e−01
Value: B
Exiguobacterium.homiense
400
300
200
100
0
A (n=14)
B (n=15)
C (n=11)
surface

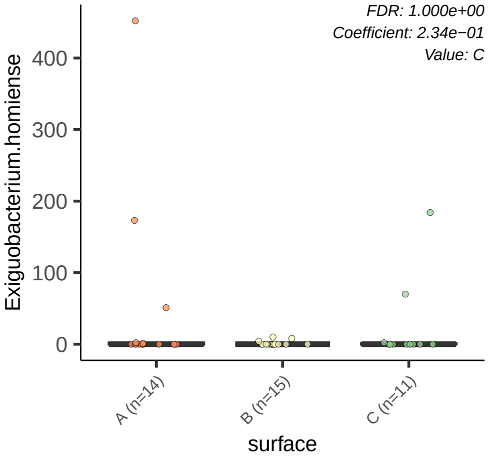

FDR: 1.000e+00
Coefficient: 2.34e−01
Value: C
Exiguobacterium.homiense
400
300
200
100
0
A (n=14)
B (n=15)
C (n=11)
surface

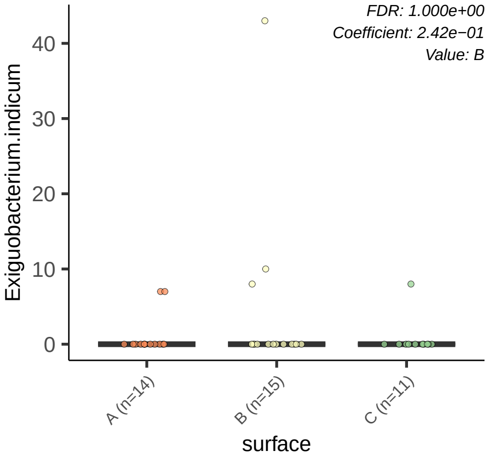

FDR: 1.000e+00
Coefficient: 2.42e−01
Value: B
Exiguobacterium.indicum
40
30
20
10
0
A (n=14)
B (n=15)
C (n=11)
surface

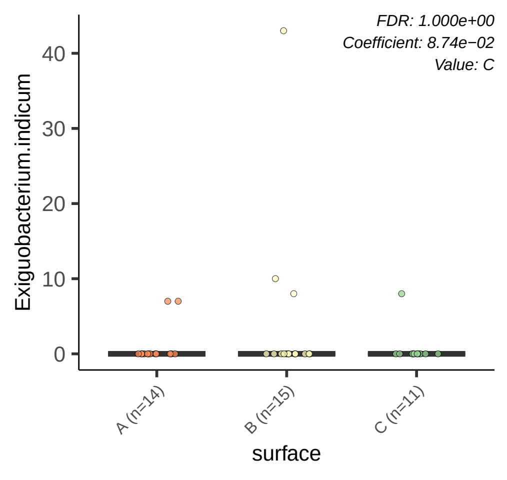
FDR: 1.000e+00
Coefficient: 8.74e−02
Value: C
Exiguobacterium.indicum
0
10
20
30
40
A (n=14)
B (n=15)
C (n=11)
surface

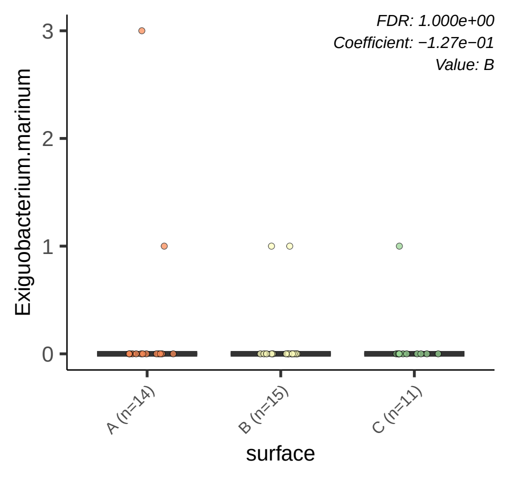

FDR: 1.000e+00
Coefficient: −1.27e−01
Value: B
Exiguobacterium.marinum
0
1
2
3
A (n=14)
B (n=15)
C (n=11)
surface

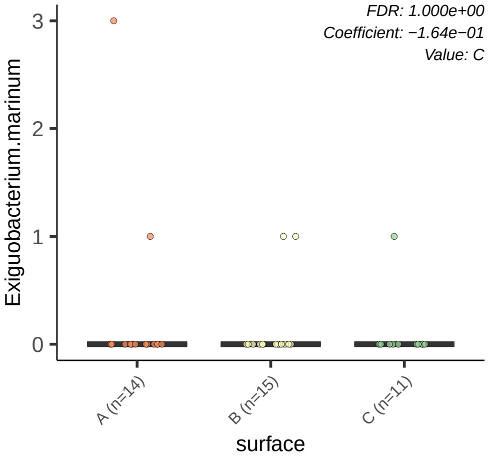

FDR: 1.000e+00
Coefficient: −1.64e−01
Value: C
Exiguobacterium.marinum
0
1
2
3
A (n=14)
B (n=15)
C (n=11)
surface

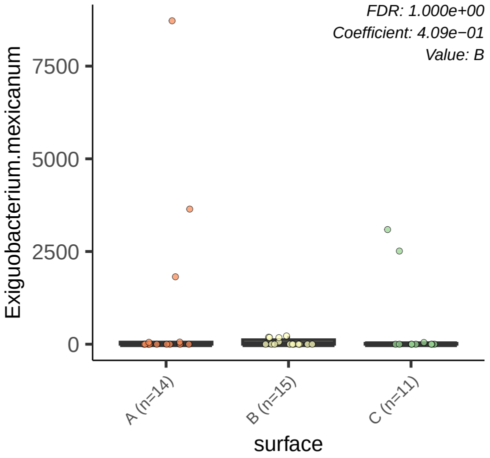

FDR: 1.000e+00
Coefficient: 4.09e−01
Value: B
Exiguobacterium.mexicanum
7500
5000
2500
0
A (n=14)
B (n=15)
C (n=11)
surface

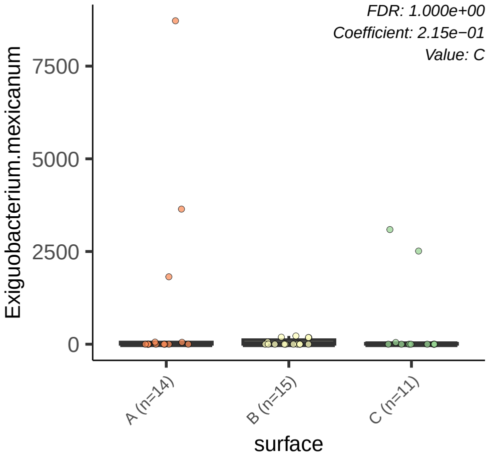
FDR: 1.000e+00
Coefficient: 2.15e−01
Value: C
Exiguobacterium.mexicanum
7500
5000
2500
0
A (n=14)
B (n=15)
C (n=11)
surface

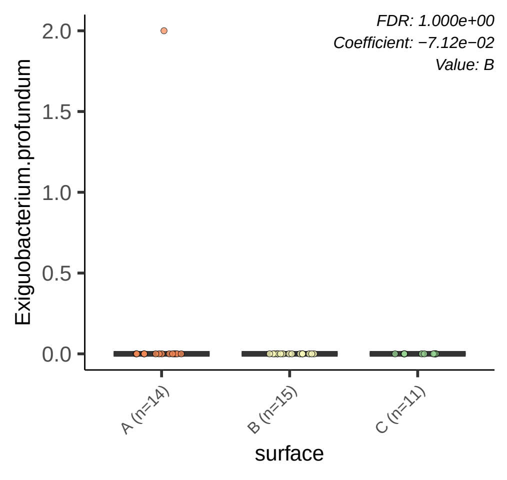
FDR: 1.000e+00
Coefficient: −7.12e−02
Value: B
Exiguobacterium.profundum
2.0
1.5
1.0
0.5
0.0
A (n=14)
B (n=15)
C (n=11)
surface

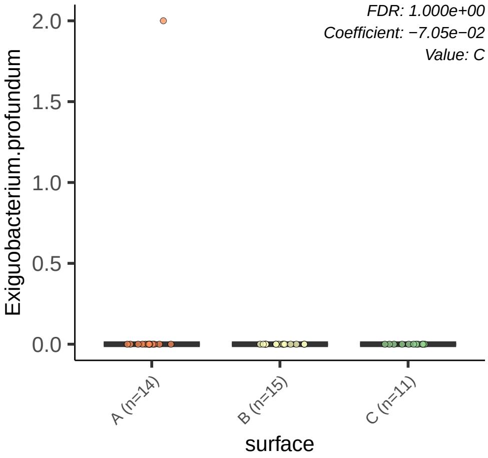
FDR: 1.000e+00
Coefficient: −7.05e−02
Value: C
Exiguobacterium.profundum
2.0
1.5
1.0
0.5
0.0
A (n=14)
B (n=15)
C (n=11)
surface

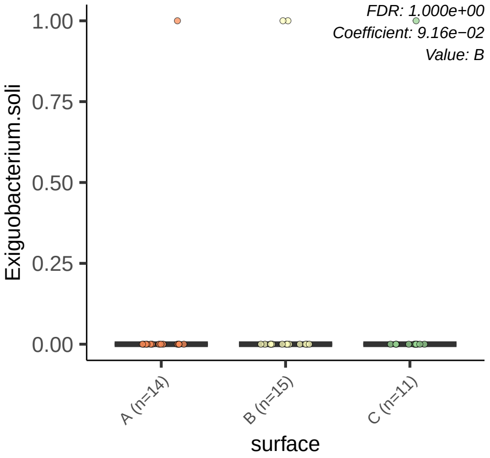
FDR: 1.000e+00
Coefficient: 9.16e−02
Value: B
Exiguobacterium.soli
1.00
0.75
0.50
0.25
0.00
A (n=14)
B (n=15)
C (n=11)
surface

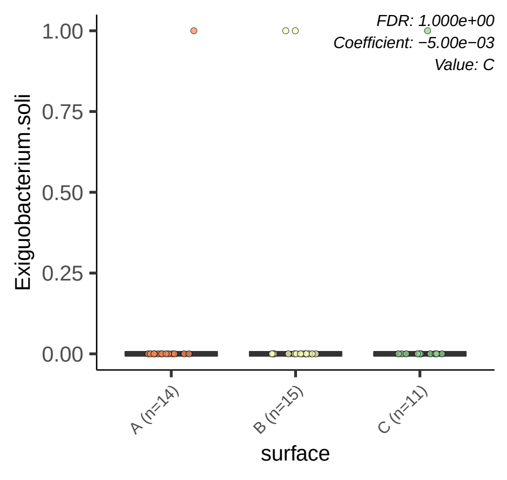
FDR: 1.000e+00
Coefficient: −5.00e−03
Value: C
Exiguobacterium.soli
1.00
0.75
0.50
0.25
0.00
A (n=14)
B (n=15)
C (n=11)
surface

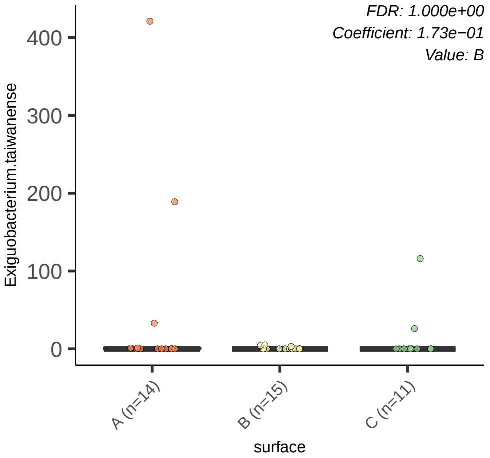

FDR: 1.000e+00
Coefficient: 1.73e−01
Value: B
Exiguobacterium.taiwanense
400
300
200
100
0
A (n=14)
B (n=15)
C (n=11)
surface

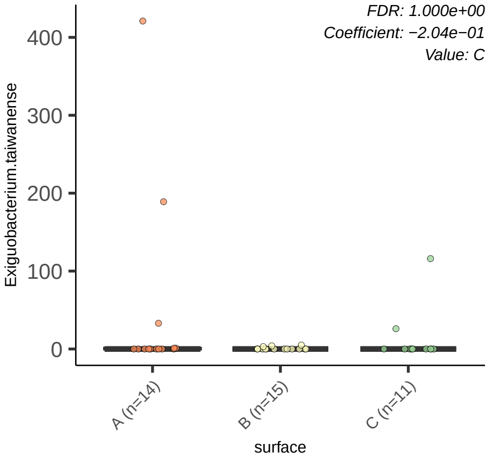

FDR: 1.000e+00
Coefficient: −2.04e−01
Value: C
Exiguobacterium.taiwanense
400
300
200
100
0
A (n=14)
B (n=15)
C (n=11)
surface

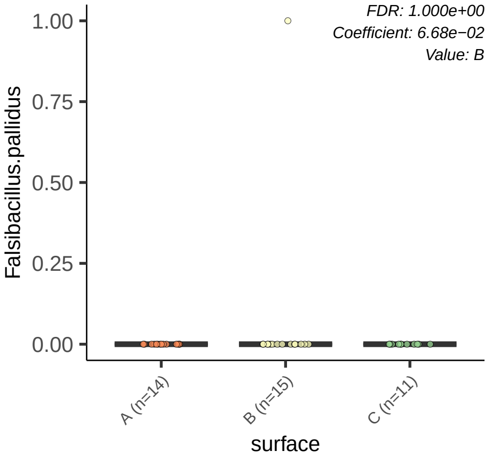

FDR: 1.000e+00
Coefficient: 6.68e−02
Value: B
Falsibacillus.pallidus
1.00
0.75
0.50
0.25
0.00
A (n=14)
B (n=15)
C (n=11)
surface

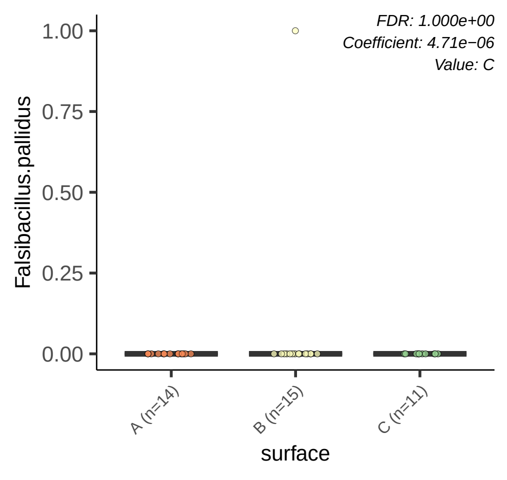
FDR: 1.000e+00
Coefficient: 4.71e−06
Value: C
Falsibacillus.pallidus
1.00
0.75
0.50
0.25
0.00
A (n=14)
B (n=15)
C (n=11)
surface

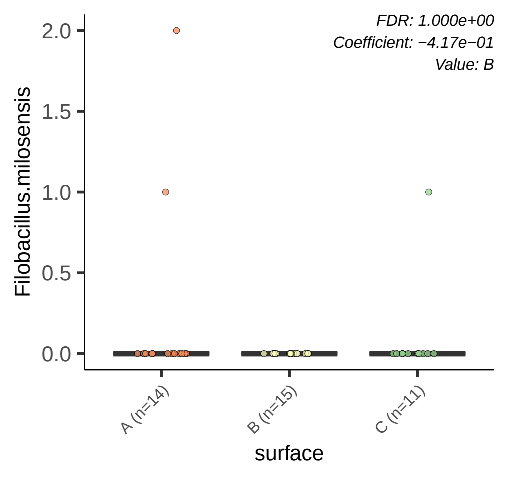
FDR: 1.000e+00
Coefficient: −4.17e−01
Value: B
Filobacillus.milosensis
2.0
1.5
1.0
0.5
0.0
A (n=14)
B (n=15)
C (n=11)
surface

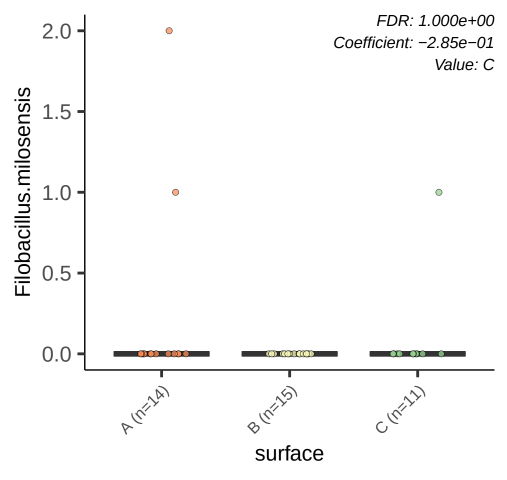
FDR: 1.000e+00
Coefficient: −2.85e−01
Value: C
Filobacillus.milosensis
2.0
1.5
1.0
0.5
0.0
A (n=14)
B (n=15)
C (n=11)
surface

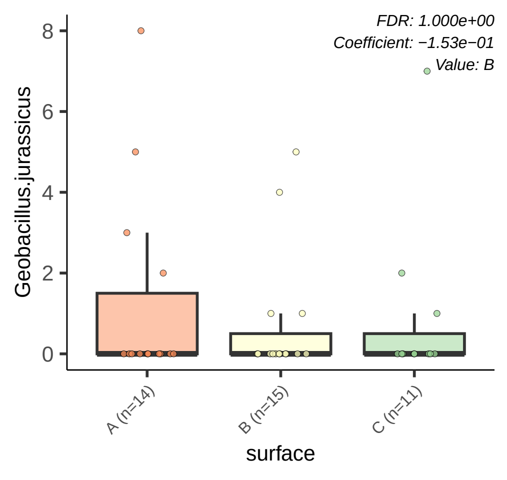
FDR: 1.000e+00
Coefficient: −1.53e−01
Value: B
Geobacillus.jurassicus
8
6
4
2
0
A (n=14)
B (n=15)
C (n=11)
surface

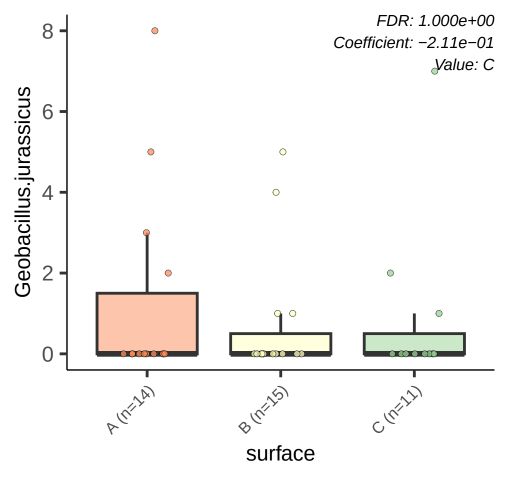
FDR: 1.000e+00
Coefficient: −2.11e−01
Value: C
Geobacillus.jurassicus
0
2
4
6
8
A (n=14)
B (n=15)
C (n=11)
surface

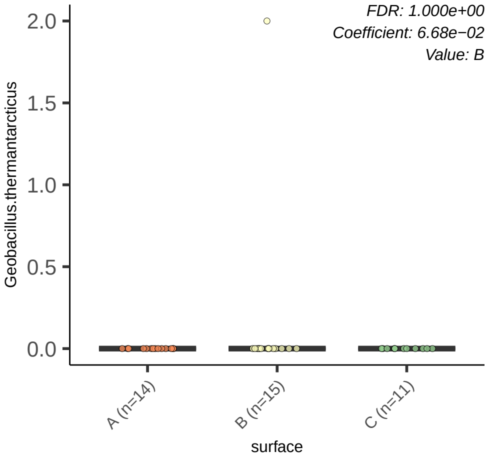

FDR: 1.000e+00
Coefficient: 6.68e−02
Value: B
Geobacillus.thermantarcticus
2.0
1.5
1.0
0.5
0.0
A (n=14)
B (n=15)
C (n=11)
surface

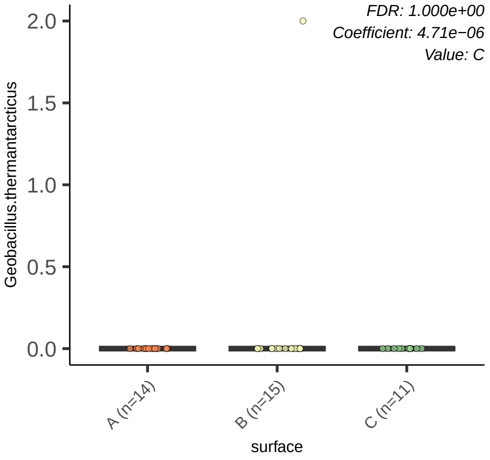
FDR: 1.000e+00
Coefficient: 4.71e−06
Value: C
Geobacillus.thermantarcticus
2.0
1.5
1.0
0.5
0.0
A (n=14)
B (n=15)
C (n=11)
surface

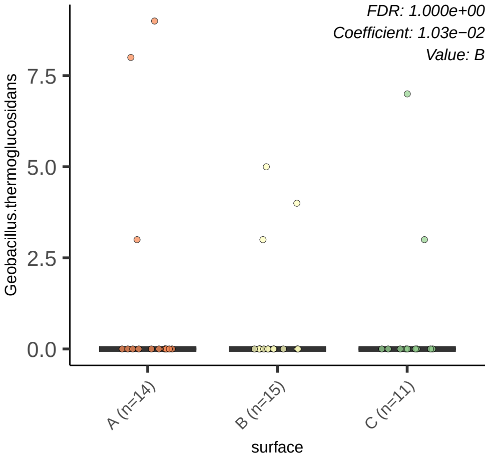
FDR: 1.000e+00
Coefficient: 1.03e−02
Value: B
Geobacillus.thermoglucosidans
7.5
5.0
2.5
0.0
A (n=14)
B (n=15)
C (n=11)
surface

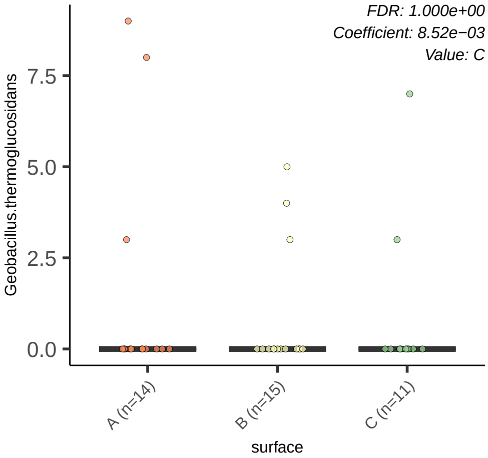
FDR: 1.000e+00
Coefficient: 8.52e−03
Value: C
Geobacillus.thermoglucosidans
7.5
5.0
2.5
0.0
A (n=14)
B (n=15)
C (n=11)
surface

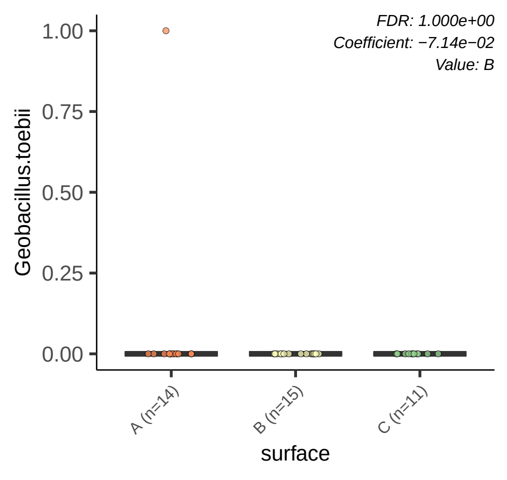
FDR: 1.000e+00
Coefficient: −7.14e−02
Value: B
Geobacillus.toebii
1.00
0.75
0.50
0.25
0.00
A (n=14)
B (n=15)
C (n=11)
surface

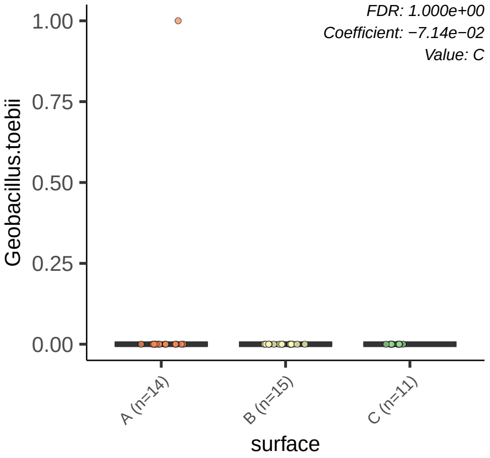
FDR: 1.000e+00
Coefficient: −7.14e−02
Value: C
Geobacillus.toebii
1.00
0.75
0.50
0.25
0.00
A (n=14)
B (n=15)
C (n=11)
surface

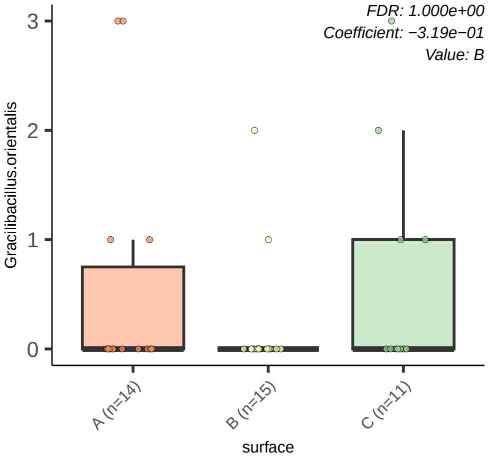

FDR: 1.000e+00
Coefficient: −3.19e−01
Value: B
Gracilibacillus.orientalis
0
1
2
3
A (n=14)
B (n=15)
C (n=11)
surface

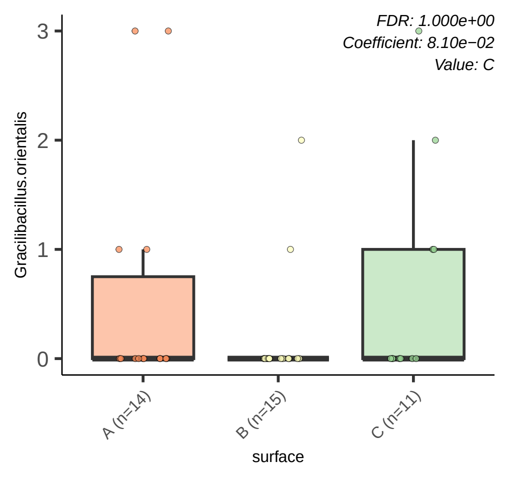

FDR: 1.000e+00
Coefficient: 8.10e−02
Value: C
Gracilibacillus.orientalis
0
1
2
3
A (n=14)
B (n=15)
C (n=11)
surface

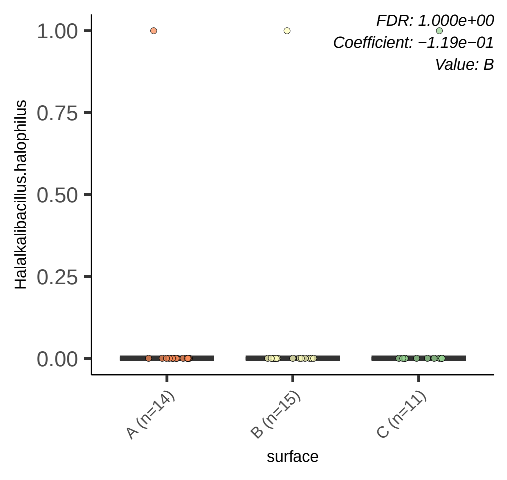

FDR: 1.000e+00
Coefficient: −1.19e−01
Value: B
1.00
0.75
0.50
0.25
0.00
Halalkalibacillus.halophilus
A (n=14)
B (n=15)
C (n=11)
surface

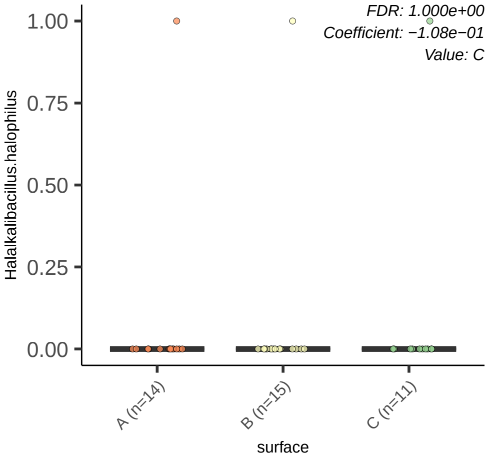
FDR: 1.000e+00
Coefficient: −1.08e−01
Value: C
Halalkalibacillus.halophilus
1.00
0.75
0.50
0.25
0.00
A (n=14)
B (n=15)
C (n=11)
surface

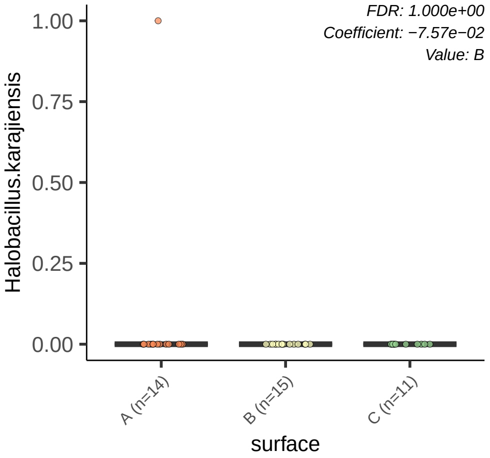

FDR: 1.000e+00
Coefficient: −7.57e−02
Value: B
Halobacillus.karajiensis
1.00
0.75
0.50
0.25
0.00
A (n=14)
B (n=15)
C (n=11)
surface

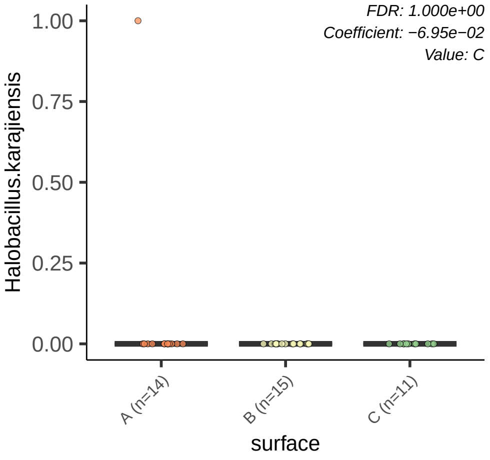
FDR: 1.000e+00
Coefficient: −6.95e−02
Value: C
Halobacillus.karajiensis
1.00
0.75
0.50
0.25
0.00
A (n=14)
B (n=15)
C (n=11)
surface

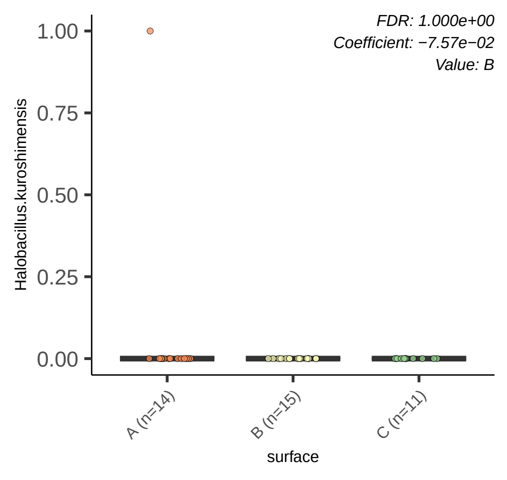

FDR: 1.000e+00
Coefficient: −7.57e−02
Value: B
1.00
0.75
0.50
0.25
0.00
Halobacillus.kuroshimensis
A (n=14)
B (n=15)
C (n=11)
surface

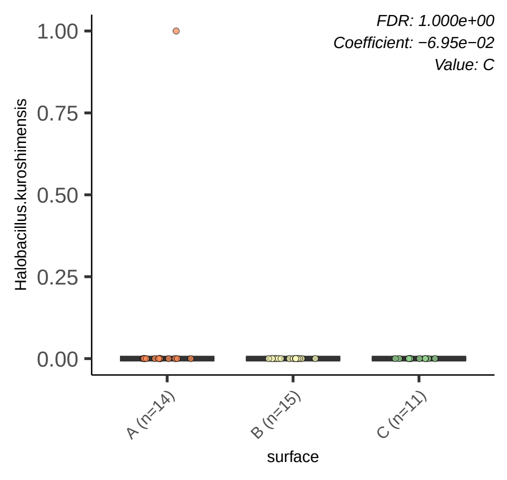
FDR: 1.000e+00
Coefficient: −6.95e−02
Value: C
1.00
0.75
0.50
0.25
0.00
Halobacillus.kuroshimensis
A (n=14)
B (n=15)
C (n=11)
surface

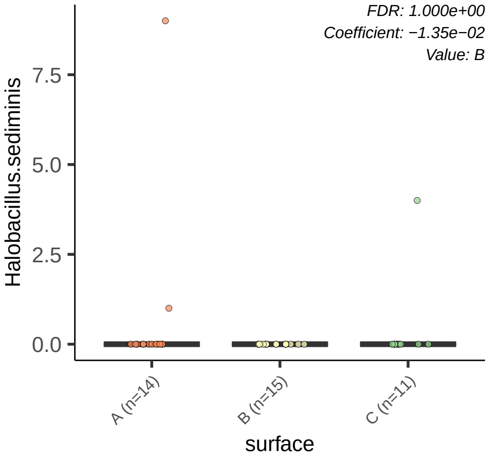

FDR: 1.000e+00
Coefficient: −1.35e−02
Value: B
Halobacillus.sedimenis
7.5
5.0
2.5
0.0
A (n=14)
B (n=15)
C (n=11)
surface

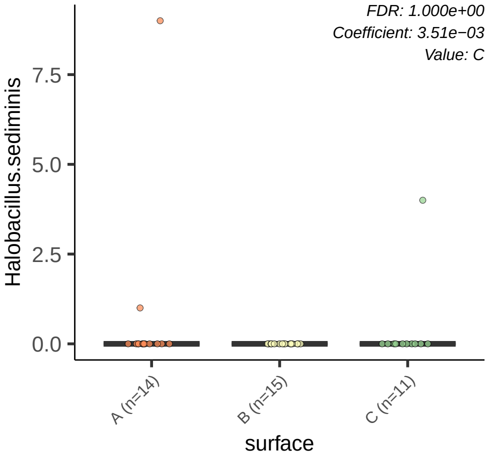
FDR: 1.000e+00
Coefficient: 3.51e−03
Value: C
Halobacillus.sedimini s
0.0
2.5
5.0
7.5
A (n=14)
B (n=15)
C (n=11)
surface

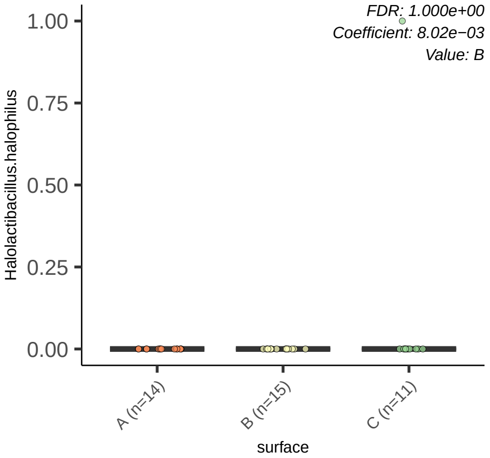
FDR: 1.000e+00
Coefficient: 8.02e−03
Value: B
1.00
0.75
0.50
0.25
0.00
Halolactibacillus.halophilus
A (n=14)
B (n=15)
C (n=11)
surface

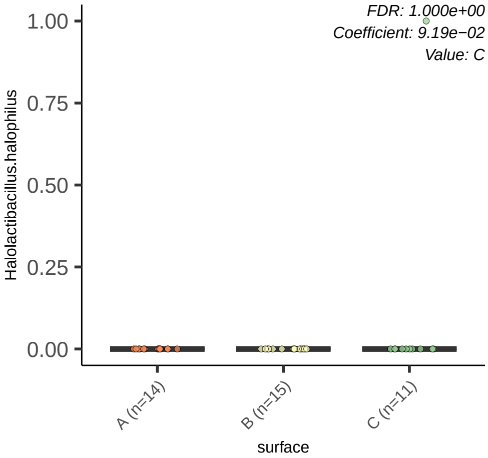

FDR: 1.000e+00
Coefficient: 9.19e−02
Value: C
1.00
0.75
0.50
0.25
0.00
Halolactibacillus.halophilus
A (n=14)
B (n=15)
C (n=11)
surface

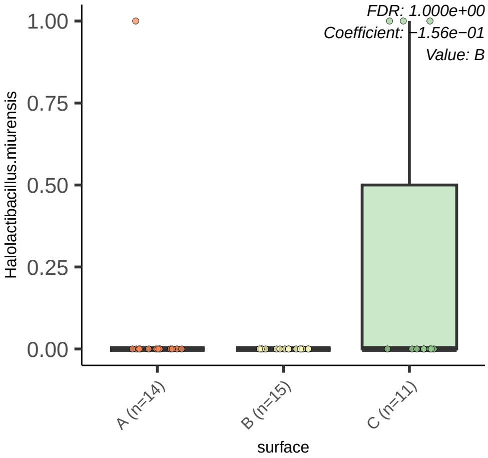
FDR: 1.000e+00
Coefficient: −1.56e−01
Value: B
Halolactibacillus.miurensis
1.00
0.75
0.50
0.25
0.00
A (n=14)
B (n=15)
C (n=11)
surface

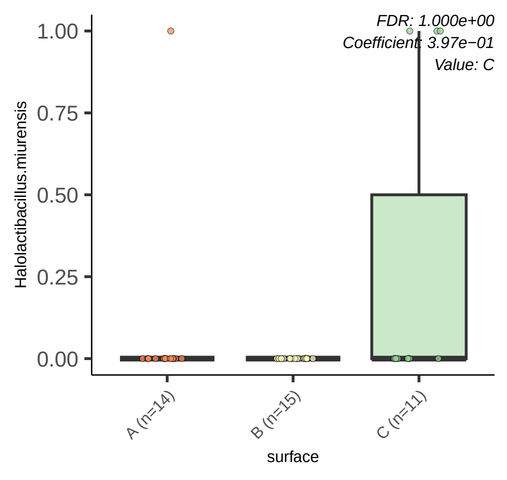

FDR: 1.000e+00
Coefficient: 3.97e−01
Value: C
Halolactibacillus.miurensis
1.00
0.75
0.50
0.25
0.00
A (n=14)
B (n=15)
C (n=11)
surface

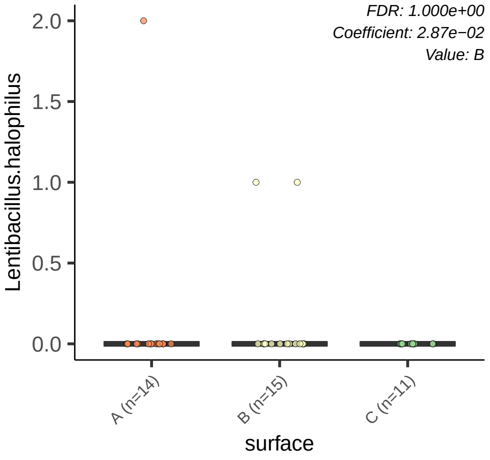

FDR: 1.000e+00
Coefficient: 2.87e−02
Value: B
Lentibacillus.halophilus
2.0
1.5
1.0
0.5
0.0
A (n=14)
B (n=15)
C (n=11)
surface

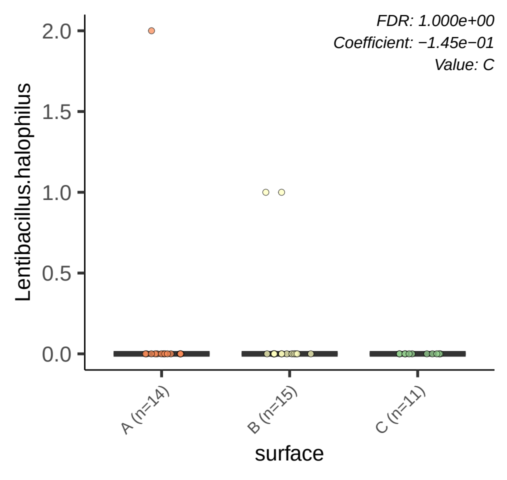

FDR: 1.000e+00
Coefficient: −1.45e−01
Value: C
Lentibacillus.halophilus
2.0
1.5
1.0
0.5
0.0
A (n=14)
B (n=15)
C (n=11)
surface

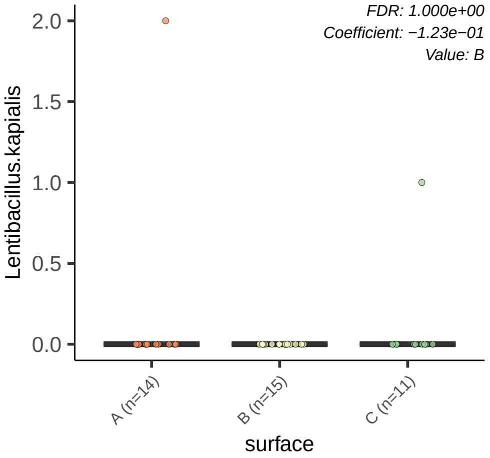
FDR: 1.000e+00
Coefficient: −1.23e−01
Value: B
Lentibacillus.kapialis
2.0
1.5
1.0
0.5
0.0
A (n=14)
B (n=15)
C (n=11)
surface

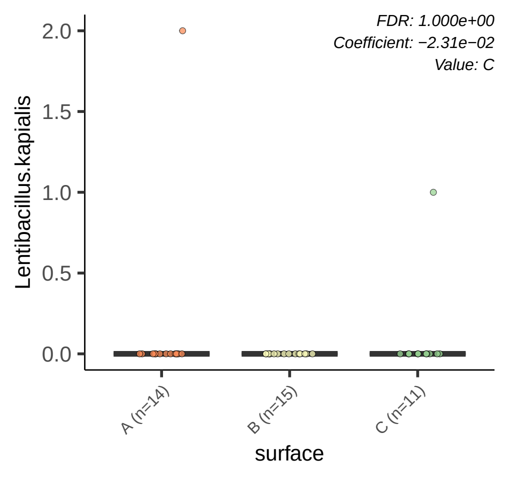

FDR: 1.000e+00
Coefficient: −2.31e−02
Value: C
Lentibacillus.kapialis
2.0
1.5
1.0
0.5
0.0
A (n=14)
B (n=15)
C (n=11)
surface

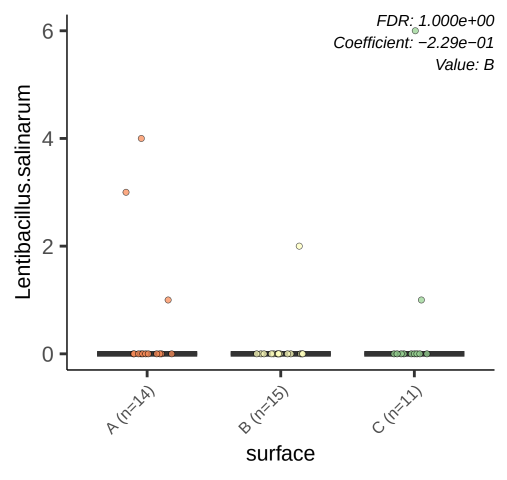
FDR: 1.000e+00
Coefficient: −2.29e−01
Value: B
Lentibacillus.salinarum
6
4
2
0
A (n=14)
B (n=15)
C (n=11)
surface

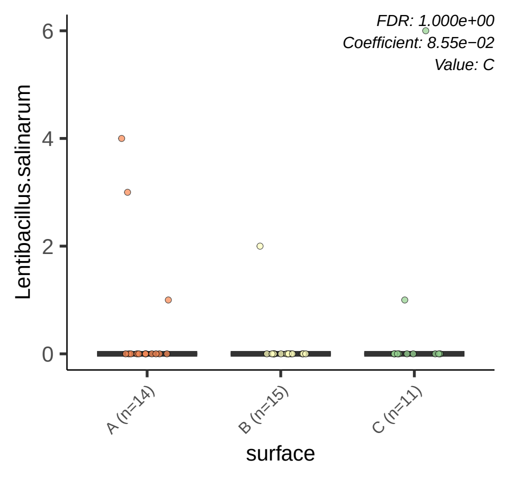

FDR: 1.000e+00
Coefficient: 8.55e−02
Value: C
Lentibacillus.salinarum
0
2
4
6
A (n=14)
B (n=15)
C (n=11)
surface

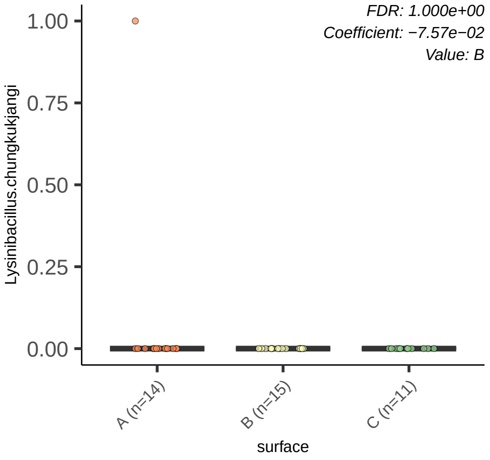
FDR: 1.000e+00
Coefficient: −7.57e−02
Value: B
Lysinibacillus.chungkukjangi
1.00
0.75
0.50
0.25
0.00
A (n=14)
B (n=15)
C (n=11)
surface

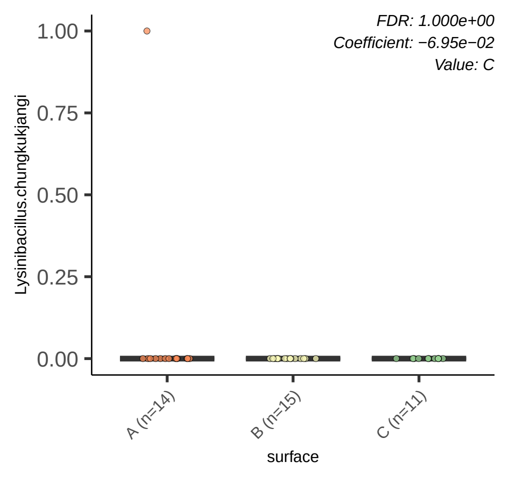

FDR: 1.000e+00
Coefficient: −6.95e−02
Value: C
Lysinibacillus.chungkukjangi
1.00
0.75
0.50
0.25
0.00
A (n=14)
B (n=15)
C (n=11)
surface

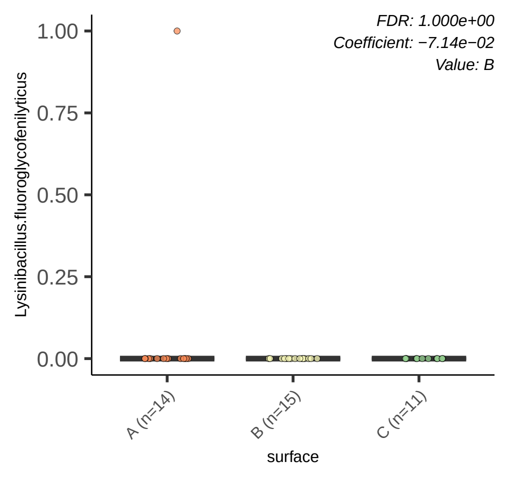

FDR: 1.000e+00
Coefficient: −7.14e−02
Value: B
Lysinibacillus.fluoroglycofenilyticus
1.00
0.75
0.50
0.25
0.00
A (n=14)
B (n=15)
C (n=11)
surface

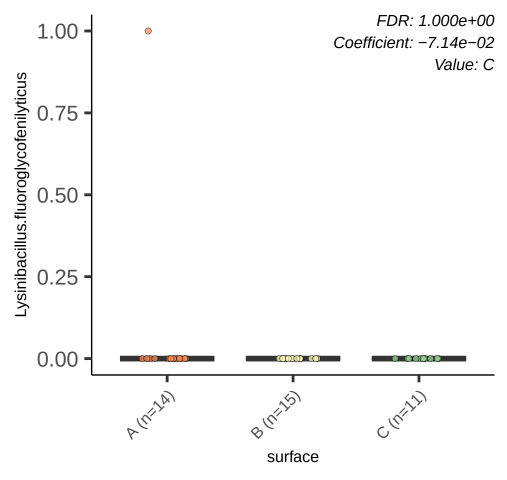

FDR: 1.000e+00
Coefficient: −7.14e−02
Value: C
1.00
0.75
0.50
0.25
0.00
Lysinibacillus.fluoroglycofenilyticus
A (n=14)
B (n=15)
C (n=11)
surface

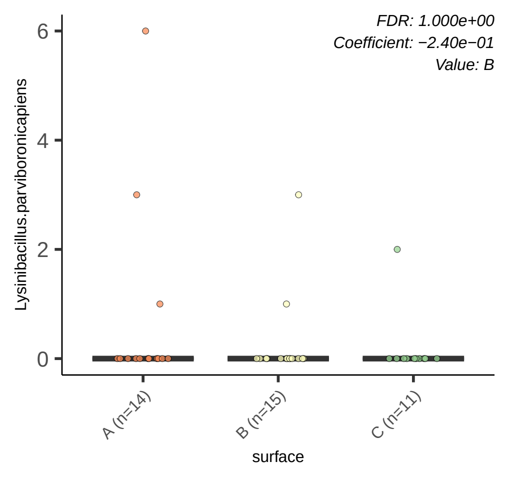
FDR: 1.000e+00
Coefficient: −2.40e−01
Value: B
Lysinibacillus.parviboronicapiens
6
4
2
0
A (n=14)
B (n=15)
C (n=11)
surface

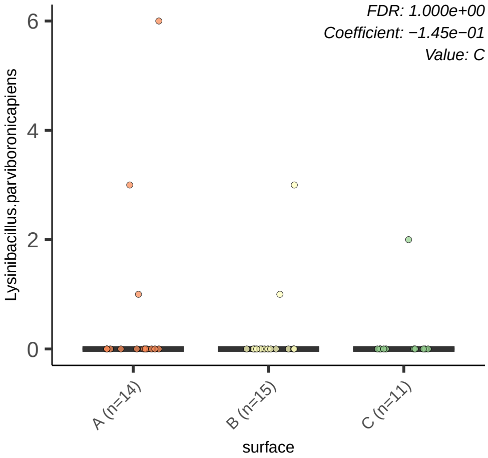

FDR: 1.000e+00
Coefficient: −1.45e−01
Value: C
Lysinibacillus.parviboronicapiens
0
2
4
6
A (n=14)
B (n=15)
C (n=11)
surface

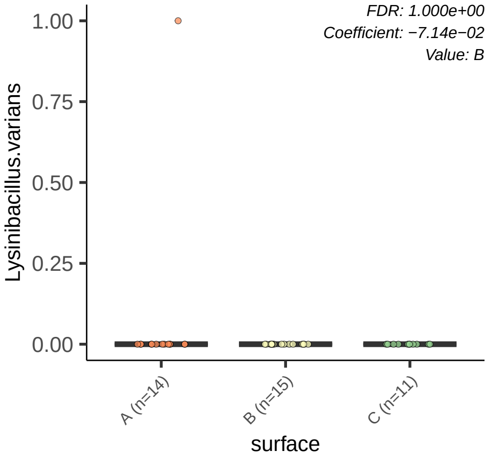
FDR: 1.000e+00
Coefficient: −7.14e−02
Value: B
Lysinibacillus.varians
1.00
0.75
0.50
0.25
0.00
A (n=14)
B (n=15)
C (n=11)
surface

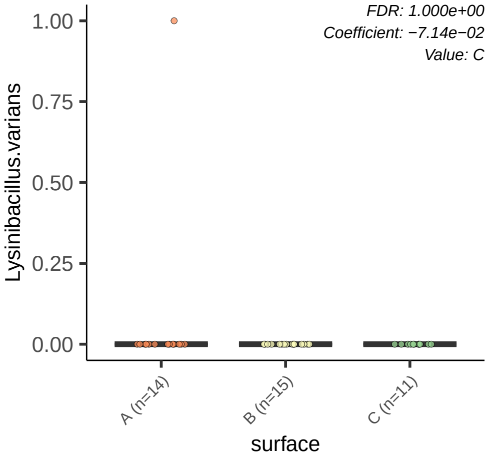
FDR: 1.000e+00
Coefficient: −7.14e−02
Value: C
Lysinibacillus.varians
1.00
0.75
0.50
0.25
0.00
A (n=14)
B (n=15)
C (n=11)
surface

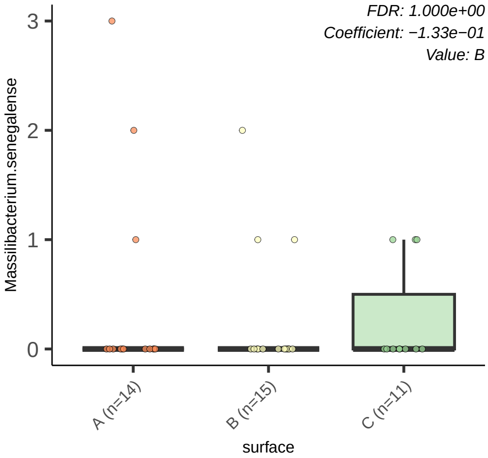
FDR: 1.000e+00
Coefficient: −1.33e−01
Value: B
Massilibacterium.senegalense
3
2
1
0
A (n=14)
B (n=15)
C (n=11)
surface

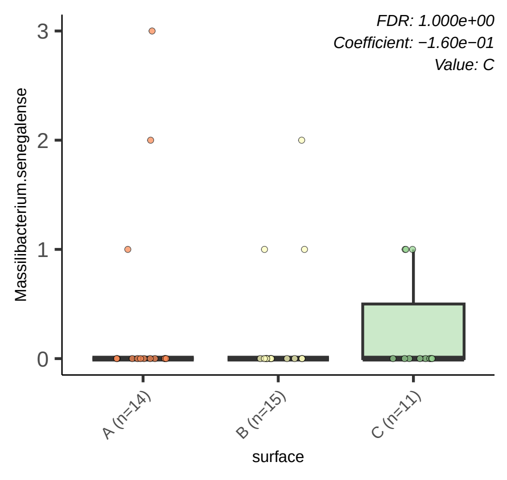

FDR: 1.000e+00
Coefficient: −1.60e−01
Value: C
Massilibacterium.senegalense
3
2
1
0
A (n=14)
B (n=15)
C (n=11)
surface

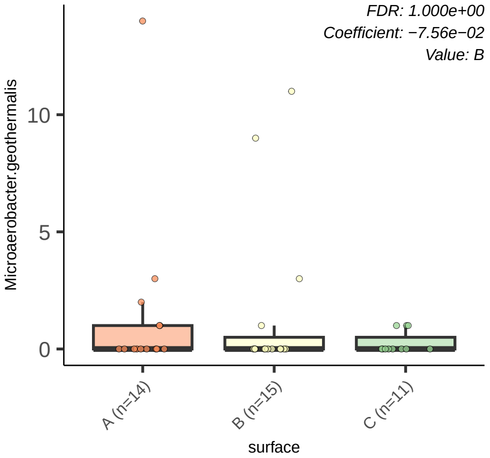

FDR: 1.000e+00
Coefficient: −7.56e−02
Value: B
Microaerobacter.geothermalis
10
5
0
A (n=14)
B (n=15)
C (n=11)
surface

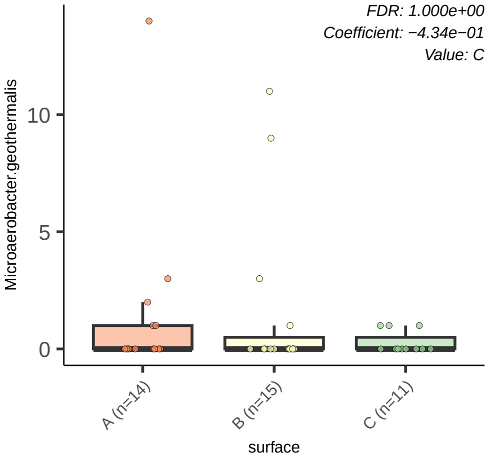
FDR: 1.000e+00
Coefficient: −4.34e−01
Value: C
Microaerobacter.geothermalis
0
5
10
A (n=14)
B (n=15)
C (n=11)
surface

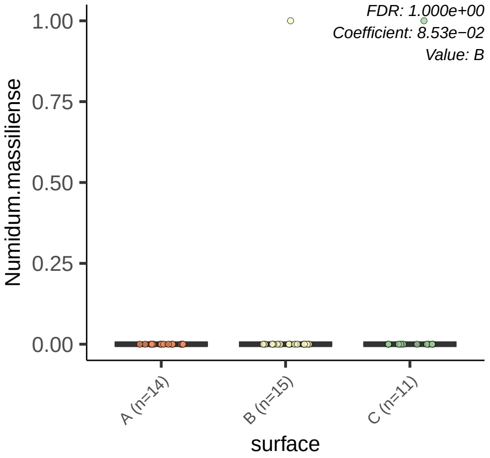

FDR: 1.000e+00
Coefficient: 8.53e−02
Value: B
1.00
0.75
0.50
0.25
0.00
Numidum.massiliense
A (n=14)
B (n=15)
C (n=11)
surface

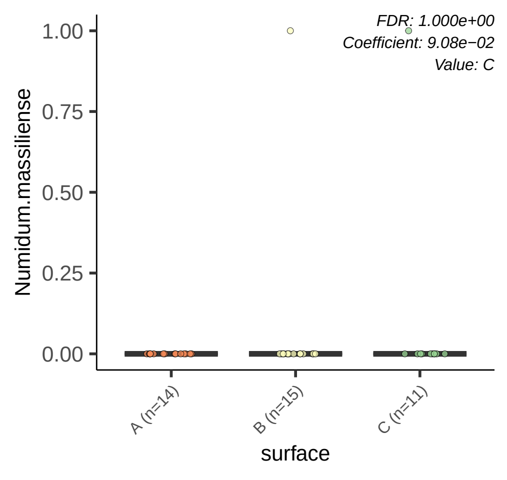
FDR: 1.000e+00
Coefficient: 9.08e−02
Value: C
Numidum.massiliense
1.00
0.75
0.50
0.25
0.00
A (n=14)
B (n=15)
C (n=11)
surface

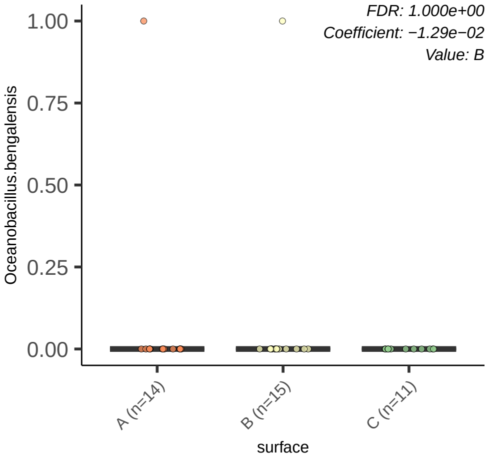

FDR: 1.000e+00
Coefficient: −1.29e−02
Value: B
Oceanobacillus.bengalensis
1.00
0.75
0.50
0.25
0.00
A (n=14)
B (n=15)
C (n=11)
surface

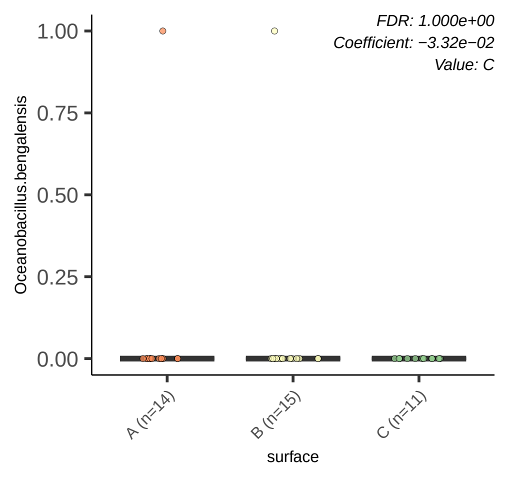

FDR: 1.000e+00
Coefficient: −3.32e−02
Value: C
Oceanobacillus.bengalensis
1.00
0.75
0.50
0.25
0.00
A (n=14)
B (n=15)
C (n=11)
surface

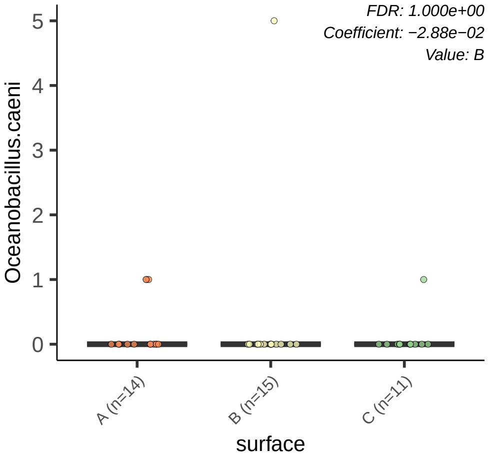

FDR: 1.000e+00
Coefficient: −2.88e−02
Value: B
Oceanobacillus.caeni
0
1
2
3
4
5
A (n=14)
B (n=15)
C (n=11)
surface

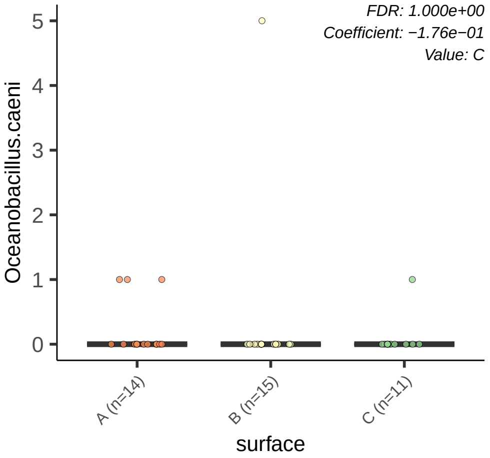

FDR: 1.000e+00
Coefficient: −1.76e−01
Value: C
Oceanobacillus.caeni
0
1
2
3
4
5
A (n=14)
B (n=15)
C (n=11)
surface

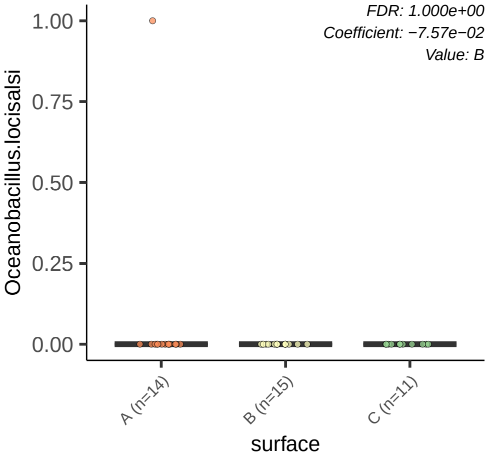
FDR: 1.000e+00
Coefficient: −7.57e−02
Value: B
Oceanobacillus.locisalsi
1.00
0.75
0.50
0.25
0.00
A (n=14)
B (n=15)
C (n=11)
surface

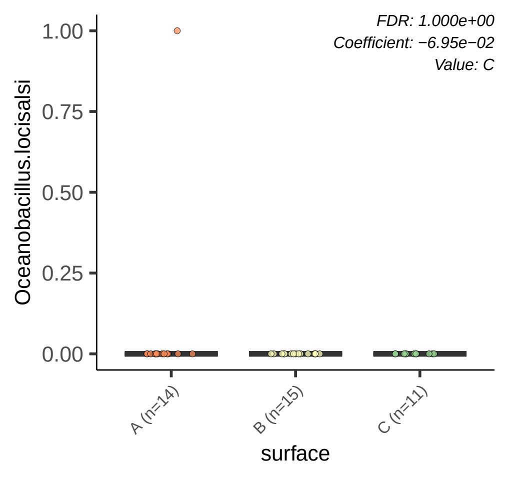

FDR: 1.000e+00
Coefficient: −6.95e−02
Value: C
Oceanobacillus.locisalsi
1.00
0.75
0.50
0.25
0.00
A (n=14)
B (n=15)
C (n=11)
surface

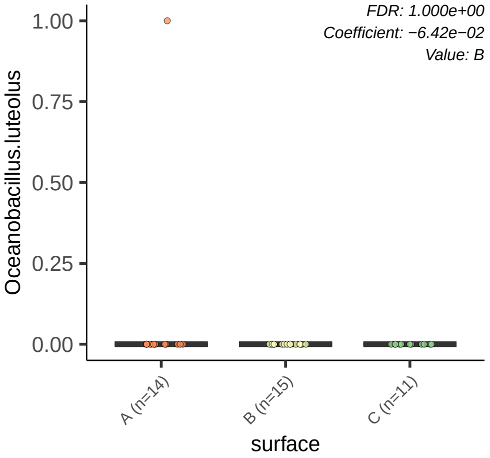
FDR: 1.000e+00
Coefficient: −6.42e−02
Value: B
Oceanobacillus.luteolus
1.00
0.75
0.50
0.25
0.00
A (n=14)
B (n=15)
C (n=11)
surface

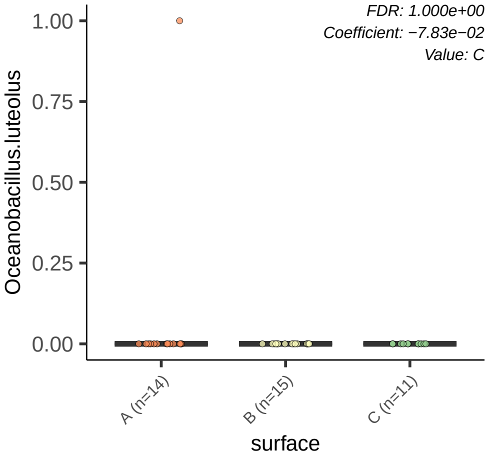

FDR: 1.000e+00
Coefficient: −7.83e−02
Value: C
Oceanobacillus.luteolus
1.00
0.75
0.50
0.25
0.00
A (n=14)
B (n=15)
C (n=11)
surface

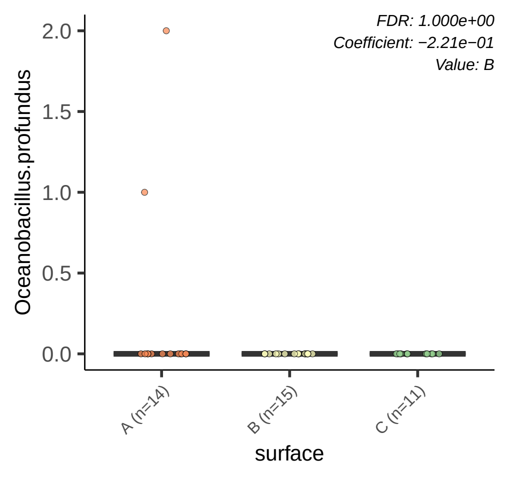
FDR: 1.000e+00
Coefficient: −2.21e−01
Value: B
Oceanobacillus.profundus
2.0
1.5
1.0
0.5
0.0
A (n=14)
B (n=15)
C (n=11)
surface

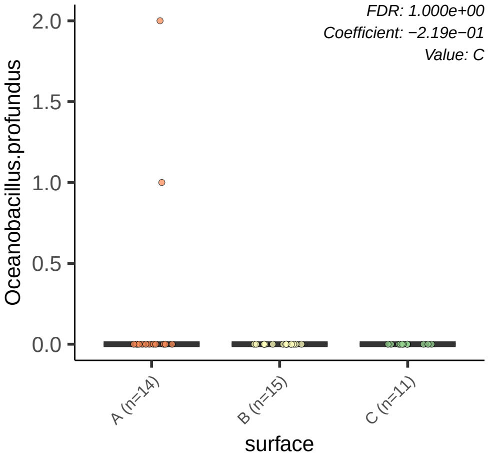

FDR: 1.000e+00
Coefficient: −2.19e−01
Value: C
Oceanobacillus.profundus
2.0
1.5
1.0
0.5
0.0
A (n=14)
B (n=15)
C (n=11)
surface

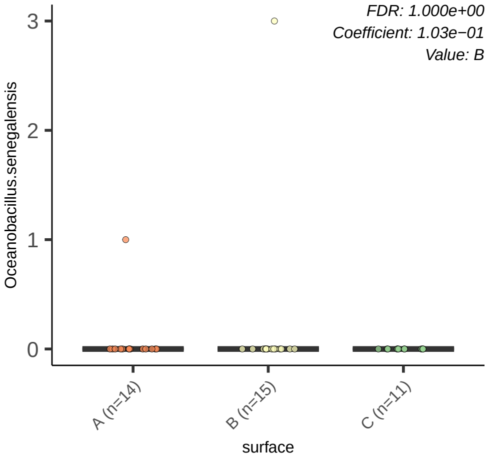
FDR: 1.000e+00
Coefficient: 1.03e−01
Value: B
Oceanobacillus.senegalensis
3
2
1
0
A (n=14)
B (n=15)
C (n=11)
surface

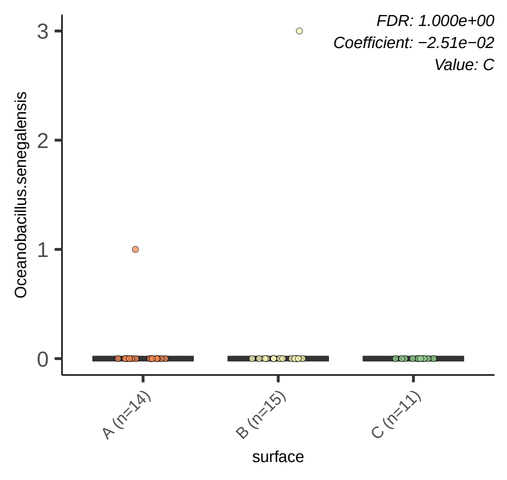
FDR: 1.000e+00
Coefficient: −2.51e−02
Value: C
Oceanobacillus.senegalensis
3
2
1
0
A (n=14)
B (n=15)
C (n=11)
surface

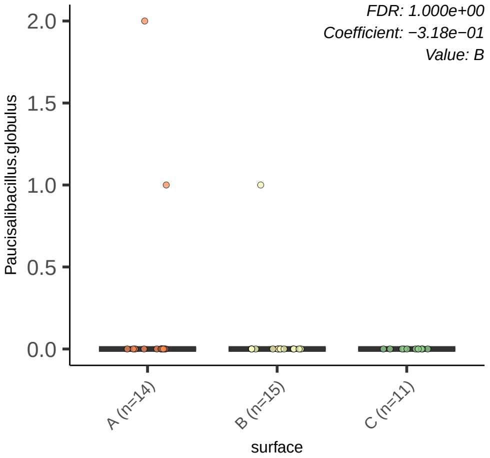
FDR: 1.000e+00
Coefficient: −3.18e−01
Value: B
Paucisalibacillus.globulus
2.0
1.5
1.0
0.5
0.0
A (n=14)
B (n=15)
C (n=11)
surface

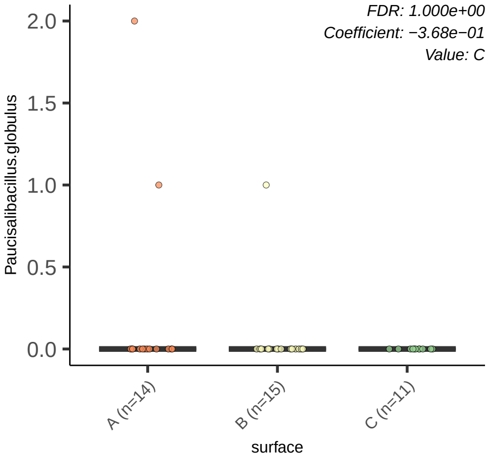
FDR: 1.000e+00
Coefficient: −3.68e−01
Value: C
Paucisalibacillus.globulus
2.0
1.5
1.0
0.5
0.0
A (n=14)
B (n=15)
C (n=11)
surface

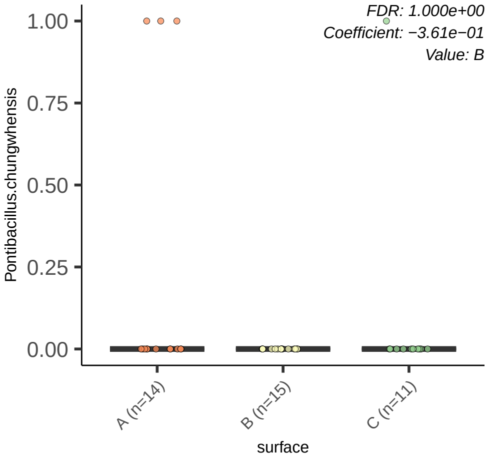

FDR: 1.000e+00
Coefficient: −3.61e−01
Value: B
1.00
0.75
0.50
0.25
0.00
Pontibacillus.chungwhensis
A (n=14)
B (n=15)
C (n=11)
surface

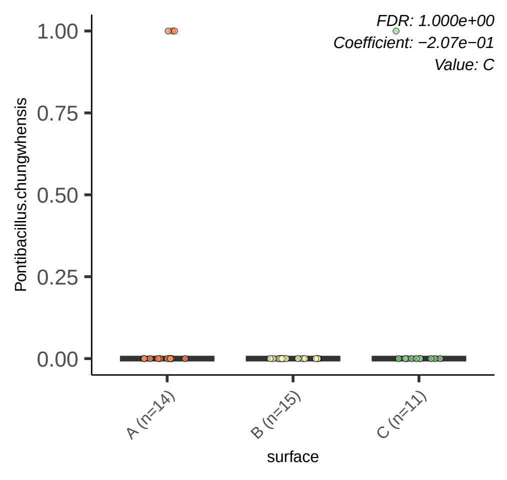

FDR: 1.000e+00
Coefficient: −2.07e−01
Value: C
1.00
0.75
0.50
0.25
0.00
Pontibacillus.chungwhensis
A (n=14)
B (n=15)
C (n=11)
surface

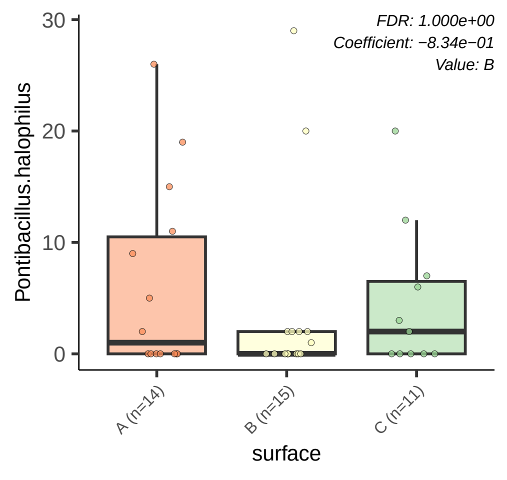

FDR: 1.000e+00
Coefficient: −8.34e−01
Value: B
Pontibacillus.halophilus
30
20
10
0
A (n=14)
B (n=15)
C (n=11)
surface

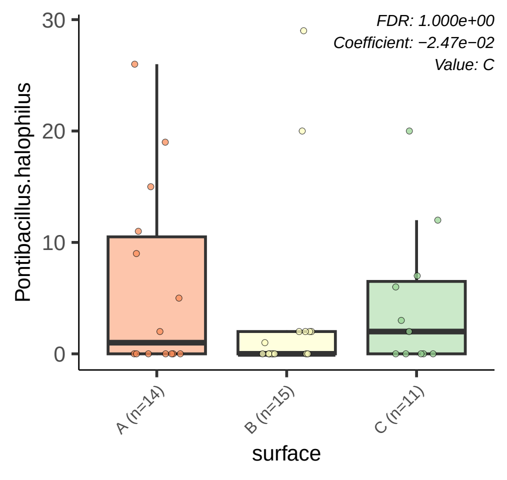

FDR: 1.000e+00
Coefficient: −2.47e−02
Value: C
Pontibacillus.halophilus
30
20
10
0
A (n=14)
B (n=15)
C (n=11)
surface

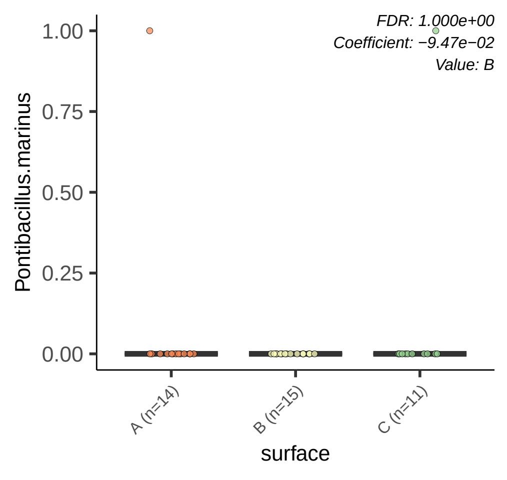
FDR: 1.000e+00
Coefficient: −9.47e−02
Value: B
Pontibacillus.marinus
1.00
0.75
0.50
0.25
0.00
A (n=14)
B (n=15)
C (n=11)
surface

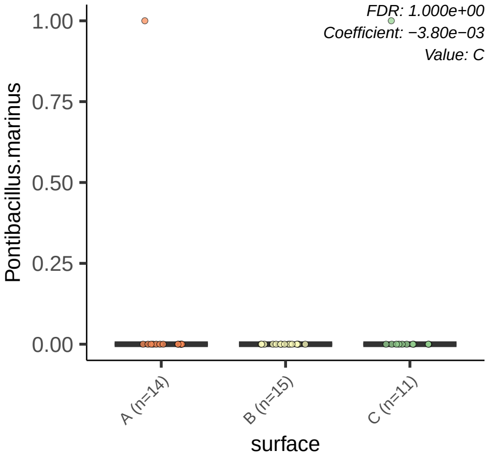

FDR: 1.000e+00
Coefficient: −3.80e−03
Value: C
Pontibacillus.marinus
1.00
0.75
0.50
0.25
0.00
A (n=14)
B (n=15)
C (n=11)
surface

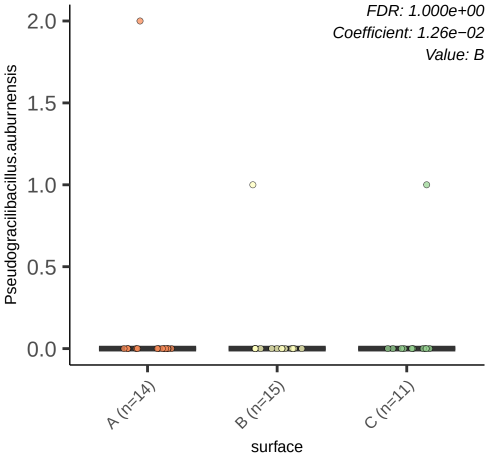

FDR: 1.000e+00
Coefficient: 1.26e−02
Value: B
Pseudogracilibacillus.auburnensis
2.0
1.5
1.0
0.5
0.0
A (n=14)
B (n=15)
C (n=11)
surface

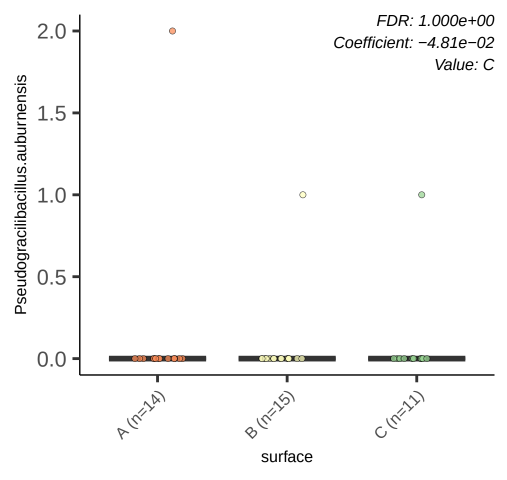

FDR: 1.000e+00
Coefficient: −4.81e−02
Value: C
Pseudogracilibacillus.auburnensis
2.0
1.5
1.0
0.5
0.0
A (n=14)
B (n=15)
C (n=11)
surface

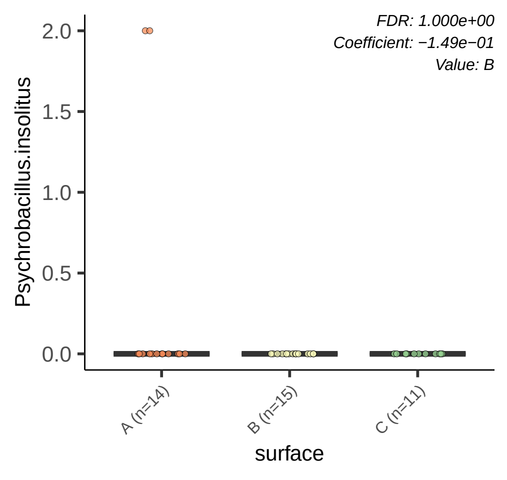
FDR: 1.000e+00
Coefficient: −1.49e−01
Value: B
Psychrobacillus.insolitus
2.0
1.5
1.0
0.5
0.0
A (n=14)
B (n=15)
C (n=11)
surface

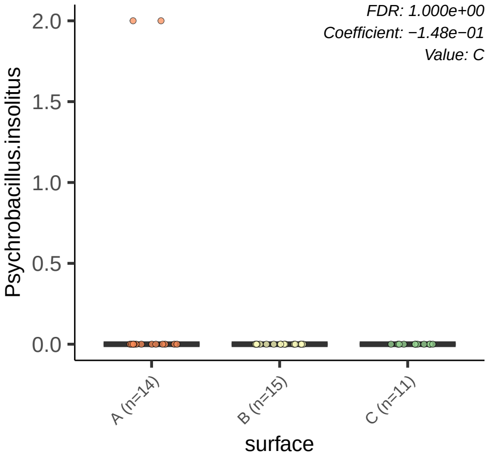
FDR: 1.000e+00
Coefficient: −1.48e−01
Value: C
Psychrobacillus.insolitus
2.0
1.5
1.0
0.5
0.0
A (n=14)
B (n=15)
C (n=11)
surface

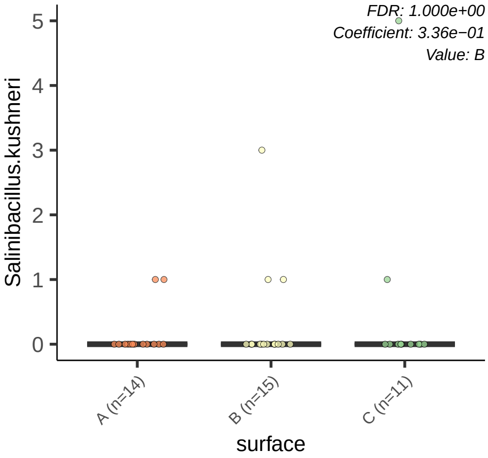

FDR: 1.000e+00
Coefficient: 3.36e−01
Value: B
Salinibacillus.kushneri
0
1
2
3
4
5
A (n=14)
B (n=15)
C (n=11)
surface

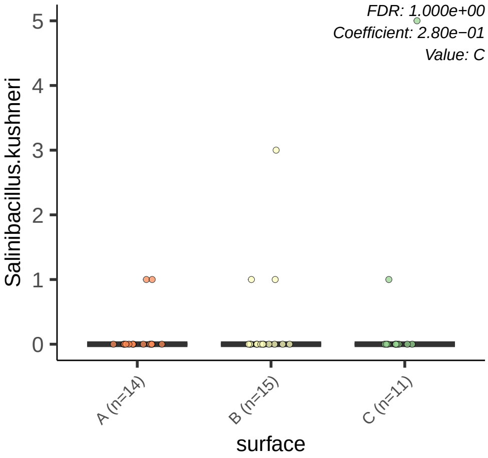

FDR: 1.000e+00
Coefficient: 2.80e−01
Value: C
Salinibacillus.kushneri
0
1
2
3
4
5
A (n=14)
B (n=15)
C (n=11)
surface

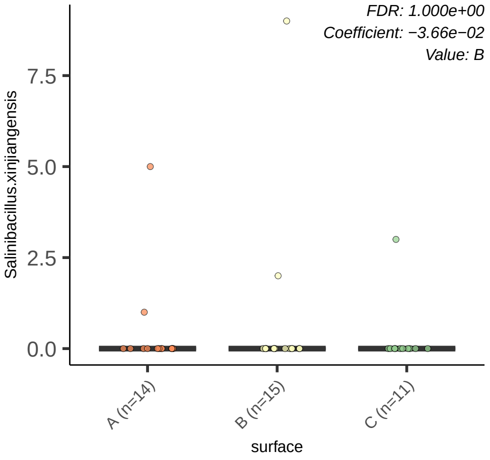

FDR: 1.000e+00
Coefficient: −3.66e−02
Value: B
Salinibacillus.xinjiangensis
7.5
5.0
2.5
0.0
A (n=14)
B (n=15)
C (n=11)
surface

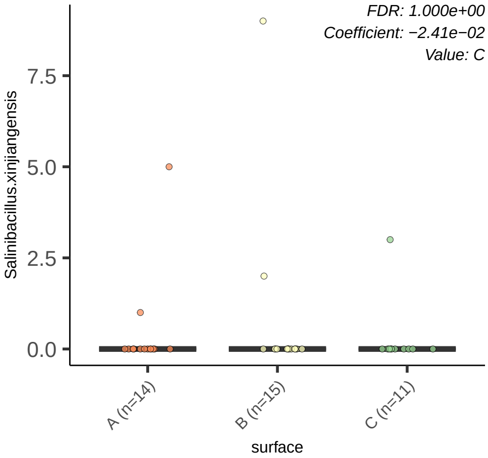

FDR: 1.000e+00
Coefficient: −2.41e−02
Value: C
Salinibacillus.xinjiangensis
7.5
5.0
2.5
0.0
A (n=14)
B (n=15)
C (n=11)
surface

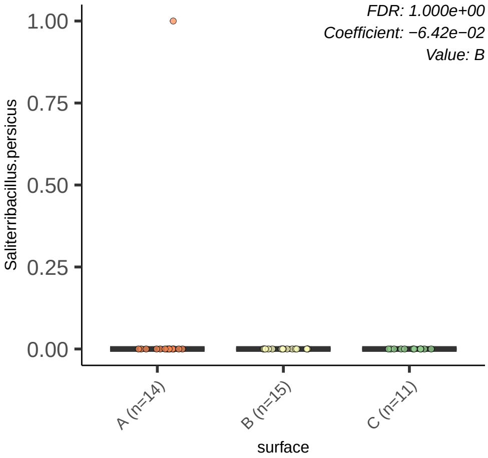

FDR: 1.000e+00
Coefficient: −6.42e−02
Value: B
Saliterribacillus.persicus
1.00
0.75
0.50
0.25
0.00
A (n=14)
B (n=15)
C (n=11)
surface

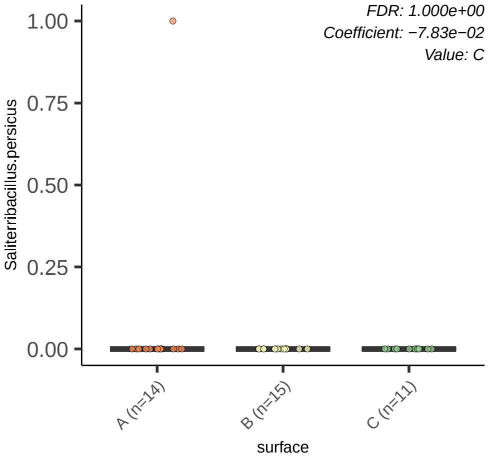

FDR: 1.000e+00
Coefficient: −7.83e−02
Value: C
1.00
0.75
0.50
0.25
0.00
Saliterribacillus.persicus
A (n=14)
B (n=15)
C (n=11)
surface

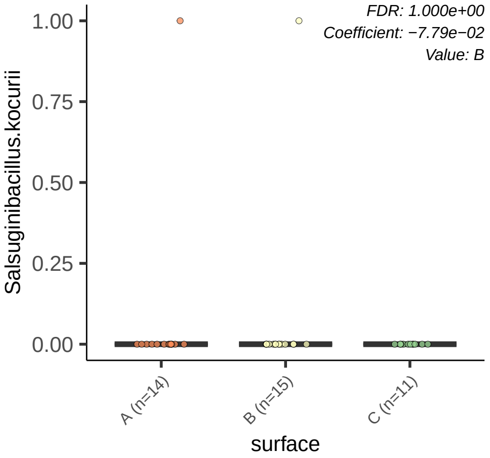

FDR: 1.000e+00
Coefficient: −7.79e−02
Value: B
Salsuginibacillus.kocurii
1.00
0.75
0.50
0.25
0.00
A (n=14)
B (n=15)
C (n=11)
surface

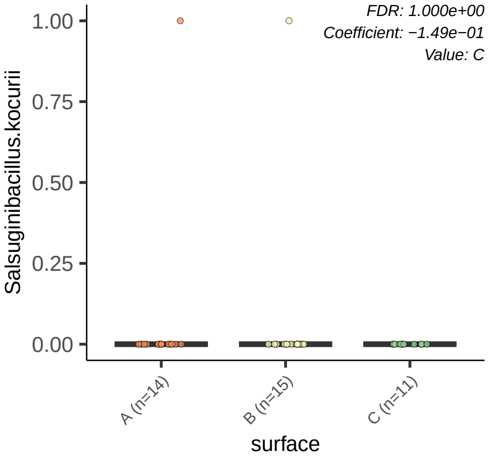

FDR: 1.000e+00
Coefficient: −1.49e−01
Value: C
Salsuginibacillus.kocurii
1.00
0.75
0.50
0.25
0.00
A (n=14)
B (n=15)
C (n=11)
surface

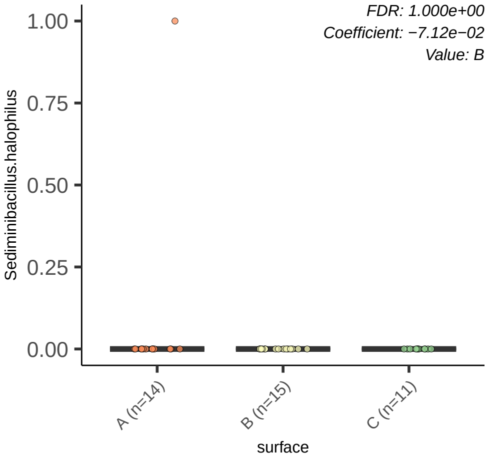
FDR: 1.000e+00
Coefficient: −7.12e−02
Value: B
Sediminibacillus.halophilus
1.00
0.75
0.50
0.25
0.00
A (n=14)
B (n=15)
C (n=11)
surface

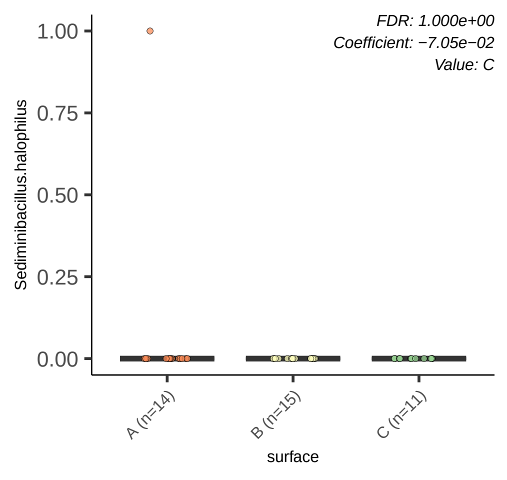
FDR: 1.000e+00
Coefficient: −7.05e−02
Value: C
1.00
0.75
0.50
0.25
0.00
Sediminibacillus.halophilus
A (n=14)
B (n=15)
C (n=11)
surface

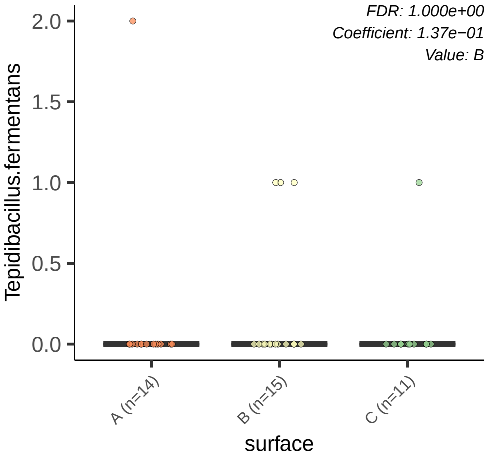
FDR: 1.000e+00
Coefficient: 1.37e−01
Value: B
Tepidibacillus.fermentans
2.0
1.5
1.0
0.5
0.0
A (n=14)
B (n=15)
C (n=11)
surface

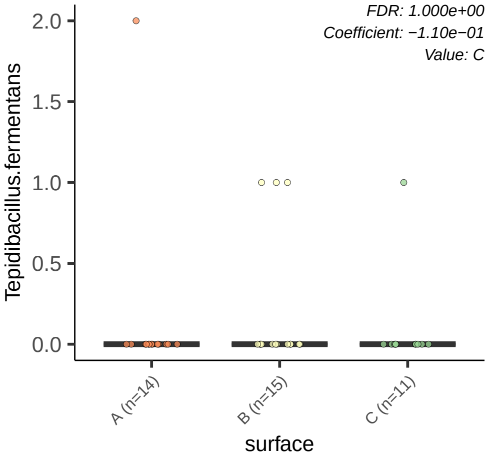

FDR: 1.000e+00
Coefficient: −1.10e−01
Value: C
Tepidibacillus.fermentans
2.0
1.5
1.0
0.5
0.0
A (n=14)
B (n=15)
C (n=11)
surface

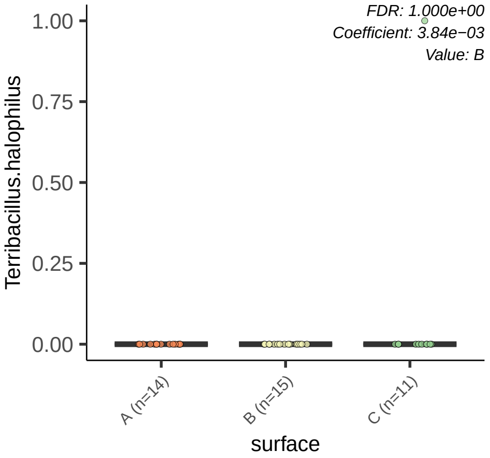
FDR: 1.000e+00
Coefficient: 3.84e−03
Value: B
Terribacillus.halophilus
1.00
0.75
0.50
0.25
0.00
A (n=14)
B (n=15)
C (n=11)
surface

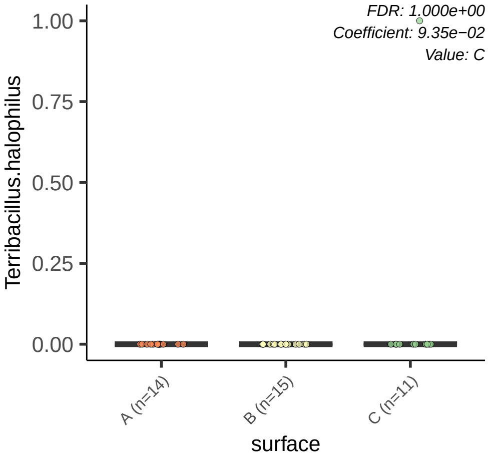

FDR: 1.000e+00
Coefficient: 9.35e−02
Value: C
Terribacillus.halophilus
1.00
0.75
0.50
0.25
0.00
A (n=14)
B (n=15)
C (n=11)
surface

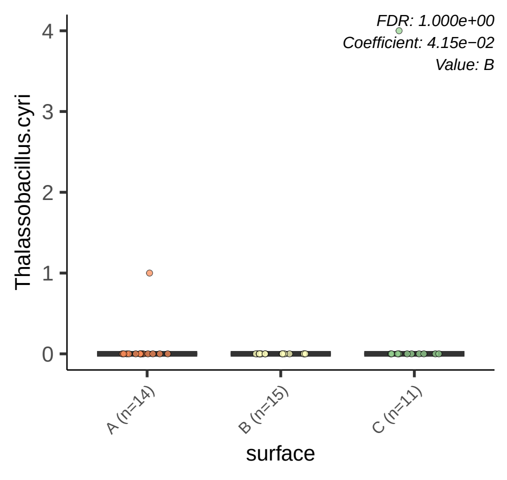

FDR: 1.000e+00
Coefficient: 4.15e−02
Value: B
Thalassobacillus.cyri
0
1
2
3
4
A (n=14)
B (n=15)
C (n=11)
surface

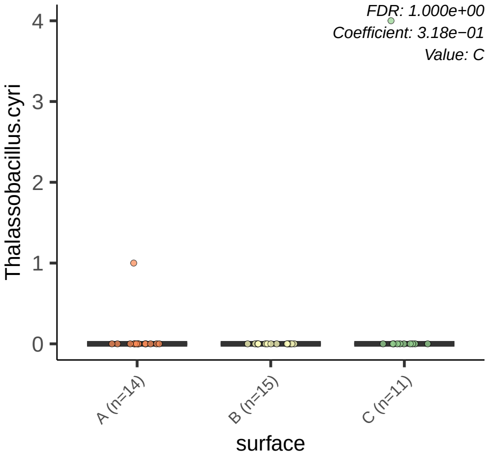
FDR: 1.000e+00
Coefficient: 3.18e−01
Value: C
Thalassobacillus.cyri
0
1
2
3
4
A (n=14)
B (n=15)
C (n=11)
surface

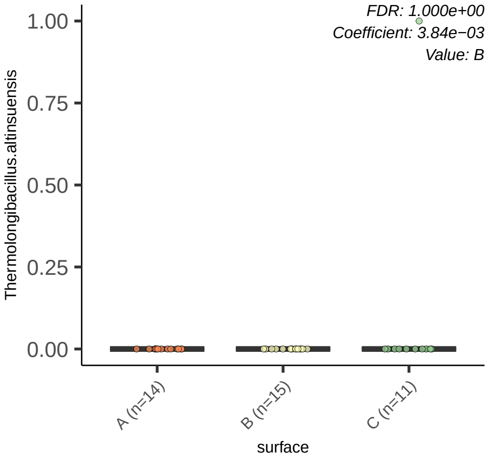

FDR: 1.000e+00
Coefficient: 3.84e−03
Value: B
1.00
0.75
0.50
0.25
0.00
Thermolongibacillus.altinsuensis
A (n=14)
B (n=15)
C (n=11)
surface

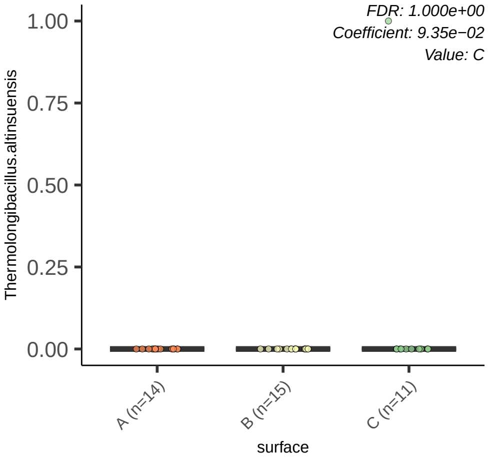
FDR: 1.000e+00
Coefficient: 9.35e−02
Value: C
Thermolongibacillus.altinsuensis
1.00
0.75
0.50
0.25
0.00
A (n=14)
B (n=15)
C (n=11)
surface

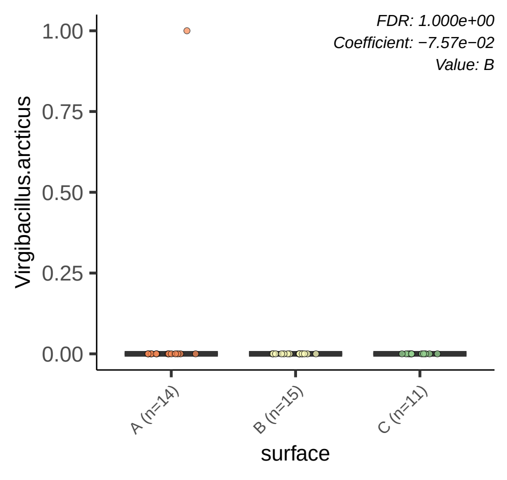

FDR: 1.000e+00
Coefficient: −7.57e−02
Value: B
Virgibacillus.arcticus
1.00
0.75
0.50
0.25
0.00
A (n=14)
B (n=15)
C (n=11)
surface

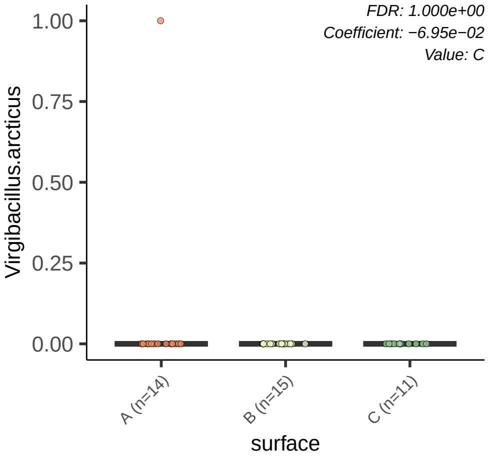
FDR: 1.000e+00
Coefficient: −6.95e−02
Value: C
Virgibacillus.arcticus
1.00
0.75
0.50
0.25
0.00
A (n=14)
B (n=15)
C (n=11)
surface

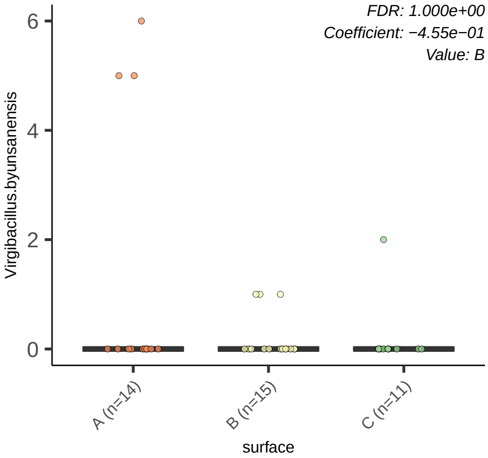

FDR: 1.000e+00
Coefficient: −4.55e−01
Value: B
Virgibacillus.byunsanensis
0
2
4
6
A (n=14)
B (n=15)
C (n=11)
surface

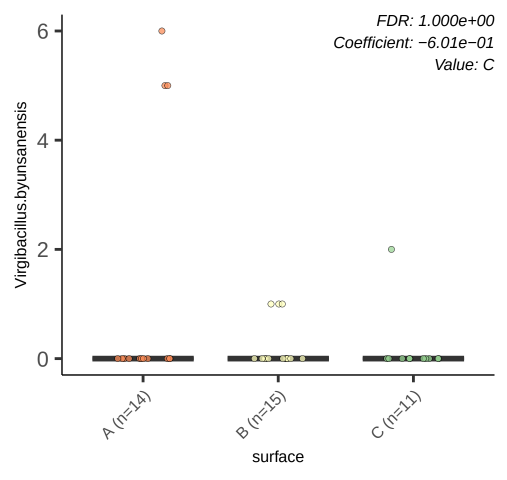

FDR: 1.000e+00
Coefficient: −6.01e−01
Value: C
Virgibacillus.byunsanensis
0
2
4
6
A (n=14)
B (n=15)
C (n=11)
surface

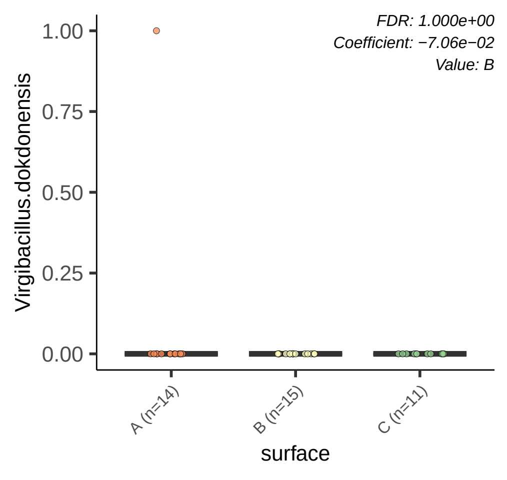

FDR: 1.000e+00
Coefficient: −7.06e−02
Value: B
Virgibacillus.dokdonensis
1.00
0.75
0.50
0.25
0.00
A (n=14)
B (n=15)
C (n=11)
surface

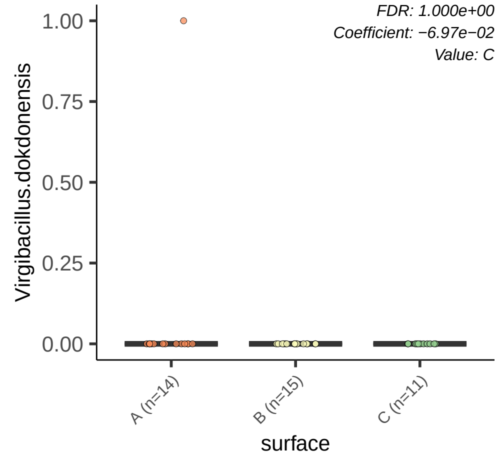

FDR: 1.000e+00
Coefficient: −6.97e−02
Value: C
Virgibacillus.dokdonensis
1.00
0.75
0.50
0.25
0.00
A (n=14)
B (n=15)
C (n=11)
surface

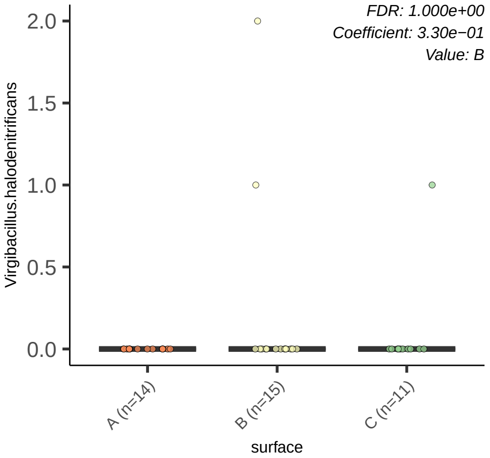

FDR: 1.000e+00
Coefficient: 3.30e−01
Value: B
Virgibacillus.halodenitrificans
2.0
1.5
1.0
0.5
0.0
A (n=14)
B (n=15)
C (n=11)
surface

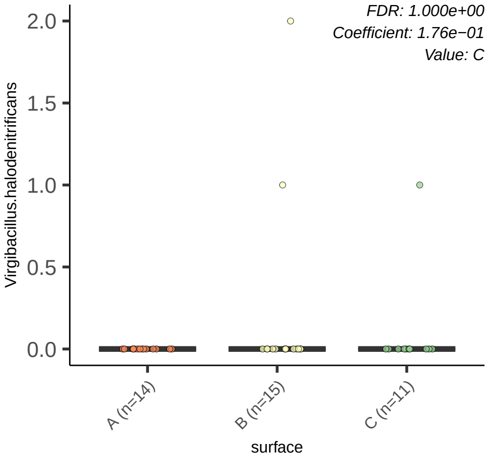
FDR: 1.000e+00
Coefficient: 1.76e−01
Value: C
Virgibacillus.halodenitrificans
2.0
1.5
1.0
0.5
0.0
A (n=14)
B (n=15)
C (n=11)
surface

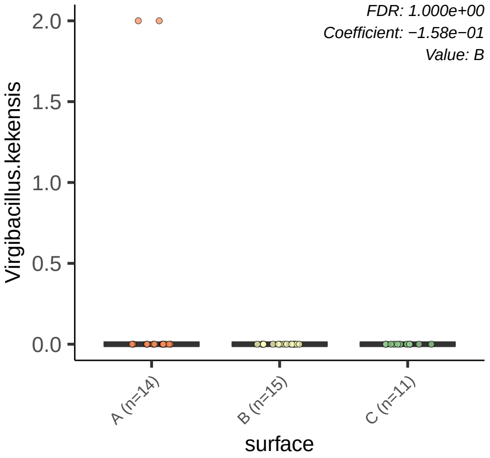
FDR: 1.000e+00
Coefficient: −1.58e−01
Value: B
Virgibacillus.kekensis
2.0
1.5
1.0
0.5
0.0
A (n=14)
B (n=15)
C (n=11)
surface

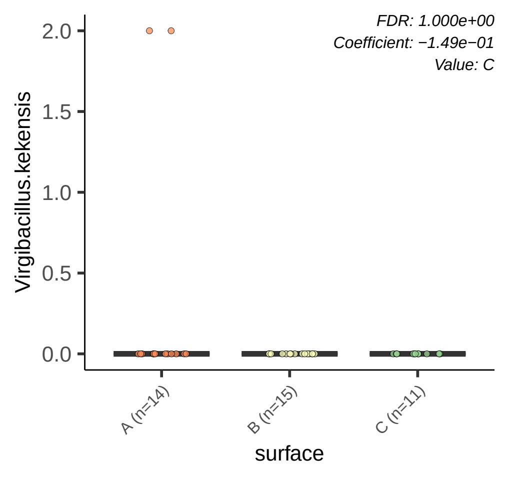
FDR: 1.000e+00
Coefficient: −1.49e−01
Value: C
Virgibacillus.kekensis
2.0
1.5
1.0
0.5
0.0
A (n=14)
B (n=15)
C (n=11)
surface

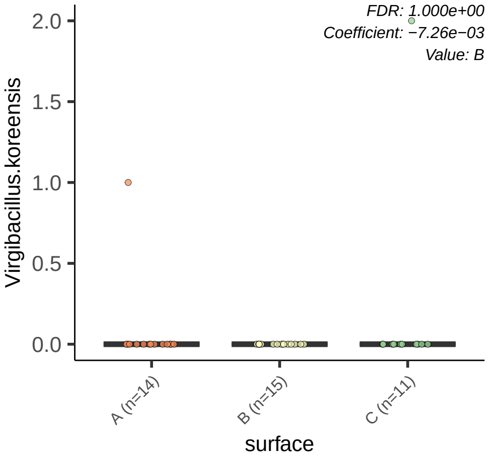

FDR: 1.000e+00
Coefficient: −7.26e−03
Value: B
Virgibacillus.koreensis
2.0
1.5
1.0
0.5
0.0
A (n=14)
B (n=15)
C (n=11)
surface

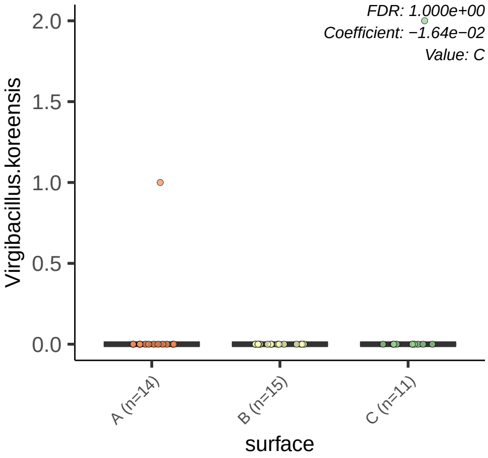

FDR: 1.000e+00
Coefficient: −1.64e−02
Value: C
Virgibacillus.koreensis
2.0
1.5
1.0
0.5
0.0
A (n=14)
B (n=15)
C (n=11)
surface

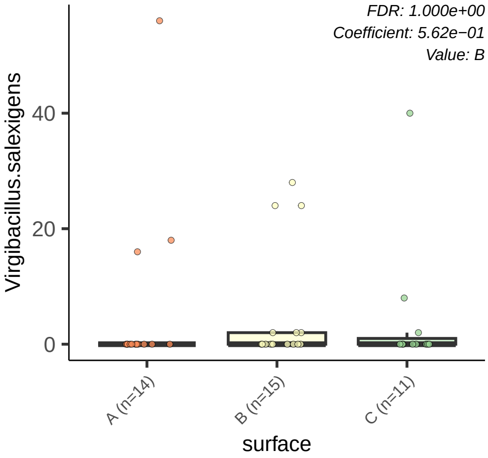
FDR: 1.000e+00
Coefficient: 5.62e−01
Value: B
Virgibacillus.salexigens
0
20
40
A (n=14)
B (n=15)
C (n=11)
surface

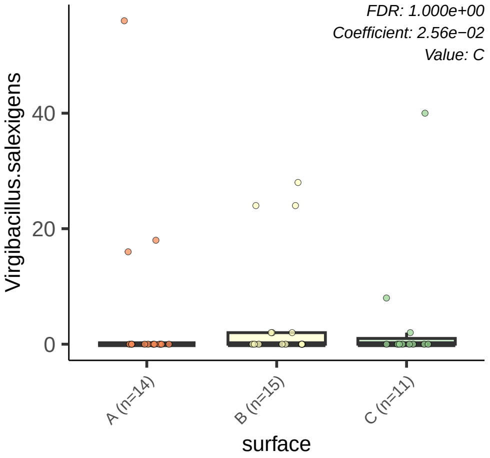

FDR: 1.000e+00
Coefficient: 2.56e−02
Value: C
Virgibacillus.salexigens
0
20
40
A (n=14)
B (n=15)
C (n=11)
surface

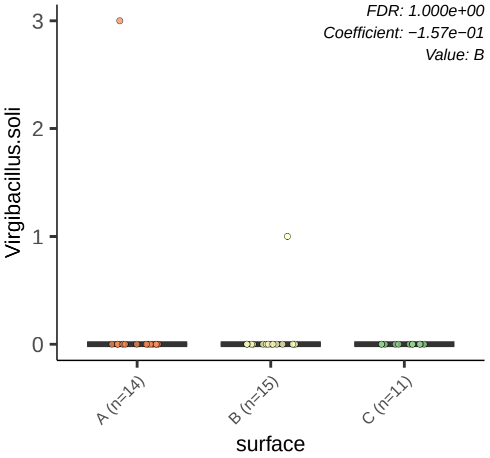
FDR: 1.000e+00
Coefficient: −1.57e−01
Value: B
Virgibacillus.soli
3
2
1
0
A (n=14)
B (n=15)
C (n=11)
surface

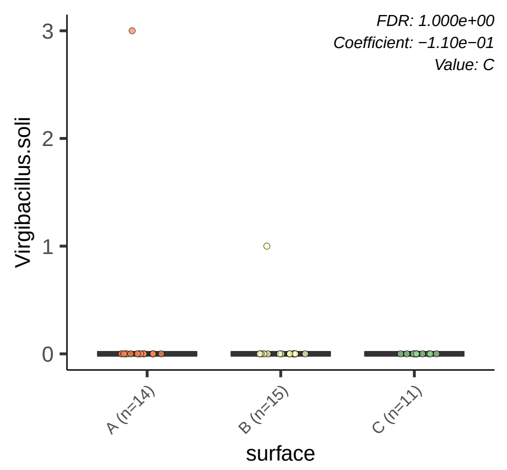
FDR: 1.000e+00
Coefficient: −1.10e−01
Value: C
Virgibacillus.soli
3
2
1
0
A (n=14)
B (n=15)
C (n=11)
surface

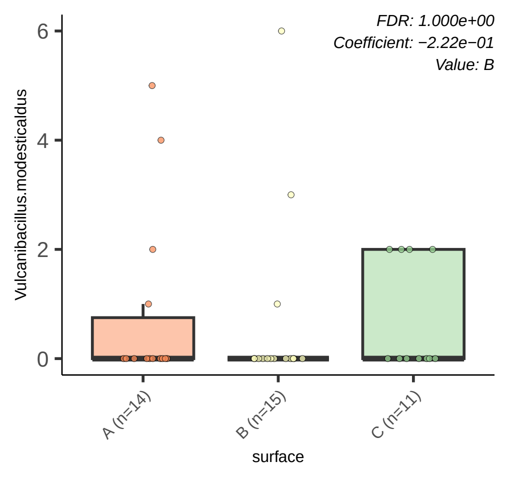
FDR: 1.000e+00
Coefficient: −2.22e−01
Value: B
6
4
2
0
Vulcanibacillus.modesticaldus
A (n=14)
B (n=15)
C (n=11)
surface

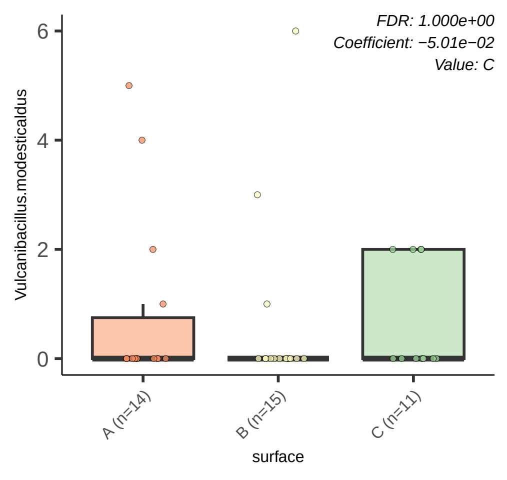

FDR: 1.000e+00
Coefficient: −5.01e−02
Value: C
Vulcanibacillus.modesticaldus
0
2
4
6
A (n=14)
B (n=15)
C (n=11)
surface

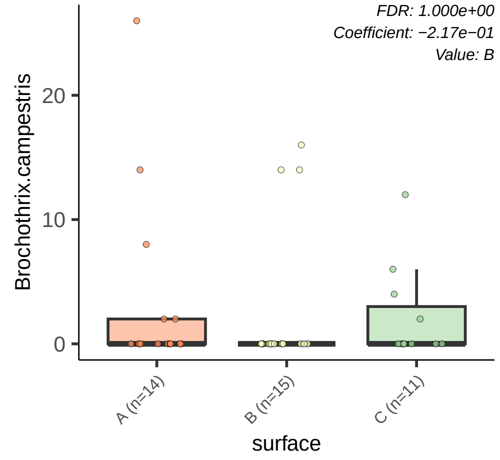

FDR: 1.000e+00
Coefficient: −2.17e−01
Value: B
Brochothrix.campestris
20
10
0
A (n=14)
B (n=15)
C (n=11)
surface

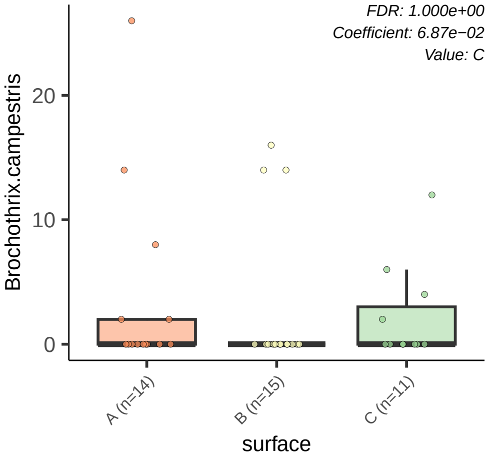
FDR: 1.000e+00
Coefficient: 6.87e−02
Value: C
Brochothrix.campestris
20
10
0
A (n=14)
B (n=15)
C (n=11)
surface

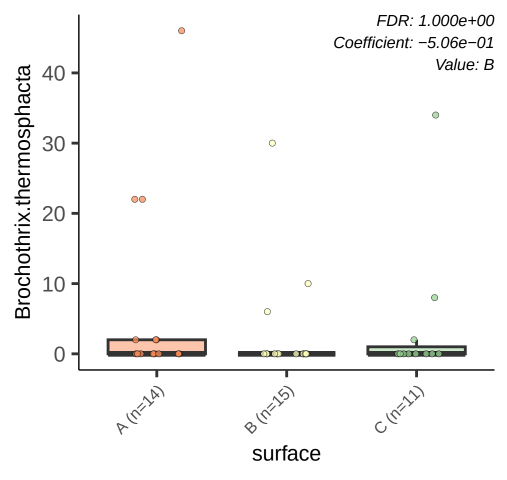

FDR: 1.000e+00
Coefficient: −5.06e−01
Value: B
Brochothrix.thermosphacta
0
10
20
30
40
A (n=14)
B (n=15)
C (n=11)
surface

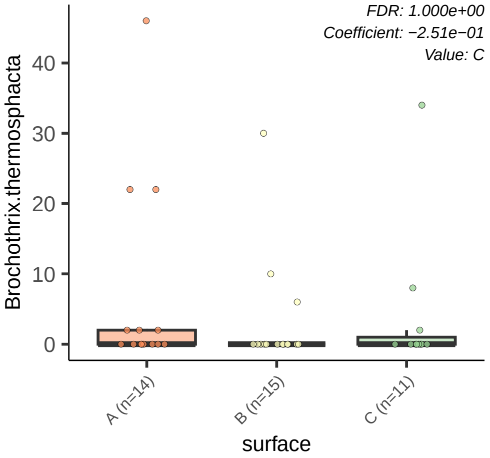

FDR: 1.000e+00
Coefficient: −2.51e−01
Value: C
Brochothrix.thermosphacta
40
30
20
10
0
A (n=14)
B (n=15)
C (n=11)
surface

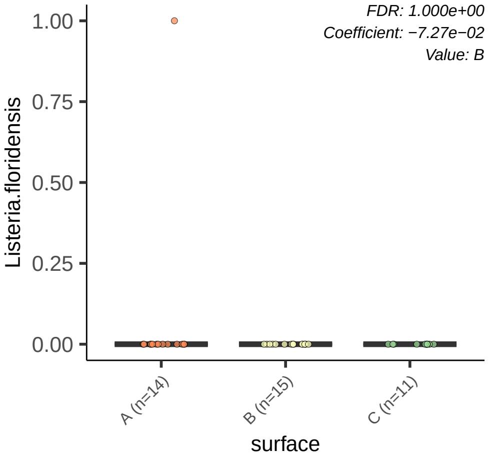

FDR: 1.000e+00
Coefficient: −7.27e−02
Value: B
Listeria.floridensis
1.00
0.75
0.50
0.25
0.00
A (n=14)
B (n=15)
C (n=11)
surface

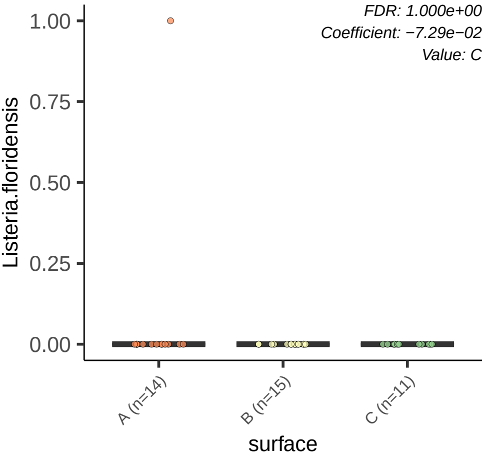
FDR: 1.000e+00
Coefficient: −7.29e−02
Value: C
Listeria.floridensis
1.00
0.75
0.50
0.25
0.00
A (n=14)
B (n=15)
C (n=11)
surface

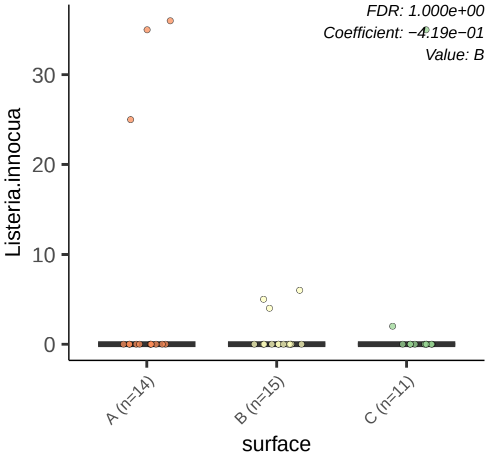
FDR: 1.000e+00
Coefficient: −4.19e−01
Value: B
Listeria.innocua
30
20
10
0
A (n=14)
B (n=15)
C (n=11)
surface

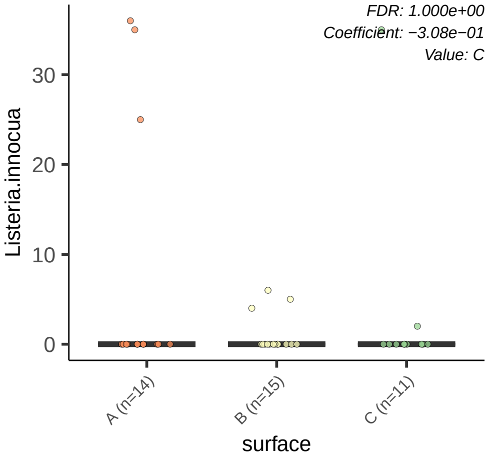

FDR: 1.000e+00
Coefficient: −3.08e−01
Value: C
Listeria.innocua
30
20
10
0
A (n=14)
B (n=15)
C (n=11)
surface

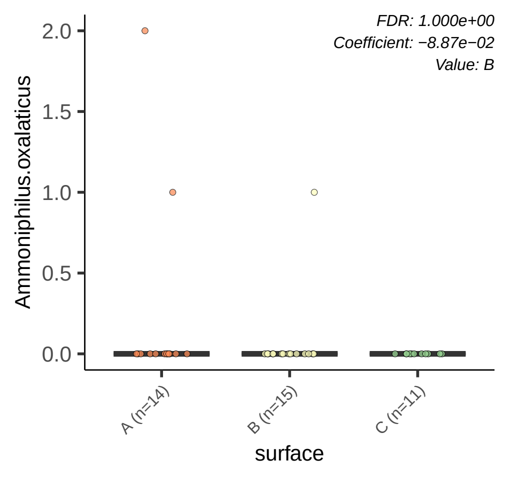
FDR: 1.000e+00
Coefficient: −8.87e−02
Value: B
Ammoniphilus.oxalaticus
2.0
1.5
1.0
0.5
0.0
A (n=14)
B (n=15)
C (n=11)
surface

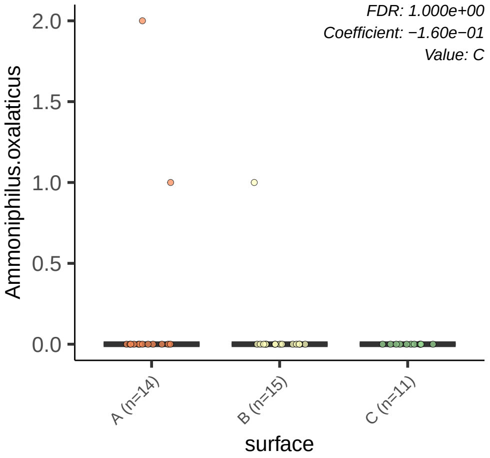
FDR: 1.000e+00
Coefficient: −1.60e−01
Value: C
Ammoniphilus.oxalaticus
2.0
1.5
1.0
0.5
0.0
A (n=14)
B (n=15)
C (n=11)
surface

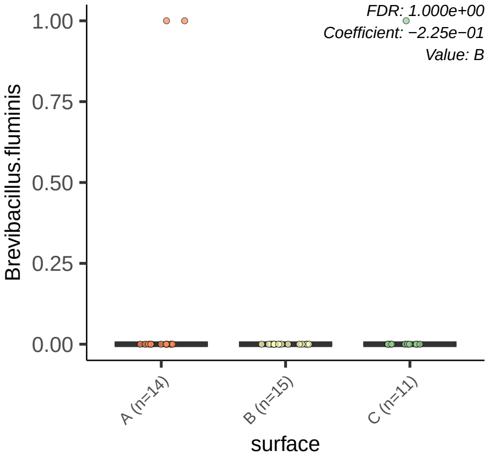

FDR: 1.000e+00
Coefficient: −2.25e−01
Value: B
Brevibacillus.fluminis
1.00
0.75
0.50
0.25
0.00
A (n=14)
B (n=15)
C (n=11)
surface

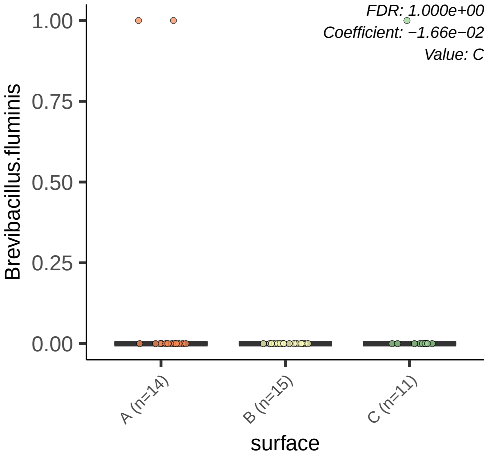

FDR: 1.000e+00
Coefficient: −1.66e−02
Value: C
Brevibacillus.fluminis
1.00
0.75
0.50
0.25
0.00
A (n=14)
B (n=15)
C (n=11)
surface

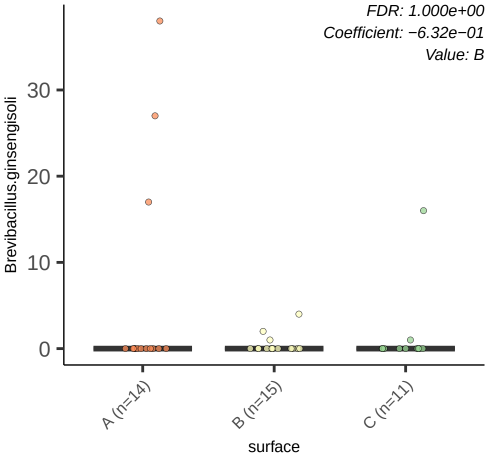

FDR: 1.000e+00
Coefficient: −6.32e−01
Value: B
Brevibacillus.ginsengisoli
30
20
10
0
A (n=14)
B (n=15)
C (n=11)
surface

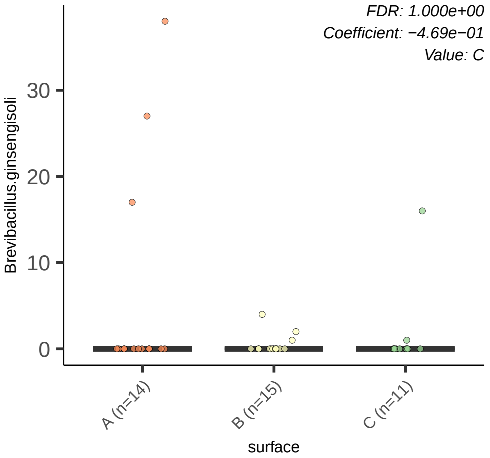
FDR: 1.000e+00
Coefficient: −4.69e−01
Value: C
Brevibacillus.ginsengisoli
30
20
10
0
A (n=14)
B (n=15)
C (n=11)
surface

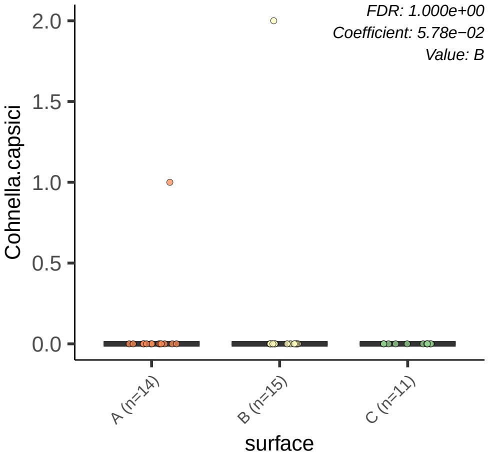
FDR: 1.000e+00
Coefficient: 5.78e−02
Value: B
Cohnella.capsici
2.0
1.5
1.0
0.5
0.0
A (n=14)
B (n=15)
C (n=11)
surface

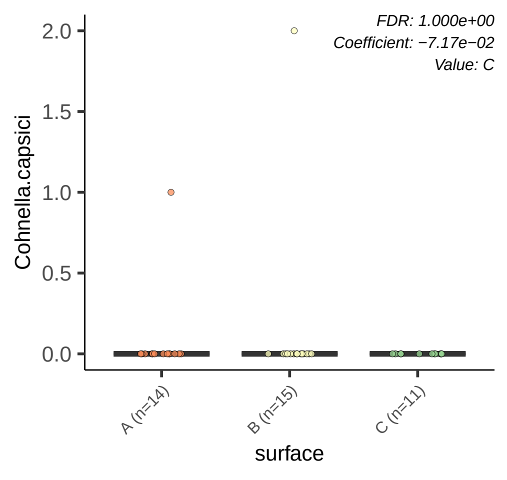

FDR: 1.000e+00
Coefficient: −7.17e−02
Value: C
Cohnella.capsici
2.0
1.5
1.0
0.5
0.0
A (n=14)
B (n=15)
C (n=11)
surface

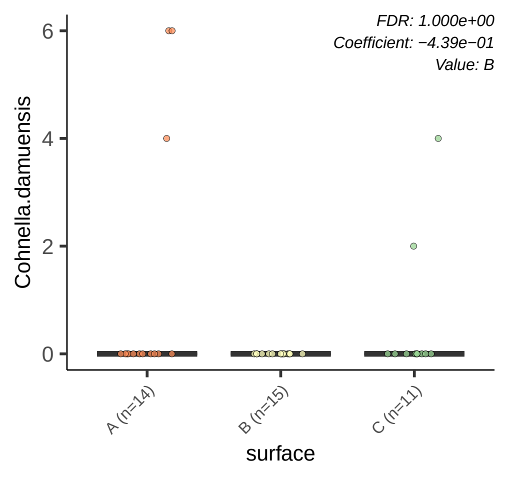
FDR: 1.000e+00
Coefficient: −4.39e−01
Value: B
Cohnella.damuensis
0
2
4
6
A (n=14)
B (n=15)
C (n=11)
surface

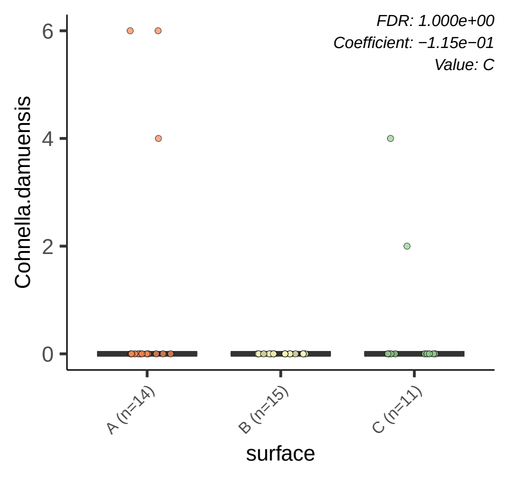
FDR: 1.000e+00
Coefficient: −1.15e−01
Value: C
Cohnella.damuensis
6
4
2
0
A (n=14)
B (n=15)
C (n=11)
surface

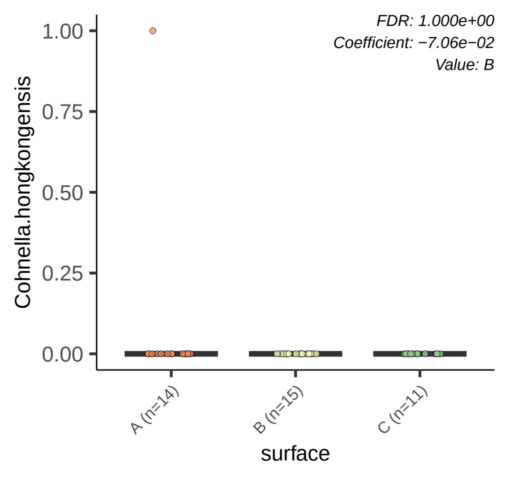
FDR: 1.000e+00
Coefficient: −7.06e−02
Value: B
Cohnella.hongkongensis
1.00
0.75
0.50
0.25
0.00
A (n=14)
B (n=15)
C (n=11)
surface

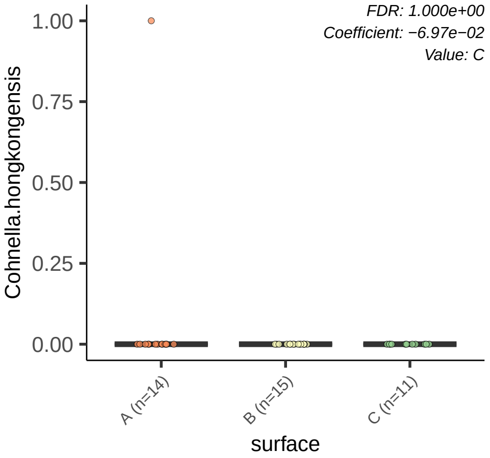

FDR: 1.000e+00
Coefficient: −6.97e−02
Value: C
Cohnella.hongkongensis
1.00
0.75
0.50
0.25
0.00
A (n=14)
B (n=15)
C (n=11)
surface

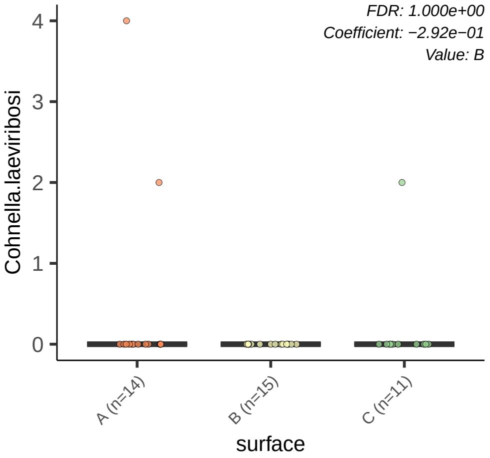

FDR: 1.000e+00
Coefficient: −2.92e−01
Value: B
Cohnella.laeviribosi
0
1
2
3
4
A (n=14)
B (n=15)
C (n=11)
surface

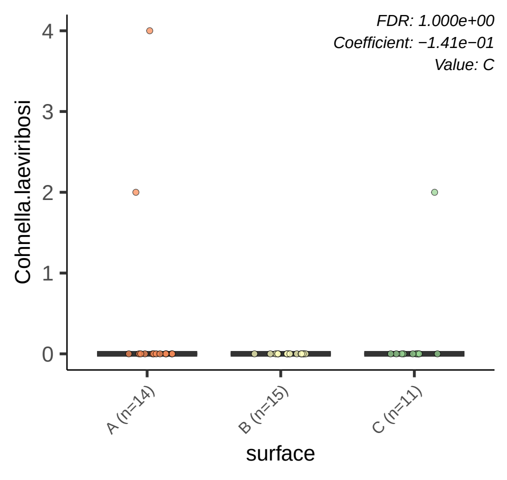
FDR: 1.000e+00
Coefficient: −1.41e−01
Value: C
Cohnella.laeviribosi
0
1
2
3
4
A (n=14)
B (n=15)
C (n=11)
surface

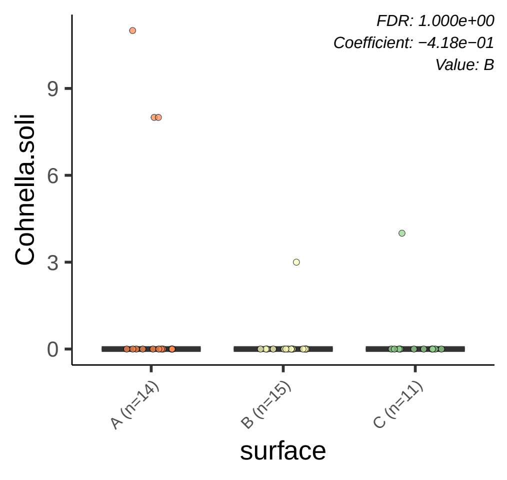
FDR: 1.000e+00
Coefficient: −4.18e−01
Value: B
Cohnella.soli
9
6
3
0
A (n=14)
B (n=15)
C (n=11)
surface

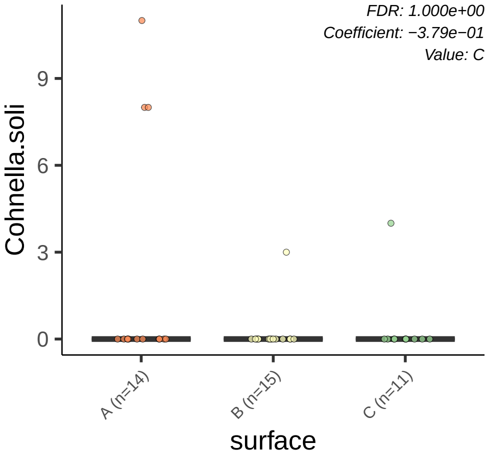
FDR: 1.000e+00
Coefficient: −3.79e−01
Value: C
Cohnella.soli
9
6
3
0
A (n=14)
B (n=15)
C (n=11)
surface

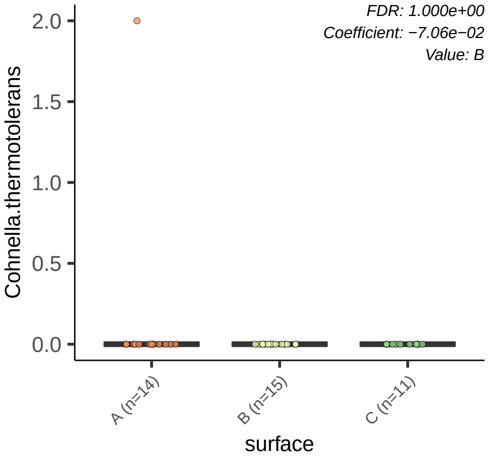
FDR: 1.000e+00
Coefficient: −7.06e−02
Value: B
Cohnella.thermotolerans
2.0
1.5
1.0
0.5
0.0
A (n=14)
B (n=15)
C (n=11)
surface

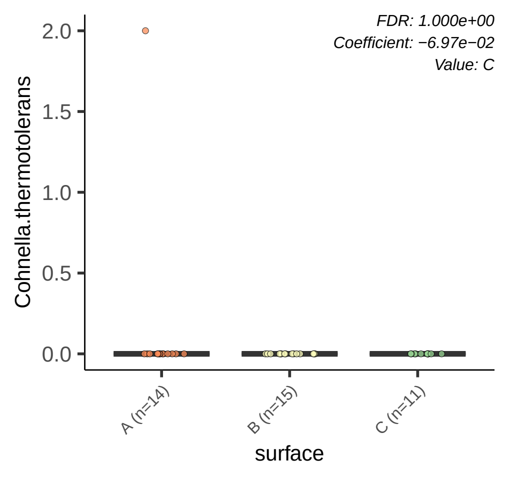
FDR: 1.000e+00
Coefficient: −6.97e−02
Value: C
Cohnella.thermotolerans
2.0
1.5
1.0
0.5
0.0
A (n=14)
B (n=15)
C (n=11)
surface

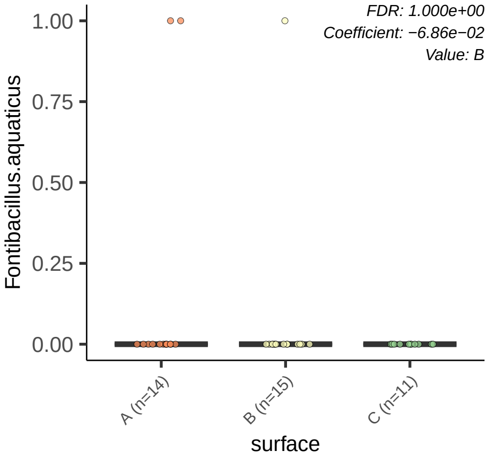
FDR: 1.000e+00
Coefficient: −6.86e−02
Value: B
Fontibacillus.aquaticus
1.00
0.75
0.50
0.25
0.00
A (n=14)
B (n=15)
C (n=11)
surface

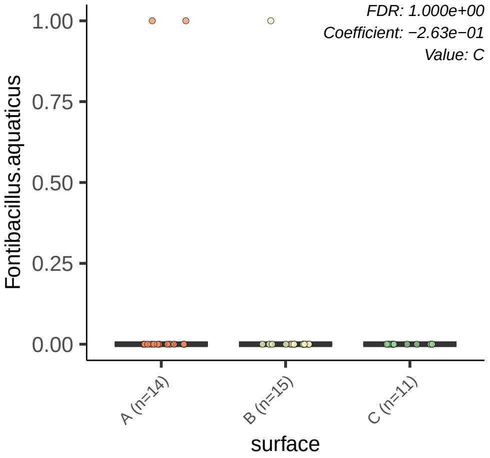

FDR: 1.000e+00
Coefficient: −2.63e−01
Value: C
1.00
0.75
0.50
0.25
0.00
Fontibacillus.aquaticus
A (n=14)
B (n=15)
C (n=11)
surface

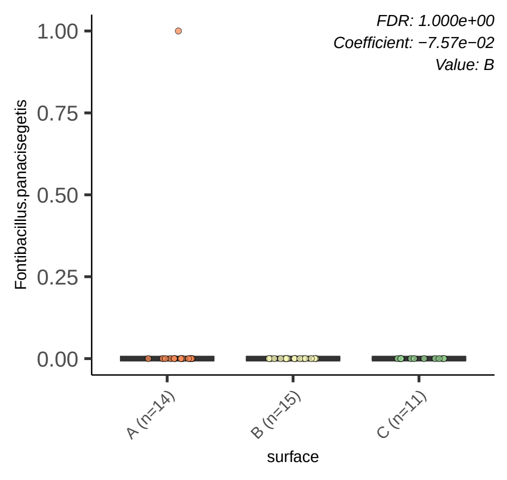
FDR: 1.000e+00
Coefficient: −7.57e−02
Value: B
Fontibacillus.panacisegetis
1.00
0.75
0.50
0.25
0.00
A (n=14)
B (n=15)
C (n=11)
surface

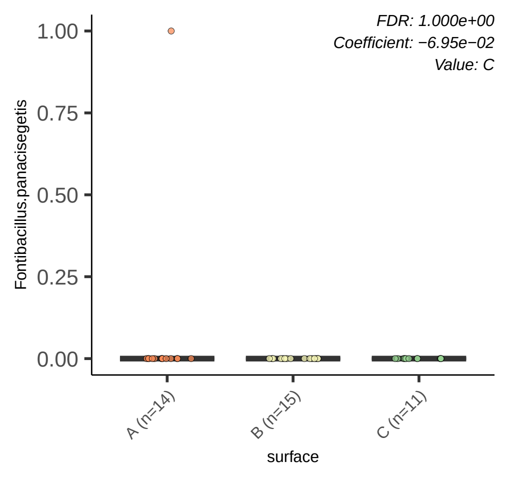

FDR: 1.000e+00
Coefficient: −6.95e−02
Value: C
Fontibacillus.panacisegetis
1.00
0.75
0.50
0.25
0.00
A (n=14)
B (n=15)
C (n=11)
surface

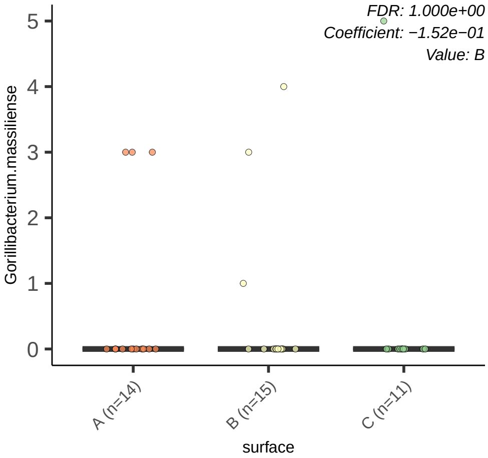
FDR: 1.000e+00
Coefficient: −1.52e−01
Value: B
Gorillibacterium.massiliense
0
1
2
3
4
5
A (n=14)
B (n=15)
C (n=11)
surface

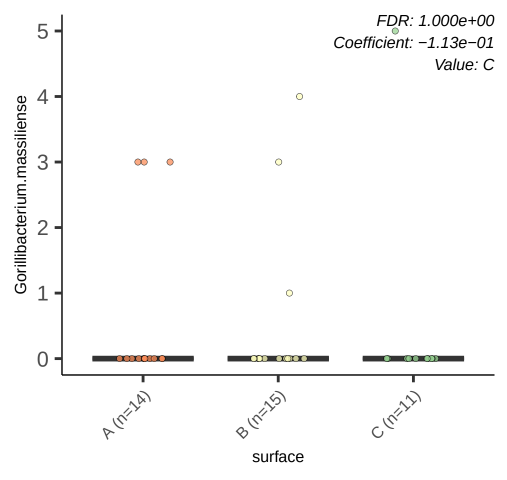
FDR: 1.000e+00
Coefficient: −1.13e−01
Value: C
Gorillibacterium.massiliense
0
1
2
3
4
5
A (n=14)
B (n=15)
C (n=11)
surface

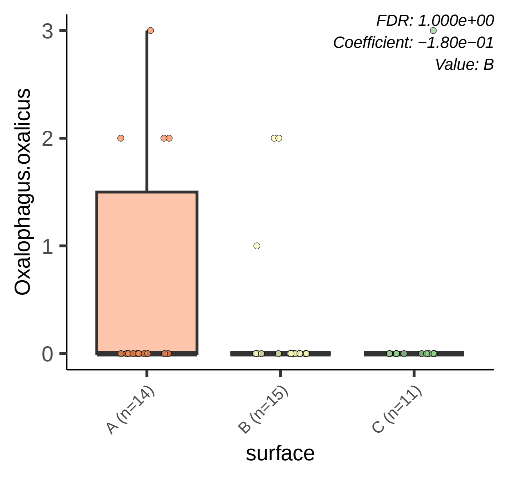
FDR: 1.000e+00
Coefficient: −1.80e−01
Value: B
Oxalophagus.oxalicus
3
2
1
0
A (n=14)
B (n=15)
C (n=11)
surface

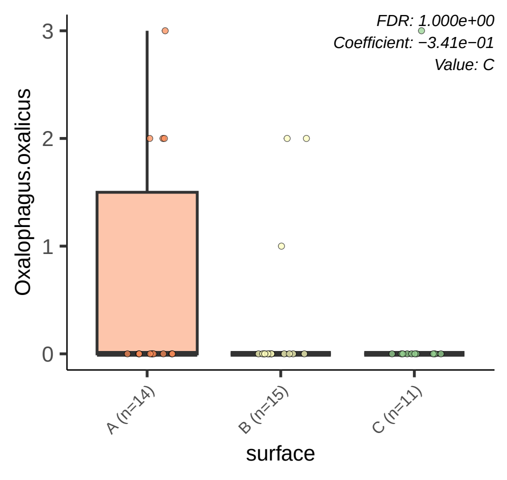

FDR: 1.000e+00
Coefficient: −3.41e−01
Value: C
Oxalophagus.oxalicus
3
2
1
0
A (n=14)
B (n=15)
C (n=11)
surface

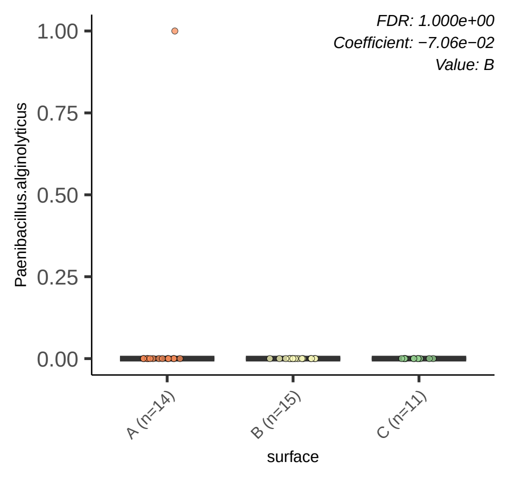

FDR: 1.000e+00
Coefficient: −7.06e−02
Value: B
Paenibacillus.alginolyticus
1.00
0.75
0.50
0.25
0.00
A (n=14)
B (n=15)
C (n=11)
surface

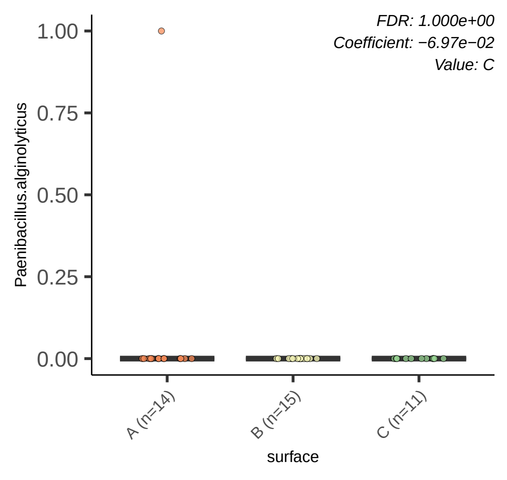
FDR: 1.000e+00
Coefficient: −6.97e−02
Value: C
Paenibacillus.alginolyticus
1.00
0.75
0.50
0.25
0.00
A (n=14)
B (n=15)
C (n=11)
surface

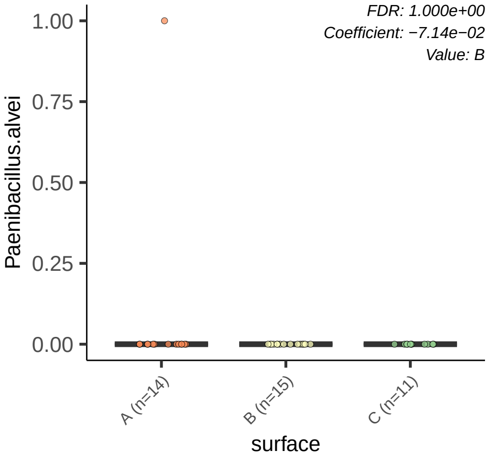
FDR: 1.000e+00
Coefficient: −7.14e−02
Value: B
Paenibacillus.alvei
1.00
0.75
0.50
0.25
0.00
A (n=14)
B (n=15)
C (n=11)
surface

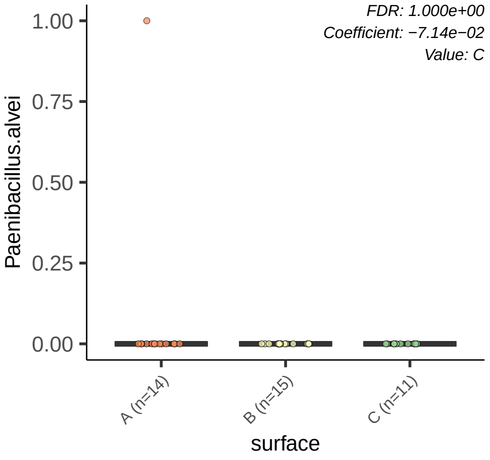

FDR: 1.000e+00
Coefficient: −7.14e−02
Value: C
Paenibacillus.alvei
1.00
0.75
0.50
0.25
0.00
A (n=14)
B (n=15)
C (n=11)
surface

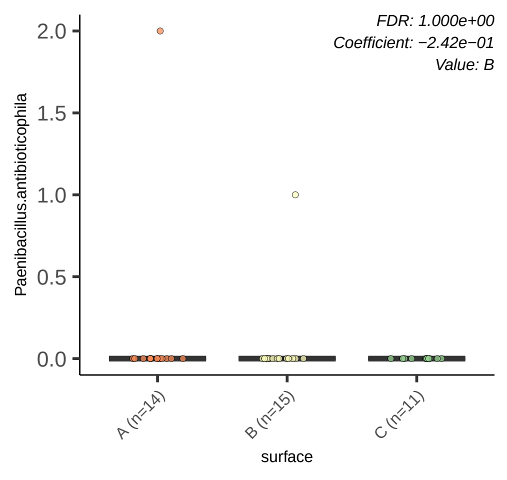

FDR: 1.000e+00
Coefficient: −2.42e−01
Value: B
Paenibacillus.antibioticophila
2.0
1.5
1.0
0.5
0.0
A (n=14)
B (n=15)
C (n=11)
surface

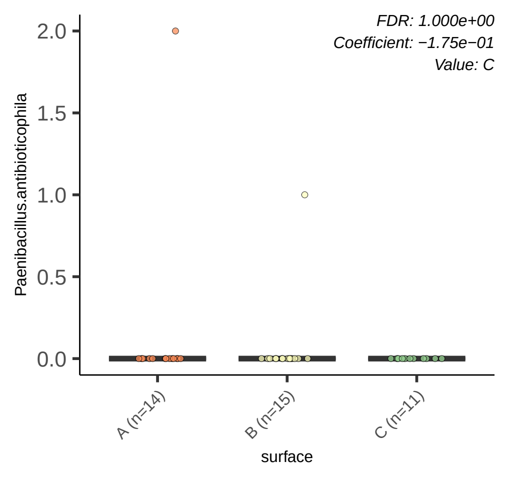
FDR: 1.000e+00
Coefficient: −1.75e−01
Value: C
Paenibacillus.antibioticophila
2.0
1.5
1.0
0.5
0.0
A (n=14)
B (n=15)
C (n=11)
surface

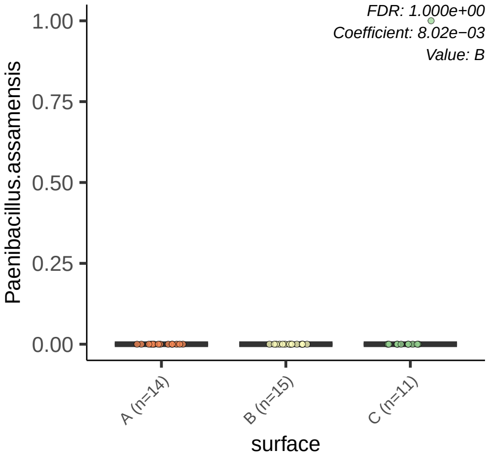

FDR: 1.000e+00
Coefficient: 8.02e−03
Value: B
Paenibacillus.assamensis
1.00
0.75
0.50
0.25
0.00
A (n=14)
B (n=15)
C (n=11)
surface

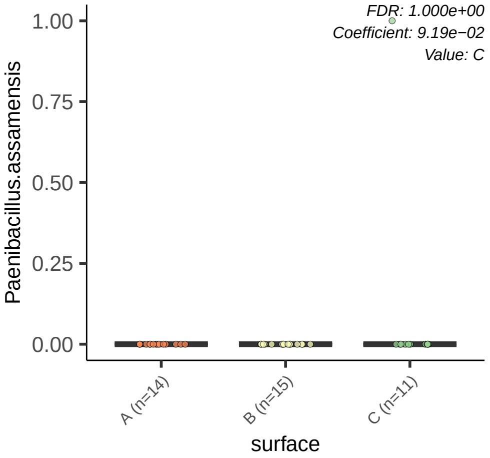

FDR: 1.000e+00
Coefficient: 9.19e−02
Value: C
Paenibacillus.assamensis
1.00
0.75
0.50
0.25
0.00
A (n=14)
B (n=15)
C (n=11)
surface

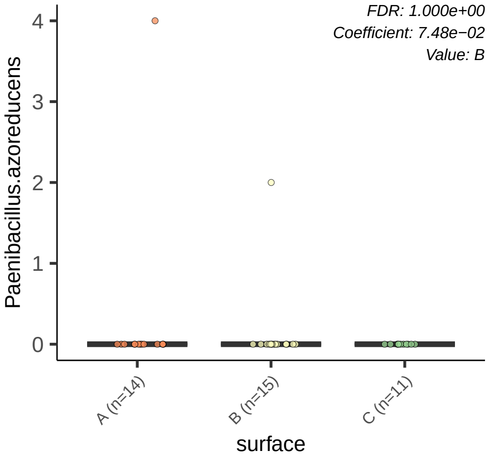

FDR: 1.000e+00
Coefficient: 7.48e−02
Value: B
Paenibacillus.azoreducens
4
3
2
1
0
A (n=14)
B (n=15)
C (n=11)
surface

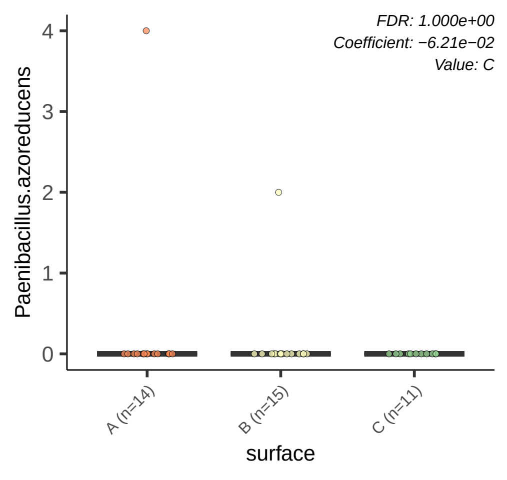
FDR: 1.000e+00
Coefficient: −6.21e−02
Value: C
Paenibacillus.azoreducens
4
3
2
1
0
A (n=14)
B (n=15)
C (n=11)
surface

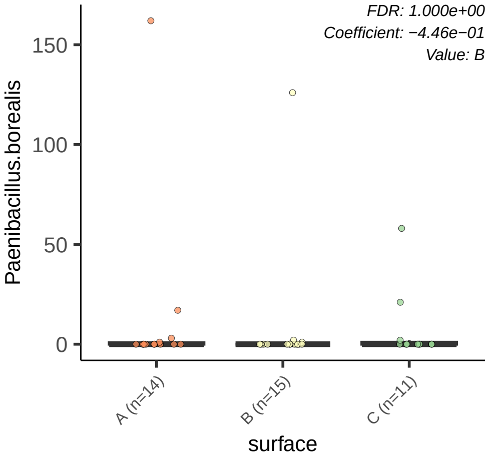

FDR: 1.000e+00
Coefficient: −4.46e−01
Value: B
Paenibacillus.borealis
150
100
50
0
A (n=14)
B (n=15)
C (n=11)
surface

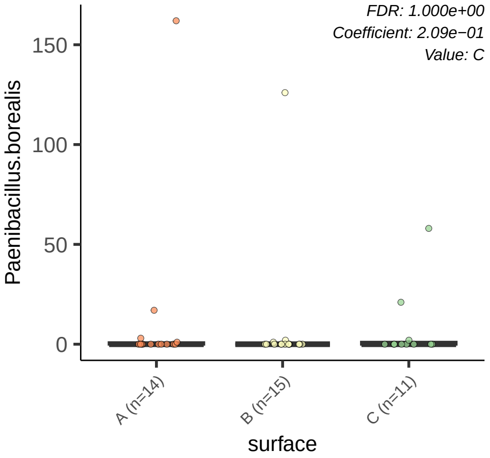
FDR: 1.000e+00
Coefficient: 2.09e−01
Value: C
Paenibacillus.borealis
150
100
50
0
A (n=14)
B (n=15)
C (n=11)
surface

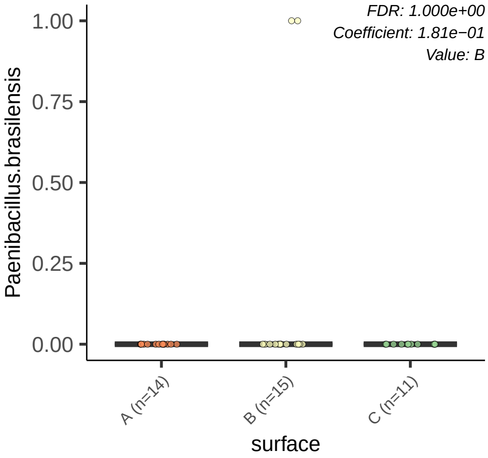

FDR: 1.000e+00
Coefficient: 1.81e−01
Value: B
1.00
0.75
0.50
0.25
0.00
Paenibacillus.brasilensis
A (n=14)
B (n=15)
C (n=11)
surface

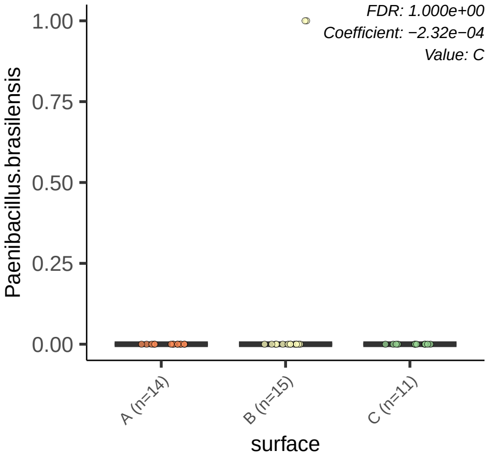
FDR: 1.000e+00
Coefficient: −2.32e−04
Value: C
Paenibacillus.brasilensis
1.00
0.75
0.50
0.25
0.00
A (n=14)
B (n=15)
C (n=11)
surface

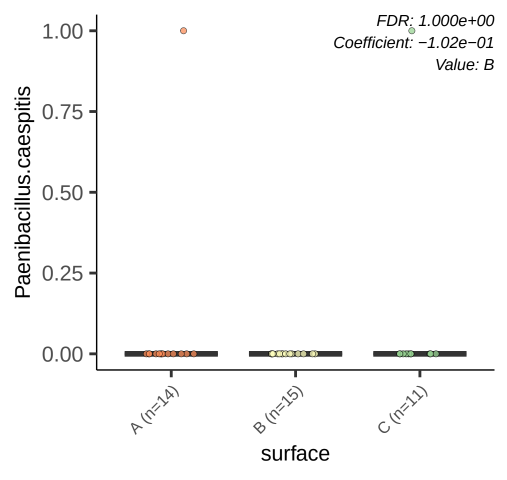

FDR: 1.000e+00
Coefficient: −1.02e−01
Value: B
Paenibacillus.caespitis
1.00
0.75
0.50
0.25
0.00
A (n=14)
B (n=15)
C (n=11)
surface

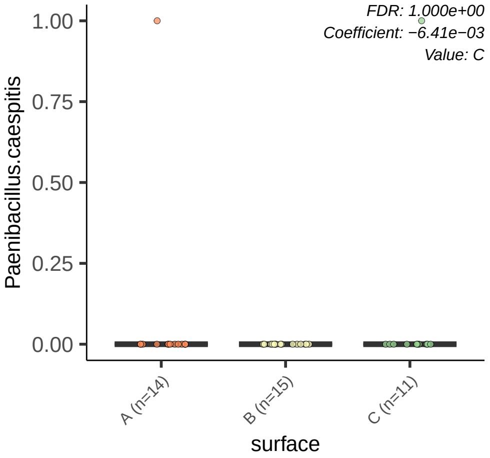

FDR: 1.000e+00
Coefficient: −6.41e−03
Value: C
Paenibacillus.caespitis
1.00
0.75
0.50
0.25
0.00
A (n=14)
B (n=15)
C (n=11)
surface

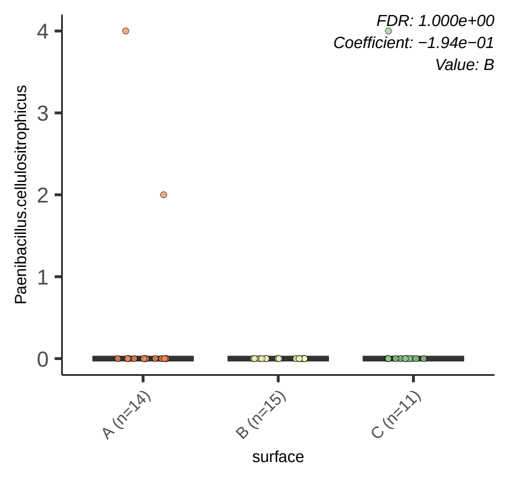

FDR: 1.000e+00
Coefficient: −1.94e−01
Value: B
Paenibacillus.cellulositrophicus
4
3
2
1
0
A (n=14)
B (n=15)
C (n=11)
surface

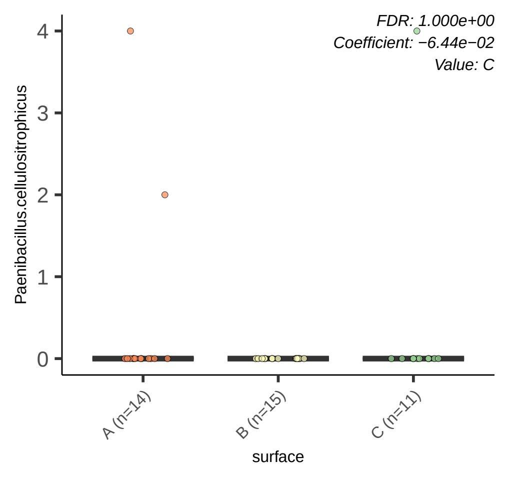

FDR: 1.000e+00
Coefficient: −6.44e−02
Value: C
Paenibacillus.cellulositrophicus
4
3
2
1
0
A (n=14)
B (n=15)
C (n=11)
surface

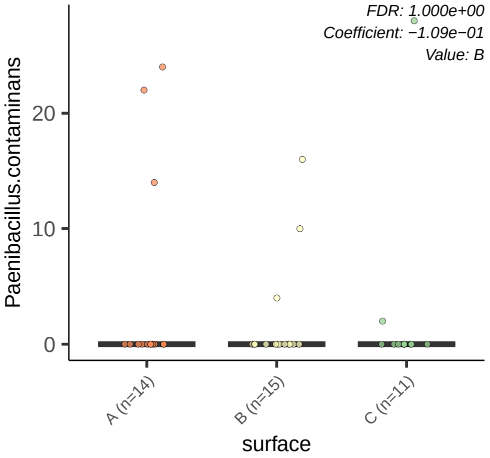

FDR: 1.000e+00
Coefficient: −1.09e−01
Value: B
Paenibacillus.contaminans
20
10
0
A (n=14)
B (n=15)
C (n=11)
surface

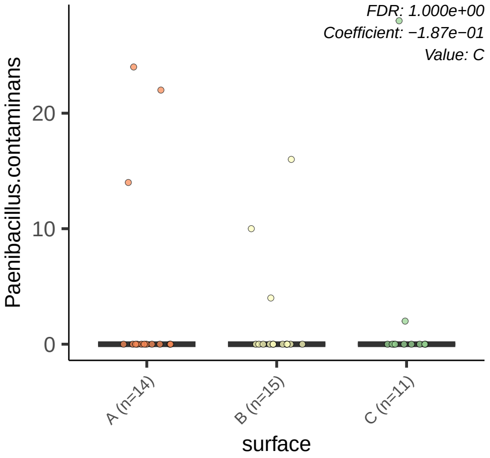

FDR: 1.000e+00
Coefficient: −1.87e−01
Value: C
Paenibacillus.contaminans
0
10
20
A (n=14)
B (n=15)
C (n=11)
surface

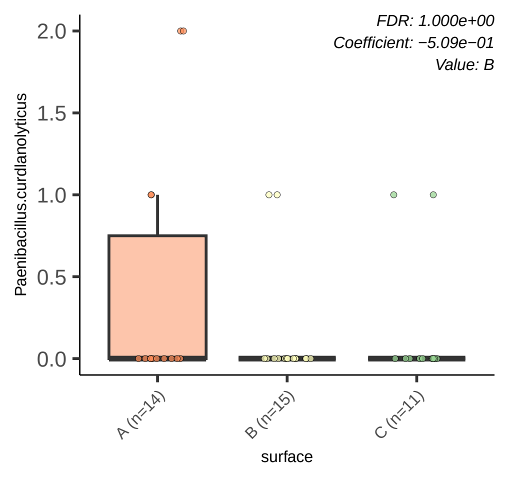

FDR: 1.000e+00
Coefficient: −5.09e−01
Value: B
Paenibacillus.curdlanolyticus
2.0
1.5
1.0
0.5
0.0
A (n=14)
B (n=15)
C (n=11)
surface

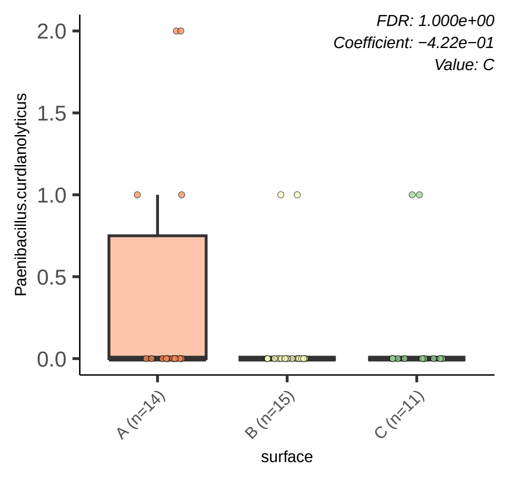

FDR: 1.000e+00
Coefficient: −4.22e−01
Value: C
Paenibacillus.curdlanolyticus
2.0
1.5
1.0
0.5
0.0
A (n=14)
B (n=15)
C (n=11)
surface

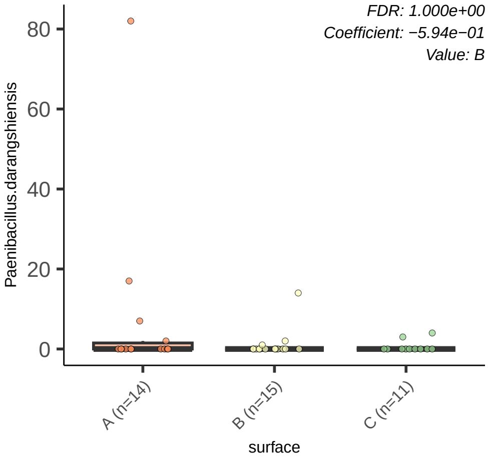
FDR: 1.000e+00
Coefficient: −5.94e−01
Value: B
80
60
40
20
0
Paenibacillus.darangshiensis
A (n=14)
B (n=15)
C (n=11)
surface

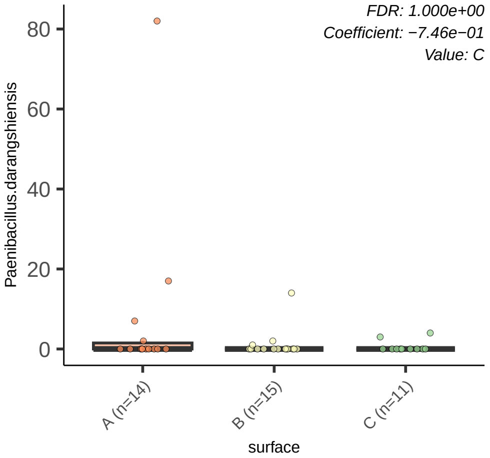

FDR: 1.000e+00
Coefficient: −7.46e−01
Value: C
Paenibacillus.darangshiensis
0
20
40
60
80
A (n=14)
B (n=15)
C (n=11)
surface

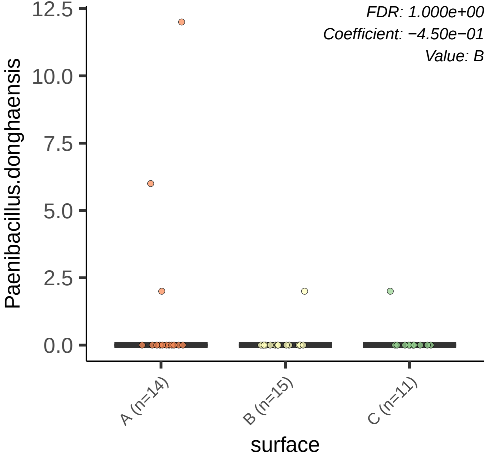
FDR: 1.000e+00
Coefficient: −4.50e−01
Value: B
Paenibacillus.donghaensis
12.5
10.0
7.5
5.0
2.5
0.0
A (n=14)
B (n=15)
C (n=11)
surface

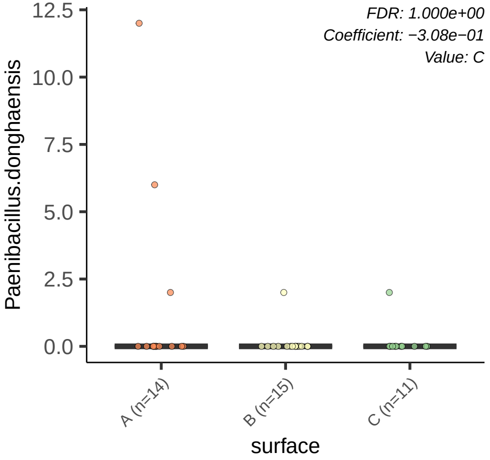

FDR: 1.000e+00
Coefficient: −3.08e−01
Value: C
Paenibacillus.donghaensis
12.5
10.0
7.5
5.0
2.5
0.0
A (n=14)
B (n=15)
C (n=11)
surface

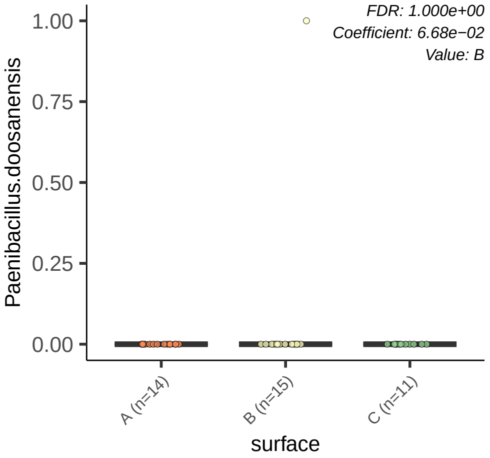

FDR: 1.000e+00
Coefficient: 6.68e−02
Value: B
Paenibacillus.doosanensis
1.00
0.75
0.50
0.25
0.00
A (n=14)
B (n=15)
C (n=11)
surface

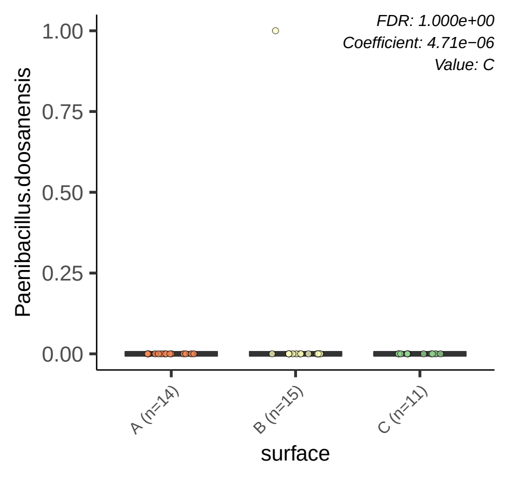
FDR: 1.000e+00
Coefficient: 4.71e−06
Value: C
Paenibacillus.doosanensis
1.00
0.75
0.50
0.25
0.00
A (n=14)
B (n=15)
C (n=11)
surface

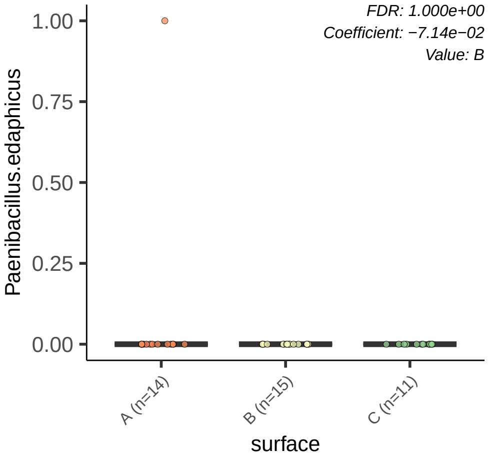
FDR: 1.000e+00
Coefficient: −7.14e−02
Value: B
Paenibacillus.edaphicus
1.00
0.75
0.50
0.25
0.00
A (n=14)
B (n=15)
C (n=11)
surface

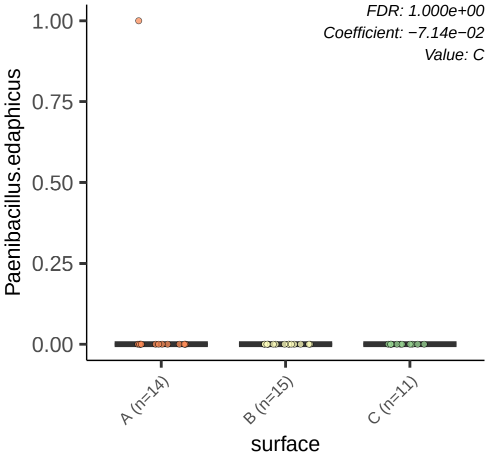

FDR: 1.000e+00
Coefficient: −7.14e−02
Value: C
Paenibacillus.edaphicus
1.00
0.75
0.50
0.25
0.00
A (n=14)
B (n=15)
C (n=11)
surface

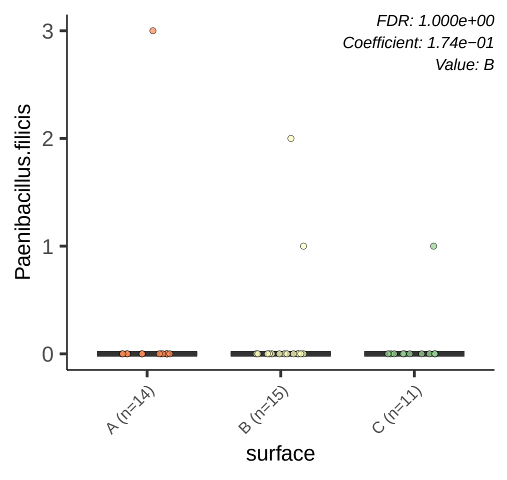
FDR: 1.000e+00
Coefficient: 1.74e−01
Value: B
Paenibacillus.filicis
3
2
1
0
A (n=14)
B (n=15)
C (n=11)
surface

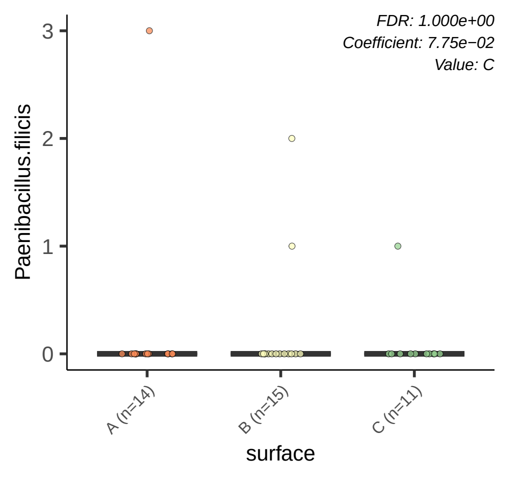

FDR: 1.000e+00
Coefficient: 7.75e−02
Value: C
Paenibacillus.filicis
0
1
2
3
A (n=14)
B (n=15)
C (n=11)
surface

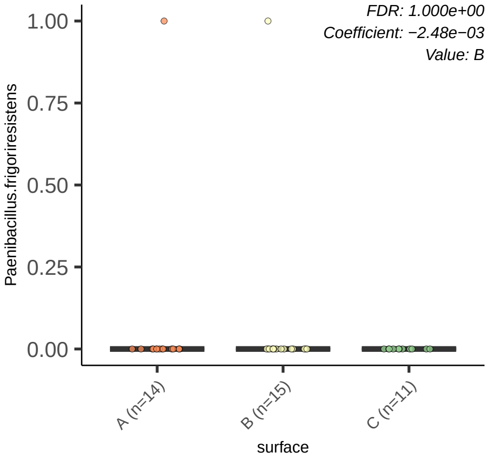

FDR: 1.000e+00
Coefficient: −2.48e−03
Value: B
1.00
0.75
0.50
0.25
0.00
Paenibacillus.frigoriresistens
A (n=14)
B (n=15)
C (n=11)
surface

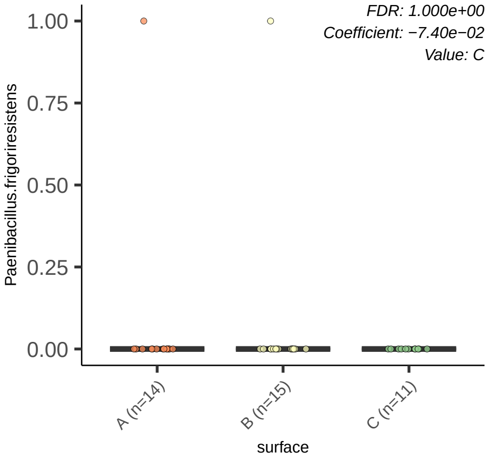
FDR: 1.000e+00
Coefficient: −7.40e−02
Value: C
1.00
0.75
0.50
0.25
0.00
Paenibacillus.frigoriresistens
A (n=14)
B (n=15)
C (n=11)
surface

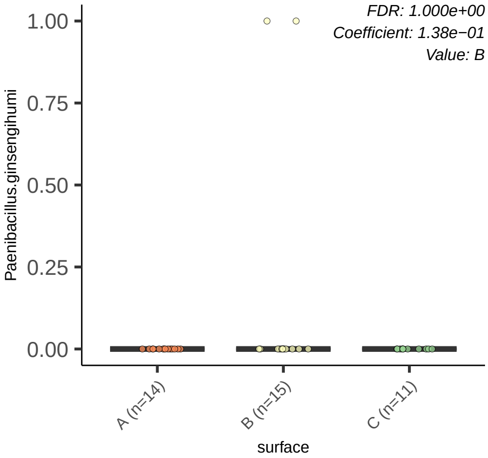
FDR: 1.000e+00
Coefficient: 1.38e−01
Value: B
Paenibacillus.ginsengihumi
1.00
0.75
0.50
0.25
0.00
A (n=14)
B (n=15)
C (n=11)
surface

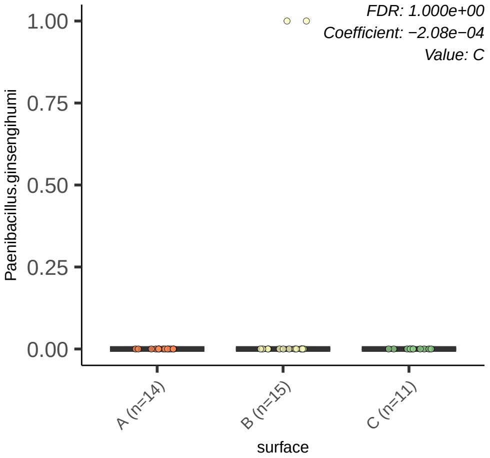
FDR: 1.000e+00
Coefficient: −2.08e−04
Value: C
1.00
0.75
0.50
0.25
0.00
Paenibacillus.ginsenghumi
A (n=14)
B (n=15)
C (n=11)
surface

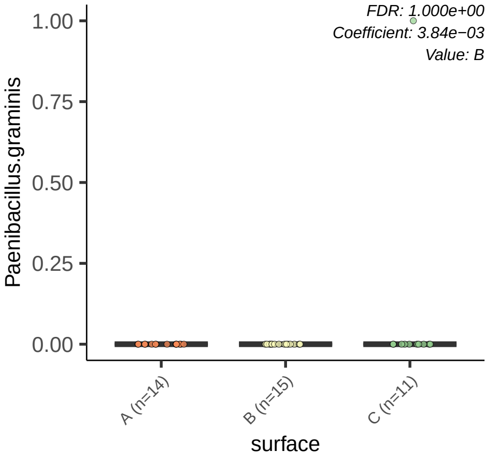
FDR: 1.000e+00
Coefficient: 3.84e−03
Value: B
Paenibacillus.graminis
1.00
0.75
0.50
0.25
0.00
A (n=14)
B (n=15)
C (n=11)
surface

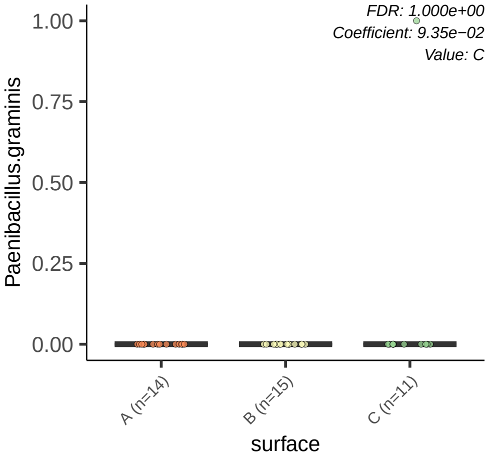

FDR: 1.000e+00
Coefficient: 9.35e−02
Value: C
Paenibacillus.graminis
1.00
0.75
0.50
0.25
0.00
A (n=14)
B (n=15)
C (n=11)
surface

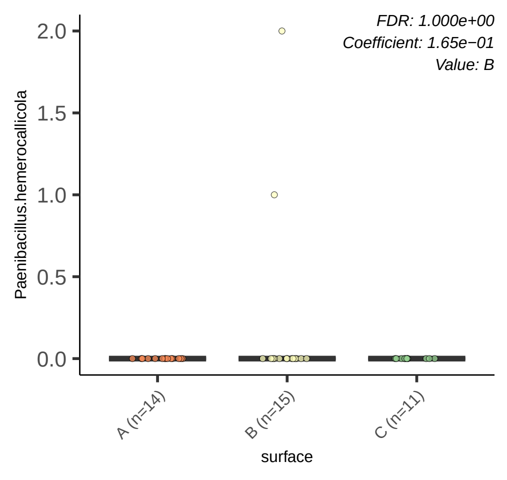
FDR: 1.000e+00
Coefficient: 1.65e−01
Value: B
2.0
1.5
1.0
0.5
0.0
Paenibacillus.hemerocallicola
A (n=14)
B (n=15)
C (n=11)
surface

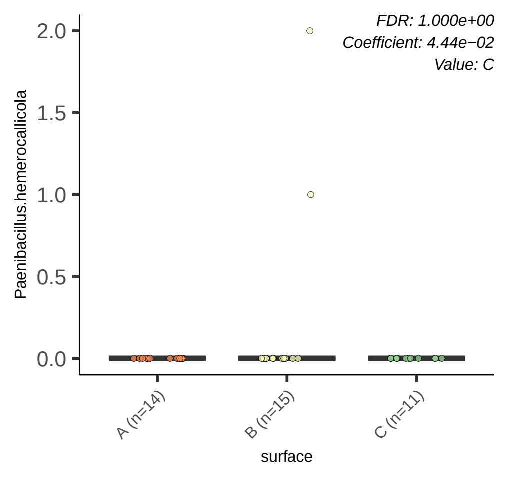

FDR: 1.000e+00
Coefficient: 4.44e−02
Value: C
Paenibacillus.hemerocallicola
2.0
1.5
1.0
0.5
0.0
A (n=14)
B (n=15)
C (n=11)
surface

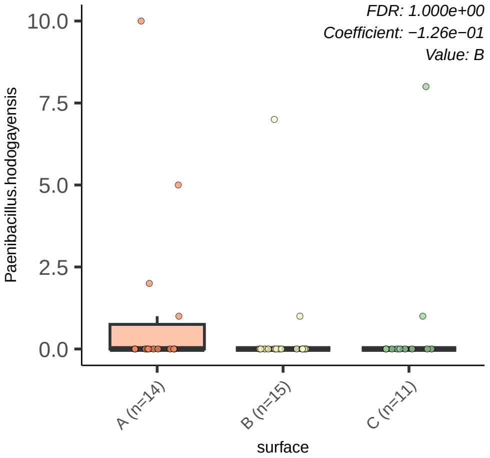

FDR: 1.000e+00
Coefficient: −1.26e−01
Value: B
10.0
7.5
5.0
2.5
0.0
Paenibacillus.hodogayensis
A (n=14)
B (n=15)
C (n=11)
surface

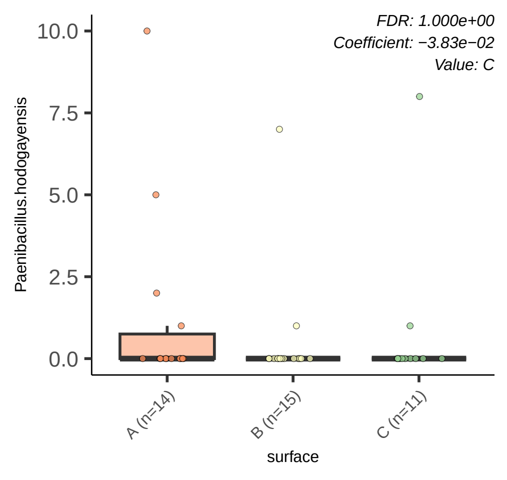

FDR: 1.000e+00
Coefficient: −3.83e−02
Value: C
10.0
7.5
5.0
2.5
0.0
Paenibacillus.hodogayensis
A (n=14)
B (n=15)
C (n=11)
surface

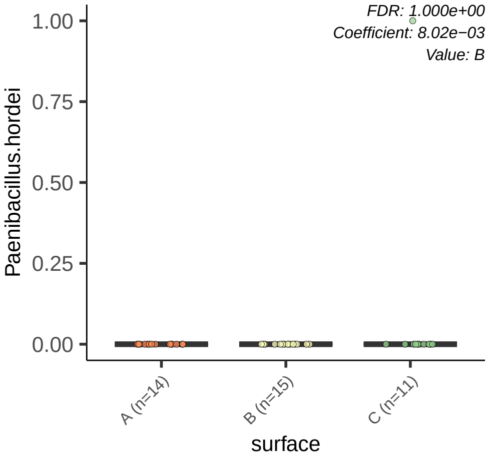
FDR: 1.000e+00
Coefficient: 8.02e−03
Value: B
Paenibacillus.hordei
1.00
0.75
0.50
0.25
0.00
A (n=14)
B (n=15)
C (n=11)
surface

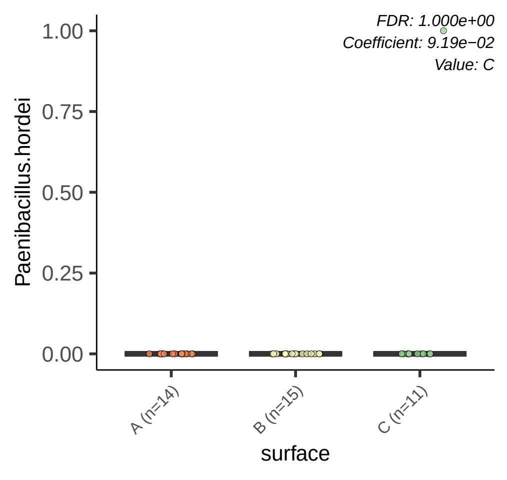
FDR: 1.000e+00
Coefficient: 9.19e−02
Value: C
Paenibacillus.hordei
1.00
0.75
0.50
0.25
0.00
A (n=14)
B (n=15)
C (n=11)
surface

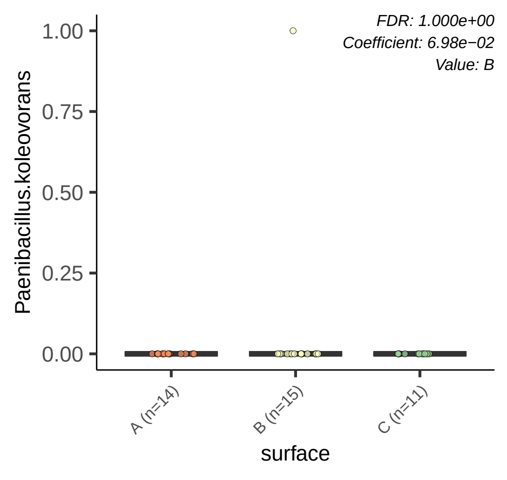
FDR: 1.000e+00
Coefficient: 6.98e−02
Value: B
Paenibacillus.koleovorans
1.00
0.75
0.50
0.25
0.00
A (n=14)
B (n=15)
C (n=11)
surface

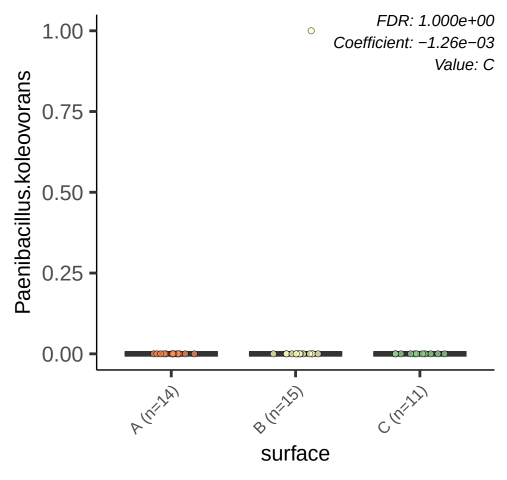

FDR: 1.000e+00
Coefficient: −1.26e−03
Value: C
Paenibacillus.koleovorans
1.00
0.75
0.50
0.25
0.00
A (n=14)
B (n=15)
C (n=11)
surface

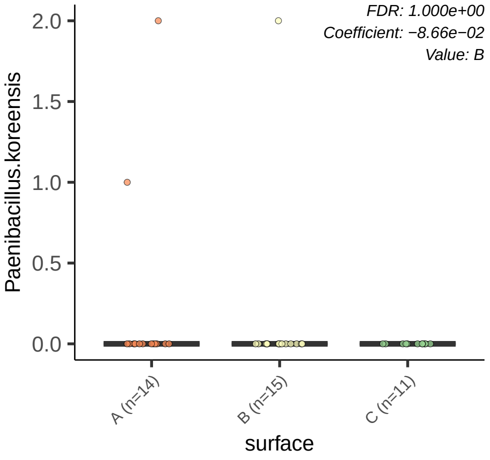
FDR: 1.000e+00
Coefficient: −8.66e−02
Value: B
Paenibacillus.koreensis
2.0
1.5
1.0
0.5
0.0
A (n=14)
B (n=15)
C (n=11)
surface

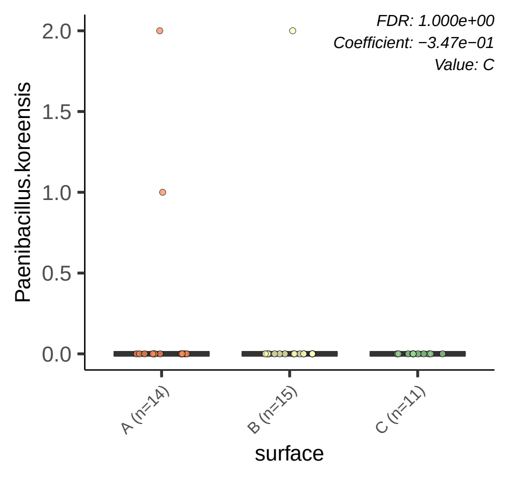
FDR: 1.000e+00
Coefficient: −3.47e−01
Value: C
Paenibacillus.koreensis
2.0
1.5
1.0
0.5
0.0
A (n=14)
B (n=15)
C (n=11)
surface

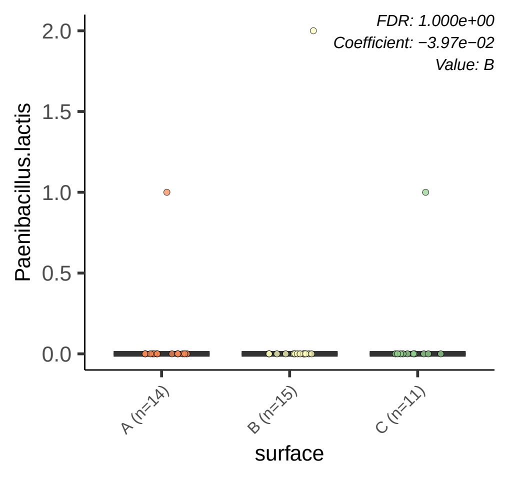
FDR: 1.000e+00
Coefficient: −3.97e−02
Value: B
Paenibacillus.lactis
2.0
1.5
1.0
0.5
0.0
A (n=14)
B (n=15)
C (n=11)
surface

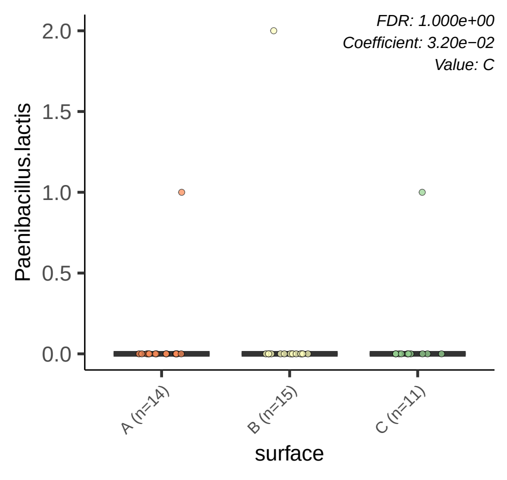

FDR: 1.000e+00
Coefficient: 3.20e−02
Value: C
Paenibacillus.lactis
2.0
1.5
1.0
0.5
0.0
A (n=14)
B (n=15)
C (n=11)
surface

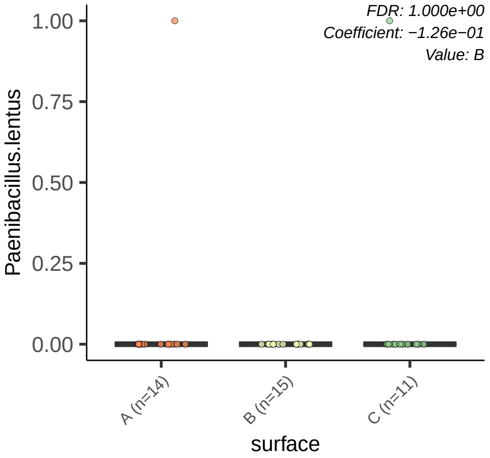

FDR: 1.000e+00
Coefficient: −1.26e−01
Value: B
Paenibacillus.lentus
1.00
0.75
0.50
0.25
0.00
A (n=14)
B (n=15)
C (n=11)
surface

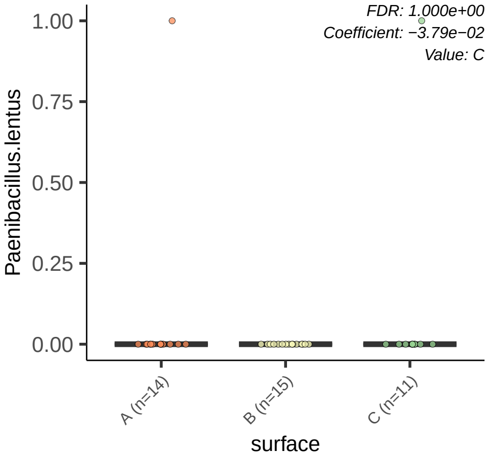
FDR: 1.000e+00
Coefficient: −3.79e−02
Value: C
Paenibacillus.lentus
1.00
0.75
0.50
0.25
0.00
A (n=14)
B (n=15)
C (n=11)
surface

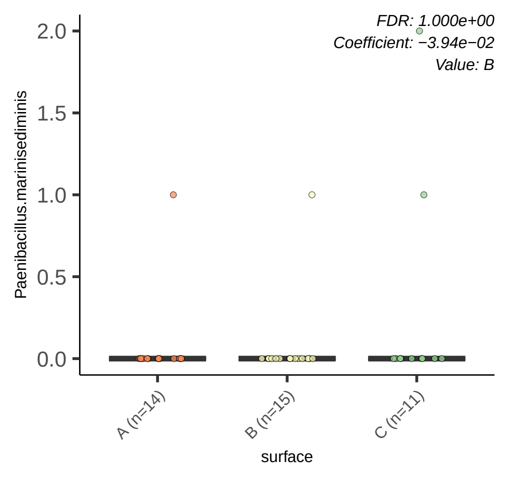
FDR: 1.000e+00
Coefficient: −3.94e−02
Value: B
Paenibacillus.marinisediminis
2.0
1.5
1.0
0.5
0.0
A (n=14)
B (n=15)
C (n=11)
surface

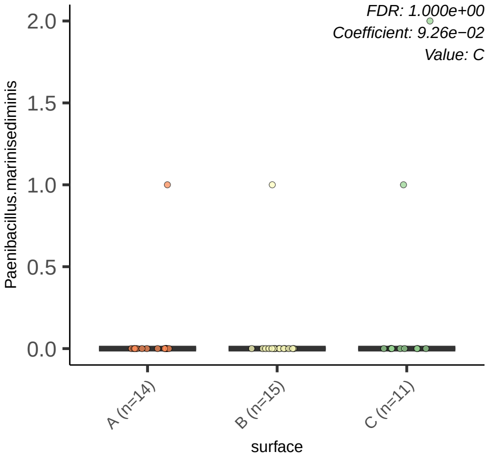
FDR: 1.000e+00
Coefficient: 9.26e−02
Value: C
Paenibacillus.marinisediminis
2.0
1.5
1.0
0.5
0.0
A (n=14)
B (n=15)
C (n=11)
surface

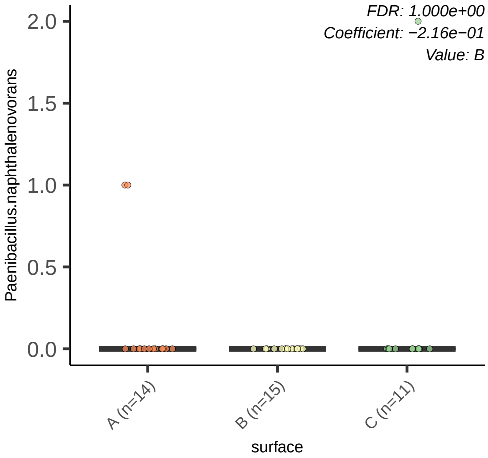
FDR: 1.000e+00
Coefficient: −2.16e−01
Value: B
Paenibacillus.naphthalenovorans
2.0
1.5
1.0
0.5
0.0
A (n=14)
B (n=15)
C (n=11)
surface

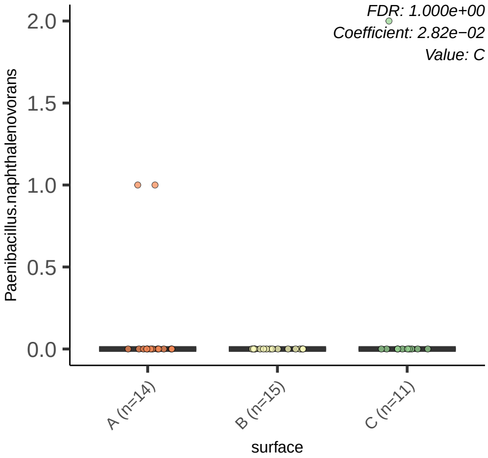

FDR: 1.000e+00
Coefficient: 2.82e−02
Value: C
Paenibacillus.naphthalenovorans
2.0
1.5
1.0
0.5
0.0
A (n=14)
B (n=15)
C (n=11)
surface

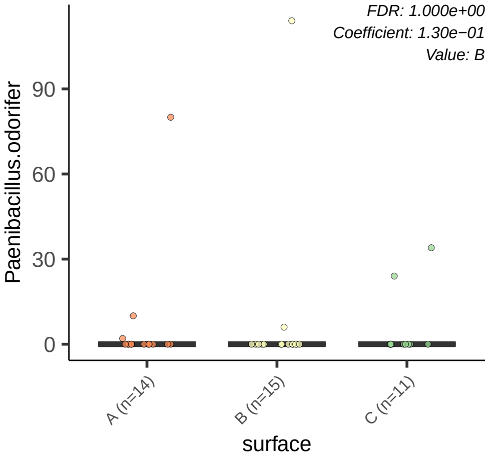

FDR: 1.000e+00
Coefficient: 1.30e−01
Value: B
Paenibacillus.odorifer
90
60
30
0
A (n=14)
B (n=15)
C (n=11)
surface

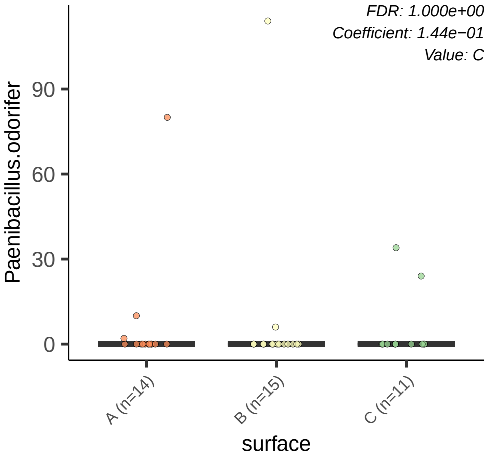
FDR: 1.000e+00
Coefficient: 1.44e−01
Value: C
Paenibacillus.odorifer
90
60
30
0
A (n=14)
B (n=15)
C (n=11)
surface

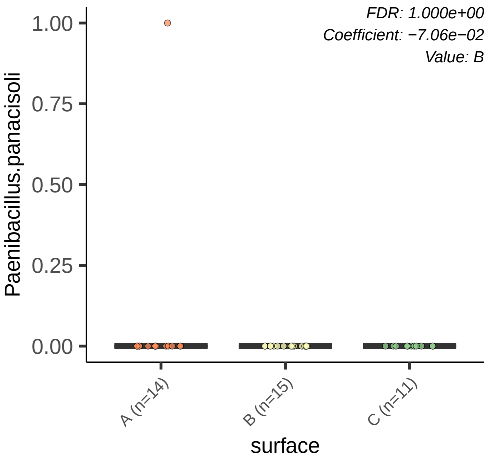

FDR: 1.000e+00
Coefficient: −7.06e−02
Value: B
Paenibacillus.panacisoli
1.00
0.75
0.50
0.25
0.00
A (n=14)
B (n=15)
C (n=11)
surface

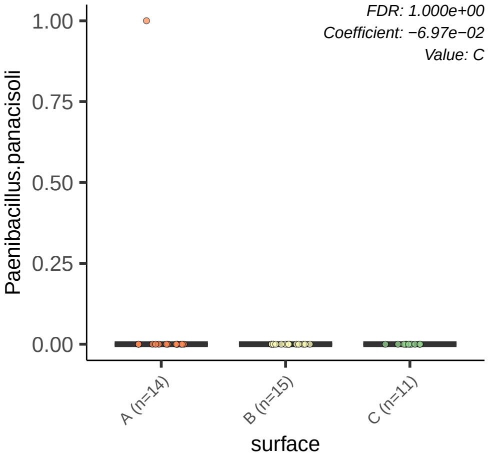
FDR: 1.000e+00
Coefficient: −6.97e−02
Value: C
Paenibacillus.panacisoli
1.00
0.75
0.50
0.25
0.00
A (n=14)
B (n=15)
C (n=11)
surface

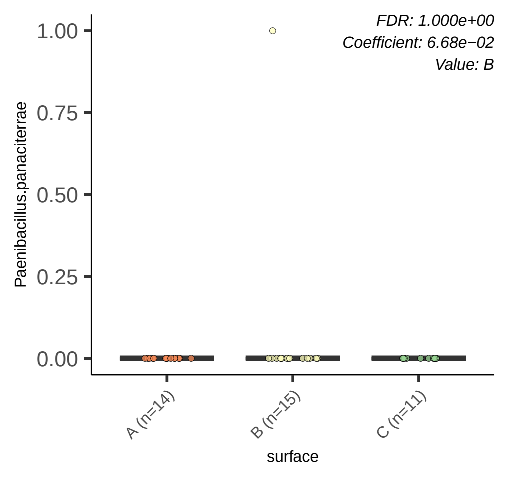
FDR: 1.000e+00
Coefficient: 6.68e−02
Value: B
1.00
0.75
0.50
0.25
0.00
Paenibacillus.panaciterrae
A (n=14)
B (n=15)
C (n=11)
surface

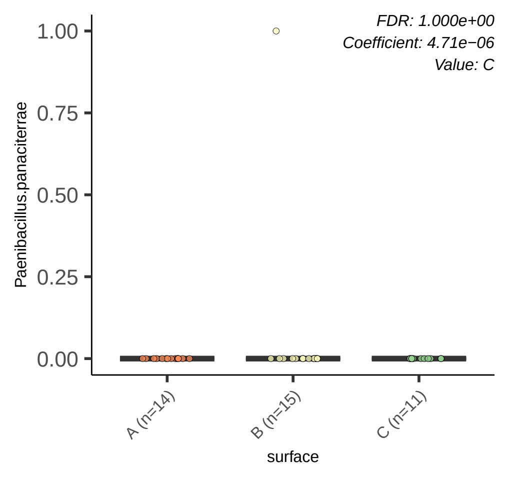

FDR: 1.000e+00
Coefficient: 4.71e−06
Value: C
1.00
0.75
0.50
0.25
0.00
Paenibacillus.panaciterrae
A (n=14)
B (n=15)
C (n=11)
surface

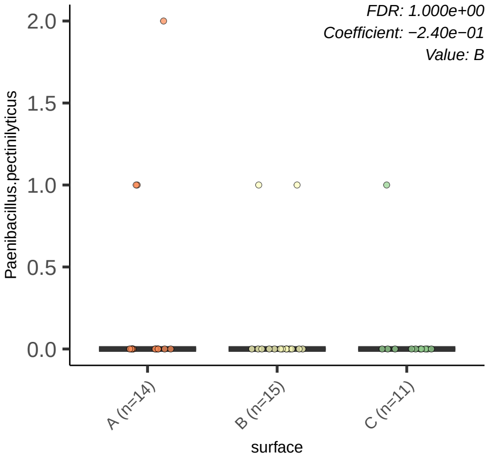
FDR: 1.000e+00
Coefficient: −2.40e−01
Value: B
Paenibacillus.pectinilyticus
2.0
1.5
1.0
0.5
0.0
A (n=14)
B (n=15)
C (n=11)
surface

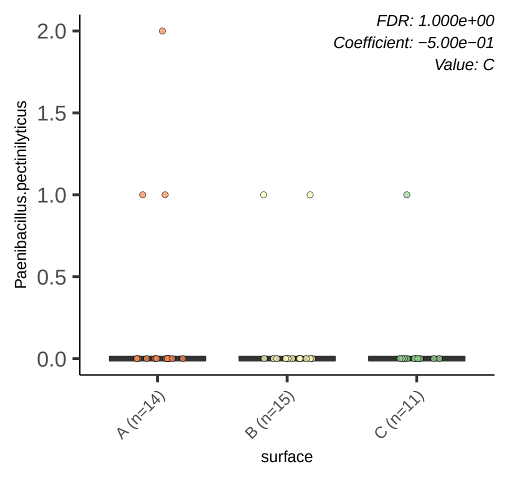

FDR: 1.000e+00
Coefficient: −5.00e−01
Value: C
Paenibacillus.pectinilyticus
2.0
1.5
1.0
0.5
0.0
A (n=14)
B (n=15)
C (n=11)
surface

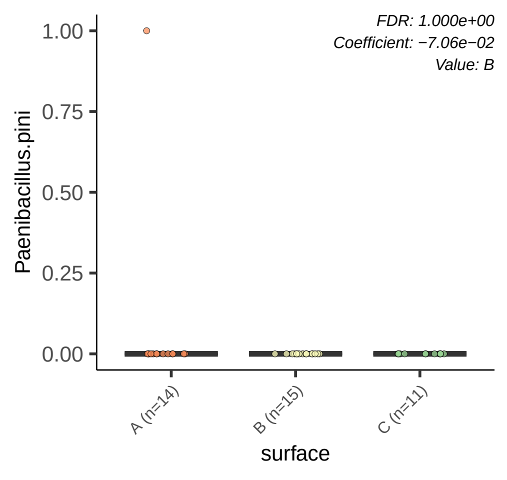

FDR: 1.000e+00
Coefficient: −7.06e−02
Value: B
Paenibacillus.pini
1.00
0.75
0.50
0.25
0.00
A (n=14)
B (n=15)
C (n=11)
surface

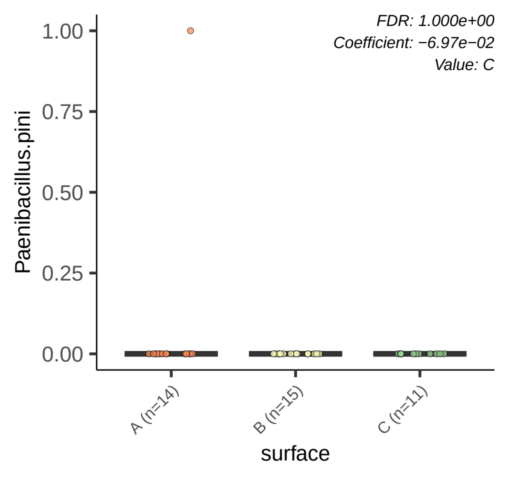
FDR: 1.000e+00
Coefficient: −6.97e−02
Value: C
Paenibacillus.pini
1.00
0.75
0.50
0.25
0.00
A (n=14)
B (n=15)
C (n=11)
surface

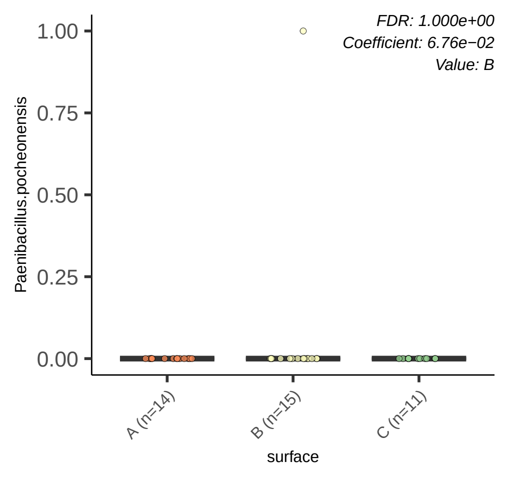
FDR: 1.000e+00
Coefficient: 6.76e−02
Value: B
Paenibacillus.pocheonensis
1.00
0.75
0.50
0.25
0.00
A (n=14)
B (n=15)
C (n=11)
surface

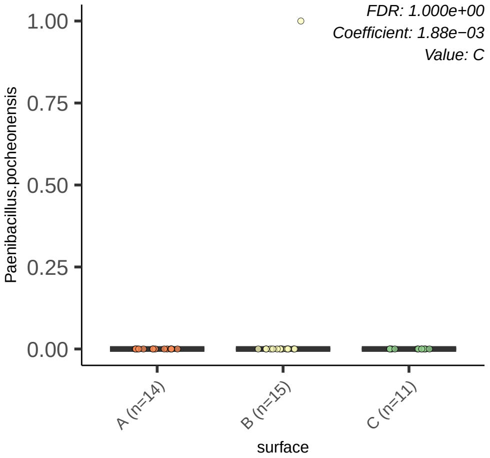

FDR: 1.000e+00
Coefficient: 1.88e−03
Value: C
Paenibacillus.pocheonensis
1.00
0.75
0.50
0.25
0.00
A (n=14)
B (n=15)
C (n=11)
surface

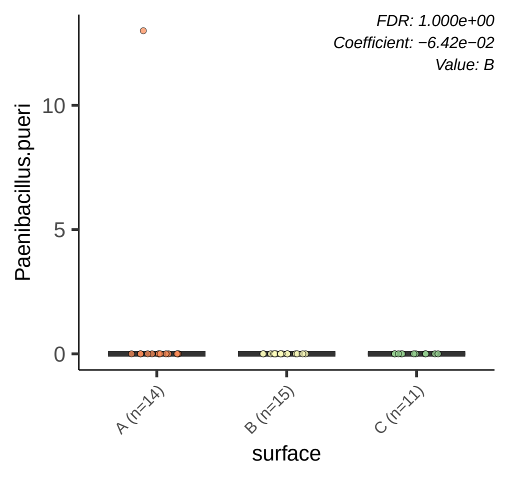
FDR: 1.000e+00
Coefficient: −6.42e−02
Value: B
Paenibacillus.pueri
10
5
0
A (n=14)
B (n=15)
C (n=11)
surface

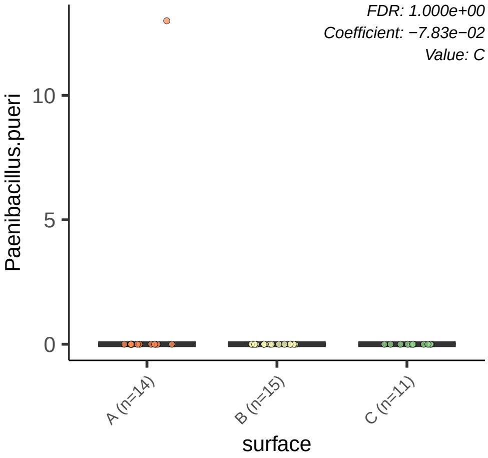
FDR: 1.000e+00
Coefficient: −7.83e−02
Value: C
Paenibacillus.pueri
10
5
0
A (n=14)
B (n=15)
C (n=11)
surface

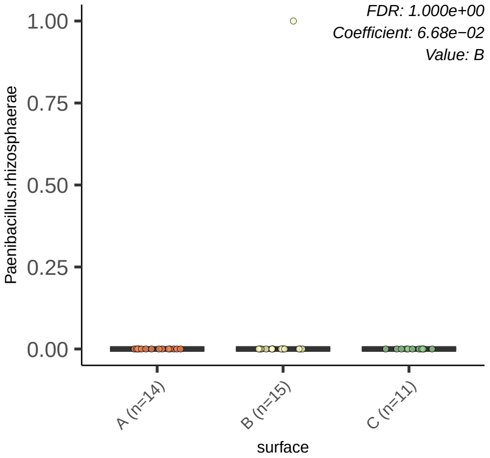

FDR: 1.000e+00
Coefficient: 6.68e−02
Value: B
Paenibacillus.rhizosphaerae
1.00
0.75
0.50
0.25
0.00
A (n=14)
B (n=15)
C (n=11)
surface

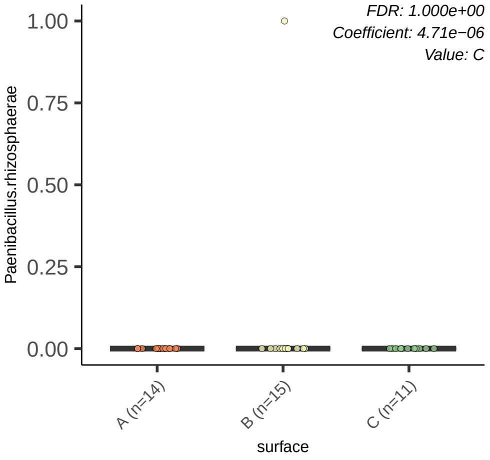
FDR: 1.000e+00
Coefficient: 4.71e−06
Value: C
1.00
0.75
0.50
0.25
0.00
Paenibacillus.rhizosphaerae
A (n=14)
B (n=15)
C (n=11)
surface

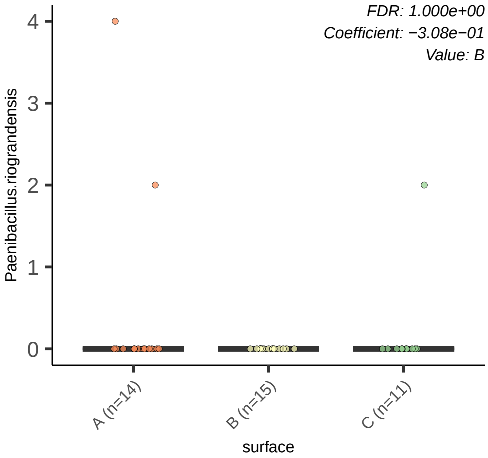
FDR: 1.000e+00
Coefficient: −3.08e−01
Value: B
Paenibacillus.riograndensis
0
1
2
3
4
A (n=14)
B (n=15)
C (n=11)
surface

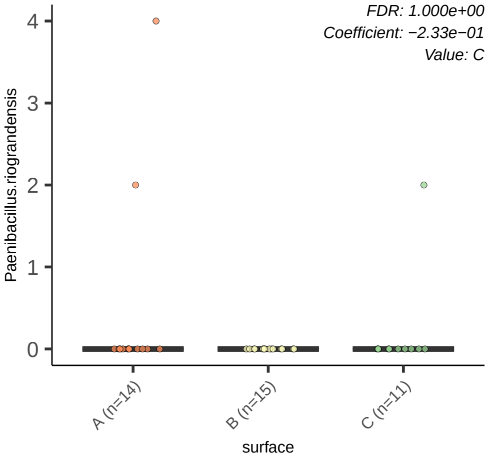
FDR: 1.000e+00
Coefficient: −2.33e−01
Value: C
Paenibacillus.riograndensis
0
1
2
3
4
A (n=14)
B (n=15)
C (n=11)
surface

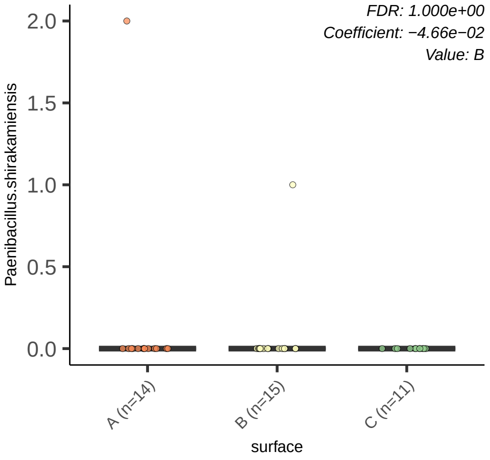
FDR: 1.000e+00
Coefficient: −4.66e−02
Value: B
Paenibacillus.shirakamiensis
2.0
1.5
1.0
0.5
0.0
A (n=14)
B (n=15)
C (n=11)
surface

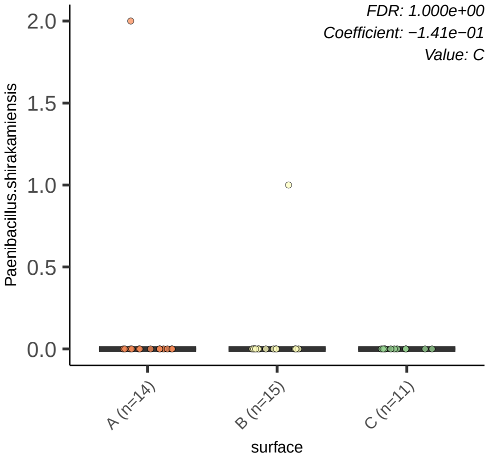
FDR: 1.000e+00
Coefficient: −1.41e−01
Value: C
Paenibacillus.shirakamiensis
2.0
1.5
1.0
0.5
0.0
A (n=14)
B (n=15)
C (n=11)
surface

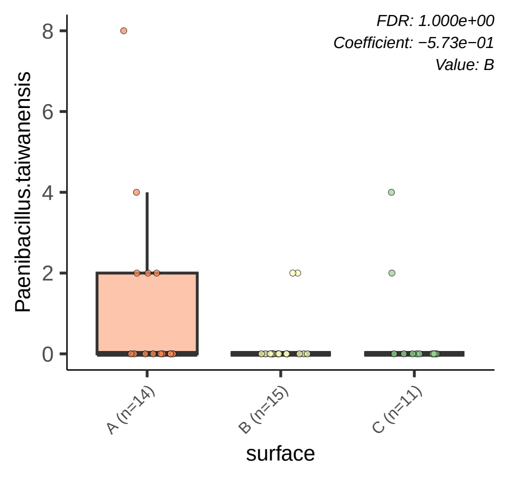
FDR: 1.000e+00
Coefficient: −5.73e−01
Value: B
Paenibacillus.taiwanensis
8
6
4
2
0
A (n=14)
B (n=15)
C (n=11)
surface

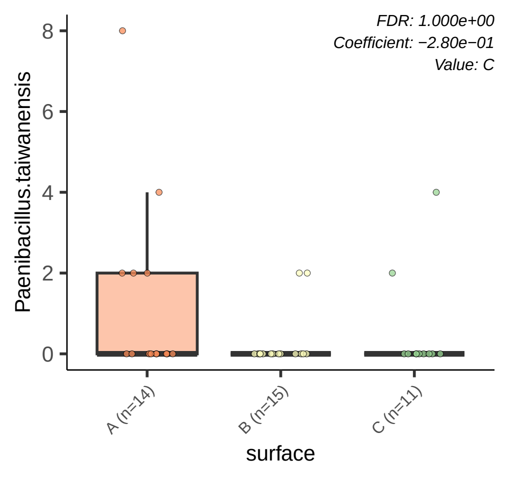
FDR: 1.000e+00
Coefficient: −2.80e−01
Value: C
Paenibacillus.taiwanensis
8
6
4
2
0
A (n=14)
B (n=15)
C (n=11)
surface

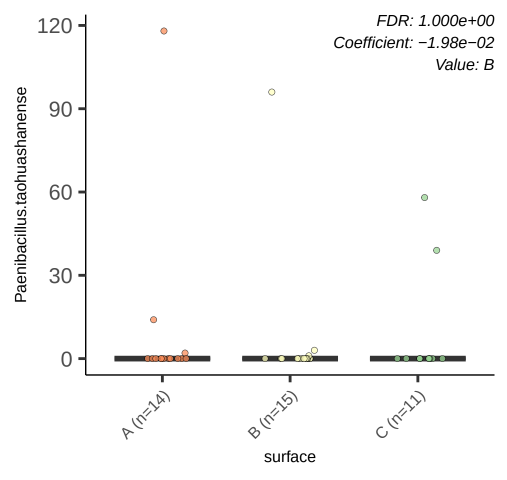
FDR: 1.000e+00
Coefficient: −1.98e−02
Value: B
Paenibacillus.taohuashanense
120
90
60
30
0
A (n=14)
B (n=15)
C (n=11)
surface

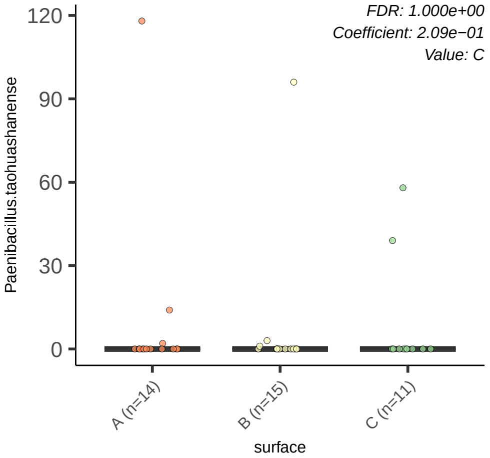
FDR: 1.000e+00
Coefficient: 2.09e−01
Value: C
Paenibacillus.taohuashanense
120
90
60
30
0
A (n=14)
B (n=15)
C (n=11)
surface

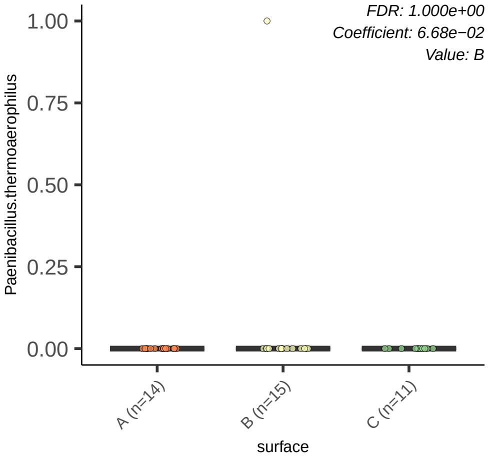
FDR: 1.000e+00
Coefficient: 6.68e−02
Value: B
Paenibacillus.thermoaerophilus
1.00
0.75
0.50
0.25
0.00
A (n=14)
B (n=15)
C (n=11)
surface

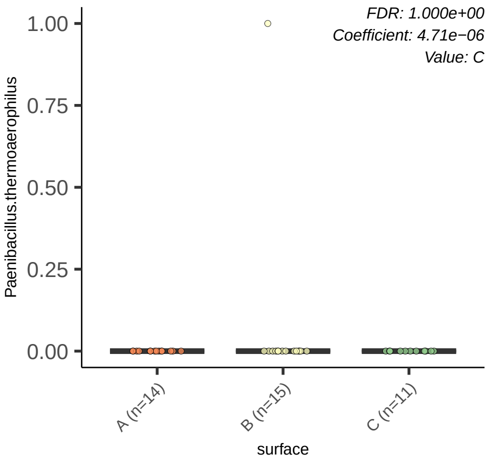
FDR: 1.000e+00
Coefficient: 4.71e−06
Value: C
1.00
0.75
0.50
0.25
0.00
Paenibacillus.thermoaerophilus
A (n=14)
B (n=15)
C (n=11)
surface

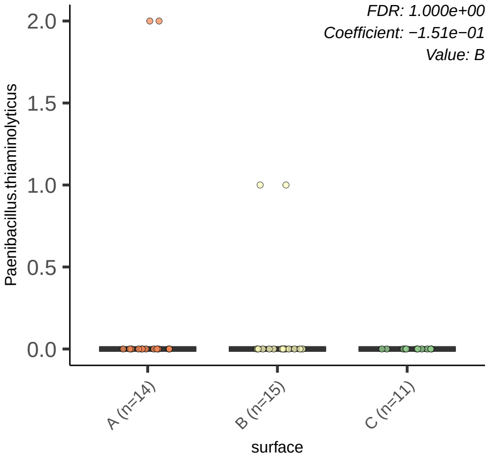
FDR: 1.000e+00
Coefficient: −1.51e−01
Value: B
Paenibacillus.thiaminolyticus
2.0
1.5
1.0
0.5
0.0
A (n=14)
B (n=15)
C (n=11)
surface

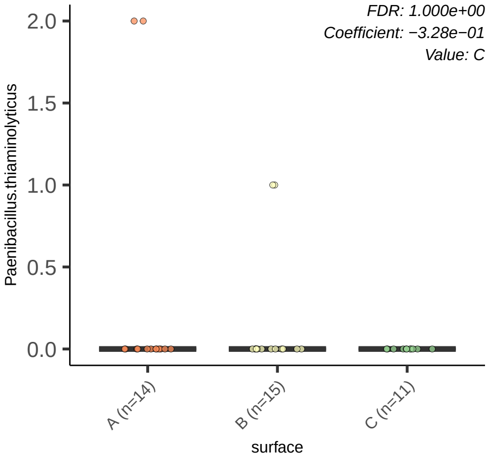

FDR: 1.000e+00
Coefficient: −3.28e−01
Value: C
Paenibacillus.thiaminolyticus
2.0
1.5
1.0
0.5
0.0
A (n=14)
B (n=15)
C (n=11)
surface

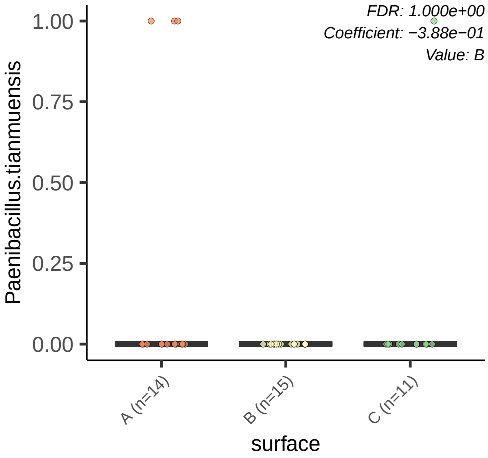

FDR: 1.000e+00
Coefficient: −3.88e−01
Value: B
Paenibacillus.tianmuensis
1.00
0.75
0.50
0.25
0.00
A (n=14)
B (n=15)
C (n=11)
surface

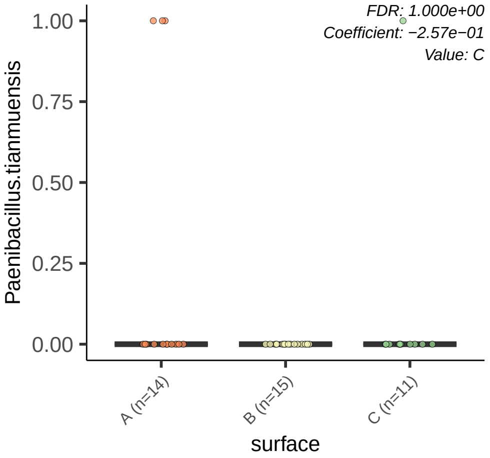
FDR: 1.000e+00
Coefficient: −2.57e−01
Value: C
Paenibacillus.tianmuensis
1.00
0.75
0.50
0.25
0.00
A (n=14)
B (n=15)
C (n=11)
surface

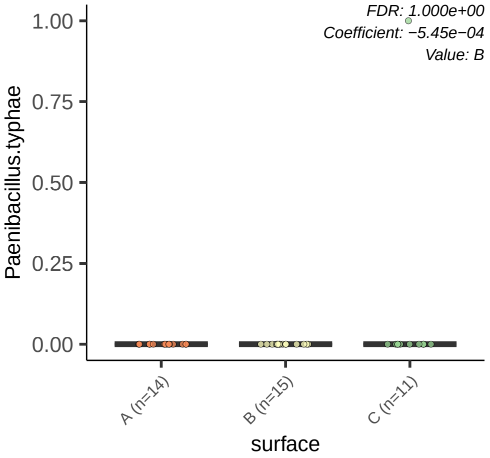
FDR: 1.000e+00
Coefficient: −5.45e−04
Value: B
Paenibacillus.typhae
1.00
0.75
0.50
0.25
0.00
A (n=14)
B (n=15)
C (n=11)
surface

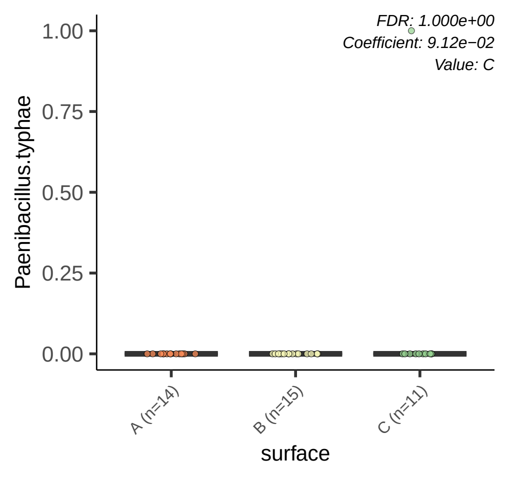

FDR: 1.000e+00
Coefficient: 9.12e−02
Value: C
Paenibacillus.typhae
1.00
0.75
0.50
0.25
0.00
A (n=14)
B (n=15)
C (n=11)
surface

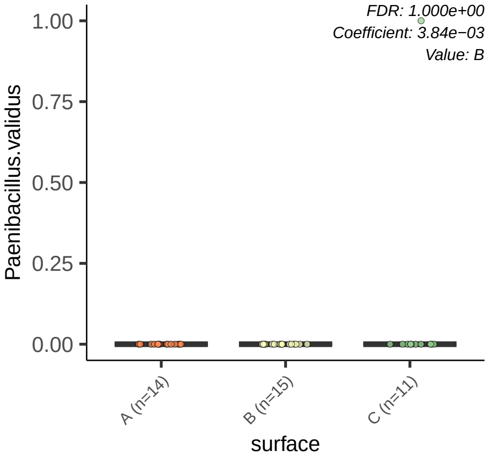
FDR: 1.000e+00
Coefficient: 3.84e−03
Value: B
Paenibacillus.validus
1.00
0.75
0.50
0.25
0.00
A (n=14)
B (n=15)
C (n=11)
surface

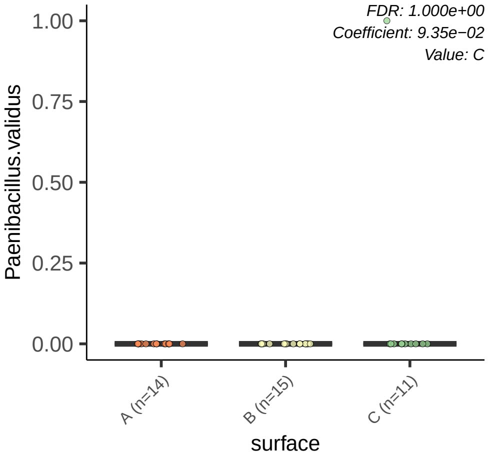
FDR: 1.000e+00
Coefficient: 9.35e−02
Value: C
Paenibacillus.validus
1.00
0.75
0.50
0.25
0.00
A (n=14)
B (n=15)
C (n=11)
surface

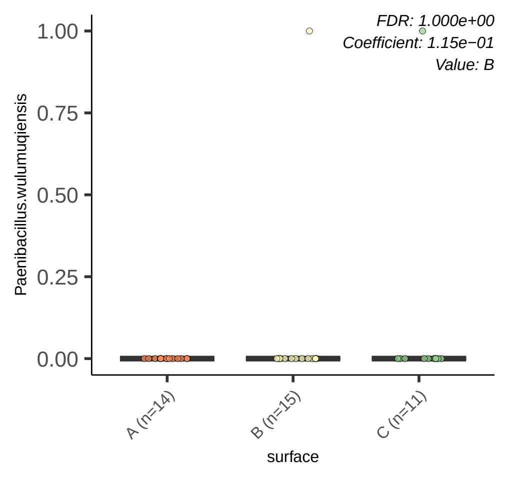

FDR: 1.000e+00
Coefficient: 1.15e−01
Value: B
1.00
0.75
0.50
0.25
0.00
Paenibacillus.wulumuqiensis
A (n=14)
B (n=15)
C (n=11)
surface

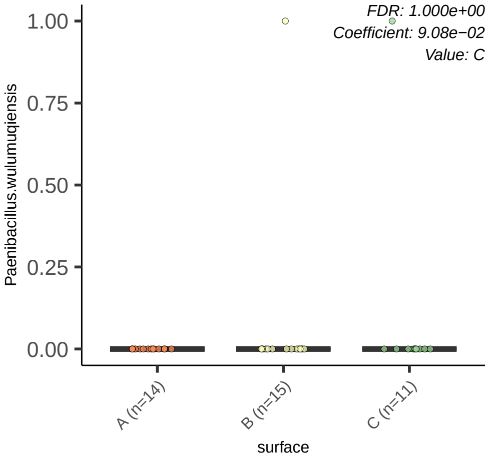

FDR: 1.000e+00
Coefficient: 9.08e−02
Value: C
1.00
0.75
0.50
0.25
0.00
Paenibacillus.wulumuqiensis
A (n=14)
B (n=15)
C (n=11)
surface

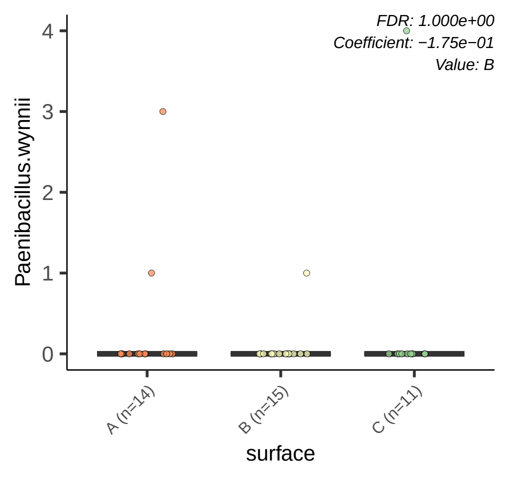

FDR: 1.000e+00
Coefficient: −1.75e−01
Value: B
Paenibacillus.wynnii
0
1
2
3
4
A (n=14)
B (n=15)
C (n=11)
surface

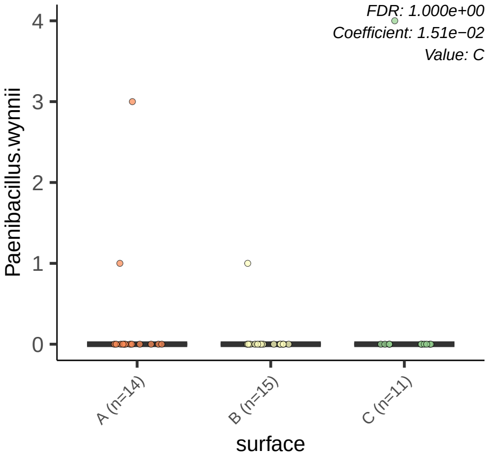

FDR: 1.000e+00
Coefficient: 1.51e−02
Value: C
Paenibacillus.wynnii
0
1
2
3
4
A (n=14)
B (n=15)
C (n=11)
surface

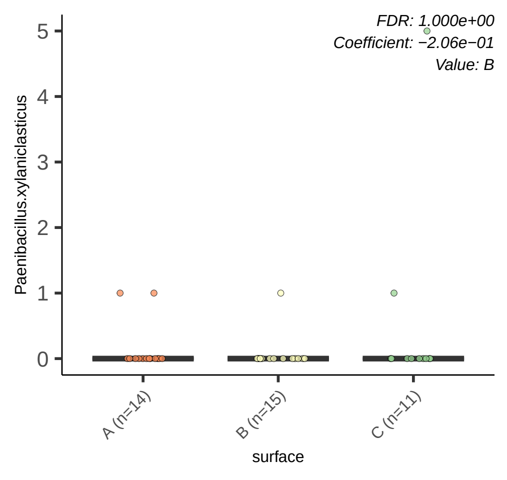

FDR: 1.000e+00
Coefficient: −2.06e−01
Value: B
Paenibacillus.xylaniclasticus
0
1
2
3
4
5
A (n=14)
B (n=15)
C (n=11)
surface

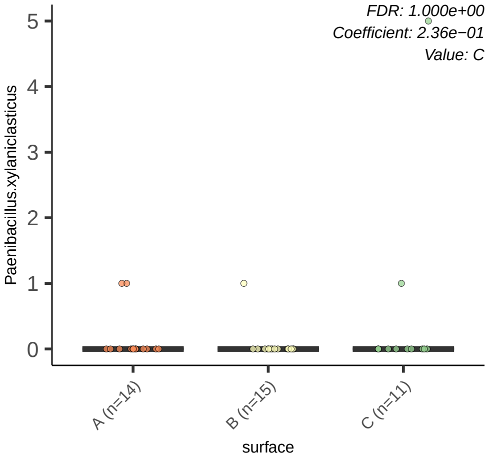
FDR: 1.000e+00
Coefficient: 2.36e−01
Value: C
Paenibacillus.xylaniclasticus
0
1
2
3
4
5
A (n=14)
B (n=15)
C (n=11)
surface

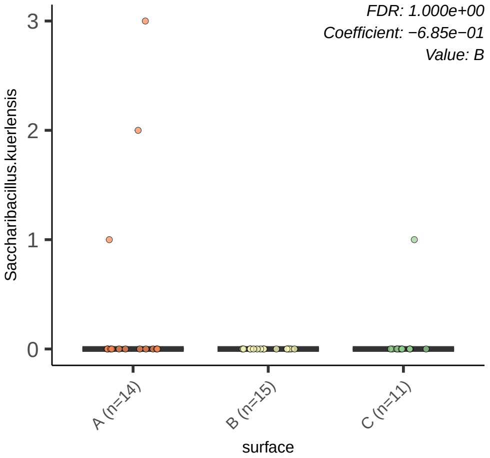
FDR: 1.000e+00
Coefficient: −6.85e−01
Value: B
Saccharibacillus.kuerlensis
0
1
2
3
A (n=14)
B (n=15)
C (n=11)
surface

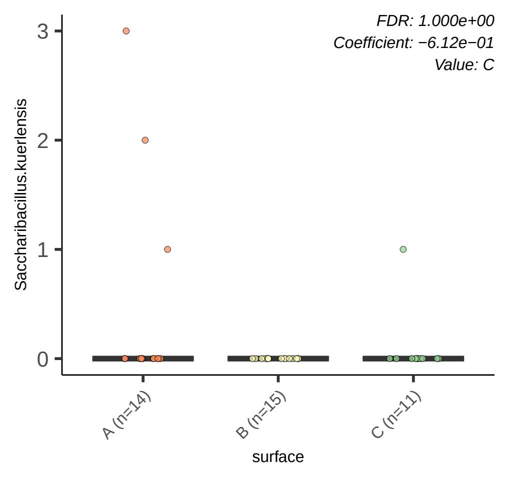
FDR: 1.000e+00
Coefficient: −6.12e−01
Value: C
Saccharibacillus.kuerlensis
3
2
1
0
A (n=14)
B (n=15)
C (n=11)
surface

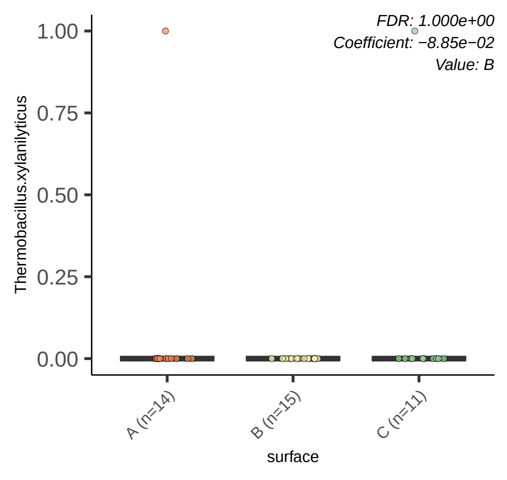
FDR: 1.000e+00
Coefficient: −8.85e−02
Value: B
1.00
0.75
0.50
0.25
0.00
Thermobacillus.xylanilyticus
A (n=14)
B (n=15)
C (n=11)
surface

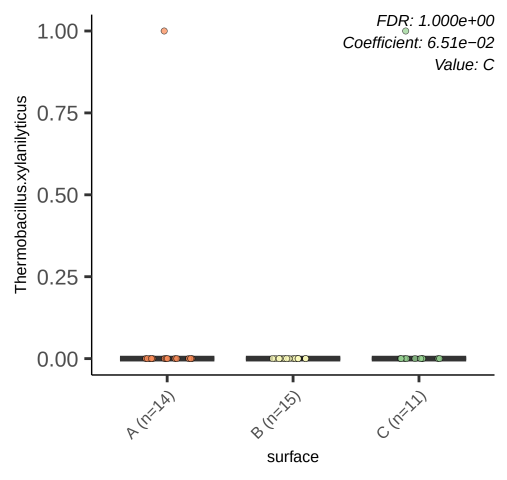
FDR: 1.000e+00
Coefficient: 6.51e−02
Value: C
1.00
0.75
0.50
0.25
0.00
Thermobacillus.xylanilyticus
A (n=14)
B (n=15)
C (n=11)
surface

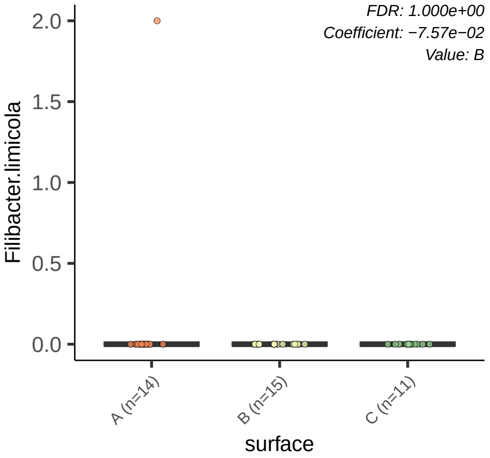

FDR: 1.000e+00
Coefficient: −7.57e−02
Value: B
Filibacter.limicola
2.0
1.5
1.0
0.5
0.0
A (n=14)
B (n=15)
C (n=11)
surface

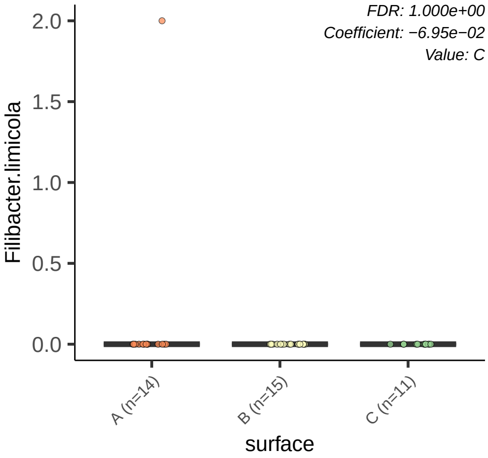
FDR: 1.000e+00
Coefficient: −6.95e−02
Value: C
Filibacter.limicola
2.0
1.5
1.0
0.5
0.0
A (n=14)
B (n=15)
C (n=11)
surface

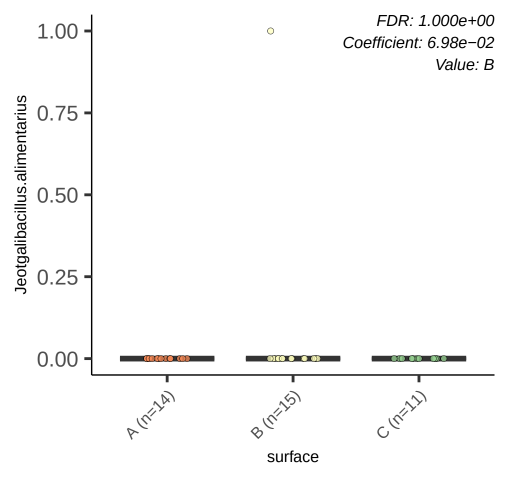
FDR: 1.000e+00
Coefficient: 6.98e−02
Value: B
Jeotgalibacillus.alimentarius
1.00
0.75
0.50
0.25
0.00
A (n=14)
B (n=15)
C (n=11)
surface

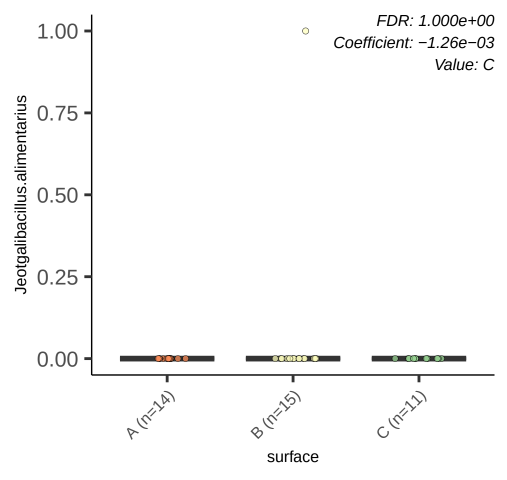

FDR: 1.000e+00
Coefficient: −1.26e−03
Value: C
1.00
0.75
0.50
0.25
0.00
Jeotgalibacillus.alimentarius
A (n=14)
B (n=15)
C (n=11)
surface

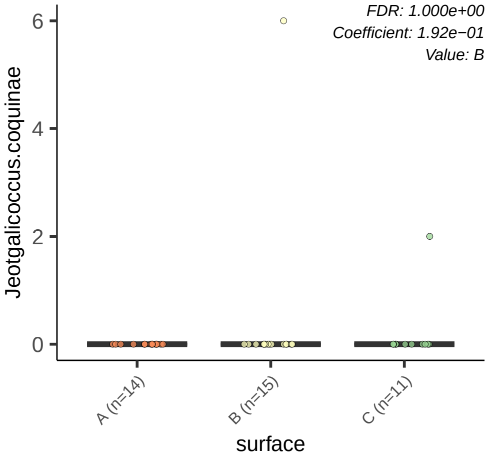

FDR: 1.000e+00
Coefficient: 1.92e−01
Value: B
Jeotgalicoccus.coquinae
6
4
2
0
A (n=14)
B (n=15)
C (n=11)
surface

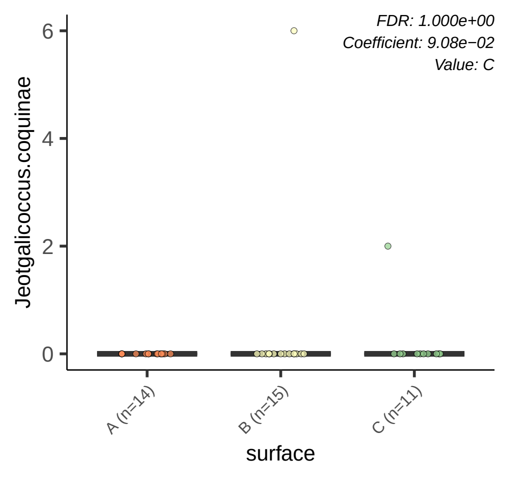
FDR: 1.000e+00
Coefficient: 9.08e−02
Value: C
Jeotgalicoccus.coquinae
6
4
2
0
A (n=14)
B (n=15)
C (n=11)
surface

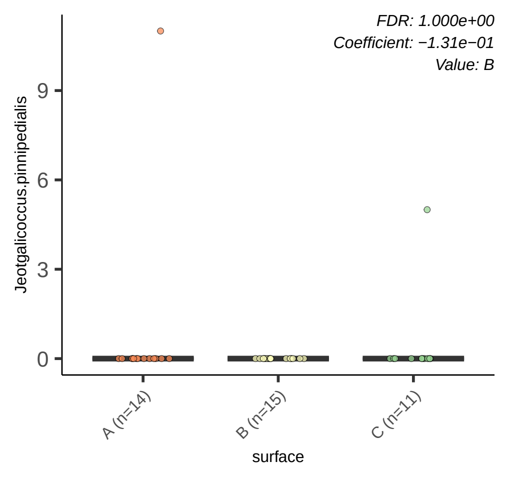
FDR: 1.000e+00
Coefficient: −1.31e−01
Value: B
Jeotgalicoccus.pinnipedialis
9
6
3
0
A (n=14)
B (n=15)
C (n=11)
surface

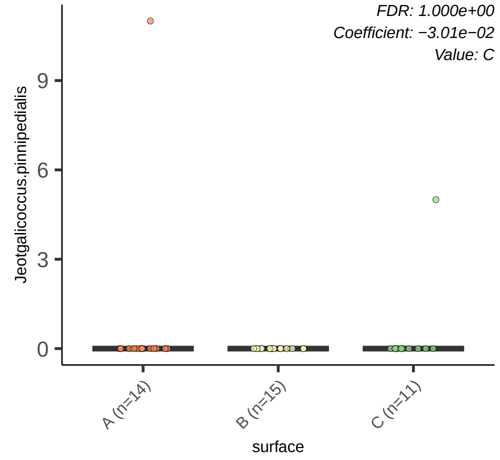
FDR: 1.000e+00
Coefficient: −3.01e−02
Value: C
Jeotgalicoccus.pinnipedialis
9
6
3
0
A (n=14)
B (n=15)
C (n=11)
surface

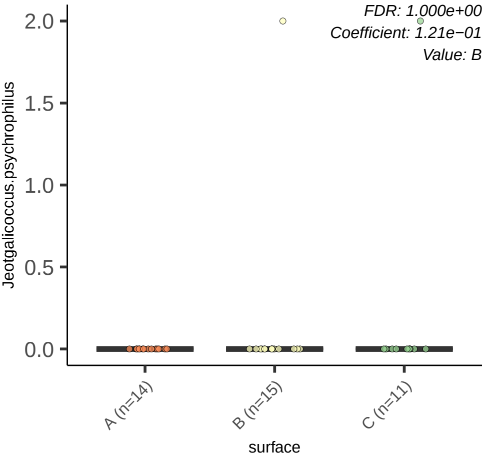

FDR: 1.000e+00
Coefficient: 1.21e−01
Value: B
Jeotgalicoccus.psychrophilus
2.0
1.5
1.0
0.5
0.0
A (n=14)
B (n=15)
C (n=11)
surface

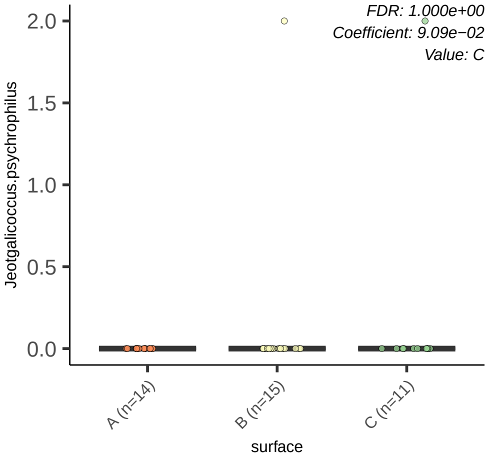

FDR: 1.000e+00
Coefficient: 9.09e−02
Value: C
2.0
1.5
1.0
0.5
0.0
Jeotgalicoccus.psychrophilus
A (n=14)
B (n=15)
C (n=11)
surface

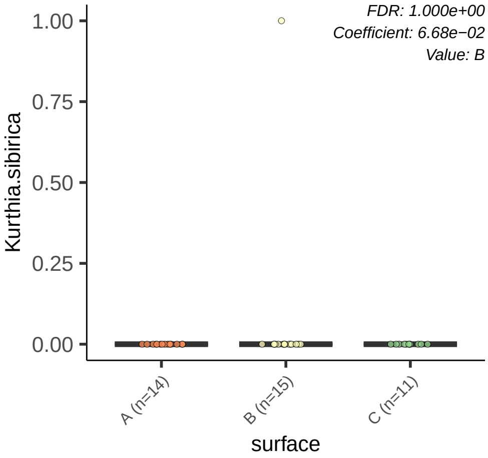

FDR: 1.000e+00
Coefficient: 6.68e−02
Value: B
Kurthia.sibirica
1.00
0.75
0.50
0.25
0.00
A (n=14)
B (n=15)
C (n=11)
surface

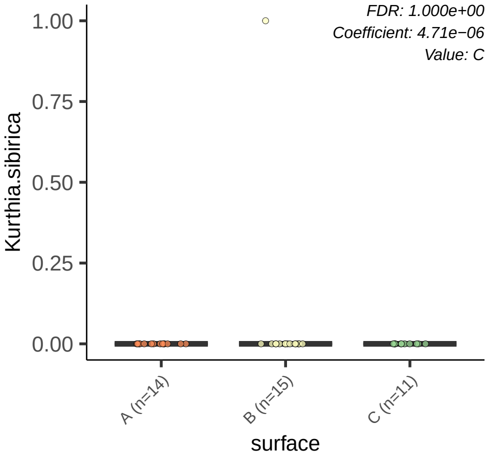
FDR: 1.000e+00
Coefficient: 4.71e−06
Value: C
1.00
0.75
0.50
0.25
0.00
Kurthia.sibirica
A (n=14)
B (n=15)
C (n=11)
surface

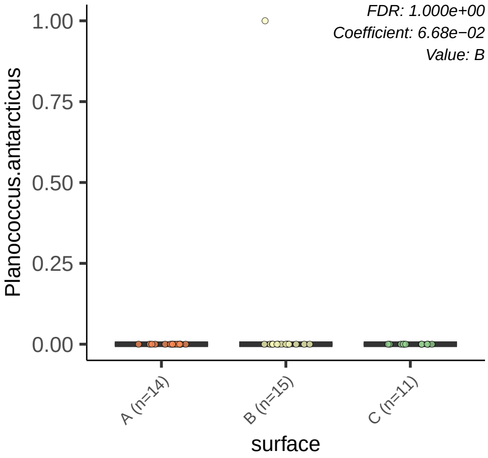
FDR: 1.000e+00
Coefficient: 6.68e−02
Value: B
Planococcus.antarcticus
1.00
0.75
0.50
0.25
0.00
A (n=14)
B (n=15)
C (n=11)
surface

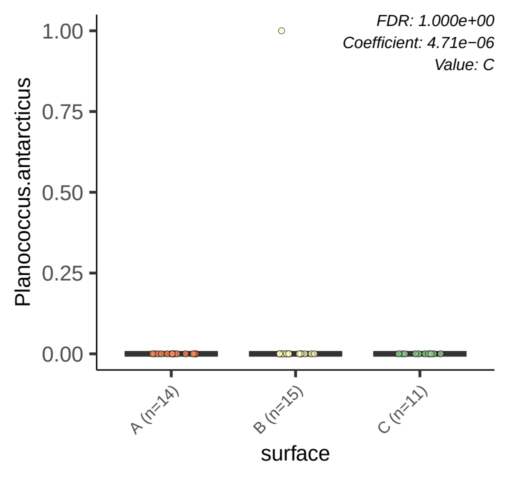
FDR: 1.000e+00
Coefficient: 4.71e−06
Value: C
Planococcus.antarcticus
1.00
0.75
0.50
0.25
0.00
A (n=14)
B (n=15)
C (n=11)
surface

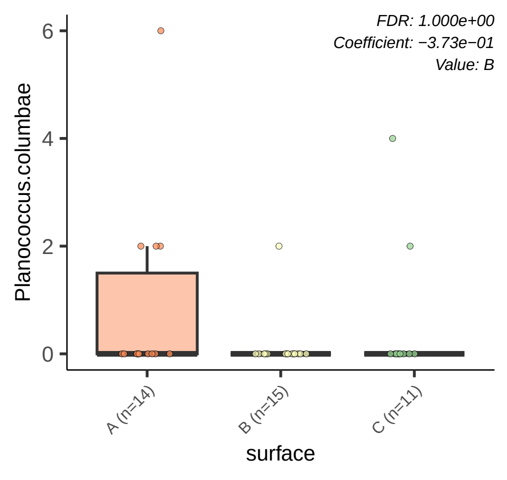
FDR: 1.000e+00
Coefficient: −3.73e−01
Value: B
Planococcus.columbae
6
4
2
0
A (n=14)
B (n=15)
C (n=11)
surface

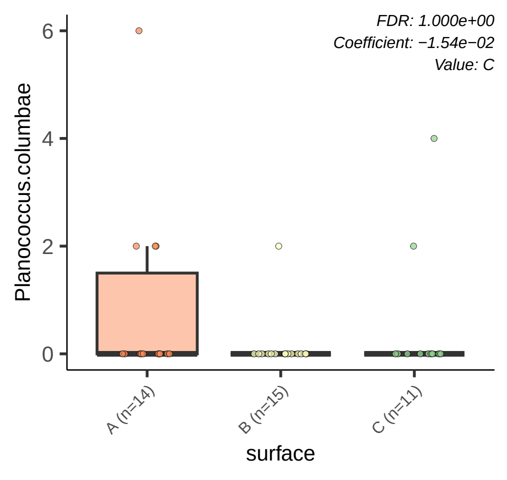

FDR: 1.000e+00
Coefficient: −1.54e−02
Value: C
Planococcus.columbae
0
2
4
6
A (n=14)
B (n=15)
C (n=11)
surface

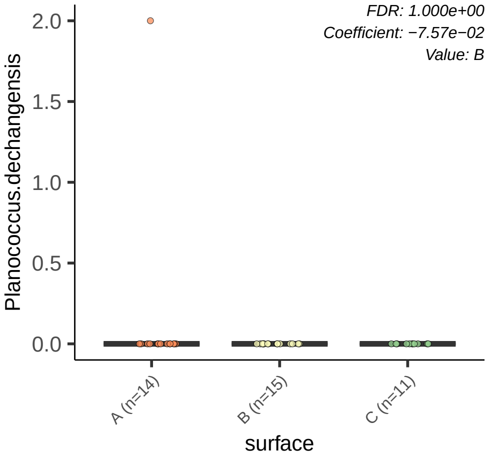
FDR: 1.000e+00
Coefficient: −7.57e−02
Value: B
Planococcus.dechangensis
2.0
1.5
1.0
0.5
0.0
A (n=14)
B (n=15)
C (n=11)
surface

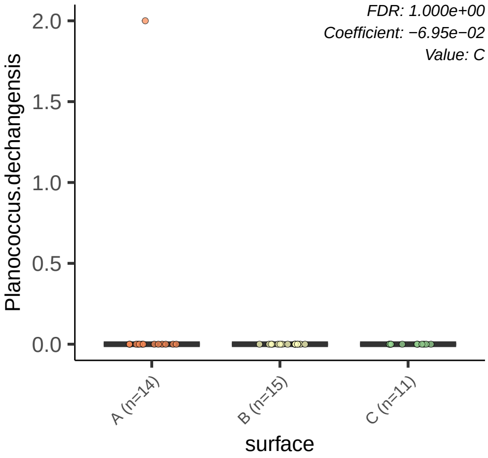
FDR: 1.000e+00
Coefficient: −6.95e−02
Value: C
Planococcus.dechangensis
2.0
1.5
1.0
0.5
0.0
A (n=14)
B (n=15)
C (n=11)
surface

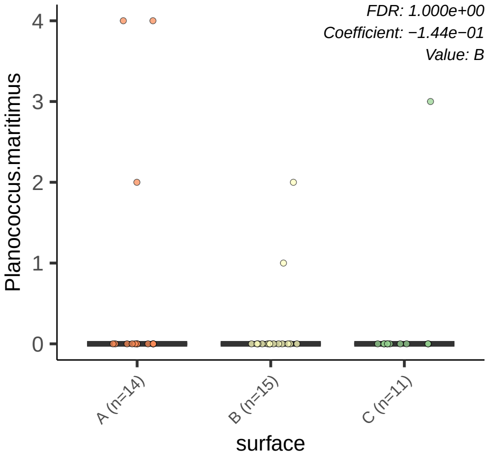
FDR: 1.000e+00
Coefficient: −1.44e−01
Value: B
Planococcus.maritimus
4
3
2
1
0
A (n=14)
B (n=15)
C (n=11)
surface

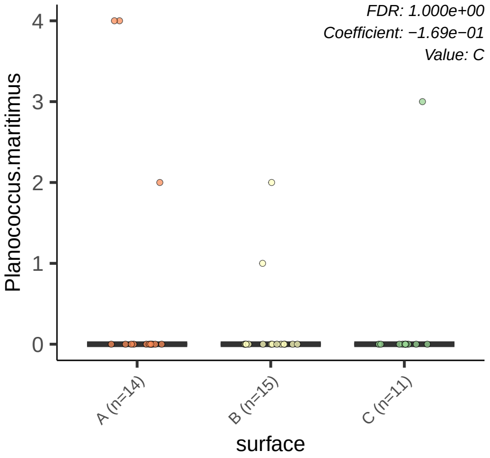
FDR: 1.000e+00
Coefficient: −1.69e−01
Value: C
Planococcus.maritimus
4
3
2
1
0
A (n=14)
B (n=15)
C (n=11)
surface

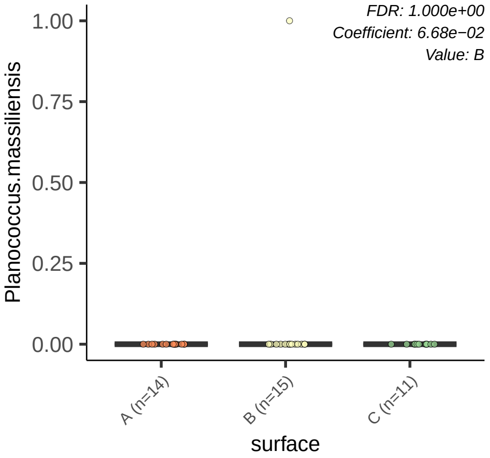
FDR: 1.000e+00
Coefficient: 6.68e−02
Value: B
Planococcus.massiliensis
1.00
0.75
0.50
0.25
0.00
A (n=14)
B (n=15)
C (n=11)
surface

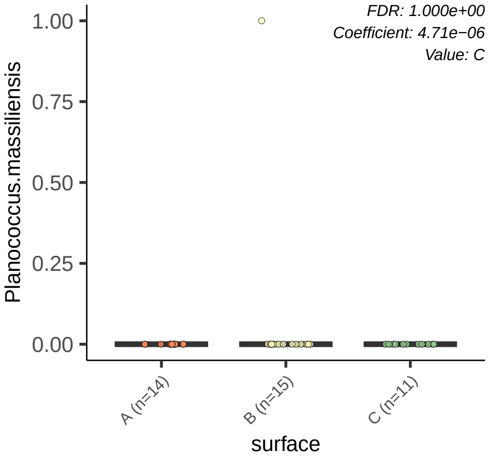
FDR: 1.000e+00
Coefficient: 4.71e−06
Value: C
Planococcus.massiliensis
1.00
0.75
0.50
0.25
0.00
A (n=14)
B (n=15)
C (n=11)
surface

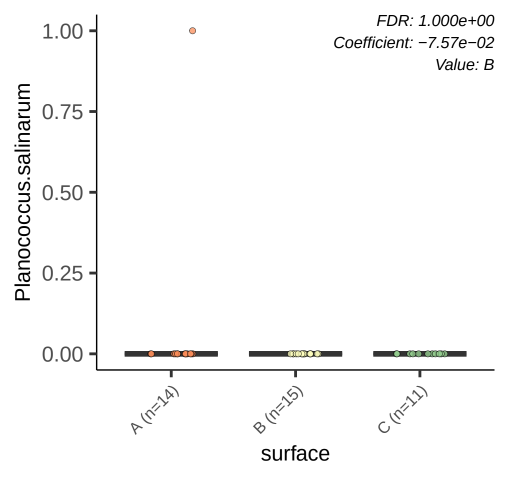
FDR: 1.000e+00
Coefficient: −7.57e−02
Value: B
Planococcus.salinarum
1.00
0.75
0.50
0.25
0.00
A (n=14)
B (n=15)
C (n=11)
surface

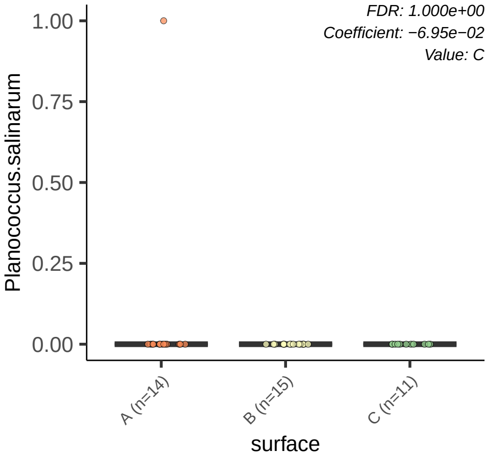
FDR: 1.000e+00
Coefficient: −6.95e−02
Value: C
Planococcus.salinarum
1.00
0.75
0.50
0.25
0.00
A (n=14)
B (n=15)
C (n=11)
surface

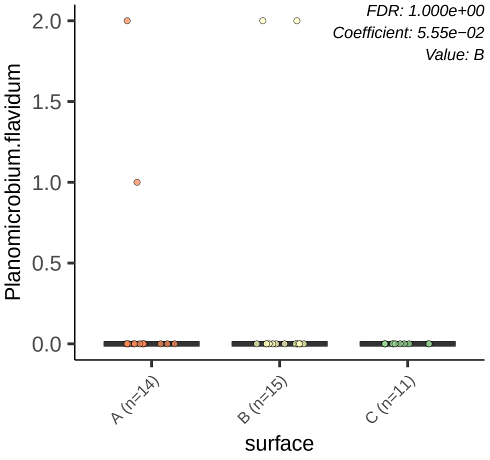
FDR: 1.000e+00
Coefficient: 5.55e−02
Value: B
Planomicrobium.flavidum
2.0
1.5
1.0
0.5
0.0
A (n=14)
B (n=15)
C (n=11)
surface

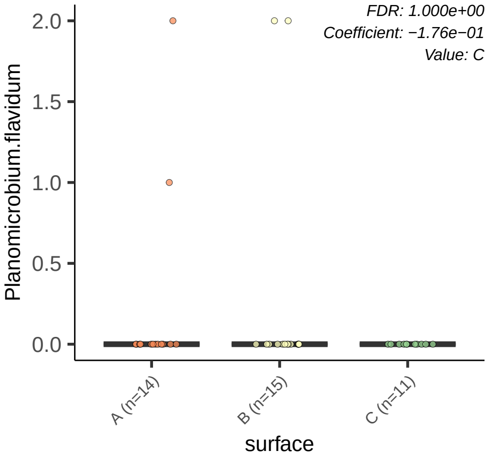

FDR: 1.000e+00
Coefficient: −1.76e−01
Value: C
2.0
1.5
1.0
0.5
0.0
Planomicrobium.flavidum
A (n=14)
B (n=15)
C (n=11)
surface

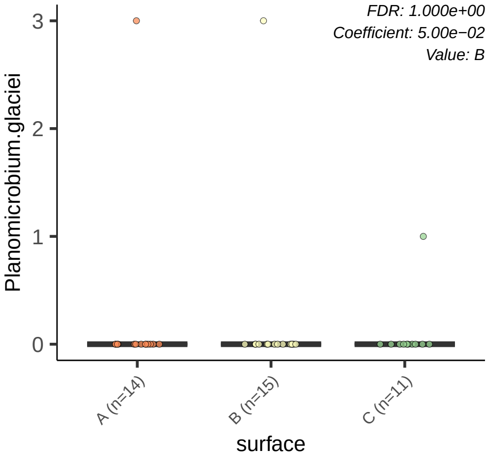

FDR: 1.000e+00
Coefficient: 5.00e−02
Value: B
Planomicrobium.glaciei
3
2
1
0
A (n=14)
B (n=15)
C (n=11)
surface

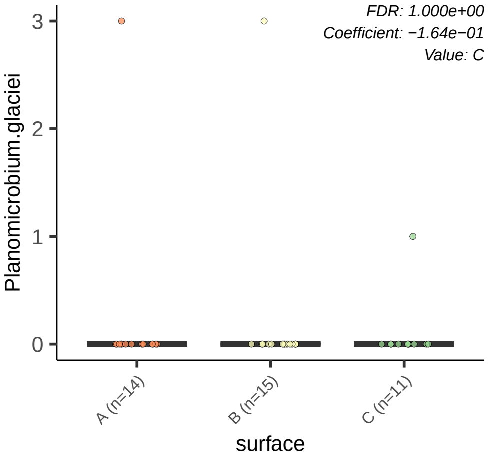
FDR: 1.000e+00
Coefficient: −1.64e−01
Value: C
Planomicrobium.glaciei
3
2
1
0
A (n=14)
B (n=15)
C (n=11)
surface

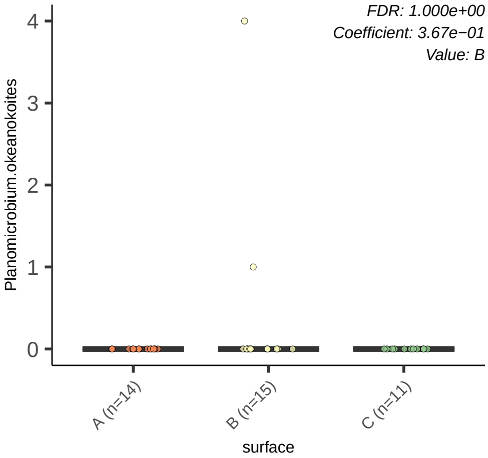

FDR: 1.000e+00
Coefficient: 3.67e−01
Value: B
Planomicrobium.okeanokoites
4
3
2
1
0
A (n=14)
B (n=15)
C (n=11)
surface

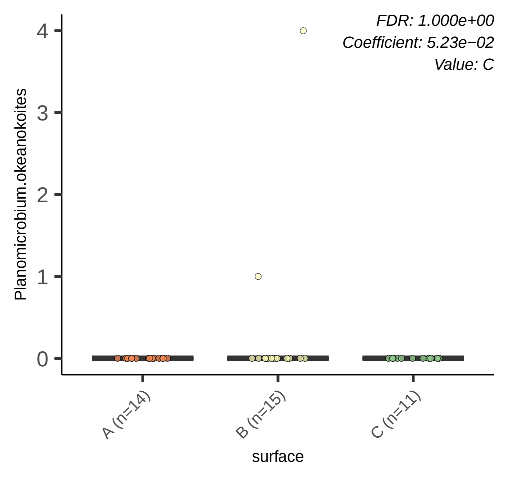
FDR: 1.000e+00
Coefficient: 5.23e−02
Value: C
Planomicrobium.okeanokoites
0
1
2
3
4
A (n=14)
B (n=15)
C (n=11)
surface

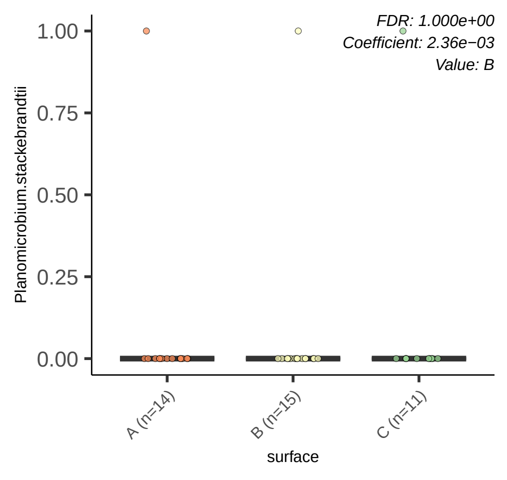
FDR: 1.000e+00
Coefficient: 2.36e−03
Value: B
1.00
0.75
0.50
0.25
0.00
Planomicrobium.stackebrandtii
A (n=14)
B (n=15)
C (n=11)
surface

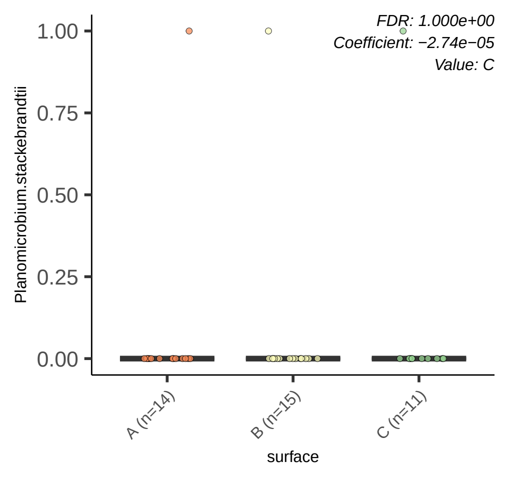
FDR: 1.000e+00
Coefficient: −2.74e−05
Value: C
Planomicrobium.stackebrandtii
1.00
0.75
0.50
0.25
0.00
A (n=14)
B (n=15)
C (n=11)
surface

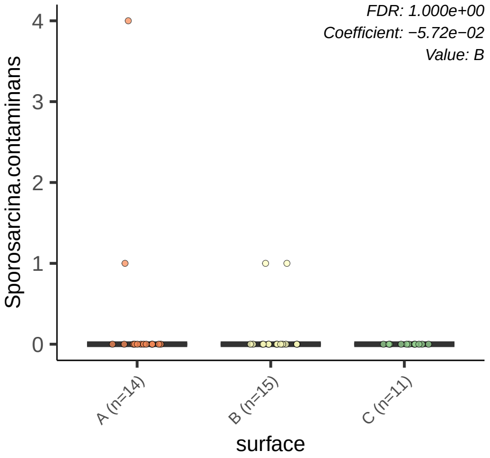

FDR: 1.000e+00
Coefficient: −5.72e−02
Value: B
Sporosarcina.contaminans
0
1
2
3
4
A (n=14)
B (n=15)
C (n=11)
surface

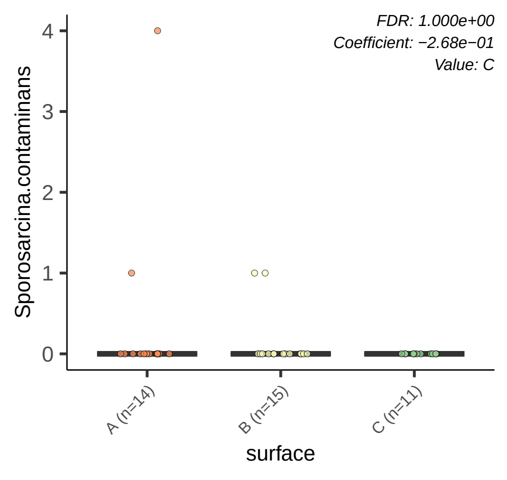
FDR: 1.000e+00
Coefficient: −2.68e−01
Value: C
Sporosarcina.contaminans
4
3
2
1
0
A (n=14)
B (n=15)
C (n=11)
surface

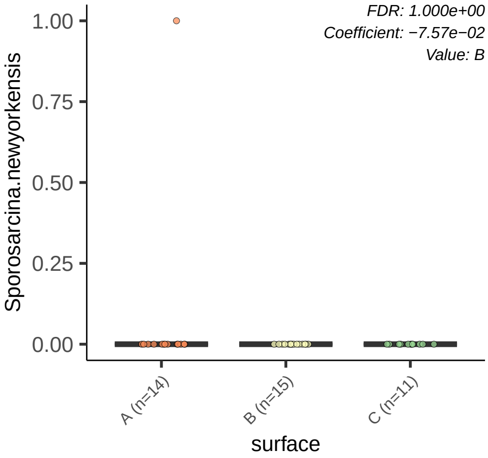

FDR: 1.000e+00
Coefficient: −7.57e−02
Value: B
Sporosarcina.newyorkensis
1.00
0.75
0.50
0.25
0.00
A (n=14)
B (n=15)
C (n=11)
surface

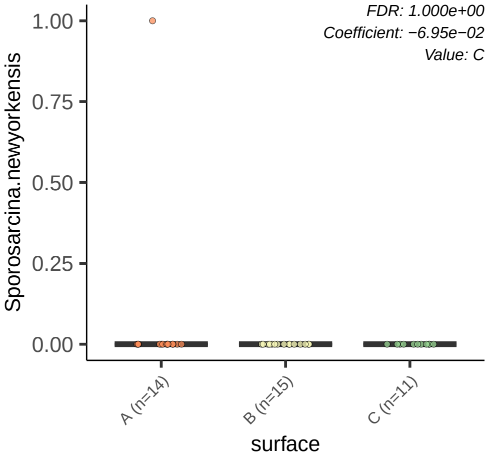
FDR: 1.000e+00
Coefficient: −6.95e−02
Value: C
Sporosarcina.newyorkensis
1.00
0.75
0.50
0.25
0.00
A (n=14)
B (n=15)
C (n=11)
surface

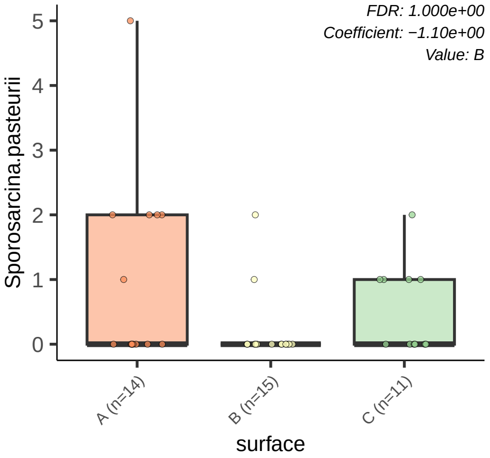

FDR: 1.000e+00
Coefficient: −1.10e+00
Value: B
Sporosarcina.pasteurii
5
4
3
2
1
0
A (n=14)
B (n=15)
C (n=11)
surface

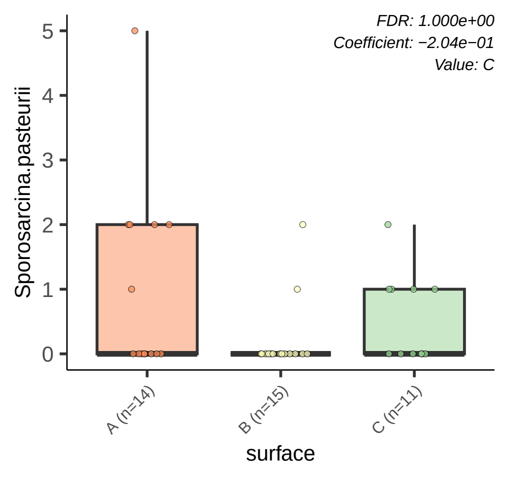
FDR: 1.000e+00
Coefficient: −2.04e−01
Value: C
Sporosarcina.pasteurii
0
1
2
3
4
5
A (n=14)
B (n=15)
C (n=11)
surface

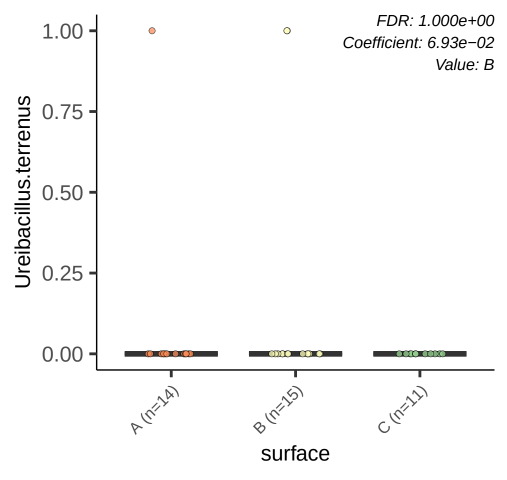

FDR: 1.000e+00
Coefficient: 6.93e−02
Value: B
Ureibacillus.terrenus
1.00
0.75
0.50
0.25
0.00
A (n=14)
B (n=15)
C (n=11)
surface

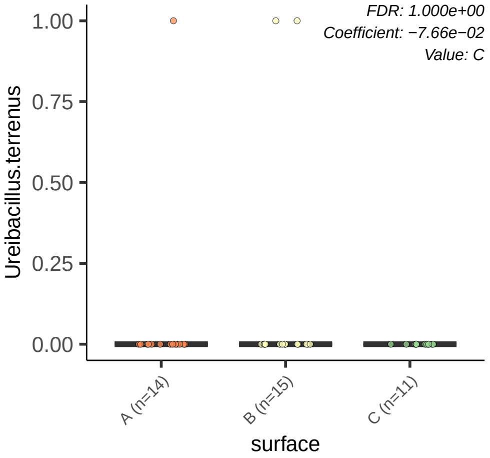

FDR: 1.000e+00
Coefficient: −7.66e−02
Value: C
Ureibacillus.terrenus
1.00
0.75
0.50
0.25
0.00
A (n=14)
B (n=15)
C (n=11)
surface

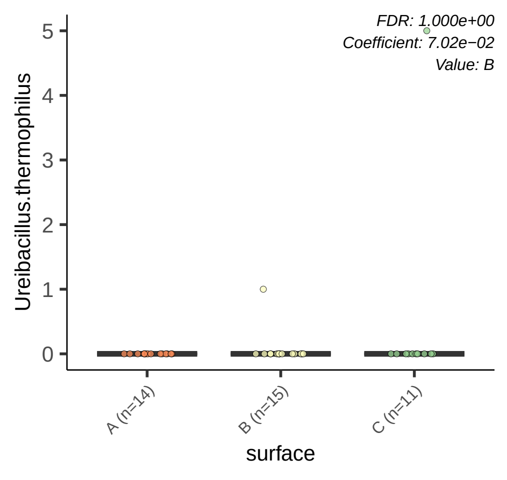
FDR: 1.000e+00
Coefficient: 7.02e−02
Value: B
Ureibacillus.thermophilus
0
1
2
3
4
5
A (n=14)
B (n=15)
C (n=11)
surface

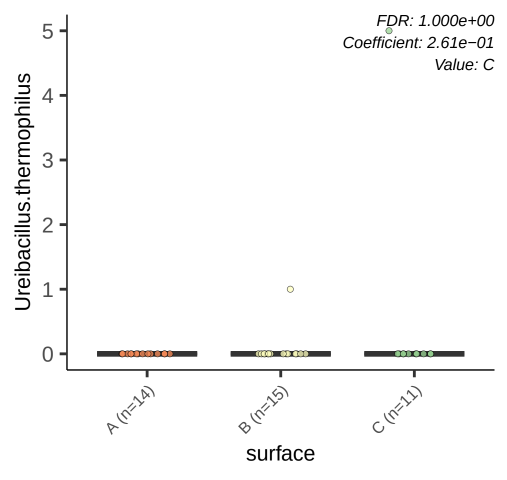
FDR: 1.000e+00
Coefficient: 2.61e−01
Value: C
Ureibacillus.thermophilus
0
1
2
3
4
5
A (n=14)
B (n=15)
C (n=11)
surface

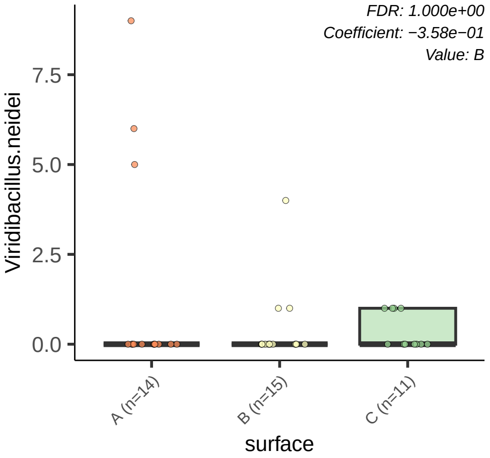

FDR: 1.000e+00
Coefficient: −3.58e−01
Value: B
Viridibacillus.neidei
7.5
5.0
2.5
0.0
A (n=14)
B (n=15)
C (n=11)
surface

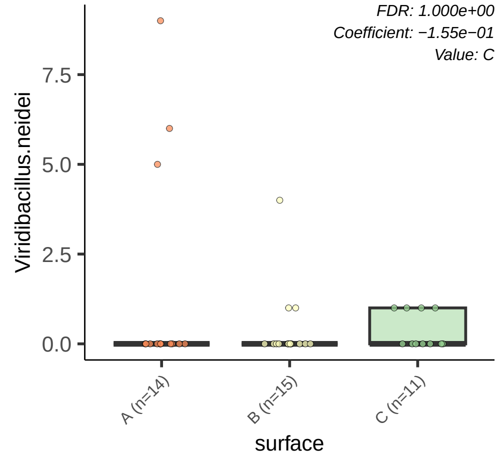

FDR: 1.000e+00
Coefficient: −1.55e−01
Value: C
Viridibacillus.neidei
7.5
5.0
2.5
0.0
A (n=14)
B (n=15)
C (n=11)
surface

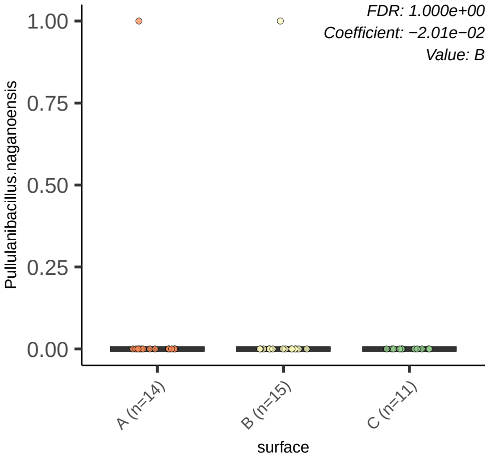
FDR: 1.000e+00
Coefficient: −2.01e−02
Value: B
1.00
0.75
0.50
0.25
0.00
Pullulanibacillus.naganoensis
A (n=14)
B (n=15)
C (n=11)
surface

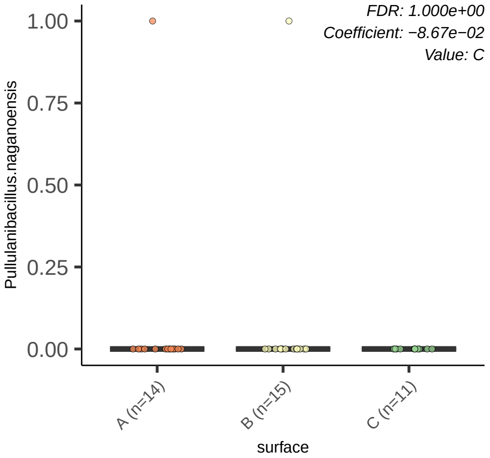

FDR: 1.000e+00
Coefficient: −8.67e−02
Value: C
Pullulanibacillus.naganoensis
1.00
0.75
0.50
0.25
0.00
A (n=14)
B (n=15)
C (n=11)
surface

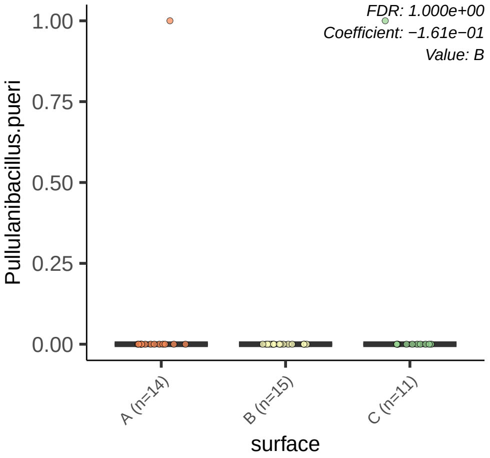

FDR: 1.000e+00
Coefficient: −1.61e−01
Value: B
Pullulanibacillus.pueri
1.00
0.75
0.50
0.25
0.00
A (n=14)
B (n=15)
C (n=11)
surface

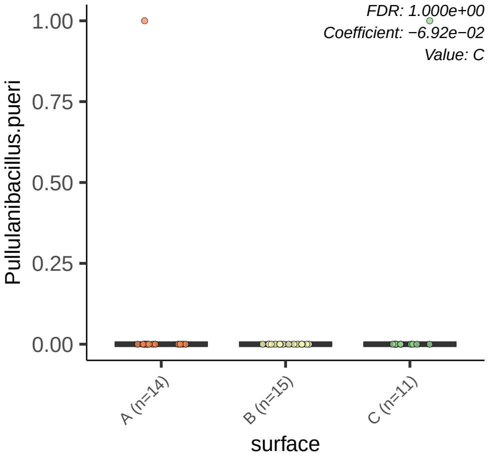
FDR: 1.000e+00
Coefficient: −6.92e−02
Value: C
Pullulanibacillus.pueri
1.00
0.75
0.50
0.25
0.00
A (n=14)
B (n=15)
C (n=11)
surface

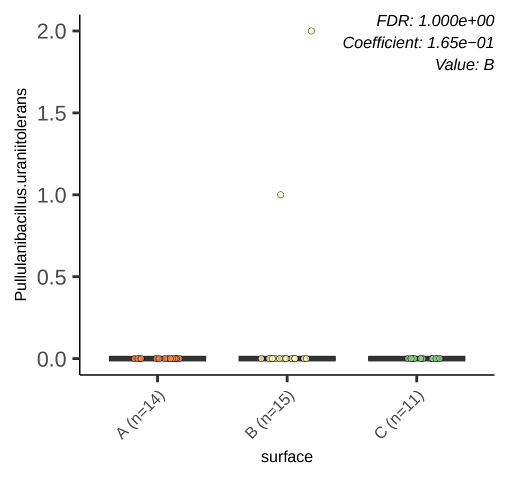

FDR: 1.000e+00
Coefficient: 1.65e−01
Value: B
Pullulanibacillus.uraniitolerans
2.0
1.5
1.0
0.5
0.0
A (n=14)
B (n=15)
C (n=11)
surface

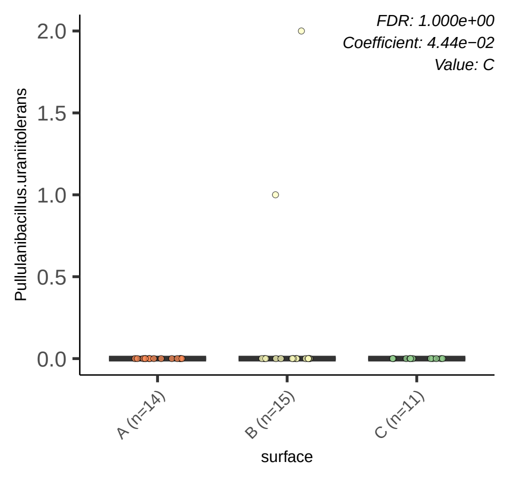
FDR: 1.000e+00
Coefficient: 4.44e−02
Value: C
Pullulanibacillus.uraniitolerans
2.0
1.5
1.0
0.5
0.0
A (n=14)
B (n=15)
C (n=11)
surface

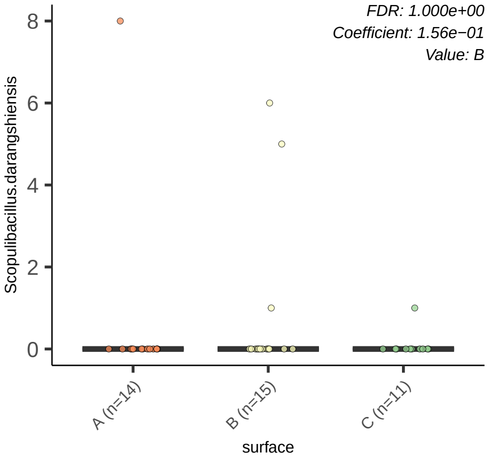
FDR: 1.000e+00
Coefficient: 1.56e−01
Value: B
Scopulibacillus.darangshiensis
8
6
4
2
0
A (n=14)
B (n=15)
C (n=11)
surface

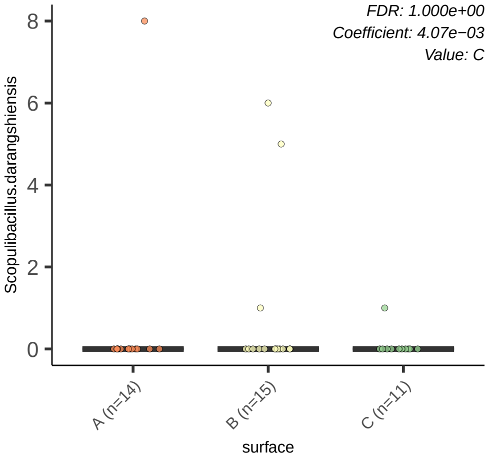
FDR: 1.000e+00
Coefficient: 4.07e−03
Value: C
Scopulibacillus.darangshiensis
8
6
4
2
0
A (n=14)
B (n=15)
C (n=11)
surface

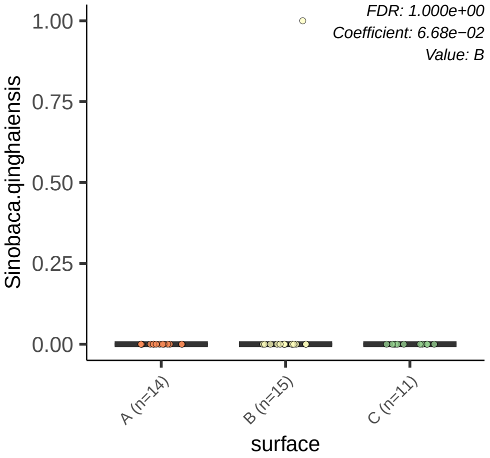
FDR: 1.000e+00
Coefficient: 6.68e−02
Value: B
Sinobaca.qinghaiensis
1.00
0.75
0.50
0.25
0.00
A (n=14)
B (n=15)
C (n=11)
surface

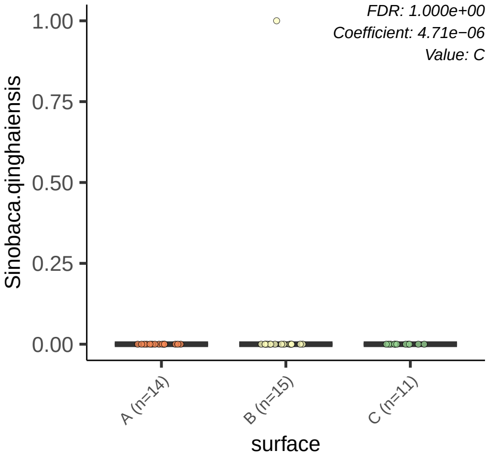

FDR: 1.000e+00
Coefficient: 4.71e−06
Value: C
Sinobaca.qinghaiensis
1.00
0.75
0.50
0.25
0.00
A (n=14)
B (n=15)
C (n=11)
surface

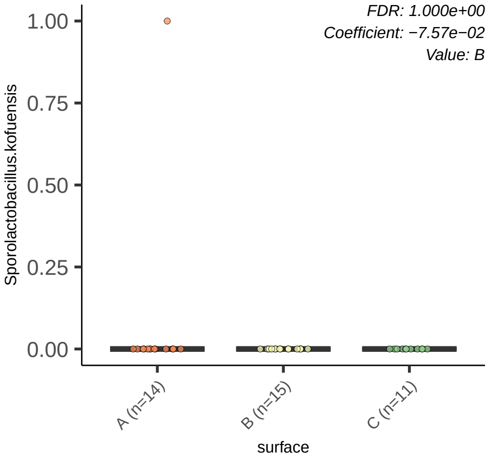

FDR: 1.000e+00
Coefficient: −7.57e−02
Value: B
Sporolactobacillus.kofuensis
1.00
0.75
0.50
0.25
0.00
A (n=14)
B (n=15)
C (n=11)
surface

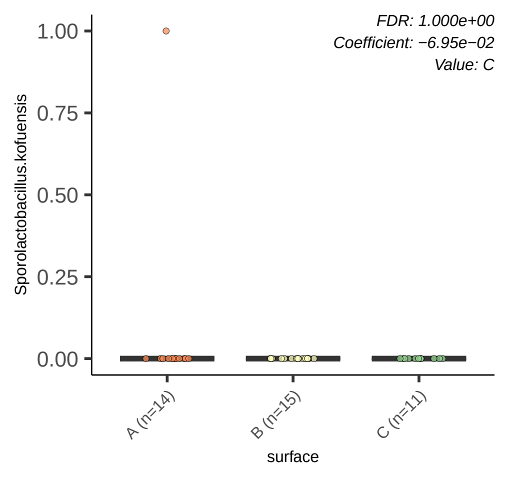

FDR: 1.000e+00
Coefficient: −6.95e−02
Value: C
1.00
0.75
0.50
0.25
0.00
Sporolactobacillus.kofuensis
A (n=14)
B (n=15)
C (n=11)
surface

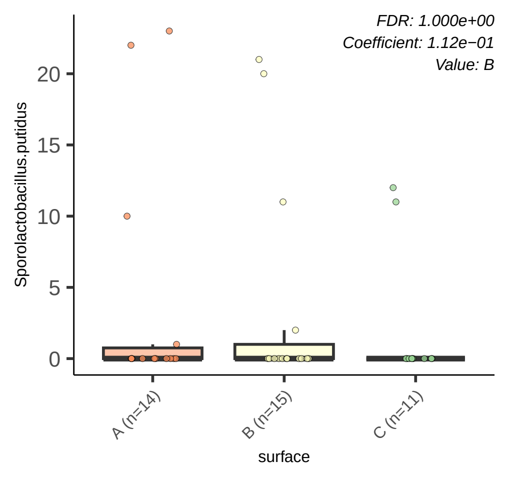
FDR: 1.000e+00
Coefficient: 1.12e−01
Value: B
Sporolactobacillus.putidus
0
5
10
15
20
A (n=14)
B (n=15)
C (n=11)
surface

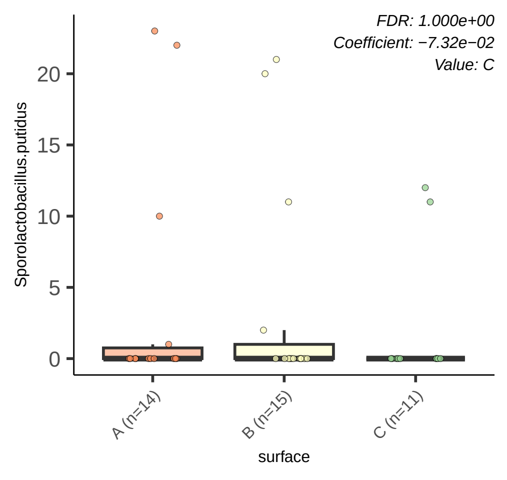
FDR: 1.000e+00
Coefficient: −7.32e−02
Value: C
Sporolactobacillus.putidus
0
5
10
15
20
A (n=14)
B (n=15)
C (n=11)
surface

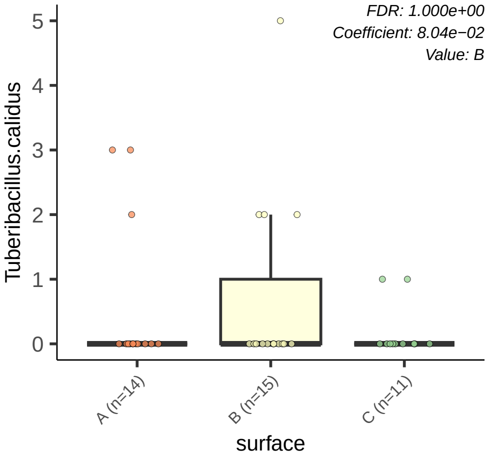

FDR: 1.000e+00
Coefficient: 8.04e−02
Value: B
Tuberibacillus.calidus
0
1
2
3
4
5
A (n=14)
B (n=15)
C (n=11)
surface

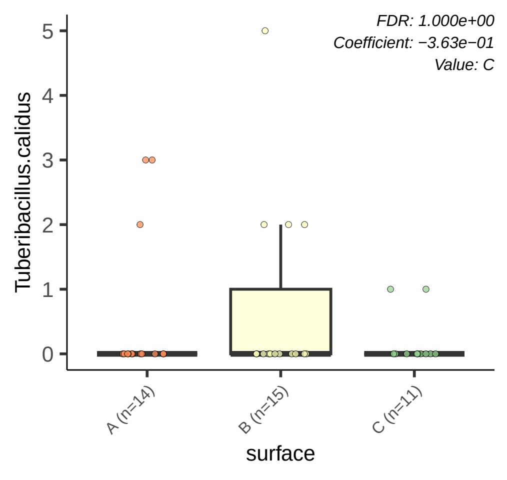
FDR: 1.000e+00
Coefficient: −3.63e−01
Value: C
Tuberibacillus.calidus
0
1
2
3
4
5
A (n=14)
B (n=15)
C (n=11)
surface

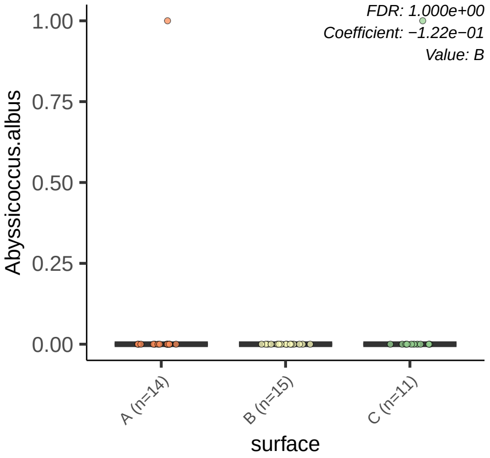
FDR: 1.000e+00
Coefficient: −1.22e−01
Value: B
Abyssicoccus.albus
1.00
0.75
0.50
0.25
0.00
A (n=14)
B (n=15)
C (n=11)
surface

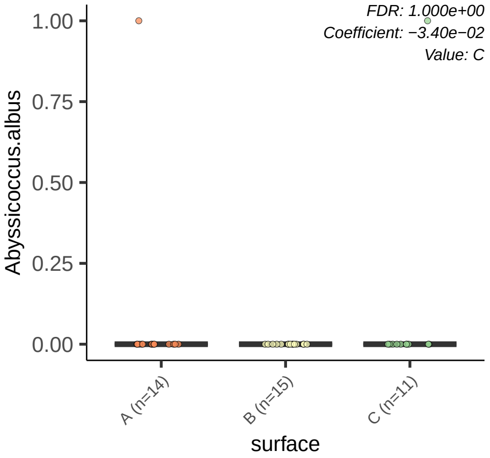
FDR: 1.000e+00
Coefficient: −3.40e−02
Value: C
Abyssicoccus.albus
1.00
0.75
0.50
0.25
0.00
A (n=14)
B (n=15)
C (n=11)
surface

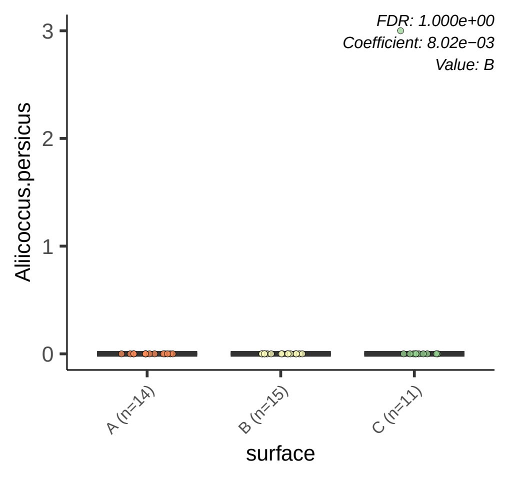

FDR: 1.000e+00
Coefficient: 8.02e−03
Value: B
Aliicoccus.persicus
3
2
1
0
A (n=14)
B (n=15)
C (n=11)
surface

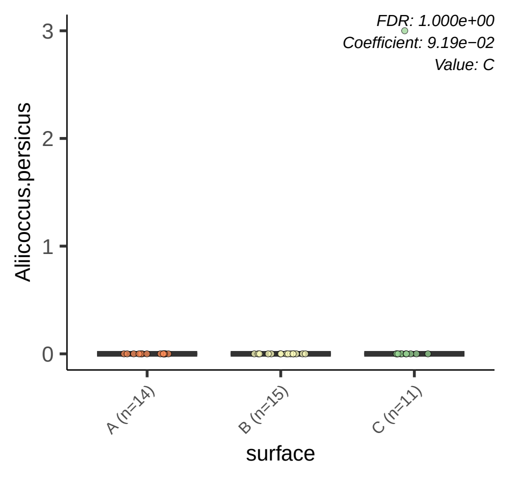
FDR: 1.000e+00
Coefficient: 9.19e−02
Value: C
Aliicoccus.persicus
3
2
1
0
A (n=14)
B (n=15)
C (n=11)
surface

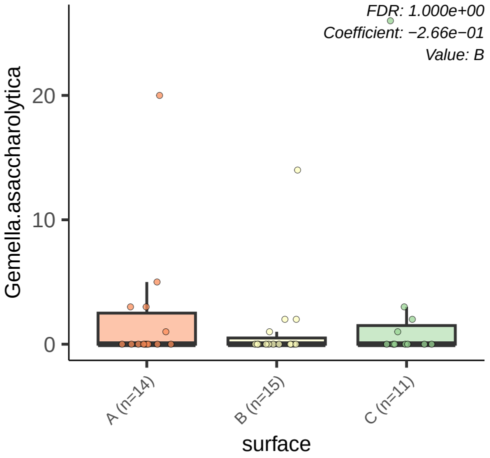

FDR: 1.000e+00
Coefficient: −2.66e−01
Value: B
Gemella.asaccharolytica
20
10
0
A (n=14)
B (n=15)
C (n=11)
surface

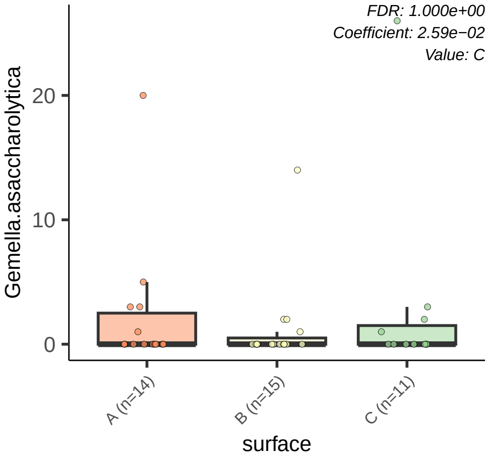

FDR: 1.000e+00
Coefficient: 2.59e−02
Value: C
Gemella.asaccharolytica
20
10
0
A (n=14)
B (n=15)
C (n=11)
surface

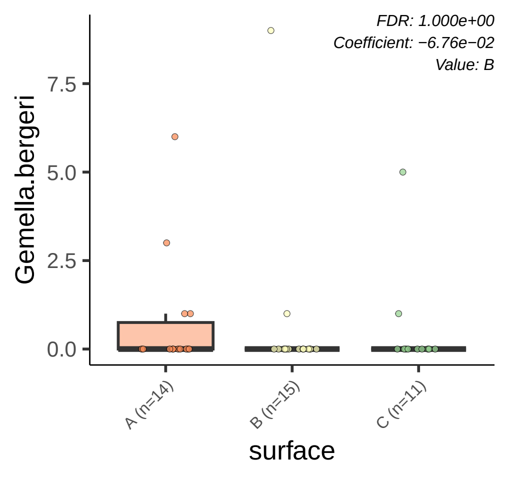
FDR: 1.000e+00
Coefficient: −6.76e−02
Value: B
Gemella.bergeri
7.5
5.0
2.5
0.0
A (n=14)
B (n=15)
C (n=11)
surface

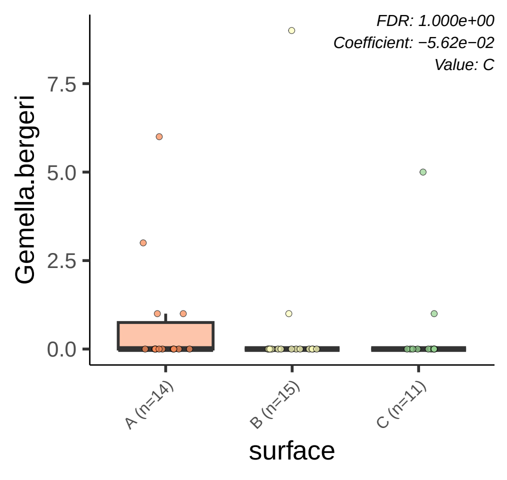
FDR: 1.000e+00
Coefficient: −5.62e−02
Value: C
Gemella.bergeri
0.0
2.5
5.0
7.5
A (n=14)
B (n=15)
C (n=11)
surface

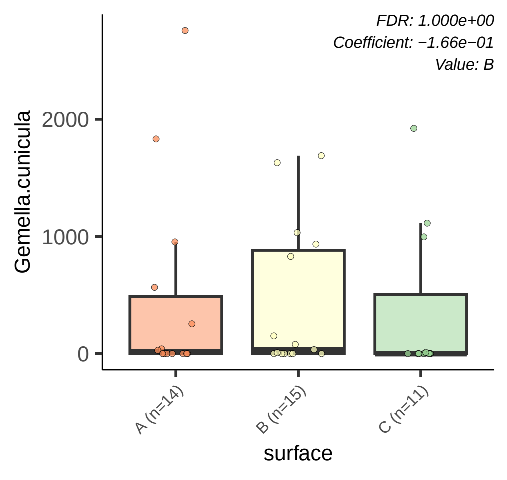
FDR: 1.000e+00
Coefficient: −1.66e−01
Value: B
Gemella.cunicula
2000
1000
0
A (n=14)
B (n=15)
C (n=11)
surface

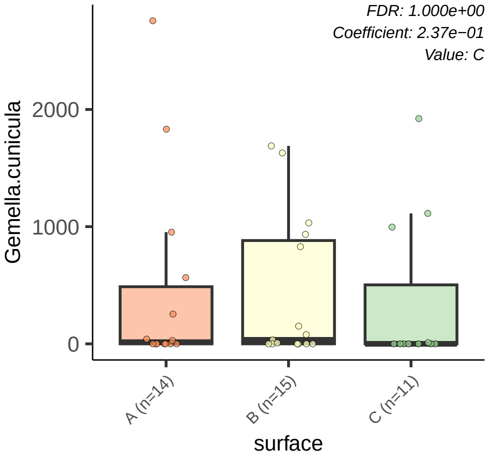
FDR: 1.000e+00
Coefficient: 2.37e−01
Value: C
2000
1000
0
Gemella.cunicula
A (n=14)
B (n=15)
C (n=11)
surface

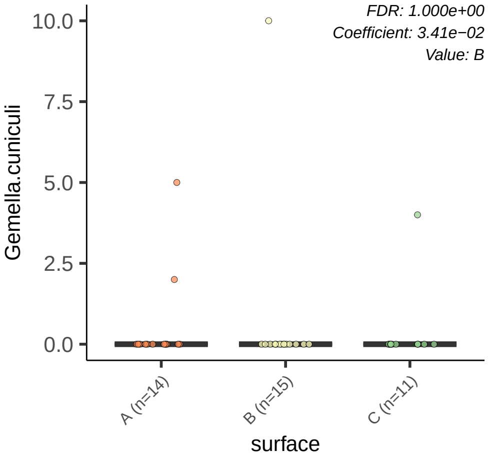

FDR: 1.000e+00
Coefficient: 3.41e−02
Value: B
Gemella.cuniculi
10.0
7.5
5.0
2.5
0.0
A (n=14)
B (n=15)
C (n=11)
surface

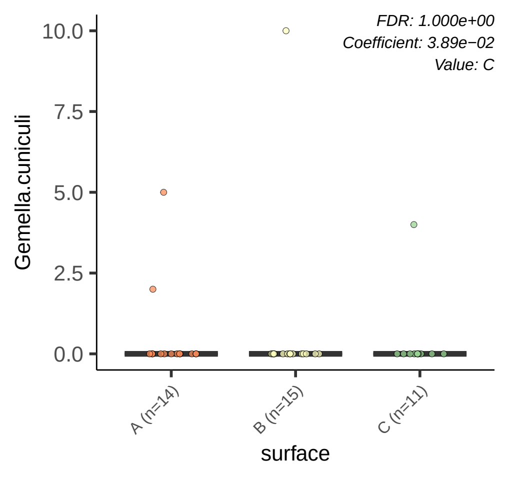
FDR: 1.000e+00
Coefficient: 3.89e−02
Value: C
Gemella.cuniculi
10.0
7.5
5.0
2.5
0.0
A (n=14)
B (n=15)
C (n=11)
surface

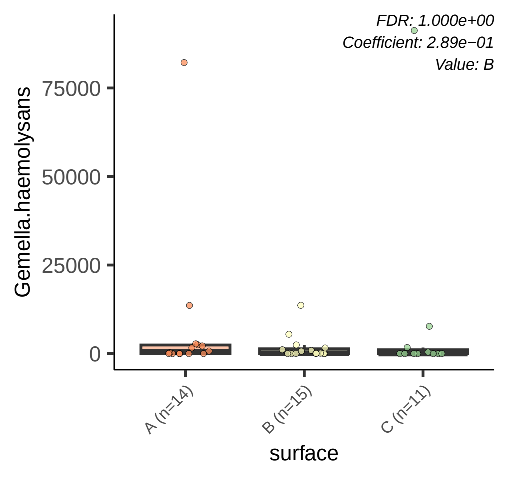
FDR: 1.000e+00
Coefficient: 2.89e−01
Value: B
Gemella.haemolysans
75000
50000
25000
0
A (n=14)
B (n=15)
C (n=11)
surface

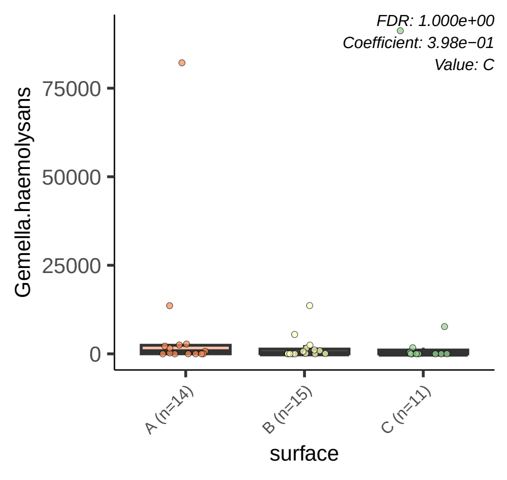

FDR: 1.000e+00
Coefficient: 3.98e−01
Value: C
Gemella.haemolysans
75000
50000
25000
0
A (n=14)
B (n=15)
C (n=11)
surface

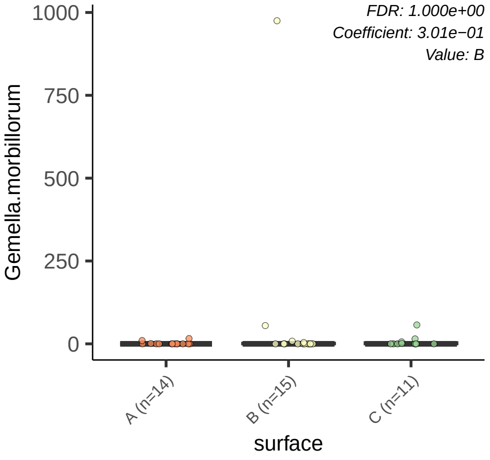
FDR: 1.000e+00
Coefficient: 3.01e−01
Value: B
Gemella.morbillorum
1000
750
500
250
0
A (n=14)
B (n=15)
C (n=11)
surface

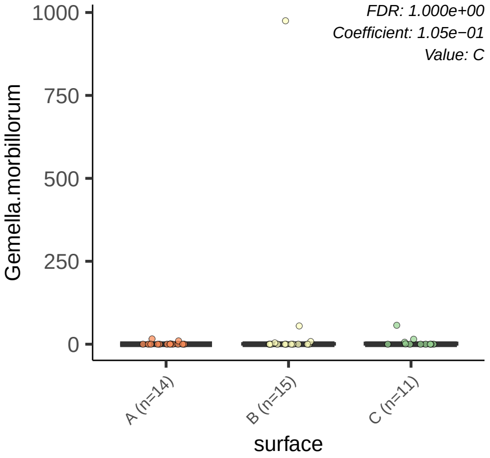
FDR: 1.000e+00
Coefficient: 1.05e−01
Value: C
Gemella.morbillorum
1000
750
500
250
0
A (n=14)
B (n=15)
C (n=11)
surface

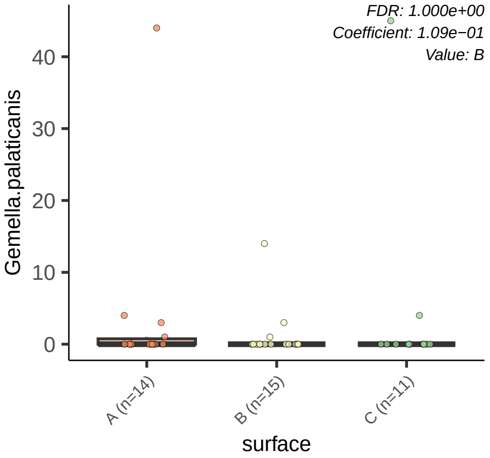
FDR: 1.000e+00
Coefficient: 1.09e−01
Value: B
Gemella.palaticanis
0
10
20
30
40
A (n=14)
B (n=15)
C (n=11)
surface

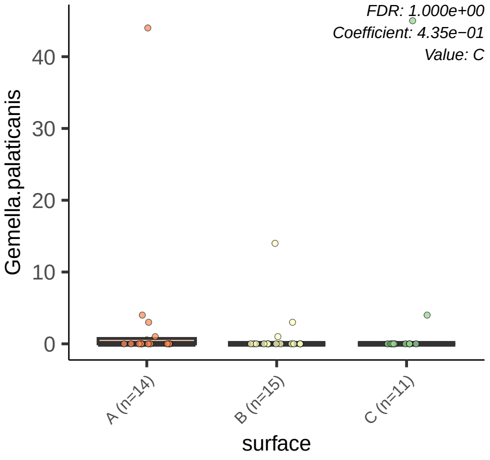

FDR: 1.000e+00
Coefficient: 4.35e−01
Value: C
Gemella.palaticanis
0
10
20
30
40
A (n=14)
B (n=15)
C (n=11)
surface

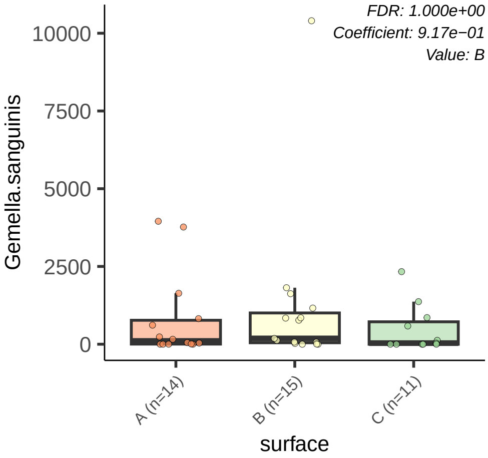

FDR: 1.000e+00
Coefficient: 9.17e−01
Value: B
10000
7500
5000
2500
0
Gemella.sanguinis
A (n=14)
B (n=15)
C (n=11)
surface

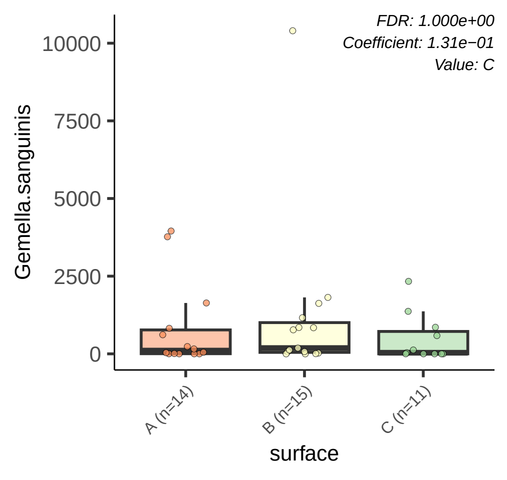
FDR: 1.000e+00
Coefficient: 1.31e−01
Value: C
10000
7500
5000
2500
0
Gemella.sanguinis
A (n=14)
B (n=15)
C (n=11)
surface

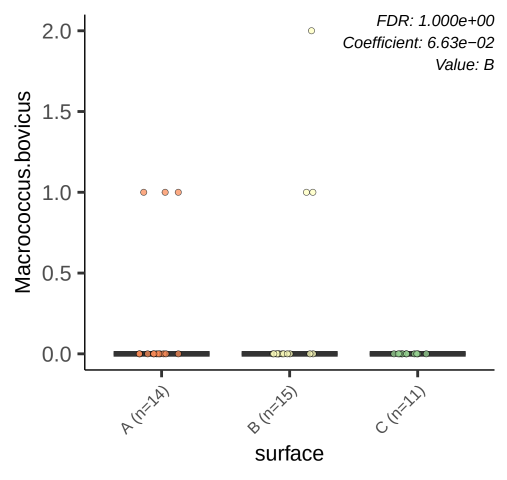

FDR: 1.000e+00
Coefficient: 6.63e−02
Value: B
Macrococcus.bovicus
2.0
1.5
1.0
0.5
0.0
A (n=14)
B (n=15)
C (n=11)
surface

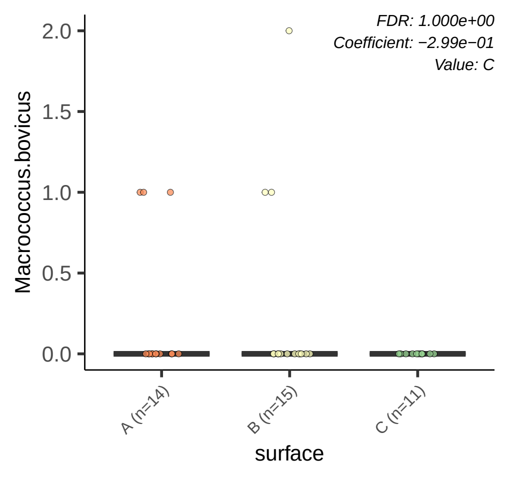
FDR: 1.000e+00
Coefficient: −2.99e−01
Value: C
Macrococcus.bovicus
2.0
1.5
1.0
0.5
0.0
A (n=14)
B (n=15)
C (n=11)
surface

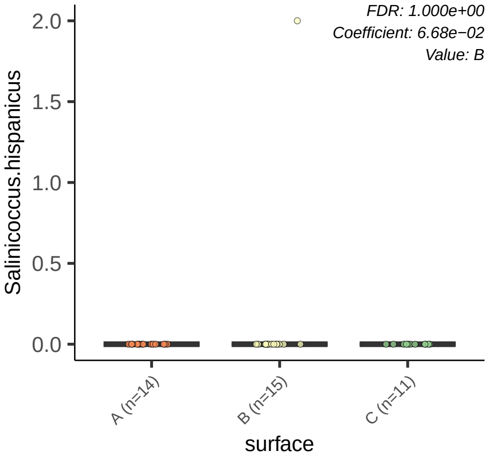
FDR: 1.000e+00
Coefficient: 6.68e−02
Value: B
Salinicoccus.hispanicus
2.0
1.5
1.0
0.5
0.0
A (n=14)
B (n=15)
C (n=11)
surface

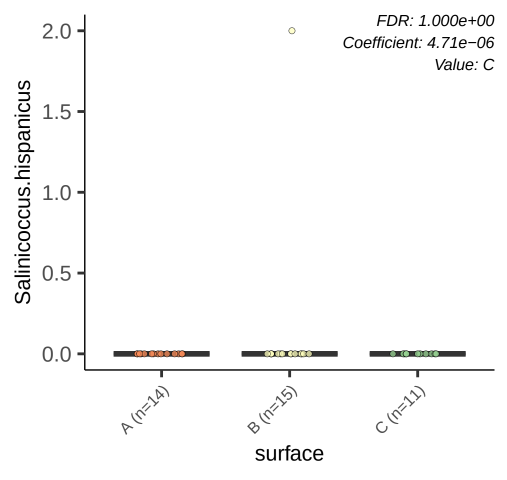
FDR: 1.000e+00
Coefficient: 4.71e−06
Value: C
Salinicoccus.hispanicus
2.0
1.5
1.0
0.5
0.0
A (n=14)
B (n=15)
C (n=11)
surface

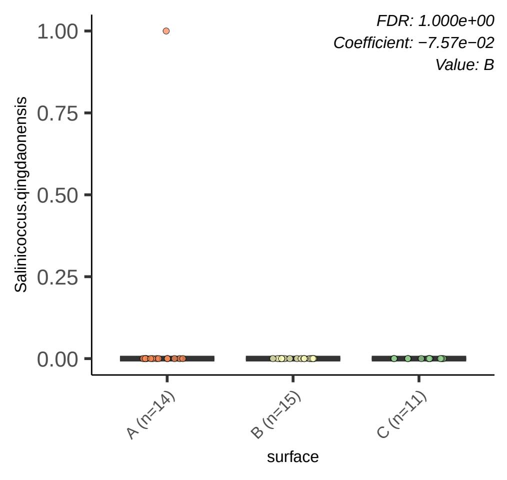
FDR: 1.000e+00
Coefficient: −7.57e−02
Value: B
Salinicoccus.qingdaonensis
1.00
0.75
0.50
0.25
0.00
A (n=14)
B (n=15)
C (n=11)
surface

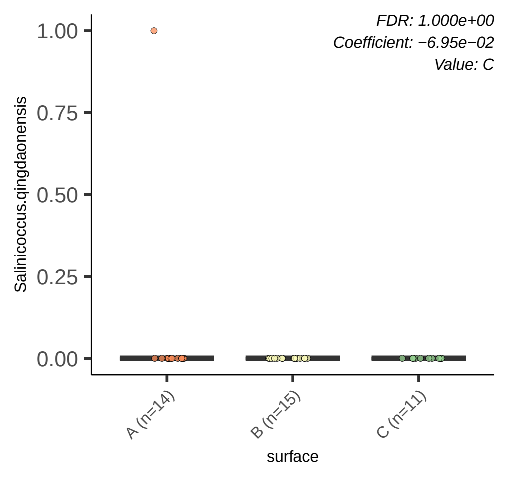
FDR: 1.000e+00
Coefficient: −6.95e−02
Value: C
Salinicoccus.qingdaonensis
1.00
0.75
0.50
0.25
0.00
A (n=14)
B (n=15)
C (n=11)
surface

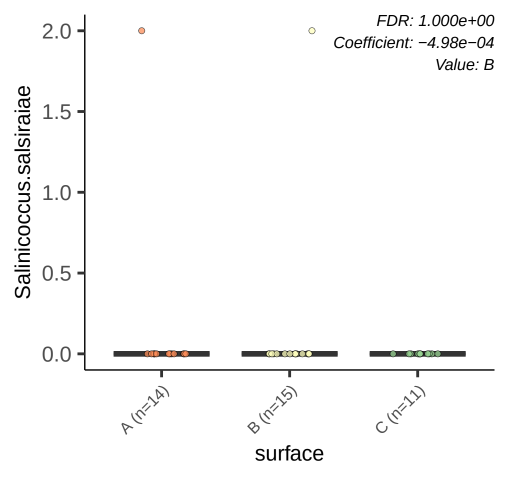
FDR: 1.000e+00
Coefficient: −4.98e−04
Value: B
Salinicoccus.salsiraiae
2.0
1.5
1.0
0.5
0.0
A (n=14)
B (n=15)
C (n=11)
surface

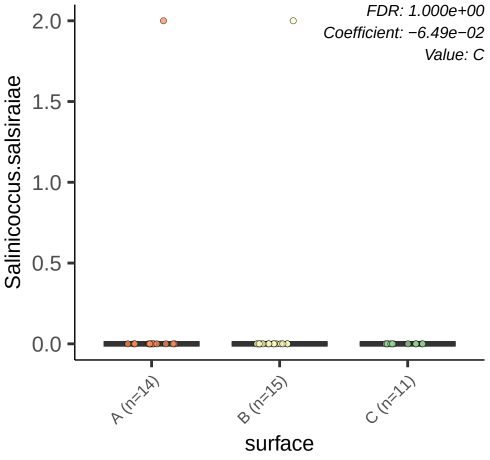
FDR: 1.000e+00
Coefficient: −6.49e−02
Value: C
Salinicoccus.salsiraiae
2.0
1.5
1.0
0.5
0.0
A (n=14)
B (n=15)
C (n=11)
surface

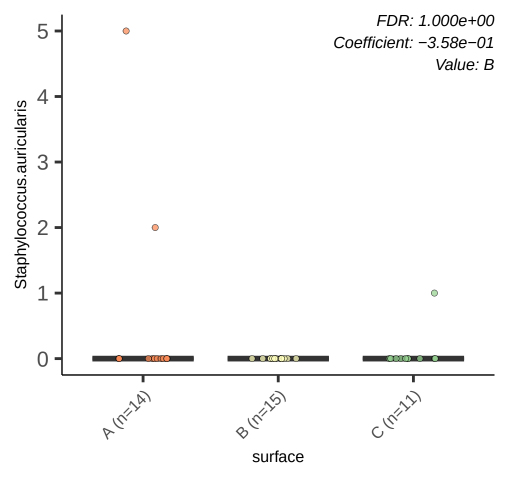

FDR: 1.000e+00
Coefficient: −3.58e−01
Value: B
Staphylococcus.auricularis
0
1
2
3
4
5
A (n=14)
B (n=15)
C (n=11)
surface

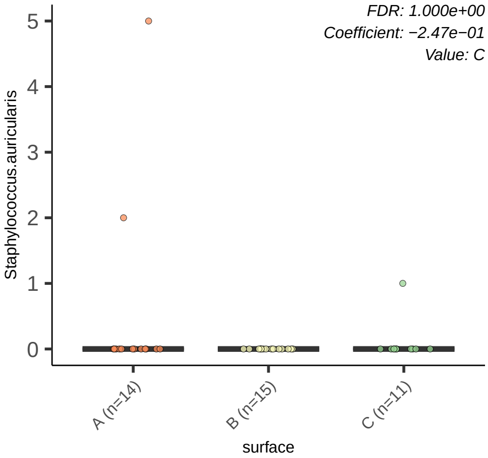
FDR: 1.000e+00
Coefficient: −2.47e−01
Value: C
Staphylococcus.auricularis
0
1
2
3
4
5
A (n=14)
B (n=15)
C (n=11)
surface

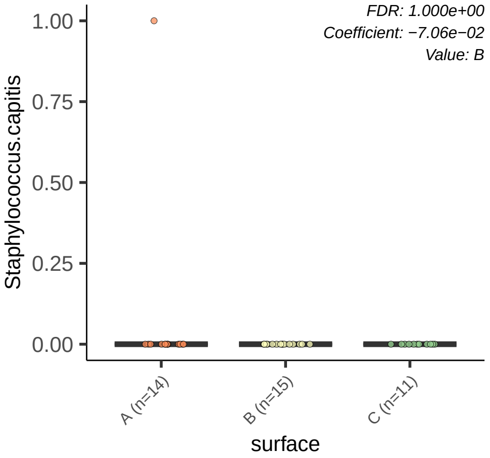
FDR: 1.000e+00
Coefficient: −7.06e−02
Value: B
Staphylococcus.capitis
1.00
0.75
0.50
0.25
0.00
A (n=14)
B (n=15)
C (n=11)
surface

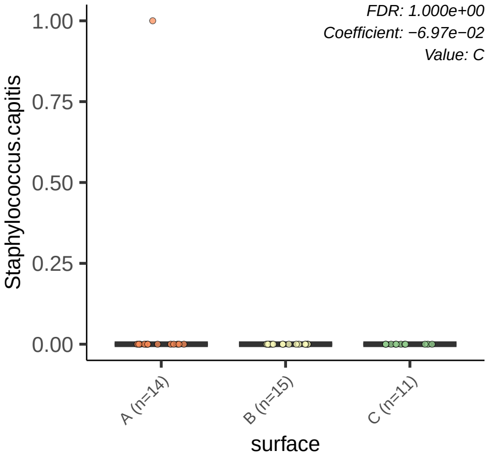

FDR: 1.000e+00
Coefficient: −6.97e−02
Value: C
Staphylococcus.capitis
1.00
0.75
0.50
0.25
0.00
A (n=14)
B (n=15)
C (n=11)
surface

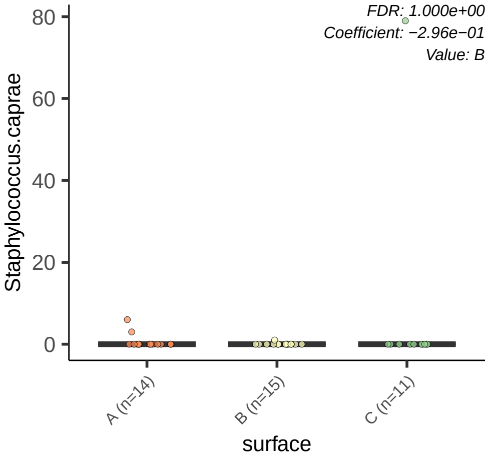
FDR: 1.000e+00
Coefficient: −2.96e−01
Value: B
Staphylococcus.caprae
80
60
40
20
0
A (n=14)
B (n=15)
C (n=11)
surface

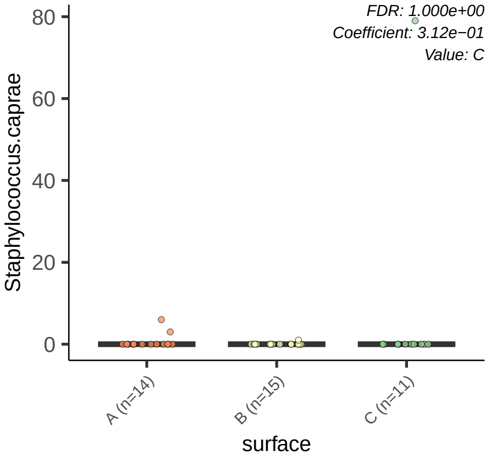
FDR: 1.000e+00
Coefficient: 3.12e−01
Value: C
Staphylococcus.caprae
80
60
40
20
0
A (n=14)
B (n=15)
C (n=11)
surface

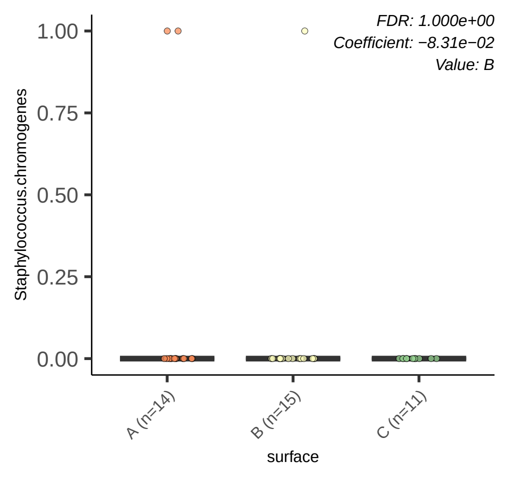
FDR: 1.000e+00
Coefficient: −8.31e−02
Value: B
Staphylococcus.chromogenes
1.00
0.75
0.50
0.25
0.00
A (n=14)
B (n=15)
C (n=11)
surface

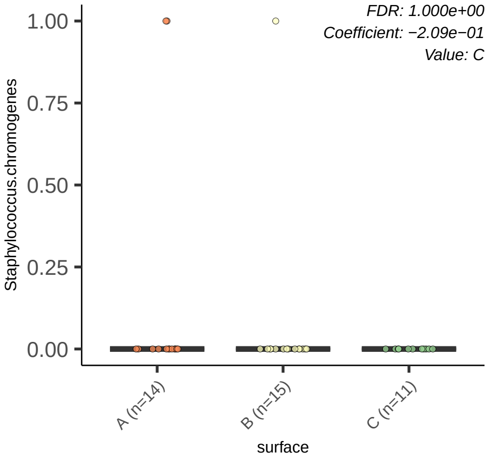
FDR: 1.000e+00
Coefficient: −2.09e−01
Value: C
1.00
0.75
0.50
0.25
0.00
Staphylococcus.chromogenes
A (n=14)
B (n=15)
C (n=11)
surface

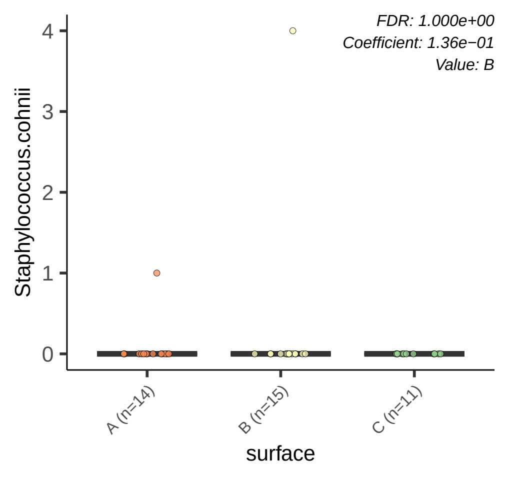

FDR: 1.000e+00
Coefficient: 1.36e−01
Value: B
Staphylococcus.cohnii
0
1
2
3
4
A (n=14)
B (n=15)
C (n=11)
surface

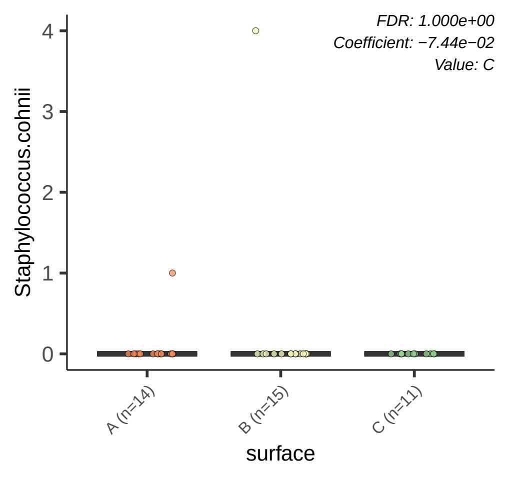

FDR: 1.000e+00
Coefficient: −7.44e−02
Value: C
Staphylococcus.cohnii
0
1
2
3
4
A (n=14)
B (n=15)
C (n=11)
surface

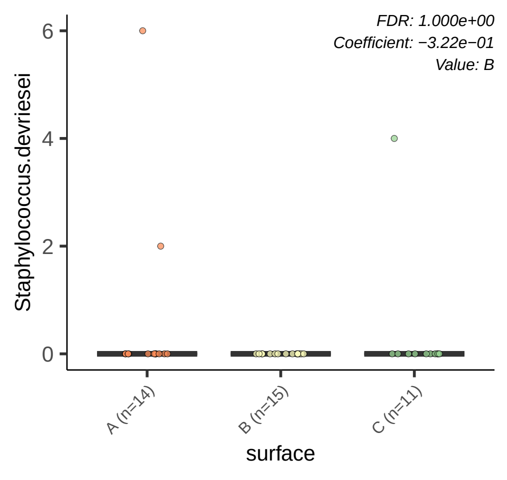
FDR: 1.000e+00
Coefficient: −3.22e−01
Value: B
Staphylococcus.devriesei
6
4
2
0
A (n=14)
B (n=15)
C (n=11)
surface

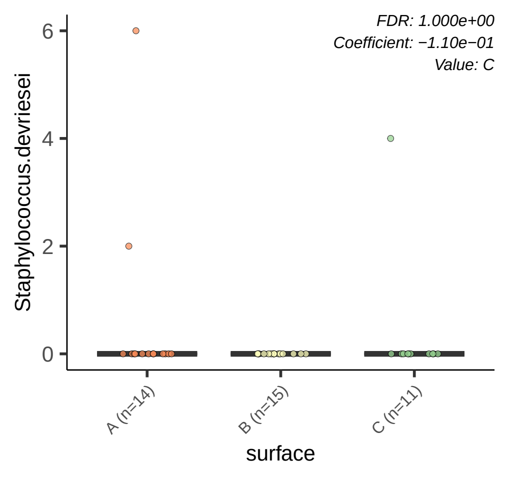
FDR: 1.000e+00
Coefficient: −1.10e−01
Value: C
Staphylococcus.devriesei
0
2
4
6
A (n=14)
B (n=15)
C (n=11)
surface

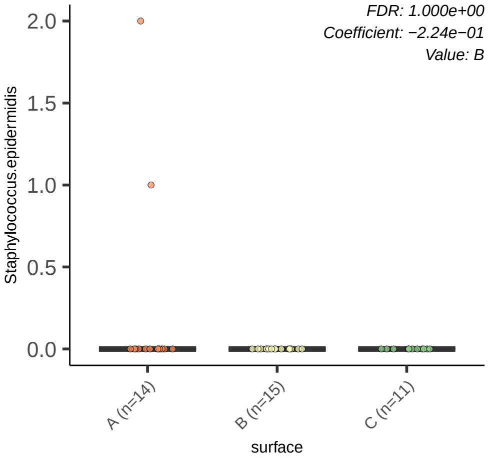
FDR: 1.000e+00
Coefficient: −2.24e−01
Value: B
Staphylococcus.epidermidis
2.0
1.5
1.0
0.5
0.0
A (n=14)
B (n=15)
C (n=11)
surface

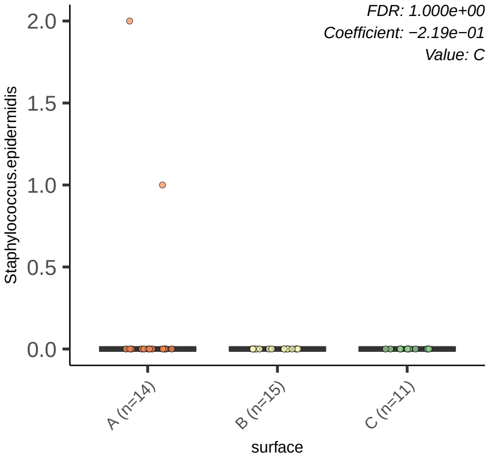
FDR: 1.000e+00
Coefficient: −2.19e−01
Value: C
Staphylococcus.epidermidis
2.0
1.5
1.0
0.5
0.0
A (n=14)
B (n=15)
C (n=11)
surface

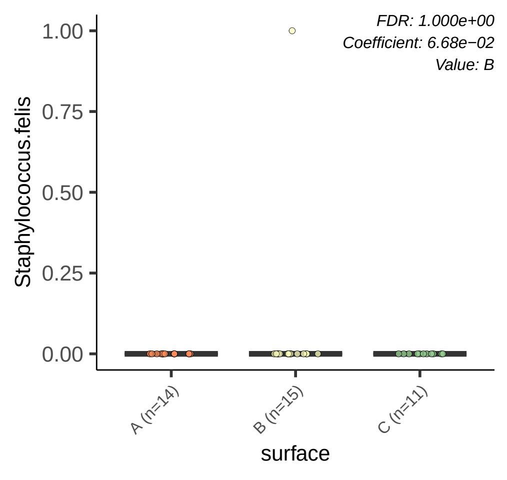

FDR: 1.000e+00
Coefficient: 6.68e−02
Value: B
Staphylococcus.felis
1.00
0.75
0.50
0.25
0.00
A (n=14)
B (n=15)
C (n=11)
surface

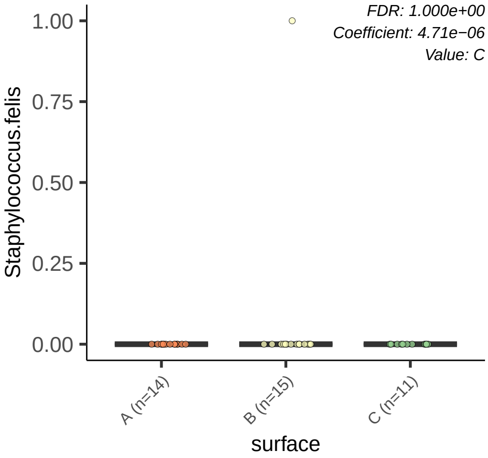
FDR: 1.000e+00
Coefficient: 4.71e−06
Value: C
Staphylococcus.felis
1.00
0.75
0.50
0.25
0.00
A (n=14)
B (n=15)
C (n=11)
surface

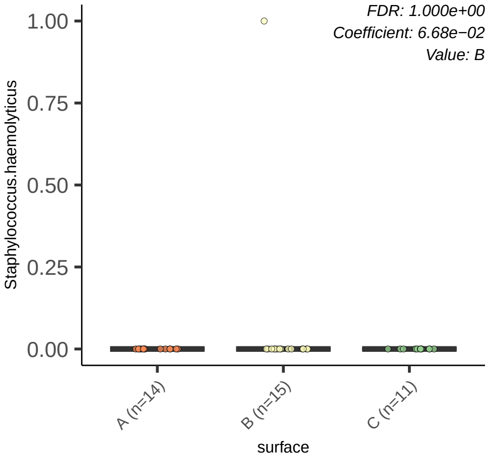

FDR: 1.000e+00
Coefficient: 6.68e−02
Value: B
Staphylococcus.haemolyticus
1.00
0.75
0.50
0.25
0.00
A (n=14)
B (n=15)
C (n=11)
surface

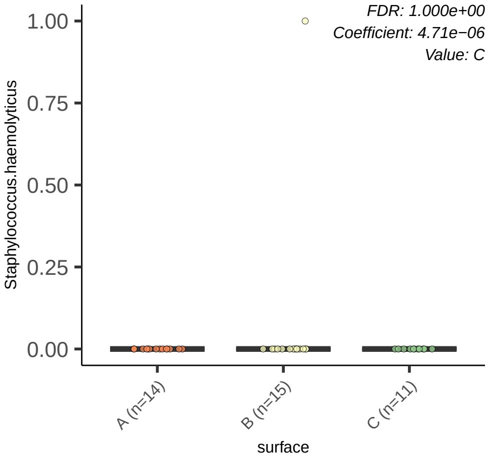

FDR: 1.000e+00
Coefficient: 4.71e−06
Value: C
1.00
0.75
0.50
0.25
0.00
Staphylococcus.haemolyticus
A (n=14)
B (n=15)
C (n=11)
surface

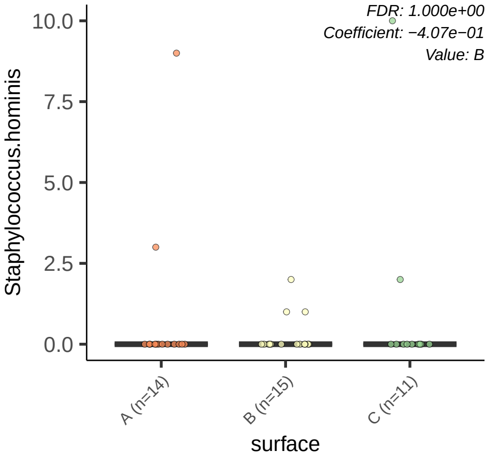
FDR: 1.000e+00
Coefficient: −4.07e−01
Value: B
Staphylococcus.hominis
10.0
7.5
5.0
2.5
0.0
A (n=14)
B (n=15)
C (n=11)
surface

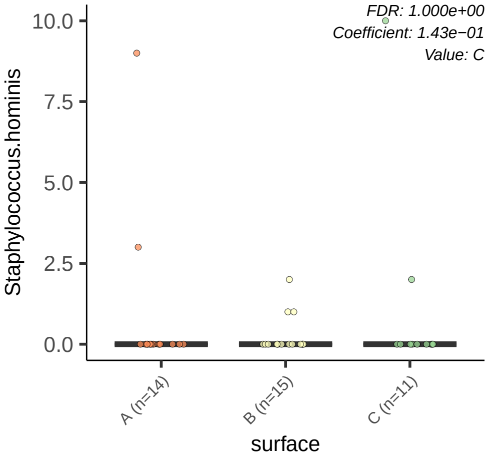
FDR: 1.000e+00
Coefficient: 1.43e−01
Value: C
Staphylococcus.hominis
10.0
7.5
5.0
2.5
0.0
A (n=14)
B (n=15)
C (n=11)
surface

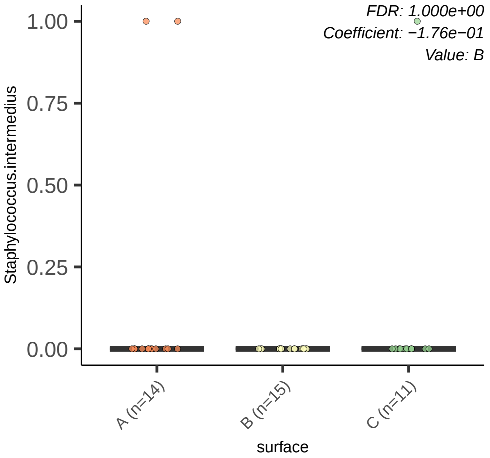
FDR: 1.000e+00
Coefficient: −1.76e−01
Value: B
Staphylococcus.intermedius
1.00
0.75
0.50
0.25
0.00
A (n=14)
B (n=15)
C (n=11)
surface

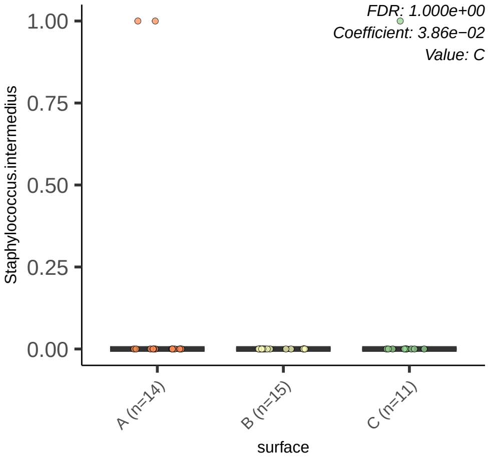
FDR: 1.000e+00
Coefficient: 3.86e−02
Value: C
Staphylococcus.intermedius
1.00
0.75
0.50
0.25
0.00
A (n=14)
B (n=15)
C (n=11)
surface

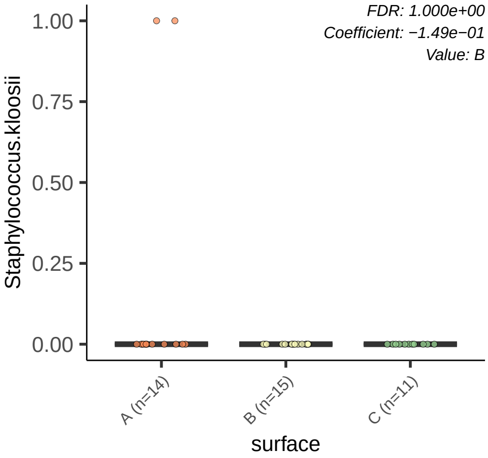

FDR: 1.000e+00
Coefficient: −1.49e−01
Value: B
Staphylococcus.kloosii
1.00
0.75
0.50
0.25
0.00
A (n=14)
B (n=15)
C (n=11)
surface

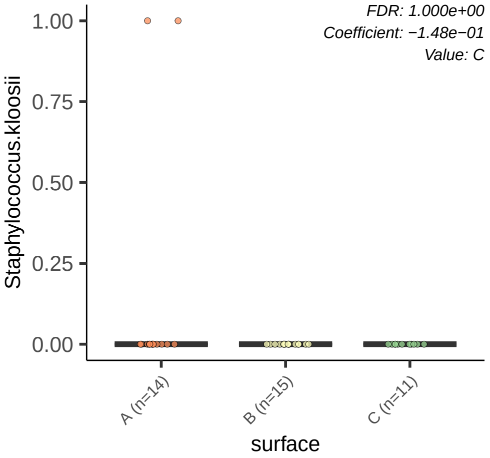
FDR: 1.000e+00
Coefficient: −1.48e−01
Value: C
Staphylococcus.kloosii
1.00
0.75
0.50
0.25
0.00
A (n=14)
B (n=15)
C (n=11)
surface

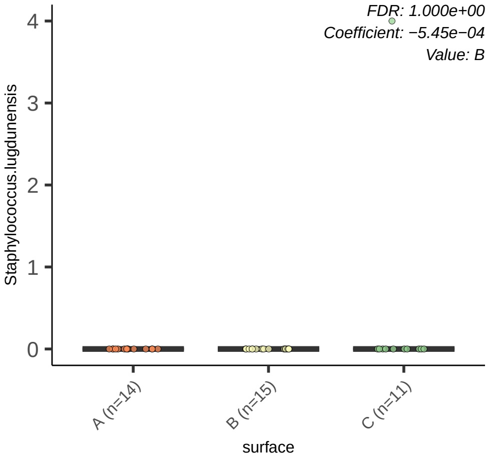
FDR: 1.000e+00
Coefficient: −5.45e−04
Value: B
Staphylococcus.lugdunensis
0
1
2
3
4
A (n=14)
B (n=15)
C (n=11)
surface

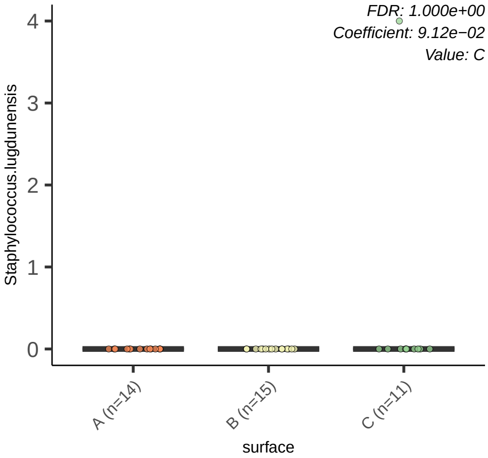
FDR: 1.000e+00
Coefficient: 9.12e−02
Value: C
Staphylococcus.lugdunensis
4
3
2
1
0
A (n=14)
B (n=15)
C (n=11)
surface

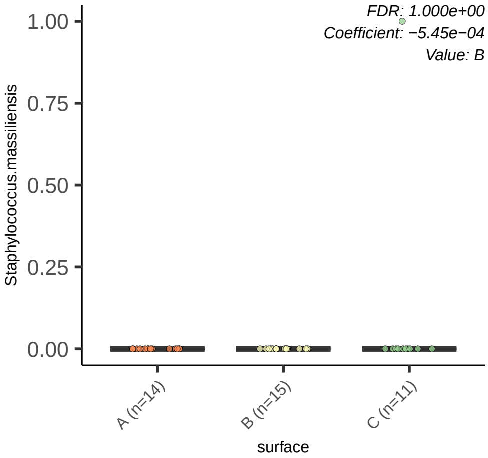

FDR: 1.000e+00
Coefficient: −5.45e−04
Value: B
1.00
0.75
0.50
0.25
0.00
Staphylococcus.massiliensis
A (n=14)
B (n=15)
C (n=11)
surface

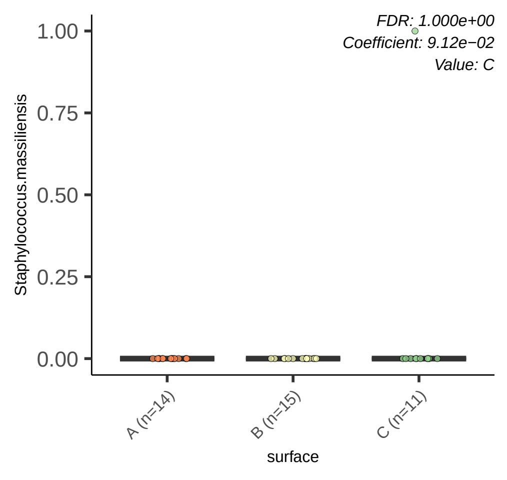
FDR: 1.000e+00
Coefficient: 9.12e−02
Value: C
1.00
0.75
0.50
0.25
0.00
Staphylococcus.massiliensis
A (n=14)
B (n=15)
C (n=11)
surface

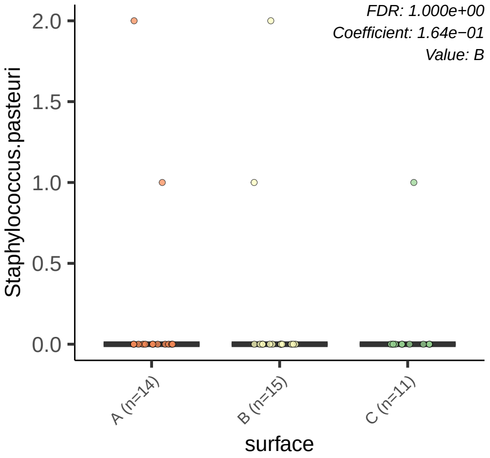

FDR: 1.000e+00
Coefficient: 1.64e−01
Value: B
Staphylococcus.pasteuri
2.0
1.5
1.0
0.5
0.0
A (n=14)
B (n=15)
C (n=11)
surface

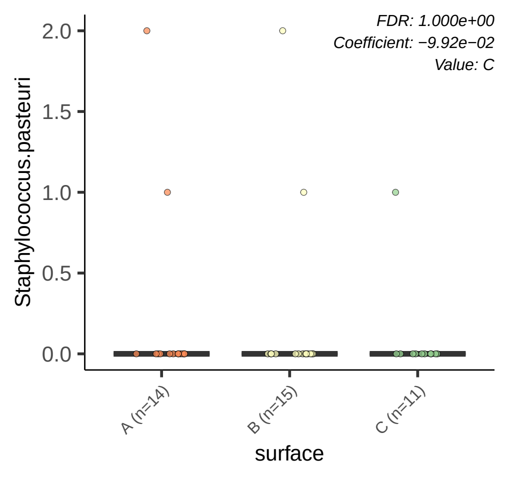
FDR: 1.000e+00
Coefficient: −9.92e−02
Value: C
Staphylococcus.pasteuri
2.0
1.5
1.0
0.5
0.0
A (n=14)
B (n=15)
C (n=11)
surface

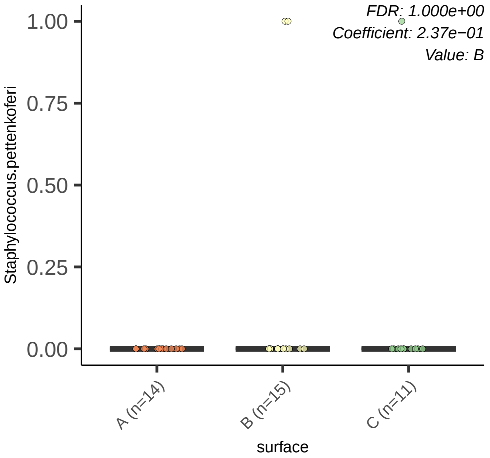

FDR: 1.000e+00
Coefficient: 2.37e−01
Value: B
1.00
0.75
0.50
0.25
0.00
Staphylococcus.pettenkoferi
A (n=14)
B (n=15)
C (n=11)
surface

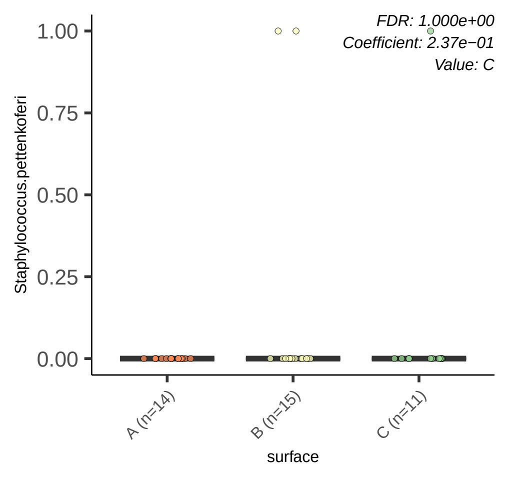
FDR: 1.000e+00
Coefficient: 2.37e−01
Value: C
1.00
0.75
0.50
0.25
0.00
Staphylococcus.pettenkoferi
A (n=14)
B (n=15)
C (n=11)
surface

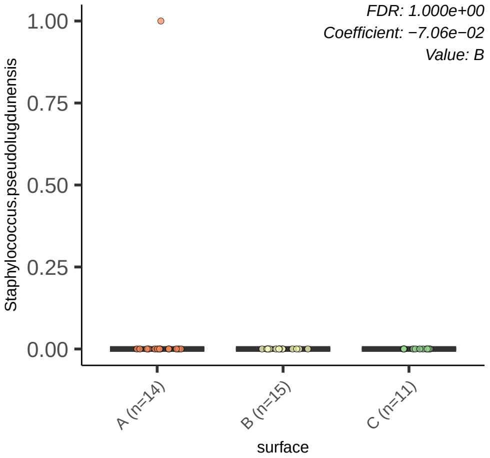
FDR: 1.000e+00
Coefficient: −7.06e−02
Value: B
Staphylococcus.pseudolugdunensis
1.00
0.75
0.50
0.25
0.00
A (n=14)
B (n=15)
C (n=11)
surface

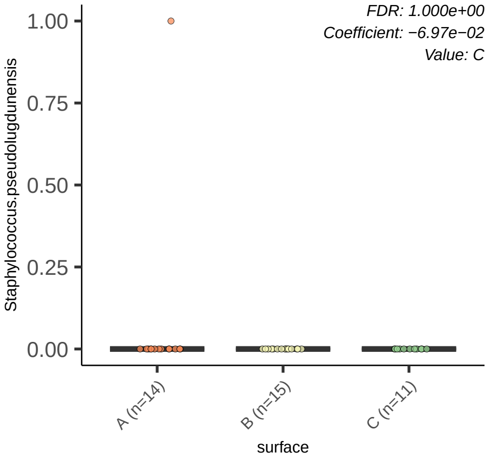
FDR: 1.000e+00
Coefficient: −6.97e−02
Value: C
Staphylococcus.pseudolugdunensis
1.00
0.75
0.50
0.25
0.00
A (n=14)
B (n=15)
C (n=11)
surface

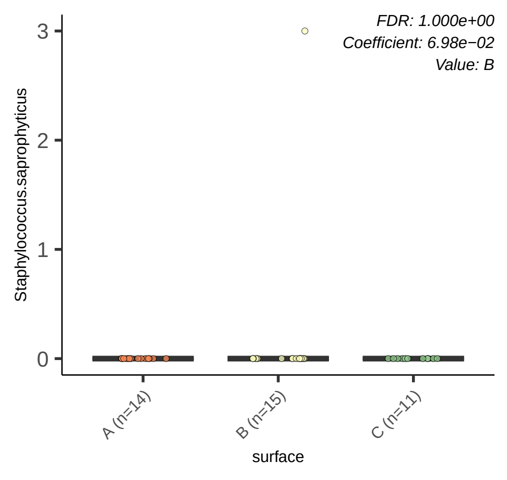
FDR: 1.000e+00
Coefficient: 6.98e−02
Value: B
Staphylococcus.saprophyticus
3
2
1
0
A (n=14)
B (n=15)
C (n=11)
surface

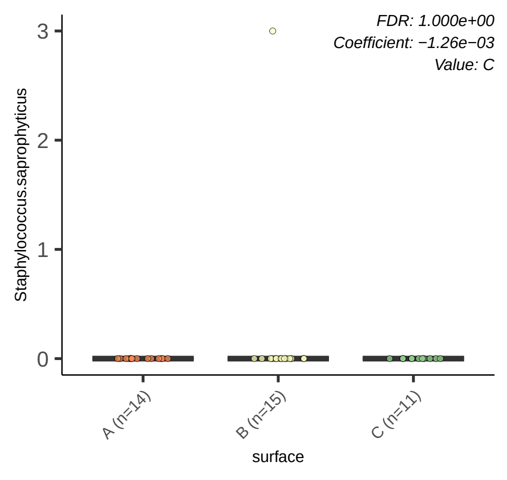
FDR: 1.000e+00
Coefficient: −1.26e−03
Value: C
Staphylococcus.saprophyticus
3
2
1
0
A (n=14)
B (n=15)
C (n=11)
surface

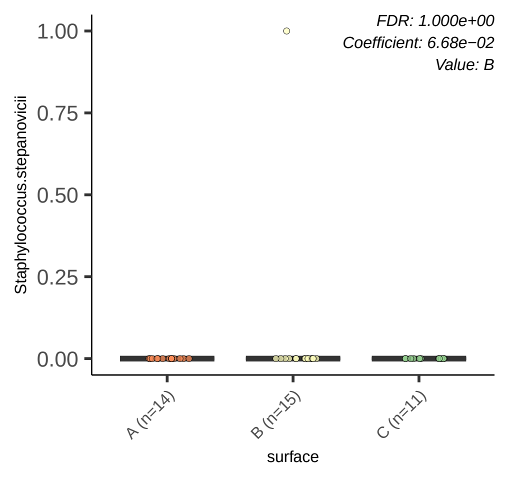
FDR: 1.000e+00
Coefficient: 6.68e−02
Value: B
Staphylococcus.stepanovicii
1.00
0.75
0.50
0.25
0.00
A (n=14)
B (n=15)
C (n=11)
surface

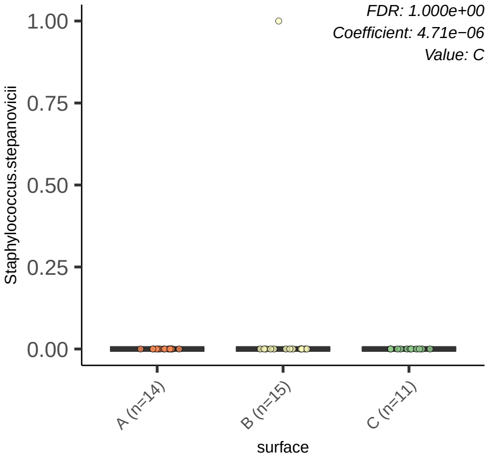

FDR: 1.000e+00
Coefficient: 4.71e−06
Value: C
Staphylococcus.stepanovicii
1.00
0.75
0.50
0.25
0.00
A (n=14)
B (n=15)
C (n=11)
surface

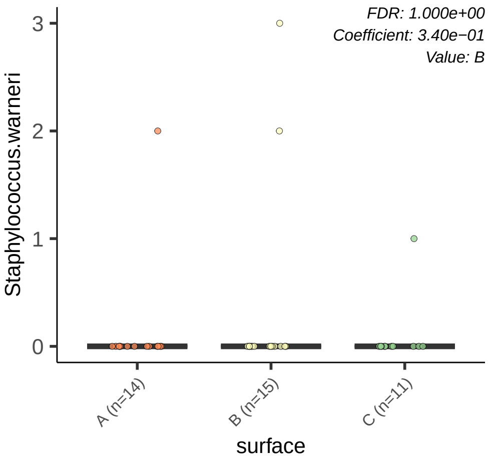

FDR: 1.000e+00
Coefficient: 3.40e−01
Value: B
Staphylococcus.warneri
3
2
1
0
A (n=14)
B (n=15)
C (n=11)
surface

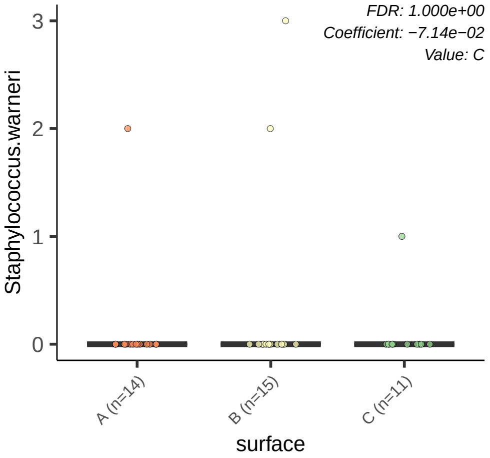

FDR: 1.000e+00
Coefficient: −7.14e−02
Value: C
Staphylococcus.warneri
0
1
2
3
A (n=14)
B (n=15)
C (n=11)
surface

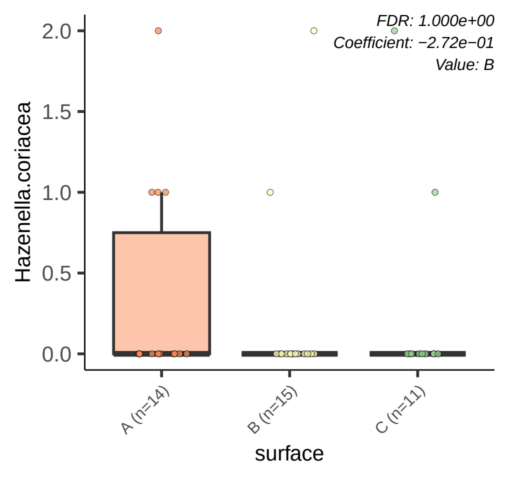
FDR: 1.000e+00
Coefficient: −2.72e−01
Value: B
Hazenella.coriacea
2.0
1.5
1.0
0.5
0.0
A (n=14)
B (n=15)
C (n=11)
surface

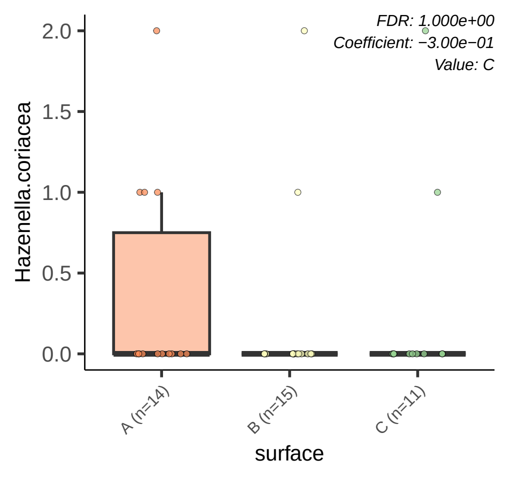
FDR: 1.000e+00
Coefficient: −3.00e−01
Value: C
Hazenella.coriacea
2.0
1.5
1.0
0.5
0.0
A (n=14)
B (n=15)
C (n=11)
surface

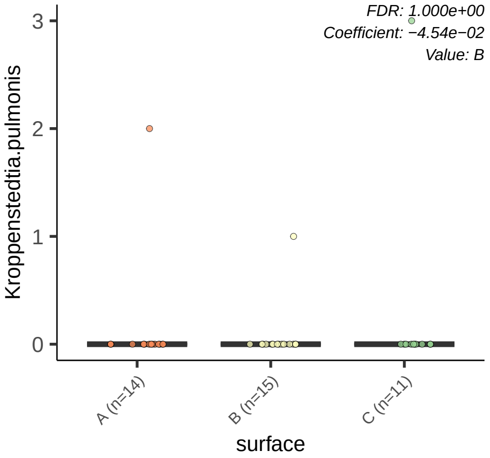
FDR: 1.000e+00
Coefficient: −4.54e−02
Value: B
Kroppenstedtia.pulmonis
0
1
2
3
A (n=14)
B (n=15)
C (n=11)
surface

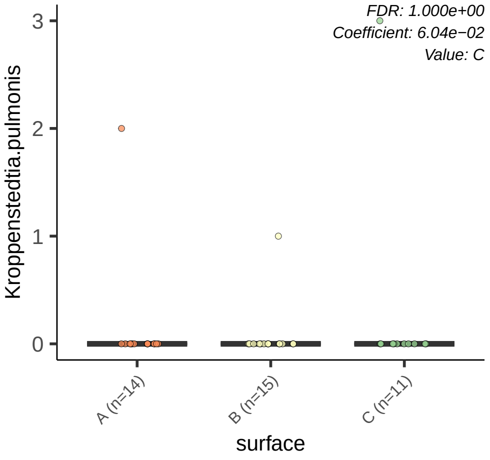
FDR: 1.000e+00
Coefficient: 6.04e−02
Value: C
Kroppenstedtia.pulmonis
0
1
2
3
A (n=14)
B (n=15)
C (n=11)
surface

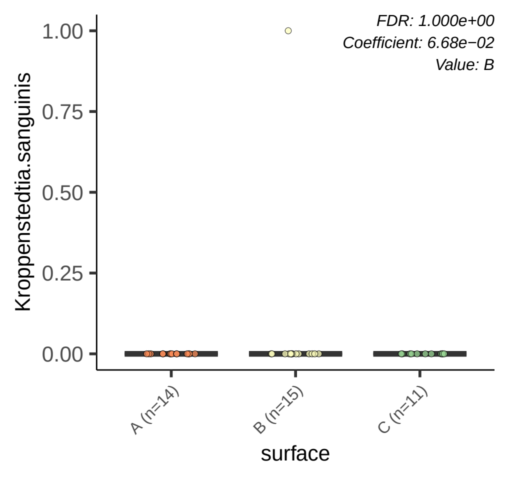
FDR: 1.000e+00
Coefficient: 6.68e−02
Value: B
Kroppenstedtia.sanguinis
1.00
0.75
0.50
0.25
0.00
A (n=14)
B (n=15)
C (n=11)
surface

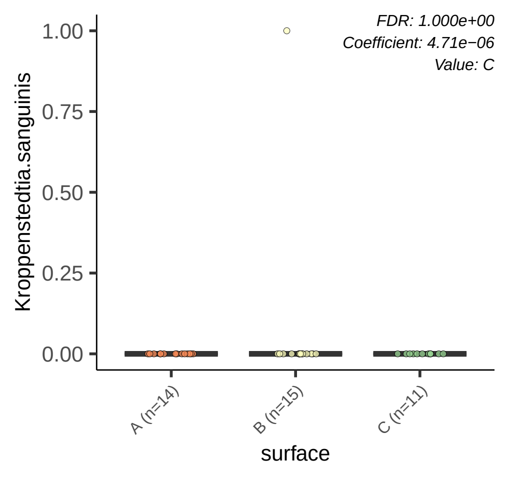

FDR: 1.000e+00
Coefficient: 4.71e−06
Value: C
Kroppenstedtia.sanguinis
1.00
0.75
0.50
0.25
0.00
A (n=14)
B (n=15)
C (n=11)
surface

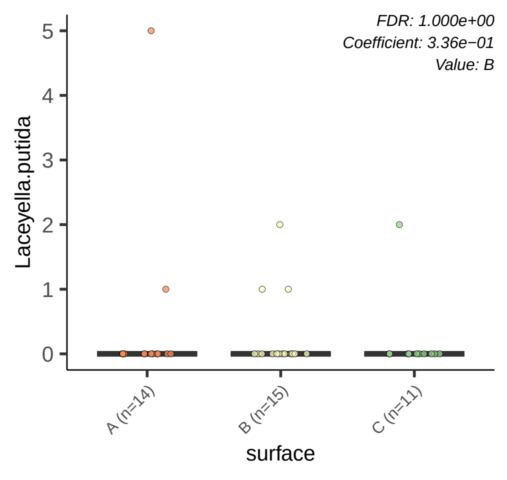
FDR: 1.000e+00
Coefficient: 3.36e−01
Value: B
Laceyella.putida
0
1
2
3
4
5
A (n=14)
B (n=15)
C (n=11)
surface

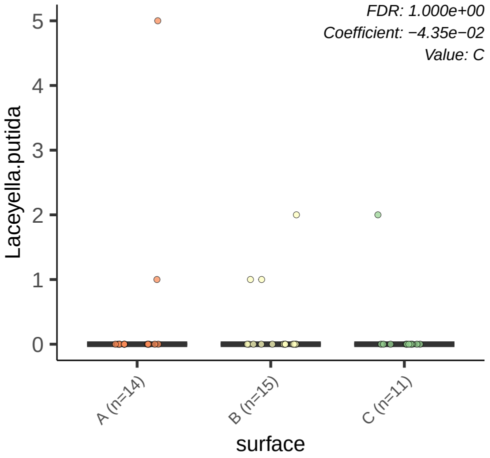
FDR: 1.000e+00
Coefficient: −4.35e−02
Value: C
Laceyella.putida
0
1
2
3
4
5
A (n=14)
B (n=15)
C (n=11)
surface

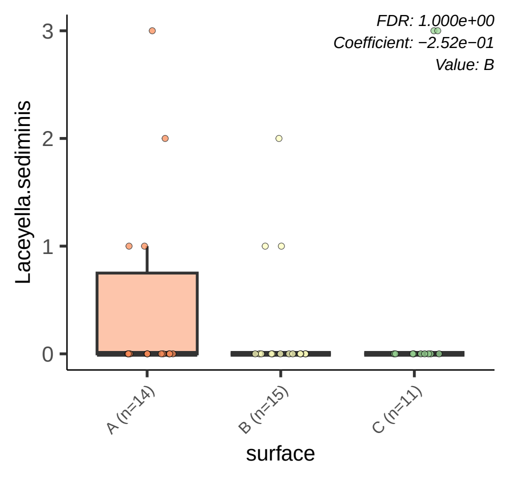

FDR: 1.000e+00
Coefficient: −2.52e−01
Value: B
Laceyella.sedimінis
0
1
2
3
A (n=14)
B (n=15)
C (n=11)
surface

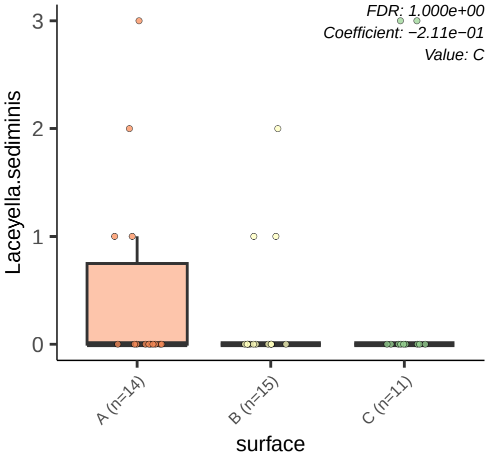

FDR: 1.000e+00
Coefficient: −2.11e−01
Value: C
Laceyella.sedimini
0
1
2
3
A (n=14)
B (n=15)
C (n=11)
surface

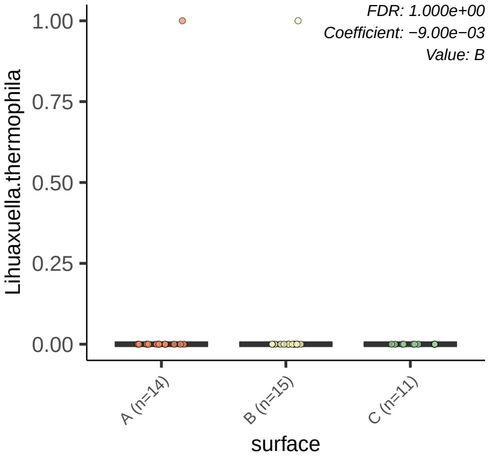
FDR: 1.000e+00
Coefficient: −9.00e−03
Value: B
Lihuaxuella.thermophila
1.00
0.75
0.50
0.25
0.00
A (n=14)
B (n=15)
C (n=11)
surface

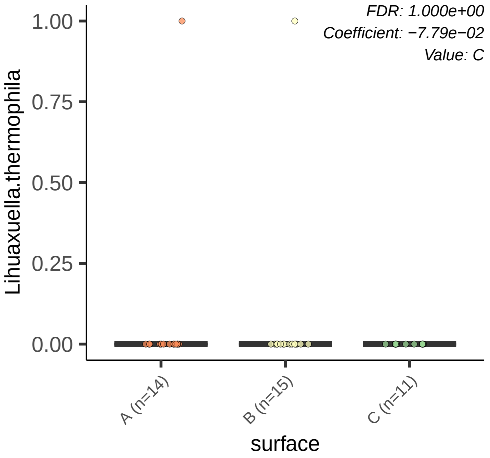
FDR: 1.000e+00
Coefficient: −7.79e−02
Value: C
Lihuaxuella.thermophila
1.00
0.75
0.50
0.25
0.00
A (n=14)
B (n=15)
C (n=11)
surface

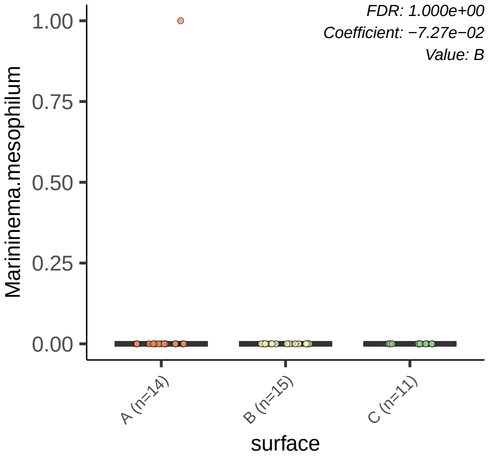
FDR: 1.000e+00
Coefficient: −7.27e−02
Value: B
Marininema.mesophilum
1.00
0.75
0.50
0.25
0.00
A (n=14)
B (n=15)
C (n=11)
surface

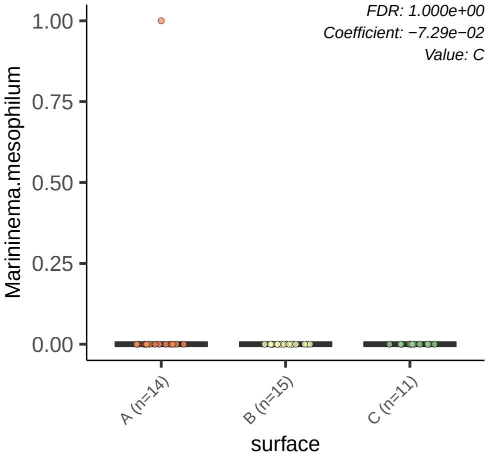
FDR: 1.000e+00
Coefficient: −7.29e−02
Value: C
Marininema.mesophilum
1.00
0.75
0.50
0.25
0.00
A (n=14)
B (n=15)
C (n=11)
surface

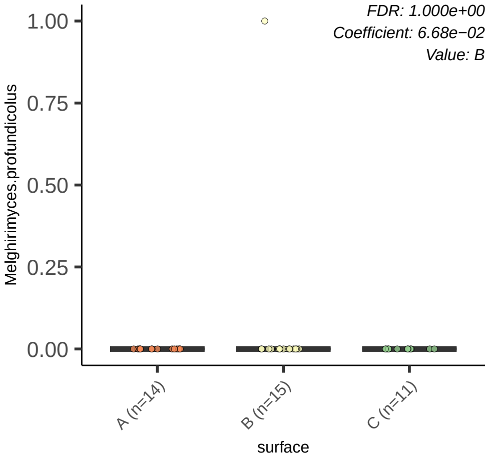
FDR: 1.000e+00
Coefficient: 6.68e−02
Value: B
Melghirimyces.profundicolus
1.00
0.75
0.50
0.25
0.00
A (n=14)
B (n=15)
C (n=11)
surface

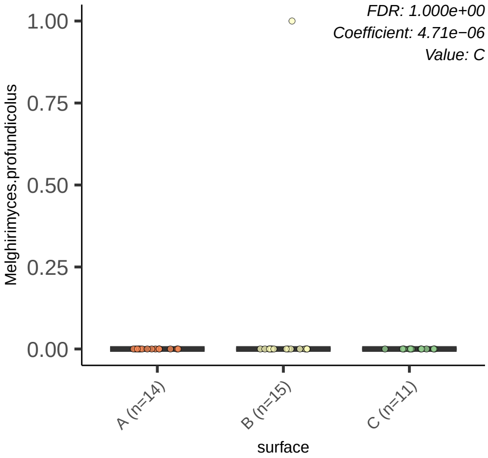
FDR: 1.000e+00
Coefficient: 4.71e−06
Value: C
1.00
0.75
0.50
0.25
0.00
Melghirimyces.profundicolus
A (n=14)
B (n=15)
C (n=11)
surface

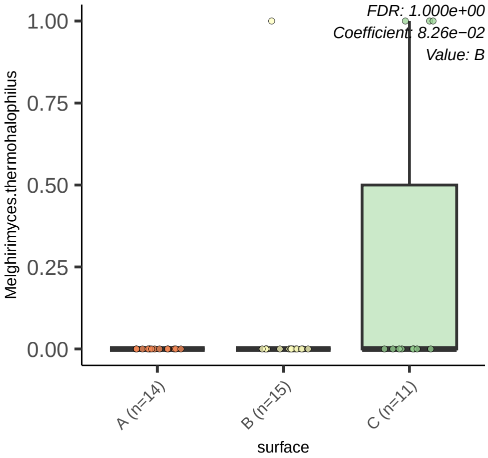

FDR: 1.000e+00
Coefficient: 8.26e−02
Value: B
1.00
0.75
0.50
0.25
0.00
Melghirimyces.thermohalophilus
A (n=14)
B (n=15)
C (n=11)
surface

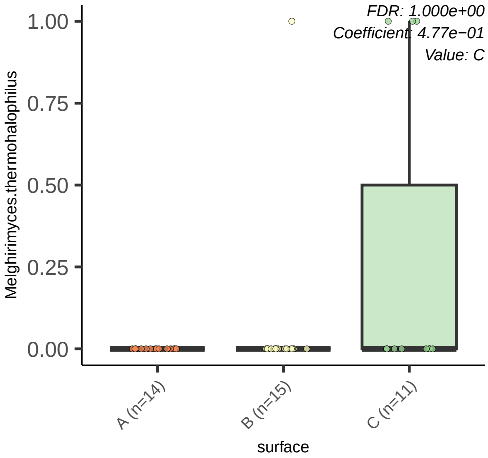

FDR: 1.000e+00
Coefficient: 4.77e−01
Value: C
Melghirimyces.thermohalophilus
1.00
0.75
0.50
0.25
0.00
A (n=14)
B (n=15)
C (n=11)
surface

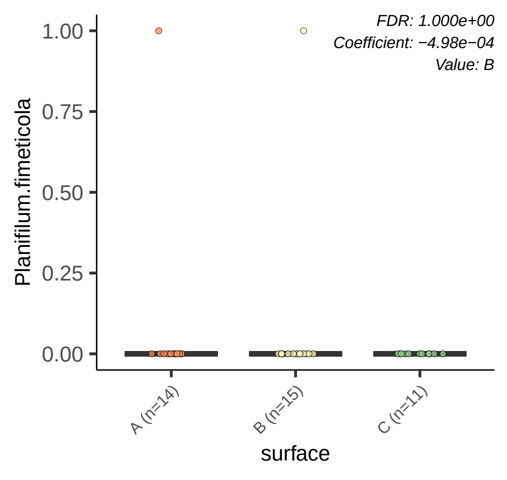
FDR: 1.000e+00
Coefficient: −4.98e−04
Value: B
Planifilum.fimeticola
1.00
0.75
0.50
0.25
0.00
A (n=14)
B (n=15)
C (n=11)
surface

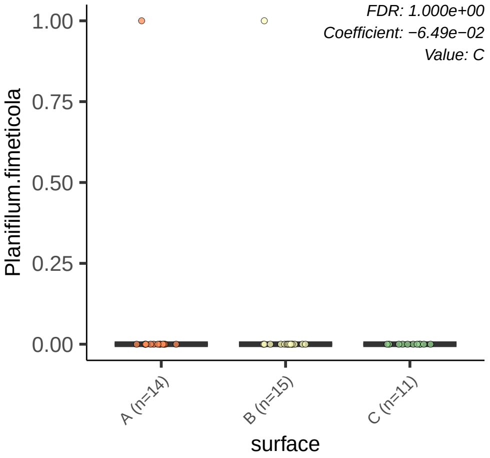

FDR: 1.000e+00
Coefficient: −6.49e−02
Value: C
Planifilum.fimeticola
1.00
0.75
0.50
0.25
0.00
A (n=14)
B (n=15)
C (n=11)
surface

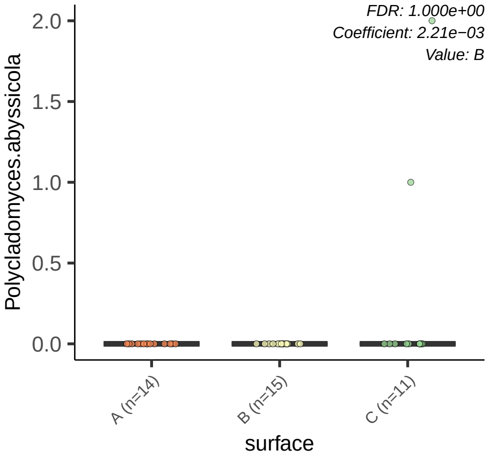
FDR: 1.000e+00
Coefficient: 2.21e−03
Value: B
Polycladomyces.abyssicola
2.0
1.5
1.0
0.5
0.0
A (n=14)
B (n=15)
C (n=11)
surface

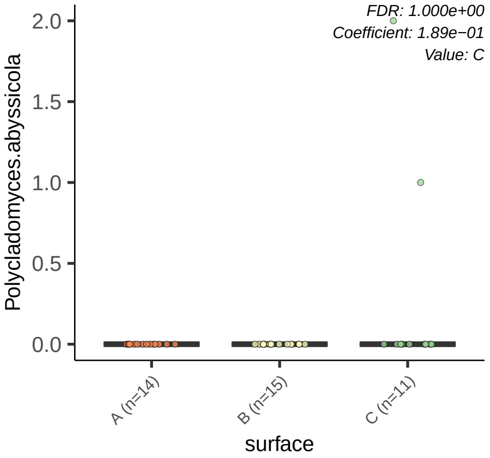

FDR: 1.000e+00
Coefficient: 1.89e−01
Value: C
Polycladomyces.abyssicola
2.0
1.5
1.0
0.5
0.0
A (n=14)
B (n=15)
C (n=11)
surface

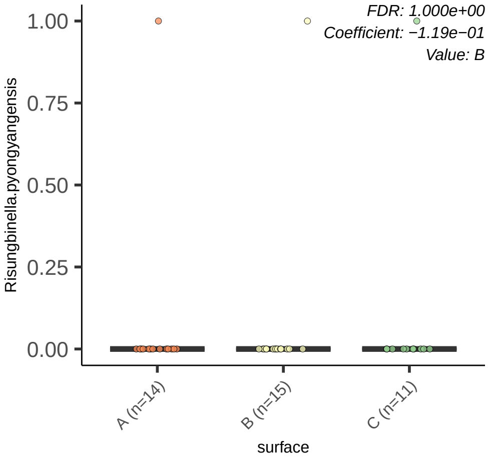
FDR: 1.000e+00
Coefficient: −1.19e−01
Value: B
Risungbinella.pyongyangensis
1.00
0.75
0.50
0.25
0.00
A (n=14)
B (n=15)
C (n=11)
surface

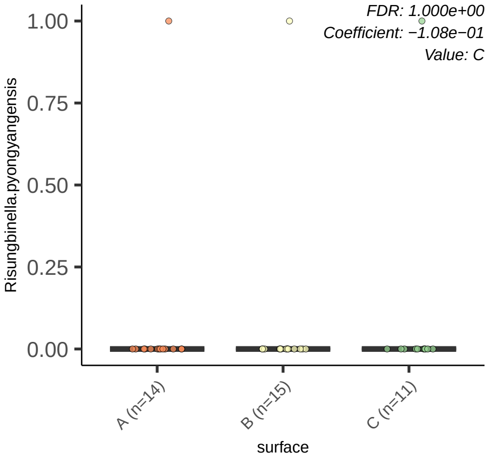
FDR: 1.000e+00
Coefficient: −1.08e−01
Value: C
1.00
0.75
0.50
0.25
0.00
Risungbinella.pyongyangensis
A (n=14)
B (n=15)
C (n=11)
surface

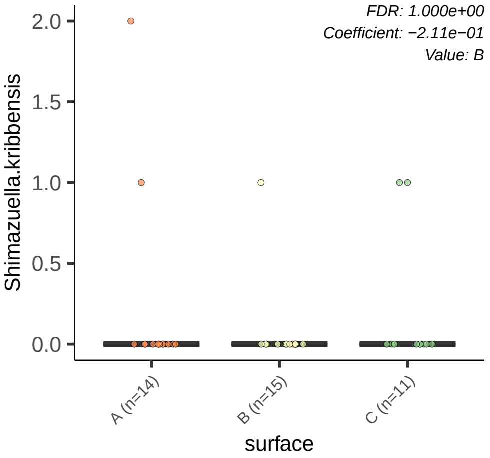
FDR: 1.000e+00
Coefficient: −2.11e−01
Value: B
Shimazuella.kribbensis
2.0
1.5
1.0
0.5
0.0
A (n=14)
B (n=15)
C (n=11)
surface

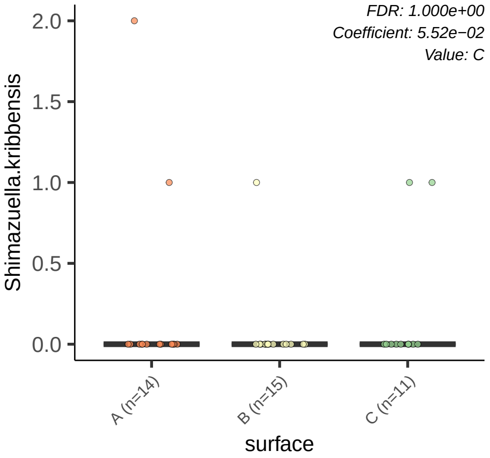
FDR: 1.000e+00
Coefficient: 5.52e−02
Value: C
Shimazuella.kribbensis
2.0
1.5
1.0
0.5
0.0
A (n=14)
B (n=15)
C (n=11)
surface

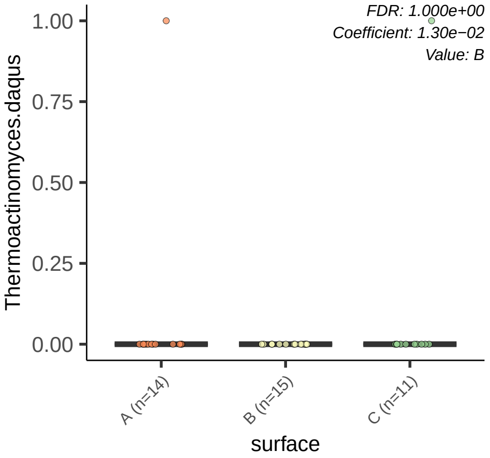
FDR: 1.000e+00
Coefficient: 1.30e−02
Value: B
Thermoactinomyces.daqus
1.00
0.75
0.50
0.25
0.00
A (n=14)
B (n=15)
C (n=11)
surface

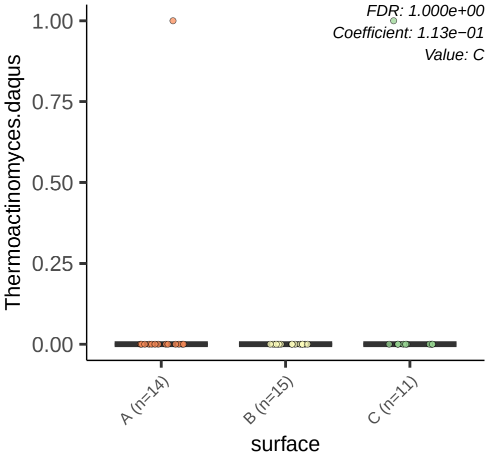

FDR: 1.000e+00
Coefficient: 1.13e−01
Value: C
Thermoactinomyces.daqus
1.00
0.75
0.50
0.25
0.00
A (n=14)
B (n=15)
C (n=11)
surface

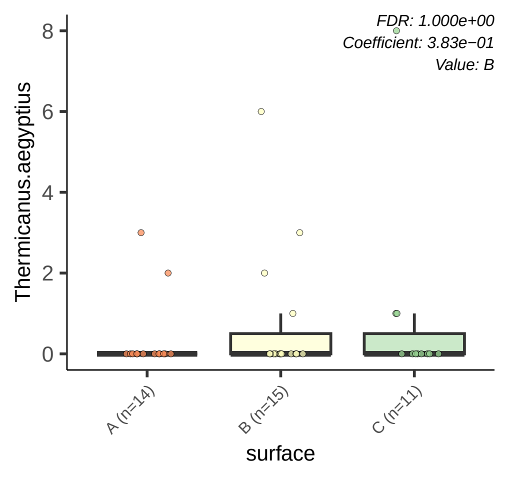
FDR: 1.000e+00
Coefficient: 3.83e−01
Value: B
Thermicanus.aegyptius
0
2
4
6
8
A (n=14)
B (n=15)
C (n=11)
surface

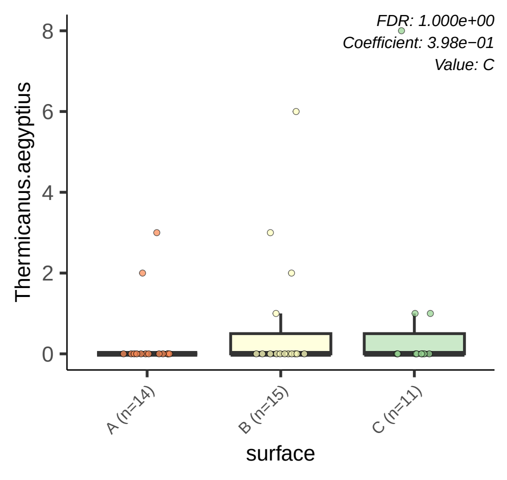
FDR: 1.000e+00
Coefficient: 3.98e−01
Value: C
Thermicanus.aegyptius
0
2
4
6
8
A (n=14)
B (n=15)
C (n=11)
surface

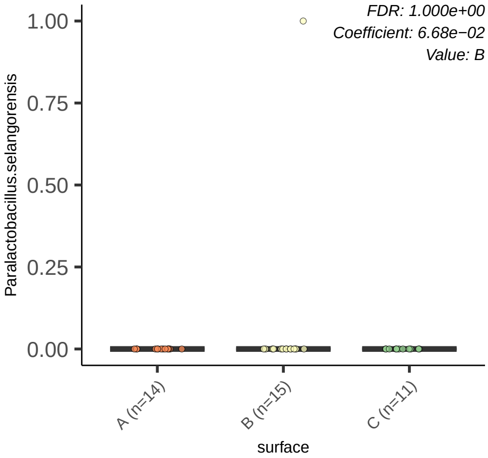
FDR: 1.000e+00
Coefficient: 6.68e−02
Value: B
Paralactobacillus.selangorensis
1.00
0.75
0.50
0.25
0.00
A (n=14)
B (n=15)
C (n=11)
surface

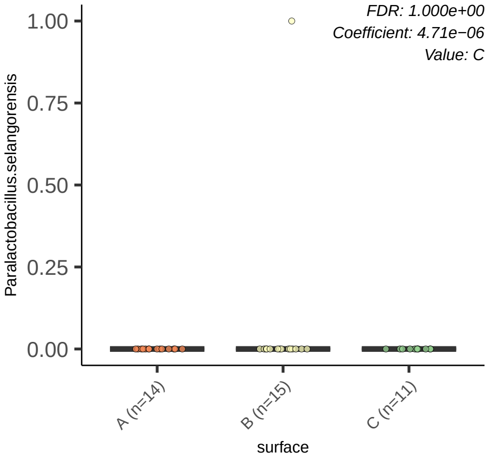

FDR: 1.000e+00
Coefficient: 4.71e−06
Value: C
Paralactobacillus.selangorensis
1.00
0.75
0.50
0.25
0.00
A (n=14)
B (n=15)
C (n=11)
surface

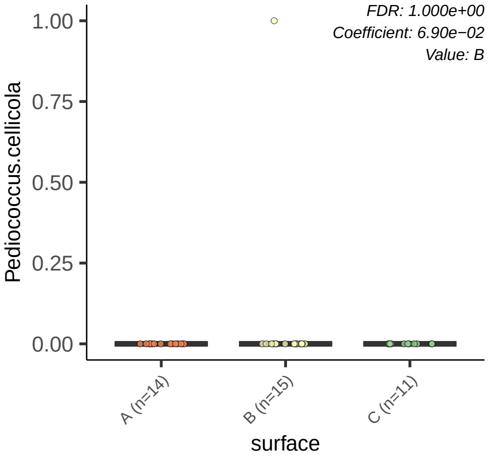
FDR: 1.000e+00
Coefficient: 6.90e−02
Value: B
Pediococcus.cellicola
1.00
0.75
0.50
0.25
0.00
A (n=14)
B (n=15)
C (n=11)
surface

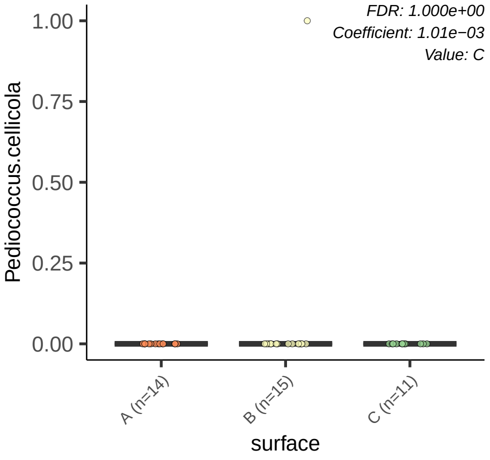
FDR: 1.000e+00
Coefficient: 1.01e−03
Value: C
Pediococcus.cellicola
1.00
0.75
0.50
0.25
0.00
A (n=14)
B (n=15)
C (n=11)
surface

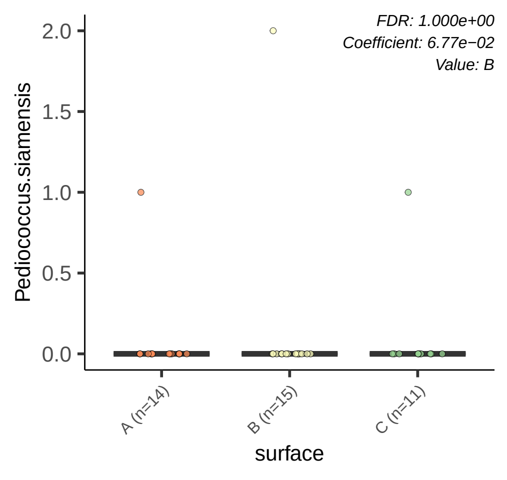
FDR: 1.000e+00
Coefficient: 6.77e−02
Value: B
Pediococcus.siamensis
2.0
1.5
1.0
0.5
0.0
A (n=14)
B (n=15)
C (n=11)
surface

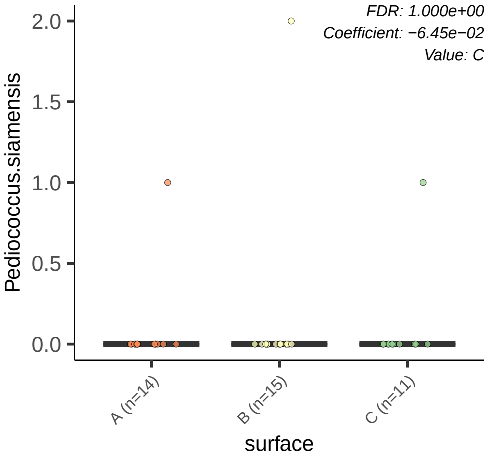
FDR: 1.000e+00
Coefficient: −6.45e−02
Value: C
Pediococcus.siamensis
2.0
1.5
1.0
0.5
0.0
A (n=14)
B (n=15)
C (n=11)
surface

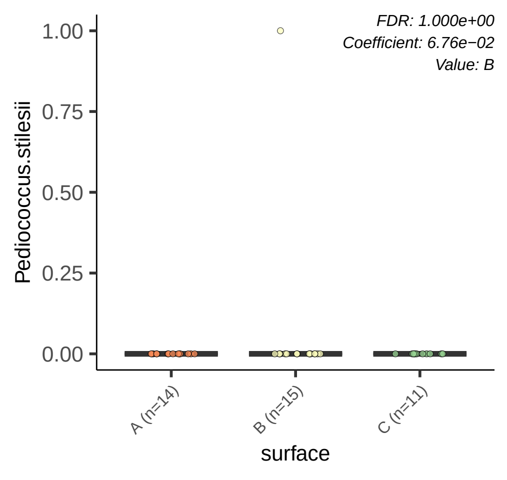
FDR: 1.000e+00
Coefficient: 6.76e−02
Value: B
Pediococcus.stilesii
1.00
0.75
0.50
0.25
0.00
A (n=14)
B (n=15)
C (n=11)
surface

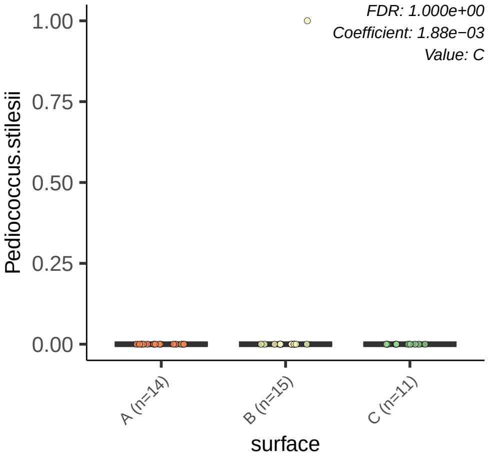

FDR: 1.000e+00
Coefficient: 1.88e−03
Value: C
1.00
0.75
0.50
0.25
0.00
Pediococcus.stilesii
A (n=14)
B (n=15)
C (n=11)
surface

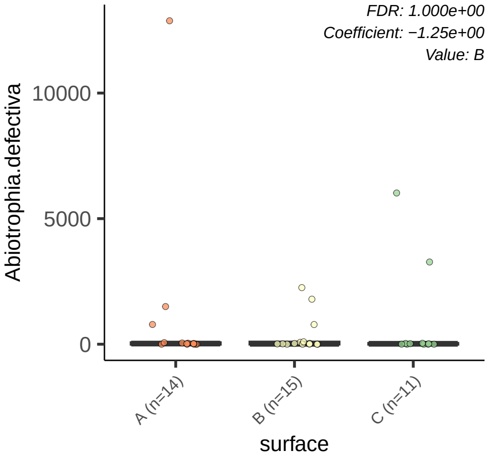

FDR: 1.000e+00
Coefficient: −1.25e+00
Value: B
Abiotrophia.defectiva
10000
5000
0
A (n=14)
B (n=15)
C (n=11)
surface

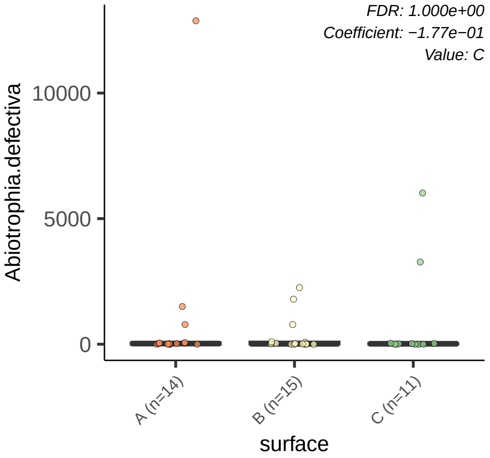

FDR: 1.000e+00
Coefficient: −1.77e−01
Value: C
Abiotrophia.defectiva
10000
5000
0
A (n=14)
B (n=15)
C (n=11)
surface

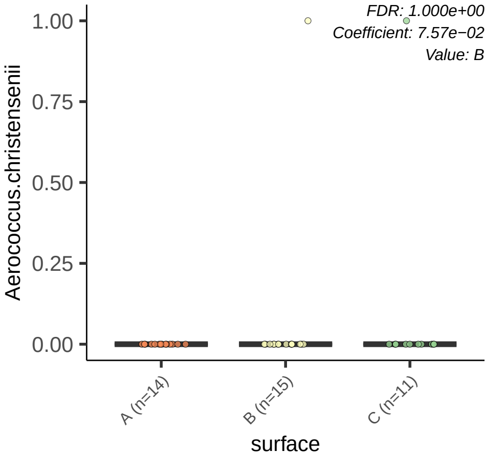
FDR: 1.000e+00
Coefficient: 7.57e−02
Value: B
1.00
0.75
0.50
0.25
0.00
Aerococcus.christensenii
A (n=14)
B (n=15)
C (n=11)
surface

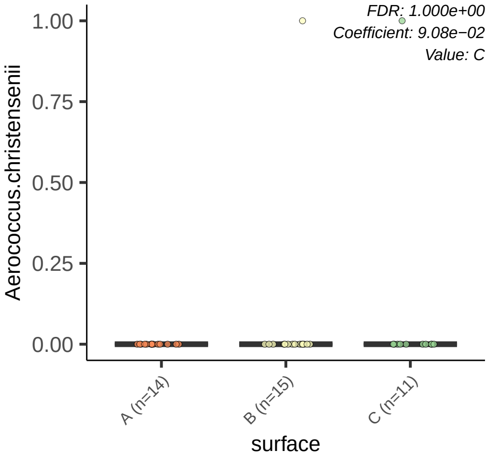

FDR: 1.000e+00
Coefficient: 9.08e−02
Value: C
Aerococcus.christensenii
1.00
0.75
0.50
0.25
0.00
A (n=14)
B (n=15)
C (n=11)
surface

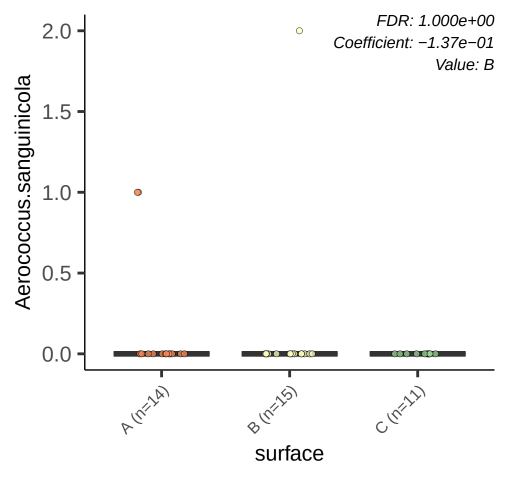
FDR: 1.000e+00
Coefficient: −1.37e−01
Value: B
Aerococcus.sanguinicola
2.0
1.5
1.0
0.5
0.0
A (n=14)
B (n=15)
C (n=11)
surface

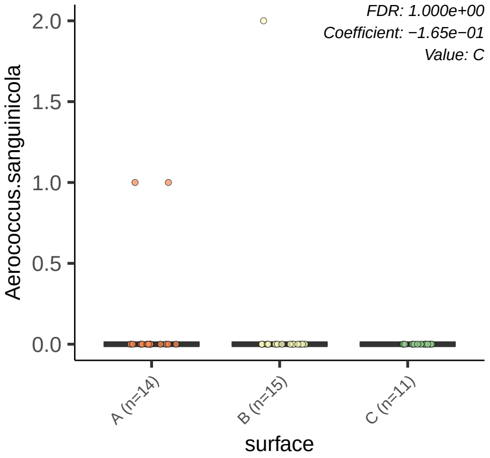

FDR: 1.000e+00
Coefficient: −1.65e−01
Value: C
Aerococcus.sanguinicola
2.0
1.5
1.0
0.5
0.0
A (n=14)
B (n=15)
C (n=11)
surface

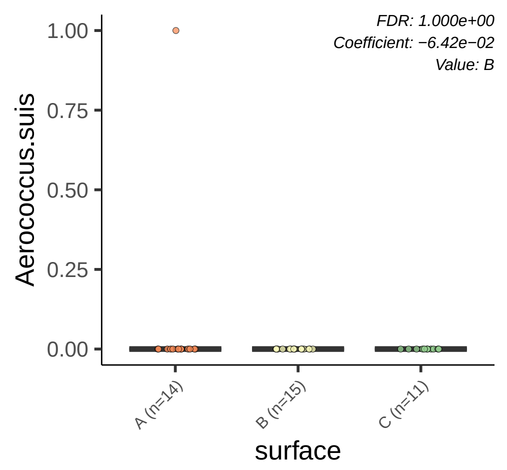

FDR: 1.000e+00
Coefficient: −6.42e−02
Value: B
Aerococcus.suis
1.00
0.75
0.50
0.25
0.00
A (n=14)
B (n=15)
C (n=11)
surface

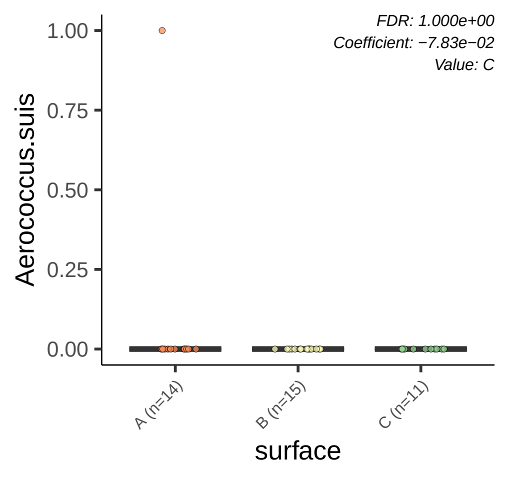
FDR: 1.000e+00
Coefficient: −7.83e−02
Value: C
Aerococcus.suis
1.00
0.75
0.50
0.25
0.00
A (n=14)
B (n=15)
C (n=11)
surface

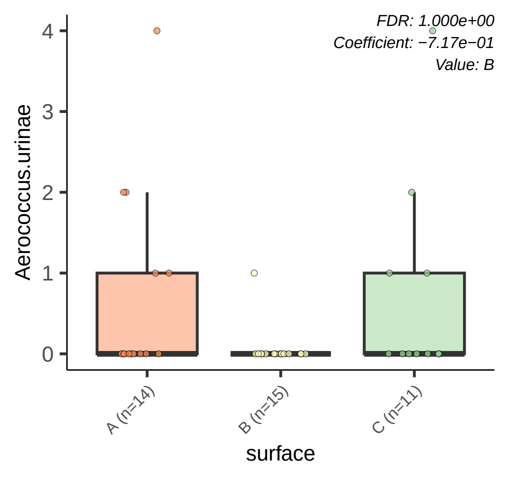
FDR: 1.000e+00
Coefficient: −7.17e−01
Value: B
Aerococcus.urinae
4
3
2
1
0
A (n=14)
B (n=15)
C (n=11)
surface

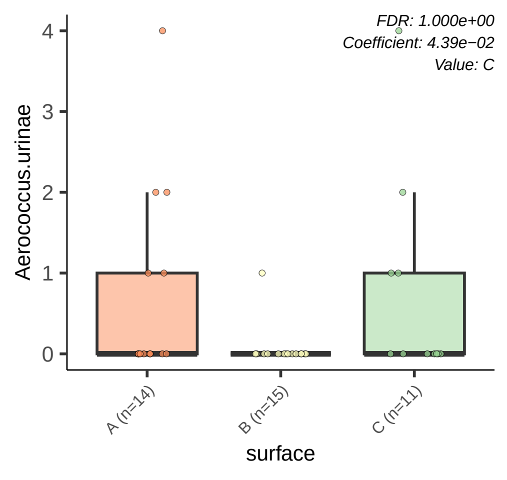
FDR: 1.000e+00
Coefficient: 4.39e−02
Value: C
Aerococcus.urinae
0
1
2
3
4
A (n=14)
B (n=15)
C (n=11)
surface

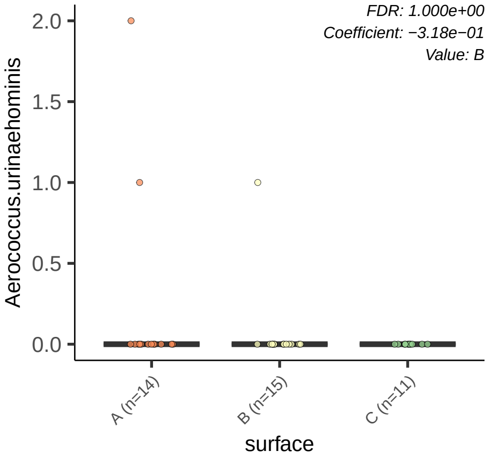
FDR: 1.000e+00
Coefficient: −3.18e−01
Value: B
Aerococcus.urinaehominis
2.0
1.5
1.0
0.5
0.0
A (n=14)
B (n=15)
C (n=11)
surface

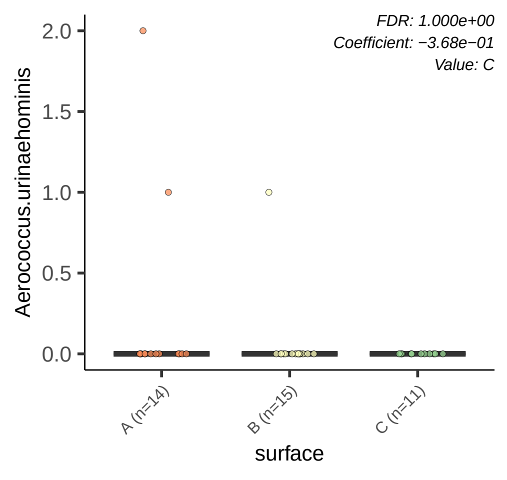

FDR: 1.000e+00
Coefficient: −3.68e−01
Value: C
Aerococcus.urinaehominis
2.0
1.5
1.0
0.5
0.0
A (n=14)
B (n=15)
C (n=11)
surface

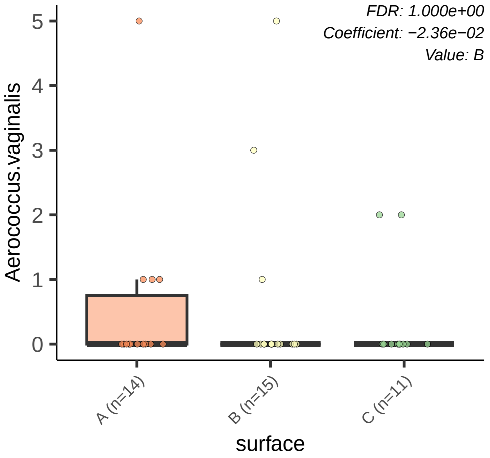

FDR: 1.000e+00
Coefficient: −2.36e−02
Value: B
Aerococcus.vaginalis
0
1
2
3
4
5
A (n=14)
B (n=15)
C (n=11)
surface

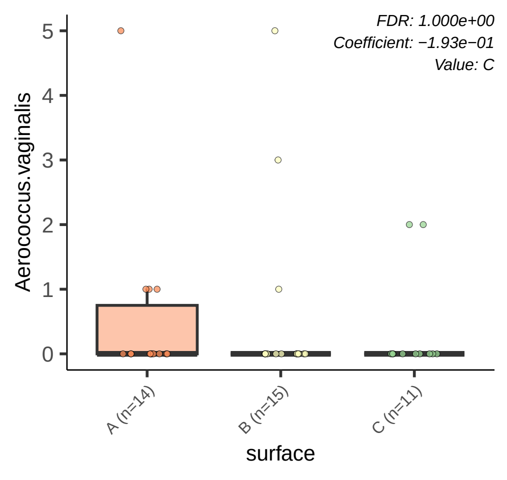
FDR: 1.000e+00
Coefficient: −1.93e−01
Value: C
Aerococcus.vaginalis
0
1
2
3
4
5
A (n=14)
B (n=15)
C (n=11)
surface

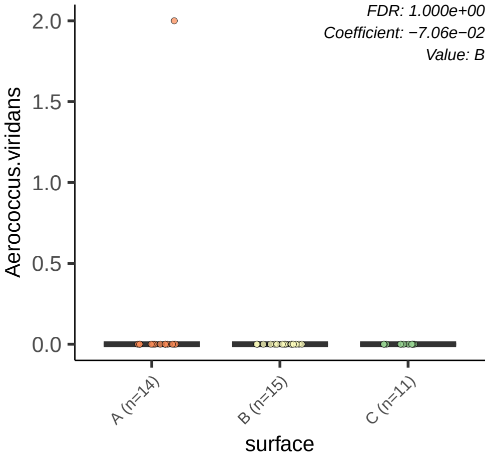
FDR: 1.000e+00
Coefficient: −7.06e−02
Value: B
Aerococcus.viridans
2.0
1.5
1.0
0.5
0.0
A (n=14)
B (n=15)
C (n=11)
surface

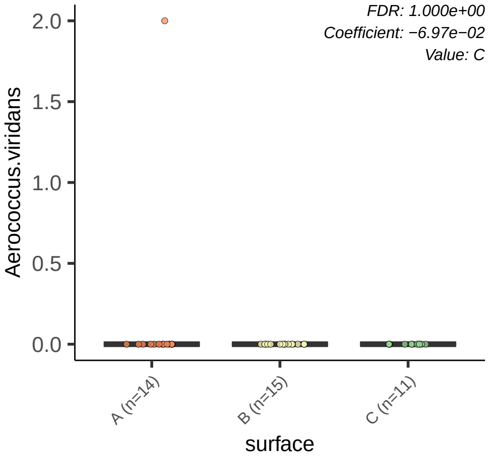

FDR: 1.000e+00
Coefficient: −6.97e−02
Value: C
Aerococcus.viridans
2.0
1.5
1.0
0.5
0.0
A (n=14)
B (n=15)
C (n=11)
surface

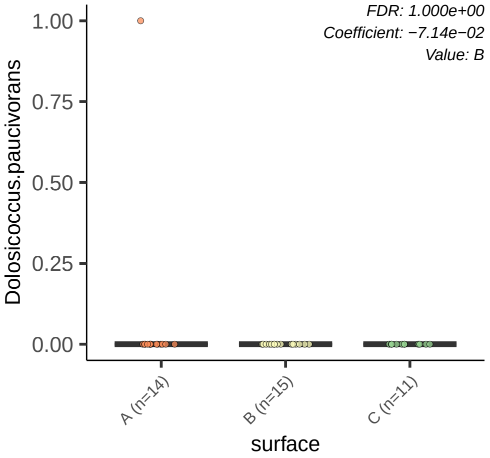

FDR: 1.000e+00
Coefficient: −7.14e−02
Value: B
Dolosicoccus.paucivorans
1.00
0.75
0.50
0.25
0.00
A (n=14)
B (n=15)
C (n=11)
surface

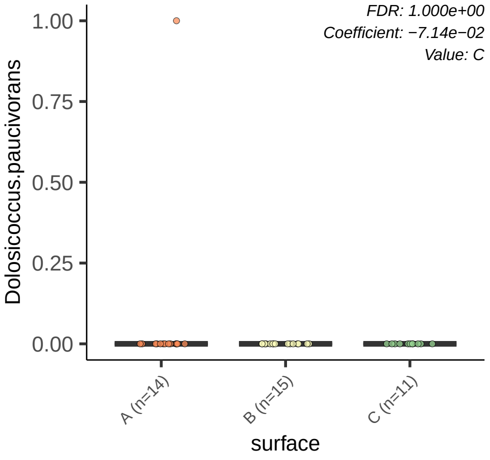

FDR: 1.000e+00
Coefficient: −7.14e−02
Value: C
Dolosicoccus.paucivorans
1.00
0.75
0.50
0.25
0.00
A (n=14)
B (n=15)
C (n=11)
surface

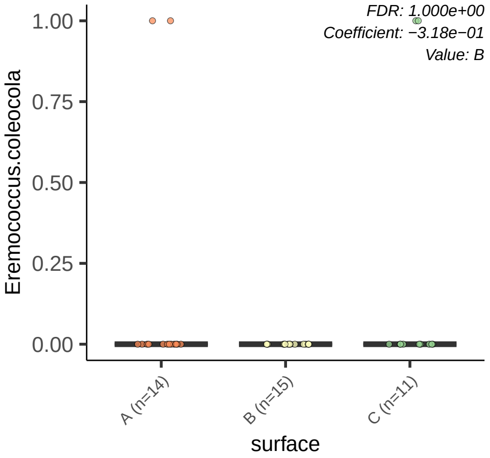

FDR: 1.000e+00
Coefficient: −3.18e−01
Value: B
Eremococcus.coleocola
1.00
0.75
0.50
0.25
0.00
A (n=14)
B (n=15)
C (n=11)
surface

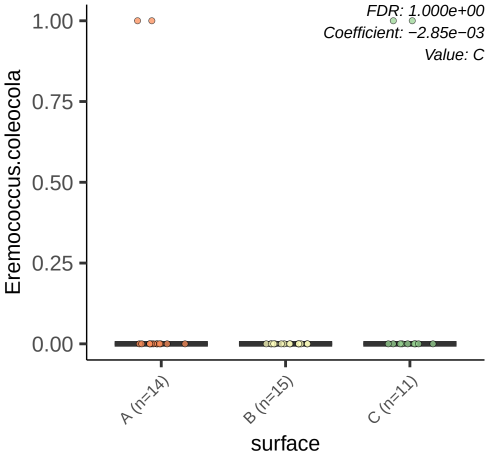

FDR: 1.000e+00
Coefficient: −2.85e−03
Value: C
Eremococcus.coleocola
1.00
0.75
0.50
0.25
0.00
A (n=14)
B (n=15)
C (n=11)
surface

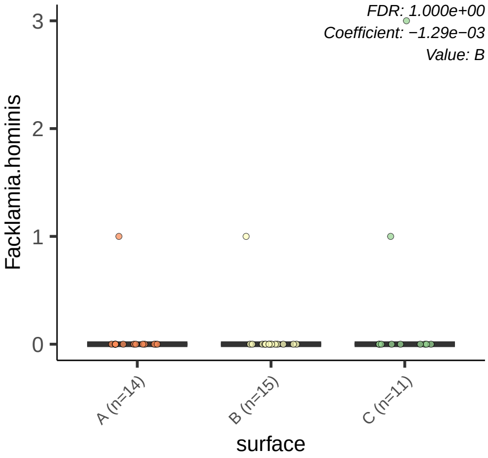
FDR: 1.000e+00
Coefficient: −1.29e−03
Value: B
Facklamia.hominis
0
1
2
3
A (n=14)
B (n=15)
C (n=11)
surface

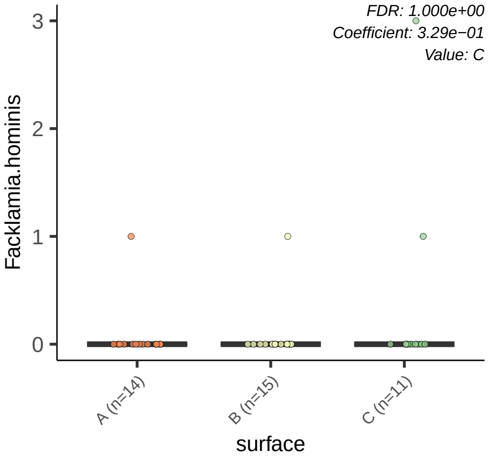
FDR: 1.000e+00
Coefficient: 3.29e−01
Value: C
Facklamia.hominis
3
2
1
0
A (n=14)
B (n=15)
C (n=11)
surface

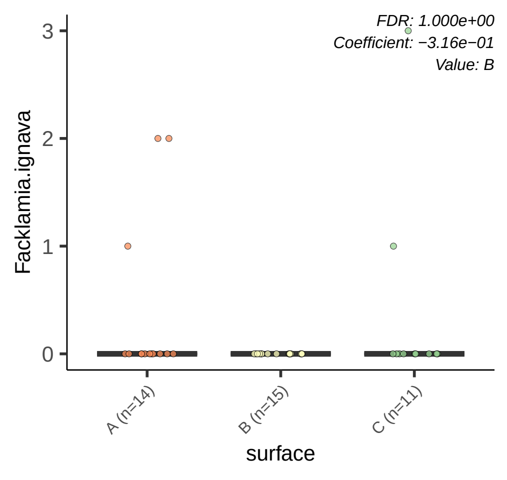
FDR: 1.000e+00
Coefficient: −3.16e−01
Value: B
Facklamia.ignava
0
1
2
3
A (n=14)
B (n=15)
C (n=11)
surface

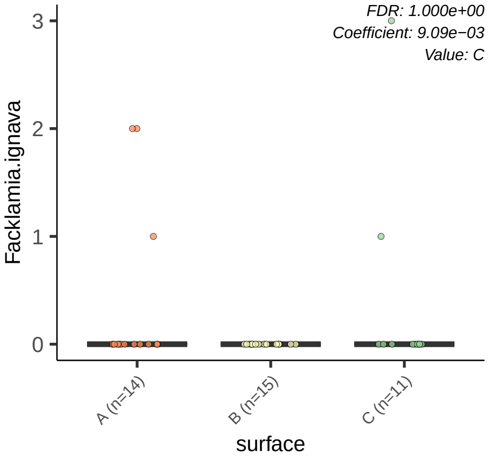
FDR: 1.000e+00
Coefficient: 9.09e−03
Value: C
Facklamia.ignava
0
1
2
3
A (n=14)
B (n=15)
C (n=11)
surface

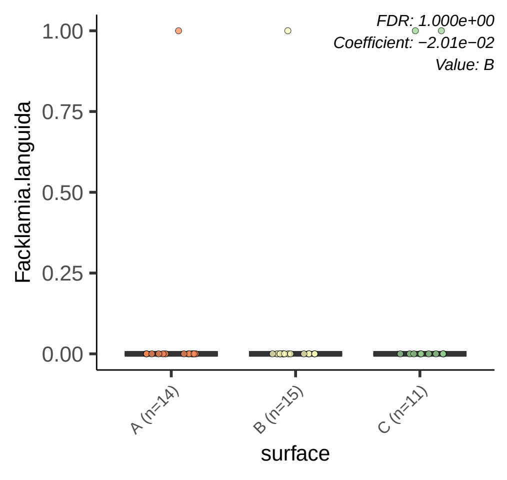

FDR: 1.000e+00
Coefficient: −2.01e−02
Value: B
1.00
0.75
0.50
0.25
0.00
Facklamia.languida
A (n=14)
B (n=15)
C (n=11)
surface

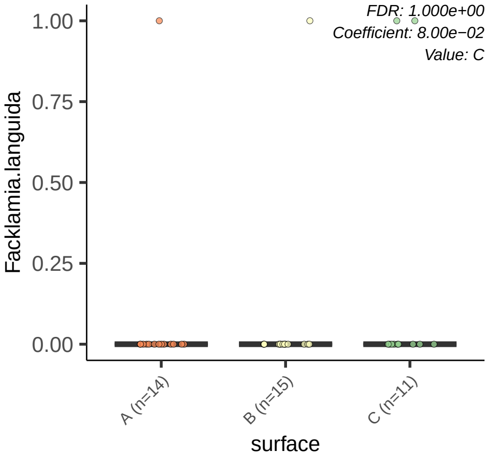
FDR: 1.000e+00
Coefficient: 8.00e−02
Value: C
Facklamia.languida
1.00
0.75
0.50
0.25
0.00
A (n=14)
B (n=15)
C (n=11)
surface

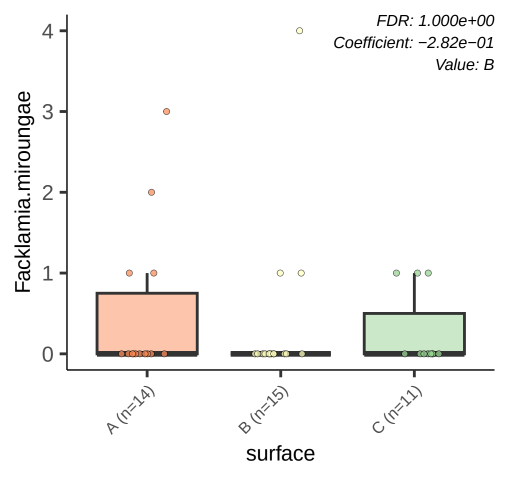

FDR: 1.000e+00
Coefficient: −2.82e−01
Value: B
Facklamia.miroungae
4
3
2
1
0
A (n=14)
B (n=15)
C (n=11)
surface

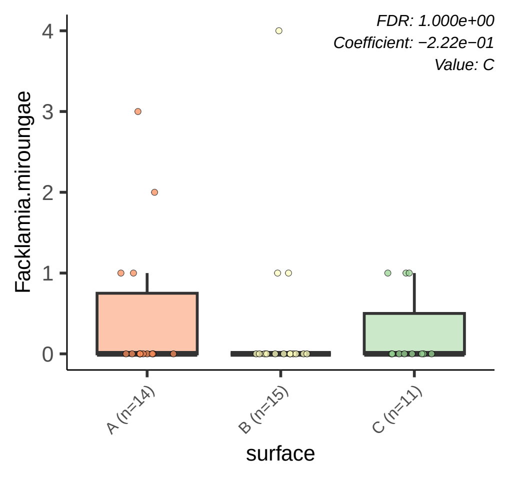

FDR: 1.000e+00
Coefficient: −2.22e−01
Value: C
Facklamia.miroungae
0
1
2
3
4
A (n=14)
B (n=15)
C (n=11)
surface

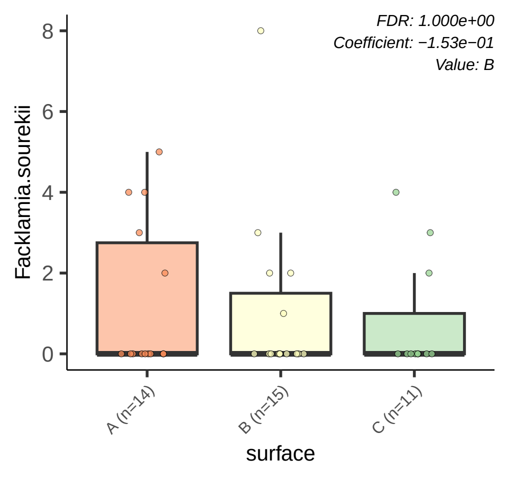

FDR: 1.000e+00
Coefficient: −1.53e−01
Value: B
Facklamia.sourekii
0
2
4
6
8
A (n=14)
B (n=15)
C (n=11)
surface

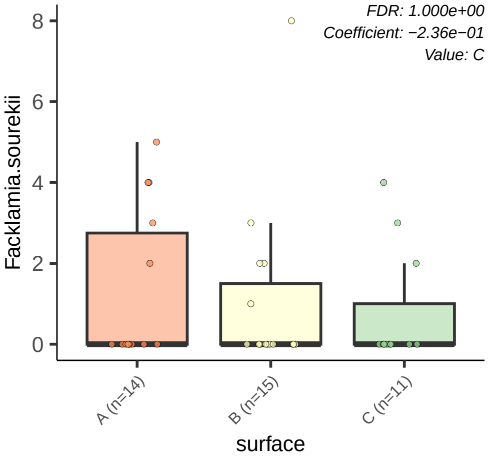

FDR: 1.000e+00
Coefficient: −2.36e−01
Value: C
Facklamia.sourekii
0
2
4
6
8
A (n=14)
B (n=15)
C (n=11)
surface

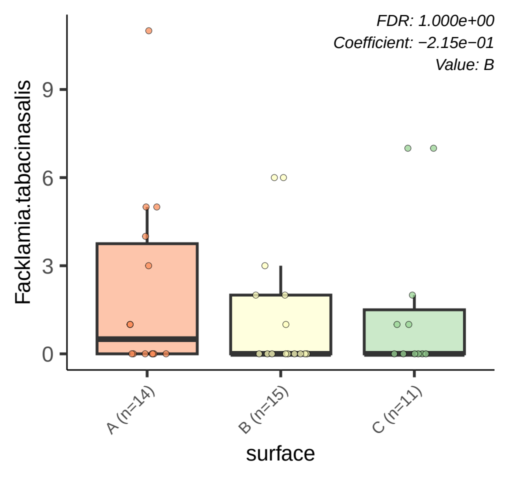

FDR: 1.000e+00
Coefficient: −2.15e−01
Value: B
Facklamia.tabacinasalis
0
3
6
9
A (n=14)
B (n=15)
C (n=11)
surface

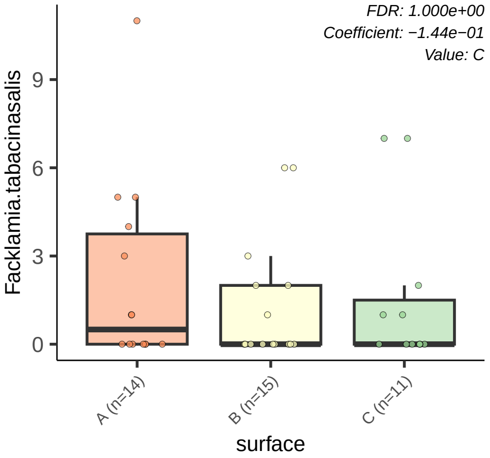
FDR: 1.000e+00
Coefficient: −1.44e−01
Value: C
Facklamia.tabacinasalis
0
3
6
9
A (n=14)
B (n=15)
C (n=11)
surface

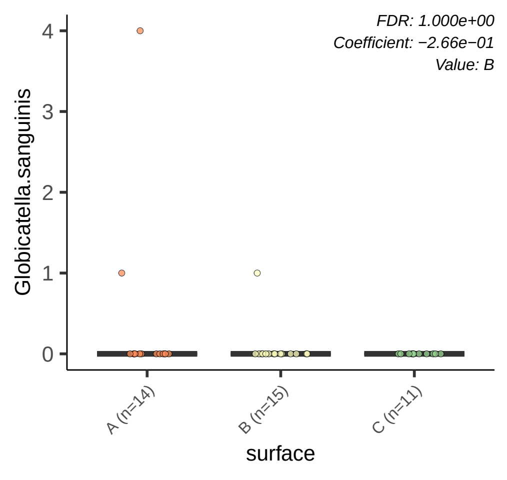
FDR: 1.000e+00
Coefficient: −2.66e−01
Value: B
Globicatella.sanguinis
4
3
2
1
0
A (n=14)
B (n=15)
C (n=11)
surface

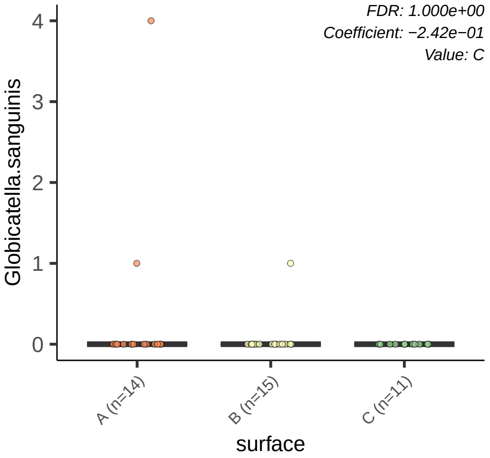
FDR: 1.000e+00
Coefficient: −2.42e−01
Value: C
Globicatella.sanguinis
4
3
2
1
0
A (n=14)
B (n=15)
C (n=11)
surface

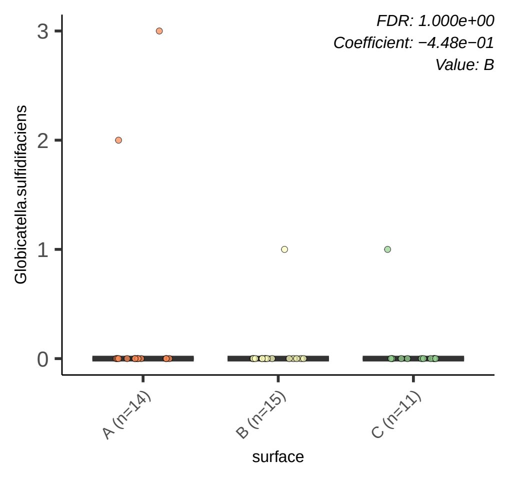

FDR: 1.000e+00
Coefficient: −4.48e−01
Value: B
Globicatella.sulfidifaciens
3
2
1
0
A (n=14)
B (n=15)
C (n=11)
surface

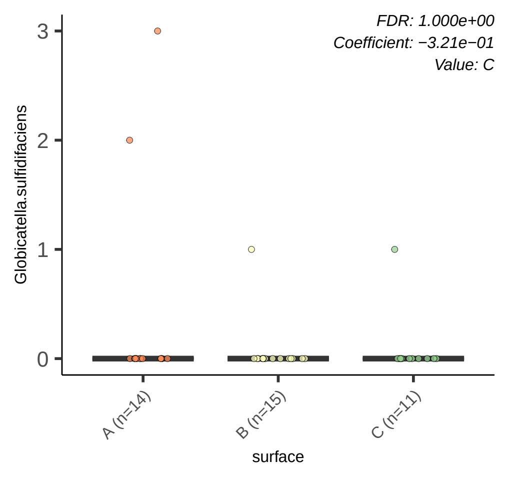
FDR: 1.000e+00
Coefficient: −3.21e−01
Value: C
Globicatella.sulfidifaciens
3
2
1
0
A (n=14)
B (n=15)
C (n=11)
surface

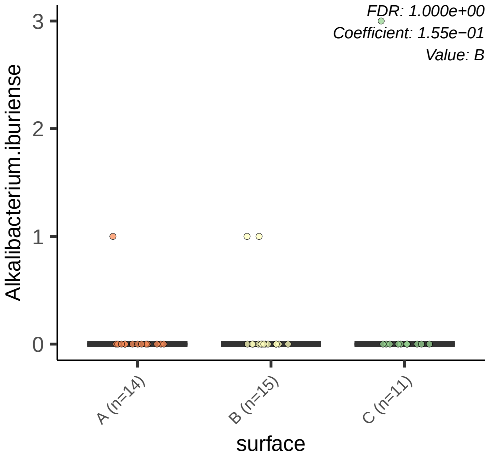
FDR: 1.000e+00
Coefficient: 1.55e−01
Value: B
Alkalibacterium.iburiense
3
2
1
0
A (n=14)
B (n=15)
C (n=11)
surface

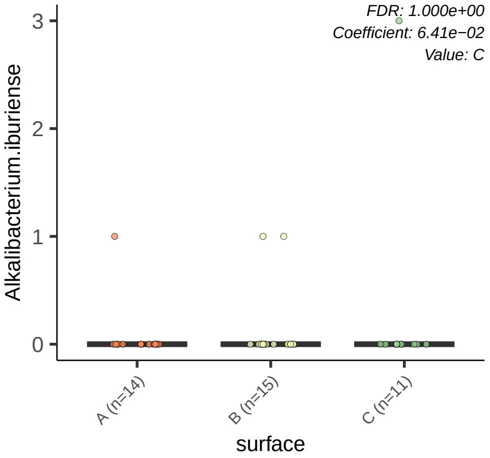

FDR: 1.000e+00
Coefficient: 6.41e−02
Value: C
Alkalibacterium.iburiense
0
1
2
3
A (n=14)
B (n=15)
C (n=11)
surface

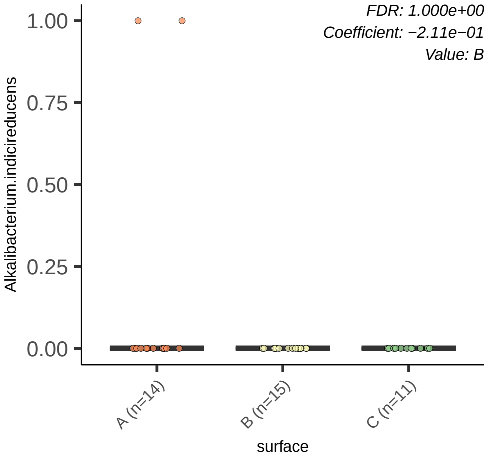
FDR: 1.000e+00
Coefficient: −2.11e−01
Value: B
Alkalibacterium.indicireducens
1.00
0.75
0.50
0.25
0.00
A (n=14)
B (n=15)
C (n=11)
surface

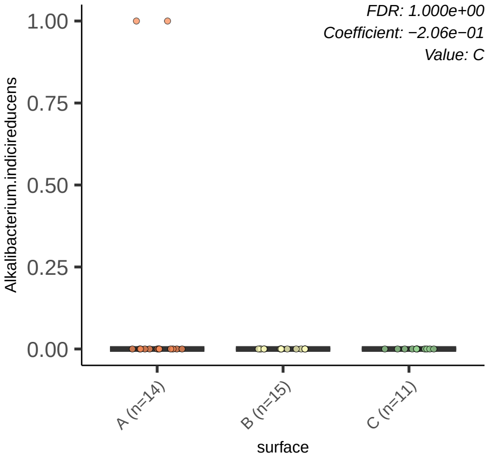

FDR: 1.000e+00
Coefficient: −2.06e−01
Value: C
1.00
0.75
0.50
0.25
0.00
Alkalibacterium.indicireducens
A (n=14)
B (n=15)
C (n=11)
surface

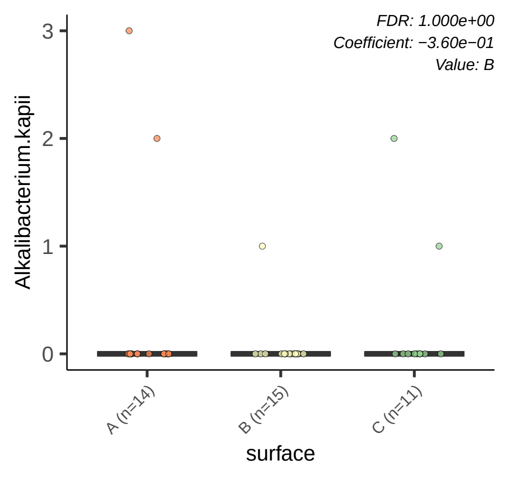
FDR: 1.000e+00
Coefficient: −3.60e−01
Value: B
Alkalibacterium.kapii
3
2
1
0
A (n=14)
B (n=15)
C (n=11)
surface

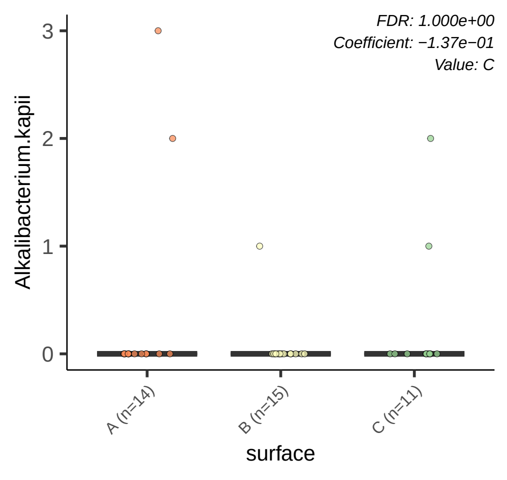

FDR: 1.000e+00
Coefficient: −1.37e−01
Value: C
Alkalibacterium.kapii
0
1
2
3
A (n=14)
B (n=15)
C (n=11)
surface

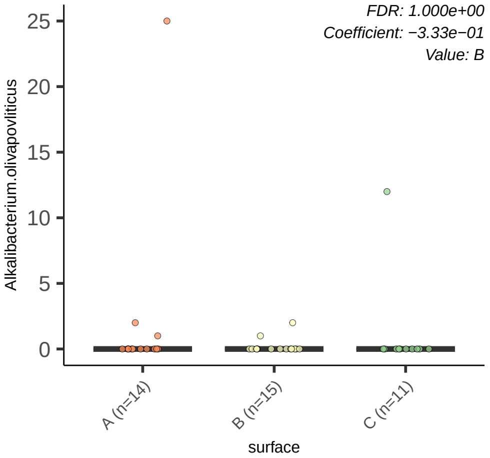

FDR: 1.000e+00
Coefficient: −3.33e−01
Value: B
Alkalibacterium.olivapovliticus
25
20
15
10
5
0
A (n=14)
B (n=15)
C (n=11)
surface

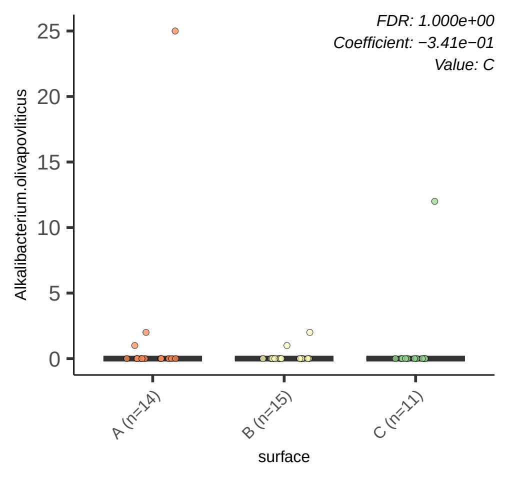
FDR: 1.000e+00
Coefficient: −3.41e−01
Value: C
Alkalibacterium.olivapovliticus
25
20
15
10
5
0
A (n=14)
B (n=15)
C (n=11)
surface

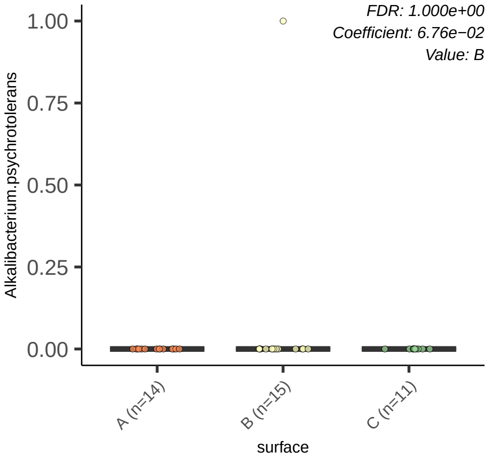
FDR: 1.000e+00
Coefficient: 6.76e−02
Value: B
Alkalibacterium.psychrotolerans
1.00
0.75
0.50
0.25
0.00
A (n=14)
B (n=15)
C (n=11)
surface

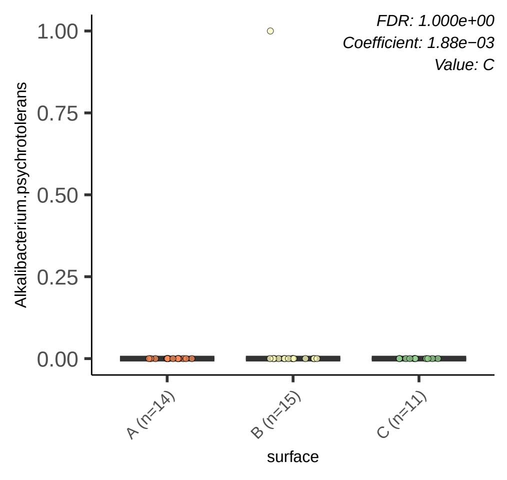
FDR: 1.000e+00
Coefficient: 1.88e−03
Value: C
Alkalibacterium.psychrotolerans
1.00
0.75
0.50
0.25
0.00
A (n=14)
B (n=15)
C (n=11)
surface

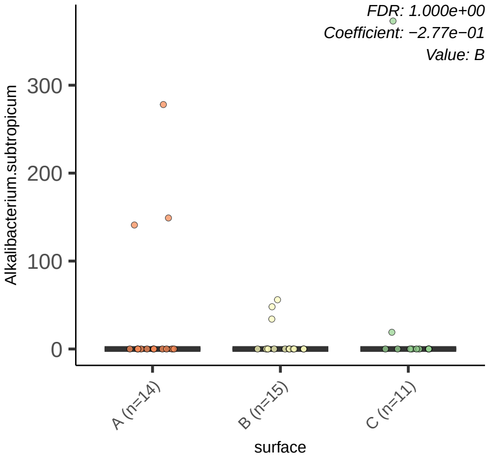

FDR: 1.000e+00
Coefficient: −2.77e−01
Value: B
Alkalibacterium.subtropicum
300
200
100
0
A (n=14)
B (n=15)
C (n=11)
surface

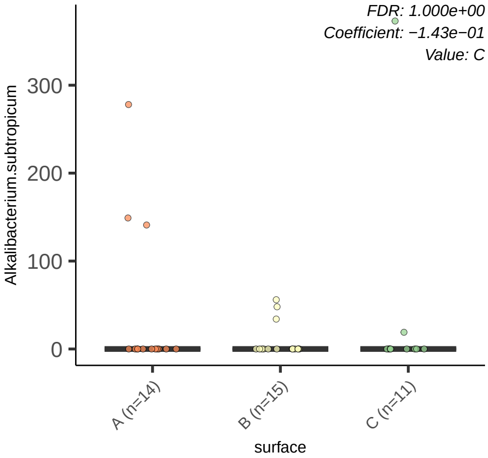

FDR: 1.000e+00
Coefficient: −1.43e−01
Value: C
Alkalibacterium.subtropicum
300
200
100
0
A (n=14)
B (n=15)
C (n=11)
surface

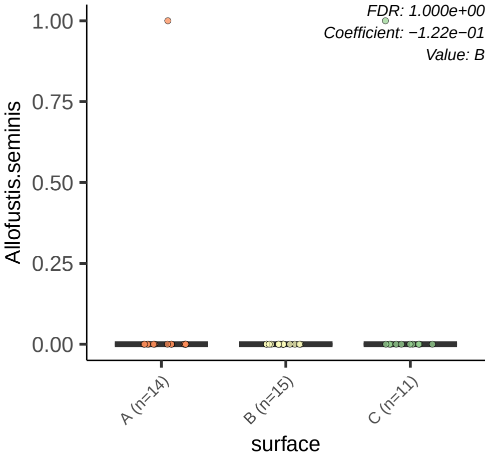
FDR: 1.000e+00
Coefficient: −1.22e−01
Value: B
Allofustis.seminis
1.00
0.75
0.50
0.25
0.00
A (n=14)
B (n=15)
C (n=11)
surface

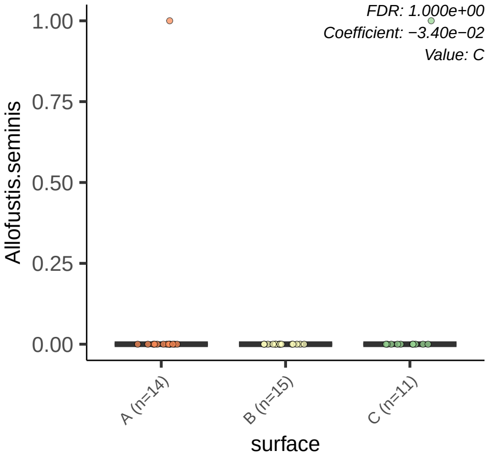

FDR: 1.000e+00
Coefficient: −3.40e−02
Value: C
Allofustis.seminis
1.00
0.75
0.50
0.25
0.00
A (n=14)
B (n=15)
C (n=11)
surface

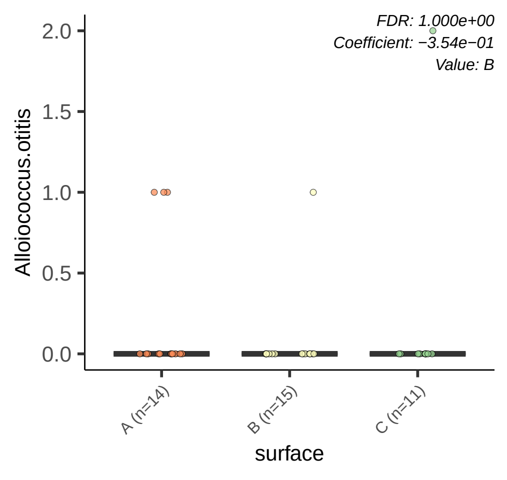
FDR: 1.000e+00
Coefficient: −3.54e−01
Value: B
Alloiococcus.otitis
2.0
1.5
1.0
0.5
0.0
A (n=14)
B (n=15)
C (n=11)
surface

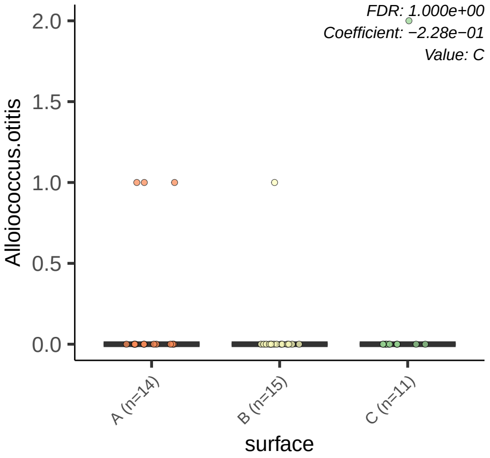

FDR: 1.000e+00
Coefficient: −2.28e−01
Value: C
Alloiococcus.otitis
2.0
1.5
1.0
0.5
0.0
A (n=14)
B (n=15)
C (n=11)
surface

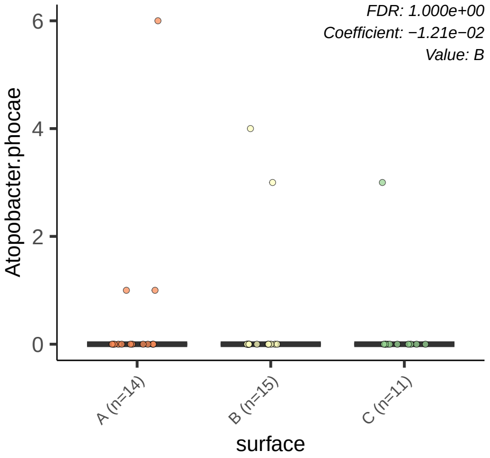
FDR: 1.000e+00
Coefficient: −1.21e−02
Value: B
Atopobacter.phocae
6
4
2
0
A (n=14)
B (n=15)
C (n=11)
surface

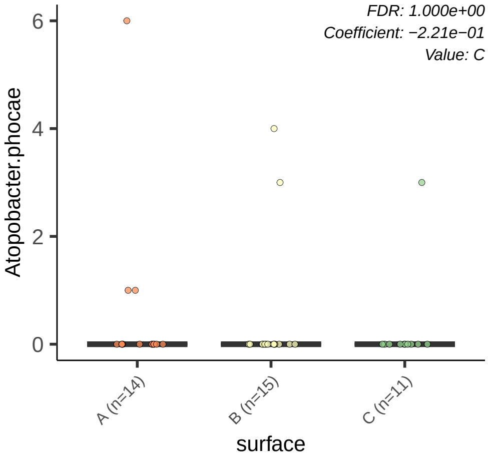

FDR: 1.000e+00
Coefficient: −2.21e−01
Value: C
Atopobacter.phocae
0
2
4
6
A (n=14)
B (n=15)
C (n=11)
surface

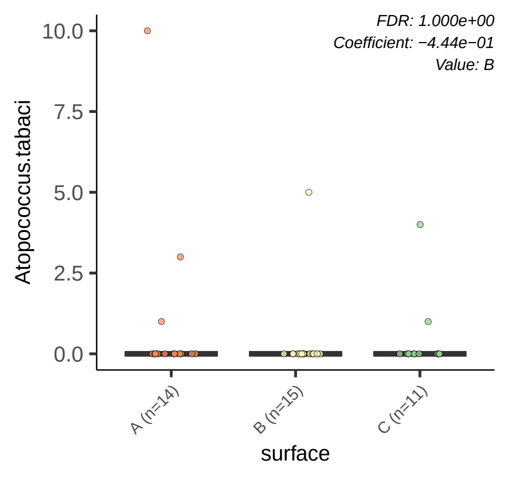

FDR: 1.000e+00
Coefficient: −4.44e−01
Value: B
Atopococcus.tabaci
10.0
7.5
5.0
2.5
0.0
A (n=14)
B (n=15)
C (n=11)
surface

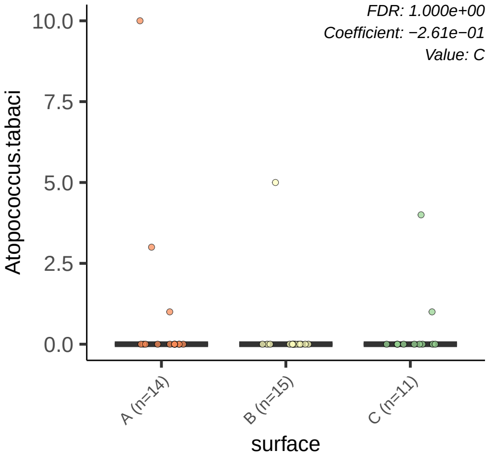

FDR: 1.000e+00
Coefficient: −2.61e−01
Value: C
Atopococcus.tabaci
10.0
7.5
5.0
2.5
0.0
A (n=14)
B (n=15)
C (n=11)
surface

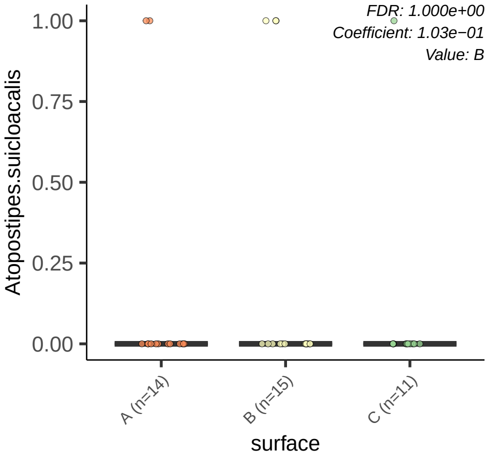

FDR: 1.000e+00
Coefficient: 1.03e−01
Value: B
Atopostipes.suicloacalis
1.00
0.75
0.50
0.25
0.00
A (n=14)
B (n=15)
C (n=11)
surface

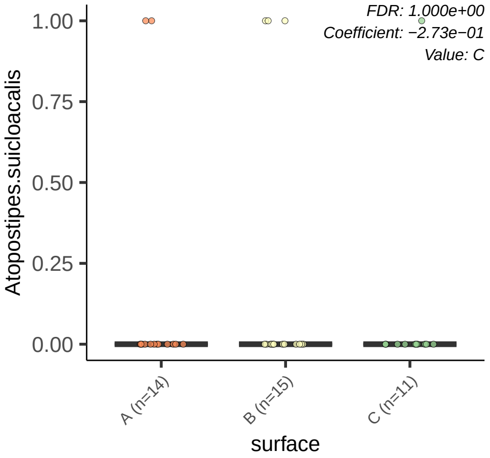

FDR: 1.000e+00
Coefficient: −2.73e−01
Value: C
Atopostipes.suicloacalis
1.00
0.75
0.50
0.25
0.00
A (n=14)
B (n=15)
C (n=11)
surface

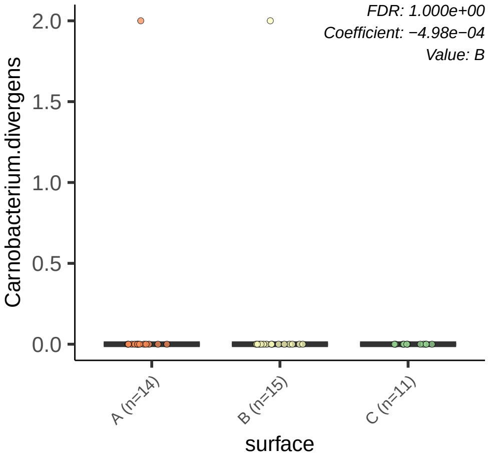
FDR: 1.000e+00
Coefficient: −4.98e−04
Value: B
Carnobacterium.divergens
2.0
1.5
1.0
0.5
0.0
A (n=14)
B (n=15)
C (n=11)
surface

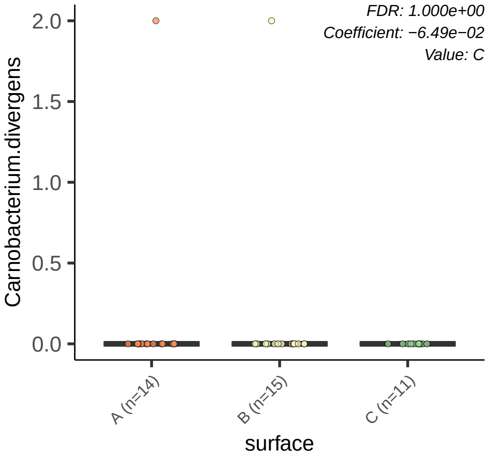
FDR: 1.000e+00
Coefficient: −6.49e−02
Value: C
Carnobacterium.divergens
2.0
1.5
1.0
0.5
0.0
A (n=14)
B (n=15)
C (n=11)
surface

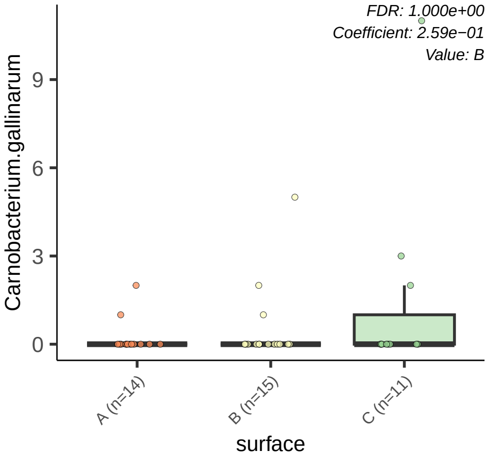
FDR: 1.000e+00
Coefficient: 2.59e−01
Value: B
Carnobacterium.gallinarum
9
6
3
0
A (n=14)
B (n=15)
C (n=11)
surface

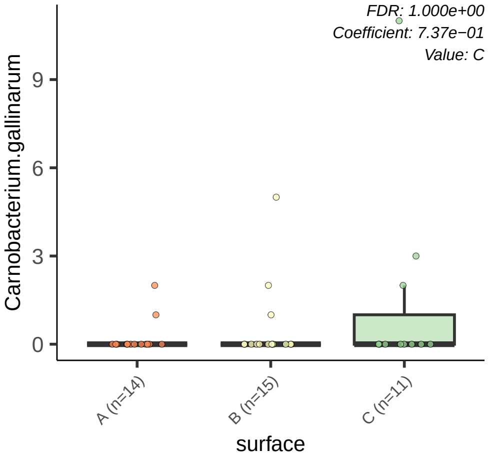

FDR: 1.000e+00
Coefficient: 7.37e−01
Value: C
Carnobacterium.gallinarum
0
3
6
9
A (n=14)
B (n=15)
C (n=11)
surface

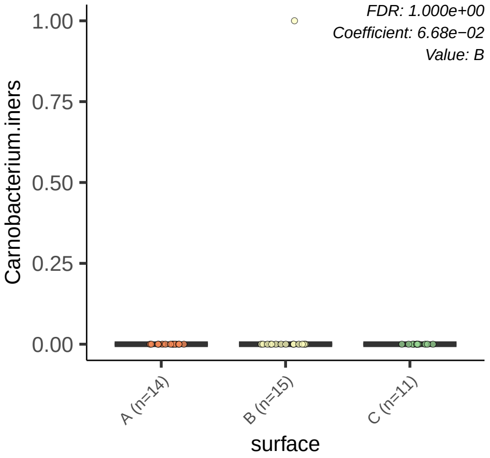
FDR: 1.000e+00
Coefficient: 6.68e−02
Value: B
Carnobacterium.iners
1.00
0.75
0.50
0.25
0.00
A (n=14)
B (n=15)
C (n=11)
surface

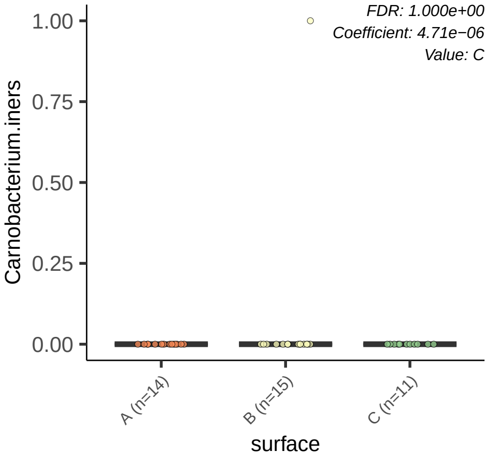

FDR: 1.000e+00
Coefficient: 4.71e−06
Value: C
Carnobacterium.iners
1.00
0.75
0.50
0.25
0.00
A (n=14)
B (n=15)
C (n=11)
surface

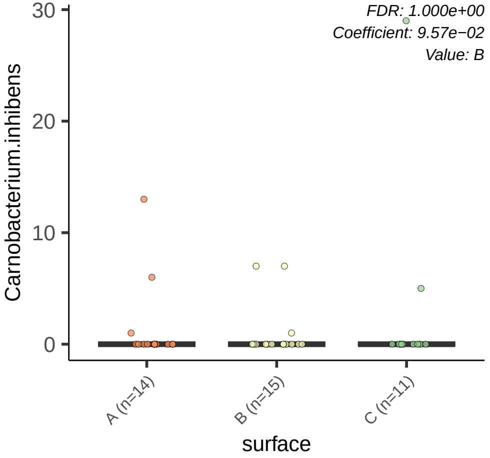

FDR: 1.000e+00
Coefficient: 9.57e−02
Value: B
Carnobacterium.inhibens
30
20
10
0
A (n=14)
B (n=15)
C (n=11)
surface

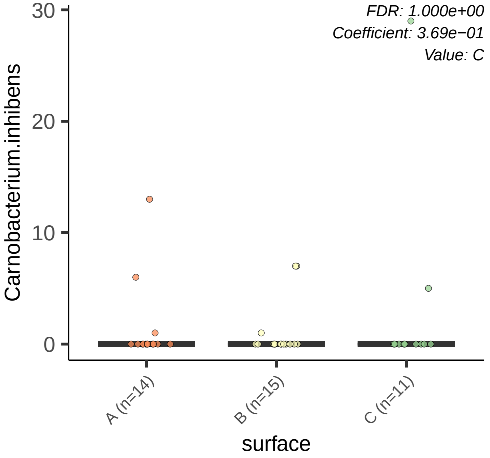

FDR: 1.000e+00
Coefficient: 3.69e−01
Value: C
Carnobacterium.inhibens
30
20
10
0
A (n=14)
B (n=15)
C (n=11)
surface

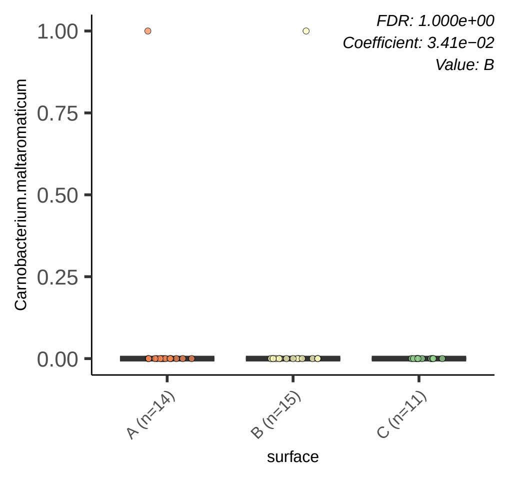

FDR: 1.000e+00
Coefficient: 3.41e−02
Value: B
1.00
0.75
0.50
0.25
0.00
Carnobacterium.maltaromaticum
A (n=14)
B (n=15)
C (n=11)
surface

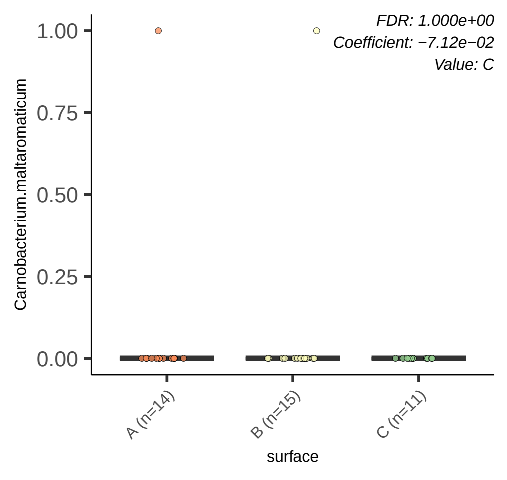
FDR: 1.000e+00
Coefficient: −7.12e−02
Value: C
Carnobacterium.maltaromaticum
1.00
0.75
0.50
0.25
0.00
A (n=14)
B (n=15)
C (n=11)
surface

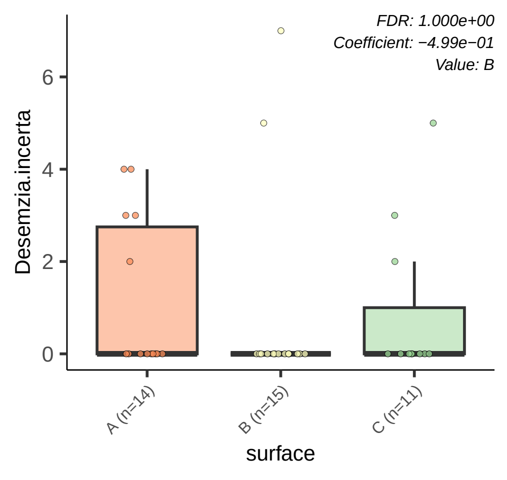
FDR: 1.000e+00
Coefficient: −4.99e−01
Value: B
Desemzia.incerta
0
2
4
6
A (n=14)
B (n=15)
C (n=11)
surface

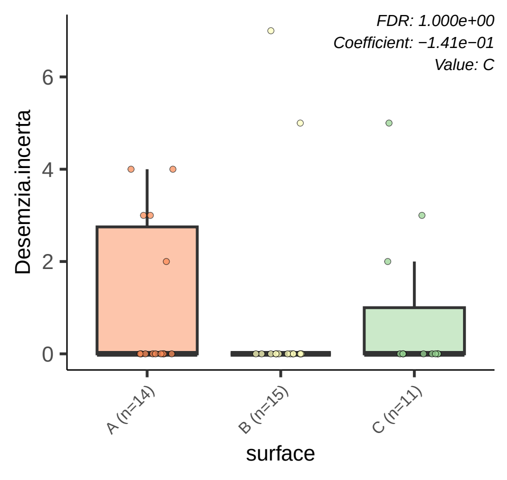
FDR: 1.000e+00
Coefficient: −1.41e−01
Value: C
Desemzia.incerta
0
2
4
6
A (n=14)
B (n=15)
C (n=11)
surface

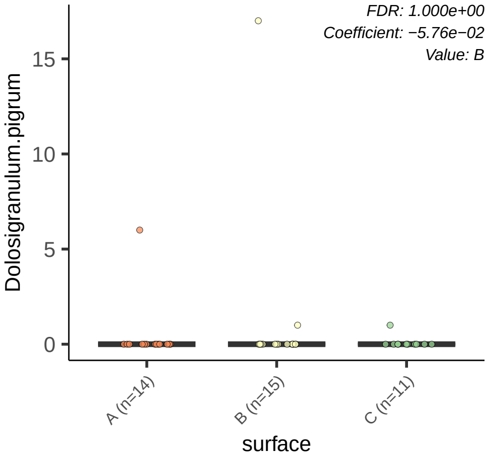
FDR: 1.000e+00
Coefficient: −5.76e−02
Value: B
Dolosigranulum.pigrum
0
5
10
15
A (n=14)
B (n=15)
C (n=11)
surface

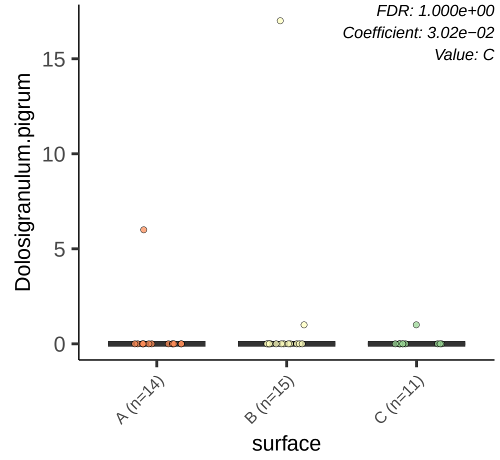
FDR: 1.000e+00
Coefficient: 3.02e−02
Value: C
Dolosigranulum.pigrum
15
10
5
0
A (n=14)
B (n=15)
C (n=11)
surface

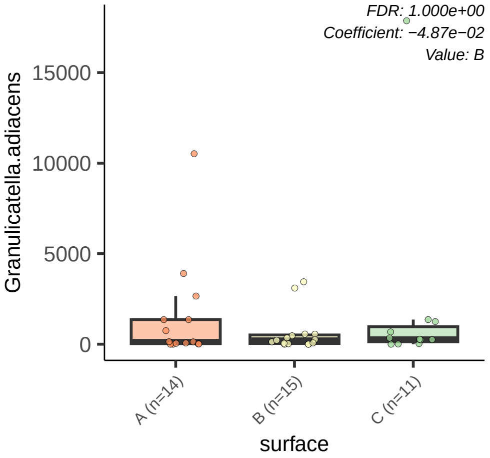

FDR: 1.000e+00
Coefficient: −4.87e−02
Value: B
Granulicatella.adiacens
15000
10000
5000
0
A (n=14)
B (n=15)
C (n=11)
surface

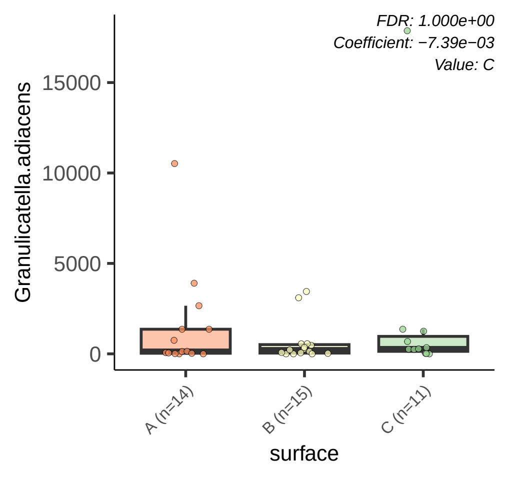
FDR: 1.000e+00
Coefficient: −7.39e−03
Value: C
Granulicatella.adiacens
15000
10000
5000
0
A (n=14)
B (n=15)
C (n=11)
surface

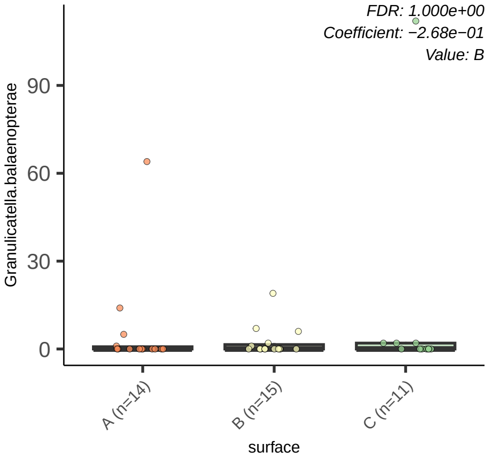

FDR: 1.000e+00
Coefficient: −2.68e−01
Value: B
90
60
30
0
Granulicatella.balaenopterae
A (n=14)
B (n=15)
C (n=11)
surface

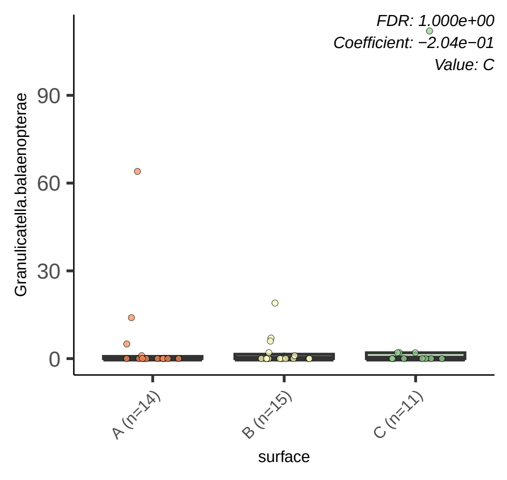

FDR: 1.000e+00
Coefficient: −2.04e−01
Value: C
90
60
30
0
Granulicatella.balaenopterae
A (n=14)
B (n=15)
C (n=11)
surface

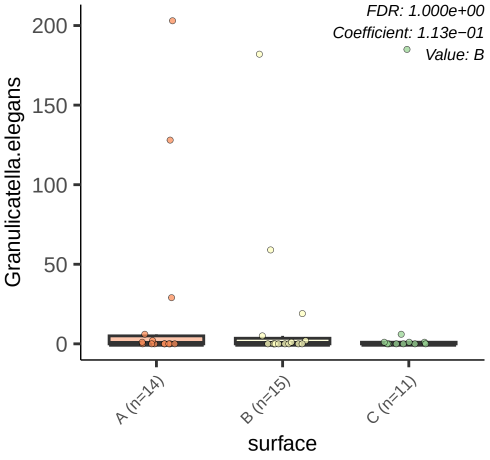

FDR: 1.000e+00
Coefficient: 1.13e−01
Value: B
Granulicatella.elegans
200
150
100
50
0
A (n=14)
B (n=15)
C (n=11)
surface

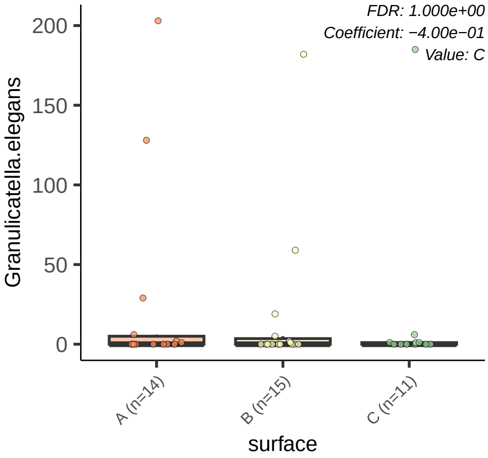

FDR: 1.000e+00
Coefficient: −4.00e−01
Value: C
Granulicatella.elegans
200
150
100
50
0
A (n=14)
B (n=15)
C (n=11)
surface

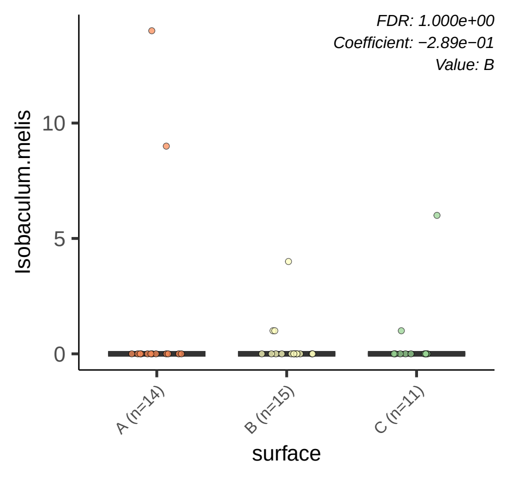
FDR: 1.000e+00
Coefficient: −2.89e−01
Value: B
Isobaculum.melis
0
5
10
A (n=14)
B (n=15)
C (n=11)
surface

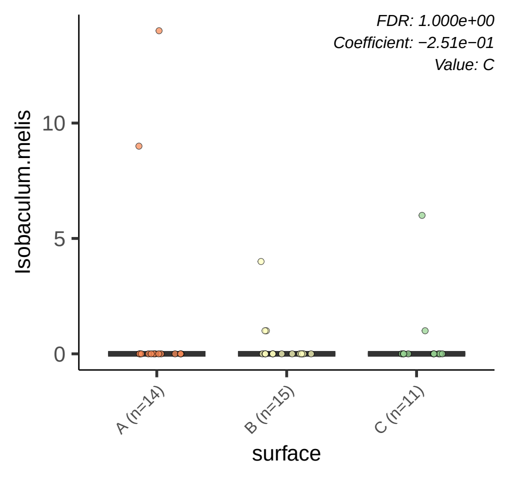
FDR: 1.000e+00
Coefficient: −2.51e−01
Value: C
Isobaculum.melis
10
5
0
A (n=14)
B (n=15)
C (n=11)
surface

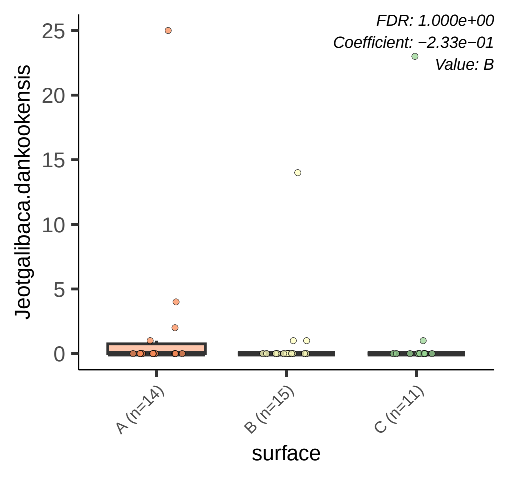

FDR: 1.000e+00
Coefficient: −2.33e−01
Value: B
Jeotgalibaca.dankookensis
0
5
10
15
20
25
A (n=14)
B (n=15)
C (n=11)
surface

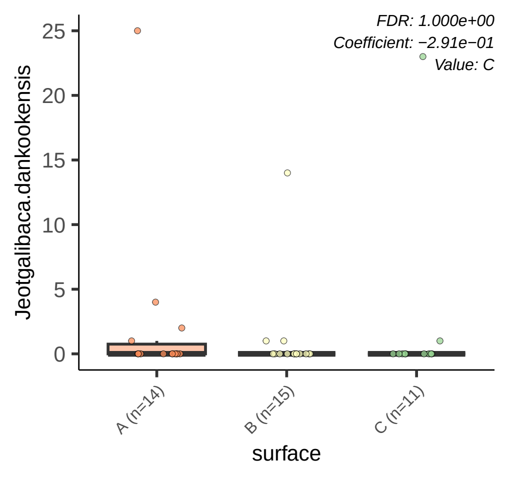

FDR: 1.000e+00
Coefficient: −2.91e−01
Value: C
Jeotgalibaca.dankookensis
0
5
10
15
20
25
A (n=14)
B (n=15)
C (n=11)
surface

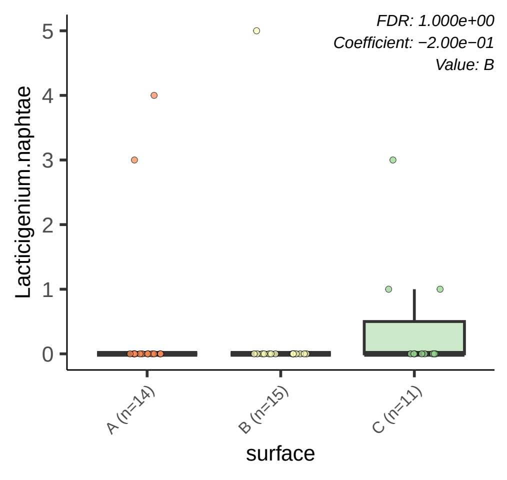

FDR: 1.000e+00
Coefficient: −2.00e−01
Value: B
Lacticigenium.naphtae
5
4
3
2
1
0
A (n=14)
B (n=15)
C (n=11)
surface

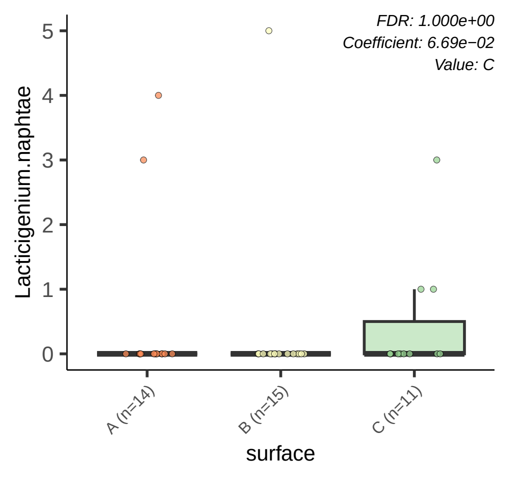

FDR: 1.000e+00
Coefficient: 6.69e−02
Value: C
Lacticigenium.naphtae
0
1
2
3
4
5
A (n=14)
B (n=15)
C (n=11)
surface

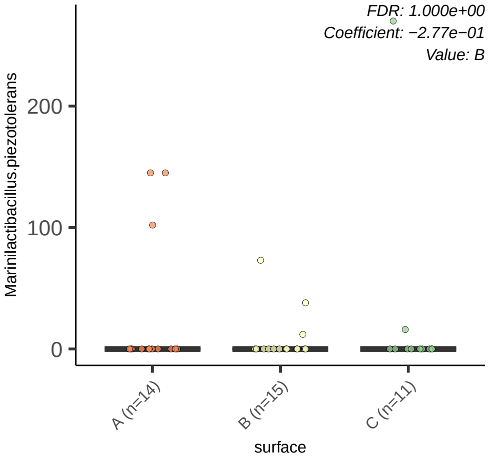

FDR: 1.000e+00
Coefficient: −2.77e−01
Value: B
Marinilactibacillus.piezotolerans
200
100
0
A (n=14)
B (n=15)
C (n=11)
surface

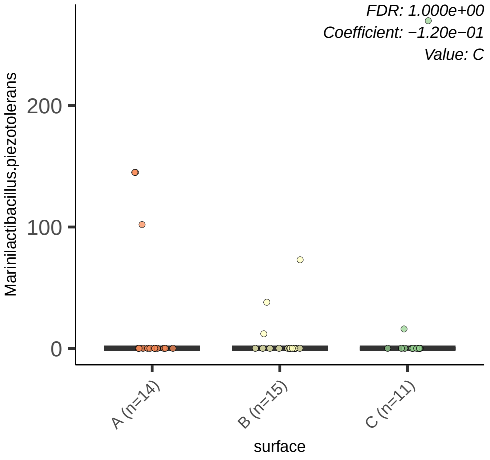
FDR: 1.000e+00
Coefficient: −1.20e−01
Value: C
Marinilactibacillus.piezotolerans
200
100
0
A (n=14)
B (n=15)
C (n=11)
surface

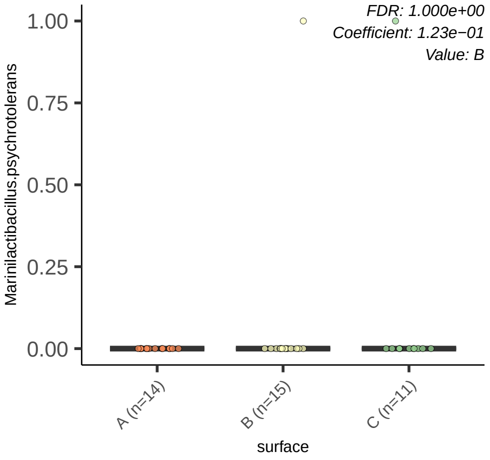
FDR: 1.000e+00
Coefficient: 1.23e−01
Value: B
Marinilactibacillus.psychrotolerans
1.00
0.75
0.50
0.25
0.00
A (n=14)
B (n=15)
C (n=11)
surface

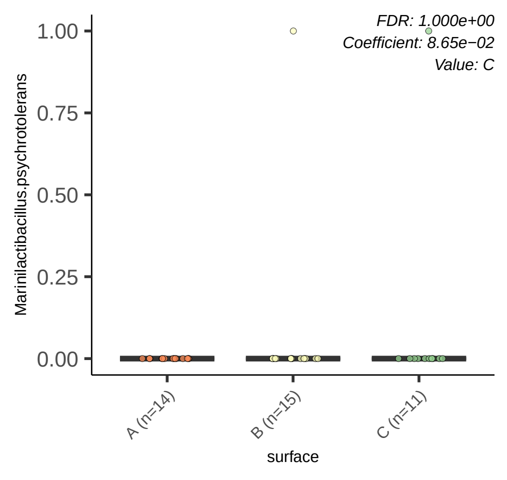
FDR: 1.000e+00
Coefficient: 8.65e−02
Value: C
Marinilactibacillus.psychrotolerans
1.00
0.75
0.50
0.25
0.00
A (n=14)
B (n=15)
C (n=11)
surface

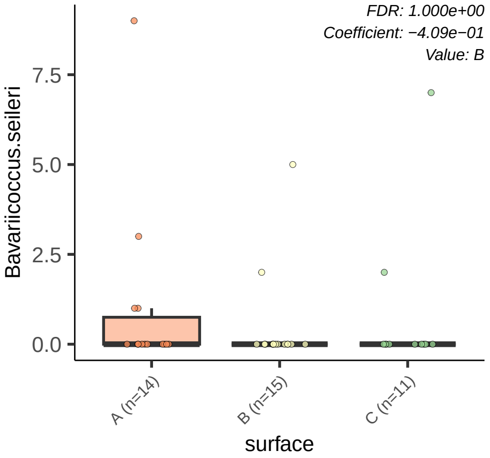
FDR: 1.000e+00
Coefficient: −4.09e−01
Value: B
Bavariicoccus.seileri
7.5
5.0
2.5
0.0
A (n=14)
B (n=15)
C (n=11)
surface

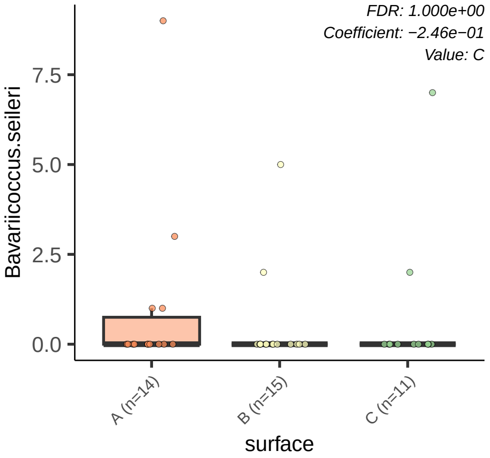

FDR: 1.000e+00
Coefficient: −2.46e−01
Value: C
Bavariicoccus.seileri
7.5
5.0
2.5
0.0
A (n=14)
B (n=15)
C (n=11)
surface

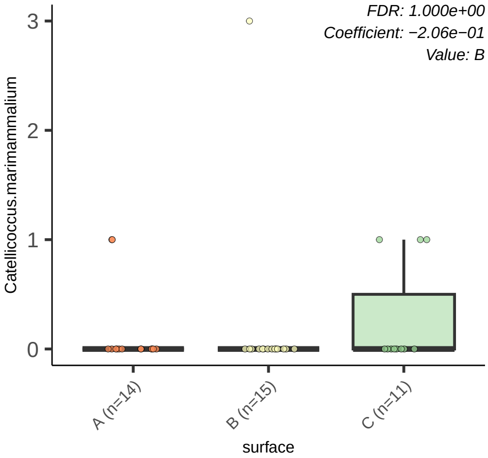

FDR: 1.000e+00
Coefficient: −2.06e−01
Value: B
Catellicoccus.marimammalium
3
2
1
0
A (n=14)
B (n=15)
C (n=11)
surface

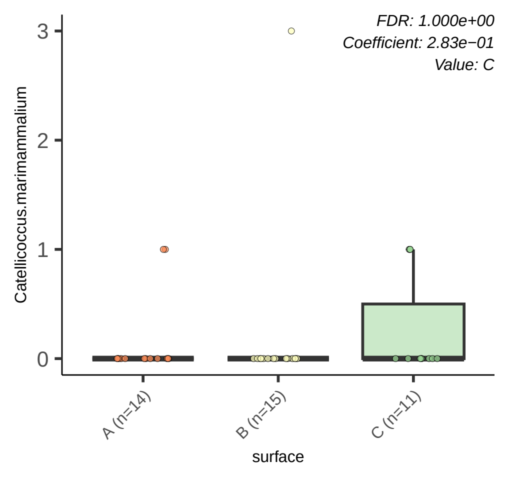
FDR: 1.000e+00
Coefficient: 2.83e−01
Value: C
Catellicoccus.marimammalium
3
2
1
0
A (n=14)
B (n=15)
C (n=11)
surface

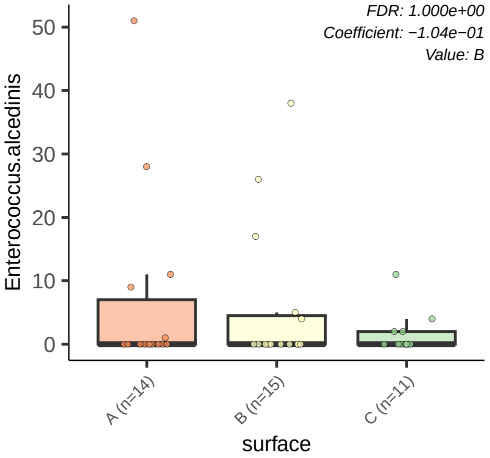
FDR: 1.000e+00
Coefficient: −1.04e−01
Value: B
Enterococcus.alcedinis
0
10
20
30
40
50
A (n=14)
B (n=15)
C (n=11)
surface

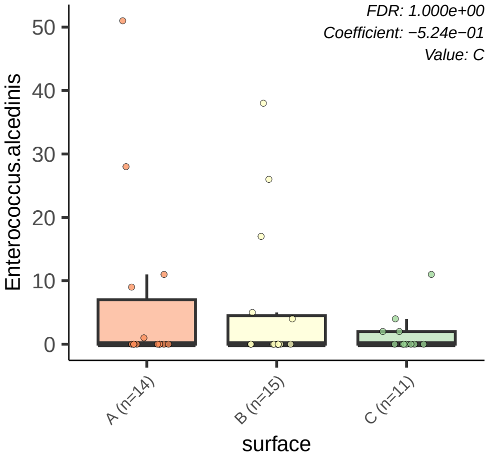
FDR: 1.000e+00
Coefficient: −5.24e−01
Value: C
Enterococcus.alcedinis
50
40
30
20
10
0
A (n=14)
B (n=15)
C (n=11)
surface

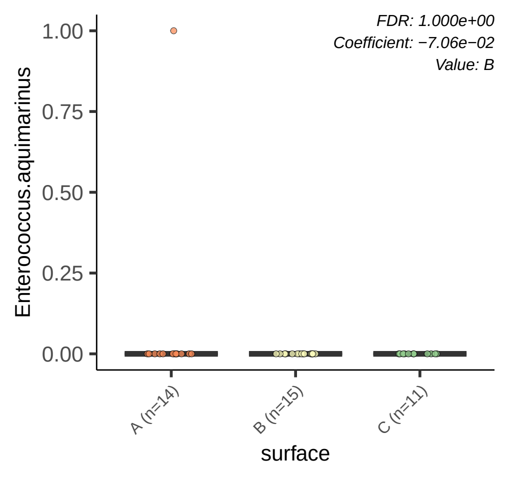

FDR: 1.000e+00
Coefficient: −7.06e−02
Value: B
Enterococcus.aquimarinus
1.00
0.75
0.50
0.25
0.00
A (n=14)
B (n=15)
C (n=11)
surface

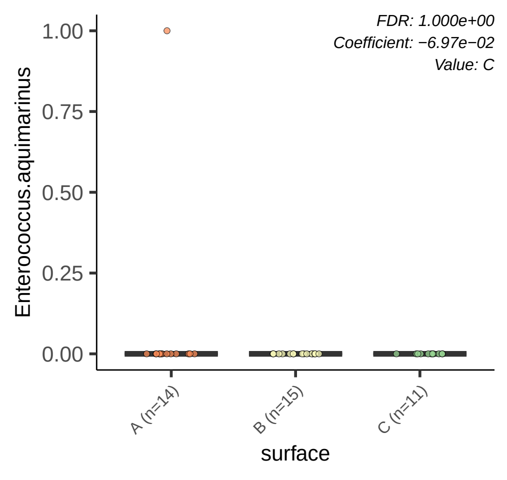

FDR: 1.000e+00
Coefficient: −6.97e−02
Value: C
Enterococcus.aquimarinus
1.00
0.75
0.50
0.25
0.00
A (n=14)
B (n=15)
C (n=11)
surface

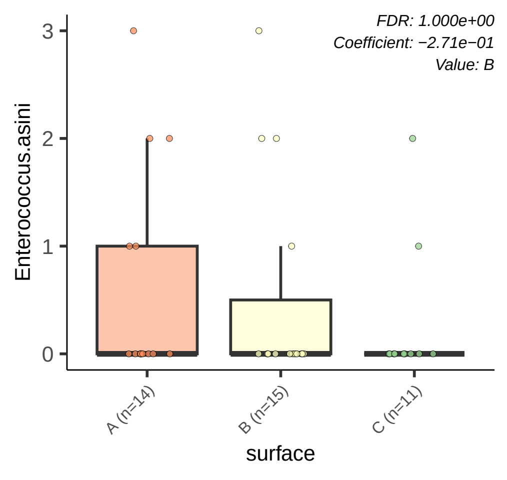

FDR: 1.000e+00
Coefficient: −2.71e−01
Value: B
Enterococcus.asini
3
2
1
0
A (n=14)
B (n=15)
C (n=11)
surface

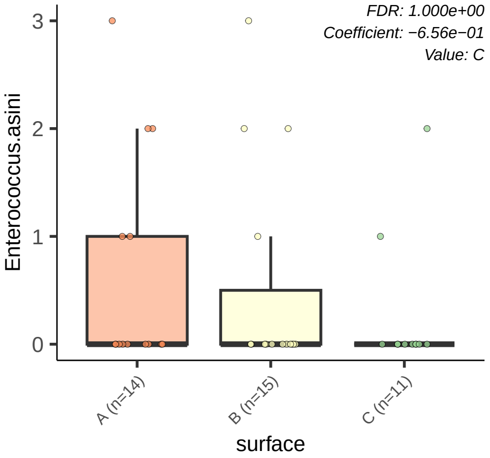
FDR: 1.000e+00
Coefficient: −6.56e−01
Value: C
Enterococcus.asini
3
2
1
0
A (n=14)
B (n=15)
C (n=11)
surface

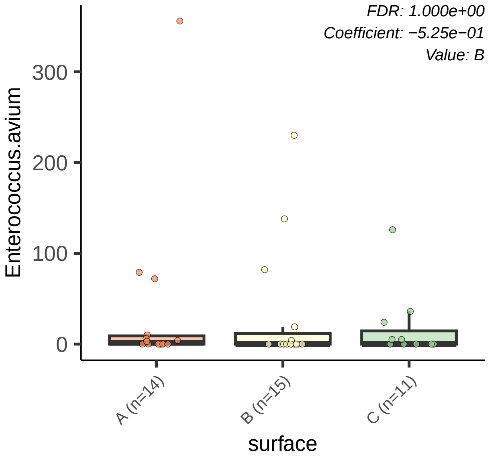
FDR: 1.000e+00
Coefficient: −5.25e−01
Value: B
Enterococcus.avium
300
200
100
0
A (n=14)
B (n=15)
C (n=11)
surface

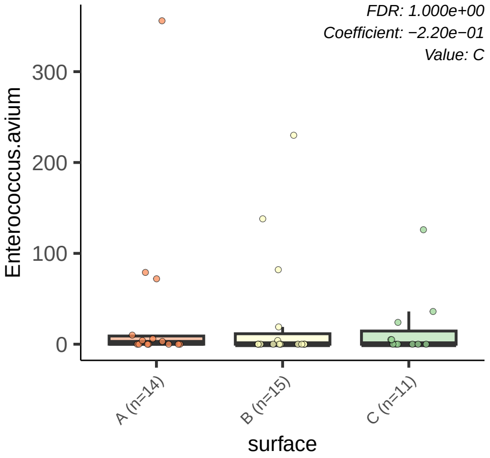
FDR: 1.000e+00
Coefficient: −2.20e−01
Value: C
Enterococcus.avium
300
200
100
0
A (n=14)
B (n=15)
C (n=11)
surface

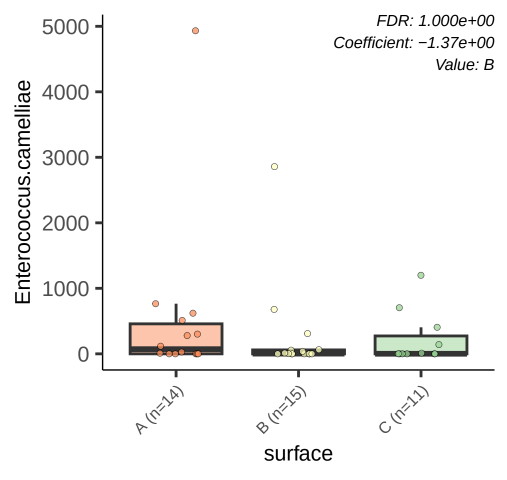
FDR: 1.000e+00
Coefficient: −1.37e+00
Value: B
Enterococcus.camelliae
5000
4000
3000
2000
1000
0
A (n=14)
B (n=15)
C (n=11)
surface

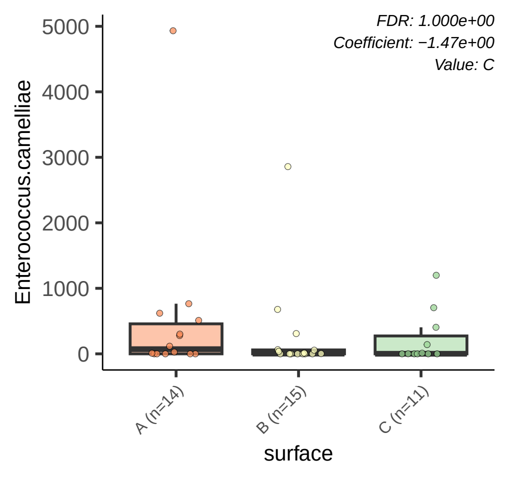
FDR: 1.000e+00
Coefficient: −1.47e+00
Value: C
Enterococcus.camelliae
5000
4000
3000
2000
1000
0
A (n=14)
B (n=15)
C (n=11)
surface

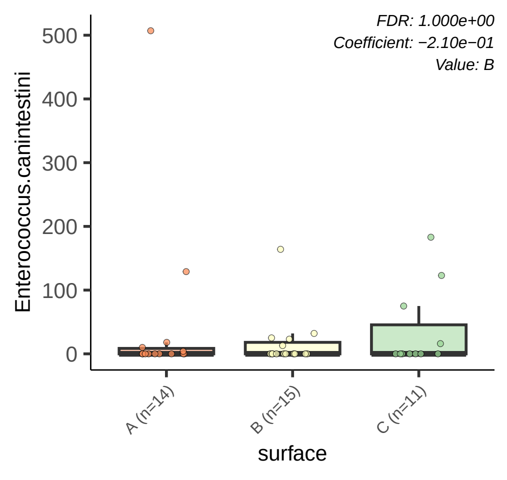

FDR: 1.000e+00
Coefficient: −2.10e−01
Value: B
Enterococcus.canintestini
500
400
300
200
100
0
A (n=14)
B (n=15)
C (n=11)
surface

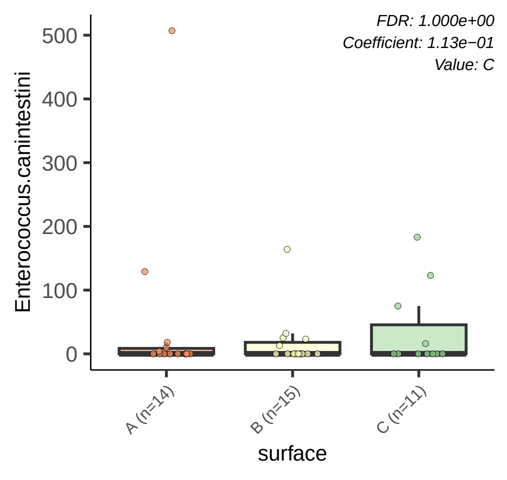
FDR: 1.000e+00
Coefficient: 1.13e−01
Value: C
Enterococcus.canintestini
500
400
300
200
100
0
A (n=14)
B (n=15)
C (n=11)
surface

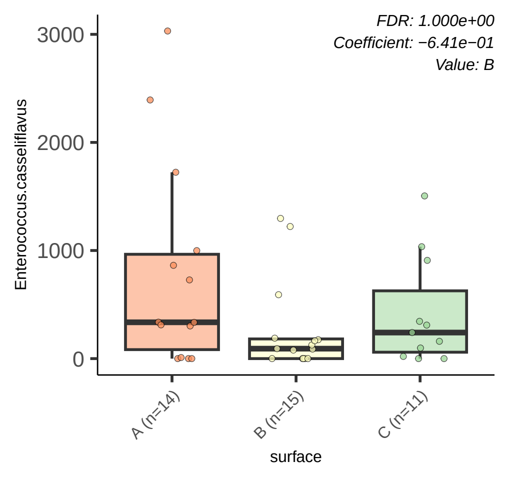

FDR: 1.000e+00
Coefficient: −6.41e−01
Value: B
3000
2000
1000
0
Enterococcus.casseliflavus
A (n=14)
B (n=15)
C (n=11)
surface

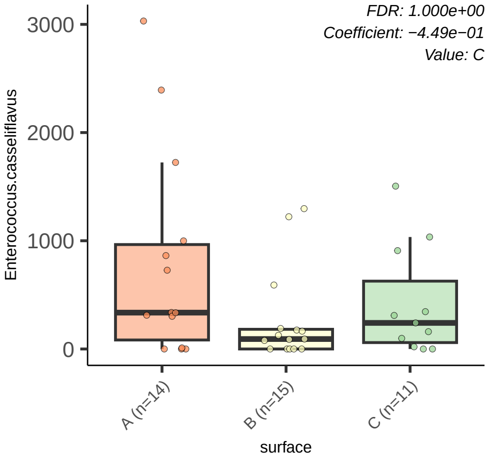

FDR: 1.000e+00
Coefficient: −4.49e−01
Value: C
Enterococcus.casseliflavus
3000
2000
1000
0
A (n=14)
B (n=15)
C (n=11)
surface

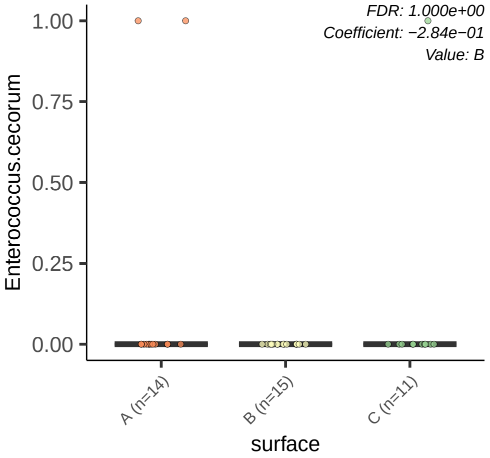
FDR: 1.000e+00
Coefficient: −2.84e−01
Value: B
Enterococcus.cecorum
1.00
0.75
0.50
0.25
0.00
A (n=14)
B (n=15)
C (n=11)
surface

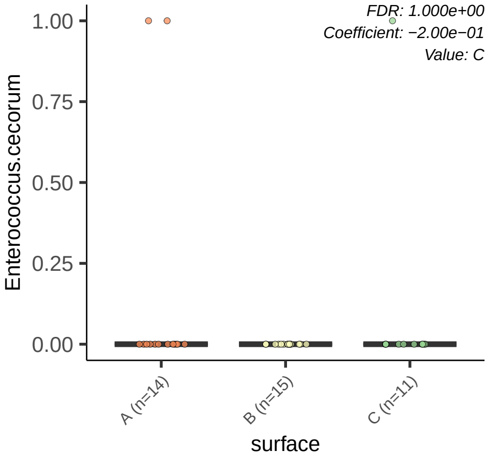

FDR: 1.000e+00
Coefficient: −2.00e−01
Value: C
Enterococcus.cecorum
1.00
0.75
0.50
0.25
0.00
A (n=14)
B (n=15)
C (n=11)
surface

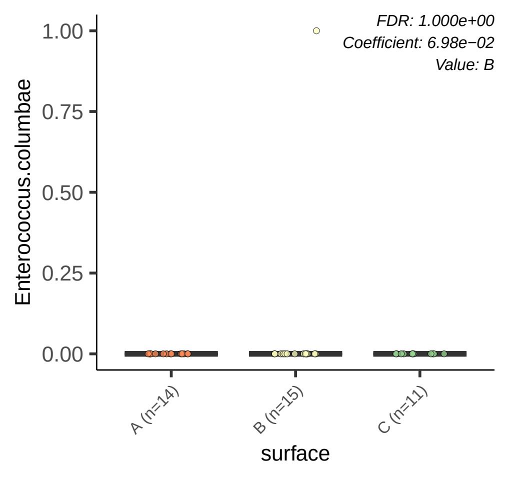
FDR: 1.000e+00
Coefficient: 6.98e−02
Value: B
Enterococcus.columbae
1.00
0.75
0.50
0.25
0.00
A (n=14)
B (n=15)
C (n=11)
surface

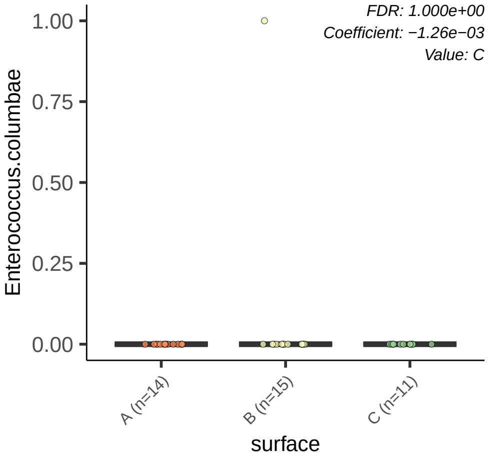

FDR: 1.000e+00
Coefficient: −1.26e−03
Value: C
Enterococcus.columbae
1.00
0.75
0.50
0.25
0.00
A (n=14)
B (n=15)
C (n=11)
surface

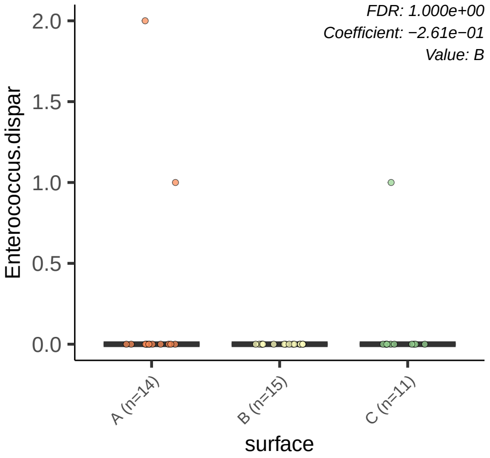

FDR: 1.000e+00
Coefficient: −2.61e−01
Value: B
Enterococcus.dispar
2.0
1.5
1.0
0.5
0.0
A (n=14)
B (n=15)
C (n=11)
surface

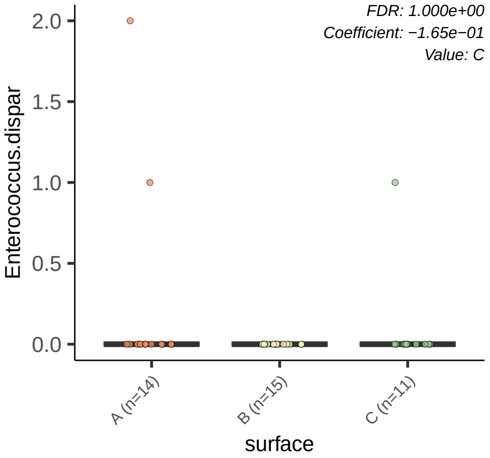
FDR: 1.000e+00
Coefficient: −1.65e−01
Value: C
Enterococcus.dispar
2.0
1.5
1.0
0.5
0.0
A (n=14)
B (n=15)
C (n=11)
surface

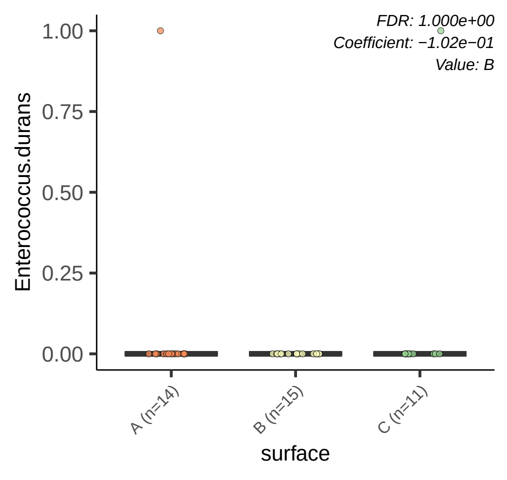
FDR: 1.000e+00
Coefficient: −1.02e−01
Value: B
Enterococcus.durans
1.00
0.75
0.50
0.25
0.00
A (n=14)
B (n=15)
C (n=11)
surface

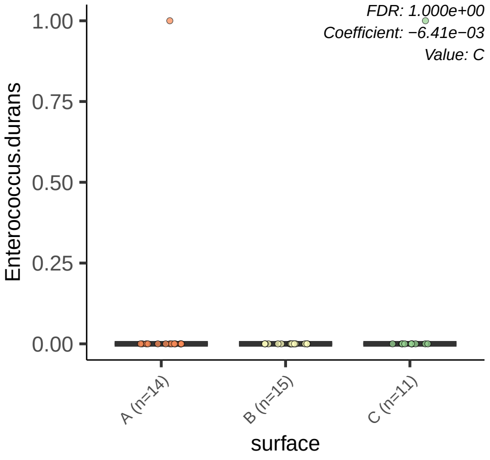
FDR: 1.000e+00
Coefficient: −6.41e−03
Value: C
Enterococcus.durans
1.00
0.75
0.50
0.25
0.00
A (n=14)
B (n=15)
C (n=11)
surface

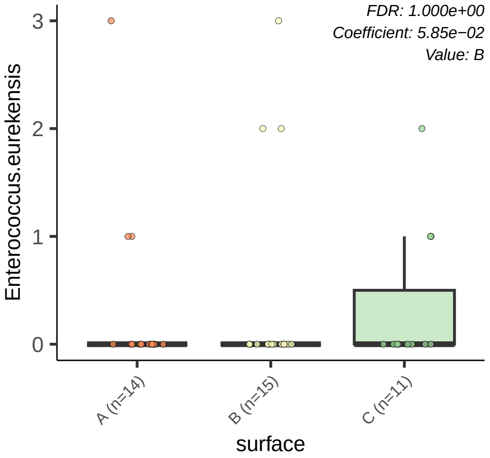

FDR: 1.000e+00
Coefficient: 5.85e−02
Value: B
Enterococcus.eurekensis
0
1
2
3
A (n=14)
B (n=15)
C (n=11)
surface

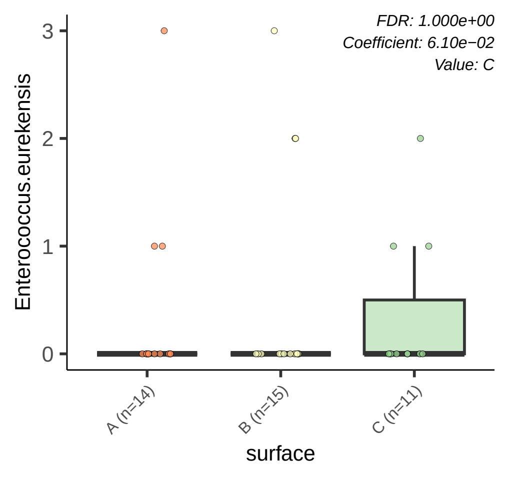

FDR: 1.000e+00
Coefficient: 6.10e−02
Value: C
Enterococcus.eurekensis
0
1
2
3
A (n=14)
B (n=15)
C (n=11)
surface

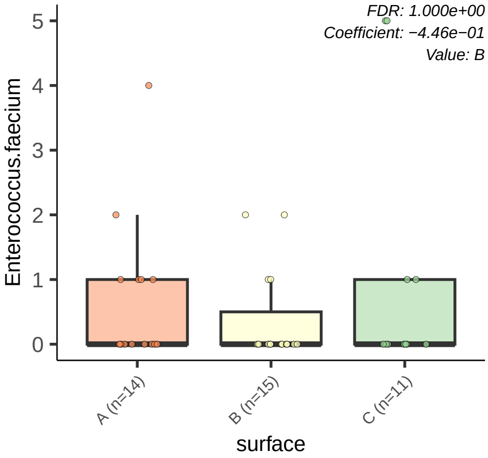
FDR: 1.000e+00
Coefficient: −4.46e−01
Value: B
Enterococcus.faecium
0
1
2
3
4
5
A (n=14)
B (n=15)
C (n=11)
surface

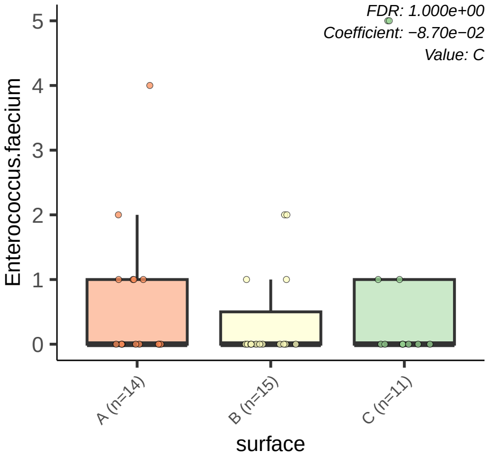

FDR: 1.000e+00
Coefficient: −8.70e−02
Value: C
Enterococcus.faecium
0
1
2
3
4
5
A (n=14)
B (n=15)
C (n=11)
surface

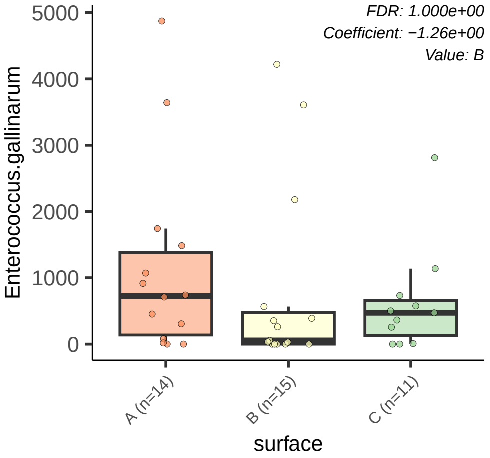

FDR: 1.000e+00
Coefficient: −1.26e+00
Value: B
Enterococcus.gallinarum
5000
4000
3000
2000
1000
0
A (n=14)
B (n=15)
C (n=11)
surface

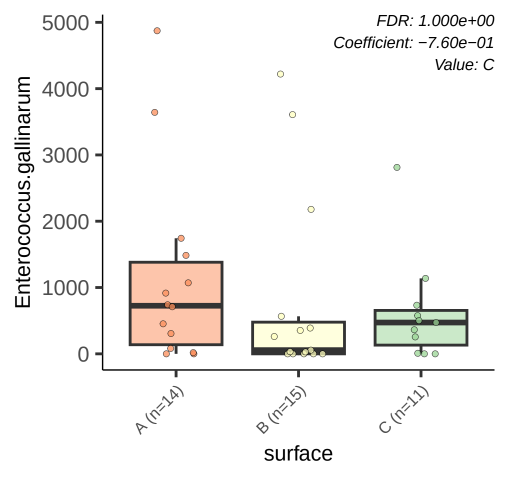

FDR: 1.000e+00
Coefficient: −7.60e−01
Value: C
Enterococcus.gallinarum
5000
4000
3000
2000
1000
0
A (n=14)
B (n=15)
C (n=11)
surface

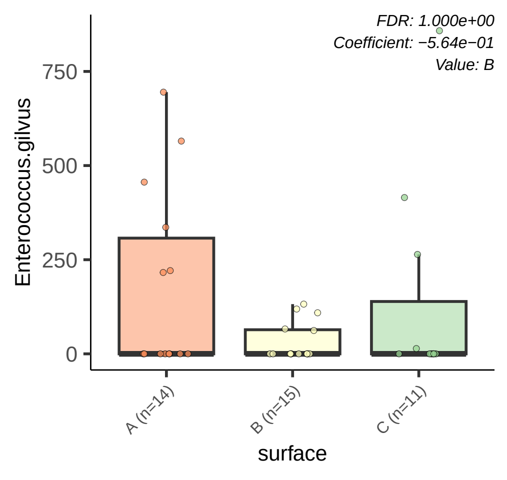
FDR: 1.000e+00
Coefficient: −5.64e−01
Value: B
Enterococcus.gilvus
750
500
250
0
A (n=14)
B (n=15)
C (n=11)
surface

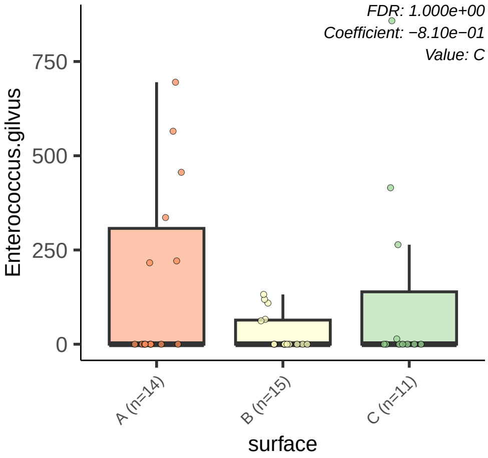

FDR: 1.000e+00
Coefficient: −8.10e−01
Value: C
Enterococcus.gilvus
750
500
250
0
A (n=14)
B (n=15)
C (n=11)
surface

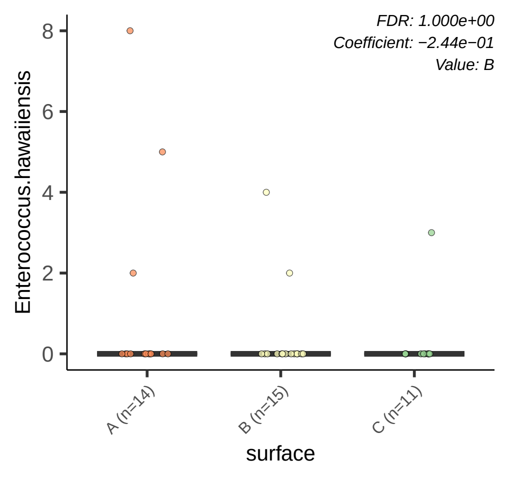
FDR: 1.000e+00
Coefficient: −2.44e−01
Value: B
Enterococcus.hawaiiensis
8
6
4
2
0
A (n=14)
B (n=15)
C (n=11)
surface

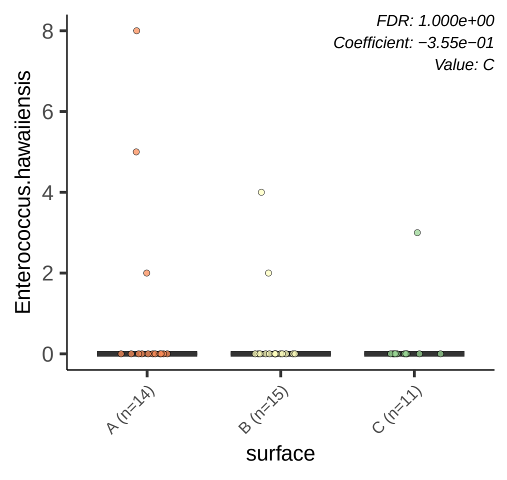

FDR: 1.000e+00
Coefficient: −3.55e−01
Value: C
Enterococcus.hawaiiensis
8
6
4
2
0
A (n=14)
B (n=15)
C (n=11)
surface

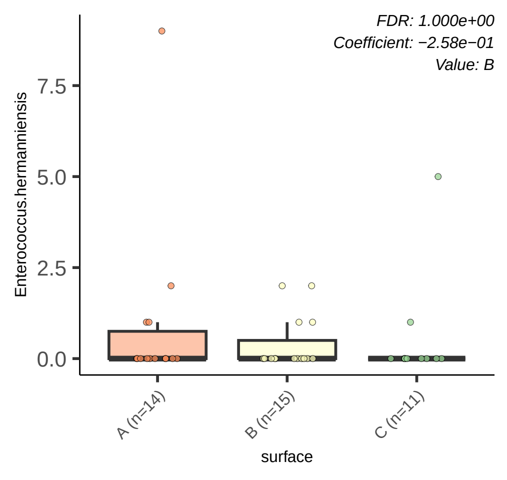

FDR: 1.000e+00
Coefficient: −2.58e−01
Value: B
Enterococcus.hermanniensis
7.5
5.0
2.5
0.0
A (n=14)
B (n=15)
C (n=11)
surface

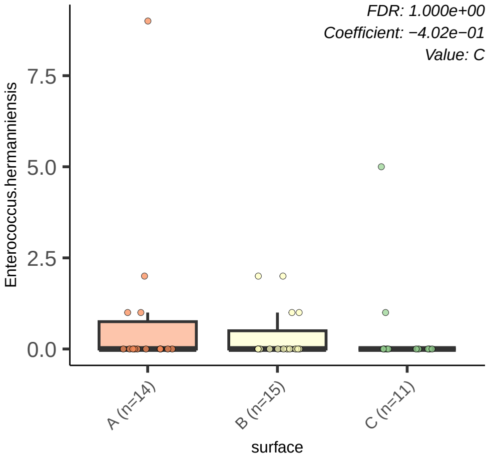

FDR: 1.000e+00
Coefficient: −4.02e−01
Value: C
Enterococcus.hermanniensis
7.5
5.0
2.5
0.0
A (n=14)
B (n=15)
C (n=11)
surface

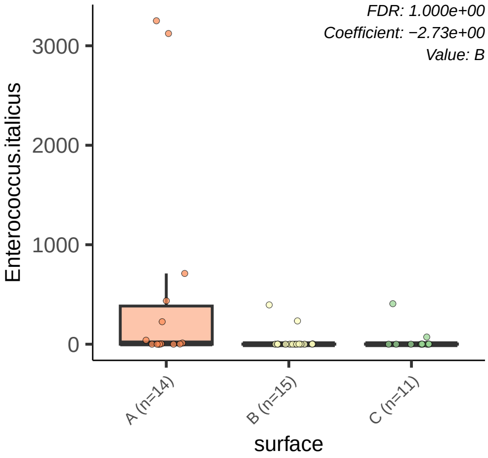
FDR: 1.000e+00
Coefficient: −2.73e+00
Value: B
Enterococcus.italicus
3000
2000
1000
0
A (n=14)
B (n=15)
C (n=11)
surface

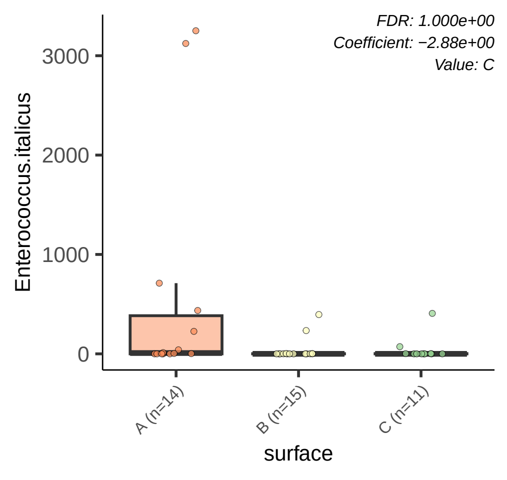

FDR: 1.000e+00
Coefficient: −2.88e+00
Value: C
Enterococcus.italicus
3000
2000
1000
0
A (n=14)
B (n=15)
C (n=11)
surface

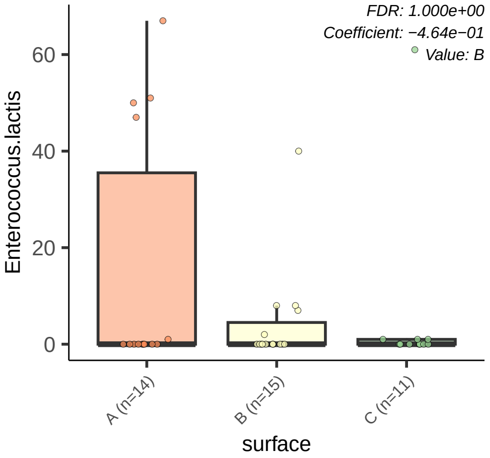
FDR: 1.000e+00
Coefficient: −4.64e−01
Value: B
Enterococcus.lactis
60
40
20
0
A (n=14)
B (n=15)
C (n=11)
surface

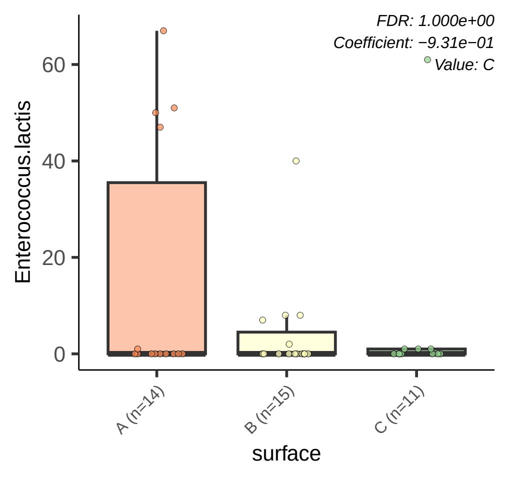

FDR: 1.000e+00
Coefficient: −9.31e−01
Value: C
Enterococcus.lactis
0
20
40
60
A (n=14)
B (n=15)
C (n=11)
surface

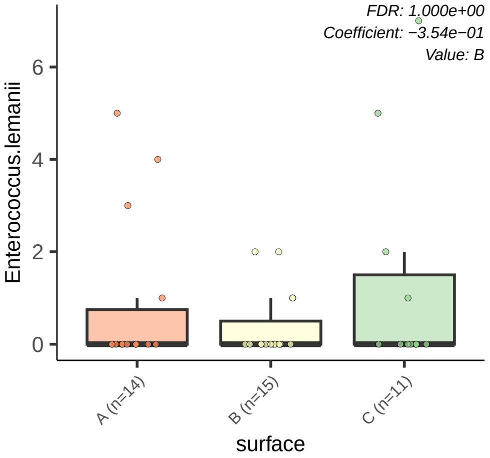

FDR: 1.000e+00
Coefficient: −3.54e−01
Value: B
Enterococcus.lemanii
0
2
4
6
A (n=14)
B (n=15)
C (n=11)
surface

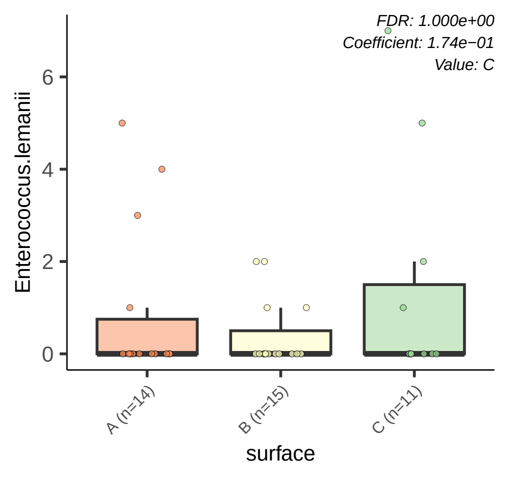

FDR: 1.000e+00
Coefficient: 1.74e−01
Value: C
Enterococcus.lemanii
0
2
4
6
A (n=14)
B (n=15)
C (n=11)
surface

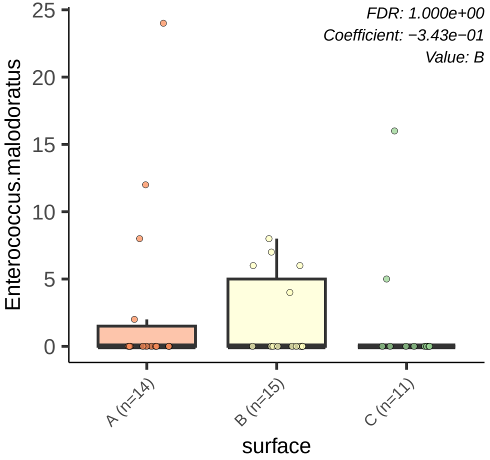

FDR: 1.000e+00
Coefficient: −3.43e−01
Value: B
Enterococcus.malodoratus
25
20
15
10
5
0
A (n=14)
B (n=15)
C (n=11)
surface

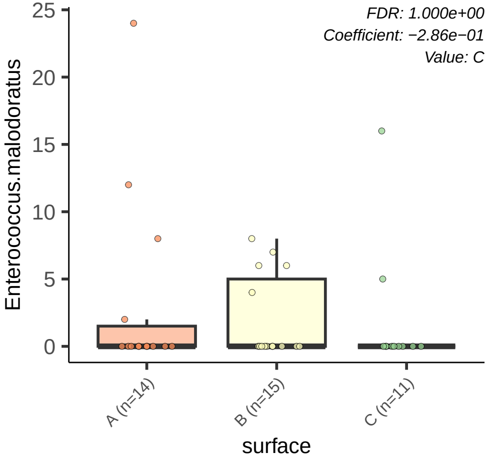
FDR: 1.000e+00
Coefficient: −2.86e−01
Value: C
Enterococcus.malodoratus
25
20
15
10
5
0
A (n=14)
B (n=15)
C (n=11)
surface

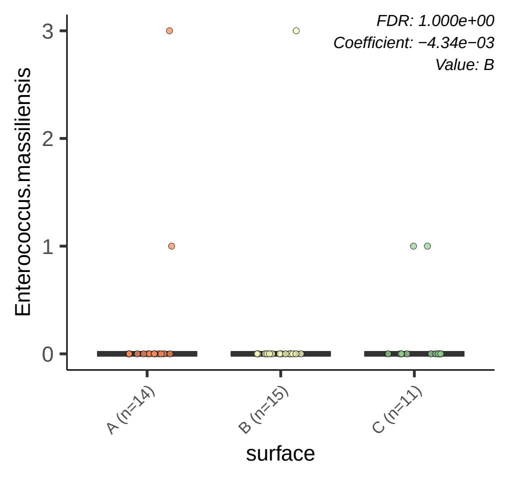
FDR: 1.000e+00
Coefficient: −4.34e−03
Value: B
Enterococcus.massiliensis
3
2
1
0
A (n=14)
B (n=15)
C (n=11)
surface

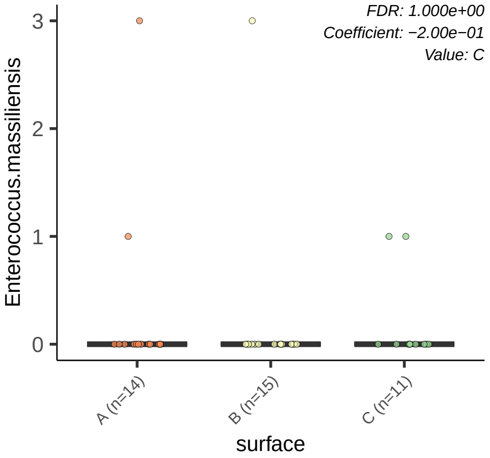
FDR: 1.000e+00
Coefficient: −2.00e−01
Value: C
Enterococcus.massiliensis
3
2
1
0
A (n=14)
B (n=15)
C (n=11)
surface

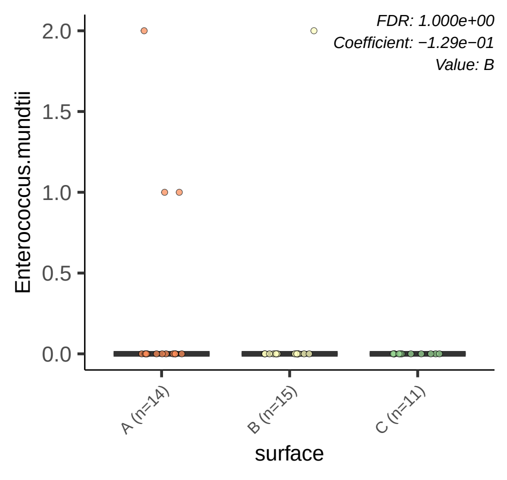
FDR: 1.000e+00
Coefficient: −1.29e−01
Value: B
Enterococcus.mundtii
2.0
1.5
1.0
0.5
0.0
A (n=14)
B (n=15)
C (n=11)
surface

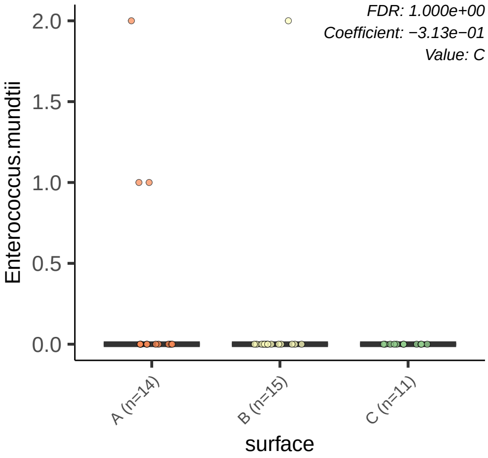
FDR: 1.000e+00
Coefficient: −3.13e−01
Value: C
Enterococcus.mundtii
2.0
1.5
1.0
0.5
0.0
A (n=14)
B (n=15)
C (n=11)
surface

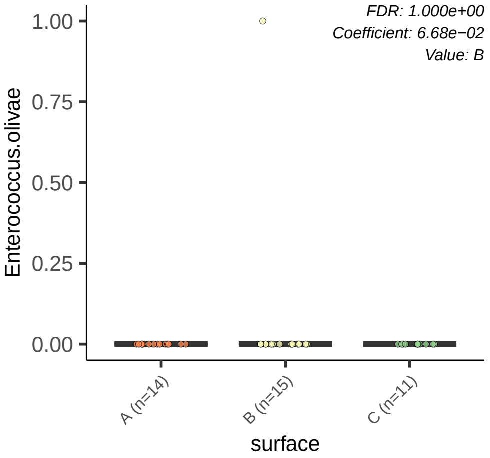
FDR: 1.000e+00
Coefficient: 6.68e−02
Value: B
Enterococcus.olivae
1.00
0.75
0.50
0.25
0.00
A (n=14)
B (n=15)
C (n=11)
surface

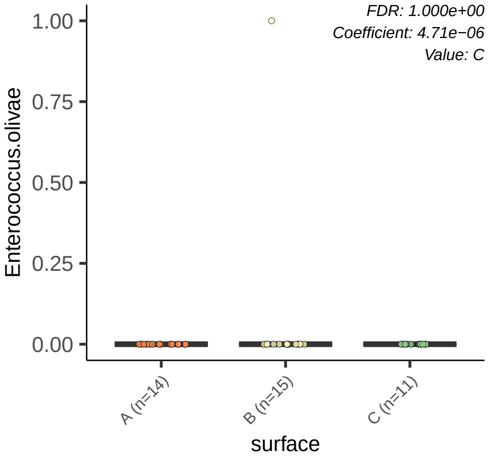

FDR: 1.000e+00
Coefficient: 4.71e−06
Value: C
Enterococcus.olivae
1.00
0.75
0.50
0.25
0.00
A (n=14)
B (n=15)
C (n=11)
surface

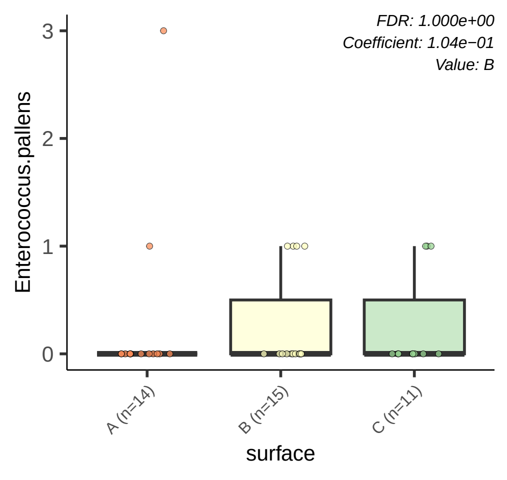
FDR: 1.000e+00
Coefficient: 1.04e−01
Value: B
Enterococcus.pallens
0
1
2
3
A (n=14)
B (n=15)
C (n=11)
surface

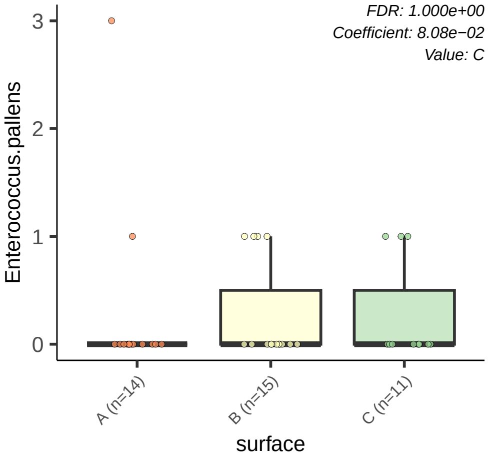

FDR: 1.000e+00
Coefficient: 8.08e−02
Value: C
Enterococcus.pallens
3
2
1
0
A (n=14)
B (n=15)
C (n=11)
surface

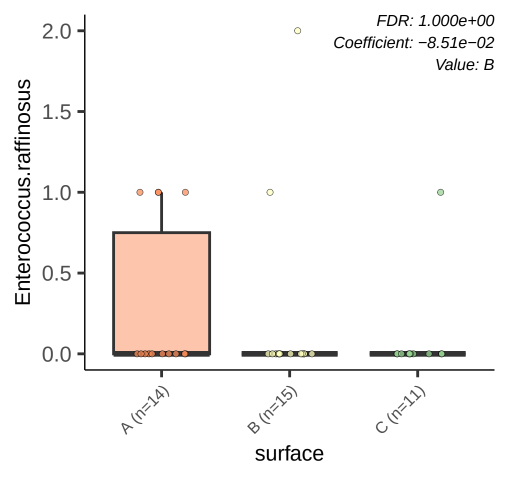
FDR: 1.000e+00
Coefficient: −8.51e−02
Value: B
Enterococcus.raffinosus
2.0
1.5
1.0
0.5
0.0
A (n=14)
B (n=15)
C (n=11)
surface

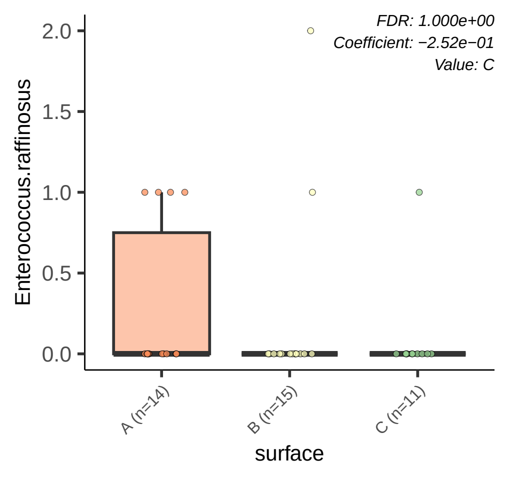

FDR: 1.000e+00
Coefficient: −2.52e−01
Value: C
Enterococcus.raffinosus
2.0
1.5
1.0
0.5
0.0
A (n=14)
B (n=15)
C (n=11)
surface

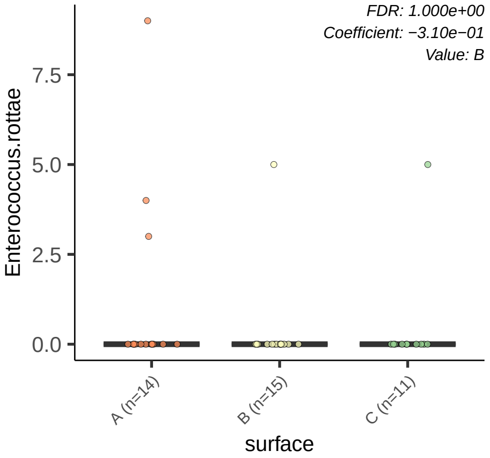
FDR: 1.000e+00
Coefficient: −3.10e−01
Value: B
Enterococcus.rottae
7.5
5.0
2.5
0.0
A (n=14)
B (n=15)
C (n=11)
surface

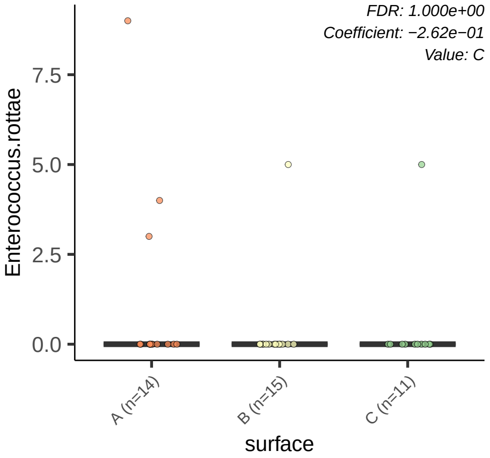

FDR: 1.000e+00
Coefficient: −2.62e−01
Value: C
Enterococcus.rottae
7.5
5.0
2.5
0.0
A (n=14)
B (n=15)
C (n=11)
surface

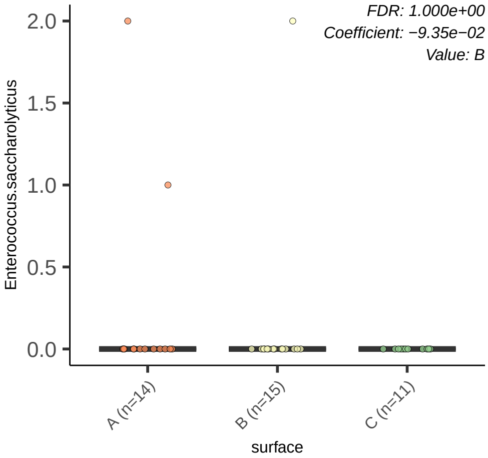

FDR: 1.000e+00
Coefficient: −9.35e−02
Value: B
Enterococcus.saccharolyticus
2.0
1.5
1.0
0.5
0.0
A (n=14)
B (n=15)
C (n=11)
surface

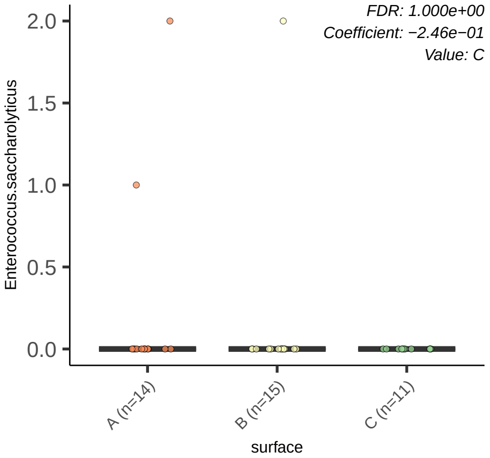
FDR: 1.000e+00
Coefficient: −2.46e−01
Value: C
Enterococcus.saccharolyticus
2.0
1.5
1.0
0.5
0.0
A (n=14)
B (n=15)
C (n=11)
surface

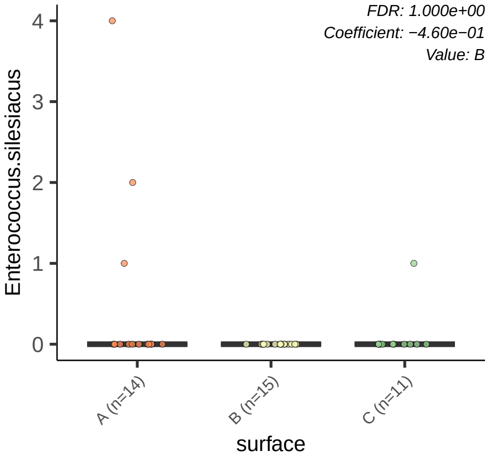
FDR: 1.000e+00
Coefficient: −4.60e−01
Value: B
Enterococcus.silesiacus
0
1
2
3
4
A (n=14)
B (n=15)
C (n=11)
surface

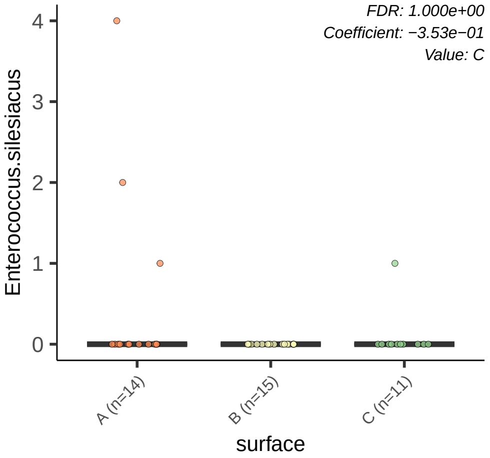
FDR: 1.000e+00
Coefficient: −3.53e−01
Value: C
Enterococcus.silesiacus
4
3
2
1
0
A (n=14)
B (n=15)
C (n=11)
surface

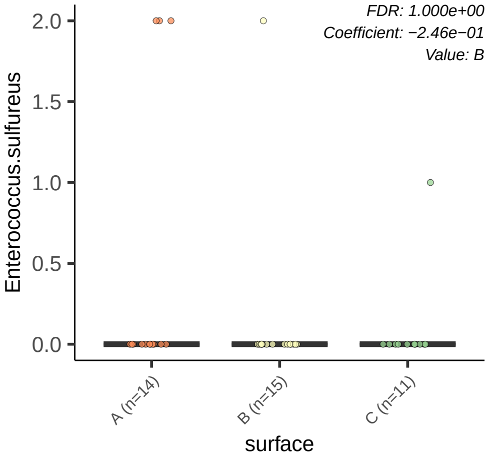

FDR: 1.000e+00
Coefficient: −2.46e−01
Value: B
Enterococcus.sulfureus
2.0
1.5
1.0
0.5
0.0
A (n=14)
B (n=15)
C (n=11)
surface

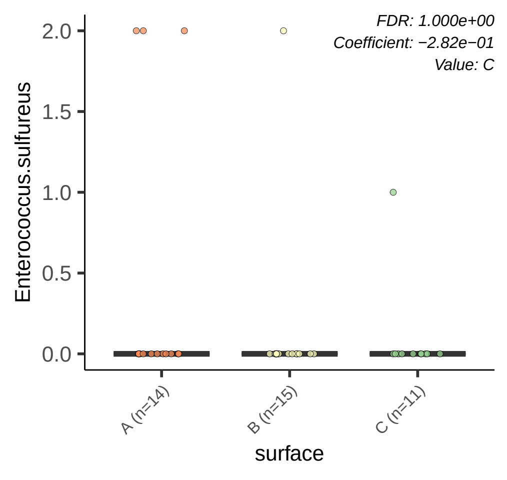

FDR: 1.000e+00
Coefficient: −2.82e−01
Value: C
Enterococcus.sulfureus
2.0
1.5
1.0
0.5
0.0
A (n=14)
B (n=15)
C (n=11)
surface

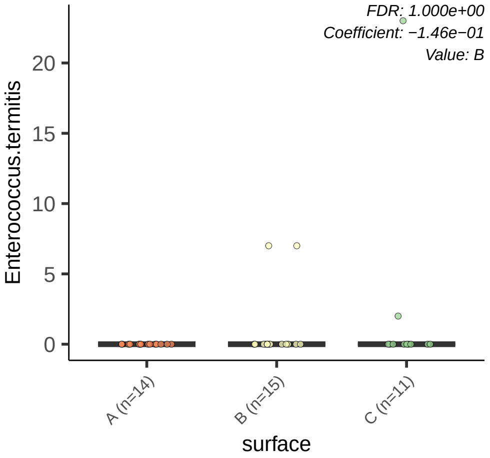
FDR: 1.000e+00
Coefficient: −1.46e−01
Value: B
Enterococcus.termitis
20
15
10
5
0
A (n=14)
B (n=15)
C (n=11)
surface

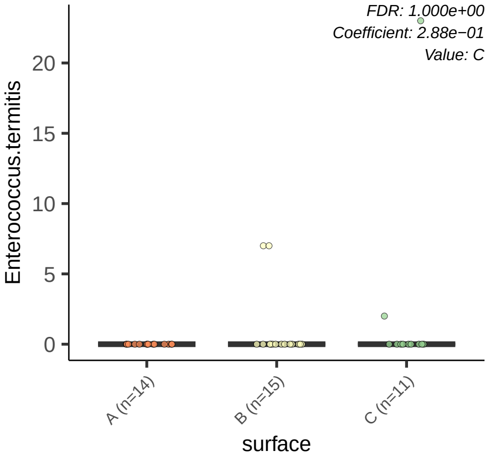
FDR: 1.000e+00
Coefficient: 2.88e−01
Value: C
Enterococcus.termitis
20
15
10
5
0
A (n=14)
B (n=15)
C (n=11)
surface

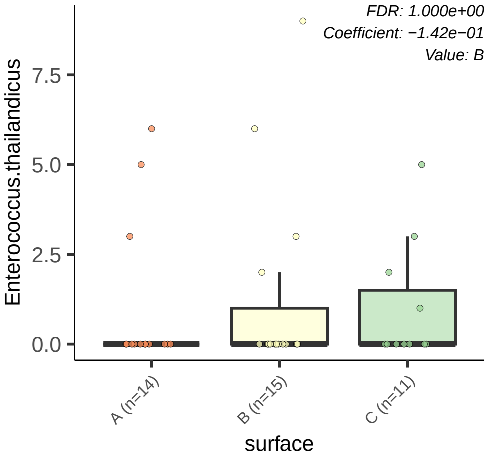

FDR: 1.000e+00
Coefficient: −1.42e−01
Value: B
Enterococcus.thailandicus
7.5
5.0
2.5
0.0
A (n=14)
B (n=15)
C (n=11)
surface

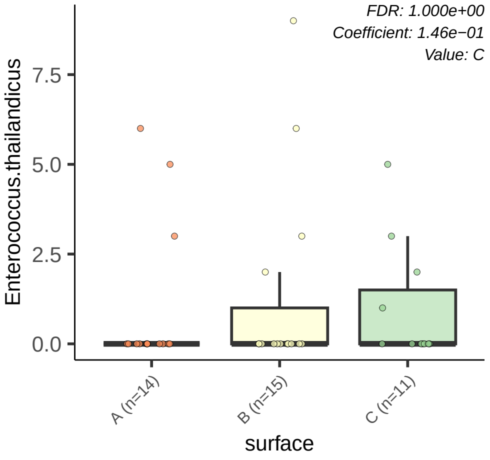

FDR: 1.000e+00
Coefficient: 1.46e−01
Value: C
Enterococcus.thailandicus
7.5
5.0
2.5
0.0
A (n=14)
B (n=15)
C (n=11)
surface

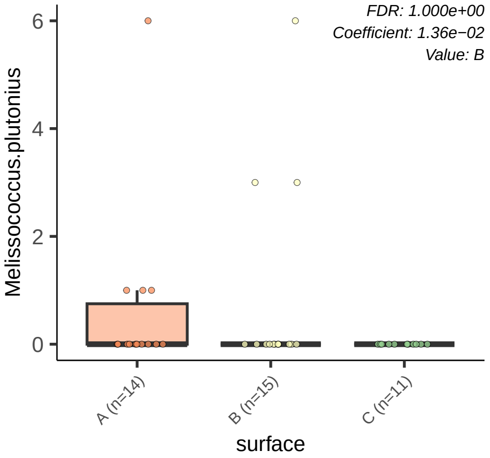
FDR: 1.000e+00
Coefficient: 1.36e−02
Value: B
Melissococcus.plutonius
6
4
2
0
A (n=14)
B (n=15)
C (n=11)
surface

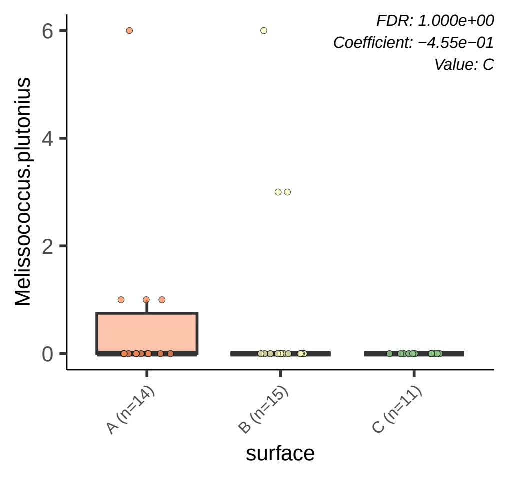

FDR: 1.000e+00
Coefficient: −4.55e−01
Value: C
Melissococcus.plutonius
6
4
2
0
A (n=14)
B (n=15)
C (n=11)
surface

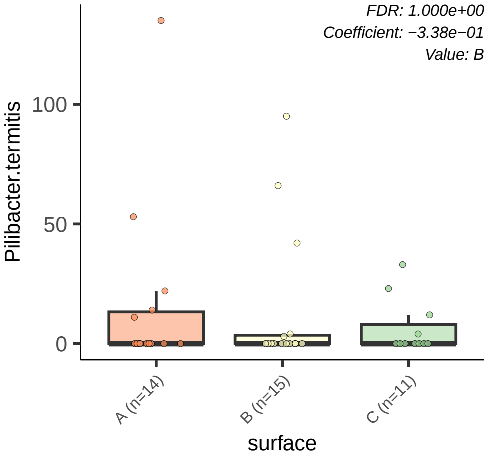
FDR: 1.000e+00
Coefficient: −3.38e−01
Value: B
Pilibacter.termitis
100
50
0
A (n=14)
B (n=15)
C (n=11)
surface

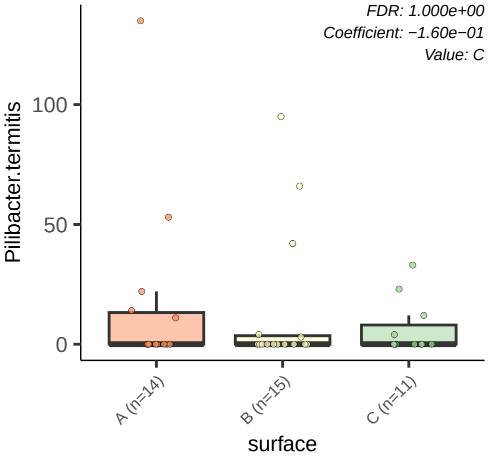

FDR: 1.000e+00
Coefficient: −1.60e−01
Value: C
Pilibacter.termitis
100
50
0
A (n=14)
B (n=15)
C (n=11)
surface

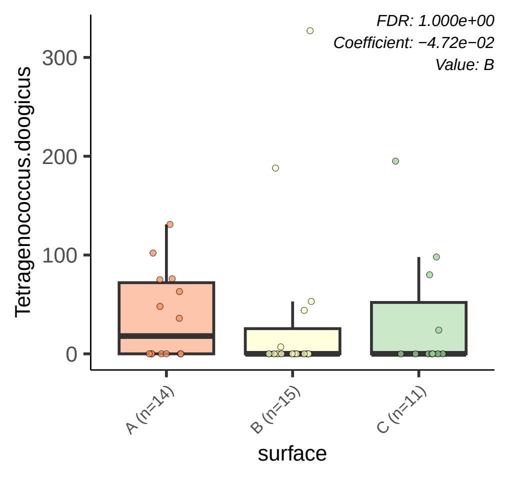

FDR: 1.000e+00
Coefficient: −4.72e−02
Value: B
Tetragenococcus.doogicus
300
200
100
0
A (n=14)
B (n=15)
C (n=11)
surface

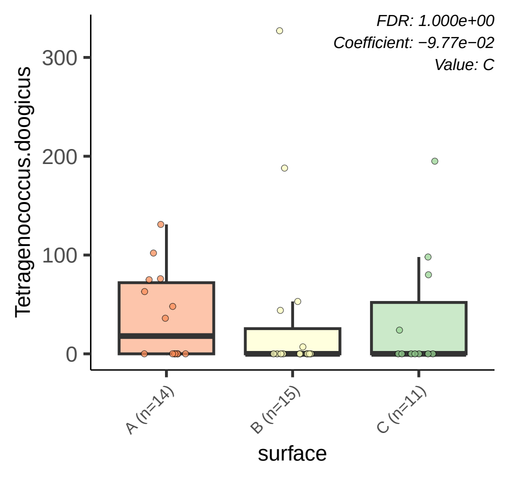

FDR: 1.000e+00
Coefficient: −9.77e−02
Value: C
Tetragenococcus.doogicus
300
200
100
0
A (n=14)
B (n=15)
C (n=11)
surface

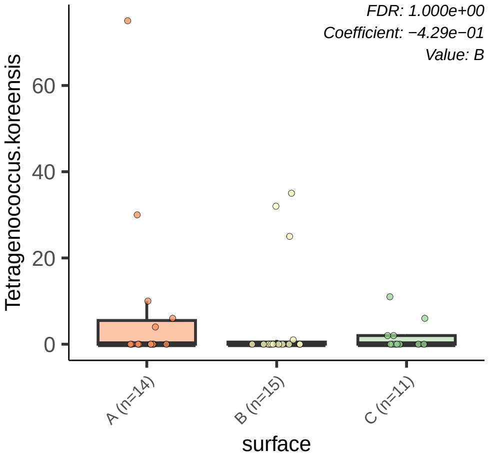

FDR: 1.000e+00
Coefficient: −4.29e−01
Value: B
Tetragenococcus.koreensis
0
20
40
60
A (n=14)
B (n=15)
C (n=11)
surface

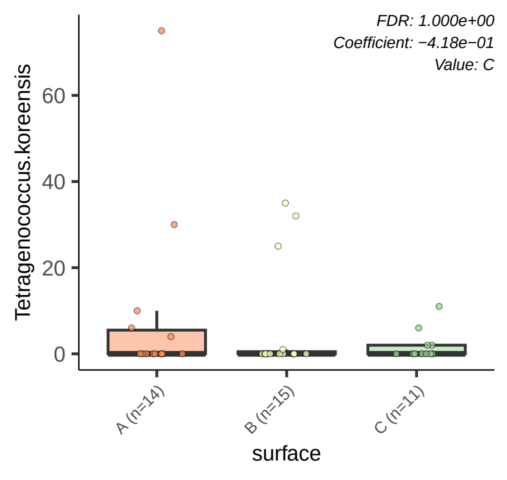

FDR: 1.000e+00
Coefficient: −4.18e−01
Value: C
Tetragenococcus.koreensis
0
20
40
60
A (n=14)
B (n=15)
C (n=11)
surface

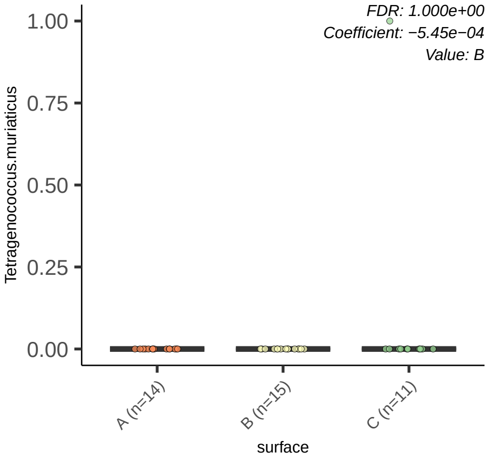
FDR: 1.000e+00
Coefficient: −5.45e−04
Value: B
Tetragenococcus.muriaticus
1.00
0.75
0.50
0.25
0.00
A (n=14)
B (n=15)
C (n=11)
surface

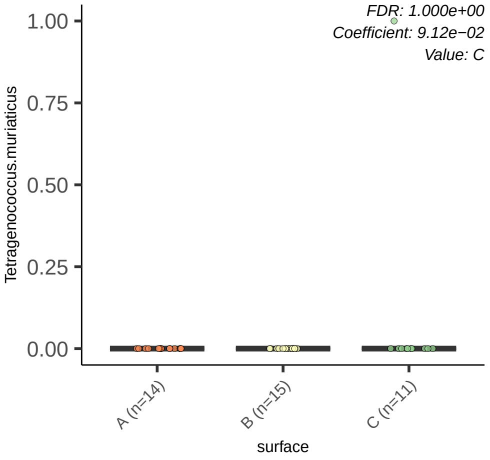
FDR: 1.000e+00
Coefficient: 9.12e−02
Value: C
1.00
0.75
0.50
0.25
0.00
Tetragenococcus.muriaticus
A (n=14)
B (n=15)
C (n=11)
surface

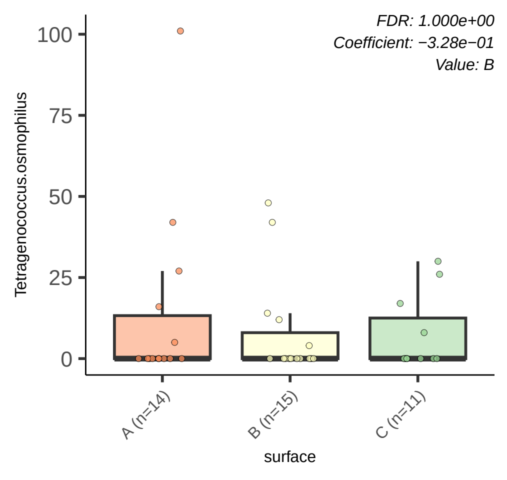

FDR: 1.000e+00
Coefficient: −3.28e−01
Value: B
Tetragenococcus.osmophilus
100
75
50
25
0
A (n=14)
B (n=15)
C (n=11)
surface

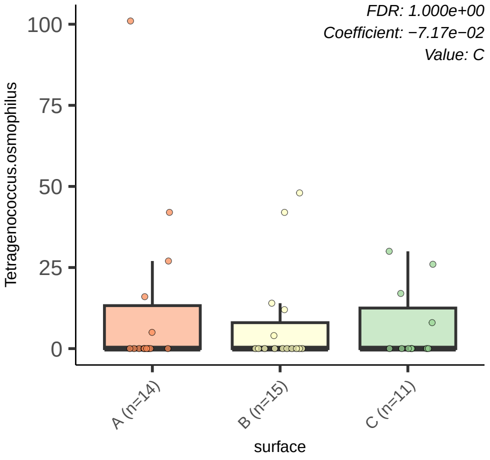

FDR: 1.000e+00
Coefficient: −7.17e−02
Value: C
Tetragenococcus.osmophilus
100
75
50
25
0
A (n=14)
B (n=15)
C (n=11)
surface

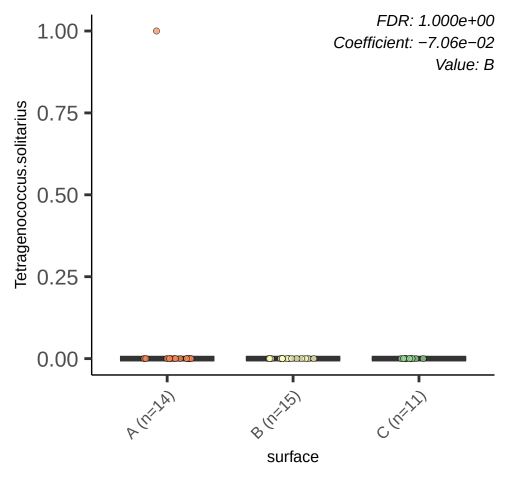

FDR: 1.000e+00
Coefficient: −7.06e−02
Value: B
Tetragenococcus.solitarius
1.00
0.75
0.50
0.25
0.00
A (n=14)
B (n=15)
C (n=11)
surface

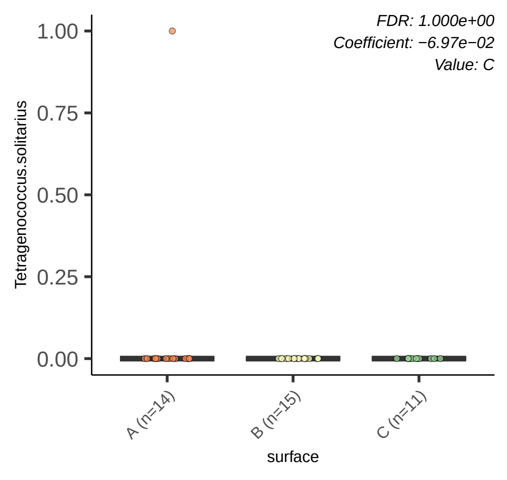
FDR: 1.000e+00
Coefficient: −6.97e−02
Value: C
Tetragenococcus.solitarius
1.00
0.75
0.50
0.25
0.00
A (n=14)
B (n=15)
C (n=11)
surface

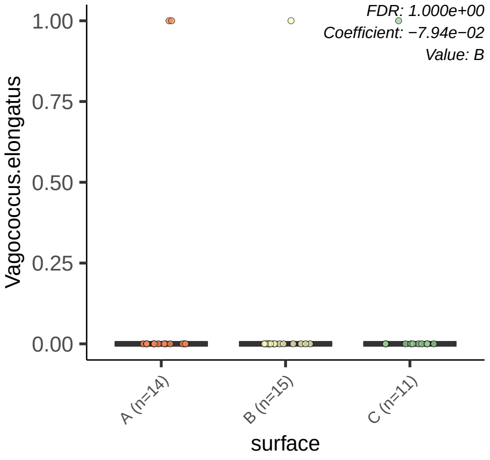

FDR: 1.000e+00
Coefficient: −7.94e−02
Value: B
Vagococcus.elongatus
1.00
0.75
0.50
0.25
0.00
A (n=14)
B (n=15)
C (n=11)
surface

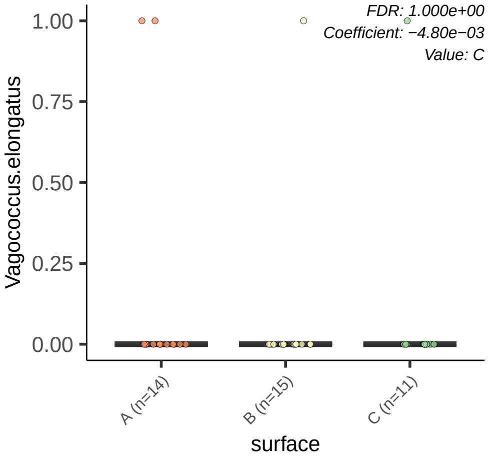
FDR: 1.000e+00
Coefficient: −4.80e−03
Value: C
Vagococcus.elongatus
1.00
0.75
0.50
0.25
0.00
A (n=14)
B (n=15)
C (n=11)
surface

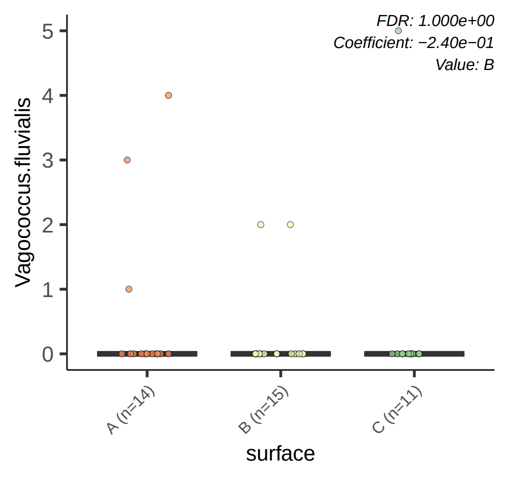

FDR: 1.000e+00
Coefficient: −2.40e−01
Value: B
Vagococcus.fluvialis
5
4
3
2
1
0
A (n=14)
B (n=15)
C (n=11)
surface

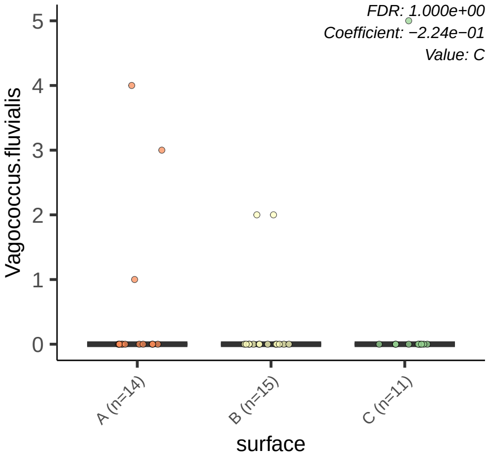
FDR: 1.000e+00
Coefficient: −2.24e−01
Value: C
Vagococcus.fluvialis
5
4
3
2
1
0
A (n=14)
B (n=15)
C (n=11)
surface

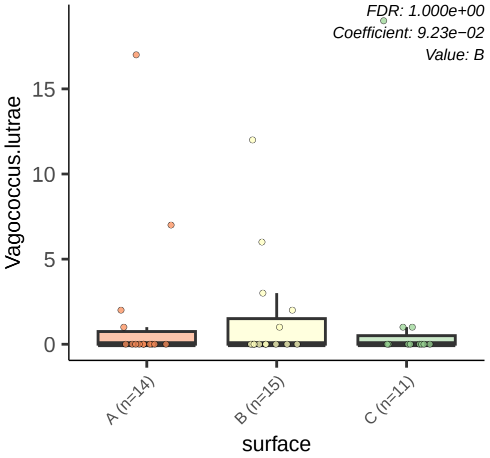

FDR: 1.000e+00
Coefficient: 9.23e−02
Value: B
Vagococcus.lutrae
15
10
5
0
A (n=14)
B (n=15)
C (n=11)
surface

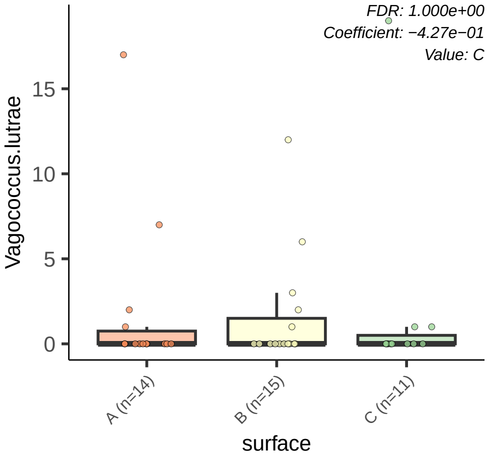

FDR: 1.000e+00
Coefficient: −4.27e−01
Value: C
Vagococcus.lutrae
15
10
5
0
A (n=14)
B (n=15)
C (n=11)
surface

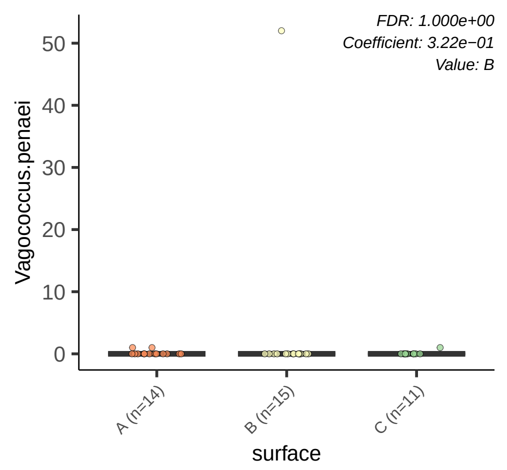
FDR: 1.000e+00
Coefficient: 3.22e−01
Value: B
Vagococcus.penaei
50
40
30
20
10
0
A (n=14)
B (n=15)
C (n=11)
surface

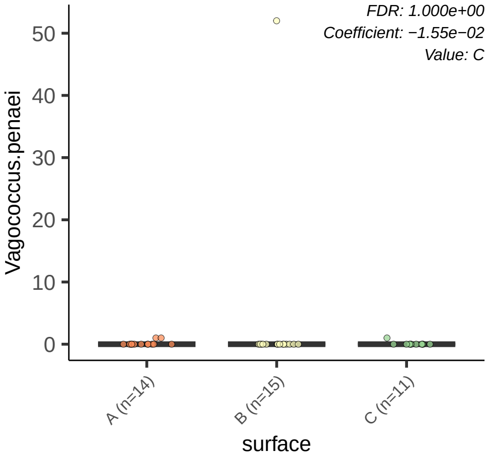
FDR: 1.000e+00
Coefficient: −1.55e−02
Value: C
Vagococcus.penaei
0
10
20
30
40
50
A (n=14)
B (n=15)
C (n=11)
surface

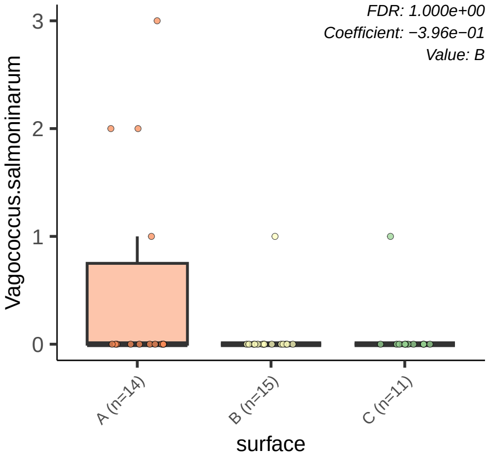
FDR: 1.000e+00
Coefficient: −3.96e−01
Value: B
Vagococcus.salmoninarum
3
2
1
0
A (n=14)
B (n=15)
C (n=11)
surface

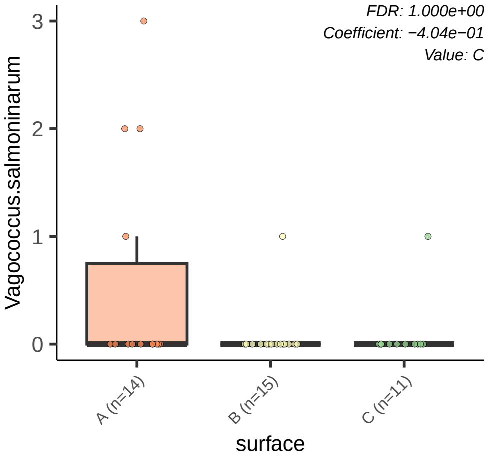
FDR: 1.000e+00
Coefficient: −4.04e−01
Value: C
Vagococcus.salmoninarum
3
2
1
0
A (n=14)
B (n=15)
C (n=11)
surface

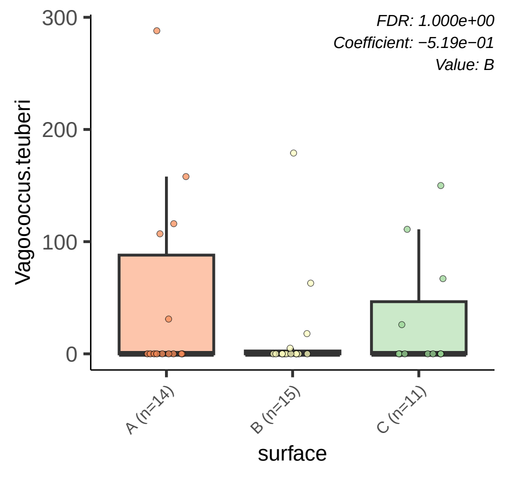
FDR: 1.000e+00
Coefficient: −5.19e−01
Value: B
Vagococcus.teuberi
300
200
100
0
A (n=14)
B (n=15)
C (n=11)
surface

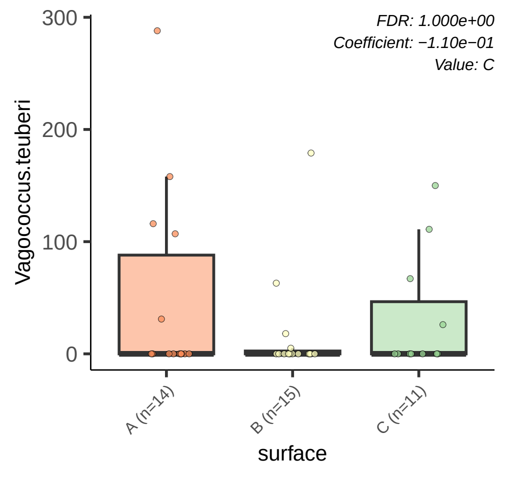

FDR: 1.000e+00
Coefficient: −1.10e−01
Value: C
300
200
100
0
Vagococcus.teuberi
A (n=14)
B (n=15)
C (n=11)
surface

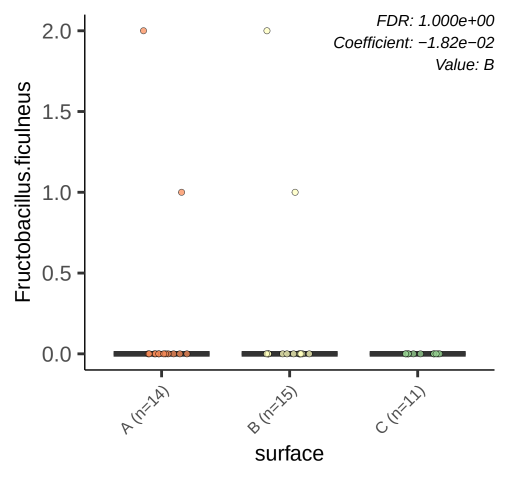
FDR: 1.000e+00
Coefficient: −1.82e−02
Value: B
Fructobacillus.ficulneus
2.0
1.5
1.0
0.5
0.0
A (n=14)
B (n=15)
C (n=11)
surface

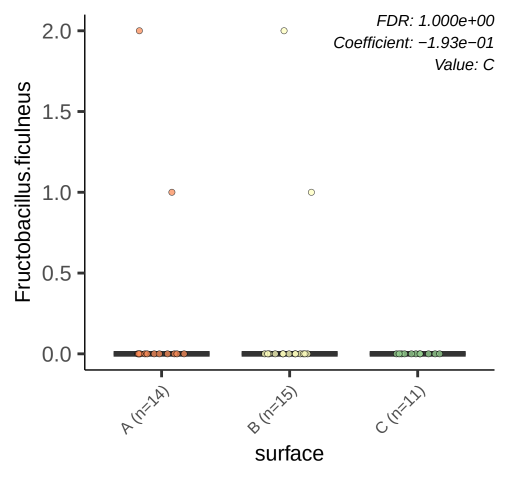
FDR: 1.000e+00
Coefficient: −1.93e−01
Value: C
Fructobacillus.ficulneus
2.0
1.5
1.0
0.5
0.0
A (n=14)
B (n=15)
C (n=11)
surface

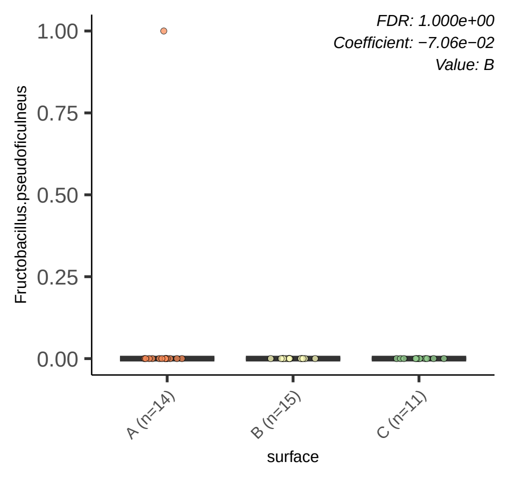
FDR: 1.000e+00
Coefficient: −7.06e−02
Value: B
Fructobacillus.pseudoficulneus
1.00
0.75
0.50
0.25
0.00
A (n=14)
B (n=15)
C (n=11)
surface

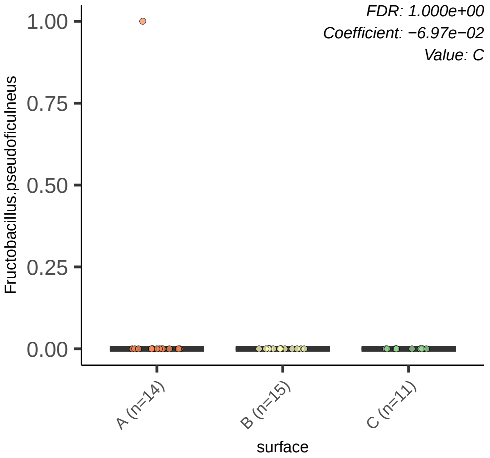
FDR: 1.000e+00
Coefficient: −6.97e−02
Value: C
Fructobacillus.pseudoficulneus
1.00
0.75
0.50
0.25
0.00
A (n=14)
B (n=15)
C (n=11)
surface

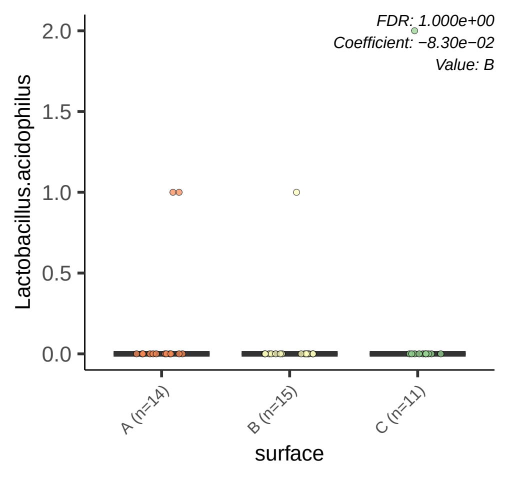
FDR: 1.000e+00
Coefficient: −8.30e−02
Value: B
Lactobacillus.acidophilus
2.0
1.5
1.0
0.5
0.0
A (n=14)
B (n=15)
C (n=11)
surface

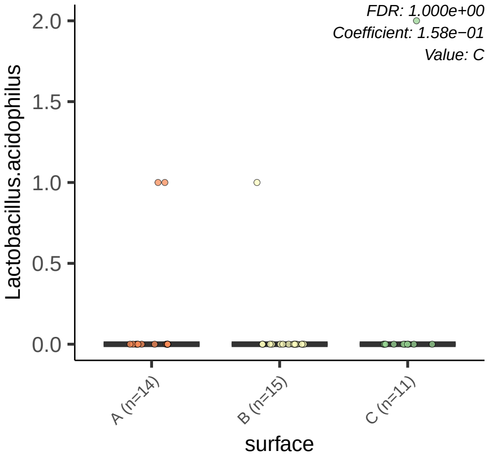

FDR: 1.000e+00
Coefficient: 1.58e−01
Value: C
Lactobacillus.acidophilus
2.0
1.5
1.0
0.5
0.0
A (n=14)
B (n=15)
C (n=11)
surface

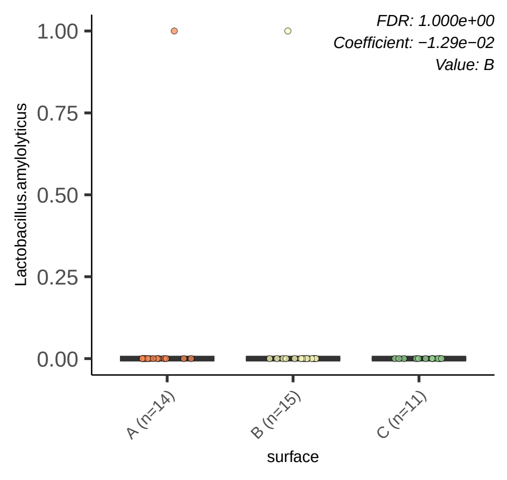
FDR: 1.000e+00
Coefficient: −1.29e−02
Value: B
Lactobacillus.amylolyticus
1.00
0.75
0.50
0.25
0.00
A (n=14)
B (n=15)
C (n=11)
surface

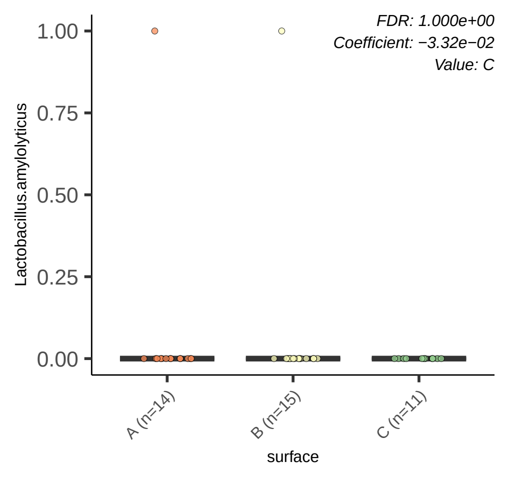
FDR: 1.000e+00
Coefficient: −3.32e−02
Value: C
1.00
0.75
0.50
0.25
0.00
Lactobacillus.amylolyticus
A (n=14)
B (n=15)
C (n=11)
surface

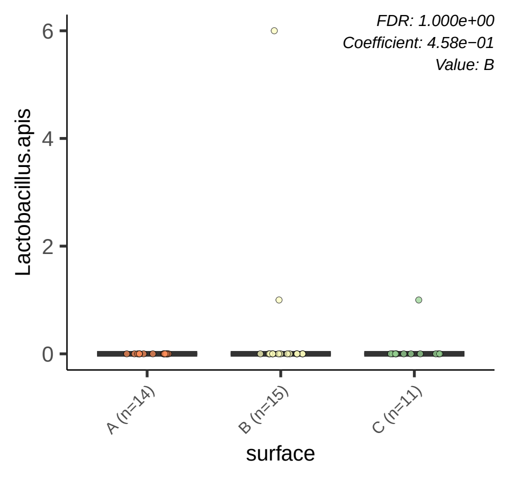
FDR: 1.000e+00
Coefficient: 4.58e−01
Value: B
Lactobacillus.apis
6
4
2
0
A (n=14)
B (n=15)
C (n=11)
surface

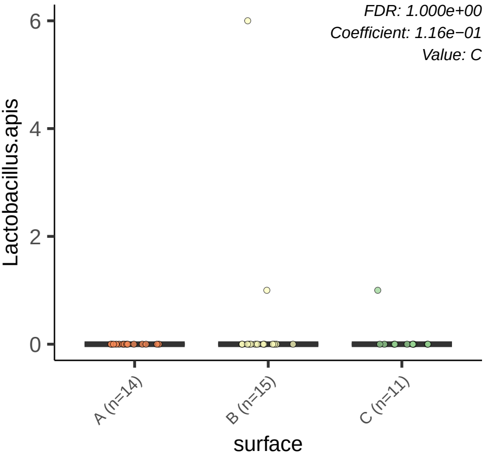
FDR: 1.000e+00
Coefficient: 1.16e−01
Value: C
Lactobacillus.apis
6
4
2
0
A (n=14)
B (n=15)
C (n=11)
surface

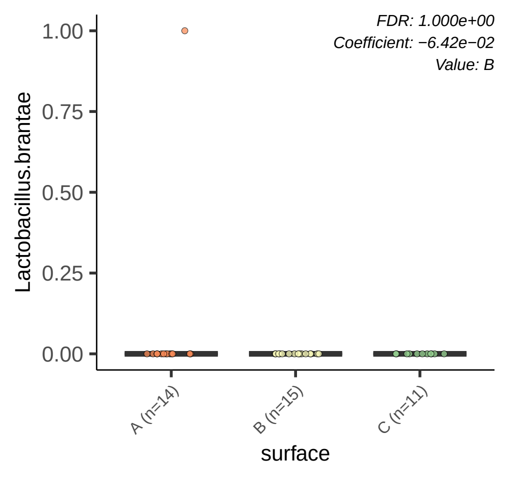
FDR: 1.000e+00
Coefficient: −6.42e−02
Value: B
Lactobacillus.brantae
1.00
0.75
0.50
0.25
0.00
A (n=14)
B (n=15)
C (n=11)
surface

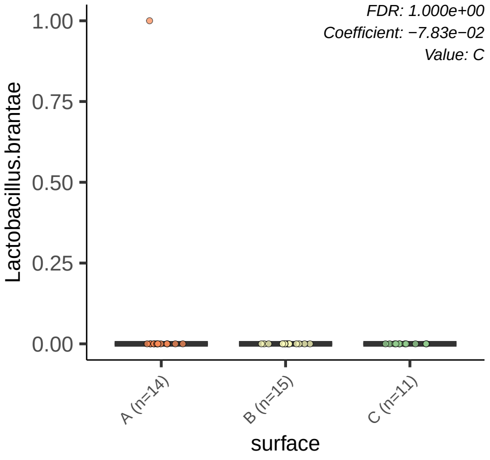
FDR: 1.000e+00
Coefficient: −7.83e−02
Value: C
Lactobacillus.brantae
1.00
0.75
0.50
0.25
0.00
A (n=14)
B (n=15)
C (n=11)
surface

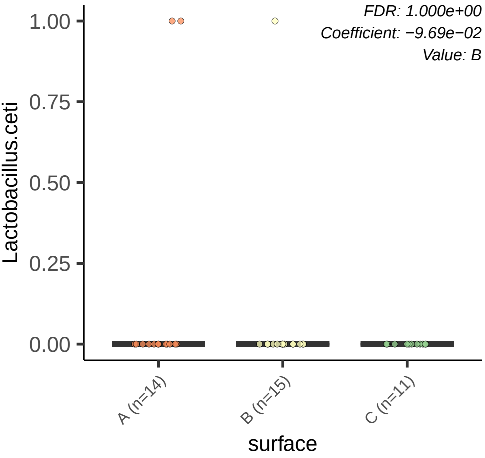

FDR: 1.000e+00
Coefficient: −9.69e−02
Value: B
Lactobacillus.ceti
1.00
0.75
0.50
0.25
0.00
A (n=14)
B (n=15)
C (n=11)
surface

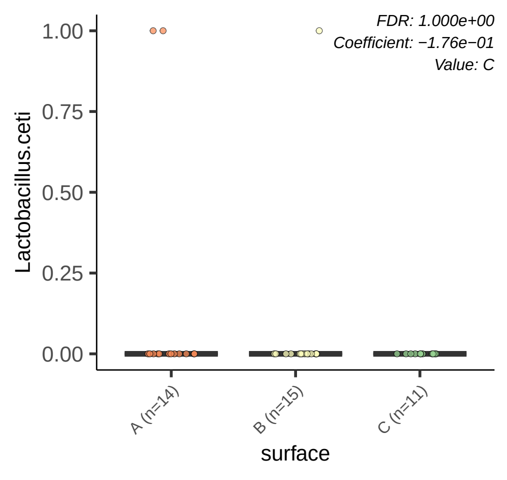

FDR: 1.000e+00
Coefficient: −1.76e−01
Value: C
Lactobacillus.ceti
1.00
0.75
0.50
0.25
0.00
A (n=14)
B (n=15)
C (n=11)
surface

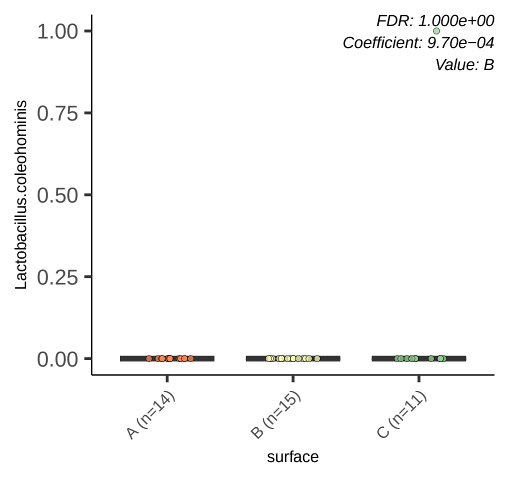

FDR: 1.000e+00
Coefficient: 9.70e−04
Value: B
1.00
0.75
0.50
0.25
0.00
Lactobacillus.coleohominis
A (n=14)
B (n=15)
C (n=11)
surface

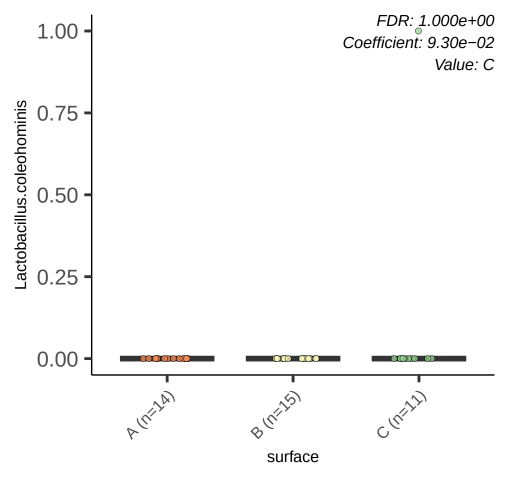
FDR: 1.000e+00
Coefficient: 9.30e−02
Value: C
1.00
0.75
0.50
0.25
0.00
Lactobacillus.coleohominis
A (n=14)
B (n=15)
C (n=11)
surface

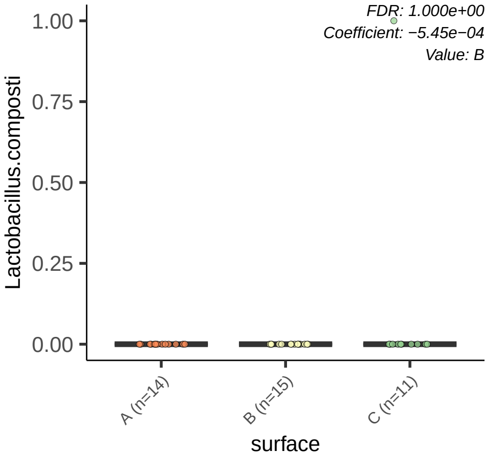

FDR: 1.000e+00
Coefficient: −5.45e−04
Value: B
Lactobacillus.composti
1.00
0.75
0.50
0.25
0.00
A (n=14)
B (n=15)
C (n=11)
surface

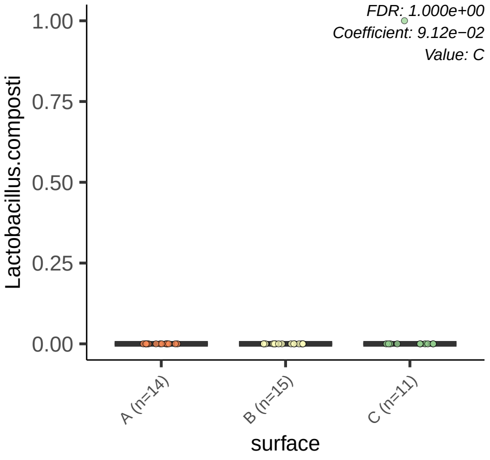
FDR: 1.000e+00
Coefficient: 9.12e−02
Value: C
Lactobacillus.composti
1.00
0.75
0.50
0.25
0.00
A (n=14)
B (n=15)
C (n=11)
surface

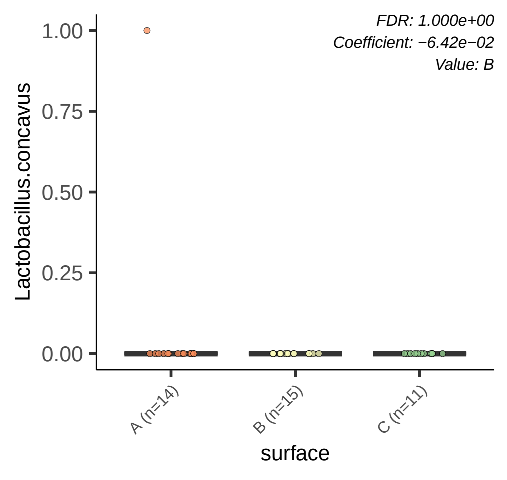
FDR: 1.000e+00
Coefficient: −6.42e−02
Value: B
Lactobacillus.concavus
1.00
0.75
0.50
0.25
0.00
A (n=14)
B (n=15)
C (n=11)
surface

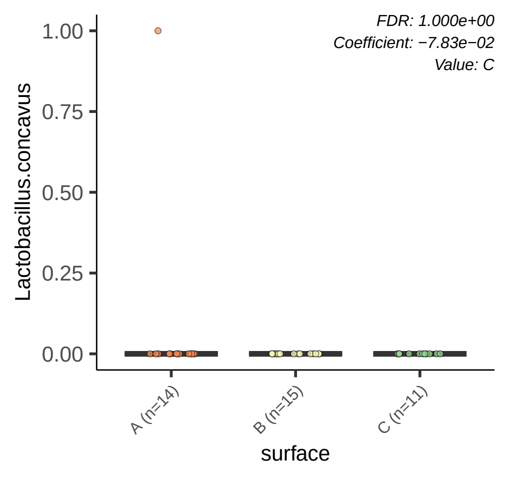

FDR: 1.000e+00
Coefficient: −7.83e−02
Value: C
Lactobacillus.concavus
1.00
0.75
0.50
0.25
0.00
A (n=14)
B (n=15)
C (n=11)
surface

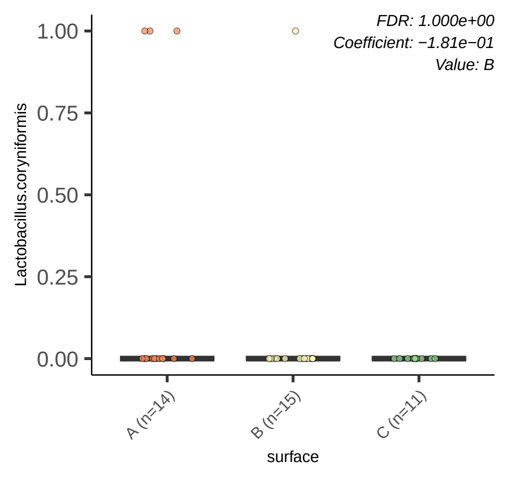
FDR: 1.000e+00
Coefficient: −1.81e−01
Value: B
Lactobacillus.coryniformis
1.00
0.75
0.50
0.25
0.00
A (n=14)
B (n=15)
C (n=11)
surface

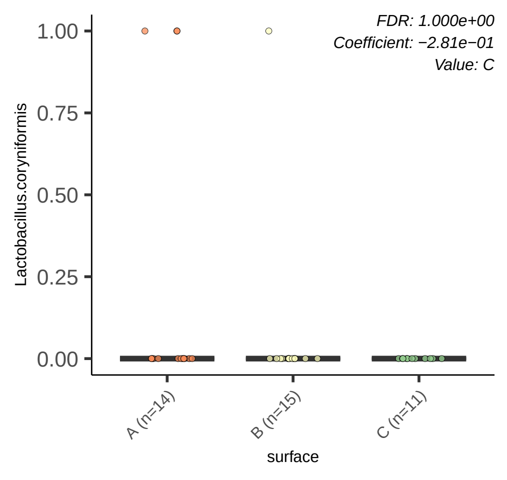
FDR: 1.000e+00
Coefficient: −2.81e−01
Value: C
Lactobacillus.coryniformis
1.00
0.75
0.50
0.25
0.00
A (n=14)
B (n=15)
C (n=11)
surface

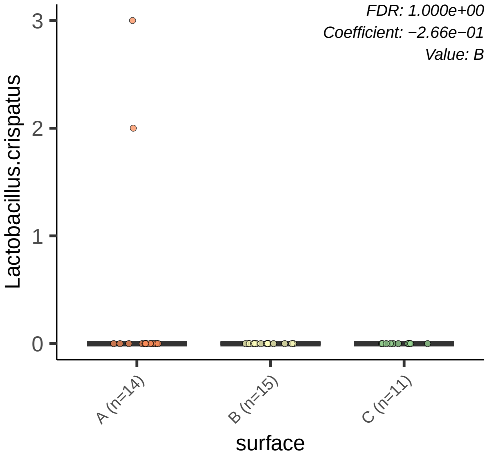
FDR: 1.000e+00
Coefficient: −2.66e−01
Value: B
Lactobacillus.crispatus
3
2
1
0
A (n=14)
B (n=15)
C (n=11)
surface

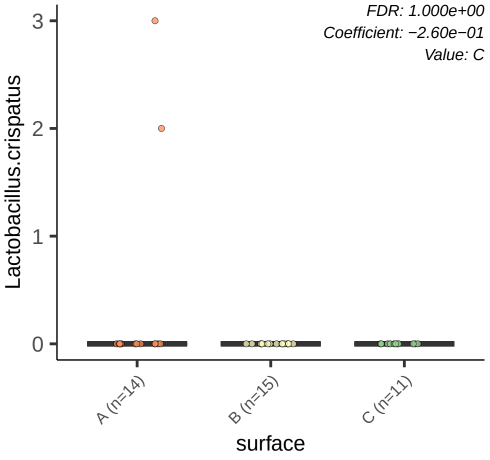
FDR: 1.000e+00
Coefficient: −2.60e−01
Value: C
Lactobacillus.crispatus
3
2
1
0
A (n=14)
B (n=15)
C (n=11)
surface

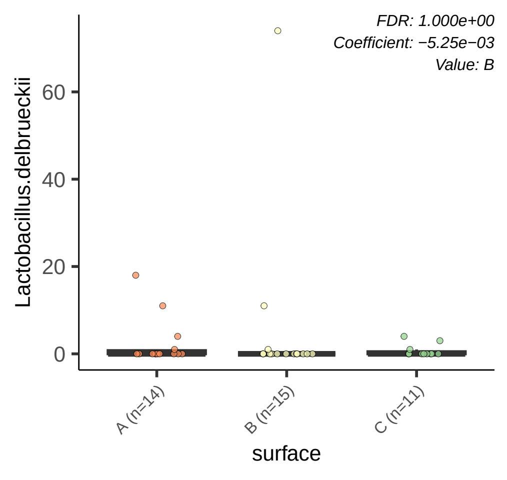

FDR: 1.000e+00
Coefficient: −5.25e−03
Value: B
Lactobacillus.delbrueckii
0
20
40
60
A (n=14)
B (n=15)
C (n=11)
surface

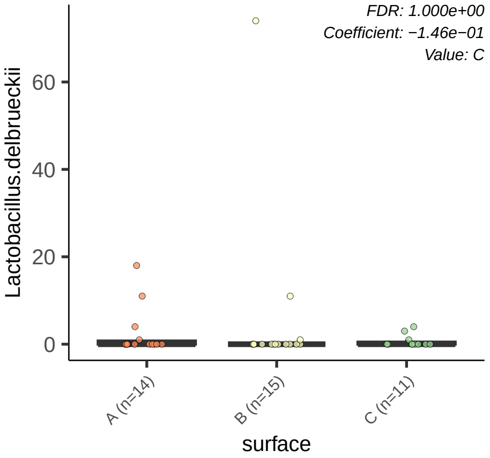
FDR: 1.000e+00
Coefficient: −1.46e−01
Value: C
Lactobacillus.delbrueckii
60
40
20
0
A (n=14)
B (n=15)
C (n=11)
surface

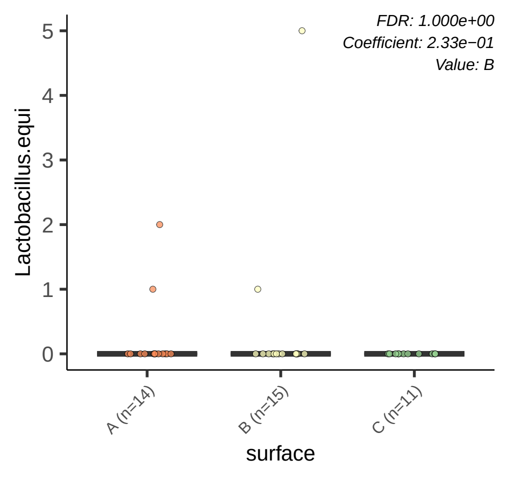

FDR: 1.000e+00
Coefficient: 2.33e−01
Value: B
Lactobacillus.equi
5
4
3
2
1
0
A (n=14)
B (n=15)
C (n=11)
surface

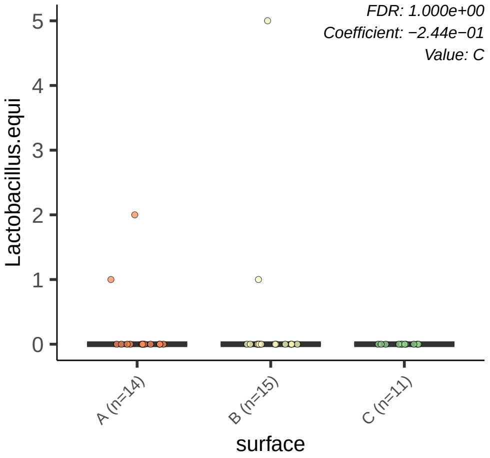
FDR: 1.000e+00
Coefficient: −2.44e−01
Value: C
Lactobacillus.equi
0
1
2
3
4
5
A (n=14)
B (n=15)
C (n=11)
surface

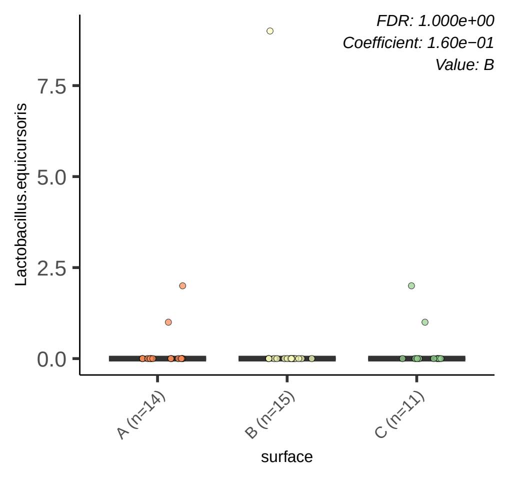

FDR: 1.000e+00
Coefficient: 1.60e−01
Value: B
Lactobacillus.equicursoris
7.5
5.0
2.5
0.0
A (n=14)
B (n=15)
C (n=11)
surface

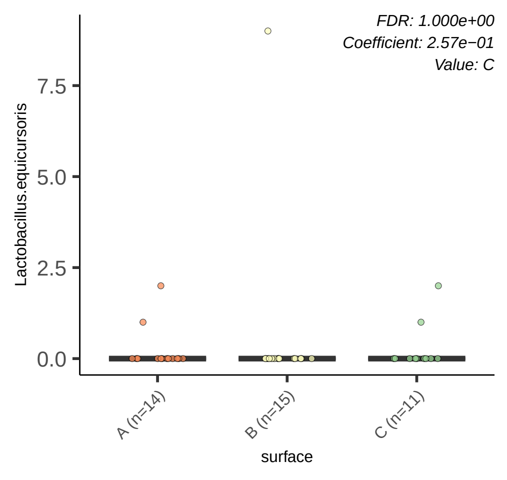

FDR: 1.000e+00
Coefficient: 2.57e−01
Value: C
Lactobacillus.equicursoris
7.5
5.0
2.5
0.0
A (n=14)
B (n=15)
C (n=11)
surface

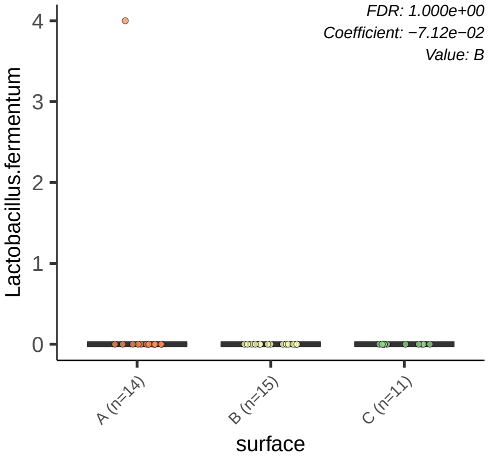

FDR: 1.000e+00
Coefficient: −7.12e−02
Value: B
Lactobacillus.fermentum
0
1
2
3
4
A (n=14)
B (n=15)
C (n=11)
surface

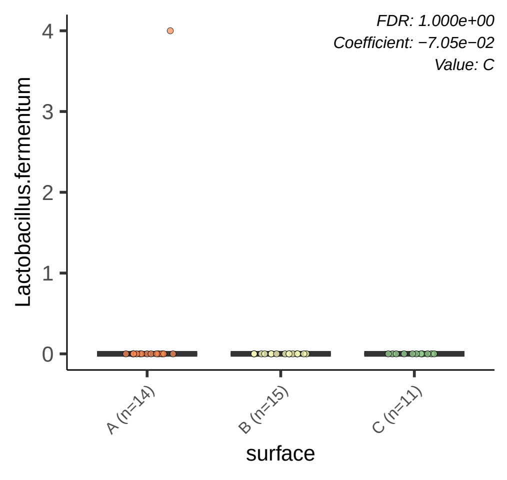
FDR: 1.000e+00
Coefficient: −7.05e−02
Value: C
Lactobacillus.fermentum
0
1
2
3
4
A (n=14)
B (n=15)
C (n=11)
surface

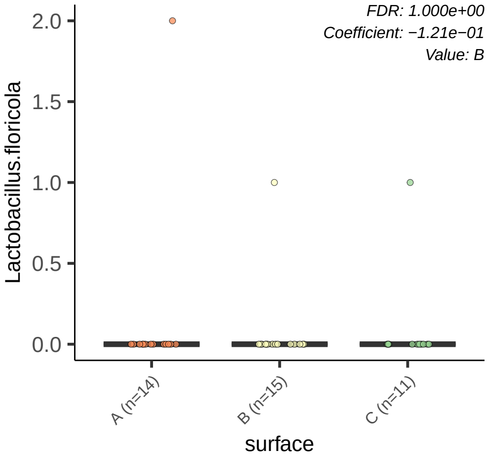
FDR: 1.000e+00
Coefficient: −1.21e−01
Value: B
Lactobacillus.floricola
2.0
1.5
1.0
0.5
0.0
A (n=14)
B (n=15)
C (n=11)
surface

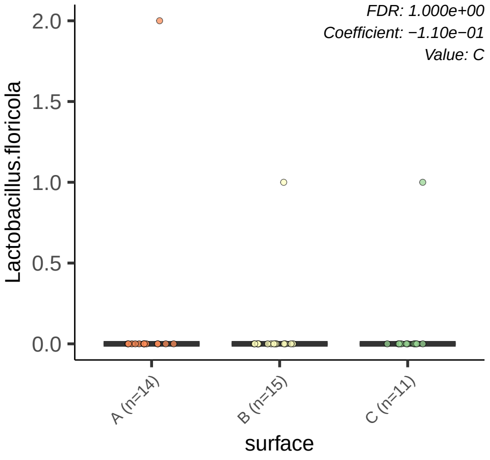
FDR: 1.000e+00
Coefficient: −1.10e−01
Value: C
Lactobacillus.floricola
2.0
1.5
1.0
0.5
0.0
A (n=14)
B (n=15)
C (n=11)
surface

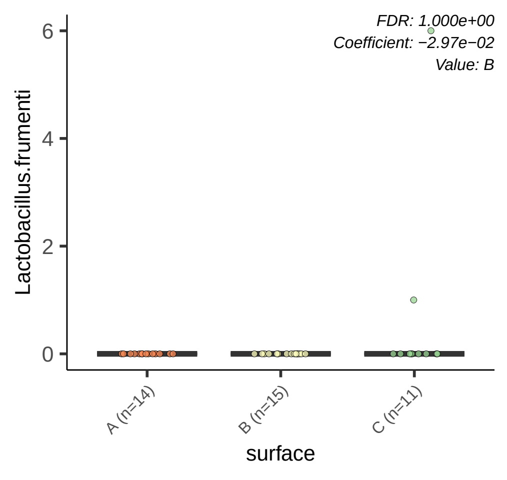
FDR: 1.000e+00
Coefficient: −2.97e−02
Value: B
Lactobacillus.frumenti
6
4
2
0
A (n=14)
B (n=15)
C (n=11)
surface

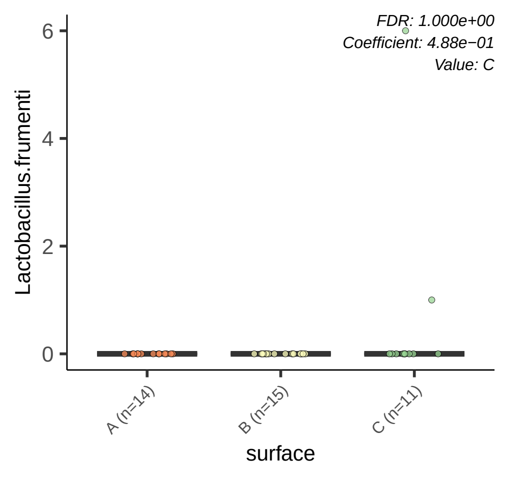
FDR: 1.000e+00
Coefficient: 4.88e−01
Value: C
Lactobacillus.frumenti
0
2
4
6
A (n=14)
B (n=15)
C (n=11)
surface

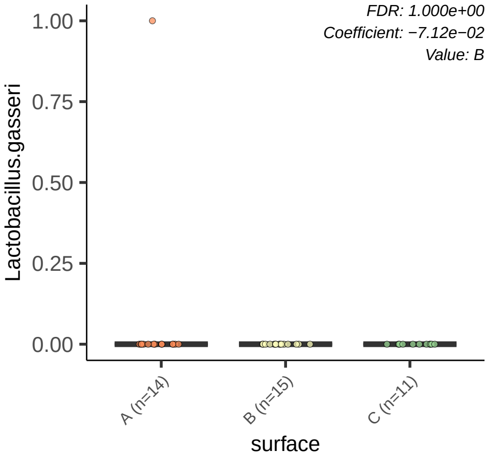
FDR: 1.000e+00
Coefficient: −7.12e−02
Value: B
Lactobacillus.gasseri
1.00
0.75
0.50
0.25
0.00
A (n=14)
B (n=15)
C (n=11)
surface

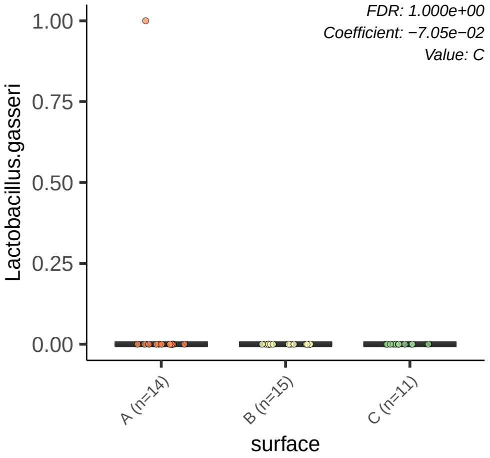

FDR: 1.000e+00
Coefficient: −7.05e−02
Value: C
Lactobacillus.gasseri
1.00
0.75
0.50
0.25
0.00
A (n=14)
B (n=15)
C (n=11)
surface

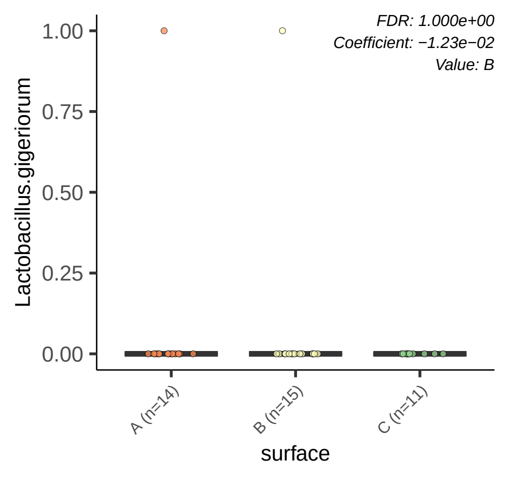

FDR: 1.000e+00
Coefficient: −1.23e−02
Value: B
1.00
0.75
0.50
0.25
0.00
Lactobacillus.gigeriorum
A (n=14)
B (n=15)
C (n=11)
surface

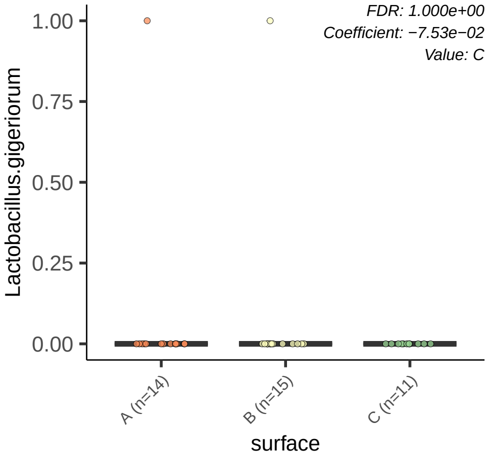
FDR: 1.000e+00
Coefficient: −7.53e−02
Value: C
Lactobacillus.gigeriorum
1.00
0.75
0.50
0.25
0.00
A (n=14)
B (n=15)
C (n=11)
surface

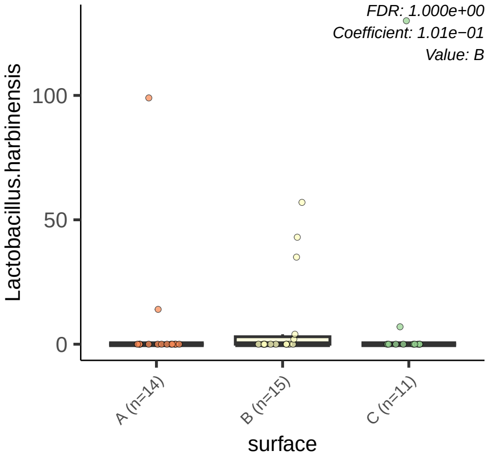

FDR: 1.000e+00
Coefficient: 1.01e−01
Value: B
Lactobacillus.harbinensis
100
50
0
A (n=14)
B (n=15)
C (n=11)
surface

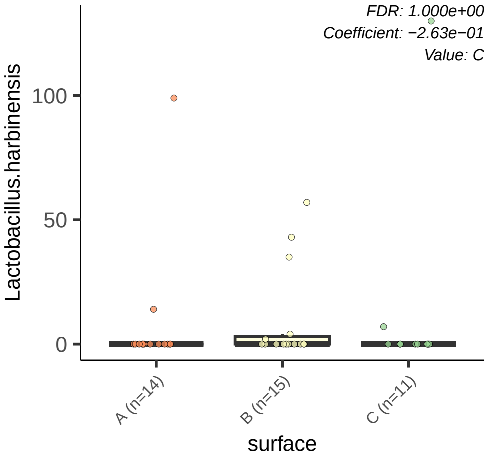
FDR: 1.000e+00
Coefficient: −2.63e−01
Value: C
Lactobacillus.harbinensis
100
50
0
A (n=14)
B (n=15)
C (n=11)
surface

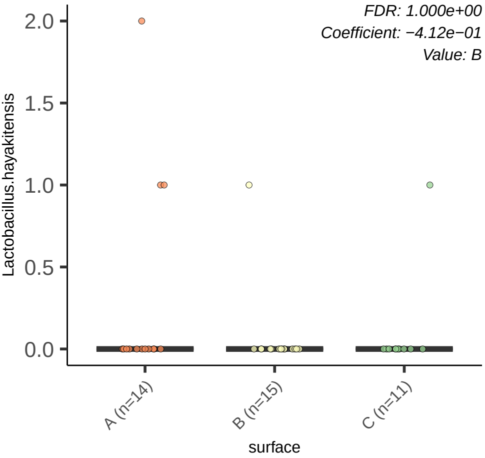
FDR: 1.000e+00
Coefficient: −4.12e−01
Value: B
Lactobacillus.hayakitensis
2.0
1.5
1.0
0.5
0.0
A (n=14)
B (n=15)
C (n=11)
surface

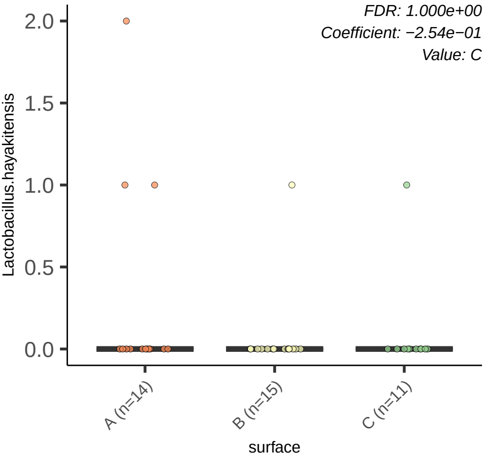
FDR: 1.000e+00
Coefficient: −2.54e−01
Value: C
Lactobacillus.hayakitensis
2.0
1.5
1.0
0.5
0.0
A (n=14)
B (n=15)
C (n=11)
surface

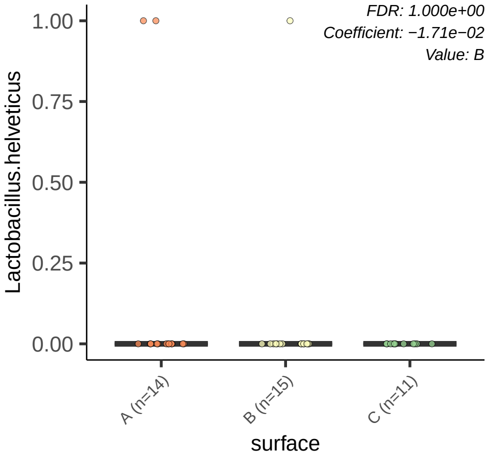

FDR: 1.000e+00
Coefficient: −1.71e−02
Value: B
Lactobacillus.helveticus
1.00
0.75
0.50
0.25
0.00
A (n=14)
B (n=15)
C (n=11)
surface

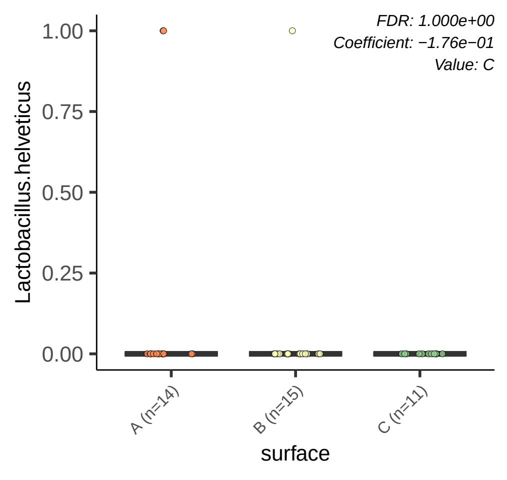
FDR: 1.000e+00
Coefficient: −1.76e−01
Value: C
Lactobacillus.helveticus
1.00
0.75
0.50
0.25
0.00
A (n=14)
B (n=15)
C (n=11)
surface

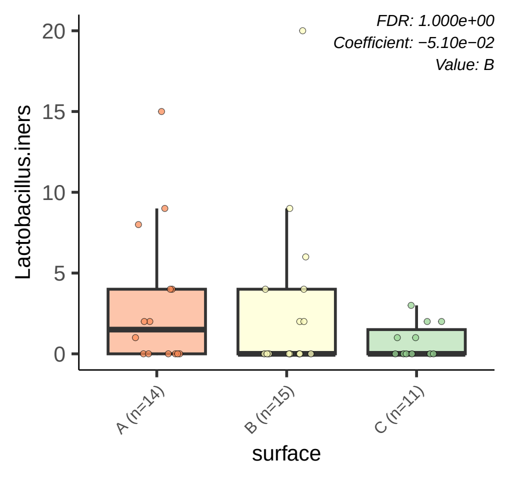
FDR: 1.000e+00
Coefficient: −5.10e−02
Value: B
Lactobacillus.iners
20
15
10
5
0
A (n=14)
B (n=15)
C (n=11)
surface

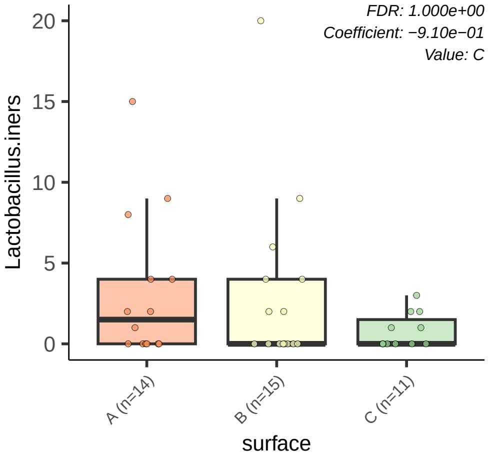

FDR: 1.000e+00
Coefficient: −9.10e−01
Value: C
Lactobacillus.iners
0
5
10
15
20
A (n=14)
B (n=15)
C (n=11)
surface

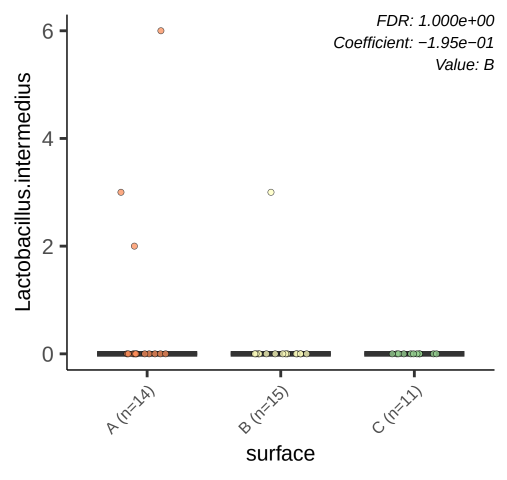

FDR: 1.000e+00
Coefficient: −1.95e−01
Value: B
Lactobacillus.intermedius
6
4
2
0
A (n=14)
B (n=15)
C (n=11)
surface

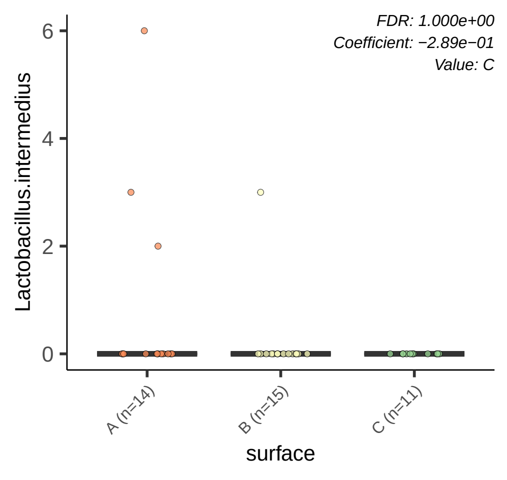
FDR: 1.000e+00
Coefficient: −2.89e−01
Value: C
Lactobacillus.intermedius
6
4
2
0
A (n=14)
B (n=15)
C (n=11)
surface

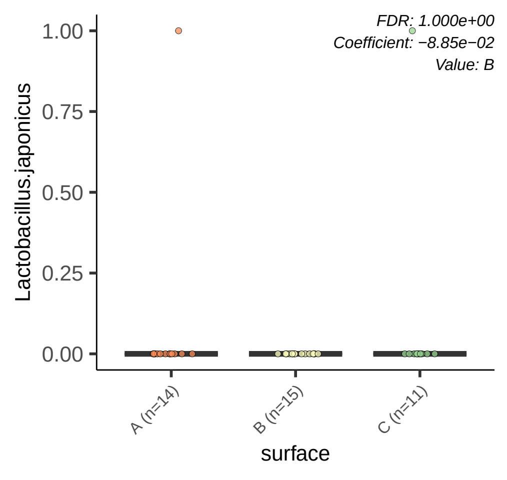

FDR: 1.000e+00
Coefficient: −8.85e−02
Value: B
Lactobacillus.japonicus
1.00
0.75
0.50
0.25
0.00
A (n=14)
B (n=15)
C (n=11)
surface

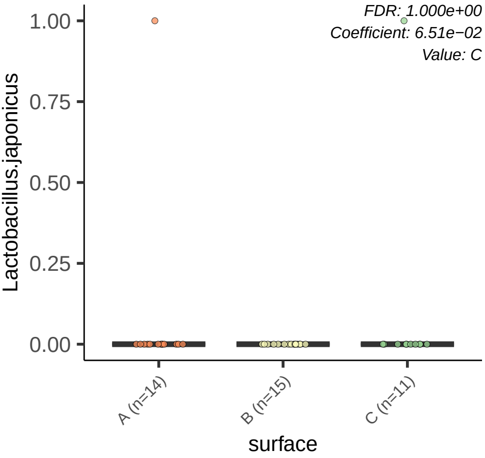
FDR: 1.000e+00
Coefficient: 6.51e−02
Value: C
Lactobacillus.japonicus
1.00
0.75
0.50
0.25
0.00
A (n=14)
B (n=15)
C (n=11)
surface

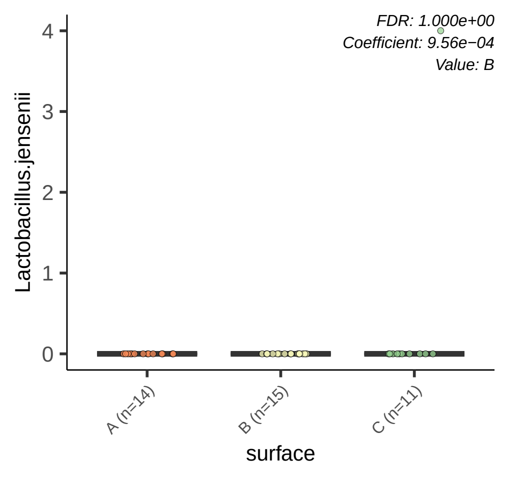
FDR: 1.000e+00
Coefficient: 9.56e−04
Value: B
Lactobacillus.jensenii
4
3
2
1
0
A (n=14)
B (n=15)
C (n=11)
surface

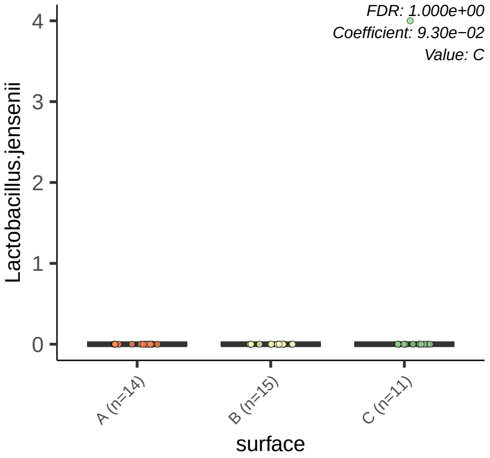

FDR: 1.000e+00
Coefficient: 9.30e−02
Value: C
Lactobacillus.jensenii
0
1
2
3
4
A (n=14)
B (n=15)
C (n=11)
surface

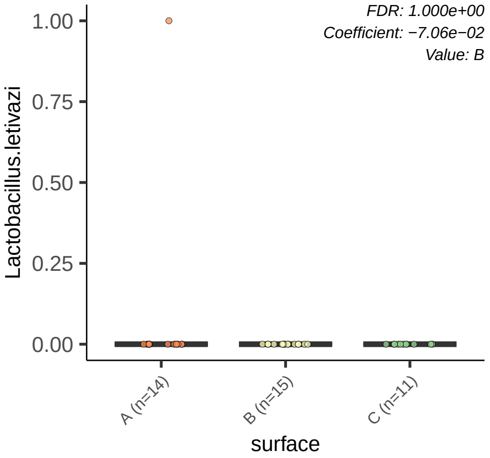
FDR: 1.000e+00
Coefficient: −7.06e−02
Value: B
Lactobacillus.letivazi
1.00
0.75
0.50
0.25
0.00
A (n=14)
B (n=15)
C (n=11)
surface

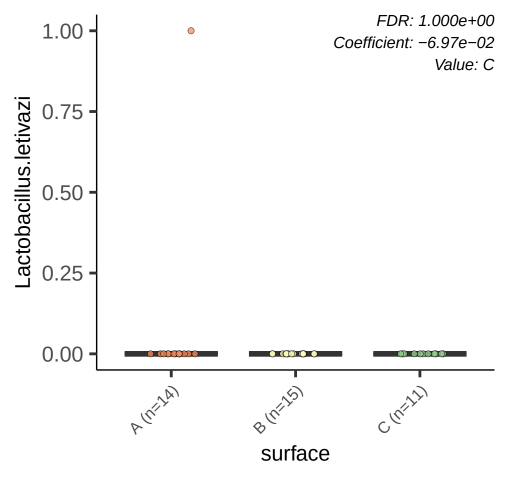

FDR: 1.000e+00
Coefficient: −6.97e−02
Value: C
Lactobacillus.letivazi
1.00
0.75
0.50
0.25
0.00
A (n=14)
B (n=15)
C (n=11)
surface

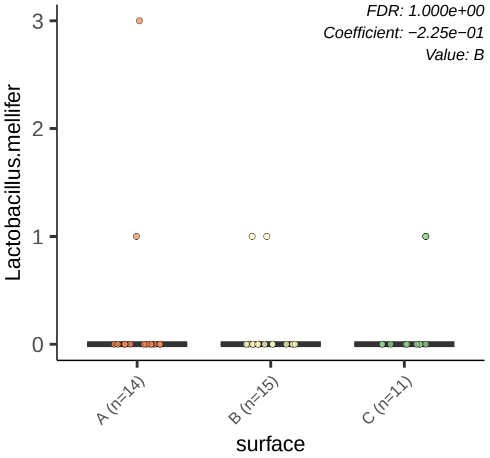
FDR: 1.000e+00
Coefficient: −2.25e−01
Value: B
Lactobacillus.mellifer
0
1
2
3
A (n=14)
B (n=15)
C (n=11)
surface

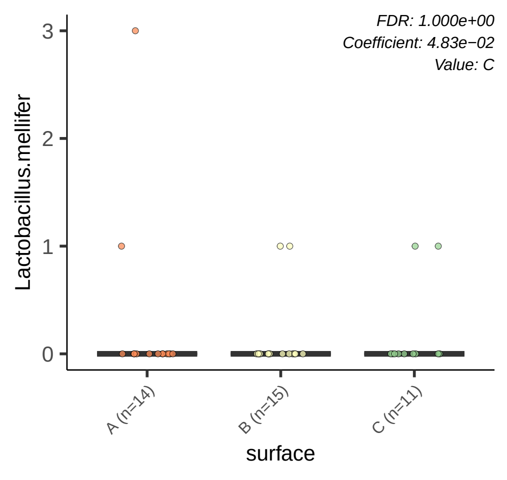

FDR: 1.000e+00
Coefficient: 4.83e−02
Value: C
Lactobacillus.mellifer
3
2
1
0
A (n=14)
B (n=15)
C (n=11)
surface

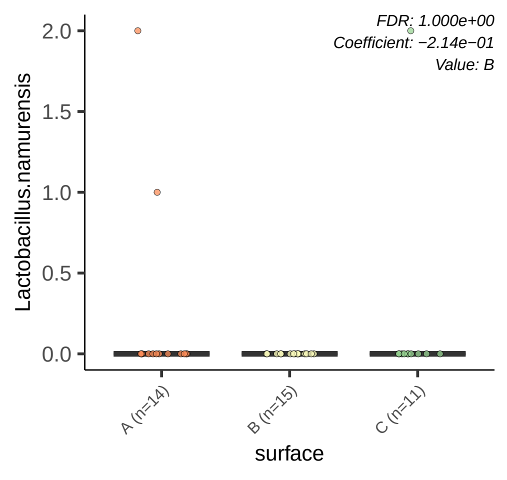

FDR: 1.000e+00
Coefficient: −2.14e−01
Value: B
Lactobacillus.namurensis
2.0
1.5
1.0
0.5
0.0
A (n=14)
B (n=15)
C (n=11)
surface

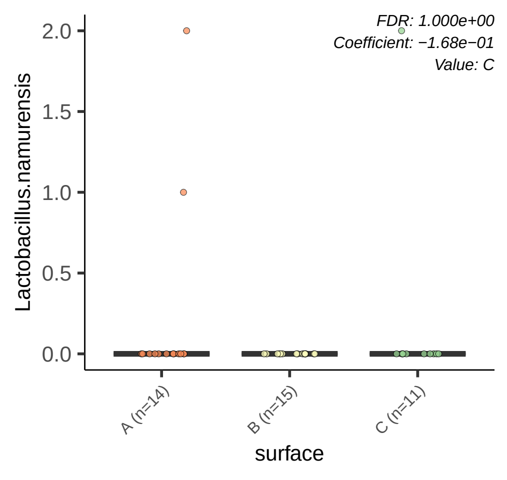

FDR: 1.000e+00
Coefficient: −1.68e−01
Value: C
Lactobacillus.namurensis
2.0
1.5
1.0
0.5
0.0
A (n=14)
B (n=15)
C (n=11)
surface

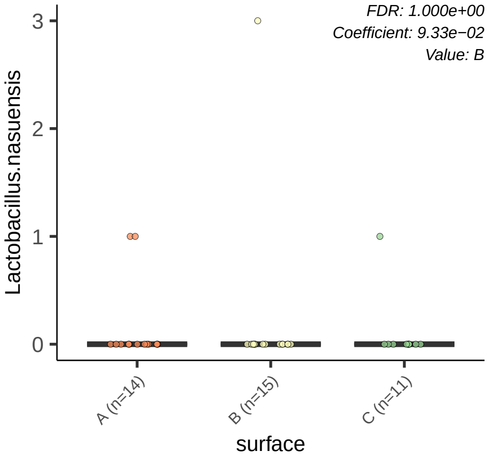
FDR: 1.000e+00
Coefficient: 9.33e−02
Value: B
Lactobacillus.nasuensis
3
2
1
0
A (n=14)
B (n=15)
C (n=11)
surface

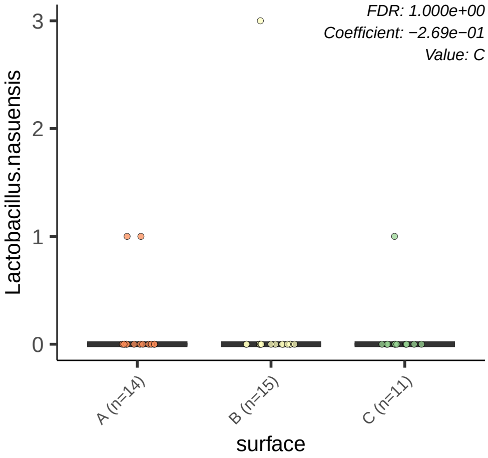

FDR: 1.000e+00
Coefficient: −2.69e−01
Value: C
Lactobacillus.nasuensis
3
2
1
0
A (n=14)
B (n=15)
C (n=11)
surface

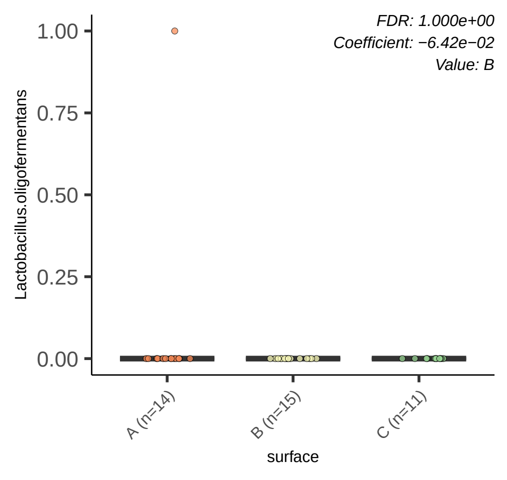
FDR: 1.000e+00
Coefficient: −6.42e−02
Value: B
Lactobacillus.oligofermentans
1.00
0.75
0.50
0.25
0.00
A (n=14)
B (n=15)
C (n=11)
surface

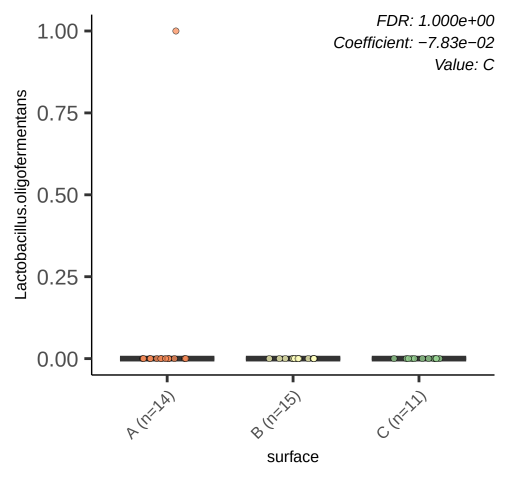
FDR: 1.000e+00
Coefficient: −7.83e−02
Value: C
Lactobacillus.oligofermentans
1.00
0.75
0.50
0.25
0.00
A (n=14)
B (n=15)
C (n=11)
surface

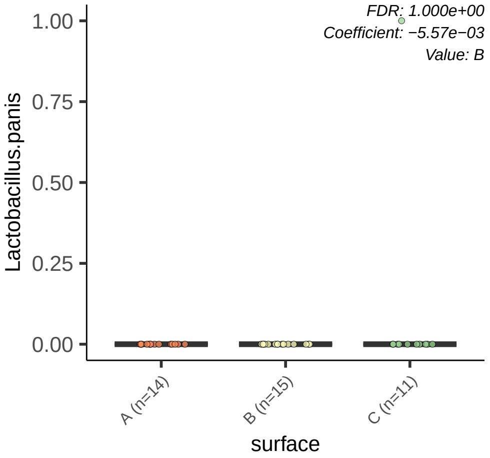
FDR: 1.000e+00
Coefficient: −5.57e−03
Value: B
Lactobacillus.panis
1.00
0.75
0.50
0.25
0.00
A (n=14)
B (n=15)
C (n=11)
surface

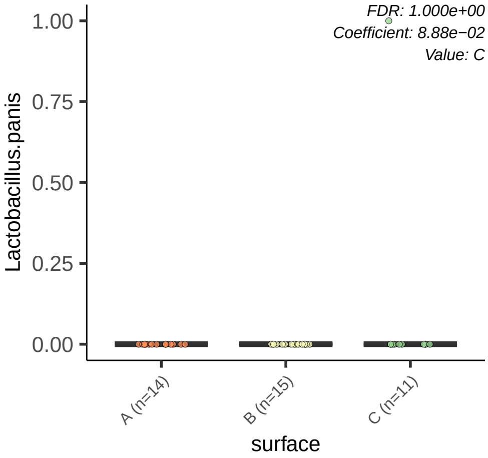

FDR: 1.000e+00
Coefficient: 8.88e−02
Value: C
Lactobacillus.panis
1.00
0.75
0.50
0.25
0.00
A (n=14)
B (n=15)
C (n=11)
surface

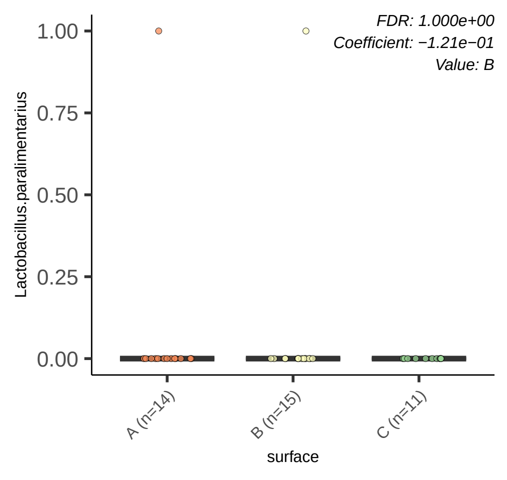
FDR: 1.000e+00
Coefficient: −1.21e−01
Value: B
Lactobacillus.paralimentarius
1.00
0.75
0.50
0.25
0.00
A (n=14)
B (n=15)
C (n=11)
surface

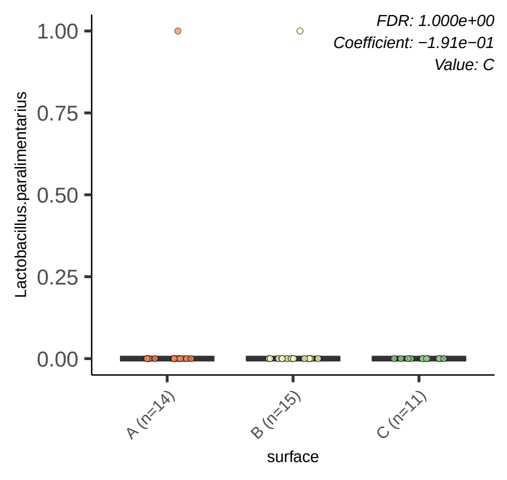

FDR: 1.000e+00
Coefficient: −1.91e−01
Value: C
Lactobacillus.paralimentarius
1.00
0.75
0.50
0.25
0.00
A (n=14)
B (n=15)
C (n=11)
surface

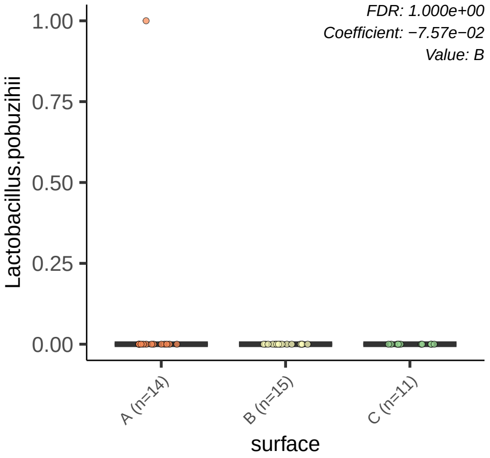
FDR: 1.000e+00
Coefficient: −7.57e−02
Value: B
Lactobacillus.pobuzihii
1.00
0.75
0.50
0.25
0.00
A (n=14)
B (n=15)
C (n=11)
surface

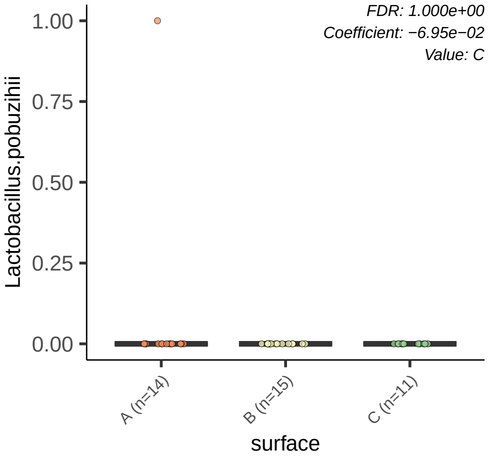
FDR: 1.000e+00
Coefficient: −6.95e−02
Value: C
Lactobacillus.pobuzihii
1.00
0.75
0.50
0.25
0.00
A (n=14)
B (n=15)
C (n=11)
surface

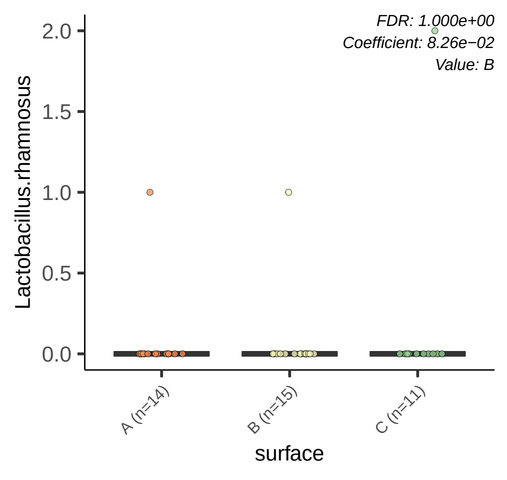
FDR: 1.000e+00
Coefficient: 8.26e−02
Value: B
Lactobacillus.rhamnosus
2.0
1.5
1.0
0.5
0.0
A (n=14)
B (n=15)
C (n=11)
surface

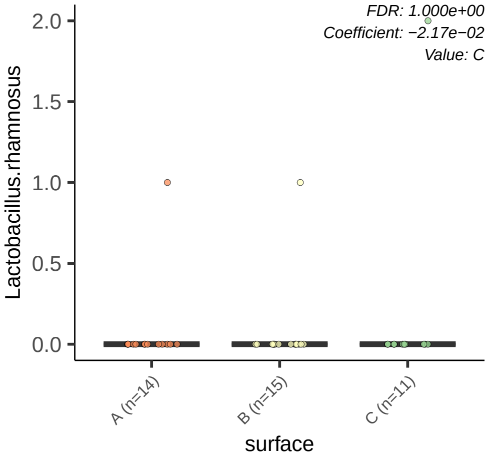
FDR: 1.000e+00
Coefficient: −2.17e−02
Value: C
Lactobacillus.rhamnosus
2.0
1.5
1.0
0.5
0.0
A (n=14)
B (n=15)
C (n=11)
surface

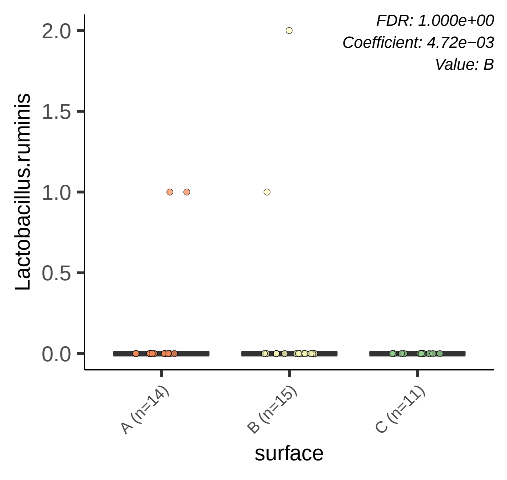
FDR: 1.000e+00
Coefficient: 4.72e−03
Value: B
Lactobacillus.ruminis
2.0
1.5
1.0
0.5
0.0
A (n=14)
B (n=15)
C (n=11)
surface

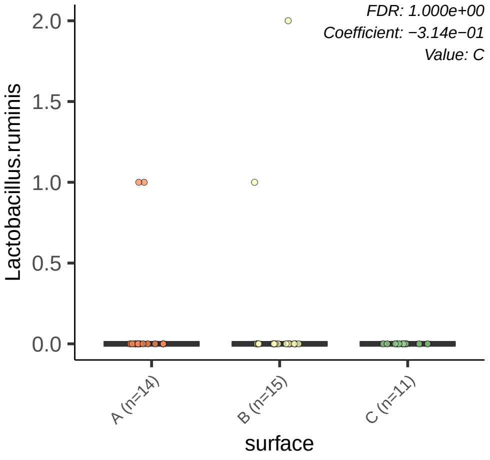
FDR: 1.000e+00
Coefficient: −3.14e−01
Value: C
Lactobacillus.ruminis
2.0
1.5
1.0
0.5
0.0
A (n=14)
B (n=15)
C (n=11)
surface

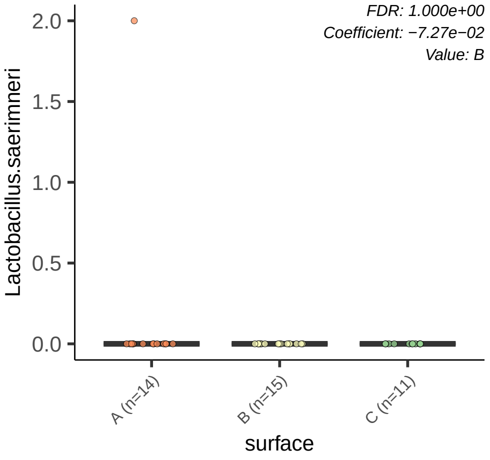
FDR: 1.000e+00
Coefficient: −7.27e−02
Value: B
Lactobacillus.saerimneri
2.0
1.5
1.0
0.5
0.0
A (n=14)
B (n=15)
C (n=11)
surface

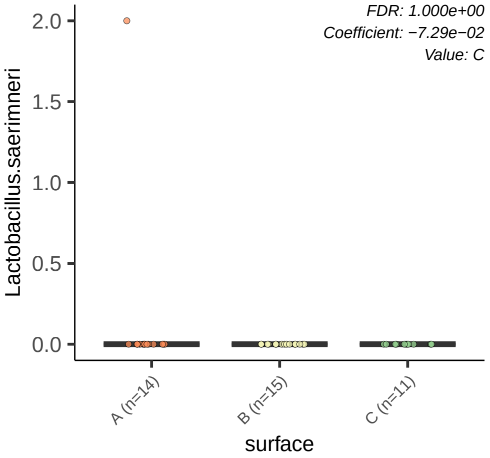
FDR: 1.000e+00
Coefficient: −7.29e−02
Value: C
Lactobacillus.saerimneri
2.0
1.5
1.0
0.5
0.0
A (n=14)
B (n=15)
C (n=11)
surface

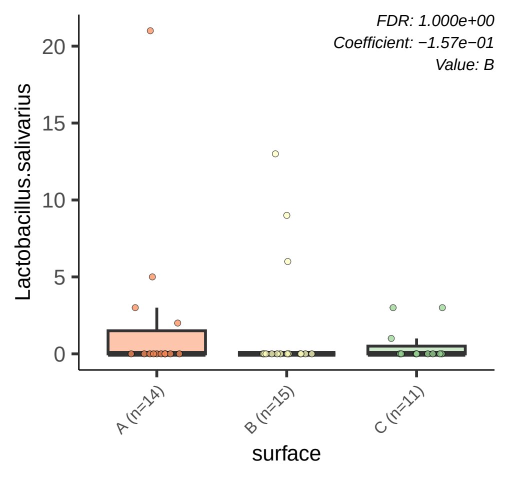
FDR: 1.000e+00
Coefficient: −1.57e−01
Value: B
Lactobacillus.salivarius
20
15
10
5
0
A (n=14)
B (n=15)
C (n=11)
surface

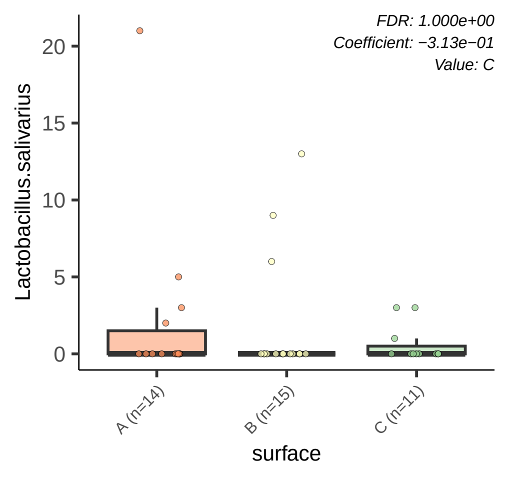
FDR: 1.000e+00
Coefficient: −3.13e−01
Value: C
Lactobacillus.salivarius
0
5
10
15
20
A (n=14)
B (n=15)
C (n=11)
surface

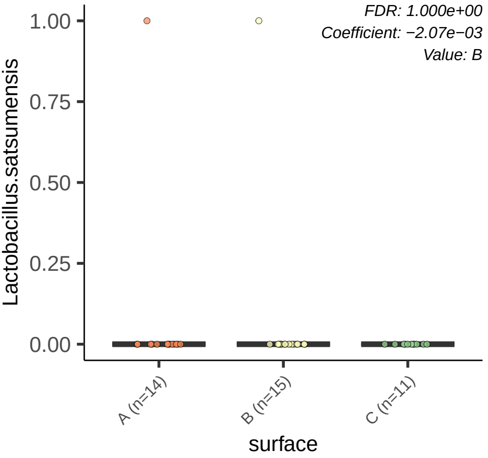
FDR: 1.000e+00
Coefficient: −2.07e−03
Value: B
Lactobacillus.satsumensis
1.00
0.75
0.50
0.25
0.00
A (n=14)
B (n=15)
C (n=11)
surface

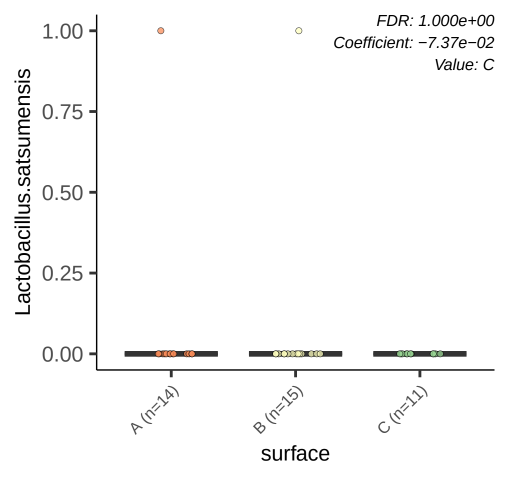

FDR: 1.000e+00
Coefficient: −7.37e−02
Value: C
Lactobacillus.satsumensis
1.00
0.75
0.50
0.25
0.00
A (n=14)
B (n=15)
C (n=11)
surface

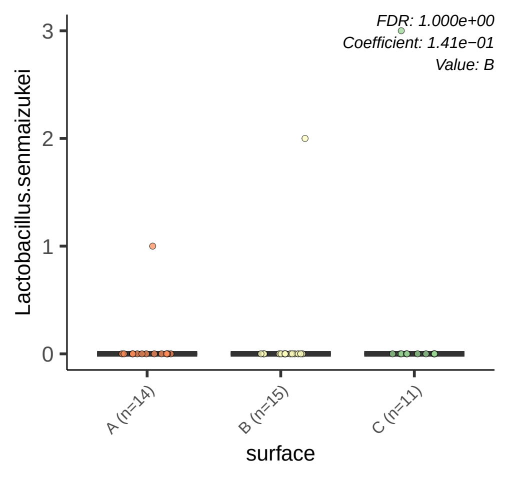
FDR: 1.000e+00
Coefficient: 1.41e−01
Value: B
Lactobacillus.senmaizukei
3
2
1
0
A (n=14)
B (n=15)
C (n=11)
surface

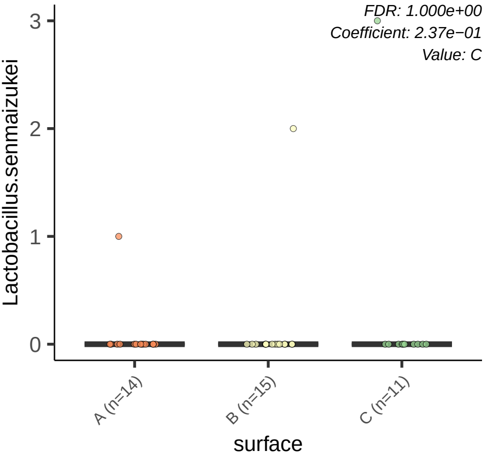

FDR: 1.000e+00
Coefficient: 2.37e−01
Value: C
Lactobacillus.senmaizukei
3
2
1
0
A (n=14)
B (n=15)
C (n=11)
surface

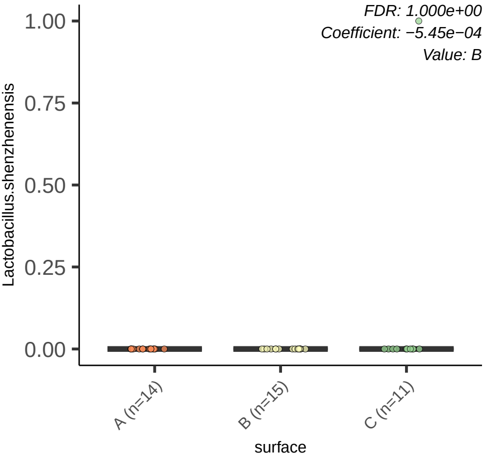
FDR: 1.000e+00
Coefficient: −5.45e−04
Value: B
Lactobacillus.shenzhenensis
1.00
0.75
0.50
0.25
0.00
A (n=14)
B (n=15)
C (n=11)
surface

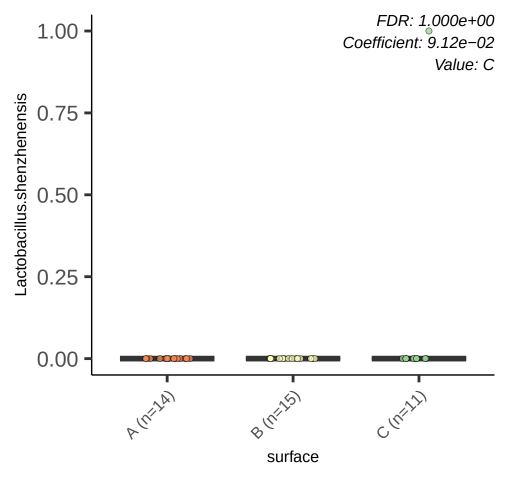

FDR: 1.000e+00
Coefficient: 9.12e−02
Value: C
1.00
0.75
0.50
0.25
0.00
Lactobacillus.shenzhenensis
A (n=14)
B (n=15)
C (n=11)
surface

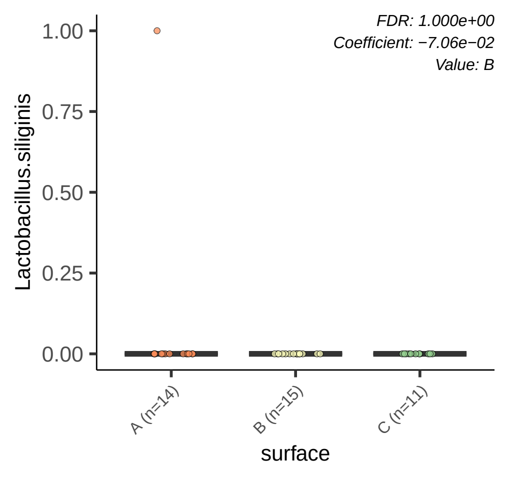
FDR: 1.000e+00
Coefficient: −7.06e−02
Value: B
Lactobacillus.siligini s
1.00
0.75
0.50
0.25
0.00
A (n=14)
B (n=15)
C (n=11)
surface

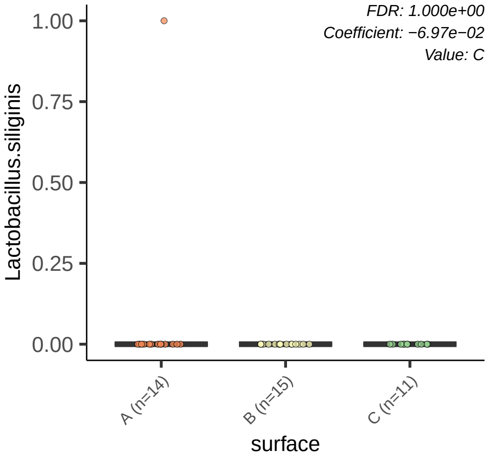
FDR: 1.000e+00
Coefficient: −6.97e−02
Value: C
Lactobacillus.siliginis
1.00
0.75
0.50
0.25
0.00
A (n=14)
B (n=15)
C (n=11)
surface

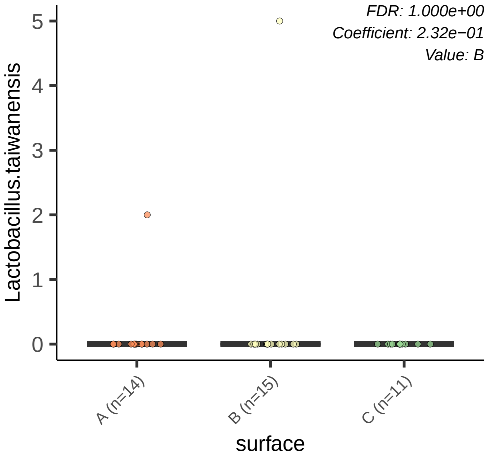
FDR: 1.000e+00
Coefficient: 2.32e−01
Value: B
Lactobacillus.taiwanensis
0
1
2
3
4
5
A (n=14)
B (n=15)
C (n=11)
surface

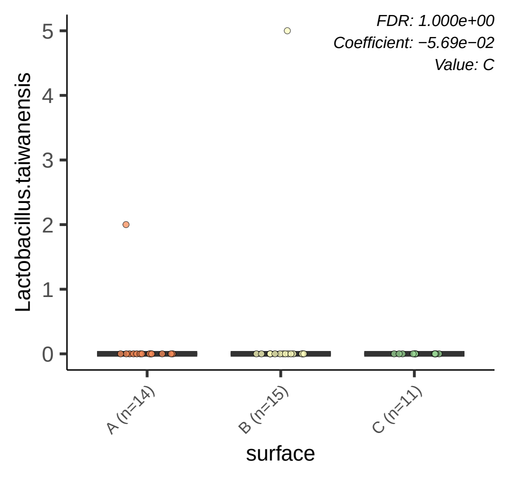
FDR: 1.000e+00
Coefficient: −5.69e−02
Value: C
Lactobacillus.taiwanensis
0
1
2
3
4
5
A (n=14)
B (n=15)
C (n=11)
surface

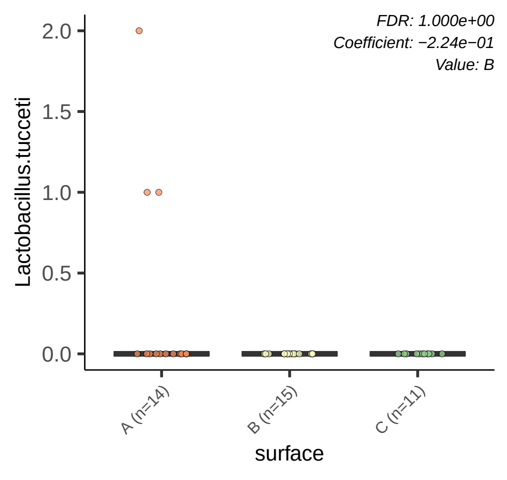
FDR: 1.000e+00
Coefficient: −2.24e−01
Value: B
Lactobacillus.tucceti
2.0
1.5
1.0
0.5
0.0
A (n=14)
B (n=15)
C (n=11)
surface

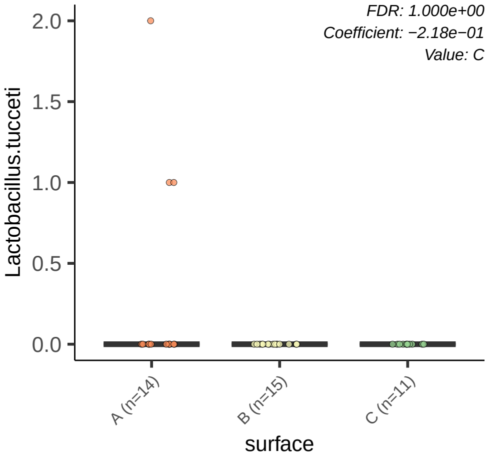
FDR: 1.000e+00
Coefficient: −2.18e−01
Value: C
Lactobacillus.tucceti
2.0
1.5
1.0
0.5
0.0
A (n=14)
B (n=15)
C (n=11)
surface

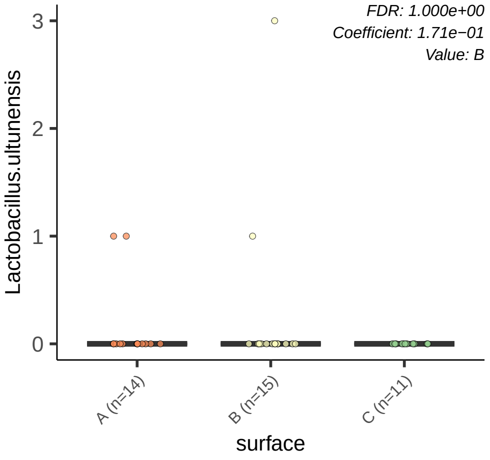
FDR: 1.000e+00
Coefficient: 1.71e−01
Value: B
Lactobacillus.ultunensis
0
1
2
3
A (n=14)
B (n=15)
C (n=11)
surface

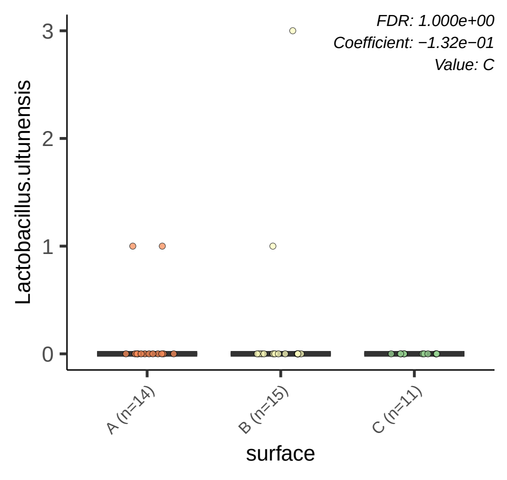

FDR: 1.000e+00
Coefficient: −1.32e−01
Value: C
Lactobacillus.ultunensis
3
2
1
0
A (n=14)
B (n=15)
C (n=11)
surface

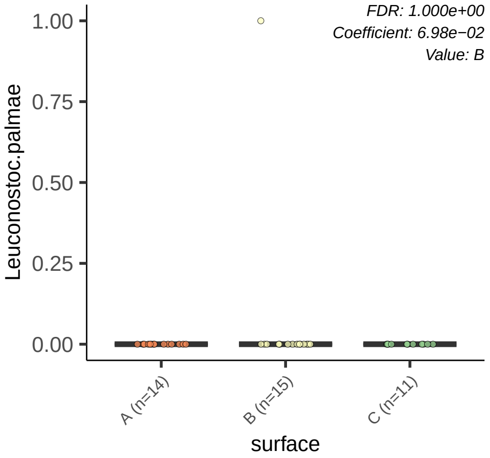
FDR: 1.000e+00
Coefficient: 6.98e−02
Value: B
1.00
0.75
0.50
0.25
0.00
Leuconostoc.palmae
A (n=14)
B (n=15)
C (n=11)
surface

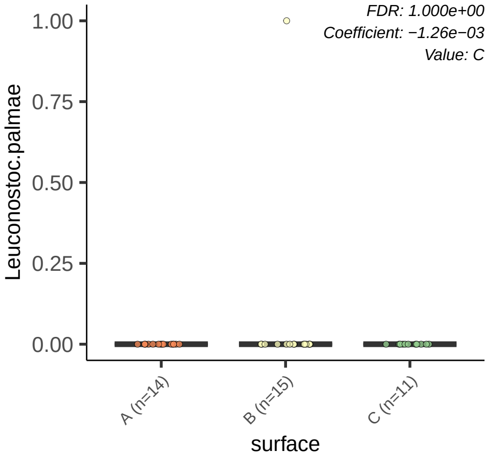

FDR: 1.000e+00
Coefficient: −1.26e−03
Value: C
Leuconostoc.palmae
1.00
0.75
0.50
0.25
0.00
A (n=14)
B (n=15)
C (n=11)
surface

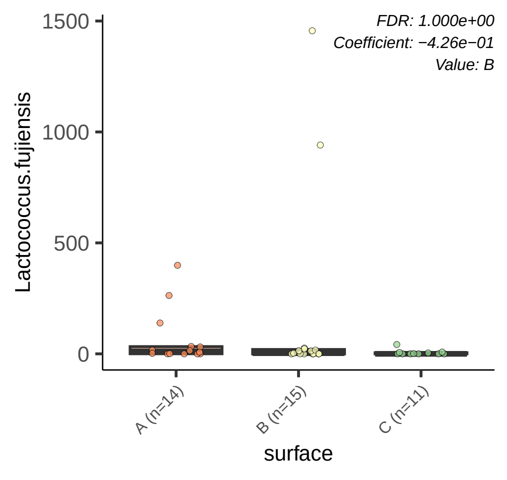

FDR: 1.000e+00
Coefficient: −4.26e−01
Value: B
Lactococcus.fujiensis
1500
1000
500
0
A (n=14)
B (n=15)
C (n=11)
surface

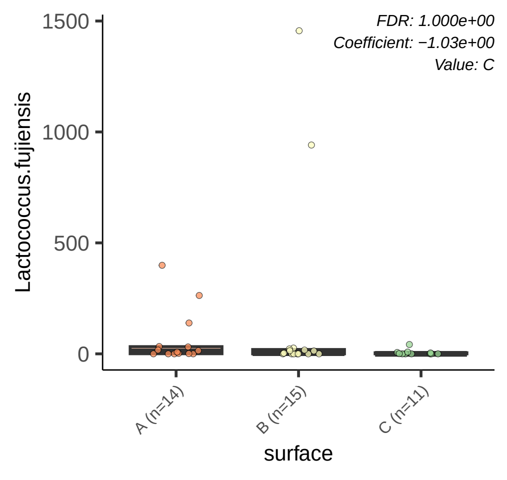
FDR: 1.000e+00
Coefficient: −1.03e+00
Value: C
Lactococcus.fujiensis
1500
1000
500
0
A (n=14)
B (n=15)
C (n=11)
surface

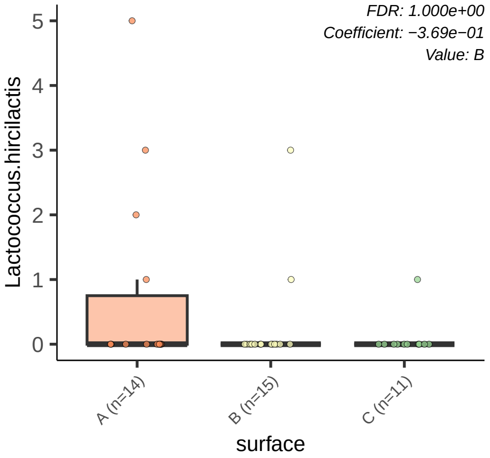
FDR: 1.000e+00
Coefficient: −3.69e−01
Value: B
Lactococcus.hircilactis
0
1
2
3
4
5
A (n=14)
B (n=15)
C (n=11)
surface

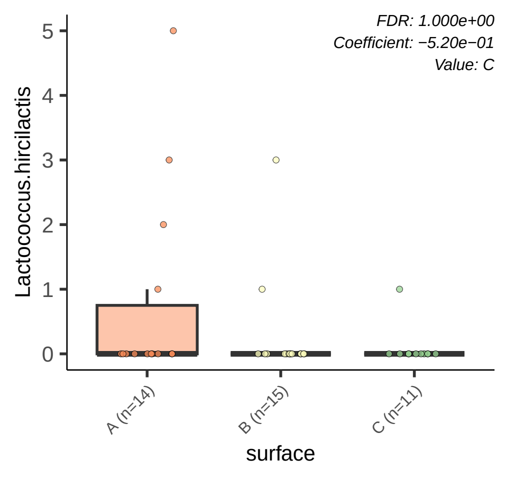
FDR: 1.000e+00
Coefficient: −5.20e−01
Value: C
Lactococcus.hircilactis
0
1
2
3
4
5
A (n=14)
B (n=15)
C (n=11)
surface

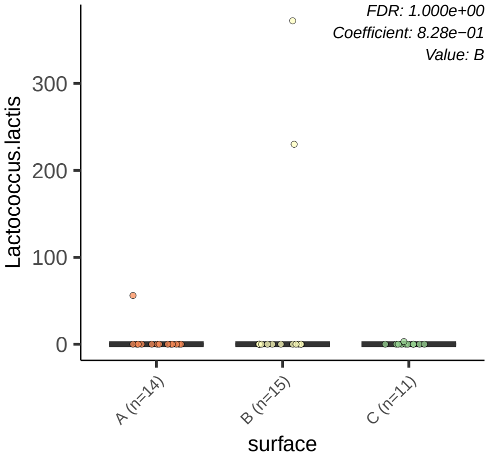

FDR: 1.000e+00
Coefficient: 8.28e−01
Value: B
Lactococcus.lactis
300
200
100
0
A (n=14)
B (n=15)
C (n=11)
surface

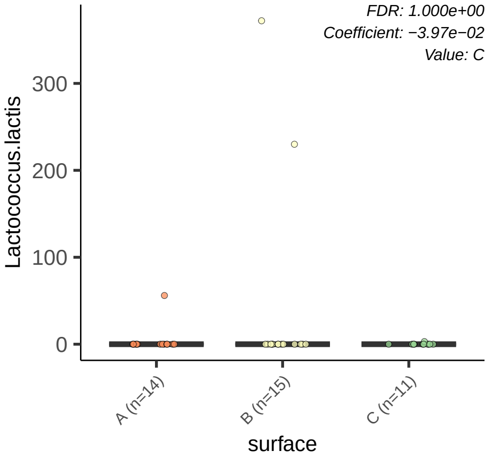
FDR: 1.000e+00
Coefficient: −3.97e−02
Value: C
Lactococcus.lactis
300
200
100
0
A (n=14)
B (n=15)
C (n=11)
surface

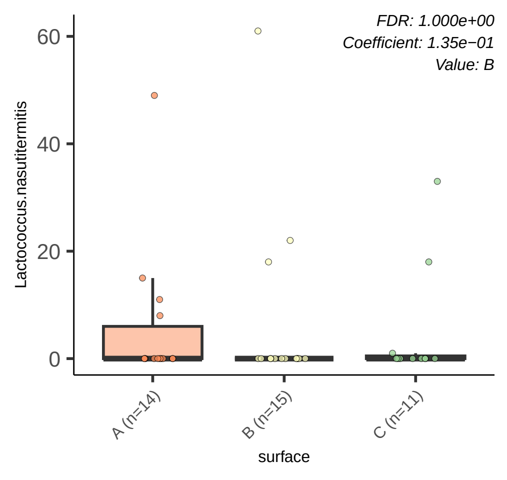

FDR: 1.000e+00
Coefficient: 1.35e−01
Value: B
Lactococcus.nasutitermitis
0
20
40
60
A (n=14)
B (n=15)
C (n=11)
surface

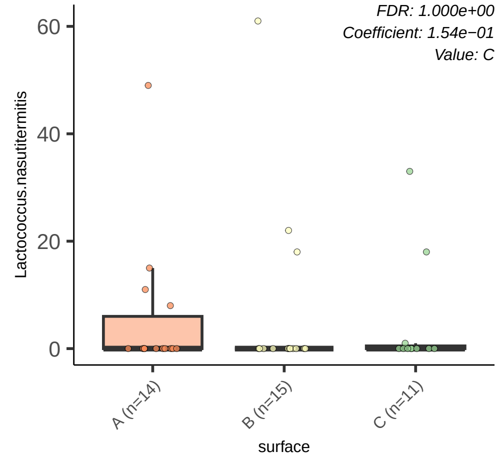

FDR: 1.000e+00
Coefficient: 1.54e−01
Value: C
Lactococcus.nasutitermitis
60
40
20
0
A (n=14)
B (n=15)
C (n=11)
surface

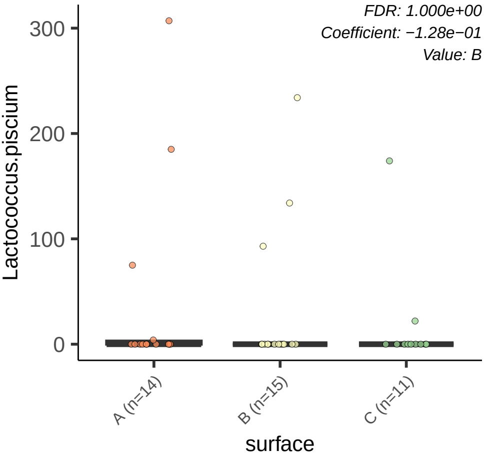
FDR: 1.000e+00
Coefficient: −1.28e−01
Value: B
Lactococcus.piscium
300
200
100
0
A (n=14)
B (n=15)
C (n=11)
surface

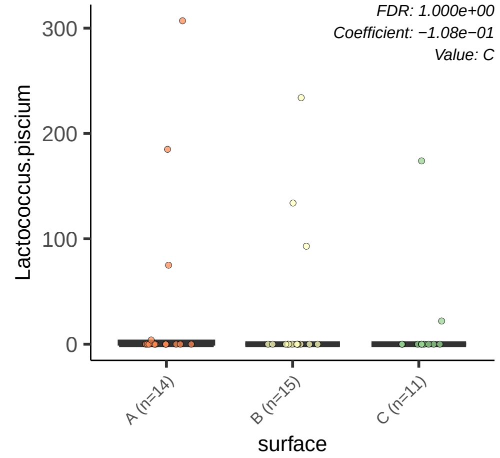

FDR: 1.000e+00
Coefficient: −1.08e−01
Value: C
Lactococcus.piscium
300
200
100
0
A (n=14)
B (n=15)
C (n=11)
surface

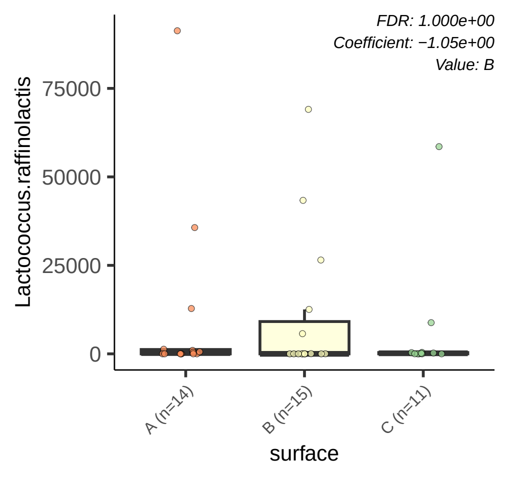
FDR: 1.000e+00
Coefficient: −1.05e+00
Value: B
Lactococcus.raffinolactis
75000
50000
25000
0
A (n=14)
B (n=15)
C (n=11)
surface

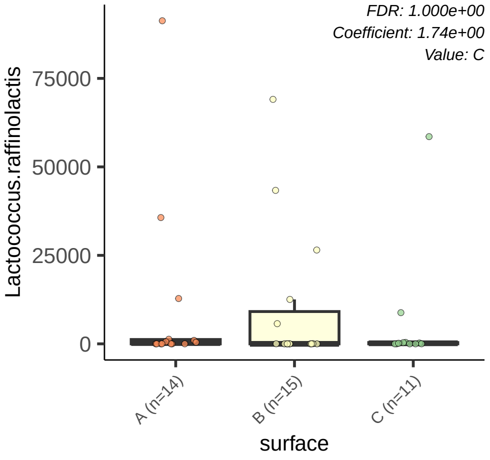

FDR: 1.000e+00
Coefficient: 1.74e+00
Value: C
Lactococcus.raffinolactis
75000
50000
25000
0
A (n=14)
B (n=15)
C (n=11)
surface

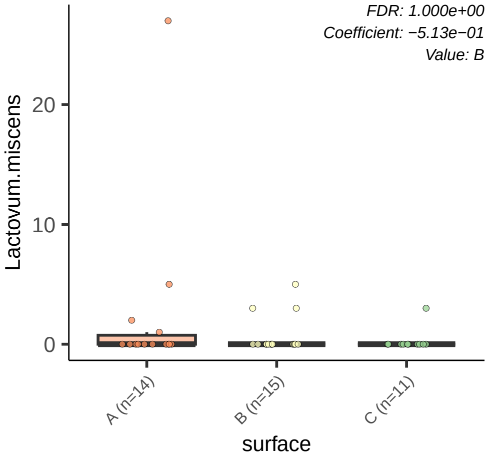
FDR: 1.000e+00
Coefficient: −5.13e−01
Value: B
Lactovum.miscens
20
10
0
A (n=14)
B (n=15)
C (n=11)
surface

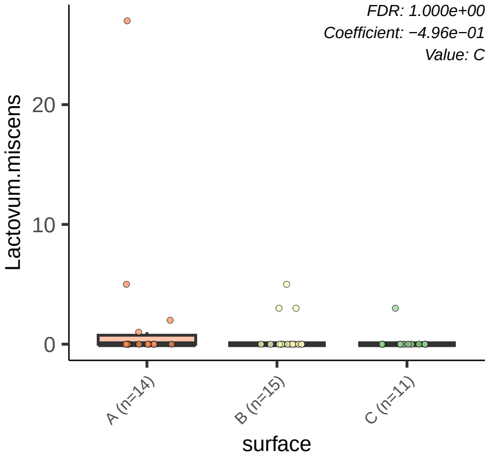

FDR: 1.000e+00
Coefficient: −4.96e−01
Value: C
Lactovum.miscens
0
10
20
A (n=14)
B (n=15)
C (n=11)
surface

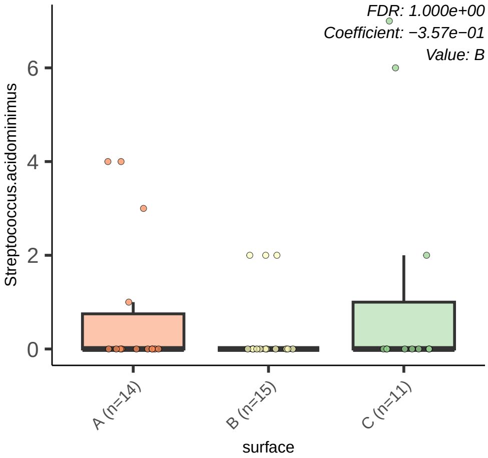
FDR: 1.000e+00
Coefficient: −3.57e−01
Value: B
Streptococcus.acidominimus
0
2
4
6
A (n=14)
B (n=15)
C (n=11)
surface

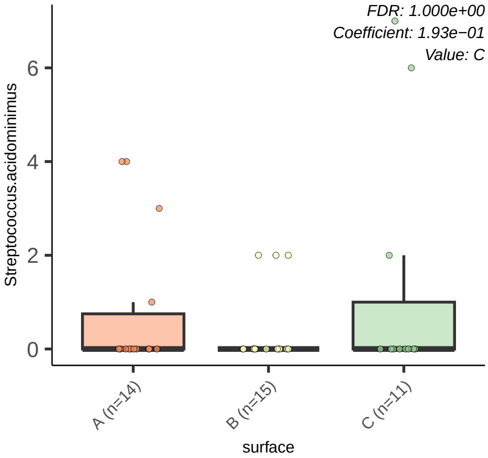

FDR: 1.000e+00
Coefficient: 1.93e−01
Value: C
Streptococcus.acidominimus
0
2
4
6
A (n=14)
B (n=15)
C (n=11)
surface

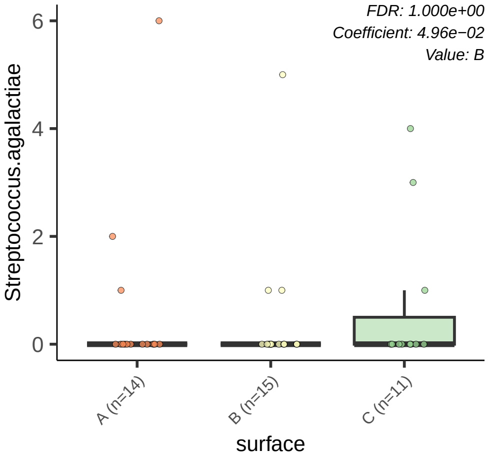
FDR: 1.000e+00
Coefficient: 4.96e−02
Value: B
Streptococcus.agalactiae
0
2
4
6
A (n=14)
B (n=15)
C (n=11)
surface

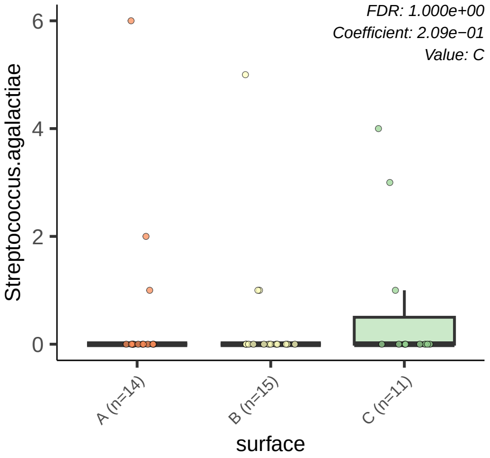
FDR: 1.000e+00
Coefficient: 2.09e−01
Value: C
Streptococcus.agalactiae
6
4
2
0
A (n=14)
B (n=15)
C (n=11)
surface

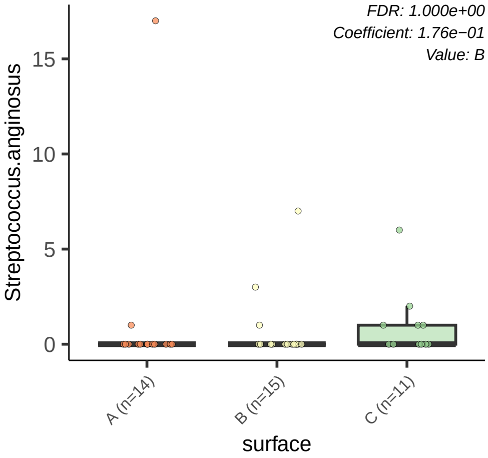

FDR: 1.000e+00
Coefficient: 1.76e−01
Value: B
Streptococcus.anginosus
15
10
5
0
A (n=14)
B (n=15)
C (n=11)
surface

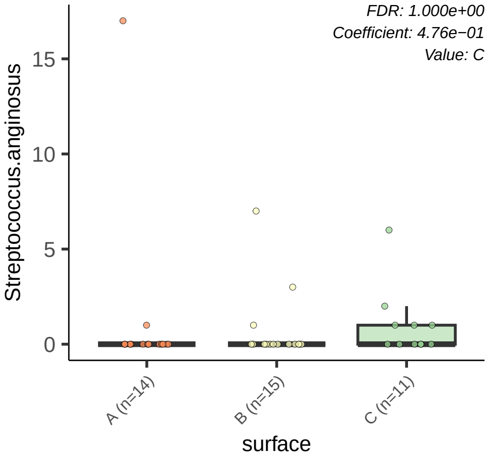
FDR: 1.000e+00
Coefficient: 4.76e−01
Value: C
Streptococcus.anginosus
0
5
10
15
A (n=14)
B (n=15)
C (n=11)
surface

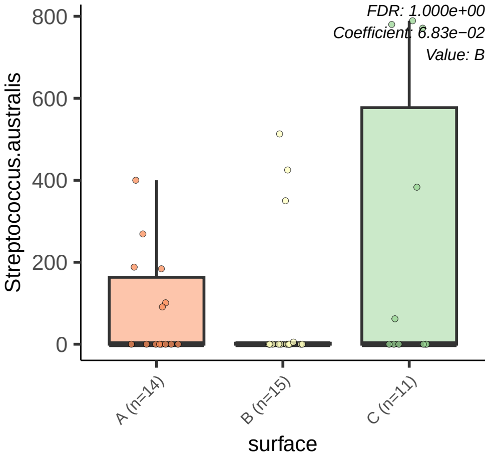

FDR: 1.000e+00
Coefficient: 6.83e−02
Value: B
Streptococcus.australis
800
600
400
200
0
A (n=14)
B (n=15)
C (n=11)
surface

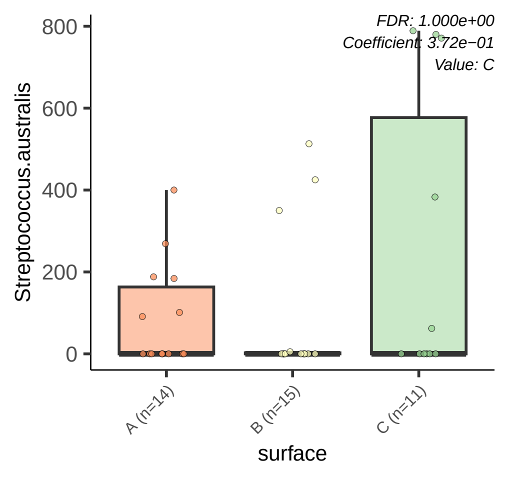

FDR: 1.000e+00
Coefficient: 3.72e−01
Value: C
Streptococcus.australis
800
600
400
200
0
A (n=14)
B (n=15)
C (n=11)
surface

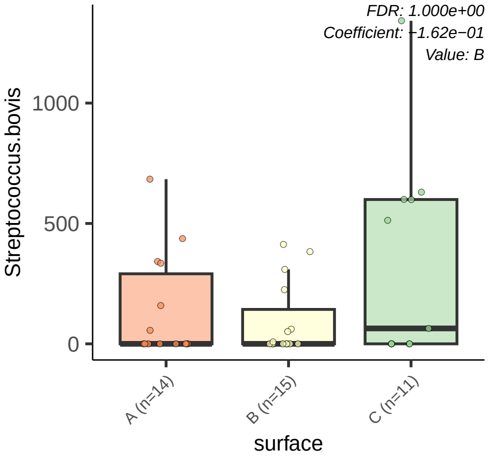

FDR: 1.000e+00
Coefficient: −1.62e−01
Value: B
Streptococcus.bovis
1000
500
0
A (n=14)
B (n=15)
C (n=11)
surface

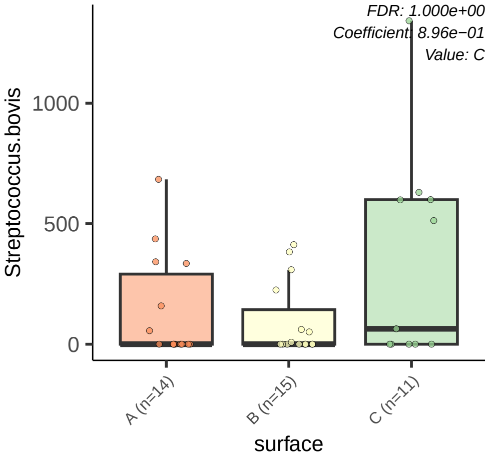
FDR: 1.000e+00
Coefficient: 8.96e−01
Value: C
Streptococcus.bovis
1000
500
0
A (n=14)
B (n=15)
C (n=11)
surface

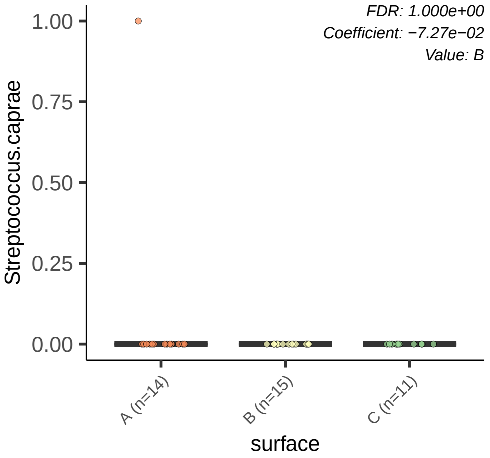

FDR: 1.000e+00
Coefficient: −7.27e−02
Value: B
1.00
0.75
0.50
0.25
0.00
Streptococcus.caprae
A (n=14)
B (n=15)
C (n=11)
surface

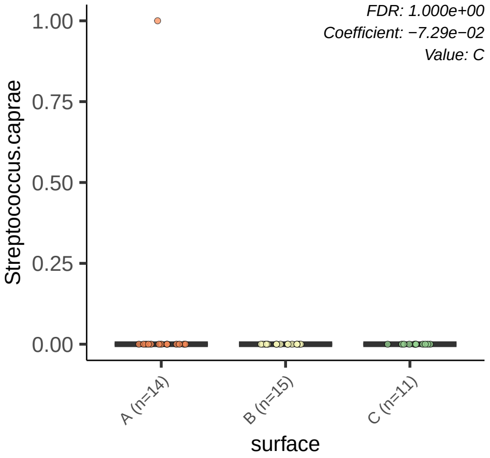

FDR: 1.000e+00
Coefficient: −7.29e−02
Value: C
Streptococcus.caprae
1.00
0.75
0.50
0.25
0.00
A (n=14)
B (n=15)
C (n=11)
surface

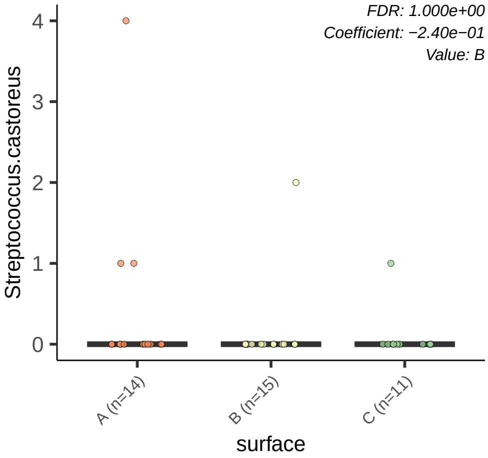

FDR: 1.000e+00
Coefficient: −2.40e−01
Value: B
Streptococcus.castoreus
0
1
2
3
4
A (n=14)
B (n=15)
C (n=11)
surface

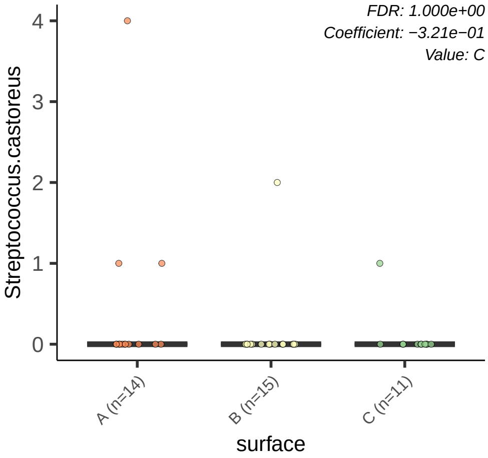
FDR: 1.000e+00
Coefficient: −3.21e−01
Value: C
Streptococcus.castoreus
0
1
2
3
4
A (n=14)
B (n=15)
C (n=11)
surface

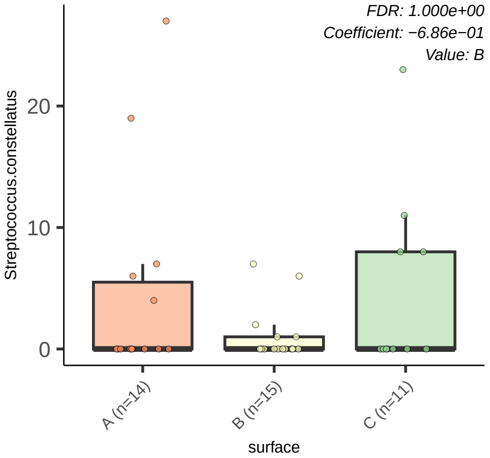

FDR: 1.000e+00
Coefficient: −6.86e−01
Value: B
Streptococcus.constellatus
20
10
0
A (n=14)
B (n=15)
C (n=11)
surface

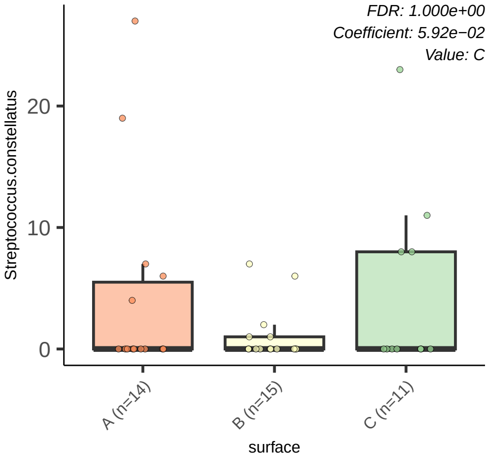
FDR: 1.000e+00
Coefficient: 5.92e−02
Value: C
Streptococcus.constellatus
0
10
20
A (n=14)
B (n=15)
C (n=11)
surface

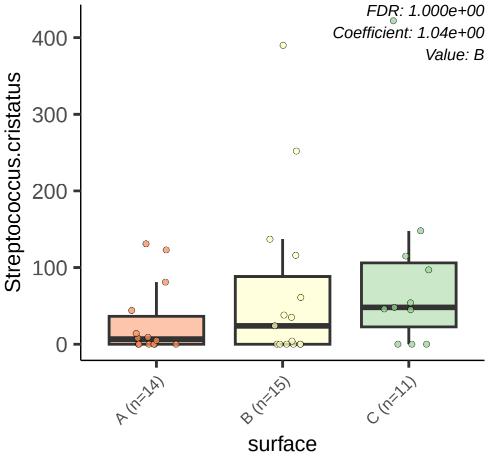

FDR: 1.000e+00
Coefficient: 1.04e+00
Value: B
Streptococcus.cristatus
400
300
200
100
0
A (n=14)
B (n=15)
C (n=11)
surface

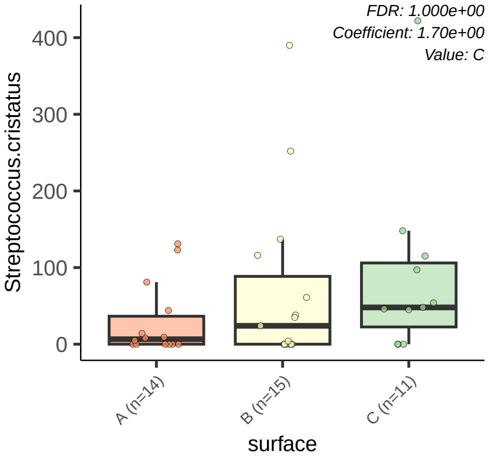
FDR: 1.000e+00
Coefficient: 1.70e+00
Value: C
Streptococcus.cristatus
400
300
200
100
0
A (n=14)
B (n=15)
C (n=11)
surface

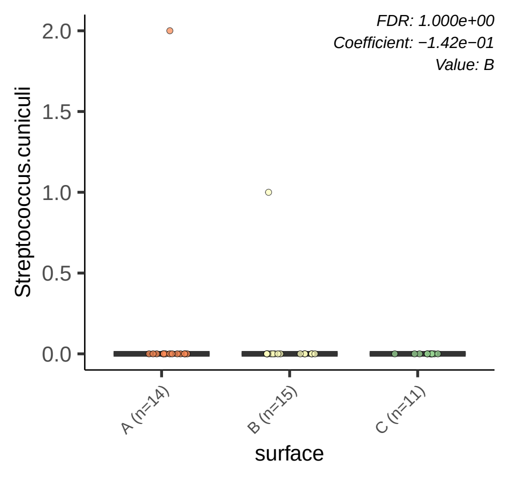
FDR: 1.000e+00
Coefficient: −1.42e−01
Value: B
Streptococcus.cuniculi
2.0
1.5
1.0
0.5
0.0
A (n=14)
B (n=15)
C (n=11)
surface

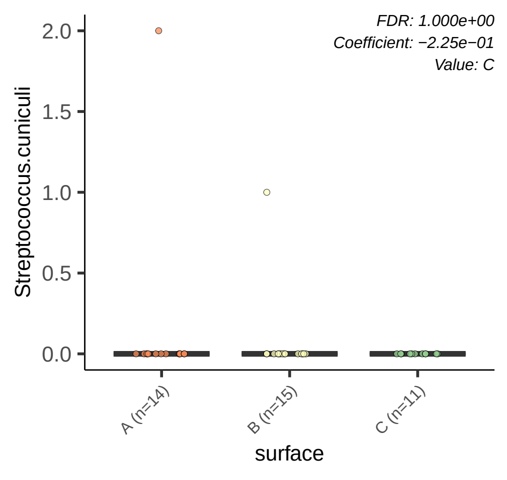
FDR: 1.000e+00
Coefficient: −2.25e−01
Value: C
Streptococcus.cuniculi
2.0
1.5
1.0
0.5
0.0
A (n=14)
B (n=15)
C (n=11)
surface

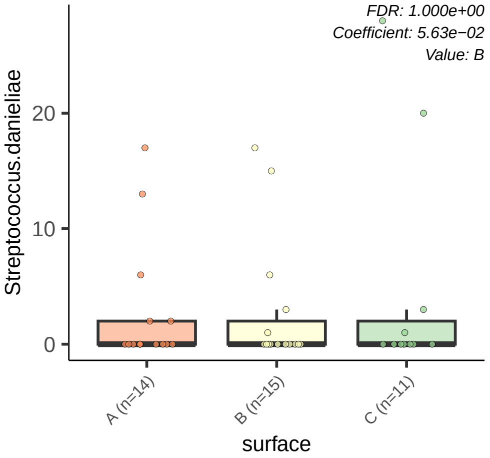

FDR: 1.000e+00
Coefficient: 5.63e−02
Value: B
Streptococcus.danieliae
0
10
20
A (n=14)
B (n=15)
C (n=11)
surface

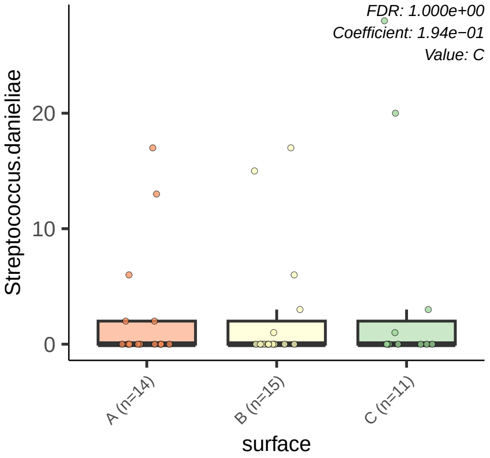
FDR: 1.000e+00
Coefficient: 1.94e−01
Value: C
Streptococcus.danieliae
20
10
0
A (n=14)
B (n=15)
C (n=11)
surface

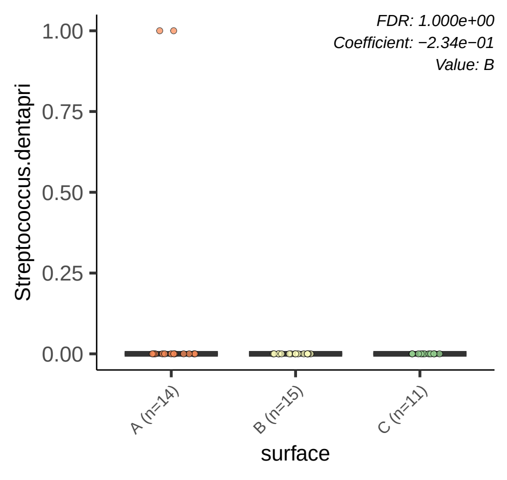
FDR: 1.000e+00
Coefficient: −2.34e−01
Value: B
1.00
0.75
0.50
0.25
0.00
Streptococcus.dentapri
A (n=14)
B (n=15)
C (n=11)
surface

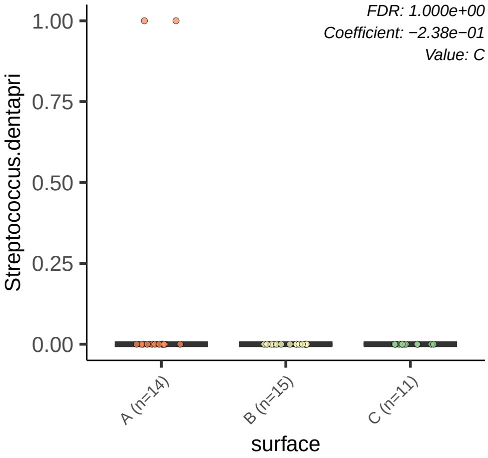

FDR: 1.000e+00
Coefficient: −2.38e−01
Value: C
Streptococcus.dentapri
1.00
0.75
0.50
0.25
0.00
A (n=14)
B (n=15)
C (n=11)
surface

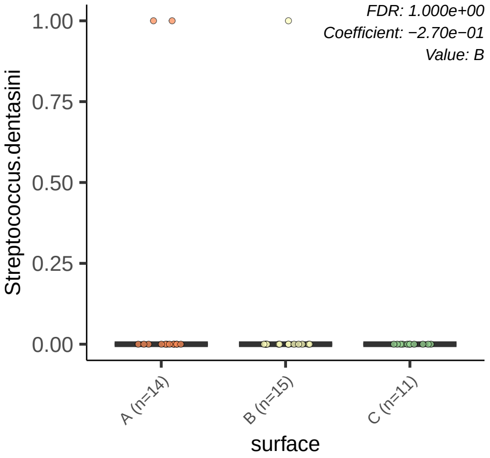
FDR: 1.000e+00
Coefficient: −2.70e−01
Value: B
Streptococcus.dentasini
1.00
0.75
0.50
0.25
0.00
A (n=14)
B (n=15)
C (n=11)
surface

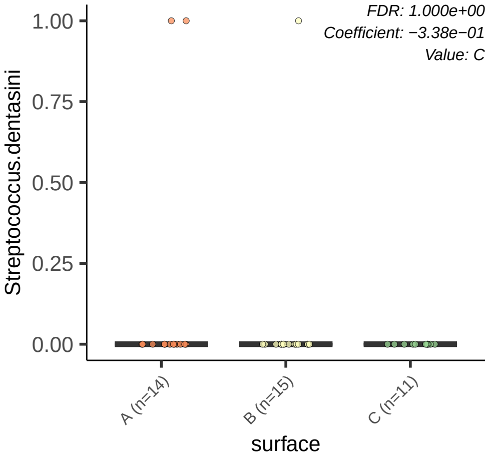

FDR: 1.000e+00
Coefficient: −3.38e−01
Value: C
Streptococcus.dentasini
1.00
0.75
0.50
0.25
0.00
A (n=14)
B (n=15)
C (n=11)
surface

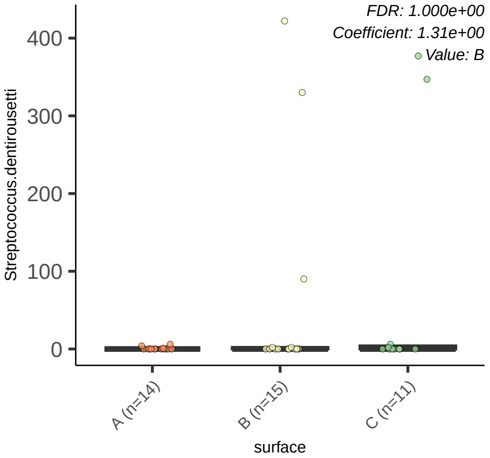
FDR: 1.000e+00
Coefficient: 1.31e+00
Value: B
Streptococcus.dentirousetti
400
300
200
100
0
A (n=14)
B (n=15)
C (n=11)
surface

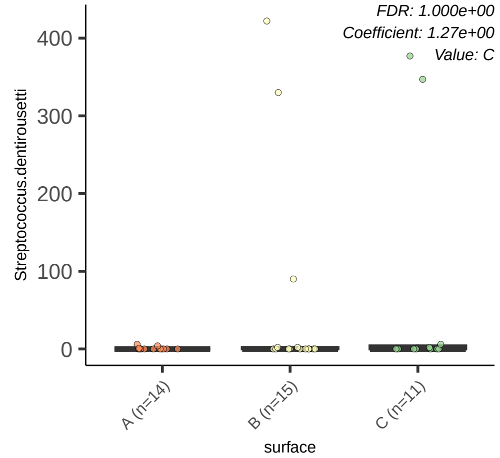
FDR: 1.000e+00
Coefficient: 1.27e+00
Value: C
Streptococcus.dentirousetti
400
300
200
100
0
A (n=14)
B (n=15)
C (n=11)
surface

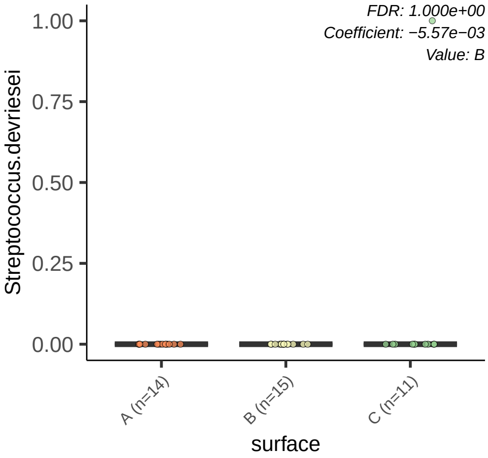

FDR: 1.000e+00
Coefficient: −5.57e−03
Value: B
Streptococcus.devriesei
1.00
0.75
0.50
0.25
0.00
A (n=14)
B (n=15)
C (n=11)
surface

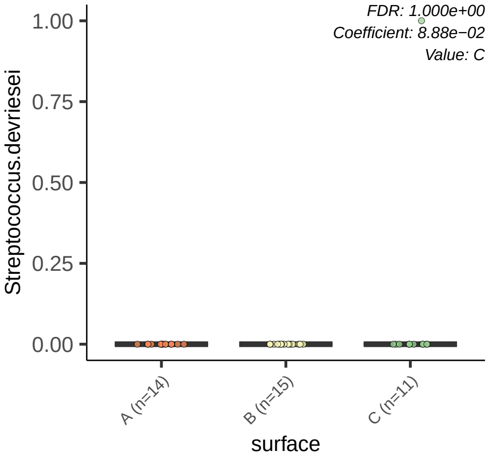
FDR: 1.000e+00
Coefficient: 8.88e−02
Value: C
Streptococcus.devriesei
1.00
0.75
0.50
0.25
0.00
A (n=14)
B (n=15)
C (n=11)
surface

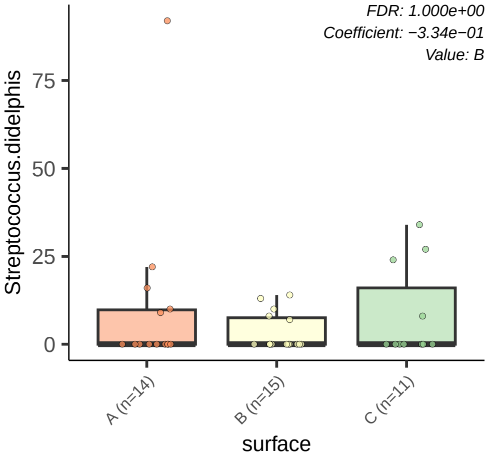
FDR: 1.000e+00
Coefficient: −3.34e−01
Value: B
Streptococcus.didelphis
0
25
50
75
A (n=14)
B (n=15)
C (n=11)
surface

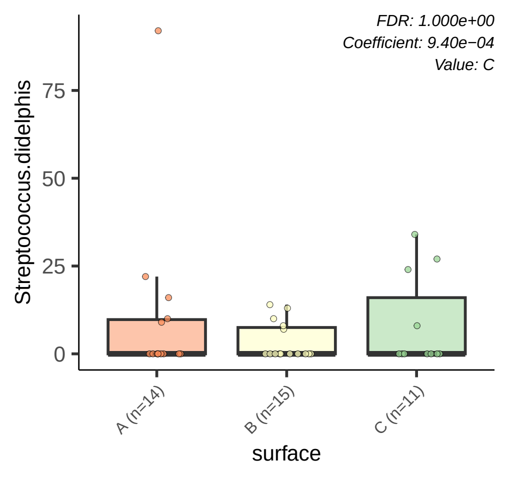

FDR: 1.000e+00
Coefficient: 9.40e−04
Value: C
Streptococcus.didelphis
0
25
50
75
A (n=14)
B (n=15)
C (n=11)
surface

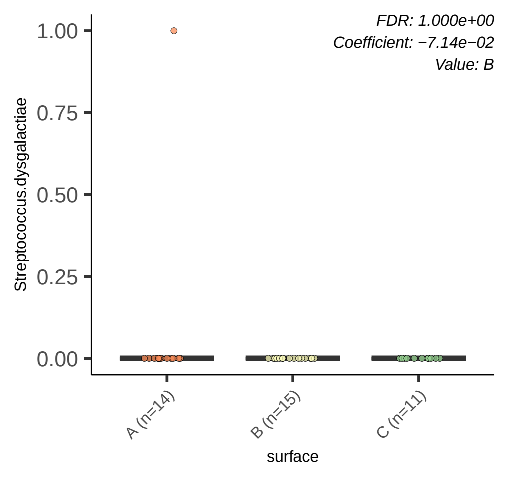
FDR: 1.000e+00
Coefficient: −7.14e−02
Value: B
Streptococcus.dysgalactiae
1.00
0.75
0.50
0.25
0.00
A (n=14)
B (n=15)
C (n=11)
surface

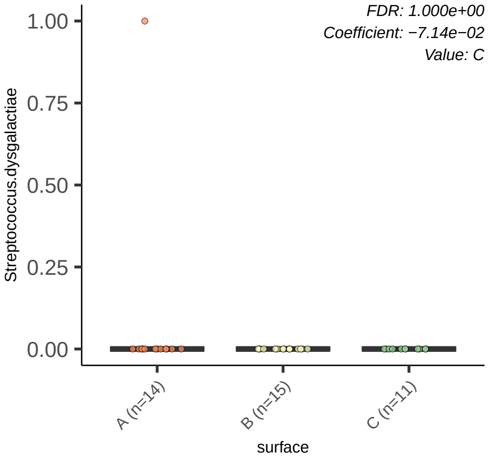
FDR: 1.000e+00
Coefficient: −7.14e−02
Value: C
Streptococcus.dysgalactiae
1.00
0.75
0.50
0.25
0.00
A (n=14)
B (n=15)
C (n=11)
surface

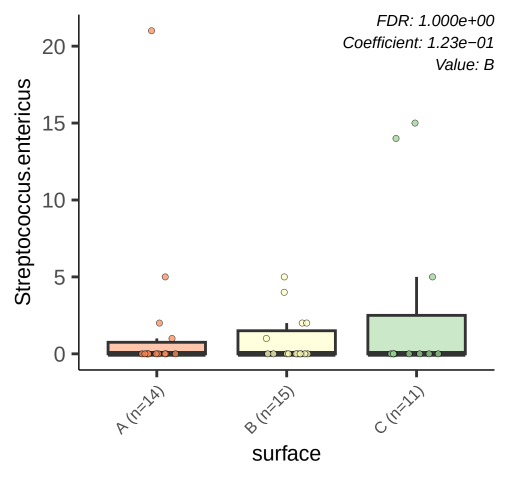
FDR: 1.000e+00
Coefficient: 1.23e−01
Value: B
Streptococcus.enterics
0
5
10
15
20
A (n=14)
B (n=15)
C (n=11)
surface

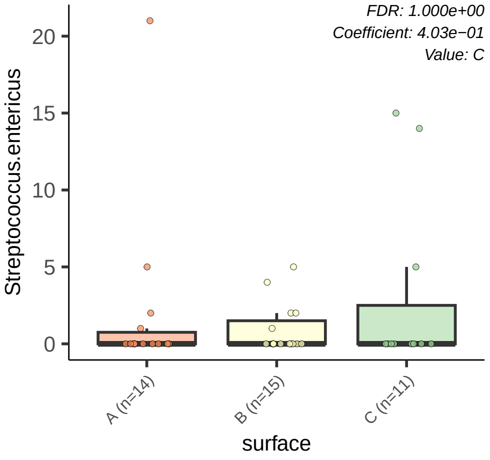

FDR: 1.000e+00
Coefficient: 4.03e−01
Value: C
Streptococcus.enterics
20
15
10
5
0
A (n=14)
B (n=15)
C (n=11)
surface

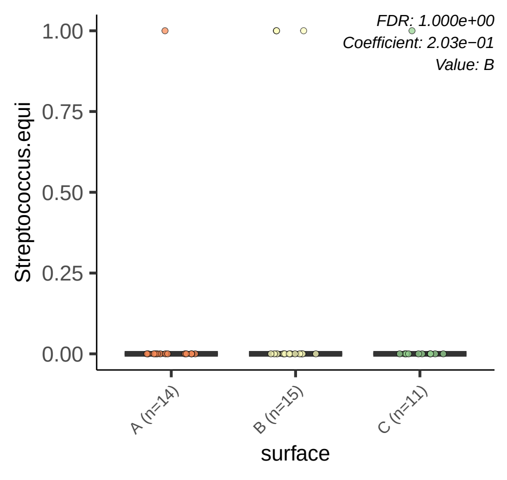

FDR: 1.000e+00
Coefficient: 2.03e−01
Value: B
Streptococcus.equi
1.00
0.75
0.50
0.25
0.00
A (n=14)
B (n=15)
C (n=11)
surface

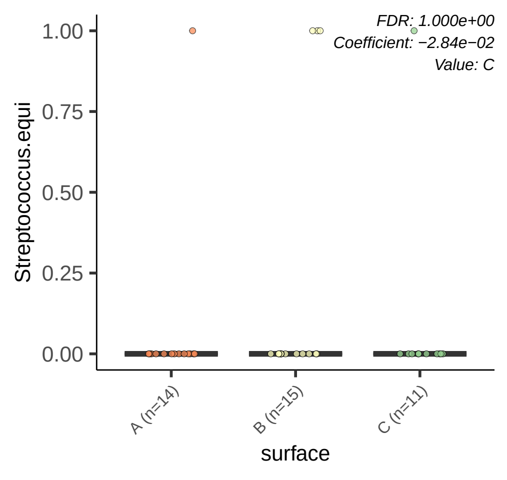

FDR: 1.000e+00
Coefficient: −2.84e−02
Value: C
Streptococcus.equi
1.00
0.75
0.50
0.25
0.00
A (n=14)
B (n=15)
C (n=11)
surface

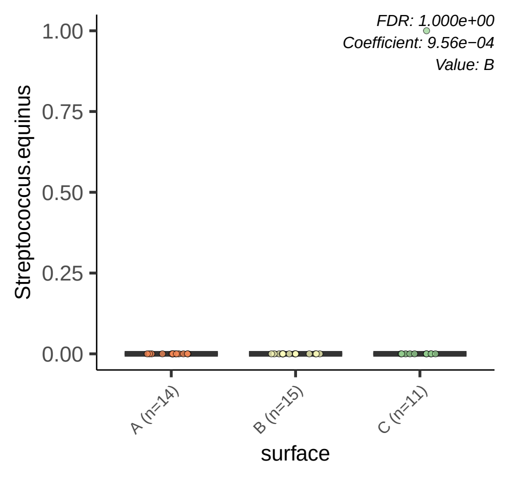
FDR: 1.000e+00
Coefficient: 9.56e−04
Value: B
Streptococcus.equinus
1.00
0.75
0.50
0.25
0.00
A (n=14)
B (n=15)
C (n=11)
surface

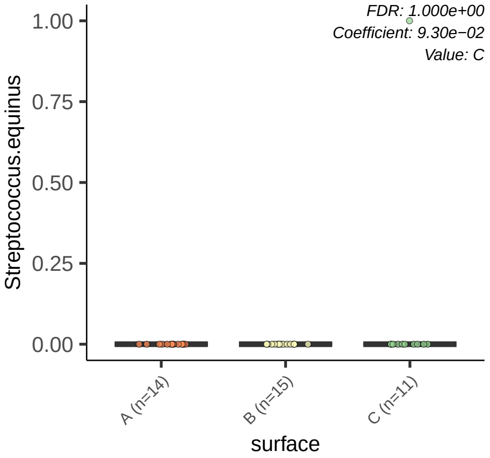

FDR: 1.000e+00
Coefficient: 9.30e−02
Value: C
Streptococcus.equinus
1.00
0.75
0.50
0.25
0.00
A (n=14)
B (n=15)
C (n=11)
surface

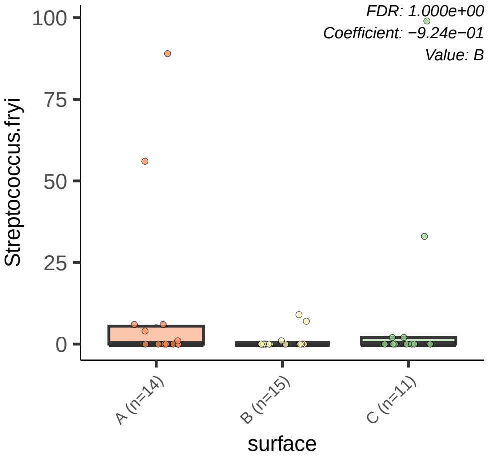

FDR: 1.000e+00
Coefficient: −9.24e−01
Value: B
Streptococcus.fryi
100
75
50
25
0
A (n=14)
B (n=15)
C (n=11)
surface

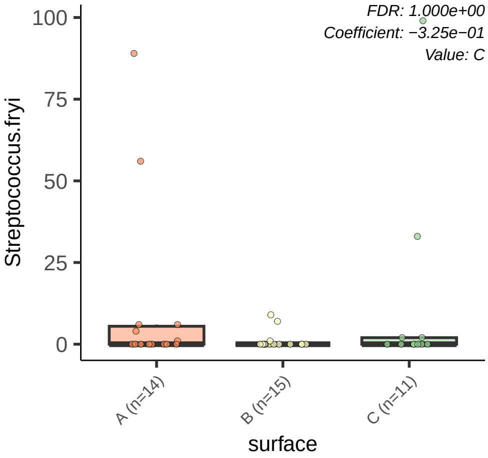

FDR: 1.000e+00
Coefficient: −3.25e−01
Value: C
Streptococcus.fryi
100
75
50
25
0
A (n=14)
B (n=15)
C (n=11)
surface

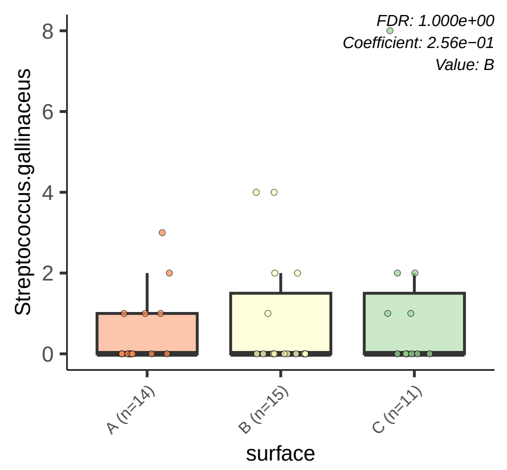
FDR: 1.000e+00
Coefficient: 2.56e−01
Value: B
Streptococcus.gallinaceus
0
2
4
6
8
A (n=14)
B (n=15)
C (n=11)
surface

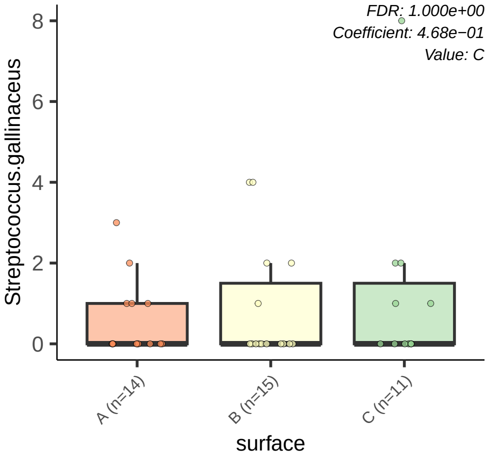
FDR: 1.000e+00
Coefficient: 4.68e−01
Value: C
Streptococcus.gallinaceus
0
2
4
6
8
A (n=14)
B (n=15)
C (n=11)
surface

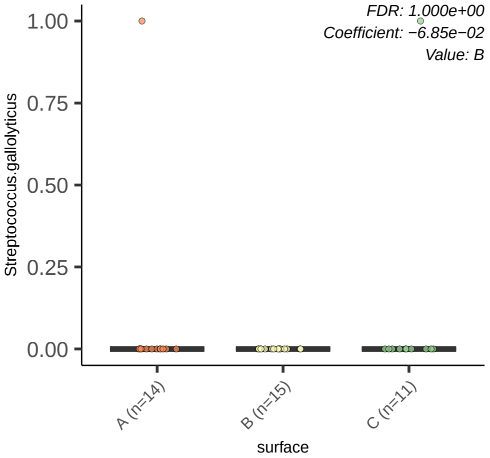
FDR: 1.000e+00
Coefficient: −6.85e−02
Value: B
Streptococcus.gallolyticus
1.00
0.75
0.50
0.25
0.00
A (n=14)
B (n=15)
C (n=11)
surface

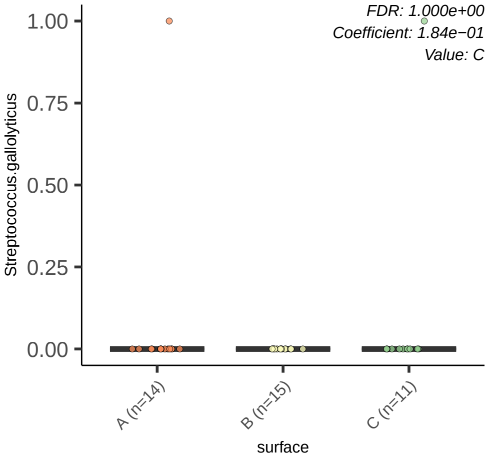

FDR: 1.000e+00
Coefficient: 1.84e−01
Value: C
Streptococcus.gallolyticus
1.00
0.75
0.50
0.25
0.00
A (n=14)
B (n=15)
C (n=11)
surface

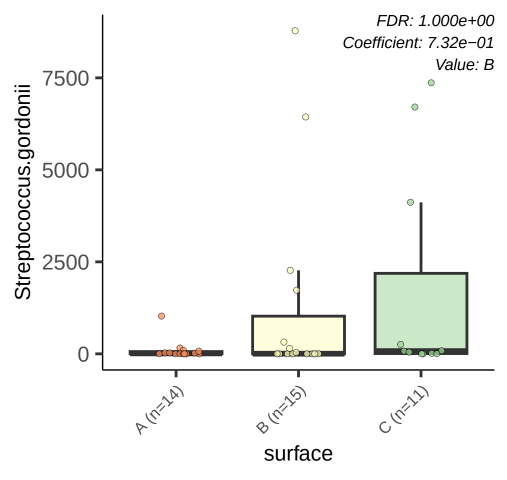
FDR: 1.000e+00
Coefficient: 7.32e−01
Value: B
Streptococcus.gordonii
7500
5000
2500
0
A (n=14)
B (n=15)
C (n=11)
surface

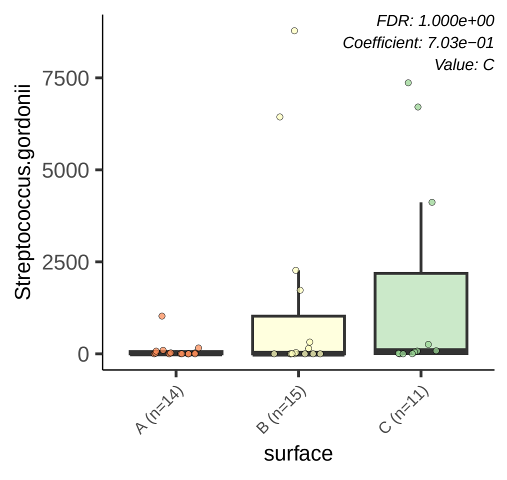
FDR: 1.000e+00
Coefficient: 7.03e−01
Value: C
Streptococcus.gordonii
7500
5000
2500
0
A (n=14)
B (n=15)
C (n=11)
surface

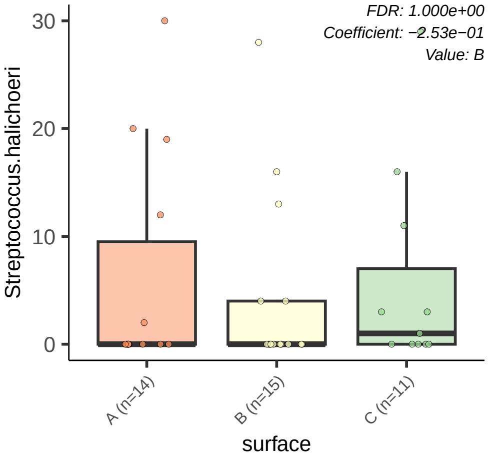

FDR: 1.000e+00
Coefficient: −2.53e−01
Value: B
Streptococcus.halichoeri
30
20
10
0
A (n=14)
B (n=15)
C (n=11)
surface

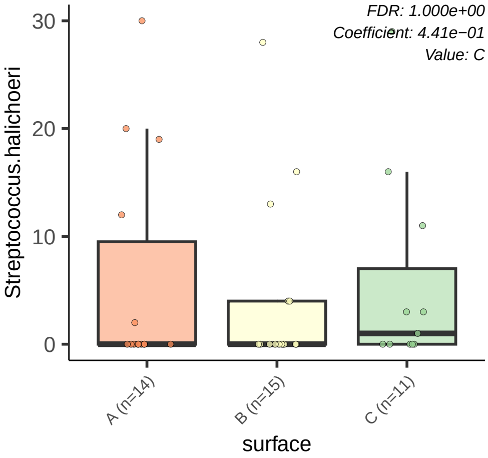

FDR: 1.000e+00
Coefficient: 4.41e−01
Value: C
Streptococcus.halichoeri
0
10
20
30
A (n=14)
B (n=15)
C (n=11)
surface

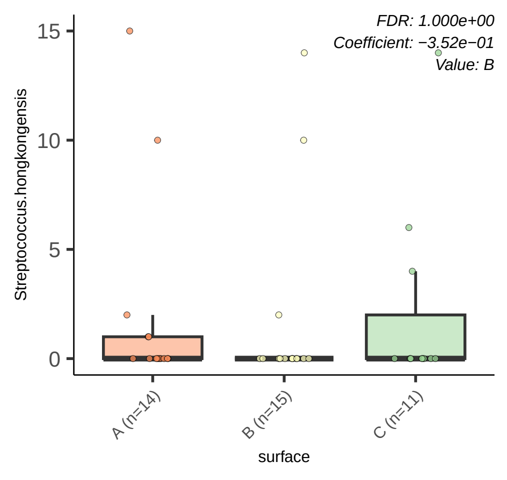

FDR: 1.000e+00
Coefficient: −3.52e−01
Value: B
Streptococcus.hongkongensis
15
10
5
0
A (n=14)
B (n=15)
C (n=11)
surface

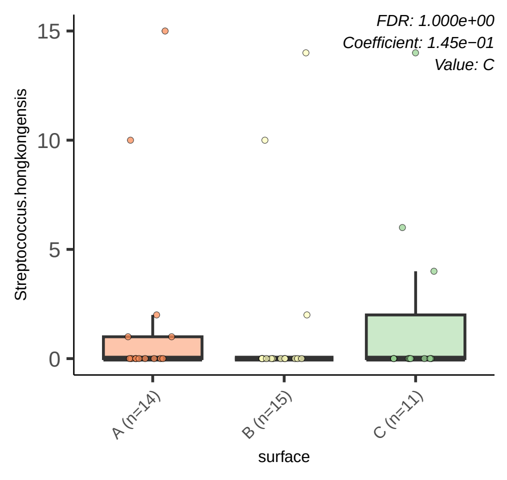
FDR: 1.000e+00
Coefficient: 1.45e−01
Value: C
Streptococcus.hongkongensis
15
10
5
0
A (n=14)
B (n=15)
C (n=11)
surface

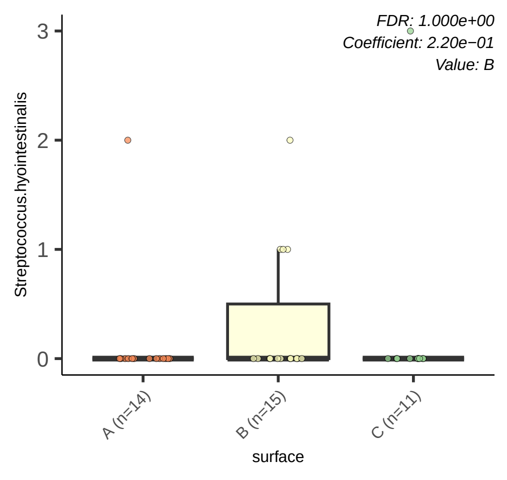

FDR: 1.000e+00
Coefficient: 2.20e−01
Value: B
Streptococcus.hyointestinalis
3
2
1
0
A (n=14)
B (n=15)
C (n=11)
surface

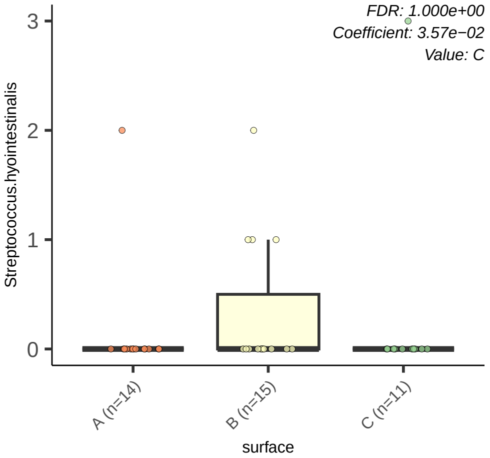

FDR: 1.000e+00
Coefficient: 3.57e−02
Value: C
Streptococcus.hyointestinalis
0
1
2
3
A (n=14)
B (n=15)
C (n=11)
surface

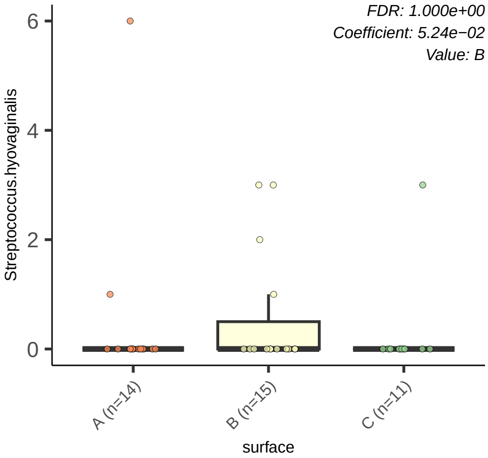

FDR: 1.000e+00
Coefficient: 5.24e−02
Value: B
Streptococcus.hyovaginalis
0
2
4
6
A (n=14)
B (n=15)
C (n=11)
surface

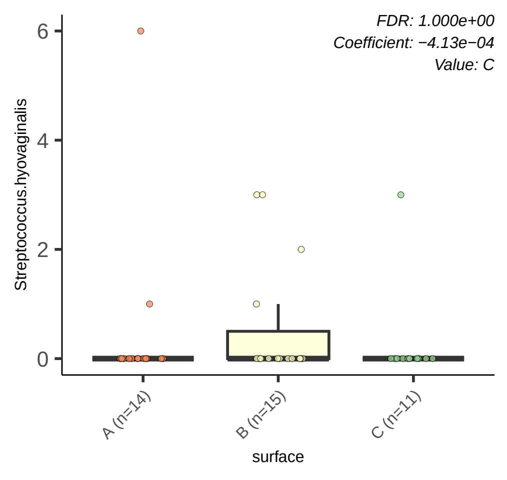
FDR: 1.000e+00
Coefficient: −4.13e−04
Value: C
Streptococcus.hyovaginalis
0
2
4
6
A (n=14)
B (n=15)
C (n=11)
surface

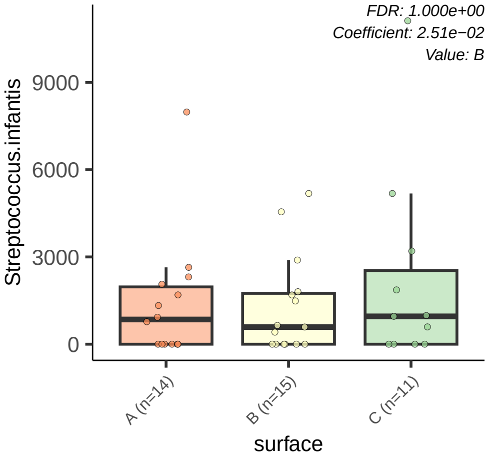

FDR: 1.000e+00
Coefficient: 2.51e−02
Value: B
Streptococcus.infantis
9000
6000
3000
0
A (n=14)
B (n=15)
C (n=11)
surface

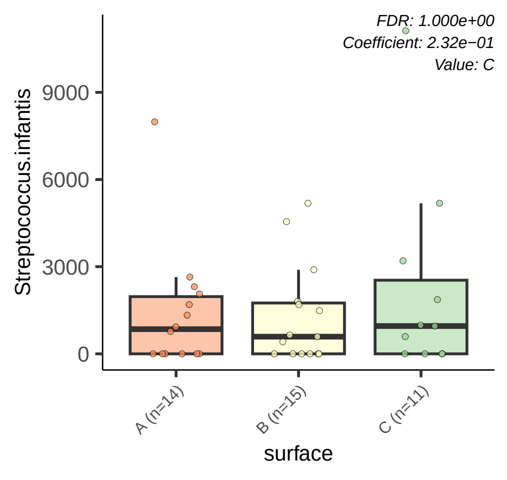
FDR: 1.000e+00
Coefficient: 2.32e−01
Value: C
Streptococcus.infantis
9000
6000
3000
0
A (n=14)
B (n=15)
C (n=11)
surface

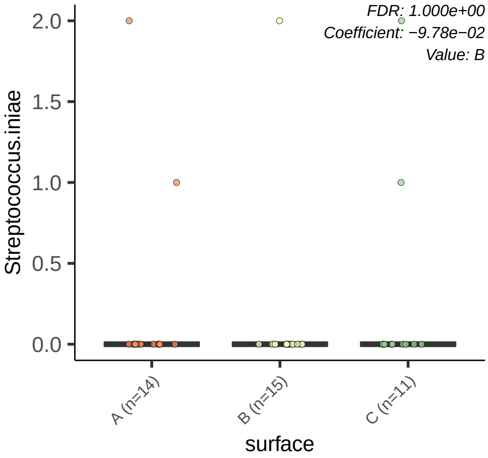
FDR: 1.000e+00
Coefficient: −9.78e−02
Value: B
Streptococcus.iniae
2.0
1.5
1.0
0.5
0.0
A (n=14)
B (n=15)
C (n=11)
surface

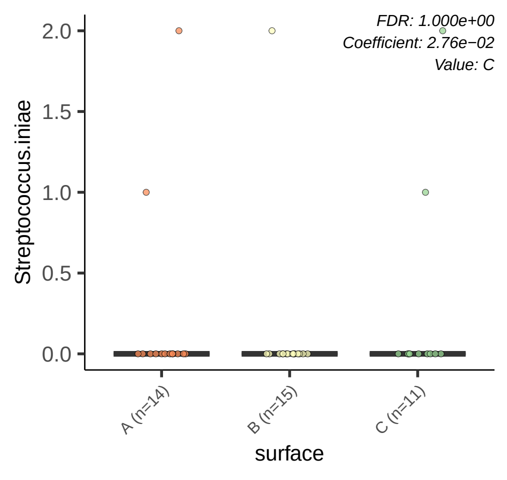
FDR: 1.000e+00
Coefficient: 2.76e−02
Value: C
Streptococcus.iniae
2.0
1.5
1.0
0.5
0.0
A (n=14)
B (n=15)
C (n=11)
surface

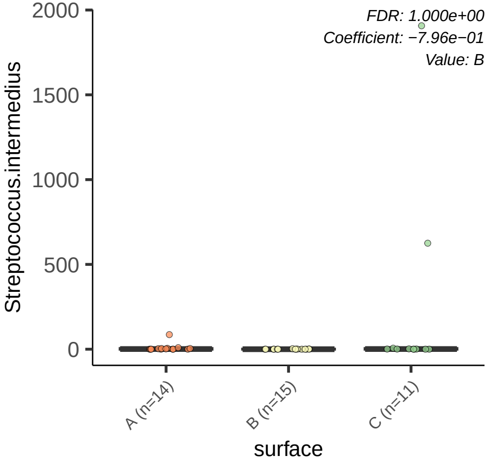
FDR: 1.000e+00
Coefficient: −7.96e−01
Value: B
2000
1500
1000
500
0
Streptococcus.intermedius
A (n=14)
B (n=15)
C (n=11)
surface

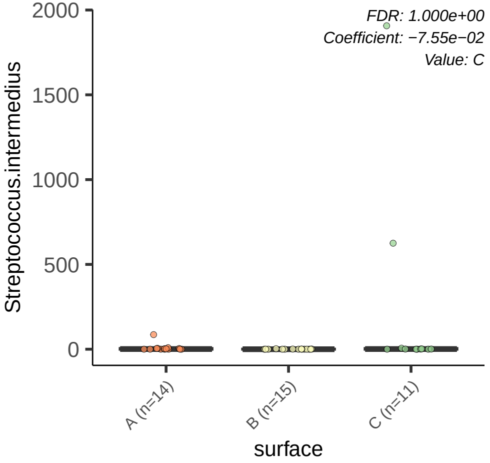
FDR: 1.000e+00
Coefficient: −7.55e−02
Value: C
Streptococcus.intermedius
2000
1500
1000
500
0
A (n=14)
B (n=15)
C (n=11)
surface

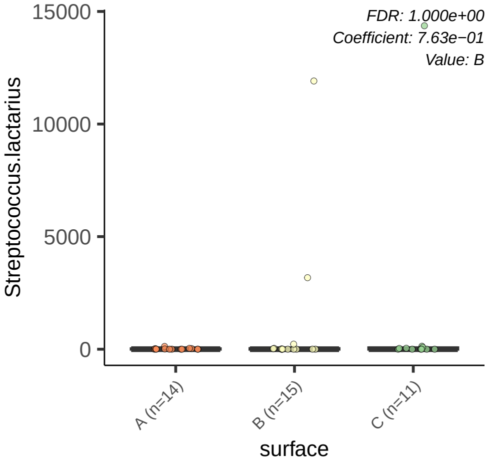
FDR: 1.000e+00
Coefficient: 7.63e−01
Value: B
Streptococcus.lactarius
15000
10000
5000
0
A (n=14)
B (n=15)
C (n=11)
surface

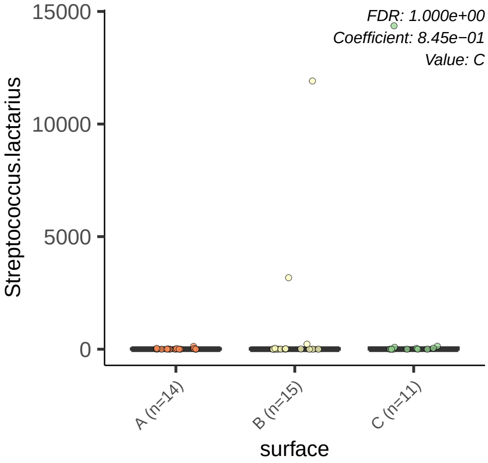
FDR: 1.000e+00
Coefficient: 8.45e−01
Value: C
15000
10000
5000
0
Streptococcus.lactarius
A (n=14)
B (n=15)
C (n=11)
surface

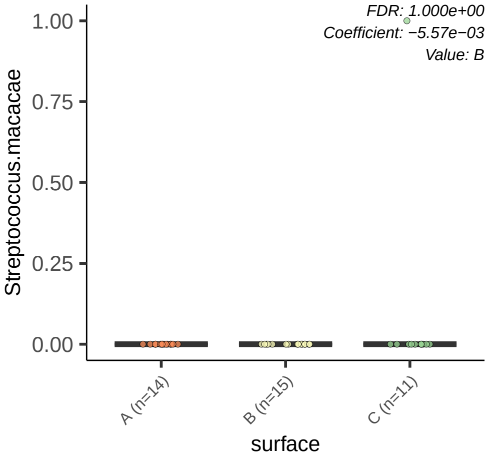
FDR: 1.000e+00
Coefficient: −5.57e−03
Value: B
Streptococcus.macacae
1.00
0.75
0.50
0.25
0.00
A (n=14)
B (n=15)
C (n=11)
surface

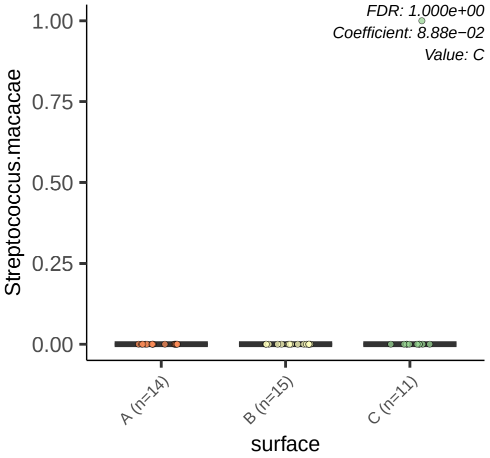
FDR: 1.000e+00
Coefficient: 8.88e−02
Value: C
Streptococcus.macacae
1.00
0.75
0.50
0.25
0.00
A (n=14)
B (n=15)
C (n=11)
surface

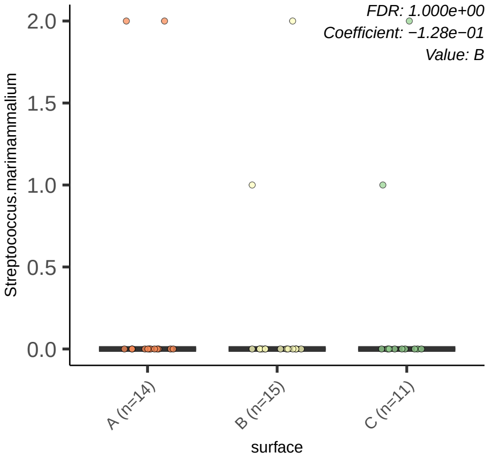
FDR: 1.000e+00
Coefficient: −1.28e−01
Value: B
Streptococcus.marimammalium
2.0
1.5
1.0
0.5
0.0
A (n=14)
B (n=15)
C (n=11)
surface

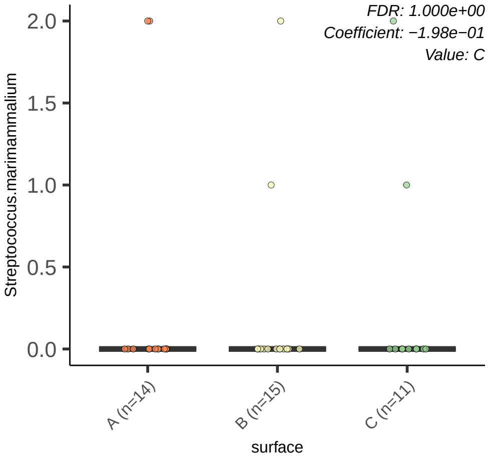
FDR: 1.000e+00
Coefficient: −1.98e−01
Value: C
Streptococcus.marimammalium
2.0
1.5
1.0
0.5
0.0
A (n=14)
B (n=15)
C (n=11)
surface

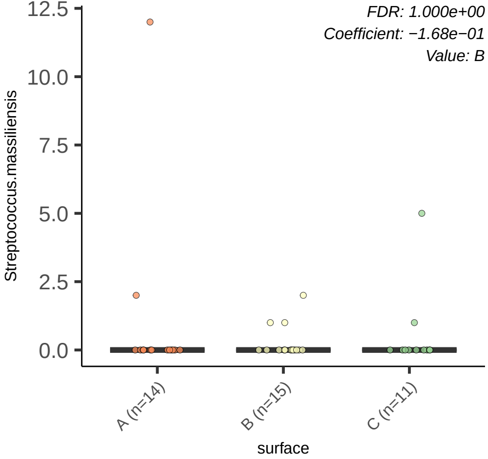

FDR: 1.000e+00
Coefficient: −1.68e−01
Value: B
Streptococcus.massiliensis
12.5
10.0
7.5
5.0
2.5
0.0
A (n=14)
B (n=15)
C (n=11)
surface

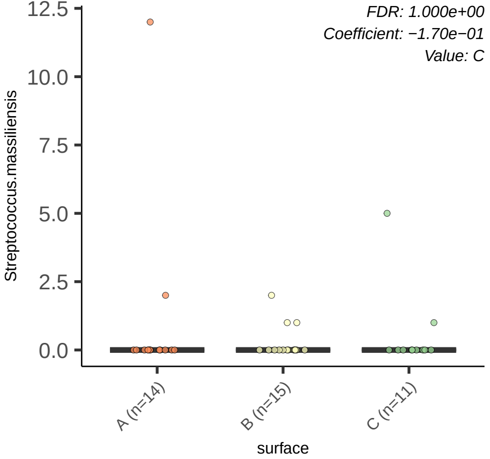
FDR: 1.000e+00
Coefficient: −1.70e−01
Value: C
12.5
10.0
7.5
5.0
2.5
0.0
Streptococcus.massiliensis
A (n=14)
B (n=15)
C (n=11)
surface

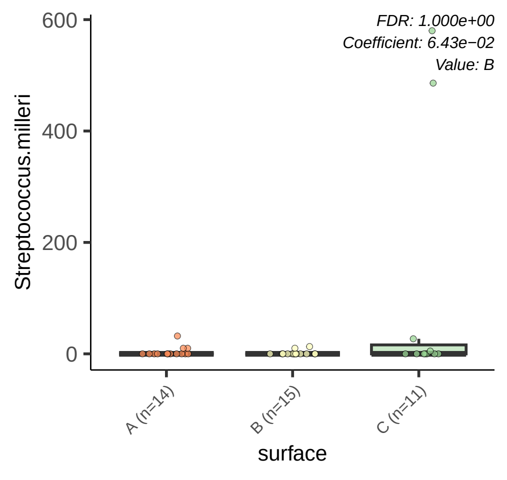

FDR: 1.000e+00
Coefficient: 6.43e−02
Value: B
Streptococcus.milleri
600
400
200
0
A (n=14)
B (n=15)
C (n=11)
surface

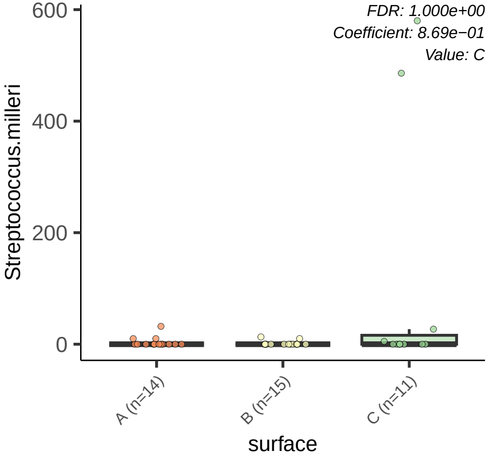

FDR: 1.000e+00
Coefficient: 8.69e−01
Value: C
Streptococcus.milleri
600
400
200
0
A (n=14)
B (n=15)
C (n=11)
surface

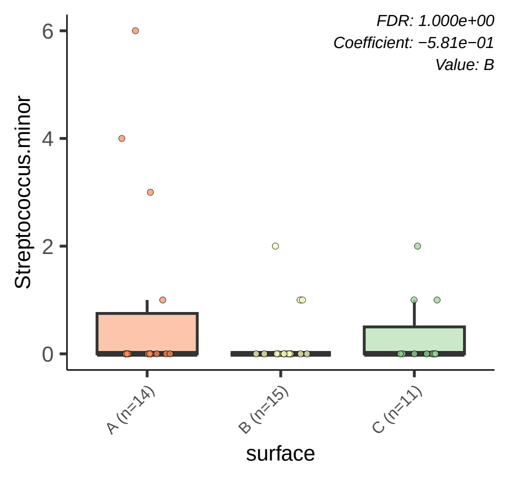
FDR: 1.000e+00
Coefficient: −5.81e−01
Value: B
Streptococcus.minor
0
2
4
6
A (n=14)
B (n=15)
C (n=11)
surface

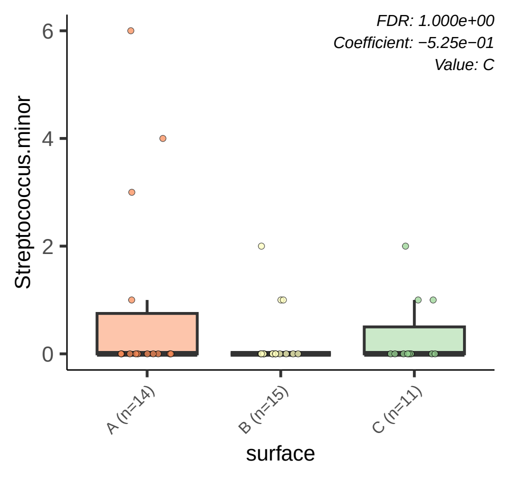

FDR: 1.000e+00
Coefficient: −5.25e−01
Value: C
Streptococcus.minor
0
2
4
6
A (n=14)
B (n=15)
C (n=11)
surface

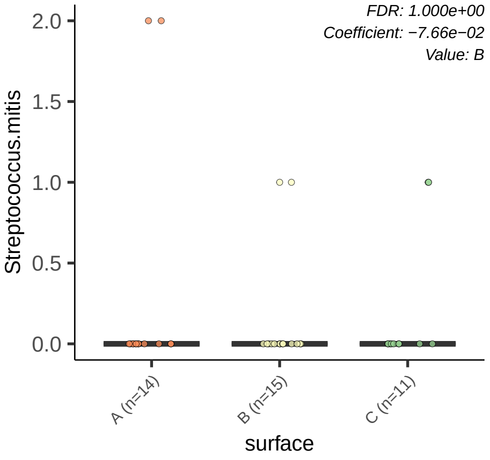
FDR: 1.000e+00
Coefficient: −7.66e−02
Value: B
Streptococcus.mitis
2.0
1.5
1.0
0.5
0.0
A (n=14)
B (n=15)
C (n=11)
surface

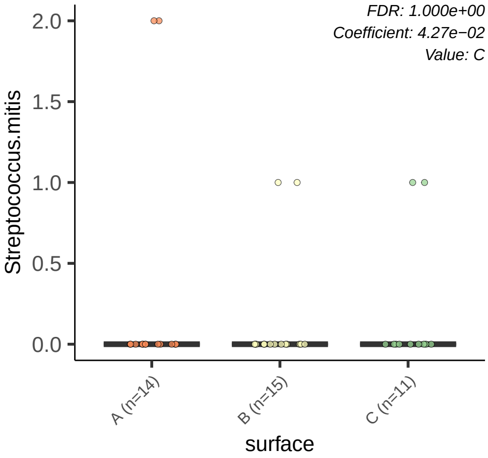
FDR: 1.000e+00
Coefficient: 4.27e−02
Value: C
Streptococcus.mitis
2.0
1.5
1.0
0.5
0.0
A (n=14)
B (n=15)
C (n=11)
surface

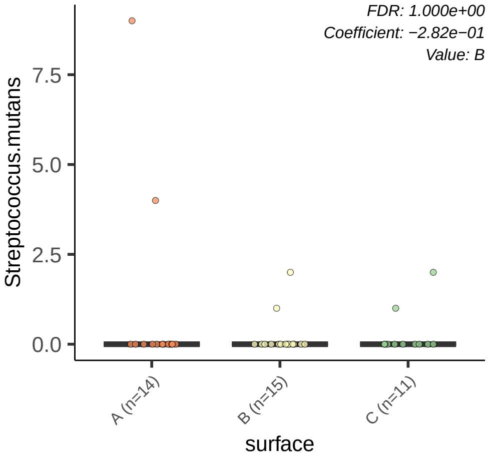
FDR: 1.000e+00
Coefficient: −2.82e−01
Value: B
Streptococcus.mutans
7.5
5.0
2.5
0.0
A (n=14)
B (n=15)
C (n=11)
surface

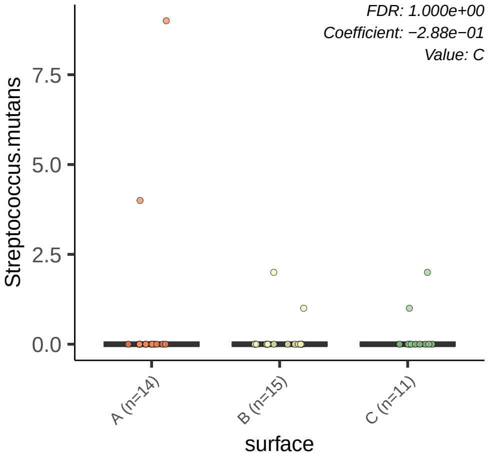

FDR: 1.000e+00
Coefficient: −2.88e−01
Value: C
Streptococcus.mutans
7.5
5.0
2.5
0.0
A (n=14)
B (n=15)
C (n=11)
surface

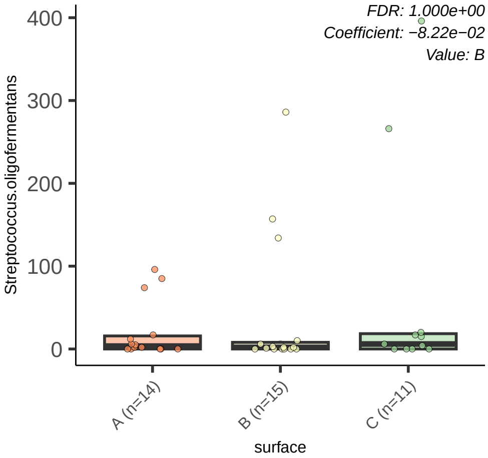

FDR: 1.000e+00
Coefficient: −8.22e−02
Value: B
Streptococcus.oligofermentans
400
300
200
100
0
A (n=14)
B (n=15)
C (n=11)
surface

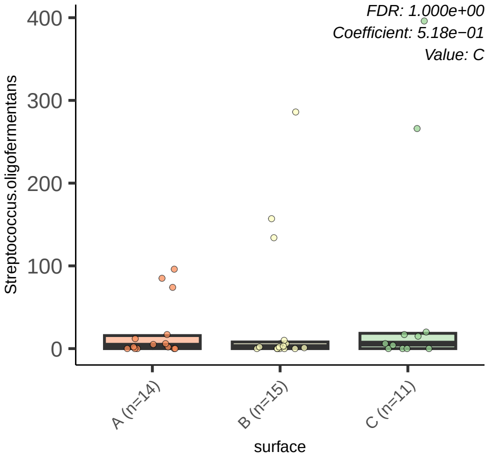
FDR: 1.000e+00
Coefficient: 5.18e−01
Value: C
Streptococcus.oligofermentans
400
300
200
100
0
A (n=14)
B (n=15)
C (n=11)
surface

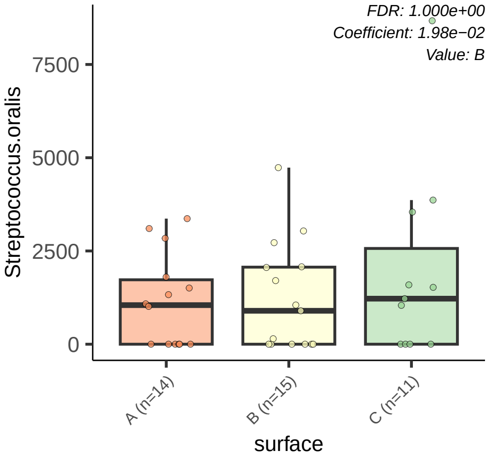

FDR: 1.000e+00
Coefficient: 1.98e−02
Value: B
Streptococcus.oralis
7500
5000
2500
0
A (n=14)
B (n=15)
C (n=11)
surface

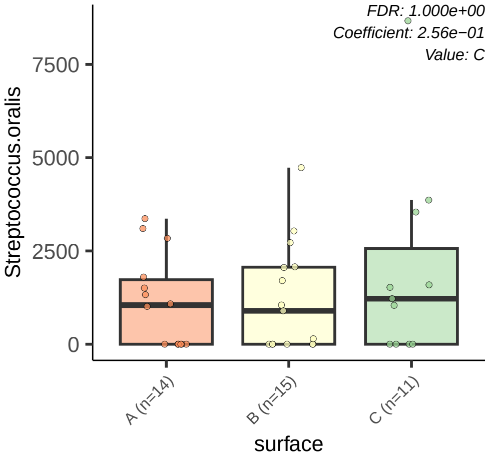

FDR: 1.000e+00
Coefficient: 2.56e−01
Value: C
Streptococcus.oralis
7500
5000
2500
0
A (n=14)
B (n=15)
C (n=11)
surface

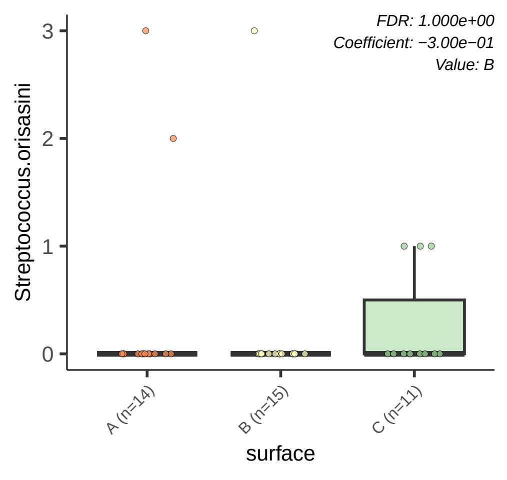

FDR: 1.000e+00
Coefficient: −3.00e−01
Value: B
Streptococcus.orisasini
3
2
1
0
A (n=14)
B (n=15)
C (n=11)
surface

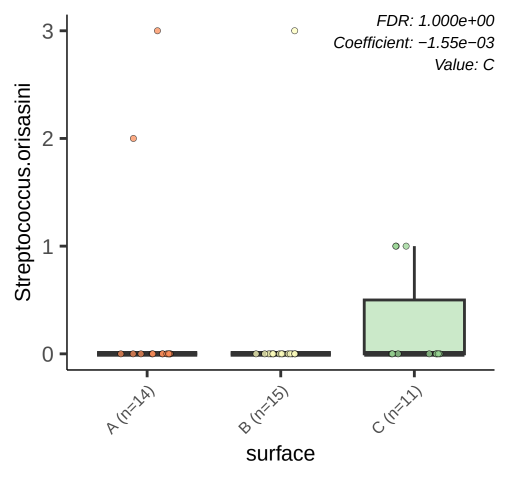

FDR: 1.000e+00
Coefficient: −1.55e−03
Value: C
Streptococcus.orisasini
3
2
1
0
A (n=14)
B (n=15)
C (n=11)
surface

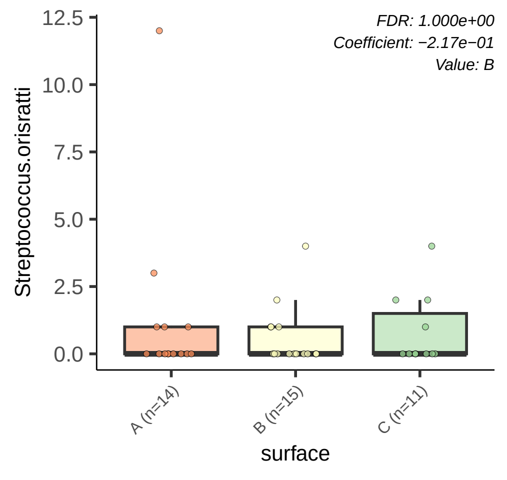

FDR: 1.000e+00
Coefficient: −2.17e−01
Value: B
Streptococcus.orisratti
12.5
10.0
7.5
5.0
2.5
0.0
A (n=14)
B (n=15)
C (n=11)
surface

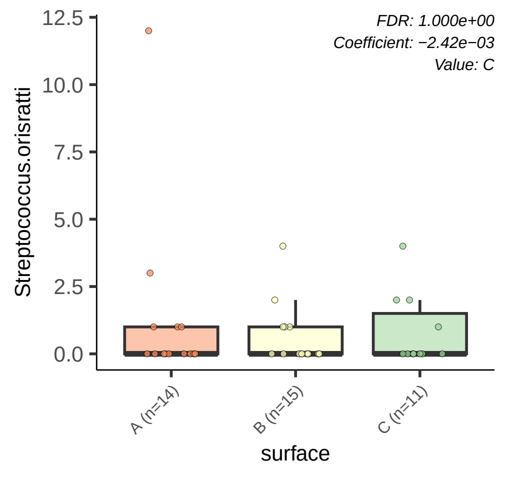

FDR: 1.000e+00
Coefficient: −2.42e−03
Value: C
Streptococcus.orisratti
12.5
10.0
7.5
5.0
2.5
0.0
A (n=14)
B (n=15)
C (n=11)
surface

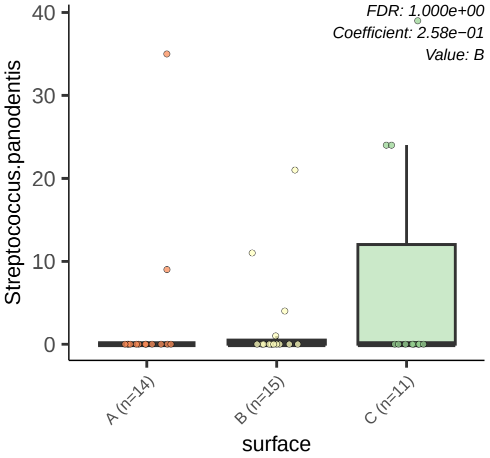
FDR: 1.000e+00
Coefficient: 2.58e−01
Value: B
Streptococcus.panodentis
40
30
20
10
0
A (n=14)
B (n=15)
C (n=11)
surface

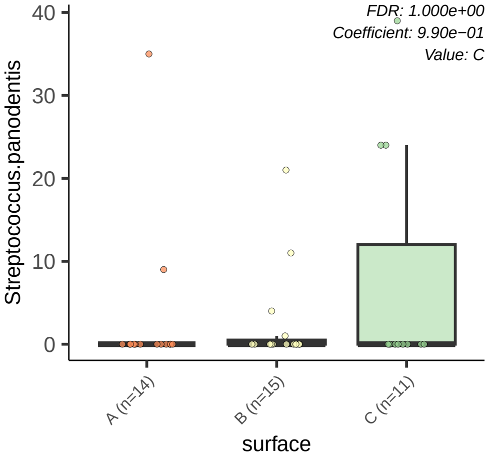
FDR: 1.000e+00
Coefficient: 9.90e−01
Value: C
Streptococcus.panodentis
40
30
20
10
0
A (n=14)
B (n=15)
C (n=11)
surface

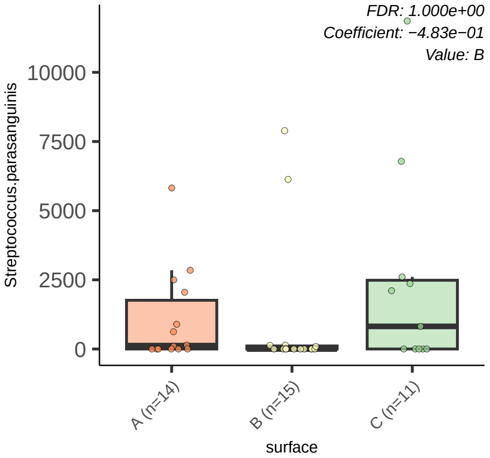
FDR: 1.000e+00
Coefficient: −4.83e−01
Value: B
Streptococcus.parasanguinis
10000
7500
5000
2500
0
A (n=14)
B (n=15)
C (n=11)
surface

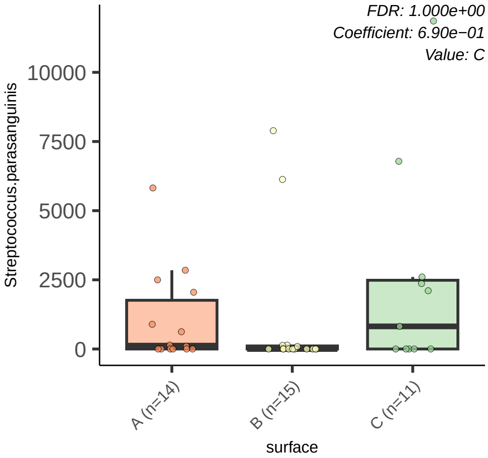

FDR: 1.000e+00
Coefficient: 6.90e−01
Value: C
10000
7500
5000
2500
0
Streptococcus.parasanguinis
A (n=14)
B (n=15)
C (n=11)
surface

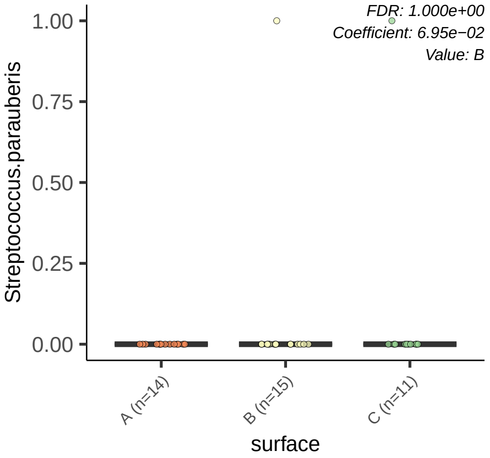

FDR: 1.000e+00
Coefficient: 6.95e−02
Value: B
Streptococcus.parauberis
1.00
0.75
0.50
0.25
0.00
A (n=14)
B (n=15)
C (n=11)
surface

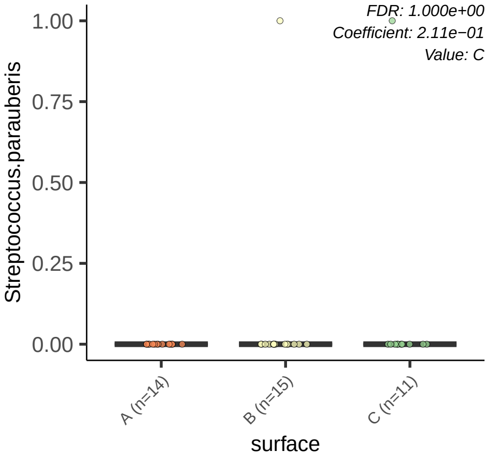

FDR: 1.000e+00
Coefficient: 2.11e−01
Value: C
Streptococcus.parauberis
1.00
0.75
0.50
0.25
0.00
A (n=14)
B (n=15)
C (n=11)
surface

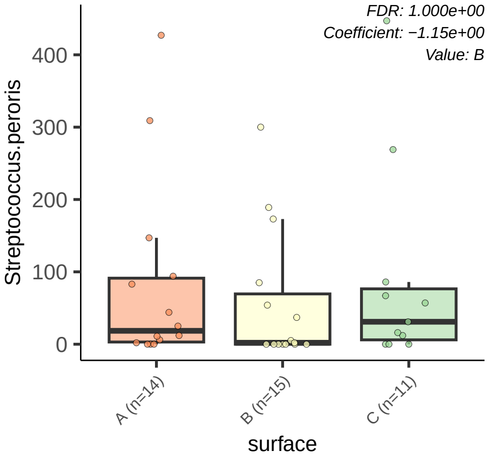
FDR: 1.000e+00
Coefficient: −1.15e+00
Value: B
Streptococcus.peroris
400
300
200
100
0
A (n=14)
B (n=15)
C (n=11)
surface

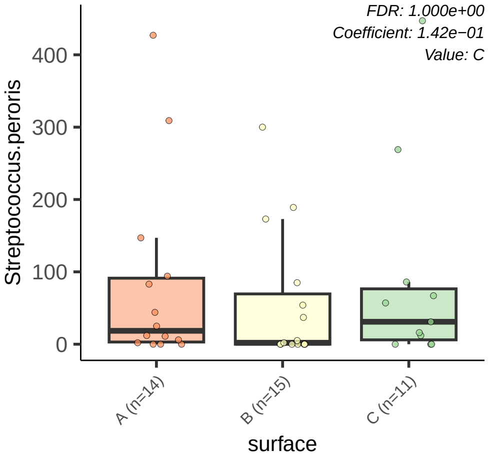

FDR: 1.000e+00
Coefficient: 1.42e−01
Value: C
Streptococcus.peroris
400
300
200
100
0
A (n=14)
B (n=15)
C (n=11)
surface

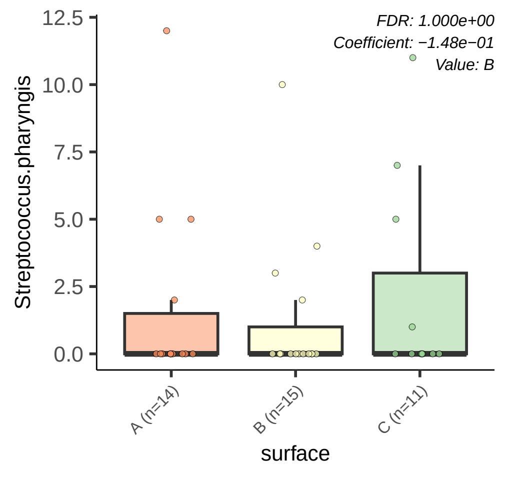
FDR: 1.000e+00
Coefficient: −1.48e−01
Value: B
Streptococcus.pharyngis
12.5
10.0
7.5
5.0
2.5
0.0
A (n=14)
B (n=15)
C (n=11)
surface

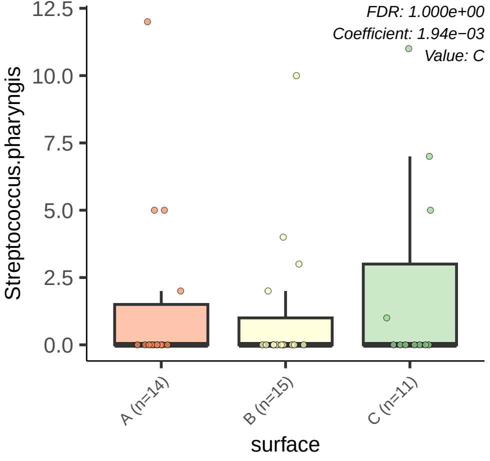
FDR: 1.000e+00
Coefficient: 1.94e−03
Value: C
Streptococcus.pharyngis
12.5
10.0
7.5
5.0
2.5
0.0
A (n=14)
B (n=15)
C (n=11)
surface

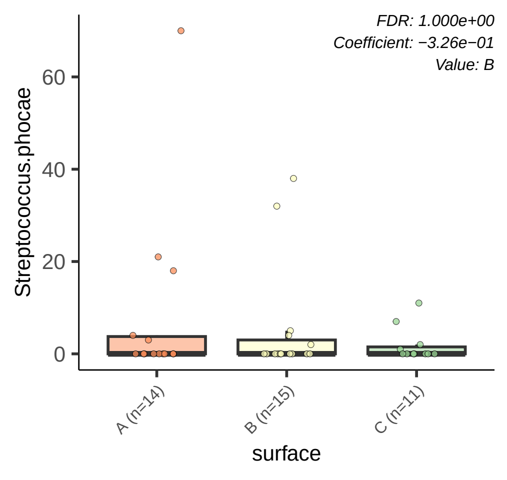
FDR: 1.000e+00
Coefficient: −3.26e−01
Value: B
Streptococcus.phocae
60
40
20
0
A (n=14)
B (n=15)
C (n=11)
surface

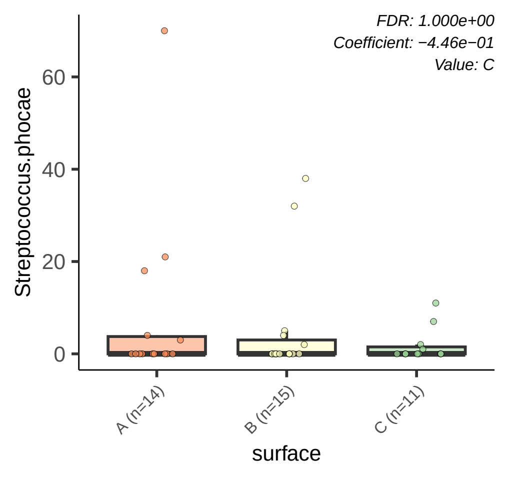
FDR: 1.000e+00
Coefficient: −4.46e−01
Value: C
Streptococcus.phocae
60
40
20
0
A (n=14)
B (n=15)
C (n=11)
surface

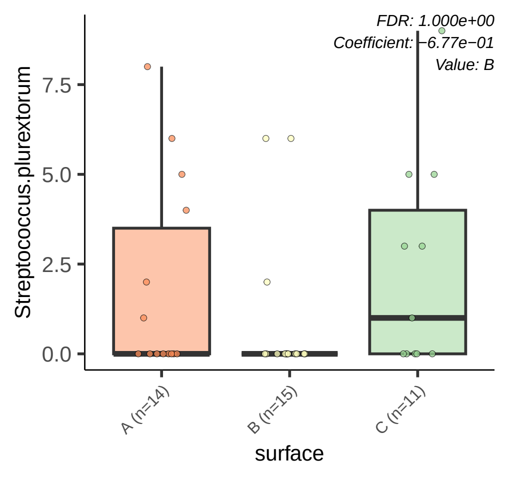
FDR: 1.000e+00
Coefficient: −6.77e−01
Value: B
Streptococcus.plurextorum
7.5
5.0
2.5
0.0
A (n=14)
B (n=15)
C (n=11)
surface

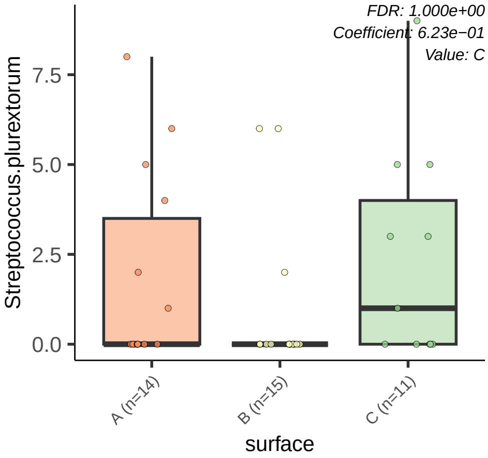
FDR: 1.000e+00
Coefficient: 6.23e−01
Value: C
Streptococcus.plurextorum
7.5
5.0
2.5
0.0
A (n=14)
B (n=15)
C (n=11)
surface

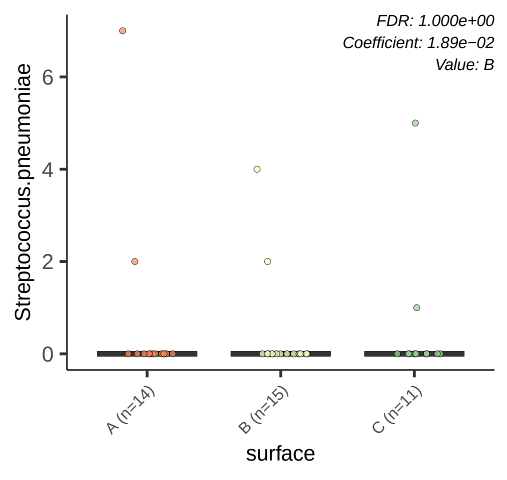
FDR: 1.000e+00
Coefficient: 1.89e−02
Value: B
Streptococcus.pneumoniae
0
2
4
6
A (n=14)
B (n=15)
C (n=11)
surface

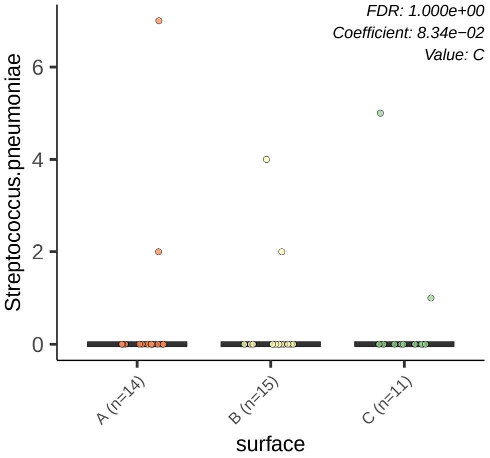
FDR: 1.000e+00
Coefficient: 8.34e−02
Value: C
Streptococcus.pneumoniae
0
2
4
6
A (n=14)
B (n=15)
C (n=11)
surface

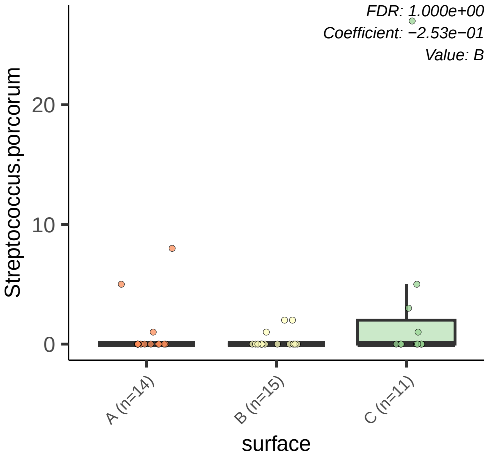

FDR: 1.000e+00
Coefficient: −2.53e−01
Value: B
Streptococcus.porcorum
20
10
0
A (n=14)
B (n=15)
C (n=11)
surface

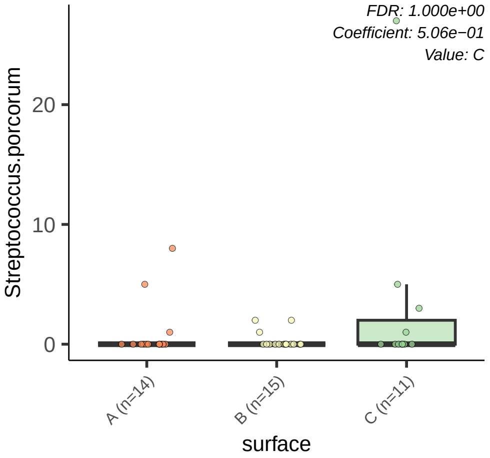
FDR: 1.000e+00
Coefficient: 5.06e−01
Value: C
Streptococcus.porcorum
20
10
0
A (n=14)
B (n=15)
C (n=11)
surface

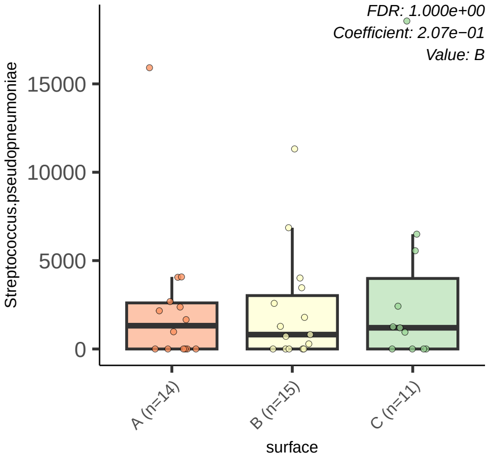

FDR: 1.000e+00
Coefficient: 2.07e−01
Value: B
Streptococcus.pseudopneumoniae
15000
10000
5000
0
A (n=14)
B (n=15)
C (n=11)
surface

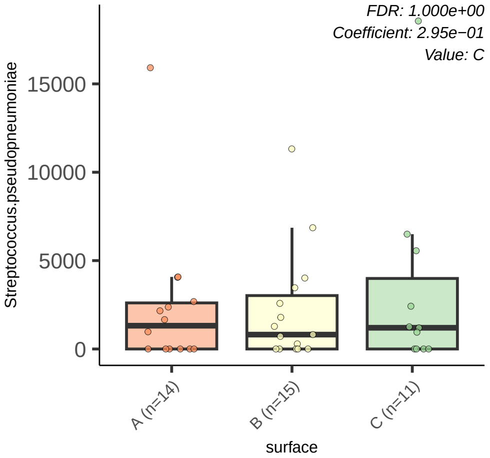
FDR: 1.000e+00
Coefficient: 2.95e−01
Value: C
Streptococcus.pseudopneumoniae
15000
10000
5000
0
A (n=14)
B (n=15)
C (n=11)
surface

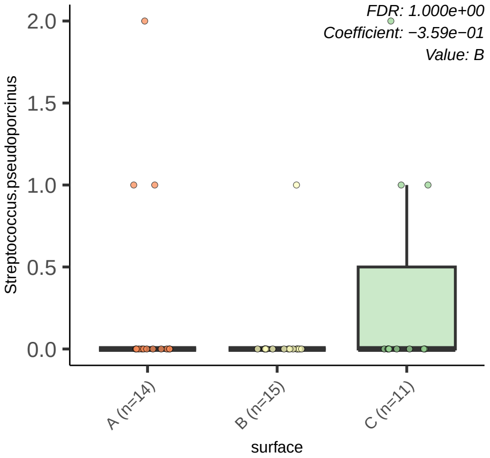

FDR: 1.000e+00
Coefficient: −3.59e−01
Value: B
Streptococcus.pseudoporcinus
2.0
1.5
1.0
0.5
0.0
A (n=14)
B (n=15)
C (n=11)
surface

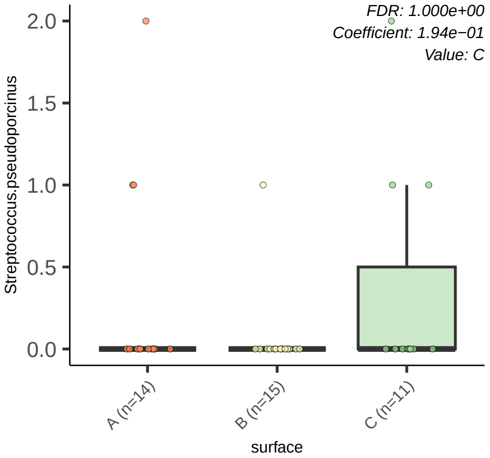
FDR: 1.000e+00
Coefficient: 1.94e−01
Value: C
Streptococcus.pseudoporcinus
2.0
1.5
1.0
0.5
0.0
A (n=14)
B (n=15)
C (n=11)
surface

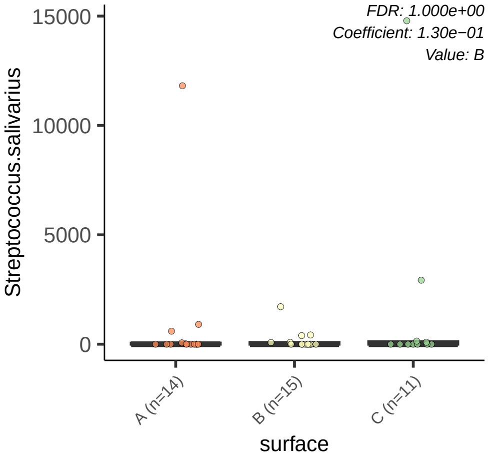

FDR: 1.000e+00
Coefficient: 1.30e−01
Value: B
15000
10000
5000
0
Streptococcus.salivarius
A (n=14)
B (n=15)
C (n=11)
surface

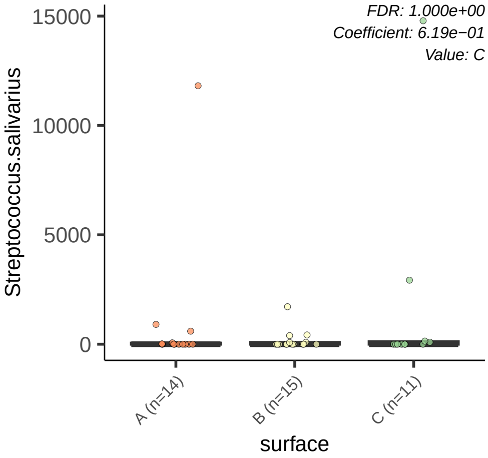
FDR: 1.000e+00
Coefficient: 6.19e−01
Value: C
15000
10000
5000
0
Streptococcus.salivarius
A (n=14)
B (n=15)
C (n=11)
surface

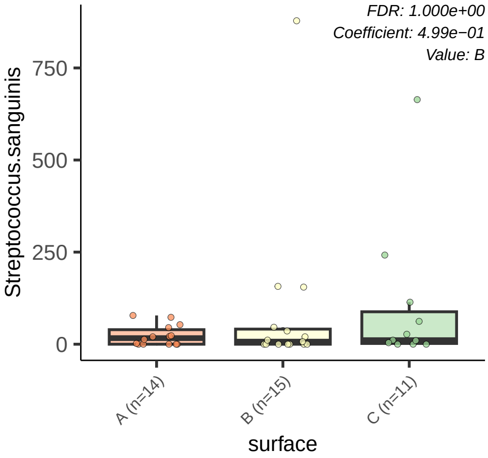
FDR: 1.000e+00
Coefficient: 4.99e−01
Value: B
Streptococcus.sanguinis
750
500
250
0
A (n=14)
B (n=15)
C (n=11)
surface

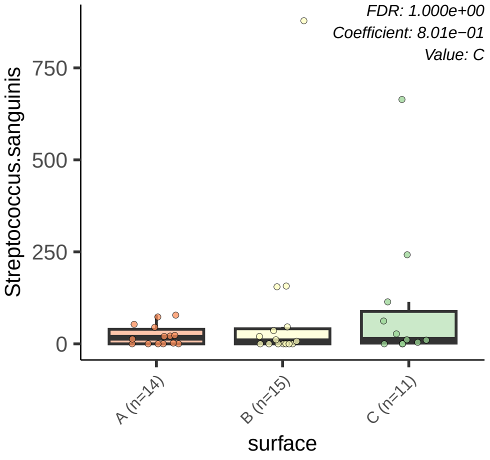
FDR: 1.000e+00
Coefficient: 8.01e−01
Value: C
Streptococcus.sanguinis
750
500
250
0
A (n=14)
B (n=15)
C (n=11)
surface

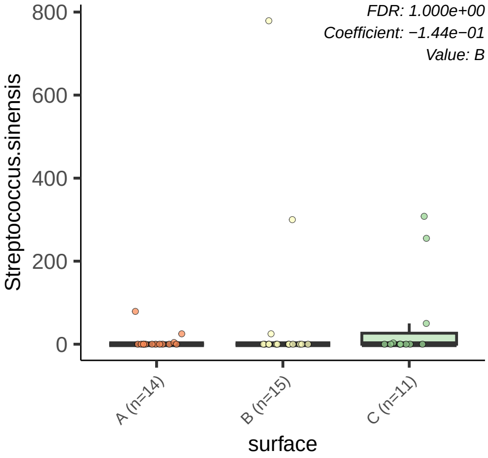

FDR: 1.000e+00
Coefficient: −1.44e−01
Value: B
Streptococcus.sinensis
800
600
400
200
0
A (n=14)
B (n=15)
C (n=11)
surface

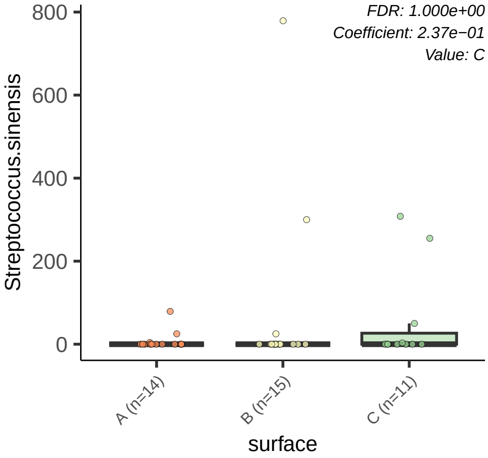

FDR: 1.000e+00
Coefficient: 2.37e−01
Value: C
Streptococcus.sinensis
800
600
400
200
0
A (n=14)
B (n=15)
C (n=11)
surface

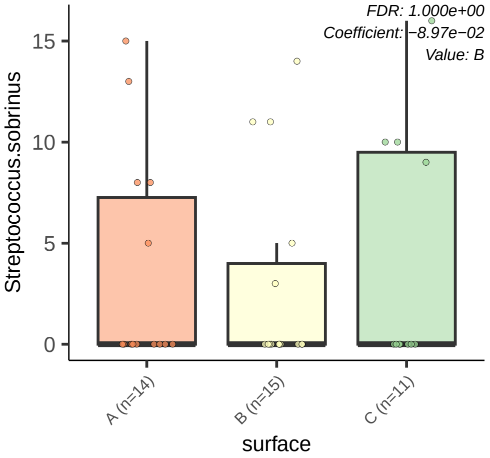
FDR: 1.000e+00
Coefficient: −8.97e−02
Value: B
Streptococcus.sobrinus
0
5
10
15
A (n=14)
B (n=15)
C (n=11)
surface

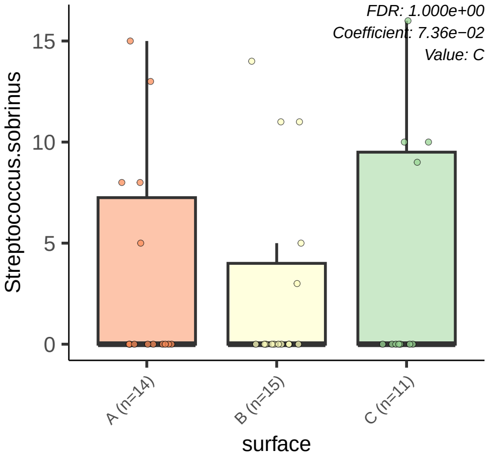
FDR: 1.000e+00
Coefficient: 7.36e−02
Value: C
Streptococcus.sobrinus
15
10
5
0
A (n=14)
B (n=15)
C (n=11)
surface

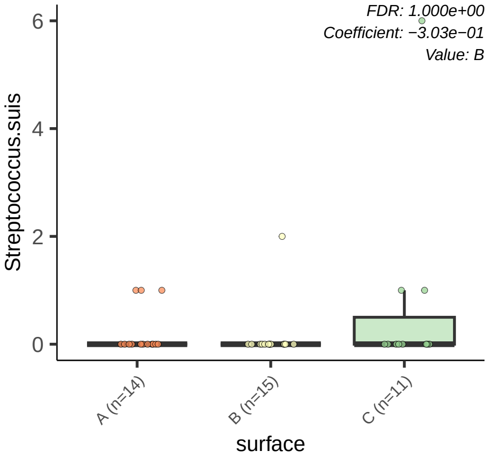
FDR: 1.000e+00
Coefficient: −3.03e−01
Value: B
Streptococcus.suis
6
4
2
0
A (n=14)
B (n=15)
C (n=11)
surface

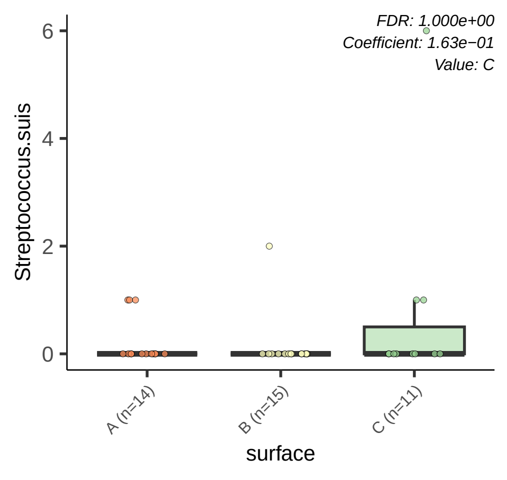

FDR: 1.000e+00
Coefficient: 1.63e−01
Value: C
Streptococcus.suis
6
4
2
0
A (n=14)
B (n=15)
C (n=11)
surface

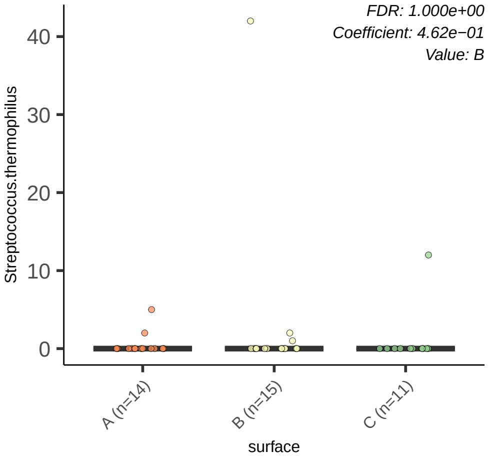
FDR: 1.000e+00
Coefficient: 4.62e−01
Value: B
Streptococcus.thermophilus
40
30
20
10
0
A (n=14)
B (n=15)
C (n=11)
surface

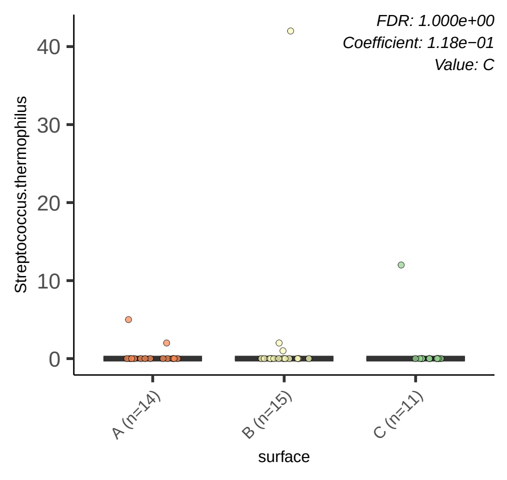
FDR: 1.000e+00
Coefficient: 1.18e−01
Value: C
Streptococcus.thermophilus
40
30
20
10
0
A (n=14)
B (n=15)
C (n=11)
surface

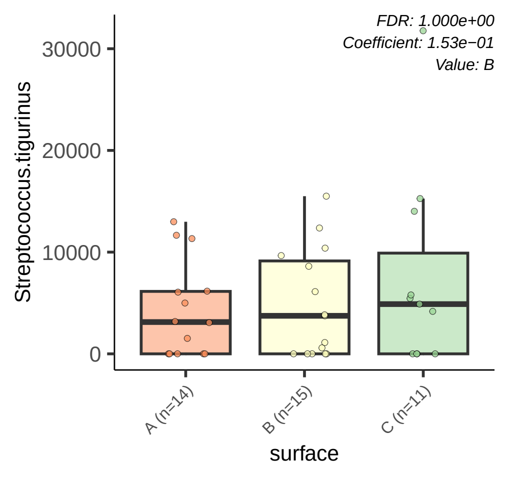

FDR: 1.000e+00
Coefficient: 1.53e−01
Value: B
Streptococcus.tigurinus
30000
20000
10000
0
A (n=14)
B (n=15)
C (n=11)
surface

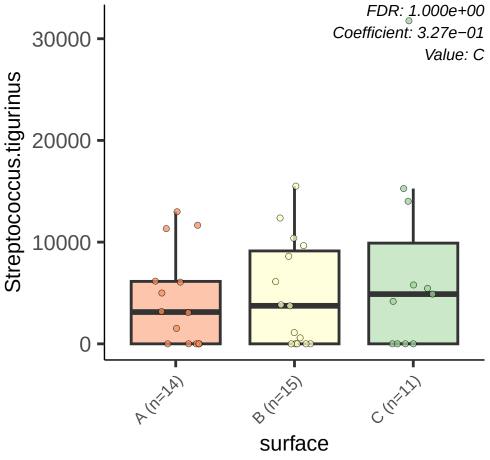

FDR: 1.000e+00
Coefficient: 3.27e−01
Value: C
30000
20000
10000
0
Streptococcus.tigurinus
A (n=14)
B (n=15)
C (n=11)
surface

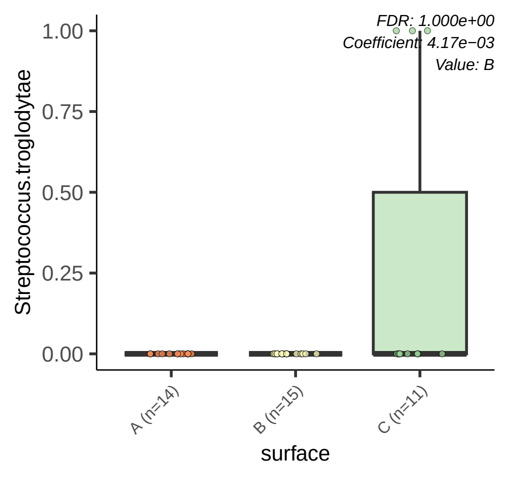

FDR: 1.000e+00
Coefficient: 4.17e−03
Value: B
Streptococcus.troglodytae
1.00
0.75
0.50
0.25
0.00
A (n=14)
B (n=15)
C (n=11)
surface

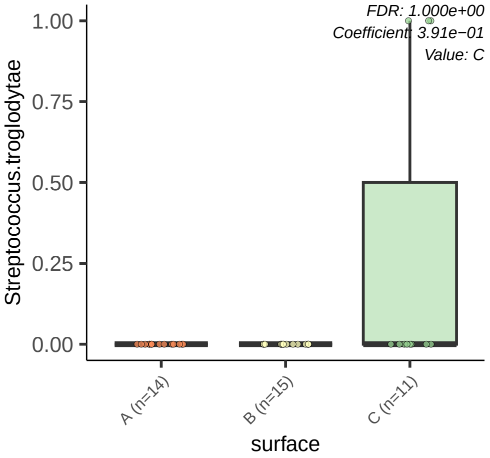
FDR: 1.000e+00
Coefficient: 3.91e−01
Value: C
Streptococcus.troglodytae
1.00
0.75
0.50
0.25
0.00
A (n=14)
B (n=15)
C (n=11)
surface

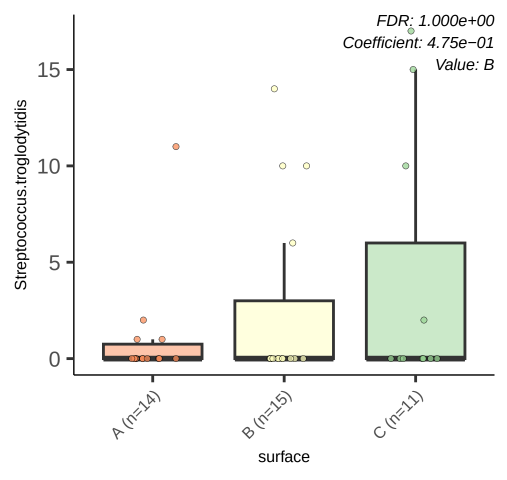
FDR: 1.000e+00
Coefficient: 4.75e−01
Value: B
Streptococcus.troglodytidis
0
5
10
15
A (n=14)
B (n=15)
C (n=11)
surface

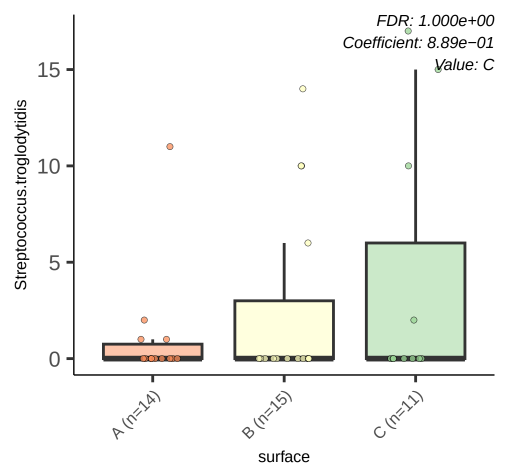
FDR: 1.000e+00
Coefficient: 8.89e−01
Value: C
Streptococcus.troglodytidis
15
10
5
0
A (n=14)
B (n=15)
C (n=11)
surface

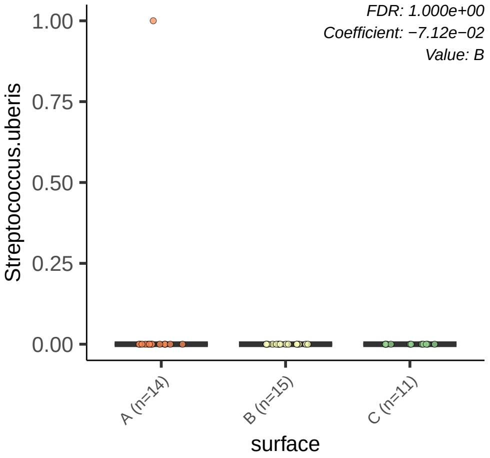

FDR: 1.000e+00
Coefficient: −7.12e−02
Value: B
Streptococcus.uberis
1.00
0.75
0.50
0.25
0.00
A (n=14)
B (n=15)
C (n=11)
surface

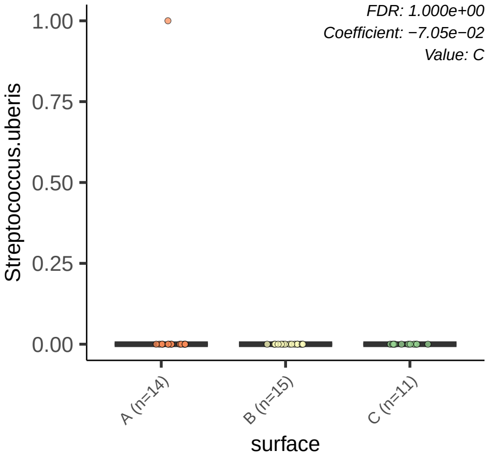
FDR: 1.000e+00
Coefficient: −7.05e−02
Value: C
Streptococcus.uberis
1.00
0.75
0.50
0.25
0.00
A (n=14)
B (n=15)
C (n=11)
surface

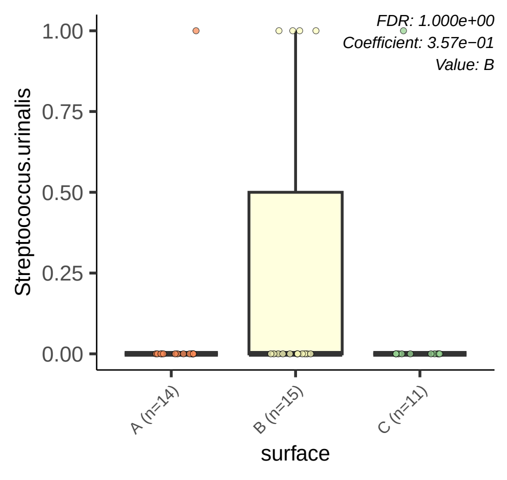
FDR: 1.000e+00
Coefficient: 3.57e−01
Value: B
Streptococcus.urinalis
1.00
0.75
0.50
0.25
0.00
A (n=14)
B (n=15)
C (n=11)
surface

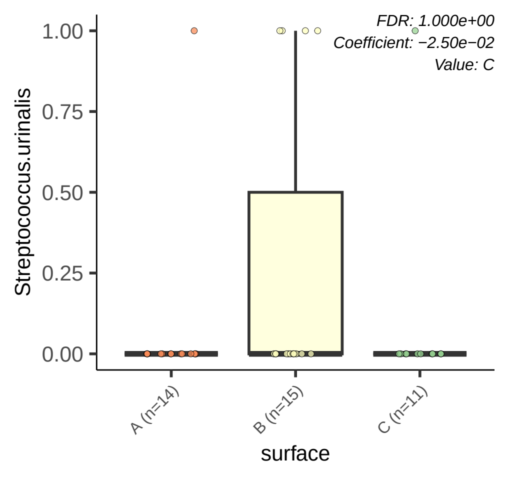
FDR: 1.000e+00
Coefficient: −2.50e−02
Value: C
Streptococcus.urinalis
1.00
0.75
0.50
0.25
0.00
A (n=14)
B (n=15)
C (n=11)
surface

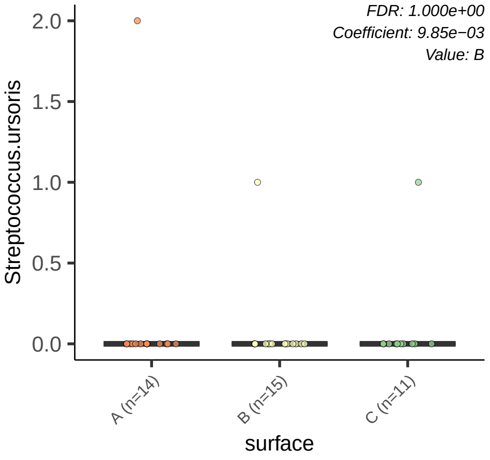
FDR: 1.000e+00
Coefficient: 9.85e−03
Value: B
Streptococcus.ursoris
2.0
1.5
1.0
0.5
0.0
A (n=14)
B (n=15)
C (n=11)
surface

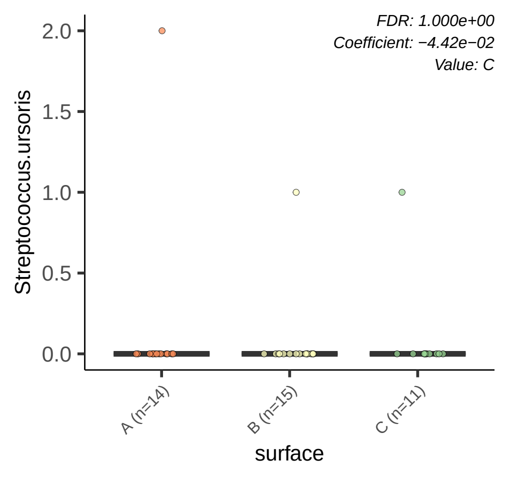
FDR: 1.000e+00
Coefficient: −4.42e−02
Value: C
Streptococcus.ursoris
2.0
1.5
1.0
0.5
0.0
A (n=14)
B (n=15)
C (n=11)
surface

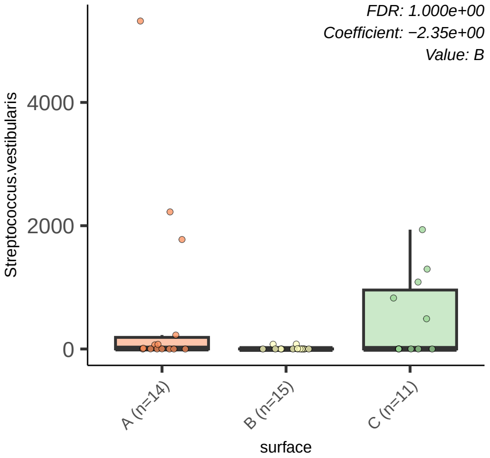

FDR: 1.000e+00
Coefficient: −2.35e+00
Value: B
Streptococcus.vestibularis
4000
2000
0
A (n=14)
B (n=15)
C (n=11)
surface

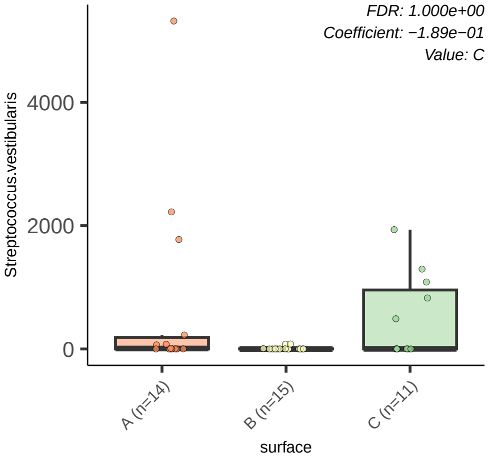

FDR: 1.000e+00
Coefficient: −1.89e−01
Value: C
Streptococcus.vestibularis
4000
2000
0
A (n=14)
B (n=15)
C (n=11)
surface

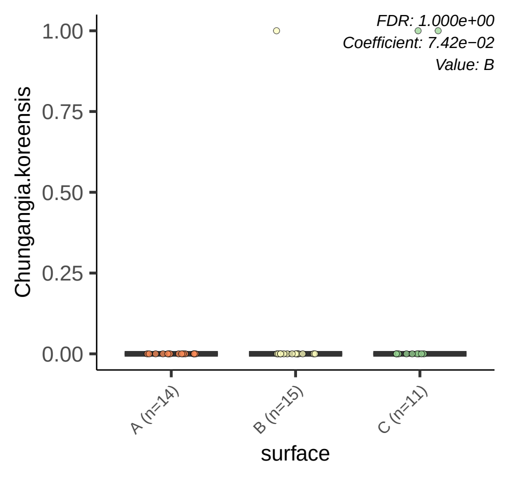

FDR: 1.000e+00
Coefficient: 7.42e−02
Value: B
Chungangia.koreensis
1.00
0.75
0.50
0.25
0.00
A (n=14)
B (n=15)
C (n=11)
surface

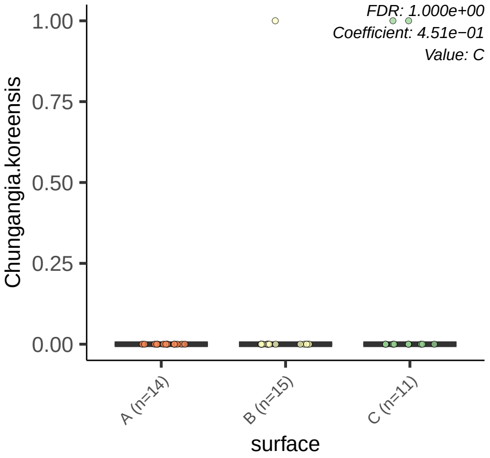
FDR: 1.000e+00
Coefficient: 4.51e−01
Value: C
Chungangia.koreensis
1.00
0.75
0.50
0.25
0.00
A (n=14)
B (n=15)
C (n=11)
surface

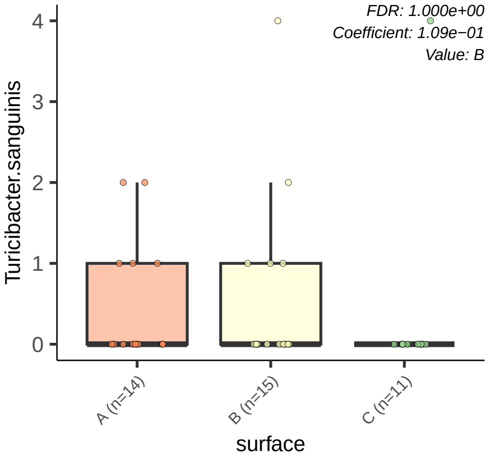
FDR: 1.000e+00
Coefficient: 1.09e−01
Value: B
Turicibacter.sanguinis
4
3
2
1
0
A (n=14)
B (n=15)
C (n=11)
surface

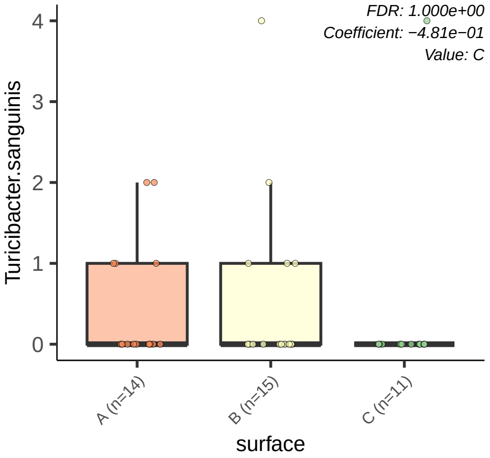
FDR: 1.000e+00
Coefficient: −4.81e−01
Value: C
Turicibacter.sanguinis
4
3
2
1
0
A (n=14)
B (n=15)
C (n=11)
surface

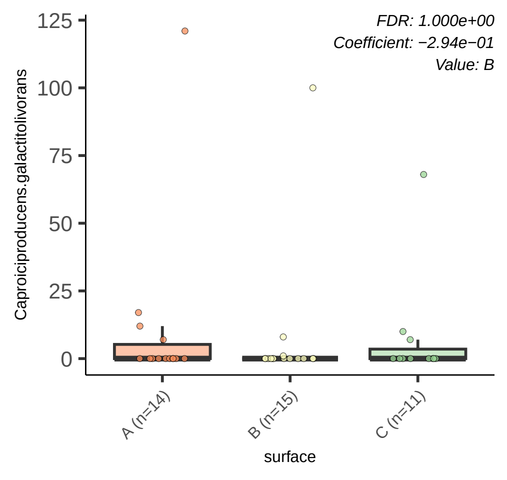
FDR: 1.000e+00
Coefficient: −2.94e−01
Value: B
Caproiciproducens.galactitolivorans
125
100
75
50
25
0
A (n=14)
B (n=15)
C (n=11)
surface

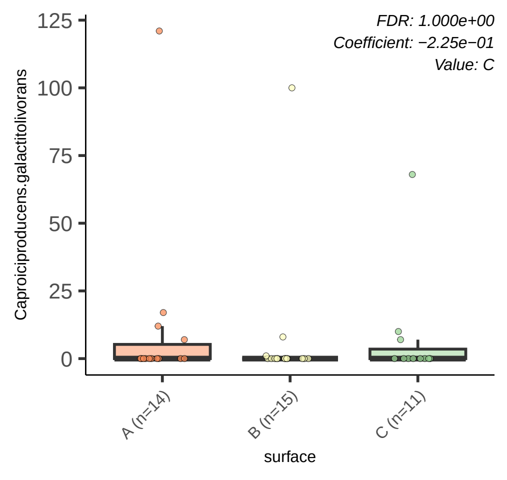
FDR: 1.000e+00
Coefficient: −2.25e−01
Value: C
Caproiciproducens.galactitolivorans
125
100
75
50
25
0
A (n=14)
B (n=15)
C (n=11)
surface

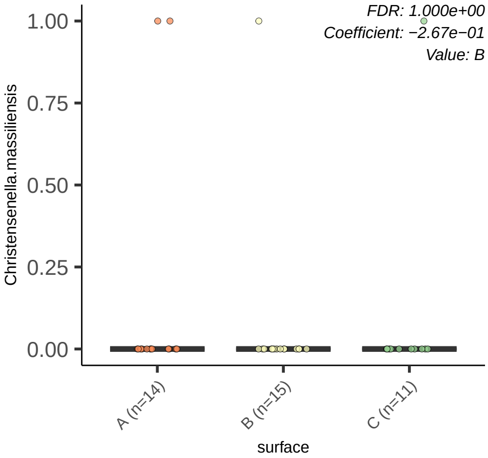
FDR: 1.000e+00
Coefficient: −2.67e−01
Value: B
Christensenella.massiliensis
1.00
0.75
0.50
0.25
0.00
A (n=14)
B (n=15)
C (n=11)
surface

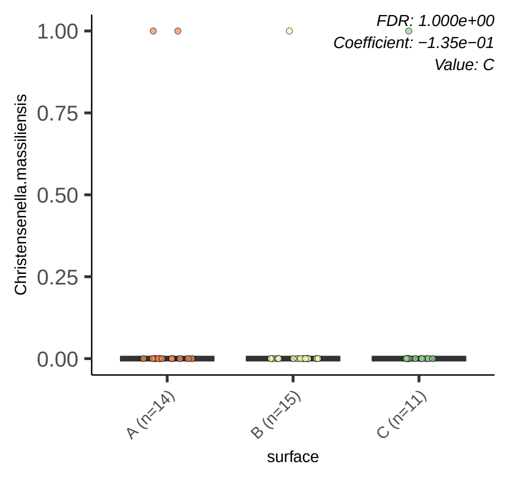

FDR: 1.000e+00
Coefficient: −1.35e−01
Value: C
1.00
0.75
0.50
0.25
0.00
Christensenella.massiliensis
A (n=14)
B (n=15)
C (n=11)
surface

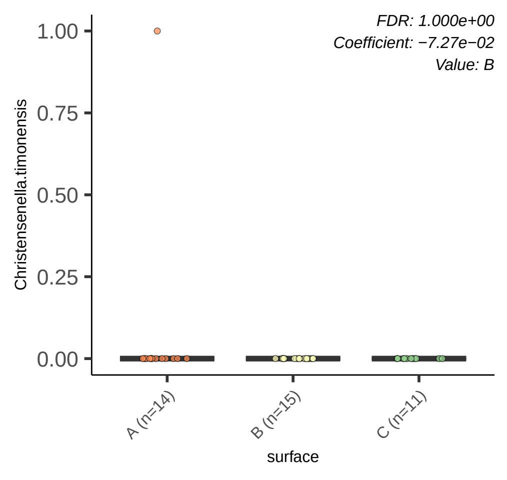

FDR: 1.000e+00
Coefficient: −7.27e−02
Value: B
Christensenella.timonensis
1.00
0.75
0.50
0.25
0.00
A (n=14)
B (n=15)
C (n=11)
surface

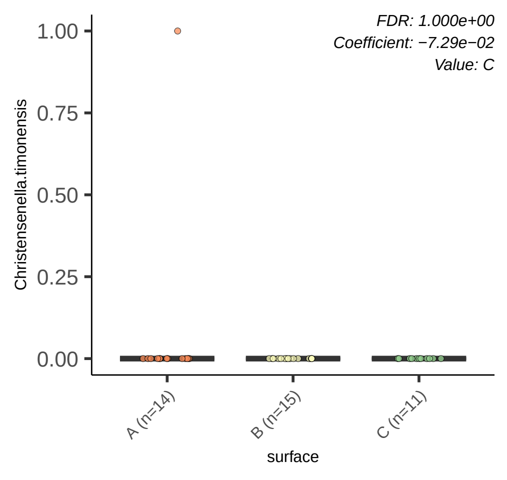
FDR: 1.000e+00
Coefficient: −7.29e−02
Value: C
1.00
0.75
0.50
0.25
0.00
Christensenella.timonensis
A (n=14)
B (n=15)
C (n=11)
surface

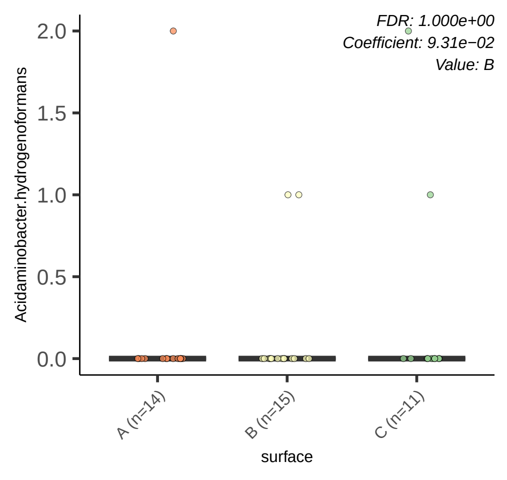

FDR: 1.000e+00
Coefficient: 9.31e−02
Value: B
Acidaminobacter.hydrogenoformans
2.0
1.5
1.0
0.5
0.0
A (n=14)
B (n=15)
C (n=11)
surface

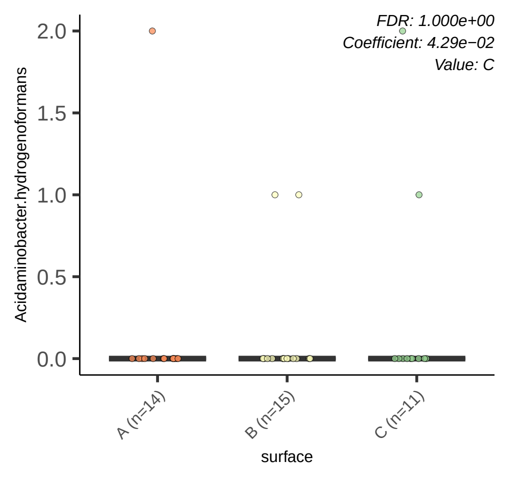
FDR: 1.000e+00
Coefficient: 4.29e−02
Value: C
Acidaminobacter.hydrogenoformans
2.0
1.5
1.0
0.5
0.0
A (n=14)
B (n=15)
C (n=11)
surface

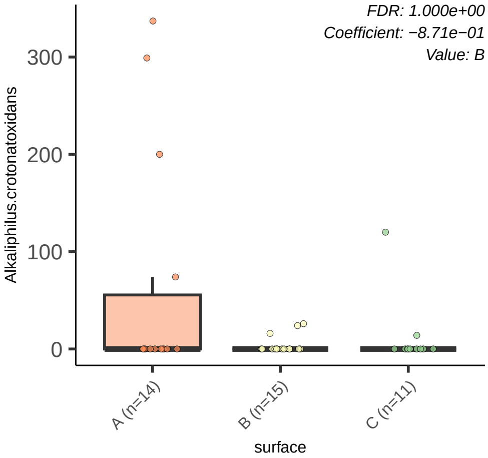

FDR: 1.000e+00
Coefficient: −8.71e−01
Value: B
Alkaliphilus.crotonatoxidans
300
200
100
0
A (n=14)
B (n=15)
C (n=11)
surface

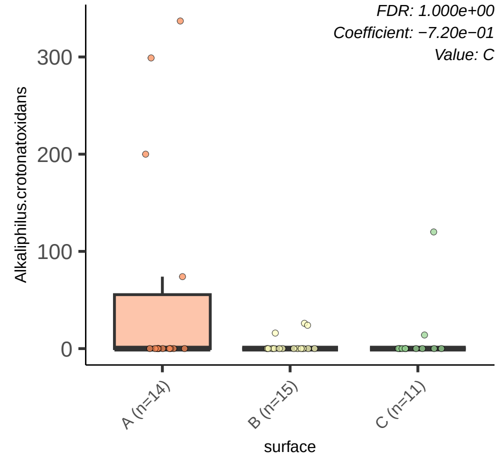
FDR: 1.000e+00
Coefficient: −7.20e−01
Value: C
Alkaliphilus.crotonatoxidans
300
200
100
0
A (n=14)
B (n=15)
C (n=11)
surface

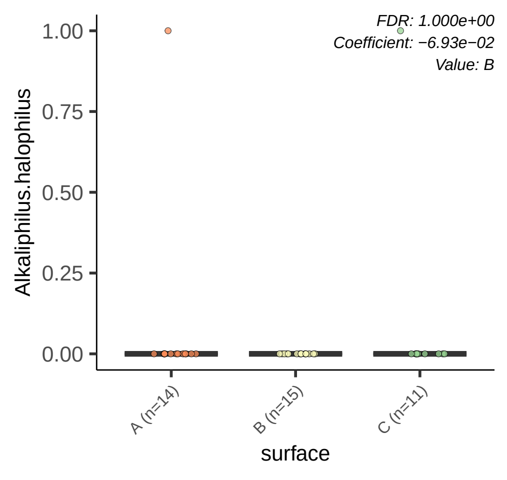

FDR: 1.000e+00
Coefficient: −6.93e−02
Value: B
Alkaliphilus.halophilus
1.00
0.75
0.50
0.25
0.00
A (n=14)
B (n=15)
C (n=11)
surface

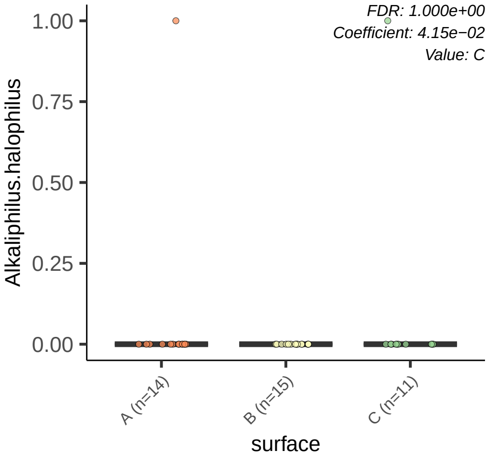
FDR: 1.000e+00
Coefficient: 4.15e−02
Value: C
Alkaliphilus.halophilus
1.00
0.75
0.50
0.25
0.00
A (n=14)
B (n=15)
C (n=11)
surface

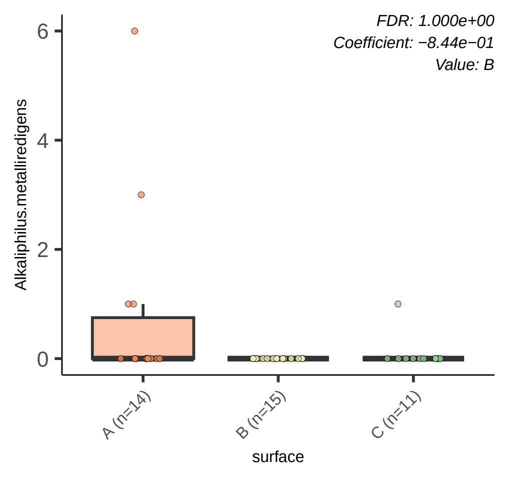
FDR: 1.000e+00
Coefficient: −8.44e−01
Value: B
Alkaliphilus.metalliredigens
6
4
2
0
A (n=14)
B (n=15)
C (n=11)
surface

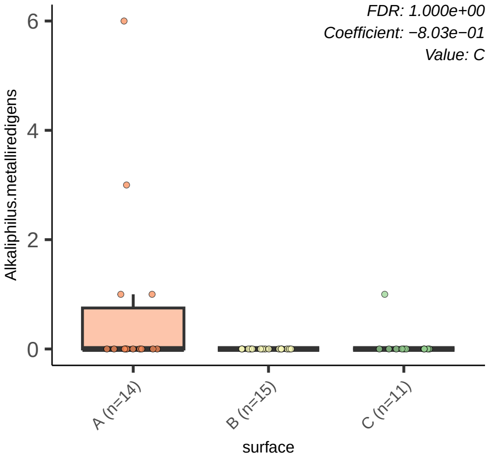

FDR: 1.000e+00
Coefficient: −8.03e−01
Value: C
Alkaliphilus.metalliredigens
0
2
4
6
A (n=14)
B (n=15)
C (n=11)
surface

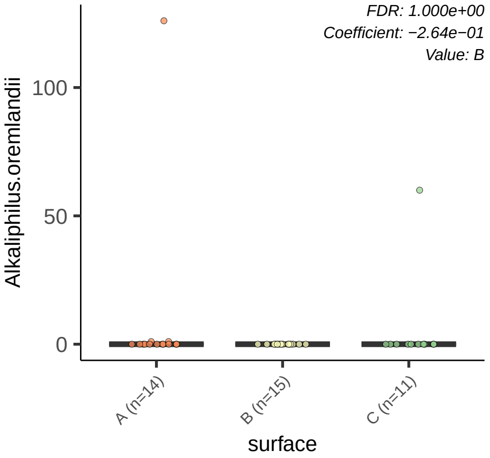
FDR: 1.000e+00
Coefficient: −2.64e−01
Value: B
Alkaliphilus.oremlandii
100
50
0
A (n=14)
B (n=15)
C (n=11)
surface

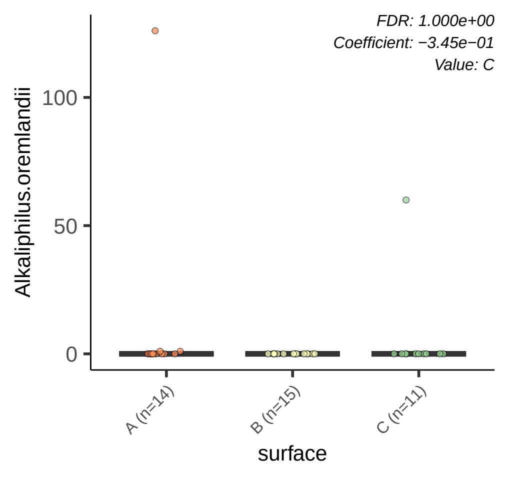
FDR: 1.000e+00
Coefficient: −3.45e−01
Value: C
Alkaliphilus.oremlandii
100
50
0
A (n=14)
B (n=15)
C (n=11)
surface

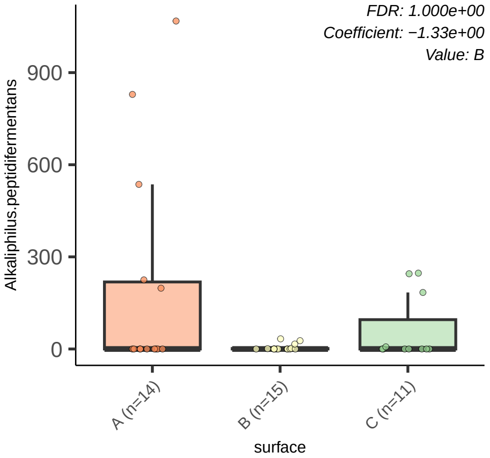
FDR: 1.000e+00
Coefficient: −1.33e+00
Value: B
Alkaliphilus.peptidifermentans
900
600
300
0
A (n=14)
B (n=15)
C (n=11)
surface

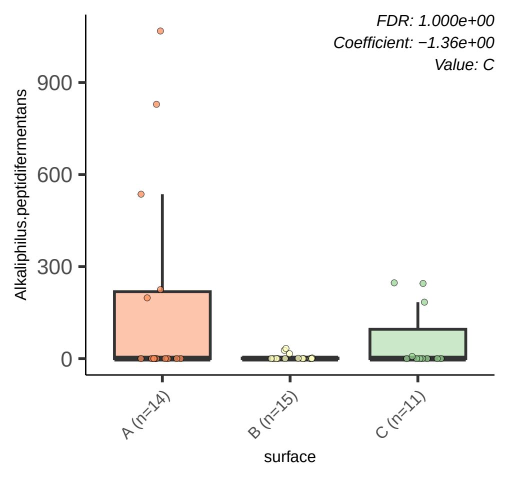
FDR: 1.000e+00
Coefficient: −1.36e+00
Value: C
Alkaliphilus.peptidifermentans
900
600
300
0
A (n=14)
B (n=15)
C (n=11)
surface

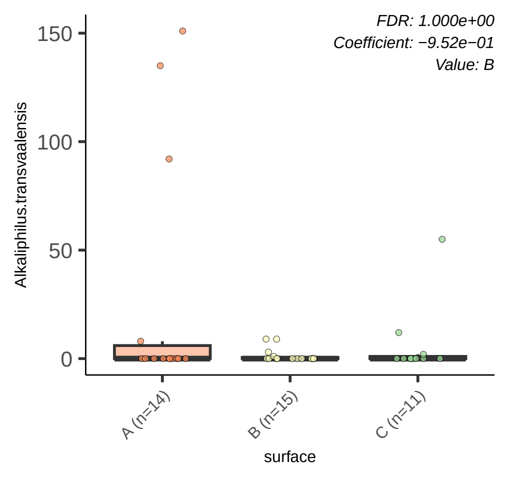
FDR: 1.000e+00
Coefficient: −9.52e−01
Value: B
150
100
50
0
Alkaliphilus.transvaalensis
A (n=14)
B (n=15)
C (n=11)
surface

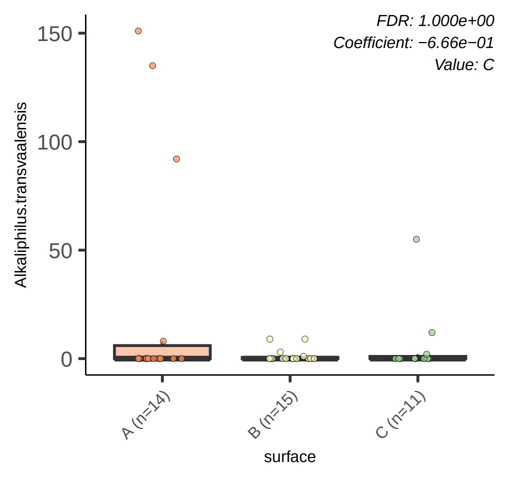
FDR: 1.000e+00
Coefficient: −6.66e−01
Value: C
150
100
50
0
Alkaliphilus.transvaalensis
A (n=14)
B (n=15)
C (n=11)
surface

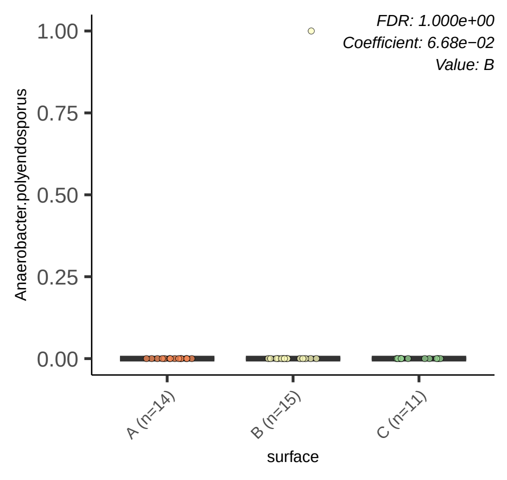
FDR: 1.000e+00
Coefficient: 6.68e−02
Value: B
Anaerobacter.polyendosporus
1.00
0.75
0.50
0.25
0.00
A (n=14)
B (n=15)
C (n=11)
surface

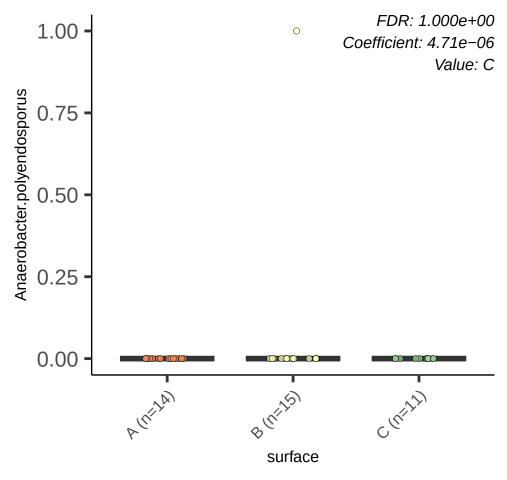

FDR: 1.000e+00
Coefficient: 4.71e−06
Value: C
1.00
0.75
0.50
0.25
0.00
Anaerobacter.polyendosporus
A (n=14)
B (n=15)
C (n=11)
surface

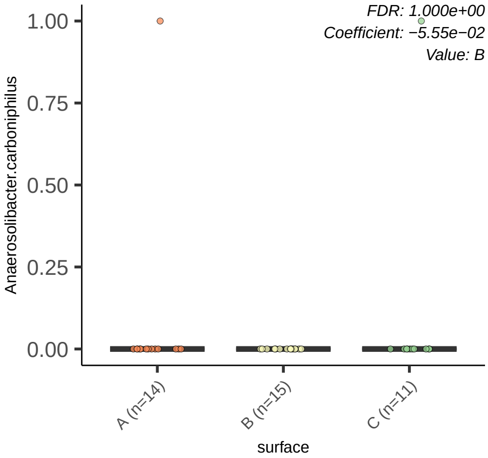
FDR: 1.000e+00
Coefficient: −5.55e−02
Value: B
Anaerosolibacter.carboniphilus
1.00
0.75
0.50
0.25
0.00
A (n=14)
B (n=15)
C (n=11)
surface

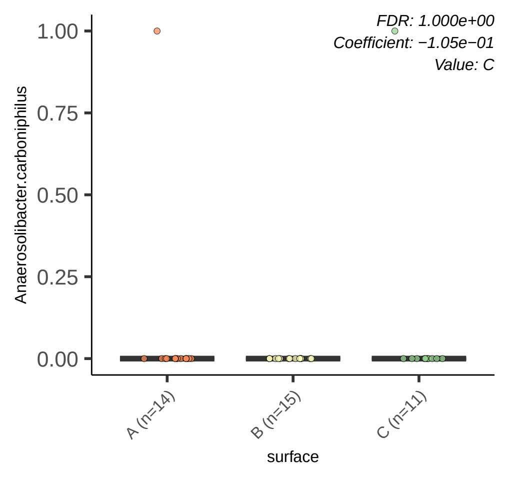

FDR: 1.000e+00
Coefficient: −1.05e−01
Value: C
Anaerosolibacter.carboniphilus
1.00
0.75
0.50
0.25
0.00
A (n=14)
B (n=15)
C (n=11)
surface

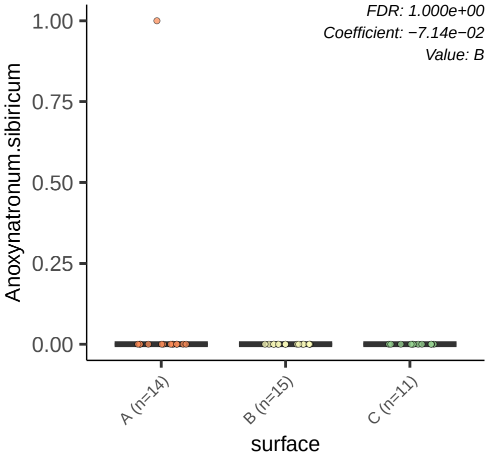
FDR: 1.000e+00
Coefficient: −7.14e−02
Value: B
Anoxynatronum.sibiricum
1.00
0.75
0.50
0.25
0.00
A (n=14)
B (n=15)
C (n=11)
surface

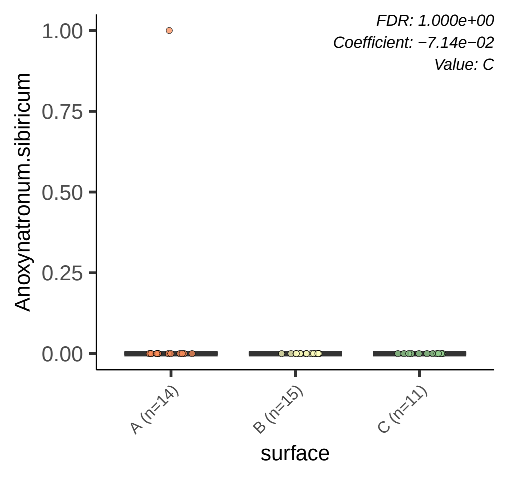
FDR: 1.000e+00
Coefficient: −7.14e−02
Value: C
Anoxynatronum.sibiricum
1.00
0.75
0.50
0.25
0.00
A (n=14)
B (n=15)
C (n=11)
surface

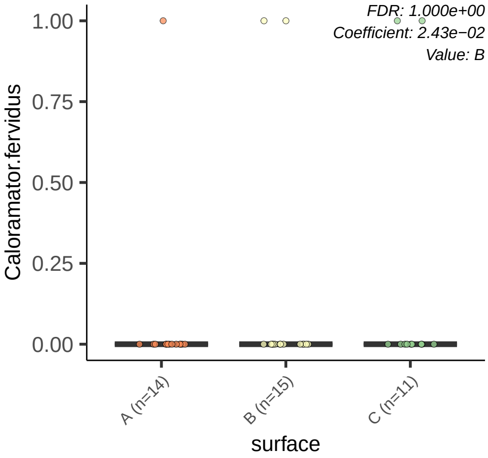

FDR: 1.000e+00
Coefficient: 2.43e−02
Value: B
Caloramator.fervidus
1.00
0.75
0.50
0.25
0.00
A (n=14)
B (n=15)
C (n=11)
surface

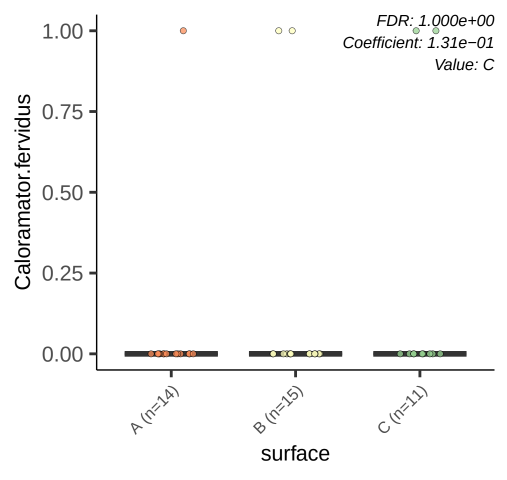
FDR: 1.000e+00
Coefficient: 1.31e−01
Value: C
Caloramator.fervidus
1.00
0.75
0.50
0.25
0.00
A (n=14)
B (n=15)
C (n=11)
surface

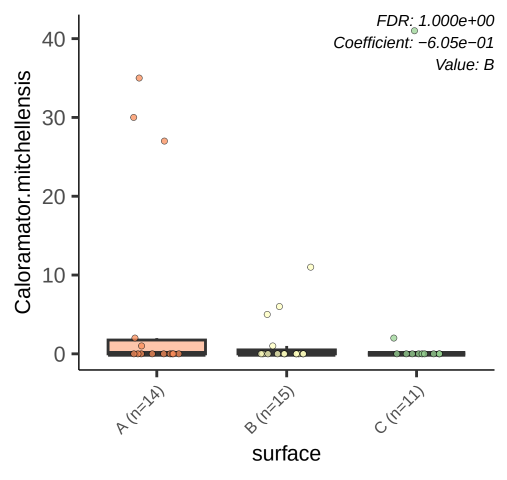

FDR: 1.000e+00
Coefficient: −6.05e−01
Value: B
Caloramator.mitchellensis
40
30
20
10
0
A (n=14)
B (n=15)
C (n=11)
surface

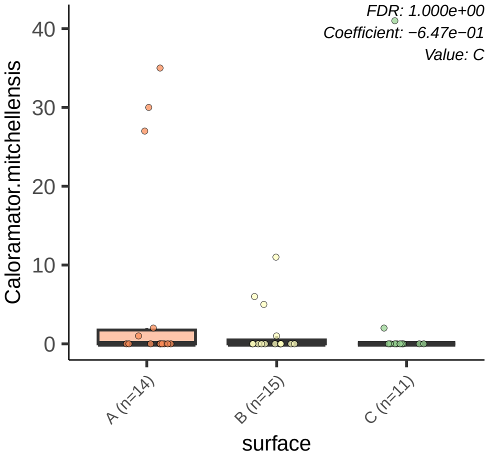
FDR: 1.000e+00
Coefficient: −6.47e−01
Value: C
Caloramator.mitchellensis
40
30
20
10
0
A (n=14)
B (n=15)
C (n=11)
surface

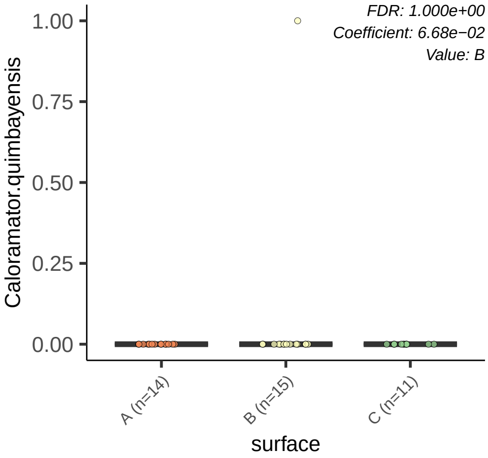

FDR: 1.000e+00
Coefficient: 6.68e−02
Value: B
Caloramator.quimbayensis
1.00
0.75
0.50
0.25
0.00
A (n=14)
B (n=15)
C (n=11)
surface

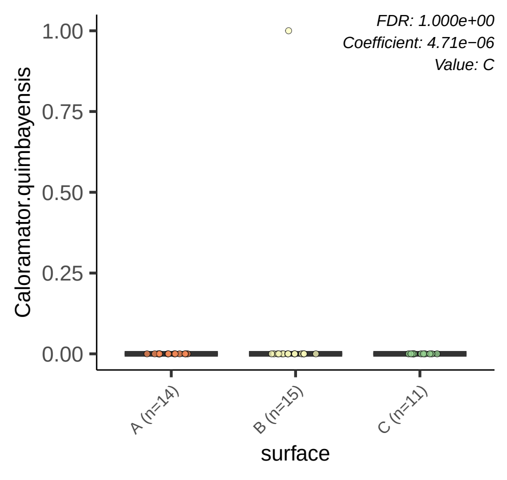
FDR: 1.000e+00
Coefficient: 4.71e−06
Value: C
Caloramator.quimbayensis
1.00
0.75
0.50
0.25
0.00
A (n=14)
B (n=15)
C (n=11)
surface

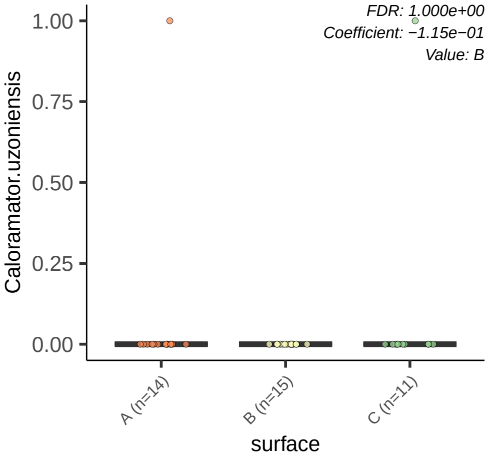
FDR: 1.000e+00
Coefficient: −1.15e−01
Value: B
Caloramator.uzoniensis
1.00
0.75
0.50
0.25
0.00
A (n=14)
B (n=15)
C (n=11)
surface

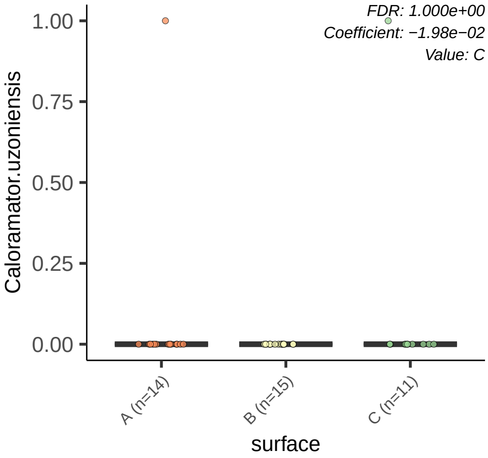

FDR: 1.000e+00
Coefficient: −1.98e−02
Value: C
Caloramator.uzoniensis
1.00
0.75
0.50
0.25
0.00
A (n=14)
B (n=15)
C (n=11)
surface

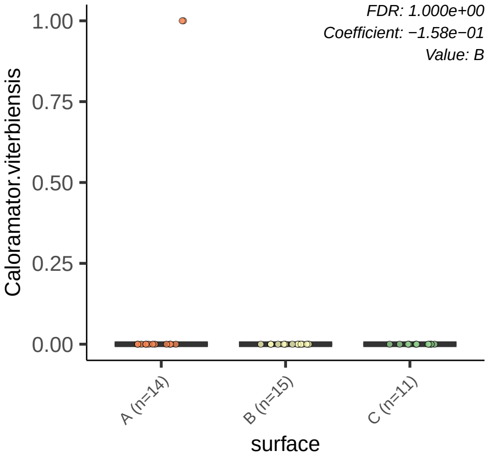
FDR: 1.000e+00
Coefficient: −1.58e−01
Value: B
Caloramator.viterbiensis
1.00
0.75
0.50
0.25
0.00
A (n=14)
B (n=15)
C (n=11)
surface

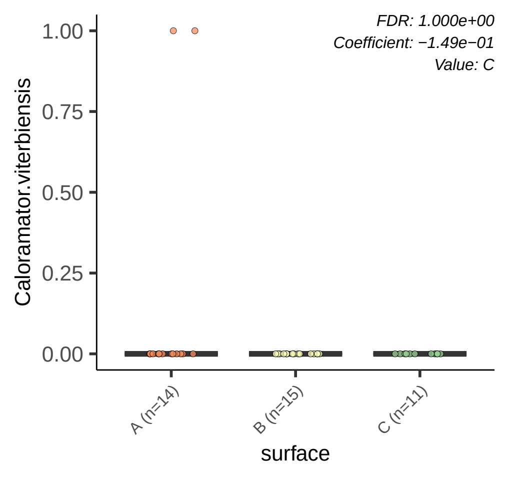
FDR: 1.000e+00
Coefficient: −1.49e−01
Value: C
Caloramator.viterbiensis
1.00
0.75
0.50
0.25
0.00
A (n=14)
B (n=15)
C (n=11)
surface

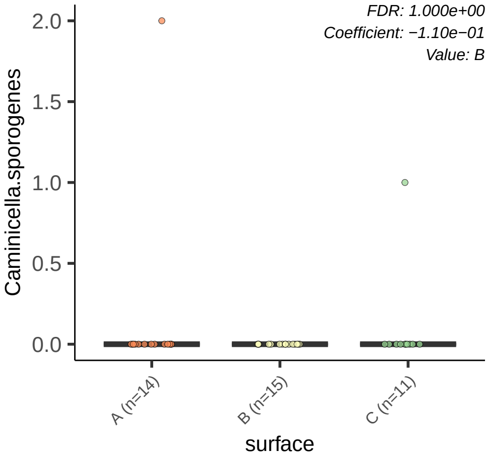
FDR: 1.000e+00
Coefficient: −1.10e−01
Value: B
Caminicella.sporogenes
2.0
1.5
1.0
0.5
0.0
A (n=14)
B (n=15)
C (n=11)
surface

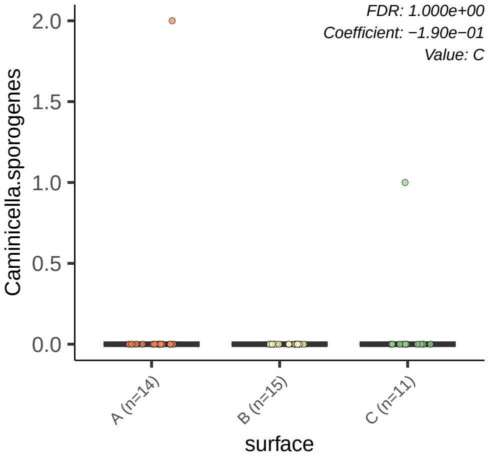
FDR: 1.000e+00
Coefficient: −1.90e−01
Value: C
Caminicella.sporogenes
2.0
1.5
1.0
0.5
0.0
A (n=14)
B (n=15)
C (n=11)
surface

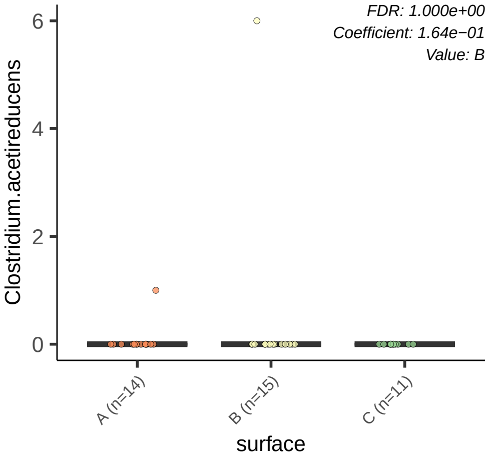
FDR: 1.000e+00
Coefficient: 1.64e−01
Value: B
Clostridium.acetireducens
0
2
4
6
A (n=14)
B (n=15)
C (n=11)
surface

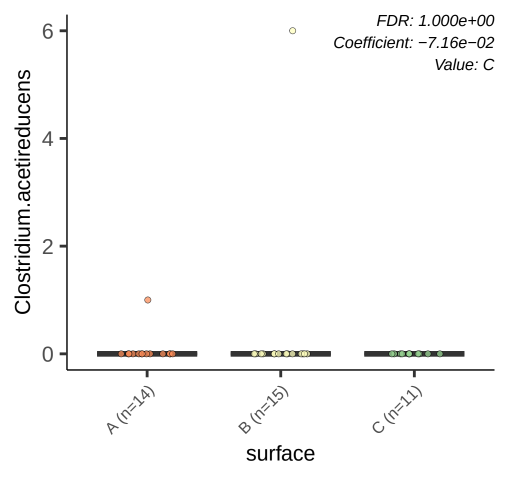
FDR: 1.000e+00
Coefficient: −7.16e−02
Value: C
Clostridium.acetireducens
6
4
2
0
A (n=14)
B (n=15)
C (n=11)
surface

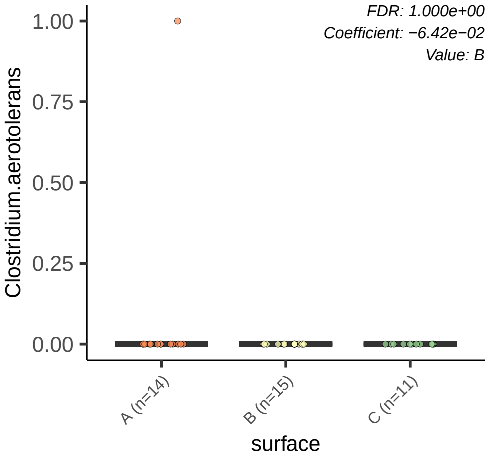
FDR: 1.000e+00
Coefficient: −6.42e−02
Value: B
Clostridium.aerotolerans
1.00
0.75
0.50
0.25
0.00
A (n=14)
B (n=15)
C (n=11)
surface

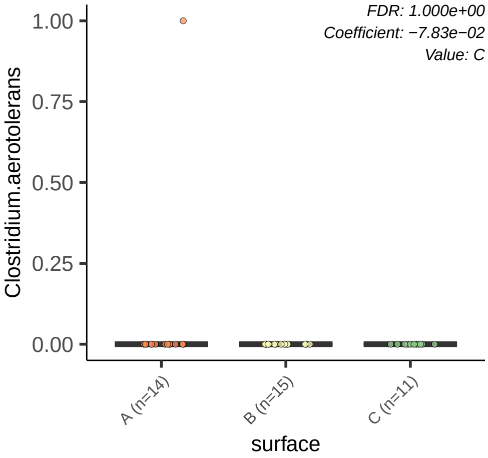
FDR: 1.000e+00
Coefficient: −7.83e−02
Value: C
Clostridium.aerotolerans
1.00
0.75
0.50
0.25
0.00
A (n=14)
B (n=15)
C (n=11)
surface

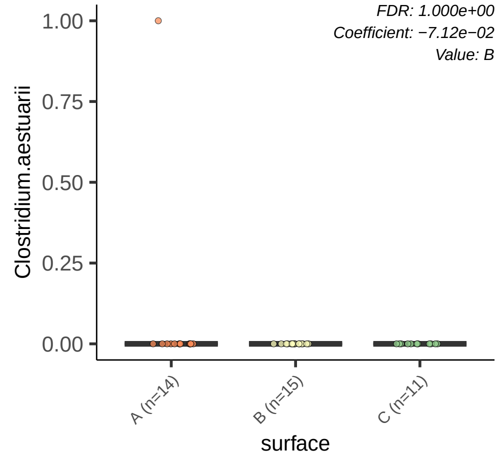
FDR: 1.000e+00
Coefficient: −7.12e−02
Value: B
Clostridium.aestuarii
1.00
0.75
0.50
0.25
0.00
A (n=14)
B (n=15)
C (n=11)
surface

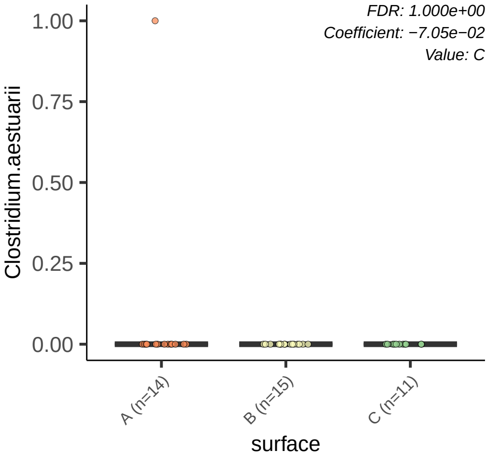

FDR: 1.000e+00
Coefficient: −7.05e−02
Value: C
Clostridium.aestuarii
1.00
0.75
0.50
0.25
0.00
A (n=14)
B (n=15)
C (n=11)
surface

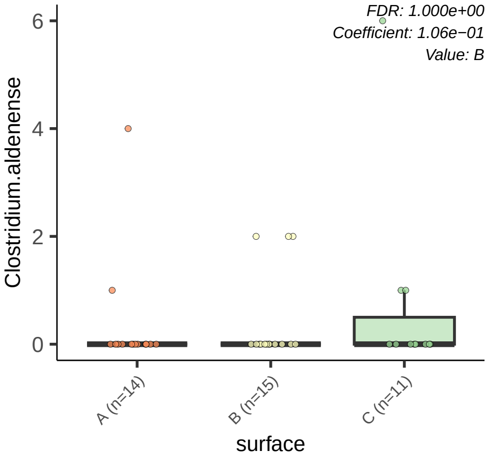
FDR: 1.000e+00
Coefficient: 1.06e−01
Value: B
Clostridium.aldenense
6
4
2
0
A (n=14)
B (n=15)
C (n=11)
surface

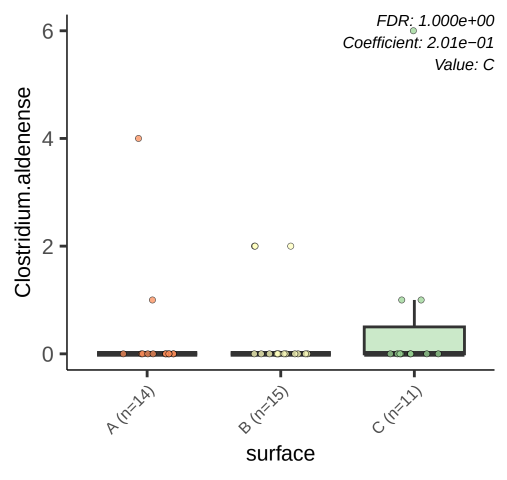

FDR: 1.000e+00
Coefficient: 2.01e−01
Value: C
Clostridium.aldenense
0
2
4
6
A (n=14)
B (n=15)
C (n=11)
surface

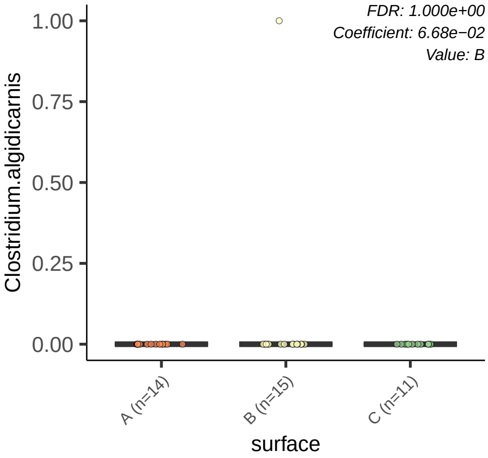
FDR: 1.000e+00
Coefficient: 6.68e−02
Value: B
Clostridium.algidicarnis
1.00
0.75
0.50
0.25
0.00
A (n=14)
B (n=15)
C (n=11)
surface

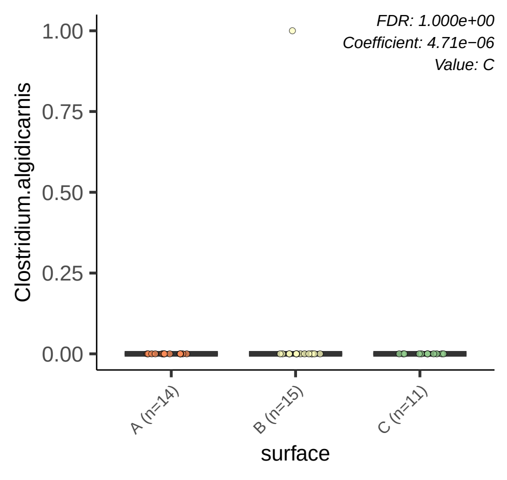
FDR: 1.000e+00
Coefficient: 4.71e−06
Value: C
Clostridium.algidicarnis
1.00
0.75
0.50
0.25
0.00
A (n=14)
B (n=15)
C (n=11)
surface

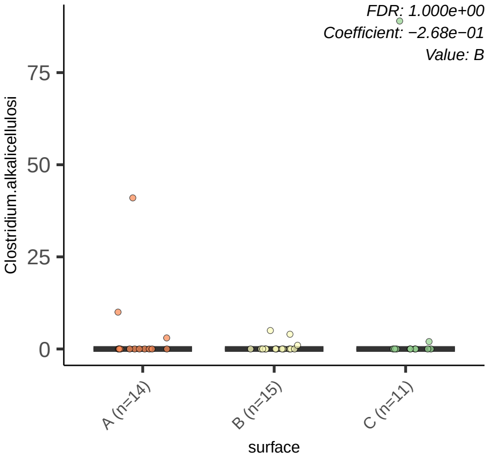
FDR: 1.000e+00
Coefficient: −2.68e−01
Value: B
Clostridium.alkalicellulosi
75
50
25
0
A (n=14)
B (n=15)
C (n=11)
surface

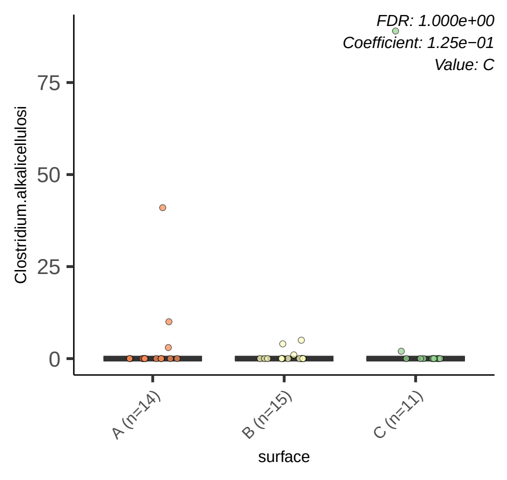
FDR: 1.000e+00
Coefficient: 1.25e−01
Value: C
Clostridium.alkalicellulosi
0
25
50
75
A (n=14)
B (n=15)
C (n=11)
surface

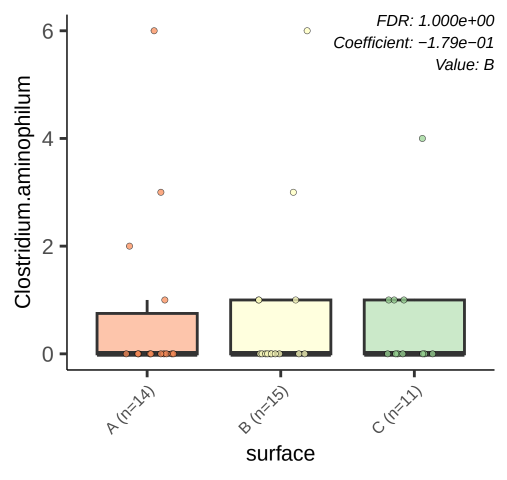

FDR: 1.000e+00
Coefficient: −1.79e−01
Value: B
Clostridium.aminophilum
0
2
4
6
A (n=14)
B (n=15)
C (n=11)
surface

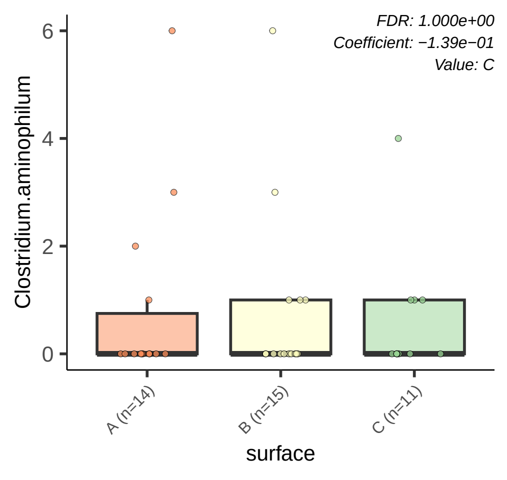

FDR: 1.000e+00
Coefficient: −1.39e−01
Value: C
Clostridium.aminophilum
0
2
4
6
A (n=14)
B (n=15)
C (n=11)
surface

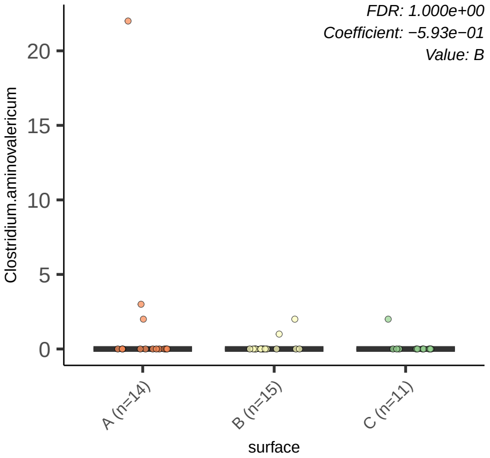

FDR: 1.000e+00
Coefficient: −5.93e−01
Value: B
Clostridium.aminovalericum
0
5
10
15
20
A (n=14)
B (n=15)
C (n=11)
surface

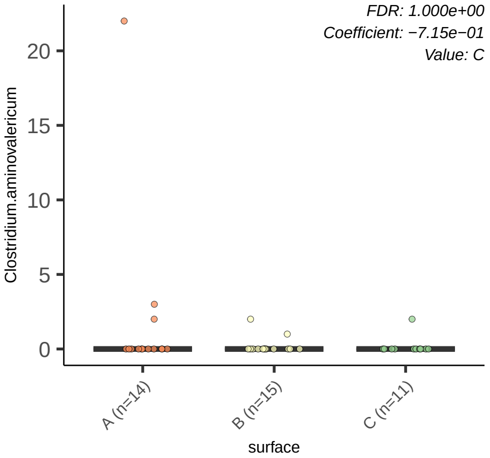
FDR: 1.000e+00
Coefficient: −7.15e−01
Value: C
Clostridium.aminovalericum
20
15
10
5
0
A (n=14)
B (n=15)
C (n=11)
surface

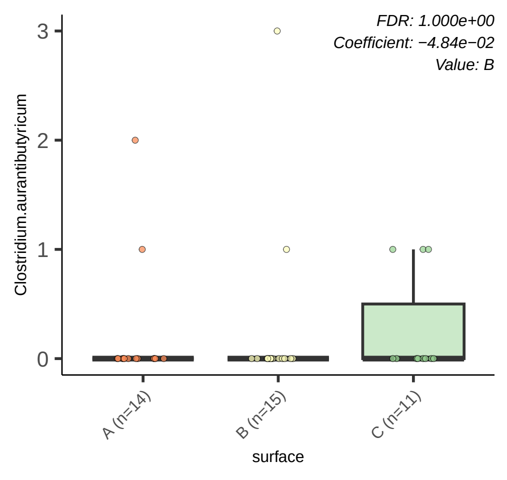

FDR: 1.000e+00
Coefficient: −4.84e−02
Value: B
Clostridium.aurantibutyricum
3
2
1
0
A (n=14)
B (n=15)
C (n=11)
surface

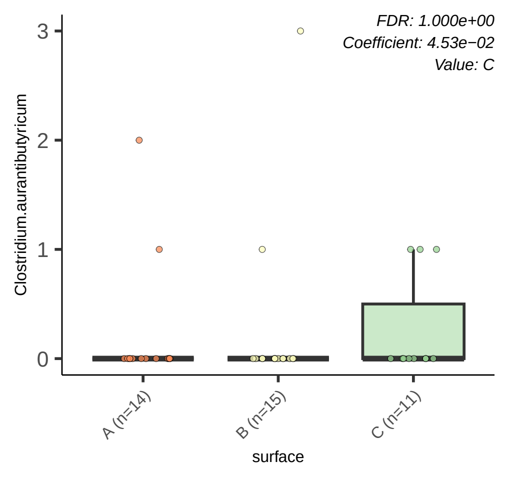
FDR: 1.000e+00
Coefficient: 4.53e−02
Value: C
Clostridium.aurantibutyricum
0
1
2
3
A (n=14)
B (n=15)
C (n=11)
surface

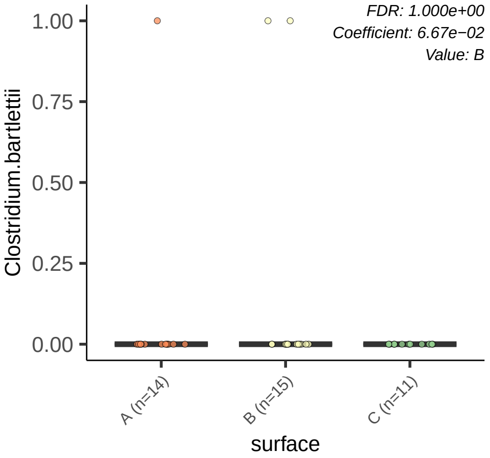

FDR: 1.000e+00
Coefficient: 6.67e−02
Value: B
Clostridium.bartlettii
1.00
0.75
0.50
0.25
0.00
A (n=14)
B (n=15)
C (n=11)
surface

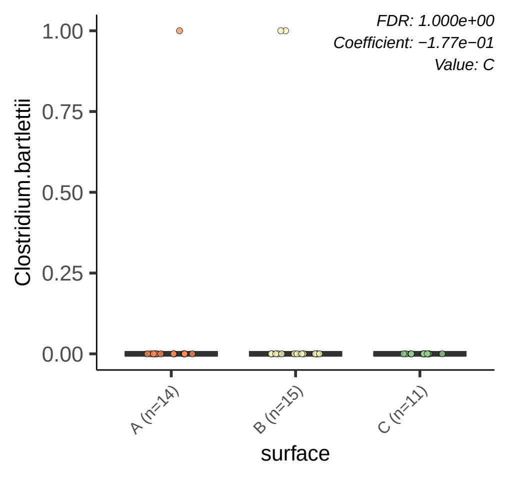
FDR: 1.000e+00
Coefficient: −1.77e−01
Value: C
Clostridium.bartlettii
1.00
0.75
0.50
0.25
0.00
A (n=14)
B (n=15)
C (n=11)
surface

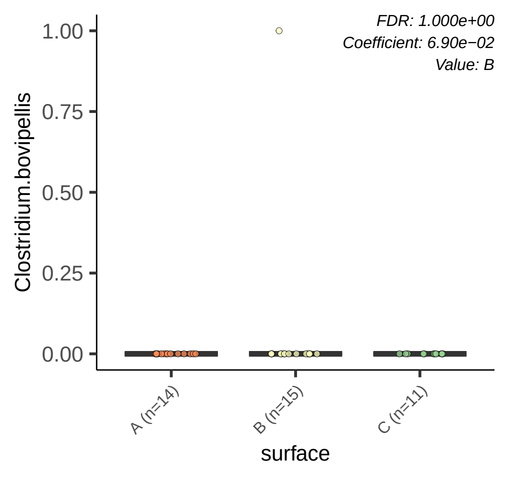
FDR: 1.000e+00
Coefficient: 6.90e−02
Value: B
Clostridium.bovipellis
1.00
0.75
0.50
0.25
0.00
A (n=14)
B (n=15)
C (n=11)
surface

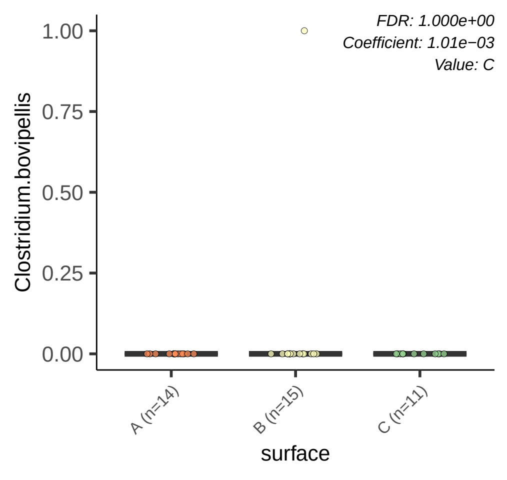
FDR: 1.000e+00
Coefficient: 1.01e−03
Value: C
Clostridium.bovipellis
1.00
0.75
0.50
0.25
0.00
A (n=14)
B (n=15)
C (n=11)
surface

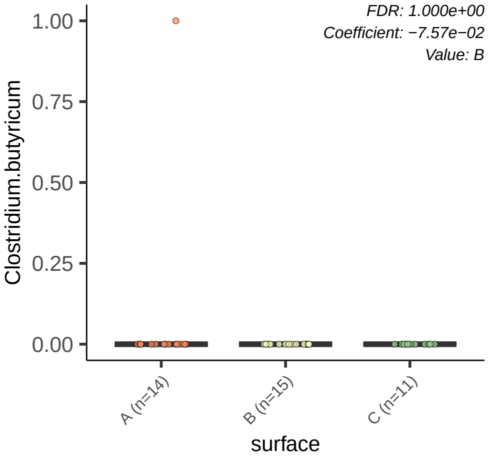
FDR: 1.000e+00
Coefficient: −7.57e−02
Value: B
Clostridium.butyricum
1.00
0.75
0.50
0.25
0.00
A (n=14)
B (n=15)
C (n=11)
surface

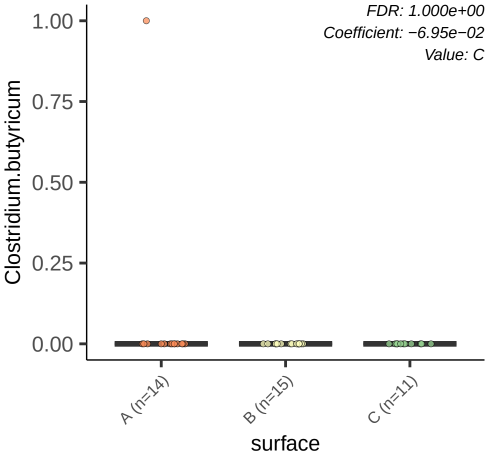

FDR: 1.000e+00
Coefficient: −6.95e−02
Value: C
Clostridium.butyricum
1.00
0.75
0.50
0.25
0.00
A (n=14)
B (n=15)
C (n=11)
surface

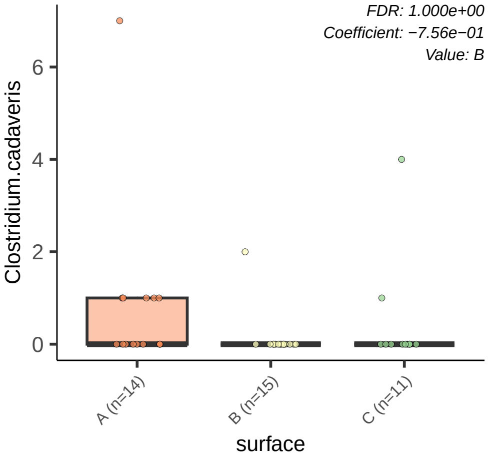
FDR: 1.000e+00
Coefficient: −7.56e−01
Value: B
Clostridium.cadaveris
0
2
4
6
A (n=14)
B (n=15)
C (n=11)
surface

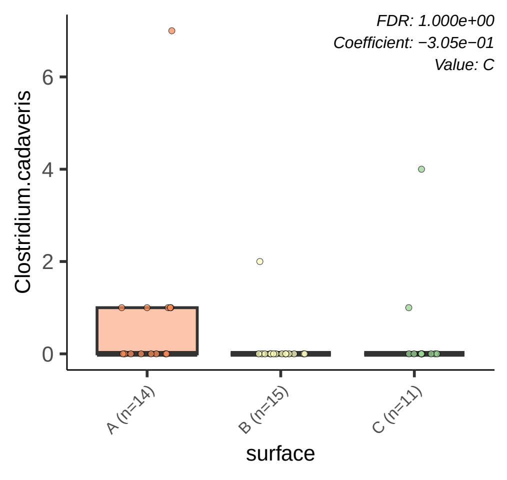

FDR: 1.000e+00
Coefficient: −3.05e−01
Value: C
Clostridium.cadaveris
0
2
4
6
A (n=14)
B (n=15)
C (n=11)
surface

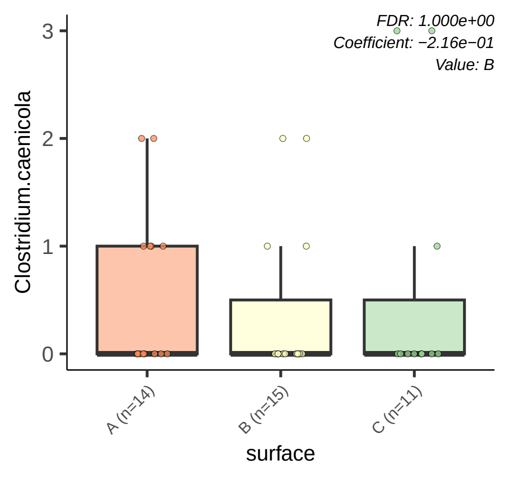

FDR: 1.000e+00
Coefficient: −2.16e−01
Value: B
Clostridium.caenicola
0
1
2
3
A (n=14)
B (n=15)
C (n=11)
surface

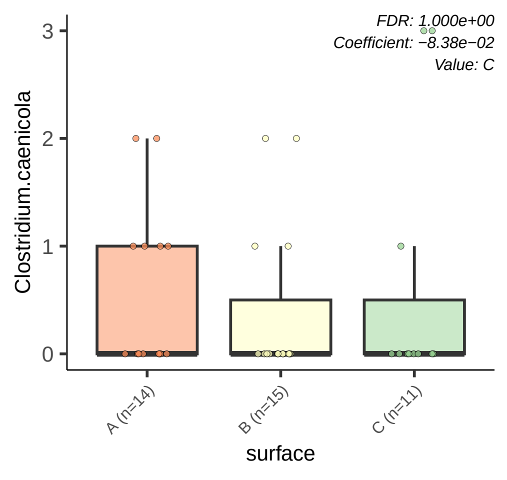
FDR: 1.000e+00
Coefficient: −8.38e−02
Value: C
Clostridium.caenicola
0
1
2
3
A (n=14)
B (n=15)
C (n=11)
surface

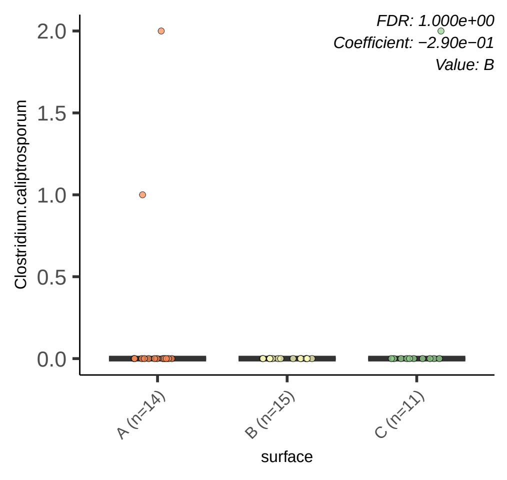

FDR: 1.000e+00
Coefficient: −2.90e−01
Value: B
Clostridium.caliptrosporum
2.0
1.5
1.0
0.5
0.0
A (n=14)
B (n=15)
C (n=11)
surface

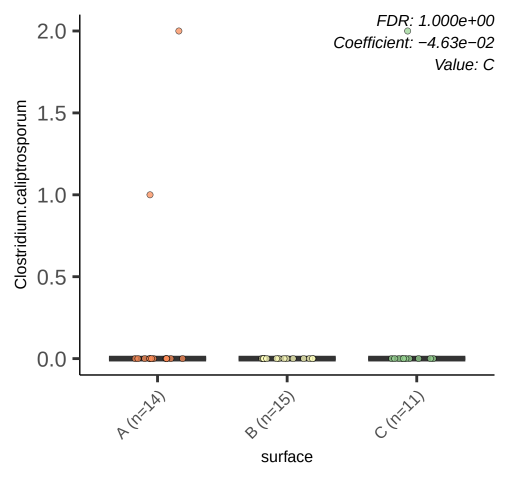

FDR: 1.000e+00
Coefficient: −4.63e−02
Value: C
Clostridium.caliptrosporum
2.0
1.5
1.0
0.5
0.0
A (n=14)
B (n=15)
C (n=11)
surface

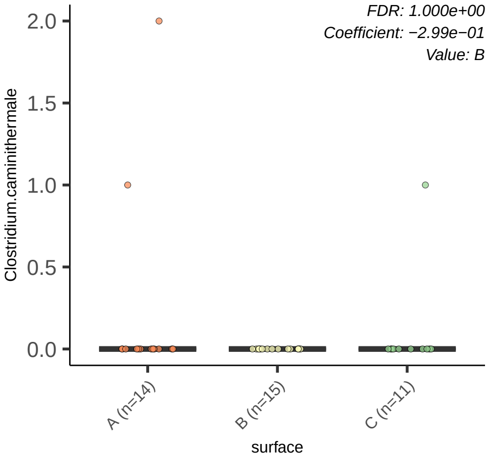
FDR: 1.000e+00
Coefficient: −2.99e−01
Value: B
Clostridium.caminithermale
2.0
1.5
1.0
0.5
0.0
A (n=14)
B (n=15)
C (n=11)
surface

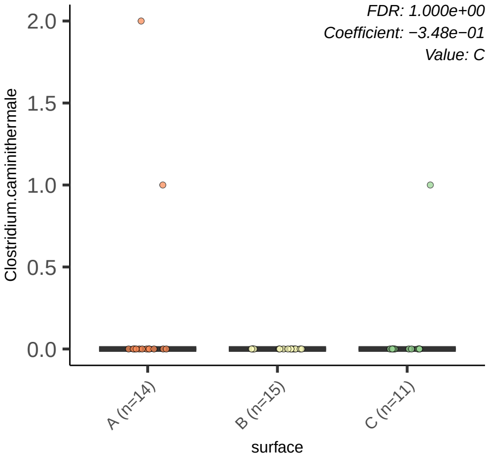
FDR: 1.000e+00
Coefficient: −3.48e−01
Value: C
Clostridium.caminithermale
2.0
1.5
1.0
0.5
0.0
A (n=14)
B (n=15)
C (n=11)
surface

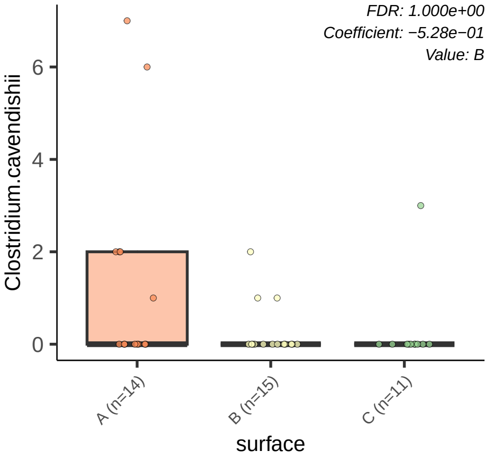
FDR: 1.000e+00
Coefficient: −5.28e−01
Value: B
Clostridium.cavendishii
0
2
4
6
A (n=14)
B (n=15)
C (n=11)
surface

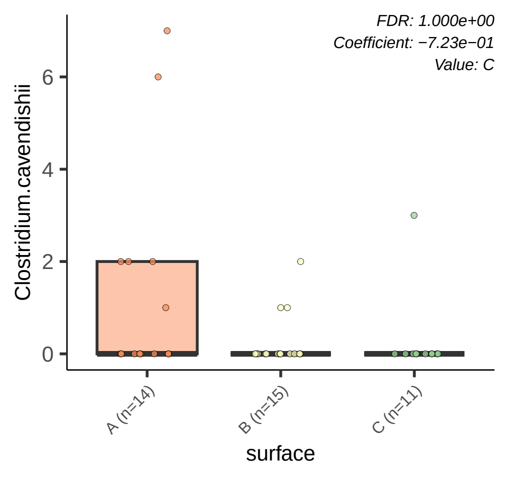
FDR: 1.000e+00
Coefficient: −7.23e−01
Value: C
Clostridium.cavendishii
0
2
4
6
A (n=14)
B (n=15)
C (n=11)
surface

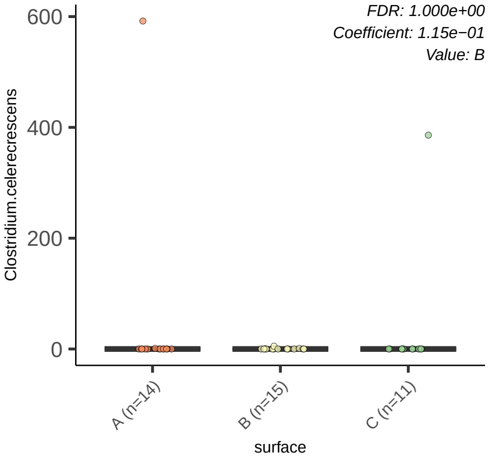

FDR: 1.000e+00
Coefficient: 1.15e−01
Value: B
Clostridium.celerecrescens
600
400
200
0
A (n=14)
B (n=15)
C (n=11)
surface

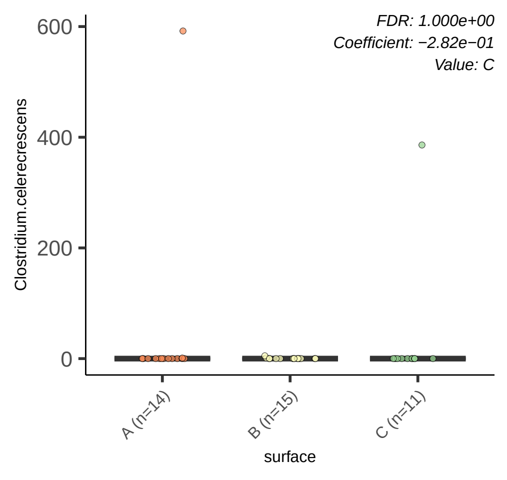
FDR: 1.000e+00
Coefficient: −2.82e−01
Value: C
600
400
200
0
Clostridium.celerecrescens
A (n=14)
B (n=15)
C (n=11)
surface

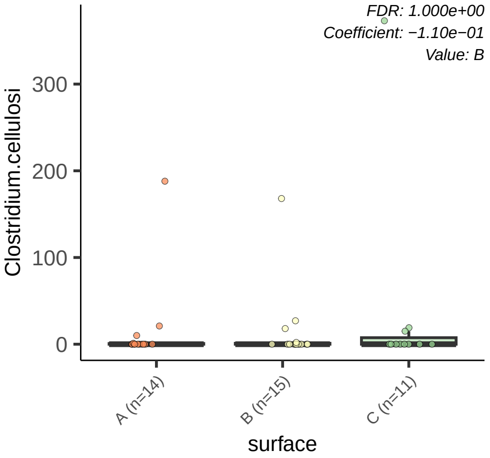
FDR: 1.000e+00
Coefficient: −1.10e−01
Value: B
Clostridium.cellulosi
300
200
100
0
A (n=14)
B (n=15)
C (n=11)
surface

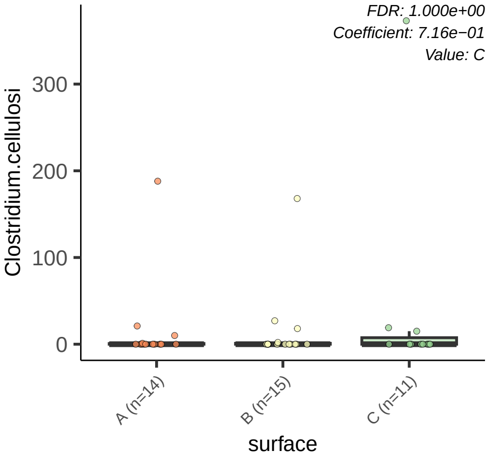

FDR: 1.000e+00
Coefficient: 7.16e−01
Value: C
Clostridium.cellulosi
300
200
100
0
A (n=14)
B (n=15)
C (n=11)
surface

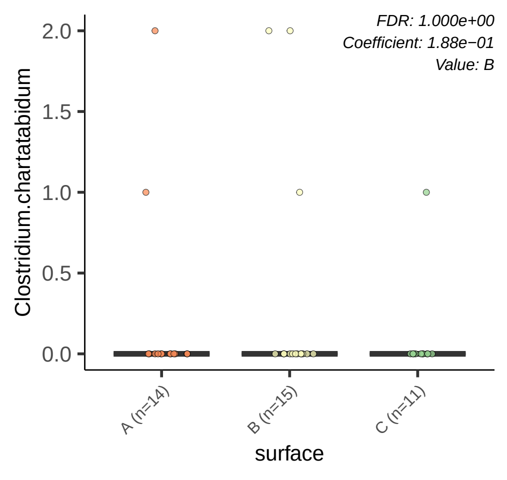
FDR: 1.000e+00
Coefficient: 1.88e−01
Value: B
Clostridium.chartatabidum
2.0
1.5
1.0
0.5
0.0
A (n=14)
B (n=15)
C (n=11)
surface

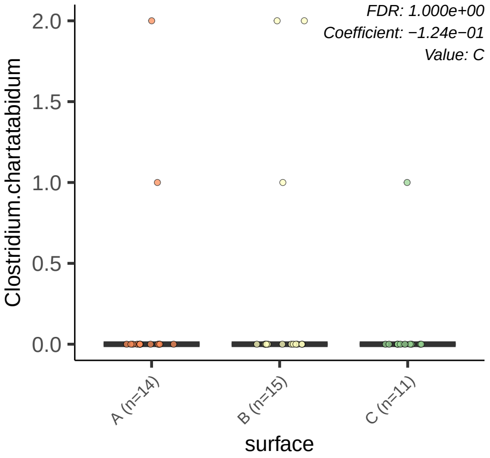
FDR: 1.000e+00
Coefficient: −1.24e−01
Value: C
Clostridium.chartatabidum
2.0
1.5
1.0
0.5
0.0
A (n=14)
B (n=15)
C (n=11)
surface

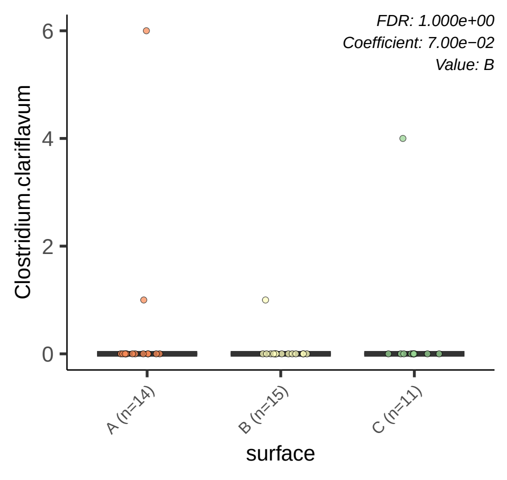
FDR: 1.000e+00
Coefficient: 7.00e−02
Value: B
Clostridium.clariflavum
0
2
4
6
A (n=14)
B (n=15)
C (n=11)
surface

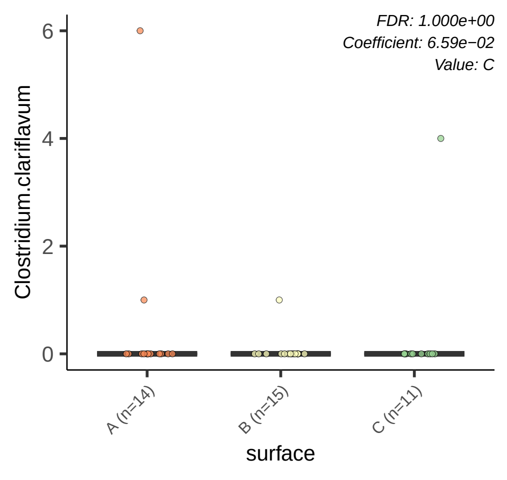
FDR: 1.000e+00
Coefficient: 6.59e−02
Value: C
Clostridium.clariflavum
6
4
2
0
A (n=14)
B (n=15)
C (n=11)
surface

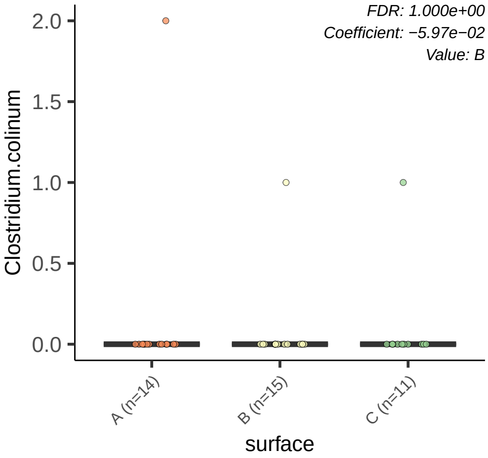

FDR: 1.000e+00
Coefficient: −5.97e−02
Value: B
Clostridium.colinum
2.0
1.5
1.0
0.5
0.0
A (n=14)
B (n=15)
C (n=11)
surface

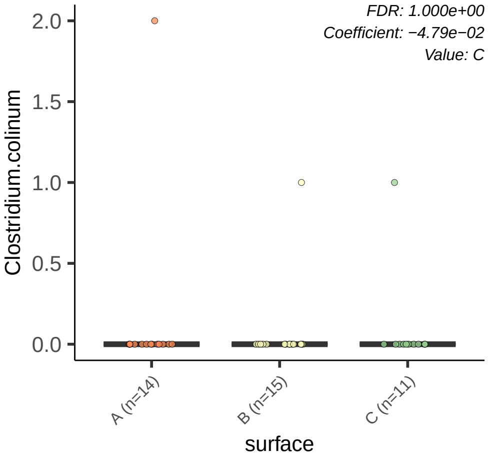
FDR: 1.000e+00
Coefficient: −4.79e−02
Value: C
Clostridium.colinum
2.0
1.5
1.0
0.5
0.0
A (n=14)
B (n=15)
C (n=11)
surface

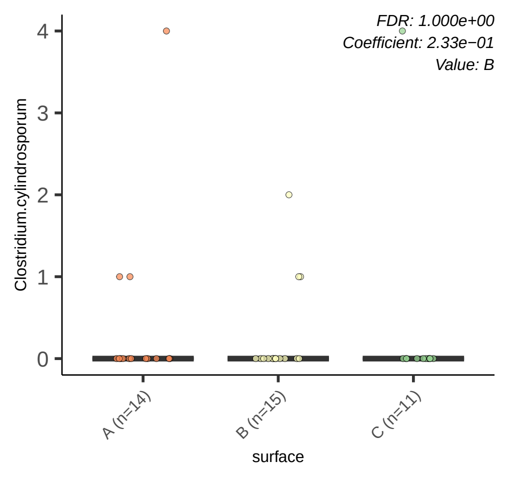
FDR: 1.000e+00
Coefficient: 2.33e−01
Value: B
Clostridium.cylindrosporum
0
1
2
3
4
A (n=14)
B (n=15)
C (n=11)
surface

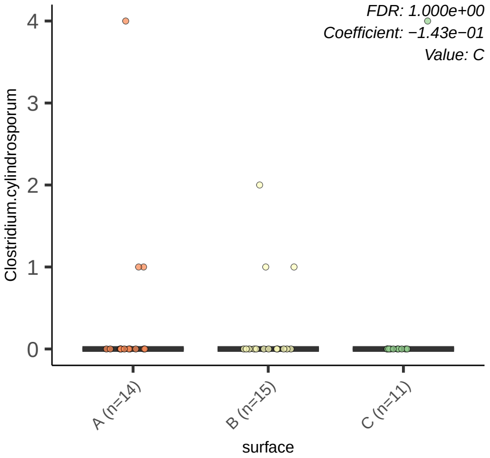

FDR: 1.000e+00
Coefficient: −1.43e−01
Value: C
Clostridium.cylindrosporum
0
1
2
3
4
A (n=14)
B (n=15)
C (n=11)
surface

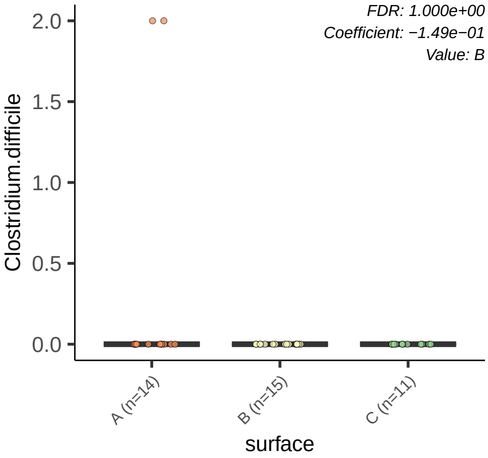
FDR: 1.000e+00
Coefficient: −1.49e−01
Value: B
Clostridium.difficile
2.0
1.5
1.0
0.5
0.0
A (n=14)
B (n=15)
C (n=11)
surface

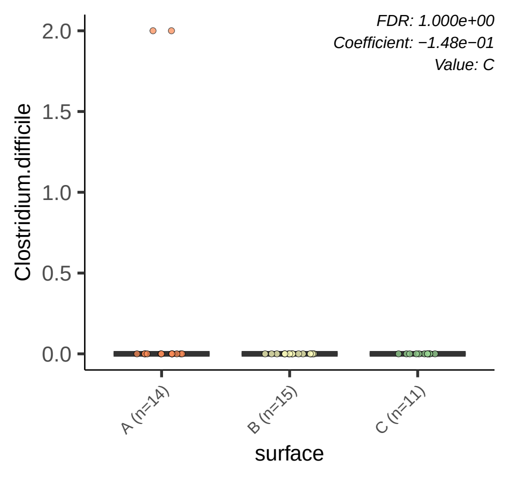

FDR: 1.000e+00
Coefficient: −1.48e−01
Value: C
Clostridium.difficile
2.0
1.5
1.0
0.5
0.0
A (n=14)
B (n=15)
C (n=11)
surface

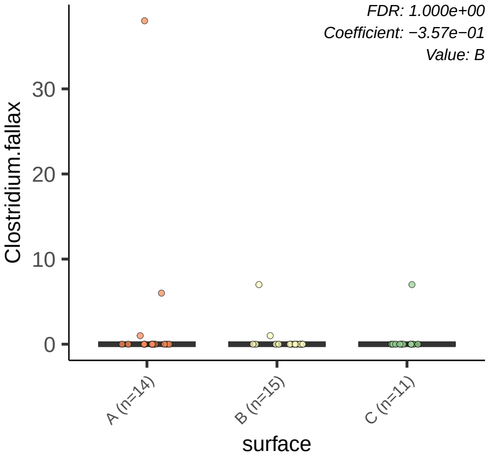
FDR: 1.000e+00
Coefficient: −3.57e−01
Value: B
Clostridium.fallax
30
20
10
0
A (n=14)
B (n=15)
C (n=11)
surface

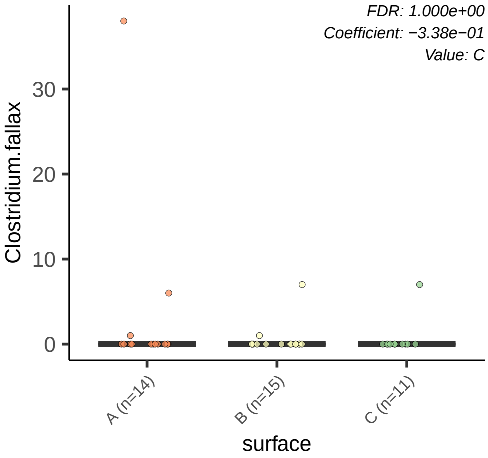

FDR: 1.000e+00
Coefficient: −3.38e−01
Value: C
Clostridium.fallax
0
10
20
30
A (n=14)
B (n=15)
C (n=11)
surface

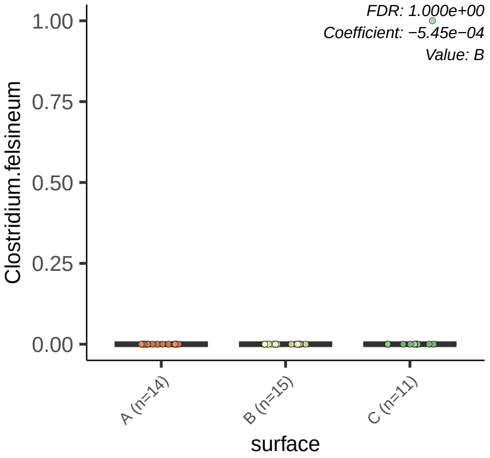
FDR: 1.000e+00
Coefficient: −5.45e−04
Value: B
Clostridium.felsineum
1.00
0.75
0.50
0.25
0.00
A (n=14)
B (n=15)
C (n=11)
surface

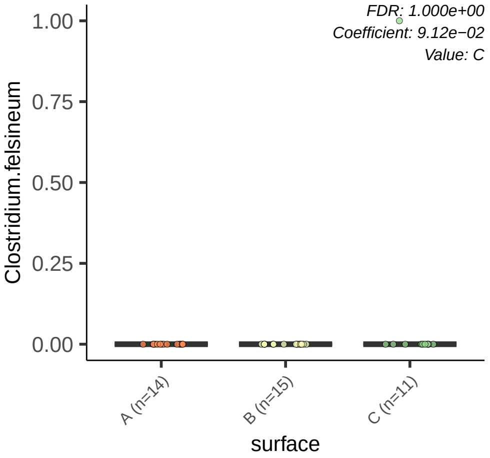
FDR: 1.000e+00
Coefficient: 9.12e−02
Value: C
1.00
0.75
0.50
0.25
0.00
Clostridium.felsineum
A (n=14)
B (n=15)
C (n=11)
surface

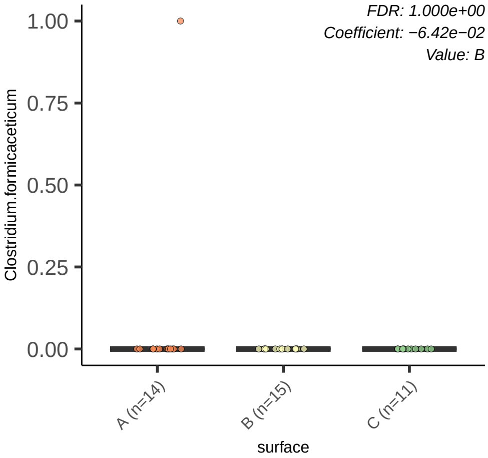
FDR: 1.000e+00
Coefficient: −6.42e−02
Value: B
Clostridium.formicaceticum
1.00
0.75
0.50
0.25
0.00
A (n=14)
B (n=15)
C (n=11)
surface

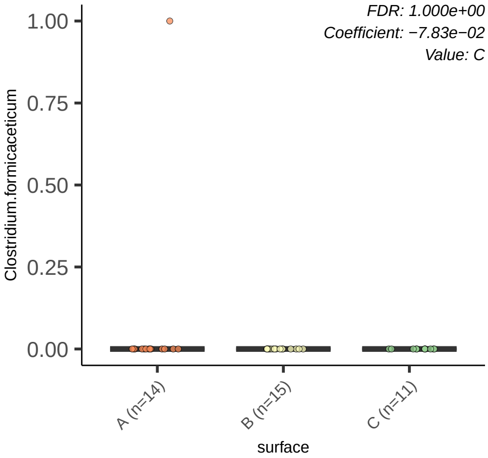

FDR: 1.000e+00
Coefficient: −7.83e−02
Value: C
1.00
0.75
0.50
0.25
0.00
Clostridium.formicaceticum
A (n=14)
B (n=15)
C (n=11)
surface

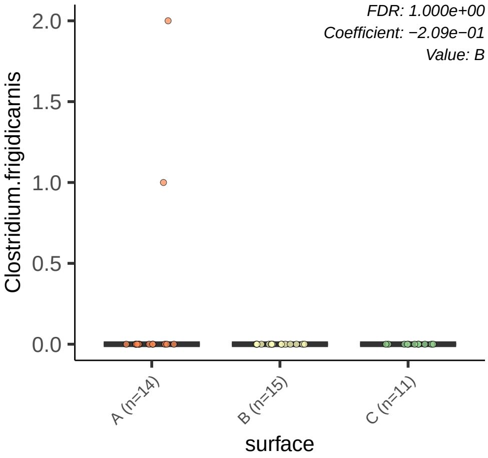
FDR: 1.000e+00
Coefficient: −2.09e−01
Value: B
Clostridium.frigidicarnis
2.0
1.5
1.0
0.5
0.0
A (n=14)
B (n=15)
C (n=11)
surface

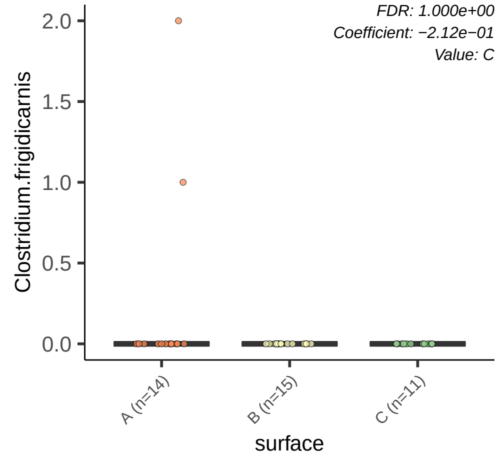
FDR: 1.000e+00
Coefficient: −2.12e−01
Value: C
Clostridium.frigidicarnis
2.0
1.5
1.0
0.5
0.0
A (n=14)
B (n=15)
C (n=11)
surface

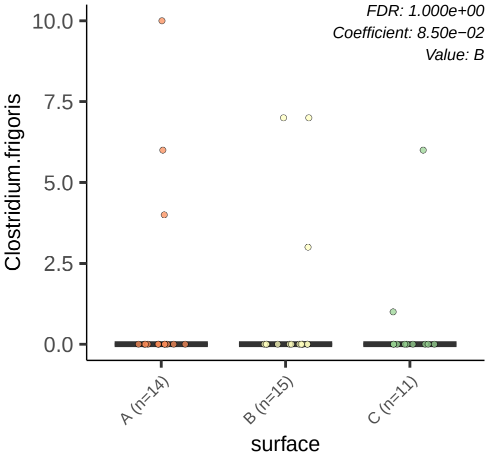

FDR: 1.000e+00
Coefficient: 8.50e−02
Value: B
Clostridium.frigoris
10.0
7.5
5.0
2.5
0.0
A (n=14)
B (n=15)
C (n=11)
surface

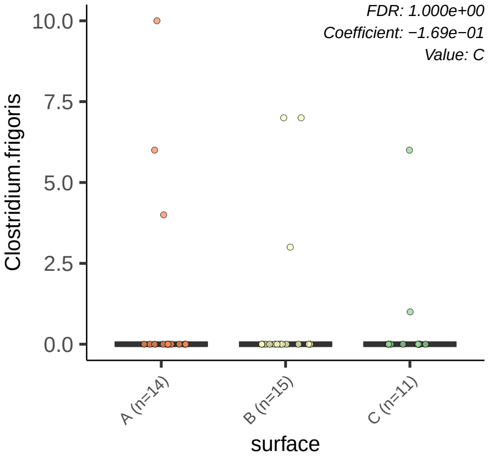

FDR: 1.000e+00
Coefficient: −1.69e−01
Value: C
Clostridium.frigoris
10.0
7.5
5.0
2.5
0.0
A (n=14)
B (n=15)
C (n=11)
surface

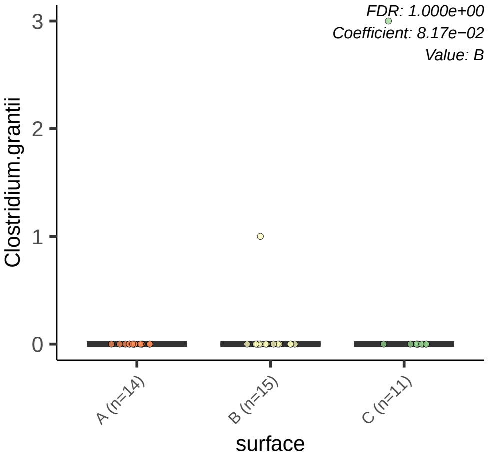
FDR: 1.000e+00
Coefficient: 8.17e−02
Value: B
Clostridium.grantii
3
2
1
0
A (n=14)
B (n=15)
C (n=11)
surface

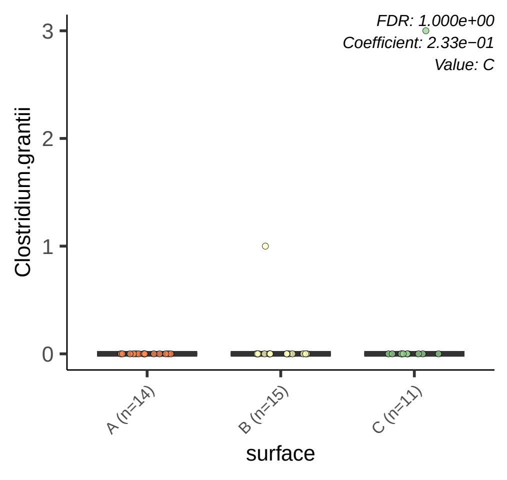
FDR: 1.000e+00
Coefficient: 2.33e−01
Value: C
Clostridium.grantii
3
2
1
0
A (n=14)
B (n=15)
C (n=11)
surface

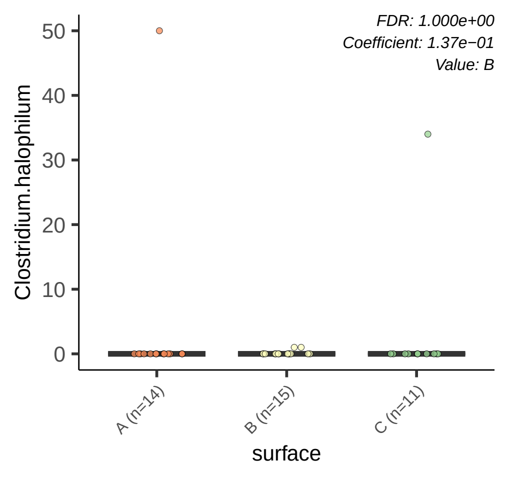
FDR: 1.000e+00
Coefficient: 1.37e−01
Value: B
Clostridium.halophilum
50
40
30
20
10
0
A (n=14)
B (n=15)
C (n=11)
surface

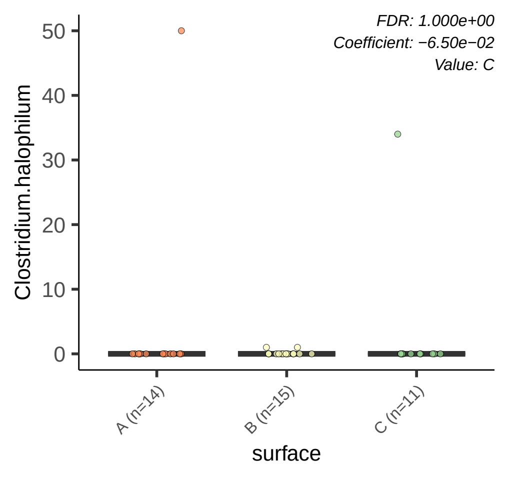

FDR: 1.000e+00
Coefficient: −6.50e−02
Value: C
Clostridium.halophilum
50
40
30
20
10
0
A (n=14)
B (n=15)
C (n=11)
surface

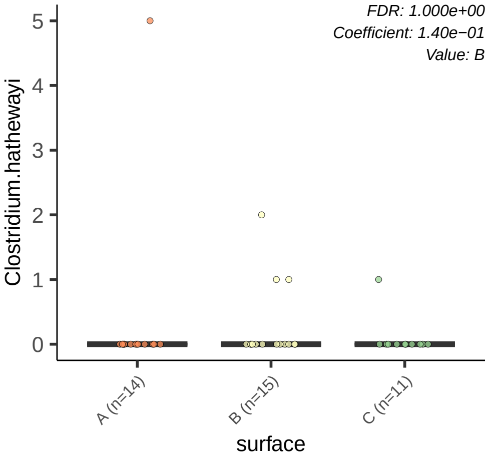
FDR: 1.000e+00
Coefficient: 1.40e−01
Value: B
Clostridium.hathewayi
0
1
2
3
4
5
A (n=14)
B (n=15)
C (n=11)
surface

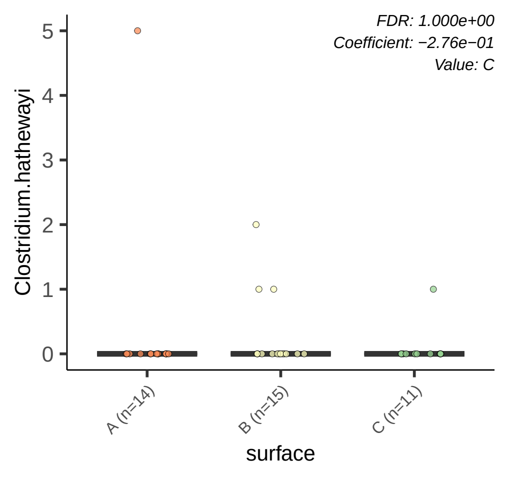

FDR: 1.000e+00
Coefficient: −2.76e−01
Value: C
Clostridium.hathewayi
5
4
3
2
1
0
A (n=14)
B (n=15)
C (n=11)
surface

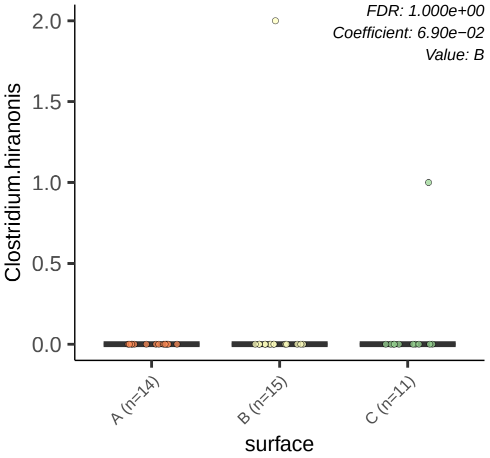
FDR: 1.000e+00
Coefficient: 6.90e−02
Value: B
Clostridium.hiranonis
2.0
1.5
1.0
0.5
0.0
A (n=14)
B (n=15)
C (n=11)
surface

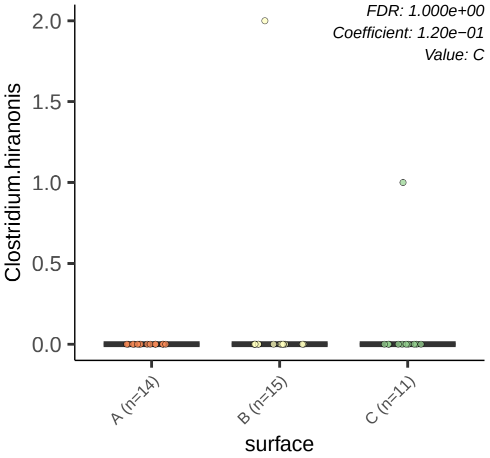

FDR: 1.000e+00
Coefficient: 1.20e−01
Value: C
Clostridium.hiranonis
2.0
1.5
1.0
0.5
0.0
A (n=14)
B (n=15)
C (n=11)
surface

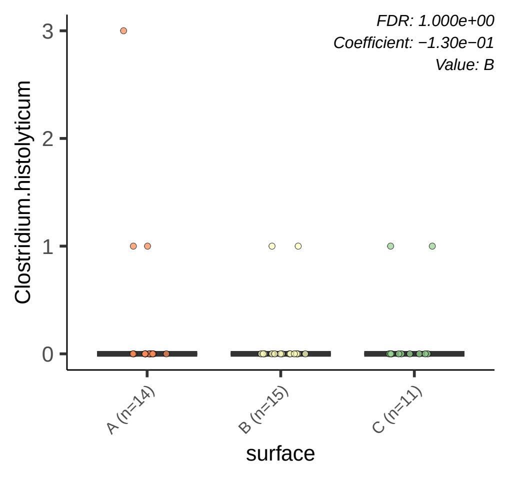

FDR: 1.000e+00
Coefficient: −1.30e−01
Value: B
Clostridium.histolyticum
3
2
1
0
A (n=14)
B (n=15)
C (n=11)
surface

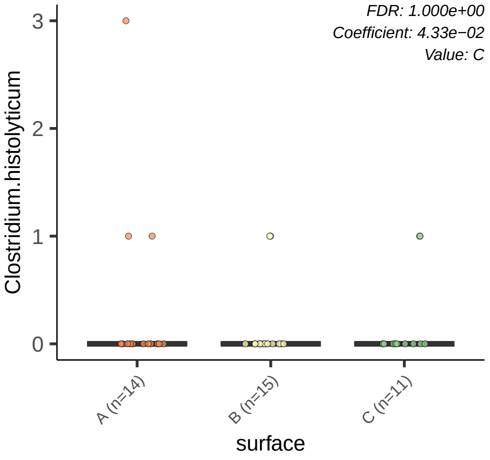

FDR: 1.000e+00
Coefficient: 4.33e−02
Value: C
Clostridium.histolyticum
3
2
1
0
A (n=14)
B (n=15)
C (n=11)
surface

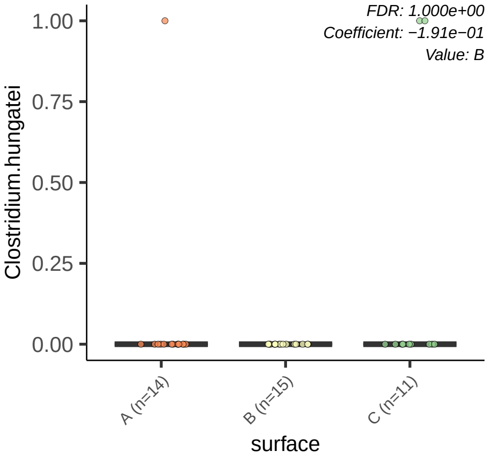
FDR: 1.000e+00
Coefficient: −1.91e−01
Value: B
Clostridium.hungatei
1.00
0.75
0.50
0.25
0.00
A (n=14)
B (n=15)
C (n=11)
surface

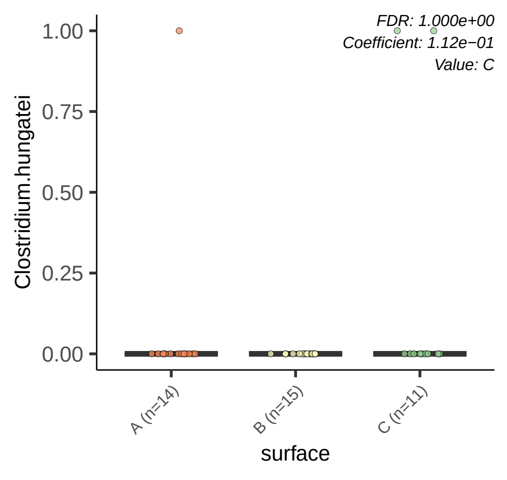
FDR: 1.000e+00
Coefficient: 1.12e−01
Value: C
Clostridium.hungatei
1.00
0.75
0.50
0.25
0.00
A (n=14)
B (n=15)
C (n=11)
surface

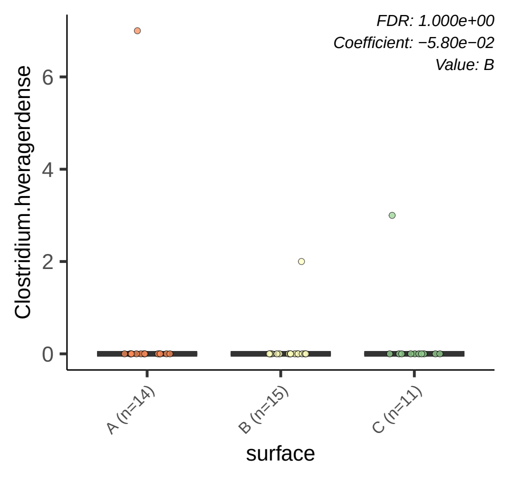

FDR: 1.000e+00
Coefficient: −5.80e−02
Value: B
Clostridium.hveragerdense
0
2
4
6
A (n=14)
B (n=15)
C (n=11)
surface

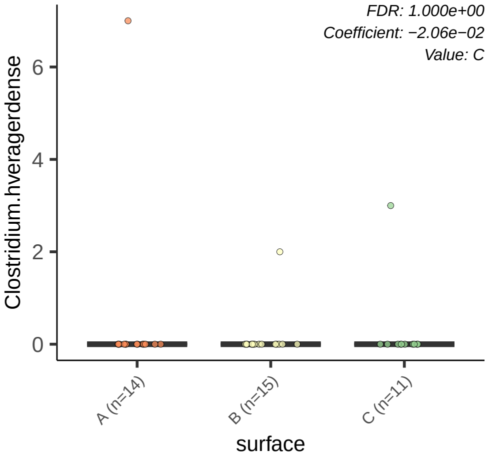

FDR: 1.000e+00
Coefficient: −2.06e−02
Value: C
Clostridium.hveragerdense
0
2
4
6
A (n=14)
B (n=15)
C (n=11)
surface

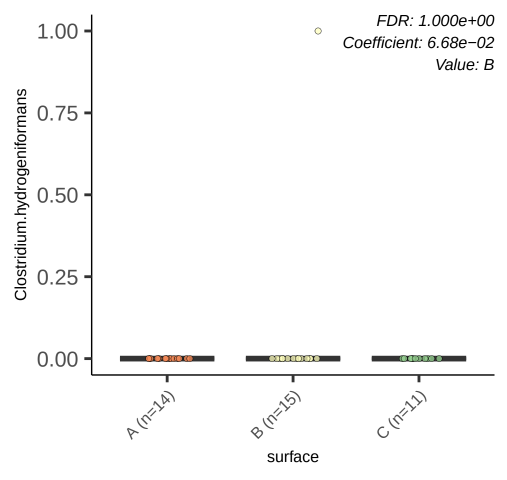
FDR: 1.000e+00
Coefficient: 6.68e−02
Value: B
Clostridium.hydrogeniformans
1.00
0.75
0.50
0.25
0.00
A (n=14)
B (n=15)
C (n=11)
surface

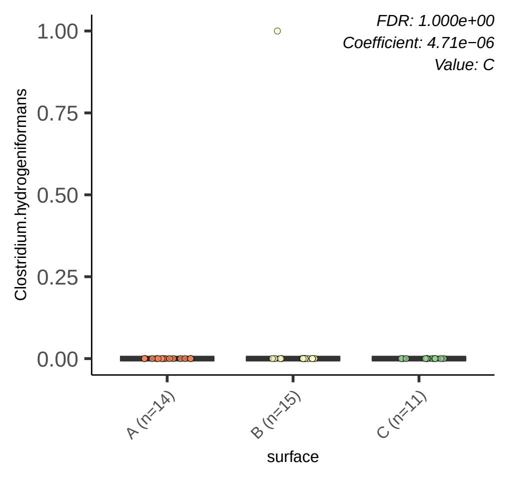
FDR: 1.000e+00
Coefficient: 4.71e−06
Value: C
1.00
0.75
0.50
0.25
0.00
Clostridium.hydrogeniformans
A (n=14)
B (n=15)
C (n=11)
surface

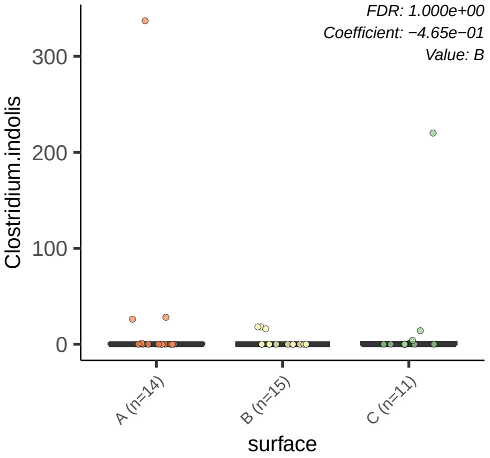
FDR: 1.000e+00
Coefficient: −4.65e−01
Value: B
Clostridium.indolis
300
200
100
0
A (n=14)
B (n=15)
C (n=11)
surface

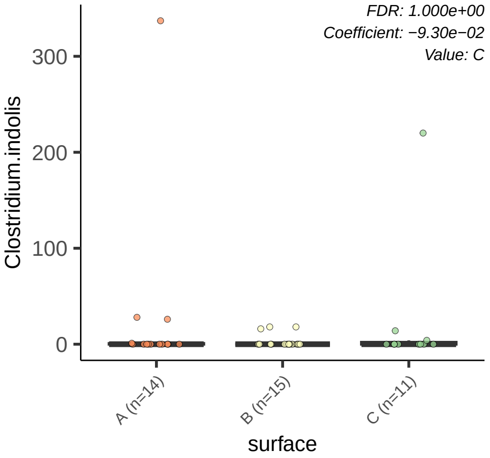

FDR: 1.000e+00
Coefficient: −9.30e−02
Value: C
Clostridium.indolis
300
200
100
0
A (n=14)
B (n=15)
C (n=11)
surface

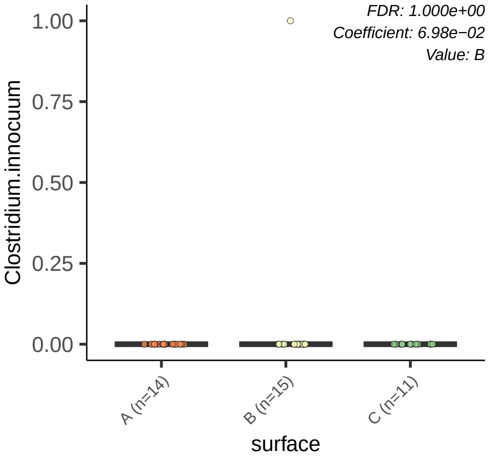

FDR: 1.000e+00
Coefficient: 6.98e−02
Value: B
Clostridium.innocuum
1.00
0.75
0.50
0.25
0.00
A (n=14)
B (n=15)
C (n=11)
surface

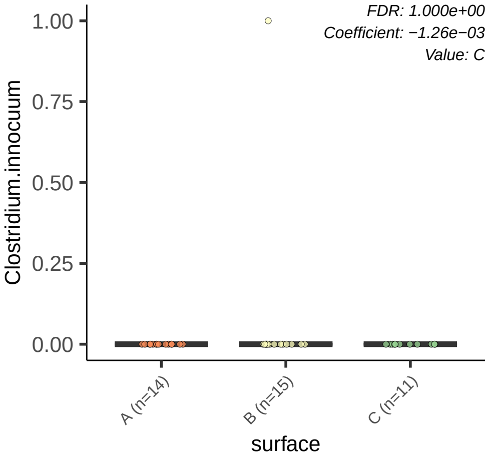
FDR: 1.000e+00
Coefficient: −1.26e−03
Value: C
Clostridium.innocuum
1.00
0.75
0.50
0.25
0.00
A (n=14)
B (n=15)
C (n=11)
surface

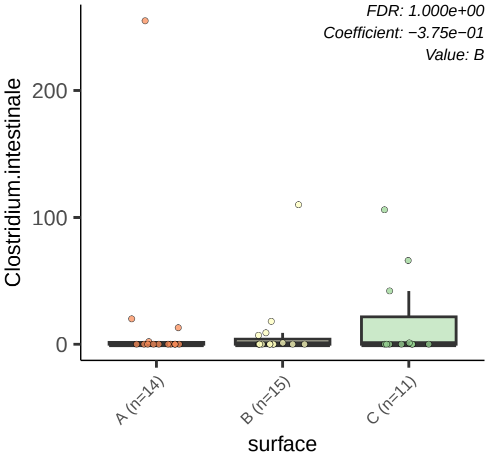

FDR: 1.000e+00
Coefficient: −3.75e−01
Value: B
Clostridium.intestinale
200
100
0
A (n=14)
B (n=15)
C (n=11)
surface

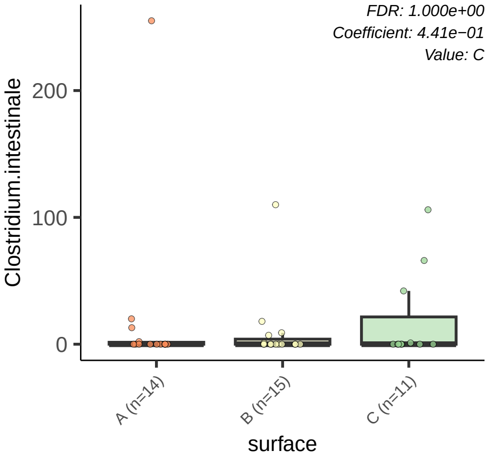
FDR: 1.000e+00
Coefficient: 4.41e−01
Value: C
Clostridium.intestinale
200
100
0
A (n=14)
B (n=15)
C (n=11)
surface

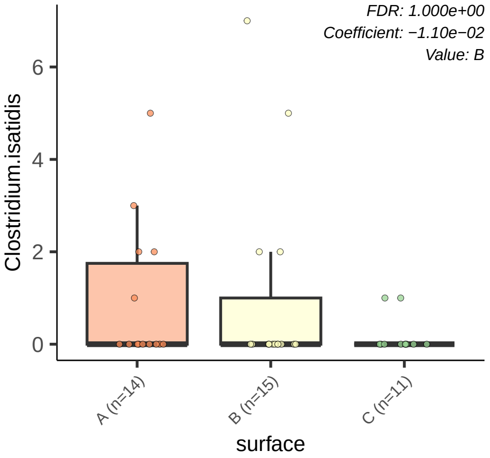
FDR: 1.000e+00
Coefficient: −1.10e−02
Value: B
Clostridium.isatidis
0
2
4
6
A (n=14)
B (n=15)
C (n=11)
surface

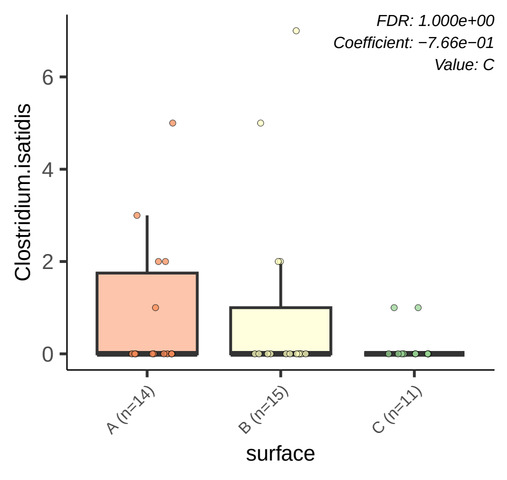

FDR: 1.000e+00
Coefficient: −7.66e−01
Value: C
Clostridium.isatidis
0
2
4
6
A (n=14)
B (n=15)
C (n=11)
surface

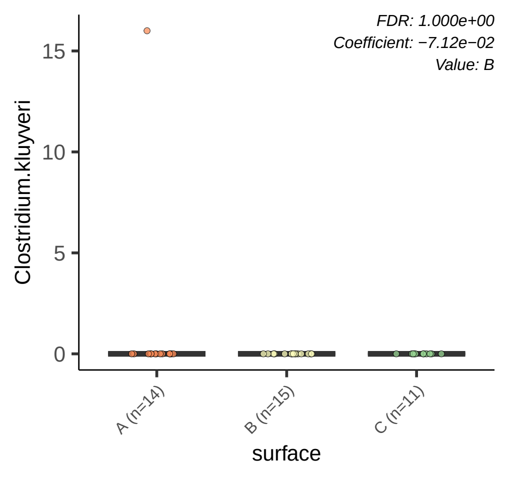

FDR: 1.000e+00
Coefficient: −7.12e−02
Value: B
Clostridium.kluyveri
15
10
5
0
A (n=14)
B (n=15)
C (n=11)
surface

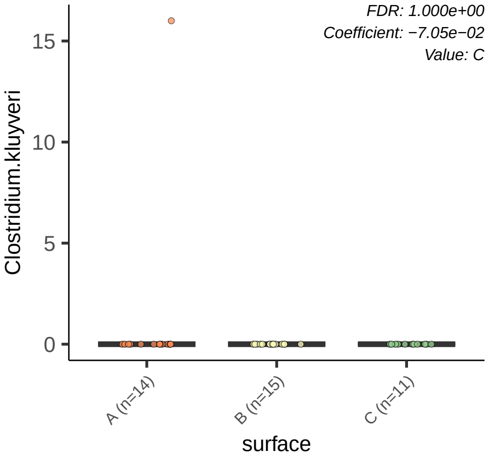

FDR: 1.000e+00
Coefficient: −7.05e−02
Value: C
Clostridium.kluyveri
15
10
5
0
A (n=14)
B (n=15)
C (n=11)
surface

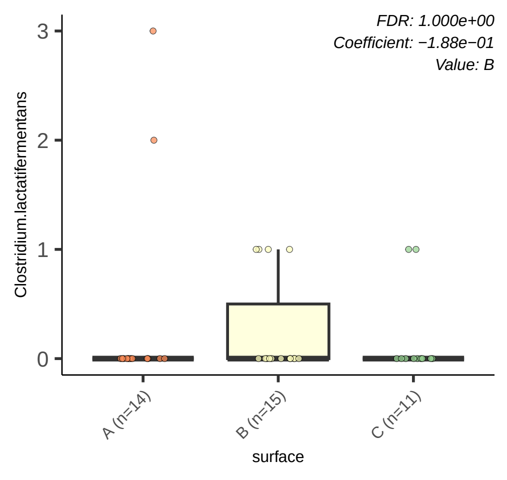
FDR: 1.000e+00
Coefficient: −1.88e−01
Value: B
Clostridium.lactatifermentans
3
2
1
0
A (n=14)
B (n=15)
C (n=11)
surface

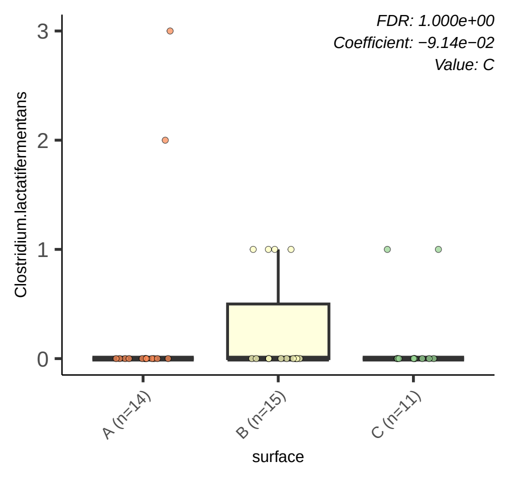
FDR: 1.000e+00
Coefficient: −9.14e−02
Value: C
Clostridium.lactatifermentans
3
2
1
0
A (n=14)
B (n=15)
C (n=11)
surface

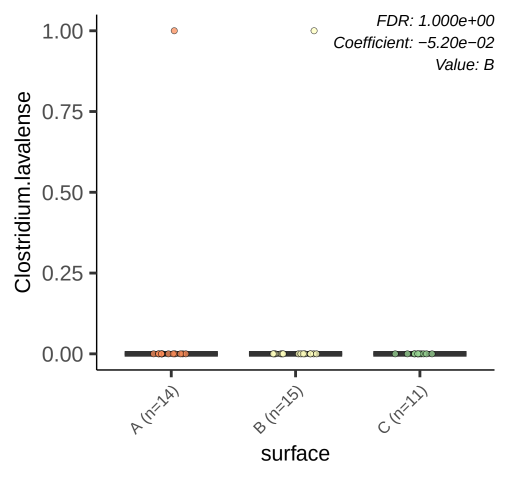
FDR: 1.000e+00
Coefficient: −5.20e−02
Value: B
Clostridium.lavalense
1.00
0.75
0.50
0.25
0.00
A (n=14)
B (n=15)
C (n=11)
surface

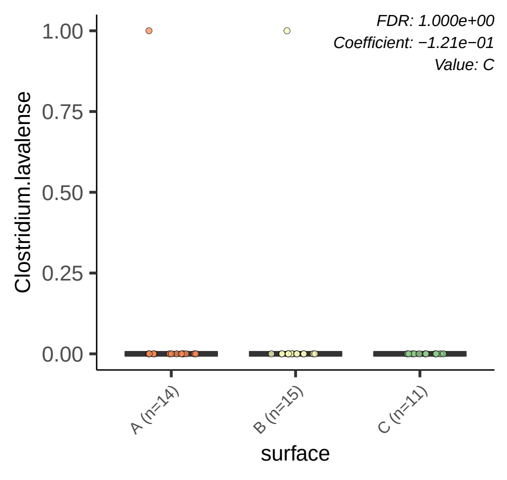
FDR: 1.000e+00
Coefficient: −1.21e−01
Value: C
Clostridium.lavalense
1.00
0.75
0.50
0.25
0.00
A (n=14)
B (n=15)
C (n=11)
surface

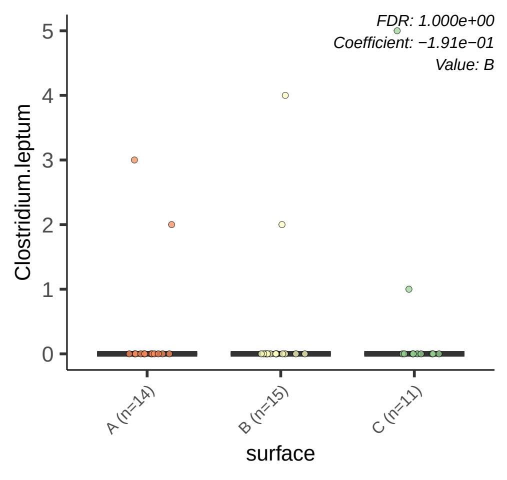
FDR: 1.000e+00
Coefficient: −1.91e−01
Value: B
Clostridium.leptum
0
1
2
3
4
5
A (n=14)
B (n=15)
C (n=11)
surface

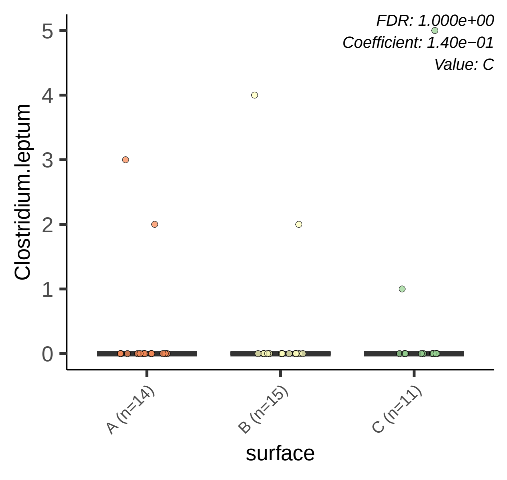
FDR: 1.000e+00
Coefficient: 1.40e−01
Value: C
Clostridium.leptum
0
1
2
3
4
5
A (n=14)
B (n=15)
C (n=11)
surface

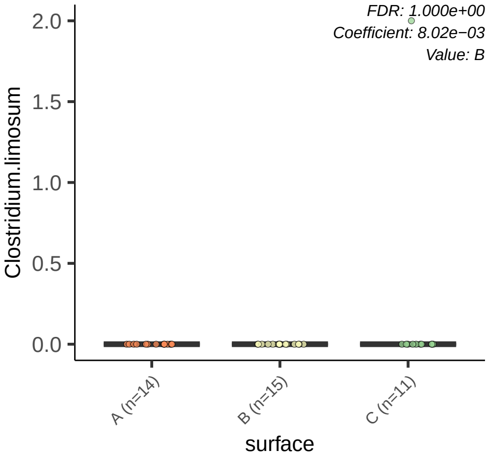
FDR: 1.000e+00
Coefficient: 8.02e−03
Value: B
Clostridium.limosum
2.0
1.5
1.0
0.5
0.0
A (n=14)
B (n=15)
C (n=11)
surface

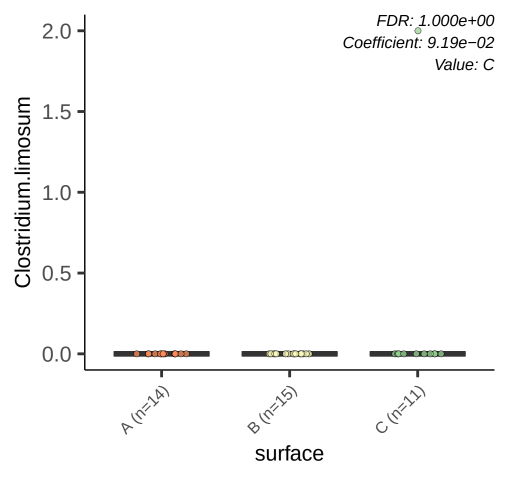
FDR: 1.000e+00
Coefficient: 9.19e−02
Value: C
Clostridium.limosum
2.0
1.5
1.0
0.5
0.0
A (n=14)
B (n=15)
C (n=11)
surface

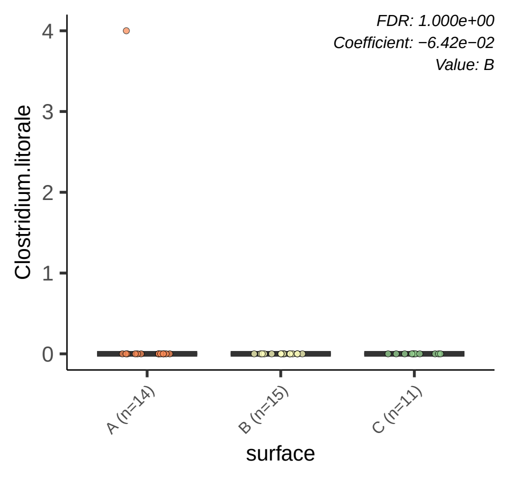
FDR: 1.000e+00
Coefficient: −6.42e−02
Value: B
Clostridium.litorale
0
1
2
3
4
A (n=14)
B (n=15)
C (n=11)
surface

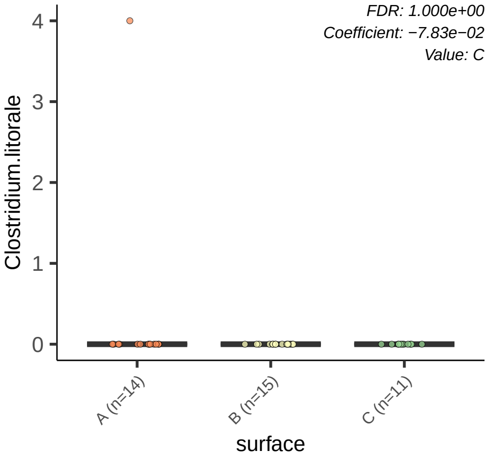
FDR: 1.000e+00
Coefficient: −7.83e−02
Value: C
Clostridium.litorale
4
3
2
1
0
A (n=14)
B (n=15)
C (n=11)
surface

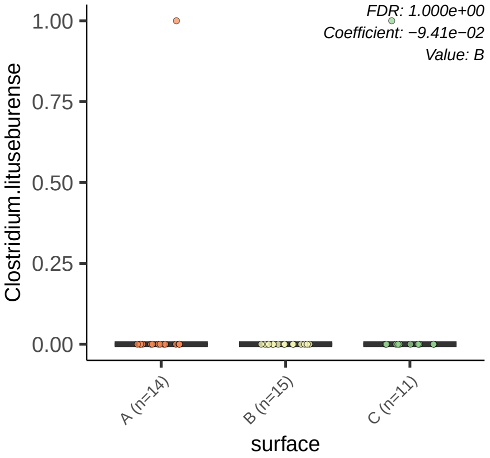
FDR: 1.000e+00
Coefficient: −9.41e−02
Value: B
Clostridium.lituseburense
1.00
0.75
0.50
0.25
0.00
A (n=14)
B (n=15)
C (n=11)
surface

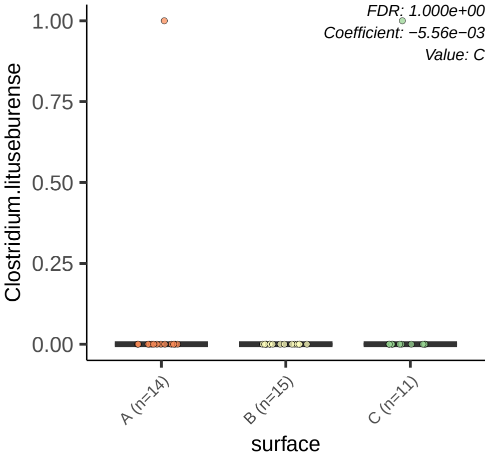
FDR: 1.000e+00
Coefficient: −5.56e−03
Value: C
Clostridium.lituseburense
1.00
0.75
0.50
0.25
0.00
A (n=14)
B (n=15)
C (n=11)
surface

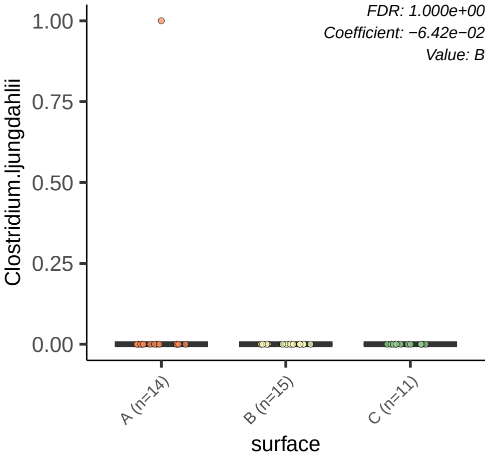
FDR: 1.000e+00
Coefficient: −6.42e−02
Value: B
Clostridium.ljungdahlii
1.00
0.75
0.50
0.25
0.00
A (n=14)
B (n=15)
C (n=11)
surface

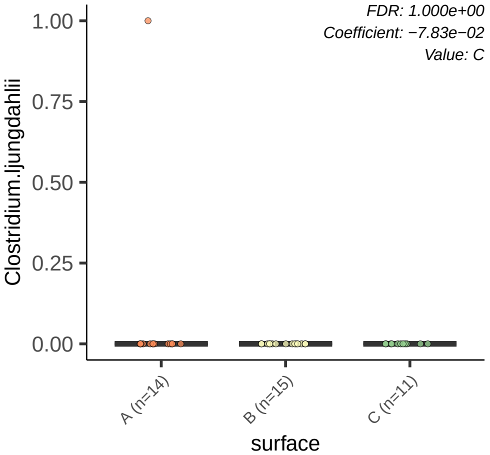

FDR: 1.000e+00
Coefficient: −7.83e−02
Value: C
Clostridium.ljungdahlii
1.00
0.75
0.50
0.25
0.00
A (n=14)
B (n=15)
C (n=11)
surface

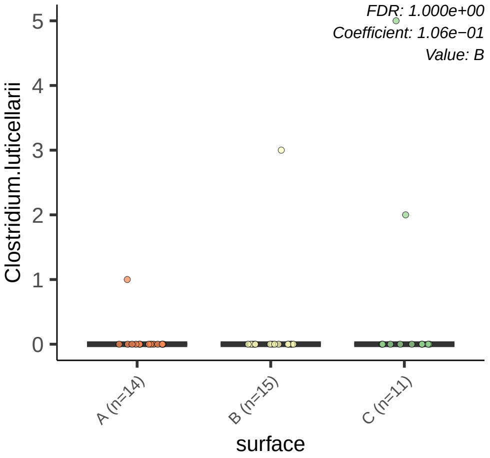

FDR: 1.000e+00
Coefficient: 1.06e−01
Value: B
Clostridium.luticellarii
5
4
3
2
1
0
A (n=14)
B (n=15)
C (n=11)
surface

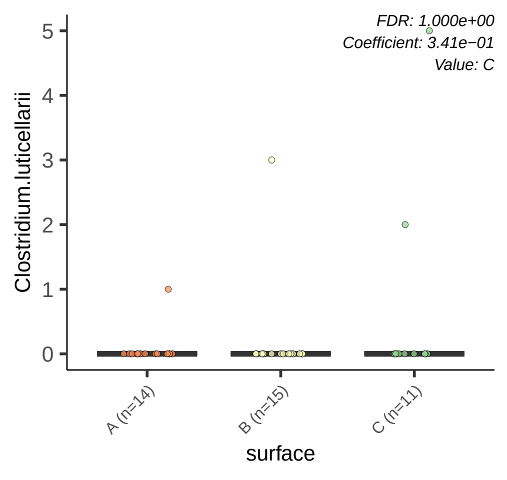
FDR: 1.000e+00
Coefficient: 3.41e−01
Value: C
Clostridium.luticellarii
0
1
2
3
4
5
A (n=14)
B (n=15)
C (n=11)
surface

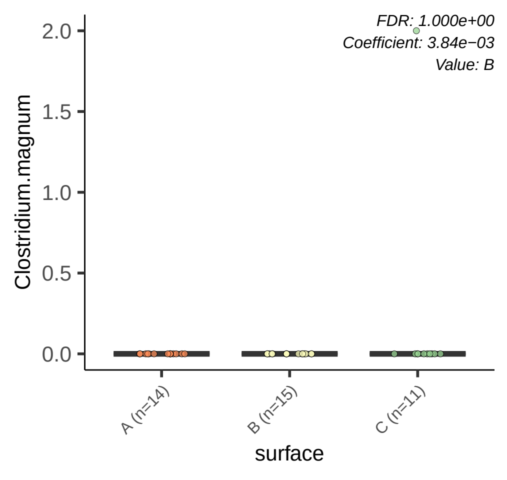
FDR: 1.000e+00
Coefficient: 3.84e−03
Value: B
Clostridium.magnum
2.0
1.5
1.0
0.5
0.0
A (n=14)
B (n=15)
C (n=11)
surface

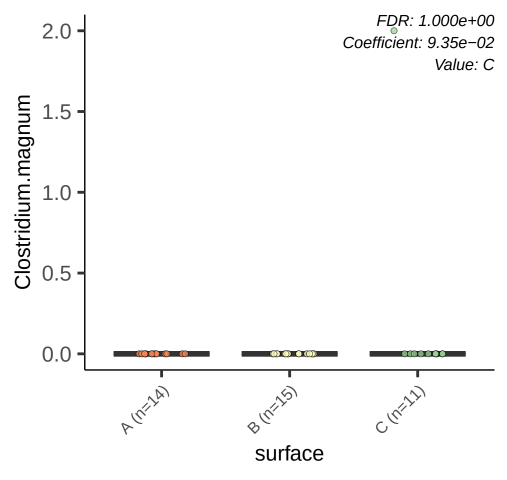
FDR: 1.000e+00
Coefficient: 9.35e−02
Value: C
Clostridium.magnum
2.0
1.5
1.0
0.5
0.0
A (n=14)
B (n=15)
C (n=11)
surface

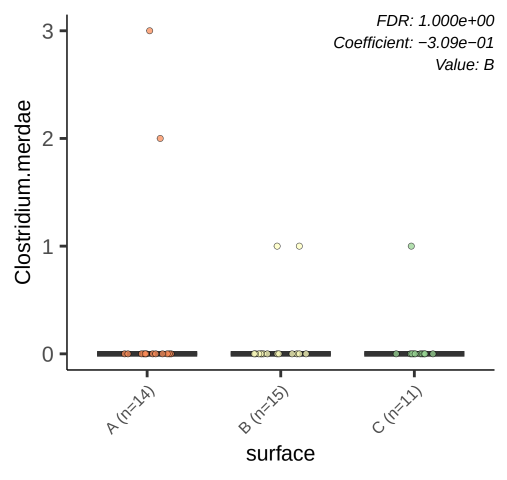

FDR: 1.000e+00
Coefficient: −3.09e−01
Value: B
Clostridium.merdae
3
2
1
0
A (n=14)
B (n=15)
C (n=11)
surface

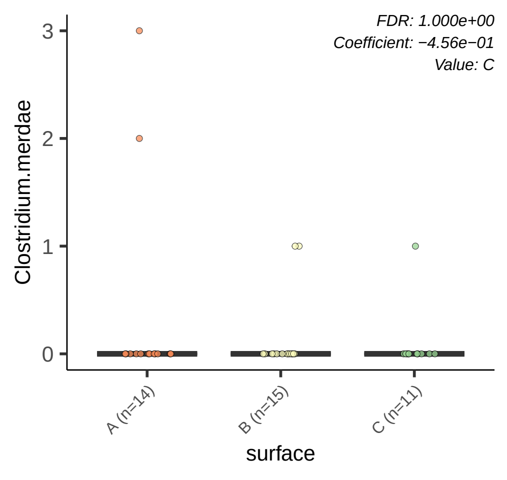
FDR: 1.000e+00
Coefficient: −4.56e−01
Value: C
Clostridium.merdae
3
2
1
0
A (n=14)
B (n=15)
C (n=11)
surface

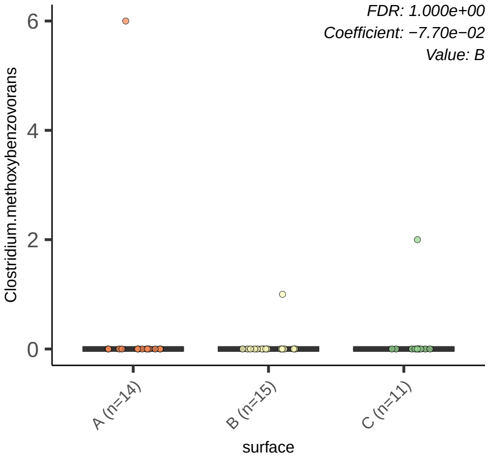

FDR: 1.000e+00
Coefficient: −7.70e−02
Value: B
Clostridium.methoxybenzovorans
0
2
4
6
A (n=14)
B (n=15)
C (n=11)
surface

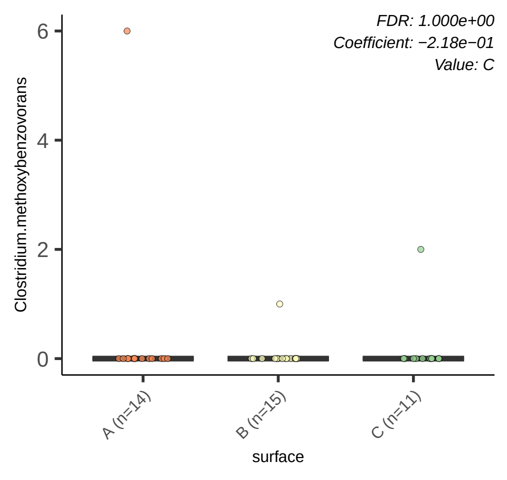
FDR: 1.000e+00
Coefficient: −2.18e−01
Value: C
Clostridium.methoxybenzovorans
6
4
2
0
A (n=14)
B (n=15)
C (n=11)
surface

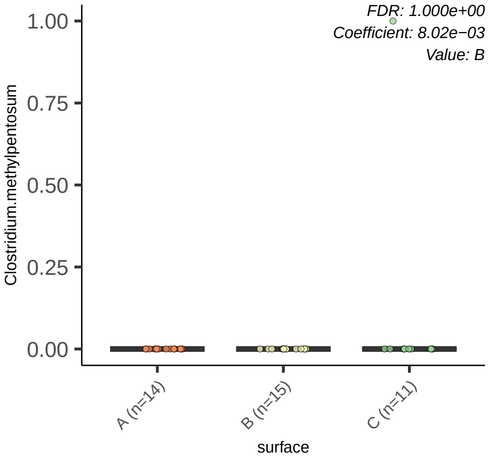

FDR: 1.000e+00
Coefficient: 8.02e−03
Value: B
1.00
0.75
0.50
0.25
0.00
Clostridium.methylpentosum
A (n=14)
B (n=15)
C (n=11)
surface

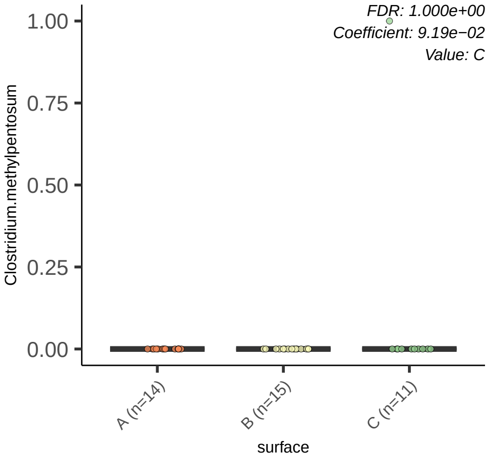

FDR: 1.000e+00
Coefficient: 9.19e−02
Value: C
1.00
0.75
0.50
0.25
0.00
Clostridium.methylpentosum
A (n=14)
B (n=15)
C (n=11)
surface

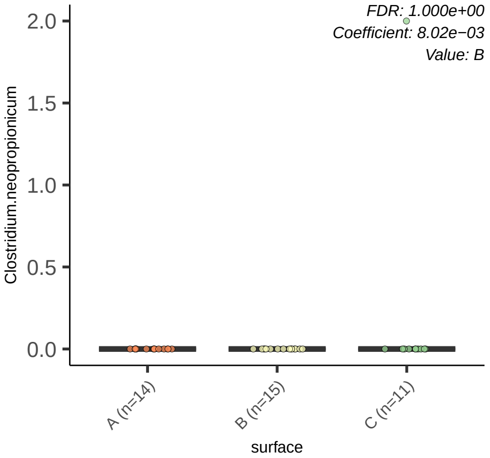
FDR: 1.000e+00
Coefficient: 8.02e−03
Value: B
Clostridium.neopropionicum
2.0
1.5
1.0
0.5
0.0
A (n=14)
B (n=15)
C (n=11)
surface

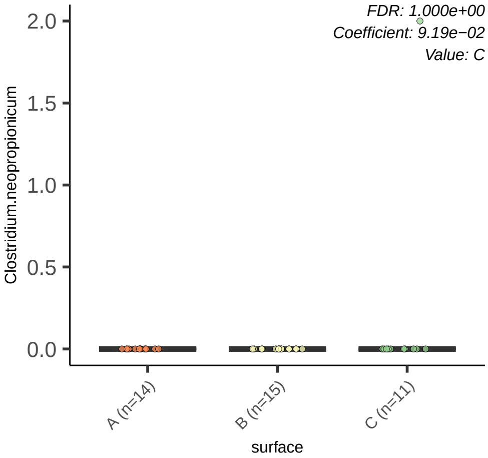
FDR: 1.000e+00
Coefficient: 9.19e−02
Value: C
Clostridium.neopropionicum
2.0
1.5
1.0
0.5
0.0
A (n=14)
B (n=15)
C (n=11)
surface

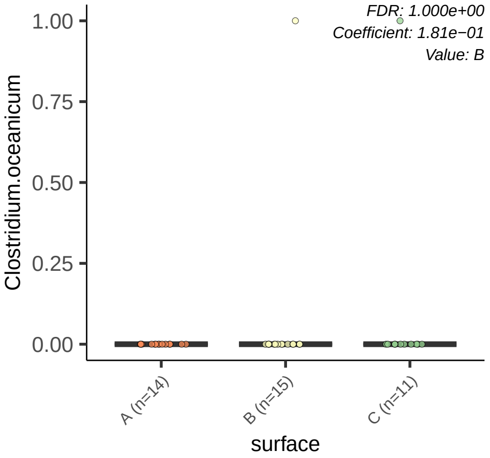

FDR: 1.000e+00
Coefficient: 1.81e−01
Value: B
Clostridium.oceanicum
1.00
0.75
0.50
0.25
0.00
A (n=14)
B (n=15)
C (n=11)
surface

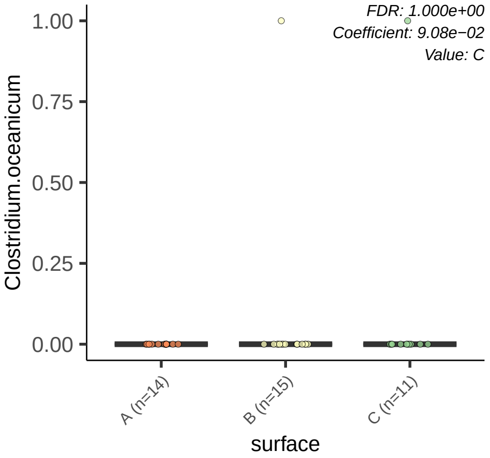
FDR: 1.000e+00
Coefficient: 9.08e−02
Value: C
Clostridium.oceanicum
1.00
0.75
0.50
0.25
0.00
A (n=14)
B (n=15)
C (n=11)
surface

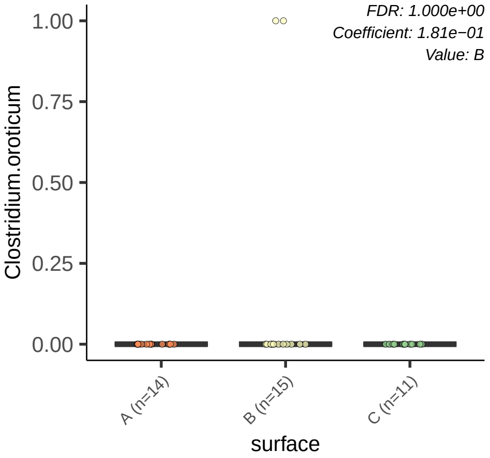
FDR: 1.000e+00
Coefficient: 1.81e−01
Value: B
Clostridium.oroticum
1.00
0.75
0.50
0.25
0.00
A (n=14)
B (n=15)
C (n=11)
surface

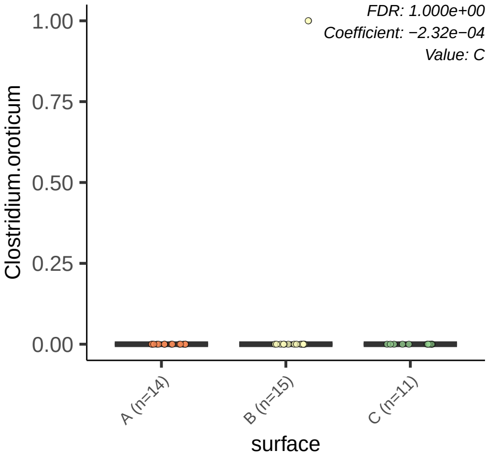
FDR: 1.000e+00
Coefficient: −2.32e−04
Value: C
Clostridium.oroticum
1.00
0.75
0.50
0.25
0.00
A (n=14)
B (n=15)
C (n=11)
surface

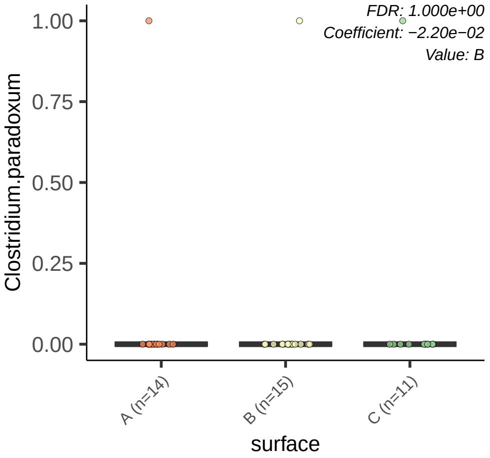

FDR: 1.000e+00
Coefficient: −2.20e−02
Value: B
Clostridium.paradoxum
1.00
0.75
0.50
0.25
0.00
A (n=14)
B (n=15)
C (n=11)
surface

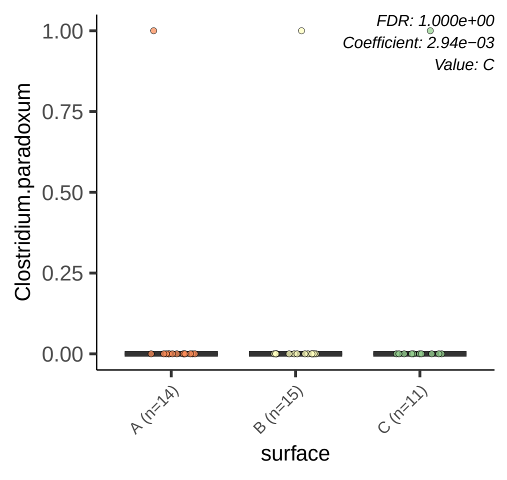
FDR: 1.000e+00
Coefficient: 2.94e−03
Value: C
1.00
0.75
0.50
0.25
0.00
Clostridium.paradoxum
A (n=14)
B (n=15)
C (n=11)
surface

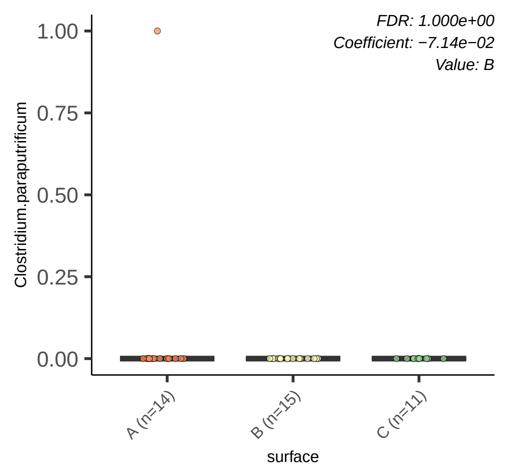

FDR: 1.000e+00
Coefficient: −7.14e−02
Value: B
1.00
0.75
0.50
0.25
0.00
Clostridium.paraputrificum
A (n=14)
B (n=15)
C (n=11)
surface

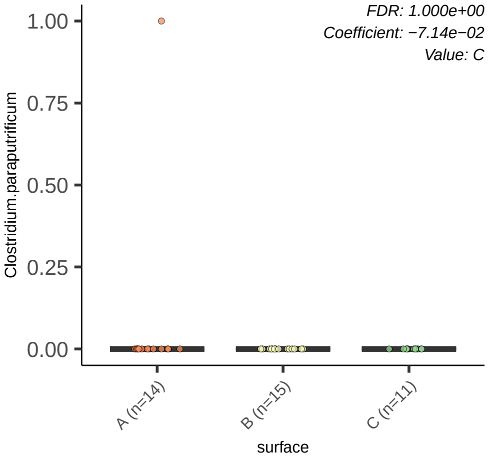
FDR: 1.000e+00
Coefficient: −7.14e−02
Value: C
1.00
0.75
0.50
0.25
0.00
Clostridium.paraputrificum
A (n=14)
B (n=15)
C (n=11)
surface

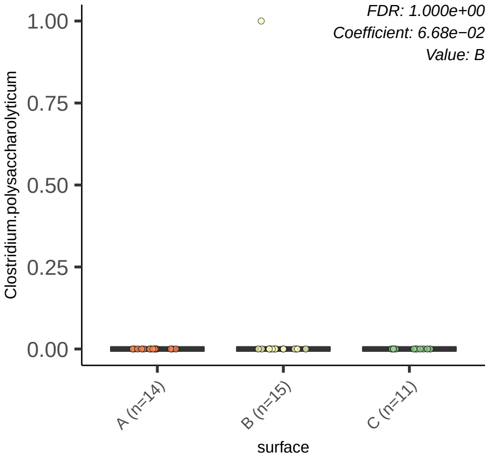

FDR: 1.000e+00
Coefficient: 6.68e−02
Value: B
Clostridium.polysaccharolyticum
1.00
0.75
0.50
0.25
0.00
A (n=14)
B (n=15)
C (n=11)
surface

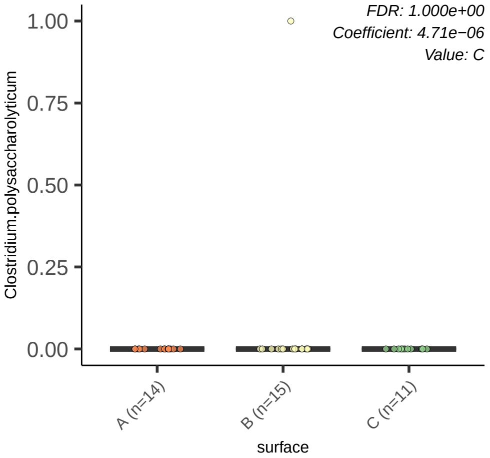
FDR: 1.000e+00
Coefficient: 4.71e−06
Value: C
Clostridium.polysaccharolyticum
1.00
0.75
0.50
0.25
0.00
A (n=14)
B (n=15)
C (n=11)
surface

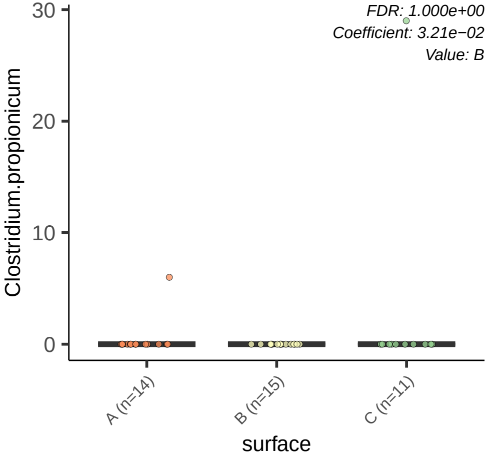
FDR: 1.000e+00
Coefficient: 3.21e−02
Value: B
Clostridium.propionicum
30
20
10
0
A (n=14)
B (n=15)
C (n=11)
surface

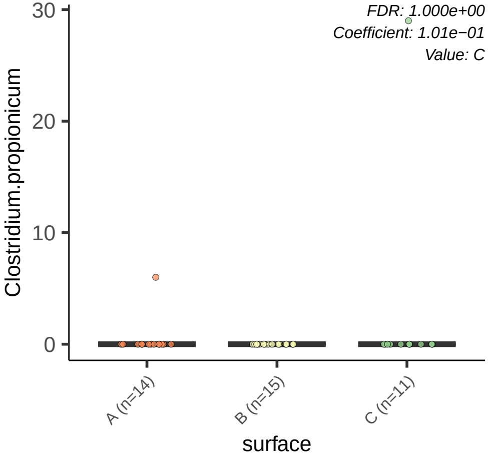

FDR: 1.000e+00
Coefficient: 1.01e−01
Value: C
Clostridium.propionicum
30
20
10
0
A (n=14)
B (n=15)
C (n=11)
surface

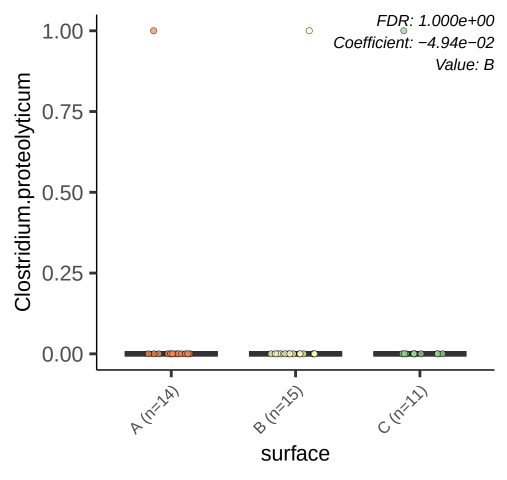
FDR: 1.000e+00
Coefficient: −4.94e−02
Value: B
Clostridium.proteolyticum
1.00
0.75
0.50
0.25
0.00
A (n=14)
B (n=15)
C (n=11)
surface

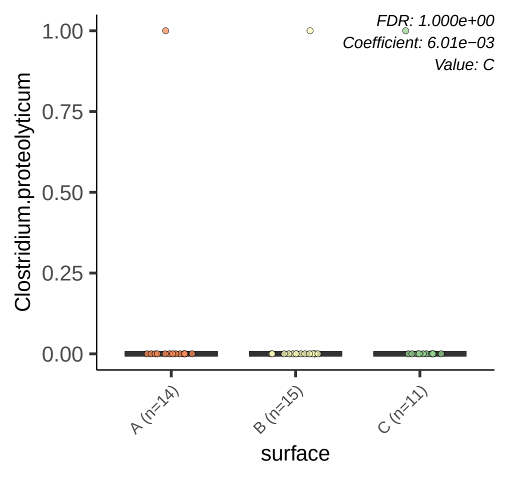
FDR: 1.000e+00
Coefficient: 6.01e−03
Value: C
Clostridium.proteolyticum
1.00
0.75
0.50
0.25
0.00
A (n=14)
B (n=15)
C (n=11)
surface

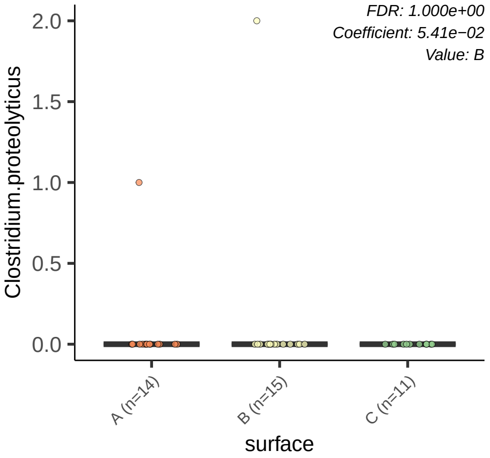
FDR: 1.000e+00
Coefficient: 5.41e−02
Value: B
Clostridium.proteolyticus
2.0
1.5
1.0
0.5
0.0
A (n=14)
B (n=15)
C (n=11)
surface

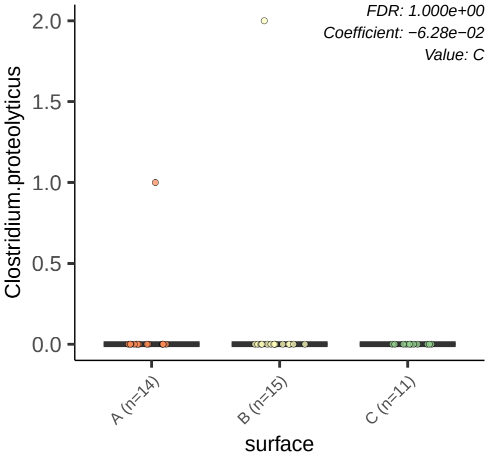
FDR: 1.000e+00
Coefficient: −6.28e−02
Value: C
Clostridium.proteolyticus
2.0
1.5
1.0
0.5
0.0
A (n=14)
B (n=15)
C (n=11)
surface

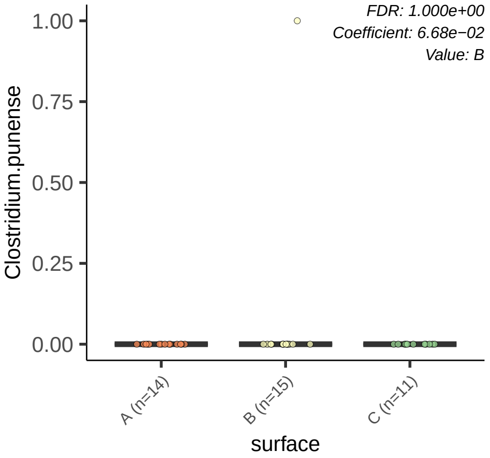
FDR: 1.000e+00
Coefficient: 6.68e−02
Value: B
Clostridium.punense
1.00
0.75
0.50
0.25
0.00
A (n=14)
B (n=15)
C (n=11)
surface

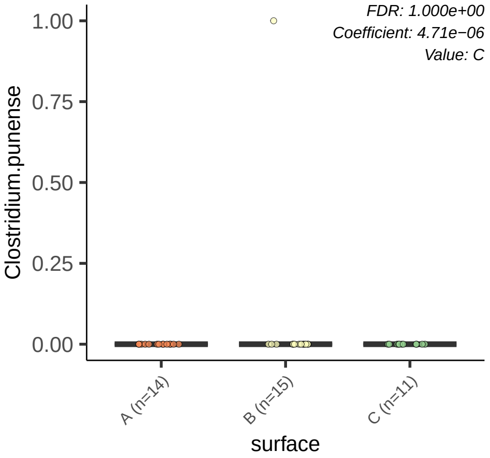

FDR: 1.000e+00
Coefficient: 4.71e−06
Value: C
Clostridium.punense
1.00
0.75
0.50
0.25
0.00
A (n=14)
B (n=15)
C (n=11)
surface

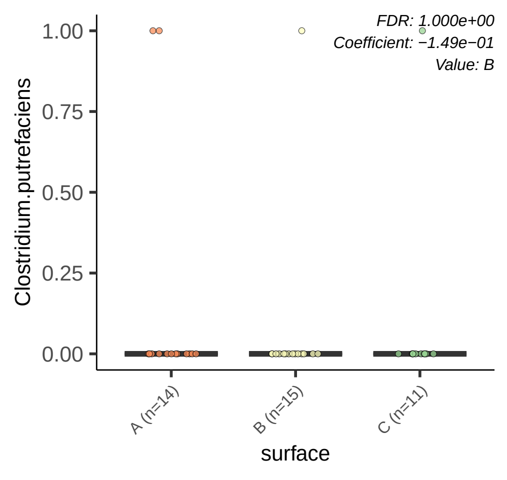
FDR: 1.000e+00
Coefficient: −1.49e−01
Value: B
Clostridium.putrefaciens
1.00
0.75
0.50
0.25
0.00
A (n=14)
B (n=15)
C (n=11)
surface

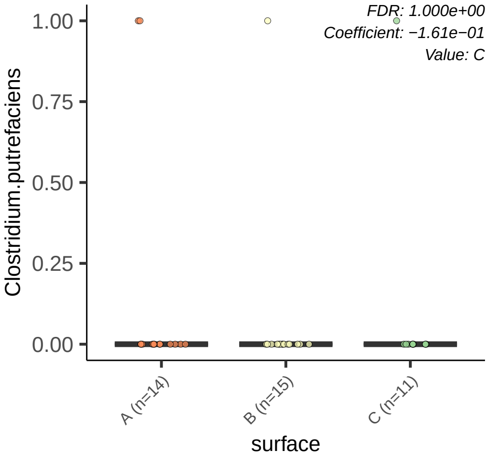

FDR: 1.000e+00
Coefficient: −1.61e−01
Value: C
Clostridium.putrefaciens
1.00
0.75
0.50
0.25
0.00
A (n=14)
B (n=15)
C (n=11)
surface

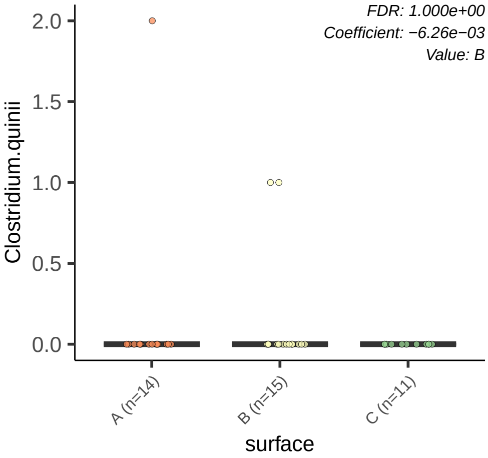

FDR: 1.000e+00
Coefficient: −6.26e−03
Value: B
Clostridium.quinii
2.0
1.5
1.0
0.5
0.0
A (n=14)
B (n=15)
C (n=11)
surface

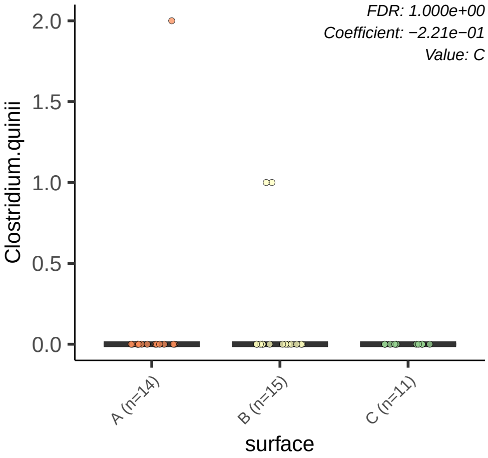
FDR: 1.000e+00
Coefficient: −2.21e−01
Value: C
Clostridium.quinii
2.0
1.5
1.0
0.5
0.0
A (n=14)
B (n=15)
C (n=11)
surface

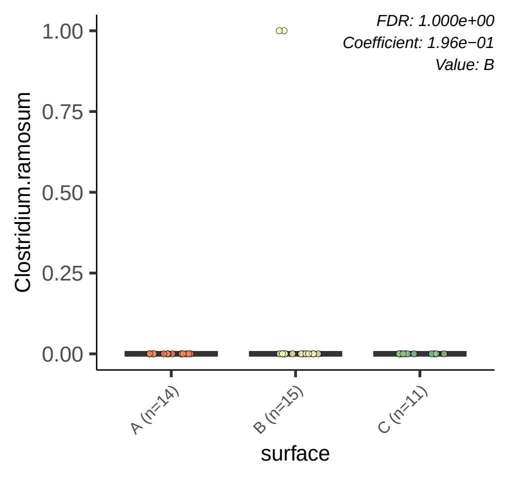
FDR: 1.000e+00
Coefficient: 1.96e−01
Value: B
Clostridium.ramosum
1.00
0.75
0.50
0.25
0.00
A (n=14)
B (n=15)
C (n=11)
surface

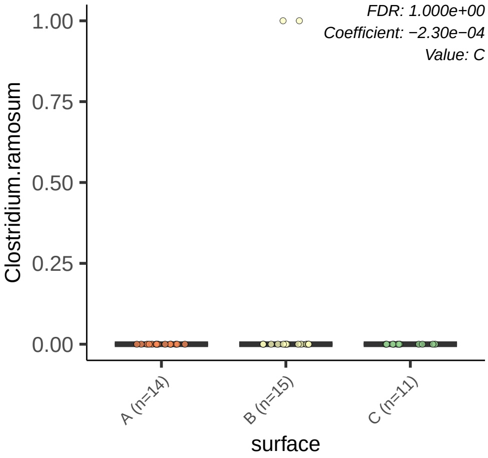

FDR: 1.000e+00
Coefficient: −2.30e−04
Value: C
Clostridium.ramosum
1.00
0.75
0.50
0.25
0.00
A (n=14)
B (n=15)
C (n=11)
surface

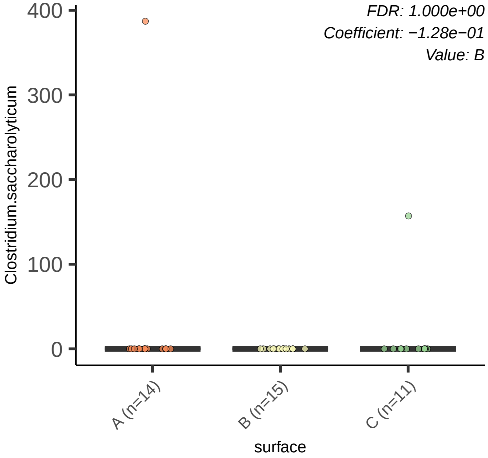
FDR: 1.000e+00
Coefficient: −1.28e−01
Value: B
Clostridium.saccharolyticum
400
300
200
100
0
A (n=14)
B (n=15)
C (n=11)
surface

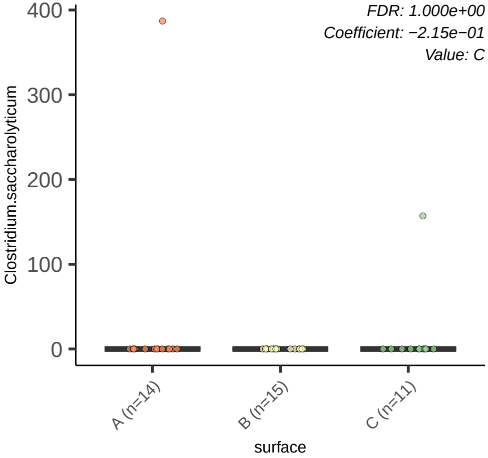
FDR: 1.000e+00
Coefficient: −2.15e−01
Value: C
Clostridium.saccharolyticum
400
300
200
100
0
A (n=14)
B (n=15)
C (n=11)
surface

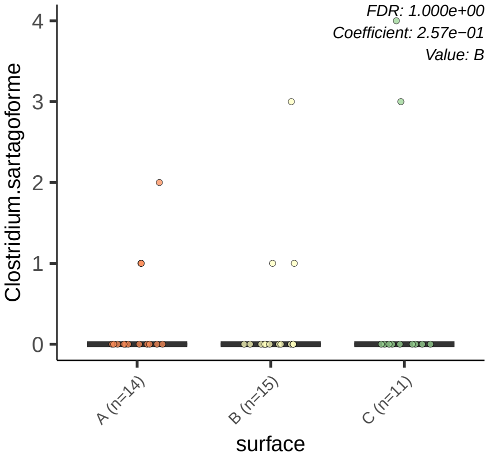
FDR: 1.000e+00
Coefficient: 2.57e−01
Value: B
Clostridium.sartagoforme
0
1
2
3
4
A (n=14)
B (n=15)
C (n=11)
surface

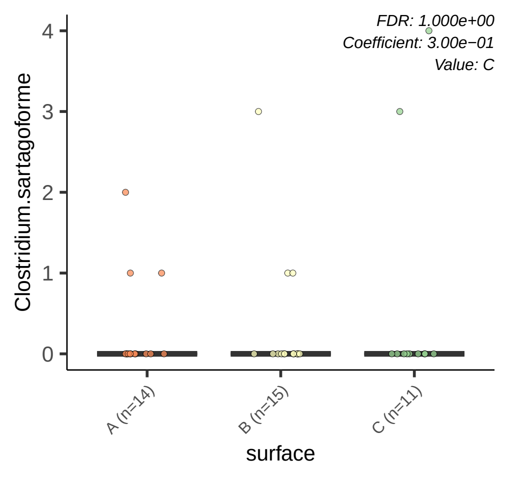
FDR: 1.000e+00
Coefficient: 3.00e−01
Value: C
Clostridium.sartagoforme
4
3
2
1
0
A (n=14)
B (n=15)
C (n=11)
surface

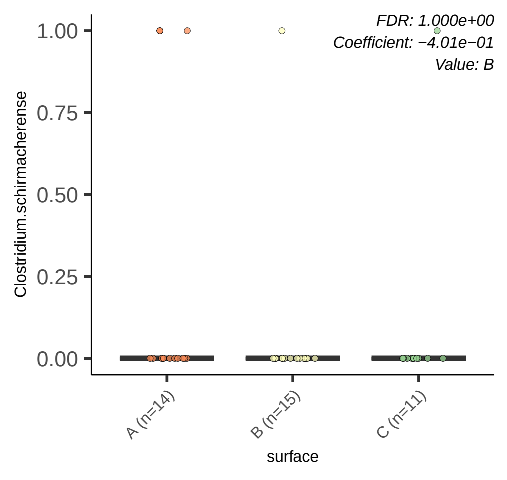
FDR: 1.000e+00
Coefficient: −4.01e−01
Value: B
Clostridium.schirmacherense
1.00
0.75
0.50
0.25
0.00
A (n=14)
B (n=15)
C (n=11)
surface

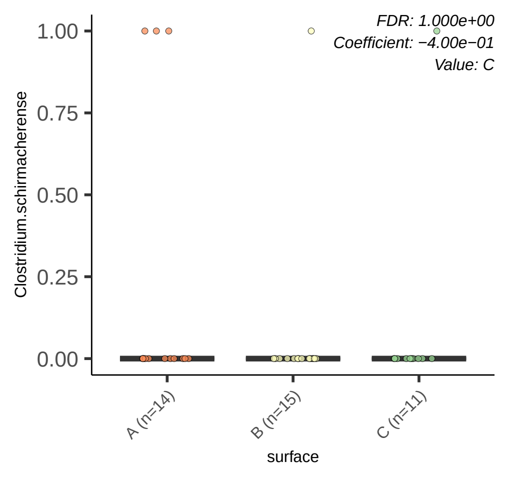
FDR: 1.000e+00
Coefficient: −4.00e−01
Value: C
Clostridium.schirmacherense
1.00
0.75
0.50
0.25
0.00
A (n=14)
B (n=15)
C (n=11)
surface

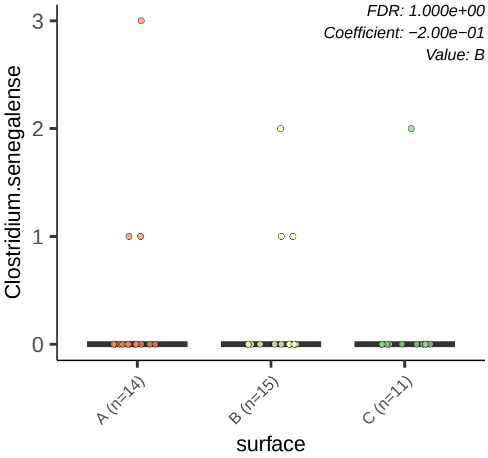

FDR: 1.000e+00
Coefficient: −2.00e−01
Value: B
Clostridium.senegalense
3
2
1
0
A (n=14)
B (n=15)
C (n=11)
surface

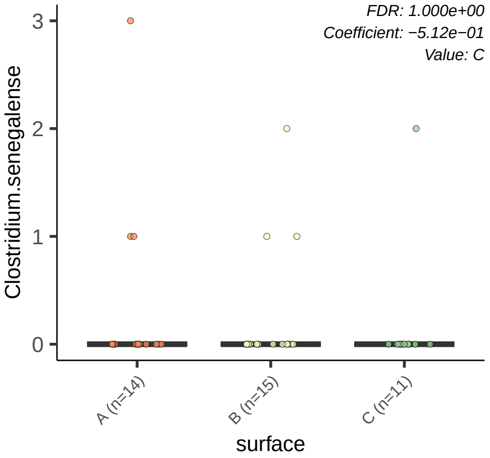
FDR: 1.000e+00
Coefficient: −5.12e−01
Value: C
Clostridium.senegalense
0
1
2
3
A (n=14)
B (n=15)
C (n=11)
surface

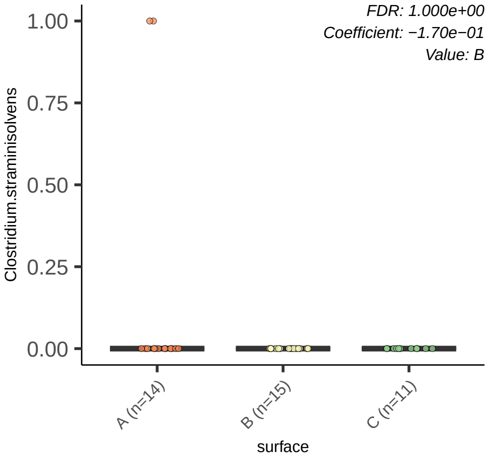
FDR: 1.000e+00
Coefficient: −1.70e−01
Value: B
Clostridium.straminisolvens
1.00
0.75
0.50
0.25
0.00
A (n=14)
B (n=15)
C (n=11)
surface

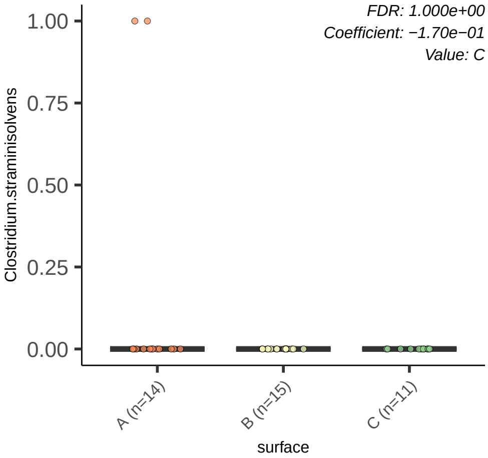
FDR: 1.000e+00
Coefficient: −1.70e−01
Value: C
Clostridium.straminisolvens
1.00
0.75
0.50
0.25
0.00
A (n=14)
B (n=15)
C (n=11)
surface

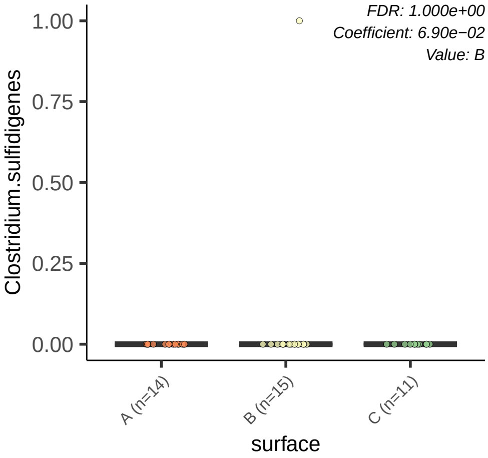

FDR: 1.000e+00
Coefficient: 6.90e−02
Value: B
Clostridium.sulfidigenes
1.00
0.75
0.50
0.25
0.00
A (n=14)
B (n=15)
C (n=11)
surface

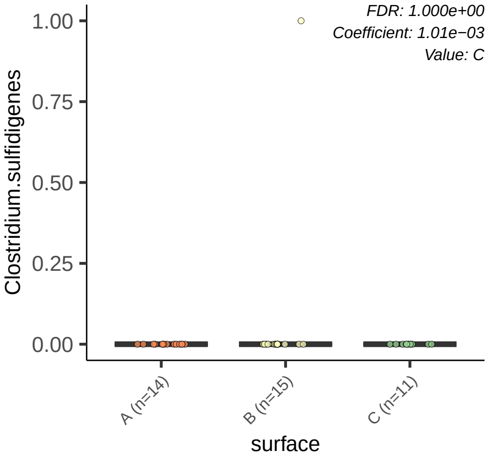
FDR: 1.000e+00
Coefficient: 1.01e−03
Value: C
Clostridium.sulfidigenes
1.00
0.75
0.50
0.25
0.00
A (n=14)
B (n=15)
C (n=11)
surface

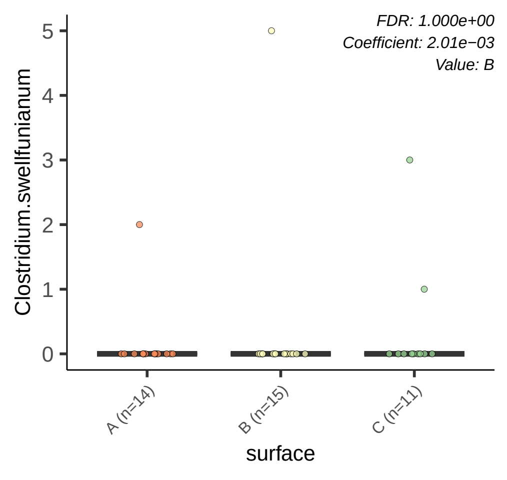

FDR: 1.000e+00
Coefficient: 2.01e−03
Value: B
Clostridium.swellfunianum
0
1
2
3
4
5
A (n=14)
B (n=15)
C (n=11)
surface

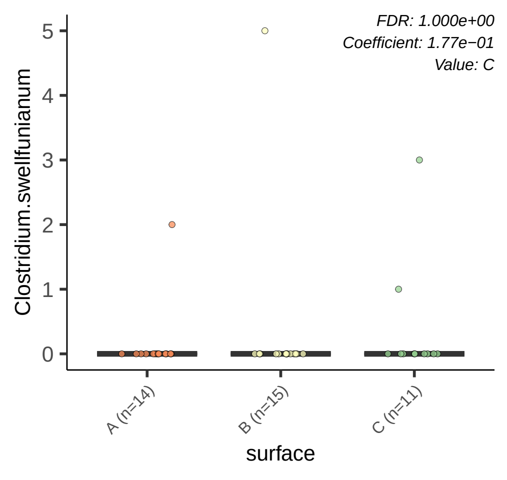
FDR: 1.000e+00
Coefficient: 1.77e−01
Value: C
Clostridium.swellfunianum
0
1
2
3
4
5
A (n=14)
B (n=15)
C (n=11)
surface

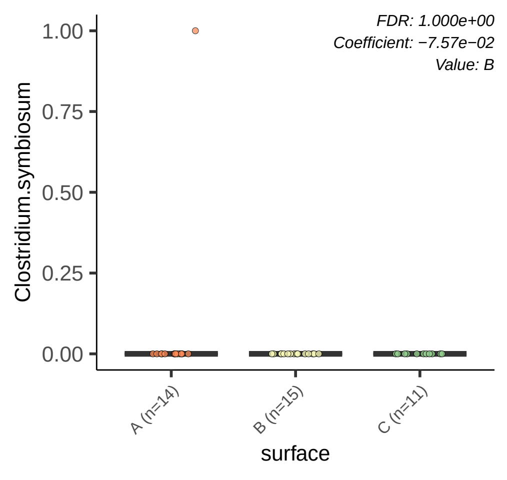
FDR: 1.000e+00
Coefficient: −7.57e−02
Value: B
Clostridium.symbiosum
1.00
0.75
0.50
0.25
0.00
A (n=14)
B (n=15)
C (n=11)
surface

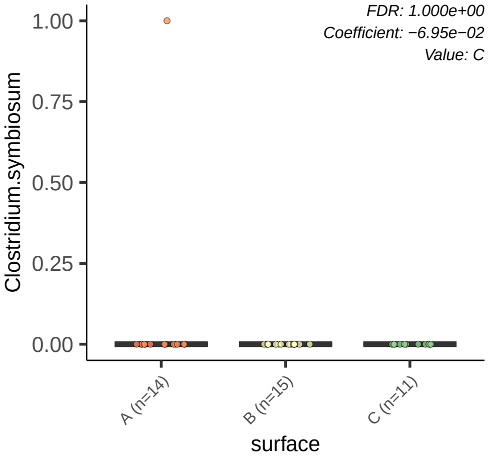
FDR: 1.000e+00
Coefficient: −6.95e−02
Value: C
Clostridium.symbiosum
1.00
0.75
0.50
0.25
0.00
A (n=14)
B (n=15)
C (n=11)
surface

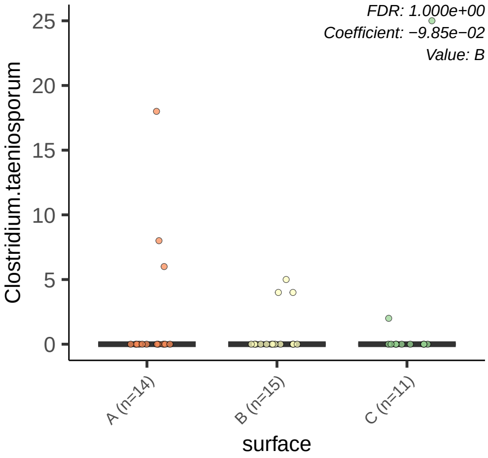

FDR: 1.000e+00
Coefficient: −9.85e−02
Value: B
Clostridium.taeniosporum
25
20
15
10
5
0
A (n=14)
B (n=15)
C (n=11)
surface

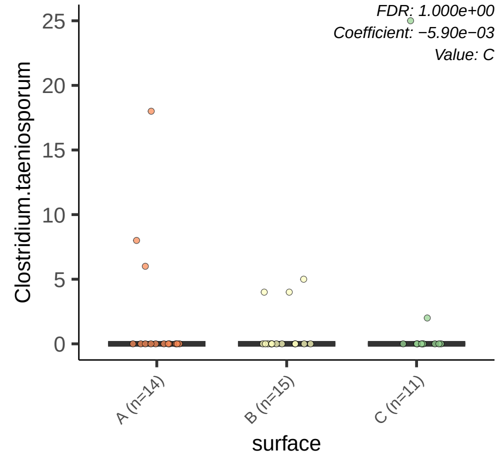

FDR: 1.000e+00
Coefficient: −5.90e−03
Value: C
Clostridium.taeniosporum
0
5
10
15
20
25
A (n=14)
B (n=15)
C (n=11)
surface

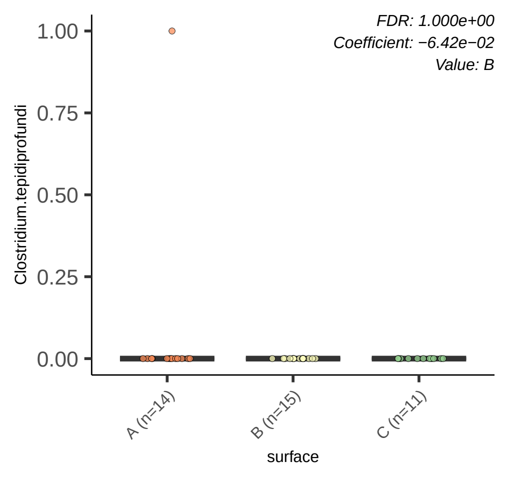
FDR: 1.000e+00
Coefficient: −6.42e−02
Value: B
1.00
0.75
0.50
0.25
0.00
Clostridium.tepidiprofundi
A (n=14)
B (n=15)
C (n=11)
surface

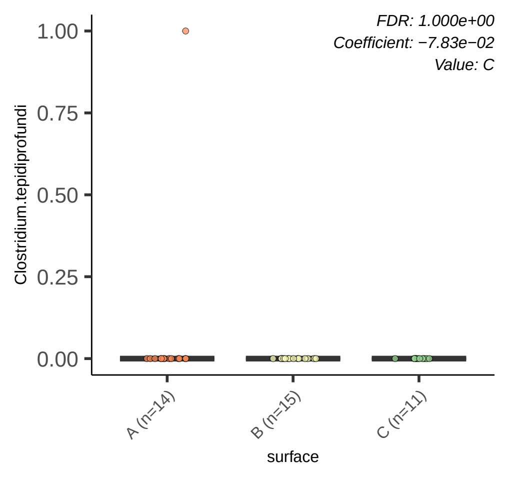
FDR: 1.000e+00
Coefficient: −7.83e−02
Value: C
Clostridium.tepidiprofundi
1.00
0.75
0.50
0.25
0.00
A (n=14)
B (n=15)
C (n=11)
surface

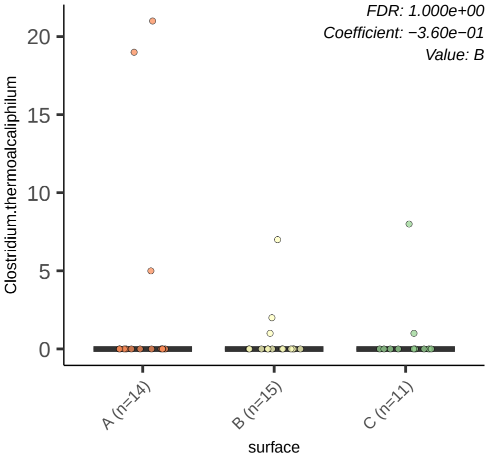
FDR: 1.000e+00
Coefficient: −3.60e−01
Value: B
Clostridium.thermoalcaliphilum
0
5
10
15
20
A (n=14)
B (n=15)
C (n=11)
surface

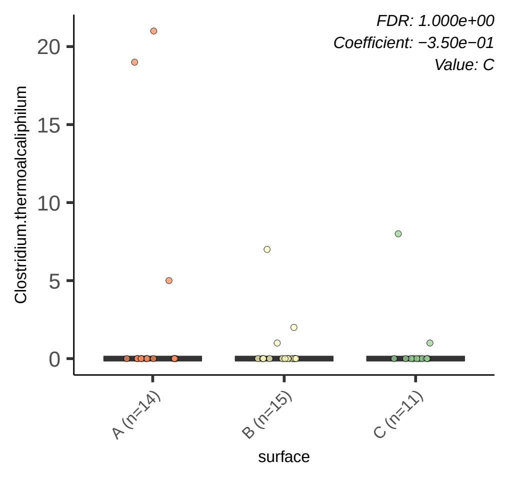

FDR: 1.000e+00
Coefficient: −3.50e−01
Value: C
Clostridium.thermoalcaliphilum
20
15
10
5
0
A (n=14)
B (n=15)
C (n=11)
surface

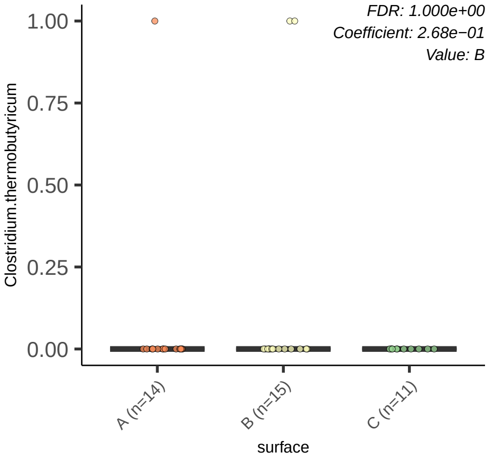

FDR: 1.000e+00
Coefficient: 2.68e−01
Value: B
1.00
0.75
0.50
0.25
0.00
Clostridium.thermobutyricum
A (n=14)
B (n=15)
C (n=11)
surface

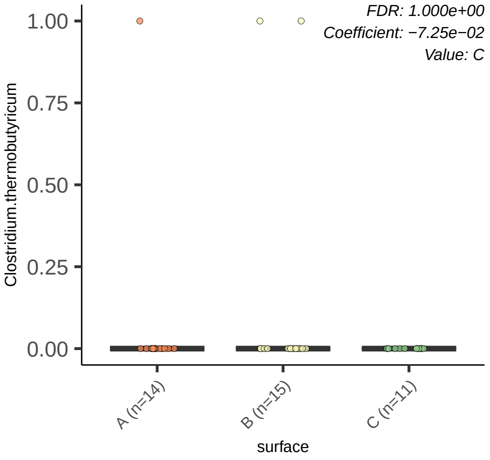

FDR: 1.000e+00
Coefficient: −7.25e−02
Value: C
1.00
0.75
0.50
0.25
0.00
Clostridium.thermobutyricum
A (n=14)
B (n=15)
C (n=11)
surface

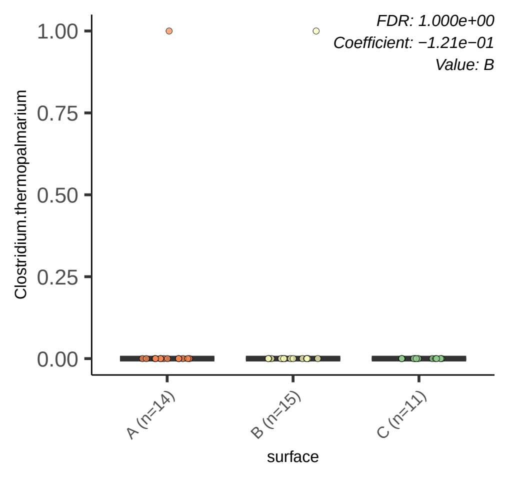

FDR: 1.000e+00
Coefficient: −1.21e−01
Value: B
Clostridium.thermopalmarium
1.00
0.75
0.50
0.25
0.00
A (n=14)
B (n=15)
C (n=11)
surface

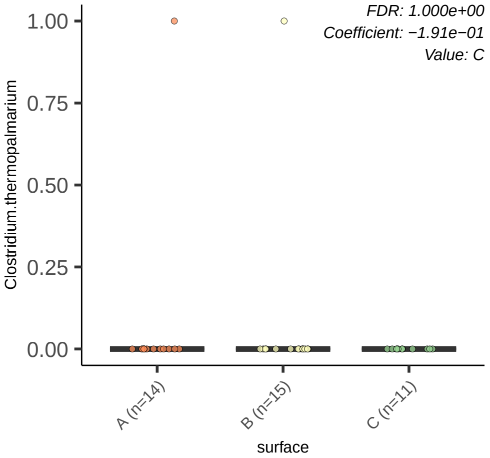

FDR: 1.000e+00
Coefficient: −1.91e−01
Value: C
Clostridium.thermopalmarium
1.00
0.75
0.50
0.25
0.00
A (n=14)
B (n=15)
C (n=11)
surface

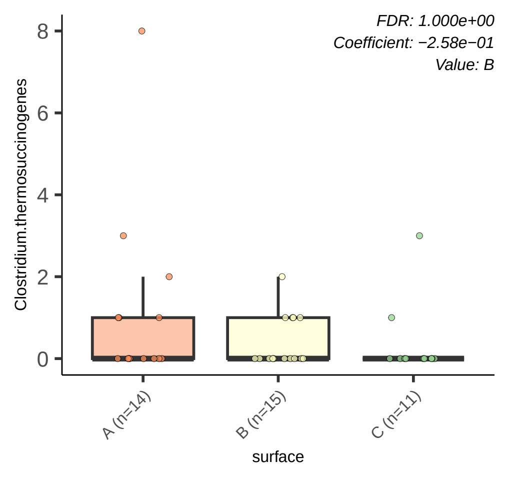
FDR: 1.000e+00
Coefficient: −2.58e−01
Value: B
Clostridium.thermosuccinogenes
0
2
4
6
8
A (n=14)
B (n=15)
C (n=11)
surface

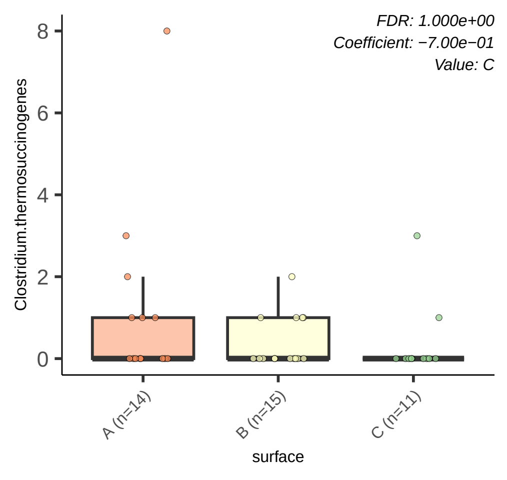
FDR: 1.000e+00
Coefficient: −7.00e−01
Value: C
Clostridium.thermosuccinogenes
0
2
4
6
8
A (n=14)
B (n=15)
C (n=11)
surface

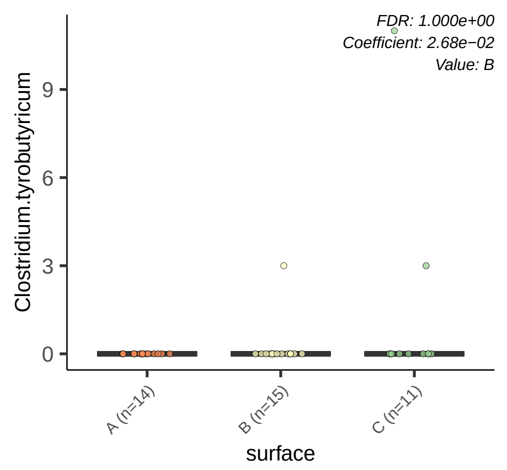
FDR: 1.000e+00
Coefficient: 2.68e−02
Value: B
Clostridium.tyrobutyricum
0
3
6
9
A (n=14)
B (n=15)
C (n=11)
surface

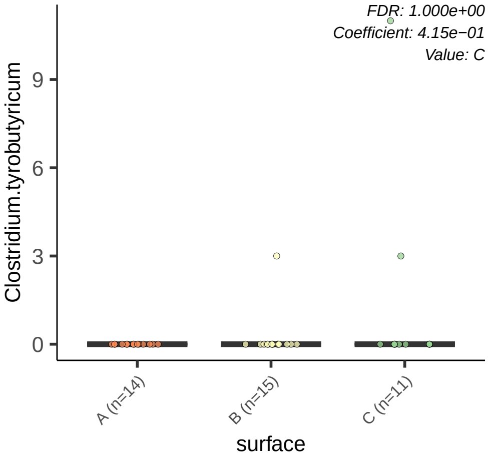
FDR: 1.000e+00
Coefficient: 4.15e−01
Value: C
Clostridium.tyrobutyricum
0
3
6
9
A (n=14)
B (n=15)
C (n=11)
surface

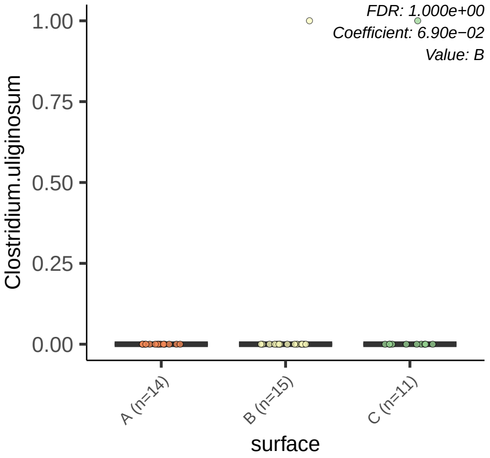

FDR: 1.000e+00
Coefficient: 6.90e−02
Value: B
Clostridium.uliginosum
1.00
0.75
0.50
0.25
0.00
A (n=14)
B (n=15)
C (n=11)
surface

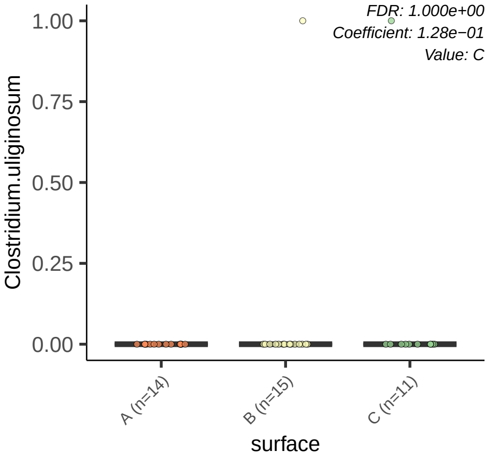
FDR: 1.000e+00
Coefficient: 1.28e−01
Value: C
Clostridium.uliginosum
1.00
0.75
0.50
0.25
0.00
A (n=14)
B (n=15)
C (n=11)
surface

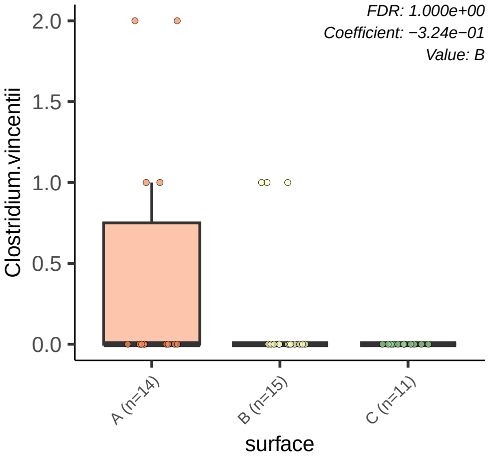
FDR: 1.000e+00
Coefficient: −3.24e−01
Value: B
Clostridium.vincentii
2.0
1.5
1.0
0.5
0.0
A (n=14)
B (n=15)
C (n=11)
surface

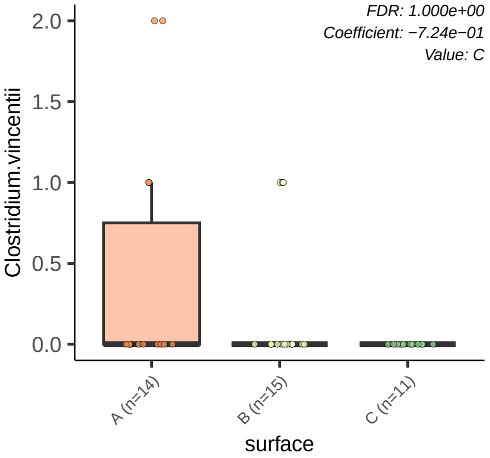

FDR: 1.000e+00
Coefficient: −7.24e−01
Value: C
Clostridium.vincentii
2.0
1.5
1.0
0.5
0.0
A (n=14)
B (n=15)
C (n=11)
surface

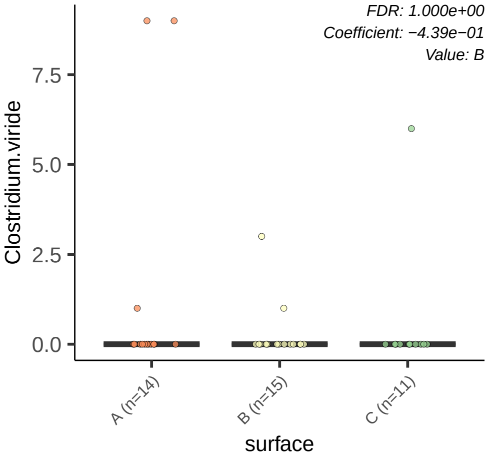
FDR: 1.000e+00
Coefficient: −4.39e−01
Value: B
Clostridium.viride
7.5
5.0
2.5
0.0
A (n=14)
B (n=15)
C (n=11)
surface

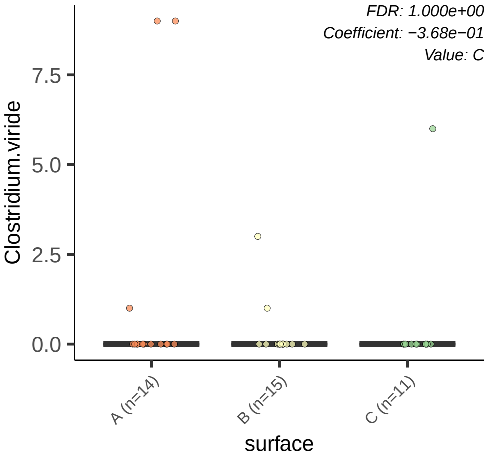
FDR: 1.000e+00
Coefficient: −3.68e−01
Value: C
Clostridium.viride
7.5
5.0
2.5
0.0
A (n=14)
B (n=15)
C (n=11)
surface

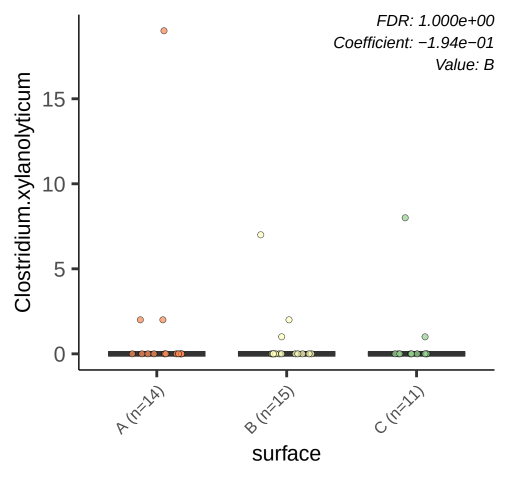

FDR: 1.000e+00
Coefficient: −1.94e−01
Value: B
Clostridium.xylanolyticum
0
5
10
15
A (n=14)
B (n=15)
C (n=11)
surface

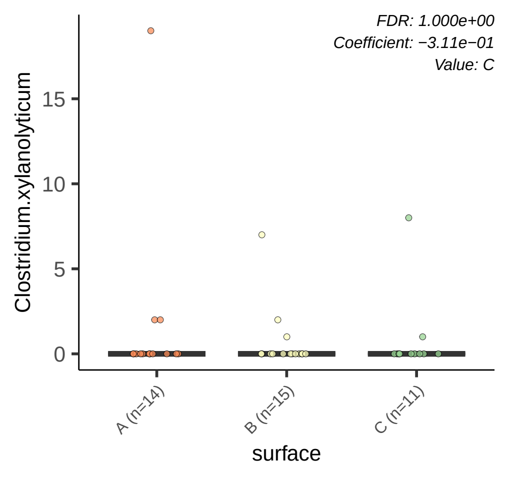

FDR: 1.000e+00
Coefficient: −3.11e−01
Value: C
Clostridium.xylanolyticum
0
5
10
15
A (n=14)
B (n=15)
C (n=11)
surface

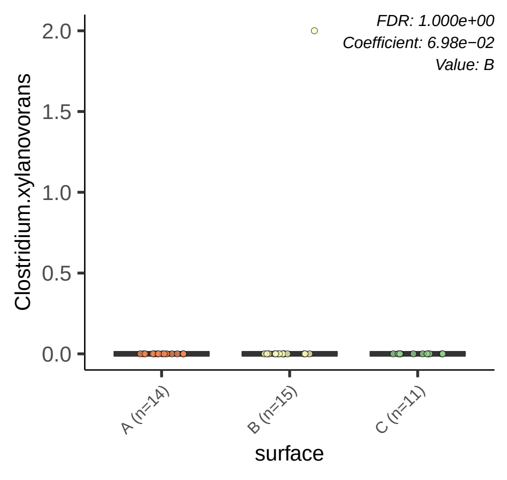
FDR: 1.000e+00
Coefficient: 6.98e−02
Value: B
Clostridium.xylanovorans
2.0
1.5
1.0
0.5
0.0
A (n=14)
B (n=15)
C (n=11)
surface

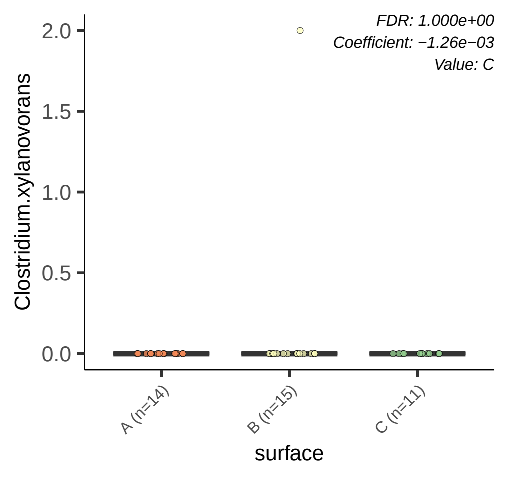

FDR: 1.000e+00
Coefficient: −1.26e−03
Value: C
Clostridium.xylanovorans
2.0
1.5
1.0
0.5
0.0
A (n=14)
B (n=15)
C (n=11)
surface

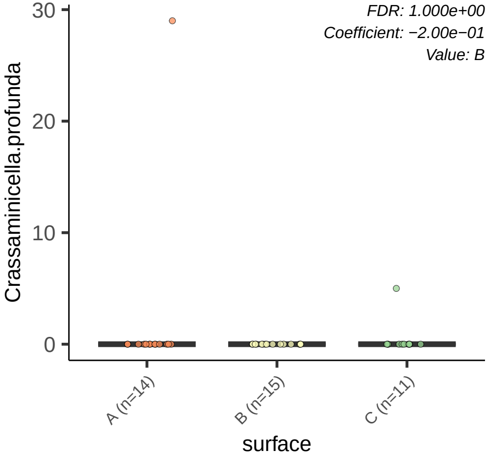

FDR: 1.000e+00
Coefficient: −2.00e−01
Value: B
Crassaminicella.profunda
30
20
10
0
A (n=14)
B (n=15)
C (n=11)
surface

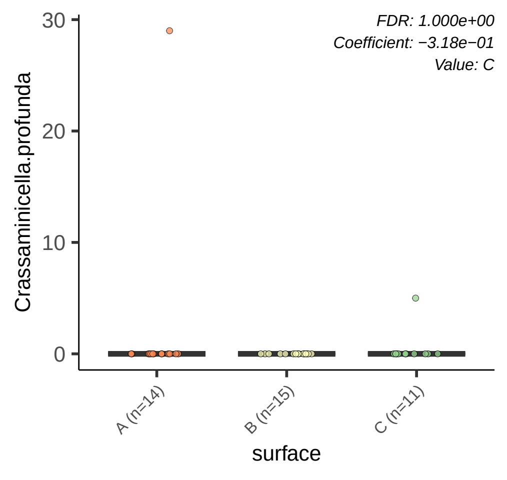

FDR: 1.000e+00
Coefficient: −3.18e−01
Value: C
Crassaminicella.profunda
30
20
10
0
A (n=14)
B (n=15)
C (n=11)
surface

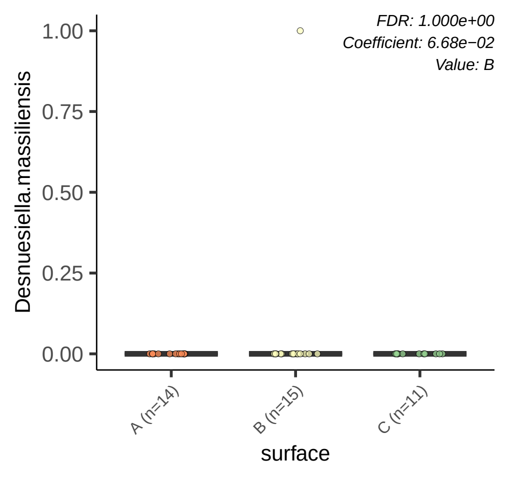
FDR: 1.000e+00
Coefficient: 6.68e−02
Value: B
Desnuesiella.massiliensis
1.00
0.75
0.50
0.25
0.00
A (n=14)
B (n=15)
C (n=11)
surface

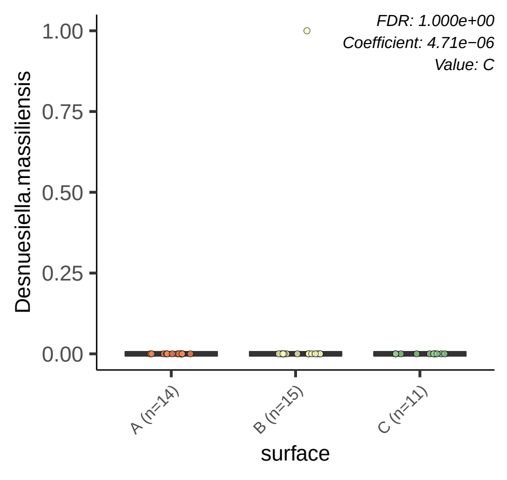
FDR: 1.000e+00
Coefficient: 4.71e−06
Value: C
Desnuesiella.massiliensis
1.00
0.75
0.50
0.25
0.00
A (n=14)
B (n=15)
C (n=11)
surface

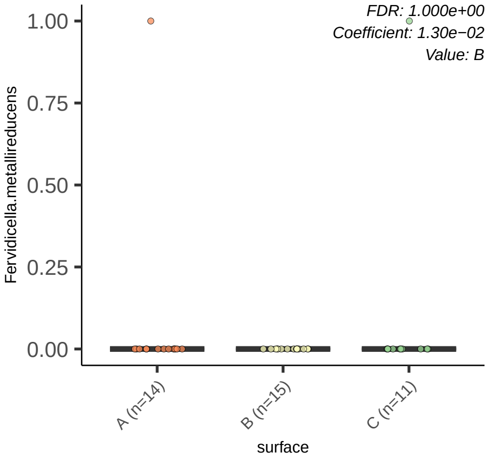

FDR: 1.000e+00
Coefficient: 1.30e−02
Value: B
Fervidicella.metallireducens
1.00
0.75
0.50
0.25
0.00
A (n=14)
B (n=15)
C (n=11)
surface

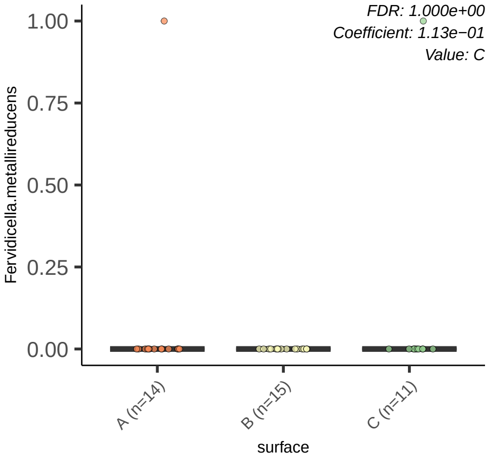
FDR: 1.000e+00
Coefficient: 1.13e−01
Value: C
1.00
0.75
0.50
0.25
0.00
Fervidicella.metallireducens
A (n=14)
B (n=15)
C (n=11)
surface

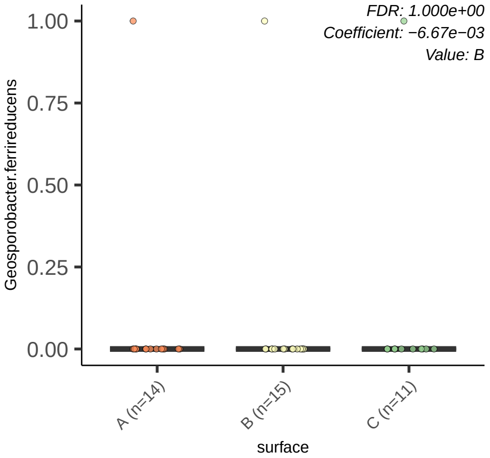
FDR: 1.000e+00
Coefficient: −6.67e−03
Value: B
Geosporobacter.ferrireducens
1.00
0.75
0.50
0.25
0.00
A (n=14)
B (n=15)
C (n=11)
surface

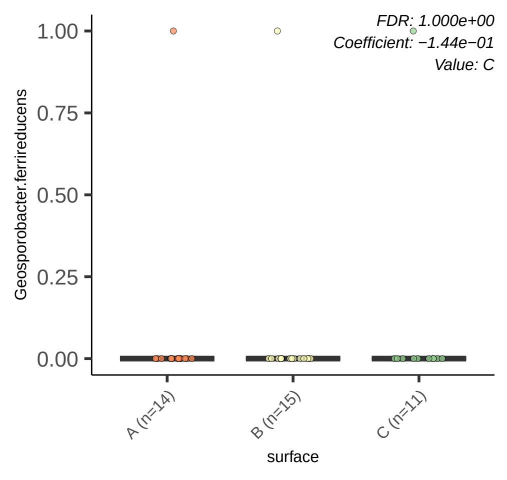

FDR: 1.000e+00
Coefficient: −1.44e−01
Value: C
1.00
0.75
0.50
0.25
0.00
Geosporobacter.ferrireducens
A (n=14)
B (n=15)
C (n=11)
surface

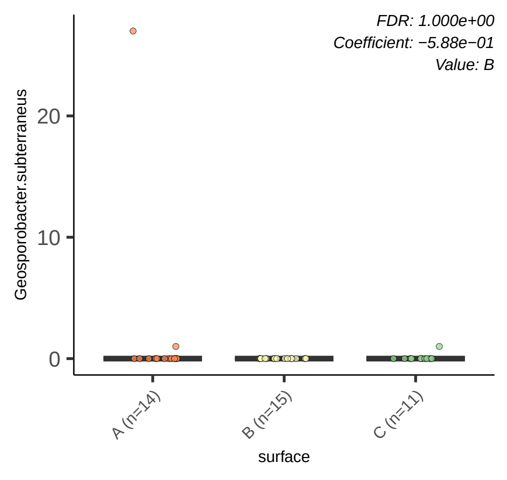
FDR: 1.000e+00
Coefficient: −5.88e−01
Value: B
Geosporobacter.subterraneus
20
10
0
A (n=14)
B (n=15)
C (n=11)
surface

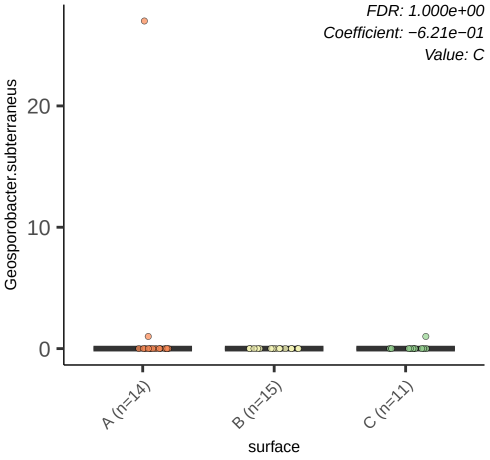

FDR: 1.000e+00
Coefficient: −6.21e−01
Value: C
Geosporobacter.subterraneus
20
10
0
A (n=14)
B (n=15)
C (n=11)
surface

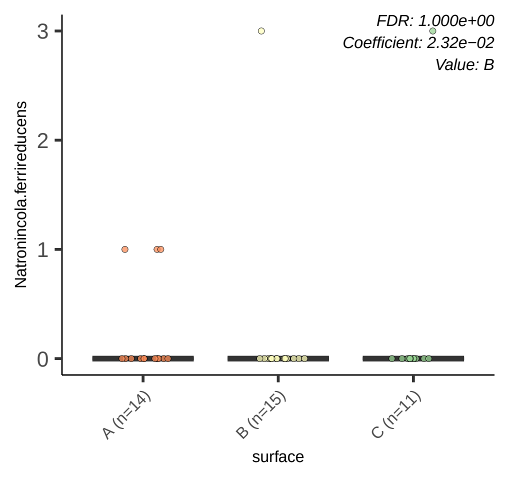
FDR: 1.000e+00
Coefficient: 2.32e−02
Value: B
Natronincola.ferrireducens
0
1
2
3
A (n=14)
B (n=15)
C (n=11)
surface

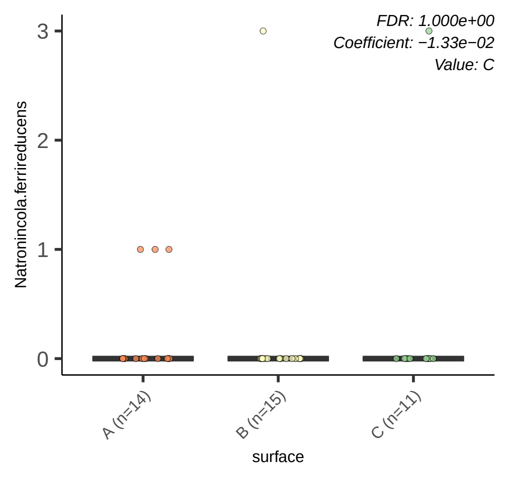

FDR: 1.000e+00
Coefficient: −1.33e−02
Value: C
Natronincola.ferrireducens
0
1
2
3
A (n=14)
B (n=15)
C (n=11)
surface

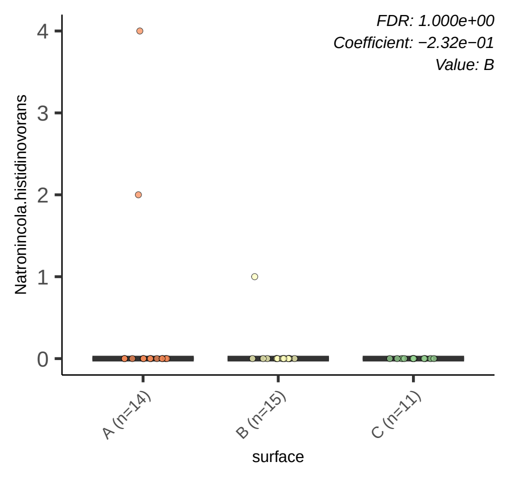
FDR: 1.000e+00
Coefficient: −2.32e−01
Value: B
Natronincola.histidinovorans
0
1
2
3
4
A (n=14)
B (n=15)
C (n=11)
surface

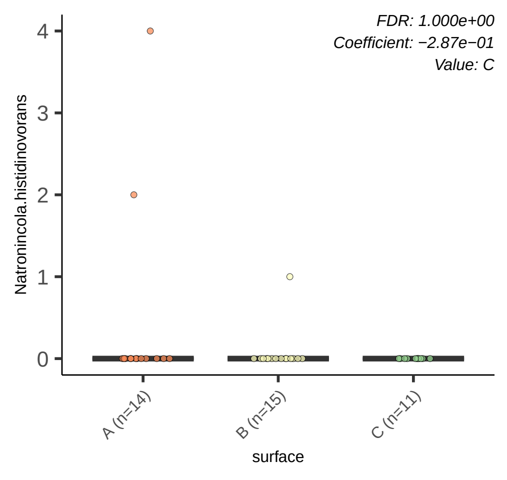
FDR: 1.000e+00
Coefficient: −2.87e−01
Value: C
Natronincola.histidinovorans
4
3
2
1
0
A (n=14)
B (n=15)
C (n=11)
surface

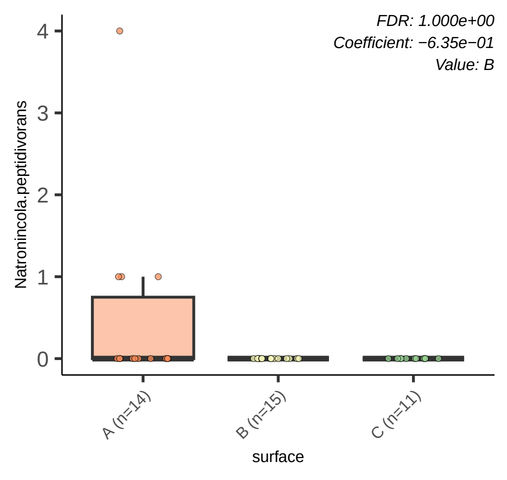
FDR: 1.000e+00
Coefficient: −6.35e−01
Value: B
Natronincola.peptidivorans
0
1
2
3
4
A (n=14)
B (n=15)
C (n=11)
surface

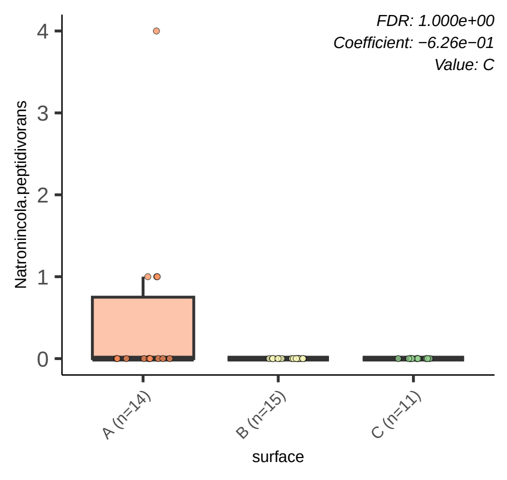
FDR: 1.000e+00
Coefficient: −6.26e−01
Value: C
Natronincola.peptidivorans
4
3
2
1
0
A (n=14)
B (n=15)
C (n=11)
surface

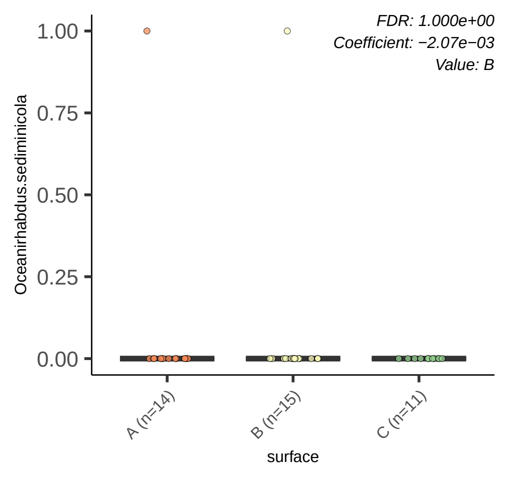

FDR: 1.000e+00
Coefficient: −2.07e−03
Value: B
Oceanirhabdus.sedimínicola
1.00
0.75
0.50
0.25
0.00
A (n=14)
B (n=15)
C (n=11)
surface

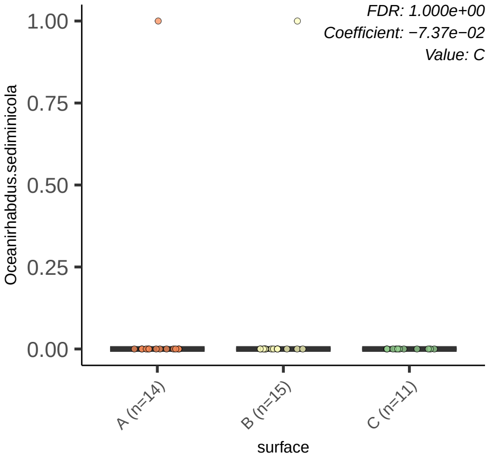
FDR: 1.000e+00
Coefficient: −7.37e−02
Value: C
Oceanirhabdus.sediminicola
1.00
0.75
0.50
0.25
0.00
A (n=14)
B (n=15)
C (n=11)
surface

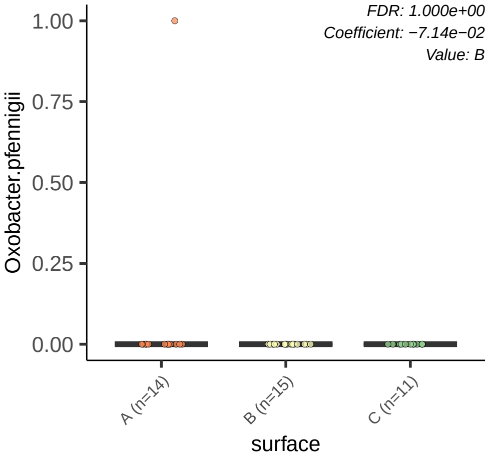
FDR: 1.000e+00
Coefficient: −7.14e−02
Value: B
Oxobacter.pfennigii
1.00
0.75
0.50
0.25
0.00
A (n=14)
B (n=15)
C (n=11)
surface

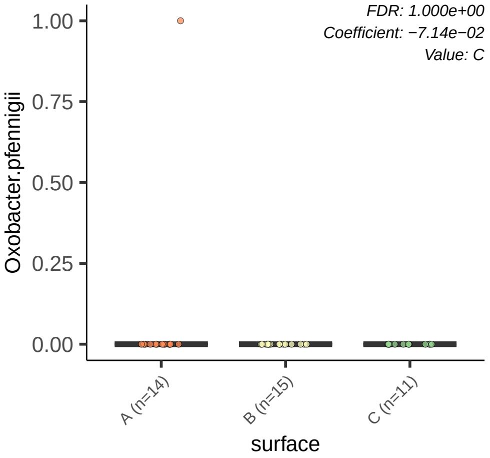

FDR: 1.000e+00
Coefficient: −7.14e−02
Value: C
Oxobacter.pfennigii
1.00
0.75
0.50
0.25
0.00
A (n=14)
B (n=15)
C (n=11)
surface

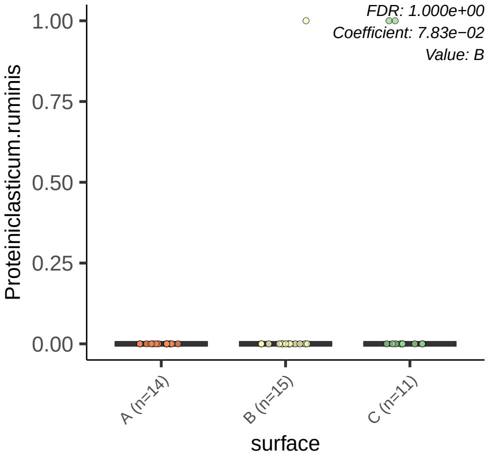

FDR: 1.000e+00
Coefficient: 7.83e−02
Value: B
Proteiniclasticum.ruminis
1.00
0.75
0.50
0.25
0.00
A (n=14)
B (n=15)
C (n=11)
surface

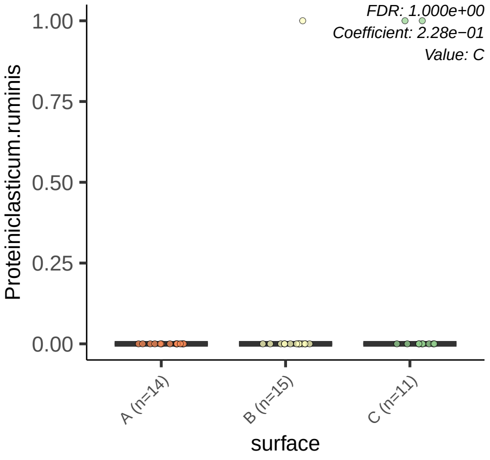

FDR: 1.000e+00
Coefficient: 2.28e−01
Value: C
Proteiniclasticum.ruminis
1.00
0.75
0.50
0.25
0.00
A (n=14)
B (n=15)
C (n=11)
surface

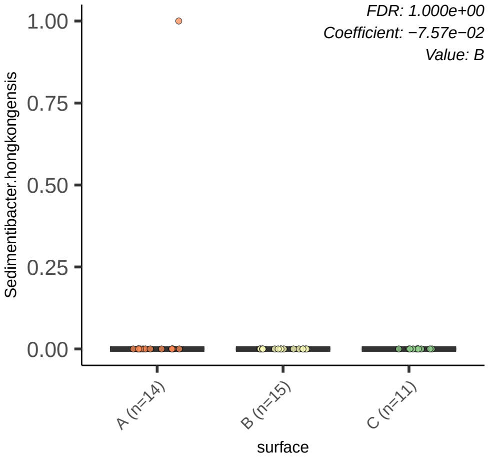

FDR: 1.000e+00
Coefficient: −7.57e−02
Value: B
1.00
0.75
0.50
0.25
0.00
Sedimentibacter.hongkongensis
A (n=14)
B (n=15)
C (n=11)
surface

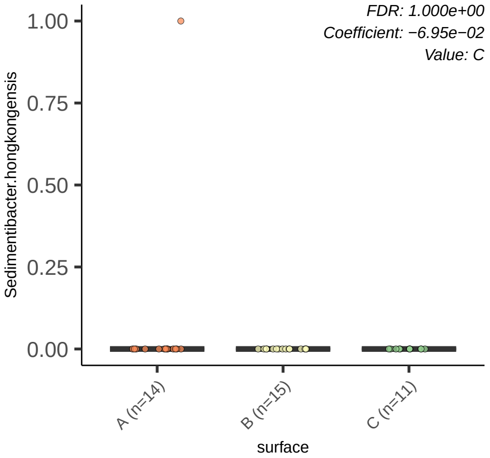
FDR: 1.000e+00
Coefficient: −6.95e−02
Value: C
1.00
0.75
0.50
0.25
0.00
Sedimentibacter.hongkongensis
A (n=14)
B (n=15)
C (n=11)
surface

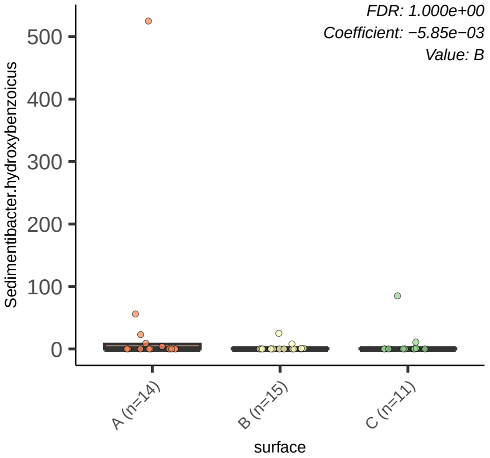

FDR: 1.000e+00
Coefficient: −5.85e−03
Value: B
Sedimentibacter.hydroxybenzoicus
500
400
300
200
100
0
A (n=14)
B (n=15)
C (n=11)
surface

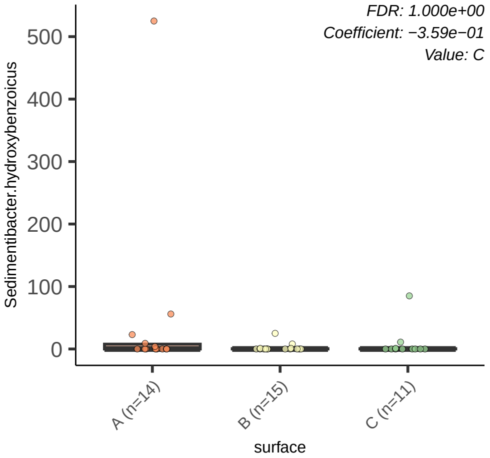

FDR: 1.000e+00
Coefficient: −3.59e−01
Value: C
Sedimentibacter.hydroxybenzoicus
500
400
300
200
100
0
A (n=14)
B (n=15)
C (n=11)
surface

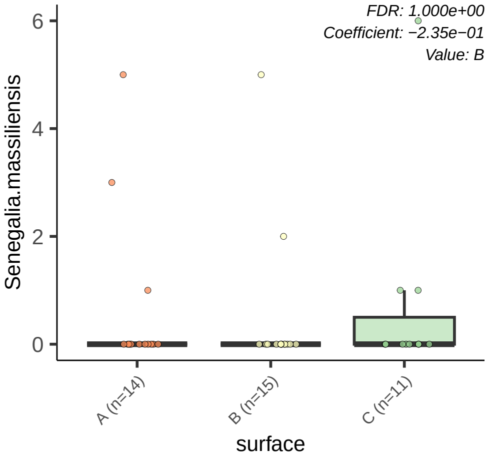
FDR: 1.000e+00
Coefficient: −2.35e−01
Value: B
Senegalia.massiliensis
0
2
4
6
A (n=14)
B (n=15)
C (n=11)
surface

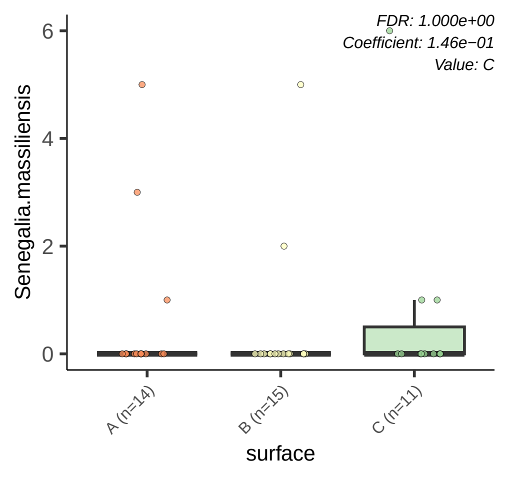
FDR: 1.000e+00
Coefficient: 1.46e−01
Value: C
Senegalia.massiliensis
0
2
4
6
A (n=14)
B (n=15)
C (n=11)
surface

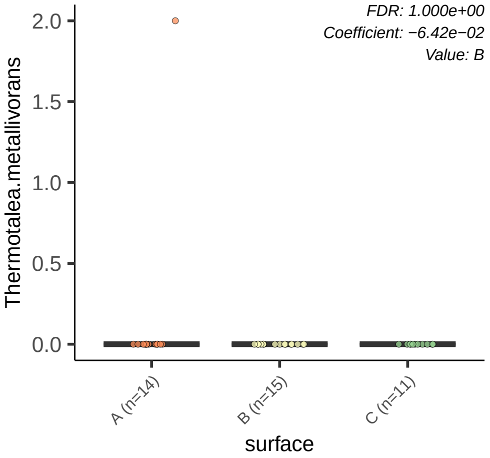
FDR: 1.000e+00
Coefficient: −6.42e−02
Value: B
Thermotalea.metallivorans
2.0
1.5
1.0
0.5
0.0
A (n=14)
B (n=15)
C (n=11)
surface

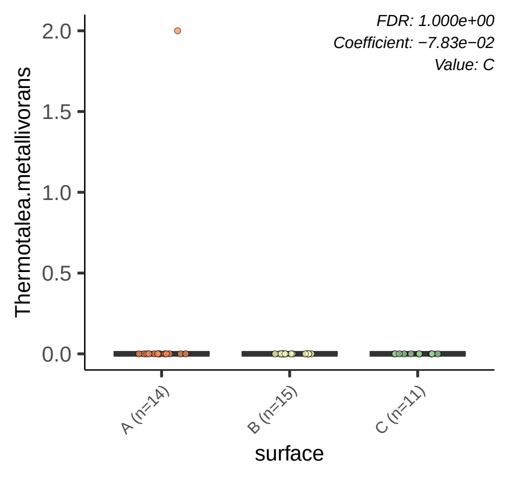

FDR: 1.000e+00
Coefficient: −7.83e−02
Value: C
Thermotalea.metallivorans
2.0
1.5
1.0
0.5
0.0
A (n=14)
B (n=15)
C (n=11)
surface

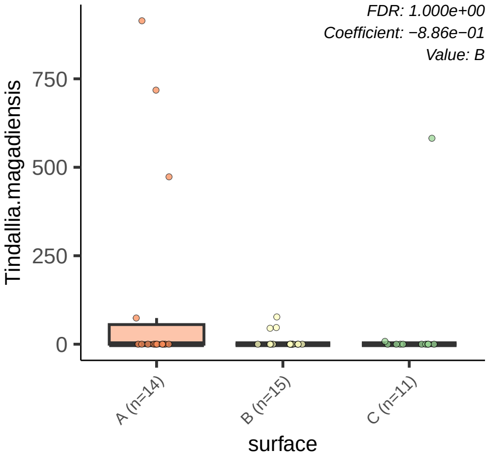

FDR: 1.000e+00
Coefficient: −8.86e−01
Value: B
Tindallia.magadiensis
750
500
250
0
A (n=14)
B (n=15)
C (n=11)
surface

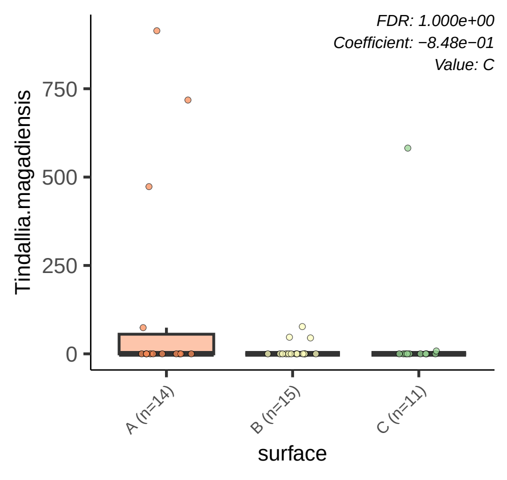

FDR: 1.000e+00
Coefficient: −8.48e−01
Value: C
Tindallia.magadiensis
750
500
250
0
A (n=14)
B (n=15)
C (n=11)
surface

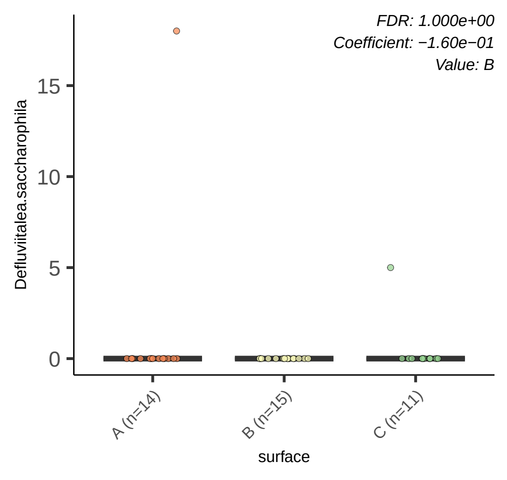
FDR: 1.000e+00
Coefficient: −1.60e−01
Value: B
Defluviitalea.saccharophila
0
5
10
15
A (n=14)
B (n=15)
C (n=11)
surface

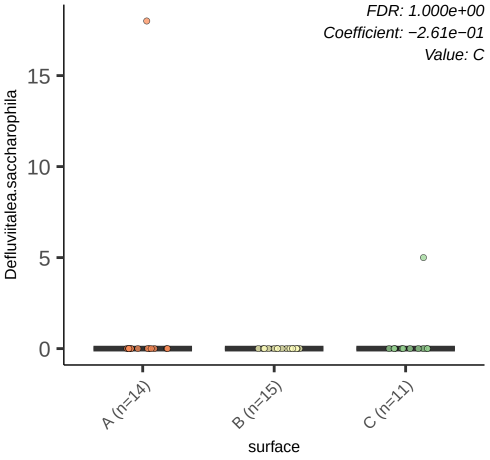

FDR: 1.000e+00
Coefficient: −2.61e−01
Value: C
Defluviitalea.saccharophila
15
10
5
0
A (n=14)
B (n=15)
C (n=11)
surface

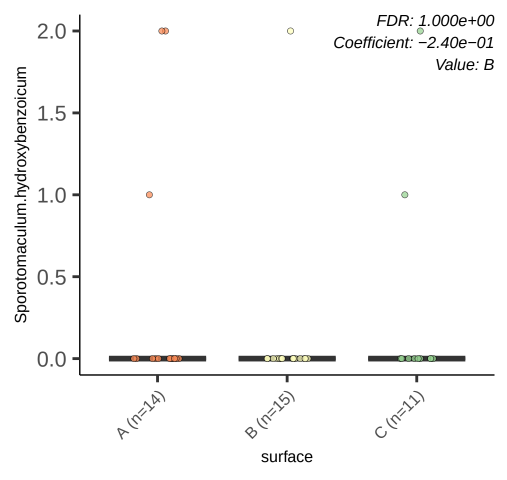

FDR: 1.000e+00
Coefficient: −2.40e−01
Value: B
Sporotomaculum.hydroxybenzoicum
2.0
1.5
1.0
0.5
0.0
A (n=14)
B (n=15)
C (n=11)
surface

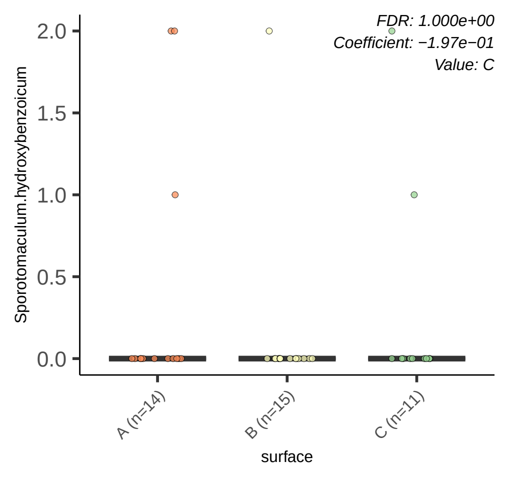

FDR: 1.000e+00
Coefficient: −1.97e−01
Value: C
Sporotomaculum.hydroxybenzoicum
2.0
1.5
1.0
0.5
0.0
A (n=14)
B (n=15)
C (n=11)
surface

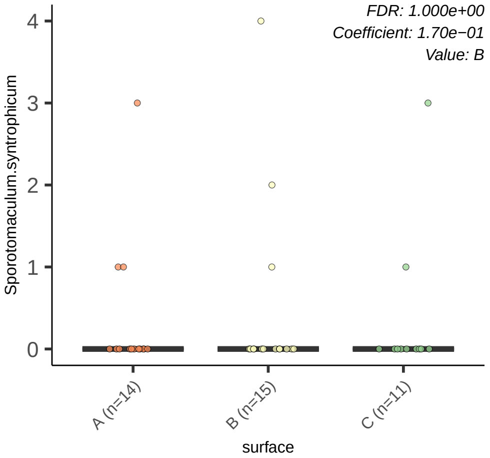
FDR: 1.000e+00
Coefficient: 1.70e−01
Value: B
Sporotomaculum.syntrophicum
4
3
2
1
0
A (n=14)
B (n=15)
C (n=11)
surface

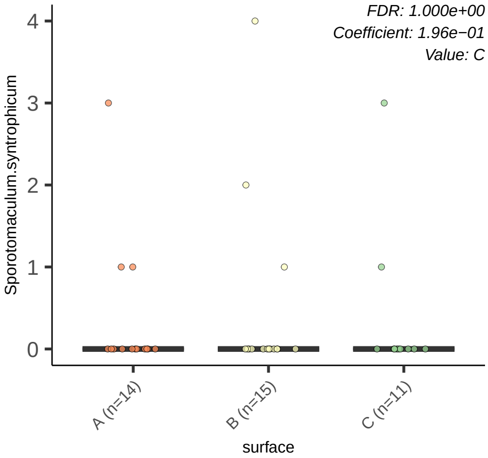
FDR: 1.000e+00
Coefficient: 1.96e−01
Value: C
Sporotomaculum.syntrophicum
0
1
2
3
4
A (n=14)
B (n=15)
C (n=11)
surface

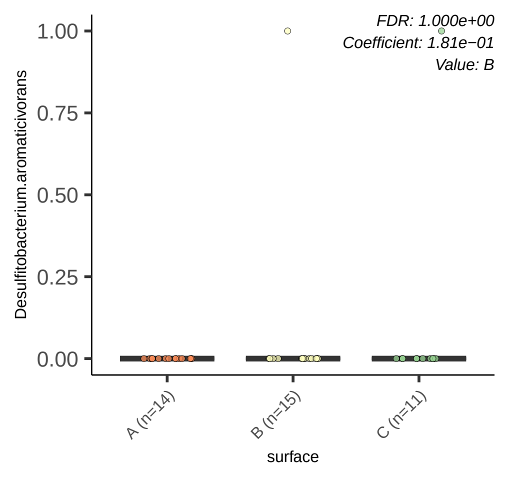

FDR: 1.000e+00
Coefficient: 1.81e−01
Value: B
Desulfitobacterium.aromaticivorans
1.00
0.75
0.50
0.25
0.00
A (n=14)
B (n=15)
C (n=11)
surface

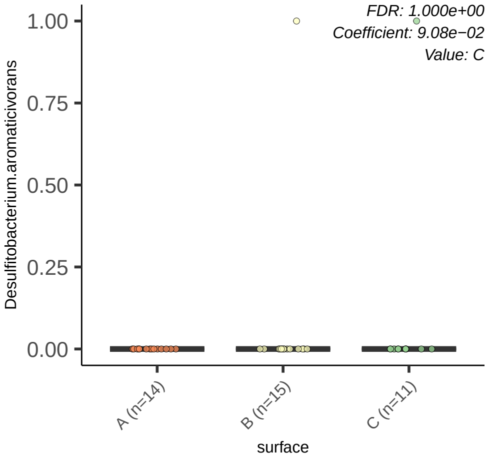

FDR: 1.000e+00
Coefficient: 9.08e−02
Value: C
Desulfitobacterium.aromaticivorans
1.00
0.75
0.50
0.25
0.00
A (n=14)
B (n=15)
C (n=11)
surface

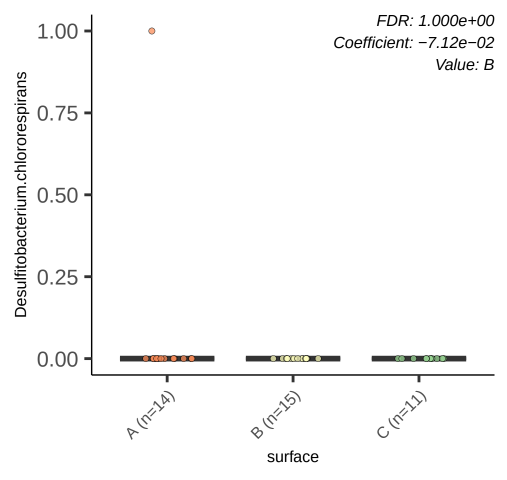

FDR: 1.000e+00
Coefficient: −7.12e−02
Value: B
Desulfitobacterium.chlororespirans
1.00
0.75
0.50
0.25
0.00
A (n=14)
B (n=15)
C (n=11)
surface

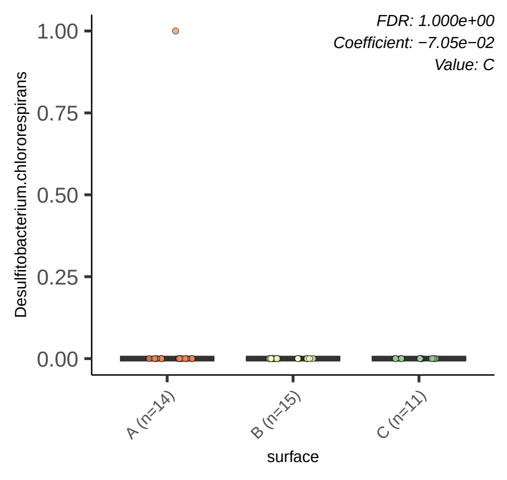

FDR: 1.000e+00
Coefficient: −7.05e−02
Value: C
Desulfitobacterium.chlororespirans
1.00
0.75
0.50
0.25
0.00
A (n=14)
B (n=15)
C (n=11)
surface

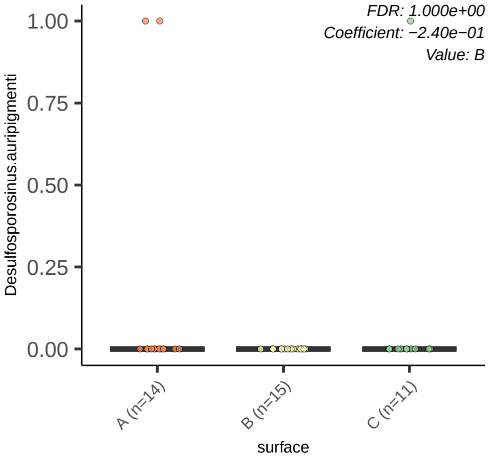

FDR: 1.000e+00
Coefficient: −2.40e−01
Value: B
Desulfosporosinus.auripigmenti
1.00
0.75
0.50
0.25
0.00
A (n=14)
B (n=15)
C (n=11)
surface

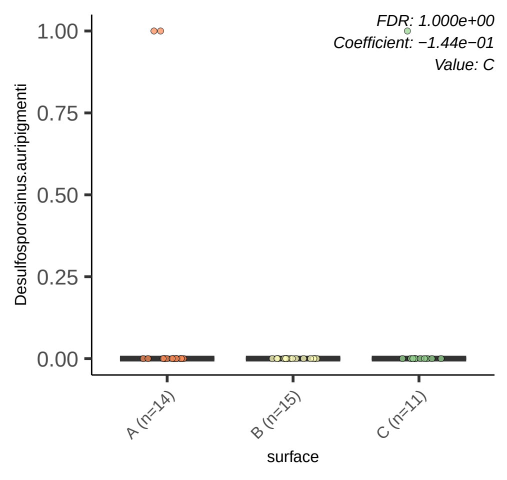

FDR: 1.000e+00
Coefficient: −1.44e−01
Value: C
1.00
0.75
0.50
0.25
0.00
Desulfosporosinus.auripigmenti
A (n=14)
B (n=15)
C (n=11)
surface

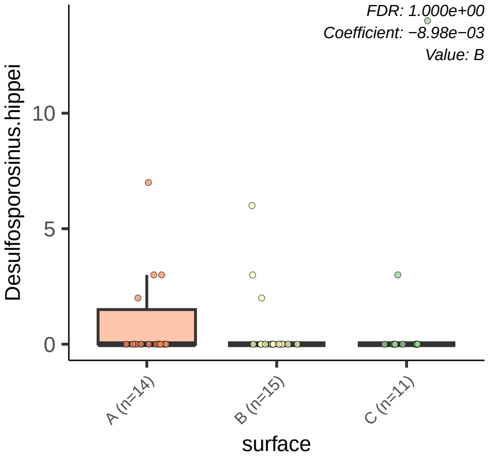

FDR: 1.000e+00
Coefficient: −8.98e−03
Value: B
Desulfosporosinus.hippei
10
5
0
A (n=14)
B (n=15)
C (n=11)
surface

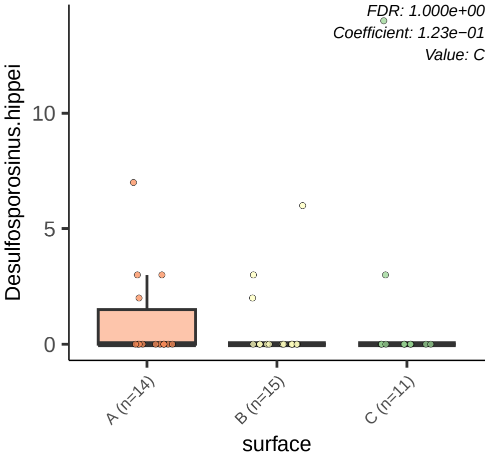
FDR: 1.000e+00
Coefficient: 1.23e−01
Value: C
Desulfosporosinus.hippei
10
5
0
A (n=14)
B (n=15)
C (n=11)
surface

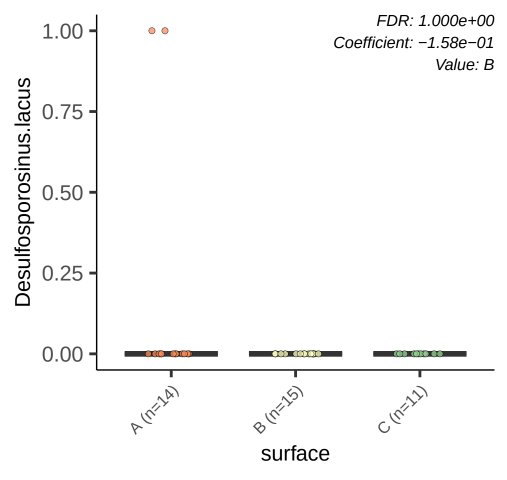
FDR: 1.000e+00
Coefficient: −1.58e−01
Value: B
Desulfosporosinus.lacus
1.00
0.75
0.50
0.25
0.00
A (n=14)
B (n=15)
C (n=11)
surface

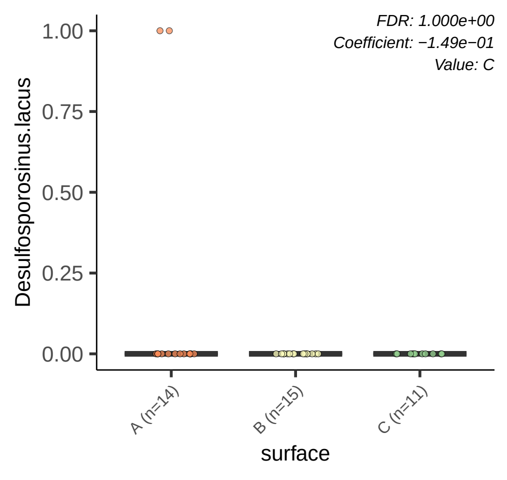
FDR: 1.000e+00
Coefficient: −1.49e−01
Value: C
Desulfosporosinus.lacus
1.00
0.75
0.50
0.25
0.00
A (n=14)
B (n=15)
C (n=11)
surface

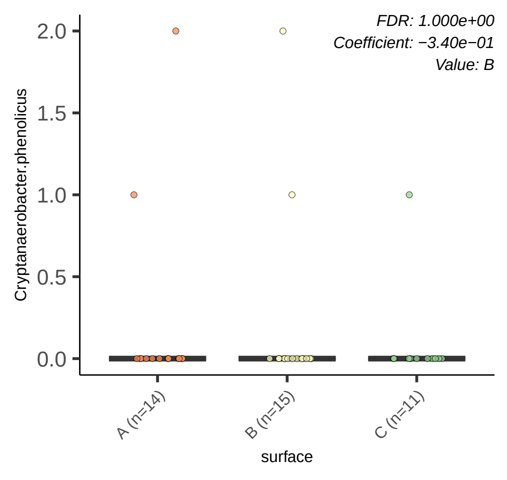

FDR: 1.000e+00
Coefficient: −3.40e−01
Value: B
Cryptanaerobacter.phenolicus
2.0
1.5
1.0
0.5
0.0
A (n=14)
B (n=15)
C (n=11)
surface

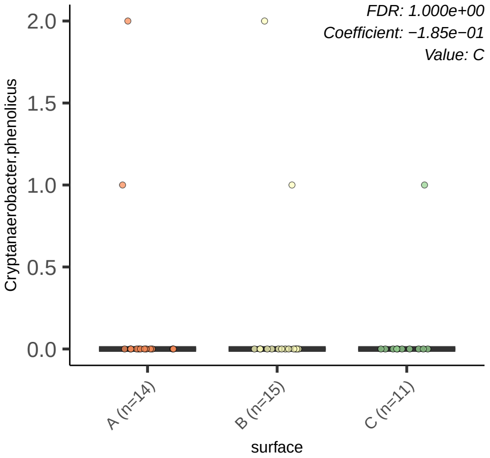

FDR: 1.000e+00
Coefficient: −1.85e−01
Value: C
Cryptanaerobacter.phenolicus
2.0
1.5
1.0
0.5
0.0
A (n=14)
B (n=15)
C (n=11)
surface

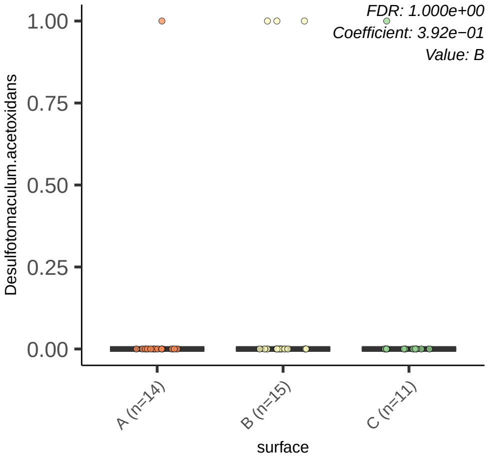
FDR: 1.000e+00
Coefficient: 3.92e−01
Value: B
1.00
0.75
0.50
0.25
0.00
Desulfotomaculum.acetoxidans
A (n=14)
B (n=15)
C (n=11)
surface

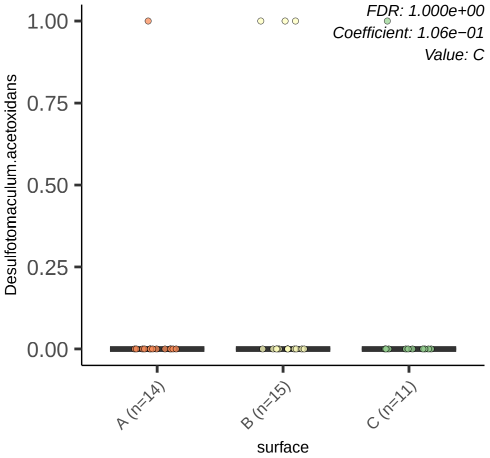

FDR: 1.000e+00
Coefficient: 1.06e−01
Value: C
Desulfotomaculum.acetoxidans
1.00
0.75
0.50
0.25
0.00
A (n=14)
B (n=15)
C (n=11)
surface

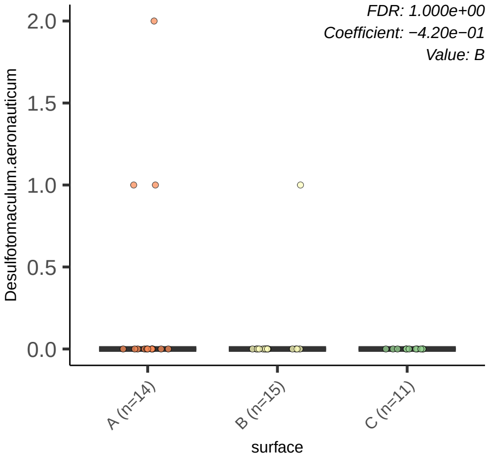
FDR: 1.000e+00
Coefficient: −4.20e−01
Value: B
Desulfotomaculum.aeronauticum
2.0
1.5
1.0
0.5
0.0
A (n=14)
B (n=15)
C (n=11)
surface

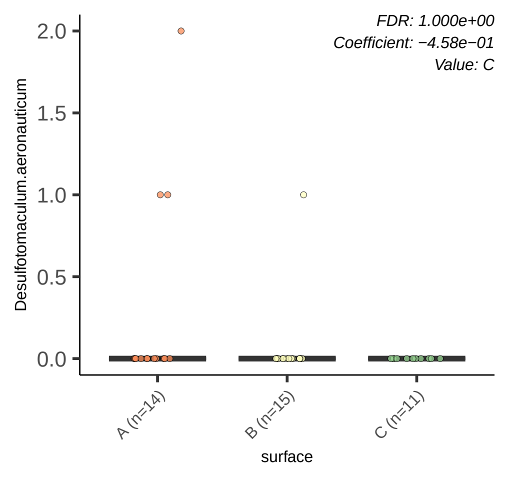
FDR: 1.000e+00
Coefficient: −4.58e−01
Value: C
Desulfotomaculum.aeronauticum
2.0
1.5
1.0
0.5
0.0
A (n=14)
B (n=15)
C (n=11)
surface

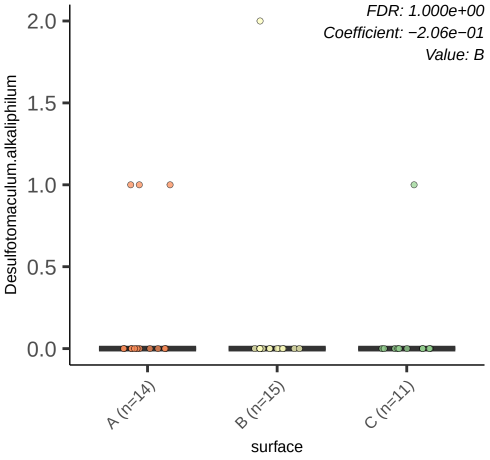

FDR: 1.000e+00
Coefficient: −2.06e−01
Value: B
Desulfotomaculum.alkaliphilum
2.0
1.5
1.0
0.5
0.0
A (n=14)
B (n=15)
C (n=11)
surface

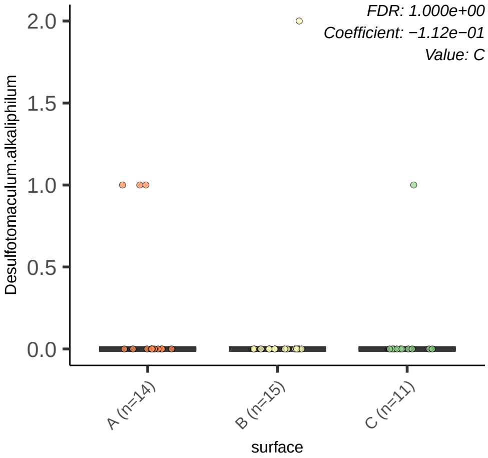
FDR: 1.000e+00
Coefficient: −1.12e−01
Value: C
Desulfotomaculum.alkaliphilum
2.0
1.5
1.0
0.5
0.0
A (n=14)
B (n=15)
C (n=11)
surface

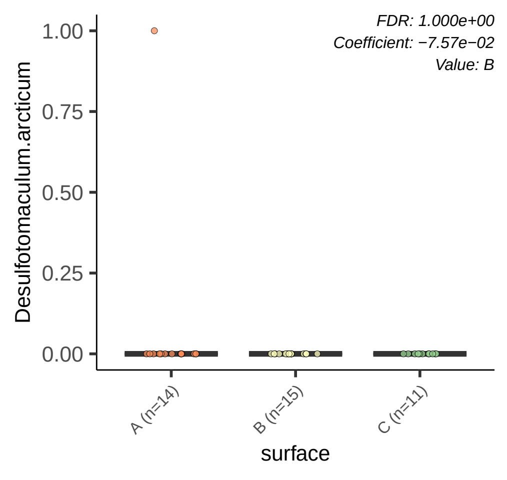
FDR: 1.000e+00
Coefficient: −7.57e−02
Value: B
Desulfotomaculum.arcticum
1.00
0.75
0.50
0.25
0.00
A (n=14)
B (n=15)
C (n=11)
surface

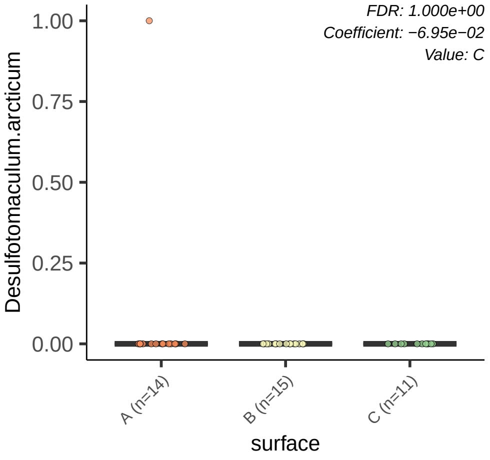
FDR: 1.000e+00
Coefficient: −6.95e−02
Value: C
Desulfotomaculum.arcticum
1.00
0.75
0.50
0.25
0.00
A (n=14)
B (n=15)
C (n=11)
surface

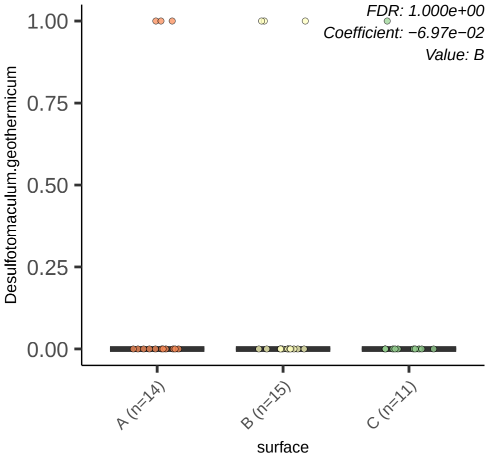
FDR: 1.000e+00
Coefficient: −6.97e−02
Value: B
Desulfotomaculum.geothermicum
1.00
0.75
0.50
0.25
0.00
A (n=14)
B (n=15)
C (n=11)
surface

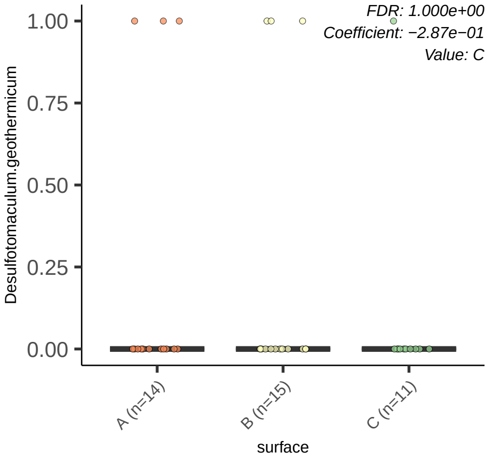

FDR: 1.000e+00
Coefficient: −2.87e−01
Value: C
Desulfotomaculum.geothermicum
1.00
0.75
0.50
0.25
0.00
A (n=14)
B (n=15)
C (n=11)
surface

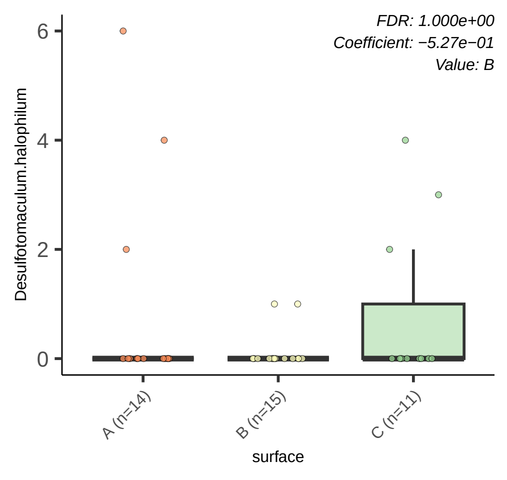

FDR: 1.000e+00
Coefficient: −5.27e−01
Value: B
Desulfotomaculum.halophilum
6
4
2
0
A (n=14)
B (n=15)
C (n=11)
surface

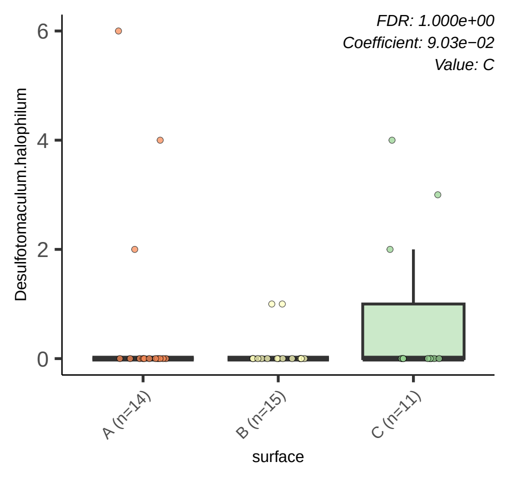
FDR: 1.000e+00
Coefficient: 9.03e−02
Value: C
Desulfotomaculum.halophilum
6
4
2
0
A (n=14)
B (n=15)
C (n=11)
surface

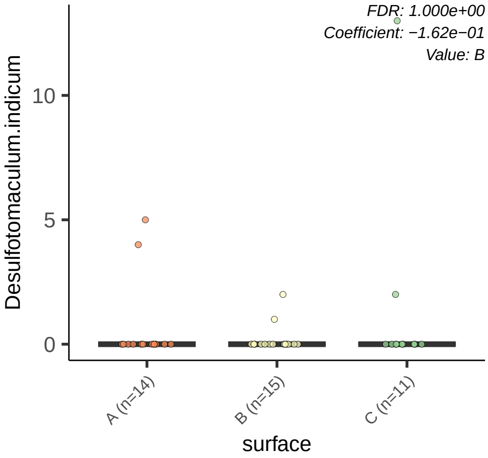

FDR: 1.000e+00
Coefficient: −1.62e−01
Value: B
Desulfotomaculum.indicum
10
5
0
A (n=14)
B (n=15)
C (n=11)
surface

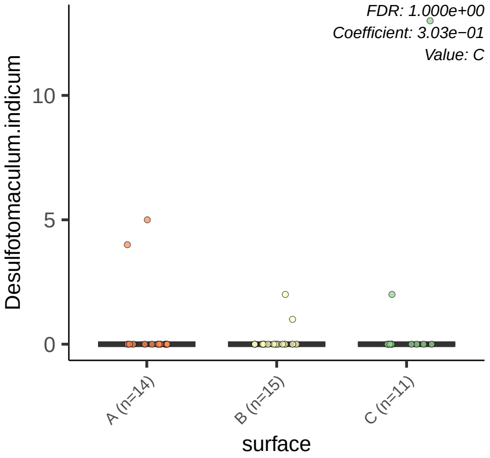
FDR: 1.000e+00
Coefficient: 3.03e−01
Value: C
Desulfotomaculum.indicum
10
5
0
A (n=14)
B (n=15)
C (n=11)
surface

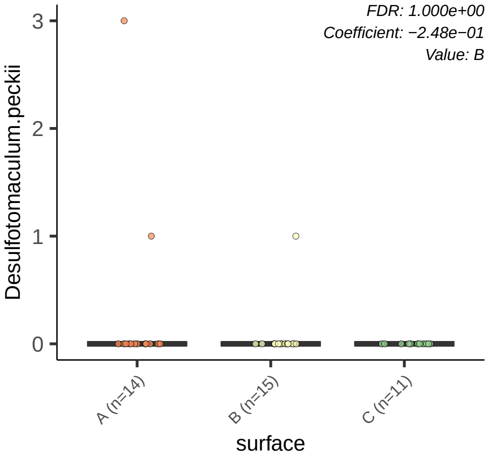
FDR: 1.000e+00
Coefficient: −2.48e−01
Value: B
Desulfotomaculum.peckii
3
2
1
0
A (n=14)
B (n=15)
C (n=11)
surface

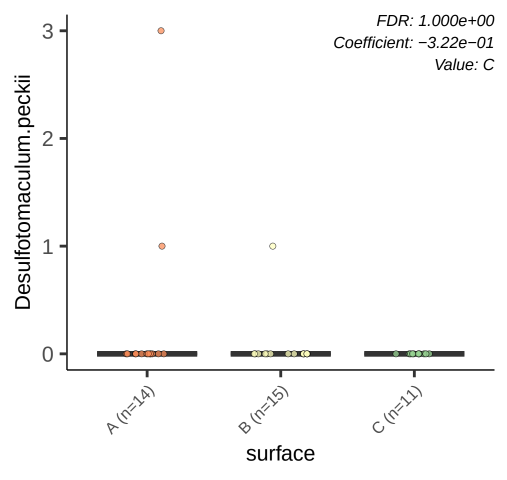

FDR: 1.000e+00
Coefficient: −3.22e−01
Value: C
Desulfotomaculum.peckii
3
2
1
0
A (n=14)
B (n=15)
C (n=11)
surface

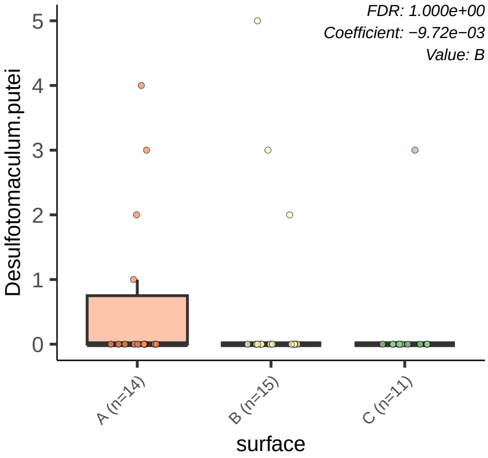

FDR: 1.000e+00
Coefficient: −9.72e−03
Value: B
Desulfotomaculum.putei
0
1
2
3
4
5
A (n=14)
B (n=15)
C (n=11)
surface

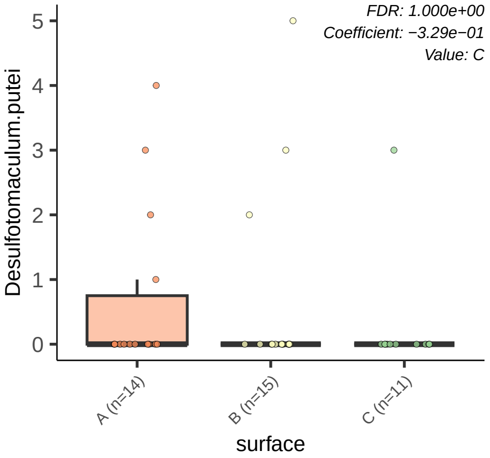

FDR: 1.000e+00
Coefficient: −3.29e−01
Value: C
Desulfotomaculum.putei
0
1
2
3
4
5
A (n=14)
B (n=15)
C (n=11)
surface

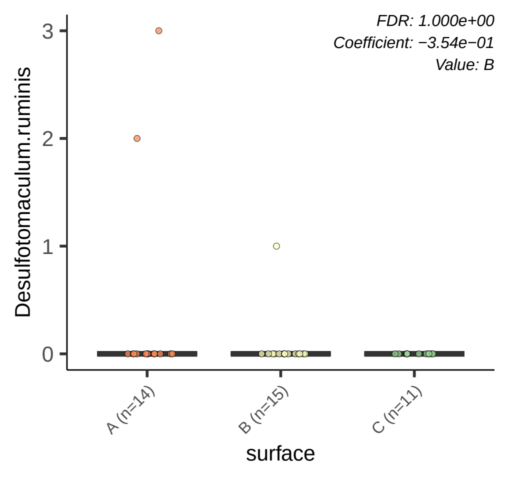

FDR: 1.000e+00
Coefficient: −3.54e−01
Value: B
Desulfotomaculum.ruminis
0
1
2
3
A (n=14)
B (n=15)
C (n=11)
surface

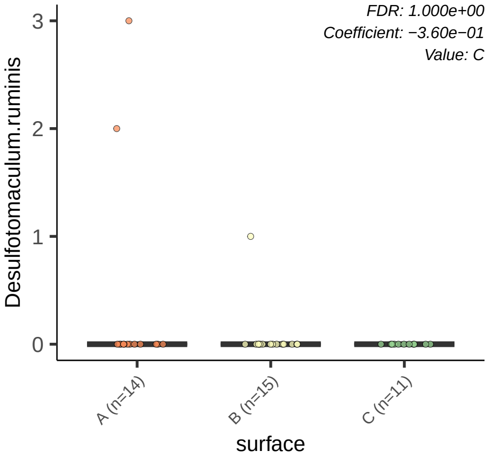
FDR: 1.000e+00
Coefficient: −3.60e−01
Value: C
Desulfotomaculum.ruminis
0
1
2
3
A (n=14)
B (n=15)
C (n=11)
surface

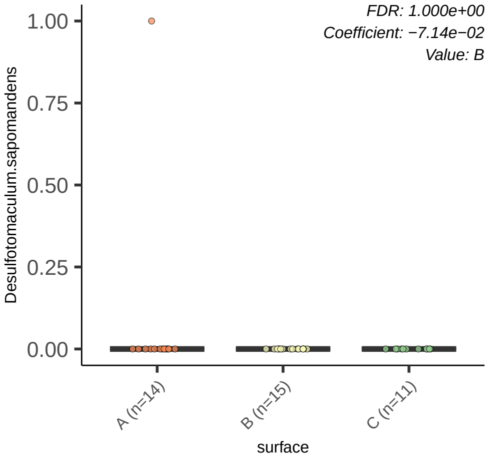
FDR: 1.000e+00
Coefficient: −7.14e−02
Value: B
Desulfotomaculum.sapomandens
1.00
0.75
0.50
0.25
0.00
A (n=14)
B (n=15)
C (n=11)
surface

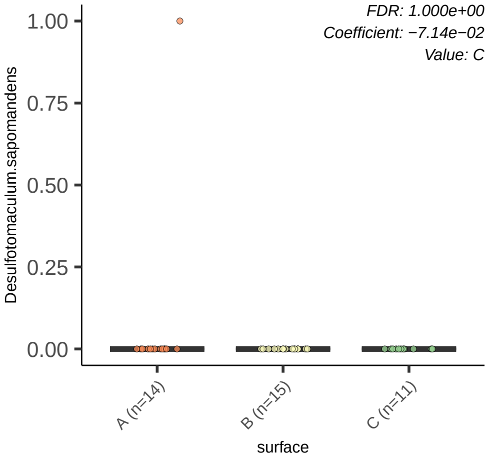
FDR: 1.000e+00
Coefficient: −7.14e−02
Value: C
Desulfotomaculum.sapomandens
1.00
0.75
0.50
0.25
0.00
A (n=14)
B (n=15)
C (n=11)
surface

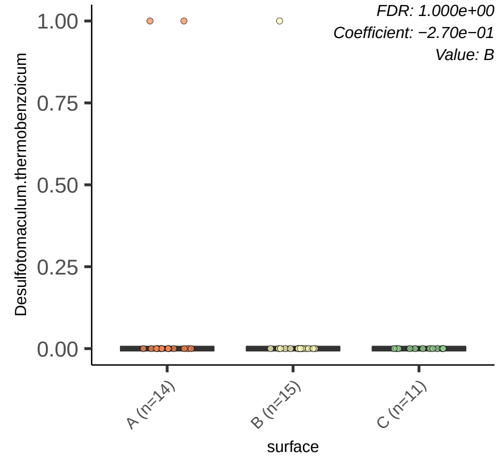

FDR: 1.000e+00
Coefficient: −2.70e−01
Value: B
1.00
0.75
0.50
0.25
0.00
Desulfotomaculum.thermobenzoicum
A (n=14)
B (n=15)
C (n=11)
surface

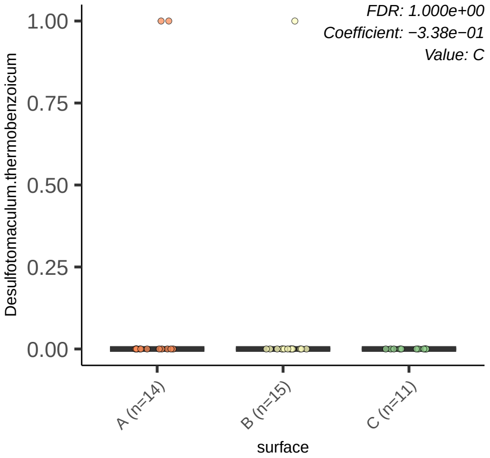
FDR: 1.000e+00
Coefficient: −3.38e−01
Value: C
Desulfotomaculum.thermobenzoicum
1.00
0.75
0.50
0.25
0.00
A (n=14)
B (n=15)
C (n=11)
surface

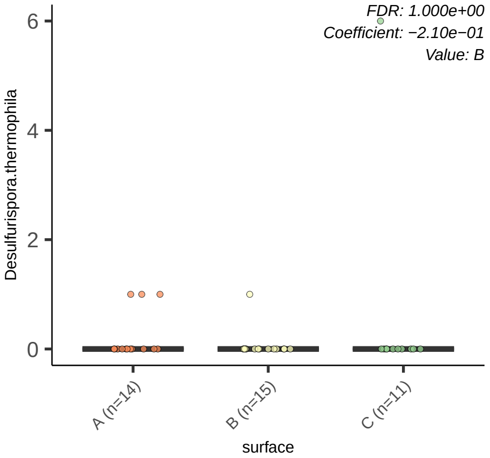
FDR: 1.000e+00
Coefficient: −2.10e−01
Value: B
Desulfurispora.thermophila
0
2
4
6
A (n=14)
B (n=15)
C (n=11)
surface

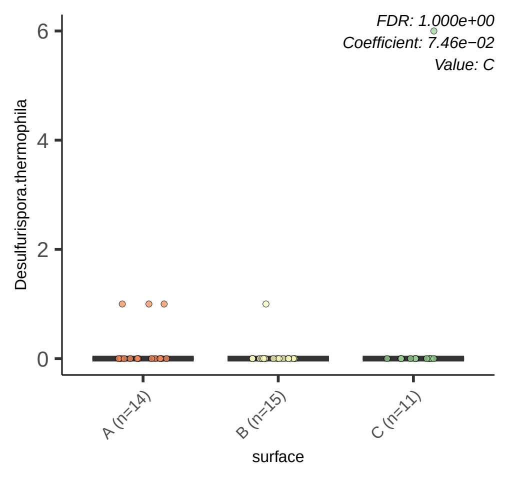

FDR: 1.000e+00
Coefficient: 7.46e−02
Value: C
Desulfurispora.thermophila
0
2
4
6
A (n=14)
B (n=15)
C (n=11)
surface

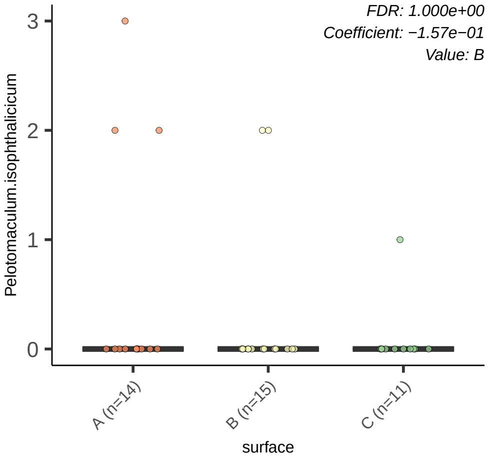
FDR: 1.000e+00
Coefficient: −1.57e−01
Value: B
Pelotomaculum.isophthalicicum
3
2
1
0
A (n=14)
B (n=15)
C (n=11)
surface

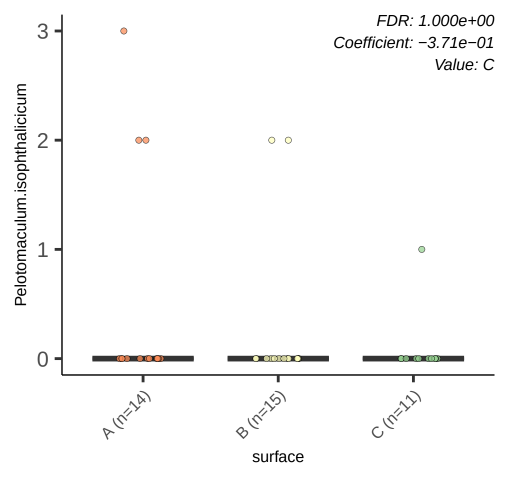

FDR: 1.000e+00
Coefficient: −3.71e−01
Value: C
Pelotomaculum.isophthalicicum
0
1
2
3
A (n=14)
B (n=15)
C (n=11)
surface

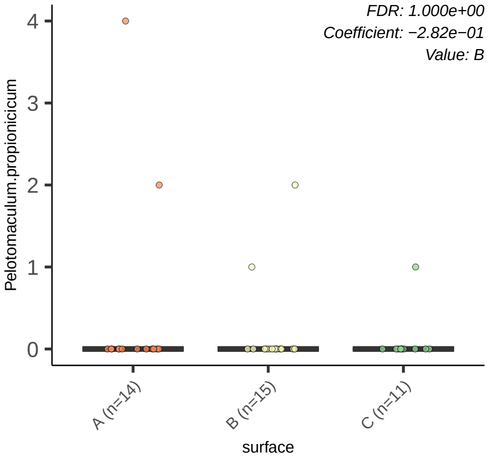
FDR: 1.000e+00
Coefficient: −2.82e−01
Value: B
Pelotomaculum.propionicicum
0
1
2
3
4
A (n=14)
B (n=15)
C (n=11)
surface

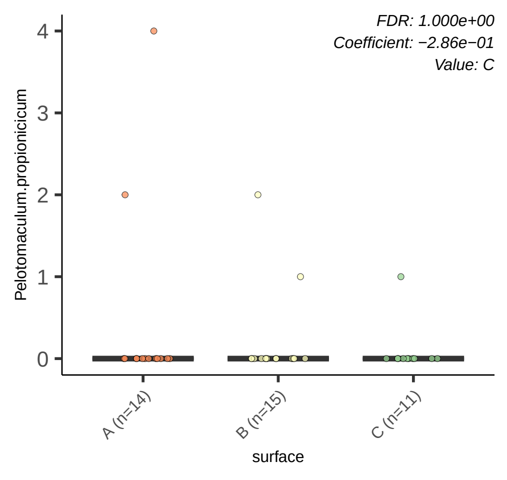

FDR: 1.000e+00
Coefficient: −2.86e−01
Value: C
Pelotomaculum.propionicicum
0
1
2
3
4
A (n=14)
B (n=15)
C (n=11)
surface

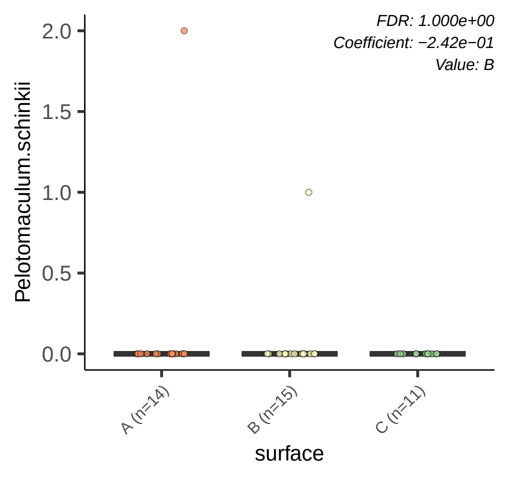
FDR: 1.000e+00
Coefficient: −2.42e−01
Value: B
Pelotomaculum.schinkii
2.0
1.5
1.0
0.5
0.0
A (n=14)
B (n=15)
C (n=11)
surface

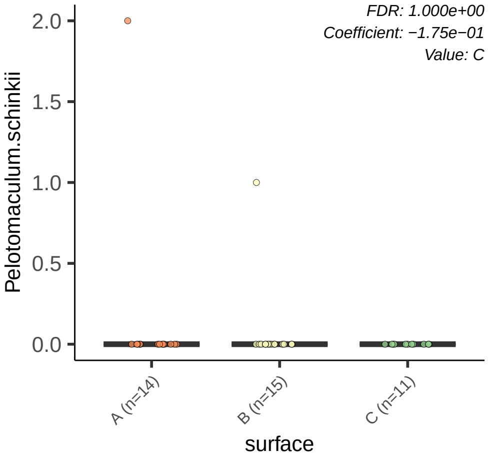
FDR: 1.000e+00
Coefficient: −1.75e−01
Value: C
Pelotomaculum.schinkii
2.0
1.5
1.0
0.5
0.0
A (n=14)
B (n=15)
C (n=11)
surface

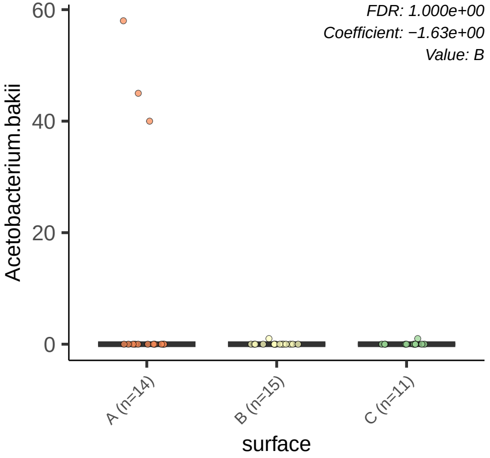

FDR: 1.000e+00
Coefficient: −1.63e+00
Value: B
Acetobacterium.bakii
60
40
20
0
A (n=14)
B (n=15)
C (n=11)
surface

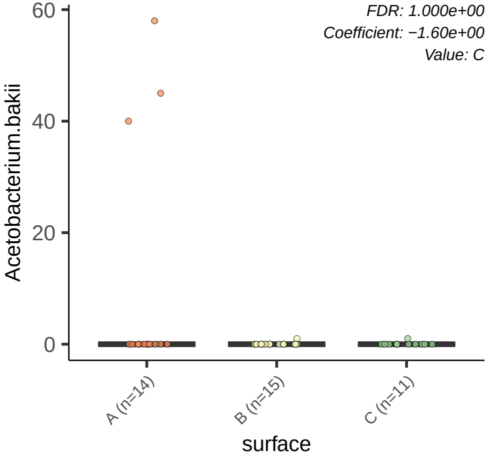
FDR: 1.000e+00
Coefficient: −1.60e+00
Value: C
Acetobacterium.bakii
60
40
20
0
A (n=14)
B (n=15)
C (n=11)
surface

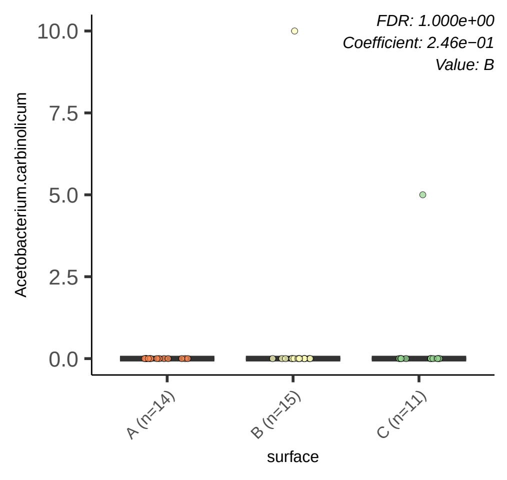
FDR: 1.000e+00
Coefficient: 2.46e−01
Value: B
Acetobacterium.carbinolicum
10.0
7.5
5.0
2.5
0.0
A (n=14)
B (n=15)
C (n=11)
surface

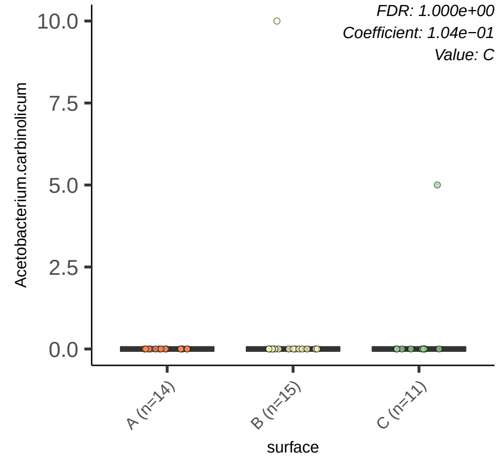
FDR: 1.000e+00
Coefficient: 1.04e−01
Value: C
10.0
7.5
5.0
2.5
0.0
Acetobacterium.carbinolicum
A (n=14)
B (n=15)
C (n=11)
surface

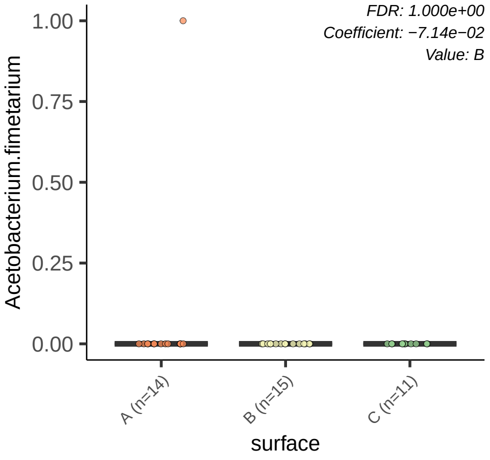
FDR: 1.000e+00
Coefficient: −7.14e−02
Value: B
Acetobacterium.fimetarium
1.00
0.75
0.50
0.25
0.00
A (n=14)
B (n=15)
C (n=11)
surface

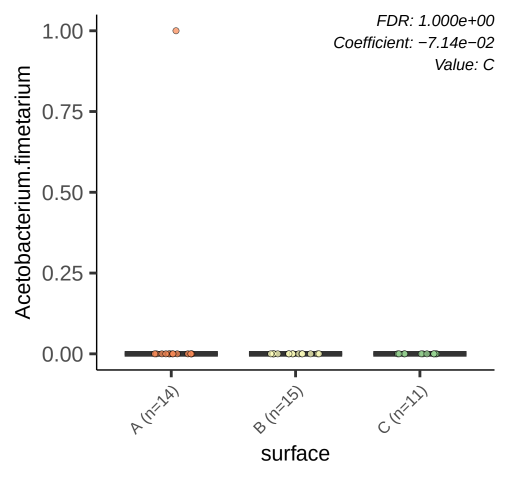
FDR: 1.000e+00
Coefficient: −7.14e−02
Value: C
Acetobacterium.fimetarium
1.00
0.75
0.50
0.25
0.00
A (n=14)
B (n=15)
C (n=11)
surface

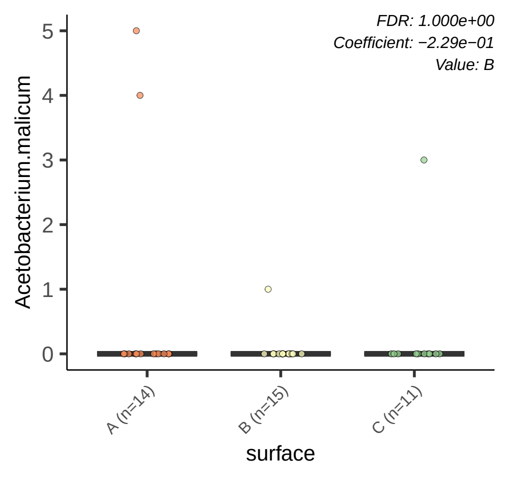
FDR: 1.000e+00
Coefficient: −2.29e−01
Value: B
Acetobacterium.malicum
0
1
2
3
4
5
A (n=14)
B (n=15)
C (n=11)
surface

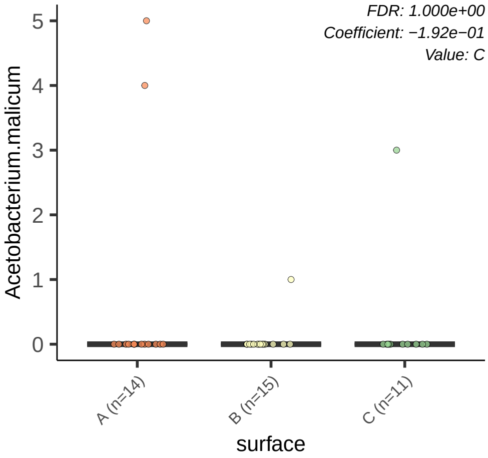

FDR: 1.000e+00
Coefficient: −1.92e−01
Value: C
Acetobacterium.malicum
0
1
2
3
4
5
A (n=14)
B (n=15)
C (n=11)
surface

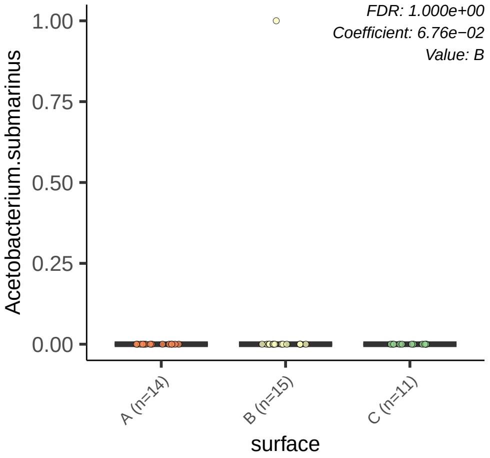

FDR: 1.000e+00
Coefficient: 6.76e−02
Value: B
Acetobacterium.submarinus
1.00
0.75
0.50
0.25
0.00
A (n=14)
B (n=15)
C (n=11)
surface

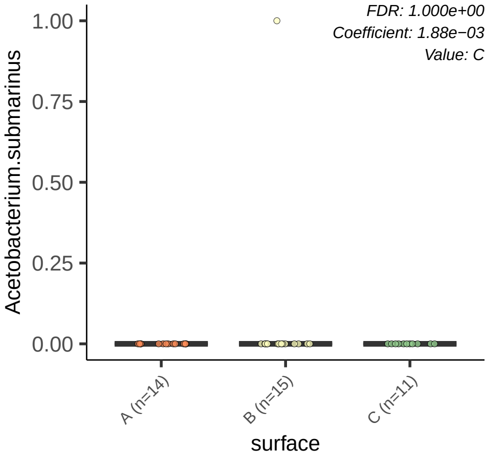
FDR: 1.000e+00
Coefficient: 1.88e−03
Value: C
Acetobacterium.submarinus
1.00
0.75
0.50
0.25
0.00
A (n=14)
B (n=15)
C (n=11)
surface

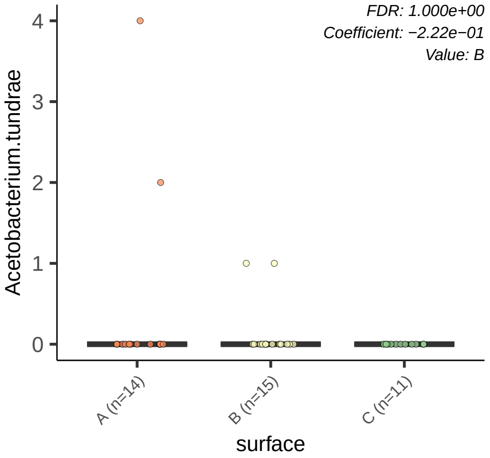

FDR: 1.000e+00
Coefficient: −2.22e−01
Value: B
Acetobacterium.tundrae
4
3
2
1
0
A (n=14)
B (n=15)
C (n=11)
surface

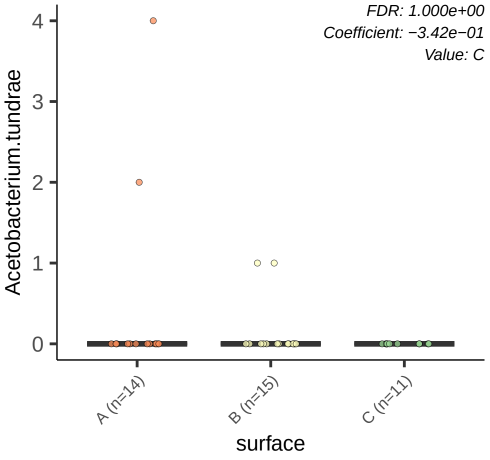
FDR: 1.000e+00
Coefficient: −3.42e−01
Value: C
Acetobacterium.tundrae
0
1
2
3
4
A (n=14)
B (n=15)
C (n=11)
surface

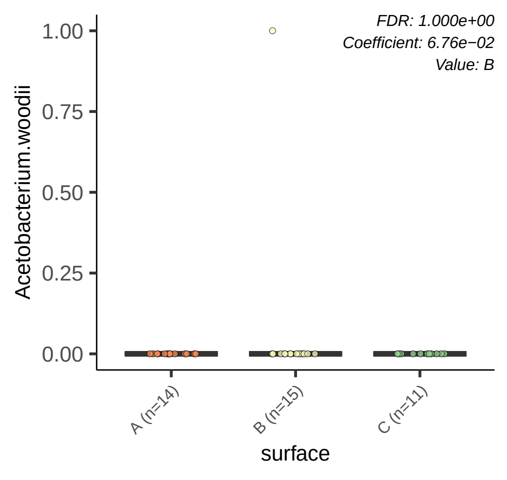

FDR: 1.000e+00
Coefficient: 6.76e−02
Value: B
Acetobacterium.woodii
1.00
0.75
0.50
0.25
0.00
A (n=14)
B (n=15)
C (n=11)
surface

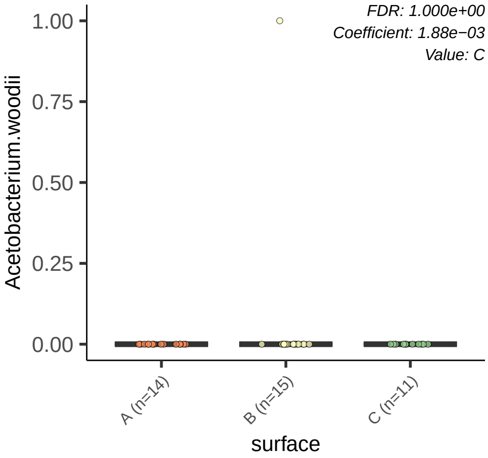
FDR: 1.000e+00
Coefficient: 1.88e−03
Value: C
Acetobacterium.woodii
1.00
0.75
0.50
0.25
0.00
A (n=14)
B (n=15)
C (n=11)
surface

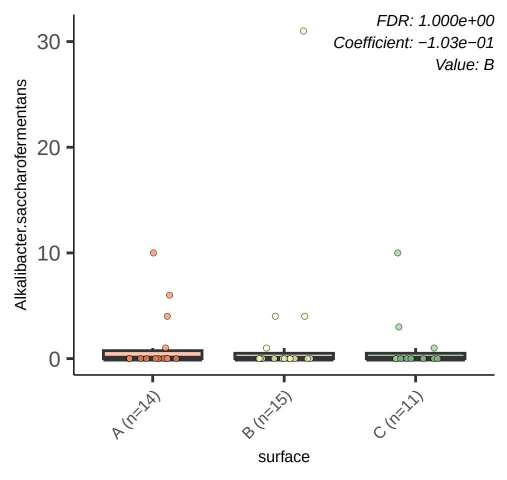

FDR: 1.000e+00
Coefficient: −1.03e−01
Value: B
Alkalibacter.saccharofermentans
30
20
10
0
A (n=14)
B (n=15)
C (n=11)
surface

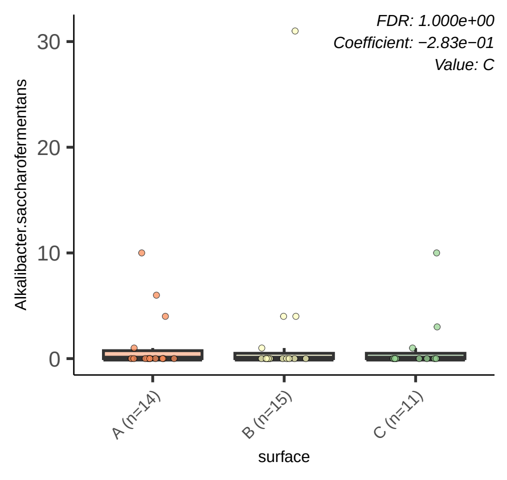
FDR: 1.000e+00
Coefficient: −2.83e−01
Value: C
30
20
10
0
Alkalibacter.saccharofermentans
A (n=14)
B (n=15)
C (n=11)
surface

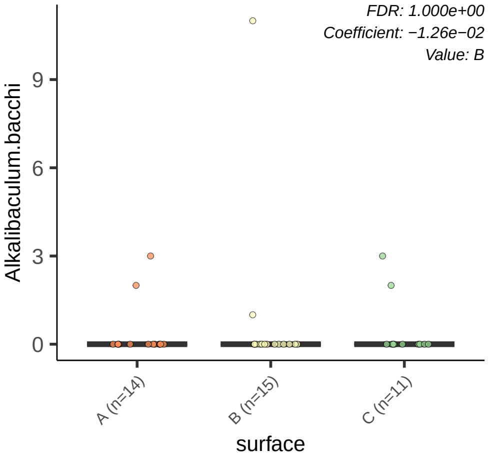

FDR: 1.000e+00
Coefficient: −1.26e−02
Value: B
Alkalibaculum.bacchi
0
3
6
9
A (n=14)
B (n=15)
C (n=11)
surface

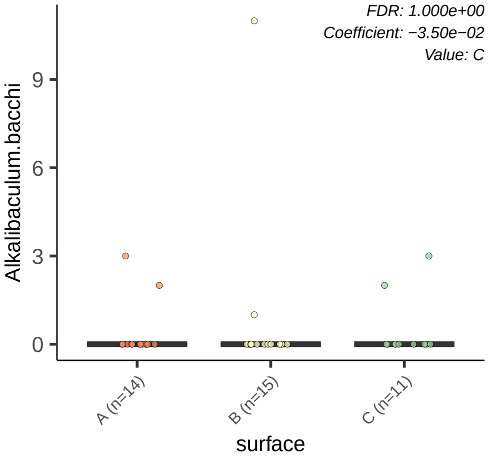

FDR: 1.000e+00
Coefficient: −3.50e−02
Value: C
Alkalibaculum.bacchi
0
3
6
9
A (n=14)
B (n=15)
C (n=11)
surface

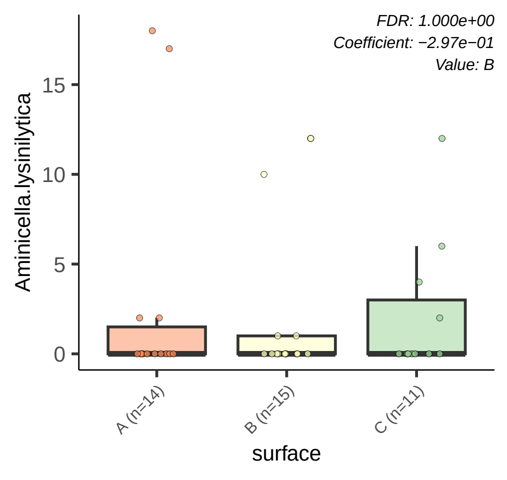
FDR: 1.000e+00
Coefficient: −2.97e−01
Value: B
Aminicella.lysinilytica
0
5
10
15
A (n=14)
B (n=15)
C (n=11)
surface

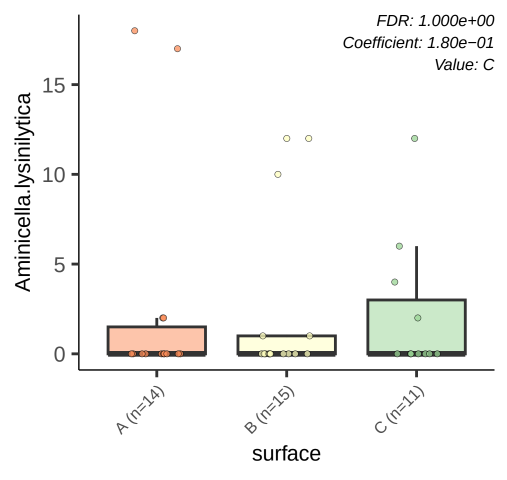

FDR: 1.000e+00
Coefficient: 1.80e−01
Value: C
Aminicella.lysinilytica
0
5
10
15
A (n=14)
B (n=15)
C (n=11)
surface

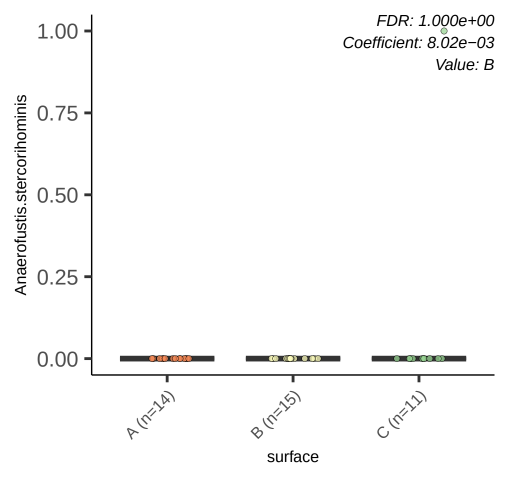
FDR: 1.000e+00
Coefficient: 8.02e−03
Value: B
1.00
0.75
0.50
0.25
0.00
Anaerofustis.stercorihominis
A (n=14)
B (n=15)
C (n=11)
surface

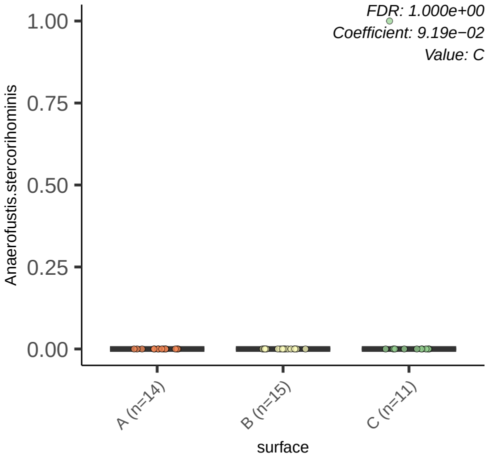
FDR: 1.000e+00
Coefficient: 9.19e−02
Value: C
Anaerofustis.stercorihominis
1.00
0.75
0.50
0.25
0.00
A (n=14)
B (n=15)
C (n=11)
surface

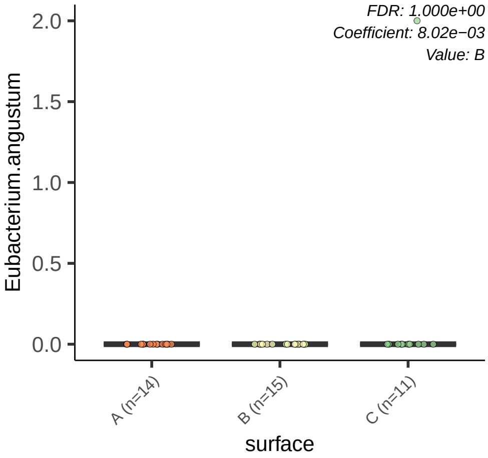

FDR: 1.000e+00
Coefficient: 8.02e−03
Value: B
Eubacterium.angustum
2.0
1.5
1.0
0.5
0.0
A (n=14)
B (n=15)
C (n=11)
surface

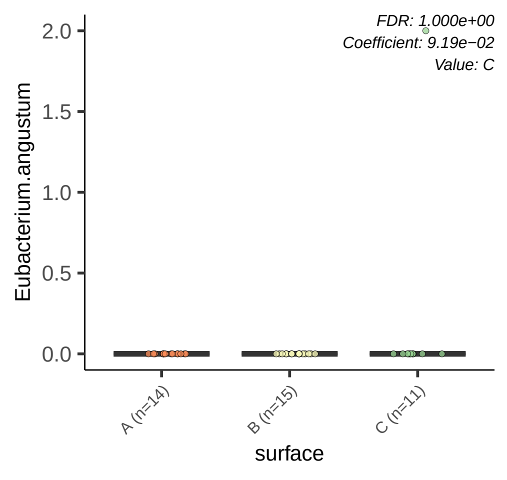

FDR: 1.000e+00
Coefficient: 9.19e−02
Value: C
Eubacterium.angustum
2.0
1.5
1.0
0.5
0.0
A (n=14)
B (n=15)
C (n=11)
surface

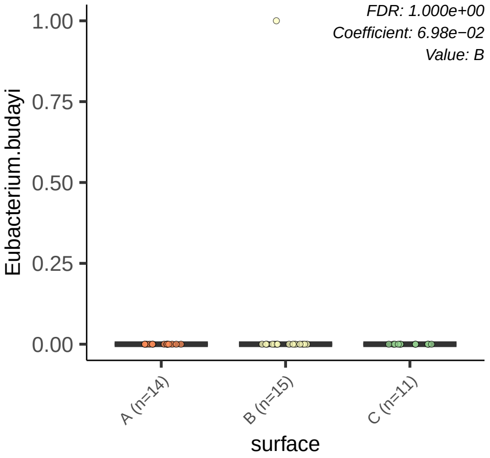
FDR: 1.000e+00
Coefficient: 6.98e−02
Value: B
1.00
0.75
0.50
0.25
0.00
Eubacterium.budayi
A (n=14)
B (n=15)
C (n=11)
surface

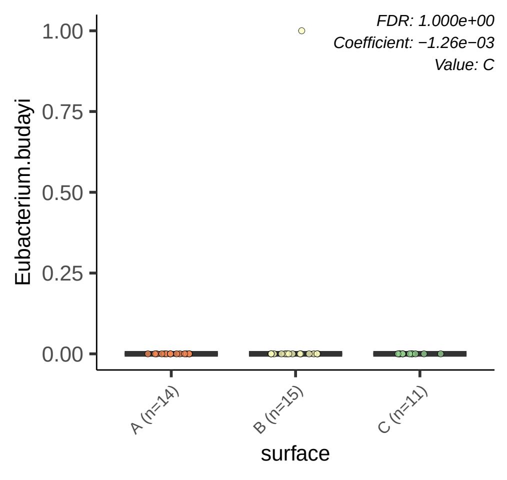
FDR: 1.000e+00
Coefficient: −1.26e−03
Value: C
Eubacterium.budayi
1.00
0.75
0.50
0.25
0.00
A (n=14)
B (n=15)
C (n=11)
surface

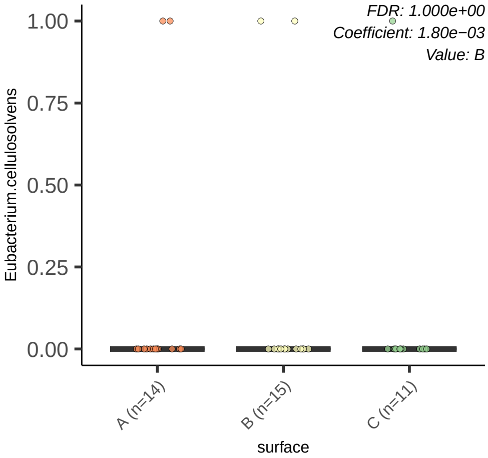

FDR: 1.000e+00
Coefficient: 1.80e−03
Value: B
1.00
0.75
0.50
0.25
0.00
Eubacterium.cellulosolvens
A (n=14)
B (n=15)
C (n=11)
surface

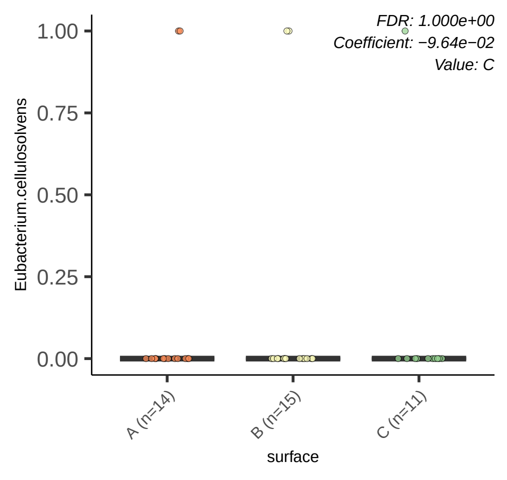
FDR: 1.000e+00
Coefficient: −9.64e−02
Value: C
Eubacterium.cellulosolvens
1.00
0.75
0.50
0.25
0.00
A (n=14)
B (n=15)
C (n=11)
surface

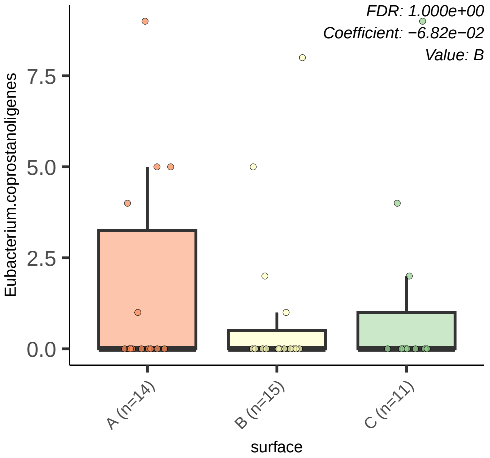
FDR: 1.000e+00
Coefficient: −6.82e−02
Value: B
Eubacterium.coprostanoligenes
7.5
5.0
2.5
0.0
A (n=14)
B (n=15)
C (n=11)
surface

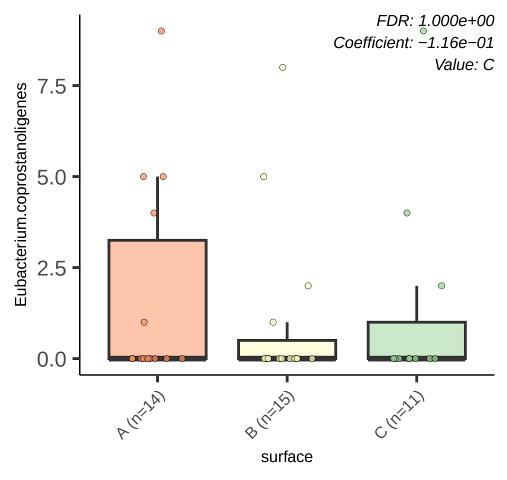
FDR: 1.000e+00
Coefficient: −1.16e−01
Value: C
Eubacterium.coprostanoligenes
0.0
2.5
5.0
7.5
A (n=14)
B (n=15)
C (n=11)
surface

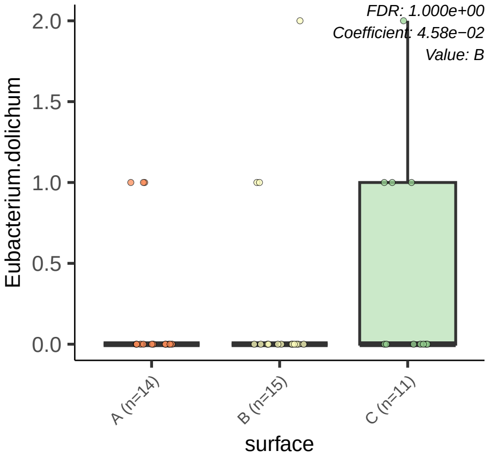

FDR: 1.000e+00
Coefficient: 4.58e−02
Value: B
Eubacterium.dolichum
2.0
1.5
1.0
0.5
0.0
A (n=14)
B (n=15)
C (n=11)
surface

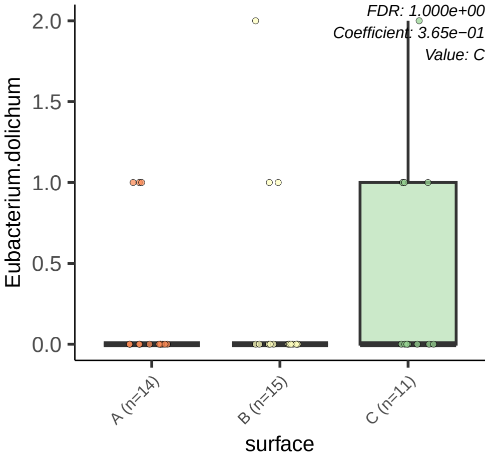

FDR: 1.000e+00
Coefficient: 3.65e−01
Value: C
Eubacterium.dolichum
2.0
1.5
1.0
0.5
0.0
A (n=14)
B (n=15)
C (n=11)
surface

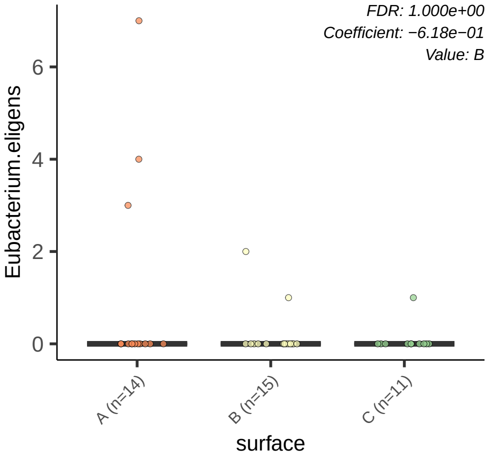
FDR: 1.000e+00
Coefficient: −6.18e−01
Value: B
Eubacterium.eligens
0
2
4
6
A (n=14)
B (n=15)
C (n=11)
surface

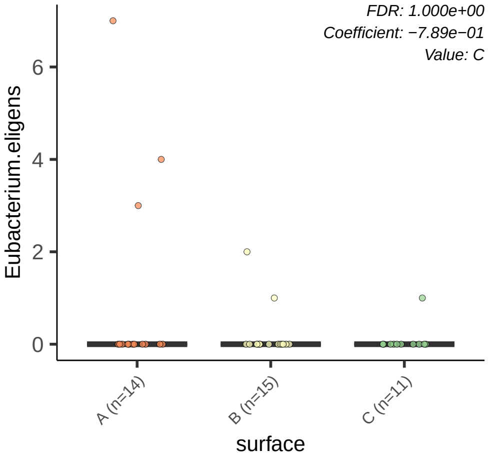
FDR: 1.000e+00
Coefficient: −7.89e−01
Value: C
Eubacterium.eligens
0
2
4
6
A (n=14)
B (n=15)
C (n=11)
surface

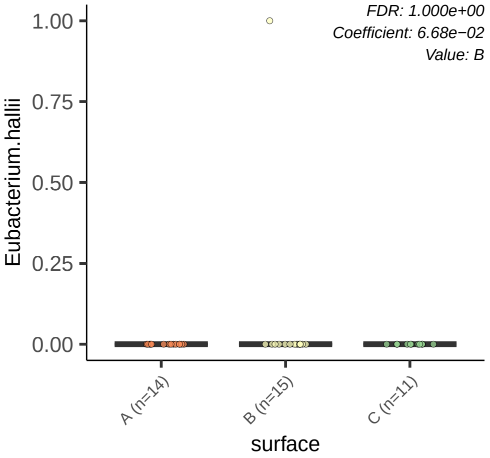
FDR: 1.000e+00
Coefficient: 6.68e−02
Value: B
Eubacterium.hallii
1.00
0.75
0.50
0.25
0.00
A (n=14)
B (n=15)
C (n=11)
surface

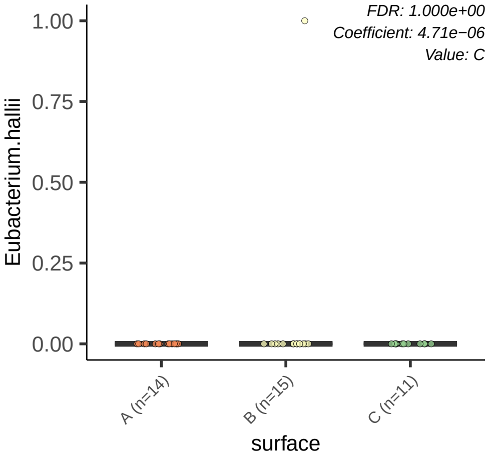
FDR: 1.000e+00
Coefficient: 4.71e−06
Value: C
1.00
0.75
0.50
0.25
0.00
Eubacterium.hallii
A (n=14)
B (n=15)
C (n=11)
surface

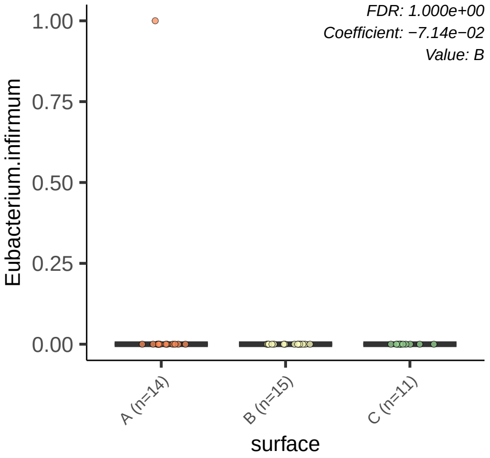
FDR: 1.000e+00
Coefficient: −7.14e−02
Value: B
Eubacterium.infirmum
1.00
0.75
0.50
0.25
0.00
A (n=14)
B (n=15)
C (n=11)
surface

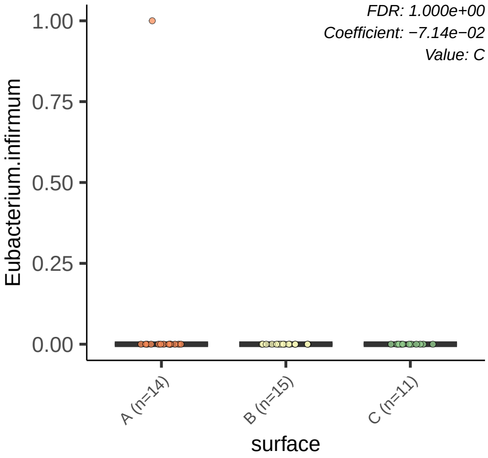
FDR: 1.000e+00
Coefficient: −7.14e−02
Value: C
Eubacterium.infirmum
1.00
0.75
0.50
0.25
0.00
A (n=14)
B (n=15)
C (n=11)
surface

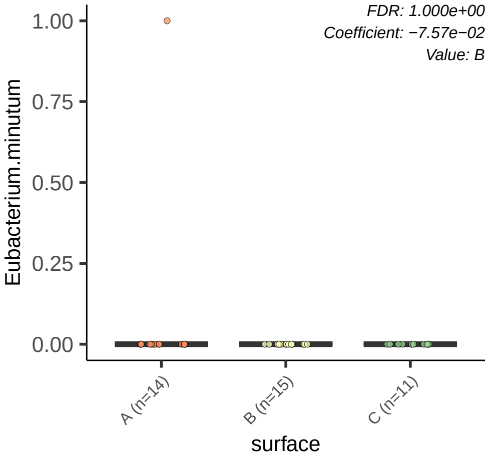

FDR: 1.000e+00
Coefficient: −7.57e−02
Value: B
Eubacterium.minutum
1.00
0.75
0.50
0.25
0.00
A (n=14)
B (n=15)
C (n=11)
surface

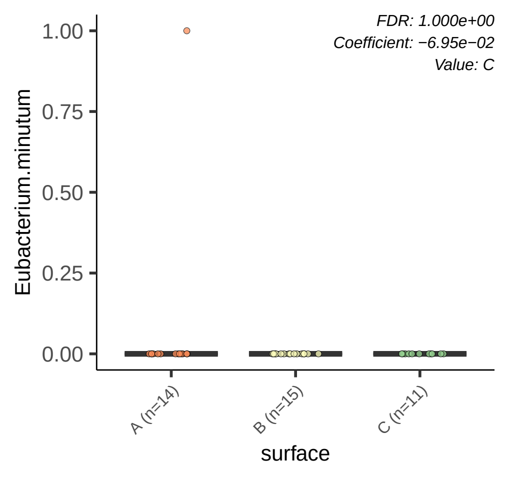

FDR: 1.000e+00
Coefficient: −6.95e−02
Value: C
Eubacterium.minutum
1.00
0.75
0.50
0.25
0.00
A (n=14)
B (n=15)
C (n=11)
surface

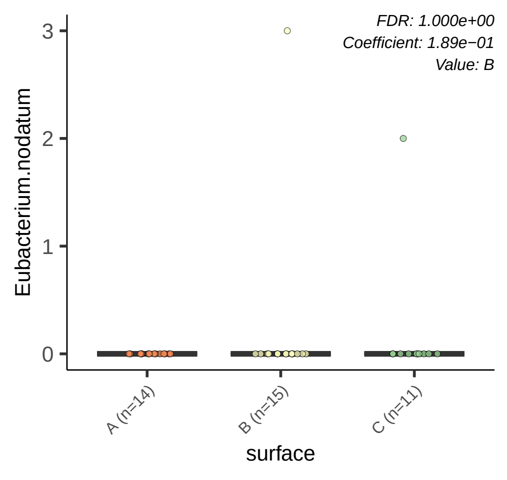
FDR: 1.000e+00
Coefficient: 1.89e−01
Value: B
Eubacterium.nodatum
3
2
1
0
A (n=14)
B (n=15)
C (n=11)
surface

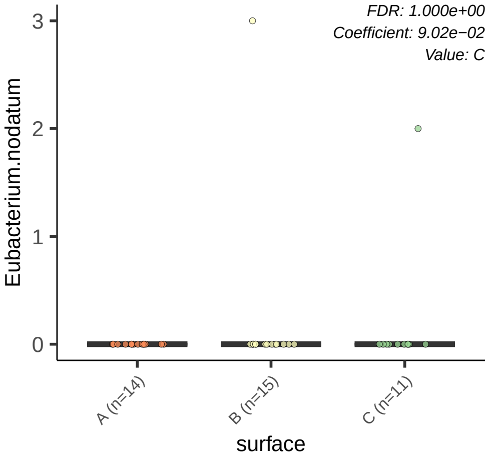
FDR: 1.000e+00
Coefficient: 9.02e−02
Value: C
Eubacterium.nodatum
0
1
2
3
A (n=14)
B (n=15)
C (n=11)
surface

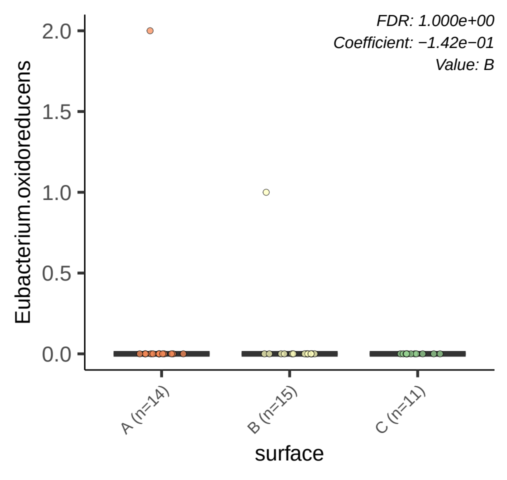
FDR: 1.000e+00
Coefficient: −1.42e−01
Value: B
Eubacterium.oxidoreducens
2.0
1.5
1.0
0.5
0.0
A (n=14)
B (n=15)
C (n=11)
surface

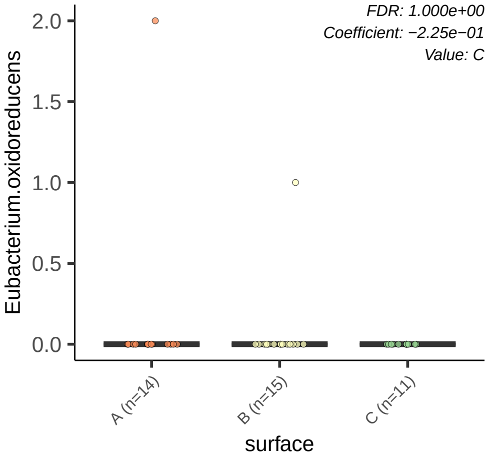

FDR: 1.000e+00
Coefficient: −2.25e−01
Value: C
Eubacterium.oxidoreducens
2.0
1.5
1.0
0.5
0.0
A (n=14)
B (n=15)
C (n=11)
surface

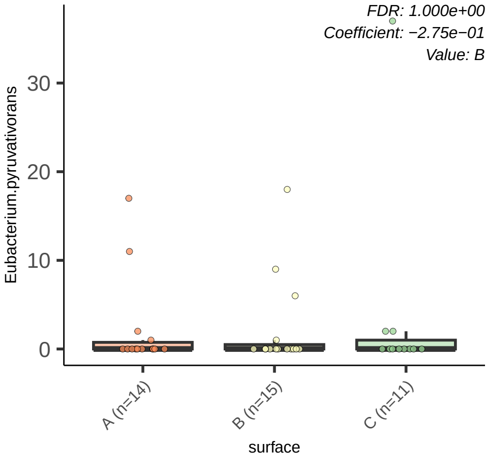

FDR: 1.000e+00
Coefficient: −2.75e−01
Value: B
Eubacterium.pyruvativorans
30
20
10
0
A (n=14)
B (n=15)
C (n=11)
surface

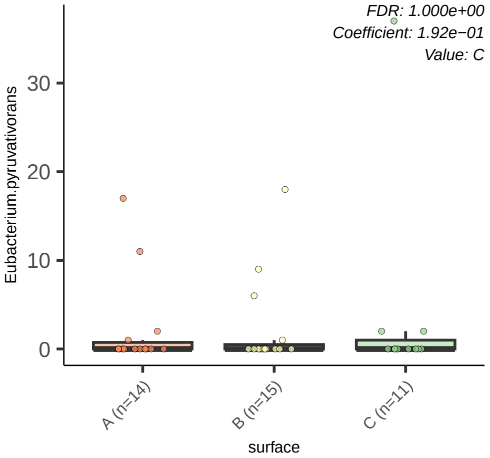

FDR: 1.000e+00
Coefficient: 1.92e−01
Value: C
Eubacterium.pyruvativorans
0
10
20
30
A (n=14)
B (n=15)
C (n=11)
surface

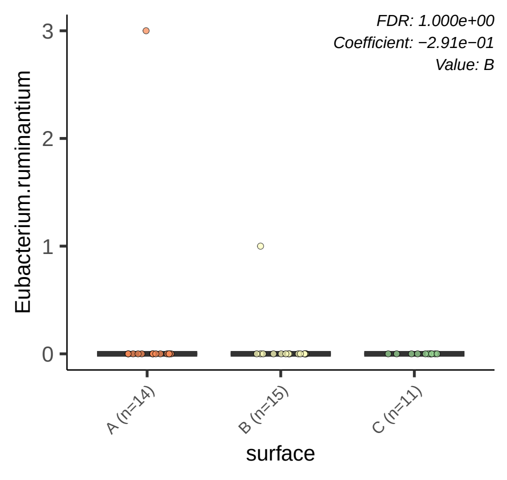

FDR: 1.000e+00
Coefficient: −2.91e−01
Value: B
Eubacterium.ruminantium
3
2
1
0
A (n=14)
B (n=15)
C (n=11)
surface

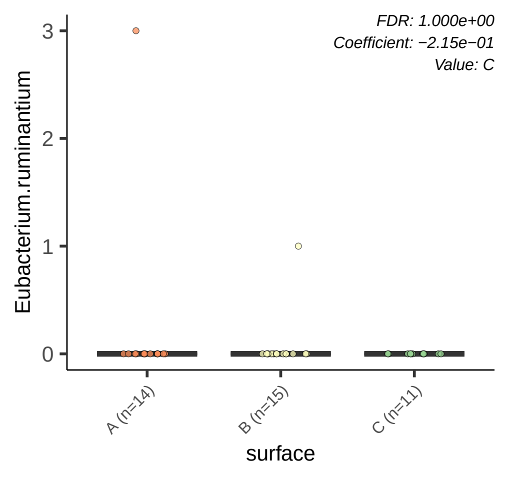
FDR: 1.000e+00
Coefficient: −2.15e−01
Value: C
Eubacterium.ruminantium
3
2
1
0
A (n=14)
B (n=15)
C (n=11)
surface

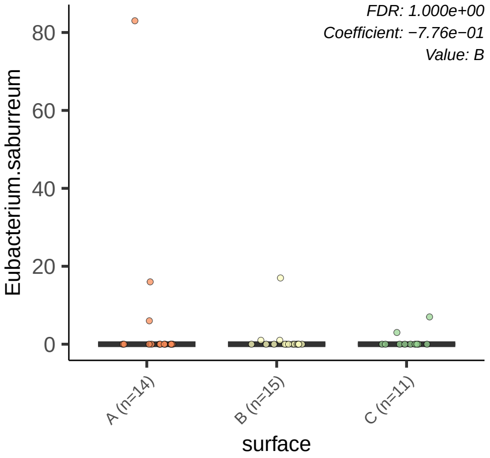
FDR: 1.000e+00
Coefficient: −7.76e−01
Value: B
Eubacterium.saburreum
80
60
40
20
0
A (n=14)
B (n=15)
C (n=11)
surface

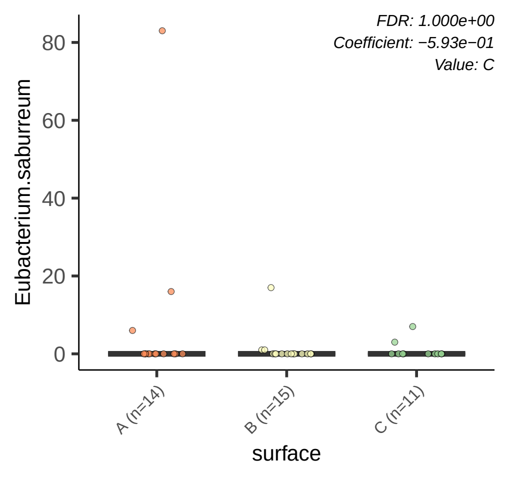

FDR: 1.000e+00
Coefficient: −5.93e−01
Value: C
Eubacterium.saburreum
0
20
40
60
80
A (n=14)
B (n=15)
C (n=11)
surface

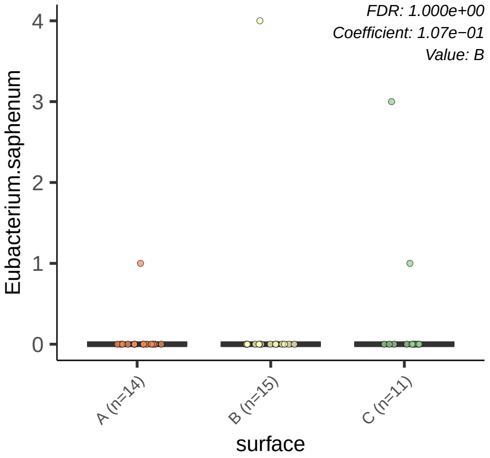

FDR: 1.000e+00
Coefficient: 1.07e−01
Value: B
Eubacterium.saphenum
0
1
2
3
4
A (n=14)
B (n=15)
C (n=11)
surface

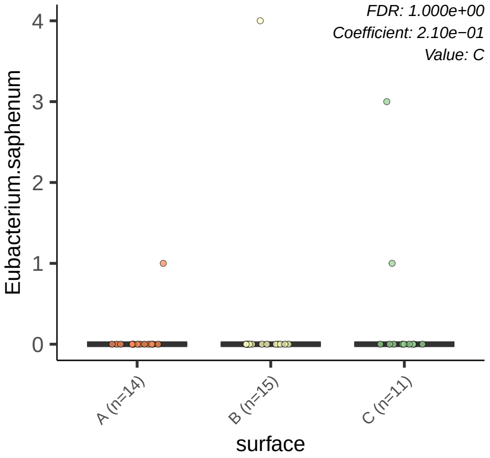

FDR: 1.000e+00
Coefficient: 2.10e−01
Value: C
Eubacterium.saphenum
4
3
2
1
0
A (n=14)
B (n=15)
C (n=11)
surface

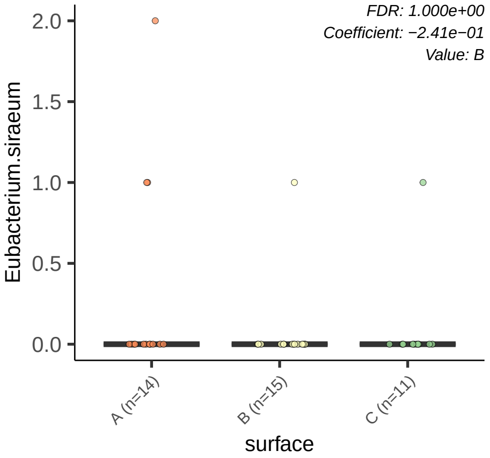

FDR: 1.000e+00
Coefficient: −2.41e−01
Value: B
Eubacterium.siraeum
2.0
1.5
1.0
0.5
0.0
A (n=14)
B (n=15)
C (n=11)
surface

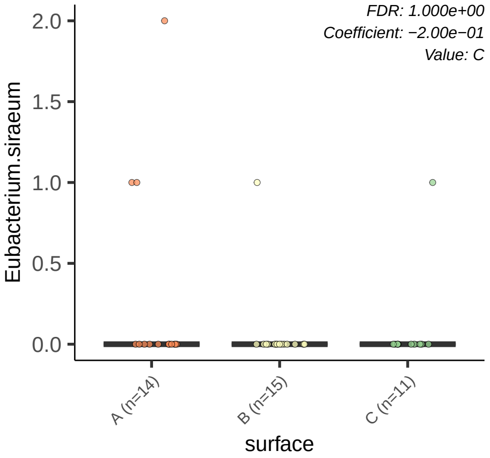

FDR: 1.000e+00
Coefficient: −2.00e−01
Value: C
Eubacterium.siraeum
2.0
1.5
1.0
0.5
0.0
A (n=14)
B (n=15)
C (n=11)
surface

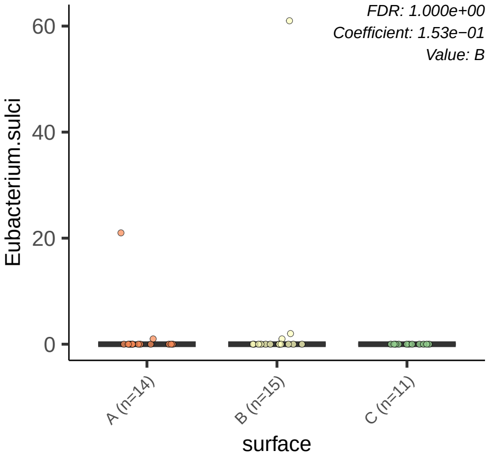

FDR: 1.000e+00
Coefficient: 1.53e−01
Value: B
Eubacterium.sulci
60
40
20
0
A (n=14)
B (n=15)
C (n=11)
surface

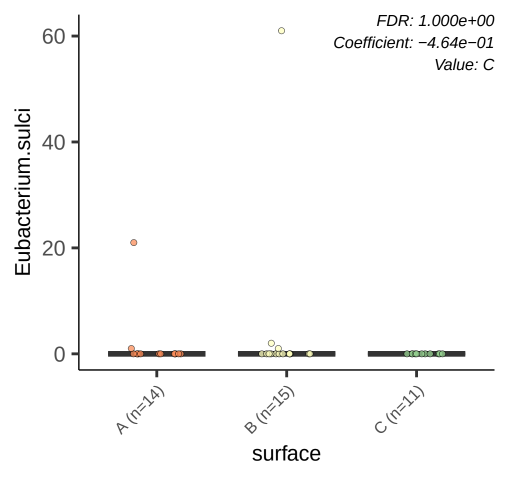

FDR: 1.000e+00
Coefficient: −4.64e−01
Value: C
Eubacterium.sulci
60
40
20
0
A (n=14)
B (n=15)
C (n=11)
surface

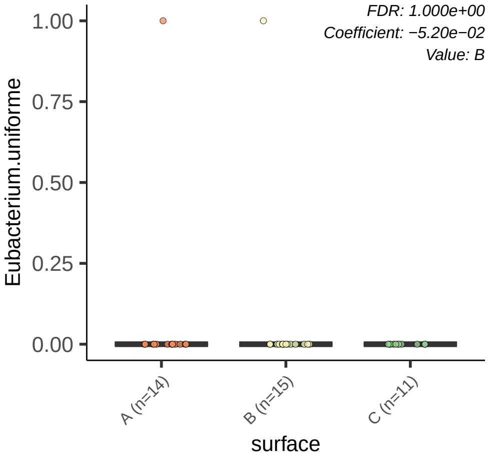
FDR: 1.000e+00
Coefficient: −5.20e−02
Value: B
Eubacterium.uniforme
1.00
0.75
0.50
0.25
0.00
A (n=14)
B (n=15)
C (n=11)
surface

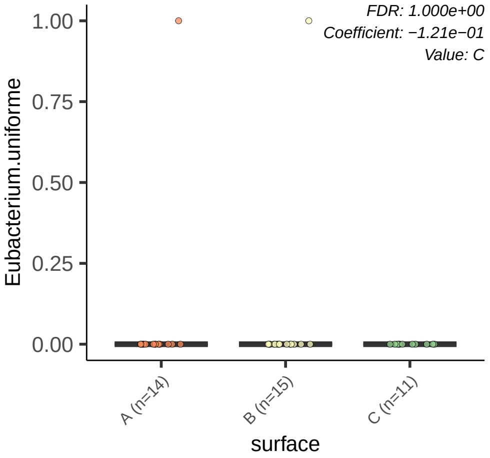

FDR: 1.000e+00
Coefficient: −1.21e−01
Value: C
Eubacterium.uniforme
1.00
0.75
0.50
0.25
0.00
A (n=14)
B (n=15)
C (n=11)
surface

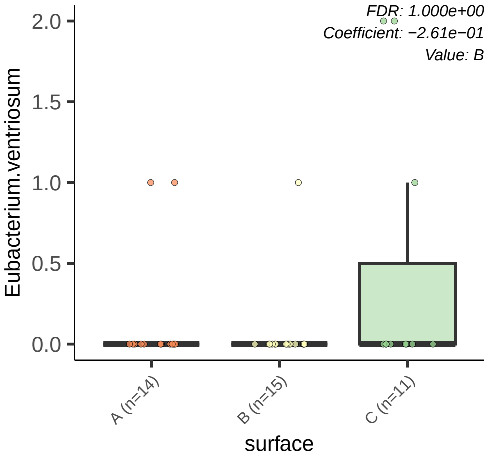

FDR: 1.000e+00
Coefficient: −2.61e−01
Value: B
Eubacterium.ventriosum
2.0
1.5
1.0
0.5
0.0
A (n=14)
B (n=15)
C (n=11)
surface

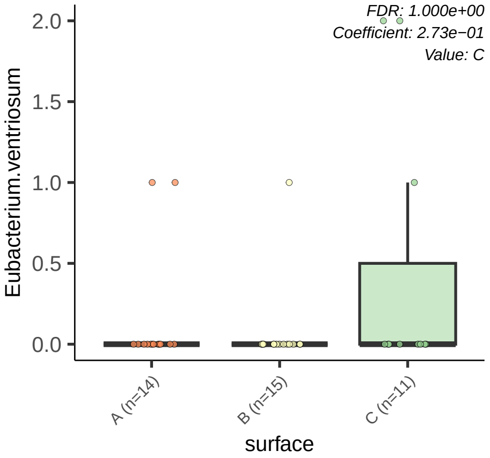

FDR: 1.000e+00
Coefficient: 2.73e−01
Value: C
Eubacterium.ventriosum
2.0
1.5
1.0
0.5
0.0
A (n=14)
B (n=15)
C (n=11)
surface

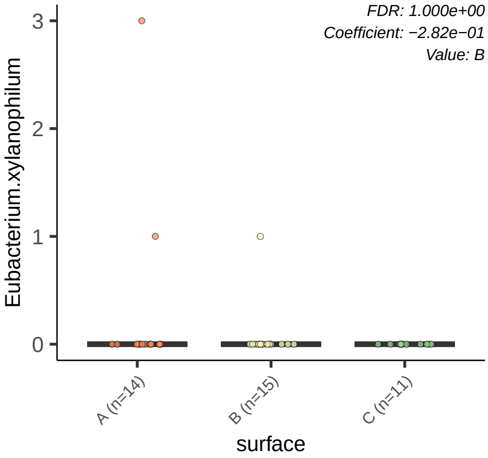

FDR: 1.000e+00
Coefficient: −2.82e−01
Value: B
Eubacterium.xylanophilum
3
2
1
0
A (n=14)
B (n=15)
C (n=11)
surface

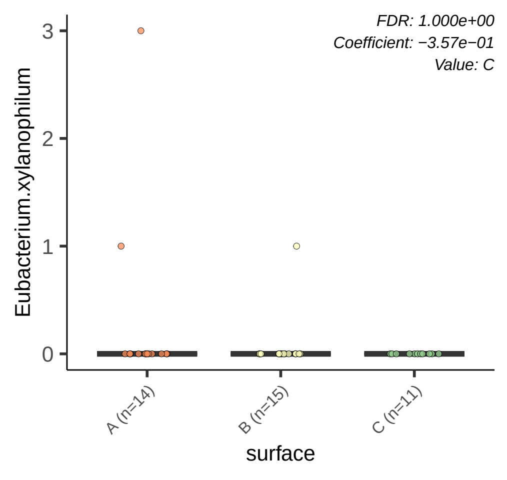
FDR: 1.000e+00
Coefficient: −3.57e−01
Value: C
Eubacterium.xylanophilum
3
2
1
0
A (n=14)
B (n=15)
C (n=11)
surface

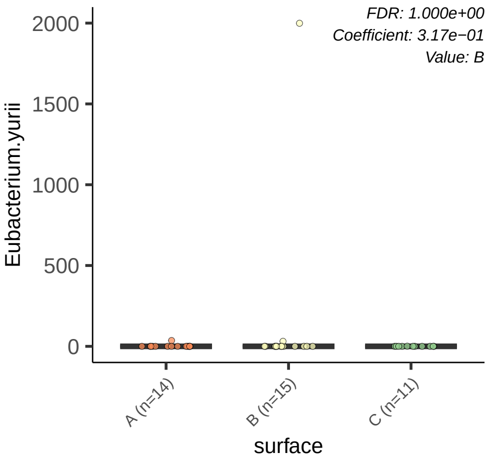

FDR: 1.000e+00
Coefficient: 3.17e−01
Value: B
2000
1500
1000
500
0
Eubacterium.yurii
A (n=14)
B (n=15)
C (n=11)
surface

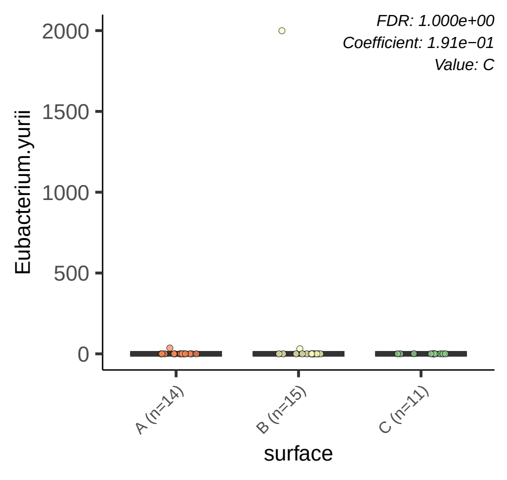
FDR: 1.000e+00
Coefficient: 1.91e−01
Value: C
Eubacterium.yurii
2000
1500
1000
500
0
A (n=14)
B (n=15)
C (n=11)
surface

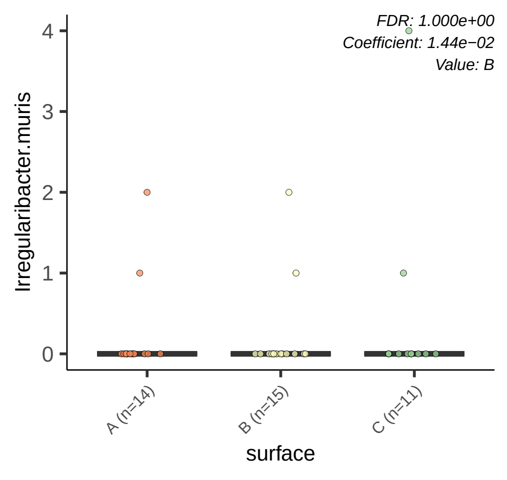
FDR: 1.000e+00
Coefficient: 1.44e−02
Value: B
Irregularibacter.muris
0
1
2
3
4
A (n=14)
B (n=15)
C (n=11)
surface

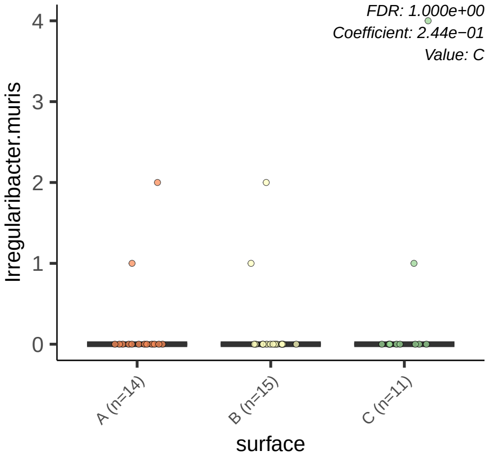
FDR: 1.000e+00
Coefficient: 2.44e−01
Value: C
Irregularibacter.muris
4
3
2
1
0
A (n=14)
B (n=15)
C (n=11)
surface

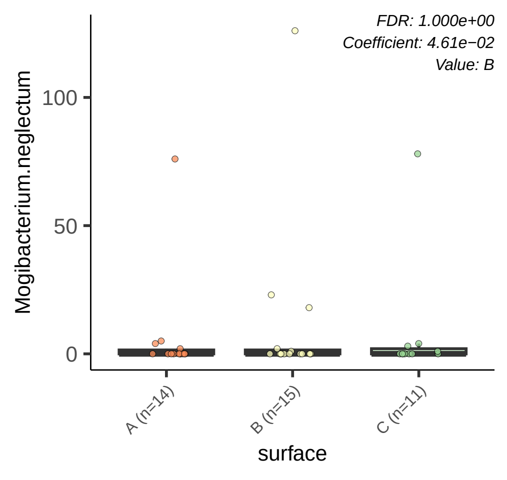
FDR: 1.000e+00
Coefficient: 4.61e−02
Value: B
Mogibacterium.neglectum
100
50
0
A (n=14)
B (n=15)
C (n=11)
surface

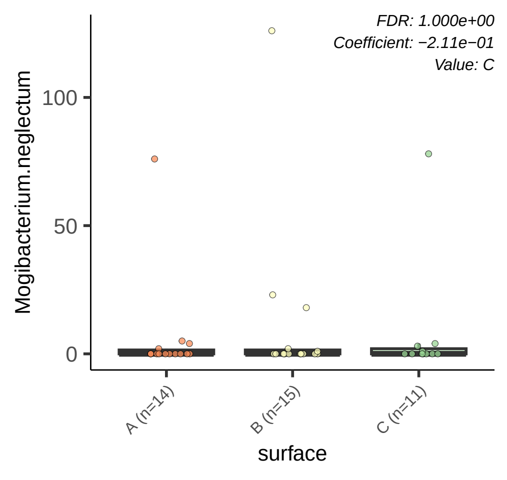
FDR: 1.000e+00
Coefficient: −2.11e−01
Value: C
Mogibacterium.neglectum
100
50
0
A (n=14)
B (n=15)
C (n=11)
surface

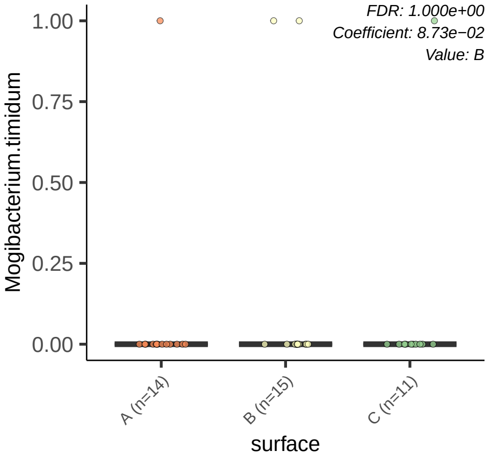
FDR: 1.000e+00
Coefficient: 8.73e−02
Value: B
Mogibacterium.timidum
1.00
0.75
0.50
0.25
0.00
A (n=14)
B (n=15)
C (n=11)
surface

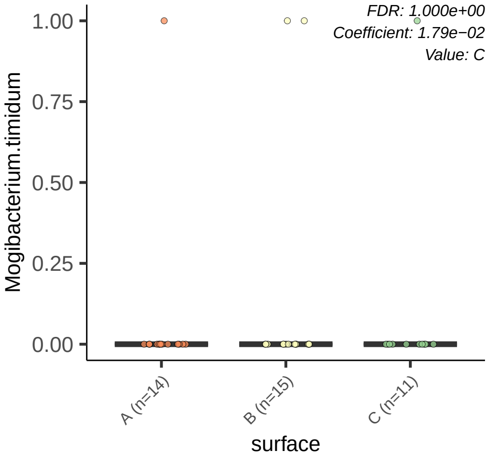
FDR: 1.000e+00
Coefficient: 1.79e−02
Value: C
Mogibacterium.timidum
1.00
0.75
0.50
0.25
0.00
A (n=14)
B (n=15)
C (n=11)
surface

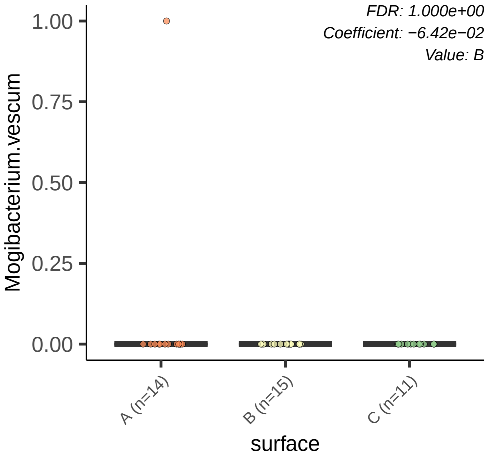
FDR: 1.000e+00
Coefficient: −6.42e−02
Value: B
Mogibacterium.vescum
1.00
0.75
0.50
0.25
0.00
A (n=14)
B (n=15)
C (n=11)
surface

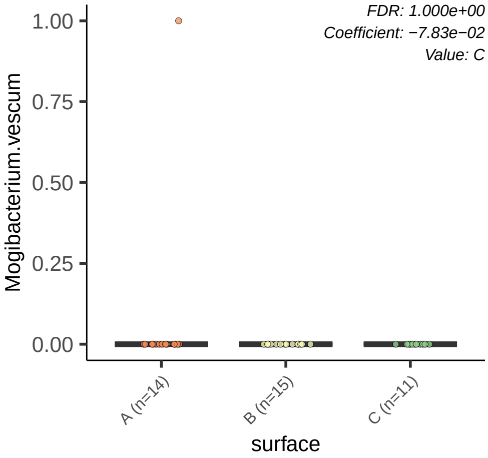

FDR: 1.000e+00
Coefficient: −7.83e−02
Value: C
Mogibacterium.vescum
1.00
0.75
0.50
0.25
0.00
A (n=14)
B (n=15)
C (n=11)
surface

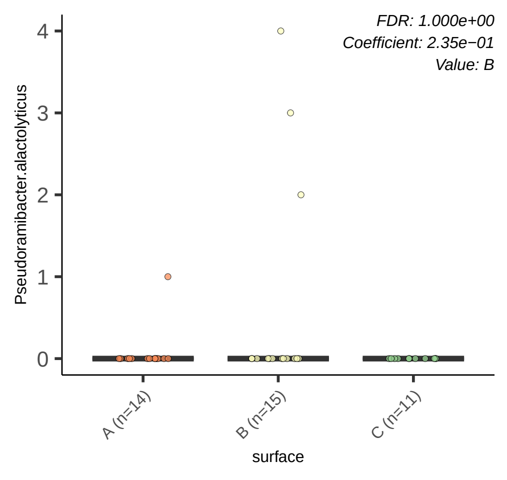

FDR: 1.000e+00
Coefficient: 2.35e−01
Value: B
Pseudoramibacter.alactolyticus
4
3
2
1
0
A (n=14)
B (n=15)
C (n=11)
surface

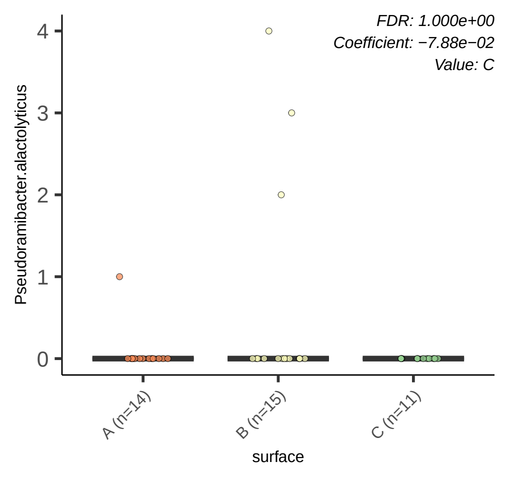
FDR: 1.000e+00
Coefficient: −7.88e−02
Value: C
Pseudoramibacter.alactolyticus
4
3
2
1
0
A (n=14)
B (n=15)
C (n=11)
surface

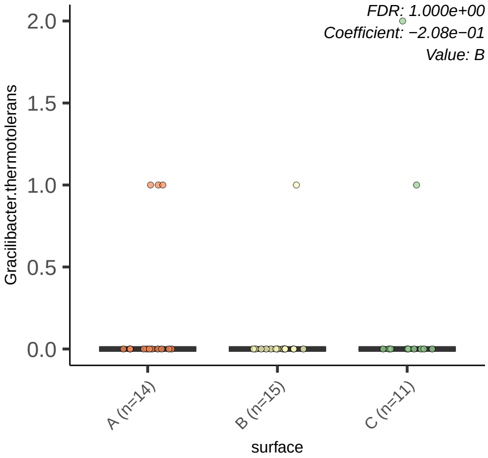
FDR: 1.000e+00
Coefficient: −2.08e−01
Value: B
Gracilibacter.thermotolerans
2.0
1.5
1.0
0.5
0.0
A (n=14)
B (n=15)
C (n=11)
surface

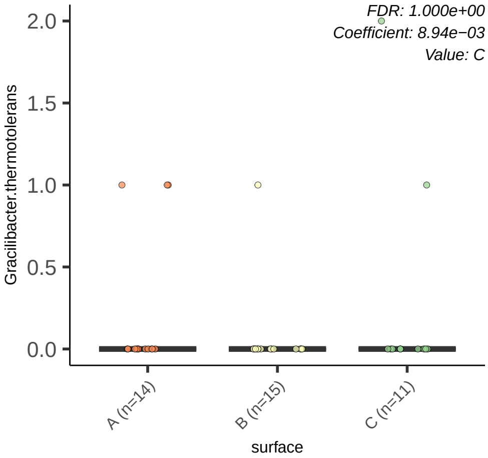
FDR: 1.000e+00
Coefficient: 8.94e−03
Value: C
Gracilibacter.thermotolerans
2.0
1.5
1.0
0.5
0.0
A (n=14)
B (n=15)
C (n=11)
surface

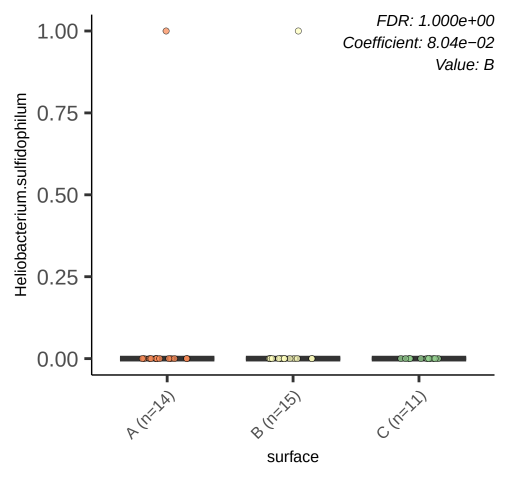

FDR: 1.000e+00
Coefficient: 8.04e−02
Value: B
1.00
0.75
0.50
0.25
0.00
Heliobacterium.sulfidophilum
A (n=14)
B (n=15)
C (n=11)
surface

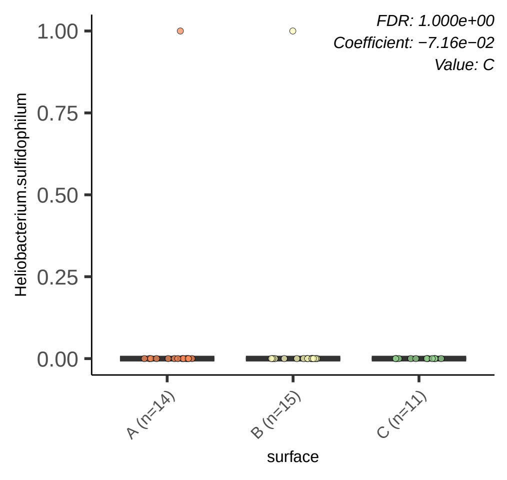

FDR: 1.000e+00
Coefficient: −7.16e−02
Value: C
Heliobacterium.sulfidophilum
1.00
0.75
0.50
0.25
0.00
A (n=14)
B (n=15)
C (n=11)
surface

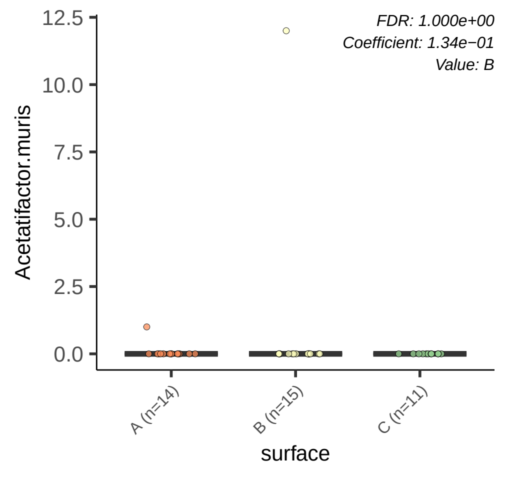

FDR: 1.000e+00
Coefficient: 1.34e−01
Value: B
12.5
10.0
7.5
5.0
2.5
0.0
Acetatifactor.muris
A (n=14)
B (n=15)
C (n=11)
surface

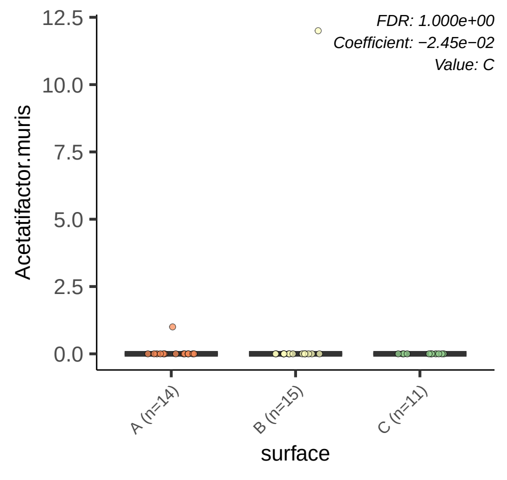

FDR: 1.000e+00
Coefficient: −2.45e−02
Value: C
Acetatifactor.muris
12.5
10.0
7.5
5.0
2.5
0.0
A (n=14)
B (n=15)
C (n=11)
surface

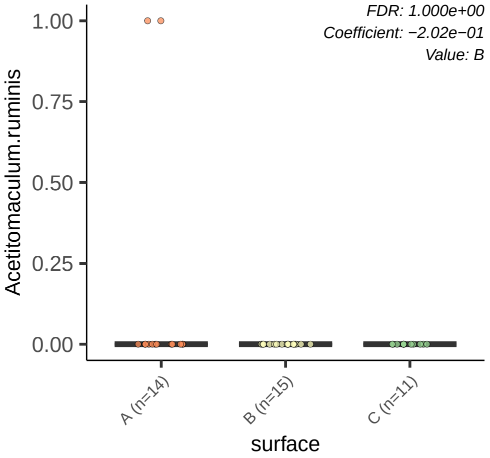
FDR: 1.000e+00
Coefficient: −2.02e−01
Value: B
Acetitomaculum.ruminis
1.00
0.75
0.50
0.25
0.00
A (n=14)
B (n=15)
C (n=11)
surface

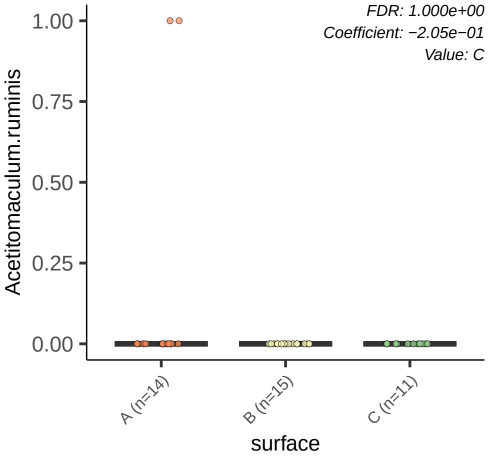

FDR: 1.000e+00
Coefficient: −2.05e−01
Value: C
Acetitomaculum.ruminis
1.00
0.75
0.50
0.25
0.00
A (n=14)
B (n=15)
C (n=11)
surface

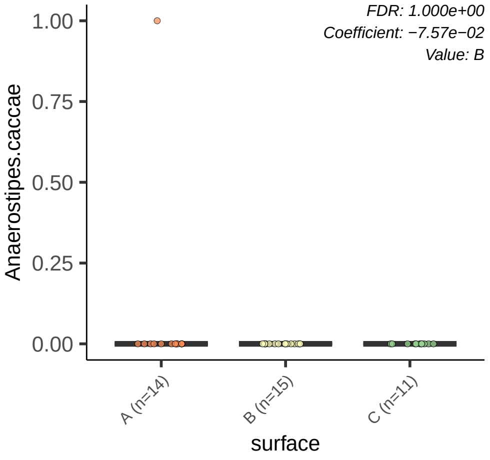
FDR: 1.000e+00
Coefficient: −7.57e−02
Value: B
Anaerostipes.caccae
1.00
0.75
0.50
0.25
0.00
A (n=14)
B (n=15)
C (n=11)
surface

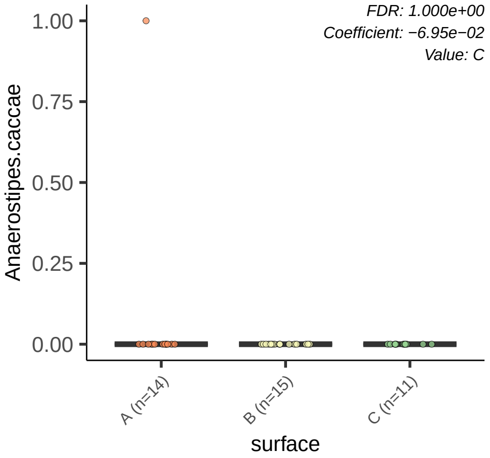
FDR: 1.000e+00
Coefficient: −6.95e−02
Value: C
Anaerostipes.caccae
1.00
0.75
0.50
0.25
0.00
A (n=14)
B (n=15)
C (n=11)
surface

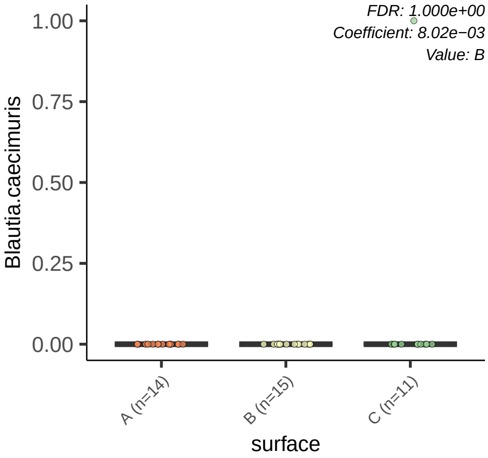
FDR: 1.000e+00
Coefficient: 8.02e−03
Value: B
Blautia.caecimuris
1.00
0.75
0.50
0.25
0.00
A (n=14)
B (n=15)
C (n=11)
surface

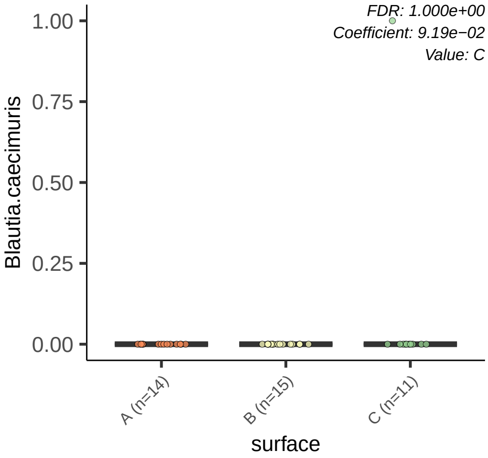
FDR: 1.000e+00
Coefficient: 9.19e−02
Value: C
Blautia.caecimuris
1.00
0.75
0.50
0.25
0.00
A (n=14)
B (n=15)
C (n=11)
surface

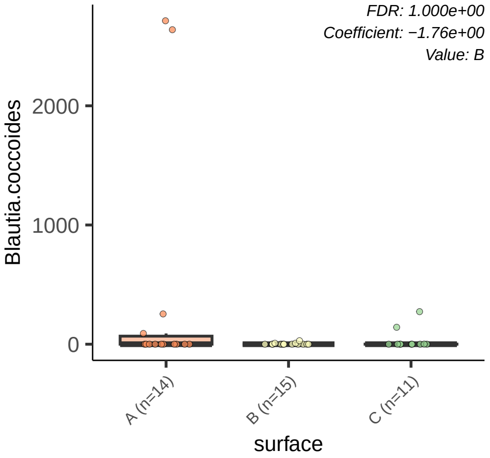
FDR: 1.000e+00
Coefficient: −1.76e+00
Value: B
Blautia.coccoides
2000
1000
0
A (n=14)
B (n=15)
C (n=11)
surface

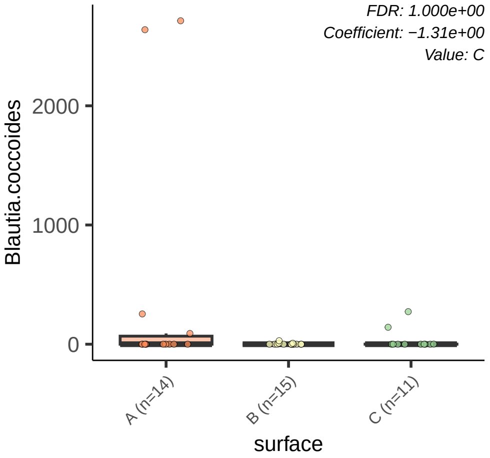

FDR: 1.000e+00
Coefficient: −1.31e+00
Value: C
Blautia.coccoides
2000
1000
0
A (n=14)
B (n=15)
C (n=11)
surface

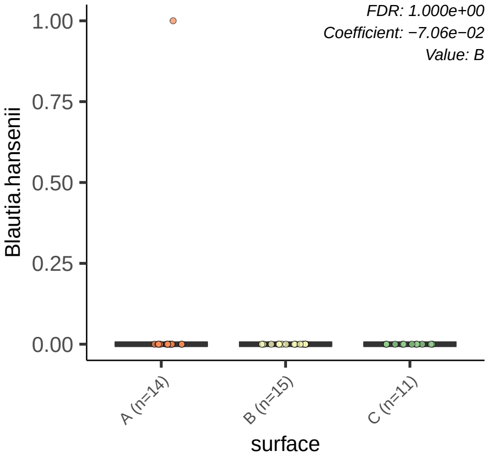
FDR: 1.000e+00
Coefficient: −7.06e−02
Value: B
Blautia.hansenii
1.00
0.75
0.50
0.25
0.00
A (n=14)
B (n=15)
C (n=11)
surface

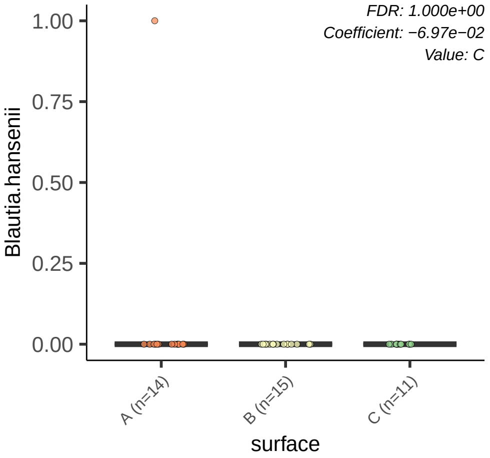
FDR: 1.000e+00
Coefficient: −6.97e−02
Value: C
Blautia.hansenii
1.00
0.75
0.50
0.25
0.00
A (n=14)
B (n=15)
C (n=11)
surface

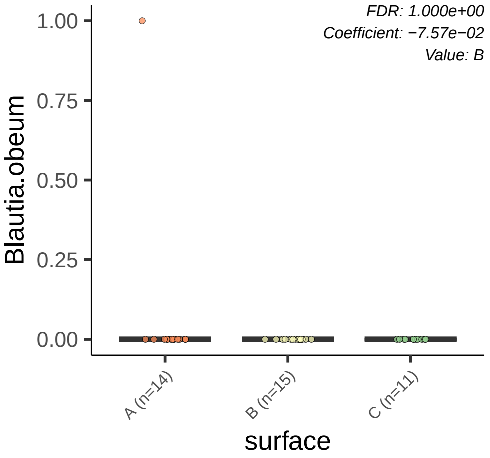
FDR: 1.000e+00
Coefficient: −7.57e−02
Value: B
Blautia.obeum
1.00
0.75
0.50
0.25
0.00
A (n=14)
B (n=15)
C (n=11)
surface

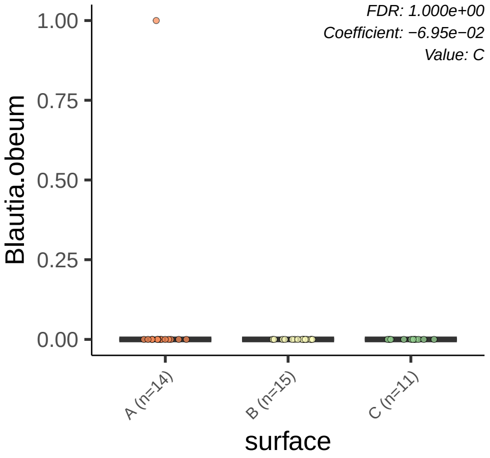
FDR: 1.000e+00
Coefficient: −6.95e−02
Value: C
Blautia.obeum
1.00
0.75
0.50
0.25
0.00
A (n=14)
B (n=15)
C (n=11)
surface

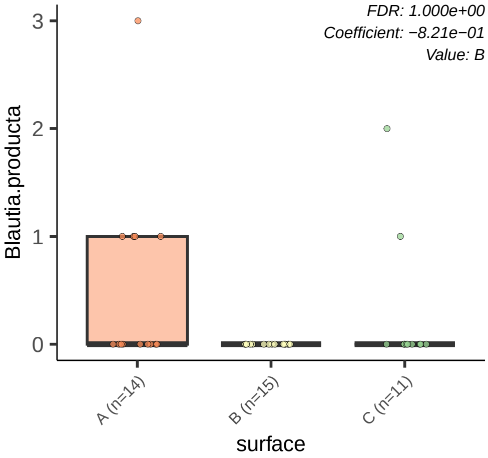

FDR: 1.000e+00
Coefficient: −8.21e−01
Value: B
Blautia.producta
3
2
1
0
A (n=14)
B (n=15)
C (n=11)
surface

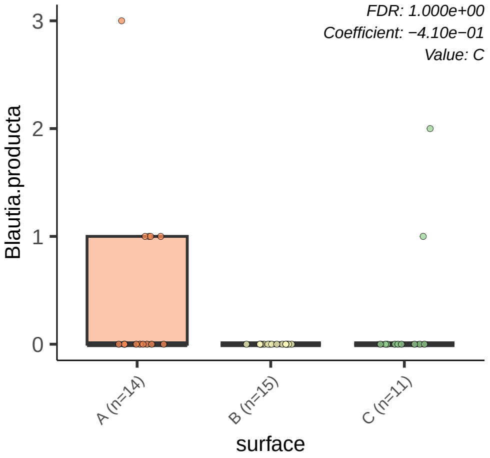
FDR: 1.000e+00
Coefficient: −4.10e−01
Value: C
Blautia.producta
3
2
1
0
A (n=14)
B (n=15)
C (n=11)
surface

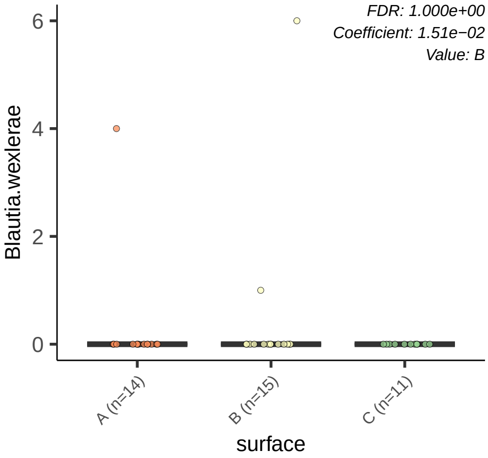

FDR: 1.000e+00
Coefficient: 1.51e−02
Value: B
Blautia.wexlerae
0
2
4
6
A (n=14)
B (n=15)
C (n=11)
surface

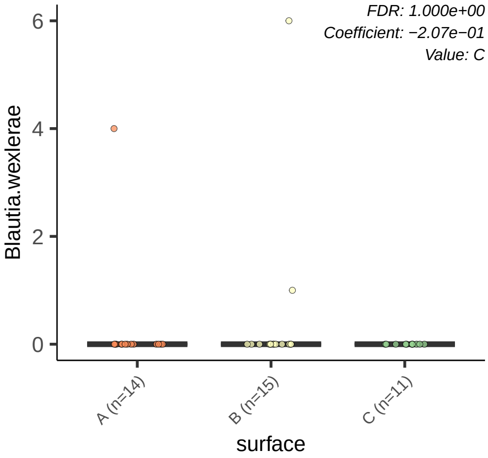

FDR: 1.000e+00
Coefficient: −2.07e−01
Value: C
Blautia.wexlerae
6
4
2
0
A (n=14)
B (n=15)
C (n=11)
surface

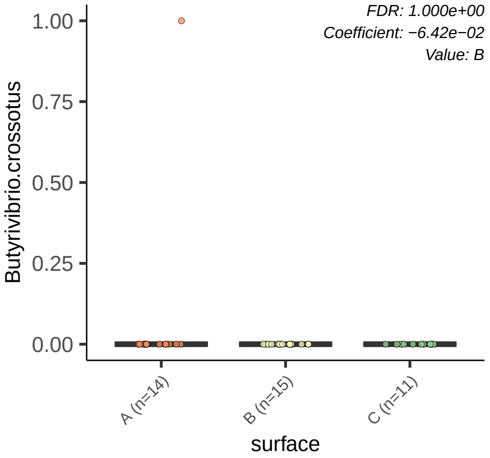
FDR: 1.000e+00
Coefficient: −6.42e−02
Value: B
Butyrivibrio.crossotus
1.00
0.75
0.50
0.25
0.00
A (n=14)
B (n=15)
C (n=11)
surface

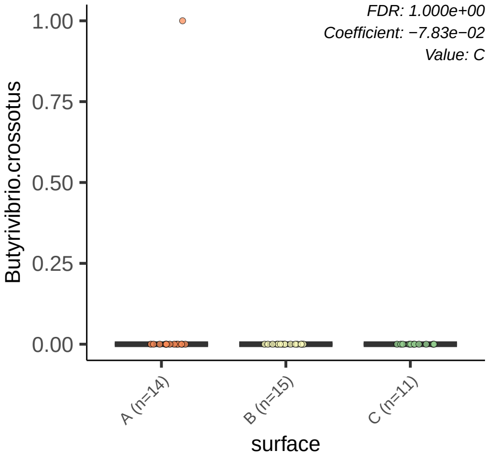
FDR: 1.000e+00
Coefficient: −7.83e−02
Value: C
Butyrivibrio.crossotus
1.00
0.75
0.50
0.25
0.00
A (n=14)
B (n=15)
C (n=11)
surface

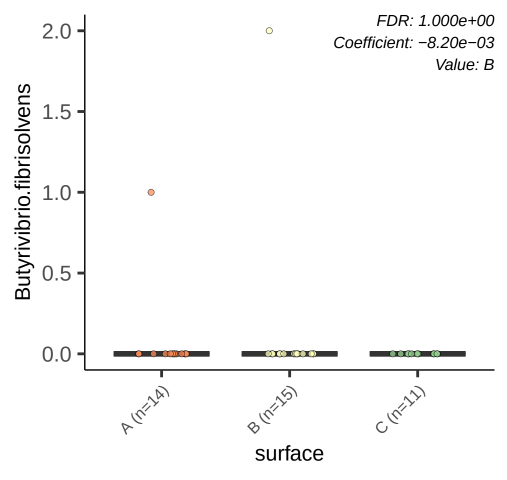

FDR: 1.000e+00
Coefficient: −8.20e−03
Value: B
Butyrivibrio.fibrisolvens
2.0
1.5
1.0
0.5
0.0
A (n=14)
B (n=15)
C (n=11)
surface

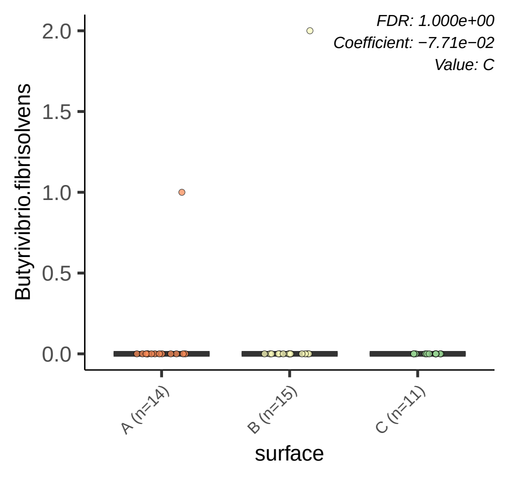

FDR: 1.000e+00
Coefficient: −7.71e−02
Value: C
2.0
1.5
1.0
0.5
0.0
Butyrivibrio.fibrisolvens
A (n=14)
B (n=15)
C (n=11)
surface

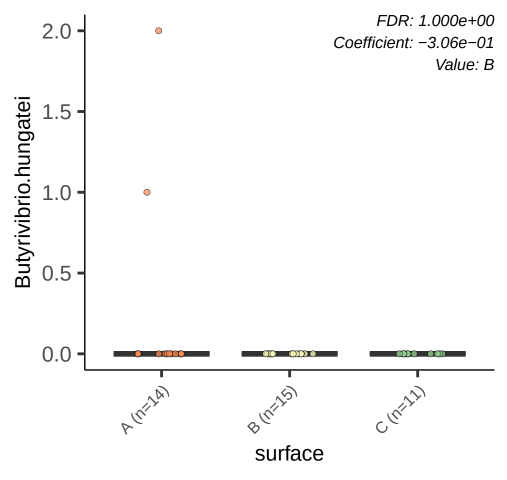
FDR: 1.000e+00
Coefficient: −3.06e−01
Value: B
Butyrivibrio.hungatei
2.0
1.5
1.0
0.5
0.0
A (n=14)
B (n=15)
C (n=11)
surface

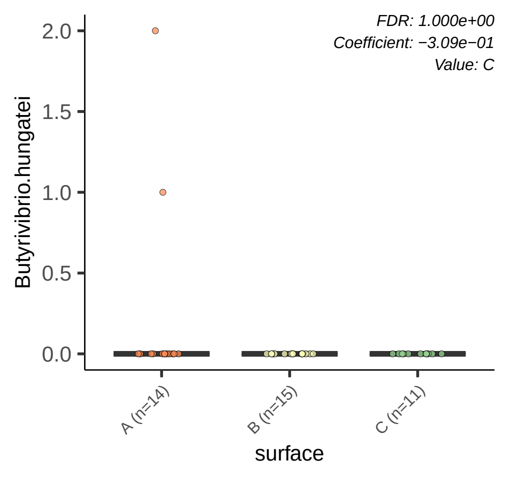
FDR: 1.000e+00
Coefficient: −3.09e−01
Value: C
Butyrivibrio.hungatei
2.0
1.5
1.0
0.5
0.0
A (n=14)
B (n=15)
C (n=11)
surface

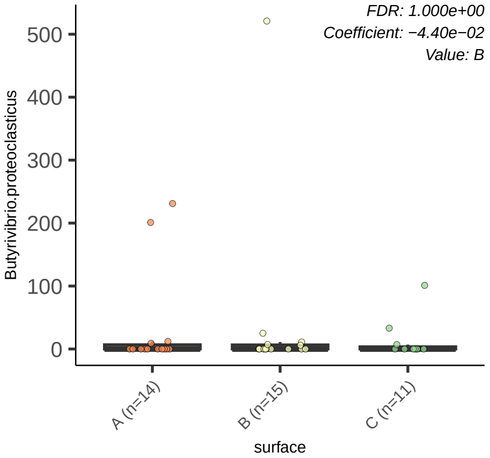
FDR: 1.000e+00
Coefficient: −4.40e−02
Value: B
Butyrivibrio.proteoclasticus
500
400
300
200
100
0
A (n=14)
B (n=15)
C (n=11)
surface

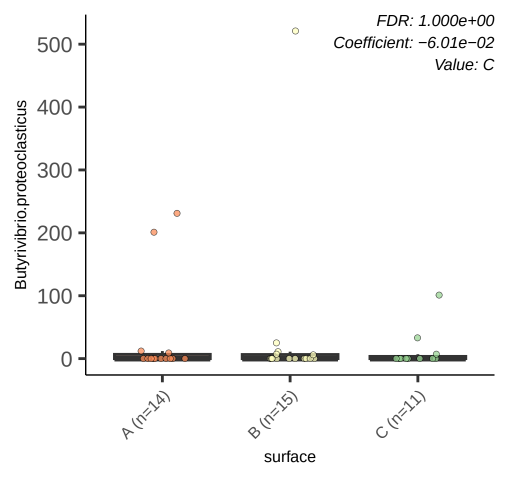
FDR: 1.000e+00
Coefficient: −6.01e−02
Value: C
Butyrivibrio.proteoclasticus
0
100
200
300
400
500
A (n=14)
B (n=15)
C (n=11)
surface

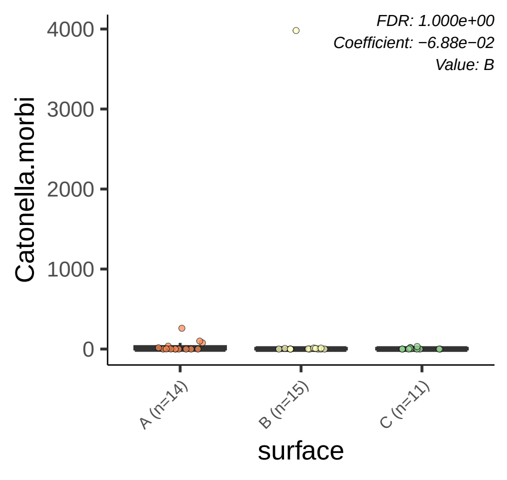
FDR: 1.000e+00
Coefficient: −6.88e−02
Value: B
Catonella.morbi
4000
3000
2000
1000
0
A (n=14)
B (n=15)
C (n=11)
surface

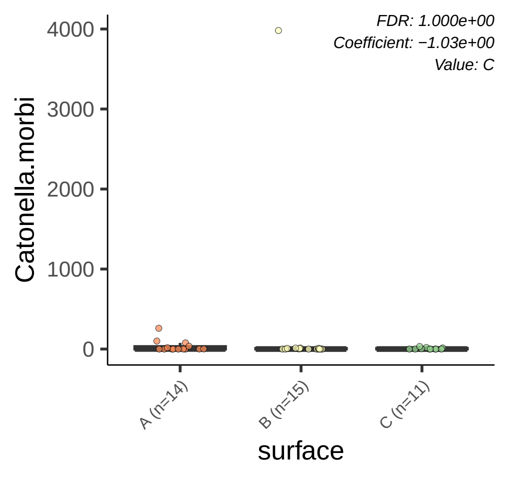
FDR: 1.000e+00
Coefficient: −1.03e+00
Value: C
Catonella.morbi
4000
3000
2000
1000
0
A (n=14)
B (n=15)
C (n=11)
surface

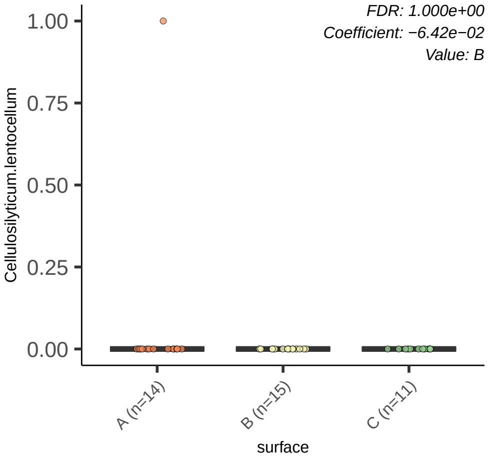
FDR: 1.000e+00
Coefficient: −6.42e−02
Value: B
1.00
0.75
0.50
0.25
0.00
Cellulosilyticum.lentocellum
A (n=14)
B (n=15)
C (n=11)
surface

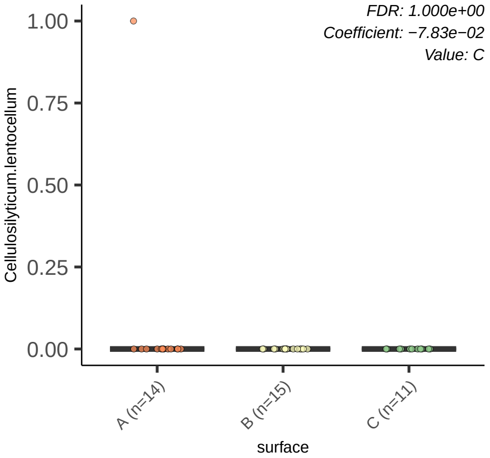
FDR: 1.000e+00
Coefficient: −7.83e−02
Value: C
Cellulosilyticum.lentocellum
1.00
0.75
0.50
0.25
0.00
A (n=14)
B (n=15)
C (n=11)
surface

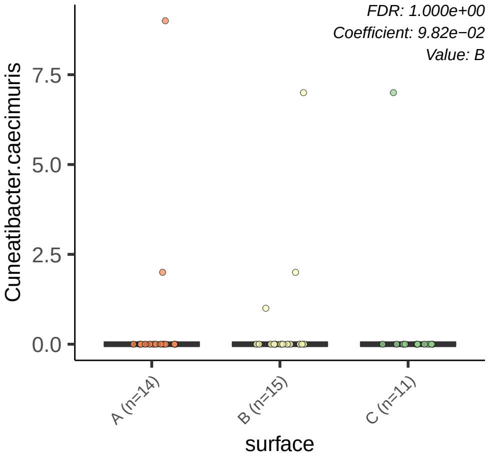
FDR: 1.000e+00
Coefficient: 9.82e−02
Value: B
Cuneatibacter.caecimuris
7.5
5.0
2.5
0.0
A (n=14)
B (n=15)
C (n=11)
surface

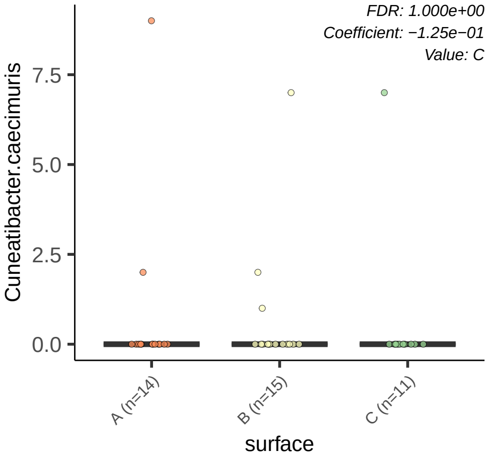
FDR: 1.000e+00
Coefficient: −1.25e−01
Value: C
Cuneatibacter.caecimuris
7.5
5.0
2.5
0.0
A (n=14)
B (n=15)
C (n=11)
surface

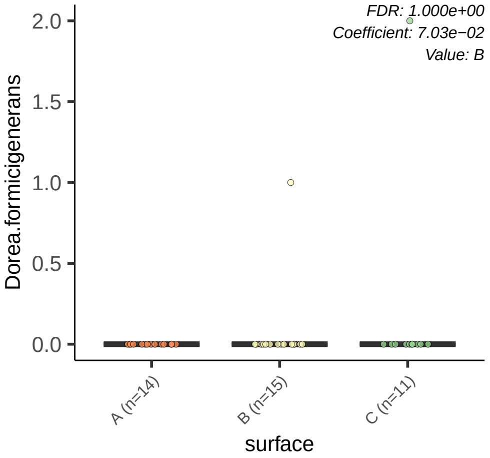
FDR: 1.000e+00
Coefficient: 7.03e−02
Value: B
Dorea.formicigenerans
2.0
1.5
1.0
0.5
0.0
A (n=14)
B (n=15)
C (n=11)
surface

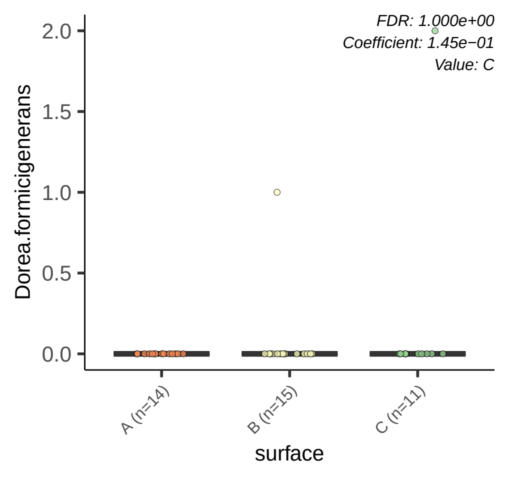
FDR: 1.000e+00
Coefficient: 1.45e−01
Value: C
Dorea.formicigenerans
2.0
1.5
1.0
0.5
0.0
A (n=14)
B (n=15)
C (n=11)
surface

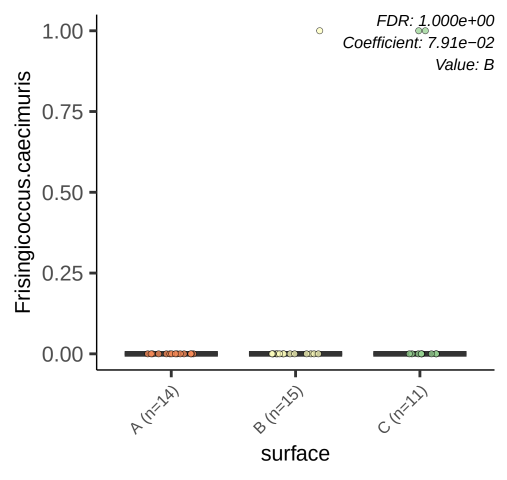
FDR: 1.000e+00
Coefficient: 7.91e−02
Value: B
Frisingicoccus.caecimuris
1.00
0.75
0.50
0.25
0.00
A (n=14)
B (n=15)
C (n=11)
surface

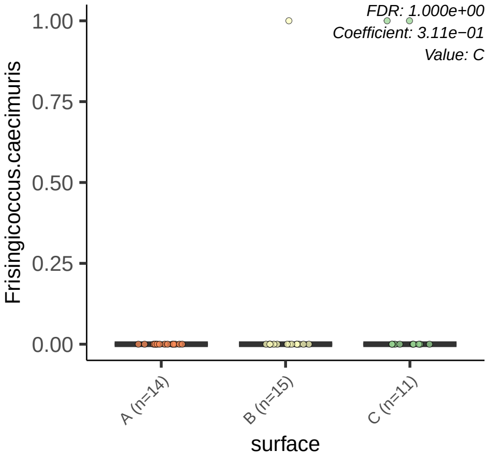

FDR: 1.000e+00
Coefficient: 3.11e−01
Value: C
Frisingicoccus.caecimuris
1.00
0.75
0.50
0.25
0.00
A (n=14)
B (n=15)
C (n=11)
surface

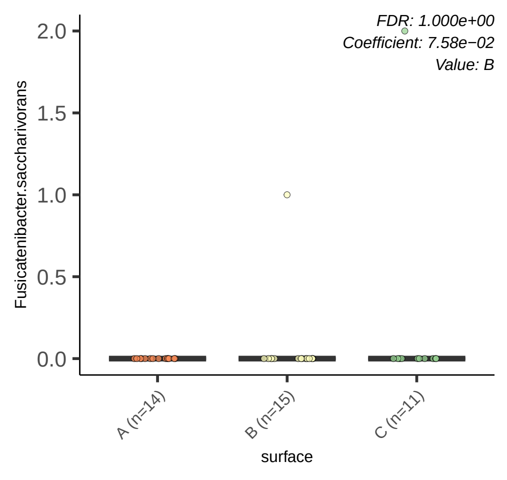
FDR: 1.000e+00
Coefficient: 7.58e−02
Value: B
Fusicatenibacter.saccharivorans
2.0
1.5
1.0
0.5
0.0
A (n=14)
B (n=15)
C (n=11)
surface

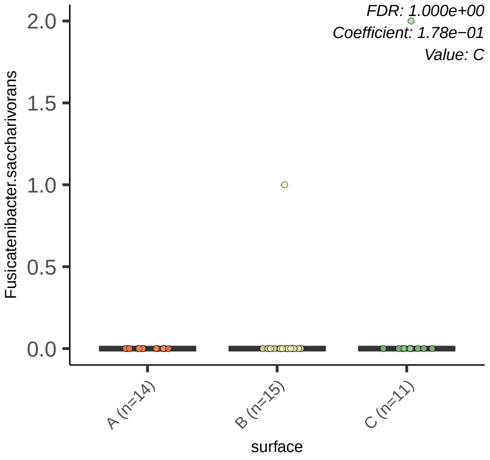
FDR: 1.000e+00
Coefficient: 1.78e−01
Value: C
Fusicatenibacter.saccharivorans
2.0
1.5
1.0
0.5
0.0
A (n=14)
B (n=15)
C (n=11)
surface

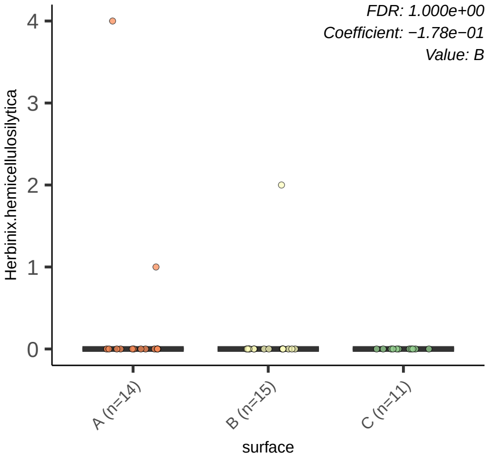

FDR: 1.000e+00
Coefficient: −1.78e−01
Value: B
Herbinix.hemicellulosilytica
0
1
2
3
4
A (n=14)
B (n=15)
C (n=11)
surface

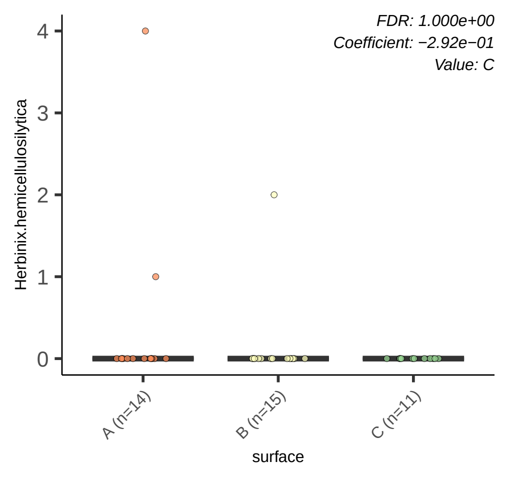
FDR: 1.000e+00
Coefficient: −2.92e−01
Value: C
Herbinix.hemicellulosilytica
0
1
2
3
4
A (n=14)
B (n=15)
C (n=11)
surface

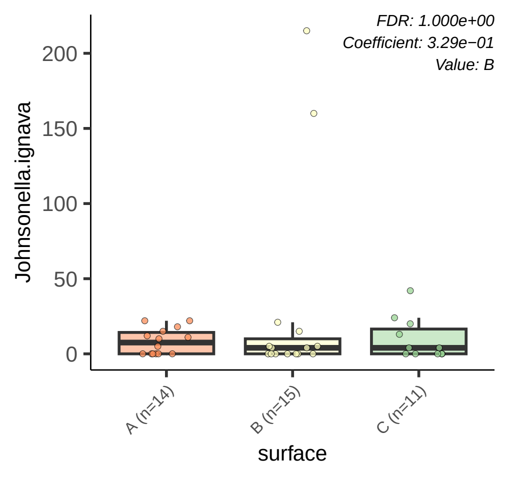

FDR: 1.000e+00
Coefficient: 3.29e−01
Value: B
Johnsonella.ignava
200
150
100
50
0
A (n=14)
B (n=15)
C (n=11)
surface

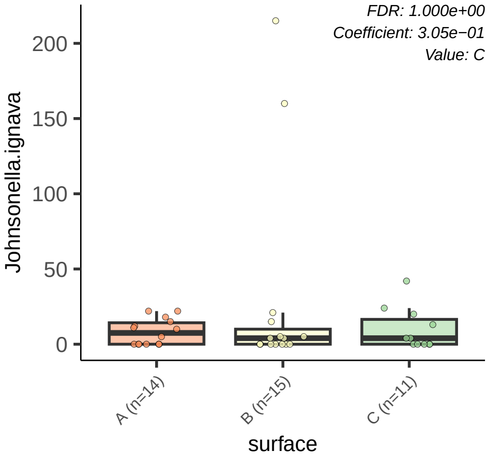
FDR: 1.000e+00
Coefficient: 3.05e−01
Value: C
Johnsonella.ignava
200
150
100
50
0
A (n=14)
B (n=15)
C (n=11)
surface

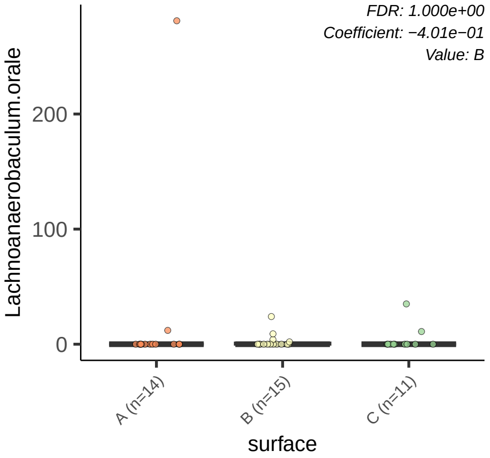
FDR: 1.000e+00
Coefficient: −4.01e−01
Value: B
Lachnoanaerobaculum.orale
200
100
0
A (n=14)
B (n=15)
C (n=11)
surface

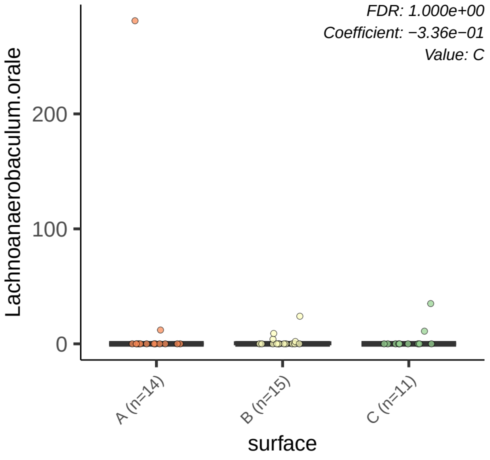
FDR: 1.000e+00
Coefficient: −3.36e−01
Value: C
Lachnoanaerobaculum.orale
200
100
0
A (n=14)
B (n=15)
C (n=11)
surface

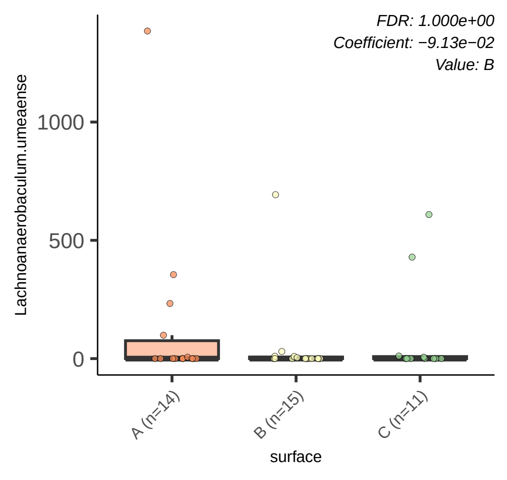
FDR: 1.000e+00
Coefficient: −9.13e−02
Value: B
Lachnoanaerobaculum.umeaense
1000
500
0
A (n=14)
B (n=15)
C (n=11)
surface

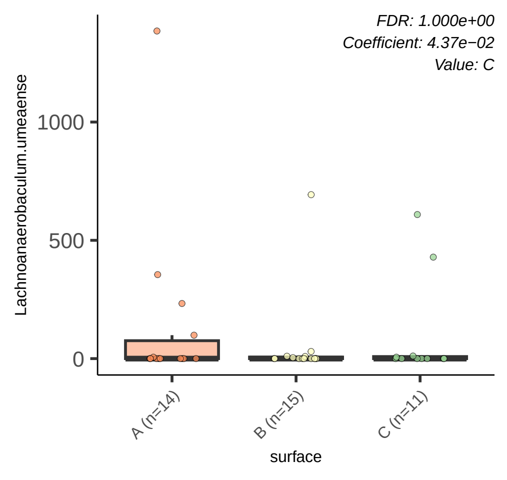
FDR: 1.000e+00
Coefficient: 4.37e−02
Value: C
Lachnoanaerobaculum.umeaense
1000
500
0
A (n=14)
B (n=15)
C (n=11)
surface

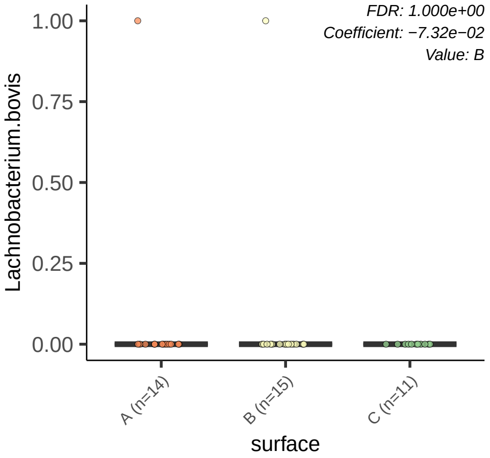
FDR: 1.000e+00
Coefficient: −7.32e−02
Value: B
Lachnobacterium.bovis
1.00
0.75
0.50
0.25
0.00
A (n=14)
B (n=15)
C (n=11)
surface

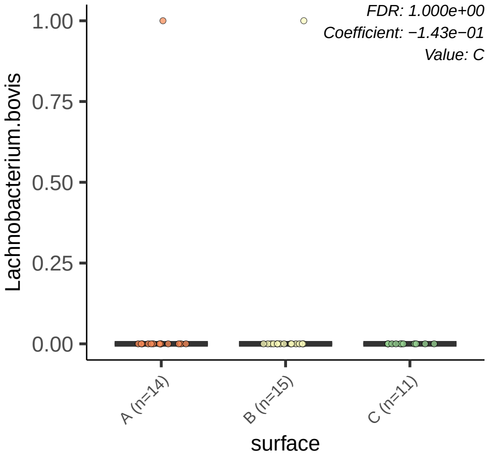

FDR: 1.000e+00
Coefficient: −1.43e−01
Value: C
Lachnobacterium.bovis
1.00
0.75
0.50
0.25
0.00
A (n=14)
B (n=15)
C (n=11)
surface

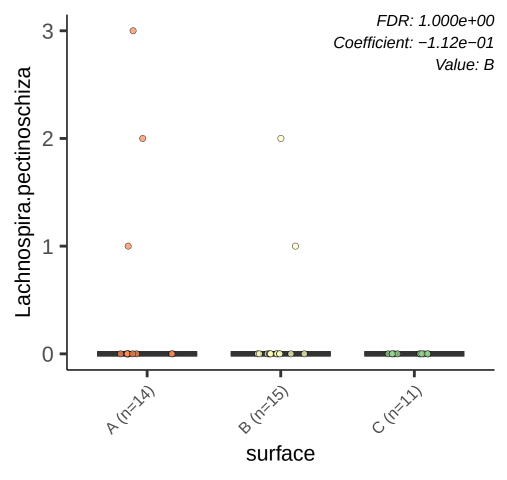
FDR: 1.000e+00
Coefficient: −1.12e−01
Value: B
Lachnospira.pectinoschiza
0
1
2
3
A (n=14)
B (n=15)
C (n=11)
surface

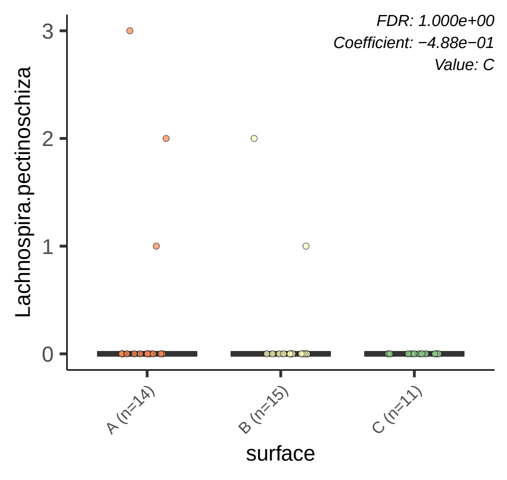

FDR: 1.000e+00
Coefficient: −4.88e−01
Value: C
Lachnospira.pectinoschiza
3
2
1
0
A (n=14)
B (n=15)
C (n=11)
surface

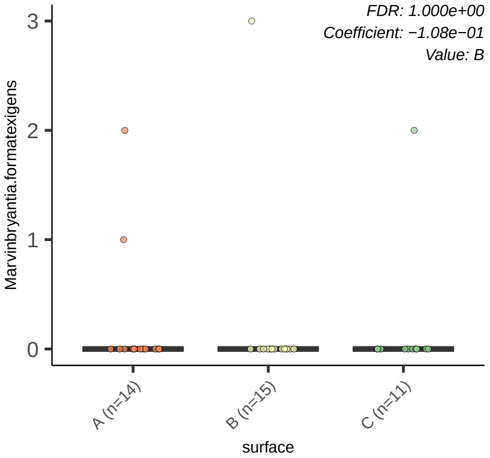

FDR: 1.000e+00
Coefficient: −1.08e−01
Value: B
Marvinbryantia.formatexigens
3
2
1
0
A (n=14)
B (n=15)
C (n=11)
surface

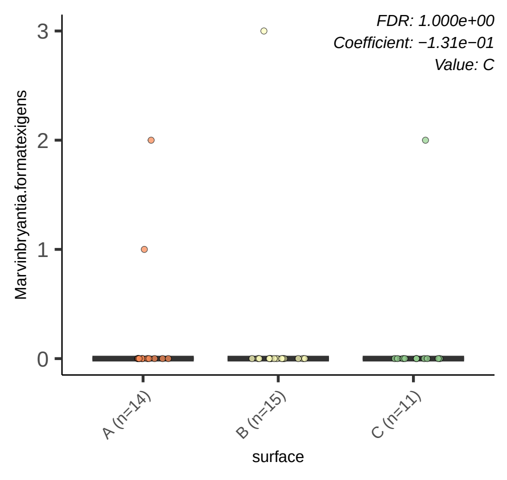

FDR: 1.000e+00
Coefficient: −1.31e−01
Value: C
Marvinbryantia.formatexigens
3
2
1
0
A (n=14)
B (n=15)
C (n=11)
surface

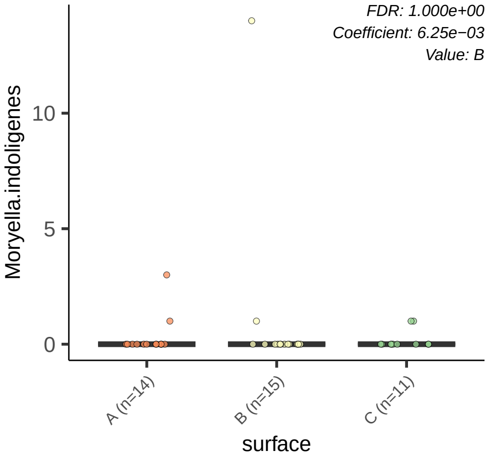
FDR: 1.000e+00
Coefficient: 6.25e−03
Value: B
Moryella.indoligenes
10
5
0
A (n=14)
B (n=15)
C (n=11)
surface

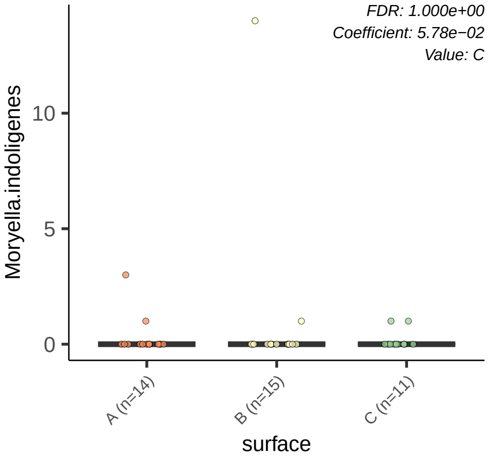

FDR: 1.000e+00
Coefficient: 5.78e−02
Value: C
Moryella.indoligenes
0
5
10
A (n=14)
B (n=15)
C (n=11)
surface

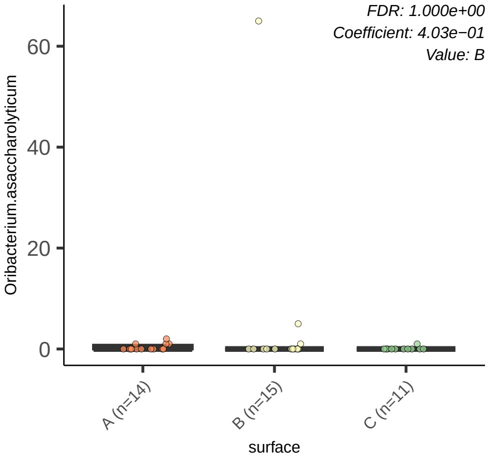

FDR: 1.000e+00
Coefficient: 4.03e−01
Value: B
Oribacterium.asaccharolyticum
0
20
40
60
A (n=14)
B (n=15)
C (n=11)
surface

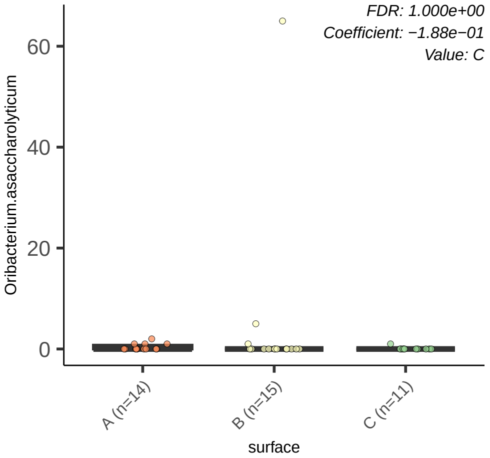
FDR: 1.000e+00
Coefficient: −1.88e−01
Value: C
Oribacterium.asaccharolyticum
60
40
20
0
A (n=14)
B (n=15)
C (n=11)
surface

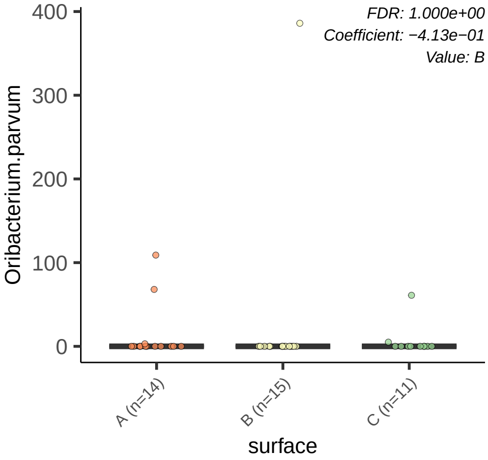
FDR: 1.000e+00
Coefficient: −4.13e−01
Value: B
Oribacterium.parvum
400
300
200
100
0
A (n=14)
B (n=15)
C (n=11)
surface

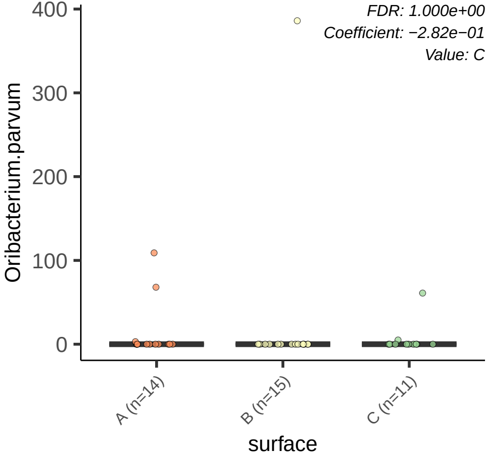
FDR: 1.000e+00
Coefficient: −2.82e−01
Value: C
Oribacterium.parvum
400
300
200
100
0
A (n=14)
B (n=15)
C (n=11)
surface

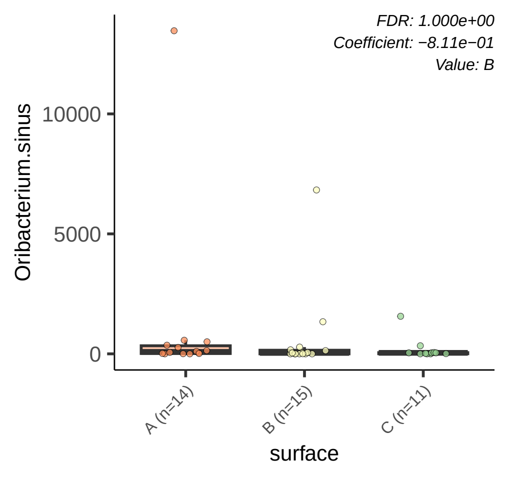
FDR: 1.000e+00
Coefficient: −8.11e−01
Value: B
Oribacterium.sinus
10000
5000
0
A (n=14)
B (n=15)
C (n=11)
surface

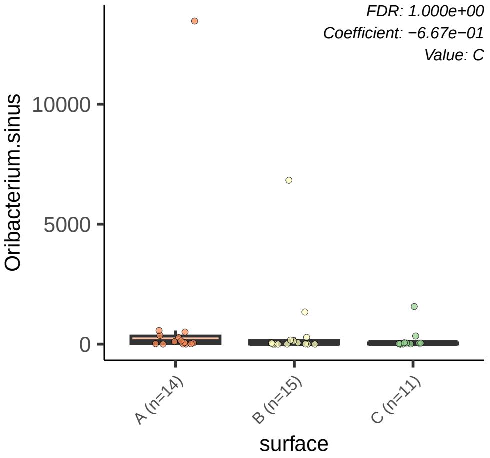

FDR: 1.000e+00
Coefficient: −6.67e−01
Value: C
Oribacterium.sinus
10000
5000
0
A (n=14)
B (n=15)
C (n=11)
surface

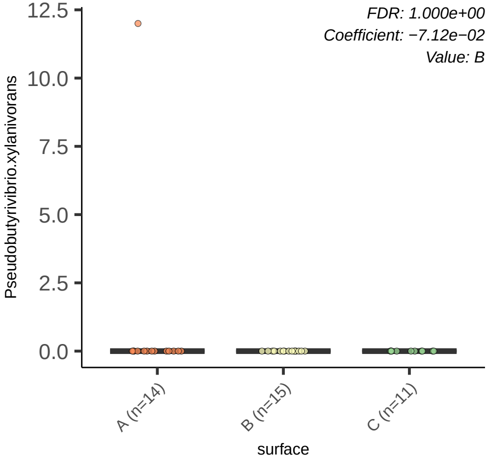
FDR: 1.000e+00
Coefficient: −7.12e−02
Value: B
Pseudobutyrivibrio.xylanivorans
12.5
10.0
7.5
5.0
2.5
0.0
A (n=14)
B (n=15)
C (n=11)
surface

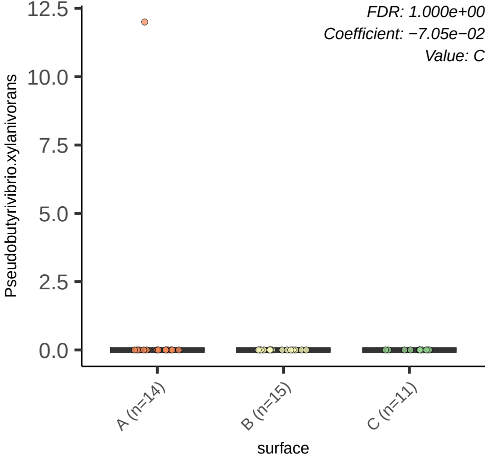

FDR: 1.000e+00
Coefficient: −7.05e−02
Value: C
Pseudobutyrivibrio.xylanivorans
12.5
10.0
7.5
5.0
2.5
0.0
A (n=14)
B (n=15)
C (n=11)
surface

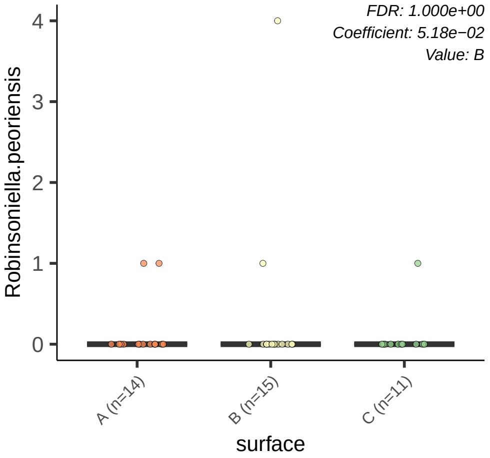
FDR: 1.000e+00
Coefficient: 5.18e−02
Value: B
Robinsoniella.peoriensis
0
1
2
3
4
A (n=14)
B (n=15)
C (n=11)
surface

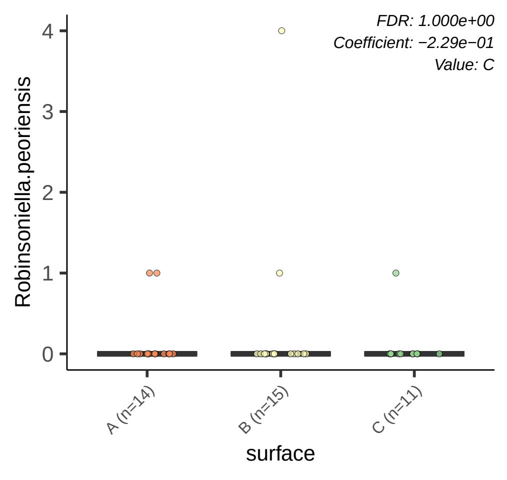
FDR: 1.000e+00
Coefficient: −2.29e−01
Value: C
Robinsoniella.peoriensis
0
1
2
3
4
A (n=14)
B (n=15)
C (n=11)
surface

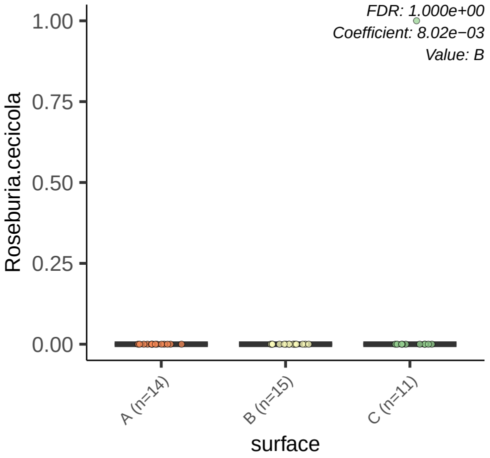
FDR: 1.000e+00
Coefficient: 8.02e−03
Value: B
Roseburia.cecicola
1.00
0.75
0.50
0.25
0.00
A (n=14)
B (n=15)
C (n=11)
surface

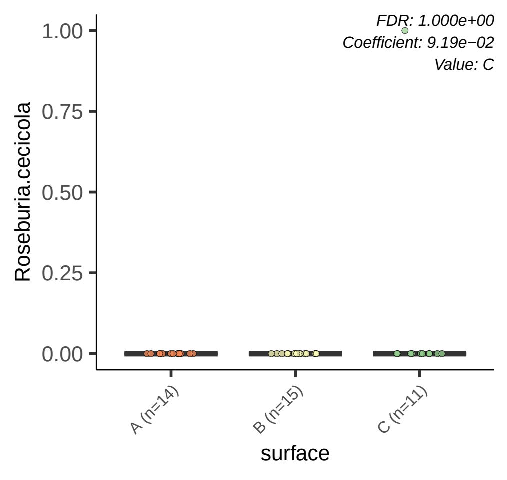

FDR: 1.000e+00
Coefficient: 9.19e−02
Value: C
Roseburia.cecicola
1.00
0.75
0.50
0.25
0.00
A (n=14)
B (n=15)
C (n=11)
surface

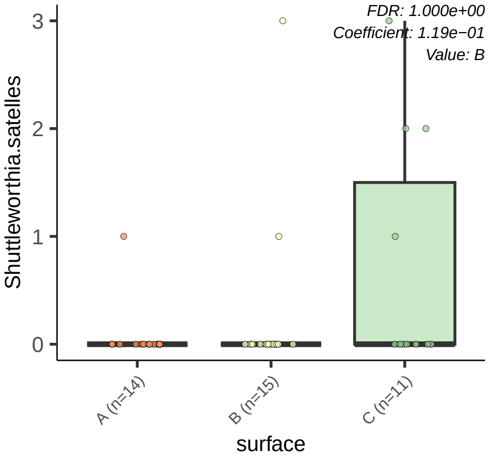
FDR: 1.000e+00
Coefficient: 1.19e−01
Value: B
Shuttleworthia.satelles
3
2
1
0
A (n=14)
B (n=15)
C (n=11)
surface

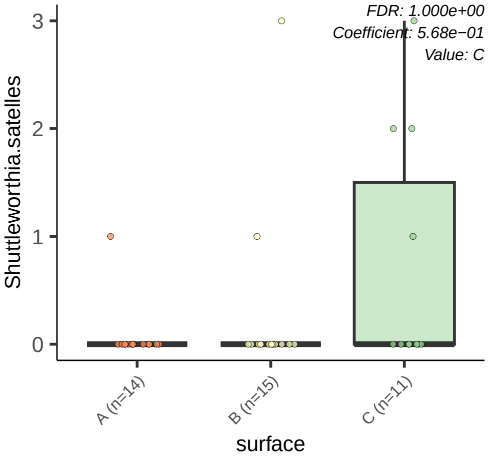

FDR: 1.000e+00
Coefficient: 5.68e−01
Value: C
Shuttleworthia.satelles
0
1
2
3
A (n=14)
B (n=15)
C (n=11)
surface

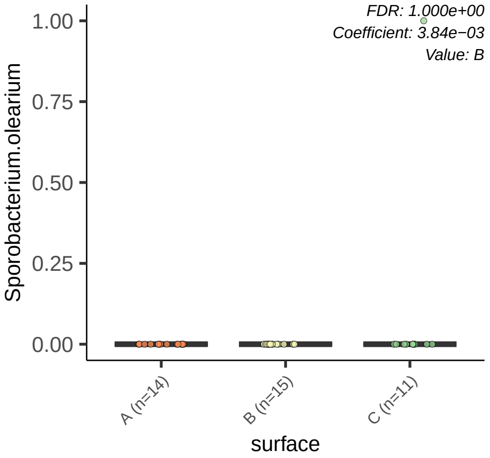
FDR: 1.000e+00
Coefficient: 3.84e−03
Value: B
Sporobacterium.olearium
1.00
0.75
0.50
0.25
0.00
A (n=14)
B (n=15)
C (n=11)
surface

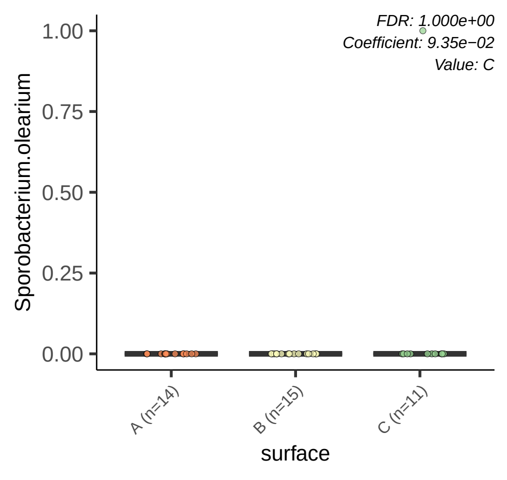
FDR: 1.000e+00
Coefficient: 9.35e−02
Value: C
Sporobacterium.olearium
1.00
0.75
0.50
0.25
0.00
A (n=14)
B (n=15)
C (n=11)
surface

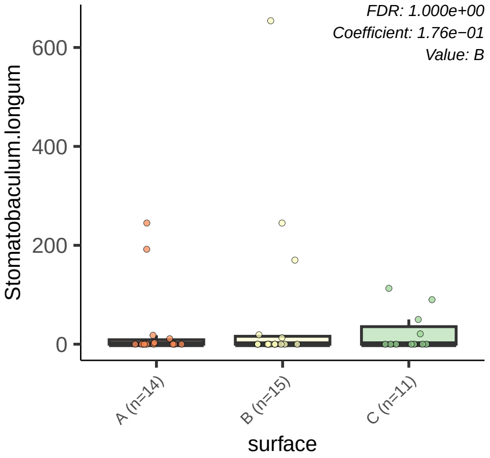

FDR: 1.000e+00
Coefficient: 1.76e−01
Value: B
Stomatobaculum.longum
600
400
200
0
A (n=14)
B (n=15)
C (n=11)
surface

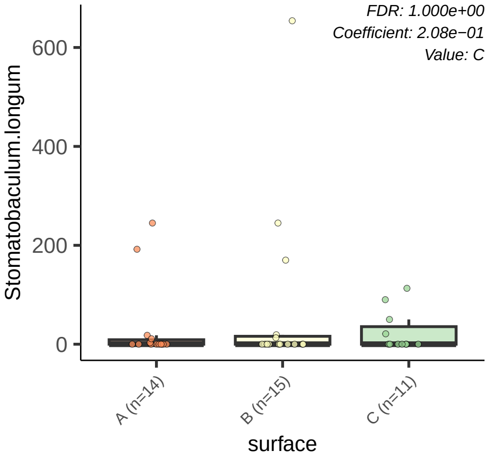

FDR: 1.000e+00
Coefficient: 2.08e−01
Value: C
Stomatobaculum.longum
600
400
200
0
A (n=14)
B (n=15)
C (n=11)
surface

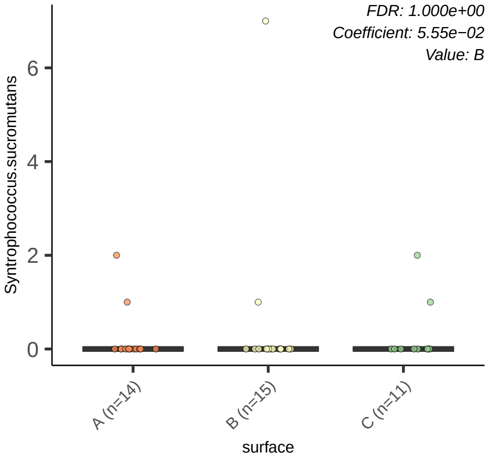
FDR: 1.000e+00
Coefficient: 5.55e−02
Value: B
Syntrophococcus.sucromutans
0
2
4
6
A (n=14)
B (n=15)
C (n=11)
surface

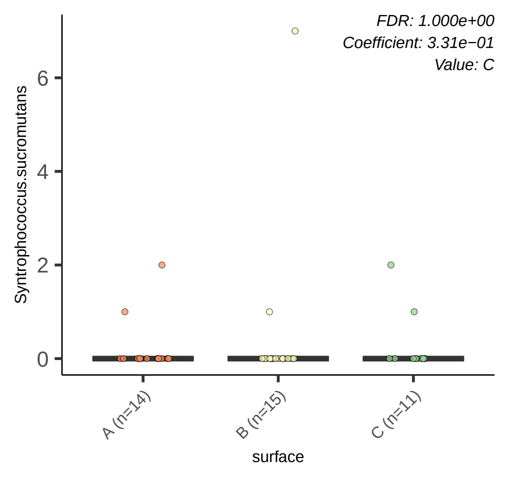

FDR: 1.000e+00
Coefficient: 3.31e−01
Value: C
Syntrophococcus.sucromutans
0
2
4
6
A (n=14)
B (n=15)
C (n=11)
surface

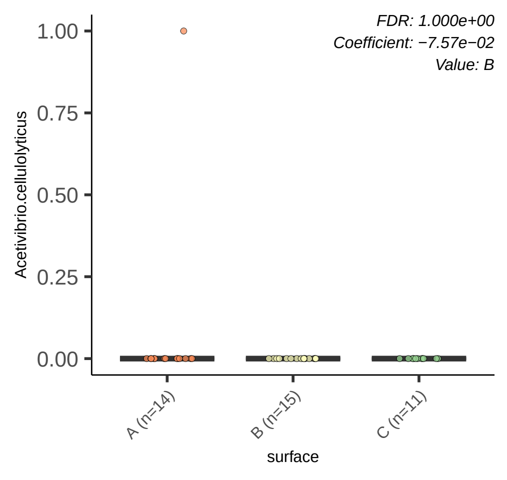

FDR: 1.000e+00
Coefficient: −7.57e−02
Value: B
1.00
0.75
0.50
0.25
0.00
Acetivibrio.cellulolyticus
A (n=14)
B (n=15)
C (n=11)
surface

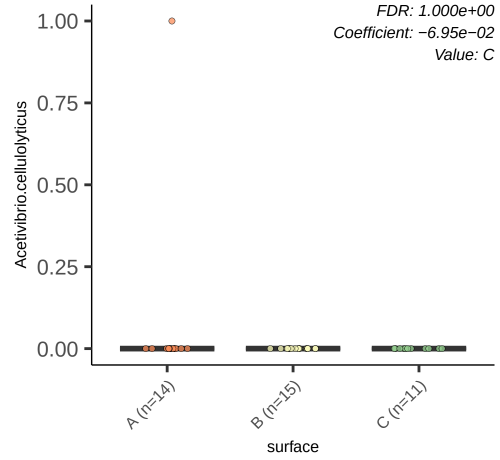
FDR: 1.000e+00
Coefficient: −6.95e−02
Value: C
Acetivibrio.cellulolyticus
1.00
0.75
0.50
0.25
0.00
A (n=14)
B (n=15)
C (n=11)
surface

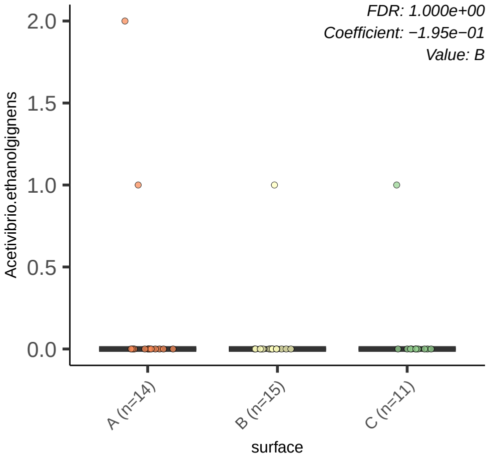

FDR: 1.000e+00
Coefficient: −1.95e−01
Value: B
Acetivibrio.ethanolgignens
2.0
1.5
1.0
0.5
0.0
A (n=14)
B (n=15)
C (n=11)
surface

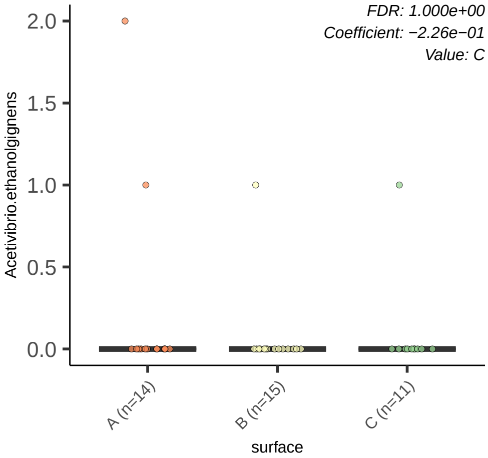
FDR: 1.000e+00
Coefficient: −2.26e−01
Value: C
Acetivibrio.ethanolgignens
2.0
1.5
1.0
0.5
0.0
A (n=14)
B (n=15)
C (n=11)
surface

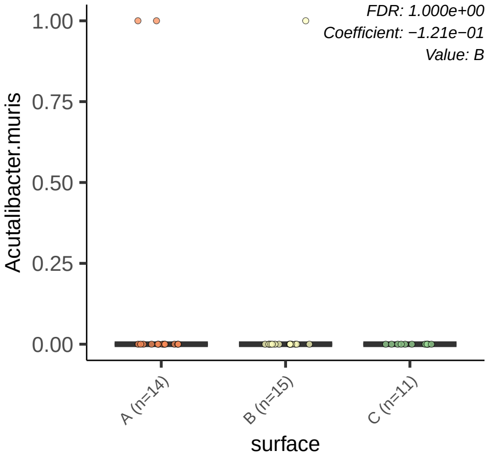

FDR: 1.000e+00
Coefficient: −1.21e−01
Value: B
Acutalibacter.muris
1.00
0.75
0.50
0.25
0.00
A (n=14)
B (n=15)
C (n=11)
surface

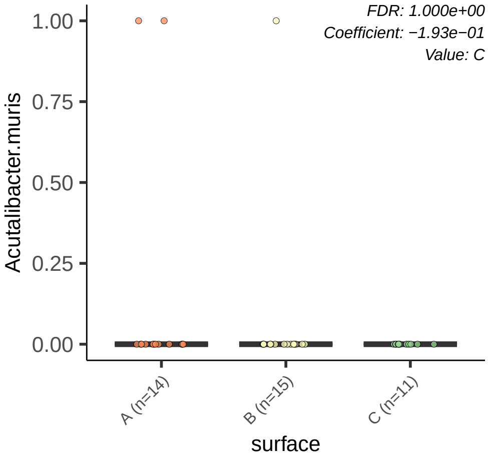

FDR: 1.000e+00
Coefficient: −1.93e−01
Value: C
Acutalibacter.muris
1.00
0.75
0.50
0.25
0.00
A (n=14)
B (n=15)
C (n=11)
surface

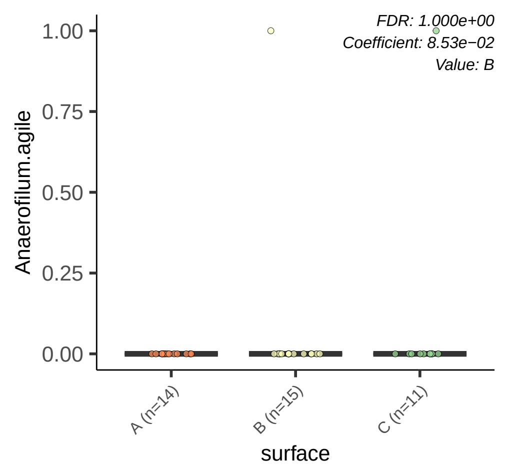

FDR: 1.000e+00
Coefficient: 8.53e−02
Value: B
Anaerofilum.agile
1.00
0.75
0.50
0.25
0.00
A (n=14)
B (n=15)
C (n=11)
surface

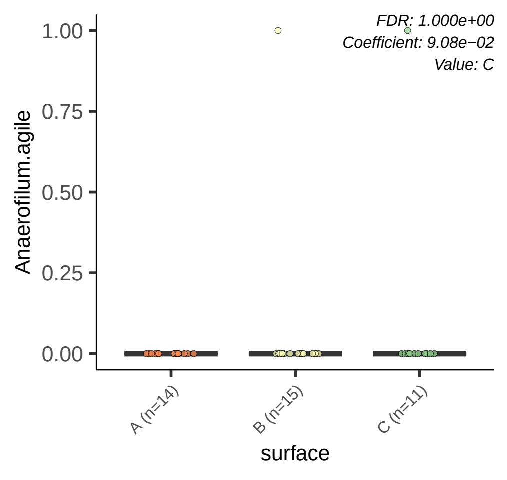
FDR: 1.000e+00
Coefficient: 9.08e−02
Value: C
Anaerofilum.agile
1.00
0.75
0.50
0.25
0.00
A (n=14)
B (n=15)
C (n=11)
surface

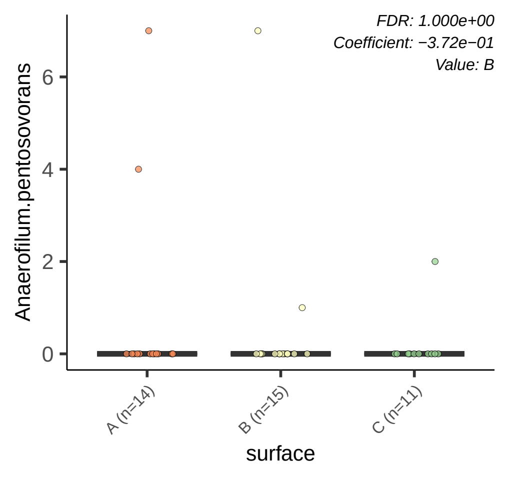

FDR: 1.000e+00
Coefficient: −3.72e−01
Value: B
Anaerofilum.pentosovorans
0
2
4
6
A (n=14)
B (n=15)
C (n=11)
surface

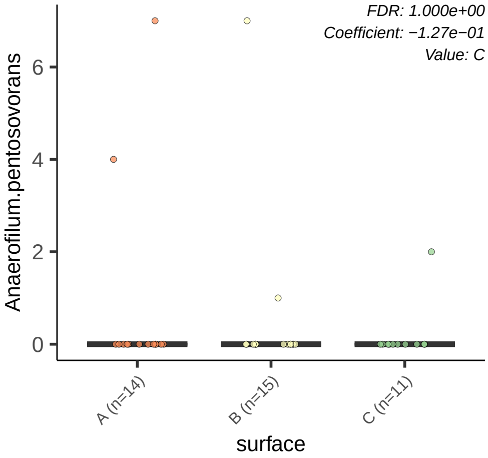

FDR: 1.000e+00
Coefficient: −1.27e−01
Value: C
Anaerofilum.pentosovorans
0
2
4
6
A (n=14)
B (n=15)
C (n=11)
surface

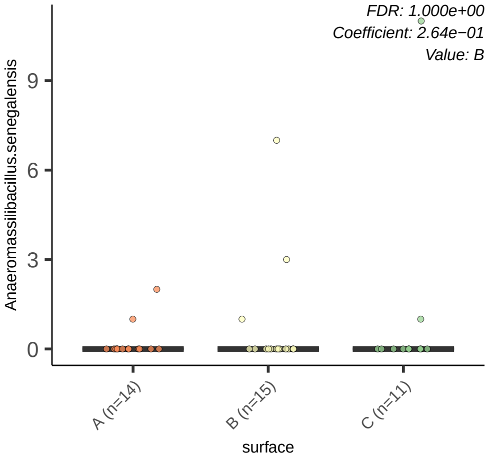
FDR: 1.000e+00
Coefficient: 2.64e−01
Value: B
Anaeromassilibacillus.senegalensis
9
6
3
0
A (n=14)
B (n=15)
C (n=11)
surface

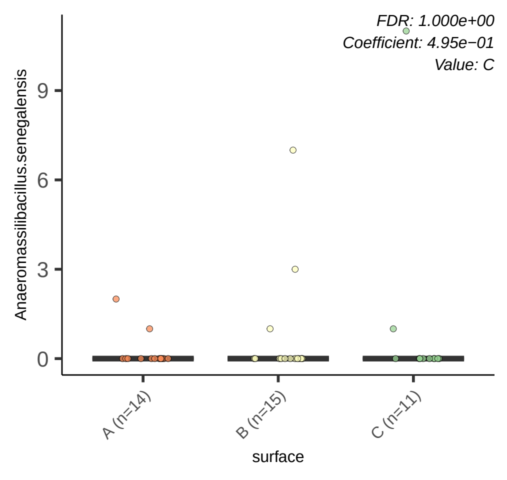
FDR: 1.000e+00
Coefficient: 4.95e−01
Value: C
Anaeromassilibacillus.senegalensis
9
6
3
0
A (n=14)
B (n=15)
C (n=11)
surface

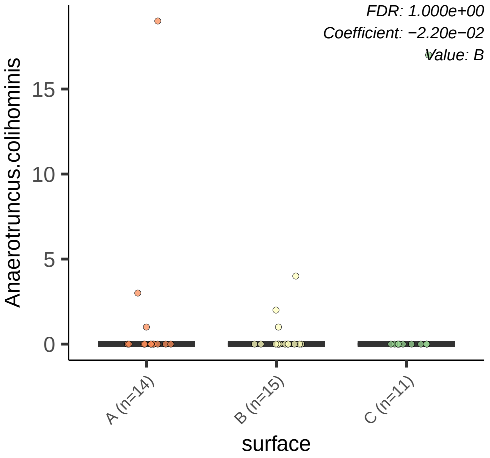

FDR: 1.000e+00
Coefficient: −2.20e−02
Value: B
Anaerotruncus.colihominis
15
10
5
0
A (n=14)
B (n=15)
C (n=11)
surface

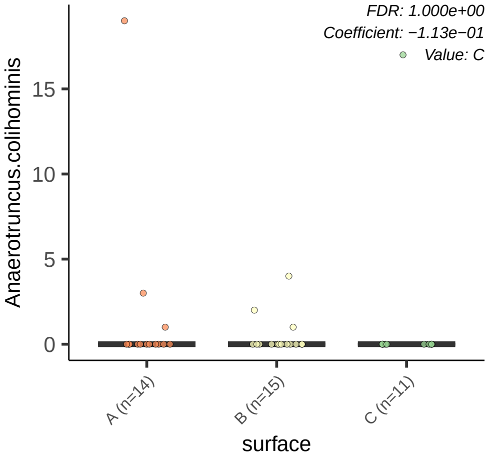
FDR: 1.000e+00
Coefficient: −1.13e−01
Value: C
Anaerotruncus.colihominis
0
5
10
15
A (n=14)
B (n=15)
C (n=11)
surface

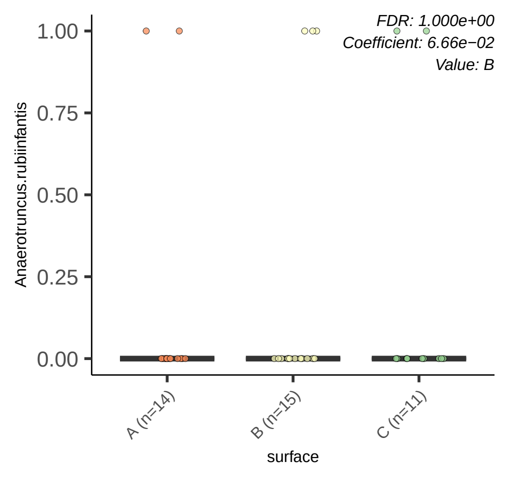

FDR: 1.000e+00
Coefficient: 6.66e−02
Value: B
Anaerotruncus.rubiinfantis
1.00
0.75
0.50
0.25
0.00
A (n=14)
B (n=15)
C (n=11)
surface

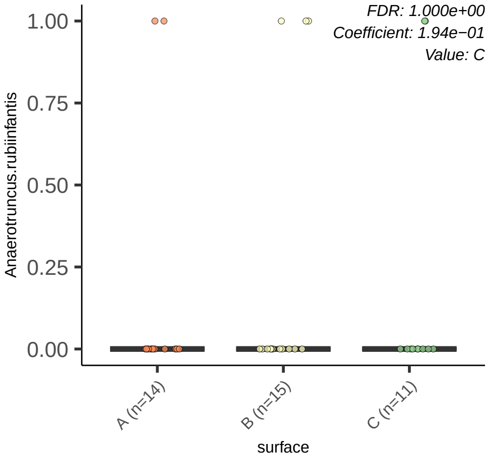

FDR: 1.000e+00
Coefficient: 1.94e−01
Value: C
Anaerotruncus.rubiinfantis
1.00
0.75
0.50
0.25
0.00
A (n=14)
B (n=15)
C (n=11)
surface

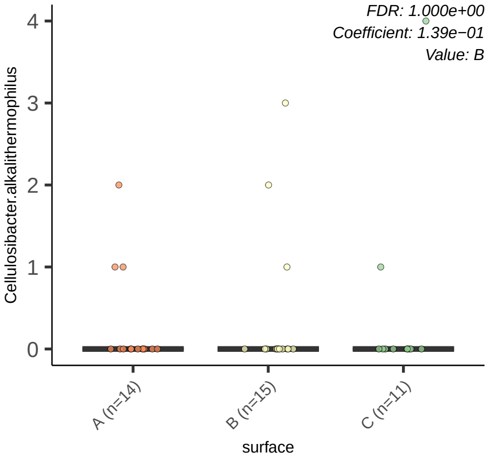
FDR: 1.000e+00
Coefficient: 1.39e−01
Value: B
Cellulosibacter.alkalithermophilus
0
1
2
3
4
A (n=14)
B (n=15)
C (n=11)
surface

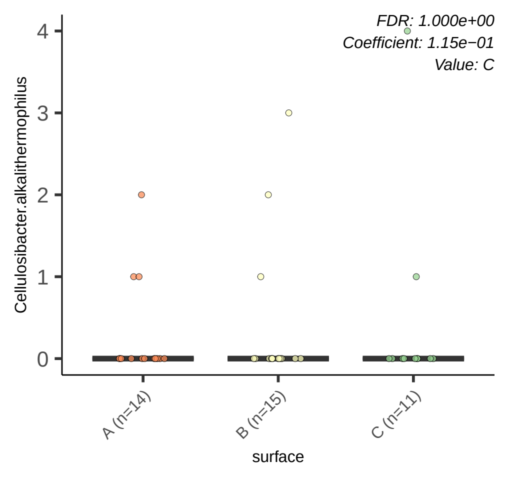
FDR: 1.000e+00
Coefficient: 1.15e−01
Value: C
Cellulosibacter.alkalithermophilus
4
3
2
1
0
A (n=14)
B (n=15)
C (n=11)
surface

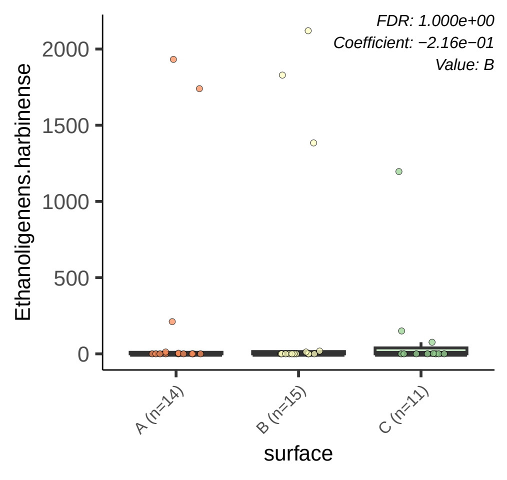
FDR: 1.000e+00
Coefficient: −2.16e−01
Value: B
Ethanoligenens.harbinense
2000
1500
1000
500
0
A (n=14)
B (n=15)
C (n=11)
surface

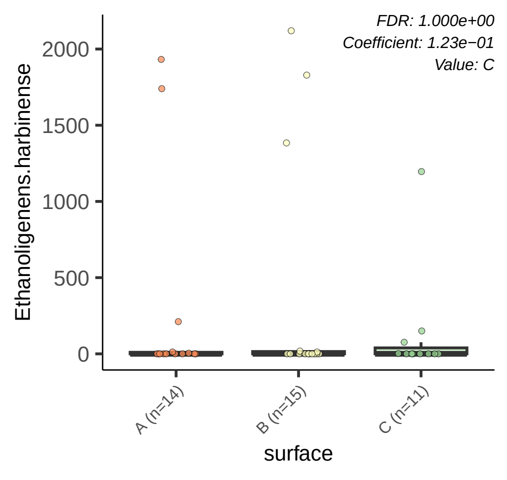
FDR: 1.000e+00
Coefficient: 1.23e−01
Value: C
Ethanoligenens.harbinense
2000
1500
1000
500
0
A (n=14)
B (n=15)
C (n=11)
surface

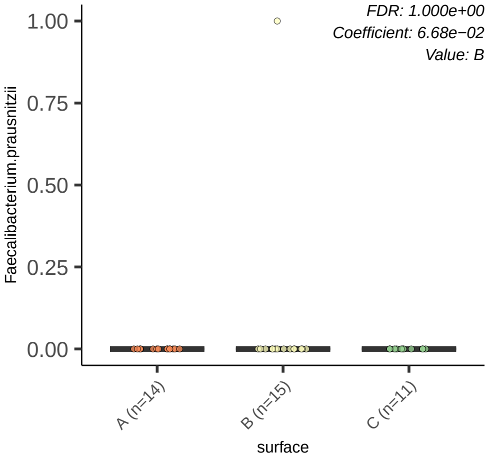
FDR: 1.000e+00
Coefficient: 6.68e−02
Value: B
1.00
0.75
0.50
0.25
0.00
Faecalibacterium.prausnitzii
A (n=14)
B (n=15)
C (n=11)
surface

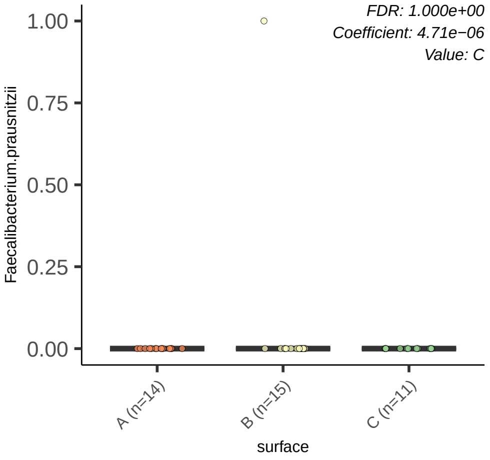
FDR: 1.000e+00
Coefficient: 4.71e−06
Value: C
Faecalibacterium.prausnitzii
1.00
0.75
0.50
0.25
0.00
A (n=14)
B (n=15)
C (n=11)
surface

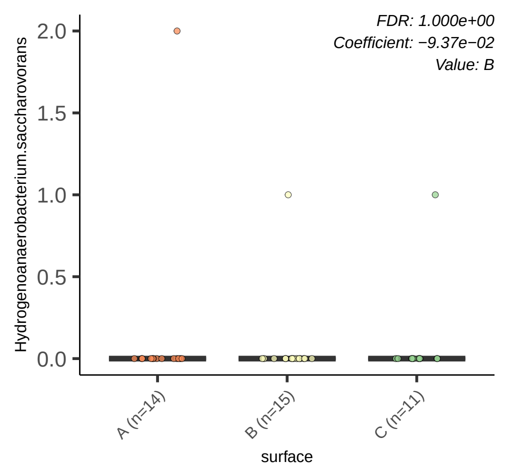

FDR: 1.000e+00
Coefficient: −9.37e−02
Value: B
Hydrogenoanaerobacterium.saccharovorans
2.0
1.5
1.0
0.5
0.0
A (n=14)
B (n=15)
C (n=11)
surface

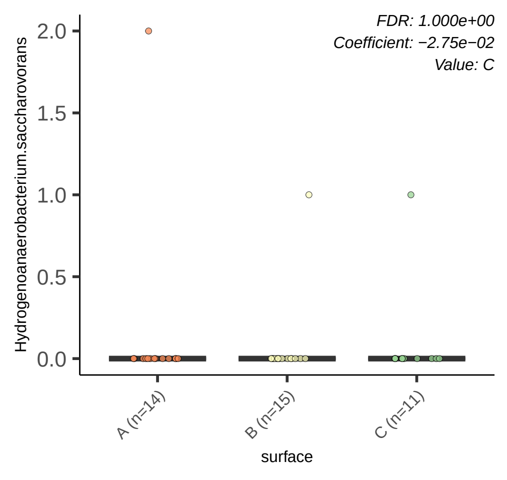

FDR: 1.000e+00
Coefficient: −2.75e−02
Value: C
Hydrogenoanaerobacterium.saccharovorans
2.0
1.5
1.0
0.5
0.0
A (n=14)
B (n=15)
C (n=11)
surface

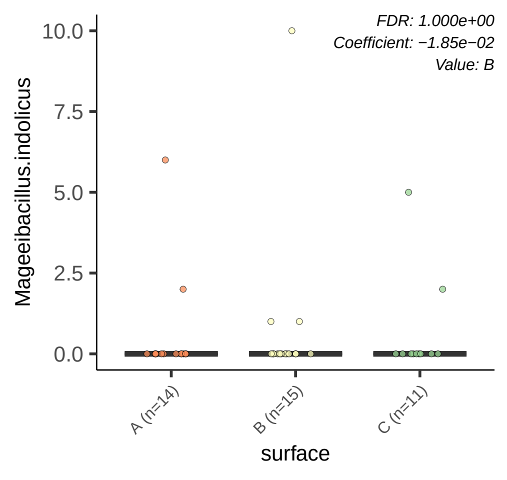

FDR: 1.000e+00
Coefficient: −1.85e−02
Value: B
Mageeibacillus.indolicus
10.0
7.5
5.0
2.5
0.0
A (n=14)
B (n=15)
C (n=11)
surface

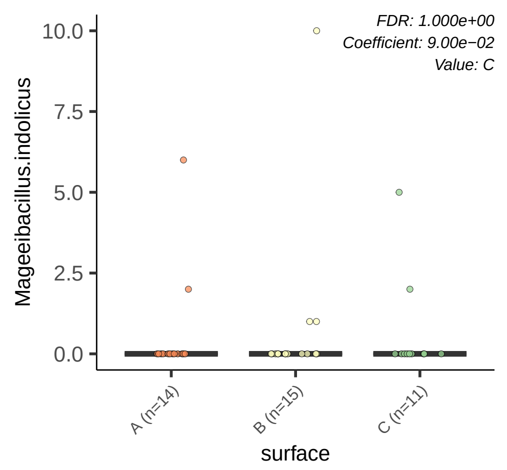
FDR: 1.000e+00
Coefficient: 9.00e−02
Value: C
Mageeibacillus.indolicus
10.0
7.5
5.0
2.5
0.0
A (n=14)
B (n=15)
C (n=11)
surface

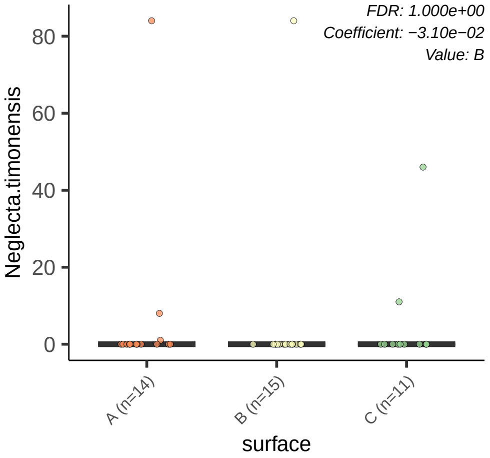
FDR: 1.000e+00
Coefficient: −3.10e−02
Value: B
Neglecta.timonensis
80
60
40
20
0
A (n=14)
B (n=15)
C (n=11)
surface

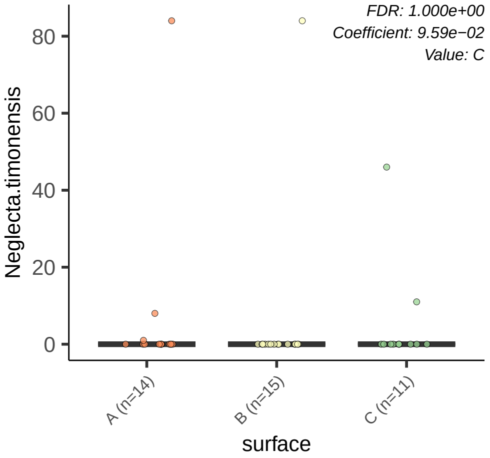
FDR: 1.000e+00
Coefficient: 9.59e−02
Value: C
Neglecta.timonensis
0
20
40
60
80
A (n=14)
B (n=15)
C (n=11)
surface

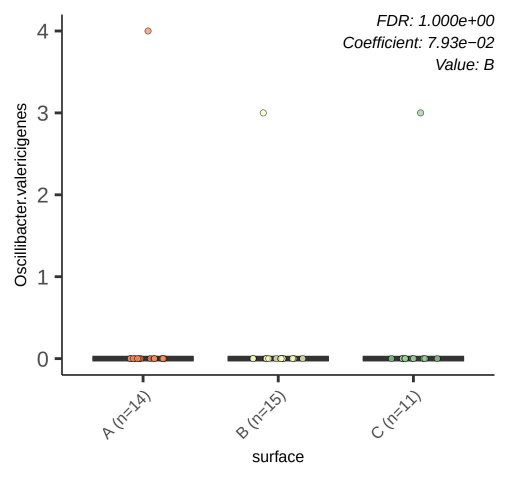

FDR: 1.000e+00
Coefficient: 7.93e−02
Value: B
Oscillibacter.valericigenes
0
1
2
3
4
A (n=14)
B (n=15)
C (n=11)
surface

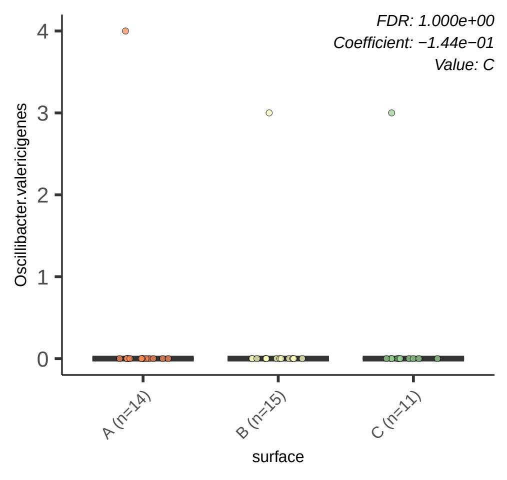

FDR: 1.000e+00
Coefficient: −1.44e−01
Value: C
Oscillibacter.valericigenes
0
1
2
3
4
A (n=14)
B (n=15)
C (n=11)
surface

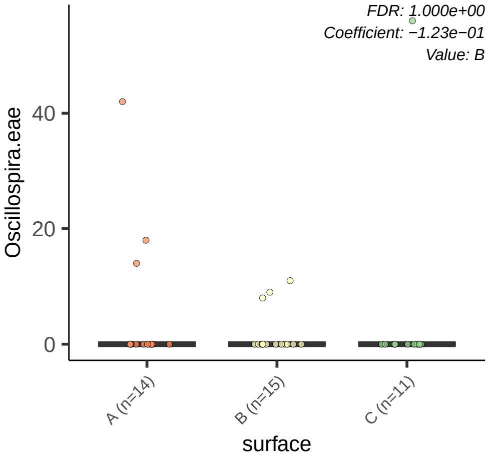

FDR: 1.000e+00
Coefficient: −1.23e−01
Value: B
Oscillospira.eae
40
20
0
A (n=14)
B (n=15)
C (n=11)
surface

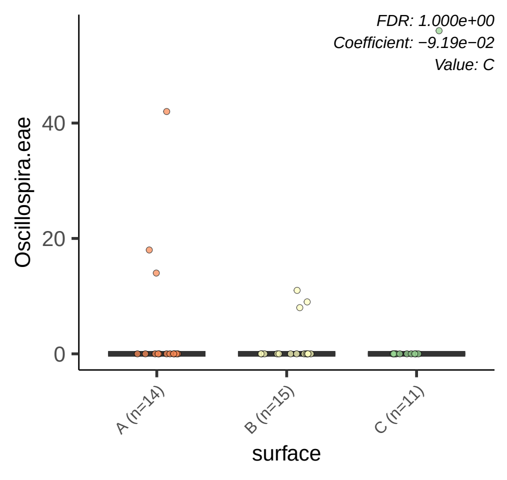

FDR: 1.000e+00
Coefficient: −9.19e−02
Value: C
Oscillospira.eae
40
20
0
A (n=14)
B (n=15)
C (n=11)
surface

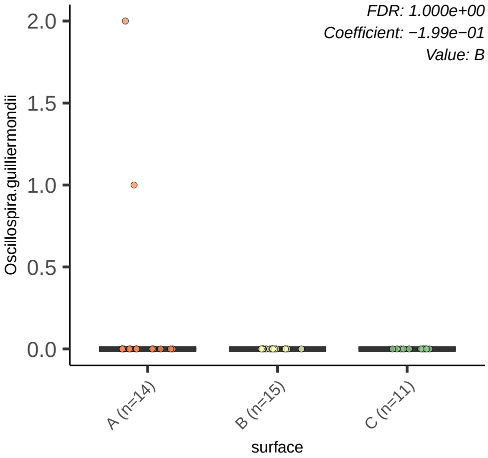
FDR: 1.000e+00
Coefficient: −1.99e−01
Value: B
Oscillospira.guilliermondii
2.0
1.5
1.0
0.5
0.0
A (n=14)
B (n=15)
C (n=11)
surface

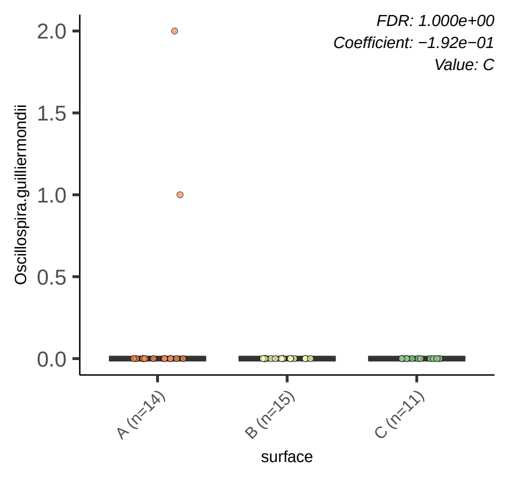

FDR: 1.000e+00
Coefficient: −1.92e−01
Value: C
Oscillospira.guilliermondii
2.0
1.5
1.0
0.5
0.0
A (n=14)
B (n=15)
C (n=11)
surface

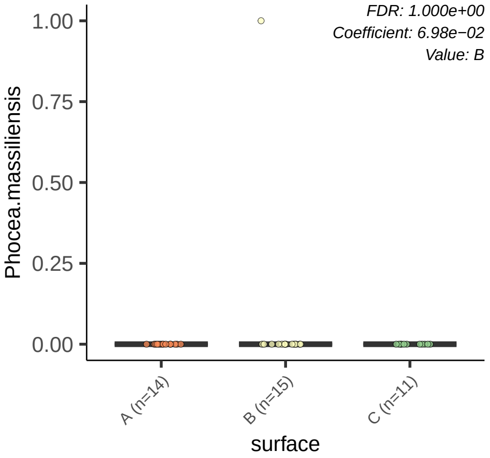
FDR: 1.000e+00
Coefficient: 6.98e−02
Value: B
Phocea.massiliensis
1.00
0.75
0.50
0.25
0.00
A (n=14)
B (n=15)
C (n=11)
surface

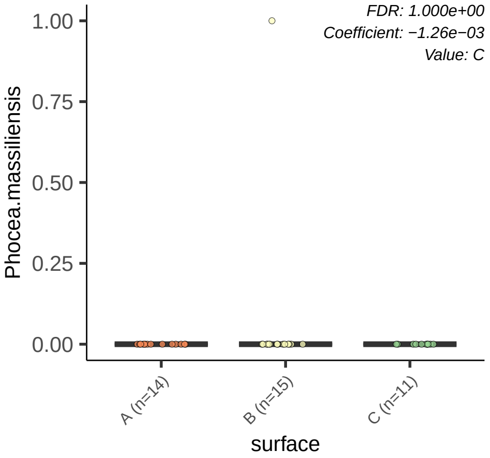
FDR: 1.000e+00
Coefficient: −1.26e−03
Value: C
Phocea.massiliensis
1.00
0.75
0.50
0.25
0.00
A (n=14)
B (n=15)
C (n=11)
surface

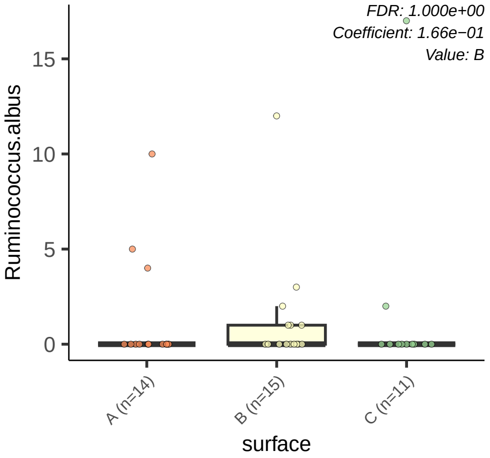

FDR: 1.000e+00
Coefficient: 1.66e−01
Value: B
Ruminococcus.albus
15
10
5
0
A (n=14)
B (n=15)
C (n=11)
surface

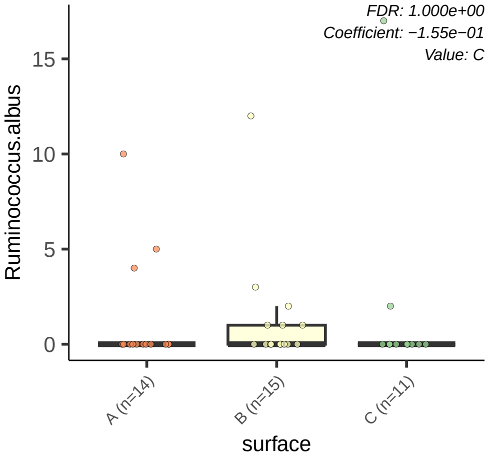
FDR: 1.000e+00
Coefficient: −1.55e−01
Value: C
Ruminococcus.albus
15
10
5
0
A (n=14)
B (n=15)
C (n=11)
surface

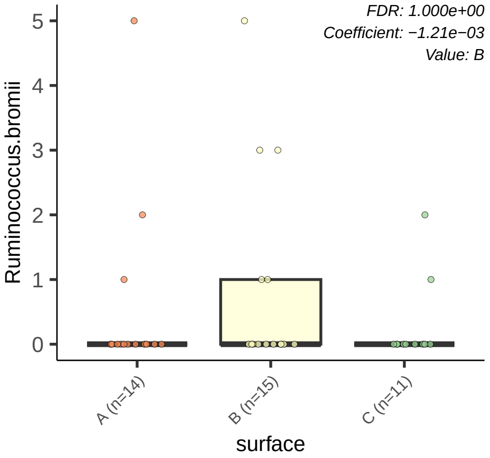

FDR: 1.000e+00
Coefficient: −1.21e−03
Value: B
Ruminococcus.bromii
0
1
2
3
4
5
A (n=14)
B (n=15)
C (n=11)
surface

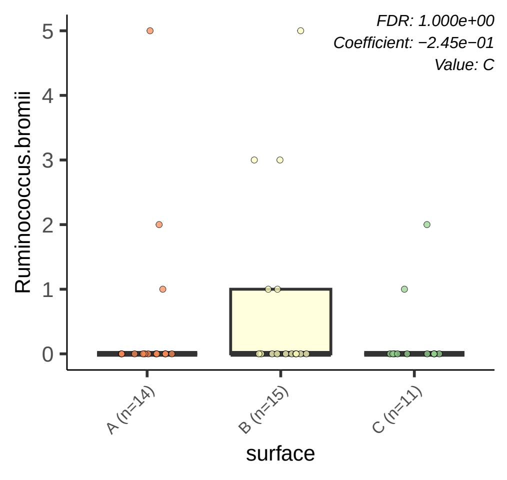
FDR: 1.000e+00
Coefficient: −2.45e−01
Value: C
Ruminococcus.bromii
0
1
2
3
4
5
A (n=14)
B (n=15)
C (n=11)
surface

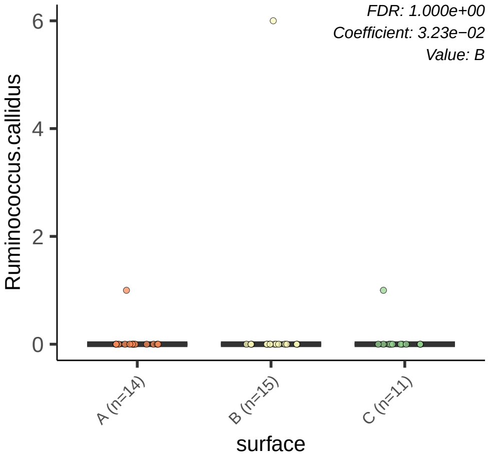
FDR: 1.000e+00
Coefficient: 3.23e−02
Value: B
Ruminococcus.callidus
6
4
2
0
A (n=14)
B (n=15)
C (n=11)
surface

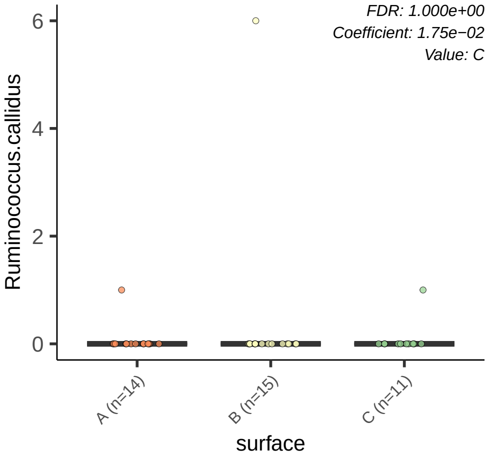

FDR: 1.000e+00
Coefficient: 1.75e−02
Value: C
Ruminococcus.callidus
0
2
4
6
A (n=14)
B (n=15)
C (n=11)
surface

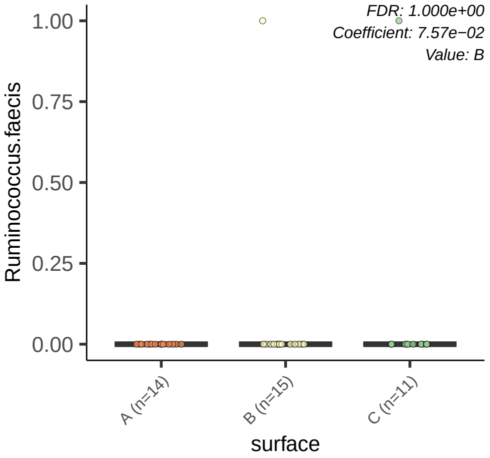
FDR: 1.000e+00
Coefficient: 7.57e−02
Value: B
Ruminococcus.faecis
1.00
0.75
0.50
0.25
0.00
A (n=14)
B (n=15)
C (n=11)
surface

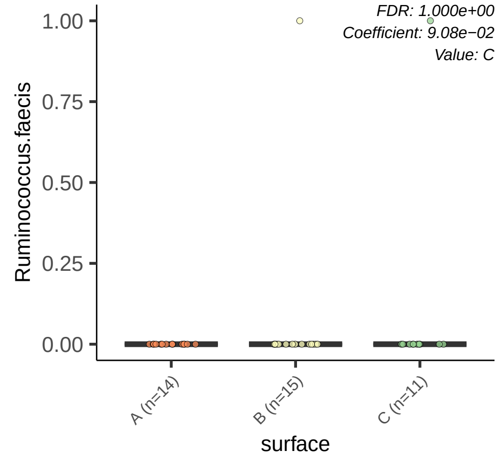

FDR: 1.000e+00
Coefficient: 9.08e−02
Value: C
Ruminococcus.faecis
1.00
0.75
0.50
0.25
0.00
A (n=14)
B (n=15)
C (n=11)
surface

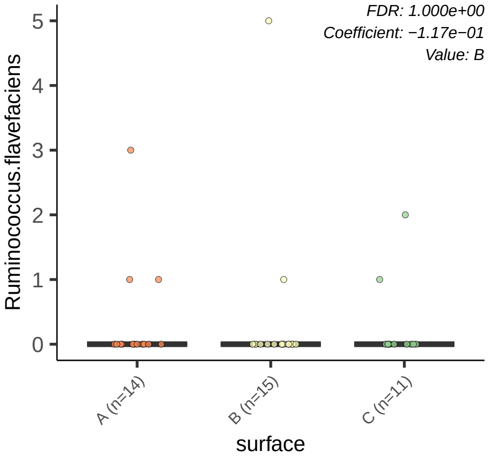
FDR: 1.000e+00
Coefficient: −1.17e−01
Value: B
Ruminococcus.flavefaciens
0
1
2
3
4
5
A (n=14)
B (n=15)
C (n=11)
surface

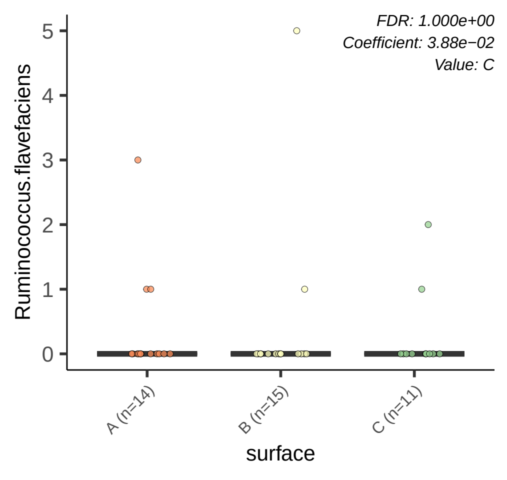
FDR: 1.000e+00
Coefficient: 3.88e−02
Value: C
Ruminococcus.flavefaciens
0
1
2
3
4
5
A (n=14)
B (n=15)
C (n=11)
surface

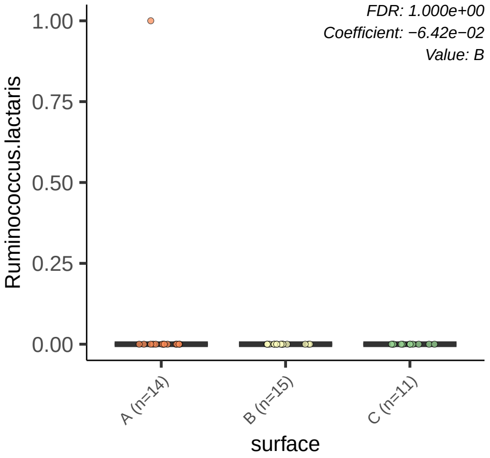
FDR: 1.000e+00
Coefficient: −6.42e−02
Value: B
Ruminococcus.lactaris
1.00
0.75
0.50
0.25
0.00
A (n=14)
B (n=15)
C (n=11)
surface

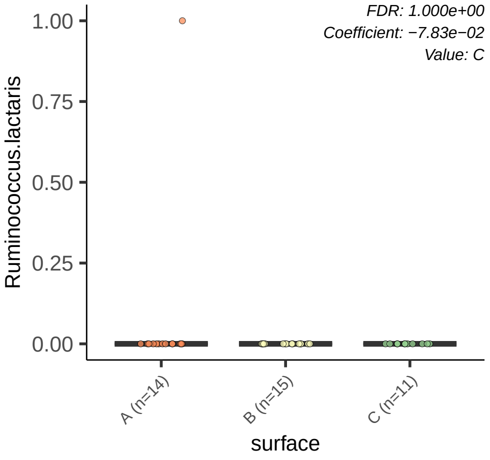

FDR: 1.000e+00
Coefficient: −7.83e−02
Value: C
Ruminococcus.lactaris
1.00
0.75
0.50
0.25
0.00
A (n=14)
B (n=15)
C (n=11)
surface

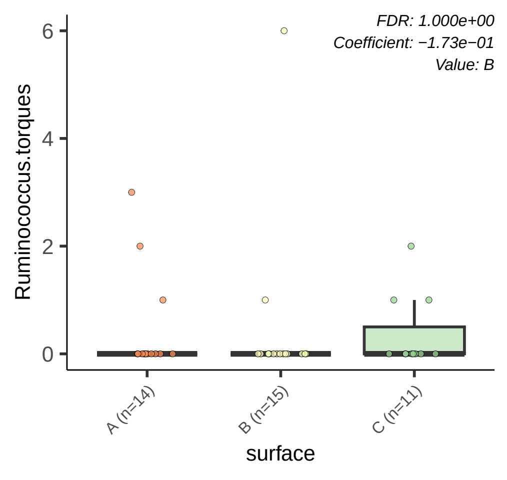

FDR: 1.000e+00
Coefficient: −1.73e−01
Value: B
Ruminococcus.torques
6
4
2
0
A (n=14)
B (n=15)
C (n=11)
surface

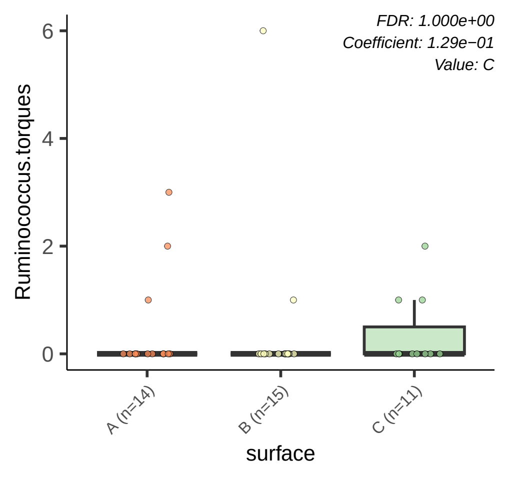
FDR: 1.000e+00
Coefficient: 1.29e−01
Value: C
Ruminococcus.torques
6
4
2
0
A (n=14)
B (n=15)
C (n=11)
surface

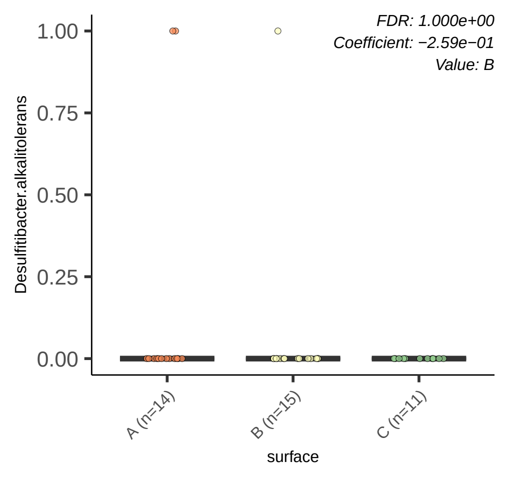
FDR: 1.000e+00
Coefficient: −2.59e−01
Value: B
1.00
0.75
0.50
0.25
0.00
Desulfitibacter.alkalitolerans
A (n=14)
B (n=15)
C (n=11)
surface

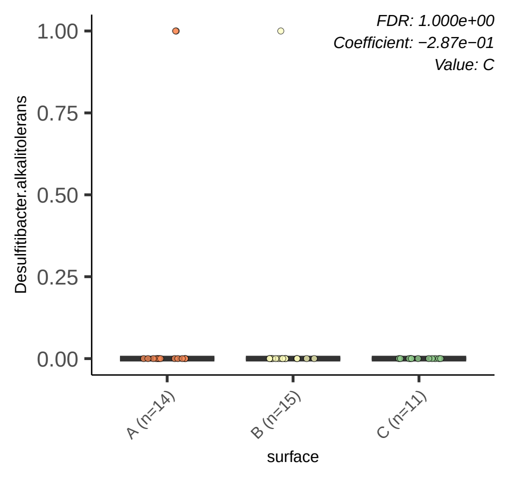

FDR: 1.000e+00
Coefficient: −2.87e−01
Value: C
Desulfitibacter.alkalitolerans
1.00
0.75
0.50
0.25
0.00
A (n=14)
B (n=15)
C (n=11)
surface

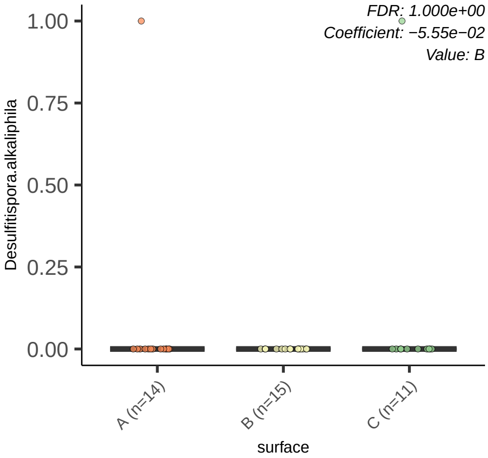

FDR: 1.000e+00
Coefficient: −5.55e−02
Value: B
1.00
0.75
0.50
0.25
0.00
Desulfitispora.alkaliphila
A (n=14)
B (n=15)
C (n=11)
surface

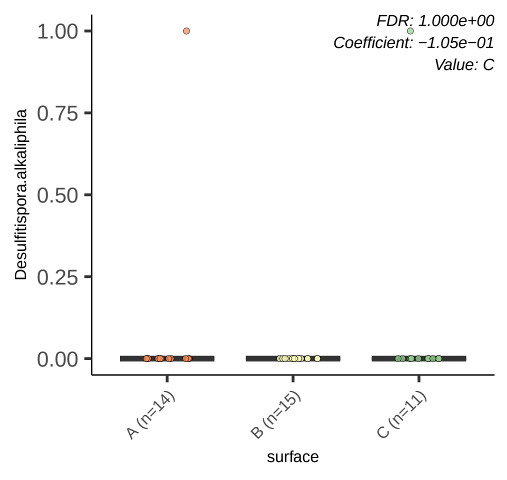
FDR: 1.000e+00
Coefficient: −1.05e−01
Value: C
1.00
0.75
0.50
0.25
0.00
Desulfitispora.alkaliphila
A (n=14)
B (n=15)
C (n=11)
surface

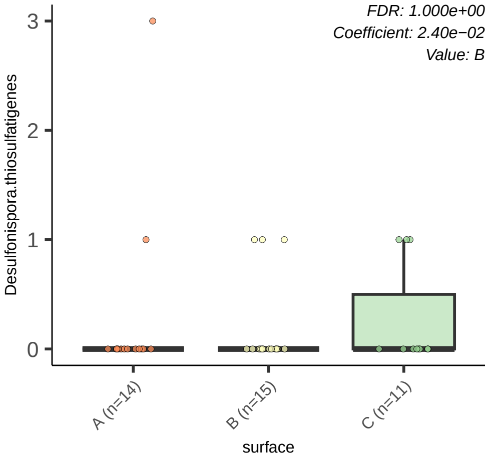
FDR: 1.000e+00
Coefficient: 2.40e−02
Value: B
Desulfonispora.thiosulfatigenes
3
2
1
0
A (n=14)
B (n=15)
C (n=11)
surface

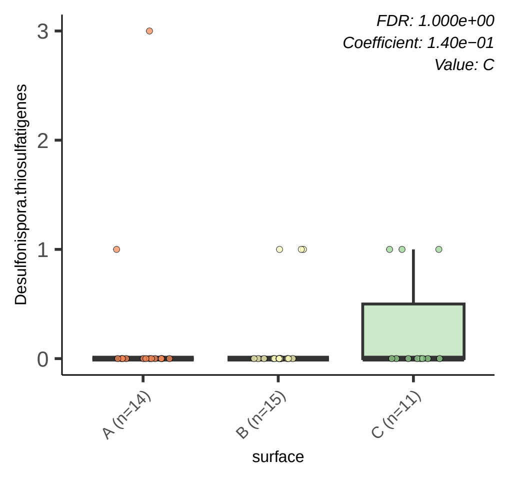

FDR: 1.000e+00
Coefficient: 1.40e−01
Value: C
Desulfonispora.thiosulfatigenes
3
2
1
0
A (n=14)
B (n=15)
C (n=11)
surface

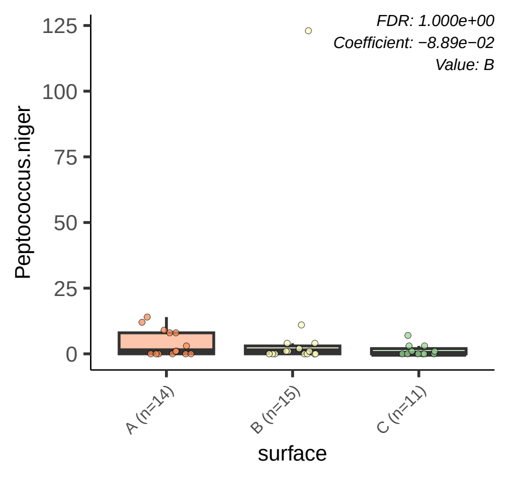

FDR: 1.000e+00
Coefficient: −8.89e−02
Value: B
Peptococcus.niger
125
100
75
50
25
0
A (n=14)
B (n=15)
C (n=11)
surface

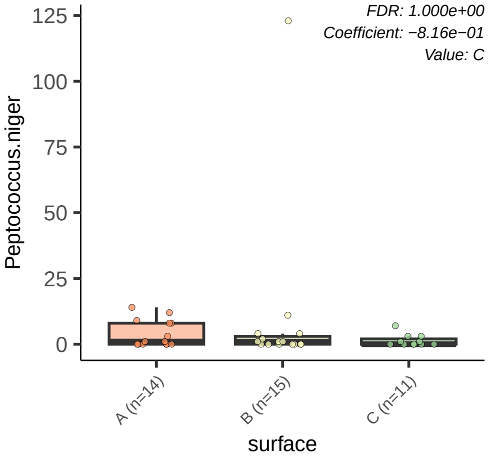

FDR: 1.000e+00
Coefficient: −8.16e−01
Value: C
Peptococcus.niger
125
100
75
50
25
0
A (n=14)
B (n=15)
C (n=11)
surface

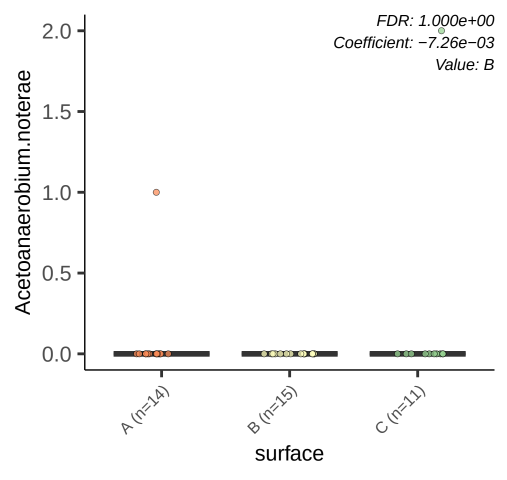

FDR: 1.000e+00
Coefficient: −7.26e−03
Value: B
Acetoanaerobium.noterae
2.0
1.5
1.0
0.5
0.0
A (n=14)
B (n=15)
C (n=11)
surface

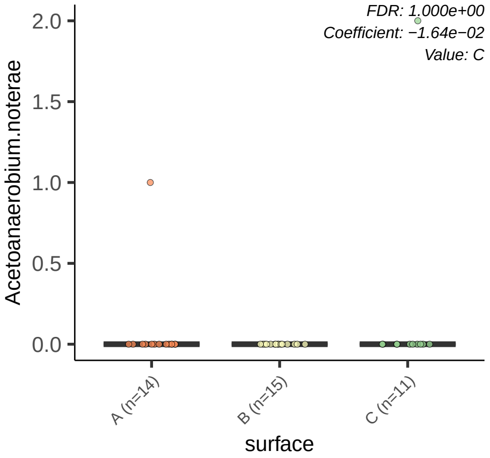

FDR: 1.000e+00
Coefficient: −1.64e−02
Value: C
Acetoanaerobium.noterae
2.0
1.5
1.0
0.5
0.0
A (n=14)
B (n=15)
C (n=11)
surface

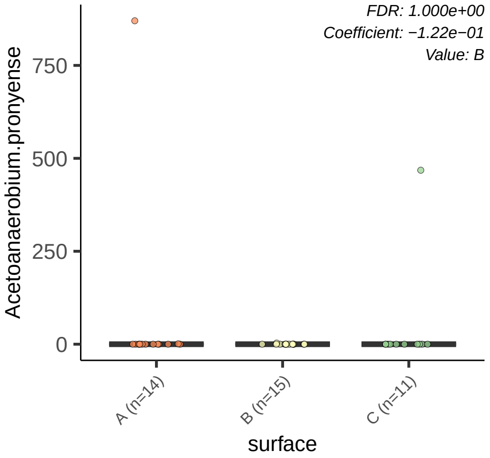

FDR: 1.000e+00
Coefficient: −1.22e−01
Value: B
Acetoanaerobium.pronyense
750
500
250
0
A (n=14)
B (n=15)
C (n=11)
surface

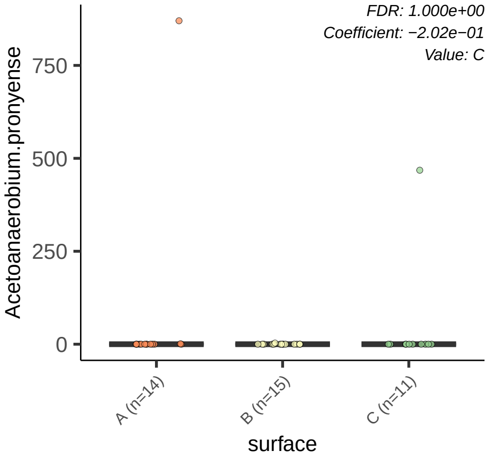
FDR: 1.000e+00
Coefficient: −2.02e−01
Value: C
Acetoanaerobium.pronyense
750
500
250
0
A (n=14)
B (n=15)
C (n=11)
surface

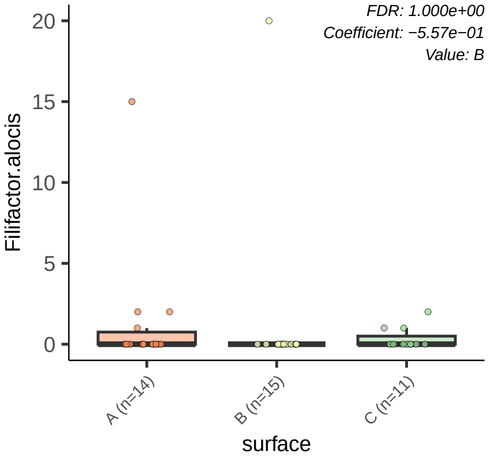

FDR: 1.000e+00
Coefficient: −5.57e−01
Value: B
Filifactor.alocis
0
5
10
15
20
A (n=14)
B (n=15)
C (n=11)
surface

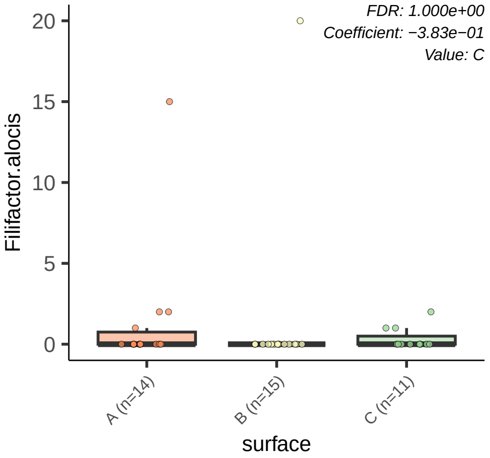

FDR: 1.000e+00
Coefficient: −3.83e−01
Value: C
Filifactor.alocis
20
15
10
5
0
A (n=14)
B (n=15)
C (n=11)
surface

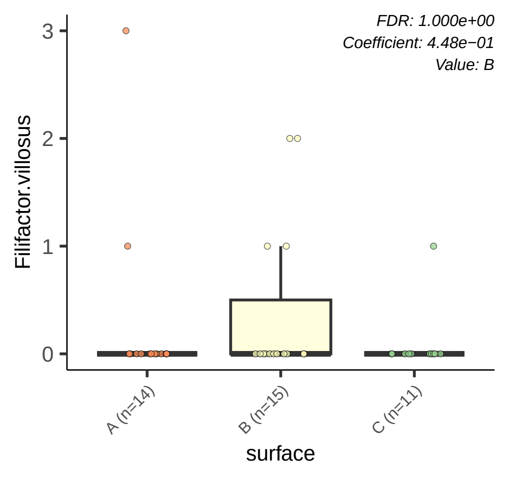

FDR: 1.000e+00
Coefficient: 4.48e−01
Value: B
Filifactor.villosus
3
2
1
0
A (n=14)
B (n=15)
C (n=11)
surface

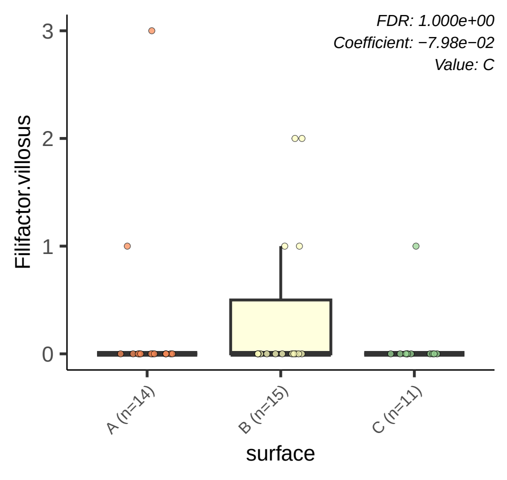
FDR: 1.000e+00
Coefficient: −7.98e−02
Value: C
Filifactor.villosus
3
2
1
0
A (n=14)
B (n=15)
C (n=11)
surface

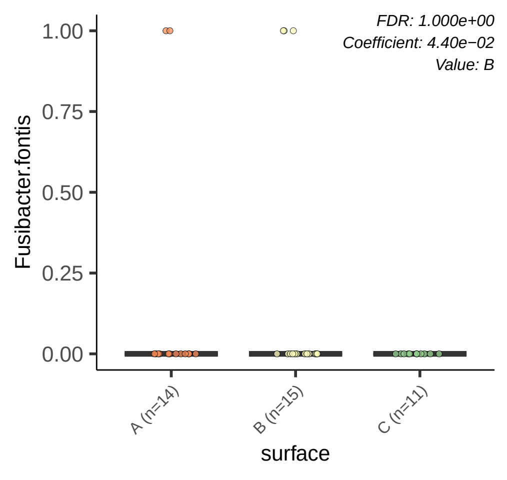

FDR: 1.000e+00
Coefficient: 4.40e−02
Value: B
Fusibacter.fontis
1.00
0.75
0.50
0.25
0.00
A (n=14)
B (n=15)
C (n=11)
surface

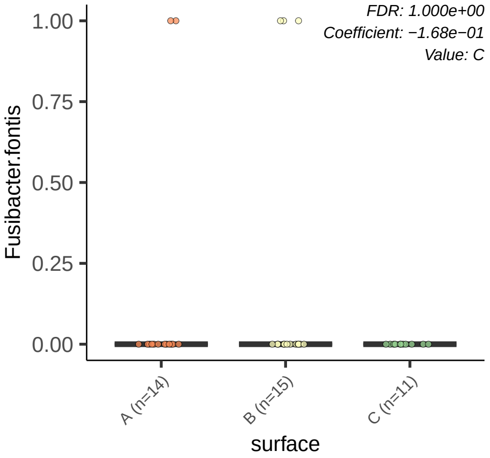
FDR: 1.000e+00
Coefficient: −1.68e−01
Value: C
Fusibacter.fontis
1.00
0.75
0.50
0.25
0.00
A (n=14)
B (n=15)
C (n=11)
surface

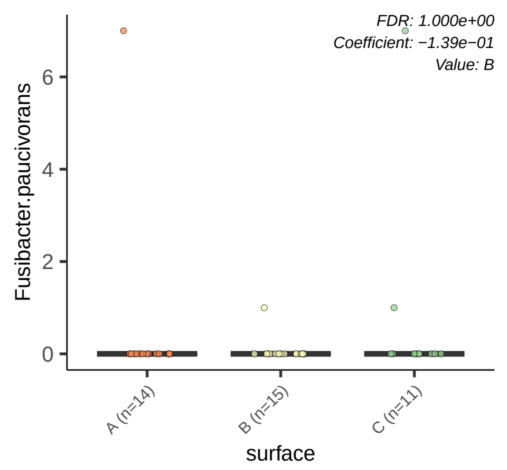

FDR: 1.000e+00
Coefficient: −1.39e−01
Value: B
Fusibacter.paucivorans
0
2
4
6
A (n=14)
B (n=15)
C (n=11)
surface

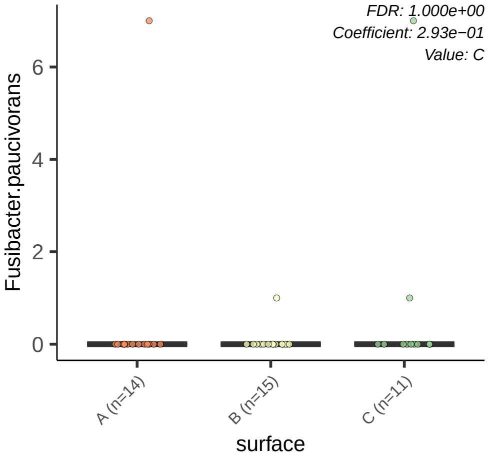

FDR: 1.000e+00
Coefficient: 2.93e−01
Value: C
Fusibacter.paucivorans
0
2
4
6
A (n=14)
B (n=15)
C (n=11)
surface

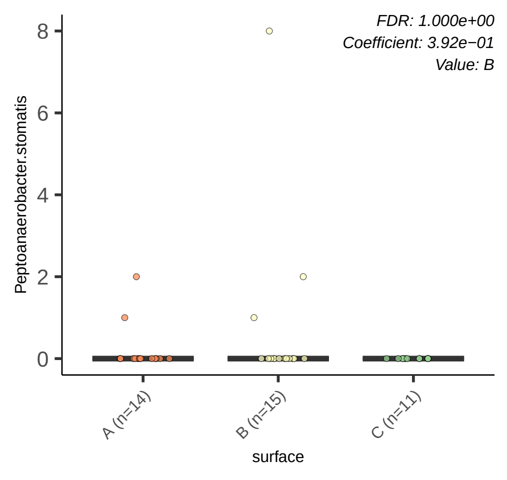

FDR: 1.000e+00
Coefficient: 3.92e−01
Value: B
Peptoanaerobacter.stomatis
0
2
4
6
8
A (n=14)
B (n=15)
C (n=11)
surface

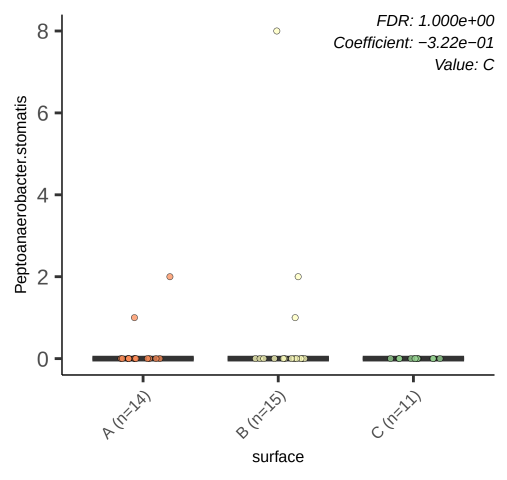
FDR: 1.000e+00
Coefficient: −3.22e−01
Value: C
Peptoanaerobacter.stomatis
8
6
4
2
0
A (n=14)
B (n=15)
C (n=11)
surface

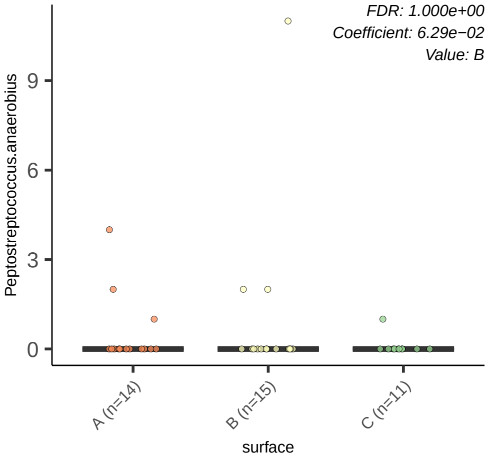
FDR: 1.000e+00
Coefficient: 6.29e−02
Value: B
Peptostreptococcus.anaerobius
0
3
6
9
A (n=14)
B (n=15)
C (n=11)
surface

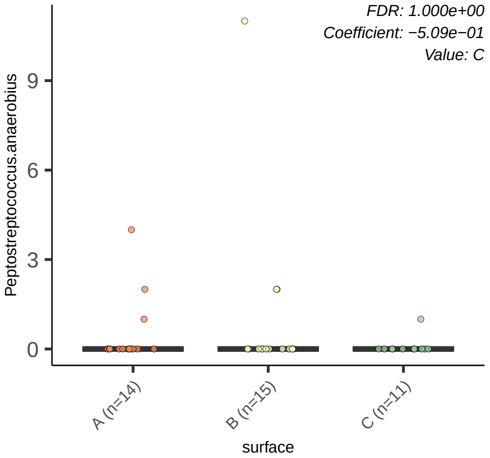
FDR: 1.000e+00
Coefficient: −5.09e−01
Value: C
Peptostreptococcus.anaerobius
9
6
3
0
A (n=14)
B (n=15)
C (n=11)
surface

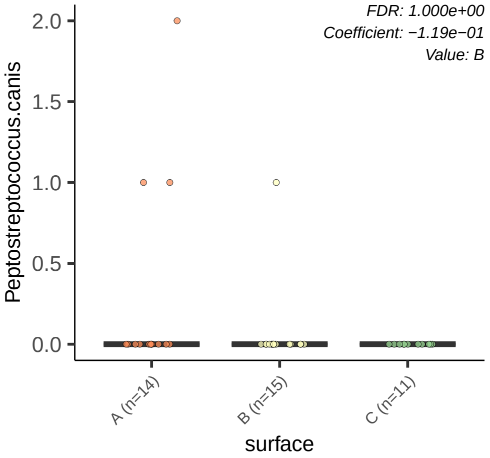
FDR: 1.000e+00
Coefficient: −1.19e−01
Value: B
Peptostreptococcus.canis
2.0
1.5
1.0
0.5
0.0
A (n=14)
B (n=15)
C (n=11)
surface

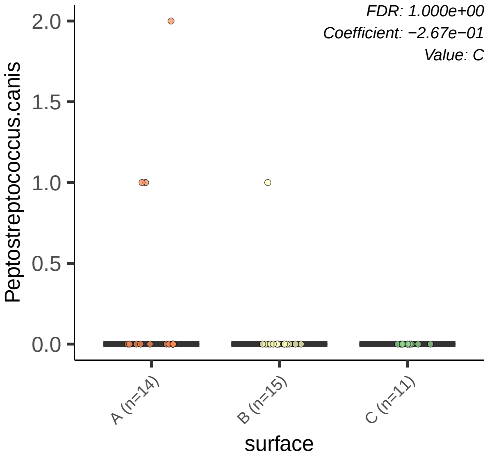
FDR: 1.000e+00
Coefficient: −2.67e−01
Value: C
Peptostreptococcus.canis
2.0
1.5
1.0
0.5
0.0
A (n=14)
B (n=15)
C (n=11)
surface

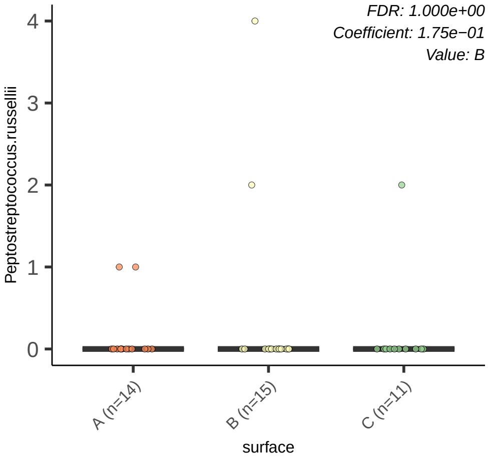
FDR: 1.000e+00
Coefficient: 1.75e−01
Value: B
Peptostreptococcus.russellii
0
1
2
3
4
A (n=14)
B (n=15)
C (n=11)
surface

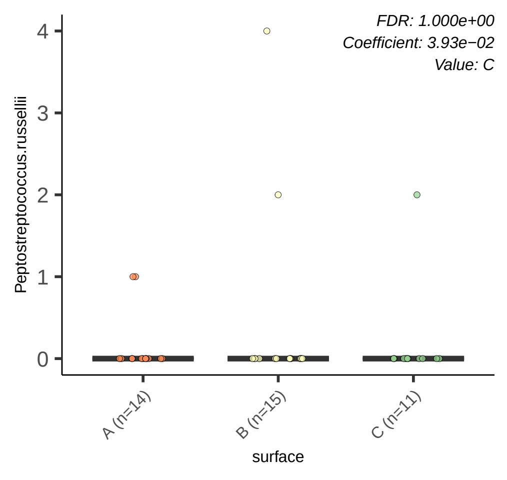

FDR: 1.000e+00
Coefficient: 3.93e−02
Value: C
Peptostreptococcus.russellii
4
3
2
1
0
A (n=14)
B (n=15)
C (n=11)
surface

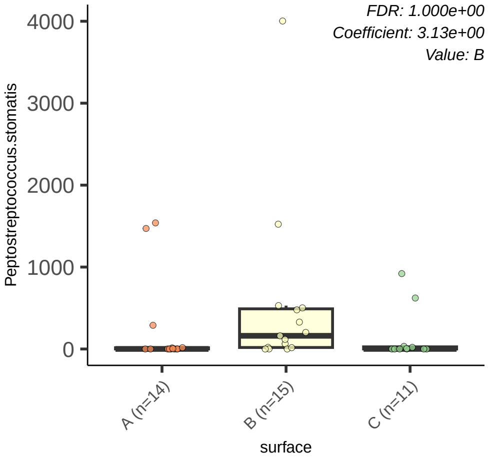

FDR: 1.000e+00
Coefficient: 3.13e+00
Value: B
Peptostreptococcus.stomatis
4000
3000
2000
1000
0
A (n=14)
B (n=15)
C (n=11)
surface

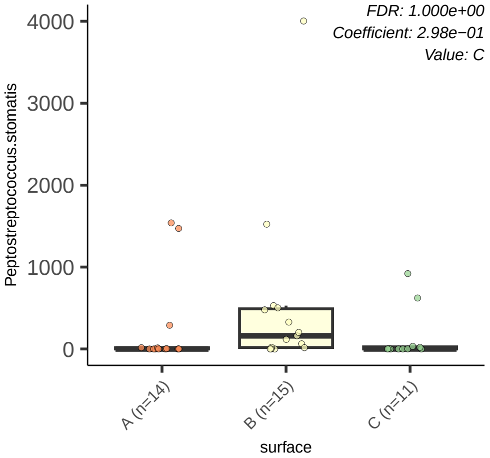
FDR: 1.000e+00
Coefficient: 2.98e−01
Value: C
Peptostreptococcus.stomatis
4000
3000
2000
1000
0
A (n=14)
B (n=15)
C (n=11)
surface

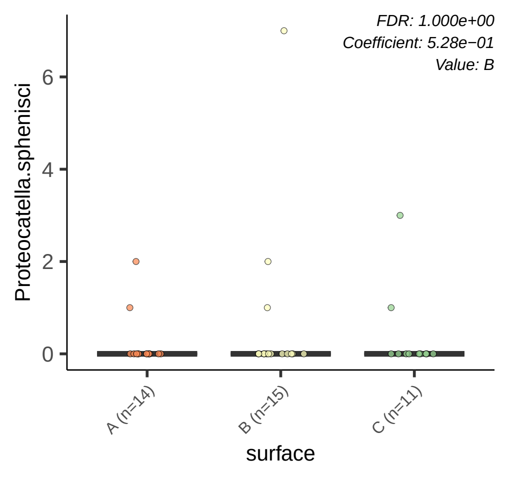

FDR: 1.000e+00
Coefficient: 5.28e−01
Value: B
Proteocatella.sphenisci
0
2
4
6
A (n=14)
B (n=15)
C (n=11)
surface

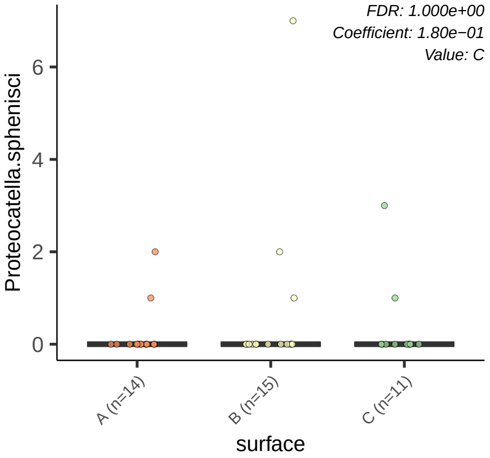
FDR: 1.000e+00
Coefficient: 1.80e−01
Value: C
Proteocatella.sphenisci
0
2
4
6
A (n=14)
B (n=15)
C (n=11)
surface

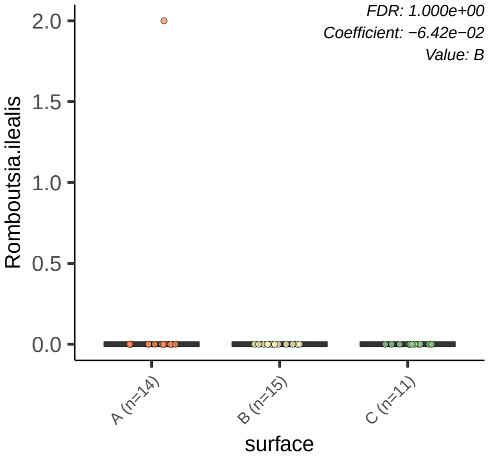
FDR: 1.000e+00
Coefficient: −6.42e−02
Value: B
Romboutsia.ilealis
2.0
1.5
1.0
0.5
0.0
A (n=14)
B (n=15)
C (n=11)
surface

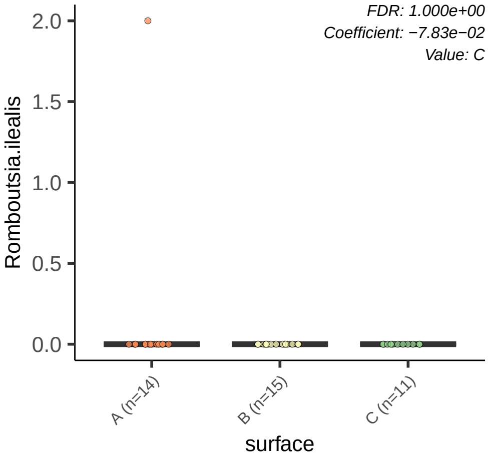
FDR: 1.000e+00
Coefficient: −7.83e−02
Value: C
Romboutsia.ilealis
2.0
1.5
1.0
0.5
0.0
A (n=14)
B (n=15)
C (n=11)
surface

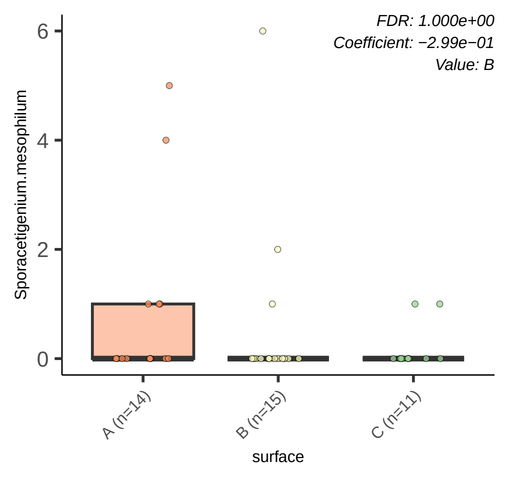

FDR: 1.000e+00
Coefficient: −2.99e−01
Value: B
Sporacetigenium.mesophilum
0
2
4
6
A (n=14)
B (n=15)
C (n=11)
surface

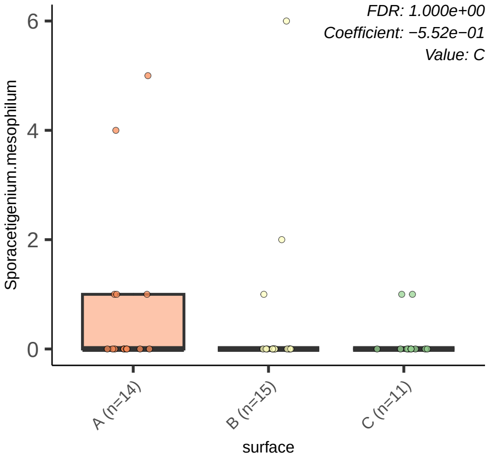

FDR: 1.000e+00
Coefficient: −5.52e−01
Value: C
Sporacetigenium.mesophilum
0
2
4
6
A (n=14)
B (n=15)
C (n=11)
surface

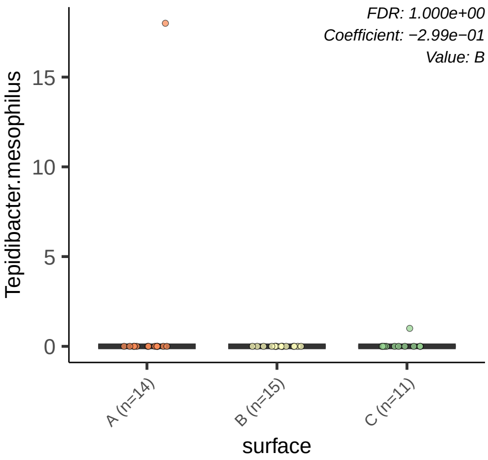
FDR: 1.000e+00
Coefficient: −2.99e−01
Value: B
Tepidibacter.mesophilus
0
5
10
15
A (n=14)
B (n=15)
C (n=11)
surface

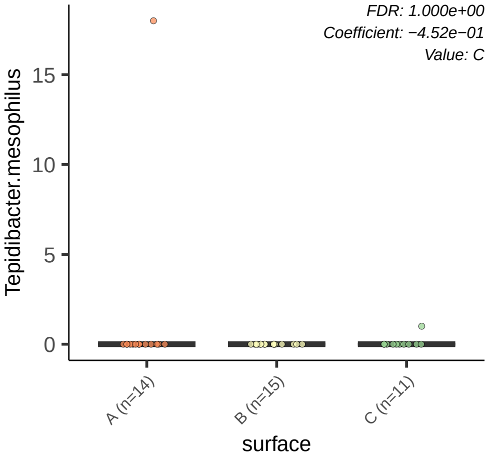

FDR: 1.000e+00
Coefficient: −4.52e−01
Value: C
Tepidibacter.mesophilus
15
10
5
0
A (n=14)
B (n=15)
C (n=11)
surface

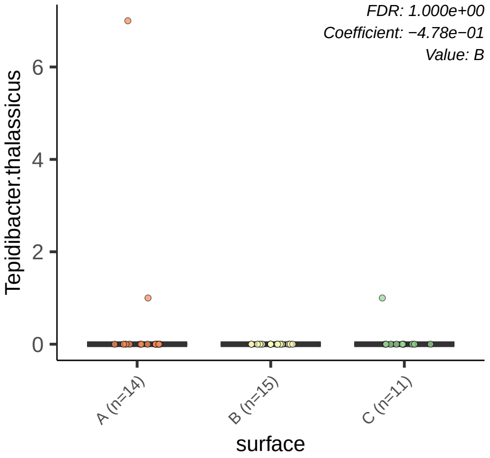

FDR: 1.000e+00
Coefficient: −4.78e−01
Value: B
Tepidibacter.thalassicus
0
2
4
6
A (n=14)
B (n=15)
C (n=11)
surface

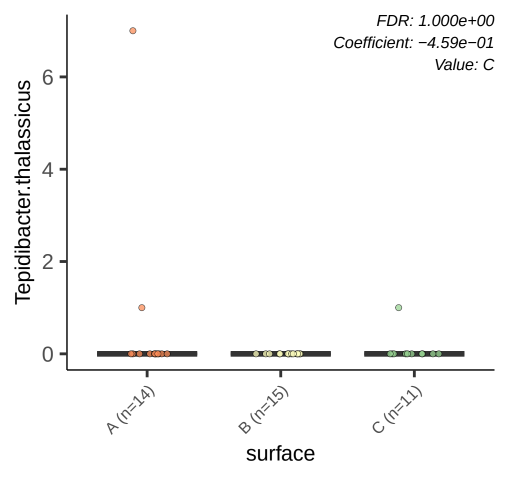
FDR: 1.000e+00
Coefficient: −4.59e−01
Value: C
Tepidibacter.thalassicus
0
2
4
6
A (n=14)
B (n=15)
C (n=11)
surface

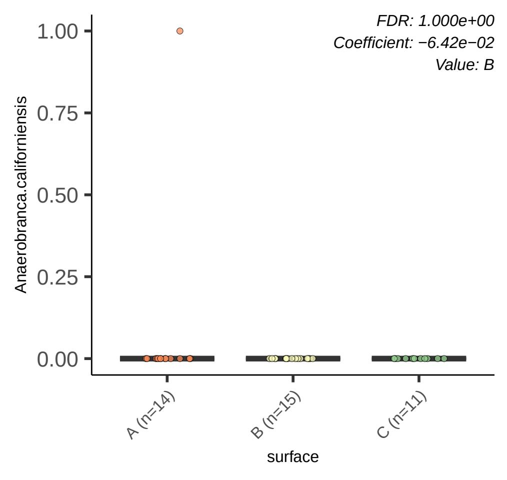
FDR: 1.000e+00
Coefficient: −6.42e−02
Value: B
Anaerobranca.californiensis
1.00
0.75
0.50
0.25
0.00
A (n=14)
B (n=15)
C (n=11)
surface

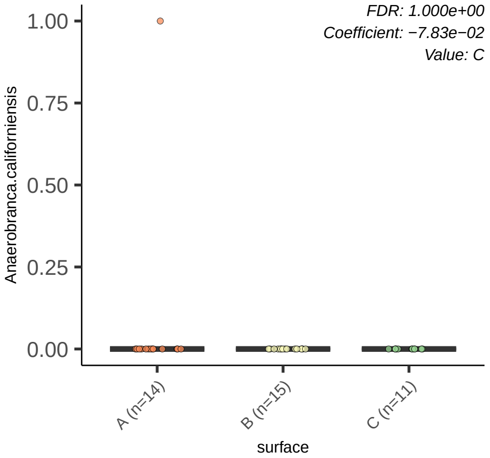

FDR: 1.000e+00
Coefficient: −7.83e−02
Value: C
1.00
0.75
0.50
0.25
0.00
Anaerobranca.californiensis
A (n=14)
B (n=15)
C (n=11)
surface

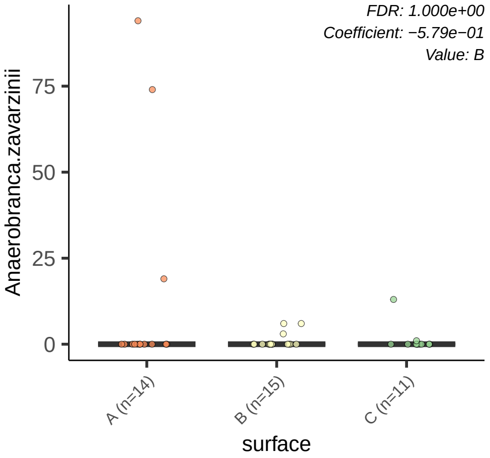

FDR: 1.000e+00
Coefficient: −5.79e−01
Value: B
Anaerobranca.zavarzinii
75
50
25
0
A (n=14)
B (n=15)
C (n=11)
surface

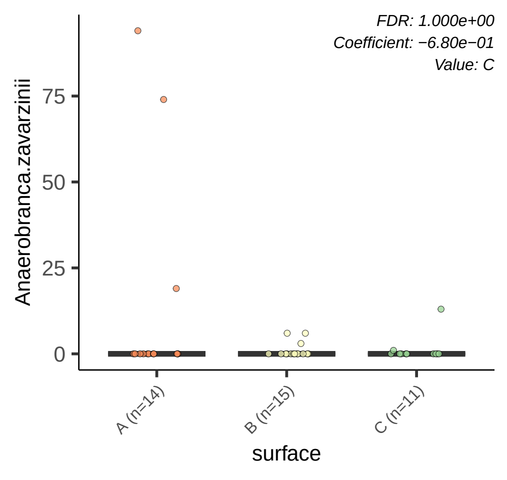
FDR: 1.000e+00
Coefficient: −6.80e−01
Value: C
Anaerobranca.zavarzinii
75
50
25
0
A (n=14)
B (n=15)
C (n=11)
surface

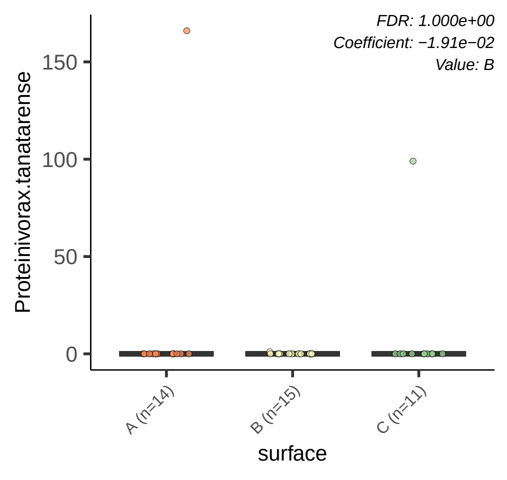
FDR: 1.000e+00
Coefficient: −1.91e−02
Value: B
Proteinivorax.tanatarense
150
100
50
0
A (n=14)
B (n=15)
C (n=11)
surface

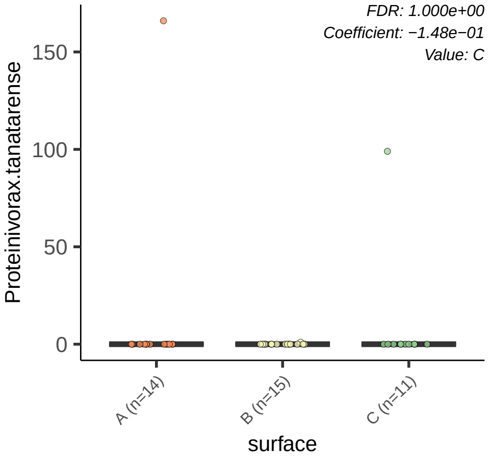

FDR: 1.000e+00
Coefficient: −1.48e−01
Value: C
Proteinivorax.tanatarense
150
100
50
0
A (n=14)
B (n=15)
C (n=11)
surface

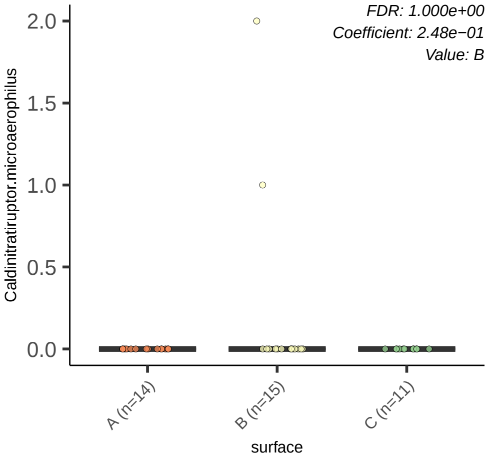

FDR: 1.000e+00
Coefficient: 2.48e−01
Value: B
Caldinitratiruptor.microaerophilus
2.0
1.5
1.0
0.5
0.0
A (n=14)
B (n=15)
C (n=11)
surface

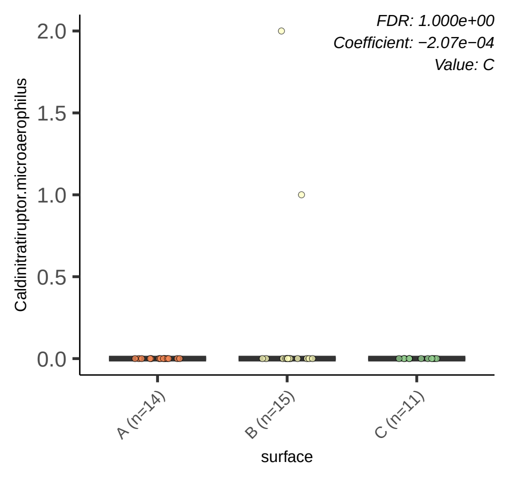
FDR: 1.000e+00
Coefficient: −2.07e−04
Value: C
Caldinitratiruptor.microaerophilus
2.0
1.5
1.0
0.5
0.0
A (n=14)
B (n=15)
C (n=11)
surface

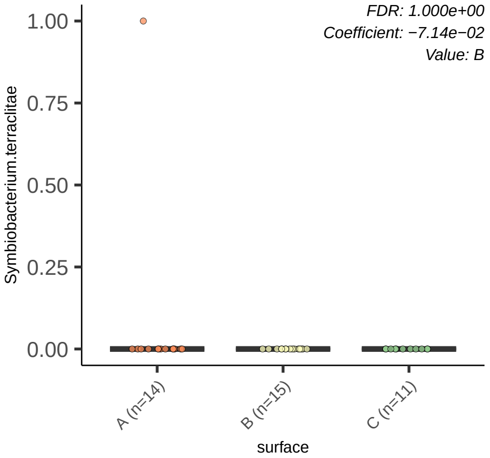
FDR: 1.000e+00
Coefficient: −7.14e−02
Value: B
Symbiobacterium.terraclitae
1.00
0.75
0.50
0.25
0.00
A (n=14)
B (n=15)
C (n=11)
surface

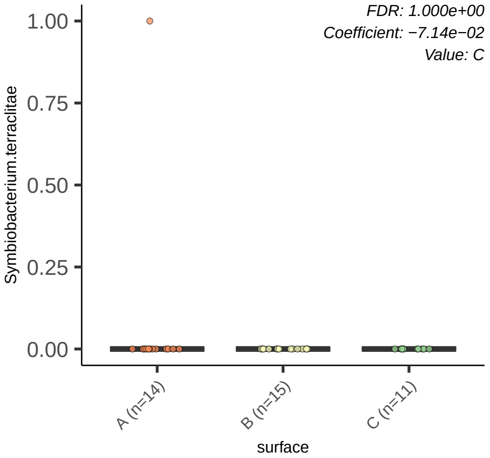
FDR: 1.000e+00
Coefficient: −7.14e−02
Value: C
Symbiobacterium.terraclitae
1.00
0.75
0.50
0.25
0.00
A (n=14)
B (n=15)
C (n=11)
surface

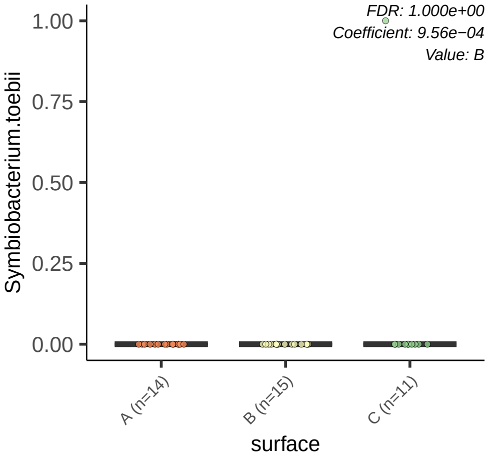
FDR: 1.000e+00
Coefficient: 9.56e−04
Value: B
Symbiobacterium.toebii
1.00
0.75
0.50
0.25
0.00
A (n=14)
B (n=15)
C (n=11)
surface

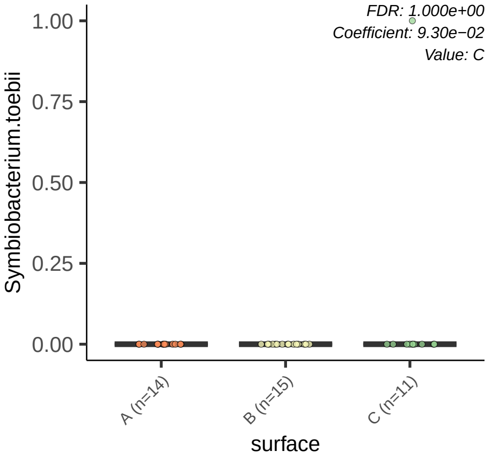

FDR: 1.000e+00
Coefficient: 9.30e−02
Value: C
Symbiobacterium.toebii
1.00
0.75
0.50
0.25
0.00
A (n=14)
B (n=15)
C (n=11)
surface

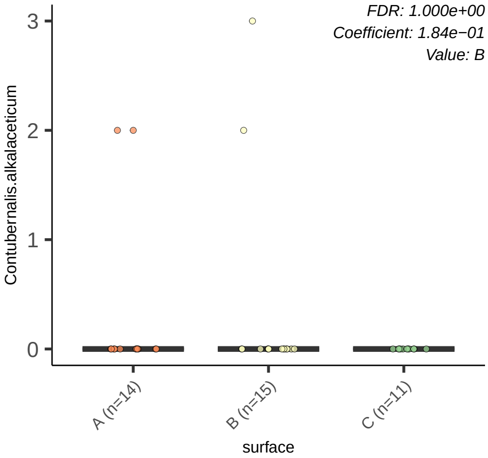
FDR: 1.000e+00
Coefficient: 1.84e−01
Value: B
Contubernalis.alkalaceticum
3
2
1
0
A (n=14)
B (n=15)
C (n=11)
surface

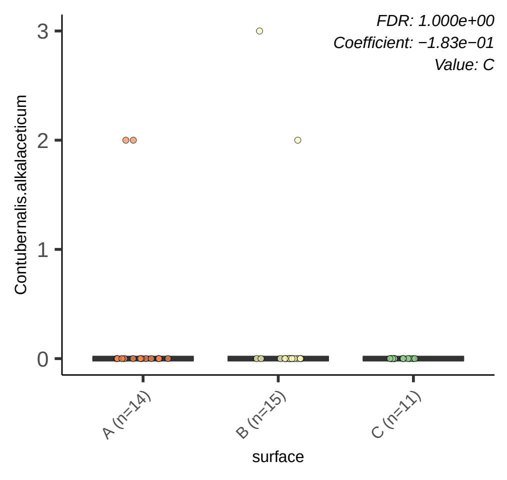
FDR: 1.000e+00
Coefficient: −1.83e−01
Value: C
Contubernalis.alkalaceticum
3
2
1
0
A (n=14)
B (n=15)
C (n=11)
surface

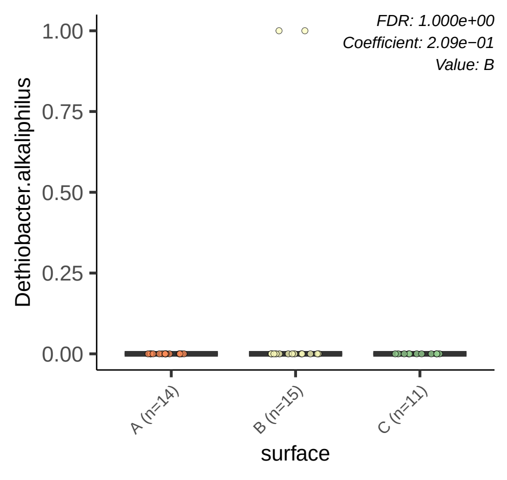

FDR: 1.000e+00
Coefficient: 2.09e−01
Value: B
Dethiobacter.alkaliphilus
1.00
0.75
0.50
0.25
0.00
A (n=14)
B (n=15)
C (n=11)
surface

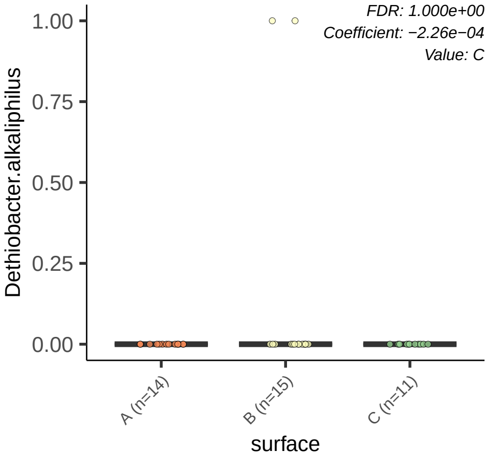

FDR: 1.000e+00
Coefficient: −2.26e−04
Value: C
Dethiobacter.alkaliphilus
1.00
0.75
0.50
0.25
0.00
A (n=14)
B (n=15)
C (n=11)
surface

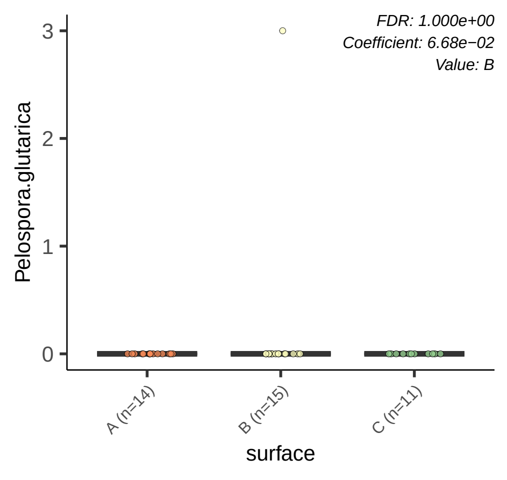

FDR: 1.000e+00
Coefficient: 6.68e−02
Value: B
Pelospora.glutarica
3
2
1
0
A (n=14)
B (n=15)
C (n=11)
surface

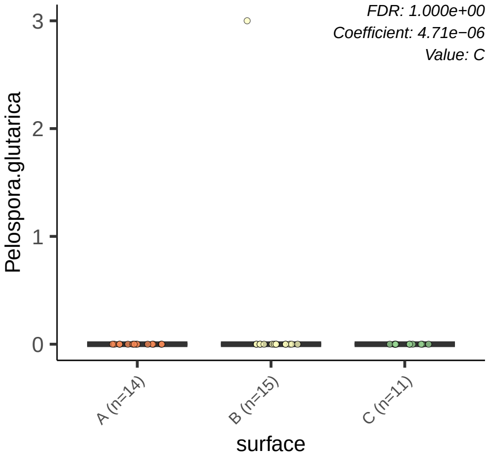

FDR: 1.000e+00
Coefficient: 4.71e−06
Value: C
Pelospora.glutarica
3
2
1
0
A (n=14)
B (n=15)
C (n=11)
surface

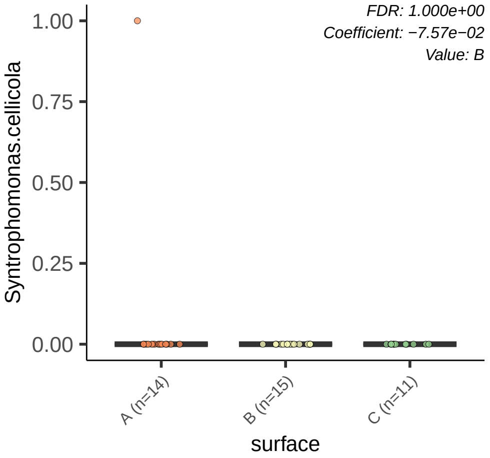
FDR: 1.000e+00
Coefficient: −7.57e−02
Value: B
Syntrophomonas.cellicola
1.00
0.75
0.50
0.25
0.00
A (n=14)
B (n=15)
C (n=11)
surface

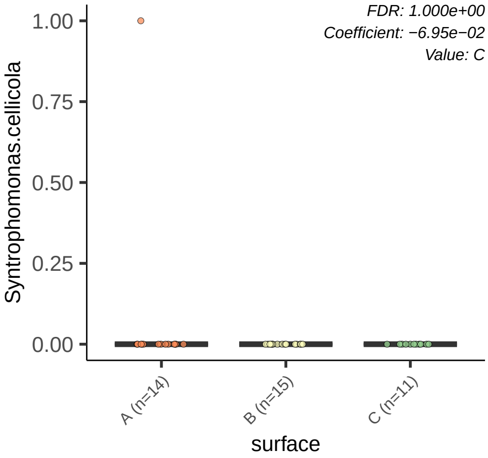
FDR: 1.000e+00
Coefficient: −6.95e−02
Value: C
Syntrophomonas.cellicola
1.00
0.75
0.50
0.25
0.00
A (n=14)
B (n=15)
C (n=11)
surface

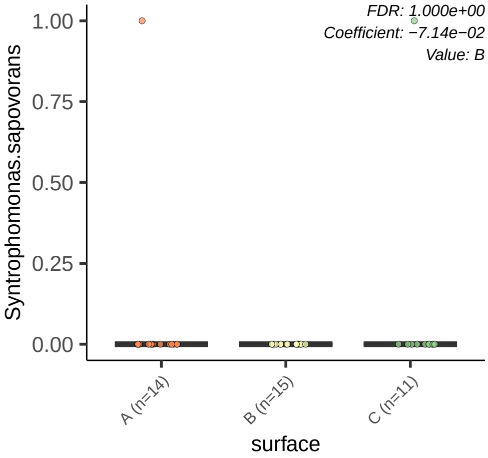
FDR: 1.000e+00
Coefficient: −7.14e−02
Value: B
Syntrophomonas.sapovorans
1.00
0.75
0.50
0.25
0.00
A (n=14)
B (n=15)
C (n=11)
surface

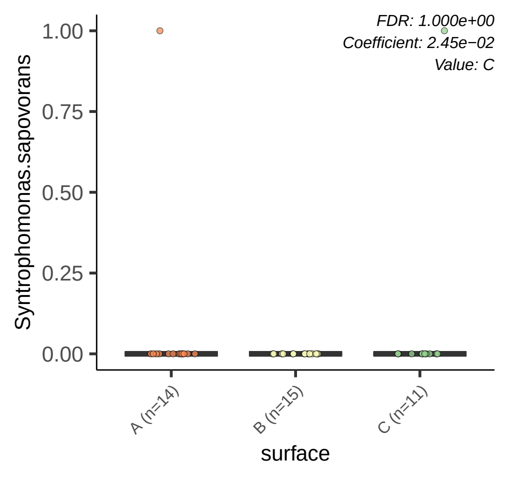
FDR: 1.000e+00
Coefficient: 2.45e−02
Value: C
Syntrophomonas.sapovorans
1.00
0.75
0.50
0.25
0.00
A (n=14)
B (n=15)
C (n=11)
surface

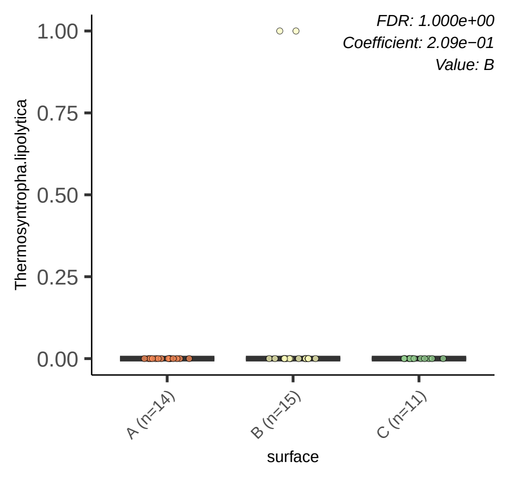
FDR: 1.000e+00
Coefficient: 2.09e−01
Value: B
Thermosyntropha.lipolytica
1.00
0.75
0.50
0.25
0.00
A (n=14)
B (n=15)
C (n=11)
surface

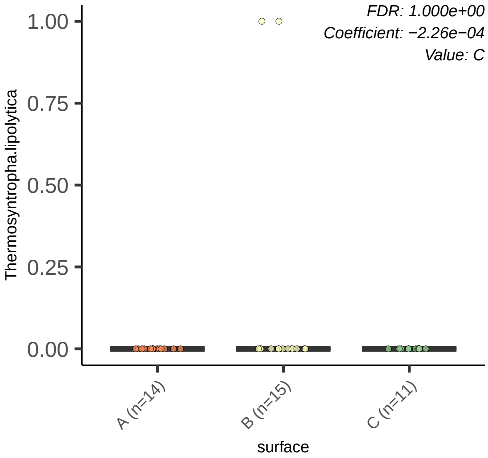
FDR: 1.000e+00
Coefficient: −2.26e−04
Value: C
Thermosyntropha.lipolytica
1.00
0.75
0.50
0.25
0.00
A (n=14)
B (n=15)
C (n=11)
surface

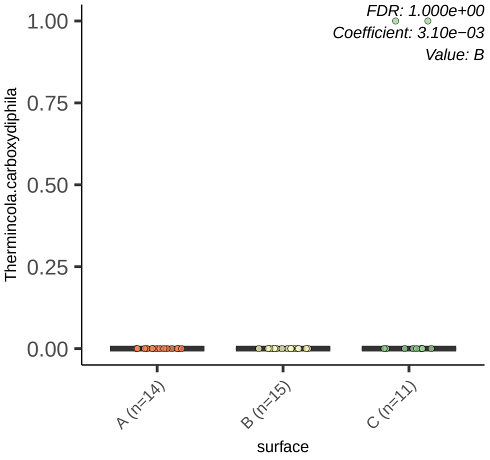

FDR: 1.000e+00
Coefficient: 3.10e−03
Value: B
1.00
0.75
0.50
0.25
0.00
Thermincola.carboxydiphila
A (n=14)
B (n=15)
C (n=11)
surface

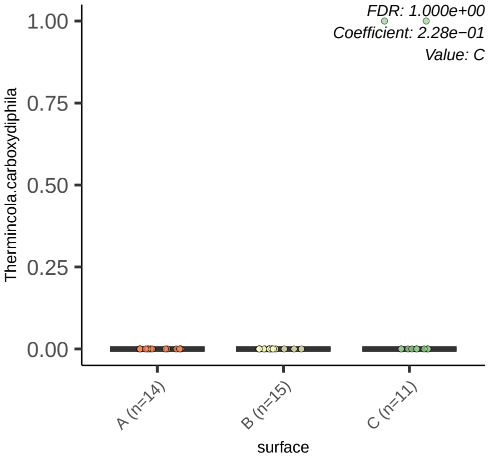

FDR: 1.000e+00
Coefficient: 2.28e−01
Value: C
Thermincola.carboxydiphila
1.00
0.75
0.50
0.25
0.00
A (n=14)
B (n=15)
C (n=11)
surface

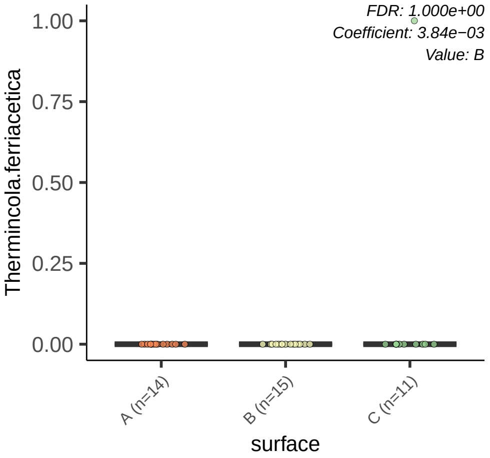

FDR: 1.000e+00
Coefficient: 3.84e−03
Value: B
Thermincola.ferriacetica
1.00
0.75
0.50
0.25
0.00
A (n=14)
B (n=15)
C (n=11)
surface

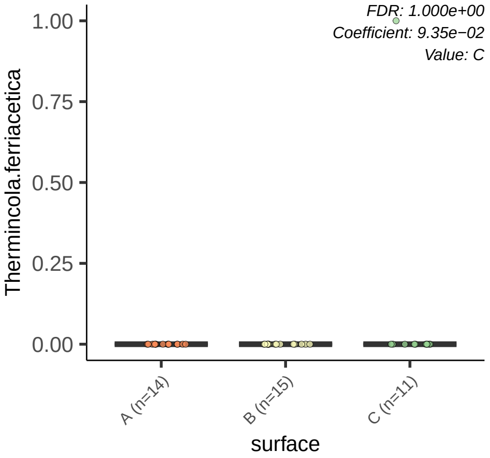
FDR: 1.000e+00
Coefficient: 9.35e−02
Value: C
Thermincola.ferriacetica
1.00
0.75
0.50
0.25
0.00
A (n=14)
B (n=15)
C (n=11)
surface

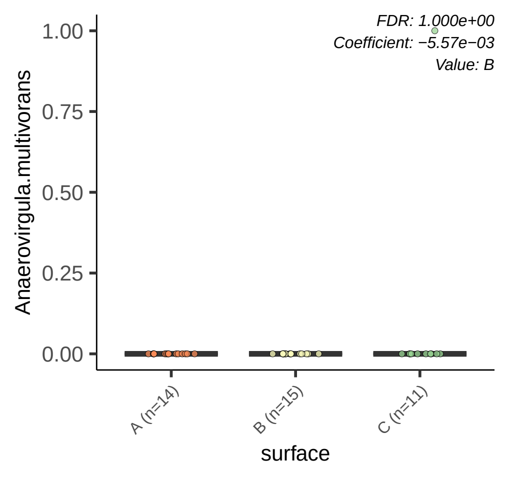

FDR: 1.000e+00
Coefficient: −5.57e−03
Value: B
Anaerovirgula.multivorans
1.00
0.75
0.50
0.25
0.00
A (n=14)
B (n=15)
C (n=11)
surface

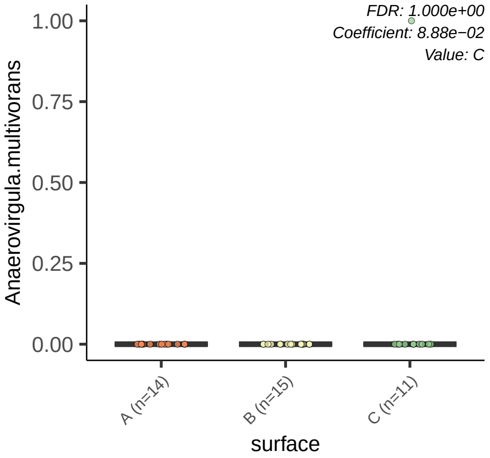

FDR: 1.000e+00
Coefficient: 8.88e−02
Value: C
Anaerovirgula.multivorans
1.00
0.75
0.50
0.25
0.00
A (n=14)
B (n=15)
C (n=11)
surface

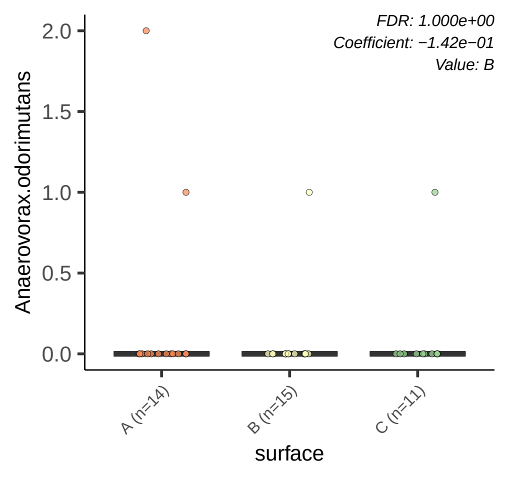
FDR: 1.000e+00
Coefficient: −1.42e−01
Value: B
Anaerovorax.odorimutans
2.0
1.5
1.0
0.5
0.0
A (n=14)
B (n=15)
C (n=11)
surface

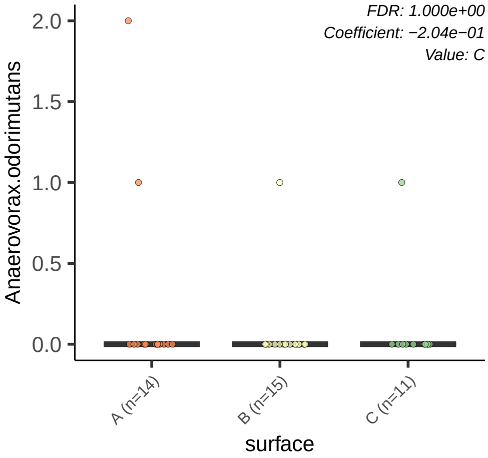
FDR: 1.000e+00
Coefficient: −2.04e−01
Value: C
Anaerovorax.odorimutans
2.0
1.5
1.0
0.5
0.0
A (n=14)
B (n=15)
C (n=11)
surface

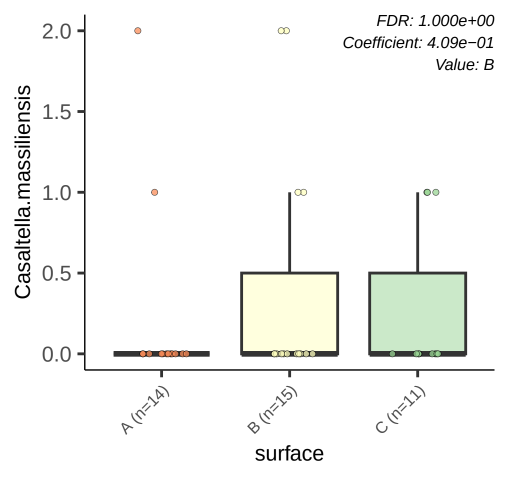

FDR: 1.000e+00
Coefficient: 4.09e−01
Value: B
Casaltella.massiliensis
2.0
1.5
1.0
0.5
0.0
A (n=14)
B (n=15)
C (n=11)
surface

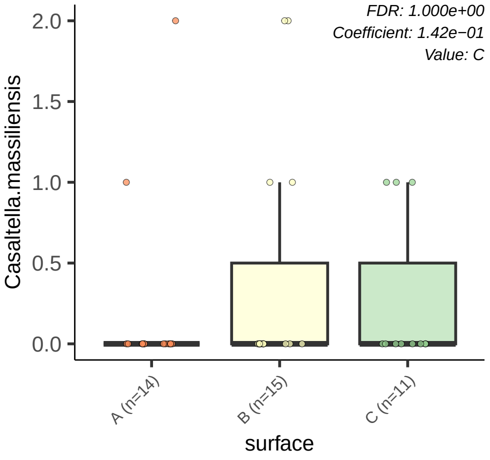
FDR: 1.000e+00
Coefficient: 1.42e−01
Value: C
Casaltella.massiliensis
2.0
1.5
1.0
0.5
0.0
A (n=14)
B (n=15)
C (n=11)
surface

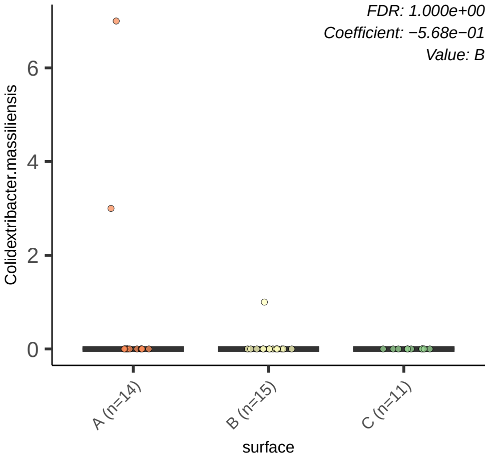
FDR: 1.000e+00
Coefficient: −5.68e−01
Value: B
Colidextribacter.massiliensis
0
2
4
6
A (n=14)
B (n=15)
C (n=11)
surface

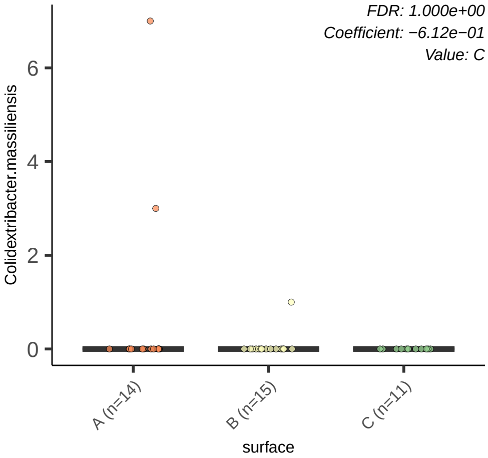

FDR: 1.000e+00
Coefficient: −6.12e−01
Value: C
Colidextribacter.massiliensis
0
2
4
6
A (n=14)
B (n=15)
C (n=11)
surface

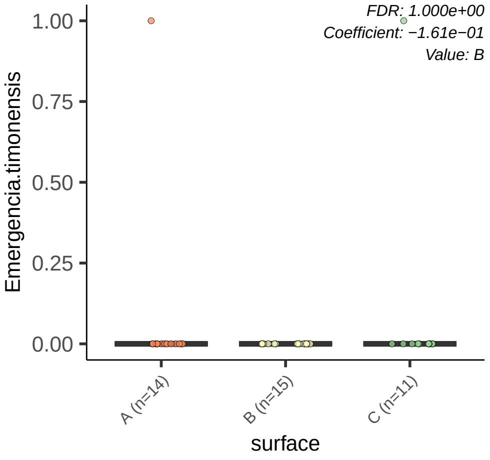
FDR: 1.000e+00
Coefficient: −1.61e−01
Value: B
Emergencia.timonensis
1.00
0.75
0.50
0.25
0.00
A (n=14)
B (n=15)
C (n=11)
surface

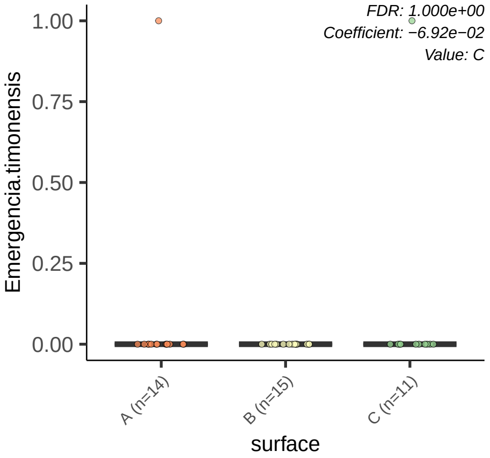

FDR: 1.000e+00
Coefficient: −6.92e−02
Value: C
Emergencia.timonensis
1.00
0.75
0.50
0.25
0.00
A (n=14)
B (n=15)
C (n=11)
surface

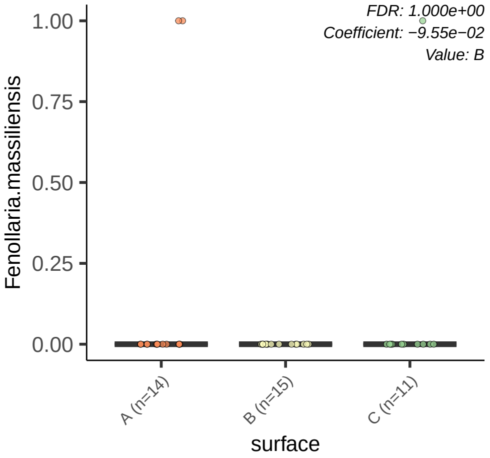
FDR: 1.000e+00
Coefficient: −9.55e−02
Value: B
Fenollaria.massiliensis
1.00
0.75
0.50
0.25
0.00
A (n=14)
B (n=15)
C (n=11)
surface

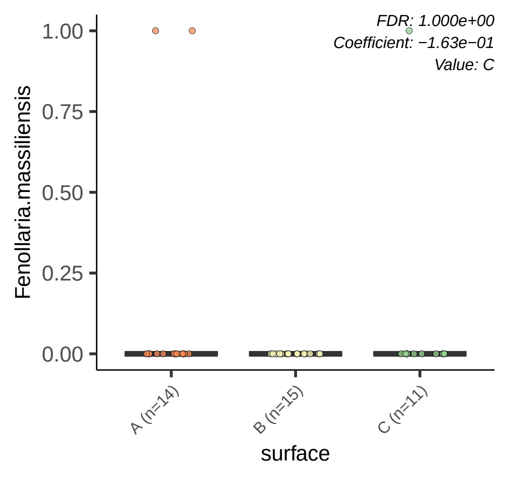
FDR: 1.000e+00
Coefficient: −1.63e−01
Value: C
Fenollaria.massiliensis
1.00
0.75
0.50
0.25
0.00
A (n=14)
B (n=15)
C (n=11)
surface

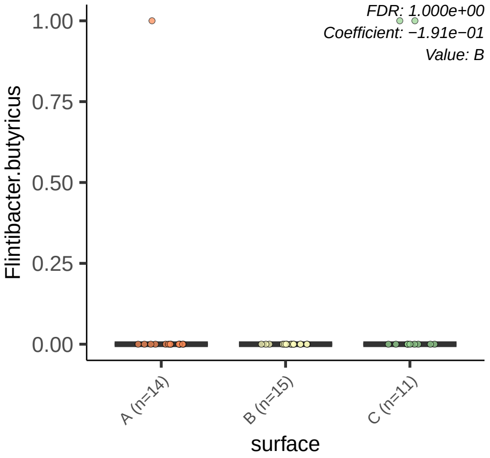

FDR: 1.000e+00
Coefficient: −1.91e−01
Value: B
Flintibacter.butyricus
1.00
0.75
0.50
0.25
0.00
A (n=14)
B (n=15)
C (n=11)
surface

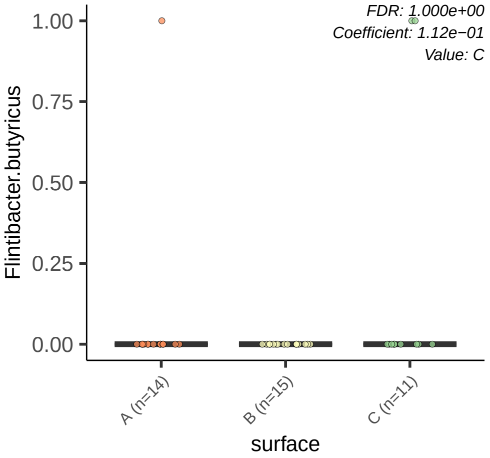
FDR: 1.000e+00
Coefficient: 1.12e−01
Value: C
Flintibacter.butyricus
1.00
0.75
0.50
0.25
0.00
A (n=14)
B (n=15)
C (n=11)
surface

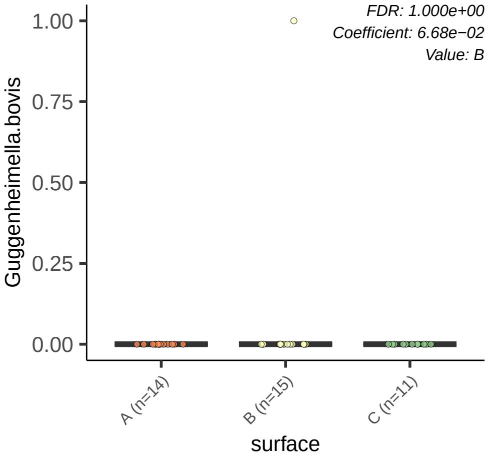
FDR: 1.000e+00
Coefficient: 6.68e−02
Value: B
Guggenheimella.bovis
1.00
0.75
0.50
0.25
0.00
A (n=14)
B (n=15)
C (n=11)
surface

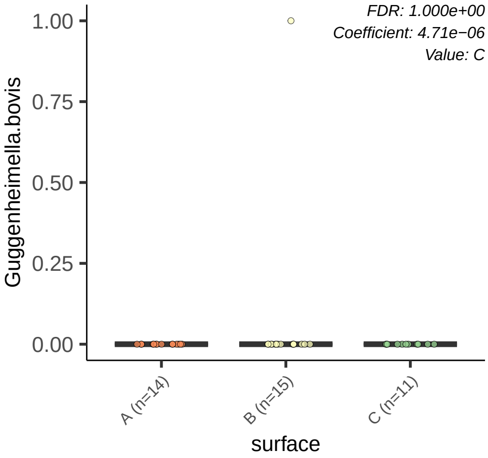
FDR: 1.000e+00
Coefficient: 4.71e−06
Value: C
Guggenheimella.bovis
1.00
0.75
0.50
0.25
0.00
A (n=14)
B (n=15)
C (n=11)
surface

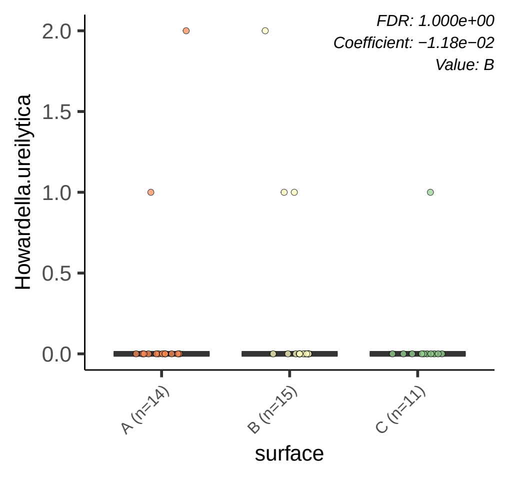

FDR: 1.000e+00
Coefficient: −1.18e−02
Value: B
Howardella.ureilytica
2.0
1.5
1.0
0.5
0.0
A (n=14)
B (n=15)
C (n=11)
surface

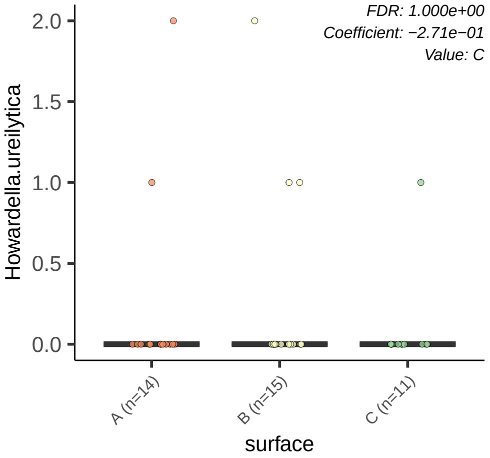
FDR: 1.000e+00
Coefficient: −2.71e−01
Value: C
Howardella.ureilytica
2.0
1.5
1.0
0.5
0.0
A (n=14)
B (n=15)
C (n=11)
surface

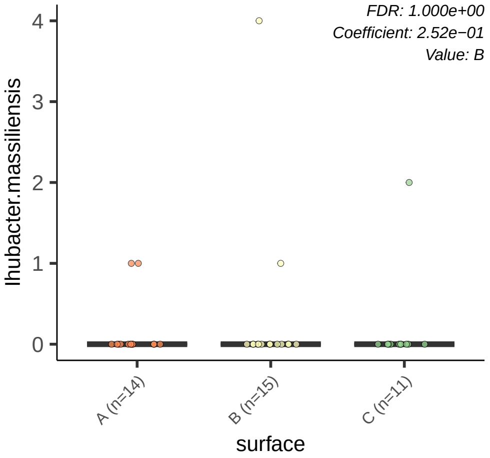
FDR: 1.000e+00
Coefficient: 2.52e−01
Value: B
Ihubacter.massiliensis
0
1
2
3
4
A (n=14)
B (n=15)
C (n=11)
surface

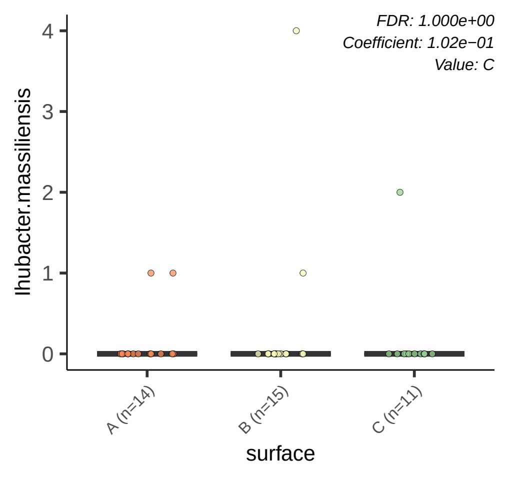

FDR: 1.000e+00
Coefficient: 1.02e−01
Value: C
Ihubacter.massiliensis
0
1
2
3
4
A (n=14)
B (n=15)
C (n=11)
surface

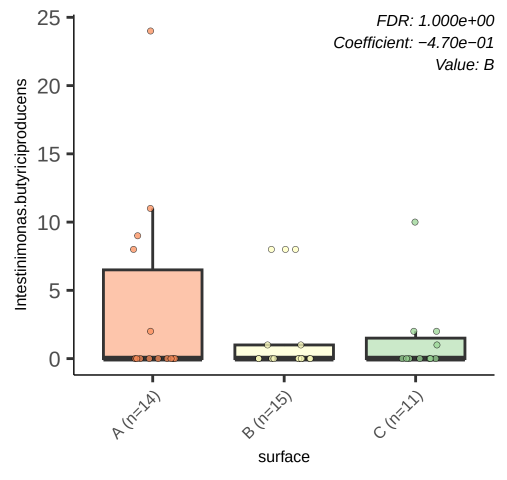
FDR: 1.000e+00
Coefficient: −4.70e−01
Value: B
Intestinimonas.butyriciproducens
25
20
15
10
5
0
A (n=14)
B (n=15)
C (n=11)
surface

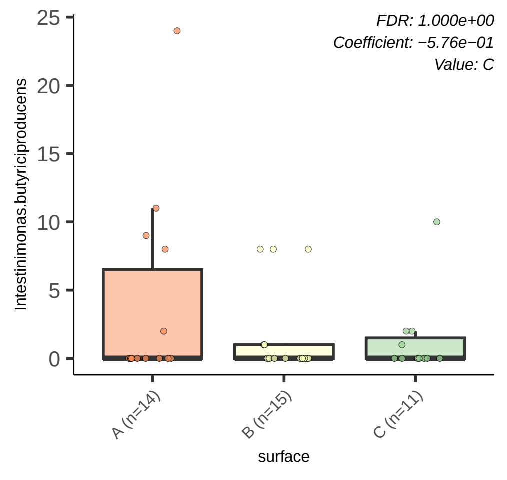
FDR: 1.000e+00
Coefficient: −5.76e−01
Value: C
Intestinimonas.butyriciproducens
25
20
15
10
5
0
A (n=14)
B (n=15)
C (n=11)
surface

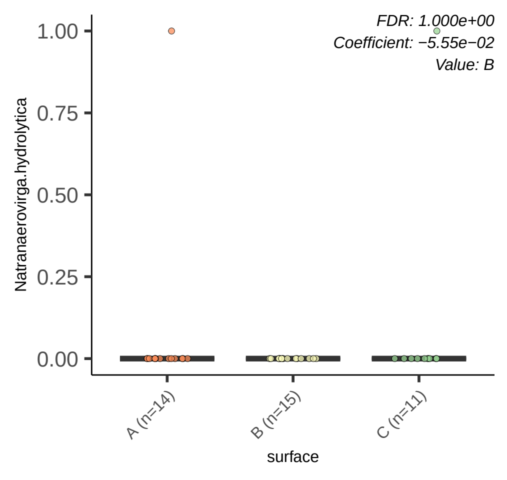

FDR: 1.000e+00
Coefficient: −5.55e−02
Value: B
Natranaerovirga.hydrolytica
1.00
0.75
0.50
0.25
0.00
A (n=14)
B (n=15)
C (n=11)
surface

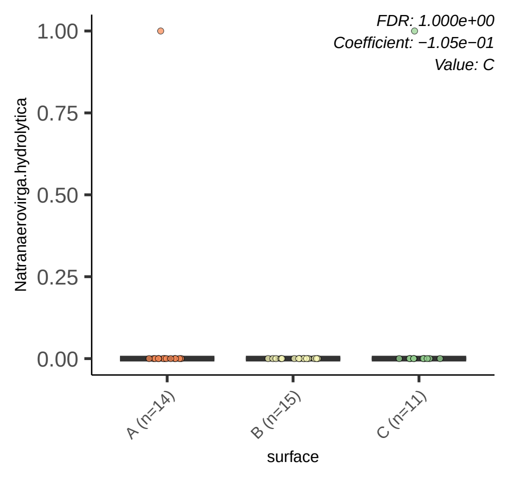
FDR: 1.000e+00
Coefficient: −1.05e−01
Value: C
Natranaerovirga.hydrolytica
1.00
0.75
0.50
0.25
0.00
A (n=14)
B (n=15)
C (n=11)
surface

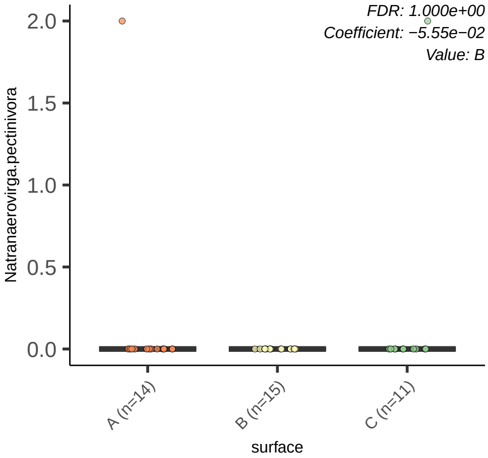

FDR: 1.000e+00
Coefficient: −5.55e−02
Value: B
Natranaerovirga.pectinivora
2.0
1.5
1.0
0.5
0.0
A (n=14)
B (n=15)
C (n=11)
surface

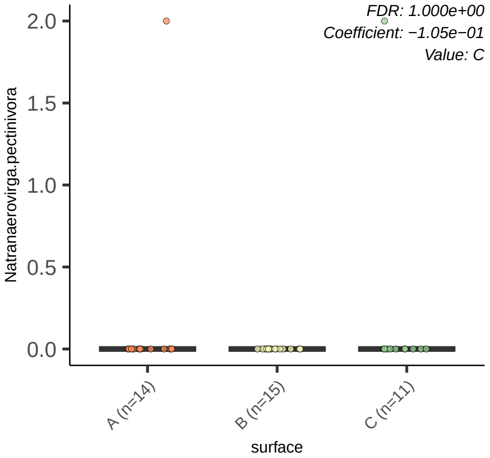

FDR: 1.000e+00
Coefficient: −1.05e−01
Value: C
Natranaerovirga.pectinivora
2.0
1.5
1.0
0.5
0.0
A (n=14)
B (n=15)
C (n=11)
surface

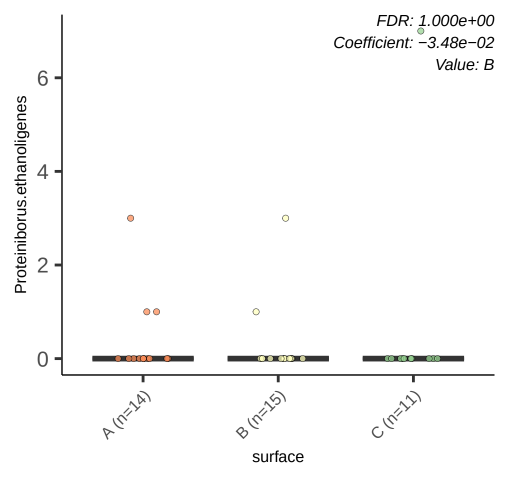
FDR: 1.000e+00
Coefficient: −3.48e−02
Value: B
Proteiniborus.ethanoligenes
0
2
4
6
A (n=14)
B (n=15)
C (n=11)
surface

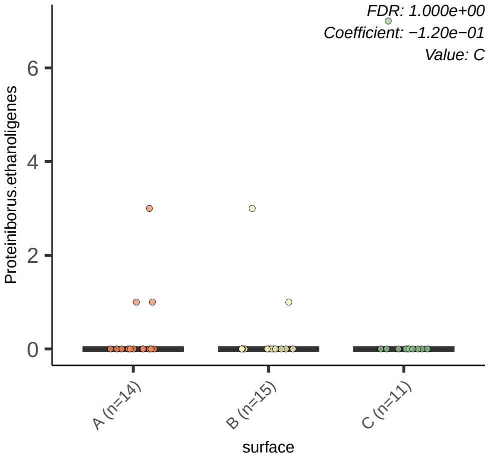
FDR: 1.000e+00
Coefficient: −1.20e−01
Value: C
Proteiniborus.ethanoligenes
0
2
4
6
A (n=14)
B (n=15)
C (n=11)
surface

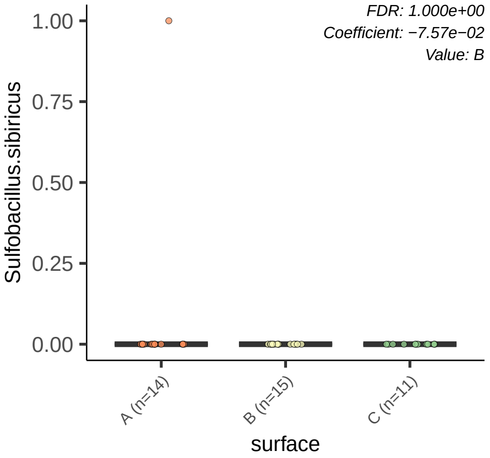

FDR: 1.000e+00
Coefficient: −7.57e−02
Value: B
Sulfobacillus.sibiricus
1.00
0.75
0.50
0.25
0.00
A (n=14)
B (n=15)
C (n=11)
surface

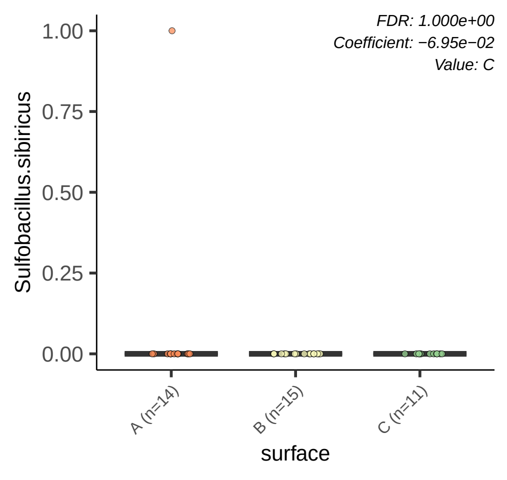
FDR: 1.000e+00
Coefficient: −6.95e−02
Value: C
Sulfobacillus.sibiricus
1.00
0.75
0.50
0.25
0.00
A (n=14)
B (n=15)
C (n=11)
surface

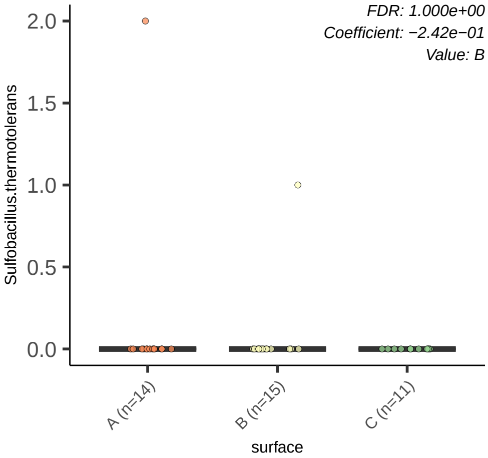
FDR: 1.000e+00
Coefficient: −2.42e−01
Value: B
Sulfobacillus.thermotolerans
2.0
1.5
1.0
0.5
0.0
A (n=14)
B (n=15)
C (n=11)
surface

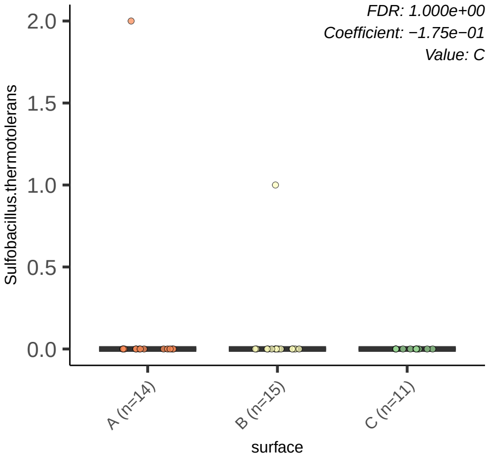

FDR: 1.000e+00
Coefficient: −1.75e−01
Value: C
Sulfobacillus.thermotolerans
2.0
1.5
1.0
0.5
0.0
A (n=14)
B (n=15)
C (n=11)
surface

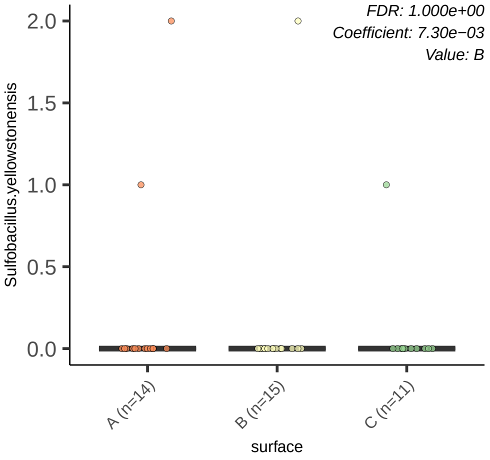
FDR: 1.000e+00
Coefficient: 7.30e−03
Value: B
Sulfobacillus.yellowstonensis
2.0
1.5
1.0
0.5
0.0
A (n=14)
B (n=15)
C (n=11)
surface

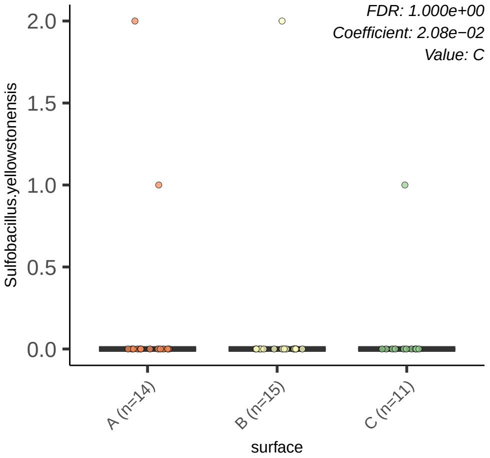
FDR: 1.000e+00
Coefficient: 2.08e−02
Value: C
Sulfobacillus.yellowstonensis
2.0
1.5
1.0
0.5
0.0
A (n=14)
B (n=15)
C (n=11)
surface

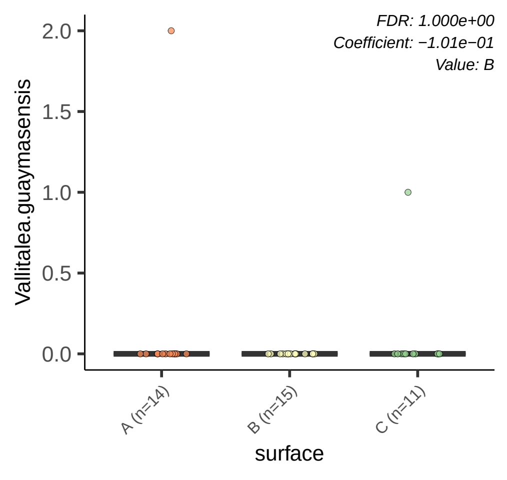

FDR: 1.000e+00
Coefficient: −1.01e−01
Value: B
Vallitalea.guaymasensis
2.0
1.5
1.0
0.5
0.0
A (n=14)
B (n=15)
C (n=11)
surface

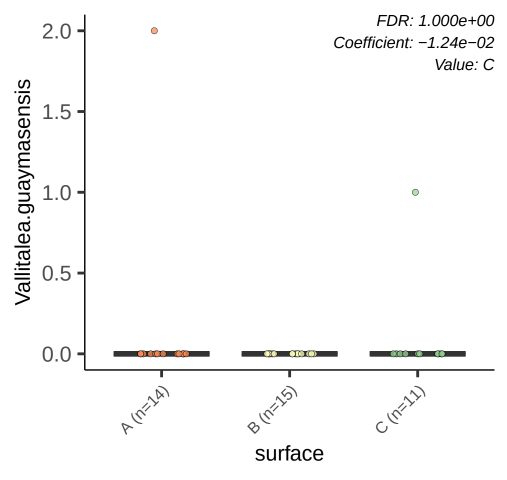

FDR: 1.000e+00
Coefficient: −1.24e−02
Value: C
Vallitalea.guaymasensis
2.0
1.5
1.0
0.5
0.0
A (n=14)
B (n=15)
C (n=11)
surface

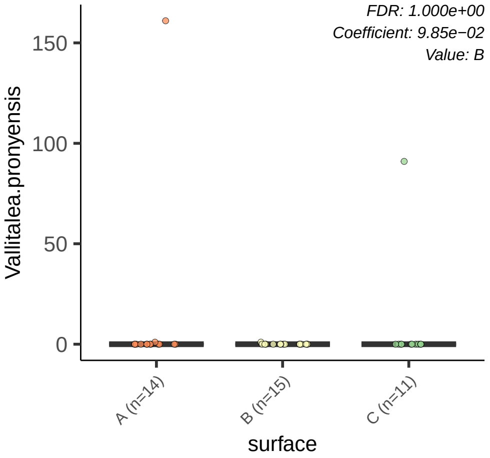
FDR: 1.000e+00
Coefficient: 9.85e−02
Value: B
Vallitalea.pronyensis
150
100
50
0
A (n=14)
B (n=15)
C (n=11)
surface

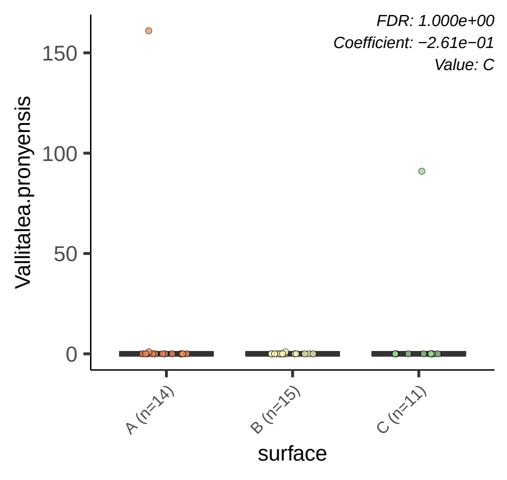

FDR: 1.000e+00
Coefficient: −2.61e−01
Value: C
Vallitalea.pronyensis
150
100
50
0
A (n=14)
B (n=15)
C (n=11)
surface

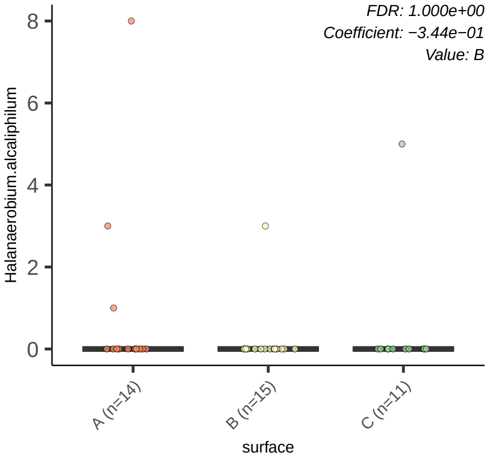
FDR: 1.000e+00
Coefficient: −3.44e−01
Value: B
Halanaerobium.alcaliphilum
8
6
4
2
0
A (n=14)
B (n=15)
C (n=11)
surface

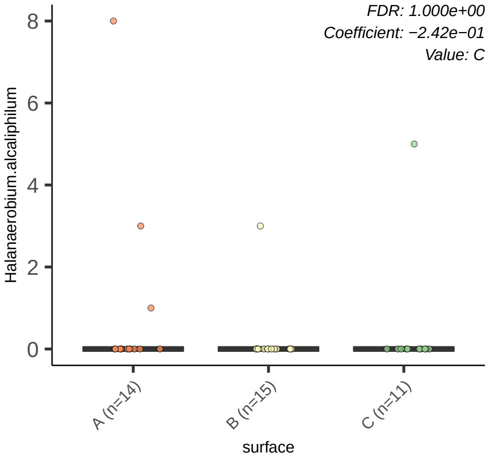

FDR: 1.000e+00
Coefficient: −2.42e−01
Value: C
Halanaerobium.alcaliphilum
8
6
4
2
0
A (n=14)
B (n=15)
C (n=11)
surface

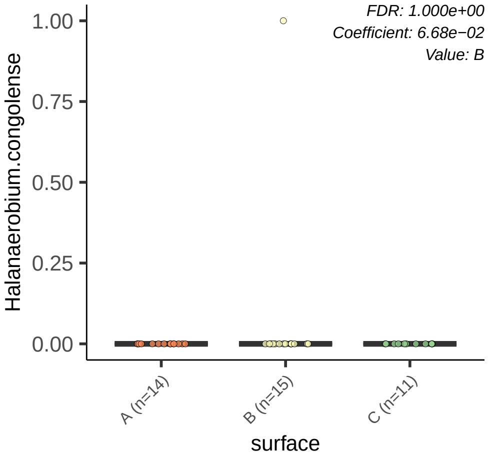

FDR: 1.000e+00
Coefficient: 6.68e−02
Value: B
Halanaerobium.congolense
1.00
0.75
0.50
0.25
0.00
A (n=14)
B (n=15)
C (n=11)
surface

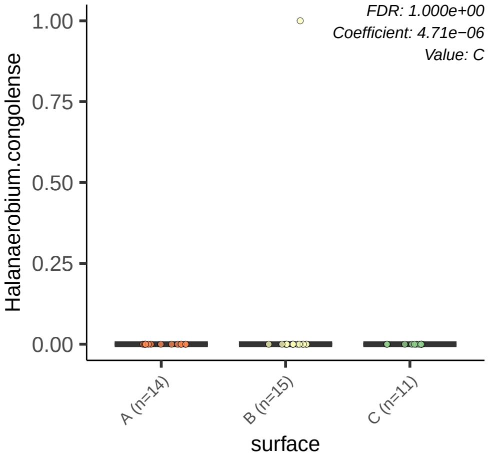
FDR: 1.000e+00
Coefficient: 4.71e−06
Value: C
Halanaerobium.congolense
1.00
0.75
0.50
0.25
0.00
A (n=14)
B (n=15)
C (n=11)
surface

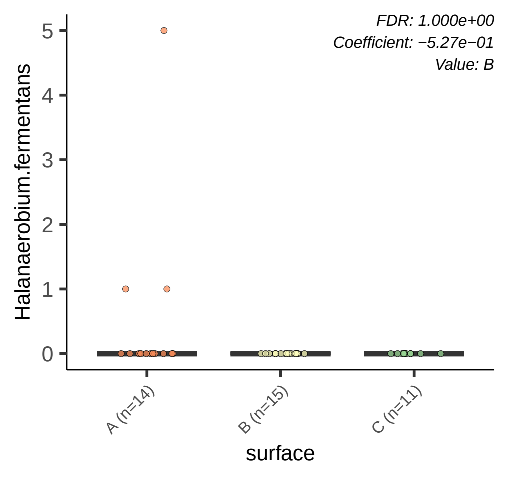
FDR: 1.000e+00
Coefficient: −5.27e−01
Value: B
Halanaerobium.fermentans
0
1
2
3
4
5
A (n=14)
B (n=15)
C (n=11)
surface

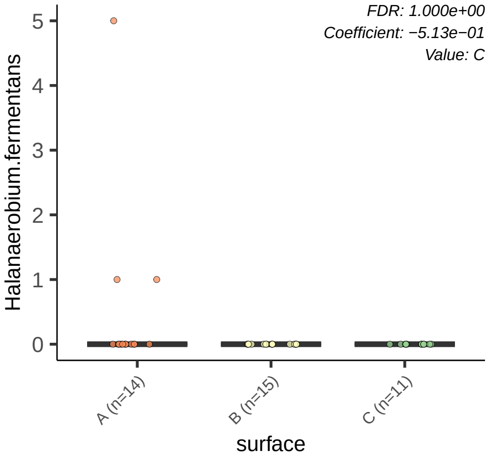
FDR: 1.000e+00
Coefficient: −5.13e−01
Value: C
Halanaerobium.fermentans
0
1
2
3
4
5
A (n=14)
B (n=15)
C (n=11)
surface

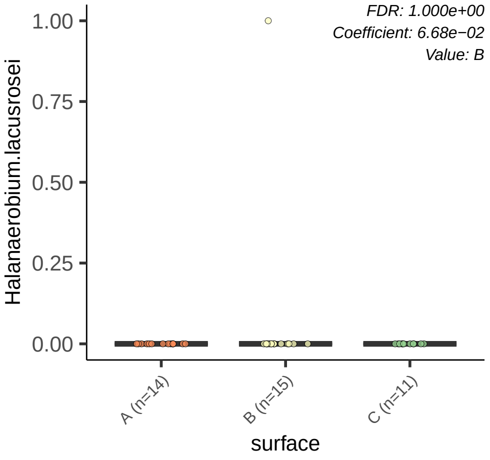
FDR: 1.000e+00
Coefficient: 6.68e−02
Value: B
Halanaerobium.lacusrosei
1.00
0.75
0.50
0.25
0.00
A (n=14)
B (n=15)
C (n=11)
surface

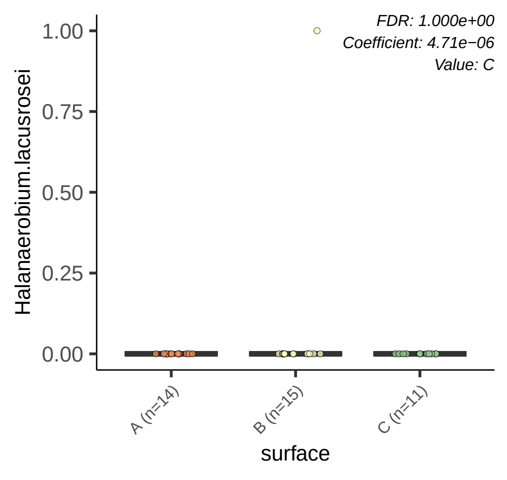
FDR: 1.000e+00
Coefficient: 4.71e−06
Value: C
Halanaerobium.lacusrosei
1.00
0.75
0.50
0.25
0.00
A (n=14)
B (n=15)
C (n=11)
surface

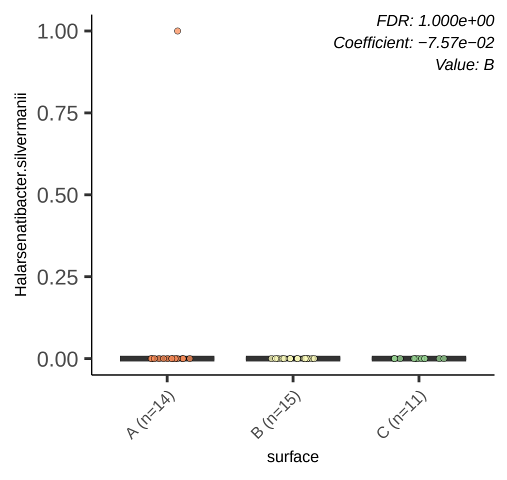
FDR: 1.000e+00
Coefficient: −7.57e−02
Value: B
Halarsenatibacter.silvermanii
1.00
0.75
0.50
0.25
0.00
A (n=14)
B (n=15)
C (n=11)
surface

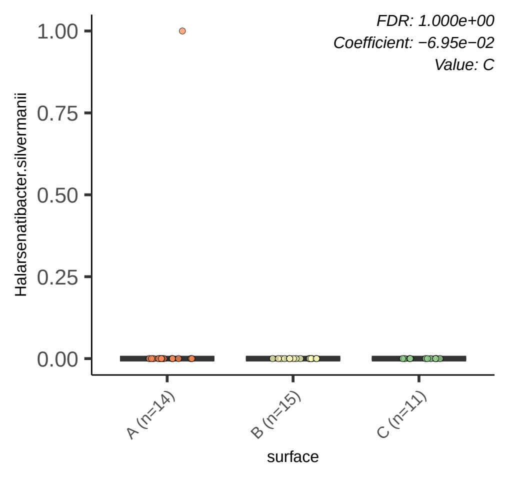
FDR: 1.000e+00
Coefficient: −6.95e−02
Value: C
Halarsenatibacter.silvermanii
1.00
0.75
0.50
0.25
0.00
A (n=14)
B (n=15)
C (n=11)
surface

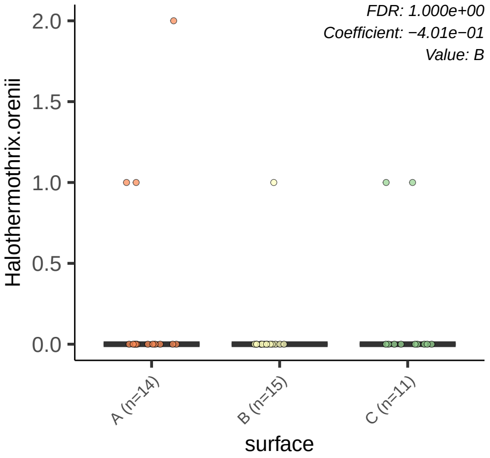

FDR: 1.000e+00
Coefficient: −4.01e−01
Value: B
Halothermothrix.orenii
2.0
1.5
1.0
0.5
0.0
A (n=14)
B (n=15)
C (n=11)
surface

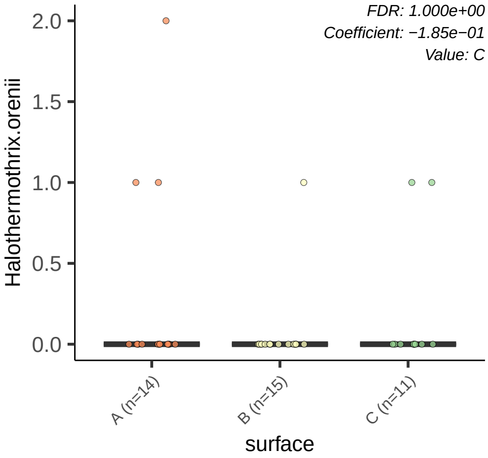

FDR: 1.000e+00
Coefficient: −1.85e−01
Value: C
Halothermothrix.orenii
2.0
1.5
1.0
0.5
0.0
A (n=14)
B (n=15)
C (n=11)
surface

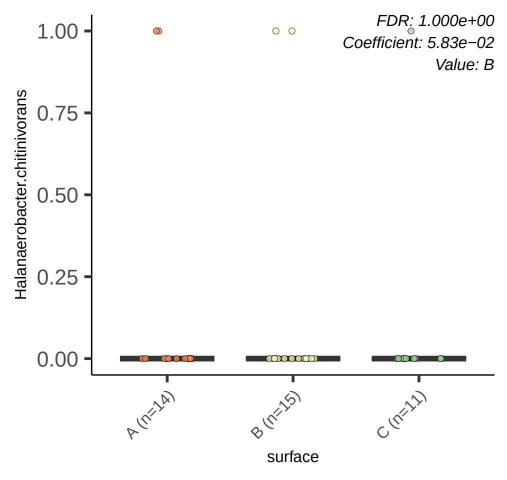

FDR: 1.000e+00
Coefficient: 5.83e−02
Value: B
Halanaerobacter.chitinivorans
1.00
0.75
0.50
0.25
0.00
A (n=14)
B (n=15)
C (n=11)
surface

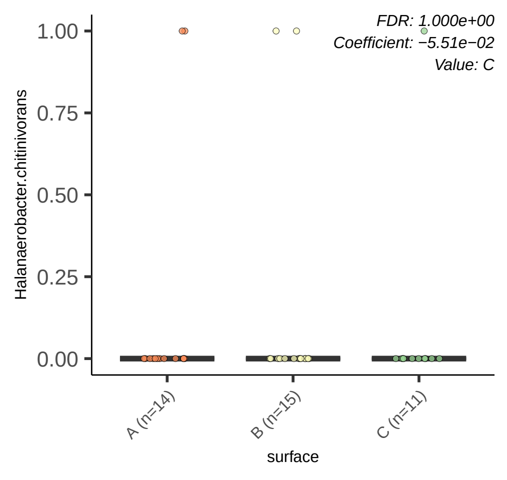
FDR: 1.000e+00
Coefficient: −5.51e−02
Value: C
Halanaerobacter.chitinivorans
1.00
0.75
0.50
0.25
0.00
A (n=14)
B (n=15)
C (n=11)
surface

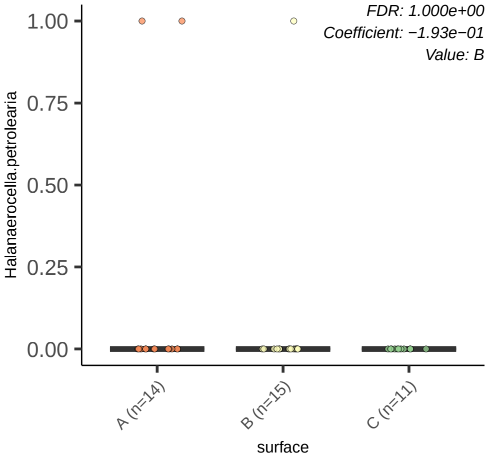

FDR: 1.000e+00
Coefficient: −1.93e−01
Value: B
1.00
0.75
0.50
0.25
0.00
Halanaerocella.petrolearia
A (n=14)
B (n=15)
C (n=11)
surface

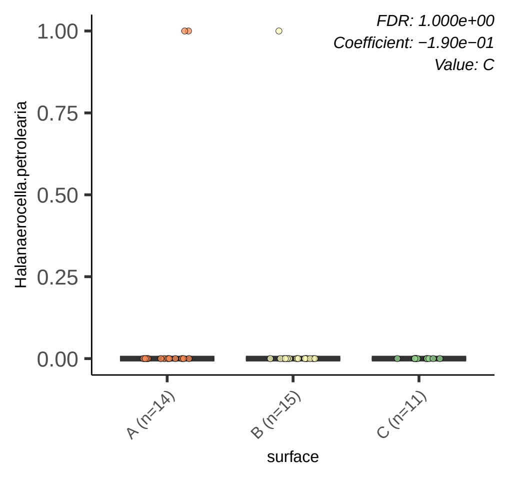
FDR: 1.000e+00
Coefficient: −1.90e−01
Value: C
1.00
0.75
0.50
0.25
0.00
Halanaerocella.petrolearia
A (n=14)
B (n=15)
C (n=11)
surface

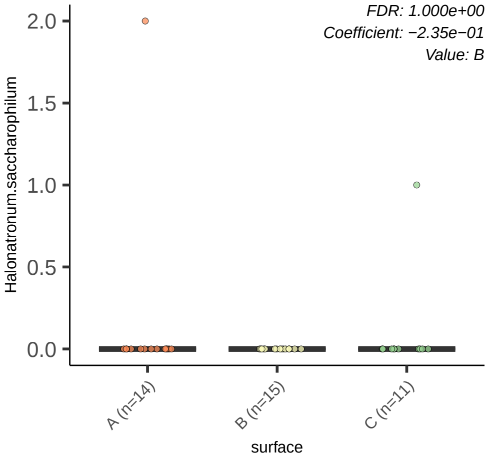
FDR: 1.000e+00
Coefficient: −2.35e−01
Value: B
Halonatronum.saccharophilum
2.0
1.5
1.0
0.5
0.0
A (n=14)
B (n=15)
C (n=11)
surface

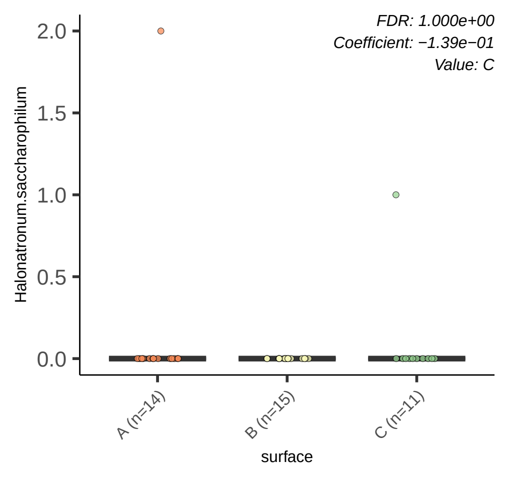

FDR: 1.000e+00
Coefficient: −1.39e−01
Value: C
Halonatronum.saccharophilum
2.0
1.5
1.0
0.5
0.0
A (n=14)
B (n=15)
C (n=11)
surface

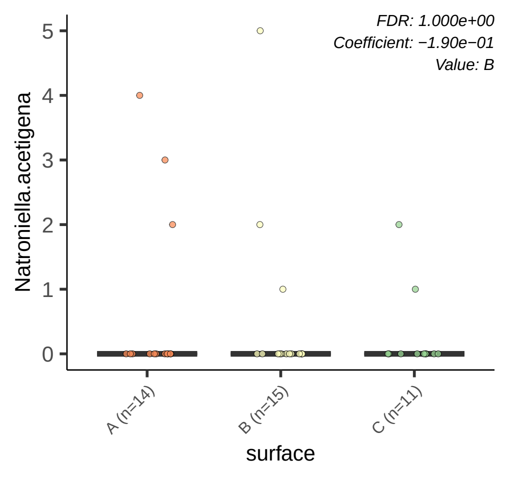
FDR: 1.000e+00
Coefficient: −1.90e−01
Value: B
Natroniella.acetigena
0
1
2
3
4
5
A (n=14)
B (n=15)
C (n=11)
surface

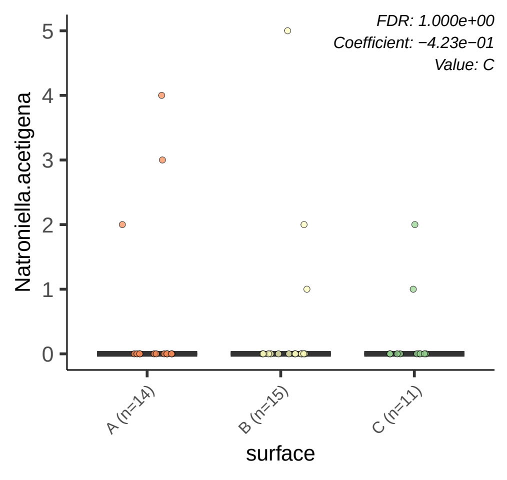

FDR: 1.000e+00
Coefficient: −4.23e−01
Value: C
Natroniella.acetigena
0
1
2
3
4
5
A (n=14)
B (n=15)
C (n=11)
surface

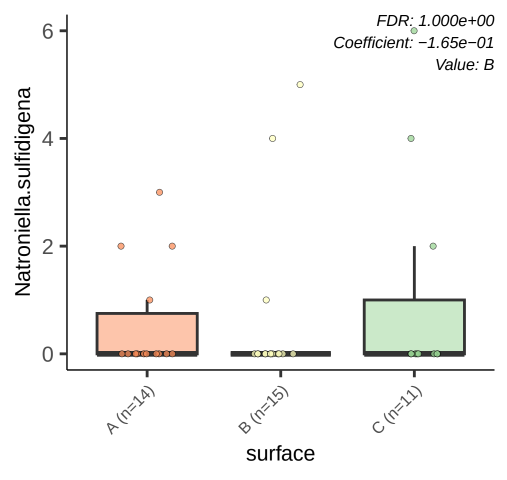

FDR: 1.000e+00
Coefficient: −1.65e−01
Value: B
Natroniella.sulfidigena
0
2
4
6
A (n=14)
B (n=15)
C (n=11)
surface

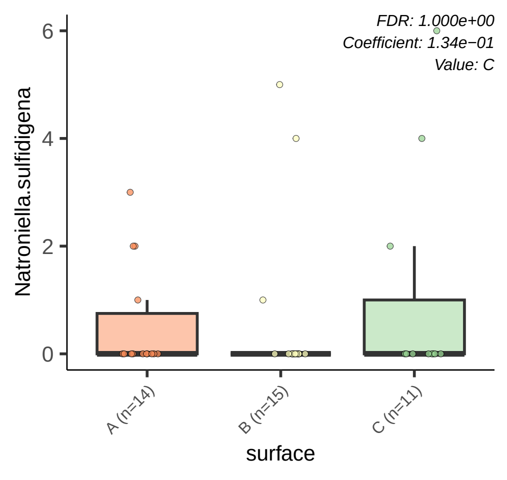
FDR: 1.000e+00
Coefficient: 1.34e−01
Value: C
Natroniella.sulfidigena
0
2
4
6
A (n=14)
B (n=15)
C (n=11)
surface

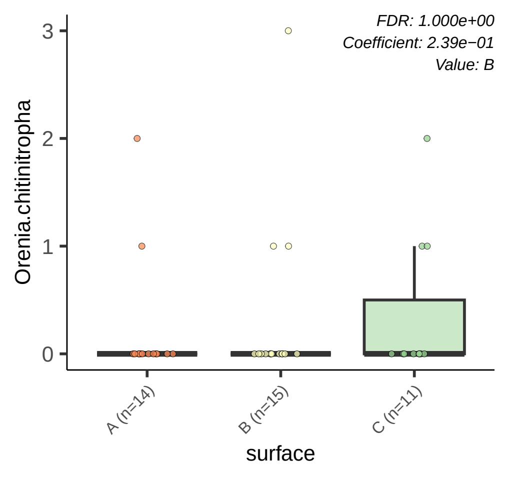
FDR: 1.000e+00
Coefficient: 2.39e−01
Value: B
Orenia.chitinitropha
0
1
2
3
A (n=14)
B (n=15)
C (n=11)
surface

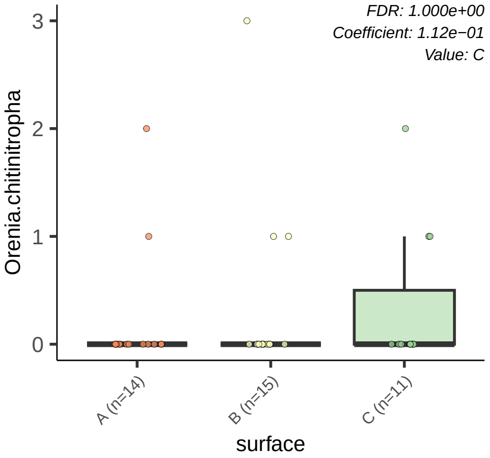
FDR: 1.000e+00
Coefficient: 1.12e−01
Value: C
Orenia.chitinitropha
3
2
1
0
A (n=14)
B (n=15)
C (n=11)
surface

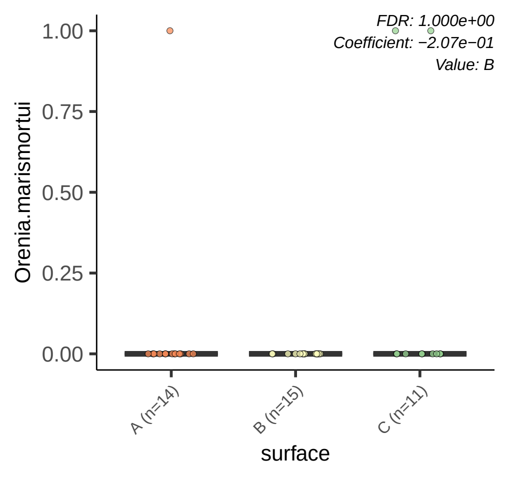

FDR: 1.000e+00
Coefficient: −2.07e−01
Value: B
Orenia.marismortui
1.00
0.75
0.50
0.25
0.00
A (n=14)
B (n=15)
C (n=11)
surface

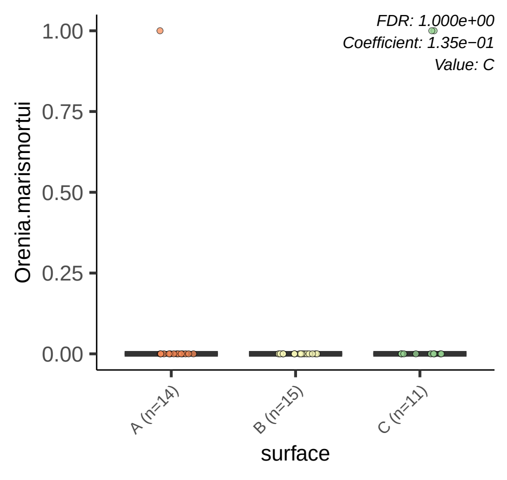

FDR: 1.000e+00
Coefficient: 1.35e−01
Value: C
Orenia.marismortui
1.00
0.75
0.50
0.25
0.00
A (n=14)
B (n=15)
C (n=11)
surface

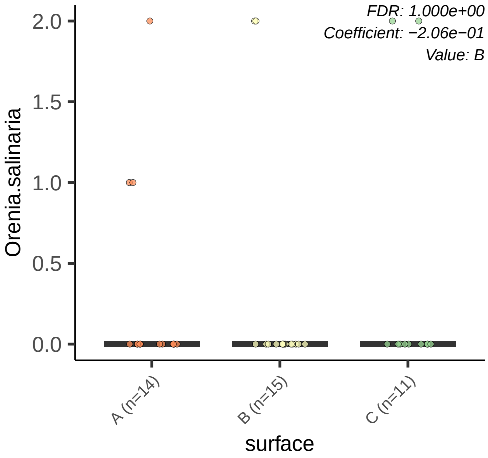
FDR: 1.000e+00
Coefficient: −2.06e−01
Value: B
Orenia.salinaria
2.0
1.5
1.0
0.5
0.0
A (n=14)
B (n=15)
C (n=11)
surface

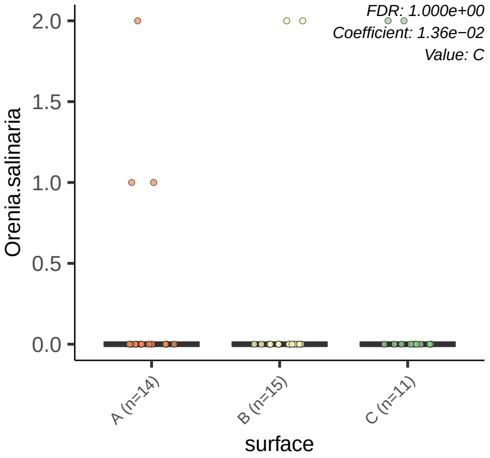

FDR: 1.000e+00
Coefficient: 1.36e−02
Value: C
2.0
1.5
1.0
0.5
0.0
Orenia.salinaria
A (n=14)
B (n=15)
C (n=11)
surface

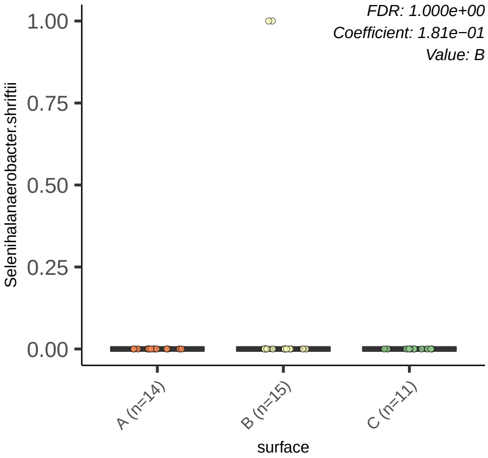
FDR: 1.000e+00
Coefficient: 1.81e−01
Value: B
Selenihalanaerobacter.shriftii
1.00
0.75
0.50
0.25
0.00
A (n=14)
B (n=15)
C (n=11)
surface

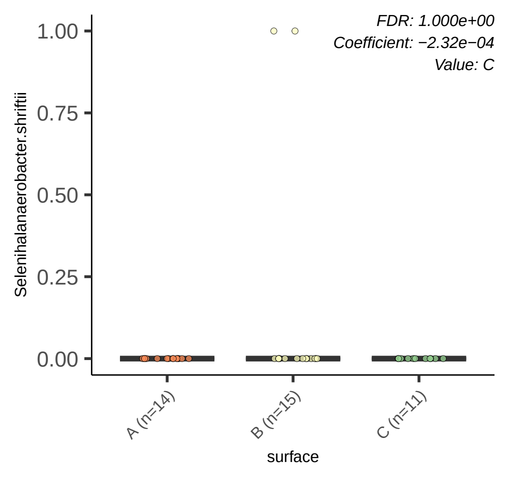

FDR: 1.000e+00
Coefficient: −2.32e−04
Value: C
Selenihalanaerobacter.shriftii
1.00
0.75
0.50
0.25
0.00
A (n=14)
B (n=15)
C (n=11)
surface

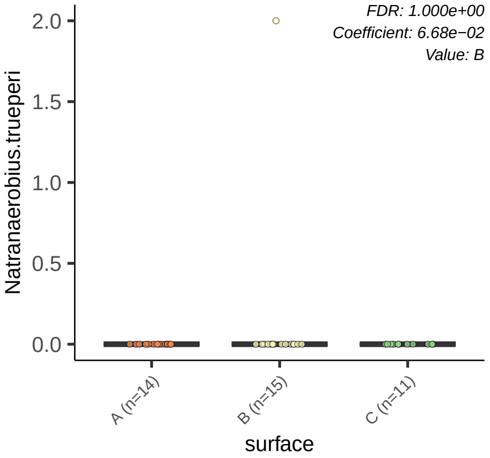
FDR: 1.000e+00
Coefficient: 6.68e−02
Value: B
Natranaerobius.trueperi
2.0
1.5
1.0
0.5
0.0
A (n=14)
B (n=15)
C (n=11)
surface

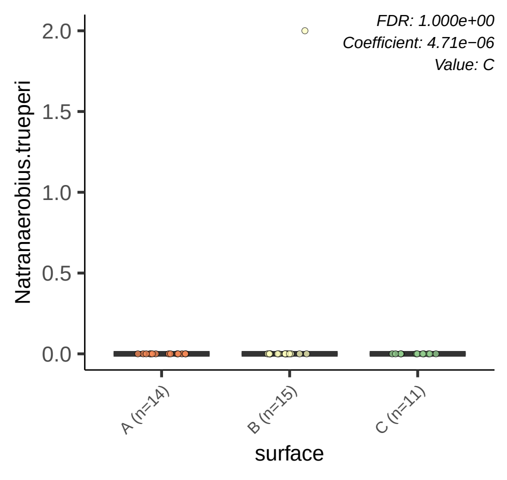
FDR: 1.000e+00
Coefficient: 4.71e−06
Value: C
Natranaerobius.trueperi
2.0
1.5
1.0
0.5
0.0
A (n=14)
B (n=15)
C (n=11)
surface

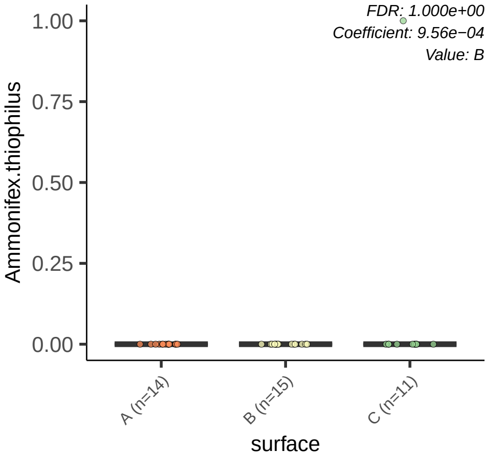
FDR: 1.000e+00
Coefficient: 9.56e−04
Value: B
Ammonifex.thiophilus
1.00
0.75
0.50
0.25
0.00
A (n=14)
B (n=15)
C (n=11)
surface

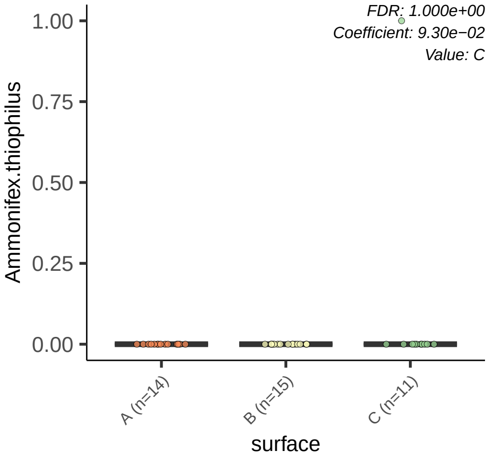

FDR: 1.000e+00
Coefficient: 9.30e−02
Value: C
Ammonifex.thiophilus
1.00
0.75
0.50
0.25
0.00
A (n=14)
B (n=15)
C (n=11)
surface

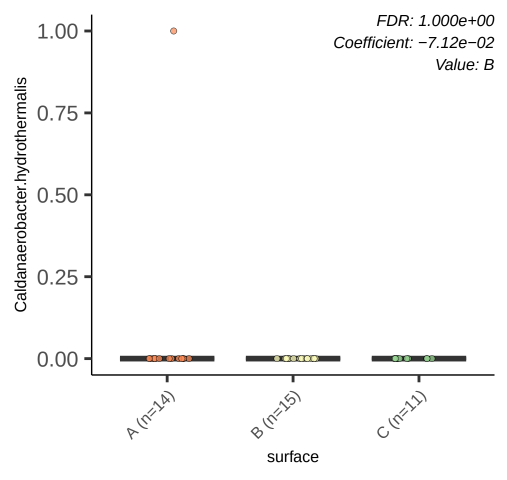
FDR: 1.000e+00
Coefficient: −7.12e−02
Value: B
1.00
0.75
0.50
0.25
0.00
Caldanaerobacter.hydrothermalis
A (n=14)
B (n=15)
C (n=11)
surface

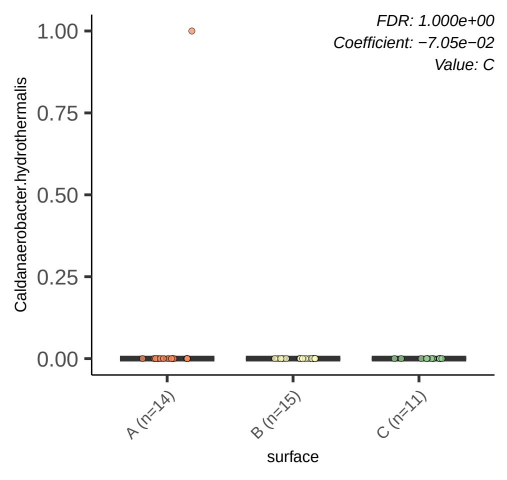
FDR: 1.000e+00
Coefficient: −7.05e−02
Value: C
1.00
0.75
0.50
0.25
0.00
Caldanaerobacter.hydrothermalis
A (n=14)
B (n=15)
C (n=11)
surface

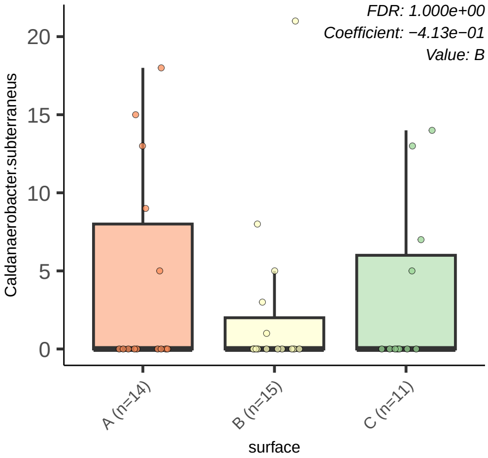

FDR: 1.000e+00
Coefficient: −4.13e−01
Value: B
Caldanaerobacter.subterraneus
0
5
10
15
20
A (n=14)
B (n=15)
C (n=11)
surface

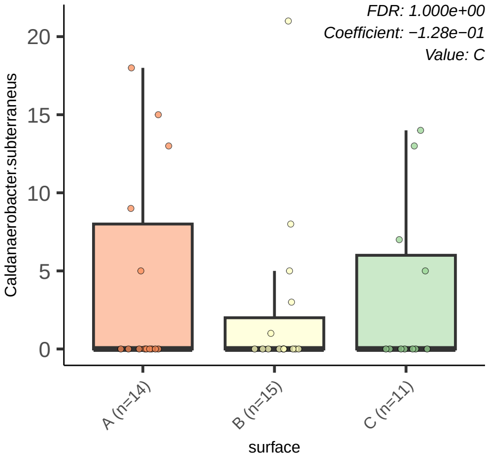
FDR: 1.000e+00
Coefficient: −1.28e−01
Value: C
Caldanaerobacter.subterraneus
0
5
10
15
20
A (n=14)
B (n=15)
C (n=11)
surface

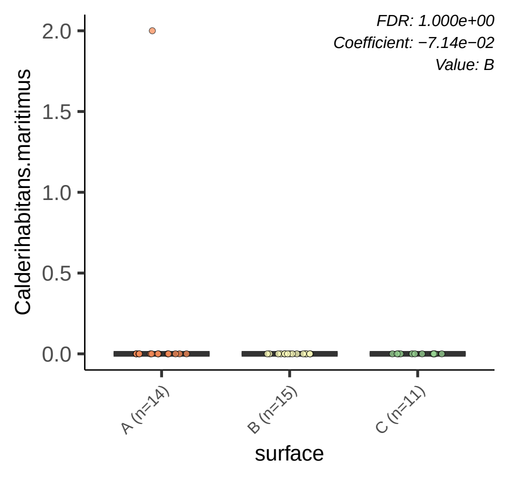
FDR: 1.000e+00
Coefficient: −7.14e−02
Value: B
Calderihabitans.maritimus
2.0
1.5
1.0
0.5
0.0
A (n=14)
B (n=15)
C (n=11)
surface

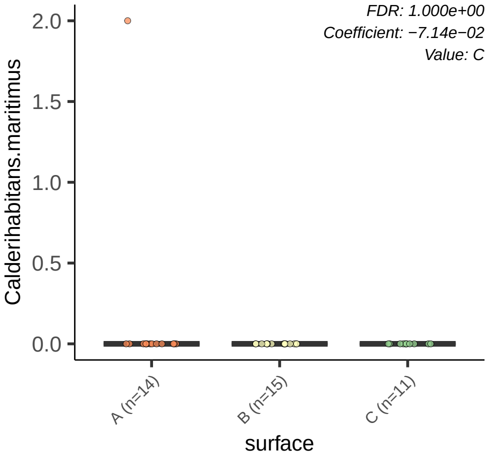

FDR: 1.000e+00
Coefficient: −7.14e−02
Value: C
Calderihabitans.maritimus
2.0
1.5
1.0
0.5
0.0
A (n=14)
B (n=15)
C (n=11)
surface

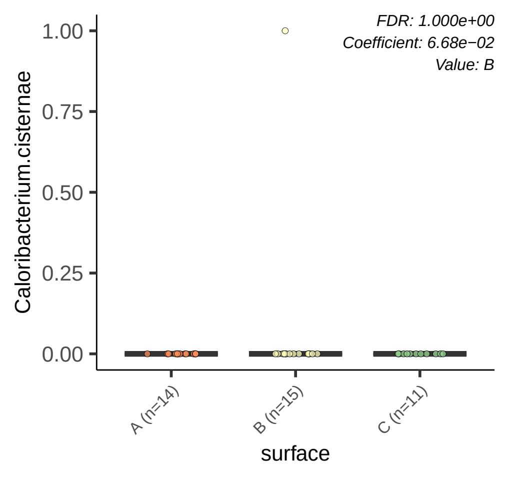
FDR: 1.000e+00
Coefficient: 6.68e−02
Value: B
Caloribacterium.cisternae
1.00
0.75
0.50
0.25
0.00
A (n=14)
B (n=15)
C (n=11)
surface

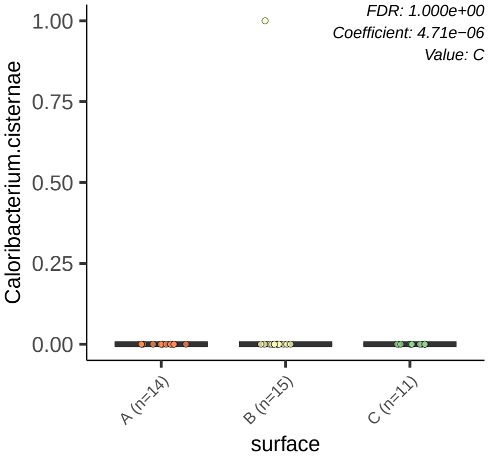

FDR: 1.000e+00
Coefficient: 4.71e−06
Value: C
Caloribacterium.cisternae
1.00
0.75
0.50
0.25
0.00
A (n=14)
B (n=15)
C (n=11)
surface

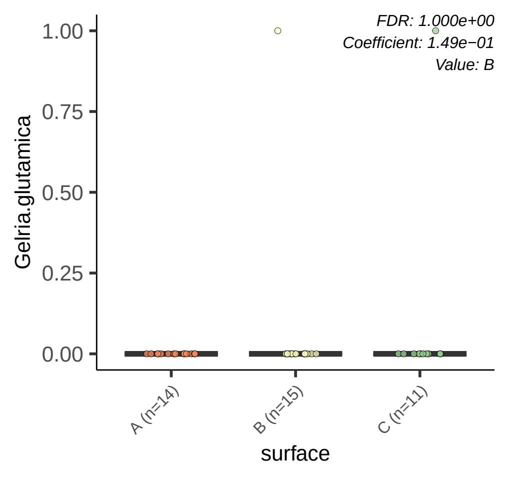
FDR: 1.000e+00
Coefficient: 1.49e−01
Value: B
Gelria.glutamica
1.00
0.75
0.50
0.25
0.00
A (n=14)
B (n=15)
C (n=11)
surface

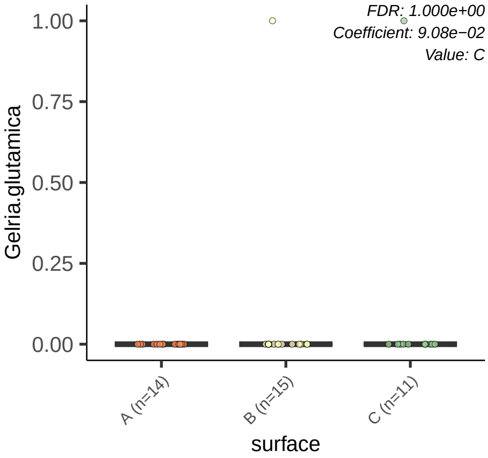

FDR: 1.000e+00
Coefficient: 9.08e−02
Value: C
Gelria.glutamica
1.00
0.75
0.50
0.25
0.00
A (n=14)
B (n=15)
C (n=11)
surface

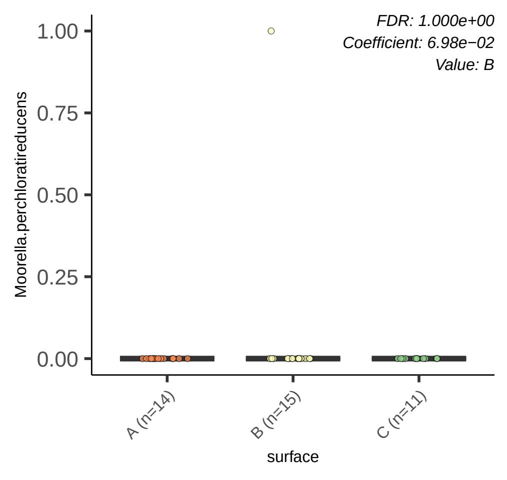
FDR: 1.000e+00
Coefficient: 6.98e−02
Value: B
Moorella.perchloratireducens
1.00
0.75
0.50
0.25
0.00
A (n=14)
B (n=15)
C (n=11)
surface

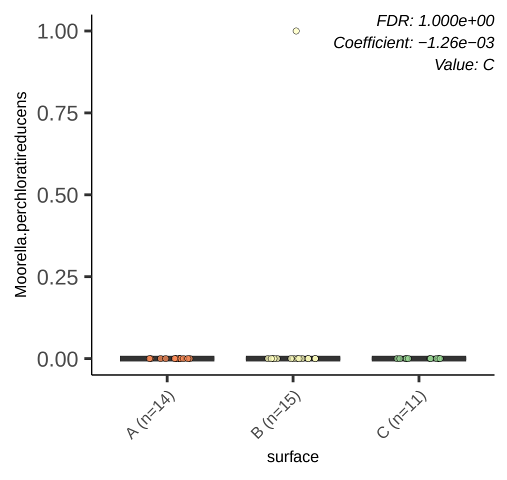

FDR: 1.000e+00
Coefficient: −1.26e−03
Value: C
Moorella.perchloratireducens
1.00
0.75
0.50
0.25
0.00
A (n=14)
B (n=15)
C (n=11)
surface

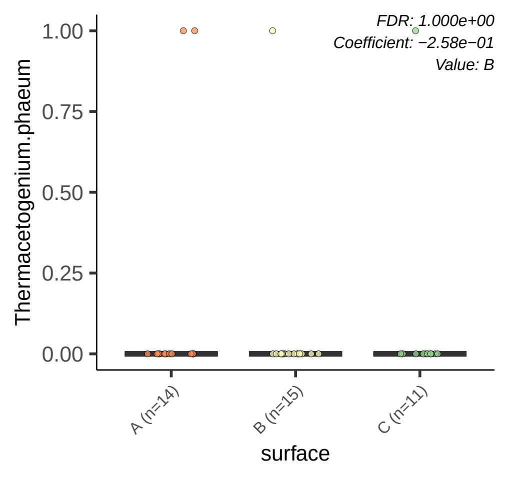

FDR: 1.000e+00
Coefficient: −2.58e−01
Value: B
Thermacetogenium.phaeum
1.00
0.75
0.50
0.25
0.00
A (n=14)
B (n=15)
C (n=11)
surface

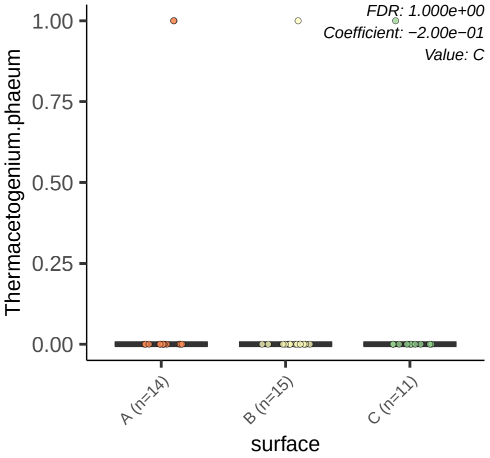

FDR: 1.000e+00
Coefficient: −2.00e−01
Value: C
Thermacetogenium.phaeum
1.00
0.75
0.50
0.25
0.00
A (n=14)
B (n=15)
C (n=11)
surface

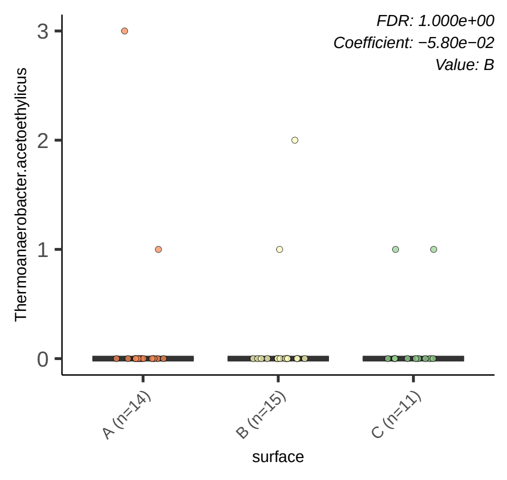

FDR: 1.000e+00
Coefficient: −5.80e−02
Value: B
Thermoanaerobacter.acetoethylicus
0
1
2
3
A (n=14)
B (n=15)
C (n=11)
surface

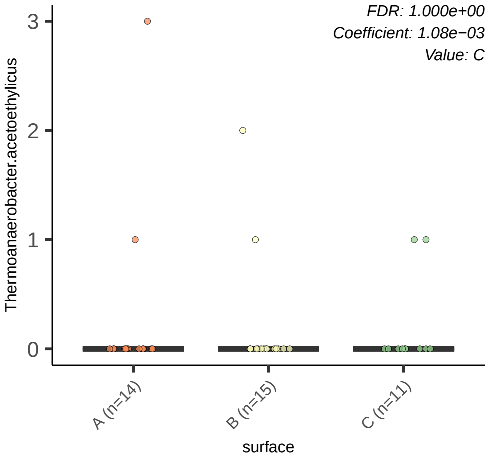
FDR: 1.000e+00
Coefficient: 1.08e−03
Value: C
Thermoanaerobacter.acetoethylicus
3
2
1
0
A (n=14)
B (n=15)
C (n=11)
surface

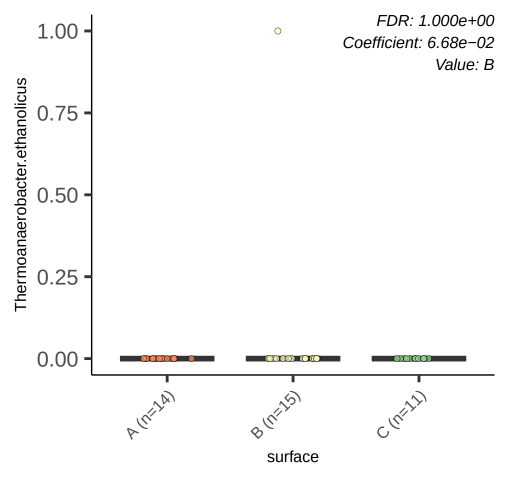
FDR: 1.000e+00
Coefficient: 6.68e−02
Value: B
Thermoanaerobacter.ethanolicus
1.00
0.75
0.50
0.25
0.00
A (n=14)
B (n=15)
C (n=11)
surface

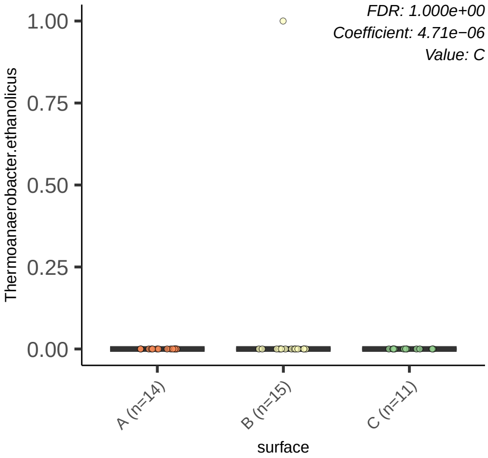

FDR: 1.000e+00
Coefficient: 4.71e−06
Value: C
Thermoanaerobacter.ethanolicus
1.00
0.75
0.50
0.25
0.00
A (n=14)
B (n=15)
C (n=11)
surface

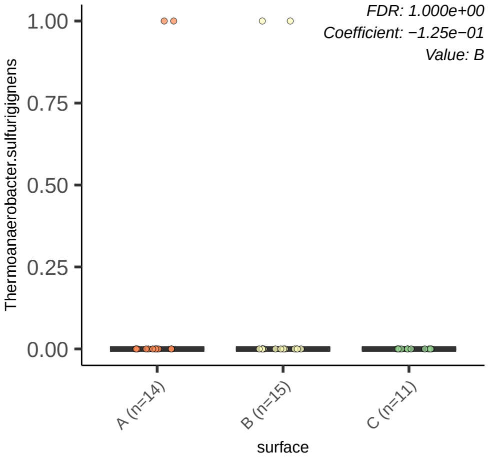
FDR: 1.000e+00
Coefficient: −1.25e−01
Value: B
Thermoanaerobacter.sulfurigignens
1.00
0.75
0.50
0.25
0.00
A (n=14)
B (n=15)
C (n=11)
surface

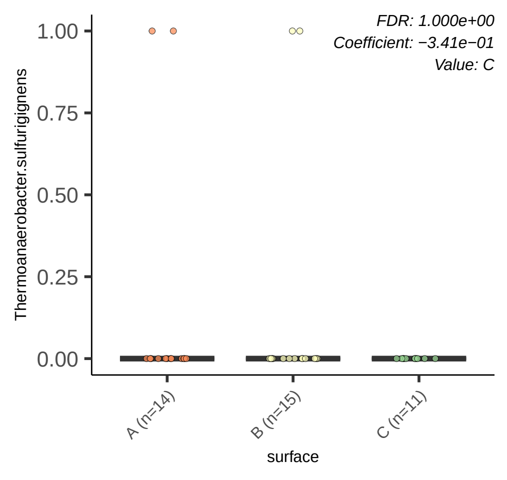
FDR: 1.000e+00
Coefficient: −3.41e−01
Value: C
Thermoanaerobacter.sulfurigignens
1.00
0.75
0.50
0.25
0.00
A (n=14)
B (n=15)
C (n=11)
surface

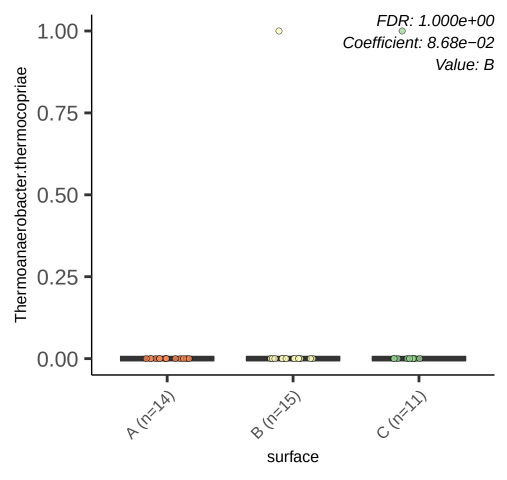

FDR: 1.000e+00
Coefficient: 8.68e−02
Value: B
Thermoanaerobacter.thermocopriae
1.00
0.75
0.50
0.25
0.00
A (n=14)
B (n=15)
C (n=11)
surface

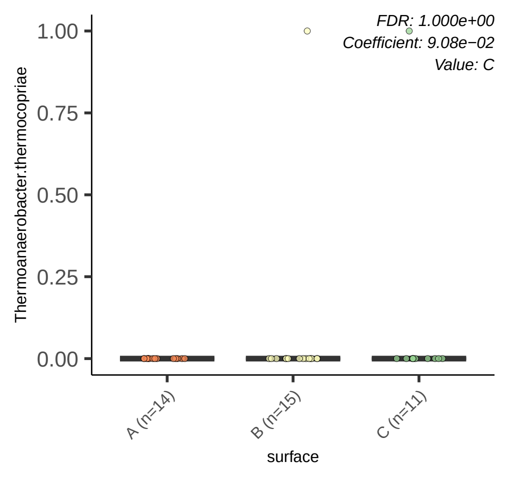

FDR: 1.000e+00
Coefficient: 9.08e−02
Value: C
Thermoanaerobacter.thermocopriae
1.00
0.75
0.50
0.25
0.00
A (n=14)
B (n=15)
C (n=11)
surface

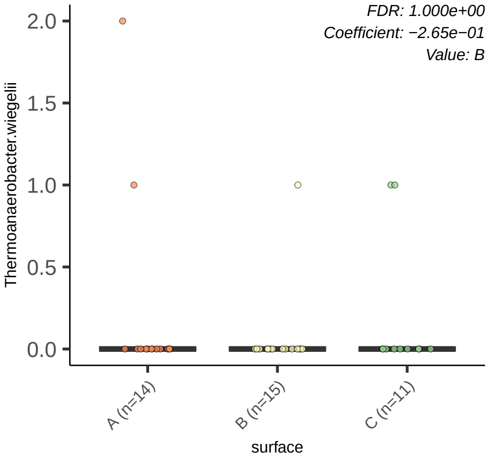
FDR: 1.000e+00
Coefficient: −2.65e−01
Value: B
Thermoanaerobacter.wiegelii
2.0
1.5
1.0
0.5
0.0
A (n=14)
B (n=15)
C (n=11)
surface

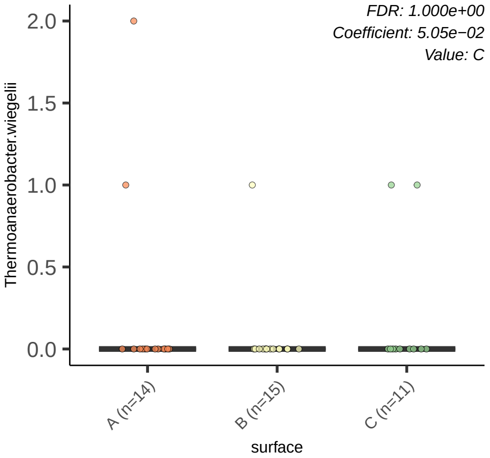
FDR: 1.000e+00
Coefficient: 5.05e−02
Value: C
Thermoanaerobacter.wiegelii
2.0
1.5
1.0
0.5
0.0
A (n=14)
B (n=15)
C (n=11)
surface

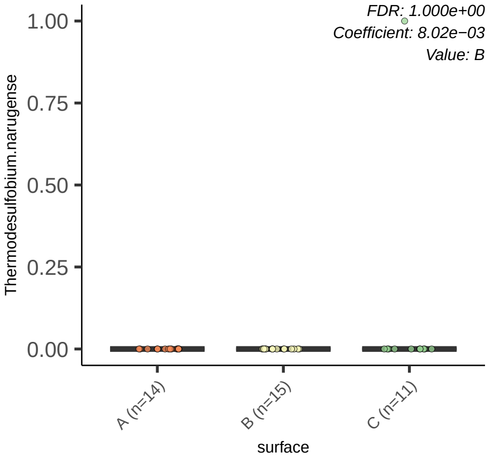
FDR: 1.000e+00
Coefficient: 8.02e−03
Value: B
1.00
0.75
0.50
0.25
0.00
Thermodesulfobium.narugense
A (n=14)
B (n=15)
C (n=11)
surface

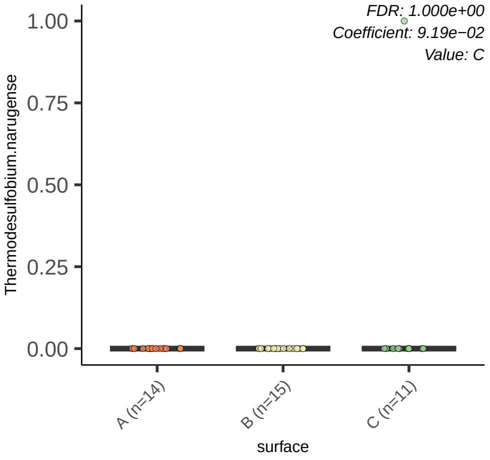
FDR: 1.000e+00
Coefficient: 9.19e−02
Value: C
1.00
0.75
0.50
0.25
0.00
Thermodesulfobium.narugense
A (n=14)
B (n=15)
C (n=11)
surface

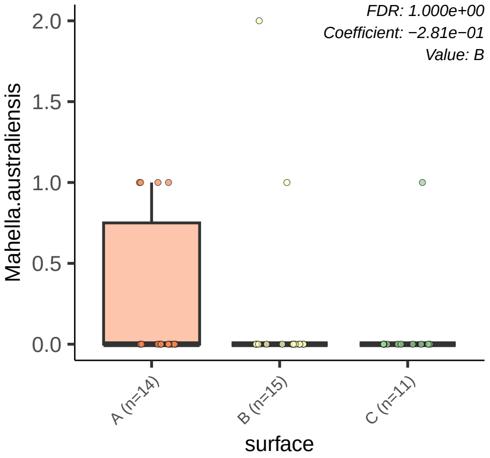

FDR: 1.000e+00
Coefficient: −2.81e−01
Value: B
Mahella.australiensis
2.0
1.5
1.0
0.5
0.0
A (n=14)
B (n=15)
C (n=11)
surface

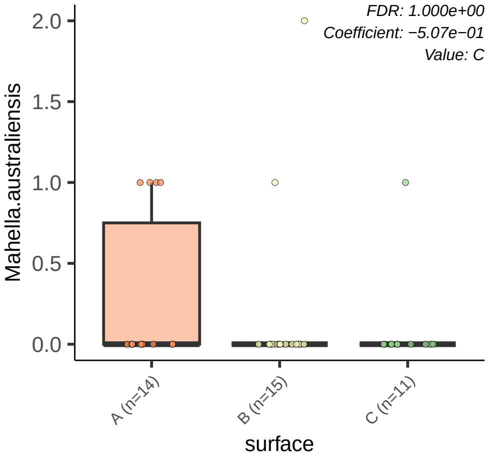

FDR: 1.000e+00
Coefficient: −5.07e−01
Value: C
Mahella.australiensis
2.0
1.5
1.0
0.5
0.0
A (n=14)
B (n=15)
C (n=11)
surface

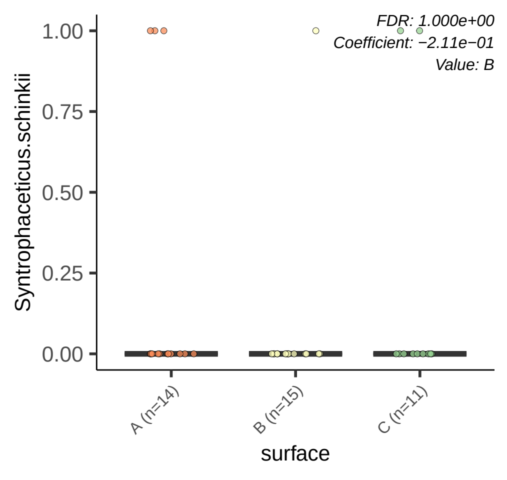
FDR: 1.000e+00
Coefficient: −2.11e−01
Value: B
Syntrophaceticus.schinkii
1.00
0.75
0.50
0.25
0.00
A (n=14)
B (n=15)
C (n=11)
surface

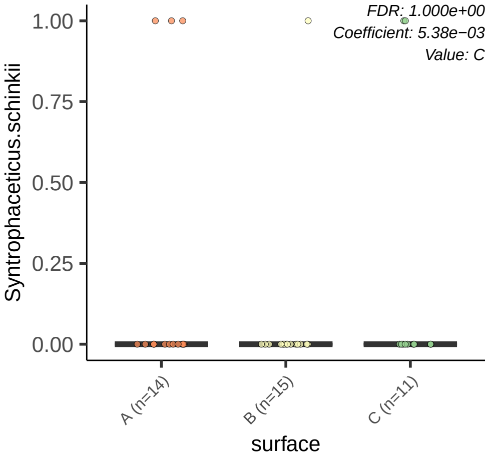
FDR: 1.000e+00
Coefficient: 5.38e−03
Value: C
Syntrophaceticus.schinkii
1.00
0.75
0.50
0.25
0.00
A (n=14)
B (n=15)
C (n=11)
surface

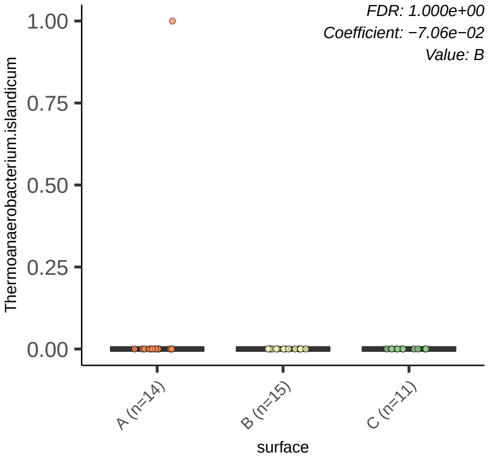
FDR: 1.000e+00
Coefficient: −7.06e−02
Value: B
Thermoanaerobacterium.islandicum
1.00
0.75
0.50
0.25
0.00
A (n=14)
B (n=15)
C (n=11)
surface

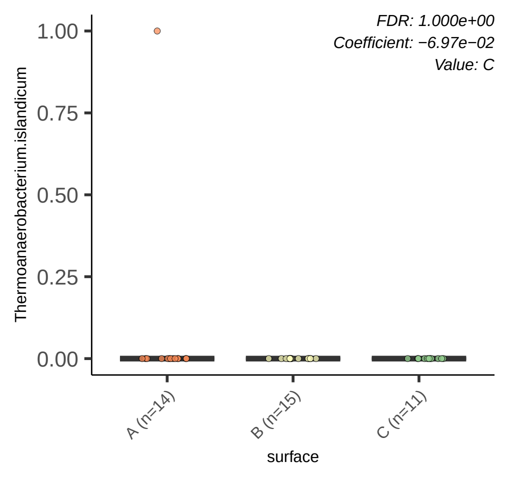
FDR: 1.000e+00
Coefficient: −6.97e−02
Value: C
Thermoanaerobacterium.islandicum
1.00
0.75
0.50
0.25
0.00
A (n=14)
B (n=15)
C (n=11)
surface

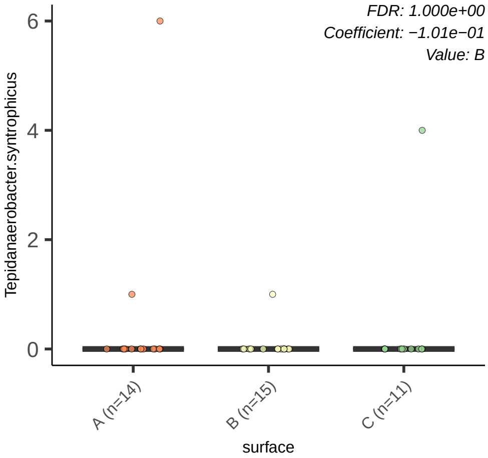
FDR: 1.000e+00
Coefficient: −1.01e−01
Value: B
Tepidanaerobacter.syntrophicus
6
4
2
0
A (n=14)
B (n=15)
C (n=11)
surface

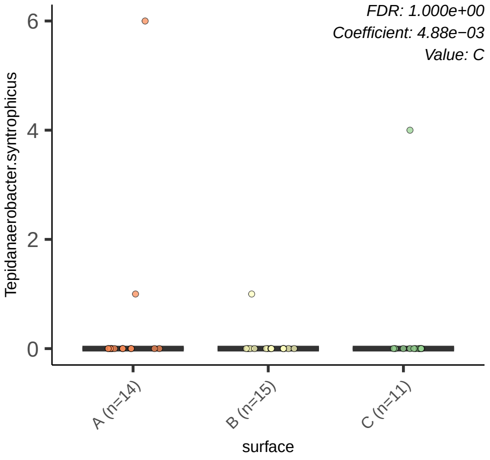
FDR: 1.000e+00
Coefficient: 4.88e−03
Value: C
Tepidanaerobacter.syntrophicus
0
2
4
6
A (n=14)
B (n=15)
C (n=11)
surface

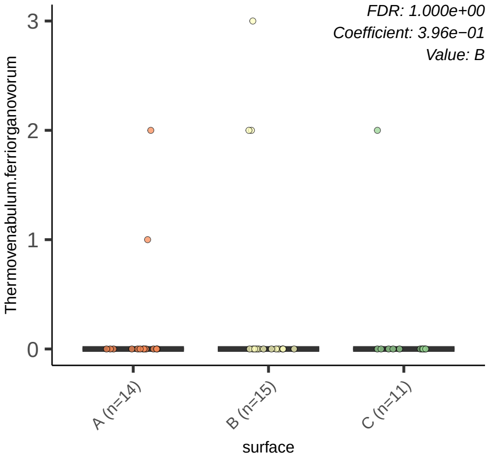

FDR: 1.000e+00
Coefficient: 3.96e−01
Value: B
Thermovenabulum.ferriorganovorum
3
2
1
0
A (n=14)
B (n=15)
C (n=11)
surface

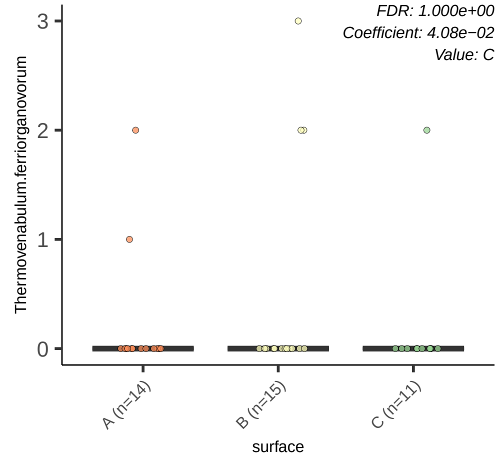

FDR: 1.000e+00
Coefficient: 4.08e−02
Value: C
Thermovenabulum.ferriorganovorum
3
2
1
0
A (n=14)
B (n=15)
C (n=11)
surface

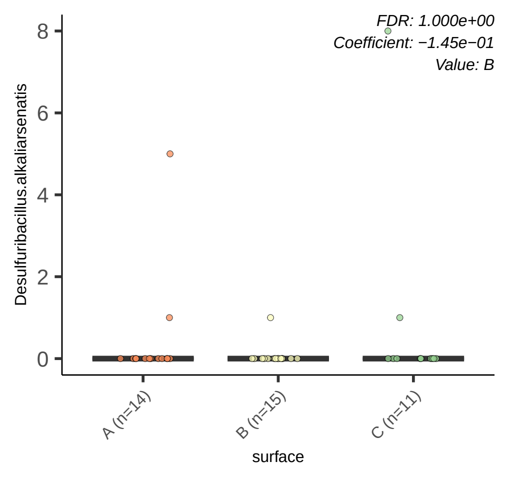
FDR: 1.000e+00
Coefficient: −1.45e−01
Value: B
Desulfuribacillus.alkaliarsenatis
0
2
4
6
8
A (n=14)
B (n=15)
C (n=11)
surface

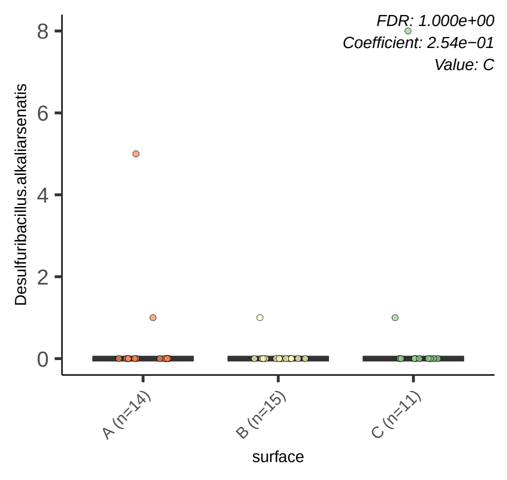
FDR: 1.000e+00
Coefficient: 2.54e−01
Value: C
Desulfuribacillus.alkaliarsenatis
0
2
4
6
8
A (n=14)
B (n=15)
C (n=11)
surface

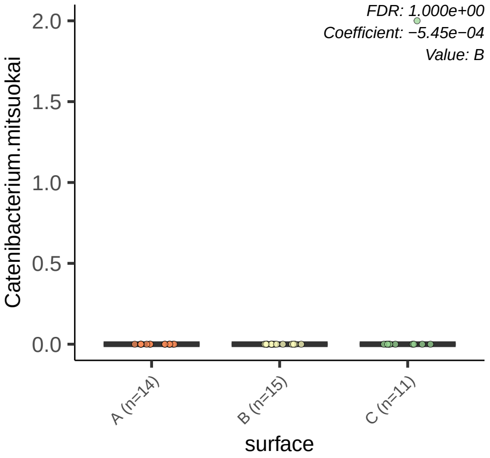

FDR: 1.000e+00
Coefficient: −5.45e−04
Value: B
Catenibacterium.mitsuokai
2.0
1.5
1.0
0.5
0.0
A (n=14)
B (n=15)
C (n=11)
surface

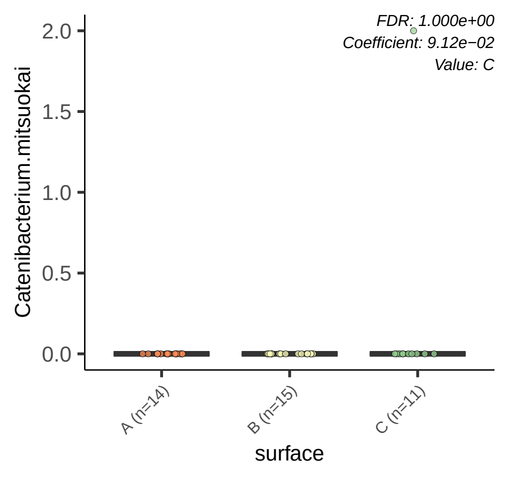

FDR: 1.000e+00
Coefficient: 9.12e−02
Value: C
Catenibacterium.mitsuokai
2.0
1.5
1.0
0.5
0.0
A (n=14)
B (n=15)
C (n=11)
surface

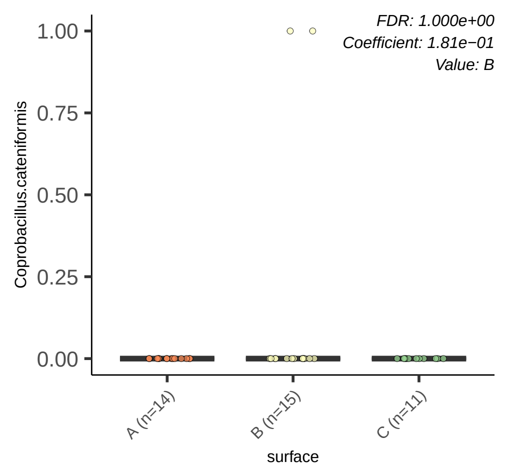

FDR: 1.000e+00
Coefficient: 1.81e−01
Value: B
Coprobacillus.cateniformis
1.00
0.75
0.50
0.25
0.00
A (n=14)
B (n=15)
C (n=11)
surface

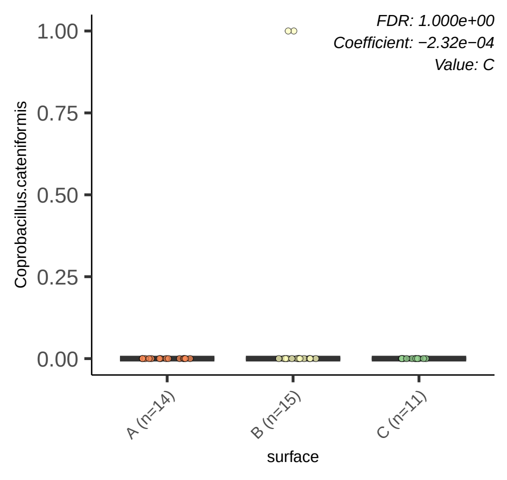
FDR: 1.000e+00
Coefficient: −2.32e−04
Value: C
Coprobacillus.cateniformis
1.00
0.75
0.50
0.25
0.00
A (n=14)
B (n=15)
C (n=11)
surface

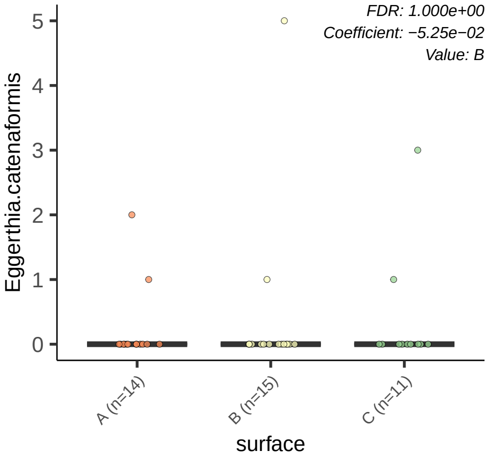
FDR: 1.000e+00
Coefficient: −5.25e−02
Value: B
Eggerthia.catenaformis
0
1
2
3
4
5
A (n=14)
B (n=15)
C (n=11)
surface

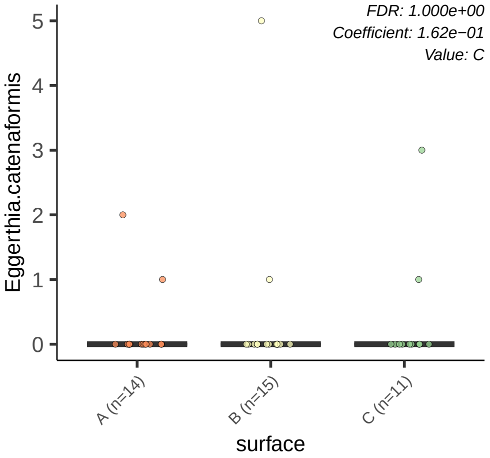
FDR: 1.000e+00
Coefficient: 1.62e−01
Value: C
Eggerthia.catenaformis
0
1
2
3
4
5
A (n=14)
B (n=15)
C (n=11)
surface

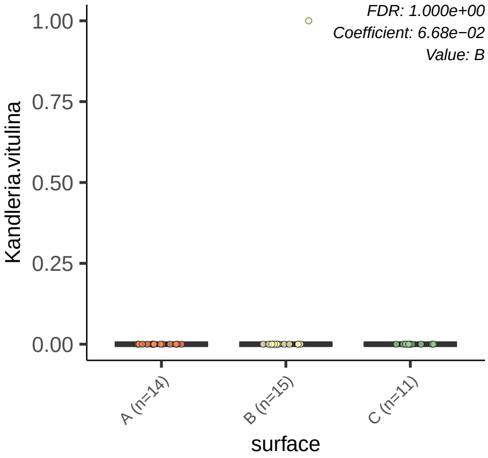

FDR: 1.000e+00
Coefficient: 6.68e−02
Value: B
Kandleria.vitulina
1.00
0.75
0.50
0.25
0.00
A (n=14)
B (n=15)
C (n=11)
surface

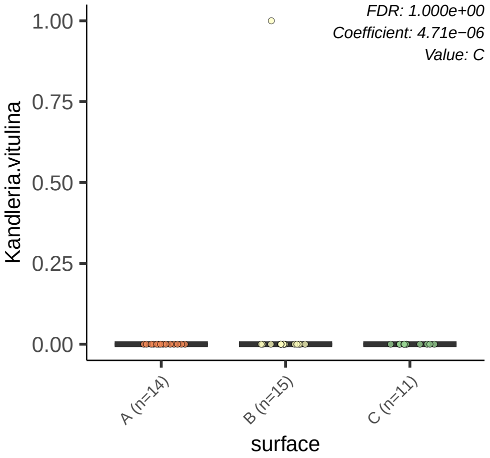
FDR: 1.000e+00
Coefficient: 4.71e−06
Value: C
Kandleria.vitulina
1.00
0.75
0.50
0.25
0.00
A (n=14)
B (n=15)
C (n=11)
surface

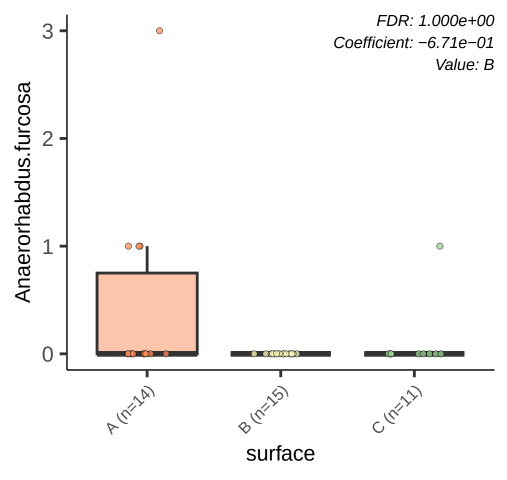

FDR: 1.000e+00
Coefficient: −6.71e−01
Value: B
Anaerorhabdus.furcosa
0
1
2
3
A (n=14)
B (n=15)
C (n=11)
surface

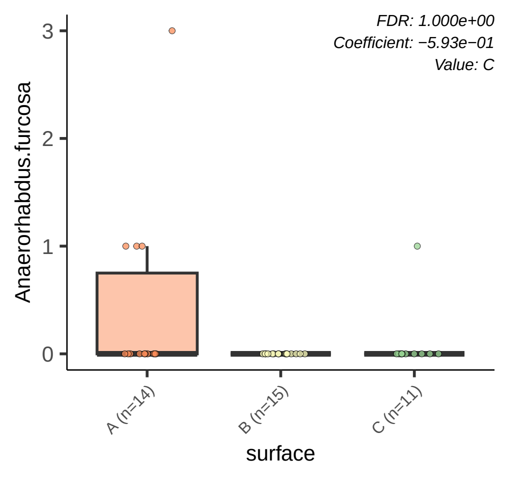
FDR: 1.000e+00
Coefficient: −5.93e−01
Value: C
Anaerorhabdus.furcosa
3
2
1
0
A (n=14)
B (n=15)
C (n=11)
surface

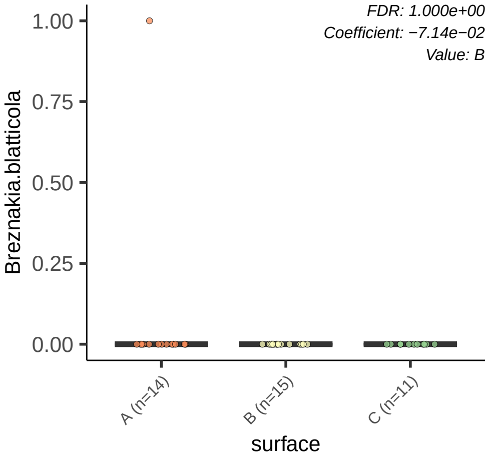

FDR: 1.000e+00
Coefficient: −7.14e−02
Value: B
Breznakia.blatticola
1.00
0.75
0.50
0.25
0.00
A (n=14)
B (n=15)
C (n=11)
surface

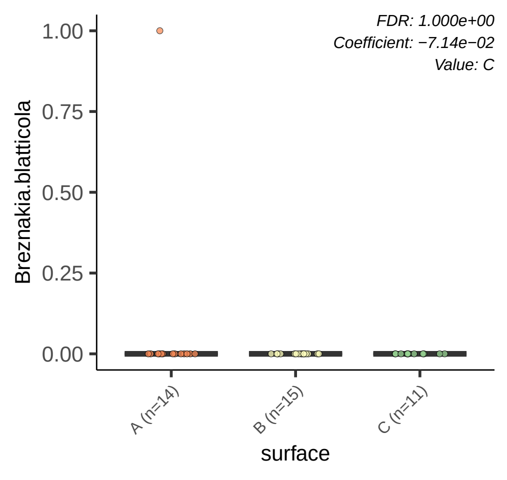
FDR: 1.000e+00
Coefficient: −7.14e−02
Value: C
Breznakia.blatticola
1.00
0.75
0.50
0.25
0.00
A (n=14)
B (n=15)
C (n=11)
surface

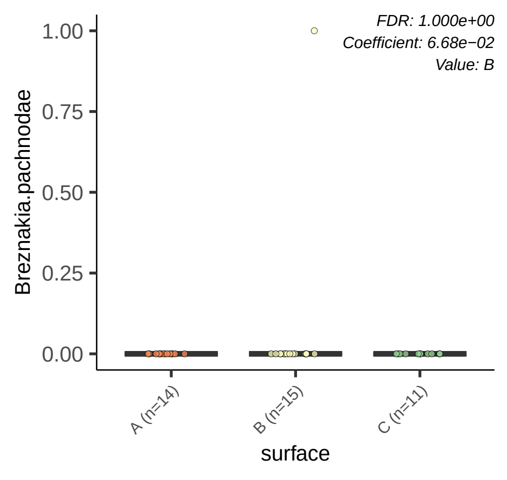

FDR: 1.000e+00
Coefficient: 6.68e−02
Value: B
1.00
0.75
0.50
0.25
0.00
Breznakia.pachnodae
A (n=14)
B (n=15)
C (n=11)
surface

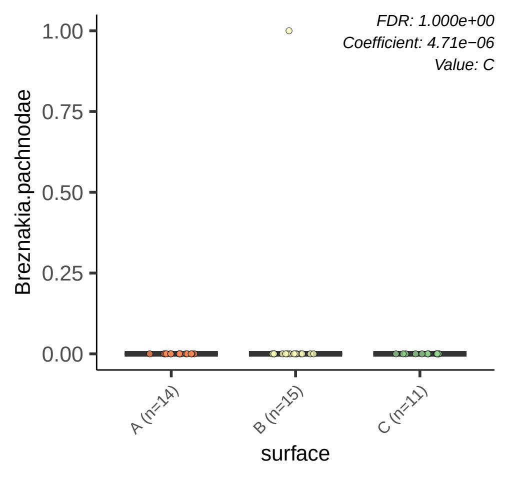
FDR: 1.000e+00
Coefficient: 4.71e−06
Value: C
Breznakia.pachnodae
1.00
0.75
0.50
0.25
0.00
A (n=14)
B (n=15)
C (n=11)
surface

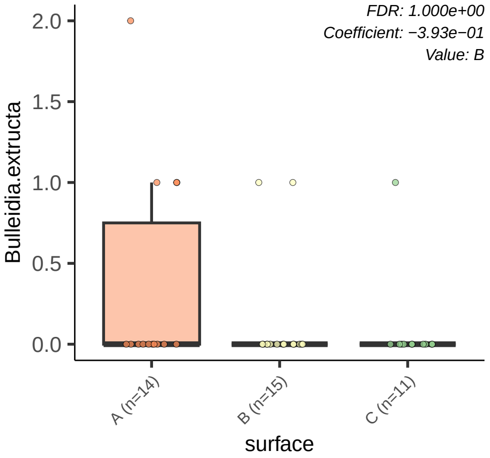
FDR: 1.000e+00
Coefficient: −3.93e−01
Value: B
Bulleidia.extructa
2.0
1.5
1.0
0.5
0.0
A (n=14)
B (n=15)
C (n=11)
surface

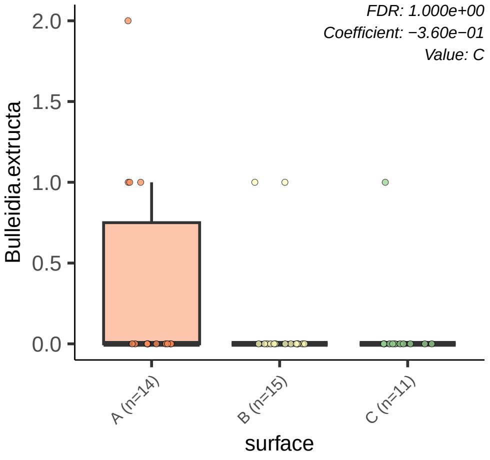
FDR: 1.000e+00
Coefficient: −3.60e−01
Value: C
Bulleidia.extructa
2.0
1.5
1.0
0.5
0.0
A (n=14)
B (n=15)
C (n=11)
surface

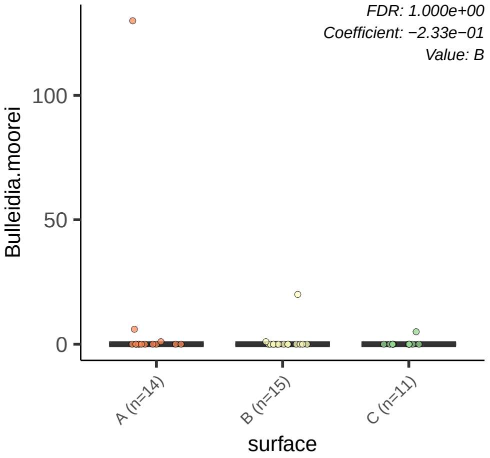

FDR: 1.000e+00
Coefficient: −2.33e−01
Value: B
Bulleidia.moorei
100
50
0
A (n=14)
B (n=15)
C (n=11)
surface

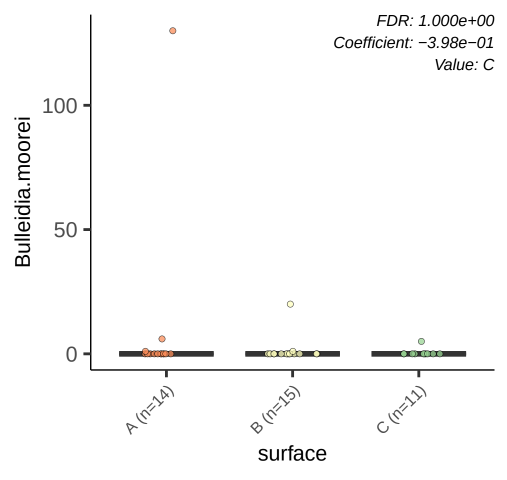

FDR: 1.000e+00
Coefficient: −3.98e−01
Value: C
Bulleidia.moorei
100
50
0
A (n=14)
B (n=15)
C (n=11)
surface

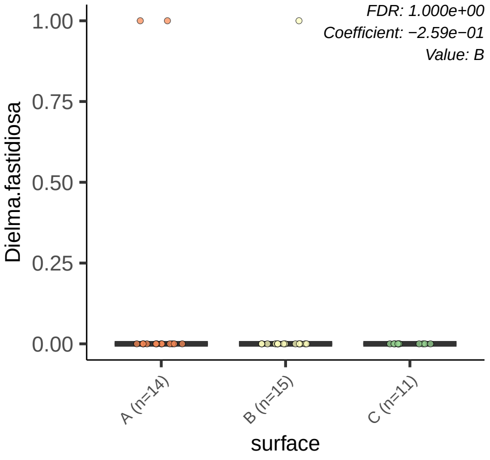
FDR: 1.000e+00
Coefficient: −2.59e−01
Value: B
Dielma.fastidiosa
1.00
0.75
0.50
0.25
0.00
A (n=14)
B (n=15)
C (n=11)
surface

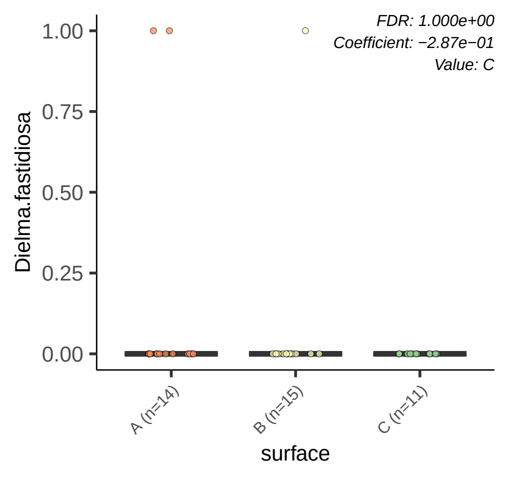

FDR: 1.000e+00
Coefficient: −2.87e−01
Value: C
Dielma.fastidiosa
1.00
0.75
0.50
0.25
0.00
A (n=14)
B (n=15)
C (n=11)
surface

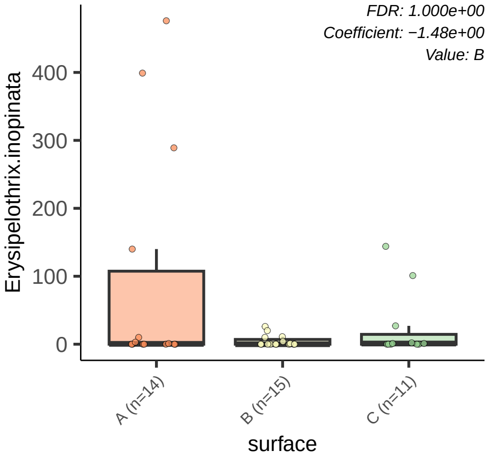
FDR: 1.000e+00
Coefficient: −1.48e+00
Value: B
Erysipelothrix.inopinata
0
100
200
300
400
A (n=14)
B (n=15)
C (n=11)
surface

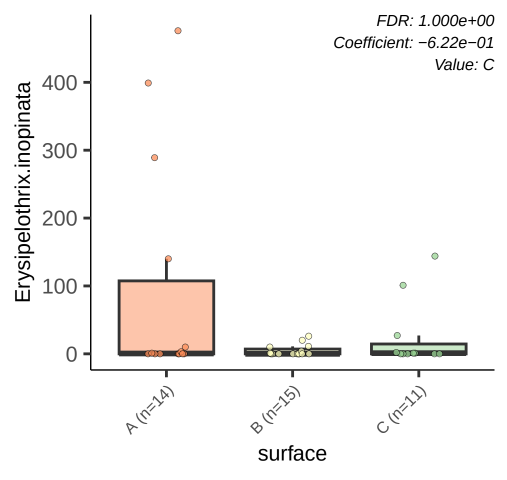

FDR: 1.000e+00
Coefficient: −6.22e−01
Value: C
Erysipelothrix.inopinata
0
100
200
300
400
A (n=14)
B (n=15)
C (n=11)
surface

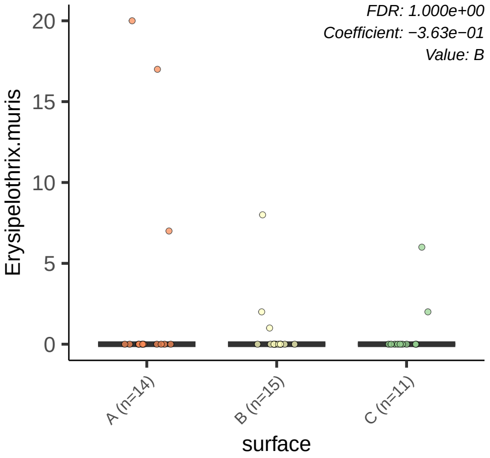
FDR: 1.000e+00
Coefficient: −3.63e−01
Value: B
Erysipelothrix.muris
20
15
10
5
0
A (n=14)
B (n=15)
C (n=11)
surface

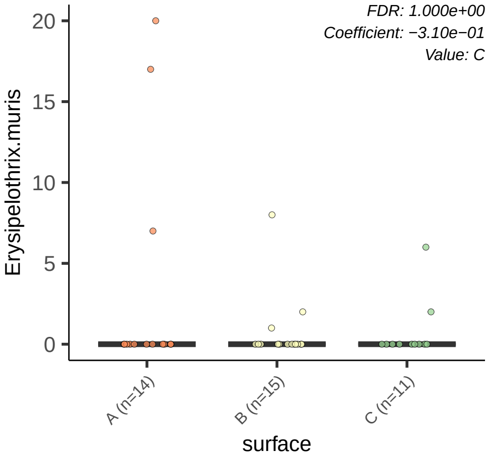
FDR: 1.000e+00
Coefficient: −3.10e−01
Value: C
Erysipelothrix.muris
0
5
10
15
20
A (n=14)
B (n=15)
C (n=11)
surface

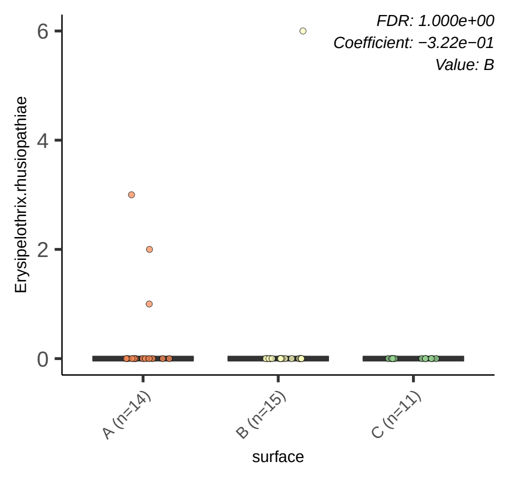
FDR: 1.000e+00
Coefficient: −3.22e−01
Value: B
Erysipelothrix.rhusiopathiae
6
4
2
0
A (n=14)
B (n=15)
C (n=11)
surface

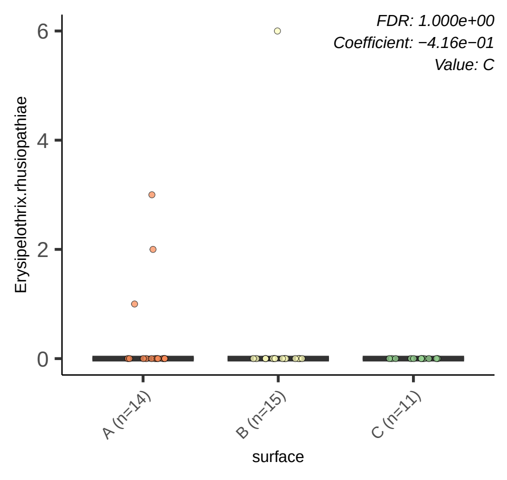
FDR: 1.000e+00
Coefficient: −4.16e−01
Value: C
Erysipelothrix.rhusiopathiae
0
2
4
6
A (n=14)
B (n=15)
C (n=11)
surface

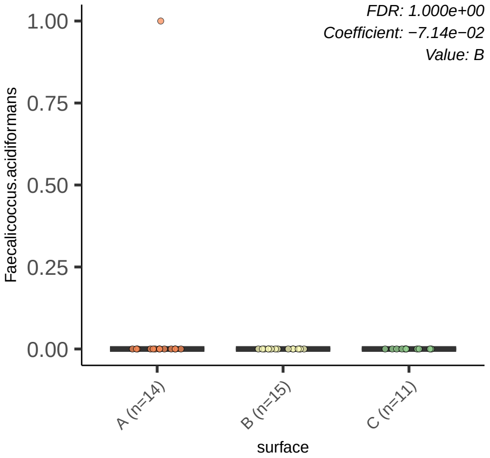
FDR: 1.000e+00
Coefficient: −7.14e−02
Value: B
Faecalicoccus.acidiformans
1.00
0.75
0.50
0.25
0.00
A (n=14)
B (n=15)
C (n=11)
surface

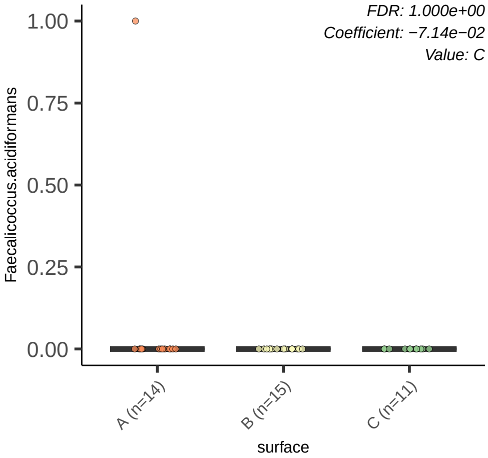
FDR: 1.000e+00
Coefficient: −7.14e−02
Value: C
Faecalicoccus.acidiformans
1.00
0.75
0.50
0.25
0.00
A (n=14)
B (n=15)
C (n=11)
surface

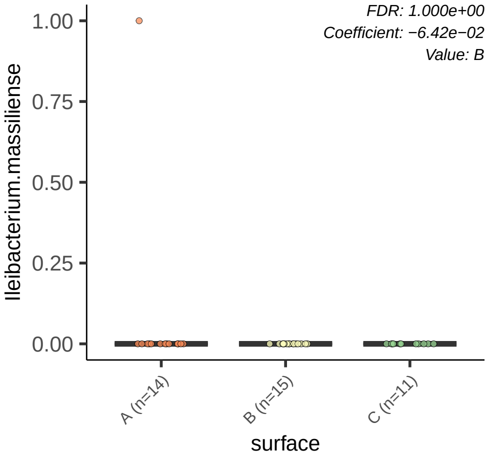

FDR: 1.000e+00
Coefficient: −6.42e−02
Value: B
Ileibacterium.massiliense
1.00
0.75
0.50
0.25
0.00
A (n=14)
B (n=15)
C (n=11)
surface

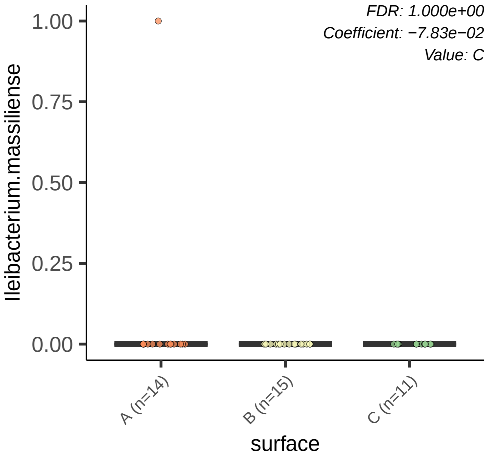
FDR: 1.000e+00
Coefficient: −7.83e−02
Value: C
Ileibacterium.massiliense
1.00
0.75
0.50
0.25
0.00
A (n=14)
B (n=15)
C (n=11)
surface

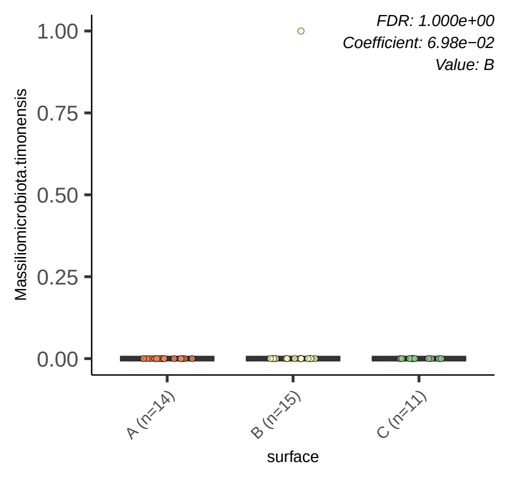

FDR: 1.000e+00
Coefficient: 6.98e−02
Value: B
Massiliomicrobiota.timonensis
1.00
0.75
0.50
0.25
0.00
A (n=14)
B (n=15)
C (n=11)
surface

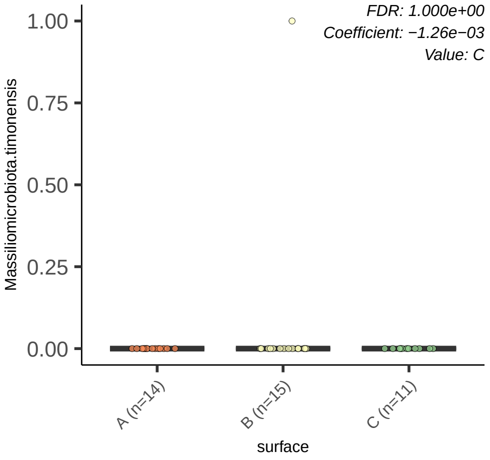

FDR: 1.000e+00
Coefficient: −1.26e−03
Value: C
Massiliomicrobiota.timonensis
1.00
0.75
0.50
0.25
0.00
A (n=14)
B (n=15)
C (n=11)
surface

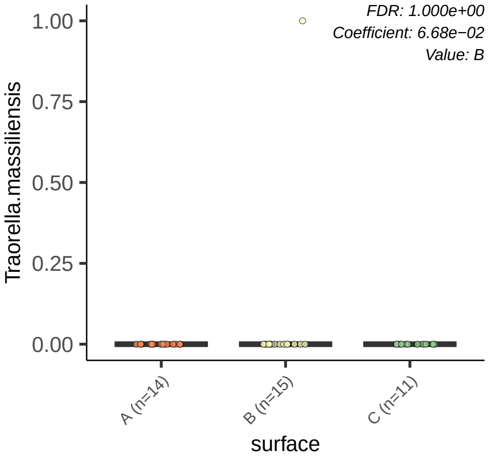
FDR: 1.000e+00
Coefficient: 6.68e−02
Value: B
Traorella.massiliensis
1.00
0.75
0.50
0.25
0.00
A (n=14)
B (n=15)
C (n=11)
surface

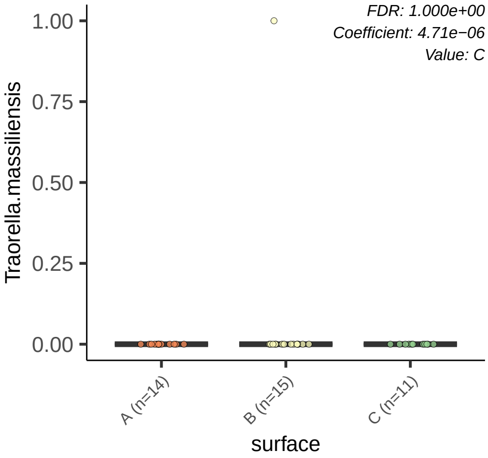
FDR: 1.000e+00
Coefficient: 4.71e−06
Value: C
Traorella.massiliensis
1.00
0.75
0.50
0.25
0.00
A (n=14)
B (n=15)
C (n=11)
surface

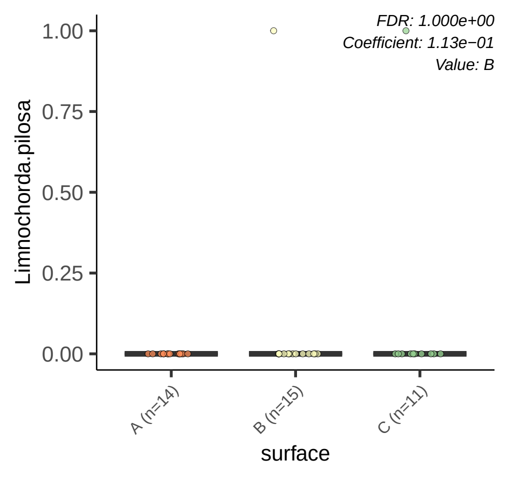
FDR: 1.000e+00
Coefficient: 1.13e−01
Value: B
Limnochorda.pilosa
1.00
0.75
0.50
0.25
0.00
A (n=14)
B (n=15)
C (n=11)
surface

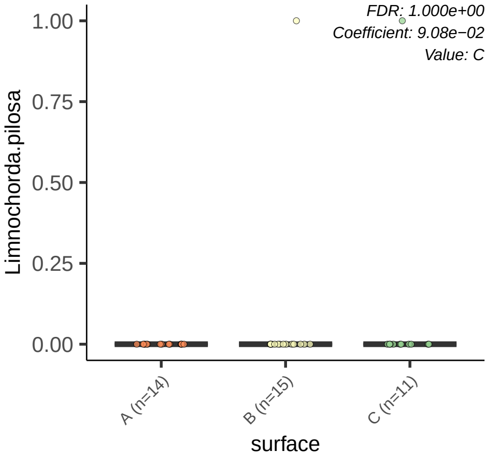
FDR: 1.000e+00
Coefficient: 9.08e−02
Value: C
Limnochorda.pilosa
1.00
0.75
0.50
0.25
0.00
A (n=14)
B (n=15)
C (n=11)
surface

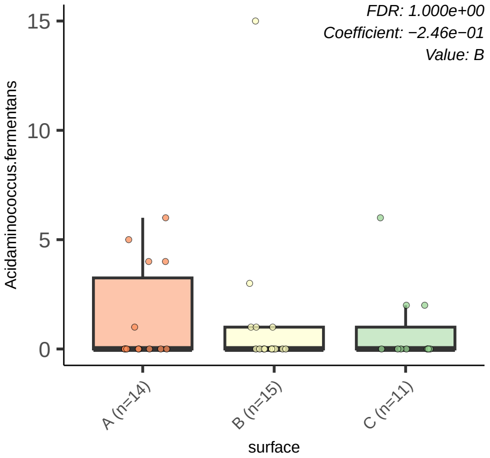

FDR: 1.000e+00
Coefficient: −2.46e−01
Value: B
Acidaminococcus.fermentans
15
10
5
0
A (n=14)
B (n=15)
C (n=11)
surface

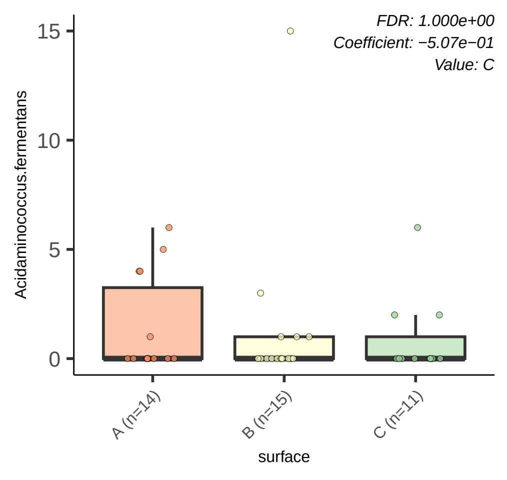

FDR: 1.000e+00
Coefficient: −5.07e−01
Value: C
Acidaminococcus.fermentans
15
10
5
0
A (n=14)
B (n=15)
C (n=11)
surface

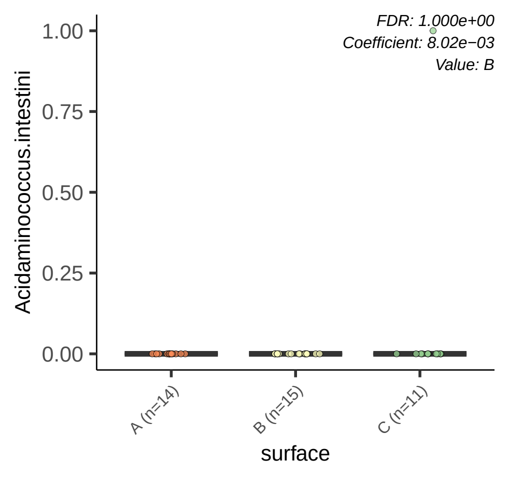
FDR: 1.000e+00
Coefficient: 8.02e−03
Value: B
Acidaminococcus.intestini
1.00
0.75
0.50
0.25
0.00
A (n=14)
B (n=15)
C (n=11)
surface

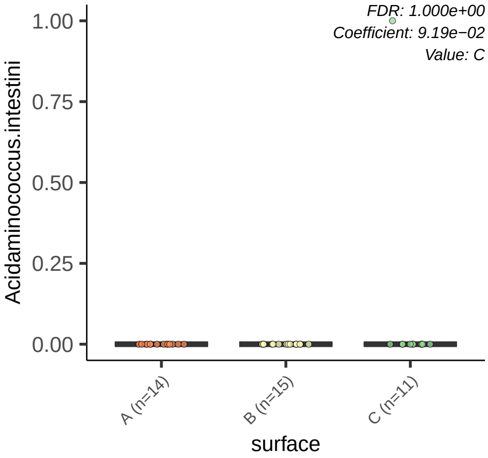

FDR: 1.000e+00
Coefficient: 9.19e−02
Value: C
Acidaminococcus.intestini
1.00
0.75
0.50
0.25
0.00
A (n=14)
B (n=15)
C (n=11)
surface

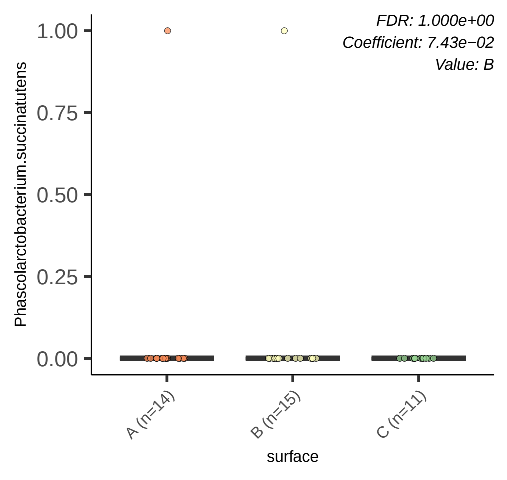
FDR: 1.000e+00
Coefficient: 7.43e−02
Value: B
Phascolarctobacterium.succinatutens
1.00
0.75
0.50
0.25
0.00
A (n=14)
B (n=15)
C (n=11)
surface

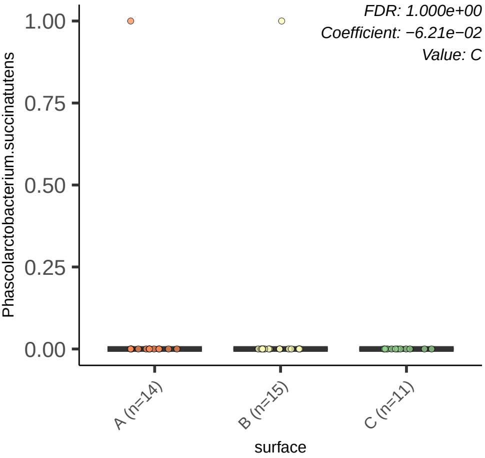
FDR: 1.000e+00
Coefficient: −6.21e−02
Value: C
1.00
0.75
0.50
0.25
0.00
Phascolarctobacterium.succinatutens
A (n=14)
B (n=15)
C (n=11)
surface

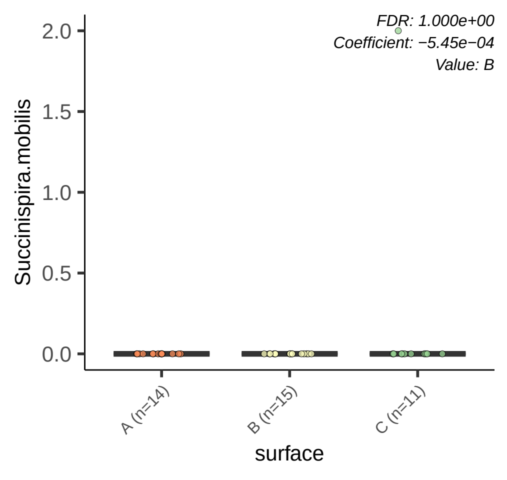

FDR: 1.000e+00
Coefficient: −5.45e−04
Value: B
Succinispira.mobilis
2.0
1.5
1.0
0.5
0.0
A (n=14)
B (n=15)
C (n=11)
surface

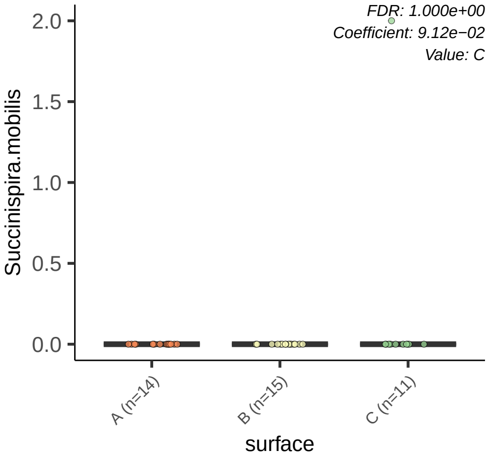
FDR: 1.000e+00
Coefficient: 9.12e−02
Value: C
2.0
1.5
1.0
0.5
0.0
Succinispira.mobilis
A (n=14)
B (n=15)
C (n=11)
surface

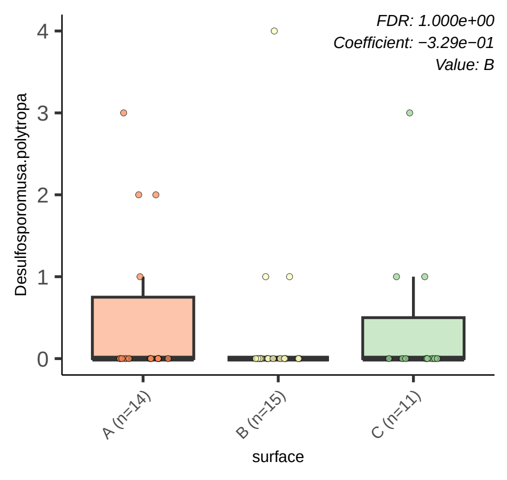
FDR: 1.000e+00
Coefficient: −3.29e−01
Value: B
Desulfosporomusa.polytropa
0
1
2
3
4
A (n=14)
B (n=15)
C (n=11)
surface

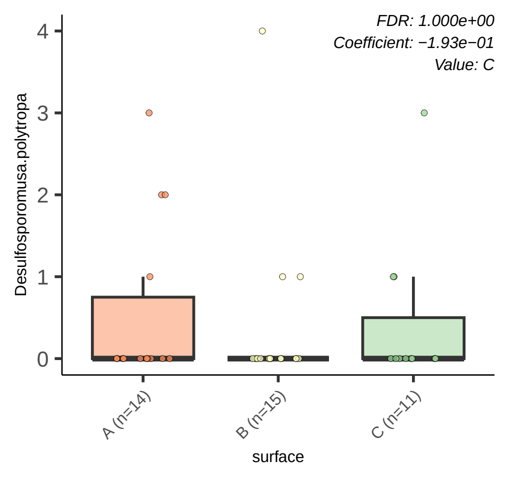

FDR: 1.000e+00
Coefficient: −1.93e−01
Value: C
Desulfosporomusa.polytropa
0
1
2
3
4
A (n=14)
B (n=15)
C (n=11)
surface

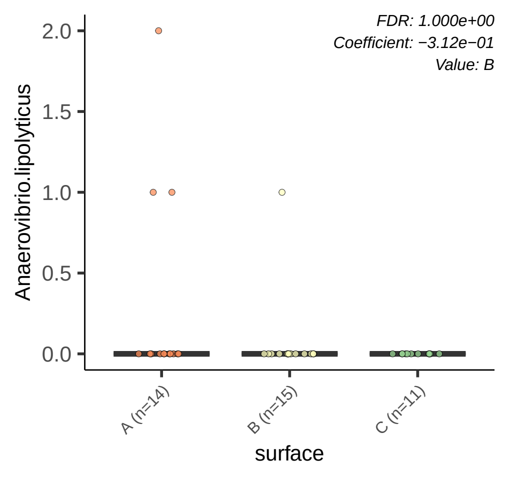

FDR: 1.000e+00
Coefficient: −3.12e−01
Value: B
Anaerovibrio.lipolyticus
2.0
1.5
1.0
0.5
0.0
A (n=14)
B (n=15)
C (n=11)
surface

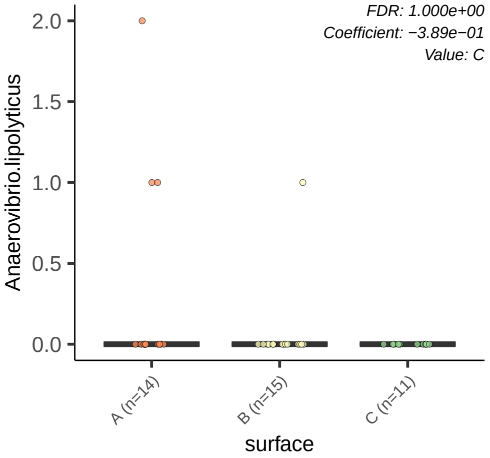
FDR: 1.000e+00
Coefficient: −3.89e−01
Value: C
Anaerovibrio.lipolyticus
2.0
1.5
1.0
0.5
0.0
A (n=14)
B (n=15)
C (n=11)
surface

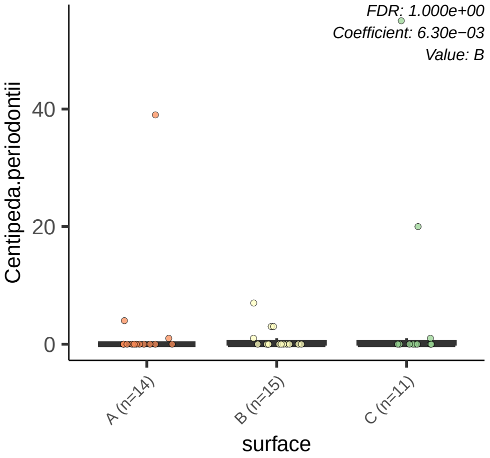
FDR: 1.000e+00
Coefficient: 6.30e−03
Value: B
Centipeda.periodontii
0
20
40
A (n=14)
B (n=15)
C (n=11)
surface

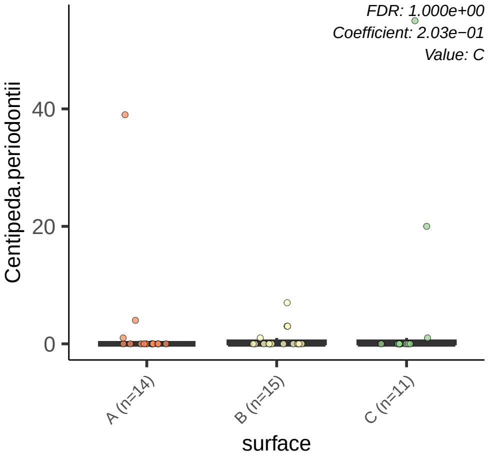

FDR: 1.000e+00
Coefficient: 2.03e−01
Value: C
Centipeda.periodontii
40
20
0
A (n=14)
B (n=15)
C (n=11)
surface

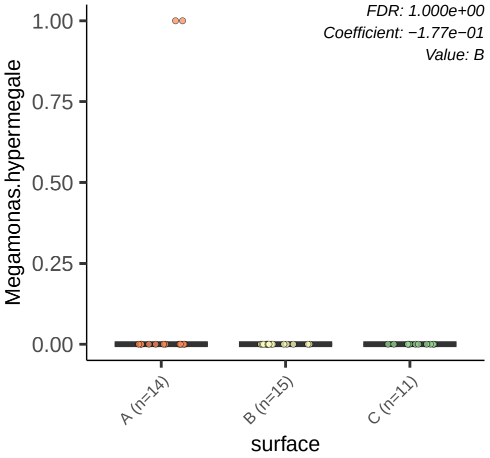
FDR: 1.000e+00
Coefficient: −1.77e−01
Value: B
Megamonas.hypermegale
1.00
0.75
0.50
0.25
0.00
A (n=14)
B (n=15)
C (n=11)
surface

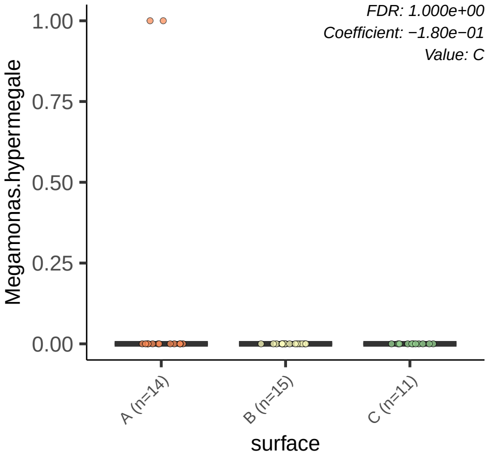
FDR: 1.000e+00
Coefficient: −1.80e−01
Value: C
Megamonas.hypermegale
1.00
0.75
0.50
0.25
0.00
A (n=14)
B (n=15)
C (n=11)
surface

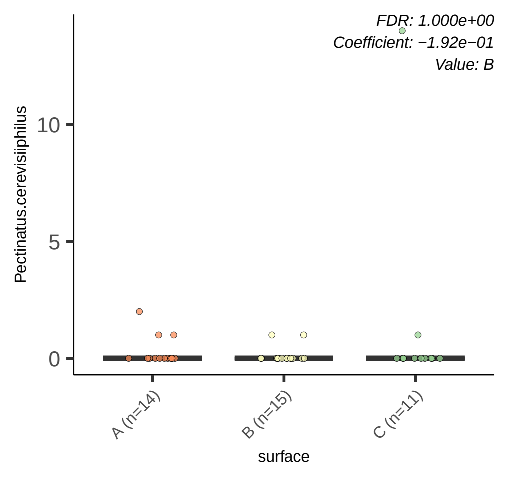
FDR: 1.000e+00
Coefficient: −1.92e−01
Value: B
Pectinatus.cerevisiiphilus
10
5
0
A (n=14)
B (n=15)
C (n=11)
surface

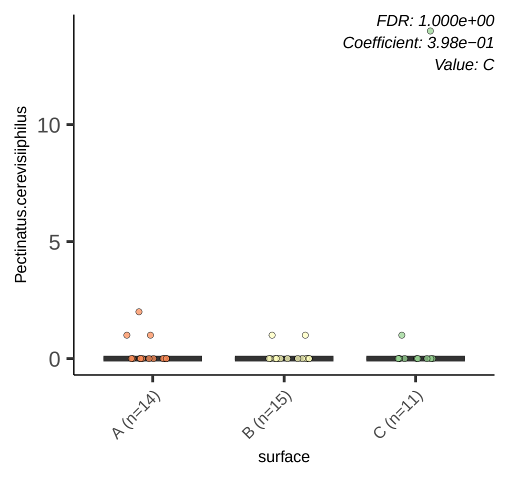
FDR: 1.000e+00
Coefficient: 3.98e−01
Value: C
Pectinatus.cerevisiiphilus
10
5
0
A (n=14)
B (n=15)
C (n=11)
surface

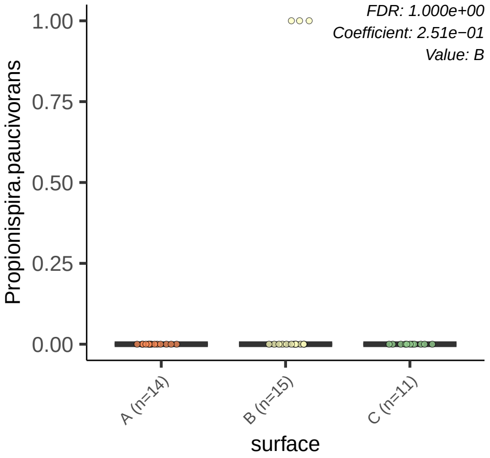
FDR: 1.000e+00
Coefficient: 2.51e−01
Value: B
Propionispira.paucivorans
1.00
0.75
0.50
0.25
0.00
A (n=14)
B (n=15)
C (n=11)
surface

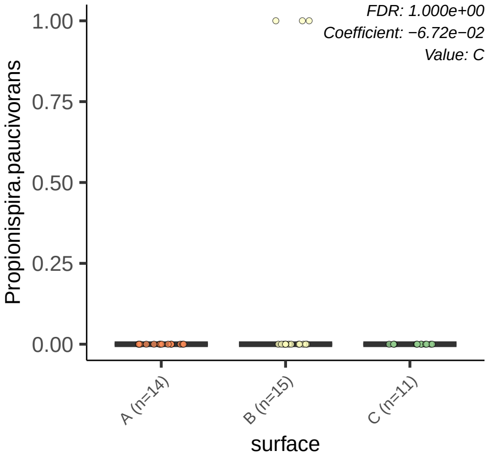
FDR: 1.000e+00
Coefficient: −6.72e−02
Value: C
Propionispira.paucivorans
1.00
0.75
0.50
0.25
0.00
A (n=14)
B (n=15)
C (n=11)
surface

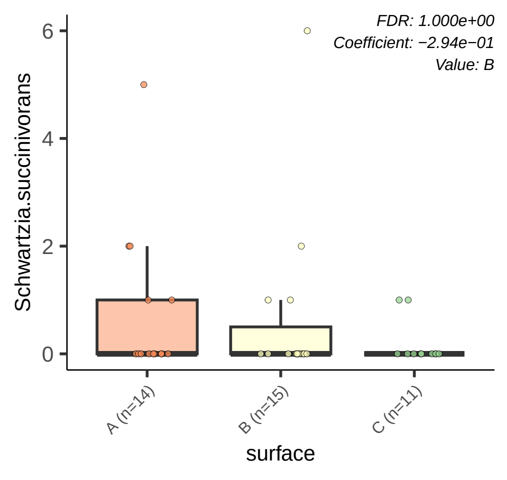
FDR: 1.000e+00
Coefficient: −2.94e−01
Value: B
Schwartzia.succinivorans
0
2
4
6
A (n=14)
B (n=15)
C (n=11)
surface

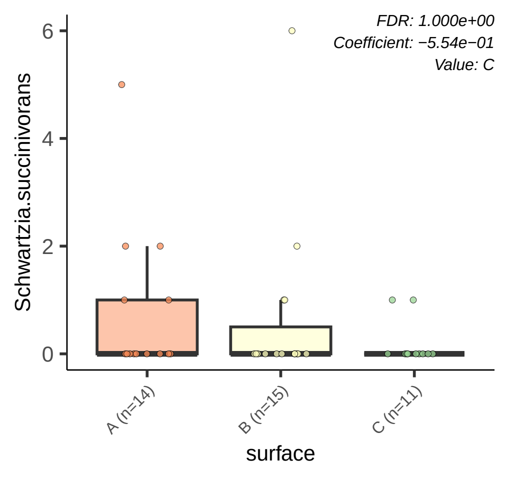

FDR: 1.000e+00
Coefficient: −5.54e−01
Value: C
Schwartzia.succinivorans
6
4
2
0
A (n=14)
B (n=15)
C (n=11)
surface

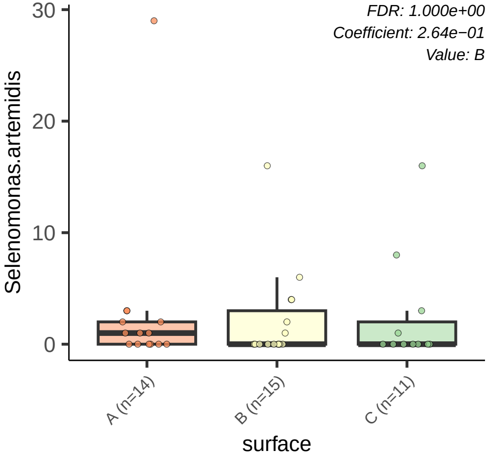
FDR: 1.000e+00
Coefficient: 2.64e−01
Value: B
Selenomonas.artemidis
30
20
10
0
A (n=14)
B (n=15)
C (n=11)
surface

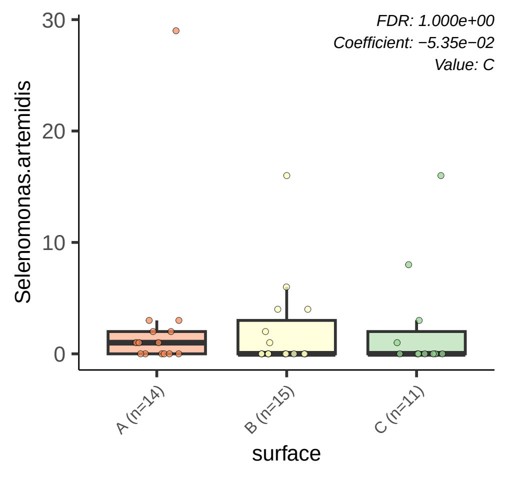

FDR: 1.000e+00
Coefficient: −5.35e−02
Value: C
Selenomonas.artemidis
30
20
10
0
A (n=14)
B (n=15)
C (n=11)
surface

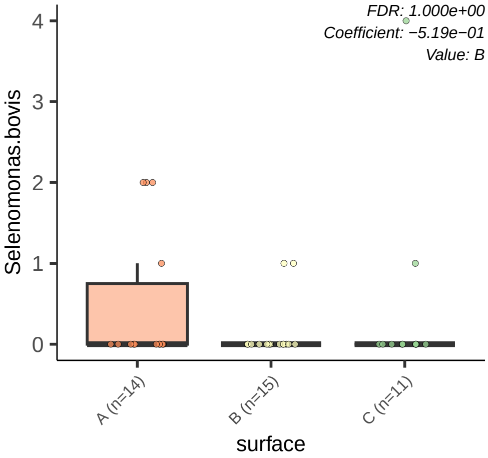
FDR: 1.000e+00
Coefficient: −5.19e−01
Value: B
Selenomonas.bovis
4
3
2
1
0
A (n=14)
B (n=15)
C (n=11)
surface

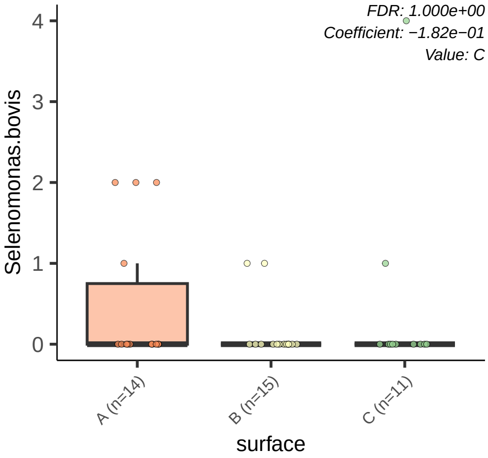

FDR: 1.000e+00
Coefficient: −1.82e−01
Value: C
Selenomonas.bovis
0
1
2
3
4
A (n=14)
B (n=15)
C (n=11)
surface

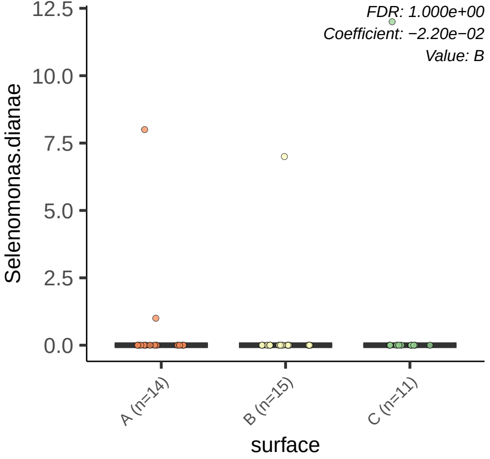

FDR: 1.000e+00
Coefficient: −2.20e−02
Value: B
Selenomonas.dianae
12.5
10.0
7.5
5.0
2.5
0.0
A (n=14)
B (n=15)
C (n=11)
surface

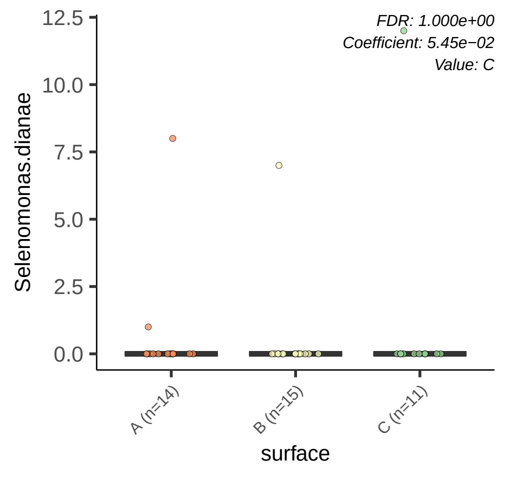

FDR: 1.000e+00
Coefficient: 5.45e−02
Value: C
Selenomonas.dianae
12.5
10.0
7.5
5.0
2.5
0.0
A (n=14)
B (n=15)
C (n=11)
surface

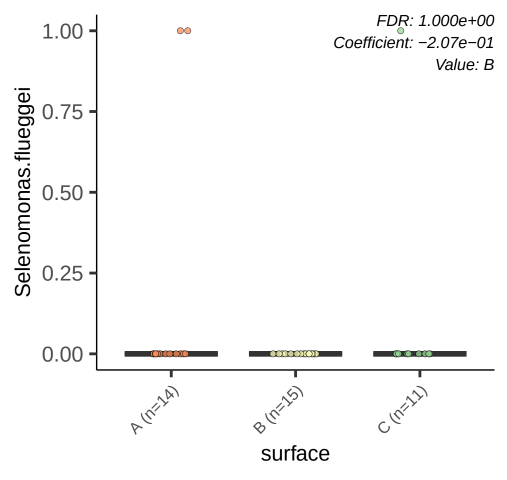
FDR: 1.000e+00
Coefficient: −2.07e−01
Value: B
Selenomonas.flueggei
1.00
0.75
0.50
0.25
0.00
A (n=14)
B (n=15)
C (n=11)
surface

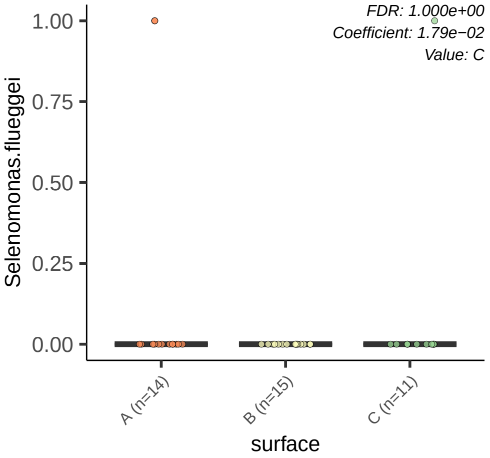

FDR: 1.000e+00
Coefficient: 1.79e−02
Value: C
Selenomonas.flueggei
1.00
0.75
0.50
0.25
0.00
A (n=14)
B (n=15)
C (n=11)
surface

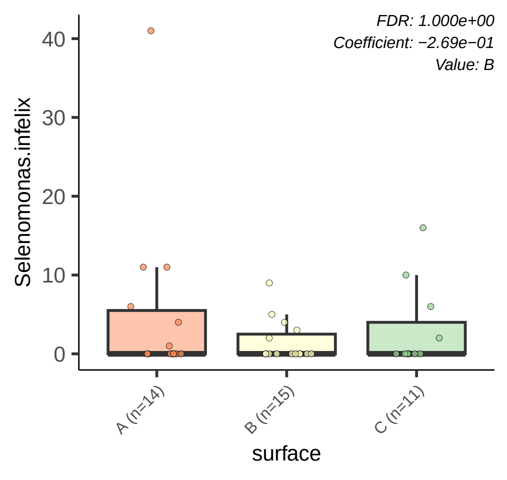
FDR: 1.000e+00
Coefficient: −2.69e−01
Value: B
Selenomonas.infelix
0
10
20
30
40
A (n=14)
B (n=15)
C (n=11)
surface

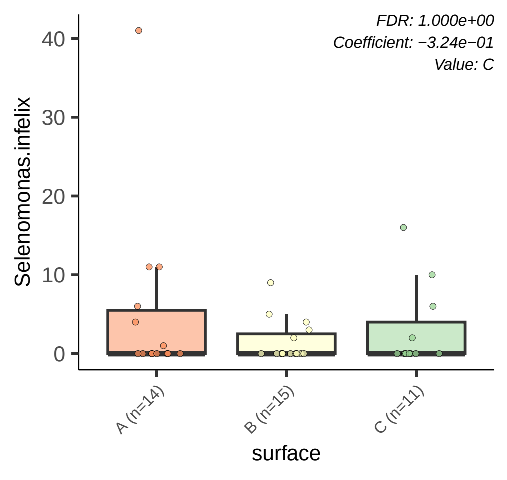

FDR: 1.000e+00
Coefficient: −3.24e−01
Value: C
Selenomonas.infelix
0
10
20
30
40
A (n=14)
B (n=15)
C (n=11)
surface

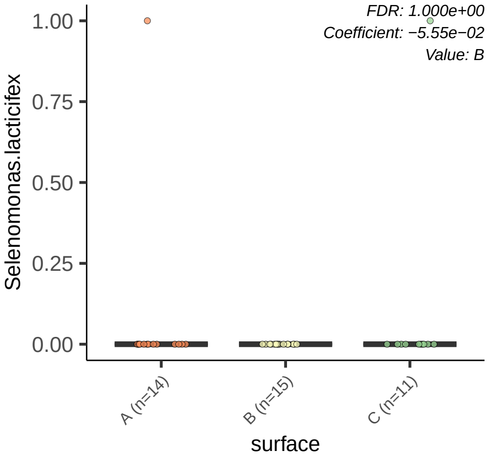

FDR: 1.000e+00
Coefficient: −5.55e−02
Value: B
Selenomonas.lacticifex
1.00
0.75
0.50
0.25
0.00
A (n=14)
B (n=15)
C (n=11)
surface

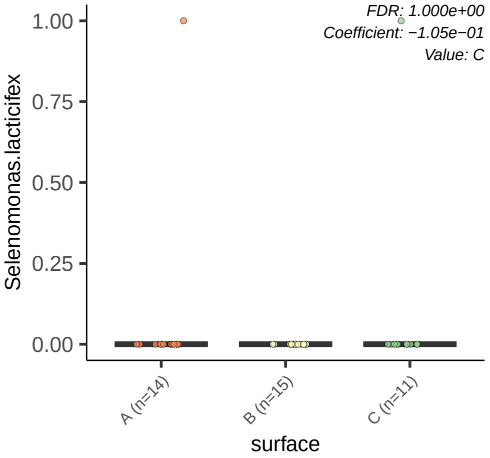
FDR: 1.000e+00
Coefficient: −1.05e−01
Value: C
Selenomonas.lacticifex
1.00
0.75
0.50
0.25
0.00
A (n=14)
B (n=15)
C (n=11)
surface

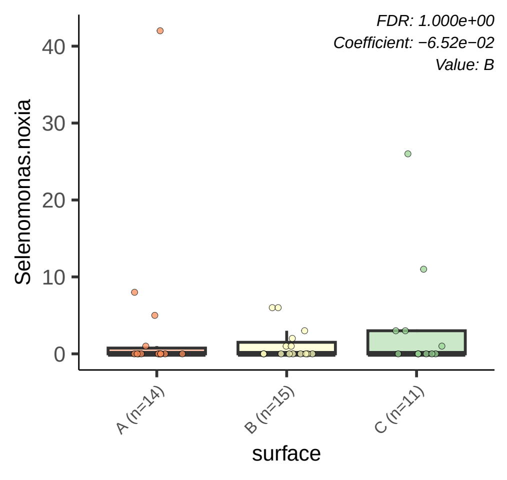

FDR: 1.000e+00
Coefficient: −6.52e−02
Value: B
Selenomonas.noxia
0
10
20
30
40
A (n=14)
B (n=15)
C (n=11)
surface

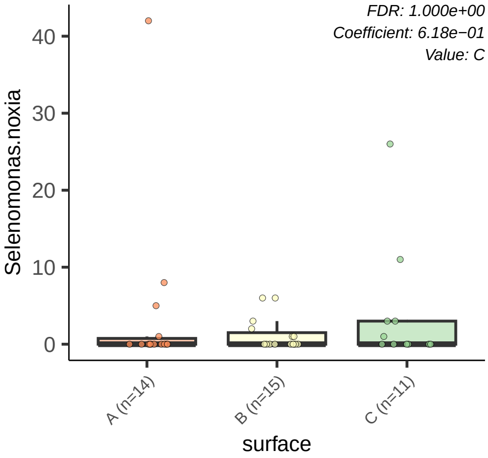
FDR: 1.000e+00
Coefficient: 6.18e−01
Value: C
Selenomonas.noxia
40
30
20
10
0
A (n=14)
B (n=15)
C (n=11)
surface

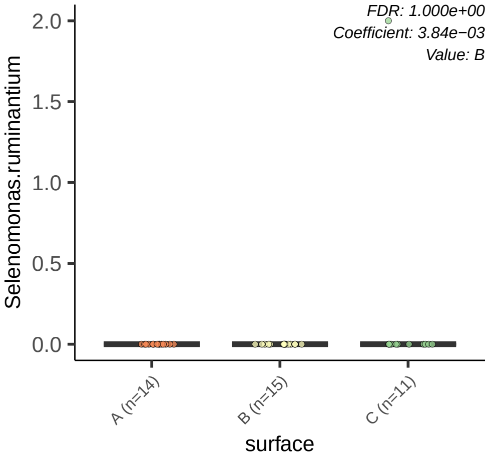

FDR: 1.000e+00
Coefficient: 3.84e−03
Value: B
Selenomonas.ruminantium
2.0
1.5
1.0
0.5
0.0
A (n=14)
B (n=15)
C (n=11)
surface

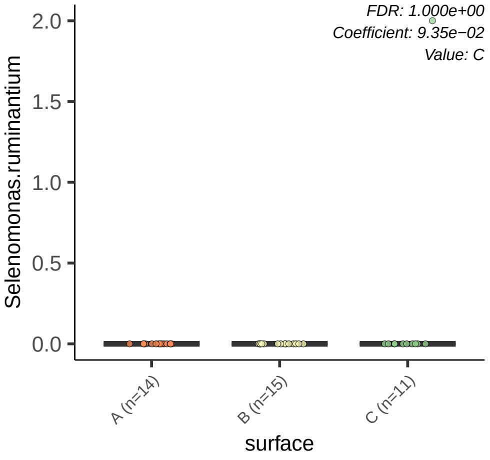
FDR: 1.000e+00
Coefficient: 9.35e−02
Value: C
Selenomonas.ruminantium
2.0
1.5
1.0
0.5
0.0
A (n=14)
B (n=15)
C (n=11)
surface

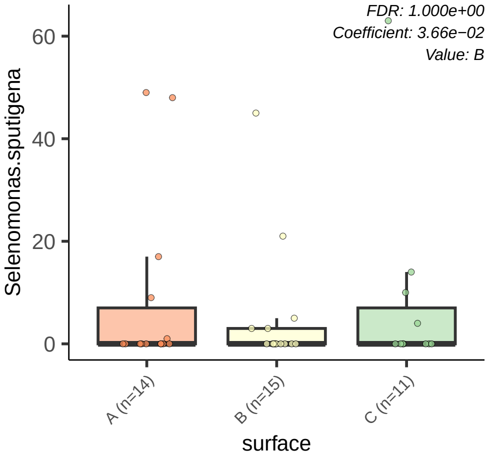
FDR: 1.000e+00
Coefficient: 3.66e−02
Value: B
Selenomonas.sputigena
0
20
40
60
A (n=14)
B (n=15)
C (n=11)
surface

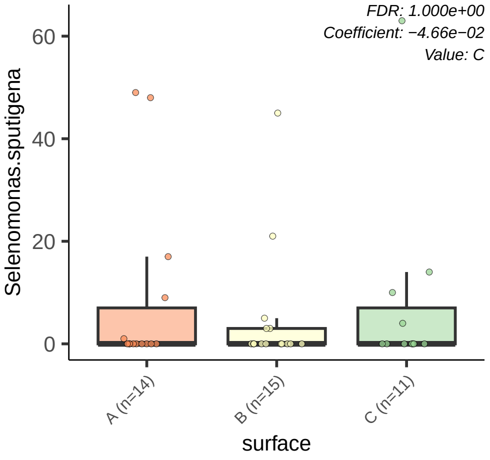

FDR: 1.000e+00
Coefficient: −4.66e−02
Value: C
Selenomonas.sputigena
0
20
40
60
A (n=14)
B (n=15)
C (n=11)
surface

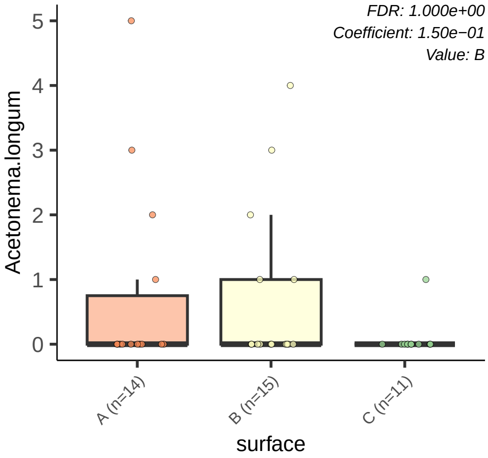

FDR: 1.000e+00
Coefficient: 1.50e−01
Value: B
Acetonema.longum
0
1
2
3
4
5
A (n=14)
B (n=15)
C (n=11)
surface

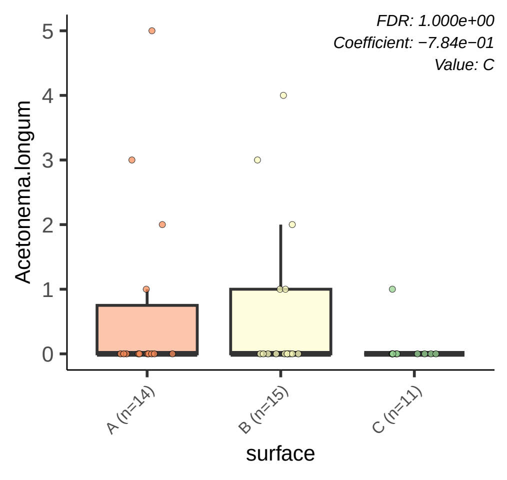

FDR: 1.000e+00
Coefficient: −7.84e−01
Value: C
Acetonema.longum
0
1
2
3
4
5
A (n=14)
B (n=15)
C (n=11)
surface

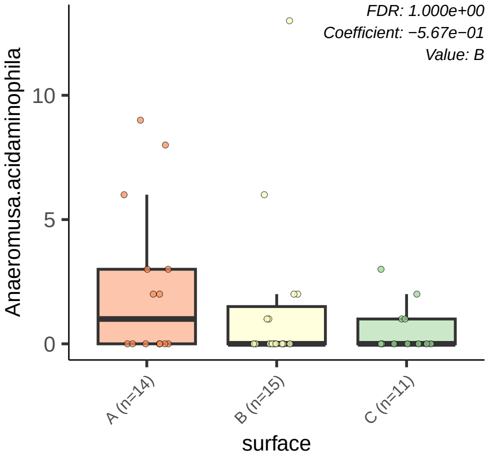

FDR: 1.000e+00
Coefficient: −5.67e−01
Value: B
Anaeromusa.acidaminophila
10
5
0
A (n=14)
B (n=15)
C (n=11)
surface

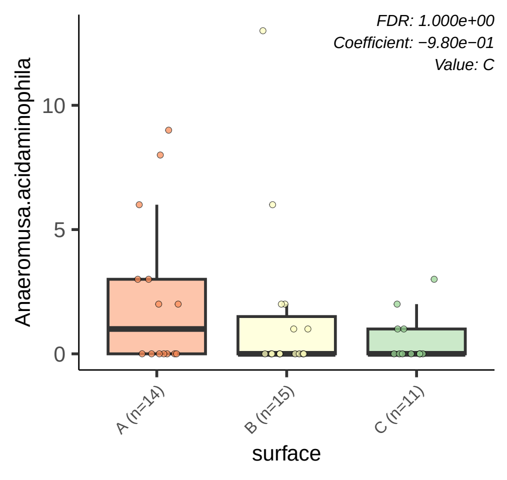
FDR: 1.000e+00
Coefficient: −9.80e−01
Value: C
Anaeromusa.acidaminophila
10
5
0
A (n=14)
B (n=15)
C (n=11)
surface

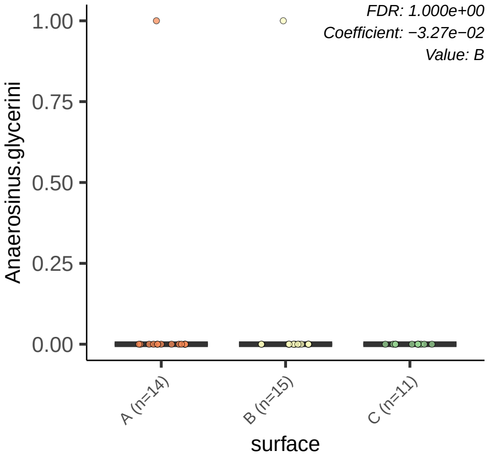
FDR: 1.000e+00
Coefficient: −3.27e−02
Value: B
Anaerosinus.glycerini
1.00
0.75
0.50
0.25
0.00
A (n=14)
B (n=15)
C (n=11)
surface

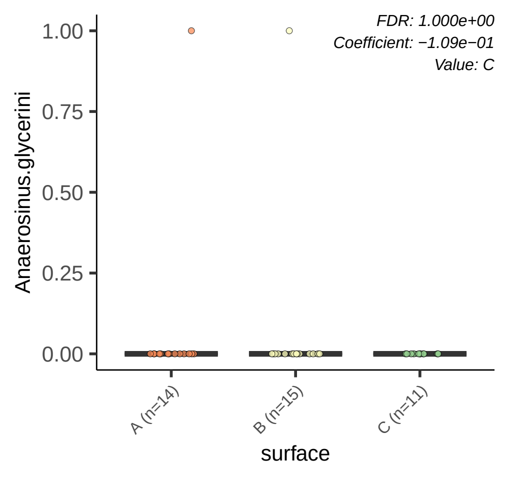

FDR: 1.000e+00
Coefficient: −1.09e−01
Value: C
Anaerosinus.glycerini
1.00
0.75
0.50
0.25
0.00
A (n=14)
B (n=15)
C (n=11)
surface

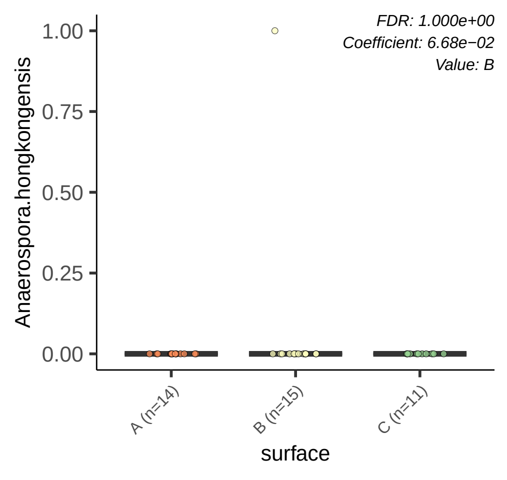
FDR: 1.000e+00
Coefficient: 6.68e−02
Value: B
Anaerospora.hongkongensis
1.00
0.75
0.50
0.25
0.00
A (n=14)
B (n=15)
C (n=11)
surface

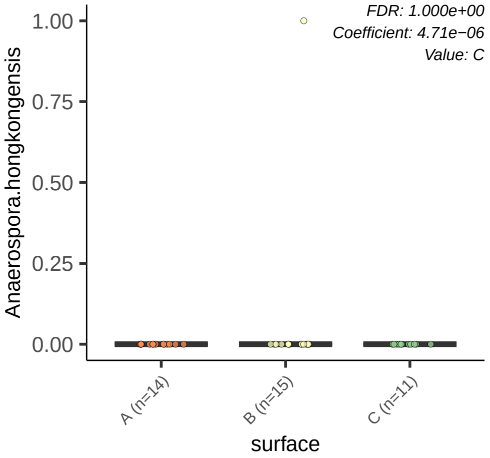
FDR: 1.000e+00
Coefficient: 4.71e−06
Value: C
Anaerospora.hongkongensis
1.00
0.75
0.50
0.25
0.00
A (n=14)
B (n=15)
C (n=11)
surface

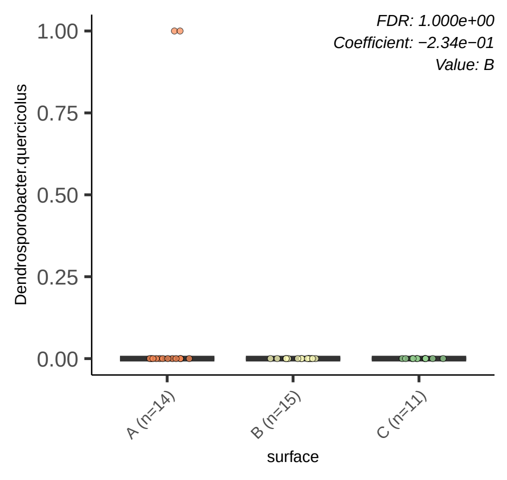

FDR: 1.000e+00
Coefficient: −2.34e−01
Value: B
1.00
0.75
0.50
0.25
0.00
Dendrosporobacter.quercicolus
A (n=14)
B (n=15)
C (n=11)
surface

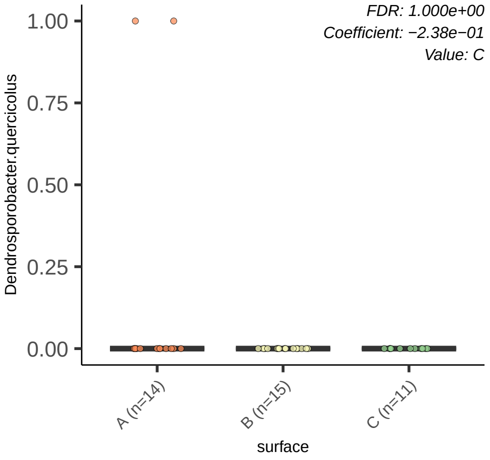
FDR: 1.000e+00
Coefficient: −2.38e−01
Value: C
Dendrosporobacter.quercicolus
1.00
0.75
0.50
0.25
0.00
A (n=14)
B (n=15)
C (n=11)
surface

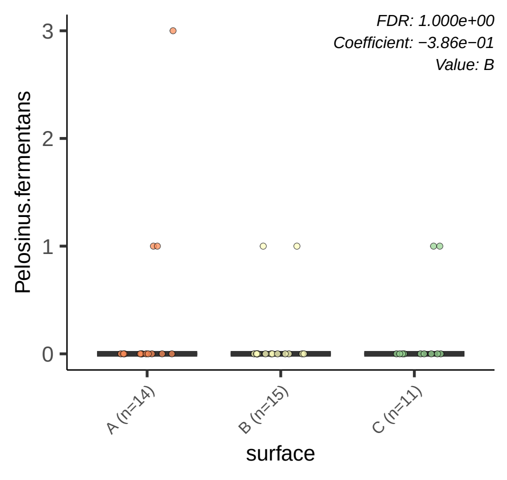
FDR: 1.000e+00
Coefficient: −3.86e−01
Value: B
Pelosinus.fermentans
0
1
2
3
A (n=14)
B (n=15)
C (n=11)
surface

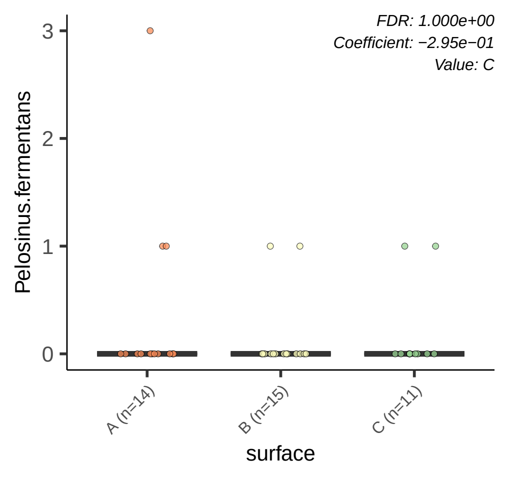
FDR: 1.000e+00
Coefficient: −2.95e−01
Value: C
Pelosinus.fermentans
0
1
2
3
A (n=14)
B (n=15)
C (n=11)
surface

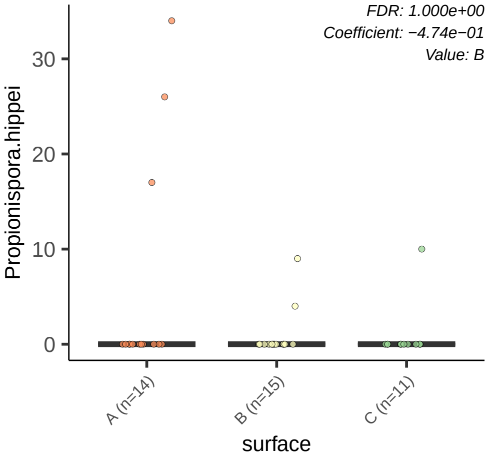
FDR: 1.000e+00
Coefficient: −4.74e−01
Value: B
Propionispora.hippei
0
10
20
30
A (n=14)
B (n=15)
C (n=11)
surface

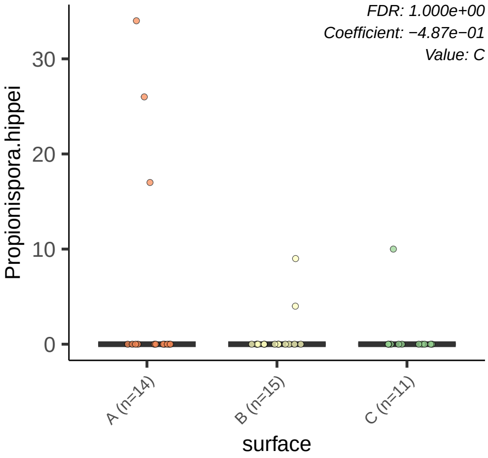

FDR: 1.000e+00
Coefficient: −4.87e−01
Value: C
Propionispora.hippei
30
20
10
0
A (n=14)
B (n=15)
C (n=11)
surface

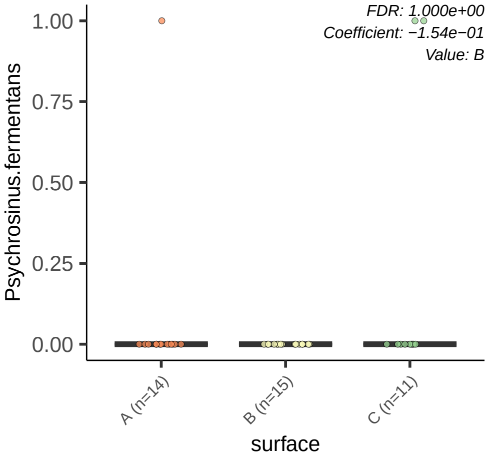

FDR: 1.000e+00
Coefficient: −1.54e−01
Value: B
Psychrosinus.fermentans
1.00
0.75
0.50
0.25
0.00
A (n=14)
B (n=15)
C (n=11)
surface

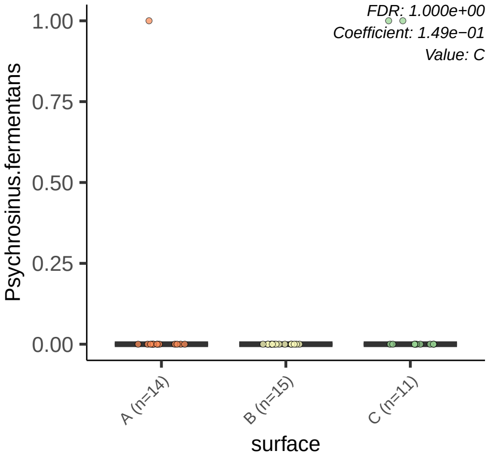

FDR: 1.000e+00
Coefficient: 1.49e−01
Value: C
Psychrosinus.fermentans
1.00
0.75
0.50
0.25
0.00
A (n=14)
B (n=15)
C (n=11)
surface

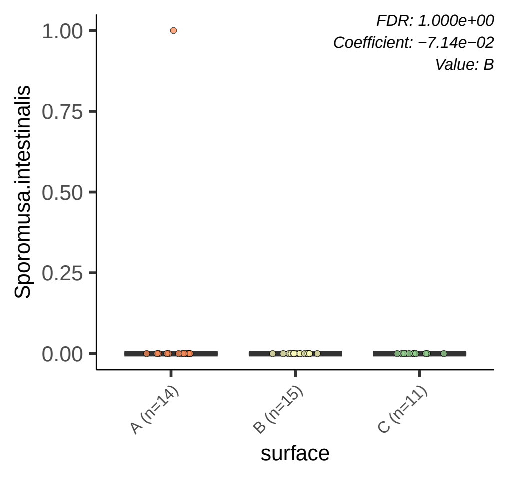
FDR: 1.000e+00
Coefficient: −7.14e−02
Value: B
Sporomusa.intestinalis
1.00
0.75
0.50
0.25
0.00
A (n=14)
B (n=15)
C (n=11)
surface

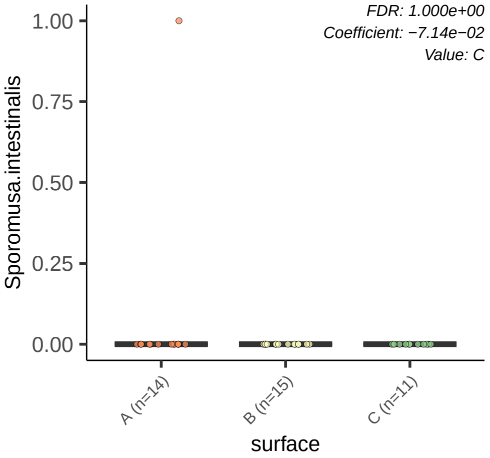
FDR: 1.000e+00
Coefficient: −7.14e−02
Value: C
Sporomusa.intestinalis
1.00
0.75
0.50
0.25
0.00
A (n=14)
B (n=15)
C (n=11)
surface

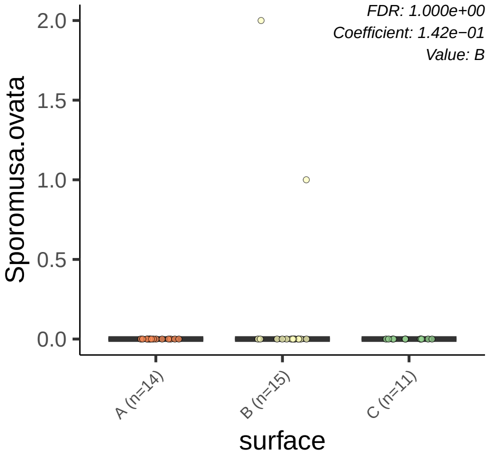

FDR: 1.000e+00
Coefficient: 1.42e−01
Value: B
Sporomusa.ovata
2.0
1.5
1.0
0.5
0.0
A (n=14)
B (n=15)
C (n=11)
surface

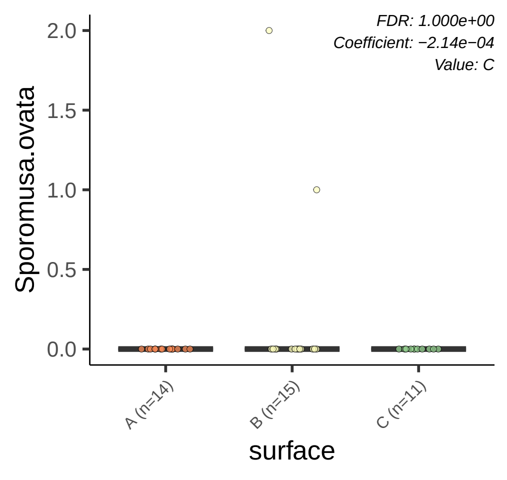
FDR: 1.000e+00
Coefficient: −2.14e−04
Value: C
Sporomusa.ovata
2.0
1.5
1.0
0.5
0.0
A (n=14)
B (n=15)
C (n=11)
surface

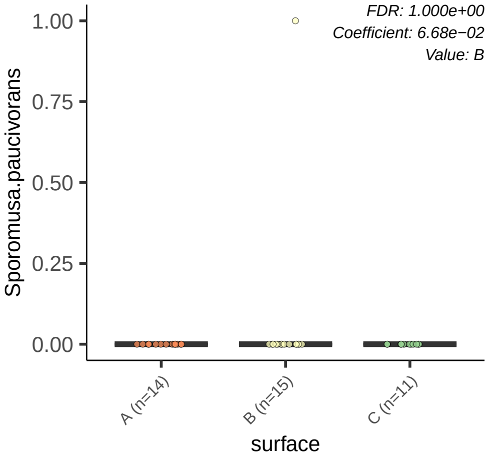
FDR: 1.000e+00
Coefficient: 6.68e−02
Value: B
Sporomusa.paucivorans
1.00
0.75
0.50
0.25
0.00
A (n=14)
B (n=15)
C (n=11)
surface

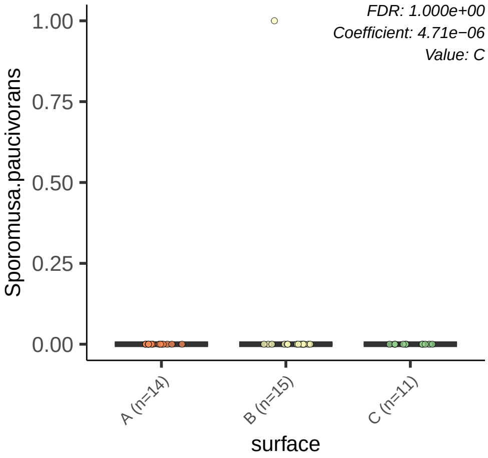

FDR: 1.000e+00
Coefficient: 4.71e−06
Value: C
Sporomusa.paucivorans
1.00
0.75
0.50
0.25
0.00
A (n=14)
B (n=15)
C (n=11)
surface

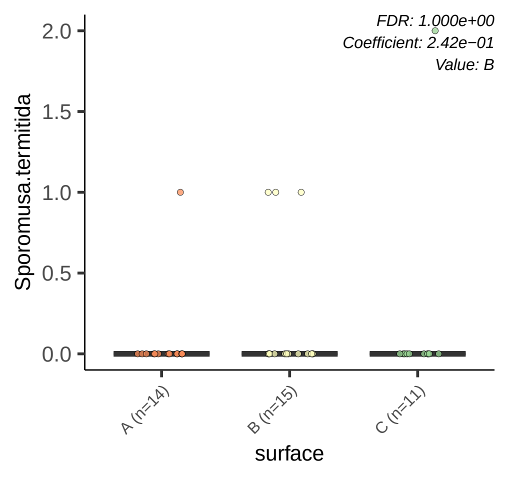
FDR: 1.000e+00
Coefficient: 2.42e−01
Value: B
Sporomusa.termitida
2.0
1.5
1.0
0.5
0.0
A (n=14)
B (n=15)
C (n=11)
surface

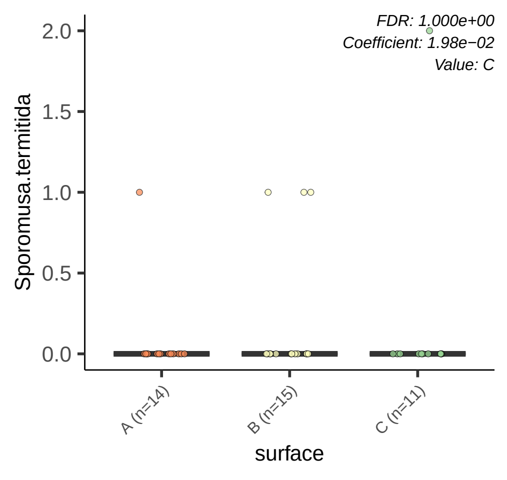
FDR: 1.000e+00
Coefficient: 1.98e−02
Value: C
Sporomusa.termitida
2.0
1.5
1.0
0.5
0.0
A (n=14)
B (n=15)
C (n=11)
surface

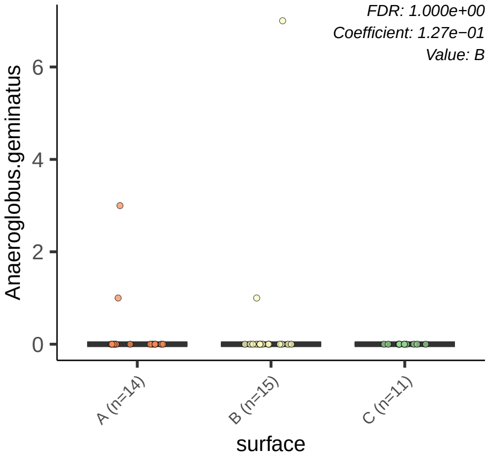
FDR: 1.000e+00
Coefficient: 1.27e−01
Value: B
Anaeroglobus.geminatus
0
2
4
6
A (n=14)
B (n=15)
C (n=11)
surface

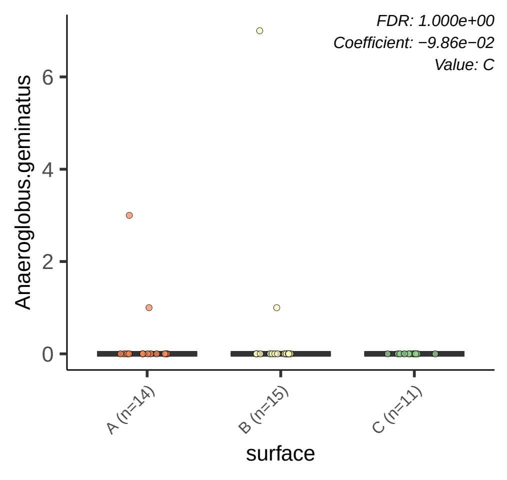
FDR: 1.000e+00
Coefficient: −9.86e−02
Value: C
Anaeroglobus.geminatus
0
2
4
6
A (n=14)
B (n=15)
C (n=11)
surface

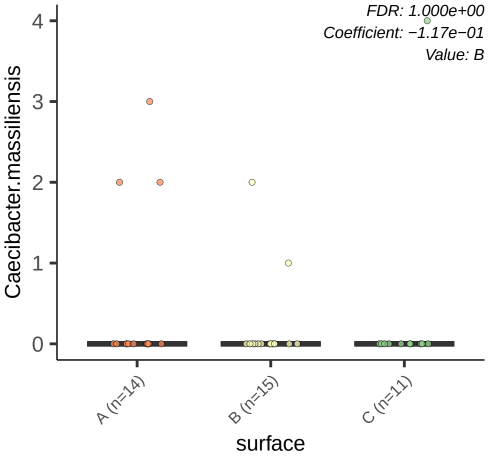

FDR: 1.000e+00
Coefficient: −1.17e−01
Value: B
Caecibacter.massiliensis
0
1
2
3
4
A (n=14)
B (n=15)
C (n=11)
surface

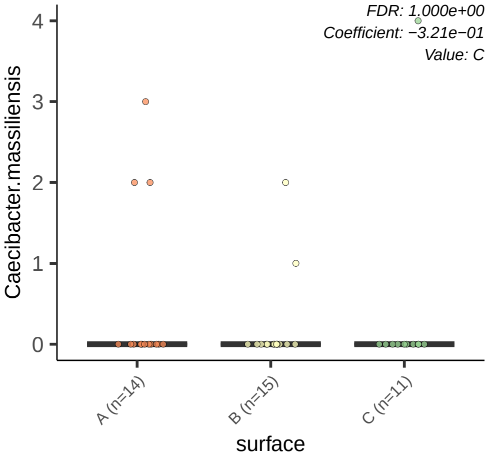
FDR: 1.000e+00
Coefficient: −3.21e−01
Value: C
Caecibacter.massiliensis
0
1
2
3
4
A (n=14)
B (n=15)
C (n=11)
surface

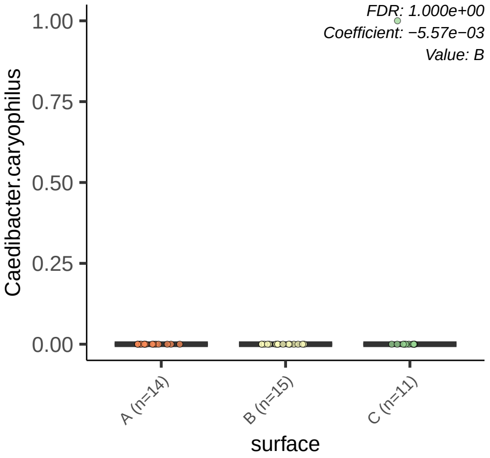
FDR: 1.000e+00
Coefficient: −5.57e−03
Value: B
Caedibacter.caryophilus
1.00
0.75
0.50
0.25
0.00
A (n=14)
B (n=15)
C (n=11)
surface

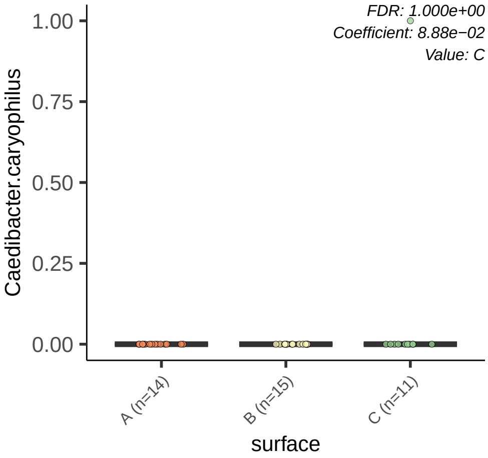
FDR: 1.000e+00
Coefficient: 8.88e−02
Value: C
Caedibacter.caryophilus
1.00
0.75
0.50
0.25
0.00
A (n=14)
B (n=15)
C (n=11)
surface

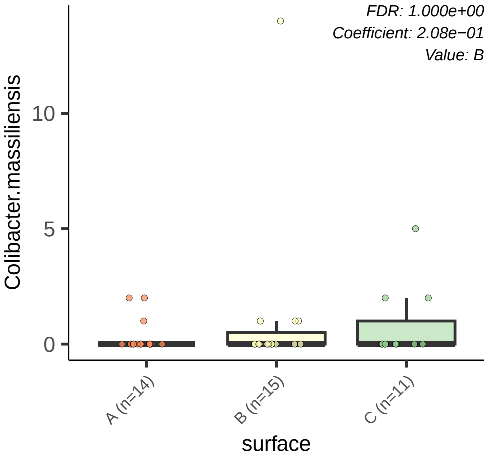

FDR: 1.000e+00
Coefficient: 2.08e−01
Value: B
Colibacter.massiliensis
10
5
0
A (n=14)
B (n=15)
C (n=11)
surface

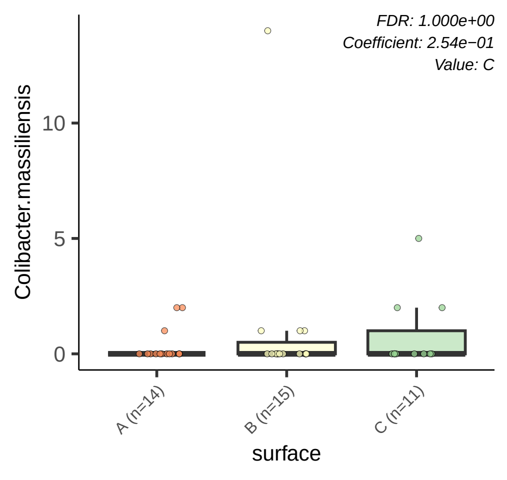

FDR: 1.000e+00
Coefficient: 2.54e−01
Value: C
Colibacter.massiliensis
0
5
10
A (n=14)
B (n=15)
C (n=11)
surface

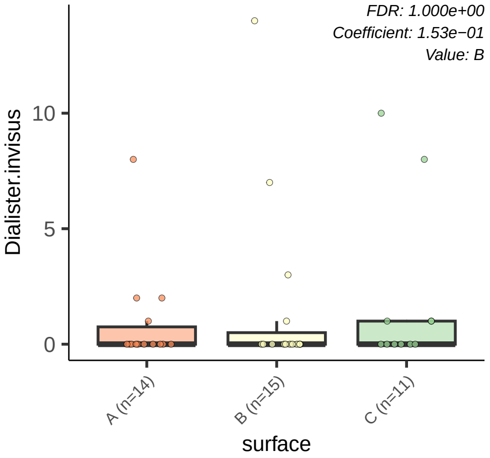
FDR: 1.000e+00
Coefficient: 1.53e−01
Value: B
Dialister.invisus
0
5
10
A (n=14)
B (n=15)
C (n=11)
surface

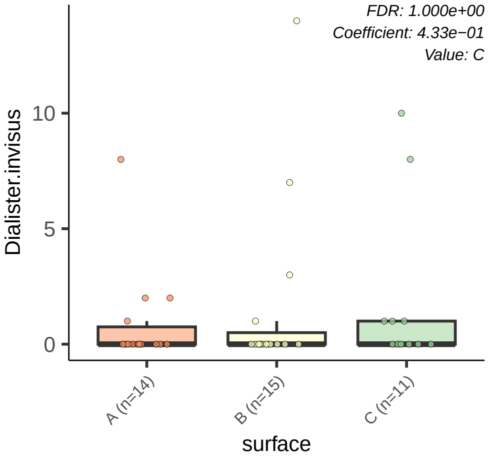
FDR: 1.000e+00
Coefficient: 4.33e−01
Value: C
Dialister.invisus
10
5
0
A (n=14)
B (n=15)
C (n=11)
surface

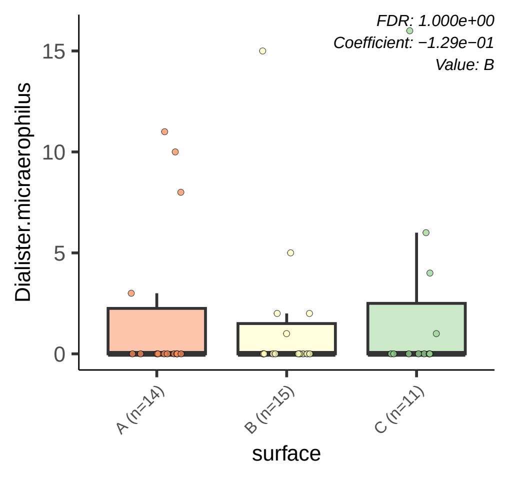
FDR: 1.000e+00
Coefficient: −1.29e−01
Value: B
Dialister.micraerophilus
15
10
5
0
A (n=14)
B (n=15)
C (n=11)
surface

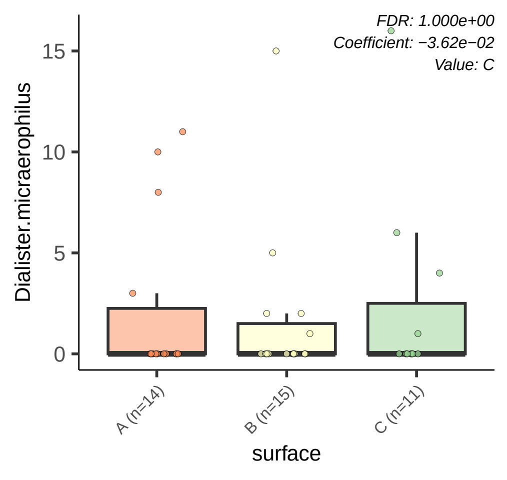

FDR: 1.000e+00
Coefficient: −3.62e−02
Value: C
Dialister.micraerophilus
15
10
5
0
A (n=14)
B (n=15)
C (n=11)
surface

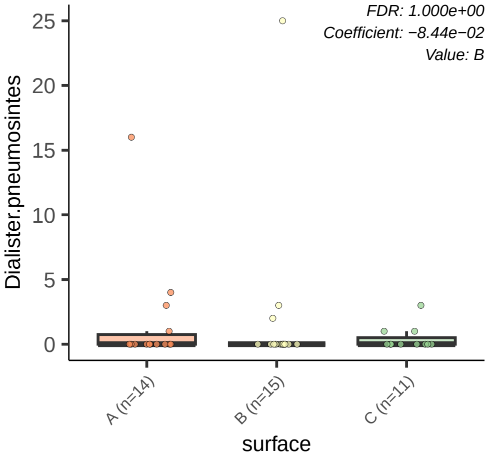

FDR: 1.000e+00
Coefficient: −8.44e−02
Value: B
Dialister.pneumosintes
0
5
10
15
20
25
A (n=14)
B (n=15)
C (n=11)
surface

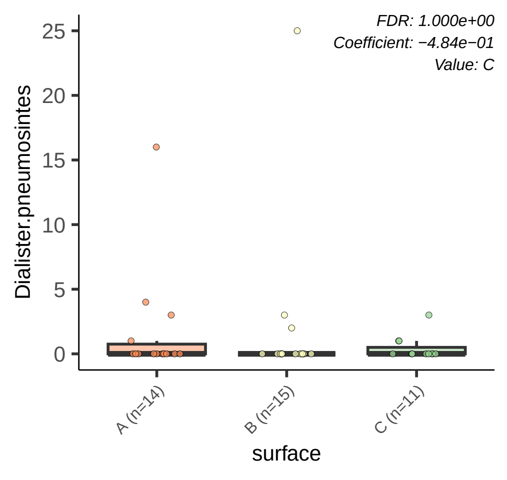

FDR: 1.000e+00
Coefficient: −4.84e−01
Value: C
Dialister.pneumosintes
0
5
10
15
20
25
A (n=14)
B (n=15)
C (n=11)
surface

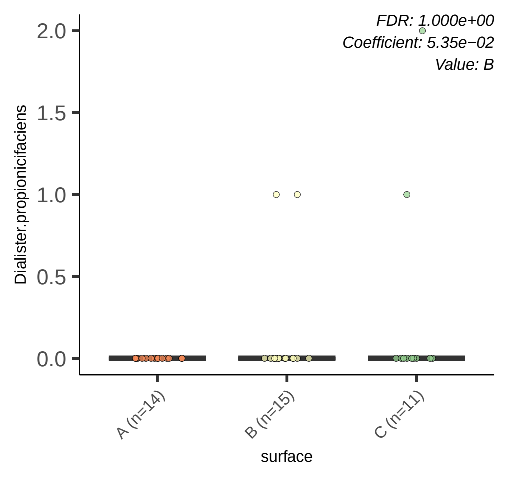
FDR: 1.000e+00
Coefficient: 5.35e−02
Value: B
Dialister.propionicifaciens
2.0
1.5
1.0
0.5
0.0
A (n=14)
B (n=15)
C (n=11)
surface

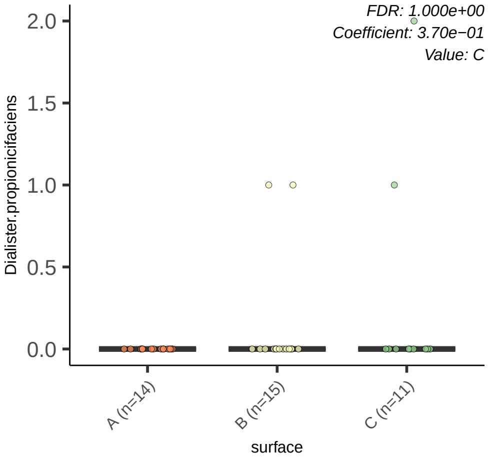
FDR: 1.000e+00
Coefficient: 3.70e−01
Value: C
Dialister.propionicifaciens
2.0
1.5
1.0
0.5
0.0
A (n=14)
B (n=15)
C (n=11)
surface

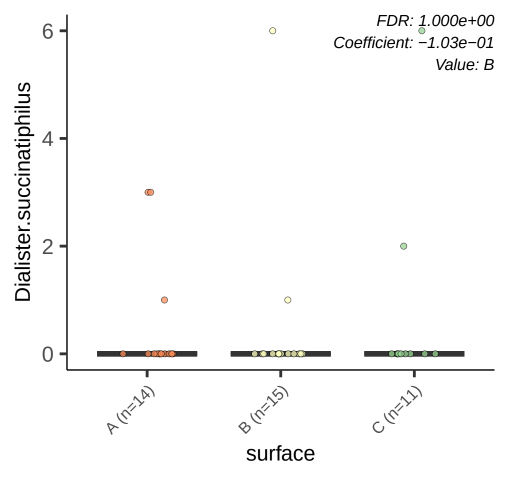

FDR: 1.000e+00
Coefficient: −1.03e−01
Value: B
Dialister.succinatiphilus
6
4
2
0
A (n=14)
B (n=15)
C (n=11)
surface

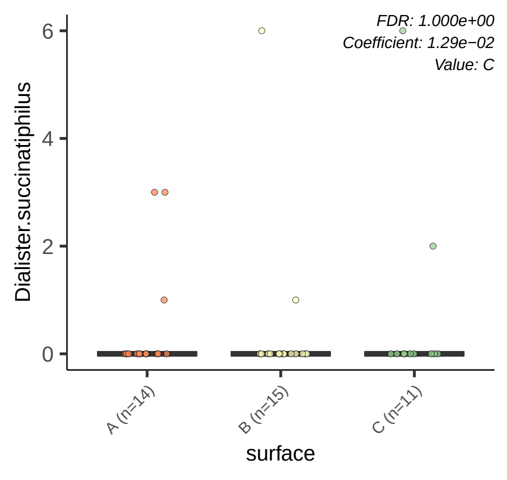

FDR: 1.000e+00
Coefficient: 1.29e−02
Value: C
Dialister.succinatiphilus
6
4
2
0
A (n=14)
B (n=15)
C (n=11)
surface

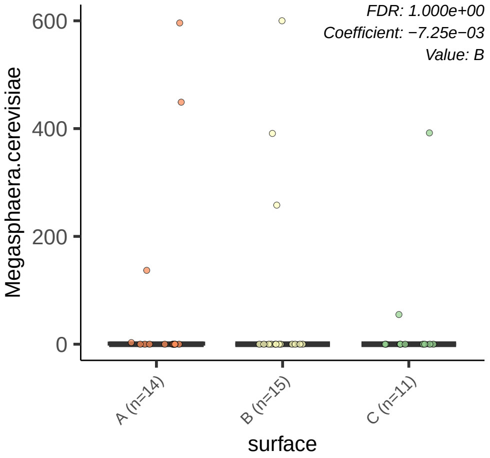
FDR: 1.000e+00
Coefficient: −7.25e−03
Value: B
Megasphaera.cerevisiae
0
200
400
600
A (n=14)
B (n=15)
C (n=11)
surface

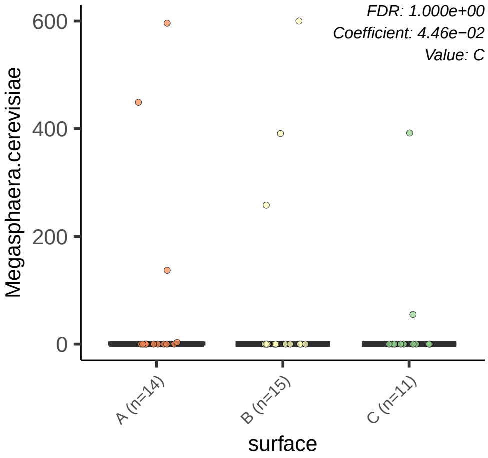

FDR: 1.000e+00
Coefficient: 4.46e−02
Value: C
Megasphaera.cerevisiae
600
400
200
0
A (n=14)
B (n=15)
C (n=11)
surface

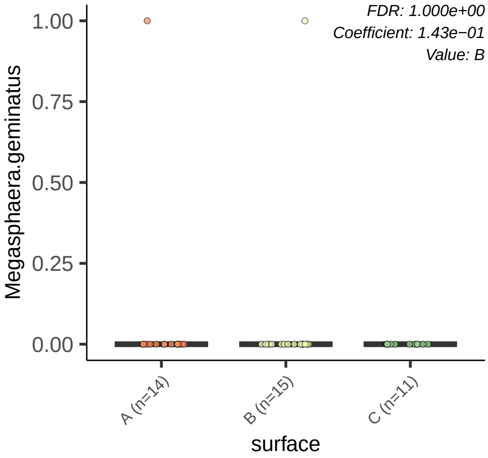

FDR: 1.000e+00
Coefficient: 1.43e−01
Value: B
Megasphaera.geminatus
1.00
0.75
0.50
0.25
0.00
A (n=14)
B (n=15)
C (n=11)
surface

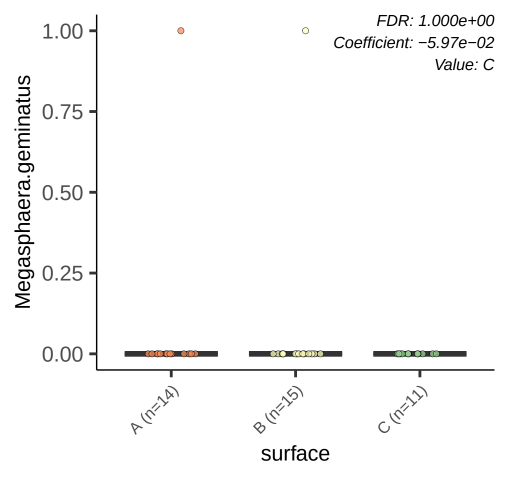

FDR: 1.000e+00
Coefficient: −5.97e−02
Value: C
Megasphaera.geminatus
1.00
0.75
0.50
0.25
0.00
A (n=14)
B (n=15)
C (n=11)
surface

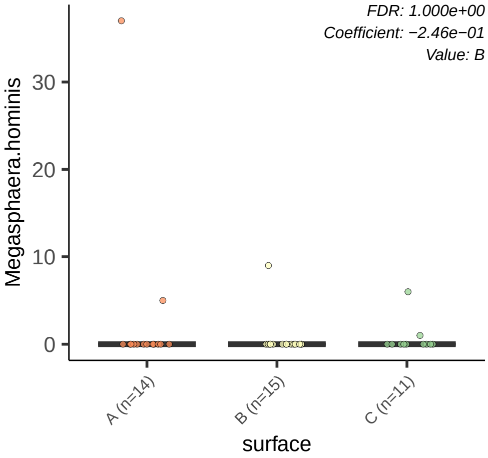
FDR: 1.000e+00
Coefficient: −2.46e−01
Value: B
Megasphaera.hominis
0
10
20
30
A (n=14)
B (n=15)
C (n=11)
surface

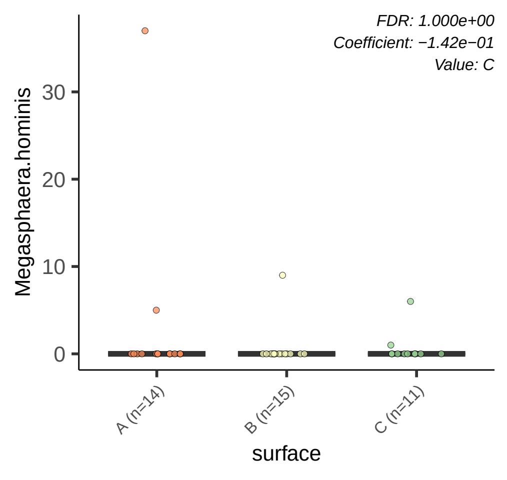
FDR: 1.000e+00
Coefficient: −1.42e−01
Value: C
Megasphaera.hominis
0
10
20
30
A (n=14)
B (n=15)
C (n=11)
surface

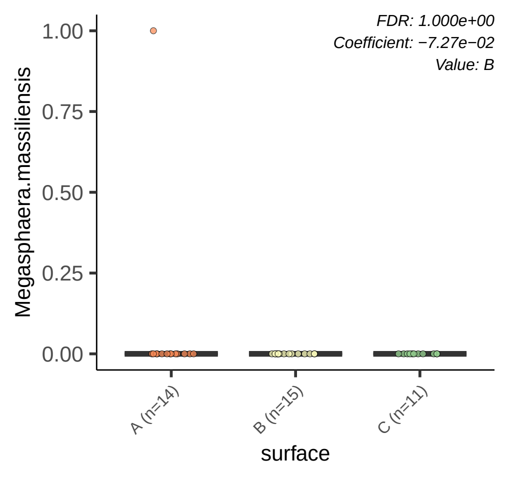

FDR: 1.000e+00
Coefficient: −7.27e−02
Value: B
Megasphaera.massiliensis
1.00
0.75
0.50
0.25
0.00
A (n=14)
B (n=15)
C (n=11)
surface

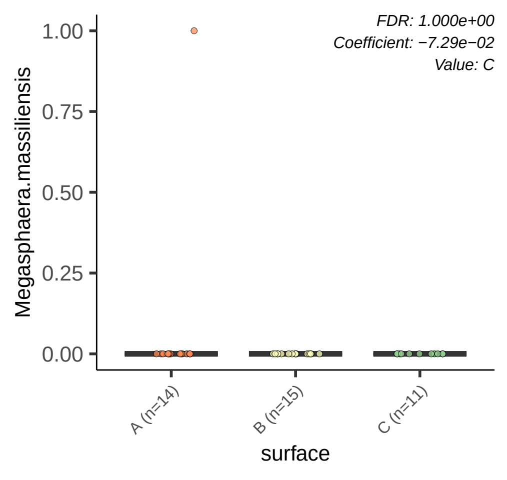

FDR: 1.000e+00
Coefficient: −7.29e−02
Value: C
Megasphaera.massiliensis
1.00
0.75
0.50
0.25
0.00
A (n=14)
B (n=15)
C (n=11)
surface

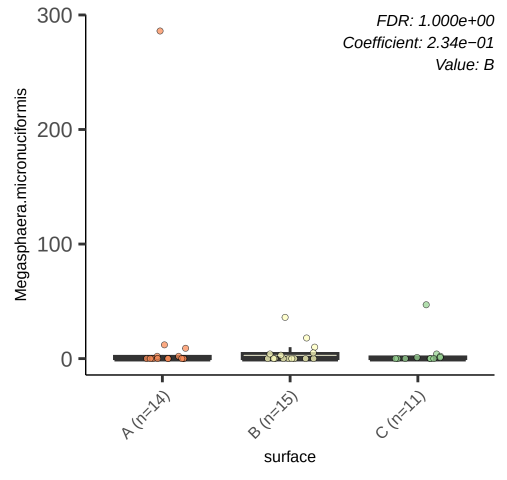
FDR: 1.000e+00
Coefficient: 2.34e−01
Value: B
300
200
100
0
Megasphaera.micronuciformis
A (n=14)
B (n=15)
C (n=11)
surface

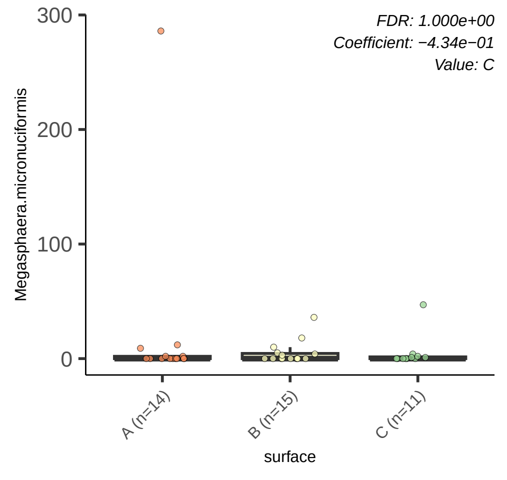
FDR: 1.000e+00
Coefficient: −4.34e−01
Value: C
Megasphaera.micronuciformis
300
200
100
0
A (n=14)
B (n=15)
C (n=11)
surface

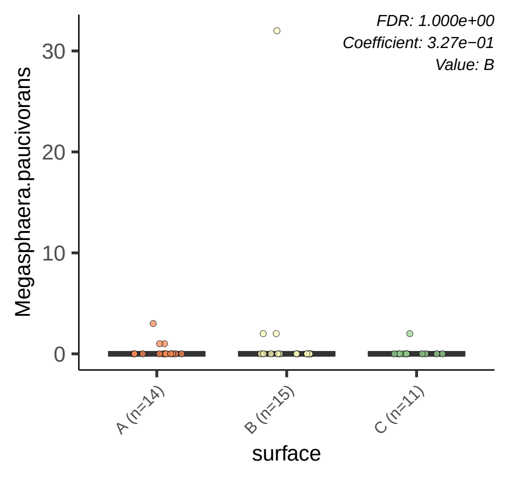

FDR: 1.000e+00
Coefficient: 3.27e−01
Value: B
Megasphaera.paucivorans
30
20
10
0
A (n=14)
B (n=15)
C (n=11)
surface

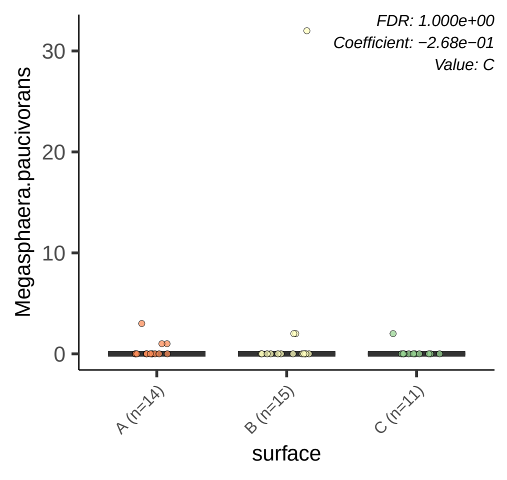
FDR: 1.000e+00
Coefficient: −2.68e−01
Value: C
Megasphaera.paucivorans
30
20
10
0
A (n=14)
B (n=15)
C (n=11)
surface

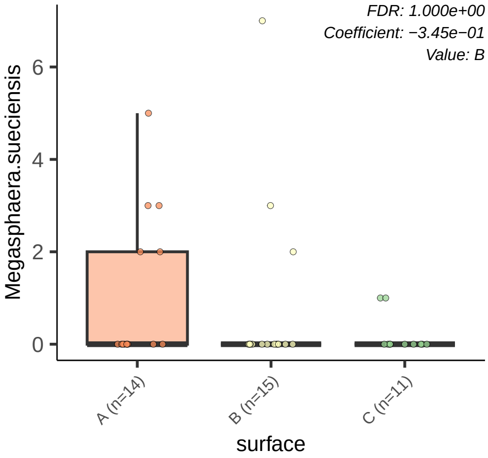

FDR: 1.000e+00
Coefficient: −3.45e−01
Value: B
Megasphaera.sueciensis
0
2
4
6
A (n=14)
B (n=15)
C (n=11)
surface

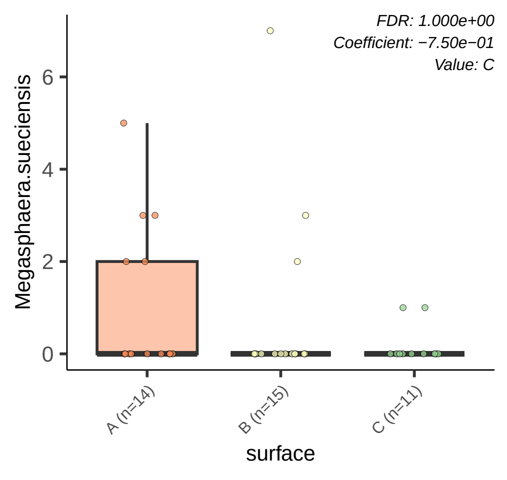
FDR: 1.000e+00
Coefficient: −7.50e−01
Value: C
Megasphaera.sueciensis
0
2
4
6
A (n=14)
B (n=15)
C (n=11)
surface

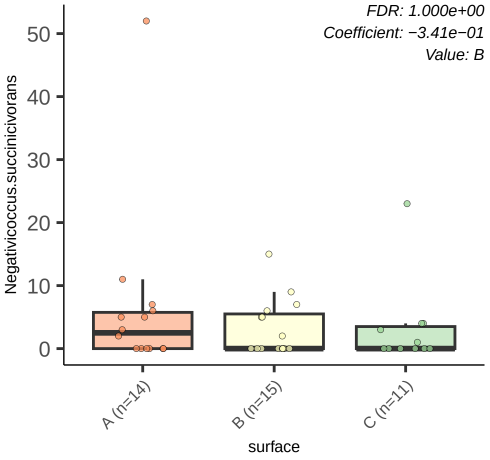

FDR: 1.000e+00
Coefficient: −3.41e−01
Value: B
Negativicoccus.succinicivorans
0
10
20
30
40
50
A (n=14)
B (n=15)
C (n=11)
surface

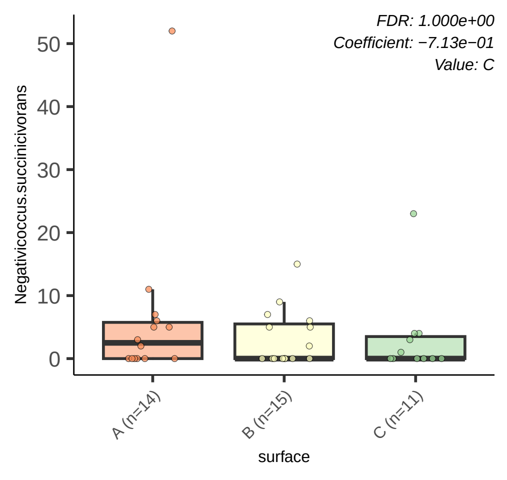
FDR: 1.000e+00
Coefficient: −7.13e−01
Value: C
Negativicoccus.succinicivorans
0
10
20
30
40
50
A (n=14)
B (n=15)
C (n=11)
surface

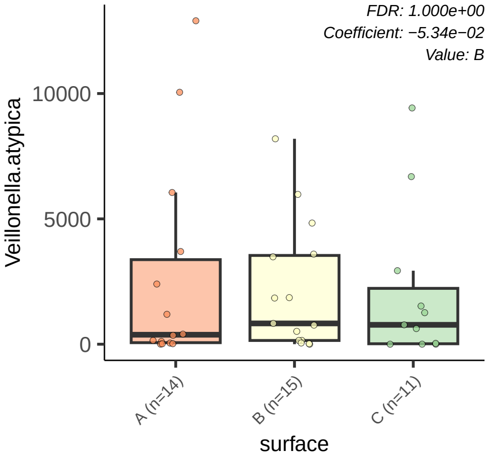

FDR: 1.000e+00
Coefficient: −5.34e−02
Value: B
Veillonella.atypica
10000
5000
0
A (n=14)
B (n=15)
C (n=11)
surface

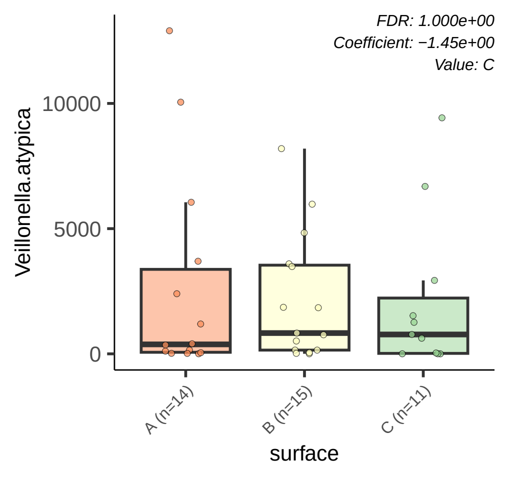
FDR: 1.000e+00
Coefficient: −1.45e+00
Value: C
Veillonella.atypica
10000
5000
0
A (n=14)
B (n=15)
C (n=11)
surface

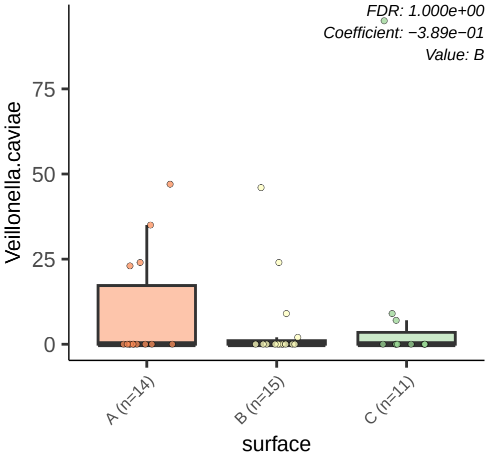
FDR: 1.000e+00
Coefficient: −3.89e−01
Value: B
Veillonella.caviae
75
50
25
0
A (n=14)
B (n=15)
C (n=11)
surface

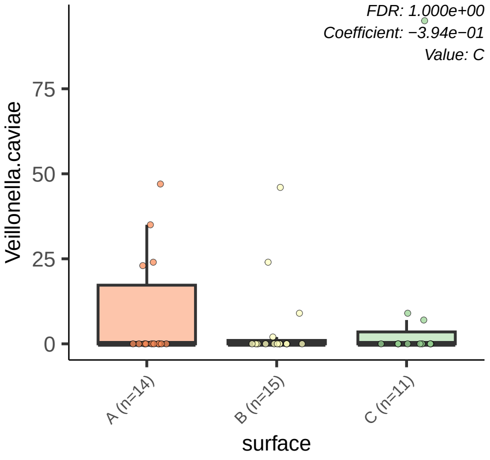
FDR: 1.000e+00
Coefficient: −3.94e−01
Value: C
Veillonella.caviae
75
50
25
0
A (n=14)
B (n=15)
C (n=11)
surface

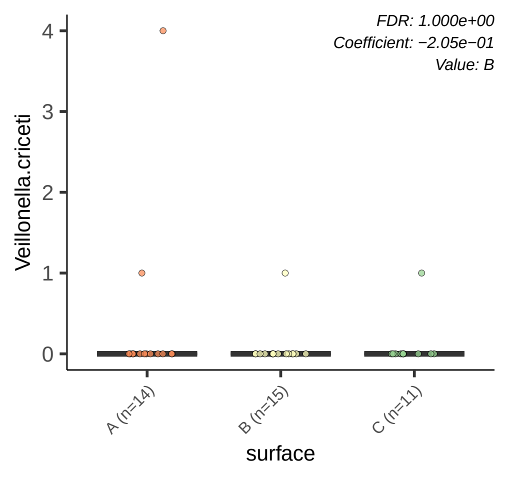

FDR: 1.000e+00
Coefficient: −2.05e−01
Value: B
Veillonella.criceti
0
1
2
3
4
A (n=14)
B (n=15)
C (n=11)
surface

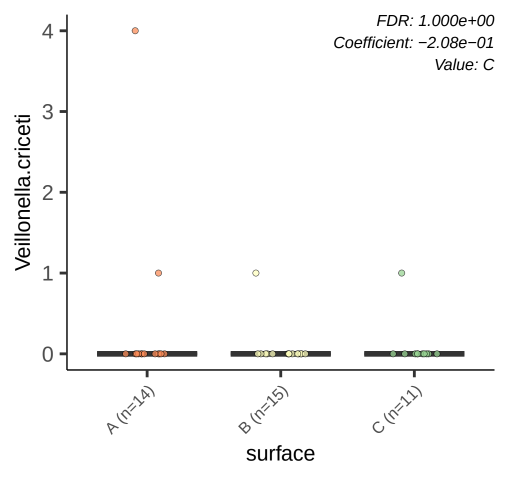

FDR: 1.000e+00
Coefficient: −2.08e−01
Value: C
Veillonella.criceti
4
3
2
1
0
A (n=14)
B (n=15)
C (n=11)
surface

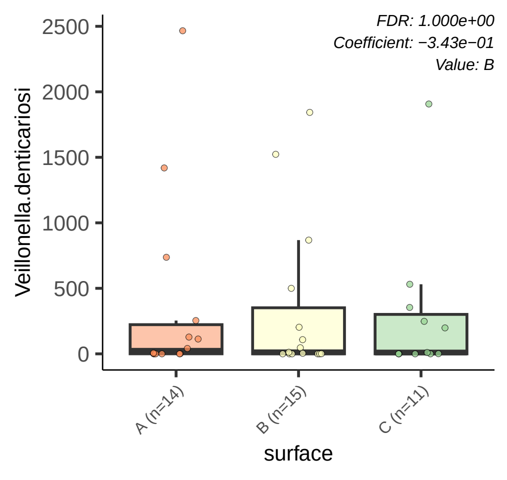

FDR: 1.000e+00
Coefficient: −3.43e−01
Value: B
Veillonella.denticariosi
2500
2000
1500
1000
500
0
A (n=14)
B (n=15)
C (n=11)
surface

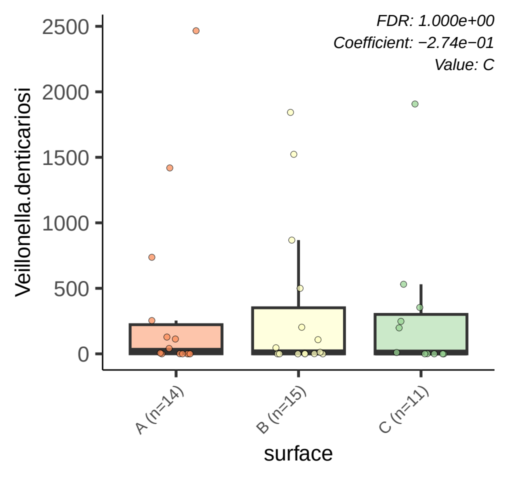

FDR: 1.000e+00
Coefficient: −2.74e−01
Value: C
Veillonella.denticariosi
2500
2000
1500
1000
500
0
A (n=14)
B (n=15)
C (n=11)
surface

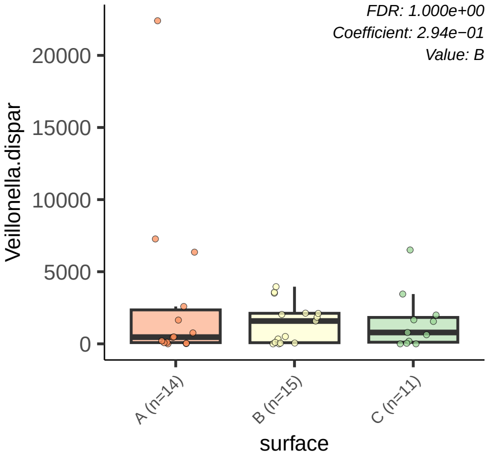
FDR: 1.000e+00
Coefficient: 2.94e−01
Value: B
20000
15000
10000
5000
0
Veillonella.dispar
A (n=14)
B (n=15)
C (n=11)
surface

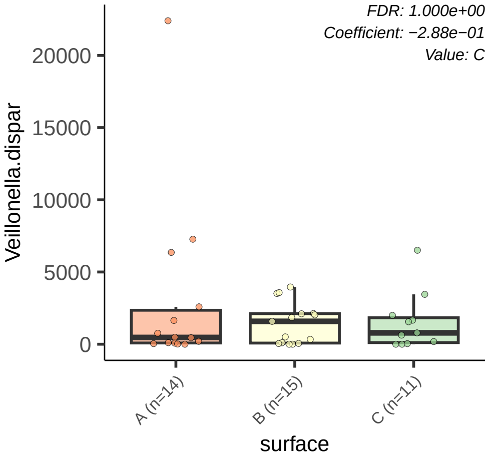
FDR: 1.000e+00
Coefficient: −2.88e−01
Value: C
Veillonella.dispar
20000
15000
10000
5000
0
A (n=14)
B (n=15)
C (n=11)
surface

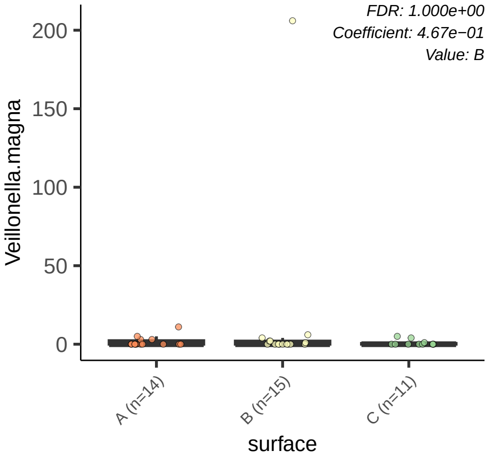

FDR: 1.000e+00
Coefficient: 4.67e−01
Value: B
Veillonella.magna
200
150
100
50
0
A (n=14)
B (n=15)
C (n=11)
surface

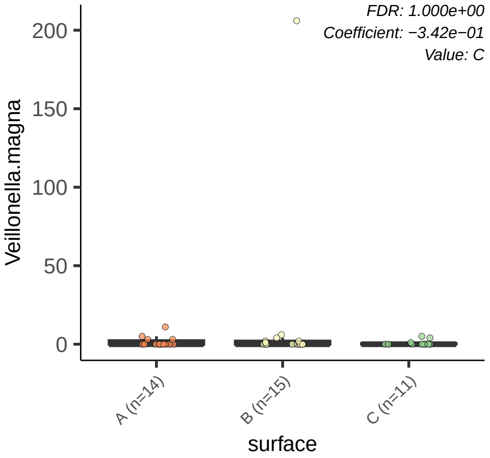
FDR: 1.000e+00
Coefficient: −3.42e−01
Value: C
Veillonella.magna
200
150
100
50
0
A (n=14)
B (n=15)
C (n=11)
surface

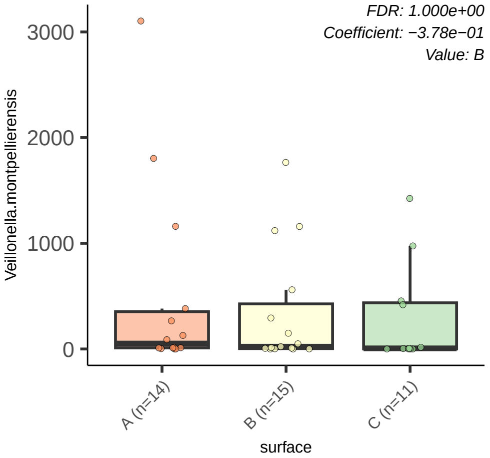

FDR: 1.000e+00
Coefficient: −3.78e−01
Value: B
Veillonella.montpellierensis
3000
2000
1000
0
A (n=14)
B (n=15)
C (n=11)
surface

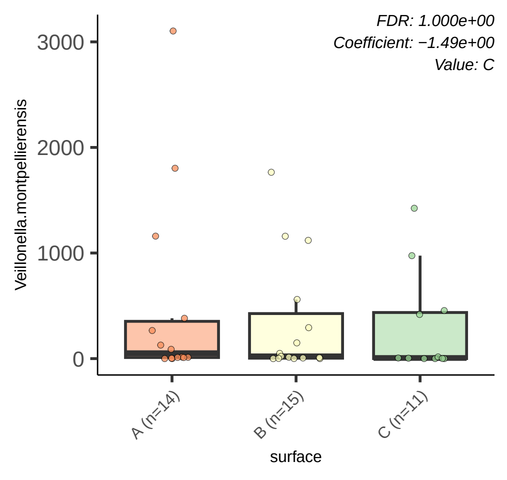
FDR: 1.000e+00
Coefficient: −1.49e+00
Value: C
3000
2000
1000
0
Veillonella.montpellierensis
A (n=14)
B (n=15)
C (n=11)
surface

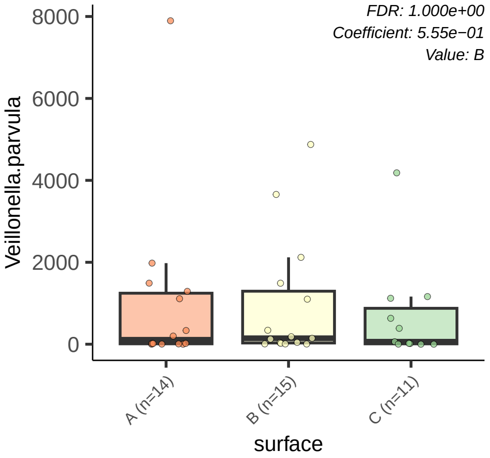

FDR: 1.000e+00
Coefficient: 5.55e−01
Value: B
8000
6000
4000
2000
0
Veillonella.parvula
A (n=14)
B (n=15)
C (n=11)
surface

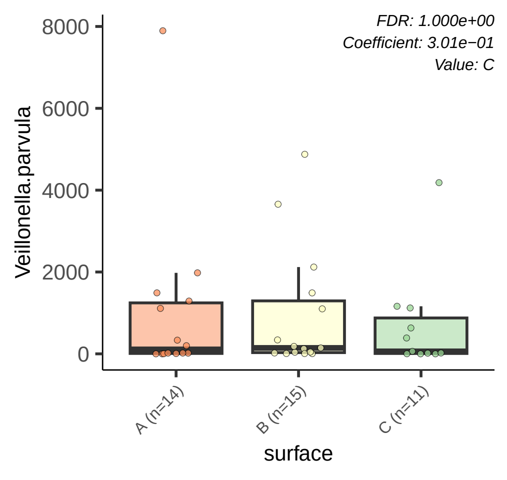

FDR: 1.000e+00
Coefficient: 3.01e−01
Value: C
Veillonella.parvula
8000
6000
4000
2000
0
A (n=14)
B (n=15)
C (n=11)
surface

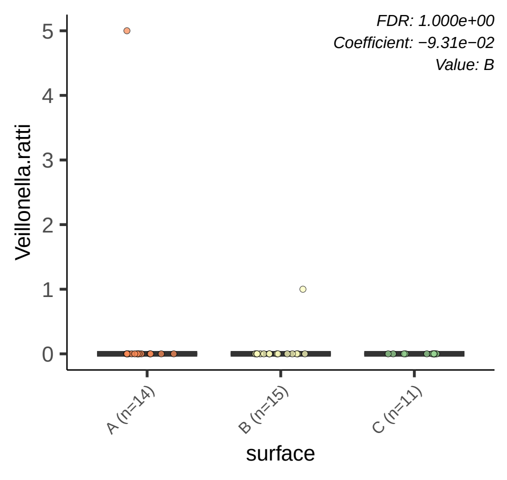
FDR: 1.000e+00
Coefficient: −9.31e−02
Value: B
Veillonella.ratti
0
1
2
3
4
5
A (n=14)
B (n=15)
C (n=11)
surface

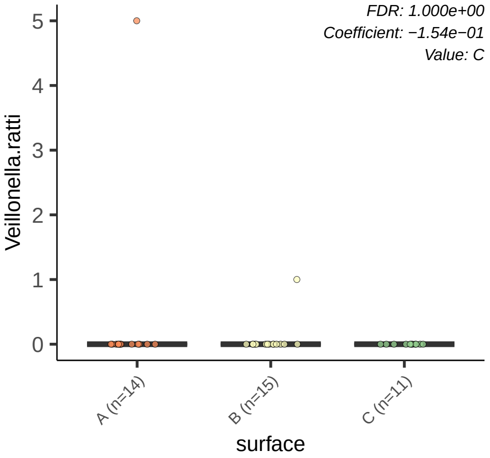
FDR: 1.000e+00
Coefficient: −1.54e−01
Value: C
Veillonella.ratti
5
4
3
2
1
0
A (n=14)
B (n=15)
C (n=11)
surface

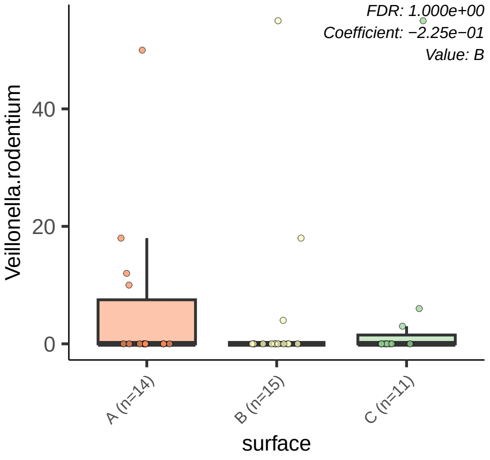
FDR: 1.000e+00
Coefficient: −2.25e−01
Value: B
Veillonella.rodentium
40
20
0
A (n=14)
B (n=15)
C (n=11)
surface

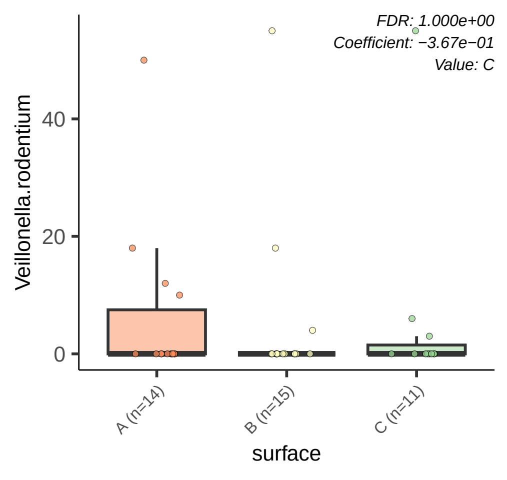

FDR: 1.000e+00
Coefficient: −3.67e−01
Value: C
Veillonella.rodentium
0
20
40
A (n=14)
B (n=15)
C (n=11)
surface

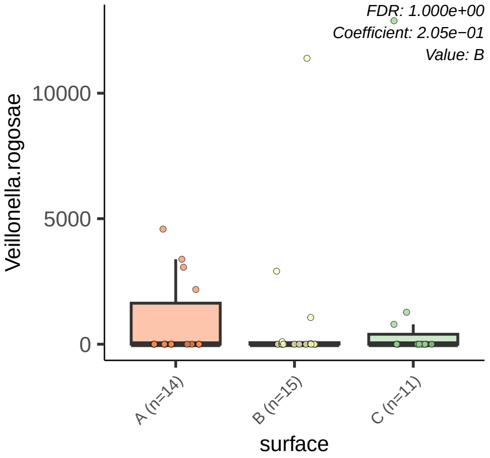

FDR: 1.000e+00
Coefficient: 2.05e−01
Value: B
Veillonella.rogosae
10000
5000
0
A (n=14)
B (n=15)
C (n=11)
surface

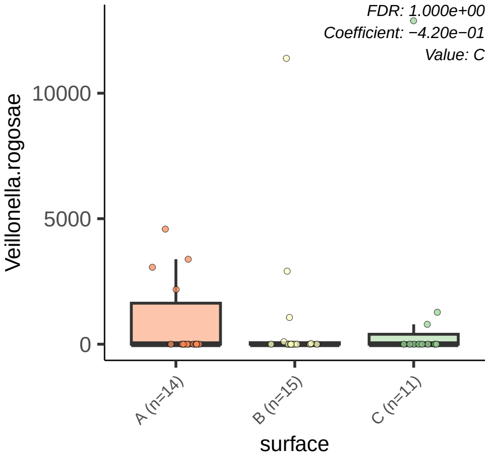

FDR: 1.000e+00
Coefficient: −4.20e−01
Value: C
Veillonella.rogosae
10000
5000
0
A (n=14)
B (n=15)
C (n=11)
surface

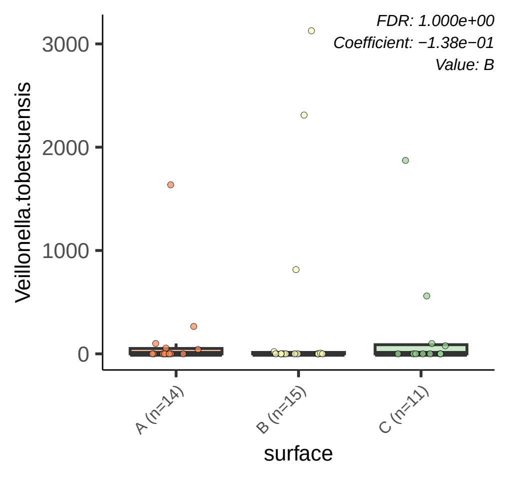
FDR: 1.000e+00
Coefficient: −1.38e−01
Value: B
Veillonella.tobetsuensis
3000
2000
1000
0
A (n=14)
B (n=15)
C (n=11)
surface

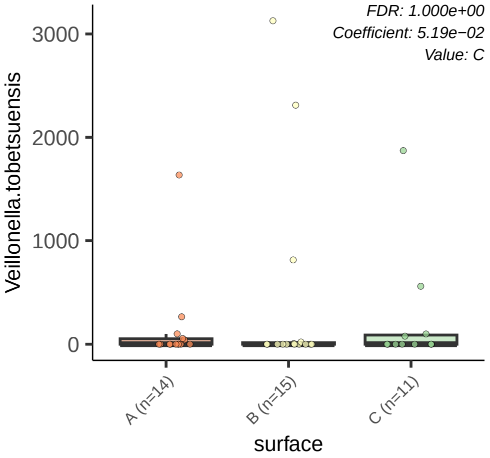

FDR: 1.000e+00
Coefficient: 5.19e−02
Value: C
Veillonella.tobetsuensis
3000
2000
1000
0
A (n=14)
B (n=15)
C (n=11)
surface

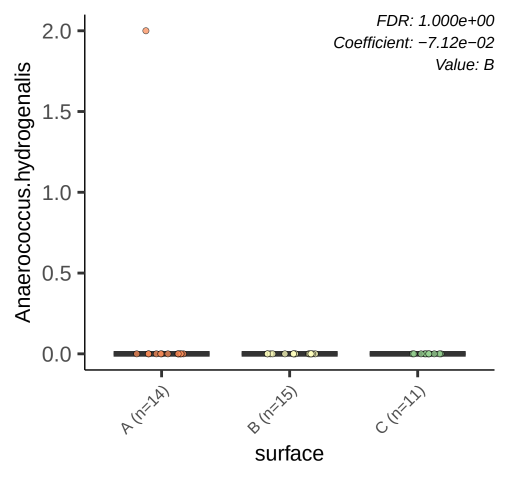

FDR: 1.000e+00
Coefficient: −7.12e−02
Value: B
Anaerococcus.hydrogenalis
2.0
1.5
1.0
0.5
0.0
A (n=14)
B (n=15)
C (n=11)
surface

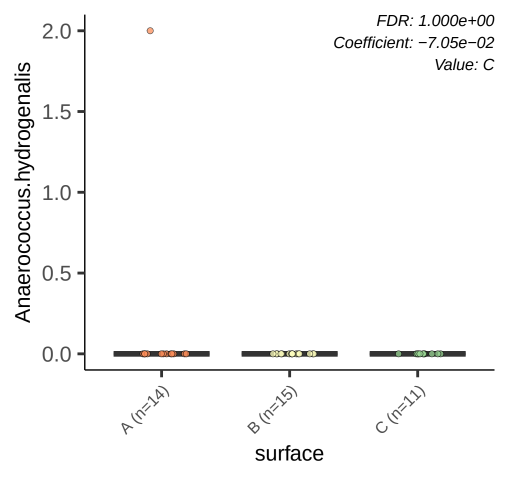

FDR: 1.000e+00
Coefficient: −7.05e−02
Value: C
Anaerococcus.hydrogenalis
2.0
1.5
1.0
0.5
0.0
A (n=14)
B (n=15)
C (n=11)
surface

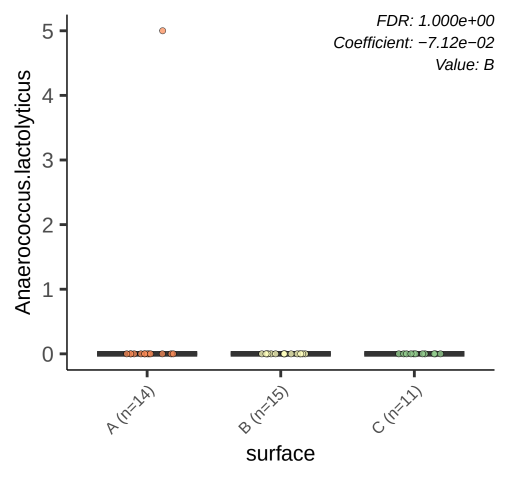
FDR: 1.000e+00
Coefficient: −7.12e−02
Value: B
Anaerococcus.lactolyticus
0
1
2
3
4
5
A (n=14)
B (n=15)
C (n=11)
surface

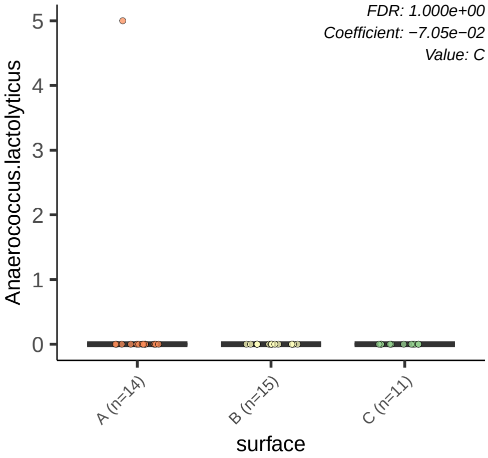
FDR: 1.000e+00
Coefficient: −7.05e−02
Value: C
Anaerococcus.lactolyticus
5
4
3
2
1
0
A (n=14)
B (n=15)
C (n=11)
surface

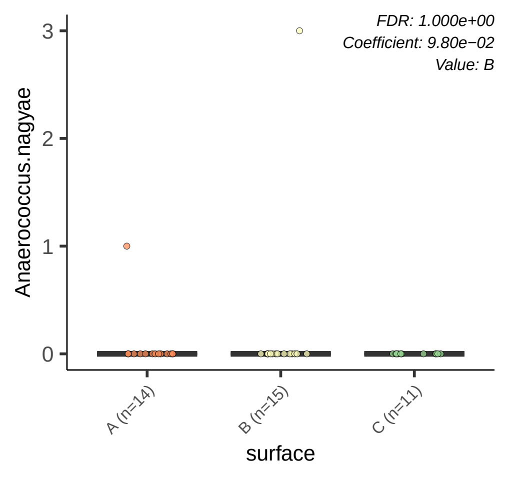

FDR: 1.000e+00
Coefficient: 9.80e−02
Value: B
Anaerococcus.nagyae
3
2
1
0
A (n=14)
B (n=15)
C (n=11)
surface

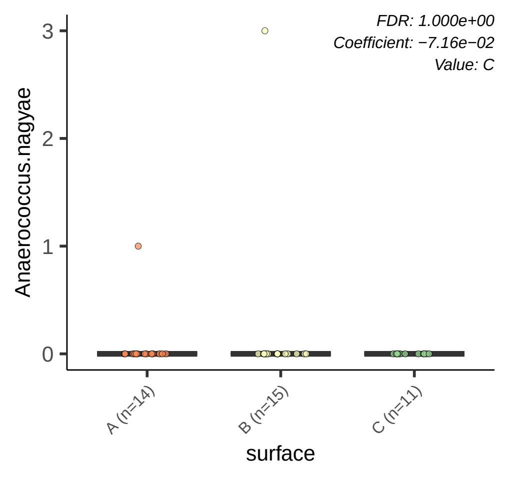

FDR: 1.000e+00
Coefficient: −7.16e−02
Value: C
Anaerococcus.nagyae
3
2
1
0
A (n=14)
B (n=15)
C (n=11)
surface

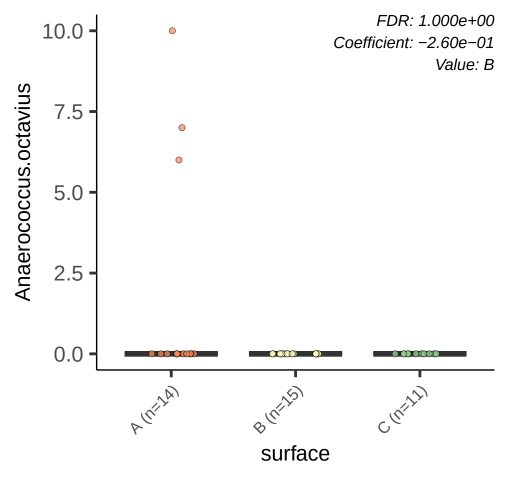
FDR: 1.000e+00
Coefficient: −2.60e−01
Value: B
Anaerococcus.octavius
10.0
7.5
5.0
2.5
0.0
A (n=14)
B (n=15)
C (n=11)
surface

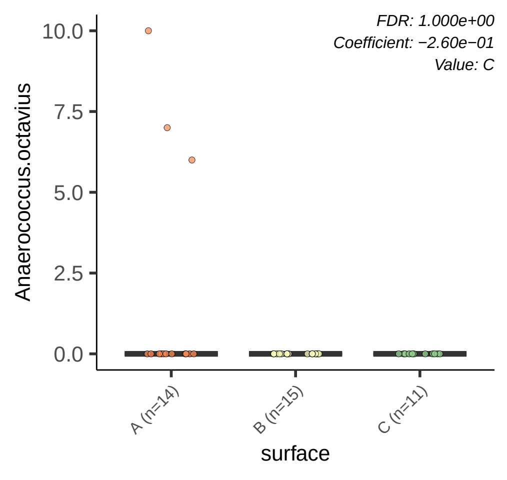
FDR: 1.000e+00
Coefficient: −2.60e−01
Value: C
Anaerococcus.octavius
10.0
7.5
5.0
2.5
0.0
A (n=14)
B (n=15)
C (n=11)
surface

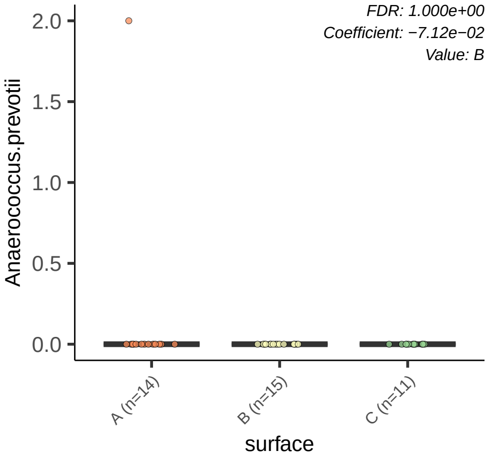
FDR: 1.000e+00
Coefficient: −7.12e−02
Value: B
Anaerococcus.prevotii
2.0
1.5
1.0
0.5
0.0
A (n=14)
B (n=15)
C (n=11)
surface

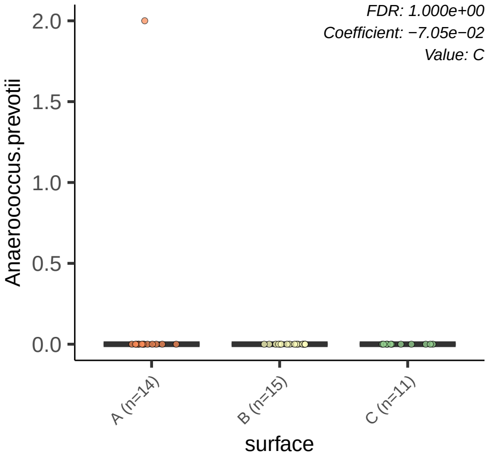
FDR: 1.000e+00
Coefficient: −7.05e−02
Value: C
Anaerococcus.prevotii
2.0
1.5
1.0
0.5
0.0
A (n=14)
B (n=15)
C (n=11)
surface

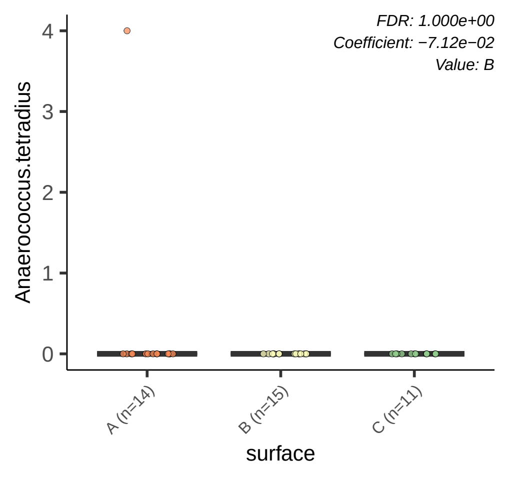
FDR: 1.000e+00
Coefficient: −7.12e−02
Value: B
Anaerococcus.tetradius
0
1
2
3
4
A (n=14)
B (n=15)
C (n=11)
surface

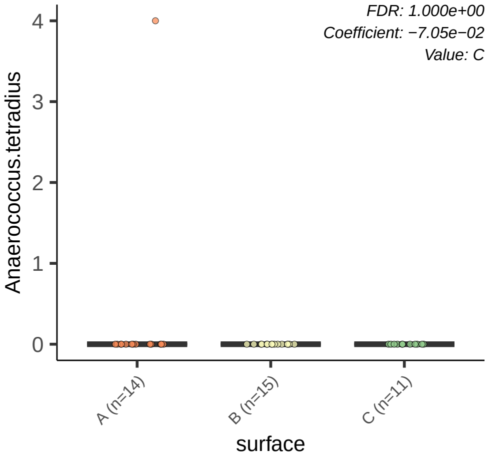

FDR: 1.000e+00
Coefficient: −7.05e−02
Value: C
Anaerococcus.tetradius
4
3
2
1
0
A (n=14)
B (n=15)
C (n=11)
surface

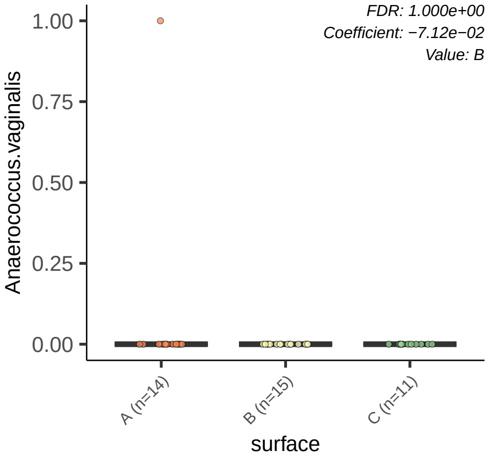
FDR: 1.000e+00
Coefficient: −7.12e−02
Value: B
Anaerococcus.vaginalis
1.00
0.75
0.50
0.25
0.00
A (n=14)
B (n=15)
C (n=11)
surface

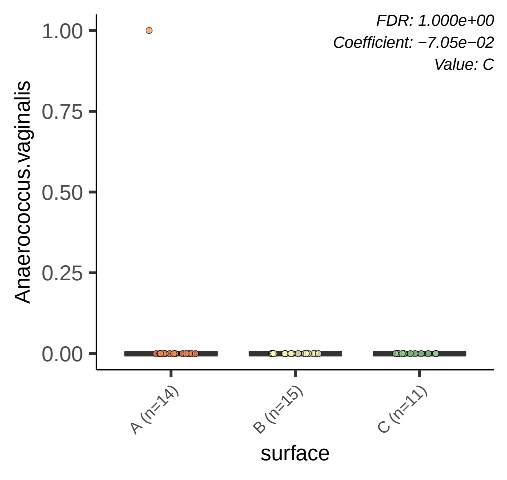
FDR: 1.000e+00
Coefficient: −7.05e−02
Value: C
Anaerococcus.vaginalis
1.00
0.75
0.50
0.25
0.00
A (n=14)
B (n=15)
C (n=11)
surface

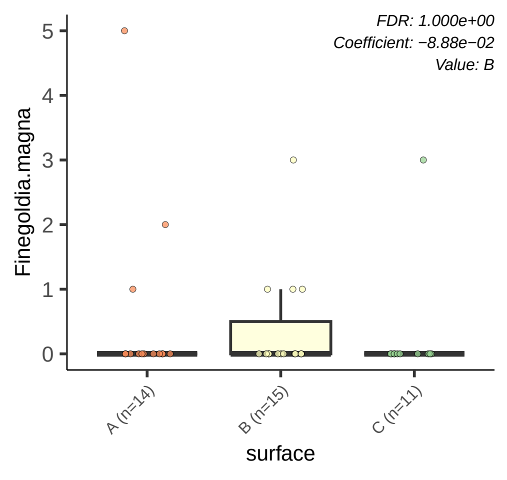
FDR: 1.000e+00
Coefficient: −8.88e−02
Value: B
Finegoldia.magna
0
1
2
3
4
5
A (n=14)
B (n=15)
C (n=11)
surface

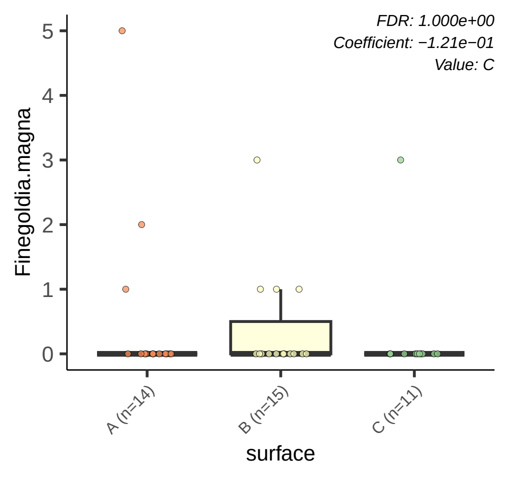
FDR: 1.000e+00
Coefficient: −1.21e−01
Value: C
Finegoldia.magna
0
1
2
3
4
5
A (n=14)
B (n=15)
C (n=11)
surface

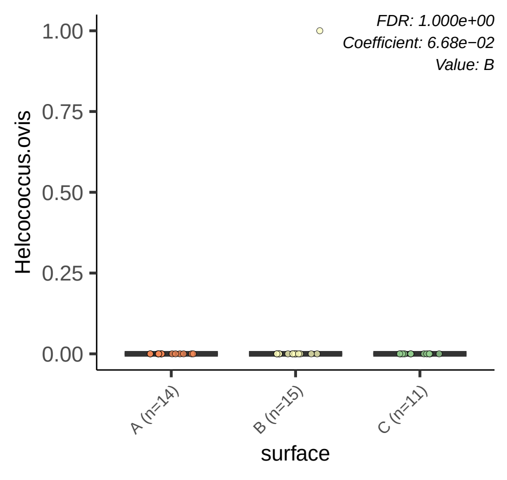
FDR: 1.000e+00
Coefficient: 6.68e−02
Value: B
Helcococcus.ovis
1.00
0.75
0.50
0.25
0.00
A (n=14)
B (n=15)
C (n=11)
surface

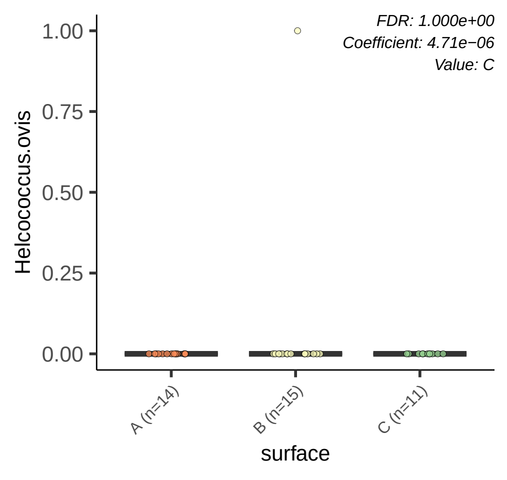

FDR: 1.000e+00
Coefficient: 4.71e−06
Value: C
Helcococcus.ovis
1.00
0.75
0.50
0.25
0.00
A (n=14)
B (n=15)
C (n=11)
surface

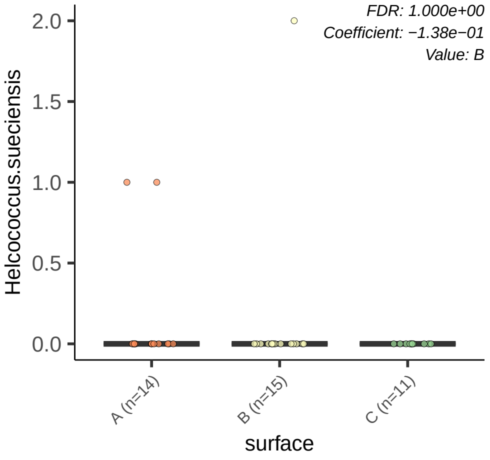
FDR: 1.000e+00
Coefficient: −1.38e−01
Value: B
Helcococcus.sueciensis
2.0
1.5
1.0
0.5
0.0
A (n=14)
B (n=15)
C (n=11)
surface

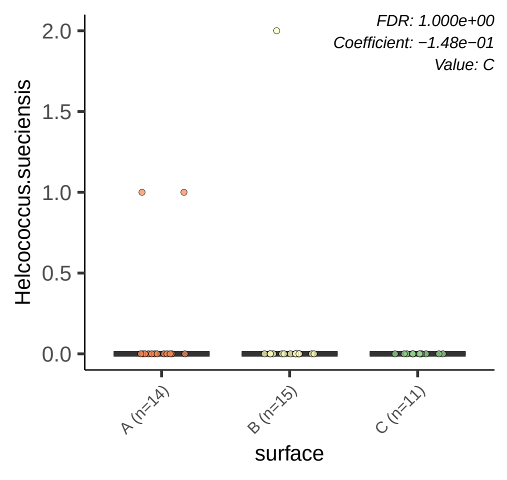
FDR: 1.000e+00
Coefficient: −1.48e−01
Value: C
Helcococcus.sueciensis
2.0
1.5
1.0
0.5
0.0
A (n=14)
B (n=15)
C (n=11)
surface

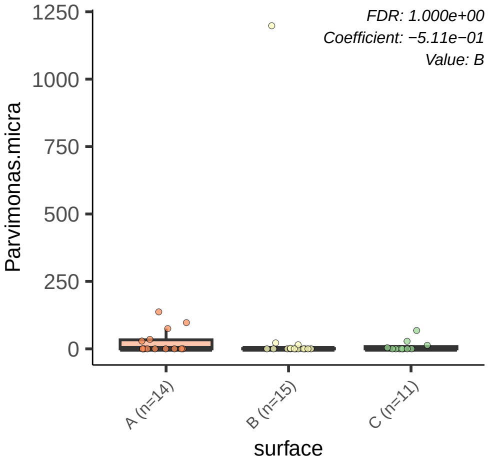
FDR: 1.000e+00
Coefficient: −5.11e−01
Value: B
Parvimonas.micra
1250
1000
750
500
250
0
A (n=14)
B (n=15)
C (n=11)
surface

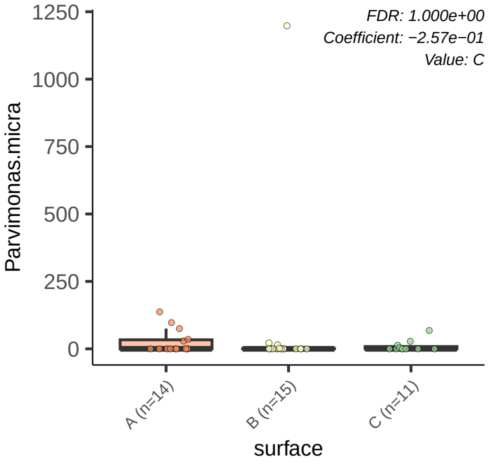

FDR: 1.000e+00
Coefficient: −2.57e−01
Value: C
Parvimonas.micra
1250
1000
750
500
250
0
A (n=14)
B (n=15)
C (n=11)
surface

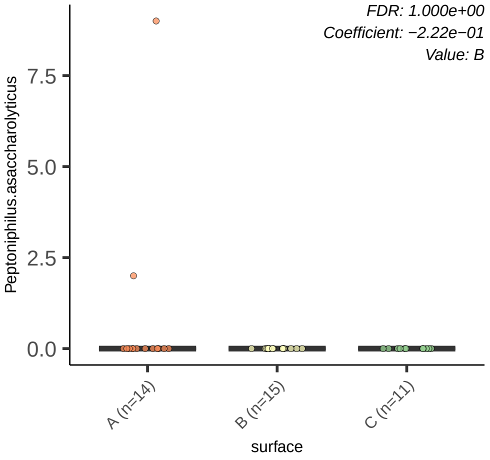
FDR: 1.000e+00
Coefficient: −2.22e−01
Value: B
Peptoniphilus.asaccharolyticus
7.5
5.0
2.5
0.0
A (n=14)
B (n=15)
C (n=11)
surface

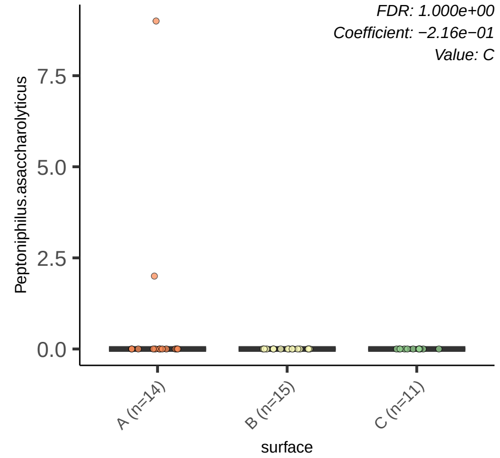

FDR: 1.000e+00
Coefficient: −2.16e−01
Value: C
Peptoniphilus.asaccharolyticus
7.5
5.0
2.5
0.0
A (n=14)
B (n=15)
C (n=11)
surface

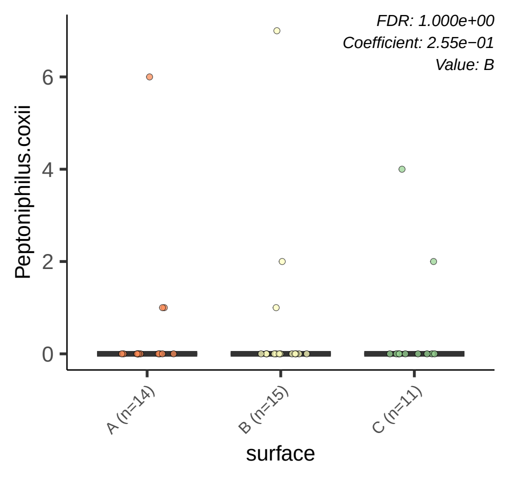

FDR: 1.000e+00
Coefficient: 2.55e−01
Value: B
Peptoniphilus.coxii
0
2
4
6
A (n=14)
B (n=15)
C (n=11)
surface

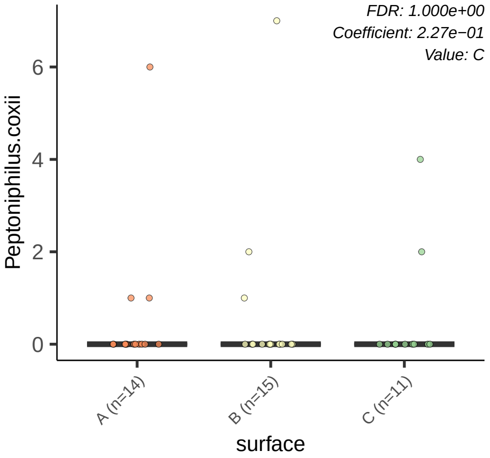
FDR: 1.000e+00
Coefficient: 2.27e−01
Value: C
Peptoniphilus.coxii
0
2
4
6
A (n=14)
B (n=15)
C (n=11)
surface

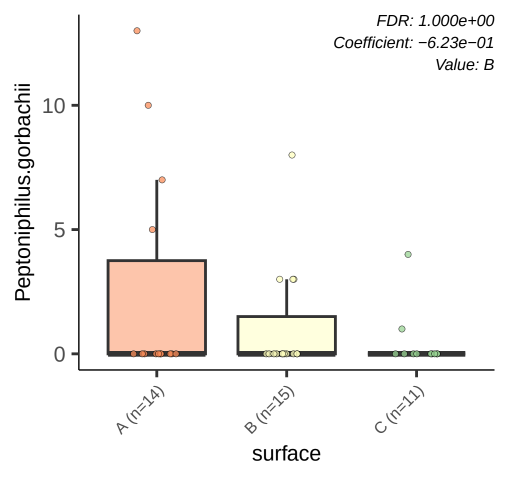
FDR: 1.000e+00
Coefficient: −6.23e−01
Value: B
Peptoniphilus.gorbachii
10
5
0
A (n=14)
B (n=15)
C (n=11)
surface

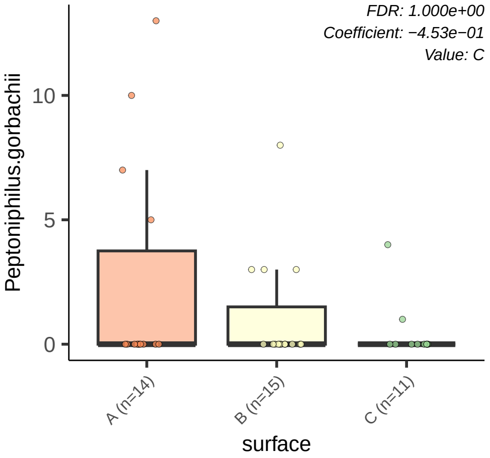
FDR: 1.000e+00
Coefficient: −4.53e−01
Value: C
Peptoniphilus.gorbachii
10
5
0
A (n=14)
B (n=15)
C (n=11)
surface

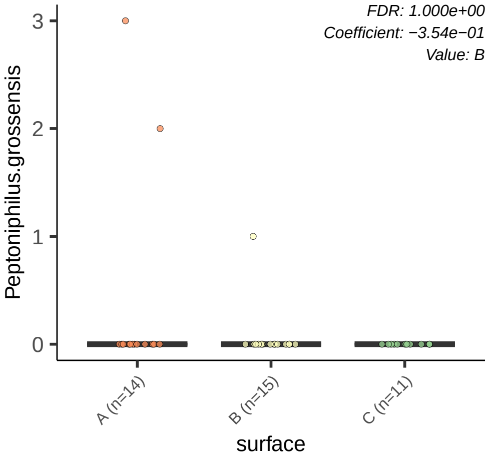
FDR: 1.000e+00
Coefficient: −3.54e−01
Value: B
Peptoniphilus.grossensis
3
2
1
0
A (n=14)
B (n=15)
C (n=11)
surface

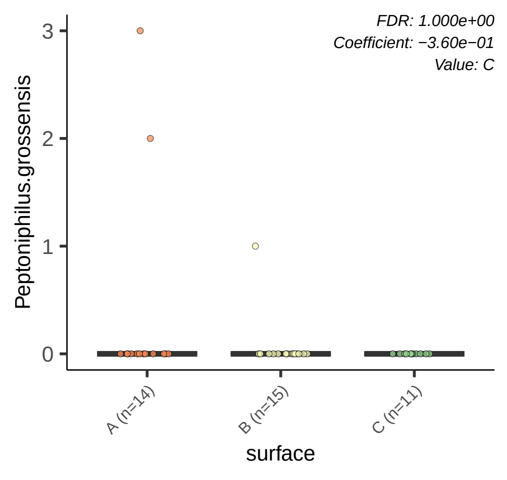

FDR: 1.000e+00
Coefficient: −3.60e−01
Value: C
Peptoniphilus.grossensis
3
2
1
0
A (n=14)
B (n=15)
C (n=11)
surface

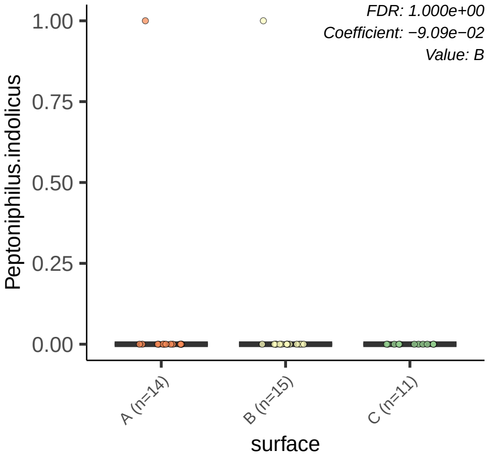

FDR: 1.000e+00
Coefficient: −9.09e−02
Value: B
Peptoniphilus.indolicus
1.00
0.75
0.50
0.25
0.00
A (n=14)
B (n=15)
C (n=11)
surface

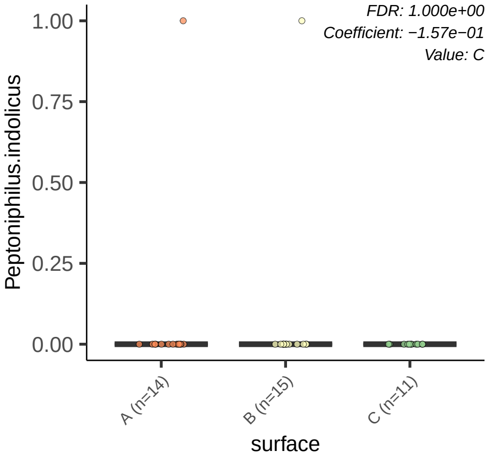
FDR: 1.000e+00
Coefficient: −1.57e−01
Value: C
Peptoniphilus.indolicus
1.00
0.75
0.50
0.25
0.00
A (n=14)
B (n=15)
C (n=11)
surface

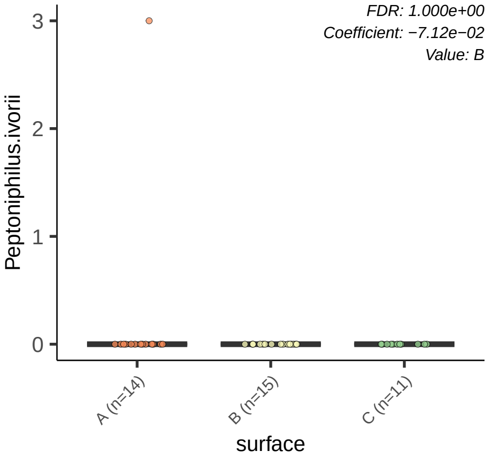
FDR: 1.000e+00
Coefficient: −7.12e−02
Value: B
Peptoniphilus.ivorii
3
2
1
0
A (n=14)
B (n=15)
C (n=11)
surface

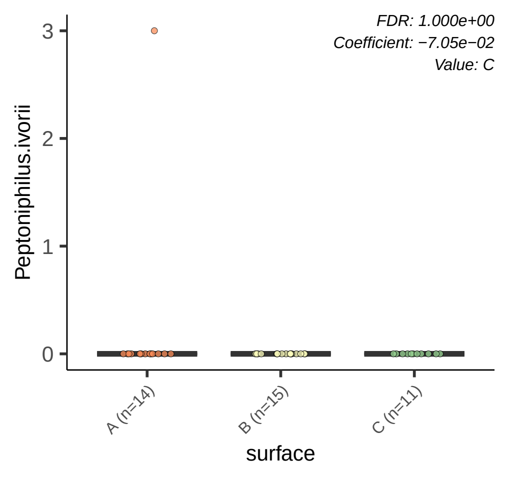

FDR: 1.000e+00
Coefficient: −7.05e−02
Value: C
Peptoniphilus.ivorii
3
2
1
0
A (n=14)
B (n=15)
C (n=11)
surface

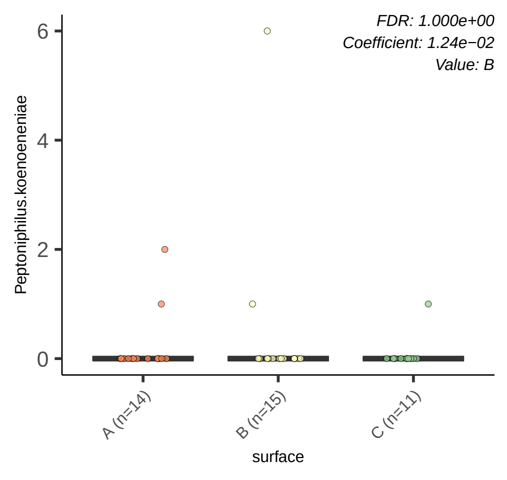

FDR: 1.000e+00
Coefficient: 1.24e−02
Value: B
Peptoniphilus.koenoeneniae
0
2
4
6
A (n=14)
B (n=15)
C (n=11)
surface

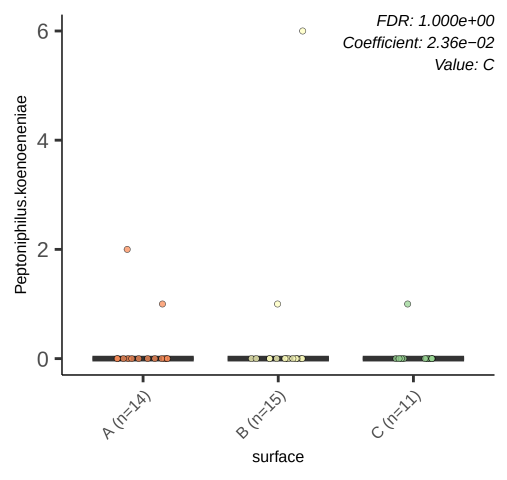
FDR: 1.000e+00
Coefficient: 2.36e−02
Value: C
Peptoniphilus.koenoeneniae
0
2
4
6
A (n=14)
B (n=15)
C (n=11)
surface

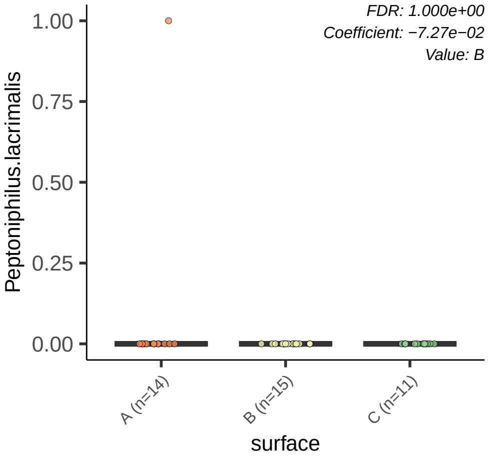
FDR: 1.000e+00
Coefficient: −7.27e−02
Value: B
Peptoniphilus.lacrimalis
1.00
0.75
0.50
0.25
0.00
A (n=14)
B (n=15)
C (n=11)
surface

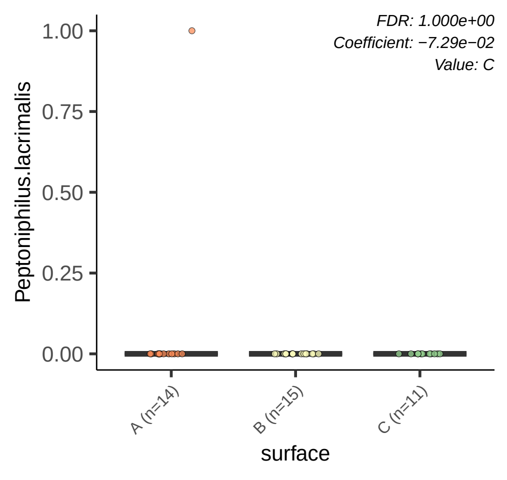
FDR: 1.000e+00
Coefficient: −7.29e−02
Value: C
Peptoniphilus.lacrimalis
1.00
0.75
0.50
0.25
0.00
A (n=14)
B (n=15)
C (n=11)
surface

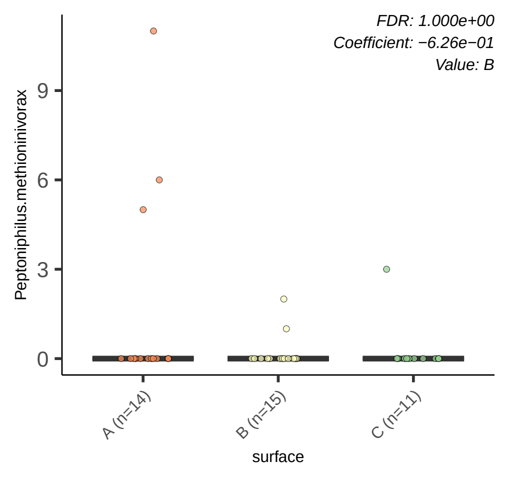

FDR: 1.000e+00
Coefficient: −6.26e−01
Value: B
Peptoniphilus.methioninivorax
9
6
3
0
A (n=14)
B (n=15)
C (n=11)
surface

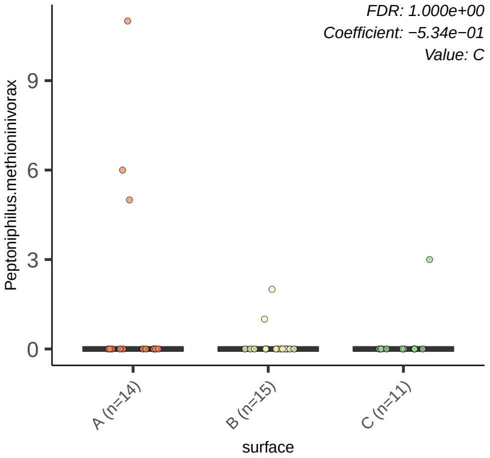
FDR: 1.000e+00
Coefficient: −5.34e−01
Value: C
Peptoniphilus.methioninivorax
9
6
3
0
A (n=14)
B (n=15)
C (n=11)
surface

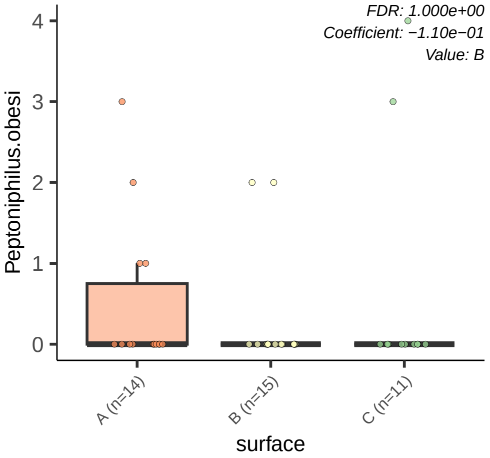

FDR: 1.000e+00
Coefficient: −1.10e−01
Value: B
Peptoniphilus.obesi
0
1
2
3
4
A (n=14)
B (n=15)
C (n=11)
surface

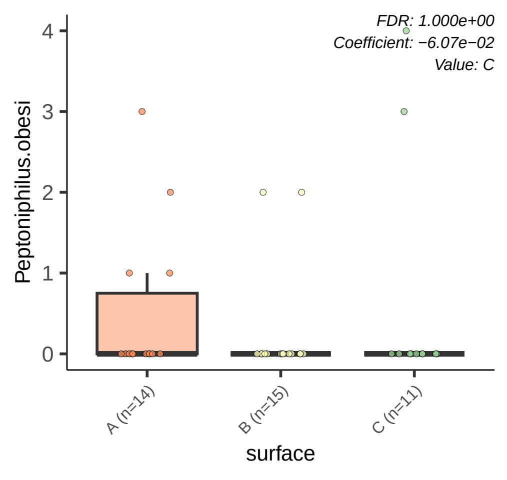
FDR: 1.000e+00
Coefficient: −6.07e−02
Value: C
Peptoniphilus.obesi
4
3
2
1
0
A (n=14)
B (n=15)
C (n=11)
surface

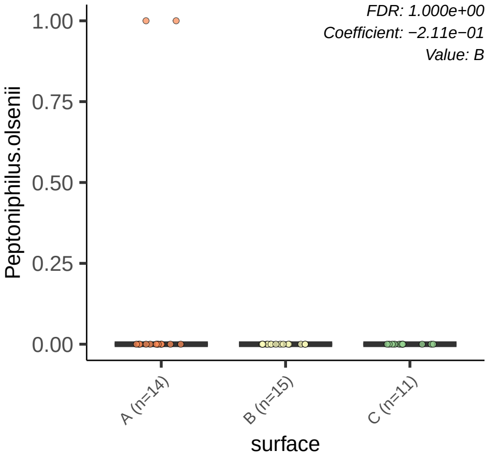

FDR: 1.000e+00
Coefficient: −2.11e−01
Value: B
Peptoniphilus.olsenii
1.00
0.75
0.50
0.25
0.00
A (n=14)
B (n=15)
C (n=11)
surface

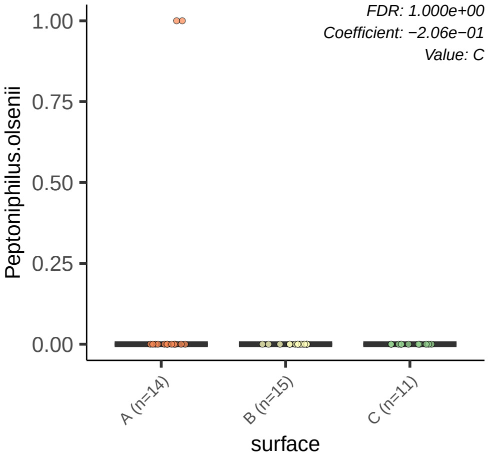

FDR: 1.000e+00
Coefficient: −2.06e−01
Value: C
1.00
0.75
0.50
0.25
0.00
Peptoniphilus.olsenii
A (n=14)
B (n=15)
C (n=11)
surface

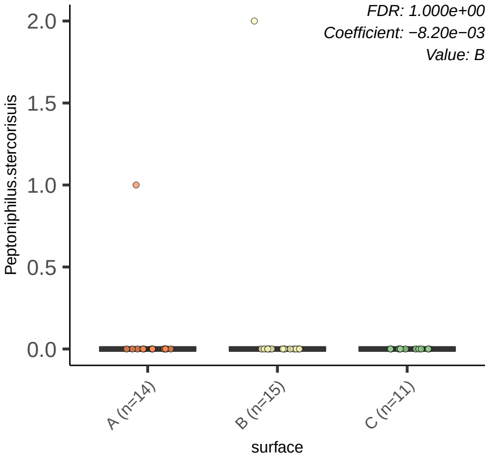
FDR: 1.000e+00
Coefficient: −8.20e−03
Value: B
Peptoniphilus.stercorisuis
2.0
1.5
1.0
0.5
0.0
A (n=14)
B (n=15)
C (n=11)
surface

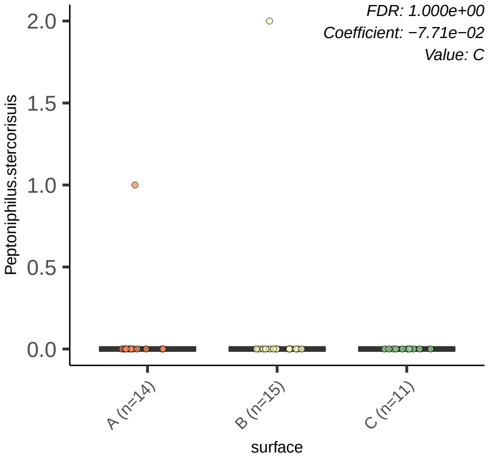

FDR: 1.000e+00
Coefficient: −7.71e−02
Value: C
Peptoniphilus.stercorisuis
2.0
1.5
1.0
0.5
0.0
A (n=14)
B (n=15)
C (n=11)
surface

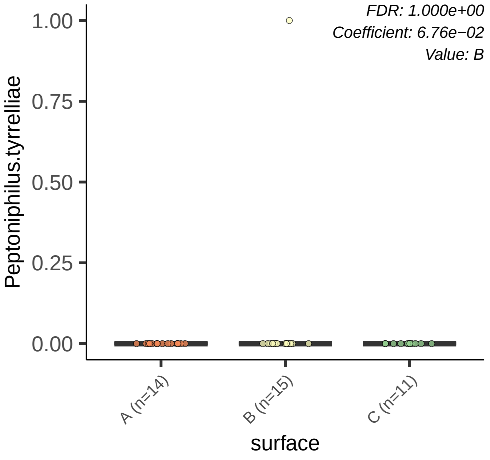

FDR: 1.000e+00
Coefficient: 6.76e−02
Value: B
Peptoniphilus.tyrrelliae
1.00
0.75
0.50
0.25
0.00
A (n=14)
B (n=15)
C (n=11)
surface

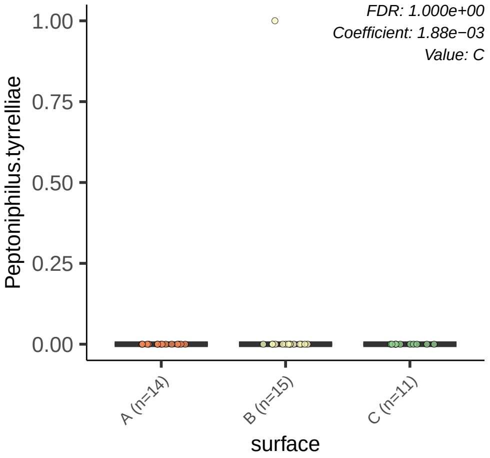
FDR: 1.000e+00
Coefficient: 1.88e−03
Value: C
Peptoniphilus.tyrrelliae
1.00
0.75
0.50
0.25
0.00
A (n=14)
B (n=15)
C (n=11)
surface

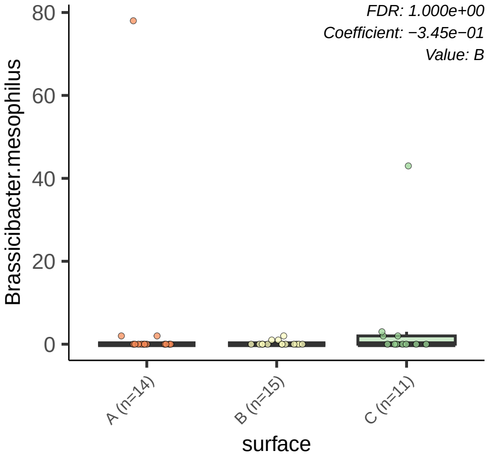
FDR: 1.000e+00
Coefficient: −3.45e−01
Value: B
Brassicibacter.mesophilus
80
60
40
20
0
A (n=14)
B (n=15)
C (n=11)
surface

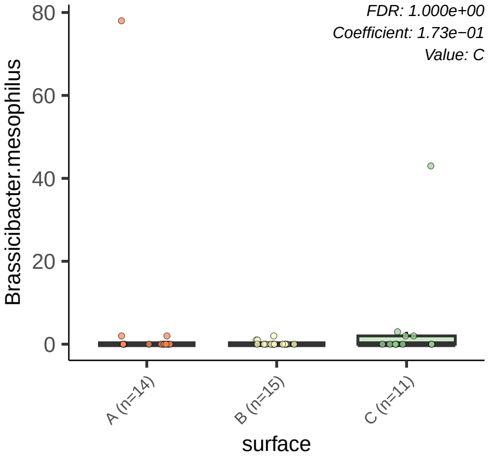
FDR: 1.000e+00
Coefficient: 1.73e−01
Value: C
Brassicibacter.mesophilus
80
60
40
20
0
A (n=14)
B (n=15)
C (n=11)
surface

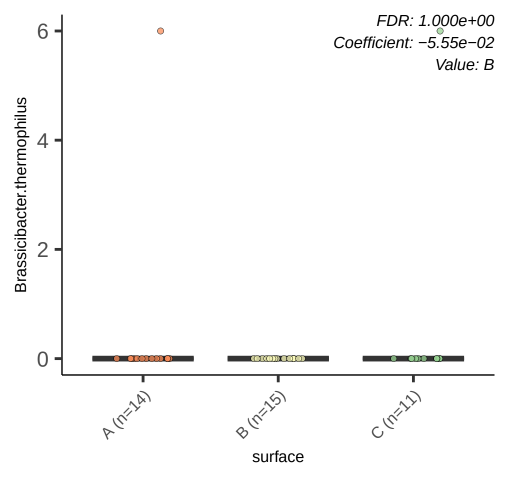
FDR: 1.000e+00
Coefficient: −5.55e−02
Value: B
Brassicibacter.thermophilus
6
4
2
0
A (n=14)
B (n=15)
C (n=11)
surface

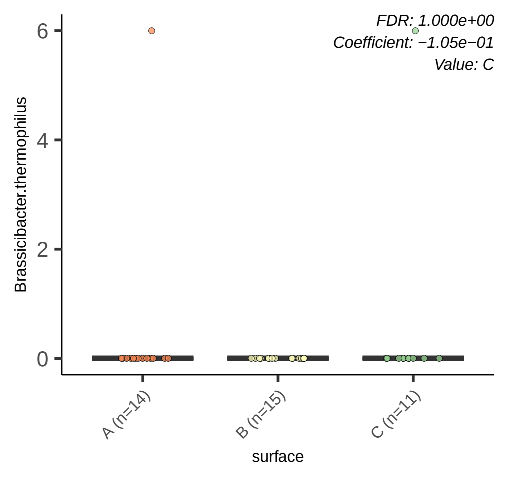
FDR: 1.000e+00
Coefficient: −1.05e−01
Value: C
Brassicibacter.thermophilus
6
4
2
0
A (n=14)
B (n=15)
C (n=11)
surface

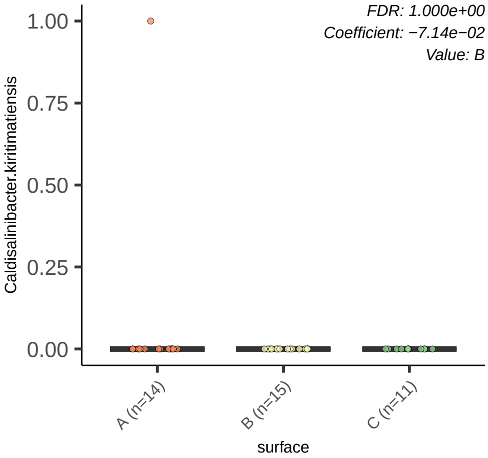
FDR: 1.000e+00
Coefficient: −7.14e−02
Value: B
Caldisalinibacter.kiritimatiensis
1.00
0.75
0.50
0.25
0.00
A (n=14)
B (n=15)
C (n=11)
surface

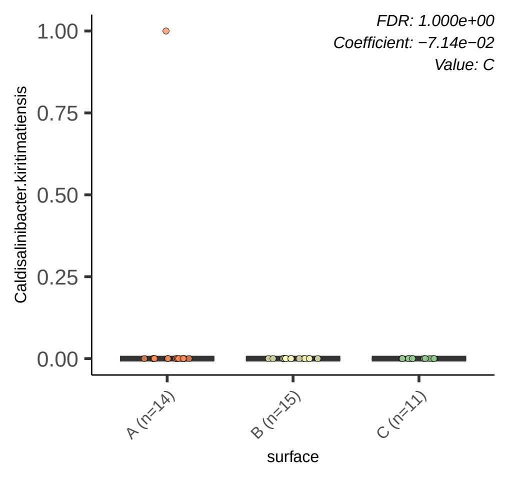

FDR: 1.000e+00
Coefficient: −7.14e−02
Value: C
Caldisalinibacter.kiritimatiensis
1.00
0.75
0.50
0.25
0.00
A (n=14)
B (n=15)
C (n=11)
surface

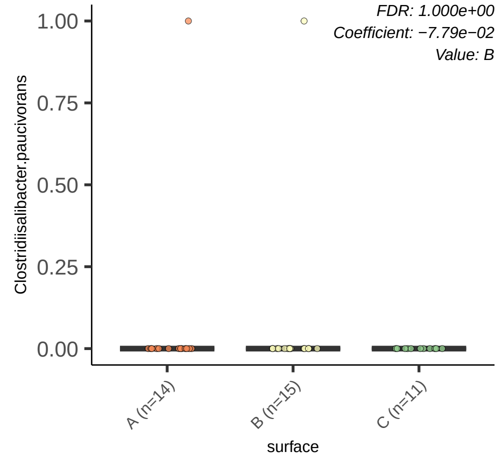
FDR: 1.000e+00
Coefficient: −7.79e−02
Value: B
Clostridiisalibacter.paucivorans
1.00
0.75
0.50
0.25
0.00
A (n=14)
B (n=15)
C (n=11)
surface

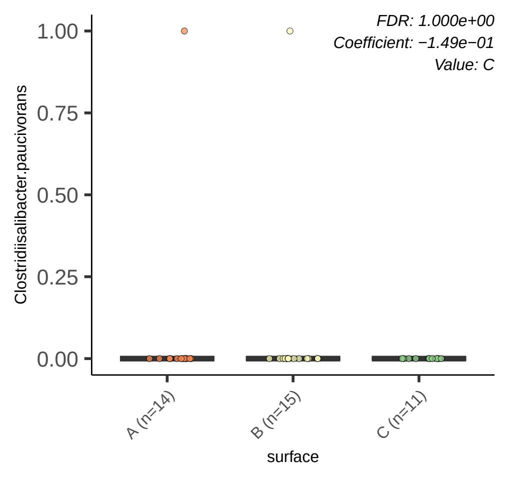

FDR: 1.000e+00
Coefficient: −1.49e−01
Value: C
1.00
0.75
0.50
0.25
0.00
Clostridiisalibacter.paucivorans
A (n=14)
B (n=15)
C (n=11)
surface

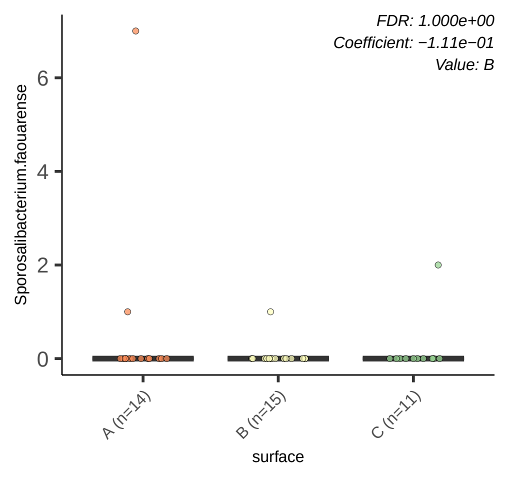

FDR: 1.000e+00
Coefficient: −1.11e−01
Value: B
Sporosalibacterium.faouarense
0
2
4
6
A (n=14)
B (n=15)
C (n=11)
surface

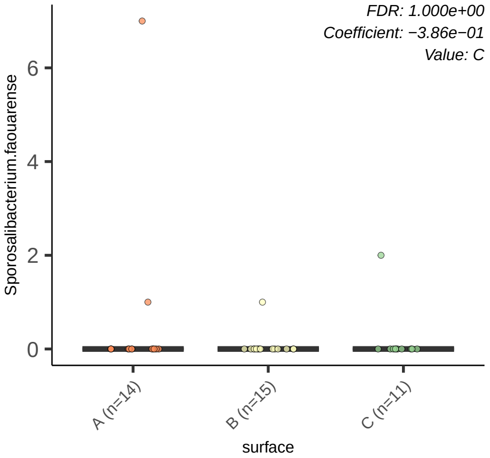
FDR: 1.000e+00
Coefficient: −3.86e−01
Value: C
Sporosalibacterium.faouarense
0
2
4
6
A (n=14)
B (n=15)
C (n=11)
surface

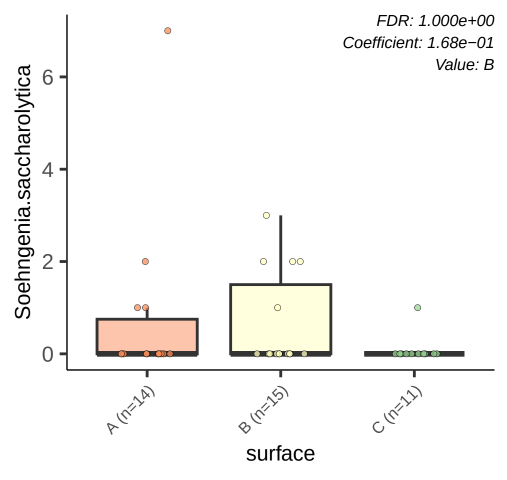

FDR: 1.000e+00
Coefficient: 1.68e−01
Value: B
Soehngenia.saccharolytica
0
2
4
6
A (n=14)
B (n=15)
C (n=11)
surface

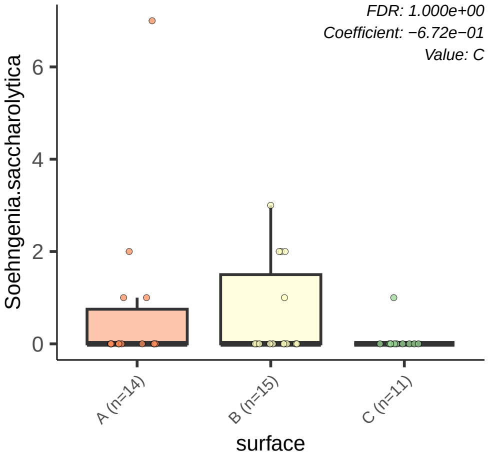

FDR: 1.000e+00
Coefficient: −6.72e−01
Value: C
Soehngenia.saccharolytica
0
2
4
6
A (n=14)
B (n=15)
C (n=11)
surface

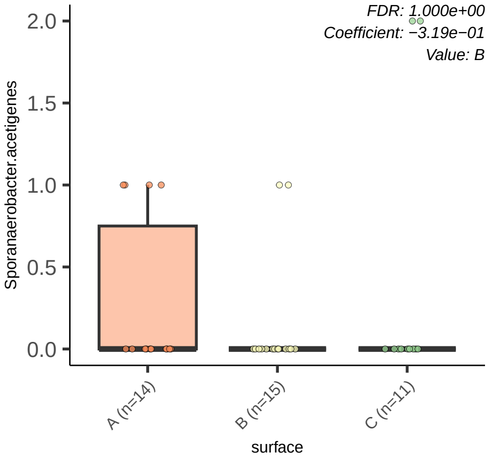

FDR: 1.000e+00
Coefficient: −3.19e−01
Value: B
Sporanaerobacter.acetigenes
2.0
1.5
1.0
0.5
0.0
A (n=14)
B (n=15)
C (n=11)
surface

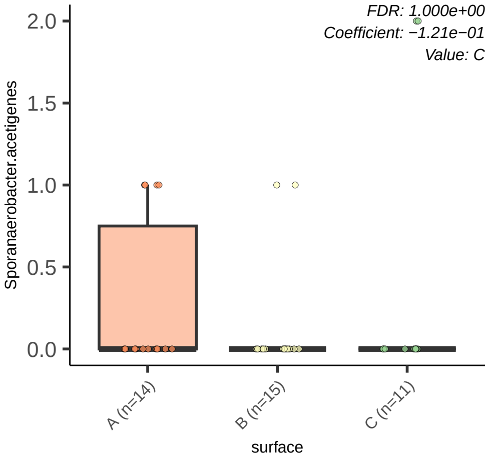
FDR: 1.000e+00
Coefficient: −1.21e−01
Value: C
2.0
1.5
1.0
0.5
0.0
Sporanaerobacter.acetigenes
A (n=14)
B (n=15)
C (n=11)
surface

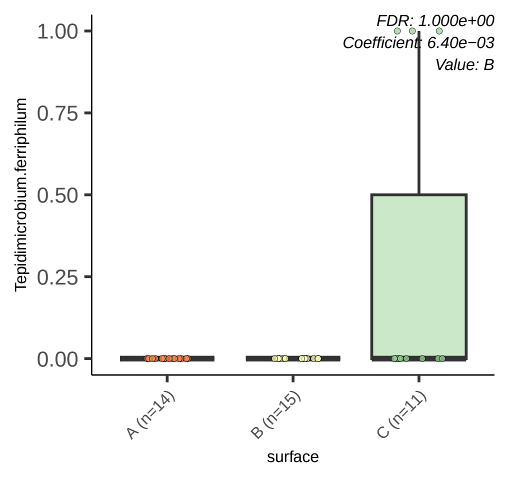

FDR: 1.000e+00
Coefficient: 6.40e−03
Value: B
1.00
0.75
0.50
0.25
0.00
Tepidimicrobium.ferriphilum
A (n=14)
B (n=15)
C (n=11)
surface

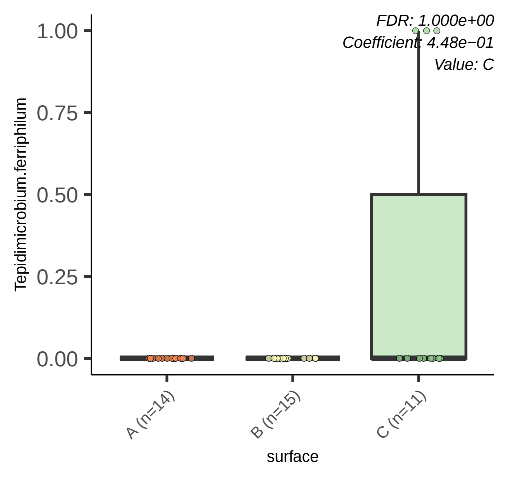
FDR: 1.000e+00
Coefficient: 4.48e−01
Value: C
Tepidimicrobium.ferriphilum
1.00
0.75
0.50
0.25
0.00
A (n=14)
B (n=15)
C (n=11)
surface

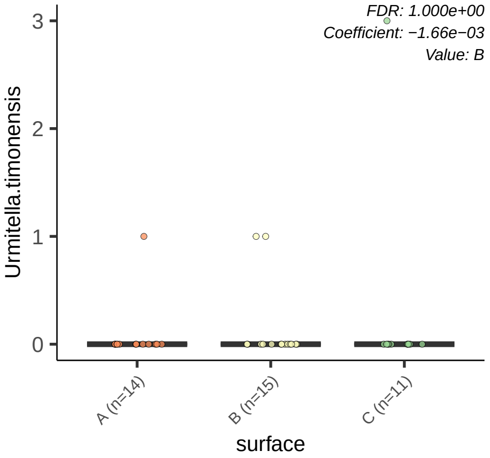
FDR: 1.000e+00
Coefficient: −1.66e−03
Value: B
Urmitella.timonensis
3
2
1
0
A (n=14)
B (n=15)
C (n=11)
surface

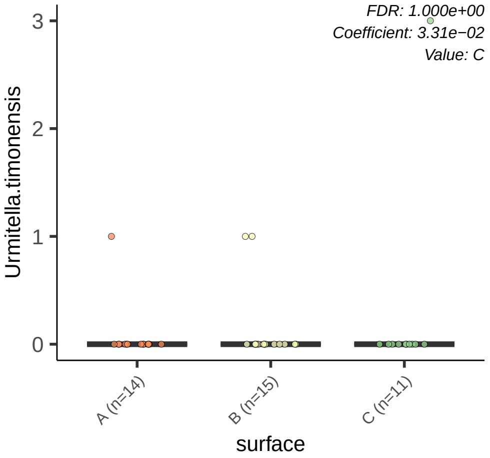
FDR: 1.000e+00
Coefficient: 3.31e−02
Value: C
Urmitella.timonensis
3
2
1
0
A (n=14)
B (n=15)
C (n=11)
surface

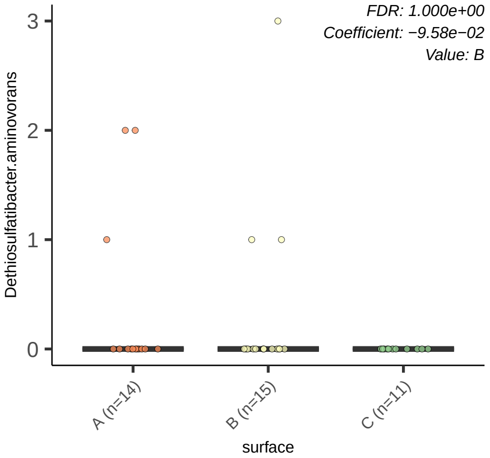
FDR: 1.000e+00
Coefficient: −9.58e−02
Value: B
Dethiosulfatibacter.aminovorans
3
2
1
0
A (n=14)
B (n=15)
C (n=11)
surface

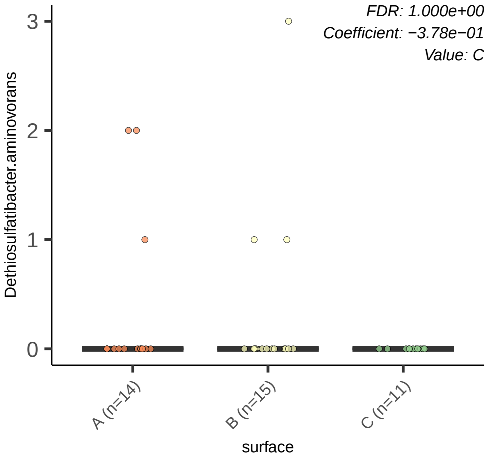
FDR: 1.000e+00
Coefficient: −3.78e−01
Value: C
Dethiosulfatibacter.aminovorans
3
2
1
0
A (n=14)
B (n=15)
C (n=11)
surface

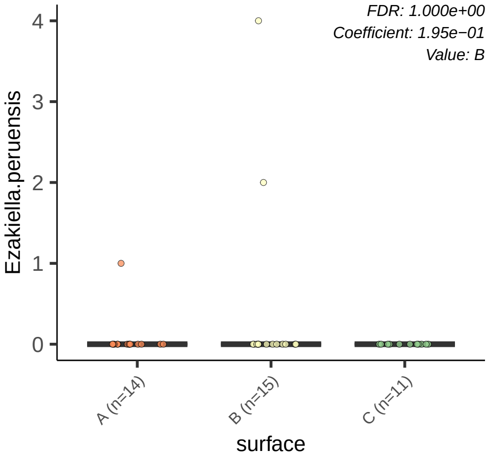
FDR: 1.000e+00
Coefficient: 1.95e−01
Value: B
Ezakiella.peruensis
0
1
2
3
4
A (n=14)
B (n=15)
C (n=11)
surface

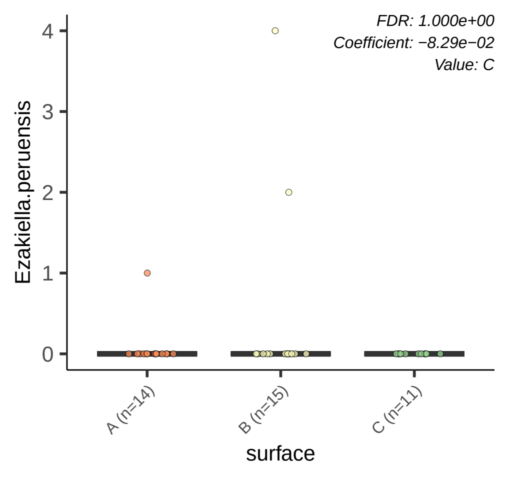

FDR: 1.000e+00
Coefficient: −8.29e−02
Value: C
Ezakiella.peruensis
0
1
2
3
4
A (n=14)
B (n=15)
C (n=11)
surface

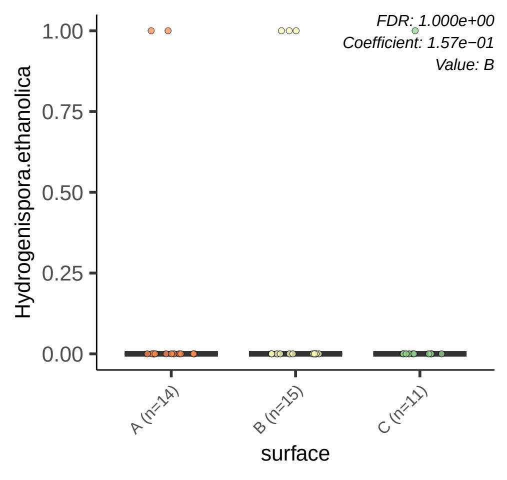

FDR: 1.000e+00
Coefficient: 1.57e−01
Value: B
Hydrogenispora.ethanolica
1.00
0.75
0.50
0.25
0.00
A (n=14)
B (n=15)
C (n=11)
surface

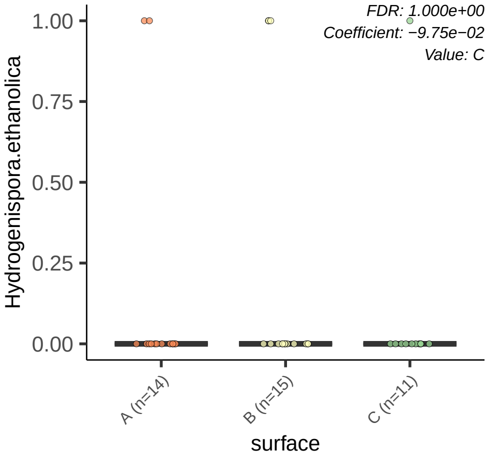

FDR: 1.000e+00
Coefficient: −9.75e−02
Value: C
Hydrogenispora.ethanolica
1.00
0.75
0.50
0.25
0.00
A (n=14)
B (n=15)
C (n=11)
surface

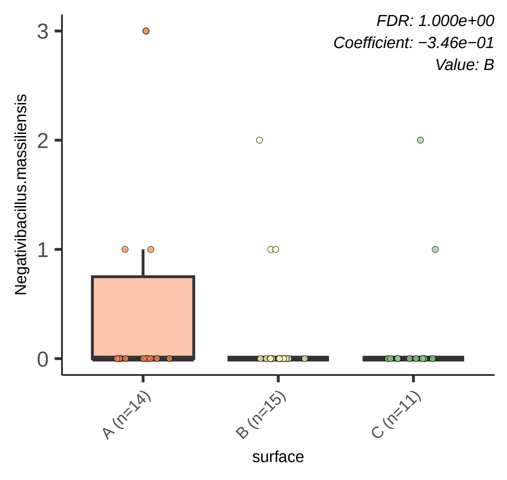

FDR: 1.000e+00
Coefficient: −3.46e−01
Value: B
Negativibacillus.massiliensis
0
1
2
3
A (n=14)
B (n=15)
C (n=11)
surface

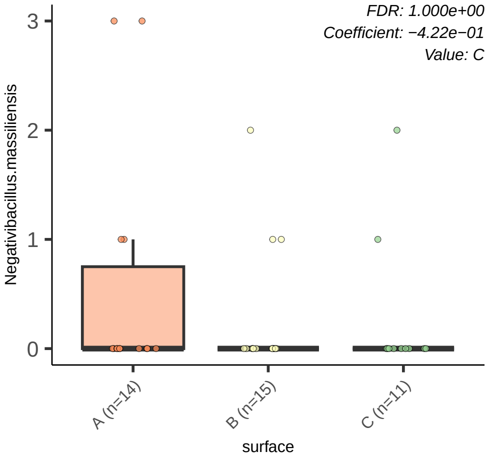
FDR: 1.000e+00
Coefficient: −4.22e−01
Value: C
Negativibacillus.massiliensis
3
2
1
0
A (n=14)
B (n=15)
C (n=11)
surface

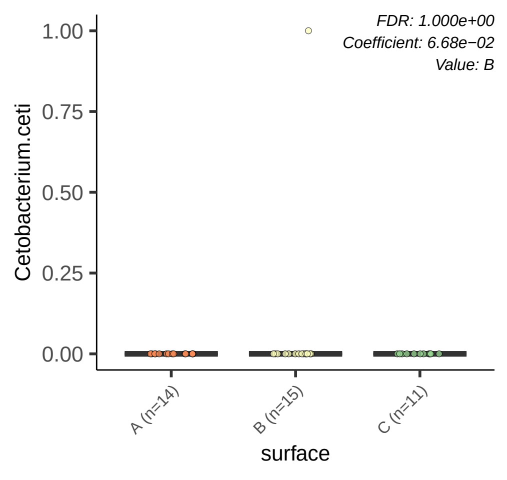

FDR: 1.000e+00
Coefficient: 6.68e−02
Value: B
Cetobacterium.ceti
1.00
0.75
0.50
0.25
0.00
A (n=14)
B (n=15)
C (n=11)
surface

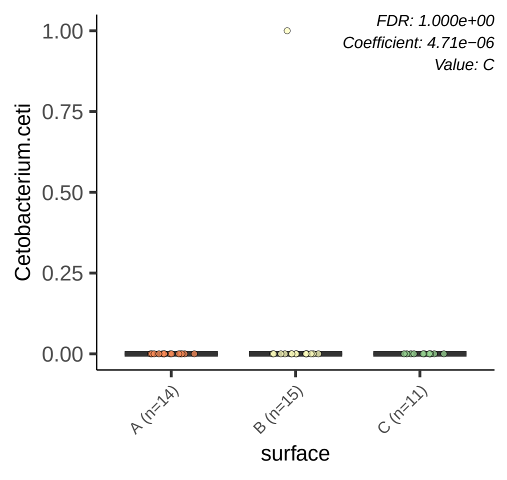
FDR: 1.000e+00
Coefficient: 4.71e−06
Value: C
Cetobacterium.ceti
1.00
0.75
0.50
0.25
0.00
A (n=14)
B (n=15)
C (n=11)
surface

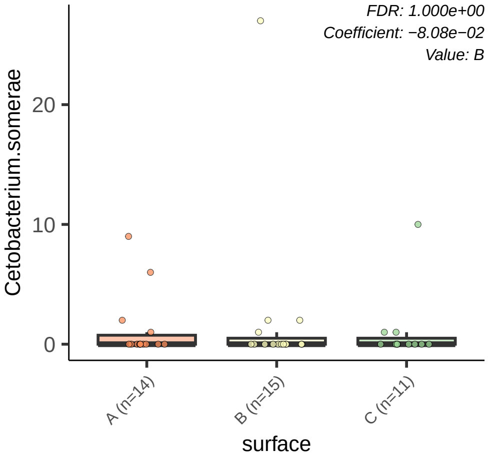

FDR: 1.000e+00
Coefficient: −8.08e−02
Value: B
Cetobacterium.somerae
20
10
0
A (n=14)
B (n=15)
C (n=11)
surface

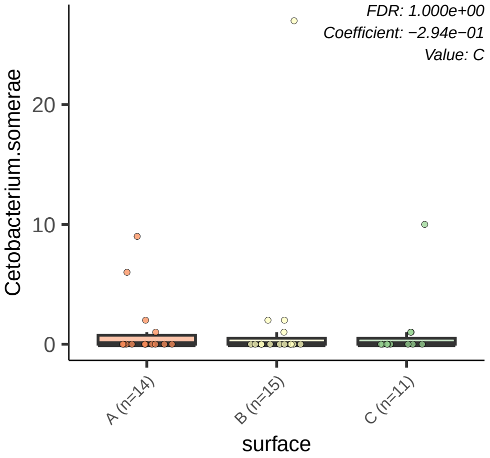
FDR: 1.000e+00
Coefficient: −2.94e−01
Value: C
Cetobacterium.somerae
0
10
20
A (n=14)
B (n=15)
C (n=11)
surface

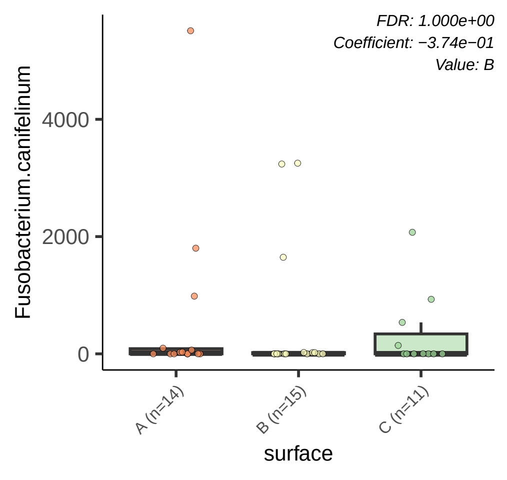
FDR: 1.000e+00
Coefficient: −3.74e−01
Value: B
Fusobacterium.canifelinum
4000
2000
0
A (n=14)
B (n=15)
C (n=11)
surface

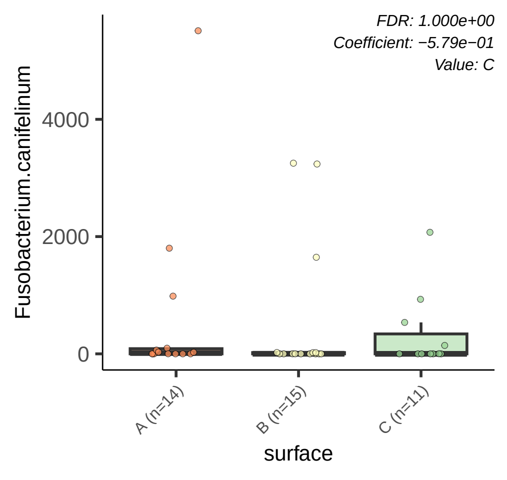
FDR: 1.000e+00
Coefficient: −5.79e−01
Value: C
Fusobacterium.canifelinum
4000
2000
0
A (n=14)
B (n=15)
C (n=11)
surface

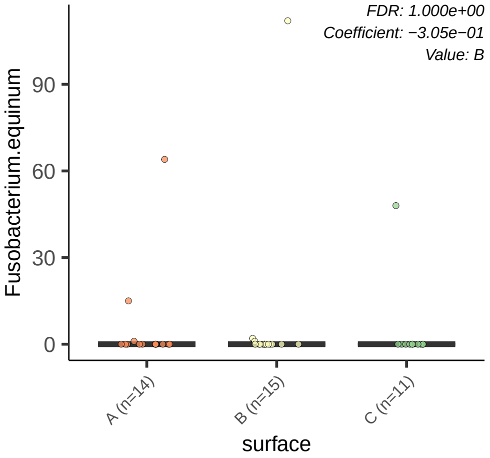
FDR: 1.000e+00
Coefficient: −3.05e−01
Value: B
Fusobacterium.equinum
90
60
30
0
A (n=14)
B (n=15)
C (n=11)
surface

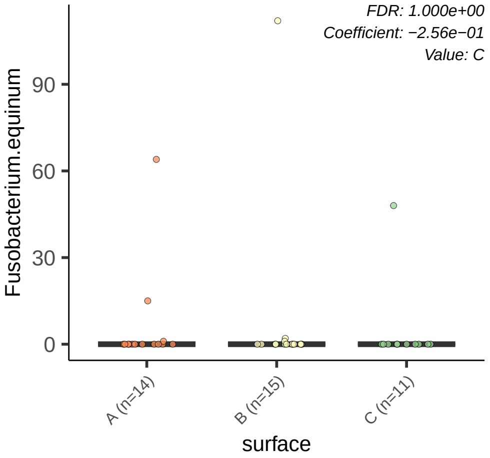
FDR: 1.000e+00
Coefficient: −2.56e−01
Value: C
Fusobacterium.equinum
90
60
30
0
A (n=14)
B (n=15)
C (n=11)
surface

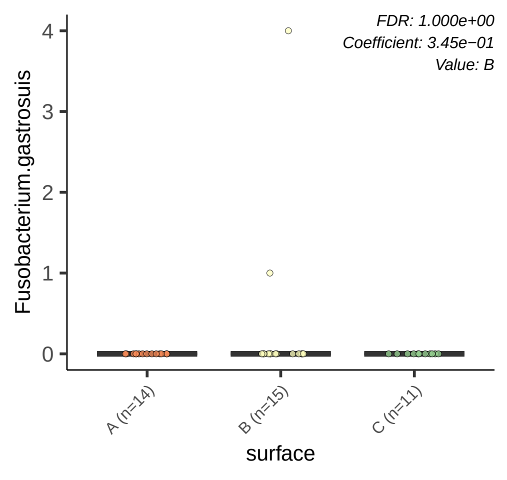

FDR: 1.000e+00
Coefficient: 3.45e−01
Value: B
Fusobacterium.gastrosuis
0
1
2
3
4
A (n=14)
B (n=15)
C (n=11)
surface

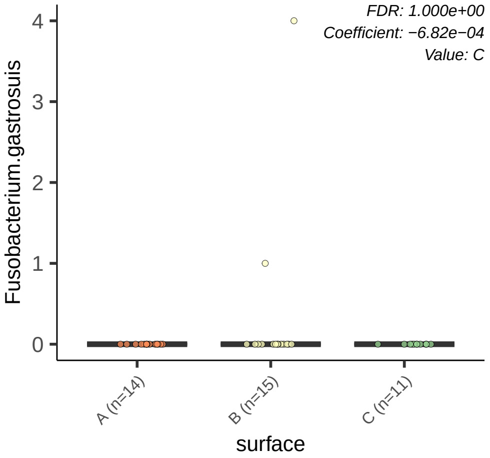

FDR: 1.000e+00
Coefficient: −6.82e−04
Value: C
Fusobacterium.gastrosuis
4
3
2
1
0
A (n=14)
B (n=15)
C (n=11)
surface

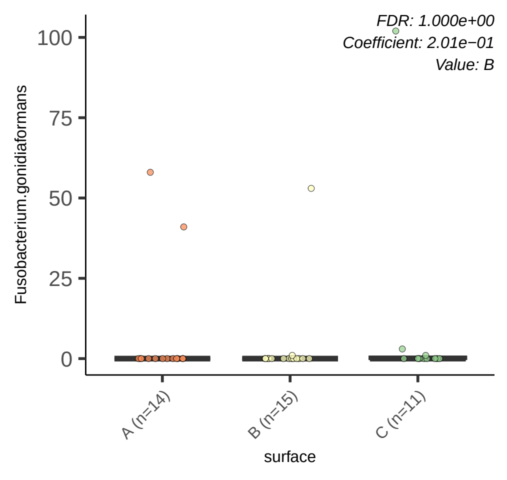

FDR: 1.000e+00
Coefficient: 2.01e−01
Value: B
100
75
50
25
0
Fusobacterium.gonidiaformans
A (n=14)
B (n=15)
C (n=11)
surface

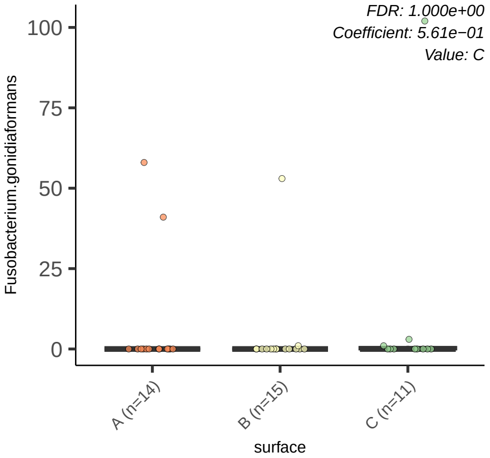

FDR: 1.000e+00
Coefficient: 5.61e−01
Value: C
Fusobacterium.gonidiaformans
100
75
50
25
0
A (n=14)
B (n=15)
C (n=11)
surface

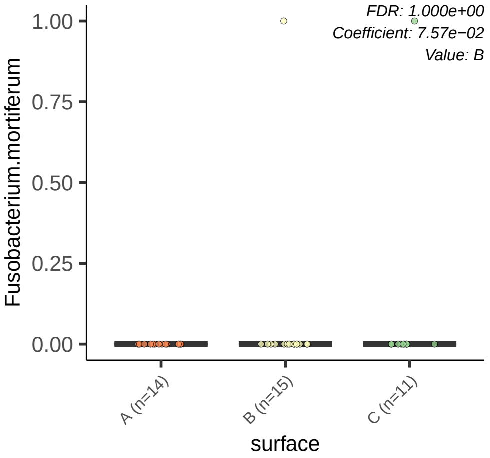
FDR: 1.000e+00
Coefficient: 7.57e−02
Value: B
Fusobacterium.mortiferum
1.00
0.75
0.50
0.25
0.00
A (n=14)
B (n=15)
C (n=11)
surface

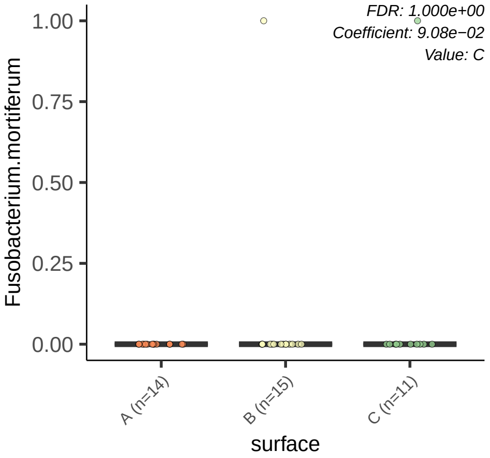

FDR: 1.000e+00
Coefficient: 9.08e−02
Value: C
Fusobacterium.mortiferum
1.00
0.75
0.50
0.25
0.00
A (n=14)
B (n=15)
C (n=11)
surface

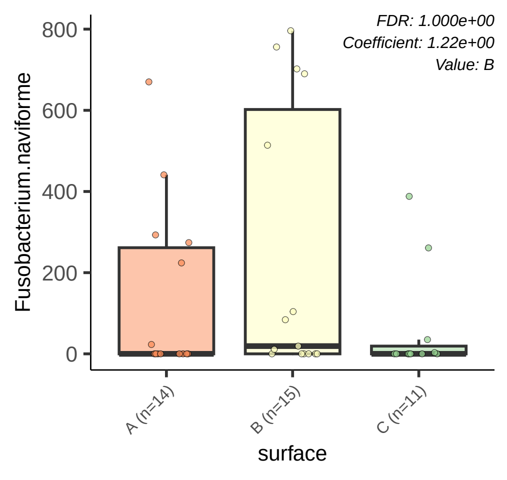
FDR: 1.000e+00
Coefficient: 1.22e+00
Value: B
Fusobacterium.naviforme
800
600
400
200
0
A (n=14)
B (n=15)
C (n=11)
surface

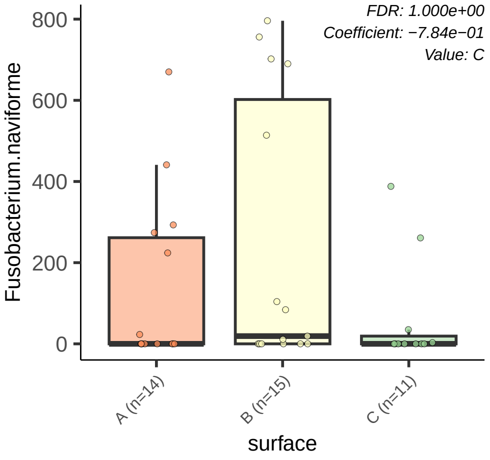

FDR: 1.000e+00
Coefficient: −7.84e−01
Value: C
Fusobacterium.naviforme
800
600
400
200
0
A (n=14)
B (n=15)
C (n=11)
surface

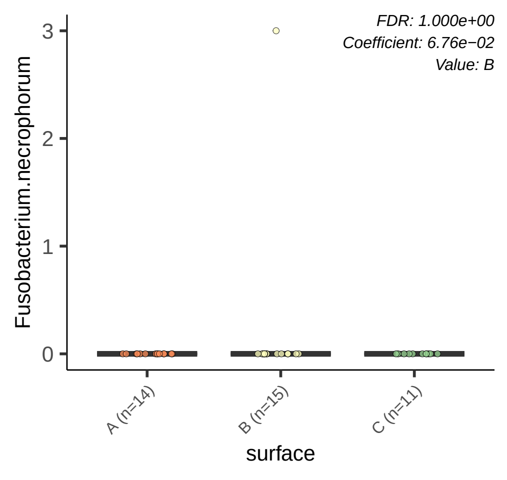
FDR: 1.000e+00
Coefficient: 6.76e−02
Value: B
Fusobacterium.necrophorum
3
2
1
0
A (n=14)
B (n=15)
C (n=11)
surface

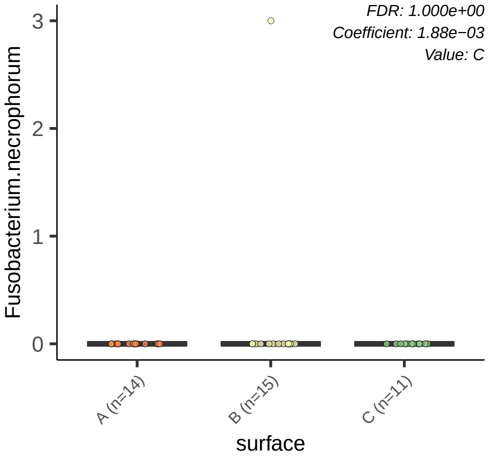

FDR: 1.000e+00
Coefficient: 1.88e−03
Value: C
Fusobacterium.necrophorum
3
2
1
0
A (n=14)
B (n=15)
C (n=11)
surface

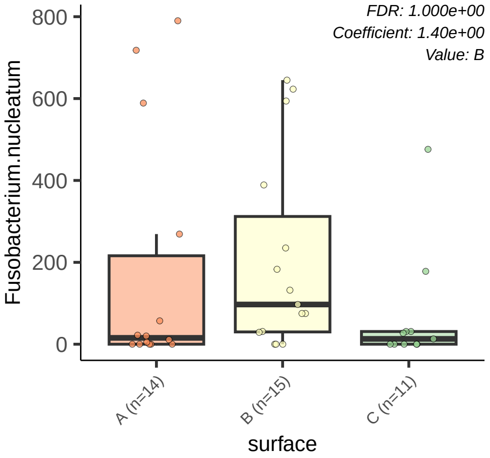
FDR: 1.000e+00
Coefficient: 1.40e+00
Value: B
Fusobacterium.nucleatum
800
600
400
200
0
A (n=14)
B (n=15)
C (n=11)
surface

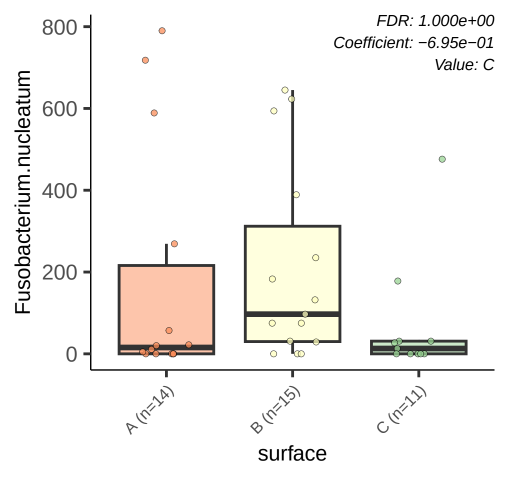

FDR: 1.000e+00
Coefficient: −6.95e−01
Value: C
Fusobacterium.nucleatum
800
600
400
200
0
A (n=14)
B (n=15)
C (n=11)
surface

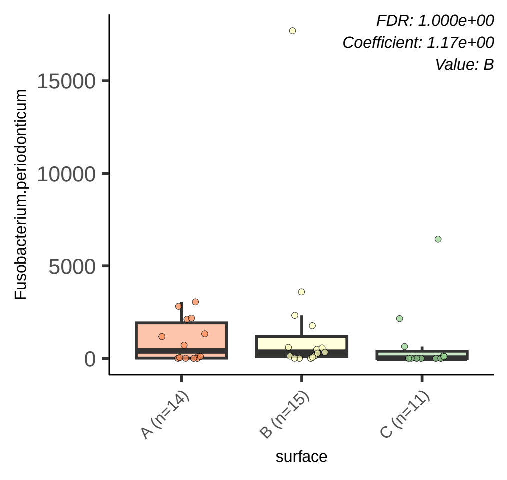
FDR: 1.000e+00
Coefficient: 1.17e+00
Value: B
Fusobacterium.periodonticum
15000
10000
5000
0
A (n=14)
B (n=15)
C (n=11)
surface

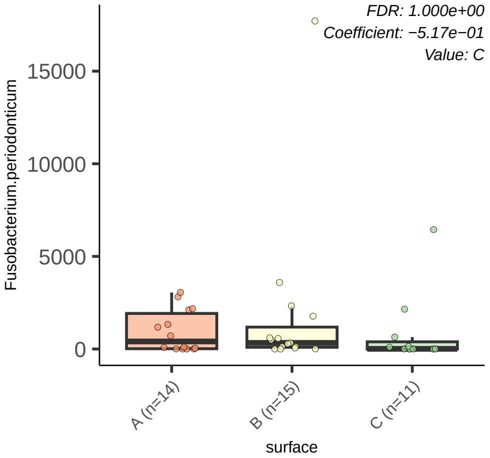

FDR: 1.000e+00
Coefficient: −5.17e−01
Value: C
15000
10000
5000
0
Fusobacterium.periodonticum
A (n=14)
B (n=15)
C (n=11)
surface

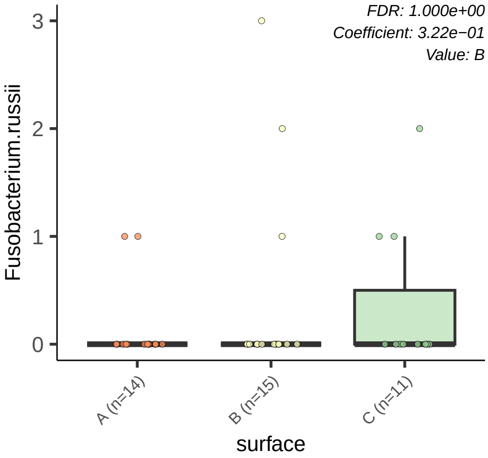
FDR: 1.000e+00
Coefficient: 3.22e−01
Value: B
Fusobacterium.russii
3
2
1
0
A (n=14)
B (n=15)
C (n=11)
surface

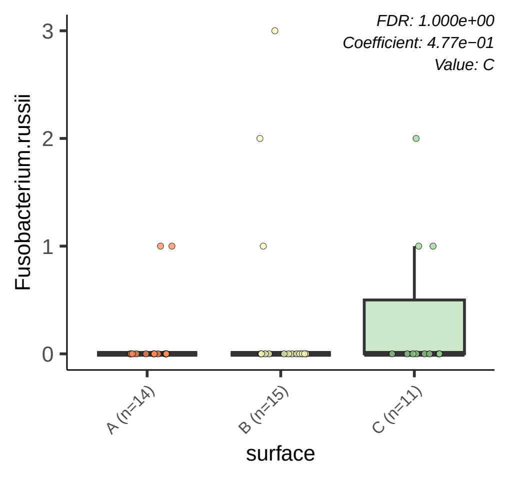

FDR: 1.000e+00
Coefficient: 4.77e−01
Value: C
Fusobacterium.russii
0
1
2
3
A (n=14)
B (n=15)
C (n=11)
surface

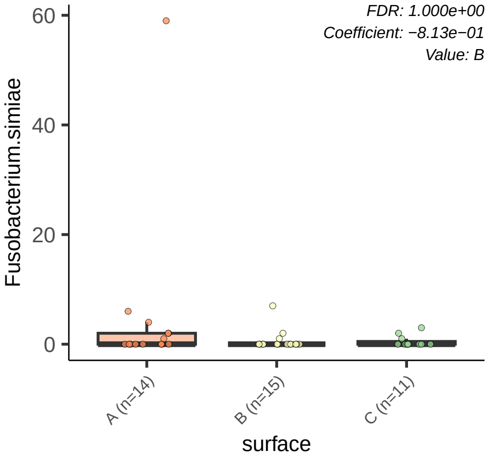
FDR: 1.000e+00
Coefficient: −8.13e−01
Value: B
Fusobacterium.simiae
60
40
20
0
A (n=14)
B (n=15)
C (n=11)
surface

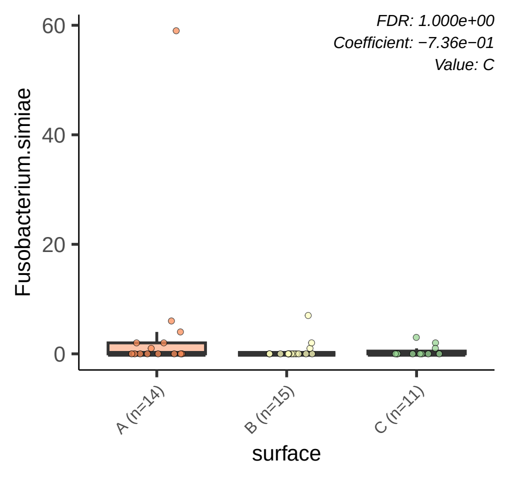
FDR: 1.000e+00
Coefficient: −7.36e−01
Value: C
Fusobacterium.simiae
0
20
40
60
A (n=14)
B (n=15)
C (n=11)
surface

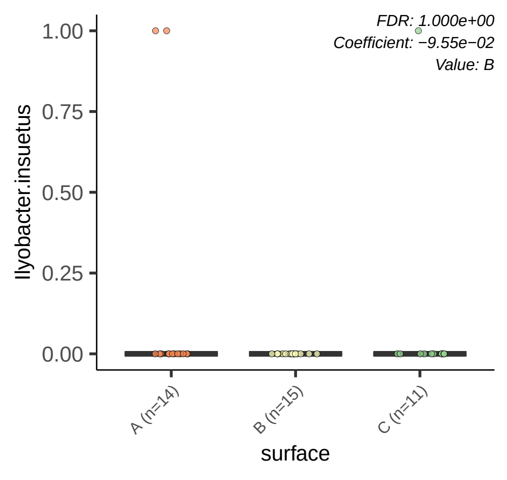
FDR: 1.000e+00
Coefficient: −9.55e−02
Value: B
Ilyobacter.insuetus
1.00
0.75
0.50
0.25
0.00
A (n=14)
B (n=15)
C (n=11)
surface

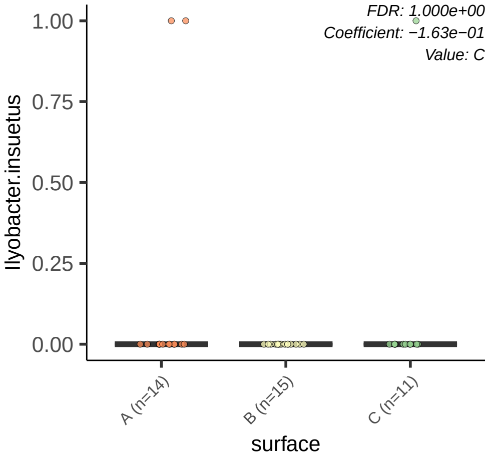
FDR: 1.000e+00
Coefficient: −1.63e−01
Value: C
Ilyobacter.insuetus
1.00
0.75
0.50
0.25
0.00
A (n=14)
B (n=15)
C (n=11)
surface

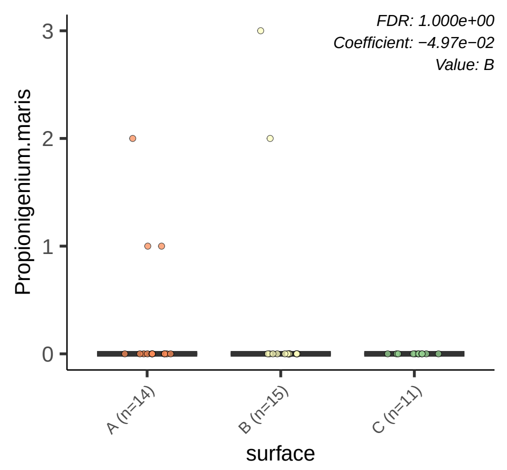

FDR: 1.000e+00
Coefficient: −4.97e−02
Value: B
Propionigenium.maris
0
1
2
3
A (n=14)
B (n=15)
C (n=11)
surface

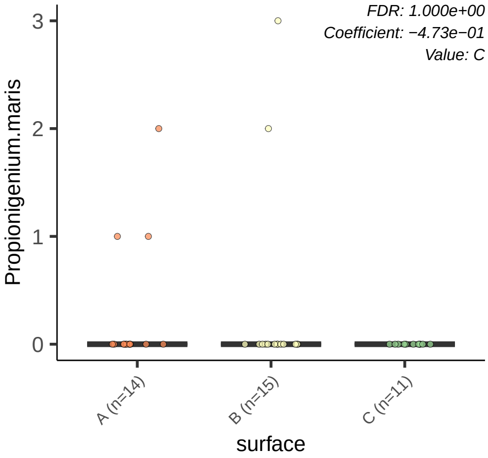
FDR: 1.000e+00
Coefficient: −4.73e−01
Value: C
Propionigenium.maris
3
2
1
0
A (n=14)
B (n=15)
C (n=11)
surface

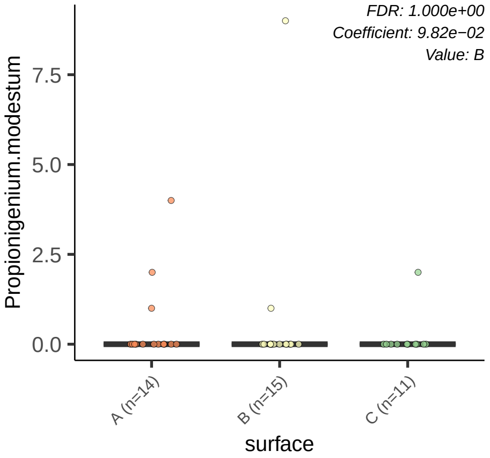
FDR: 1.000e+00
Coefficient: 9.82e−02
Value: B
Propionigenium.modestum
0.0
2.5
5.0
7.5
A (n=14)
B (n=15)
C (n=11)
surface

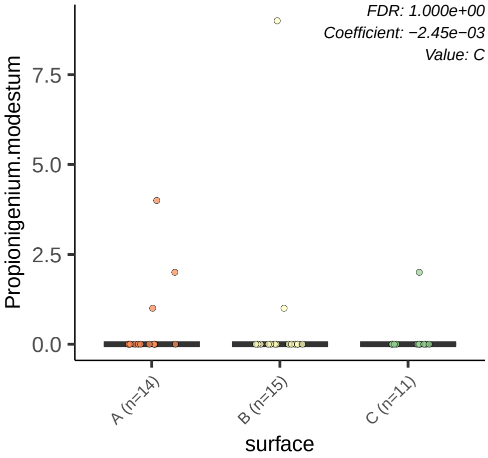

FDR: 1.000e+00
Coefficient: −2.45e−03
Value: C
Propionigenium.modestum
7.5
5.0
2.5
0.0
A (n=14)
B (n=15)
C (n=11)
surface

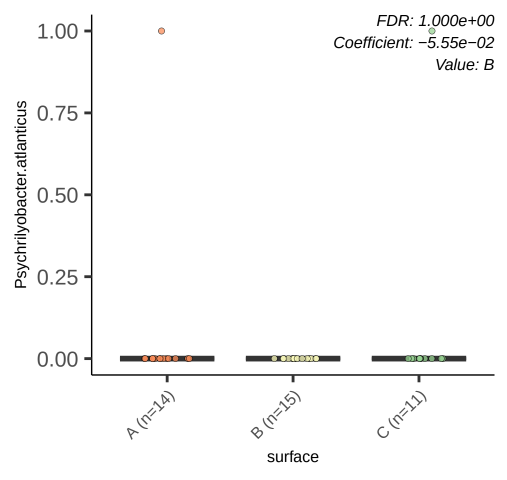

FDR: 1.000e+00
Coefficient: −5.55e−02
Value: B
1.00
0.75
0.50
0.25
0.00
Psychrilyobacter.atlanticus
A (n=14)
B (n=15)
C (n=11)
surface

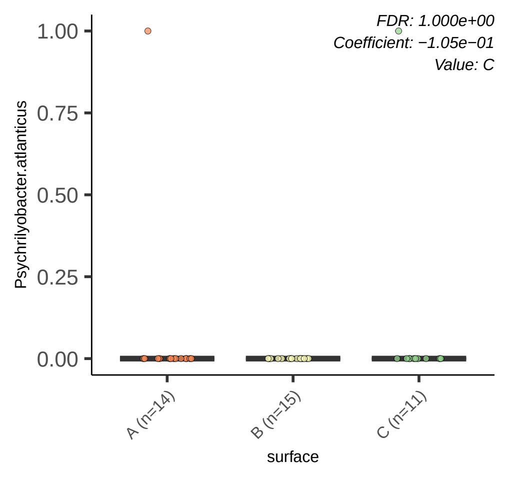

FDR: 1.000e+00
Coefficient: −1.05e−01
Value: C
Psychrilyobacter.atlanticus
1.00
0.75
0.50
0.25
0.00
A (n=14)
B (n=15)
C (n=11)
surface

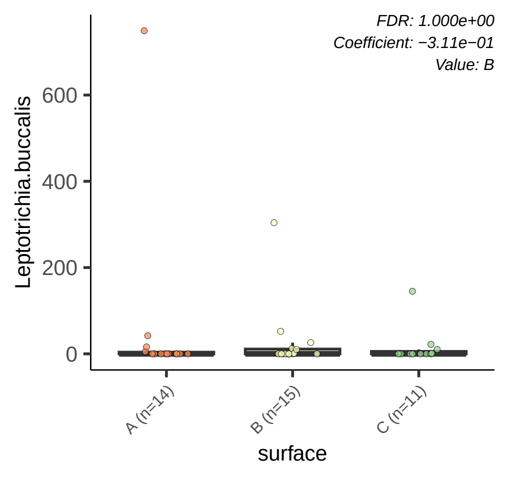
FDR: 1.000e+00
Coefficient: −3.11e−01
Value: B
Leptotrichia.buccalis
0
200
400
600
A (n=14)
B (n=15)
C (n=11)
surface

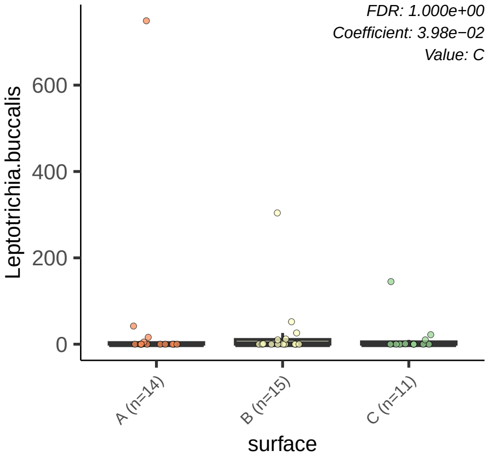
FDR: 1.000e+00
Coefficient: 3.98e−02
Value: C
Leptotrichia.buccalis
600
400
200
0
A (n=14)
B (n=15)
C (n=11)
surface

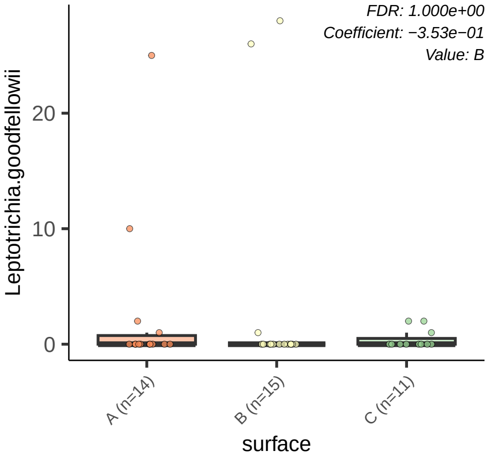

FDR: 1.000e+00
Coefficient: −3.53e−01
Value: B
Leptotrichia.goodfellowii
0
10
20
A (n=14)
B (n=15)
C (n=11)
surface

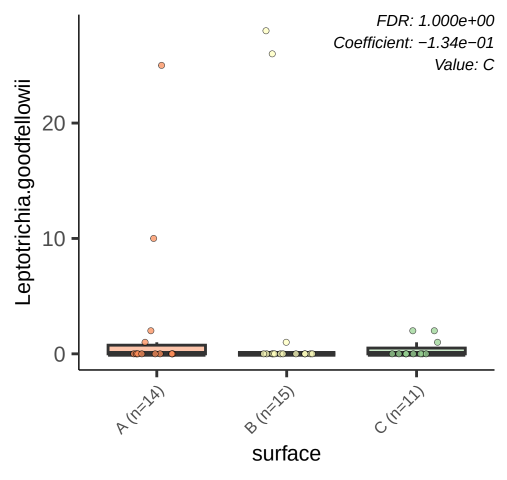

FDR: 1.000e+00
Coefficient: −1.34e−01
Value: C
Leptotrichia.goodfellowii
20
10
0
A (n=14)
B (n=15)
C (n=11)
surface

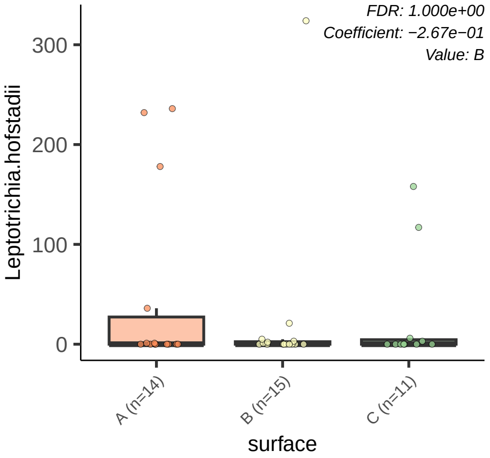

FDR: 1.000e+00
Coefficient: −2.67e−01
Value: B
Leptotrichia.hofstadii
300
200
100
0
A (n=14)
B (n=15)
C (n=11)
surface

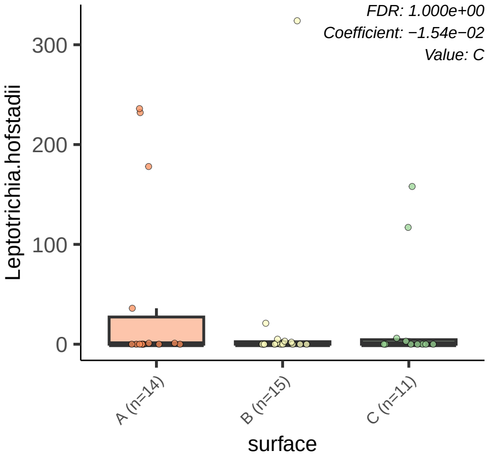

FDR: 1.000e+00
Coefficient: −1.54e−02
Value: C
Leptotrichia.hofstadii
300
200
100
0
A (n=14)
B (n=15)
C (n=11)
surface

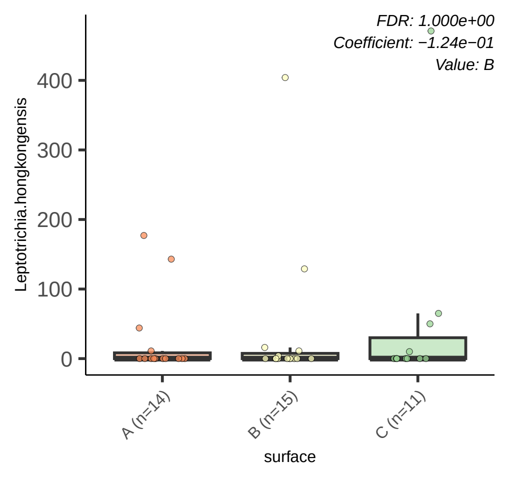

FDR: 1.000e+00
Coefficient: −1.24e−01
Value: B
Leptotrichia.hongkongensis
400
300
200
100
0
A (n=14)
B (n=15)
C (n=11)
surface

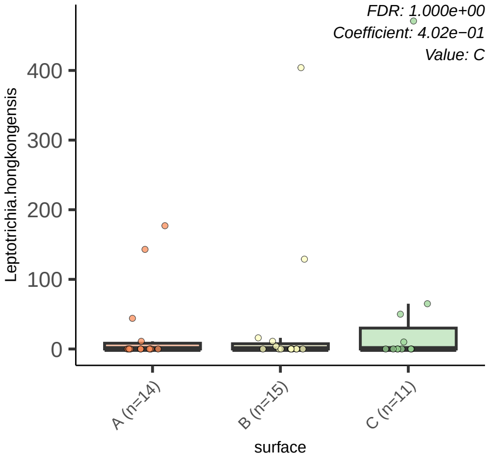

FDR: 1.000e+00
Coefficient: 4.02e−01
Value: C
Leptotrichia.hongkongensis
400
300
200
100
0
A (n=14)
B (n=15)
C (n=11)
surface

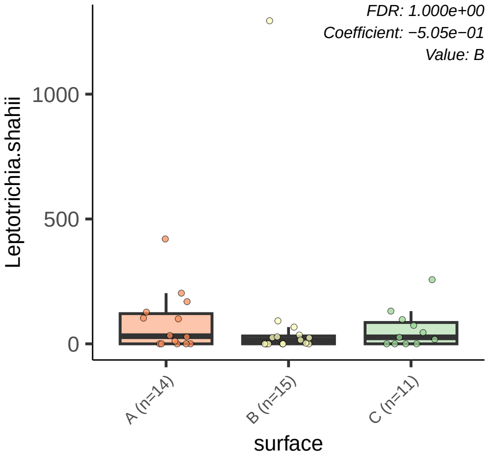

FDR: 1.000e+00
Coefficient: −5.05e−01
Value: B
Leptotrichia.shahii
1000
500
0
A (n=14)
B (n=15)
C (n=11)
surface

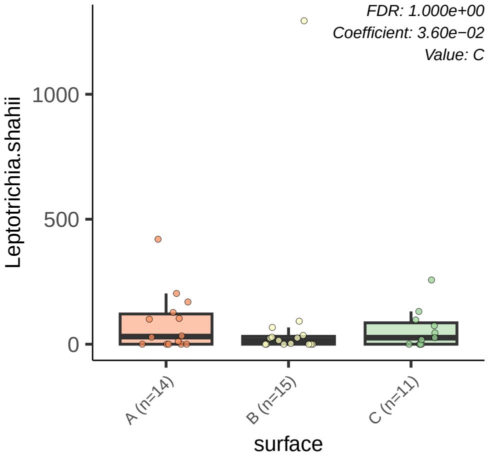
FDR: 1.000e+00
Coefficient: 3.60e−02
Value: C
Leptotrichia.shahii
1000
500
0
A (n=14)
B (n=15)
C (n=11)
surface

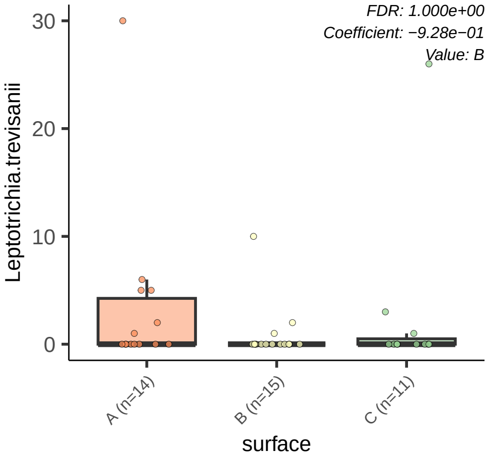

FDR: 1.000e+00
Coefficient: −9.28e−01
Value: B
Leptotrichia.trevisanii
30
20
10
0
A (n=14)
B (n=15)
C (n=11)
surface

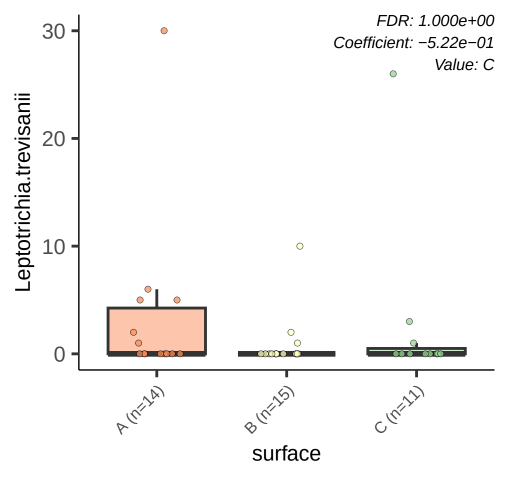
FDR: 1.000e+00
Coefficient: −5.22e−01
Value: C
Leptotrichia.trevisanii
30
20
10
0
A (n=14)
B (n=15)
C (n=11)
surface

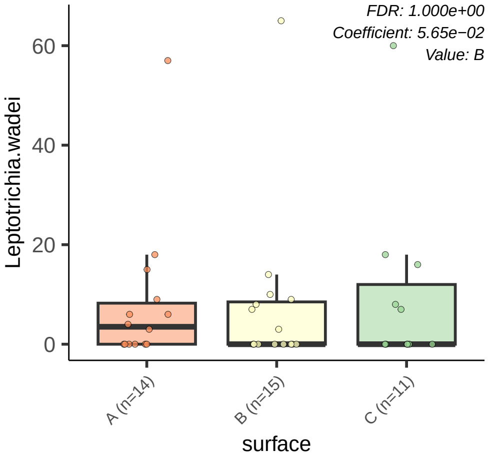

FDR: 1.000e+00
Coefficient: 5.65e−02
Value: B
Leptotrichia.wadei
0
20
40
60
A (n=14)
B (n=15)
C (n=11)
surface

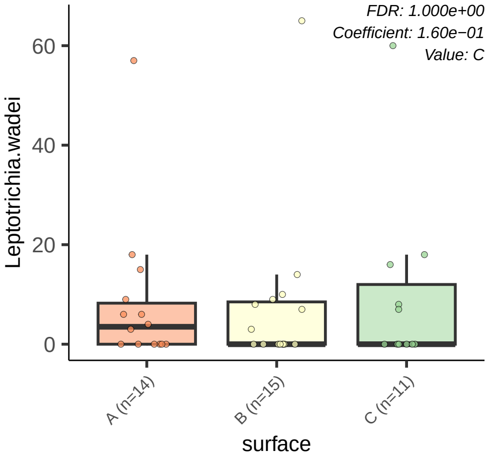

FDR: 1.000e+00
Coefficient: 1.60e−01
Value: C
Leptotrichia.wadei
0
20
40
60
A (n=14)
B (n=15)
C (n=11)
surface

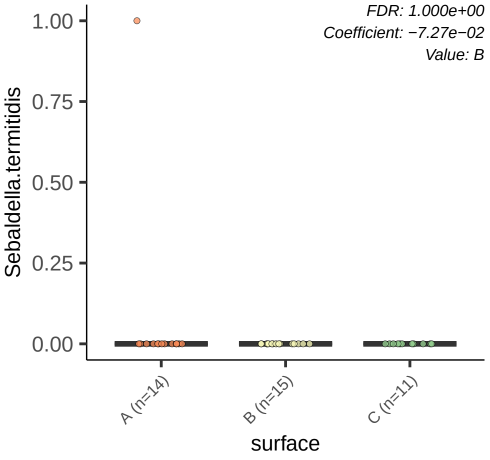
FDR: 1.000e+00
Coefficient: −7.27e−02
Value: B
Sebaldella.termitidis
1.00
0.75
0.50
0.25
0.00
A (n=14)
B (n=15)
C (n=11)
surface

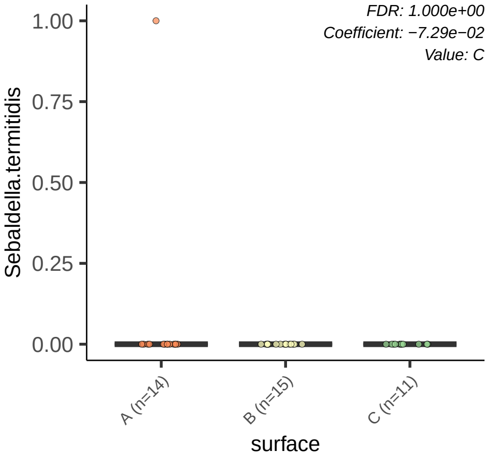
FDR: 1.000e+00
Coefficient: −7.29e−02
Value: C
Sebaldella.termitidis
1.00
0.75
0.50
0.25
0.00
A (n=14)
B (n=15)
C (n=11)
surface

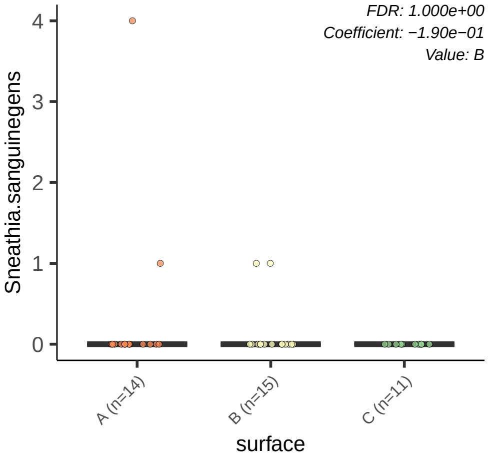
FDR: 1.000e+00
Coefficient: −1.90e−01
Value: B
Sneathia.sanguinegens
4
3
2
1
0
A (n=14)
B (n=15)
C (n=11)
surface

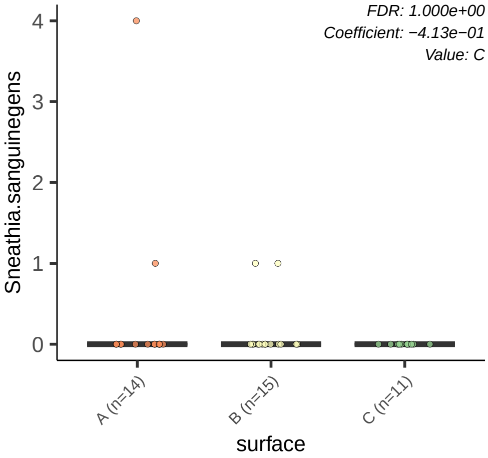
FDR: 1.000e+00
Coefficient: −4.13e−01
Value: C
Sneathia.sanguinegens
4
3
2
1
0
A (n=14)
B (n=15)
C (n=11)
surface

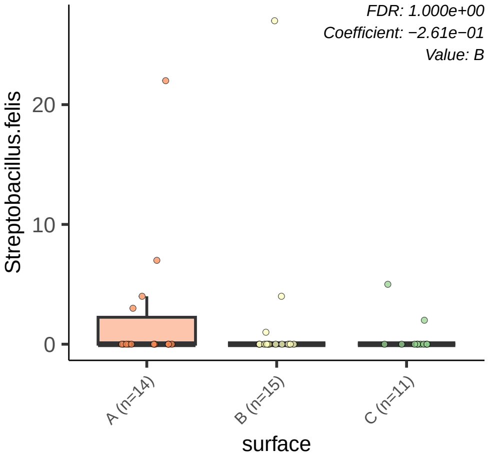
FDR: 1.000e+00
Coefficient: −2.61e−01
Value: B
Streptobacillus.felis
20
10
0
A (n=14)
B (n=15)
C (n=11)
surface

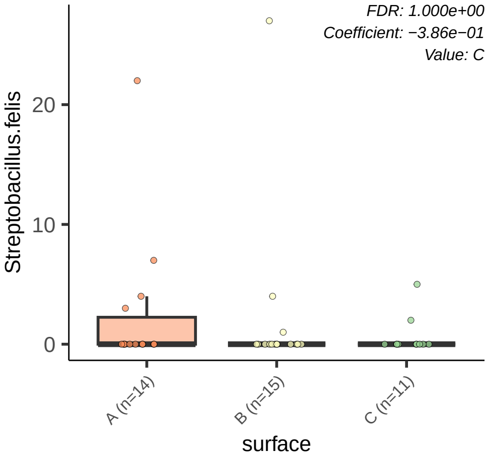
FDR: 1.000e+00
Coefficient: −3.86e−01
Value: C
Streptobacillus.felis
0
10
20
A (n=14)
B (n=15)
C (n=11)
surface

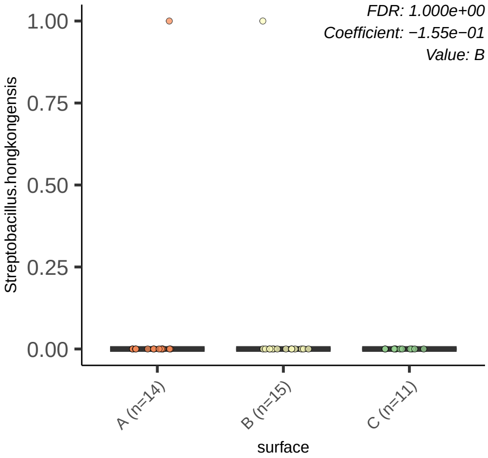

FDR: 1.000e+00
Coefficient: −1.55e−01
Value: B
Streptobacillus.hongkongensis
1.00
0.75
0.50
0.25
0.00
A (n=14)
B (n=15)
C (n=11)
surface

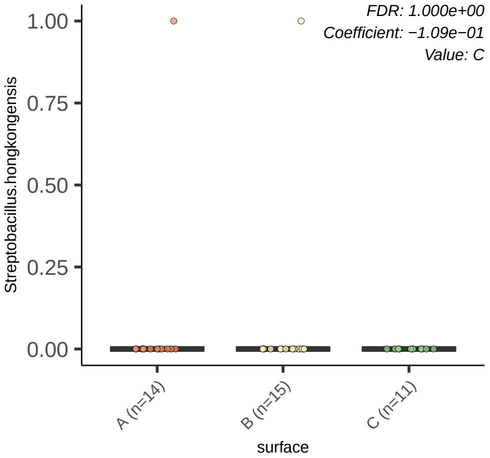
FDR: 1.000e+00
Coefficient: −1.09e−01
Value: C
1.00
0.75
0.50
0.25
0.00
Streptobacillus.hongkongensis
A (n=14)
B (n=15)
C (n=11)
surface

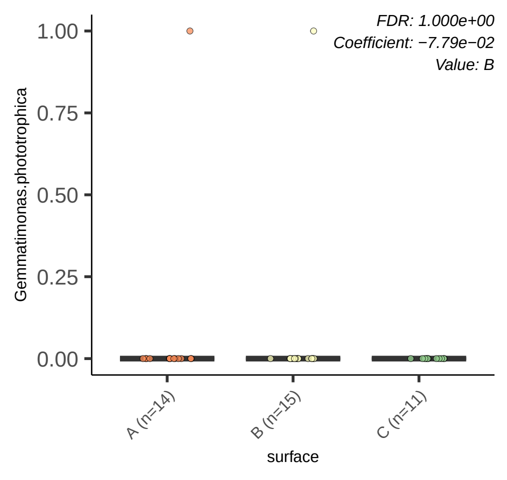
FDR: 1.000e+00
Coefficient: −7.79e−02
Value: B
Gemmatimonas.phototrophica
1.00
0.75
0.50
0.25
0.00
A (n=14)
B (n=15)
C (n=11)
surface

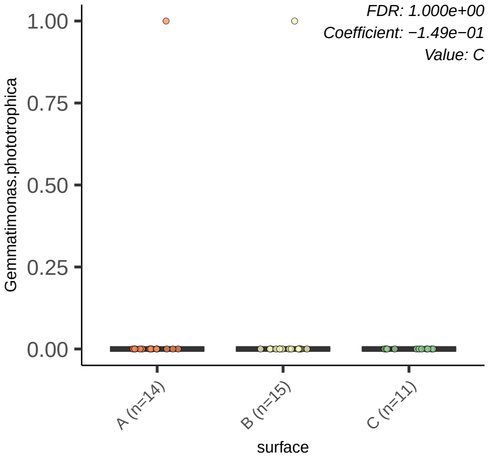
FDR: 1.000e+00
Coefficient: −1.49e−01
Value: C
Gemmatimonas.phototrophica
1.00
0.75
0.50
0.25
0.00
A (n=14)
B (n=15)
C (n=11)
surface

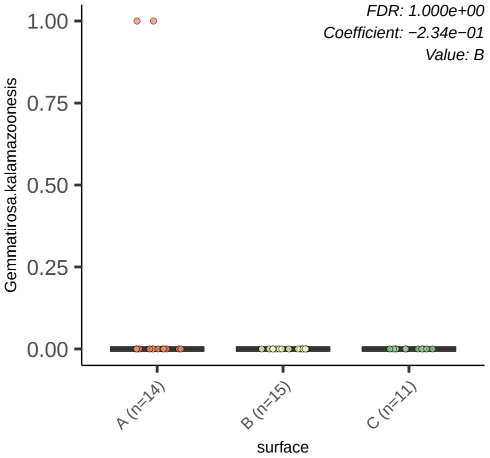

FDR: 1.000e+00
Coefficient: −2.34e−01
Value: B
Gemmatirosa.kalamazoonesis
1.00
0.75
0.50
0.25
0.00
A (n=14)
B (n=15)
C (n=11)
surface

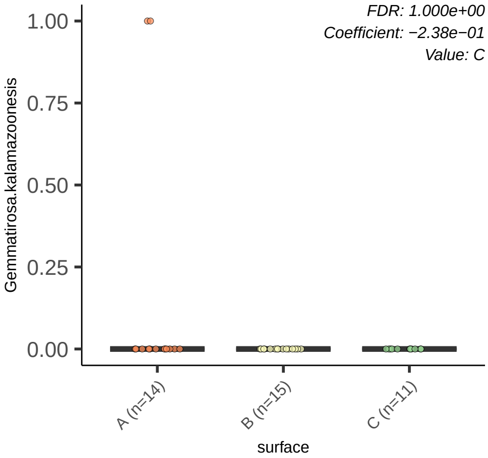
FDR: 1.000e+00
Coefficient: −2.38e−01
Value: C
Gemmatirosa.kalamazoonesis
1.00
0.75
0.50
0.25
0.00
A (n=14)
B (n=15)
C (n=11)
surface

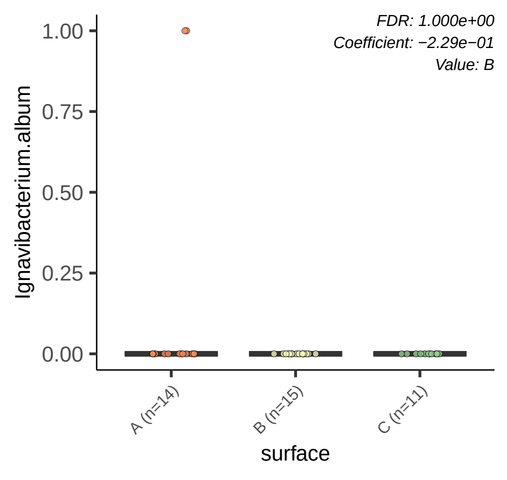

FDR: 1.000e+00
Coefficient: −2.29e−01
Value: B
Ignavibacterium.album
1.00
0.75
0.50
0.25
0.00
A (n=14)
B (n=15)
C (n=11)
surface

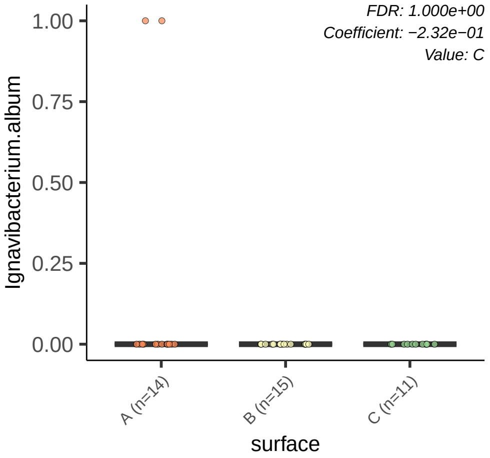
FDR: 1.000e+00
Coefficient: −2.32e−01
Value: C
Ignavibacterium.album
1.00
0.75
0.50
0.25
0.00
A (n=14)
B (n=15)
C (n=11)
surface

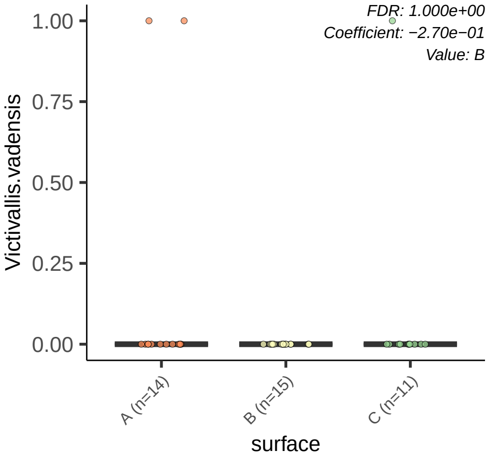

FDR: 1.000e+00
Coefficient: −2.70e−01
Value: B
1.00
0.75
0.50
0.25
0.00
Victivallis.vadensis
A (n=14)
B (n=15)
C (n=11)
surface

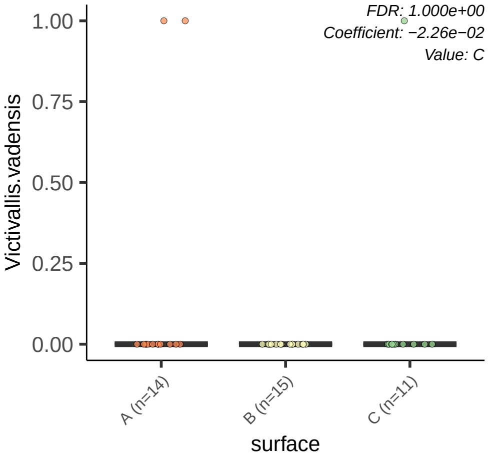

FDR: 1.000e+00
Coefficient: −2.26e−02
Value: C
Victivallis.vadensis
1.00
0.75
0.50
0.25
0.00
A (n=14)
B (n=15)
C (n=11)
surface

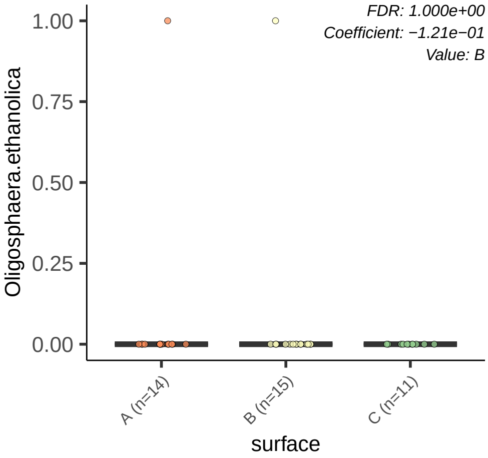

FDR: 1.000e+00
Coefficient: −1.21e−01
Value: B
Oligosphaera.ethanolica
1.00
0.75
0.50
0.25
0.00
A (n=14)
B (n=15)
C (n=11)
surface

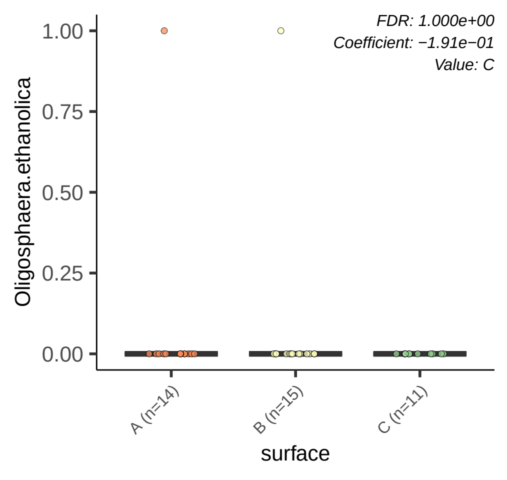
FDR: 1.000e+00
Coefficient: −1.91e−01
Value: C
Oligosphaera.ethanolica
1.00
0.75
0.50
0.25
0.00
A (n=14)
B (n=15)
C (n=11)
surface

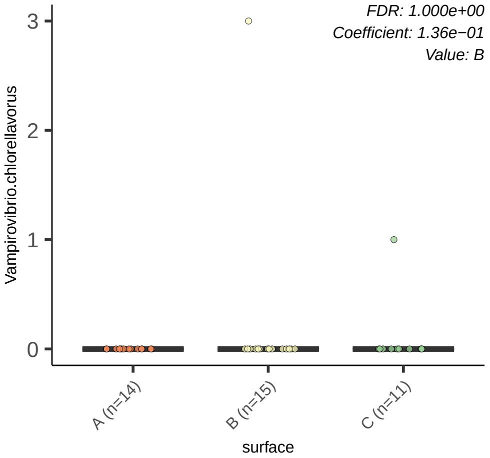
FDR: 1.000e+00
Coefficient: 1.36e−01
Value: B
Vampirovibrio.chlorellavorus
3
2
1
0
A (n=14)
B (n=15)
C (n=11)
surface

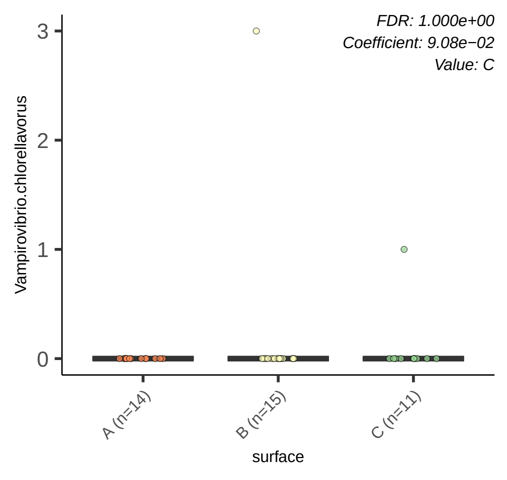

FDR: 1.000e+00
Coefficient: 9.08e−02
Value: C
Vampirovibrio.chlorellavorus
3
2
1
0
A (n=14)
B (n=15)
C (n=11)
surface

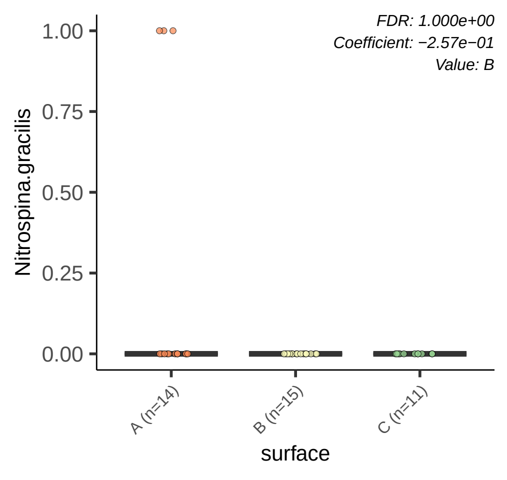
FDR: 1.000e+00
Coefficient: −2.57e−01
Value: B
Nitrospina.gracilis
1.00
0.75
0.50
0.25
0.00
A (n=14)
B (n=15)
C (n=11)
surface

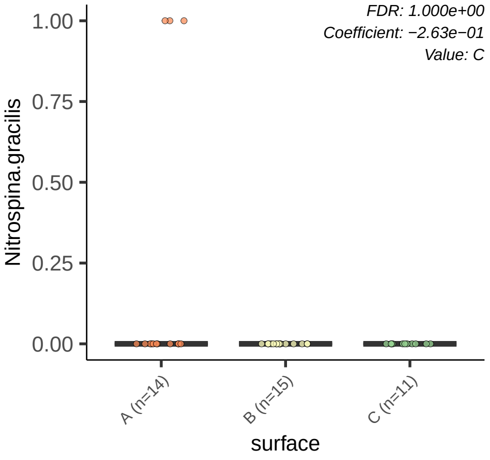

FDR: 1.000e+00
Coefficient: −2.63e−01
Value: C
Nitrospina.gracilis
1.00
0.75
0.50
0.25
0.00
A (n=14)
B (n=15)
C (n=11)
surface

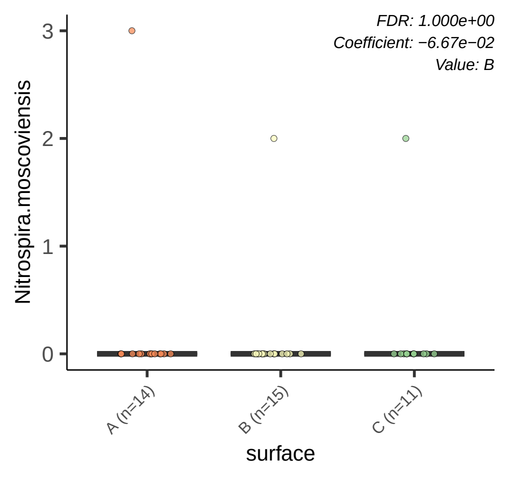
FDR: 1.000e+00
Coefficient: −6.67e−02
Value: B
Nitrospira.moscoviensis
3
2
1
0
A (n=14)
B (n=15)
C (n=11)
surface

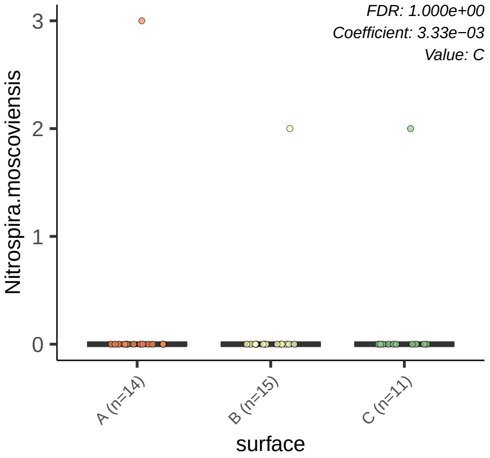
FDR: 1.000e+00
Coefficient: 3.33e−03
Value: C
Nitrospira.moscoviensis
3
2
1
0
A (n=14)
B (n=15)
C (n=11)
surface

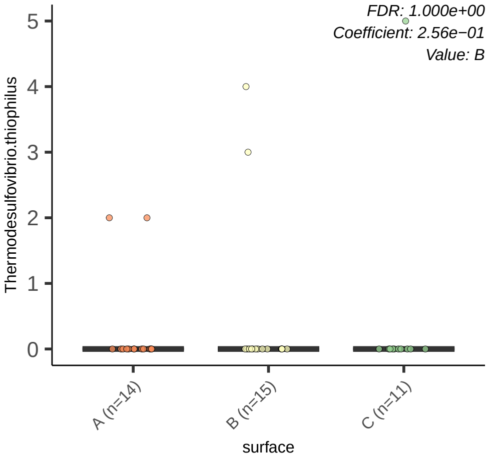
FDR: 1.000e+00
Coefficient: 2.56e−01
Value: B
Thermodesulfovibrio.thiophilus
0
1
2
3
4
5
A (n=14)
B (n=15)
C (n=11)
surface

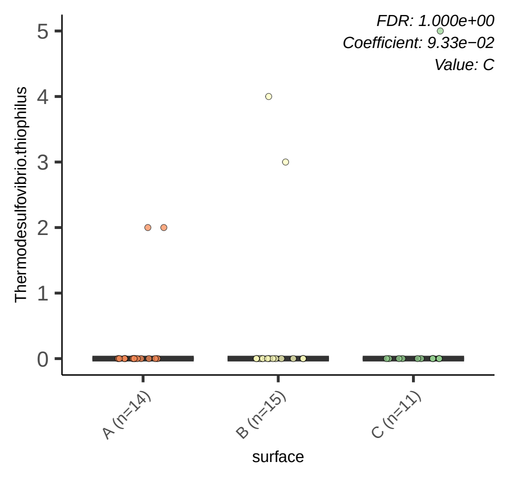

FDR: 1.000e+00
Coefficient: 9.33e−02
Value: C
Thermodesulfovibrio.thiophilus
0
1
2
3
4
5
A (n=14)
B (n=15)
C (n=11)
surface

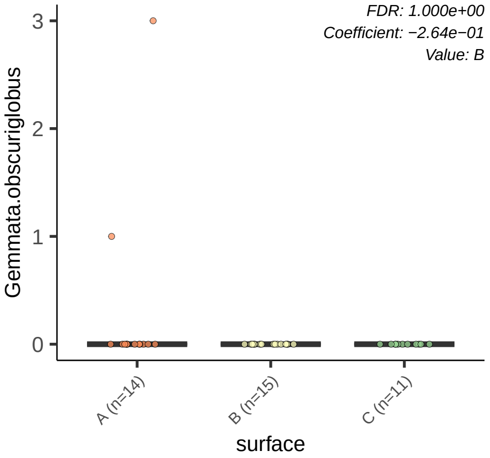

FDR: 1.000e+00
Coefficient: −2.64e−01
Value: B
Gemmata.obscuriglobus
3
2
1
0
A (n=14)
B (n=15)
C (n=11)
surface

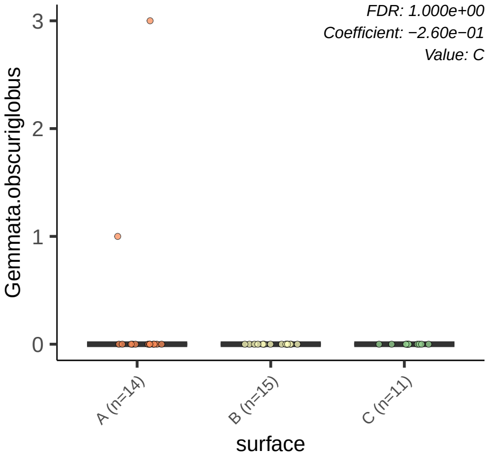
FDR: 1.000e+00
Coefficient: −2.60e−01
Value: C
Gemmata.obscuriglobus
3
2
1
0
A (n=14)
B (n=15)
C (n=11)
surface

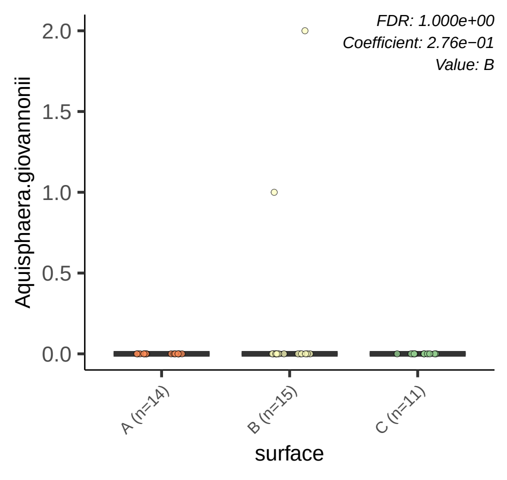

FDR: 1.000e+00
Coefficient: 2.76e−01
Value: B
Aquisphaera.giovannonii
2.0
1.5
1.0
0.5
0.0
A (n=14)
B (n=15)
C (n=11)
surface

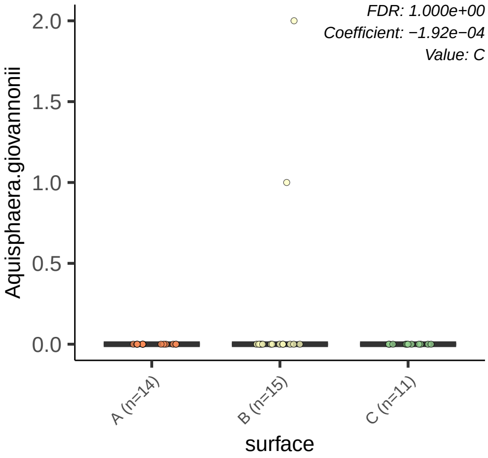

FDR: 1.000e+00
Coefficient: −1.92e−04
Value: C
Aquisphaera.giovannonii
2.0
1.5
1.0
0.5
0.0
A (n=14)
B (n=15)
C (n=11)
surface

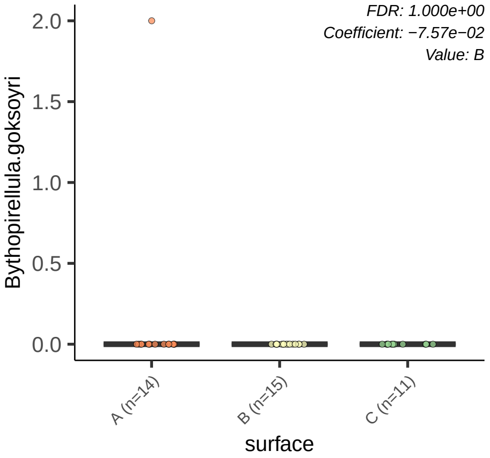

FDR: 1.000e+00
Coefficient: −7.57e−02
Value: B
Bythopirellula.goksoyri
2.0
1.5
1.0
0.5
0.0
A (n=14)
B (n=15)
C (n=11)
surface

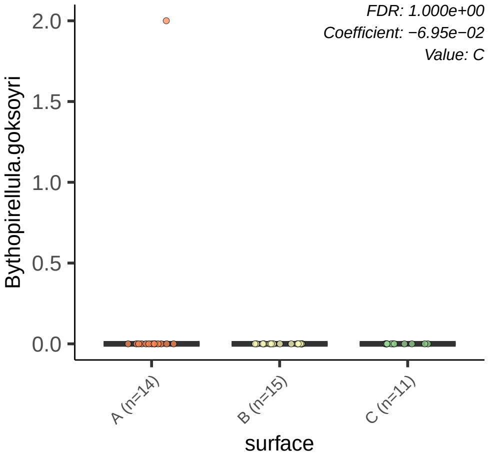
FDR: 1.000e+00
Coefficient: −6.95e−02
Value: C
Bythopirellula.goksoyri
2.0
1.5
1.0
0.5
0.0
A (n=14)
B (n=15)
C (n=11)
surface

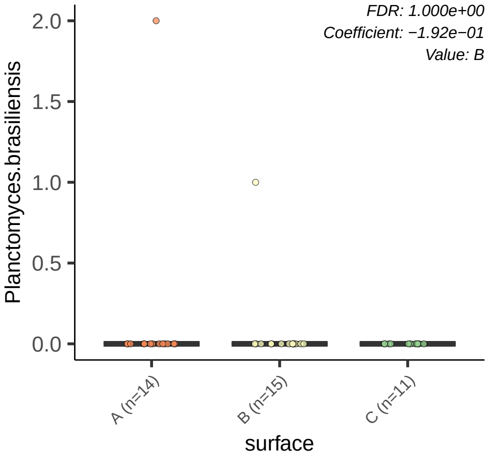
FDR: 1.000e+00
Coefficient: −1.92e−01
Value: B
Planctomyces.brasiliensis
2.0
1.5
1.0
0.5
0.0
A (n=14)
B (n=15)
C (n=11)
surface

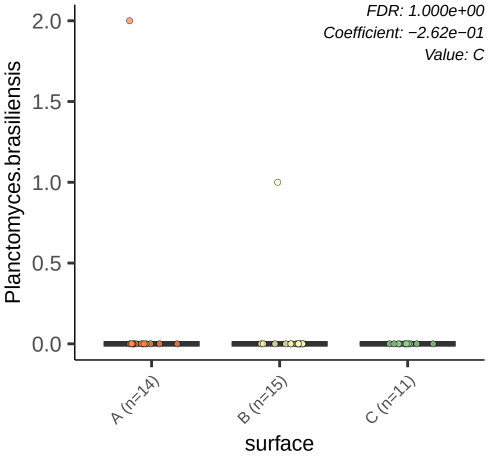

FDR: 1.000e+00
Coefficient: −2.62e−01
Value: C
Planctomyces.brasiliensis
2.0
1.5
1.0
0.5
0.0
A (n=14)
B (n=15)
C (n=11)
surface

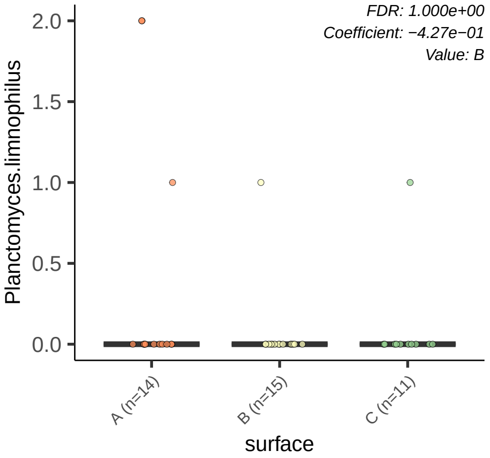

FDR: 1.000e+00
Coefficient: −4.27e−01
Value: B
Planctomyces.limnophilus
2.0
1.5
1.0
0.5
0.0
A (n=14)
B (n=15)
C (n=11)
surface

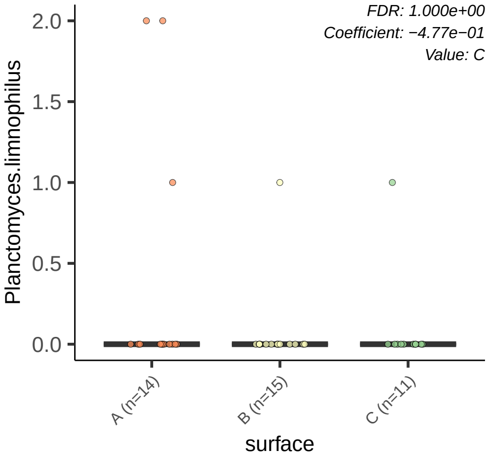

FDR: 1.000e+00
Coefficient: −4.77e−01
Value: C
Planctomyces.limnophilus
2.0
1.5
1.0
0.5
0.0
A (n=14)
B (n=15)
C (n=11)
surface

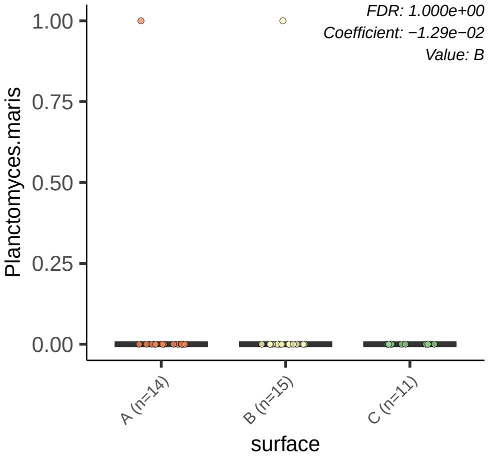
FDR: 1.000e+00
Coefficient: −1.29e−02
Value: B
1.00
0.75
0.50
0.25
0.00
Planctomyces.maris
A (n=14)
B (n=15)
C (n=11)
surface

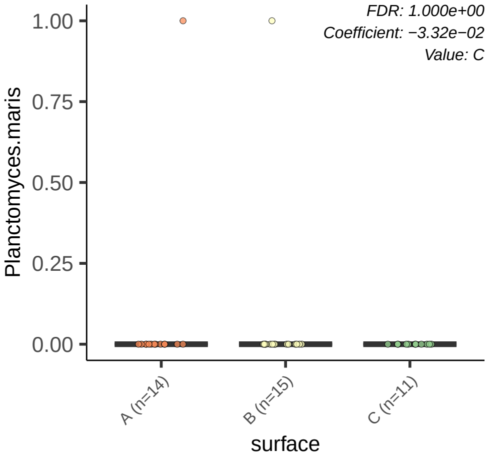

FDR: 1.000e+00
Coefficient: −3.32e−02
Value: C
Planctomyces.maris
1.00
0.75
0.50
0.25
0.00
A (n=14)
B (n=15)
C (n=11)
surface

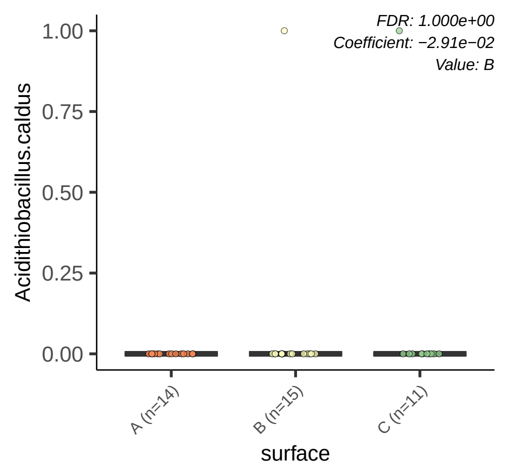

FDR: 1.000e+00
Coefficient: −2.91e−02
Value: B
Acidithiobacillus.caldus
1.00
0.75
0.50
0.25
0.00
A (n=14)
B (n=15)
C (n=11)
surface

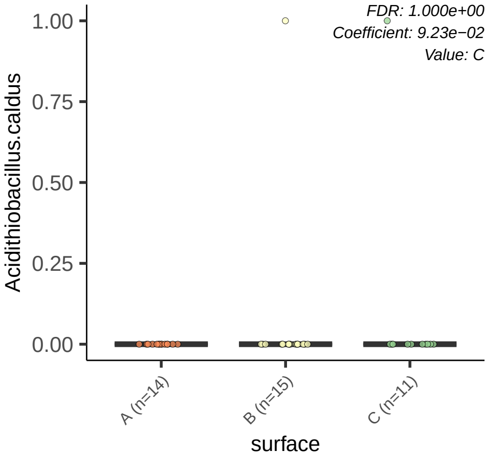
FDR: 1.000e+00
Coefficient: 9.23e−02
Value: C
1.00
0.75
0.50
0.25
0.00
Acidithiobacillus.caldus
A (n=14)
B (n=15)
C (n=11)
surface

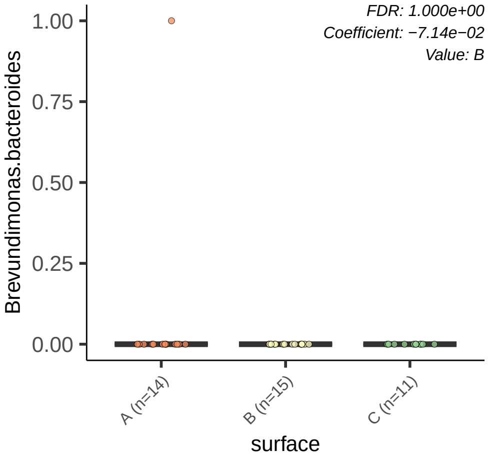

FDR: 1.000e+00
Coefficient: −7.14e−02
Value: B
Brevundimonas.bacteroides
1.00
0.75
0.50
0.25
0.00
A (n=14)
B (n=15)
C (n=11)
surface

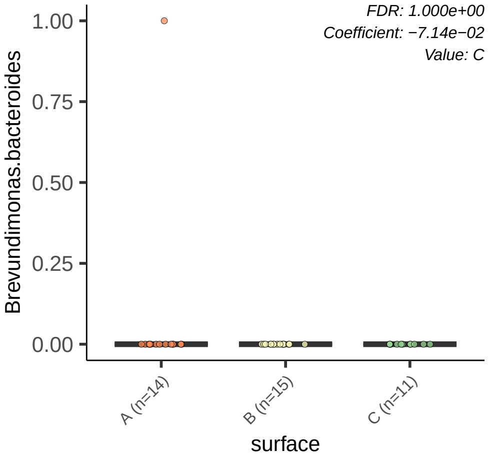

FDR: 1.000e+00
Coefficient: −7.14e−02
Value: C
Brevundimonas.bacteroides
1.00
0.75
0.50
0.25
0.00
A (n=14)
B (n=15)
C (n=11)
surface

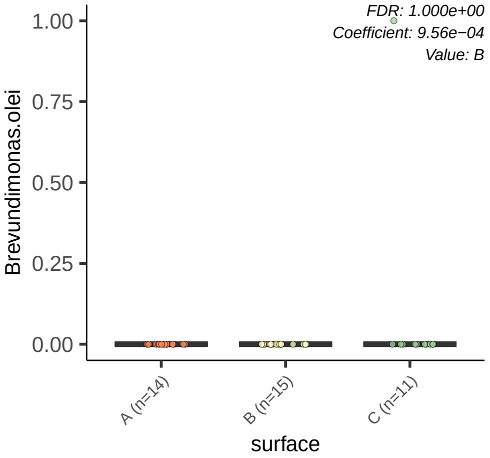
FDR: 1.000e+00
Coefficient: 9.56e−04
Value: B
Brevundimonas.olei
1.00
0.75
0.50
0.25
0.00
A (n=14)
B (n=15)
C (n=11)
surface

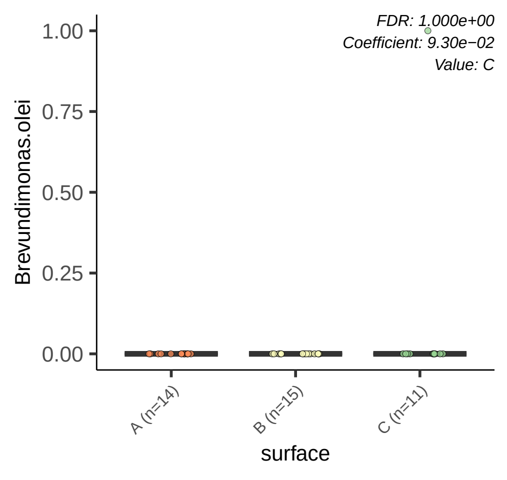

FDR: 1.000e+00
Coefficient: 9.30e−02
Value: C
Brevundimonas.olei
1.00
0.75
0.50
0.25
0.00
A (n=14)
B (n=15)
C (n=11)
surface

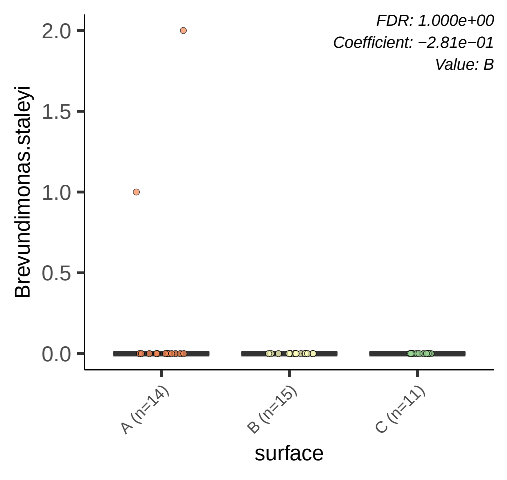

FDR: 1.000e+00
Coefficient: −2.81e−01
Value: B
Brevundimonas.staleyi
2.0
1.5
1.0
0.5
0.0
A (n=14)
B (n=15)
C (n=11)
surface

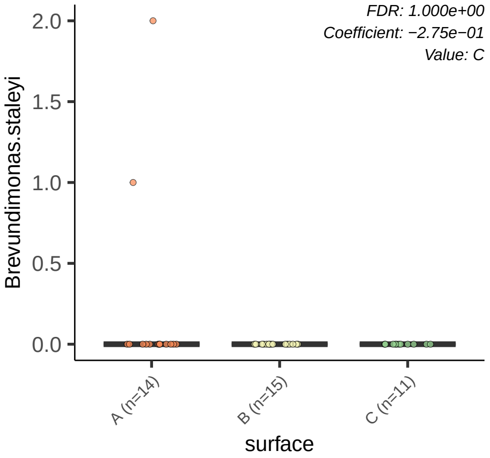
FDR: 1.000e+00
Coefficient: −2.75e−01
Value: C
Brevundimonas.staleyi
2.0
1.5
1.0
0.5
0.0
A (n=14)
B (n=15)
C (n=11)
surface

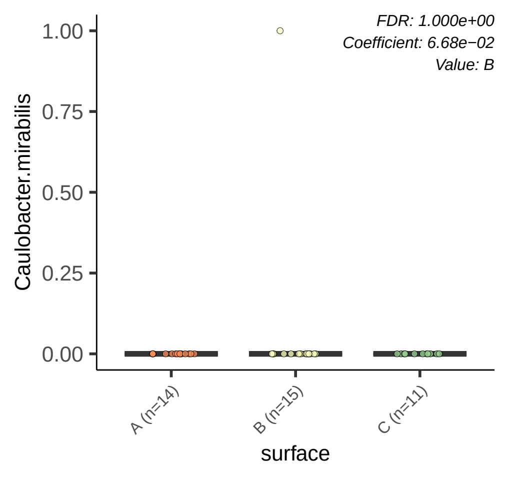
FDR: 1.000e+00
Coefficient: 6.68e−02
Value: B
Caulobacter.mirabilis
1.00
0.75
0.50
0.25
0.00
A (n=14)
B (n=15)
C (n=11)
surface

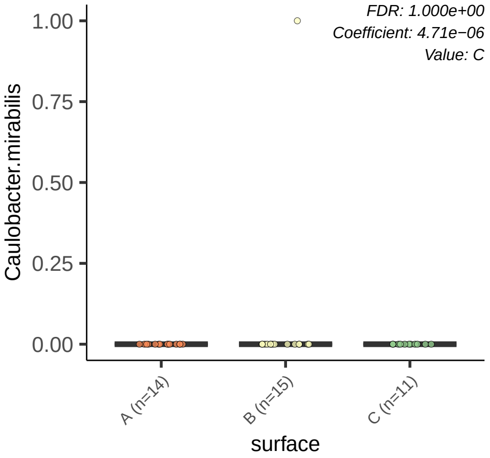
FDR: 1.000e+00
Coefficient: 4.71e−06
Value: C
Caulobacter.mirabilis
1.00
0.75
0.50
0.25
0.00
A (n=14)
B (n=15)
C (n=11)
surface

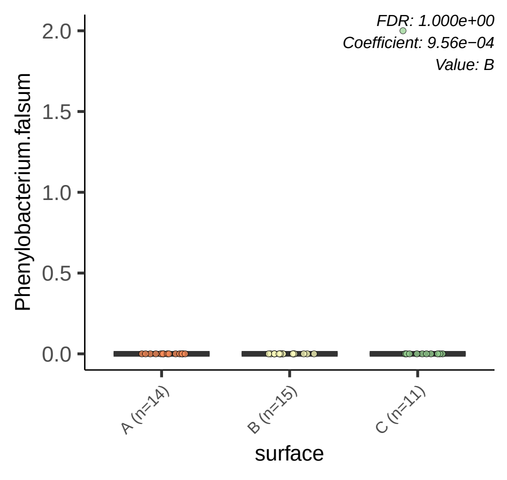
FDR: 1.000e+00
Coefficient: 9.56e−04
Value: B
Phenylobacterium.falsum
2.0
1.5
1.0
0.5
0.0
A (n=14)
B (n=15)
C (n=11)
surface

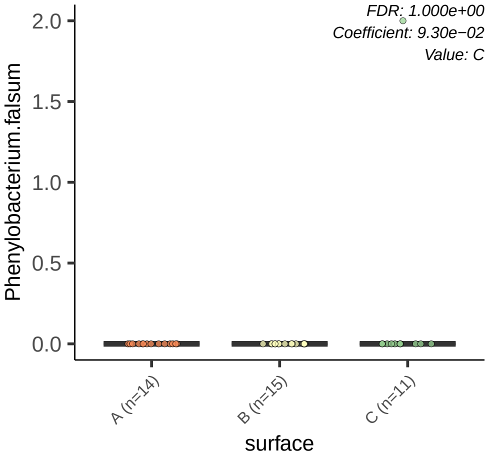
FDR: 1.000e+00
Coefficient: 9.30e−02
Value: C
Phenylobacterium.falsum
2.0
1.5
1.0
0.5
0.0
A (n=14)
B (n=15)
C (n=11)
surface

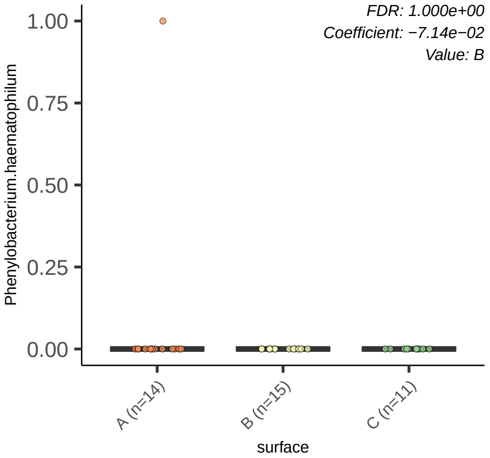
FDR: 1.000e+00
Coefficient: −7.14e−02
Value: B
1.00
0.75
0.50
0.25
0.00
Phenylobacterium.haematophilum
A (n=14)
B (n=15)
C (n=11)
surface

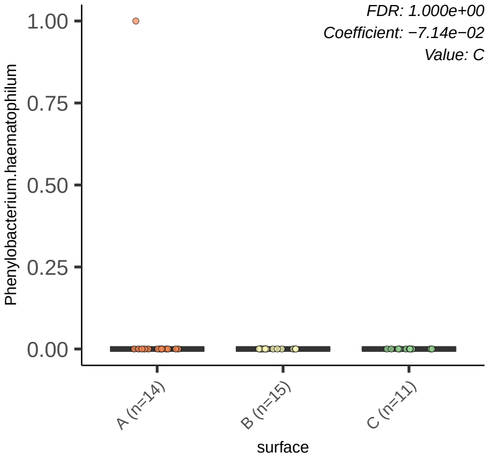

FDR: 1.000e+00
Coefficient: −7.14e−02
Value: C
Phenylobacterium.haematophilum
1.00
0.75
0.50
0.25
0.00
A (n=14)
B (n=15)
C (n=11)
surface

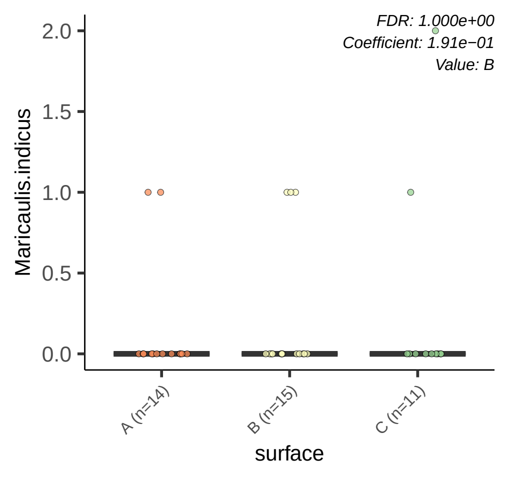
FDR: 1.000e+00
Coefficient: 1.91e−01
Value: B
Maricaulis.indicus
2.0
1.5
1.0
0.5
0.0
A (n=14)
B (n=15)
C (n=11)
surface

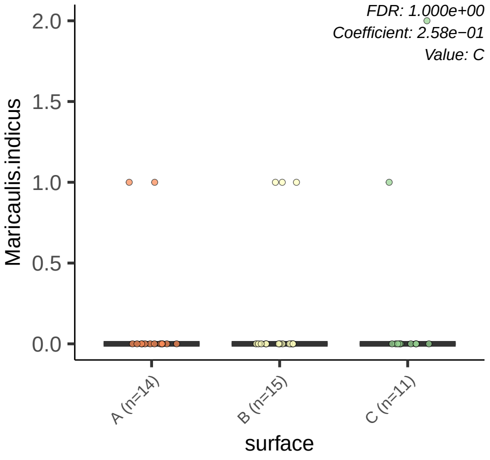

FDR: 1.000e+00
Coefficient: 2.58e−01
Value: C
Maricaulis.indicus
2.0
1.5
1.0
0.5
0.0
A (n=14)
B (n=15)
C (n=11)
surface

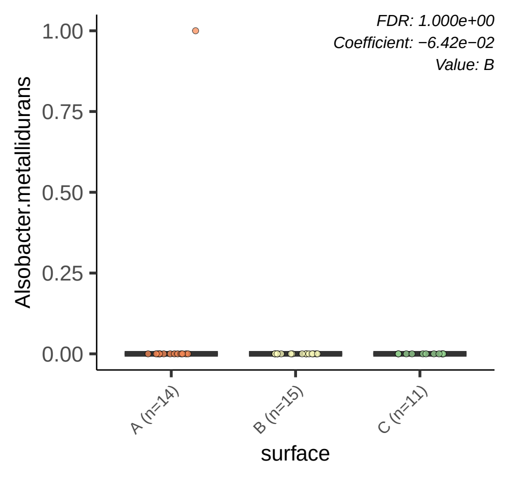

FDR: 1.000e+00
Coefficient: −6.42e−02
Value: B
Alsobacter.metallidurans
1.00
0.75
0.50
0.25
0.00
A (n=14)
B (n=15)
C (n=11)
surface

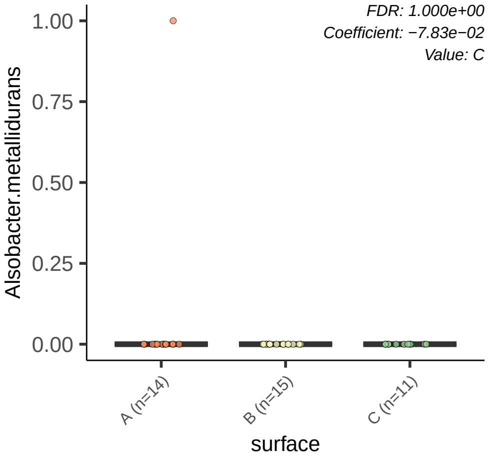

FDR: 1.000e+00
Coefficient: −7.83e−02
Value: C
Alsobacter.metallidurans
1.00
0.75
0.50
0.25
0.00
A (n=14)
B (n=15)
C (n=11)
surface

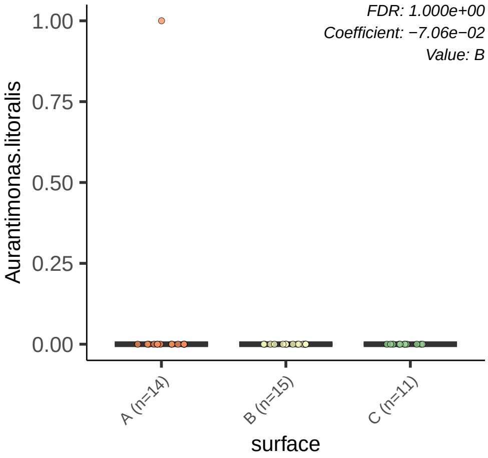

FDR: 1.000e+00
Coefficient: −7.06e−02
Value: B
Aurantimonas.litoralis
1.00
0.75
0.50
0.25
0.00
A (n=14)
B (n=15)
C (n=11)
surface

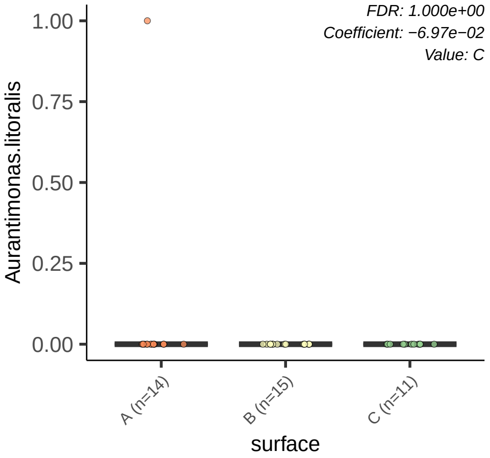

FDR: 1.000e+00
Coefficient: −6.97e−02
Value: C
Aurantimonas.litoralis
1.00
0.75
0.50
0.25
0.00
A (n=14)
B (n=15)
C (n=11)
surface

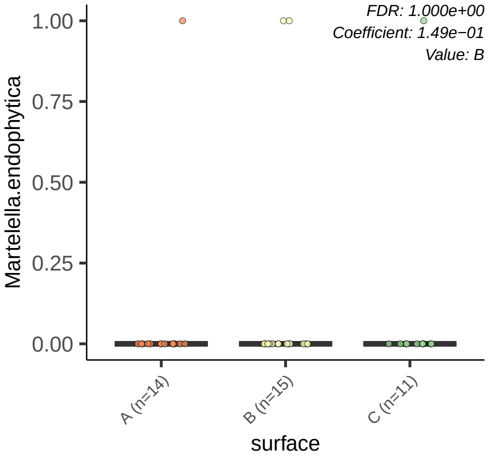
FDR: 1.000e+00
Coefficient: 1.49e−01
Value: B
Martelella.endophytica
1.00
0.75
0.50
0.25
0.00
A (n=14)
B (n=15)
C (n=11)
surface

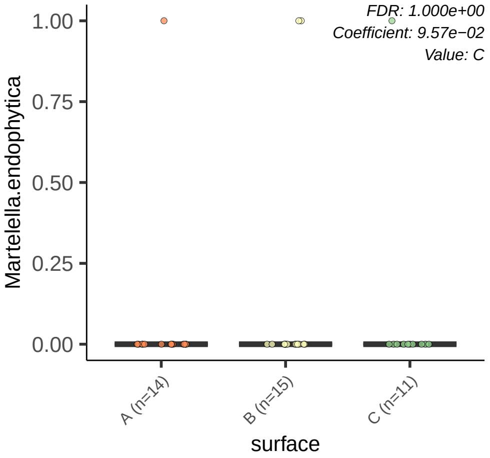
FDR: 1.000e+00
Coefficient: 9.57e−02
Value: C
Martelella.endophytica
1.00
0.75
0.50
0.25
0.00
A (n=14)
B (n=15)
C (n=11)
surface

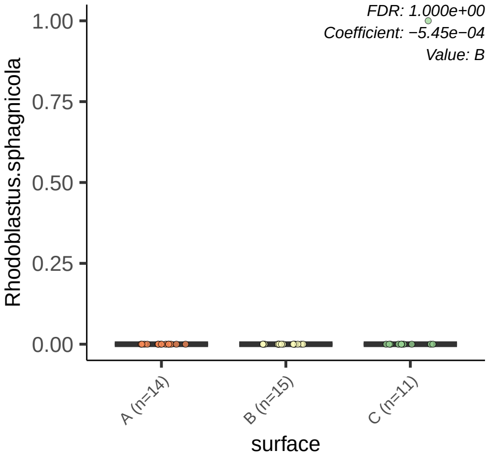
FDR: 1.000e+00
Coefficient: −5.45e−04
Value: B
Rhodoblastus.sphagnicola
1.00
0.75
0.50
0.25
0.00
A (n=14)
B (n=15)
C (n=11)
surface

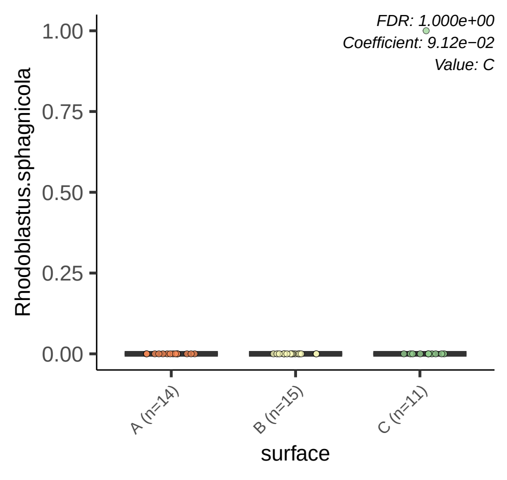
FDR: 1.000e+00
Coefficient: 9.12e−02
Value: C
Rhodoblastus.sphagnicola
1.00
0.75
0.50
0.25
0.00
A (n=14)
B (n=15)
C (n=11)
surface

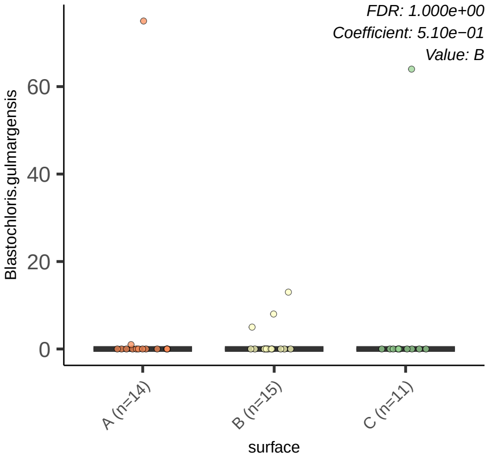
FDR: 1.000e+00
Coefficient: 5.10e−01
Value: B
Blastochloris.gulmargensis
0
20
40
60
A (n=14)
B (n=15)
C (n=11)
surface

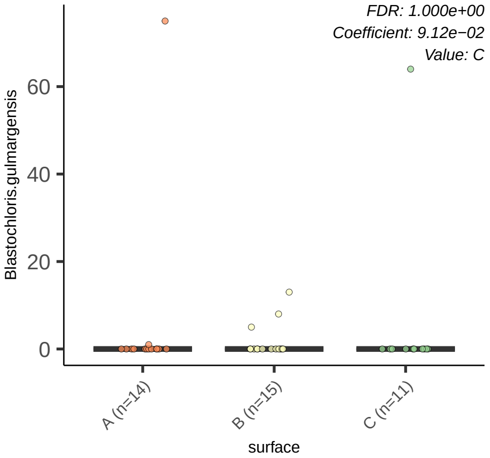
FDR: 1.000e+00
Coefficient: 9.12e−02
Value: C
Blastochloris.gulmargensis
0
20
40
60
A (n=14)
B (n=15)
C (n=11)
surface

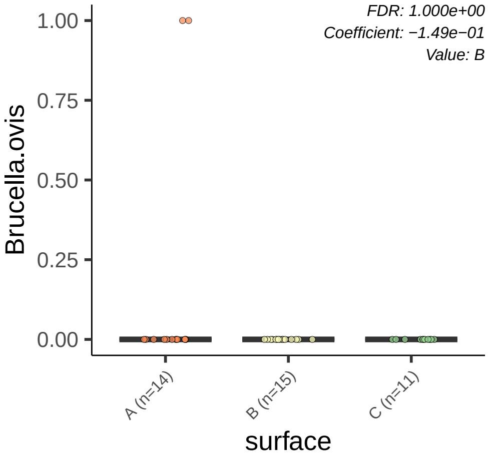
FDR: 1.000e+00
Coefficient: −1.49e−01
Value: B
Brucella.ovis
1.00
0.75
0.50
0.25
0.00
A (n=14)
B (n=15)
C (n=11)
surface

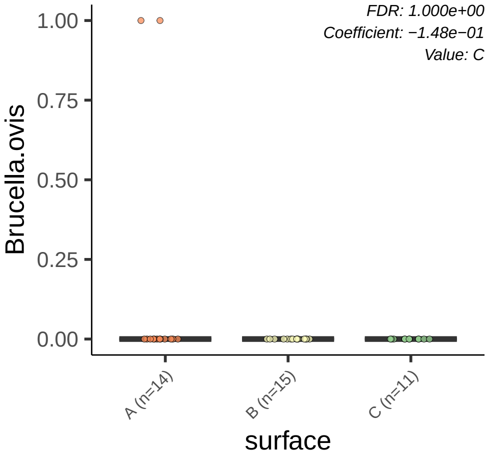

FDR: 1.000e+00
Coefficient: −1.48e−01
Value: C
1.00
0.75
0.50
0.25
0.00
Brucella.ovis
A (n=14)
B (n=15)
C (n=11)
surface

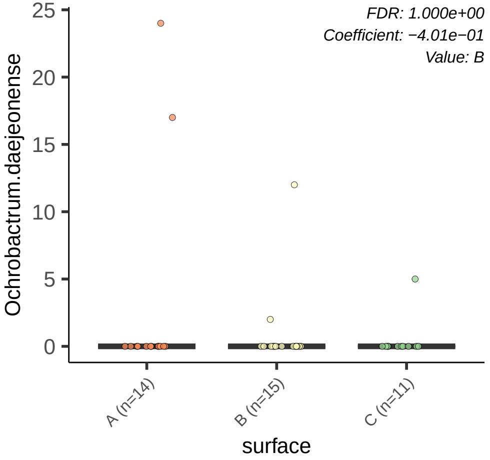

FDR: 1.000e+00
Coefficient: −4.01e−01
Value: B
Ochrobactrum.daejeonense
25
20
15
10
5
0
A (n=14)
B (n=15)
C (n=11)
surface

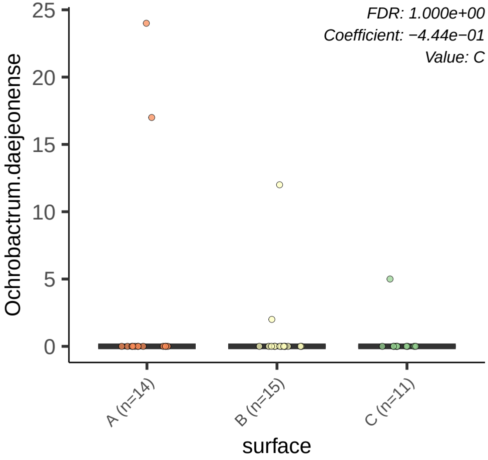
FDR: 1.000e+00
Coefficient: −4.44e−01
Value: C
Ochrobactrum.daejeonense
25
20
15
10
5
0
A (n=14)
B (n=15)
C (n=11)
surface

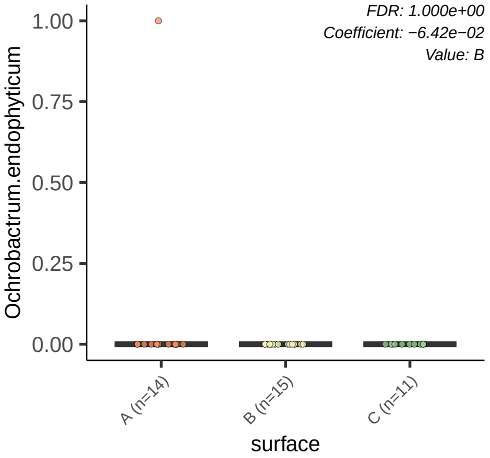
FDR: 1.000e+00
Coefficient: −6.42e−02
Value: B
Ochrobactrum.endophyticum
1.00
0.75
0.50
0.25
0.00
A (n=14)
B (n=15)
C (n=11)
surface

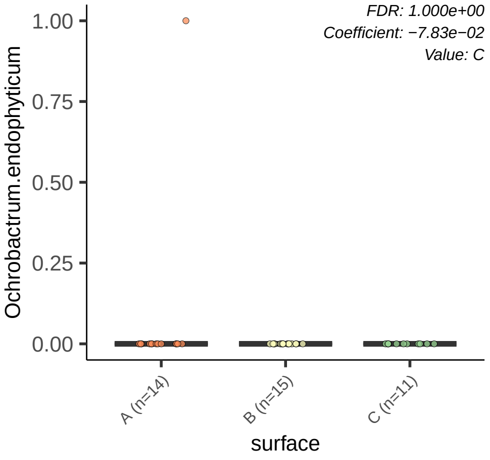

FDR: 1.000e+00
Coefficient: −7.83e−02
Value: C
Ochrobactrum.endophyticum
1.00
0.75
0.50
0.25
0.00
A (n=14)
B (n=15)
C (n=11)
surface

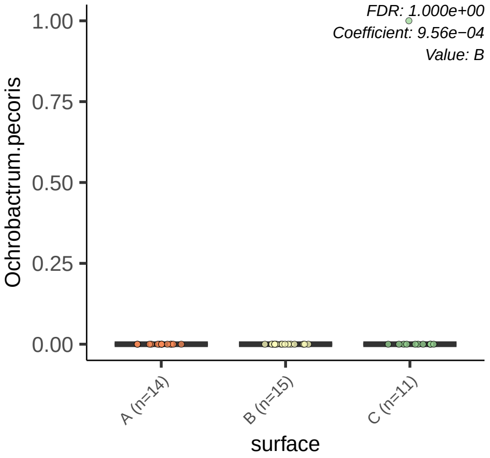

FDR: 1.000e+00
Coefficient: 9.56e−04
Value: B
Ochrobactrum.pecoris
1.00
0.75
0.50
0.25
0.00
A (n=14)
B (n=15)
C (n=11)
surface

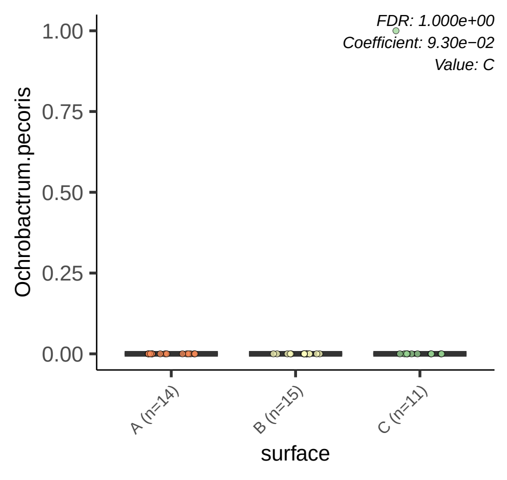
FDR: 1.000e+00
Coefficient: 9.30e−02
Value: C
Ochrobactrum.pecoris
1.00
0.75
0.50
0.25
0.00
A (n=14)
B (n=15)
C (n=11)
surface

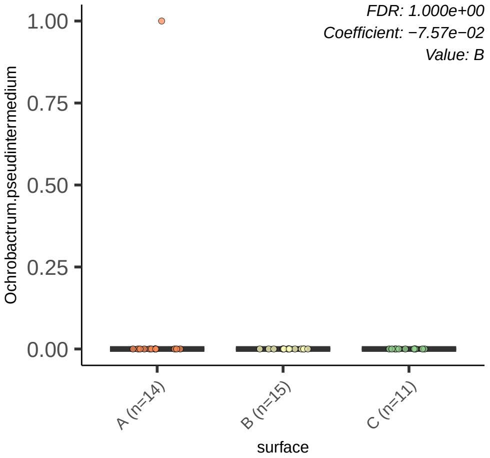
FDR: 1.000e+00
Coefficient: −7.57e−02
Value: B
Ochrobactrum.pseudintermedium
1.00
0.75
0.50
0.25
0.00
A (n=14)
B (n=15)
C (n=11)
surface

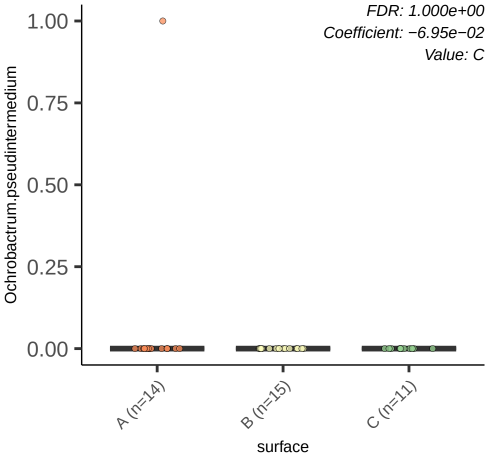
FDR: 1.000e+00
Coefficient: −6.95e−02
Value: C
1.00
0.75
0.50
0.25
0.00
Ochrobactrum.pseudintermedium
A (n=14)
B (n=15)
C (n=11)
surface

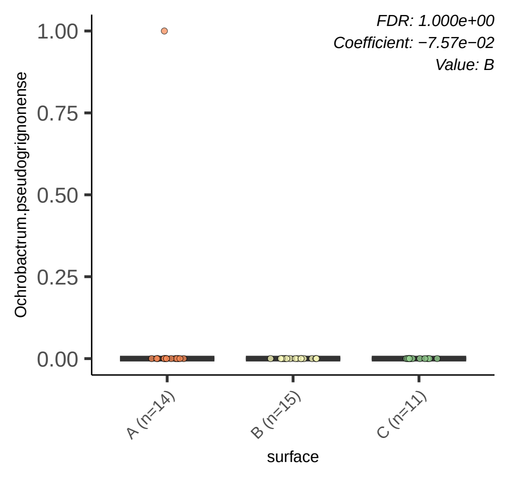
FDR: 1.000e+00
Coefficient: −7.57e−02
Value: B
Ochrobactrum.pseudogrignonense
1.00
0.75
0.50
0.25
0.00
A (n=14)
B (n=15)
C (n=11)
surface

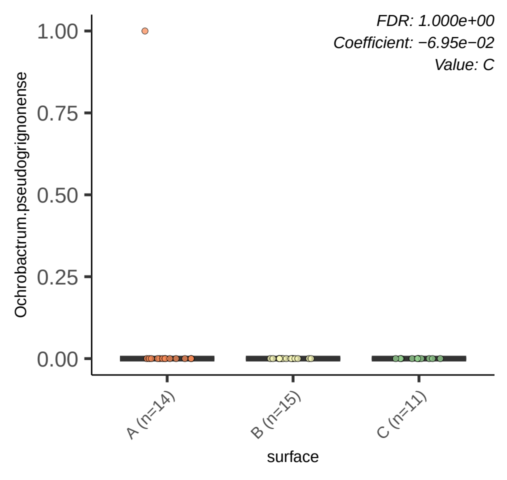

FDR: 1.000e+00
Coefficient: −6.95e−02
Value: C
Ochrobactrum.pseudogrignonense
1.00
0.75
0.50
0.25
0.00
A (n=14)
B (n=15)
C (n=11)
surface

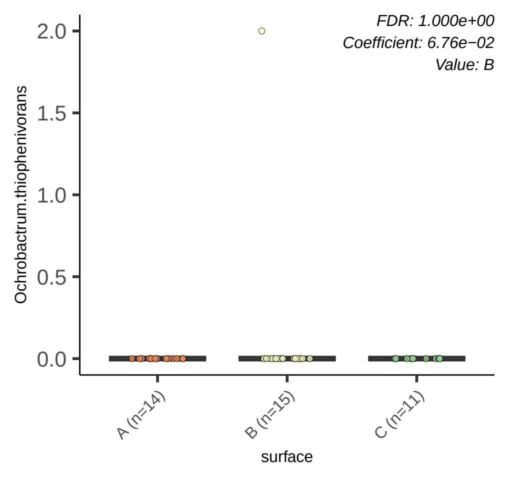
FDR: 1.000e+00
Coefficient: 6.76e−02
Value: B
Ochrobactrum.thiophenivorans
2.0
1.5
1.0
0.5
0.0
A (n=14)
B (n=15)
C (n=11)
surface

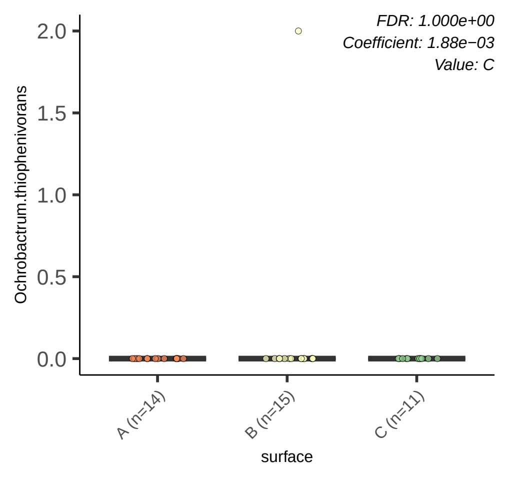
FDR: 1.000e+00
Coefficient: 1.88e−03
Value: C
Ochrobactrum.thiophenivorans
2.0
1.5
1.0
0.5
0.0
A (n=14)
B (n=15)
C (n=11)
surface

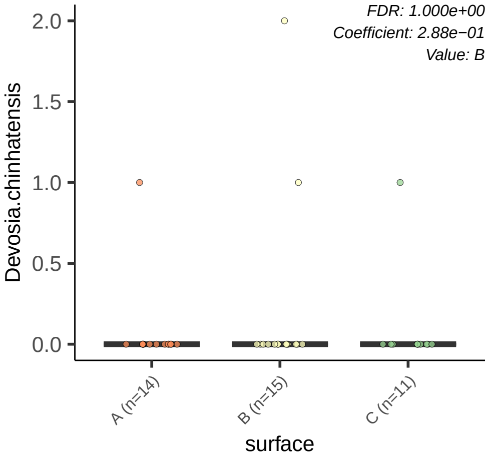
FDR: 1.000e+00
Coefficient: 2.88e−01
Value: B
Devosia.chinhatensis
2.0
1.5
1.0
0.5
0.0
A (n=14)
B (n=15)
C (n=11)
surface

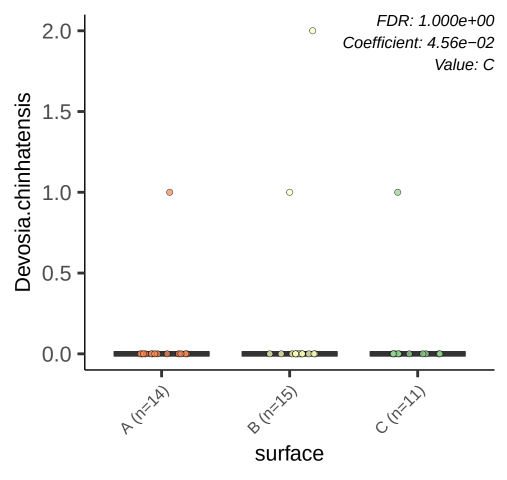
FDR: 1.000e+00
Coefficient: 4.56e−02
Value: C
Devosia.chinhatensis
2.0
1.5
1.0
0.5
0.0
A (n=14)
B (n=15)
C (n=11)
surface

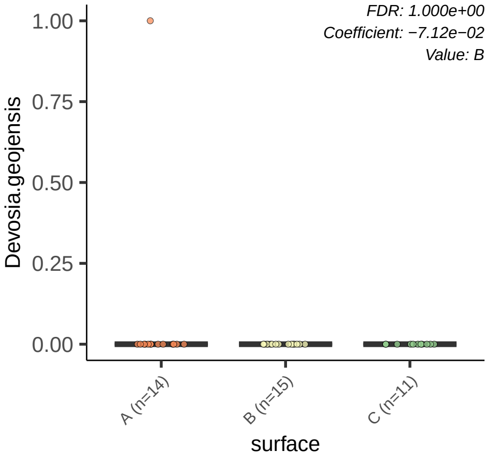
FDR: 1.000e+00
Coefficient: −7.12e−02
Value: B
Devosia.geojensis
1.00
0.75
0.50
0.25
0.00
A (n=14)
B (n=15)
C (n=11)
surface

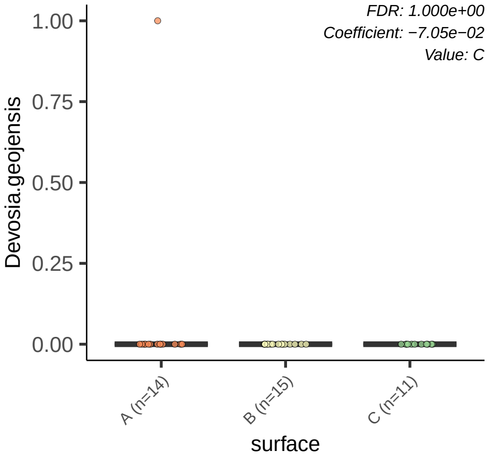
FDR: 1.000e+00
Coefficient: −7.05e−02
Value: C
Devosia.geojensis
1.00
0.75
0.50
0.25
0.00
A (n=14)
B (n=15)
C (n=11)
surface

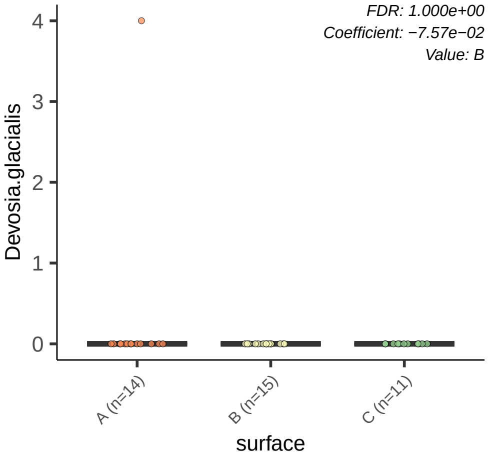

FDR: 1.000e+00
Coefficient: −7.57e−02
Value: B
Devosia.glacialis
0
1
2
3
4
A (n=14)
B (n=15)
C (n=11)
surface

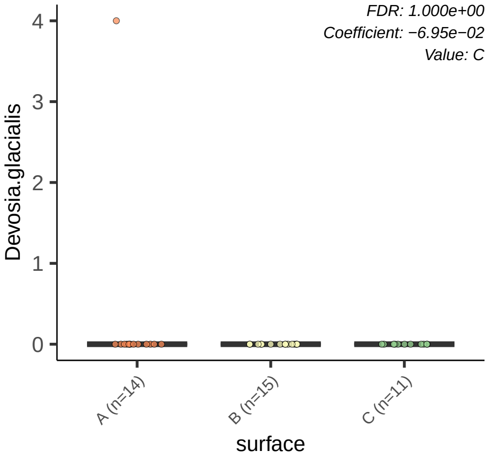

FDR: 1.000e+00
Coefficient: −6.95e−02
Value: C
Devosia.glacialis
0
1
2
3
4
A (n=14)
B (n=15)
C (n=11)
surface

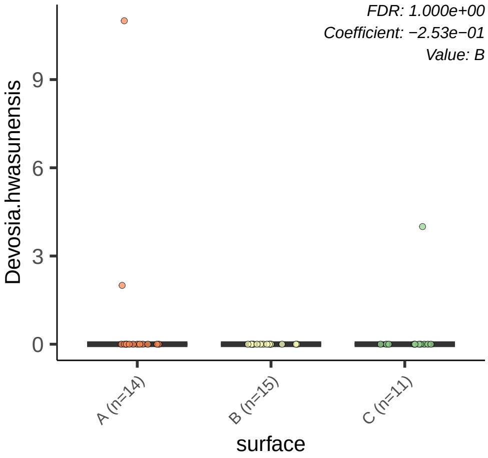
FDR: 1.000e+00
Coefficient: −2.53e−01
Value: B
Devosia.hwasunensis
0
3
6
9
A (n=14)
B (n=15)
C (n=11)
surface

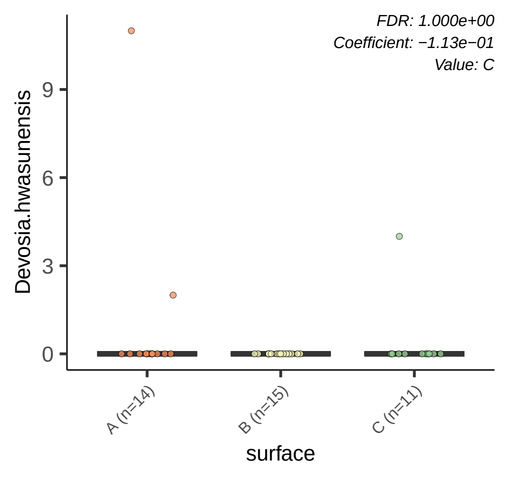
FDR: 1.000e+00
Coefficient: −1.13e−01
Value: C
Devosia.hwasunensis
9
6
3
0
A (n=14)
B (n=15)
C (n=11)
surface

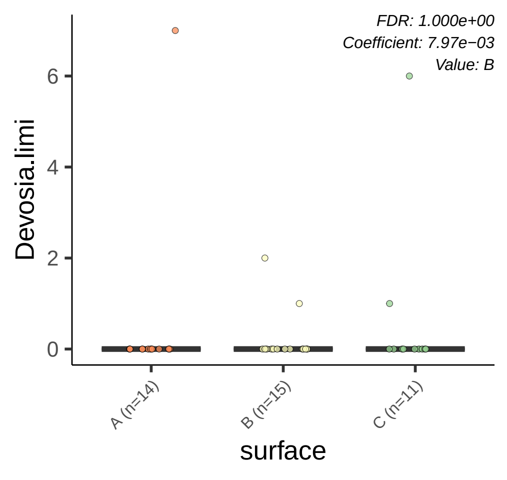
FDR: 1.000e+00
Coefficient: 7.97e−03
Value: B
Devosia.limi
0
2
4
6
A (n=14)
B (n=15)
C (n=11)
surface

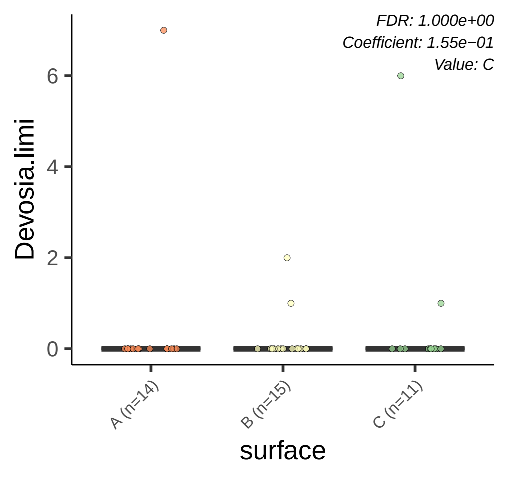

FDR: 1.000e+00
Coefficient: 1.55e−01
Value: C
Devosia.limi
0
2
4
6
A (n=14)
B (n=15)
C (n=11)
surface

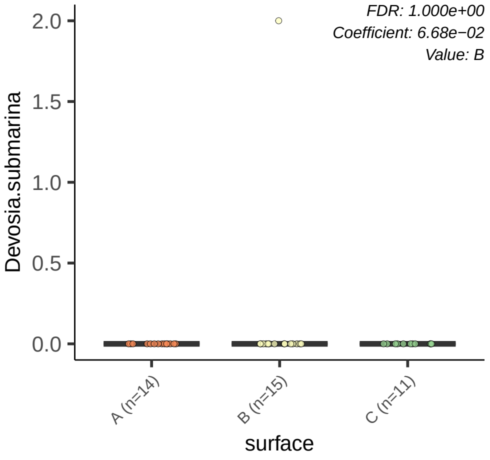
FDR: 1.000e+00
Coefficient: 6.68e−02
Value: B
Devosia.submarina
2.0
1.5
1.0
0.5
0.0
A (n=14)
B (n=15)
C (n=11)
surface

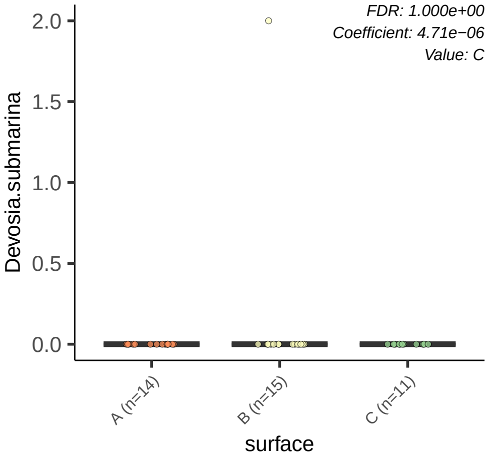
FDR: 1.000e+00
Coefficient: 4.71e−06
Value: C
Devosia.submarina
2.0
1.5
1.0
0.5
0.0
A (n=14)
B (n=15)
C (n=11)
surface

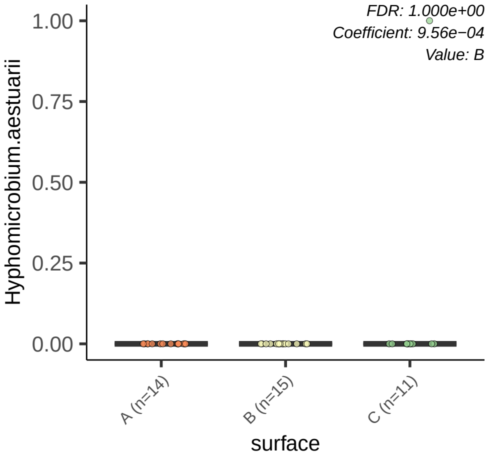

FDR: 1.000e+00
Coefficient: 9.56e−04
Value: B
Hyphomicrobium.aestuarii
1.00
0.75
0.50
0.25
0.00
A (n=14)
B (n=15)
C (n=11)
surface

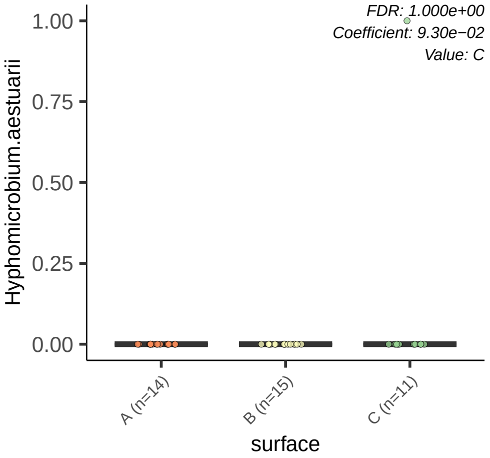
FDR: 1.000e+00
Coefficient: 9.30e−02
Value: C
Hyphomicrobium.aestuarii
1.00
0.75
0.50
0.25
0.00
A (n=14)
B (n=15)
C (n=11)
surface

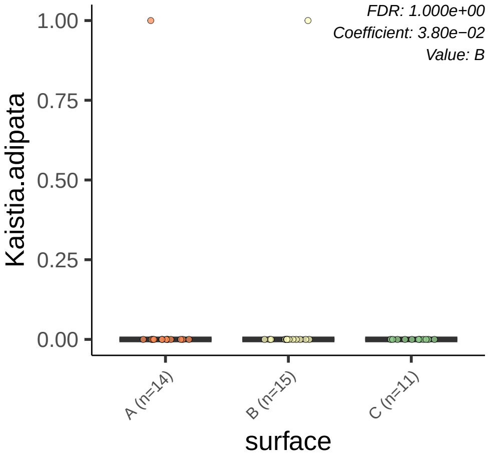
FDR: 1.000e+00
Coefficient: 3.80e−02
Value: B
Kaistia.adipata
1.00
0.75
0.50
0.25
0.00
A (n=14)
B (n=15)
C (n=11)
surface

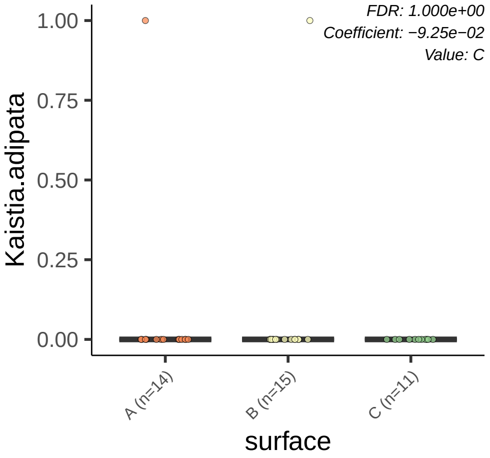
FDR: 1.000e+00
Coefficient: −9.25e−02
Value: C
Kaistia.adipata
1.00
0.75
0.50
0.25
0.00
A (n=14)
B (n=15)
C (n=11)
surface

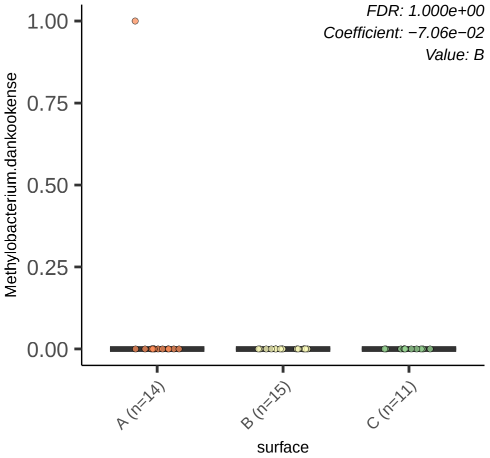
FDR: 1.000e+00
Coefficient: −7.06e−02
Value: B
Methylobacterium.dankookense
1.00
0.75
0.50
0.25
0.00
A (n=14)
B (n=15)
C (n=11)
surface

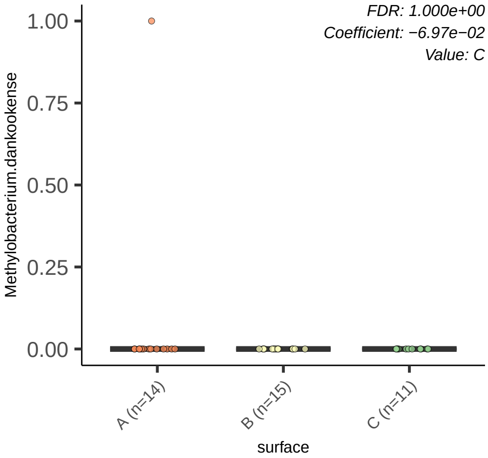
FDR: 1.000e+00
Coefficient: −6.97e−02
Value: C
Methylobacterium.dankookense
1.00
0.75
0.50
0.25
0.00
A (n=14)
B (n=15)
C (n=11)
surface

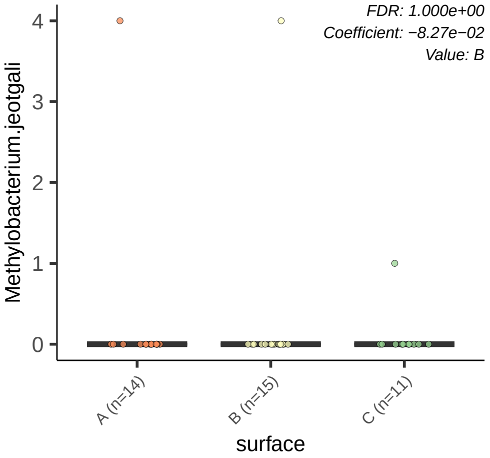

FDR: 1.000e+00
Coefficient: −8.27e−02
Value: B
Methylobacterium.jeotgali
0
1
2
3
4
A (n=14)
B (n=15)
C (n=11)
surface

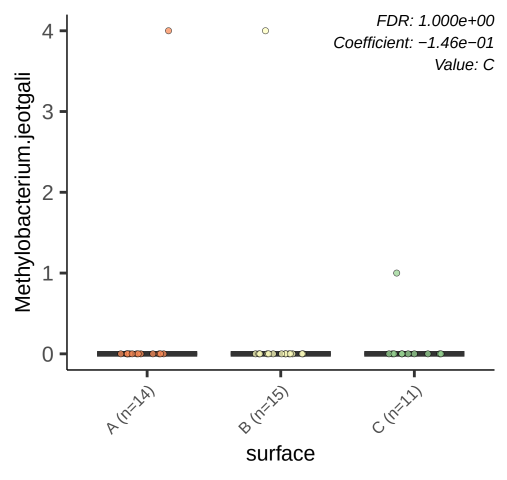
FDR: 1.000e+00
Coefficient: −1.46e−01
Value: C
Methylobacterium.jeotgali
0
1
2
3
4
A (n=14)
B (n=15)
C (n=11)
surface

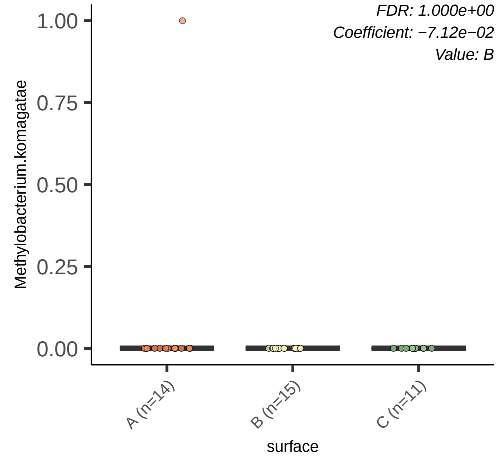

FDR: 1.000e+00
Coefficient: −7.12e−02
Value: B
Methylobacterium.komagatae
1.00
0.75
0.50
0.25
0.00
A (n=14)
B (n=15)
C (n=11)
surface

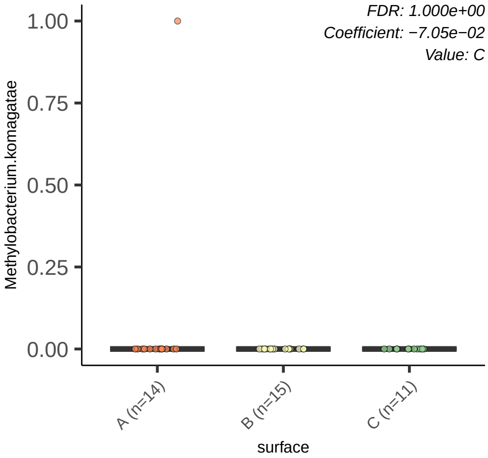

FDR: 1.000e+00
Coefficient: −7.05e−02
Value: C
Methylobacterium.komagatae
1.00
0.75
0.50
0.25
0.00
A (n=14)
B (n=15)
C (n=11)
surface

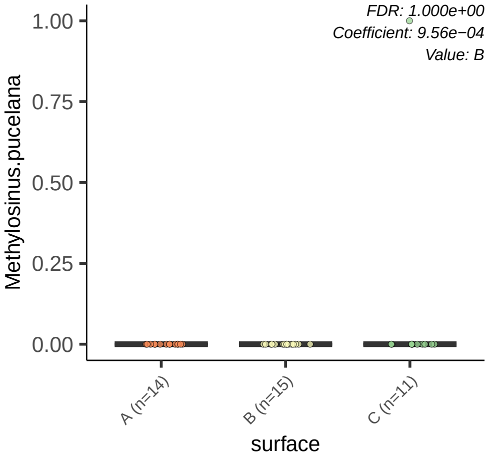
FDR: 1.000e+00
Coefficient: 9.56e−04
Value: B
Methylosinus.pucelana
1.00
0.75
0.50
0.25
0.00
A (n=14)
B (n=15)
C (n=11)
surface

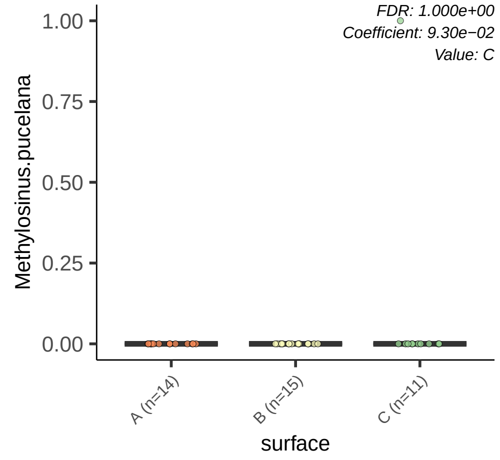
FDR: 1.000e+00
Coefficient: 9.30e−02
Value: C
Methylosinus.pucelana
1.00
0.75
0.50
0.25
0.00
A (n=14)
B (n=15)
C (n=11)
surface

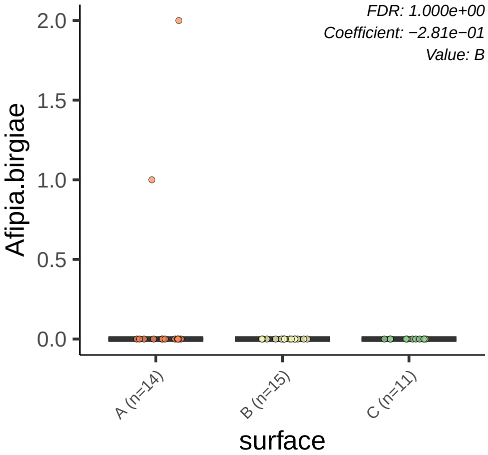

FDR: 1.000e+00
Coefficient: −2.81e−01
Value: B
Afipia.birgiae
2.0
1.5
1.0
0.5
0.0
A (n=14)
B (n=15)
C (n=11)
surface

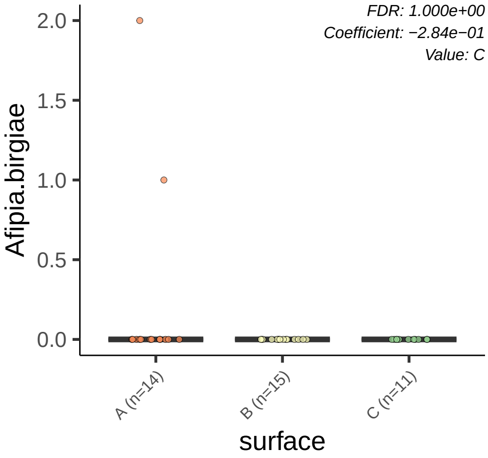

FDR: 1.000e+00
Coefficient: −2.84e−01
Value: C
Afipia.birgiae
2.0
1.5
1.0
0.5
0.0
A (n=14)
B (n=15)
C (n=11)
surface

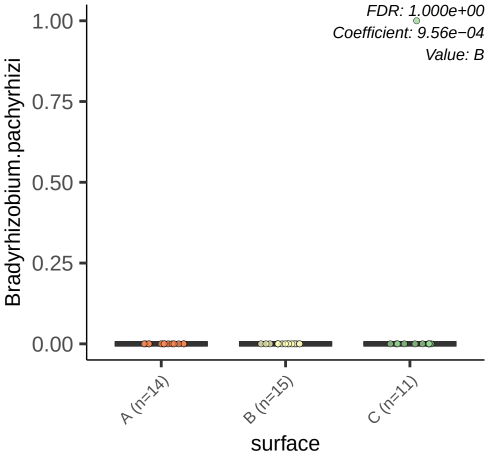
FDR: 1.000e+00
Coefficient: 9.56e−04
Value: B
Bradyrhizobium.pachyrhizi
1.00
0.75
0.50
0.25
0.00
A (n=14)
B (n=15)
C (n=11)
surface

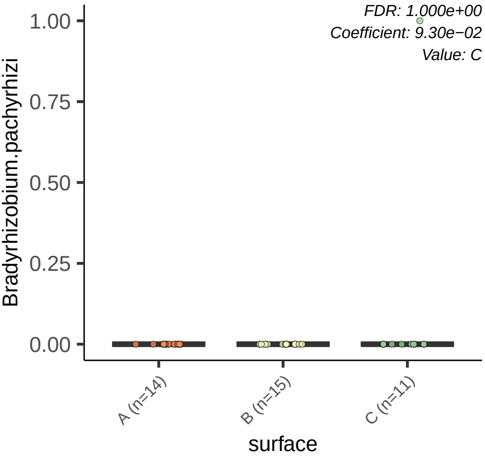
FDR: 1.000e+00
Coefficient: 9.30e−02
Value: C
Bradyrhizobium.pachyrhizi
1.00
0.75
0.50
0.25
0.00
A (n=14)
B (n=15)
C (n=11)
surface

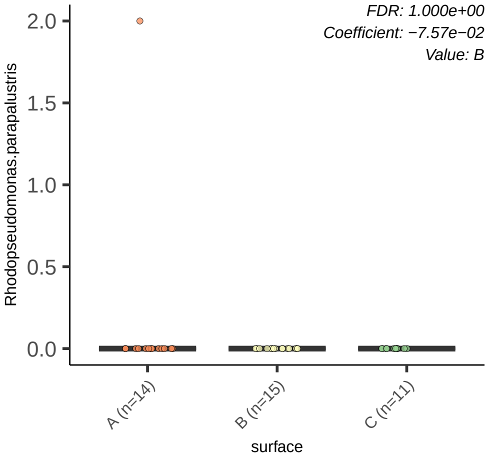
FDR: 1.000e+00
Coefficient: −7.57e−02
Value: B
Rhodopseudomonas.parapalustris
2.0
1.5
1.0
0.5
0.0
A (n=14)
B (n=15)
C (n=11)
surface

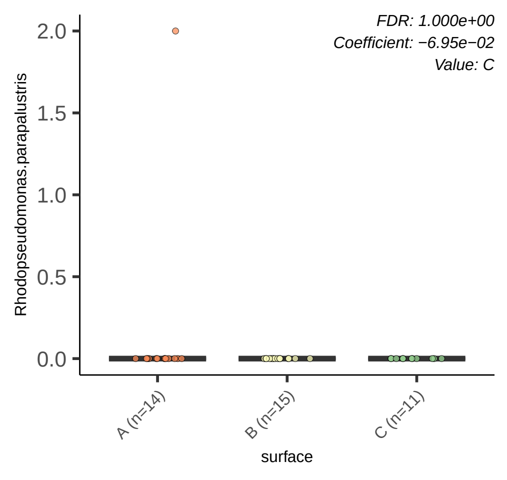
FDR: 1.000e+00
Coefficient: −6.95e−02
Value: C
Rhodopseudomonas.parapalustris
2.0
1.5
1.0
0.5
0.0
A (n=14)
B (n=15)
C (n=11)
surface

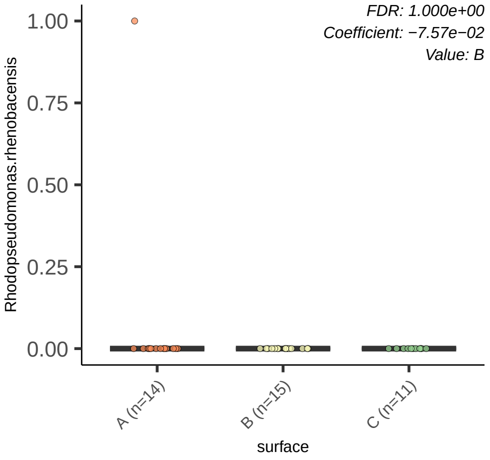
FDR: 1.000e+00
Coefficient: −7.57e−02
Value: B
Rhodopseudomonas.rhenobacensis
1.00
0.75
0.50
0.25
0.00
A (n=14)
B (n=15)
C (n=11)
surface

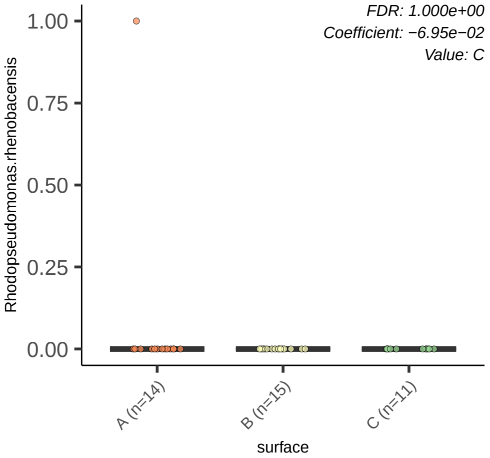

FDR: 1.000e+00
Coefficient: −6.95e−02
Value: C
Rhodopseudomonas.rhenobacensis
1.00
0.75
0.50
0.25
0.00
A (n=14)
B (n=15)
C (n=11)
surface

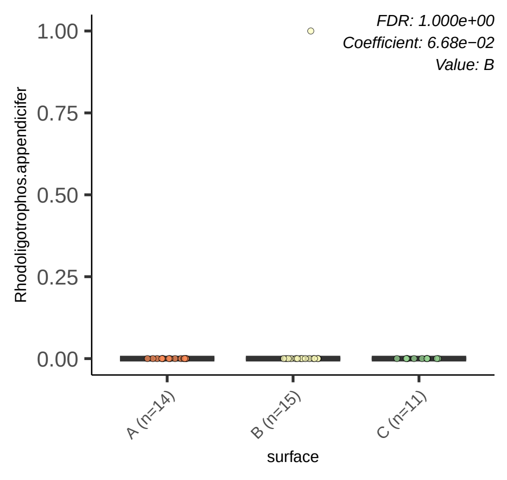
FDR: 1.000e+00
Coefficient: 6.68e−02
Value: B
Rhodoligotrophos.appendicifer
1.00
0.75
0.50
0.25
0.00
A (n=14)
B (n=15)
C (n=11)
surface

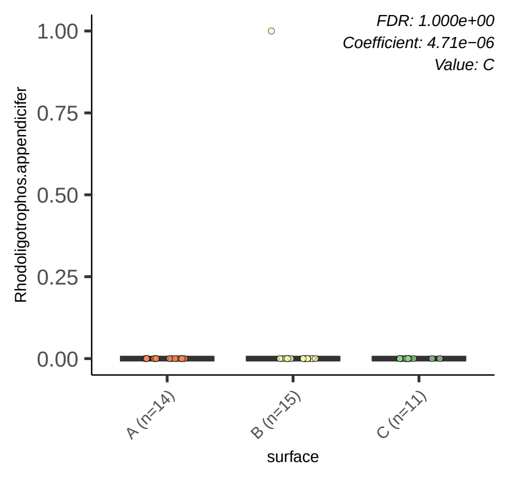

FDR: 1.000e+00
Coefficient: 4.71e−06
Value: C
Rhodoligotrophos.appendicifer
1.00
0.75
0.50
0.25
0.00
A (n=14)
B (n=15)
C (n=11)
surface

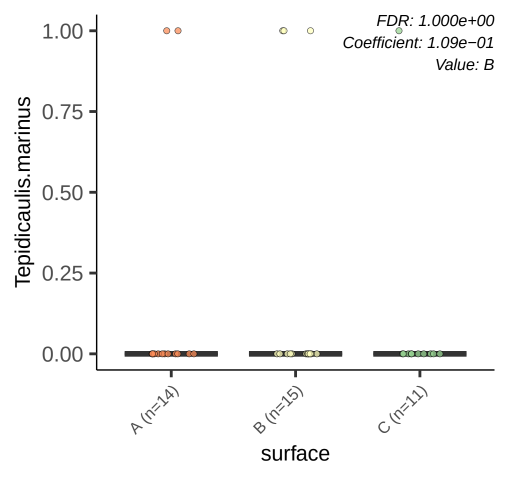

FDR: 1.000e+00
Coefficient: 1.09e−01
Value: B
1.00
0.75
0.50
0.25
0.00
Tepidicaulis.marinus
A (n=14)
B (n=15)
C (n=11)
surface

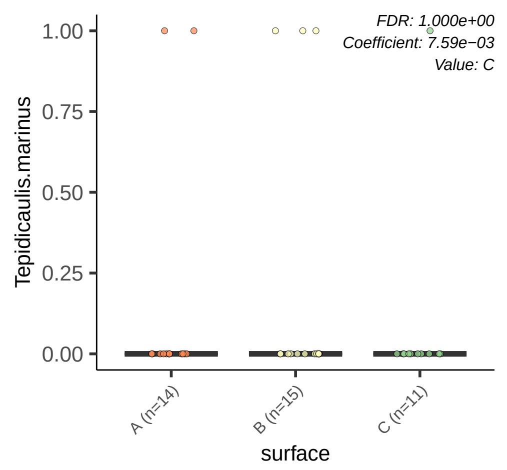
FDR: 1.000e+00
Coefficient: 7.59e−03
Value: C
Tepidicaulis.marinus
1.00
0.75
0.50
0.25
0.00
A (n=14)
B (n=15)
C (n=11)
surface

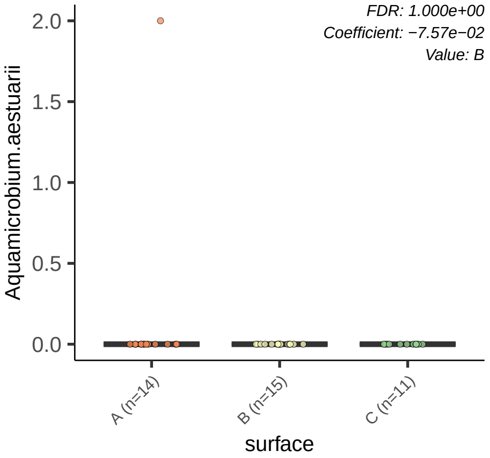
FDR: 1.000e+00
Coefficient: −7.57e−02
Value: B
Aquamicrobium.aestuarii
2.0
1.5
1.0
0.5
0.0
A (n=14)
B (n=15)
C (n=11)
surface

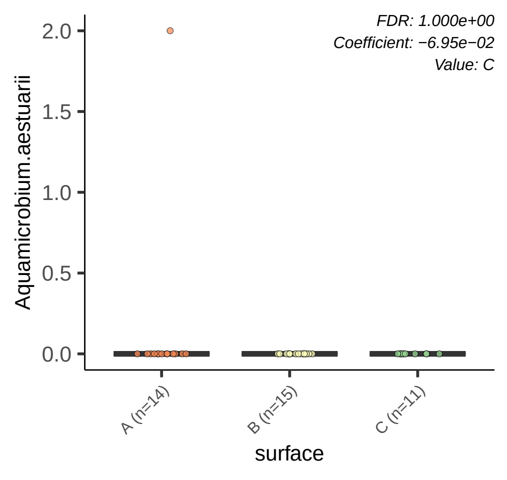

FDR: 1.000e+00
Coefficient: −6.95e−02
Value: C
Aquamicrobium.aestuarii
2.0
1.5
1.0
0.5
0.0
A (n=14)
B (n=15)
C (n=11)
surface

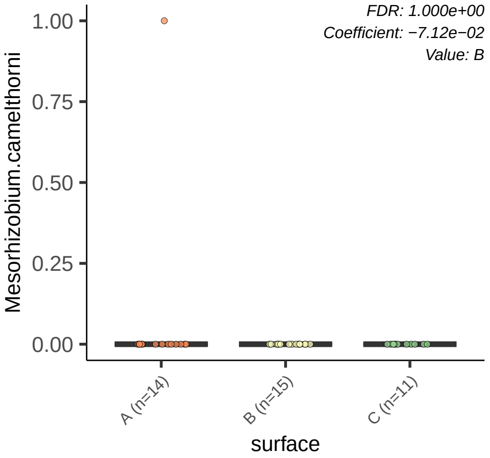

FDR: 1.000e+00
Coefficient: −7.12e−02
Value: B
Mesorhizobium.camelthorni
1.00
0.75
0.50
0.25
0.00
A (n=14)
B (n=15)
C (n=11)
surface

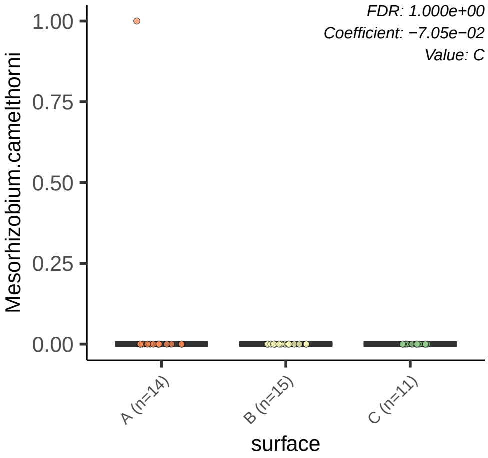

FDR: 1.000e+00
Coefficient: −7.05e−02
Value: C
Mesorhizobium.camelthorni
1.00
0.75
0.50
0.25
0.00
A (n=14)
B (n=15)
C (n=11)
surface

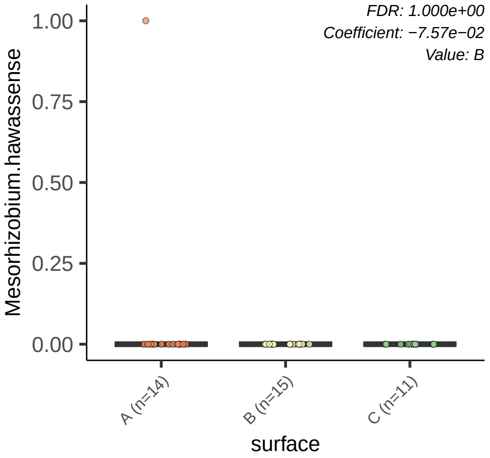

FDR: 1.000e+00
Coefficient: −7.57e−02
Value: B
Mesorhizobium.hawassense
1.00
0.75
0.50
0.25
0.00
A (n=14)
B (n=15)
C (n=11)
surface

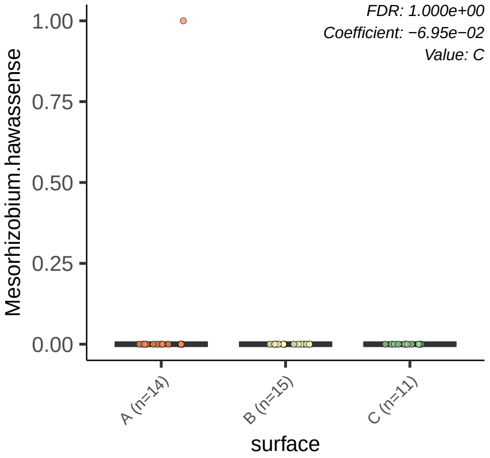

FDR: 1.000e+00
Coefficient: −6.95e−02
Value: C
Mesorhizobium.hawassense
1.00
0.75
0.50
0.25
0.00
A (n=14)
B (n=15)
C (n=11)
surface

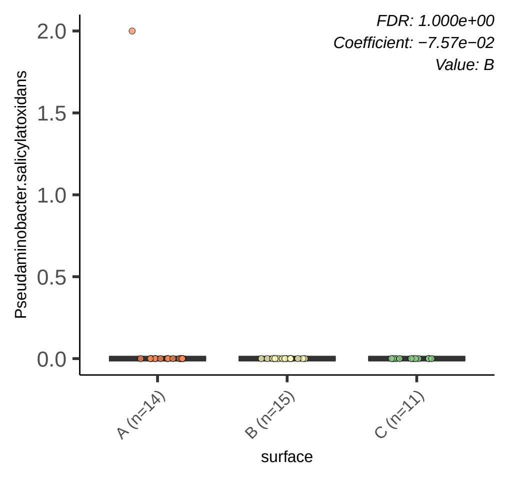

FDR: 1.000e+00
Coefficient: −7.57e−02
Value: B
Pseudaminobacter.salicylatoxidans
2.0
1.5
1.0
0.5
0.0
A (n=14)
B (n=15)
C (n=11)
surface

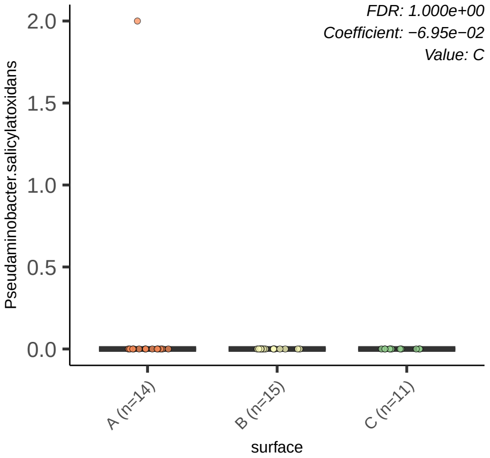
FDR: 1.000e+00
Coefficient: −6.95e−02
Value: C
Pseudaminobacter.salicylatoxidans
2.0
1.5
1.0
0.5
0.0
A (n=14)
B (n=15)
C (n=11)
surface

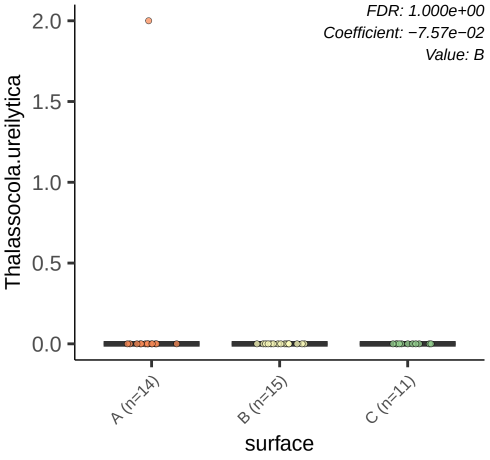

FDR: 1.000e+00
Coefficient: −7.57e−02
Value: B
Thalassocola.ureilytica
2.0
1.5
1.0
0.5
0.0
A (n=14)
B (n=15)
C (n=11)
surface

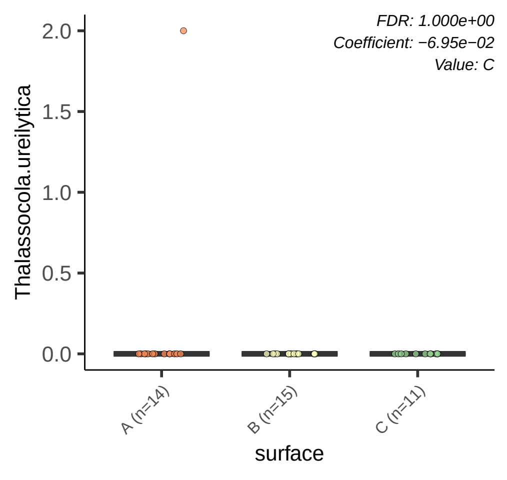
FDR: 1.000e+00
Coefficient: −6.95e−02
Value: C
Thalassocola.ureilytica
2.0
1.5
1.0
0.5
0.0
A (n=14)
B (n=15)
C (n=11)
surface

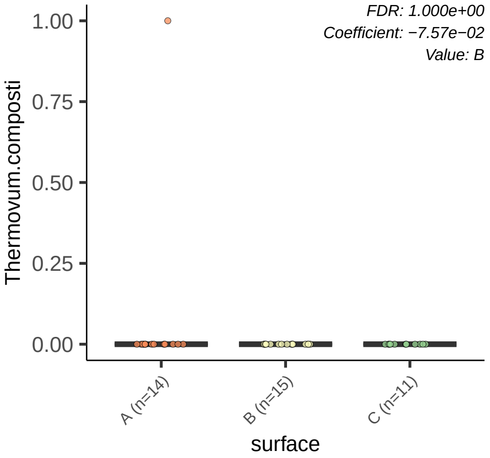

FDR: 1.000e+00
Coefficient: −7.57e−02
Value: B
Thermovum.composti
1.00
0.75
0.50
0.25
0.00
A (n=14)
B (n=15)
C (n=11)
surface

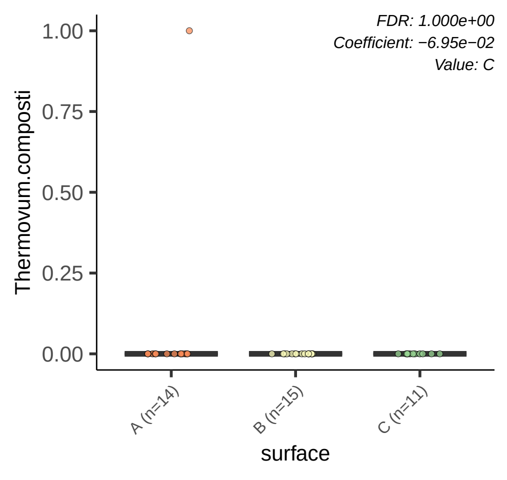
FDR: 1.000e+00
Coefficient: −6.95e−02
Value: C
Thermovum.composti
1.00
0.75
0.50
0.25
0.00
A (n=14)
B (n=15)
C (n=11)
surface

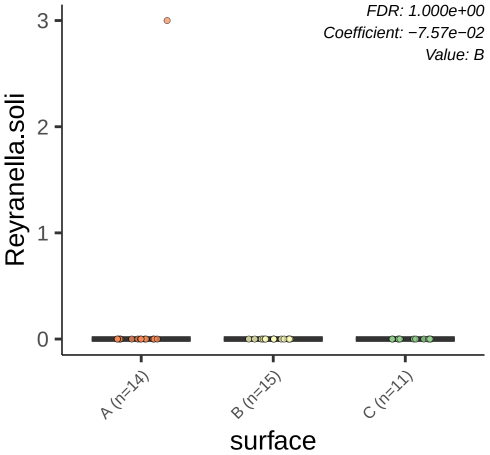
FDR: 1.000e+00
Coefficient: −7.57e−02
Value: B
Reyranella.soli
3
2
1
0
A (n=14)
B (n=15)
C (n=11)
surface

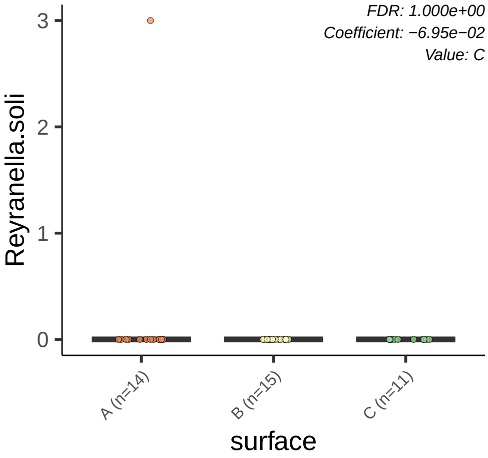

FDR: 1.000e+00
Coefficient: −6.95e−02
Value: C
Reyranella.soli
3
2
1
0
A (n=14)
B (n=15)
C (n=11)
surface

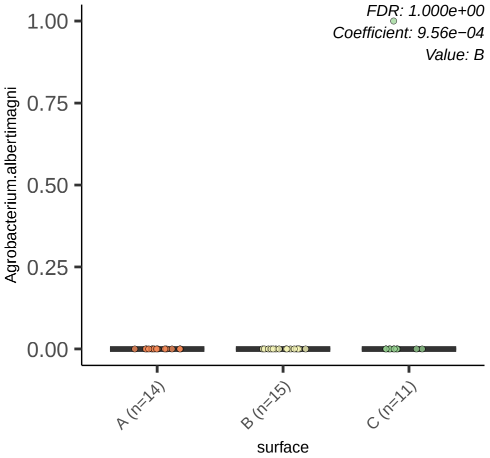

FDR: 1.000e+00
Coefficient: 9.56e−04
Value: B
1.00
0.75
0.50
0.25
0.00
Agrobacterium.albertimagni
A (n=14)
B (n=15)
C (n=11)
surface

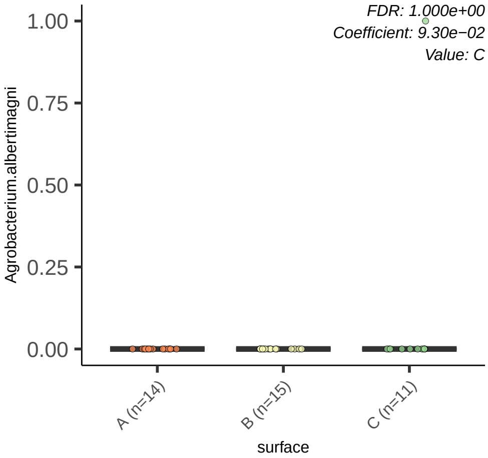
FDR: 1.000e+00
Coefficient: 9.30e−02
Value: C
Agrobacterium.albertimagni
1.00
0.75
0.50
0.25
0.00
A (n=14)
B (n=15)
C (n=11)
surface

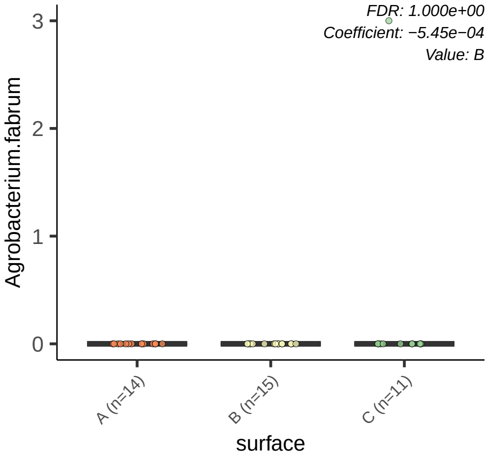
FDR: 1.000e+00
Coefficient: −5.45e−04
Value: B
Agrobacterium.fabrum
3
2
1
0
A (n=14)
B (n=15)
C (n=11)
surface

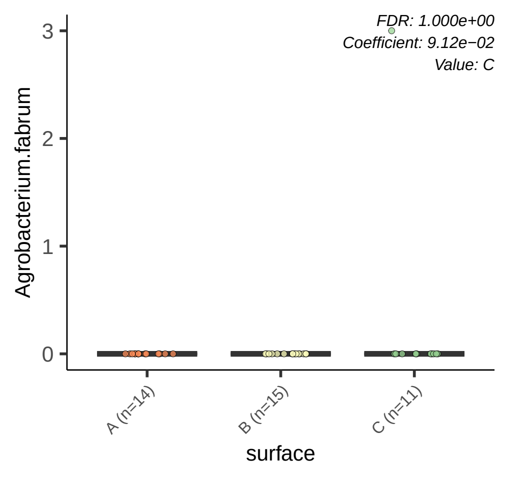
FDR: 1.000e+00
Coefficient: 9.12e−02
Value: C
Agrobacterium.fabrum
3
2
1
0
A (n=14)
B (n=15)
C (n=11)
surface

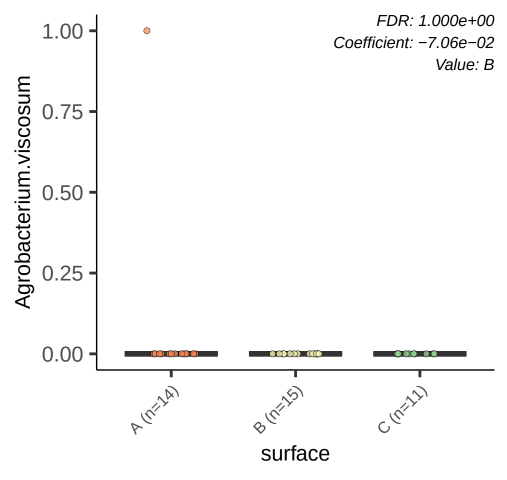

FDR: 1.000e+00
Coefficient: −7.06e−02
Value: B
Agrobacterium.viscosum
1.00
0.75
0.50
0.25
0.00
A (n=14)
B (n=15)
C (n=11)
surface

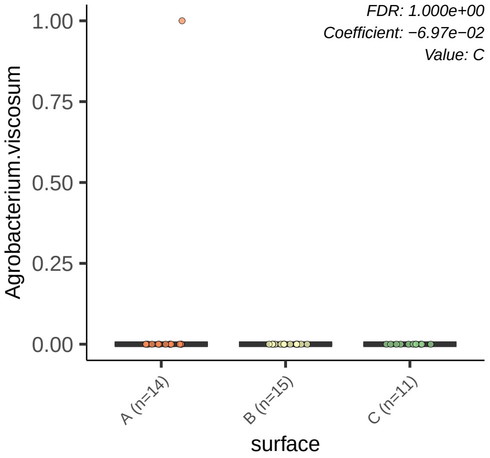
FDR: 1.000e+00
Coefficient: −6.97e−02
Value: C
Agrobacterium.viscosum
1.00
0.75
0.50
0.25
0.00
A (n=14)
B (n=15)
C (n=11)
surface

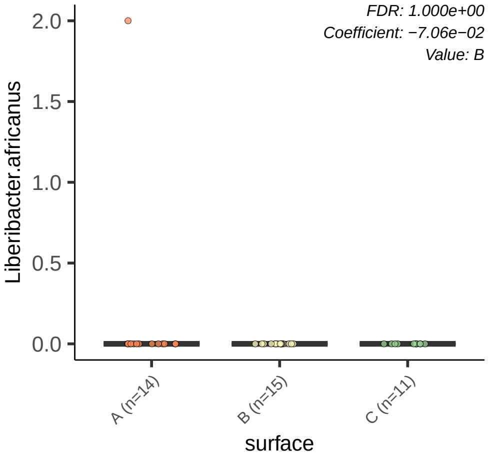

FDR: 1.000e+00
Coefficient: −7.06e−02
Value: B
Liberibacter.africanus
2.0
1.5
1.0
0.5
0.0
A (n=14)
B (n=15)
C (n=11)
surface

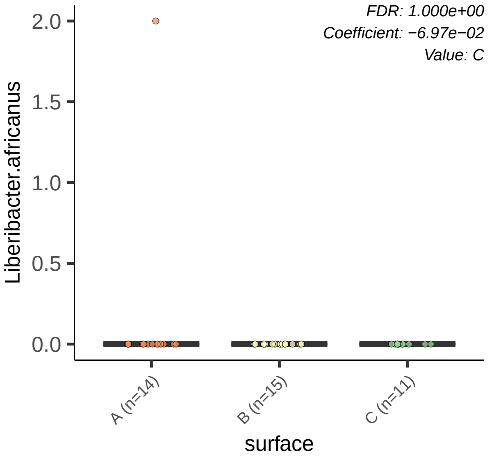
FDR: 1.000e+00
Coefficient: −6.97e−02
Value: C
Liberibacter.africanus
2.0
1.5
1.0
0.5
0.0
A (n=14)
B (n=15)
C (n=11)
surface

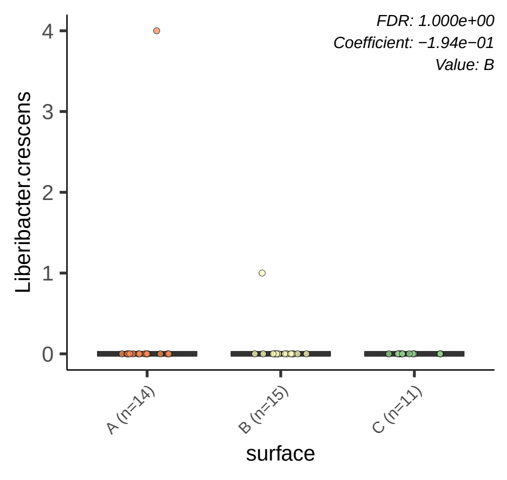

FDR: 1.000e+00
Coefficient: −1.94e−01
Value: B
Liberibacter.crescens
0
1
2
3
4
A (n=14)
B (n=15)
C (n=11)
surface

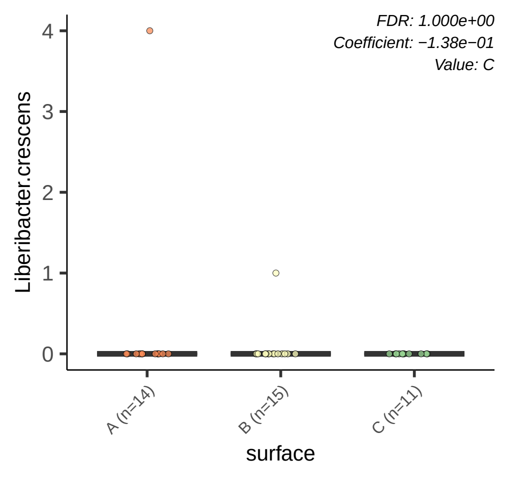

FDR: 1.000e+00
Coefficient: −1.38e−01
Value: C
Liberibacter.crescens
0
1
2
3
4
A (n=14)
B (n=15)
C (n=11)
surface

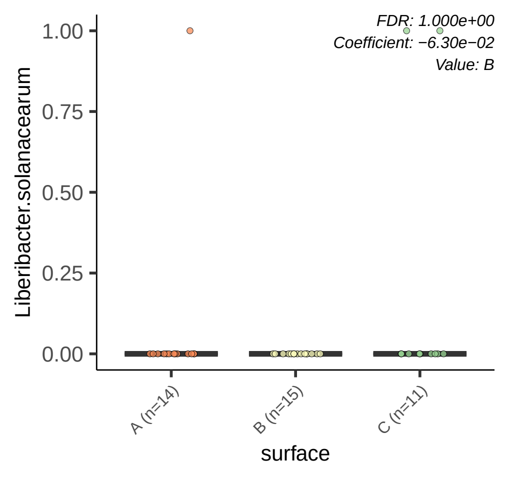
FDR: 1.000e+00
Coefficient: −6.30e−02
Value: B
Liberibacter.solanacearum
1.00
0.75
0.50
0.25
0.00
A (n=14)
B (n=15)
C (n=11)
surface

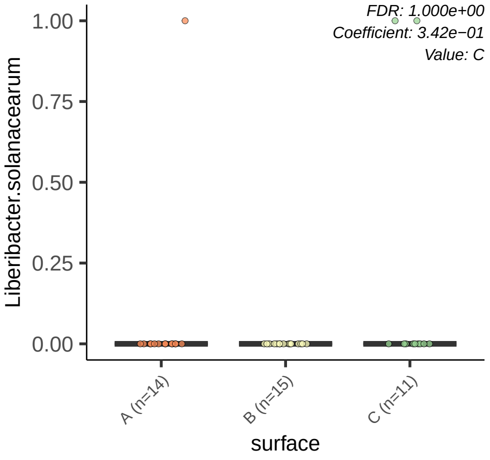

FDR: 1.000e+00
Coefficient: 3.42e−01
Value: C
Liberibacter.solanacearum
1.00
0.75
0.50
0.25
0.00
A (n=14)
B (n=15)
C (n=11)
surface

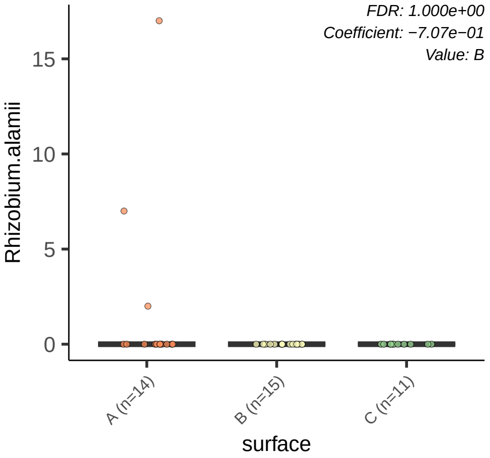
FDR: 1.000e+00
Coefficient: −7.07e−01
Value: B
Rhizobium.alamii
0
5
10
15
A (n=14)
B (n=15)
C (n=11)
surface

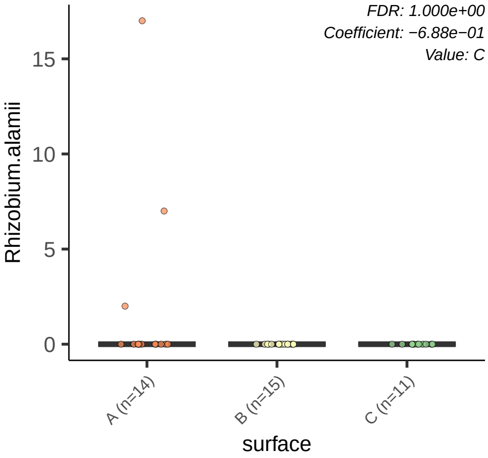
FDR: 1.000e+00
Coefficient: −6.88e−01
Value: C
Rhizobium.alamii
15
10
5
0
A (n=14)
B (n=15)
C (n=11)
surface

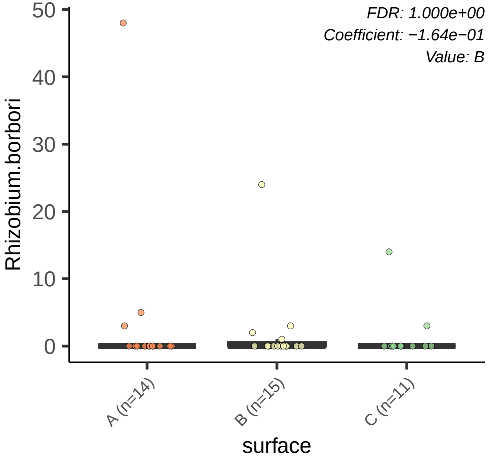

FDR: 1.000e+00
Coefficient: −1.64e−01
Value: B
Rhizobium.borbori
50
40
30
20
10
0
A (n=14)
B (n=15)
C (n=11)
surface

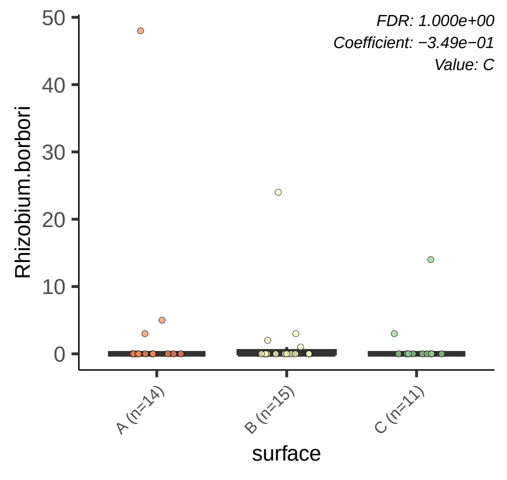
FDR: 1.000e+00
Coefficient: −3.49e−01
Value: C
Rhizobium.borbori
50
40
30
20
10
0
A (n=14)
B (n=15)
C (n=11)
surface

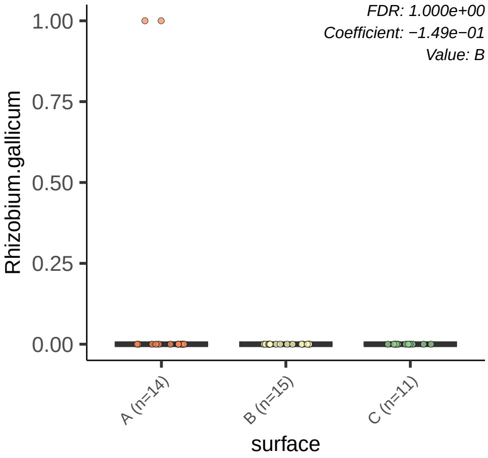

FDR: 1.000e+00
Coefficient: −1.49e−01
Value: B
Rhizobium.gallicum
1.00
0.75
0.50
0.25
0.00
A (n=14)
B (n=15)
C (n=11)
surface

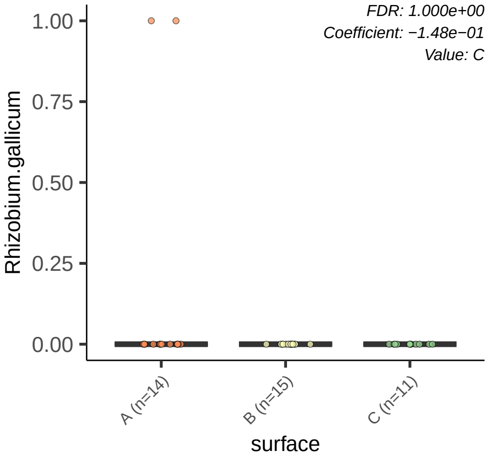
FDR: 1.000e+00
Coefficient: −1.48e−01
Value: C
Rhizobium.gallicum
1.00
0.75
0.50
0.25
0.00
A (n=14)
B (n=15)
C (n=11)
surface

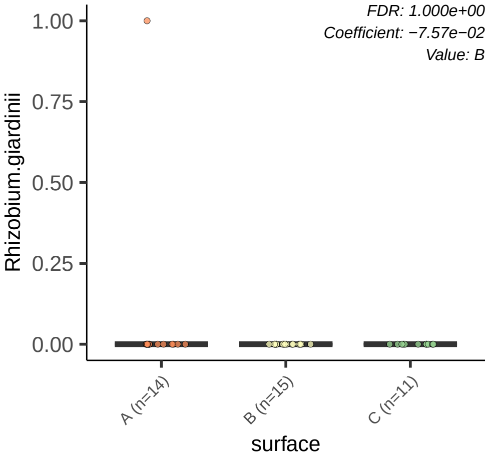
FDR: 1.000e+00
Coefficient: −7.57e−02
Value: B
Rhizobium.giardinii
1.00
0.75
0.50
0.25
0.00
A (n=14)
B (n=15)
C (n=11)
surface

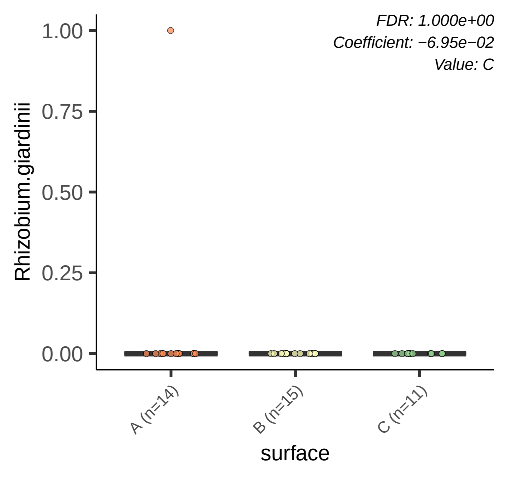
FDR: 1.000e+00
Coefficient: −6.95e−02
Value: C
Rhizobium.giardinii
1.00
0.75
0.50
0.25
0.00
A (n=14)
B (n=15)
C (n=11)
surface

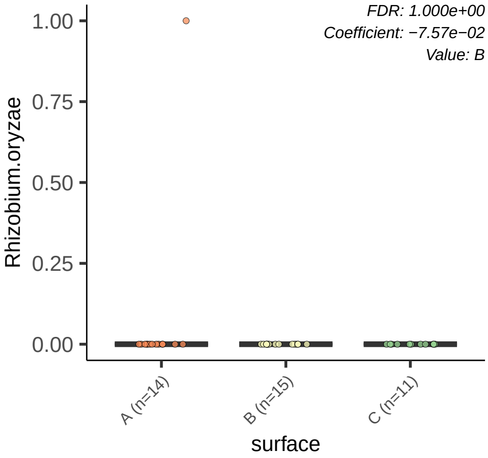
FDR: 1.000e+00
Coefficient: −7.57e−02
Value: B
Rhizobium.oryzae
1.00
0.75
0.50
0.25
0.00
A (n=14)
B (n=15)
C (n=11)
surface

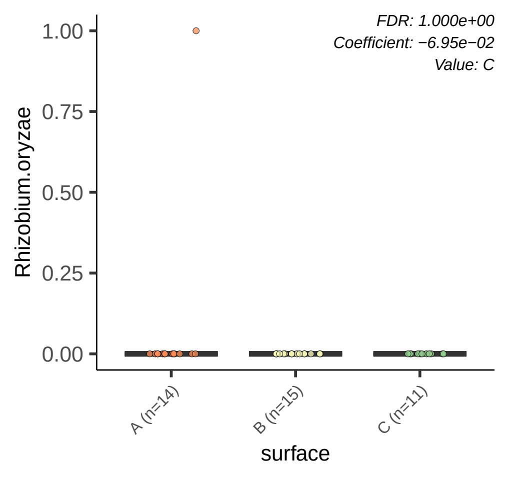
FDR: 1.000e+00
Coefficient: −6.95e−02
Value: C
Rhizobium.oryzae
1.00
0.75
0.50
0.25
0.00
A (n=14)
B (n=15)
C (n=11)
surface

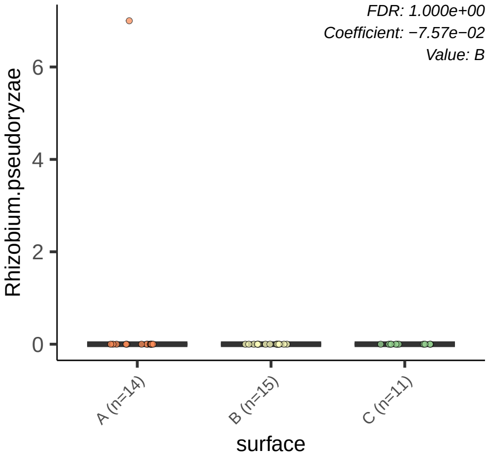
FDR: 1.000e+00
Coefficient: −7.57e−02
Value: B
Rhizobium.pseudoryzae
0
2
4
6
A (n=14)
B (n=15)
C (n=11)
surface

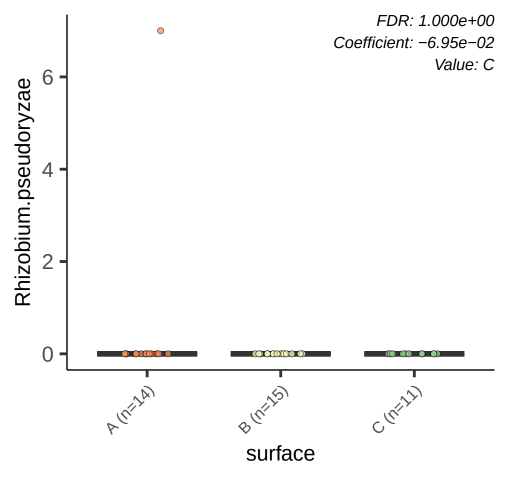

FDR: 1.000e+00
Coefficient: −6.95e−02
Value: C
Rhizobium.pseudoryzae
0
2
4
6
A (n=14)
B (n=15)
C (n=11)
surface

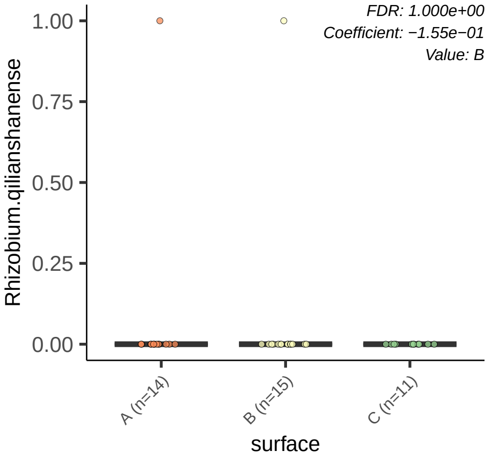

FDR: 1.000e+00
Coefficient: −1.55e−01
Value: B
Rhizobium.qilianshanense
1.00
0.75
0.50
0.25
0.00
A (n=14)
B (n=15)
C (n=11)
surface

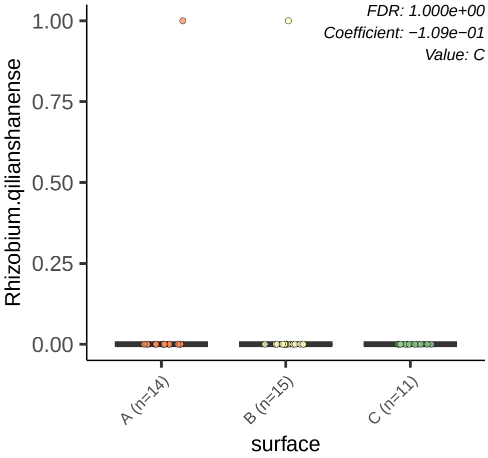

FDR: 1.000e+00
Coefficient: −1.09e−01
Value: C
Rhizobium.qilianshanense
1.00
0.75
0.50
0.25
0.00
A (n=14)
B (n=15)
C (n=11)
surface

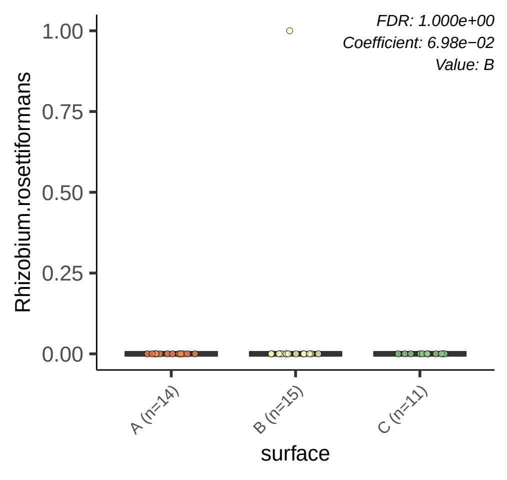
FDR: 1.000e+00
Coefficient: 6.98e−02
Value: B
Rhizobium.rosettiformans
1.00
0.75
0.50
0.25
0.00
A (n=14)
B (n=15)
C (n=11)
surface

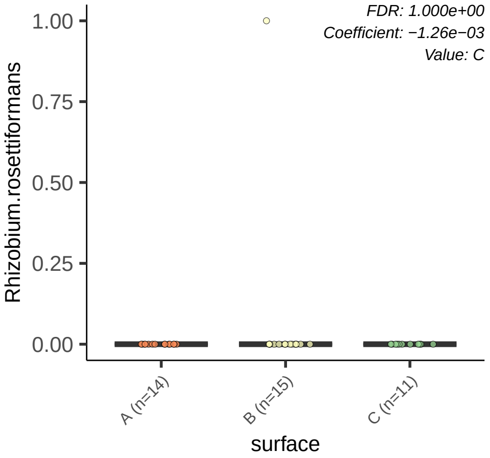
FDR: 1.000e+00
Coefficient: −1.26e−03
Value: C
Rhizobium.rosettiformans
1.00
0.75
0.50
0.25
0.00
A (n=14)
B (n=15)
C (n=11)
surface

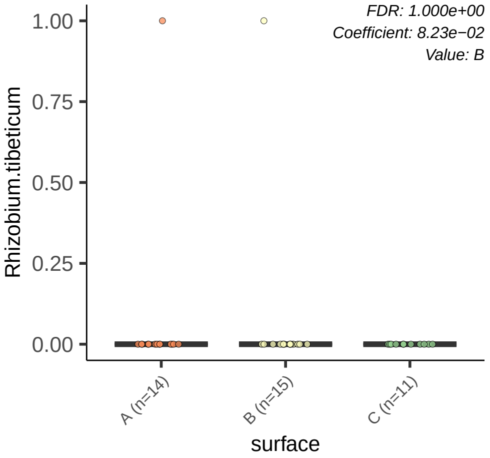
FDR: 1.000e+00
Coefficient: 8.23e−02
Value: B
Rhizobium.tibeticum
1.00
0.75
0.50
0.25
0.00
A (n=14)
B (n=15)
C (n=11)
surface

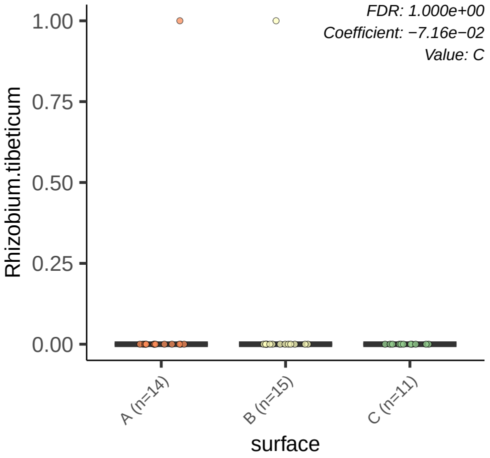
FDR: 1.000e+00
Coefficient: −7.16e−02
Value: C
Rhizobium.tibeticum
1.00
0.75
0.50
0.25
0.00
A (n=14)
B (n=15)
C (n=11)
surface

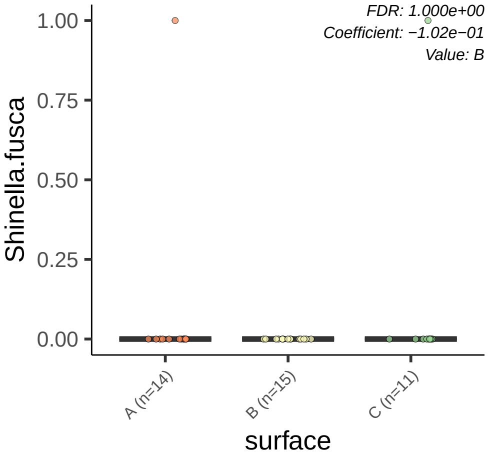

FDR: 1.000e+00
Coefficient: −1.02e−01
Value: B
Shinella.fusca
1.00
0.75
0.50
0.25
0.00
A (n=14)
B (n=15)
C (n=11)
surface

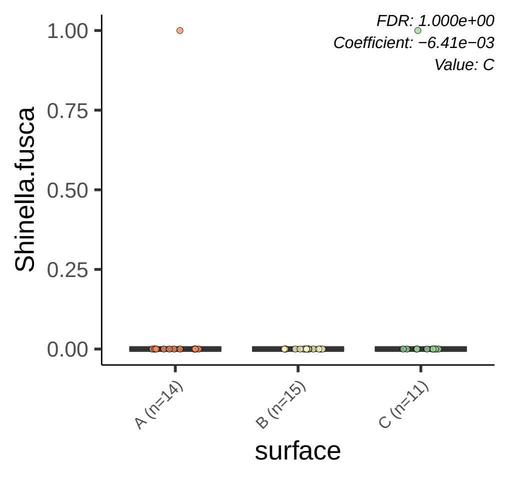

FDR: 1.000e+00
Coefficient: −6.41e−03
Value: C
Shinella.fusca
1.00
0.75
0.50
0.25
0.00
A (n=14)
B (n=15)
C (n=11)
surface

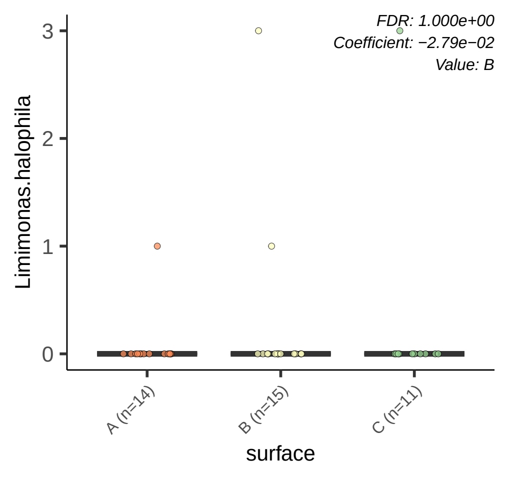
FDR: 1.000e+00
Coefficient: −2.79e−02
Value: B
Limimonas.halophila
3
2
1
0
A (n=14)
B (n=15)
C (n=11)
surface

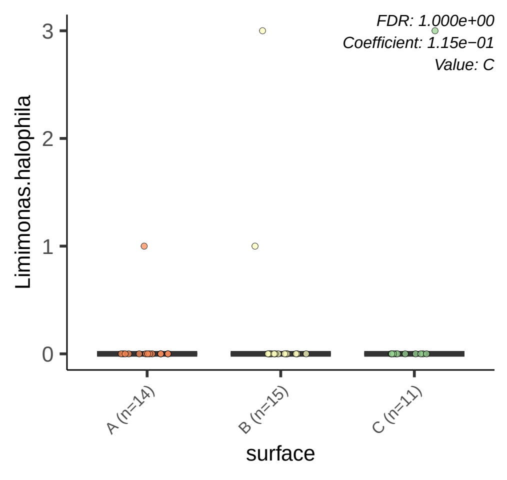
FDR: 1.000e+00
Coefficient: 1.15e−01
Value: C
Limimonas.halophila
0
1
2
3
A (n=14)
B (n=15)
C (n=11)
surface

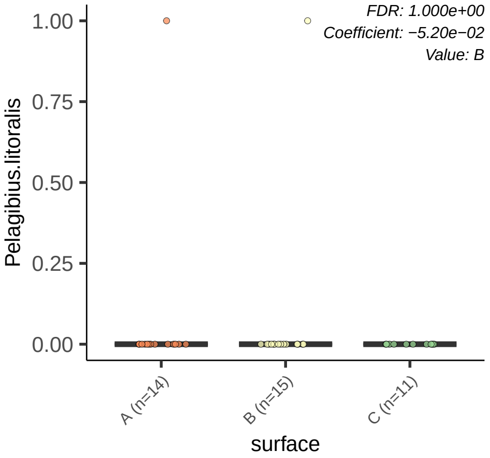
FDR: 1.000e+00
Coefficient: −5.20e−02
Value: B
Pelagibius.litoralis
1.00
0.75
0.50
0.25
0.00
A (n=14)
B (n=15)
C (n=11)
surface

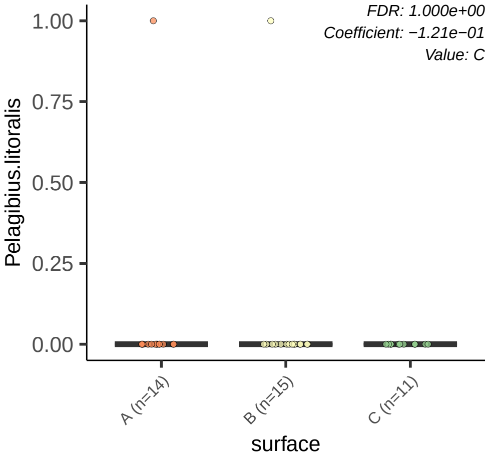

FDR: 1.000e+00
Coefficient: −1.21e−01
Value: C
Pelagibius.litoralis
1.00
0.75
0.50
0.25
0.00
A (n=14)
B (n=15)
C (n=11)
surface

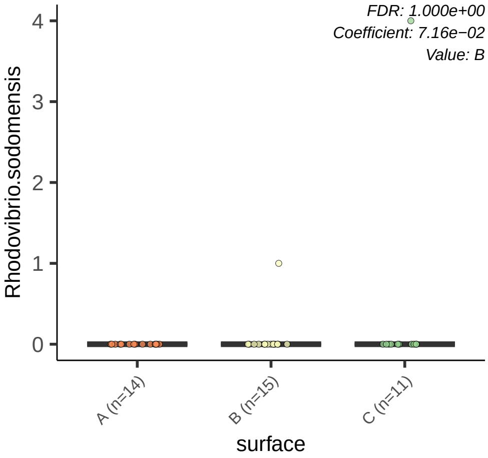

FDR: 1.000e+00
Coefficient: 7.16e−02
Value: B
Rhodovibrio.sodomensis
4
3
2
1
0
A (n=14)
B (n=15)
C (n=11)
surface

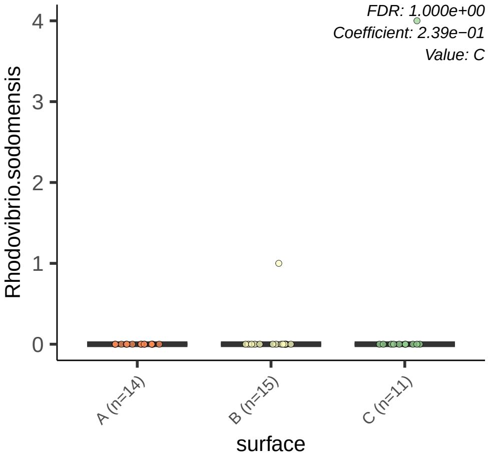
FDR: 1.000e+00
Coefficient: 2.39e−01
Value: C
Rhodovibrio.sodomensis
0
1
2
3
4
A (n=14)
B (n=15)
C (n=11)
surface

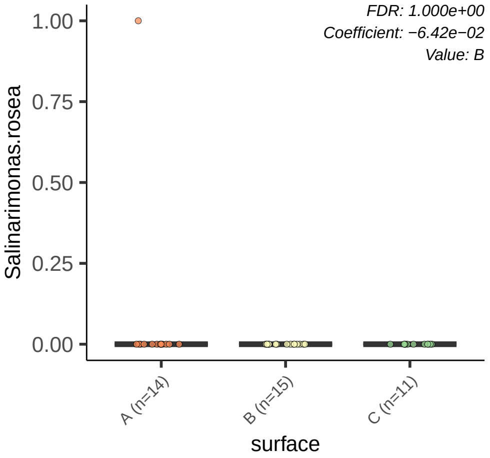

FDR: 1.000e+00
Coefficient: −6.42e−02
Value: B
Salinarimonas.rosea
1.00
0.75
0.50
0.25
0.00
A (n=14)
B (n=15)
C (n=11)
surface

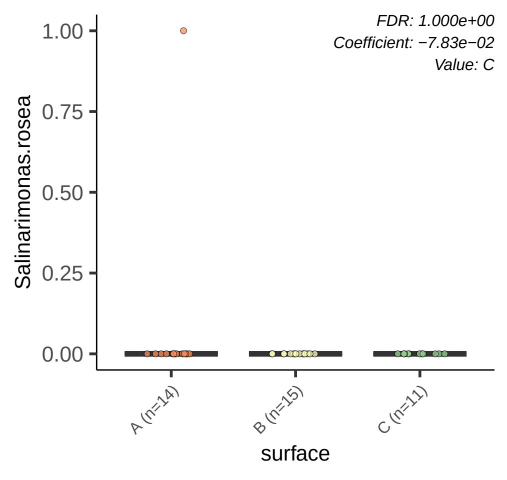
FDR: 1.000e+00
Coefficient: −7.83e−02
Value: C
Salinarimonas.rosea
1.00
0.75
0.50
0.25
0.00
A (n=14)
B (n=15)
C (n=11)
surface

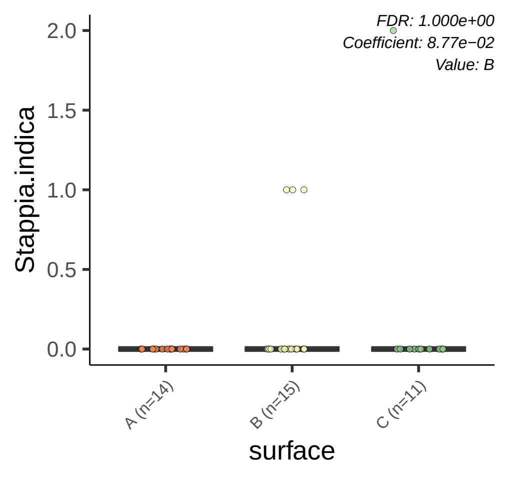
FDR: 1.000e+00
Coefficient: 8.77e−02
Value: B
Stappia.indica
2.0
1.5
1.0
0.5
0.0
A (n=14)
B (n=15)
C (n=11)
surface

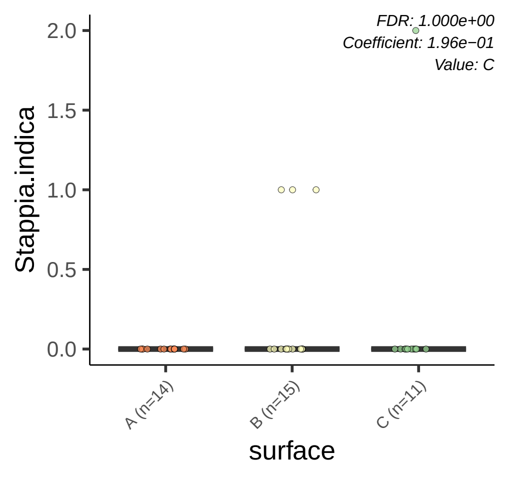
FDR: 1.000e+00
Coefficient: 1.96e−01
Value: C
Stappia.indica
2.0
1.5
1.0
0.5
0.0
A (n=14)
B (n=15)
C (n=11)
surface

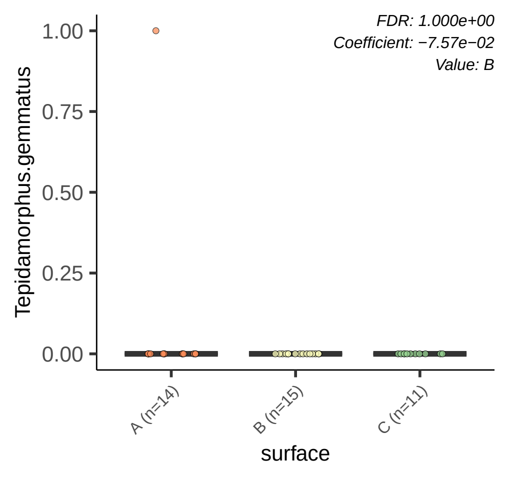
FDR: 1.000e+00
Coefficient: −7.57e−02
Value: B
Tepidamorphus.gemmatus
1.00
0.75
0.50
0.25
0.00
A (n=14)
B (n=15)
C (n=11)
surface

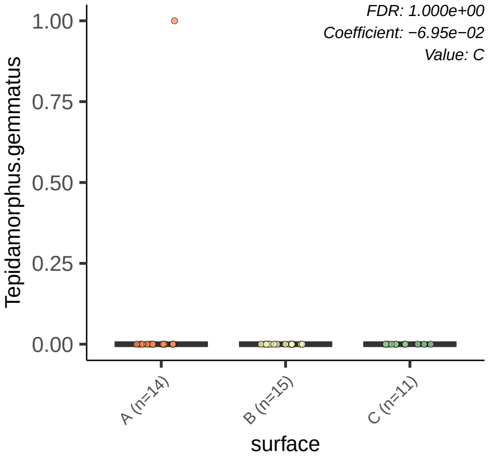
FDR: 1.000e+00
Coefficient: −6.95e−02
Value: C
Tepidamorphus.gemmatus
1.00
0.75
0.50
0.25
0.00
A (n=14)
B (n=15)
C (n=11)
surface

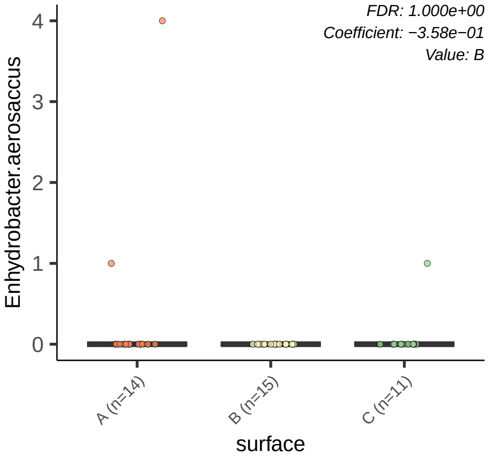

FDR: 1.000e+00
Coefficient: −3.58e−01
Value: B
Enhydrobacter.aerosaccus
0
1
2
3
4
A (n=14)
B (n=15)
C (n=11)
surface

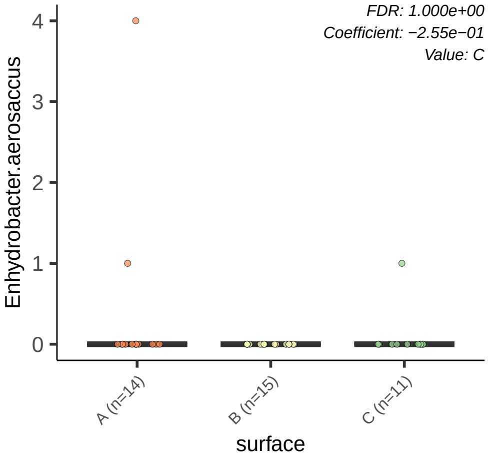
FDR: 1.000e+00
Coefficient: −2.55e−01
Value: C
Enhydrobacter.aerosaccus
4
3
2
1
0
A (n=14)
B (n=15)
C (n=11)
surface

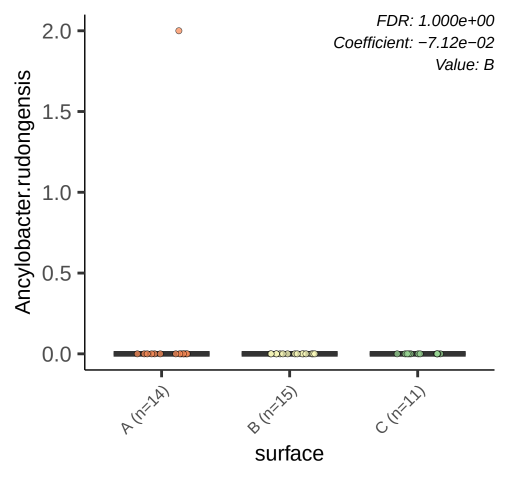
FDR: 1.000e+00
Coefficient: −7.12e−02
Value: B
Ancylobacter.rudongensis
2.0
1.5
1.0
0.5
0.0
A (n=14)
B (n=15)
C (n=11)
surface

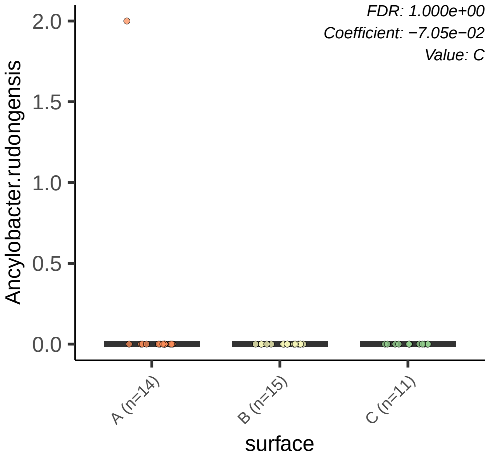
FDR: 1.000e+00
Coefficient: −7.05e−02
Value: C
Ancylobacter.rudongensis
2.0
1.5
1.0
0.5
0.0
A (n=14)
B (n=15)
C (n=11)
surface

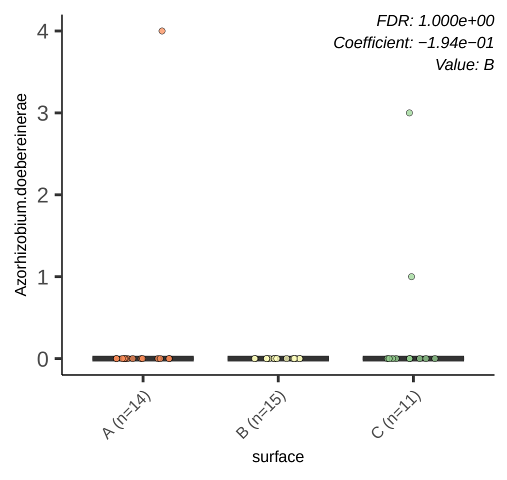
FDR: 1.000e+00
Coefficient: −1.94e−01
Value: B
Azorhizobium.doebereinerae
4
3
2
1
0
A (n=14)
B (n=15)
C (n=11)
surface

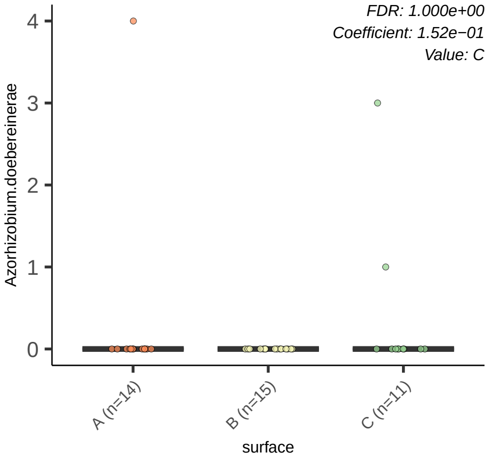

FDR: 1.000e+00
Coefficient: 1.52e−01
Value: C
Azorhizobium.doebereinerae
0
1
2
3
4
A (n=14)
B (n=15)
C (n=11)
surface

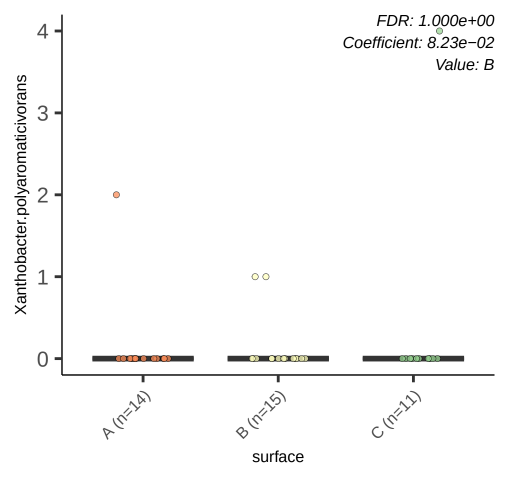

FDR: 1.000e+00
Coefficient: 8.23e−02
Value: B
Xanthobacter.polyaromaticivorans
0
1
2
3
4
A (n=14)
B (n=15)
C (n=11)
surface

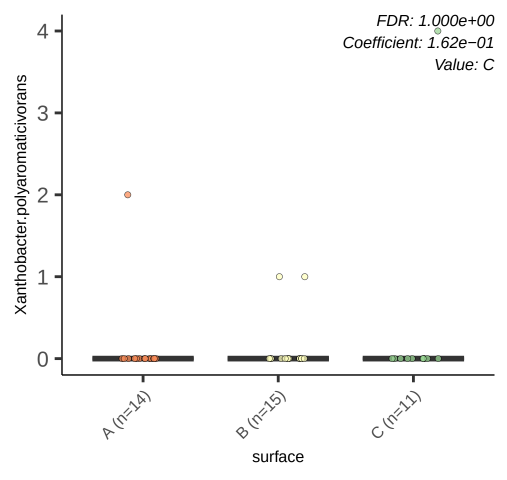
FDR: 1.000e+00
Coefficient: 1.62e−01
Value: C
Xanthobacter.polyaromaticivorans
4
3
2
1
0
A (n=14)
B (n=15)
C (n=11)
surface

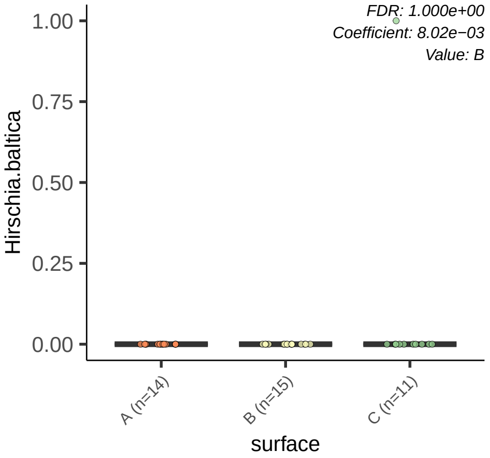

FDR: 1.000e+00
Coefficient: 8.02e−03
Value: B
Hirschia.baltica
1.00
0.75
0.50
0.25
0.00
A (n=14)
B (n=15)
C (n=11)
surface

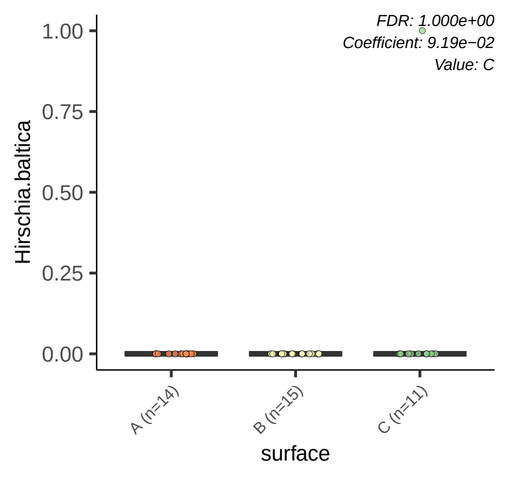
FDR: 1.000e+00
Coefficient: 9.19e−02
Value: C
1.00
0.75
0.50
0.25
0.00
Hirschia.baltica
A (n=14)
B (n=15)
C (n=11)
surface

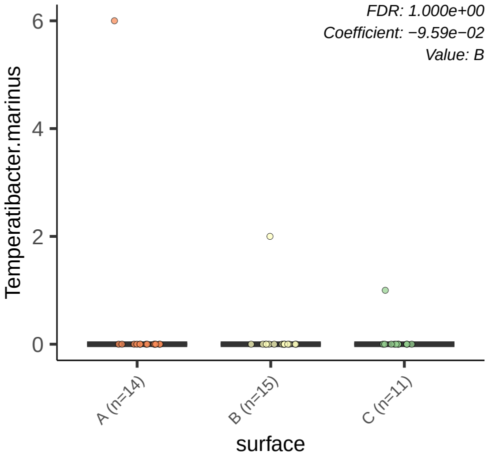

FDR: 1.000e+00
Coefficient: −9.59e−02
Value: B
Temperatibacter.marinus
6
4
2
0
A (n=14)
B (n=15)
C (n=11)
surface

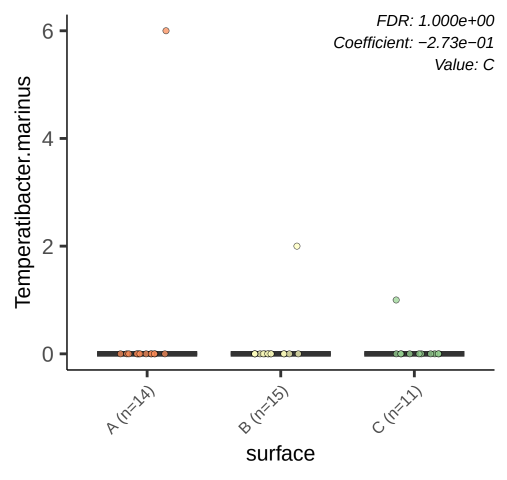

FDR: 1.000e+00
Coefficient: −2.73e−01
Value: C
Temperatibacter.marinus
6
4
2
0
A (n=14)
B (n=15)
C (n=11)
surface

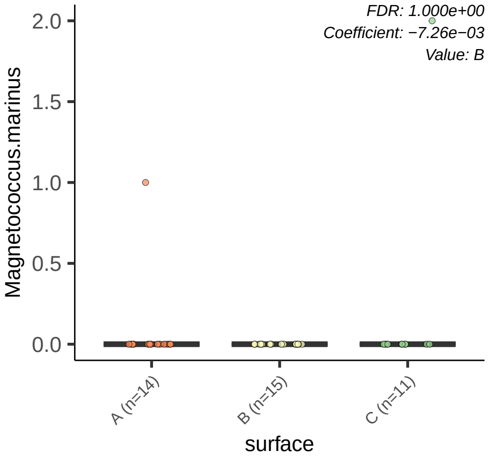
FDR: 1.000e+00
Coefficient: −7.26e−03
Value: B
Magnetococcus.marinus
2.0
1.5
1.0
0.5
0.0
A (n=14)
B (n=15)
C (n=11)
surface

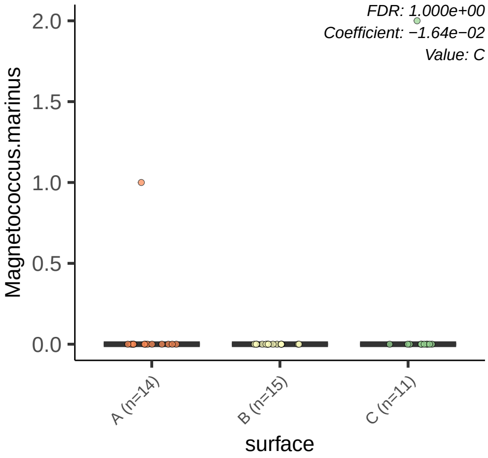

FDR: 1.000e+00
Coefficient: −1.64e−02
Value: C
Magnetococcus.marinus
2.0
1.5
1.0
0.5
0.0
A (n=14)
B (n=15)
C (n=11)
surface

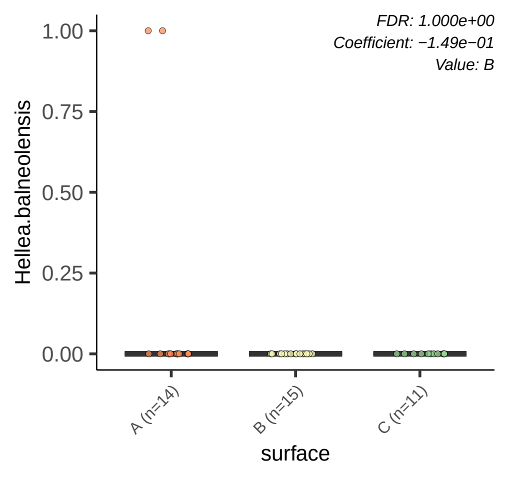

FDR: 1.000e+00
Coefficient: −1.49e−01
Value: B
Hellea.balneolensis
1.00
0.75
0.50
0.25
0.00
A (n=14)
B (n=15)
C (n=11)
surface

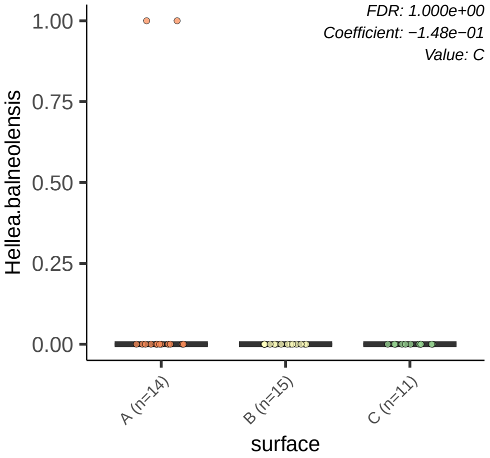

FDR: 1.000e+00
Coefficient: −1.48e−01
Value: C
Hellea.balneolensis
1.00
0.75
0.50
0.25
0.00
A (n=14)
B (n=15)
C (n=11)
surface

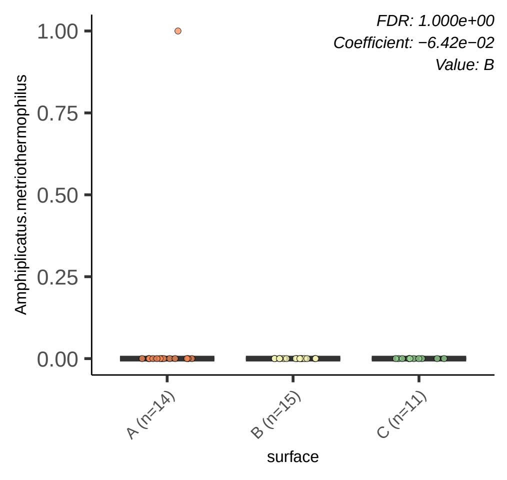
FDR: 1.000e+00
Coefficient: −6.42e−02
Value: B
Amphiplicatus.metriothermophilus
1.00
0.75
0.50
0.25
0.00
A (n=14)
B (n=15)
C (n=11)
surface

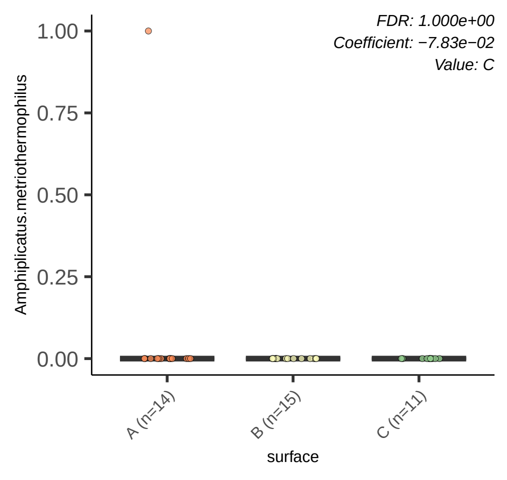

FDR: 1.000e+00
Coefficient: −7.83e−02
Value: C
Amphiplicatus.metriothermophilus
1.00
0.75
0.50
0.25
0.00
A (n=14)
B (n=15)
C (n=11)
surface

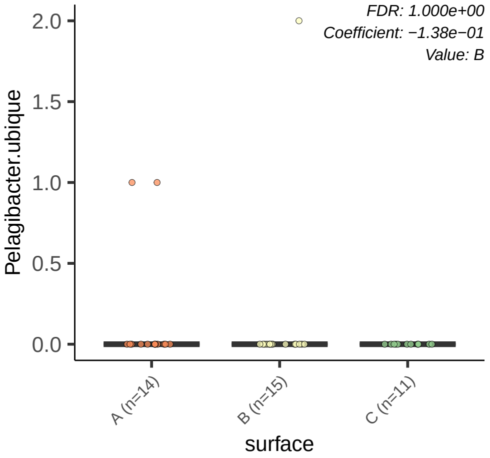

FDR: 1.000e+00
Coefficient: −1.38e−01
Value: B
Pelagibacter.ubique
2.0
1.5
1.0
0.5
0.0
A (n=14)
B (n=15)
C (n=11)
surface

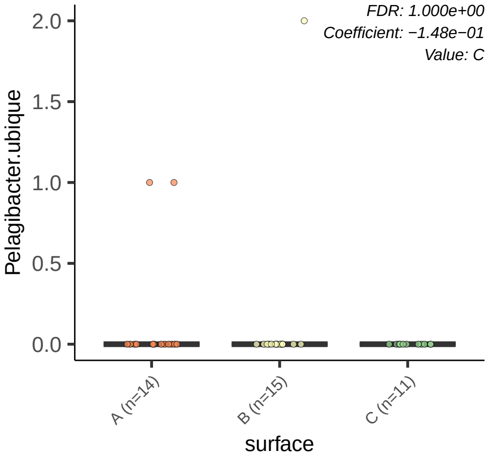

FDR: 1.000e+00
Coefficient: −1.48e−01
Value: C
Pelagibacter.ubique
2.0
1.5
1.0
0.5
0.0
A (n=14)
B (n=15)
C (n=11)
surface

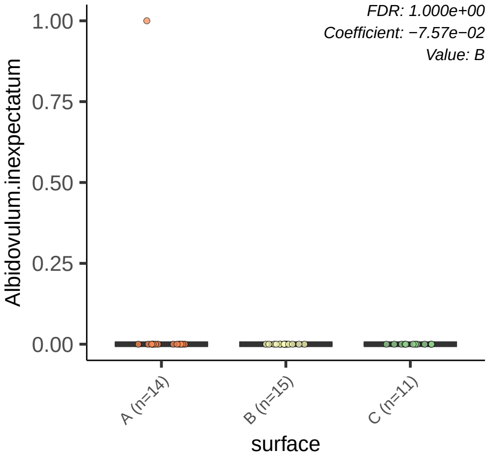

FDR: 1.000e+00
Coefficient: −7.57e−02
Value: B
Albidovulum.inexpectatum
1.00
0.75
0.50
0.25
0.00
A (n=14)
B (n=15)
C (n=11)
surface

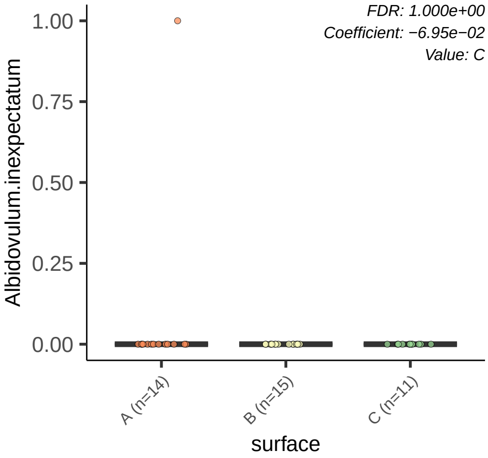
FDR: 1.000e+00
Coefficient: −6.95e−02
Value: C
Albidovulum.inexpectatum
1.00
0.75
0.50
0.25
0.00
A (n=14)
B (n=15)
C (n=11)
surface

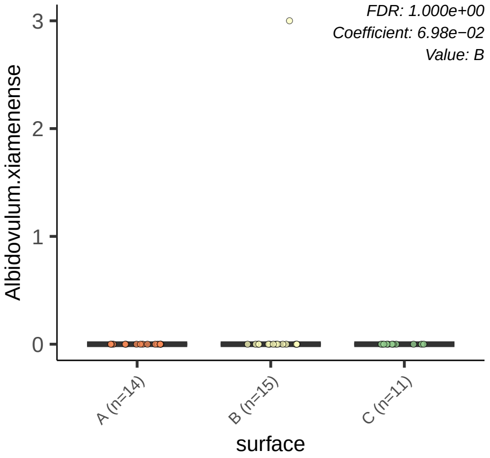
FDR: 1.000e+00
Coefficient: 6.98e−02
Value: B
Albidovulum.xiamenense
0
1
2
3
A (n=14)
B (n=15)
C (n=11)
surface

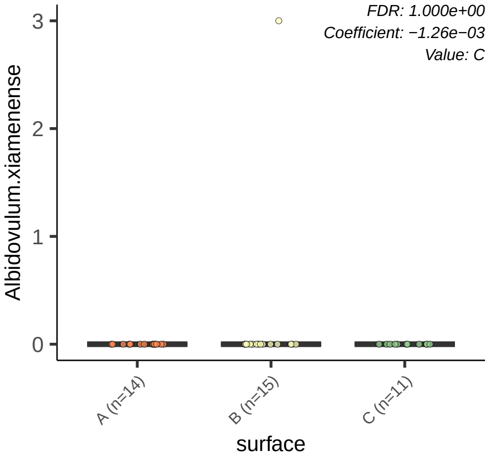
FDR: 1.000e+00
Coefficient: −1.26e−03
Value: C
Albidovulum.xiamenense
3
2
1
0
A (n=14)
B (n=15)
C (n=11)
surface

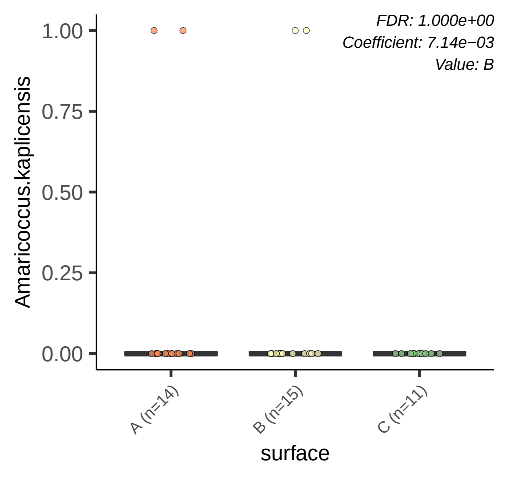
FDR: 1.000e+00
Coefficient: 7.14e−03
Value: B
Amaricoccus.kaplicensis
1.00
0.75
0.50
0.25
0.00
A (n=14)
B (n=15)
C (n=11)
surface

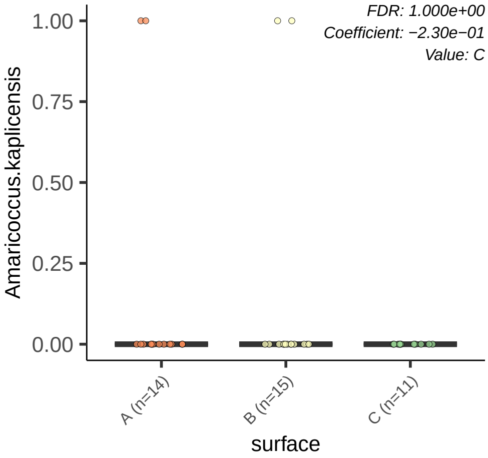

FDR: 1.000e+00
Coefficient: −2.30e−01
Value: C
Amaricoccus.kaplicensis
1.00
0.75
0.50
0.25
0.00
A (n=14)
B (n=15)
C (n=11)
surface

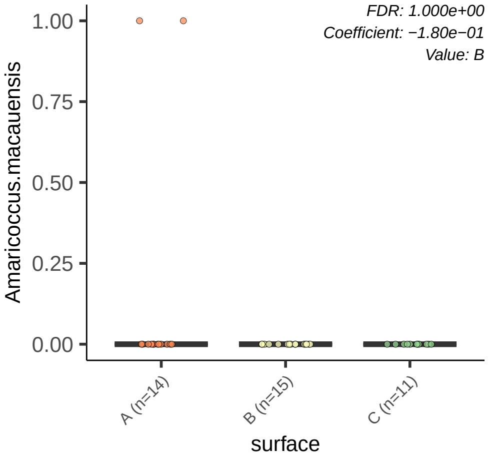
FDR: 1.000e+00
Coefficient: −1.80e−01
Value: B
Amaricoccus.macauensis
1.00
0.75
0.50
0.25
0.00
A (n=14)
B (n=15)
C (n=11)
surface

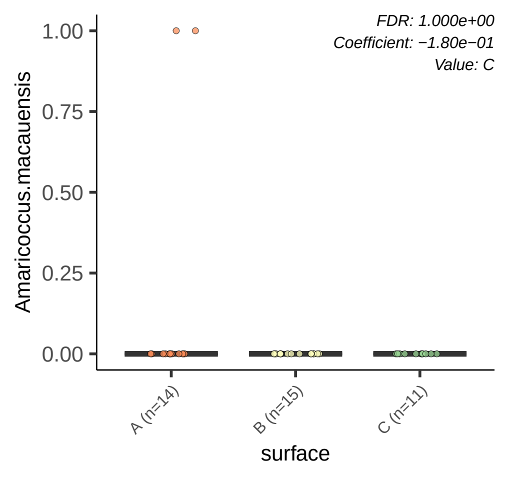

FDR: 1.000e+00
Coefficient: −1.80e−01
Value: C
Amaricoccus.macauensis
1.00
0.75
0.50
0.25
0.00
A (n=14)
B (n=15)
C (n=11)
surface

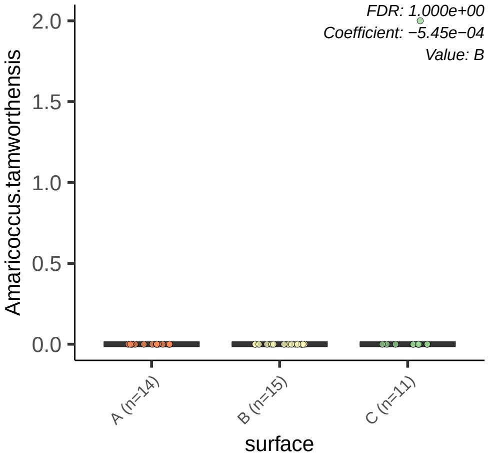

FDR: 1.000e+00
Coefficient: −5.45e−04
Value: B
Amaricoccus.tamworthensis
2.0
1.5
1.0
0.5
0.0
A (n=14)
B (n=15)
C (n=11)
surface

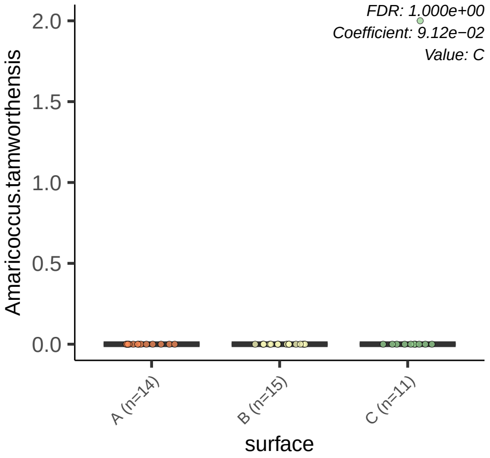
FDR: 1.000e+00
Coefficient: 9.12e−02
Value: C
Amaricoccus.tamworthensis
2.0
1.5
1.0
0.5
0.0
A (n=14)
B (n=15)
C (n=11)
surface

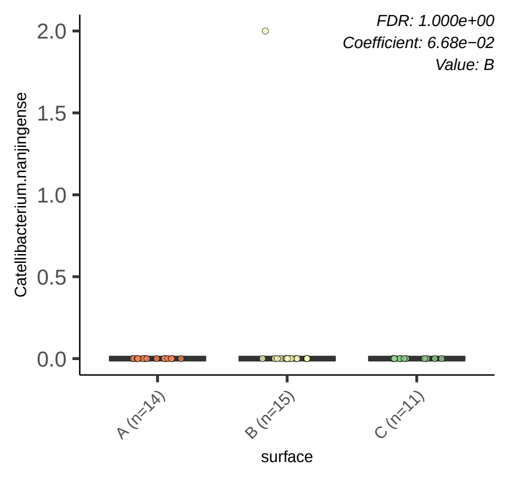
FDR: 1.000e+00
Coefficient: 6.68e−02
Value: B
Catellibacterium.nanjingense
2.0
1.5
1.0
0.5
0.0
A (n=14)
B (n=15)
C (n=11)
surface

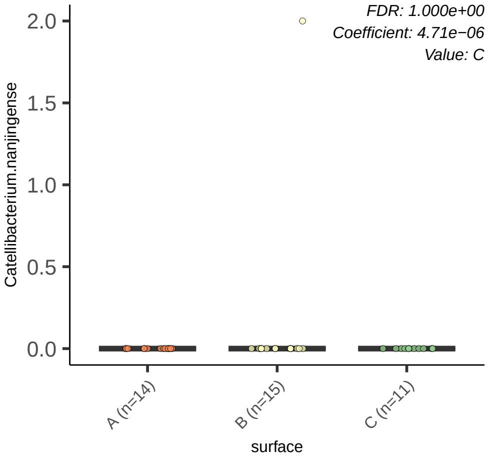

FDR: 1.000e+00
Coefficient: 4.71e−06
Value: C
Catellibacterium.nanjingense
2.0
1.5
1.0
0.5
0.0
A (n=14)
B (n=15)
C (n=11)
surface

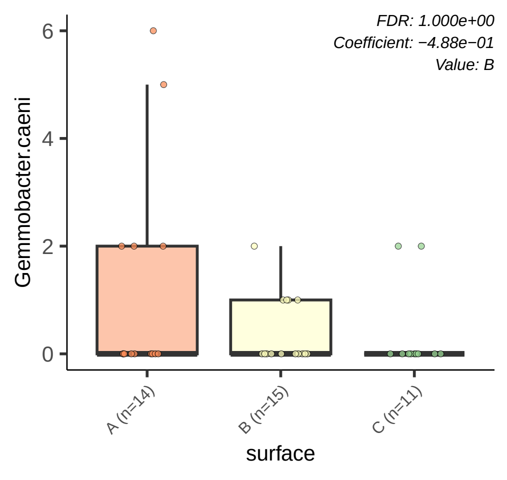

FDR: 1.000e+00
Coefficient: −4.88e−01
Value: B
Gemmobacter.caeni
6
4
2
0
A (n=14)
B (n=15)
C (n=11)
surface

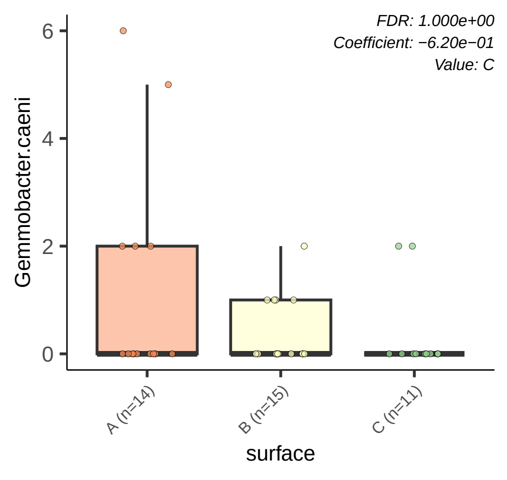

FDR: 1.000e+00
Coefficient: −6.20e−01
Value: C
Gemmobacter.caeni
0
2
4
6
A (n=14)
B (n=15)
C (n=11)
surface

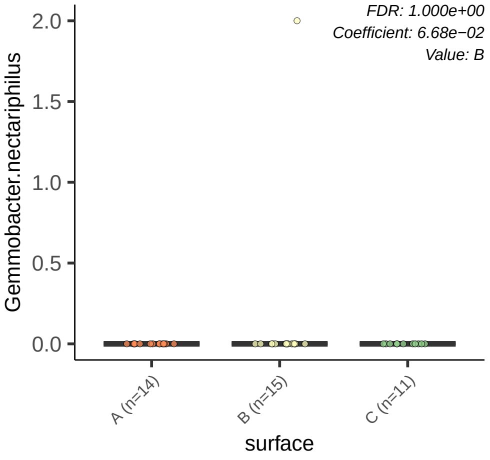
FDR: 1.000e+00
Coefficient: 6.68e−02
Value: B
Gemmobacter.nectariphilus
2.0
1.5
1.0
0.5
0.0
A (n=14)
B (n=15)
C (n=11)
surface

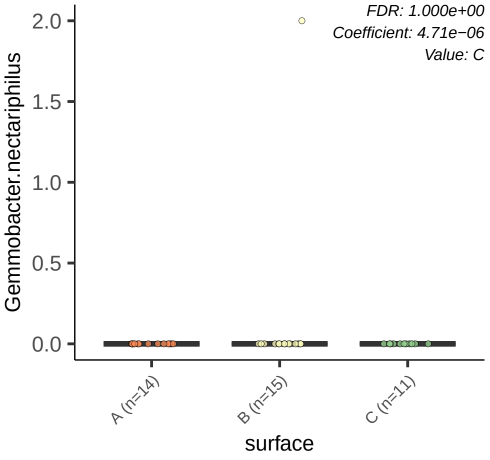
FDR: 1.000e+00
Coefficient: 4.71e−06
Value: C
Gemmobacter.nectariphilus
2.0
1.5
1.0
0.5
0.0
A (n=14)
B (n=15)
C (n=11)
surface

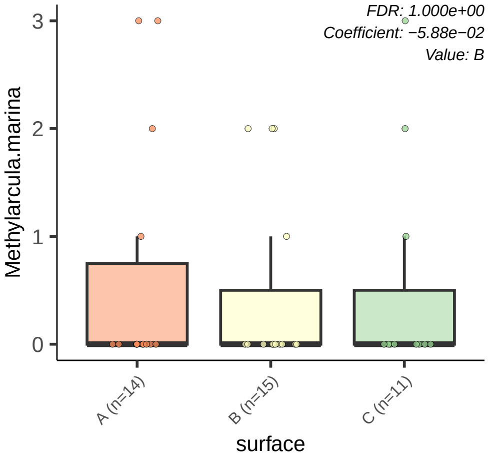

FDR: 1.000e+00
Coefficient: −5.88e−02
Value: B
Methylarcula.marina
0
1
2
3
A (n=14)
B (n=15)
C (n=11)
surface

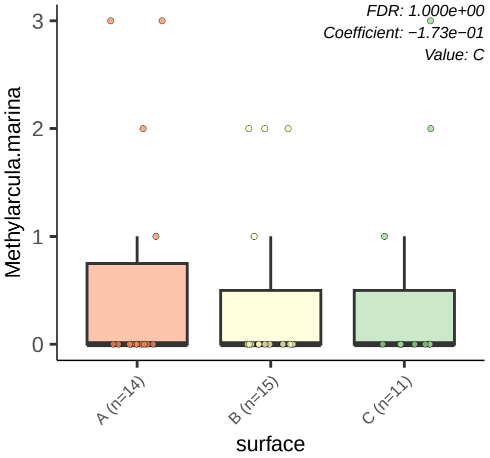
FDR: 1.000e+00
Coefficient: −1.73e−01
Value: C
Methylarcula.marina
0
1
2
3
A (n=14)
B (n=15)
C (n=11)
surface

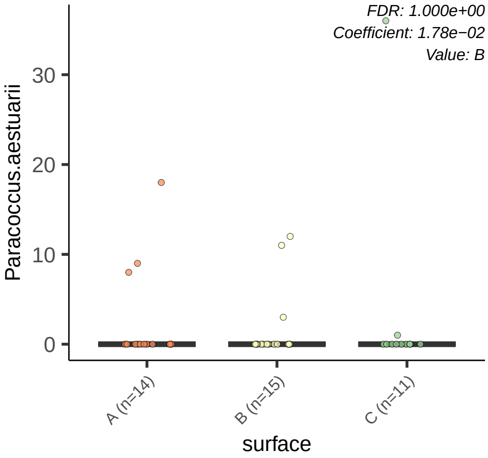
FDR: 1.000e+00
Coefficient: 1.78e−02
Value: B
Paracoccus.aestuarii
0
10
20
30
A (n=14)
B (n=15)
C (n=11)
surface

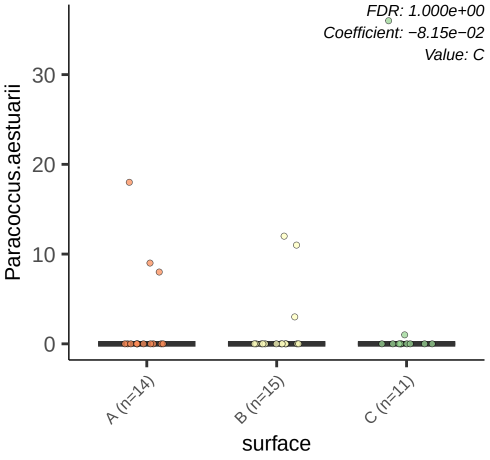
FDR: 1.000e+00
Coefficient: −8.15e−02
Value: C
Paracoccus.aestuarii
30
20
10
0
A (n=14)
B (n=15)
C (n=11)
surface

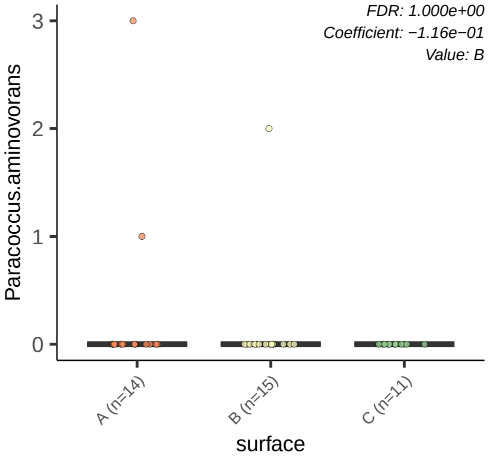
FDR: 1.000e+00
Coefficient: −1.16e−01
Value: B
Paracoccus.aminovorans
3
2
1
0
A (n=14)
B (n=15)
C (n=11)
surface

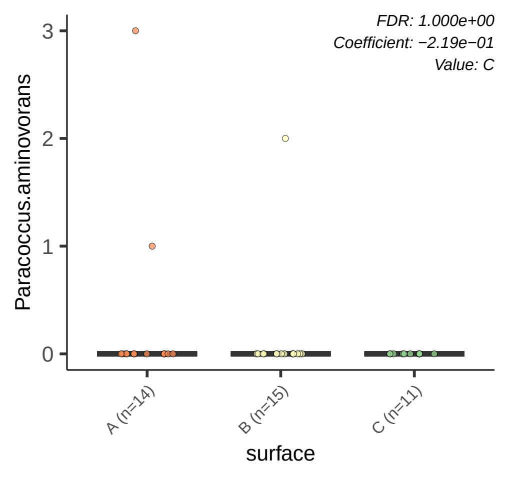
FDR: 1.000e+00
Coefficient: −2.19e−01
Value: C
Paracoccus.aminovorans
3
2
1
0
A (n=14)
B (n=15)
C (n=11)
surface

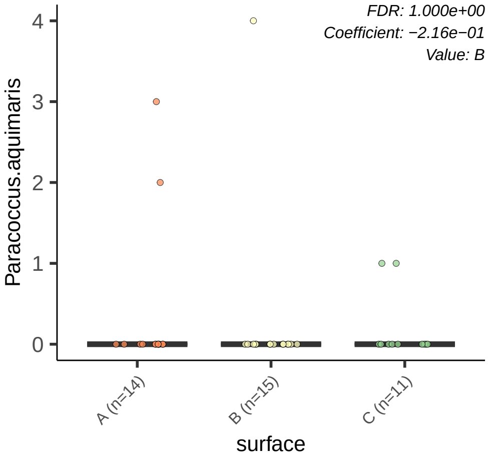
FDR: 1.000e+00
Coefficient: −2.16e−01
Value: B
Paracoccus.aquimaris
4
3
2
1
0
A (n=14)
B (n=15)
C (n=11)
surface

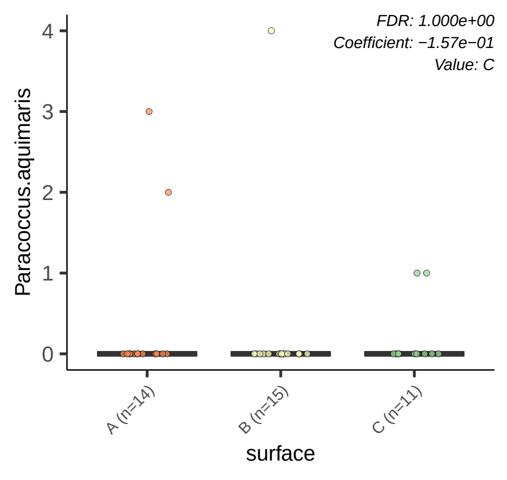
FDR: 1.000e+00
Coefficient: −1.57e−01
Value: C
Paracoccus.aquimaris
4
3
2
1
0
A (n=14)
B (n=15)
C (n=11)
surface

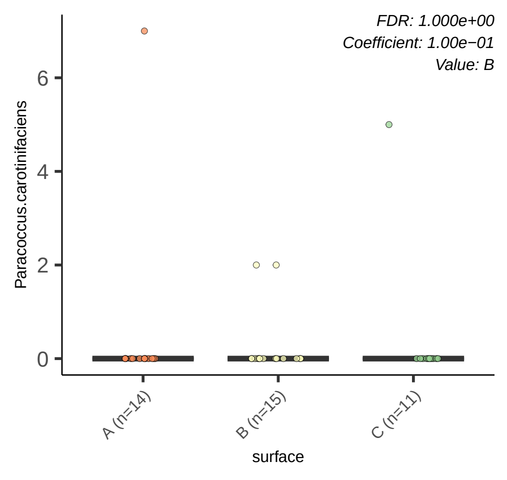
FDR: 1.000e+00
Coefficient: 1.00e−01
Value: B
Paracoccus.carotinifaciens
0
2
4
6
A (n=14)
B (n=15)
C (n=11)
surface

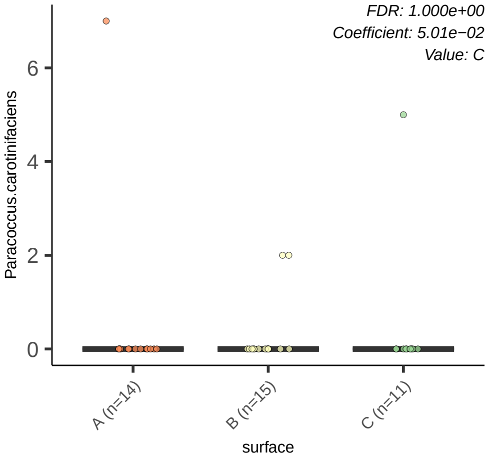
FDR: 1.000e+00
Coefficient: 5.01e−02
Value: C
Paracoccus.carotinifaciens
0
2
4
6
A (n=14)
B (n=15)
C (n=11)
surface

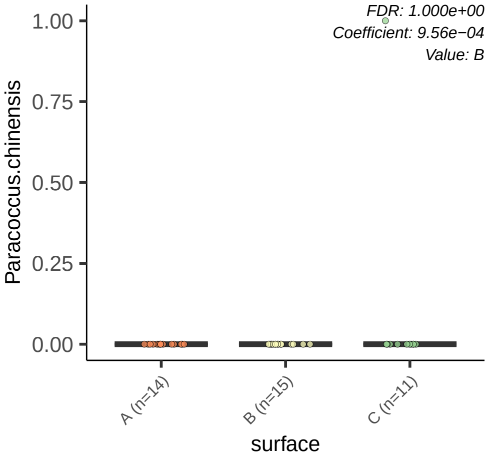
FDR: 1.000e+00
Coefficient: 9.56e−04
Value: B
Paracoccus.chinensis
1.00
0.75
0.50
0.25
0.00
A (n=14)
B (n=15)
C (n=11)
surface

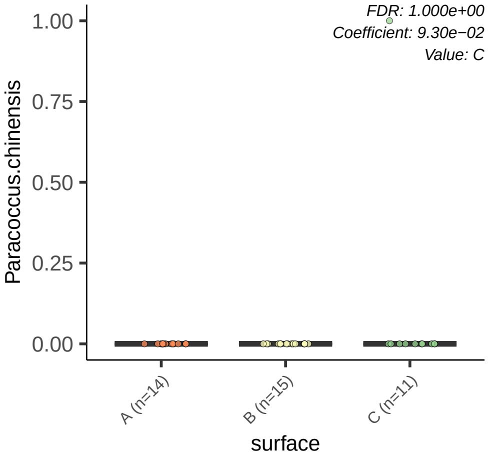
FDR: 1.000e+00
Coefficient: 9.30e−02
Value: C
Paracoccus.chinensis
1.00
0.75
0.50
0.25
0.00
A (n=14)
B (n=15)
C (n=11)
surface

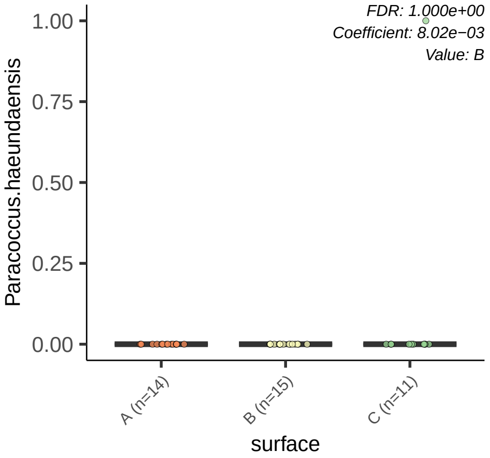
FDR: 1.000e+00
Coefficient: 8.02e−03
Value: B
Paracoccus.haeundaensis
1.00
0.75
0.50
0.25
0.00
A (n=14)
B (n=15)
C (n=11)
surface

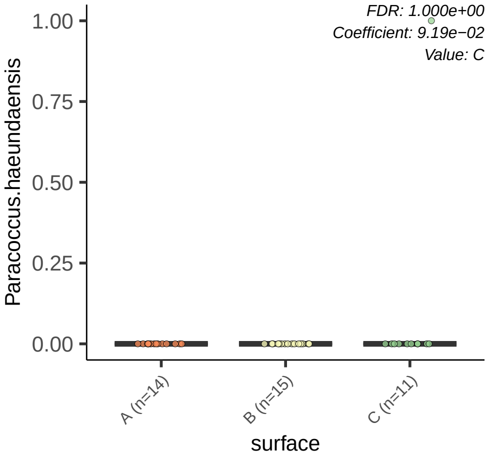
FDR: 1.000e+00
Coefficient: 9.19e−02
Value: C
Paracoccus.haeundaensis
1.00
0.75
0.50
0.25
0.00
A (n=14)
B (n=15)
C (n=11)
surface

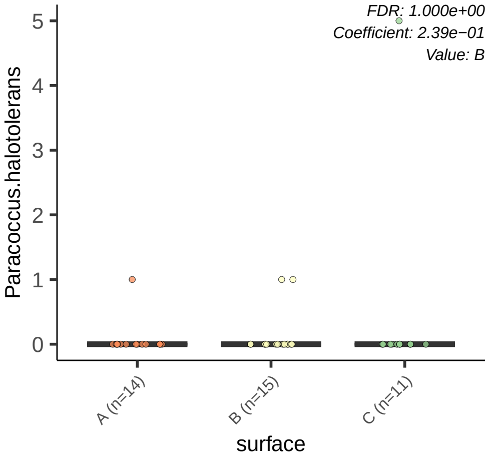
FDR: 1.000e+00
Coefficient: 2.39e−01
Value: B
Paracoccus.halotolerans
0
1
2
3
4
5
A (n=14)
B (n=15)
C (n=11)
surface

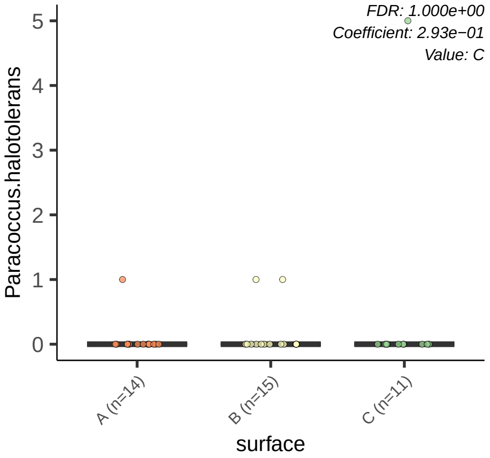

FDR: 1.000e+00
Coefficient: 2.93e−01
Value: C
Paracoccus.halotolerans
0
1
2
3
4
5
A (n=14)
B (n=15)
C (n=11)
surface

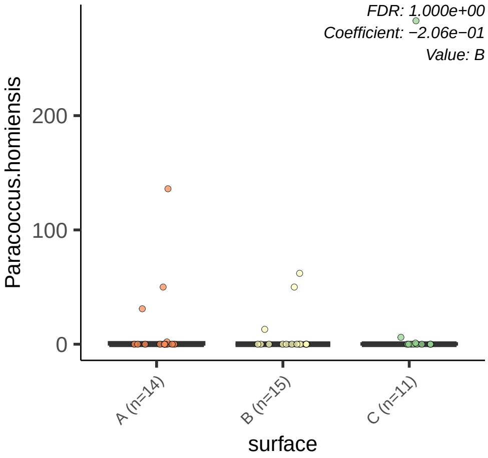

FDR: 1.000e+00
Coefficient: −2.06e−01
Value: B
Paracoccus.homiensis
200
100
0
A (n=14)
B (n=15)
C (n=11)
surface

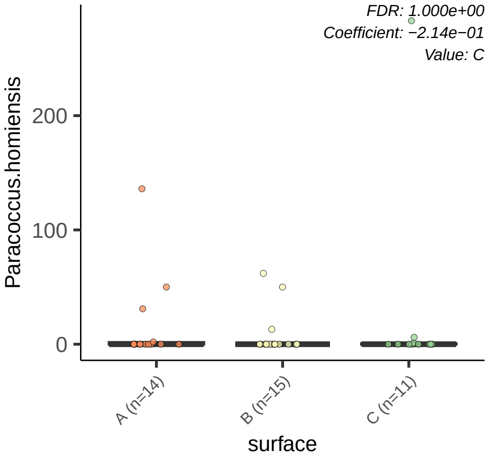
FDR: 1.000e+00
Coefficient: −2.14e−01
Value: C
Paracoccus.homiensis
200
100
0
A (n=14)
B (n=15)
C (n=11)
surface

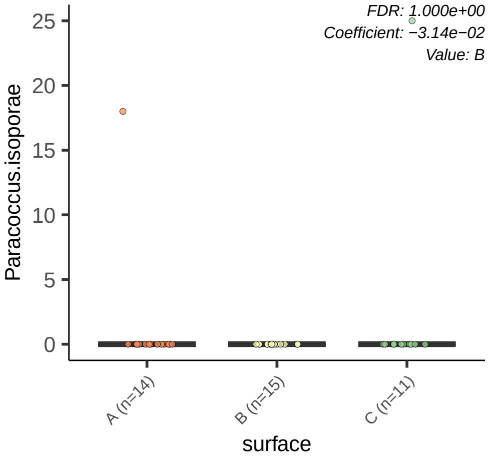
FDR: 1.000e+00
Coefficient: −3.14e−02
Value: B
Paracoccus.isoporae
25
20
15
10
5
0
A (n=14)
B (n=15)
C (n=11)
surface

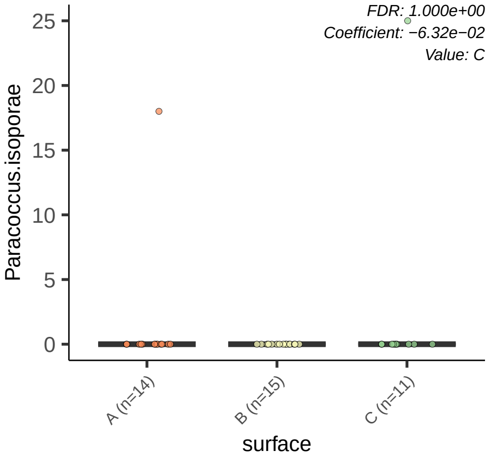
FDR: 1.000e+00
Coefficient: −6.32e−02
Value: C
Paracoccus.isoporae
25
20
15
10
5
0
A (n=14)
B (n=15)
C (n=11)
surface

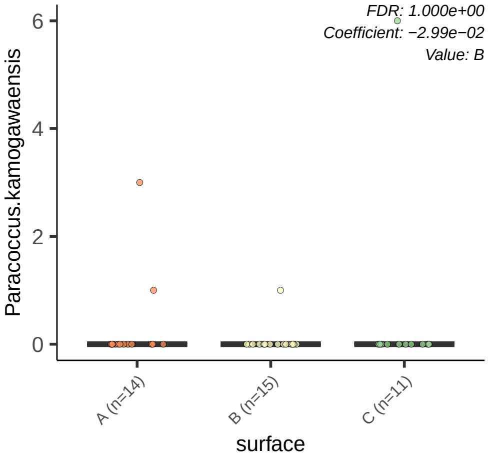
FDR: 1.000e+00
Coefficient: −2.99e−02
Value: B
Paracoccus.kamogawaensis
6
4
2
0
A (n=14)
B (n=15)
C (n=11)
surface

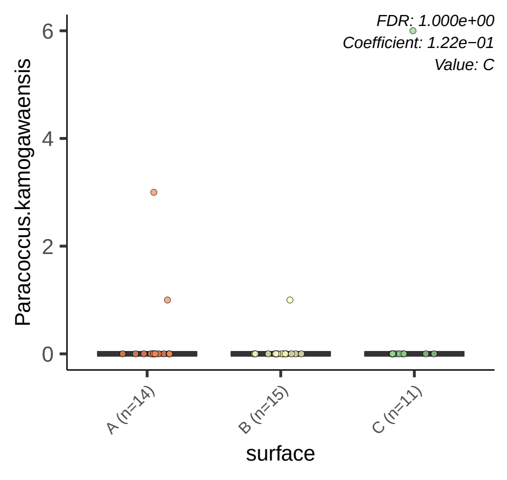

FDR: 1.000e+00
Coefficient: 1.22e−01
Value: C
Paracoccus.kamogawaensis
0
2
4
6
A (n=14)
B (n=15)
C (n=11)
surface

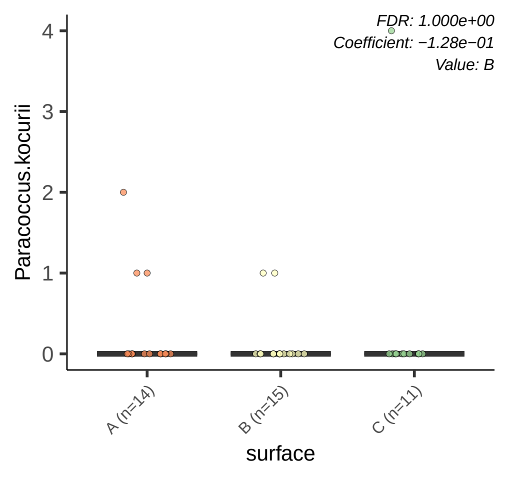
FDR: 1.000e+00
Coefficient: −1.28e−01
Value: B
Paracoccus.kocurii
0
1
2
3
4
A (n=14)
B (n=15)
C (n=11)
surface

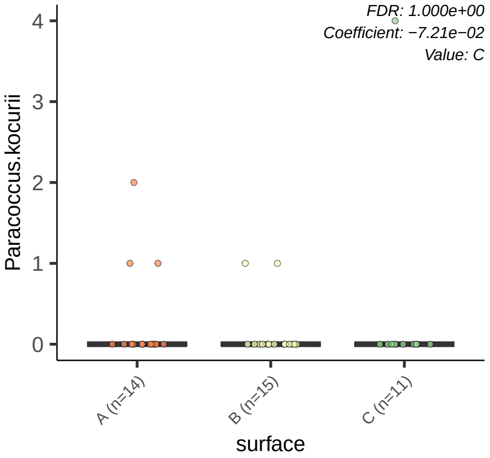
FDR: 1.000e+00
Coefficient: −7.21e−02
Value: C
Paracoccus.kocurii
0
1
2
3
4
A (n=14)
B (n=15)
C (n=11)
surface

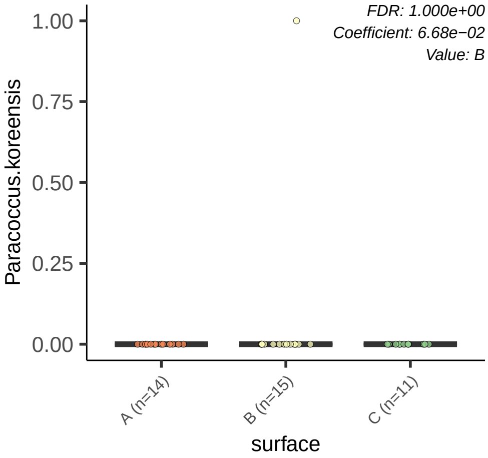
FDR: 1.000e+00
Coefficient: 6.68e−02
Value: B
Paracoccus.koreensis
1.00
0.75
0.50
0.25
0.00
A (n=14)
B (n=15)
C (n=11)
surface

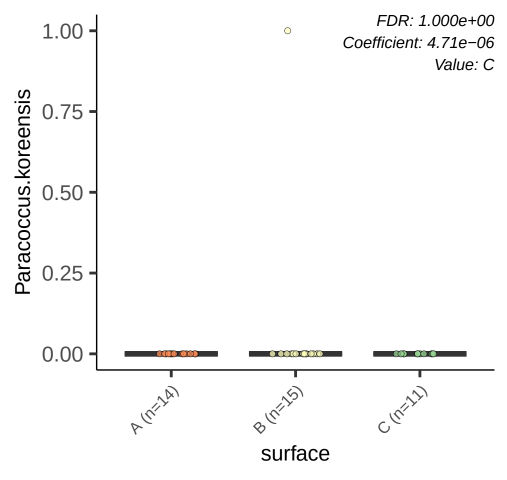
FDR: 1.000e+00
Coefficient: 4.71e−06
Value: C
Paracoccus.koreensis
1.00
0.75
0.50
0.25
0.00
A (n=14)
B (n=15)
C (n=11)
surface

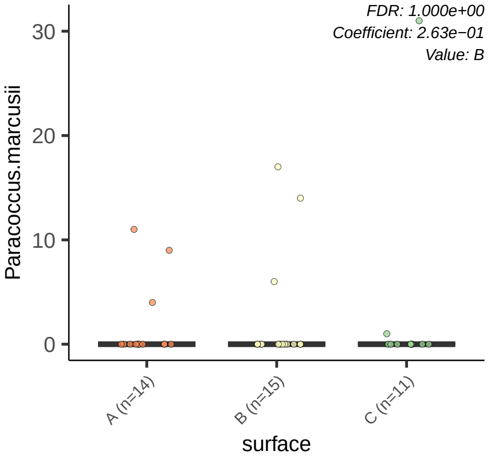

FDR: 1.000e+00
Coefficient: 2.63e−01
Value: B
Paracoccus.marcusii
30
20
10
0
A (n=14)
B (n=15)
C (n=11)
surface

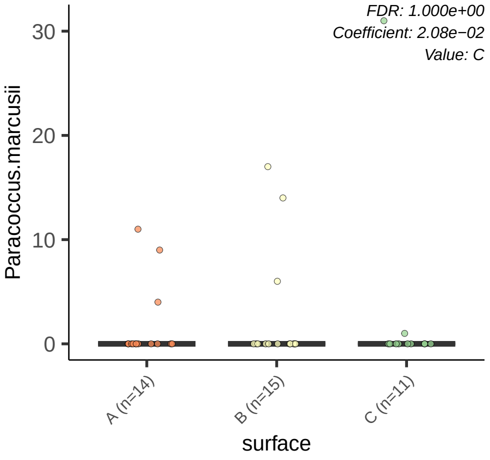

FDR: 1.000e+00
Coefficient: 2.08e−02
Value: C
Paracoccus.marcusii
30
20
10
0
A (n=14)
B (n=15)
C (n=11)
surface

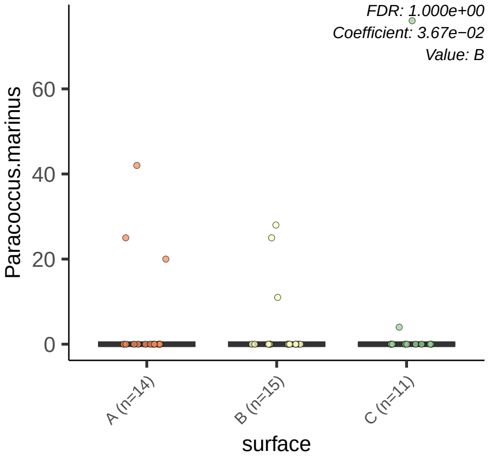
FDR: 1.000e+00
Coefficient: 3.67e−02
Value: B
Paracoccus.marinus
60
40
20
0
A (n=14)
B (n=15)
C (n=11)
surface

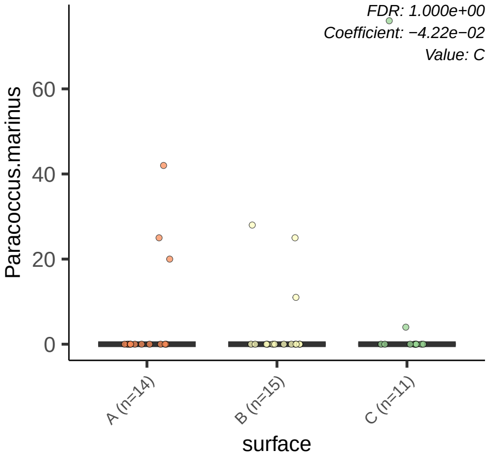
FDR: 1.000e+00
Coefficient: −4.22e−02
Value: C
Paracoccus.marinus
60
40
20
0
A (n=14)
B (n=15)
C (n=11)
surface

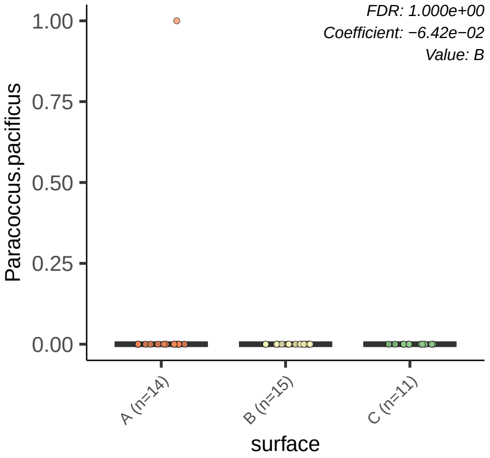

FDR: 1.000e+00
Coefficient: −6.42e−02
Value: B
Paracoccus.pacificus
1.00
0.75
0.50
0.25
0.00
A (n=14)
B (n=15)
C (n=11)
surface

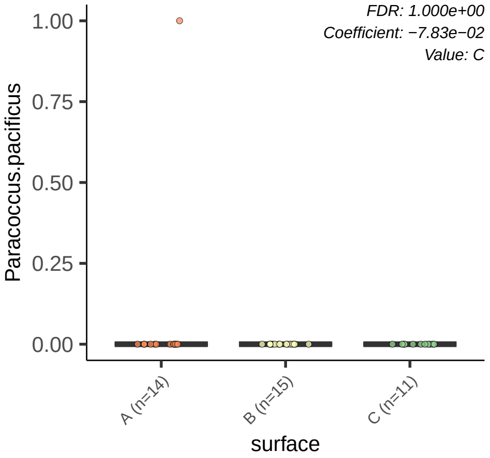
FDR: 1.000e+00
Coefficient: −7.83e−02
Value: C
Paracoccus.pacificus
1.00
0.75
0.50
0.25
0.00
A (n=14)
B (n=15)
C (n=11)
surface

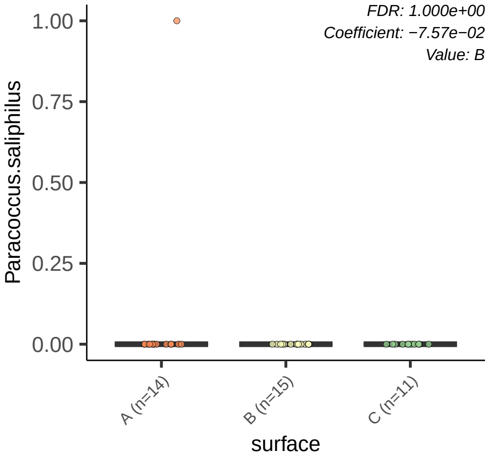
FDR: 1.000e+00
Coefficient: −7.57e−02
Value: B
Paracoccus.saliphilus
1.00
0.75
0.50
0.25
0.00
A (n=14)
B (n=15)
C (n=11)
surface

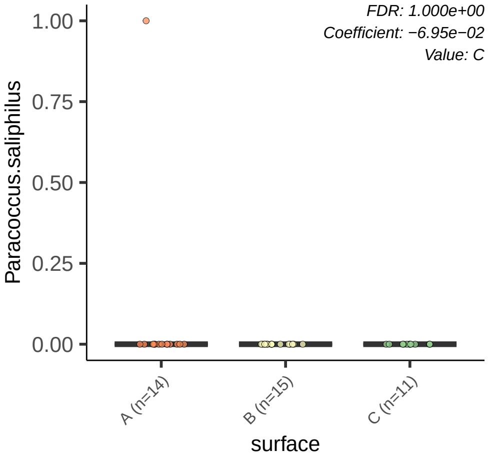
FDR: 1.000e+00
Coefficient: −6.95e−02
Value: C
Paracoccus.saliphilus
1.00
0.75
0.50
0.25
0.00
A (n=14)
B (n=15)
C (n=11)
surface

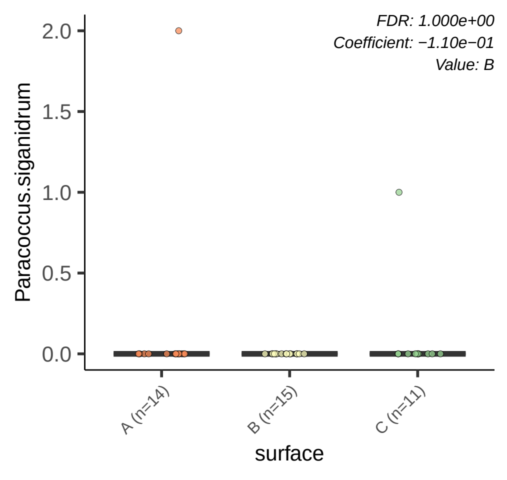
FDR: 1.000e+00
Coefficient: −1.10e−01
Value: B
Paracoccus.siganidrum
2.0
1.5
1.0
0.5
0.0
A (n=14)
B (n=15)
C (n=11)
surface

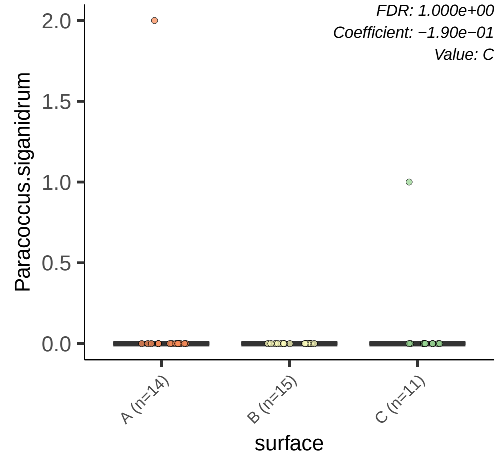

FDR: 1.000e+00
Coefficient: −1.90e−01
Value: C
Paracoccus.siganidrum
2.0
1.5
1.0
0.5
0.0
A (n=14)
B (n=15)
C (n=11)
surface

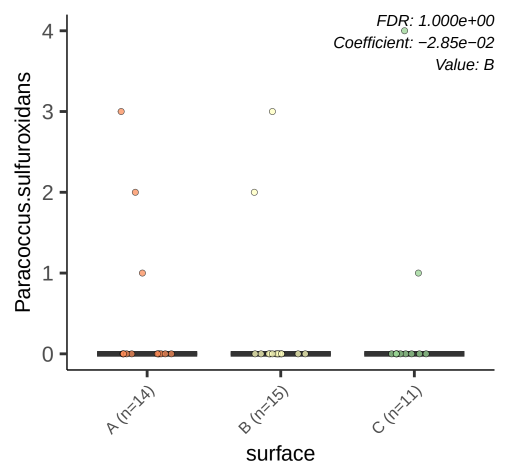
FDR: 1.000e+00
Coefficient: −2.85e−02
Value: B
Paracoccus.sulfuroxidans
0
1
2
3
4
A (n=14)
B (n=15)
C (n=11)
surface

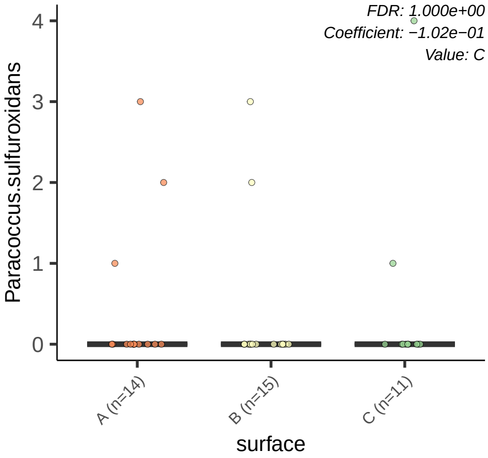
FDR: 1.000e+00
Coefficient: −1.02e−01
Value: C
Paracoccus.sulfuroxidans
0
1
2
3
4
A (n=14)
B (n=15)
C (n=11)
surface

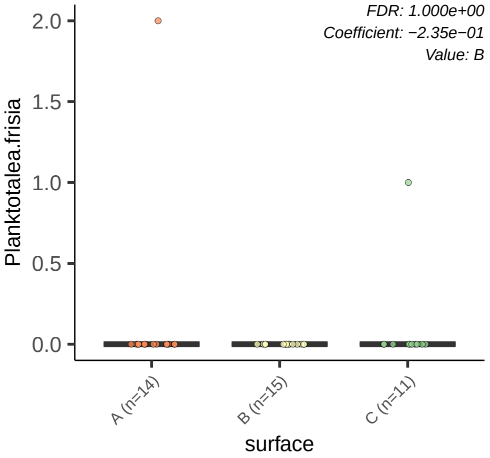
FDR: 1.000e+00
Coefficient: −2.35e−01
Value: B
Planktotalea.frisia
2.0
1.5
1.0
0.5
0.0
A (n=14)
B (n=15)
C (n=11)
surface

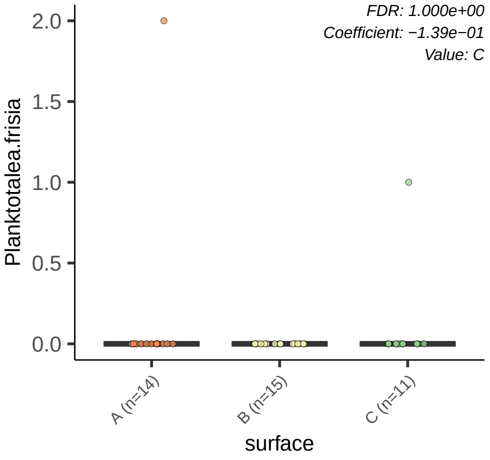

FDR: 1.000e+00
Coefficient: −1.39e−01
Value: C
Planktotalea.frisia
2.0
1.5
1.0
0.5
0.0
A (n=14)
B (n=15)
C (n=11)
surface

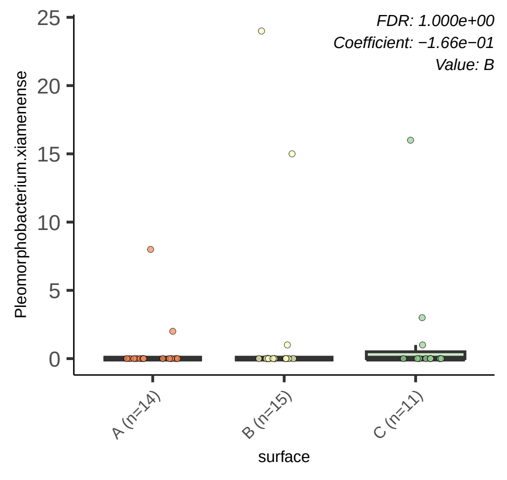

FDR: 1.000e+00
Coefficient: −1.66e−01
Value: B
Pleomorphobacterium.xiamenense
25
20
15
10
5
0
A (n=14)
B (n=15)
C (n=11)
surface

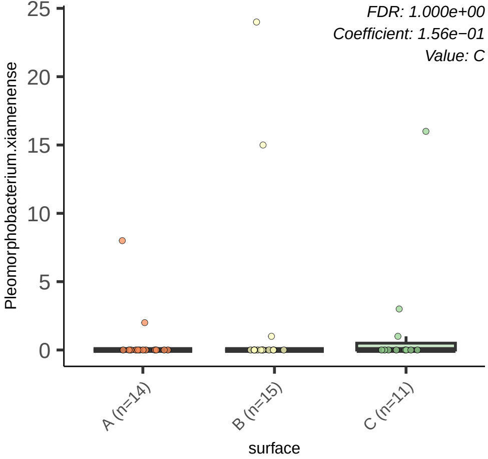
FDR: 1.000e+00
Coefficient: 1.56e−01
Value: C
Pleomorphobacterium.xiamenense
25
20
15
10
5
0
A (n=14)
B (n=15)
C (n=11)
surface

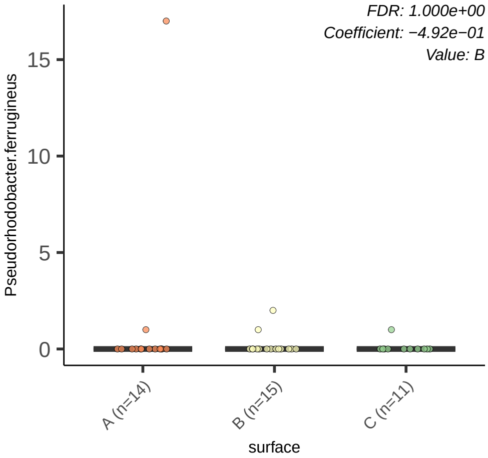
FDR: 1.000e+00
Coefficient: −4.92e−01
Value: B
Pseudorhodobacter.ferugineus
0
5
10
15
A (n=14)
B (n=15)
C (n=11)
surface

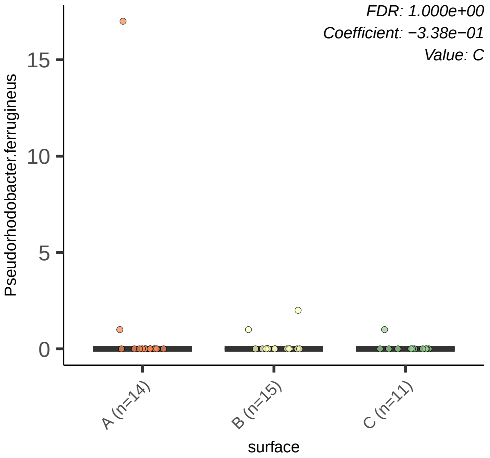

FDR: 1.000e+00
Coefficient: −3.38e−01
Value: C
Pseudorhodobacter.ferrugineus
15
10
5
0
A (n=14)
B (n=15)
C (n=11)
surface

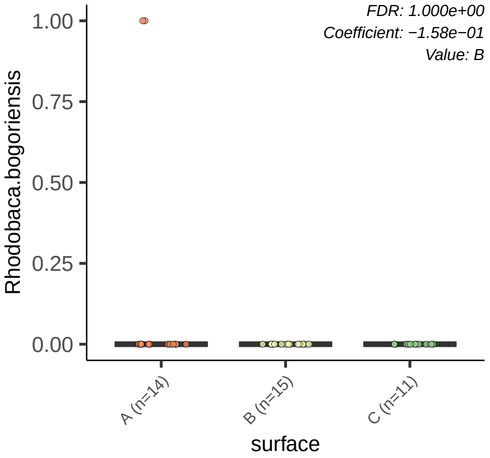
FDR: 1.000e+00
Coefficient: −1.58e−01
Value: B
Rhodobaca.bogoriensis
1.00
0.75
0.50
0.25
0.00
A (n=14)
B (n=15)
C (n=11)
surface

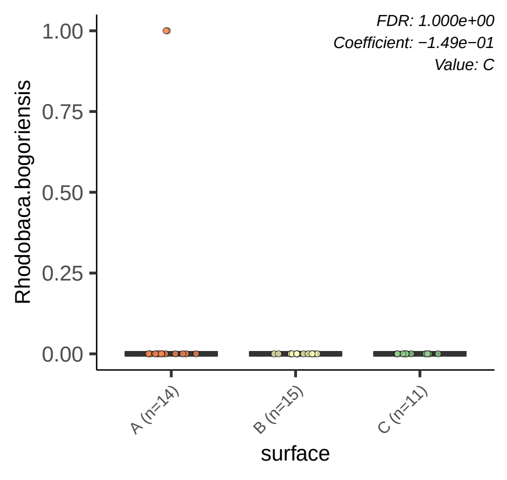
FDR: 1.000e+00
Coefficient: −1.49e−01
Value: C
Rhodobaca.bogoriensis
1.00
0.75
0.50
0.25
0.00
A (n=14)
B (n=15)
C (n=11)
surface

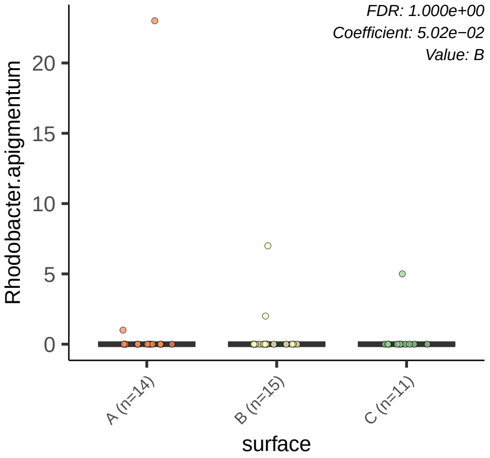
FDR: 1.000e+00
Coefficient: 5.02e−02
Value: B
Rhodobacter.apigmentum
0
5
10
15
20
A (n=14)
B (n=15)
C (n=11)
surface

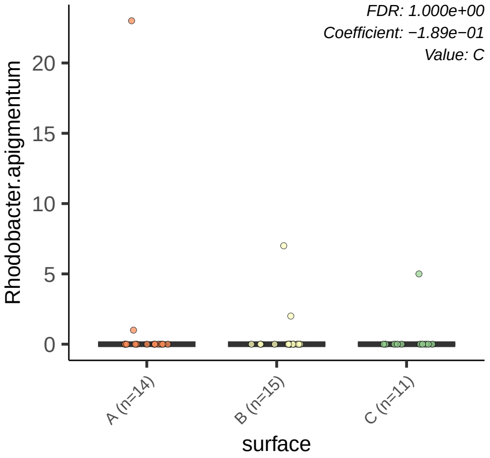
FDR: 1.000e+00
Coefficient: −1.89e−01
Value: C
Rhodobacter.apigmentum
20
15
10
5
0
A (n=14)
B (n=15)
C (n=11)
surface

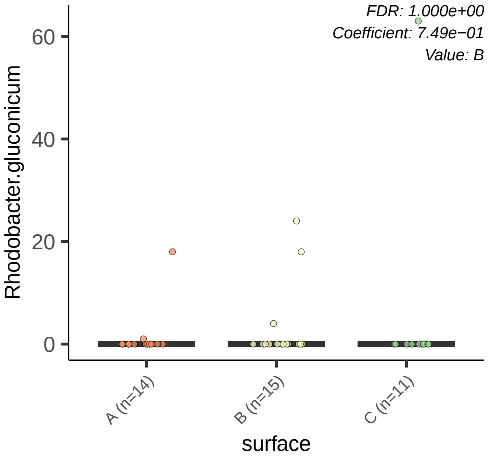
FDR: 1.000e+00
Coefficient: 7.49e−01
Value: B
Rhodobacter.gluconicum
0
20
40
60
A (n=14)
B (n=15)
C (n=11)
surface

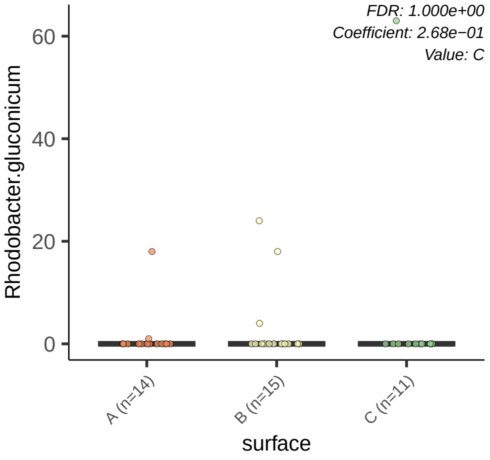
FDR: 1.000e+00
Coefficient: 2.68e−01
Value: C
Rhodobacter.gluconicum
0
20
40
60
A (n=14)
B (n=15)
C (n=11)
surface

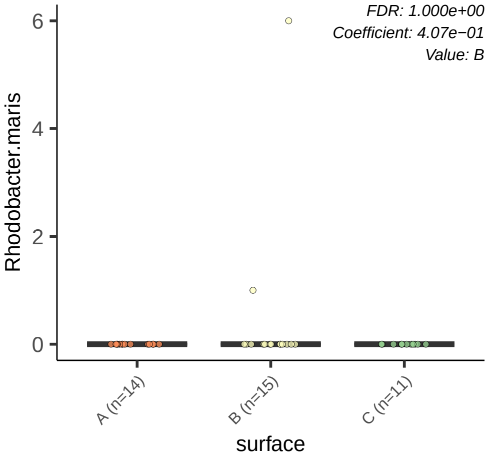
FDR: 1.000e+00
Coefficient: 4.07e−01
Value: B
Rhodobacter.maris
0
2
4
6
A (n=14)
B (n=15)
C (n=11)
surface

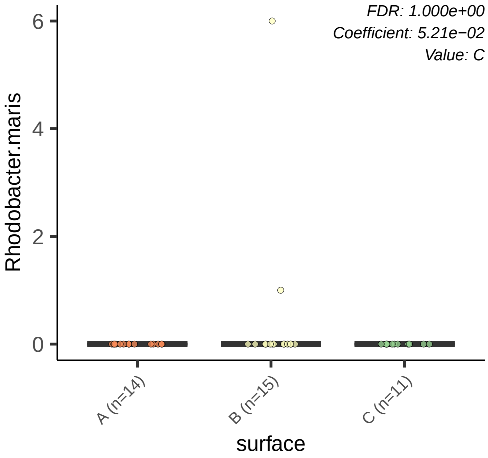

FDR: 1.000e+00
Coefficient: 5.21e−02
Value: C
Rhodobacter.maris
6
4
2
0
A (n=14)
B (n=15)
C (n=11)
surface

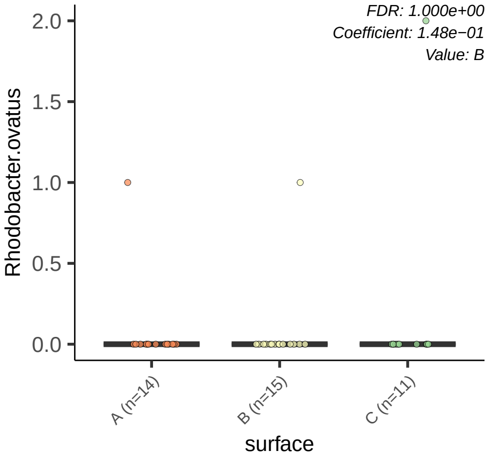
FDR: 1.000e+00
Coefficient: 1.48e−01
Value: B
Rhodobacter.ovatus
2.0
1.5
1.0
0.5
0.0
A (n=14)
B (n=15)
C (n=11)
surface

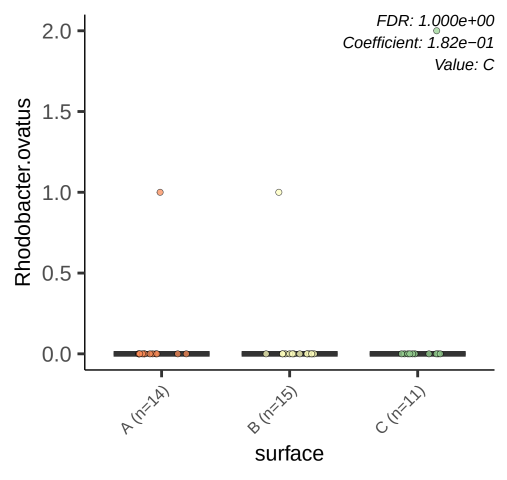
FDR: 1.000e+00
Coefficient: 1.82e−01
Value: C
Rhodobacter.ovatus
2.0
1.5
1.0
0.5
0.0
A (n=14)
B (n=15)
C (n=11)
surface

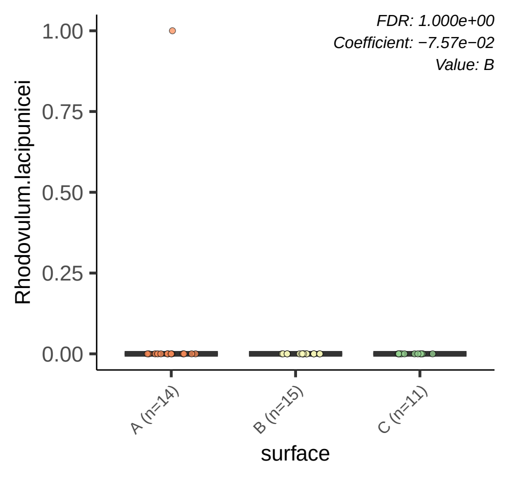

FDR: 1.000e+00
Coefficient: −7.57e−02
Value: B
Rhodovulum.lacipunicei
1.00
0.75
0.50
0.25
0.00
A (n=14)
B (n=15)
C (n=11)
surface

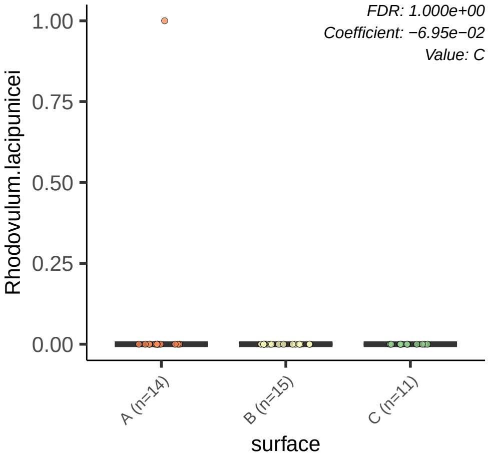

FDR: 1.000e+00
Coefficient: −6.95e−02
Value: C
Rhodovulum.lacipunicei
1.00
0.75
0.50
0.25
0.00
A (n=14)
B (n=15)
C (n=11)
surface

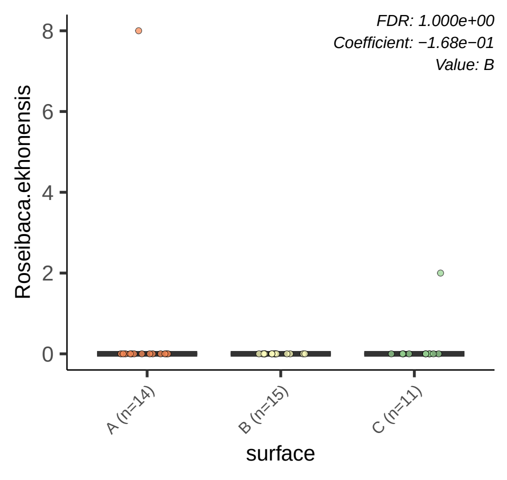

FDR: 1.000e+00
Coefficient: −1.68e−01
Value: B
Roseibaca.ekhonensis
8
6
4
2
0
A (n=14)
B (n=15)
C (n=11)
surface

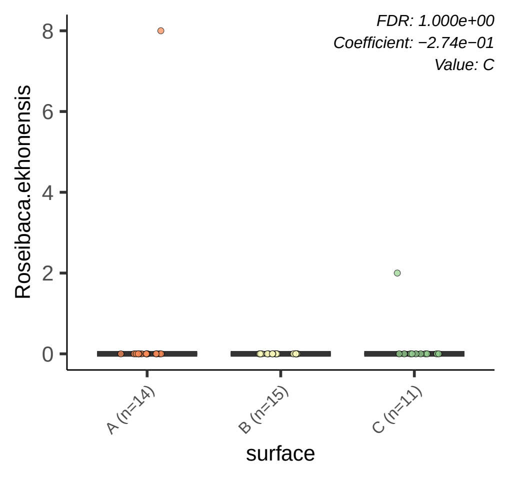
FDR: 1.000e+00
Coefficient: −2.74e−01
Value: C
Roseibaca.ekhonensis
8
6
4
2
0
A (n=14)
B (n=15)
C (n=11)
surface

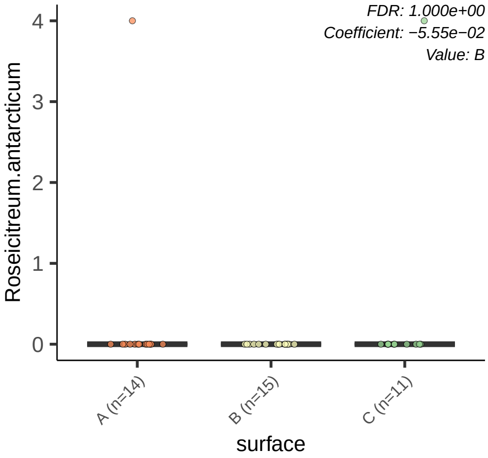

FDR: 1.000e+00
Coefficient: −5.55e−02
Value: B
Roseicitreum.antarcticum
0
1
2
3
4
A (n=14)
B (n=15)
C (n=11)
surface

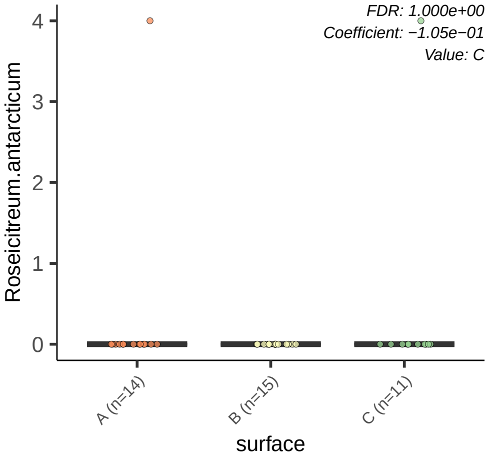
FDR: 1.000e+00
Coefficient: −1.05e−01
Value: C
Roseicitreum.antarcticum
0
1
2
3
4
A (n=14)
B (n=15)
C (n=11)
surface

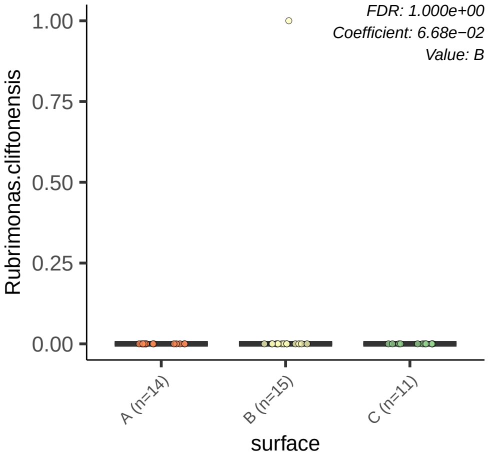
FDR: 1.000e+00
Coefficient: 6.68e−02
Value: B
Rubrimonas.cliftonensis
1.00
0.75
0.50
0.25
0.00
A (n=14)
B (n=15)
C (n=11)
surface

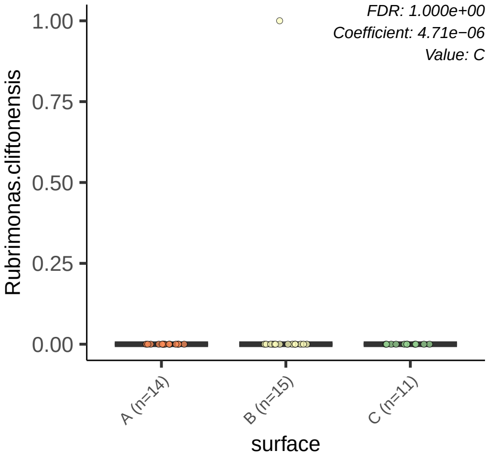
FDR: 1.000e+00
Coefficient: 4.71e−06
Value: C
Rubrimonas.cliftonensis
1.00
0.75
0.50
0.25
0.00
A (n=14)
B (n=15)
C (n=11)
surface

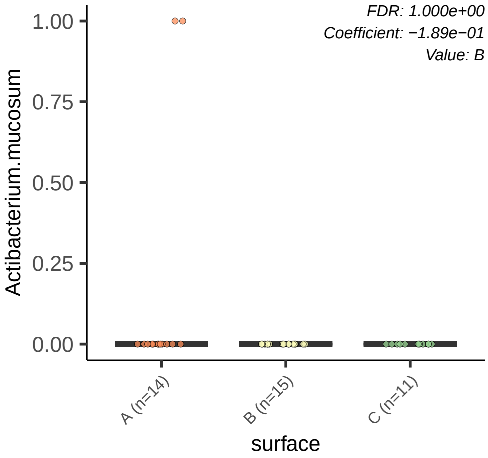
FDR: 1.000e+00
Coefficient: −1.89e−01
Value: B
Actibacterium.mucosum
1.00
0.75
0.50
0.25
0.00
A (n=14)
B (n=15)
C (n=11)
surface

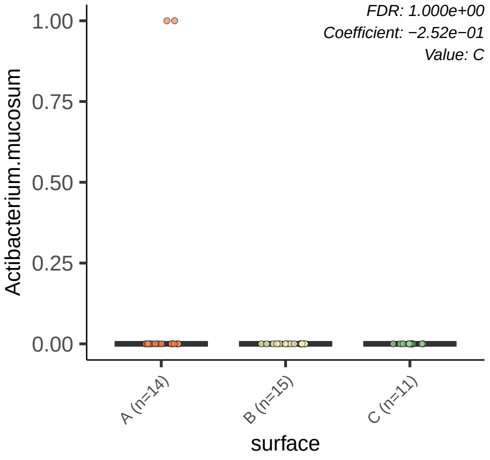

FDR: 1.000e+00
Coefficient: −2.52e−01
Value: C
Actibacterium.mucosum
1.00
0.75
0.50
0.25
0.00
A (n=14)
B (n=15)
C (n=11)
surface

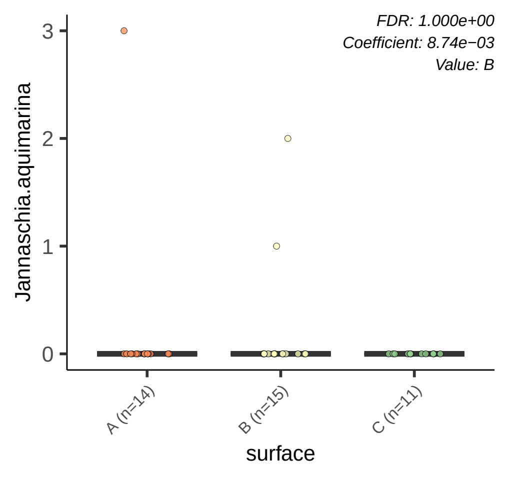
FDR: 1.000e+00
Coefficient: 8.74e−03
Value: B
Jannaschia.aquimarina
3
2
1
0
A (n=14)
B (n=15)
C (n=11)
surface

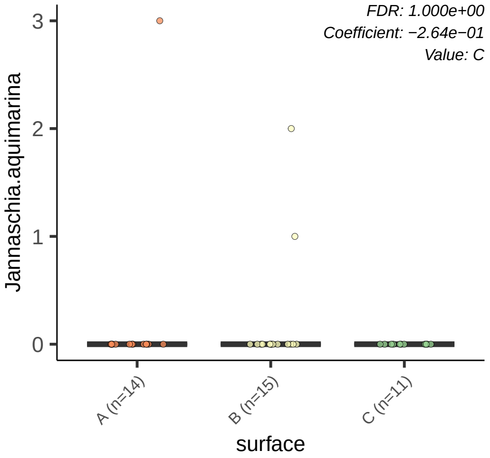
FDR: 1.000e+00
Coefficient: −2.64e−01
Value: C
Jannaschia.aquimarina
3
2
1
0
A (n=14)
B (n=15)
C (n=11)
surface

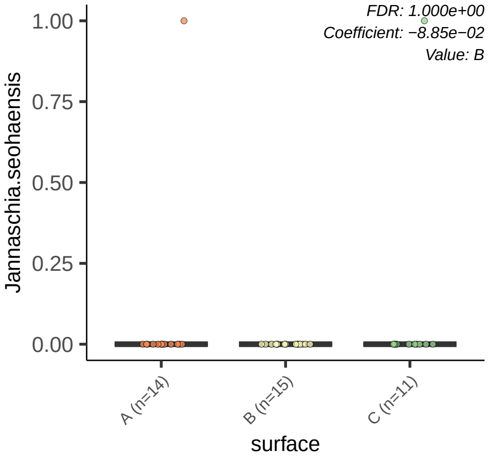
FDR: 1.000e+00
Coefficient: −8.85e−02
Value: B
Jannaschia.seohaensis
1.00
0.75
0.50
0.25
0.00
A (n=14)
B (n=15)
C (n=11)
surface

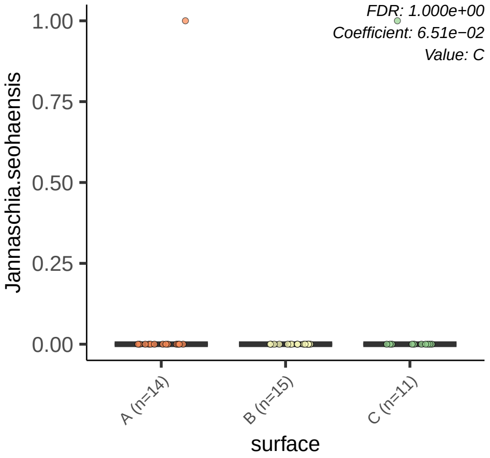

FDR: 1.000e+00
Coefficient: 6.51e−02
Value: C
Jannaschia.seohaensis
1.00
0.75
0.50
0.25
0.00
A (n=14)
B (n=15)
C (n=11)
surface

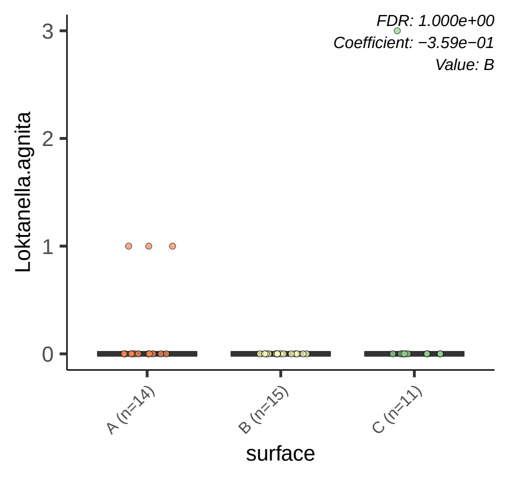

FDR: 1.000e+00
Coefficient: −3.59e−01
Value: B
Loktanella.agnita
0
1
2
3
A (n=14)
B (n=15)
C (n=11)
surface

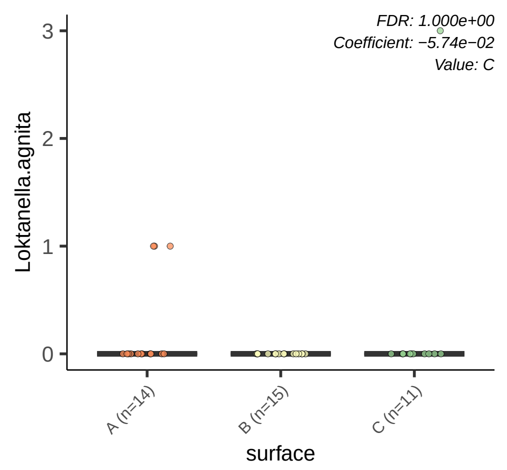

FDR: 1.000e+00
Coefficient: −5.74e−02
Value: C
3
2
1
0
Loktanella.agnita
A (n=14)
B (n=15)
C (n=11)
surface

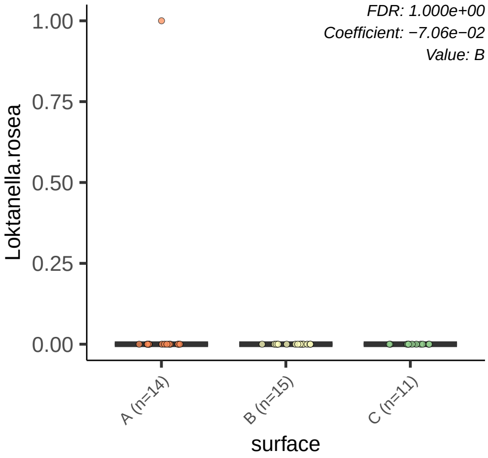

FDR: 1.000e+00
Coefficient: −7.06e−02
Value: B
Loktanella.rosea
1.00
0.75
0.50
0.25
0.00
A (n=14)
B (n=15)
C (n=11)
surface

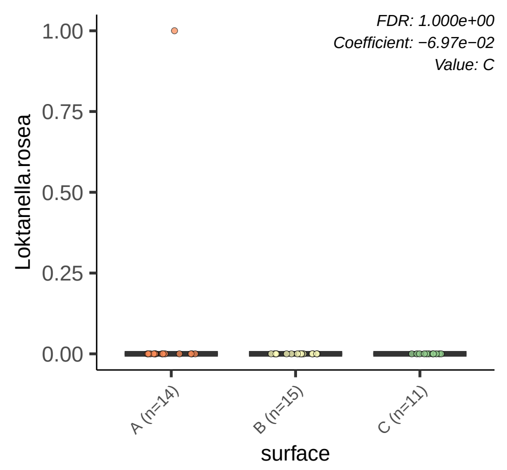
FDR: 1.000e+00
Coefficient: −6.97e−02
Value: C
Loktanella.rosea
1.00
0.75
0.50
0.25
0.00
A (n=14)
B (n=15)
C (n=11)
surface

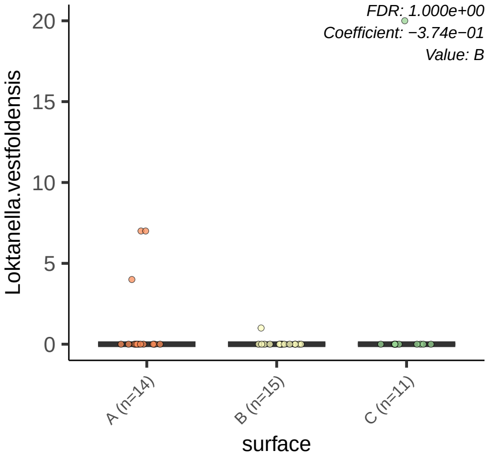

FDR: 1.000e+00
Coefficient: −3.74e−01
Value: B
Loktanella.vestfoldensis
20
15
10
5
0
A (n=14)
B (n=15)
C (n=11)
surface

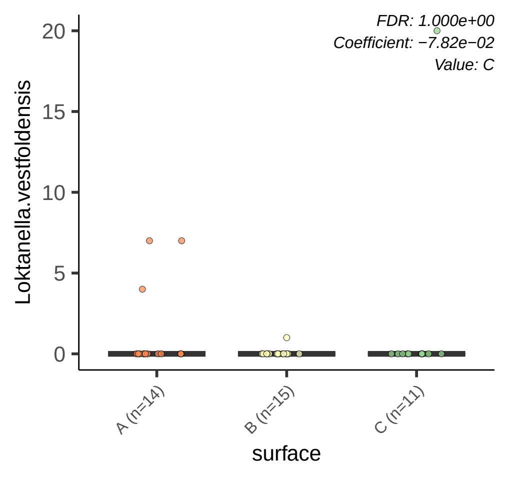
FDR: 1.000e+00
Coefficient: −7.82e−02
Value: C
Loktanella.vestfoldensis
20
15
10
5
0
A (n=14)
B (n=15)
C (n=11)
surface

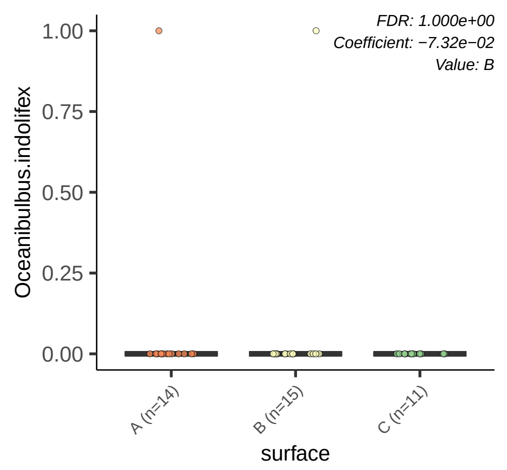
FDR: 1.000e+00
Coefficient: −7.32e−02
Value: B
Oceanibulbus.indolifex
1.00
0.75
0.50
0.25
0.00
A (n=14)
B (n=15)
C (n=11)
surface

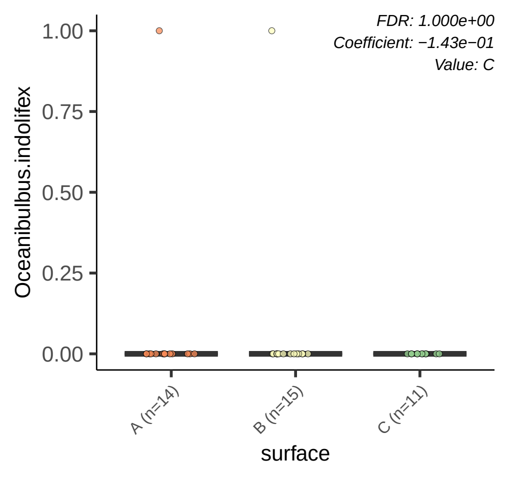
FDR: 1.000e+00
Coefficient: −1.43e−01
Value: C
Oceanibulbus.indolifex
1.00
0.75
0.50
0.25
0.00
A (n=14)
B (n=15)
C (n=11)
surface

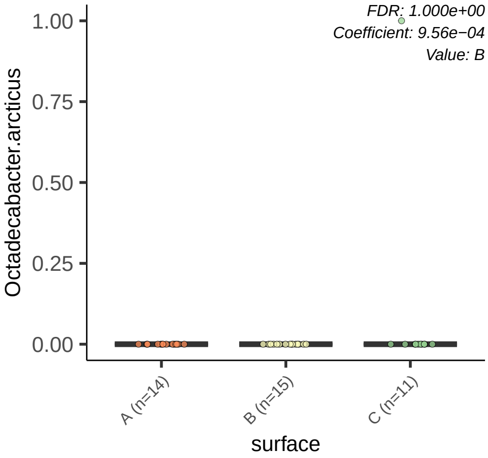
FDR: 1.000e+00
Coefficient: 9.56e−04
Value: B
Octadecabacter.arcticus
1.00
0.75
0.50
0.25
0.00
A (n=14)
B (n=15)
C (n=11)
surface

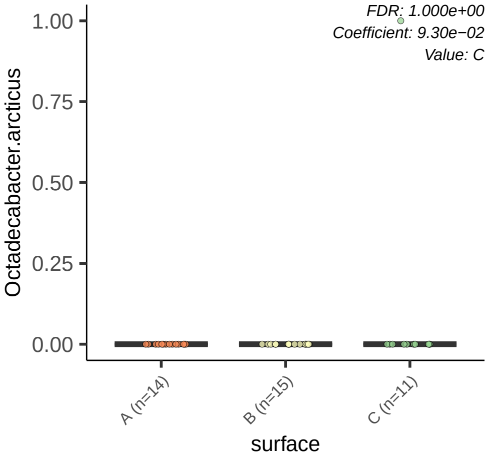
FDR: 1.000e+00
Coefficient: 9.30e−02
Value: C
Octadecabacter.arcticus
1.00
0.75
0.50
0.25
0.00
A (n=14)
B (n=15)
C (n=11)
surface

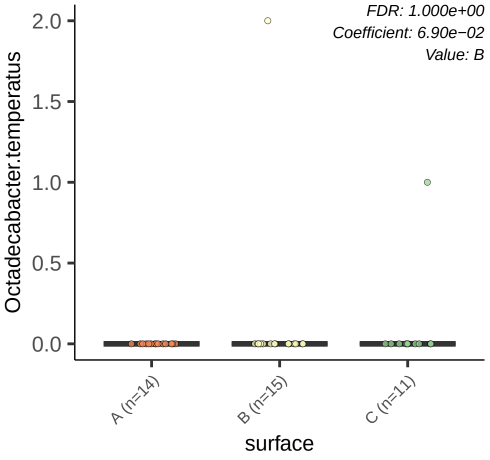
FDR: 1.000e+00
Coefficient: 6.90e−02
Value: B
Octadecabacter.temperatus
2.0
1.5
1.0
0.5
0.0
A (n=14)
B (n=15)
C (n=11)
surface

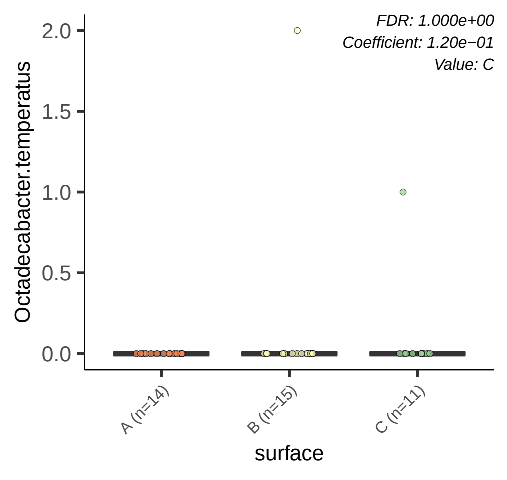

FDR: 1.000e+00
Coefficient: 1.20e−01
Value: C
Octadecabacter.temperatus
2.0
1.5
1.0
0.5
0.0
A (n=14)
B (n=15)
C (n=11)
surface

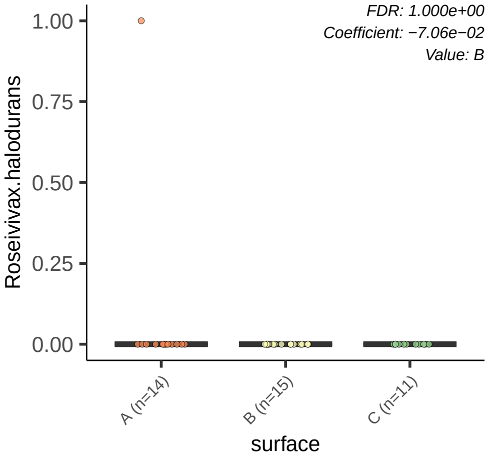
FDR: 1.000e+00
Coefficient: −7.06e−02
Value: B
Roseivivax.halodurans
1.00
0.75
0.50
0.25
0.00
A (n=14)
B (n=15)
C (n=11)
surface

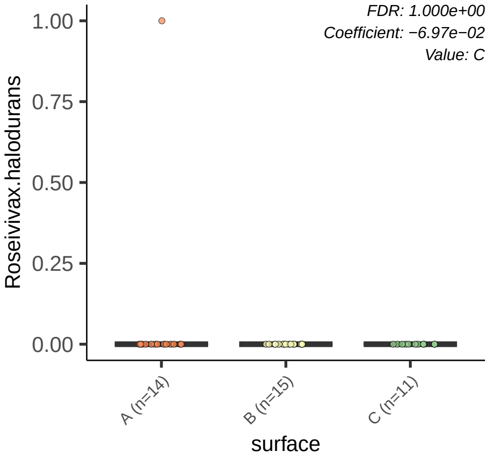

FDR: 1.000e+00
Coefficient: −6.97e−02
Value: C
Roseivivax.halodurans
1.00
0.75
0.50
0.25
0.00
A (n=14)
B (n=15)
C (n=11)
surface

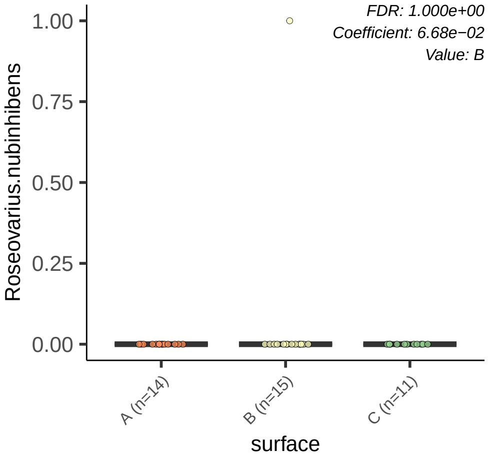
FDR: 1.000e+00
Coefficient: 6.68e−02
Value: B
Roseovarius.nubinhibens
1.00
0.75
0.50
0.25
0.00
A (n=14)
B (n=15)
C (n=11)
surface

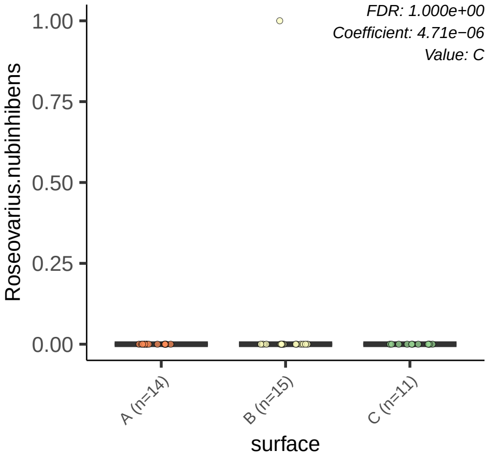
FDR: 1.000e+00
Coefficient: 4.71e−06
Value: C
Roseovarius.nubinhibens
1.00
0.75
0.50
0.25
0.00
A (n=14)
B (n=15)
C (n=11)
surface

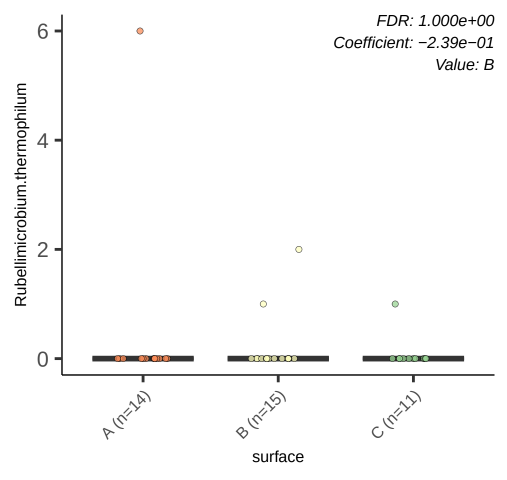

FDR: 1.000e+00
Coefficient: −2.39e−01
Value: B
Rubellimicrobium.thermophilum
6
4
2
0
A (n=14)
B (n=15)
C (n=11)
surface

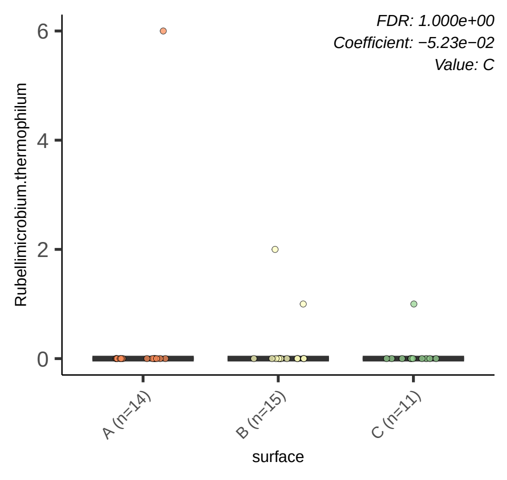

FDR: 1.000e+00
Coefficient: −5.23e−02
Value: C
Rubellimicrobium.thermophilum
6
4
2
0
A (n=14)
B (n=15)
C (n=11)
surface

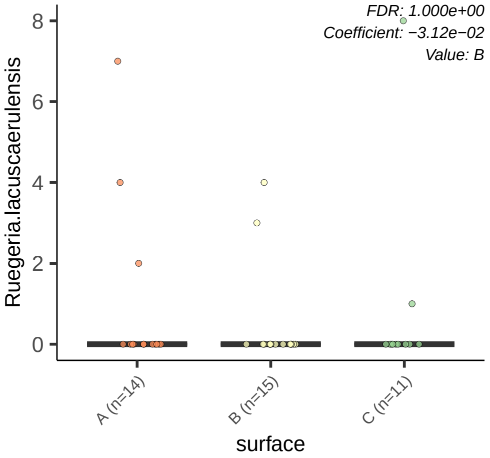

FDR: 1.000e+00
Coefficient: −3.12e−02
Value: B
Ruegeria.lacuscaerulensis
0
2
4
6
8
A (n=14)
B (n=15)
C (n=11)
surface

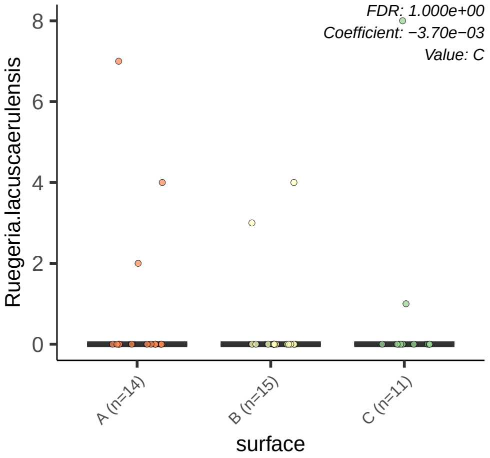

FDR: 1.000e+00
Coefficient: −3.70e−03
Value: C
Ruegeria.lacuscaerulensis
0
2
4
6
8
A (n=14)
B (n=15)
C (n=11)
surface

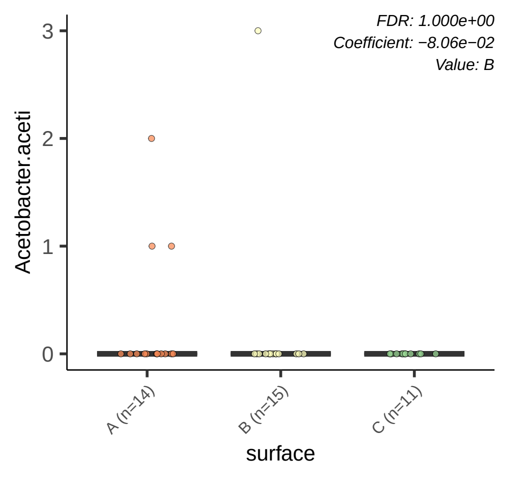
FDR: 1.000e+00
Coefficient: −8.06e−02
Value: B
Acetobacter.aceti
3
2
1
0
A (n=14)
B (n=15)
C (n=11)
surface

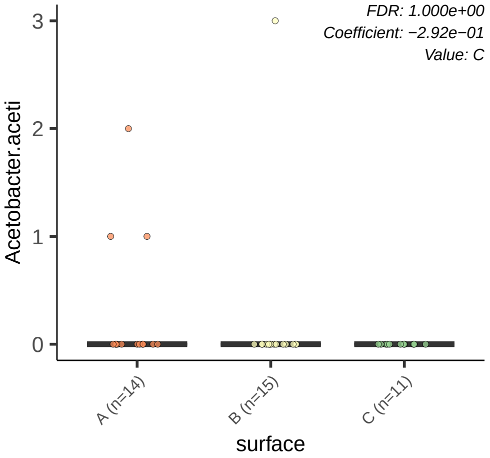
FDR: 1.000e+00
Coefficient: −2.92e−01
Value: C
Acetobacter.aceti
3
2
1
0
A (n=14)
B (n=15)
C (n=11)
surface

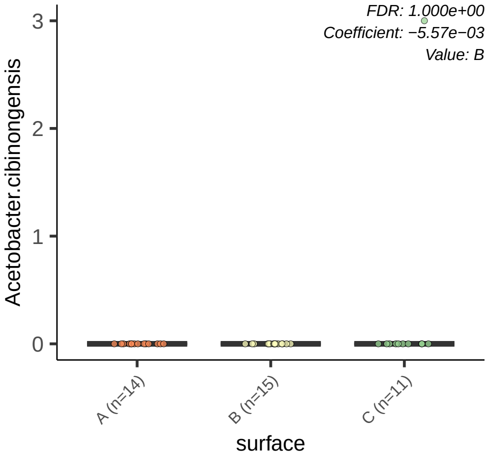
FDR: 1.000e+00
Coefficient: −5.57e−03
Value: B
Acetobacter.cibinongensis
3
2
1
0
A (n=14)
B (n=15)
C (n=11)
surface

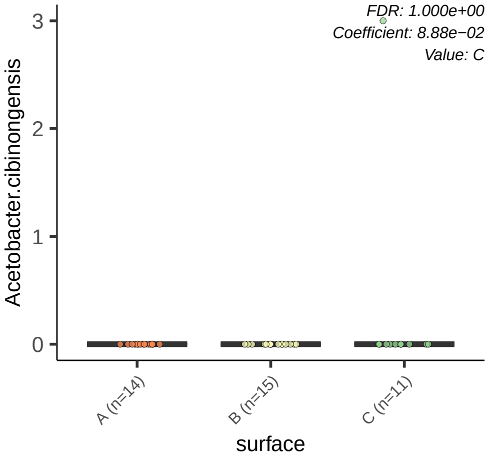
FDR: 1.000e+00
Coefficient: 8.88e−02
Value: C
Acetobacter.cibinongensis
0
1
2
3
A (n=14)
B (n=15)
C (n=11)
surface

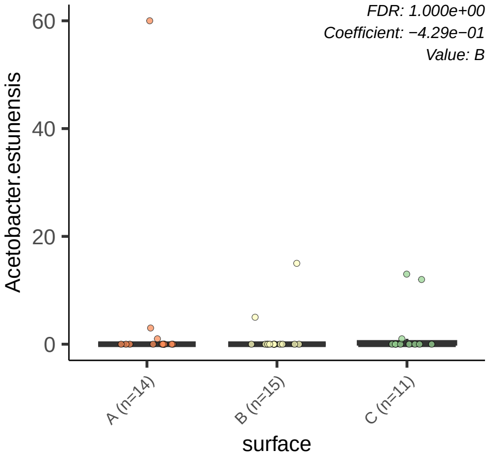
FDR: 1.000e+00
Coefficient: −4.29e−01
Value: B
Acetobacter.estunensis
60
40
20
0
A (n=14)
B (n=15)
C (n=11)
surface

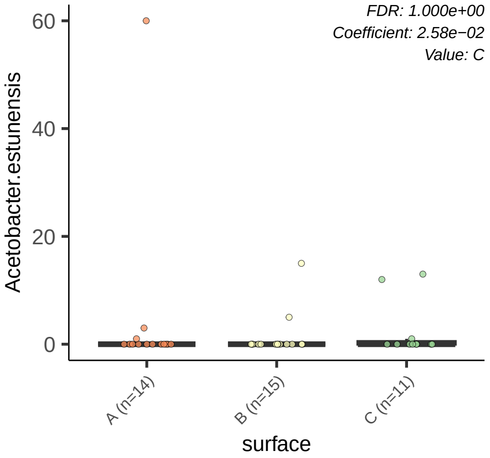
FDR: 1.000e+00
Coefficient: 2.58e−02
Value: C
60
40
20
0
Acetobacter.estunensis
A (n=14)
B (n=15)
C (n=11)
surface

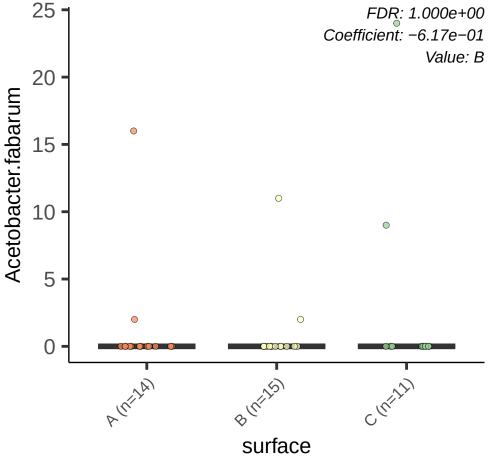
FDR: 1.000e+00
Coefficient: −6.17e−01
Value: B
Acetobacter.fabarum
25
20
15
10
5
0
A (n=14)
B (n=15)
C (n=11)
surface

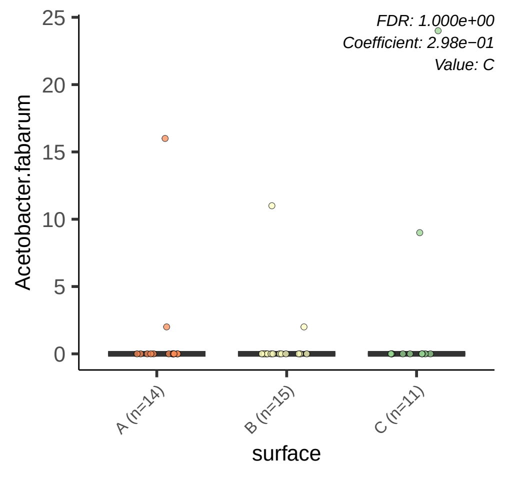

FDR: 1.000e+00
Coefficient: 2.98e−01
Value: C
Acetobacter.fabarum
25
20
15
10
5
0
A (n=14)
B (n=15)
C (n=11)
surface

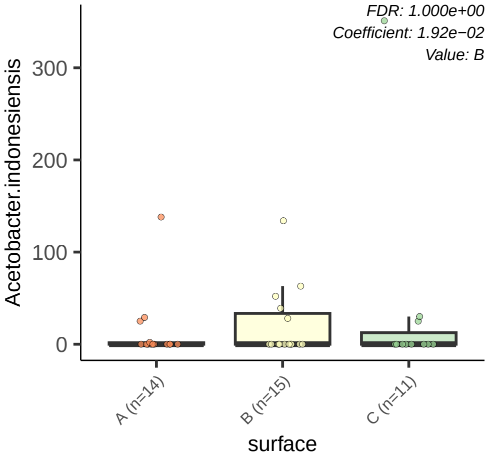

FDR: 1.000e+00
Coefficient: 1.92e−02
Value: B
Acetobacter.indonesiensis
300
200
100
0
A (n=14)
B (n=15)
C (n=11)
surface

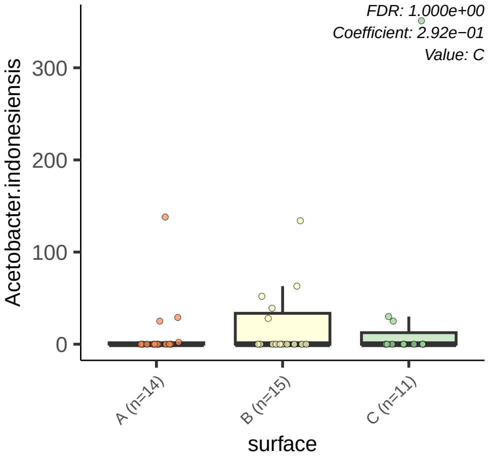
FDR: 1.000e+00
Coefficient: 2.92e−01
Value: C
Acetobacter.indonesiensis
300
200
100
0
A (n=14)
B (n=15)
C (n=11)
surface

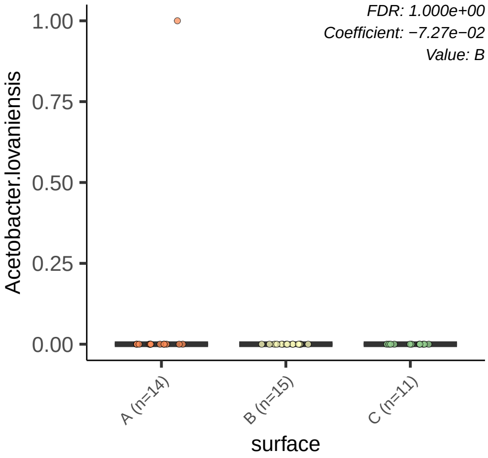

FDR: 1.000e+00
Coefficient: −7.27e−02
Value: B
Acetobacter.lovaniensis
1.00
0.75
0.50
0.25
0.00
A (n=14)
B (n=15)
C (n=11)
surface

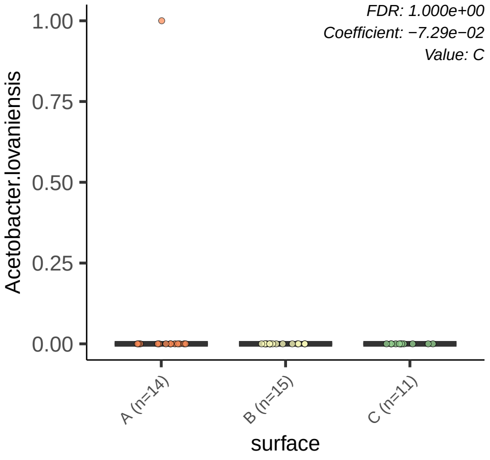
FDR: 1.000e+00
Coefficient: −7.29e−02
Value: C
Acetobacter.lovaniensis
1.00
0.75
0.50
0.25
0.00
A (n=14)
B (n=15)
C (n=11)
surface

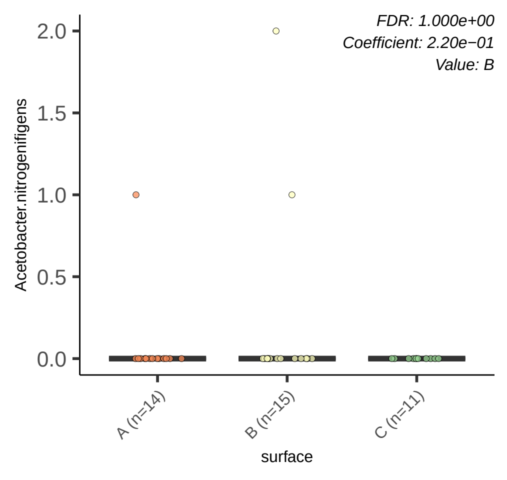
FDR: 1.000e+00
Coefficient: 2.20e−01
Value: B
Acetobacter.nitrogenifigens
2.0
1.5
1.0
0.5
0.0
A (n=14)
B (n=15)
C (n=11)
surface

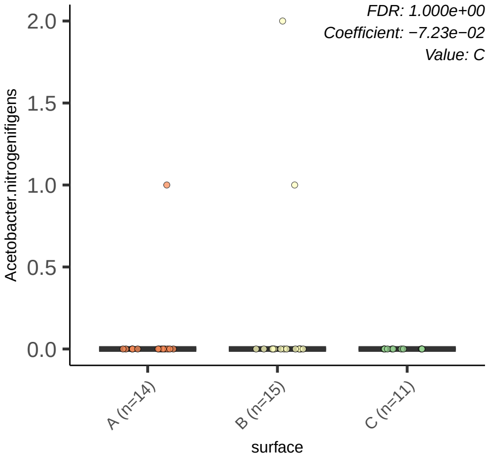
FDR: 1.000e+00
Coefficient: −7.23e−02
Value: C
Acetobacter.nitrogenifigens
2.0
1.5
1.0
0.5
0.0
A (n=14)
B (n=15)
C (n=11)
surface

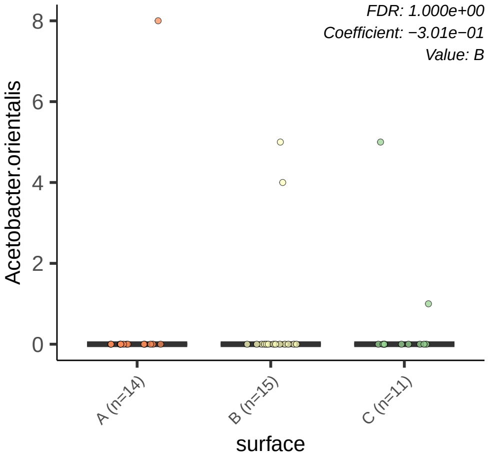

FDR: 1.000e+00
Coefficient: −3.01e−01
Value: B
Acetobacter.orientalis
8
6
4
2
0
A (n=14)
B (n=15)
C (n=11)
surface

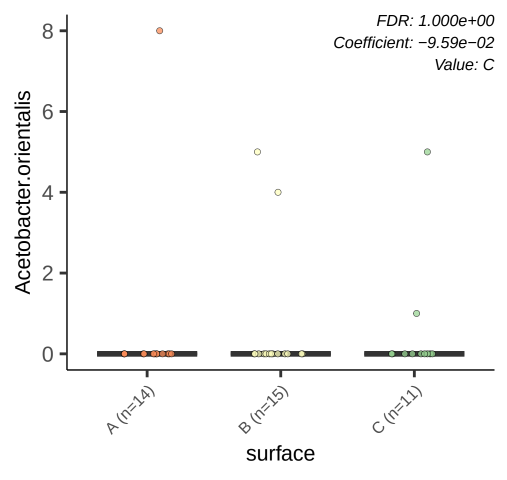

FDR: 1.000e+00
Coefficient: −9.59e−02
Value: C
Acetobacter.orientalis
0
2
4
6
8
A (n=14)
B (n=15)
C (n=11)
surface

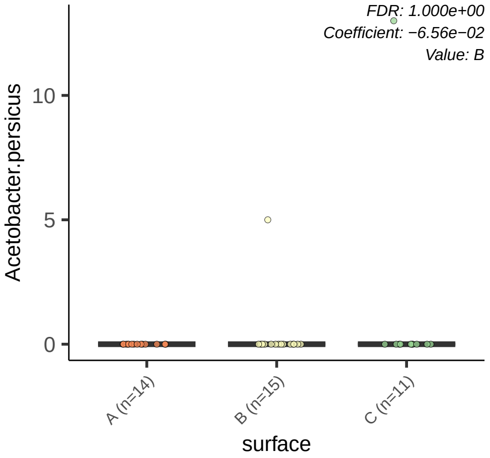
FDR: 1.000e+00
Coefficient: −6.56e−02
Value: B
Acetobacter.persicus
10
5
0
A (n=14)
B (n=15)
C (n=11)
surface

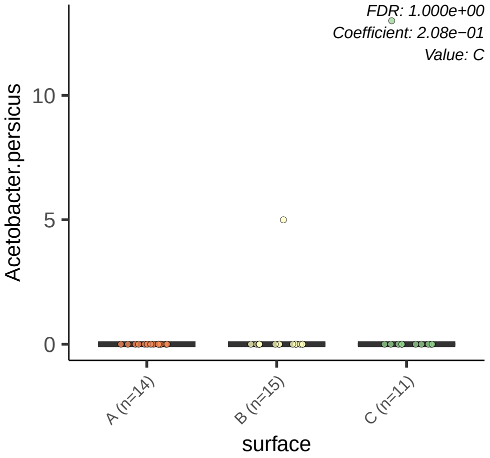

FDR: 1.000e+00
Coefficient: 2.08e−01
Value: C
Acetobacter.persicus
0
5
10
A (n=14)
B (n=15)
C (n=11)
surface

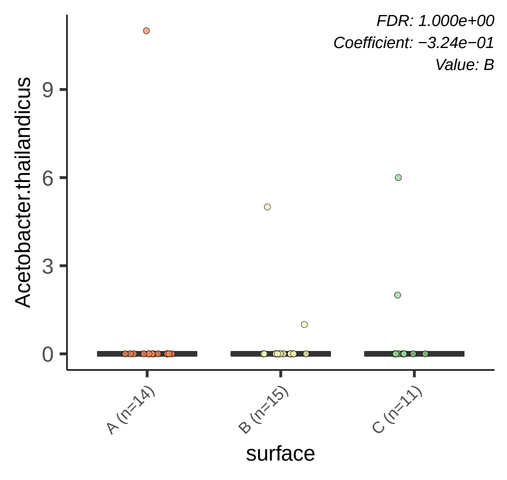

FDR: 1.000e+00
Coefficient: −3.24e−01
Value: B
Acetobacter.thailandicus
9
6
3
0
A (n=14)
B (n=15)
C (n=11)
surface

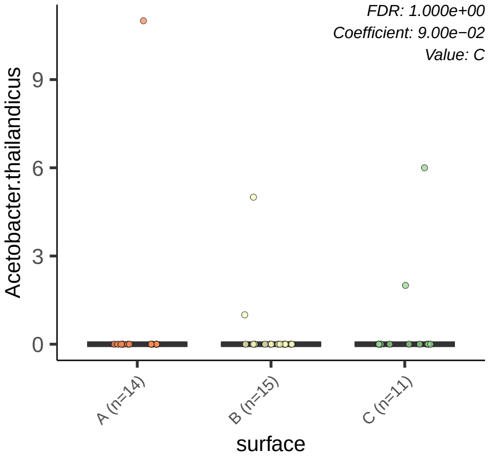
FDR: 1.000e+00
Coefficient: 9.00e−02
Value: C
Acetobacter.thailandicus
0
3
6
9
A (n=14)
B (n=15)
C (n=11)
surface

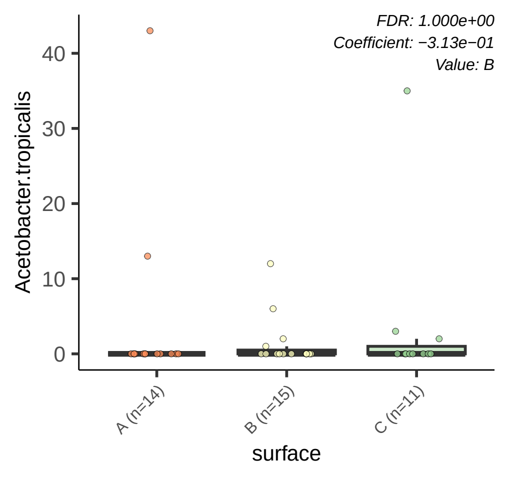
FDR: 1.000e+00
Coefficient: −3.13e−01
Value: B
Acetobacter.tropicalis
0
10
20
30
40
A (n=14)
B (n=15)
C (n=11)
surface

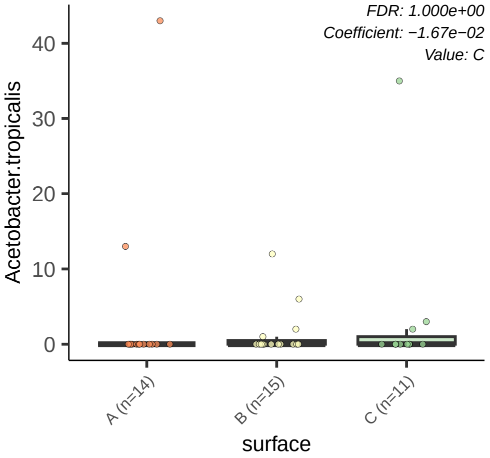
FDR: 1.000e+00
Coefficient: −1.67e−02
Value: C
Acetobacter.tropicalis
40
30
20
10
0
A (n=14)
B (n=15)
C (n=11)
surface

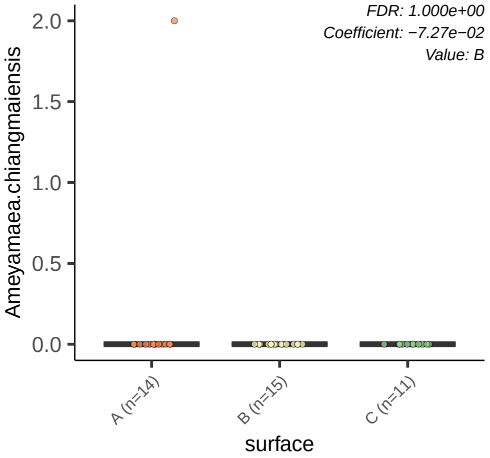
FDR: 1.000e+00
Coefficient: −7.27e−02
Value: B
Ameyamaea.chiangmaiensis
2.0
1.5
1.0
0.5
0.0
A (n=14)
B (n=15)
C (n=11)
surface

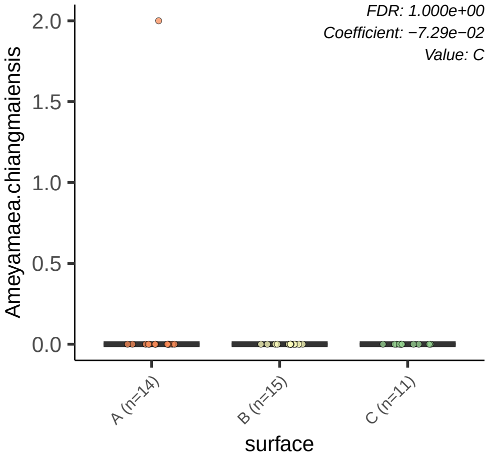
FDR: 1.000e+00
Coefficient: −7.29e−02
Value: C
Ameyamaea.chiangmaiensis
2.0
1.5
1.0
0.5
0.0
A (n=14)
B (n=15)
C (n=11)
surface

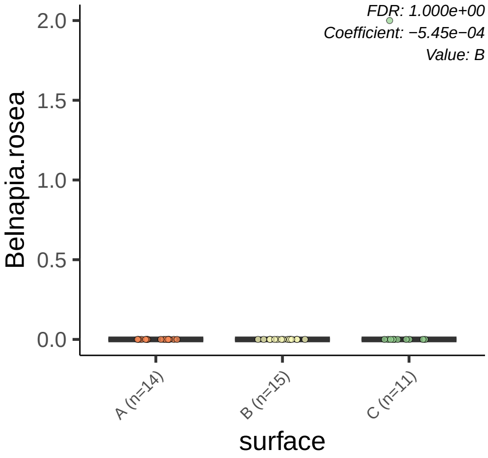

FDR: 1.000e+00
Coefficient: −5.45e−04
Value: B
Belnapia.rosea
2.0
1.5
1.0
0.5
0.0
A (n=14)
B (n=15)
C (n=11)
surface

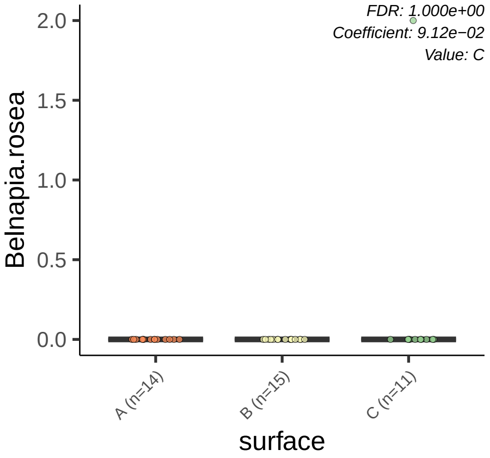
FDR: 1.000e+00
Coefficient: 9.12e−02
Value: C
Belnapia.rosea
2.0
1.5
1.0
0.5
0.0
A (n=14)
B (n=15)
C (n=11)
surface

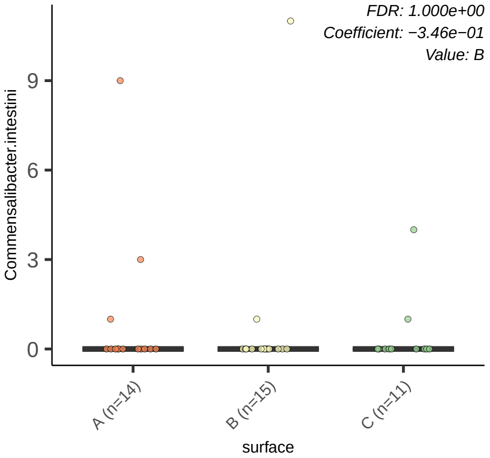
FDR: 1.000e+00
Coefficient: −3.46e−01
Value: B
Commensalibacter.intestini
9
6
3
0
A (n=14)
B (n=15)
C (n=11)
surface

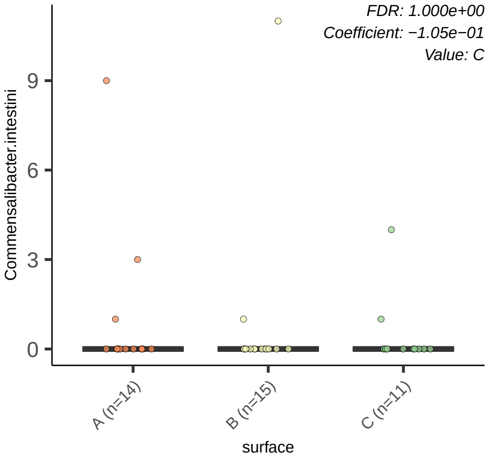
FDR: 1.000e+00
Coefficient: −1.05e−01
Value: C
Commensalibacter.intestini
0
3
6
9
A (n=14)
B (n=15)
C (n=11)
surface

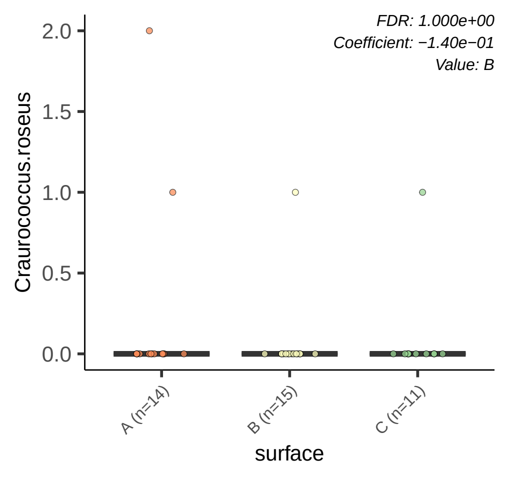
FDR: 1.000e+00
Coefficient: −1.40e−01
Value: B
Craurococcus.roseus
2.0
1.5
1.0
0.5
0.0
A (n=14)
B (n=15)
C (n=11)
surface

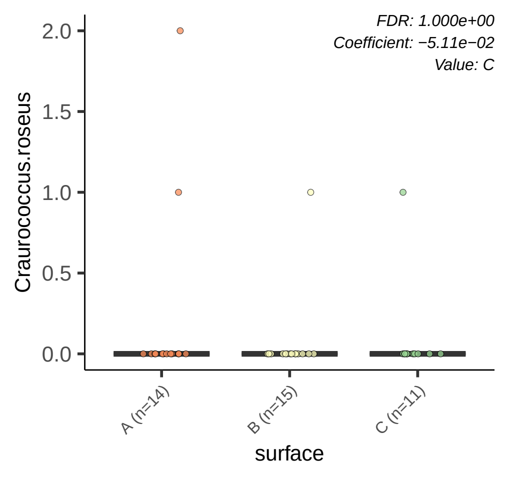
FDR: 1.000e+00
Coefficient: −5.11e−02
Value: C
Craurococcus.roseus
2.0
1.5
1.0
0.5
0.0
A (n=14)
B (n=15)
C (n=11)
surface

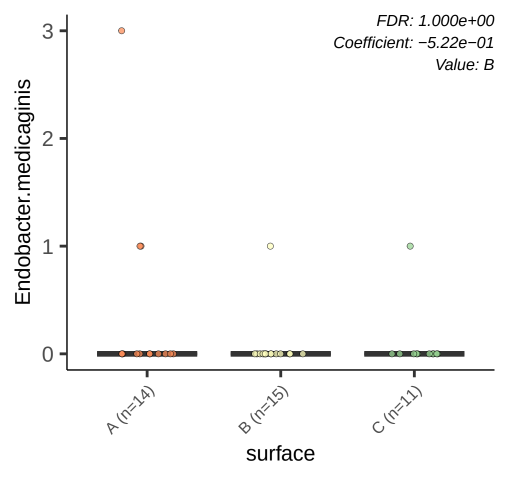

FDR: 1.000e+00
Coefficient: −5.22e−01
Value: B
Endobacter.medicaginis
0
1
2
3
A (n=14)
B (n=15)
C (n=11)
surface

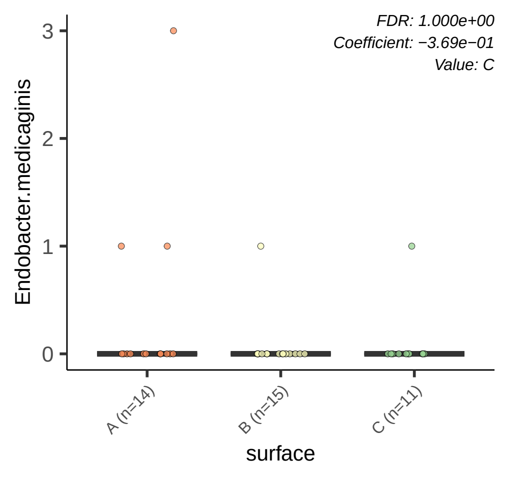

FDR: 1.000e+00
Coefficient: −3.69e−01
Value: C
Endobacter.medicaginis
3
2
1
0
A (n=14)
B (n=15)
C (n=11)
surface

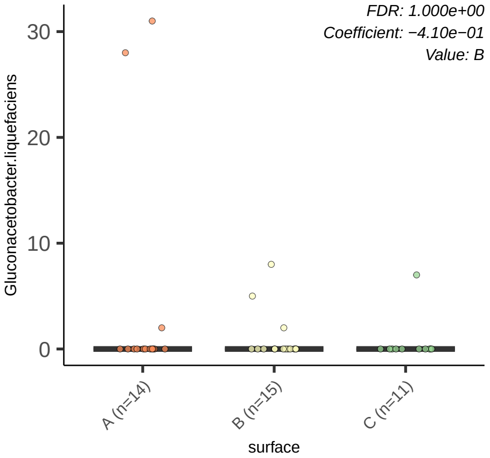
FDR: 1.000e+00
Coefficient: −4.10e−01
Value: B
Gluconacetobacter.liquefaciens
30
20
10
0
A (n=14)
B (n=15)
C (n=11)
surface

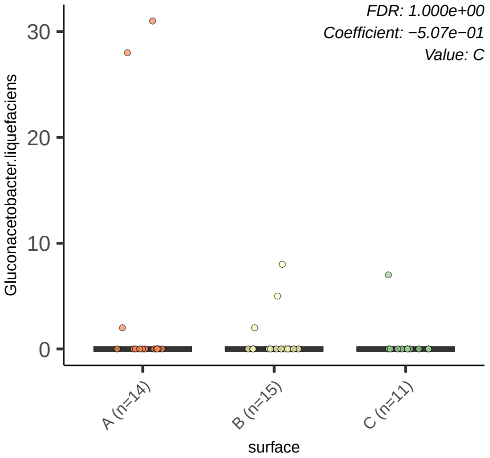

FDR: 1.000e+00
Coefficient: −5.07e−01
Value: C
Gluconacetobacter.liquefaciens
30
20
10
0
A (n=14)
B (n=15)
C (n=11)
surface

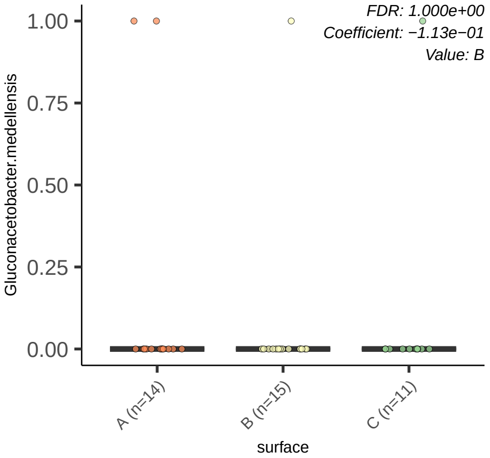

FDR: 1.000e+00
Coefficient: −1.13e−01
Value: B
Gluconacetobacter.medellensis
1.00
0.75
0.50
0.25
0.00
A (n=14)
B (n=15)
C (n=11)
surface

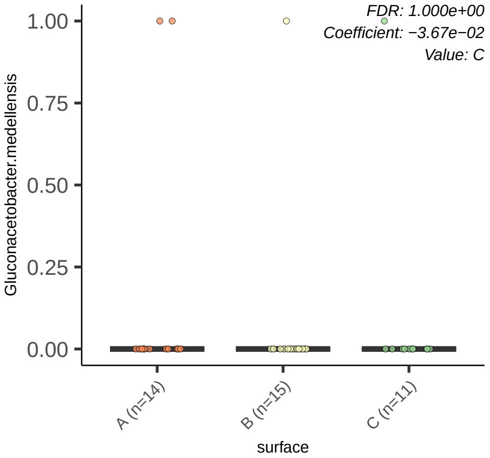
FDR: 1.000e+00
Coefficient: −3.67e−02
Value: C
Gluconacetobacter.medellensis
1.00
0.75
0.50
0.25
0.00
A (n=14)
B (n=15)
C (n=11)
surface

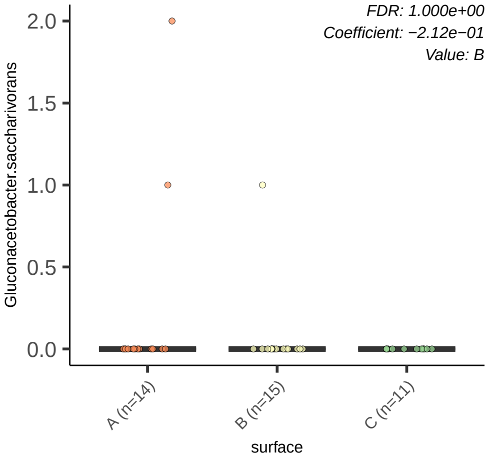
FDR: 1.000e+00
Coefficient: −2.12e−01
Value: B
Gluconacetobacter.saccharivorans
2.0
1.5
1.0
0.5
0.0
A (n=14)
B (n=15)
C (n=11)
surface

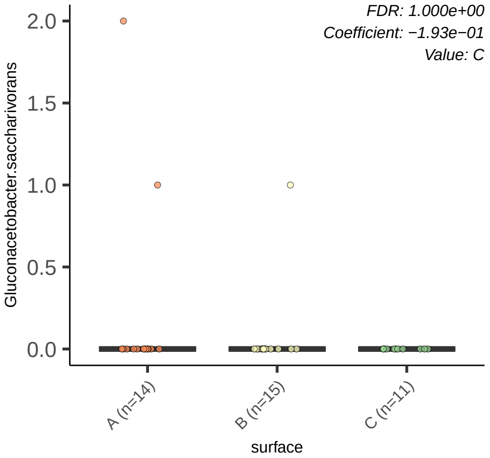
FDR: 1.000e+00
Coefficient: −1.93e−01
Value: C
Gluconacetobacter.saccharivorans
2.0
1.5
1.0
0.5
0.0
A (n=14)
B (n=15)
C (n=11)
surface

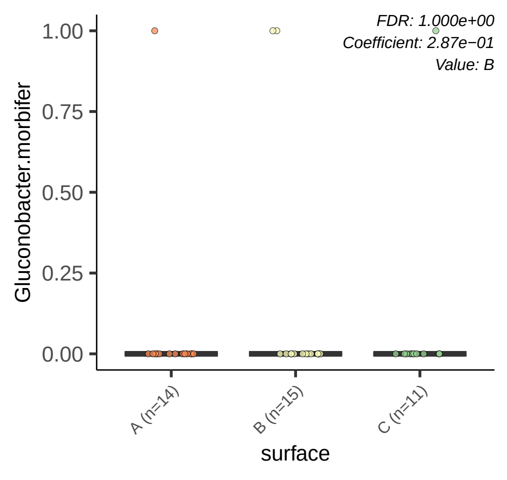

FDR: 1.000e+00
Coefficient: 2.87e−01
Value: B
Gluconobacter.morbifer
1.00
0.75
0.50
0.25
0.00
A (n=14)
B (n=15)
C (n=11)
surface

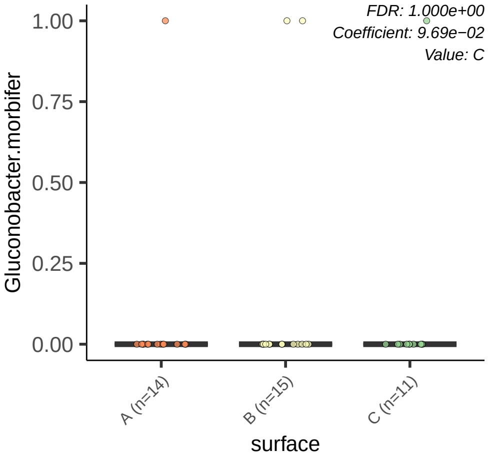
FDR: 1.000e+00
Coefficient: 9.69e−02
Value: C
Gluconobacter.morbifer
1.00
0.75
0.50
0.25
0.00
A (n=14)
B (n=15)
C (n=11)
surface

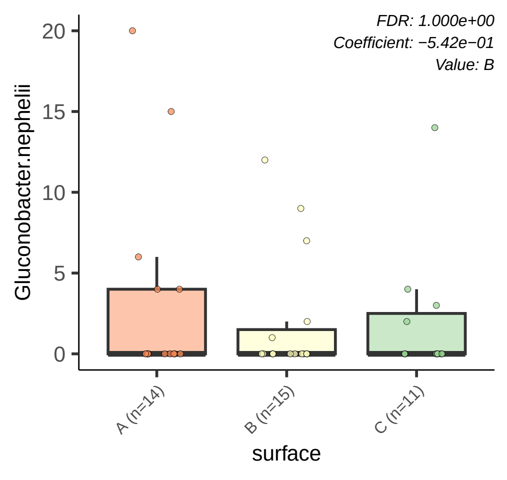

FDR: 1.000e+00
Coefficient: −5.42e−01
Value: B
Gluconobacter.nephelii
20
15
10
5
0
A (n=14)
B (n=15)
C (n=11)
surface

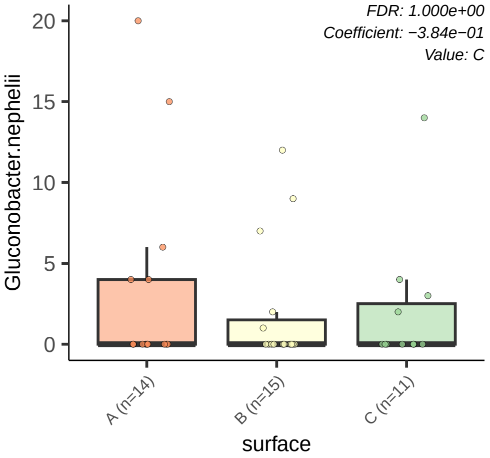
FDR: 1.000e+00
Coefficient: −3.84e−01
Value: C
Gluconobacter.nephelii
20
15
10
5
0
A (n=14)
B (n=15)
C (n=11)
surface

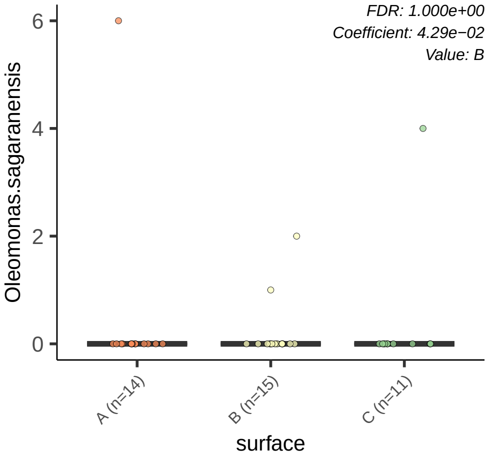

FDR: 1.000e+00
Coefficient: 4.29e−02
Value: B
Oleomonas.sagaranensis
0
2
4
6
A (n=14)
B (n=15)
C (n=11)
surface

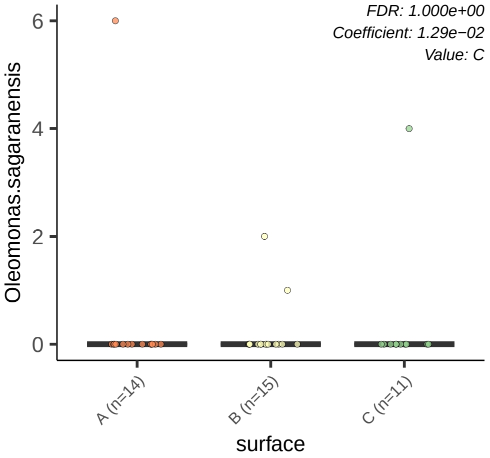

FDR: 1.000e+00
Coefficient: 1.29e−02
Value: C
Oleomonas.sagaranensis
6
4
2
0
A (n=14)
B (n=15)
C (n=11)
surface

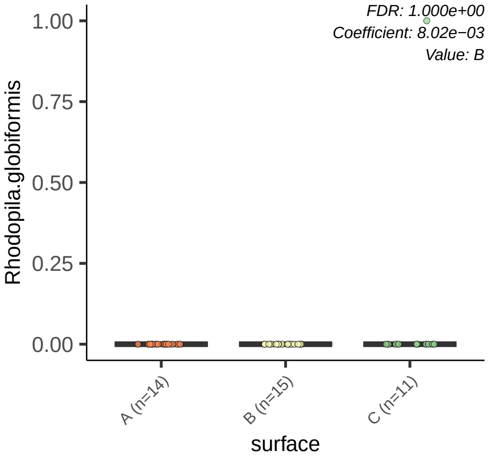

FDR: 1.000e+00
Coefficient: 8.02e−03
Value: B
Rhodopila.globiformis
1.00
0.75
0.50
0.25
0.00
A (n=14)
B (n=15)
C (n=11)
surface

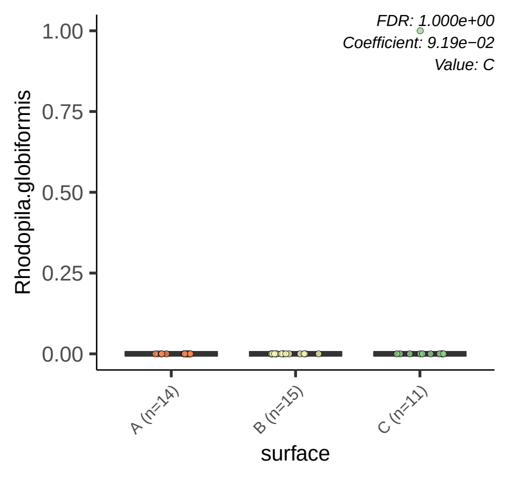
FDR: 1.000e+00
Coefficient: 9.19e−02
Value: C
Rhodopila.globiformis
1.00
0.75
0.50
0.25
0.00
A (n=14)
B (n=15)
C (n=11)
surface

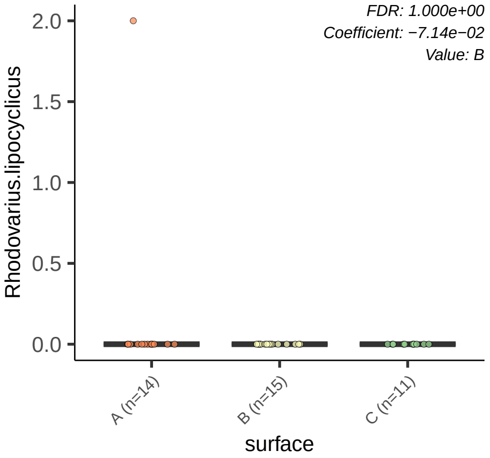
FDR: 1.000e+00
Coefficient: −7.14e−02
Value: B
Rhodovarius.lipocyclicus
2.0
1.5
1.0
0.5
0.0
A (n=14)
B (n=15)
C (n=11)
surface

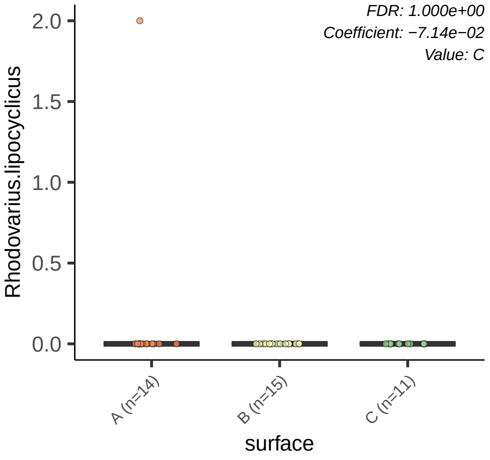
FDR: 1.000e+00
Coefficient: −7.14e−02
Value: C
Rhodovarius.lipocyclicus
2.0
1.5
1.0
0.5
0.0
A (n=14)
B (n=15)
C (n=11)
surface

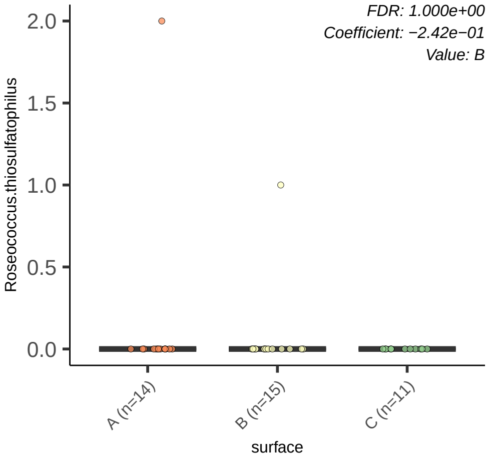
FDR: 1.000e+00
Coefficient: −2.42e−01
Value: B
Roseococcus.thiosulfatophilus
2.0
1.5
1.0
0.5
0.0
A (n=14)
B (n=15)
C (n=11)
surface

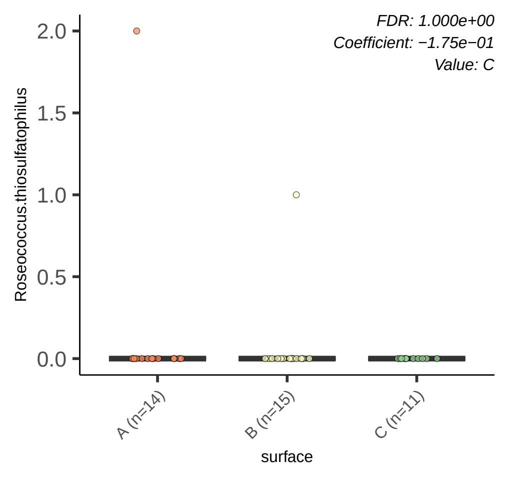

FDR: 1.000e+00
Coefficient: −1.75e−01
Value: C
Roseococcus.thiosulfatophilus
2.0
1.5
1.0
0.5
0.0
A (n=14)
B (n=15)
C (n=11)
surface

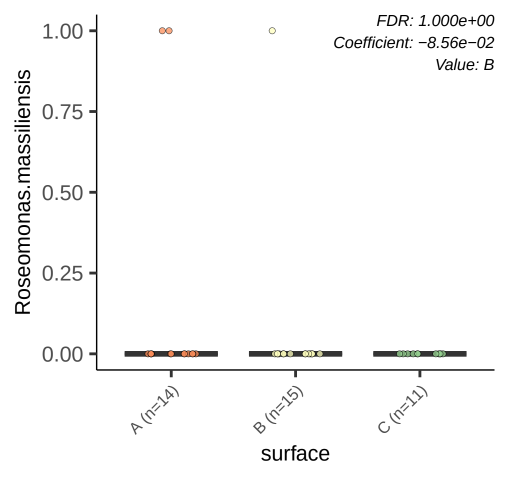
FDR: 1.000e+00
Coefficient: −8.56e−02
Value: B
Roseomonas.massiliensis
1.00
0.75
0.50
0.25
0.00
A (n=14)
B (n=15)
C (n=11)
surface

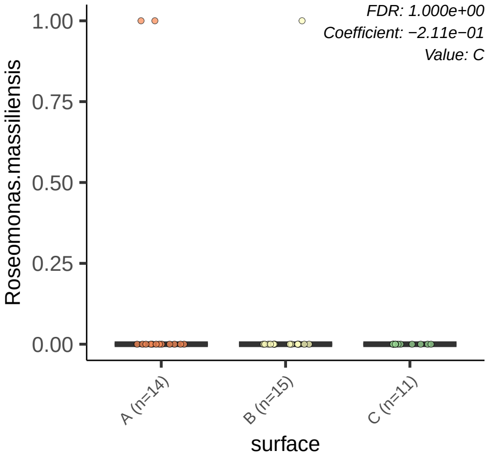

FDR: 1.000e+00
Coefficient: −2.11e−01
Value: C
Roseomonas.massiliensis
1.00
0.75
0.50
0.25
0.00
A (n=14)
B (n=15)
C (n=11)
surface

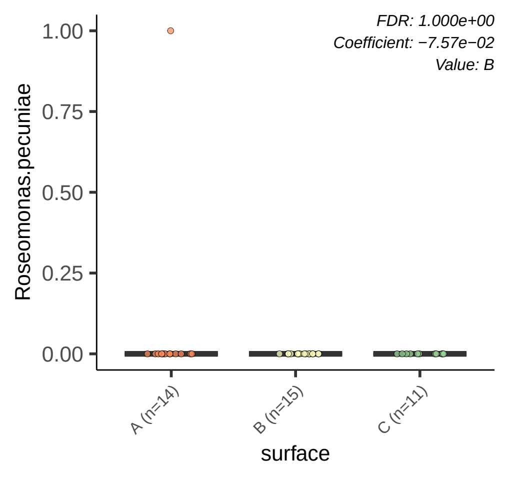
FDR: 1.000e+00
Coefficient: −7.57e−02
Value: B
Roseomonas.pecuniae
1.00
0.75
0.50
0.25
0.00
A (n=14)
B (n=15)
C (n=11)
surface

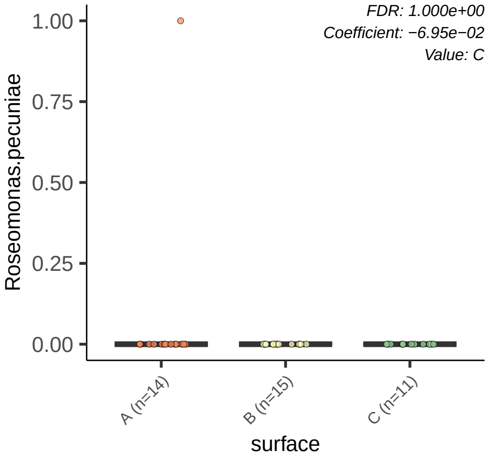

FDR: 1.000e+00
Coefficient: −6.95e−02
Value: C
Roseomonas.pecuniae
1.00
0.75
0.50
0.25
0.00
A (n=14)
B (n=15)
C (n=11)
surface

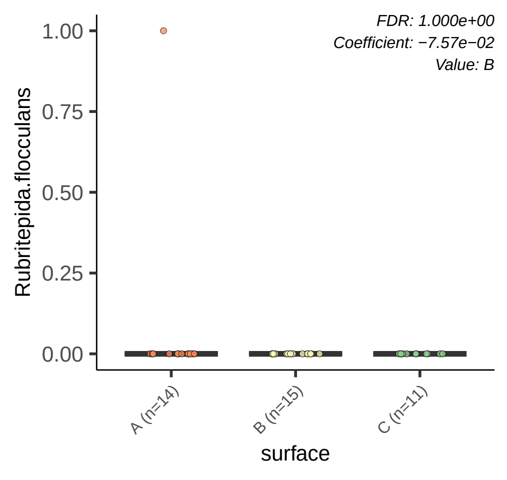
FDR: 1.000e+00
Coefficient: −7.57e−02
Value: B
Rubritepida.flocculans
1.00
0.75
0.50
0.25
0.00
A (n=14)
B (n=15)
C (n=11)
surface

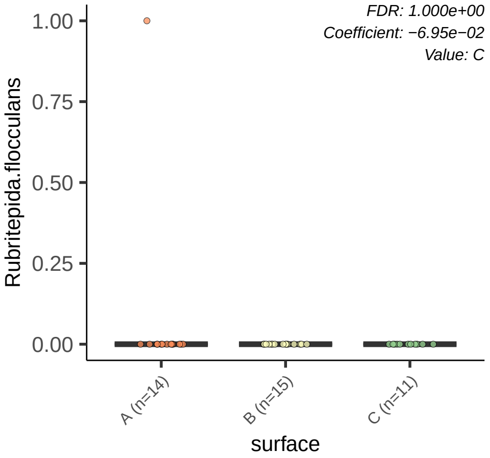
FDR: 1.000e+00
Coefficient: −6.95e−02
Value: C
Rubritepida.flocculans
1.00
0.75
0.50
0.25
0.00
A (n=14)
B (n=15)
C (n=11)
surface

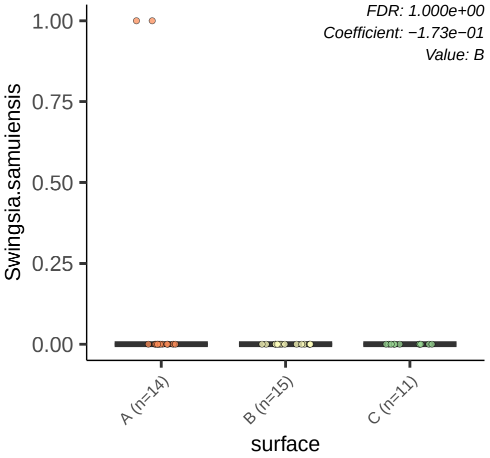
FDR: 1.000e+00
Coefficient: −1.73e−01
Value: B
Swingsia.samuiensis
1.00
0.75
0.50
0.25
0.00
A (n=14)
B (n=15)
C (n=11)
surface

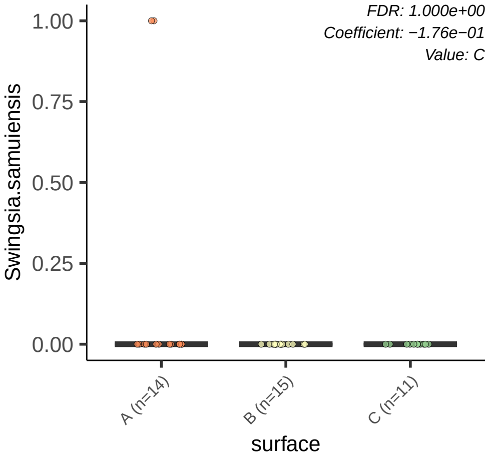
FDR: 1.000e+00
Coefficient: −1.76e−01
Value: C
Swingsia.samuiensis
1.00
0.75
0.50
0.25
0.00
A (n=14)
B (n=15)
C (n=11)
surface

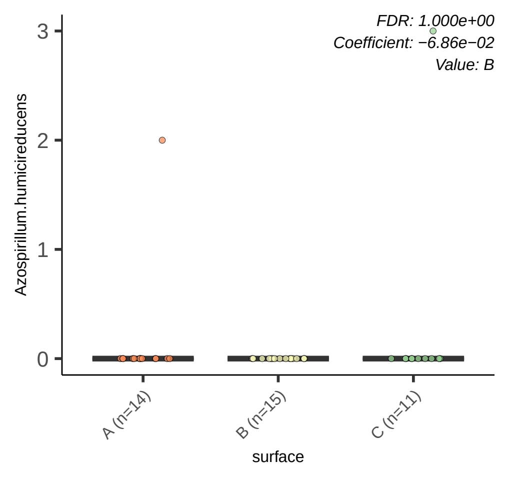
FDR: 1.000e+00
Coefficient: −6.86e−02
Value: B
Azospirillum.humicireducens
3
2
1
0
A (n=14)
B (n=15)
C (n=11)
surface

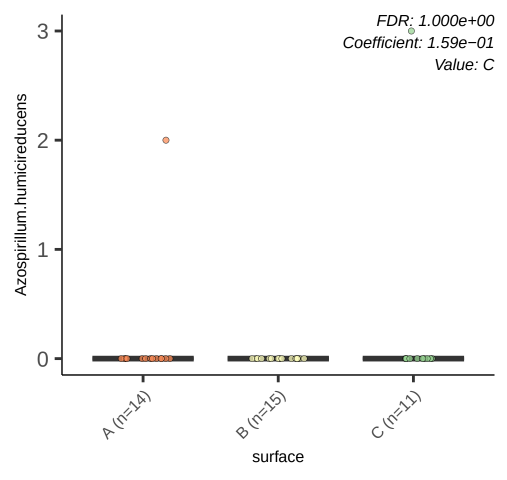

FDR: 1.000e+00
Coefficient: 1.59e−01
Value: C
Azospirillum.humicireducens
3
2
1
0
A (n=14)
B (n=15)
C (n=11)
surface

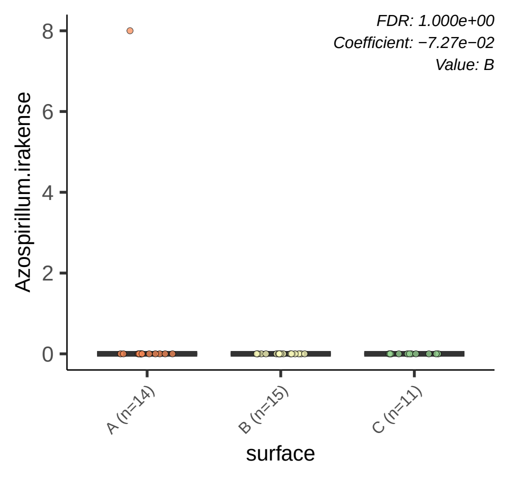
FDR: 1.000e+00
Coefficient: −7.27e−02
Value: B
Azospirillum.irakense
8
6
4
2
0
A (n=14)
B (n=15)
C (n=11)
surface

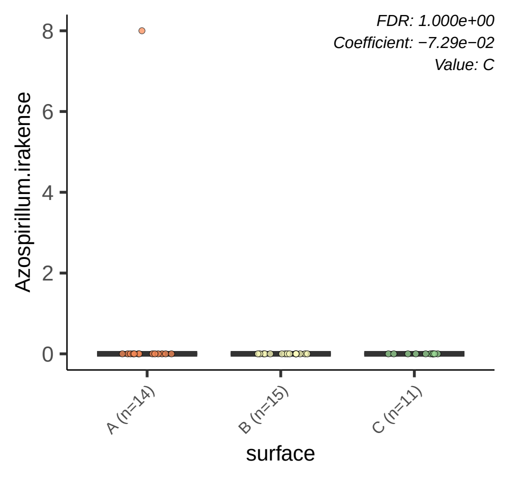

FDR: 1.000e+00
Coefficient: −7.29e−02
Value: C
Azospirillum.irakense
8
6
4
2
0
A (n=14)
B (n=15)
C (n=11)
surface

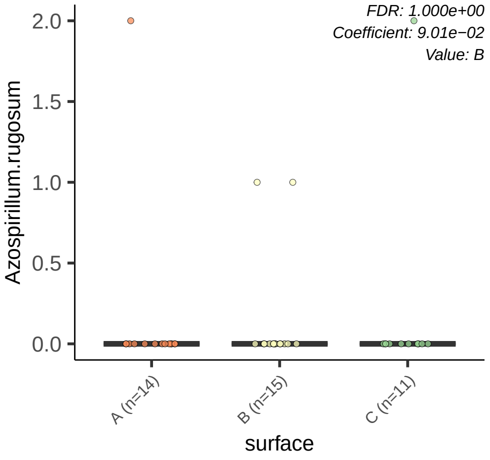

FDR: 1.000e+00
Coefficient: 9.01e−02
Value: B
Azospirillum.rugosum
2.0
1.5
1.0
0.5
0.0
A (n=14)
B (n=15)
C (n=11)
surface

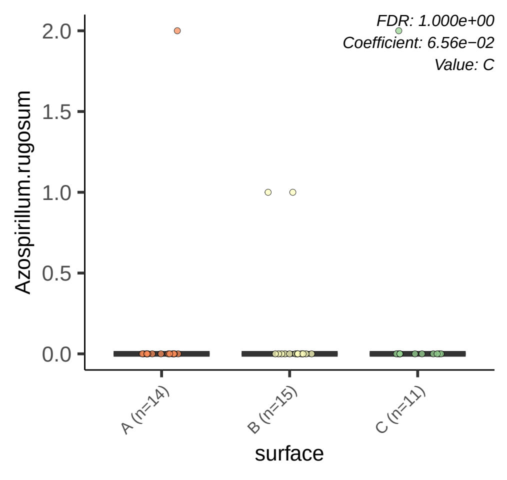

FDR: 1.000e+00
Coefficient: 6.56e−02
Value: C
Azospirillum.rugosum
2.0
1.5
1.0
0.5
0.0
A (n=14)
B (n=15)
C (n=11)
surface

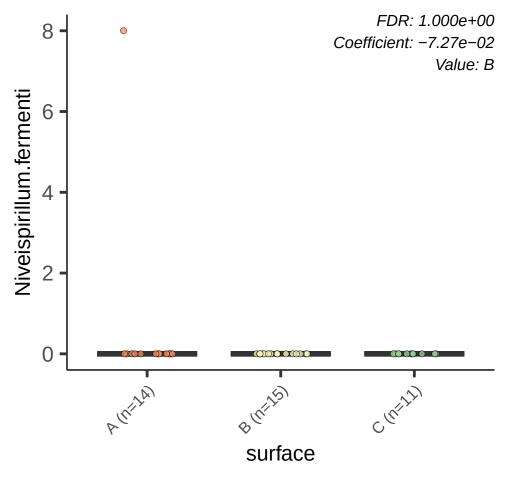

FDR: 1.000e+00
Coefficient: −7.27e−02
Value: B
Niveispirillum.fermenti
0
2
4
6
8
A (n=14)
B (n=15)
C (n=11)
surface

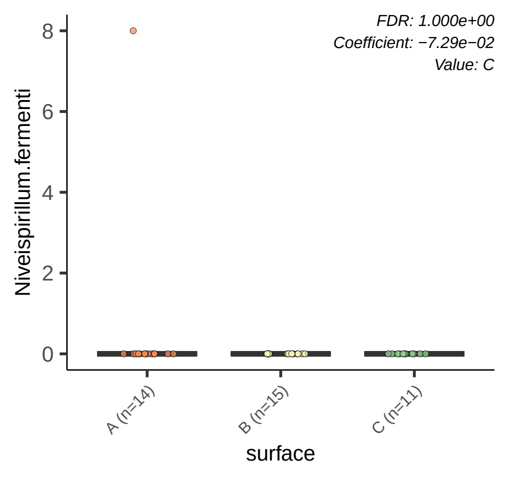

FDR: 1.000e+00
Coefficient: −7.29e−02
Value: C
Niveispirillum.fermenti
8
6
4
2
0
A (n=14)
B (n=15)
C (n=11)
surface

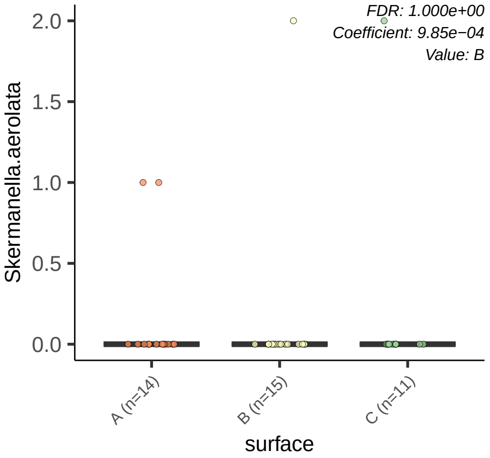

FDR: 1.000e+00
Coefficient: 9.85e−04
Value: B
Skermanella.aerolata
2.0
1.5
1.0
0.5
0.0
A (n=14)
B (n=15)
C (n=11)
surface

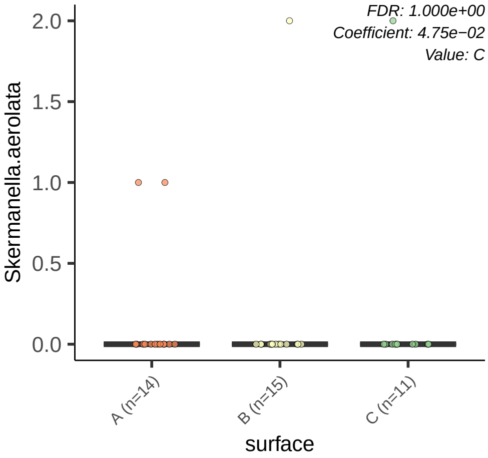
FDR: 1.000e+00
Coefficient: 4.75e−02
Value: C
Skermanella.aerolata
2.0
1.5
1.0
0.5
0.0
A (n=14)
B (n=15)
C (n=11)
surface

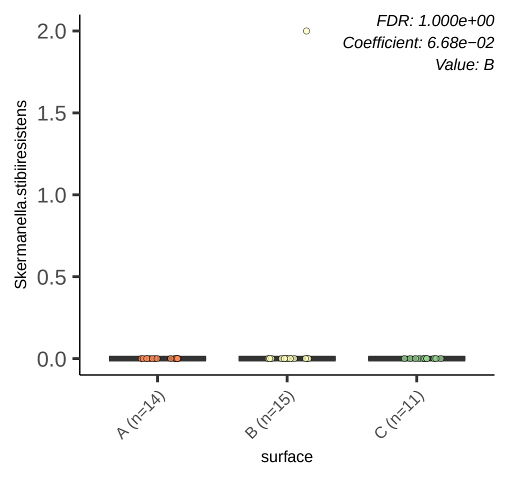

FDR: 1.000e+00
Coefficient: 6.68e−02
Value: B
Skermanella.stibiiresistens
2.0
1.5
1.0
0.5
0.0
A (n=14)
B (n=15)
C (n=11)
surface

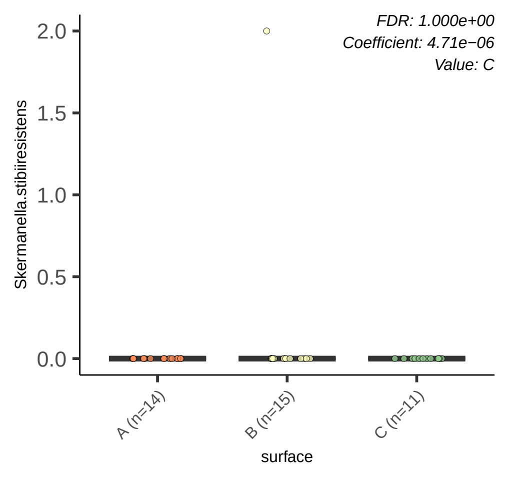
FDR: 1.000e+00
Coefficient: 4.71e−06
Value: C
Skermanella.stibiiresistens
2.0
1.5
1.0
0.5
0.0
A (n=14)
B (n=15)
C (n=11)
surface

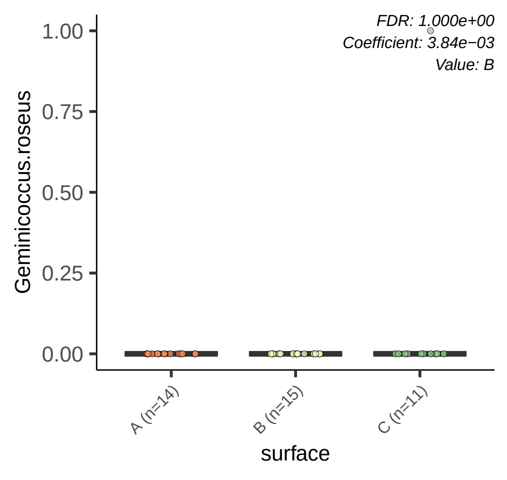
FDR: 1.000e+00
Coefficient: 3.84e−03
Value: B
Geminicoccus.roseus
1.00
0.75
0.50
0.25
0.00
A (n=14)
B (n=15)
C (n=11)
surface

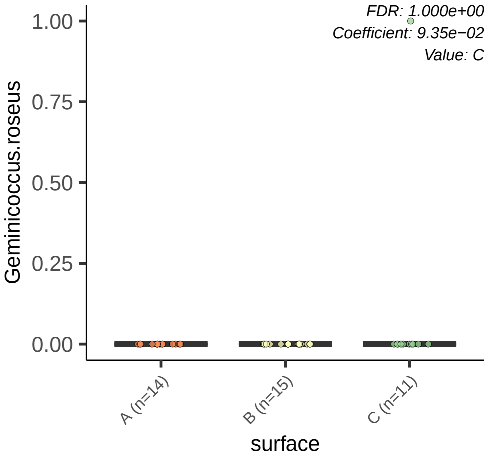

FDR: 1.000e+00
Coefficient: 9.35e−02
Value: C
Geminicoccus.roseus
1.00
0.75
0.50
0.25
0.00
A (n=14)
B (n=15)
C (n=11)
surface

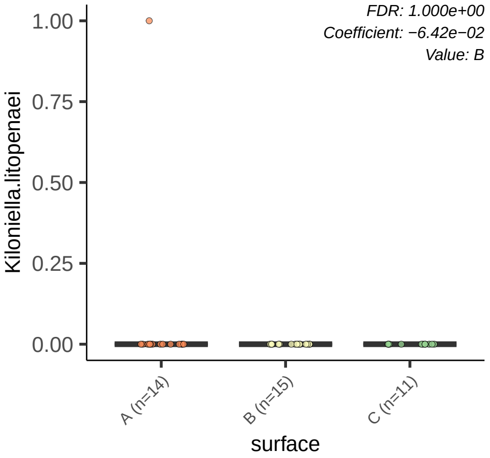
FDR: 1.000e+00
Coefficient: −6.42e−02
Value: B
Kiloniella.litopenaei
1.00
0.75
0.50
0.25
0.00
A (n=14)
B (n=15)
C (n=11)
surface

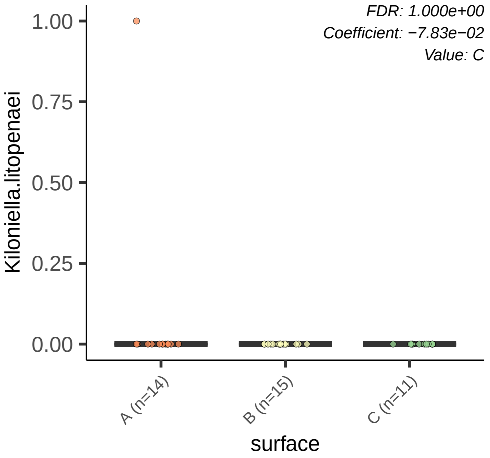
FDR: 1.000e+00
Coefficient: −7.83e−02
Value: C
Kiloniella.litopenaei
1.00
0.75
0.50
0.25
0.00
A (n=14)
B (n=15)
C (n=11)
surface

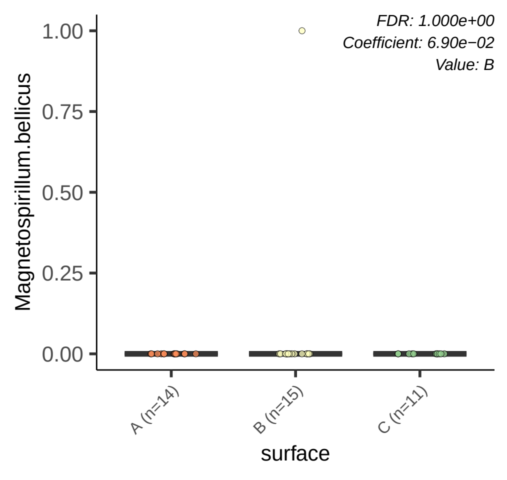
FDR: 1.000e+00
Coefficient: 6.90e−02
Value: B
Magnetospirillum.bellicus
1.00
0.75
0.50
0.25
0.00
A (n=14)
B (n=15)
C (n=11)
surface

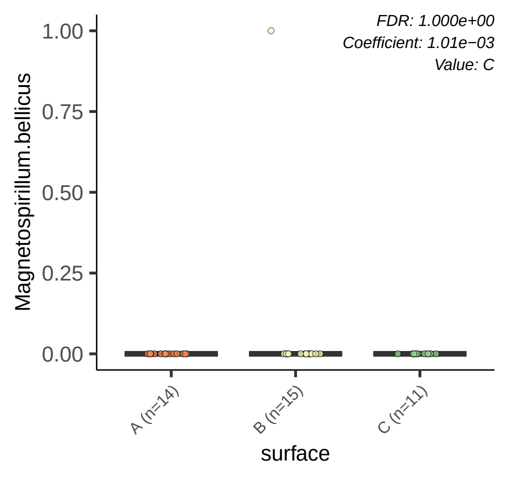
FDR: 1.000e+00
Coefficient: 1.01e−03
Value: C
Magnetospirillum.bellicus
1.00
0.75
0.50
0.25
0.00
A (n=14)
B (n=15)
C (n=11)
surface

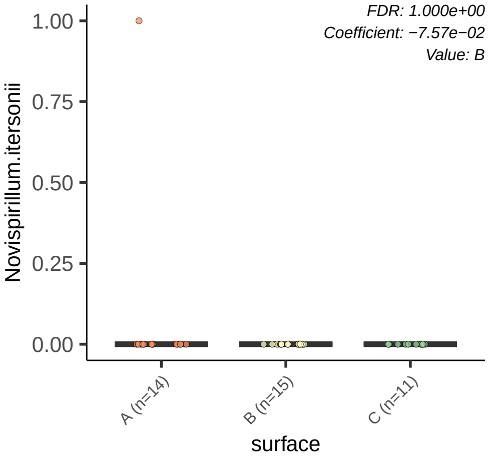
FDR: 1.000e+00
Coefficient: −7.57e−02
Value: B
Novispirillum.itersonii
1.00
0.75
0.50
0.25
0.00
A (n=14)
B (n=15)
C (n=11)
surface

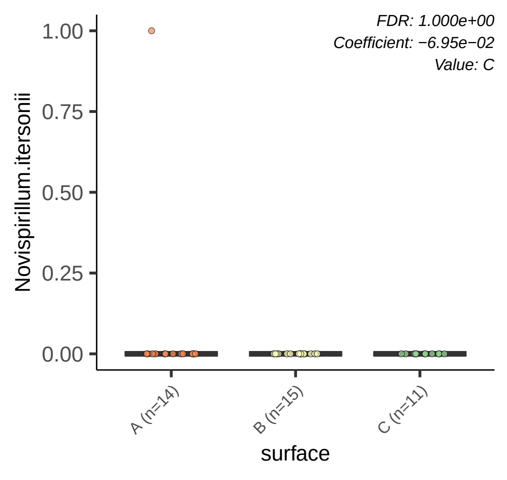
FDR: 1.000e+00
Coefficient: −6.95e−02
Value: C
Novispirillum.itersonii
1.00
0.75
0.50
0.25
0.00
A (n=14)
B (n=15)
C (n=11)
surface

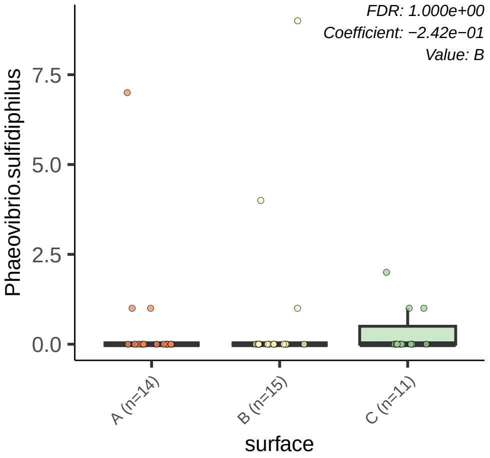
FDR: 1.000e+00
Coefficient: −2.42e−01
Value: B
Phaeovibrio.sulfidiphilus
7.5
5.0
2.5
0.0
A (n=14)
B (n=15)
C (n=11)
surface

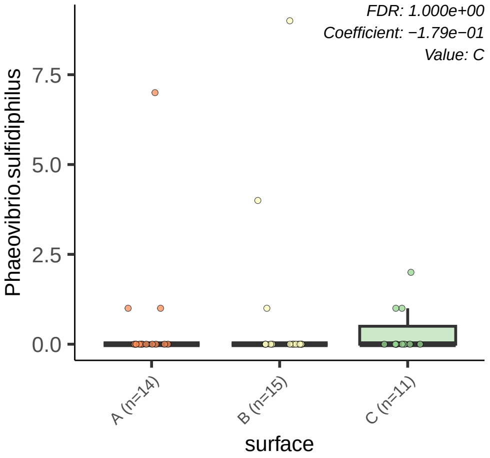

FDR: 1.000e+00
Coefficient: −1.79e−01
Value: C
Phaeovibrio.sulfidiphilus
7.5
5.0
2.5
0.0
A (n=14)
B (n=15)
C (n=11)
surface

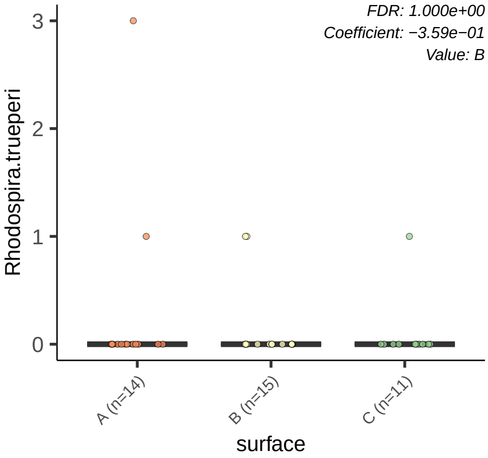
FDR: 1.000e+00
Coefficient: −3.59e−01
Value: B
Rhodospira.trueperi
3
2
1
0
A (n=14)
B (n=15)
C (n=11)
surface

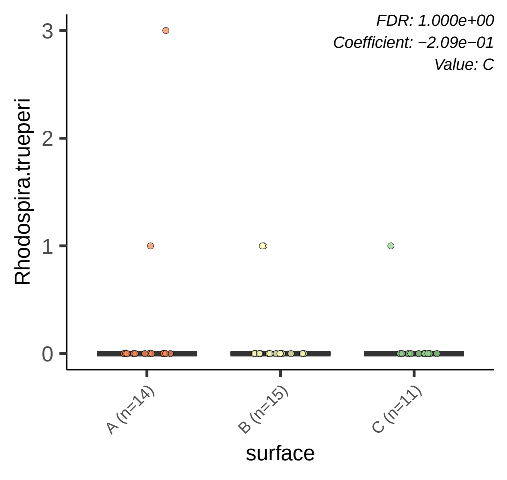
FDR: 1.000e+00
Coefficient: −2.09e−01
Value: C
Rhodospira.trueperi
0
1
2
3
A (n=14)
B (n=15)
C (n=11)
surface

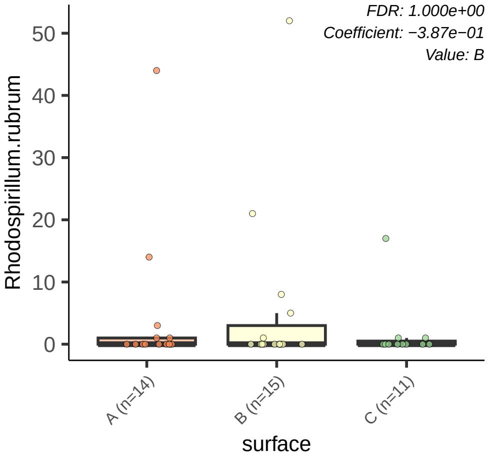

FDR: 1.000e+00
Coefficient: −3.87e−01
Value: B
Rhodospirillum.rubrum
0
10
20
30
40
50
A (n=14)
B (n=15)
C (n=11)
surface

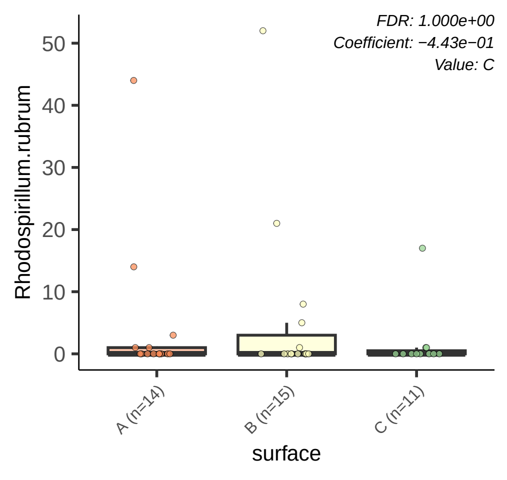

FDR: 1.000e+00
Coefficient: −4.43e−01
Value: C
Rhodospirillum.rubrum
50
40
30
20
10
0
A (n=14)
B (n=15)
C (n=11)
surface

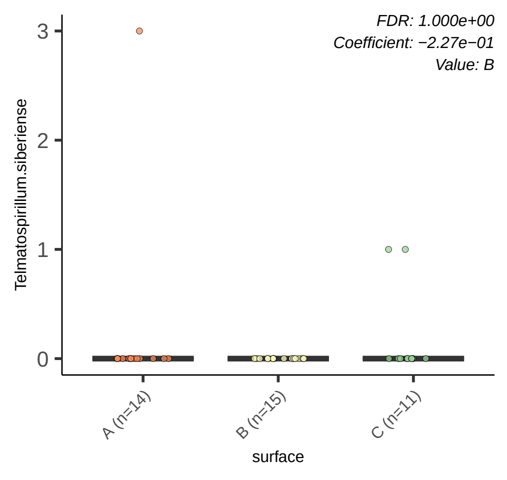

FDR: 1.000e+00
Coefficient: −2.27e−01
Value: B
Telmatospirillum.siberiense
3
2
1
0
A (n=14)
B (n=15)
C (n=11)
surface

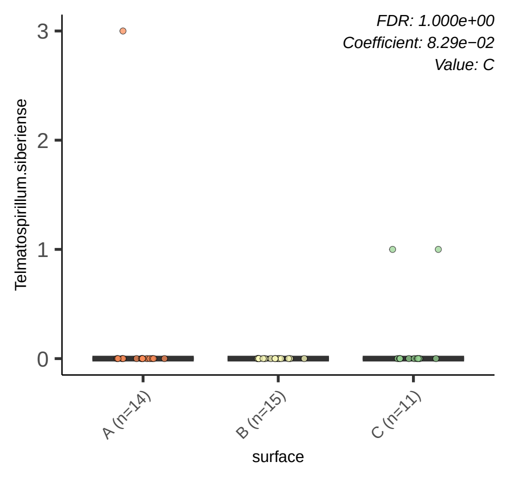
FDR: 1.000e+00
Coefficient: 8.29e−02
Value: C
Telmatospirillum.siberiense
3
2
1
0
A (n=14)
B (n=15)
C (n=11)
surface

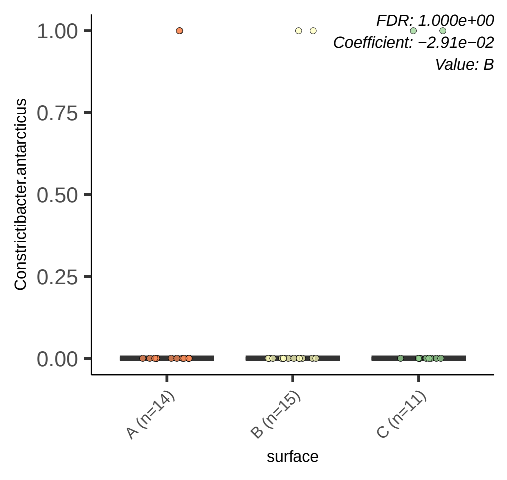

FDR: 1.000e+00
Coefficient: −2.91e−02
Value: B
1.00
0.75
0.50
0.25
0.00
Constrictibacter.antarcticus
A (n=14)
B (n=15)
C (n=11)
surface

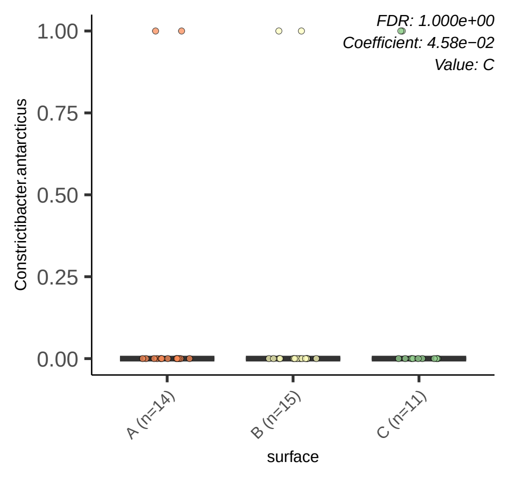
FDR: 1.000e+00
Coefficient: 4.58e−02
Value: C
Constrictibacter.antarcticus
1.00
0.75
0.50
0.25
0.00
A (n=14)
B (n=15)
C (n=11)
surface

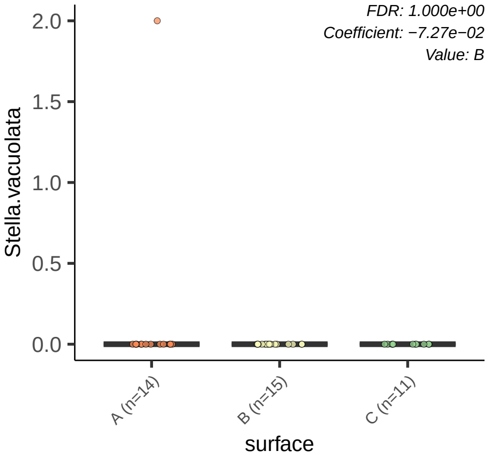

FDR: 1.000e+00
Coefficient: −7.27e−02
Value: B
Stella.vacuolata
2.0
1.5
1.0
0.5
0.0
A (n=14)
B (n=15)
C (n=11)
surface

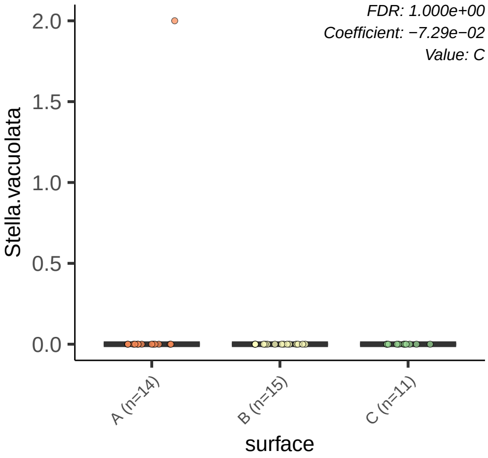
FDR: 1.000e+00
Coefficient: −7.29e−02
Value: C
Stella.vacuolata
2.0
1.5
1.0
0.5
0.0
A (n=14)
B (n=15)
C (n=11)
surface

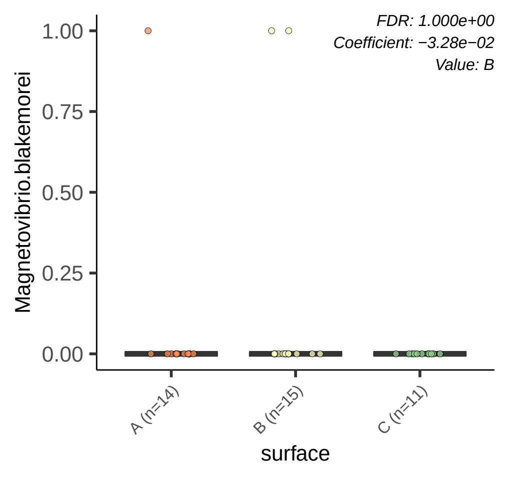
FDR: 1.000e+00
Coefficient: −3.28e−02
Value: B
Magnetovibrio.blakemorei
1.00
0.75
0.50
0.25
0.00
A (n=14)
B (n=15)
C (n=11)
surface

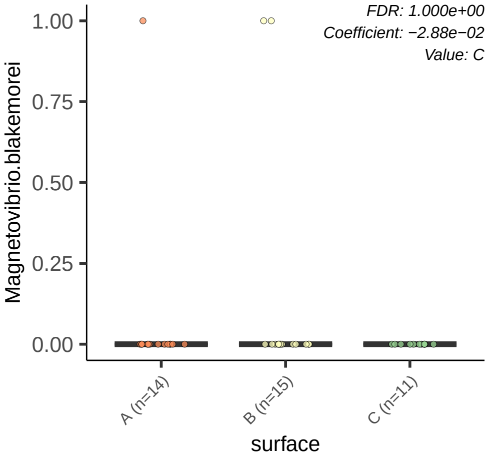

FDR: 1.000e+00
Coefficient: −2.88e−02
Value: C
Magnetovibrio.blakemorei
1.00
0.75
0.50
0.25
0.00
A (n=14)
B (n=15)
C (n=11)
surface

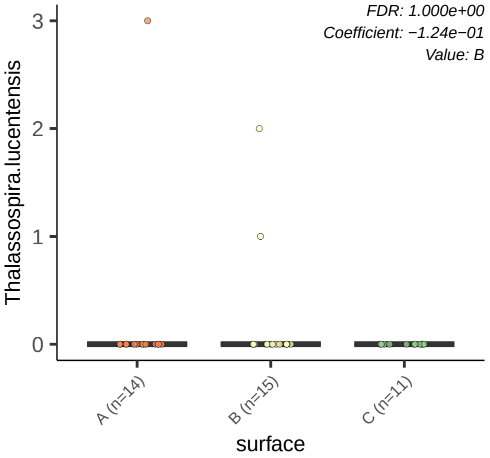
FDR: 1.000e+00
Coefficient: −1.24e−01
Value: B
Thalassospira.lucentensis
3
2
1
0
A (n=14)
B (n=15)
C (n=11)
surface

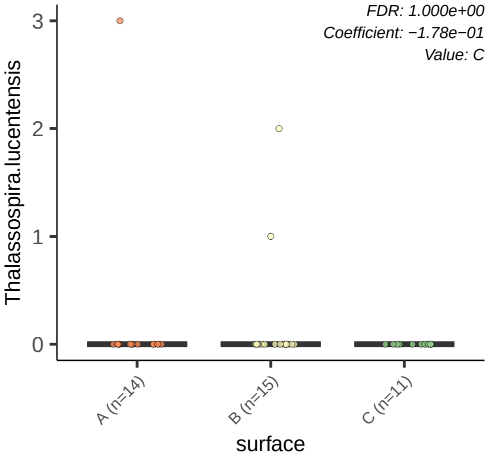

FDR: 1.000e+00
Coefficient: −1.78e−01
Value: C
Thalassospira.lucentensis
0
1
2
3
A (n=14)
B (n=15)
C (n=11)
surface

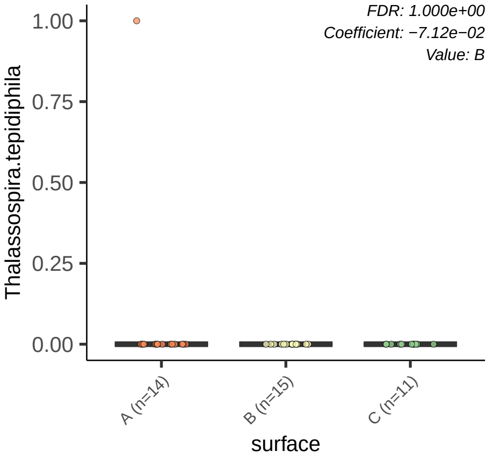

FDR: 1.000e+00
Coefficient: −7.12e−02
Value: B
Thalassospira.tepidiphila
1.00
0.75
0.50
0.25
0.00
A (n=14)
B (n=15)
C (n=11)
surface

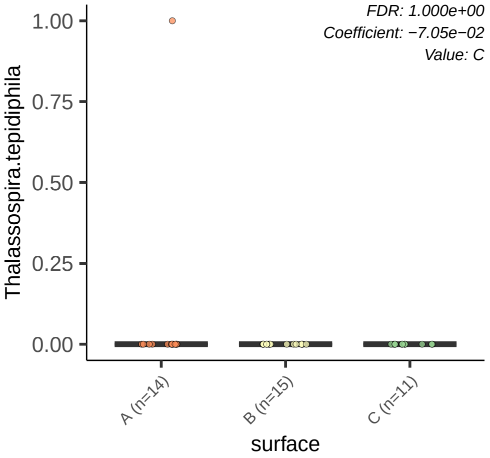

FDR: 1.000e+00
Coefficient: −7.05e−02
Value: C
Thalassospira.tepidiphila
1.00
0.75
0.50
0.25
0.00
A (n=14)
B (n=15)
C (n=11)
surface

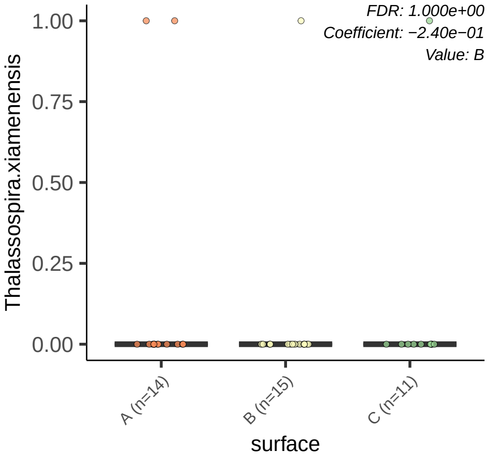
FDR: 1.000e+00
Coefficient: −2.40e−01
Value: B
Thalassospira.xiamenensis
1.00
0.75
0.50
0.25
0.00
A (n=14)
B (n=15)
C (n=11)
surface

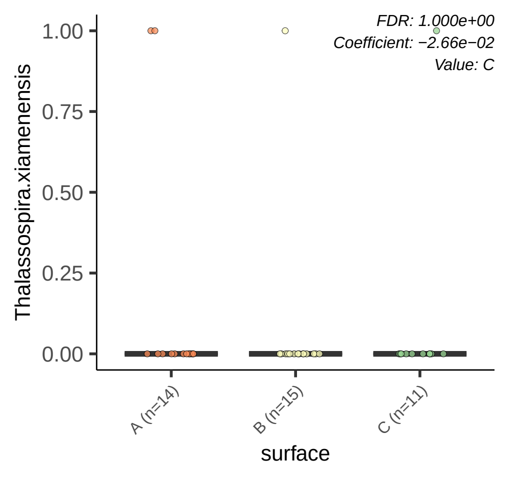
FDR: 1.000e+00
Coefficient: −2.66e−02
Value: C
Thalassospira.xiamenensis
1.00
0.75
0.50
0.25
0.00
A (n=14)
B (n=15)
C (n=11)
surface

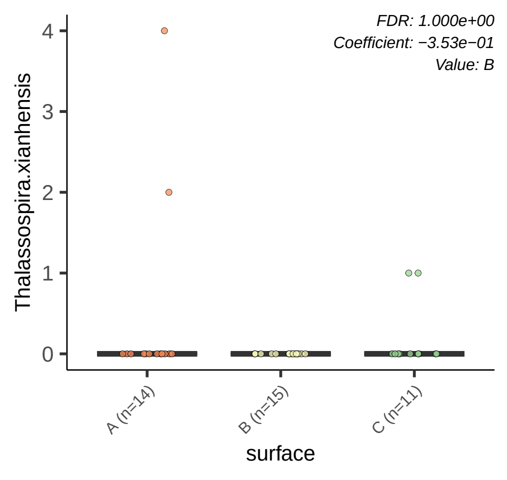

FDR: 1.000e+00
Coefficient: −3.53e−01
Value: B
Thalassospira.xianhensis
0
1
2
3
4
A (n=14)
B (n=15)
C (n=11)
surface

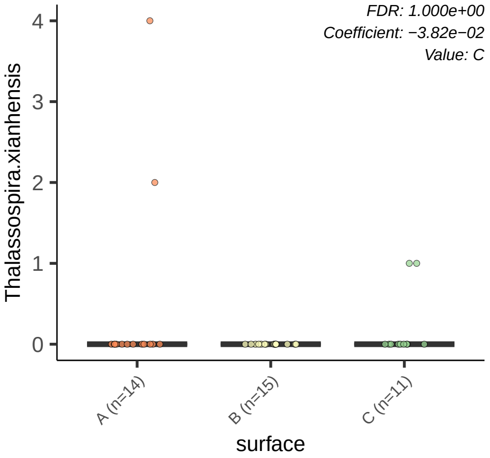
FDR: 1.000e+00
Coefficient: −3.82e−02
Value: C
Thalassospira.xianhensis
0
1
2
3
4
A (n=14)
B (n=15)
C (n=11)
surface

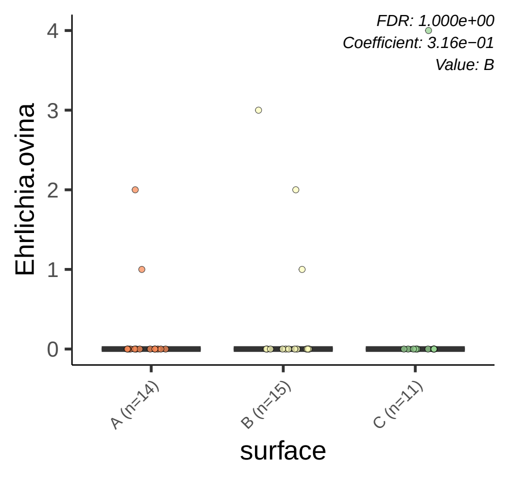

FDR: 1.000e+00
Coefficient: 3.16e−01
Value: B
Ehrlichia.ovina
0
1
2
3
4
A (n=14)
B (n=15)
C (n=11)
surface

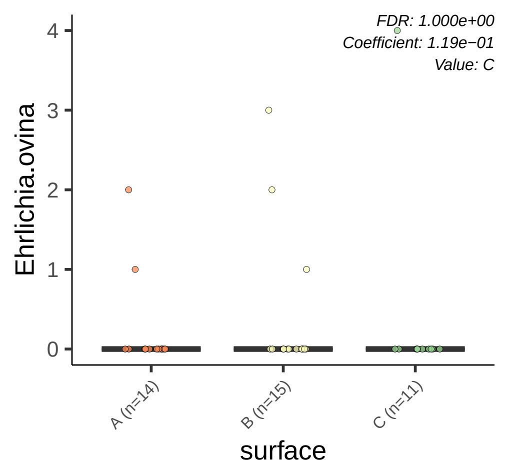
FDR: 1.000e+00
Coefficient: 1.19e−01
Value: C
Ehrlichia.ovina
0
1
2
3
4
A (n=14)
B (n=15)
C (n=11)
surface

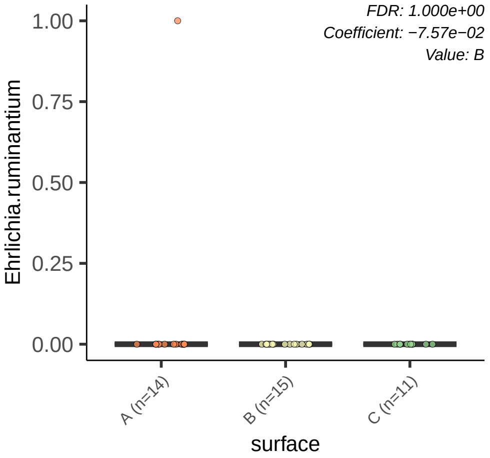
FDR: 1.000e+00
Coefficient: −7.57e−02
Value: B
Ehrlichia.ruminantium
1.00
0.75
0.50
0.25
0.00
A (n=14)
B (n=15)
C (n=11)
surface

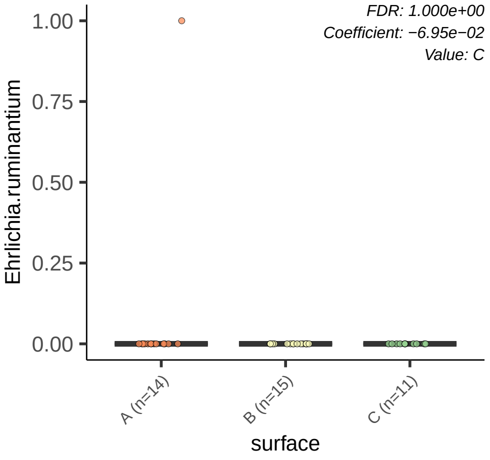
FDR: 1.000e+00
Coefficient: −6.95e−02
Value: C
Ehrlichia.ruminantium
1.00
0.75
0.50
0.25
0.00
A (n=14)
B (n=15)
C (n=11)
surface

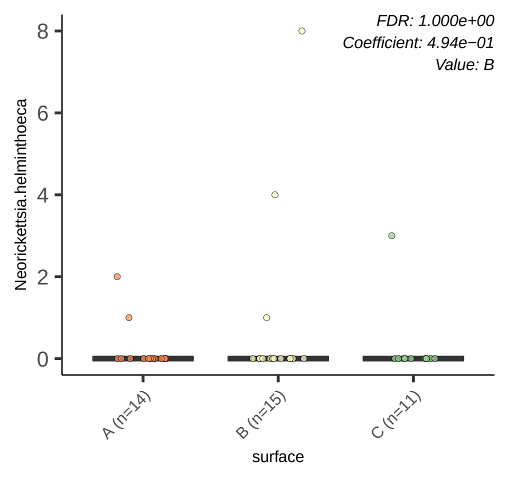
FDR: 1.000e+00
Coefficient: 4.94e−01
Value: B
Neoricketsia.helminthoeca
0
2
4
6
8
A (n=14)
B (n=15)
C (n=11)
surface

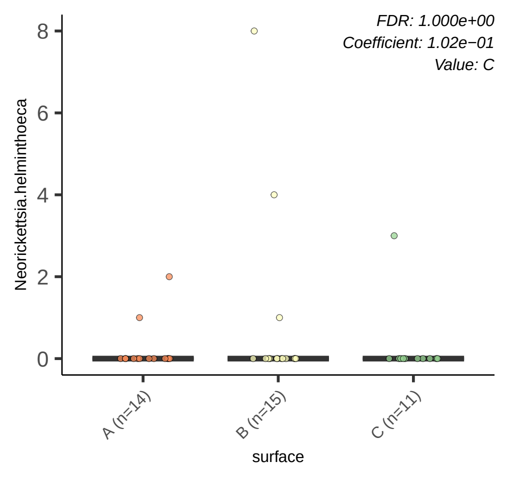
FDR: 1.000e+00
Coefficient: 1.02e−01
Value: C
Neorickettsia.helminthoeca
0
2
4
6
8
A (n=14)
B (n=15)
C (n=11)
surface

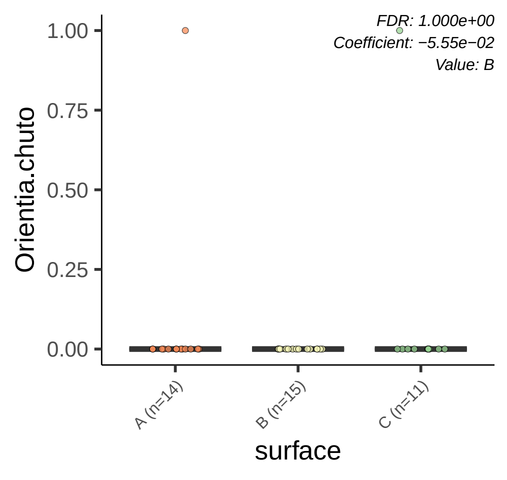

FDR: 1.000e+00
Coefficient: −5.55e−02
Value: B
Orientia.chuto
1.00
0.75
0.50
0.25
0.00
A (n=14)
B (n=15)
C (n=11)
surface

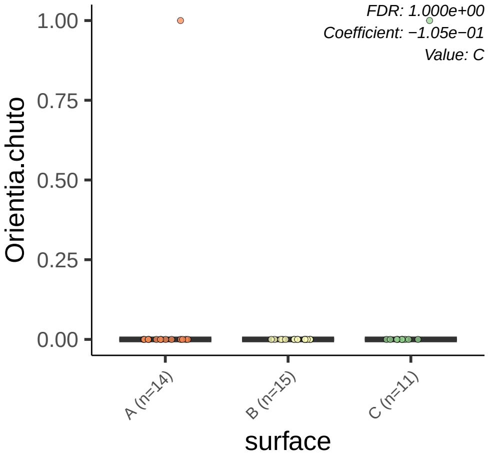
FDR: 1.000e+00
Coefficient: −1.05e−01
Value: C
Orientia.chuto
1.00
0.75
0.50
0.25
0.00
A (n=14)
B (n=15)
C (n=11)
surface

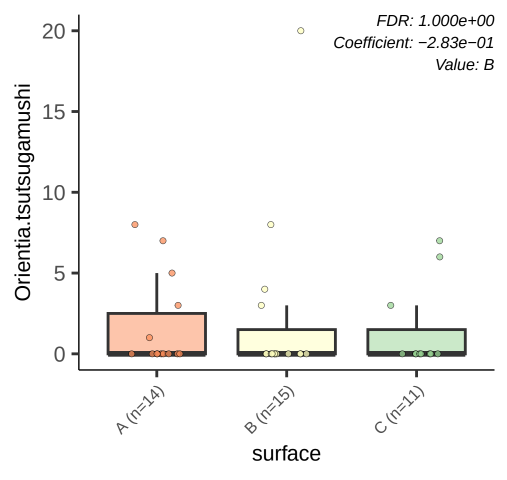
FDR: 1.000e+00
Coefficient: −2.83e−01
Value: B
Orientia.tsutsugamushi
0
5
10
15
20
A (n=14)
B (n=15)
C (n=11)
surface

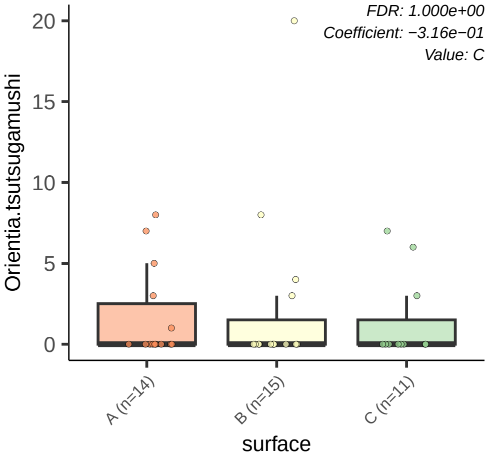

FDR: 1.000e+00
Coefficient: −3.16e−01
Value: C
Orientia.tsutsugamushi
20
15
10
5
0
A (n=14)
B (n=15)
C (n=11)
surface

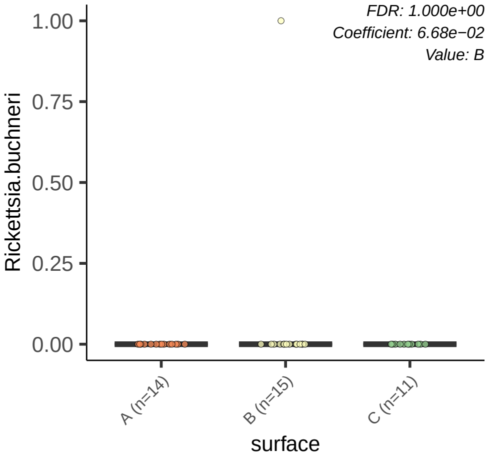

FDR: 1.000e+00
Coefficient: 6.68e−02
Value: B
Rickettsia.buchneri
1.00
0.75
0.50
0.25
0.00
A (n=14)
B (n=15)
C (n=11)
surface

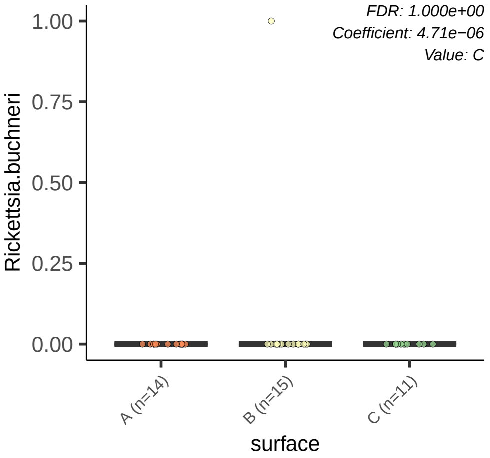
FDR: 1.000e+00
Coefficient: 4.71e−06
Value: C
Rickettsia.buchneri
1.00
0.75
0.50
0.25
0.00
A (n=14)
B (n=15)
C (n=11)
surface

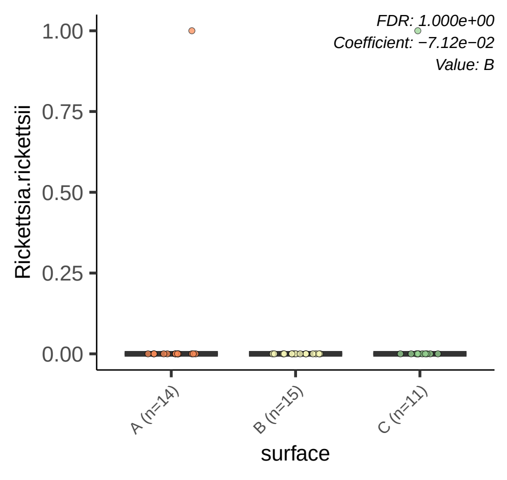

FDR: 1.000e+00
Coefficient: −7.12e−02
Value: B
Rickettsia.rickettsii
1.00
0.75
0.50
0.25
0.00
A (n=14)
B (n=15)
C (n=11)
surface

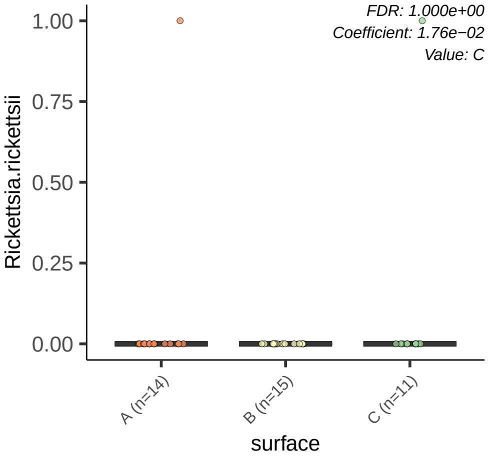
FDR: 1.000e+00
Coefficient: 1.76e−02
Value: C
Rickettsia.rickettsii
1.00
0.75
0.50
0.25
0.00
A (n=14)
B (n=15)
C (n=11)
surface

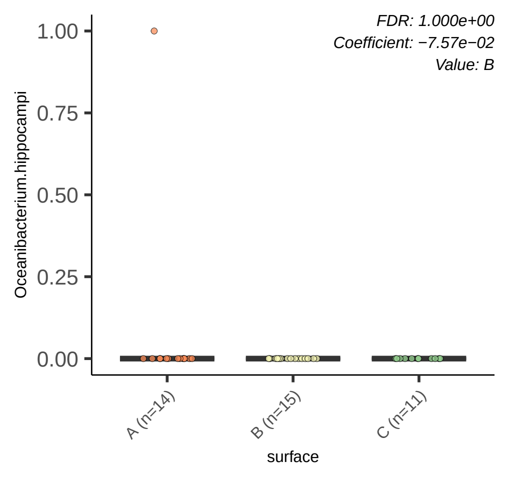
FDR: 1.000e+00
Coefficient: −7.57e−02
Value: B
Oceanibacterium.hippocampi
1.00
0.75
0.50
0.25
0.00
A (n=14)
B (n=15)
C (n=11)
surface

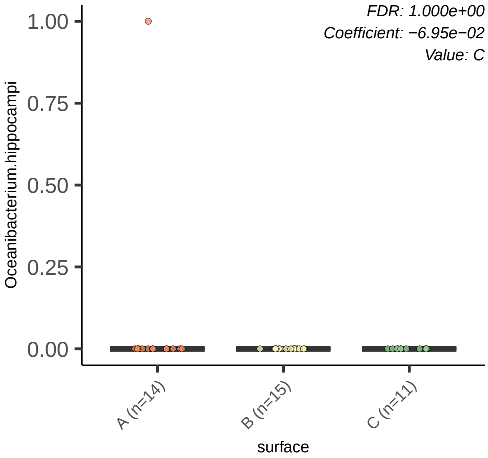

FDR: 1.000e+00
Coefficient: −6.95e−02
Value: C
1.00
0.75
0.50
0.25
0.00
Oceanibacterium.hippocampi
A (n=14)
B (n=15)
C (n=11)
surface

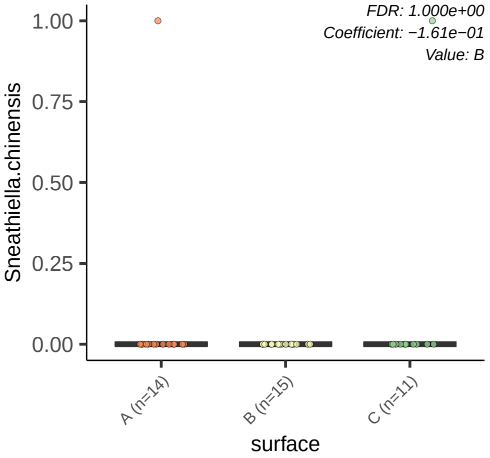

FDR: 1.000e+00
Coefficient: −1.61e−01
Value: B
Sneathiella.chinensis
1.00
0.75
0.50
0.25
0.00
A (n=14)
B (n=15)
C (n=11)
surface

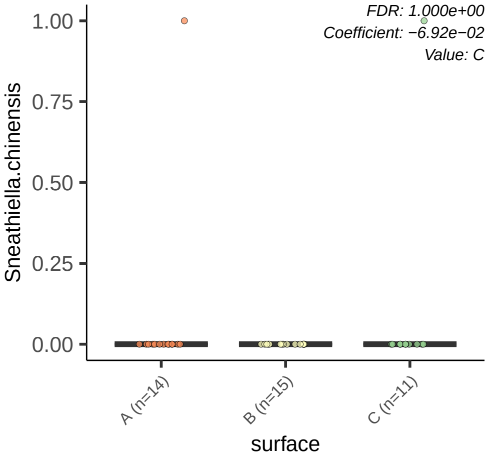

FDR: 1.000e+00
Coefficient: −6.92e−02
Value: C
Sneathiella.chinensis
1.00
0.75
0.50
0.25
0.00
A (n=14)
B (n=15)
C (n=11)
surface

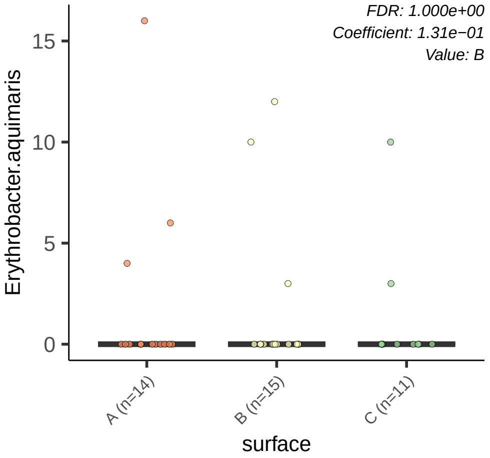

FDR: 1.000e+00
Coefficient: 1.31e−01
Value: B
Erythrobacter.aquimaris
15
10
5
0
A (n=14)
B (n=15)
C (n=11)
surface

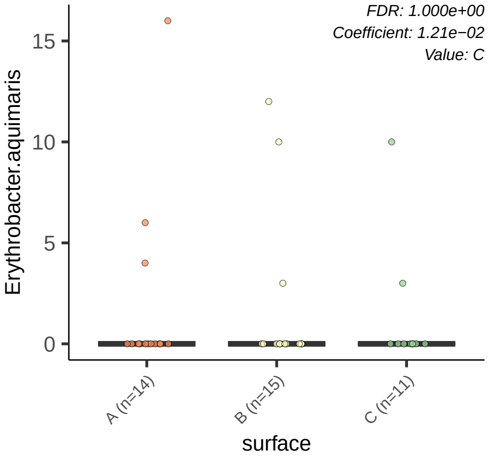
FDR: 1.000e+00
Coefficient: 1.21e−02
Value: C
Erythrobacter.aquimaris
15
10
5
0
A (n=14)
B (n=15)
C (n=11)
surface

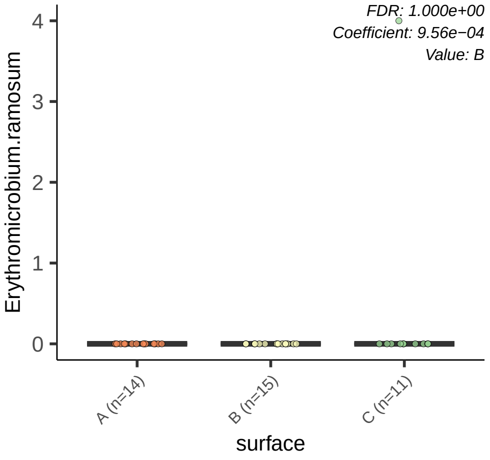

FDR: 1.000e+00
Coefficient: 9.56e−04
Value: B
Erythromicrobium.ramosum
4
3
2
1
0
A (n=14)
B (n=15)
C (n=11)
surface

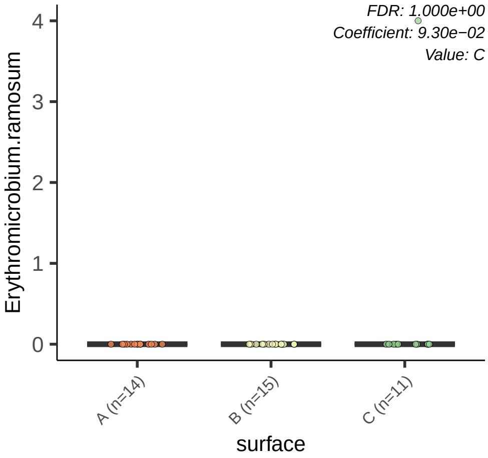
FDR: 1.000e+00
Coefficient: 9.30e−02
Value: C
Erythromicrobium.ramosum
0
1
2
3
4
A (n=14)
B (n=15)
C (n=11)
surface

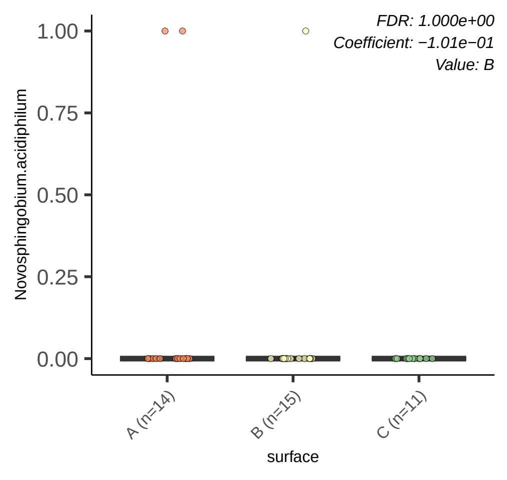

FDR: 1.000e+00
Coefficient: −1.01e−01
Value: B
Novosphingobium.acidiphilum
1.00
0.75
0.50
0.25
0.00
A (n=14)
B (n=15)
C (n=11)
surface

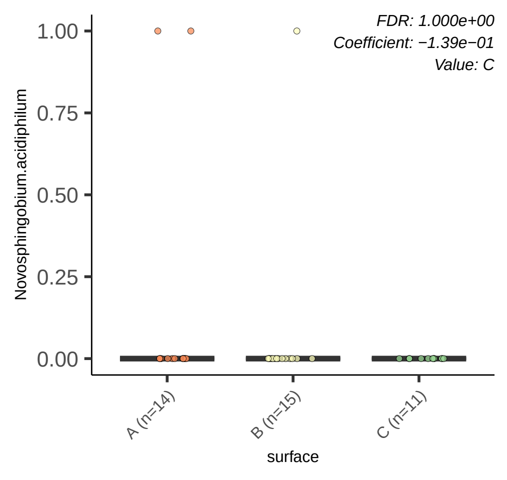
FDR: 1.000e+00
Coefficient: −1.39e−01
Value: C
Novosphingobium.acidiphilum
1.00
0.75
0.50
0.25
0.00
A (n=14)
B (n=15)
C (n=11)
surface

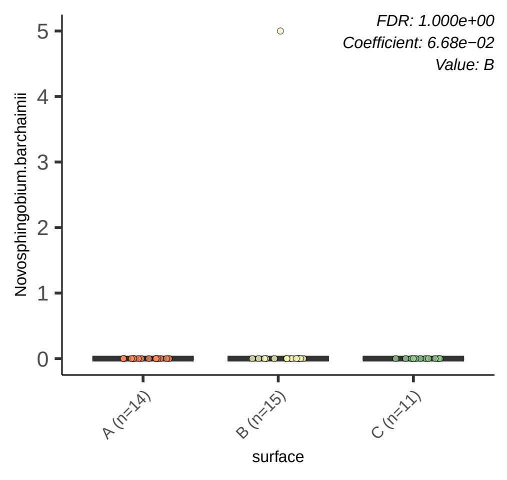

FDR: 1.000e+00
Coefficient: 6.68e−02
Value: B
Novosphingobium.barchaimii
0
1
2
3
4
5
A (n=14)
B (n=15)
C (n=11)
surface

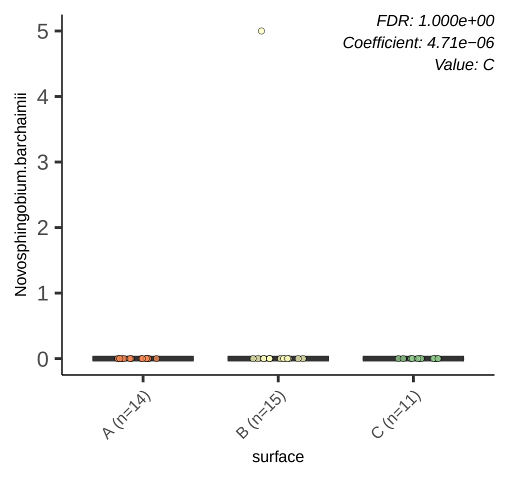
FDR: 1.000e+00
Coefficient: 4.71e−06
Value: C
Novosphingobium.barchaimii
0
1
2
3
4
5
A (n=14)
B (n=15)
C (n=11)
surface

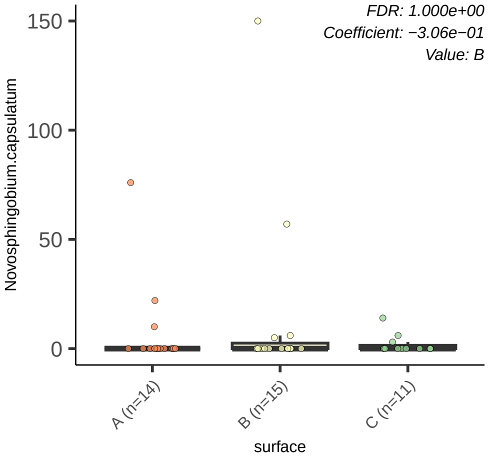
FDR: 1.000e+00
Coefficient: −3.06e−01
Value: B
150
100
50
0
Novosphingobium.capsulatum
A (n=14)
B (n=15)
C (n=11)
surface

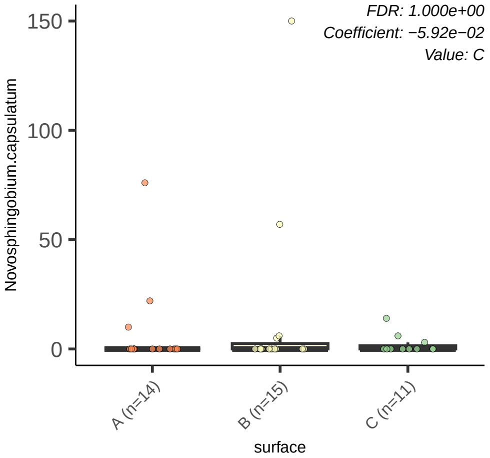

FDR: 1.000e+00
Coefficient: −5.92e−02
Value: C
Novosphingobium.capsulatum
150
100
50
0
A (n=14)
B (n=15)
C (n=11)
surface

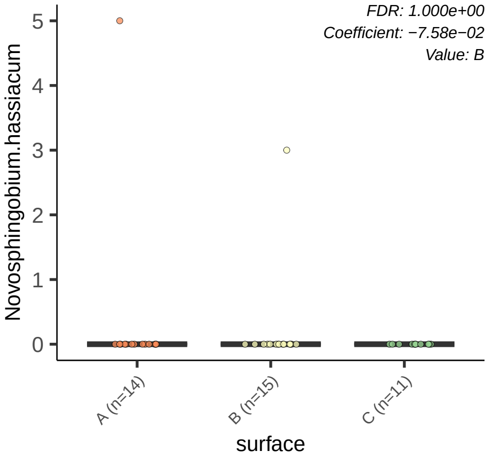
FDR: 1.000e+00
Coefficient: −7.58e−02
Value: B
Novosphingobium.hassiacum
5
4
3
2
1
0
A (n=14)
B (n=15)
C (n=11)
surface

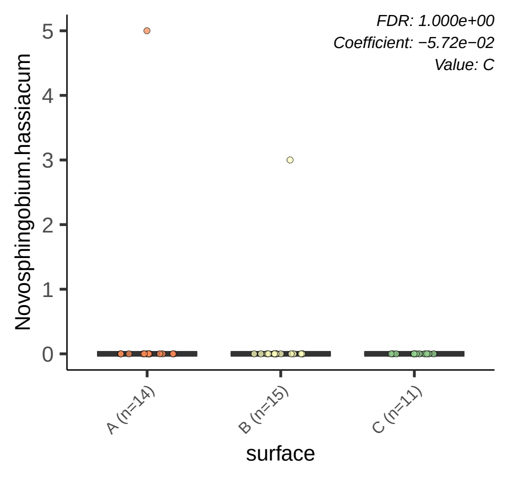

FDR: 1.000e+00
Coefficient: −5.72e−02
Value: C
Novosphingobium.hassiacum
0
1
2
3
4
5
A (n=14)
B (n=15)
C (n=11)
surface

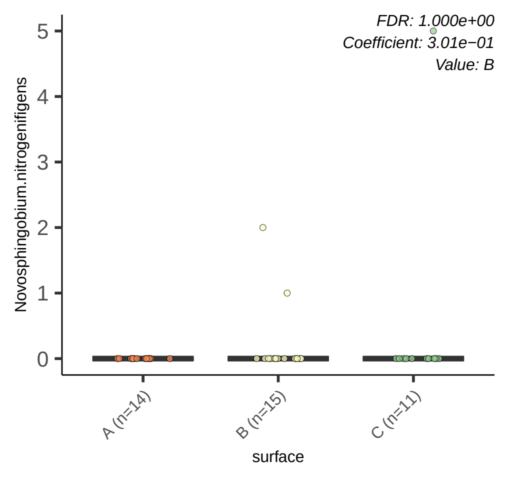

FDR: 1.000e+00
Coefficient: 3.01e−01
Value: B
Novosphingobium.nitrogenifigens
0
1
2
3
4
5
A (n=14)
B (n=15)
C (n=11)
surface

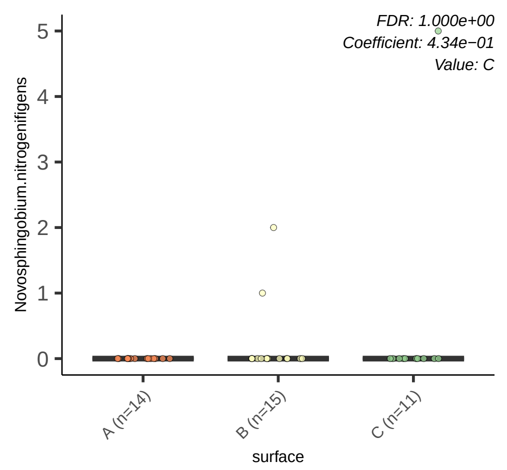

FDR: 1.000e+00
Coefficient: 4.34e−01
Value: C
Novosphingobium.nitrogenifigens
0
1
2
3
4
5
A (n=14)
B (n=15)
C (n=11)
surface

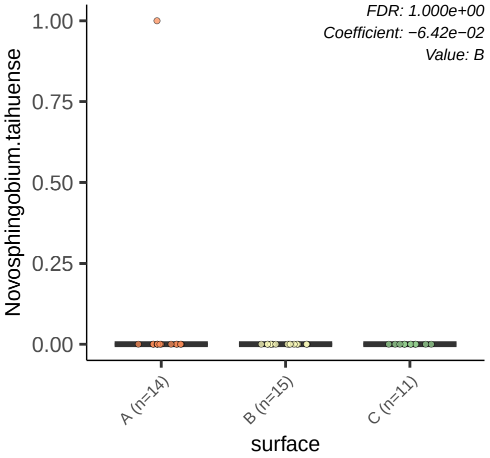
FDR: 1.000e+00
Coefficient: −6.42e−02
Value: B
Novosphingobium.taihuense
1.00
0.75
0.50
0.25
0.00
A (n=14)
B (n=15)
C (n=11)
surface

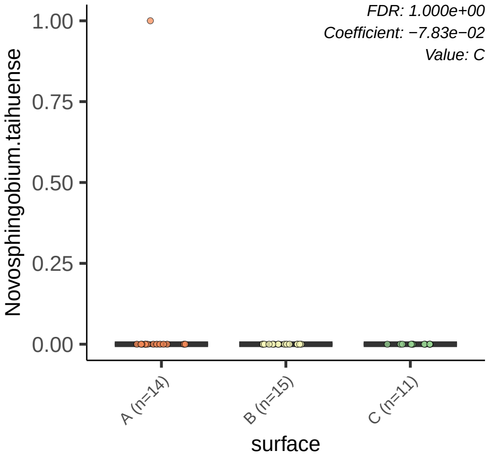
FDR: 1.000e+00
Coefficient: −7.83e−02
Value: C
Novosphingobium.taihuense
1.00
0.75
0.50
0.25
0.00
A (n=14)
B (n=15)
C (n=11)
surface

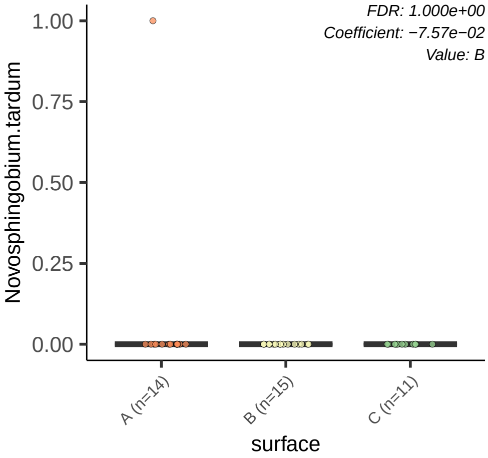

FDR: 1.000e+00
Coefficient: −7.57e−02
Value: B
Novosphingobium.tardum
1.00
0.75
0.50
0.25
0.00
A (n=14)
B (n=15)
C (n=11)
surface

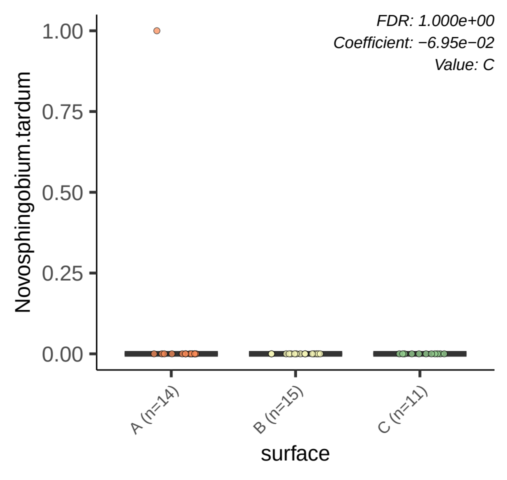
FDR: 1.000e+00
Coefficient: −6.95e−02
Value: C
Novosphingobium.tardum
1.00
0.75
0.50
0.25
0.00
A (n=14)
B (n=15)
C (n=11)
surface

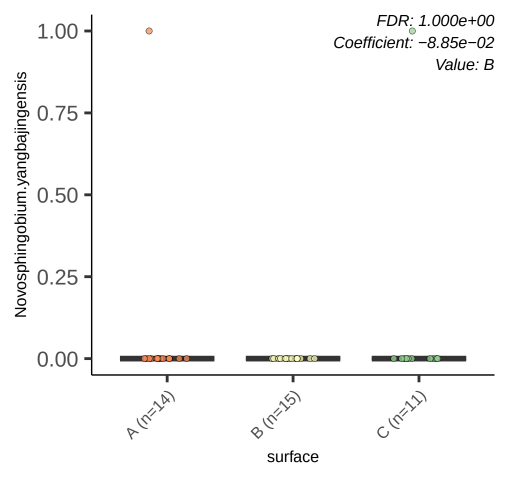

FDR: 1.000e+00
Coefficient: −8.85e−02
Value: B
Novosphingobium.yangbajingensis
1.00
0.75
0.50
0.25
0.00
A (n=14)
B (n=15)
C (n=11)
surface

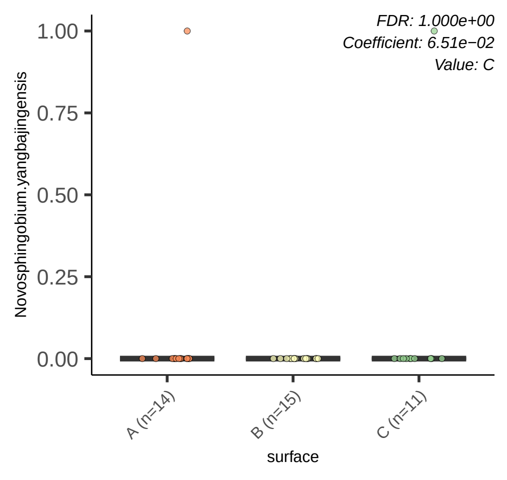
FDR: 1.000e+00
Coefficient: 6.51e−02
Value: C
1.00
0.75
0.50
0.25
0.00
Novosphingobium.yangbajingensis
A (n=14)
B (n=15)
C (n=11)
surface

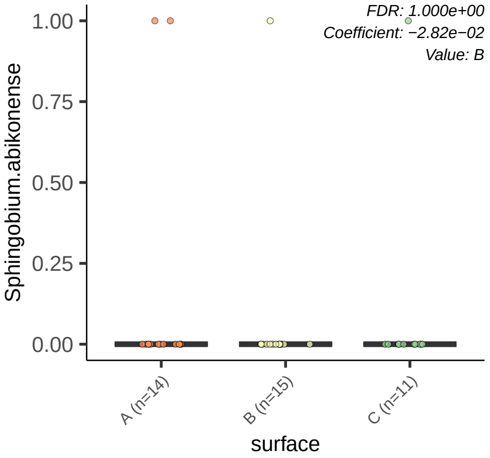
FDR: 1.000e+00
Coefficient: −2.82e−02
Value: B
Sphingobium.abikonense
1.00
0.75
0.50
0.25
0.00
A (n=14)
B (n=15)
C (n=11)
surface

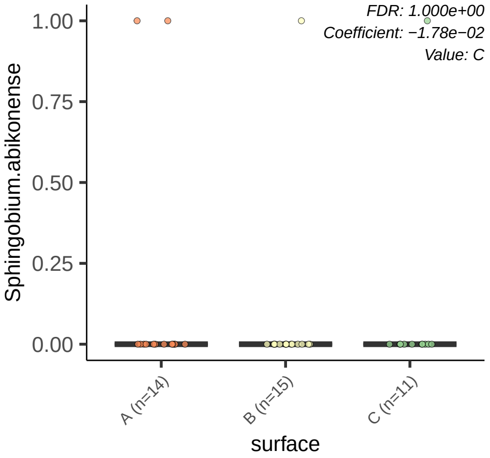

FDR: 1.000e+00
Coefficient: −1.78e−02
Value: C
Sphingobium.abikonense
1.00
0.75
0.50
0.25
0.00
A (n=14)
B (n=15)
C (n=11)
surface

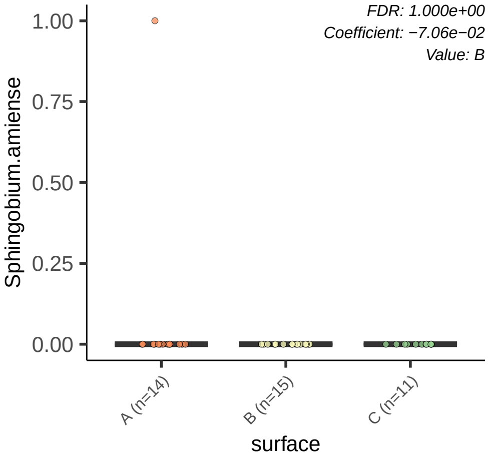
FDR: 1.000e+00
Coefficient: −7.06e−02
Value: B
Sphingobium.amiense
1.00
0.75
0.50
0.25
0.00
A (n=14)
B (n=15)
C (n=11)
surface

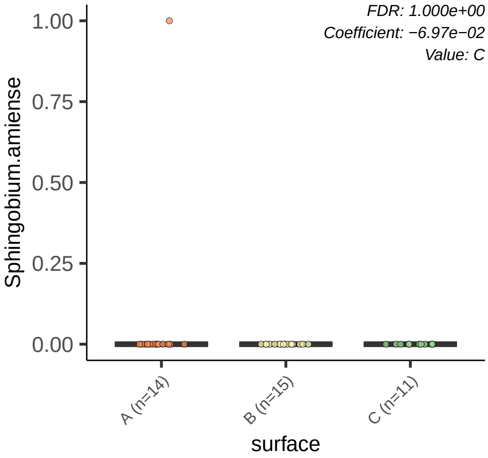
FDR: 1.000e+00
Coefficient: −6.97e−02
Value: C
Sphingobium.amiense
1.00
0.75
0.50
0.25
0.00
A (n=14)
B (n=15)
C (n=11)
surface

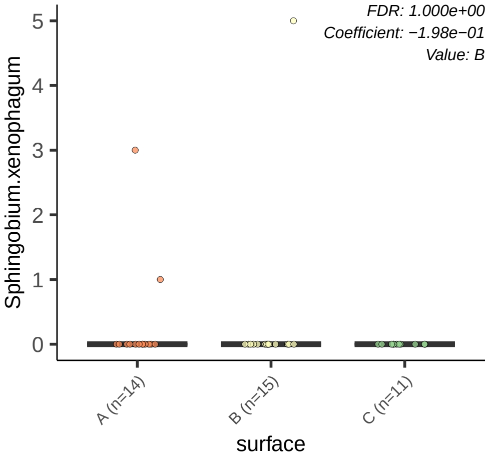

FDR: 1.000e+00
Coefficient: −1.98e−01
Value: B
Sphingobium.xenophagum
0
1
2
3
4
5
A (n=14)
B (n=15)
C (n=11)
surface

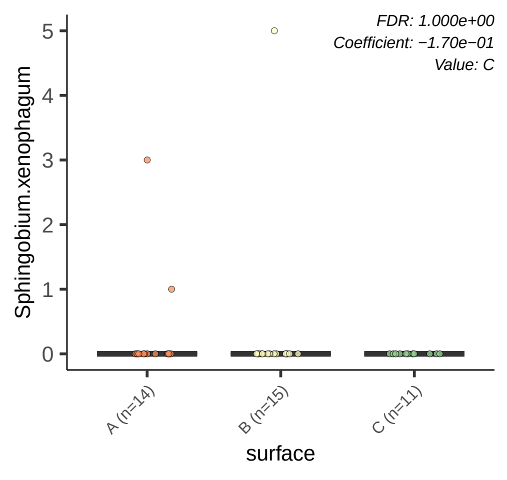
FDR: 1.000e+00
Coefficient: −1.70e−01
Value: C
Sphingobium.xenophagum
5
4
3
2
1
0
A (n=14)
B (n=15)
C (n=11)
surface

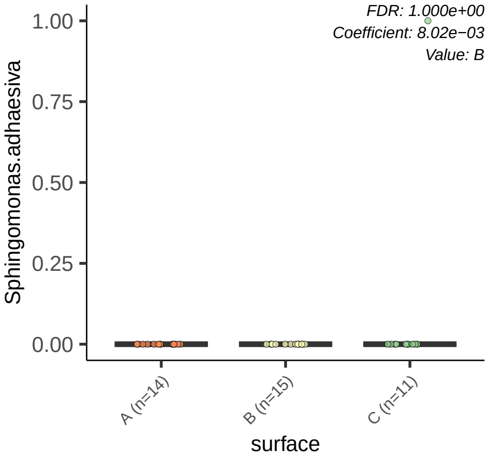
FDR: 1.000e+00
Coefficient: 8.02e−03
Value: B
Sphingomonas.adhaesiva
1.00
0.75
0.50
0.25
0.00
A (n=14)
B (n=15)
C (n=11)
surface

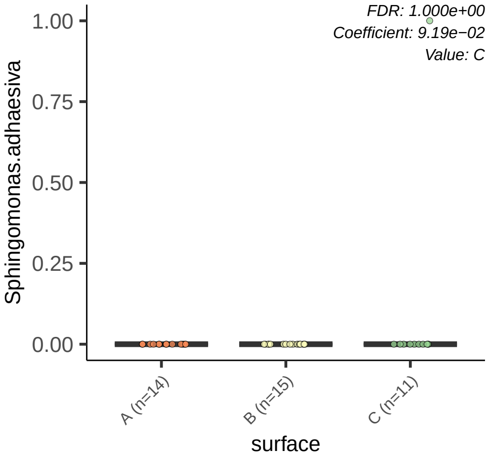
FDR: 1.000e+00
Coefficient: 9.19e−02
Value: C
Sphingomonas.adhaesiva
1.00
0.75
0.50
0.25
0.00
A (n=14)
B (n=15)
C (n=11)
surface

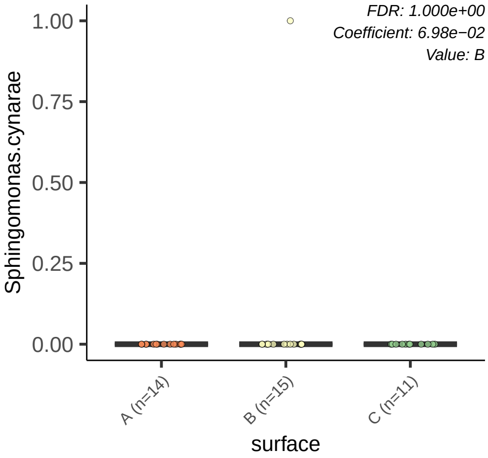
FDR: 1.000e+00
Coefficient: 6.98e−02
Value: B
Sphingomonas.cynarae
1.00
0.75
0.50
0.25
0.00
A (n=14)
B (n=15)
C (n=11)
surface

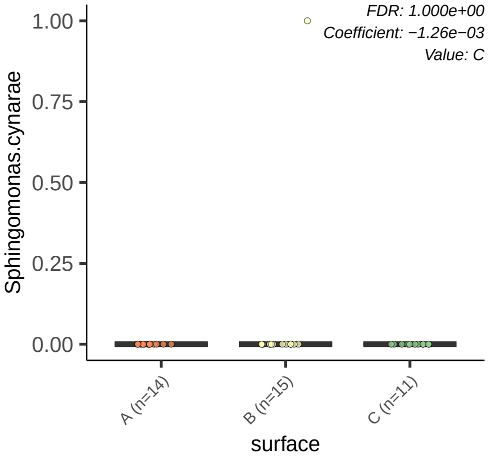
FDR: 1.000e+00
Coefficient: −1.26e−03
Value: C
Sphingomonas.cynarae
1.00
0.75
0.50
0.25
0.00
A (n=14)
B (n=15)
C (n=11)
surface

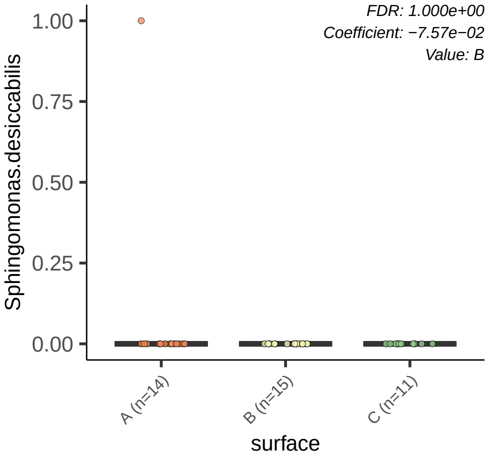
FDR: 1.000e+00
Coefficient: −7.57e−02
Value: B
Sphingomonas.desiccabilis
1.00
0.75
0.50
0.25
0.00
A (n=14)
B (n=15)
C (n=11)
surface

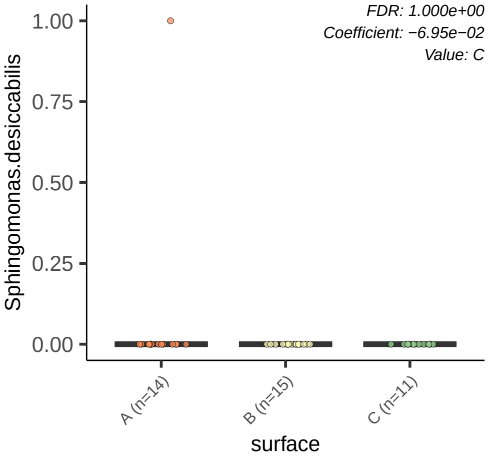

FDR: 1.000e+00
Coefficient: −6.95e−02
Value: C
Sphingomonas.desiccabilis
1.00
0.75
0.50
0.25
0.00
A (n=14)
B (n=15)
C (n=11)
surface

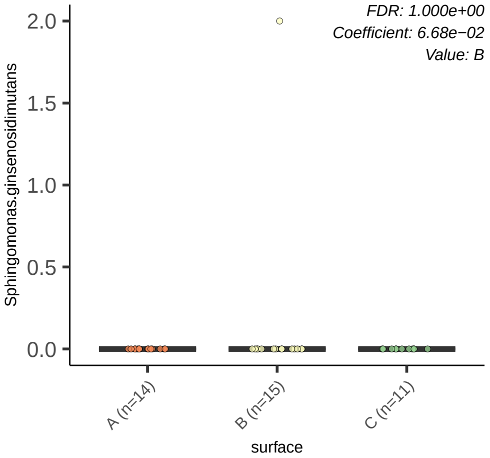
FDR: 1.000e+00
Coefficient: 6.68e−02
Value: B
Sphingomonas.ginsenosidimutans
2.0
1.5
1.0
0.5
0.0
A (n=14)
B (n=15)
C (n=11)
surface

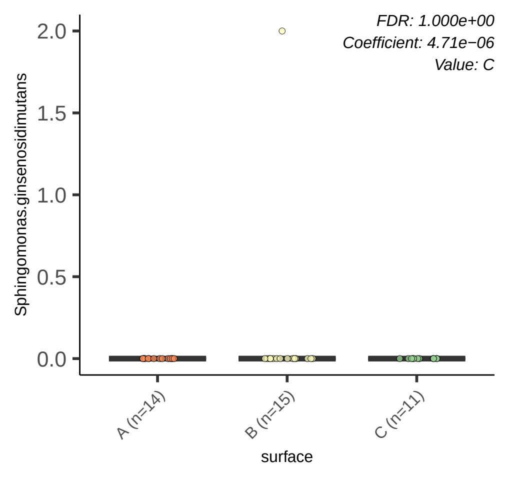

FDR: 1.000e+00
Coefficient: 4.71e−06
Value: C
Sphingomonas.ginsenosidimutans
2.0
1.5
1.0
0.5
0.0
A (n=14)
B (n=15)
C (n=11)
surface

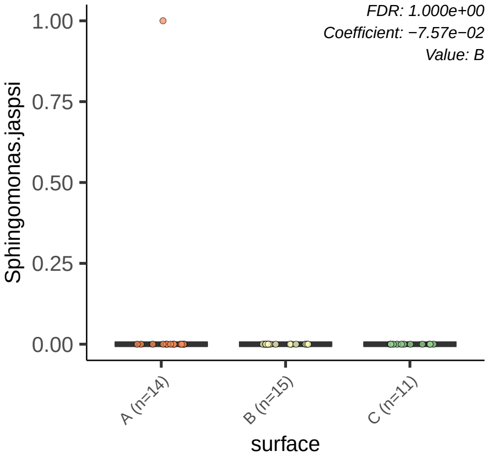
FDR: 1.000e+00
Coefficient: −7.57e−02
Value: B
Sphingomonas.jaspsi
1.00
0.75
0.50
0.25
0.00
A (n=14)
B (n=15)
C (n=11)
surface

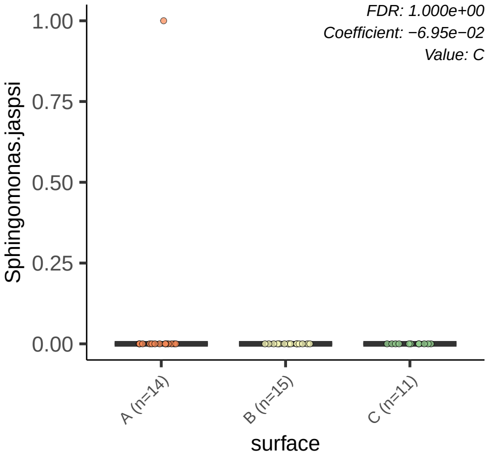
FDR: 1.000e+00
Coefficient: −6.95e−02
Value: C
Sphingomonas.jaspsi
1.00
0.75
0.50
0.25
0.00
A (n=14)
B (n=15)
C (n=11)
surface

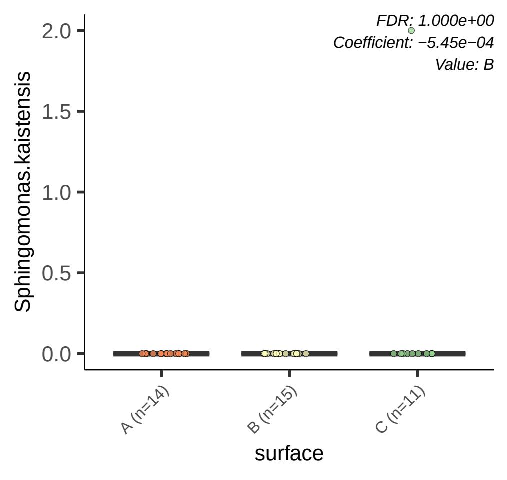

FDR: 1.000e+00
Coefficient: −5.45e−04
Value: B
Sphingomonas.kaistensis
2.0
1.5
1.0
0.5
0.0
A (n=14)
B (n=15)
C (n=11)
surface

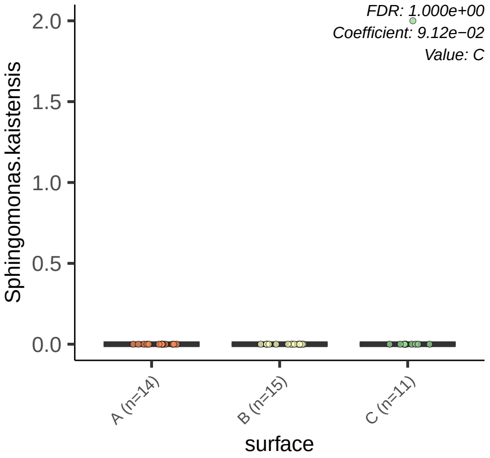

FDR: 1.000e+00
Coefficient: 9.12e−02
Value: C
Sphingomonas.kaistensis
2.0
1.5
1.0
0.5
0.0
A (n=14)
B (n=15)
C (n=11)
surface

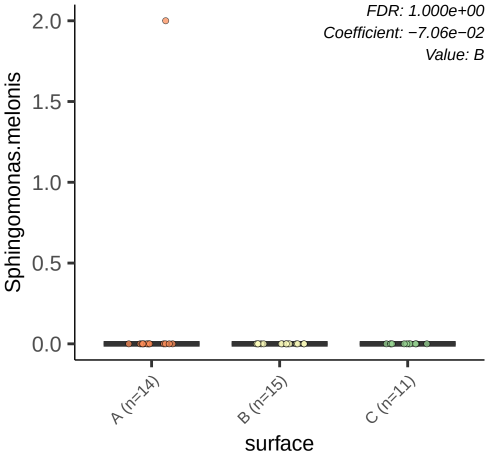
FDR: 1.000e+00
Coefficient: −7.06e−02
Value: B
Sphingomonas.melonis
2.0
1.5
1.0
0.5
0.0
A (n=14)
B (n=15)
C (n=11)
surface

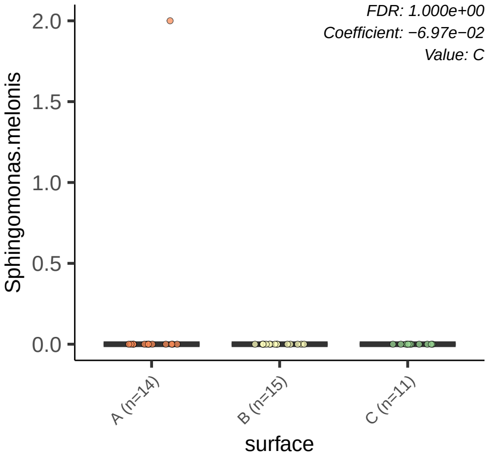
FDR: 1.000e+00
Coefficient: −6.97e−02
Value: C
Sphingomonas.melonis
2.0
1.5
1.0
0.5
0.0
A (n=14)
B (n=15)
C (n=11)
surface

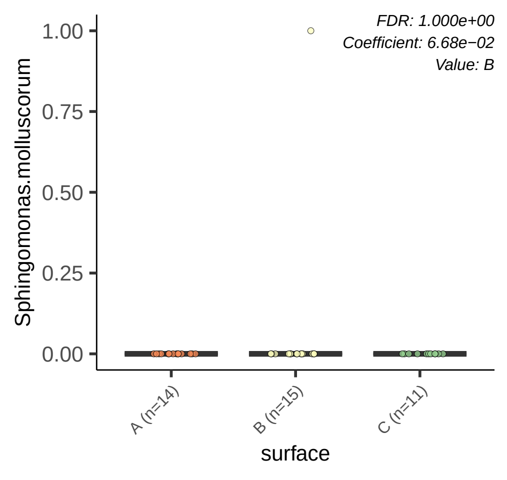
FDR: 1.000e+00
Coefficient: 6.68e−02
Value: B
Sphingomonas.molluscorum
1.00
0.75
0.50
0.25
0.00
A (n=14)
B (n=15)
C (n=11)
surface

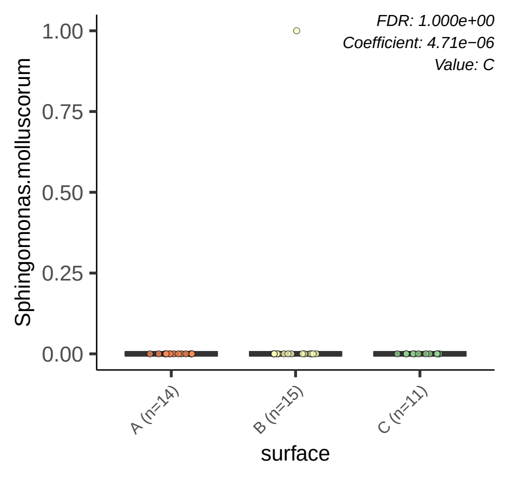

FDR: 1.000e+00
Coefficient: 4.71e−06
Value: C
Sphingomonas.molluscorum
1.00
0.75
0.50
0.25
0.00
A (n=14)
B (n=15)
C (n=11)
surface

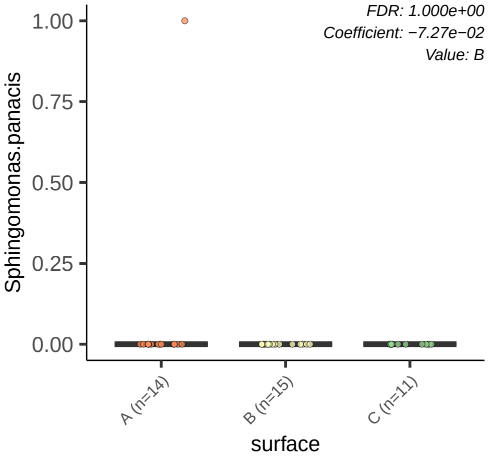

FDR: 1.000e+00
Coefficient: −7.27e−02
Value: B
Sphingomonas.panacis
1.00
0.75
0.50
0.25
0.00
A (n=14)
B (n=15)
C (n=11)
surface

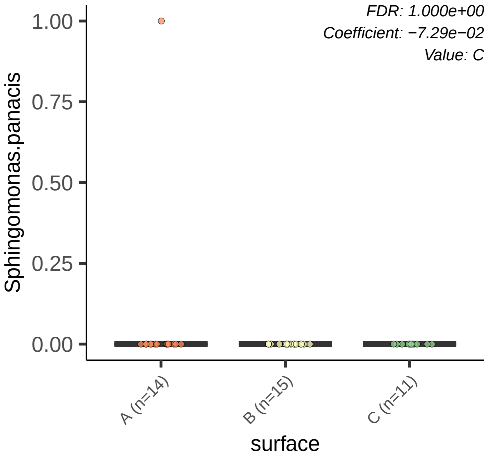
FDR: 1.000e+00
Coefficient: −7.29e−02
Value: C
Sphingomonas.panacis
1.00
0.75
0.50
0.25
0.00
A (n=14)
B (n=15)
C (n=11)
surface

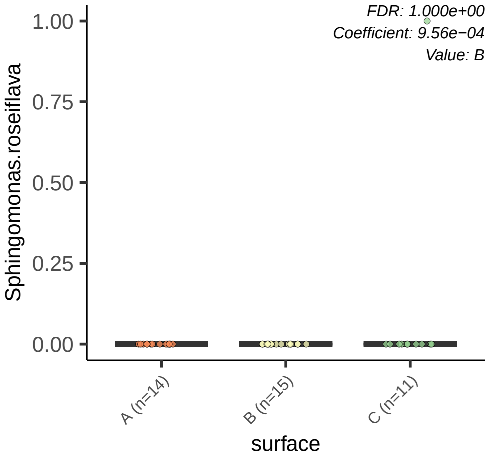
FDR: 1.000e+00
Coefficient: 9.56e−04
Value: B
Sphingomonas.roseiflava
1.00
0.75
0.50
0.25
0.00
A (n=14)
B (n=15)
C (n=11)
surface

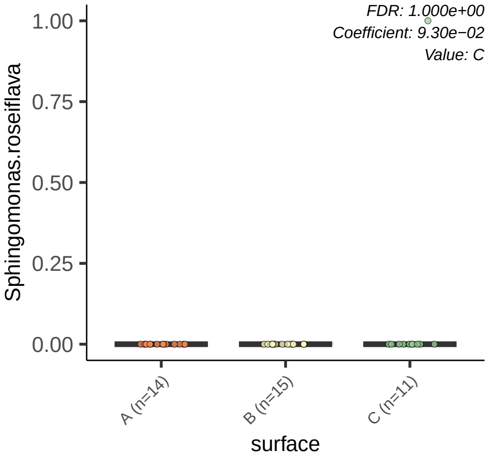

FDR: 1.000e+00
Coefficient: 9.30e−02
Value: C
Sphingomonas.roseiflava
1.00
0.75
0.50
0.25
0.00
A (n=14)
B (n=15)
C (n=11)
surface

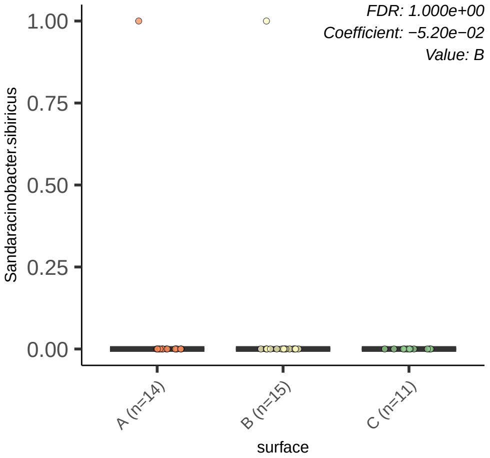
FDR: 1.000e+00
Coefficient: −5.20e−02
Value: B
Sandaracinobacter.sibiricus
1.00
0.75
0.50
0.25
0.00
A (n=14)
B (n=15)
C (n=11)
surface

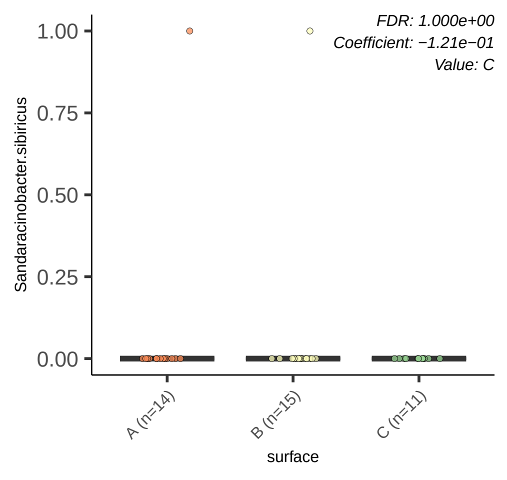

FDR: 1.000e+00
Coefficient: −1.21e−01
Value: C
Sandaracinobacter.sibiricus
1.00
0.75
0.50
0.25
0.00
A (n=14)
B (n=15)
C (n=11)
surface

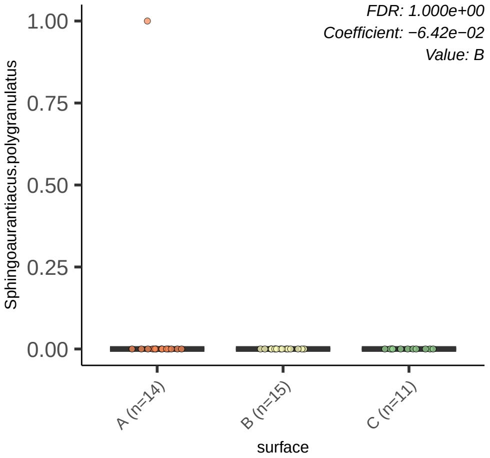
FDR: 1.000e+00
Coefficient: −6.42e−02
Value: B
Sphingoaurantiacus.polygranulatus
1.00
0.75
0.50
0.25
0.00
A (n=14)
B (n=15)
C (n=11)
surface

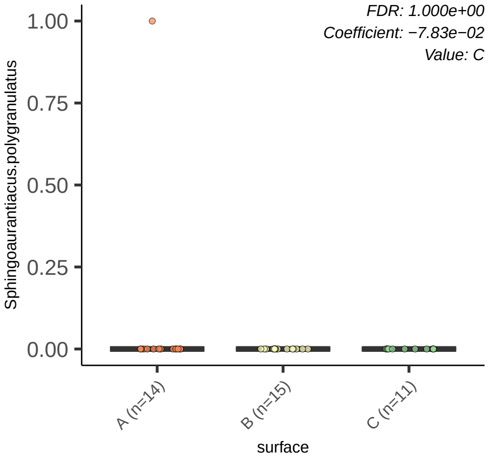
FDR: 1.000e+00
Coefficient: −7.83e−02
Value: C
Sphingoaurantiacus.polygranulatus
1.00
0.75
0.50
0.25
0.00
A (n=14)
B (n=15)
C (n=11)
surface

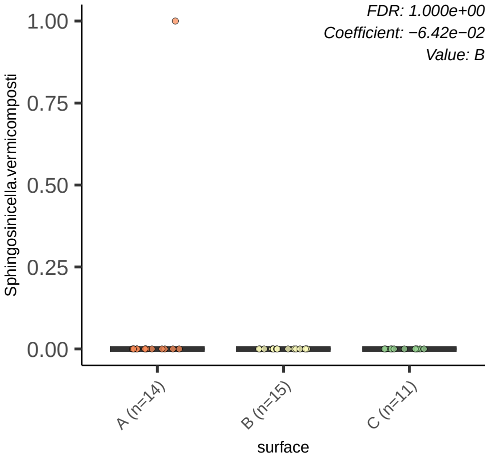
FDR: 1.000e+00
Coefficient: −6.42e−02
Value: B
1.00
0.75
0.50
0.25
0.00
Sphingosinicella.vermicomposti
A (n=14)
B (n=15)
C (n=11)
surface

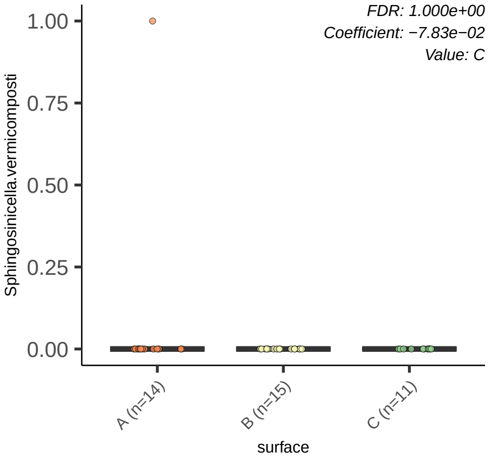
FDR: 1.000e+00
Coefficient: −7.83e−02
Value: C
Sphingosinicella.vermicomposti
1.00
0.75
0.50
0.25
0.00
A (n=14)
B (n=15)
C (n=11)
surface

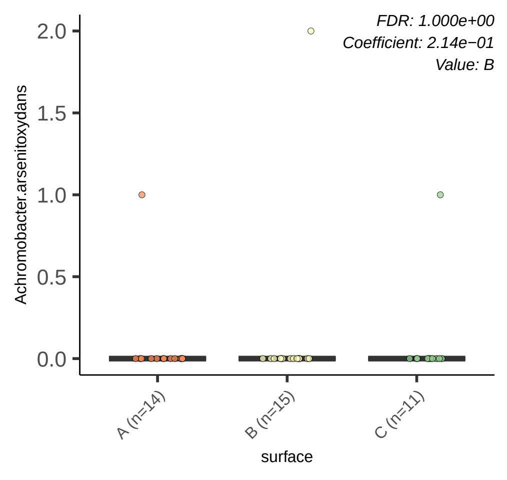
FDR: 1.000e+00
Coefficient: 2.14e−01
Value: B
Achromobacter.arsenitoxydans
2.0
1.5
1.0
0.5
0.0
A (n=14)
B (n=15)
C (n=11)
surface

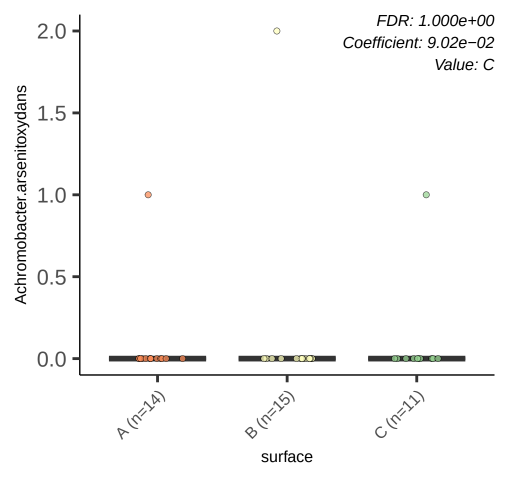
FDR: 1.000e+00
Coefficient: 9.02e−02
Value: C
Achromobacter.arsenitoxydans
2.0
1.5
1.0
0.5
0.0
A (n=14)
B (n=15)
C (n=11)
surface

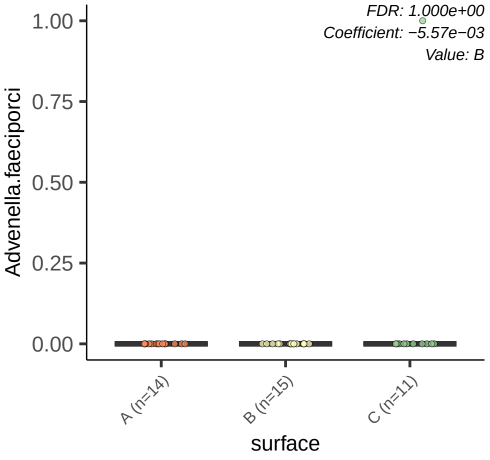
FDR: 1.000e+00
Coefficient: −5.57e−03
Value: B
Advenella.faeciporci
1.00
0.75
0.50
0.25
0.00
A (n=14)
B (n=15)
C (n=11)
surface

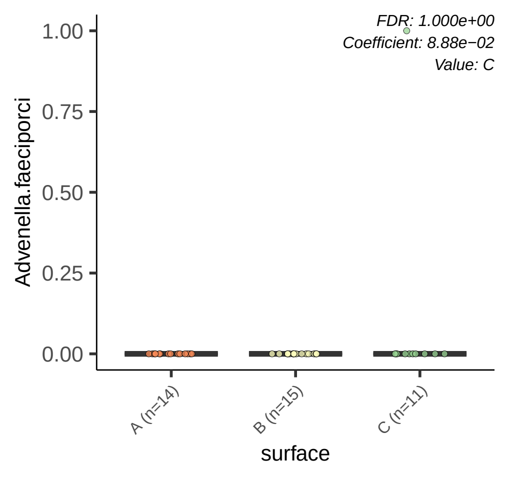
FDR: 1.000e+00
Coefficient: 8.88e−02
Value: C
Advenella.faeciporci
1.00
0.75
0.50
0.25
0.00
A (n=14)
B (n=15)
C (n=11)
surface

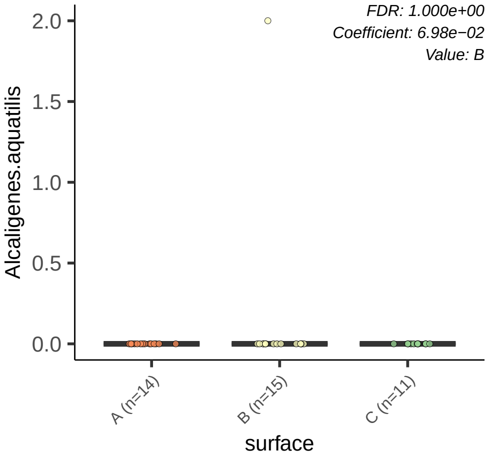
FDR: 1.000e+00
Coefficient: 6.98e−02
Value: B
Alcaligenes.aquatilis
2.0
1.5
1.0
0.5
0.0
A (n=14)
B (n=15)
C (n=11)
surface

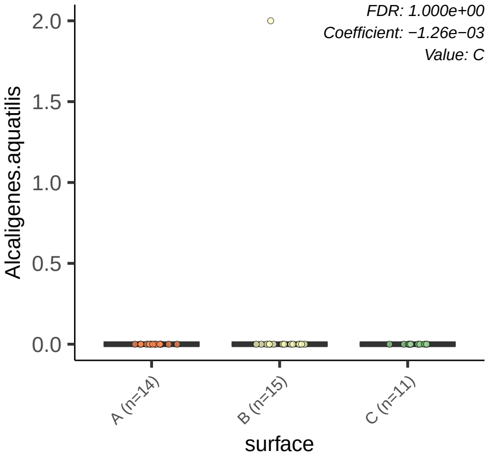

FDR: 1.000e+00
Coefficient: −1.26e−03
Value: C
Alcaligenes.aquatilis
2.0
1.5
1.0
0.5
0.0
A (n=14)
B (n=15)
C (n=11)
surface

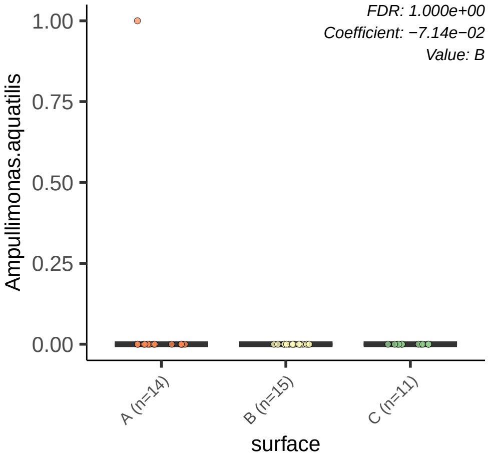
FDR: 1.000e+00
Coefficient: −7.14e−02
Value: B
Ampullimonas.aquatilis
1.00
0.75
0.50
0.25
0.00
A (n=14)
B (n=15)
C (n=11)
surface

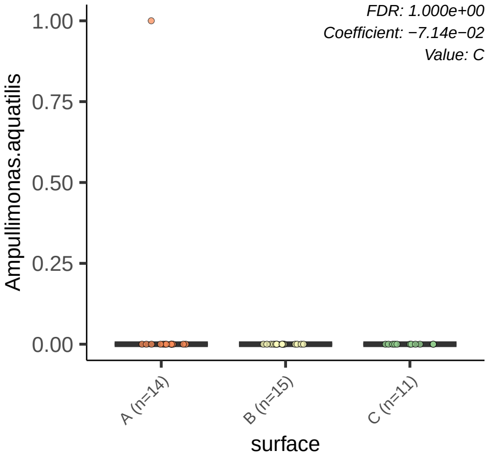
FDR: 1.000e+00
Coefficient: −7.14e−02
Value: C
Ampullimonas.aquatilis
1.00
0.75
0.50
0.25
0.00
A (n=14)
B (n=15)
C (n=11)
surface

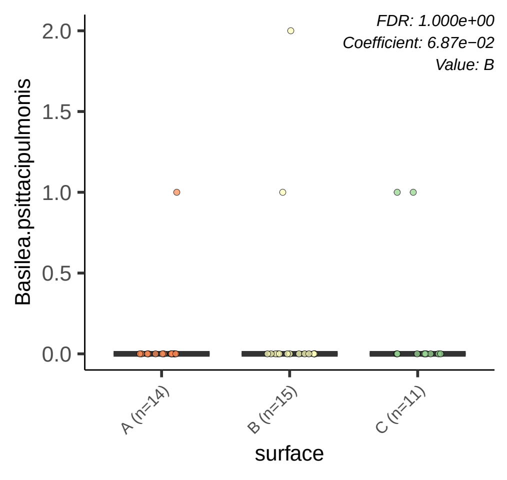
FDR: 1.000e+00
Coefficient: 6.87e−02
Value: B
Basilea.psittacipulmonis
2.0
1.5
1.0
0.5
0.0
A (n=14)
B (n=15)
C (n=11)
surface

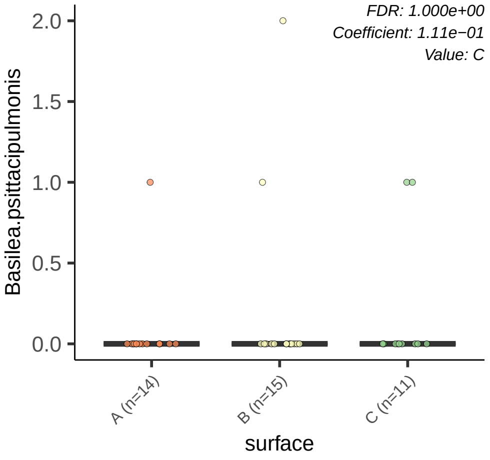
FDR: 1.000e+00
Coefficient: 1.11e−01
Value: C
Basilea.psittacipulmonis
2.0
1.5
1.0
0.5
0.0
A (n=14)
B (n=15)
C (n=11)
surface

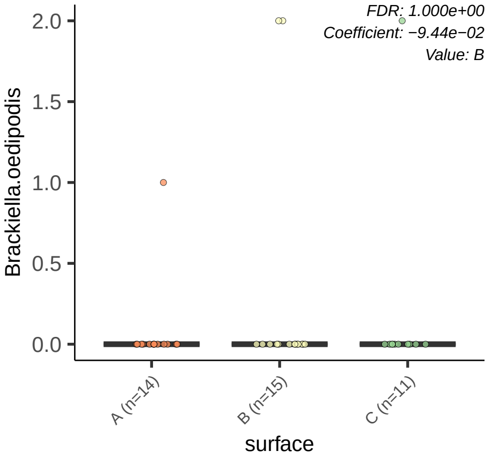

FDR: 1.000e+00
Coefficient: −9.44e−02
Value: B
Brackiella.oedipodis
2.0
1.5
1.0
0.5
0.0
A (n=14)
B (n=15)
C (n=11)
surface

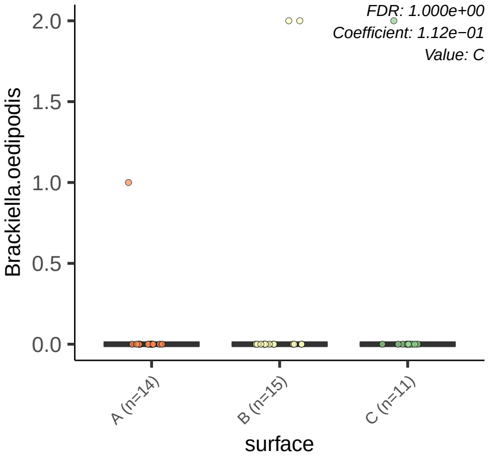

FDR: 1.000e+00
Coefficient: 1.12e−01
Value: C
Brackiella.oedipodis
2.0
1.5
1.0
0.5
0.0
A (n=14)
B (n=15)
C (n=11)
surface

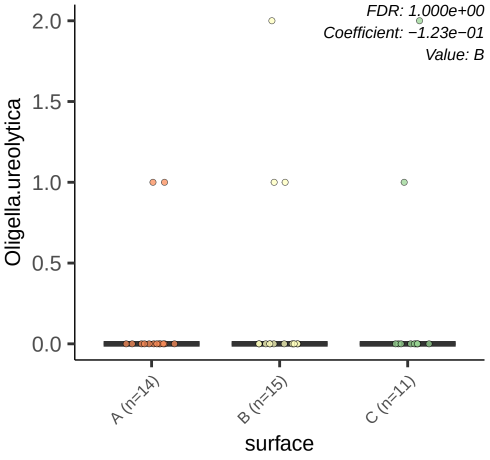
FDR: 1.000e+00
Coefficient: −1.23e−01
Value: B
Oligella.ureolytica
2.0
1.5
1.0
0.5
0.0
A (n=14)
B (n=15)
C (n=11)
surface

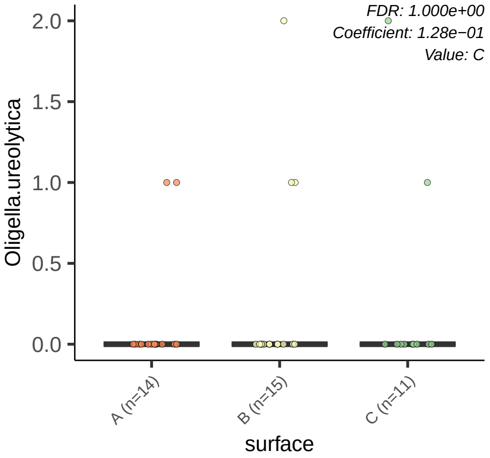

FDR: 1.000e+00
Coefficient: 1.28e−01
Value: C
Oligella.ureolytica
2.0
1.5
1.0
0.5
0.0
A (n=14)
B (n=15)
C (n=11)
surface

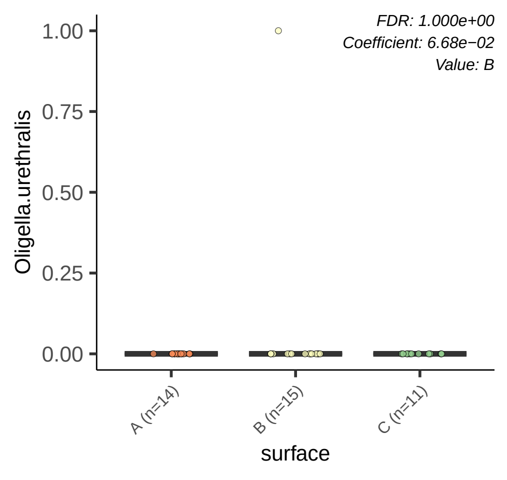

FDR: 1.000e+00
Coefficient: 6.68e−02
Value: B
1.00
0.75
0.50
0.25
0.00
Oligella.urethralis
A (n=14)
B (n=15)
C (n=11)
surface

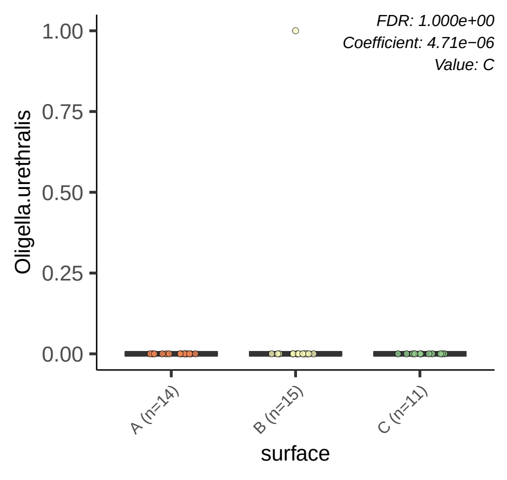
FDR: 1.000e+00
Coefficient: 4.71e−06
Value: C
1.00
0.75
0.50
0.25
0.00
Oligella.urethralis
A (n=14)
B (n=15)
C (n=11)
surface

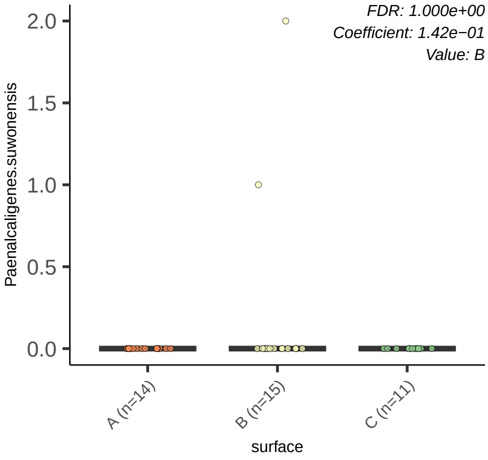

FDR: 1.000e+00
Coefficient: 1.42e−01
Value: B
Paenalcaligenes.suwonensis
2.0
1.5
1.0
0.5
0.0
A (n=14)
B (n=15)
C (n=11)
surface

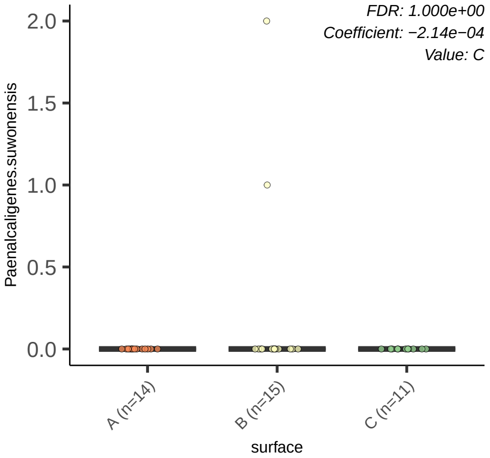
FDR: 1.000e+00
Coefficient: −2.14e−04
Value: C
2.0
1.5
1.0
0.5
0.0
Paenalcaligenes.suwonensis
A (n=14)
B (n=15)
C (n=11)
surface

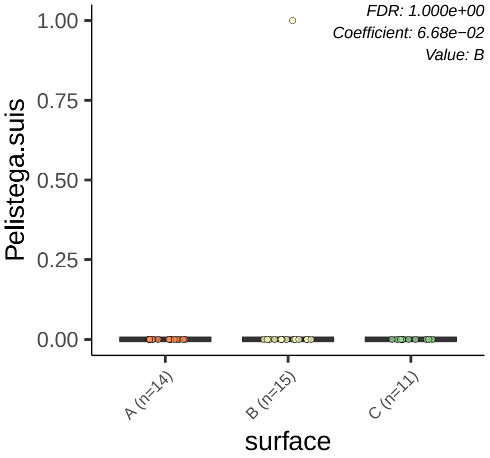

FDR: 1.000e+00
Coefficient: 6.68e−02
Value: B
Pelistega.suis
1.00
0.75
0.50
0.25
0.00
A (n=14)
B (n=15)
C (n=11)
surface

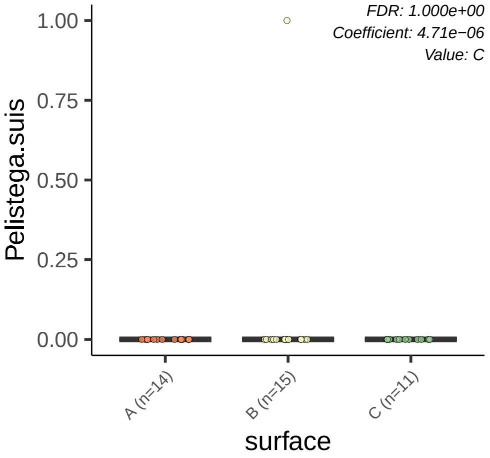
FDR: 1.000e+00
Coefficient: 4.71e−06
Value: C
Pelistega.suis
1.00
0.75
0.50
0.25
0.00
A (n=14)
B (n=15)
C (n=11)
surface

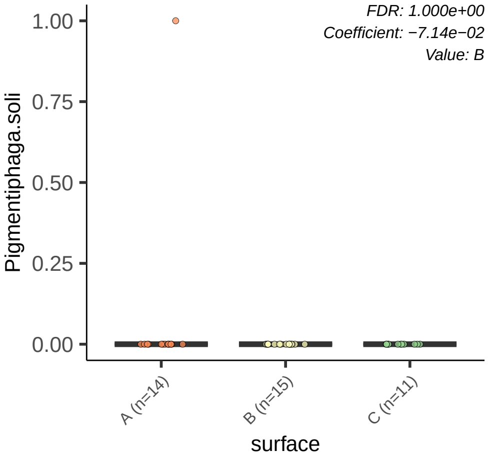

FDR: 1.000e+00
Coefficient: −7.14e−02
Value: B
Pigmentiphaga.soli
1.00
0.75
0.50
0.25
0.00
A (n=14)
B (n=15)
C (n=11)
surface

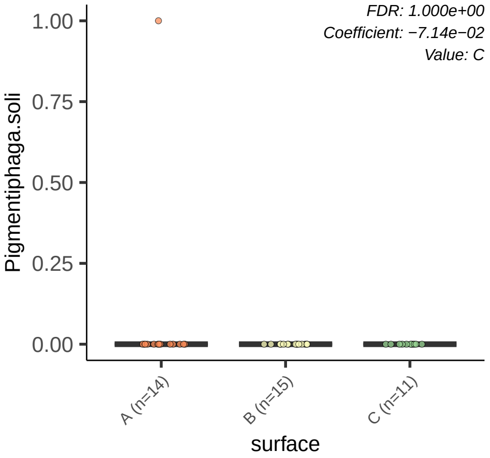
FDR: 1.000e+00
Coefficient: −7.14e−02
Value: C
Pigmentiphaga.soli
1.00
0.75
0.50
0.25
0.00
A (n=14)
B (n=15)
C (n=11)
surface

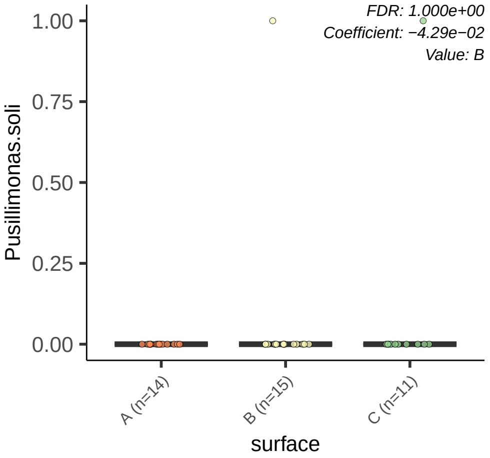
FDR: 1.000e+00
Coefficient: −4.29e−02
Value: B
Pusillimonas.soli
1.00
0.75
0.50
0.25
0.00
A (n=14)
B (n=15)
C (n=11)
surface

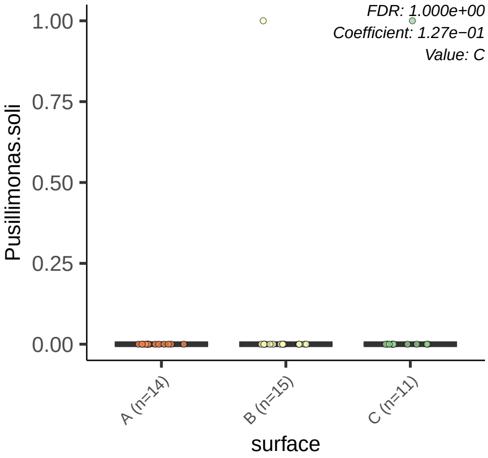

FDR: 1.000e+00
Coefficient: 1.27e−01
Value: C
Pusillimonas.soli
1.00
0.75
0.50
0.25
0.00
A (n=14)
B (n=15)
C (n=11)
surface

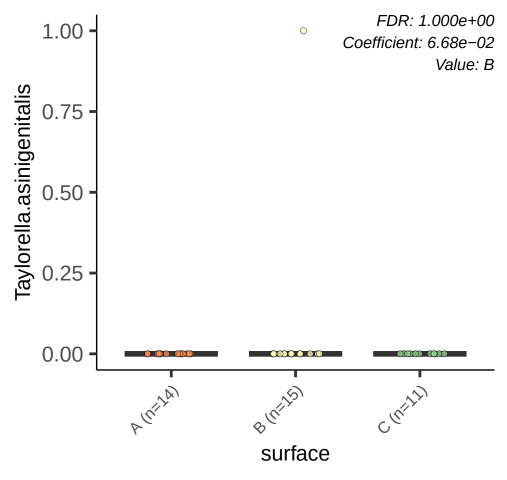

FDR: 1.000e+00
Coefficient: 6.68e−02
Value: B
Taylorella.asinigenitalis
1.00
0.75
0.50
0.25
0.00
A (n=14)
B (n=15)
C (n=11)
surface

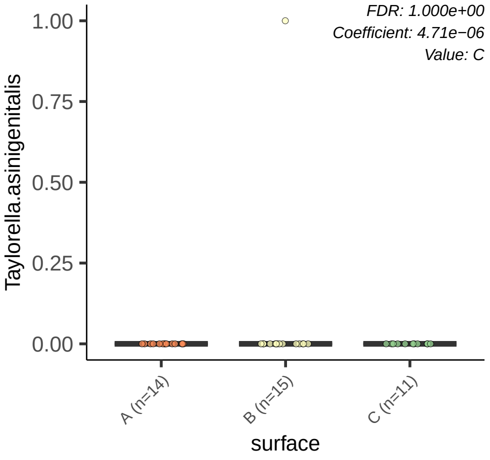
FDR: 1.000e+00
Coefficient: 4.71e−06
Value: C
Taylorella.asinigenitalis
1.00
0.75
0.50
0.25
0.00
A (n=14)
B (n=15)
C (n=11)
surface

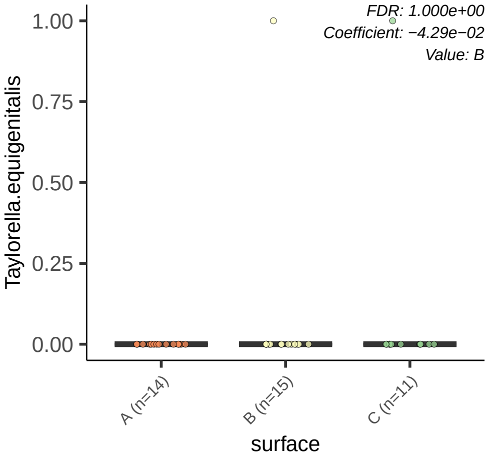

FDR: 1.000e+00
Coefficient: −4.29e−02
Value: B
Taylorella.equigenitalis
1.00
0.75
0.50
0.25
0.00
A (n=14)
B (n=15)
C (n=11)
surface

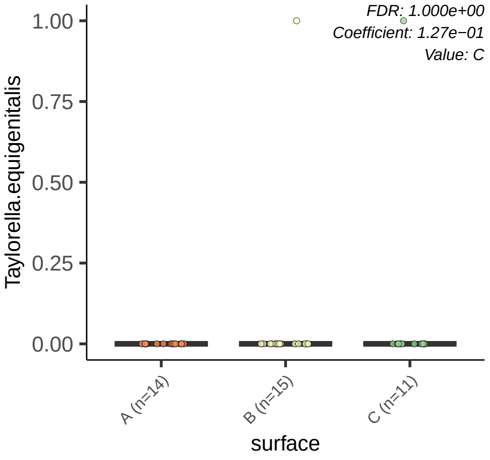

FDR: 1.000e+00
Coefficient: 1.27e−01
Value: C
Taylorella.equigenitalis
1.00
0.75
0.50
0.25
0.00
A (n=14)
B (n=15)
C (n=11)
surface

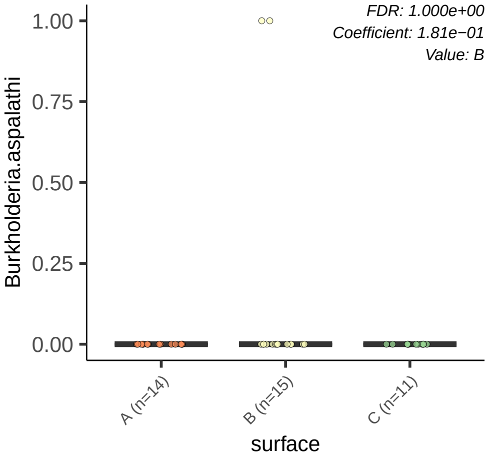

FDR: 1.000e+00
Coefficient: 1.81e−01
Value: B
Burkholderia.aspalathi
1.00
0.75
0.50
0.25
0.00
A (n=14)
B (n=15)
C (n=11)
surface

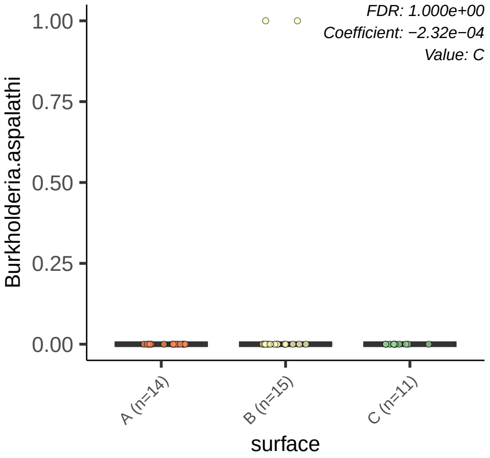

FDR: 1.000e+00
Coefficient: −2.32e−04
Value: C
Burkholderia.aspalathi
1.00
0.75
0.50
0.25
0.00
A (n=14)
B (n=15)
C (n=11)
surface

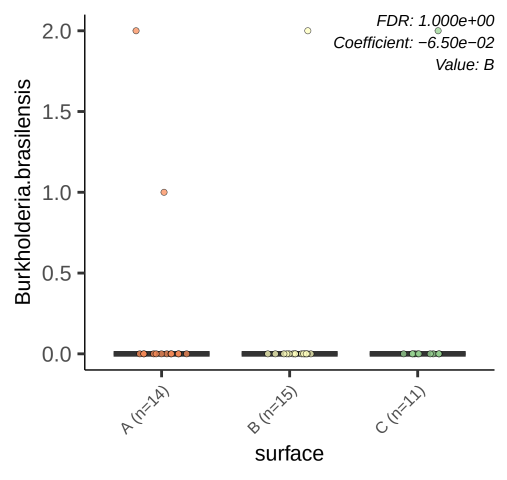

FDR: 1.000e+00
Coefficient: −6.50e−02
Value: B
Burkholderia.brasilensis
2.0
1.5
1.0
0.5
0.0
A (n=14)
B (n=15)
C (n=11)
surface

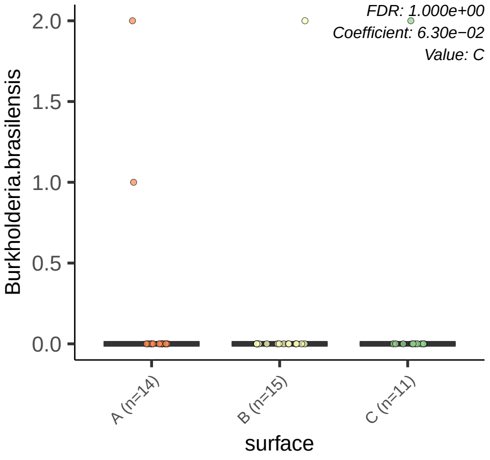

FDR: 1.000e+00
Coefficient: 6.30e−02
Value: C
Burkholderia.brasilensis
2.0
1.5
1.0
0.5
0.0
A (n=14)
B (n=15)
C (n=11)
surface

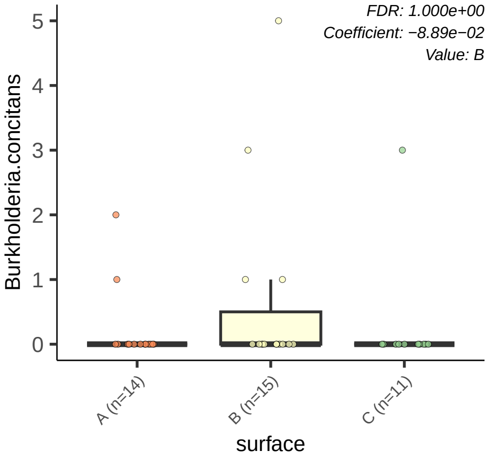
FDR: 1.000e+00
Coefficient: −8.89e−02
Value: B
Burkholderia.concitans
0
1
2
3
4
5
A (n=14)
B (n=15)
C (n=11)
surface

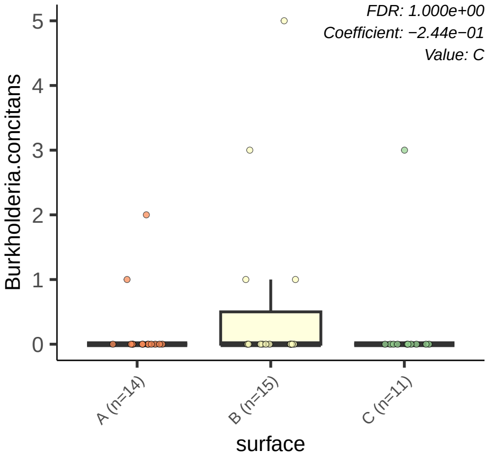
FDR: 1.000e+00
Coefficient: −2.44e−01
Value: C
Burkholderia.concitans
0
1
2
3
4
5
A (n=14)
B (n=15)
C (n=11)
surface

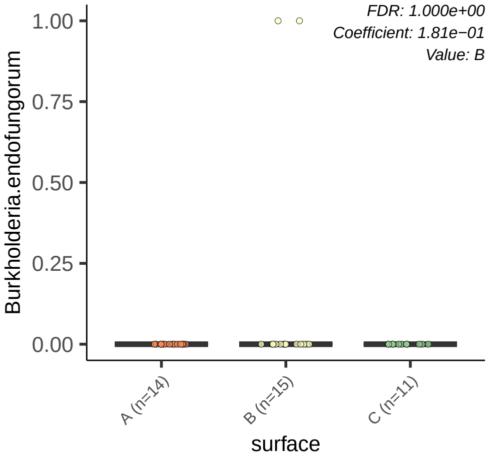

FDR: 1.000e+00
Coefficient: 1.81e−01
Value: B
Burkholderia.endofungorum
1.00
0.75
0.50
0.25
0.00
A (n=14)
B (n=15)
C (n=11)
surface

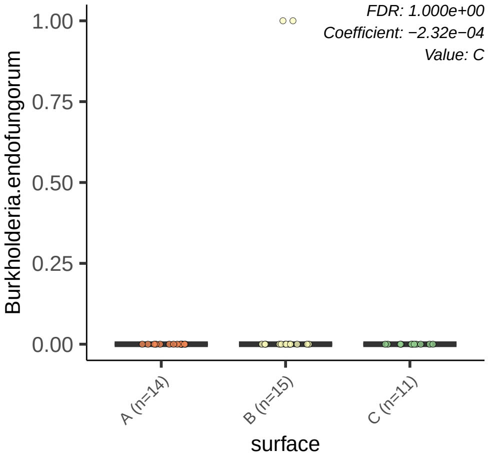
FDR: 1.000e+00
Coefficient: −2.32e−04
Value: C
Burkholderia.endofungorum
1.00
0.75
0.50
0.25
0.00
A (n=14)
B (n=15)
C (n=11)
surface

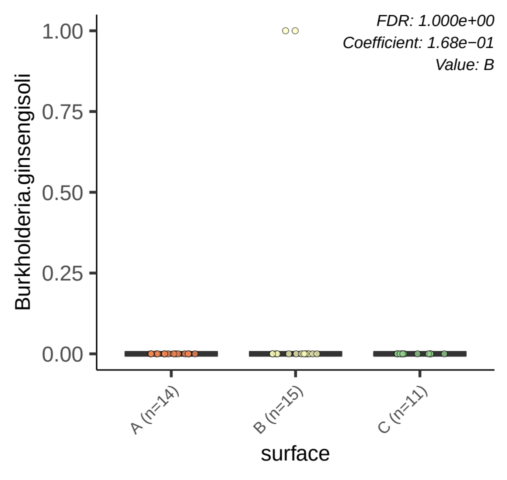

FDR: 1.000e+00
Coefficient: 1.68e−01
Value: B
Burkholderia.ginsengisoli
1.00
0.75
0.50
0.25
0.00
A (n=14)
B (n=15)
C (n=11)
surface

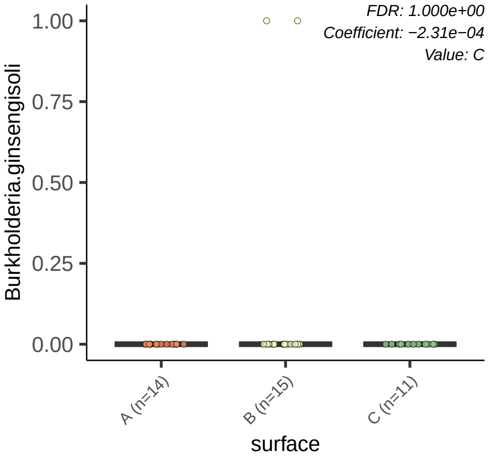

FDR: 1.000e+00
Coefficient: −2.31e−04
Value: C
Burkholderia.ginsengisoli
1.00
0.75
0.50
0.25
0.00
A (n=14)
B (n=15)
C (n=11)
surface

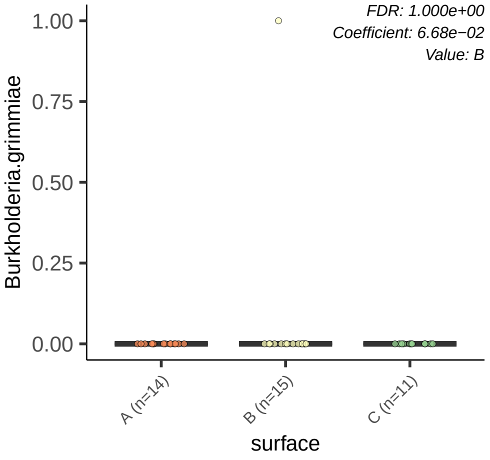
FDR: 1.000e+00
Coefficient: 6.68e−02
Value: B
Burkholderia.grimmiae
1.00
0.75
0.50
0.25
0.00
A (n=14)
B (n=15)
C (n=11)
surface

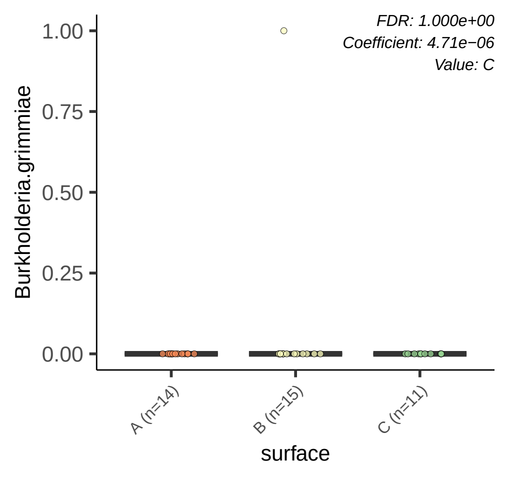

FDR: 1.000e+00
Coefficient: 4.71e−06
Value: C
Burkholderia.grimmiae
1.00
0.75
0.50
0.25
0.00
A (n=14)
B (n=15)
C (n=11)
surface

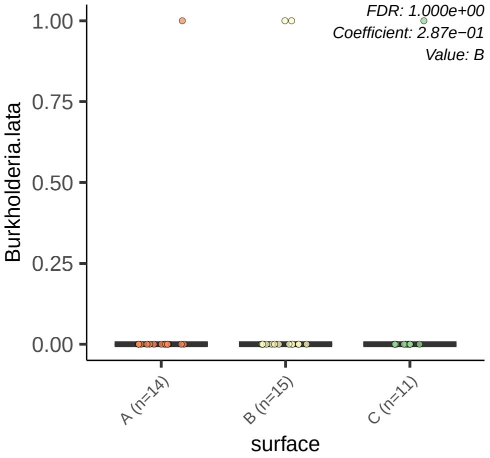

FDR: 1.000e+00
Coefficient: 2.87e−01
Value: B
1.00
0.75
0.50
0.25
0.00
Burkholderia.lata
A (n=14)
B (n=15)
C (n=11)
surface

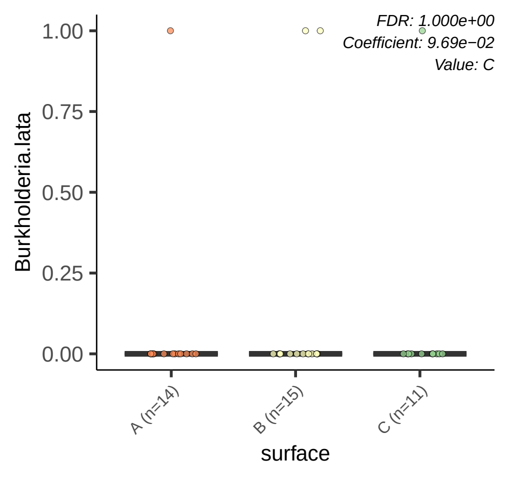
FDR: 1.000e+00
Coefficient: 9.69e−02
Value: C
1.00
0.75
0.50
0.25
0.00
Burkholderia.lata
A (n=14)
B (n=15)
C (n=11)
surface

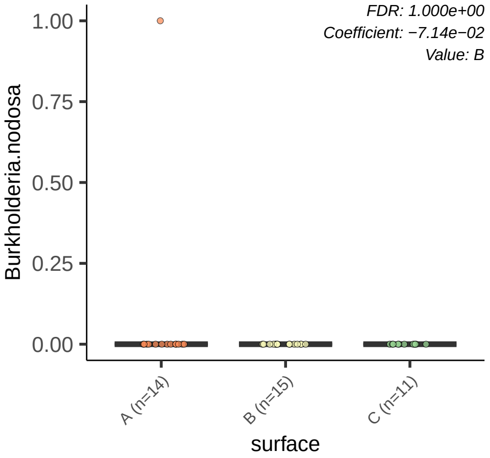

FDR: 1.000e+00
Coefficient: −7.14e−02
Value: B
Burkholderia.nodosa
1.00
0.75
0.50
0.25
0.00
A (n=14)
B (n=15)
C (n=11)
surface

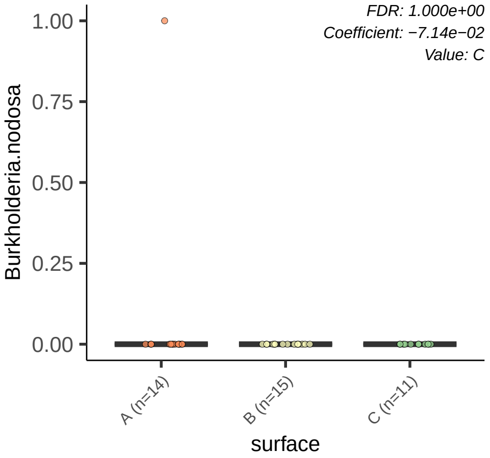
FDR: 1.000e+00
Coefficient: −7.14e−02
Value: C
Burkholderia.nodosa
1.00
0.75
0.50
0.25
0.00
A (n=14)
B (n=15)
C (n=11)
surface

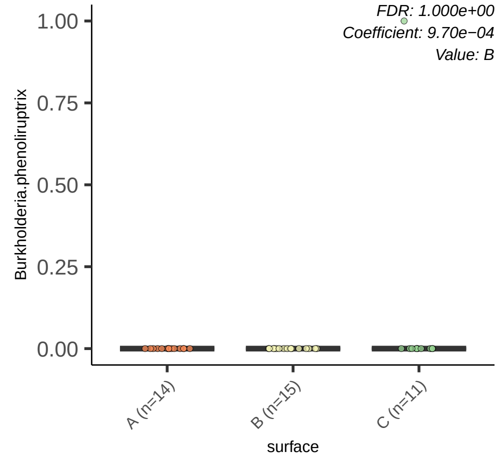
FDR: 1.000e+00
Coefficient: 9.70e−04
Value: B
1.00
0.75
0.50
0.25
0.00
Burkholderia.phenoliruptrix
A (n=14)
B (n=15)
C (n=11)
surface

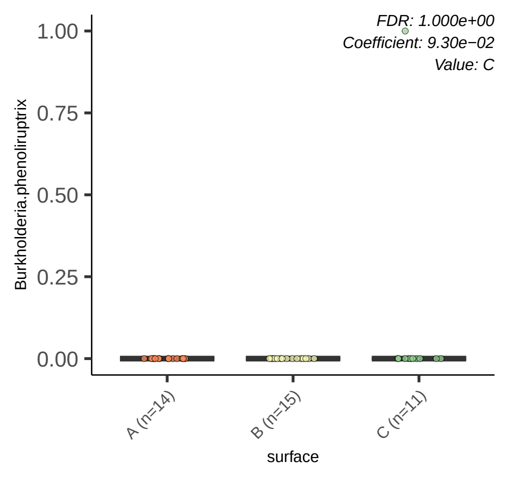
FDR: 1.000e+00
Coefficient: 9.30e−02
Value: C
1.00
0.75
0.50
0.25
0.00
Burkholderia.phenoliruptrix
A (n=14)
B (n=15)
C (n=11)
surface

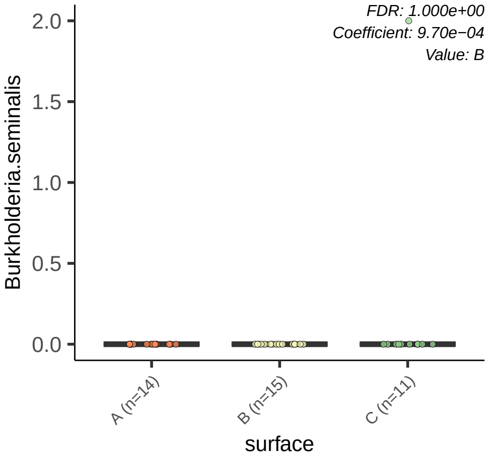
FDR: 1.000e+00
Coefficient: 9.70e−04
Value: B
Burkholderia.seminalis
2.0
1.5
1.0
0.5
0.0
A (n=14)
B (n=15)
C (n=11)
surface

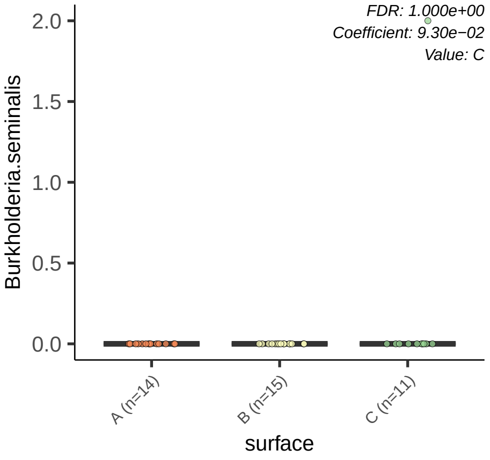
FDR: 1.000e+00
Coefficient: 9.30e−02
Value: C
Burkholderia.seminalis
2.0
1.5
1.0
0.5
0.0
A (n=14)
B (n=15)
C (n=11)
surface

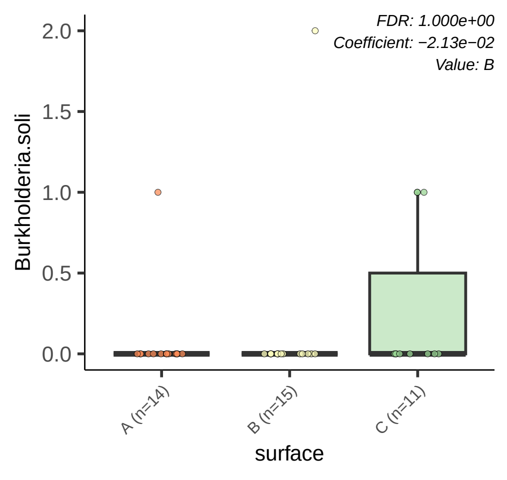

FDR: 1.000e+00
Coefficient: −2.13e−02
Value: B
Burkholderia.soli
2.0
1.5
1.0
0.5
0.0
A (n=14)
B (n=15)
C (n=11)
surface

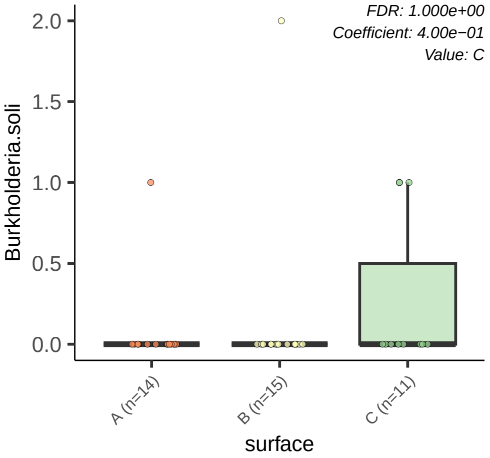
FDR: 1.000e+00
Coefficient: 4.00e−01
Value: C
Burkholderia.soli
2.0
1.5
1.0
0.5
0.0
A (n=14)
B (n=15)
C (n=11)
surface

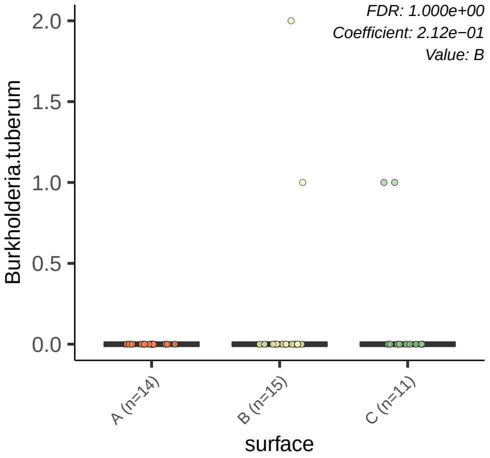
FDR: 1.000e+00
Coefficient: 2.12e−01
Value: B
Burkholderia.tuberum
2.0
1.5
1.0
0.5
0.0
A (n=14)
B (n=15)
C (n=11)
surface

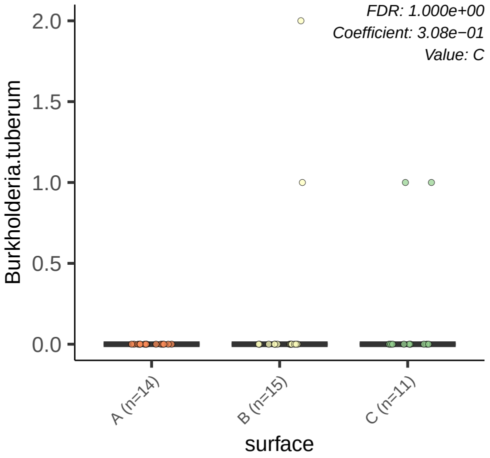

FDR: 1.000e+00
Coefficient: 3.08e−01
Value: C
Burkholderia.tuberum
2.0
1.5
1.0
0.5
0.0
A (n=14)
B (n=15)
C (n=11)
surface

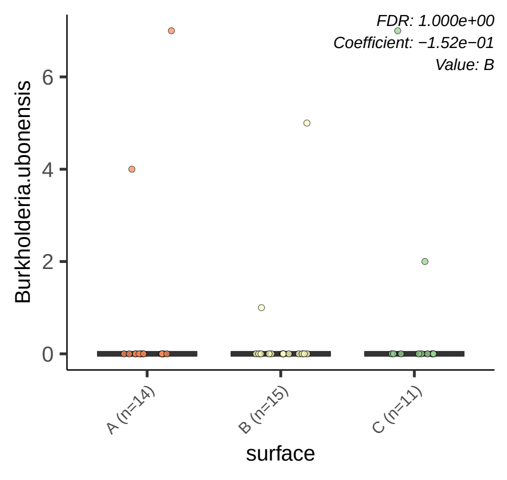

FDR: 1.000e+00
Coefficient: −1.52e−01
Value: B
Burkholderia.ubonensis
0
2
4
6
A (n=14)
B (n=15)
C (n=11)
surface

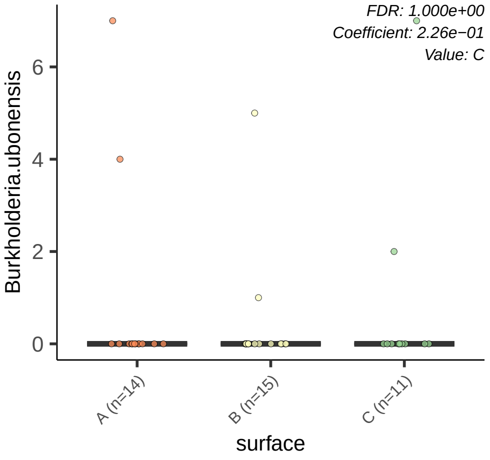

FDR: 1.000e+00
Coefficient: 2.26e−01
Value: C
Burkholderia.ubonensis
0
2
4
6
A (n=14)
B (n=15)
C (n=11)
surface

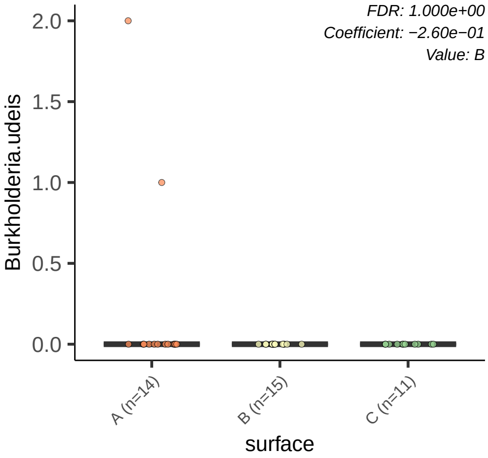

FDR: 1.000e+00
Coefficient: −2.60e−01
Value: B
Burkholderia.udeis
2.0
1.5
1.0
0.5
0.0
A (n=14)
B (n=15)
C (n=11)
surface

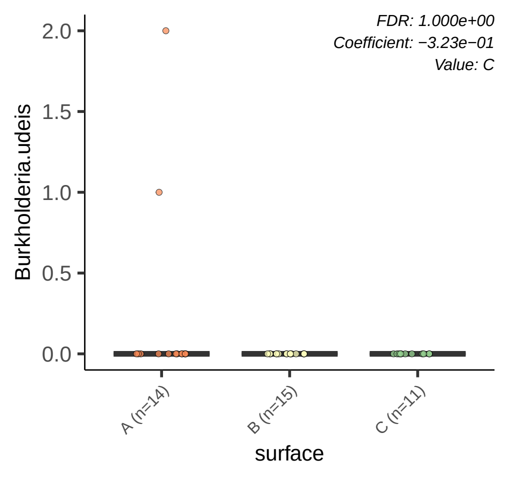

FDR: 1.000e+00
Coefficient: −3.23e−01
Value: C
Burkholderia.udeis
2.0
1.5
1.0
0.5
0.0
A (n=14)
B (n=15)
C (n=11)
surface

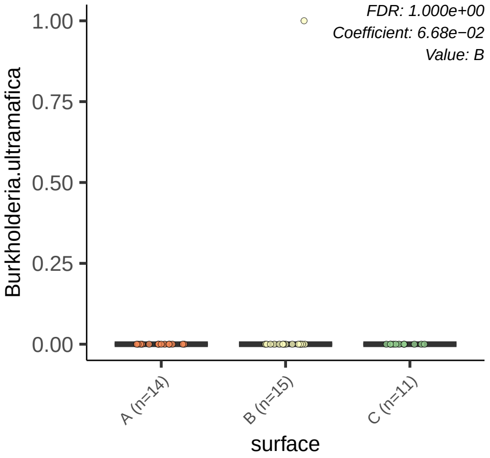

FDR: 1.000e+00
Coefficient: 6.68e−02
Value: B
Burkholderia.ultramafica
1.00
0.75
0.50
0.25
0.00
A (n=14)
B (n=15)
C (n=11)
surface

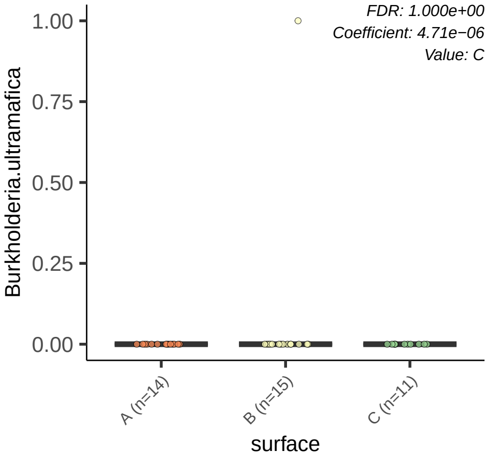
FDR: 1.000e+00
Coefficient: 4.71e−06
Value: C
Burkholderia.ultramafica
1.00
0.75
0.50
0.25
0.00
A (n=14)
B (n=15)
C (n=11)
surface

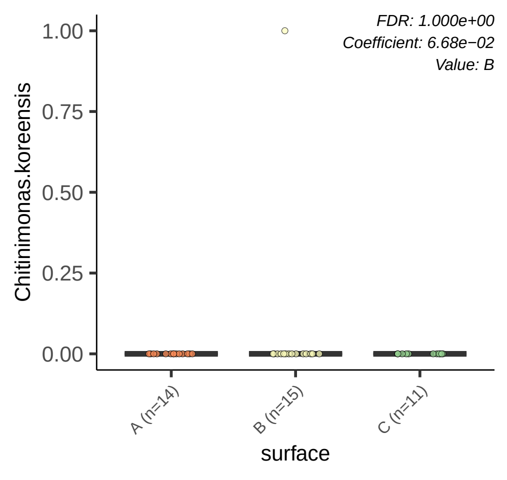
FDR: 1.000e+00
Coefficient: 6.68e−02
Value: B
Chitinimonas.koreensis
1.00
0.75
0.50
0.25
0.00
A (n=14)
B (n=15)
C (n=11)
surface

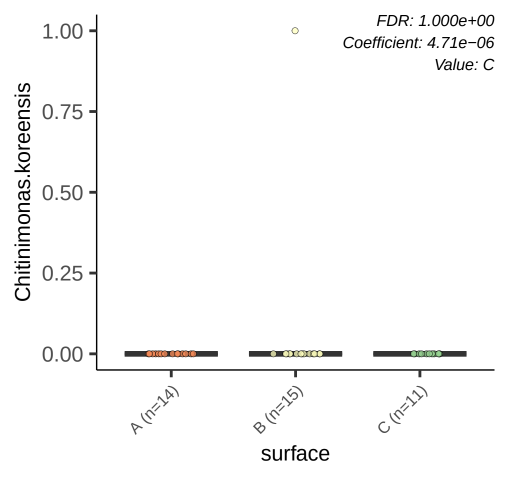
FDR: 1.000e+00
Coefficient: 4.71e−06
Value: C
Chitinimonas.koreensis
1.00
0.75
0.50
0.25
0.00
A (n=14)
B (n=15)
C (n=11)
surface

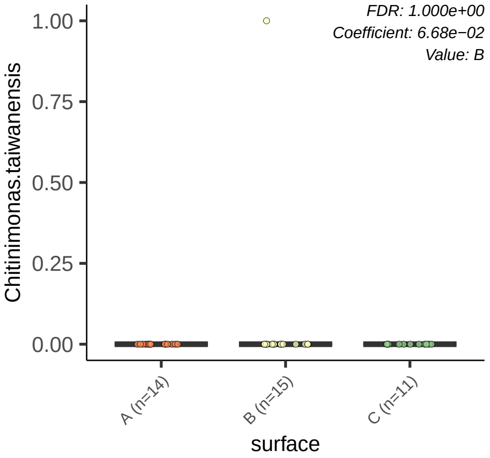
FDR: 1.000e+00
Coefficient: 6.68e−02
Value: B
Chitinimonas.taiwanensis
1.00
0.75
0.50
0.25
0.00
A (n=14)
B (n=15)
C (n=11)
surface

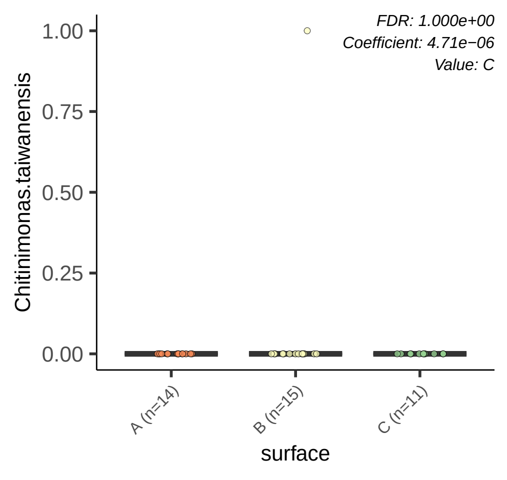
FDR: 1.000e+00
Coefficient: 4.71e−06
Value: C
Chitinimonas.taiwanensis
1.00
0.75
0.50
0.25
0.00
A (n=14)
B (n=15)
C (n=11)
surface

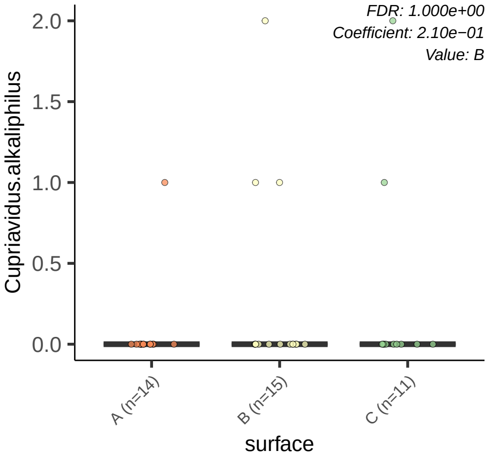

FDR: 1.000e+00
Coefficient: 2.10e−01
Value: B
Cupriavidus.alkaliphilus
2.0
1.5
1.0
0.5
0.0
A (n=14)
B (n=15)
C (n=11)
surface

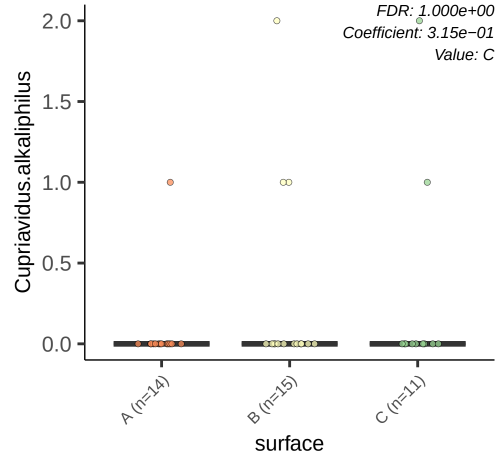

FDR: 1.000e+00
Coefficient: 3.15e−01
Value: C
Cupriavidus.alkaliphilus
2.0
1.5
1.0
0.5
0.0
A (n=14)
B (n=15)
C (n=11)
surface

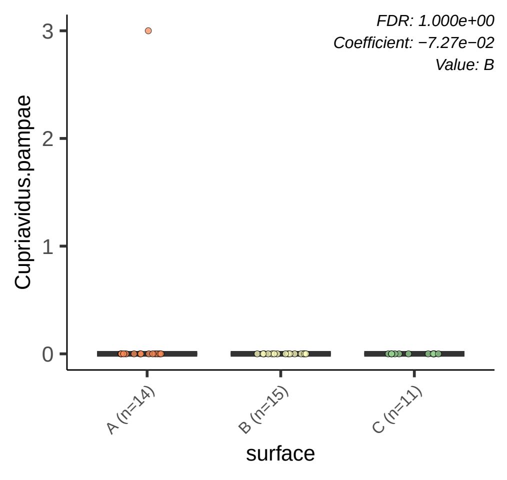

FDR: 1.000e+00
Coefficient: −7.27e−02
Value: B
Cupriavidus.pampae
3
2
1
0
A (n=14)
B (n=15)
C (n=11)
surface

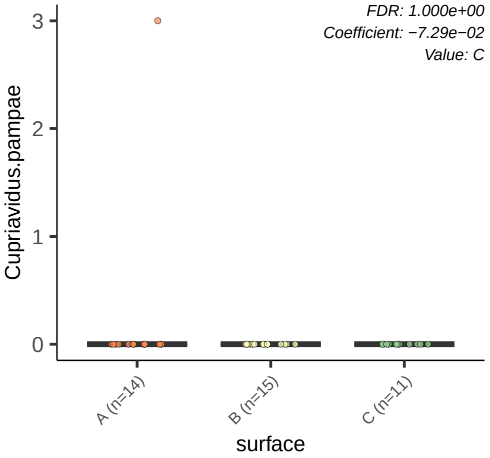
FDR: 1.000e+00
Coefficient: −7.29e−02
Value: C
Cupriavidus.pampae
3
2
1
0
A (n=14)
B (n=15)
C (n=11)
surface

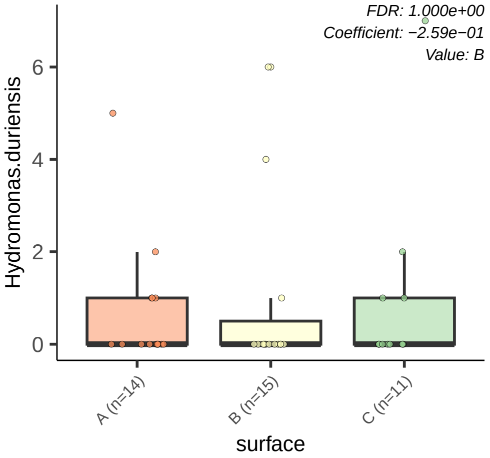

FDR: 1.000e+00
Coefficient: −2.59e−01
Value: B
Hydromonas.duriensis
0
2
4
6
A (n=14)
B (n=15)
C (n=11)
surface

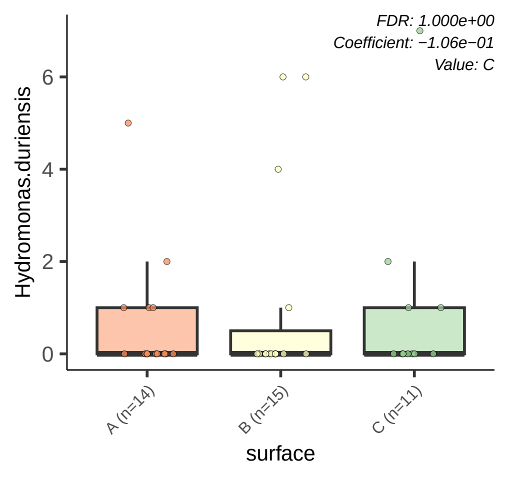
FDR: 1.000e+00
Coefficient: −1.06e−01
Value: C
Hydromonas.duriensis
0
2
4
6
A (n=14)
B (n=15)
C (n=11)
surface

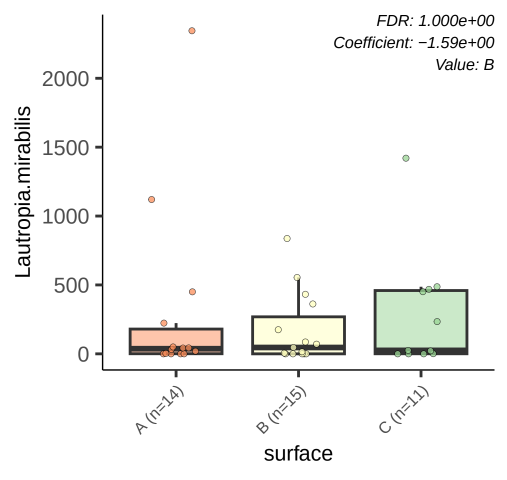

FDR: 1.000e+00
Coefficient: −1.59e+00
Value: B
Lautropia.mirabilis
2000
1500
1000
500
0
A (n=14)
B (n=15)
C (n=11)
surface

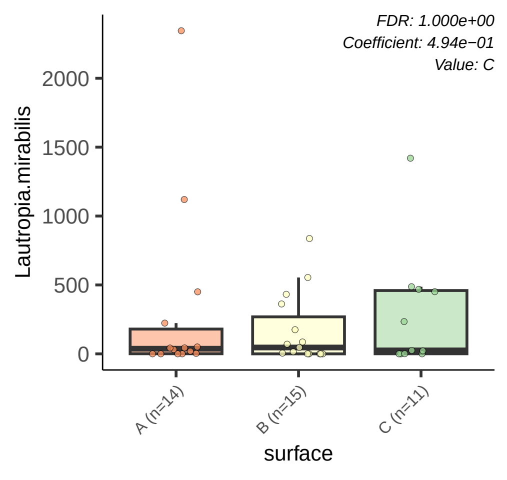

FDR: 1.000e+00
Coefficient: 4.94e−01
Value: C
Lautropia.mirabilis
2000
1500
1000
500
0
A (n=14)
B (n=15)
C (n=11)
surface

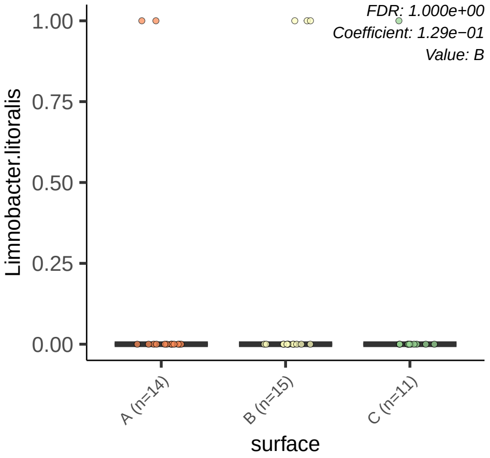

FDR: 1.000e+00
Coefficient: 1.29e−01
Value: B
Limnobacter.litoralis
1.00
0.75
0.50
0.25
0.00
A (n=14)
B (n=15)
C (n=11)
surface

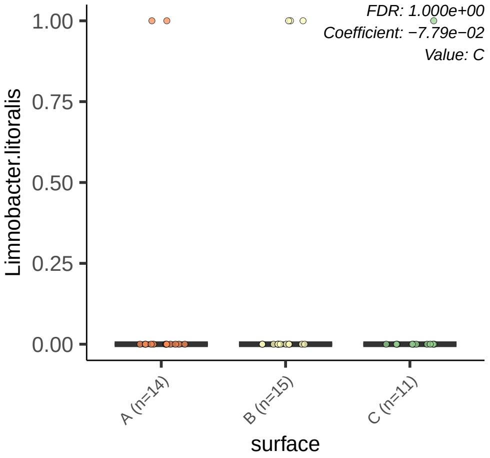
FDR: 1.000e+00
Coefficient: −7.79e−02
Value: C
Limnobacter.litoralis
1.00
0.75
0.50
0.25
0.00
A (n=14)
B (n=15)
C (n=11)
surface

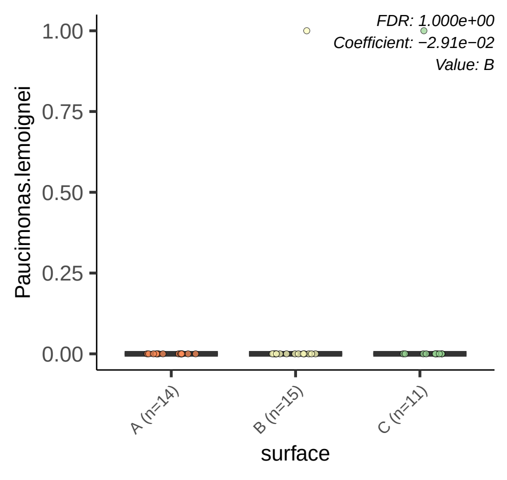
FDR: 1.000e+00
Coefficient: −2.91e−02
Value: B
Paucimonas.lemoignei
1.00
0.75
0.50
0.25
0.00
A (n=14)
B (n=15)
C (n=11)
surface

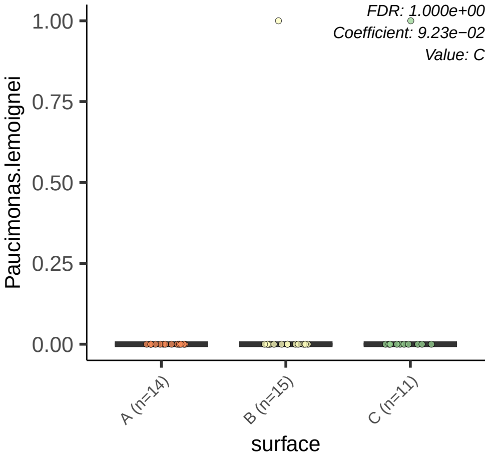
FDR: 1.000e+00
Coefficient: 9.23e−02
Value: C
Paucimonas.lemoignei
1.00
0.75
0.50
0.25
0.00
A (n=14)
B (n=15)
C (n=11)
surface

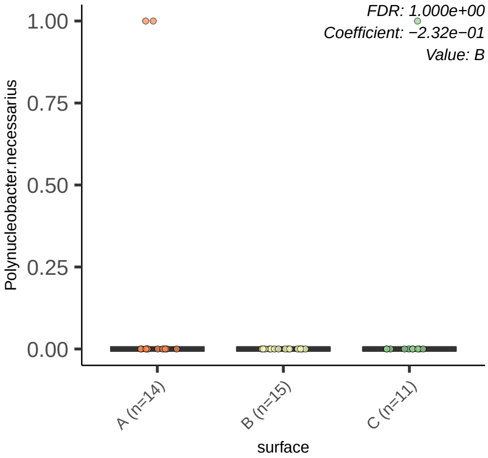
FDR: 1.000e+00
Coefficient: −2.32e−01
Value: B
Polynucleobacter.necessarius
1.00
0.75
0.50
0.25
0.00
A (n=14)
B (n=15)
C (n=11)
surface

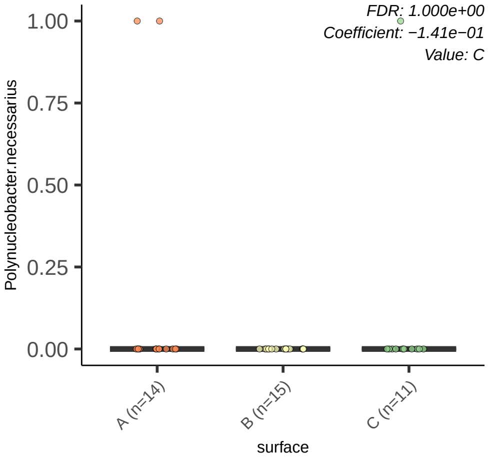
FDR: 1.000e+00
Coefficient: −1.41e−01
Value: C
Polynucleobacter.necessarius
1.00
0.75
0.50
0.25
0.00
A (n=14)
B (n=15)
C (n=11)
surface

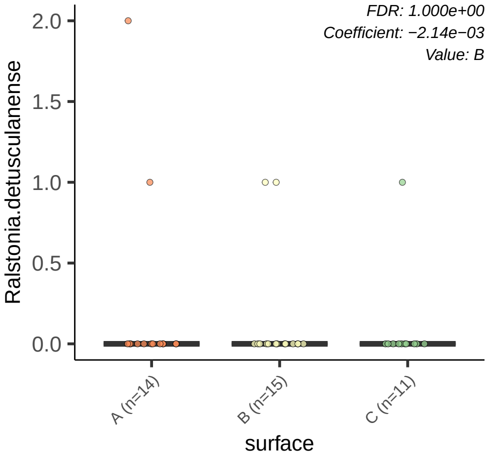

FDR: 1.000e+00
Coefficient: −2.14e−03
Value: B
Ralstonia.detusculanense
2.0
1.5
1.0
0.5
0.0
A (n=14)
B (n=15)
C (n=11)
surface

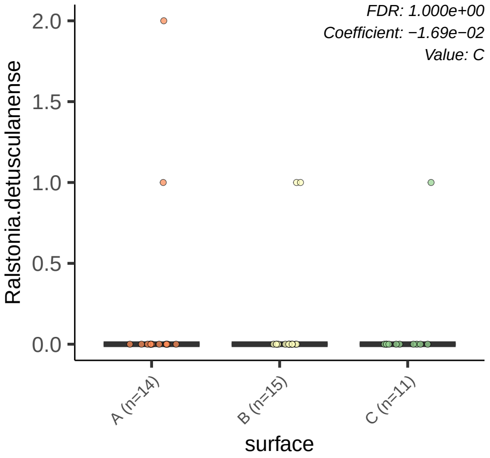
FDR: 1.000e+00
Coefficient: −1.69e−02
Value: C
Ralstonia.detusculanense
2.0
1.5
1.0
0.5
0.0
A (n=14)
B (n=15)
C (n=11)
surface

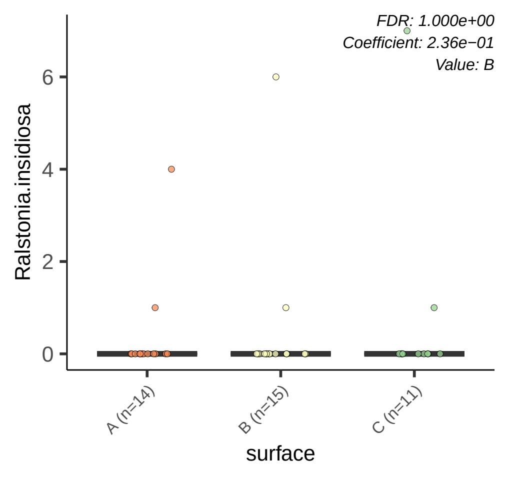
FDR: 1.000e+00
Coefficient: 2.36e−01
Value: B
Ralstonia.insidiosa
0
2
4
6
A (n=14)
B (n=15)
C (n=11)
surface

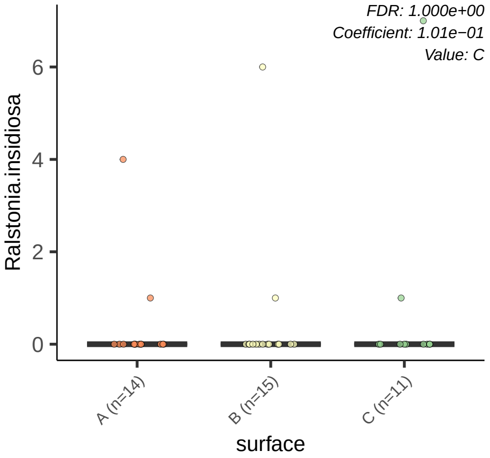
FDR: 1.000e+00
Coefficient: 1.01e−01
Value: C
Ralstonia.insidiosa
0
2
4
6
A (n=14)
B (n=15)
C (n=11)
surface

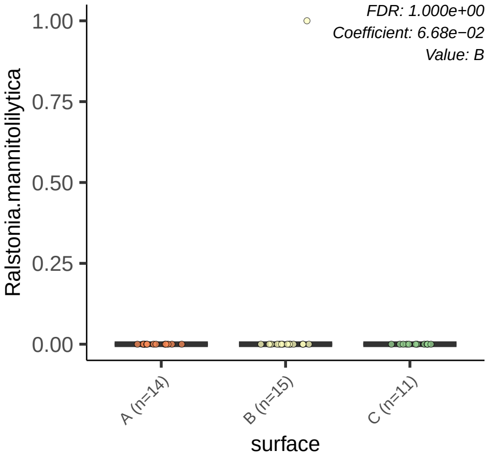
FDR: 1.000e+00
Coefficient: 6.68e−02
Value: B
Ralstonia.mannitolilytica
1.00
0.75
0.50
0.25
0.00
A (n=14)
B (n=15)
C (n=11)
surface

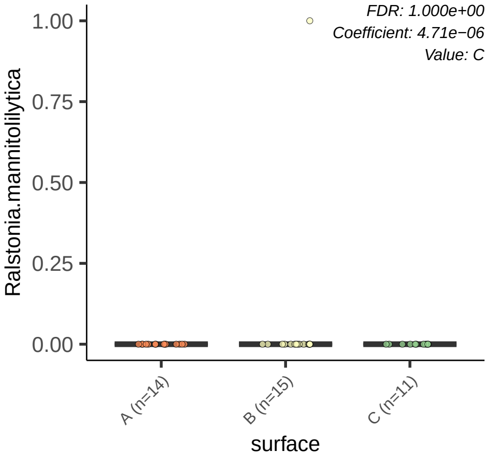
FDR: 1.000e+00
Coefficient: 4.71e−06
Value: C
Ralstonia.mannitolilytica
1.00
0.75
0.50
0.25
0.00
A (n=14)
B (n=15)
C (n=11)
surface

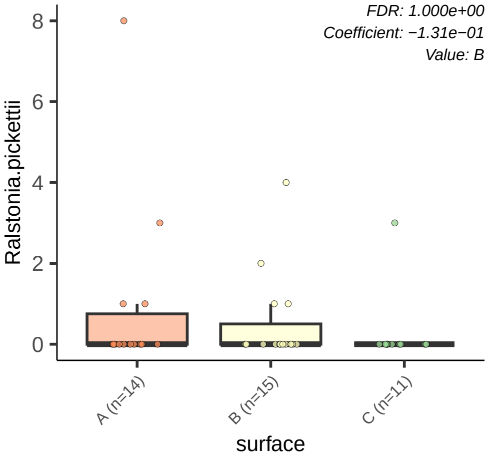
FDR: 1.000e+00
Coefficient: −1.31e−01
Value: B
Ralstonia.pickettii
8
6
4
2
0
A (n=14)
B (n=15)
C (n=11)
surface

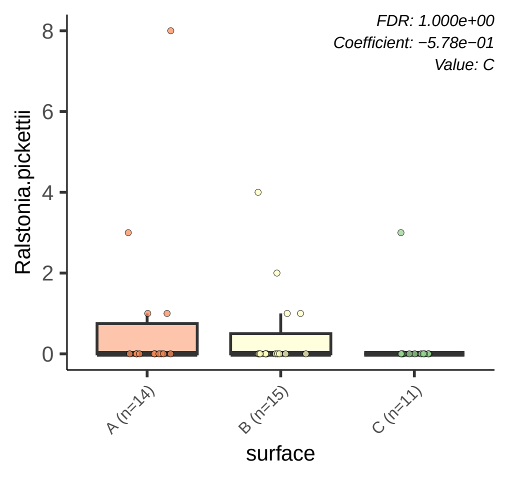
FDR: 1.000e+00
Coefficient: −5.78e−01
Value: C
Ralstonia.pickettii
8
6
4
2
0
A (n=14)
B (n=15)
C (n=11)
surface

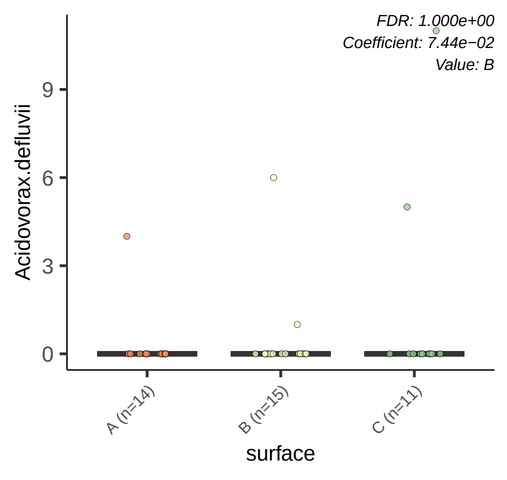

FDR: 1.000e+00
Coefficient: 7.44e−02
Value: B
Acidovorax.defluvii
0
3
6
9
A (n=14)
B (n=15)
C (n=11)
surface

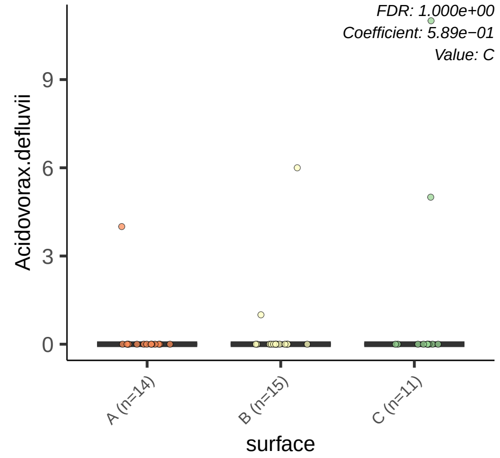

FDR: 1.000e+00
Coefficient: 5.89e−01
Value: C
Acidovorax.defluvii
9
6
3
0
A (n=14)
B (n=15)
C (n=11)
surface

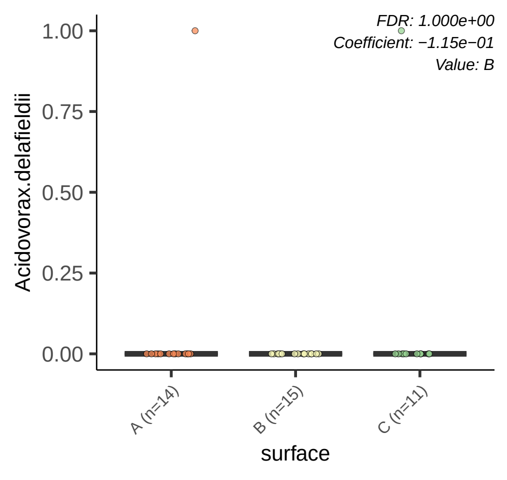
FDR: 1.000e+00
Coefficient: −1.15e−01
Value: B
Acidovorax.delafieldii
1.00
0.75
0.50
0.25
0.00
A (n=14)
B (n=15)
C (n=11)
surface

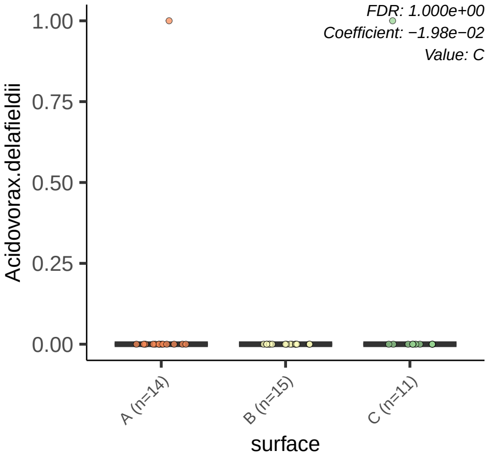
FDR: 1.000e+00
Coefficient: −1.98e−02
Value: C
1.00
0.75
0.50
0.25
0.00
Acidovorax.delafieldii
A (n=14)
B (n=15)
C (n=11)
surface

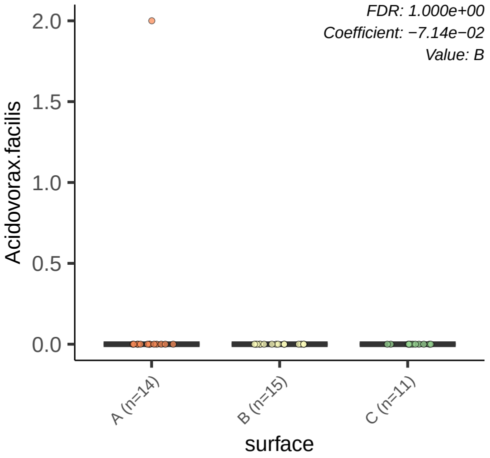
FDR: 1.000e+00
Coefficient: −7.14e−02
Value: B
Acidovorax.facilis
2.0
1.5
1.0
0.5
0.0
A (n=14)
B (n=15)
C (n=11)
surface

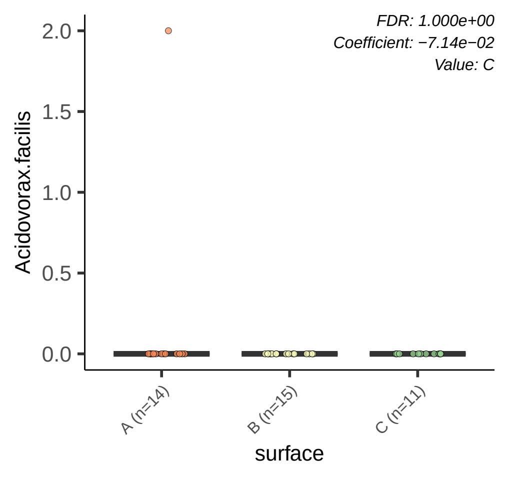

FDR: 1.000e+00
Coefficient: −7.14e−02
Value: C
2.0
1.5
1.0
0.5
0.0
Acidovorax.facilis
A (n=14)
B (n=15)
C (n=11)
surface

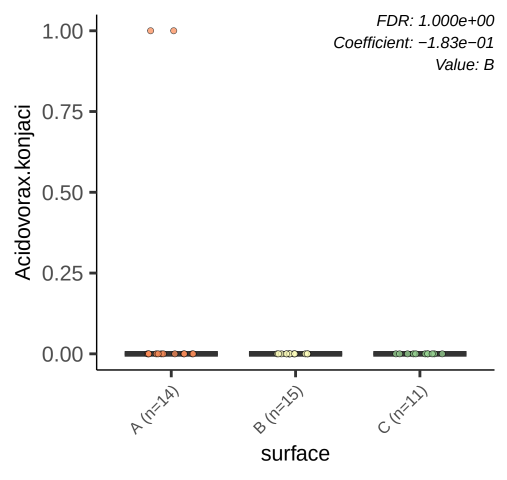
FDR: 1.000e+00
Coefficient: −1.83e−01
Value: B
Acidovorax.konjaci
1.00
0.75
0.50
0.25
0.00
A (n=14)
B (n=15)
C (n=11)
surface

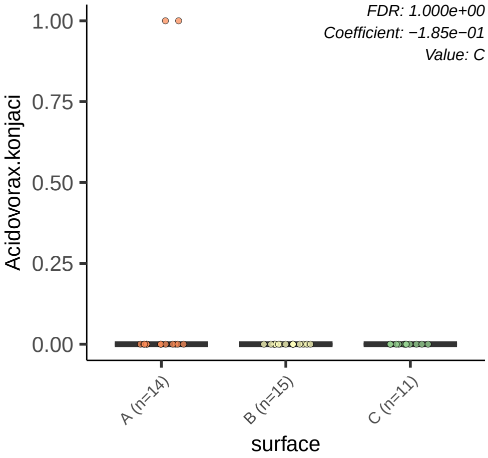
FDR: 1.000e+00
Coefficient: −1.85e−01
Value: C
Acidovorax.konjaci
1.00
0.75
0.50
0.25
0.00
A (n=14)
B (n=15)
C (n=11)
surface

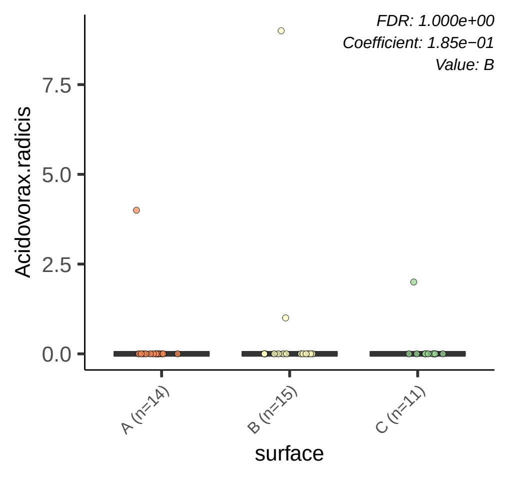

FDR: 1.000e+00
Coefficient: 1.85e−01
Value: B
Acidovorax.radicis
0.0
2.5
5.0
7.5
A (n=14)
B (n=15)
C (n=11)
surface

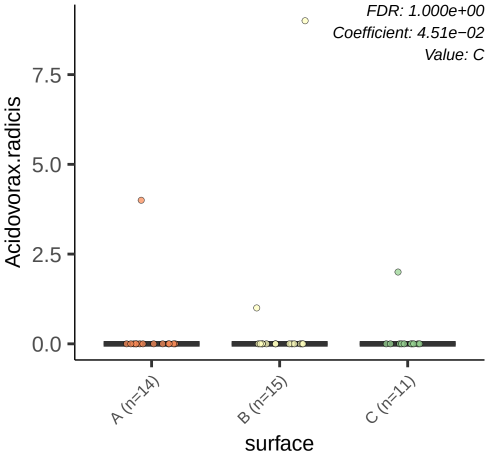

FDR: 1.000e+00
Coefficient: 4.51e−02
Value: C
Acidovorax.radicis
7.5
5.0
2.5
0.0
A (n=14)
B (n=15)
C (n=11)
surface

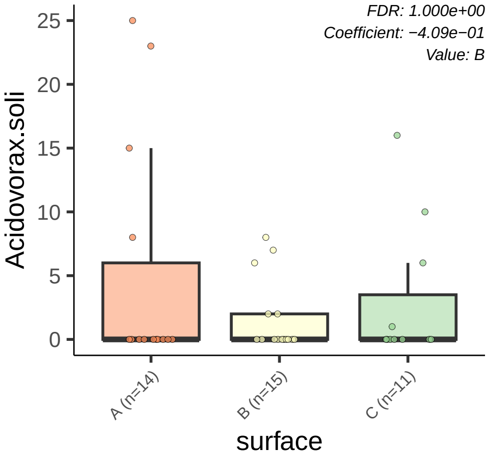

FDR: 1.000e+00
Coefficient: −4.09e−01
Value: B
Acidovorax.soli
0
5
10
15
20
25
A (n=14)
B (n=15)
C (n=11)
surface

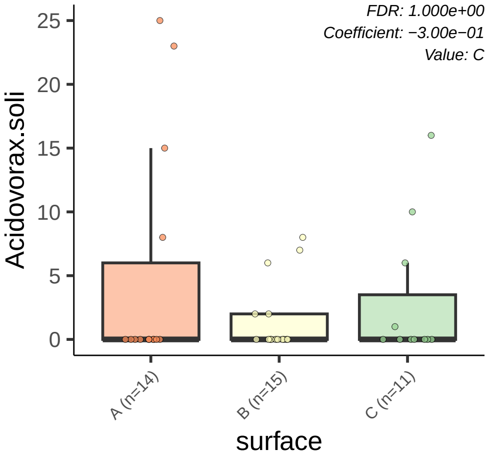
FDR: 1.000e+00
Coefficient: −3.00e−01
Value: C
Acidovorax.soli
0
5
10
15
20
25
A (n=14)
B (n=15)
C (n=11)
surface

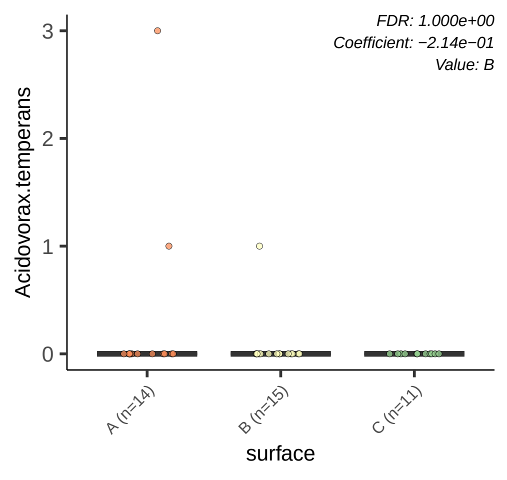

FDR: 1.000e+00
Coefficient: −2.14e−01
Value: B
Acidovorax.temperans
3
2
1
0
A (n=14)
B (n=15)
C (n=11)
surface

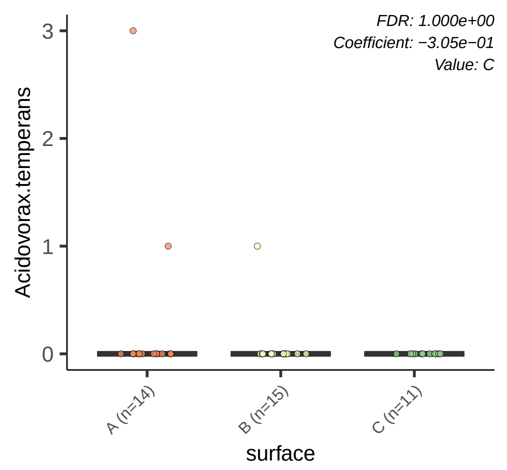

FDR: 1.000e+00
Coefficient: −3.05e−01
Value: C
Acidovorax.temperans
3
2
1
0
A (n=14)
B (n=15)
C (n=11)
surface

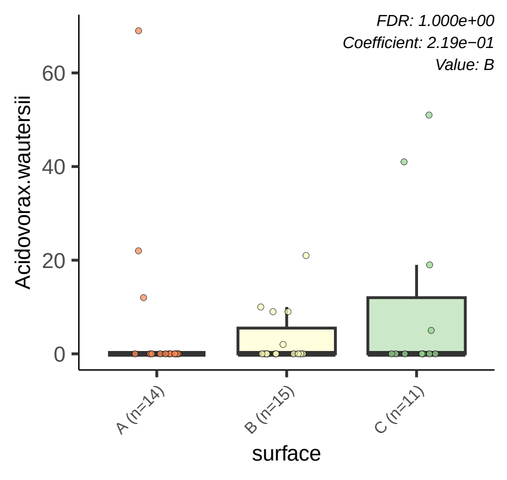

FDR: 1.000e+00
Coefficient: 2.19e−01
Value: B
Acidovorax.wautersii
0
20
40
60
A (n=14)
B (n=15)
C (n=11)
surface

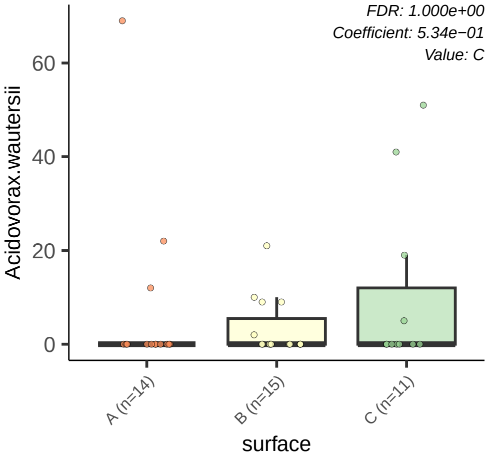
FDR: 1.000e+00
Coefficient: 5.34e−01
Value: C
Acidovorax.wautersii
0
20
40
60
A (n=14)
B (n=15)
C (n=11)
surface

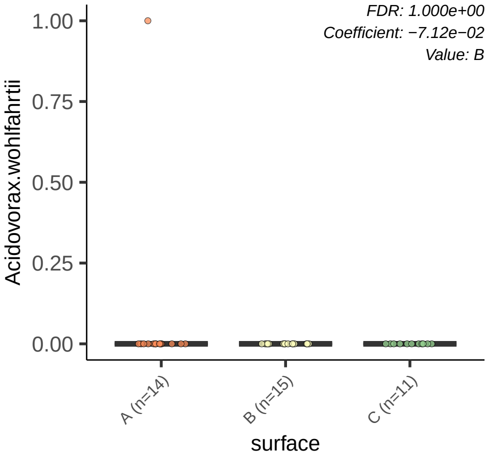

FDR: 1.000e+00
Coefficient: −7.12e−02
Value: B
Acidovorax.wohlfahrtii
1.00
0.75
0.50
0.25
0.00
A (n=14)
B (n=15)
C (n=11)
surface

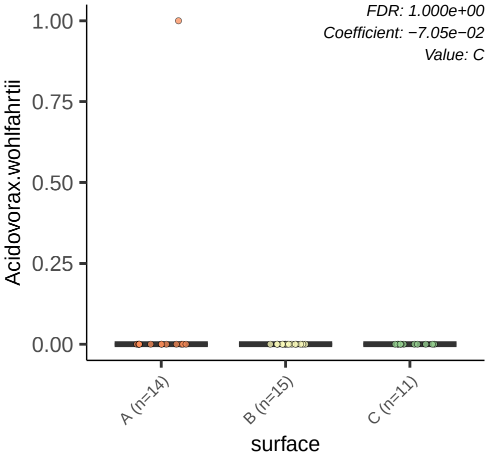
FDR: 1.000e+00
Coefficient: −7.05e−02
Value: C
Acidovorax.wohlfahrtii
1.00
0.75
0.50
0.25
0.00
A (n=14)
B (n=15)
C (n=11)
surface

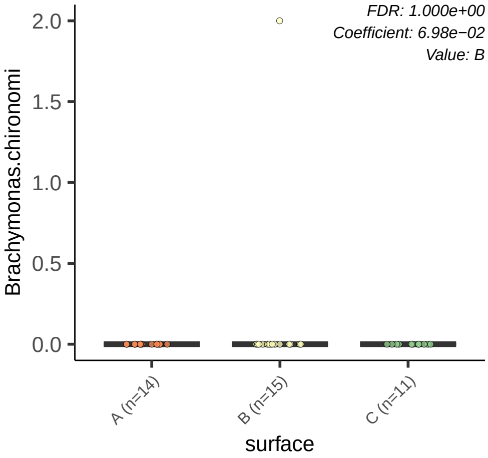
FDR: 1.000e+00
Coefficient: 6.98e−02
Value: B
Brachymonas.chironomi
2.0
1.5
1.0
0.5
0.0
A (n=14)
B (n=15)
C (n=11)
surface

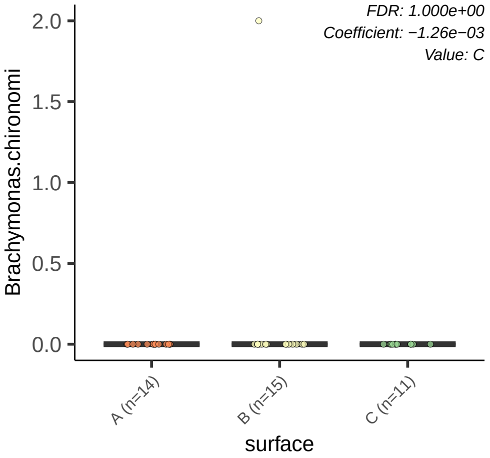
FDR: 1.000e+00
Coefficient: −1.26e−03
Value: C
Brachymonas.chironomi
2.0
1.5
1.0
0.5
0.0
A (n=14)
B (n=15)
C (n=11)
surface

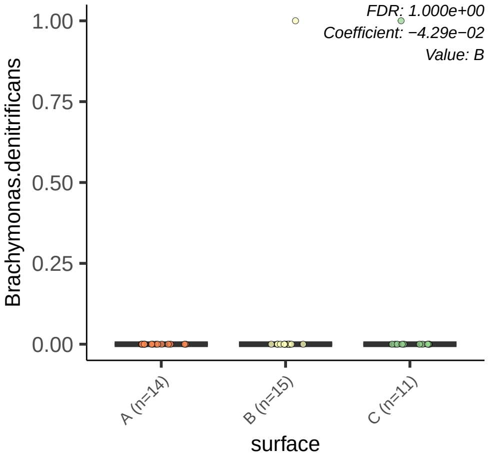
FDR: 1.000e+00
Coefficient: −4.29e−02
Value: B
1.00
0.75
0.50
0.25
0.00
Brachymonas.denitrificans
A (n=14)
B (n=15)
C (n=11)
surface

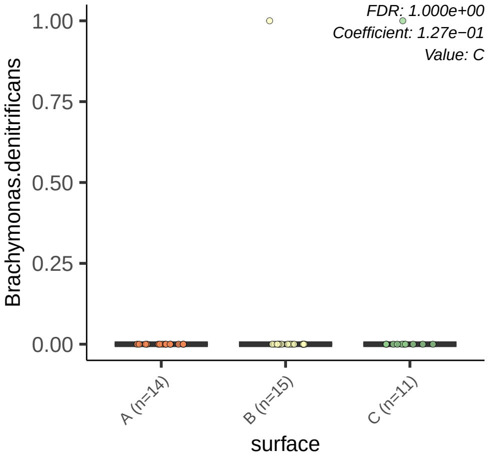

FDR: 1.000e+00
Coefficient: 1.27e−01
Value: C
Brachymonas.denitrificans
1.00
0.75
0.50
0.25
0.00
A (n=14)
B (n=15)
C (n=11)
surface

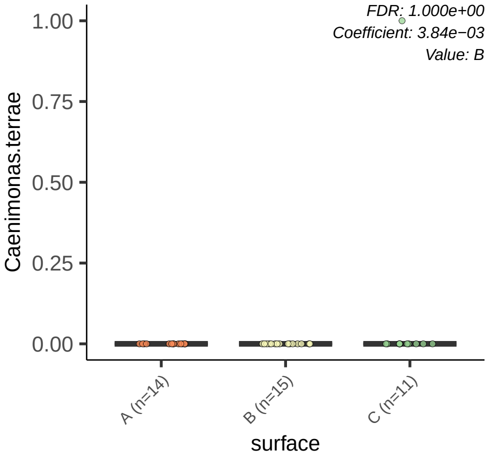

FDR: 1.000e+00
Coefficient: 3.84e−03
Value: B
Caenimonas.terrae
1.00
0.75
0.50
0.25
0.00
A (n=14)
B (n=15)
C (n=11)
surface

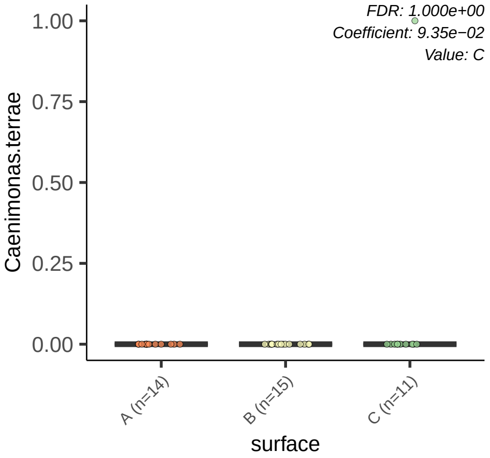
FDR: 1.000e+00
Coefficient: 9.35e−02
Value: C
Caenimonas.terrae
1.00
0.75
0.50
0.25
0.00
A (n=14)
B (n=15)
C (n=11)
surface

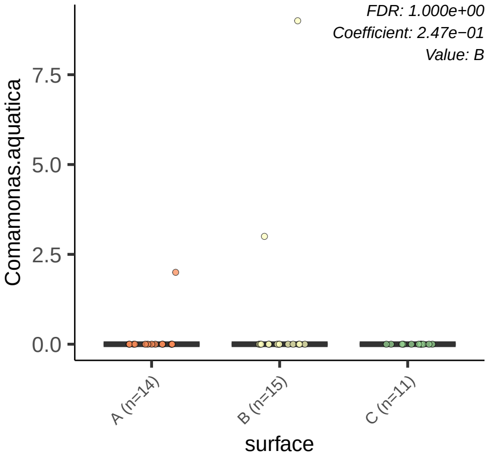

FDR: 1.000e+00
Coefficient: 2.47e−01
Value: B
Comamonas.aquatica
7.5
5.0
2.5
0.0
A (n=14)
B (n=15)
C (n=11)
surface

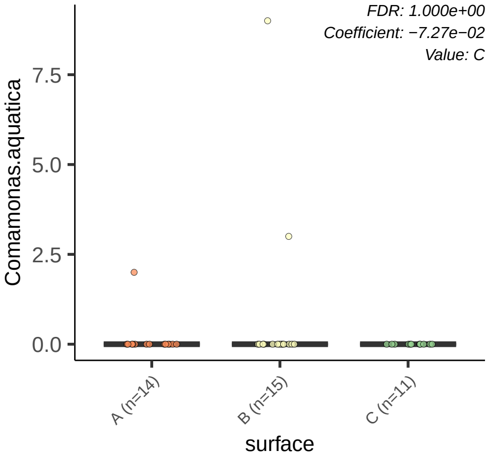
FDR: 1.000e+00
Coefficient: −7.27e−02
Value: C
Comamonas.aquatica
7.5
5.0
2.5
0.0
A (n=14)
B (n=15)
C (n=11)
surface

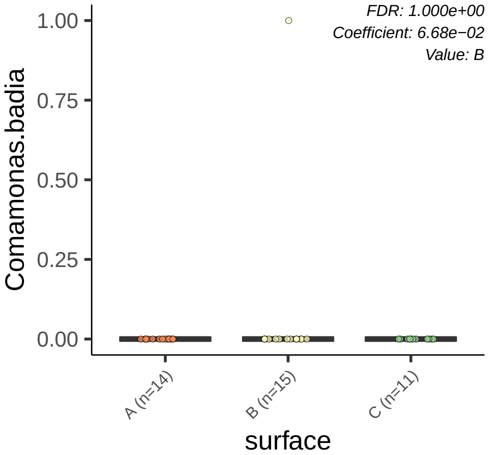
FDR: 1.000e+00
Coefficient: 6.68e−02
Value: B
Comamonas.badia
1.00
0.75
0.50
0.25
0.00
A (n=14)
B (n=15)
C (n=11)
surface

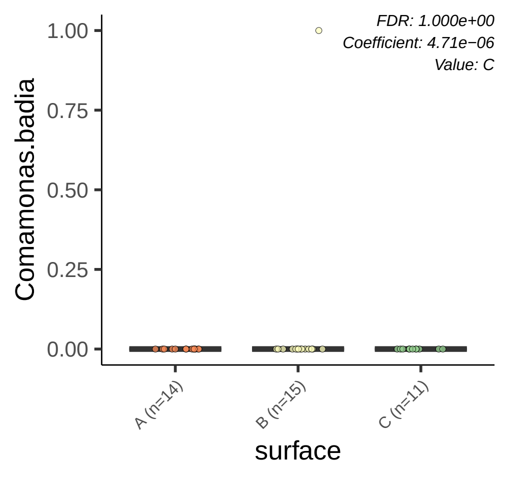
FDR: 1.000e+00
Coefficient: 4.71e−06
Value: C
Comamonas.badia
1.00
0.75
0.50
0.25
0.00
A (n=14)
B (n=15)
C (n=11)
surface

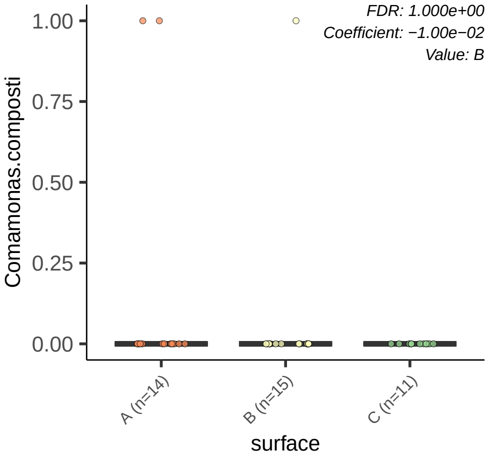
FDR: 1.000e+00
Coefficient: −1.00e−02
Value: B
Comamonas.composti
1.00
0.75
0.50
0.25
0.00
A (n=14)
B (n=15)
C (n=11)
surface

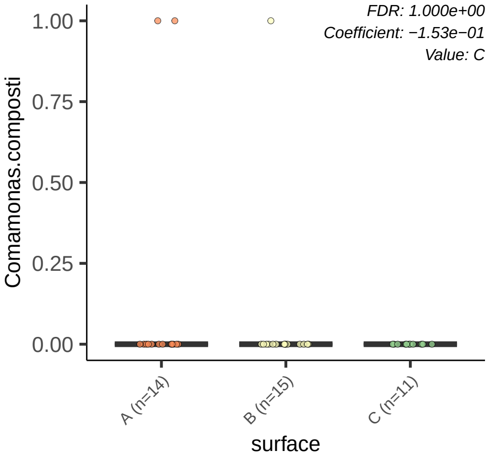
FDR: 1.000e+00
Coefficient: −1.53e−01
Value: C
Comamonas.composti
1.00
0.75
0.50
0.25
0.00
A (n=14)
B (n=15)
C (n=11)
surface

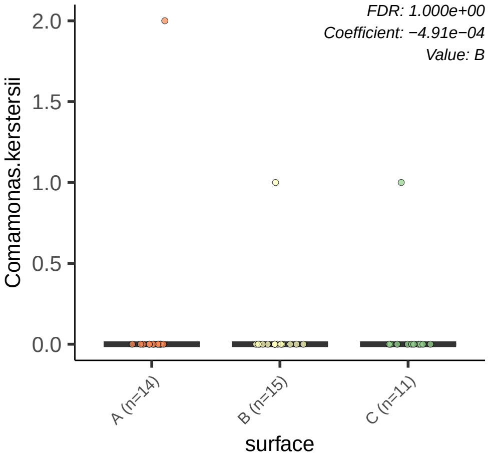
FDR: 1.000e+00
Coefficient: −4.91e−04
Value: B
Comamonas.kerstersii
2.0
1.5
1.0
0.5
0.0
A (n=14)
B (n=15)
C (n=11)
surface

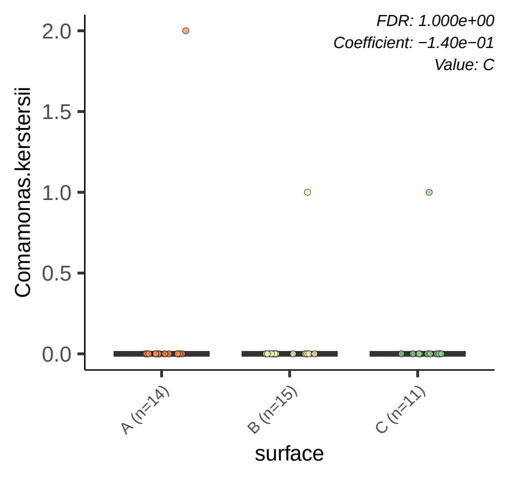
FDR: 1.000e+00
Coefficient: −1.40e−01
Value: C
Comamonas.kerstersii
2.0
1.5
1.0
0.5
0.0
A (n=14)
B (n=15)
C (n=11)
surface

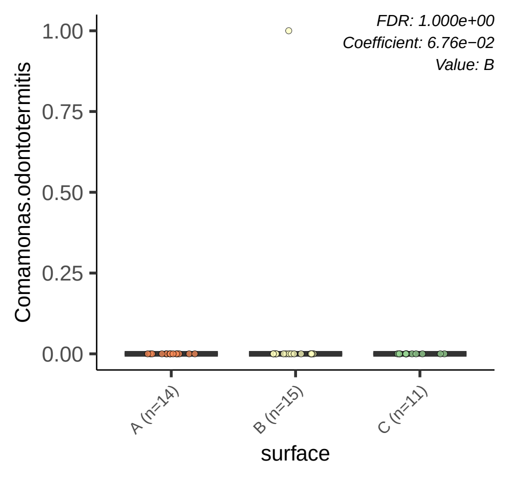

FDR: 1.000e+00
Coefficient: 6.76e−02
Value: B
Comamonas.odontotermitis
1.00
0.75
0.50
0.25
0.00
A (n=14)
B (n=15)
C (n=11)
surface

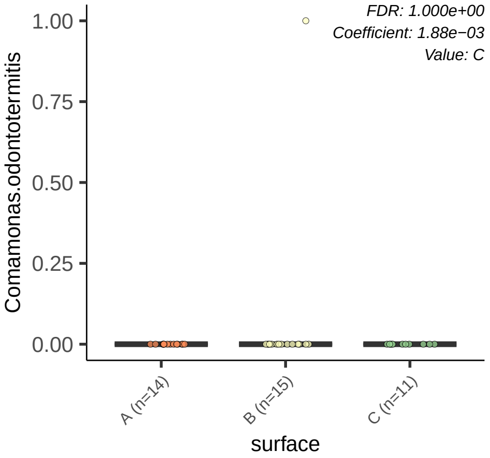
FDR: 1.000e+00
Coefficient: 1.88e−03
Value: C
Comamonas.odontotermitis
1.00
0.75
0.50
0.25
0.00
A (n=14)
B (n=15)
C (n=11)
surface

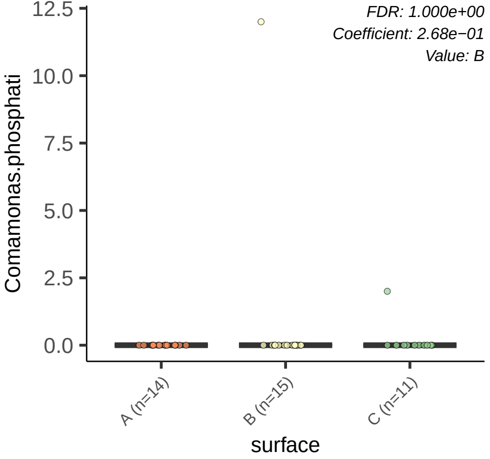
FDR: 1.000e+00
Coefficient: 2.68e−01
Value: B
Comamonas.phosphati
12.5
10.0
7.5
5.0
2.5
0.0
A (n=14)
B (n=15)
C (n=11)
surface

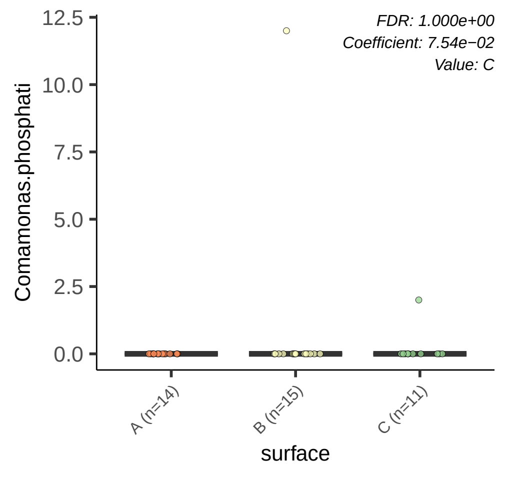

FDR: 1.000e+00
Coefficient: 7.54e−02
Value: C
Comamonas.phosphati
12.5
10.0
7.5
5.0
2.5
0.0
A (n=14)
B (n=15)
C (n=11)
surface

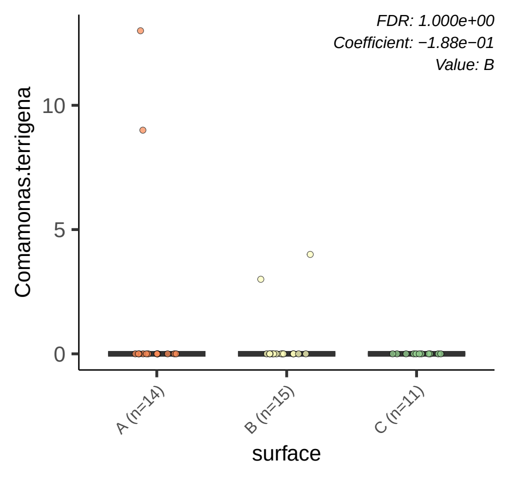

FDR: 1.000e+00
Coefficient: −1.88e−01
Value: B
Comamonas.terrigena
10
5
0
A (n=14)
B (n=15)
C (n=11)
surface

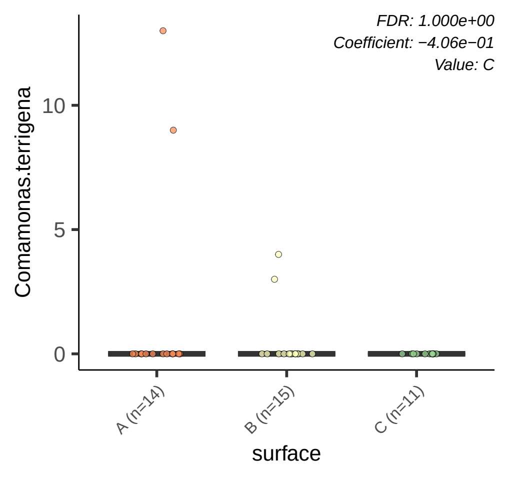
FDR: 1.000e+00
Coefficient: −4.06e−01
Value: C
Comamonas.terrigena
10
5
0
A (n=14)
B (n=15)
C (n=11)
surface

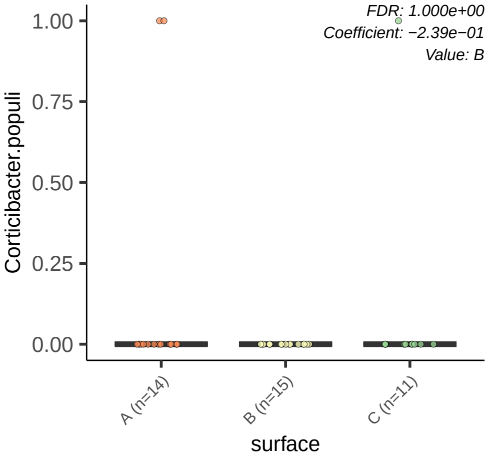
FDR: 1.000e+00
Coefficient: −2.39e−01
Value: B
Corticibacter.populi
1.00
0.75
0.50
0.25
0.00
A (n=14)
B (n=15)
C (n=11)
surface

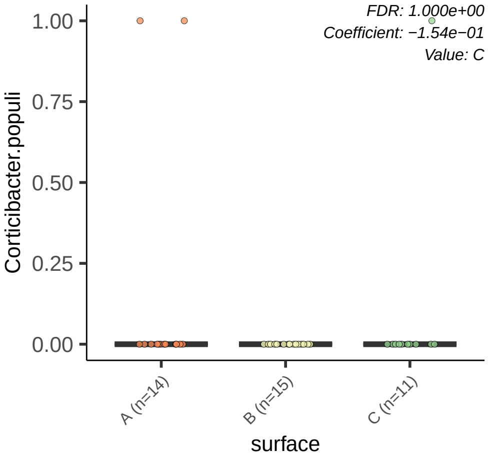

FDR: 1.000e+00
Coefficient: −1.54e−01
Value: C
Corticibacter.populi
1.00
0.75
0.50
0.25
0.00
A (n=14)
B (n=15)
C (n=11)
surface

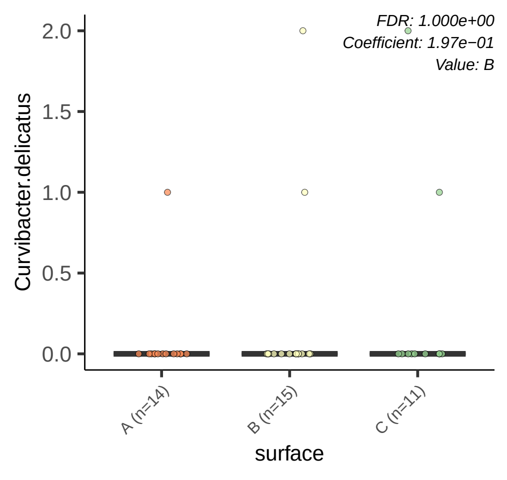
FDR: 1.000e+00
Coefficient: 1.97e−01
Value: B
Curvibacter.delicatus
0.0
0.5
1.0
1.5
2.0
A (n=14)
B (n=15)
C (n=11)
surface

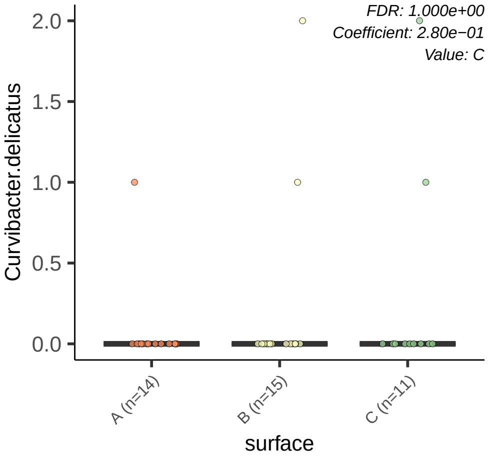
FDR: 1.000e+00
Coefficient: 2.80e−01
Value: C
Curvibacter.delicatus
2.0
1.5
1.0
0.5
0.0
A (n=14)
B (n=15)
C (n=11)
surface

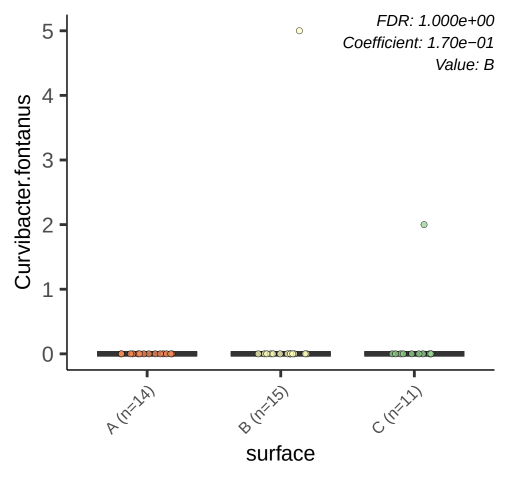
FDR: 1.000e+00
Coefficient: 1.70e−01
Value: B
Curvibacter.fontanus
0
1
2
3
4
5
A (n=14)
B (n=15)
C (n=11)
surface

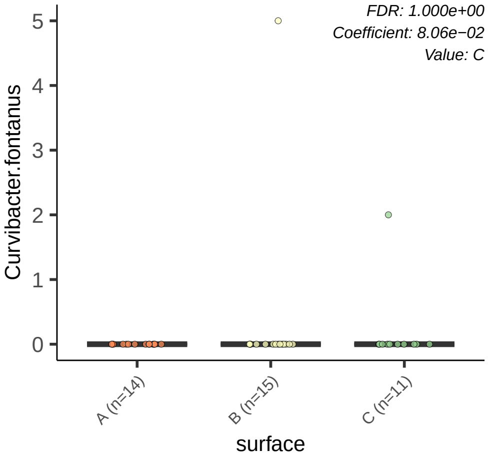

FDR: 1.000e+00
Coefficient: 8.06e−02
Value: C
Curvibacter.fontanus
0
1
2
3
4
5
A (n=14)
B (n=15)
C (n=11)
surface

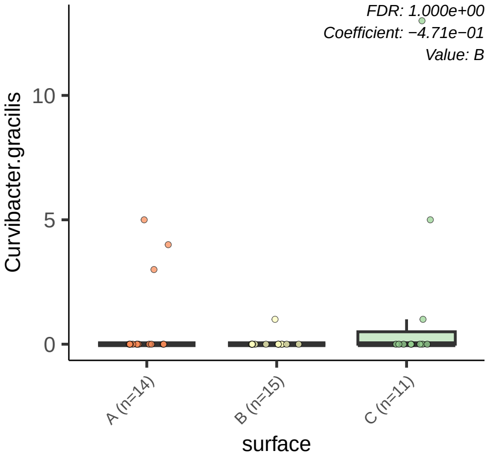
FDR: 1.000e+00
Coefficient: −4.71e−01
Value: B
Curvibacter.gracilis
10
5
0
A (n=14)
B (n=15)
C (n=11)
surface

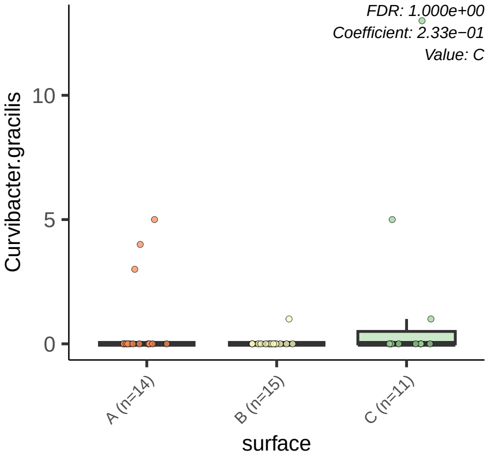

FDR: 1.000e+00
Coefficient: 2.33e−01
Value: C
Curvibacter.gracilis
10
5
0
A (n=14)
B (n=15)
C (n=11)
surface

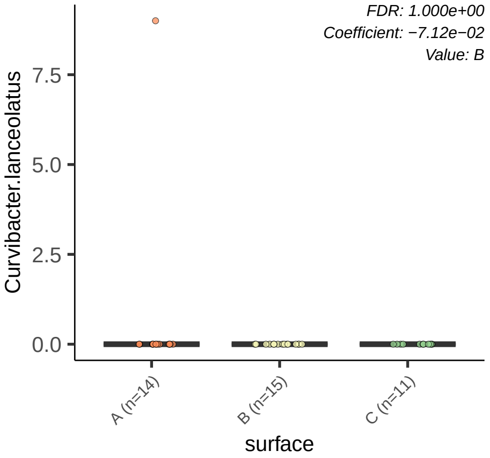

FDR: 1.000e+00
Coefficient: −7.12e−02
Value: B
Curvibacter.lanceolatus
7.5
5.0
2.5
0.0
A (n=14)
B (n=15)
C (n=11)
surface

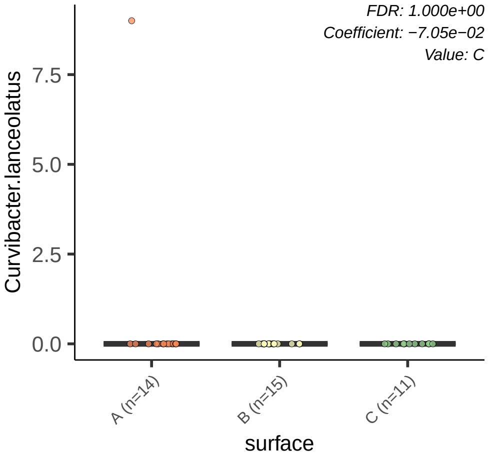

FDR: 1.000e+00
Coefficient: −7.05e−02
Value: C
Curvibacter.lanceolatus
7.5
5.0
2.5
0.0
A (n=14)
B (n=15)
C (n=11)
surface

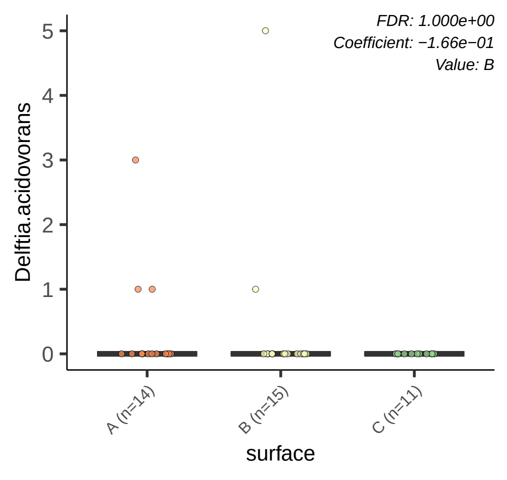
FDR: 1.000e+00
Coefficient: −1.66e−01
Value: B
Delftia.acidovorans
0
1
2
3
4
5
A (n=14)
B (n=15)
C (n=11)
surface

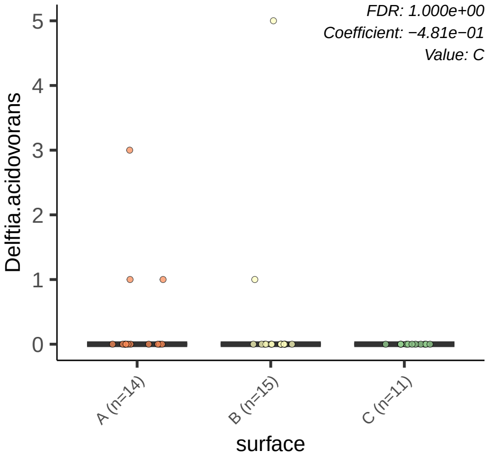
FDR: 1.000e+00
Coefficient: −4.81e−01
Value: C
Delftia.acidovorans
5
4
3
2
1
0
A (n=14)
B (n=15)
C (n=11)
surface

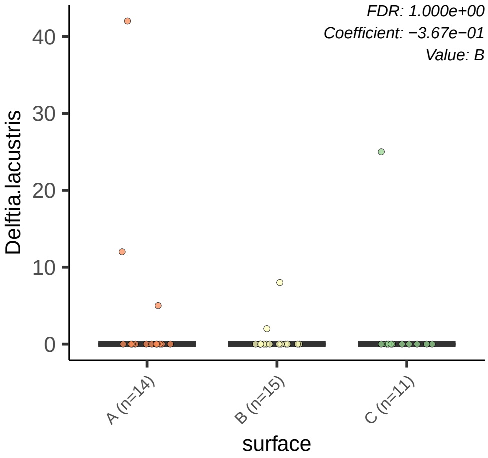

FDR: 1.000e+00
Coefficient: −3.67e−01
Value: B
Delftia.lacustris
40
30
20
10
0
A (n=14)
B (n=15)
C (n=11)
surface

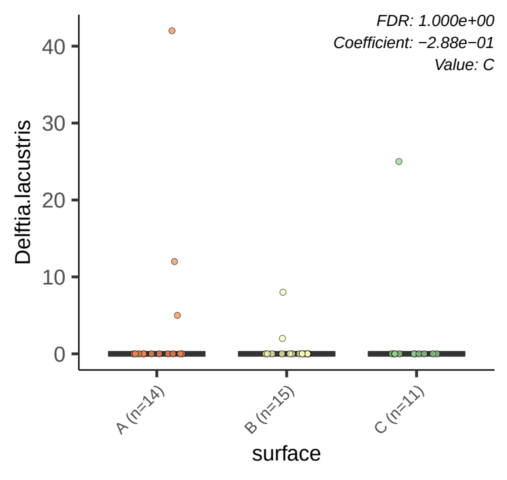

FDR: 1.000e+00
Coefficient: −2.88e−01
Value: C
Delftia.lacustris
0
10
20
30
40
A (n=14)
B (n=15)
C (n=11)
surface

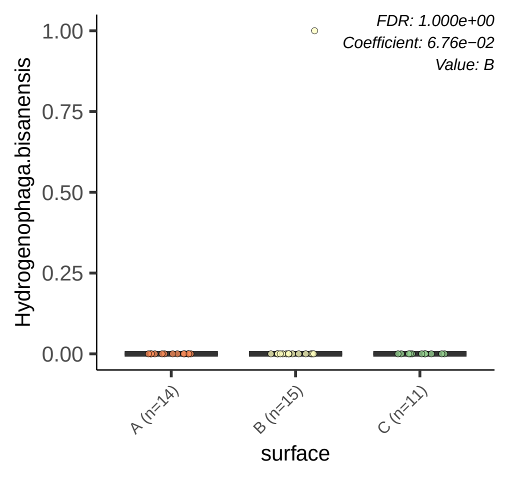
FDR: 1.000e+00
Coefficient: 6.76e−02
Value: B
Hydrogenophaga.bisanensis
1.00
0.75
0.50
0.25
0.00
A (n=14)
B (n=15)
C (n=11)
surface

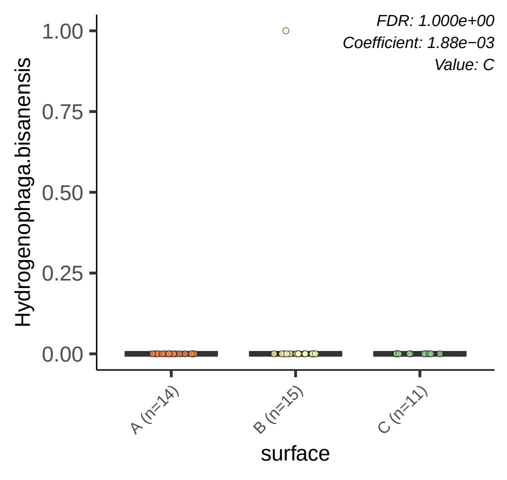
FDR: 1.000e+00
Coefficient: 1.88e−03
Value: C
Hydrogenophaga.bisanensis
1.00
0.75
0.50
0.25
0.00
A (n=14)
B (n=15)
C (n=11)
surface

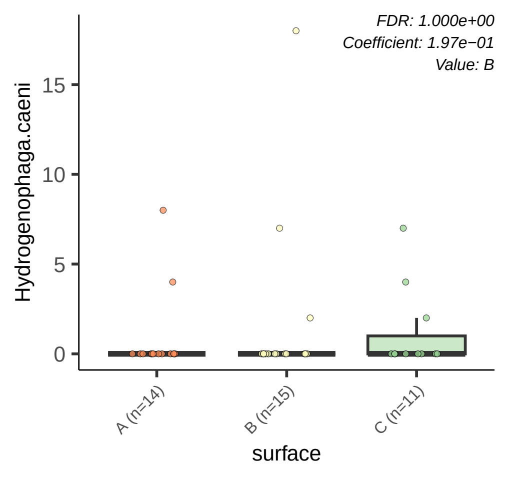

FDR: 1.000e+00
Coefficient: 1.97e−01
Value: B
Hydrogenophaga.caeni
15
10
5
0
A (n=14)
B (n=15)
C (n=11)
surface

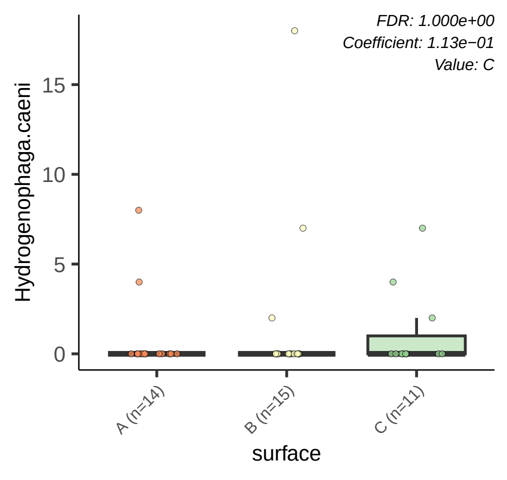
FDR: 1.000e+00
Coefficient: 1.13e−01
Value: C
Hydrogenophaga.caeni
15
10
5
0
A (n=14)
B (n=15)
C (n=11)
surface

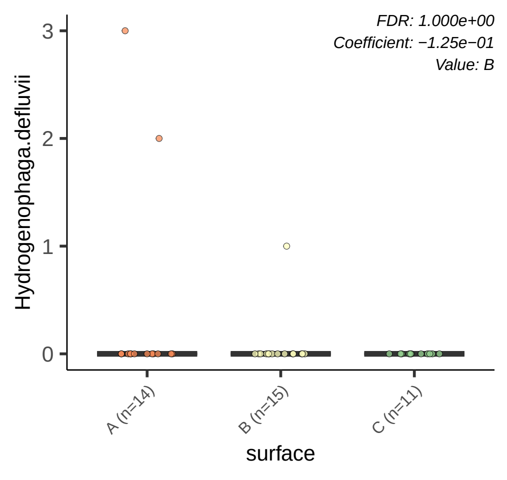

FDR: 1.000e+00
Coefficient: −1.25e−01
Value: B
Hydrogenophaga.defluvii
3
2
1
0
A (n=14)
B (n=15)
C (n=11)
surface

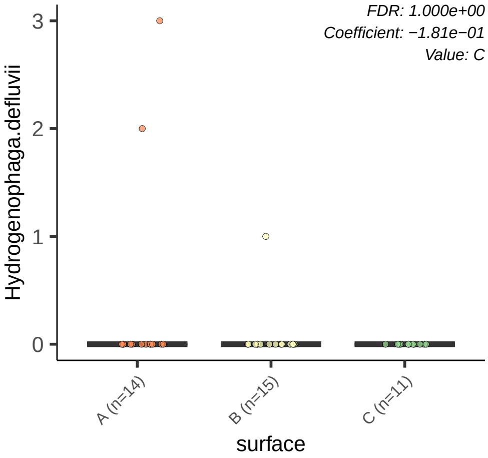
FDR: 1.000e+00
Coefficient: −1.81e−01
Value: C
Hydrogenophaga.defluvii
3
2
1
0
A (n=14)
B (n=15)
C (n=11)
surface

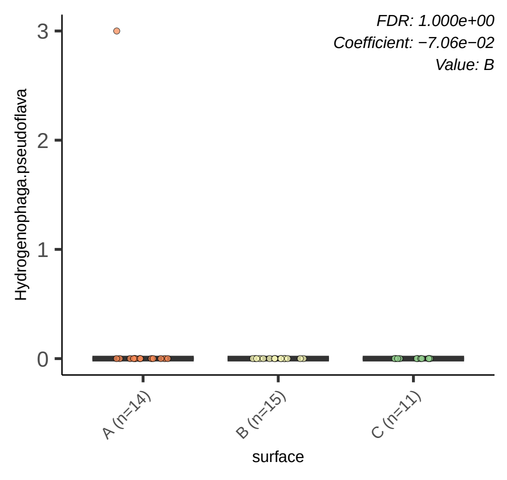

FDR: 1.000e+00
Coefficient: −7.06e−02
Value: B
Hydrogenophaga.pseudoflava
3
2
1
0
A (n=14)
B (n=15)
C (n=11)
surface

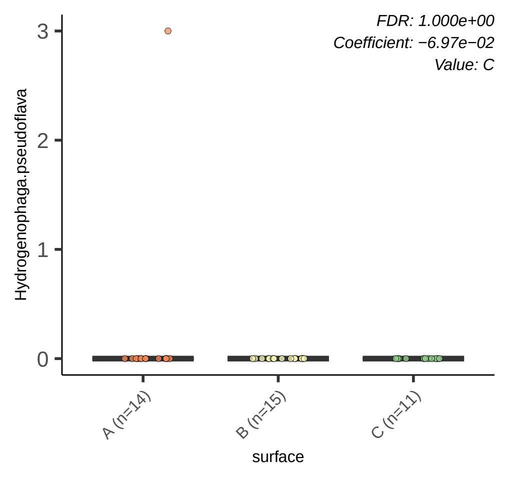
FDR: 1.000e+00
Coefficient: −6.97e−02
Value: C
Hydrogenophaga.pseudoflava
3
2
1
0
A (n=14)
B (n=15)
C (n=11)
surface

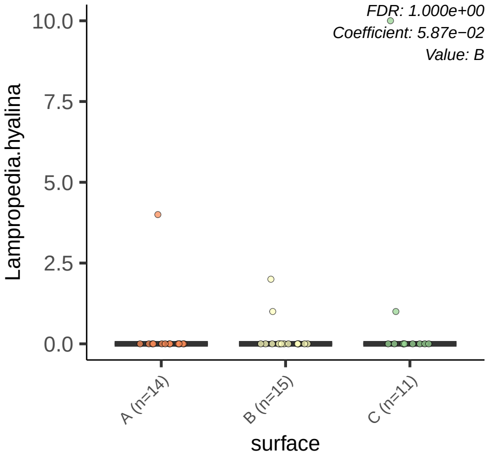
FDR: 1.000e+00
Coefficient: 5.87e−02
Value: B
Lampropedia.hyalina
10.0
7.5
5.0
2.5
0.0
A (n=14)
B (n=15)
C (n=11)
surface

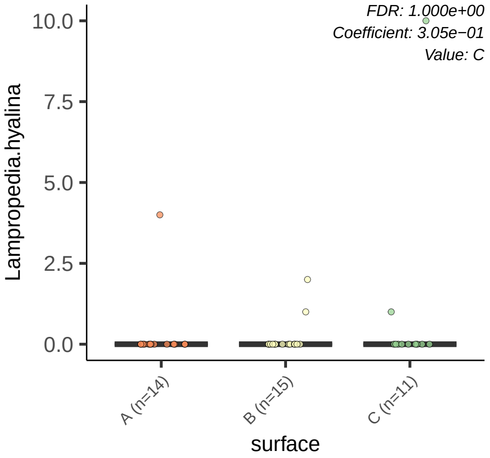

FDR: 1.000e+00
Coefficient: 3.05e−01
Value: C
Lampropedia.hyalina
10.0
7.5
5.0
2.5
0.0
A (n=14)
B (n=15)
C (n=11)
surface

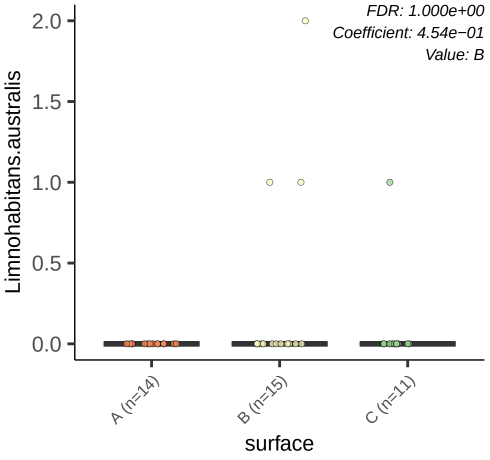
FDR: 1.000e+00
Coefficient: 4.54e−01
Value: B
Limnohabitans.australis
2.0
1.5
1.0
0.5
0.0
A (n=14)
B (n=15)
C (n=11)
surface

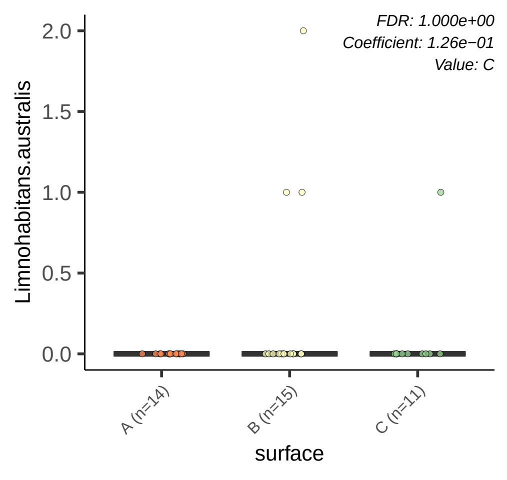
FDR: 1.000e+00
Coefficient: 1.26e−01
Value: C
Limnohabitans.australis
2.0
1.5
1.0
0.5
0.0
A (n=14)
B (n=15)
C (n=11)
surface

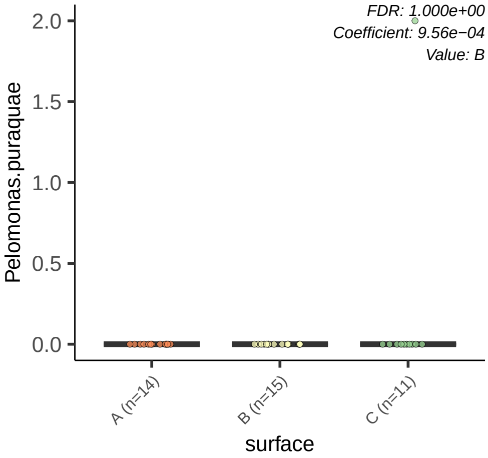

FDR: 1.000e+00
Coefficient: 9.56e−04
Value: B
Pelomonas.puraquae
2.0
1.5
1.0
0.5
0.0
A (n=14)
B (n=15)
C (n=11)
surface

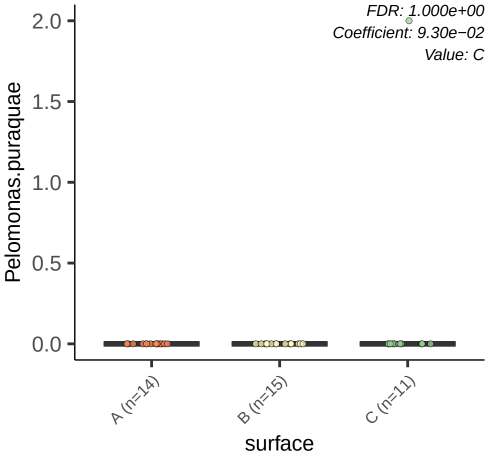
FDR: 1.000e+00
Coefficient: 9.30e−02
Value: C
Pelomonas.puraquae
2.0
1.5
1.0
0.5
0.0
A (n=14)
B (n=15)
C (n=11)
surface

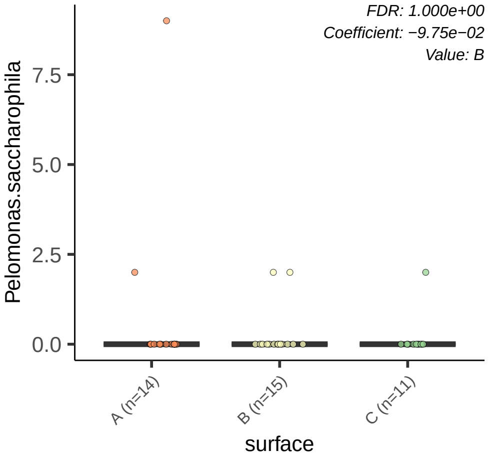
FDR: 1.000e+00
Coefficient: −9.75e−02
Value: B
Pelomonas.saccharophila
7.5
5.0
2.5
0.0
A (n=14)
B (n=15)
C (n=11)
surface

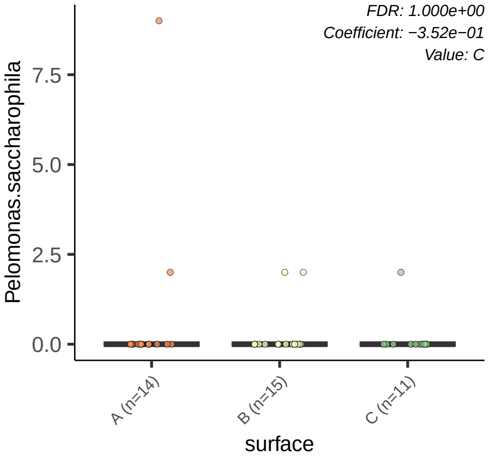
FDR: 1.000e+00
Coefficient: −3.52e−01
Value: C
Pelomonas.saccharophila
7.5
5.0
2.5
0.0
A (n=14)
B (n=15)
C (n=11)
surface

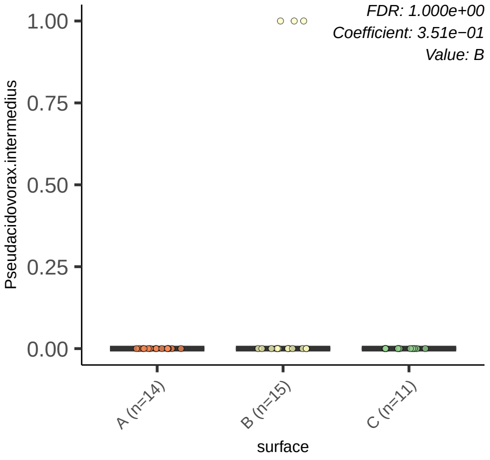

FDR: 1.000e+00
Coefficient: 3.51e−01
Value: B
Pseudacidovorax.intermedius
1.00
0.75
0.50
0.25
0.00
A (n=14)
B (n=15)
C (n=11)
surface

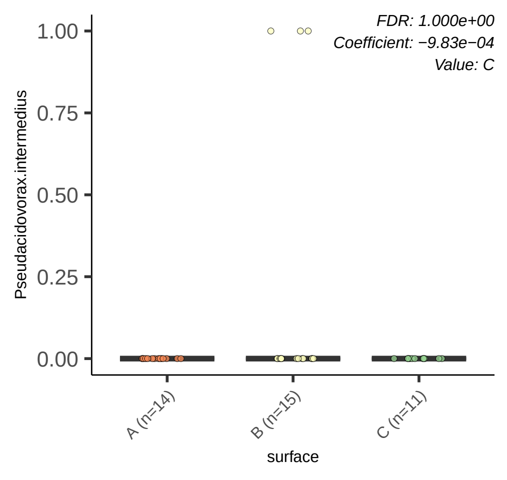

FDR: 1.000e+00
Coefficient: −9.83e−04
Value: C
1.00
0.75
0.50
0.25
0.00
Pseudacidovorax.intermedius
A (n=14)
B (n=15)
C (n=11)
surface

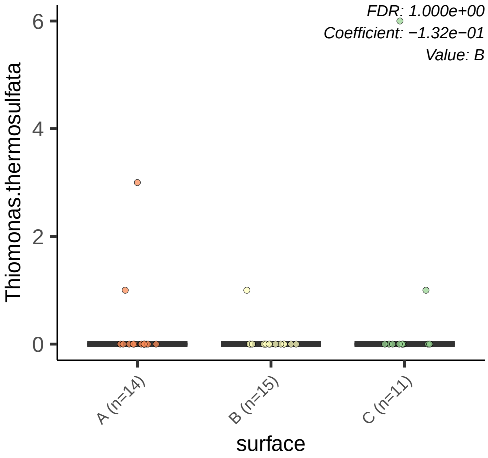
FDR: 1.000e+00
Coefficient: −1.32e−01
Value: B
Thiomonas.thermosulfata
0
2
4
6
A (n=14)
B (n=15)
C (n=11)
surface

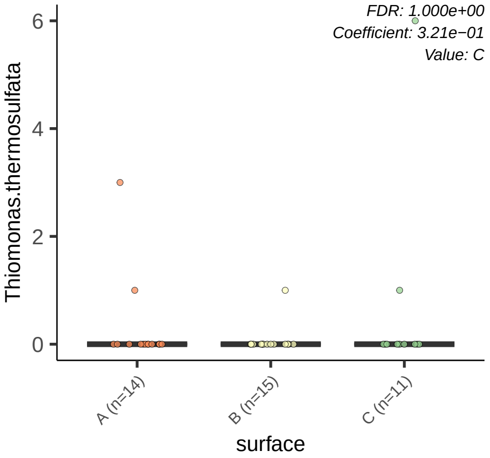
FDR: 1.000e+00
Coefficient: 3.21e−01
Value: C
Thiomonas.thermosulfata
6
4
2
0
A (n=14)
B (n=15)
C (n=11)
surface

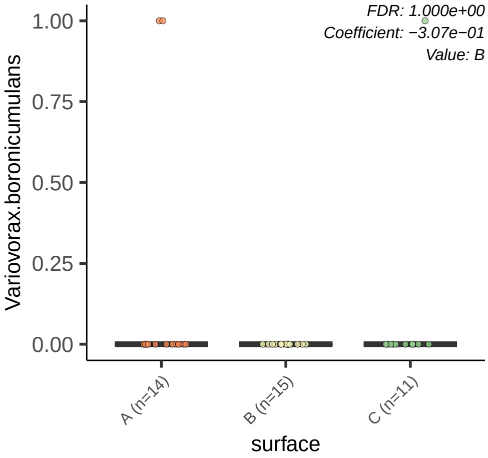
FDR: 1.000e+00
Coefficient: −3.07e−01
Value: B
Variovorax.boronicumulans
1.00
0.75
0.50
0.25
0.00
A (n=14)
B (n=15)
C (n=11)
surface

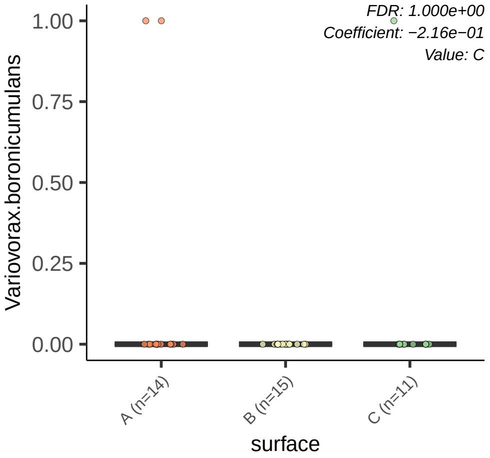
FDR: 1.000e+00
Coefficient: −2.16e−01
Value: C
Variovorax.boronicumulans
1.00
0.75
0.50
0.25
0.00
A (n=14)
B (n=15)
C (n=11)
surface

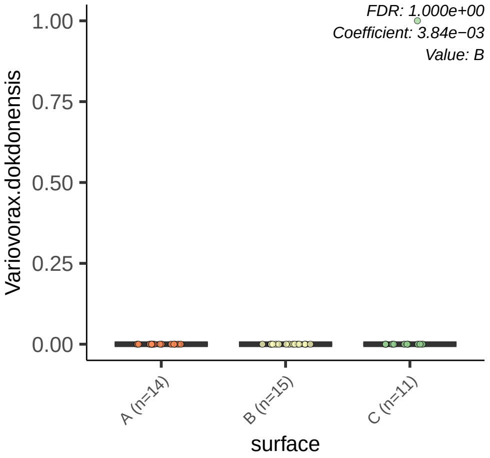
FDR: 1.000e+00
Coefficient: 3.84e−03
Value: B
Variovorax.dokdonensis
1.00
0.75
0.50
0.25
0.00
A (n=14)
B (n=15)
C (n=11)
surface

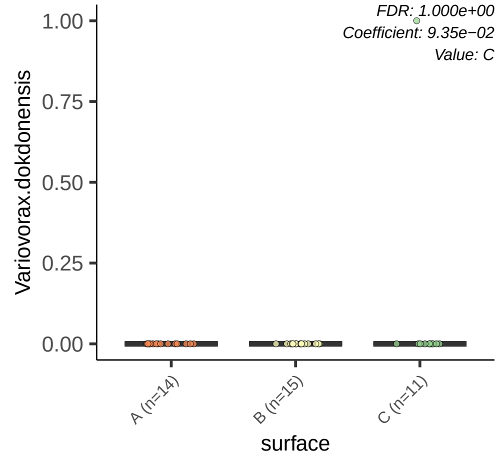

FDR: 1.000e+00
Coefficient: 9.35e−02
Value: C
Variovorax.dokdonensis
1.00
0.75
0.50
0.25
0.00
A (n=14)
B (n=15)
C (n=11)
surface

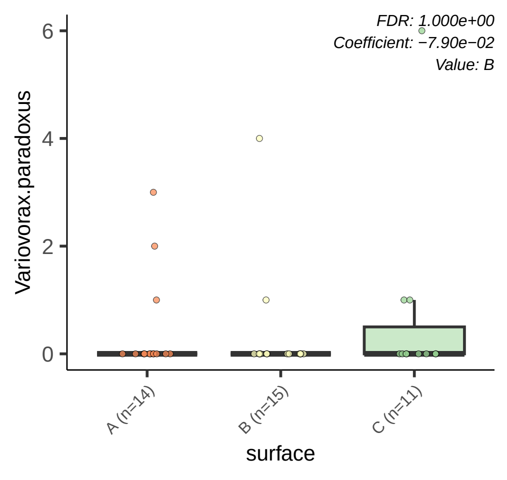
FDR: 1.000e+00
Coefficient: −7.90e−02
Value: B
Variovorax.paradoxus
0
2
4
6
A (n=14)
B (n=15)
C (n=11)
surface

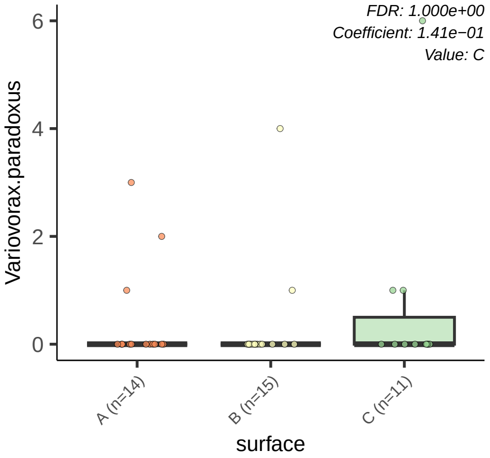
FDR: 1.000e+00
Coefficient: 1.41e−01
Value: C
Variovorax.paradoxus
0
2
4
6
A (n=14)
B (n=15)
C (n=11)
surface

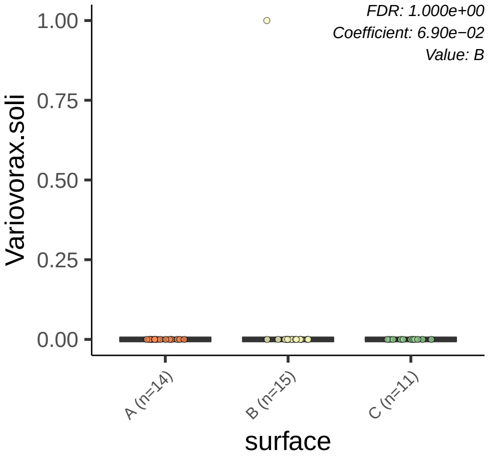

FDR: 1.000e+00
Coefficient: 6.90e−02
Value: B
Variovorax.soli
1.00
0.75
0.50
0.25
0.00
A (n=14)
B (n=15)
C (n=11)
surface

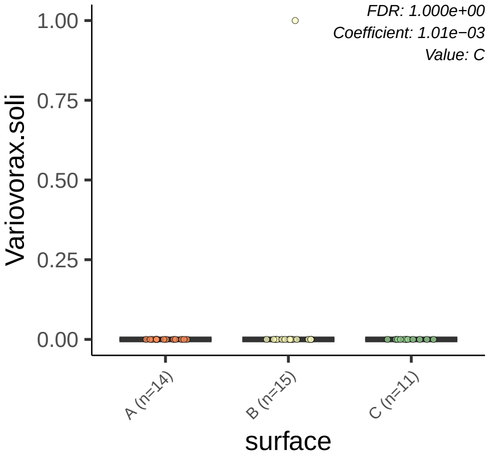
FDR: 1.000e+00
Coefficient: 1.01e−03
Value: C
Variovorax.soli
1.00
0.75
0.50
0.25
0.00
A (n=14)
B (n=15)
C (n=11)
surface

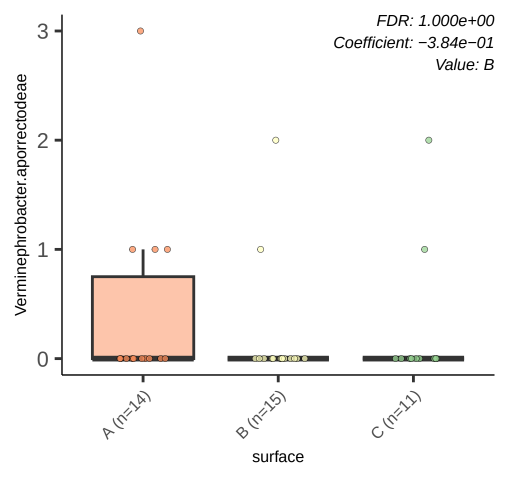

FDR: 1.000e+00
Coefficient: −3.84e−01
Value: B
Verminephrobacter.aporrectodeae
3
2
1
0
A (n=14)
B (n=15)
C (n=11)
surface

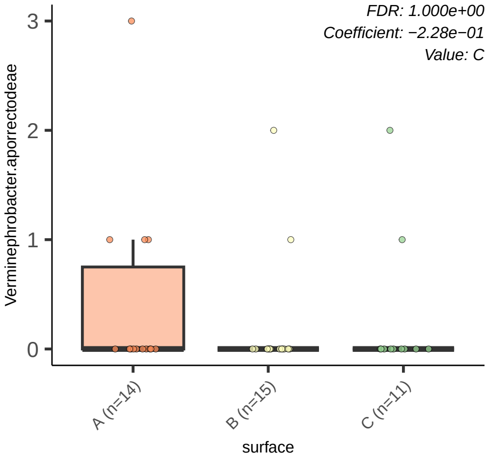

FDR: 1.000e+00
Coefficient: −2.28e−01
Value: C
Verminephrobacter.aporrectodeae
3
2
1
0
A (n=14)
B (n=15)
C (n=11)
surface

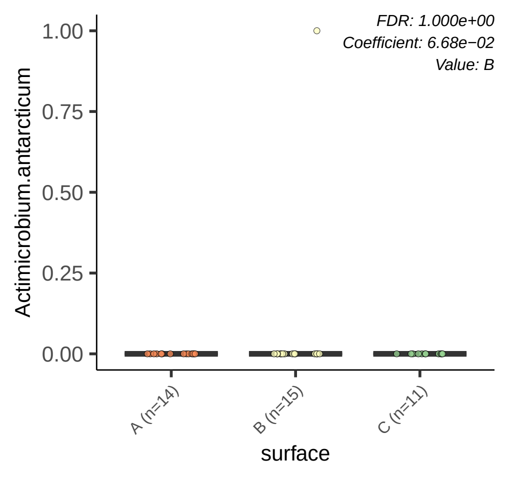

FDR: 1.000e+00
Coefficient: 6.68e−02
Value: B
Actimicrobium.antarcticum
1.00
0.75
0.50
0.25
0.00
A (n=14)
B (n=15)
C (n=11)
surface

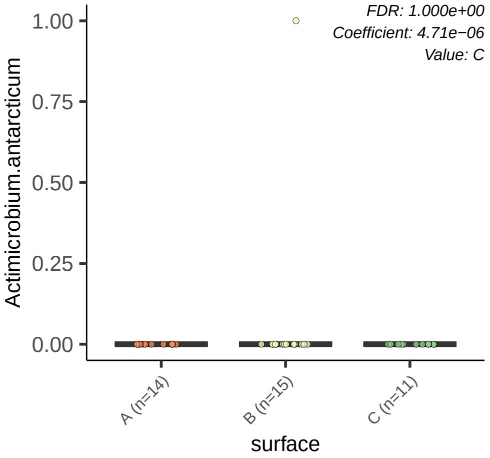
FDR: 1.000e+00
Coefficient: 4.71e−06
Value: C
Actimicrobium.antarcticum
1.00
0.75
0.50
0.25
0.00
A (n=14)
B (n=15)
C (n=11)
surface

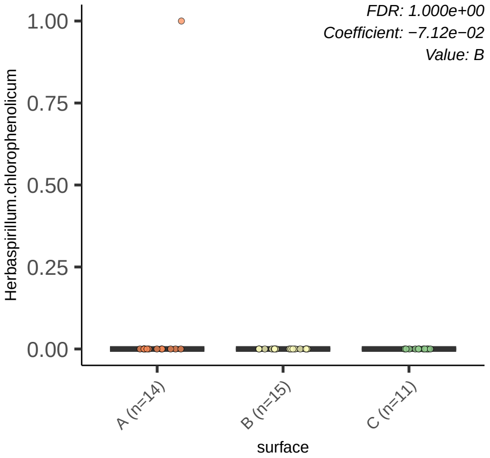
FDR: 1.000e+00
Coefficient: −7.12e−02
Value: B
Herbaspirillum.chlorophenolicum
1.00
0.75
0.50
0.25
0.00
A (n=14)
B (n=15)
C (n=11)
surface

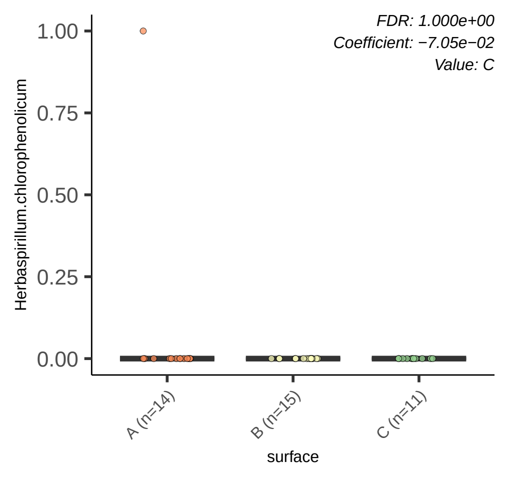
FDR: 1.000e+00
Coefficient: −7.05e−02
Value: C
Herbaspirillum.chlorophenolicum
1.00
0.75
0.50
0.25
0.00
A (n=14)
B (n=15)
C (n=11)
surface

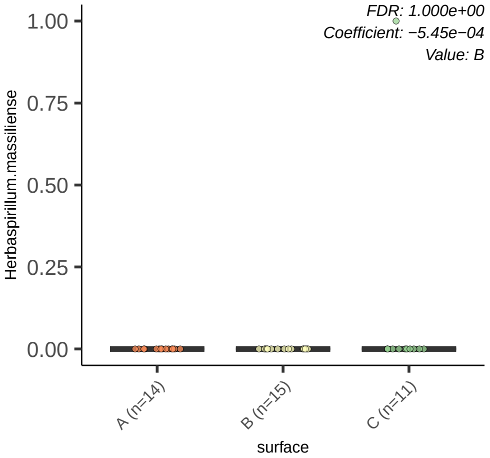

FDR: 1.000e+00
Coefficient: −5.45e−04
Value: B
1.00
0.75
0.50
0.25
0.00
Herbaspirillum.massiliense
A (n=14)
B (n=15)
C (n=11)
surface

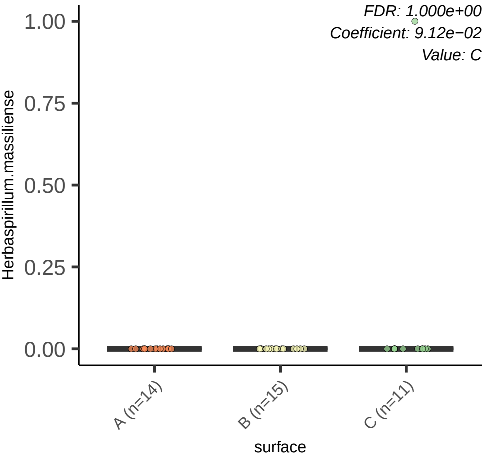
FDR: 1.000e+00
Coefficient: 9.12e−02
Value: C
Herbaspirillum.massiliense
1.00
0.75
0.50
0.25
0.00
A (n=14)
B (n=15)
C (n=11)
surface

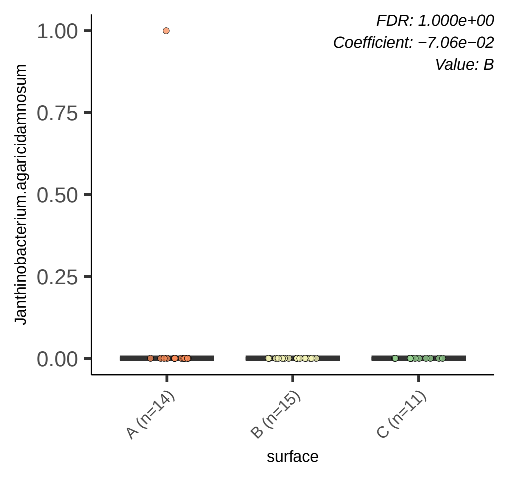

FDR: 1.000e+00
Coefficient: −7.06e−02
Value: B
Janthinobacterium.agaricidamnosum
1.00
0.75
0.50
0.25
0.00
A (n=14)
B (n=15)
C (n=11)
surface

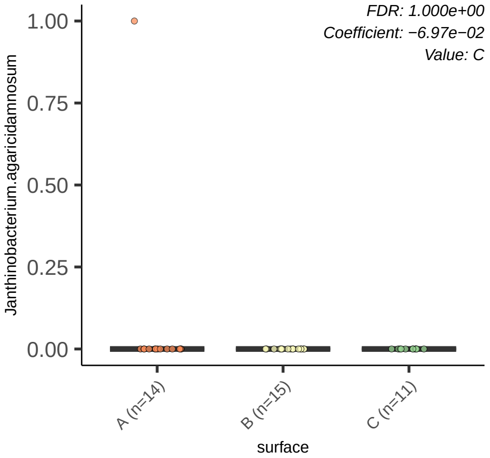
FDR: 1.000e+00
Coefficient: −6.97e−02
Value: C
Janthinobacterium.agaricidamnosum
1.00
0.75
0.50
0.25
0.00
A (n=14)
B (n=15)
C (n=11)
surface

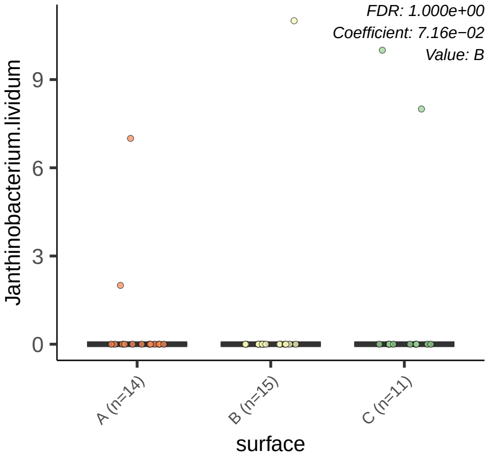
FDR: 1.000e+00
Coefficient: 7.16e−02
Value: B
Janthinobacterium.lividum
0
3
6
9
A (n=14)
B (n=15)
C (n=11)
surface

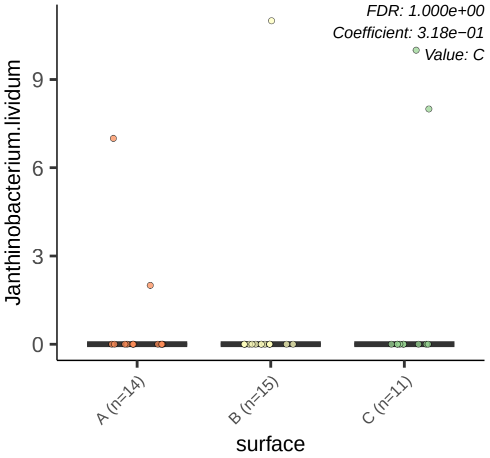
FDR: 1.000e+00
Coefficient: 3.18e−01
Value: C
Janthinobacterium.lividum
0
3
6
9
A (n=14)
B (n=15)
C (n=11)
surface

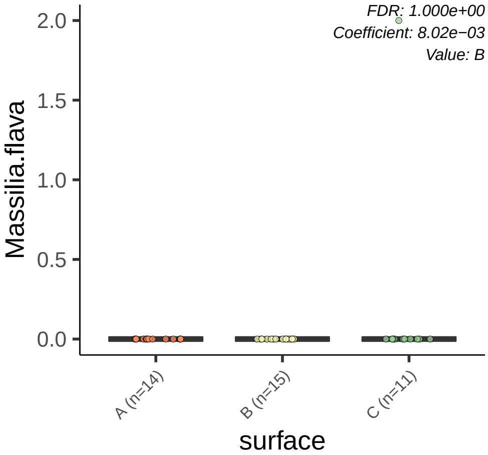
FDR: 1.000e+00
Coefficient: 8.02e−03
Value: B
Massilia.flava
2.0
1.5
1.0
0.5
0.0
A (n=14)
B (n=15)
C (n=11)
surface

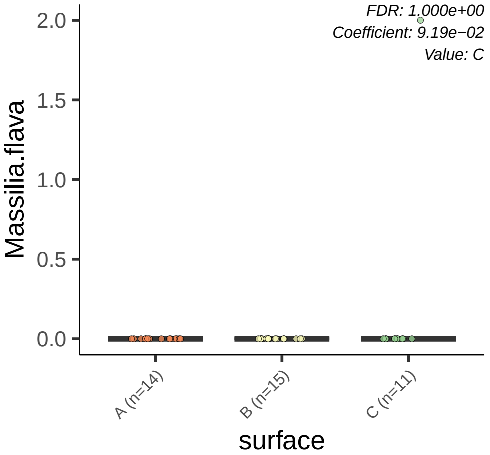
FDR: 1.000e+00
Coefficient: 9.19e−02
Value: C
Massilia.flava
2.0
1.5
1.0
0.5
0.0
A (n=14)
B (n=15)
C (n=11)
surface

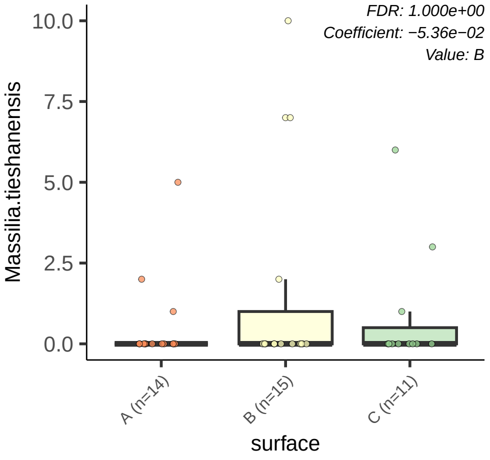
FDR: 1.000e+00
Coefficient: −5.36e−02
Value: B
Massilia.tieshanensis
10.0
7.5
5.0
2.5
0.0
A (n=14)
B (n=15)
C (n=11)
surface

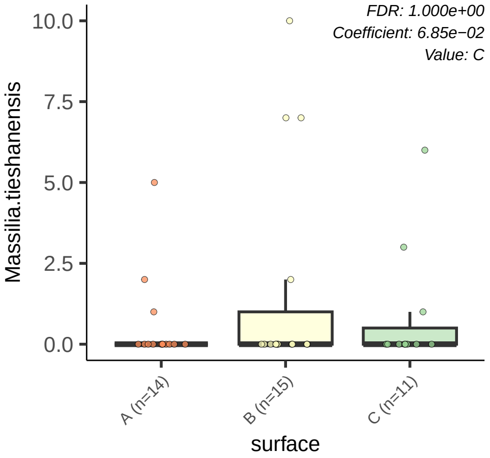

FDR: 1.000e+00
Coefficient: 6.85e−02
Value: C
Massilia.tieshanensis
10.0
7.5
5.0
2.5
0.0
A (n=14)
B (n=15)
C (n=11)
surface

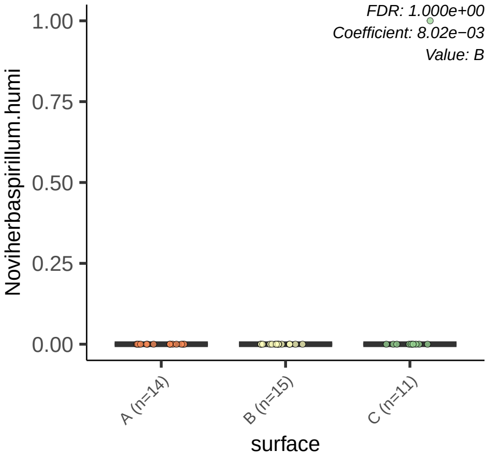

FDR: 1.000e+00
Coefficient: 8.02e−03
Value: B
Noviherbaspirillum.humi
1.00
0.75
0.50
0.25
0.00
A (n=14)
B (n=15)
C (n=11)
surface

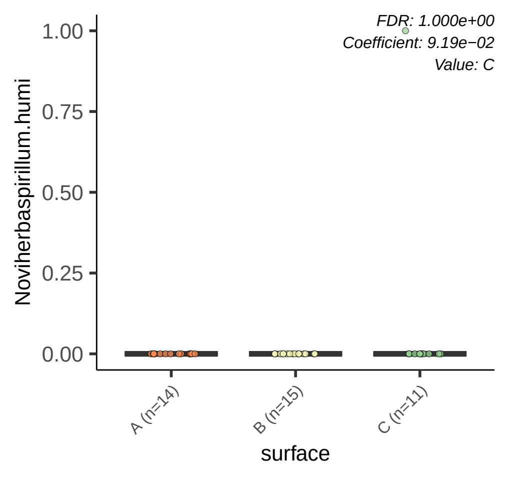

FDR: 1.000e+00
Coefficient: 9.19e−02
Value: C
Noviherbaspirillum.humi
1.00
0.75
0.50
0.25
0.00
A (n=14)
B (n=15)
C (n=11)
surface

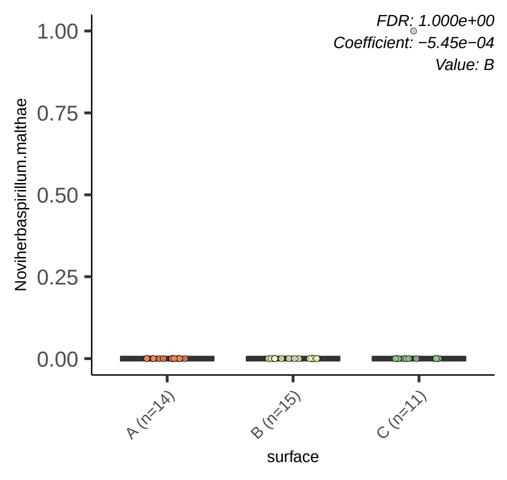
FDR: 1.000e+00
Coefficient: −5.45e−04
Value: B
1.00
0.75
0.50
0.25
0.00
Noviherbaspirillum.malthae
A (n=14)
B (n=15)
C (n=11)
surface

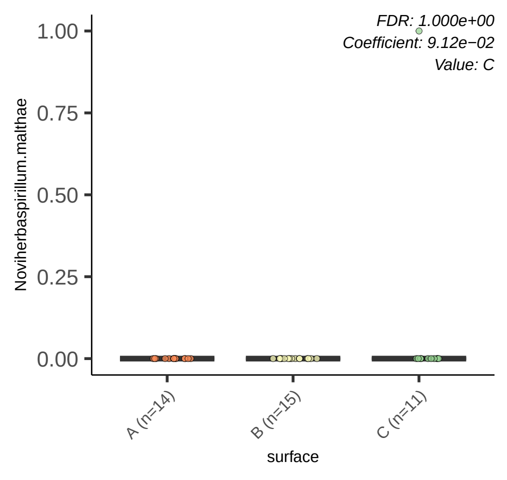
FDR: 1.000e+00
Coefficient: 9.12e−02
Value: C
Noviherbaspirillum.malthae
1.00
0.75
0.50
0.25
0.00
A (n=14)
B (n=15)
C (n=11)
surface

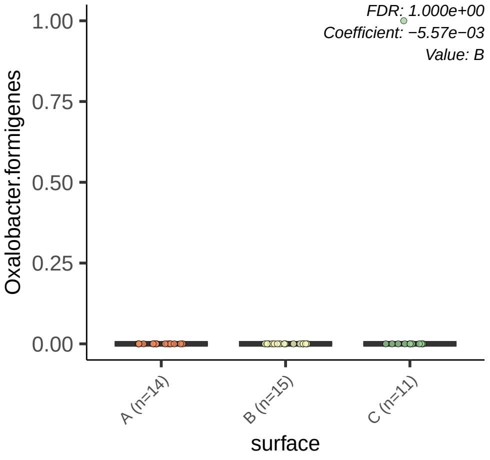

FDR: 1.000e+00
Coefficient: −5.57e−03
Value: B
Oxalobacter.formigenes
1.00
0.75
0.50
0.25
0.00
A (n=14)
B (n=15)
C (n=11)
surface

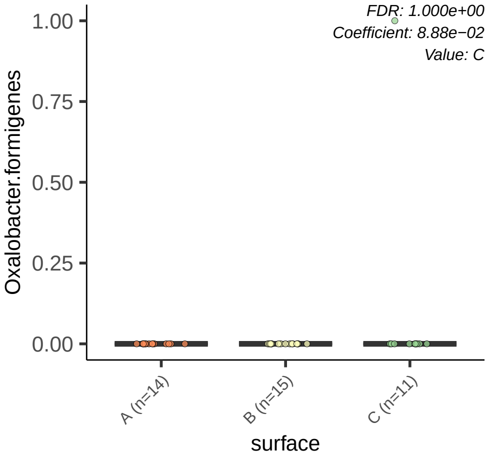

FDR: 1.000e+00
Coefficient: 8.88e−02
Value: C
Oxalobacter.formigenes
1.00
0.75
0.50
0.25
0.00
A (n=14)
B (n=15)
C (n=11)
surface

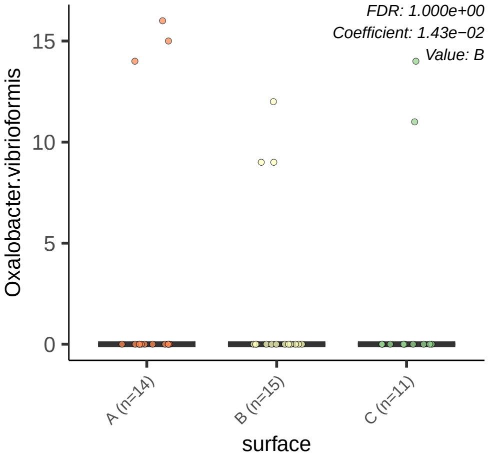

FDR: 1.000e+00
Coefficient: 1.43e−02
Value: B
Oxalobacter.vibrioformis
0
5
10
15
A (n=14)
B (n=15)
C (n=11)
surface

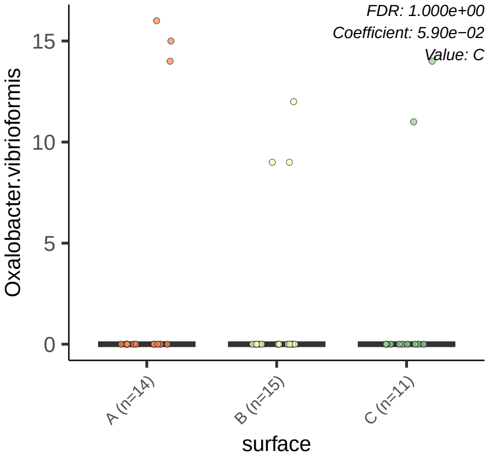
FDR: 1.000e+00
Coefficient: 5.90e−02
Value: C
Oxalobacter.vibrioformis
15
10
5
0
A (n=14)
B (n=15)
C (n=11)
surface

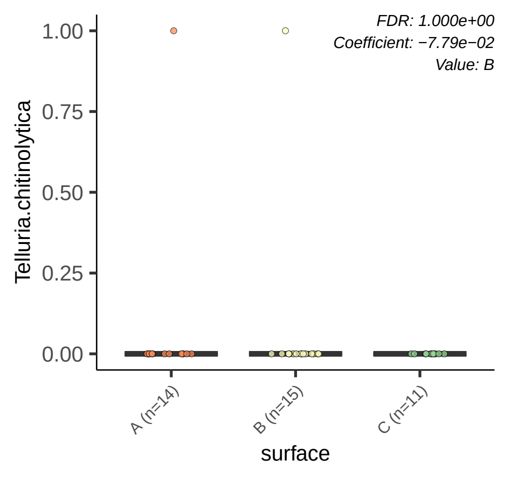
FDR: 1.000e+00
Coefficient: −7.79e−02
Value: B
Telluria.chitinolytica
1.00
0.75
0.50
0.25
0.00
A (n=14)
B (n=15)
C (n=11)
surface

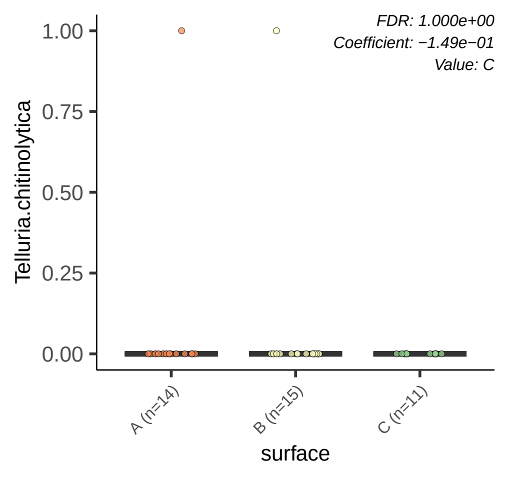

FDR: 1.000e+00
Coefficient: −1.49e−01
Value: C
Telluria.chitinolytica
1.00
0.75
0.50
0.25
0.00
A (n=14)
B (n=15)
C (n=11)
surface

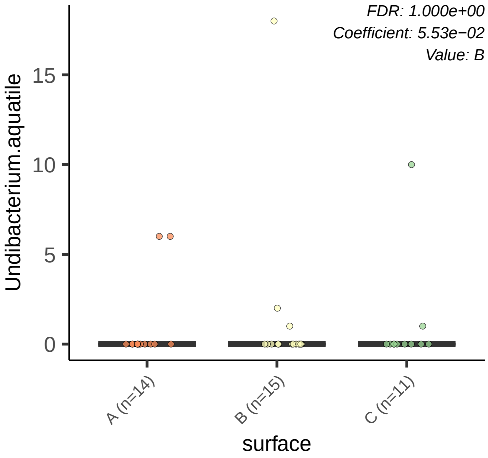
FDR: 1.000e+00
Coefficient: 5.53e−02
Value: B
Undibacterium.aquatile
0
5
10
15
A (n=14)
B (n=15)
C (n=11)
surface

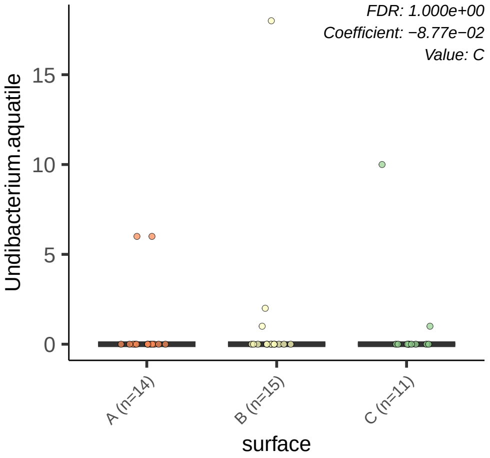
FDR: 1.000e+00
Coefficient: −8.77e−02
Value: C
Undibacterium.aquatile
15
10
5
0
A (n=14)
B (n=15)
C (n=11)
surface

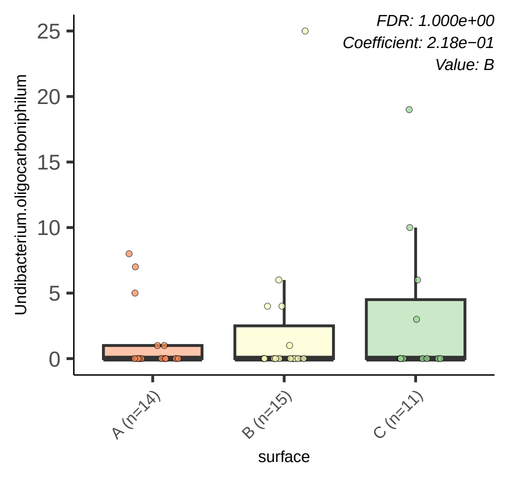
FDR: 1.000e+00
Coefficient: 2.18e−01
Value: B
Undibacterium.oligocarbonophilum
25
20
15
10
5
0
A (n=14)
B (n=15)
C (n=11)
surface

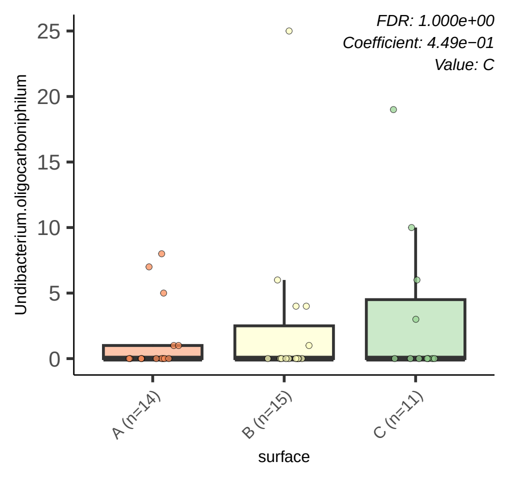

FDR: 1.000e+00
Coefficient: 4.49e−01
Value: C
Undibacterium.oligocarboniphilum
0
5
10
15
20
25
A (n=14)
B (n=15)
C (n=11)
surface

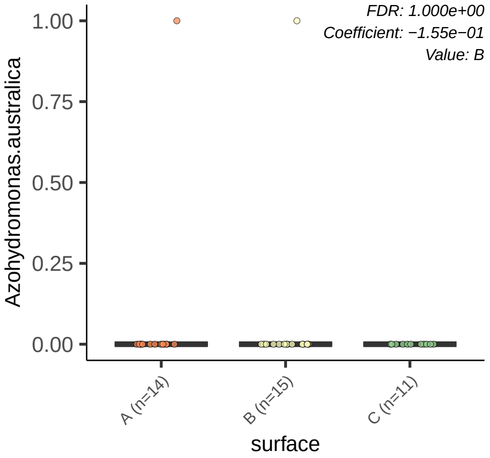

FDR: 1.000e+00
Coefficient: −1.55e−01
Value: B
Azohydromonas.australica
1.00
0.75
0.50
0.25
0.00
A (n=14)
B (n=15)
C (n=11)
surface

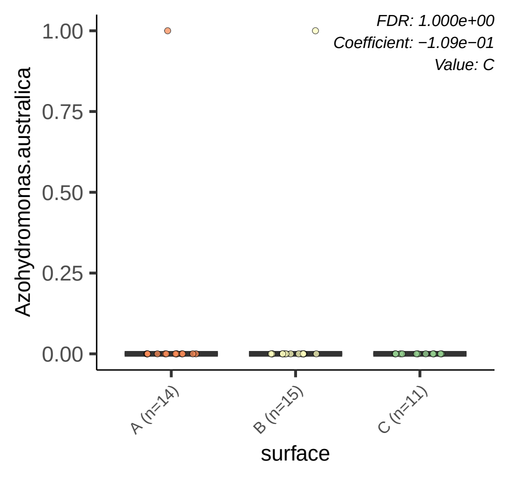
FDR: 1.000e+00
Coefficient: −1.09e−01
Value: C
Azohydromonas.australica
1.00
0.75
0.50
0.25
0.00
A (n=14)
B (n=15)
C (n=11)
surface

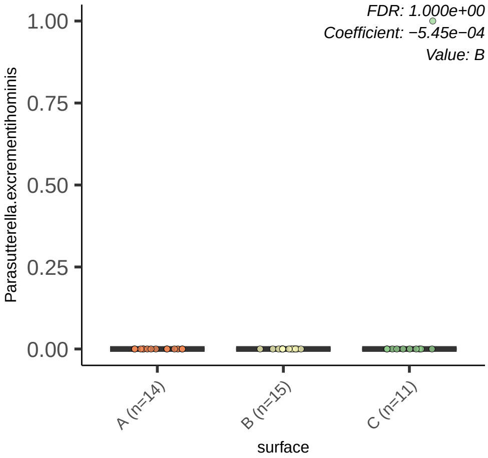
FDR: 1.000e+00
Coefficient: −5.45e−04
Value: B
Parasutterella.excrementihominis
1.00
0.75
0.50
0.25
0.00
A (n=14)
B (n=15)
C (n=11)
surface

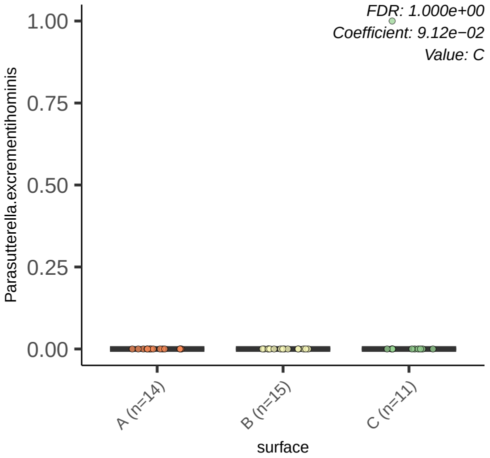
FDR: 1.000e+00
Coefficient: 9.12e−02
Value: C
Parasutterella.excrementihominis
1.00
0.75
0.50
0.25
0.00
A (n=14)
B (n=15)
C (n=11)
surface

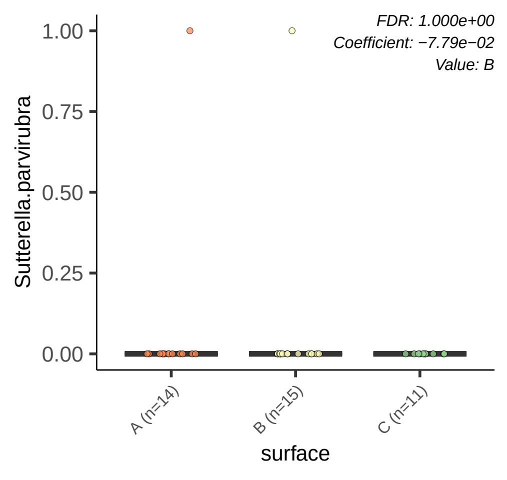
FDR: 1.000e+00
Coefficient: −7.79e−02
Value: B
Sutterella.parvirubra
1.00
0.75
0.50
0.25
0.00
A (n=14)
B (n=15)
C (n=11)
surface

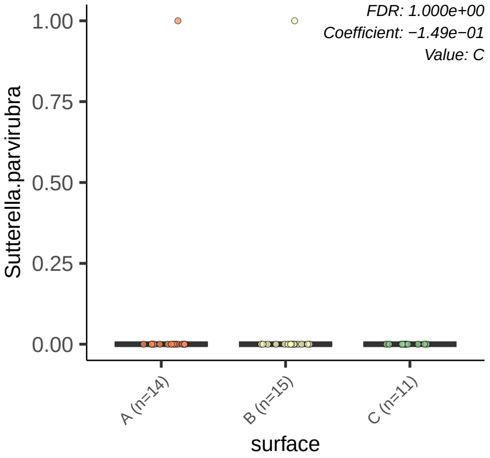
FDR: 1.000e+00
Coefficient: −1.49e−01
Value: C
Sutterella.parvirubra
1.00
0.75
0.50
0.25
0.00
A (n=14)
B (n=15)
C (n=11)
surface

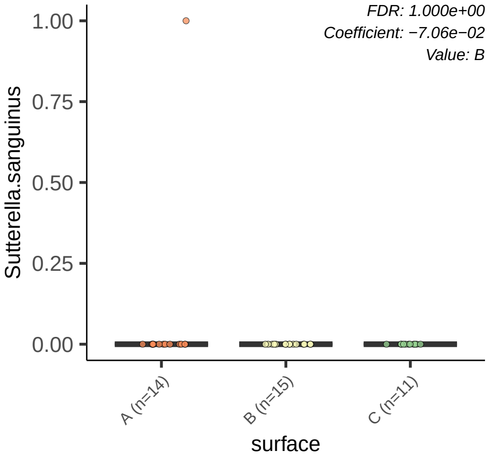
FDR: 1.000e+00
Coefficient: −7.06e−02
Value: B
Sutterella.sanguinus
1.00
0.75
0.50
0.25
0.00
A (n=14)
B (n=15)
C (n=11)
surface

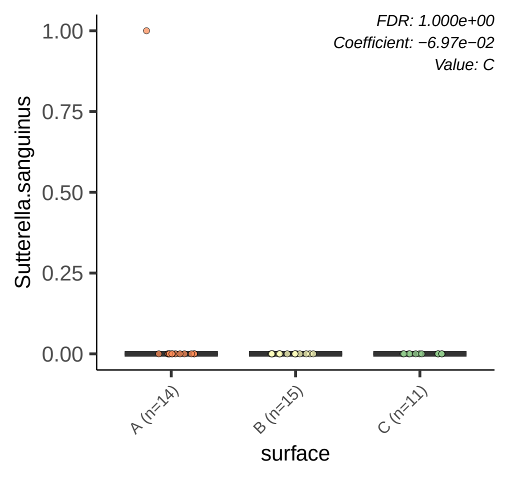
FDR: 1.000e+00
Coefficient: −6.97e−02
Value: C
Sutterella.sanguinus
1.00
0.75
0.50
0.25
0.00
A (n=14)
B (n=15)
C (n=11)
surface

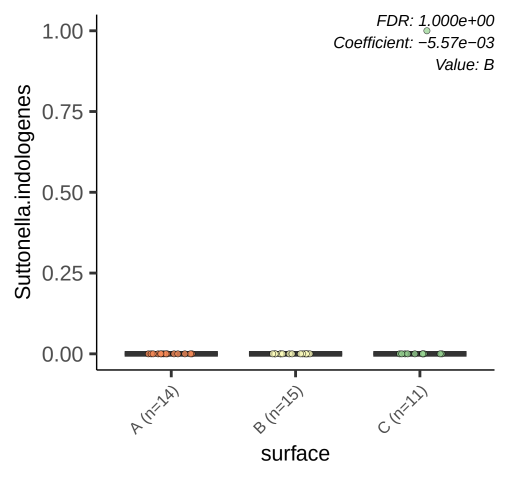
FDR: 1.000e+00
Coefficient: −5.57e−03
Value: B
1.00
0.75
0.50
0.25
0.00
Suttonella.indologenes
A (n=14)
B (n=15)
C (n=11)
surface

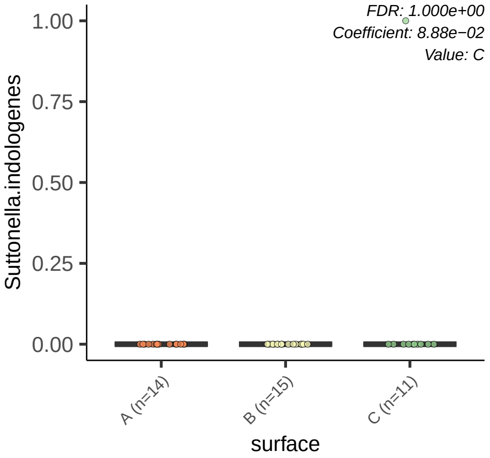
FDR: 1.000e+00
Coefficient: 8.88e−02
Value: C
Suttonella.indologenes
1.00
0.75
0.50
0.25
0.00
A (n=14)
B (n=15)
C (n=11)
surface

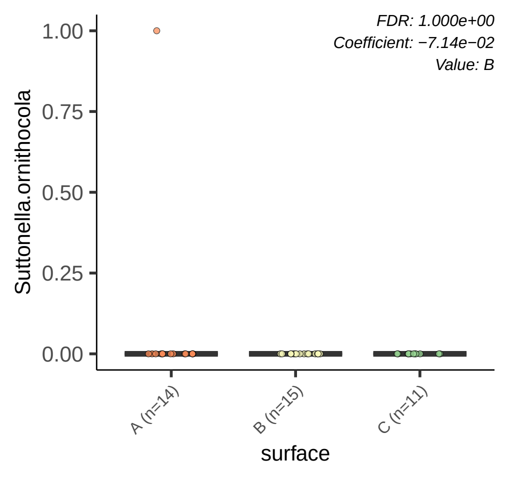

FDR: 1.000e+00
Coefficient: −7.14e−02
Value: B
Suttonella.ornithocola
1.00
0.75
0.50
0.25
0.00
A (n=14)
B (n=15)
C (n=11)
surface

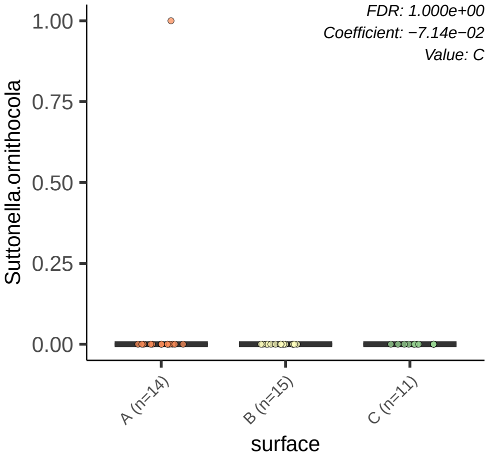

FDR: 1.000e+00
Coefficient: −7.14e−02
Value: C
Suttonella.ornithocola
1.00
0.75
0.50
0.25
0.00
A (n=14)
B (n=15)
C (n=11)
surface

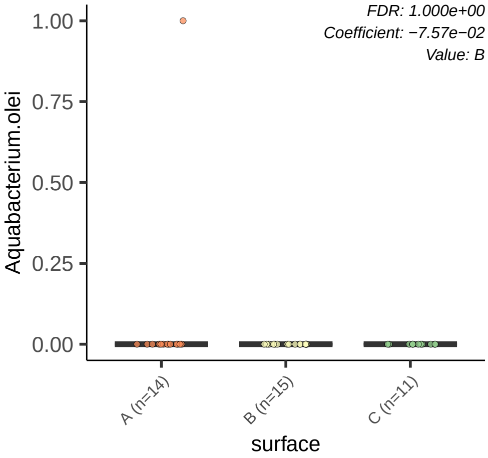

FDR: 1.000e+00
Coefficient: −7.57e−02
Value: B
Aquabacterium.olei
1.00
0.75
0.50
0.25
0.00
A (n=14)
B (n=15)
C (n=11)
surface

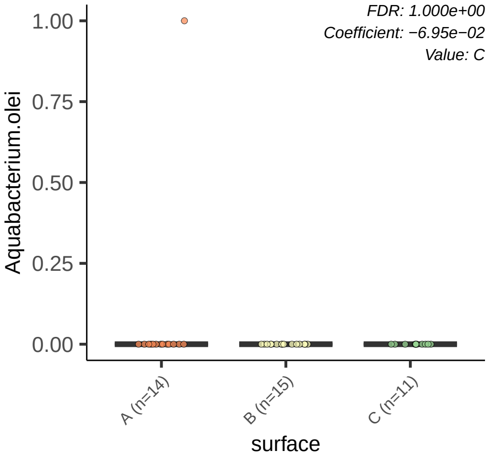
FDR: 1.000e+00
Coefficient: −6.95e−02
Value: C
Aquabacterium.olei
1.00
0.75
0.50
0.25
0.00
A (n=14)
B (n=15)
C (n=11)
surface

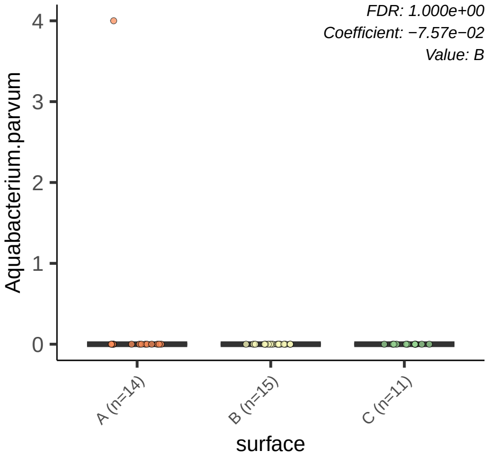
FDR: 1.000e+00
Coefficient: −7.57e−02
Value: B
Aquabacterium.parvum
0
1
2
3
4
A (n=14)
B (n=15)
C (n=11)
surface

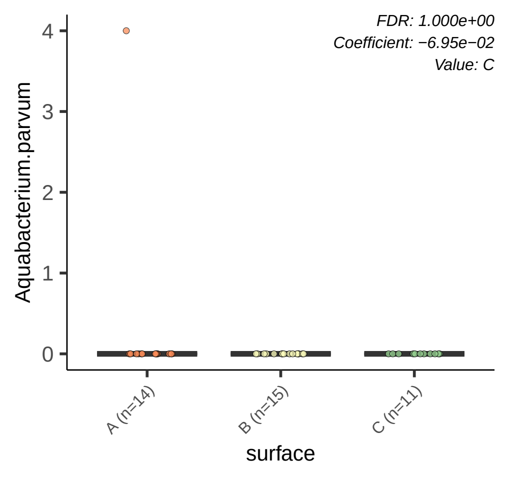
FDR: 1.000e+00
Coefficient: −6.95e−02
Value: C
Aquabacterium.parvum
4
3
2
1
0
A (n=14)
B (n=15)
C (n=11)
surface

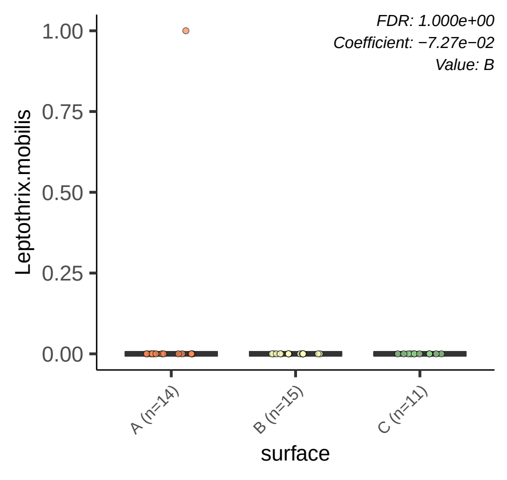
FDR: 1.000e+00
Coefficient: −7.27e−02
Value: B
Leptothrix.mobilis
1.00
0.75
0.50
0.25
0.00
A (n=14)
B (n=15)
C (n=11)
surface

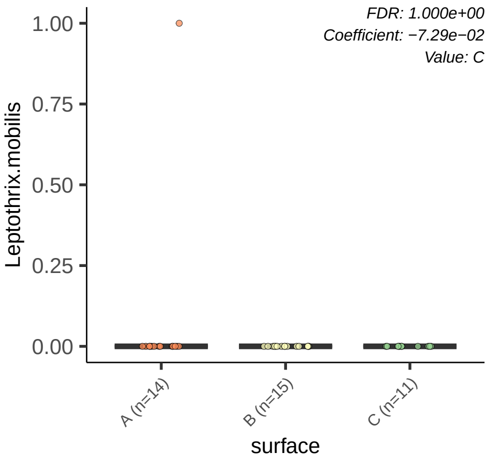

FDR: 1.000e+00
Coefficient: −7.29e−02
Value: C
Leptothrix.mobilis
1.00
0.75
0.50
0.25
0.00
A (n=14)
B (n=15)
C (n=11)
surface

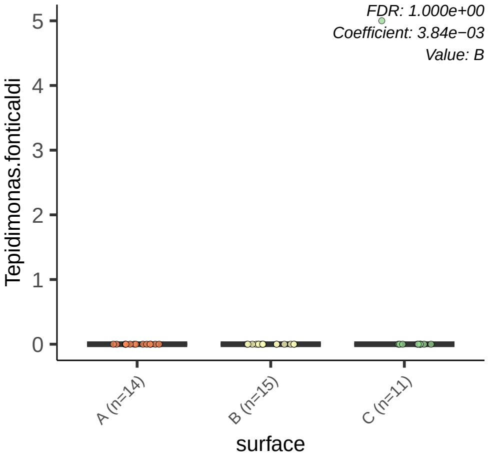

FDR: 1.000e+00
Coefficient: 3.84e−03
Value: B
Tepidimonas.fonticaldi
0
1
2
3
4
5
A (n=14)
B (n=15)
C (n=11)
surface

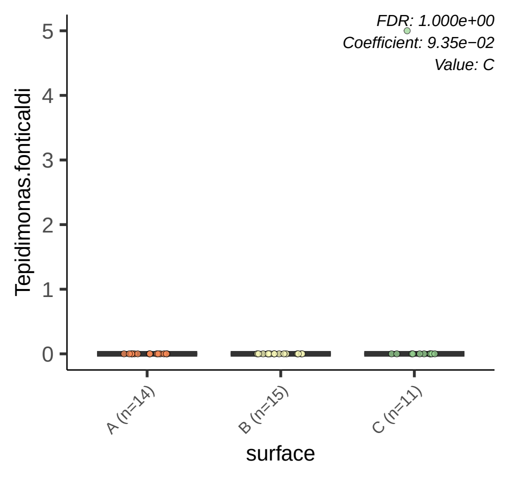
FDR: 1.000e+00
Coefficient: 9.35e−02
Value: C
Tepidimonas.fonticaldi
0
1
2
3
4
5
A (n=14)
B (n=15)
C (n=11)
surface

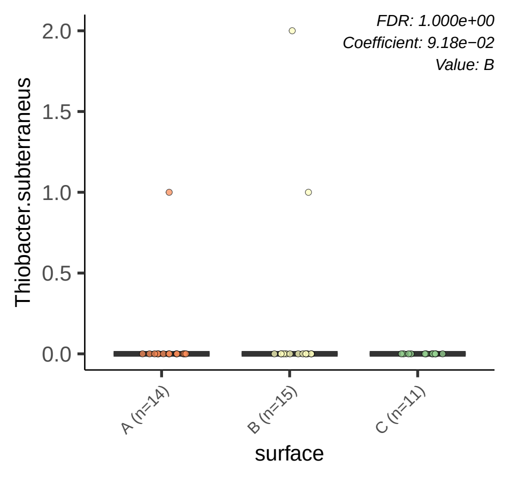
FDR: 1.000e+00
Coefficient: 9.18e−02
Value: B
Thiobacter.subterraneus
2.0
1.5
1.0
0.5
0.0
A (n=14)
B (n=15)
C (n=11)
surface

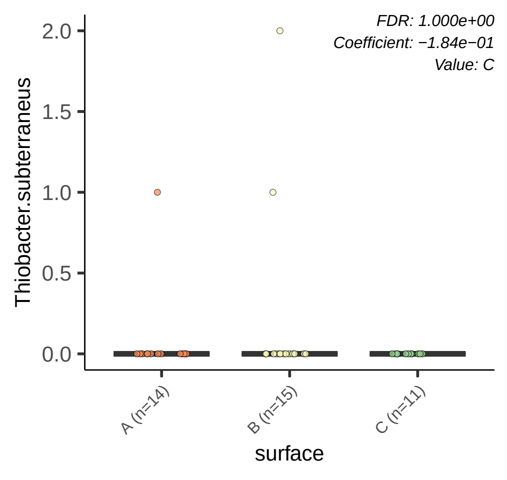

FDR: 1.000e+00
Coefficient: −1.84e−01
Value: C
Thiobacter.subterraneus
2.0
1.5
1.0
0.5
0.0
A (n=14)
B (n=15)
C (n=11)
surface

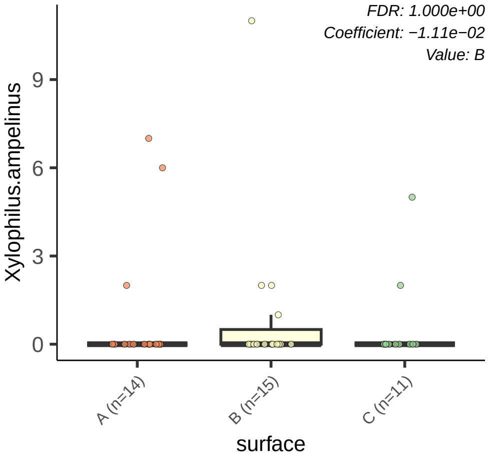
FDR: 1.000e+00
Coefficient: −1.11e−02
Value: B
Xylophilus.ampelinus
9
6
3
0
A (n=14)
B (n=15)
C (n=11)
surface

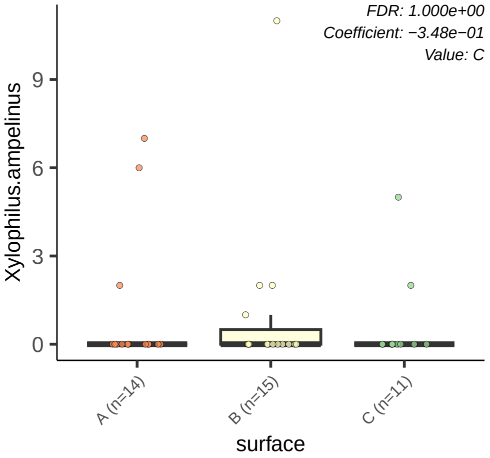
FDR: 1.000e+00
Coefficient: −3.48e−01
Value: C
Xylophilus.ampelinus
0
3
6
9
A (n=14)
B (n=15)
C (n=11)
surface

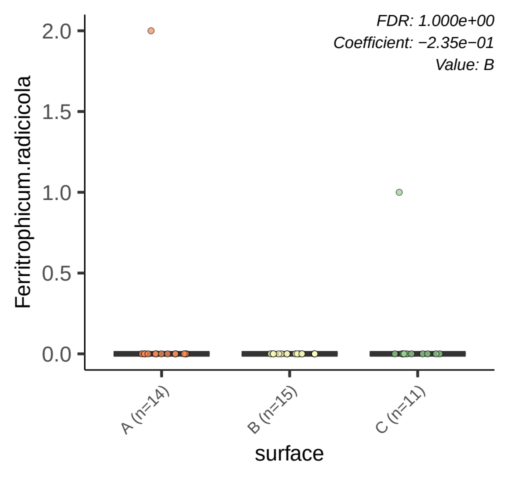
FDR: 1.000e+00
Coefficient: −2.35e−01
Value: B
Ferritrophicum.radicicola
2.0
1.5
1.0
0.5
0.0
A (n=14)
B (n=15)
C (n=11)
surface

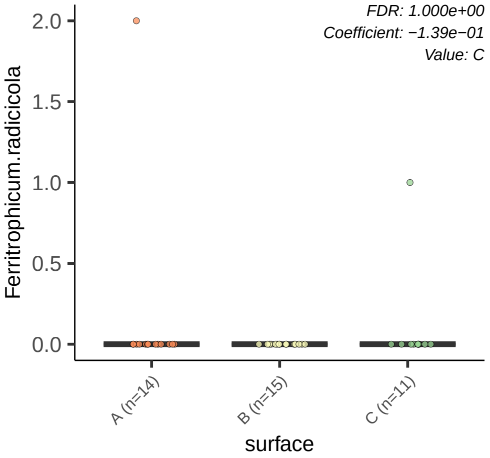

FDR: 1.000e+00
Coefficient: −1.39e−01
Value: C
Ferritrophicum.radicicola
2.0
1.5
1.0
0.5
0.0
A (n=14)
B (n=15)
C (n=11)
surface

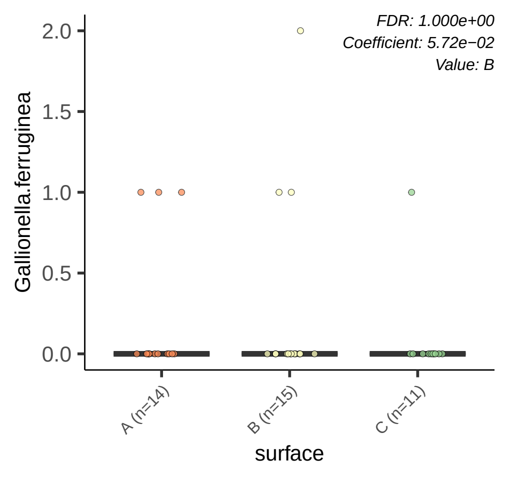

FDR: 1.000e+00
Coefficient: 5.72e−02
Value: B
Gallionella.ferruginea
2.0
1.5
1.0
0.5
0.0
A (n=14)
B (n=15)
C (n=11)
surface

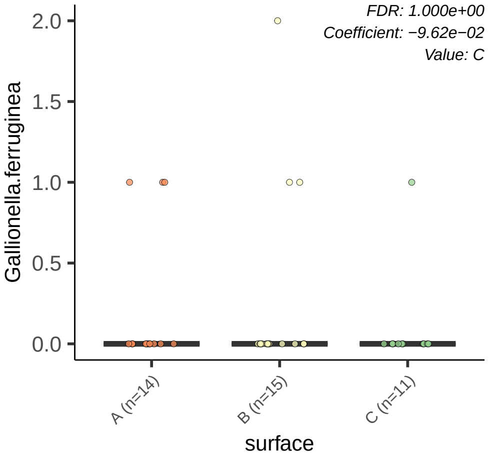

FDR: 1.000e+00
Coefficient: −9.62e−02
Value: C
Gallionella.ferruginea
2.0
1.5
1.0
0.5
0.0
A (n=14)
B (n=15)
C (n=11)
surface

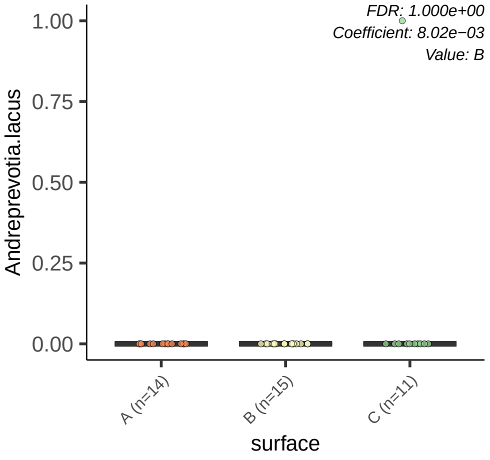

FDR: 1.000e+00
Coefficient: 8.02e−03
Value: B
Andreprevotia.lacus
1.00
0.75
0.50
0.25
0.00
A (n=14)
B (n=15)
C (n=11)
surface

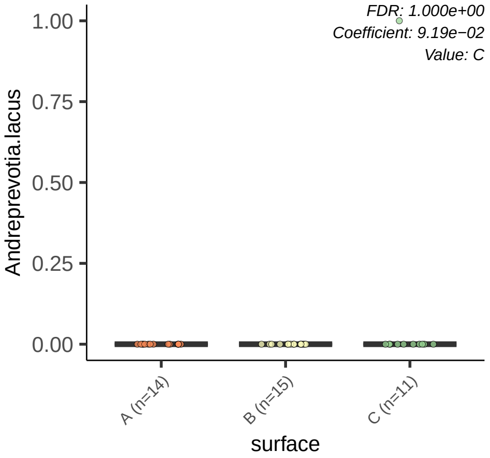

FDR: 1.000e+00
Coefficient: 9.19e−02
Value: C
Andreprevotia.lacus
1.00
0.75
0.50
0.25
0.00
A (n=14)
B (n=15)
C (n=11)
surface

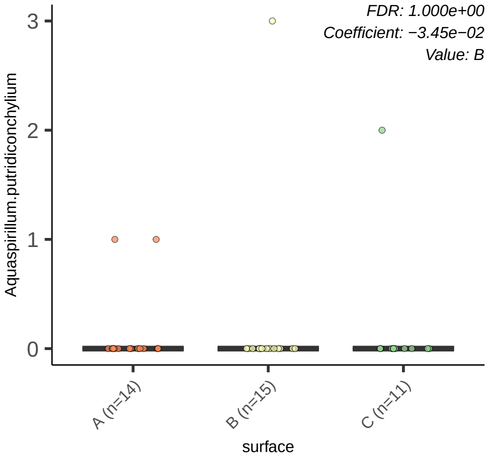

FDR: 1.000e+00
Coefficient: −3.45e−02
Value: B
Aquaspirillum.putridiconchylium
0
1
2
3
A (n=14)
B (n=15)
C (n=11)
surface

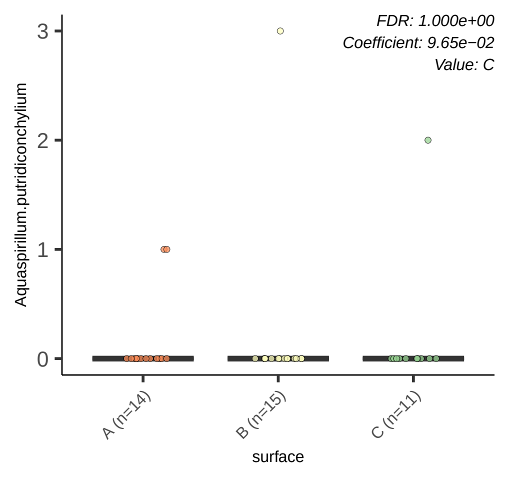
FDR: 1.000e+00
Coefficient: 9.65e−02
Value: C
Aquaspirillum.putridiconchylium
0
1
2
3
A (n=14)
B (n=15)
C (n=11)
surface

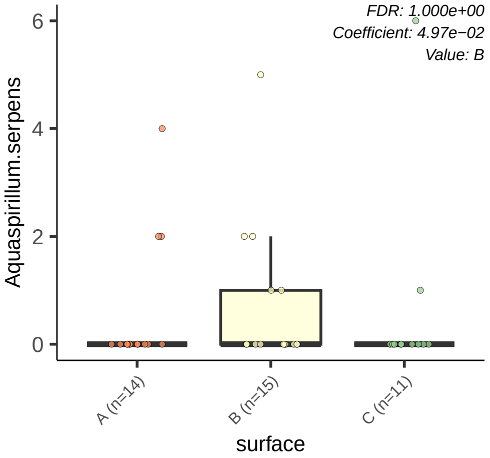
FDR: 1.000e+00
Coefficient: 4.97e−02
Value: B
Aquaspirillum.serpens
0
2
4
6
A (n=14)
B (n=15)
C (n=11)
surface

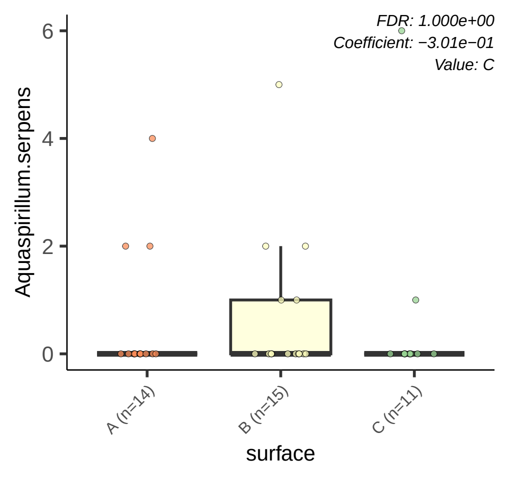

FDR: 1.000e+00
Coefficient: −3.01e−01
Value: C
Aquaspirillum.serpens
0
2
4
6
A (n=14)
B (n=15)
C (n=11)
surface

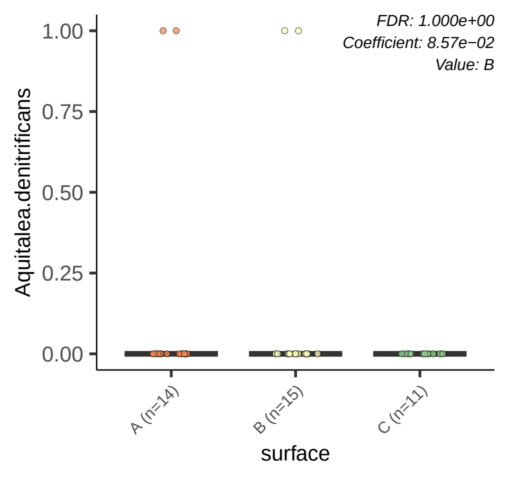
FDR: 1.000e+00
Coefficient: 8.57e−02
Value: B
Aquitalea.denitrificans
1.00
0.75
0.50
0.25
0.00
A (n=14)
B (n=15)
C (n=11)
surface

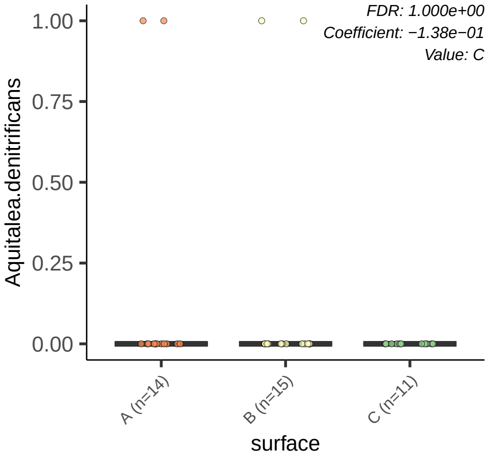

FDR: 1.000e+00
Coefficient: −1.38e−01
Value: C
Aquitalea.denitrificans
1.00
0.75
0.50
0.25
0.00
A (n=14)
B (n=15)
C (n=11)
surface

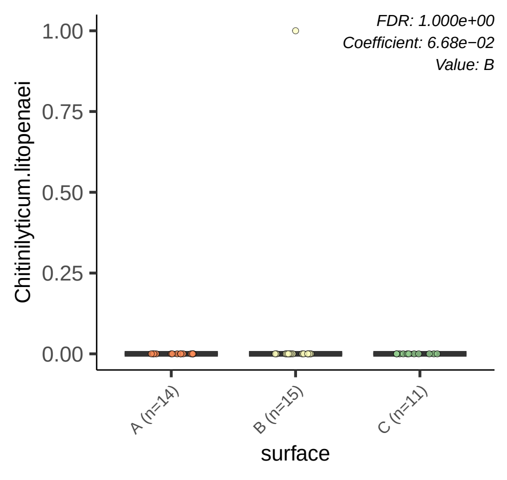

FDR: 1.000e+00
Coefficient: 6.68e−02
Value: B
Chitinilyticum.litopenaei
1.00
0.75
0.50
0.25
0.00
A (n=14)
B (n=15)
C (n=11)
surface

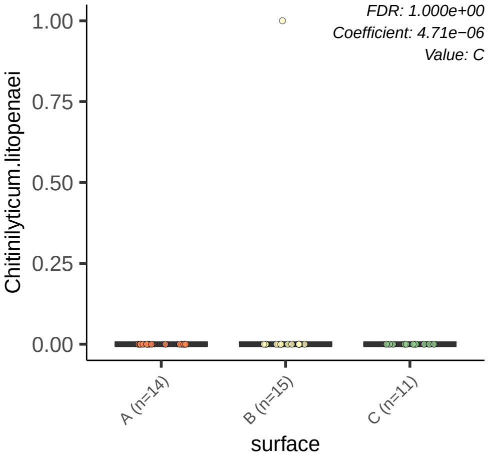
FDR: 1.000e+00
Coefficient: 4.71e−06
Value: C
Chitinilyticum.litopenaei
1.00
0.75
0.50
0.25
0.00
A (n=14)
B (n=15)
C (n=11)
surface

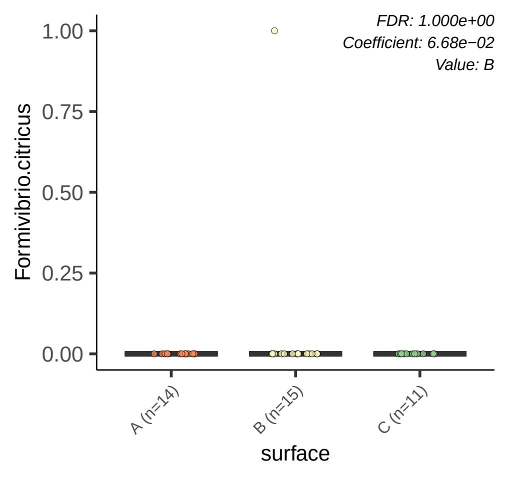
FDR: 1.000e+00
Coefficient: 6.68e−02
Value: B
Formivibrio.citricus
1.00
0.75
0.50
0.25
0.00
A (n=14)
B (n=15)
C (n=11)
surface

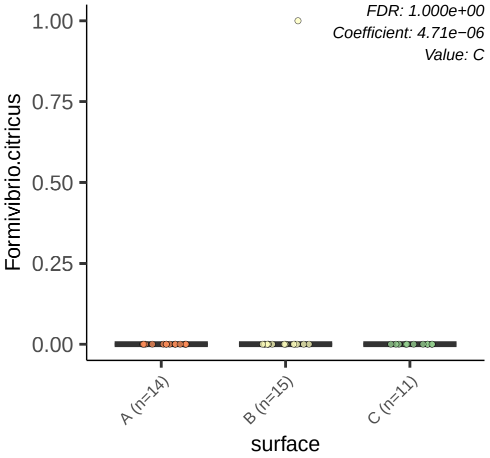
FDR: 1.000e+00
Coefficient: 4.71e−06
Value: C
Formivibrio.citricus
1.00
0.75
0.50
0.25
0.00
A (n=14)
B (n=15)
C (n=11)
surface

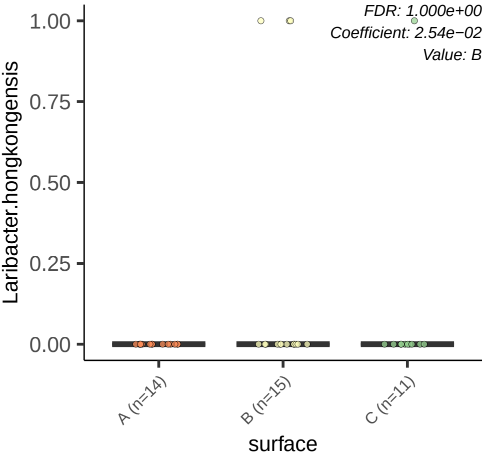
FDR: 1.000e+00
Coefficient: 2.54e−02
Value: B
Laribacter.hongkongensis
1.00
0.75
0.50
0.25
0.00
A (n=14)
B (n=15)
C (n=11)
surface

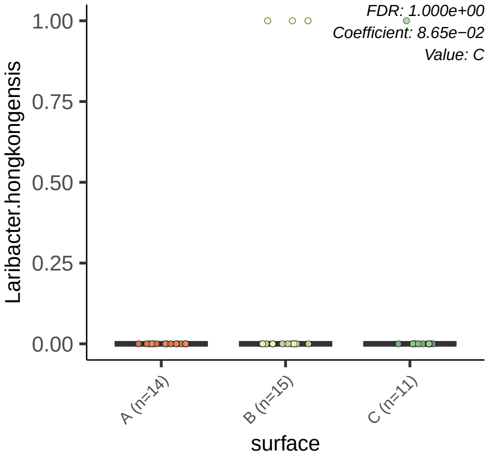
FDR: 1.000e+00
Coefficient: 8.65e−02
Value: C
Laribacter.hongkongensis
1.00
0.75
0.50
0.25
0.00
A (n=14)
B (n=15)
C (n=11)
surface

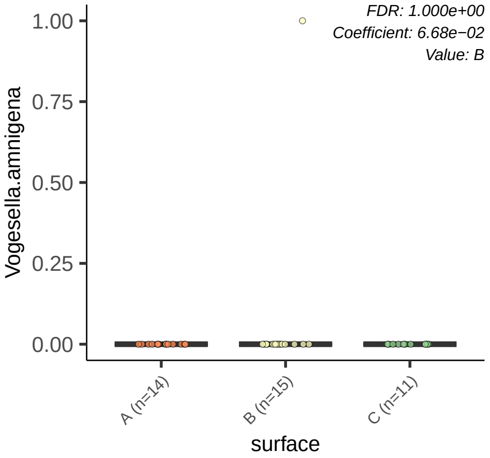
FDR: 1.000e+00
Coefficient: 6.68e−02
Value: B
Vogesella.amnigena
1.00
0.75
0.50
0.25
0.00
A (n=14)
B (n=15)
C (n=11)
surface

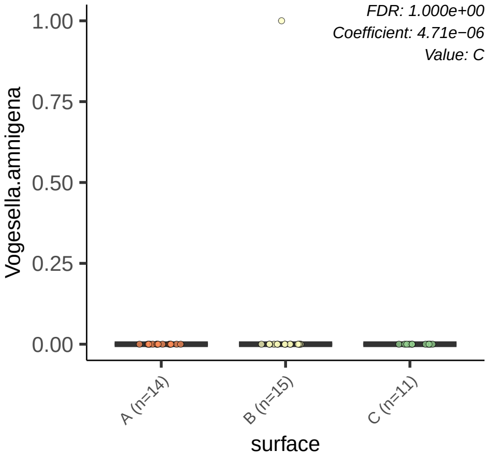

FDR: 1.000e+00
Coefficient: 4.71e−06
Value: C
Vogesella.amnigena
1.00
0.75
0.50
0.25
0.00
A (n=14)
B (n=15)
C (n=11)
surface

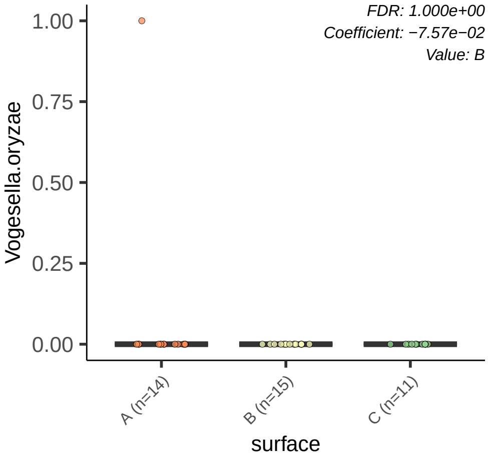

FDR: 1.000e+00
Coefficient: −7.57e−02
Value: B
Vogesella.oryzae
1.00
0.75
0.50
0.25
0.00
A (n=14)
B (n=15)
C (n=11)
surface

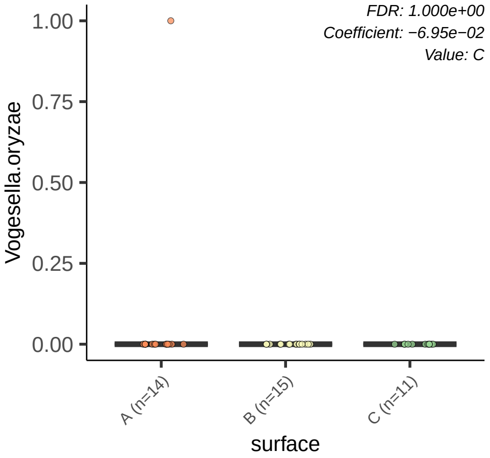

FDR: 1.000e+00
Coefficient: −6.95e−02
Value: C
Vogesella.oryzae
1.00
0.75
0.50
0.25
0.00
A (n=14)
B (n=15)
C (n=11)
surface

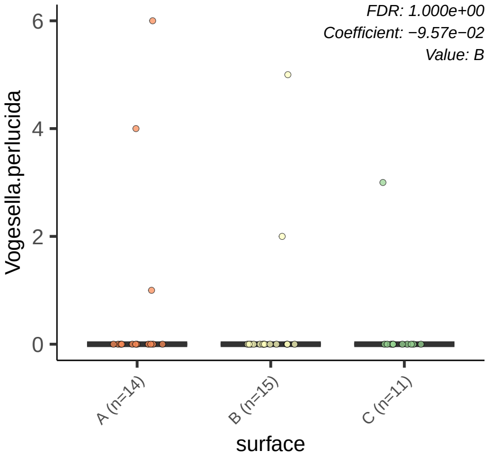

FDR: 1.000e+00
Coefficient: −9.57e−02
Value: B
Vogesella.perlucida
0
2
4
6
A (n=14)
B (n=15)
C (n=11)
surface

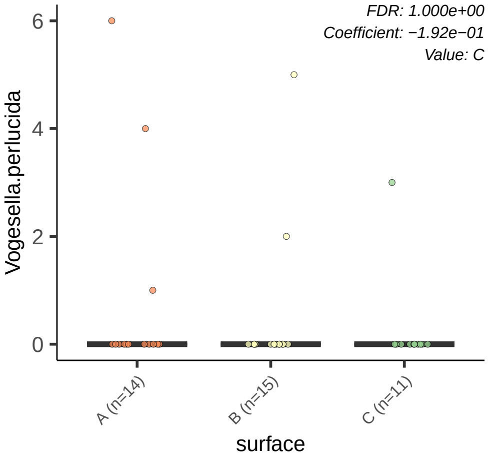
FDR: 1.000e+00
Coefficient: −1.92e−01
Value: C
Vogesella.perlucida
0
2
4
6
A (n=14)
B (n=15)
C (n=11)
surface

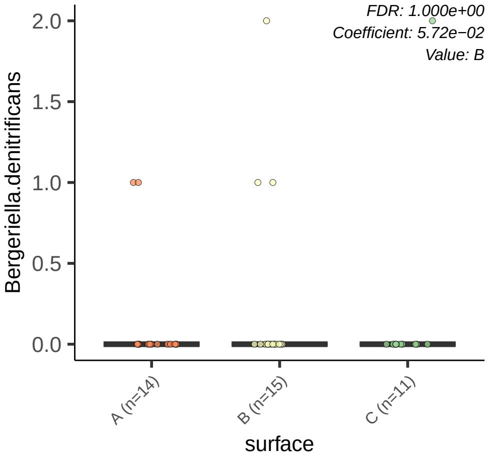
FDR: 1.000e+00
Coefficient: 5.72e−02
Value: B
Bergeriella.denitrificans
2.0
1.5
1.0
0.5
0.0
A (n=14)
B (n=15)
C (n=11)
surface

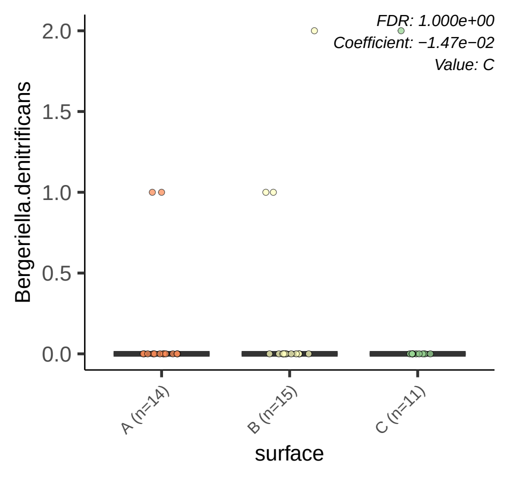
FDR: 1.000e+00
Coefficient: −1.47e−02
Value: C
Bergeriella.denitrificans
2.0
1.5
1.0
0.5
0.0
A (n=14)
B (n=15)
C (n=11)
surface

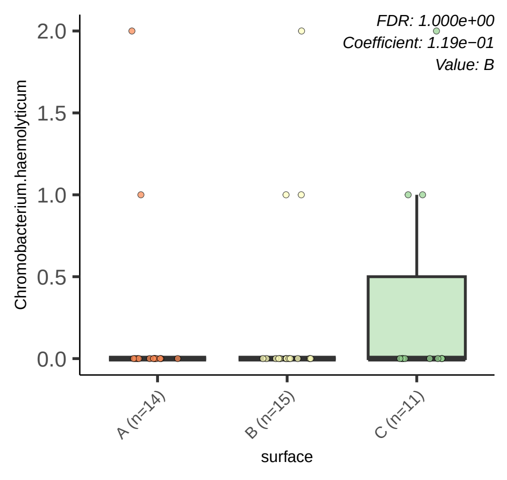
FDR: 1.000e+00
Coefficient: 1.19e−01
Value: B
Chromobacterium.haemolyticum
2.0
1.5
1.0
0.5
0.0
A (n=14)
B (n=15)
C (n=11)
surface

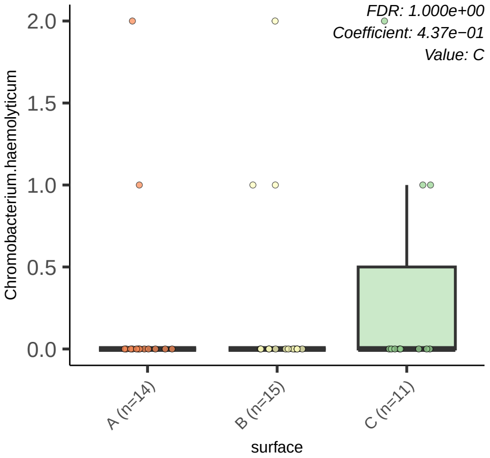
FDR: 1.000e+00
Coefficient: 4.37e−01
Value: C
2.0
1.5
1.0
0.5
0.0
Chromobacterium.haemolyticum
A (n=14)
B (n=15)
C (n=11)
surface

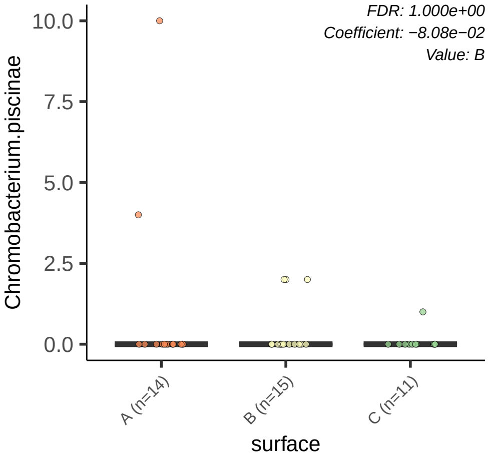

FDR: 1.000e+00
Coefficient: −8.08e−02
Value: B
Chromobacterium.piscinae
10.0
7.5
5.0
2.5
0.0
A (n=14)
B (n=15)
C (n=11)
surface

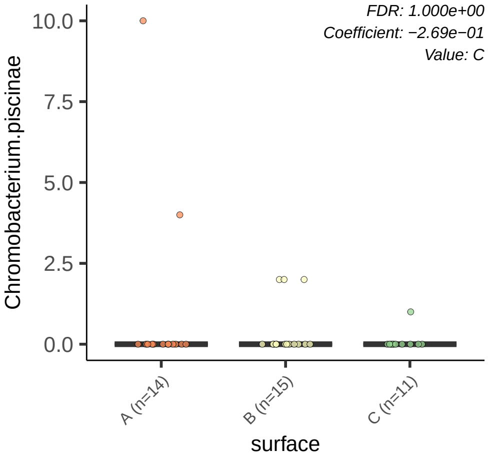

FDR: 1.000e+00
Coefficient: −2.69e−01
Value: C
Chromobacterium.piscinae
10.0
7.5
5.0
2.5
0.0
A (n=14)
B (n=15)
C (n=11)
surface

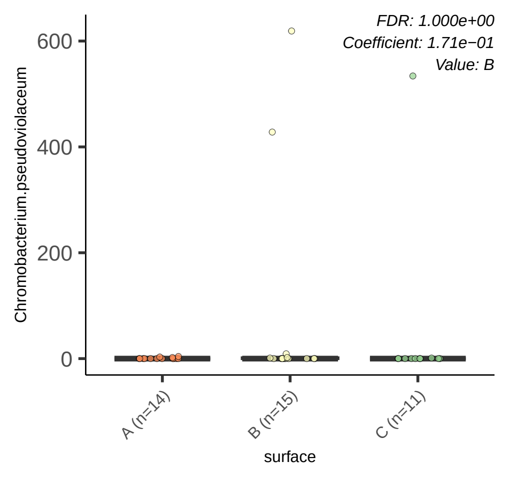

FDR: 1.000e+00
Coefficient: 1.71e−01
Value: B
600
400
200
0
Chromobacterium.pseudoviolaceum
A (n=14)
B (n=15)
C (n=11)
surface

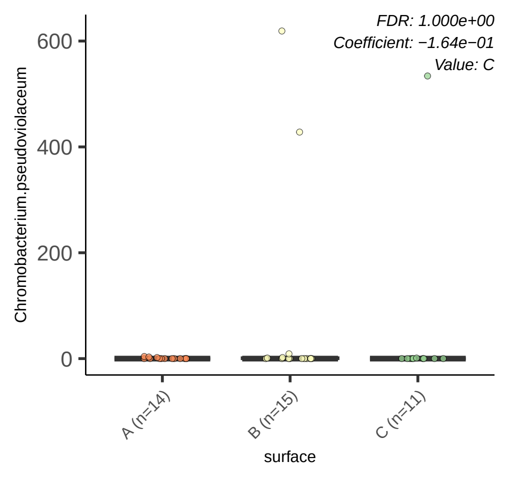
FDR: 1.000e+00
Coefficient: −1.64e−01
Value: C
Chromobacterium.pseudoviolaceum
600
400
200
0
A (n=14)
B (n=15)
C (n=11)
surface

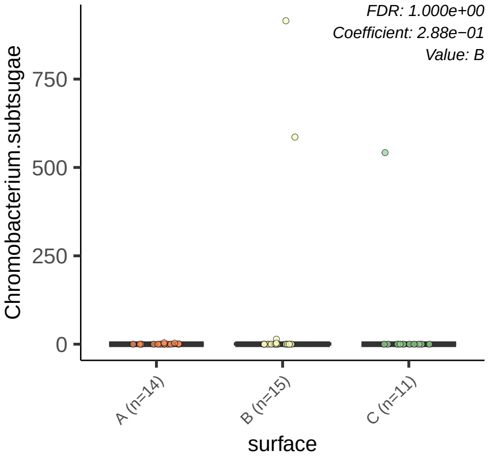
FDR: 1.000e+00
Coefficient: 2.88e−01
Value: B
Chromobacterium.subtsugae
750
500
250
0
A (n=14)
B (n=15)
C (n=11)
surface

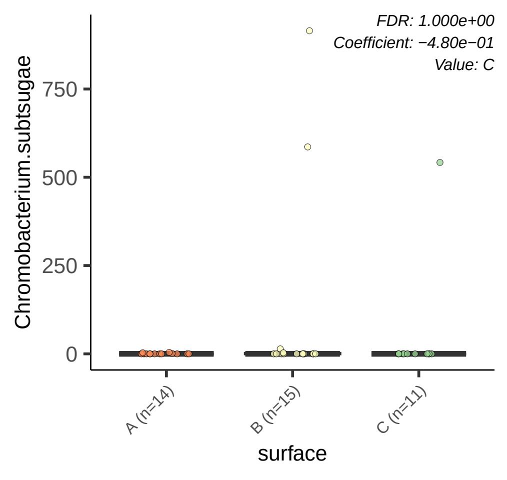
FDR: 1.000e+00
Coefficient: −4.80e−01
Value: C
Chromobacterium.subtsugae
750
500
250
0
A (n=14)
B (n=15)
C (n=11)
surface

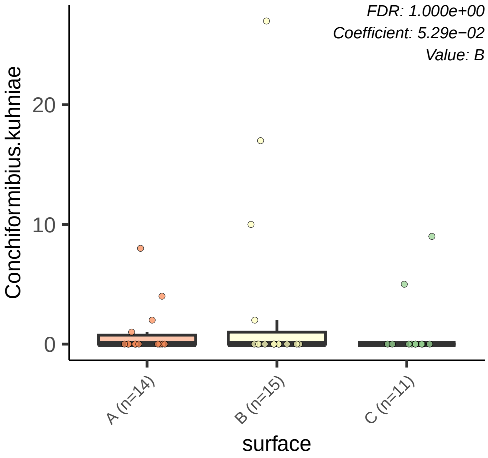
FDR: 1.000e+00
Coefficient: 5.29e−02
Value: B
Conchiformibius.kuhniae
20
10
0
A (n=14)
B (n=15)
C (n=11)
surface

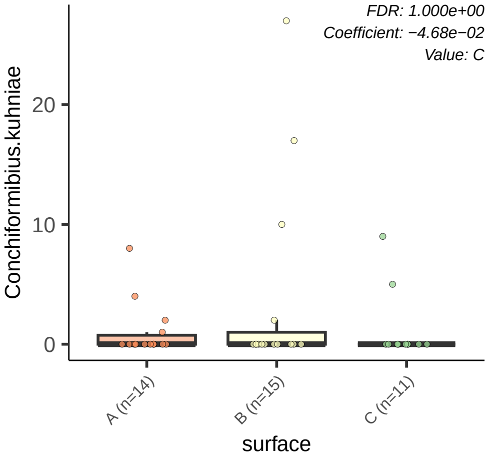
FDR: 1.000e+00
Coefficient: −4.68e−02
Value: C
Conchiformibius.kuhniae
20
10
0
A (n=14)
B (n=15)
C (n=11)
surface

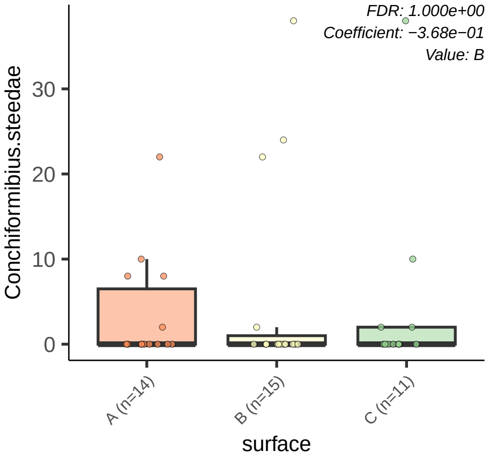
FDR: 1.000e+00
Coefficient: −3.68e−01
Value: B
Conchiformibius.steedae
30
20
10
0
A (n=14)
B (n=15)
C (n=11)
surface

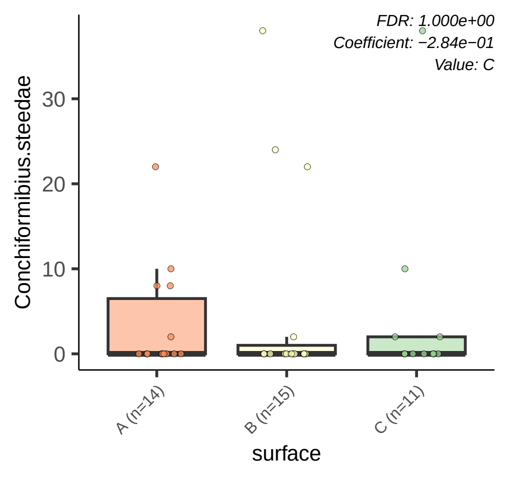
FDR: 1.000e+00
Coefficient: −2.84e−01
Value: C
Conchiformibius.steedae
0
10
20
30
A (n=14)
B (n=15)
C (n=11)
surface

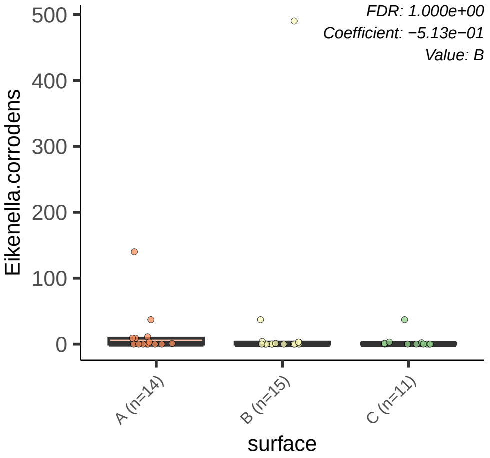
FDR: 1.000e+00
Coefficient: −5.13e−01
Value: B
Eikenella.corrodens
500
400
300
200
100
0
A (n=14)
B (n=15)
C (n=11)
surface

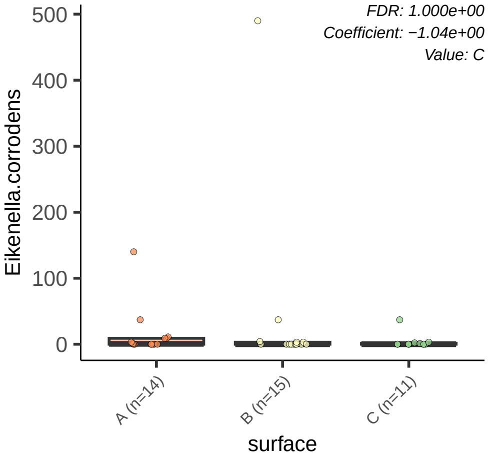
FDR: 1.000e+00
Coefficient: −1.04e+00
Value: C
Eikenella.corrodens
500
400
300
200
100
0
A (n=14)
B (n=15)
C (n=11)
surface

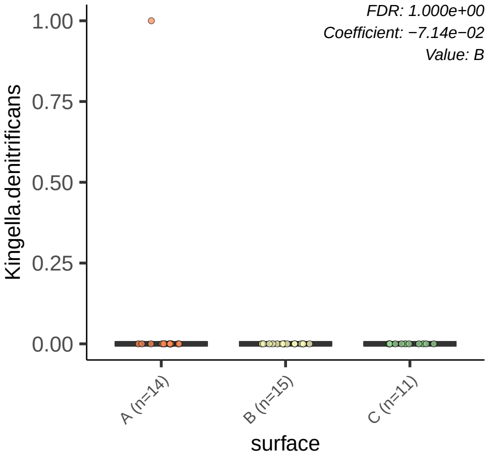
FDR: 1.000e+00
Coefficient: −7.14e−02
Value: B
Kingella.denitrificans
1.00
0.75
0.50
0.25
0.00
A (n=14)
B (n=15)
C (n=11)
surface

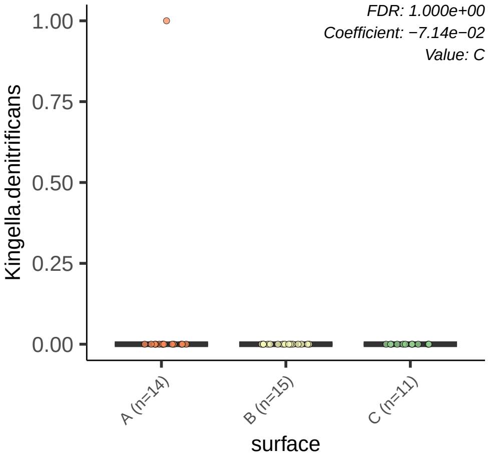

FDR: 1.000e+00
Coefficient: −7.14e−02
Value: C
Kingella.denitrificans
1.00
0.75
0.50
0.25
0.00
A (n=14)
B (n=15)
C (n=11)
surface

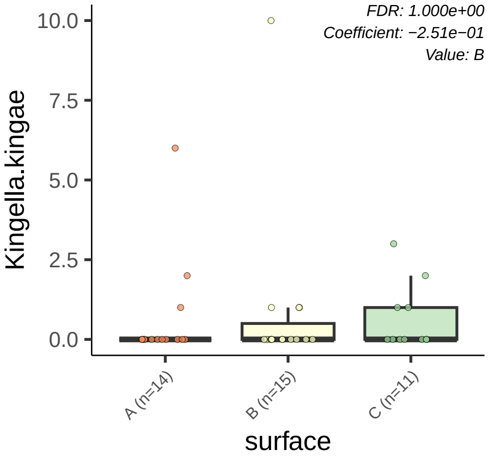
FDR: 1.000e+00
Coefficient: −2.51e−01
Value: B
Kingella.kingae
10.0
7.5
5.0
2.5
0.0
A (n=14)
B (n=15)
C (n=11)
surface

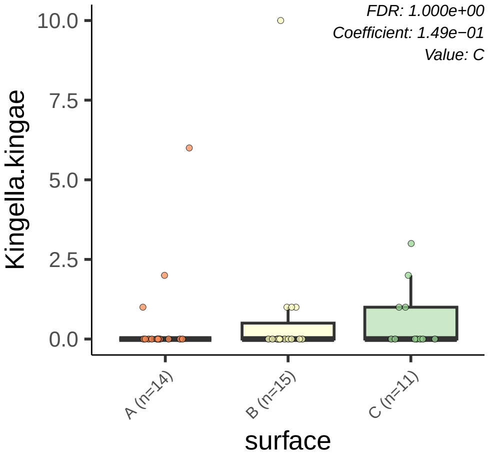

FDR: 1.000e+00
Coefficient: 1.49e−01
Value: C
Kingella.kingae
10.0
7.5
5.0
2.5
0.0
A (n=14)
B (n=15)
C (n=11)
surface

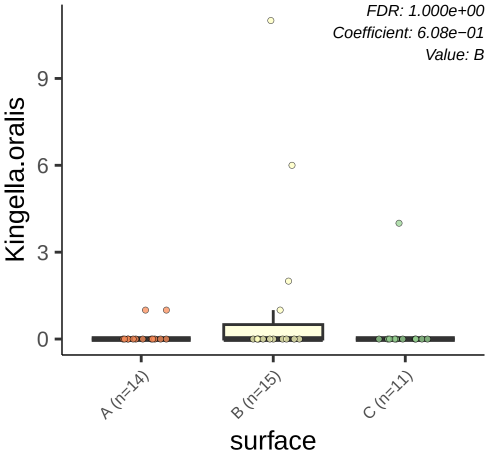

FDR: 1.000e+00
Coefficient: 6.08e−01
Value: B
Kingella.oralis
0
3
6
9
A (n=14)
B (n=15)
C (n=11)
surface

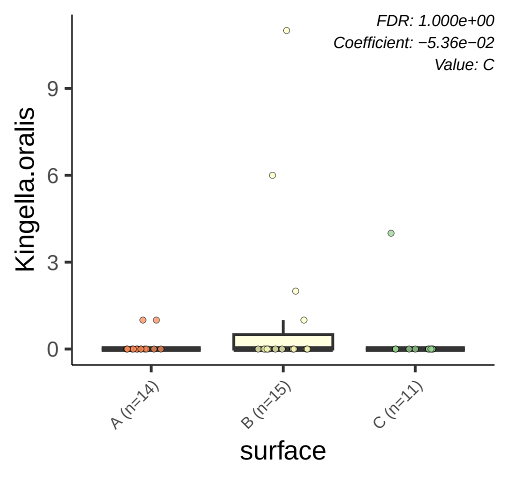

FDR: 1.000e+00
Coefficient: −5.36e−02
Value: C
Kingella.oralis
0
3
6
9
A (n=14)
B (n=15)
C (n=11)
surface

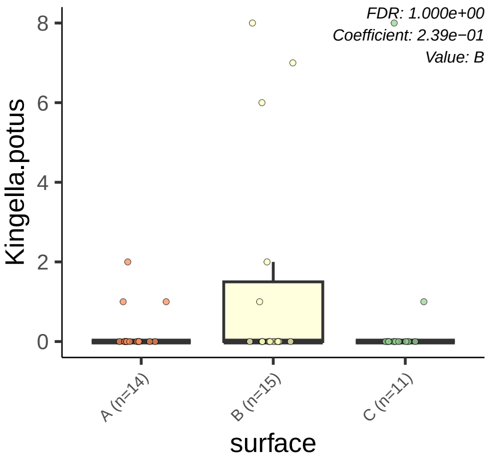

FDR: 1.000e+00
Coefficient: 2.39e−01
Value: B
Kingella.potus
0
2
4
6
8
A (n=14)
B (n=15)
C (n=11)
surface

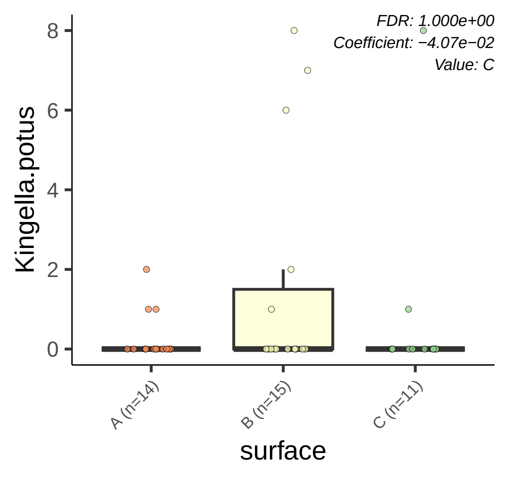
FDR: 1.000e+00
Coefficient: −4.07e−02
Value: C
Kingella.potus
0
2
4
6
8
A (n=14)
B (n=15)
C (n=11)
surface

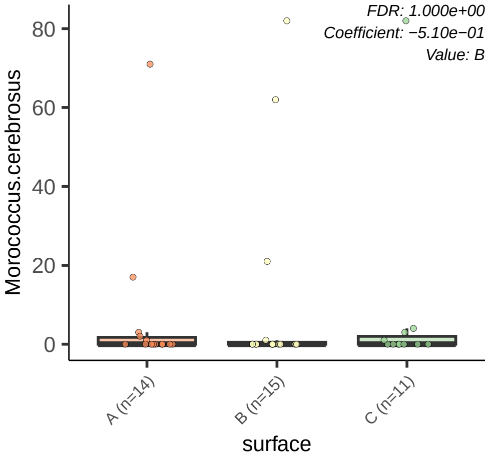
FDR: 1.000e+00
Coefficient: −5.10e−01
Value: B
Morococcus.cerebrosus
0
20
40
60
80
A (n=14)
B (n=15)
C (n=11)
surface

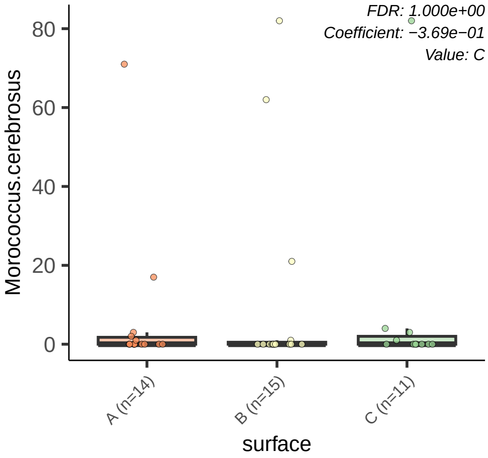

FDR: 1.000e+00
Coefficient: −3.69e−01
Value: C
Morococcus.cerebrosus
80
60
40
20
0
A (n=14)
B (n=15)
C (n=11)
surface

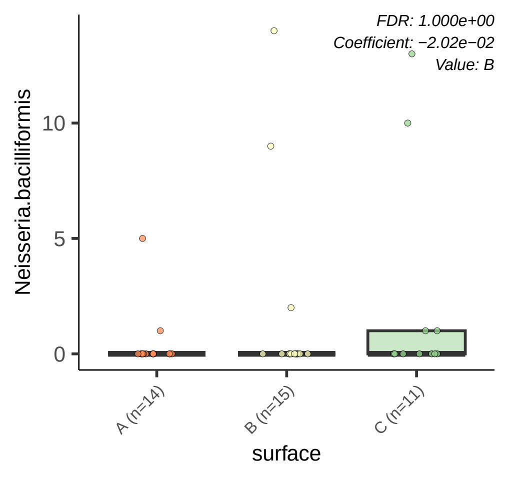

FDR: 1.000e+00
Coefficient: −2.02e−02
Value: B
Neisseria.bacilliformis
0
5
10
A (n=14)
B (n=15)
C (n=11)
surface

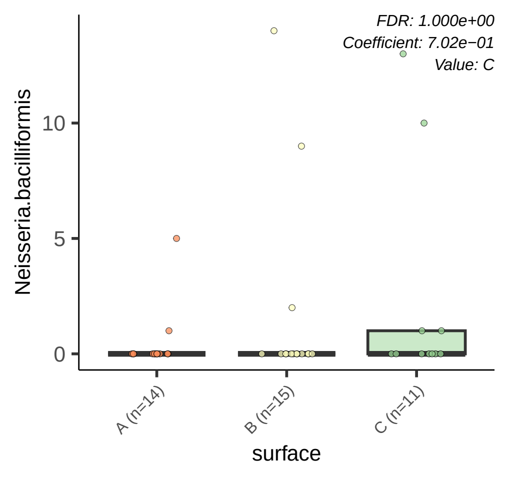

FDR: 1.000e+00
Coefficient: 7.02e−01
Value: C
Neisseria.bacilliformis
0
5
10
A (n=14)
B (n=15)
C (n=11)
surface

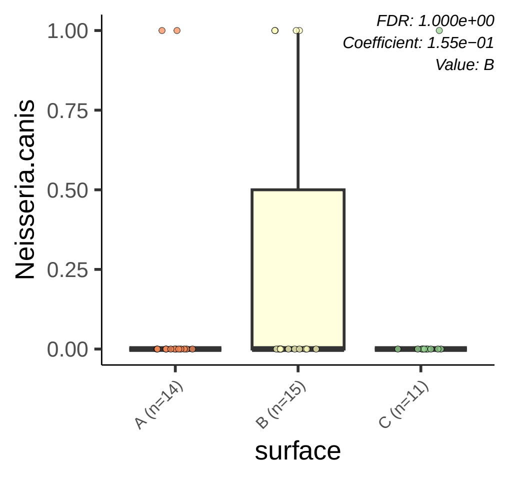
FDR: 1.000e+00
Coefficient: 1.55e−01
Value: B
Neisseria.canis
1.00
0.75
0.50
0.25
0.00
A (n=14)
B (n=15)
C (n=11)
surface

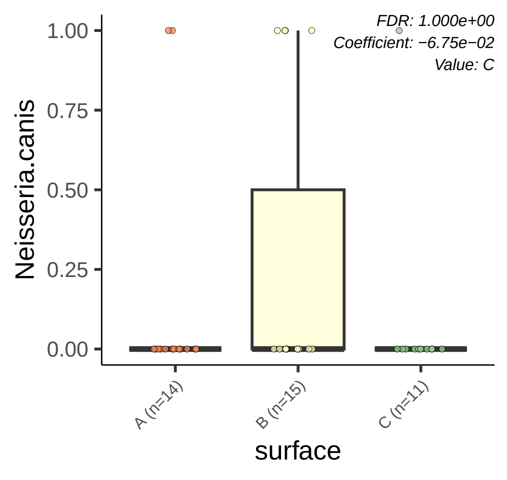
FDR: 1.000e+00
Coefficient: −6.75e−02
Value: C
Neisseria.canis
1.00
0.75
0.50
0.25
0.00
A (n=14)
B (n=15)
C (n=11)
surface

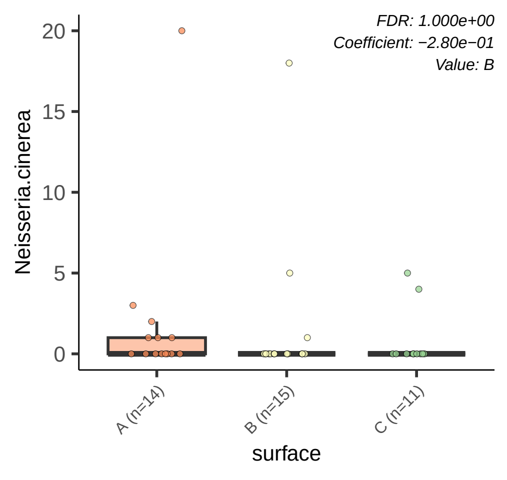
FDR: 1.000e+00
Coefficient: −2.80e−01
Value: B
Neisseria.cinerea
20
15
10
5
0
A (n=14)
B (n=15)
C (n=11)
surface

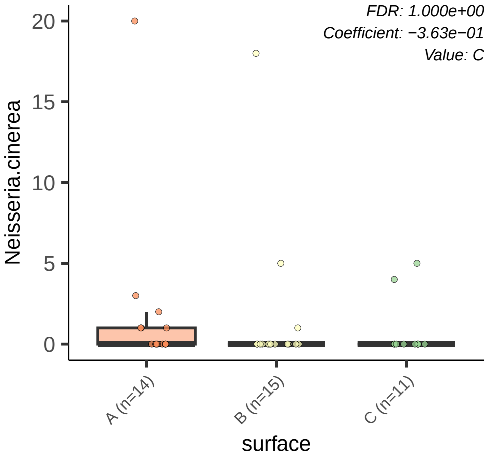

FDR: 1.000e+00
Coefficient: −3.63e−01
Value: C
Neisseria.cinerea
20
15
10
5
0
A (n=14)
B (n=15)
C (n=11)
surface

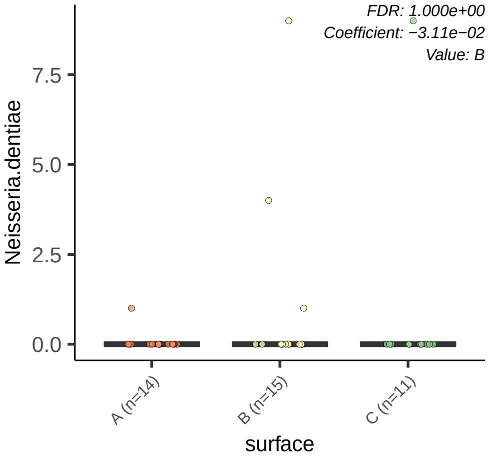

FDR: 1.000e+00
Coefficient: −3.11e−02
Value: B
Neisseria.dentiae
7.5
5.0
2.5
0.0
A (n=14)
B (n=15)
C (n=11)
surface

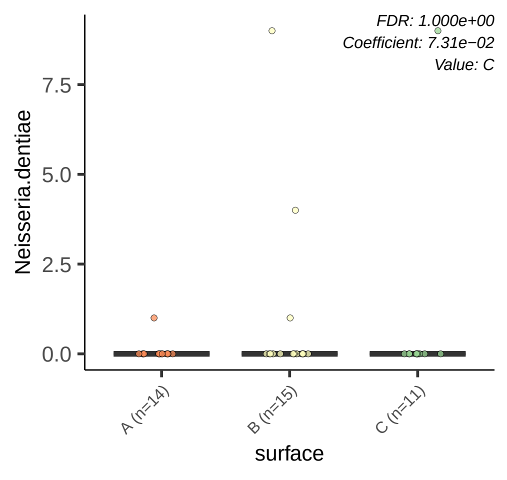

FDR: 1.000e+00
Coefficient: 7.31e−02
Value: C
Neisseria.dentiae
0.0
2.5
5.0
7.5
A (n=14)
B (n=15)
C (n=11)
surface

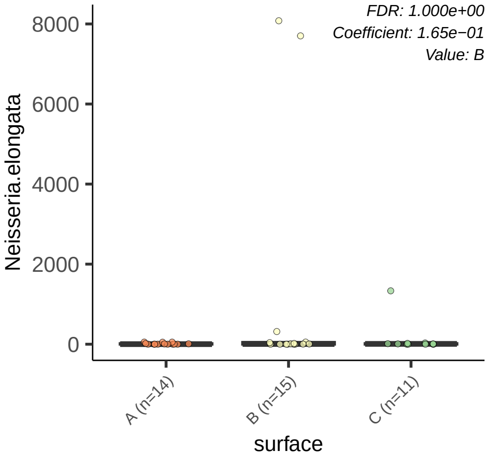
FDR: 1.000e+00
Coefficient: 1.65e−01
Value: B
8000
6000
4000
2000
0
Neisseria.elongata
A (n=14)
B (n=15)
C (n=11)
surface

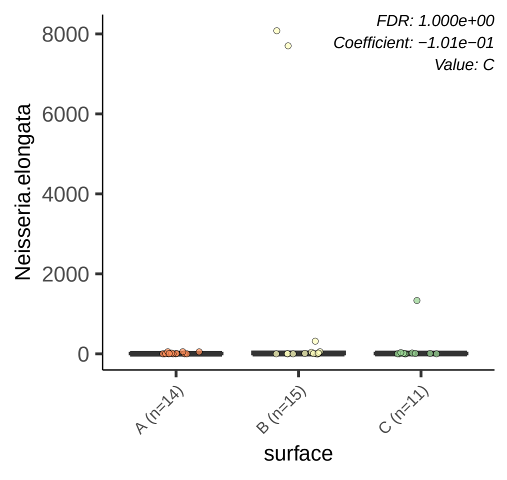
FDR: 1.000e+00
Coefficient: −1.01e−01
Value: C
Neisseria.elongata
8000
6000
4000
2000
0
A (n=14)
B (n=15)
C (n=11)
surface

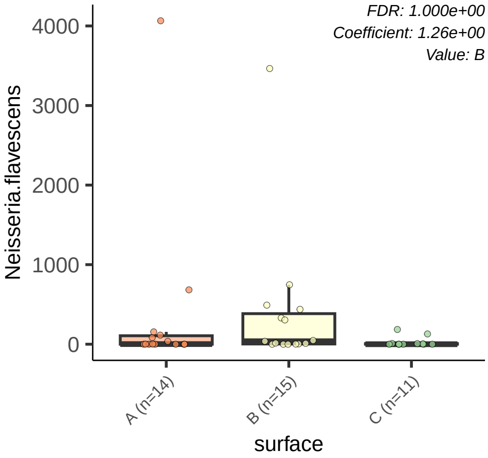
FDR: 1.000e+00
Coefficient: 1.26e+00
Value: B
Neisseria.flavescens
0
1000
2000
3000
4000
A (n=14)
B (n=15)
C (n=11)
surface

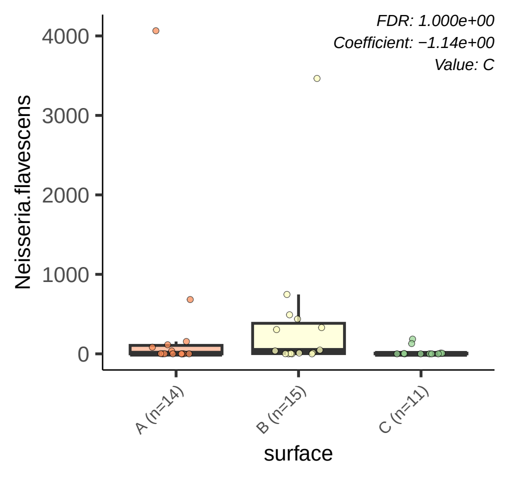

FDR: 1.000e+00
Coefficient: −1.14e+00
Value: C
Neisseria.flavescens
4000
3000
2000
1000
0
A (n=14)
B (n=15)
C (n=11)
surface

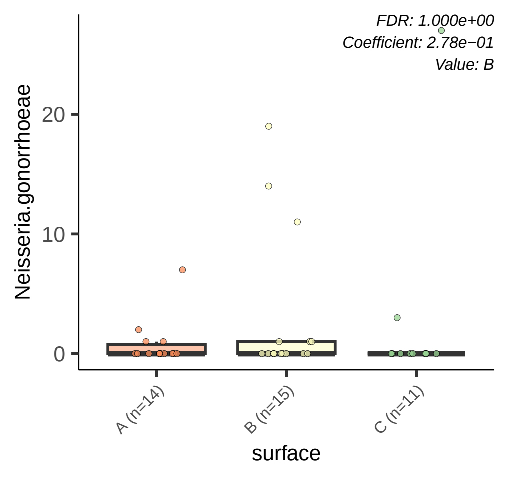

FDR: 1.000e+00
Coefficient: 2.78e−01
Value: B
Neisseria.gonorrhoeae
20
10
0
A (n=14)
B (n=15)
C (n=11)
surface

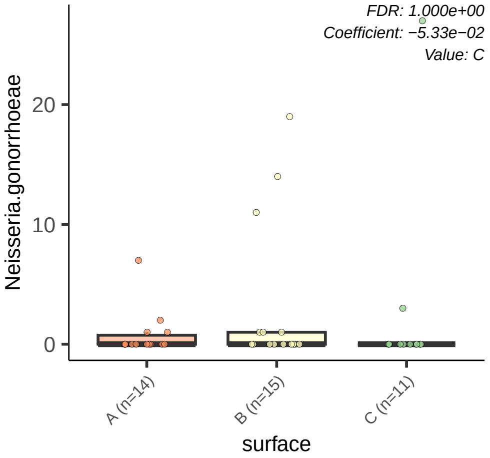

FDR: 1.000e+00
Coefficient: −5.33e−02
Value: C
Neisseria.gonorrhoeae
20
10
0
A (n=14)
B (n=15)
C (n=11)
surface

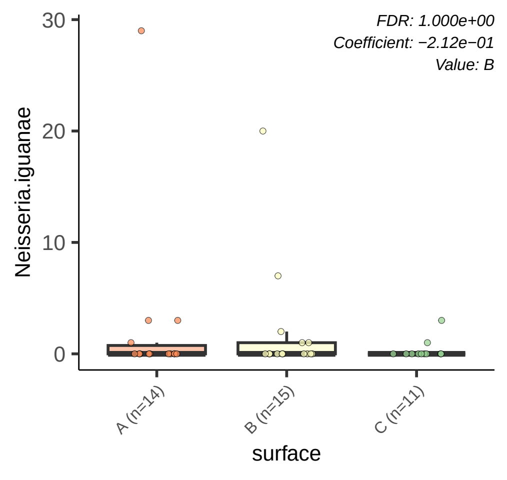
FDR: 1.000e+00
Coefficient: −2.12e−01
Value: B
Neisseria.iguanae
30
20
10
0
A (n=14)
B (n=15)
C (n=11)
surface

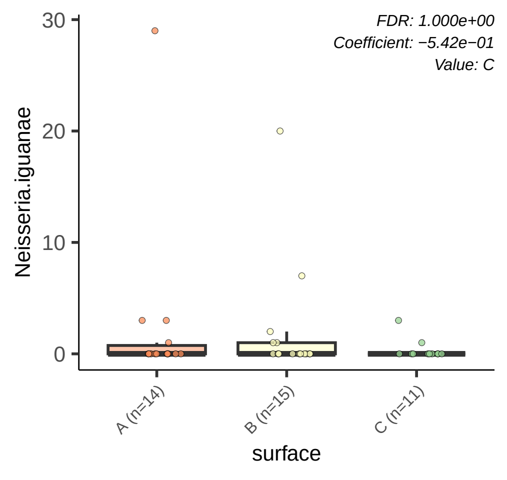
FDR: 1.000e+00
Coefficient: −5.42e−01
Value: C
Neisseria.iguanae
30
20
10
0
A (n=14)
B (n=15)
C (n=11)
surface

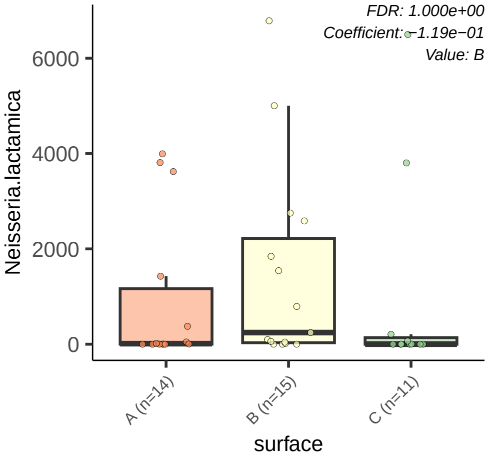
FDR: 1.000e+00
Coefficient: −1.19e−01
Value: B
6000
4000
2000
0
Neisseria.lactamica
A (n=14)
B (n=15)
C (n=11)
surface

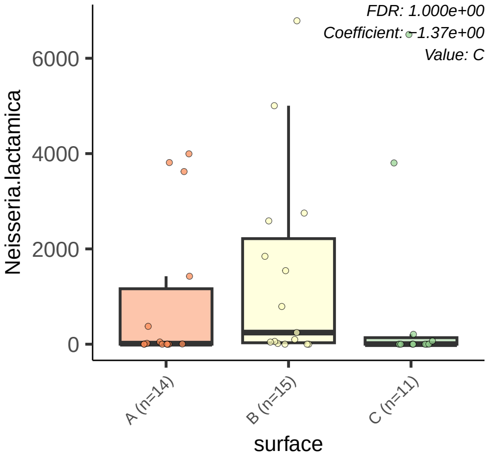

FDR: 1.000e+00
Coefficient: -1.37e+00
Value: C
6000
4000
2000
0
Neisseria.lactamica
A (n=14)
B (n=15)
C (n=11)
surface

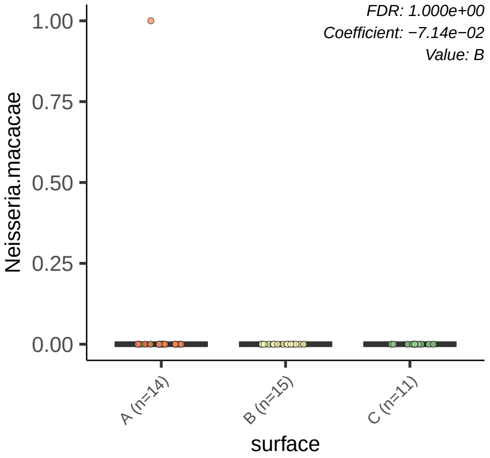
FDR: 1.000e+00
Coefficient: −7.14e−02
Value: B
1.00
0.75
0.50
0.25
0.00
Neisseria.macacae
A (n=14)
B (n=15)
C (n=11)
surface

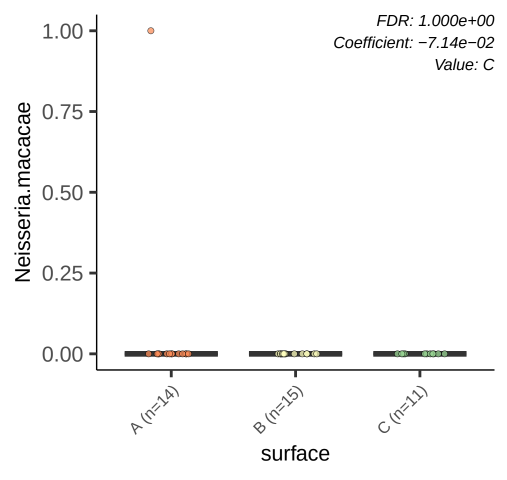

FDR: 1.000e+00
Coefficient: −7.14e−02
Value: C
Neisseria.macacae
1.00
0.75
0.50
0.25
0.00
A (n=14)
B (n=15)
C (n=11)
surface

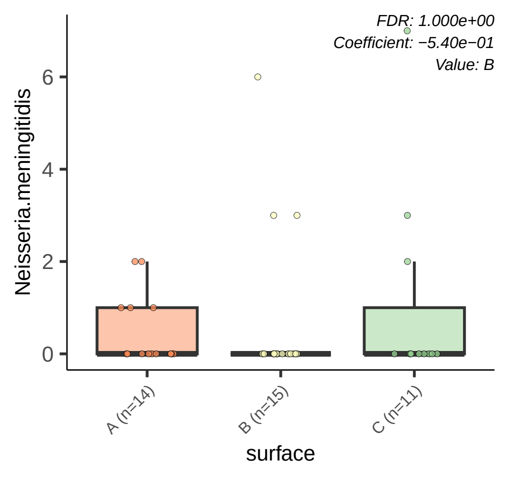
FDR: 1.000e+00
Coefficient: −5.40e−01
Value: B
Neisseria.meningitidis
0
2
4
6
A (n=14)
B (n=15)
C (n=11)
surface

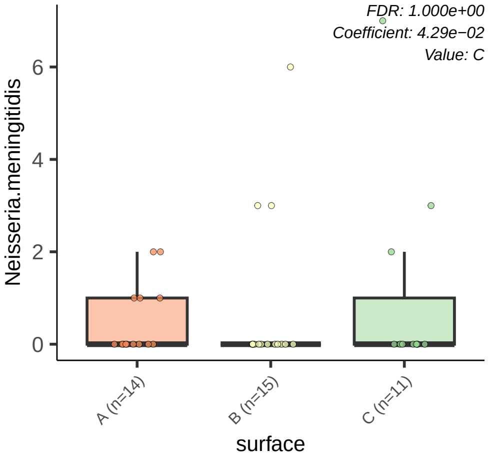

FDR: 1.000e+00
Coefficient: 4.29e−02
Value: C
Neisseria.meningitidis
0
2
4
6
A (n=14)
B (n=15)
C (n=11)
surface

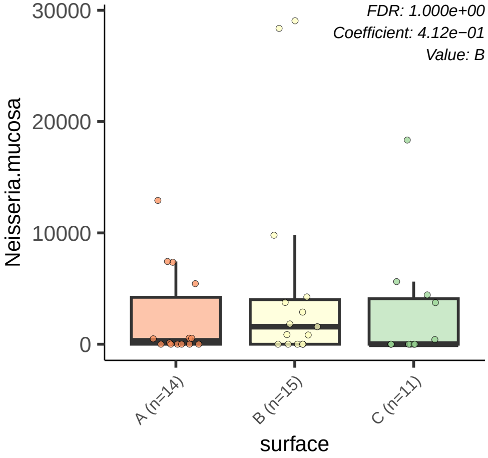

FDR: 1.000e+00
Coefficient: 4.12e−01
Value: B
30000
20000
10000
0
Neisseria.mucosa
A (n=14)
B (n=15)
C (n=11)
surface

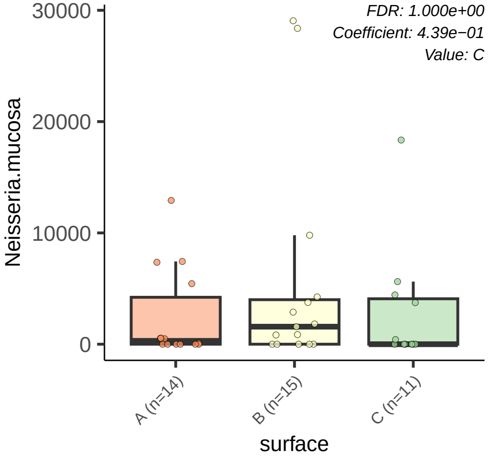
FDR: 1.000e+00
Coefficient: 4.39e−01
Value: C
Neisseria.mucosa
30000
20000
10000
0
A (n=14)
B (n=15)
C (n=11)
surface

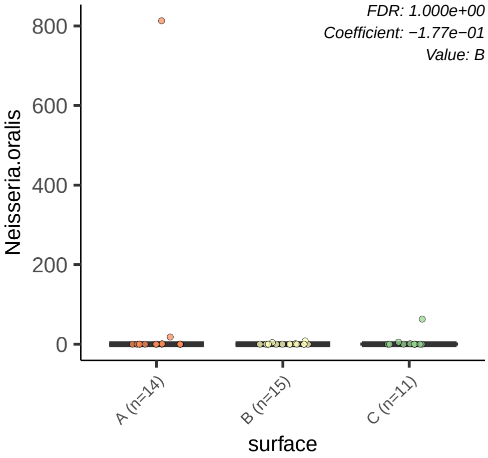
FDR: 1.000e+00
Coefficient: −1.77e−01
Value: B
Neisseria.oralis
800
600
400
200
0
A (n=14)
B (n=15)
C (n=11)
surface

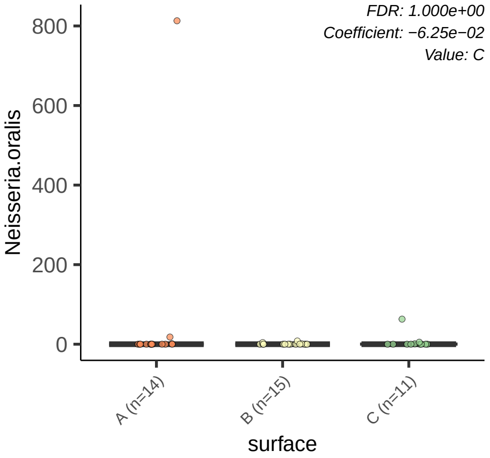
FDR: 1.000e+00
Coefficient: −6.25e−02
Value: C
Neisseria.oralis
800
600
400
200
0
A (n=14)
B (n=15)
C (n=11)
surface

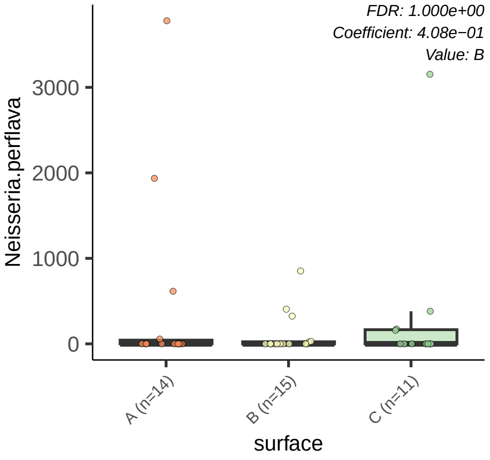

FDR: 1.000e+00
Coefficient: 4.08e−01
Value: B
Neisseria.perflava
3000
2000
1000
0
A (n=14)
B (n=15)
C (n=11)
surface

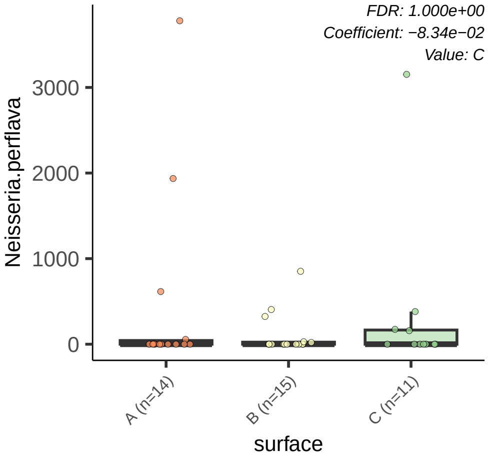

FDR: 1.000e+00
Coefficient: −8.34e−02
Value: C
Neisseria.perflava
3000
2000
1000
0
A (n=14)
B (n=15)
C (n=11)
surface

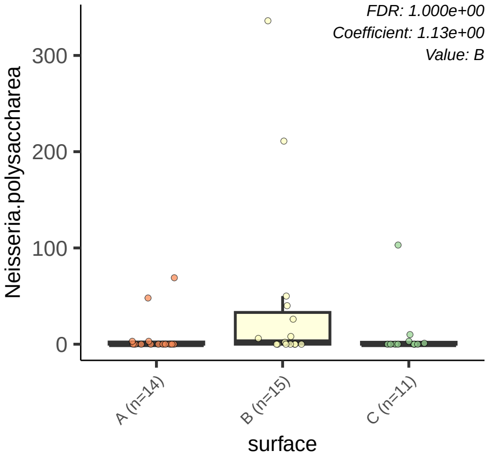

FDR: 1.000e+00
Coefficient: 1.13e+00
Value: B
Neisseria.polysaccharea
300
200
100
0
A (n=14)
B (n=15)
C (n=11)
surface

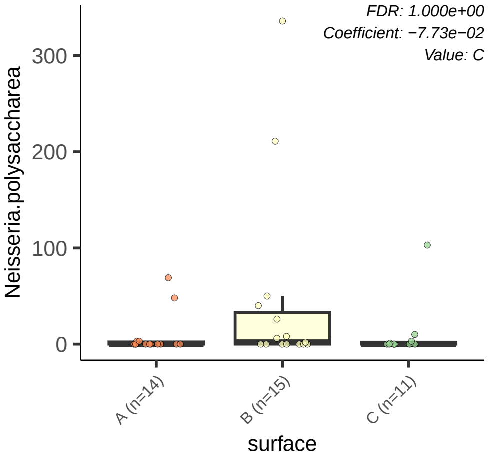
FDR: 1.000e+00
Coefficient: −7.73e−02
Value: C
Neisseria.polysaccharea
300
200
100
0
A (n=14)
B (n=15)
C (n=11)
surface

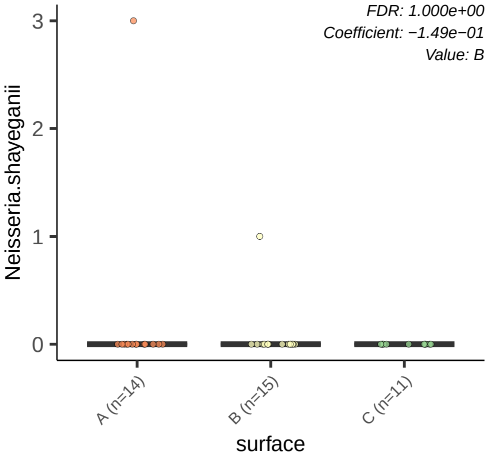
FDR: 1.000e+00
Coefficient: −1.49e−01
Value: B
Neisseria.shayeganii
3
2
1
0
A (n=14)
B (n=15)
C (n=11)
surface

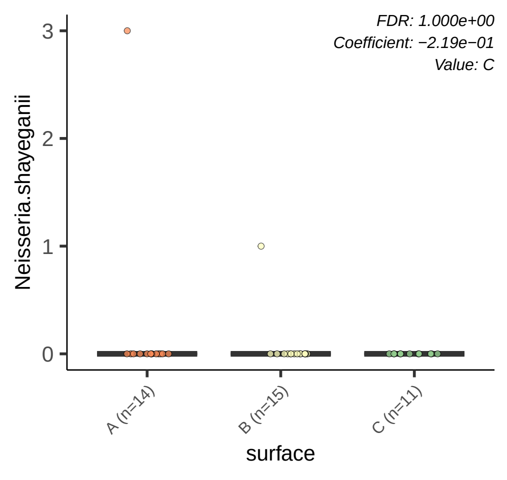

FDR: 1.000e+00
Coefficient: −2.19e−01
Value: C
Neisseria.shayeganii
0
1
2
3
A (n=14)
B (n=15)
C (n=11)
surface

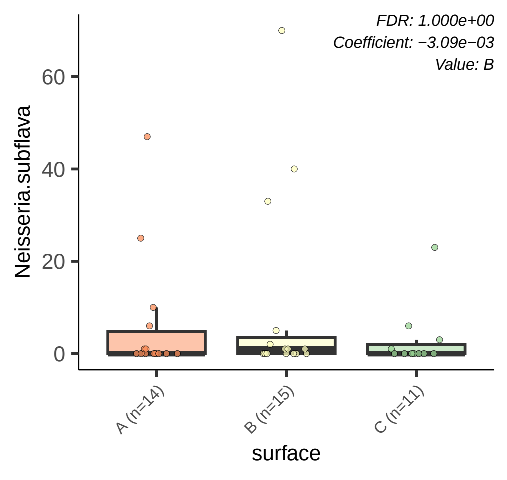

FDR: 1.000e+00
Coefficient: −3.09e−03
Value: B
Neisseria.subflava
0
20
40
60
A (n=14)
B (n=15)
C (n=11)
surface

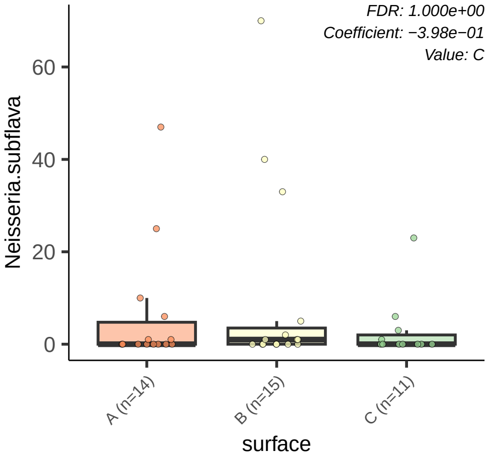

FDR: 1.000e+00
Coefficient: −3.98e−01
Value: C
Neisseria.subflava
60
40
20
0
A (n=14)
B (n=15)
C (n=11)
surface

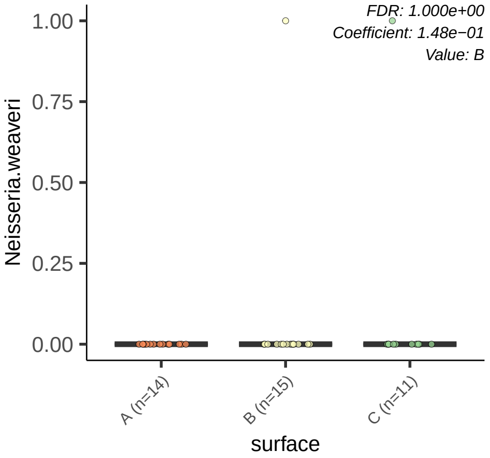
FDR: 1.000e+00
Coefficient: 1.48e−01
Value: B
1.00
0.75
0.50
0.25
0.00
Neisseria.weaveri
A (n=14)
B (n=15)
C (n=11)
surface

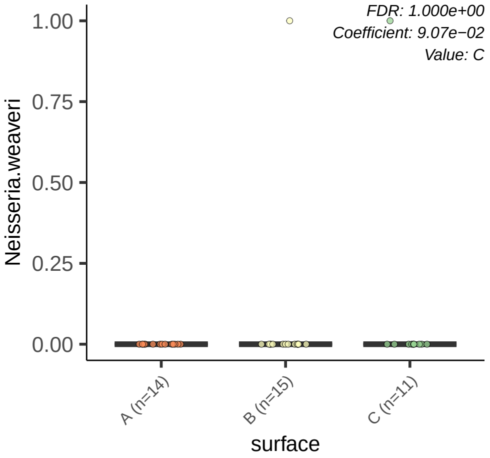
FDR: 1.000e+00
Coefficient: 9.07e−02
Value: C
Neisseria.weaveri
1.00
0.75
0.50
0.25
0.00
A (n=14)
B (n=15)
C (n=11)
surface

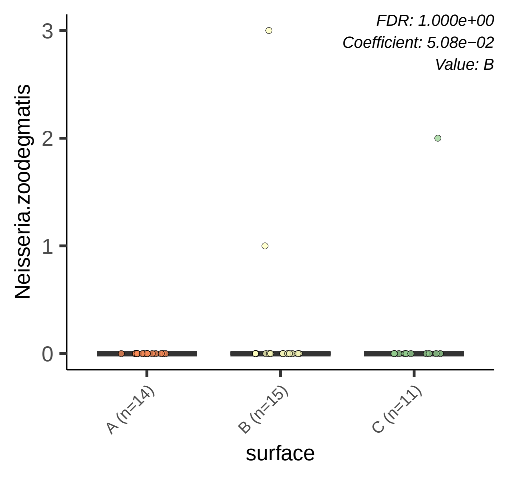
FDR: 1.000e+00
Coefficient: 5.08e−02
Value: B
Neisseria.zoodegmatis
0
1
2
3
A (n=14)
B (n=15)
C (n=11)
surface

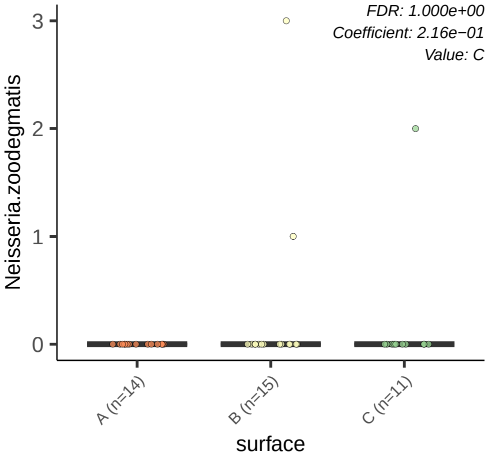

FDR: 1.000e+00
Coefficient: 2.16e−01
Value: C
Neisseria.zoodegmatis
0
1
2
3
A (n=14)
B (n=15)
C (n=11)
surface

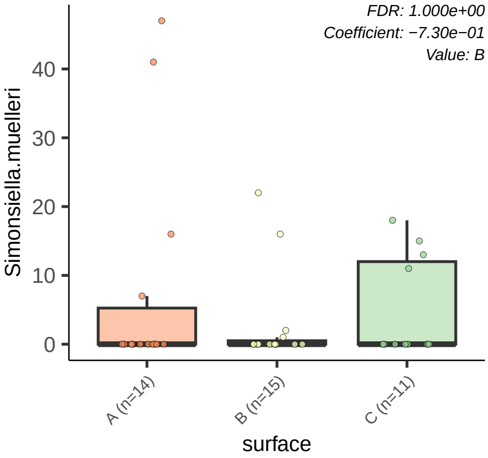
FDR: 1.000e+00
Coefficient: −7.30e−01
Value: B
Simonsiella.muelleri
0
10
20
30
40
A (n=14)
B (n=15)
C (n=11)
surface

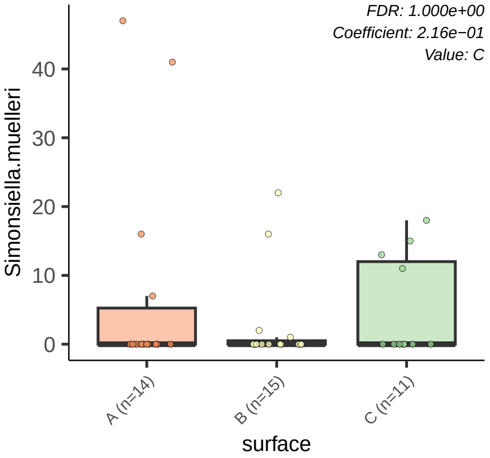

FDR: 1.000e+00
Coefficient: 2.16e−01
Value: C
Simonsiella.muelleri
40
30
20
10
0
A (n=14)
B (n=15)
C (n=11)
surface

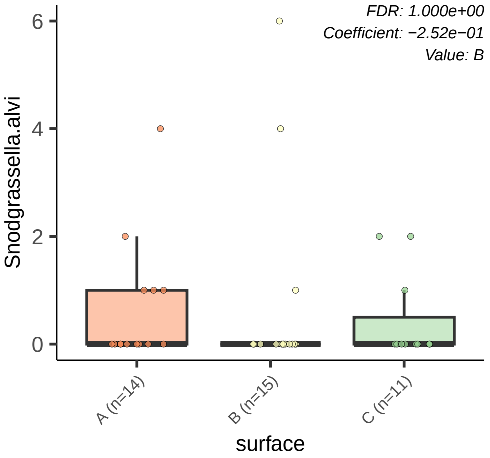
FDR: 1.000e+00
Coefficient: −2.52e−01
Value: B
Snodgrassella.alvi
0
2
4
6
A (n=14)
B (n=15)
C (n=11)
surface

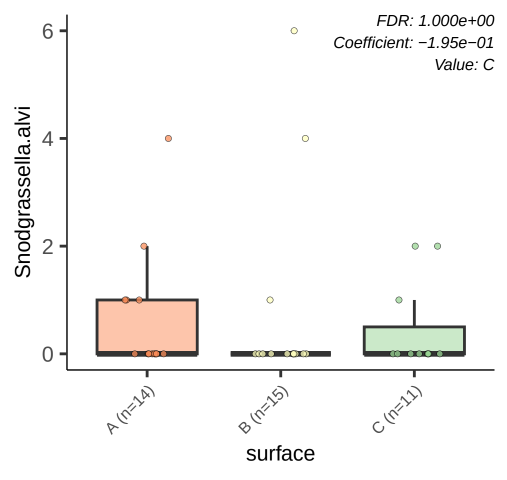

FDR: 1.000e+00
Coefficient: −1.95e−01
Value: C
Snodgrassella.alvi
0
2
4
6
A (n=14)
B (n=15)
C (n=11)
surface

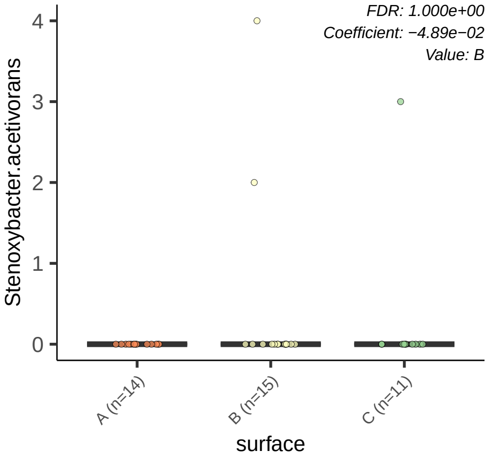
FDR: 1.000e+00
Coefficient: −4.89e−02
Value: B
Stenoxybacter.acetivorans
4
3
2
1
0
A (n=14)
B (n=15)
C (n=11)
surface

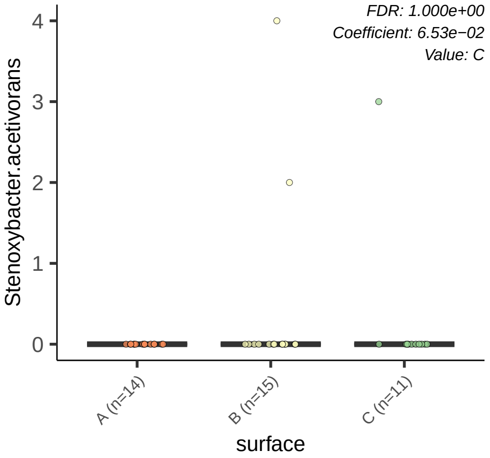

FDR: 1.000e+00
Coefficient: 6.53e−02
Value: C
Stenoxybacter.acetivorans
0
1
2
3
4
A (n=14)
B (n=15)
C (n=11)
surface

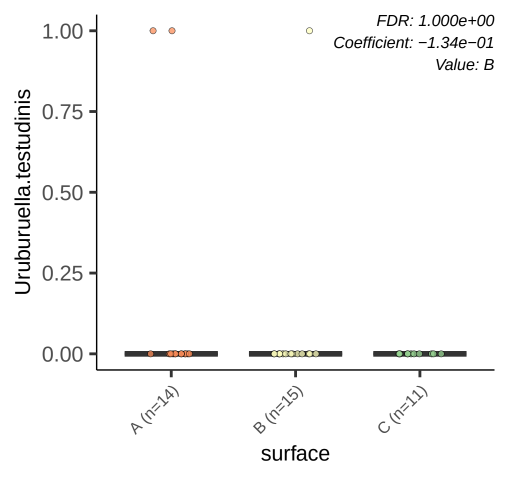
FDR: 1.000e+00
Coefficient: −1.34e−01
Value: B
Uruburuella.testudinis
1.00
0.75
0.50
0.25
0.00
A (n=14)
B (n=15)
C (n=11)
surface

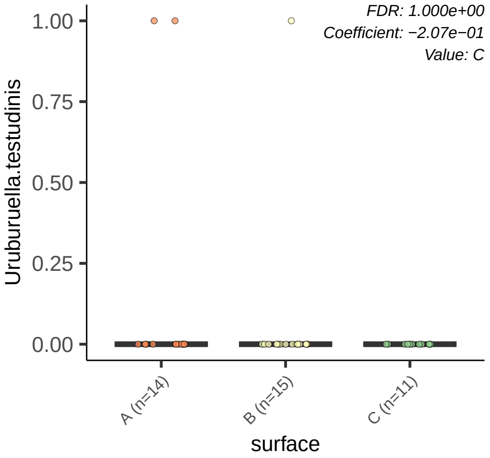
FDR: 1.000e+00
Coefficient: −2.07e−01
Value: C
Uruburuella.testudinis
1.00
0.75
0.50
0.25
0.00
A (n=14)
B (n=15)
C (n=11)
surface

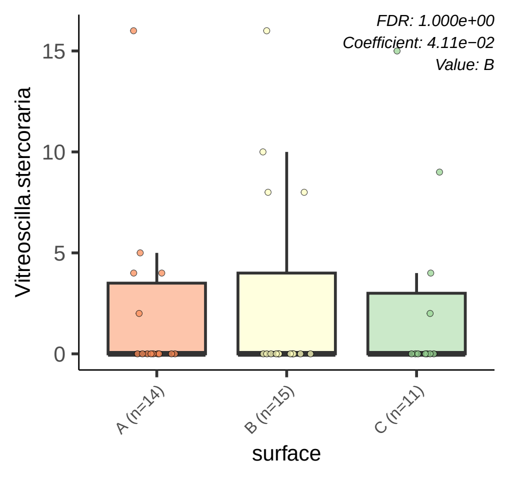
FDR: 1.000e+00
Coefficient: 4.11e−02
Value: B
Vitreoscilla.stercoraria
0
5
10
15
A (n=14)
B (n=15)
C (n=11)
surface

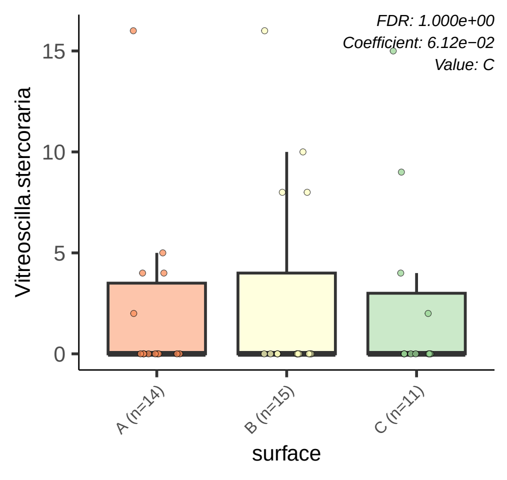

FDR: 1.000e+00
Coefficient: 6.12e−02
Value: C
Vitreoscilla.stercoraria
0
5
10
15
A (n=14)
B (n=15)
C (n=11)
surface

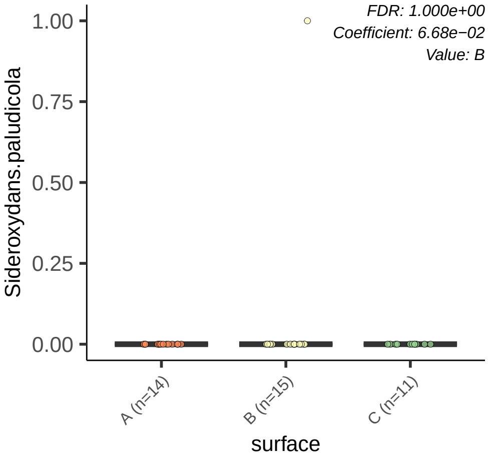

FDR: 1.000e+00
Coefficient: 6.68e−02
Value: B
Sideroxydans.paludicola
1.00
0.75
0.50
0.25
0.00
A (n=14)
B (n=15)
C (n=11)
surface

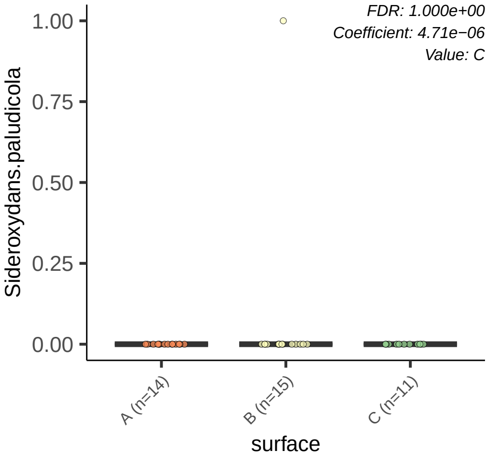

FDR: 1.000e+00
Coefficient: 4.71e−06
Value: C
Sideroxydans.paludicola
1.00
0.75
0.50
0.25
0.00
A (n=14)
B (n=15)
C (n=11)
surface

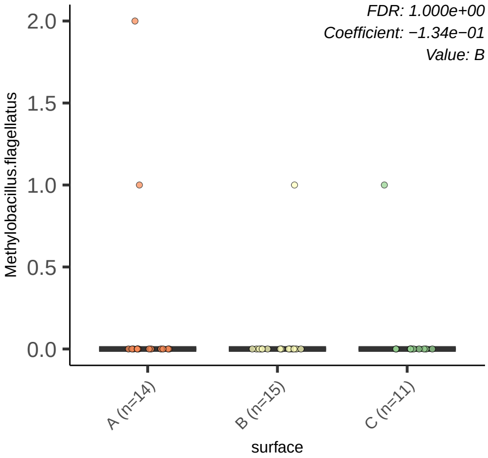
FDR: 1.000e+00
Coefficient: −1.34e−01
Value: B
Methylobacillus.flagellatus
2.0
1.5
1.0
0.5
0.0
A (n=14)
B (n=15)
C (n=11)
surface

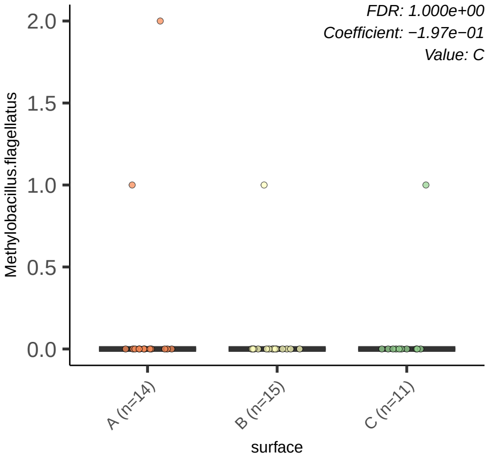
FDR: 1.000e+00
Coefficient: −1.97e−01
Value: C
Methylobacillus.flagellatus
2.0
1.5
1.0
0.5
0.0
A (n=14)
B (n=15)
C (n=11)
surface

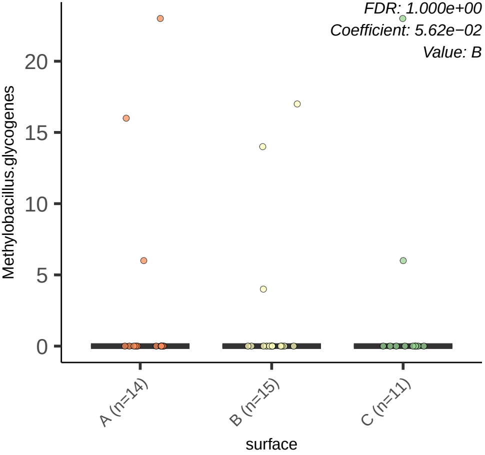
FDR: 1.000e+00
Coefficient: 5.62e−02
Value: B
Methylobacillus.glycogenes
0
5
10
15
20
A (n=14)
B (n=15)
C (n=11)
surface

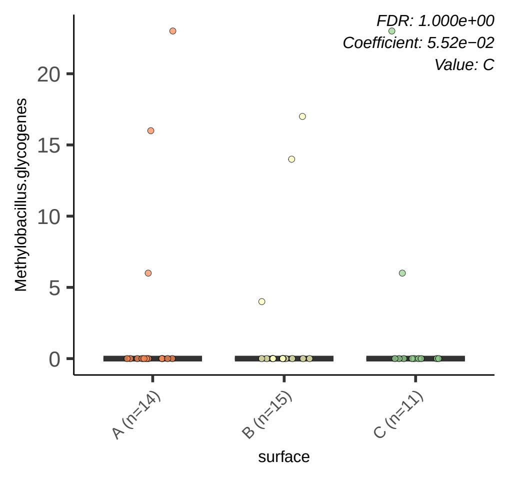

FDR: 1.000e+00
Coefficient: 5.52e−02
Value: C
Methylobacillus.glycogenes
20
15
10
5
0
A (n=14)
B (n=15)
C (n=11)
surface

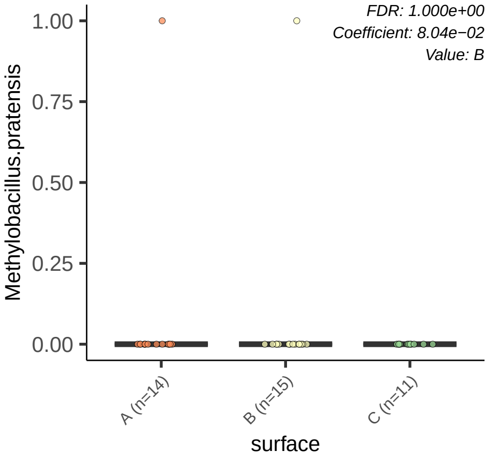

FDR: 1.000e+00
Coefficient: 8.04e−02
Value: B
Methylobacillus.pratensis
1.00
0.75
0.50
0.25
0.00
A (n=14)
B (n=15)
C (n=11)
surface

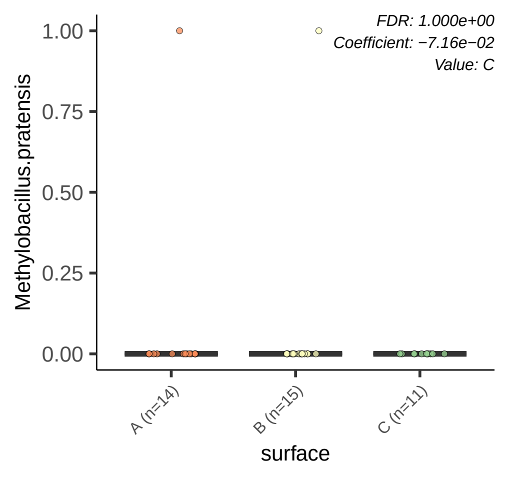
FDR: 1.000e+00
Coefficient: −7.16e−02
Value: C
Methylobacillus.pratensis
1.00
0.75
0.50
0.25
0.00
A (n=14)
B (n=15)
C (n=11)
surface

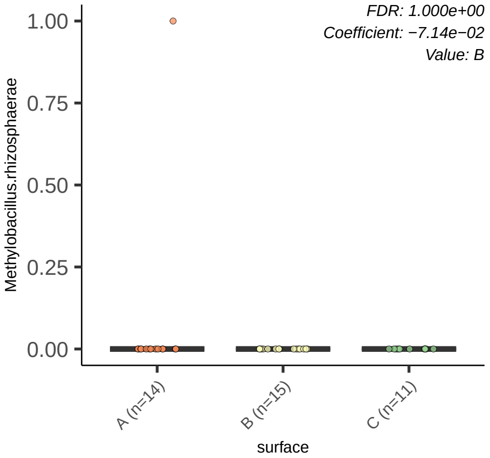
FDR: 1.000e+00
Coefficient: −7.14e−02
Value: B
Methylobacillus.rhizosphaerae
1.00
0.75
0.50
0.25
0.00
A (n=14)
B (n=15)
C (n=11)
surface

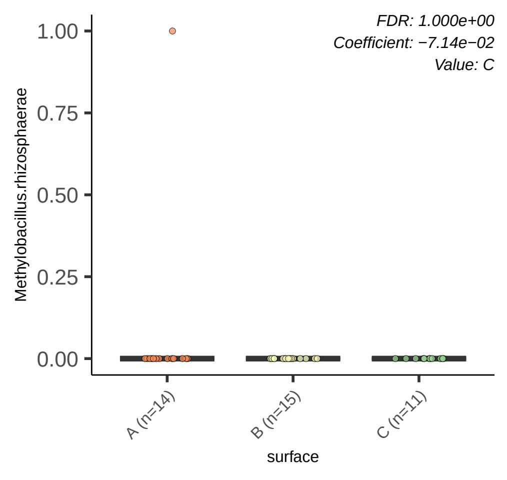
FDR: 1.000e+00
Coefficient: −7.14e−02
Value: C
Methylobacillus.rhizosphaerae
1.00
0.75
0.50
0.25
0.00
A (n=14)
B (n=15)
C (n=11)
surface

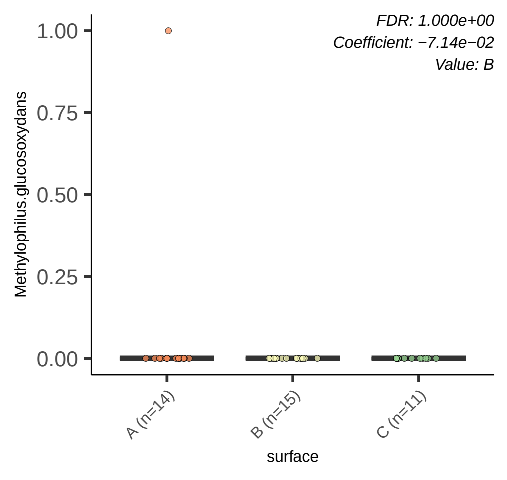

FDR: 1.000e+00
Coefficient: −7.14e−02
Value: B
Methylophilus.glucosoxydans
1.00
0.75
0.50
0.25
0.00
A (n=14)
B (n=15)
C (n=11)
surface

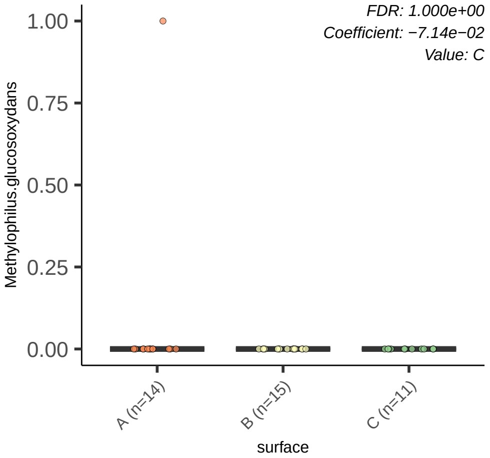

FDR: 1.000e+00
Coefficient: −7.14e−02
Value: C
1.00
0.75
0.50
0.25
0.00
Methylophilus.glucosoxydans
A (n=14)
B (n=15)
C (n=11)
surface

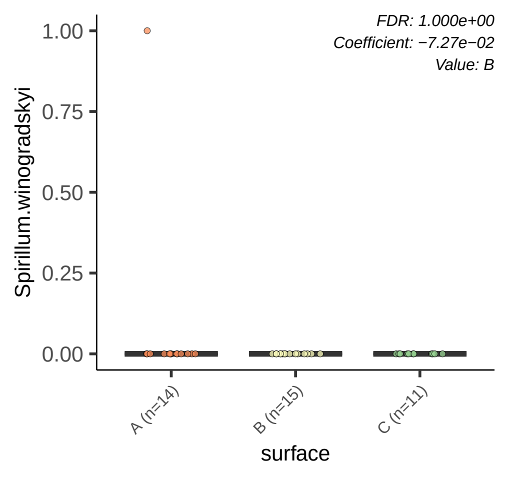

FDR: 1.000e+00
Coefficient: −7.27e−02
Value: B
Spirillum.winogradskyi
1.00
0.75
0.50
0.25
0.00
A (n=14)
B (n=15)
C (n=11)
surface

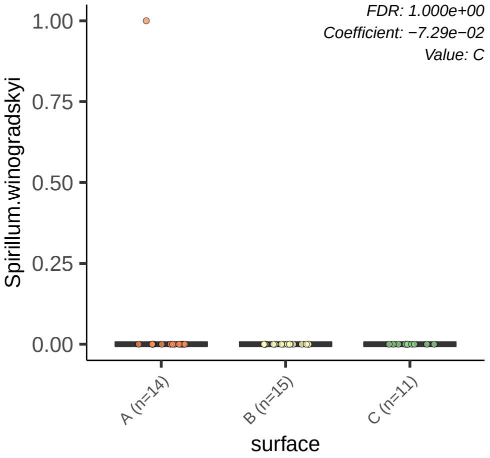
FDR: 1.000e+00
Coefficient: −7.29e−02
Value: C
Spirillum.winogradskyi
1.00
0.75
0.50
0.25
0.00
A (n=14)
B (n=15)
C (n=11)
surface

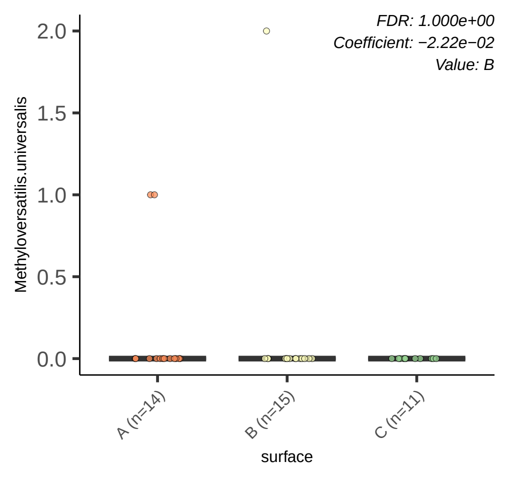

FDR: 1.000e+00
Coefficient: −2.22e−02
Value: B
Methyloversatilis.universalis
2.0
1.5
1.0
0.5
0.0
A (n=14)
B (n=15)
C (n=11)
surface

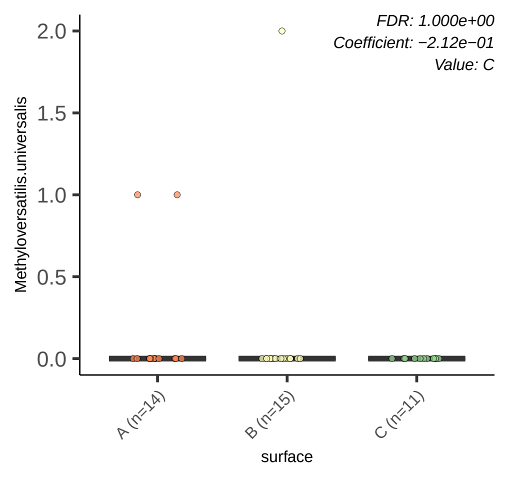
FDR: 1.000e+00
Coefficient: −2.12e−01
Value: C
Methyloversatilis.universalis
2.0
1.5
1.0
0.5
0.0
A (n=14)
B (n=15)
C (n=11)
surface

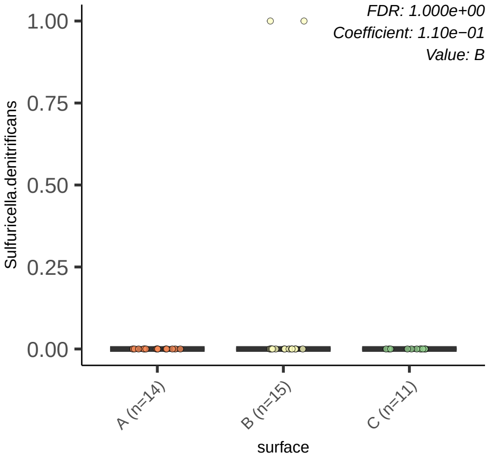
FDR: 1.000e+00
Coefficient: 1.10e−01
Value: B
1.00
0.75
0.50
0.25
0.00
Sulfuricella.denitrificans
A (n=14)
B (n=15)
C (n=11)
surface

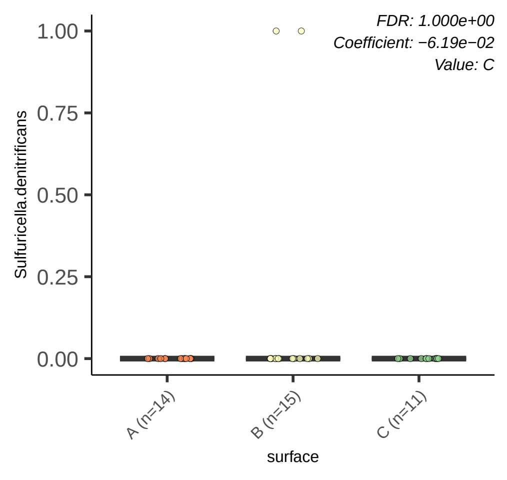

FDR: 1.000e+00
Coefficient: −6.19e−02
Value: C
1.00
0.75
0.50
0.25
0.00
Sulfuricella.denitrificans
A (n=14)
B (n=15)
C (n=11)
surface

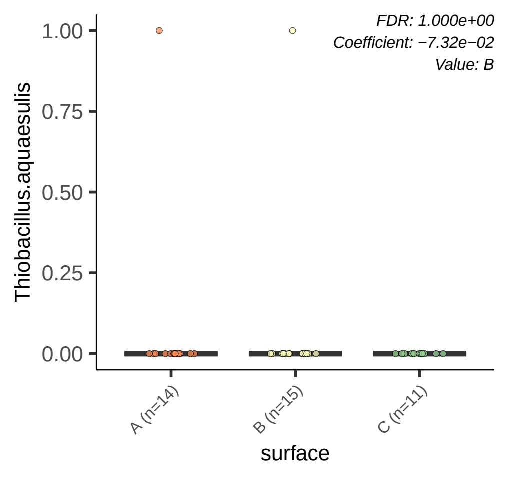
FDR: 1.000e+00
Coefficient: −7.32e−02
Value: B
Thiobacillus.aquaesulis
1.00
0.75
0.50
0.25
0.00
A (n=14)
B (n=15)
C (n=11)
surface

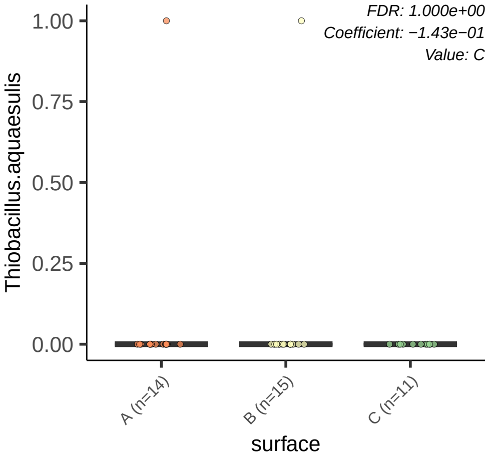
FDR: 1.000e+00
Coefficient: −1.43e−01
Value: C
Thiobacillus.aquaesulis
1.00
0.75
0.50
0.25
0.00
A (n=14)
B (n=15)
C (n=11)
surface

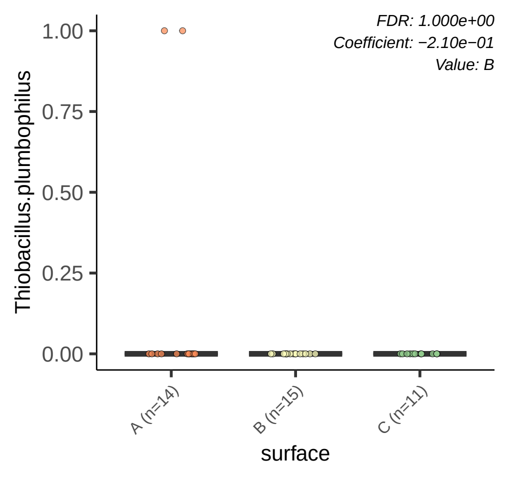
FDR: 1.000e+00
Coefficient: −2.10e−01
Value: B
Thiobacillus.plumbophilus
1.00
0.75
0.50
0.25
0.00
A (n=14)
B (n=15)
C (n=11)
surface

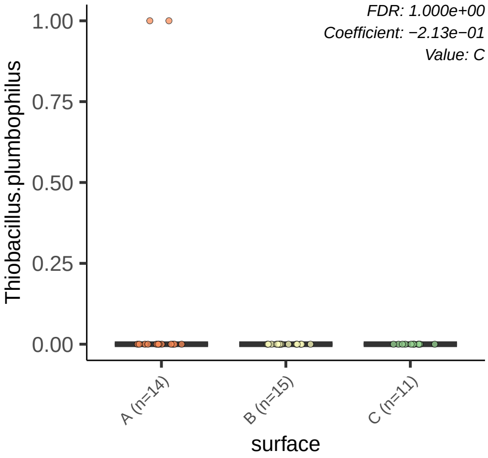
FDR: 1.000e+00
Coefficient: −2.13e−01
Value: C
Thiobacillus.plumbophilus
1.00
0.75
0.50
0.25
0.00
A (n=14)
B (n=15)
C (n=11)
surface

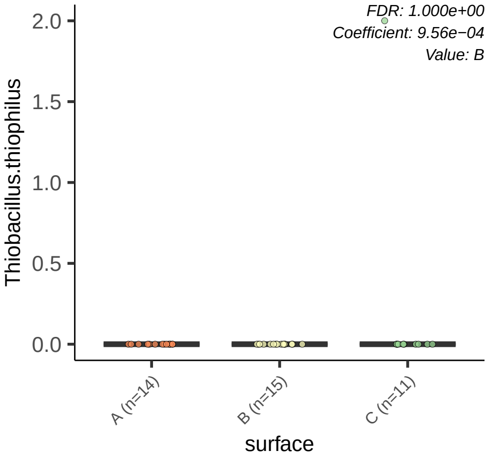
FDR: 1.000e+00
Coefficient: 9.56e−04
Value: B
Thiobacillus.thiophilus
2.0
1.5
1.0
0.5
0.0
A (n=14)
B (n=15)
C (n=11)
surface

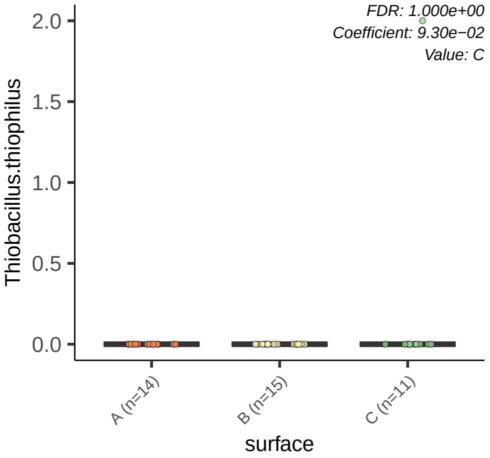
FDR: 1.000e+00
Coefficient: 9.30e−02
Value: C
Thiobacillus.thiophilus
2.0
1.5
1.0
0.5
0.0
A (n=14)
B (n=15)
C (n=11)
surface

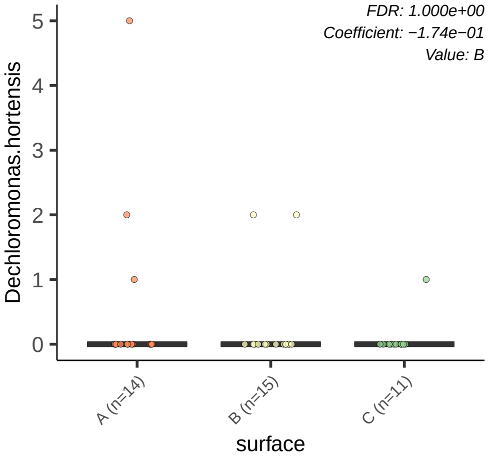
FDR: 1.000e+00
Coefficient: −1.74e−01
Value: B
Dechloromonas.hortensis
0
1
2
3
4
5
A (n=14)
B (n=15)
C (n=11)
surface

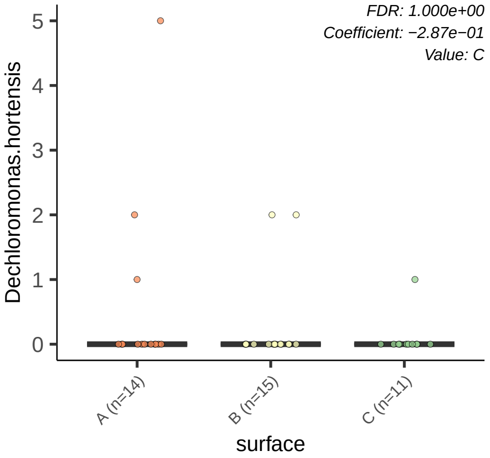

FDR: 1.000e+00
Coefficient: −2.87e−01
Value: C
Dechloromonas.hortensis
0
1
2
3
4
5
A (n=14)
B (n=15)
C (n=11)
surface

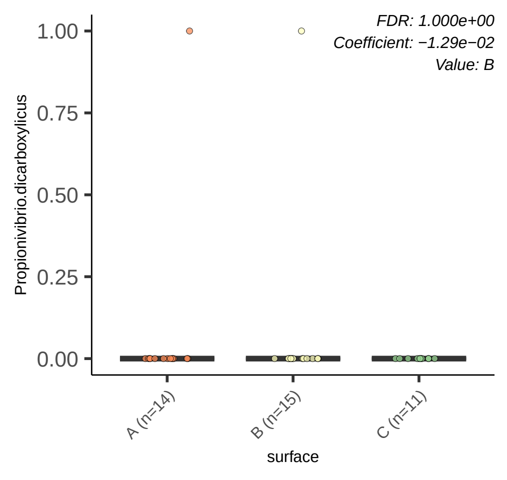

FDR: 1.000e+00
Coefficient: −1.29e−02
Value: B
1.00
0.75
0.50
0.25
0.00
Propionivibrio.dicarboxylicus
A (n=14)
B (n=15)
C (n=11)
surface

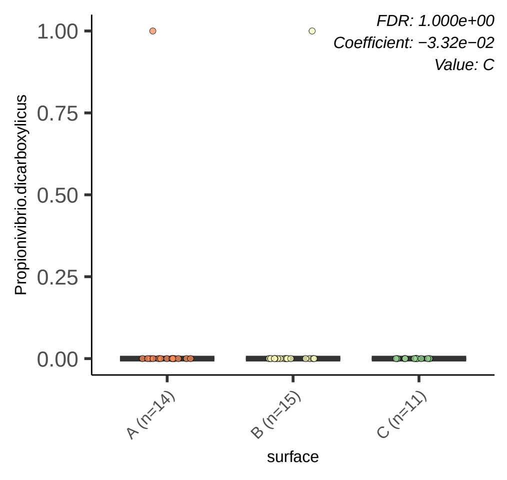
FDR: 1.000e+00
Coefficient: −3.32e−02
Value: C
Propionivibrio.dicarboxylicus
1.00
0.75
0.50
0.25
0.00
A (n=14)
B (n=15)
C (n=11)
surface

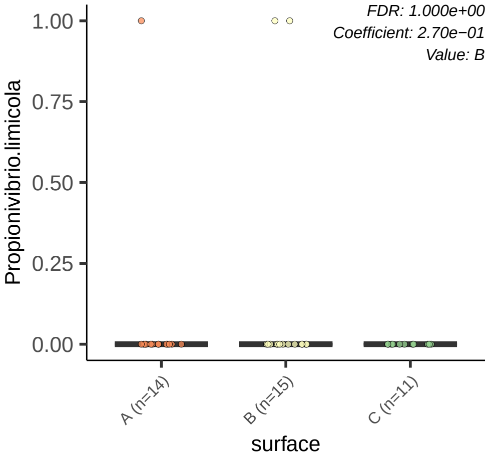
FDR: 1.000e+00
Coefficient: 2.70e−01
Value: B
Propionivibrio.limicola
1.00
0.75
0.50
0.25
0.00
A (n=14)
B (n=15)
C (n=11)
surface

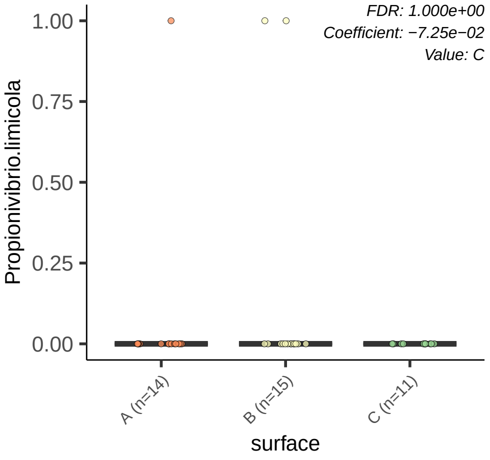
FDR: 1.000e+00
Coefficient: −7.25e−02
Value: C
Propionivibrio.limicola
1.00
0.75
0.50
0.25
0.00
A (n=14)
B (n=15)
C (n=11)
surface

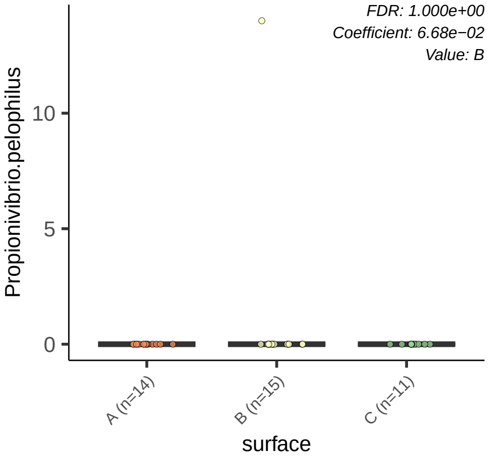

FDR: 1.000e+00
Coefficient: 6.68e−02
Value: B
Propionivibrio.pelophilus
0
5
10
A (n=14)
B (n=15)
C (n=11)
surface

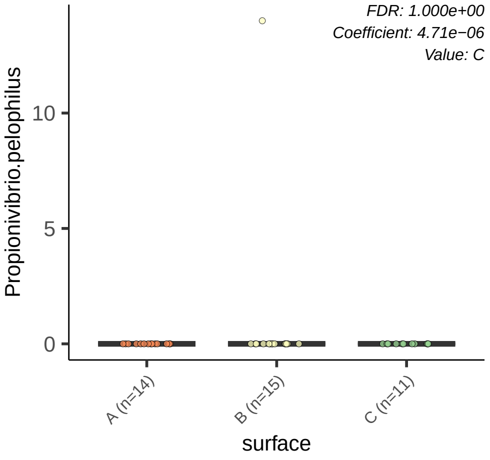
FDR: 1.000e+00
Coefficient: 4.71e−06
Value: C
Propionivibrio.pelophilus
10
5
0
A (n=14)
B (n=15)
C (n=11)
surface

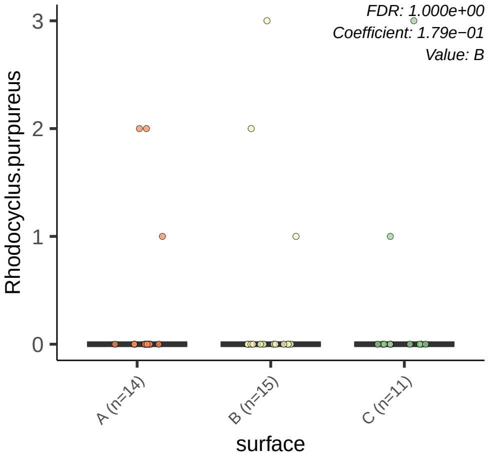
FDR: 1.000e+00
Coefficient: 1.79e−01
Value: B
Rhodocyclus.purpureus
3
2
1
0
A (n=14)
B (n=15)
C (n=11)
surface

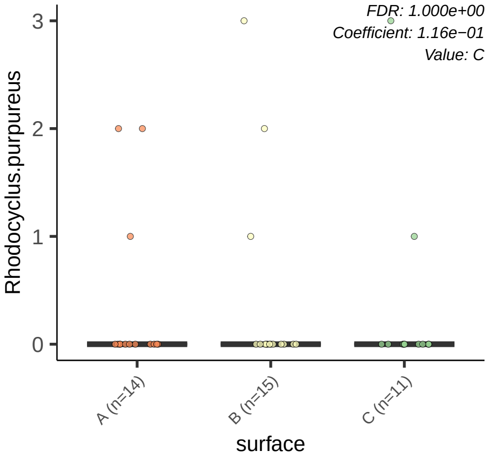
FDR: 1.000e+00
Coefficient: 1.16e−01
Value: C
Rhodocyclus.purpureus
0
1
2
3
A (n=14)
B (n=15)
C (n=11)
surface

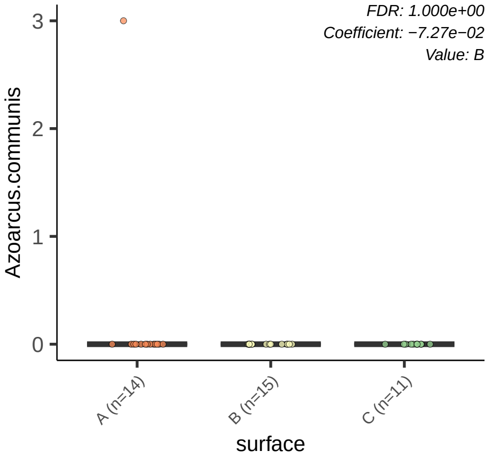
FDR: 1.000e+00
Coefficient: −7.27e−02
Value: B
Azoarcus.communis
3
2
1
0
A (n=14)
B (n=15)
C (n=11)
surface

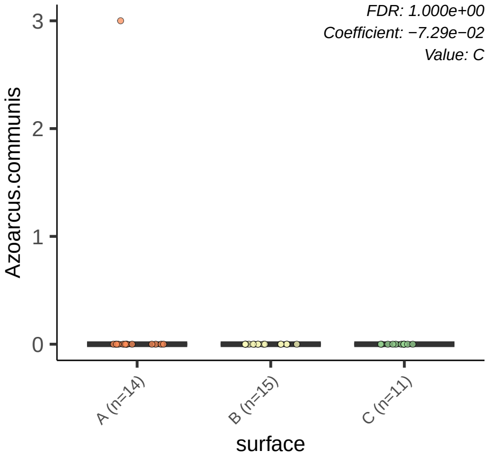
FDR: 1.000e+00
Coefficient: −7.29e−02
Value: C
Azoarcus.communis
3
2
1
0
A (n=14)
B (n=15)
C (n=11)
surface

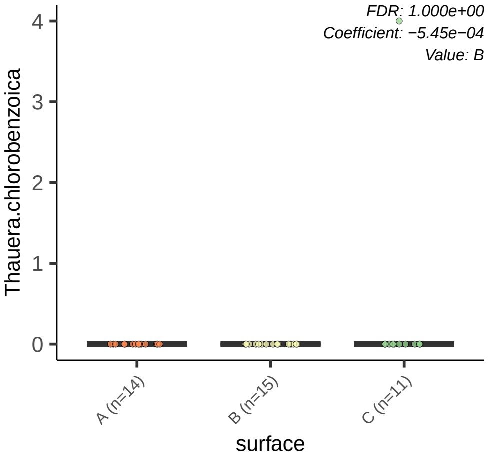
FDR: 1.000e+00
Coefficient: −5.45e−04
Value: B
Thauera.chlorobenzoica
0
1
2
3
4
A (n=14)
B (n=15)
C (n=11)
surface

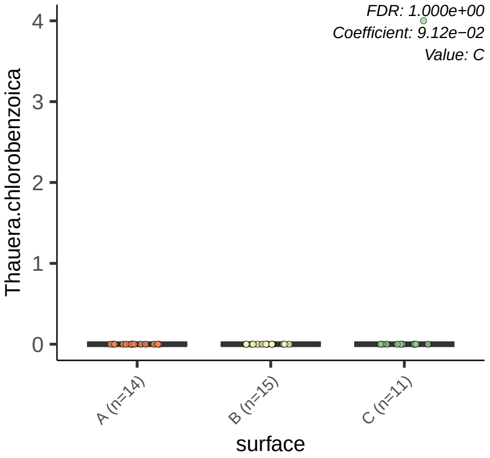
FDR: 1.000e+00
Coefficient: 9.12e−02
Value: C
Thauera.chlorobenzoica
0
1
2
3
4
A (n=14)
B (n=15)
C (n=11)
surface

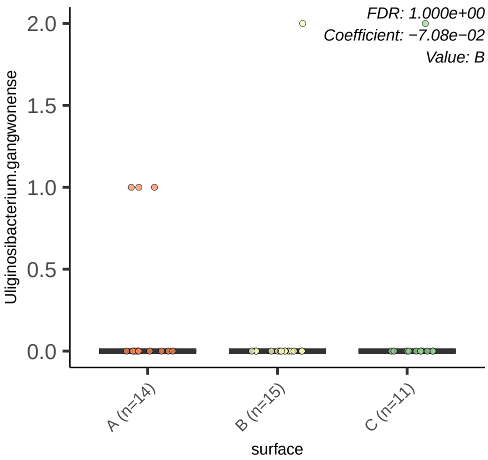

FDR: 1.000e+00
Coefficient: −7.08e−02
Value: B
Uliginosibacterium.gangwonense
2.0
1.5
1.0
0.5
0.0
A (n=14)
B (n=15)
C (n=11)
surface

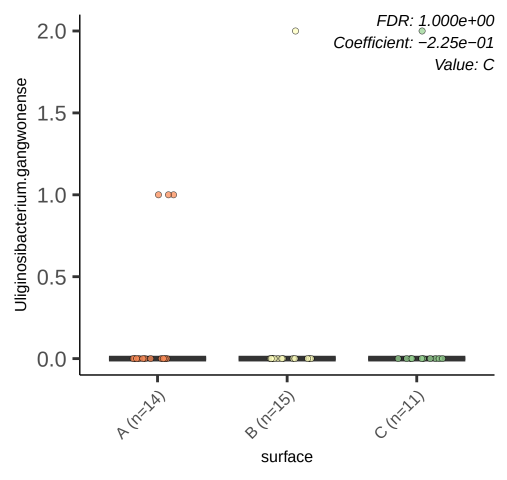
FDR: 1.000e+00
Coefficient: −2.25e−01
Value: C
Uliginosibacterium.gangwonense
2.0
1.5
1.0
0.5
0.0
A (n=14)
B (n=15)
C (n=11)
surface

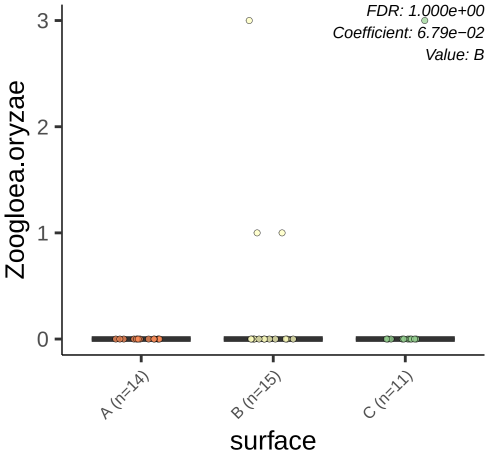
FDR: 1.000e+00
Coefficient: 6.79e−02
Value: B
Zoogloea.oryzae
3
2
1
0
A (n=14)
B (n=15)
C (n=11)
surface

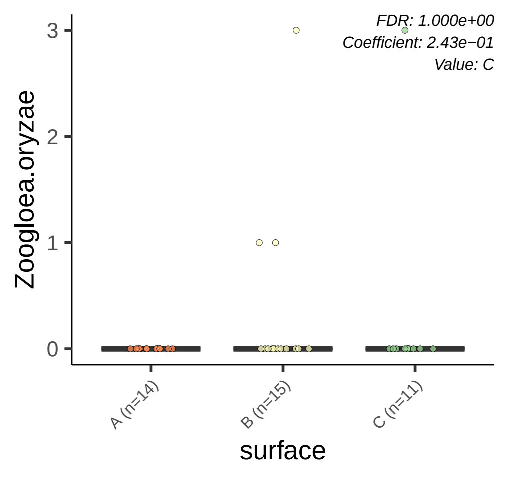

FDR: 1.000e+00
Coefficient: 2.43e−01
Value: C
Zoogloea.oryzae
3
2
1
0
A (n=14)
B (n=15)
C (n=11)
surface

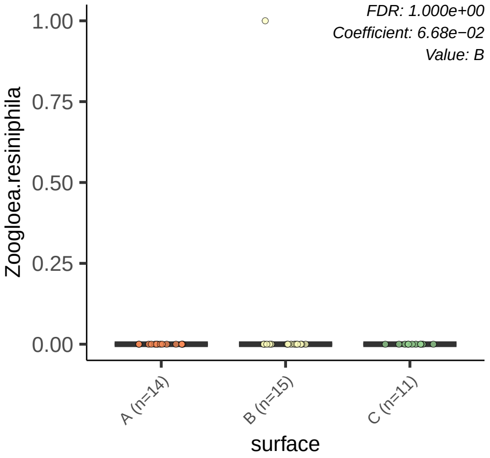
FDR: 1.000e+00
Coefficient: 6.68e−02
Value: B
Zoogloea.resiniphila
1.00
0.75
0.50
0.25
0.00
A (n=14)
B (n=15)
C (n=11)
surface

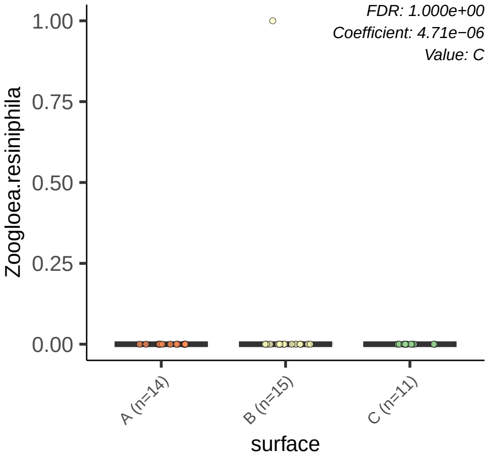
FDR: 1.000e+00
Coefficient: 4.71e−06
Value: C
Zoogloea.resiniphila
1.00
0.75
0.50
0.25
0.00
A (n=14)
B (n=15)
C (n=11)
surface

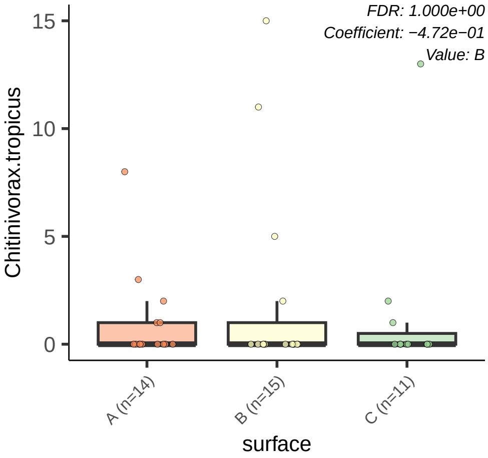

FDR: 1.000e+00
Coefficient: −4.72e−01
Value: B
Chitinivorax.tropicus
15
10
5
0
A (n=14)
B (n=15)
C (n=11)
surface

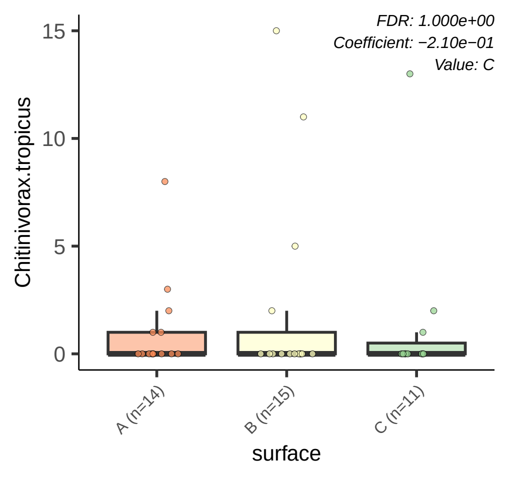
FDR: 1.000e+00
Coefficient: −2.10e−01
Value: C
Chitinivorax.tropicus
15
10
5
0
A (n=14)
B (n=15)
C (n=11)
surface

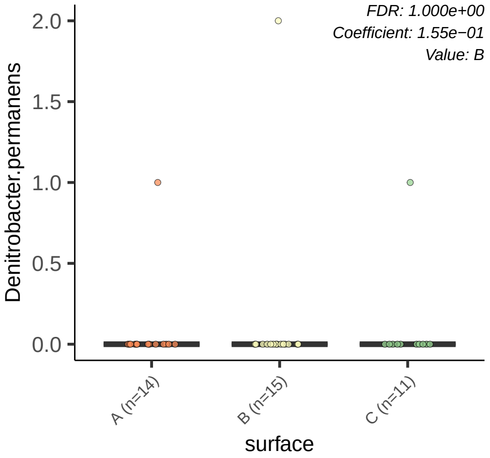
FDR: 1.000e+00
Coefficient: 1.55e−01
Value: B
Denitrobacter.permanens
2.0
1.5
1.0
0.5
0.0
A (n=14)
B (n=15)
C (n=11)
surface

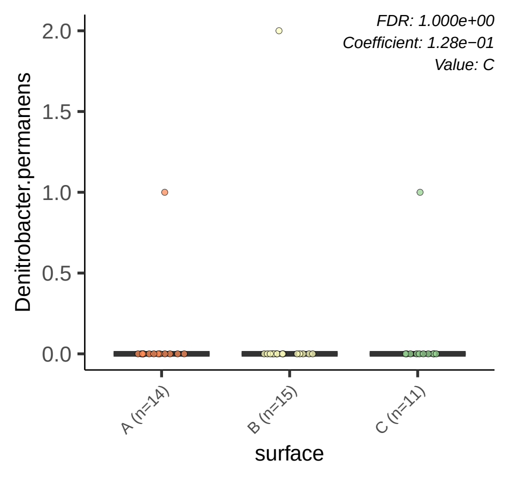

FDR: 1.000e+00
Coefficient: 1.28e−01
Value: C
Denitrobacter.permanens
2.0
1.5
1.0
0.5
0.0
A (n=14)
B (n=15)
C (n=11)
surface

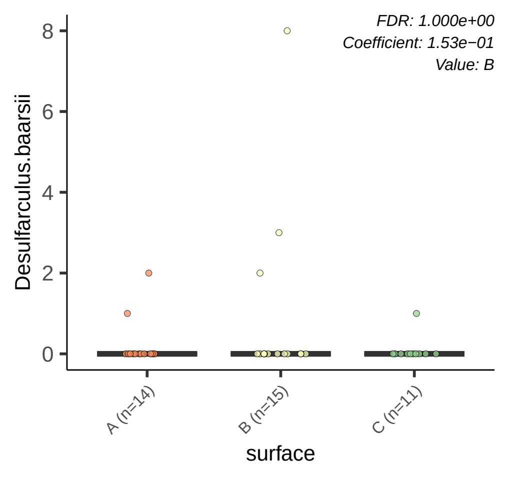

FDR: 1.000e+00
Coefficient: 1.53e−01
Value: B
Desulfarculus.baarsii
0
2
4
6
8
A (n=14)
B (n=15)
C (n=11)
surface

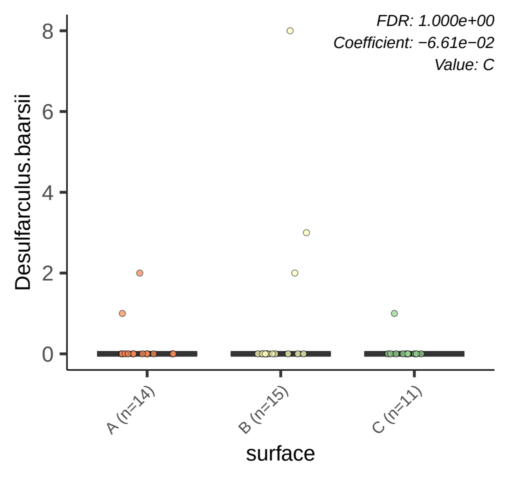
FDR: 1.000e+00
Coefficient: −6.61e−02
Value: C
Desulfarculus.baarsii
0
2
4
6
8
A (n=14)
B (n=15)
C (n=11)
surface

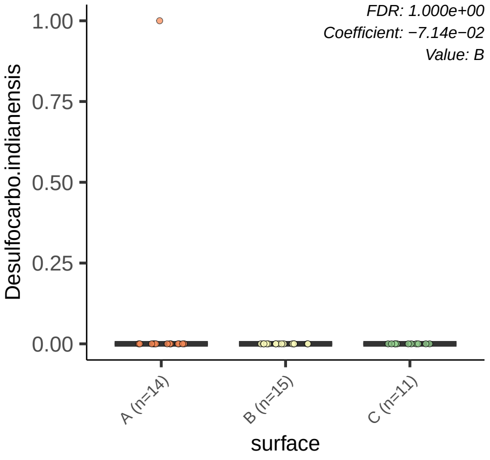
FDR: 1.000e+00
Coefficient: −7.14e−02
Value: B
Desulfocarbo.indianensis
1.00
0.75
0.50
0.25
0.00
A (n=14)
B (n=15)
C (n=11)
surface

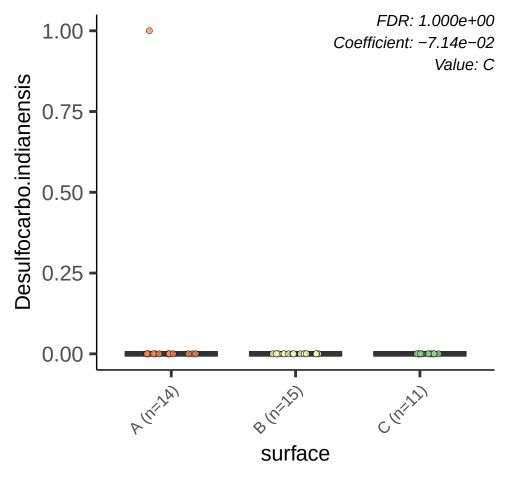
FDR: 1.000e+00
Coefficient: −7.14e−02
Value: C
Desulfocarbo.indianensis
1.00
0.75
0.50
0.25
0.00
A (n=14)
B (n=15)
C (n=11)
surface

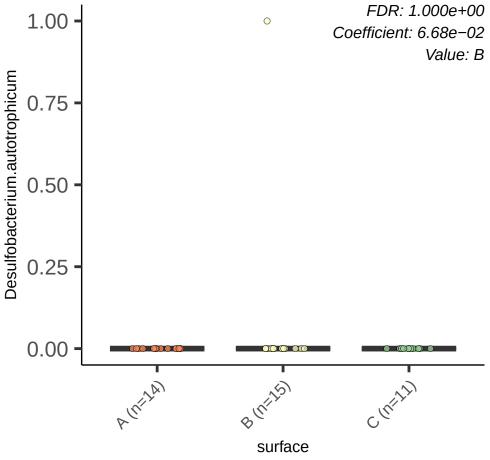
FDR: 1.000e+00
Coefficient: 6.68e−02
Value: B
Desulfobacterium.autotrophicum
1.00
0.75
0.50
0.25
0.00
A (n=14)
B (n=15)
C (n=11)
surface

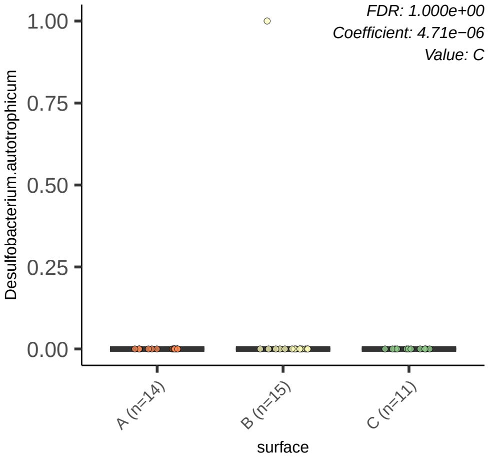
FDR: 1.000e+00
Coefficient: 4.71e−06
Value: C
Desulfobacterium.autotrophicum
1.00
0.75
0.50
0.25
0.00
A (n=14)
B (n=15)
C (n=11)
surface

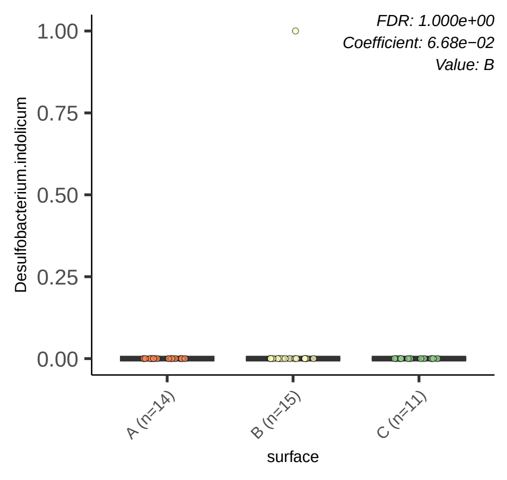

FDR: 1.000e+00
Coefficient: 6.68e−02
Value: B
1.00
0.75
0.50
0.25
0.00
Desulfobacterium.indolicum
A (n=14)
B (n=15)
C (n=11)
surface

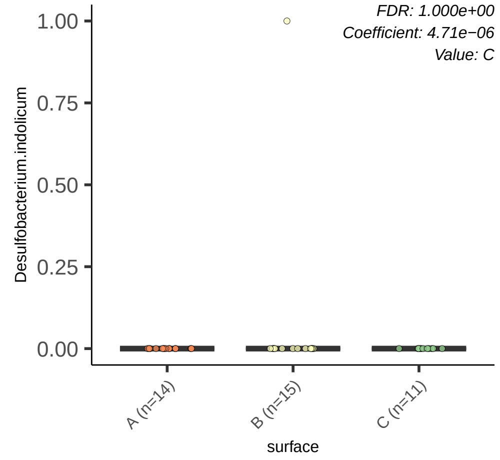

FDR: 1.000e+00
Coefficient: 4.71e−06
Value: C
1.00
0.75
0.50
0.25
0.00
Desulfobacterium.indolicum
A (n=14)
B (n=15)
C (n=11)
surface

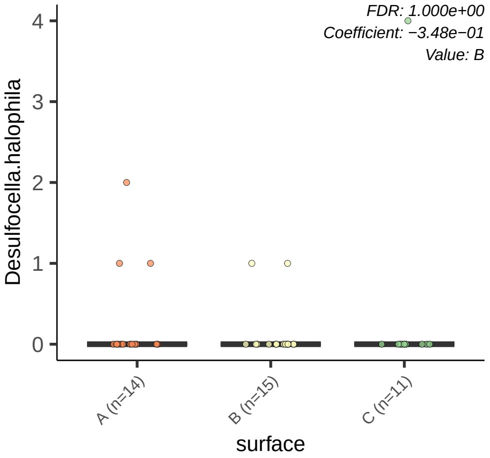

FDR: 1.000e+00
Coefficient: −3.48e−01
Value: B
Desulfocella.halophila
4
3
2
1
0
A (n=14)
B (n=15)
C (n=11)
surface

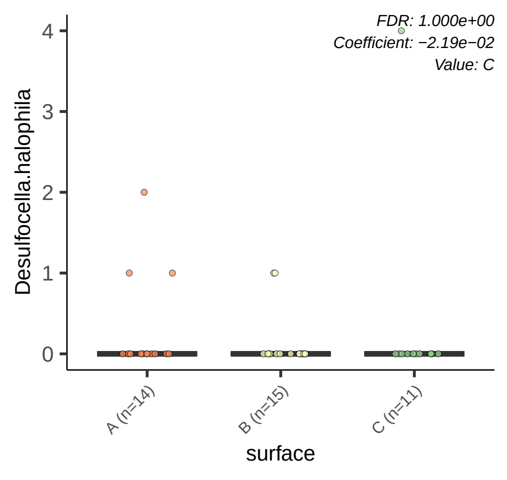

FDR: 1.000e+00
Coefficient: −2.19e−02
Value: C
Desulfocella.halophila
0
1
2
3
4
A (n=14)
B (n=15)
C (n=11)
surface

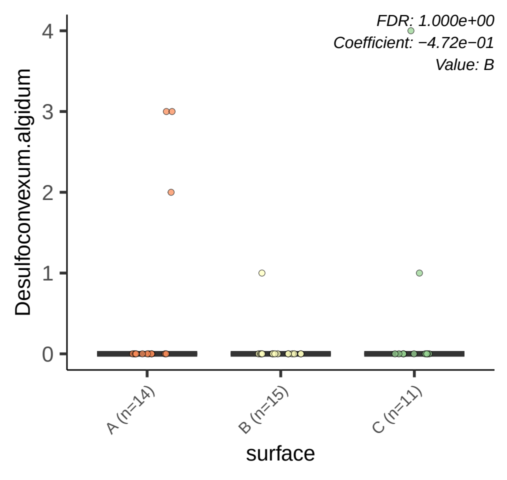
FDR: 1.000e+00
Coefficient: −4.72e−01
Value: B
Desulfoconvexum.algidum
4
3
2
1
0
A (n=14)
B (n=15)
C (n=11)
surface

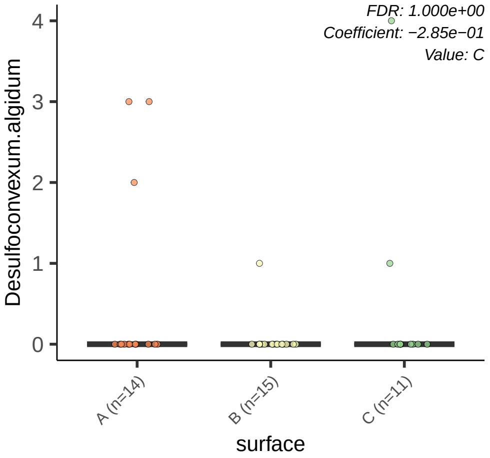
FDR: 1.000e+00
Coefficient: −2.85e−01
Value: C
Desulfoconvexum.algidum
0
1
2
3
4
A (n=14)
B (n=15)
C (n=11)
surface

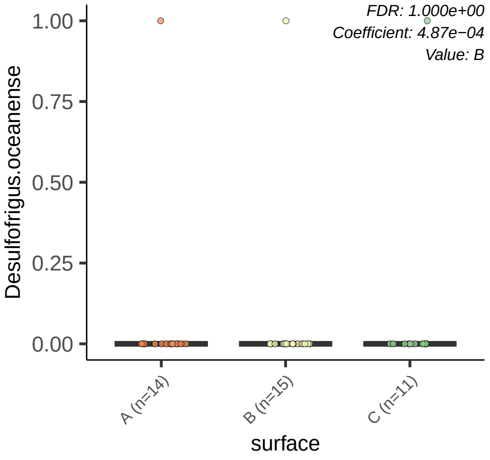

FDR: 1.000e+00
Coefficient: 4.87e−04
Value: B
Desulfofrigus.oceanense
1.00
0.75
0.50
0.25
0.00
A (n=14)
B (n=15)
C (n=11)
surface

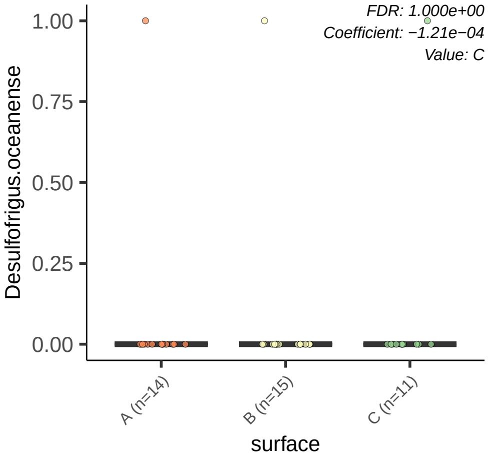
FDR: 1.000e+00
Coefficient: −1.21e−04
Value: C
Desulfofrigus.oceanense
1.00
0.75
0.50
0.25
0.00
A (n=14)
B (n=15)
C (n=11)
surface

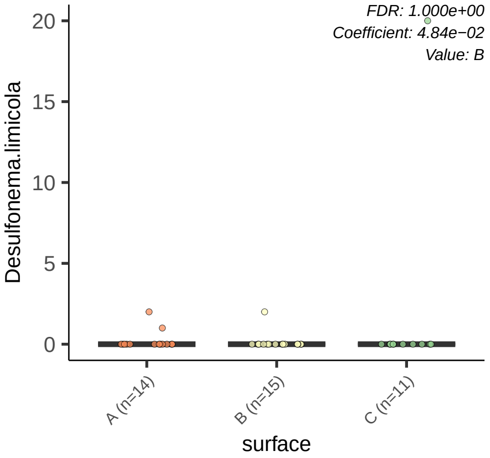
FDR: 1.000e+00
Coefficient: 4.84e−02
Value: B
Desulfonema.limicola
20
15
10
5
0
A (n=14)
B (n=15)
C (n=11)
surface

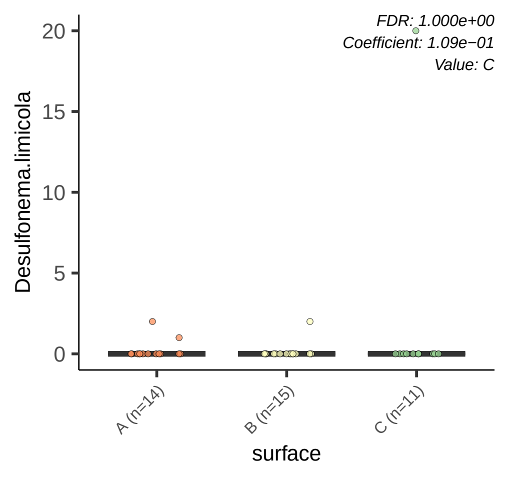

FDR: 1.000e+00
Coefficient: 1.09e−01
Value: C
Desulfonema.limicola
20
15
10
5
0
A (n=14)
B (n=15)
C (n=11)
surface

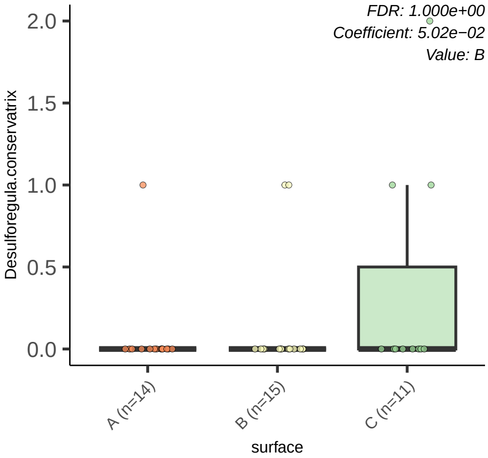

FDR: 1.000e+00
Coefficient: 5.02e−02
Value: B
Desulforegula.conservatrix
2.0
1.5
1.0
0.5
0.0
A (n=14)
B (n=15)
C (n=11)
surface

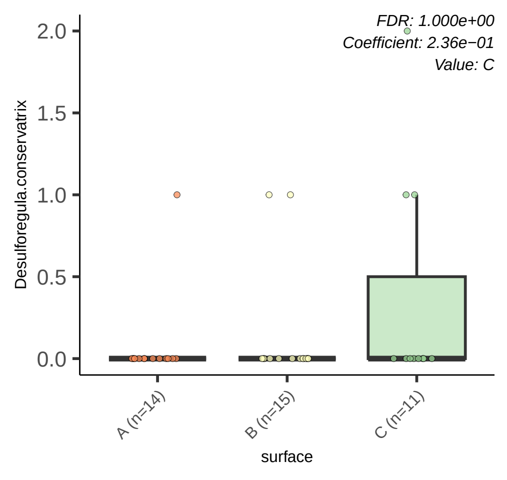
FDR: 1.000e+00
Coefficient: 2.36e−01
Value: C
Desulforegula.conservatrix
2.0
1.5
1.0
0.5
0.0
A (n=14)
B (n=15)
C (n=11)
surface

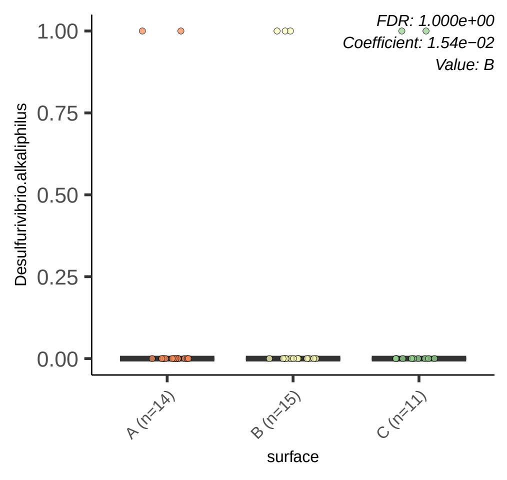

FDR: 1.000e+00
Coefficient: 1.54e−02
Value: B
1.00
0.75
0.50
0.25
0.00
Desulfurivibrio.alkaliphilus
A (n=14)
B (n=15)
C (n=11)
surface

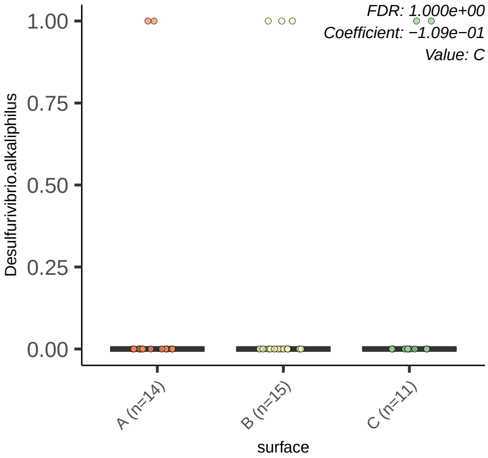

FDR: 1.000e+00
Coefficient: −1.09e−01
Value: C
Desulfurivibrio.alkaliphilus
1.00
0.75
0.50
0.25
0.00
A (n=14)
B (n=15)
C (n=11)
surface

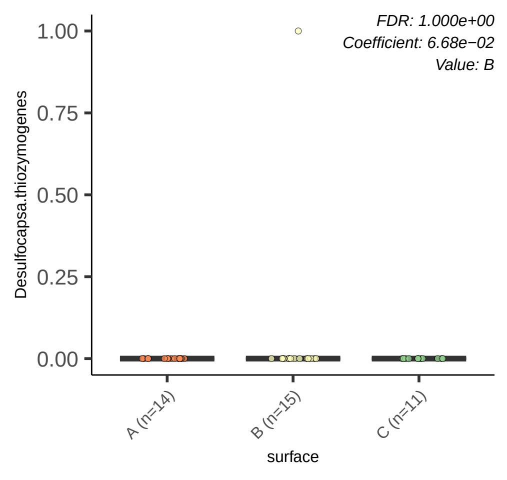
FDR: 1.000e+00
Coefficient: 6.68e−02
Value: B
Desulfocapsa.thiozymogenes
1.00
0.75
0.50
0.25
0.00
A (n=14)
B (n=15)
C (n=11)
surface

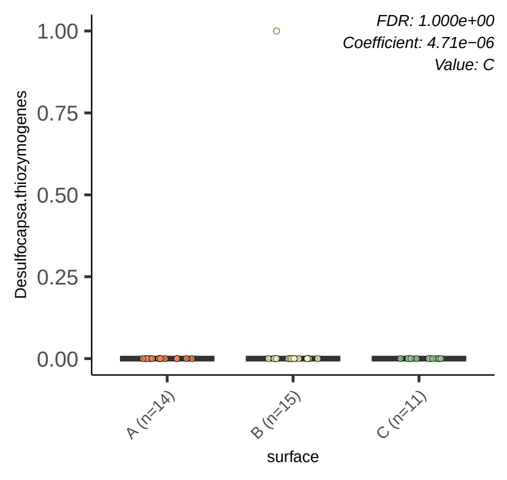
FDR: 1.000e+00
Coefficient: 4.71e−06
Value: C
1.00
0.75
0.50
0.25
0.00
Desulfocapsa.thiozymogenes
A (n=14)
B (n=15)
C (n=11)
surface

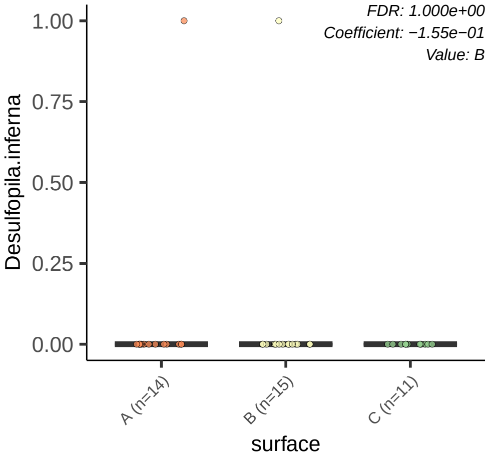
FDR: 1.000e+00
Coefficient: −1.55e−01
Value: B
Desulfopila.inferna
1.00
0.75
0.50
0.25
0.00
A (n=14)
B (n=15)
C (n=11)
surface

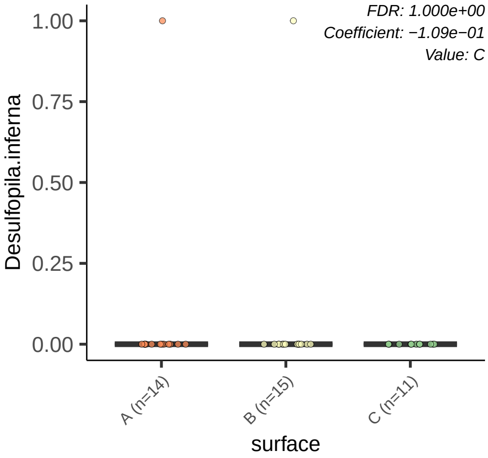

FDR: 1.000e+00
Coefficient: −1.09e−01
Value: C
Desulfopila.inferna
1.00
0.75
0.50
0.25
0.00
A (n=14)
B (n=15)
C (n=11)
surface

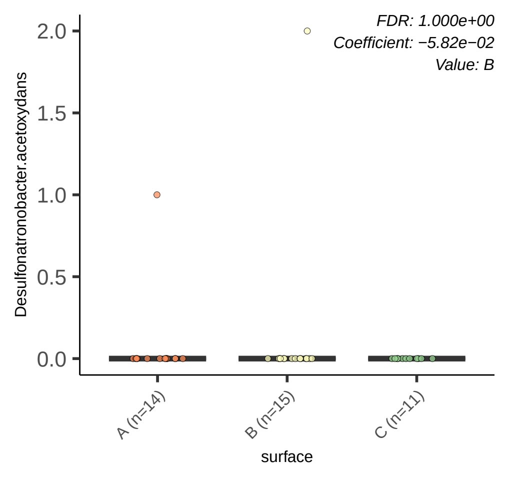

FDR: 1.000e+00
Coefficient: −5.82e−02
Value: B
Desulfonatronobacter.acetoxydans
2.0
1.5
1.0
0.5
0.0
A (n=14)
B (n=15)
C (n=11)
surface

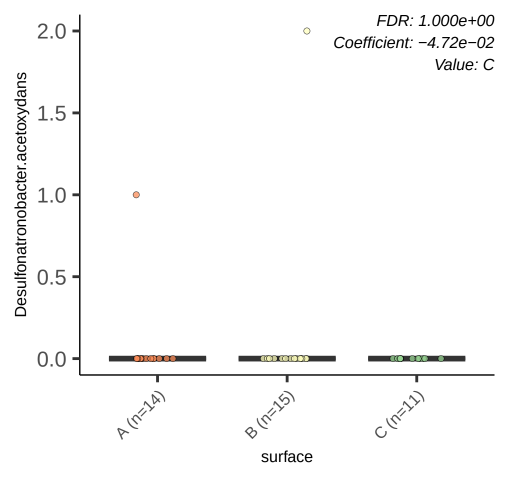

FDR: 1.000e+00
Coefficient: −4.72e−02
Value: C
Desulfonatronobacter.acetoxydans
2.0
1.5
1.0
0.5
0.0
A (n=14)
B (n=15)
C (n=11)
surface

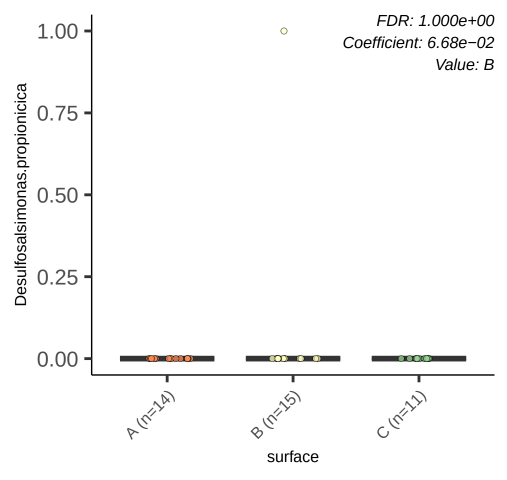

FDR: 1.000e+00
Coefficient: 6.68e−02
Value: B
Desulfosalsimonas.propionicica
1.00
0.75
0.50
0.25
0.00
A (n=14)
B (n=15)
C (n=11)
surface

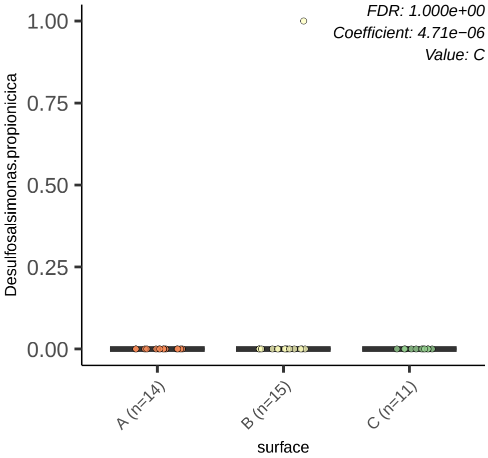
FDR: 1.000e+00
Coefficient: 4.71e−06
Value: C
Desulfosalsimonas.propionicica
1.00
0.75
0.50
0.25
0.00
A (n=14)
B (n=15)
C (n=11)
surface

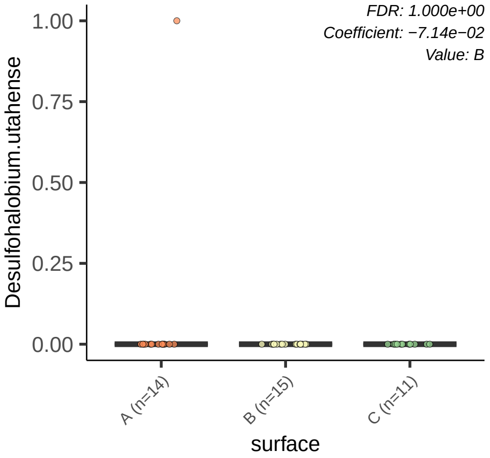

FDR: 1.000e+00
Coefficient: −7.14e−02
Value: B
Desulfohalobium.utahense
1.00
0.75
0.50
0.25
0.00
A (n=14)
B (n=15)
C (n=11)
surface

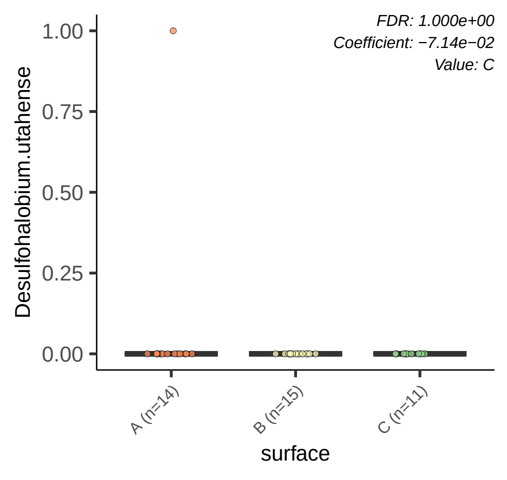
FDR: 1.000e+00
Coefficient: −7.14e−02
Value: C
Desulfohalobium.utahense
1.00
0.75
0.50
0.25
0.00
A (n=14)
B (n=15)
C (n=11)
surface

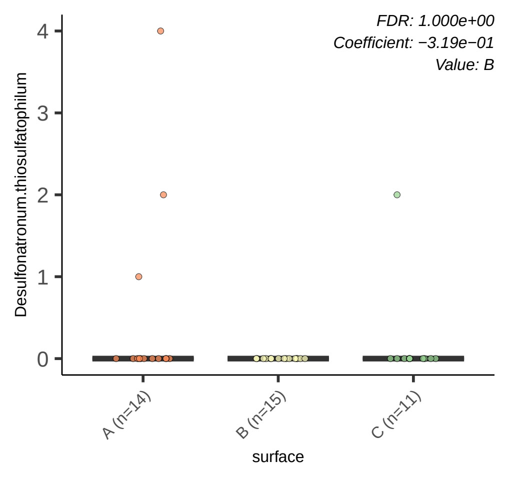

FDR: 1.000e+00
Coefficient: −3.19e−01
Value: B
Desulfonatronum.thiosulfatophilum
4
3
2
1
0
A (n=14)
B (n=15)
C (n=11)
surface

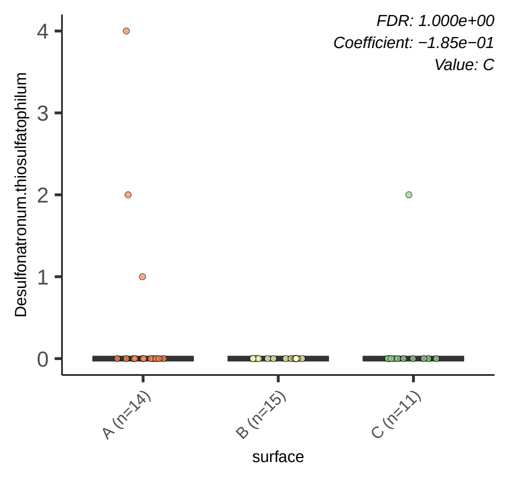
FDR: 1.000e+00
Coefficient: −1.85e−01
Value: C
Desulfonatronum.thiosulfatophilum
0
1
2
3
4
A (n=14)
B (n=15)
C (n=11)
surface

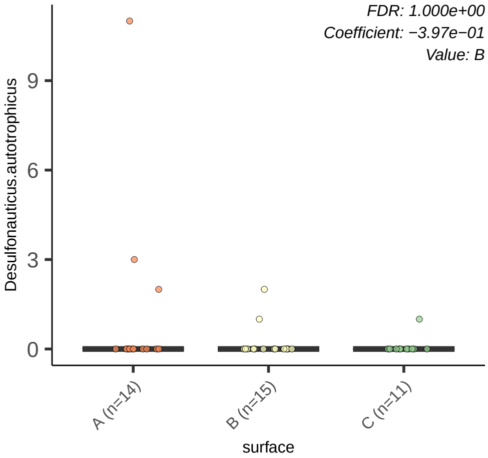

FDR: 1.000e+00
Coefficient: −3.97e−01
Value: B
Desulfonauticus.autotrophicus
0
3
6
9
A (n=14)
B (n=15)
C (n=11)
surface

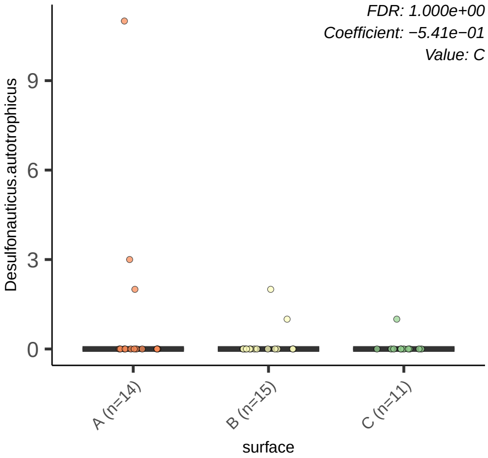

FDR: 1.000e+00
Coefficient: −5.41e−01
Value: C
Desulfonauticus.autotrophicus
0
3
6
9
A (n=14)
B (n=15)
C (n=11)
surface

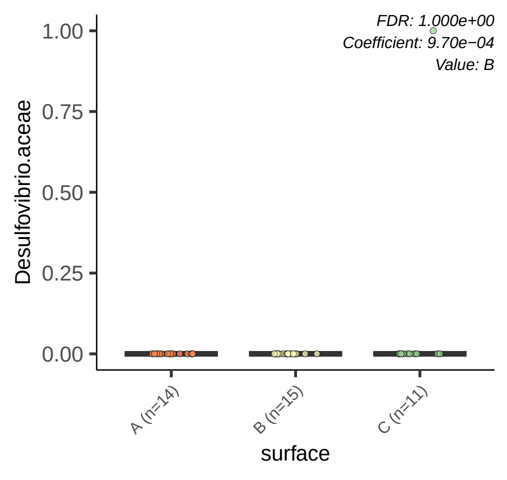
FDR: 1.000e+00
Coefficient: 9.70e−04
Value: B
Desulfovibrio.aceae
1.00
0.75
0.50
0.25
0.00
A (n=14)
B (n=15)
C (n=11)
surface

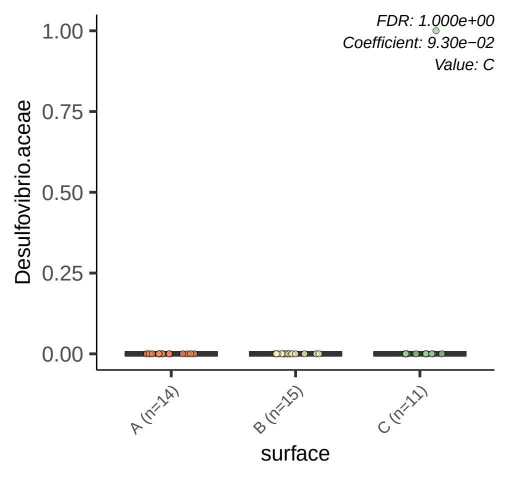
FDR: 1.000e+00
Coefficient: 9.30e−02
Value: C
Desulfovibrio.aceae
1.00
0.75
0.50
0.25
0.00
A (n=14)
B (n=15)
C (n=11)
surface

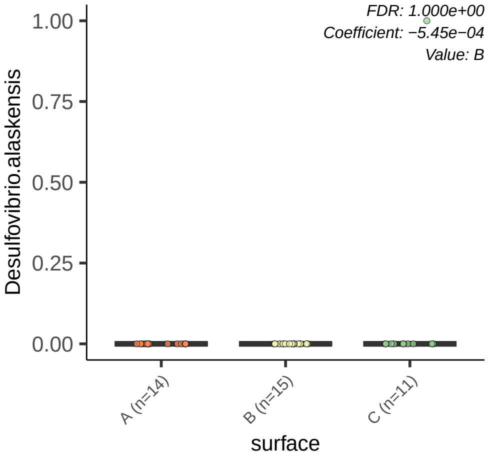
FDR: 1.000e+00
Coefficient: −5.45e−04
Value: B
Desulfovibrio.alaskensis
1.00
0.75
0.50
0.25
0.00
A (n=14)
B (n=15)
C (n=11)
surface

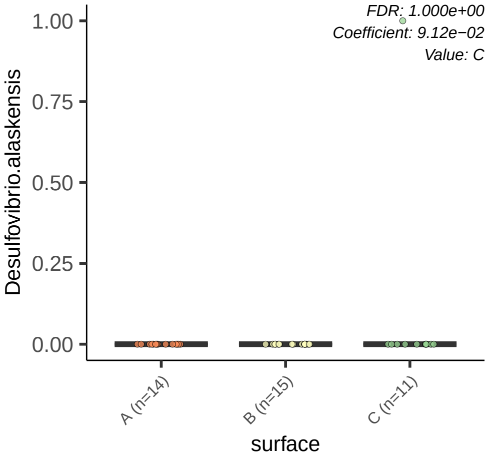

FDR: 1.000e+00
Coefficient: 9.12e−02
Value: C
Desulfovibrio.alaskensis
1.00
0.75
0.50
0.25
0.00
A (n=14)
B (n=15)
C (n=11)
surface

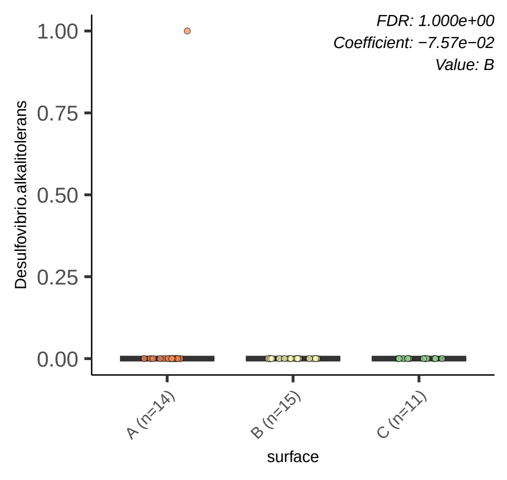
FDR: 1.000e+00
Coefficient: −7.57e−02
Value: B
Desulfovibrio.alkalitolerans
1.00
0.75
0.50
0.25
0.00
A (n=14)
B (n=15)
C (n=11)
surface

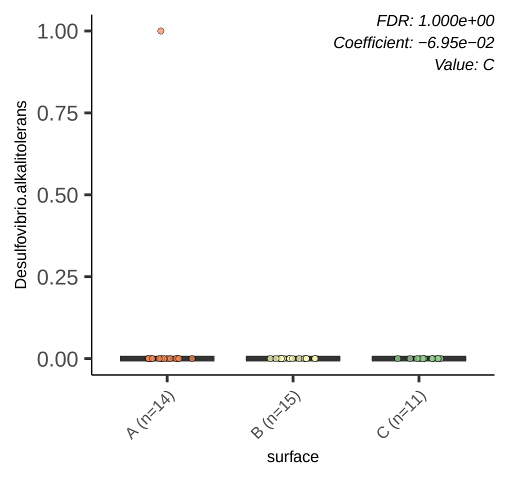

FDR: 1.000e+00
Coefficient: −6.95e−02
Value: C
Desulfovibrio.alkalitolerans
1.00
0.75
0.50
0.25
0.00
A (n=14)
B (n=15)
C (n=11)
surface

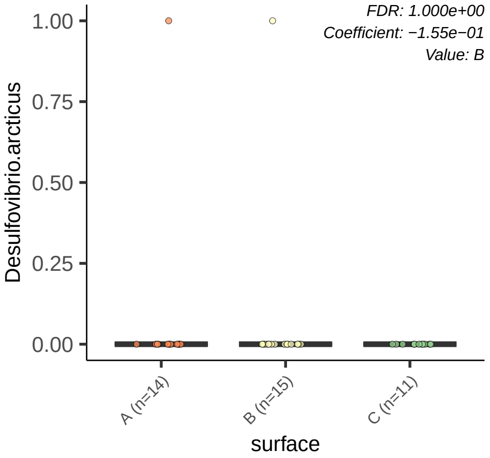
FDR: 1.000e+00
Coefficient: −1.55e−01
Value: B
1.00
0.75
0.50
0.25
0.00
Desulfovibrio.arcticus
A (n=14)
B (n=15)
C (n=11)
surface

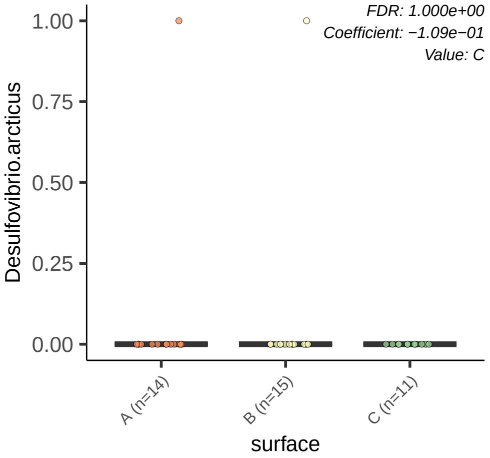
FDR: 1.000e+00
Coefficient: −1.09e−01
Value: C
Desulfovibrio.arcticus
1.00
0.75
0.50
0.25
0.00
A (n=14)
B (n=15)
C (n=11)
surface

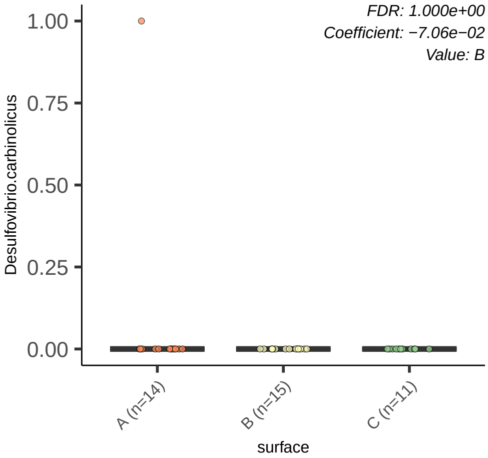

FDR: 1.000e+00
Coefficient: −7.06e−02
Value: B
Desulfovibrio.carbinolicus
1.00
0.75
0.50
0.25
0.00
A (n=14)
B (n=15)
C (n=11)
surface

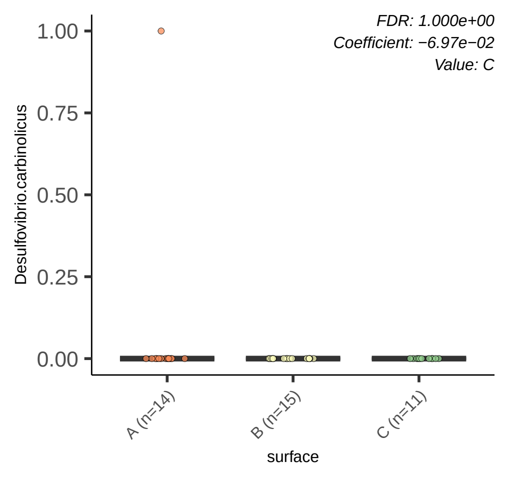
FDR: 1.000e+00
Coefficient: −6.97e−02
Value: C
Desulfovibrio.carbinolicus
1.00
0.75
0.50
0.25
0.00
A (n=14)
B (n=15)
C (n=11)
surface

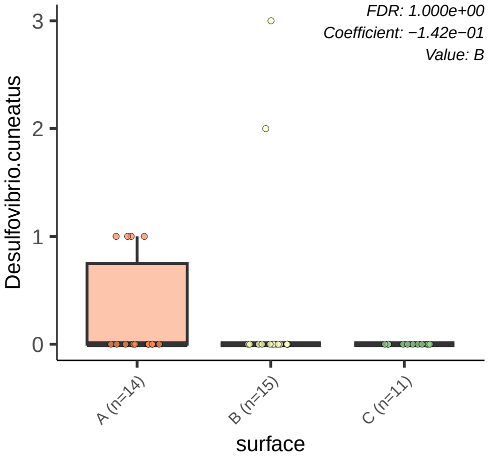

FDR: 1.000e+00
Coefficient: −1.42e−01
Value: B
Desulfovibrio.cuneatus
3
2
1
0
A (n=14)
B (n=15)
C (n=11)
surface

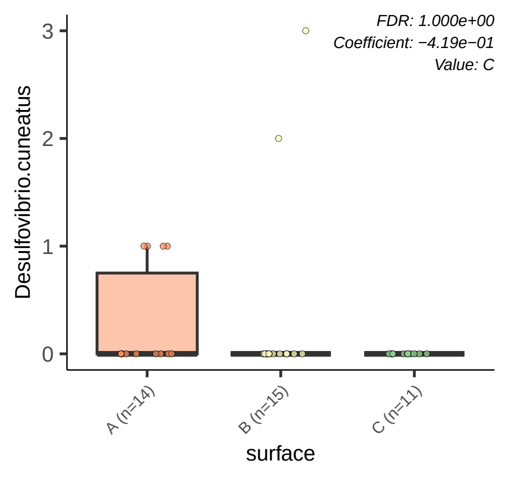
FDR: 1.000e+00
Coefficient: −4.19e−01
Value: C
Desulfovibrio.cuneatus
3
2
1
0
A (n=14)
B (n=15)
C (n=11)
surface

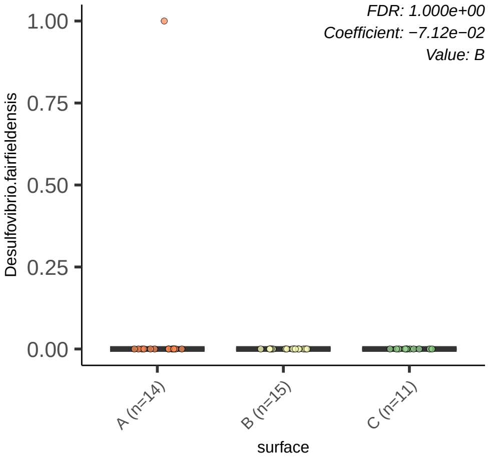
FDR: 1.000e+00
Coefficient: −7.12e−02
Value: B
1.00
0.75
0.50
0.25
0.00
Desulfovibrio.fairfieldensis
A (n=14)
B (n=15)
C (n=11)
surface

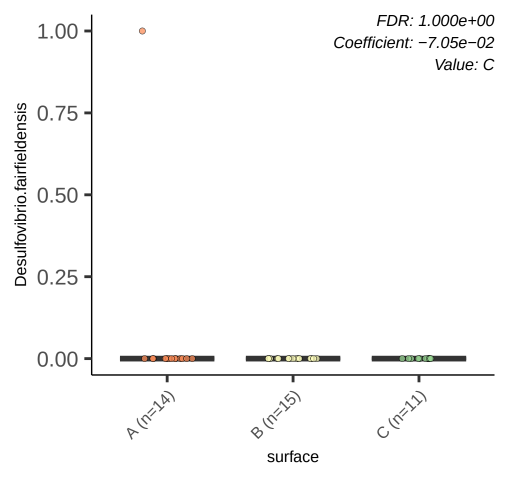

FDR: 1.000e+00
Coefficient: −7.05e−02
Value: C
Desulfovibrio.fairfieldensis
1.00
0.75
0.50
0.25
0.00
A (n=14)
B (n=15)
C (n=11)
surface

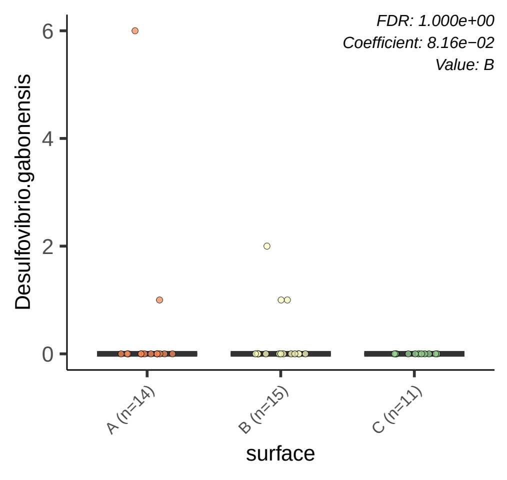
FDR: 1.000e+00
Coefficient: 8.16e−02
Value: B
Desulfovibrio.gabonensis
0
2
4
6
A (n=14)
B (n=15)
C (n=11)
surface

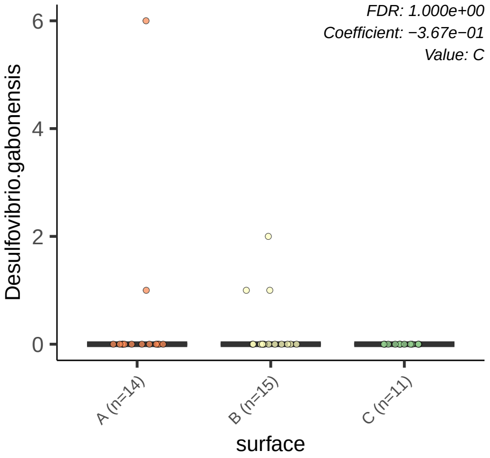

FDR: 1.000e+00
Coefficient: −3.67e−01
Value: C
Desulfovibrio.gabonensis
6
4
2
0
A (n=14)
B (n=15)
C (n=11)
surface

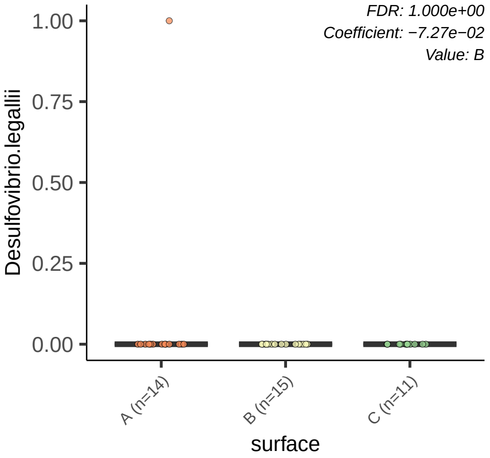

FDR: 1.000e+00
Coefficient: −7.27e−02
Value: B
Desulfovibrio.legallii
1.00
0.75
0.50
0.25
0.00
A (n=14)
B (n=15)
C (n=11)
surface

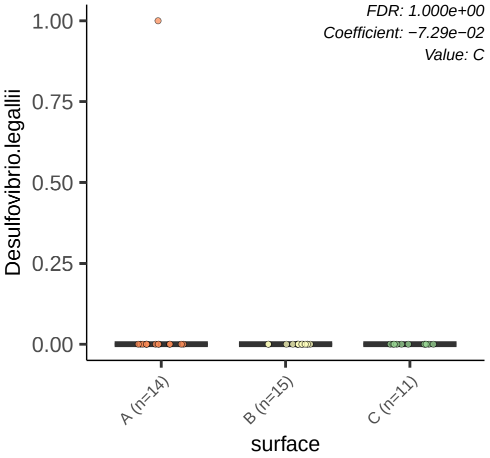
FDR: 1.000e+00
Coefficient: −7.29e−02
Value: C
Desulfovibrio.legallii
1.00
0.75
0.50
0.25
0.00
A (n=14)
B (n=15)
C (n=11)
surface

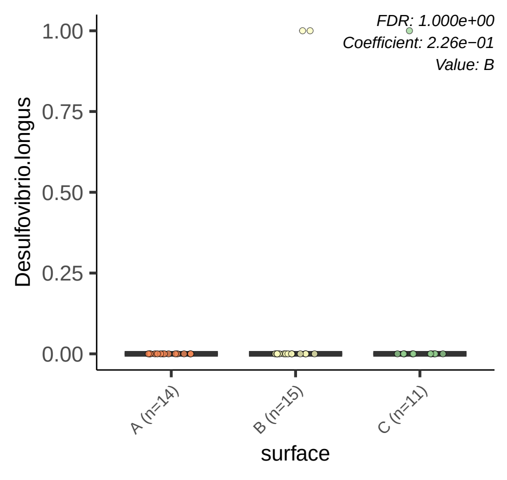
FDR: 1.000e+00
Coefficient: 2.26e−01
Value: B
Desulfovibrio.longus
1.00
0.75
0.50
0.25
0.00
A (n=14)
B (n=15)
C (n=11)
surface

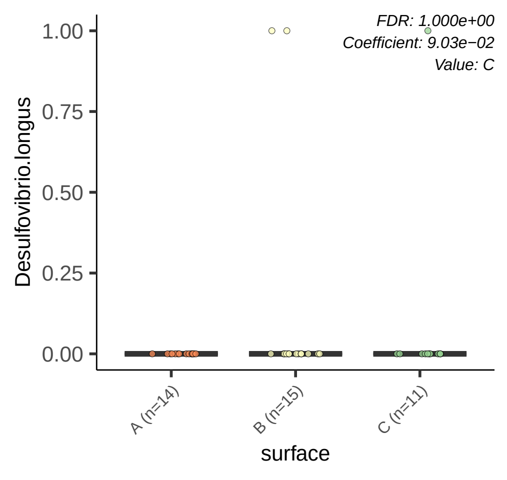
FDR: 1.000e+00
Coefficient: 9.03e−02
Value: C
Desulfovibrio.longus
1.00
0.75
0.50
0.25
0.00
A (n=14)
B (n=15)
C (n=11)
surface

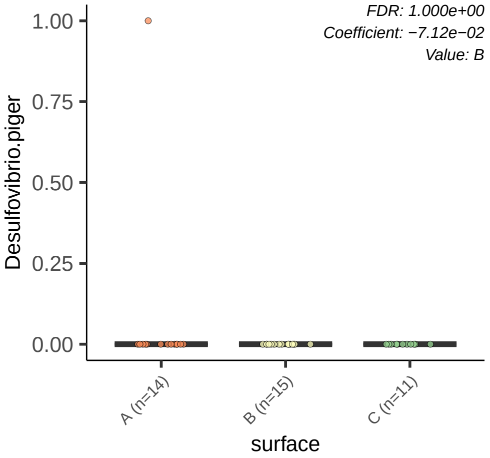
FDR: 1.000e+00
Coefficient: −7.12e−02
Value: B
Desulfovibrio.piger
1.00
0.75
0.50
0.25
0.00
A (n=14)
B (n=15)
C (n=11)
surface

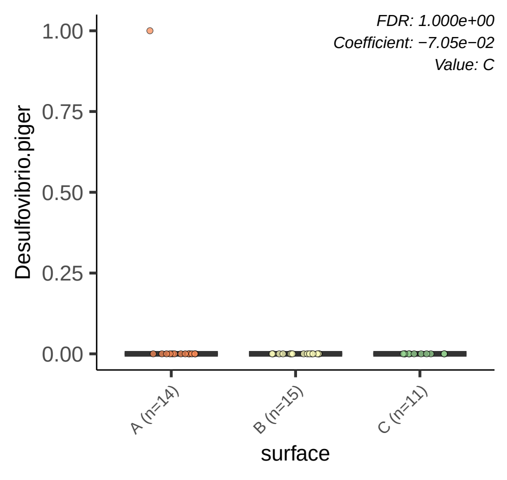
FDR: 1.000e+00
Coefficient: −7.05e−02
Value: C
Desulfovibrio.piger
1.00
0.75
0.50
0.25
0.00
A (n=14)
B (n=15)
C (n=11)
surface

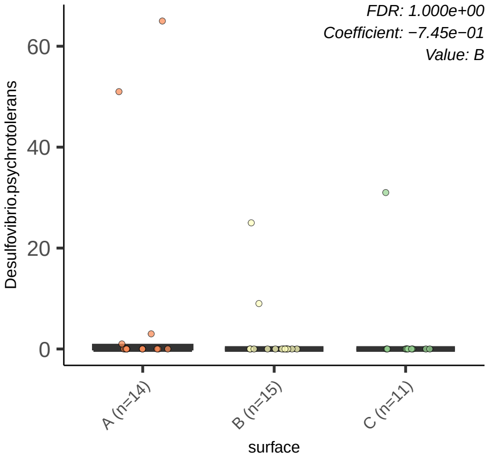

FDR: 1.000e+00
Coefficient: −7.45e−01
Value: B
Desulfovibrio.psychrotolerans
60
40
20
0
A (n=14)
B (n=15)
C (n=11)
surface

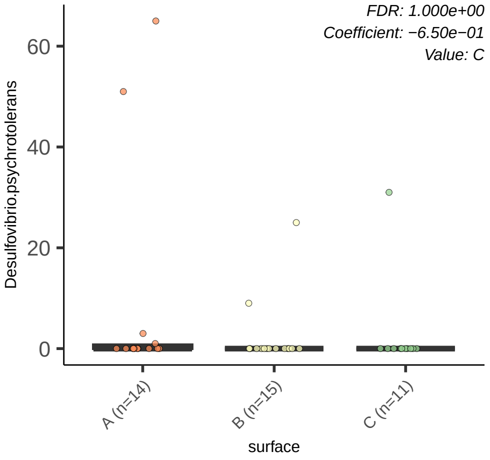
FDR: 1.000e+00
Coefficient: −6.50e−01
Value: C
Desulfovibrio.psychrotolerans
60
40
20
0
A (n=14)
B (n=15)
C (n=11)
surface

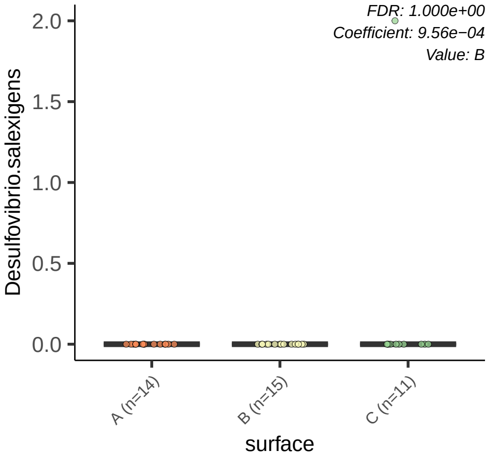

FDR: 1.000e+00
Coefficient: 9.56e−04
Value: B
Desulfovibrio.salexigens
2.0
1.5
1.0
0.5
0.0
A (n=14)
B (n=15)
C (n=11)
surface

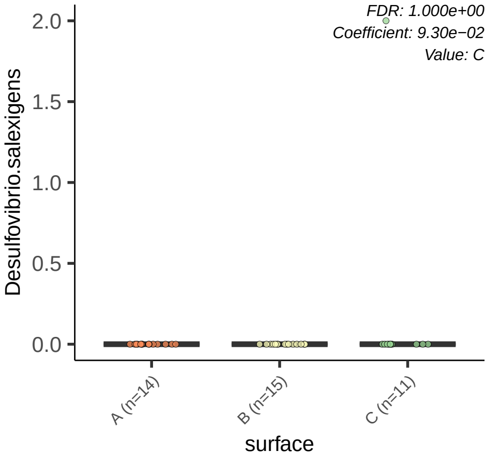

FDR: 1.000e+00
Coefficient: 9.30e−02
Value: C
Desulfovibrio.salexigens
2.0
1.5
1.0
0.5
0.0
A (n=14)
B (n=15)
C (n=11)
surface

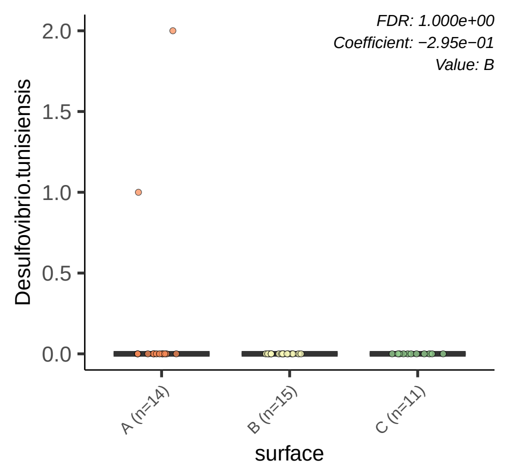
FDR: 1.000e+00
Coefficient: −2.95e−01
Value: B
Desulfovibrio.tunisiensis
2.0
1.5
1.0
0.5
0.0
A (n=14)
B (n=15)
C (n=11)
surface

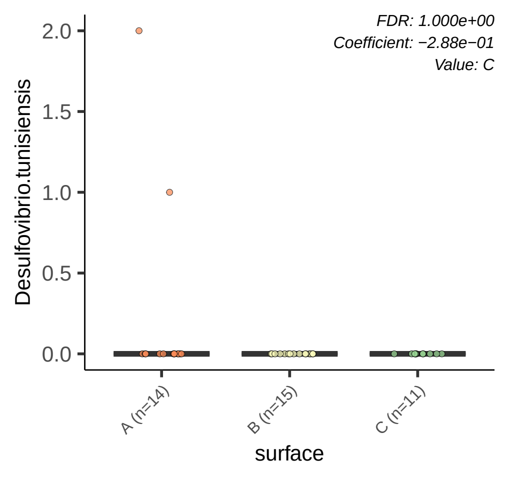

FDR: 1.000e+00
Coefficient: −2.88e−01
Value: C
Desulfovibrio.tunisiensis
2.0
1.5
1.0
0.5
0.0
A (n=14)
B (n=15)
C (n=11)
surface

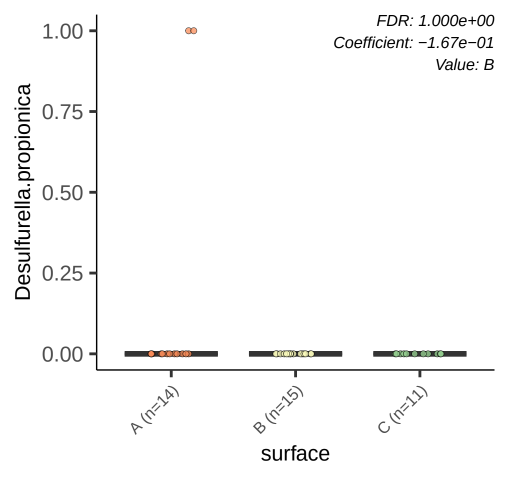
FDR: 1.000e+00
Coefficient: −1.67e−01
Value: B
Desulfurella.propionica
1.00
0.75
0.50
0.25
0.00
A (n=14)
B (n=15)
C (n=11)
surface

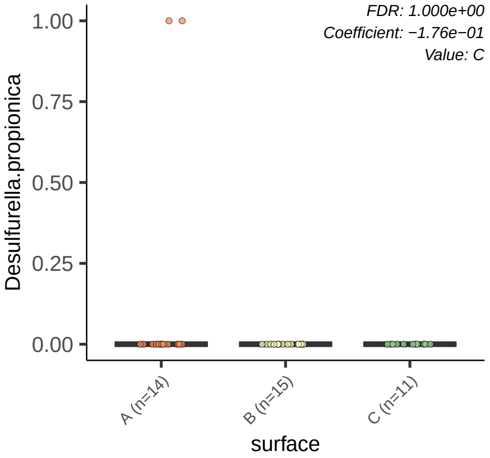
FDR: 1.000e+00
Coefficient: −1.76e−01
Value: C
Desulfurella.propionica
1.00
0.75
0.50
0.25
0.00
A (n=14)
B (n=15)
C (n=11)
surface

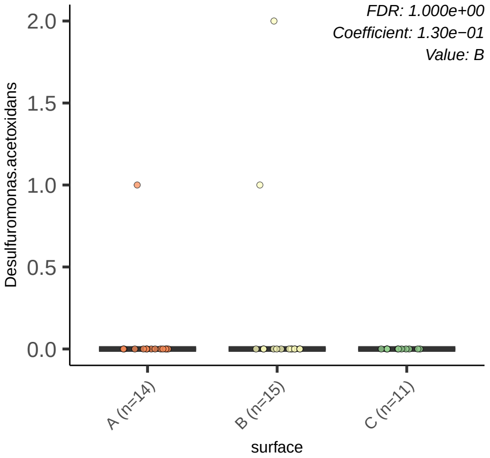

FDR: 1.000e+00
Coefficient: 1.30e−01
Value: B
Desulfuromonas.acetoxidans
2.0
1.5
1.0
0.5
0.0
A (n=14)
B (n=15)
C (n=11)
surface

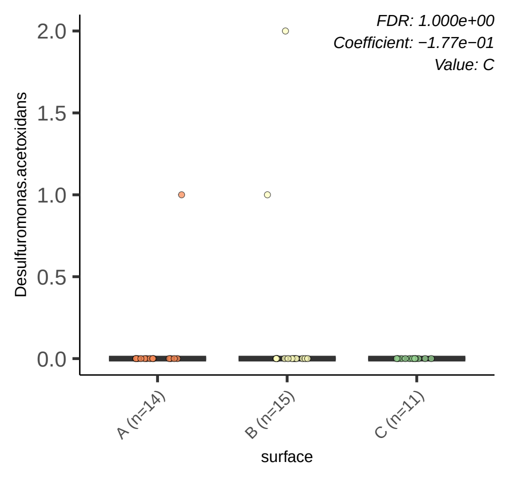

FDR: 1.000e+00
Coefficient: −1.77e−01
Value: C
Desulfuromonas.acetoxidans
2.0
1.5
1.0
0.5
0.0
A (n=14)
B (n=15)
C (n=11)
surface

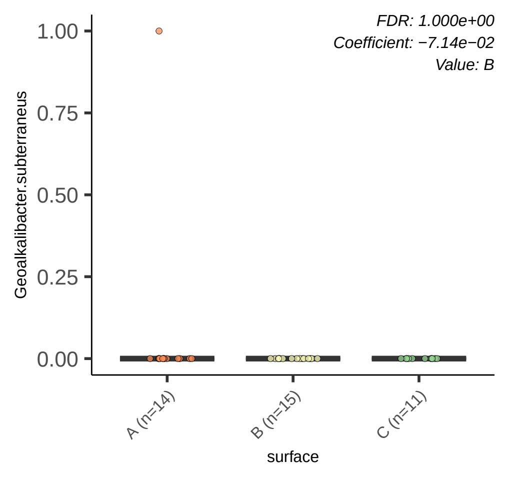
FDR: 1.000e+00
Coefficient: −7.14e−02
Value: B
Geoalkalibacter.subterraneus
1.00
0.75
0.50
0.25
0.00
A (n=14)
B (n=15)
C (n=11)
surface

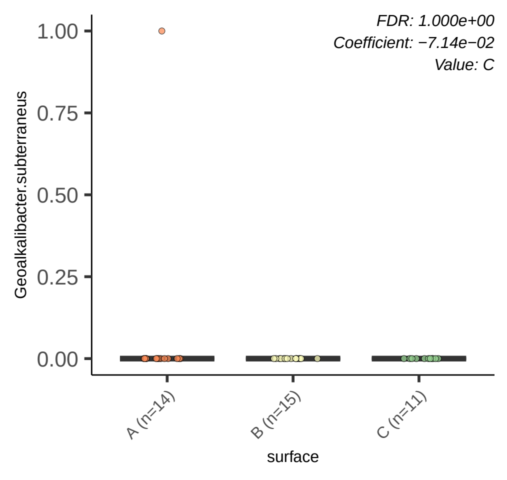
FDR: 1.000e+00
Coefficient: −7.14e−02
Value: C
Geoalkalibacter.subterraneus
1.00
0.75
0.50
0.25
0.00
A (n=14)
B (n=15)
C (n=11)
surface

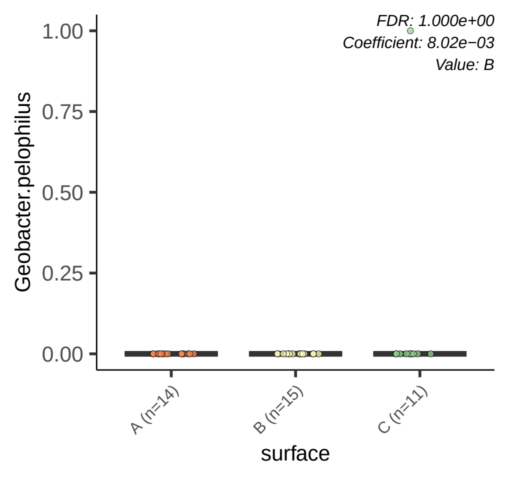

FDR: 1.000e+00
Coefficient: 8.02e−03
Value: B
Geobacter.pelophilus
1.00
0.75
0.50
0.25
0.00
A (n=14)
B (n=15)
C (n=11)
surface

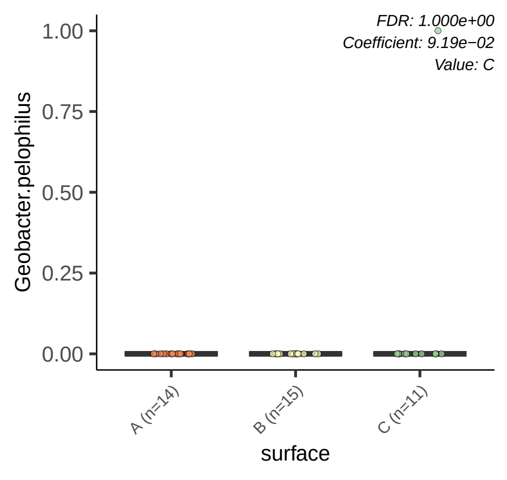
FDR: 1.000e+00
Coefficient: 9.19e−02
Value: C
1.00
0.75
0.50
0.25
0.00
Geobacter.pelophilus
A (n=14)
B (n=15)
C (n=11)
surface

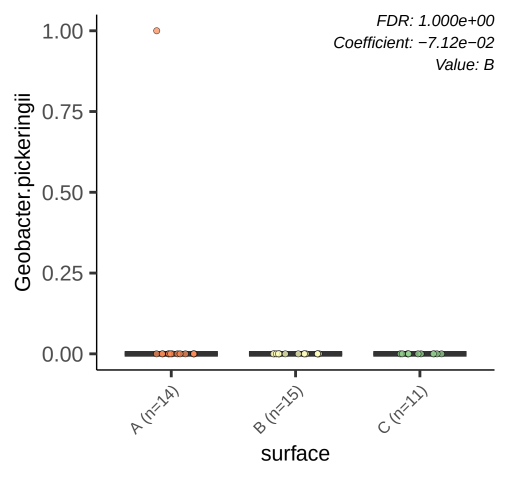
FDR: 1.000e+00
Coefficient: −7.12e−02
Value: B
1.00
0.75
0.50
0.25
0.00
Geobacter.pickeringii
A (n=14)
B (n=15)
C (n=11)
surface

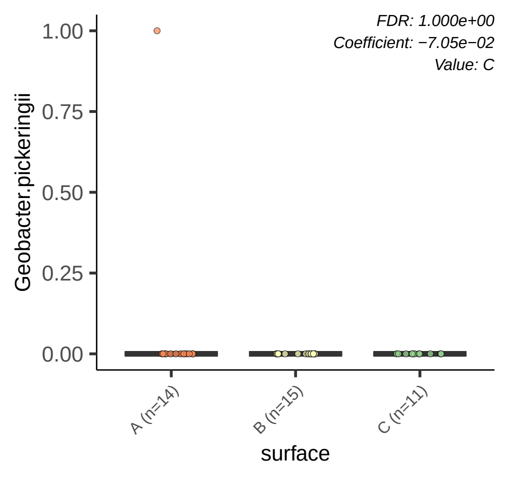
FDR: 1.000e+00
Coefficient: −7.05e−02
Value: C
Geobacter.pickeringii
1.00
0.75
0.50
0.25
0.00
A (n=14)
B (n=15)
C (n=11)
surface

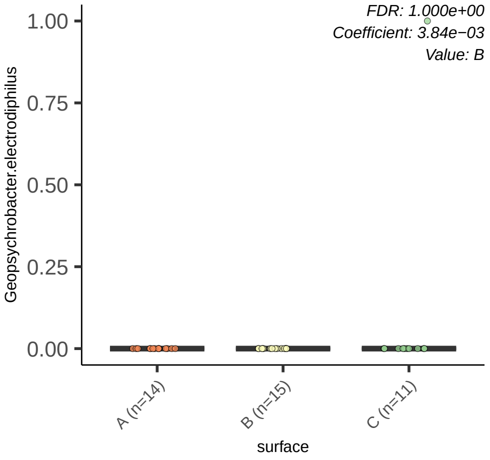
FDR: 1.000e+00
Coefficient: 3.84e−03
Value: B
Geopsychrobacter.electrodiphilus
1.00
0.75
0.50
0.25
0.00
A (n=14)
B (n=15)
C (n=11)
surface

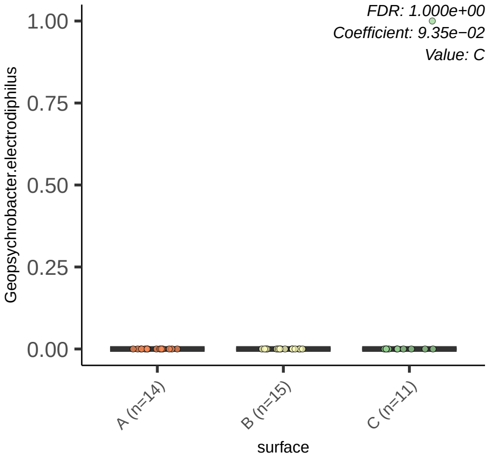
FDR: 1.000e+00
Coefficient: 9.35e−02
Value: C
Geopsychrobacter.electrodiphilus
1.00
0.75
0.50
0.25
0.00
A (n=14)
B (n=15)
C (n=11)
surface

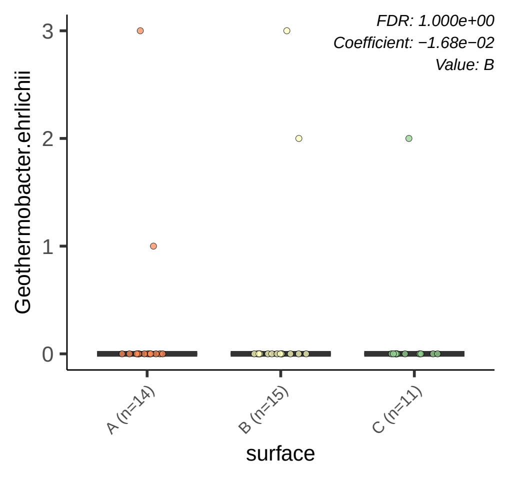

FDR: 1.000e+00
Coefficient: −1.68e−02
Value: B
Geothermobacter.ehrlichii
3
2
1
0
A (n=14)
B (n=15)
C (n=11)
surface

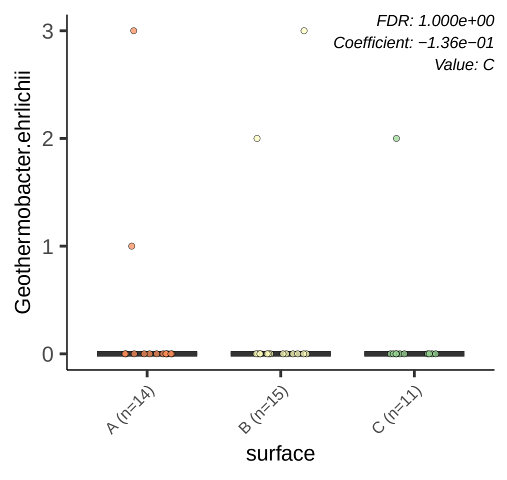
FDR: 1.000e+00
Coefficient: −1.36e−01
Value: C
Geothermobacter.ehrlichii
3
2
1
0
A (n=14)
B (n=15)
C (n=11)
surface

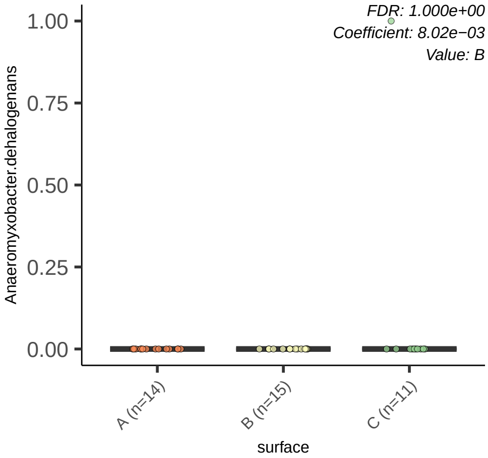

FDR: 1.000e+00
Coefficient: 8.02e−03
Value: B
Anaeromyxobacter.dehalogenans
1.00
0.75
0.50
0.25
0.00
A (n=14)
B (n=15)
C (n=11)
surface

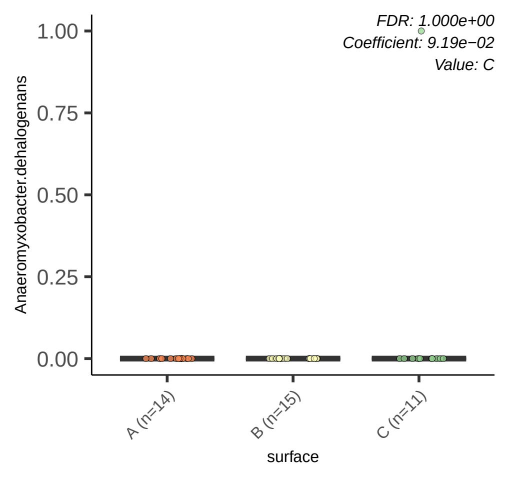
FDR: 1.000e+00
Coefficient: 9.19e−02
Value: C
1.00
0.75
0.50
0.25
0.00
Anaeromyxobacter.dehalogenans
A (n=14)
B (n=15)
C (n=11)
surface

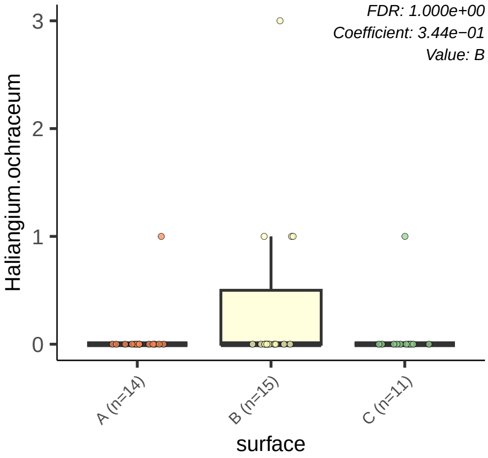
FDR: 1.000e+00
Coefficient: 3.44e−01
Value: B
Haliangium.ochraceum
3
2
1
0
A (n=14)
B (n=15)
C (n=11)
surface

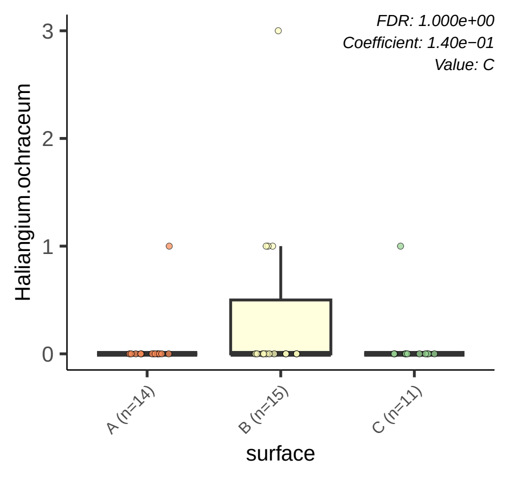

FDR: 1.000e+00
Coefficient: 1.40e−01
Value: C
Haliangium.ochraceum
0
1
2
3
A (n=14)
B (n=15)
C (n=11)
surface

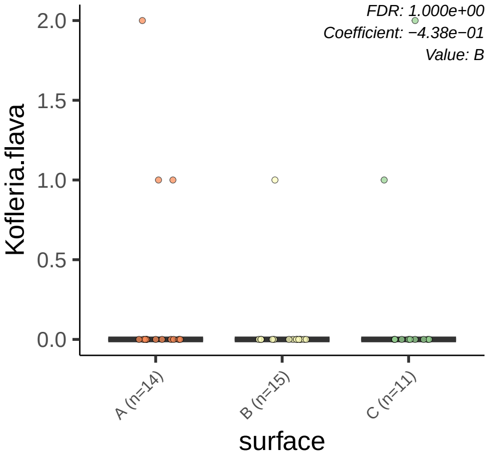
FDR: 1.000e+00
Coefficient: −4.38e−01
Value: B
Kofleria.flava
2.0
1.5
1.0
0.5
0.0
A (n=14)
B (n=15)
C (n=11)
surface

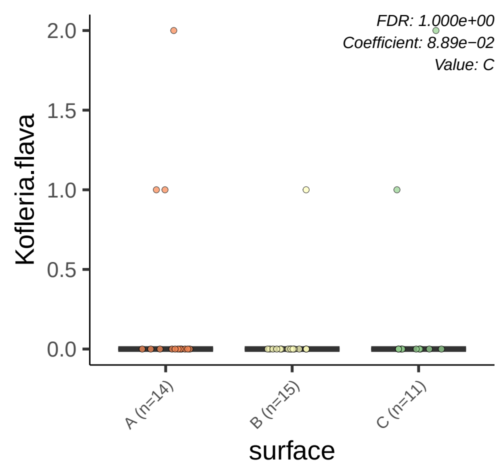
FDR: 1.000e+00
Coefficient: 8.89e−02
Value: C
Kofleria.flava
2.0
1.5
1.0
0.5
0.0
A (n=14)
B (n=15)
C (n=11)
surface

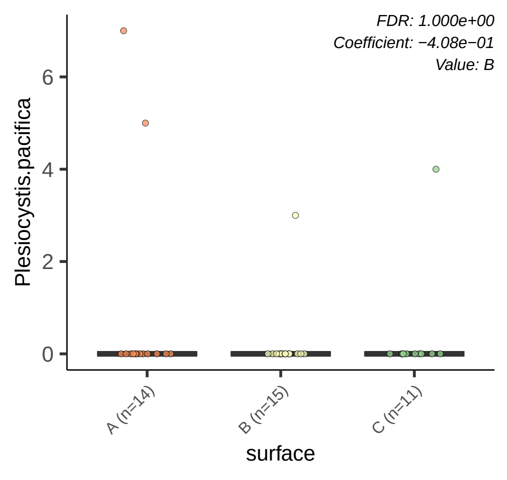
FDR: 1.000e+00
Coefficient: −4.08e−01
Value: B
Plesiocystis.pacifica
0
2
4
6
A (n=14)
B (n=15)
C (n=11)
surface

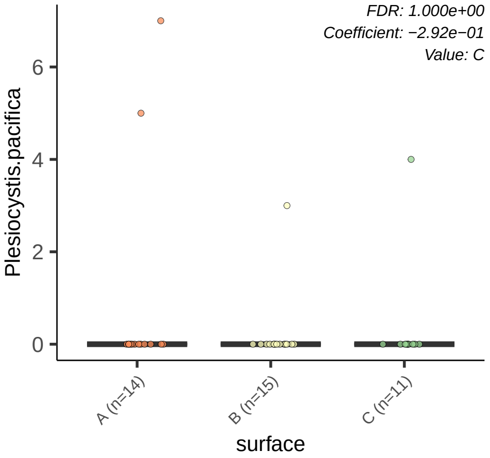
FDR: 1.000e+00
Coefficient: −2.92e−01
Value: C
Plesiocystis.pacifica
0
2
4
6
A (n=14)
B (n=15)
C (n=11)
surface

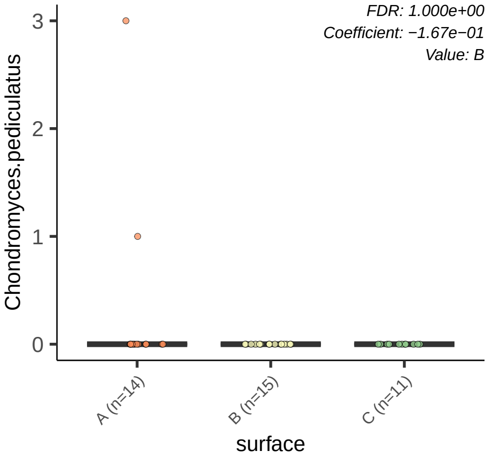
FDR: 1.000e+00
Coefficient: −1.67e−01
Value: B
Chondromyces.pediculatus
3
2
1
0
A (n=14)
B (n=15)
C (n=11)
surface

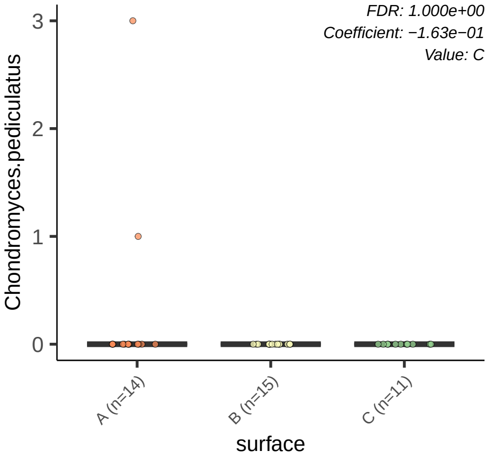

FDR: 1.000e+00
Coefficient: −1.63e−01
Value: C
Chondromyces.pediculatus
3
2
1
0
A (n=14)
B (n=15)
C (n=11)
surface

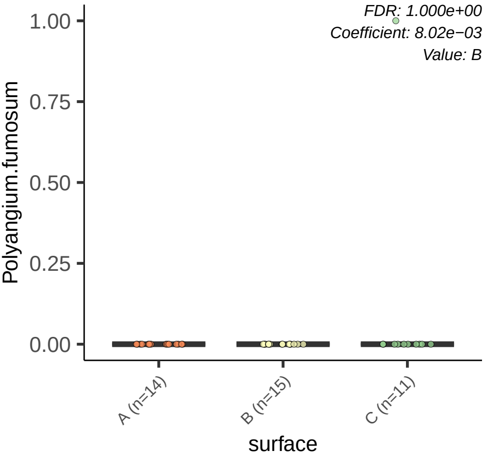
FDR: 1.000e+00
Coefficient: 8.02e−03
Value: B
Polyangium.fumosum
1.00
0.75
0.50
0.25
0.00
A (n=14)
B (n=15)
C (n=11)
surface

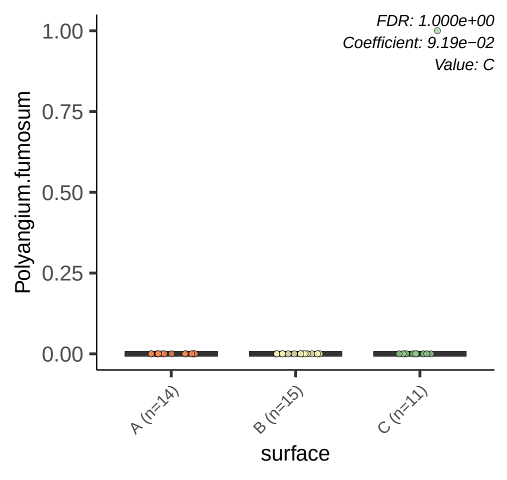

FDR: 1.000e+00
Coefficient: 9.19e−02
Value: C
Polyangium.fumosum
1.00
0.75
0.50
0.25
0.00
A (n=14)
B (n=15)
C (n=11)
surface

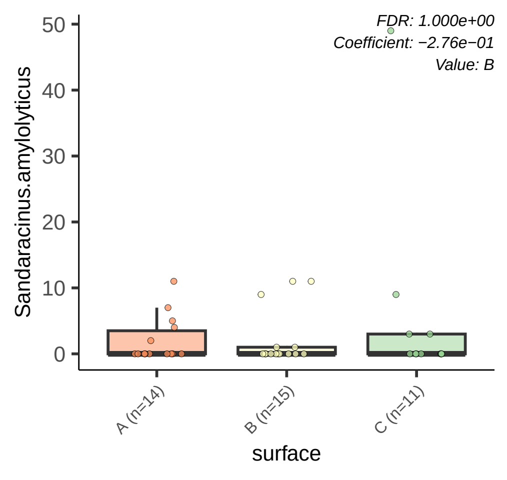

FDR: 1.000e+00
Coefficient: −2.76e−01
Value: B
Sandaracinus.amylolyticus
50
40
30
20
10
0
A (n=14)
B (n=15)
C (n=11)
surface

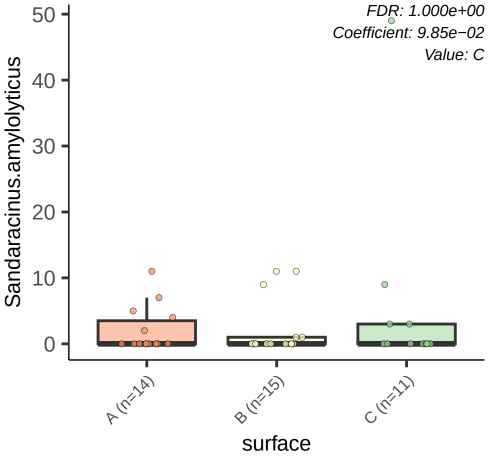

FDR: 1.000e+00
Coefficient: 9.85e−02
Value: C
Sandaracinus.amylolyticus
50
40
30
20
10
0
A (n=14)
B (n=15)
C (n=11)
surface

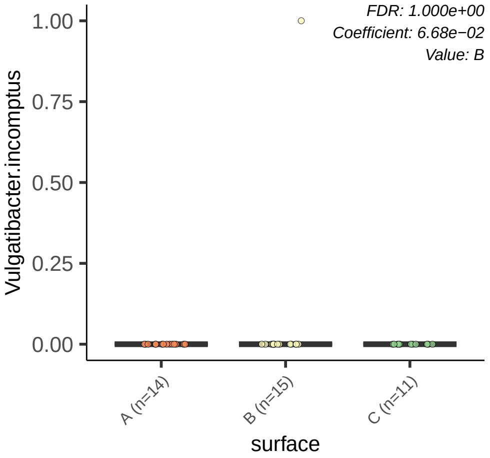
FDR: 1.000e+00
Coefficient: 6.68e−02
Value: B
Vulgatibacter.incomptus
1.00
0.75
0.50
0.25
0.00
A (n=14)
B (n=15)
C (n=11)
surface

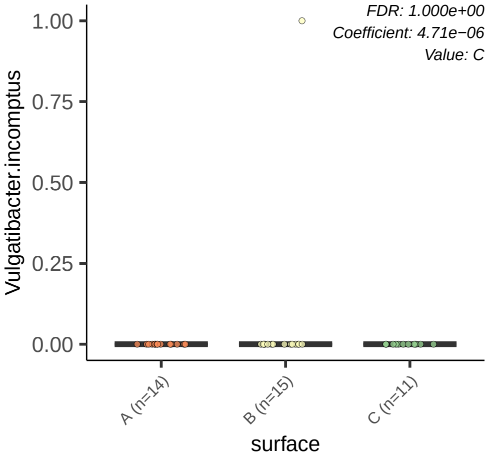
FDR: 1.000e+00
Coefficient: 4.71e−06
Value: C
Vulgatibacter.incomptus
1.00
0.75
0.50
0.25
0.00
A (n=14)
B (n=15)
C (n=11)
surface

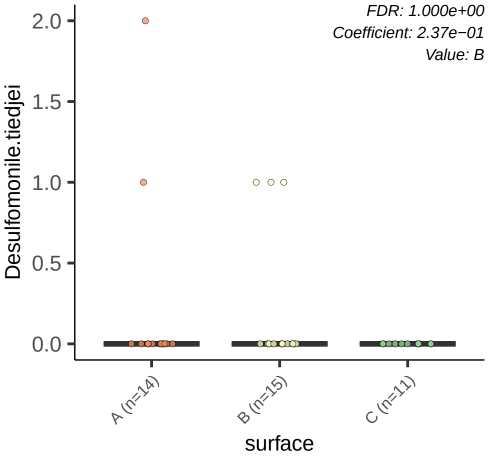

FDR: 1.000e+00
Coefficient: 2.37e−01
Value: B
Desulfomonile.tiedjei
2.0
1.5
1.0
0.5
0.0
A (n=14)
B (n=15)
C (n=11)
surface

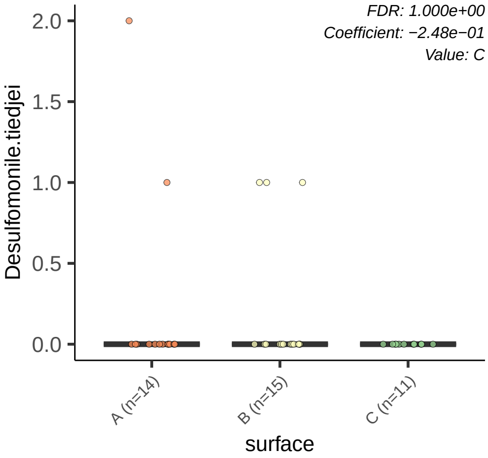
FDR: 1.000e+00
Coefficient: −2.48e−01
Value: C
Desulfomonile.tiedjei
2.0
1.5
1.0
0.5
0.0
A (n=14)
B (n=15)
C (n=11)
surface

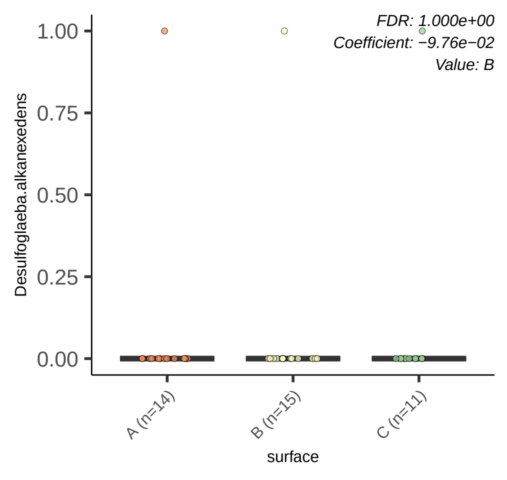
FDR: 1.000e+00
Coefficient: −9.76e−02
Value: B
Desulfoglaeba.alkanexedens
1.00
0.75
0.50
0.25
0.00
A (n=14)
B (n=15)
C (n=11)
surface

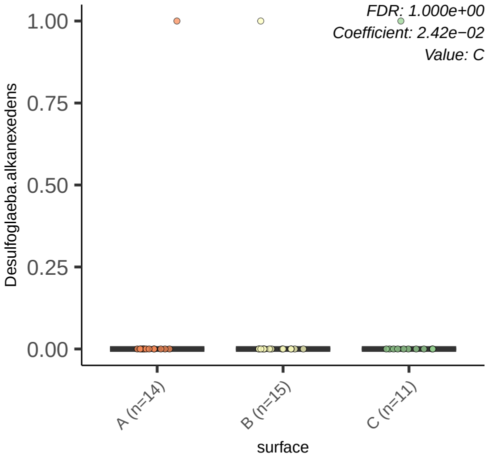

FDR: 1.000e+00
Coefficient: 2.42e−02
Value: C
Desulfoglaeba.alkanexedens
1.00
0.75
0.50
0.25
0.00
A (n=14)
B (n=15)
C (n=11)
surface

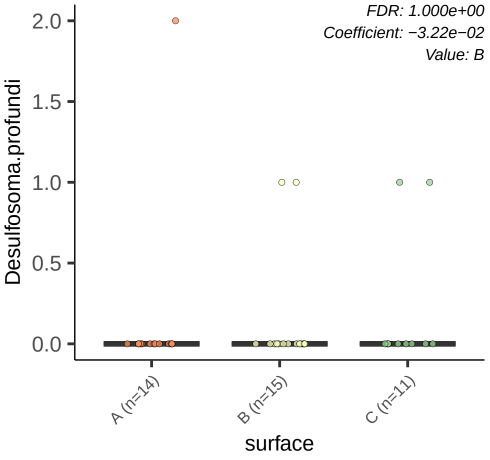

FDR: 1.000e+00
Coefficient: −3.22e−02
Value: B
Desulfosoma.profundi
2.0
1.5
1.0
0.5
0.0
A (n=14)
B (n=15)
C (n=11)
surface

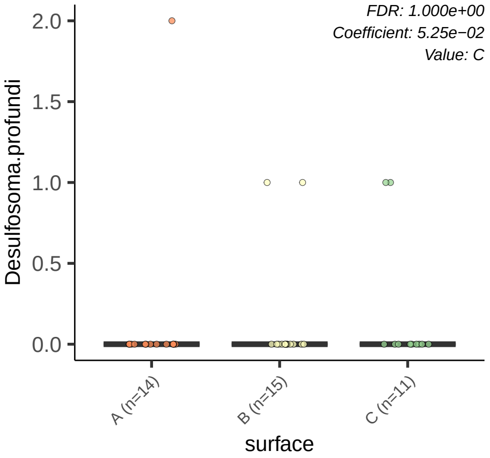
FDR: 1.000e+00
Coefficient: 5.25e−02
Value: C
Desulfosoma.profundi
2.0
1.5
1.0
0.5
0.0
A (n=14)
B (n=15)
C (n=11)
surface

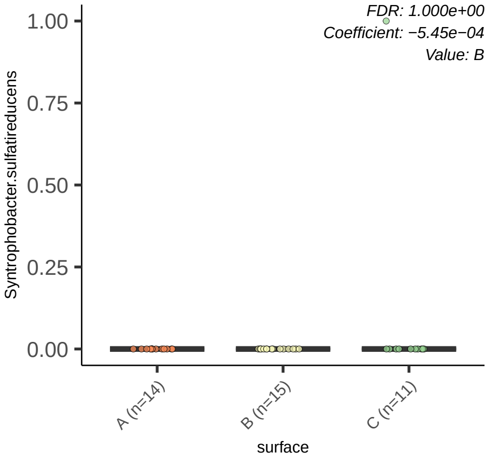
FDR: 1.000e+00
Coefficient: −5.45e−04
Value: B
Syntrophobacter.sulfatireducens
1.00
0.75
0.50
0.25
0.00
A (n=14)
B (n=15)
C (n=11)
surface

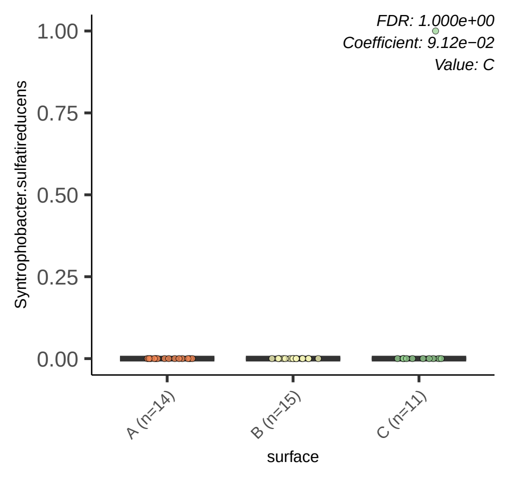
FDR: 1.000e+00
Coefficient: 9.12e−02
Value: C
Syntrophobacter.sulfatireducens
1.00
0.75
0.50
0.25
0.00
A (n=14)
B (n=15)
C (n=11)
surface

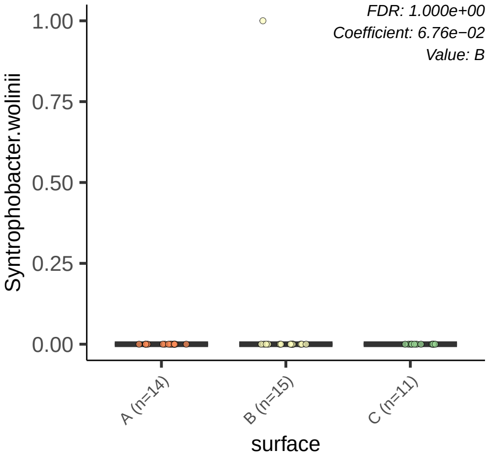
FDR: 1.000e+00
Coefficient: 6.76e−02
Value: B
Syntrophobacter.wolinii
1.00
0.75
0.50
0.25
0.00
A (n=14)
B (n=15)
C (n=11)
surface

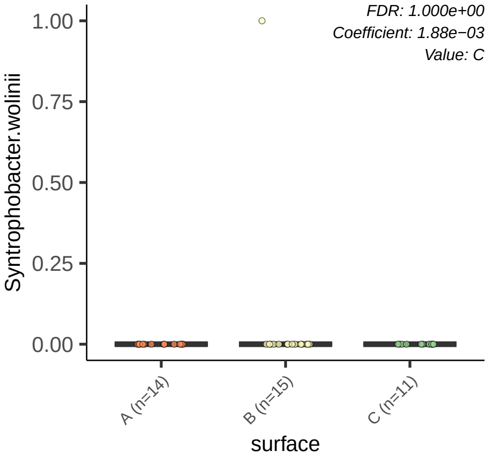
FDR: 1.000e+00
Coefficient: 1.88e−03
Value: C
Syntrophobacter.wolinii
1.00
0.75
0.50
0.25
0.00
A (n=14)
B (n=15)
C (n=11)
surface

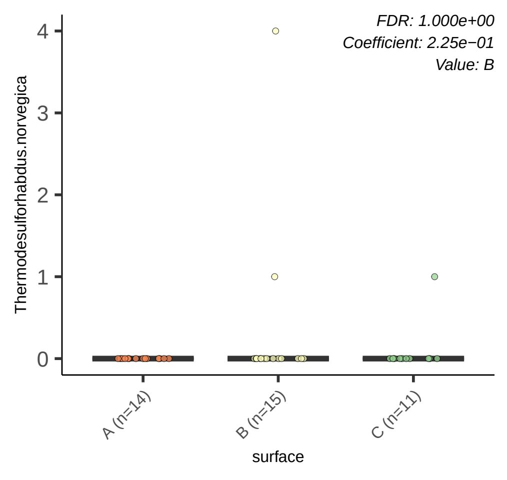
FDR: 1.000e+00
Coefficient: 2.25e−01
Value: B
Thermodesulforhabdus.norvegica
0
1
2
3
4
A (n=14)
B (n=15)
C (n=11)
surface

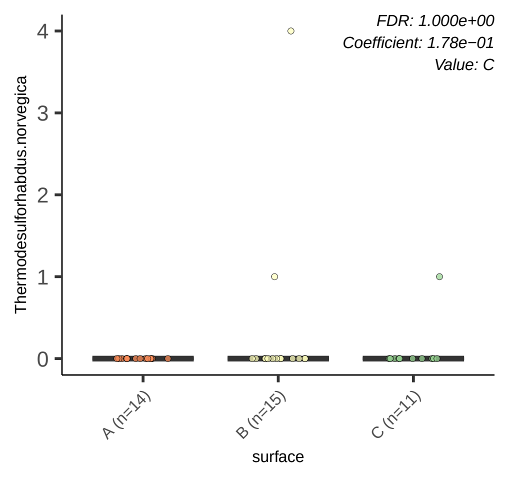

FDR: 1.000e+00
Coefficient: 1.78e−01
Value: C
Thermodesulforhabdus.norvegica
4
3
2
1
0
A (n=14)
B (n=15)
C (n=11)
surface

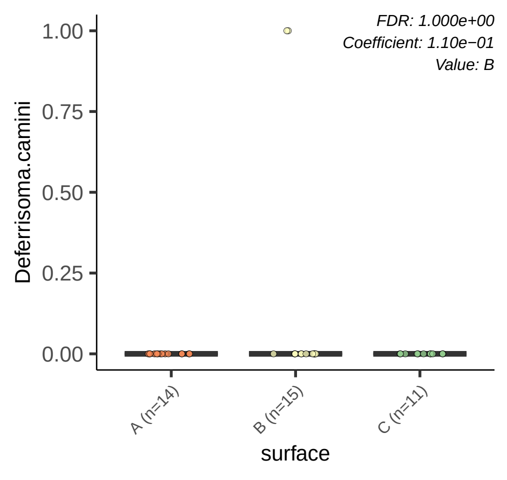
FDR: 1.000e+00
Coefficient: 1.10e−01
Value: B
Deferrisoma.camini
1.00
0.75
0.50
0.25
0.00
A (n=14)
B (n=15)
C (n=11)
surface

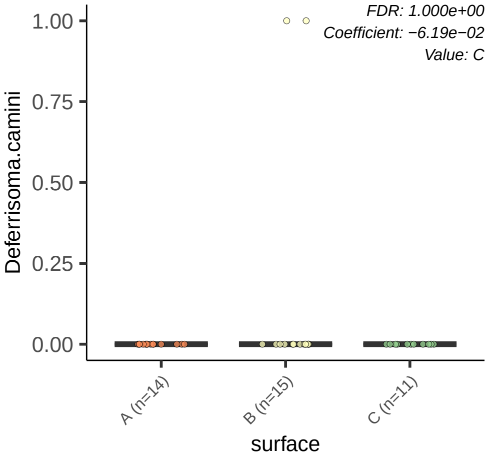

FDR: 1.000e+00
Coefficient: −6.19e−02
Value: C
Deferrisoma.camini
1.00
0.75
0.50
0.25
0.00
A (n=14)
B (n=15)
C (n=11)
surface

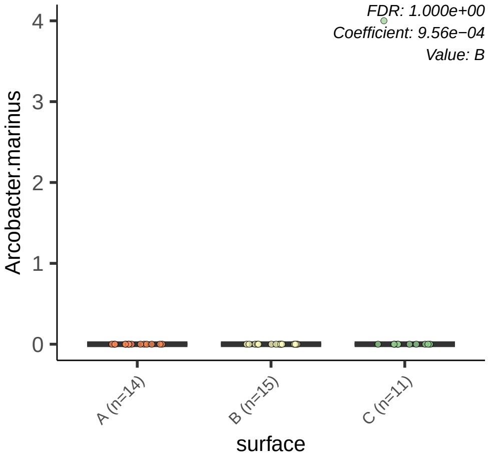
FDR: 1.000e+00
Coefficient: 9.56e−04
Value: B
Arcobacter.marinus
4
3
2
1
0
A (n=14)
B (n=15)
C (n=11)
surface

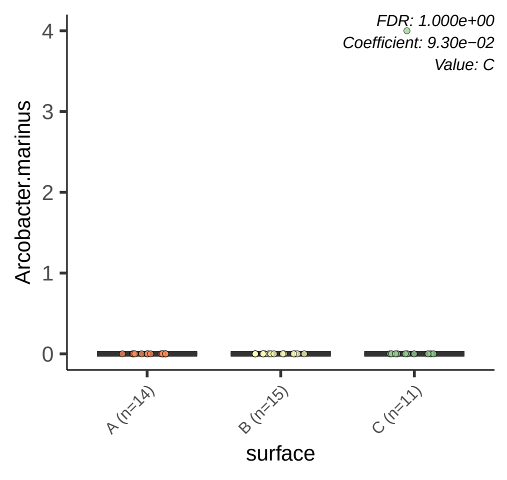

FDR: 1.000e+00
Coefficient: 9.30e−02
Value: C
Arcobacter.marinus
0
1
2
3
4
A (n=14)
B (n=15)
C (n=11)
surface

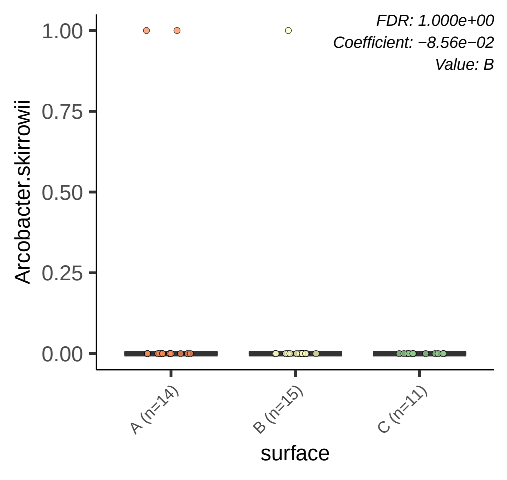

FDR: 1.000e+00
Coefficient: −8.56e−02
Value: B
Arcobacter.skirrowii
1.00
0.75
0.50
0.25
0.00
A (n=14)
B (n=15)
C (n=11)
surface

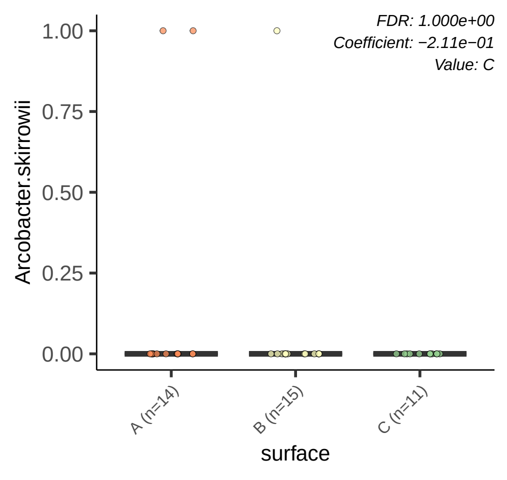
FDR: 1.000e+00
Coefficient: −2.11e−01
Value: C
Arcobacter.skirrowii
1.00
0.75
0.50
0.25
0.00
A (n=14)
B (n=15)
C (n=11)
surface

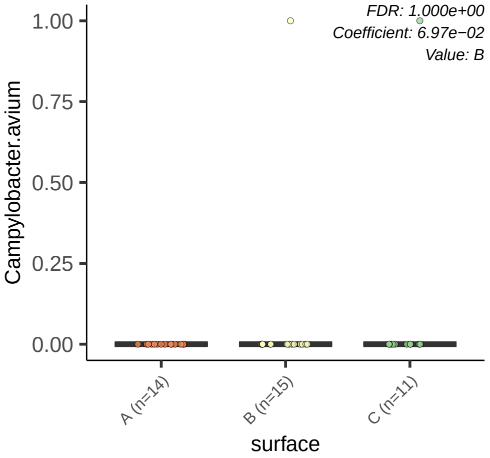

FDR: 1.000e+00
Coefficient: 6.97e−02
Value: B
Campylobacter.avium
1.00
0.75
0.50
0.25
0.00
A (n=14)
B (n=15)
C (n=11)
surface

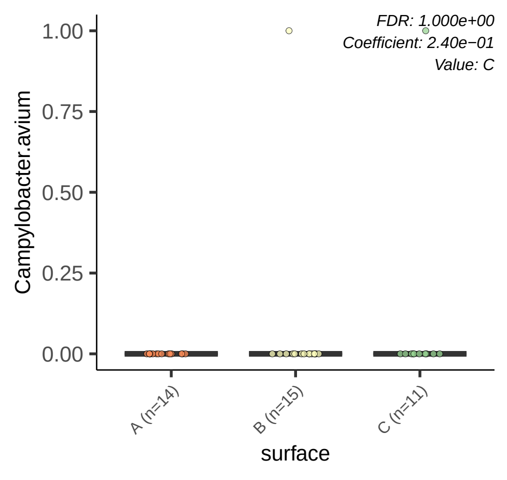
FDR: 1.000e+00
Coefficient: 2.40e−01
Value: C
Campylobacter.avium
1.00
0.75
0.50
0.25
0.00
A (n=14)
B (n=15)
C (n=11)
surface

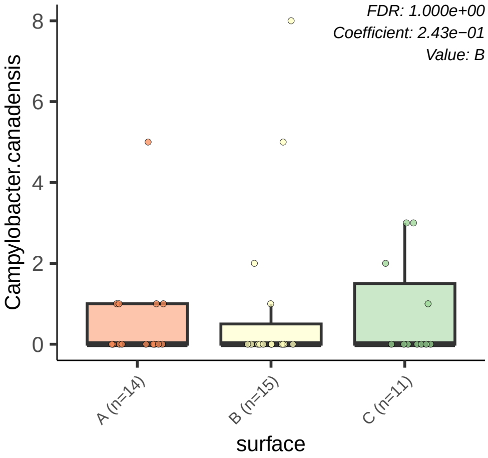
FDR: 1.000e+00
Coefficient: 2.43e−01
Value: B
Campylobacter.canadensis
8
6
4
2
0
A (n=14)
B (n=15)
C (n=11)
surface

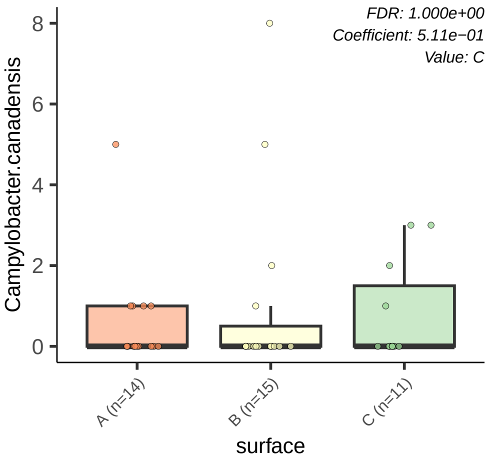

FDR: 1.000e+00
Coefficient: 5.11e−01
Value: C
Campylobacter.canadensis
0
2
4
6
8
A (n=14)
B (n=15)
C (n=11)
surface

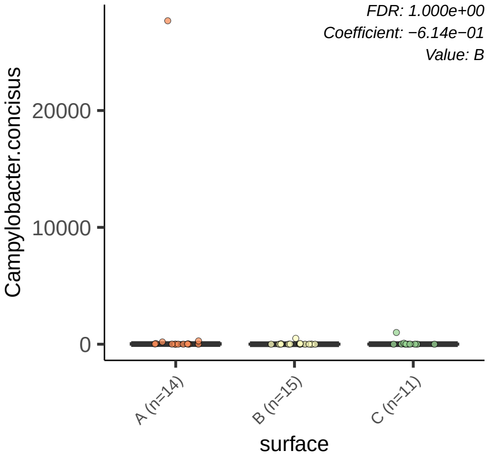
FDR: 1.000e+00
Coefficient: −6.14e−01
Value: B
Campylobacter.concisus
20000
10000
0
A (n=14)
B (n=15)
C (n=11)
surface

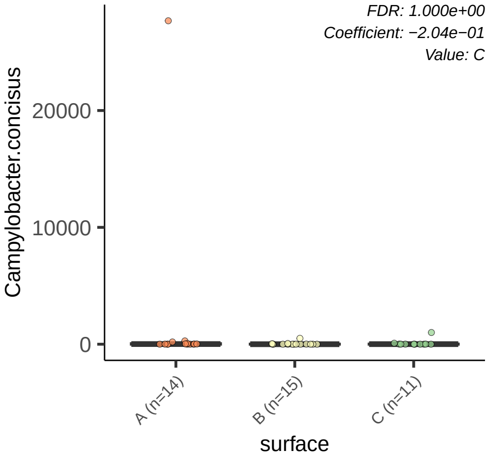

FDR: 1.000e+00
Coefficient: −2.04e−01
Value: C
Campylobacter.concisus
20000
10000
0
A (n=14)
B (n=15)
C (n=11)
surface

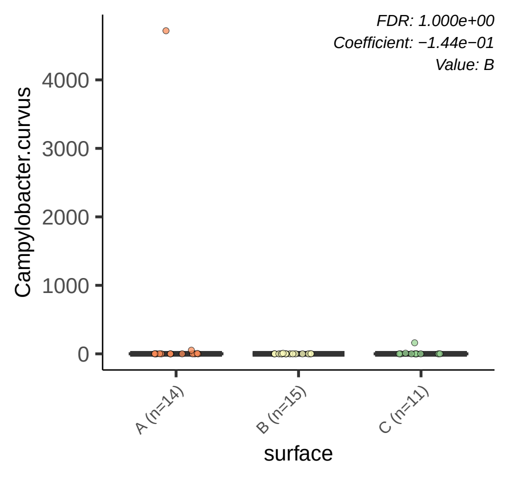

FDR: 1.000e+00
Coefficient: −1.44e−01
Value: B
Campylobacter.curvus
4000
3000
2000
1000
0
A (n=14)
B (n=15)
C (n=11)
surface

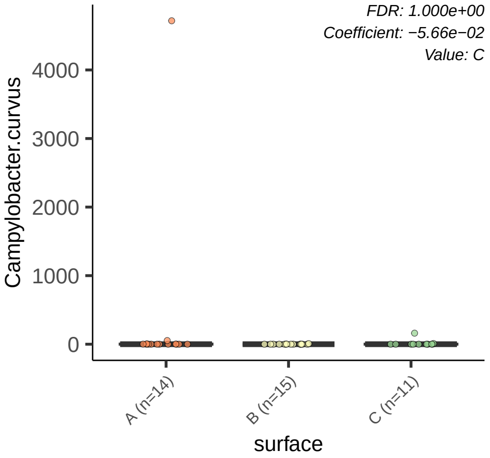
FDR: 1.000e+00
Coefficient: −5.66e−02
Value: C
Campylobacter.curvus
4000
3000
2000
1000
0
A (n=14)
B (n=15)
C (n=11)
surface

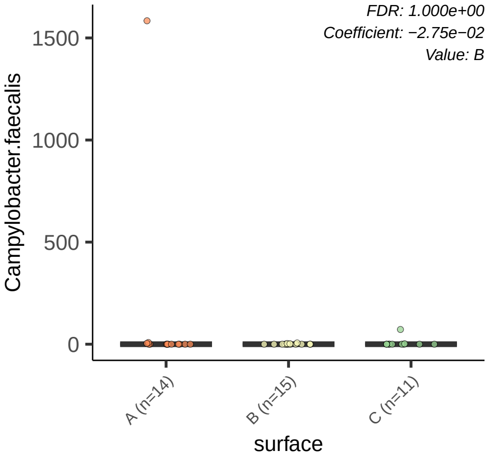
FDR: 1.000e+00
Coefficient: −2.75e−02
Value: B
Campylobacter.faecalis
1500
1000
500
0
A (n=14)
B (n=15)
C (n=11)
surface

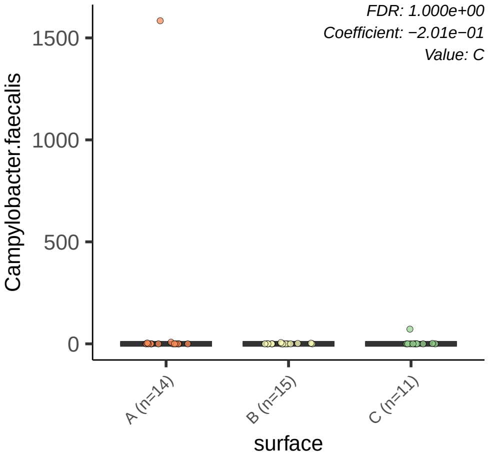

FDR: 1.000e+00
Coefficient: −2.01e−01
Value: C
1500
1000
500
0
Campylobacter.faecalis
A (n=14)
B (n=15)
C (n=11)
surface

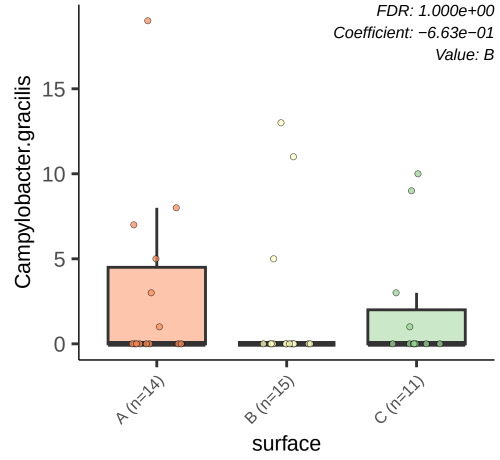

FDR: 1.000e+00
Coefficient: −6.63e−01
Value: B
Campylobacter.gracilis
15
10
5
0
A (n=14)
B (n=15)
C (n=11)
surface

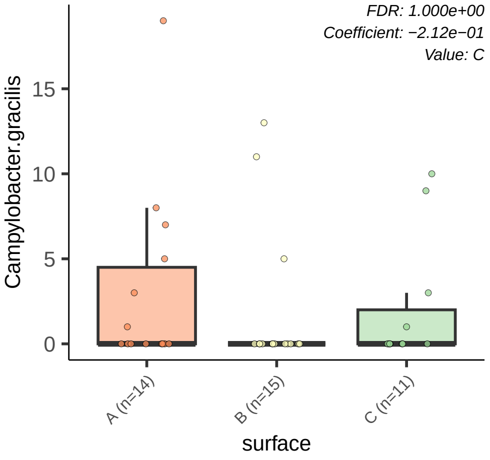

FDR: 1.000e+00
Coefficient: −2.12e−01
Value: C
Campylobacter.gracilis
0
5
10
15
A (n=14)
B (n=15)
C (n=11)
surface

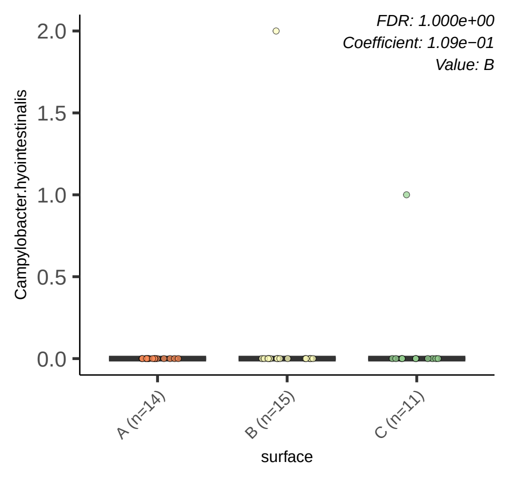

FDR: 1.000e+00
Coefficient: 1.09e−01
Value: B
Campylobacter.hyointestinalis
2.0
1.5
1.0
0.5
0.0
A (n=14)
B (n=15)
C (n=11)
surface

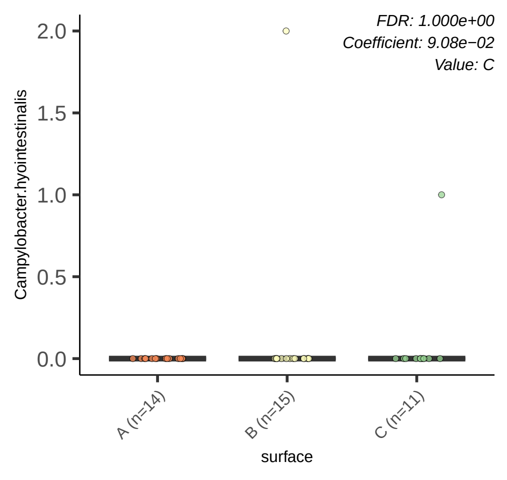
FDR: 1.000e+00
Coefficient: 9.08e−02
Value: C
2.0
1.5
1.0
0.5
0.0
Campylobacter.hyointestinalis
A (n=14)
B (n=15)
C (n=11)
surface

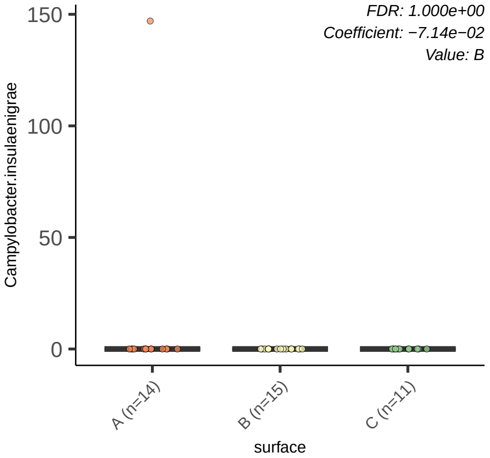

FDR: 1.000e+00
Coefficient: −7.14e−02
Value: B
Campylobacter.insulaenigrae
150
100
50
0
A (n=14)
B (n=15)
C (n=11)
surface

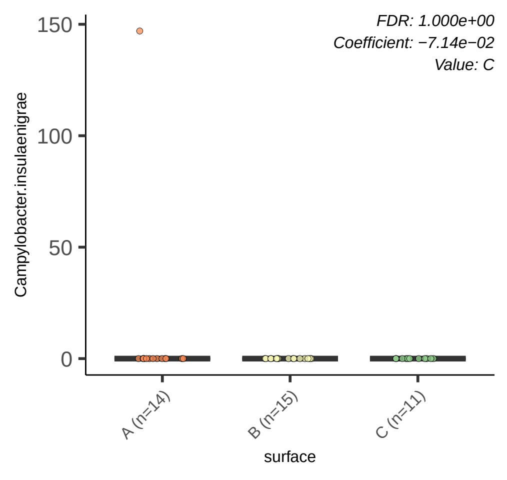
FDR: 1.000e+00
Coefficient: −7.14e−02
Value: C
Campylobacter.insulaenigrae
150
100
50
0
A (n=14)
B (n=15)
C (n=11)
surface

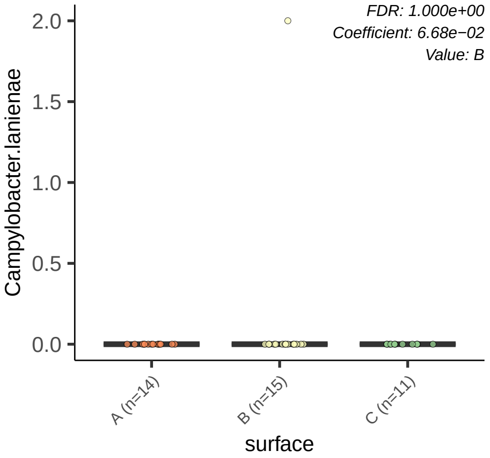

FDR: 1.000e+00
Coefficient: 6.68e−02
Value: B
Campylobacter.lanienae
2.0
1.5
1.0
0.5
0.0
A (n=14)
B (n=15)
C (n=11)
surface

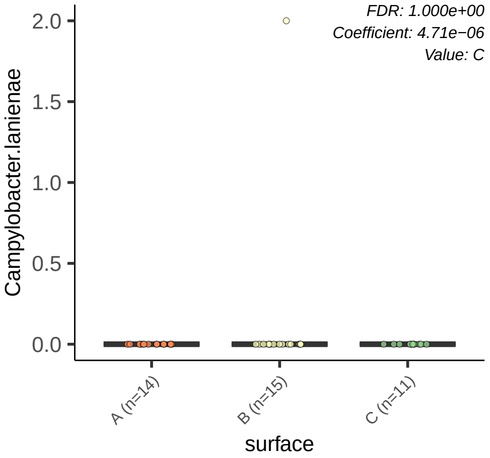
FDR: 1.000e+00
Coefficient: 4.71e−06
Value: C
Campylobacter.lanienae
2.0
1.5
1.0
0.5
0.0
A (n=14)
B (n=15)
C (n=11)
surface

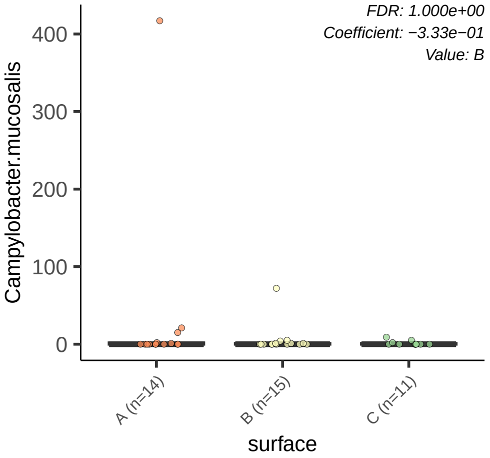
FDR: 1.000e+00
Coefficient: −3.33e−01
Value: B
Campylobacter.mucosalis
400
300
200
100
0
A (n=14)
B (n=15)
C (n=11)
surface

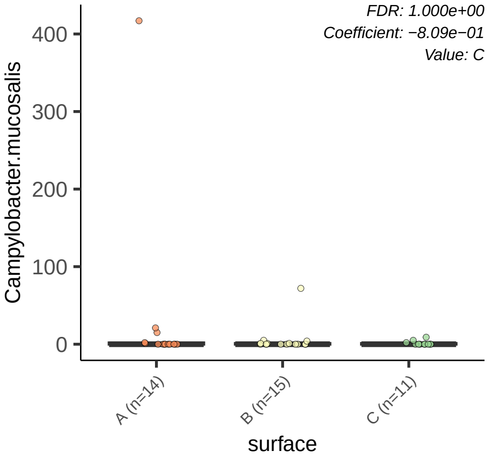
FDR: 1.000e+00
Coefficient: −8.09e−01
Value: C
Campylobacter.mucosalis
400
300
200
100
0
A (n=14)
B (n=15)
C (n=11)
surface

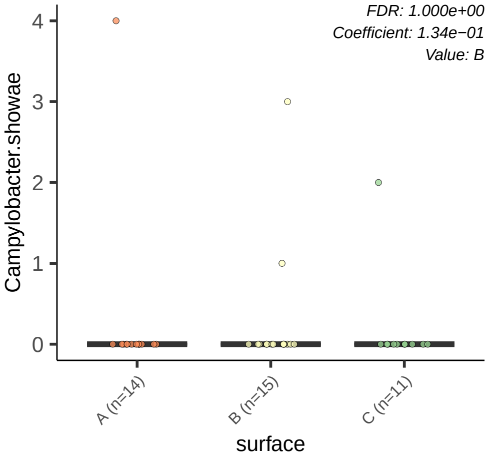
FDR: 1.000e+00
Coefficient: 1.34e−01
Value: B
Campylobacter.showae
0
1
2
3
4
A (n=14)
B (n=15)
C (n=11)
surface

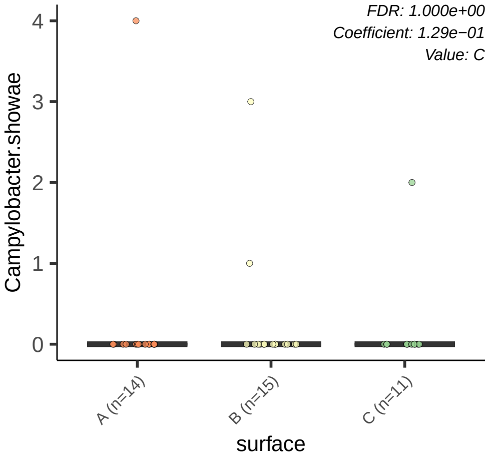

FDR: 1.000e+00
Coefficient: 1.29e−01
Value: C
Campylobacter.showae
0
1
2
3
4
A (n=14)
B (n=15)
C (n=11)
surface

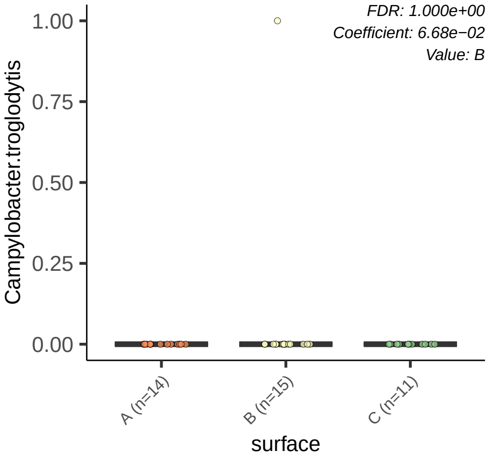
FDR: 1.000e+00
Coefficient: 6.68e−02
Value: B
Campylobacter.troglodytis
1.00
0.75
0.50
0.25
0.00
A (n=14)
B (n=15)
C (n=11)
surface

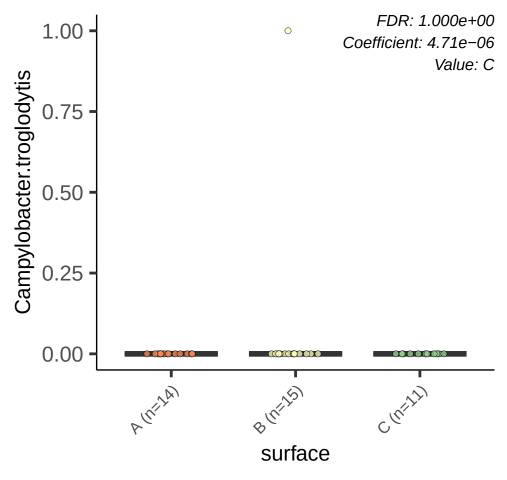
FDR: 1.000e+00
Coefficient: 4.71e−06
Value: C
Campylobacter.troglodytis
1.00
0.75
0.50
0.25
0.00
A (n=14)
B (n=15)
C (n=11)
surface

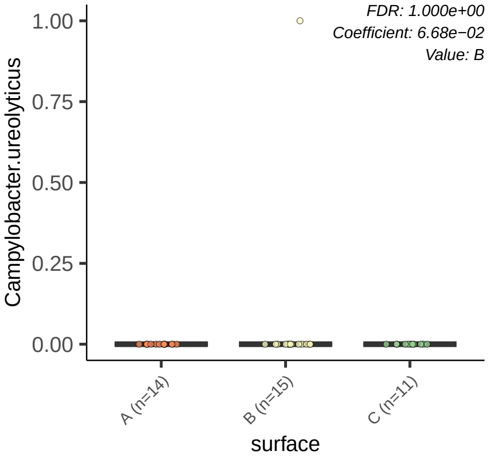

FDR: 1.000e+00
Coefficient: 6.68e−02
Value: B
Campylobacter.ureolyticus
1.00
0.75
0.50
0.25
0.00
A (n=14)
B (n=15)
C (n=11)
surface

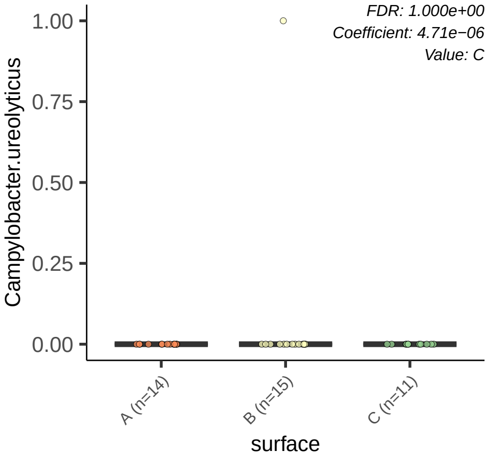
FDR: 1.000e+00
Coefficient: 4.71e−06
Value: C
Campylobacter.ureolyticus
1.00
0.75
0.50
0.25
0.00
A (n=14)
B (n=15)
C (n=11)
surface

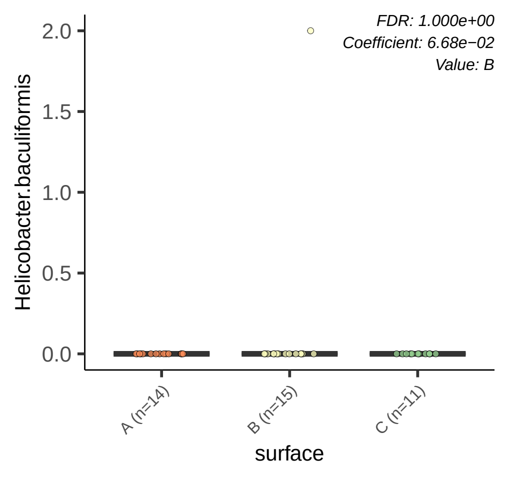
FDR: 1.000e+00
Coefficient: 6.68e−02
Value: B
Helicobacter.baculiformis
2.0
1.5
1.0
0.5
0.0
A (n=14)
B (n=15)
C (n=11)
surface

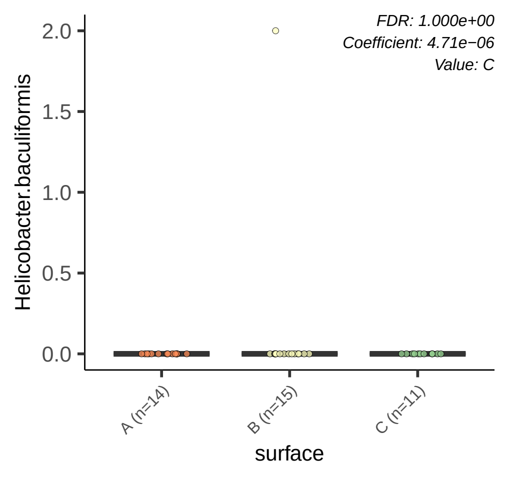
FDR: 1.000e+00
Coefficient: 4.71e−06
Value: C
Helicobacter.baculiformis
2.0
1.5
1.0
0.5
0.0
A (n=14)
B (n=15)
C (n=11)
surface

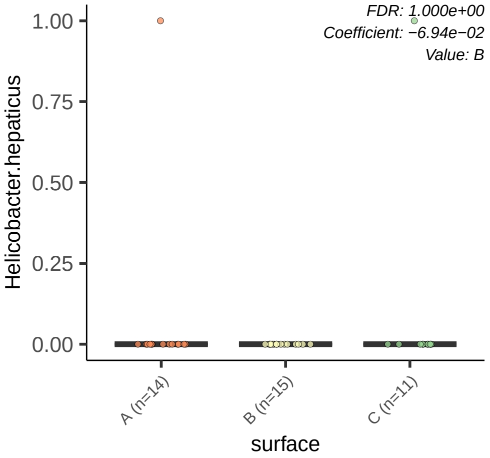

FDR: 1.000e+00
Coefficient: −6.94e−02
Value: B
Helicobacter.hepaticus
1.00
0.75
0.50
0.25
0.00
A (n=14)
B (n=15)
C (n=11)
surface

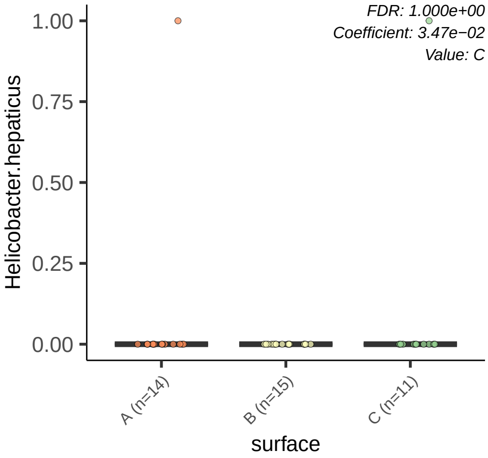
FDR: 1.000e+00
Coefficient: 3.47e−02
Value: C
Helicobacter.hepaticus
1.00
0.75
0.50
0.25
0.00
A (n=14)
B (n=15)
C (n=11)
surface

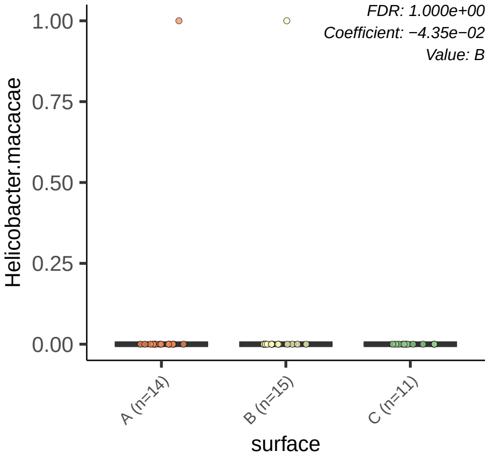

FDR: 1.000e+00
Coefficient: −4.35e−02
Value: B
Helicobacter.macacae
1.00
0.75
0.50
0.25
0.00
A (n=14)
B (n=15)
C (n=11)
surface

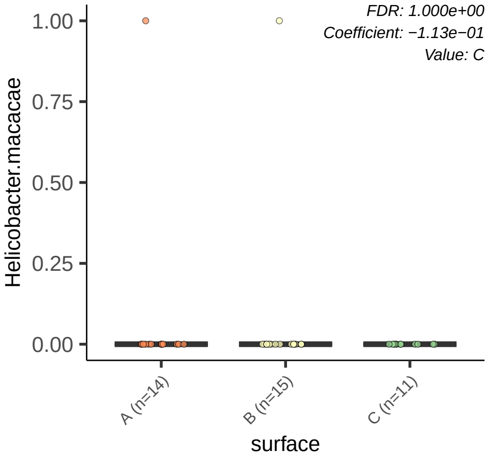
FDR: 1.000e+00
Coefficient: −1.13e−01
Value: C
Helicobacter.macacae
1.00
0.75
0.50
0.25
0.00
A (n=14)
B (n=15)
C (n=11)
surface

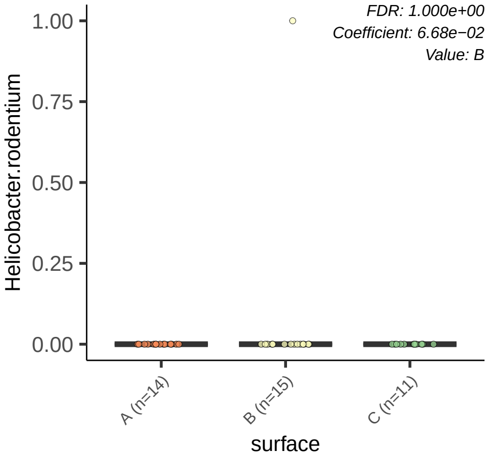
FDR: 1.000e+00
Coefficient: 6.68e−02
Value: B
1.00
0.75
0.50
0.25
0.00
Helicobacter.rodentium
A (n=14)
B (n=15)
C (n=11)
surface

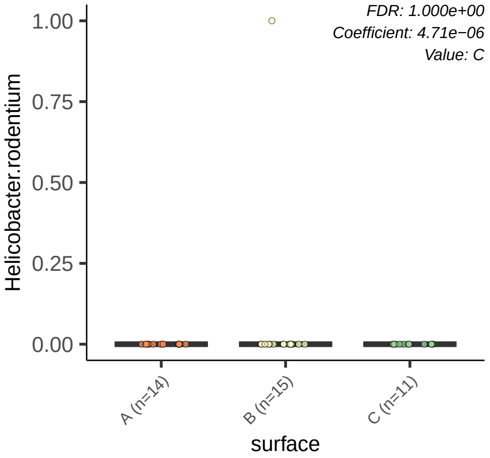
FDR: 1.000e+00
Coefficient: 4.71e−06
Value: C
Helicobacter.rodentium
1.00
0.75
0.50
0.25
0.00
A (n=14)
B (n=15)
C (n=11)
surface

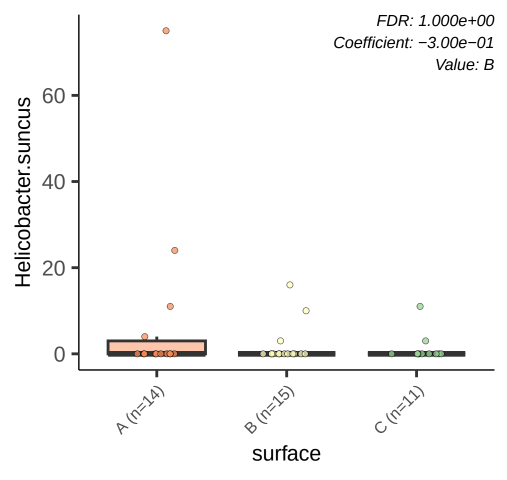

FDR: 1.000e+00
Coefficient: −3.00e−01
Value: B
Helicobacter.suncus
0
20
40
60
A (n=14)
B (n=15)
C (n=11)
surface

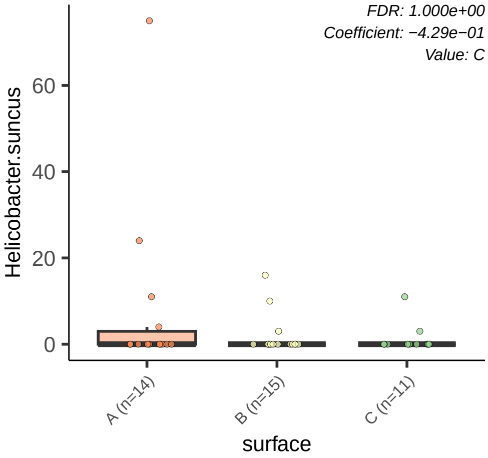

FDR: 1.000e+00
Coefficient: −4.29e−01
Value: C
Helicobacter.suncus
0
20
40
60
A (n=14)
B (n=15)
C (n=11)
surface

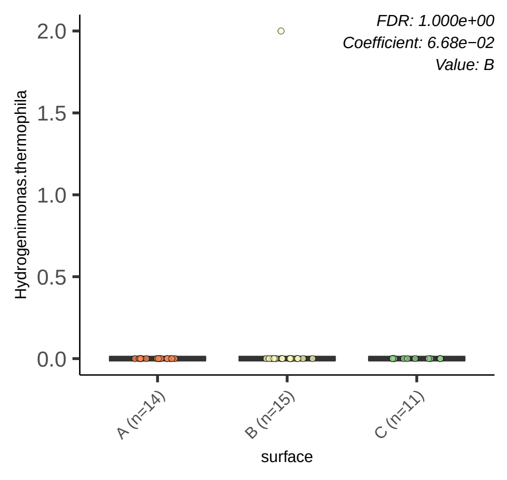
FDR: 1.000e+00
Coefficient: 6.68e−02
Value: B
Hydrogenimonas.thermophila
2.0
1.5
1.0
0.5
0.0
A (n=14)
B (n=15)
C (n=11)
surface

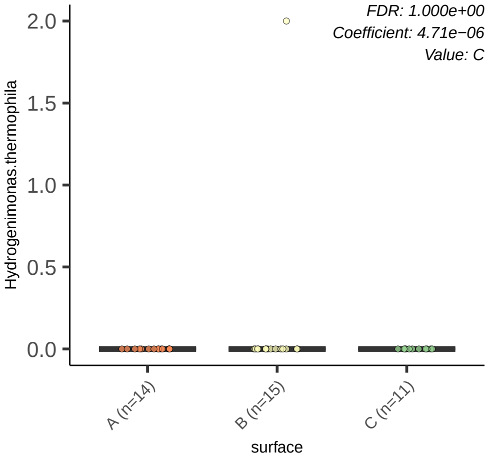
FDR: 1.000e+00
Coefficient: 4.71e−06
Value: C
Hydrogenimonas.thermophila
2.0
1.5
1.0
0.5
0.0
A (n=14)
B (n=15)
C (n=11)
surface

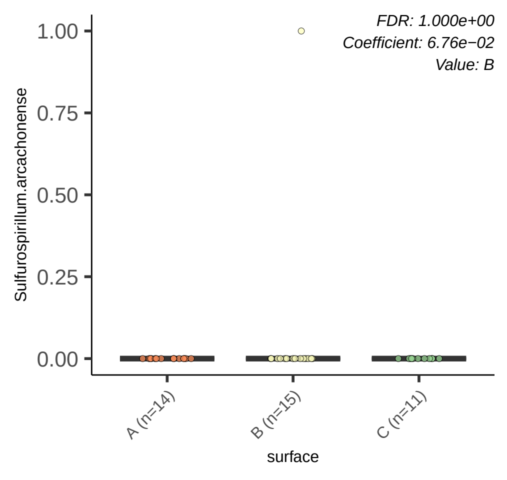

FDR: 1.000e+00
Coefficient: 6.76e−02
Value: B
Sulfurospirillum.arcachonense
1.00
0.75
0.50
0.25
0.00
A (n=14)
B (n=15)
C (n=11)
surface

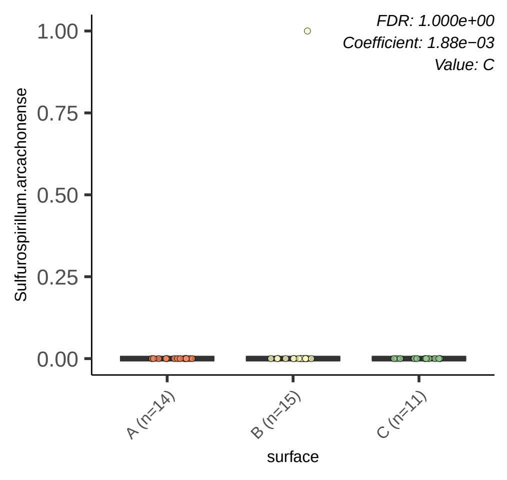

FDR: 1.000e+00
Coefficient: 1.88e−03
Value: C
Sulfurospirillum.arcachonense
1.00
0.75
0.50
0.25
0.00
A (n=14)
B (n=15)
C (n=11)
surface

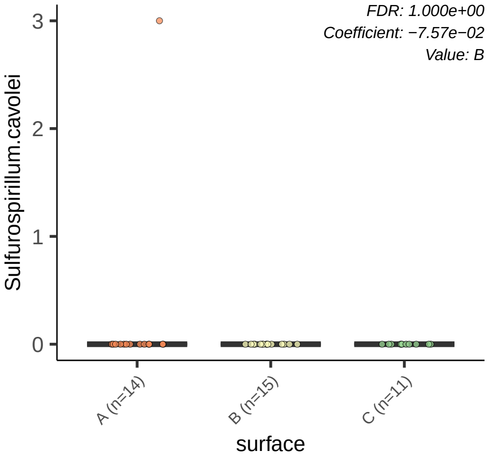

FDR: 1.000e+00
Coefficient: −7.57e−02
Value: B
Sulfurospirillum.cavolei
0
1
2
3
A (n=14)
B (n=15)
C (n=11)
surface

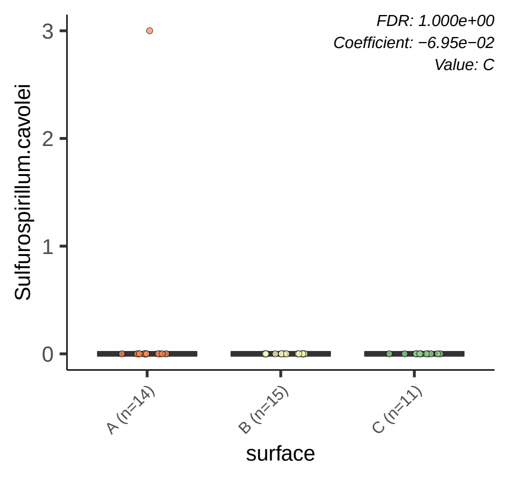

FDR: 1.000e+00
Coefficient: −6.95e−02
Value: C
Sulfurospirillum.cavolei
3
2
1
0
A (n=14)
B (n=15)
C (n=11)
surface

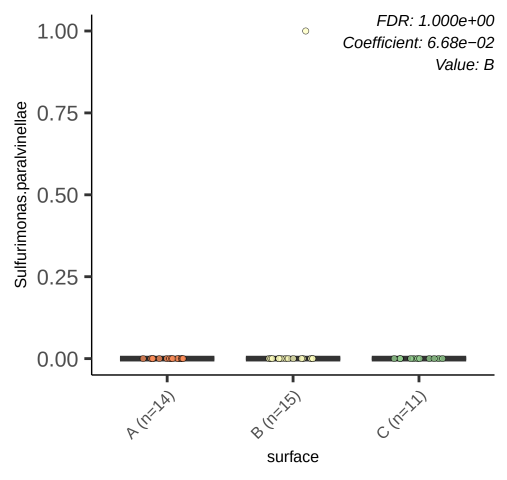
FDR: 1.000e+00
Coefficient: 6.68e−02
Value: B
Sulfurimonas.paralvinellae
1.00
0.75
0.50
0.25
0.00
A (n=14)
B (n=15)
C (n=11)
surface

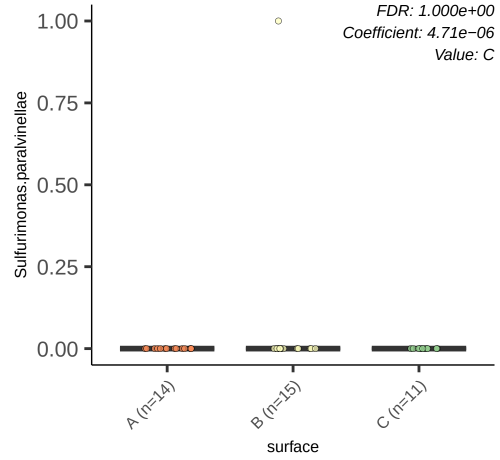

FDR: 1.000e+00
Coefficient: 4.71e−06
Value: C
1.00
0.75
0.50
0.25
0.00
Sulfurimonas.paralvinellae
A (n=14)
B (n=15)
C (n=11)
surface

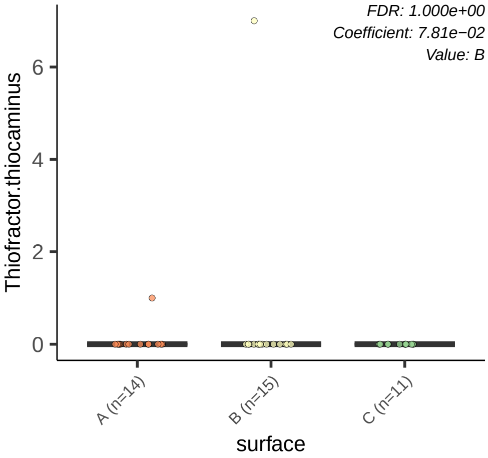

FDR: 1.000e+00
Coefficient: 7.81e−02
Value: B
Thiofractor.thiocaminus
0
2
4
6
A (n=14)
B (n=15)
C (n=11)
surface

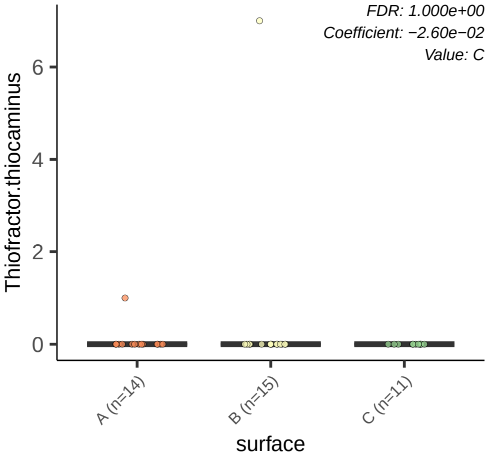
FDR: 1.000e+00
Coefficient: −2.60e−02
Value: C
Thiofractor.thiocaminus
0
2
4
6
A (n=14)
B (n=15)
C (n=11)
surface

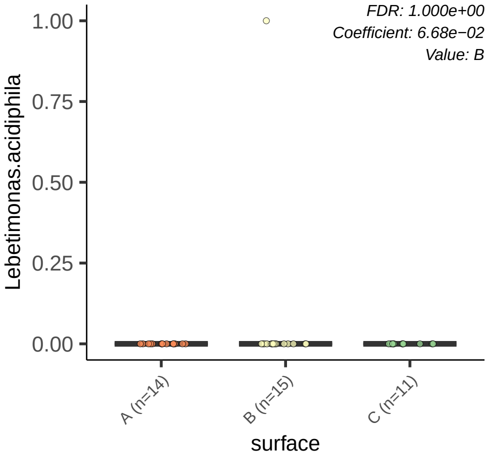

FDR: 1.000e+00
Coefficient: 6.68e−02
Value: B
Lebetimonas.acidiphila
1.00
0.75
0.50
0.25
0.00
A (n=14)
B (n=15)
C (n=11)
surface

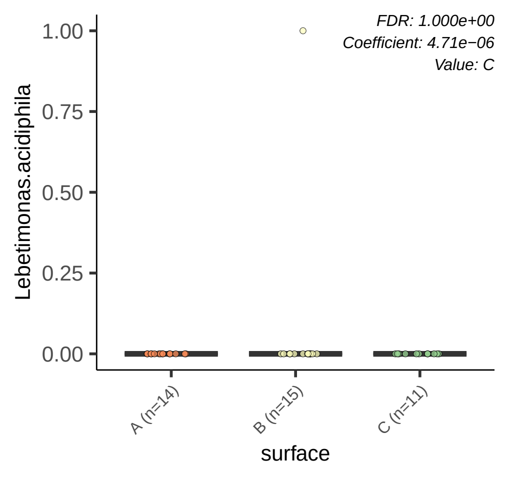

FDR: 1.000e+00
Coefficient: 4.71e−06
Value: C
Lebetimonas.acidiphila
1.00
0.75
0.50
0.25
0.00
A (n=14)
B (n=15)
C (n=11)
surface

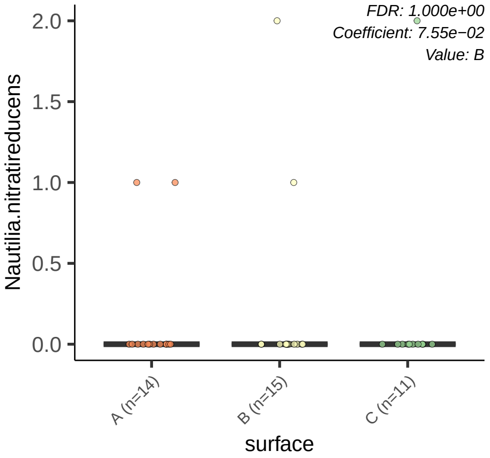
FDR: 1.000e+00
Coefficient: 7.55e−02
Value: B
Nautilia.nitratireducens
2.0
1.5
1.0
0.5
0.0
A (n=14)
B (n=15)
C (n=11)
surface

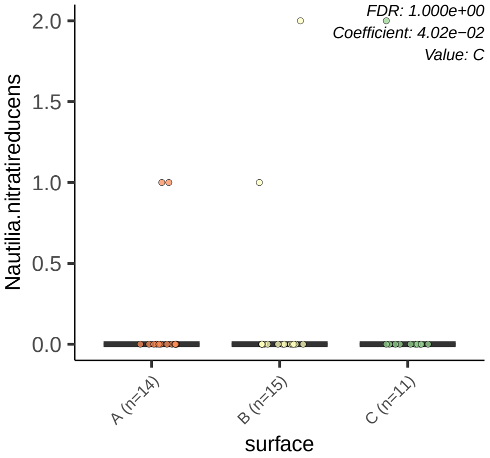
FDR: 1.000e+00
Coefficient: 4.02e−02
Value: C
Nautilia.nitratireducens
2.0
1.5
1.0
0.5
0.0
A (n=14)
B (n=15)
C (n=11)
surface

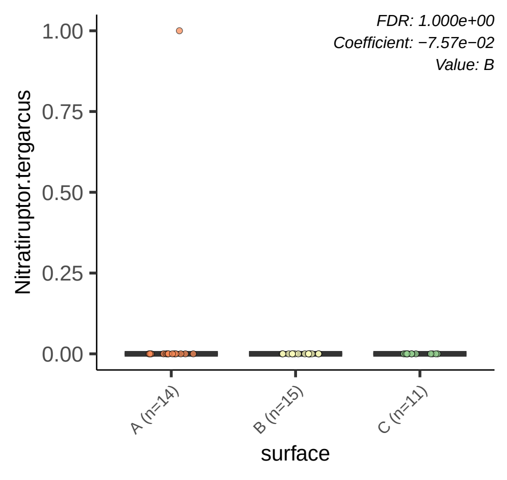
FDR: 1.000e+00
Coefficient: −7.57e−02
Value: B
Nitratiruptor.tergarcus
1.00
0.75
0.50
0.25
0.00
A (n=14)
B (n=15)
C (n=11)
surface

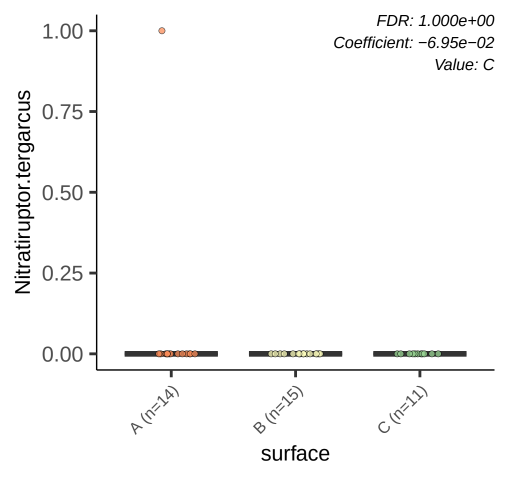

FDR: 1.000e+00
Coefficient: −6.95e−02
Value: C
Nitratiruptor.tergarcus
1.00
0.75
0.50
0.25
0.00
A (n=14)
B (n=15)
C (n=11)
surface

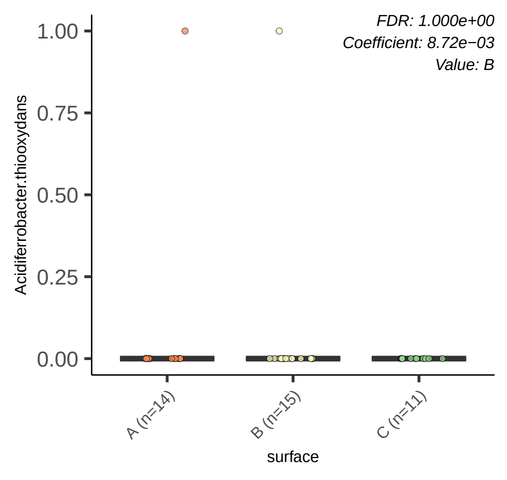

FDR: 1.000e+00
Coefficient: 8.72e−03
Value: B
Acidiferrobacter.thiooxydans
1.00
0.75
0.50
0.25
0.00
A (n=14)
B (n=15)
C (n=11)
surface

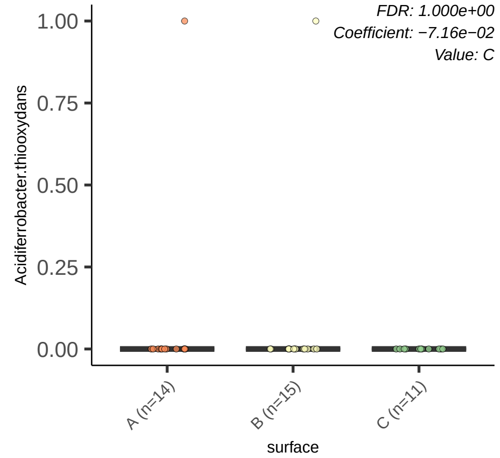
FDR: 1.000e+00
Coefficient: −7.16e−02
Value: C
Acidiferrobacter.thiooxydans
1.00
0.75
0.50
0.25
0.00
A (n=14)
B (n=15)
C (n=11)
surface

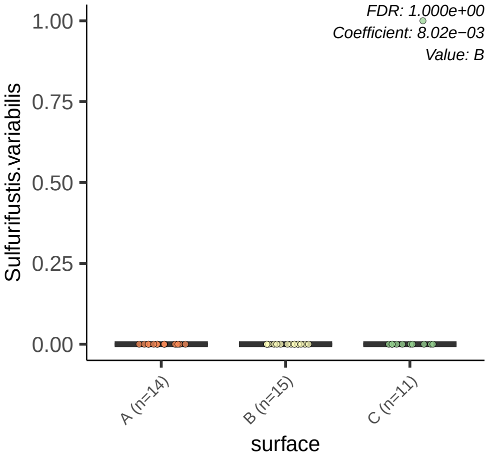

FDR: 1.000e+00
Coefficient: 8.02e−03
Value: B
Sulfurifustis.variabilis
1.00
0.75
0.50
0.25
0.00
A (n=14)
B (n=15)
C (n=11)
surface

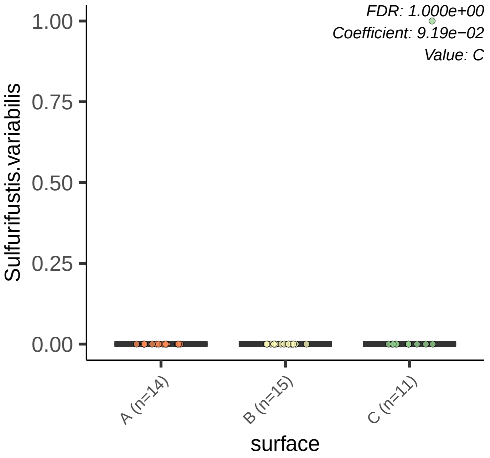
FDR: 1.000e+00
Coefficient: 9.19e−02
Value: C
Sulfurifustis.variabilis
1.00
0.75
0.50
0.25
0.00
A (n=14)
B (n=15)
C (n=11)
surface

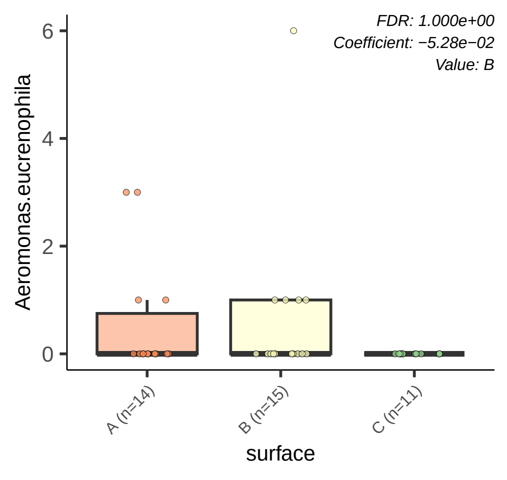
FDR: 1.000e+00
Coefficient: −5.28e−02
Value: B
Aeromonas.eucrenophila
0
2
4
6
A (n=14)
B (n=15)
C (n=11)
surface

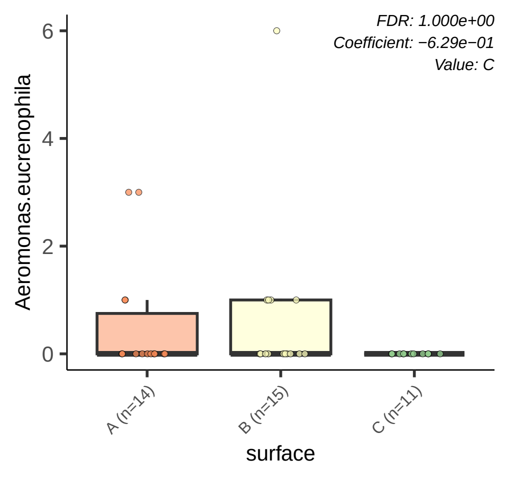
FDR: 1.000e+00
Coefficient: −6.29e−01
Value: C
Aeromonas.eucrenophila
0
2
4
6
A (n=14)
B (n=15)
C (n=11)
surface

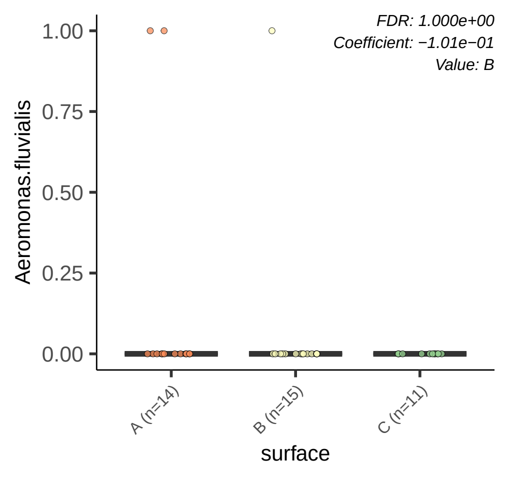
FDR: 1.000e+00
Coefficient: −1.01e−01
Value: B
Aeromonas.fluvialis
1.00
0.75
0.50
0.25
0.00
A (n=14)
B (n=15)
C (n=11)
surface

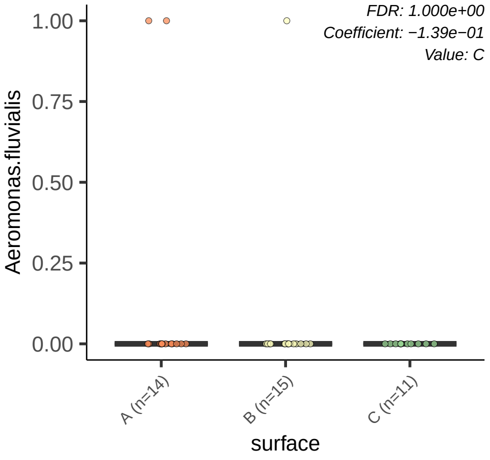

FDR: 1.000e+00
Coefficient: −1.39e−01
Value: C
Aeromonas.fluvialis
1.00
0.75
0.50
0.25
0.00
A (n=14)
B (n=15)
C (n=11)
surface

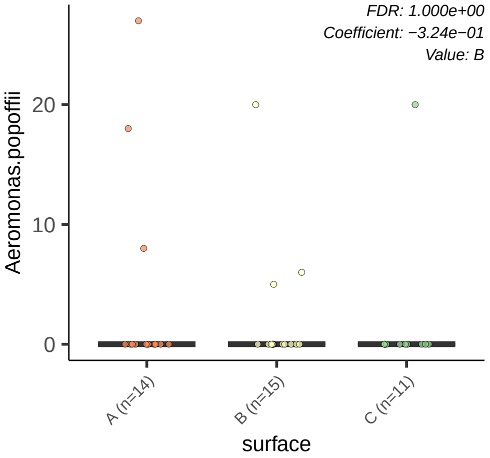

FDR: 1.000e+00
Coefficient: −3.24e−01
Value: B
Aeromonas.popoffii
0
10
20
A (n=14)
B (n=15)
C (n=11)
surface

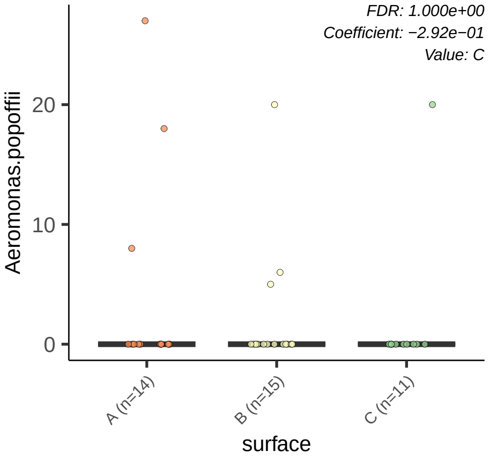

FDR: 1.000e+00
Coefficient: −2.92e−01
Value: C
Aeromonas.popoffii
0
10
20
A (n=14)
B (n=15)
C (n=11)
surface

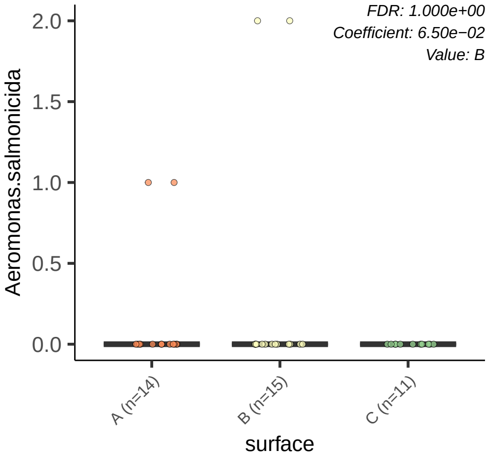
FDR: 1.000e+00
Coefficient: 6.50e−02
Value: B
Aeromonas.salmonicida
2.0
1.5
1.0
0.5
0.0
A (n=14)
B (n=15)
C (n=11)
surface

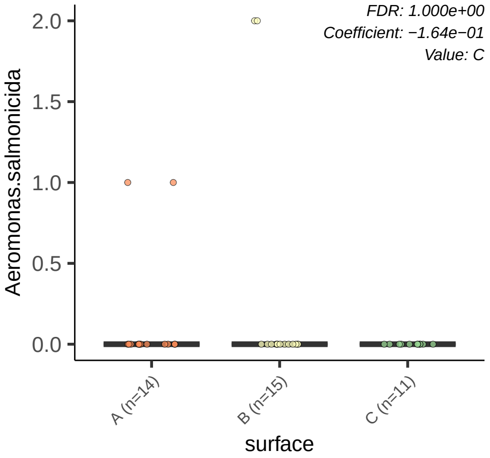
FDR: 1.000e+00
Coefficient: −1.64e−01
Value: C
Aeromonas.salmonicida
2.0
1.5
1.0
0.5
0.0
A (n=14)
B (n=15)
C (n=11)
surface

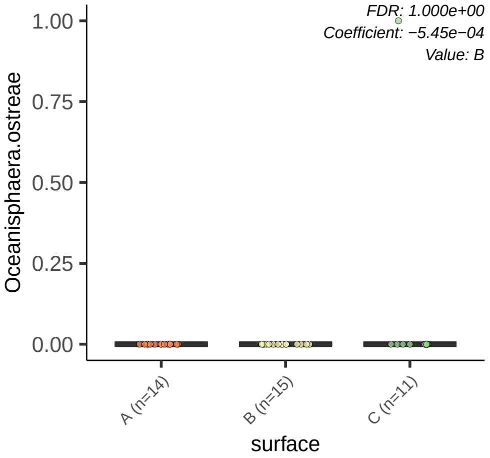

FDR: 1.000e+00
Coefficient: −5.45e−04
Value: B
Oceanisphaera.ostreae
1.00
0.75
0.50
0.25
0.00
A (n=14)
B (n=15)
C (n=11)
surface

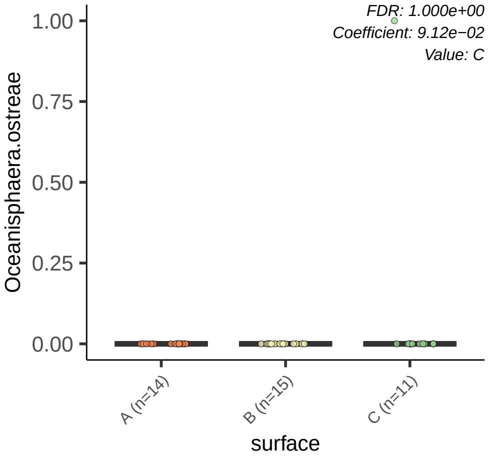
FDR: 1.000e+00
Coefficient: 9.12e−02
Value: C
Oceanisphaera.ostreae
1.00
0.75
0.50
0.25
0.00
A (n=14)
B (n=15)
C (n=11)
surface

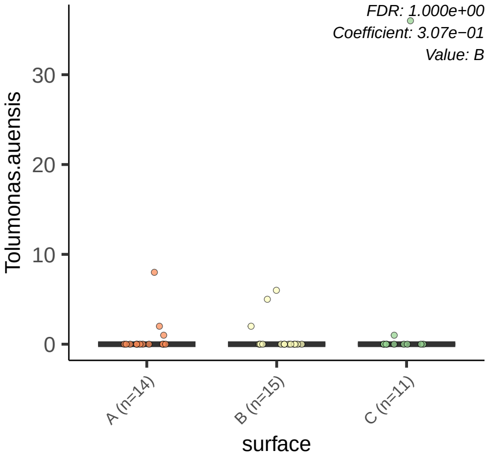

FDR: 1.000e+00
Coefficient: 3.07e−01
Value: B
Tolumonas.auensis
0
10
20
30
A (n=14)
B (n=15)
C (n=11)
surface

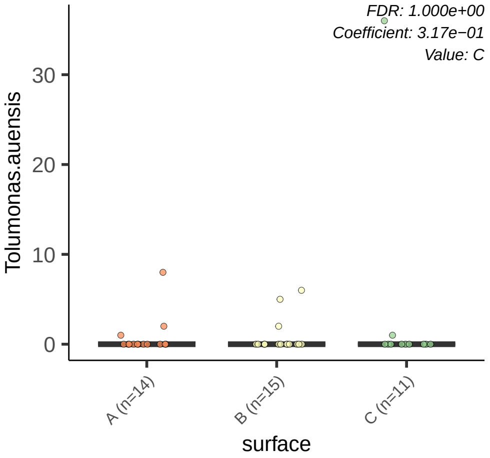

FDR: 1.000e+00
Coefficient: 3.17e−01
Value: C
Tolumonas.auensis
0
10
20
30
A (n=14)
B (n=15)
C (n=11)
surface

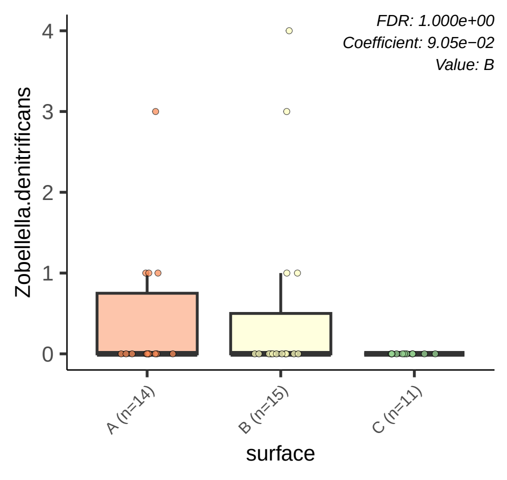

FDR: 1.000e+00
Coefficient: 9.05e−02
Value: B
Zobellella.denitrificans
0
1
2
3
4
A (n=14)
B (n=15)
C (n=11)
surface

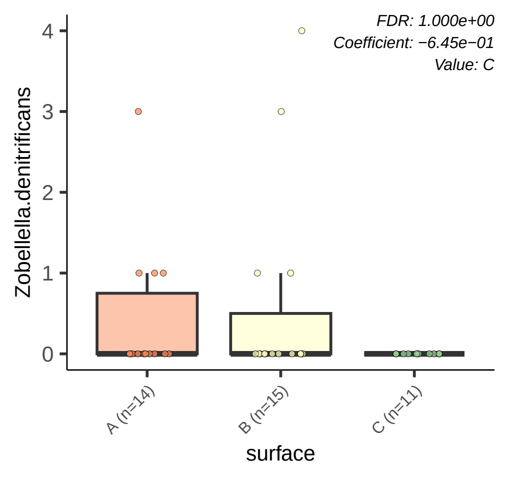

FDR: 1.000e+00
Coefficient: −6.45e−01
Value: C
Zobellella.denitrificans
0
1
2
3
4
A (n=14)
B (n=15)
C (n=11)
surface

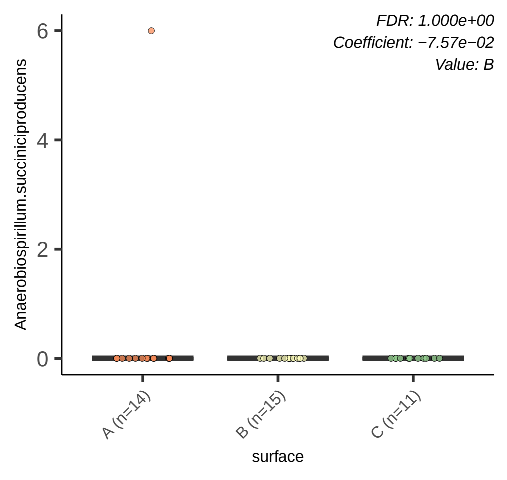
FDR: 1.000e+00
Coefficient: −7.57e−02
Value: B
Anaerobiospirillum.succiniciproducens
6
4
2
0
A (n=14)
B (n=15)
C (n=11)
surface

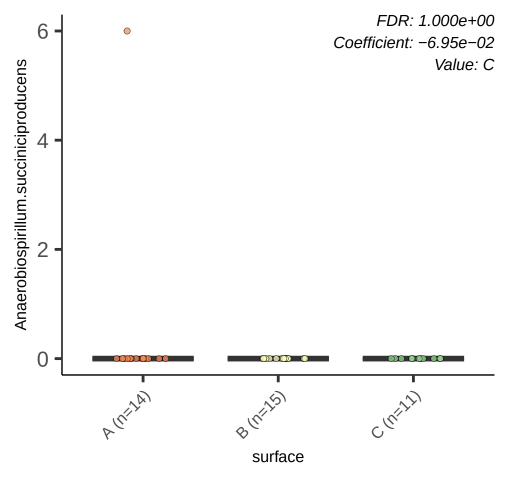
FDR: 1.000e+00
Coefficient: −6.95e−02
Value: C
Anaerobiospirillum.succiniciproducens
6
4
2
0
A (n=14)
B (n=15)
C (n=11)
surface

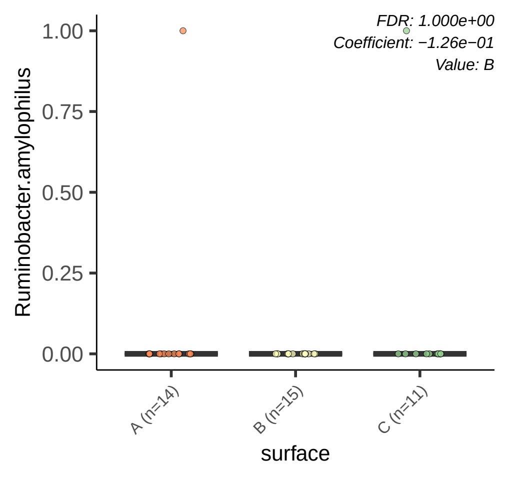
FDR: 1.000e+00
Coefficient: −1.26e−01
Value: B
Ruminobacter.amylophilus
1.00
0.75
0.50
0.25
0.00
A (n=14)
B (n=15)
C (n=11)
surface

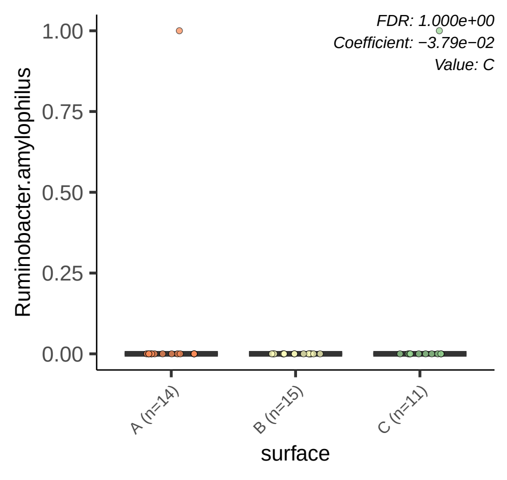

FDR: 1.000e+00
Coefficient: −3.79e−02
Value: C
Ruminobacter.amylophilus
1.00
0.75
0.50
0.25
0.00
A (n=14)
B (n=15)
C (n=11)
surface

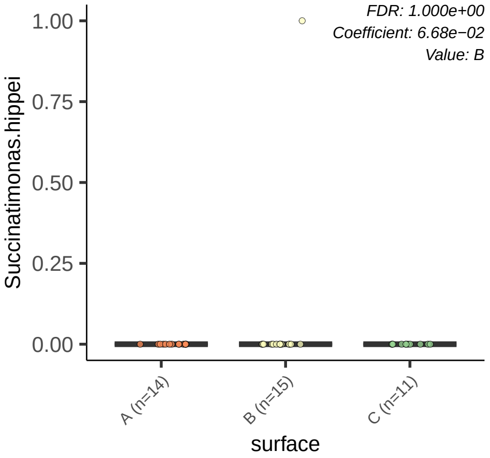
FDR: 1.000e+00
Coefficient: 6.68e−02
Value: B
1.00
0.75
0.50
0.25
0.00
Succinatimonas.hippei
A (n=14)
B (n=15)
C (n=11)
surface

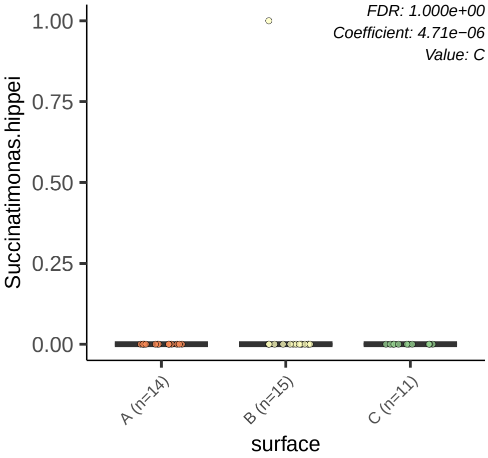

FDR: 1.000e+00
Coefficient: 4.71e−06
Value: C
Succinatimonas.hippei
1.00
0.75
0.50
0.25
0.00
A (n=14)
B (n=15)
C (n=11)
surface

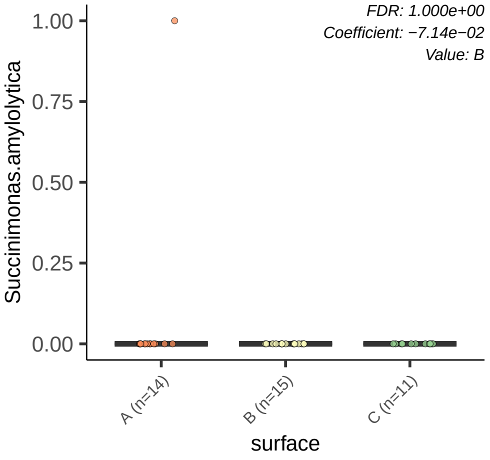

FDR: 1.000e+00
Coefficient: −7.14e−02
Value: B
Succinimonas.amylolytica
1.00
0.75
0.50
0.25
0.00
A (n=14)
B (n=15)
C (n=11)
surface

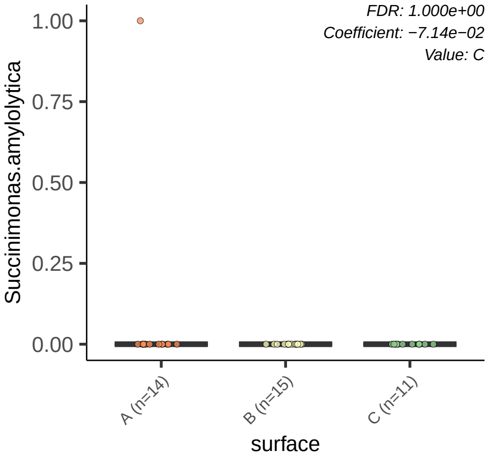

FDR: 1.000e+00
Coefficient: −7.14e−02
Value: C
Succinimonas.amylolytica
1.00
0.75
0.50
0.25
0.00
A (n=14)
B (n=15)
C (n=11)
surface

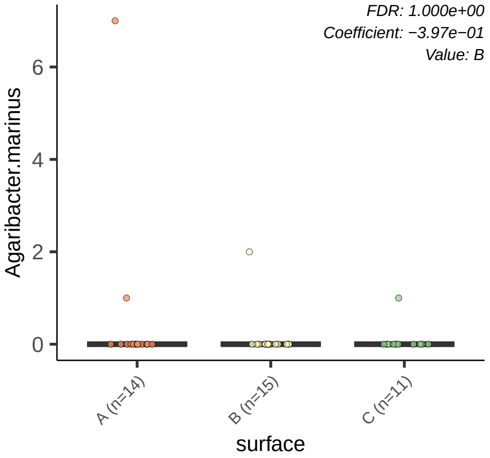

FDR: 1.000e+00
Coefficient: −3.97e−01
Value: B
Agaribacter.marinus
0
2
4
6
A (n=14)
B (n=15)
C (n=11)
surface

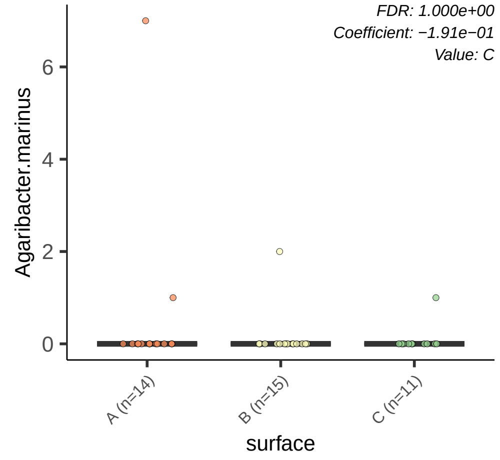

FDR: 1.000e+00
Coefficient: −1.91e−01
Value: C
Agaribacter.marinus
0
2
4
6
A (n=14)
B (n=15)
C (n=11)
surface

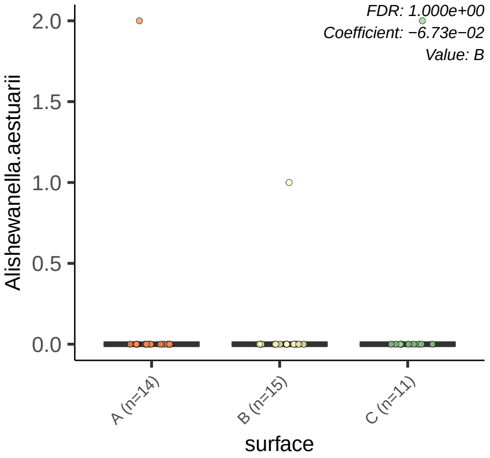

FDR: 1.000e+00
Coefficient: −6.73e−02
Value: B
Alishewanella.aestuarii
2.0
1.5
1.0
0.5
0.0
A (n=14)
B (n=15)
C (n=11)
surface

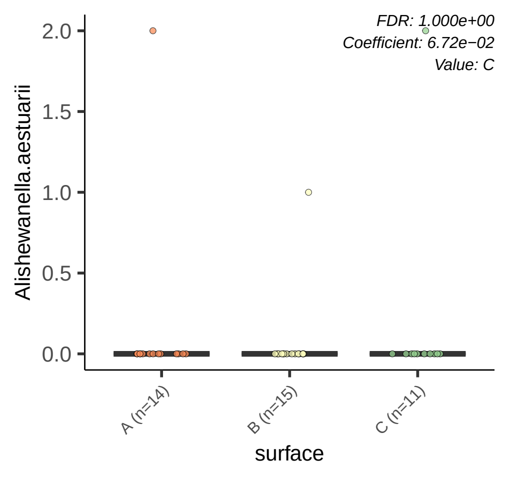
FDR: 1.000e+00
Coefficient: 6.72e−02
Value: C
Alishewanella.aestuarii
2.0
1.5
1.0
0.5
0.0
A (n=14)
B (n=15)
C (n=11)
surface

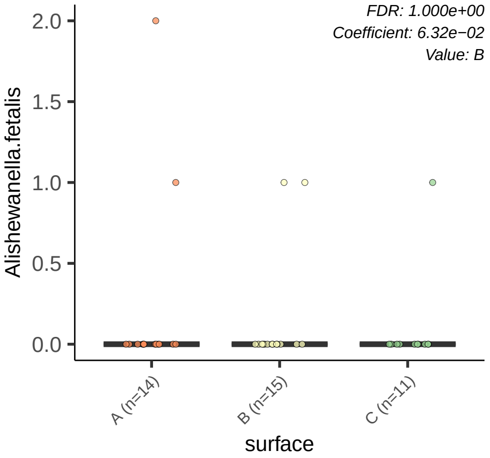

FDR: 1.000e+00
Coefficient: 6.32e−02
Value: B
Alishewanella.fetalis
2.0
1.5
1.0
0.5
0.0
A (n=14)
B (n=15)
C (n=11)
surface

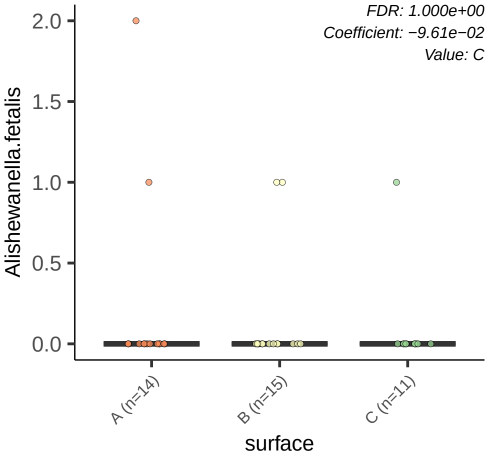

FDR: 1.000e+00
Coefficient: −9.61e−02
Value: C
Alishewanella.fetalis
2.0
1.5
1.0
0.5
0.0
A (n=14)
B (n=15)
C (n=11)
surface

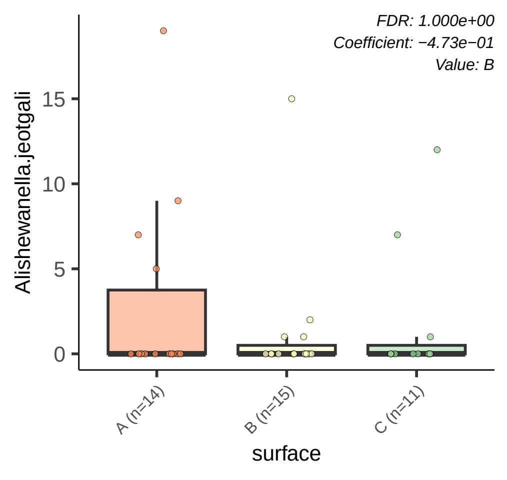
FDR: 1.000e+00
Coefficient: −4.73e−01
Value: B
Alishewanella.jeotgali
0
5
10
15
A (n=14)
B (n=15)
C (n=11)
surface

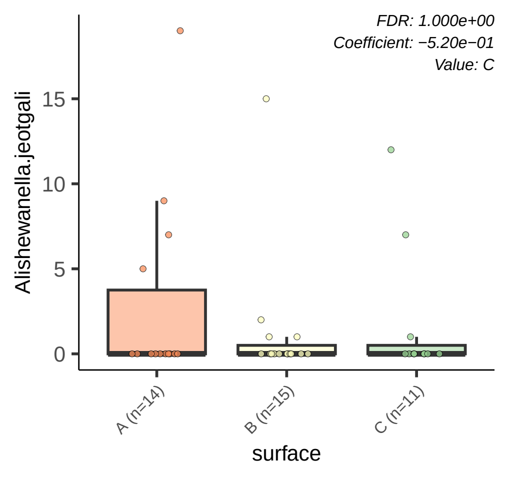
FDR: 1.000e+00
Coefficient: −5.20e−01
Value: C
Alishewanella.jeotgali
0
5
10
15
A (n=14)
B (n=15)
C (n=11)
surface

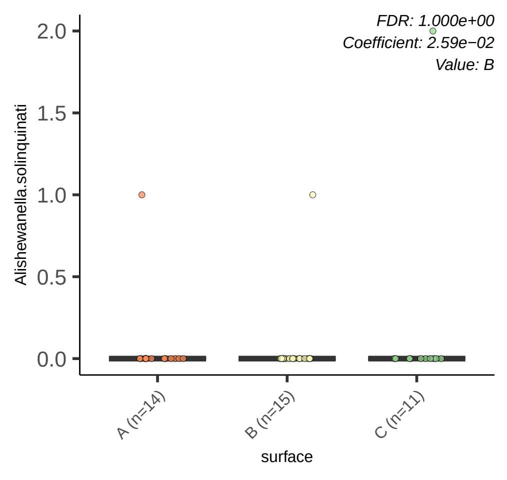
FDR: 1.000e+00
Coefficient: 2.59e−02
Value: B
Alishewanella.solinquinati
2.0
1.5
1.0
0.5
0.0
A (n=14)
B (n=15)
C (n=11)
surface

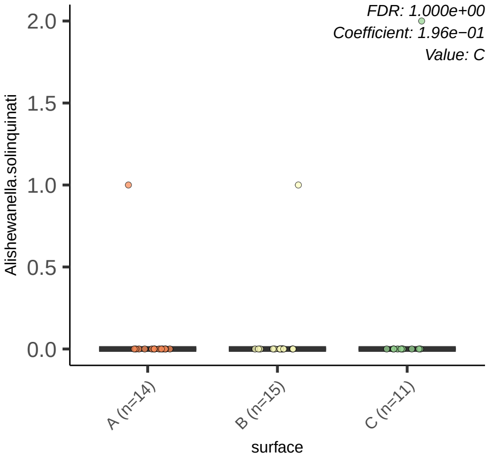

FDR: 1.000e+00
Coefficient: 1.96e−01
Value: C
Alishewanella.solinquinati
2.0
1.5
1.0
0.5
0.0
A (n=14)
B (n=15)
C (n=11)
surface

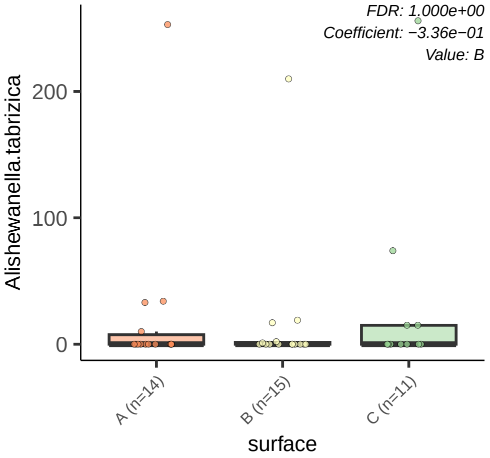
FDR: 1.000e+00
Coefficient: −3.36e−01
Value: B
Alishewanella.tabrizica
200
100
0
A (n=14)
B (n=15)
C (n=11)
surface

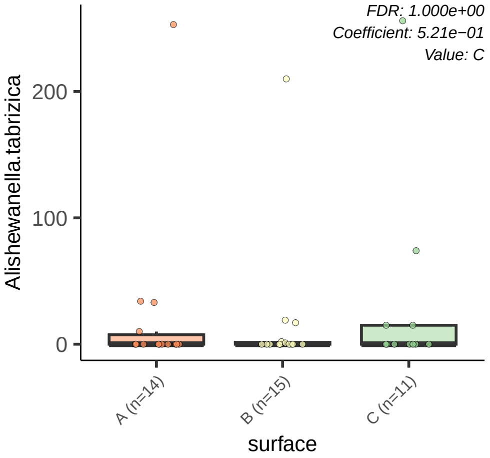
FDR: 1.000e+00
Coefficient: 5.21e−01
Value: C
Alishewanella.tabrizica
200
100
0
A (n=14)
B (n=15)
C (n=11)
surface

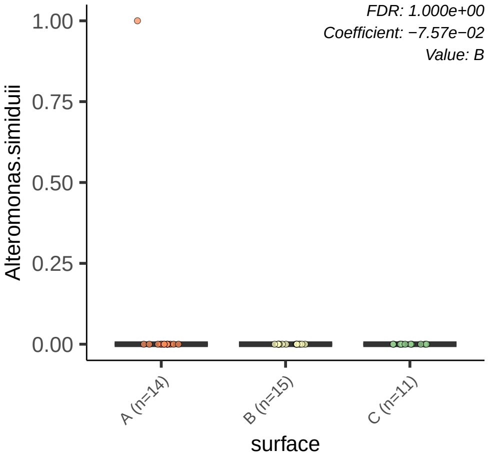

FDR: 1.000e+00
Coefficient: −7.57e−02
Value: B
Alteromonas.simiduii
1.00
0.75
0.50
0.25
0.00
A (n=14)
B (n=15)
C (n=11)
surface

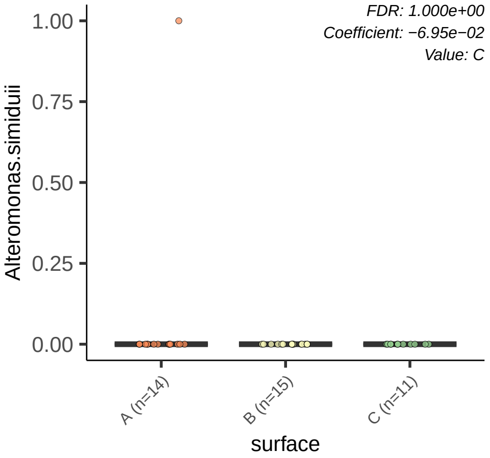
FDR: 1.000e+00
Coefficient: −6.95e−02
Value: C
1.00
0.75
0.50
0.25
0.00
Alteromonas.simiduii
A (n=14)
B (n=15)
C (n=11)
surface

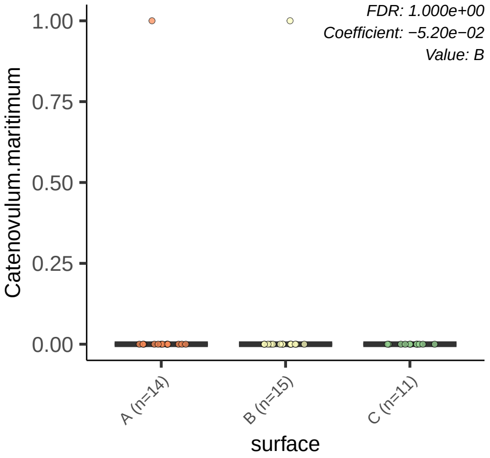

FDR: 1.000e+00
Coefficient: −5.20e−02
Value: B
Catenovulum.maritimum
1.00
0.75
0.50
0.25
0.00
A (n=14)
B (n=15)
C (n=11)
surface

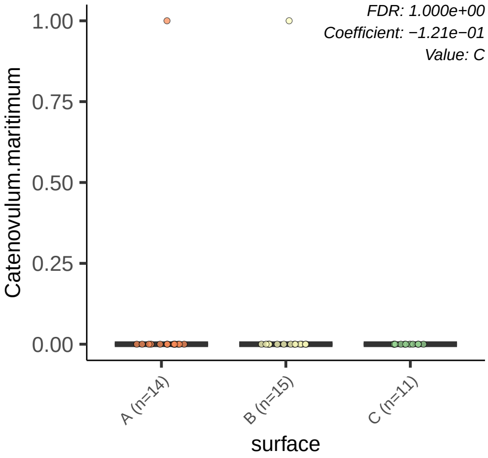
FDR: 1.000e+00
Coefficient: −1.21e−01
Value: C
Catenovulum.maritimum
1.00
0.75
0.50
0.25
0.00
A (n=14)
B (n=15)
C (n=11)
surface

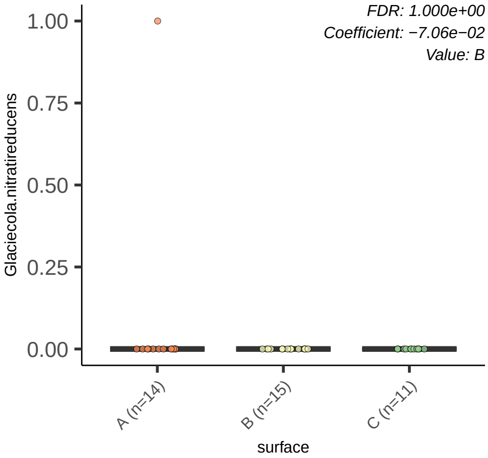

FDR: 1.000e+00
Coefficient: −7.06e−02
Value: B
Glaciecola.nitratireducens
1.00
0.75
0.50
0.25
0.00
A (n=14)
B (n=15)
C (n=11)
surface

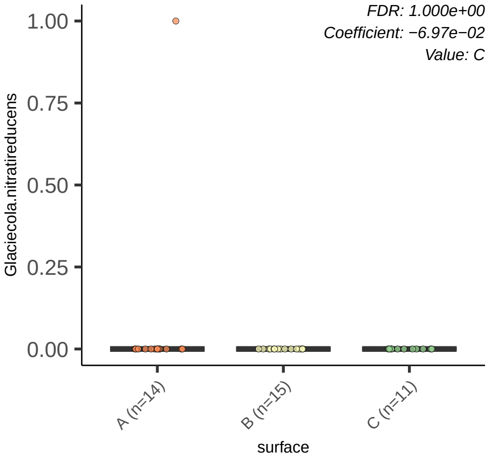
FDR: 1.000e+00
Coefficient: −6.97e−02
Value: C
1.00
0.75
0.50
0.25
0.00
Glaciecola.nitratireducens
A (n=14)
B (n=15)
C (n=11)
surface

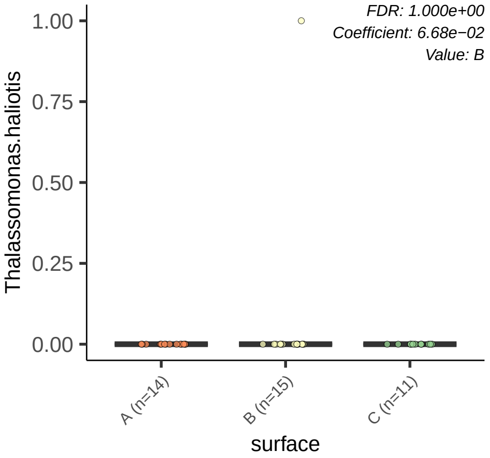

FDR: 1.000e+00
Coefficient: 6.68e−02
Value: B
Thalassomonas.haliotis
1.00
0.75
0.50
0.25
0.00
A (n=14)
B (n=15)
C (n=11)
surface

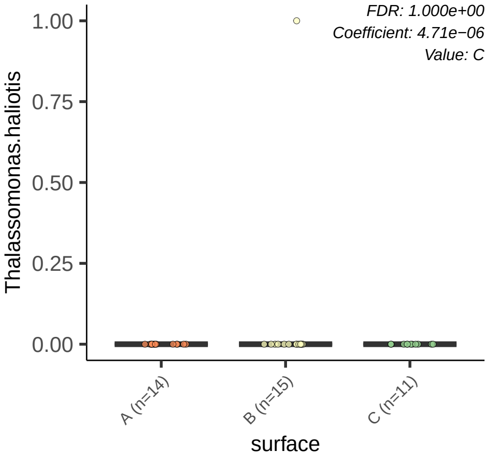

FDR: 1.000e+00
Coefficient: 4.71e−06
Value: C
Thalassomonas.haliotis
1.00
0.75
0.50
0.25
0.00
A (n=14)
B (n=15)
C (n=11)
surface

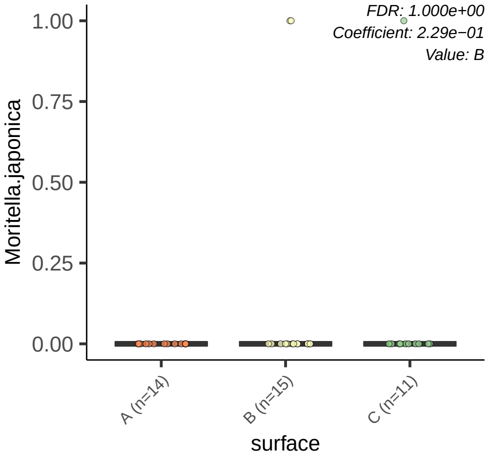
FDR: 1.000e+00
Coefficient: 2.29e−01
Value: B
Moritella.japonica
1.00
0.75
0.50
0.25
0.00
A (n=14)
B (n=15)
C (n=11)
surface

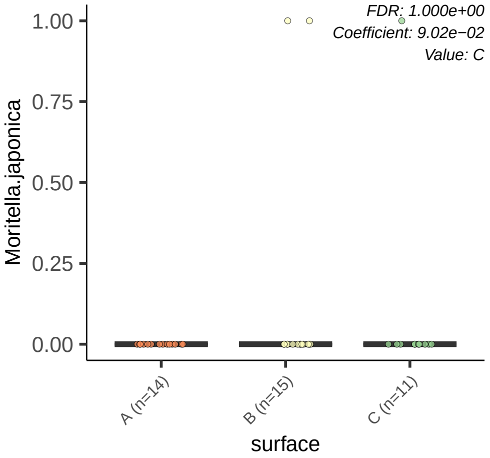
FDR: 1.000e+00
Coefficient: 9.02e−02
Value: C
Moritella.japonica
1.00
0.75
0.50
0.25
0.00
A (n=14)
B (n=15)
C (n=11)
surface

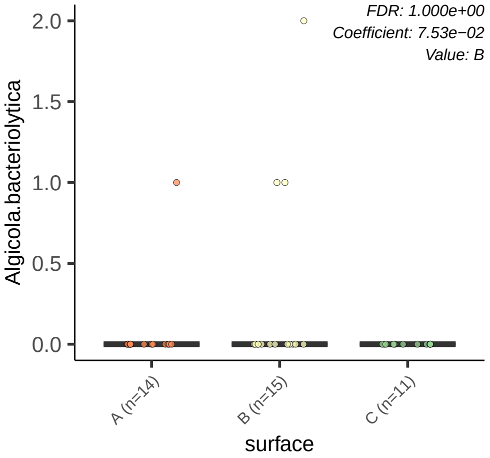
FDR: 1.000e+00
Coefficient: 7.53e−02
Value: B
Algicola.bacteriolytica
2.0
1.5
1.0
0.5
0.0
A (n=14)
B (n=15)
C (n=11)
surface

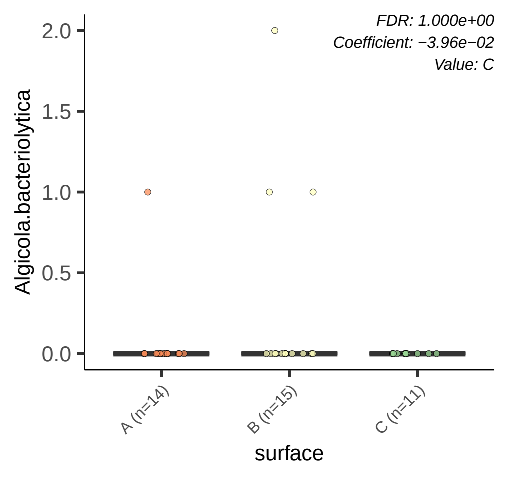
FDR: 1.000e+00
Coefficient: −3.96e−02
Value: C
Algicola.bacteriolytica
2.0
1.5
1.0
0.5
0.0
A (n=14)
B (n=15)
C (n=11)
surface

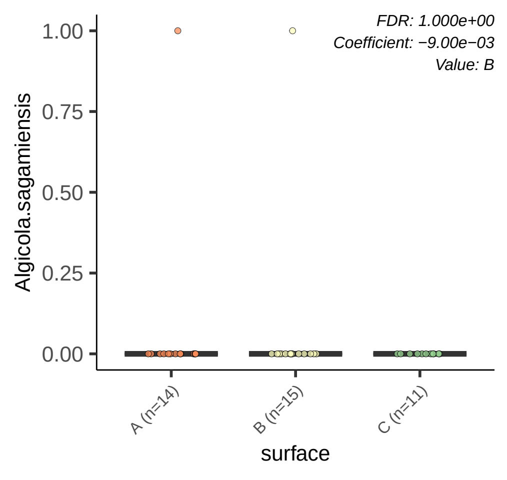
FDR: 1.000e+00
Coefficient: −9.00e−03
Value: B
Algicola.sagamiensis
1.00
0.75
0.50
0.25
0.00
A (n=14)
B (n=15)
C (n=11)
surface

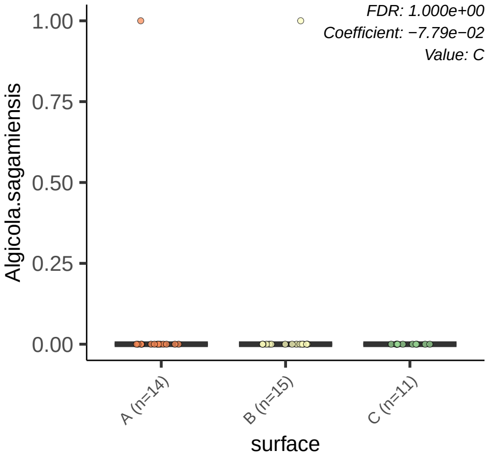
FDR: 1.000e+00
Coefficient: −7.79e−02
Value: C
Algicola.sagamiensis
1.00
0.75
0.50
0.25
0.00
A (n=14)
B (n=15)
C (n=11)
surface

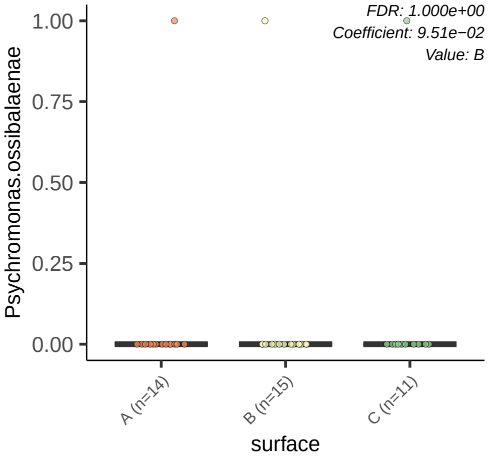

FDR: 1.000e+00
Coefficient: 9.51e−02
Value: B
Psychromonas.ossibalaenae
1.00
0.75
0.50
0.25
0.00
A (n=14)
B (n=15)
C (n=11)
surface

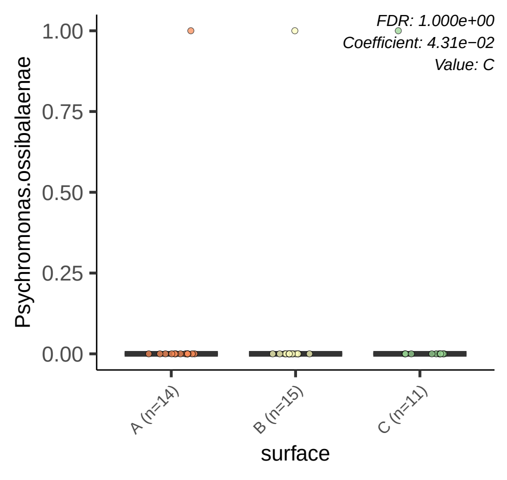

FDR: 1.000e+00
Coefficient: 4.31e−02
Value: C
Psychromonas.ossibalaenae
1.00
0.75
0.50
0.25
0.00
A (n=14)
B (n=15)
C (n=11)
surface

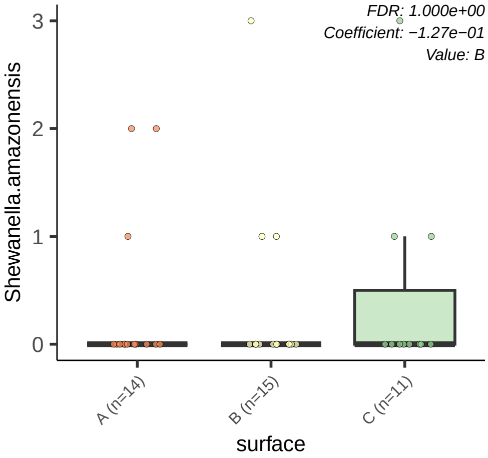

FDR: 1.000e+00
Coefficient: −1.27e−01
Value: B
Shewanella.amazonensis
0
1
2
3
A (n=14)
B (n=15)
C (n=11)
surface

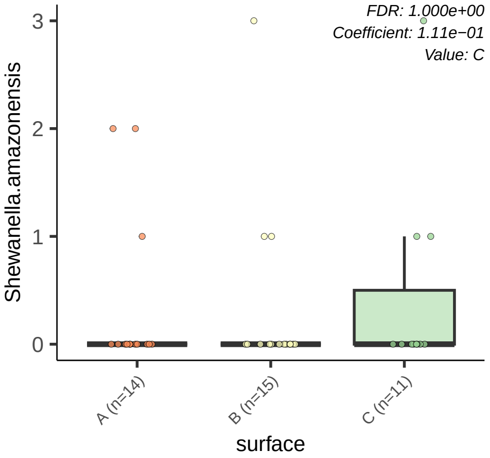

FDR: 1.000e+00
Coefficient: 1.11e−01
Value: C
Shewanella.amazonensis
0
1
2
3
A (n=14)
B (n=15)
C (n=11)
surface

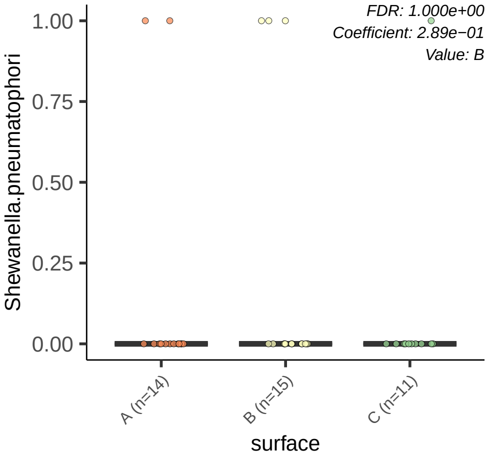
FDR: 1.000e+00
Coefficient: 2.89e−01
Value: B
Shewanella.pneumatophori
1.00
0.75
0.50
0.25
0.00
A (n=14)
B (n=15)
C (n=11)
surface

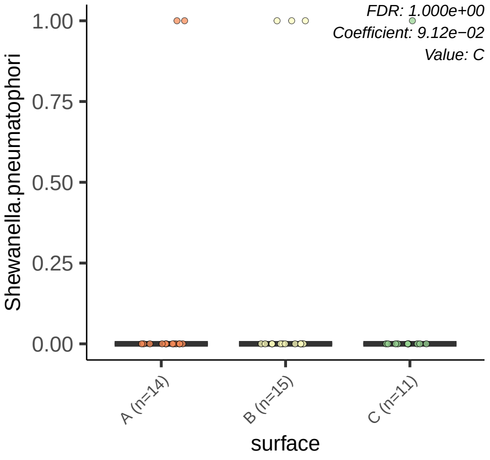

FDR: 1.000e+00
Coefficient: 9.12e−02
Value: C
Shewanella.pneumatophori
1.00
0.75
0.50
0.25
0.00
A (n=14)
B (n=15)
C (n=11)
surface

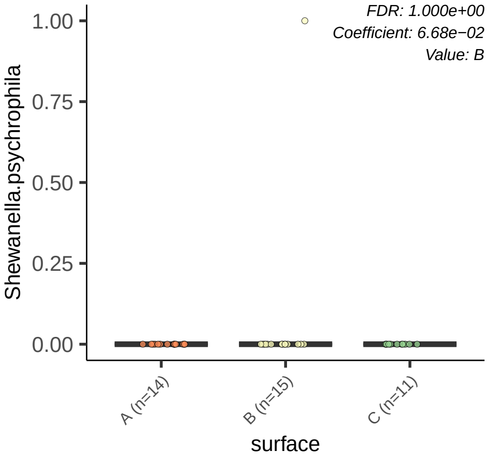
FDR: 1.000e+00
Coefficient: 6.68e−02
Value: B
Shewanella.psychrophila
1.00
0.75
0.50
0.25
0.00
A (n=14)
B (n=15)
C (n=11)
surface

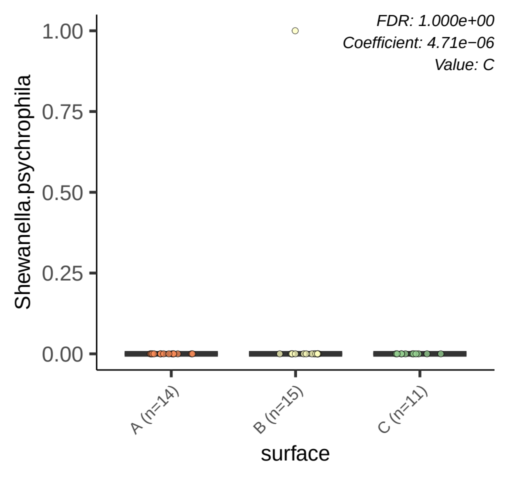
FDR: 1.000e+00
Coefficient: 4.71e−06
Value: C
Shewanella.psychrophila
1.00
0.75
0.50
0.25
0.00
A (n=14)
B (n=15)
C (n=11)
surface

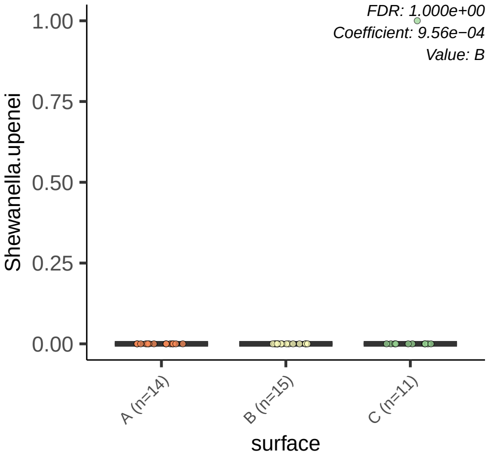

FDR: 1.000e+00
Coefficient: 9.56e−04
Value: B
1.00
0.75
0.50
0.25
0.00
Shewanella.upenei
A (n=14)
B (n=15)
C (n=11)
surface

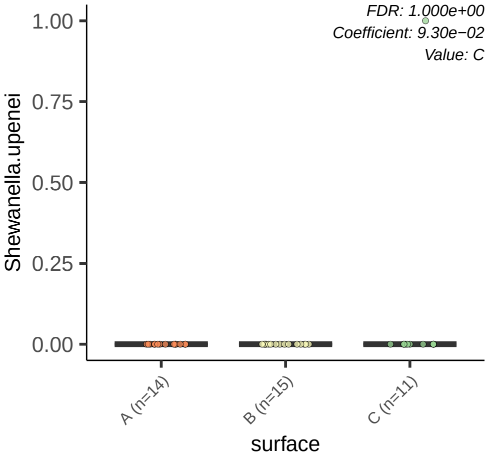
FDR: 1.000e+00
Coefficient: 9.30e−02
Value: C
Shewanella.upenei
1.00
0.75
0.50
0.25
0.00
A (n=14)
B (n=15)
C (n=11)
surface

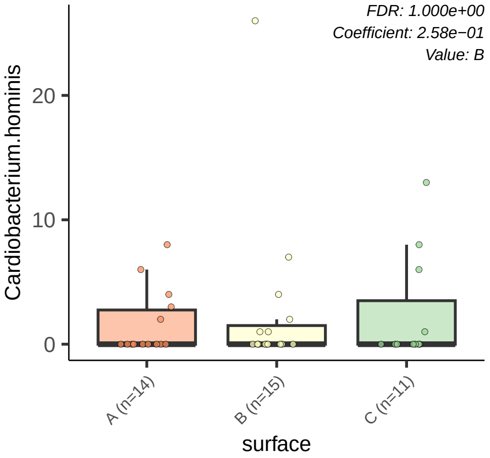

FDR: 1.000e+00
Coefficient: 2.58e−01
Value: B
Cardiobacterium.hominis
0
10
20
A (n=14)
B (n=15)
C (n=11)
surface

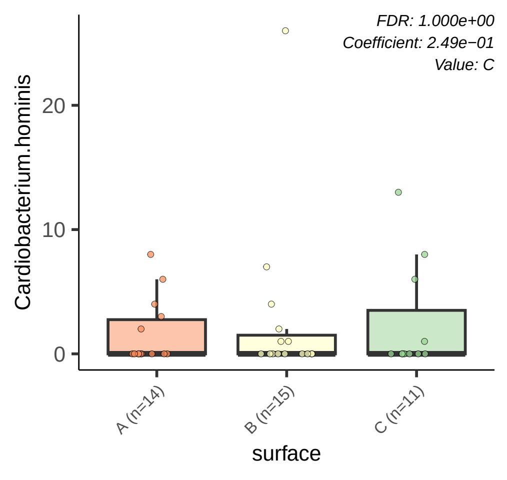
FDR: 1.000e+00
Coefficient: 2.49e−01
Value: C
Cardiobacterium.hominis
20
10
0
A (n=14)
B (n=15)
C (n=11)
surface

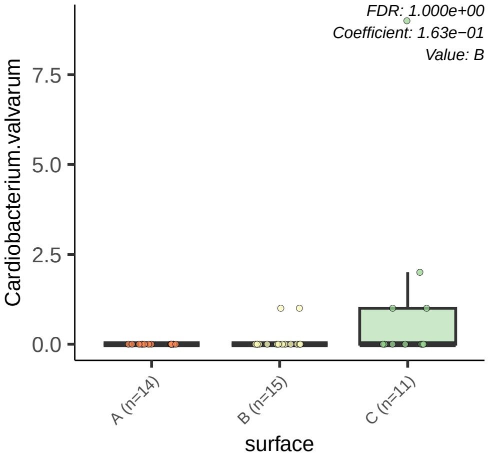
FDR: 1.000e+00
Coefficient: 1.63e−01
Value: B
Cardiobacterium.valvarum
7.5
5.0
2.5
0.0
A (n=14)
B (n=15)
C (n=11)
surface

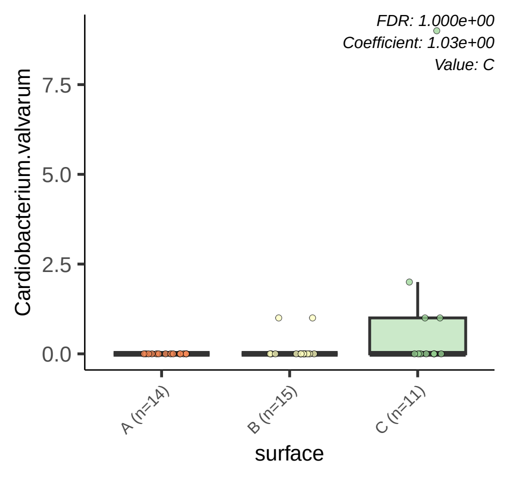
FDR: 1.000e+00
Coefficient: 1.03e+00
Value: C
Cardiobacterium.valvarum
7.5
5.0
2.5
0.0
A (n=14)
B (n=15)
C (n=11)
surface

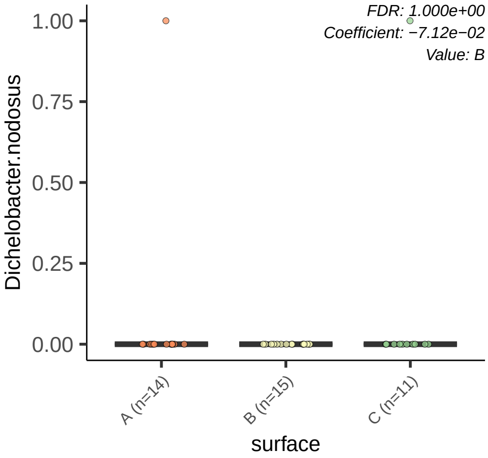

FDR: 1.000e+00
Coefficient: −7.12e−02
Value: B
Dichelobacter.nodosus
1.00
0.75
0.50
0.25
0.00
A (n=14)
B (n=15)
C (n=11)
surface

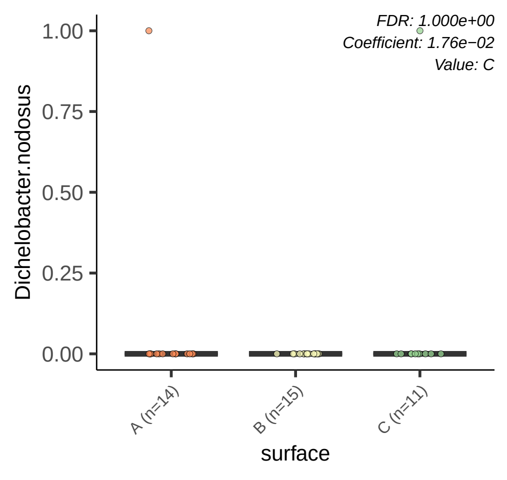

FDR: 1.000e+00
Coefficient: 1.76e−02
Value: C
Dichelobacter.nodosus
1.00
0.75
0.50
0.25
0.00
A (n=14)
B (n=15)
C (n=11)
surface

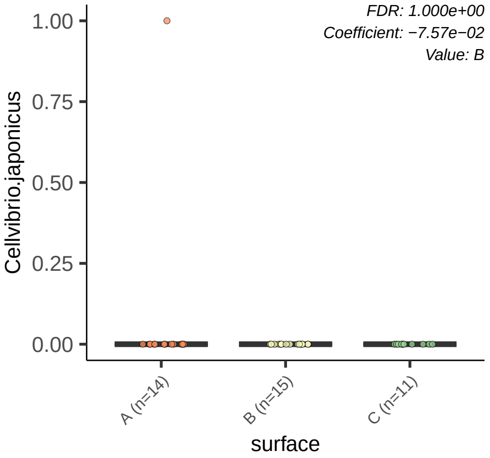
FDR: 1.000e+00
Coefficient: −7.57e−02
Value: B
Cellvibrio.japonicus
1.00
0.75
0.50
0.25
0.00
A (n=14)
B (n=15)
C (n=11)
surface

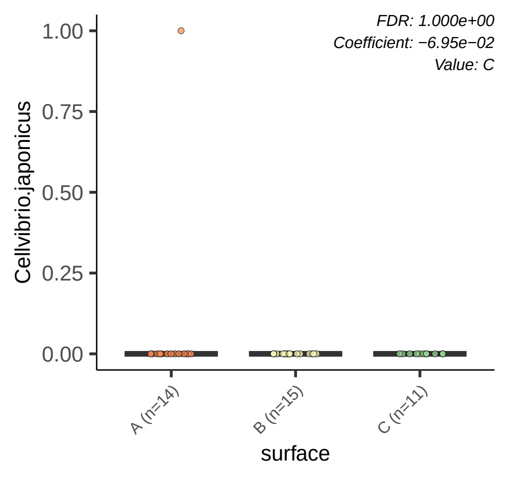
FDR: 1.000e+00
Coefficient: −6.95e−02
Value: C
1.00
0.75
0.50
0.25
0.00
Cellvibrio.japonicus
A (n=14)
B (n=15)
C (n=11)
surface

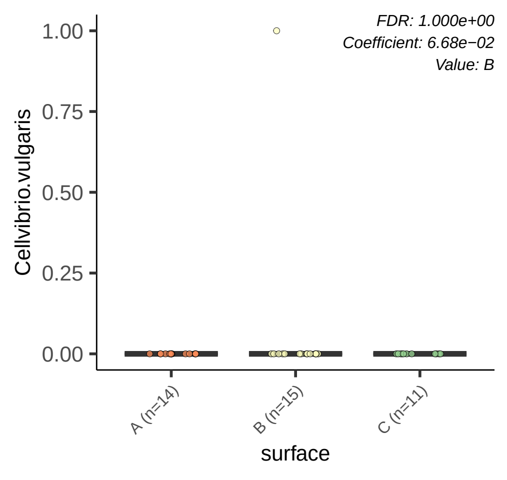
FDR: 1.000e+00
Coefficient: 6.68e−02
Value: B
Cellvibrio.vulgaris
1.00
0.75
0.50
0.25
0.00
A (n=14)
B (n=15)
C (n=11)
surface

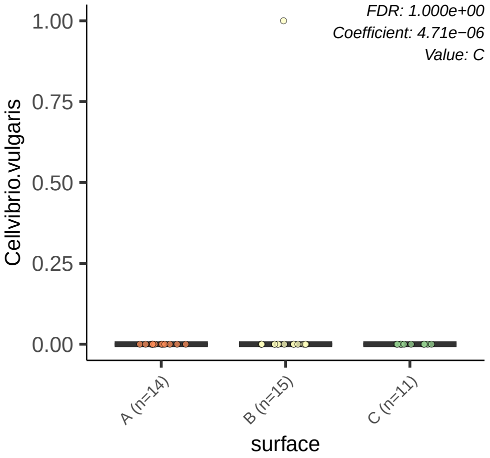
FDR: 1.000e+00
Coefficient: 4.71e−06
Value: C
Cellvibrio.vulgaris
1.00
0.75
0.50
0.25
0.00
A (n=14)
B (n=15)
C (n=11)
surface

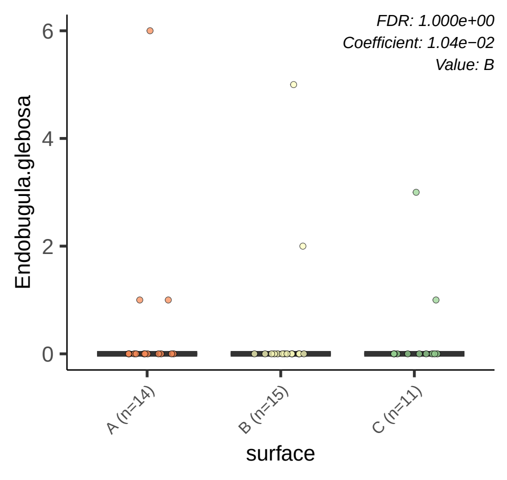
FDR: 1.000e+00
Coefficient: 1.04e−02
Value: B
Endobugula.glebosa
0
2
4
6
A (n=14)
B (n=15)
C (n=11)
surface

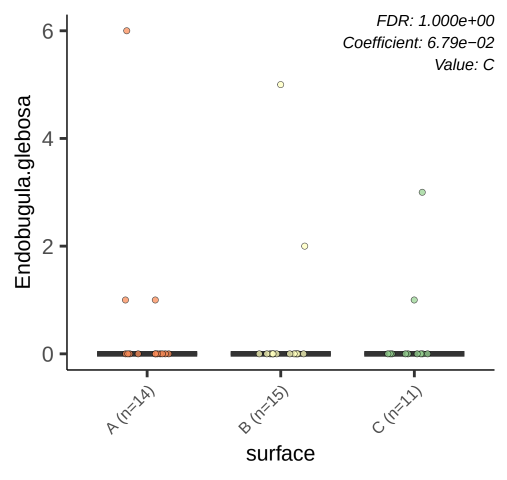
FDR: 1.000e+00
Coefficient: 6.79e−02
Value: C
Endobugula.glebosa
0
2
4
6
A (n=14)
B (n=15)
C (n=11)
surface

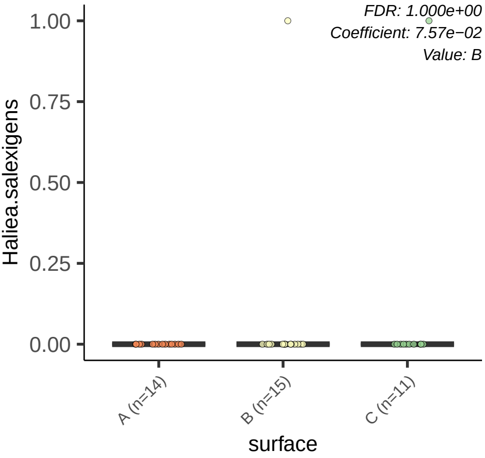
FDR: 1.000e+00
Coefficient: 7.57e−02
Value: B
Haliea.salexigens
1.00
0.75
0.50
0.25
0.00
A (n=14)
B (n=15)
C (n=11)
surface

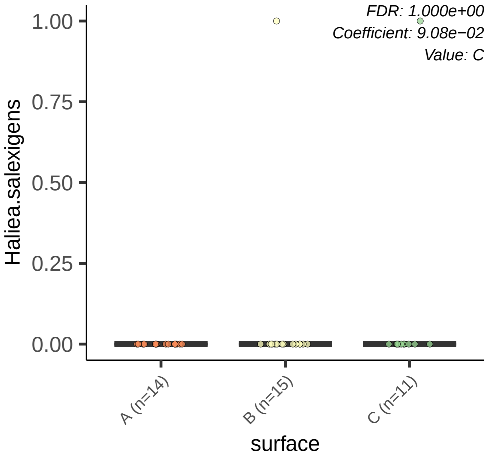
FDR: 1.000e+00
Coefficient: 9.08e−02
Value: C
Haliea.salexigens
1.00
0.75
0.50
0.25
0.00
A (n=14)
B (n=15)
C (n=11)
surface

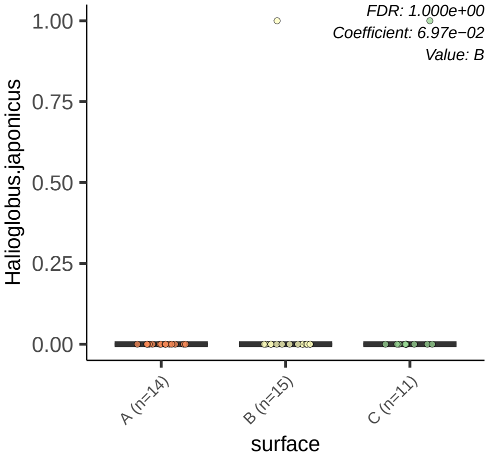
FDR: 1.000e+00
Coefficient: 6.97e−02
Value: B
Halioglobus.japonicus
1.00
0.75
0.50
0.25
0.00
A (n=14)
B (n=15)
C (n=11)
surface

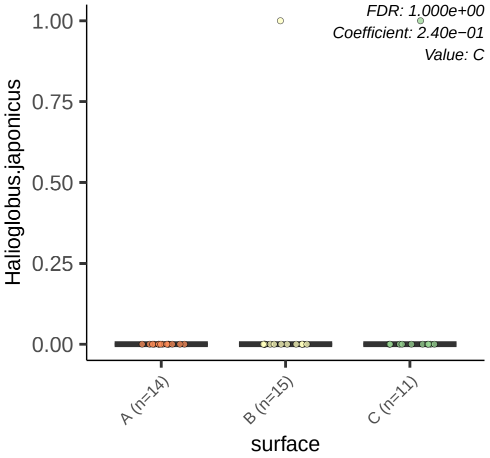

FDR: 1.000e+00
Coefficient: 2.40e−01
Value: C
Halioglobus.japonicus
1.00
0.75
0.50
0.25
0.00
A (n=14)
B (n=15)
C (n=11)
surface

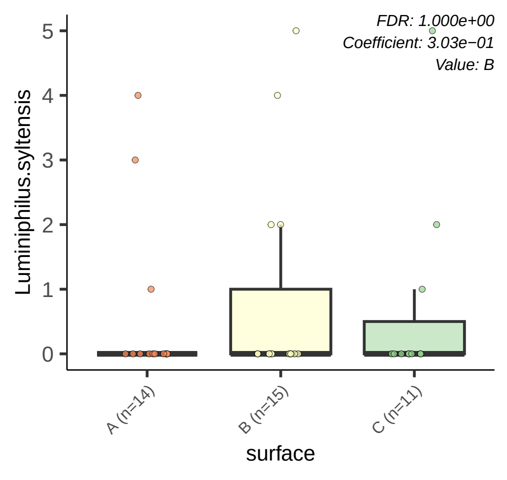
FDR: 1.000e+00
Coefficient: 3.03e−01
Value: B
Luminiphilus.syltensis
0
1
2
3
4
5
A (n=14)
B (n=15)
C (n=11)
surface

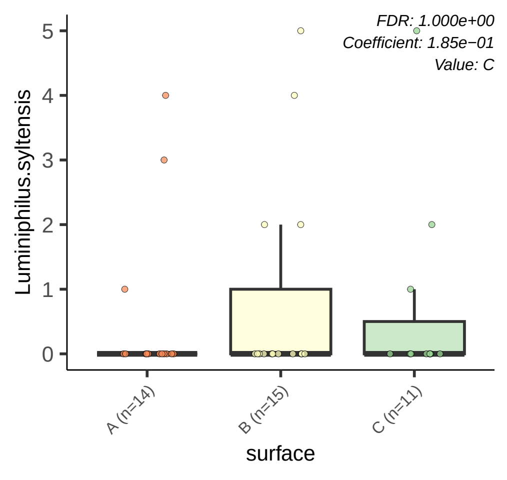

FDR: 1.000e+00
Coefficient: 1.85e−01
Value: C
Luminiphilus.syltensis
5
4
3
2
1
0
A (n=14)
B (n=15)
C (n=11)
surface

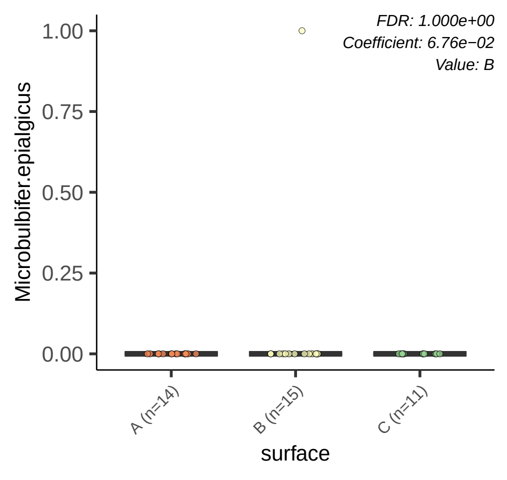
FDR: 1.000e+00
Coefficient: 6.76e−02
Value: B
Microbulbifer.epialgicus
1.00
0.75
0.50
0.25
0.00
A (n=14)
B (n=15)
C (n=11)
surface

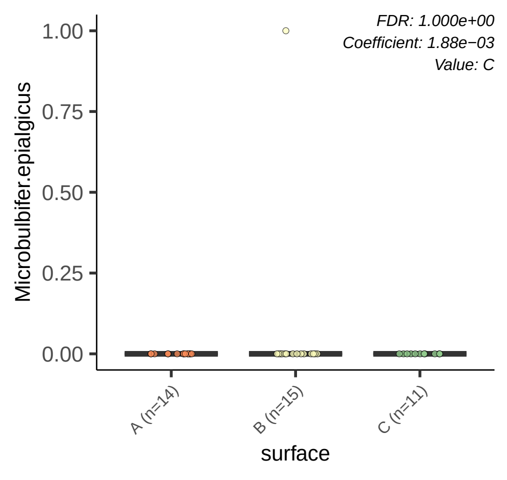
FDR: 1.000e+00
Coefficient: 1.88e−03
Value: C
Microbulbifer.epialgicus
1.00
0.75
0.50
0.25
0.00
A (n=14)
B (n=15)
C (n=11)
surface

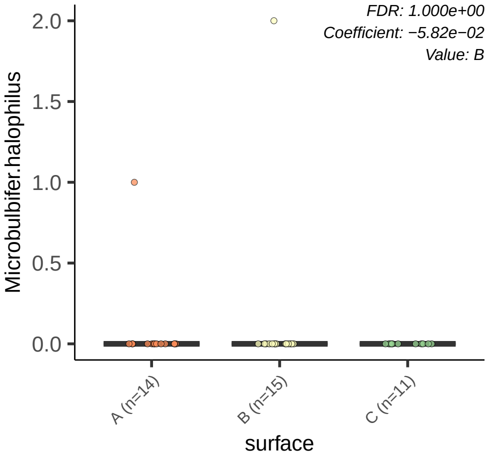
FDR: 1.000e+00
Coefficient: −5.82e−02
Value: B
Microbulbifer.halophilus
2.0
1.5
1.0
0.5
0.0
A (n=14)
B (n=15)
C (n=11)
surface

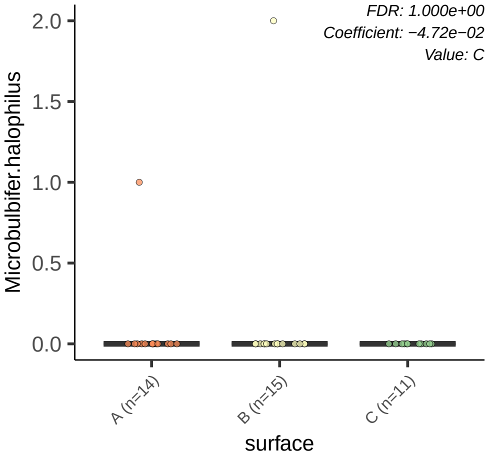
FDR: 1.000e+00
Coefficient: −4.72e−02
Value: C
Microbulbifer.halophilus
2.0
1.5
1.0
0.5
0.0
A (n=14)
B (n=15)
C (n=11)
surface

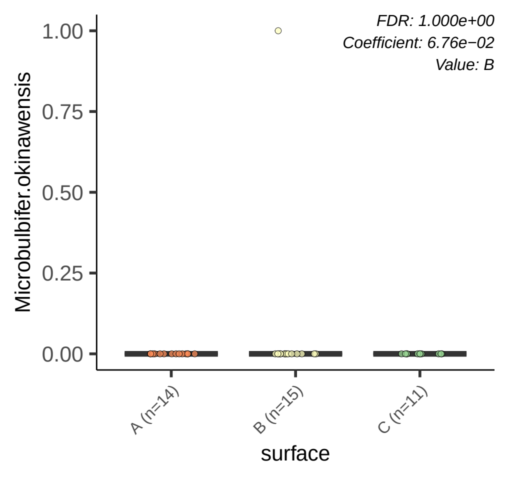

FDR: 1.000e+00
Coefficient: 6.76e−02
Value: B
Microbulbifer.okinawensis
1.00
0.75
0.50
0.25
0.00
A (n=14)
B (n=15)
C (n=11)
surface

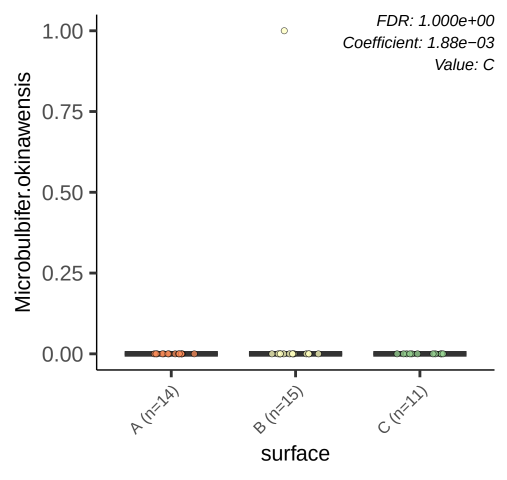

FDR: 1.000e+00
Coefficient: 1.88e−03
Value: C
Microbulbifer.okinawensis
1.00
0.75
0.50
0.25
0.00
A (n=14)
B (n=15)
C (n=11)
surface

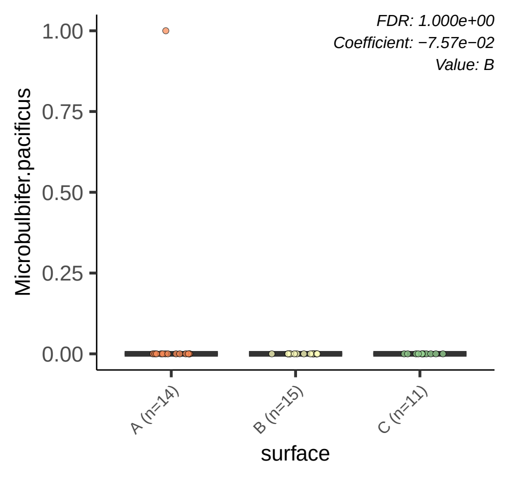
FDR: 1.000e+00
Coefficient: −7.57e−02
Value: B
Microbulbifer.pacificus
1.00
0.75
0.50
0.25
0.00
A (n=14)
B (n=15)
C (n=11)
surface

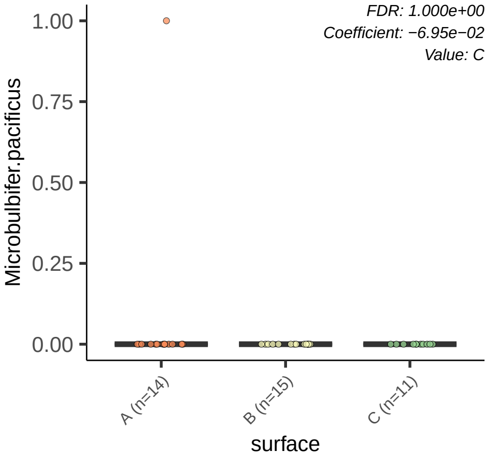
FDR: 1.000e+00
Coefficient: −6.95e−02
Value: C
Microbulbifer.pacificus
1.00
0.75
0.50
0.25
0.00
A (n=14)
B (n=15)
C (n=11)
surface

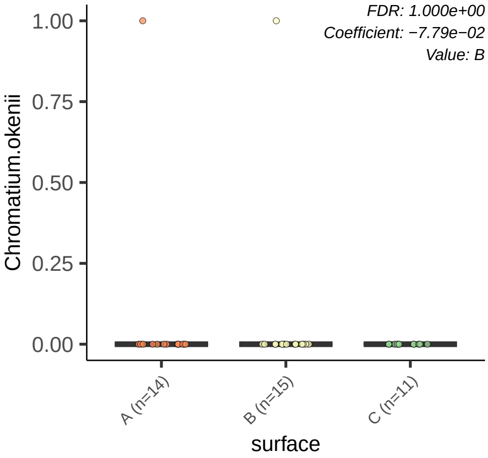
FDR: 1.000e+00
Coefficient: −7.79e−02
Value: B
Chromatium.okenii
1.00
0.75
0.50
0.25
0.00
A (n=14)
B (n=15)
C (n=11)
surface

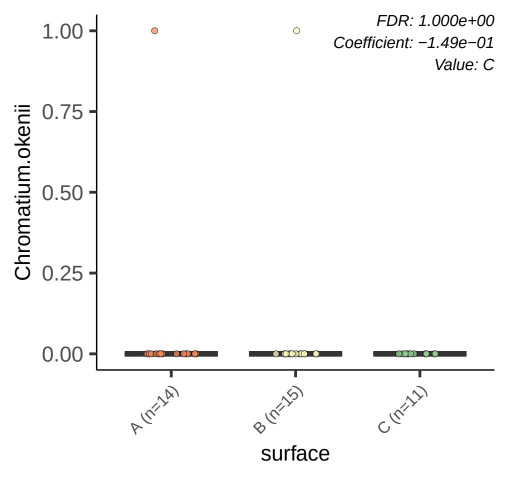
FDR: 1.000e+00
Coefficient: −1.49e−01
Value: C
Chromatium.okenii
1.00
0.75
0.50
0.25
0.00
A (n=14)
B (n=15)
C (n=11)
surface

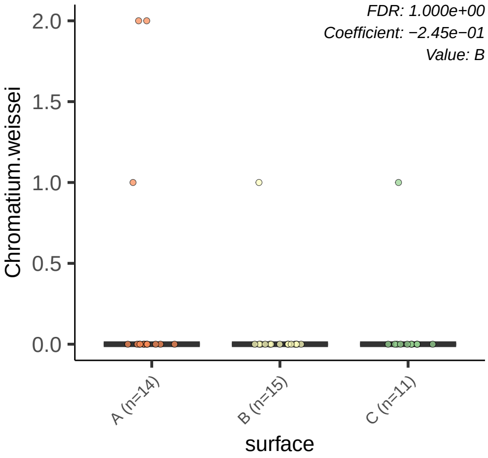
FDR: 1.000e+00
Coefficient: −2.45e−01
Value: B
Chromatium.weissei
2.0
1.5
1.0
0.5
0.0
A (n=14)
B (n=15)
C (n=11)
surface

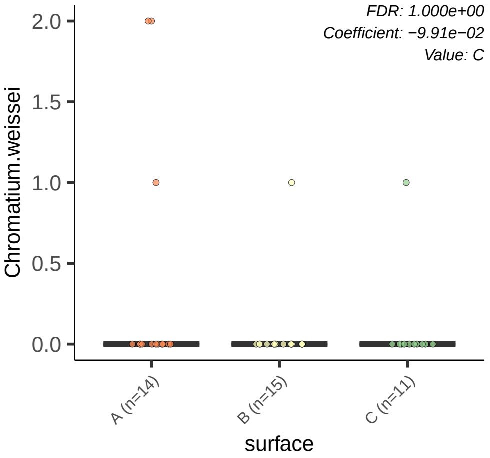

FDR: 1.000e+00
Coefficient: −9.91e−02
Value: C
Chromatium.weissei
2.0
1.5
1.0
0.5
0.0
A (n=14)
B (n=15)
C (n=11)
surface

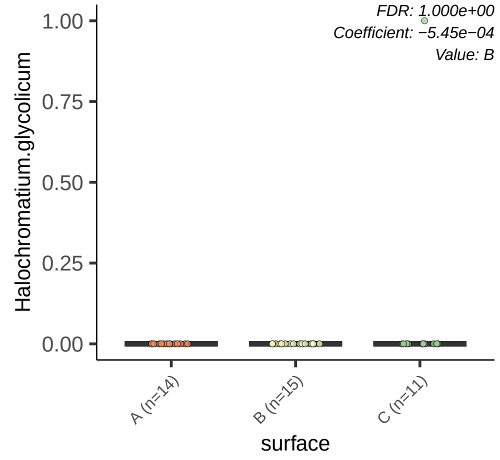
FDR: 1.000e+00
Coefficient: −5.45e−04
Value: B
Halochromatium.glycolicum
1.00
0.75
0.50
0.25
0.00
A (n=14)
B (n=15)
C (n=11)
surface

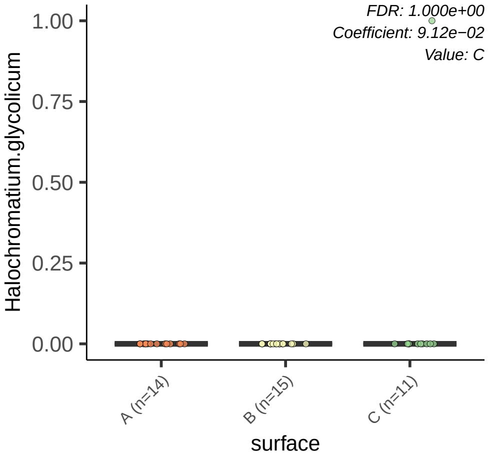
FDR: 1.000e+00
Coefficient: 9.12e−02
Value: C
Halochromatium.glycolicum
1.00
0.75
0.50
0.25
0.00
A (n=14)
B (n=15)
C (n=11)
surface

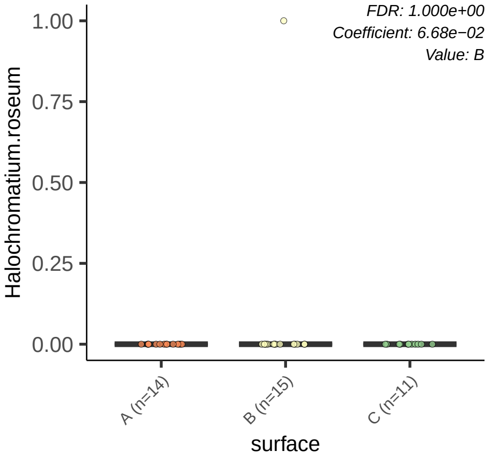
FDR: 1.000e+00
Coefficient: 6.68e−02
Value: B
1.00
0.75
0.50
0.25
0.00
Halochromatium.roseum
A (n=14)
B (n=15)
C (n=11)
surface

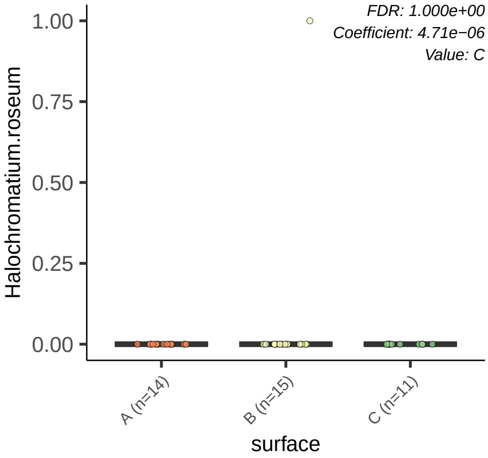

FDR: 1.000e+00
Coefficient: 4.71e−06
Value: C
Halochromatium.roseum
1.00
0.75
0.50
0.25
0.00
A (n=14)
B (n=15)
C (n=11)
surface

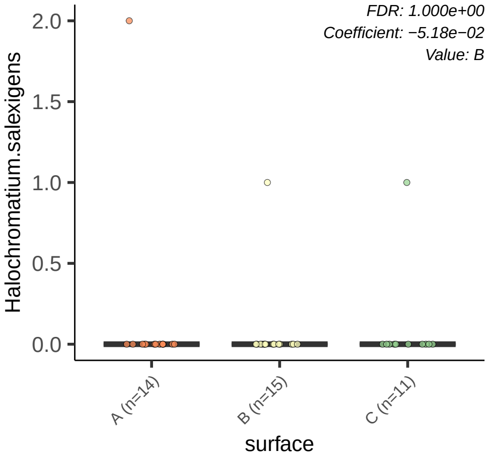

FDR: 1.000e+00
Coefficient: −5.18e−02
Value: B
Halochromatium.salexigens
2.0
1.5
1.0
0.5
0.0
A (n=14)
B (n=15)
C (n=11)
surface

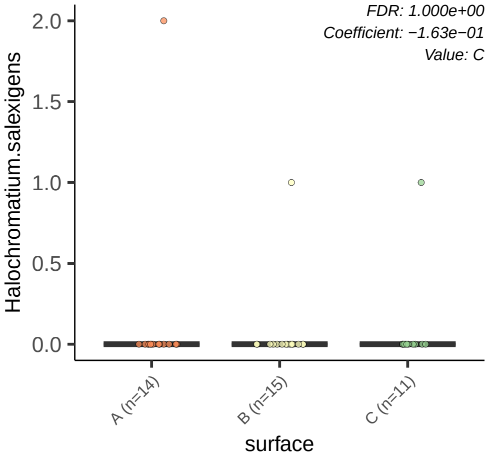
FDR: 1.000e+00
Coefficient: −1.63e−01
Value: C
Halochromatium.salexigens
2.0
1.5
1.0
0.5
0.0
A (n=14)
B (n=15)
C (n=11)
surface

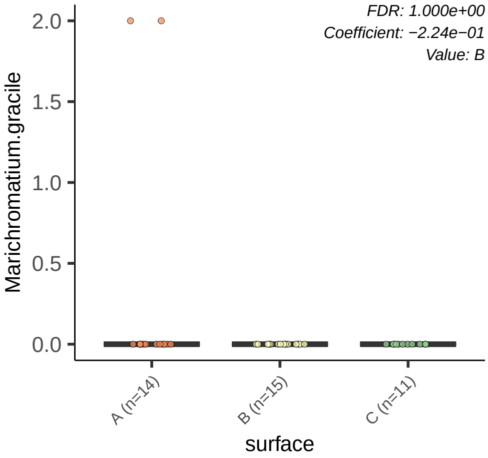
FDR: 1.000e+00
Coefficient: −2.24e−01
Value: B
Marichromatium.gracile
2.0
1.5
1.0
0.5
0.0
A (n=14)
B (n=15)
C (n=11)
surface

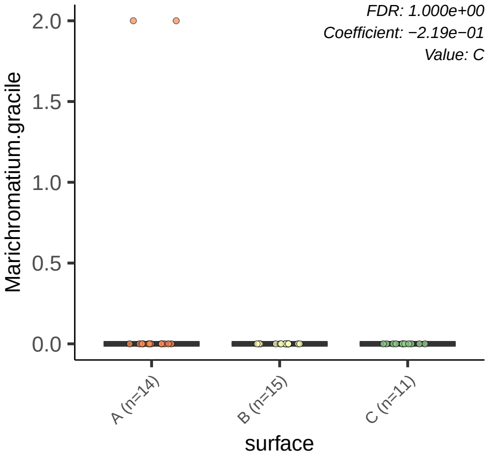
FDR: 1.000e+00
Coefficient: −2.19e−01
Value: C
Marichromatium.gracile
2.0
1.5
1.0
0.5
0.0
A (n=14)
B (n=15)
C (n=11)
surface

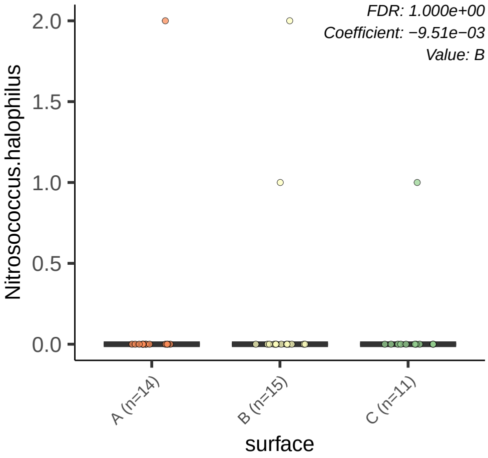

FDR: 1.000e+00
Coefficient: −9.51e−03
Value: B
Nitrosococcus.halophilus
2.0
1.5
1.0
0.5
0.0
A (n=14)
B (n=15)
C (n=11)
surface

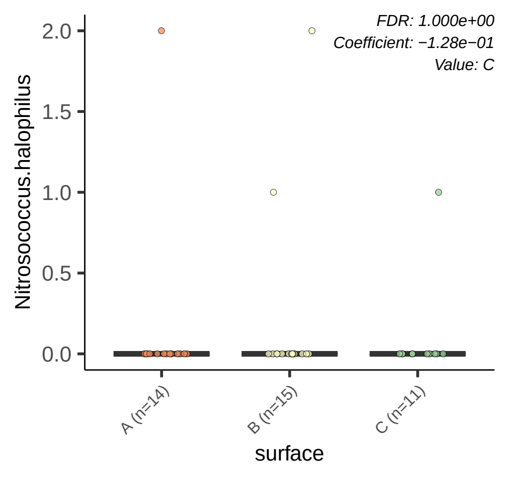
FDR: 1.000e+00
Coefficient: −1.28e−01
Value: C
Nitrosococcus.halophilus
2.0
1.5
1.0
0.5
0.0
A (n=14)
B (n=15)
C (n=11)
surface

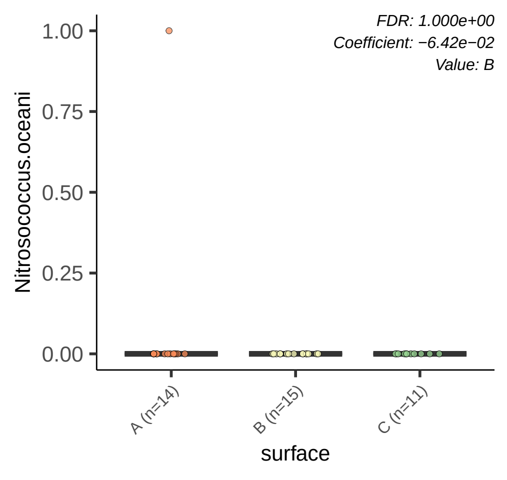
FDR: 1.000e+00
Coefficient: −6.42e−02
Value: B
Nitrosococcus.oceani
1.00
0.75
0.50
0.25
0.00
A (n=14)
B (n=15)
C (n=11)
surface

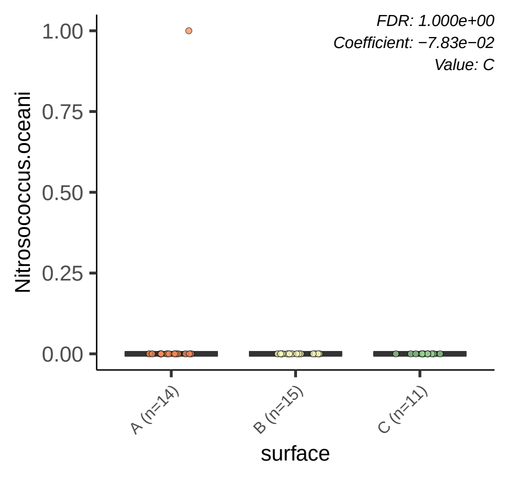
FDR: 1.000e+00
Coefficient: −7.83e−02
Value: C
Nitrosococcus.oceani
1.00
0.75
0.50
0.25
0.00
A (n=14)
B (n=15)
C (n=11)
surface

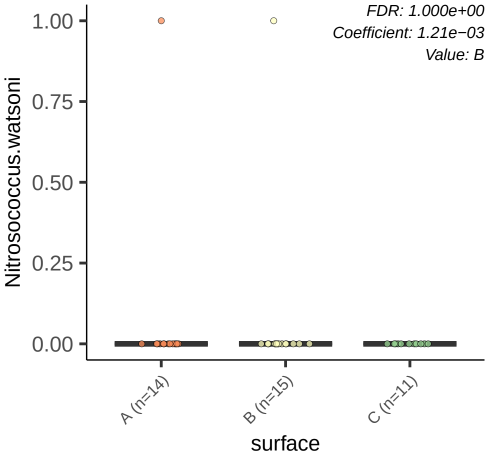

FDR: 1.000e+00
Coefficient: 1.21e−03
Value: B
Nitrosococcus.watsoni
1.00
0.75
0.50
0.25
0.00
A (n=14)
B (n=15)
C (n=11)
surface

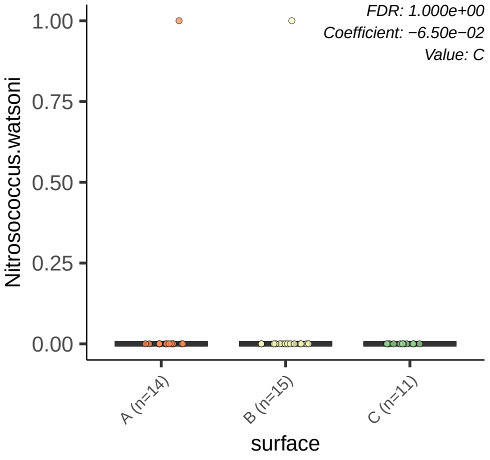

FDR: 1.000e+00
Coefficient: −6.50e−02
Value: C
Nitrosococcus.watsoni
1.00
0.75
0.50
0.25
0.00
A (n=14)
B (n=15)
C (n=11)
surface

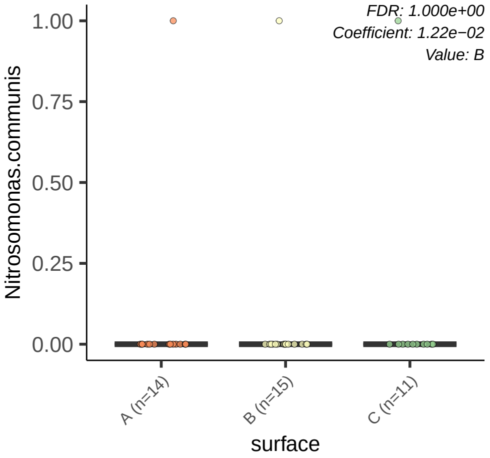
FDR: 1.000e+00
Coefficient: 1.22e−02
Value: B
Nitrosomonas.communis
1.00
0.75
0.50
0.25
0.00
A (n=14)
B (n=15)
C (n=11)
surface

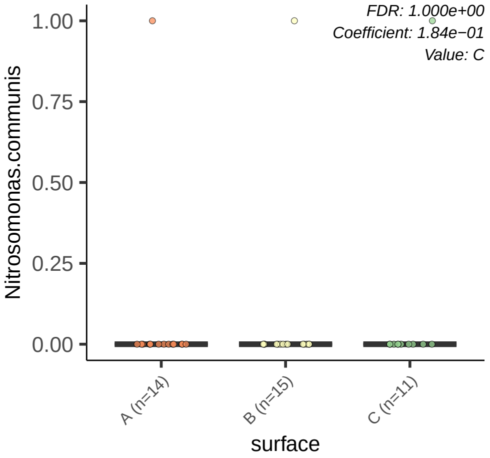
FDR: 1.000e+00
Coefficient: 1.84e−01
Value: C
Nitrosomonas.communis
1.00
0.75
0.50
0.25
0.00
A (n=14)
B (n=15)
C (n=11)
surface

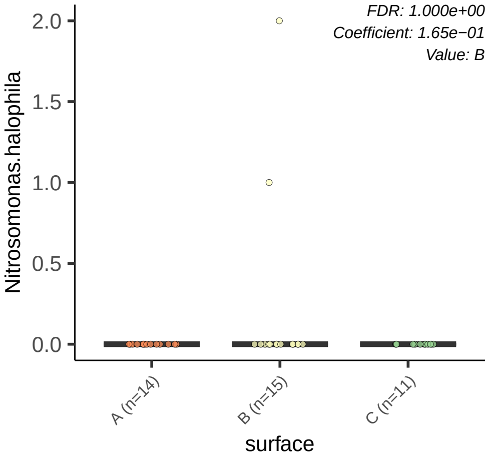

FDR: 1.000e+00
Coefficient: 1.65e−01
Value: B
Nitrosomonas.halophila
2.0
1.5
1.0
0.5
0.0
A (n=14)
B (n=15)
C (n=11)
surface

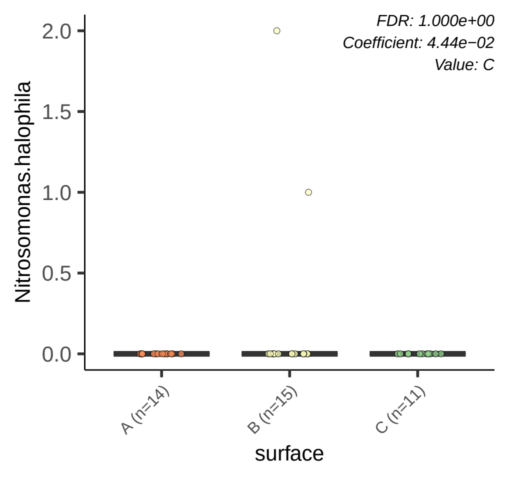
FDR: 1.000e+00
Coefficient: 4.44e−02
Value: C
Nitrosomonas.halophila
2.0
1.5
1.0
0.5
0.0
A (n=14)
B (n=15)
C (n=11)
surface

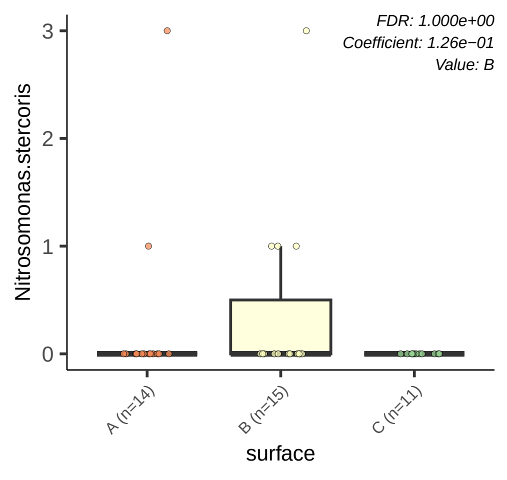

FDR: 1.000e+00
Coefficient: 1.26e−01
Value: B
Nitrosomonas.stercoris
0
1
2
3
A (n=14)
B (n=15)
C (n=11)
surface

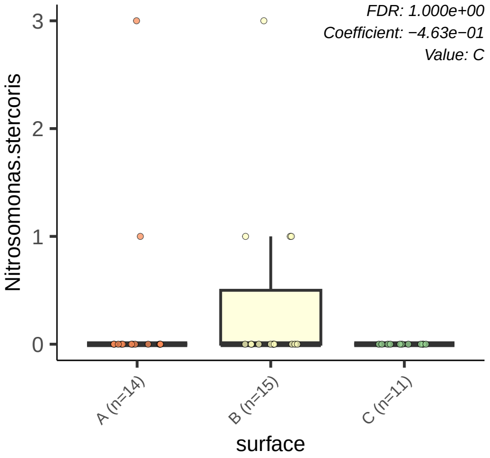
FDR: 1.000e+00
Coefficient: −4.63e−01
Value: C
Nitrosomonas.stercoris
3
2
1
0
A (n=14)
B (n=15)
C (n=11)
surface

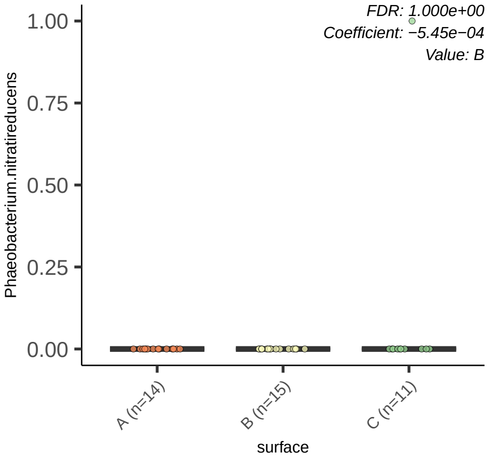

FDR: 1.000e+00
Coefficient: −5.45e−04
Value: B
Phaeobacterium.nitratireducens
1.00
0.75
0.50
0.25
0.00
A (n=14)
B (n=15)
C (n=11)
surface

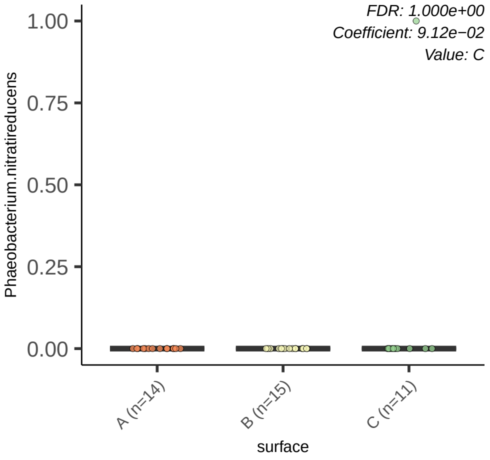

FDR: 1.000e+00
Coefficient: 9.12e−02
Value: C
Phaeobacterium.nitratireducens
1.00
0.75
0.50
0.25
0.00
A (n=14)
B (n=15)
C (n=11)
surface

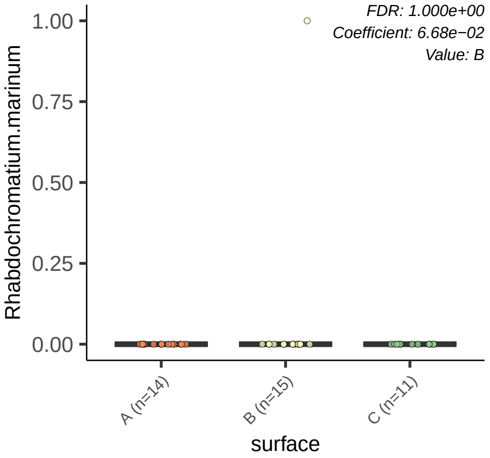
FDR: 1.000e+00
Coefficient: 6.68e−02
Value: B
Rhabdochromatium.marinum
1.00
0.75
0.50
0.25
0.00
A (n=14)
B (n=15)
C (n=11)
surface

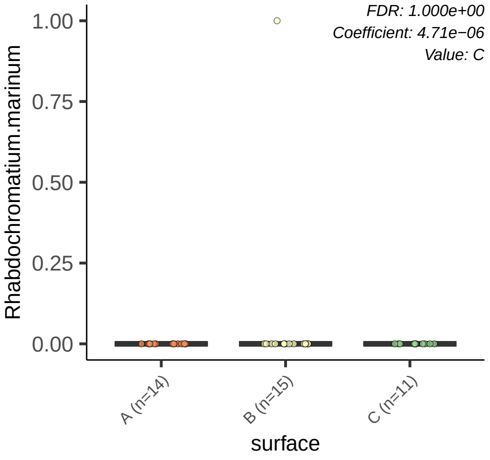
FDR: 1.000e+00
Coefficient: 4.71e−06
Value: C
Rhabdochromatium.marinum
1.00
0.75
0.50
0.25
0.00
A (n=14)
B (n=15)
C (n=11)
surface

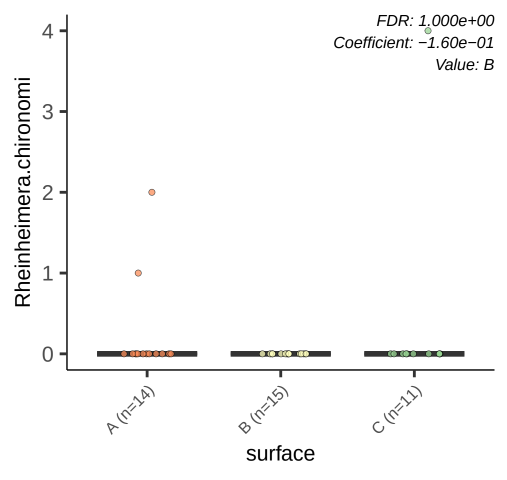

FDR: 1.000e+00
Coefficient: −1.60e−01
Value: B
Rheinheimera.chironomi
4
3
2
1
0
A (n=14)
B (n=15)
C (n=11)
surface

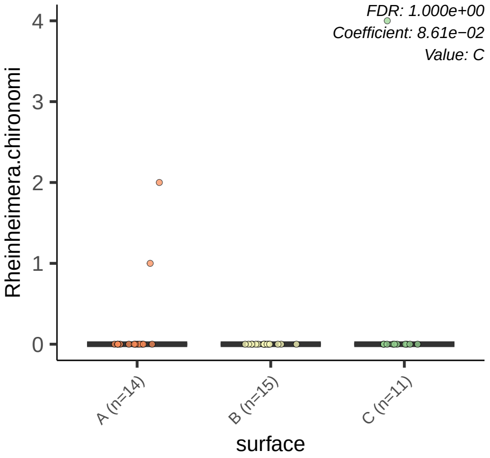

FDR: 1.000e+00
Coefficient: 8.61e−02
Value: C
Rheinheimera.chironomi
0
1
2
3
4
A (n=14)
B (n=15)
C (n=11)
surface

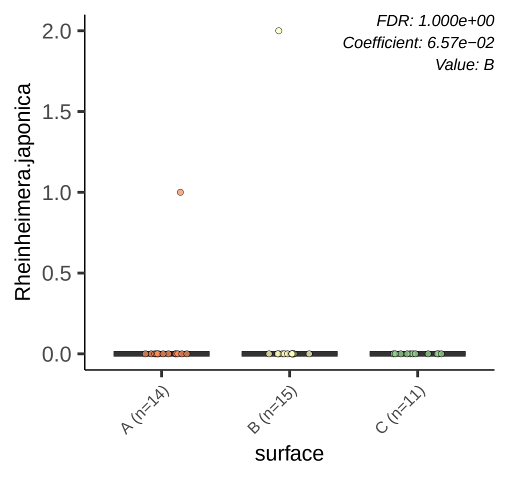
FDR: 1.000e+00
Coefficient: 6.57e−02
Value: B
Rheinheimera.japonica
2.0
1.5
1.0
0.5
0.0
A (n=14)
B (n=15)
C (n=11)
surface

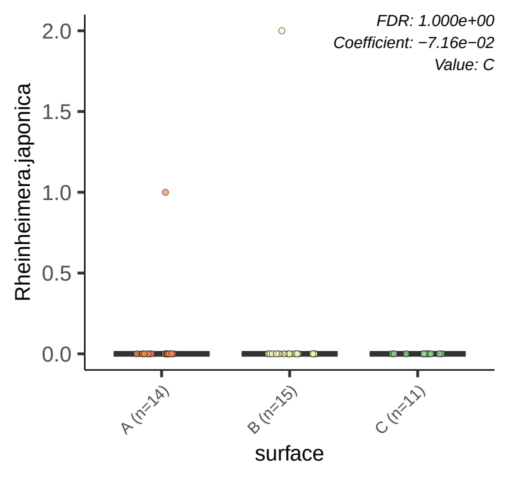
FDR: 1.000e+00
Coefficient: −7.16e−02
Value: C
Rheinheimera.japonica
2.0
1.5
1.0
0.5
0.0
A (n=14)
B (n=15)
C (n=11)
surface

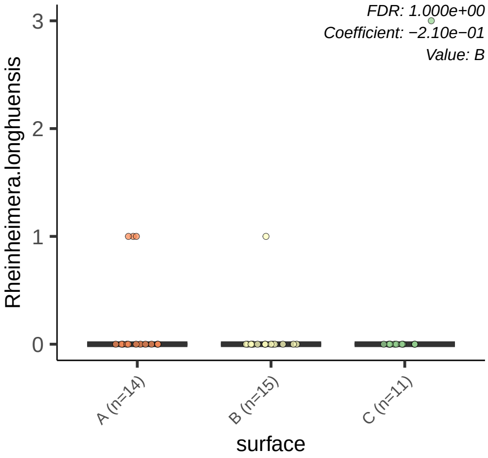

FDR: 1.000e+00
Coefficient: −2.10e−01
Value: B
Rheinheimera.longhuensis
3
2
1
0
A (n=14)
B (n=15)
C (n=11)
surface

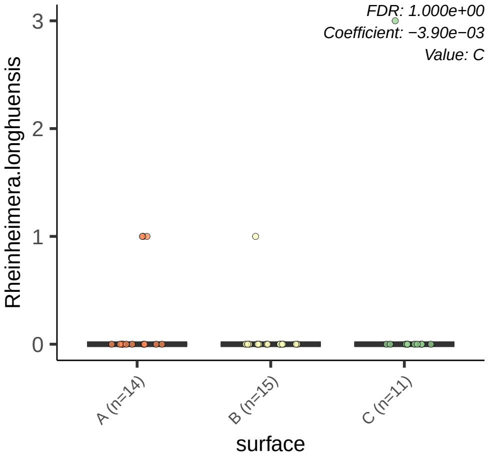
FDR: 1.000e+00
Coefficient: −3.90e−03
Value: C
Rheinheimera.longhuensis
0
1
2
3
A (n=14)
B (n=15)
C (n=11)
surface

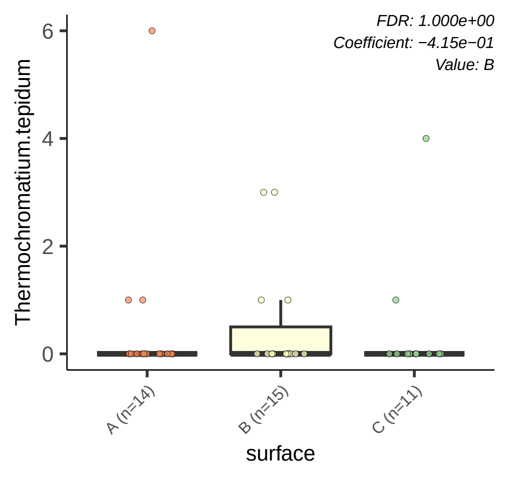

FDR: 1.000e+00
Coefficient: −4.15e−01
Value: B
Thermochromatium.tepidum
0
2
4
6
A (n=14)
B (n=15)
C (n=11)
surface

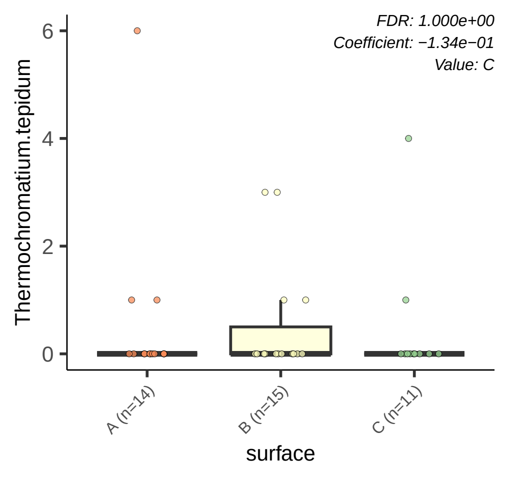
FDR: 1.000e+00
Coefficient: −1.34e−01
Value: C
Thermochromatium.tepidum
0
2
4
6
A (n=14)
B (n=15)
C (n=11)
surface

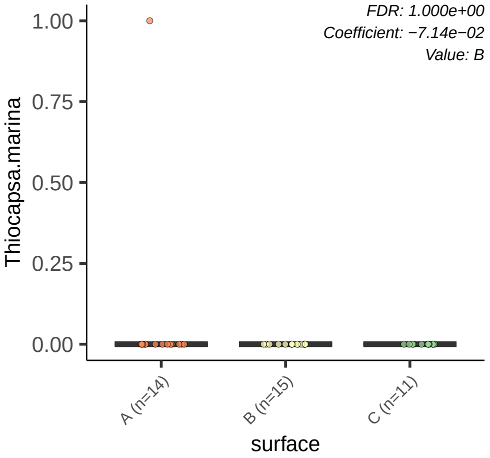
FDR: 1.000e+00
Coefficient: −7.14e−02
Value: B
Thiocapsa.marina
1.00
0.75
0.50
0.25
0.00
A (n=14)
B (n=15)
C (n=11)
surface

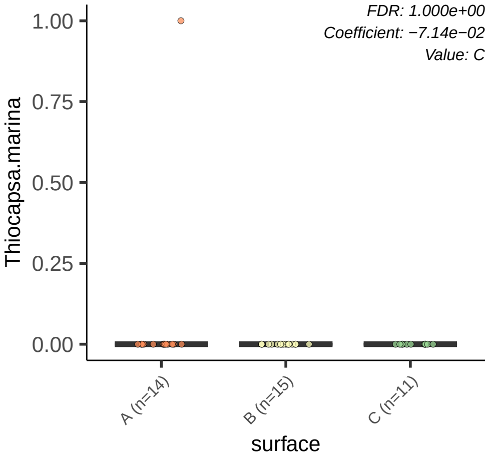
FDR: 1.000e+00
Coefficient: −7.14e−02
Value: C
Thiocapsa.marina
1.00
0.75
0.50
0.25
0.00
A (n=14)
B (n=15)
C (n=11)
surface

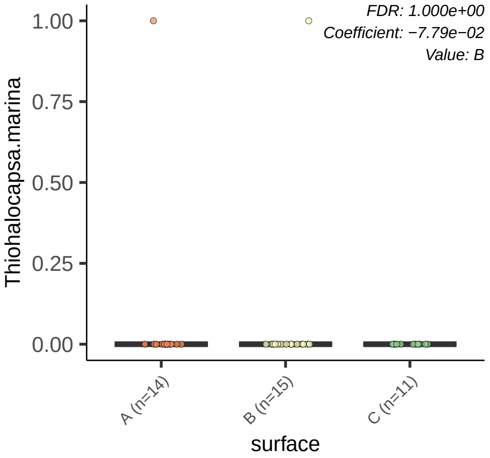
FDR: 1.000e+00
Coefficient: −7.79e−02
Value: B
Thiohalocapsa.marina
1.00
0.75
0.50
0.25
0.00
A (n=14)
B (n=15)
C (n=11)
surface

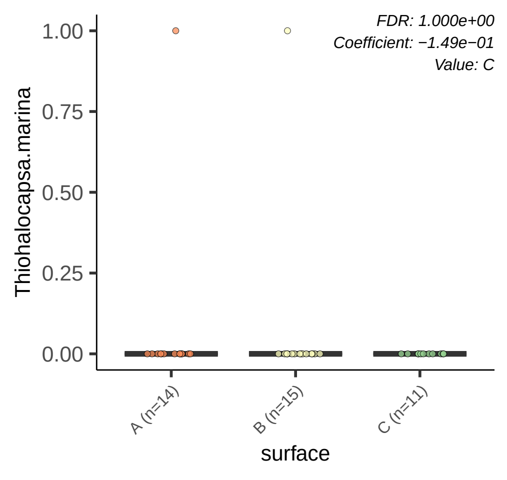
FDR: 1.000e+00
Coefficient: −1.49e−01
Value: C
Thiohalocapsa.marina
1.00
0.75
0.50
0.25
0.00
A (n=14)
B (n=15)
C (n=11)
surface

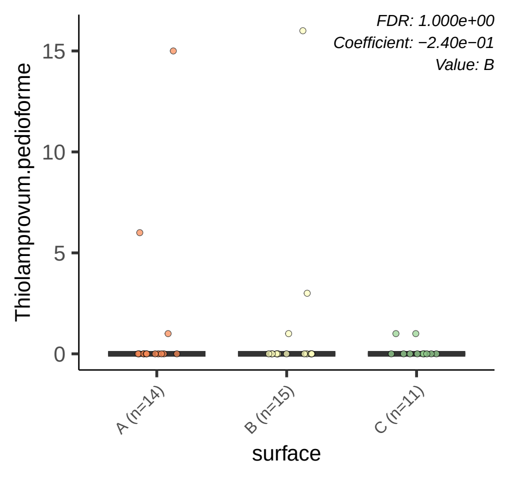

FDR: 1.000e+00
Coefficient: −2.40e−01
Value: B
Thiolamprovum.pedioforme
15
10
5
0
A (n=14)
B (n=15)
C (n=11)
surface

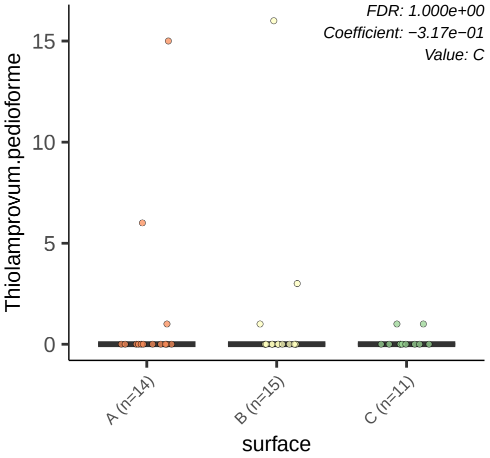

FDR: 1.000e+00
Coefficient: −3.17e−01
Value: C
Thiolamprovum.pediforme
15
10
5
0
A (n=14)
B (n=15)
C (n=11)
surface

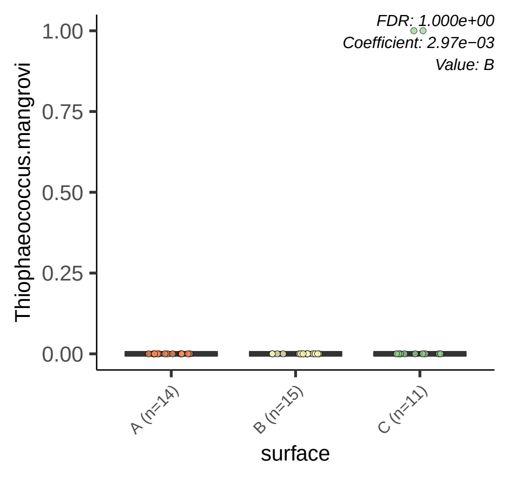
FDR: 1.000e+00
Coefficient: 2.97e−03
Value: B
Thiophaeococcus.mangrovi
1.00
0.75
0.50
0.25
0.00
A (n=14)
B (n=15)
C (n=11)
surface

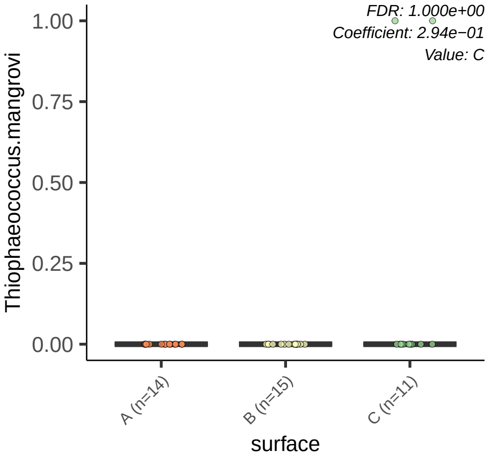
FDR: 1.000e+00
Coefficient: 2.94e−01
Value: C
Thiophaeococcus.mangrovi
1.00
0.75
0.50
0.25
0.00
A (n=14)
B (n=15)
C (n=11)
surface

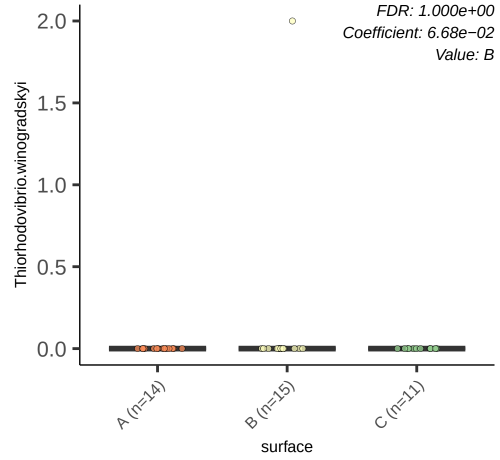
FDR: 1.000e+00
Coefficient: 6.68e−02
Value: B
Thiorhodovibrio.winogradskyi
2.0
1.5
1.0
0.5
0.0
A (n=14)
B (n=15)
C (n=11)
surface

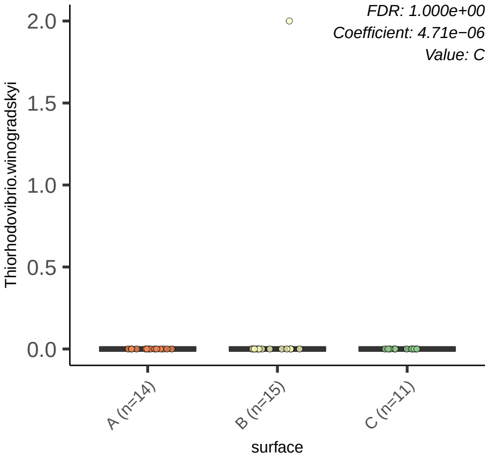

FDR: 1.000e+00
Coefficient: 4.71e−06
Value: C
Thiorhodovibrio.winogradskyi
2.0
1.5
1.0
0.5
0.0
A (n=14)
B (n=15)
C (n=11)
surface

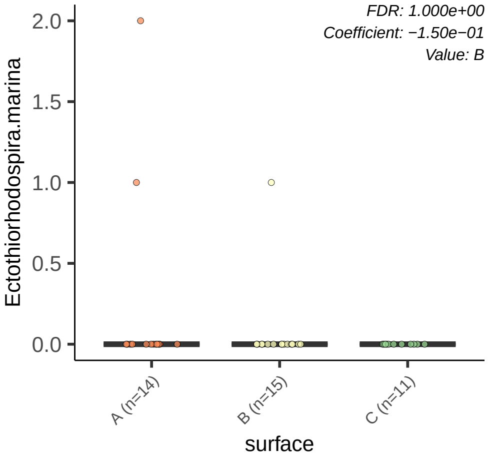
FDR: 1.000e+00
Coefficient: −1.50e−01
Value: B
Ectothiorhodospira.marina
2.0
1.5
1.0
0.5
0.0
A (n=14)
B (n=15)
C (n=11)
surface

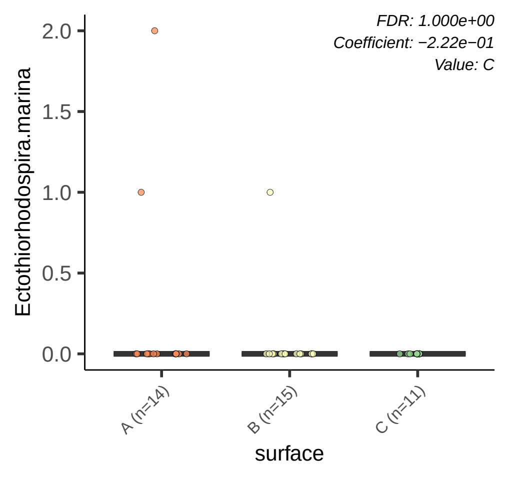

FDR: 1.000e+00
Coefficient: −2.22e−01
Value: C
Ectothiorhodospira.marina
2.0
1.5
1.0
0.5
0.0
A (n=14)
B (n=15)
C (n=11)
surface

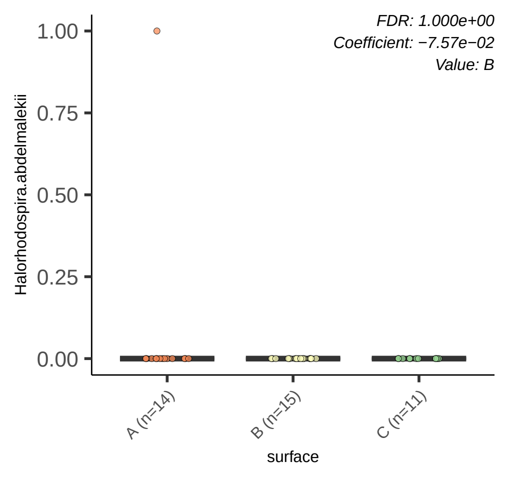

FDR: 1.000e+00
Coefficient: −7.57e−02
Value: B
Halorhodospira.abdelmalekii
1.00
0.75
0.50
0.25
0.00
A (n=14)
B (n=15)
C (n=11)
surface

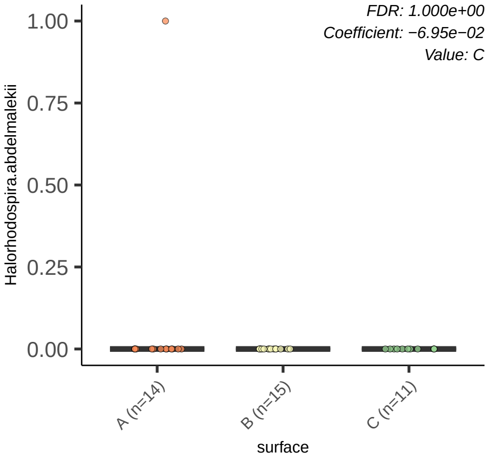

FDR: 1.000e+00
Coefficient: −6.95e−02
Value: C
Halorhodospira.abdelmalekii
1.00
0.75
0.50
0.25
0.00
A (n=14)
B (n=15)
C (n=11)
surface

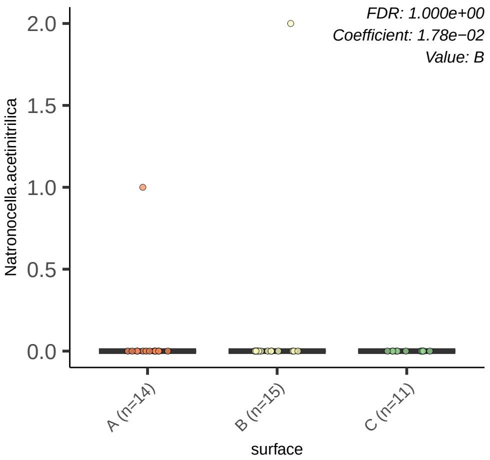

FDR: 1.000e+00
Coefficient: 1.78e−02
Value: B
Natronocella.acetinitrilica
2.0
1.5
1.0
0.5
0.0
A (n=14)
B (n=15)
C (n=11)
surface

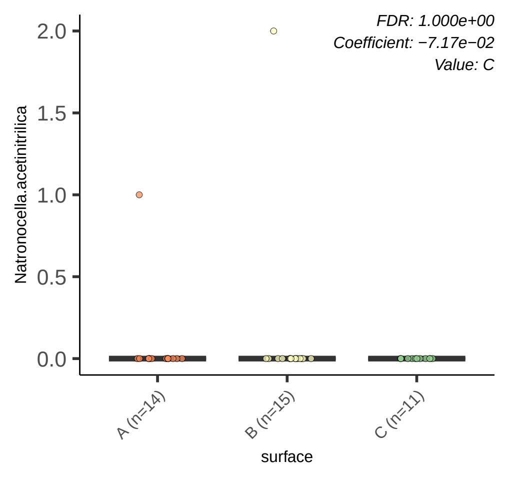
FDR: 1.000e+00
Coefficient: −7.17e−02
Value: C
Natronocella.acetinitrilica
2.0
1.5
1.0
0.5
0.0
A (n=14)
B (n=15)
C (n=11)
surface

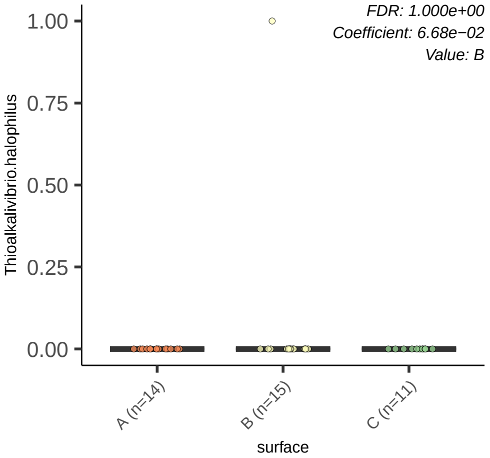

FDR: 1.000e+00
Coefficient: 6.68e−02
Value: B
1.00
0.75
0.50
0.25
0.00
Thioalkalivibrio.halophilus
A (n=14)
B (n=15)
C (n=11)
surface

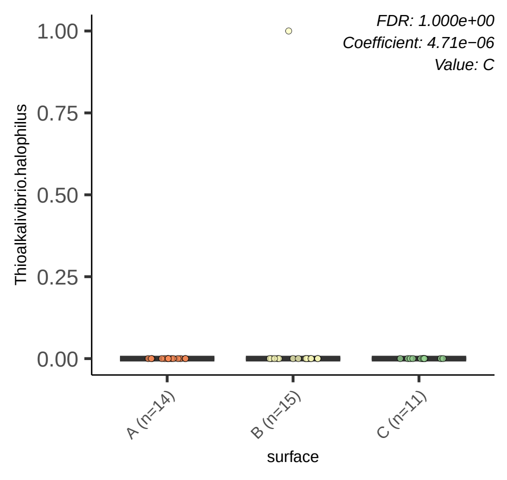
FDR: 1.000e+00
Coefficient: 4.71e−06
Value: C
Thioalkalivibrio.halophilus
1.00
0.75
0.50
0.25
0.00
A (n=14)
B (n=15)
C (n=11)
surface

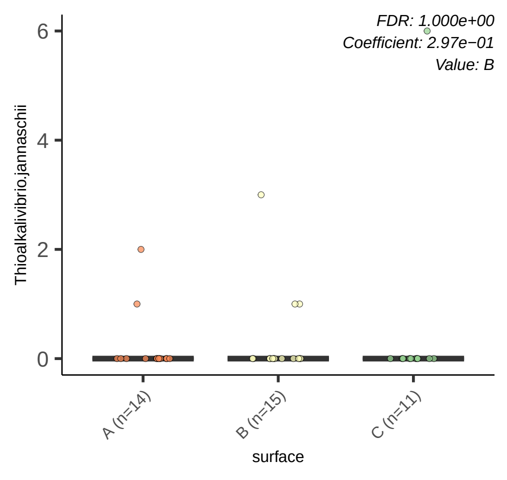
FDR: 1.000e+00
Coefficient: 2.97e−01
Value: B
Thioalkalivibrio.jannaschii
0
2
4
6
A (n=14)
B (n=15)
C (n=11)
surface

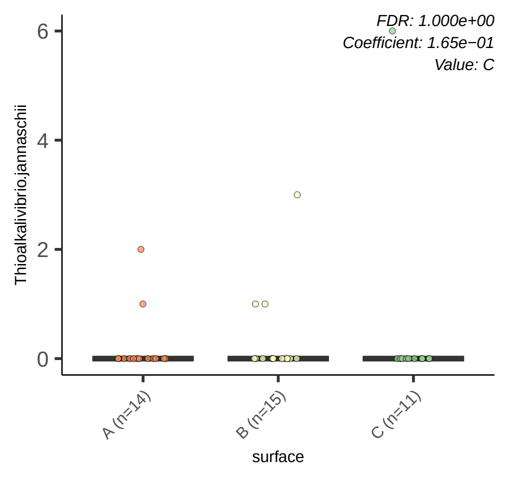
FDR: 1.000e+00
Coefficient: 1.65e−01
Value: C
Thioalkalivibrio.jannaschii
6
4
2
0
A (n=14)
B (n=15)
C (n=11)
surface

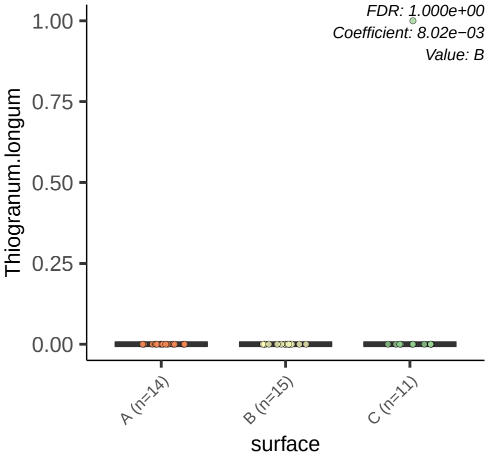
FDR: 1.000e+00
Coefficient: 8.02e−03
Value: B
Thiogranum.longum
1.00
0.75
0.50
0.25
0.00
A (n=14)
B (n=15)
C (n=11)
surface

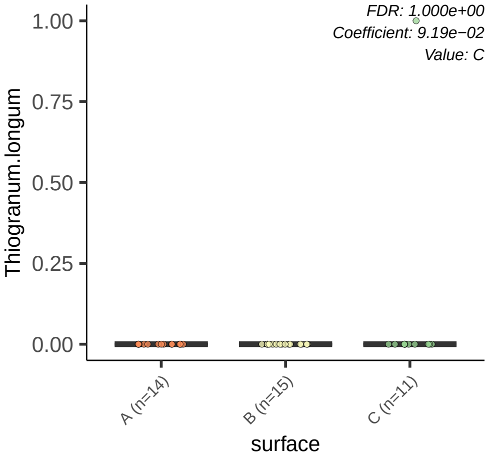
FDR: 1.000e+00
Coefficient: 9.19e−02
Value: C
Thiogranum.longum
1.00
0.75
0.50
0.25
0.00
A (n=14)
B (n=15)
C (n=11)
surface

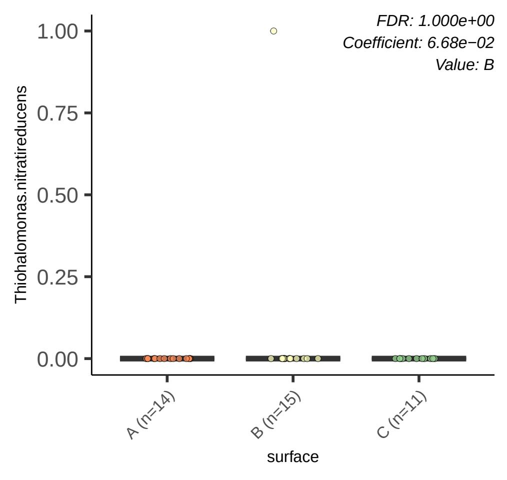
FDR: 1.000e+00
Coefficient: 6.68e−02
Value: B
Thiohalomonas.nitratireducens
1.00
0.75
0.50
0.25
0.00
A (n=14)
B (n=15)
C (n=11)
surface

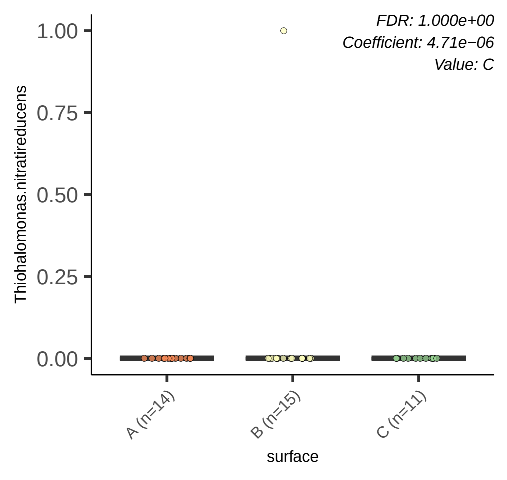
FDR: 1.000e+00
Coefficient: 4.71e−06
Value: C
Thiohalomonas.nitratireducens
1.00
0.75
0.50
0.25
0.00
A (n=14)
B (n=15)
C (n=11)
surface

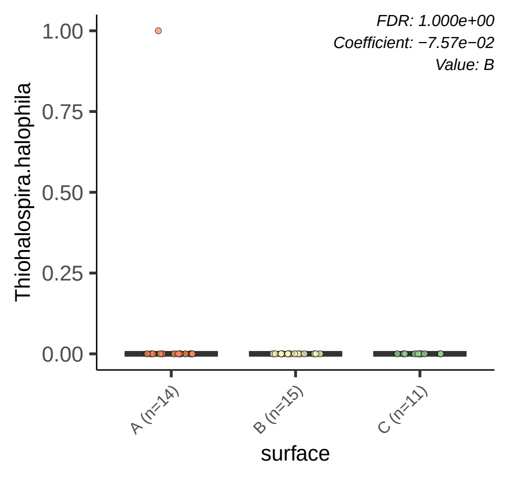
FDR: 1.000e+00
Coefficient: −7.57e−02
Value: B
Thiohalospira.halophila
1.00
0.75
0.50
0.25
0.00
A (n=14)
B (n=15)
C (n=11)
surface

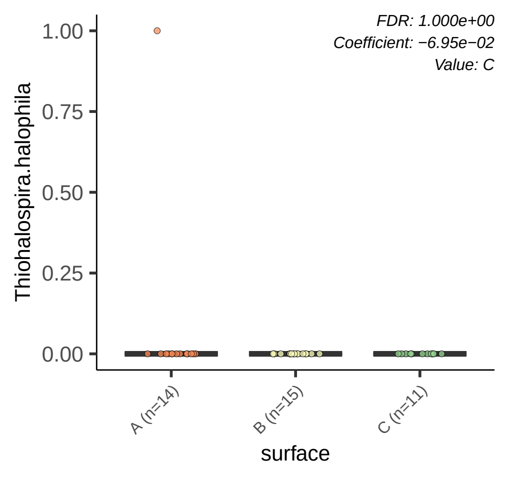

FDR: 1.000e+00
Coefficient: −6.95e−02
Value: C
Thiohalospira.halophila
1.00
0.75
0.50
0.25
0.00
A (n=14)
B (n=15)
C (n=11)
surface

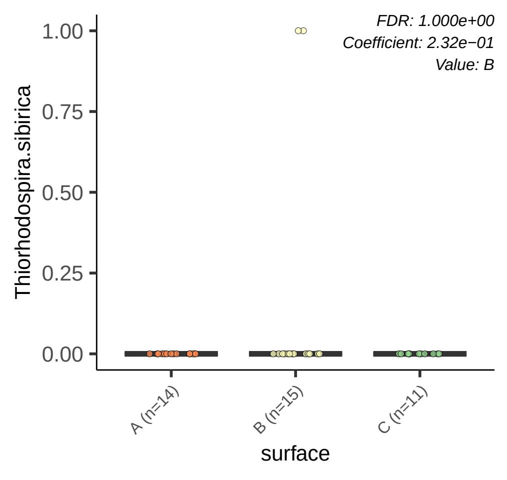

FDR: 1.000e+00
Coefficient: 2.32e−01
Value: B
Thiorhodospira.sibirica
1.00
0.75
0.50
0.25
0.00
A (n=14)
B (n=15)
C (n=11)
surface

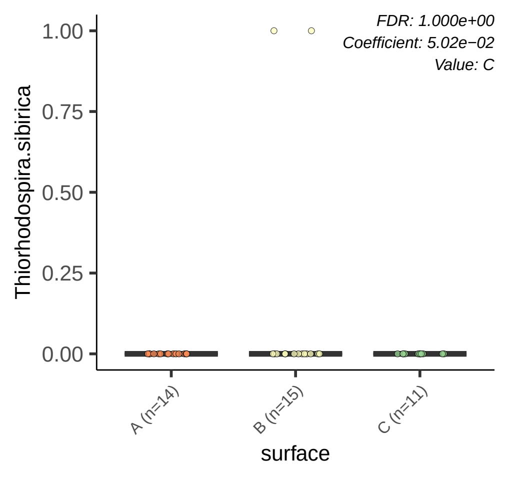
FDR: 1.000e+00
Coefficient: 5.02e−02
Value: C
Thiorhodospira.sibirica
1.00
0.75
0.50
0.25
0.00
A (n=14)
B (n=15)
C (n=11)
surface

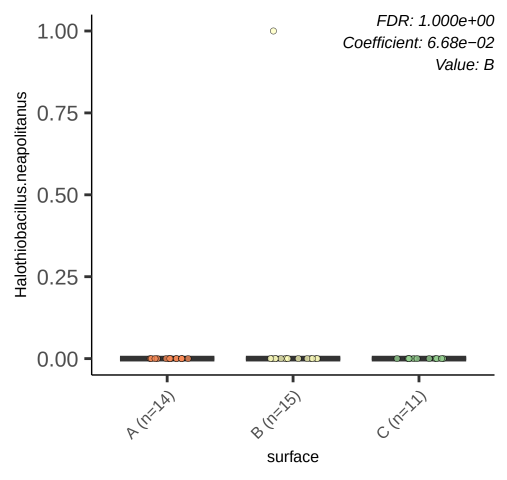

FDR: 1.000e+00
Coefficient: 6.68e−02
Value: B
Halothiobacillus.neapolitanus
1.00
0.75
0.50
0.25
0.00
A (n=14)
B (n=15)
C (n=11)
surface

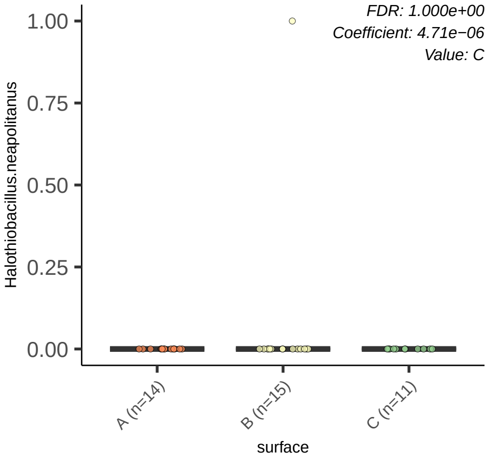

FDR: 1.000e+00
Coefficient: 4.71e−06
Value: C
Halothiobacillus.neapolitanus
1.00
0.75
0.50
0.25
0.00
A (n=14)
B (n=15)
C (n=11)
surface

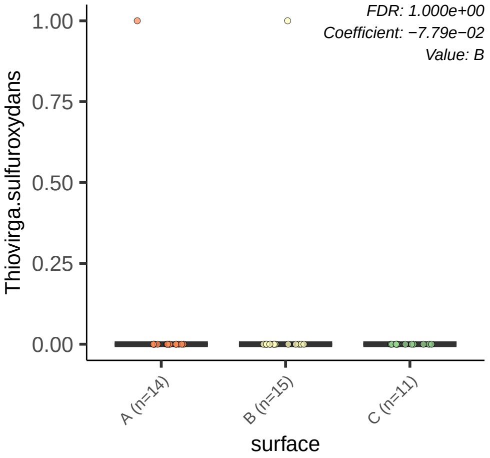

FDR: 1.000e+00
Coefficient: −7.79e−02
Value: B
Thiovirga.sulfuroxydans
1.00
0.75
0.50
0.25
0.00
A (n=14)
B (n=15)
C (n=11)
surface

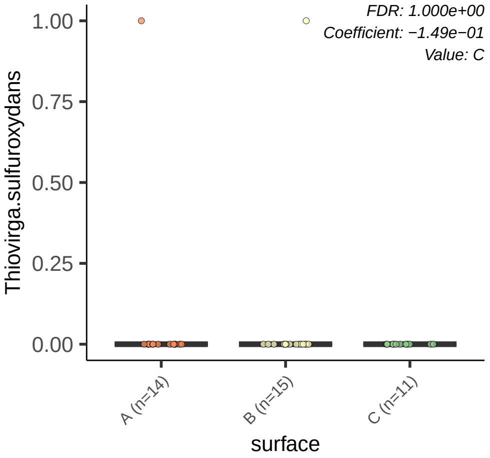
FDR: 1.000e+00
Coefficient: −1.49e−01
Value: C
Thiovirga.sulfuroxydans
1.00
0.75
0.50
0.25
0.00
A (n=14)
B (n=15)
C (n=11)
surface

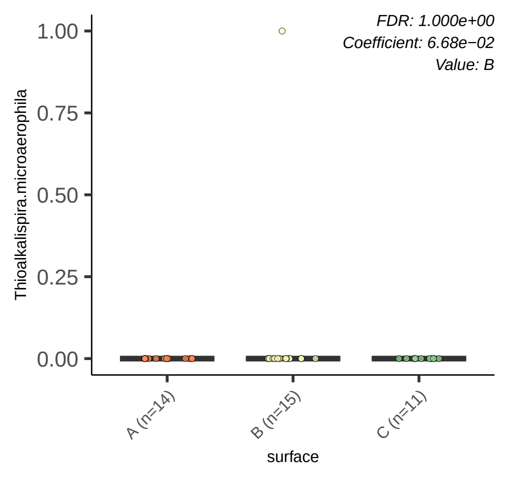

FDR: 1.000e+00
Coefficient: 6.68e−02
Value: B
Thioalkalispira.microaerophila
1.00
0.75
0.50
0.25
0.00
A (n=14)
B (n=15)
C (n=11)
surface

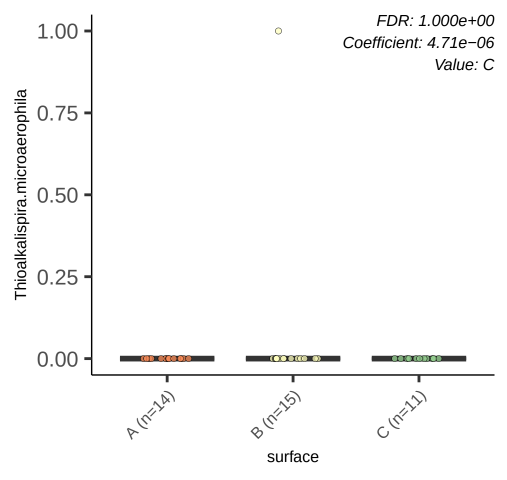
FDR: 1.000e+00
Coefficient: 4.71e−06
Value: C
1.00
0.75
0.50
0.25
0.00
Thioalkalispira.microaerophila
A (n=14)
B (n=15)
C (n=11)
surface

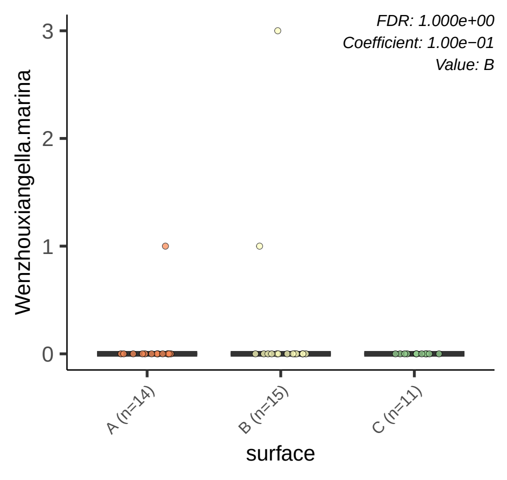
FDR: 1.000e+00
Coefficient: 1.00e−01
Value: B
Wenzhouxiangella.marina
0
1
2
3
A (n=14)
B (n=15)
C (n=11)
surface

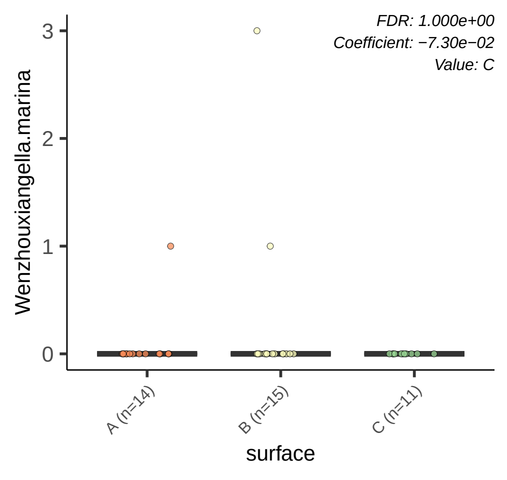

FDR: 1.000e+00
Coefficient: −7.30e−02
Value: C
Wenzhouxiangella.marina
3
2
1
0
A (n=14)
B (n=15)
C (n=11)
surface

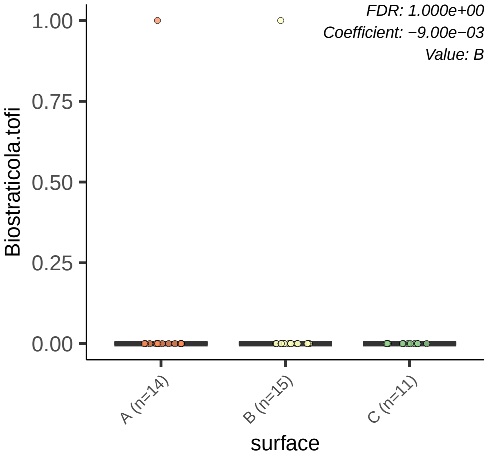
FDR: 1.000e+00
Coefficient: −9.00e−03
Value: B
Biostraticola.tofi
1.00
0.75
0.50
0.25
0.00
A (n=14)
B (n=15)
C (n=11)
surface

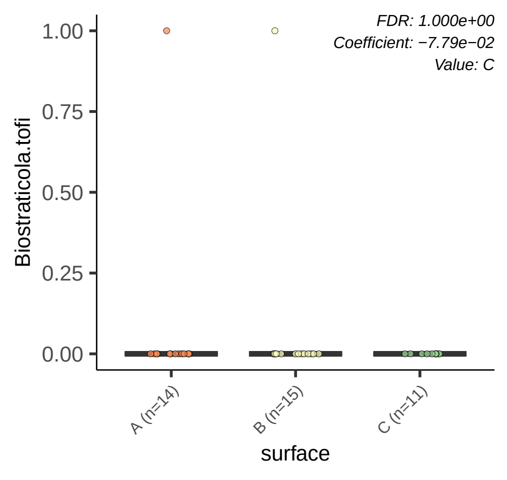
FDR: 1.000e+00
Coefficient: −7.79e−02
Value: C
Biostraticola.tofi
1.00
0.75
0.50
0.25
0.00
A (n=14)
B (n=15)
C (n=11)
surface

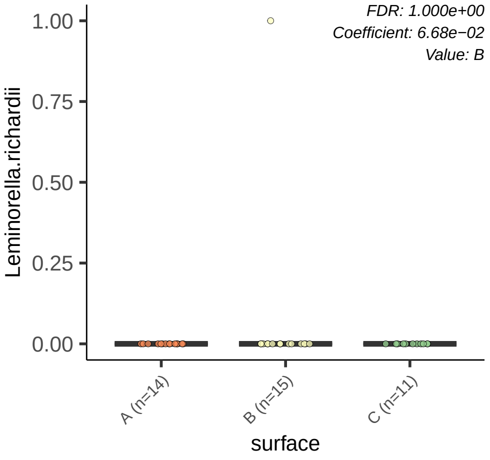

FDR: 1.000e+00
Coefficient: 6.68e−02
Value: B
Leminorella.richardii
1.00
0.75
0.50
0.25
0.00
A (n=14)
B (n=15)
C (n=11)
surface

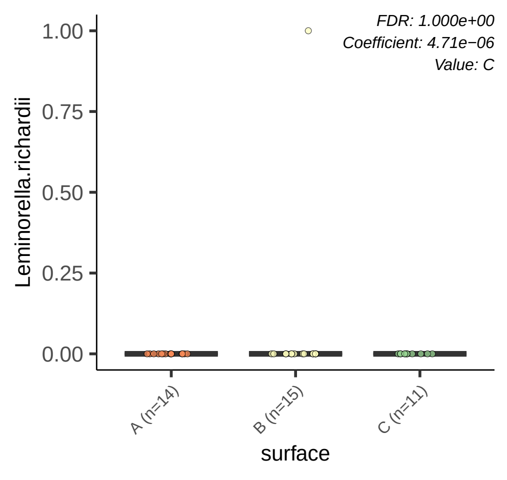
FDR: 1.000e+00
Coefficient: 4.71e−06
Value: C
Leminorella.richardii
1.00
0.75
0.50
0.25
0.00
A (n=14)
B (n=15)
C (n=11)
surface

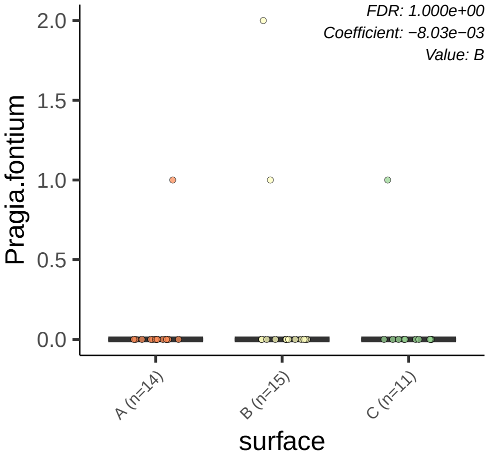

FDR: 1.000e+00
Coefficient: −8.03e−03
Value: B
Pragia.fontium
2.0
1.5
1.0
0.5
0.0
A (n=14)
B (n=15)
C (n=11)
surface

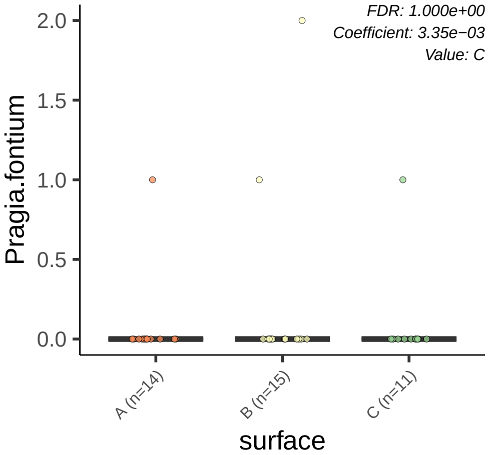

FDR: 1.000e+00
Coefficient: 3.35e−03
Value: C
Pragia.fontium
2.0
1.5
1.0
0.5
0.0
A (n=14)
B (n=15)
C (n=11)
surface

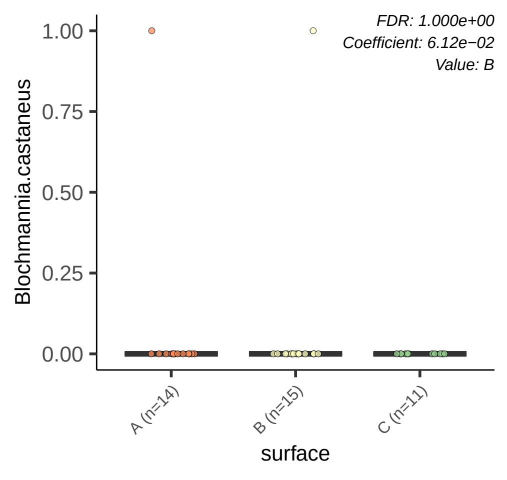
FDR: 1.000e+00
Coefficient: 6.12e−02
Value: B
Blochmannia.castaneus
1.00
0.75
0.50
0.25
0.00
A (n=14)
B (n=15)
C (n=11)
surface

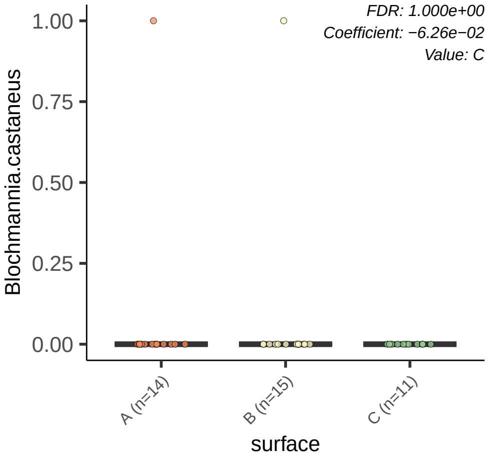
FDR: 1.000e+00
Coefficient: −6.26e−02
Value: C
1.00
0.75
0.50
0.25
0.00
Blochmannia.castaneus
A (n=14)
B (n=15)
C (n=11)
surface

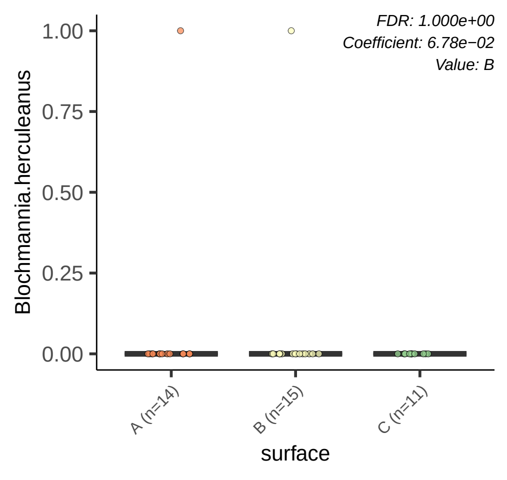

FDR: 1.000e+00
Coefficient: 6.78e−02
Value: B
Blochmannia.herculeanus
1.00
0.75
0.50
0.25
0.00
A (n=14)
B (n=15)
C (n=11)
surface

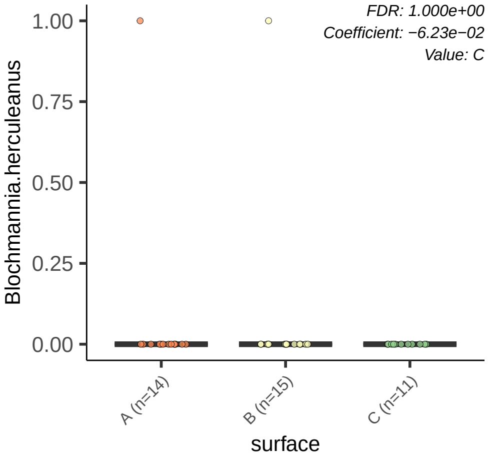
FDR: 1.000e+00
Coefficient: −6.23e−02
Value: C
Blochmannia.herculeanus
1.00
0.75
0.50
0.25
0.00
A (n=14)
B (n=15)
C (n=11)
surface

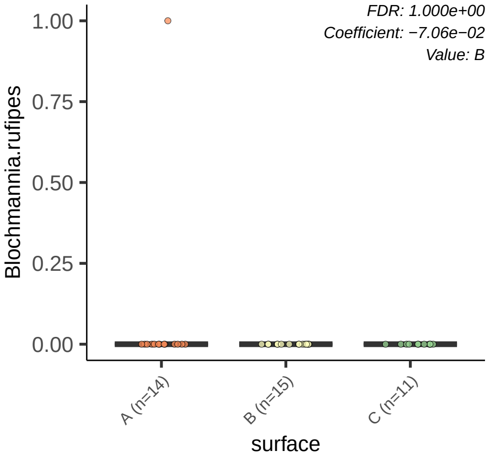

FDR: 1.000e+00
Coefficient: −7.06e−02
Value: B
Blochmannia.rufipes
1.00
0.75
0.50
0.25
0.00
A (n=14)
B (n=15)
C (n=11)
surface

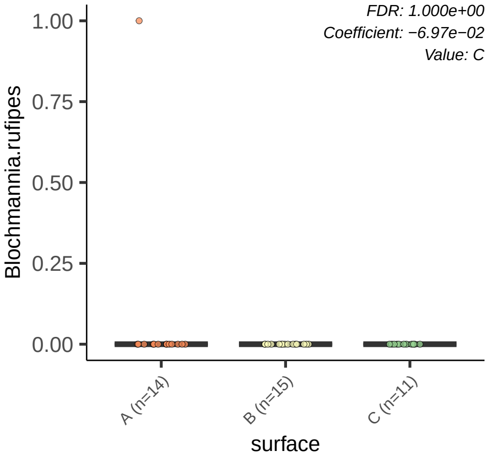
FDR: 1.000e+00
Coefficient: −6.97e−02
Value: C
Blochmannia.rufipes
1.00
0.75
0.50
0.25
0.00
A (n=14)
B (n=15)
C (n=11)
surface

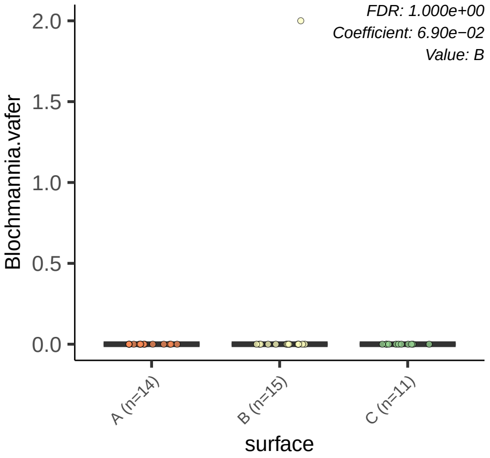

FDR: 1.000e+00
Coefficient: 6.90e−02
Value: B
Blochmannia.vafer
2.0
1.5
1.0
0.5
0.0
A (n=14)
B (n=15)
C (n=11)
surface

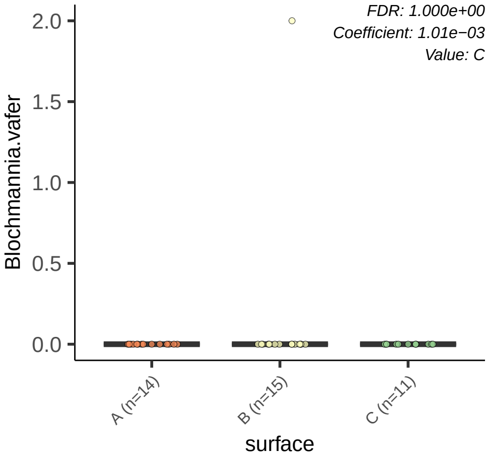

FDR: 1.000e+00
Coefficient: 1.01e−03
Value: C
Blochmannia.vafer
2.0
1.5
1.0
0.5
0.0
A (n=14)
B (n=15)
C (n=11)
surface

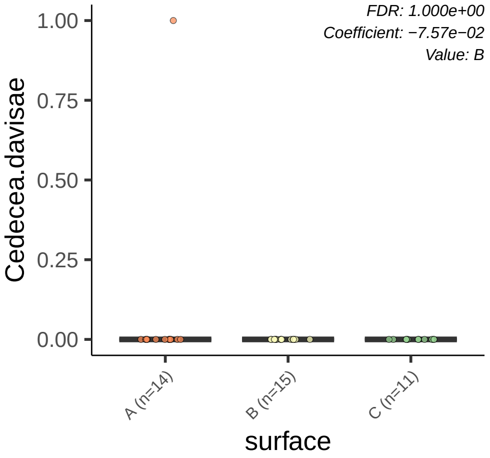
FDR: 1.000e+00
Coefficient: −7.57e−02
Value: B
Cedecea.davisae
1.00
0.75
0.50
0.25
0.00
A (n=14)
B (n=15)
C (n=11)
surface

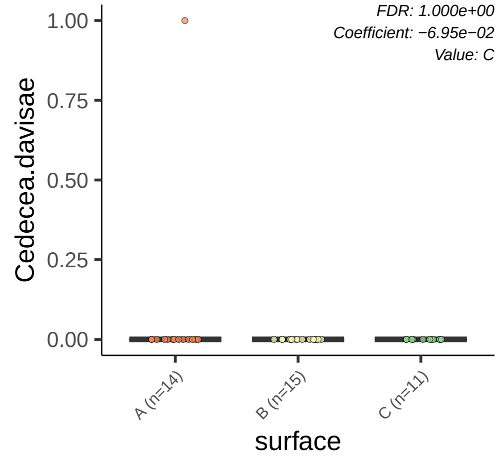
FDR: 1.000e+00
Coefficient: −6.95e−02
Value: C
Cedecea.davisae
1.00
0.75
0.50
0.25
0.00
A (n=14)
B (n=15)
C (n=11)
surface

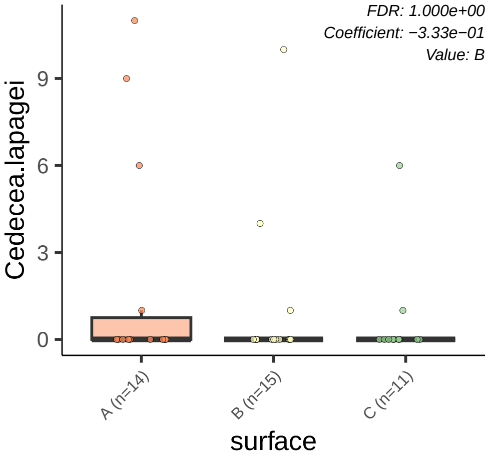
FDR: 1.000e+00
Coefficient: −3.33e−01
Value: B
Cedecea.lapagei
0
3
6
9
A (n=14)
B (n=15)
C (n=11)
surface

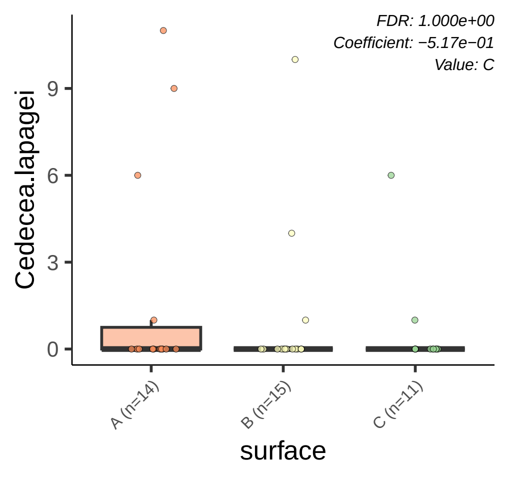

FDR: 1.000e+00
Coefficient: −5.17e−01
Value: C
Cedecea.lapagei
0
3
6
9
A (n=14)
B (n=15)
C (n=11)
surface

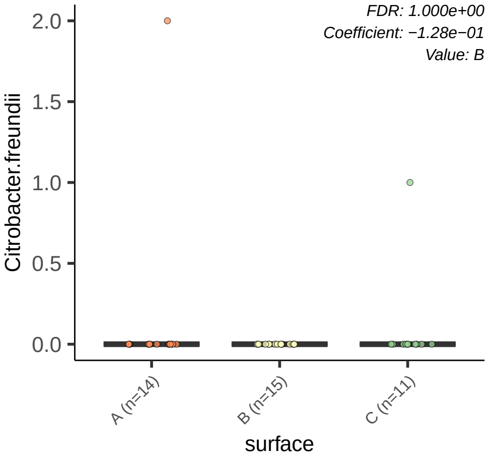
FDR: 1.000e+00
Coefficient: −1.28e−01
Value: B
Citrobacter.freundii
2.0
1.5
1.0
0.5
0.0
A (n=14)
B (n=15)
C (n=11)
surface

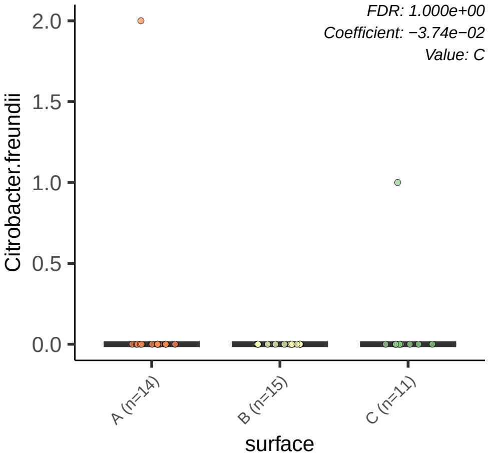
FDR: 1.000e+00
Coefficient: −3.74e−02
Value: C
2.0
1.5
1.0
0.5
0.0
Citrobacter.freundii
A (n=14)
B (n=15)
C (n=11)
surface

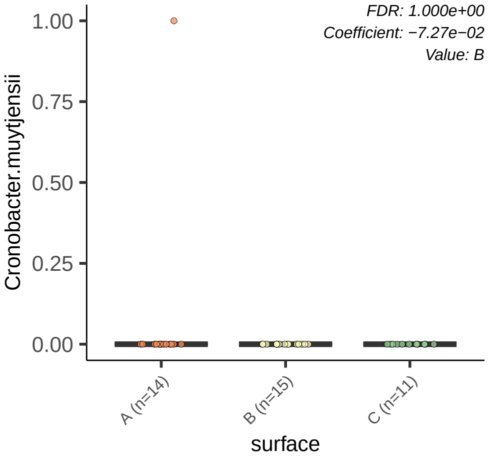
FDR: 1.000e+00
Coefficient: −7.27e−02
Value: B
Cronobacter.muytjensii
1.00
0.75
0.50
0.25
0.00
A (n=14)
B (n=15)
C (n=11)
surface

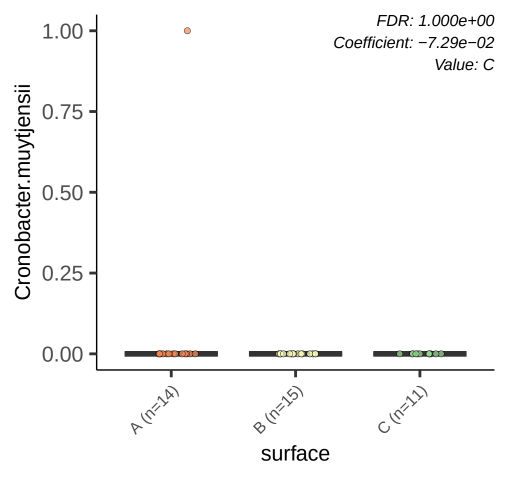

FDR: 1.000e+00
Coefficient: −7.29e−02
Value: C
Cronobacter.muytjensii
1.00
0.75
0.50
0.25
0.00
A (n=14)
B (n=15)
C (n=11)
surface

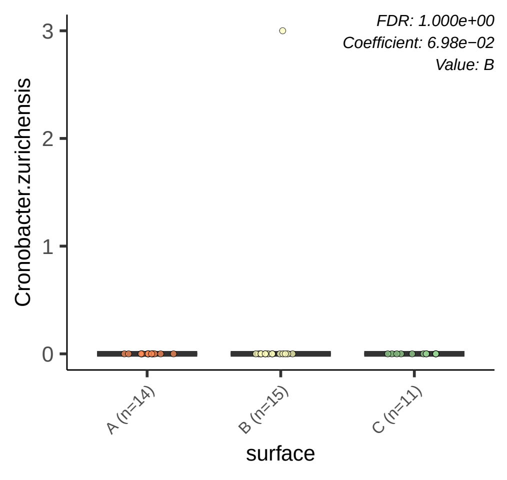
FDR: 1.000e+00
Coefficient: 6.98e−02
Value: B
Cronobacter.zurichensis
3
2
1
0
A (n=14)
B (n=15)
C (n=11)
surface

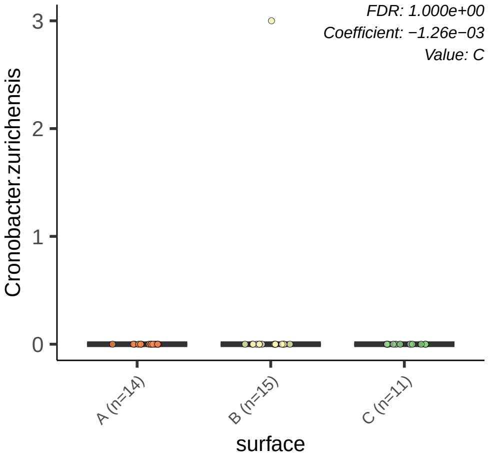
FDR: 1.000e+00
Coefficient: −1.26e−03
Value: C
Cronobacter.zurichensis
3
2
1
0
A (n=14)
B (n=15)
C (n=11)
surface

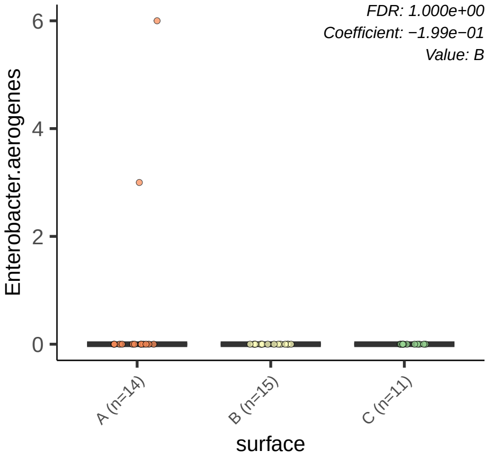

FDR: 1.000e+00
Coefficient: −1.99e−01
Value: B
Enterobacter.aerogenes
6
4
2
0
A (n=14)
B (n=15)
C (n=11)
surface

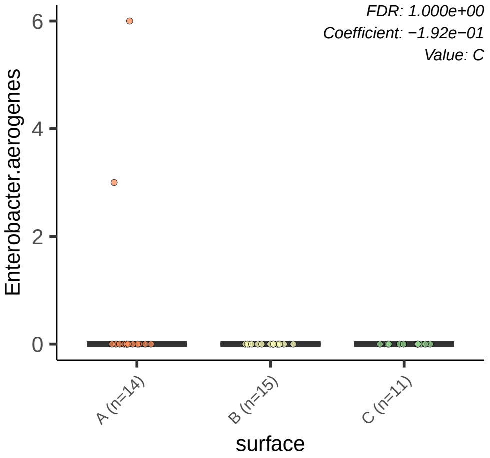
FDR: 1.000e+00
Coefficient: −1.92e−01
Value: C
Enterobacter.aerogenes
0
2
4
6
A (n=14)
B (n=15)
C (n=11)
surface

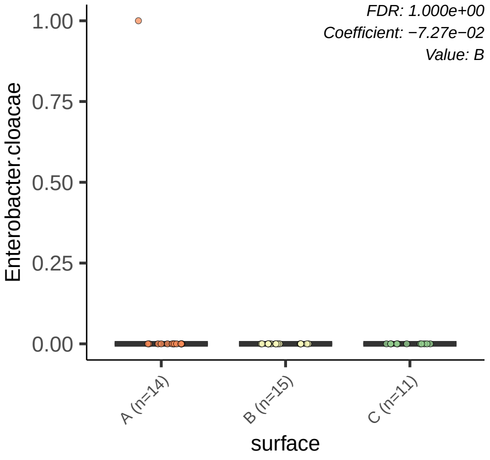
FDR: 1.000e+00
Coefficient: −7.27e−02
Value: B
Enterobacter.cloacae
1.00
0.75
0.50
0.25
0.00
A (n=14)
B (n=15)
C (n=11)
surface

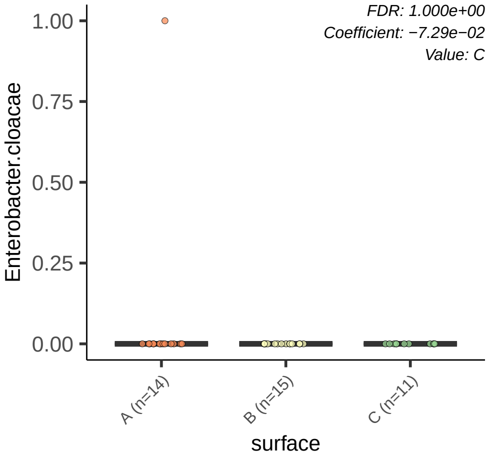
FDR: 1.000e+00
Coefficient: −7.29e−02
Value: C
Enterobacter.cloacae
1.00
0.75
0.50
0.25
0.00
A (n=14)
B (n=15)
C (n=11)
surface

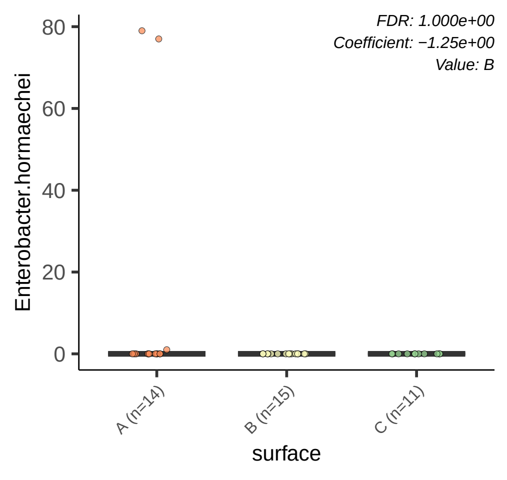

FDR: 1.000e+00
Coefficient: −1.25e+00
Value: B
Enterobacter.hormaechei
80
60
40
20
0
A (n=14)
B (n=15)
C (n=11)
surface

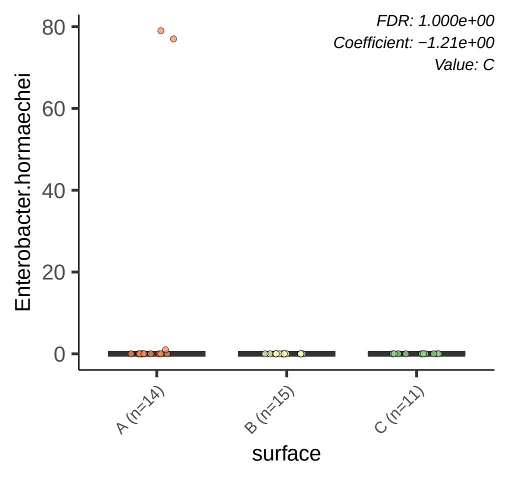
FDR: 1.000e+00
Coefficient: −1.21e+00
Value: C
Enterobacter.hormaechei
80
60
40
20
0
A (n=14)
B (n=15)
C (n=11)
surface

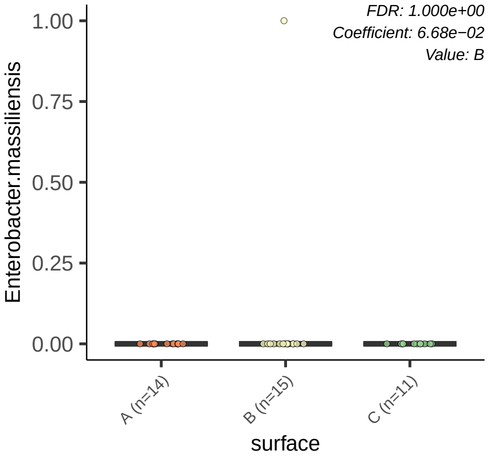

FDR: 1.000e+00
Coefficient: 6.68e−02
Value: B
Enterobacter.massiliensis
1.00
0.75
0.50
0.25
0.00
A (n=14)
B (n=15)
C (n=11)
surface

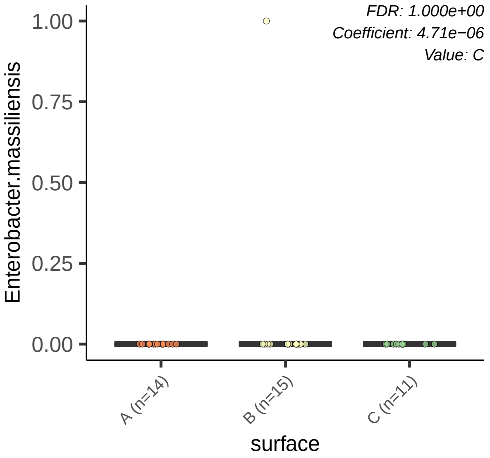

FDR: 1.000e+00
Coefficient: 4.71e−06
Value: C
Enterobacter.massiliensis
1.00
0.75
0.50
0.25
0.00
A (n=14)
B (n=15)
C (n=11)
surface

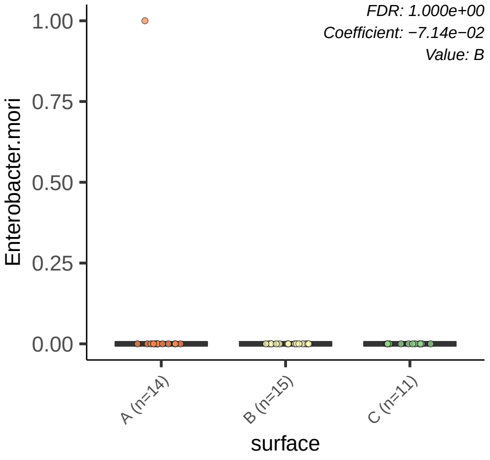

FDR: 1.000e+00
Coefficient: −7.14e−02
Value: B
Enterobacter.mori
1.00
0.75
0.50
0.25
0.00
A (n=14)
B (n=15)
C (n=11)
surface

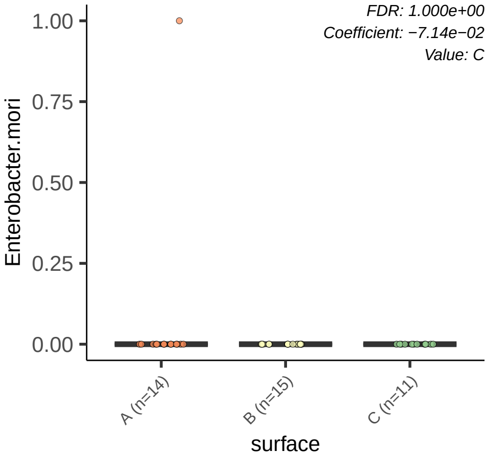
FDR: 1.000e+00
Coefficient: −7.14e−02
Value: C
Enterobacter.mori
1.00
0.75
0.50
0.25
0.00
A (n=14)
B (n=15)
C (n=11)
surface

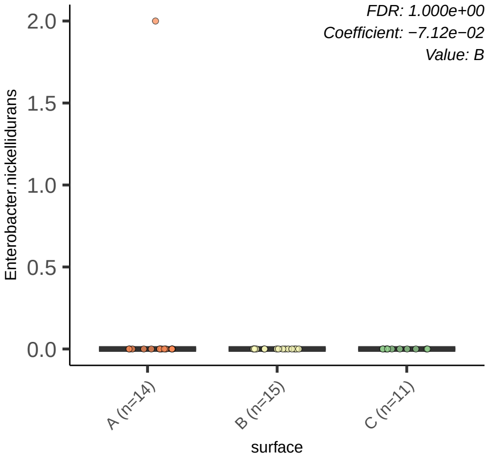
FDR: 1.000e+00
Coefficient: −7.12e−02
Value: B
Enterobacter.nickellidurans
2.0
1.5
1.0
0.5
0.0
A (n=14)
B (n=15)
C (n=11)
surface

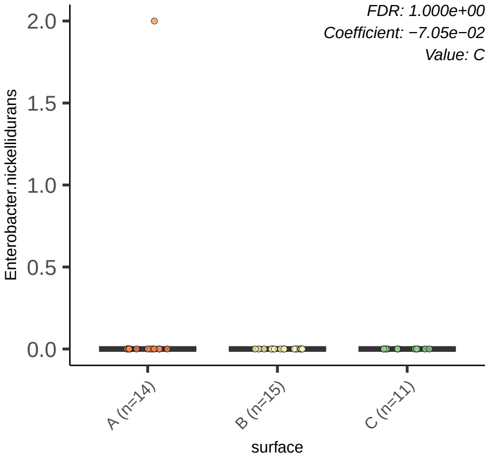
FDR: 1.000e+00
Coefficient: −7.05e−02
Value: C
Enterobacter.nickellidurans
2.0
1.5
1.0
0.5
0.0
A (n=14)
B (n=15)
C (n=11)
surface

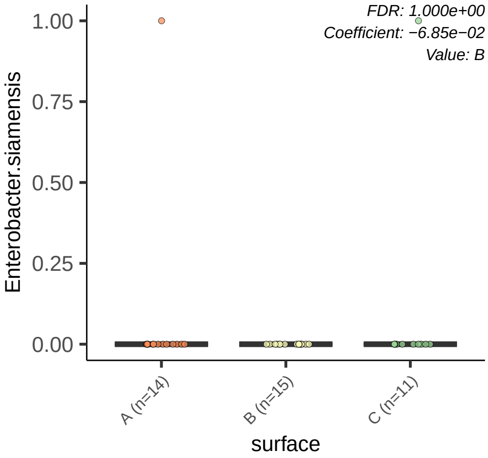

FDR: 1.000e+00
Coefficient: −6.85e−02
Value: B
Enterobacter.siamensis
1.00
0.75
0.50
0.25
0.00
A (n=14)
B (n=15)
C (n=11)
surface

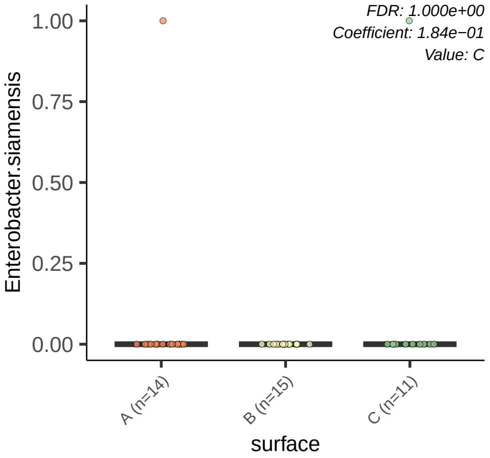
FDR: 1.000e+00
Coefficient: 1.84e−01
Value: C
Enterobacter.siamensis
1.00
0.75
0.50
0.25
0.00
A (n=14)
B (n=15)
C (n=11)
surface

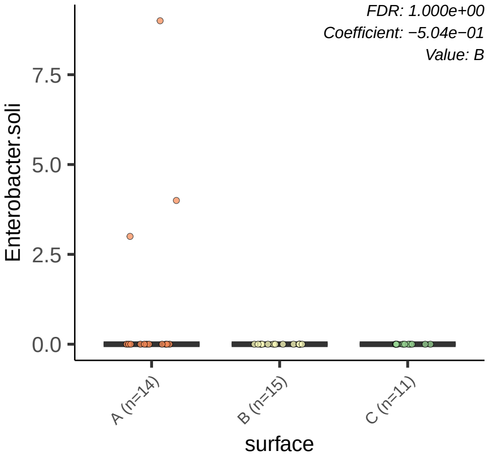
FDR: 1.000e+00
Coefficient: −5.04e−01
Value: B
Enterobacter.soli
7.5
5.0
2.5
0.0
A (n=14)
B (n=15)
C (n=11)
surface

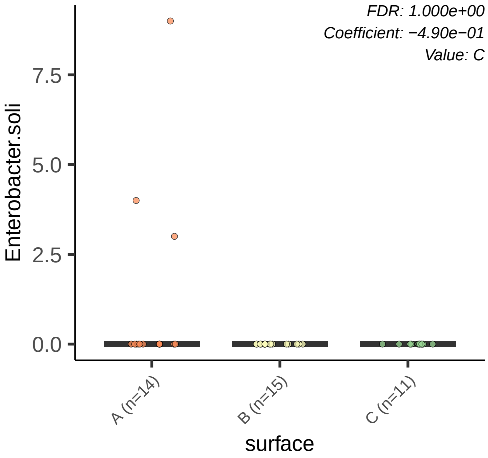

FDR: 1.000e+00
Coefficient: −4.90e−01
Value: C
Enterobacter.soli
7.5
5.0
2.5
0.0
A (n=14)
B (n=15)
C (n=11)
surface

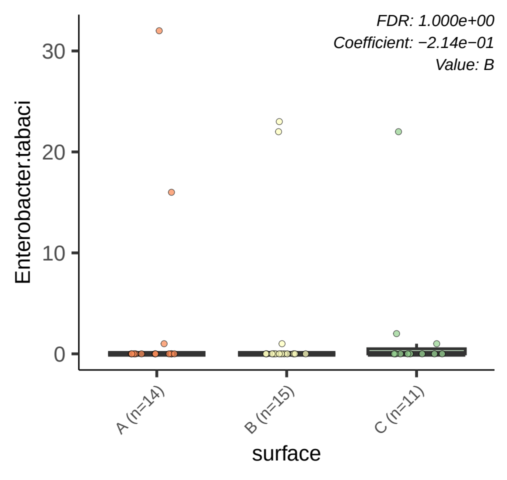

FDR: 1.000e+00
Coefficient: −2.14e−01
Value: B
Enterobacter.tabaci
0
10
20
30
A (n=14)
B (n=15)
C (n=11)
surface

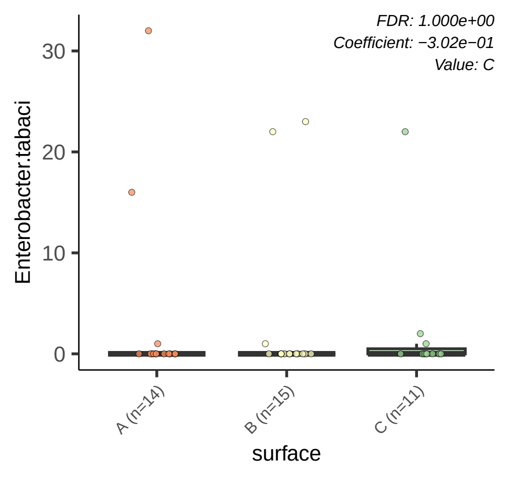

FDR: 1.000e+00
Coefficient: −3.02e−01
Value: C
Enterobacter.tabaci
0
10
20
30
A (n=14)
B (n=15)
C (n=11)
surface

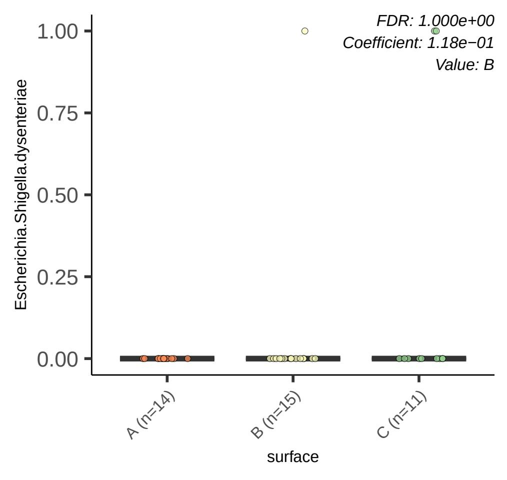
FDR: 1.000e+00
Coefficient: 1.18e−01
Value: B
Escherichia.Shigella.dysenteriae
1.00
0.75
0.50
0.25
0.00
A (n=14)
B (n=15)
C (n=11)
surface

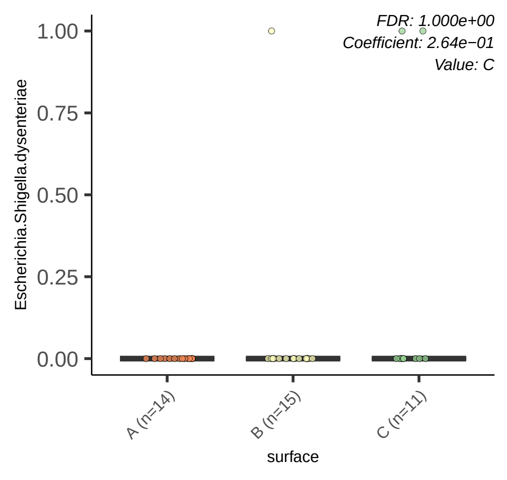
FDR: 1.000e+00
Coefficient: 2.64e−01
Value: C
1.00
0.75
0.50
0.25
0.00
Escherichia.Shigella.dysenteriae
A (n=14)
B (n=15)
C (n=11)
surface

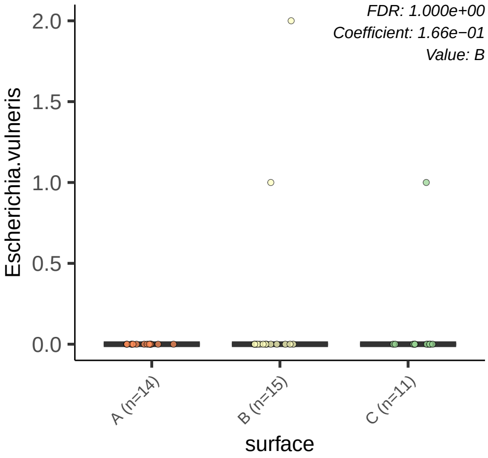
FDR: 1.000e+00
Coefficient: 1.66e−01
Value: B
Escherichia.vulneris
2.0
1.5
1.0
0.5
0.0
A (n=14)
B (n=15)
C (n=11)
surface

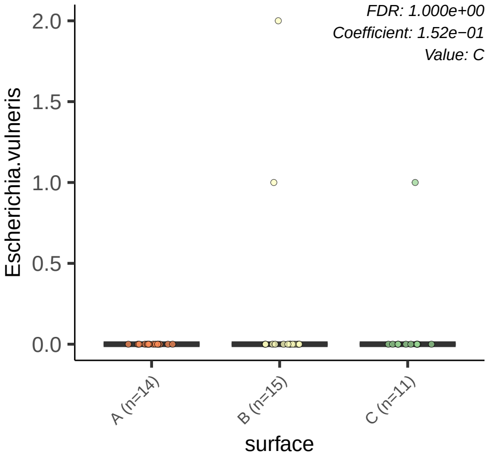
FDR: 1.000e+00
Coefficient: 1.52e−01
Value: C
2.0
1.5
1.0
0.5
0.0
Escherichia.vulneris
A (n=14)
B (n=15)
C (n=11)
surface

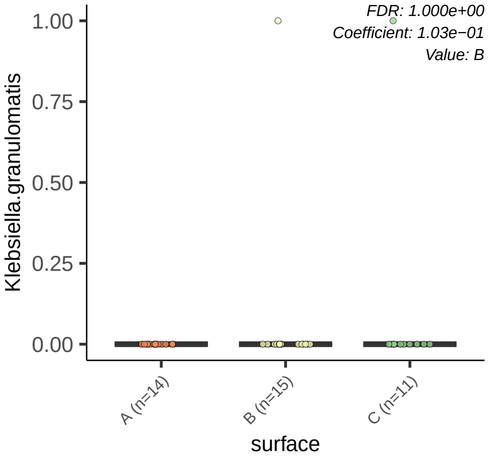
FDR: 1.000e+00
Coefficient: 1.03e−01
Value: B
Klebsiella.granulomatis
1.00
0.75
0.50
0.25
0.00
A (n=14)
B (n=15)
C (n=11)
surface

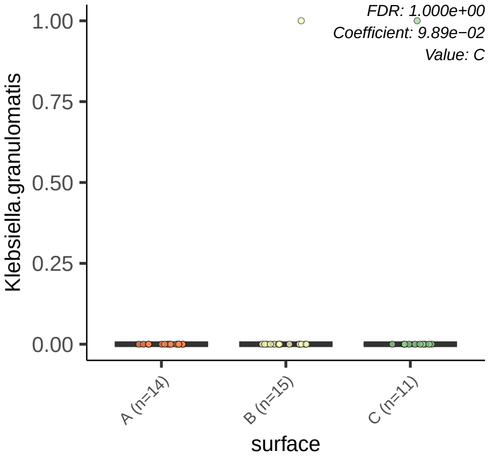

FDR: 1.000e+00
Coefficient: 9.89e−02
Value: C
Klebsiella.granulomatis
1.00
0.75
0.50
0.25
0.00
A (n=14)
B (n=15)
C (n=11)
surface

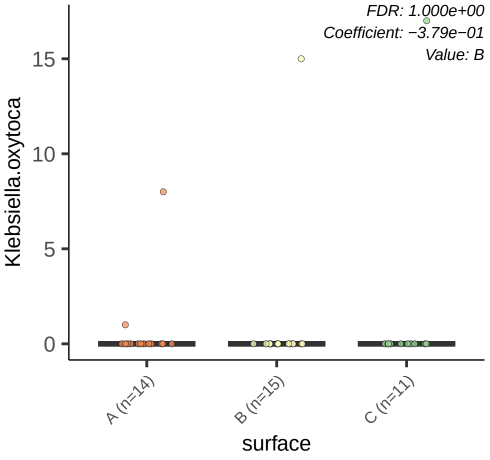

FDR: 1.000e+00
Coefficient: −3.79e−01
Value: B
Klebsiella.oxytoca
0
5
10
15
A (n=14)
B (n=15)
C (n=11)
surface

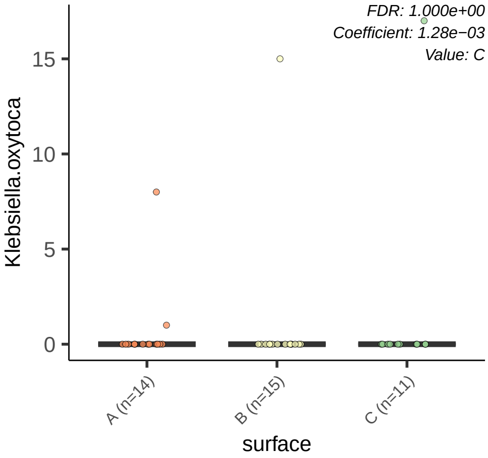
FDR: 1.000e+00
Coefficient: 1.28e−03
Value: C
Klebsiella.oxytoca
15
10
5
0
A (n=14)
B (n=15)
C (n=11)
surface

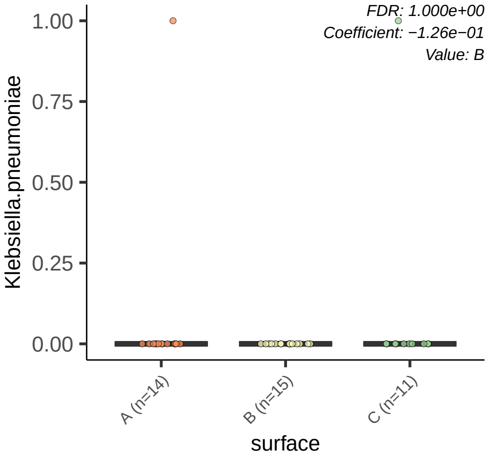
FDR: 1.000e+00
Coefficient: −1.26e−01
Value: B
Klebsiella.pneumoniae
1.00
0.75
0.50
0.25
0.00
A (n=14)
B (n=15)
C (n=11)
surface

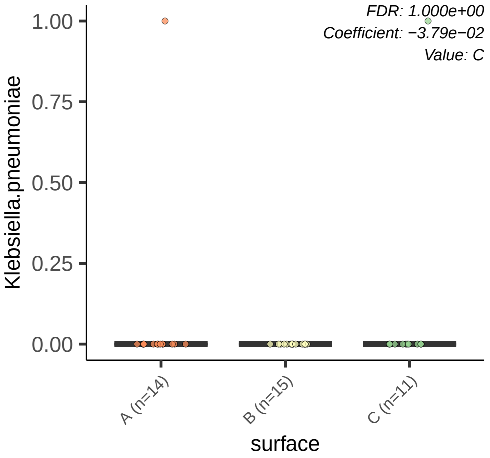
FDR: 1.000e+00
Coefficient: −3.79e−02
Value: C
Klebsiella.pneumoniae
1.00
0.75
0.50
0.25
0.00
A (n=14)
B (n=15)
C (n=11)
surface

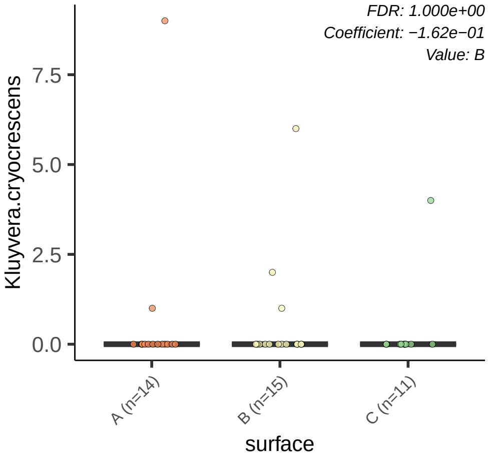

FDR: 1.000e+00
Coefficient: −1.62e−01
Value: B
Kluyvera.cryocrescens
7.5
5.0
2.5
0.0
A (n=14)
B (n=15)
C (n=11)
surface

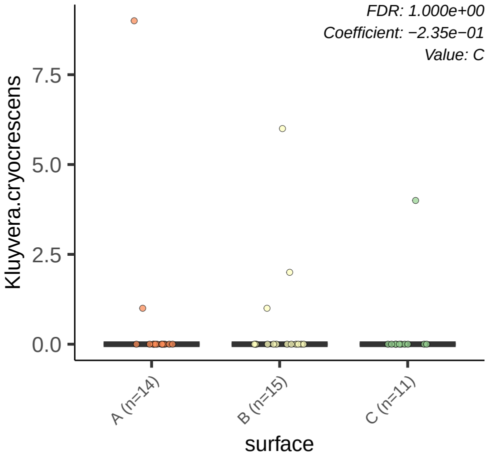
FDR: 1.000e+00
Coefficient: −2.35e−01
Value: C
Kluyvera.cryocrescens
7.5
5.0
2.5
0.0
A (n=14)
B (n=15)
C (n=11)
surface

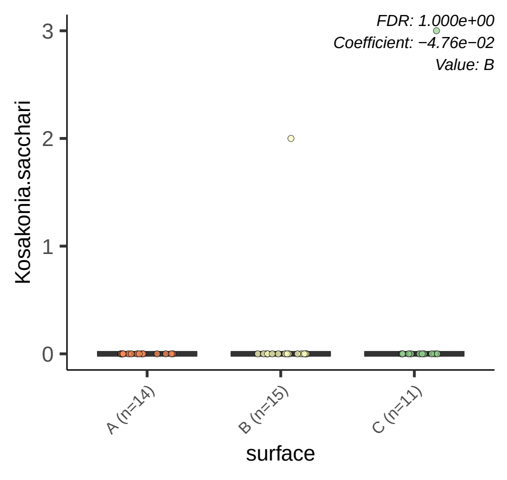
FDR: 1.000e+00
Coefficient: −4.76e−02
Value: B
Kosakonia.sacchari
3
2
1
0
A (n=14)
B (n=15)
C (n=11)
surface

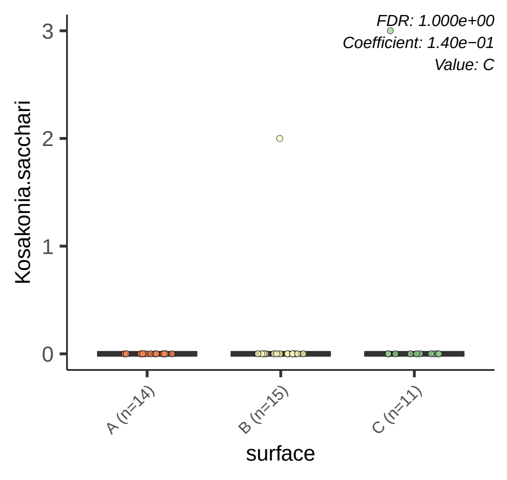
FDR: 1.000e+00
Coefficient: 1.40e−01
Value: C
Kosakonia.sacchari
0
1
2
3
A (n=14)
B (n=15)
C (n=11)
surface

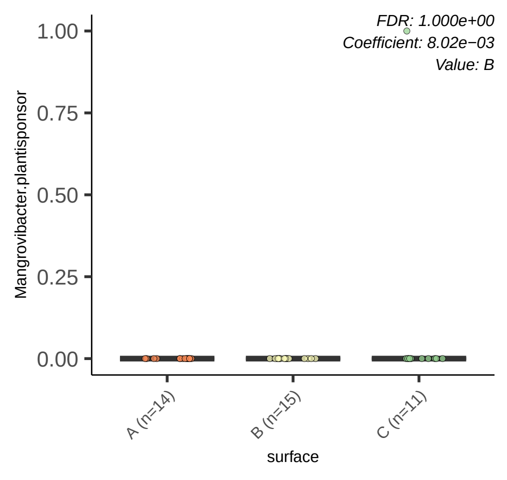
FDR: 1.000e+00
Coefficient: 8.02e−03
Value: B
1.00
0.75
0.50
0.25
0.00
Mangrovibacter.plantisponsor
A (n=14)
B (n=15)
C (n=11)
surface

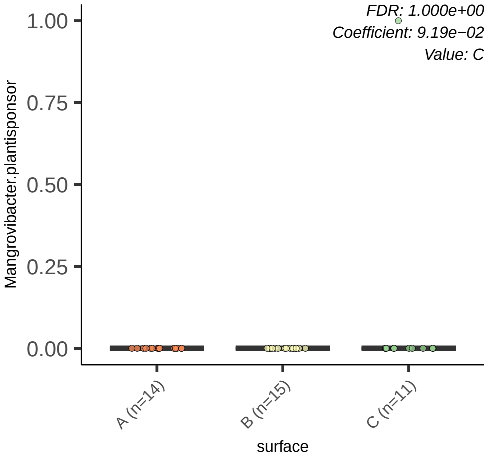

FDR: 1.000e+00
Coefficient: 9.19e−02
Value: C
Mangrovibacter.plantisponsor
1.00
0.75
0.50
0.25
0.00
A (n=14)
B (n=15)
C (n=11)
surface

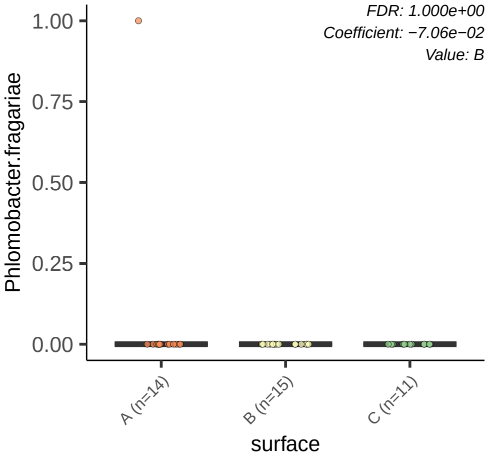
FDR: 1.000e+00
Coefficient: −7.06e−02
Value: B
Phlomobacter.fragariae
1.00
0.75
0.50
0.25
0.00
A (n=14)
B (n=15)
C (n=11)
surface

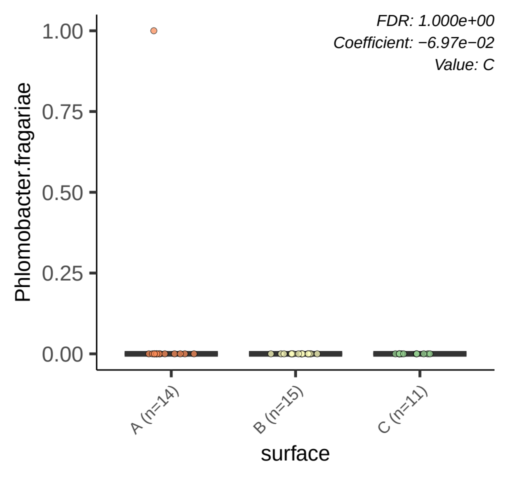
FDR: 1.000e+00
Coefficient: −6.97e−02
Value: C
Phlomobacter.fragariae
1.00
0.75
0.50
0.25
0.00
A (n=14)
B (n=15)
C (n=11)
surface

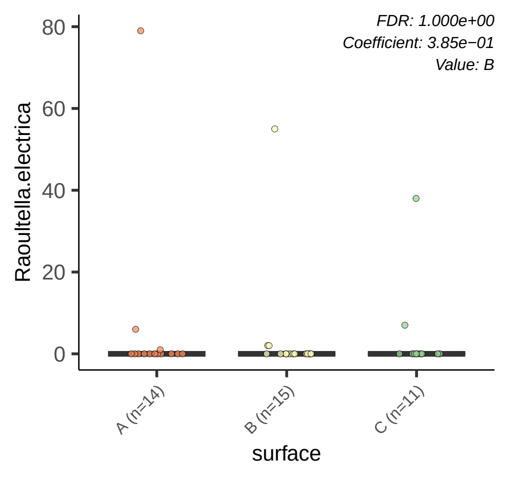

FDR: 1.000e+00
Coefficient: 3.85e−01
Value: B
Raoultella.electrica
0
20
40
60
80
A (n=14)
B (n=15)
C (n=11)
surface

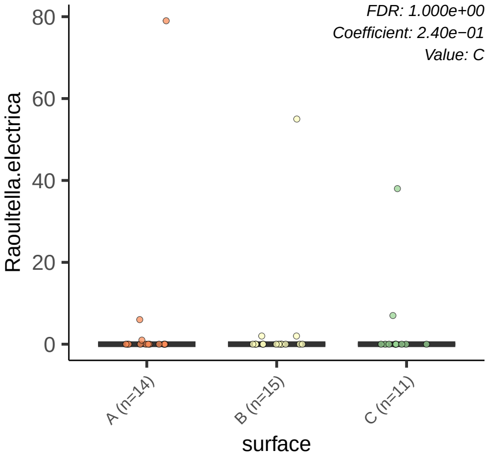
FDR: 1.000e+00
Coefficient: 2.40e−01
Value: C
Raoultella.electrica
0
20
40
60
80
A (n=14)
B (n=15)
C (n=11)
surface

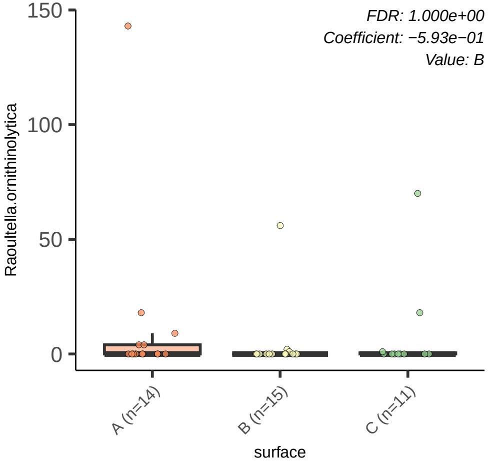
FDR: 1.000e+00
Coefficient: −5.93e−01
Value: B
Raoultella.ornithinolytica
150
100
50
0
A (n=14)
B (n=15)
C (n=11)
surface

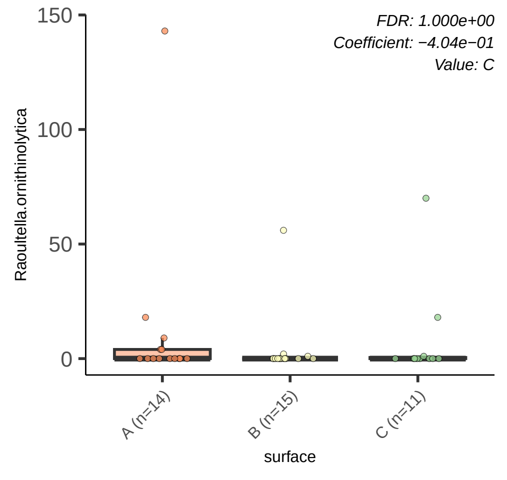
FDR: 1.000e+00
Coefficient: −4.04e−01
Value: C
150
100
50
0
Raoultella.ornithinolytica
A (n=14)
B (n=15)
C (n=11)
surface

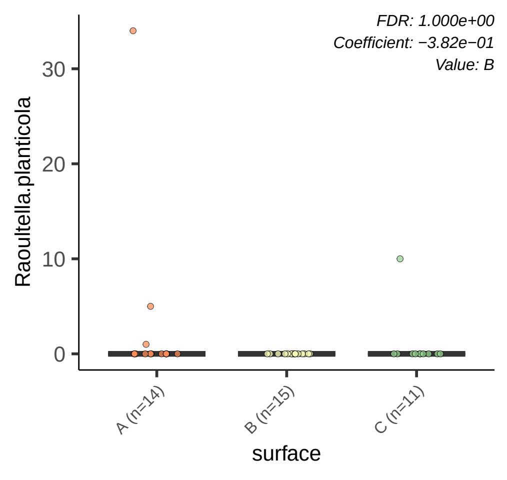
FDR: 1.000e+00
Coefficient: −3.82e−01
Value: B
Raoultella.planticola
30
20
10
0
A (n=14)
B (n=15)
C (n=11)
surface

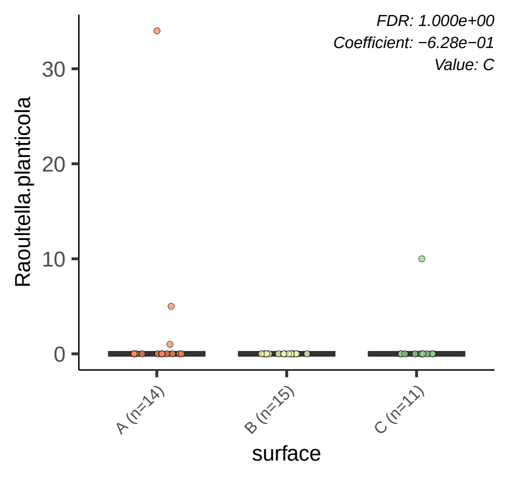
FDR: 1.000e+00
Coefficient: −6.28e−01
Value: C
Raoultella.planticola
30
20
10
0
A (n=14)
B (n=15)
C (n=11)
surface

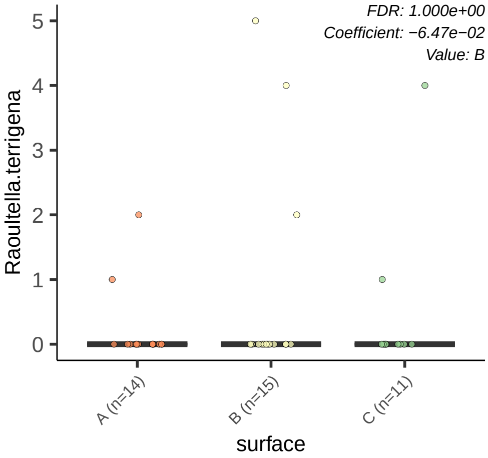
FDR: 1.000e+00
Coefficient: −6.47e−02
Value: B
Raoultella.terrigena
0
1
2
3
4
5
A (n=14)
B (n=15)
C (n=11)
surface

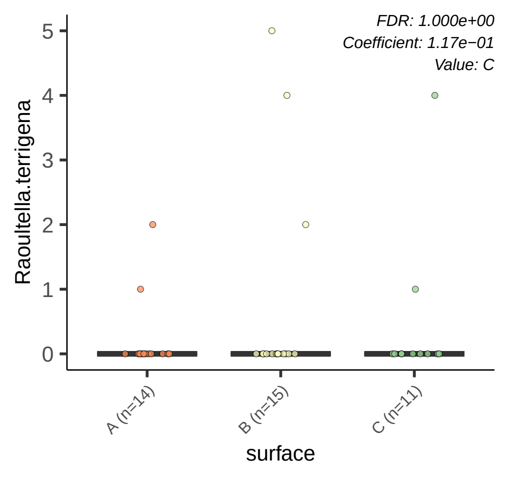
FDR: 1.000e+00
Coefficient: 1.17e−01
Value: C
Raoultella.terrigena
5
4
3
2
1
0
A (n=14)
B (n=15)
C (n=11)
surface

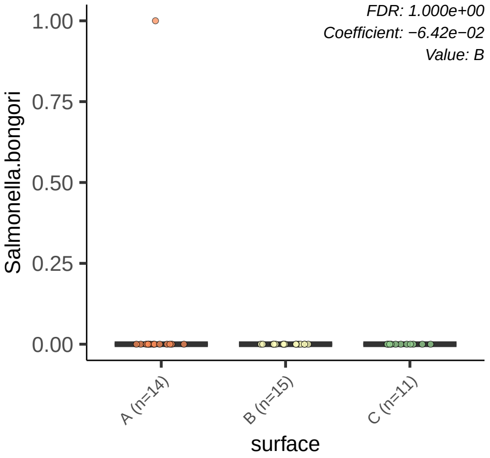
FDR: 1.000e+00
Coefficient: −6.42e−02
Value: B
Salmonella.bongori
1.00
0.75
0.50
0.25
0.00
A (n=14)
B (n=15)
C (n=11)
surface

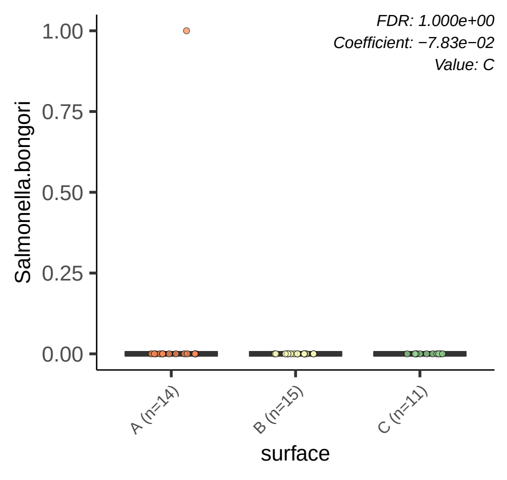
FDR: 1.000e+00
Coefficient: −7.83e−02
Value: C
Salmonella.bongori
1.00
0.75
0.50
0.25
0.00
A (n=14)
B (n=15)
C (n=11)
surface

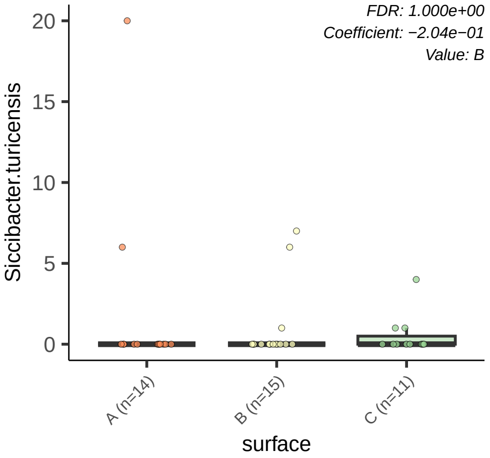
FDR: 1.000e+00
Coefficient: −2.04e−01
Value: B
Siccibacter.turicensis
20
15
10
5
0
A (n=14)
B (n=15)
C (n=11)
surface

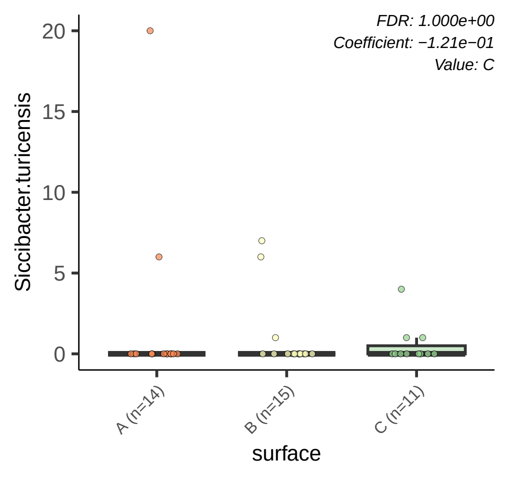

FDR: 1.000e+00
Coefficient: −1.21e−01
Value: C
Siccibacter.turicensis
20
15
10
5
0
A (n=14)
B (n=15)
C (n=11)
surface

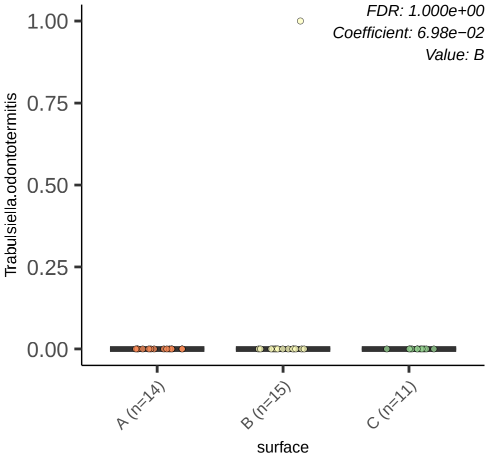
FDR: 1.000e+00
Coefficient: 6.98e−02
Value: B
1.00
0.75
0.50
0.25
0.00
Trabulsiella.odontotermitis
A (n=14)
B (n=15)
C (n=11)
surface

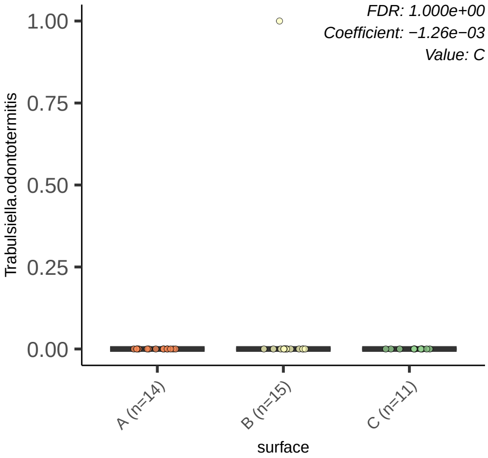

FDR: 1.000e+00
Coefficient: −1.26e−03
Value: C
1.00
0.75
0.50
0.25
0.00
Trabulsiella.odontotermitis
A (n=14)
B (n=15)
C (n=11)
surface

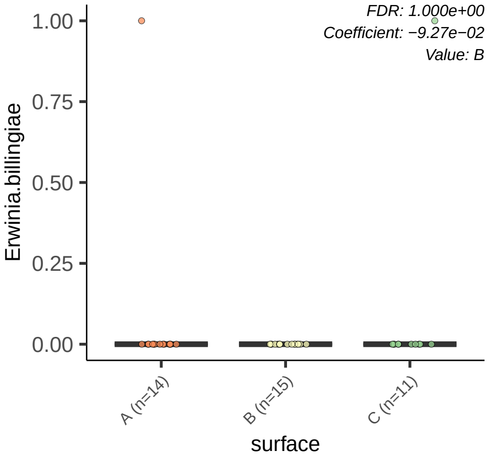

FDR: 1.000e+00
Coefficient: −9.27e−02
Value: B
Erwinia.billingiae
1.00
0.75
0.50
0.25
0.00
A (n=14)
B (n=15)
C (n=11)
surface

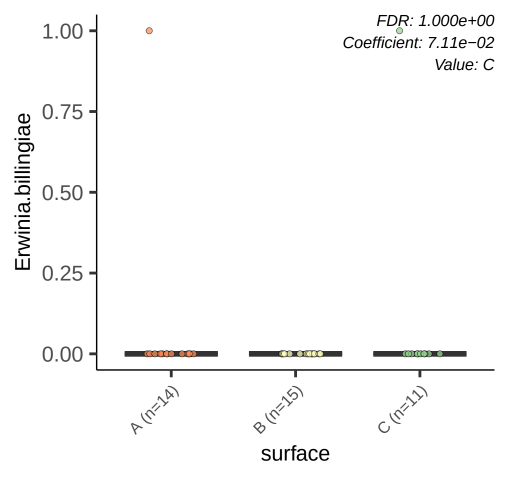
FDR: 1.000e+00
Coefficient: 7.11e−02
Value: C
1.00
0.75
0.50
0.25
0.00
Erwinia.billingiae
A (n=14)
B (n=15)
C (n=11)
surface

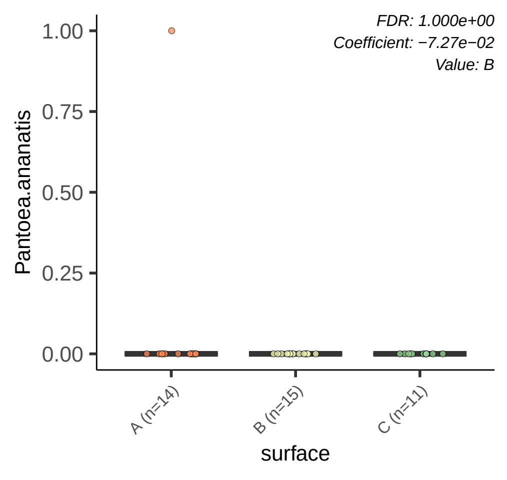

FDR: 1.000e+00
Coefficient: −7.27e−02
Value: B
1.00
0.75
0.50
0.25
0.00
Pantoea.ananatis
A (n=14)
B (n=15)
C (n=11)
surface

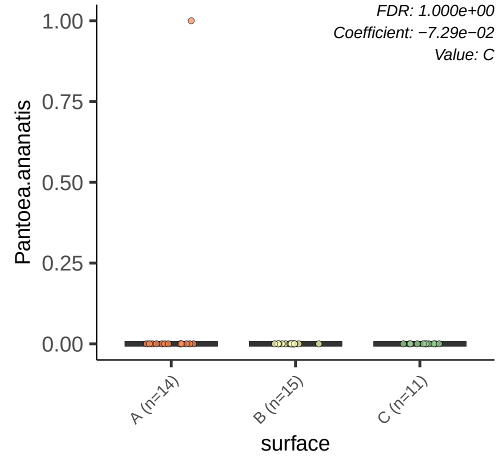

FDR: 1.000e+00
Coefficient: −7.29e−02
Value: C
1.00
0.75
0.50
0.25
0.00
Pantoea.ananatis
A (n=14)
B (n=15)
C (n=11)
surface

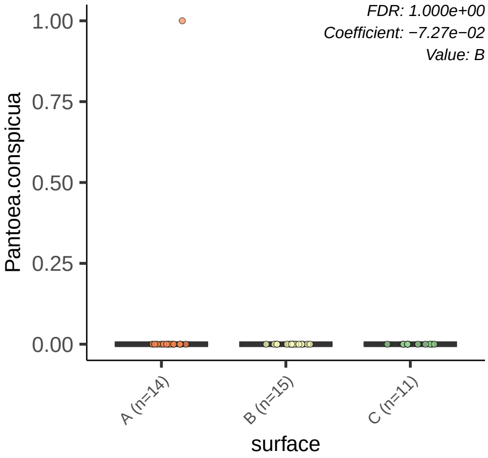

FDR: 1.000e+00
Coefficient: −7.27e−02
Value: B
Pantoea.conspicua
1.00
0.75
0.50
0.25
0.00
A (n=14)
B (n=15)
C (n=11)
surface

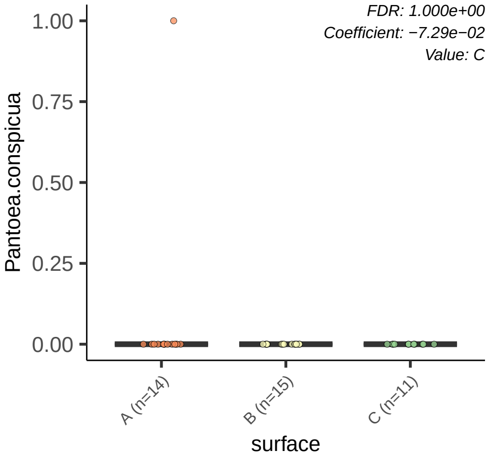

FDR: 1.000e+00
Coefficient: −7.29e−02
Value: C
Pantoea.conspicua
1.00
0.75
0.50
0.25
0.00
A (n=14)
B (n=15)
C (n=11)
surface

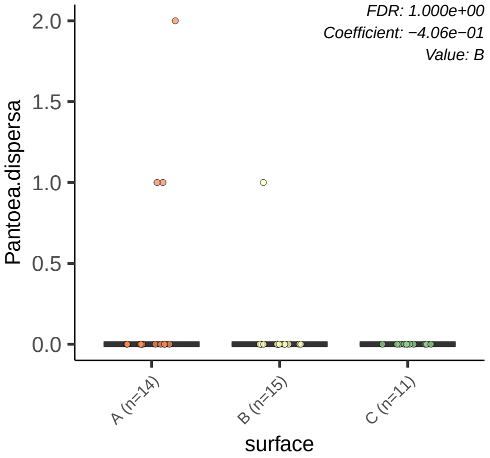
FDR: 1.000e+00
Coefficient: −4.06e−01
Value: B
Pantoea.dispersa
2.0
1.5
1.0
0.5
0.0
A (n=14)
B (n=15)
C (n=11)
surface

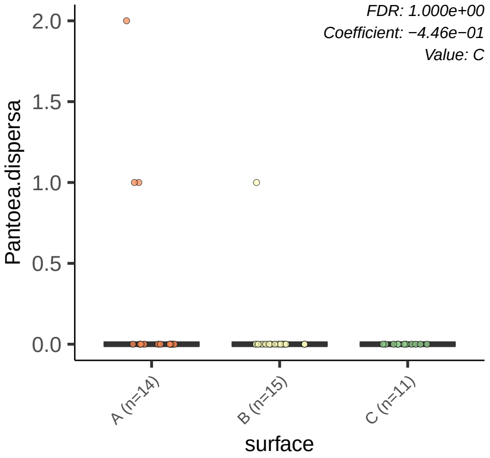
FDR: 1.000e+00
Coefficient: −4.46e−01
Value: C
Pantoea.dispersa
2.0
1.5
1.0
0.5
0.0
A (n=14)
B (n=15)
C (n=11)
surface

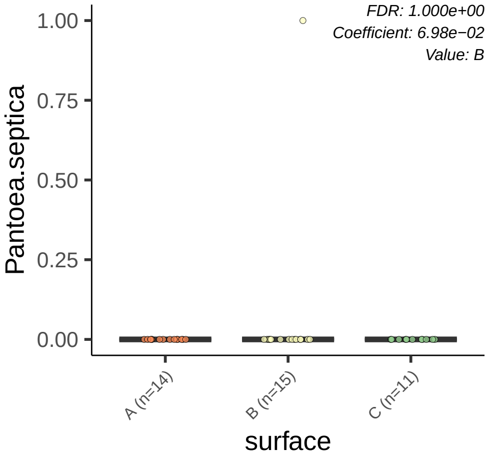
FDR: 1.000e+00
Coefficient: 6.98e−02
Value: B
Pantoea.septica
1.00
0.75
0.50
0.25
0.00
A (n=14)
B (n=15)
C (n=11)
surface

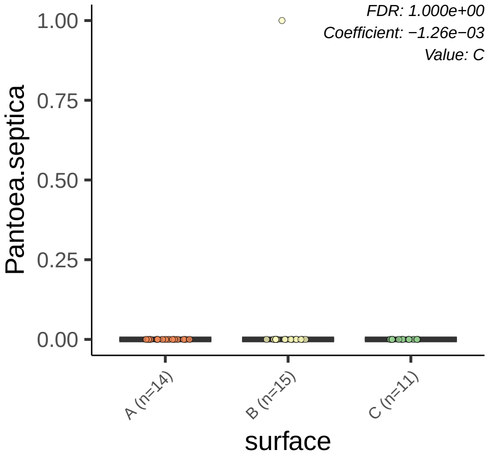

FDR: 1.000e+00
Coefficient: −1.26e−03
Value: C
Pantoea.septica
1.00
0.75
0.50
0.25
0.00
A (n=14)
B (n=15)
C (n=11)
surface

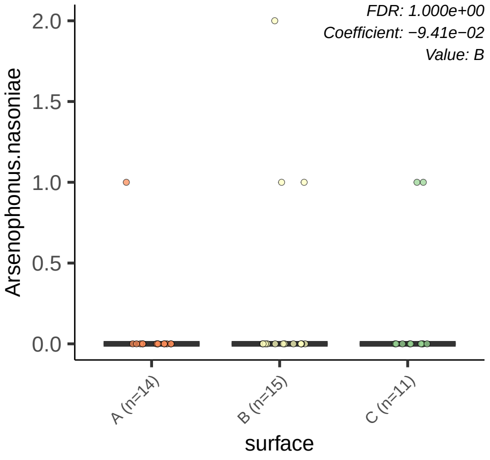

FDR: 1.000e+00
Coefficient: −9.41e−02
Value: B
Arsenophonus.nasoniae
2.0
1.5
1.0
0.5
0.0
A (n=14)
B (n=15)
C (n=11)
surface

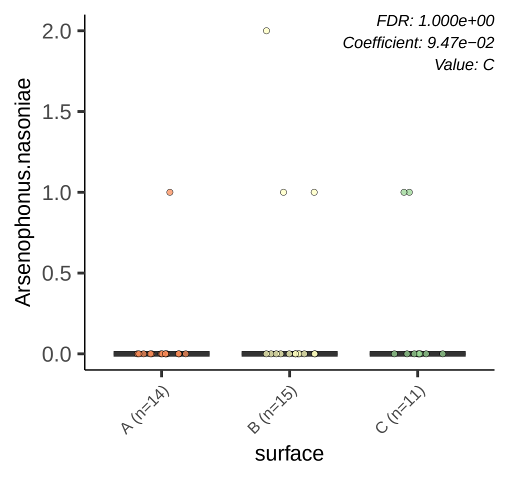

FDR: 1.000e+00
Coefficient: 9.47e−02
Value: C
Arsenophonus.nasoniae
2.0
1.5
1.0
0.5
0.0
A (n=14)
B (n=15)
C (n=11)
surface

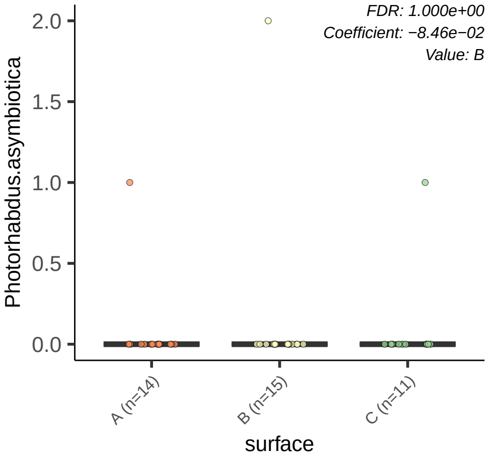

FDR: 1.000e+00
Coefficient: −8.46e−02
Value: B
Photorhabdus.asymbiotica
2.0
1.5
1.0
0.5
0.0
A (n=14)
B (n=15)
C (n=11)
surface

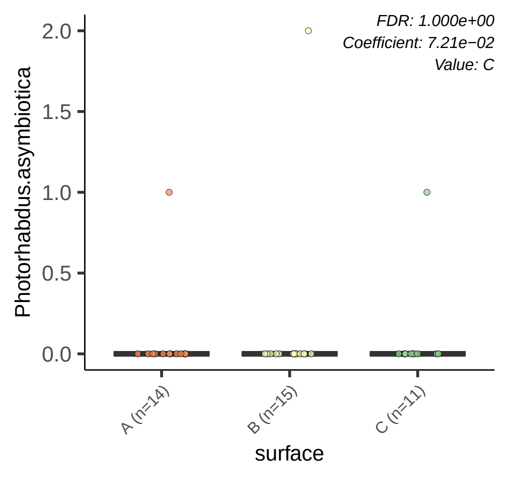
FDR: 1.000e+00
Coefficient: 7.21e−02
Value: C
Photorhabdus.asymbiotica
2.0
1.5
1.0
0.5
0.0
A (n=14)
B (n=15)
C (n=11)
surface

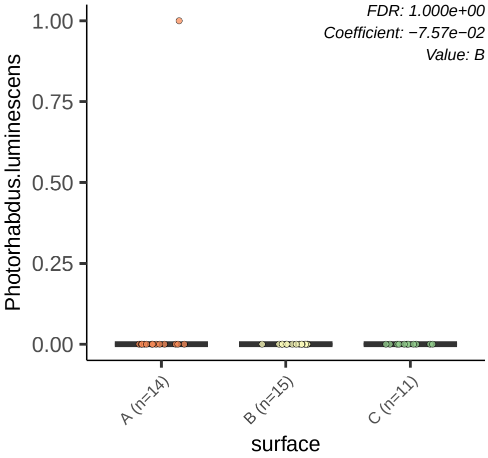

FDR: 1.000e+00
Coefficient: −7.57e−02
Value: B
Photorhabdus.luminescens
1.00
0.75
0.50
0.25
0.00
A (n=14)
B (n=15)
C (n=11)
surface

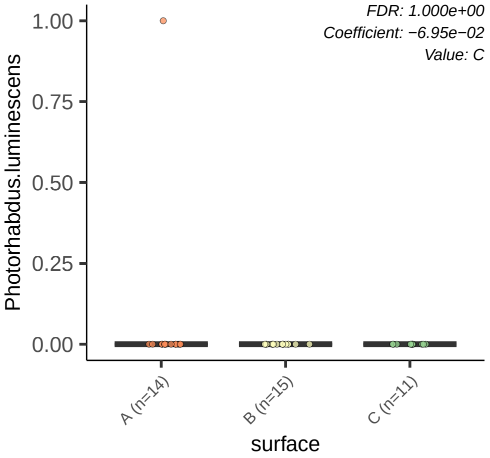

FDR: 1.000e+00
Coefficient: −6.95e−02
Value: C
Photorhabdus.luminescens
1.00
0.75
0.50
0.25
0.00
A (n=14)
B (n=15)
C (n=11)
surface

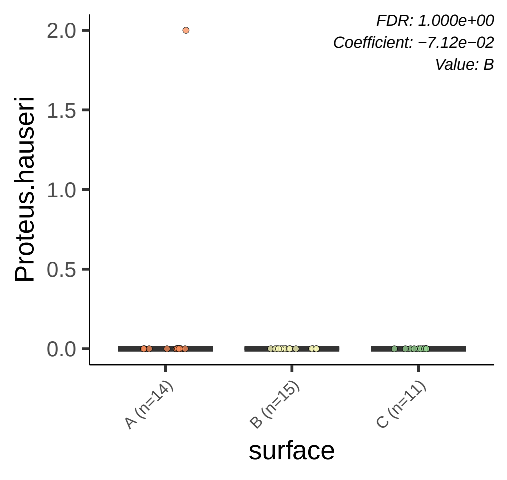

FDR: 1.000e+00
Coefficient: −7.12e−02
Value: B
Proteus.hauseri
2.0
1.5
1.0
0.5
0.0
A (n=14)
B (n=15)
C (n=11)
surface

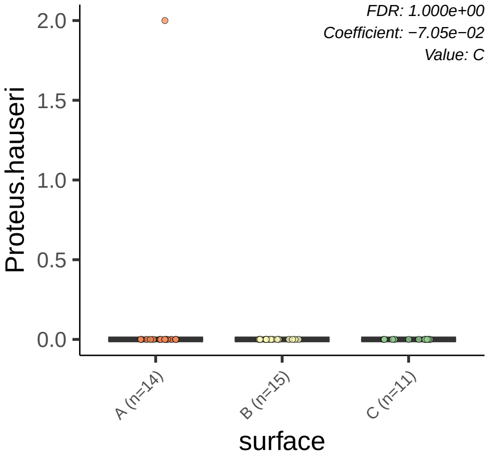

FDR: 1.000e+00
Coefficient: −7.05e−02
Value: C
Proteus.hauseri
2.0
1.5
1.0
0.5
0.0
A (n=14)
B (n=15)
C (n=11)
surface

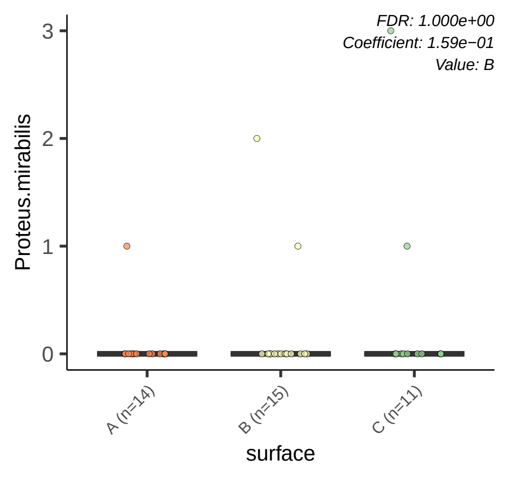

FDR: 1.000e+00
Coefficient: 1.59e−01
Value: B
Proteus.mirabilis
0
1
2
3
A (n=14)
B (n=15)
C (n=11)
surface

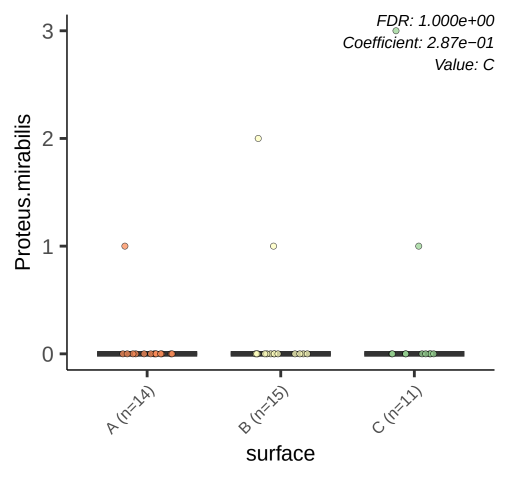

FDR: 1.000e+00
Coefficient: 2.87e−01
Value: C
Proteus.mirabilis
0
1
2
3
A (n=14)
B (n=15)
C (n=11)
surface

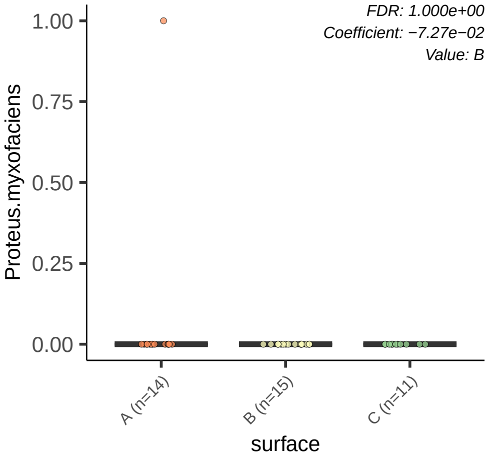
FDR: 1.000e+00
Coefficient: −7.27e−02
Value: B
Proteus.myxofaciens
1.00
0.75
0.50
0.25
0.00
A (n=14)
B (n=15)
C (n=11)
surface

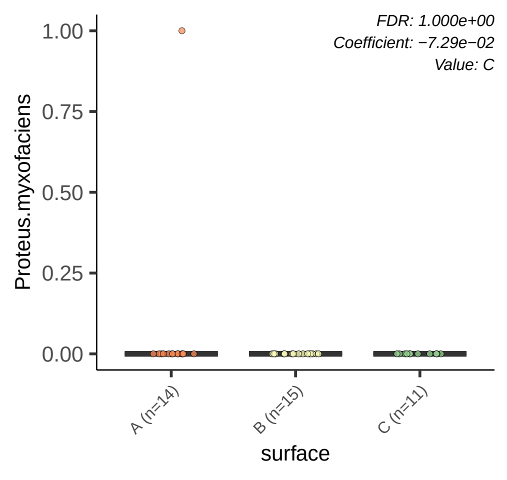
FDR: 1.000e+00
Coefficient: −7.29e−02
Value: C
Proteus.myxofaciens
1.00
0.75
0.50
0.25
0.00
A (n=14)
B (n=15)
C (n=11)
surface

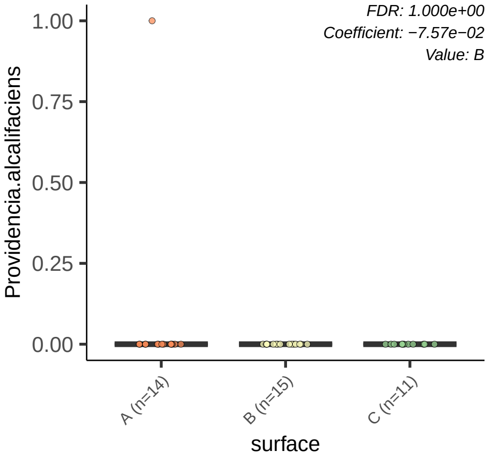
FDR: 1.000e+00
Coefficient: −7.57e−02
Value: B
Providencia.alcalifaciens
1.00
0.75
0.50
0.25
0.00
A (n=14)
B (n=15)
C (n=11)
surface

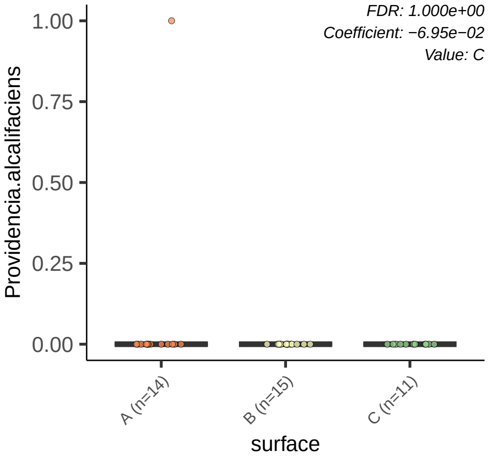
FDR: 1.000e+00
Coefficient: −6.95e−02
Value: C
Providencia.alcalifaciens
1.00
0.75
0.50
0.25
0.00
A (n=14)
B (n=15)
C (n=11)
surface

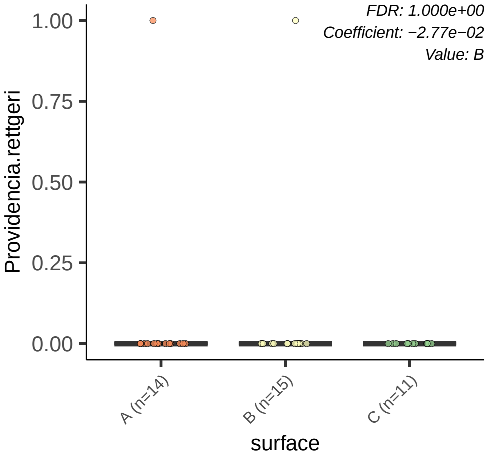
FDR: 1.000e+00
Coefficient: −2.77e−02
Value: B
Providencia.retgeri
1.00
0.75
0.50
0.25
0.00
A (n=14)
B (n=15)
C (n=11)
surface

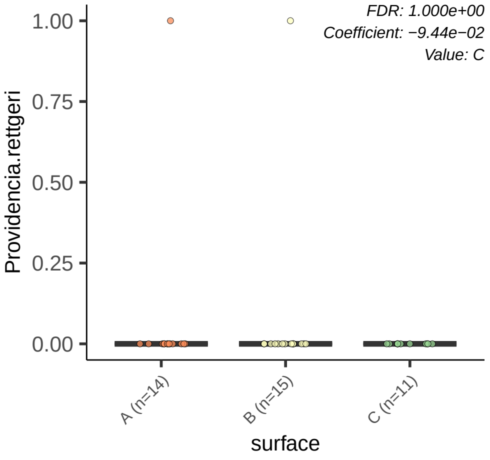
FDR: 1.000e+00
Coefficient: −9.44e−02
Value: C
Providencia.retgeri
1.00
0.75
0.50
0.25
0.00
A (n=14)
B (n=15)
C (n=11)
surface

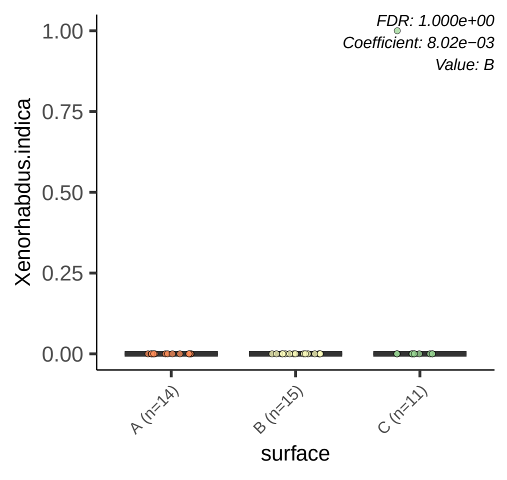

FDR: 1.000e+00
Coefficient: 8.02e−03
Value: B
1.00
0.75
0.50
0.25
0.00
Xenorhabdus.indica
A (n=14)
B (n=15)
C (n=11)
surface

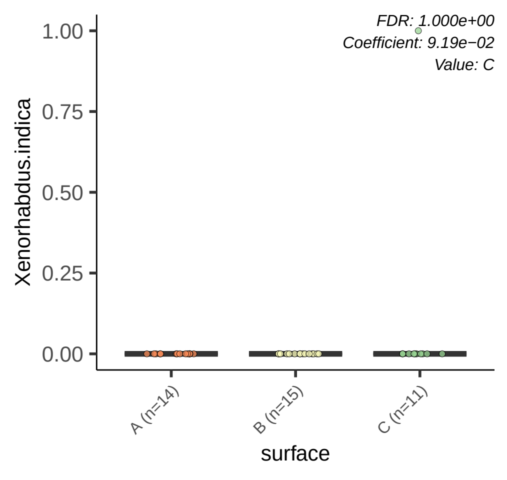
FDR: 1.000e+00
Coefficient: 9.19e−02
Value: C
Xenorhabdus.indica
1.00
0.75
0.50
0.25
0.00
A (n=14)
B (n=15)
C (n=11)
surface

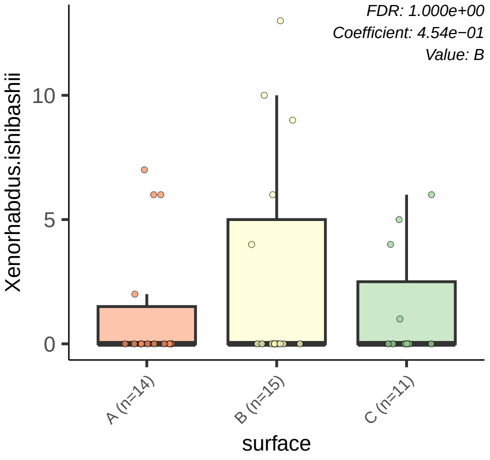

FDR: 1.000e+00
Coefficient: 4.54e−01
Value: B
Xenorhabdus.ishibashii
0
5
10
A (n=14)
B (n=15)
C (n=11)
surface

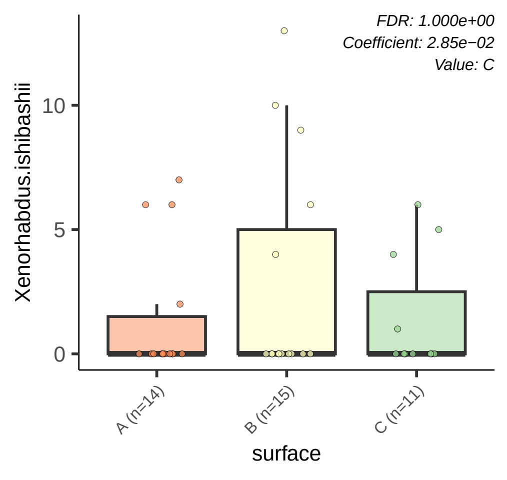

FDR: 1.000e+00
Coefficient: 2.85e−02
Value: C
Xenorhabdus.ishibashii
0
5
10
A (n=14)
B (n=15)
C (n=11)
surface

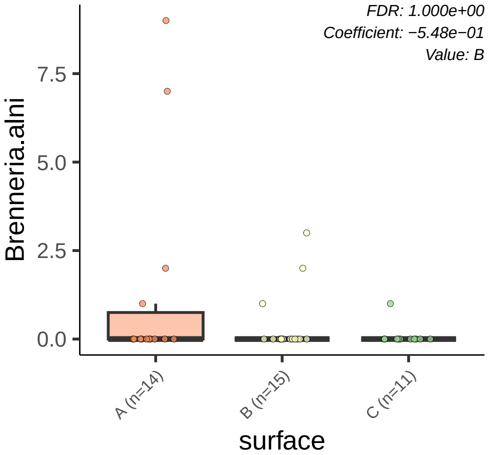
FDR: 1.000e+00
Coefficient: −5.48e−01
Value: B
Brenneria.alni
7.5
5.0
2.5
0.0
A (n=14)
B (n=15)
C (n=11)
surface

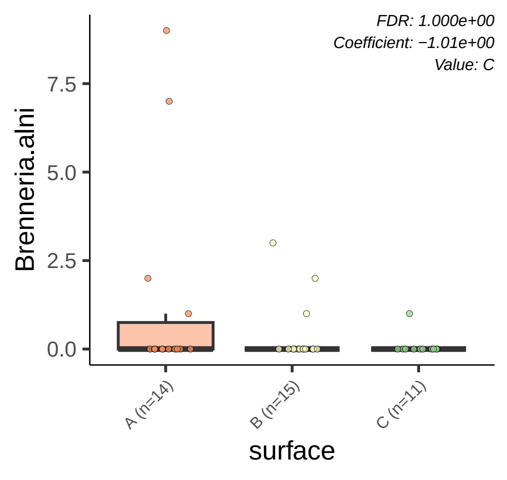

FDR: 1.000e+00
Coefficient: −1.01e+00
Value: C
Brenneria.alni
7.5
5.0
2.5
0.0
A (n=14)
B (n=15)
C (n=11)
surface

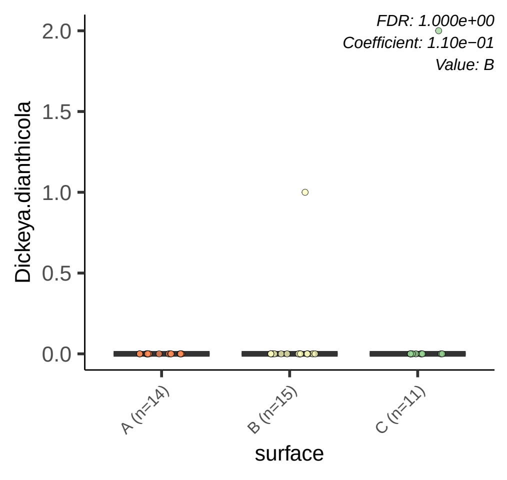
FDR: 1.000e+00
Coefficient: 1.10e−01
Value: B
Dickeya.dianthicola
2.0
1.5
1.0
0.5
0.0
A (n=14)
B (n=15)
C (n=11)
surface

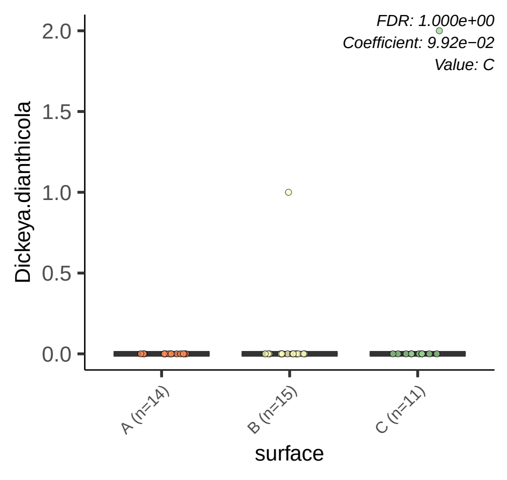
FDR: 1.000e+00
Coefficient: 9.92e−02
Value: C
Dickeya.dianthicola
2.0
1.5
1.0
0.5
0.0
A (n=14)
B (n=15)
C (n=11)
surface

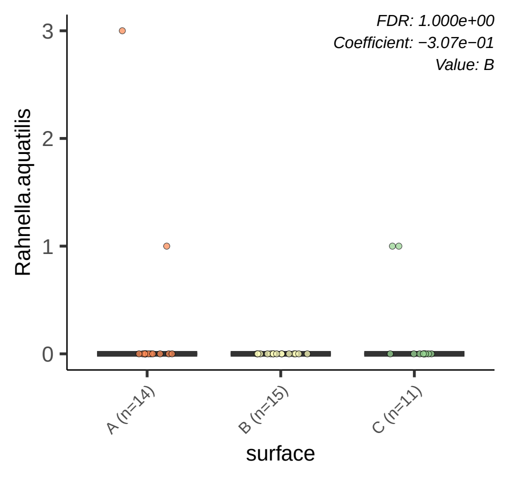
FDR: 1.000e+00
Coefficient: −3.07e−01
Value: B
Rahnella.aquatilis
3
2
1
0
A (n=14)
B (n=15)
C (n=11)
surface

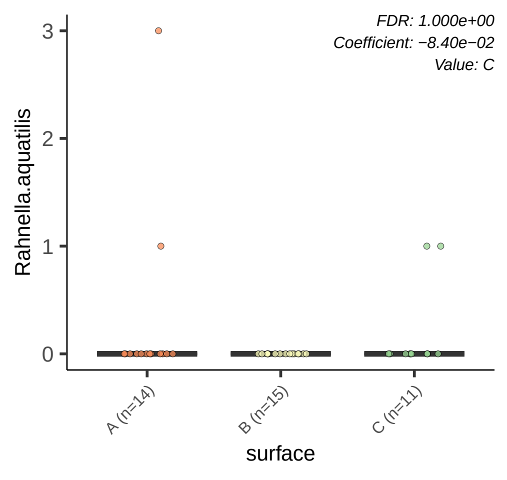
FDR: 1.000e+00
Coefficient: −8.40e−02
Value: C
Rahnella.aquatilis
3
2
1
0
A (n=14)
B (n=15)
C (n=11)
surface

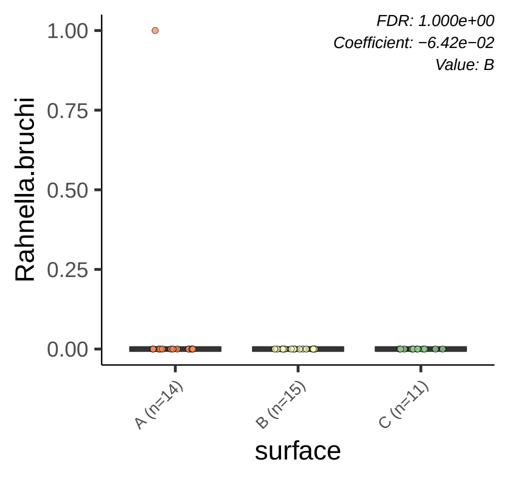
FDR: 1.000e+00
Coefficient: −6.42e−02
Value: B
Rahnella.bruchi
1.00
0.75
0.50
0.25
0.00
A (n=14)
B (n=15)
C (n=11)
surface

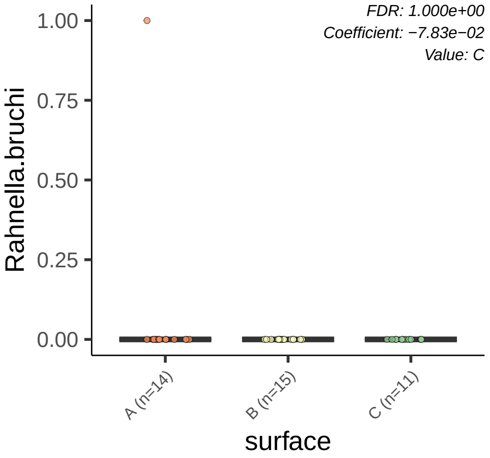
FDR: 1.000e+00
Coefficient: −7.83e−02
Value: C
Rahnella.bruchi
1.00
0.75
0.50
0.25
0.00
A (n=14)
B (n=15)
C (n=11)
surface

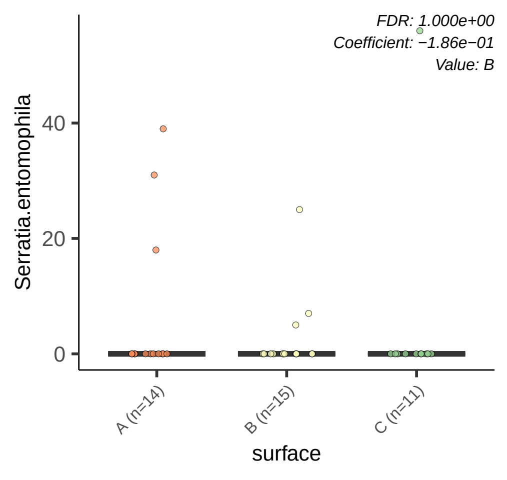
FDR: 1.000e+00
Coefficient: −1.86e−01
Value: B
Serratia.entomophila
40
20
0
A (n=14)
B (n=15)
C (n=11)
surface

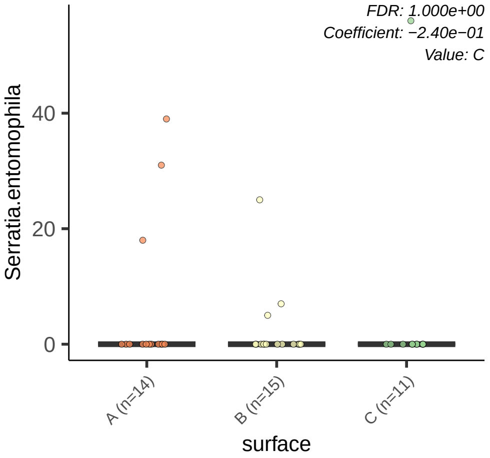

FDR: 1.000e+00
Coefficient: −2.40e−01
Value: C
Serratia.entomophila
0
20
40
A (n=14)
B (n=15)
C (n=11)
surface

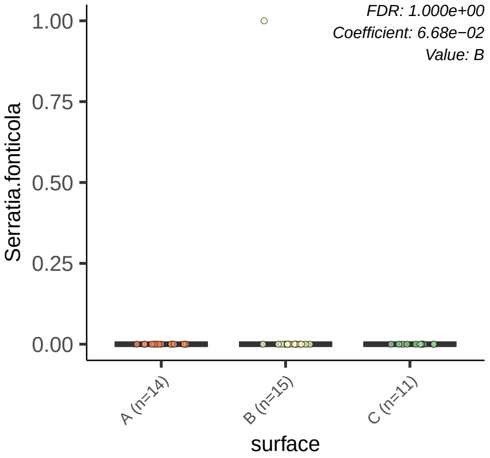
FDR: 1.000e+00
Coefficient: 6.68e−02
Value: B
Serratia.fonticola
1.00
0.75
0.50
0.25
0.00
A (n=14)
B (n=15)
C (n=11)
surface

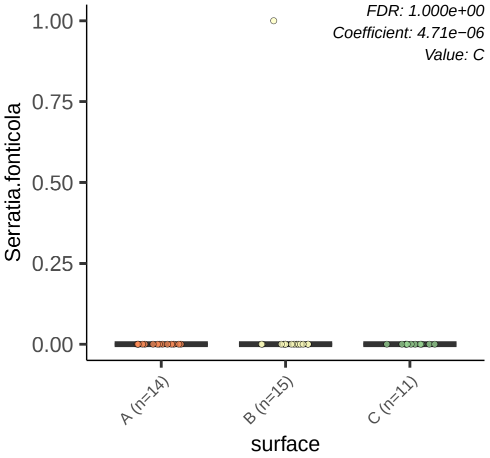

FDR: 1.000e+00
Coefficient: 4.71e−06
Value: C
Serratia.fonticola
1.00
0.75
0.50
0.25
0.00
A (n=14)
B (n=15)
C (n=11)
surface

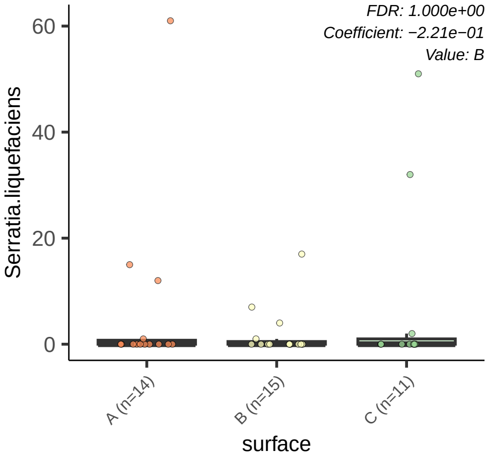
FDR: 1.000e+00
Coefficient: −2.21e−01
Value: B
Serratia.liquefaciens
60
40
20
0
A (n=14)
B (n=15)
C (n=11)
surface

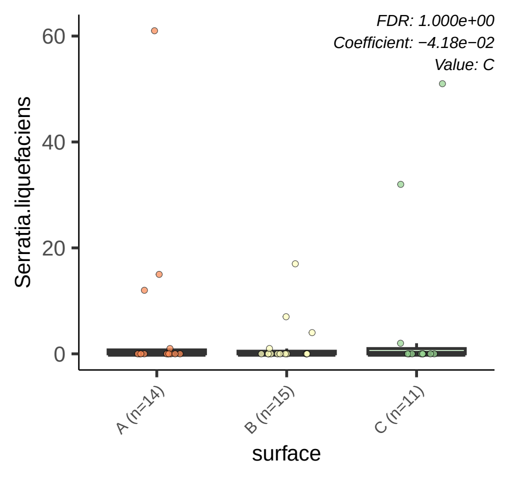
FDR: 1.000e+00
Coefficient: −4.18e−02
Value: C
Serratia.liquefaciens
60
40
20
0
A (n=14)
B (n=15)
C (n=11)
surface

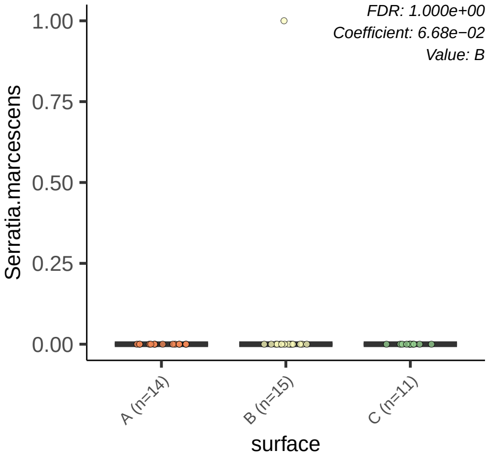
FDR: 1.000e+00
Coefficient: 6.68e−02
Value: B
Serratia.marcescens
1.00
0.75
0.50
0.25
0.00
A (n=14)
B (n=15)
C (n=11)
surface

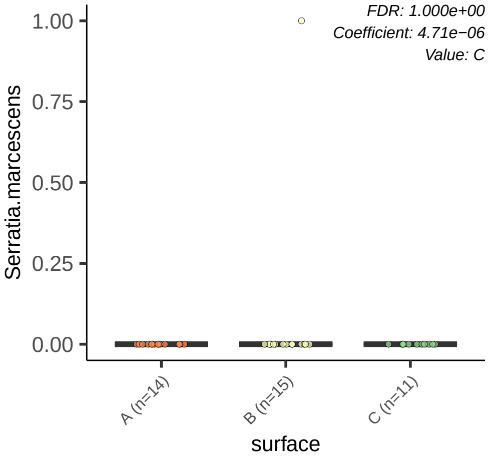

FDR: 1.000e+00
Coefficient: 4.71e−06
Value: C
Serratia.marcescens
1.00
0.75
0.50
0.25
0.00
A (n=14)
B (n=15)
C (n=11)
surface

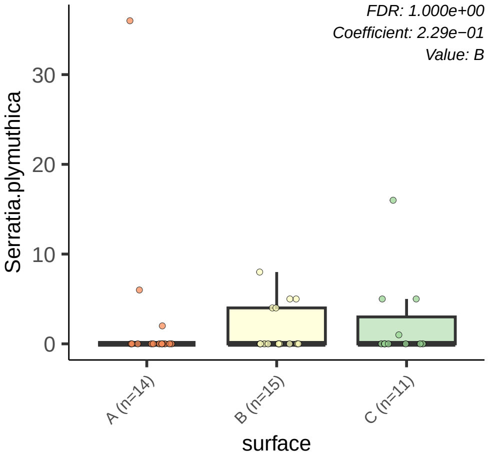

FDR: 1.000e+00
Coefficient: 2.29e−01
Value: B
Serratia.plymuthica
30
20
10
0
A (n=14)
B (n=15)
C (n=11)
surface

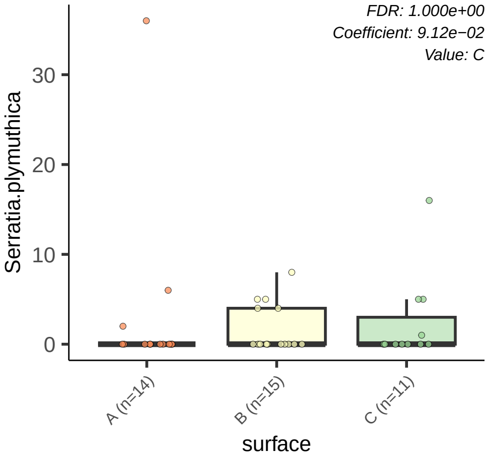
FDR: 1.000e+00
Coefficient: 9.12e−02
Value: C
Serratia.plymuthica
0
10
20
30
A (n=14)
B (n=15)
C (n=11)
surface

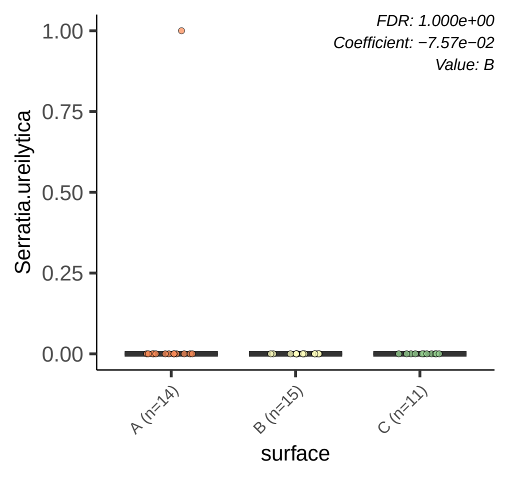
FDR: 1.000e+00
Coefficient: −7.57e−02
Value: B
Serratia.ureilytica
1.00
0.75
0.50
0.25
0.00
A (n=14)
B (n=15)
C (n=11)
surface

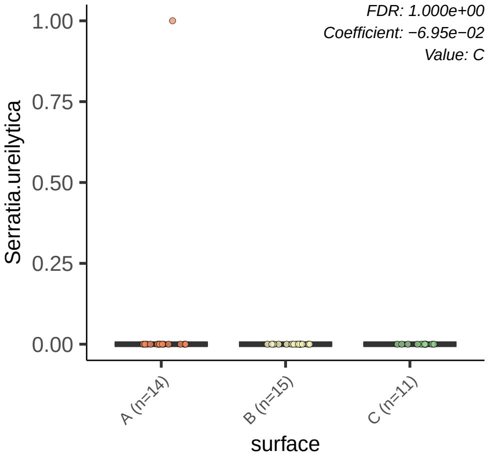

FDR: 1.000e+00
Coefficient: −6.95e−02
Value: C
Serratia.ureilytica
1.00
0.75
0.50
0.25
0.00
A (n=14)
B (n=15)
C (n=11)
surface

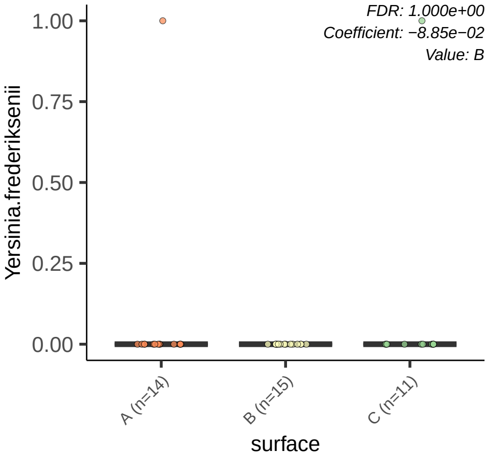

FDR: 1.000e+00
Coefficient: −8.85e−02
Value: B
Yersinia.frederiksenii
1.00
0.75
0.50
0.25
0.00
A (n=14)
B (n=15)
C (n=11)
surface

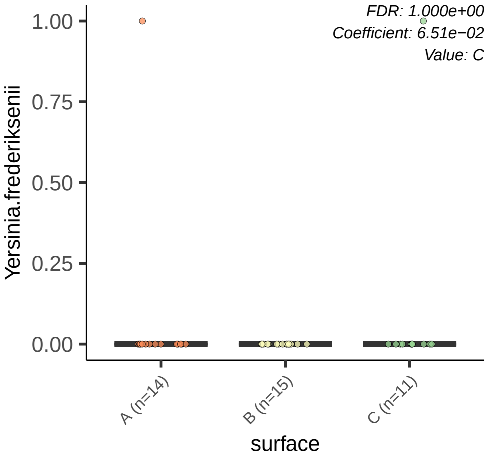
FDR: 1.000e+00
Coefficient: 6.51e−02
Value: C
Yersinia.frederiksenii
1.00
0.75
0.50
0.25
0.00
A (n=14)
B (n=15)
C (n=11)
surface

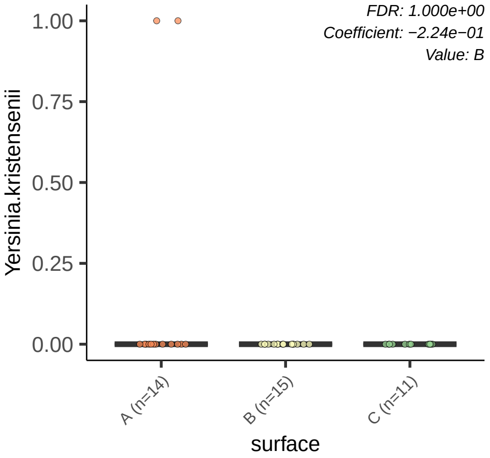
FDR: 1.000e+00
Coefficient: −2.24e−01
Value: B
Yersinia.kristensenii
1.00
0.75
0.50
0.25
0.00
A (n=14)
B (n=15)
C (n=11)
surface

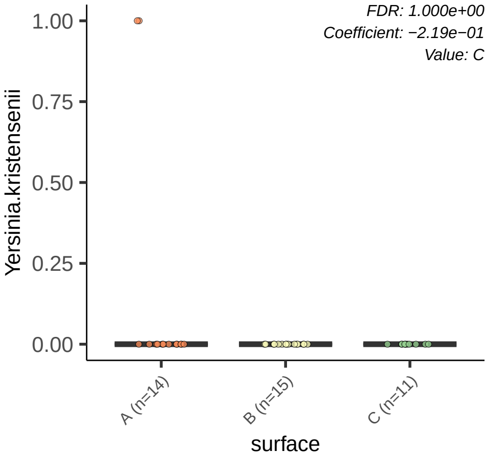
FDR: 1.000e+00
Coefficient: −2.19e−01
Value: C
Yersinia.kristensenii
1.00
0.75
0.50
0.25
0.00
A (n=14)
B (n=15)
C (n=11)
surface

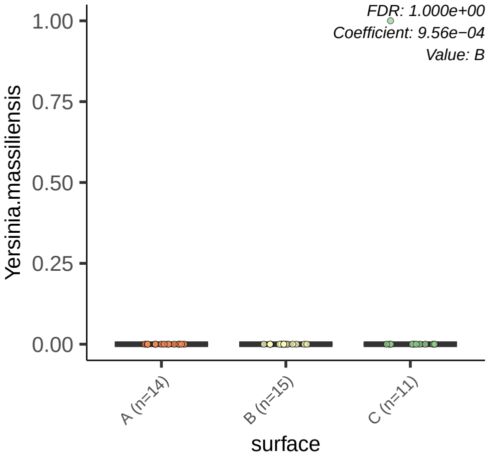
FDR: 1.000e+00
Coefficient: 9.56e−04
Value: B
Yersinia.massiliensis
1.00
0.75
0.50
0.25
0.00
A (n=14)
B (n=15)
C (n=11)
surface

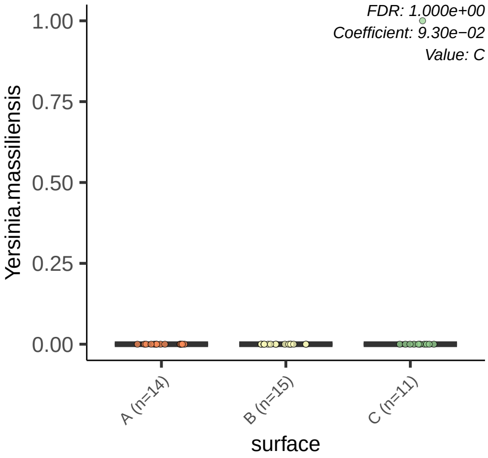

FDR: 1.000e+00
Coefficient: 9.30e−02
Value: C
Yersinia.massiliensis
1.00
0.75
0.50
0.25
0.00
A (n=14)
B (n=15)
C (n=11)
surface

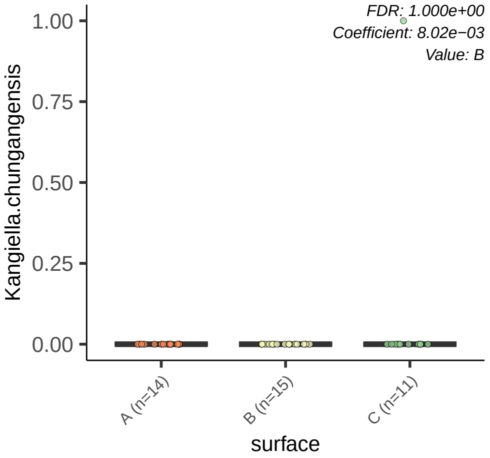

FDR: 1.000e+00
Coefficient: 8.02e−03
Value: B
Kangiella.chungangensis
1.00
0.75
0.50
0.25
0.00
A (n=14)
B (n=15)
C (n=11)
surface

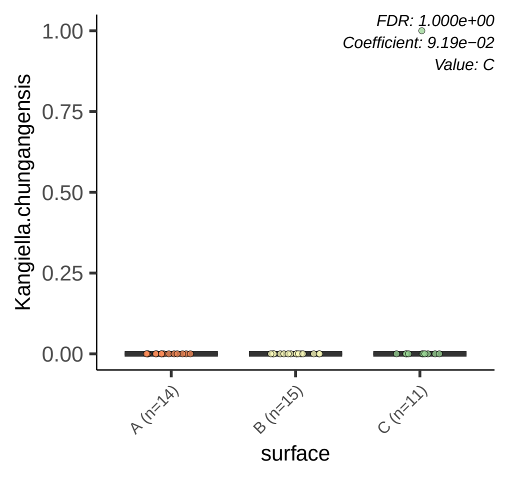
FDR: 1.000e+00
Coefficient: 9.19e−02
Value: C
Kangiella.chungangensis
1.00
0.75
0.50
0.25
0.00
A (n=14)
B (n=15)
C (n=11)
surface

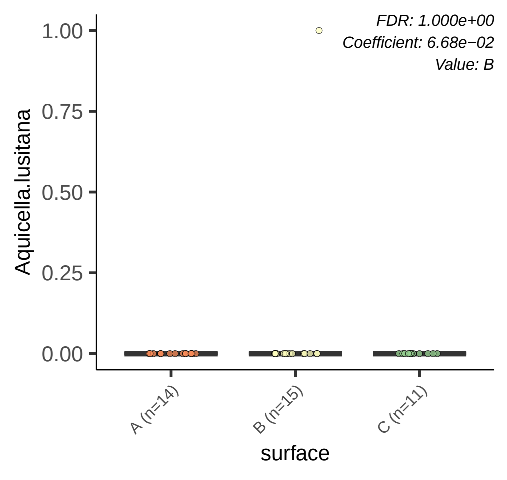

FDR: 1.000e+00
Coefficient: 6.68e−02
Value: B
Aquicella.lusitana
1.00
0.75
0.50
0.25
0.00
A (n=14)
B (n=15)
C (n=11)
surface

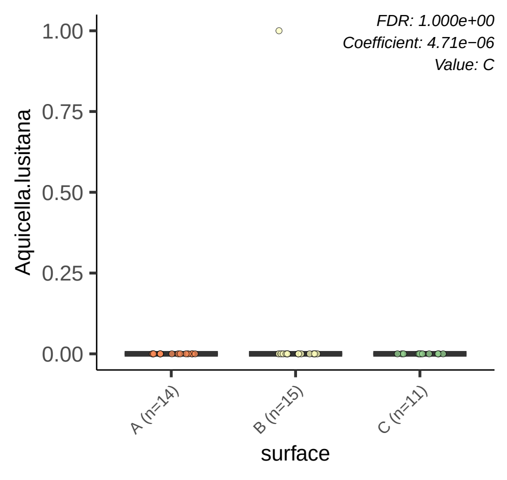
FDR: 1.000e+00
Coefficient: 4.71e−06
Value: C
Aquicella.lusitana
1.00
0.75
0.50
0.25
0.00
A (n=14)
B (n=15)
C (n=11)
surface

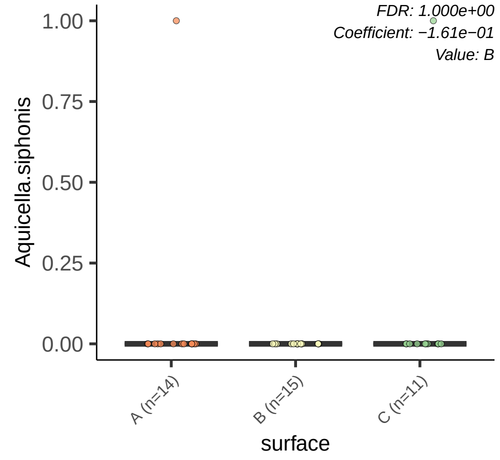

FDR: 1.000e+00
Coefficient: −1.61e−01
Value: B
1.00
0.75
0.50
0.25
0.00
Aquicella.siphonis
A (n=14)
B (n=15)
C (n=11)
surface

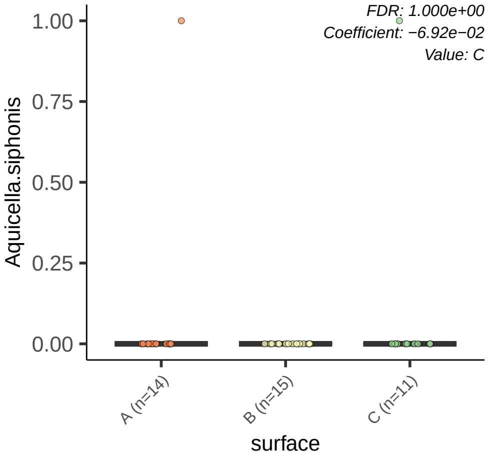

FDR: 1.000e+00
Coefficient: −6.92e−02
Value: C
Aquicella.siphonis
1.00
0.75
0.50
0.25
0.00
A (n=14)
B (n=15)
C (n=11)
surface

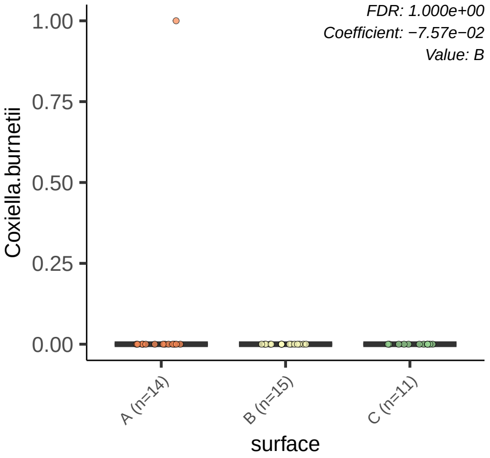
FDR: 1.000e+00
Coefficient: −7.57e−02
Value: B
Coxiella.burnetii
1.00
0.75
0.50
0.25
0.00
A (n=14)
B (n=15)
C (n=11)
surface

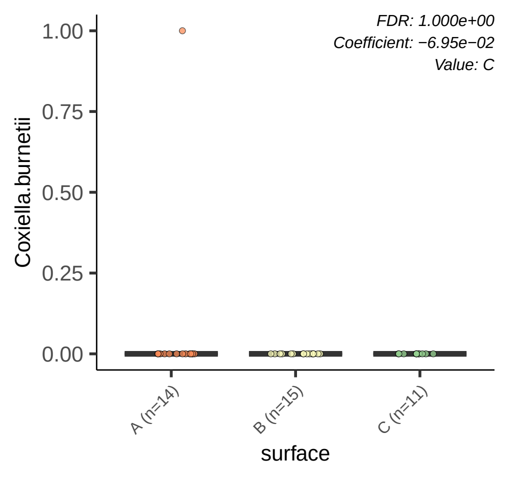

FDR: 1.000e+00
Coefficient: −6.95e−02
Value: C
Coxiella.burnetii
1.00
0.75
0.50
0.25
0.00
A (n=14)
B (n=15)
C (n=11)
surface

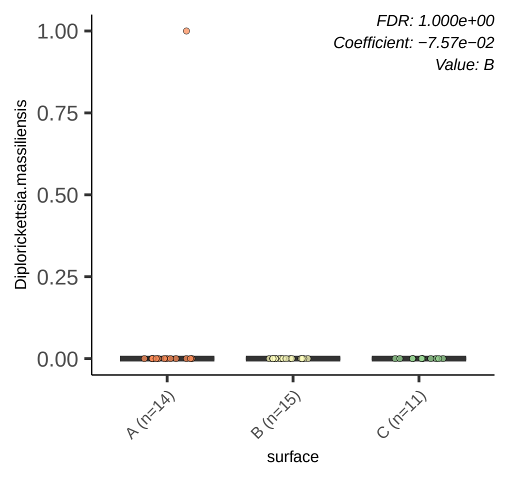
FDR: 1.000e+00
Coefficient: −7.57e−02
Value: B
Diplorickettsia.massiliensis
1.00
0.75
0.50
0.25
0.00
A (n=14)
B (n=15)
C (n=11)
surface

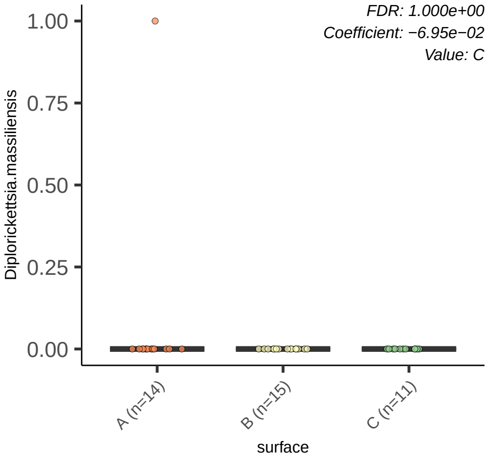

FDR: 1.000e+00
Coefficient: −6.95e−02
Value: C
1.00
0.75
0.50
0.25
0.00
Diplorickettsia.massiliensis
A (n=14)
B (n=15)
C (n=11)
surface

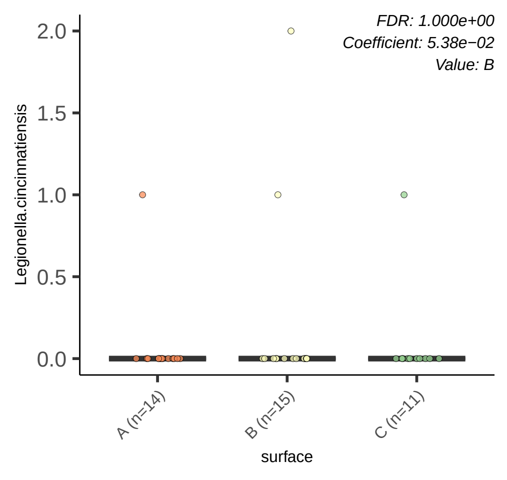

FDR: 1.000e+00
Coefficient: 5.38e−02
Value: B
Legionella.cincinnatiensis
2.0
1.5
1.0
0.5
0.0
A (n=14)
B (n=15)
C (n=11)
surface

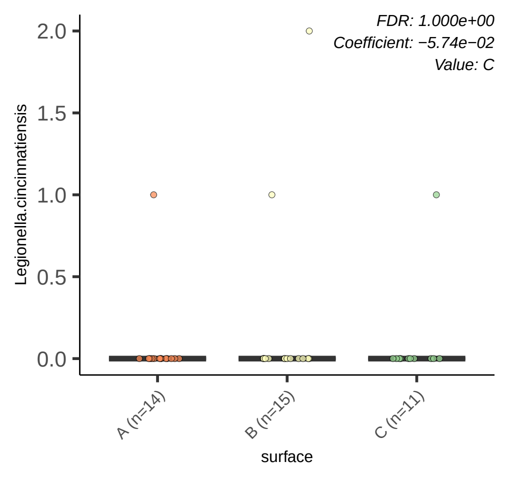
FDR: 1.000e+00
Coefficient: −5.74e−02
Value: C
Legionella.cincinnatiensis
2.0
1.5
1.0
0.5
0.0
A (n=14)
B (n=15)
C (n=11)
surface

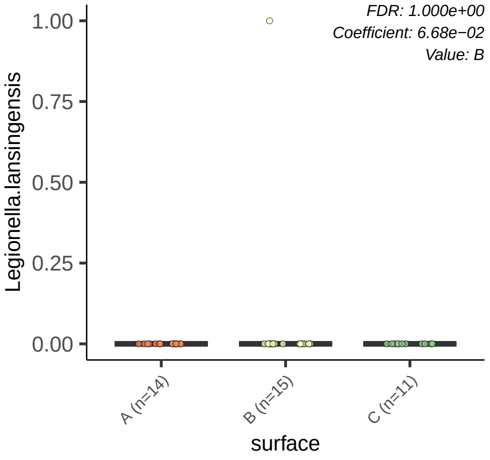

FDR: 1.000e+00
Coefficient: 6.68e−02
Value: B
1.00
0.75
0.50
0.25
0.00
Legionella.lansingensis
A (n=14)
B (n=15)
C (n=11)
surface

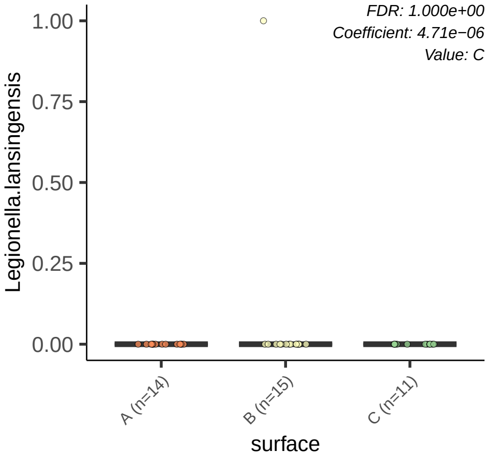
FDR: 1.000e+00
Coefficient: 4.71e−06
Value: C
Legionella.lansingensis
1.00
0.75
0.50
0.25
0.00
A (n=14)
B (n=15)
C (n=11)
surface

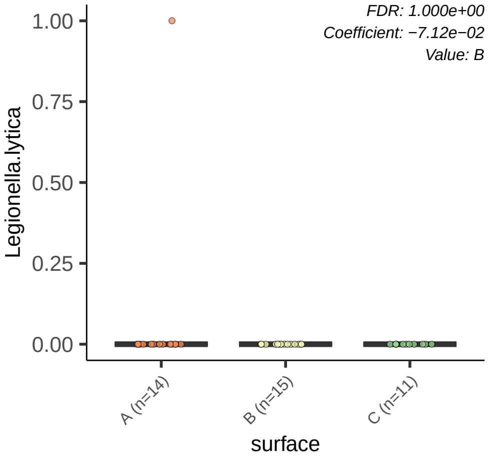

FDR: 1.000e+00
Coefficient: −7.12e−02
Value: B
Legionella.lytica
1.00
0.75
0.50
0.25
0.00
A (n=14)
B (n=15)
C (n=11)
surface

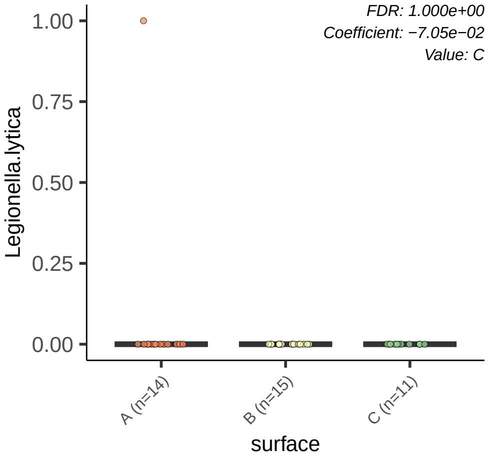
FDR: 1.000e+00
Coefficient: −7.05e−02
Value: C
Legionella.lytica
1.00
0.75
0.50
0.25
0.00
A (n=14)
B (n=15)
C (n=11)
surface

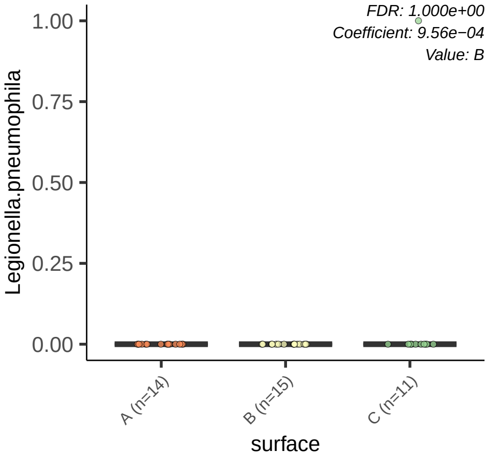
FDR: 1.000e+00
Coefficient: 9.56e−04
Value: B
Legionella.pneumophila
1.00
0.75
0.50
0.25
0.00
A (n=14)
B (n=15)
C (n=11)
surface

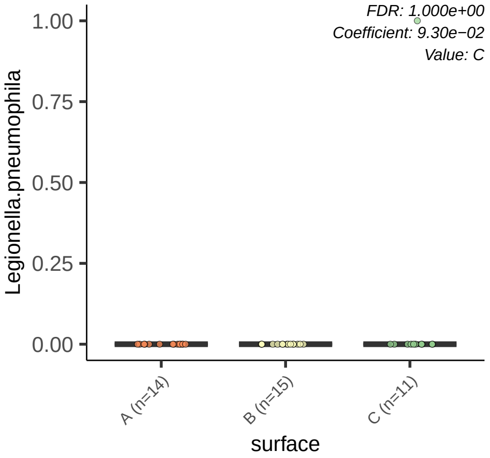

FDR: 1.000e+00
Coefficient: 9.30e−02
Value: C
Legionella.pneumophila
1.00
0.75
0.50
0.25
0.00
A (n=14)
B (n=15)
C (n=11)
surface

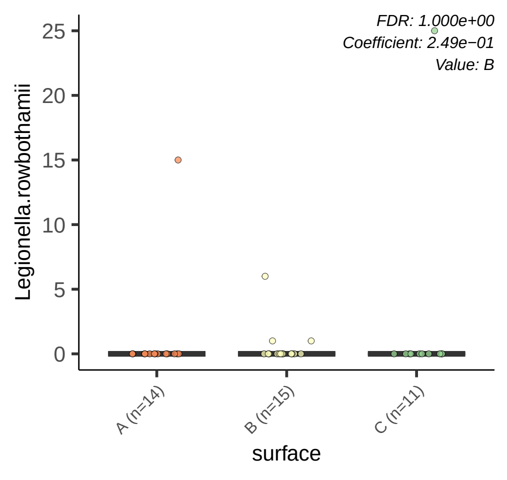
FDR: 1.000e+00
Coefficient: 2.49e−01
Value: B
Legionella.rowbothamii
0
5
10
15
20
25
A (n=14)
B (n=15)
C (n=11)
surface

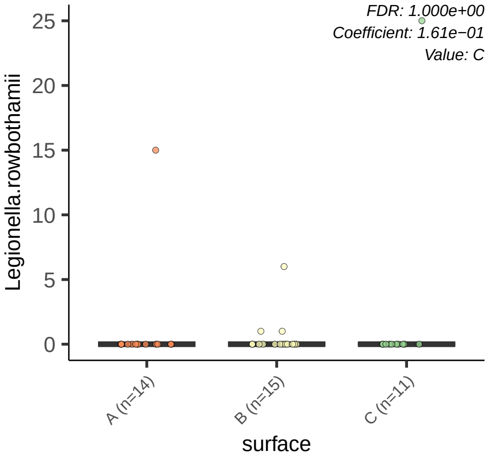

FDR: 1.000e+00
Coefficient: 1.61e−01
Value: C
Legionella.rowbothamii
25
20
15
10
5
0
A (n=14)
B (n=15)
C (n=11)
surface

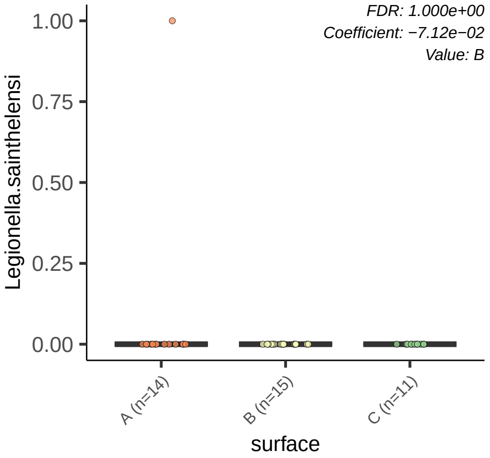
FDR: 1.000e+00
Coefficient: −7.12e−02
Value: B
Legionella.sainthelensi
1.00
0.75
0.50
0.25
0.00
A (n=14)
B (n=15)
C (n=11)
surface

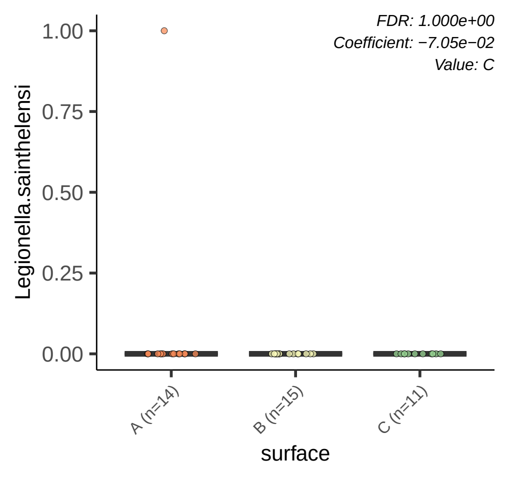
FDR: 1.000e+00
Coefficient: −7.05e−02
Value: C
Legionella.sainthelensi
1.00
0.75
0.50
0.25
0.00
A (n=14)
B (n=15)
C (n=11)
surface

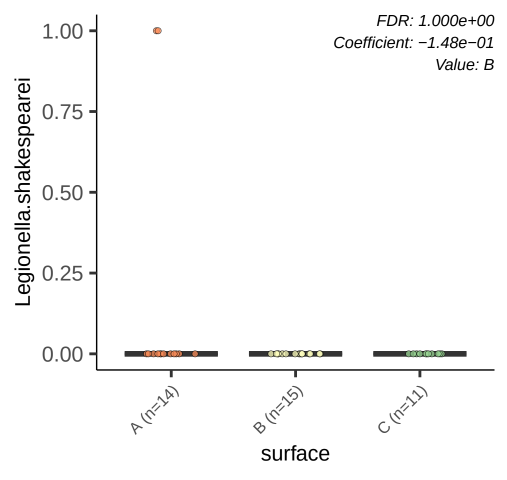

FDR: 1.000e+00
Coefficient: −1.48e−01
Value: B
Legionella.shakespearei
1.00
0.75
0.50
0.25
0.00
A (n=14)
B (n=15)
C (n=11)
surface

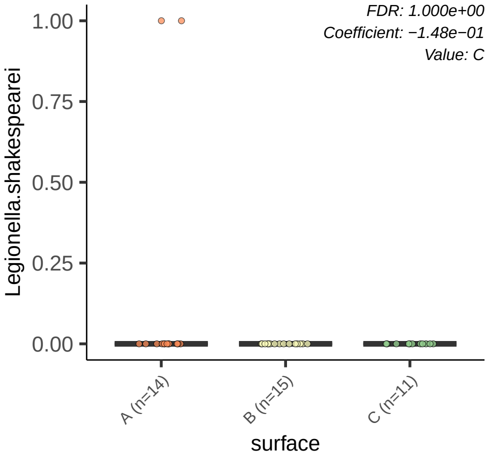

FDR: 1.000e+00
Coefficient: −1.48e−01
Value: C
Legionella.shakespearei
1.00
0.75
0.50
0.25
0.00
A (n=14)
B (n=15)
C (n=11)
surface

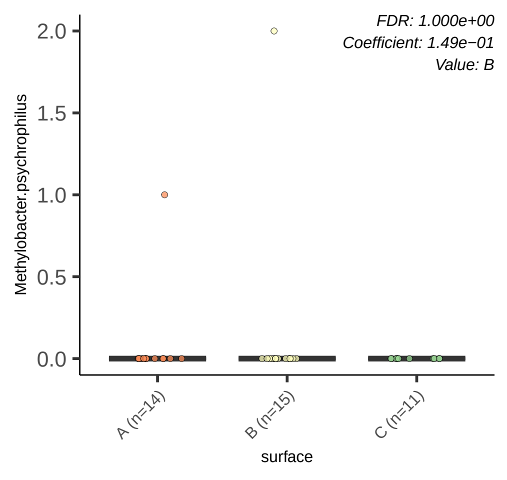

FDR: 1.000e+00
Coefficient: 1.49e−01
Value: B
Methylobacter.psychrophilus
2.0
1.5
1.0
0.5
0.0
A (n=14)
B (n=15)
C (n=11)
surface

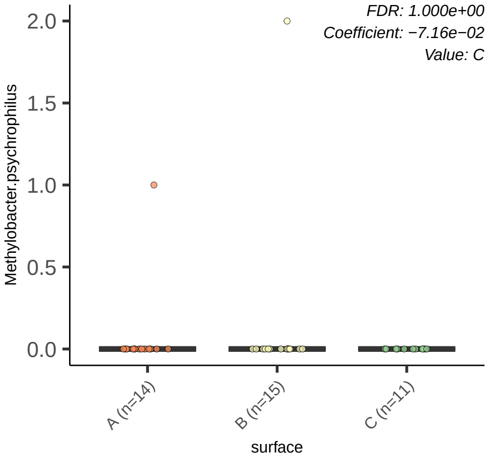
FDR: 1.000e+00
Coefficient: −7.16e−02
Value: C
Methylobacter.psychrophilus
2.0
1.5
1.0
0.5
0.0
A (n=14)
B (n=15)
C (n=11)
surface

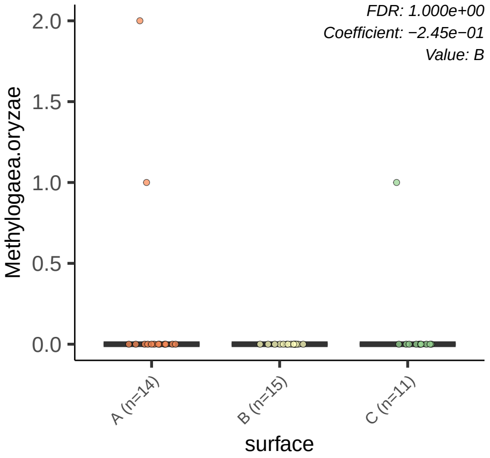

FDR: 1.000e+00
Coefficient: −2.45e−01
Value: B
Methylogaea.oryzae
2.0
1.5
1.0
0.5
0.0
A (n=14)
B (n=15)
C (n=11)
surface

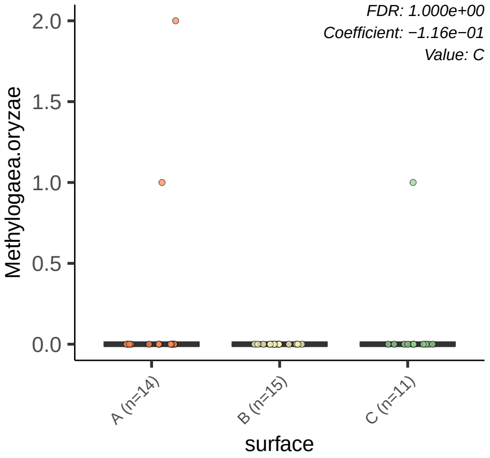

FDR: 1.000e+00
Coefficient: −1.16e−01
Value: C
Methylogaea.oryzae
2.0
1.5
1.0
0.5
0.0
A (n=14)
B (n=15)
C (n=11)
surface

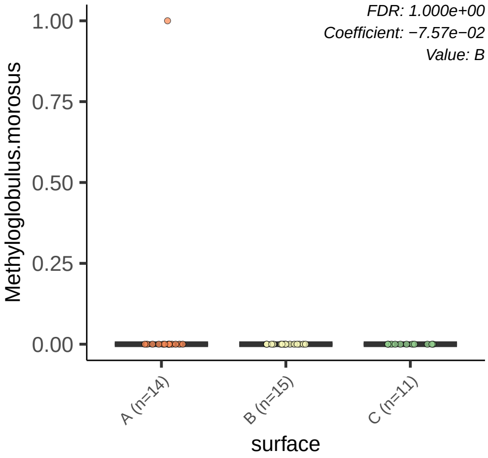
FDR: 1.000e+00
Coefficient: −7.57e−02
Value: B
Methyloglobulus.morosus
1.00
0.75
0.50
0.25
0.00
A (n=14)
B (n=15)
C (n=11)
surface

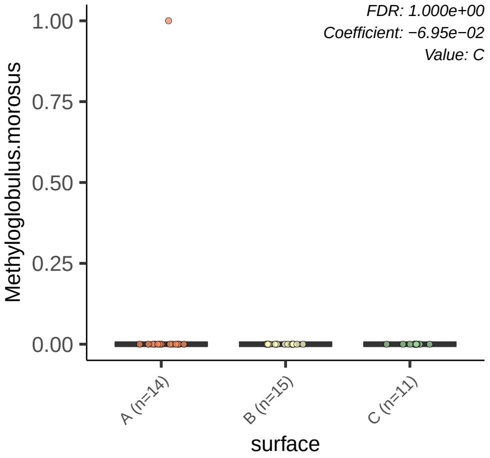

FDR: 1.000e+00
Coefficient: −6.95e−02
Value: C
Methyloglobulus.morosus
1.00
0.75
0.50
0.25
0.00
A (n=14)
B (n=15)
C (n=11)
surface

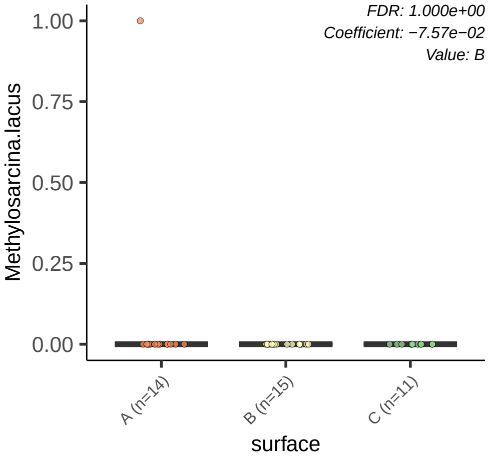

FDR: 1.000e+00
Coefficient: −7.57e−02
Value: B
Methylosarcina.lacus
1.00
0.75
0.50
0.25
0.00
A (n=14)
B (n=15)
C (n=11)
surface

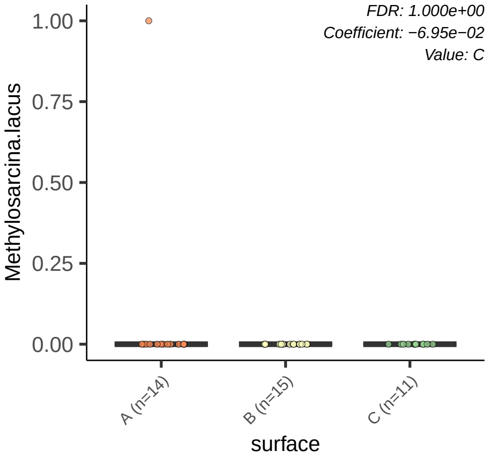

FDR: 1.000e+00
Coefficient: −6.95e−02
Value: C
Methylosarcina.lacus
1.00
0.75
0.50
0.25
0.00
A (n=14)
B (n=15)
C (n=11)
surface

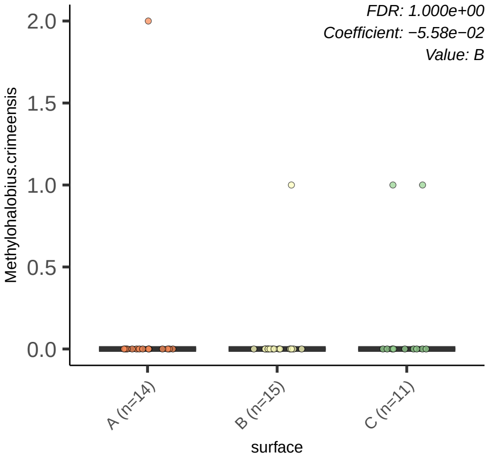
FDR: 1.000e+00
Coefficient: −5.58e−02
Value: B
Methylohalobius.crimeensis
2.0
1.5
1.0
0.5
0.0
A (n=14)
B (n=15)
C (n=11)
surface

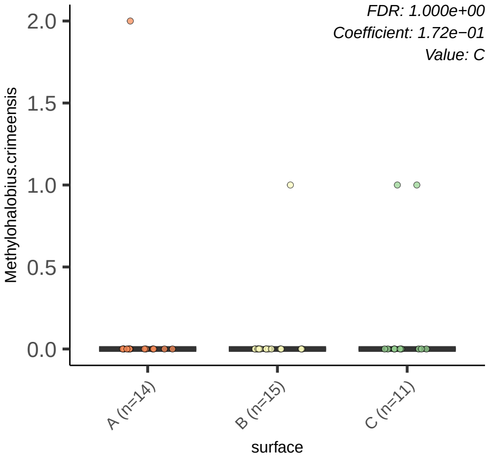

FDR: 1.000e+00
Coefficient: 1.72e−01
Value: C
Methylohalobius.crimeensis
2.0
1.5
1.0
0.5
0.0
A (n=14)
B (n=15)
C (n=11)
surface

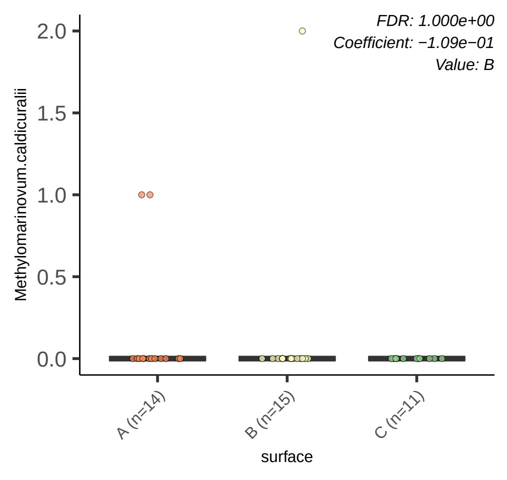
FDR: 1.000e+00
Coefficient: −1.09e−01
Value: B
Methylomarinovum.caldicuralii
2.0
1.5
1.0
0.5
0.0
A (n=14)
B (n=15)
C (n=11)
surface

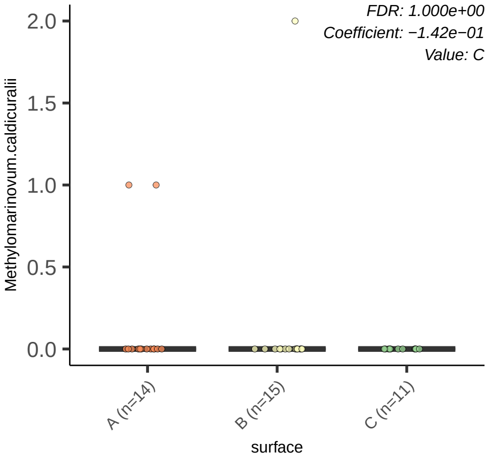
FDR: 1.000e+00
Coefficient: −1.42e−01
Value: C
2.0
1.5
1.0
0.5
0.0
Methylomarinovum.caldicuralii
A (n=14)
B (n=15)
C (n=11)
surface

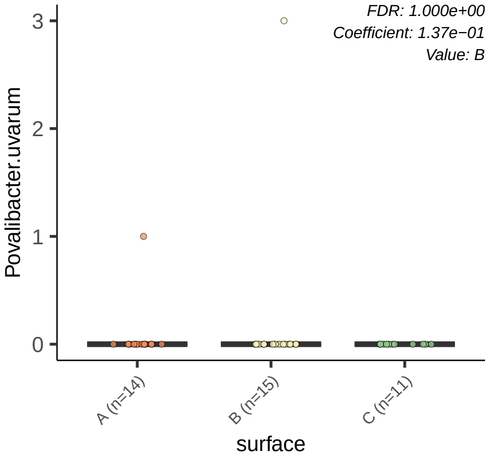

FDR: 1.000e+00
Coefficient: 1.37e−01
Value: B
Povalibacter.uvarum
3
2
1
0
A (n=14)
B (n=15)
C (n=11)
surface

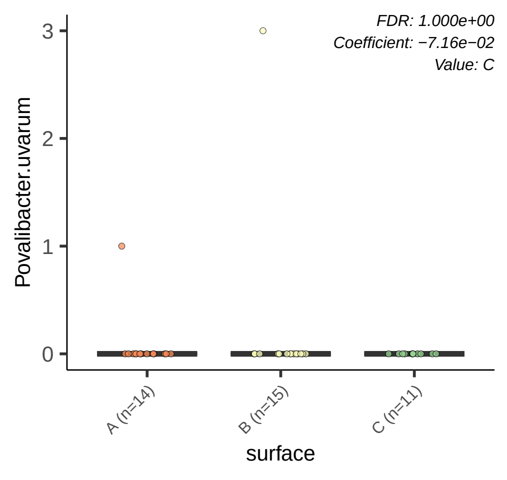
FDR: 1.000e+00
Coefficient: −7.16e−02
Value: C
Povalibacter.uvarum
0
1
2
3
A (n=14)
B (n=15)
C (n=11)
surface

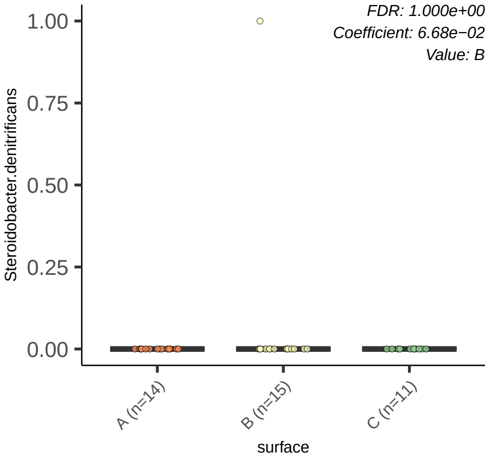
FDR: 1.000e+00
Coefficient: 6.68e−02
Value: B
Steroidobacter.denitrificans
1.00
0.75
0.50
0.25
0.00
A (n=14)
B (n=15)
C (n=11)
surface

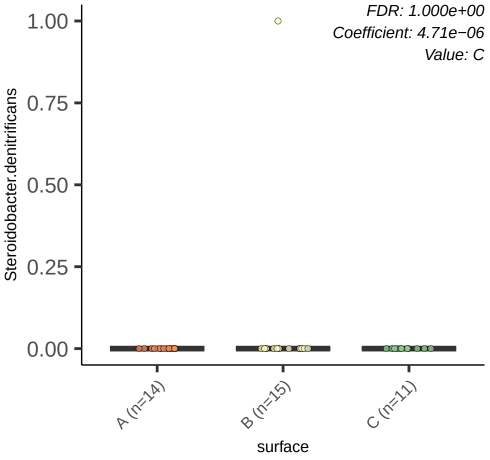
FDR: 1.000e+00
Coefficient: 4.71e−06
Value: C
Steroidobacter.denitrificans
1.00
0.75
0.50
0.25
0.00
A (n=14)
B (n=15)
C (n=11)
surface

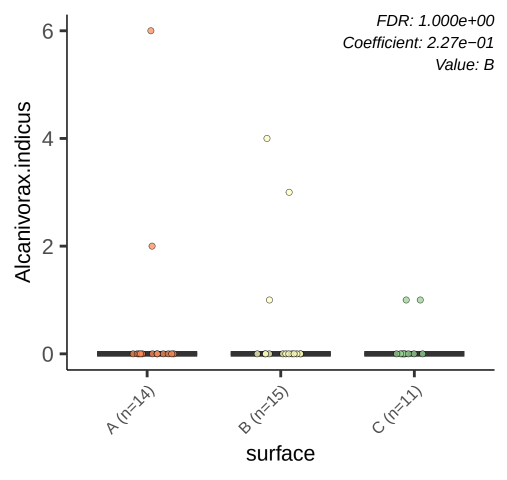
FDR: 1.000e+00
Coefficient: 2.27e−01
Value: B
Alcanivorax.indicus
6
4
2
0
A (n=14)
B (n=15)
C (n=11)
surface

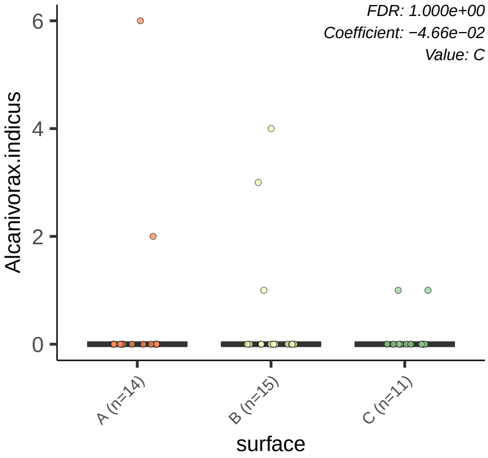

FDR: 1.000e+00
Coefficient: −4.66e−02
Value: C
Alcanivorax.indicus
6
4
2
0
A (n=14)
B (n=15)
C (n=11)
surface

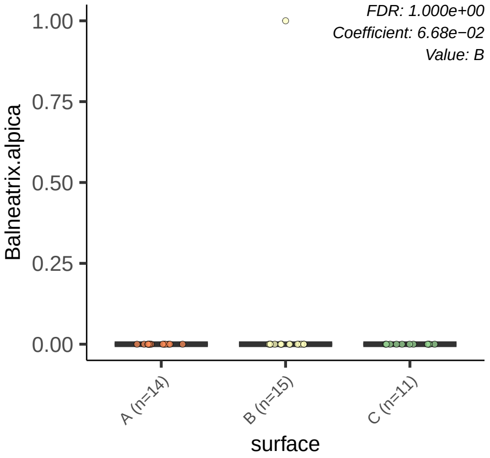

FDR: 1.000e+00
Coefficient: 6.68e−02
Value: B
Balneatrix.alpica
1.00
0.75
0.50
0.25
0.00
A (n=14)
B (n=15)
C (n=11)
surface

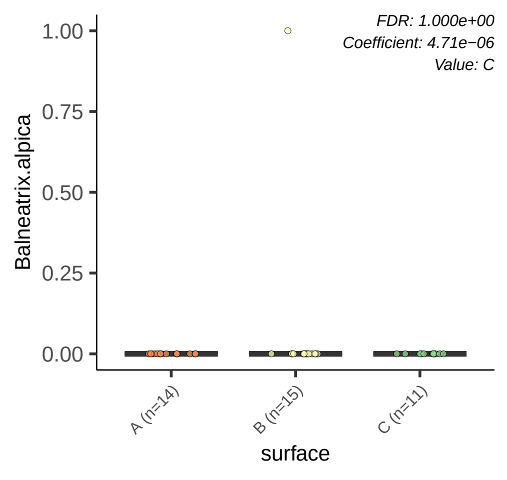
FDR: 1.000e+00
Coefficient: 4.71e−06
Value: C
Balneatrix.alpica
1.00
0.75
0.50
0.25
0.00
A (n=14)
B (n=15)
C (n=11)
surface

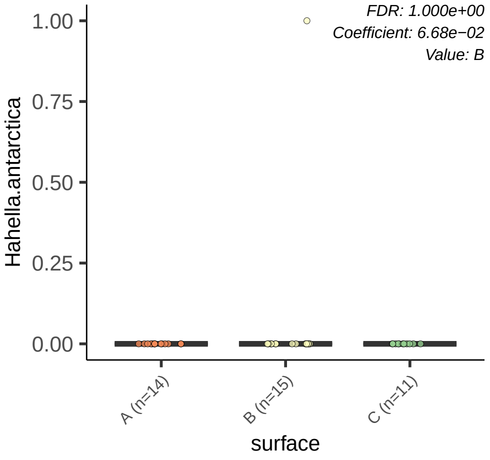

FDR: 1.000e+00
Coefficient: 6.68e−02
Value: B
Hahella.antarctica
1.00
0.75
0.50
0.25
0.00
A (n=14)
B (n=15)
C (n=11)
surface

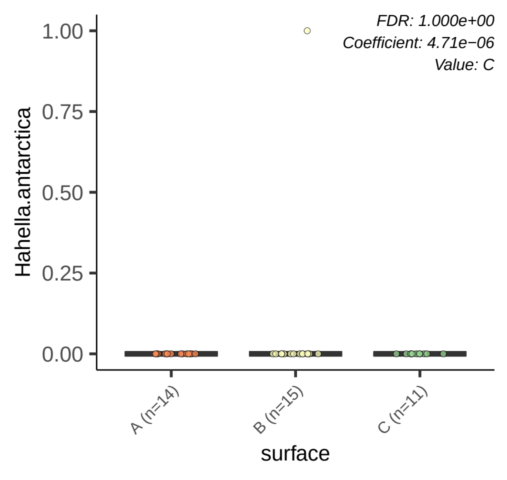
FDR: 1.000e+00
Coefficient: 4.71e−06
Value: C
Hahella.antarctica
1.00
0.75
0.50
0.25
0.00
A (n=14)
B (n=15)
C (n=11)
surface

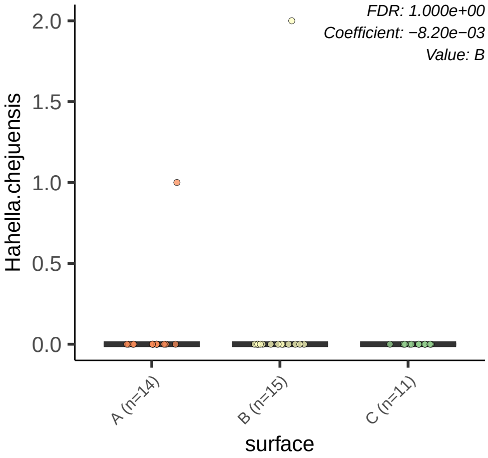
FDR: 1.000e+00
Coefficient: −8.20e−03
Value: B
Hahella.chejuensis
2.0
1.5
1.0
0.5
0.0
A (n=14)
B (n=15)
C (n=11)
surface

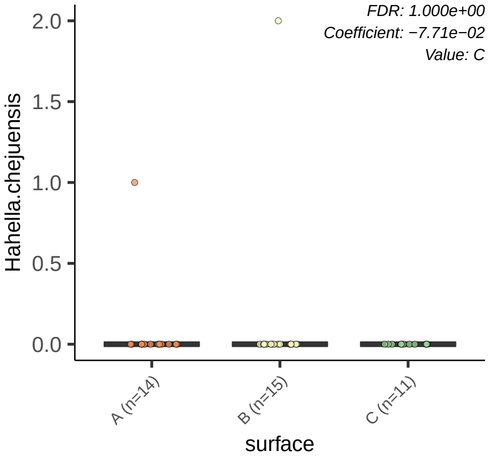
FDR: 1.000e+00
Coefficient: −7.71e−02
Value: C
Hahella.chejuensis
2.0
1.5
1.0
0.5
0.0
A (n=14)
B (n=15)
C (n=11)
surface

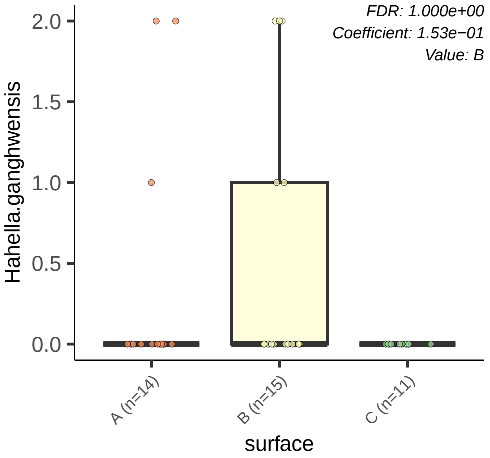

FDR: 1.000e+00
Coefficient: 1.53e−01
Value: B
Hahella.ganghwensis
2.0
1.5
1.0
0.5
0.0
A (n=14)
B (n=15)
C (n=11)
surface

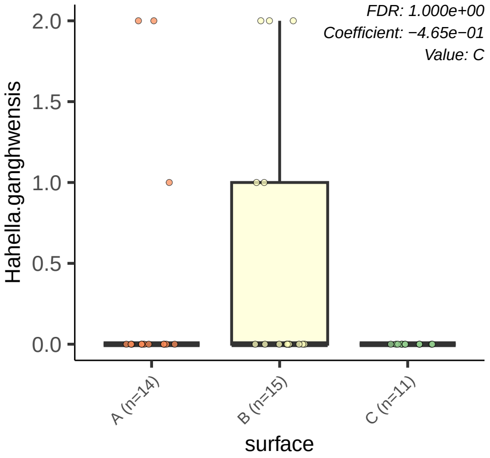
FDR: 1.000e+00
Coefficient: −4.65e−01
Value: C
Hahella.ganghwensis
2.0
1.5
1.0
0.5
0.0
A (n=14)
B (n=15)
C (n=11)
surface

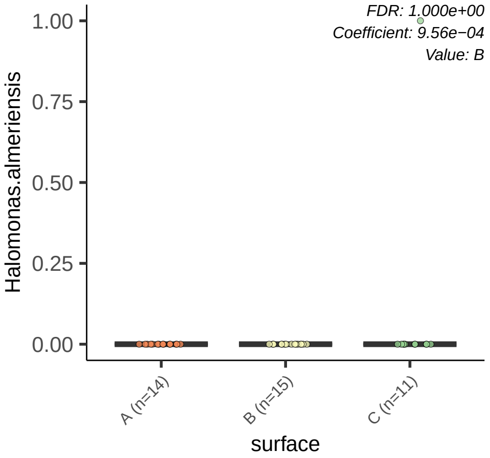
FDR: 1.000e+00
Coefficient: 9.56e−04
Value: B
Halomonas.almeriensis
1.00
0.75
0.50
0.25
0.00
A (n=14)
B (n=15)
C (n=11)
surface

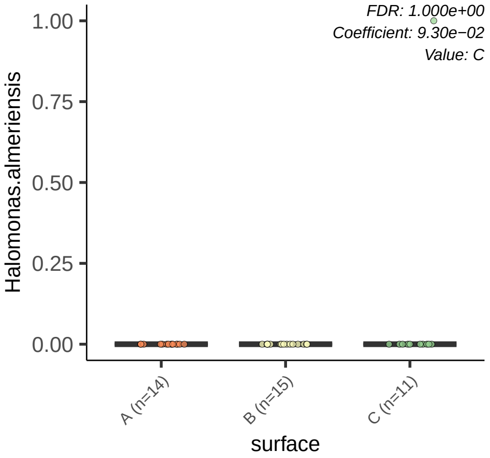
FDR: 1.000e+00
Coefficient: 9.30e−02
Value: C
Halomonas.almeriensis
1.00
0.75
0.50
0.25
0.00
A (n=14)
B (n=15)
C (n=11)
surface

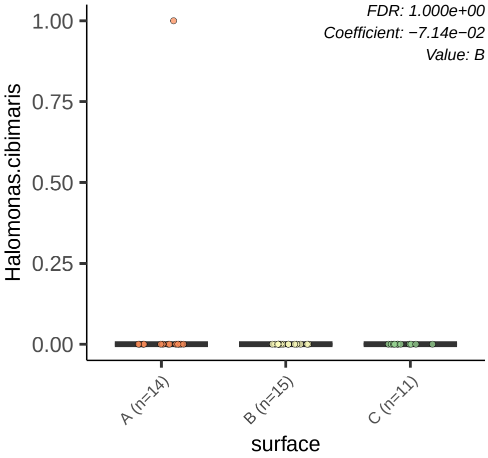
FDR: 1.000e+00
Coefficient: −7.14e−02
Value: B
Halomonas.cibimaris
1.00
0.75
0.50
0.25
0.00
A (n=14)
B (n=15)
C (n=11)
surface

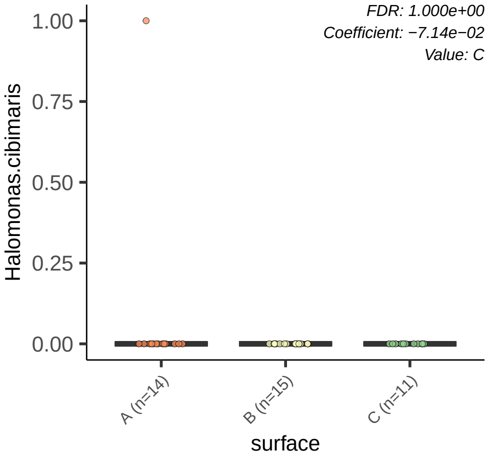

FDR: 1.000e+00
Coefficient: −7.14e−02
Value: C
1.00
0.75
0.50
0.25
0.00
Halomonas.cibimaris
A (n=14)
B (n=15)
C (n=11)
surface

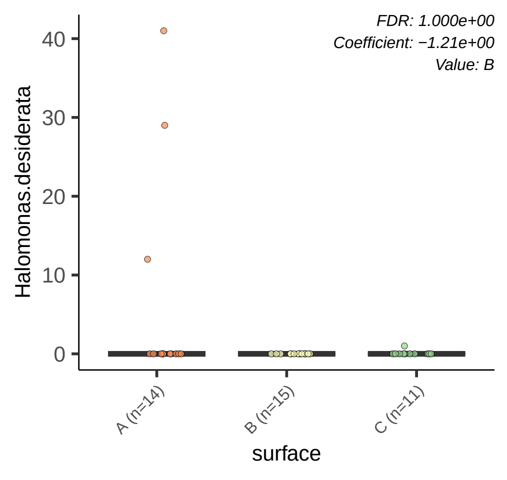

FDR: 1.000e+00
Coefficient: −1.21e+00
Value: B
Halomonas.desiderata
40
30
20
10
0
A (n=14)
B (n=15)
C (n=11)
surface

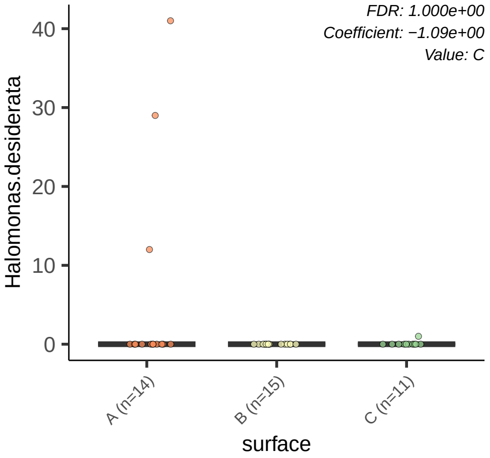
FDR: 1.000e+00
Coefficient: −1.09e+00
Value: C
Halomonas.desiderata
0
10
20
30
40
A (n=14)
B (n=15)
C (n=11)
surface

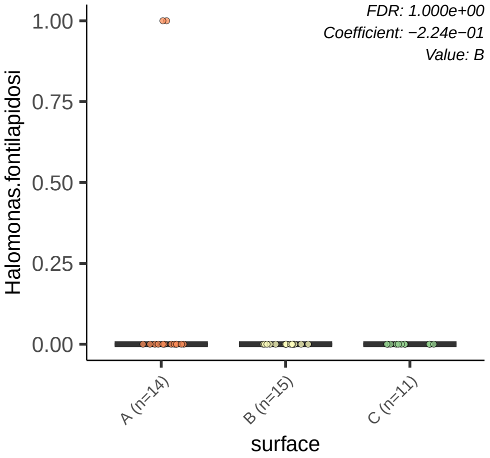
FDR: 1.000e+00
Coefficient: −2.24e−01
Value: B
Halomonas.fontilapidosi
1.00
0.75
0.50
0.25
0.00
A (n=14)
B (n=15)
C (n=11)
surface

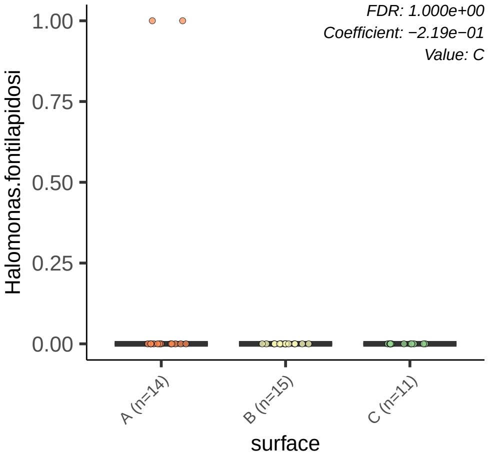
FDR: 1.000e+00
Coefficient: −2.19e−01
Value: C
Halomonas.fontilapidosi
1.00
0.75
0.50
0.25
0.00
A (n=14)
B (n=15)
C (n=11)
surface

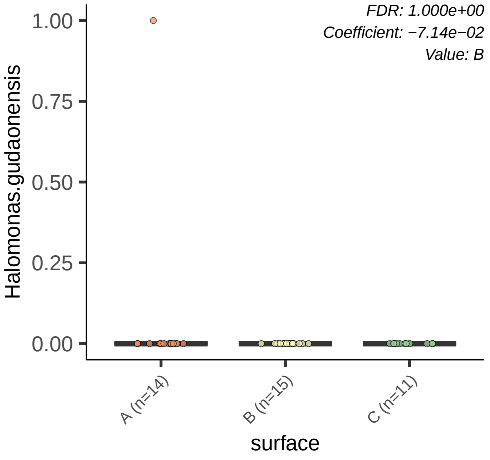
FDR: 1.000e+00
Coefficient: −7.14e−02
Value: B
Halomonas.gudaonensis
1.00
0.75
0.50
0.25
0.00
A (n=14)
B (n=15)
C (n=11)
surface

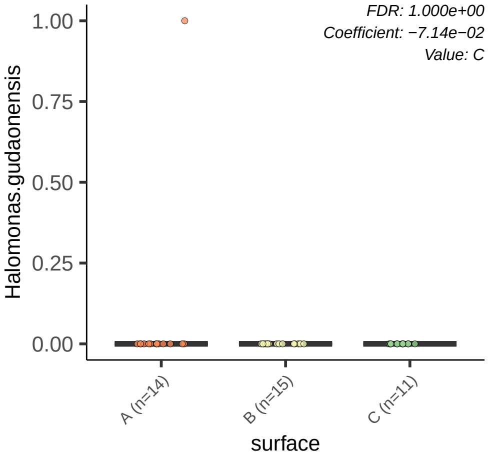

FDR: 1.000e+00
Coefficient: −7.14e−02
Value: C
Halomonas.gudaonensis
1.00
0.75
0.50
0.25
0.00
A (n=14)
B (n=15)
C (n=11)
surface

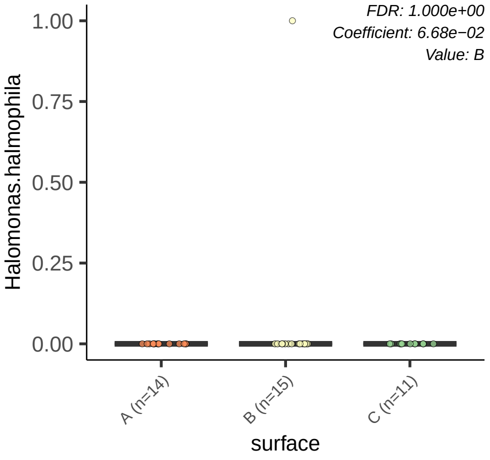

FDR: 1.000e+00
Coefficient: 6.68e−02
Value: B
Halomonas.halmophila
1.00
0.75
0.50
0.25
0.00
A (n=14)
B (n=15)
C (n=11)
surface

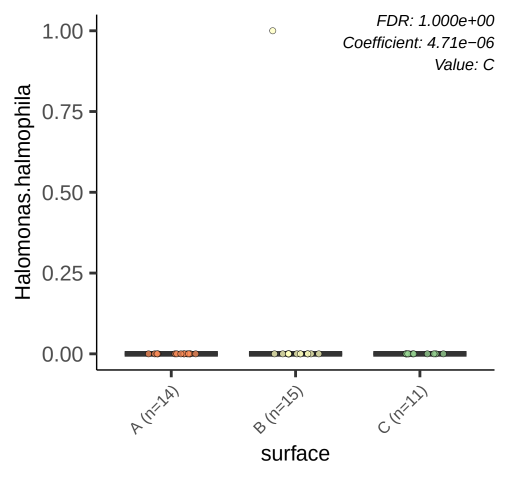
FDR: 1.000e+00
Coefficient: 4.71e−06
Value: C
Halomonas.halmophila
1.00
0.75
0.50
0.25
0.00
A (n=14)
B (n=15)
C (n=11)
surface

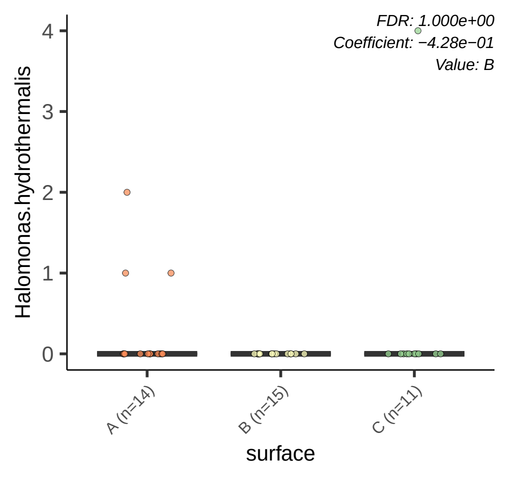

FDR: 1.000e+00
Coefficient: −4.28e−01
Value: B
Halomonas.hydrothermalis
0
1
2
3
4
A (n=14)
B (n=15)
C (n=11)
surface

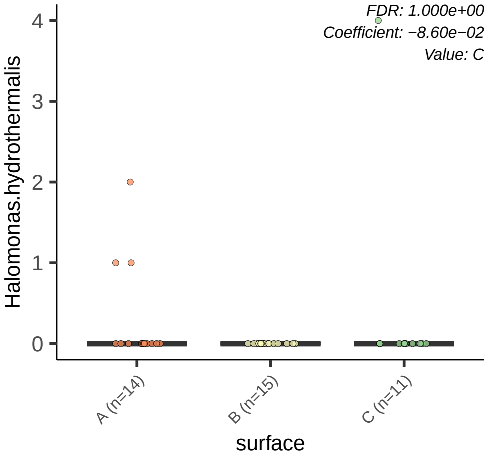

FDR: 1.000e+00
Coefficient: −8.60e−02
Value: C
Halomonas.hydrothermalis
0
1
2
3
4
A (n=14)
B (n=15)
C (n=11)
surface

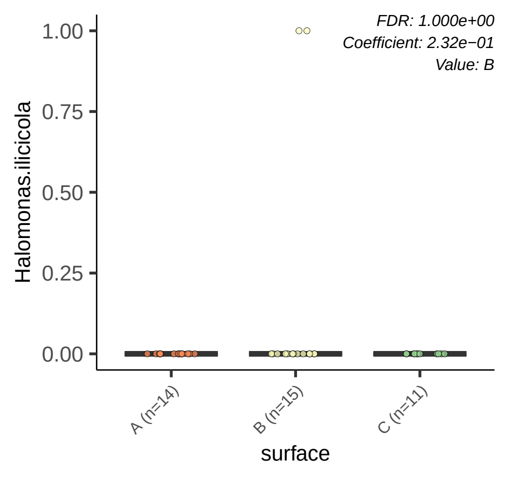

FDR: 1.000e+00
Coefficient: 2.32e−01
Value: B
Halomonas.ilicicola
1.00
0.75
0.50
0.25
0.00
A (n=14)
B (n=15)
C (n=11)
surface

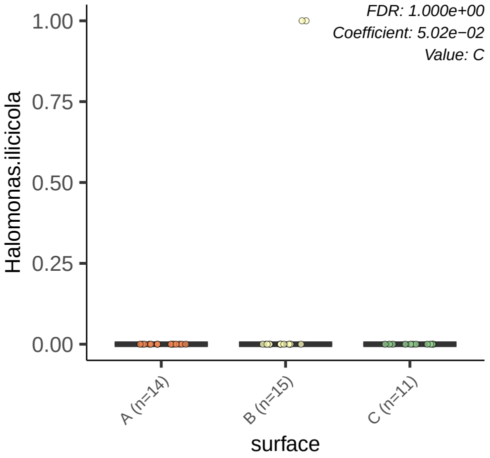

FDR: 1.000e+00
Coefficient: 5.02e−02
Value: C
Halomonas.ilicicola
1.00
0.75
0.50
0.25
0.00
A (n=14)
B (n=15)
C (n=11)
surface

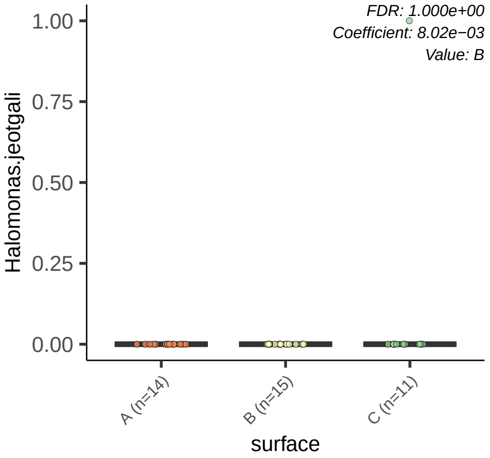

FDR: 1.000e+00
Coefficient: 8.02e−03
Value: B
Halomonas.jeotgali
1.00
0.75
0.50
0.25
0.00
A (n=14)
B (n=15)
C (n=11)
surface

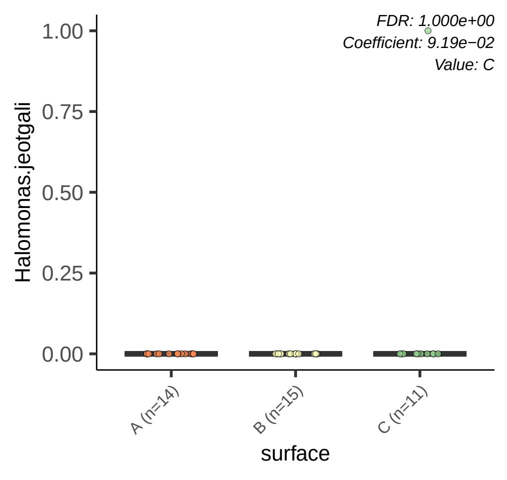
FDR: 1.000e+00
Coefficient: 9.19e−02
Value: C
Halomonas.jeotgali
1.00
0.75
0.50
0.25
0.00
A (n=14)
B (n=15)
C (n=11)
surface

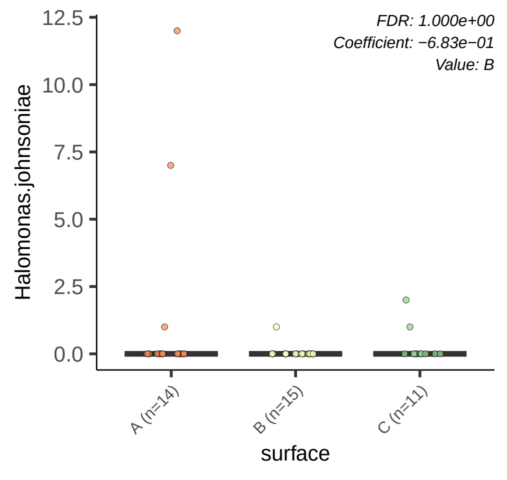
FDR: 1.000e+00
Coefficient: −6.83e−01
Value: B
Halomonas.johnsoniae
12.5
10.0
7.5
5.0
2.5
0.0
A (n=14)
B (n=15)
C (n=11)
surface

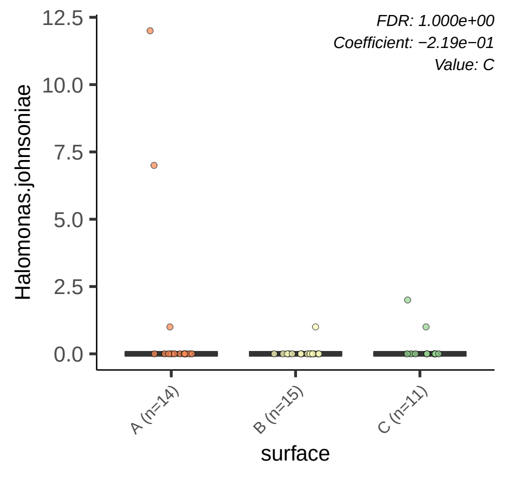
FDR: 1.000e+00
Coefficient: −2.19e−01
Value: C
Halomonas.johnsoniae
12.5
10.0
7.5
5.0
2.5
0.0
A (n=14)
B (n=15)
C (n=11)
surface

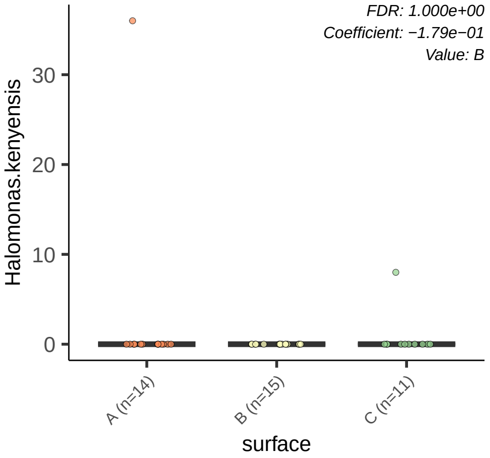

FDR: 1.000e+00
Coefficient: −1.79e−01
Value: B
Halomonas.kenyensis
30
20
10
0
A (n=14)
B (n=15)
C (n=11)
surface

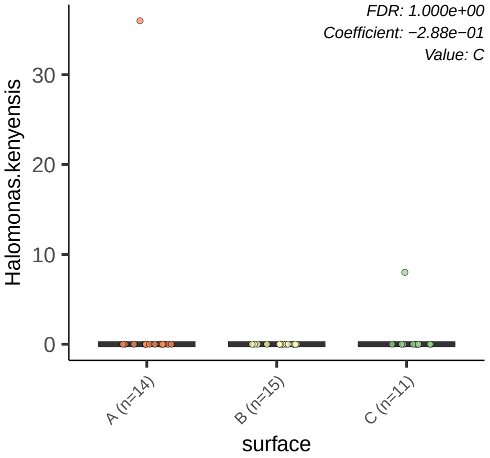

FDR: 1.000e+00
Coefficient: −2.88e−01
Value: C
Halomonas.kenyensis
0
10
20
30
A (n=14)
B (n=15)
C (n=11)
surface

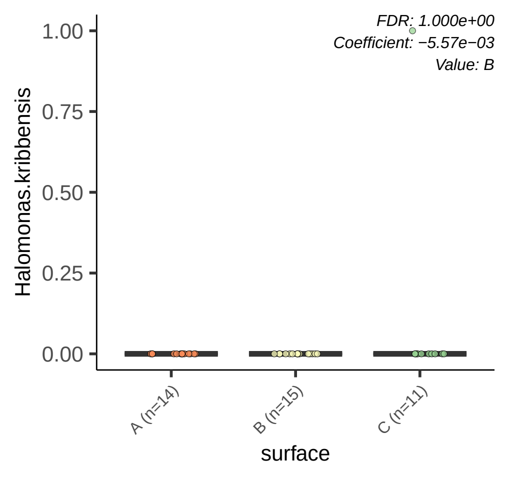
FDR: 1.000e+00
Coefficient: −5.57e−03
Value: B
Halomonas.kribbensis
1.00
0.75
0.50
0.25
0.00
A (n=14)
B (n=15)
C (n=11)
surface

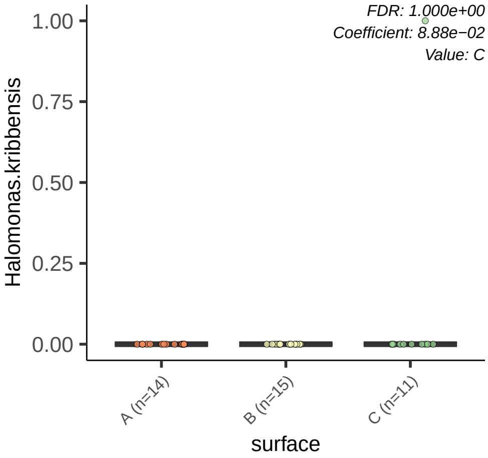
FDR: 1.000e+00
Coefficient: 8.88e−02
Value: C
Halomonas.kribbensis
1.00
0.75
0.50
0.25
0.00
A (n=14)
B (n=15)
C (n=11)
surface

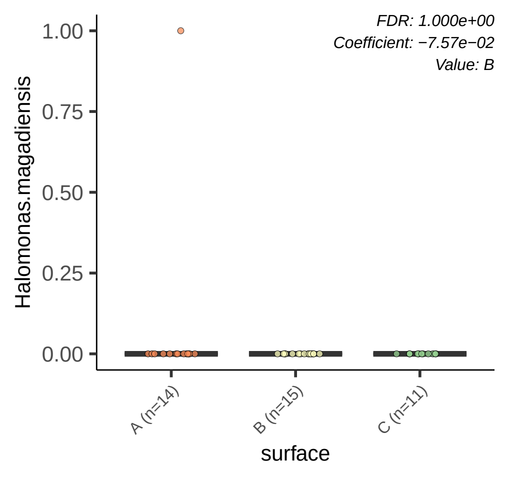
FDR: 1.000e+00
Coefficient: −7.57e−02
Value: B
Halomonas.magadiensis
1.00
0.75
0.50
0.25
0.00
A (n=14)
B (n=15)
C (n=11)
surface

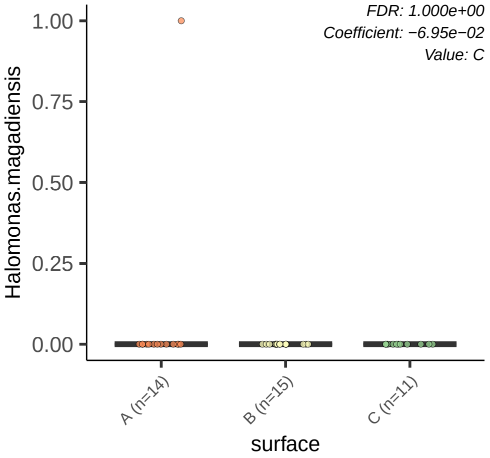

FDR: 1.000e+00
Coefficient: −6.95e−02
Value: C
Halomonas.magadiensis
1.00
0.75
0.50
0.25
0.00
A (n=14)
B (n=15)
C (n=11)
surface

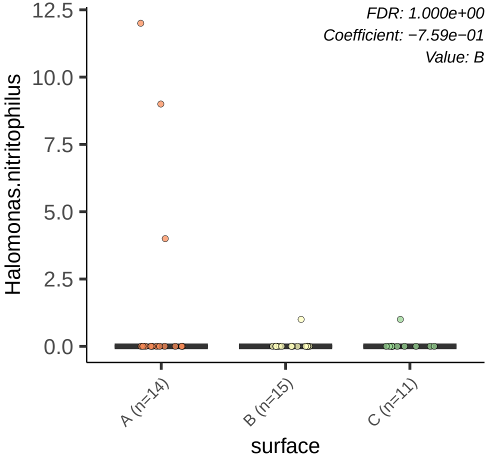

FDR: 1.000e+00
Coefficient: −7.59e−01
Value: B
Halomonas.nitritophilus
12.5
10.0
7.5
5.0
2.5
0.0
A (n=14)
B (n=15)
C (n=11)
surface

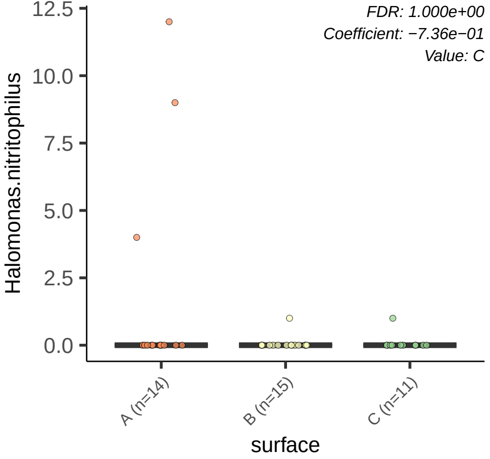

FDR: 1.000e+00
Coefficient: −7.36e−01
Value: C
Halomonas.nitritophilus
12.5
10.0
7.5
5.0
2.5
0.0
A (n=14)
B (n=15)
C (n=11)
surface

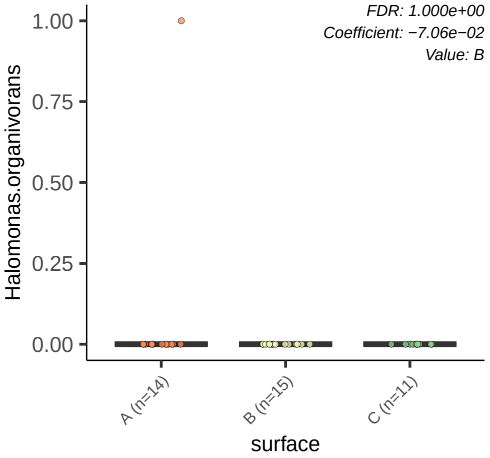
FDR: 1.000e+00
Coefficient: −7.06e−02
Value: B
Halomonas.organivorans
1.00
0.75
0.50
0.25
0.00
A (n=14)
B (n=15)
C (n=11)
surface

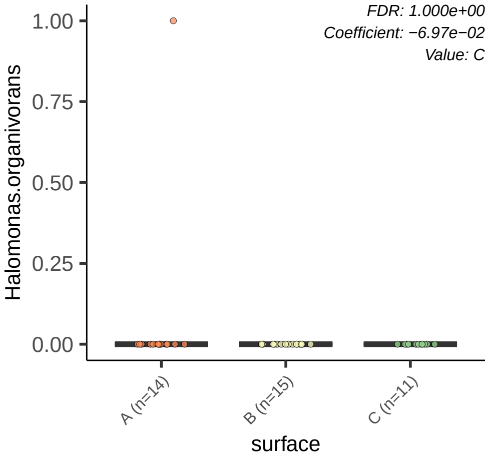

FDR: 1.000e+00
Coefficient: −6.97e−02
Value: C
Halomonas.organivorans
1.00
0.75
0.50
0.25
0.00
A (n=14)
B (n=15)
C (n=11)
surface

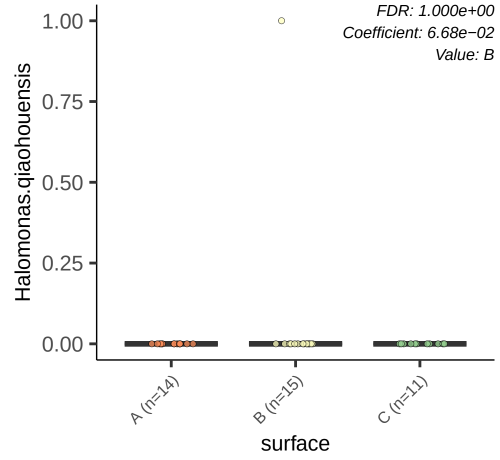
FDR: 1.000e+00
Coefficient: 6.68e−02
Value: B
Halomonas.qiaohouensis
1.00
0.75
0.50
0.25
0.00
A (n=14)
B (n=15)
C (n=11)
surface

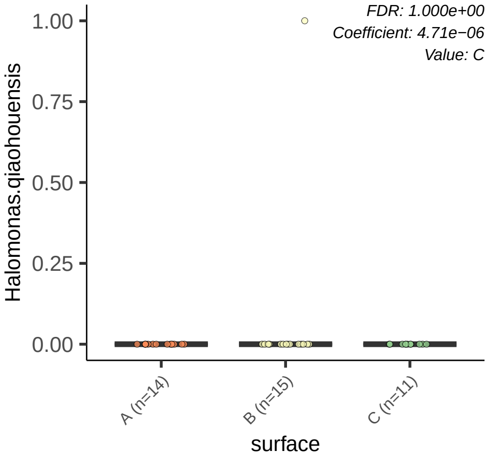
FDR: 1.000e+00
Coefficient: 4.71e−06
Value: C
Halomonas.qiaohouensis
1.00
0.75
0.50
0.25
0.00
A (n=14)
B (n=15)
C (n=11)
surface

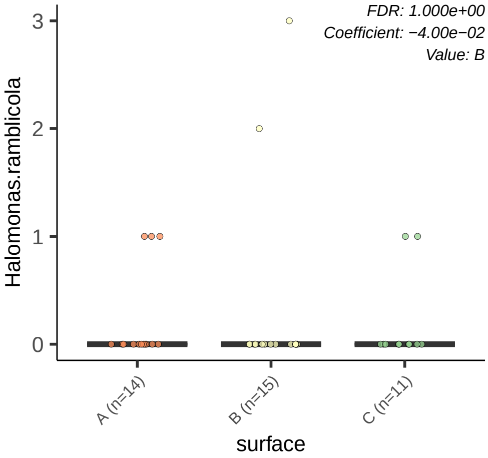

FDR: 1.000e+00
Coefficient: −4.00e−02
Value: B
Halomonas.ramblicola
3
2
1
0
A (n=14)
B (n=15)
C (n=11)
surface

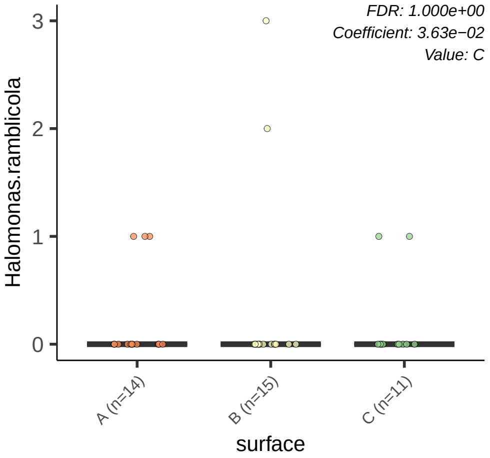

FDR: 1.000e+00
Coefficient: 3.63e−02
Value: C
Halomonas.ramblicola
3
2
1
0
A (n=14)
B (n=15)
C (n=11)
surface

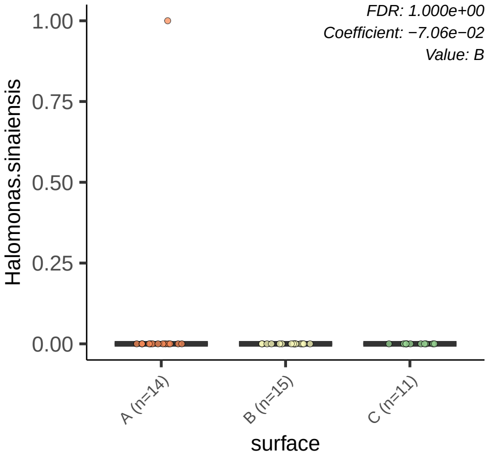

FDR: 1.000e+00
Coefficient: −7.06e−02
Value: B
Halomonas.sinaiensis
1.00
0.75
0.50
0.25
0.00
A (n=14)
B (n=15)
C (n=11)
surface

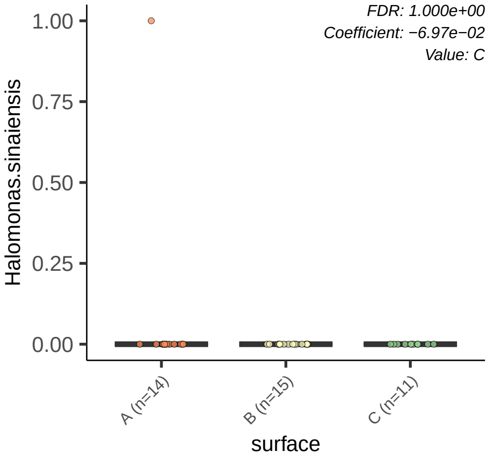
FDR: 1.000e+00
Coefficient: −6.97e−02
Value: C
Halomonas.sinaiensis
1.00
0.75
0.50
0.25
0.00
A (n=14)
B (n=15)
C (n=11)
surface

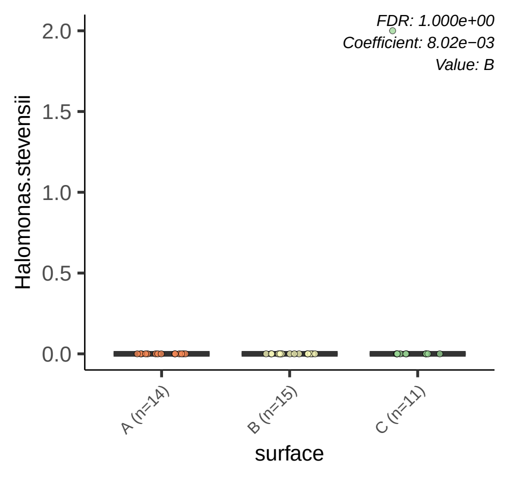

FDR: 1.000e+00
Coefficient: 8.02e−03
Value: B
Halomonas.stevensii
2.0
1.5
1.0
0.5
0.0
A (n=14)
B (n=15)
C (n=11)
surface

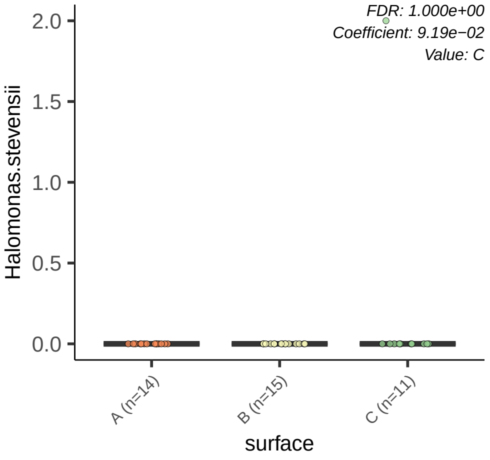
FDR: 1.000e+00
Coefficient: 9.19e−02
Value: C
Halomonas.stevensii
2.0
1.5
1.0
0.5
0.0
A (n=14)
B (n=15)
C (n=11)
surface

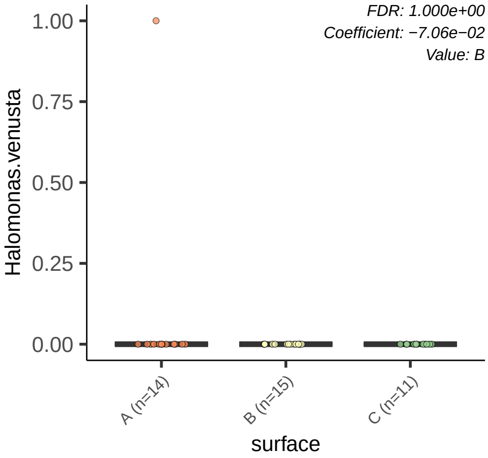
FDR: 1.000e+00
Coefficient: −7.06e−02
Value: B
Halomonas.venusta
1.00
0.75
0.50
0.25
0.00
A (n=14)
B (n=15)
C (n=11)
surface

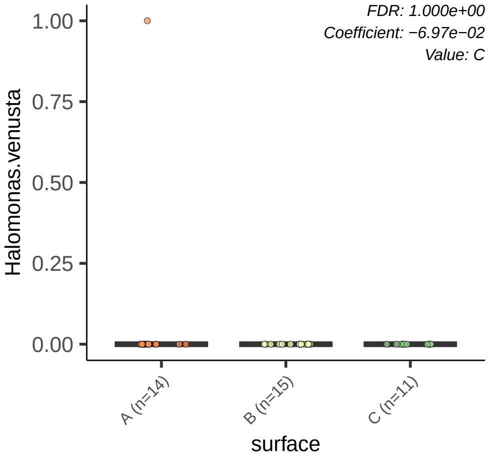
FDR: 1.000e+00
Coefficient: −6.97e−02
Value: C
Halomonas.venusta
1.00
0.75
0.50
0.25
0.00
A (n=14)
B (n=15)
C (n=11)
surface

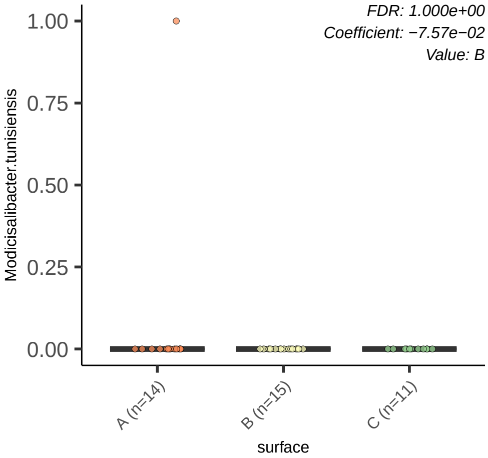
FDR: 1.000e+00
Coefficient: −7.57e−02
Value: B
1.00
0.75
0.50
0.25
0.00
Modicisalibacter.tunisiensis
A (n=14)
B (n=15)
C (n=11)
surface

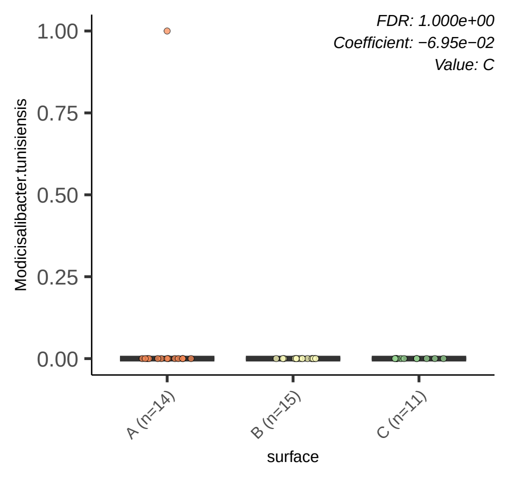

FDR: 1.000e+00
Coefficient: −6.95e−02
Value: C
Modicisalibacter.tunisiensis
1.00
0.75
0.50
0.25
0.00
A (n=14)
B (n=15)
C (n=11)
surface

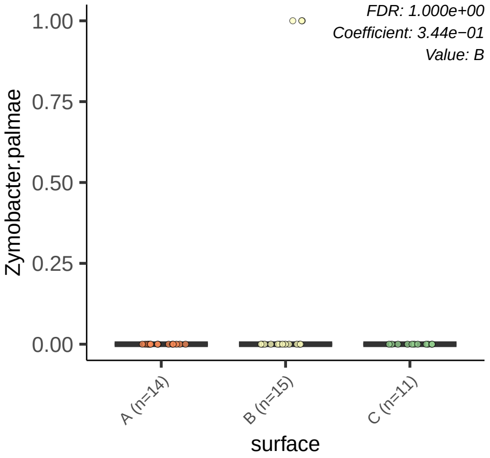

FDR: 1.000e+00
Coefficient: 3.44e−01
Value: B
Zymobacter.palmae
1.00
0.75
0.50
0.25
0.00
A (n=14)
B (n=15)
C (n=11)
surface

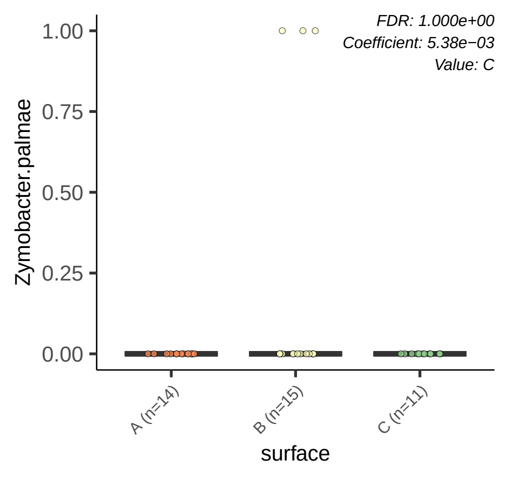
FDR: 1.000e+00
Coefficient: 5.38e−03
Value: C
Zymobacter.palmae
1.00
0.75
0.50
0.25
0.00
A (n=14)
B (n=15)
C (n=11)
surface

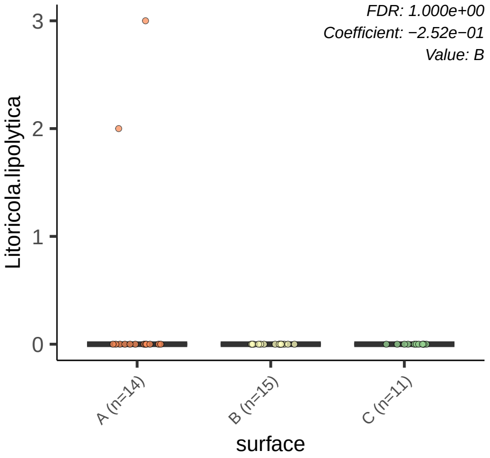
FDR: 1.000e+00
Coefficient: −2.52e−01
Value: B
Litoricola.lipolytica
3
2
1
0
A (n=14)
B (n=15)
C (n=11)
surface

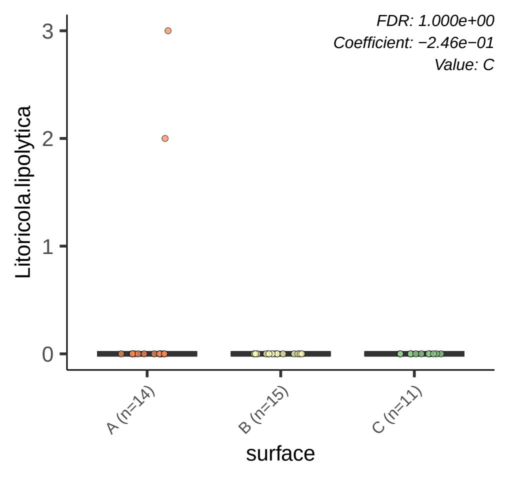
FDR: 1.000e+00
Coefficient: −2.46e−01
Value: C
Litoricola.lipolytica
3
2
1
0
A (n=14)
B (n=15)
C (n=11)
surface

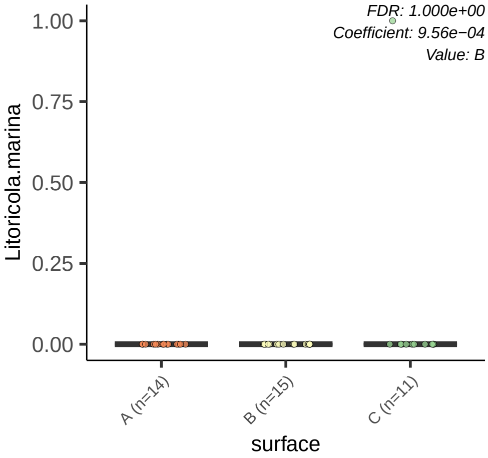

FDR: 1.000e+00
Coefficient: 9.56e−04
Value: B
Litoricola.marina
1.00
0.75
0.50
0.25
0.00
A (n=14)
B (n=15)
C (n=11)
surface

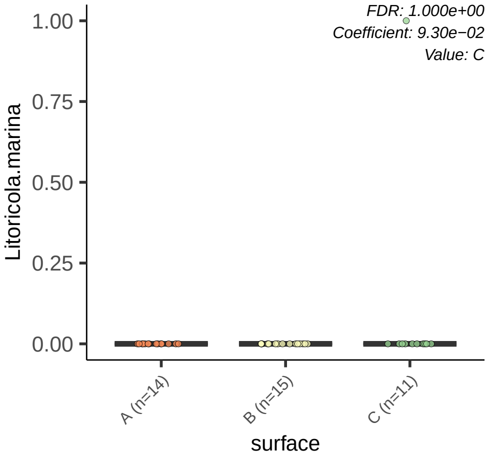
FDR: 1.000e+00
Coefficient: 9.30e−02
Value: C
Litoricola.marina
1.00
0.75
0.50
0.25
0.00
A (n=14)
B (n=15)
C (n=11)
surface

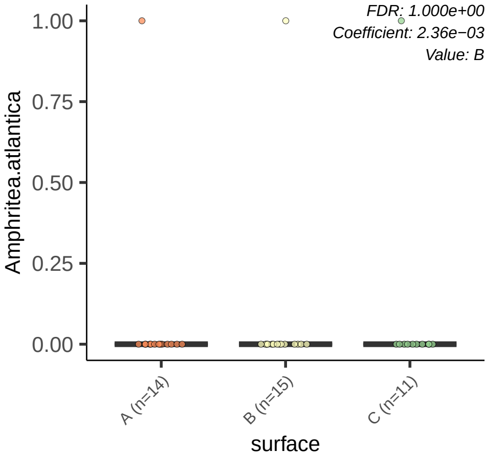

FDR: 1.000e+00
Coefficient: 2.36e−03
Value: B
Amphritea.atlantica
1.00
0.75
0.50
0.25
0.00
A (n=14)
B (n=15)
C (n=11)
surface

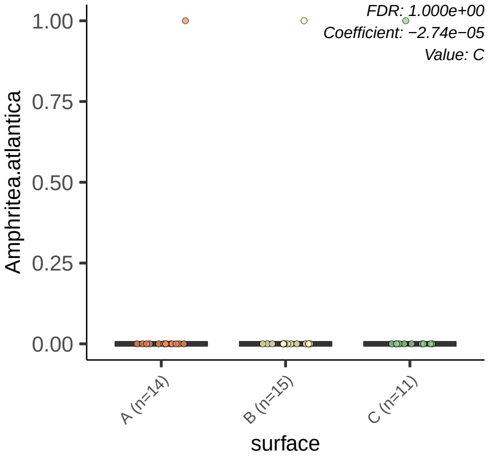
FDR: 1.000e+00
Coefficient: −2.74e−05
Value: C
Amphritea.atlantica
1.00
0.75
0.50
0.25
0.00
A (n=14)
B (n=15)
C (n=11)
surface

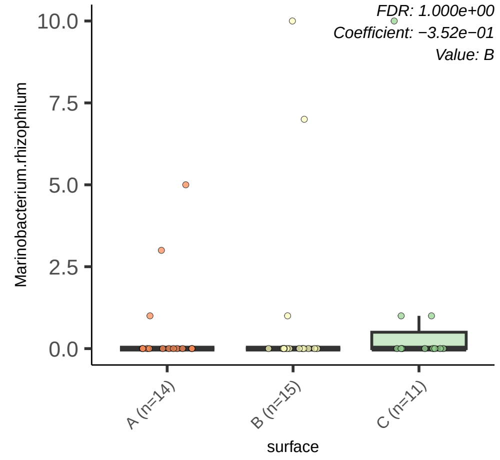
FDR: 1.000e+00
Coefficient: −3.52e−01
Value: B
Marinobacterium.rhizophilum
10.0
7.5
5.0
2.5
0.0
A (n=14)
B (n=15)
C (n=11)
surface

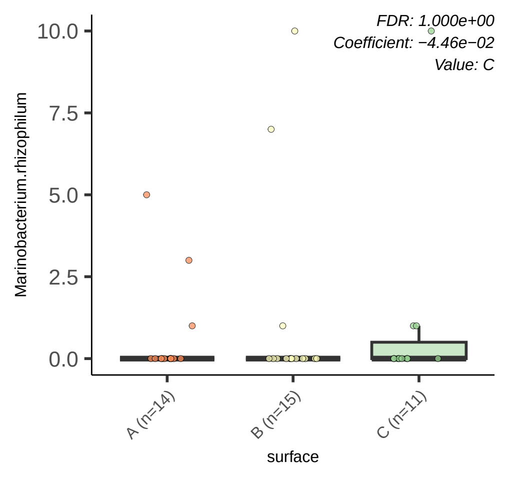

FDR: 1.000e+00
Coefficient: −4.46e−02
Value: C
Marinobacterium.rhizophilum
10.0
7.5
5.0
2.5
0.0
A (n=14)
B (n=15)
C (n=11)
surface

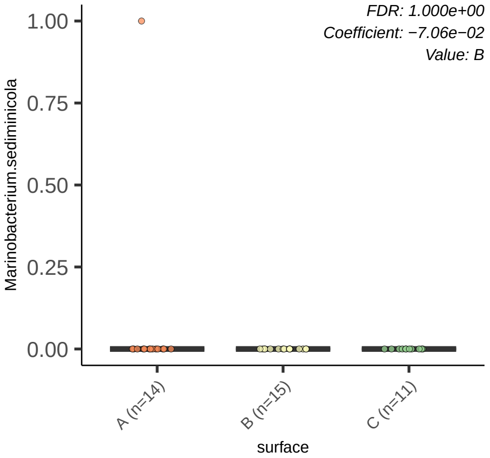
FDR: 1.000e+00
Coefficient: −7.06e−02
Value: B
Marinobacterium.sedimincola
1.00
0.75
0.50
0.25
0.00
A (n=14)
B (n=15)
C (n=11)
surface

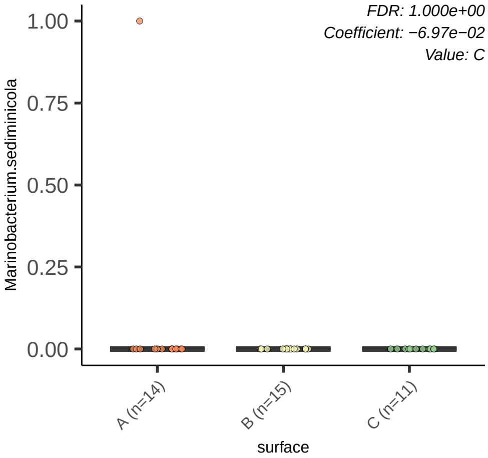
FDR: 1.000e+00
Coefficient: −6.97e−02
Value: C
1.00
0.75
0.50
0.25
0.00
Marinobacterium.sediminicola
A (n=14)
B (n=15)
C (n=11)
surface

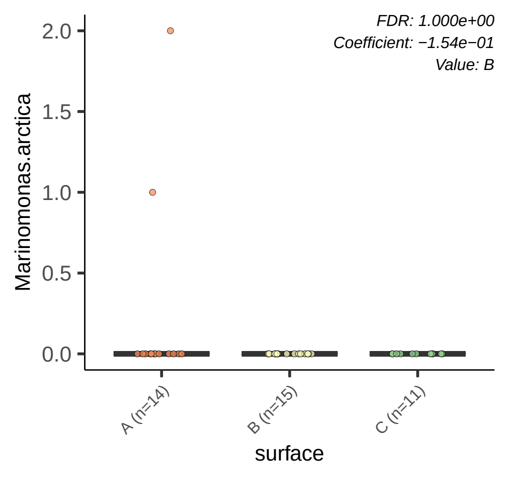
FDR: 1.000e+00
Coefficient: −1.54e−01
Value: B
Marinomonas.arctica
2.0
1.5
1.0
0.5
0.0
A (n=14)
B (n=15)
C (n=11)
surface

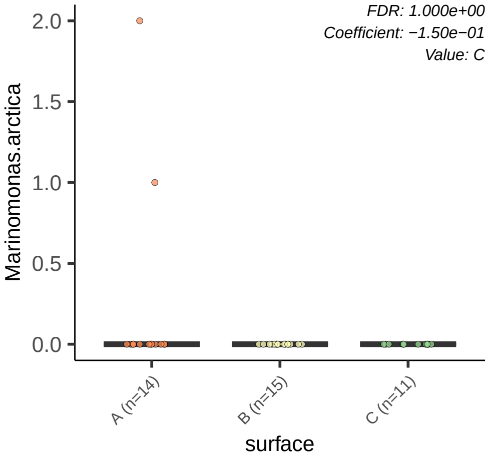
FDR: 1.000e+00
Coefficient: −1.50e−01
Value: C
Marinomonas.arctica
2.0
1.5
1.0
0.5
0.0
A (n=14)
B (n=15)
C (n=11)
surface

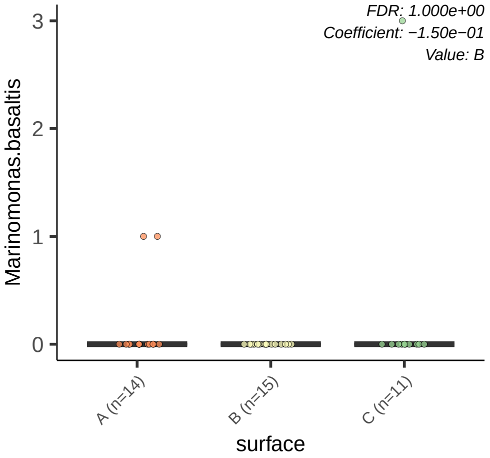
FDR: 1.000e+00
Coefficient: −1.50e−01
Value: B
Marinomonas.basaltis
0
1
2
3
A (n=14)
B (n=15)
C (n=11)
surface

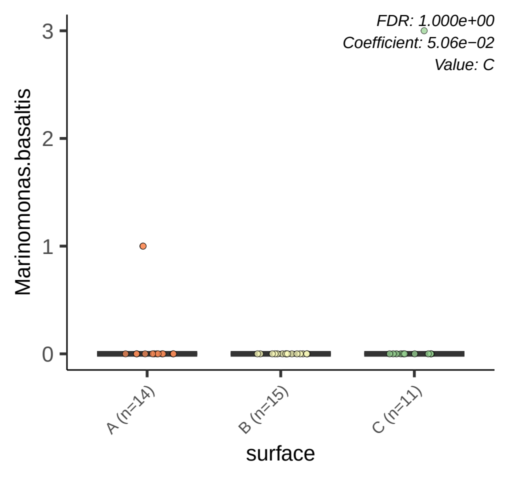
FDR: 1.000e+00
Coefficient: 5.06e−02
Value: C
Marinomonas.basaltis
3
2
1
0
A (n=14)
B (n=15)
C (n=11)
surface

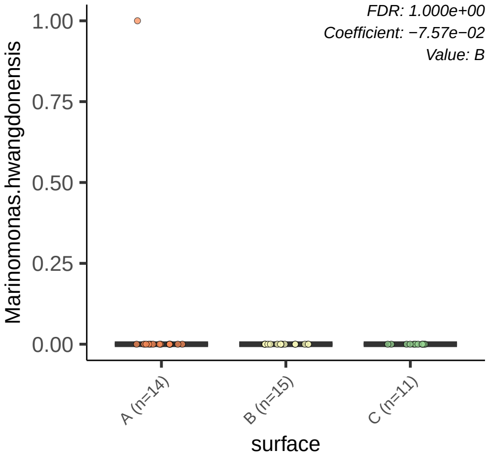

FDR: 1.000e+00
Coefficient: −7.57e−02
Value: B
Marinomonas.hwangdonensis
1.00
0.75
0.50
0.25
0.00
A (n=14)
B (n=15)
C (n=11)
surface

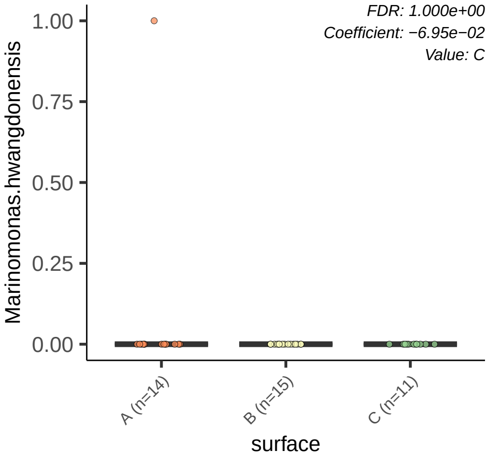
FDR: 1.000e+00
Coefficient: −6.95e−02
Value: C
Marinomonas.hwangdonensis
1.00
0.75
0.50
0.25
0.00
A (n=14)
B (n=15)
C (n=11)
surface

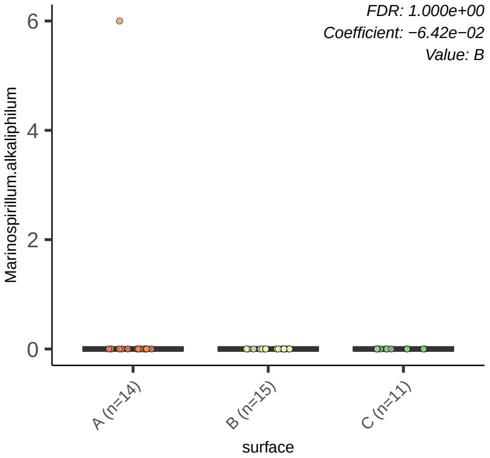
FDR: 1.000e+00
Coefficient: −6.42e−02
Value: B
Marinospirillum.alkaliphilum
6
4
2
0
A (n=14)
B (n=15)
C (n=11)
surface

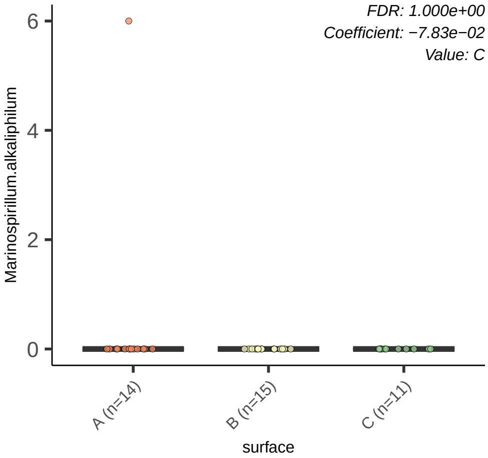

FDR: 1.000e+00
Coefficient: −7.83e−02
Value: C
Marinospirillum.alkaliphilum
6
4
2
0
A (n=14)
B (n=15)
C (n=11)
surface

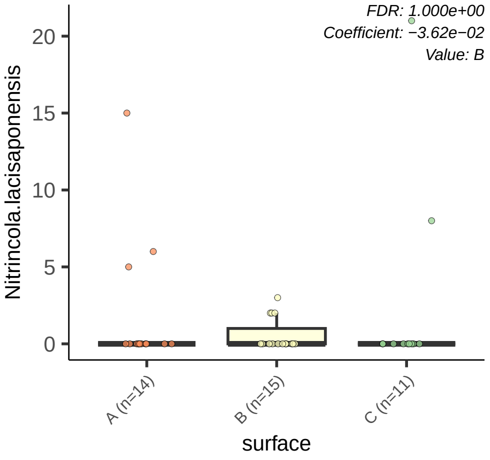

FDR: 1.000e+00
Coefficient: −3.62e−02
Value: B
Nitrincola.lacisaponensis
20
15
10
5
0
A (n=14)
B (n=15)
C (n=11)
surface

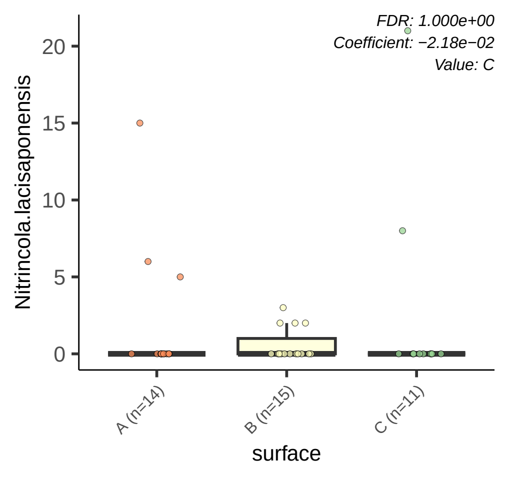
FDR: 1.000e+00
Coefficient: −2.18e−02
Value: C
Nitrincola.lacisaponensis
20
15
10
5
0
A (n=14)
B (n=15)
C (n=11)
surface

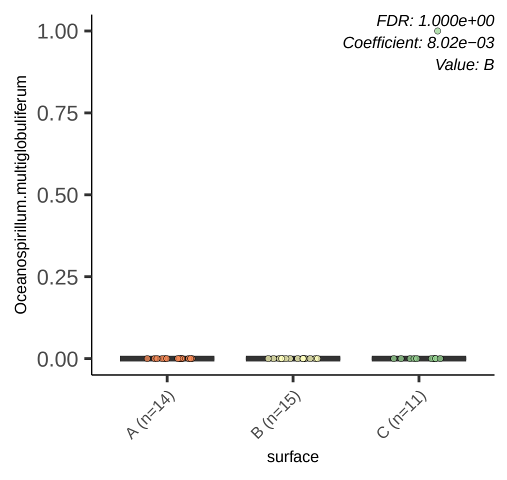

FDR: 1.000e+00
Coefficient: 8.02e−03
Value: B
Oceanospirillum.multiglobuliferum
1.00
0.75
0.50
0.25
0.00
A (n=14)
B (n=15)
C (n=11)
surface

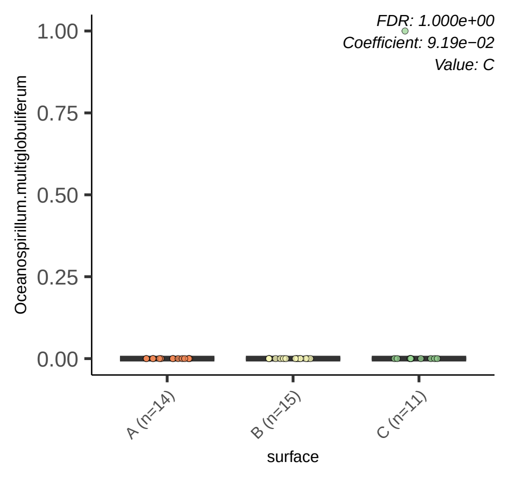
FDR: 1.000e+00
Coefficient: 9.19e−02
Value: C
Oceanospirillum.multiglobuliferum
1.00
0.75
0.50
0.25
0.00
A (n=14)
B (n=15)
C (n=11)
surface

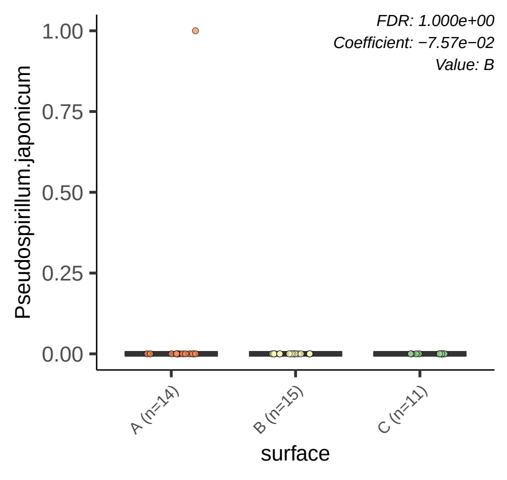
FDR: 1.000e+00
Coefficient: −7.57e−02
Value: B
Pseudospirillum.japonicum
1.00
0.75
0.50
0.25
0.00
A (n=14)
B (n=15)
C (n=11)
surface

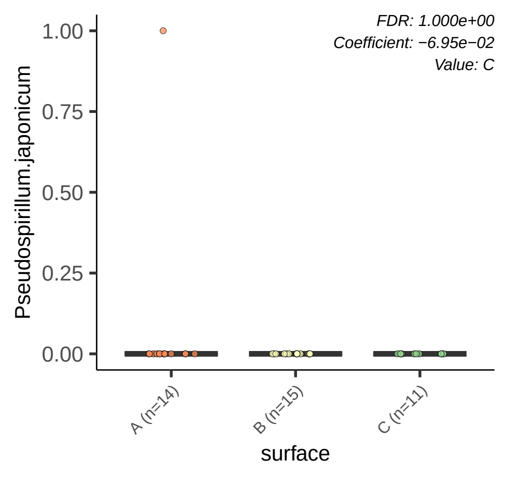

FDR: 1.000e+00
Coefficient: −6.95e−02
Value: C
Pseudospirillum.japonicum
1.00
0.75
0.50
0.25
0.00
A (n=14)
B (n=15)
C (n=11)
surface

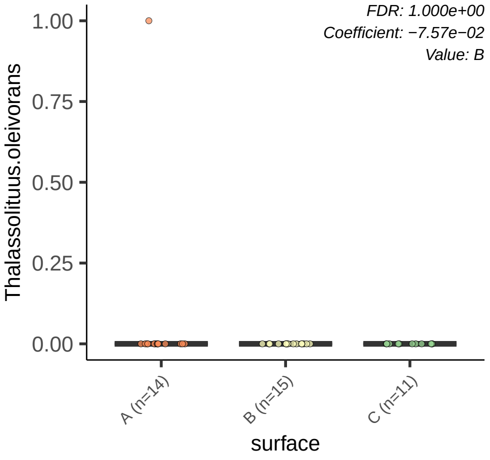

FDR: 1.000e+00
Coefficient: −7.57e−02
Value: B
Thalassolituus.oleivorans
1.00
0.75
0.50
0.25
0.00
A (n=14)
B (n=15)
C (n=11)
surface

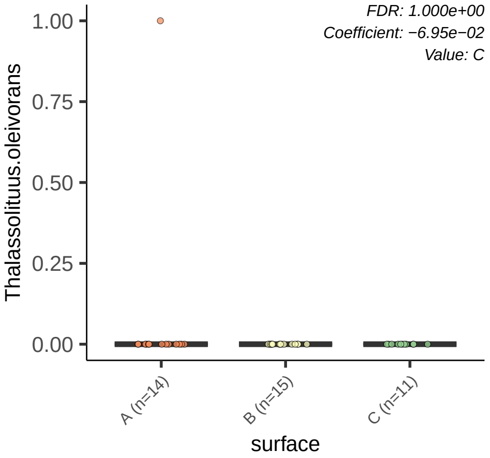
FDR: 1.000e+00
Coefficient: −6.95e−02
Value: C
Thalassolituus.oleivorans
1.00
0.75
0.50
0.25
0.00
A (n=14)
B (n=15)
C (n=11)
surface

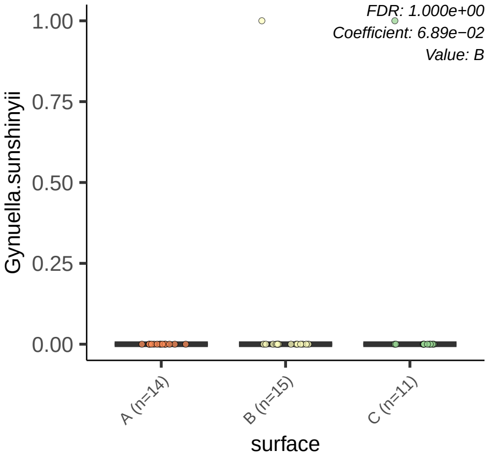
FDR: 1.000e+00
Coefficient: 6.89e−02
Value: B
Gynuella.sunshinyii
1.00
0.75
0.50
0.25
0.00
A (n=14)
B (n=15)
C (n=11)
surface

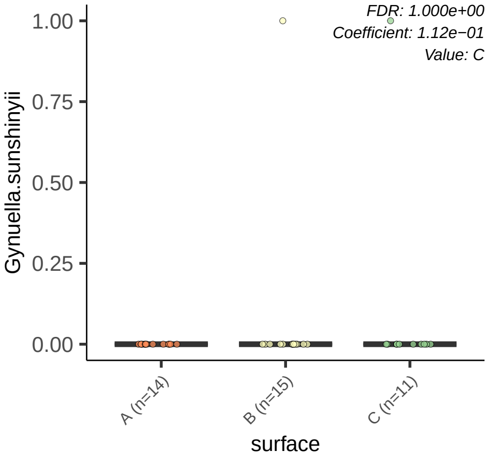
FDR: 1.000e+00
Coefficient: 1.12e−01
Value: C
Gynuella.sunshinyii
1.00
0.75
0.50
0.25
0.00
A (n=14)
B (n=15)
C (n=11)
surface

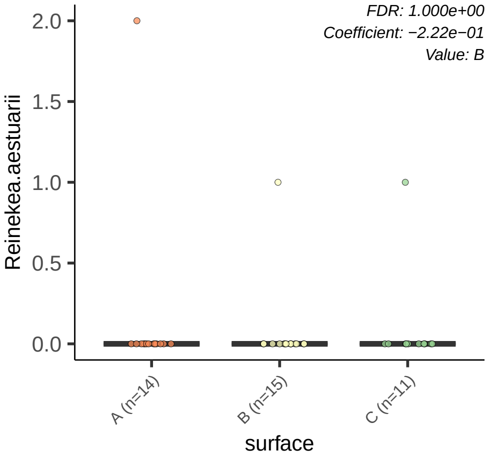
FDR: 1.000e+00
Coefficient: −2.22e−01
Value: B
Reinekea.aestuarii
2.0
1.5
1.0
0.5
0.0
A (n=14)
B (n=15)
C (n=11)
surface

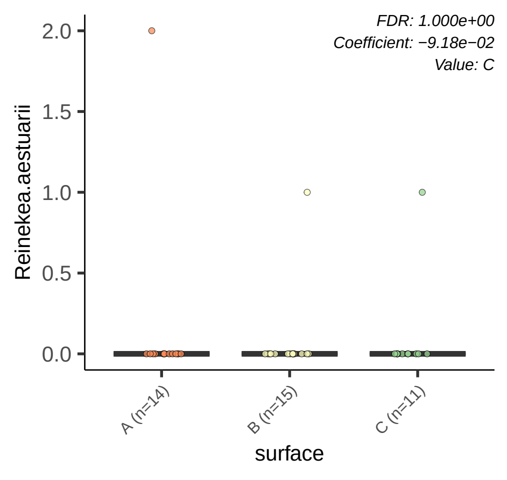
FDR: 1.000e+00
Coefficient: −9.18e−02
Value: C
Reinekea.aestuarii
2.0
1.5
1.0
0.5
0.0
A (n=14)
B (n=15)
C (n=11)
surface

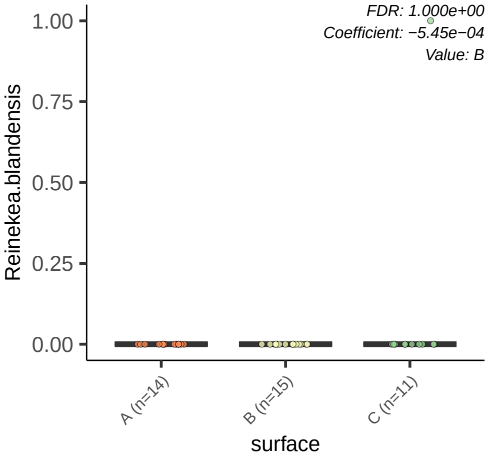
FDR: 1.000e+00
Coefficient: −5.45e−04
Value: B
Reinekea.blandensis
1.00
0.75
0.50
0.25
0.00
A (n=14)
B (n=15)
C (n=11)
surface

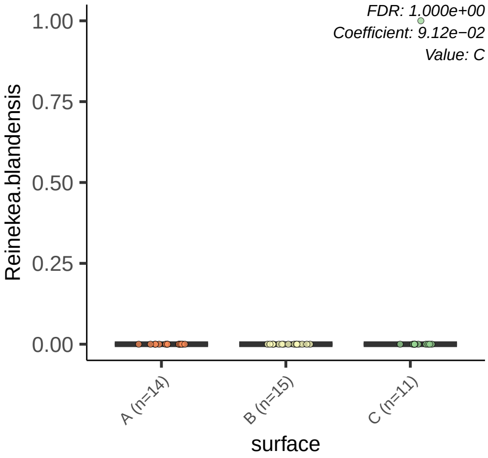

FDR: 1.000e+00
Coefficient: 9.12e−02
Value: C
Reinekea.blandensis
1.00
0.75
0.50
0.25
0.00
A (n=14)
B (n=15)
C (n=11)
surface

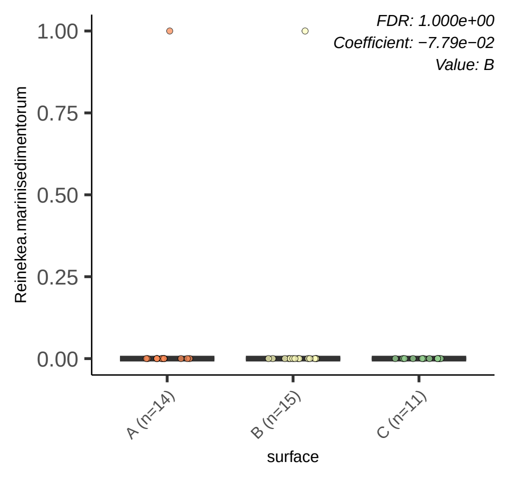
FDR: 1.000e+00
Coefficient: −7.79e−02
Value: B
Reinekea.marinisedimentorum
1.00
0.75
0.50
0.25
0.00
A (n=14)
B (n=15)
C (n=11)
surface

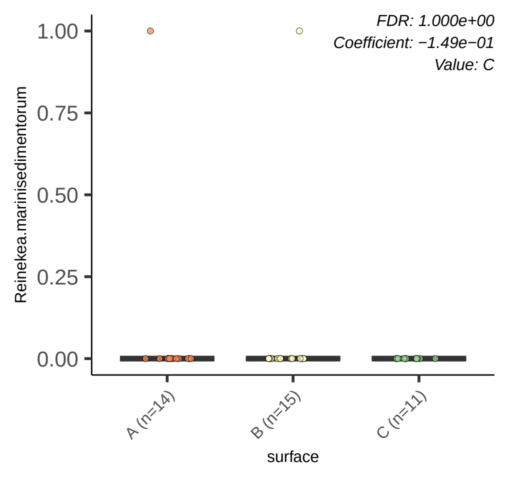
FDR: 1.000e+00
Coefficient: −1.49e−01
Value: C
Reinekea.marinisedimentorum
1.00
0.75
0.50
0.25
0.00
A (n=14)
B (n=15)
C (n=11)
surface

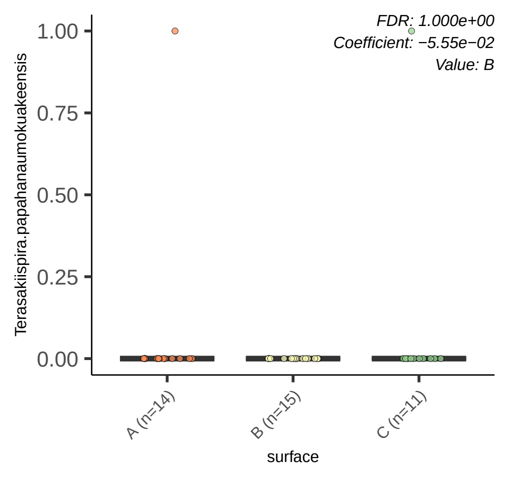

FDR: 1.000e+00
Coefficient: −5.55e−02
Value: B
Terasakiispira.papahanaumokuakeensis
1.00
0.75
0.50
0.25
0.00
A (n=14)
B (n=15)
C (n=11)
surface

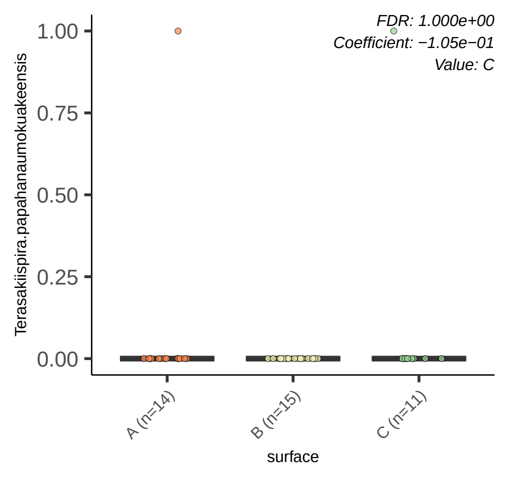
FDR: 1.000e+00
Coefficient: −1.05e−01
Value: C
Terasakiispira.papahanaumokuakeensis
1.00
0.75
0.50
0.25
0.00
A (n=14)
B (n=15)
C (n=11)
surface

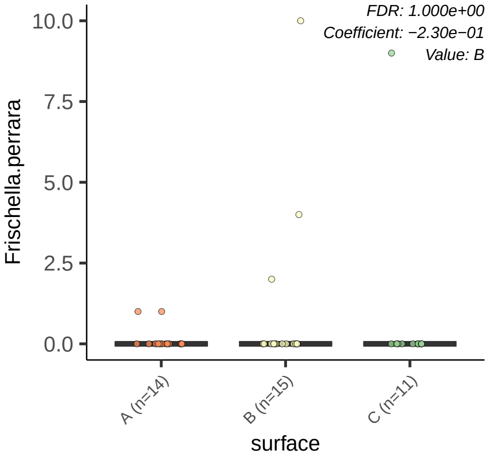
FDR: 1.000e+00
Coefficient: −2.30e−01
Value: B
Frischella.perrara
10.0
7.5
5.0
2.5
0.0
A (n=14)
B (n=15)
C (n=11)
surface

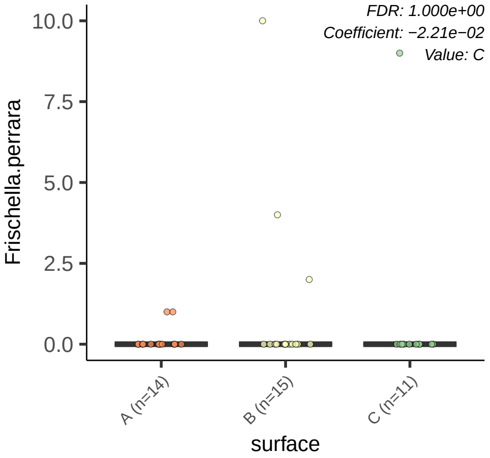
FDR: 1.000e+00
Coefficient: −2.21e−02
Value: C
Frischella.perrara
10.0
7.5
5.0
2.5
0.0
A (n=14)
B (n=15)
C (n=11)
surface

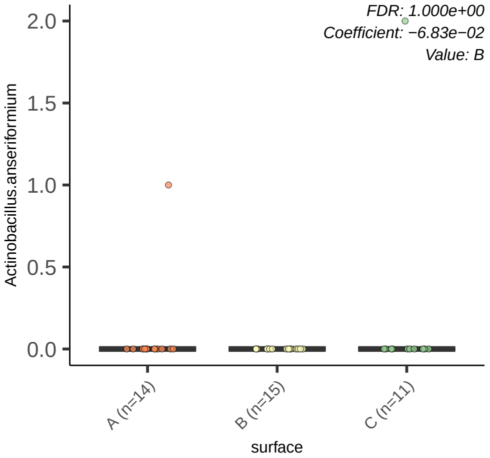

FDR: 1.000e+00
Coefficient: −6.83e−02
Value: B
Actinobacillus.anseriformium
2.0
1.5
1.0
0.5
0.0
A (n=14)
B (n=15)
C (n=11)
surface

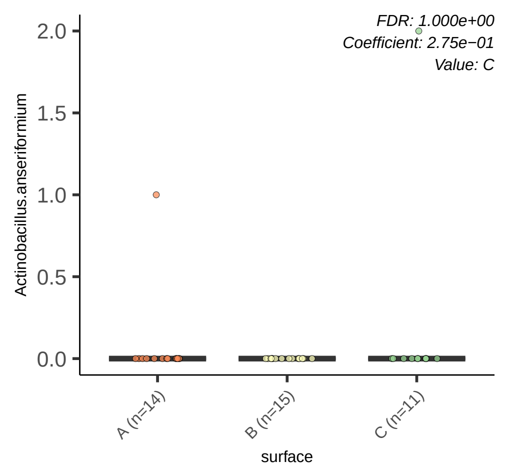
FDR: 1.000e+00
Coefficient: 2.75e−01
Value: C
Actinobacillus.anseriformium
2.0
1.5
1.0
0.5
0.0
A (n=14)
B (n=15)
C (n=11)
surface

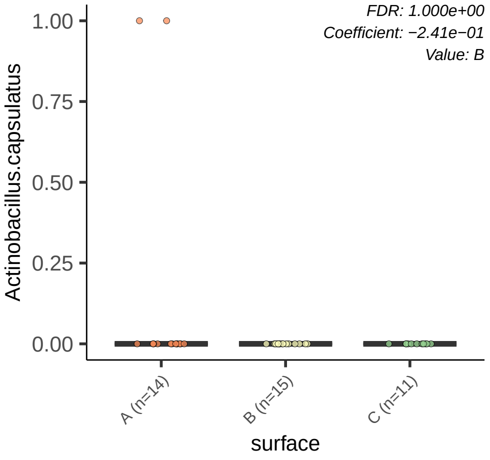

FDR: 1.000e+00
Coefficient: −2.41e−01
Value: B
Actinobacillus.capsulatus
1.00
0.75
0.50
0.25
0.00
A (n=14)
B (n=15)
C (n=11)
surface

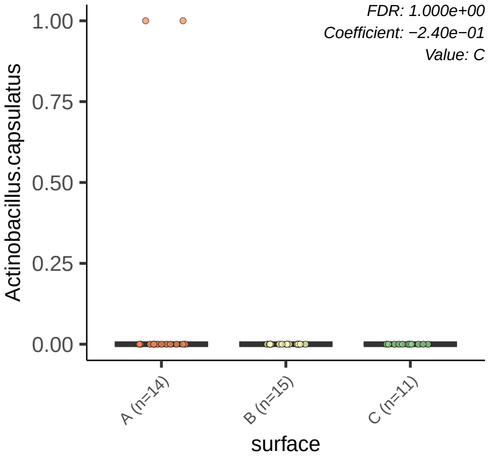

FDR: 1.000e+00
Coefficient: −2.40e−01
Value: C
Actinobacillus.capsulatus
1.00
0.75
0.50
0.25
0.00
A (n=14)
B (n=15)
C (n=11)
surface

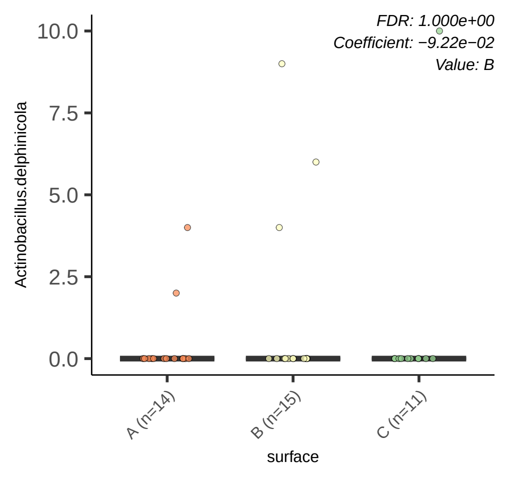
FDR: 1.000e+00
Coefficient: −9.22e−02
Value: B
10.0
7.5
5.0
2.5
0.0
Actinobacillus.delphinicola
A (n=14)
B (n=15)
C (n=11)
surface

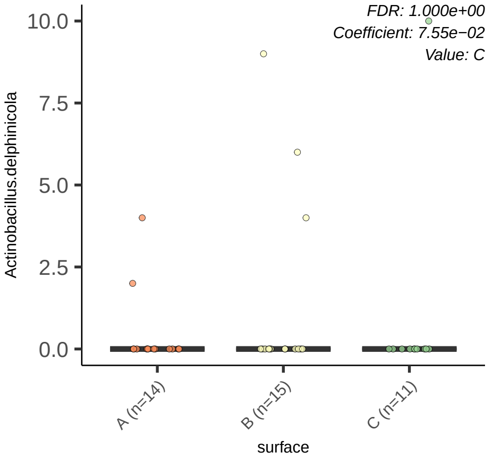

FDR: 1.000e+00
Coefficient: 7.55e−02
Value: C
10.0
7.5
5.0
2.5
0.0
Actinobacillus.delphinicola
A (n=14)
B (n=15)
C (n=11)
surface

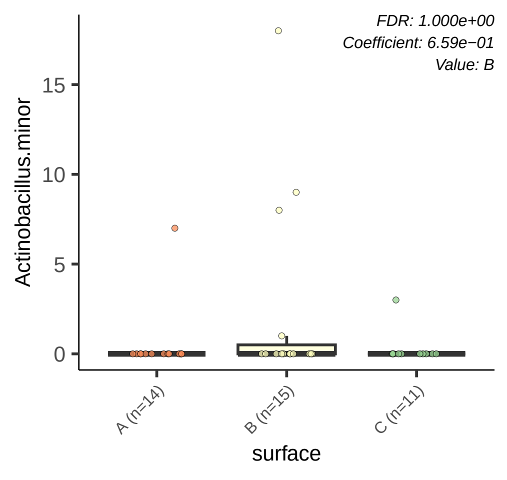
FDR: 1.000e+00
Coefficient: 6.59e−01
Value: B
Actinobacillus.minor
0
5
10
15
A (n=14)
B (n=15)
C (n=11)
surface

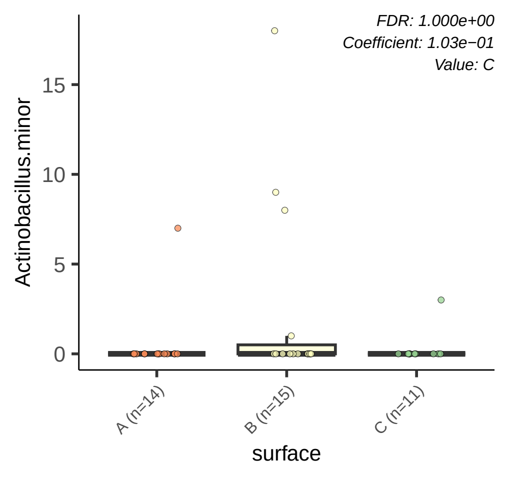
FDR: 1.000e+00
Coefficient: 1.03e−01
Value: C
Actinobacillus.minor
0
5
10
15
A (n=14)
B (n=15)
C (n=11)
surface

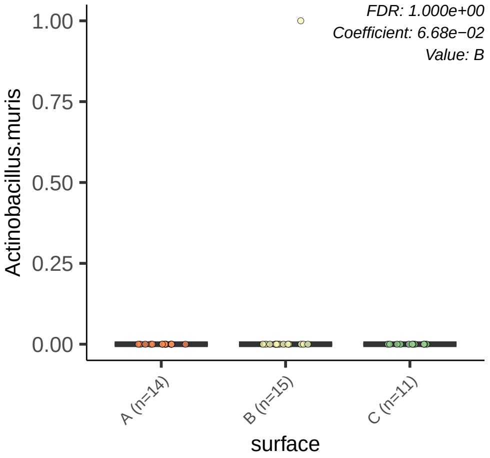
FDR: 1.000e+00
Coefficient: 6.68e−02
Value: B
Actinobacillus.muris
1.00
0.75
0.50
0.25
0.00
A (n=14)
B (n=15)
C (n=11)
surface

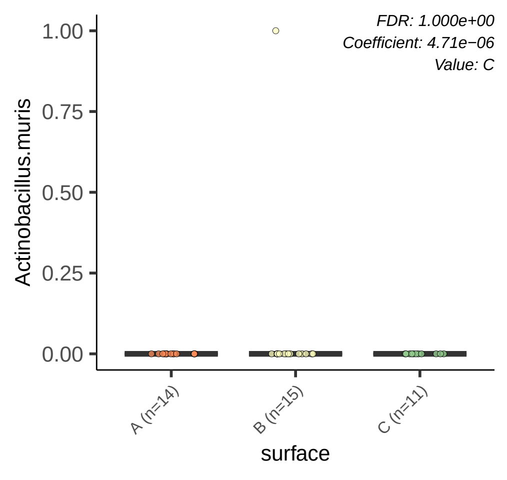
FDR: 1.000e+00
Coefficient: 4.71e−06
Value: C
Actinobacillus.muris
1.00
0.75
0.50
0.25
0.00
A (n=14)
B (n=15)
C (n=11)
surface

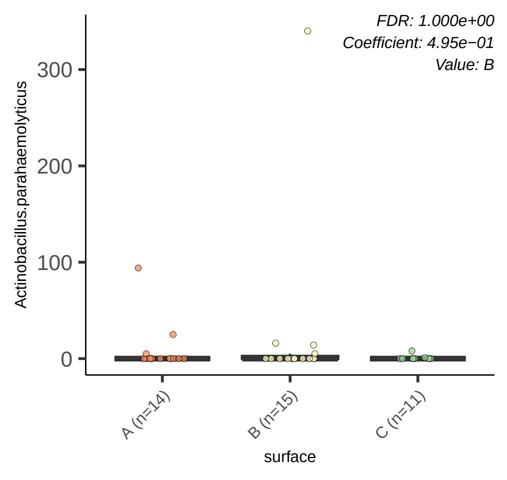
FDR: 1.000e+00
Coefficient: 4.95e−01
Value: B
Actinobacillus.parahaemolyticus
300
200
100
0
A (n=14)
B (n=15)
C (n=11)
surface

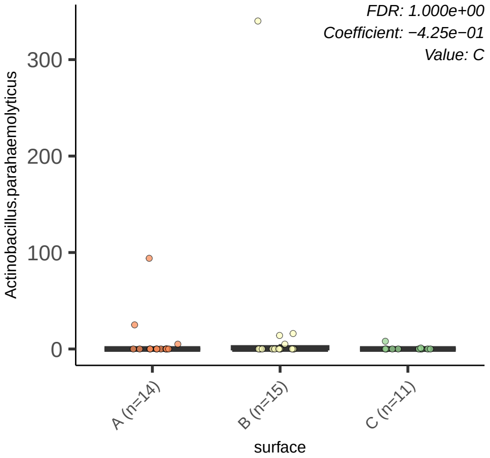

FDR: 1.000e+00
Coefficient: −4.25e−01
Value: C
Actinobacillus.parahaemolyticus
300
200
100
0
A (n=14)
B (n=15)
C (n=11)
surface

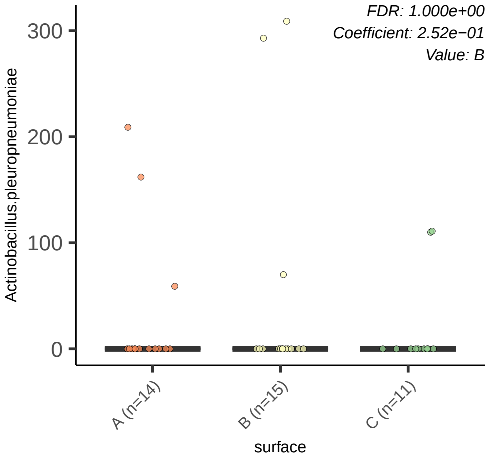
FDR: 1.000e+00
Coefficient: 2.52e−01
Value: B
Actinobacillus.pleuropneumoniae
300
200
100
0
A (n=14)
B (n=15)
C (n=11)
surface

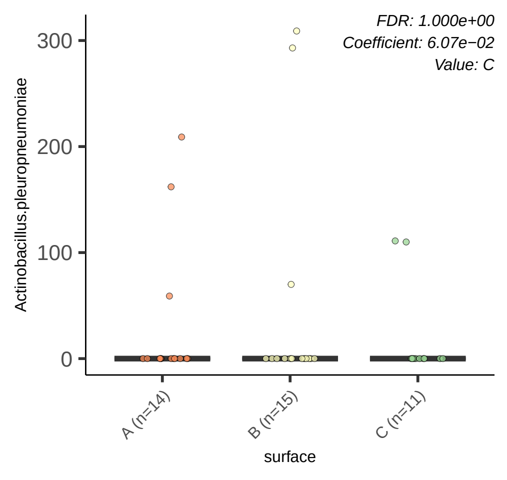
FDR: 1.000e+00
Coefficient: 6.07e−02
Value: C
Actinobacillus.pleuropneumoniae
300
200
100
0
A (n=14)
B (n=15)
C (n=11)
surface

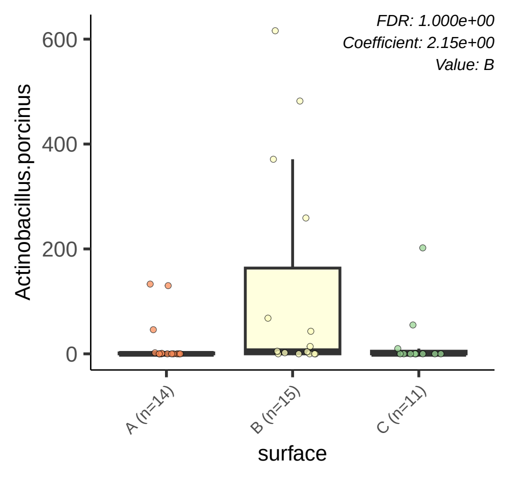
FDR: 1.000e+00
Coefficient: 2.15e+00
Value: B
Actinobacillus.porcinus
0
200
400
600
A (n=14)
B (n=15)
C (n=11)
surface

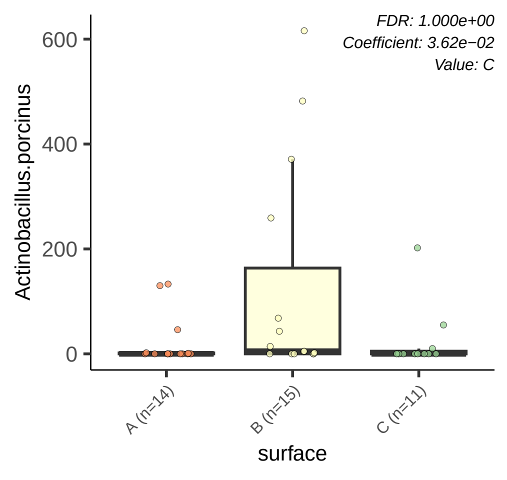

FDR: 1.000e+00
Coefficient: 3.62e−02
Value: C
Actinobacillus.porcinus
600
400
200
0
A (n=14)
B (n=15)
C (n=11)
surface

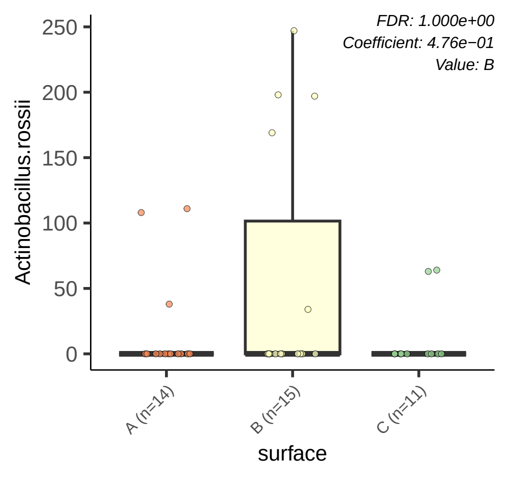
FDR: 1.000e+00
Coefficient: 4.76e−01
Value: B
Actinobacillus.rossii
250
200
150
100
50
0
A (n=14)
B (n=15)
C (n=11)
surface

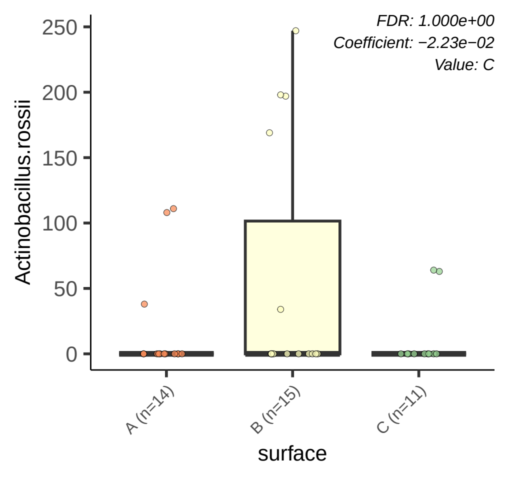
FDR: 1.000e+00
Coefficient: −2.23e−02
Value: C
Actinobacillus.rossii
250
200
150
100
50
0
A (n=14)
B (n=15)
C (n=11)
surface

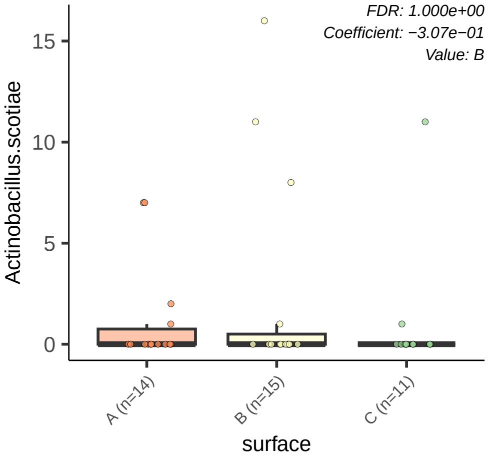

FDR: 1.000e+00
Coefficient: −3.07e−01
Value: B
Actinobacillus.scotiae
15
10
5
0
A (n=14)
B (n=15)
C (n=11)
surface

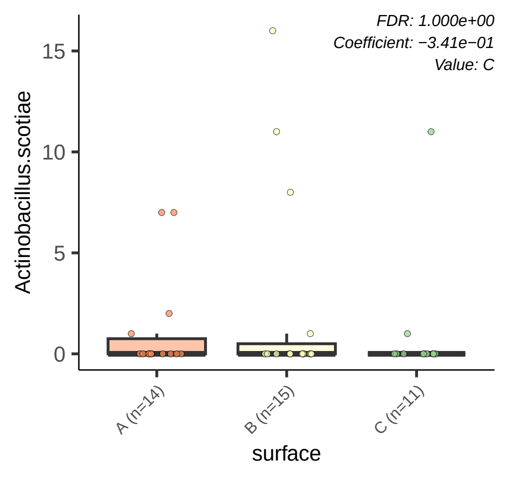
FDR: 1.000e+00
Coefficient: −3.41e−01
Value: C
Actinobacillus.scotiae
15
10
5
0
A (n=14)
B (n=15)
C (n=11)
surface

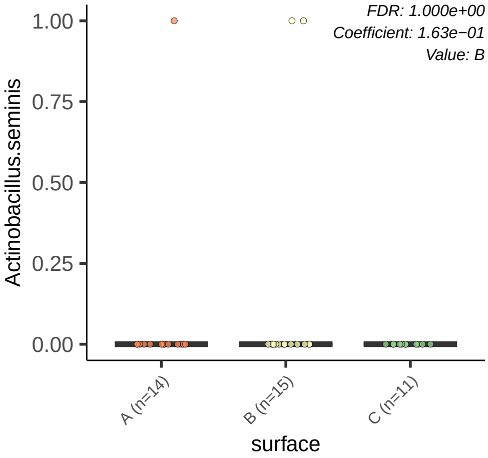

FDR: 1.000e+00
Coefficient: 1.63e−01
Value: B
Actinobacillus.seminis
1.00
0.75
0.50
0.25
0.00
A (n=14)
B (n=15)
C (n=11)
surface

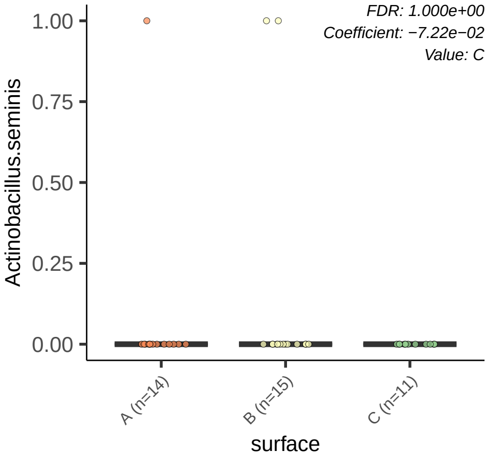

FDR: 1.000e+00
Coefficient: −7.22e−02
Value: C
Actinobacillus.seminis
1.00
0.75
0.50
0.25
0.00
A (n=14)
B (n=15)
C (n=11)
surface

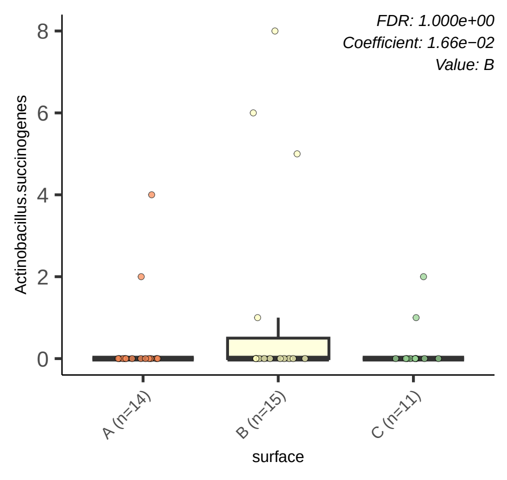
FDR: 1.000e+00
Coefficient: 1.66e−02
Value: B
Actinobacillus.succinogenes
0
2
4
6
8
A (n=14)
B (n=15)
C (n=11)
surface

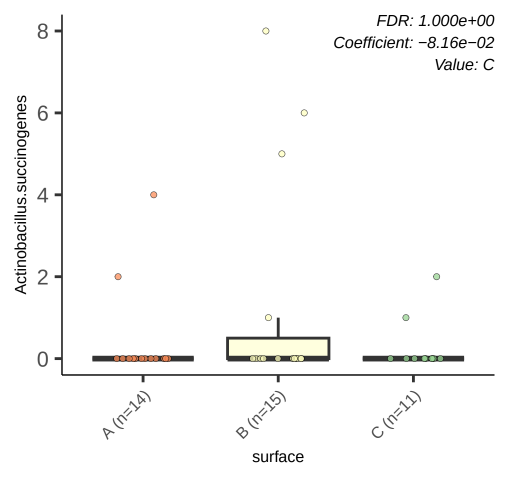
FDR: 1.000e+00
Coefficient: −8.16e−02
Value: C
Actinobacillus.succinogenes
0
2
4
6
8
A (n=14)
B (n=15)
C (n=11)
surface

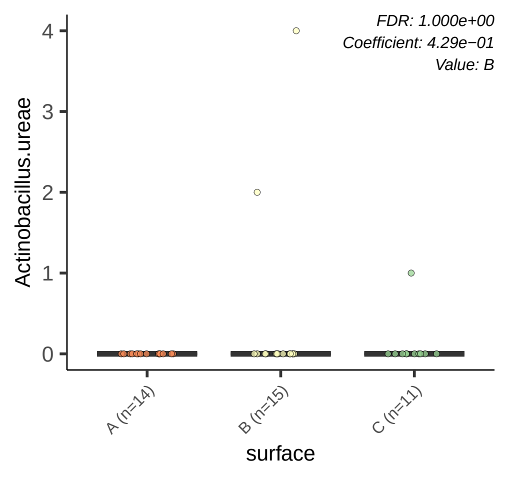
FDR: 1.000e+00
Coefficient: 4.29e−01
Value: B
Actinobacillus.ureae
0
1
2
3
4
A (n=14)
B (n=15)
C (n=11)
surface

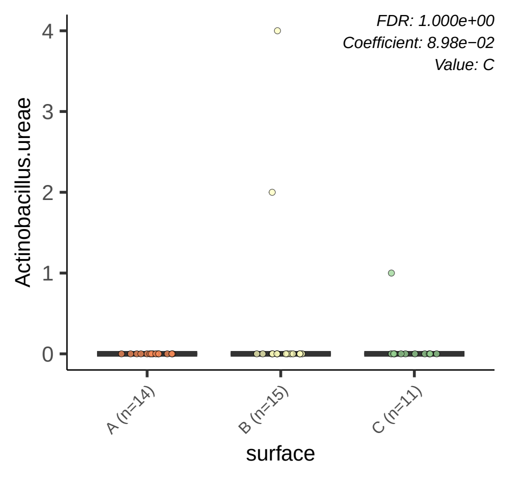
FDR: 1.000e+00
Coefficient: 8.98e−02
Value: C
Actinobacillus.ureae
4
3
2
1
0
A (n=14)
B (n=15)
C (n=11)
surface

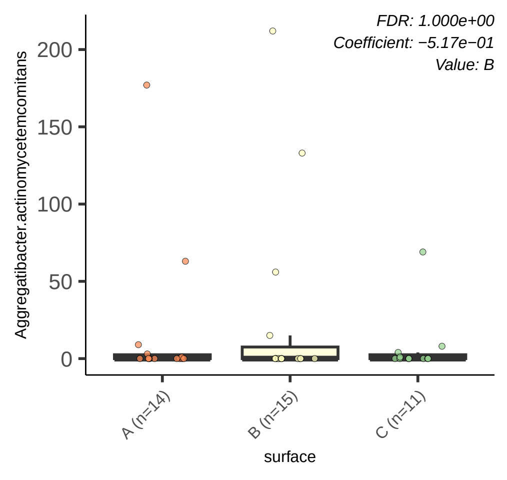
FDR: 1.000e+00
Coefficient: −5.17e−01
Value: B
Aggregatibacter.actinomycetemcomitans
200
150
100
50
0
A (n=14)
B (n=15)
C (n=11)
surface

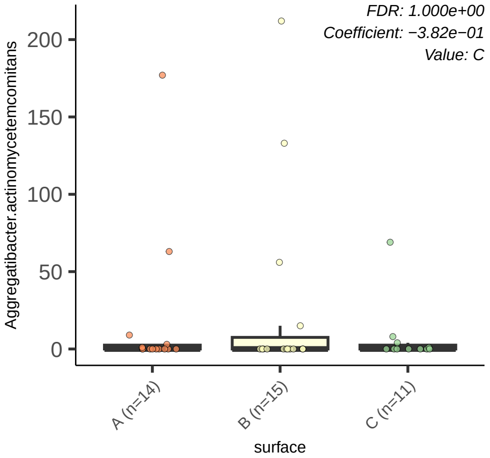

FDR: 1.000e+00
Coefficient: −3.82e−01
Value: C
Aggregatibacter.actinomycetemcomitans
200
150
100
50
0
A (n=14)
B (n=15)
C (n=11)
surface

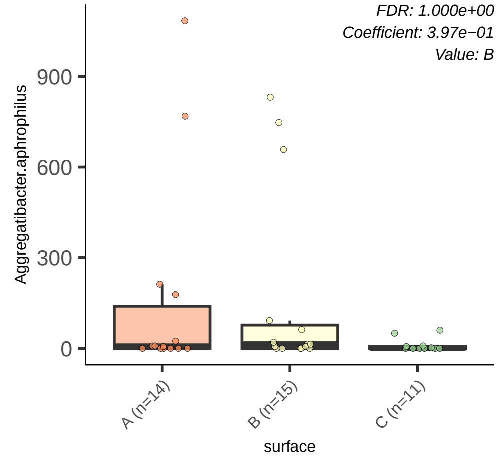

FDR: 1.000e+00
Coefficient: 3.97e−01
Value: B
Aggregatibacter.aphrophilus
900
600
300
0
A (n=14)
B (n=15)
C (n=11)
surface

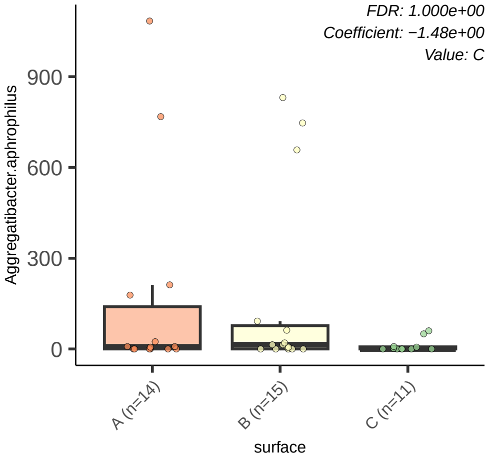
FDR: 1.000e+00
Coefficient: −1.48e+00
Value: C
Aggregatibacter.aphrophilus
900
600
300
0
A (n=14)
B (n=15)
C (n=11)
surface

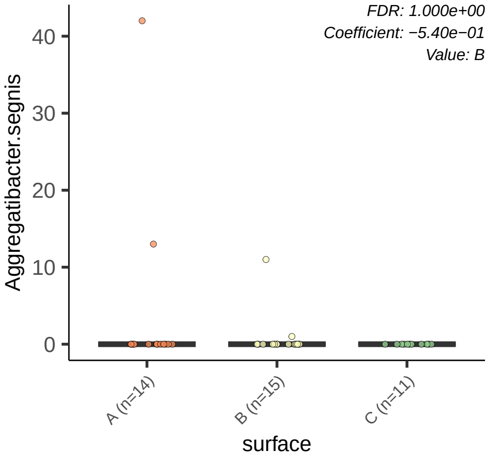

FDR: 1.000e+00
Coefficient: −5.40e−01
Value: B
Aggregatibacter.segnis
40
30
20
10
0
A (n=14)
B (n=15)
C (n=11)
surface

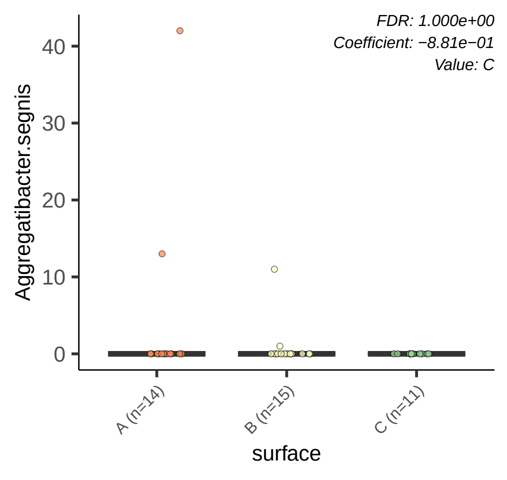

FDR: 1.000e+00
Coefficient: −8.81e−01
Value: C
Aggregatibacter.segnis
40
30
20
10
0
A (n=14)
B (n=15)
C (n=11)
surface

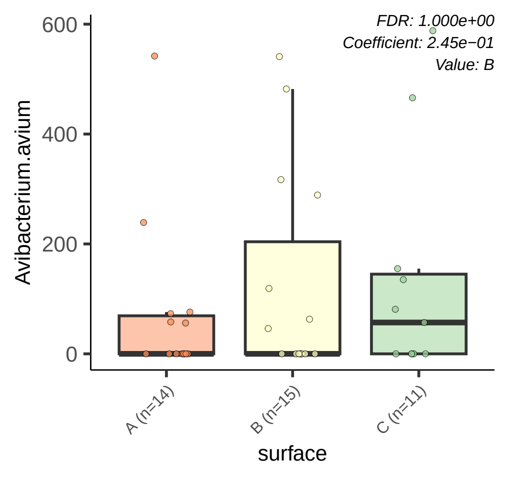
FDR: 1.000e+00
Coefficient: 2.45e−01
Value: B
600
400
200
0
Avibacterium.avium
A (n=14)
B (n=15)
C (n=11)
surface

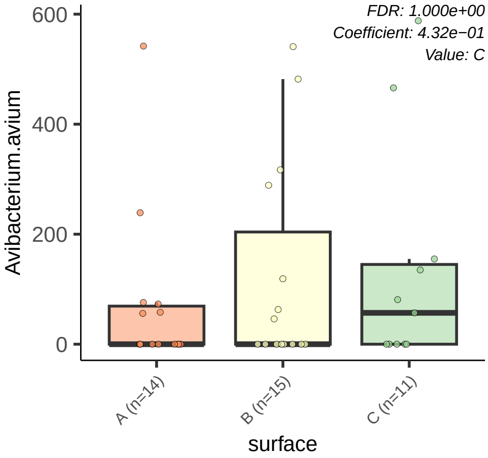
FDR: 1.000e+00
Coefficient: 4.32e−01
Value: C
Avibacterium.avium
600
400
200
0
A (n=14)
B (n=15)
C (n=11)
surface

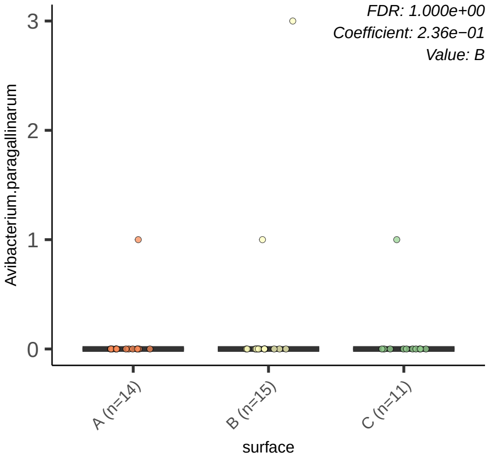

FDR: 1.000e+00
Coefficient: 2.36e−01
Value: B
Avibacterium.paragallinarum
3
2
1
0
A (n=14)
B (n=15)
C (n=11)
surface

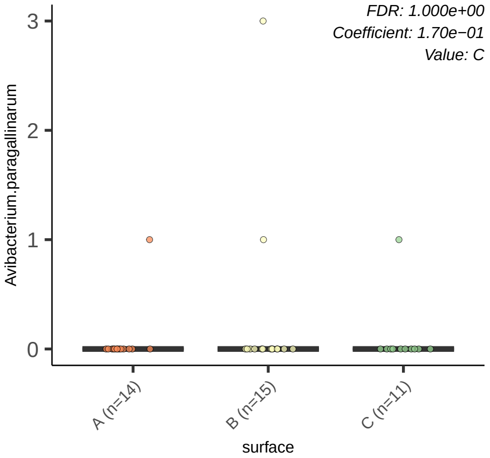
FDR: 1.000e+00
Coefficient: 1.70e−01
Value: C
Avibacterium.paragallinarum
3
2
1
0
A (n=14)
B (n=15)
C (n=11)
surface

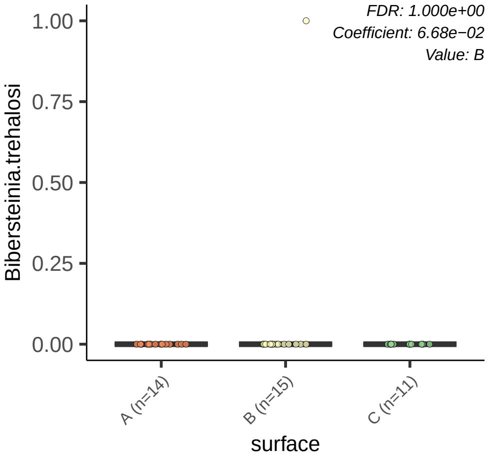

FDR: 1.000e+00
Coefficient: 6.68e−02
Value: B
Bibersteinia.trehalosi
1.00
0.75
0.50
0.25
0.00
A (n=14)
B (n=15)
C (n=11)
surface

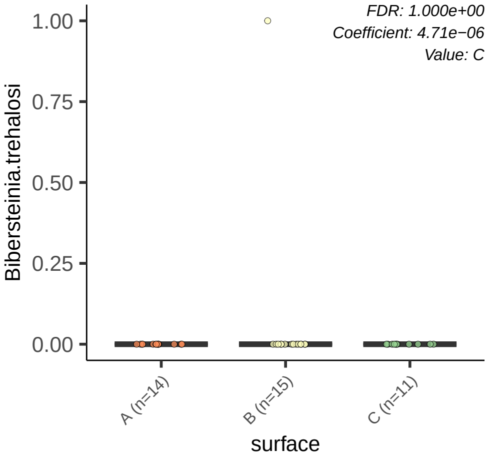
FDR: 1.000e+00
Coefficient: 4.71e−06
Value: C
Bibersteinia.trehalosi
1.00
0.75
0.50
0.25
0.00
A (n=14)
B (n=15)
C (n=11)
surface

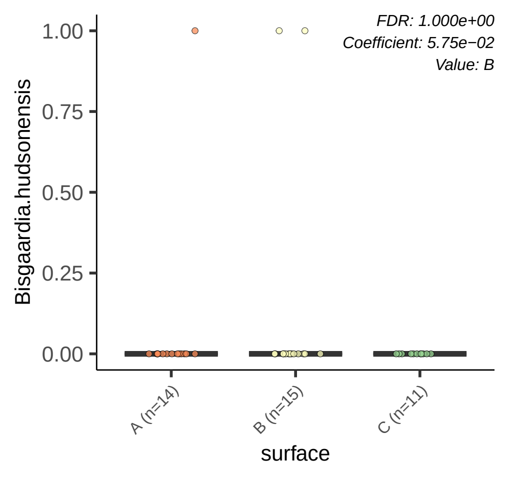
FDR: 1.000e+00
Coefficient: 5.75e−02
Value: B
Bisgaardia.hudsonensis
1.00
0.75
0.50
0.25
0.00
A (n=14)
B (n=15)
C (n=11)
surface

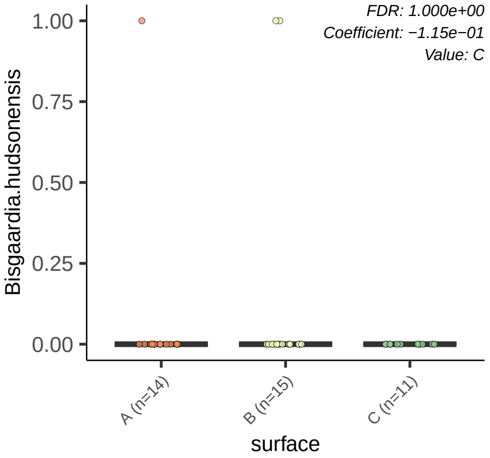

FDR: 1.000e+00
Coefficient: −1.15e−01
Value: C
Bisgaardia.hudsonensis
1.00
0.75
0.50
0.25
0.00
A (n=14)
B (n=15)
C (n=11)
surface

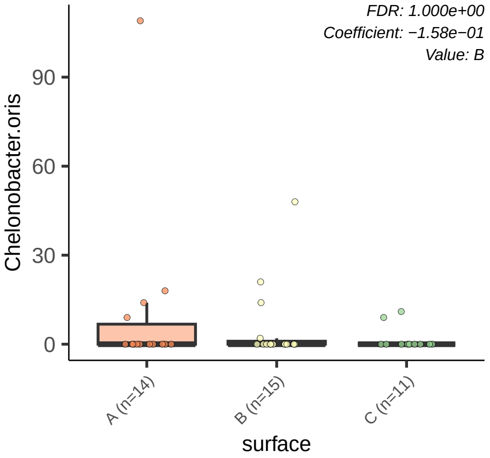

FDR: 1.000e+00
Coefficient: −1.58e−01
Value: B
Chelonobacter.oris
90
60
30
0
A (n=14)
B (n=15)
C (n=11)
surface

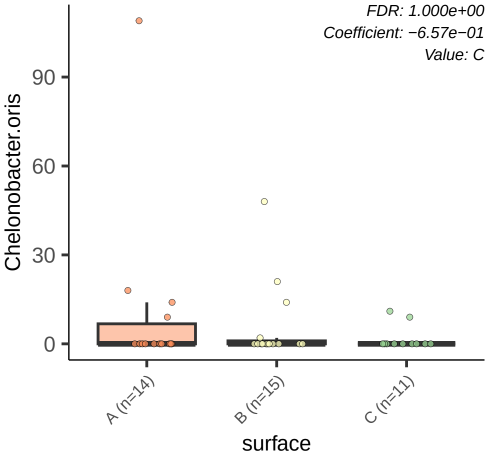

FDR: 1.000e+00
Coefficient: −6.57e−01
Value: C
Chelonobacter.oris
0
30
60
90
A (n=14)
B (n=15)
C (n=11)
surface

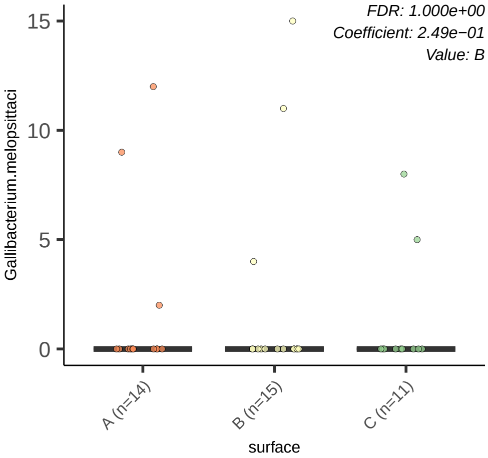
FDR: 1.000e+00
Coefficient: 2.49e−01
Value: B
Gallibacterium.melopsittaci
15
10
5
0
A (n=14)
B (n=15)
C (n=11)
surface

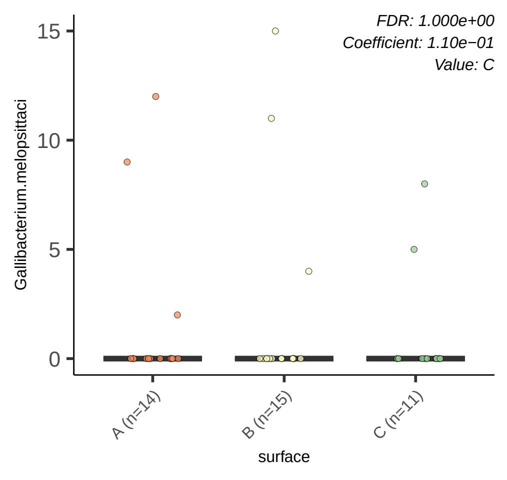
FDR: 1.000e+00
Coefficient: 1.10e−01
Value: C
Gallibacterium.melopsittaci
15
10
5
0
A (n=14)
B (n=15)
C (n=11)
surface

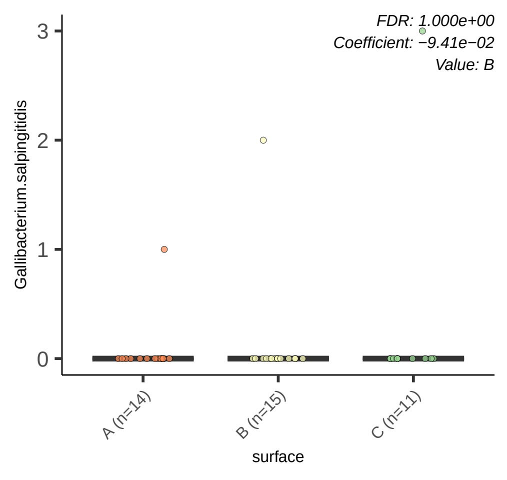
FDR: 1.000e+00
Coefficient: −9.41e−02
Value: B
Gallibacterium.salpingitidis
0
1
2
3
A (n=14)
B (n=15)
C (n=11)
surface

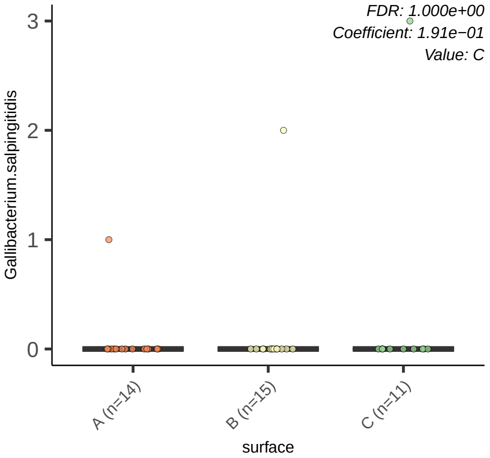
FDR: 1.000e+00
Coefficient: 1.91e−01
Value: C
Gallibacterium.salpingitidis
3
2
1
0
A (n=14)
B (n=15)
C (n=11)
surface

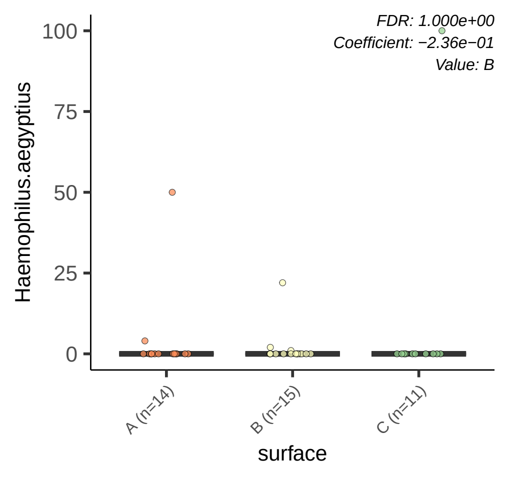
FDR: 1.000e+00
Coefficient: −2.36e−01
Value: B
Haemophilus.aegyptius
100
75
50
25
0
A (n=14)
B (n=15)
C (n=11)
surface

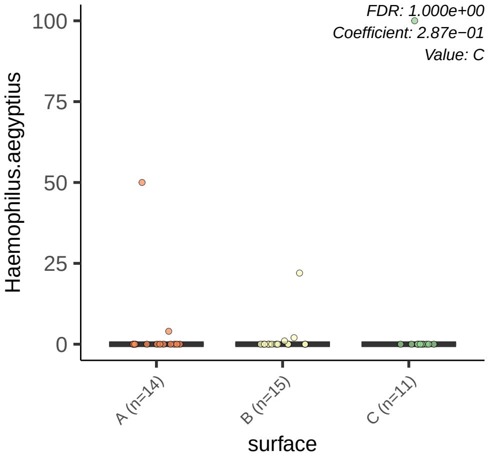
FDR: 1.000e+00
Coefficient: 2.87e−01
Value: C
Haemophilus.aegyptius
100
75
50
25
0
A (n=14)
B (n=15)
C (n=11)
surface

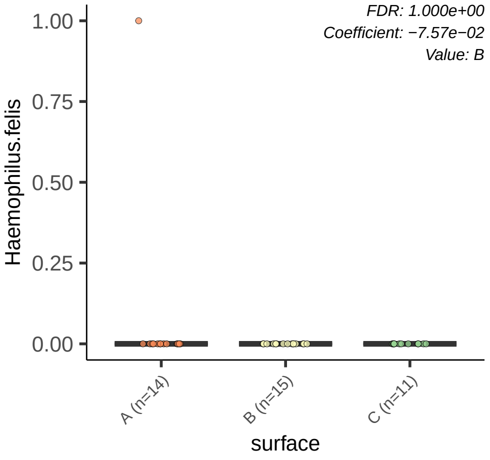

FDR: 1.000e+00
Coefficient: −7.57e−02
Value: B
Haemophilus.felis
1.00
0.75
0.50
0.25
0.00
A (n=14)
B (n=15)
C (n=11)
surface

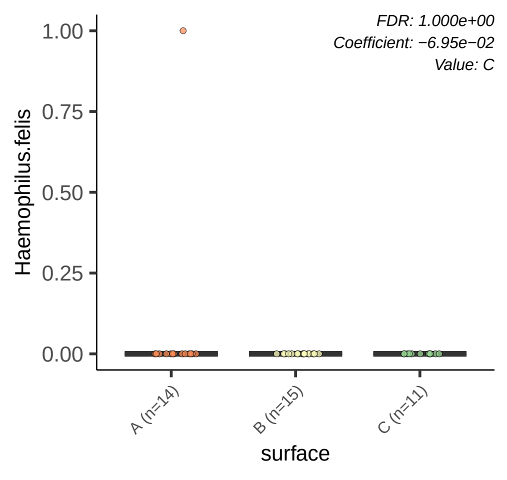
FDR: 1.000e+00
Coefficient: −6.95e−02
Value: C
Haemophilus.felis
1.00
0.75
0.50
0.25
0.00
A (n=14)
B (n=15)
C (n=11)
surface

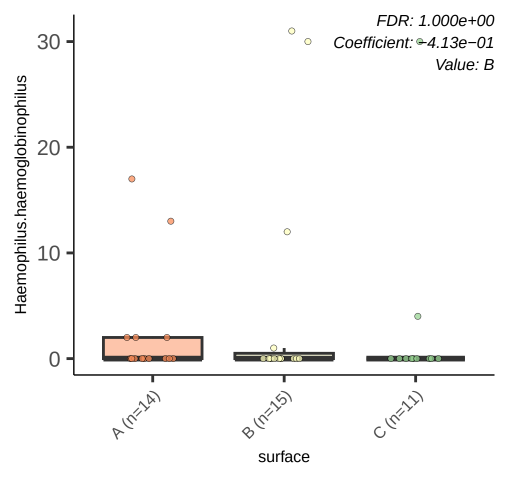
FDR: 1.000e+00
Coefficient: -4.13e-01
Value: B
Haemophilus.haemoglobinophilus
30
20
10
0
A (n=14)
B (n=15)
C (n=11)
surface

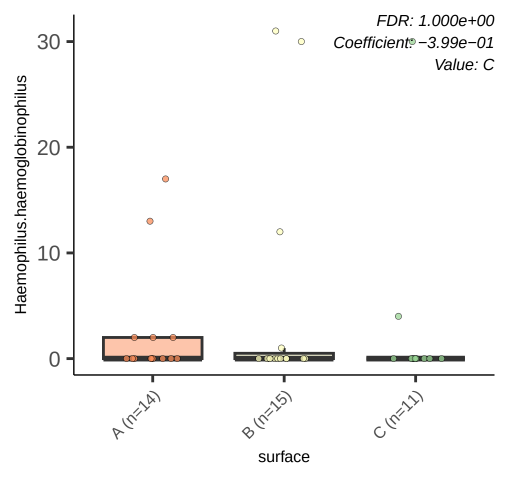

FDR: 1.000e+00
Coefficient: −3.99e−01
Value: C
Haemophilus.haemoglobinophilus
30
20
10
0
A (n=14)
B (n=15)
C (n=11)
surface

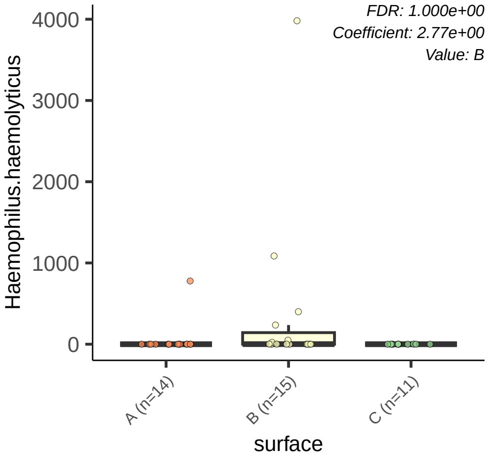
FDR: 1.000e+00
Coefficient: 2.77e+00
Value: B
Haemophilus.haemolyticus
4000
3000
2000
1000
0
A (n=14)
B (n=15)
C (n=11)
surface

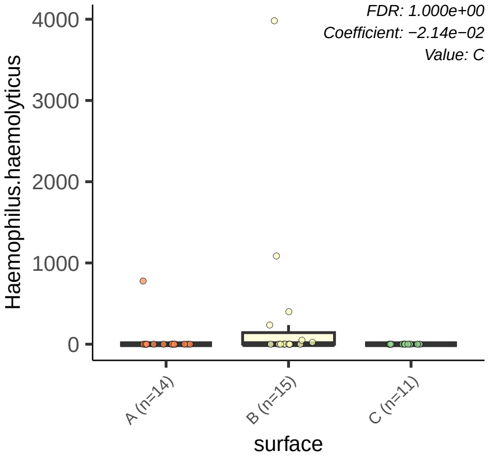
FDR: 1.000e+00
Coefficient: −2.14e−02
Value: C
Haemophilus.haemolyticus
4000
3000
2000
1000
0
A (n=14)
B (n=15)
C (n=11)
surface

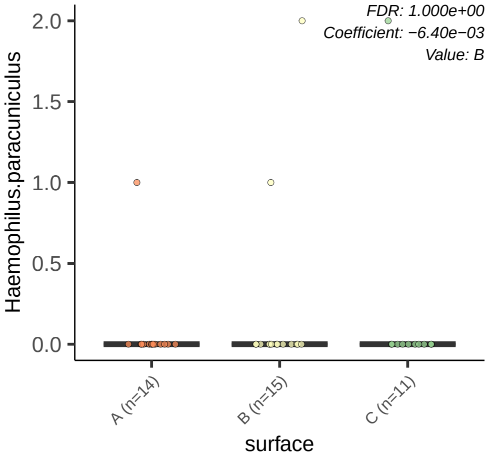

FDR: 1.000e+00
Coefficient: −6.40e−03
Value: B
Haemophilus.paracuniculus
2.0
1.5
1.0
0.5
0.0
A (n=14)
B (n=15)
C (n=11)
surface

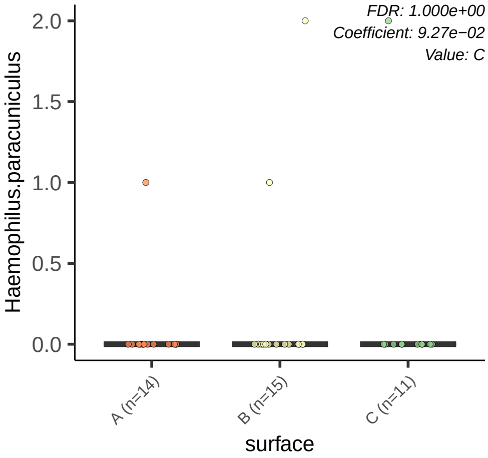

FDR: 1.000e+00
Coefficient: 9.27e−02
Value: C
Haemophilus.paracuniculus
2.0
1.5
1.0
0.5
0.0
A (n=14)
B (n=15)
C (n=11)
surface

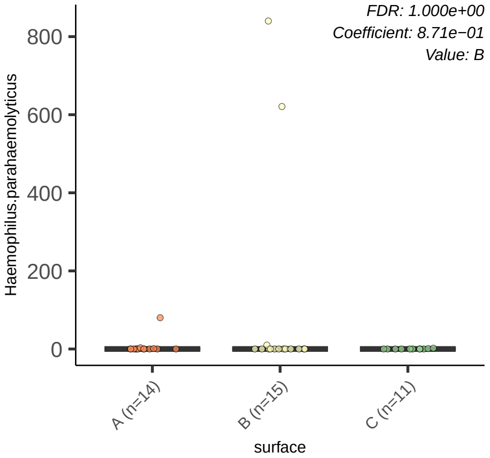

FDR: 1.000e+00
Coefficient: 8.71e−01
Value: B
Haemophilus.parahaemolyticus
800
600
400
200
0
A (n=14)
B (n=15)
C (n=11)
surface

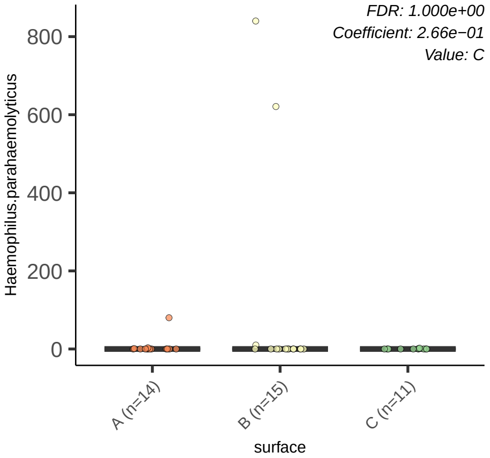

FDR: 1.000e+00
Coefficient: 2.66e−01
Value: C
Haemophilus.parahaemolyticus
800
600
400
200
0
A (n=14)
B (n=15)
C (n=11)
surface

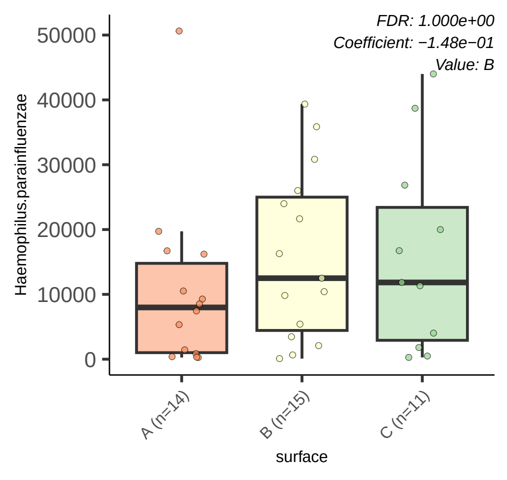
FDR: 1.000e+00
Coefficient: −1.48e−01
Value: B
Haemophilus.parainfluenzae
50000
40000
30000
20000
10000
0
A (n=14)
B (n=15)
C (n=11)
surface

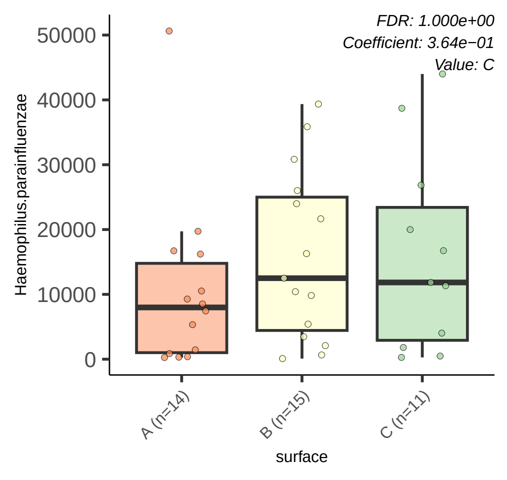
FDR: 1.000e+00
Coefficient: 3.64e−01
Value: C
Haemophilus.parainfluenzae
50000
40000
30000
20000
10000
0
A (n=14)
B (n=15)
C (n=11)
surface

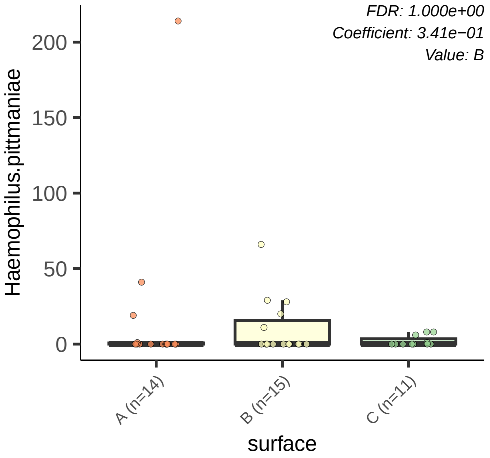
FDR: 1.000e+00
Coefficient: 3.41e−01
Value: B
Haemophilus.pittmaniae
200
150
100
50
0
A (n=14)
B (n=15)
C (n=11)
surface

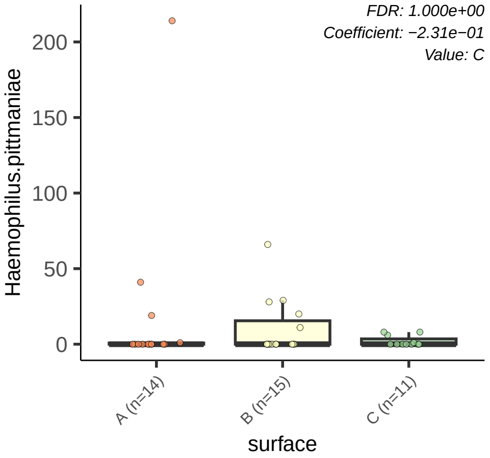

FDR: 1.000e+00
Coefficient: −2.31e−01
Value: C
Haemophilus.pittmaniae
200
150
100
50
0
A (n=14)
B (n=15)
C (n=11)
surface

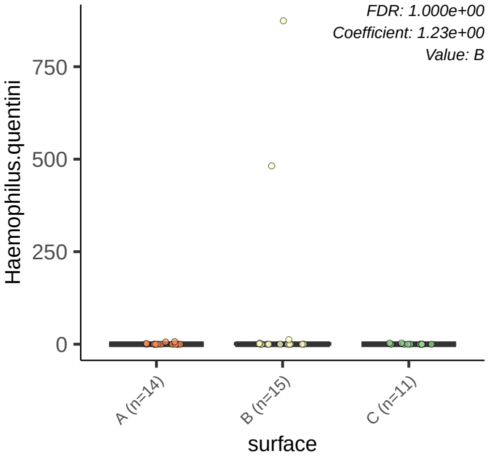
FDR: 1.000e+00
Coefficient: 1.23e+00
Value: B
Haemophilus.quentini
750
500
250
0
A (n=14)
B (n=15)
C (n=11)
surface

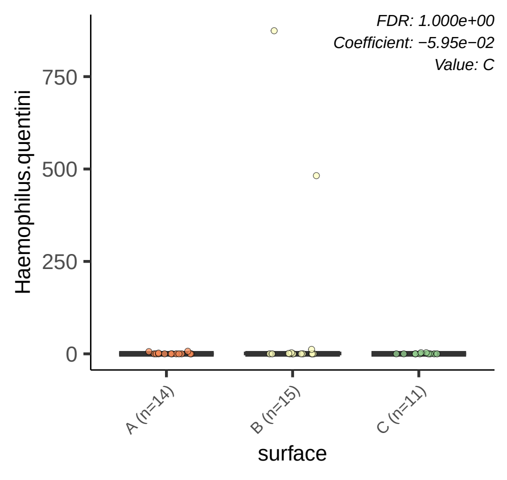
FDR: 1.000e+00
Coefficient: −5.95e−02
Value: C
Haemophilus.quentini
750
500
250
0
A (n=14)
B (n=15)
C (n=11)
surface

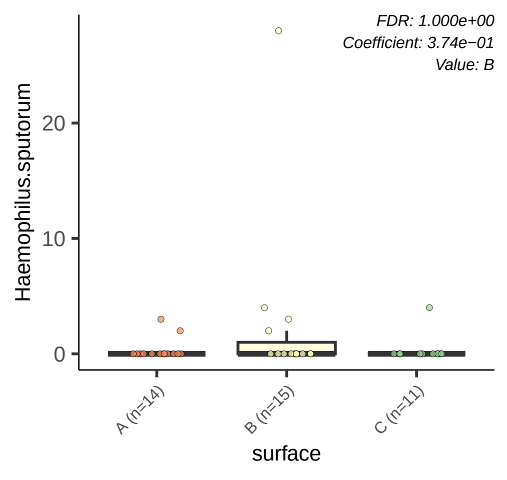
FDR: 1.000e+00
Coefficient: 3.74e−01
Value: B
Haemophilus.sputorum
0
10
20
A (n=14)
B (n=15)
C (n=11)
surface

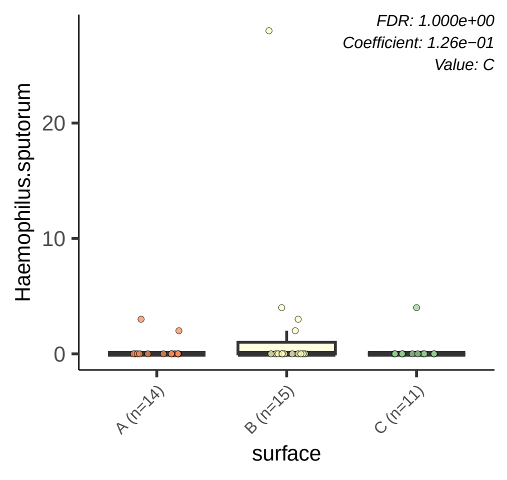
FDR: 1.000e+00
Coefficient: 1.26e−01
Value: C
Haemophilus.sputorum
0
10
20
A (n=14)
B (n=15)
C (n=11)
surface

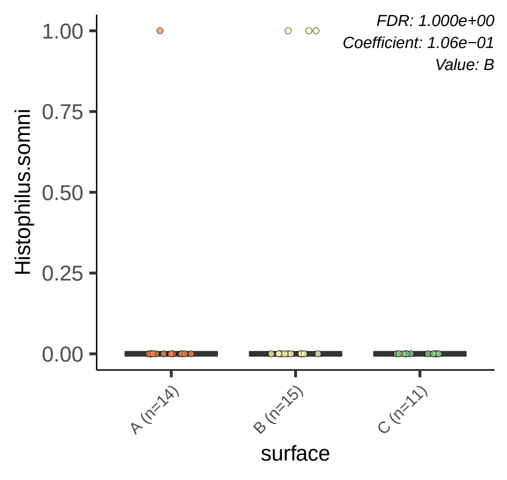
FDR: 1.000e+00
Coefficient: 1.06e−01
Value: B
Histophilus.somni
1.00
0.75
0.50
0.25
0.00
A (n=14)
B (n=15)
C (n=11)
surface

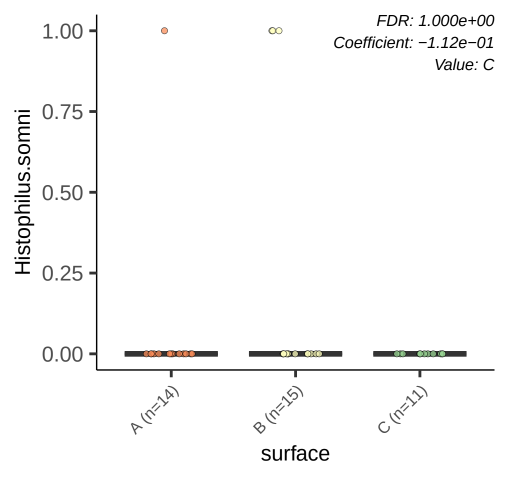

FDR: 1.000e+00
Coefficient: −1.12e−01
Value: C
Histophilus.somni
1.00
0.75
0.50
0.25
0.00
A (n=14)
B (n=15)
C (n=11)
surface

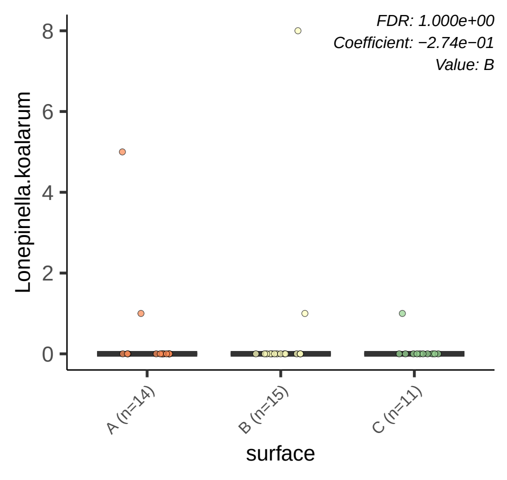
FDR: 1.000e+00
Coefficient: −2.74e−01
Value: B
Lonepinella.koalarum
8
6
4
2
0
A (n=14)
B (n=15)
C (n=11)
surface

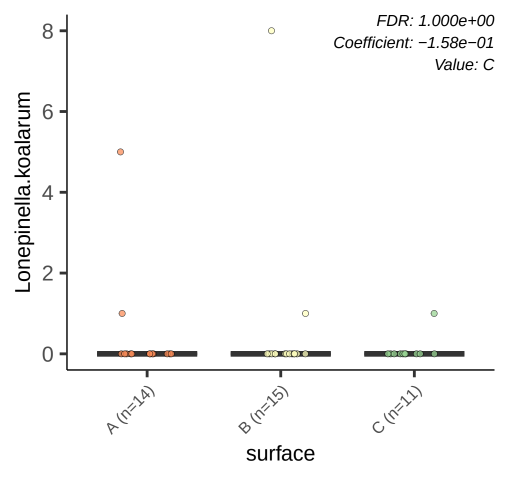

FDR: 1.000e+00
Coefficient: −1.58e−01
Value: C
Lonepinella.koalarum
8
6
4
2
0
A (n=14)
B (n=15)
C (n=11)
surface

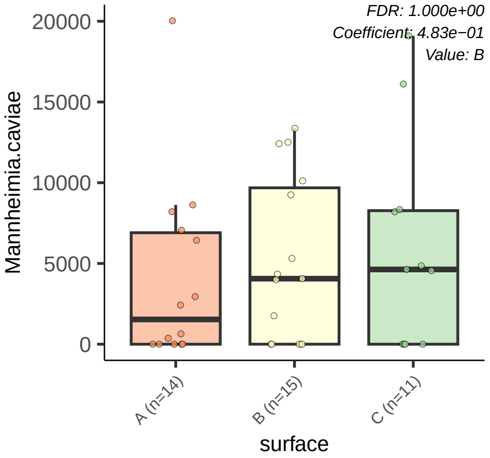
FDR: 1.000e+00
Coefficient: 4.83e−01
Value: B
Mannheimia.caviae
20000
15000
10000
5000
0
A (n=14)
B (n=15)
C (n=11)
surface

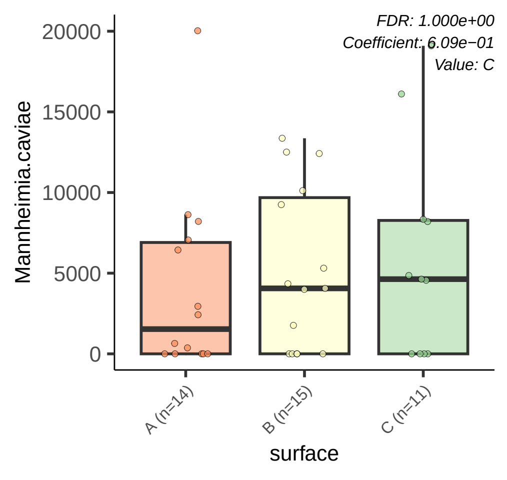

FDR: 1.000e+00
Coefficient: 6.09e−01
Value: C
Mannheimia.caviae
20000
15000
10000
5000
0
A (n=14)
B (n=15)
C (n=11)
surface

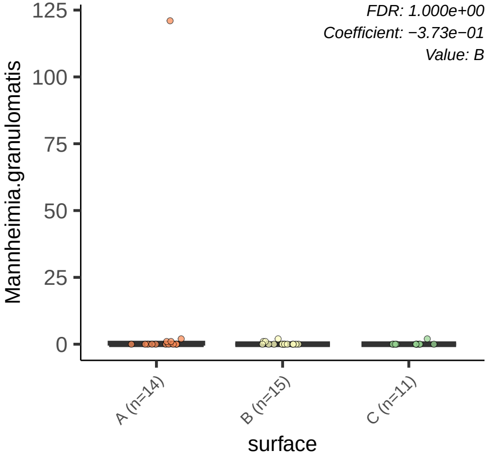
FDR: 1.000e+00
Coefficient: −3.73e−01
Value: B
Mannheimia.granulomatis
125
100
75
50
25
0
A (n=14)
B (n=15)
C (n=11)
surface

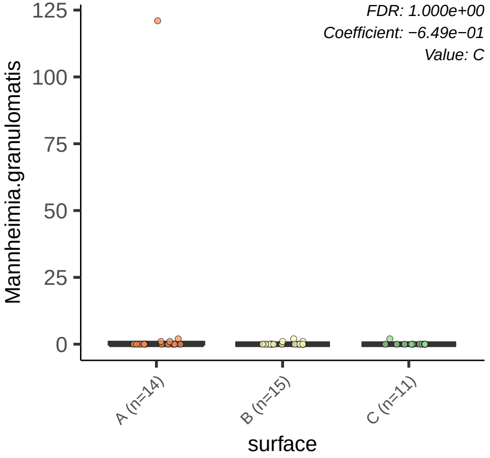
FDR: 1.000e+00
Coefficient: −6.49e−01
Value: C
Mannheimia.granulomatis
125
100
75
50
25
0
A (n=14)
B (n=15)
C (n=11)
surface

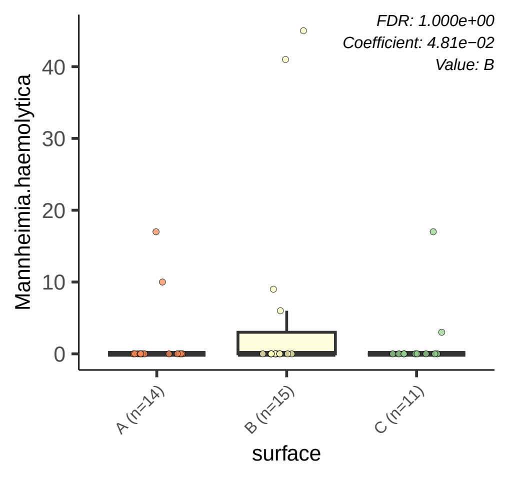
FDR: 1.000e+00
Coefficient: 4.81e−02
Value: B
Mannheimia.haemolytica
0
10
20
30
40
A (n=14)
B (n=15)
C (n=11)
surface

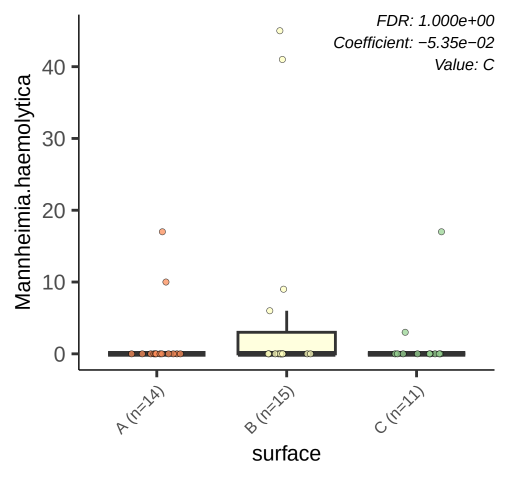

FDR: 1.000e+00
Coefficient: −5.35e−02
Value: C
Mannheimia.haemolytica
40
30
20
10
0
A (n=14)
B (n=15)
C (n=11)
surface

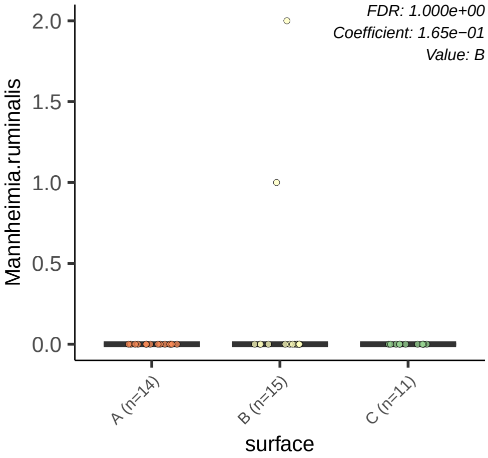
FDR: 1.000e+00
Coefficient: 1.65e−01
Value: B
Mannheimia.ruminalis
2.0
1.5
1.0
0.5
0.0
A (n=14)
B (n=15)
C (n=11)
surface

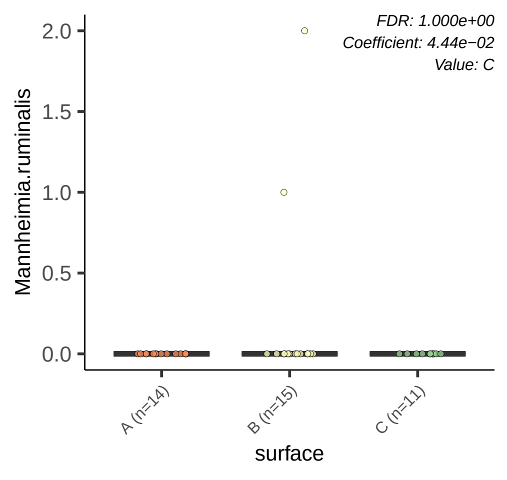

FDR: 1.000e+00
Coefficient: 4.44e−02
Value: C
Mannheimia.ruminalis
2.0
1.5
1.0
0.5
0.0
A (n=14)
B (n=15)
C (n=11)
surface

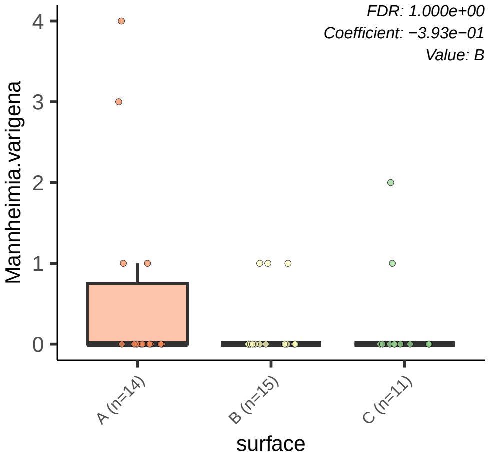

FDR: 1.000e+00
Coefficient: −3.93e−01
Value: B
Mannheimia.varigena
0
1
2
3
4
A (n=14)
B (n=15)
C (n=11)
surface

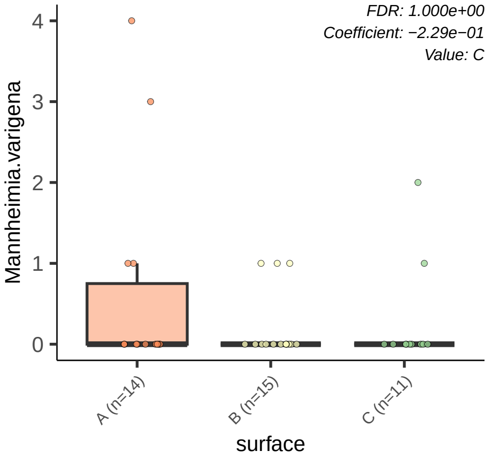
FDR: 1.000e+00
Coefficient: −2.29e−01
Value: C
Mannheimia.varigena
0
1
2
3
4
A (n=14)
B (n=15)
C (n=11)
surface

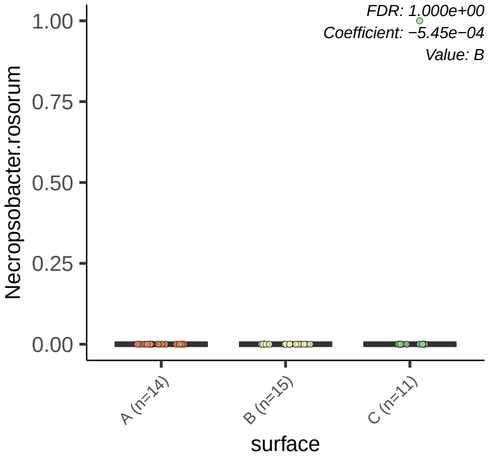

FDR: 1.000e+00
Coefficient: −5.45e−04
Value: B
Necropsobacter.rosorum
1.00
0.75
0.50
0.25
0.00
A (n=14)
B (n=15)
C (n=11)
surface

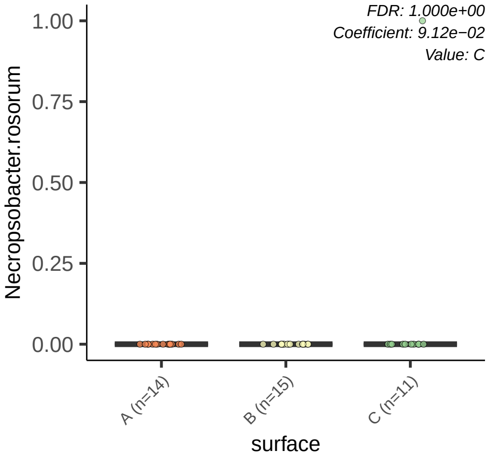
FDR: 1.000e+00
Coefficient: 9.12e−02
Value: C
Necropsobacter.rosorum
1.00
0.75
0.50
0.25
0.00
A (n=14)
B (n=15)
C (n=11)
surface

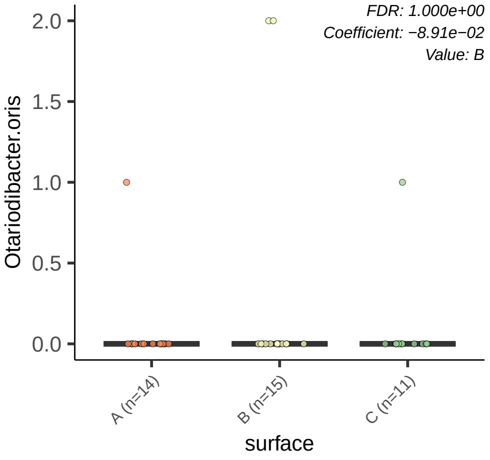
FDR: 1.000e+00
Coefficient: −8.91e−02
Value: B
Otariodibacter.oris
2.0
1.5
1.0
0.5
0.0
A (n=14)
B (n=15)
C (n=11)
surface

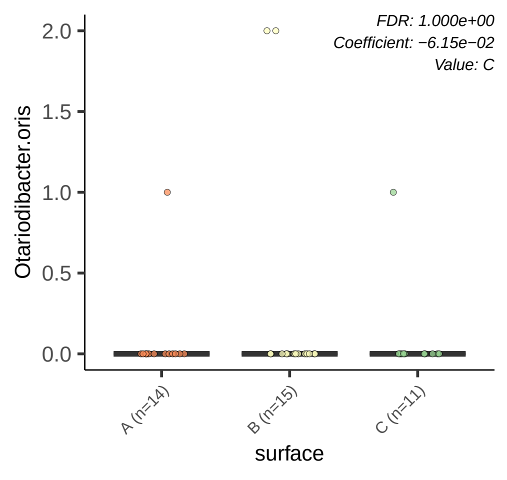
FDR: 1.000e+00
Coefficient: −6.15e−02
Value: C
Otariodibacter.oris
2.0
1.5
1.0
0.5
0.0
A (n=14)
B (n=15)
C (n=11)
surface

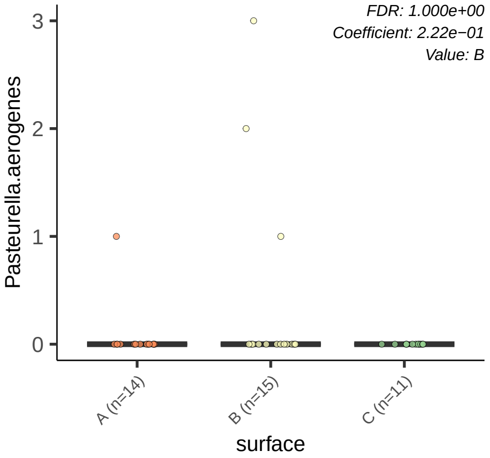

FDR: 1.000e+00
Coefficient: 2.22e−01
Value: B
Pasteurella.aerogenes
0
1
2
3
A (n=14)
B (n=15)
C (n=11)
surface

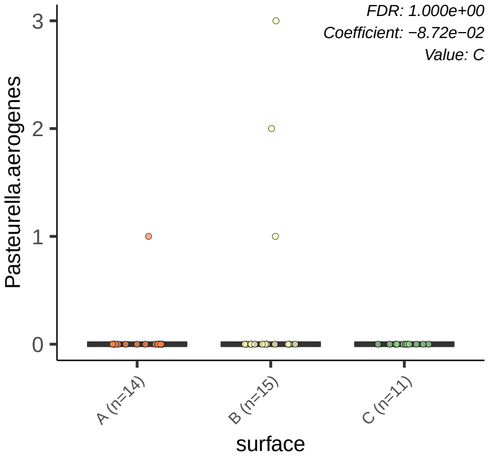

FDR: 1.000e+00
Coefficient: −8.72e−02
Value: C
Pasteurella.aerogenes
0
1
2
3
A (n=14)
B (n=15)
C (n=11)
surface

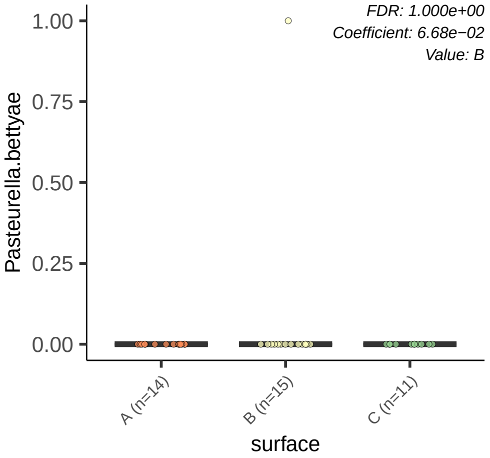

FDR: 1.000e+00
Coefficient: 6.68e−02
Value: B
Pasteurella.bettyae
1.00
0.75
0.50
0.25
0.00
A (n=14)
B (n=15)
C (n=11)
surface

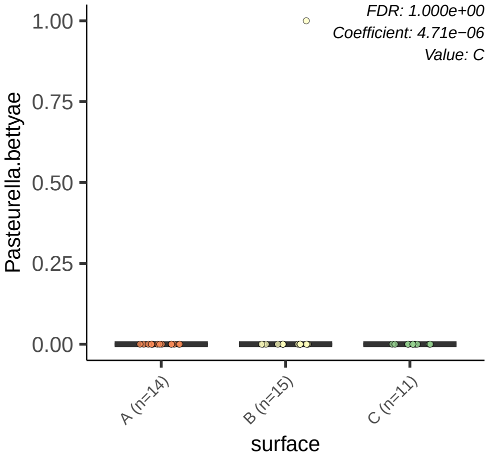
FDR: 1.000e+00
Coefficient: 4.71e−06
Value: C
Pasteurella.bettyae
1.00
0.75
0.50
0.25
0.00
A (n=14)
B (n=15)
C (n=11)
surface

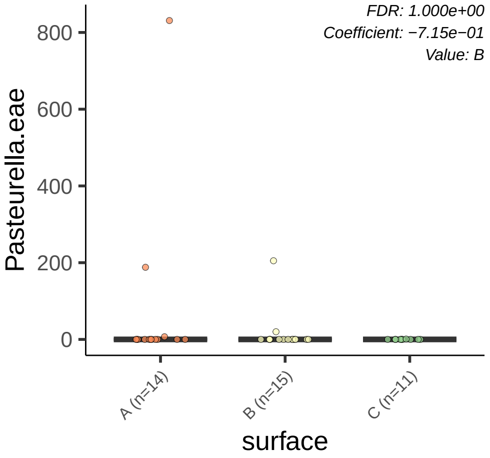
FDR: 1.000e+00
Coefficient: −7.15e−01
Value: B
Pasteurella.eae
800
600
400
200
0
A (n=14)
B (n=15)
C (n=11)
surface

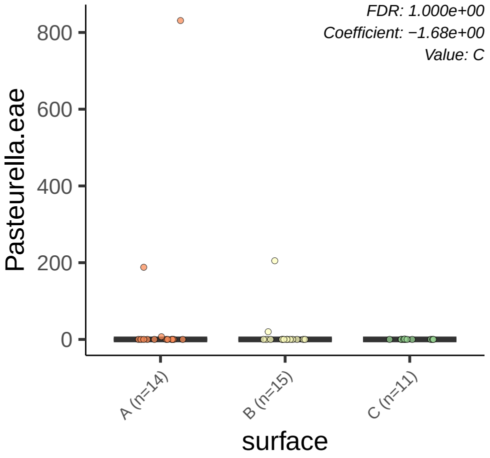

FDR: 1.000e+00
Coefficient: −1.68e+00
Value: C
Pasteurella.eae
800
600
400
200
0
A (n=14)
B (n=15)
C (n=11)
surface

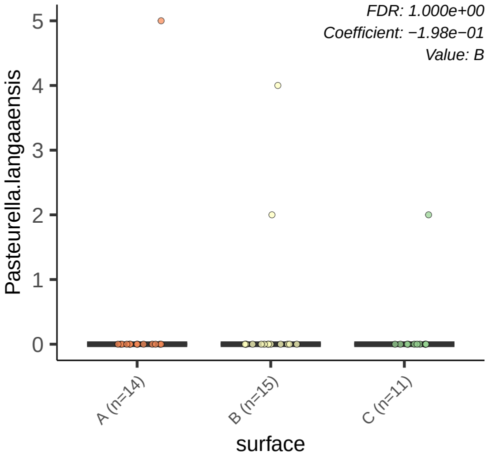
FDR: 1.000e+00
Coefficient: −1.98e−01
Value: B
Pasteurella.langaaensis
0
1
2
3
4
5
A (n=14)
B (n=15)
C (n=11)
surface

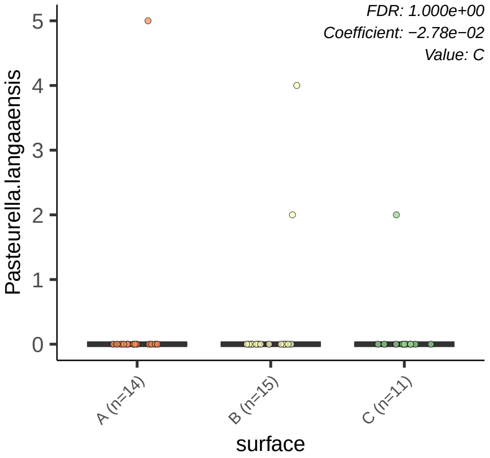
FDR: 1.000e+00
Coefficient: −2.78e−02
Value: C
Pasteurella.langaaensis
5
4
3
2
1
0
A (n=14)
B (n=15)
C (n=11)
surface

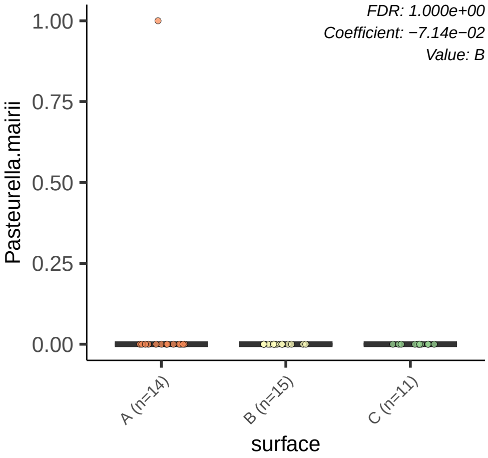

FDR: 1.000e+00
Coefficient: −7.14e−02
Value: B
Pasteurella.mairii
1.00
0.75
0.50
0.25
0.00
A (n=14)
B (n=15)
C (n=11)
surface

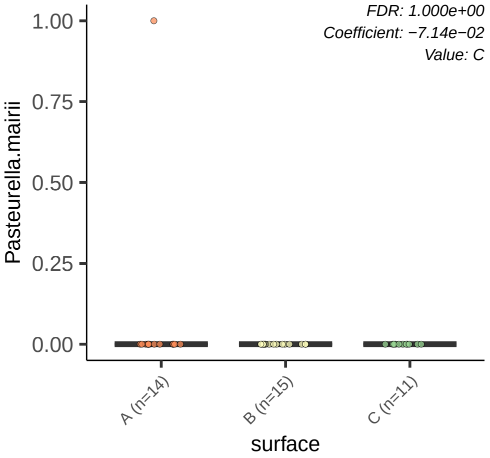

FDR: 1.000e+00
Coefficient: −7.14e−02
Value: C
Pasteurella.mairii
1.00
0.75
0.50
0.25
0.00
A (n=14)
B (n=15)
C (n=11)
surface

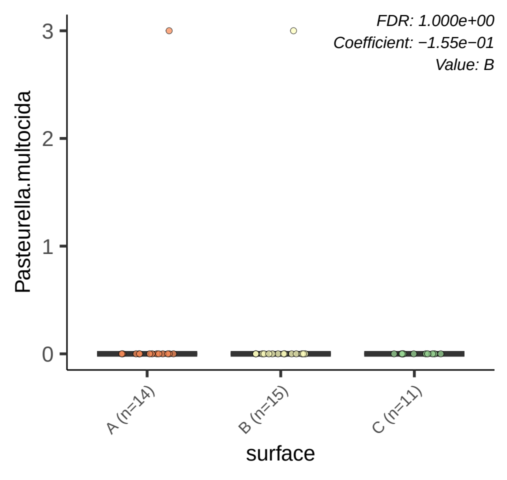
FDR: 1.000e+00
Coefficient: −1.55e−01
Value: B
Pasteurella.multocida
0
1
2
3
A (n=14)
B (n=15)
C (n=11)
surface

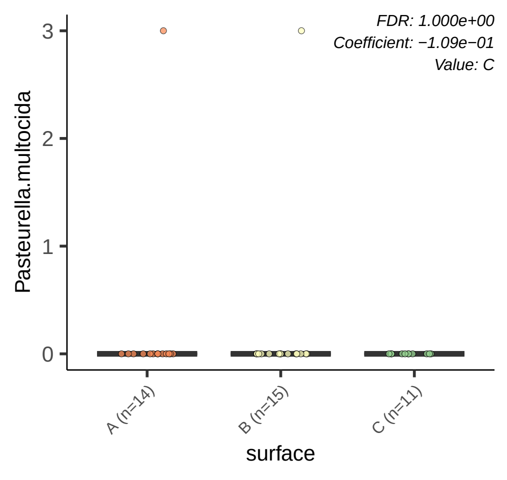
FDR: 1.000e+00
Coefficient: −1.09e−01
Value: C
Pasteurella.multocida
3
2
1
0
A (n=14)
B (n=15)
C (n=11)
surface

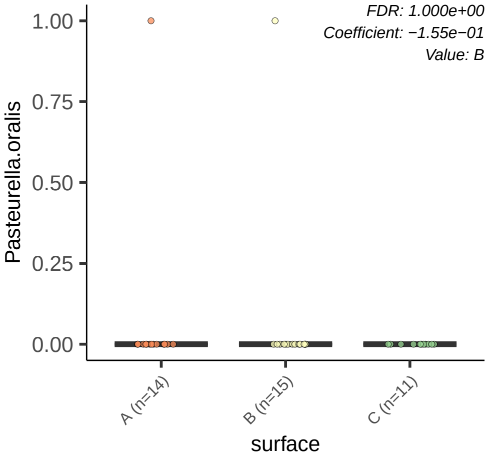
FDR: 1.000e+00
Coefficient: −1.55e−01
Value: B
Pasteurella.oralis
1.00
0.75
0.50
0.25
0.00
A (n=14)
B (n=15)
C (n=11)
surface

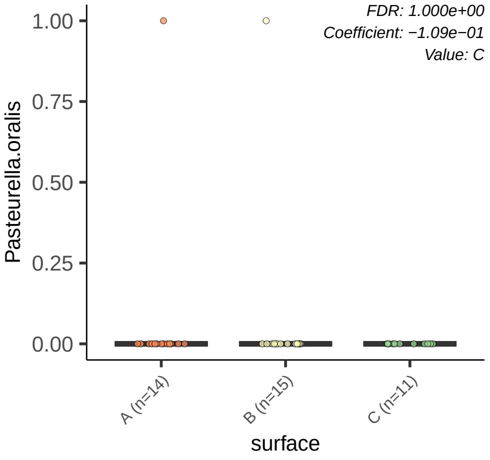
FDR: 1.000e+00
Coefficient: −1.09e−01
Value: C
Pasteurella.oralis
1.00
0.75
0.50
0.25
0.00
A (n=14)
B (n=15)
C (n=11)
surface

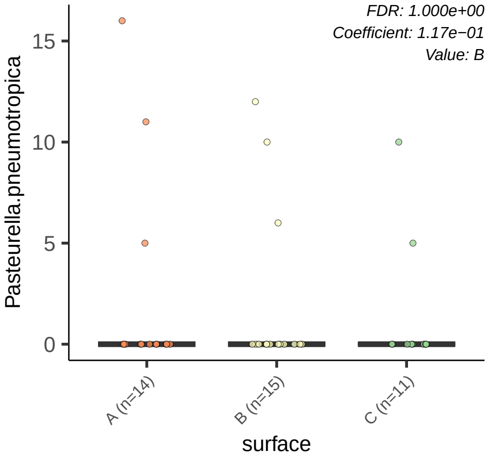

FDR: 1.000e+00
Coefficient: 1.17e−01
Value: B
Pasteurella.pneumotropica
15
10
5
0
A (n=14)
B (n=15)
C (n=11)
surface

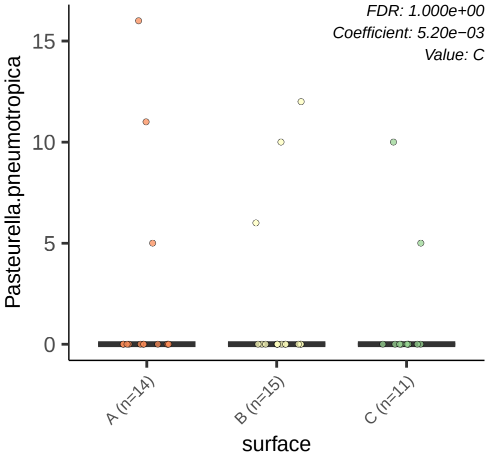
FDR: 1.000e+00
Coefficient: 5.20e−03
Value: C
Pasteurella.pneumotropica
15
10
5
0
A (n=14)
B (n=15)
C (n=11)
surface

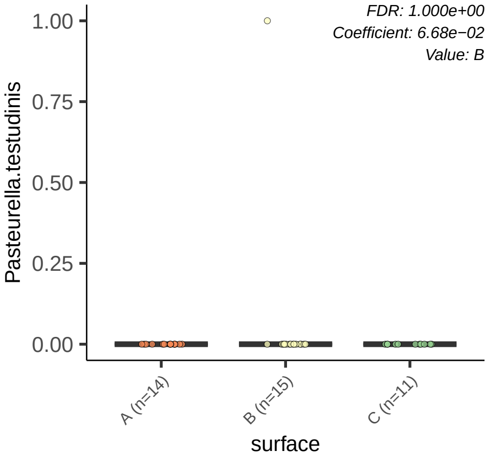
FDR: 1.000e+00
Coefficient: 6.68e−02
Value: B
Pasteurella.testudinis
1.00
0.75
0.50
0.25
0.00
A (n=14)
B (n=15)
C (n=11)
surface

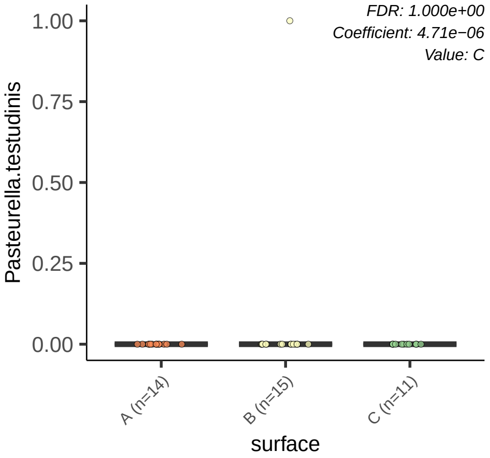

FDR: 1.000e+00
Coefficient: 4.71e−06
Value: C
Pasteurella.testudinis
1.00
0.75
0.50
0.25
0.00
A (n=14)
B (n=15)
C (n=11)
surface

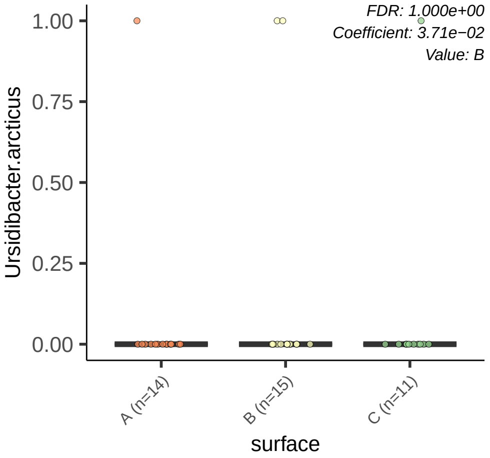
FDR: 1.000e+00
Coefficient: 3.71e−02
Value: B
Ursidibacter.arcticus
1.00
0.75
0.50
0.25
0.00
A (n=14)
B (n=15)
C (n=11)
surface

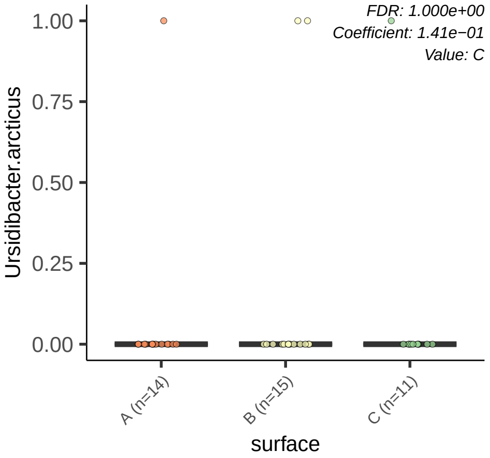

FDR: 1.000e+00
Coefficient: 1.41e−01
Value: C
Ursidibacter.arcticus
1.00
0.75
0.50
0.25
0.00
A (n=14)
B (n=15)
C (n=11)
surface

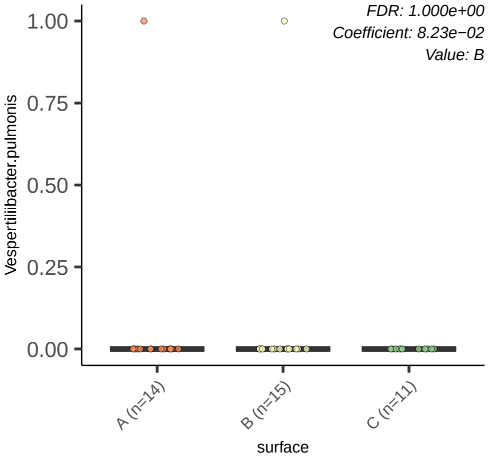
FDR: 1.000e+00
Coefficient: 8.23e−02
Value: B
Vespertiliibacter.pulmonis
1.00
0.75
0.50
0.25
0.00
A (n=14)
B (n=15)
C (n=11)
surface

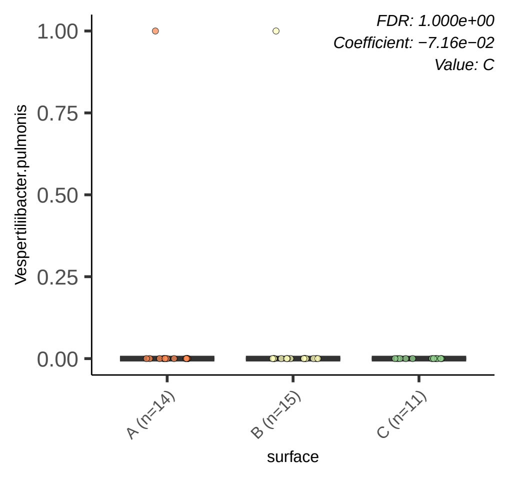

FDR: 1.000e+00
Coefficient: −7.16e−02
Value: C
Vespertiliibacter.pulmonis
1.00
0.75
0.50
0.25
0.00
A (n=14)
B (n=15)
C (n=11)
surface

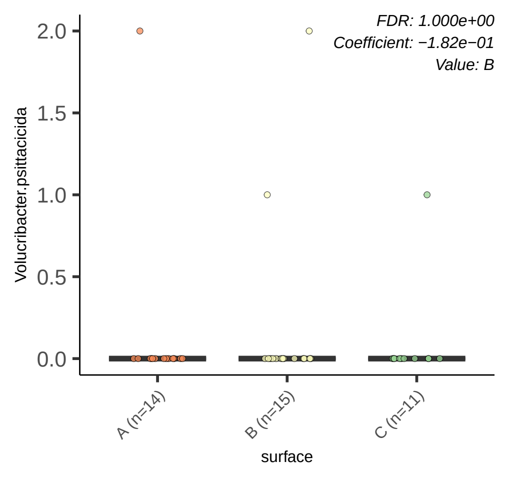
FDR: 1.000e+00
Coefficient: −1.82e−01
Value: B
Volucribacter.psittacicida
2.0
1.5
1.0
0.5
0.0
A (n=14)
B (n=15)
C (n=11)
surface

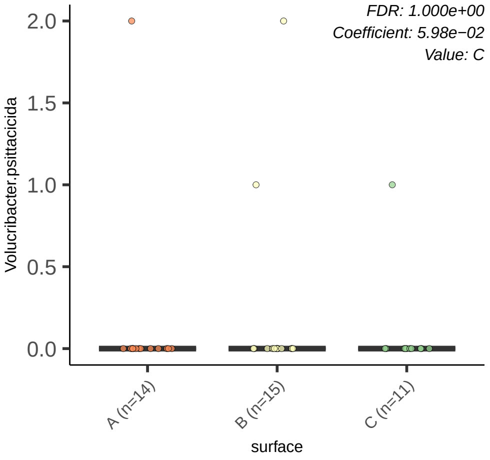
FDR: 1.000e+00
Coefficient: 5.98e−02
Value: C
Volucribacter.psittacicida
2.0
1.5
1.0
0.5
0.0
A (n=14)
B (n=15)
C (n=11)
surface

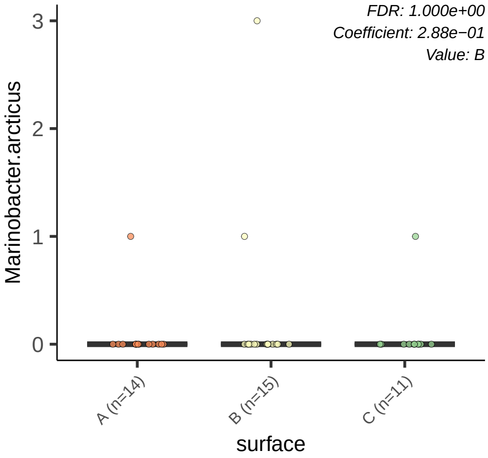
FDR: 1.000e+00
Coefficient: 2.88e−01
Value: B
Marinobacter.arcticus
3
2
1
0
A (n=14)
B (n=15)
C (n=11)
surface

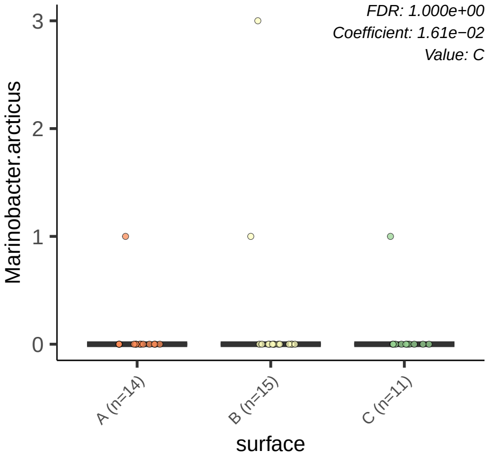
FDR: 1.000e+00
Coefficient: 1.61e−02
Value: C
Marinobacter.arcticus
0
1
2
3
A (n=14)
B (n=15)
C (n=11)
surface

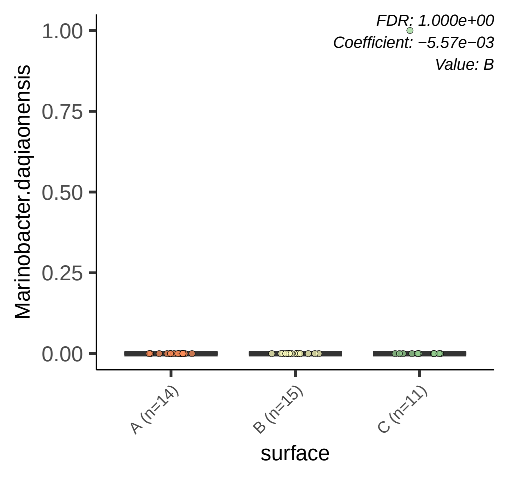
FDR: 1.000e+00
Coefficient: −5.57e−03
Value: B
Marinobacter.daqiaonensis
1.00
0.75
0.50
0.25
0.00
A (n=14)
B (n=15)
C (n=11)
surface

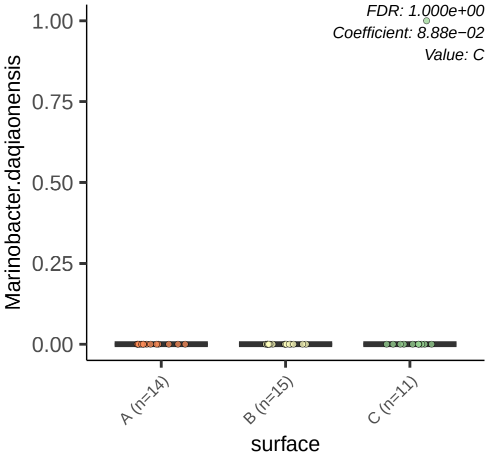
FDR: 1.000e+00
Coefficient: 8.88e−02
Value: C
Marinobacter.daqiaonensis
1.00
0.75
0.50
0.25
0.00
A (n=14)
B (n=15)
C (n=11)
surface

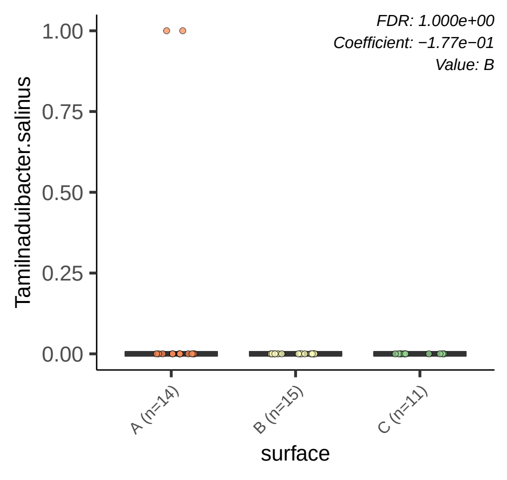

FDR: 1.000e+00
Coefficient: −1.77e−01
Value: B
Tamilnaduibacter.salinus
1.00
0.75
0.50
0.25
0.00
A (n=14)
B (n=15)
C (n=11)
surface

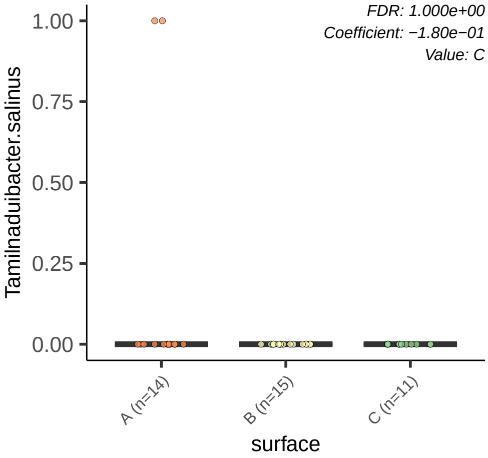

FDR: 1.000e+00
Coefficient: −1.80e−01
Value: C
Tamilnaduibacter.salinus
1.00
0.75
0.50
0.25
0.00
A (n=14)
B (n=15)
C (n=11)
surface

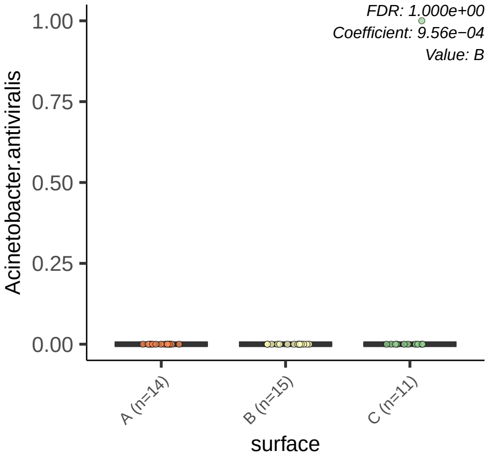
FDR: 1.000e+00
Coefficient: 9.56e−04
Value: B
Acinetobacter.antiviralis
1.00
0.75
0.50
0.25
0.00
A (n=14)
B (n=15)
C (n=11)
surface

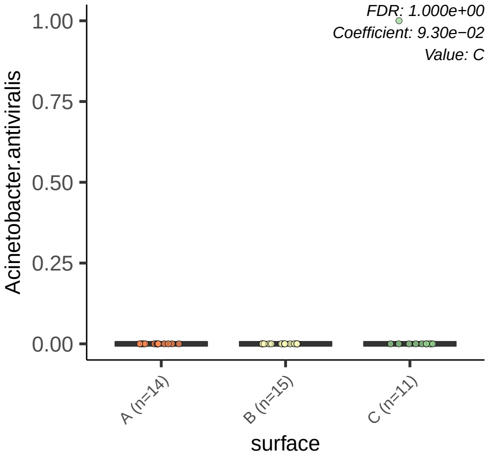
FDR: 1.000e+00
Coefficient: 9.30e−02
Value: C
Acinetobacter.antiviralis
1.00
0.75
0.50
0.25
0.00
A (n=14)
B (n=15)
C (n=11)
surface

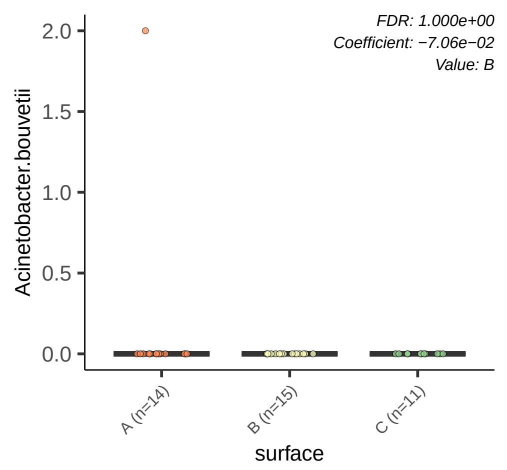
FDR: 1.000e+00
Coefficient: −7.06e−02
Value: B
Acinetobacter.bouvetii
2.0
1.5
1.0
0.5
0.0
A (n=14)
B (n=15)
C (n=11)
surface

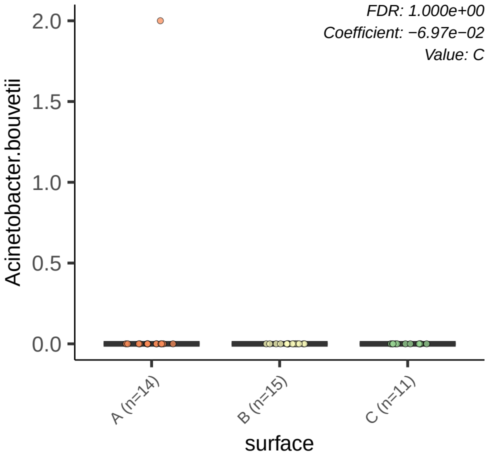
FDR: 1.000e+00
Coefficient: −6.97e−02
Value: C
Acinetobacter.bouvetii
2.0
1.5
1.0
0.5
0.0
A (n=14)
B (n=15)
C (n=11)
surface

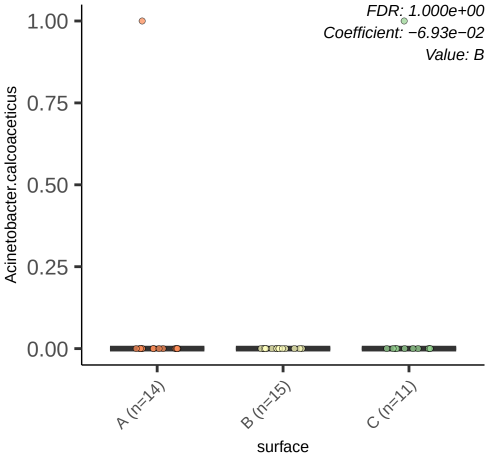
FDR: 1.000e+00
Coefficient: −6.93e−02
Value: B
Acinetobacter.calcoaceticus
1.00
0.75
0.50
0.25
0.00
A (n=14)
B (n=15)
C (n=11)
surface

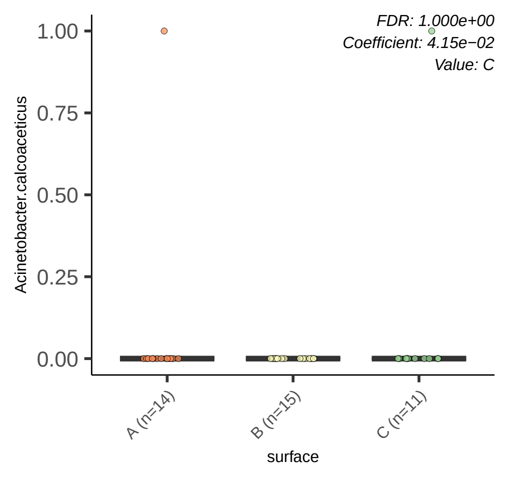

FDR: 1.000e+00
Coefficient: 4.15e−02
Value: C
1.00
0.75
0.50
0.25
0.00
Acinetobacter.calcoaceticus
A (n=14)
B (n=15)
C (n=11)
surface

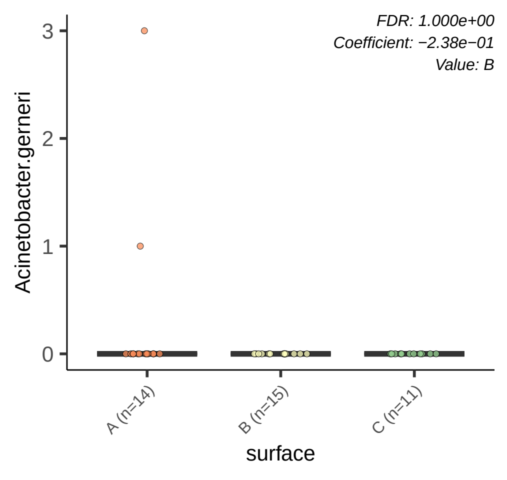
FDR: 1.000e+00
Coefficient: −2.38e−01
Value: B
Acinetobacter.gerneri
3
2
1
0
A (n=14)
B (n=15)
C (n=11)
surface

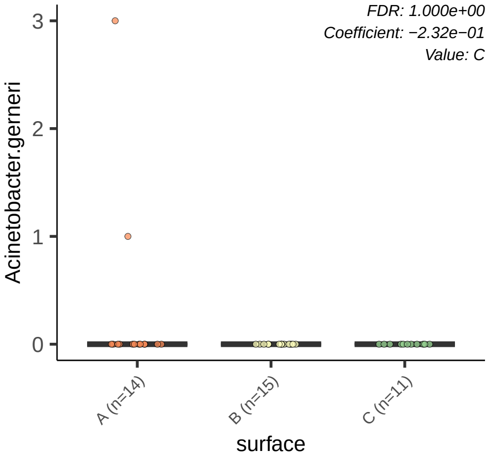
FDR: 1.000e+00
Coefficient: −2.32e−01
Value: C
Acinetobacter.gerneri
3
2
1
0
A (n=14)
B (n=15)
C (n=11)
surface

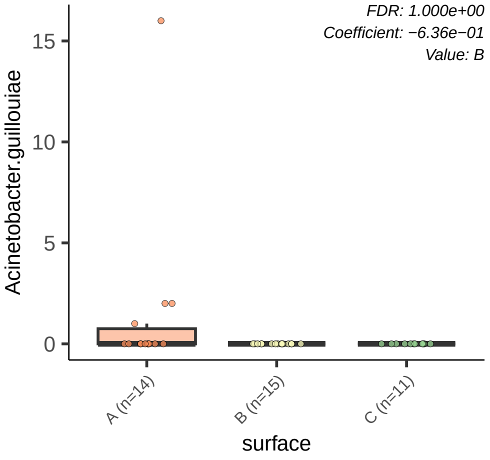

FDR: 1.000e+00
Coefficient: −6.36e−01
Value: B
Acinetobacter.guillouiae
15
10
5
0
A (n=14)
B (n=15)
C (n=11)
surface

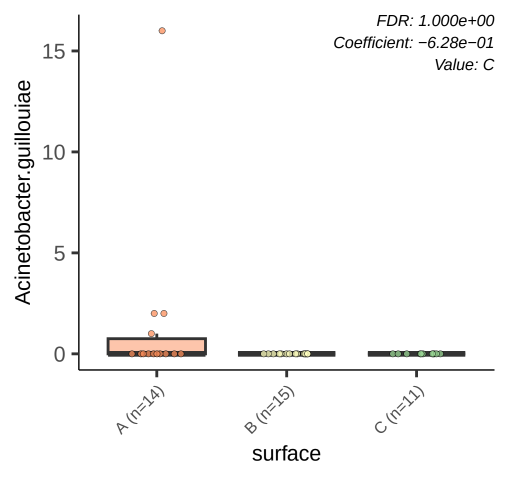
FDR: 1.000e+00
Coefficient: −6.28e−01
Value: C
Acinetobacter.guillouiae
15
10
5
0
A (n=14)
B (n=15)
C (n=11)
surface

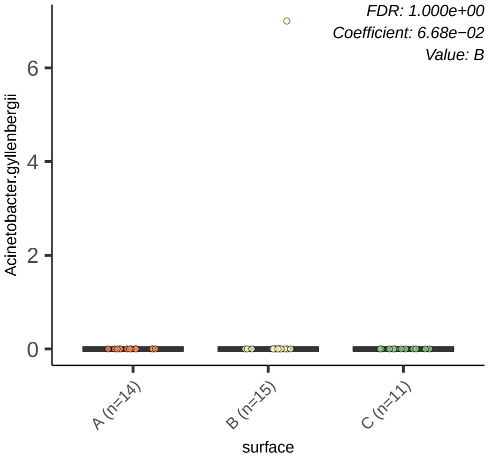

FDR: 1.000e+00
Coefficient: 6.68e−02
Value: B
Acinetobacter.gyllenbergii
0
2
4
6
A (n=14)
B (n=15)
C (n=11)
surface

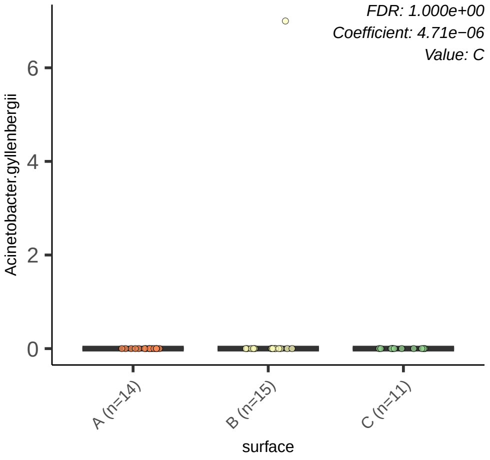

FDR: 1.000e+00
Coefficient: 4.71e−06
Value: C
Acinetobacter.gyllenbergii
0
2
4
6
A (n=14)
B (n=15)
C (n=11)
surface

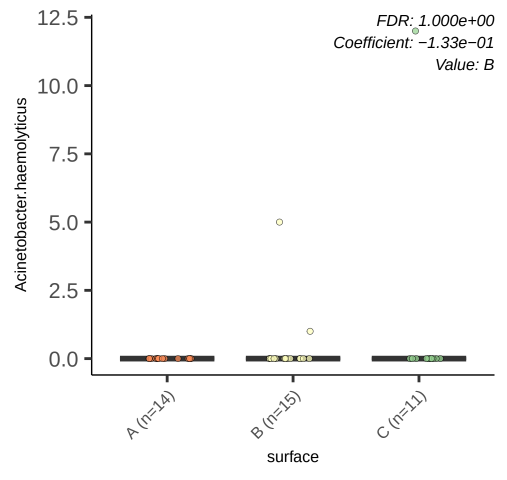
FDR: 1.000e+00
Coefficient: −1.33e−01
Value: B
Acinetobacter.haemolyticus
12.5
10.0
7.5
5.0
2.5
0.0
A (n=14)
B (n=15)
C (n=11)
surface

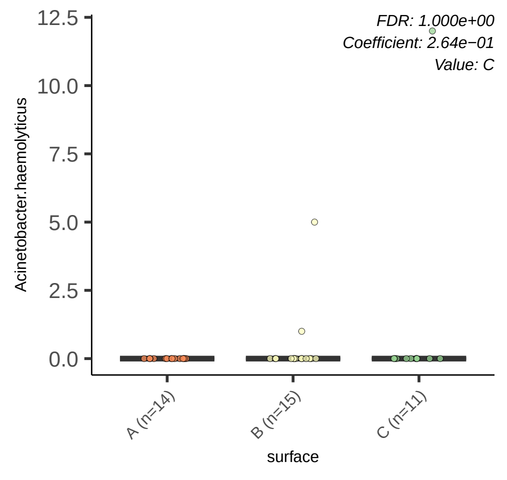

FDR: 1.000e+00
Coefficient: 2.64e−01
Value: C
Acinetobacter.haemolyticus
12.5
10.0
7.5
5.0
2.5
0.0
A (n=14)
B (n=15)
C (n=11)
surface

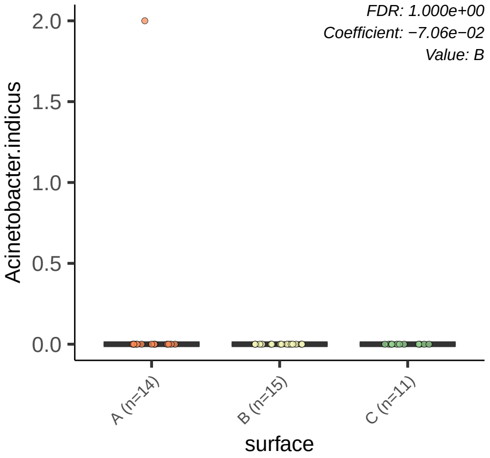
FDR: 1.000e+00
Coefficient: −7.06e−02
Value: B
Acinetobacter.indicus
2.0
1.5
1.0
0.5
0.0
A (n=14)
B (n=15)
C (n=11)
surface

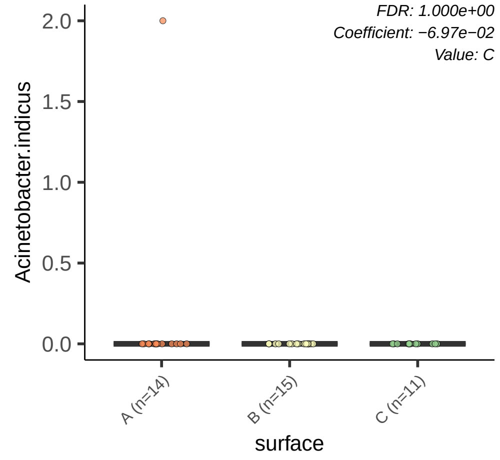

FDR: 1.000e+00
Coefficient: −6.97e−02
Value: C
Acinetobacter.indicus
2.0
1.5
1.0
0.5
0.0
A (n=14)
B (n=15)
C (n=11)
surface

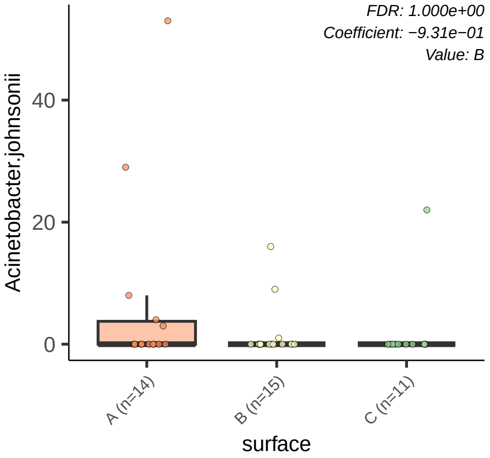
FDR: 1.000e+00
Coefficient: −9.31e−01
Value: B
Acinetobacter.johnsonii
40
20
0
A (n=14)
B (n=15)
C (n=11)
surface

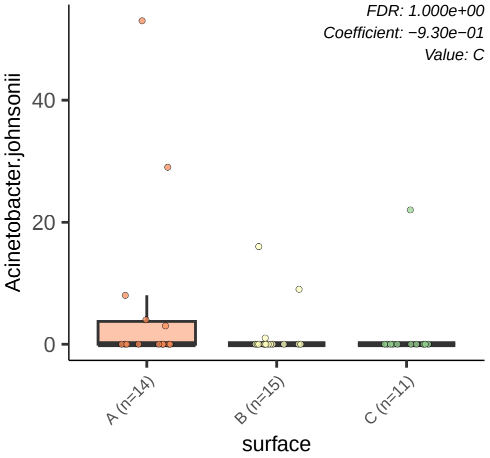
FDR: 1.000e+00
Coefficient: −9.30e−01
Value: C
Acinetobacter.johnsonii
0
20
40
A (n=14)
B (n=15)
C (n=11)
surface

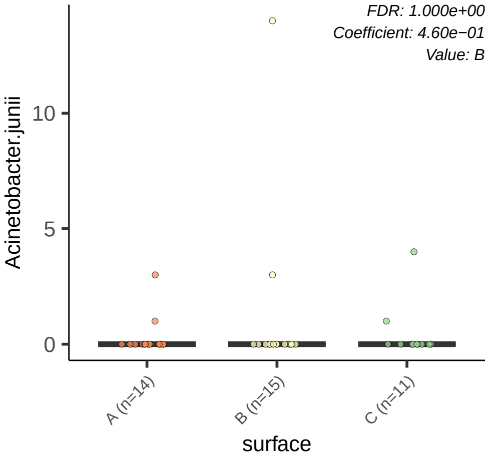

FDR: 1.000e+00
Coefficient: 4.60e−01
Value: B
Acinetobacter.junii
10
5
0
A (n=14)
B (n=15)
C (n=11)
surface

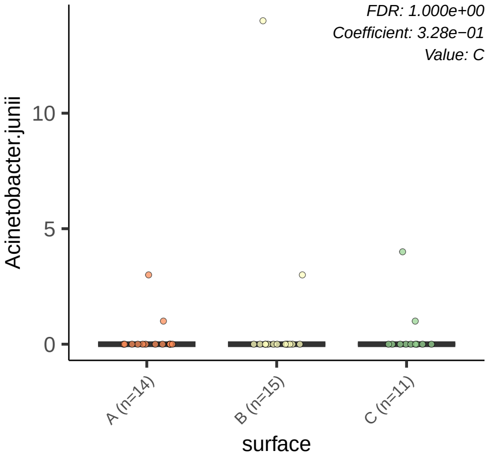

FDR: 1.000e+00
Coefficient: 3.28e−01
Value: C
Acinetobacter.junii
10
5
0
A (n=14)
B (n=15)
C (n=11)
surface

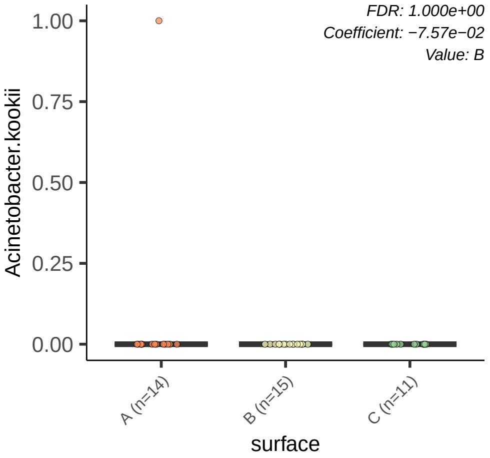
FDR: 1.000e+00
Coefficient: −7.57e−02
Value: B
Acinetobacter.kookii
1.00
0.75
0.50
0.25
0.00
A (n=14)
B (n=15)
C (n=11)
surface

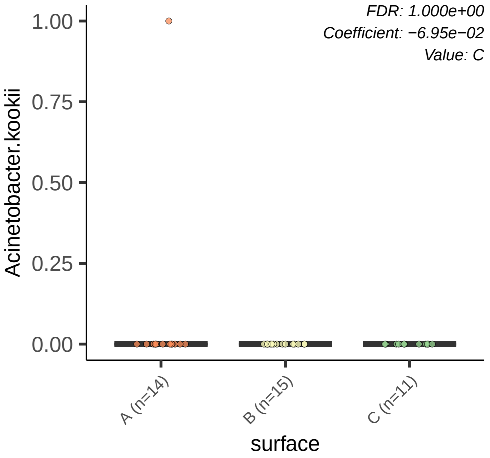

FDR: 1.000e+00
Coefficient: −6.95e−02
Value: C
1.00
0.75
0.50
0.25
0.00
Acinetobacter.kookii
A (n=14)
B (n=15)
C (n=11)
surface

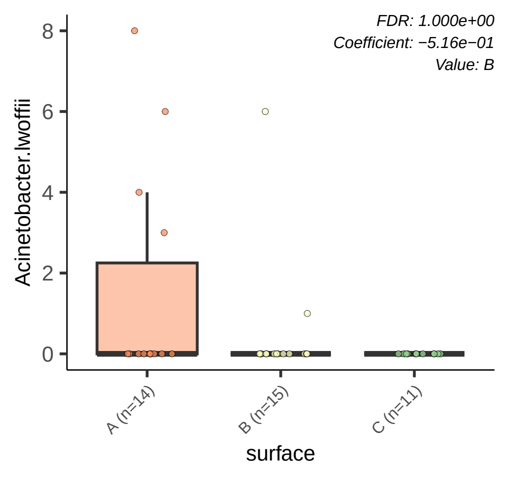
FDR: 1.000e+00
Coefficient: −5.16e−01
Value: B
Acinetobacter.lwoffii
8
6
4
2
0
A (n=14)
B (n=15)
C (n=11)
surface

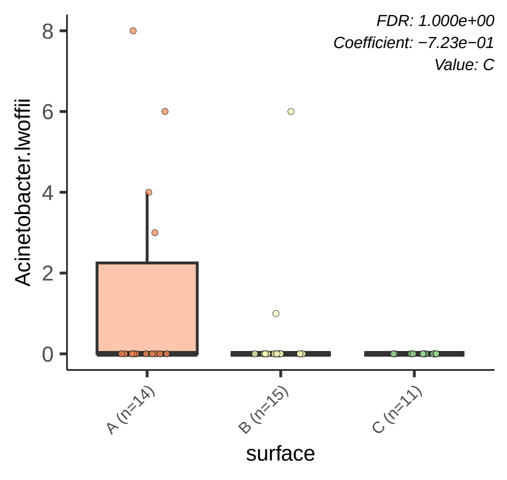

FDR: 1.000e+00
Coefficient: −7.23e−01
Value: C
Acinetobacter.lwoffii
8
6
4
2
0
A (n=14)
B (n=15)
C (n=11)
surface

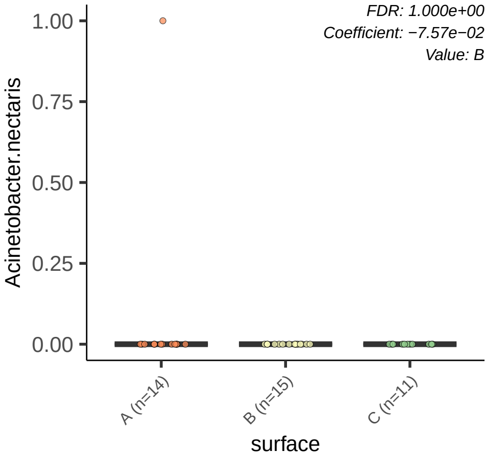

FDR: 1.000e+00
Coefficient: −7.57e−02
Value: B
Acinetobacter.nectaris
1.00
0.75
0.50
0.25
0.00
A (n=14)
B (n=15)
C (n=11)
surface

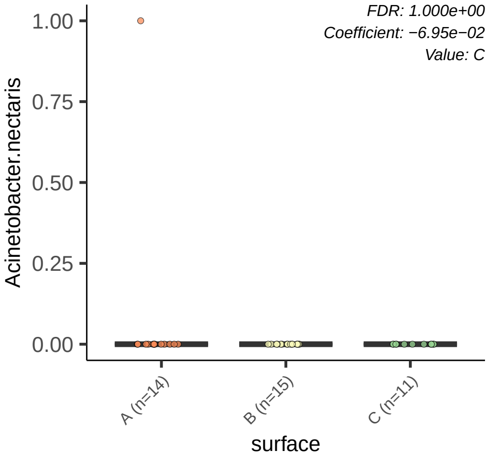
FDR: 1.000e+00
Coefficient: −6.95e−02
Value: C
Acinetobacter.nectaris
1.00
0.75
0.50
0.25
0.00
A (n=14)
B (n=15)
C (n=11)
surface

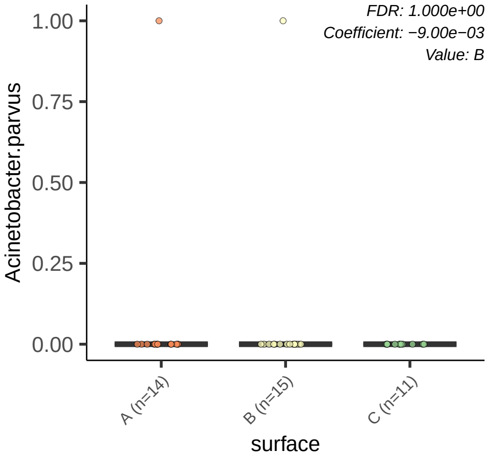

FDR: 1.000e+00
Coefficient: −9.00e−03
Value: B
Acinetobacter.parvus
1.00
0.75
0.50
0.25
0.00
A (n=14)
B (n=15)
C (n=11)
surface

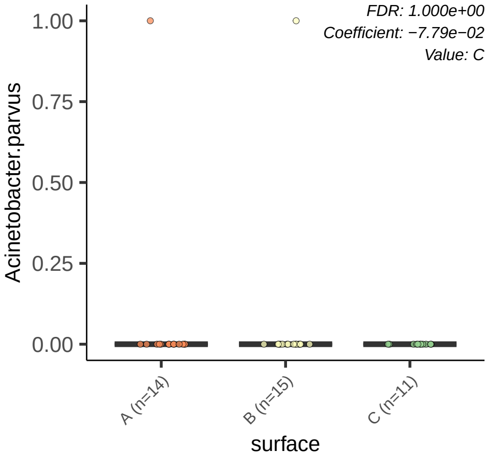
FDR: 1.000e+00
Coefficient: −7.79e−02
Value: C
Acinetobacter.parvus
1.00
0.75
0.50
0.25
0.00
A (n=14)
B (n=15)
C (n=11)
surface

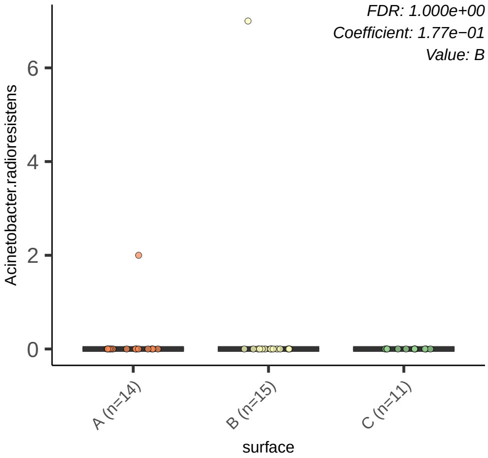
FDR: 1.000e+00
Coefficient: 1.77e−01
Value: B
Acinetobacter.radioresistens
0
2
4
6
A (n=14)
B (n=15)
C (n=11)
surface

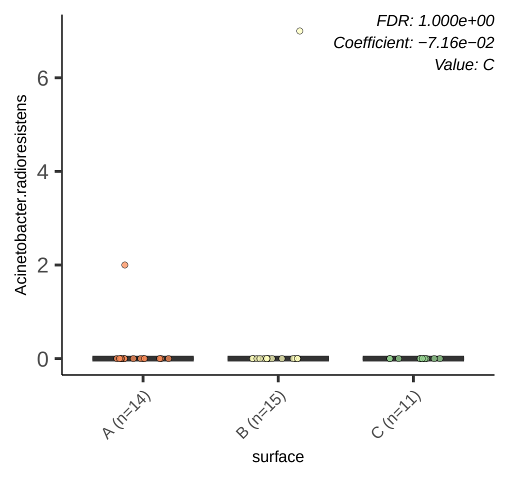

FDR: 1.000e+00
Coefficient: −7.16e−02
Value: C
Acinetobacter.radioresistens
0
2
4
6
A (n=14)
B (n=15)
C (n=11)
surface

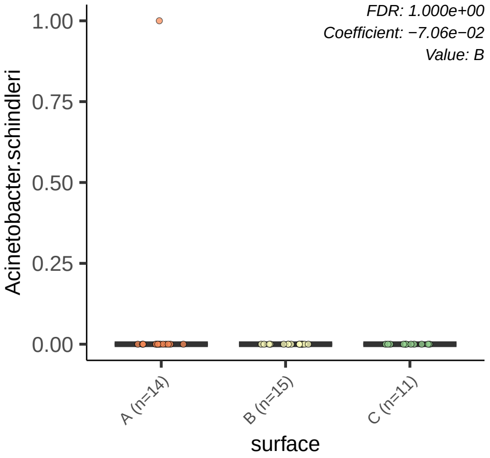
FDR: 1.000e+00
Coefficient: −7.06e−02
Value: B
Acinetobacter.schindleri
1.00
0.75
0.50
0.25
0.00
A (n=14)
B (n=15)
C (n=11)
surface

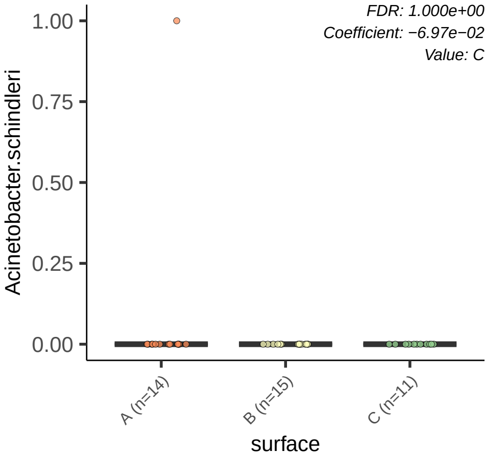

FDR: 1.000e+00
Coefficient: −6.97e−02
Value: C
Acinetobacter.schindleri
1.00
0.75
0.50
0.25
0.00
A (n=14)
B (n=15)
C (n=11)
surface

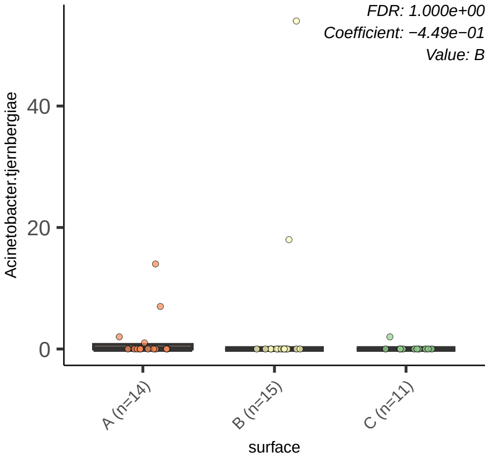
FDR: 1.000e+00
Coefficient: −4.49e−01
Value: B
Acinetobacter.tjernbergiae
40
20
0
A (n=14)
B (n=15)
C (n=11)
surface

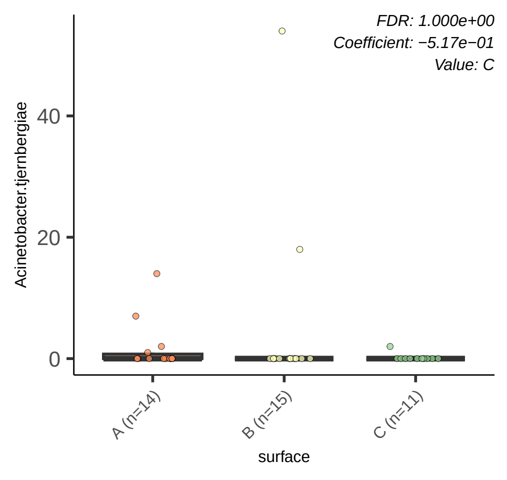
FDR: 1.000e+00
Coefficient: −5.17e−01
Value: C
Acinetobacter.tjernbergiae
40
20
0
A (n=14)
B (n=15)
C (n=11)
surface

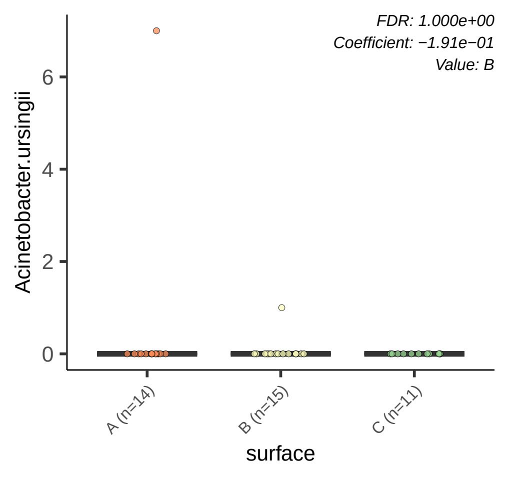
FDR: 1.000e+00
Coefficient: −1.91e−01
Value: B
Acinetobacter.ursingii
0
2
4
6
A (n=14)
B (n=15)
C (n=11)
surface

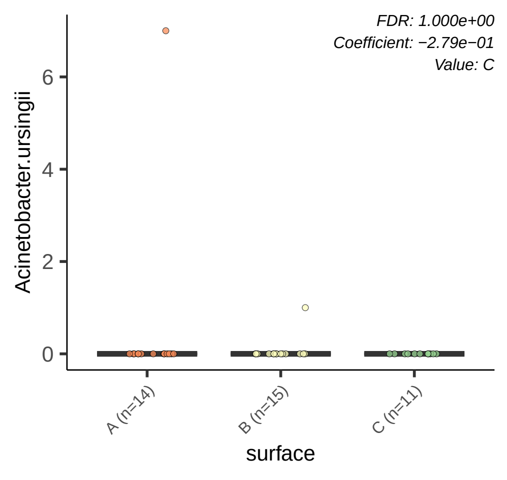

FDR: 1.000e+00
Coefficient: −2.79e−01
Value: C
Acinetobacter.ursingii
0
2
4
6
A (n=14)
B (n=15)
C (n=11)
surface

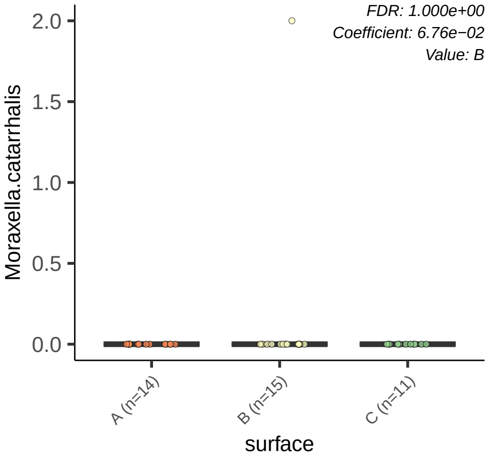
FDR: 1.000e+00
Coefficient: 6.76e−02
Value: B
Moraxella.catarrhalis
2.0
1.5
1.0
0.5
0.0
A (n=14)
B (n=15)
C (n=11)
surface

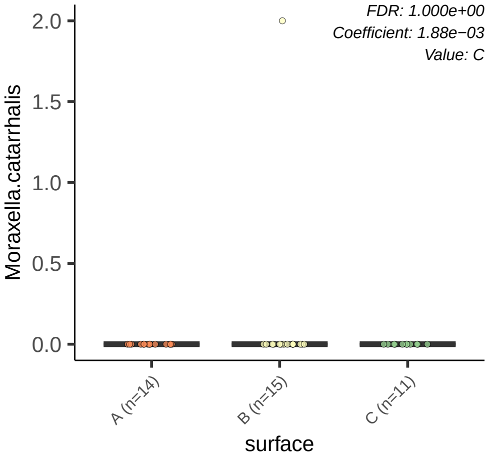
FDR: 1.000e+00
Coefficient: 1.88e−03
Value: C
Moraxella.catarrhalis
2.0
1.5
1.0
0.5
0.0
A (n=14)
B (n=15)
C (n=11)
surface

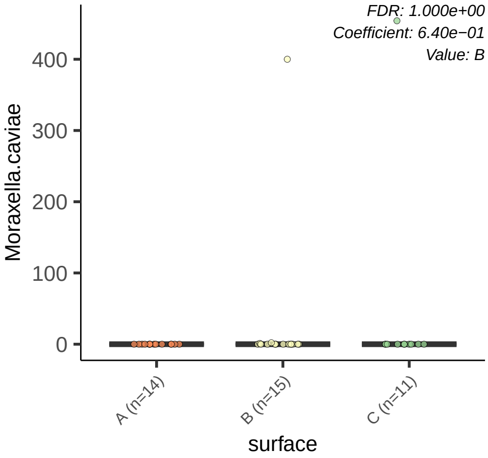
FDR: 1.000e+00
Coefficient: 6.40e−01
Value: B
Moraxella.caviae
400
300
200
100
0
A (n=14)
B (n=15)
C (n=11)
surface

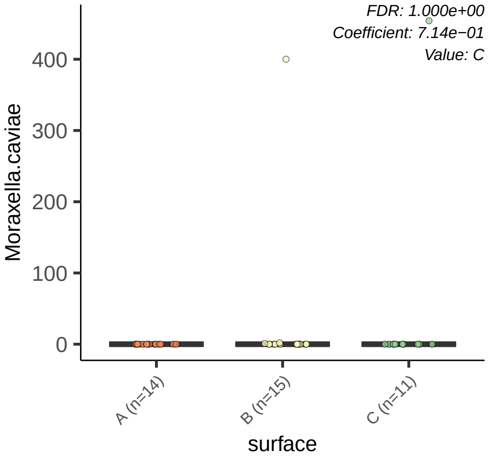
FDR: 1.000e+00
Coefficient: 7.14e−01
Value: C
Moraxella.caviae
400
300
200
100
0
A (n=14)
B (n=15)
C (n=11)
surface

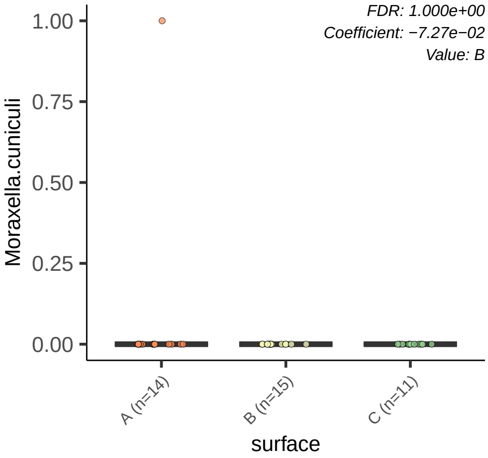

FDR: 1.000e+00
Coefficient: −7.27e−02
Value: B
Moraxella.cuniculi
1.00
0.75
0.50
0.25
0.00
A (n=14)
B (n=15)
C (n=11)
surface

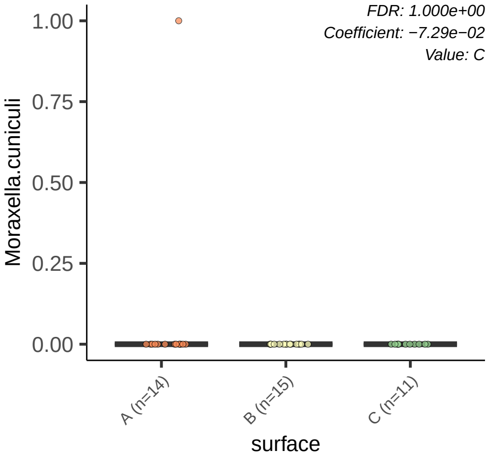
FDR: 1.000e+00
Coefficient: −7.29e−02
Value: C
Moraxella.cuniculi
1.00
0.75
0.50
0.25
0.00
A (n=14)
B (n=15)
C (n=11)
surface

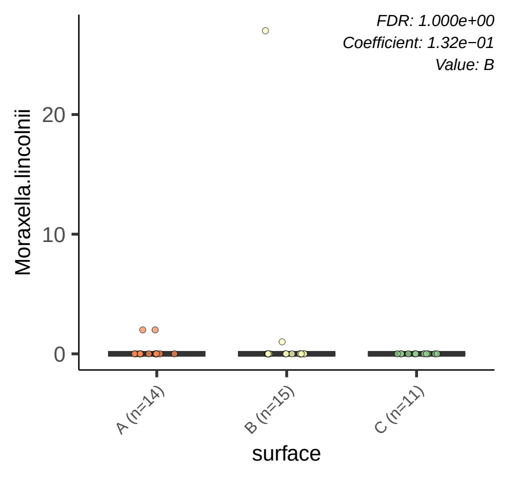

FDR: 1.000e+00
Coefficient: 1.32e−01
Value: B
Moraxella.lincolnii
20
10
0
A (n=14)
B (n=15)
C (n=11)
surface

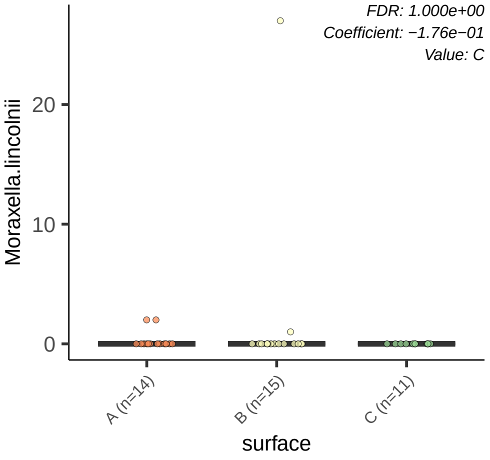
FDR: 1.000e+00
Coefficient: −1.76e−01
Value: C
Moraxella.lincolnii
20
10
0
A (n=14)
B (n=15)
C (n=11)
surface

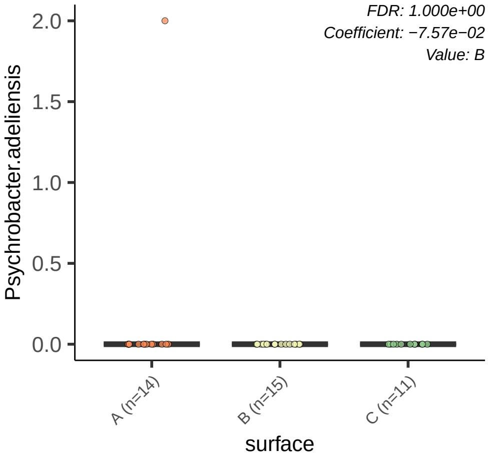

FDR: 1.000e+00
Coefficient: −7.57e−02
Value: B
Psychrobacter.adeliensis
2.0
1.5
1.0
0.5
0.0
A (n=14)
B (n=15)
C (n=11)
surface

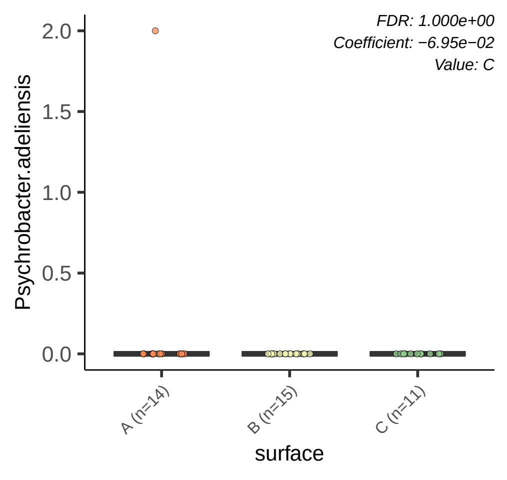
FDR: 1.000e+00
Coefficient: −6.95e−02
Value: C
Psychrobacter.adeliensis
2.0
1.5
1.0
0.5
0.0
A (n=14)
B (n=15)
C (n=11)
surface

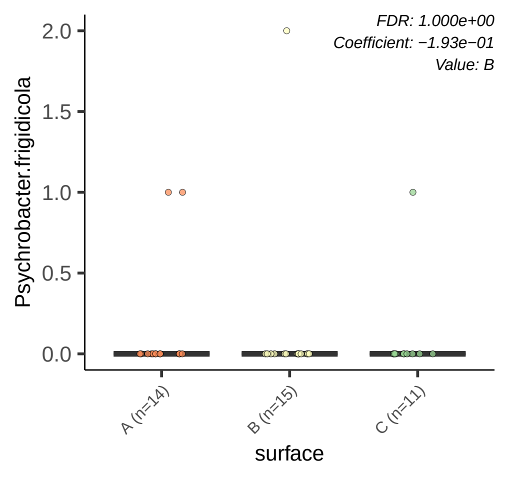

FDR: 1.000e+00
Coefficient: −1.93e−01
Value: B
Psychrobacter.frigidicola
2.0
1.5
1.0
0.5
0.0
A (n=14)
B (n=15)
C (n=11)
surface

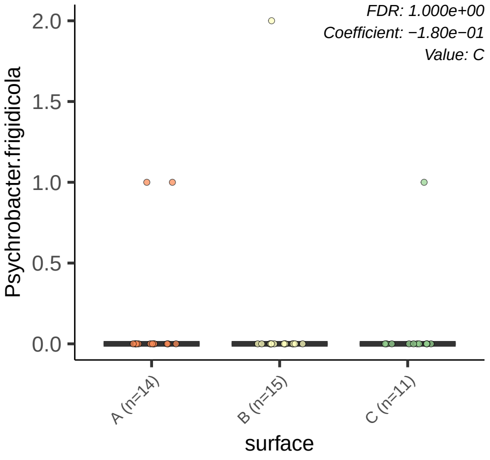
FDR: 1.000e+00
Coefficient: −1.80e−01
Value: C
Psychrobacter.frigidicola
2.0
1.5
1.0
0.5
0.0
A (n=14)
B (n=15)
C (n=11)
surface

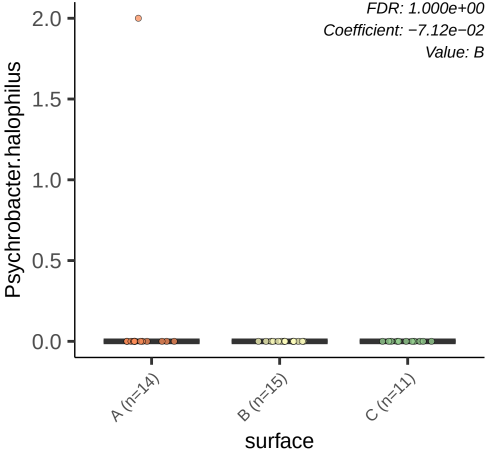
FDR: 1.000e+00
Coefficient: −7.12e−02
Value: B
Psychrobacter.halophilus
2.0
1.5
1.0
0.5
0.0
A (n=14)
B (n=15)
C (n=11)
surface

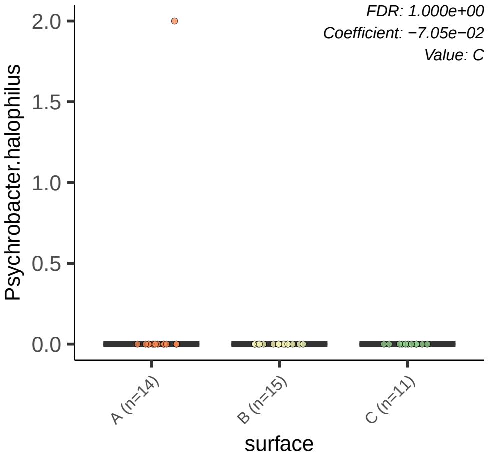
FDR: 1.000e+00
Coefficient: −7.05e−02
Value: C
Psychrobacter.halophilus
2.0
1.5
1.0
0.5
0.0
A (n=14)
B (n=15)
C (n=11)
surface

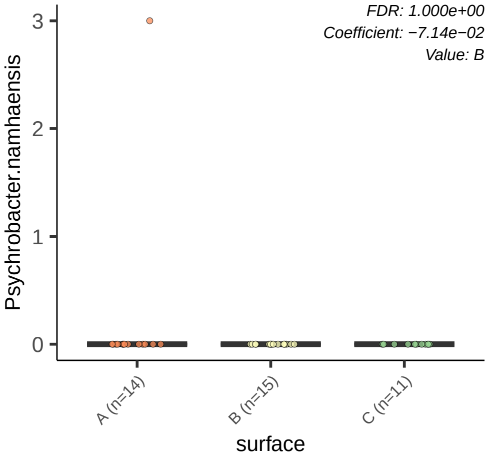
FDR: 1.000e+00
Coefficient: −7.14e−02
Value: B
Psychrobacter.namhaensis
3
2
1
0
A (n=14)
B (n=15)
C (n=11)
surface

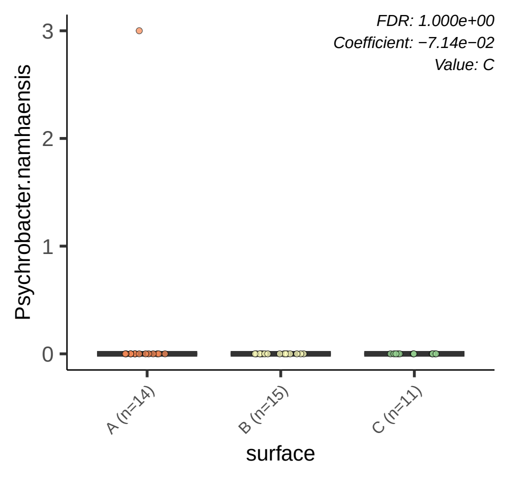
FDR: 1.000e+00
Coefficient: −7.14e−02
Value: C
Psychrobacter.namhaensis
3
2
1
0
A (n=14)
B (n=15)
C (n=11)
surface

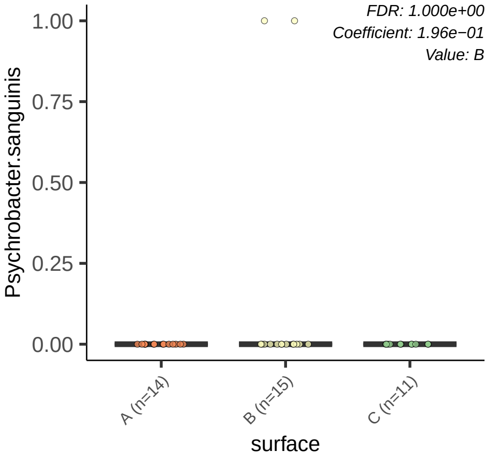
FDR: 1.000e+00
Coefficient: 1.96e−01
Value: B
Psychrobacter.sanguinis
1.00
0.75
0.50
0.25
0.00
A (n=14)
B (n=15)
C (n=11)
surface

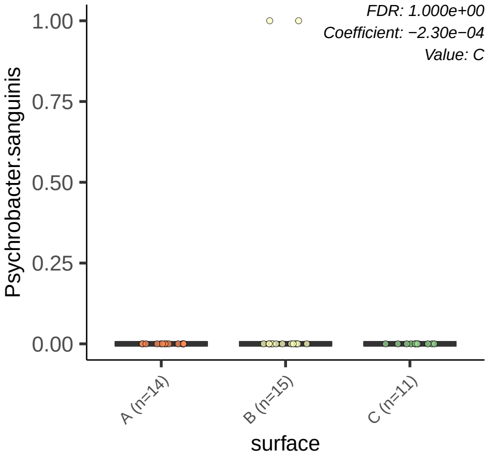

FDR: 1.000e+00
Coefficient: −2.30e−04
Value: C
Psychrobacter.sanguinis
1.00
0.75
0.50
0.25
0.00
A (n=14)
B (n=15)
C (n=11)
surface

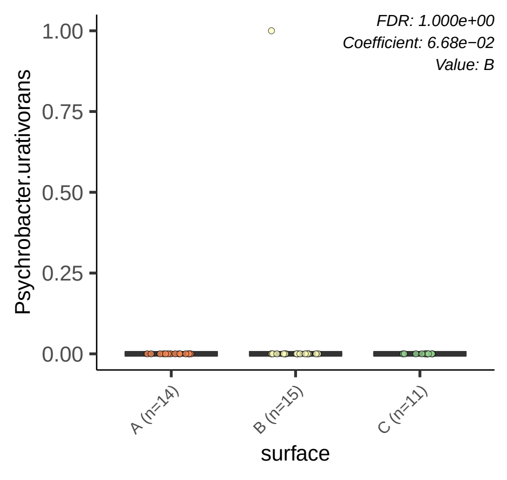
FDR: 1.000e+00
Coefficient: 6.68e−02
Value: B
Psychrobacter.urativorans
1.00
0.75
0.50
0.25
0.00
A (n=14)
B (n=15)
C (n=11)
surface

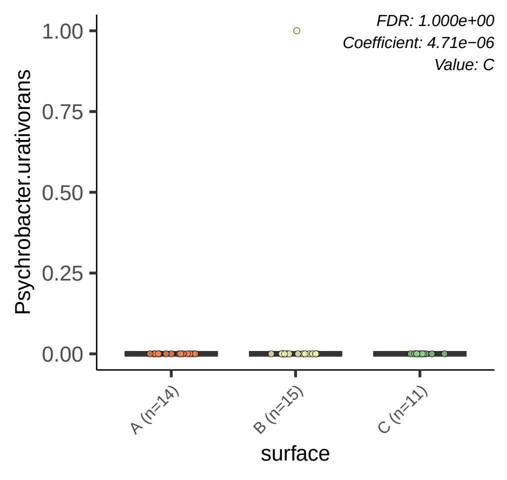
FDR: 1.000e+00
Coefficient: 4.71e−06
Value: C
Psychrobacter.urativorans
1.00
0.75
0.50
0.25
0.00
A (n=14)
B (n=15)
C (n=11)
surface

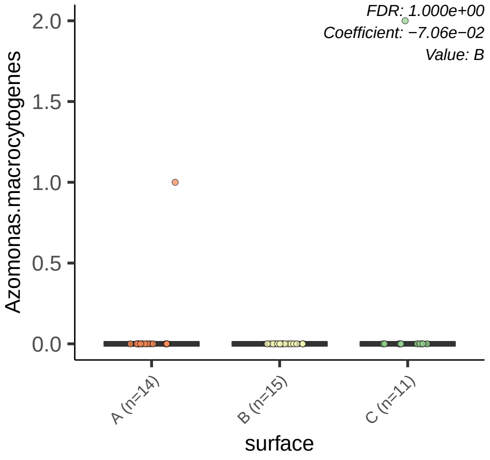
FDR: 1.000e+00
Coefficient: −7.06e−02
Value: B
Azomonas.macrocytogenes
2.0
1.5
1.0
0.5
0.0
A (n=14)
B (n=15)
C (n=11)
surface

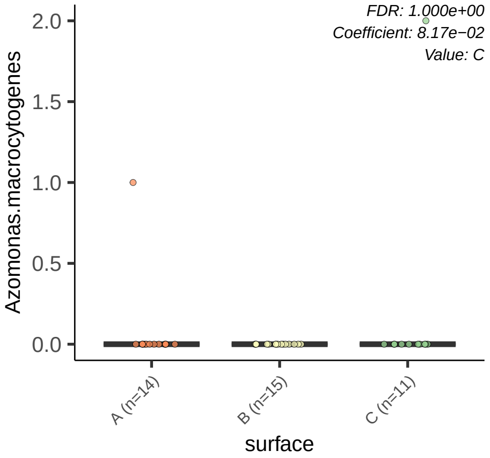
FDR: 1.000e+00
Coefficient: 8.17e−02
Value: C
Azomonas.macrocytogenes
2.0
1.5
1.0
0.5
0.0
A (n=14)
B (n=15)
C (n=11)
surface

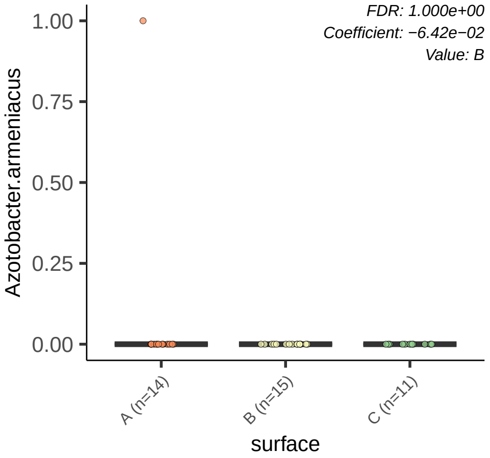

FDR: 1.000e+00
Coefficient: −6.42e−02
Value: B
Azotobacter.armeniacus
1.00
0.75
0.50
0.25
0.00
A (n=14)
B (n=15)
C (n=11)
surface

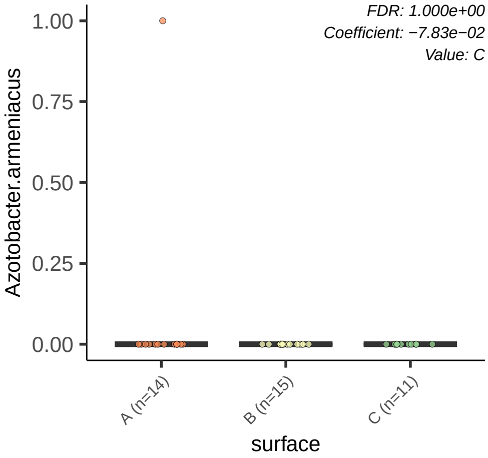
FDR: 1.000e+00
Coefficient: −7.83e−02
Value: C
Azotobacter.armeniacus
1.00
0.75
0.50
0.25
0.00
A (n=14)
B (n=15)
C (n=11)
surface

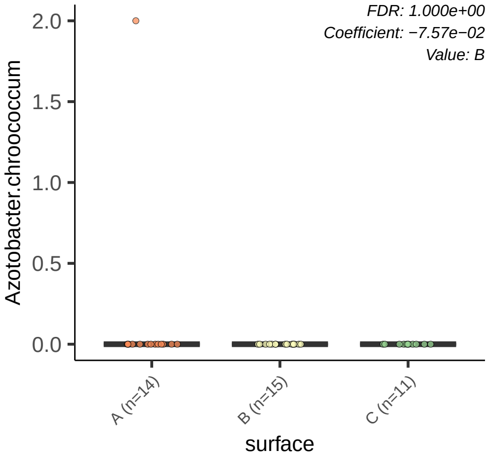

FDR: 1.000e+00
Coefficient: −7.57e−02
Value: B
Azotobacter.chroococcum
2.0
1.5
1.0
0.5
0.0
A (n=14)
B (n=15)
C (n=11)
surface

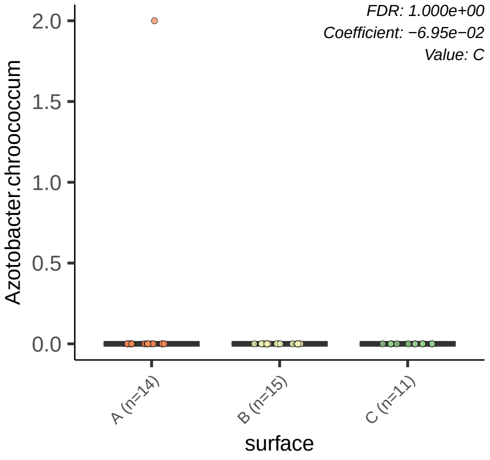
FDR: 1.000e+00
Coefficient: −6.95e−02
Value: C
Azotobacter.chroococcum
2.0
1.5
1.0
0.5
0.0
A (n=14)
B (n=15)
C (n=11)
surface

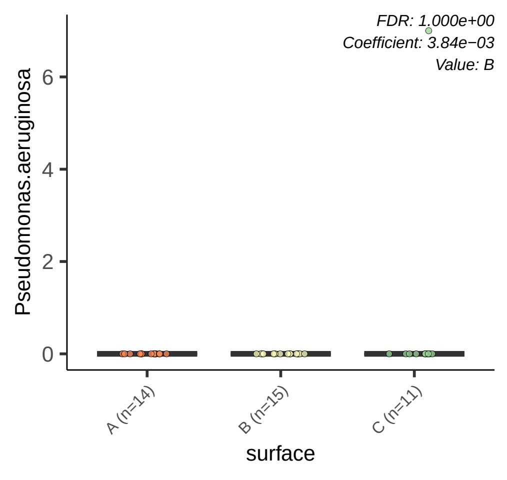
FDR: 1.000e+00
Coefficient: 3.84e−03
Value: B
Pseudomonas.aeruginosa
0
2
4
6
A (n=14)
B (n=15)
C (n=11)
surface

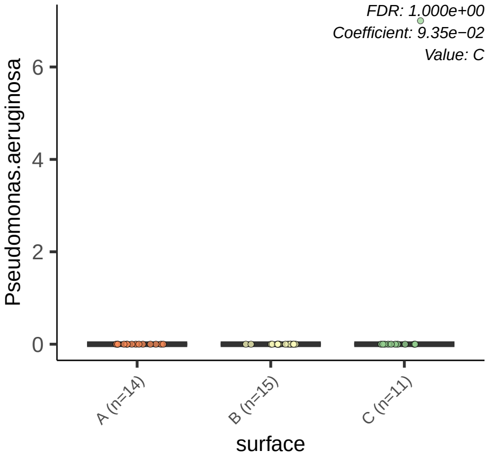
FDR: 1.000e+00
Coefficient: 9.35e−02
Value: C
Pseudomonas.aeruginosa
6
4
2
0
A (n=14)
B (n=15)
C (n=11)
surface

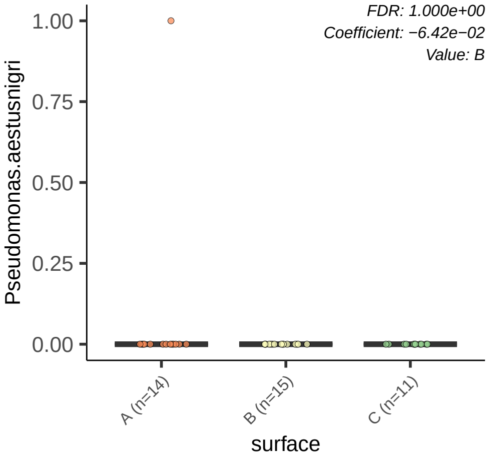
FDR: 1.000e+00
Coefficient: −6.42e−02
Value: B
Pseudomonas.aestusnigri
1.00
0.75
0.50
0.25
0.00
A (n=14)
B (n=15)
C (n=11)
surface

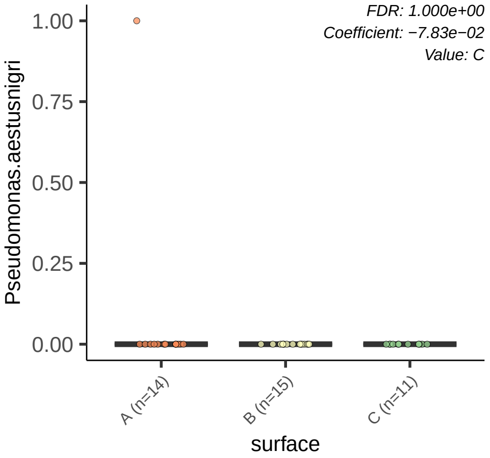

FDR: 1.000e+00
Coefficient: −7.83e−02
Value: C
Pseudomonas.aestusnigri
1.00
0.75
0.50
0.25
0.00
A (n=14)
B (n=15)
C (n=11)
surface

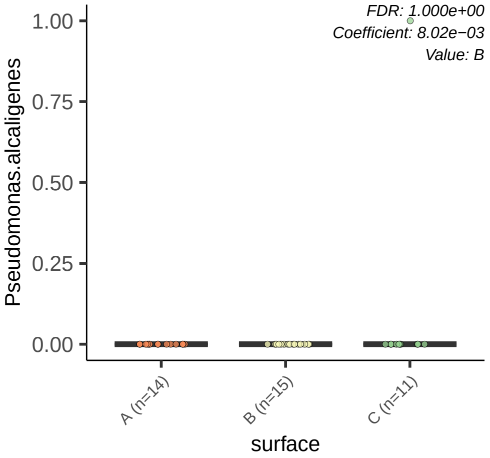
FDR: 1.000e+00
Coefficient: 8.02e−03
Value: B
Pseudomonas.alcaligenes
1.00
0.75
0.50
0.25
0.00
A (n=14)
B (n=15)
C (n=11)
surface

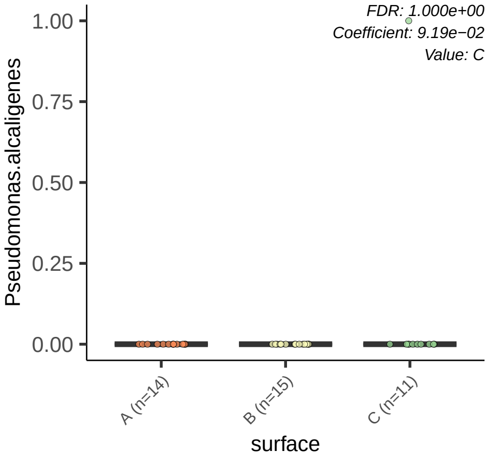
FDR: 1.000e+00
Coefficient: 9.19e−02
Value: C
Pseudomonas.alcaligenes
1.00
0.75
0.50
0.25
0.00
A (n=14)
B (n=15)
C (n=11)
surface

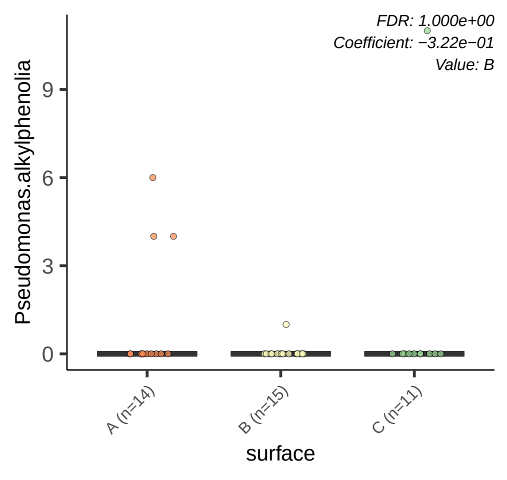

FDR: 1.000e+00
Coefficient: −3.22e−01
Value: B
Pseudomonas.alkylphenolia
0
3
6
9
A (n=14)
B (n=15)
C (n=11)
surface

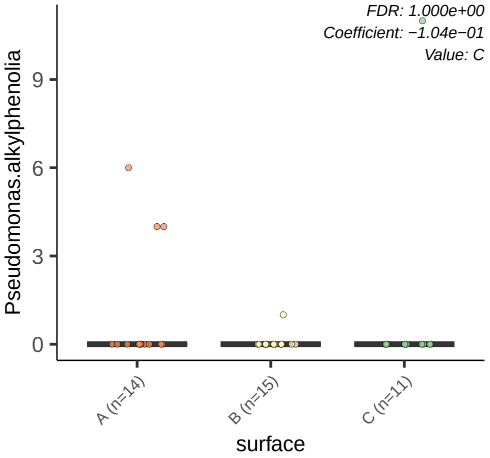

FDR: 1.000e+00
Coefficient: −1.04e−01
Value: C
Pseudomonas.alkylphenolia
9
6
3
0
A (n=14)
B (n=15)
C (n=11)
surface

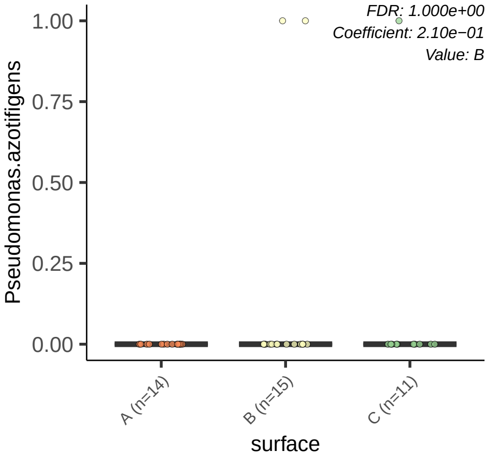

FDR: 1.000e+00
Coefficient: 2.10e−01
Value: B
Pseudomonas.azotifigens
1.00
0.75
0.50
0.25
0.00
A (n=14)
B (n=15)
C (n=11)
surface

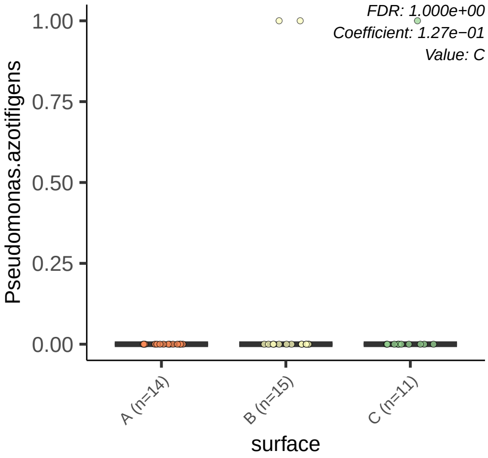

FDR: 1.000e+00
Coefficient: 1.27e−01
Value: C
Pseudomonas.azotifigens
1.00
0.75
0.50
0.25
0.00
A (n=14)
B (n=15)
C (n=11)
surface

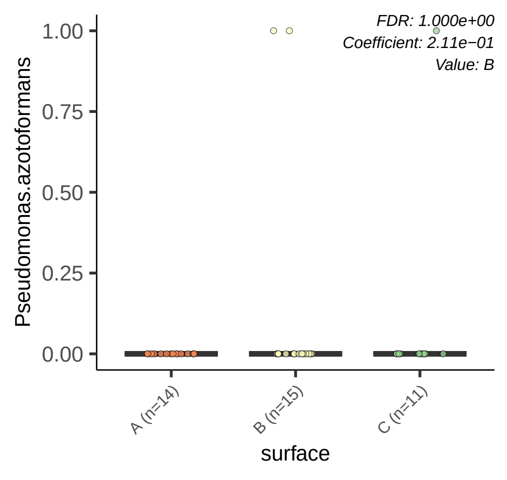
FDR: 1.000e+00
Coefficient: 2.11e−01
Value: B
Pseudomonas.azotoformans
1.00
0.75
0.50
0.25
0.00
A (n=14)
B (n=15)
C (n=11)
surface

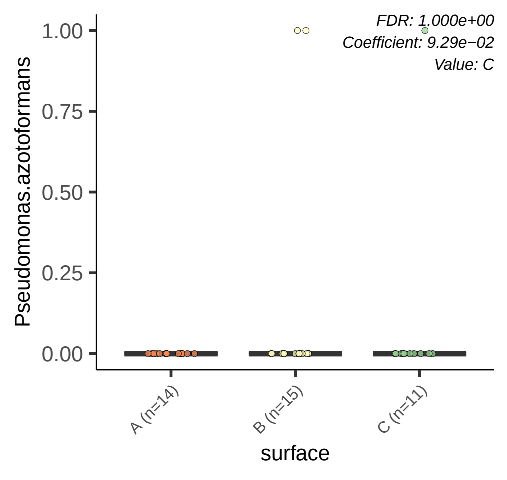
FDR: 1.000e+00
Coefficient: 9.29e−02
Value: C
Pseudomonas.azotoformans
1.00
0.75
0.50
0.25
0.00
A (n=14)
B (n=15)
C (n=11)
surface

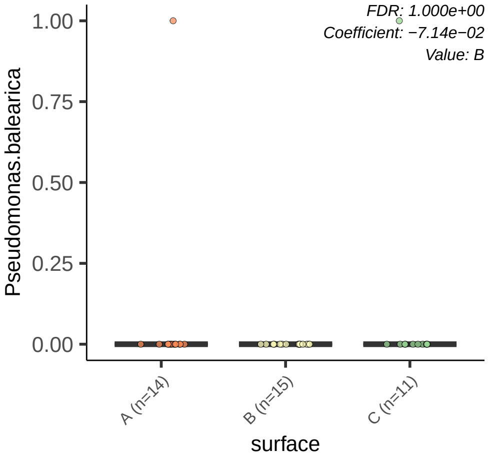
FDR: 1.000e+00
Coefficient: −7.14e−02
Value: B
Pseudomonas.balearica
1.00
0.75
0.50
0.25
0.00
A (n=14)
B (n=15)
C (n=11)
surface

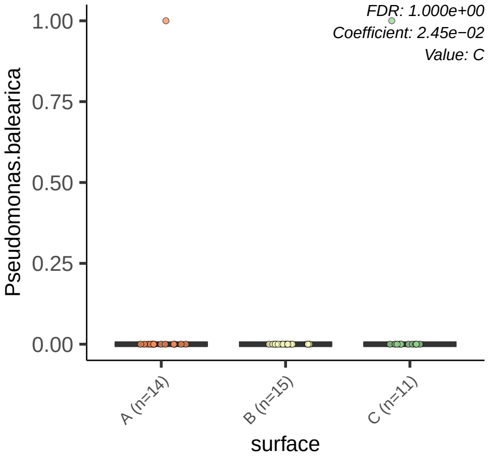

FDR: 1.000e+00
Coefficient: 2.45e−02
Value: C
Pseudomonas.balearica
1.00
0.75
0.50
0.25
0.00
A (n=14)
B (n=15)
C (n=11)
surface

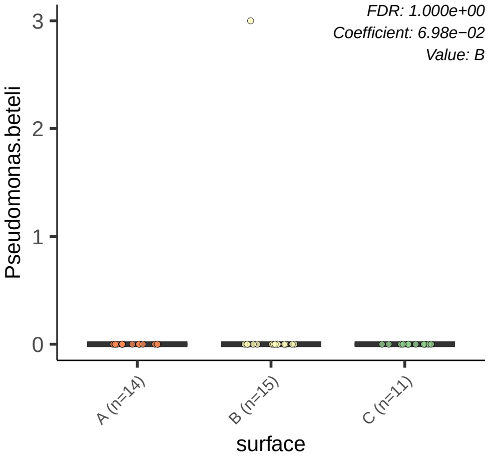
FDR: 1.000e+00
Coefficient: 6.98e−02
Value: B
Pseudomonas.beteli
3
2
1
0
A (n=14)
B (n=15)
C (n=11)
surface

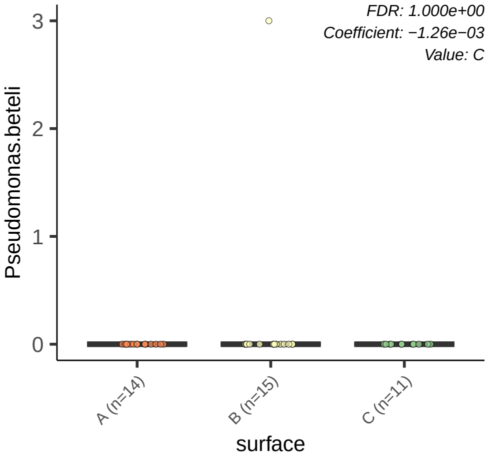
FDR: 1.000e+00
Coefficient: −1.26e−03
Value: C
Pseudomonas.beteli
3
2
1
0
A (n=14)
B (n=15)
C (n=11)
surface

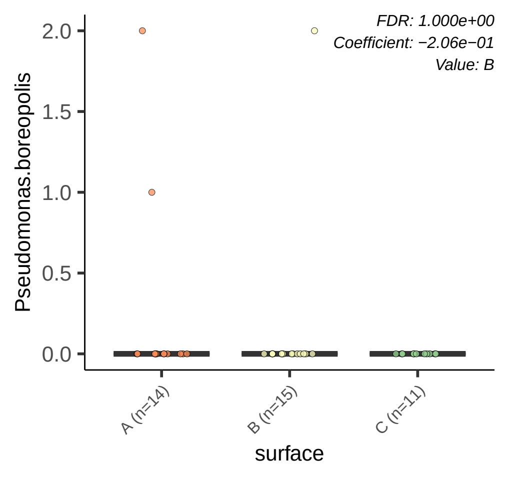
FDR: 1.000e+00
Coefficient: −2.06e−01
Value: B
Pseudomonas.boreopolis
2.0
1.5
1.0
0.5
0.0
A (n=14)
B (n=15)
C (n=11)
surface

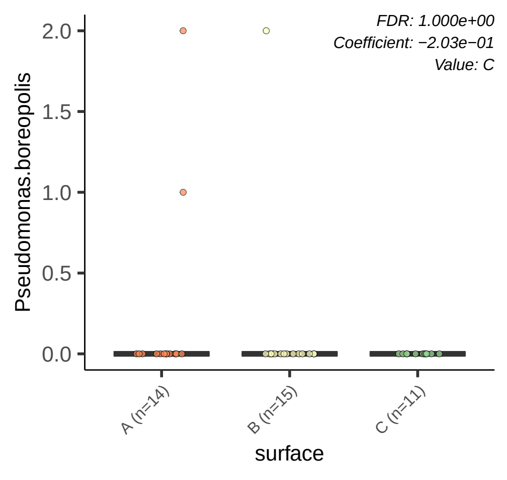

FDR: 1.000e+00
Coefficient: −2.03e−01
Value: C
Pseudomonas.boreopolis
2.0
1.5
1.0
0.5
0.0
A (n=14)
B (n=15)
C (n=11)
surface

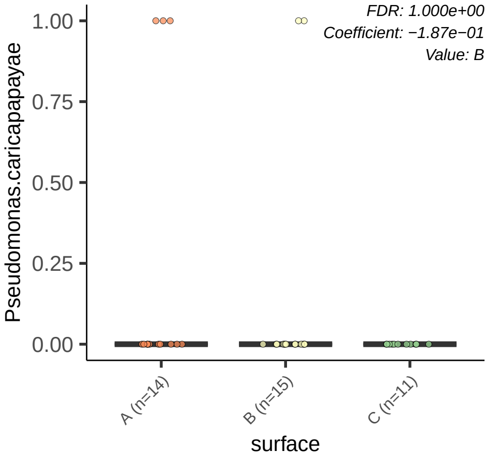
FDR: 1.000e+00
Coefficient: −1.87e−01
Value: B
Pseudomonas.caricapapayae
1.00
0.75
0.50
0.25
0.00
A (n=14)
B (n=15)
C (n=11)
surface

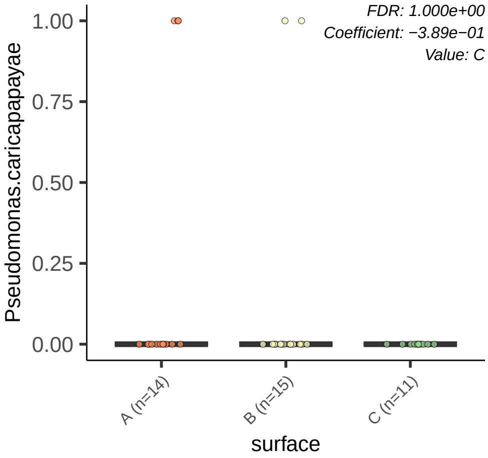
FDR: 1.000e+00
Coefficient: −3.89e−01
Value: C
Pseudomonas.caricapapayae
1.00
0.75
0.50
0.25
0.00
A (n=14)
B (n=15)
C (n=11)
surface

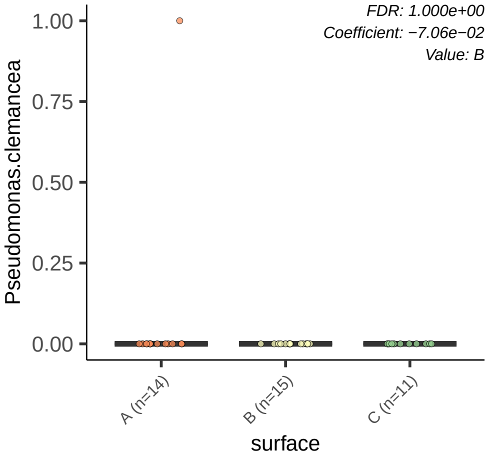

FDR: 1.000e+00
Coefficient: −7.06e−02
Value: B
Pseudomonas.clemancea
1.00
0.75
0.50
0.25
0.00
A (n=14)
B (n=15)
C (n=11)
surface

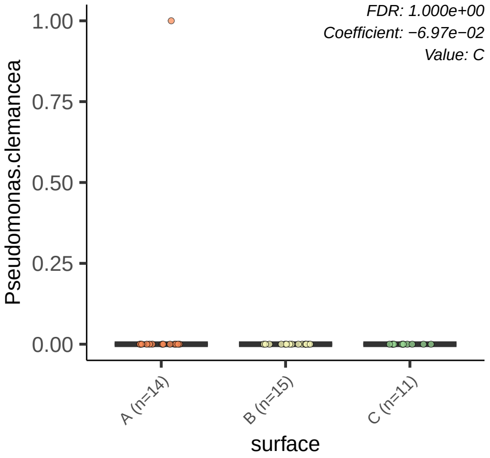
FDR: 1.000e+00
Coefficient: −6.97e−02
Value: C
Pseudomonas.clemancea
1.00
0.75
0.50
0.25
0.00
A (n=14)
B (n=15)
C (n=11)
surface

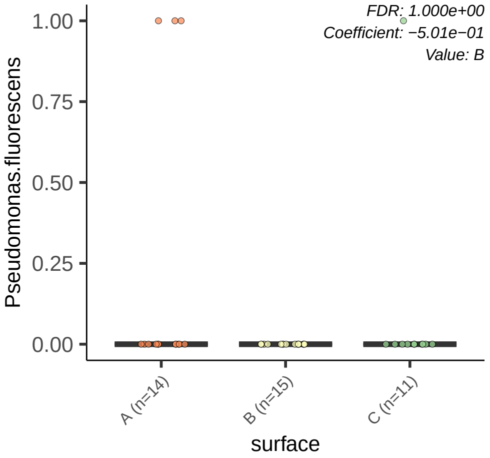

FDR: 1.000e+00
Coefficient: −5.01e−01
Value: B
Pseudomonas.fluorescens
1.00
0.75
0.50
0.25
0.00
A (n=14)
B (n=15)
C (n=11)
surface

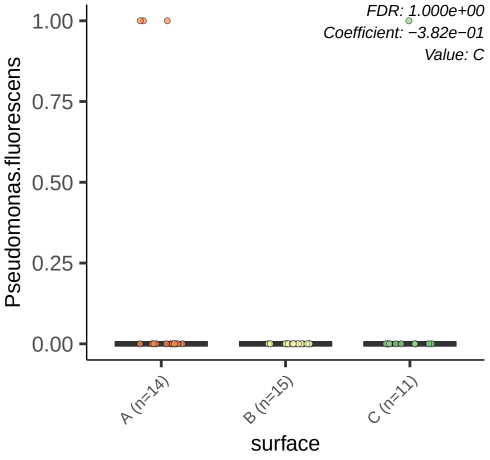

FDR: 1.000e+00
Coefficient: −3.82e−01
Value: C
Pseudomonas.fluorescens
1.00
0.75
0.50
0.25
0.00
A (n=14)
B (n=15)
C (n=11)
surface

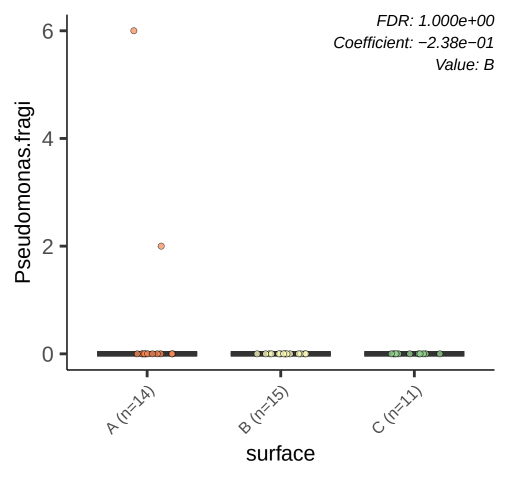
FDR: 1.000e+00
Coefficient: −2.38e−01
Value: B
Pseudomonas.fragi
6
4
2
0
A (n=14)
B (n=15)
C (n=11)
surface

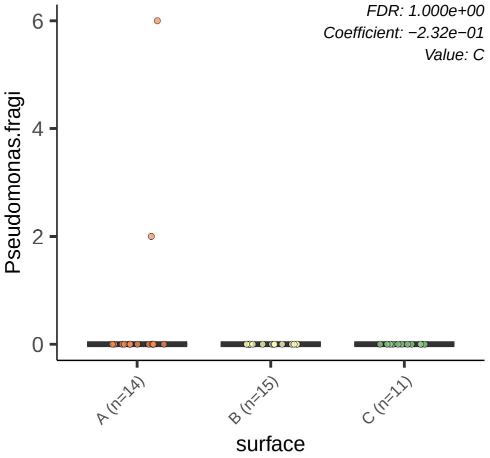
FDR: 1.000e+00
Coefficient: −2.32e−01
Value: C
Pseudomonas.fragi
6
4
2
0
A (n=14)
B (n=15)
C (n=11)
surface

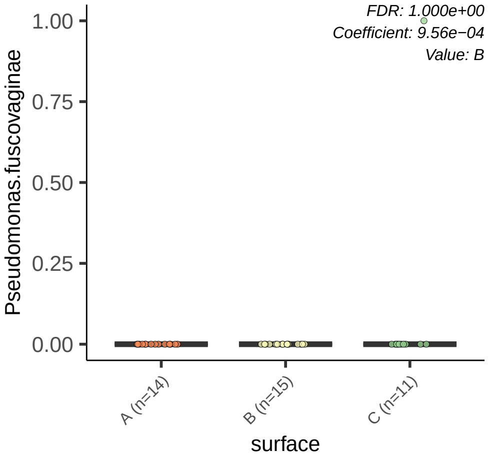

FDR: 1.000e+00
Coefficient: 9.56e−04
Value: B
Pseudomonas.fuscovaginae
1.00
0.75
0.50
0.25
0.00
A (n=14)
B (n=15)
C (n=11)
surface

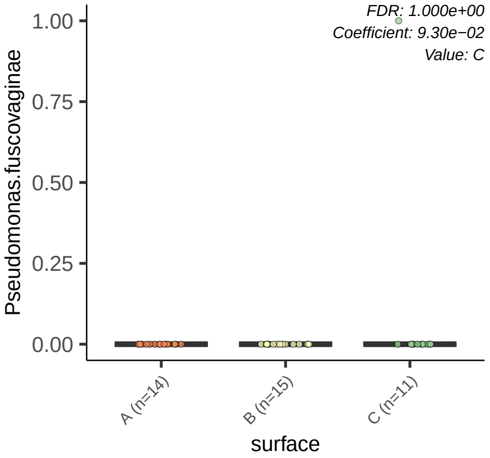
FDR: 1.000e+00
Coefficient: 9.30e−02
Value: C
Pseudomonas.fuscovaginae
1.00
0.75
0.50
0.25
0.00
A (n=14)
B (n=15)
C (n=11)
surface

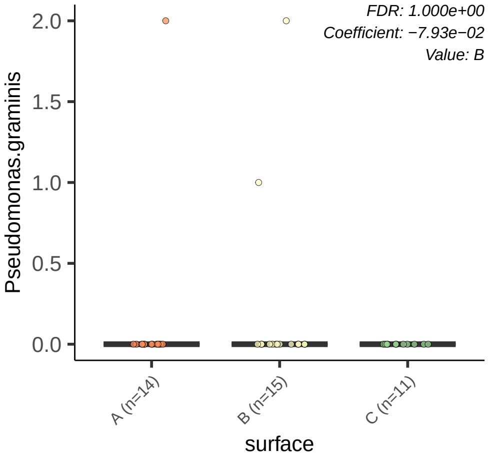
FDR: 1.000e+00
Coefficient: −7.93e−02
Value: B
Pseudomonas.graminis
2.0
1.5
1.0
0.5
0.0
A (n=14)
B (n=15)
C (n=11)
surface

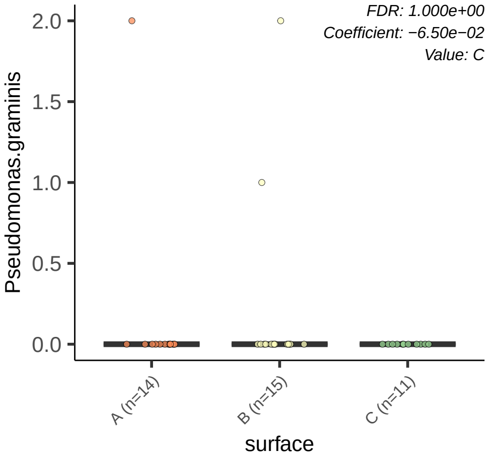
FDR: 1.000e+00
Coefficient: −6.50e−02
Value: C
Pseudomonas.graminis
2.0
1.5
1.0
0.5
0.0
A (n=14)
B (n=15)
C (n=11)
surface

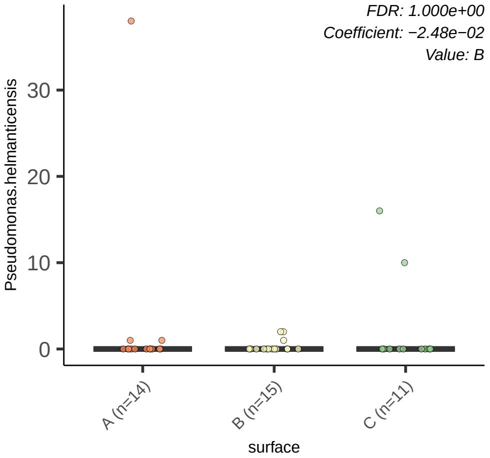
FDR: 1.000e+00
Coefficient: −2.48e−02
Value: B
Pseudomonas.helmanticensis
30
20
10
0
A (n=14)
B (n=15)
C (n=11)
surface

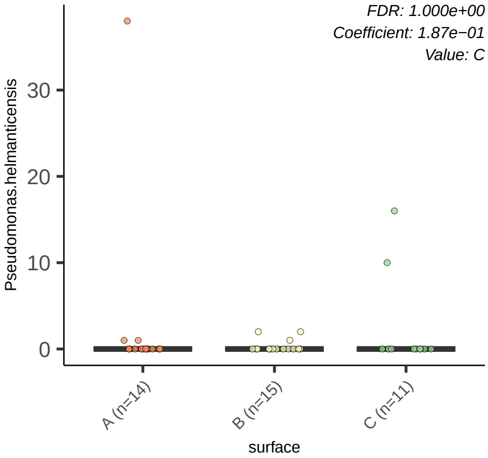
FDR: 1.000e+00
Coefficient: 1.87e−01
Value: C
Pseudomonas.helmanticensis
30
20
10
0
A (n=14)
B (n=15)
C (n=11)
surface

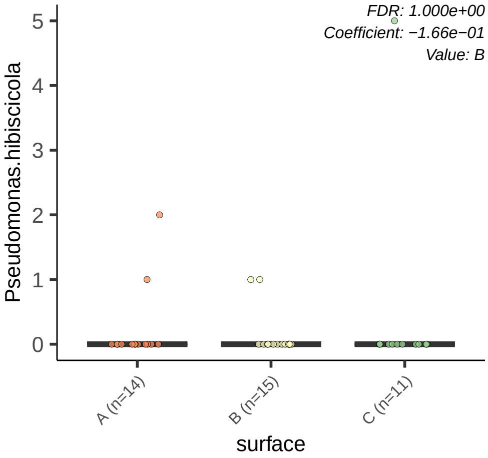
FDR: 1.000e+00
Coefficient: −1.66e−01
Value: B
Pseudomonas.hibiscicola
0
1
2
3
4
5
A (n=14)
B (n=15)
C (n=11)
surface

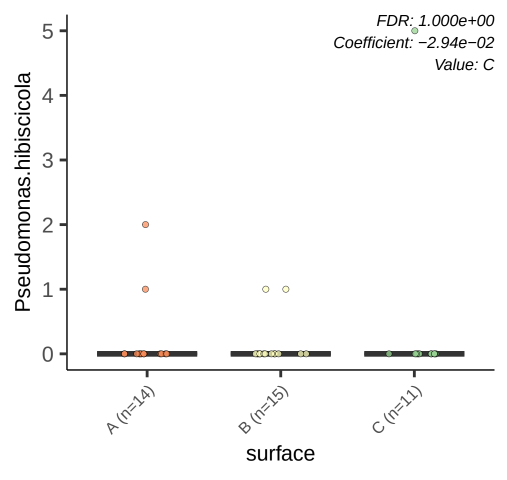
FDR: 1.000e+00
Coefficient: −2.94e−02
Value: C
Pseudomonas.hibiscicola
5
4
3
2
1
0
A (n=14)
B (n=15)
C (n=11)
surface

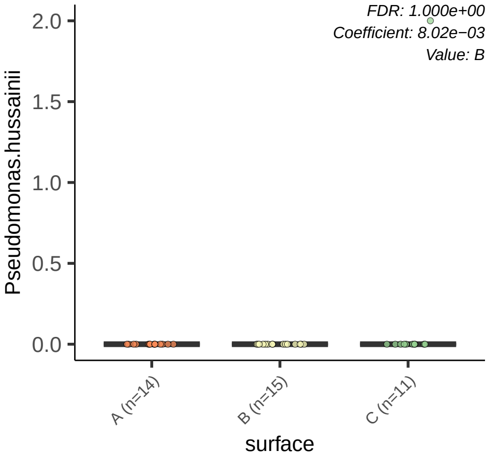

FDR: 1.000e+00
Coefficient: 8.02e−03
Value: B
Pseudomonas.hussainii
2.0
1.5
1.0
0.5
0.0
A (n=14)
B (n=15)
C (n=11)
surface

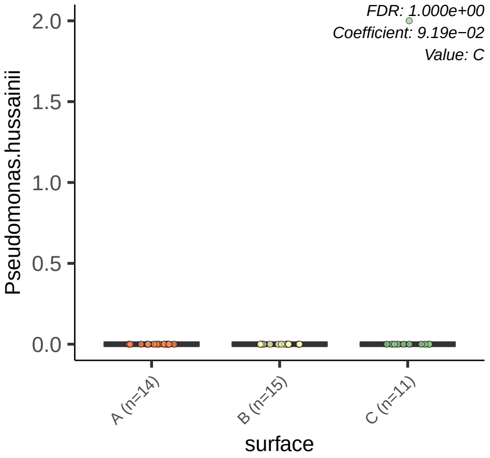

FDR: 1.000e+00
Coefficient: 9.19e−02
Value: C
Pseudomonas.hussainii
2.0
1.5
1.0
0.5
0.0
A (n=14)
B (n=15)
C (n=11)
surface

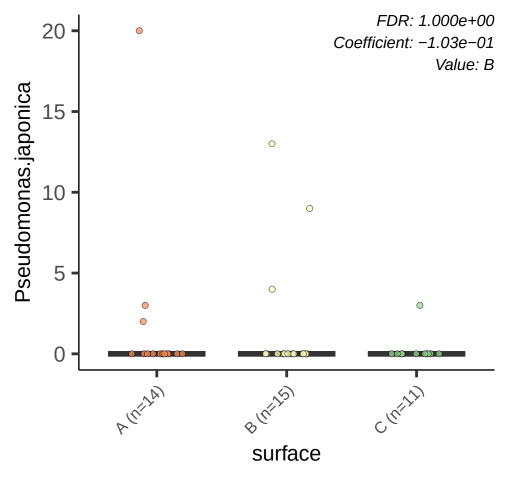
FDR: 1.000e+00
Coefficient: −1.03e−01
Value: B
Pseudomonas.japonica
20
15
10
5
0
A (n=14)
B (n=15)
C (n=11)
surface

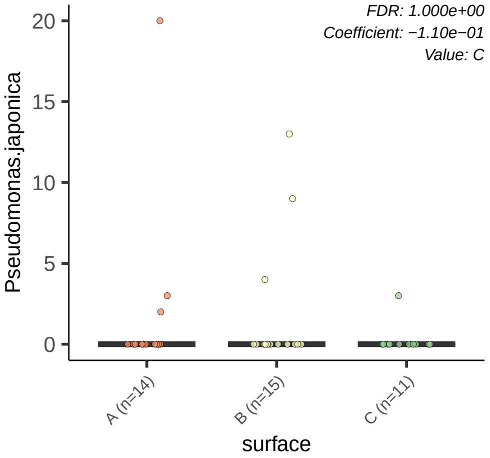
FDR: 1.000e+00
Coefficient: −1.10e−01
Value: C
Pseudomonas.japonica
0
5
10
15
20
A (n=14)
B (n=15)
C (n=11)
surface

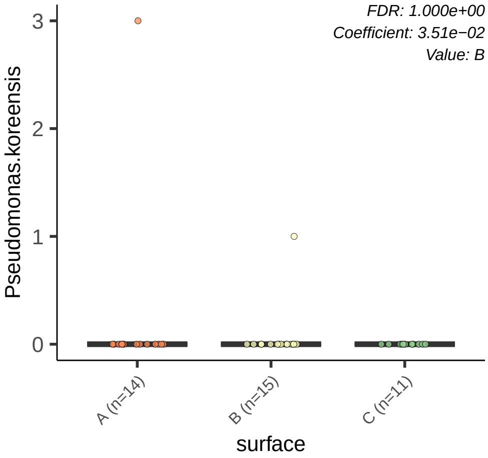
FDR: 1.000e+00
Coefficient: 3.51e−02
Value: B
Pseudomonas.koreensis
3
2
1
0
A (n=14)
B (n=15)
C (n=11)
surface

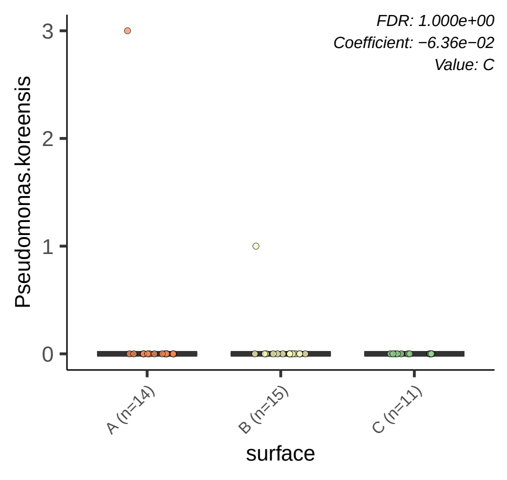

FDR: 1.000e+00
Coefficient: −6.36e−02
Value: C
Pseudomonas.koreensis
3
2
1
0
A (n=14)
B (n=15)
C (n=11)
surface

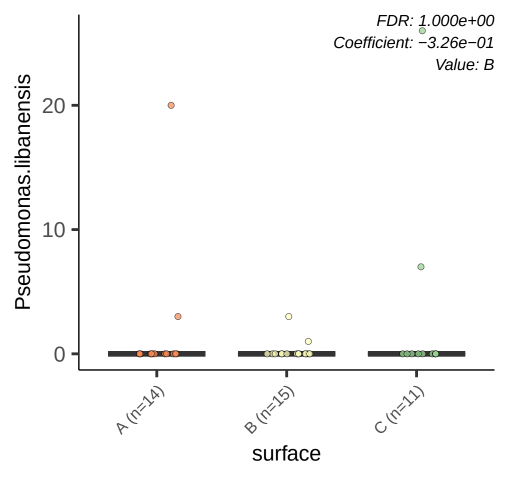
FDR: 1.000e+00
Coefficient: −3.26e−01
Value: B
Pseudomonas.libanensis
20
10
0
A (n=14)
B (n=15)
C (n=11)
surface

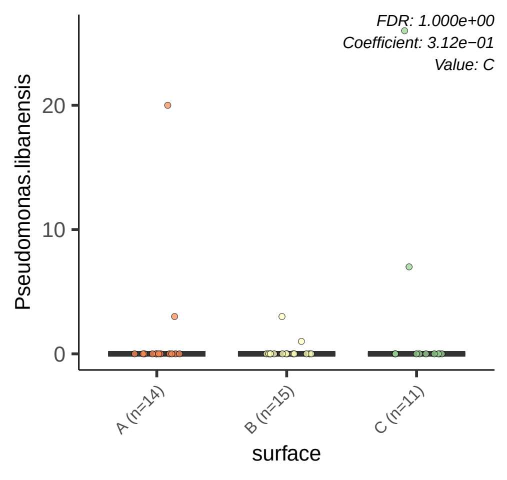
FDR: 1.000e+00
Coefficient: 3.12e−01
Value: C
Pseudomonas.libanensis
20
10
0
A (n=14)
B (n=15)
C (n=11)
surface

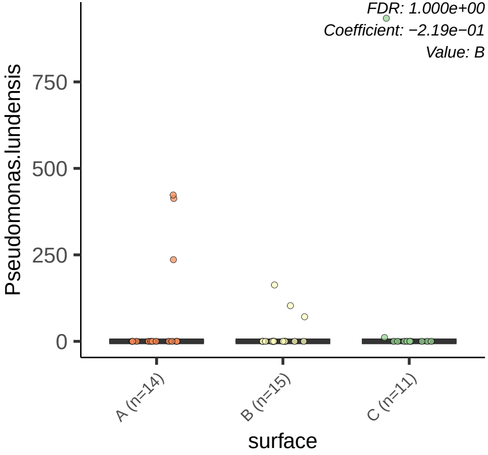
FDR: 1.000e+00
Coefficient: −2.19e−01
Value: B
Pseudomonas.lundensis
750
500
250
0
A (n=14)
B (n=15)
C (n=11)
surface

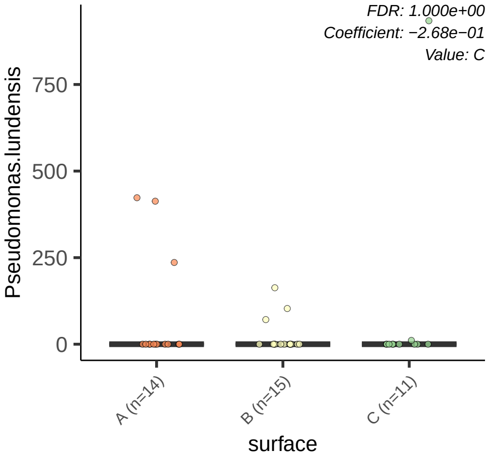

FDR: 1.000e+00
Coefficient: −2.68e−01
Value: C
Pseudomonas.lundensis
750
500
250
0
A (n=14)
B (n=15)
C (n=11)
surface

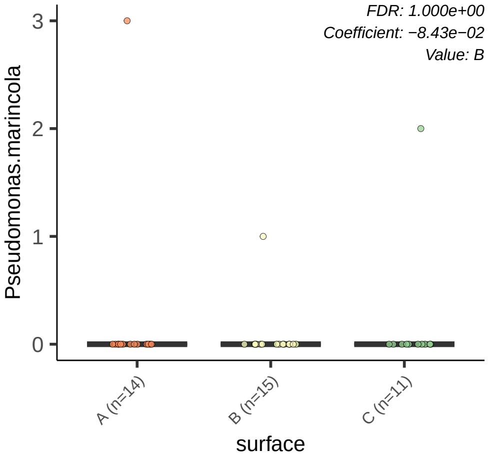
FDR: 1.000e+00
Coefficient: −8.43e−02
Value: B
Pseudomonas.marincola
3
2
1
0
A (n=14)
B (n=15)
C (n=11)
surface

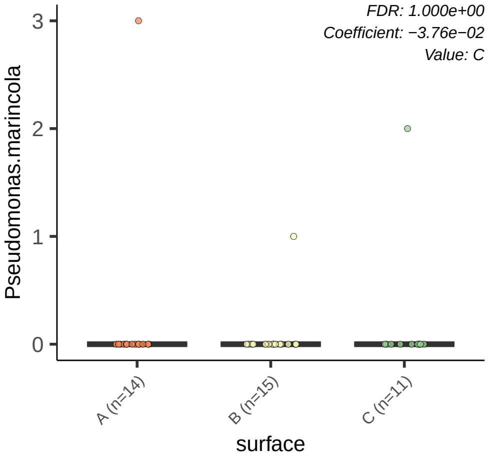
FDR: 1.000e+00
Coefficient: −3.76e−02
Value: C
Pseudomonas.marincola
0
1
2
3
A (n=14)
B (n=15)
C (n=11)
surface

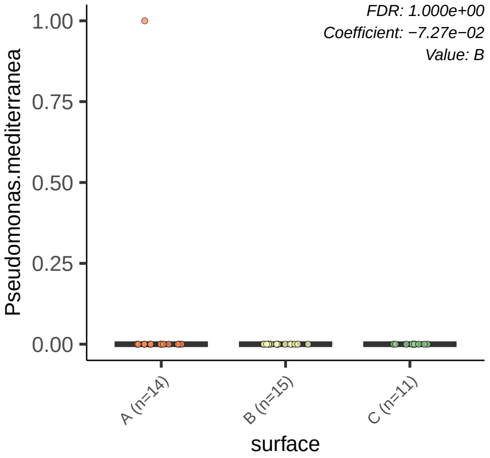
FDR: 1.000e+00
Coefficient: −7.27e−02
Value: B
Pseudomonas.mediterranea
1.00
0.75
0.50
0.25
0.00
A (n=14)
B (n=15)
C (n=11)
surface

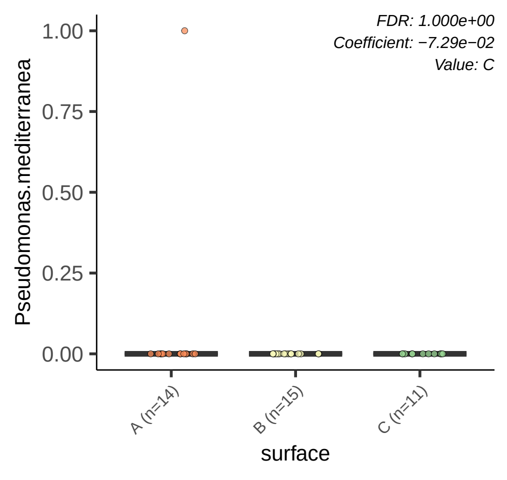

FDR: 1.000e+00
Coefficient: −7.29e−02
Value: C
Pseudomonas.mediterranea
1.00
0.75
0.50
0.25
0.00
A (n=14)
B (n=15)
C (n=11)
surface

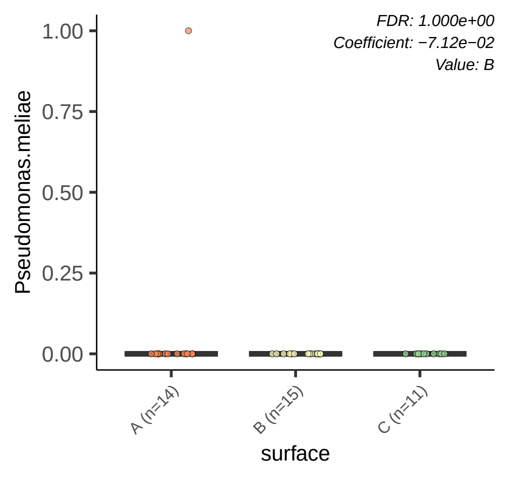
FDR: 1.000e+00
Coefficient: −7.12e−02
Value: B
Pseudomonas.meliae
1.00
0.75
0.50
0.25
0.00
A (n=14)
B (n=15)
C (n=11)
surface

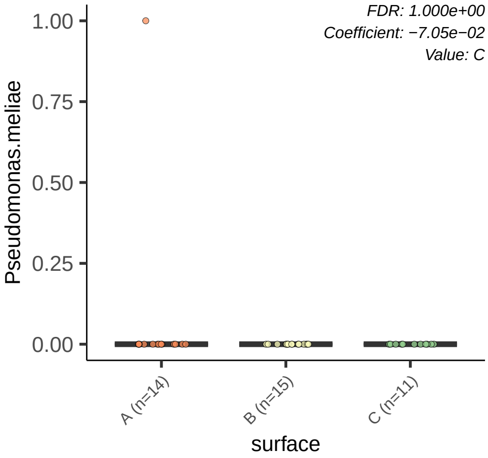

FDR: 1.000e+00
Coefficient: −7.05e−02
Value: C
1.00
0.75
0.50
0.25
0.00
Pseudomonas.meliae
A (n=14)
B (n=15)
C (n=11)
surface

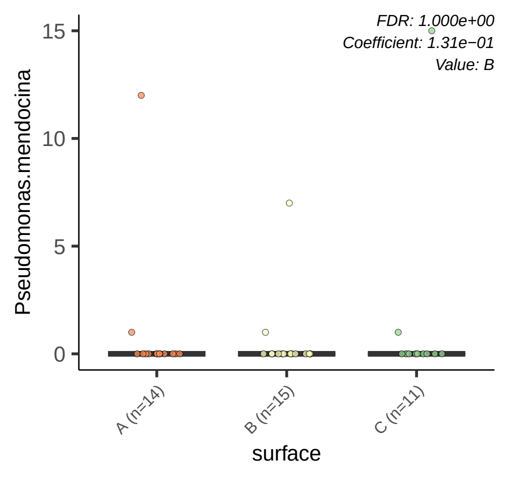
FDR: 1.000e+00
Coefficient: 1.31e−01
Value: B
Pseudomonas.mendocina
15
10
5
0
A (n=14)
B (n=15)
C (n=11)
surface

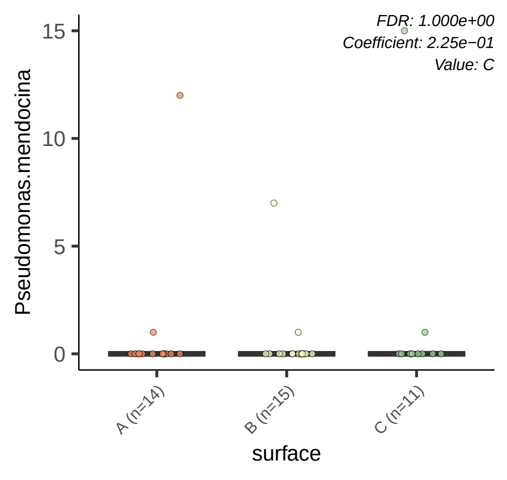
FDR: 1.000e+00
Coefficient: 2.25e−01
Value: C
Pseudomonas.mendocina
15
10
5
0
A (n=14)
B (n=15)
C (n=11)
surface

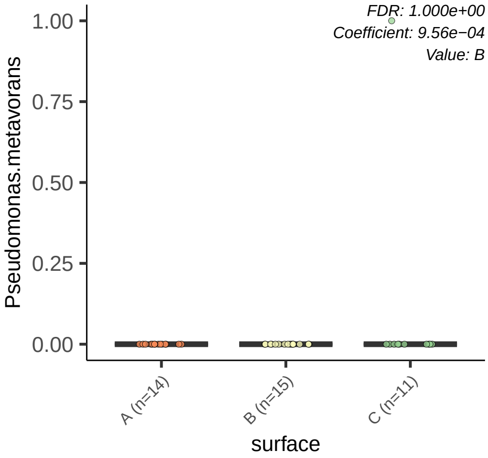
FDR: 1.000e+00
Coefficient: 9.56e−04
Value: B
Pseudomonas.metavorans
1.00
0.75
0.50
0.25
0.00
A (n=14)
B (n=15)
C (n=11)
surface

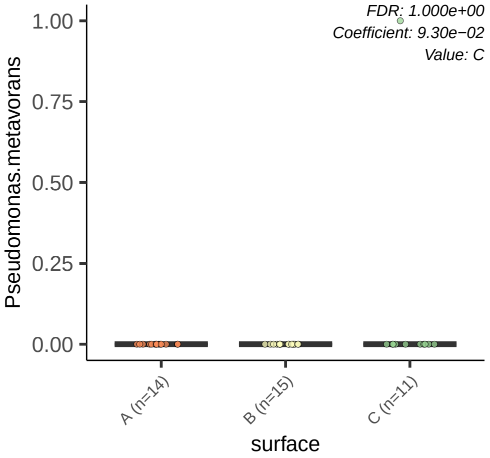
FDR: 1.000e+00
Coefficient: 9.30e−02
Value: C
Pseudomonas.metavorans
1.00
0.75
0.50
0.25
0.00
A (n=14)
B (n=15)
C (n=11)
surface

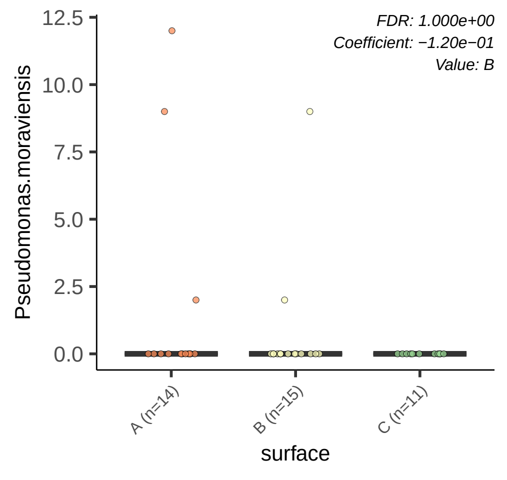

FDR: 1.000e+00
Coefficient: −1.20e−01
Value: B
Pseudomonas.moraviensis
12.5
10.0
7.5
5.0
2.5
0.0
A (n=14)
B (n=15)
C (n=11)
surface

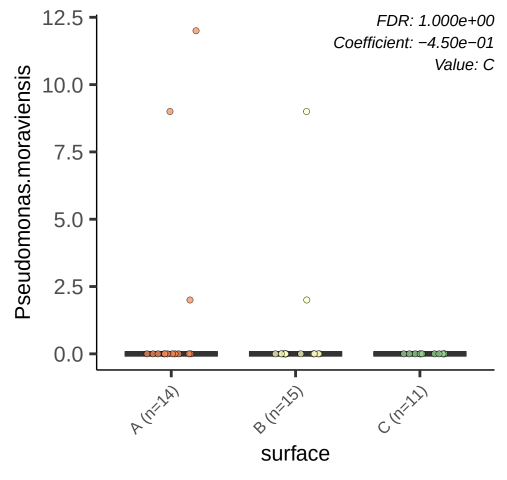
FDR: 1.000e+00
Coefficient: −4.50e−01
Value: C
Pseudomonas.moraviensis
12.5
10.0
7.5
5.0
2.5
0.0
A (n=14)
B (n=15)
C (n=11)
surface

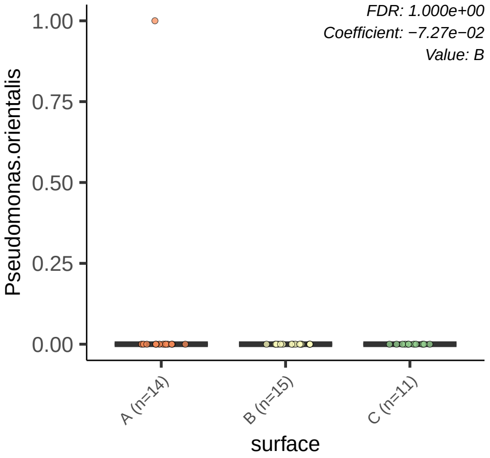
FDR: 1.000e+00
Coefficient: −7.27e−02
Value: B
Pseudomonas.orientalis
1.00
0.75
0.50
0.25
0.00
A (n=14)
B (n=15)
C (n=11)
surface

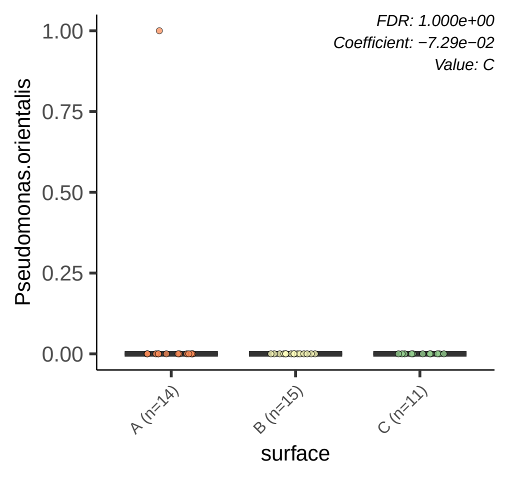
FDR: 1.000e+00
Coefficient: −7.29e−02
Value: C
Pseudomonas.orientalis
1.00
0.75
0.50
0.25
0.00
A (n=14)
B (n=15)
C (n=11)
surface

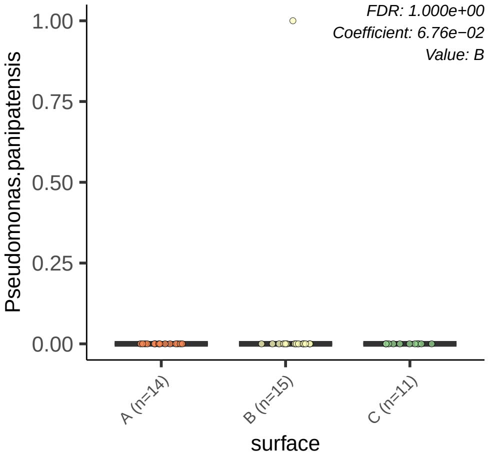

FDR: 1.000e+00
Coefficient: 6.76e−02
Value: B
Pseudomonas.panipatensis
1.00
0.75
0.50
0.25
0.00
A (n=14)
B (n=15)
C (n=11)
surface

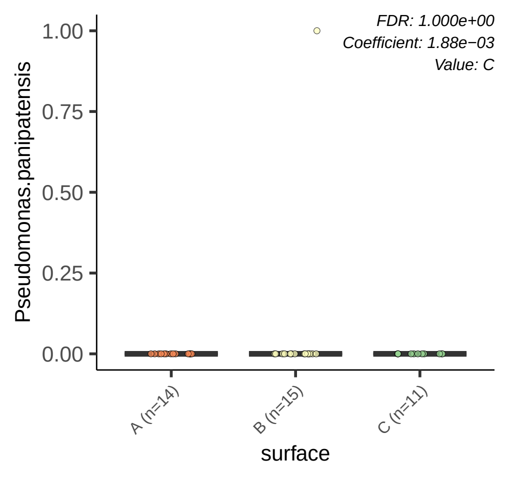

FDR: 1.000e+00
Coefficient: 1.88e−03
Value: C
Pseudomonas.panipatensis
1.00
0.75
0.50
0.25
0.00
A (n=14)
B (n=15)
C (n=11)
surface

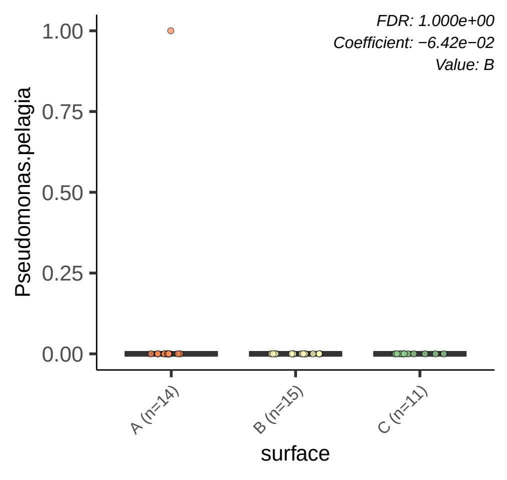

FDR: 1.000e+00
Coefficient: −6.42e−02
Value: B
Pseudomonas.pelagia
1.00
0.75
0.50
0.25
0.00
A (n=14)
B (n=15)
C (n=11)
surface

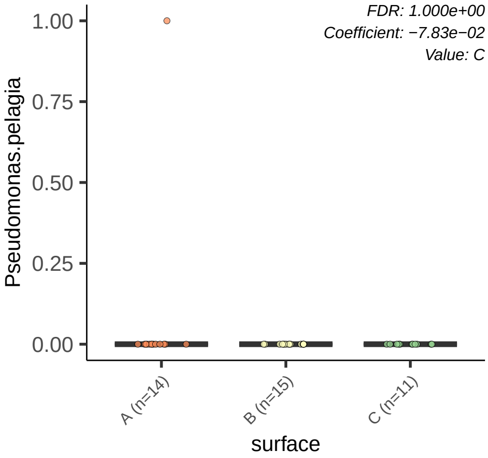
FDR: 1.000e+00
Coefficient: −7.83e−02
Value: C
Pseudomonas.pelagia
1.00
0.75
0.50
0.25
0.00
A (n=14)
B (n=15)
C (n=11)
surface

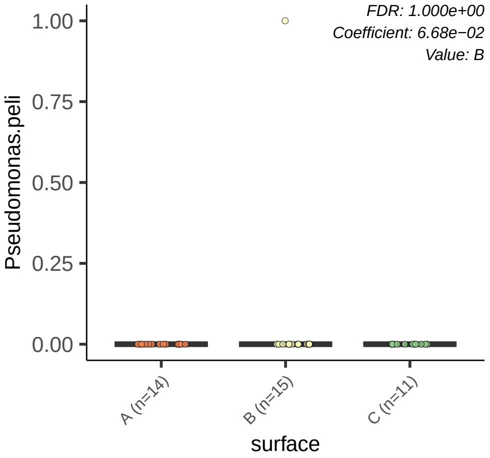
FDR: 1.000e+00
Coefficient: 6.68e−02
Value: B
Pseudomonas.peli
1.00
0.75
0.50
0.25
0.00
A (n=14)
B (n=15)
C (n=11)
surface

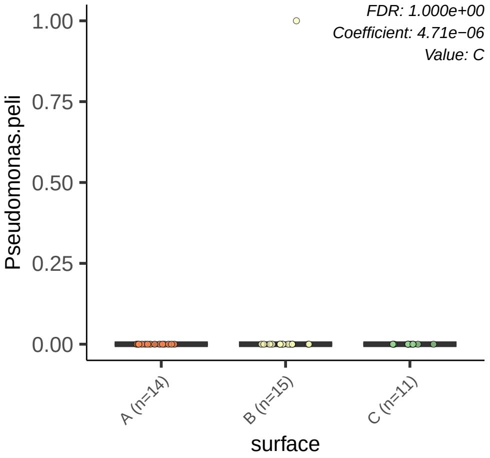
FDR: 1.000e+00
Coefficient: 4.71e−06
Value: C
Pseudomonas.peli
1.00
0.75
0.50
0.25
0.00
A (n=14)
B (n=15)
C (n=11)
surface

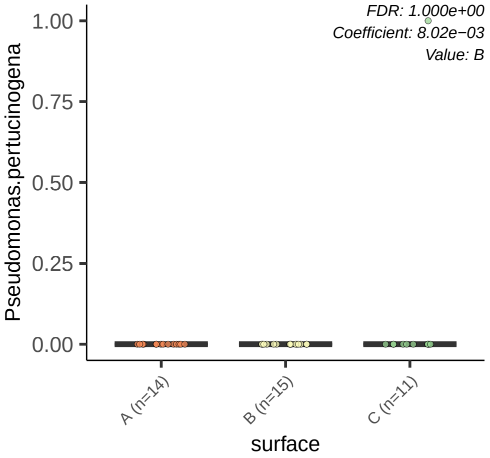

FDR: 1.000e+00
Coefficient: 8.02e−03
Value: B
Pseudomonas.pertucinogena
1.00
0.75
0.50
0.25
0.00
A (n=14)
B (n=15)
C (n=11)
surface

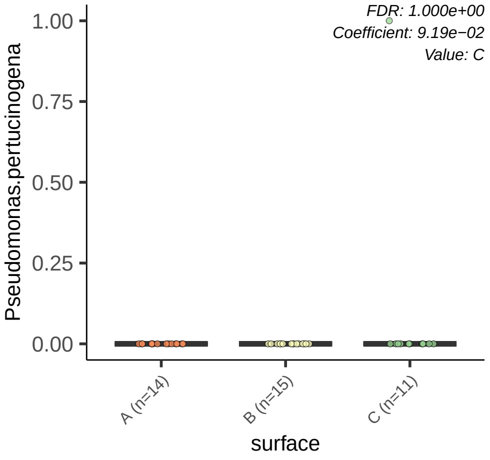
FDR: 1.000e+00
Coefficient: 9.19e−02
Value: C
Pseudomonas.pertucinogena
1.00
0.75
0.50
0.25
0.00
A (n=14)
B (n=15)
C (n=11)
surface

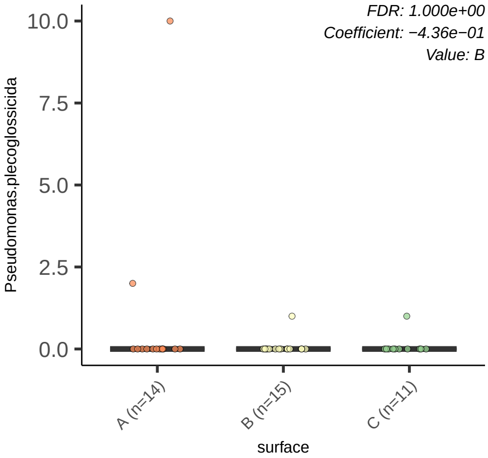
FDR: 1.000e+00
Coefficient: −4.36e−01
Value: B
Pseudomonas.plecoglossicida
10.0
7.5
5.0
2.5
0.0
A (n=14)
B (n=15)
C (n=11)
surface

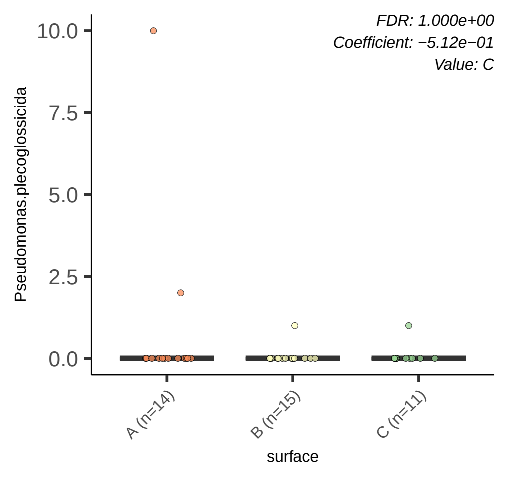
FDR: 1.000e+00
Coefficient: −5.12e−01
Value: C
Pseudomonas.plecoglossicida
10.0
7.5
5.0
2.5
0.0
A (n=14)
B (n=15)
C (n=11)
surface

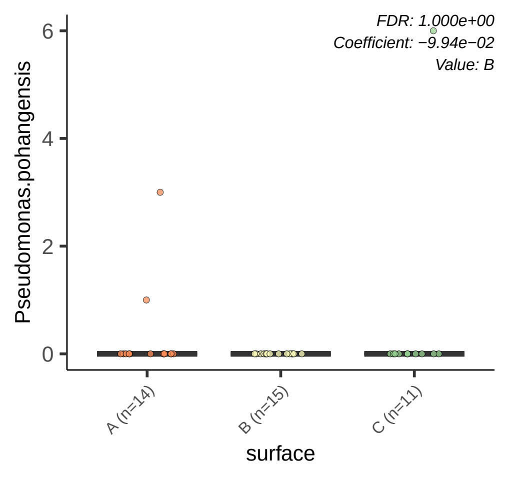
FDR: 1.000e+00
Coefficient: −9.94e−02
Value: B
Pseudomonas.pohangensis
6
4
2
0
A (n=14)
B (n=15)
C (n=11)
surface

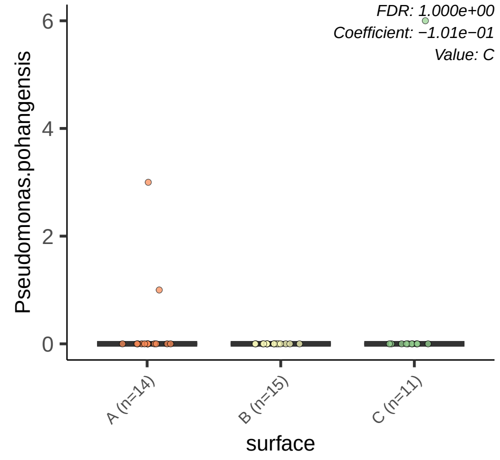
FDR: 1.000e+00
Coefficient: −1.01e−01
Value: C
Pseudomonas.pohangensis
6
4
2
0
A (n=14)
B (n=15)
C (n=11)
surface

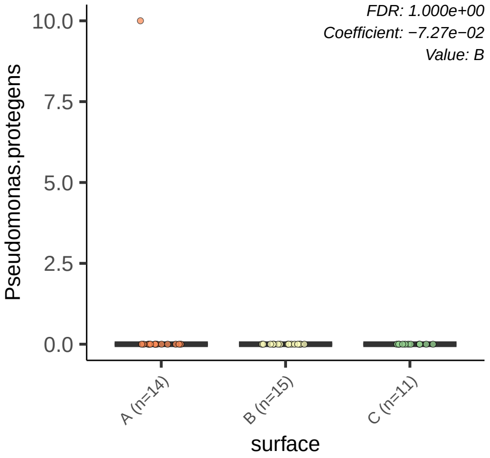
FDR: 1.000e+00
Coefficient: −7.27e−02
Value: B
Pseudomonas.protegens
10.0
7.5
5.0
2.5
0.0
A (n=14)
B (n=15)
C (n=11)
surface

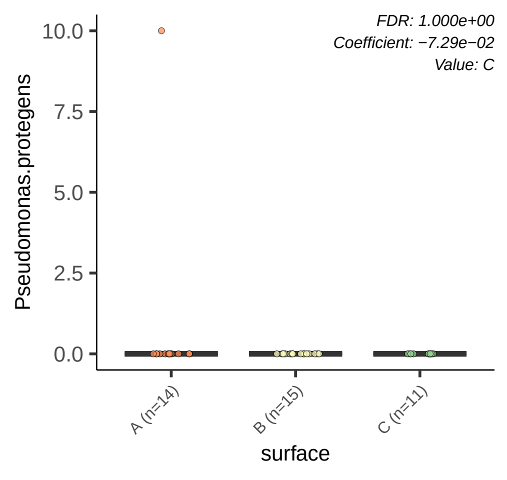

FDR: 1.000e+00
Coefficient: −7.29e−02
Value: C
Pseudomonas.protegens
10.0
7.5
5.0
2.5
0.0
A (n=14)
B (n=15)
C (n=11)
surface

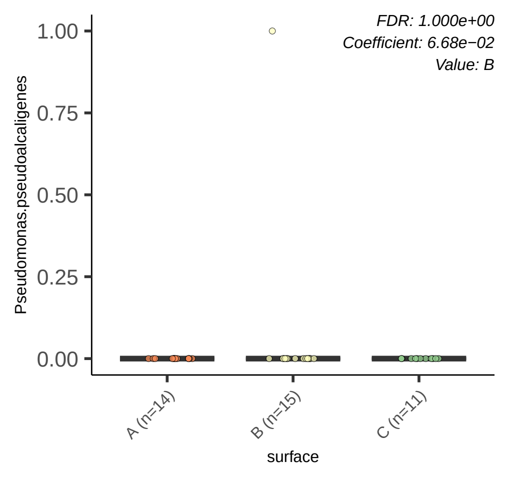

FDR: 1.000e+00
Coefficient: 6.68e−02
Value: B
1.00
0.75
0.50
0.25
0.00
Pseudomonas.pseudoalcaligenes
A (n=14)
B (n=15)
C (n=11)
surface

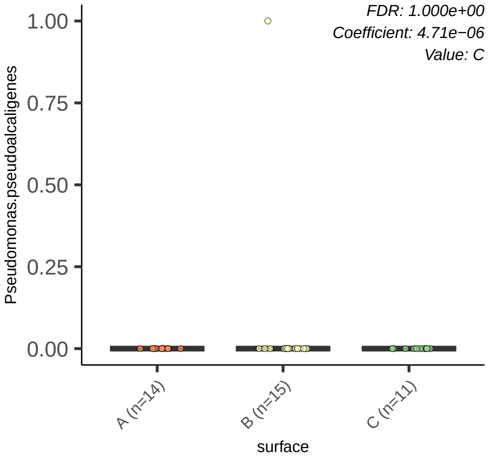
FDR: 1.000e+00
Coefficient: 4.71e−06
Value: C
Pseudomonas.pseudoalcaligenes
1.00
0.75
0.50
0.25
0.00
A (n=14)
B (n=15)
C (n=11)
surface

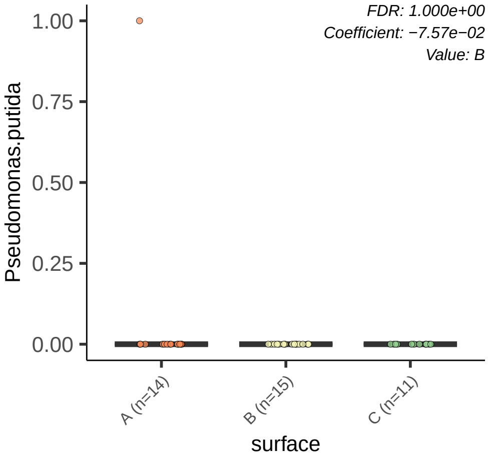

FDR: 1.000e+00
Coefficient: −7.57e−02
Value: B
Pseudomonas.putida
1.00
0.75
0.50
0.25
0.00
A (n=14)
B (n=15)
C (n=11)
surface

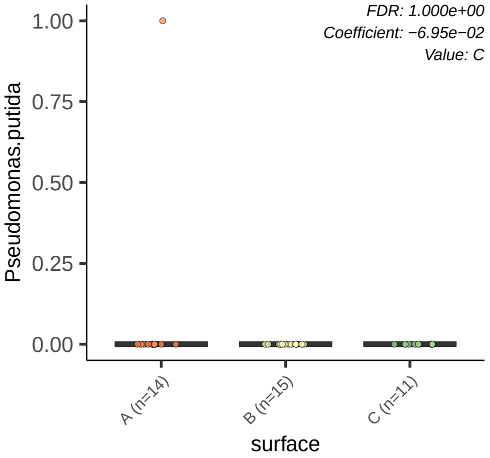

FDR: 1.000e+00
Coefficient: −6.95e−02
Value: C
Pseudomonas.putida
1.00
0.75
0.50
0.25
0.00
A (n=14)
B (n=15)
C (n=11)
surface

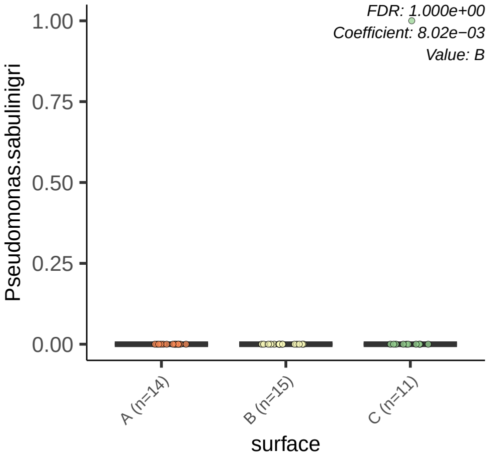
FDR: 1.000e+00
Coefficient: 8.02e−03
Value: B
Pseudomonas.sabulinigri
1.00
0.75
0.50
0.25
0.00
A (n=14)
B (n=15)
C (n=11)
surface

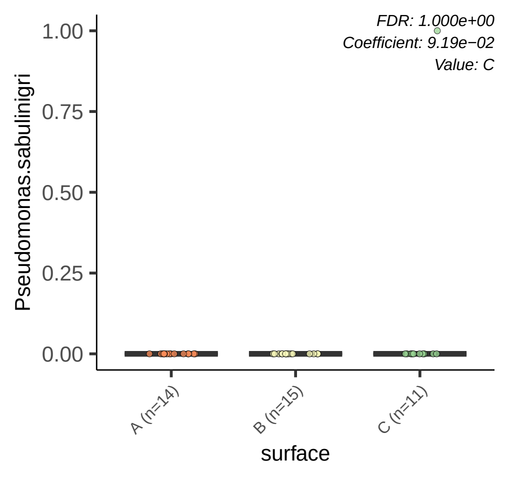

FDR: 1.000e+00
Coefficient: 9.19e−02
Value: C
Pseudomonas.sabulinigri
1.00
0.75
0.50
0.25
0.00
A (n=14)
B (n=15)
C (n=11)
surface

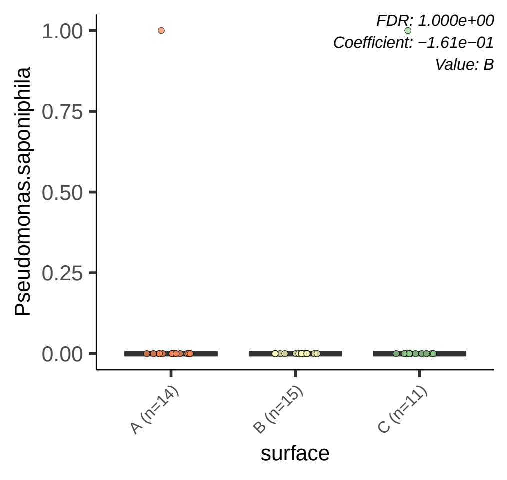

FDR: 1.000e+00
Coefficient: −1.61e−01
Value: B
Pseudomonas.saponiphila
1.00
0.75
0.50
0.25
0.00
A (n=14)
B (n=15)
C (n=11)
surface

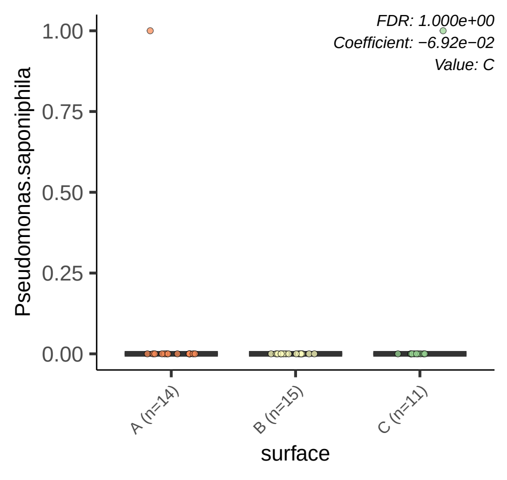

FDR: 1.000e+00
Coefficient: −6.92e−02
Value: C
Pseudomonas.saponiphila
1.00
0.75
0.50
0.25
0.00
A (n=14)
B (n=15)
C (n=11)
surface

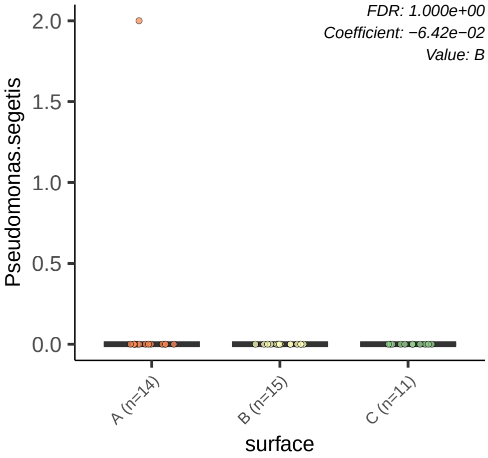

FDR: 1.000e+00
Coefficient: −6.42e−02
Value: B
Pseudomonas.segetis
2.0
1.5
1.0
0.5
0.0
A (n=14)
B (n=15)
C (n=11)
surface

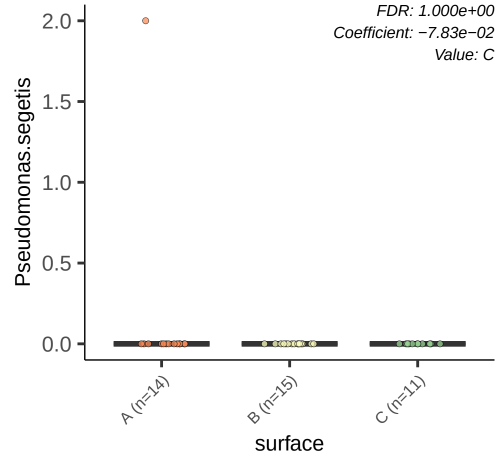
FDR: 1.000e+00
Coefficient: −7.83e−02
Value: C
Pseudomonas.segetis
2.0
1.5
1.0
0.5
0.0
A (n=14)
B (n=15)
C (n=11)
surface

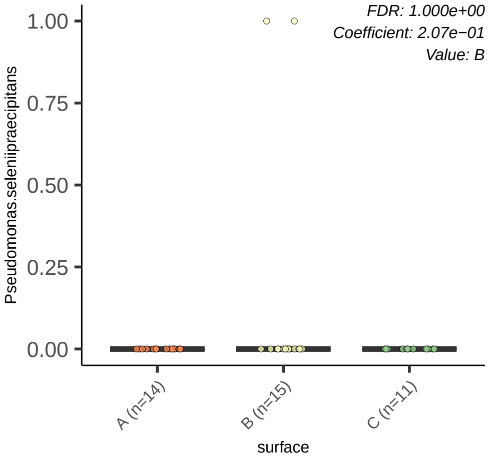

FDR: 1.000e+00
Coefficient: 2.07e−01
Value: B
Pseudomonas.seleniipraecipitans
1.00
0.75
0.50
0.25
0.00
A (n=14)
B (n=15)
C (n=11)
surface

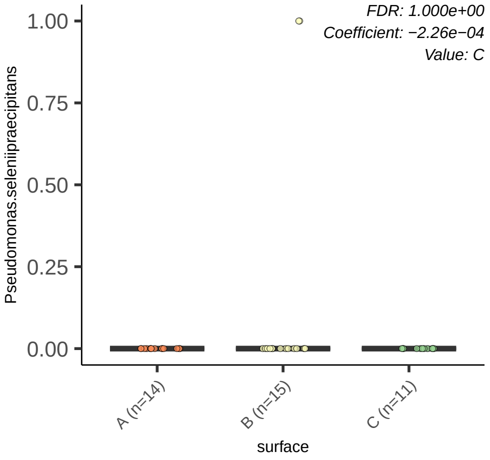

FDR: 1.000e+00
Coefficient: −2.26e−04
Value: C
Pseudomonas.seleniipraecipitans
1.00
0.75
0.50
0.25
0.00
A (n=14)
B (n=15)
C (n=11)
surface

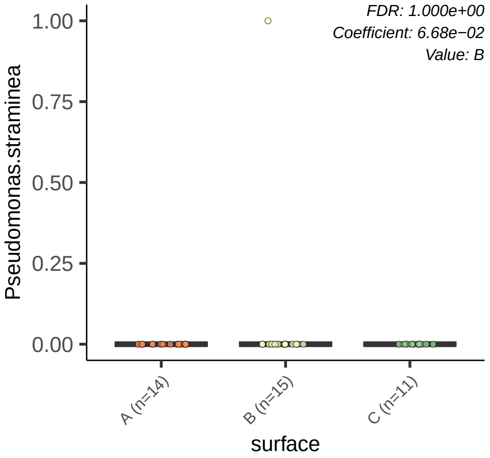
FDR: 1.000e+00
Coefficient: 6.68e−02
Value: B
Pseudomonas.straminea
1.00
0.75
0.50
0.25
0.00
A (n=14)
B (n=15)
C (n=11)
surface

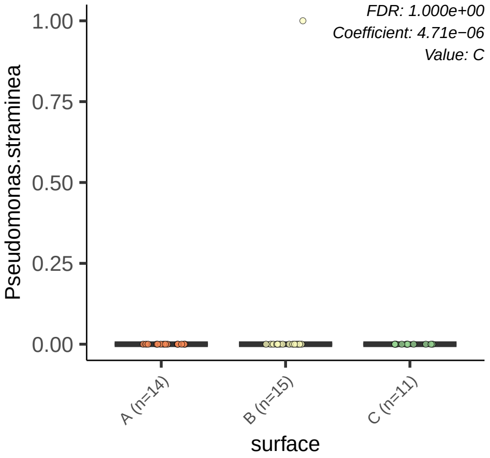
FDR: 1.000e+00
Coefficient: 4.71e−06
Value: C
Pseudomonas.straminea
1.00
0.75
0.50
0.25
0.00
A (n=14)
B (n=15)
C (n=11)
surface

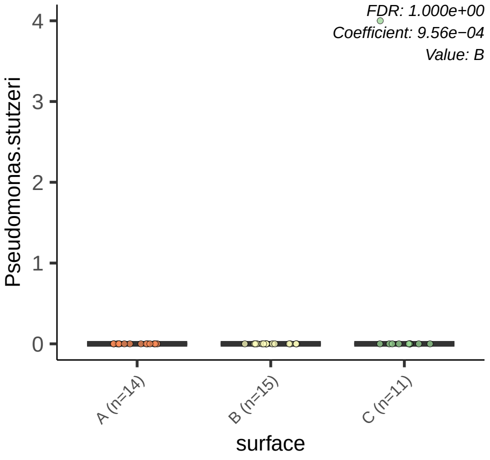

FDR: 1.000e+00
Coefficient: 9.56e−04
Value: B
Pseudomonas.stutzeri
4
3
2
1
0
A (n=14)
B (n=15)
C (n=11)
surface

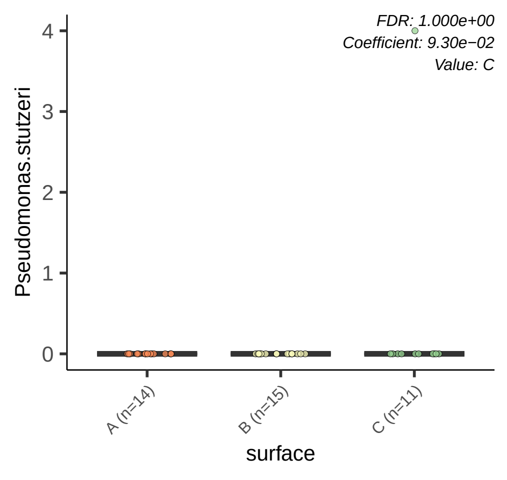

FDR: 1.000e+00
Coefficient: 9.30e−02
Value: C
Pseudomonas.stutzeri
4
3
2
1
0
A (n=14)
B (n=15)
C (n=11)
surface

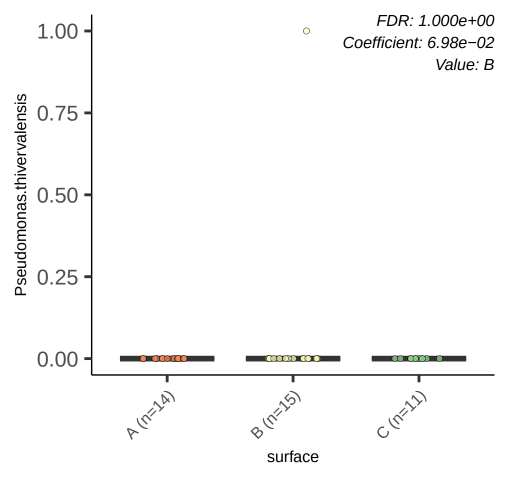

FDR: 1.000e+00
Coefficient: 6.98e−02
Value: B
1.00
0.75
0.50
0.25
0.00
Pseudomonas.thivervalensis
A (n=14)
B (n=15)
C (n=11)
surface

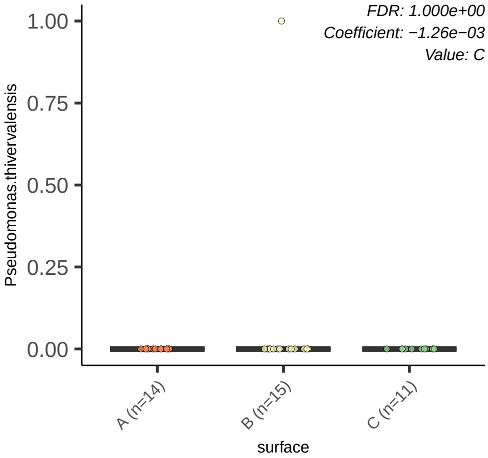
FDR: 1.000e+00
Coefficient: −1.26e−03
Value: C
Pseudomonas.thivervalensis
1.00
0.75
0.50
0.25
0.00
A (n=14)
B (n=15)
C (n=11)
surface

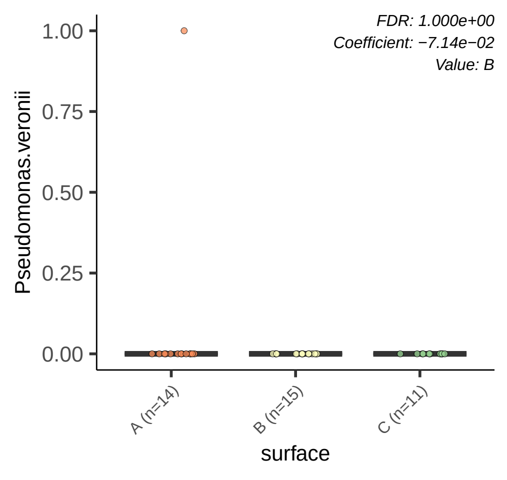

FDR: 1.000e+00
Coefficient: −7.14e−02
Value: B
Pseudomonas.veronii
1.00
0.75
0.50
0.25
0.00
A (n=14)
B (n=15)
C (n=11)
surface

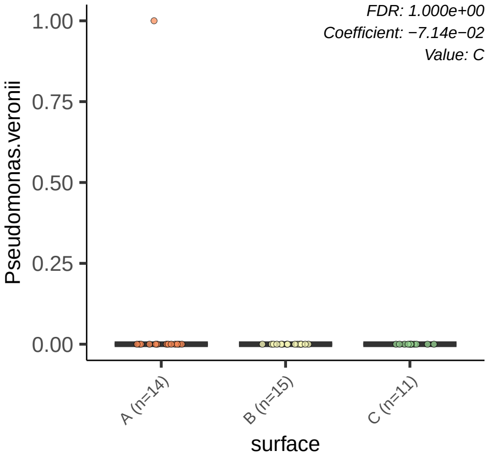

FDR: 1.000e+00
Coefficient: −7.14e−02
Value: C
Pseudomonas.veronii
1.00
0.75
0.50
0.25
0.00
A (n=14)
B (n=15)
C (n=11)
surface

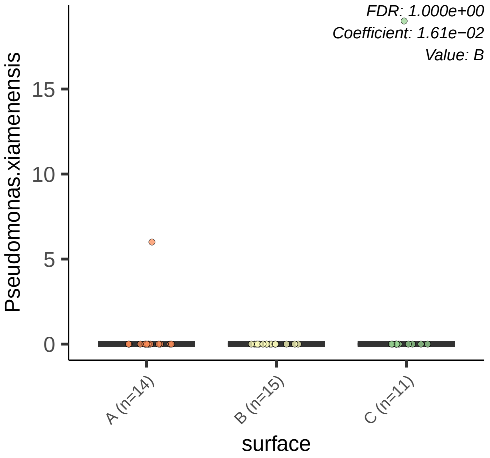
FDR: 1.000e+00
Coefficient: 1.61e−02
Value: B
Pseudomonas.xiamenensis
15
10
5
0
A (n=14)
B (n=15)
C (n=11)
surface

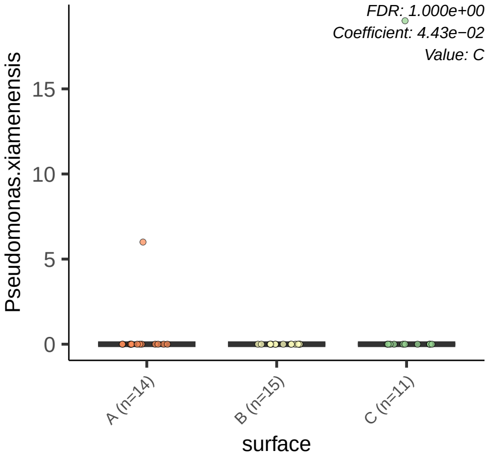

FDR: 1.000e+00
Coefficient: 4.43e−02
Value: C
Pseudomonas.xiamenensis
15
10
5
0
A (n=14)
B (n=15)
C (n=11)
surface

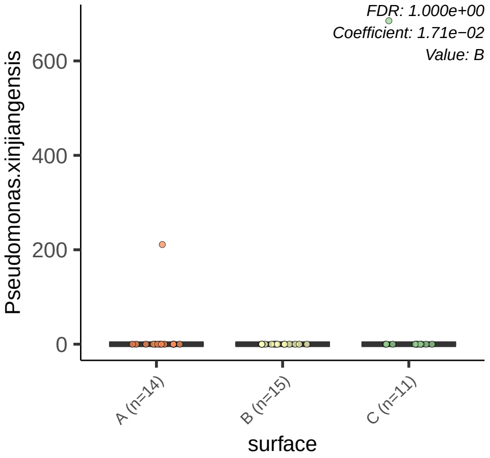
FDR: 1.000e+00
Coefficient: 1.71e−02
Value: B
Pseudomonas.xinjiangensis
600
400
200
0
A (n=14)
B (n=15)
C (n=11)
surface

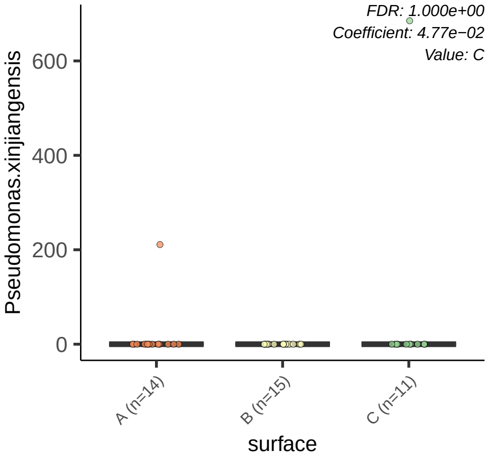
FDR: 1.000e+00
Coefficient: 4.77e−02
Value: C
Pseudomonas.xinjiangensis
600
400
200
0
A (n=14)
B (n=15)
C (n=11)
surface

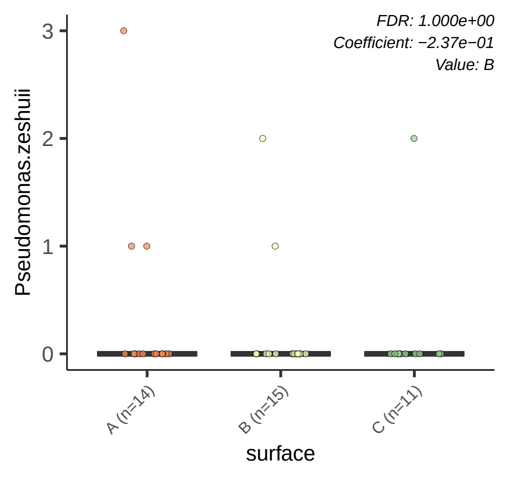

FDR: 1.000e+00
Coefficient: −2.37e−01
Value: B
Pseudomonas.zeshuii
3
2
1
0
A (n=14)
B (n=15)
C (n=11)
surface

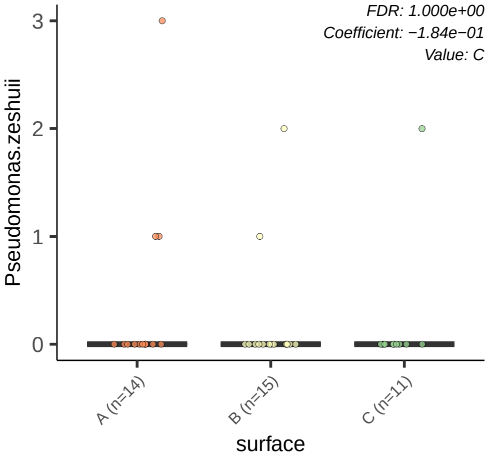
FDR: 1.000e+00
Coefficient: −1.84e−01
Value: C
Pseudomonas.zeshuii
3
2
1
0
A (n=14)
B (n=15)
C (n=11)
surface

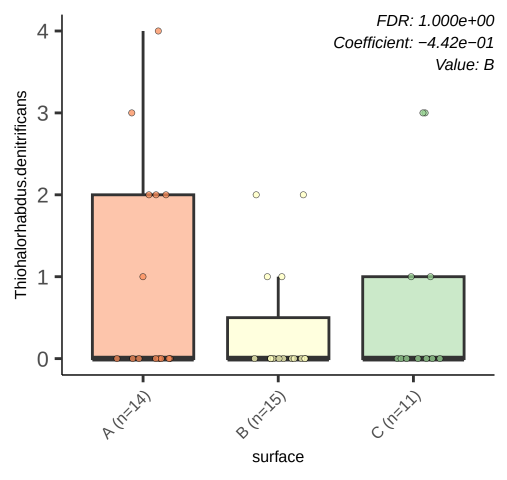

FDR: 1.000e+00
Coefficient: −4.42e−01
Value: B
Thiohalorhabdus.denitrificans
0
1
2
3
4
A (n=14)
B (n=15)
C (n=11)
surface

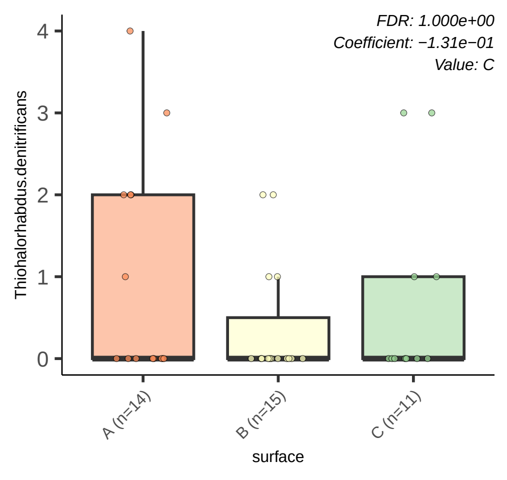

FDR: 1.000e+00
Coefficient: −1.31e−01
Value: C
Thiohalorhabdus.denitrificans
0
1
2
3
4
A (n=14)
B (n=15)
C (n=11)
surface

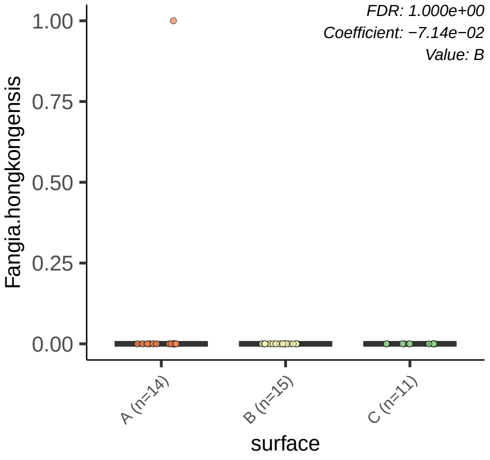
FDR: 1.000e+00
Coefficient: −7.14e−02
Value: B
Fangia.hongkongensis
1.00
0.75
0.50
0.25
0.00
A (n=14)
B (n=15)
C (n=11)
surface

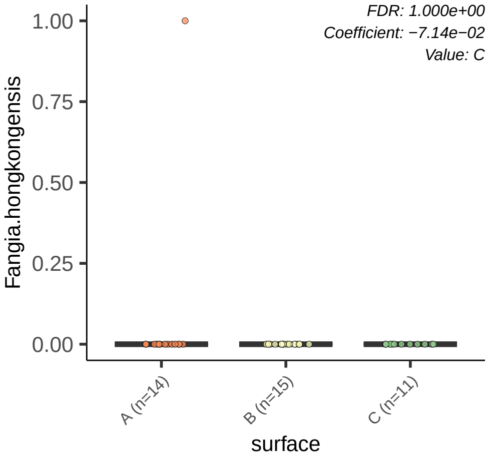

FDR: 1.000e+00
Coefficient: −7.14e−02
Value: C
Fangia.hongkongensis
1.00
0.75
0.50
0.25
0.00
A (n=14)
B (n=15)
C (n=11)
surface

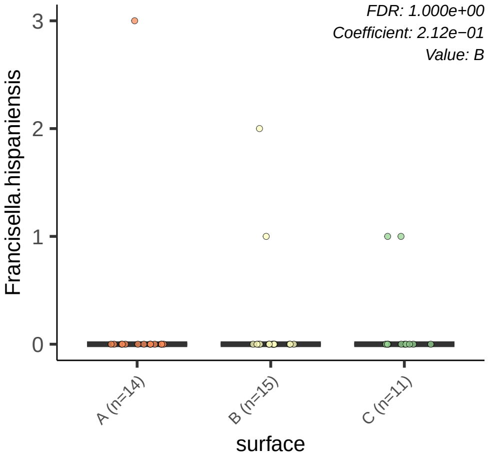

FDR: 1.000e+00
Coefficient: 2.12e−01
Value: B
Francisella.hispaniensis
0
1
2
3
A (n=14)
B (n=15)
C (n=11)
surface

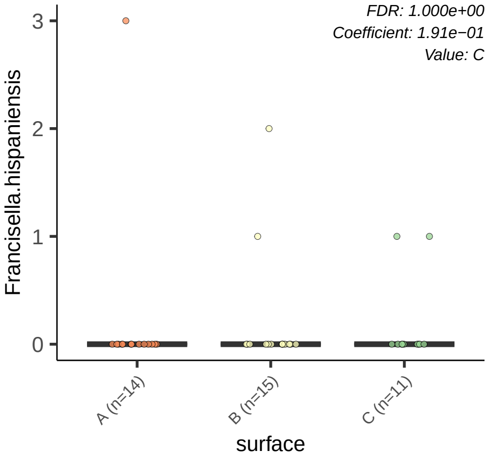
FDR: 1.000e+00
Coefficient: 1.91e−01
Value: C
Francisella.hispaniensis
3
2
1
0
A (n=14)
B (n=15)
C (n=11)
surface

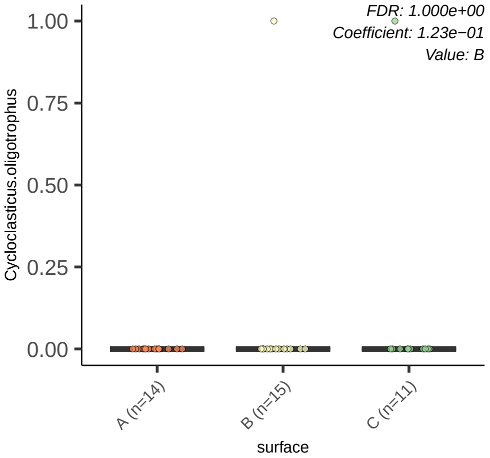

FDR: 1.000e+00
Coefficient: 1.23e−01
Value: B
Cycloclasticus.oligotrophus
1.00
0.75
0.50
0.25
0.00
A (n=14)
B (n=15)
C (n=11)
surface

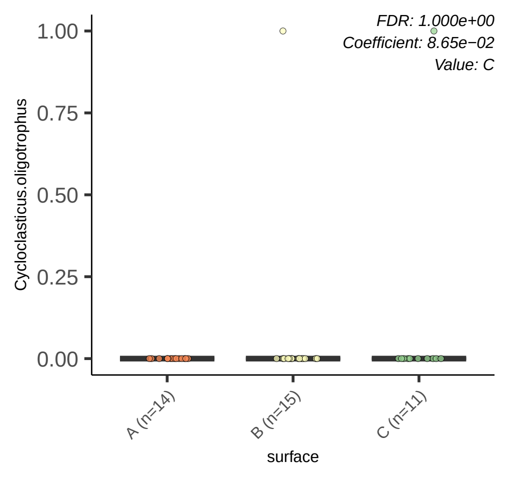
FDR: 1.000e+00
Coefficient: 8.65e−02
Value: C
Cycloclasticus.oligotrophus
1.00
0.75
0.50
0.25
0.00
A (n=14)
B (n=15)
C (n=11)
surface

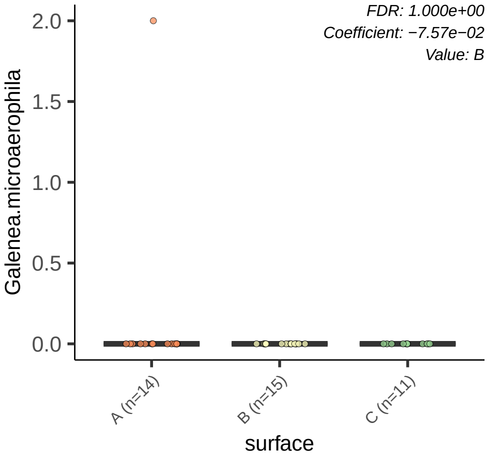
FDR: 1.000e+00
Coefficient: −7.57e−02
Value: B
Galenea.microaerophila
2.0
1.5
1.0
0.5
0.0
A (n=14)
B (n=15)
C (n=11)
surface

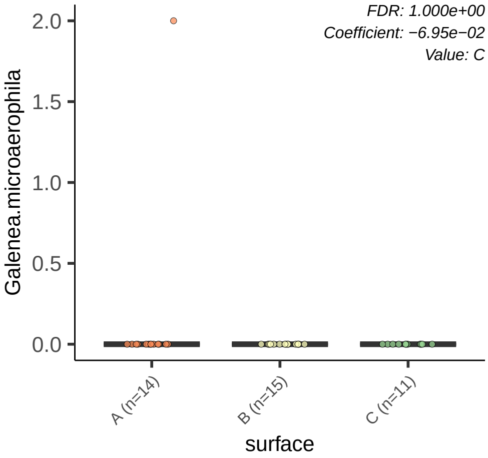
FDR: 1.000e+00
Coefficient: −6.95e−02
Value: C
Galenea.microaerophila
2.0
1.5
1.0
0.5
0.0
A (n=14)
B (n=15)
C (n=11)
surface

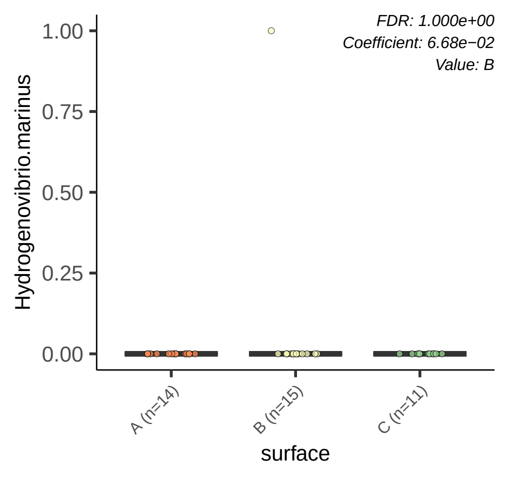
FDR: 1.000e+00
Coefficient: 6.68e−02
Value: B
Hydrogenovibrio.marinus
1.00
0.75
0.50
0.25
0.00
A (n=14)
B (n=15)
C (n=11)
surface

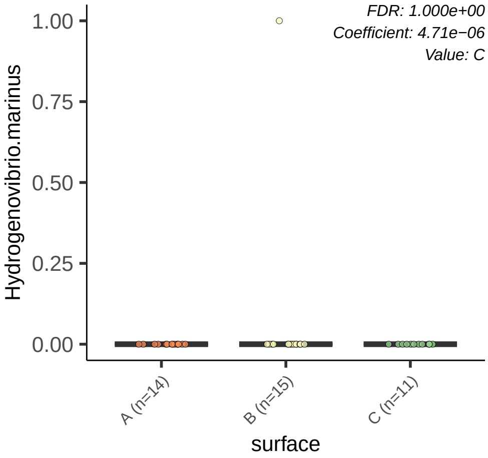

FDR: 1.000e+00
Coefficient: 4.71e−06
Value: C
Hydrogenovibrio.marinus
1.00
0.75
0.50
0.25
0.00
A (n=14)
B (n=15)
C (n=11)
surface

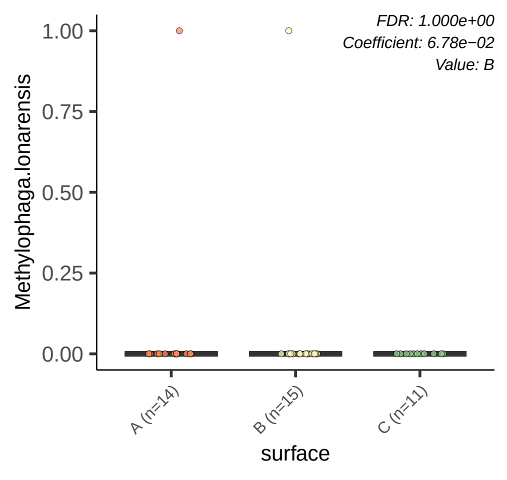
FDR: 1.000e+00
Coefficient: 6.78e−02
Value: B
Methylophaga.lonarensis
1.00
0.75
0.50
0.25
0.00
A (n=14)
B (n=15)
C (n=11)
surface

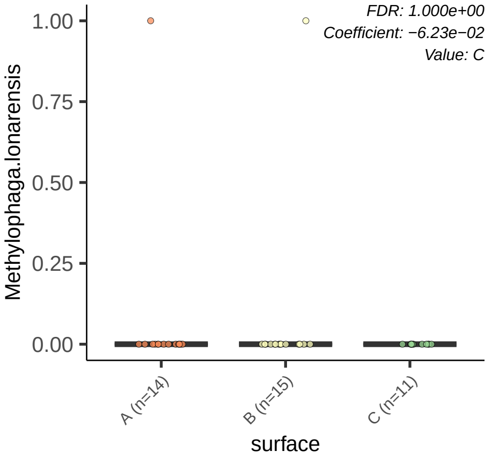
FDR: 1.000e+00
Coefficient: −6.23e−02
Value: C
Methylophaga.lonarensis
1.00
0.75
0.50
0.25
0.00
A (n=14)
B (n=15)
C (n=11)
surface

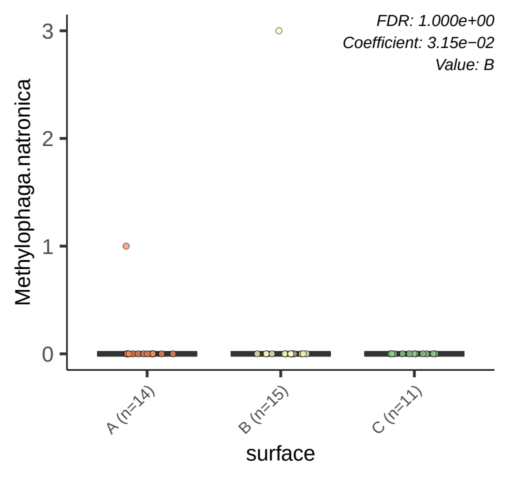
FDR: 1.000e+00
Coefficient: 3.15e−02
Value: B
Methylophaga.natronica
0
1
2
3
A (n=14)
B (n=15)
C (n=11)
surface

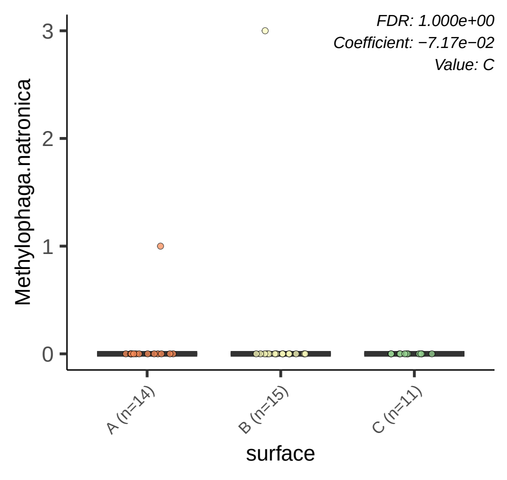
FDR: 1.000e+00
Coefficient: −7.17e−02
Value: C
Methylophaga.natronica
3
2
1
0
A (n=14)
B (n=15)
C (n=11)
surface

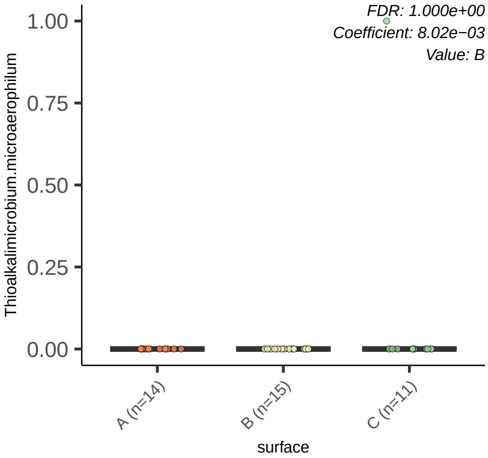
FDR: 1.000e+00
Coefficient: 8.02e−03
Value: B
Thioalkalimicrobium.microaerophilum
1.00
0.75
0.50
0.25
0.00
A (n=14)
B (n=15)
C (n=11)
surface

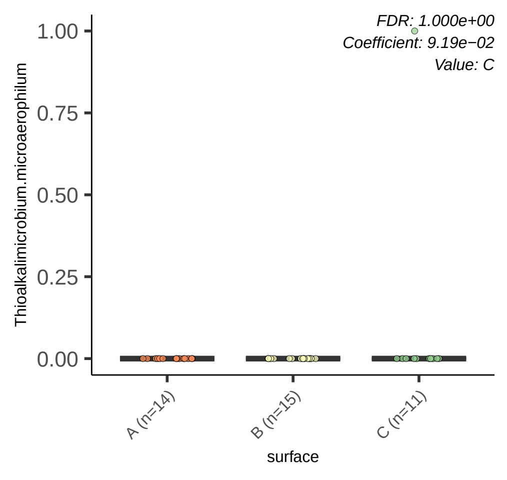
FDR: 1.000e+00
Coefficient: 9.19e−02
Value: C
1.00
0.75
0.50
0.25
0.00
Thioalkalimicrobium.microaerophilum
A (n=14)
B (n=15)
C (n=11)
surface

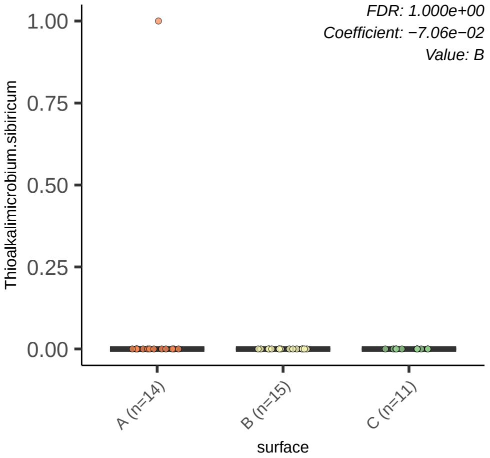
FDR: 1.000e+00
Coefficient: −7.06e−02
Value: B
Thioalkalimicrobium.sibiricum
1.00
0.75
0.50
0.25
0.00
A (n=14)
B (n=15)
C (n=11)
surface

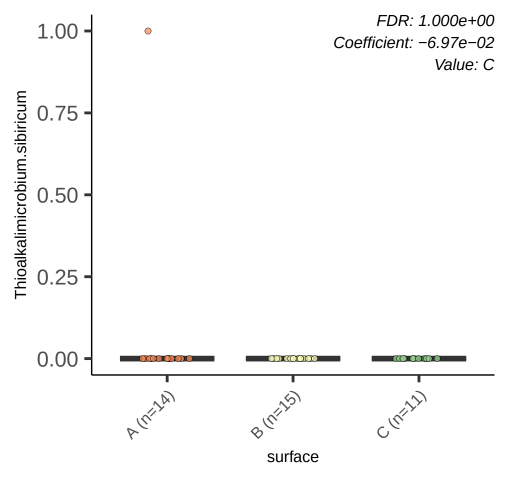
FDR: 1.000e+00
Coefficient: −6.97e−02
Value: C
Thioalkalimicrobium.sibiricum
1.00
0.75
0.50
0.25
0.00
A (n=14)
B (n=15)
C (n=11)
surface

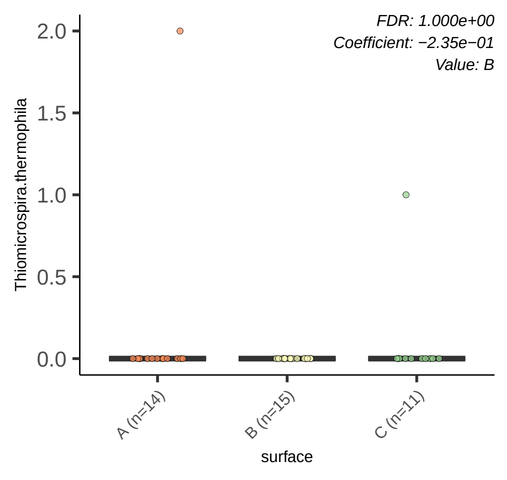

FDR: 1.000e+00
Coefficient: −2.35e−01
Value: B
Thiomicrospira.thermophila
2.0
1.5
1.0
0.5
0.0
A (n=14)
B (n=15)
C (n=11)
surface

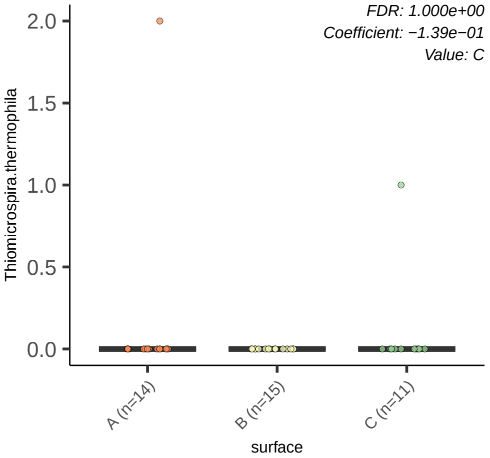
FDR: 1.000e+00
Coefficient: −1.39e−01
Value: C
Thiomicrospira.thermophila
2.0
1.5
1.0
0.5
0.0
A (n=14)
B (n=15)
C (n=11)
surface

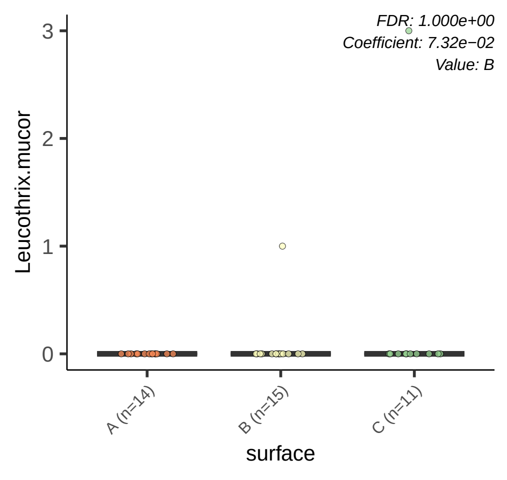
FDR: 1.000e+00
Coefficient: 7.32e−02
Value: B
Leucothrix.mucor
3
2
1
0
A (n=14)
B (n=15)
C (n=11)
surface

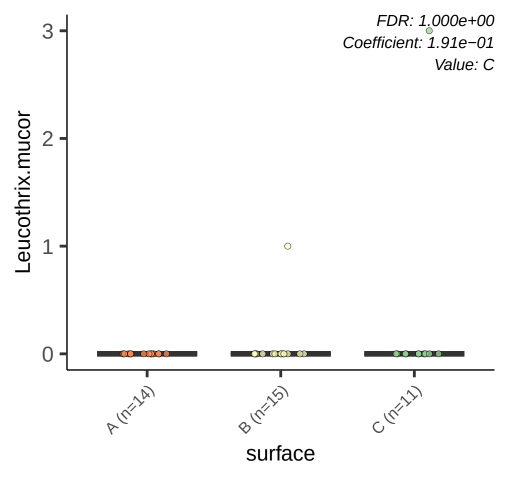
FDR: 1.000e+00
Coefficient: 1.91e−01
Value: C
Leucothrix.mucor
3
2
1
0
A (n=14)
B (n=15)
C (n=11)
surface

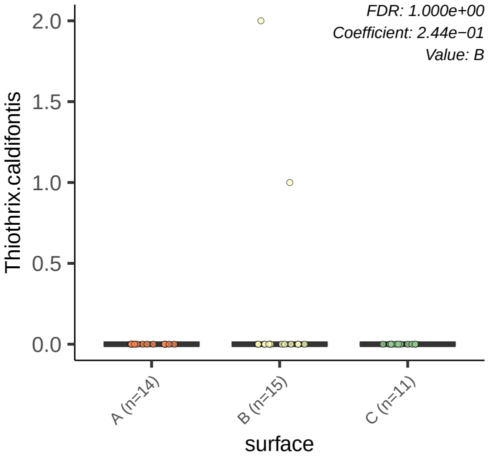

FDR: 1.000e+00
Coefficient: 2.44e−01
Value: B
Thiothrix.caldifontis
2.0
1.5
1.0
0.5
0.0
A (n=14)
B (n=15)
C (n=11)
surface

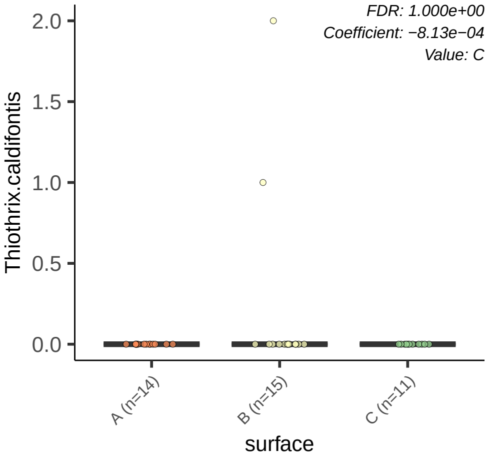

FDR: 1.000e+00
Coefficient: −8.13e−04
Value: C
Thiothrix.caldifontis
2.0
1.5
1.0
0.5
0.0
A (n=14)
B (n=15)
C (n=11)
surface

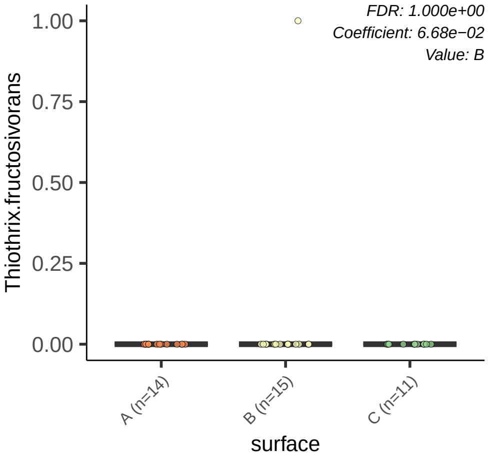

FDR: 1.000e+00
Coefficient: 6.68e−02
Value: B
Thiothrix.fructosivorans
1.00
0.75
0.50
0.25
0.00
A (n=14)
B (n=15)
C (n=11)
surface

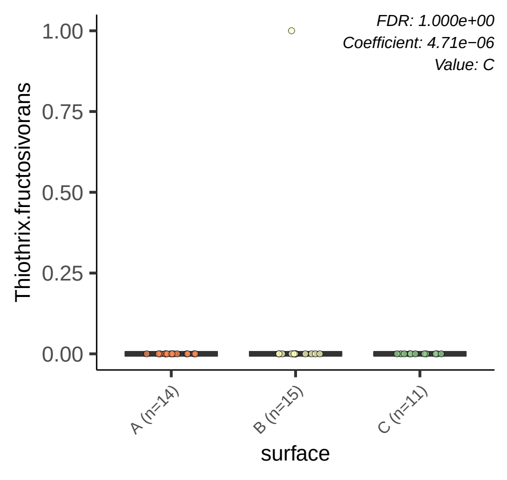
FDR: 1.000e+00
Coefficient: 4.71e−06
Value: C
Thiothrix.fructosivorans
1.00
0.75
0.50
0.25
0.00
A (n=14)
B (n=15)
C (n=11)
surface

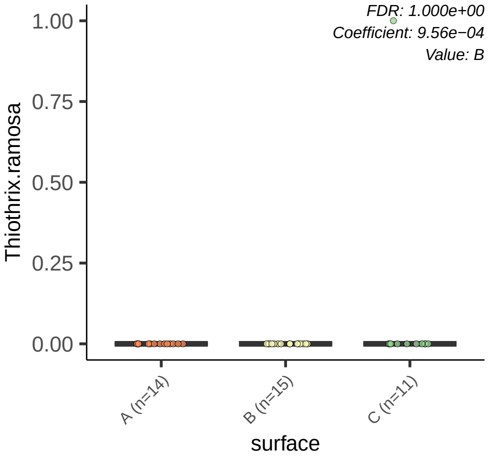
FDR: 1.000e+00
Coefficient: 9.56e−04
Value: B
Thiothrix.ramosa
1.00
0.75
0.50
0.25
0.00
A (n=14)
B (n=15)
C (n=11)
surface

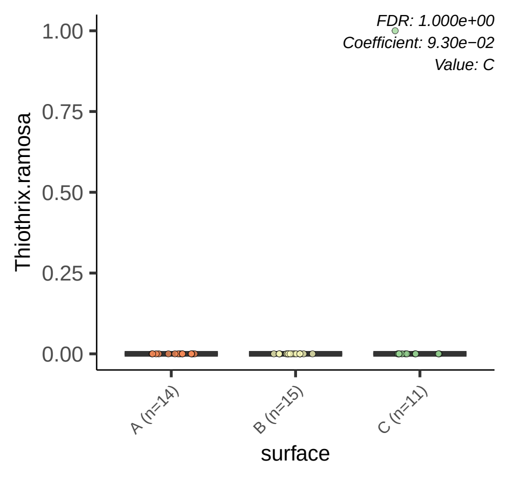
FDR: 1.000e+00
Coefficient: 9.30e−02
Value: C
Thiothrix.ramosa
1.00
0.75
0.50
0.25
0.00
A (n=14)
B (n=15)
C (n=11)
surface

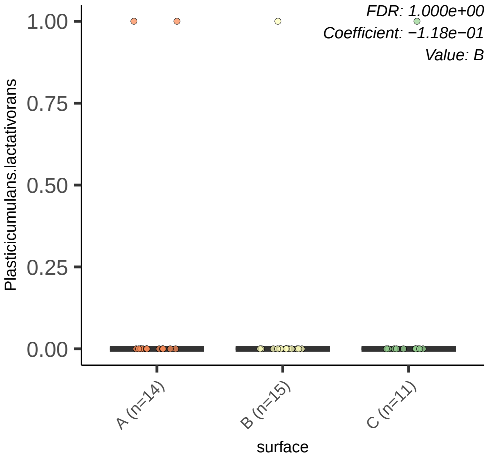
FDR: 1.000e+00
Coefficient: −1.18e−01
Value: B
Plasticicumulans.lactativorans
1.00
0.75
0.50
0.25
0.00
A (n=14)
B (n=15)
C (n=11)
surface

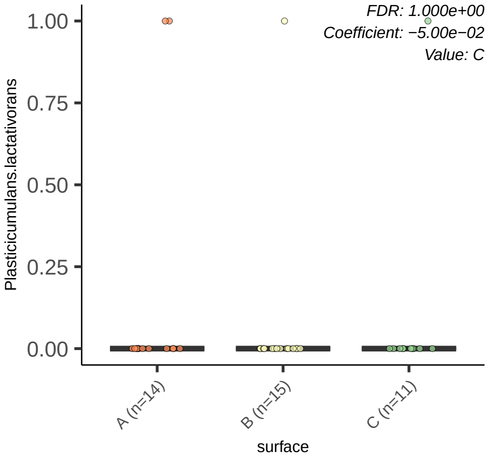

FDR: 1.000e+00
Coefficient: −5.00e−02
Value: C
Plasticicumulans.lactativorans
1.00
0.75
0.50
0.25
0.00
A (n=14)
B (n=15)
C (n=11)
surface

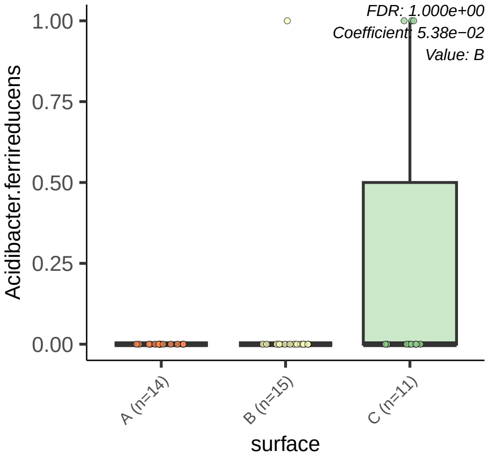

FDR: 1.000e+00
Coefficient: 5.38e−02
Value: B
Acidibacter.ferrireducens
1.00
0.75
0.50
0.25
0.00
A (n=14)
B (n=15)
C (n=11)
surface

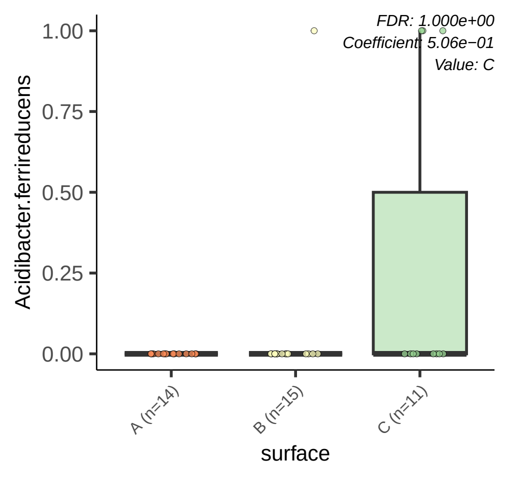
FDR: 1.000e+00
Coefficient: 5.06e−01
Value: C
1.00
0.75
0.50
0.25
0.00
Acidibacter.ferrireducens
A (n=14)
B (n=15)
C (n=11)
surface

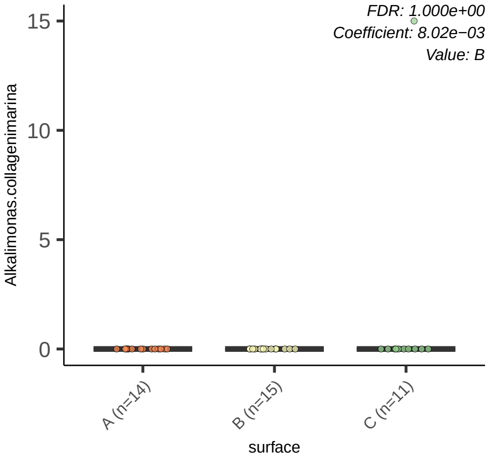
FDR: 1.000e+00
Coefficient: 8.02e−03
Value: B
Alkalimonas.collagenimarina
15
10
5
0
A (n=14)
B (n=15)
C (n=11)
surface

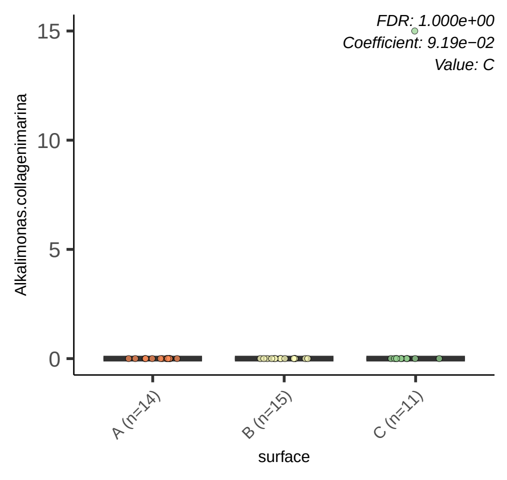

FDR: 1.000e+00
Coefficient: 9.19e−02
Value: C
15
10
5
0
Alkalimonas.collagenimarina
A (n=14)
B (n=15)
C (n=11)
surface

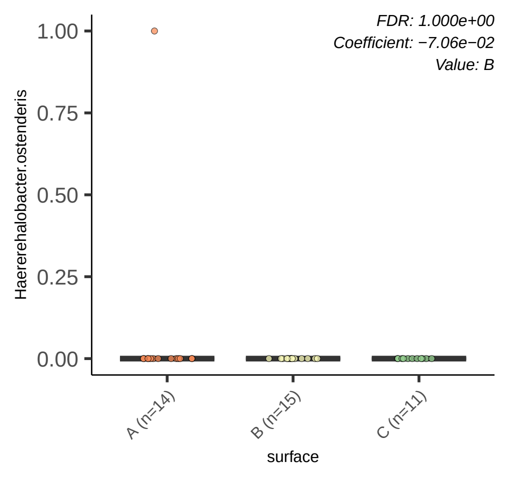
FDR: 1.000e+00
Coefficient: −7.06e−02
Value: B
Haererehalobacter.ostenderis
1.00
0.75
0.50
0.25
0.00
A (n=14)
B (n=15)
C (n=11)
surface

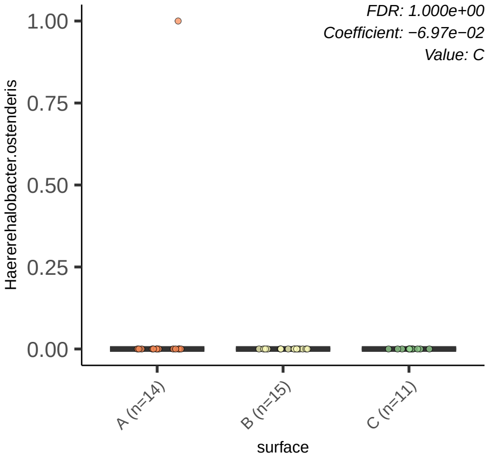

FDR: 1.000e+00
Coefficient: −6.97e−02
Value: C
1.00
0.75
0.50
0.25
0.00
Haererehalobacter.ostenderis
A (n=14)
B (n=15)
C (n=11)
surface

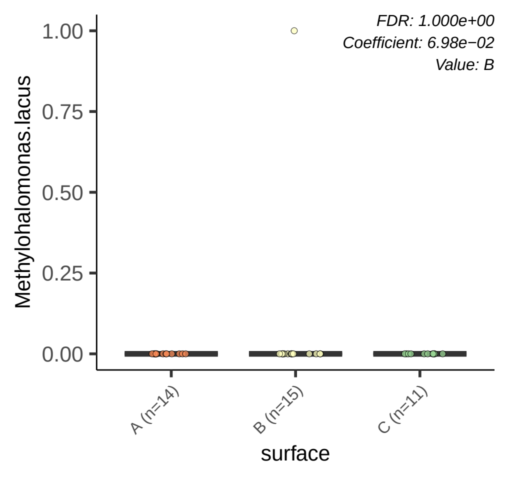
FDR: 1.000e+00
Coefficient: 6.98e−02
Value: B
Methylohalomonas.lacus
1.00
0.75
0.50
0.25
0.00
A (n=14)
B (n=15)
C (n=11)
surface

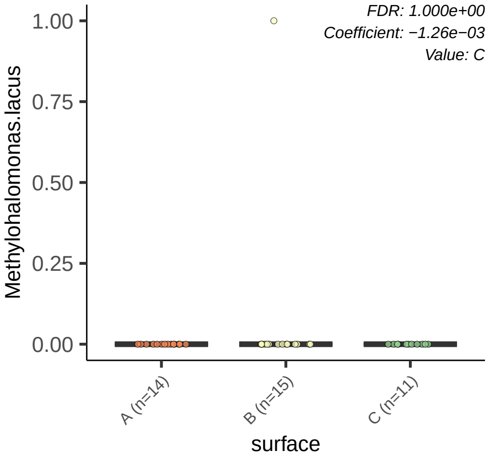

FDR: 1.000e+00
Coefficient: −1.26e−03
Value: C
Methylohalomonas.lacus
1.00
0.75
0.50
0.25
0.00
A (n=14)
B (n=15)
C (n=11)
surface

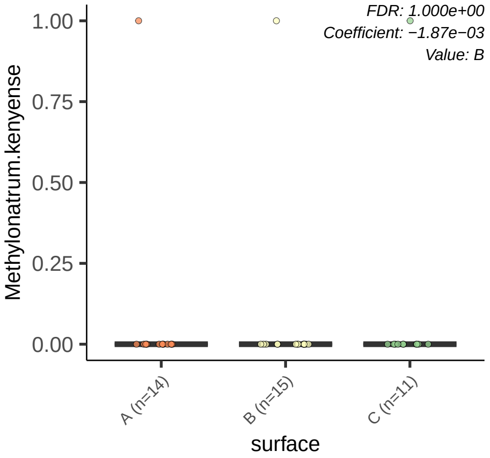

FDR: 1.000e+00
Coefficient: −1.87e−03
Value: B
Methylonatrum.kenyense
1.00
0.75
0.50
0.25
0.00
A (n=14)
B (n=15)
C (n=11)
surface

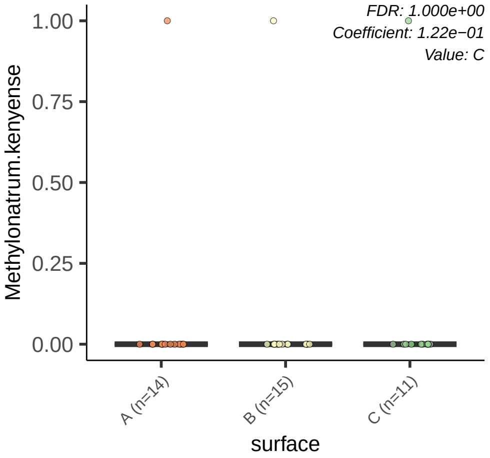
FDR: 1.000e+00
Coefficient: 1.22e−01
Value: C
Methylonatrum.kenyense
1.00
0.75
0.50
0.25
0.00
A (n=14)
B (n=15)
C (n=11)
surface

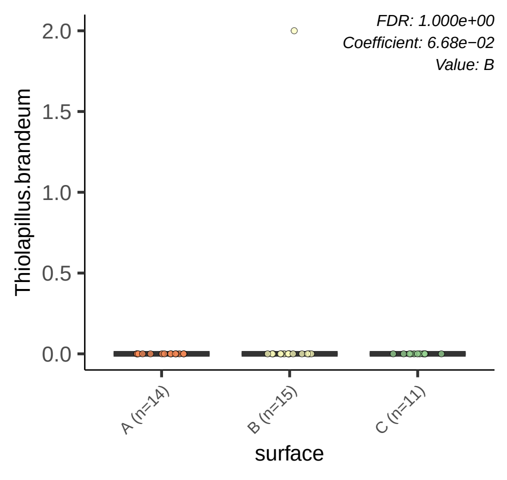
FDR: 1.000e+00
Coefficient: 6.68e−02
Value: B
Thiolapillus.brandeum
2.0
1.5
1.0
0.5
0.0
A (n=14)
B (n=15)
C (n=11)
surface

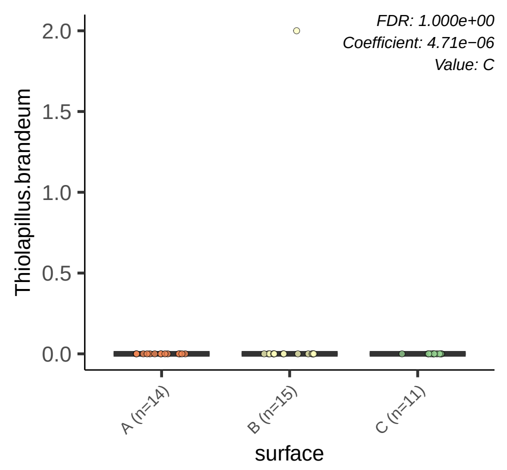

FDR: 1.000e+00
Coefficient: 4.71e−06
Value: C
Thiolapillus.brandeum
2.0
1.5
1.0
0.5
0.0
A (n=14)
B (n=15)
C (n=11)
surface

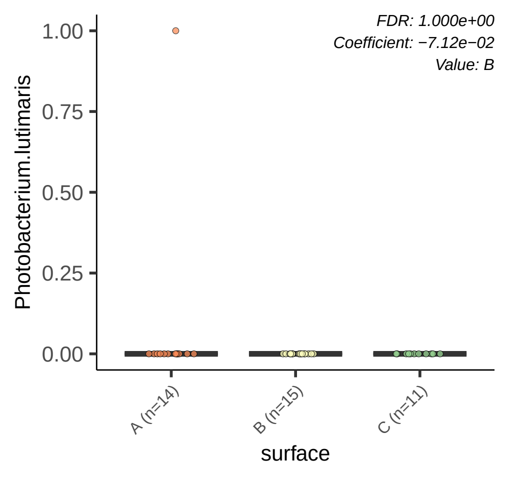

FDR: 1.000e+00
Coefficient: −7.12e−02
Value: B
Photobacterium.lutimaris
1.00
0.75
0.50
0.25
0.00
A (n=14)
B (n=15)
C (n=11)
surface

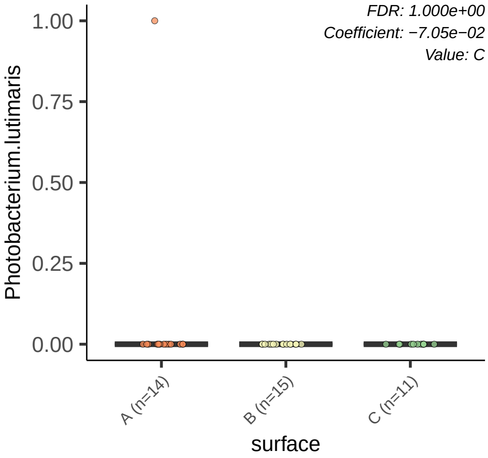
FDR: 1.000e+00
Coefficient: −7.05e−02
Value: C
Photobacterium.lutimaris
1.00
0.75
0.50
0.25
0.00
A (n=14)
B (n=15)
C (n=11)
surface

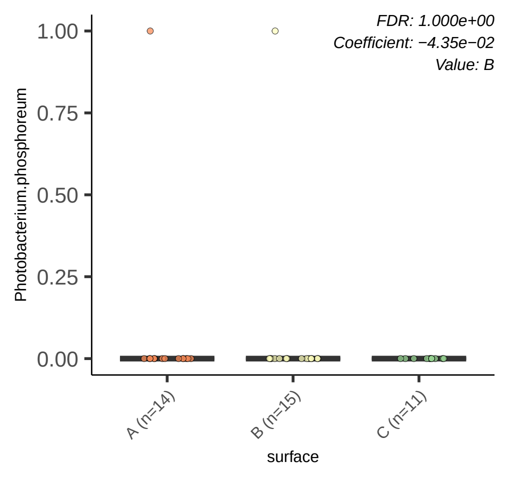

FDR: 1.000e+00
Coefficient: −4.35e−02
Value: B
Photobacterium.phosphoreum
1.00
0.75
0.50
0.25
0.00
A (n=14)
B (n=15)
C (n=11)
surface

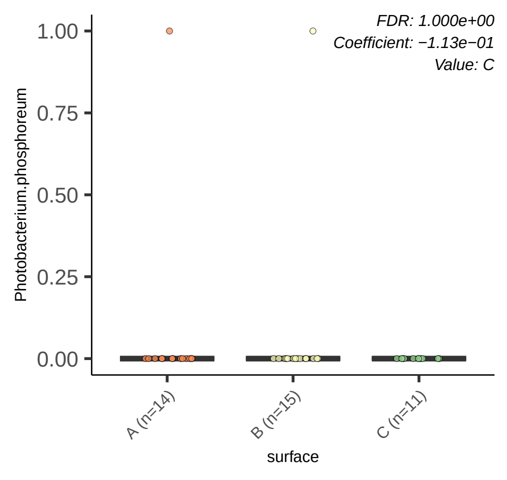
FDR: 1.000e+00
Coefficient: −1.13e−01
Value: C
1.00
0.75
0.50
0.25
0.00
Photobacterium.phosphoreum
A (n=14)
B (n=15)
C (n=11)
surface

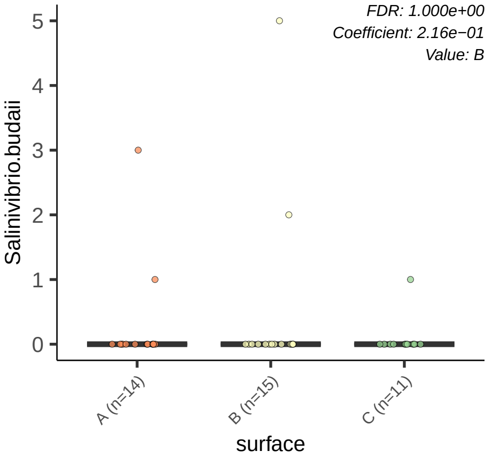
FDR: 1.000e+00
Coefficient: 2.16e−01
Value: B
Salinivibrio.budaii
0
1
2
3
4
5
A (n=14)
B (n=15)
C (n=11)
surface

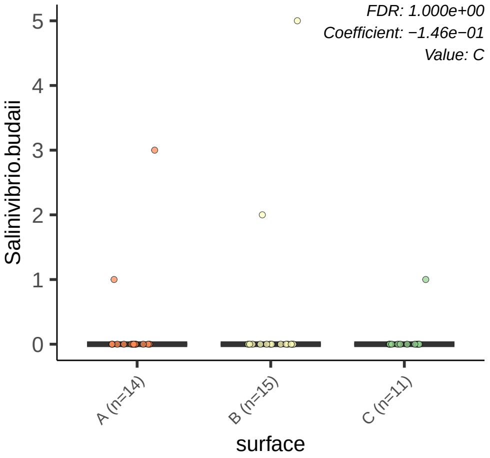
FDR: 1.000e+00
Coefficient: −1.46e−01
Value: C
Salinivibrio.budaii
0
1
2
3
4
5
A (n=14)
B (n=15)
C (n=11)
surface

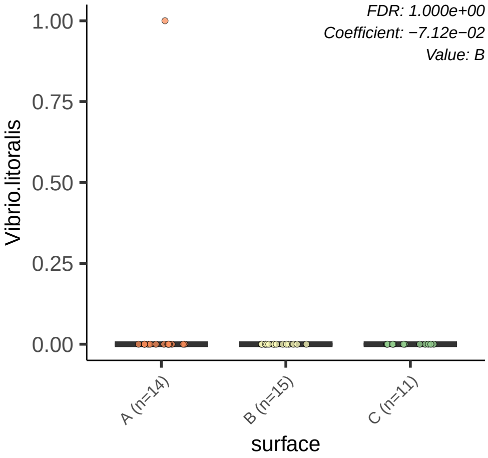
FDR: 1.000e+00
Coefficient: −7.12e−02
Value: B
Vibrio.litoralis
1.00
0.75
0.50
0.25
0.00
A (n=14)
B (n=15)
C (n=11)
surface

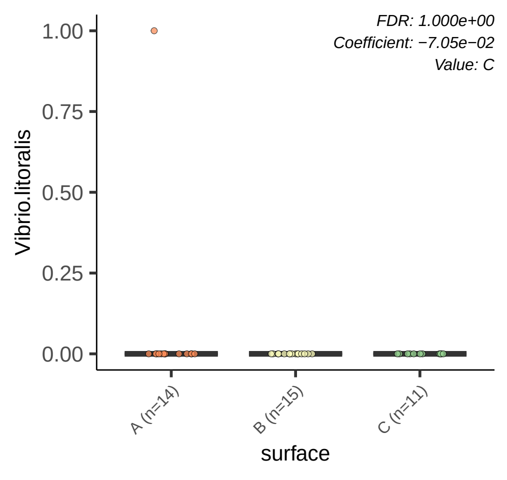

FDR: 1.000e+00
Coefficient: −7.05e−02
Value: C
1.00
0.75
0.50
0.25
0.00
Vibrio.litoralis
A (n=14)
B (n=15)
C (n=11)
surface

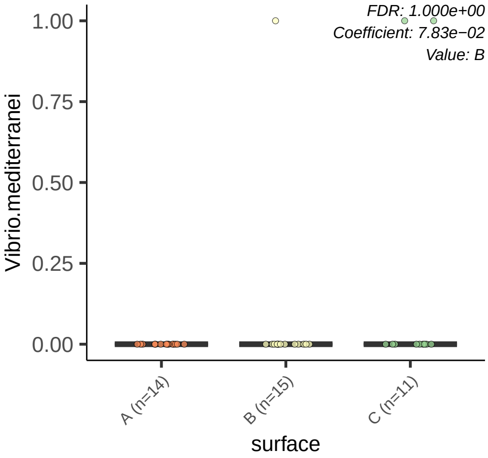

FDR: 1.000e+00
Coefficient: 7.83e−02
Value: B
Vibrio.mediterranei
1.00
0.75
0.50
0.25
0.00
A (n=14)
B (n=15)
C (n=11)
surface

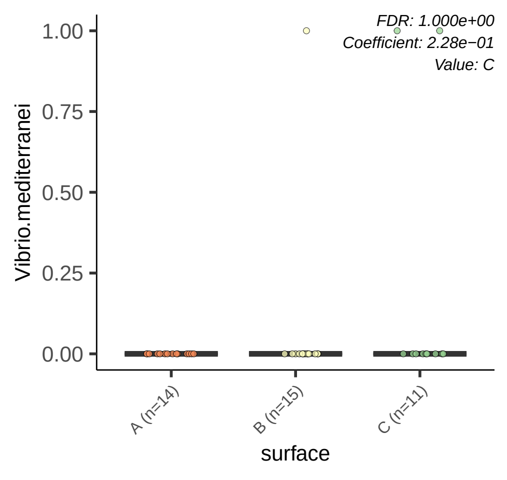

FDR: 1.000e+00
Coefficient: 2.28e−01
Value: C
1.00
0.75
0.50
0.25
0.00
Vibrio.mediterranei
A (n=14)
B (n=15)
C (n=11)
surface

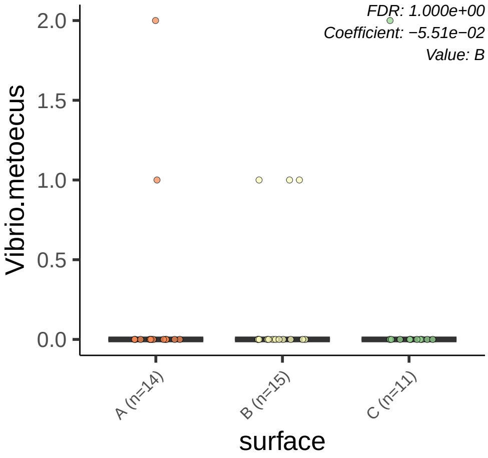

FDR: 1.000e+00
Coefficient: −5.51e−02
Value: B
Vibrio.metoecus
2.0
1.5
1.0
0.5
0.0
A (n=14)
B (n=15)
C (n=11)
surface

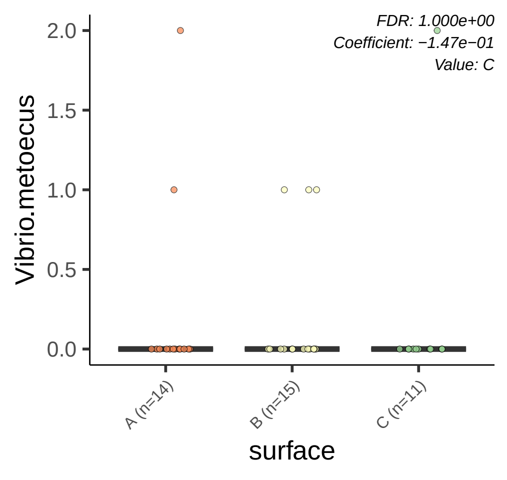
FDR: 1.000e+00
Coefficient: −1.47e−01
Value: C
Vibrio.metoecus
2.0
1.5
1.0
0.5
0.0
A (n=14)
B (n=15)
C (n=11)
surface

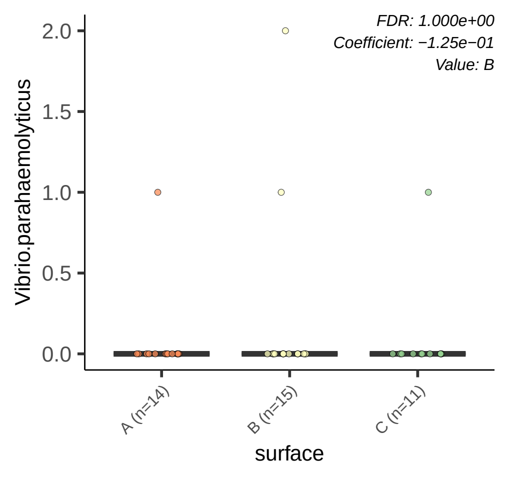
FDR: 1.000e+00
Coefficient: −1.25e−01
Value: B
Vibrio.parahaemolyticus
2.0
1.5
1.0
0.5
0.0
A (n=14)
B (n=15)
C (n=11)
surface

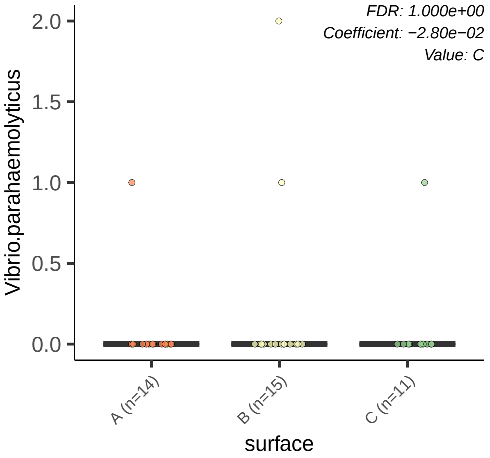

FDR: 1.000e+00
Coefficient: −2.80e−02
Value: C
Vibrio.parahaemolyticus
2.0
1.5
1.0
0.5
0.0
A (n=14)
B (n=15)
C (n=11)
surface

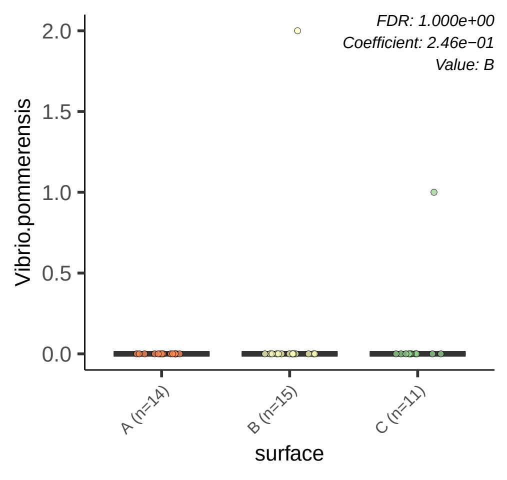
FDR: 1.000e+00
Coefficient: 2.46e−01
Value: B
Vibrio.pommerensis
2.0
1.5
1.0
0.5
0.0
A (n=14)
B (n=15)
C (n=11)
surface

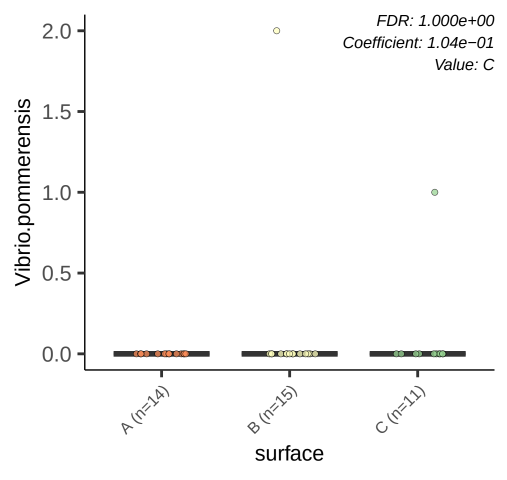
FDR: 1.000e+00
Coefficient: 1.04e−01
Value: C
Vibrio.pommerensis
2.0
1.5
1.0
0.5
0.0
A (n=14)
B (n=15)
C (n=11)
surface

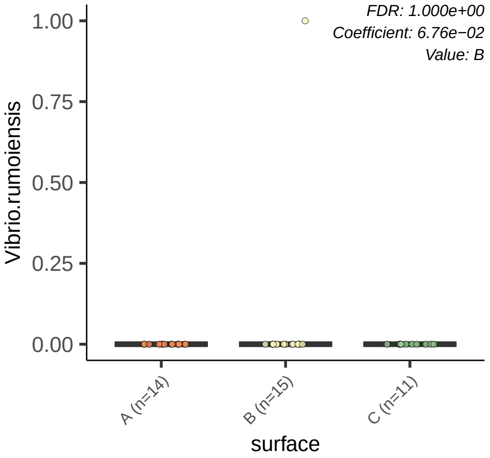

FDR: 1.000e+00
Coefficient: 6.76e−02
Value: B
Vibrio.rumoiensis
1.00
0.75
0.50
0.25
0.00
A (n=14)
B (n=15)
C (n=11)
surface

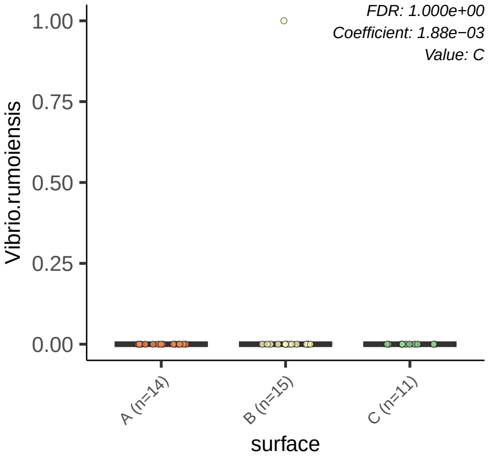

FDR: 1.000e+00
Coefficient: 1.88e−03
Value: C
Vibrio.rumoiensis
1.00
0.75
0.50
0.25
0.00
A (n=14)
B (n=15)
C (n=11)
surface

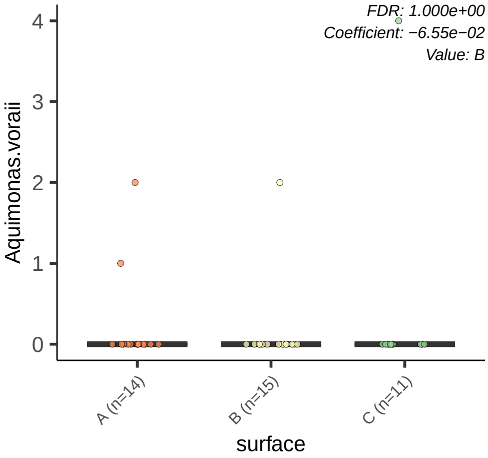

FDR: 1.000e+00
Coefficient: −6.55e−02
Value: B
Aquimonas.voraii
0
1
2
3
4
A (n=14)
B (n=15)
C (n=11)
surface

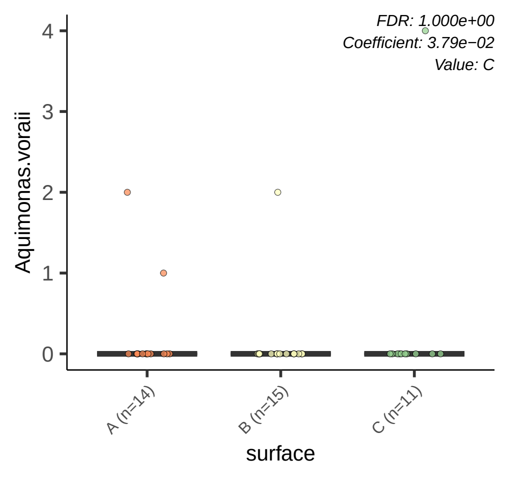

FDR: 1.000e+00
Coefficient: 3.79e−02
Value: C
Aquimonas.voraii
0
1
2
3
4
A (n=14)
B (n=15)
C (n=11)
surface

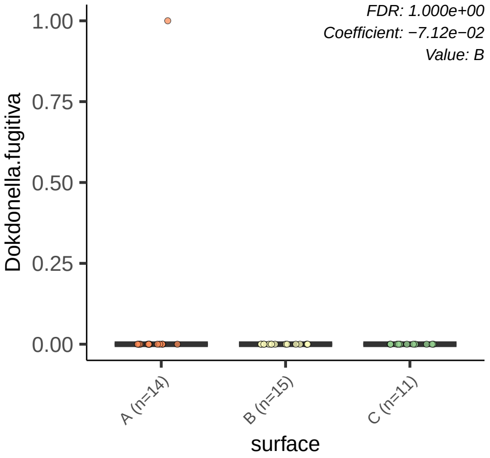
FDR: 1.000e+00
Coefficient: −7.12e−02
Value: B
Dokdonella.fugitiva
1.00
0.75
0.50
0.25
0.00
A (n=14)
B (n=15)
C (n=11)
surface

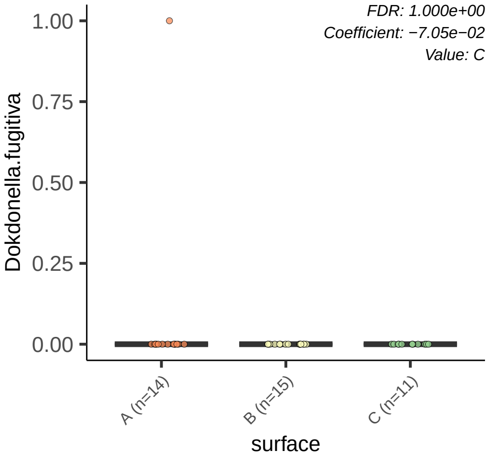
FDR: 1.000e+00
Coefficient: −7.05e−02
Value: C
Dokdonella.fugitiva
1.00
0.75
0.50
0.25
0.00
A (n=14)
B (n=15)
C (n=11)
surface

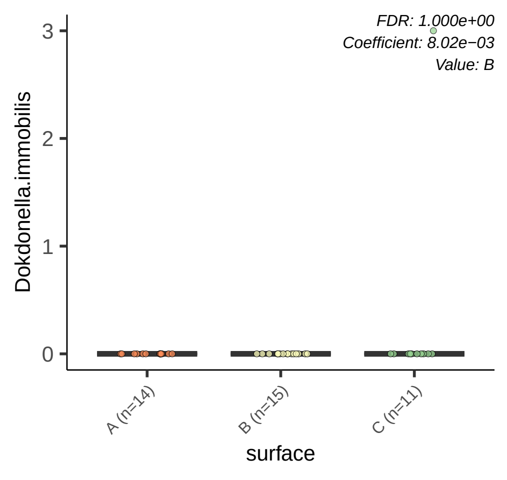

FDR: 1.000e+00
Coefficient: 8.02e−03
Value: B
Dokdonella.immobilis
0
1
2
3
A (n=14)
B (n=15)
C (n=11)
surface

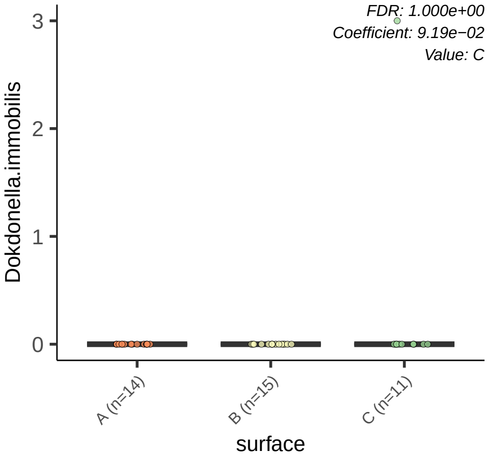
FDR: 1.000e+00
Coefficient: 9.19e−02
Value: C
Dokdonella.immobilis
3
2
1
0
A (n=14)
B (n=15)
C (n=11)
surface

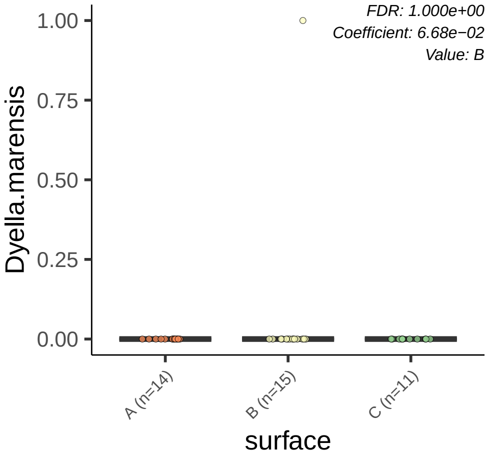

FDR: 1.000e+00
Coefficient: 6.68e−02
Value: B
Dyella.marensis
1.00
0.75
0.50
0.25
0.00
A (n=14)
B (n=15)
C (n=11)
surface

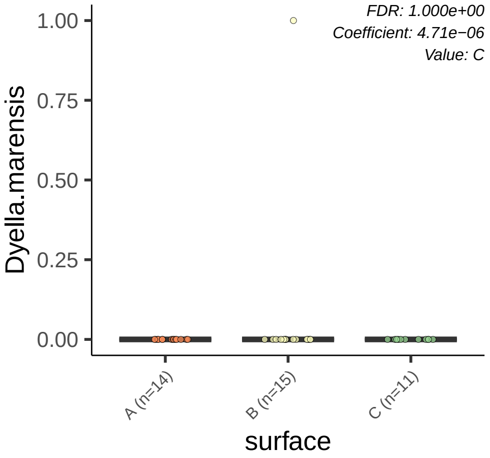
FDR: 1.000e+00
Coefficient: 4.71e−06
Value: C
Dyella.marensis
1.00
0.75
0.50
0.25
0.00
A (n=14)
B (n=15)
C (n=11)
surface

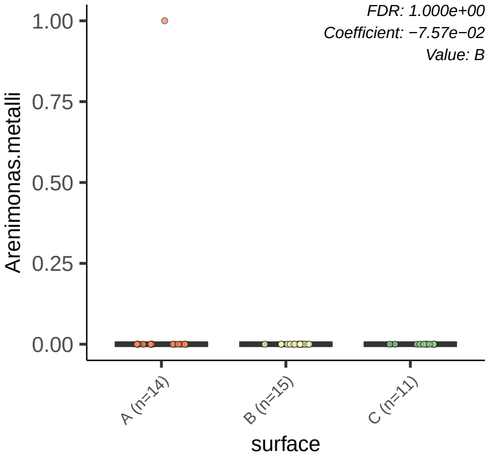

FDR: 1.000e+00
Coefficient: −7.57e−02
Value: B
Arenimonas.metalli
1.00
0.75
0.50
0.25
0.00
A (n=14)
B (n=15)
C (n=11)
surface

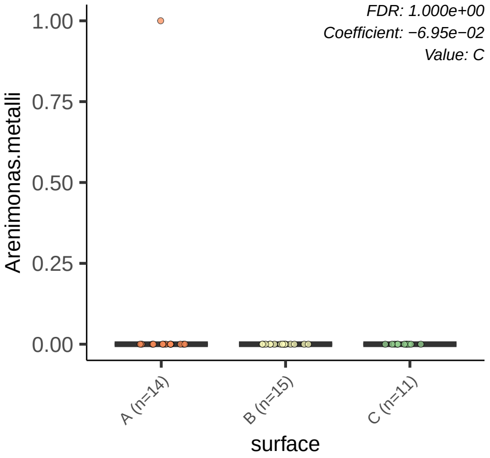
FDR: 1.000e+00
Coefficient: −6.95e−02
Value: C
1.00
0.75
0.50
0.25
0.00
Arenimonas.metalli
A (n=14)
B (n=15)
C (n=11)
surface

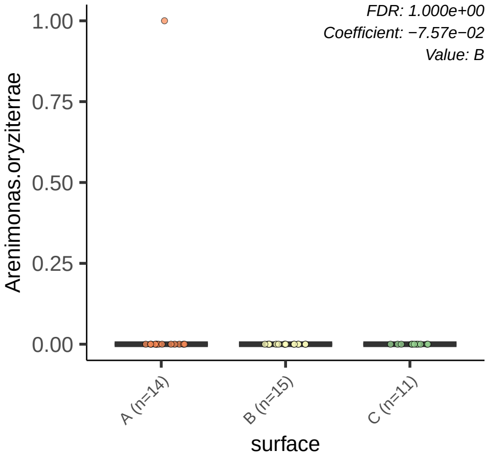
FDR: 1.000e+00
Coefficient: −7.57e−02
Value: B
Arenimonas.oryziterrae
1.00
0.75
0.50
0.25
0.00
A (n=14)
B (n=15)
C (n=11)
surface

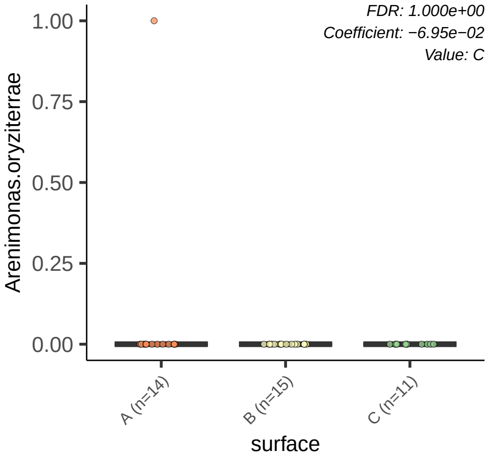

FDR: 1.000e+00
Coefficient: −6.95e−02
Value: C
Arenimonas.oryziterrae
1.00
0.75
0.50
0.25
0.00
A (n=14)
B (n=15)
C (n=11)
surface

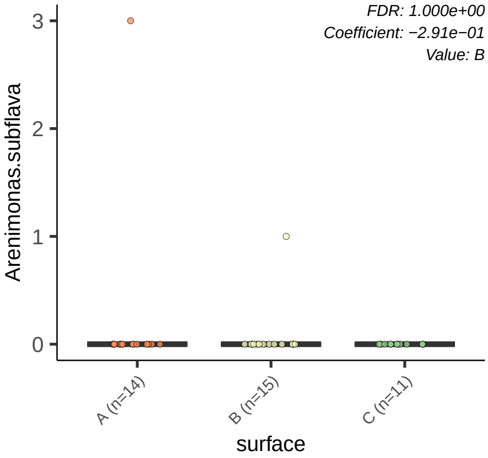

FDR: 1.000e+00
Coefficient: −2.91e−01
Value: B
Arenimonas.subflava
3
2
1
0
A (n=14)
B (n=15)
C (n=11)
surface

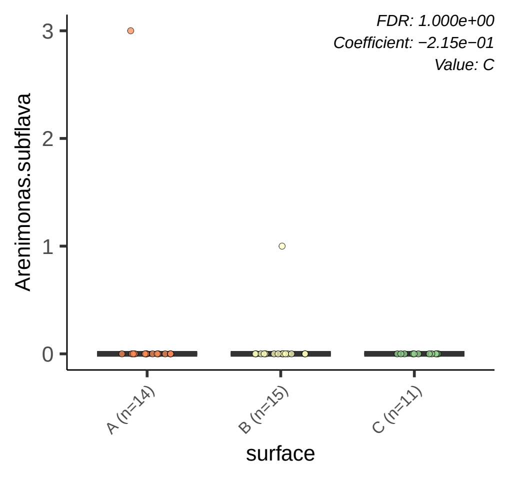
FDR: 1.000e+00
Coefficient: −2.15e−01
Value: C
Arenimonas.subflava
3
2
1
0
A (n=14)
B (n=15)
C (n=11)
surface

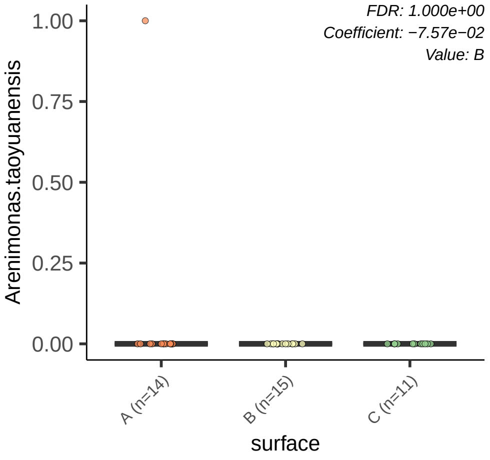
FDR: 1.000e+00
Coefficient: −7.57e−02
Value: B
Arenimonas.taoyuanensis
1.00
0.75
0.50
0.25
0.00
A (n=14)
B (n=15)
C (n=11)
surface

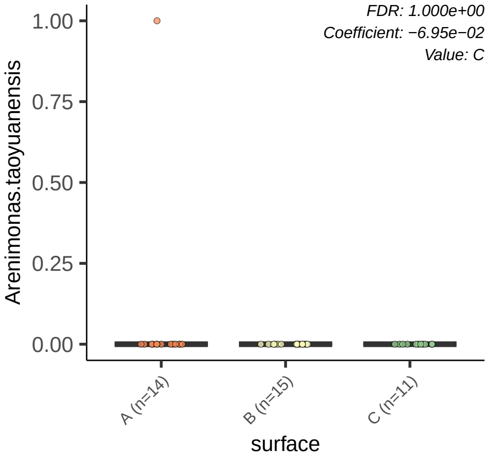

FDR: 1.000e+00
Coefficient: −6.95e−02
Value: C
Arenimonas.taoyuanensis
1.00
0.75
0.50
0.25
0.00
A (n=14)
B (n=15)
C (n=11)
surface

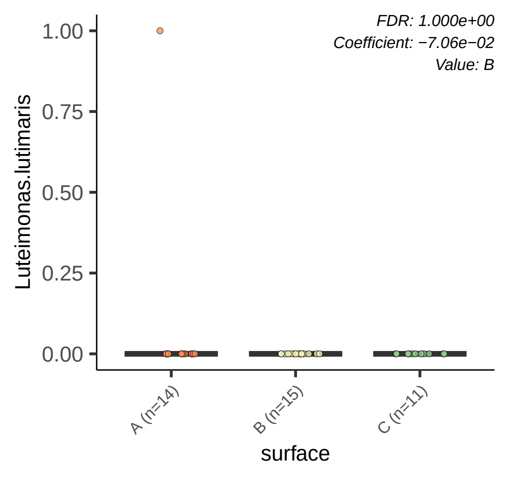
FDR: 1.000e+00
Coefficient: −7.06e−02
Value: B
Luteimonas.lutimaris
1.00
0.75
0.50
0.25
0.00
A (n=14)
B (n=15)
C (n=11)
surface

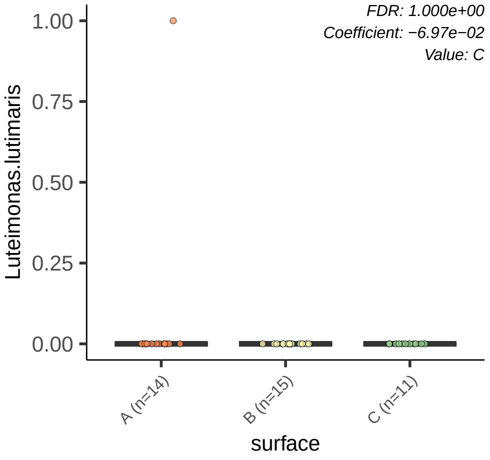
FDR: 1.000e+00
Coefficient: −6.97e−02
Value: C
Luteimonas.lutimaris
1.00
0.75
0.50
0.25
0.00
A (n=14)
B (n=15)
C (n=11)
surface

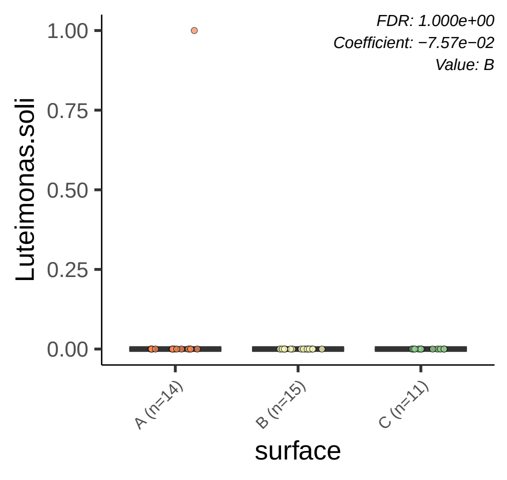

FDR: 1.000e+00
Coefficient: −7.57e−02
Value: B
Luteimonas.soli
1.00
0.75
0.50
0.25
0.00
A (n=14)
B (n=15)
C (n=11)
surface

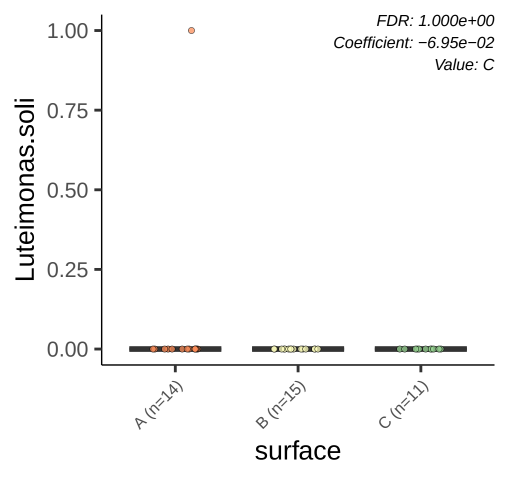
FDR: 1.000e+00
Coefficient: −6.95e−02
Value: C
Luteimonas.soli
1.00
0.75
0.50
0.25
0.00
A (n=14)
B (n=15)
C (n=11)
surface

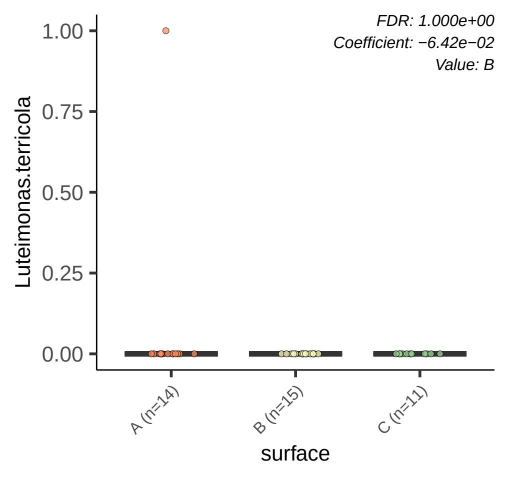
FDR: 1.000e+00
Coefficient: −6.42e−02
Value: B
Luteimonas.terricola
1.00
0.75
0.50
0.25
0.00
A (n=14)
B (n=15)
C (n=11)
surface

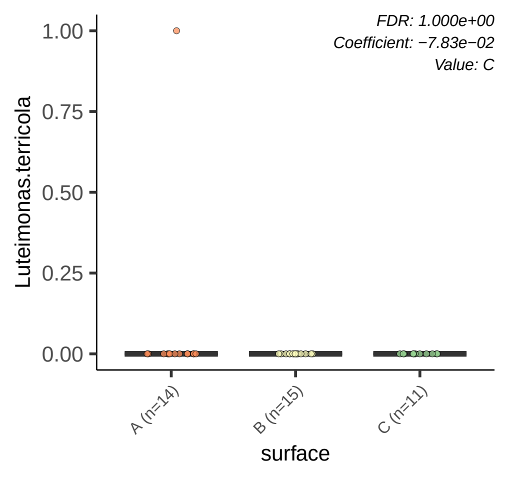
FDR: 1.000e+00
Coefficient: −7.83e−02
Value: C
Luteimonas.terricola
1.00
0.75
0.50
0.25
0.00
A (n=14)
B (n=15)
C (n=11)
surface

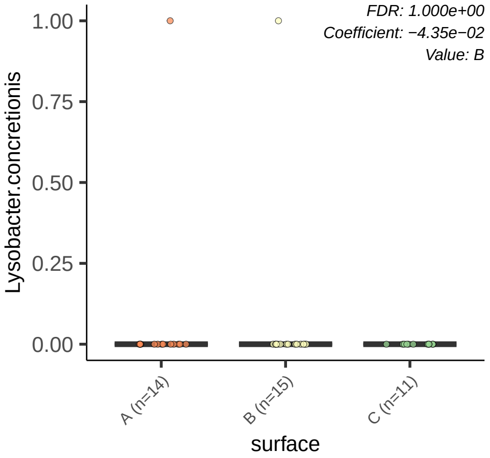
FDR: 1.000e+00
Coefficient: −4.35e−02
Value: B
Lysobacter.concretionis
1.00
0.75
0.50
0.25
0.00
A (n=14)
B (n=15)
C (n=11)
surface

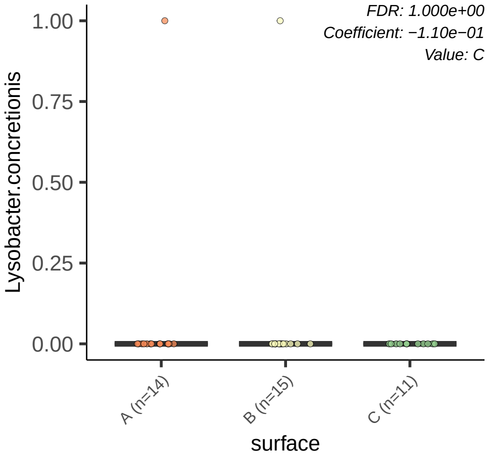
FDR: 1.000e+00
Coefficient: −1.10e−01
Value: C
Lysobacter.concretionis
1.00
0.75
0.50
0.25
0.00
A (n=14)
B (n=15)
C (n=11)
surface

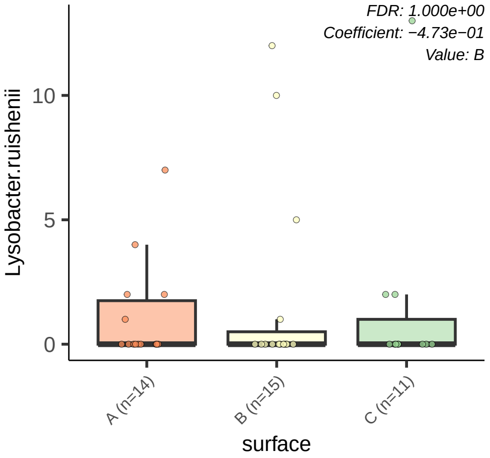
FDR: 1.000e+00
Coefficient: −4.73e−01
Value: B
Lysobacter.ruishenii
0
5
10
A (n=14)
B (n=15)
C (n=11)
surface

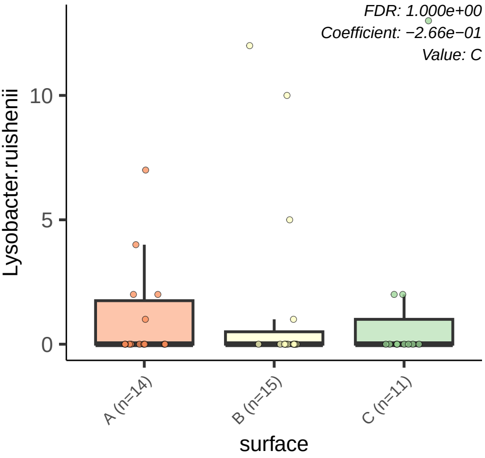

FDR: 1.000e+00
Coefficient: −2.66e−01
Value: C
Lysobacter.ruishenii
10
5
0
A (n=14)
B (n=15)
C (n=11)
surface

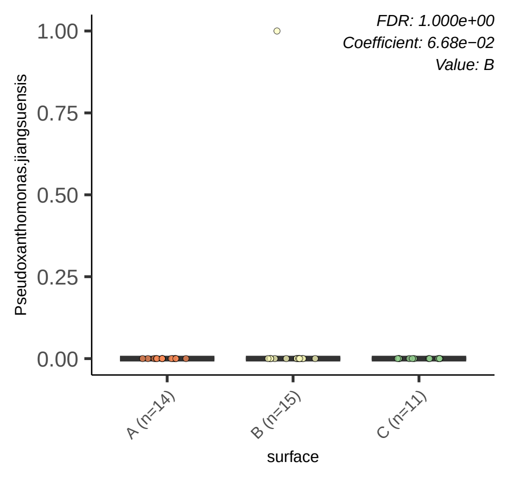
FDR: 1.000e+00
Coefficient: 6.68e−02
Value: B
Pseudoxanthomonas.jiangsuensis
1.00
0.75
0.50
0.25
0.00
A (n=14)
B (n=15)
C (n=11)
surface

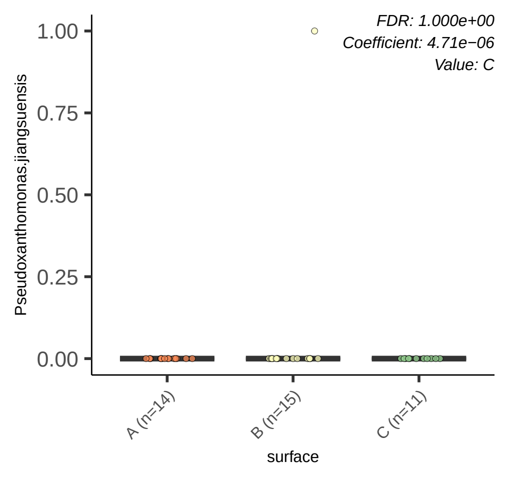
FDR: 1.000e+00
Coefficient: 4.71e−06
Value: C
Pseudoxanthomonas.jiangsuensis
1.00
0.75
0.50
0.25
0.00
A (n=14)
B (n=15)
C (n=11)
surface

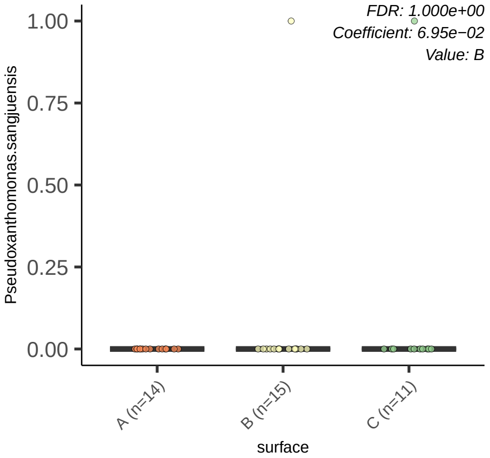

FDR: 1.000e+00
Coefficient: 6.95e−02
Value: B
Pseudoxanthomonas.sangjuensis
1.00
0.75
0.50
0.25
0.00
A (n=14)
B (n=15)
C (n=11)
surface

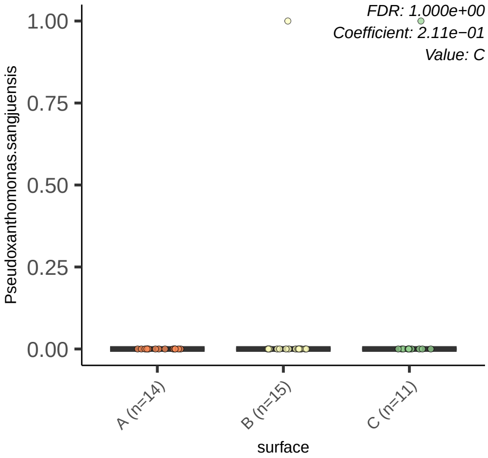
FDR: 1.000e+00
Coefficient: 2.11e−01
Value: C
Pseudoxanthomonas.sangjuensis
1.00
0.75
0.50
0.25
0.00
A (n=14)
B (n=15)
C (n=11)
surface

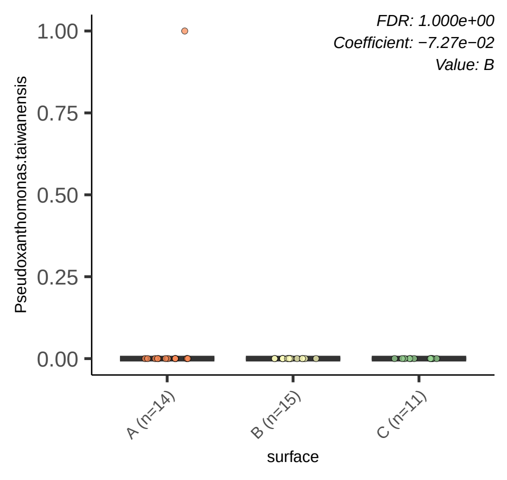
FDR: 1.000e+00
Coefficient: −7.27e−02
Value: B
Pseudoxanthomonas.taiwanensis
1.00
0.75
0.50
0.25
0.00
A (n=14)
B (n=15)
C (n=11)
surface

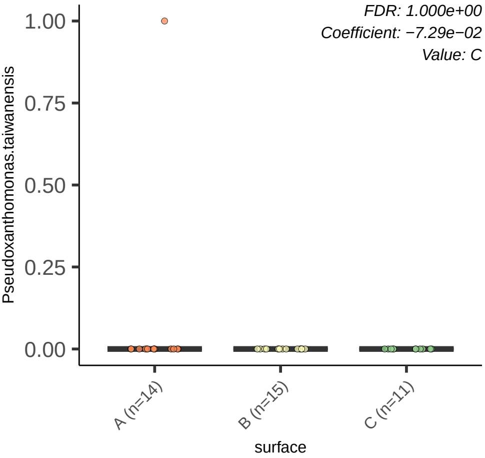

FDR: 1.000e+00
Coefficient: −7.29e−02
Value: C
Pseudoxanthomonas.taiwanensis
1.00
0.75
0.50
0.25
0.00
A (n=14)
B (n=15)
C (n=11)
surface

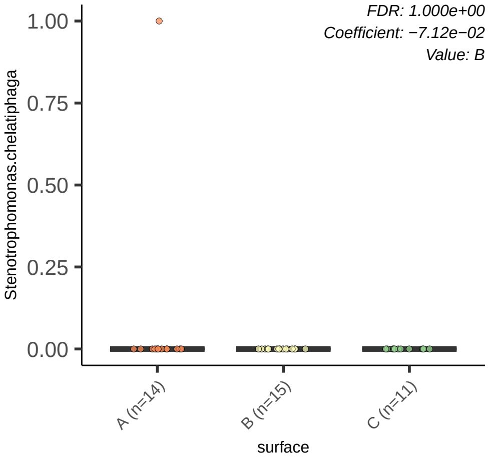
FDR: 1.000e+00
Coefficient: −7.12e−02
Value: B
Stenotrophomonas.chelatiphaga
1.00
0.75
0.50
0.25
0.00
A (n=14)
B (n=15)
C (n=11)
surface

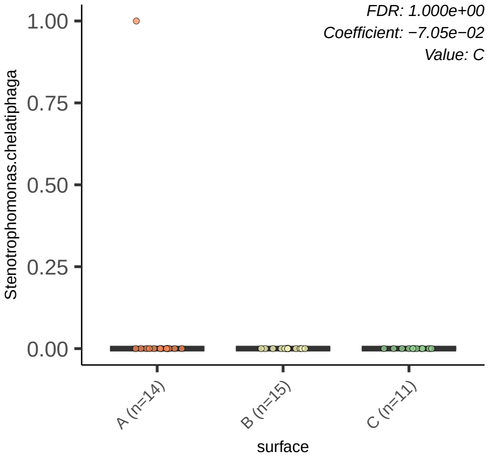
FDR: 1.000e+00
Coefficient: −7.05e−02
Value: C
1.00
0.75
0.50
0.25
0.00
Stenotrophomonas.chelatiphaga
A (n=14)
B (n=15)
C (n=11)
surface

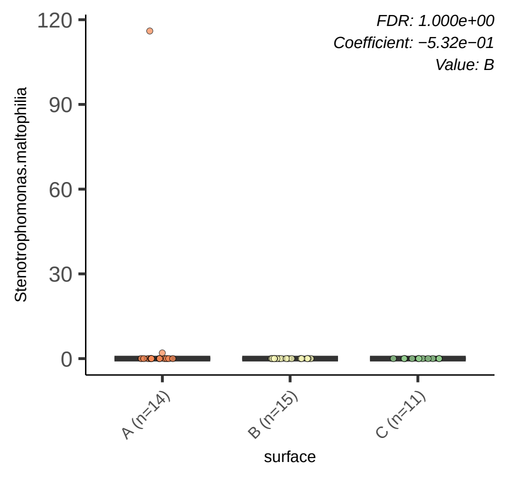
FDR: 1.000e+00
Coefficient: −5.32e−01
Value: B
Stenotrophomonas.maltophilia
120
90
60
30
0
A (n=14)
B (n=15)
C (n=11)
surface

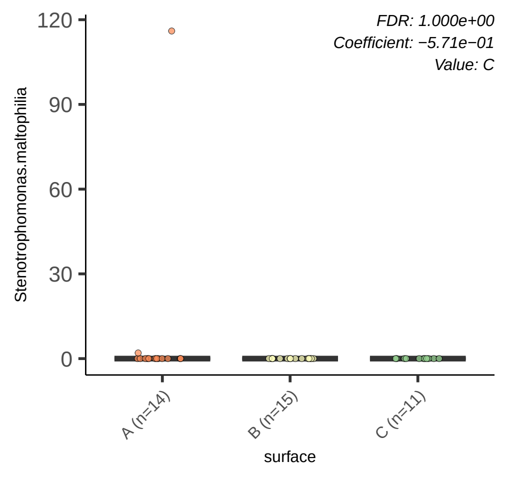
FDR: 1.000e+00
Coefficient: −5.71e−01
Value: C
120
90
60
30
0
Stenotrophomonas.maltophilia
A (n=14)
B (n=15)
C (n=11)
surface

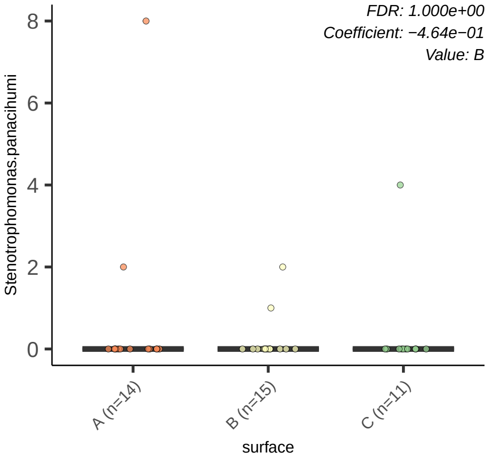

FDR: 1.000e+00
Coefficient: −4.64e−01
Value: B
Stenotrophomonas.panacihumi
8
6
4
2
0
A (n=14)
B (n=15)
C (n=11)
surface

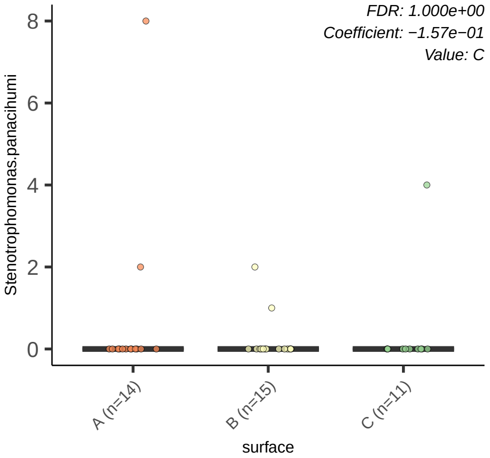

FDR: 1.000e+00
Coefficient: −1.57e−01
Value: C
Stenotrophomonas.panacihumi
8
6
4
2
0
A (n=14)
B (n=15)
C (n=11)
surface

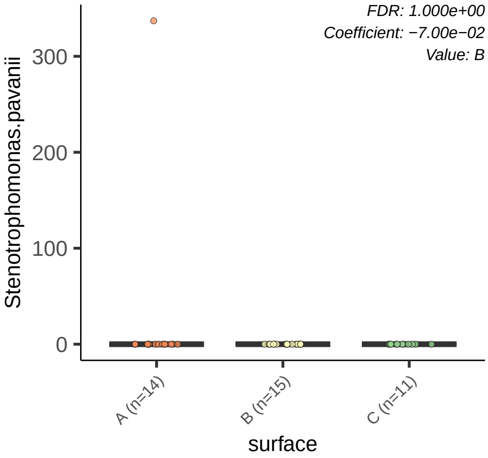
FDR: 1.000e+00
Coefficient: −7.00e−02
Value: B
Stenotrophomonas.pavanii
300
200
100
0
A (n=14)
B (n=15)
C (n=11)
surface

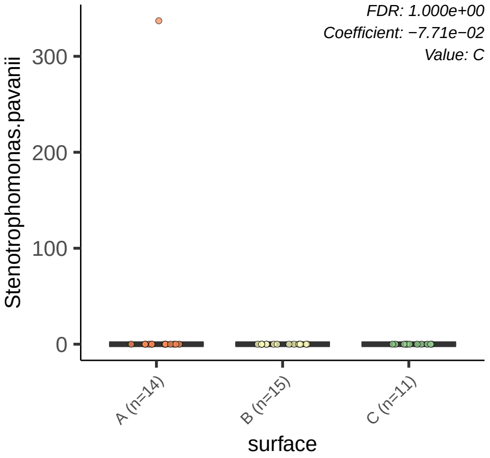

FDR: 1.000e+00
Coefficient: −7.71e−02
Value: C
Stenotrophomonas.pavanii
300
200
100
0
A (n=14)
B (n=15)
C (n=11)
surface

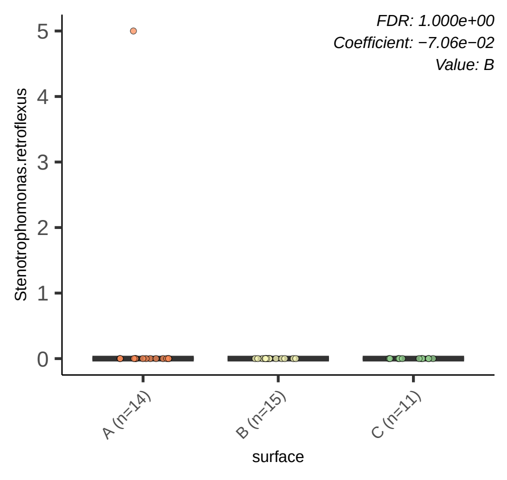
FDR: 1.000e+00
Coefficient: −7.06e−02
Value: B
Stenotrophomonas.retroflexus
0
1
2
3
4
5
A (n=14)
B (n=15)
C (n=11)
surface

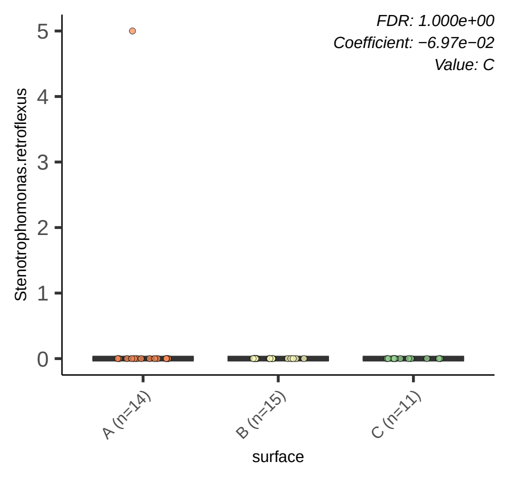
FDR: 1.000e+00
Coefficient: −6.97e−02
Value: C
Stenotrophomonas.retroflexus
5
4
3
2
1
0
A (n=14)
B (n=15)
C (n=11)
surface

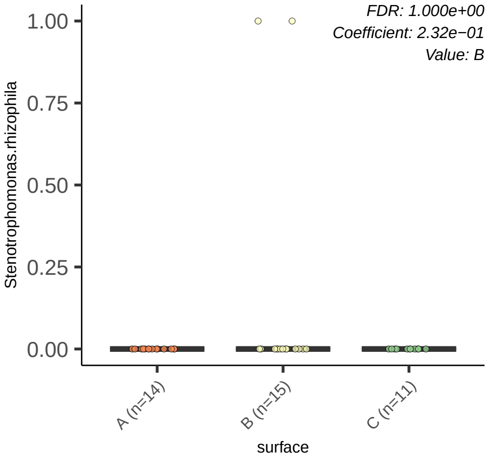
FDR: 1.000e+00
Coefficient: 2.32e−01
Value: B
1.00
0.75
0.50
0.25
0.00
Stenotrophomonas.rhizophila
A (n=14)
B (n=15)
C (n=11)
surface

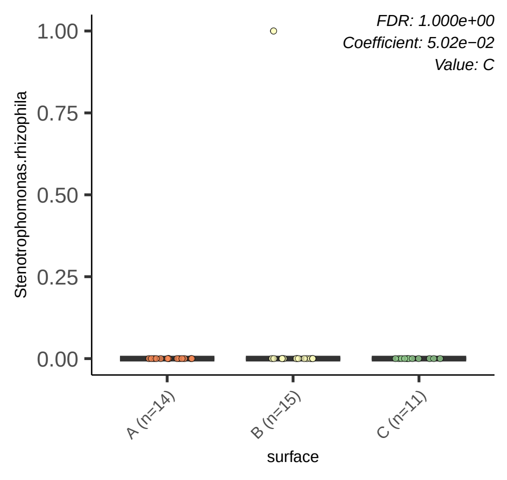
FDR: 1.000e+00
Coefficient: 5.02e−02
Value: C
Stenotrophomonas.rhizophila
1.00
0.75
0.50
0.25
0.00
A (n=14)
B (n=15)
C (n=11)
surface

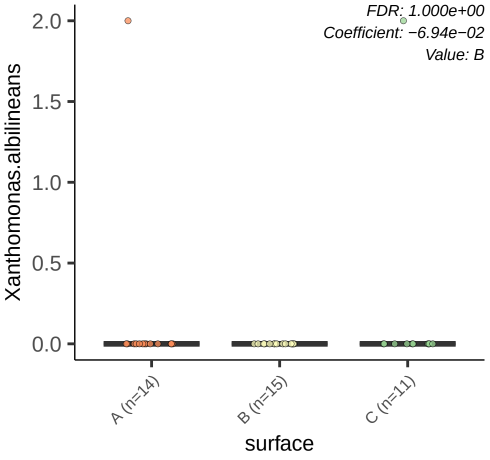

FDR: 1.000e+00
Coefficient: −6.94e−02
Value: B
Xanthomonas.albilineans
2.0
1.5
1.0
0.5
0.0
A (n=14)
B (n=15)
C (n=11)
surface

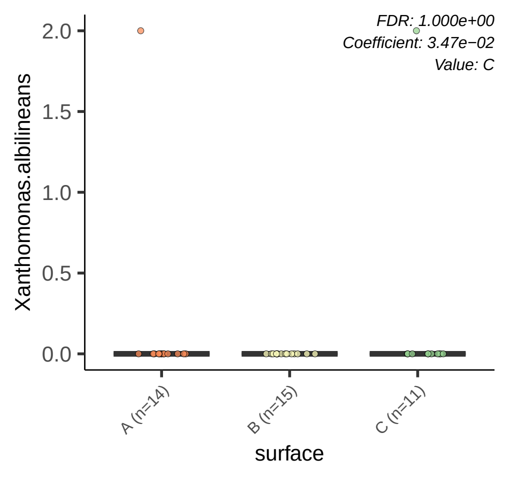
FDR: 1.000e+00
Coefficient: 3.47e−02
Value: C
Xanthomonas.albilineans
2.0
1.5
1.0
0.5
0.0
A (n=14)
B (n=15)
C (n=11)
surface

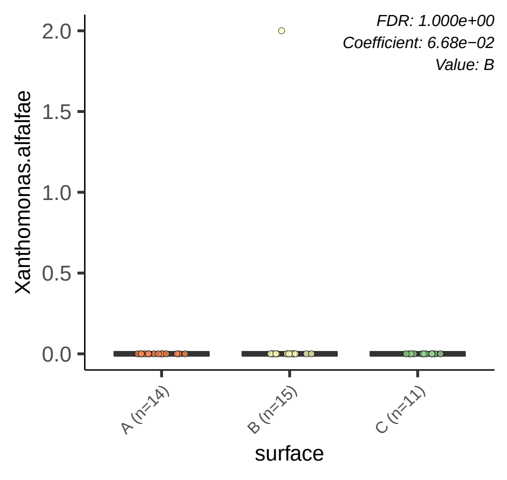
FDR: 1.000e+00
Coefficient: 6.68e−02
Value: B
Xanthomonas.alfalfae
2.0
1.5
1.0
0.5
0.0
A (n=14)
B (n=15)
C (n=11)
surface

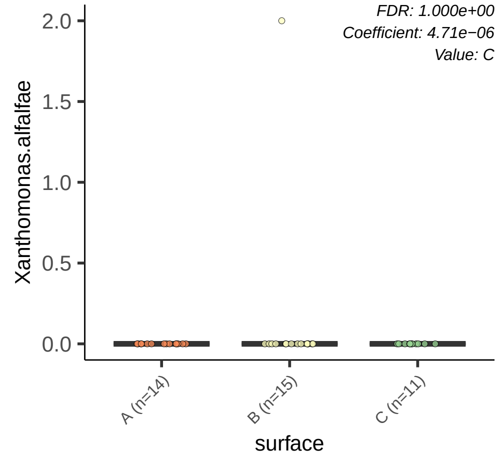
FDR: 1.000e+00
Coefficient: 4.71e−06
Value: C
Xanthomonas.alfalfae
2.0
1.5
1.0
0.5
0.0
A (n=14)
B (n=15)
C (n=11)
surface

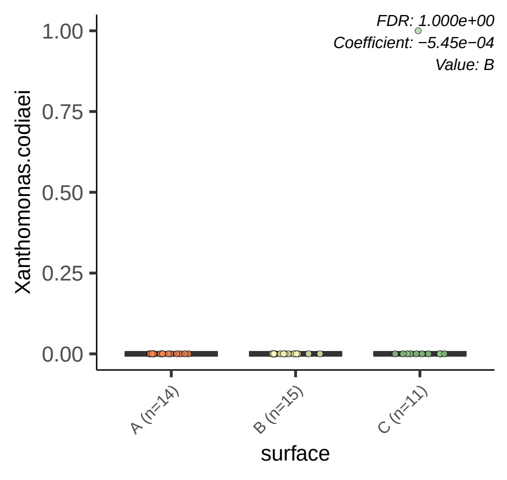
FDR: 1.000e+00
Coefficient: −5.45e−04
Value: B
Xanthomonas.codiaei
1.00
0.75
0.50
0.25
0.00
A (n=14)
B (n=15)
C (n=11)
surface

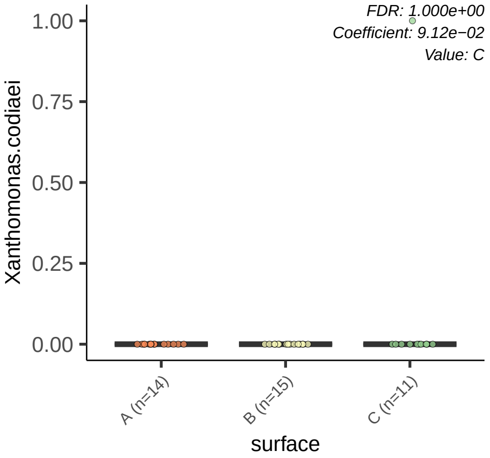
FDR: 1.000e+00
Coefficient: 9.12e−02
Value: C
Xanthomonas.codiaei
1.00
0.75
0.50
0.25
0.00
A (n=14)
B (n=15)
C (n=11)
surface

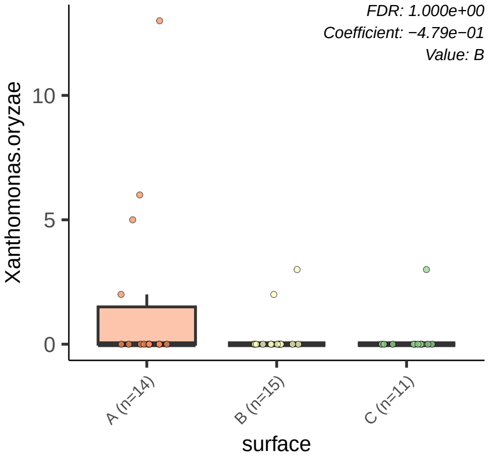
FDR: 1.000e+00
Coefficient: −4.79e−01
Value: B
Xanthomonas.oryzae
10
5
0
A (n=14)
B (n=15)
C (n=11)
surface

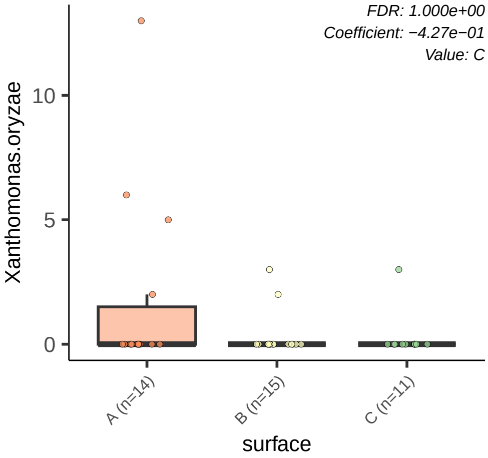
FDR: 1.000e+00
Coefficient: −4.27e−01
Value: C
Xanthomonas.oryzae
10
5
0
A (n=14)
B (n=15)
C (n=11)
surface

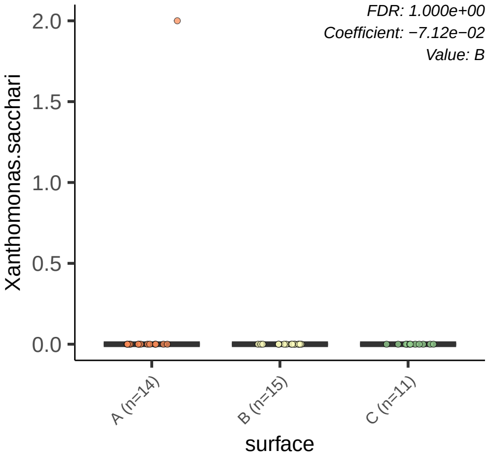
FDR: 1.000e+00
Coefficient: −7.12e−02
Value: B
Xanthomonas.sacchari
2.0
1.5
1.0
0.5
0.0
A (n=14)
B (n=15)
C (n=11)
surface

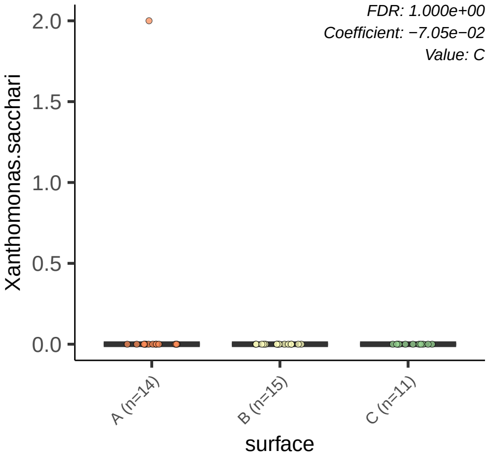
FDR: 1.000e+00
Coefficient: −7.05e−02
Value: C
Xanthomonas.sacchari
2.0
1.5
1.0
0.5
0.0
A (n=14)
B (n=15)
C (n=11)
surface

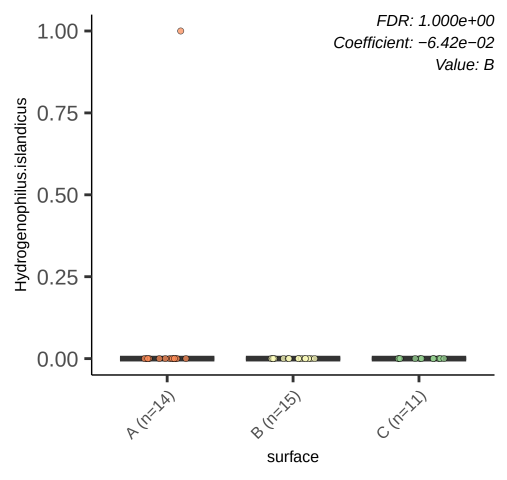
FDR: 1.000e+00
Coefficient: −6.42e−02
Value: B
1.00
0.75
0.50
0.25
0.00
Hydrogenophilus.islandicus
A (n=14)
B (n=15)
C (n=11)
surface

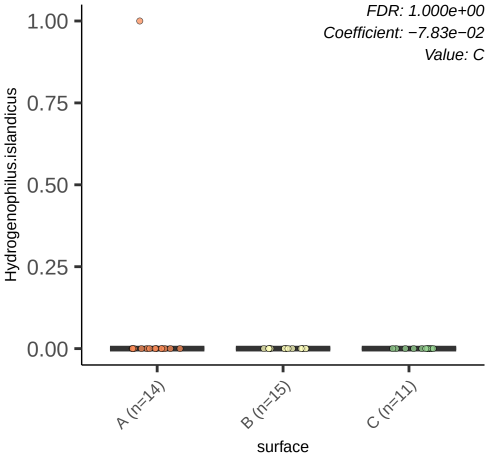
FDR: 1.000e+00
Coefficient: −7.83e−02
Value: C
1.00
0.75
0.50
0.25
0.00
Hydrogenophilus.islandicus
A (n=14)
B (n=15)
C (n=11)
surface

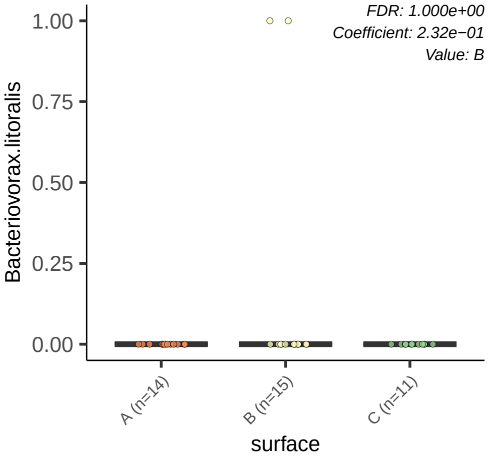

FDR: 1.000e+00
Coefficient: 2.32e−01
Value: B
Bacteriovorax.litoralis
1.00
0.75
0.50
0.25
0.00
A (n=14)
B (n=15)
C (n=11)
surface

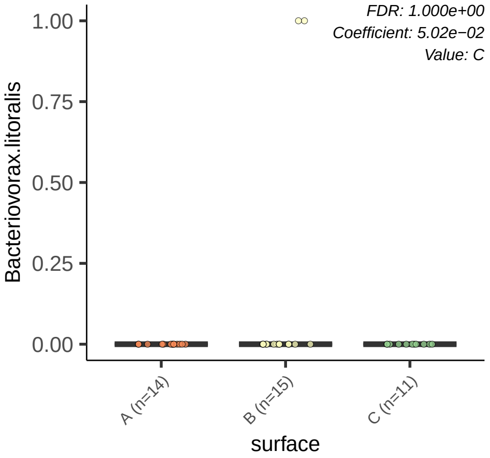

FDR: 1.000e+00
Coefficient: 5.02e−02
Value: C
1.00
0.75
0.50
0.25
0.00
Bacteriovorax.litoralis
A (n=14)
B (n=15)
C (n=11)
surface

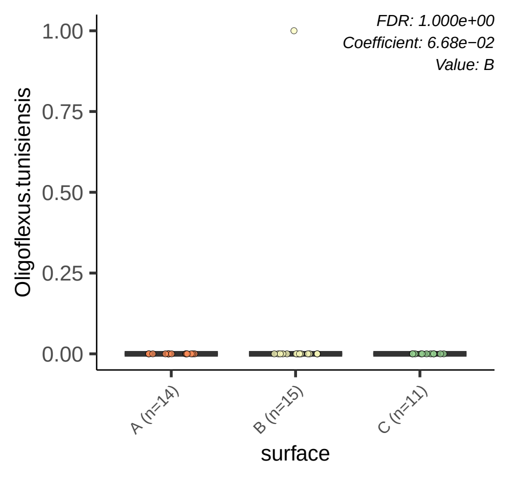
FDR: 1.000e+00
Coefficient: 6.68e−02
Value: B
Oligoflexus.tunisiensis
1.00
0.75
0.50
0.25
0.00
A (n=14)
B (n=15)
C (n=11)
surface

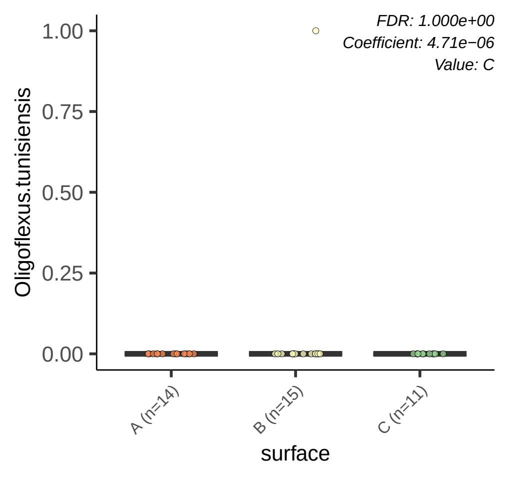
FDR: 1.000e+00
Coefficient: 4.71e−06
Value: C
Oligoflexus.tunisiensis
1.00
0.75
0.50
0.25
0.00
A (n=14)
B (n=15)
C (n=11)
surface

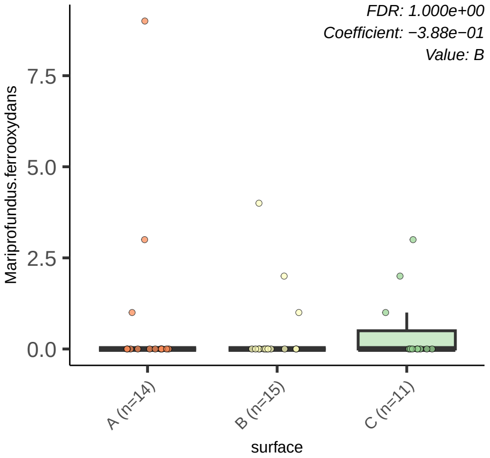

FDR: 1.000e+00
Coefficient: −3.88e−01
Value: B
Mariprofundus.ferrooxydans
7.5
5.0
2.5
0.0
A (n=14)
B (n=15)
C (n=11)
surface

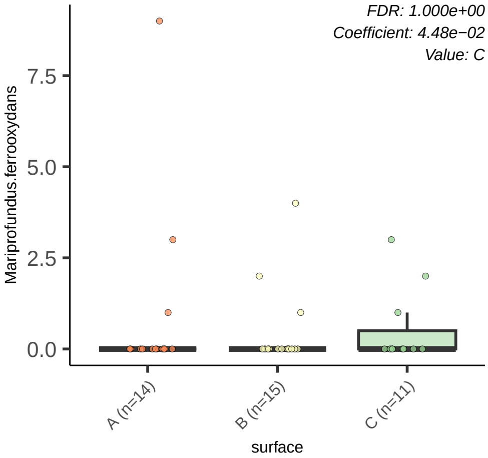

FDR: 1.000e+00
Coefficient: 4.48e−02
Value: C
Mariprofundus.ferrooxydans
7.5
5.0
2.5
0.0
A (n=14)
B (n=15)
C (n=11)
surface

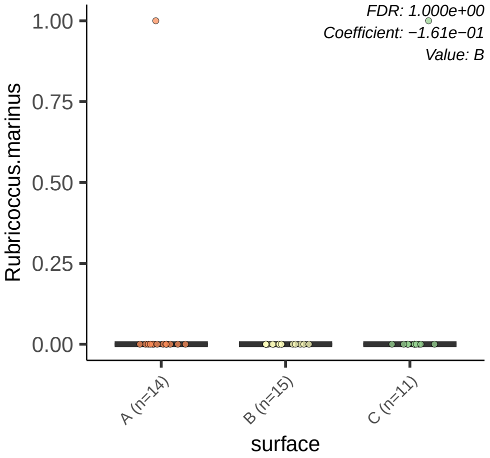
FDR: 1.000e+00
Coefficient: −1.61e−01
Value: B
Rubricoccus.marinus
1.00
0.75
0.50
0.25
0.00
A (n=14)
B (n=15)
C (n=11)
surface

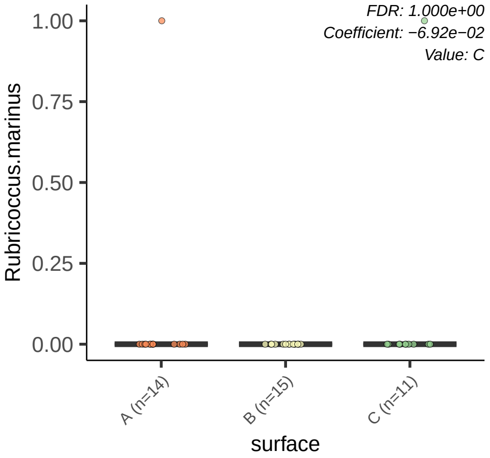

FDR: 1.000e+00
Coefficient: −6.92e−02
Value: C
Rubricoccus.marinus
1.00
0.75
0.50
0.25
0.00
A (n=14)
B (n=15)
C (n=11)
surface

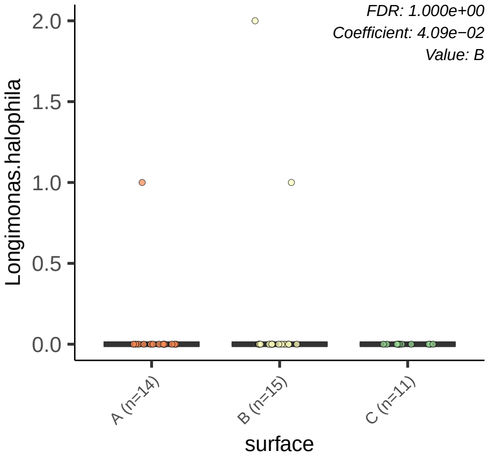
FDR: 1.000e+00
Coefficient: 4.09e−02
Value: B
2.0
1.5
1.0
0.5
0.0
Longimonas.halophila
A (n=14)
B (n=15)
C (n=11)
surface

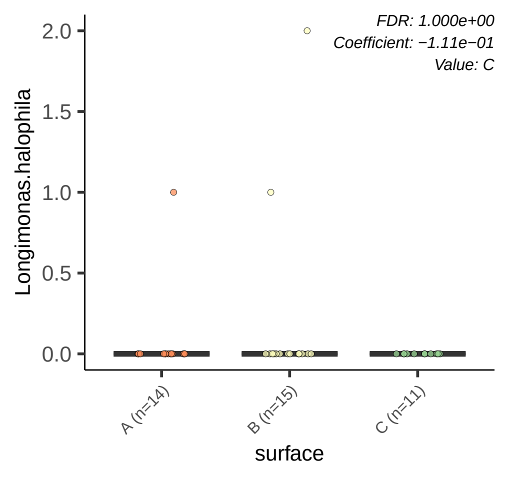
FDR: 1.000e+00
Coefficient: −1.11e−01
Value: C
Longimonas.halophila
2.0
1.5
1.0
0.5
0.0
A (n=14)
B (n=15)
C (n=11)
surface

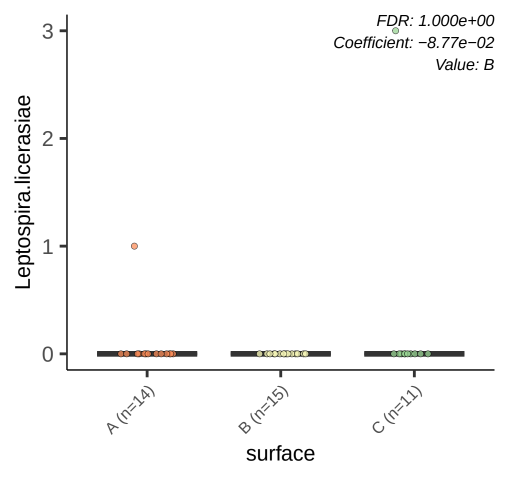

FDR: 1.000e+00
Coefficient: −8.77e−02
Value: B
Leptospira.licerasiae
0
1
2
3
A (n=14)
B (n=15)
C (n=11)
surface

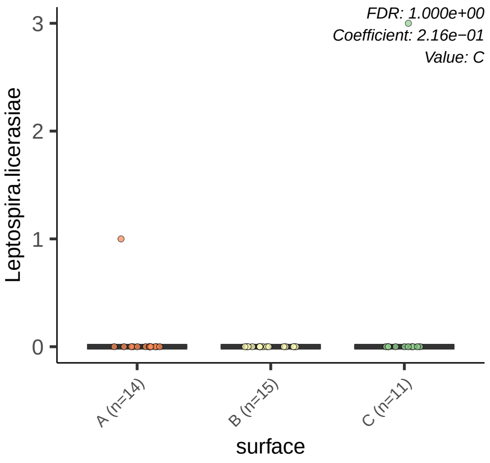
FDR: 1.000e+00
Coefficient: 2.16e−01
Value: C
Leptospira.licerasiae
0
1
2
3
A (n=14)
B (n=15)
C (n=11)
surface

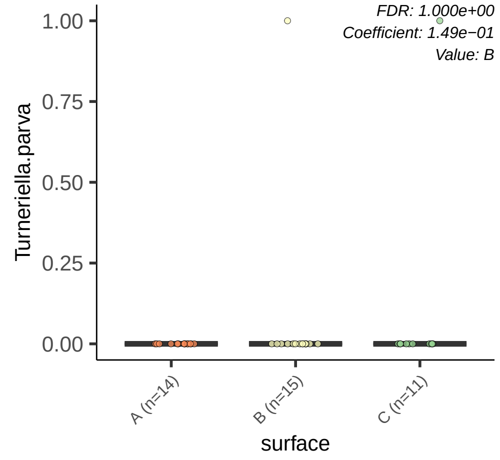
FDR: 1.000e+00
Coefficient: 1.49e−01
Value: B
Turneriella.parva
1.00
0.75
0.50
0.25
0.00
A (n=14)
B (n=15)
C (n=11)
surface

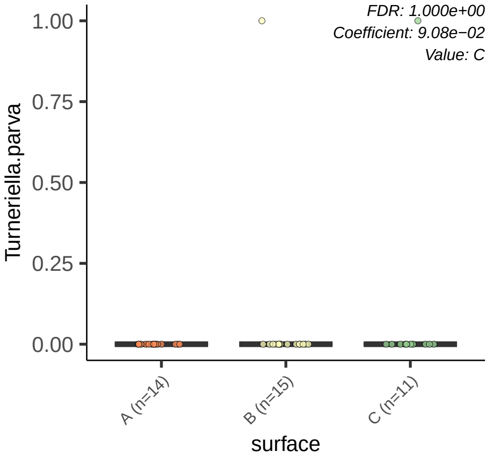

FDR: 1.000e+00
Coefficient: 9.08e−02
Value: C
Turneriella.parva
1.00
0.75
0.50
0.25
0.00
A (n=14)
B (n=15)
C (n=11)
surface

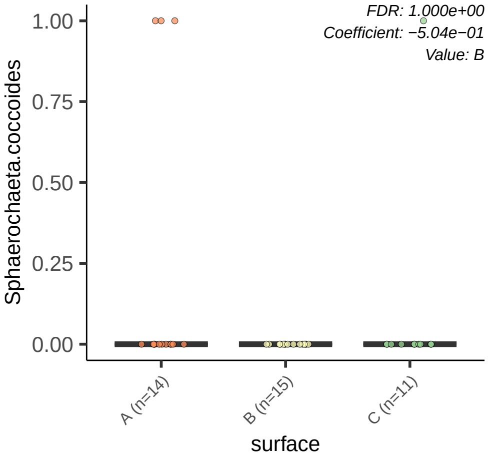

FDR: 1.000e+00
Coefficient: −5.04e−01
Value: B
Sphaerochaeta.coccoides
1.00
0.75
0.50
0.25
0.00
A (n=14)
B (n=15)
C (n=11)
surface

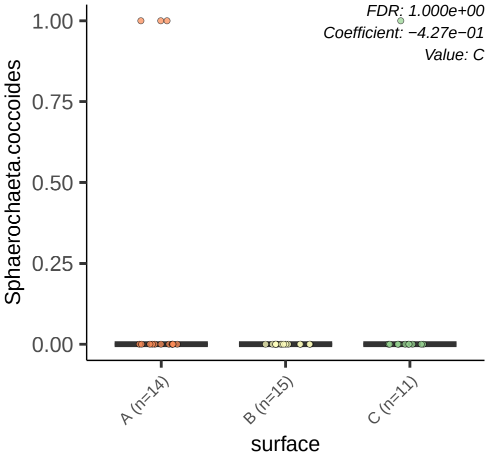
FDR: 1.000e+00
Coefficient: −4.27e−01
Value: C
Sphaerochaeta.coccoides
1.00
0.75
0.50
0.25
0.00
A (n=14)
B (n=15)
C (n=11)
surface

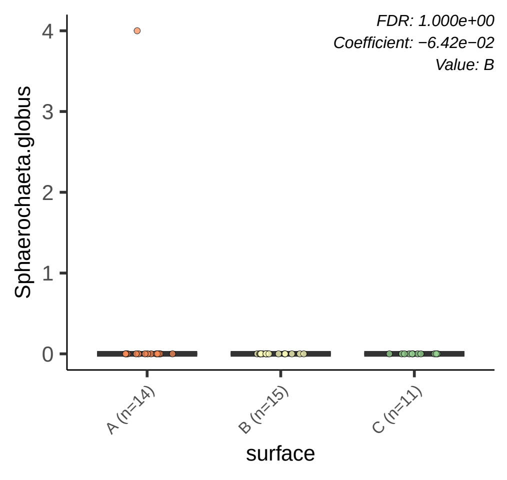

FDR: 1.000e+00
Coefficient: −6.42e−02
Value: B
Sphaerochaeta.globus
0
1
2
3
4
A (n=14)
B (n=15)
C (n=11)
surface

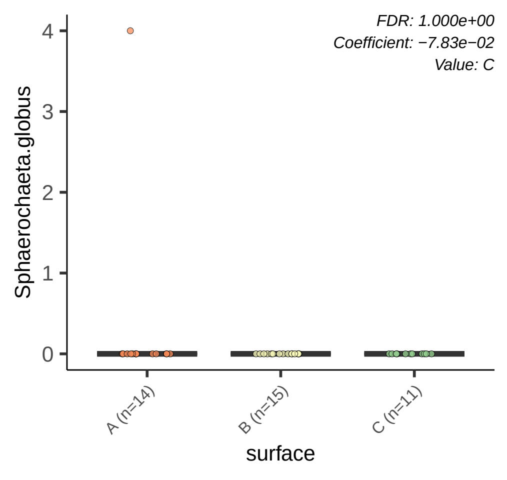
FDR: 1.000e+00
Coefficient: −7.83e−02
Value: C
Sphaerochaeta.globus
4
3
2
1
0
A (n=14)
B (n=15)
C (n=11)
surface

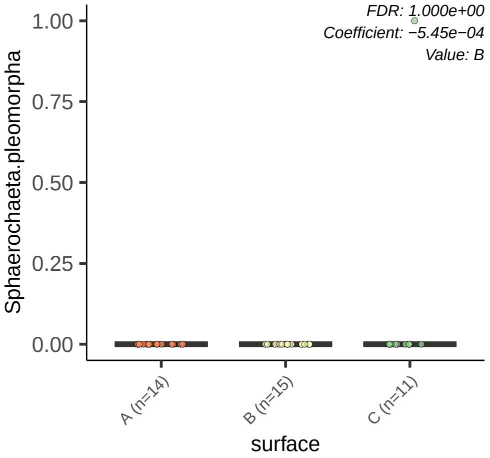
FDR: 1.000e+00
Coefficient: −5.45e−04
Value: B
Sphaerochaeta.pleomorpha
1.00
0.75
0.50
0.25
0.00
A (n=14)
B (n=15)
C (n=11)
surface

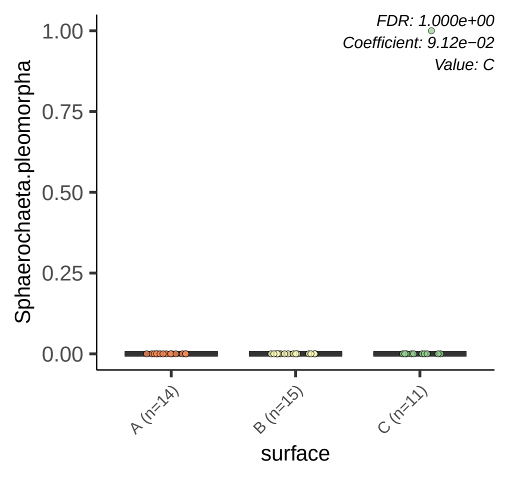

FDR: 1.000e+00
Coefficient: 9.12e−02
Value: C
Sphaerochaeta.pleomorpha
1.00
0.75
0.50
0.25
0.00
A (n=14)
B (n=15)
C (n=11)
surface

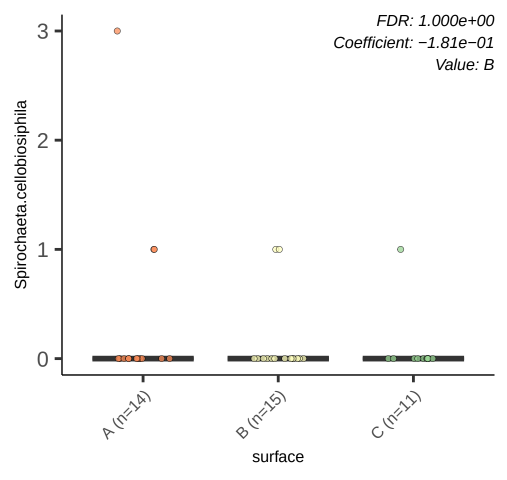

FDR: 1.000e+00
Coefficient: −1.81e−01
Value: B
Spirochaeta.cellobiosiphila
3
2
1
0
A (n=14)
B (n=15)
C (n=11)
surface

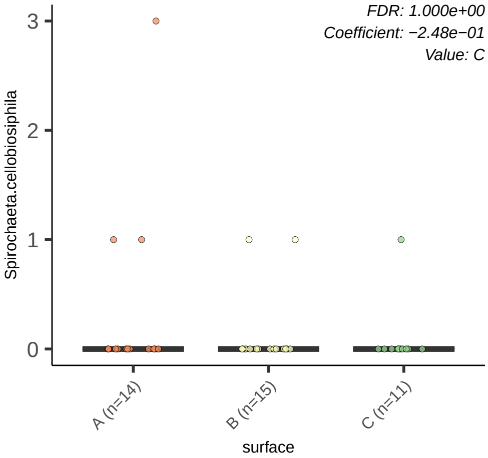

FDR: 1.000e+00
Coefficient: −2.48e−01
Value: C
Spirochaeta.cellobiosiphila
3
2
1
0
A (n=14)
B (n=15)
C (n=11)
surface

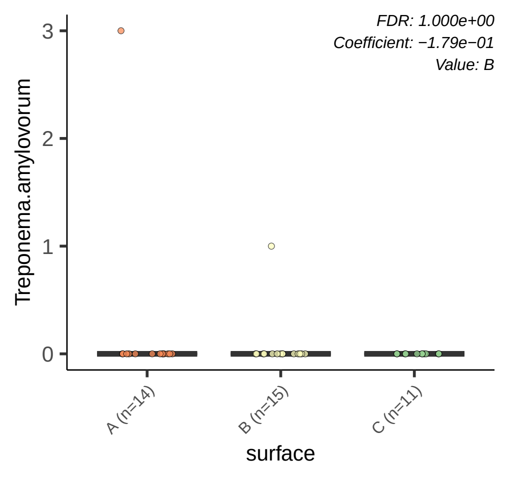
FDR: 1.000e+00
Coefficient: −1.79e−01
Value: B
Treponema.amylovorum
3
2
1
0
A (n=14)
B (n=15)
C (n=11)
surface

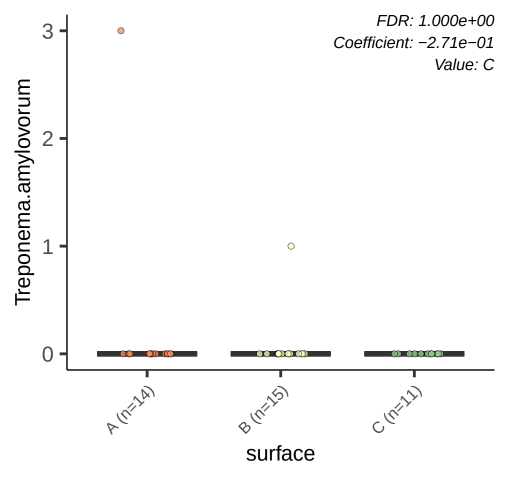
FDR: 1.000e+00
Coefficient: −2.71e−01
Value: C
Treponema.amylovorum
3
2
1
0
A (n=14)
B (n=15)
C (n=11)
surface

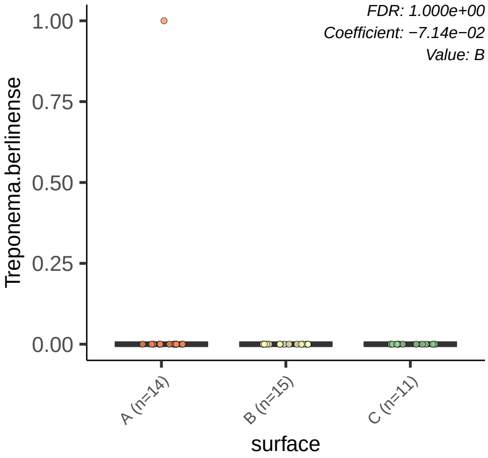

FDR: 1.000e+00
Coefficient: −7.14e−02
Value: B
Treponema.berlinense
1.00
0.75
0.50
0.25
0.00
A (n=14)
B (n=15)
C (n=11)
surface

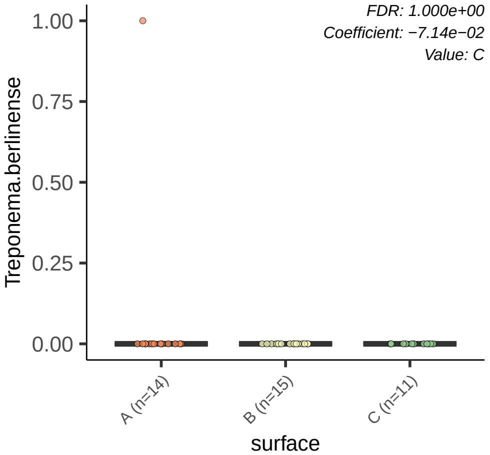
FDR: 1.000e+00
Coefficient: −7.14e−02
Value: C
Treponema.berlinense
1.00
0.75
0.50
0.25
0.00
A (n=14)
B (n=15)
C (n=11)
surface

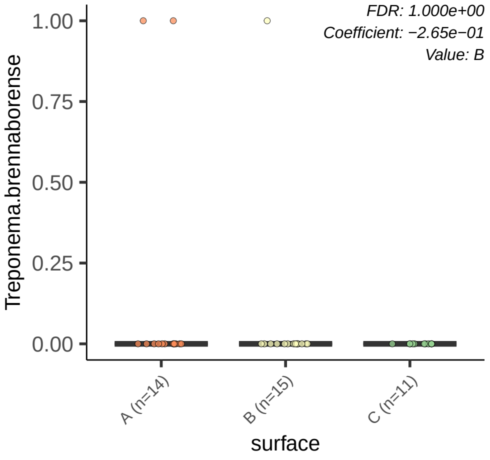
FDR: 1.000e+00
Coefficient: −2.65e−01
Value: B
Treponema.brennaborense
1.00
0.75
0.50
0.25
0.00
A (n=14)
B (n=15)
C (n=11)
surface

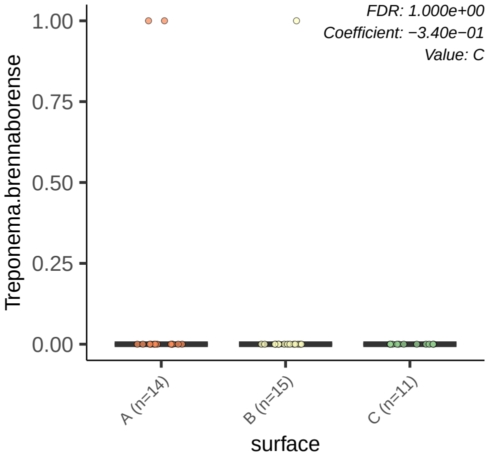
FDR: 1.000e+00
Coefficient: −3.40e−01
Value: C
Treponema.brennaborense
1.00
0.75
0.50
0.25
0.00
A (n=14)
B (n=15)
C (n=11)
surface

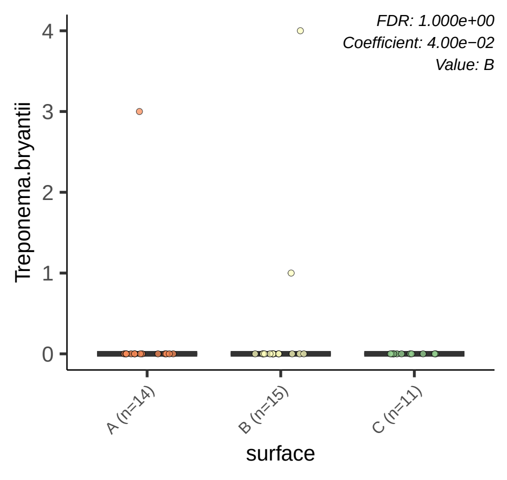

FDR: 1.000e+00
Coefficient: 4.00e−02
Value: B
Treponema.bryantii
0
1
2
3
4
A (n=14)
B (n=15)
C (n=11)
surface

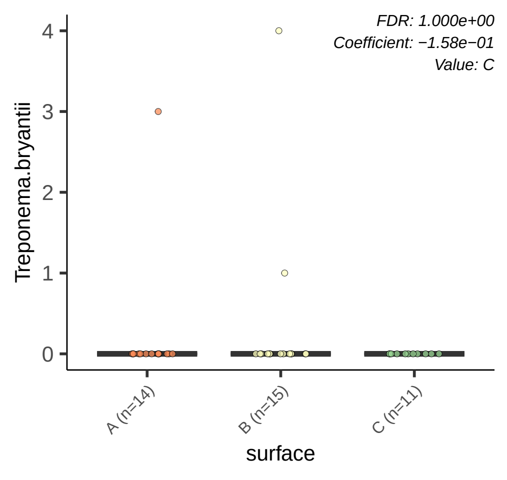
FDR: 1.000e+00
Coefficient: −1.58e−01
Value: C
Treponema.bryantii
0
1
2
3
4
A (n=14)
B (n=15)
C (n=11)
surface

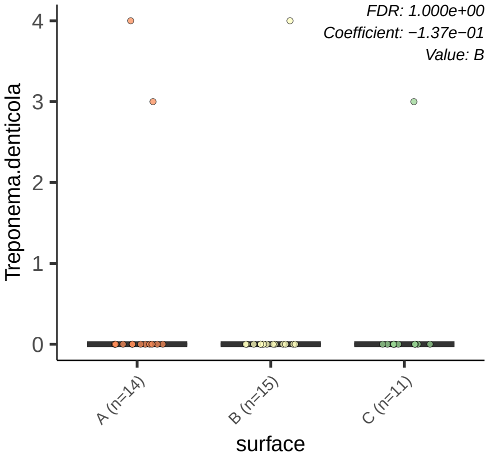
FDR: 1.000e+00
Coefficient: −1.37e−01
Value: B
Treponema.denticola
4
3
2
1
0
A (n=14)
B (n=15)
C (n=11)
surface

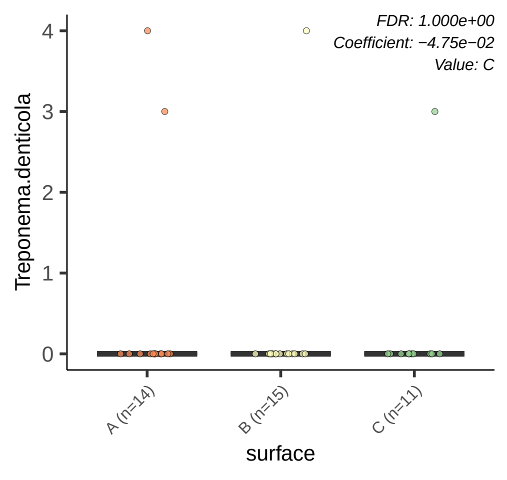
FDR: 1.000e+00
Coefficient: −4.75e−02
Value: C
Treponema.denticola
0
1
2
3
4
A (n=14)
B (n=15)
C (n=11)
surface

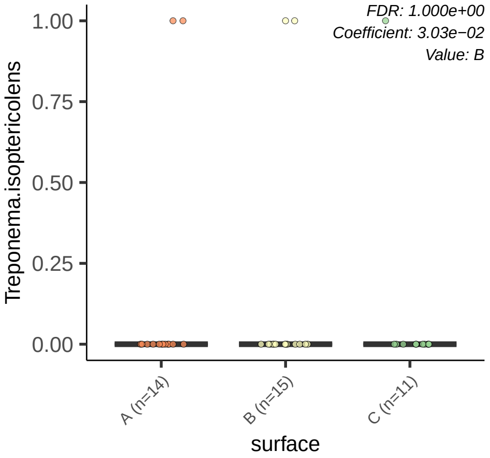
FDR: 1.000e+00
Coefficient: 3.03e−02
Value: B
Treponema.isoptericolens
1.00
0.75
0.50
0.25
0.00
A (n=14)
B (n=15)
C (n=11)
surface

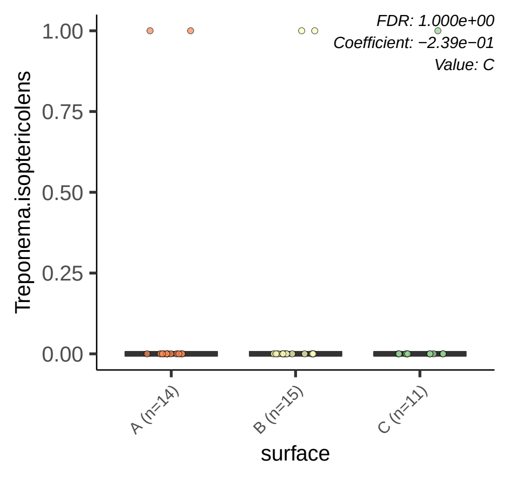

FDR: 1.000e+00
Coefficient: −2.39e−01
Value: C
Treponema.isoptericolens
1.00
0.75
0.50
0.25
0.00
A (n=14)
B (n=15)
C (n=11)
surface

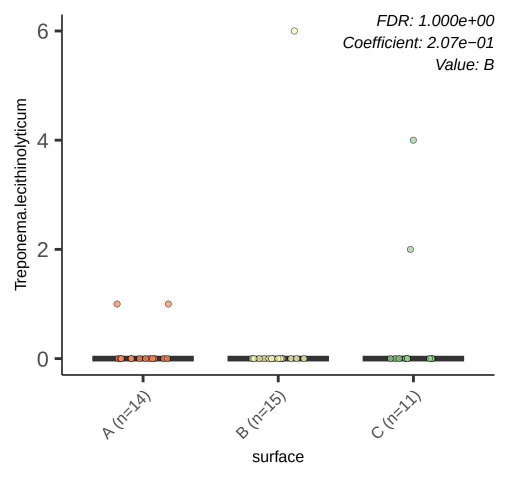

FDR: 1.000e+00
Coefficient: 2.07e−01
Value: B
Treponema.lecithinolyticum
0
2
4
6
A (n=14)
B (n=15)
C (n=11)
surface

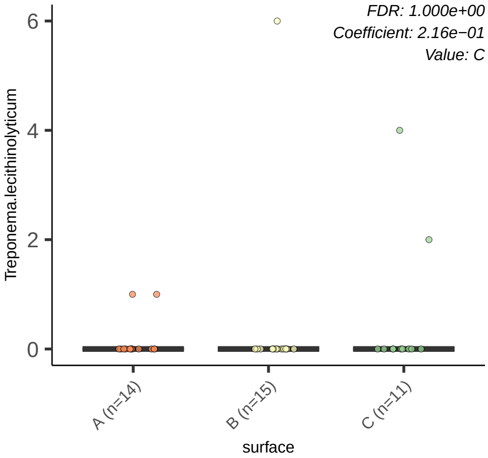

FDR: 1.000e+00
Coefficient: 2.16e−01
Value: C
Treponema.lecithinolyticum
0
2
4
6
A (n=14)
B (n=15)
C (n=11)
surface

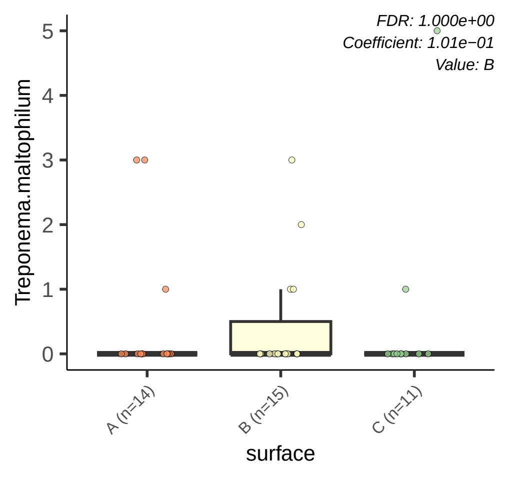

FDR: 1.000e+00
Coefficient: 1.01e−01
Value: B
Treponema.maltophilum
0
1
2
3
4
5
A (n=14)
B (n=15)
C (n=11)
surface

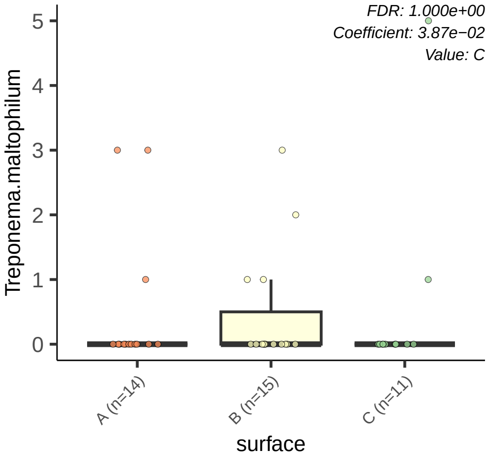

FDR: 1.000e+00
Coefficient: 3.87e−02
Value: C
Treponema.maltophilum
0
1
2
3
4
5
A (n=14)
B (n=15)
C (n=11)
surface

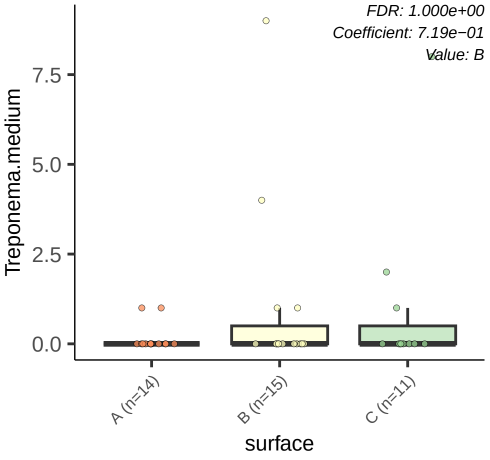

FDR: 1.000e+00
Coefficient: 7.19e−01
Value: B
Treponema.medium
7.5
5.0
2.5
0.0
A (n=14)
B (n=15)
C (n=11)
surface

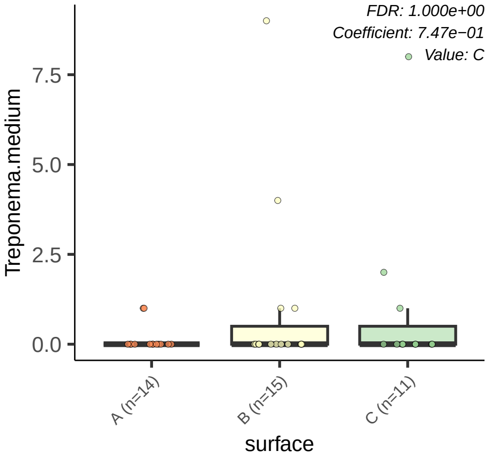
FDR: 1.000e+00
Coefficient: 7.47e−01
Value: C
Treponema.medium
0.0
2.5
5.0
7.5
A (n=14)
B (n=15)
C (n=11)
surface

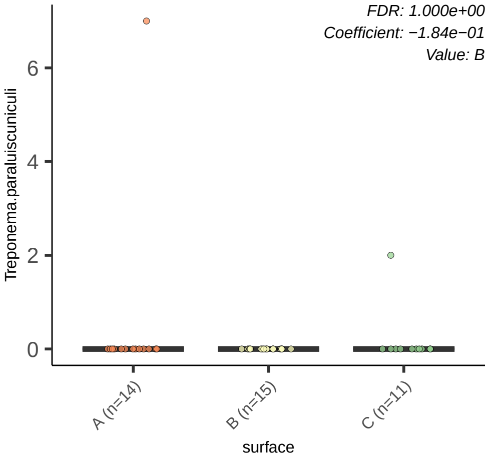

FDR: 1.000e+00
Coefficient: −1.84e−01
Value: B
Treponema.paraluiscuniculi
6
4
2
0
A (n=14)
B (n=15)
C (n=11)
surface

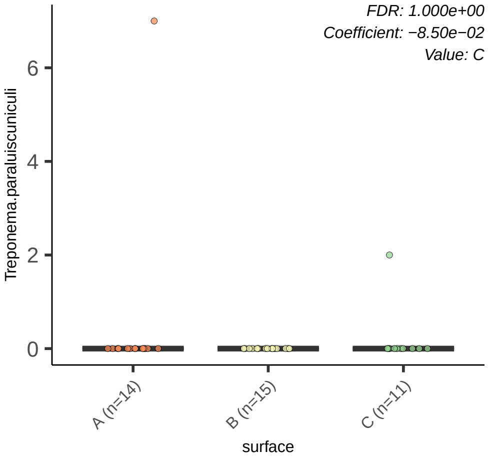
FDR: 1.000e+00
Coefficient: −8.50e−02
Value: C
Treponema.paraluiscuniculi
0
2
4
6
A (n=14)
B (n=15)
C (n=11)
surface

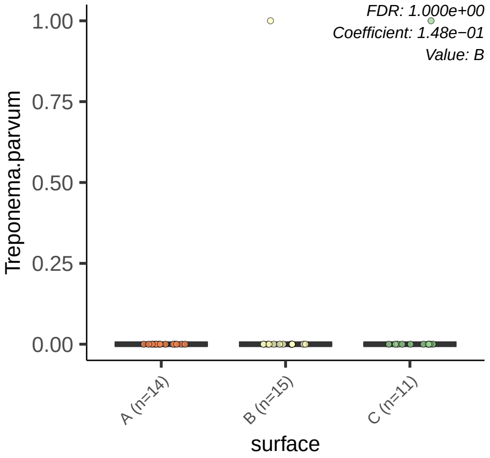
FDR: 1.000e+00
Coefficient: 1.48e−01
Value: B
Treponema.parvum
1.00
0.75
0.50
0.25
0.00
A (n=14)
B (n=15)
C (n=11)
surface

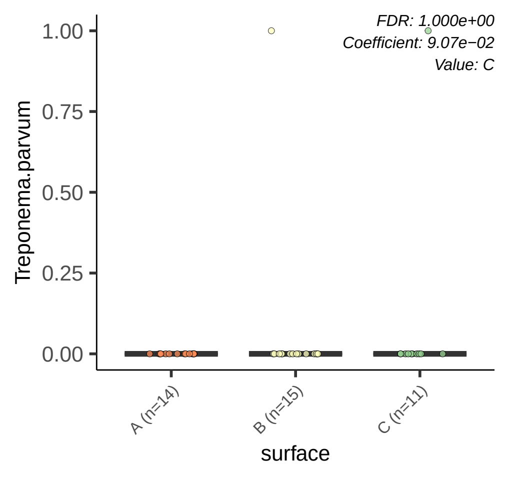
FDR: 1.000e+00
Coefficient: 9.07e−02
Value: C
Treponema.parvum
1.00
0.75
0.50
0.25
0.00
A (n=14)
B (n=15)
C (n=11)
surface

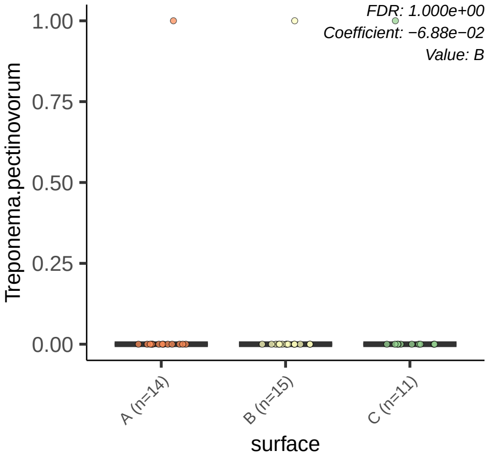
FDR: 1.000e+00
Coefficient: −6.88e−02
Value: B
Treponema.pectinovorum
1.00
0.75
0.50
0.25
0.00
A (n=14)
B (n=15)
C (n=11)
surface

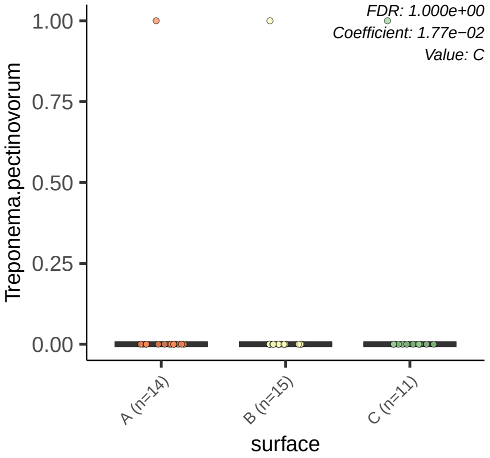

FDR: 1.000e+00
Coefficient: 1.77e−02
Value: C
Treponema.pectinovorum
1.00
0.75
0.50
0.25
0.00
A (n=14)
B (n=15)
C (n=11)
surface

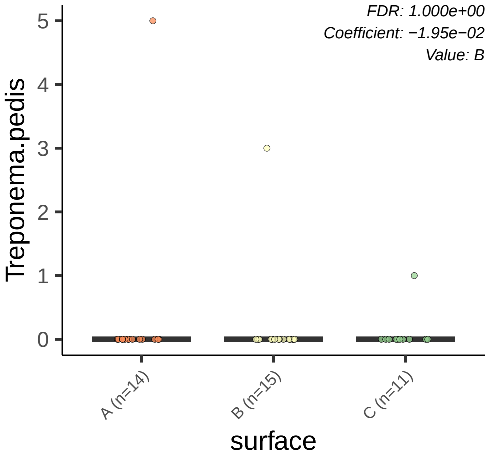

FDR: 1.000e+00
Coefficient: −1.95e−02
Value: B
Treponema.pedis
5
4
3
2
1
0
A (n=14)
B (n=15)
C (n=11)
surface

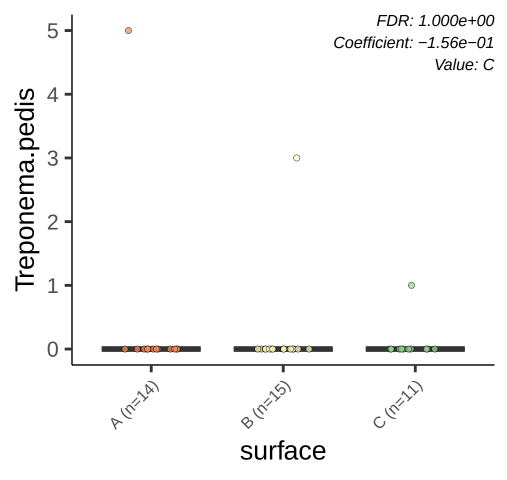
FDR: 1.000e+00
Coefficient: −1.56e−01
Value: C
Treponema.pedis
5
4
3
2
1
0
A (n=14)
B (n=15)
C (n=11)
surface

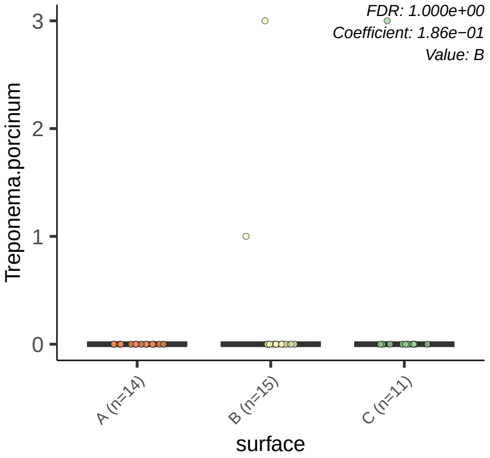
FDR: 1.000e+00
Coefficient: 1.86e−01
Value: B
Treponema.porcinum
3
2
1
0
A (n=14)
B (n=15)
C (n=11)
surface

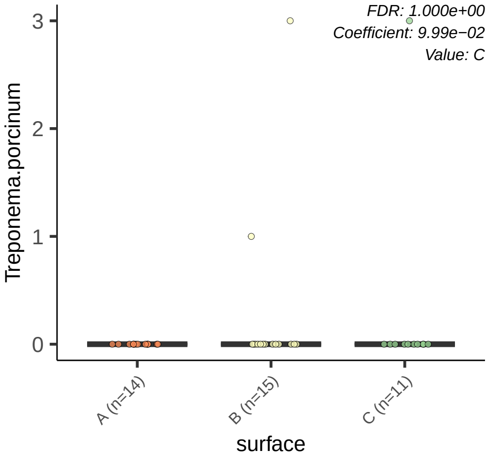

FDR: 1.000e+00
Coefficient: 9.99e−02
Value: C
Treponema.porcinum
3
2
1
0
A (n=14)
B (n=15)
C (n=11)
surface

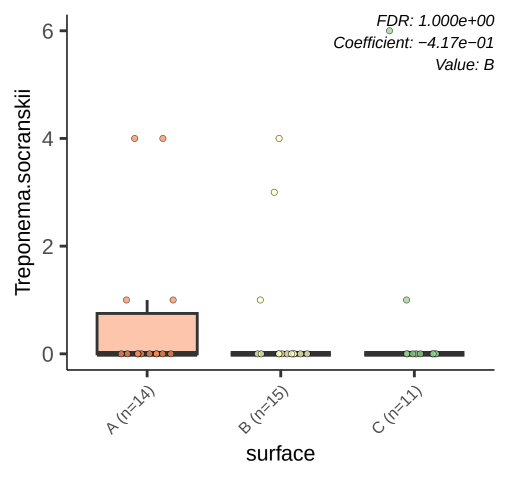
FDR: 1.000e+00
Coefficient: −4.17e−01
Value: B
Treponema.socranskii
6
4
2
0
A (n=14)
B (n=15)
C (n=11)
surface

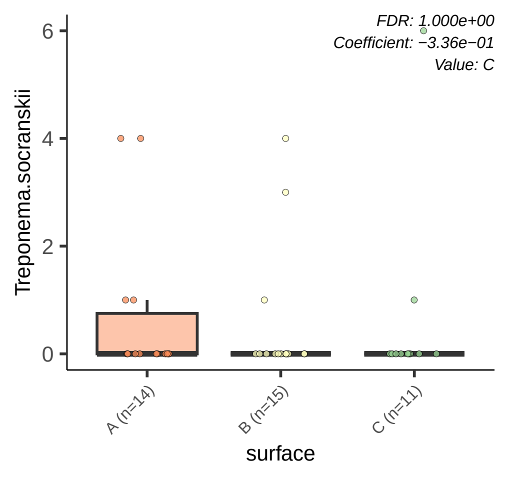
FDR: 1.000e+00
Coefficient: −3.36e−01
Value: C
Treponema.socranskii
6
4
2
0
A (n=14)
B (n=15)
C (n=11)
surface

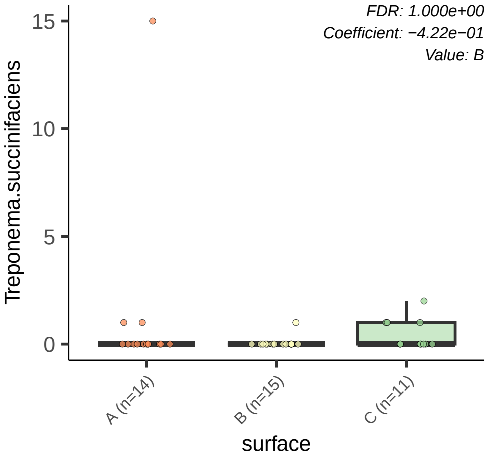

FDR: 1.000e+00
Coefficient: −4.22e−01
Value: B
Treponema.succinifaciens
15
10
5
0
A (n=14)
B (n=15)
C (n=11)
surface

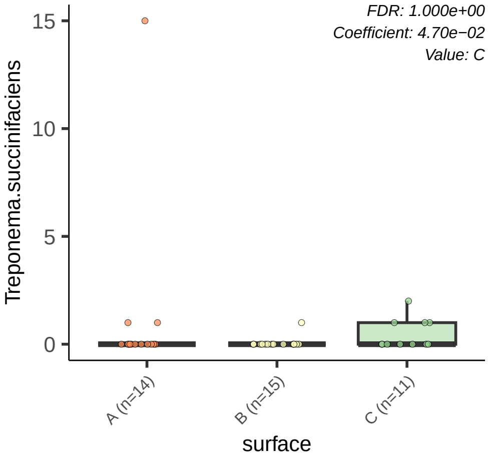

FDR: 1.000e+00
Coefficient: 4.70e−02
Value: C
Treponema.succinifaciens
15
10
5
0
A (n=14)
B (n=15)
C (n=11)
surface

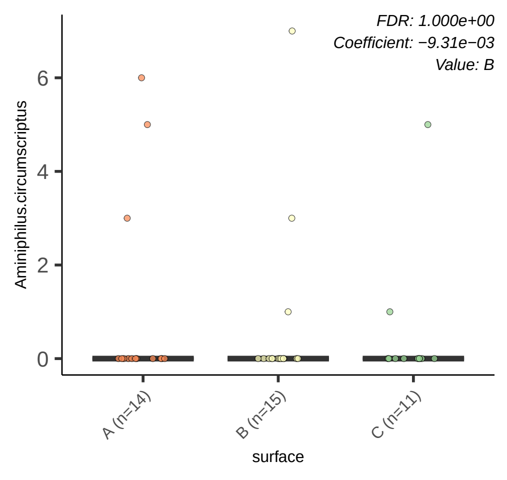
FDR: 1.000e+00
Coefficient: −9.31e−03
Value: B
Aminiphilus.circumscriptus
0
2
4
6
A (n=14)
B (n=15)
C (n=11)
surface

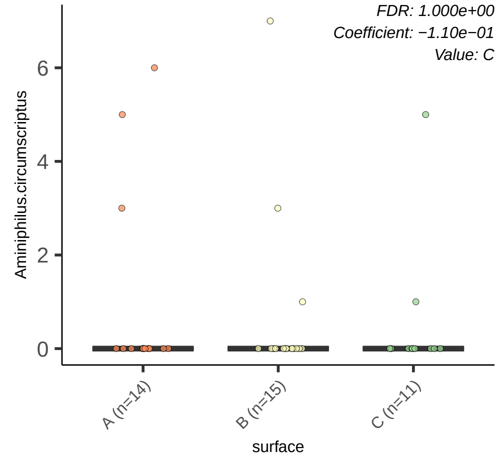
FDR: 1.000e+00
Coefficient: −1.10e−01
Value: C
Aminiphilus.circumscriptus
0
2
4
6
A (n=14)
B (n=15)
C (n=11)
surface

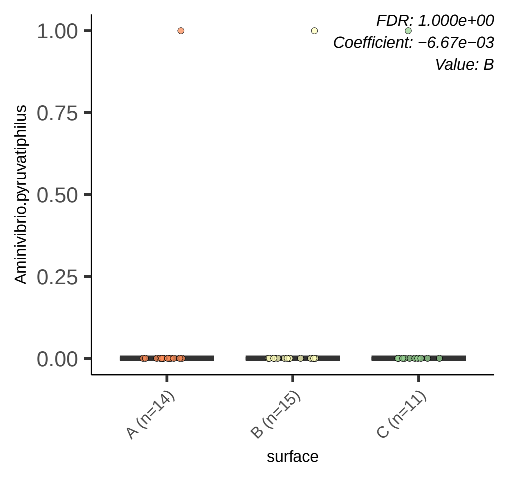
FDR: 1.000e+00
Coefficient: −6.67e−03
Value: B
1.00
0.75
0.50
0.25
0.00
Aminivibrio.pyruvatiphilus
A (n=14)
B (n=15)
C (n=11)
surface

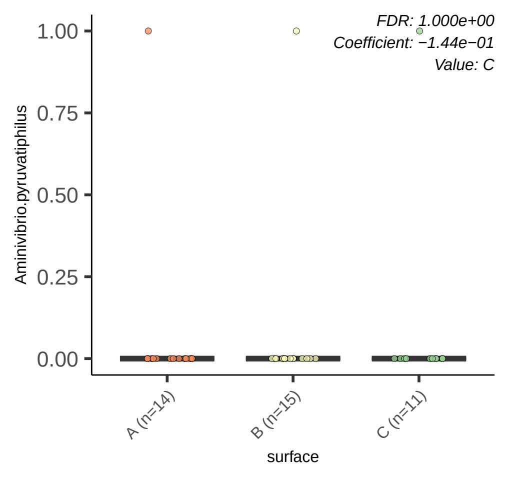
FDR: 1.000e+00
Coefficient: −1.44e−01
Value: C
Aminivibrio.pyruvatiphilus
1.00
0.75
0.50
0.25
0.00
A (n=14)
B (n=15)
C (n=11)
surface

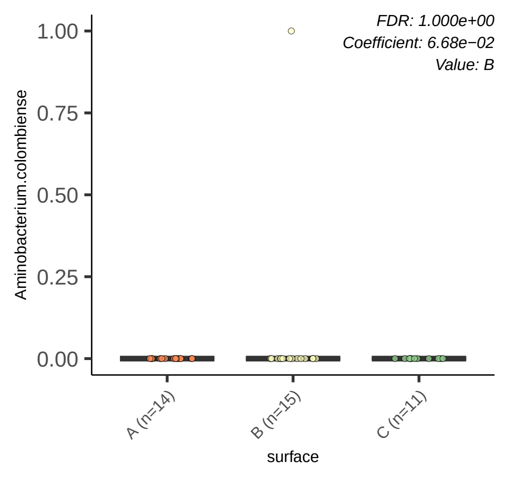

FDR: 1.000e+00
Coefficient: 6.68e−02
Value: B
1.00
0.75
0.50
0.25
0.00
Aminobacterium.colombiense
A (n=14)
B (n=15)
C (n=11)
surface

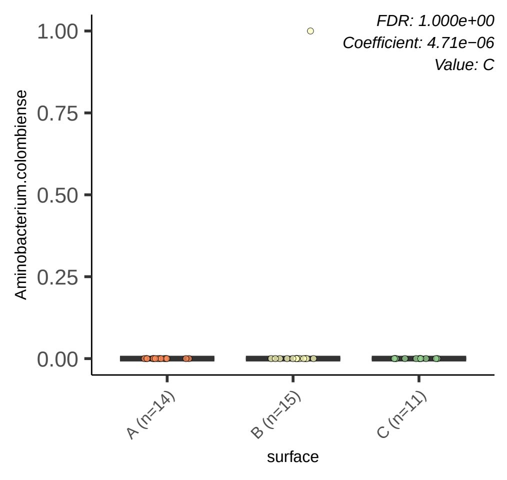

FDR: 1.000e+00
Coefficient: 4.71e−06
Value: C
Aminobacterium.colombiense
1.00
0.75
0.50
0.25
0.00
A (n=14)
B (n=15)
C (n=11)
surface

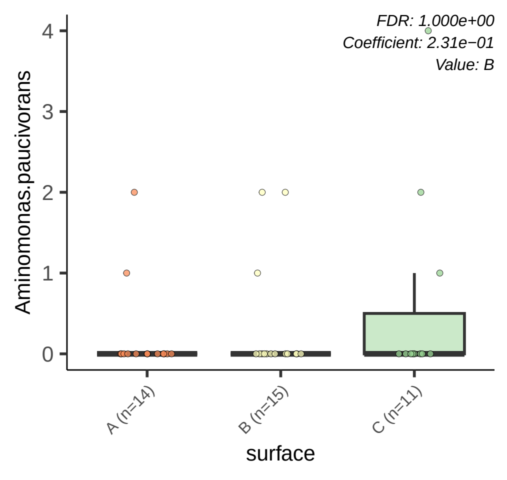
FDR: 1.000e+00
Coefficient: 2.31e−01
Value: B
Aminomonas.paucivorans
0
1
2
3
4
A (n=14)
B (n=15)
C (n=11)
surface

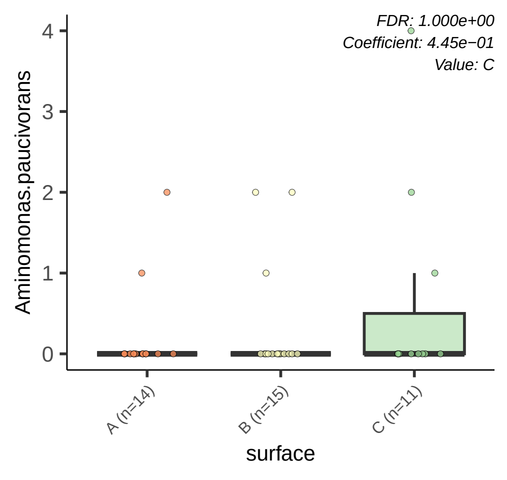

FDR: 1.000e+00
Coefficient: 4.45e−01
Value: C
Aminomonas.paucivorans
0
1
2
3
4
A (n=14)
B (n=15)
C (n=11)
surface

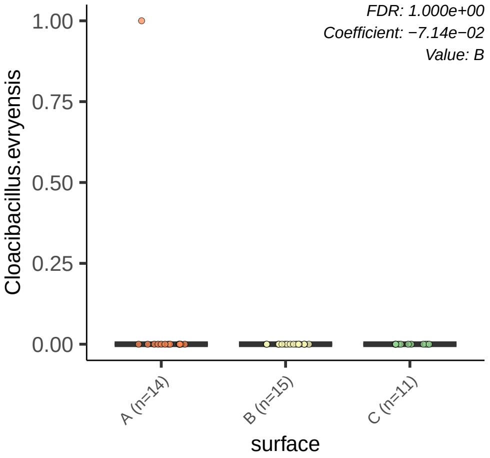

FDR: 1.000e+00
Coefficient: −7.14e−02
Value: B
Cloacibacillus.evryensis
1.00
0.75
0.50
0.25
0.00
A (n=14)
B (n=15)
C (n=11)
surface

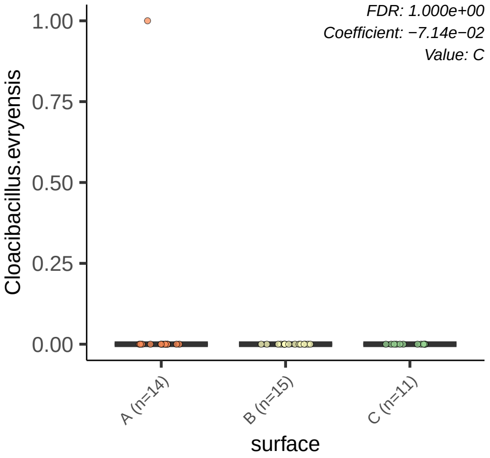

FDR: 1.000e+00
Coefficient: −7.14e−02
Value: C
Cloacibacillus.evryensis
1.00
0.75
0.50
0.25
0.00
A (n=14)
B (n=15)
C (n=11)
surface

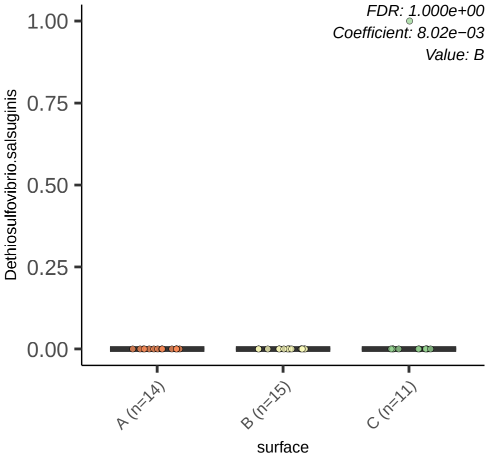
FDR: 1.000e+00
Coefficient: 8.02e−03
Value: B
Dethiosulfovibrio.salsuginis
1.00
0.75
0.50
0.25
0.00
A (n=14)
B (n=15)
C (n=11)
surface

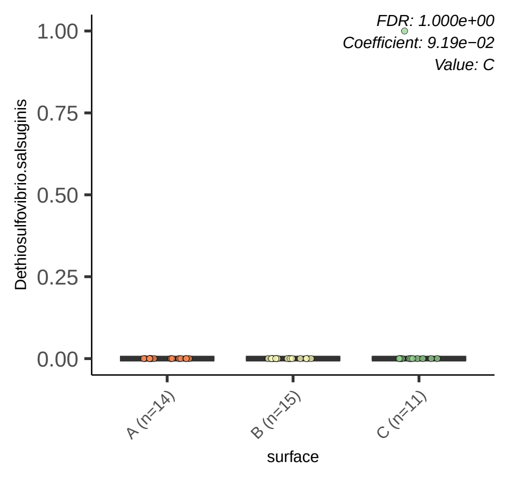

FDR: 1.000e+00
Coefficient: 9.19e−02
Value: C
1.00
0.75
0.50
0.25
0.00
Dethiosulfovibrio.salsuginis
A (n=14)
B (n=15)
C (n=11)
surface

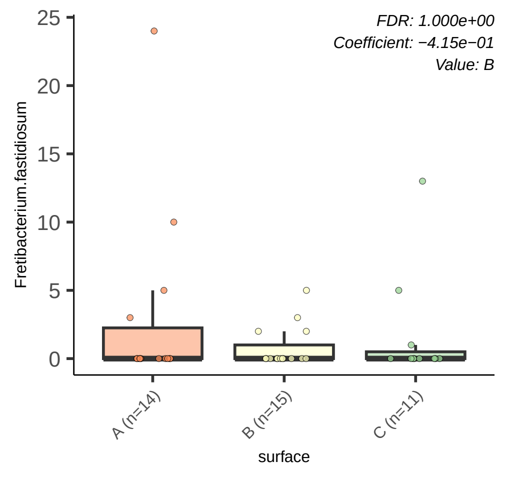
FDR: 1.000e+00
Coefficient: −4.15e−01
Value: B
Fretibacterium.fastidiosum
25
20
15
10
5
0
A (n=14)
B (n=15)
C (n=11)
surface

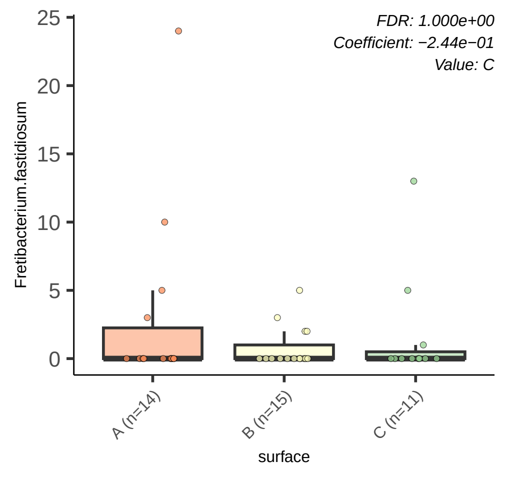

FDR: 1.000e+00
Coefficient: −2.44e−01
Value: C
Fretibacterium.fastidiosum
25
20
15
10
5
0
A (n=14)
B (n=15)
C (n=11)
surface

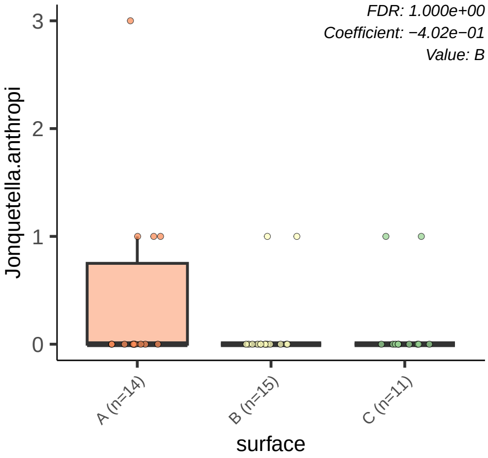
FDR: 1.000e+00
Coefficient: −4.02e−01
Value: B
Jonquetella.anthropi
3
2
1
0
A (n=14)
B (n=15)
C (n=11)
surface

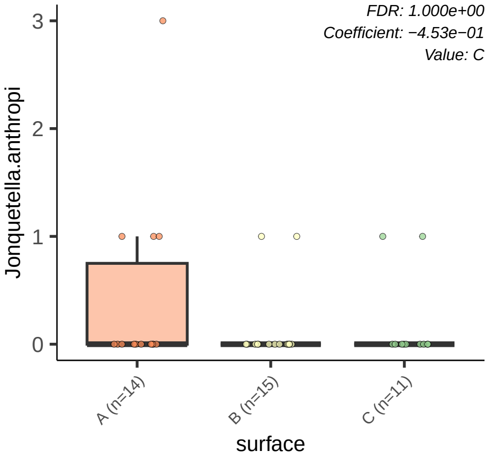
FDR: 1.000e+00
Coefficient: −4.53e−01
Value: C
Jonquetella.anthropi
3
2
1
0
A (n=14)
B (n=15)
C (n=11)
surface

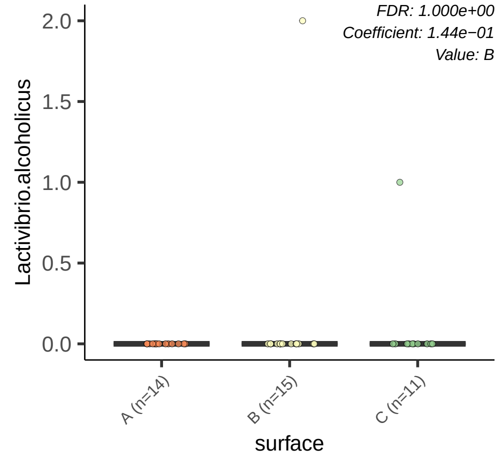
FDR: 1.000e+00
Coefficient: 1.44e−01
Value: B
Lactivibrio.alcoholicus
2.0
1.5
1.0
0.5
0.0
A (n=14)
B (n=15)
C (n=11)
surface

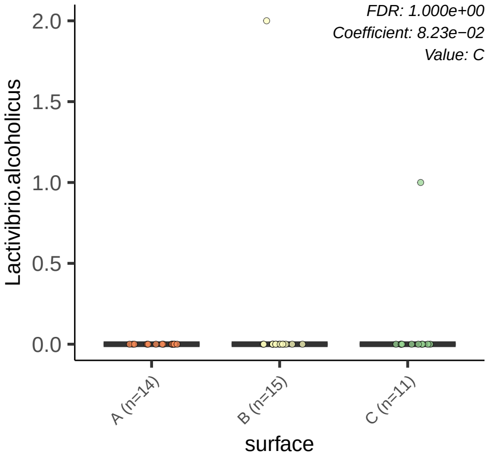

FDR: 1.000e+00
Coefficient: 8.23e−02
Value: C
Lactivibrio.alcoholicus
2.0
1.5
1.0
0.5
0.0
A (n=14)
B (n=15)
C (n=11)
surface

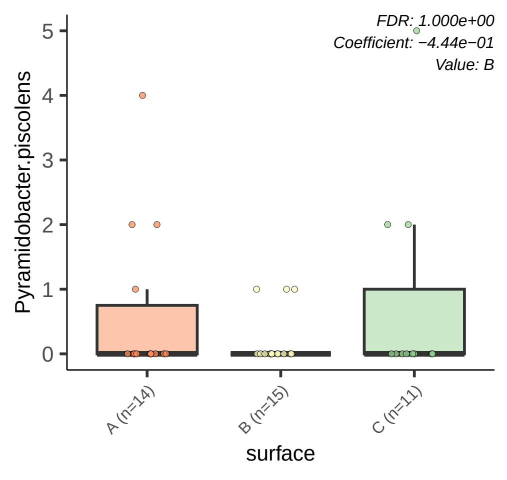

FDR: 1.000e+00
Coefficient: −4.44e−01
Value: B
Pyramidobacter.piscolens
0
1
2
3
4
5
A (n=14)
B (n=15)
C (n=11)
surface

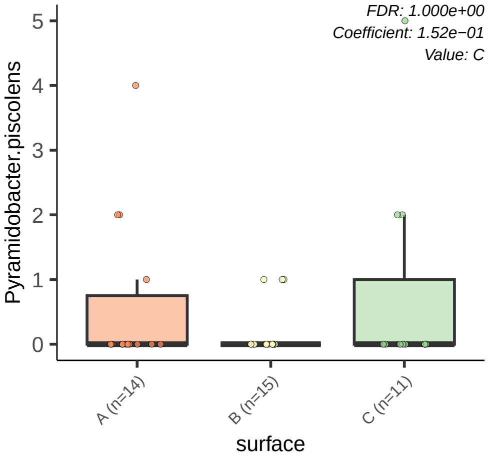
FDR: 1.000e+00
Coefficient: 1.52e−01
Value: C
Pyramidobacter.piscolens
0
1
2
3
4
5
A (n=14)
B (n=15)
C (n=11)
surface

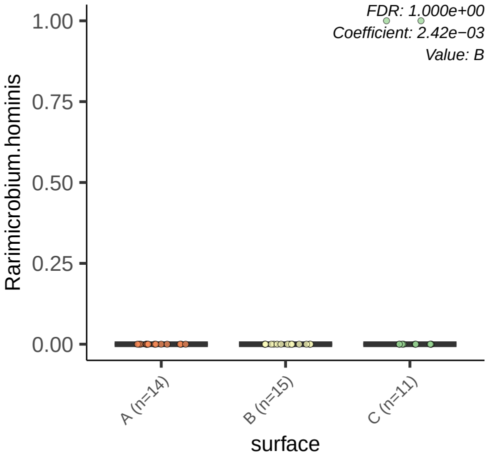

FDR: 1.000e+00
Coefficient: 2.42e−03
Value: B
Rarimicrobium.hominis
1.00
0.75
0.50
0.25
0.00
A (n=14)
B (n=15)
C (n=11)
surface

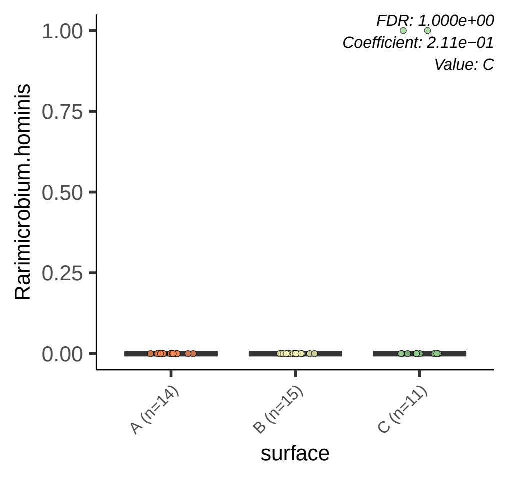

FDR: 1.000e+00
Coefficient: 2.11e−01
Value: C
Rarimicrobium.hominis
1.00
0.75
0.50
0.25
0.00
A (n=14)
B (n=15)
C (n=11)
surface

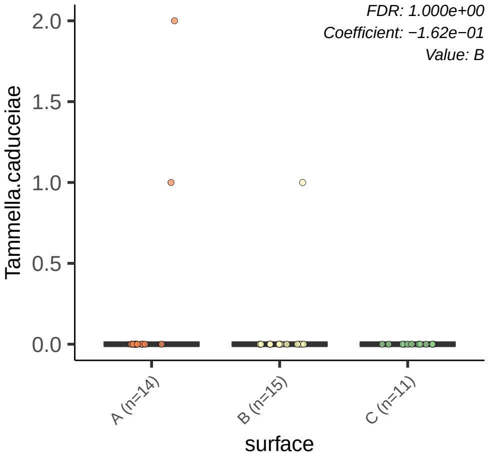
FDR: 1.000e+00
Coefficient: −1.62e−01
Value: B
Tammella.caduceiae
2.0
1.5
1.0
0.5
0.0
A (n=14)
B (n=15)
C (n=11)
surface

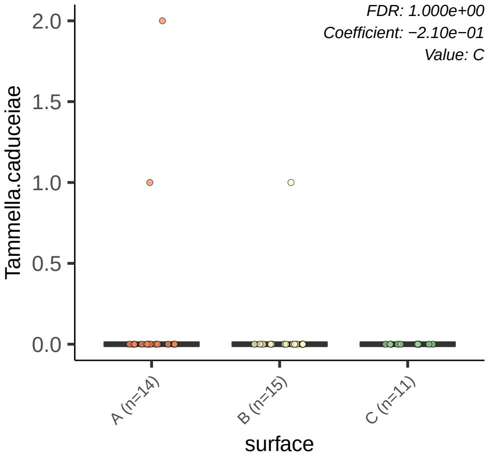
FDR: 1.000e+00
Coefficient: −2.10e−01
Value: C
Tammella.caduceiae
2.0
1.5
1.0
0.5
0.0
A (n=14)
B (n=15)
C (n=11)
surface

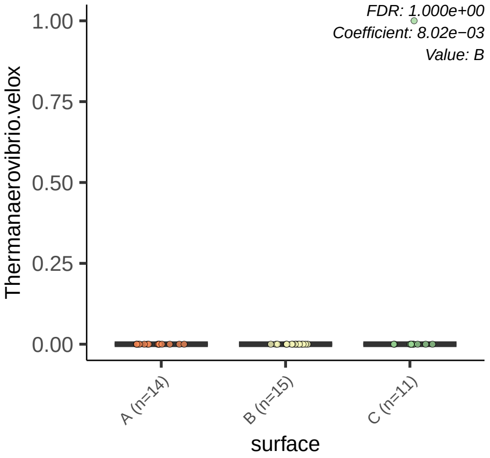
FDR: 1.000e+00
Coefficient: 8.02e−03
Value: B
Thermanaerovibrio.velox
1.00
0.75
0.50
0.25
0.00
A (n=14)
B (n=15)
C (n=11)
surface

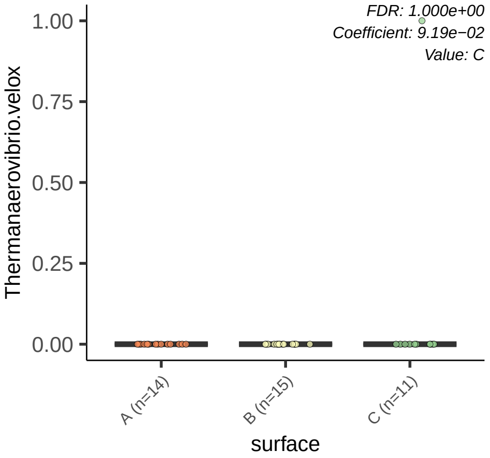
FDR: 1.000e+00
Coefficient: 9.19e−02
Value: C
Thermanaerovibrio.velox
1.00
0.75
0.50
0.25
0.00
A (n=14)
B (n=15)
C (n=11)
surface

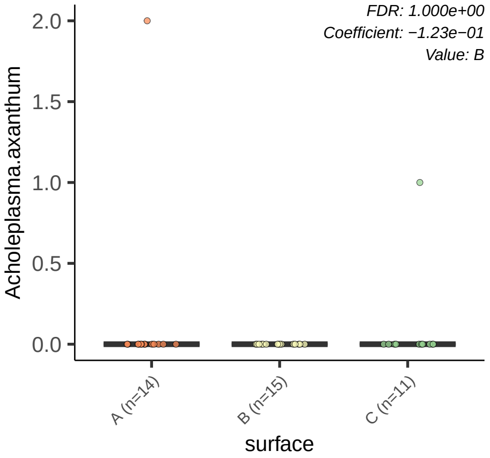
FDR: 1.000e+00
Coefficient: −1.23e−01
Value: B
Acholeplasma.axanthum
2.0
1.5
1.0
0.5
0.0
A (n=14)
B (n=15)
C (n=11)
surface

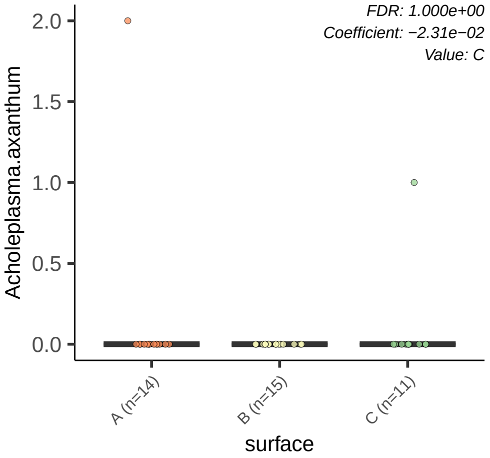

FDR: 1.000e+00
Coefficient: −2.31e−02
Value: C
Acholeplasma.axanthum
2.0
1.5
1.0
0.5
0.0
A (n=14)
B (n=15)
C (n=11)
surface

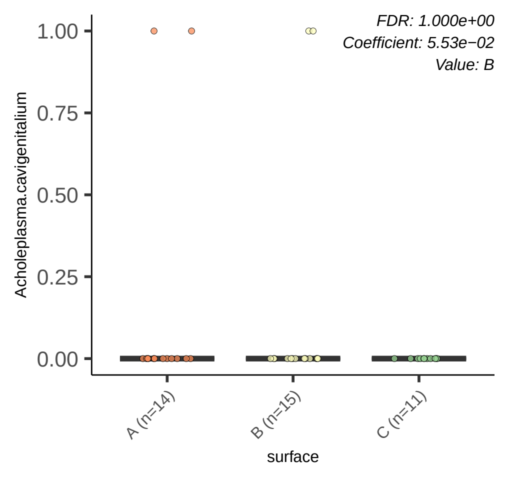
FDR: 1.000e+00
Coefficient: 5.53e−02
Value: B
Acholeplasma.cavigenitalium
1.00
0.75
0.50
0.25
0.00
A (n=14)
B (n=15)
C (n=11)
surface

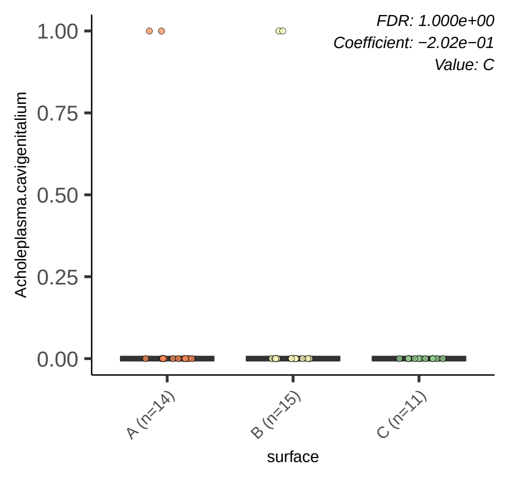

FDR: 1.000e+00
Coefficient: −2.02e−01
Value: C
1.00
0.75
0.50
0.25
0.00
Acholeplasma.cavigenitalium
A (n=14)
B (n=15)
C (n=11)
surface

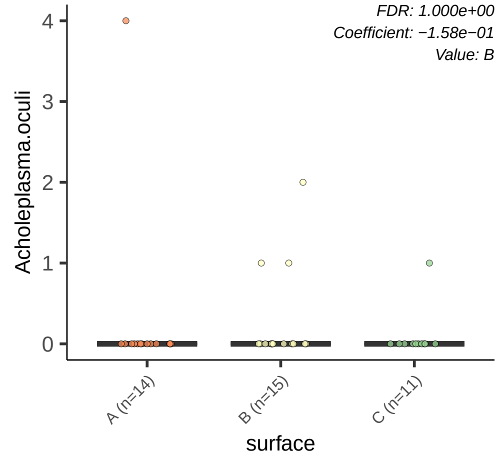
FDR: 1.000e+00
Coefficient: −1.58e−01
Value: B
Acholeplasma.oculi
0
1
2
3
4
A (n=14)
B (n=15)
C (n=11)
surface

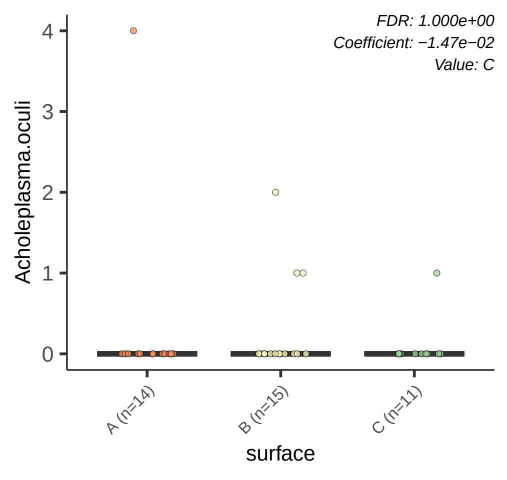

FDR: 1.000e+00
Coefficient: −1.47e−02
Value: C
Acholeplasma.oculi
4
3
2
1
0
A (n=14)
B (n=15)
C (n=11)
surface

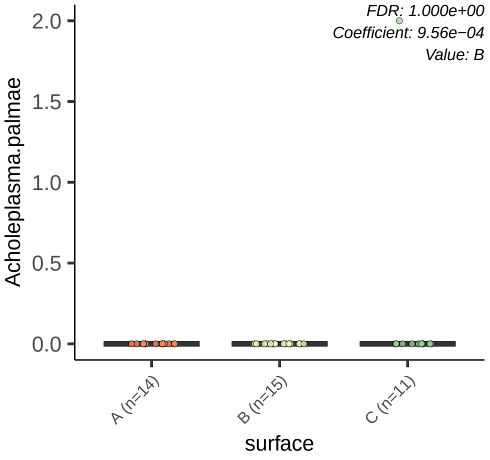
FDR: 1.000e+00
Coefficient: 9.56e−04
Value: B
Acholeplasma.palmae
2.0
1.5
1.0
0.5
0.0
A (n=14)
B (n=15)
C (n=11)
surface

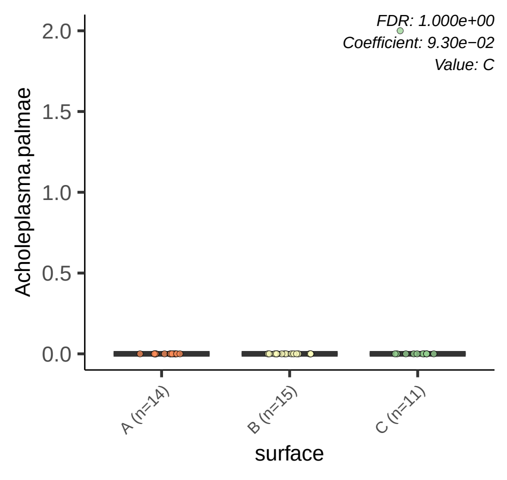
FDR: 1.000e+00
Coefficient: 9.30e−02
Value: C
Acholeplasma.palmae
2.0
1.5
1.0
0.5
0.0
A (n=14)
B (n=15)
C (n=11)
surface

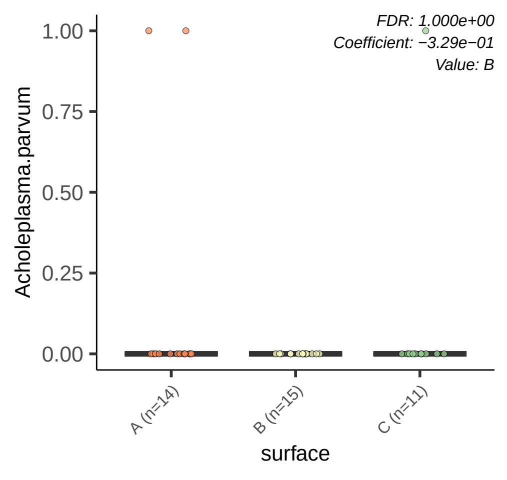

FDR: 1.000e+00
Coefficient: −3.29e−01
Value: B
Acholeplasma.parvum
1.00
0.75
0.50
0.25
0.00
A (n=14)
B (n=15)
C (n=11)
surface

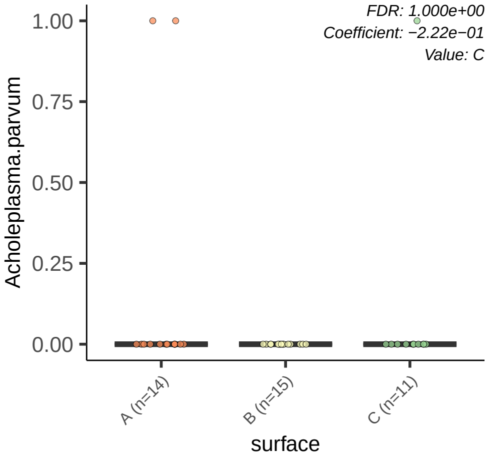

FDR: 1.000e+00
Coefficient: −2.22e−01
Value: C
Acholeplasma.parvum
1.00
0.75
0.50
0.25
0.00
A (n=14)
B (n=15)
C (n=11)
surface

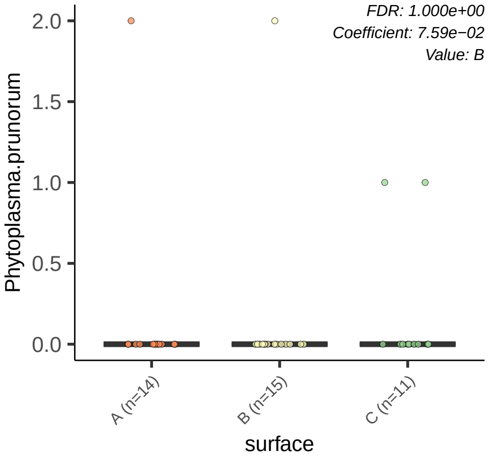
FDR: 1.000e+00
Coefficient: 7.59e−02
Value: B
Phytoplasma.prunorum
2.0
1.5
1.0
0.5
0.0
A (n=14)
B (n=15)
C (n=11)
surface

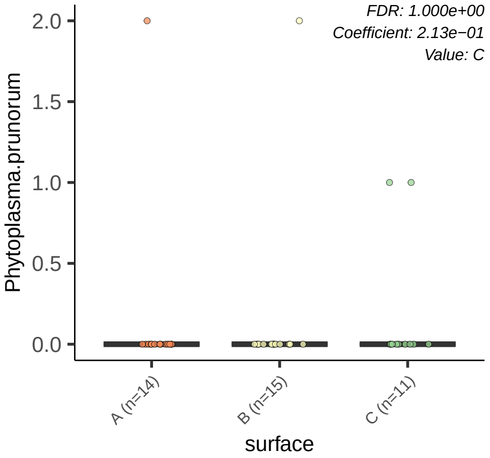
FDR: 1.000e+00
Coefficient: 2.13e−01
Value: C
Phytoplasma.prunorum
2.0
1.5
1.0
0.5
0.0
A (n=14)
B (n=15)
C (n=11)
surface

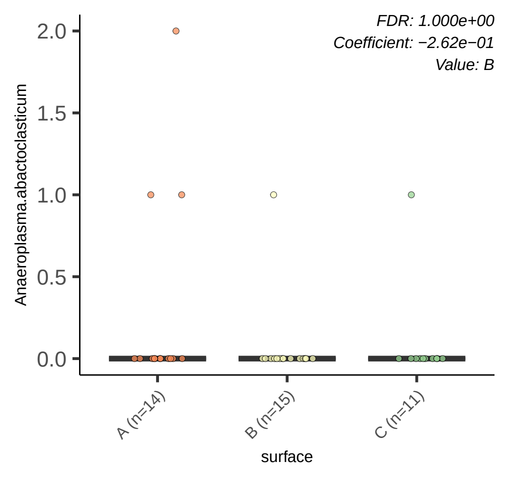
FDR: 1.000e+00
Coefficient: −2.62e−01
Value: B
Anaeroplasma.abactoclasticum
2.0
1.5
1.0
0.5
0.0
A (n=14)
B (n=15)
C (n=11)
surface

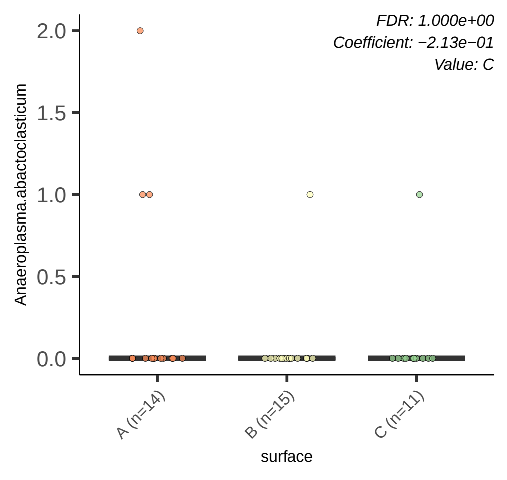

FDR: 1.000e+00
Coefficient: −2.13e−01
Value: C
Anaeroplasma.abactoclasticum
2.0
1.5
1.0
0.5
0.0
A (n=14)
B (n=15)
C (n=11)
surface

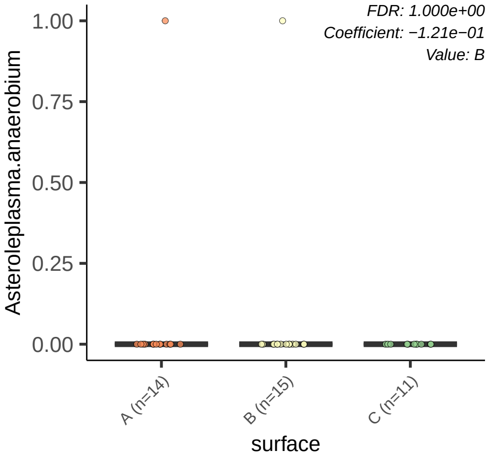

FDR: 1.000e+00
Coefficient: −1.21e−01
Value: B
Asteroleplasma.anaerobium
1.00
0.75
0.50
0.25
0.00
A (n=14)
B (n=15)
C (n=11)
surface

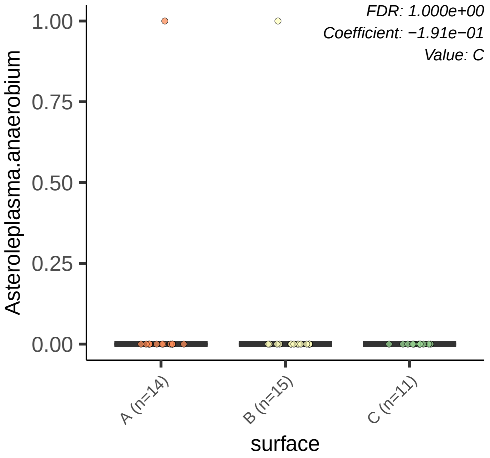

FDR: 1.000e+00
Coefficient: −1.91e−01
Value: C
Asteroleplasma.anaerobium
1.00
0.75
0.50
0.25
0.00
A (n=14)
B (n=15)
C (n=11)
surface

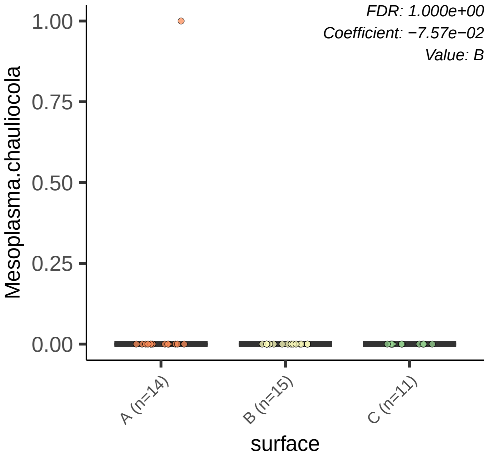
FDR: 1.000e+00
Coefficient: −7.57e−02
Value: B
Mesoplasma.chauliocola
1.00
0.75
0.50
0.25
0.00
A (n=14)
B (n=15)
C (n=11)
surface

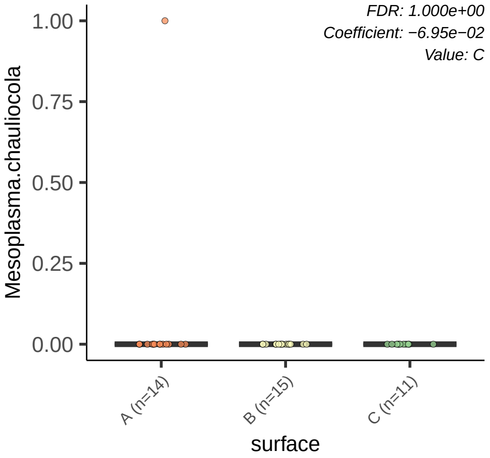
FDR: 1.000e+00
Coefficient: −6.95e−02
Value: C
Mesoplasma.chauliocola
1.00
0.75
0.50
0.25
0.00
A (n=14)
B (n=15)
C (n=11)
surface

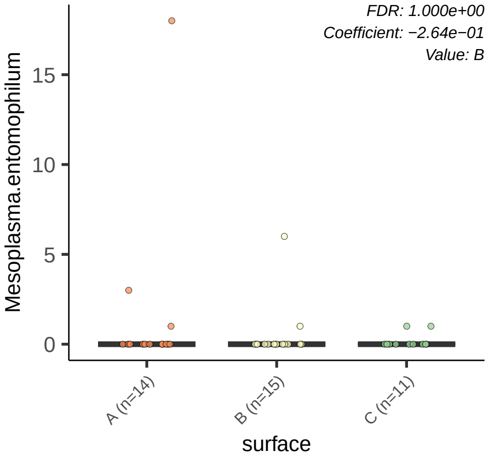

FDR: 1.000e+00
Coefficient: −2.64e−01
Value: B
Mesoplasma.entomophilum
0
5
10
15
A (n=14)
B (n=15)
C (n=11)
surface

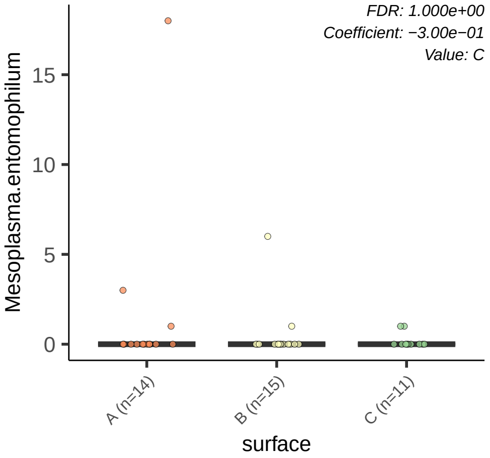
FDR: 1.000e+00
Coefficient: −3.00e−01
Value: C
Mesoplasma.entomophilum
0
5
10
15
A (n=14)
B (n=15)
C (n=11)
surface

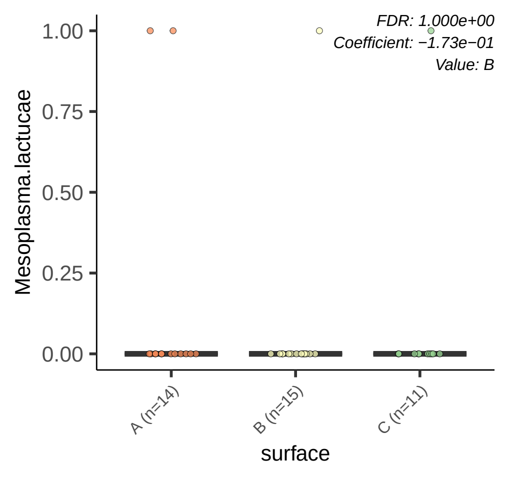
FDR: 1.000e+00
Coefficient: −1.73e−01
Value: B
Mesoplasma.lactucae
1.00
0.75
0.50
0.25
0.00
A (n=14)
B (n=15)
C (n=11)
surface

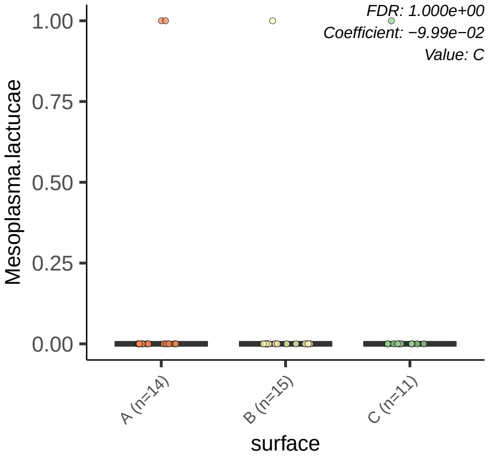
FDR: 1.000e+00
Coefficient: −9.99e−02
Value: C
Mesoplasma.lactucae
1.00
0.75
0.50
0.25
0.00
A (n=14)
B (n=15)
C (n=11)
surface

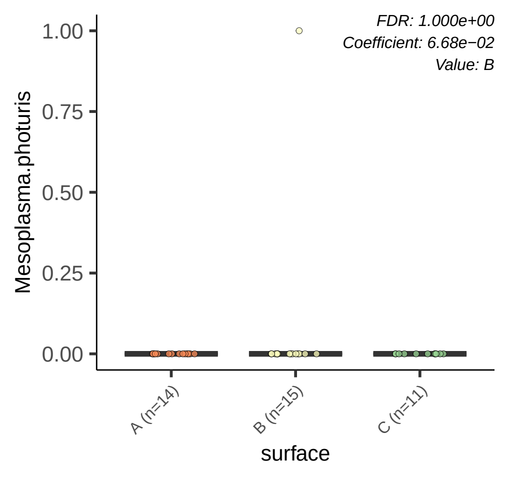
FDR: 1.000e+00
Coefficient: 6.68e−02
Value: B
Mesoplasma.photuris
1.00
0.75
0.50
0.25
0.00
A (n=14)
B (n=15)
C (n=11)
surface

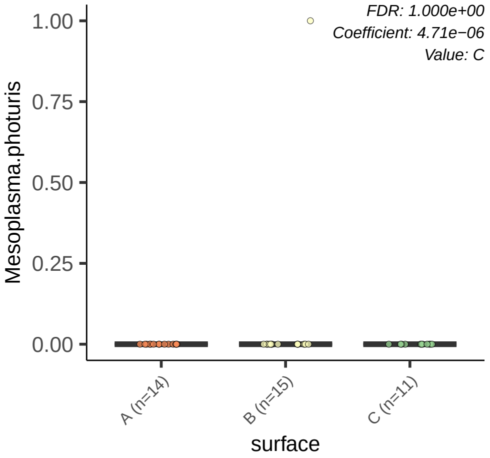

FDR: 1.000e+00
Coefficient: 4.71e−06
Value: C
Mesoplasma.photuris
1.00
0.75
0.50
0.25
0.00
A (n=14)
B (n=15)
C (n=11)
surface

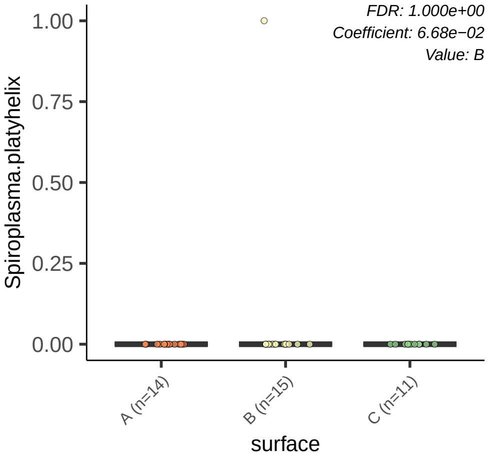

FDR: 1.000e+00
Coefficient: 6.68e−02
Value: B
Spiroplasma.platyhelix
1.00
0.75
0.50
0.25
0.00
A (n=14)
B (n=15)
C (n=11)
surface

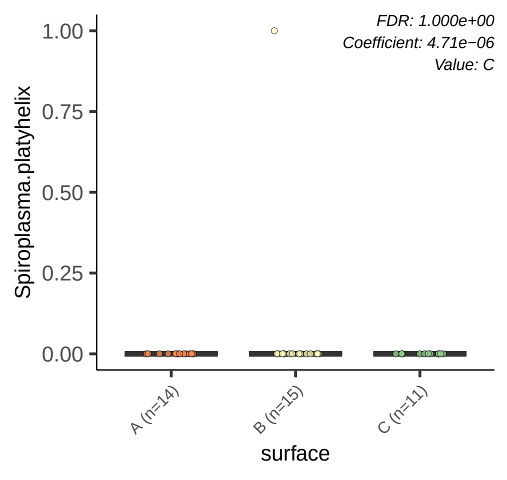

FDR: 1.000e+00
Coefficient: 4.71e−06
Value: C
Spiroplasma.platyhelix
1.00
0.75
0.50
0.25
0.00
A (n=14)
B (n=15)
C (n=11)
surface

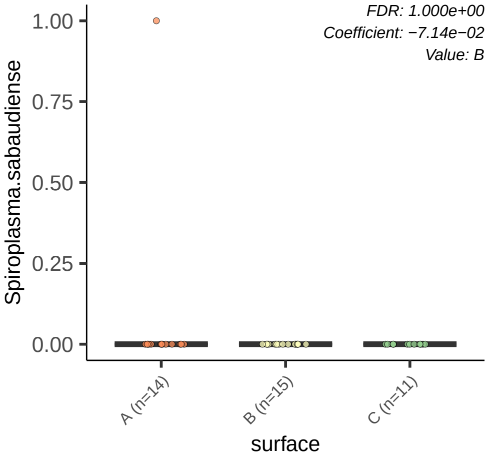

FDR: 1.000e+00
Coefficient: −7.14e−02
Value: B
Spiroplasma.sabaudiense
1.00
0.75
0.50
0.25
0.00
A (n=14)
B (n=15)
C (n=11)
surface

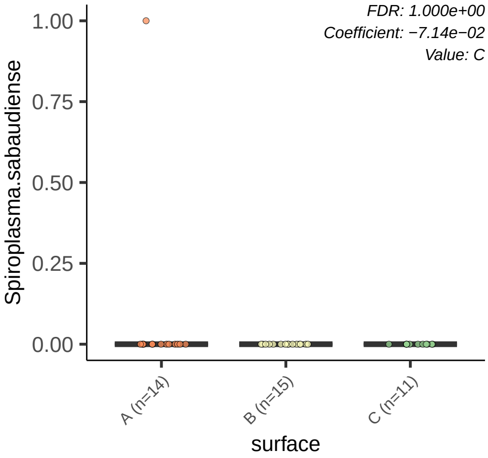
FDR: 1.000e+00
Coefficient: −7.14e−02
Value: C
Spiroplasma.sabaudiense
1.00
0.75
0.50
0.25
0.00
A (n=14)
B (n=15)
C (n=11)
surface

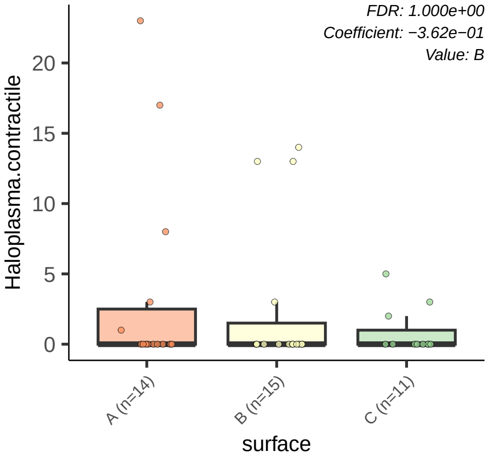

FDR: 1.000e+00
Coefficient: −3.62e−01
Value: B
Haloplasma.contractile
0
5
10
15
20
A (n=14)
B (n=15)
C (n=11)
surface

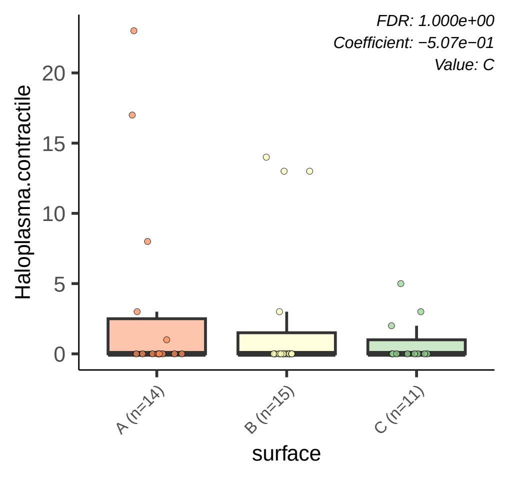

FDR: 1.000e+00
Coefficient: −5.07e−01
Value: C
Haloplasma.contractile
0
5
10
15
20
A (n=14)
B (n=15)
C (n=11)
surface

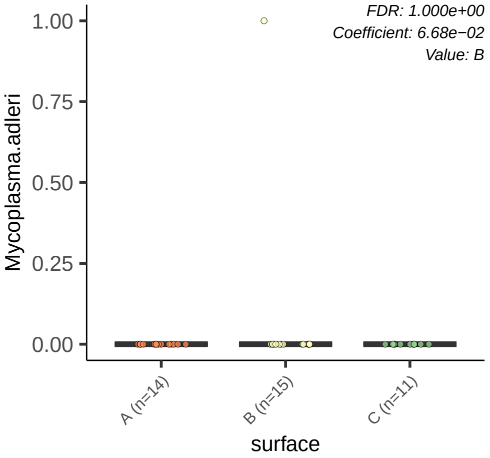

FDR: 1.000e+00
Coefficient: 6.68e−02
Value: B
Mycoplasma.adleri
1.00
0.75
0.50
0.25
0.00
A (n=14)
B (n=15)
C (n=11)
surface

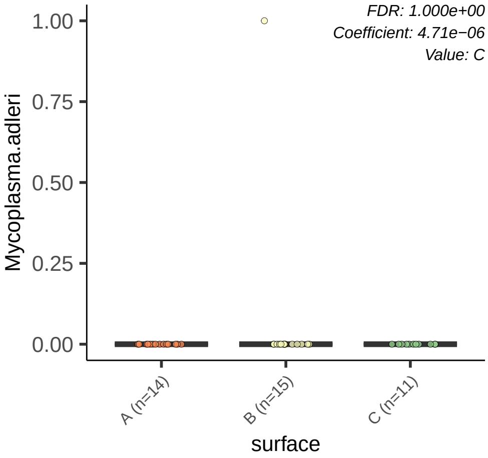
FDR: 1.000e+00
Coefficient: 4.71e−06
Value: C
Mycoplasma.adleri
1.00
0.75
0.50
0.25
0.00
A (n=14)
B (n=15)
C (n=11)
surface

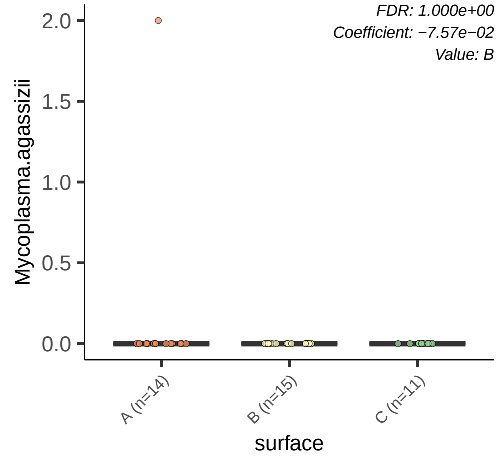

FDR: 1.000e+00
Coefficient: −7.57e−02
Value: B
Mycoplasma.agassizii
2.0
1.5
1.0
0.5
0.0
A (n=14)
B (n=15)
C (n=11)
surface

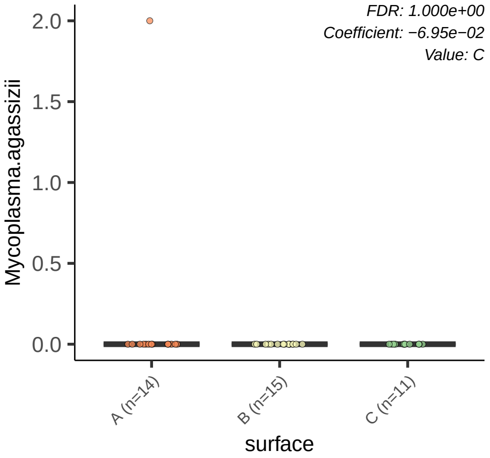
FDR: 1.000e+00
Coefficient: −6.95e−02
Value: C
Mycoplasma.agassizii
2.0
1.5
1.0
0.5
0.0
A (n=14)
B (n=15)
C (n=11)
surface

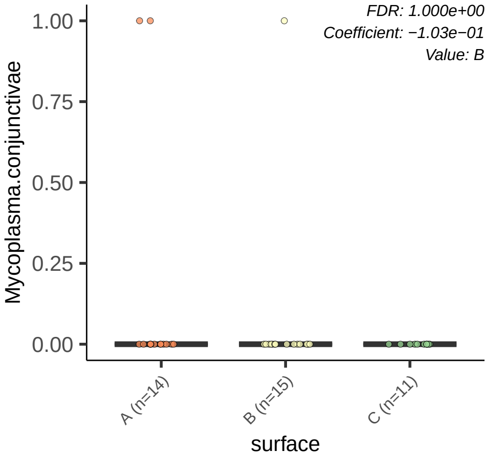
FDR: 1.000e+00
Coefficient: −1.03e−01
Value: B
Mycoplasma.conjunctivae
1.00
0.75
0.50
0.25
0.00
A (n=14)
B (n=15)
C (n=11)
surface

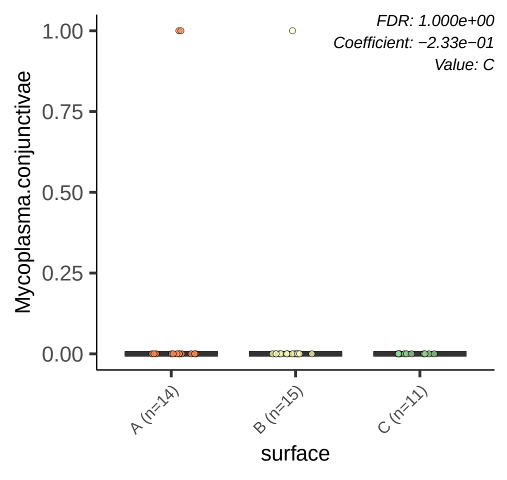
FDR: 1.000e+00
Coefficient: −2.33e−01
Value: C
Mycoplasma.conjunctivae
1.00
0.75
0.50
0.25
0.00
A (n=14)
B (n=15)
C (n=11)
surface

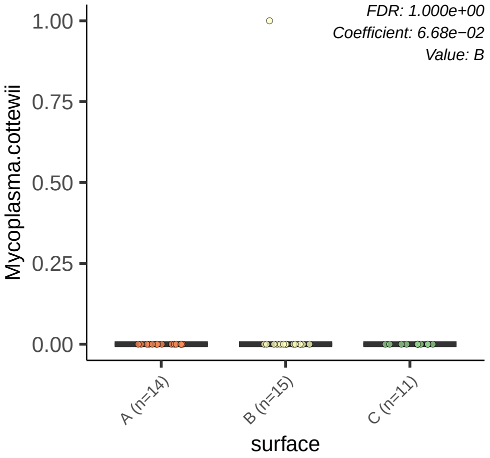
FDR: 1.000e+00
Coefficient: 6.68e−02
Value: B
Mycoplasma.cottewii
1.00
0.75
0.50
0.25
0.00
A (n=14)
B (n=15)
C (n=11)
surface

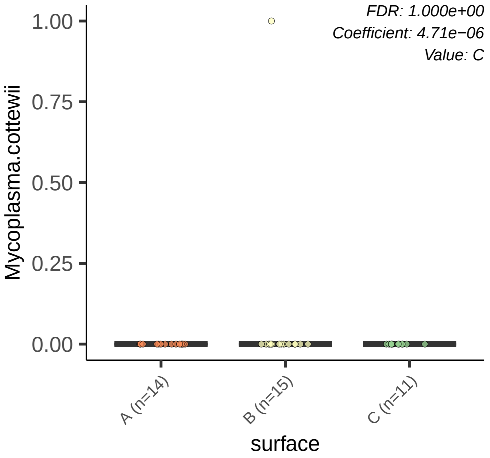

FDR: 1.000e+00
Coefficient: 4.71e−06
Value: C
Mycoplasma.cottewii
1.00
0.75
0.50
0.25
0.00
A (n=14)
B (n=15)
C (n=11)
surface

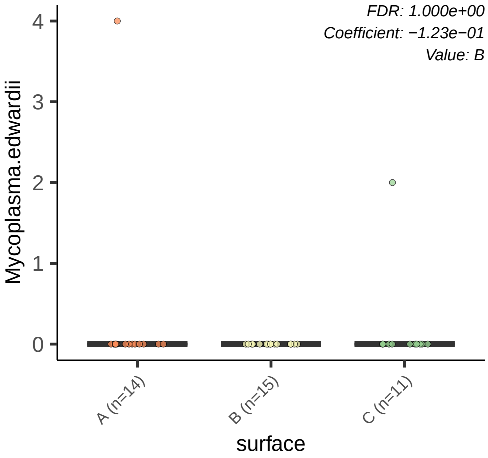
FDR: 1.000e+00
Coefficient: −1.23e−01
Value: B
Mycoplasma.edwardii
4
3
2
1
0
A (n=14)
B (n=15)
C (n=11)
surface

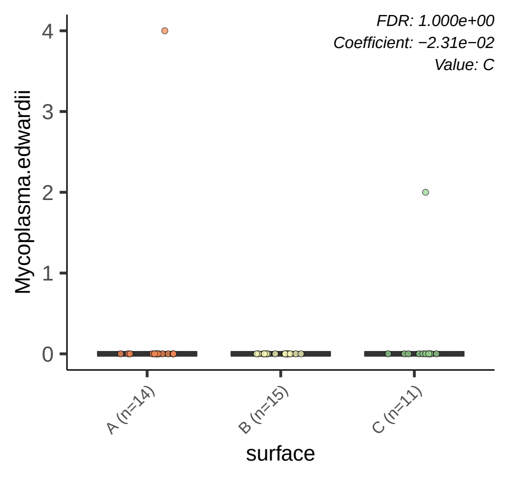
FDR: 1.000e+00
Coefficient: −2.31e−02
Value: C
Mycoplasma.edwardii
0
1
2
3
4
A (n=14)
B (n=15)
C (n=11)
surface

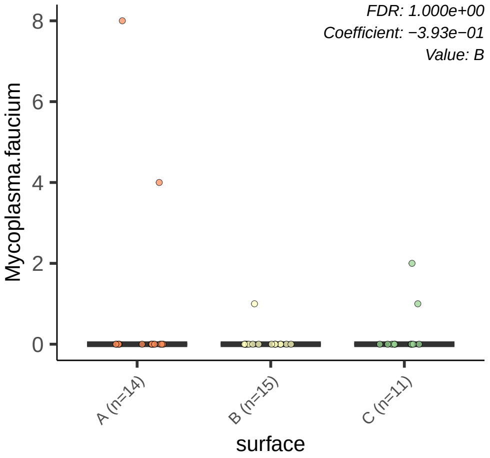
FDR: 1.000e+00
Coefficient: −3.93e−01
Value: B
Mycoplasma.faucium
8
6
4
2
0
A (n=14)
B (n=15)
C (n=11)
surface

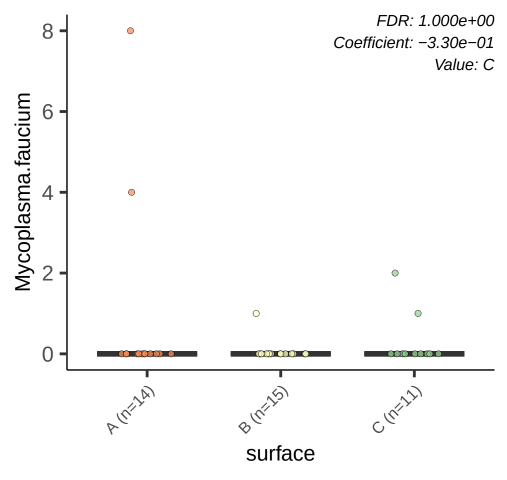
FDR: 1.000e+00
Coefficient: −3.30e−01
Value: C
Mycoplasma.faucium
8
6
4
2
0
A (n=14)
B (n=15)
C (n=11)
surface

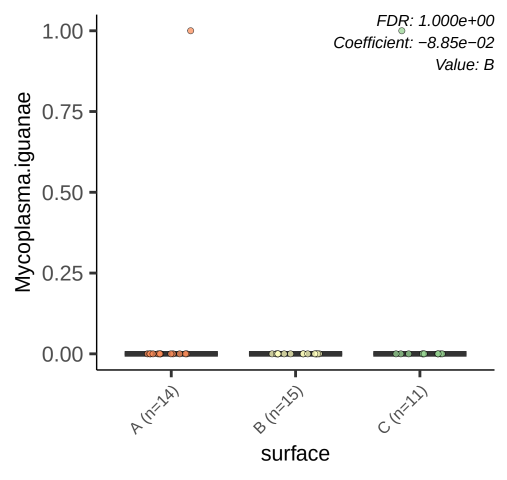
FDR: 1.000e+00
Coefficient: −8.85e−02
Value: B
Mycoplasma.iguanae
1.00
0.75
0.50
0.25
0.00
A (n=14)
B (n=15)
C (n=11)
surface

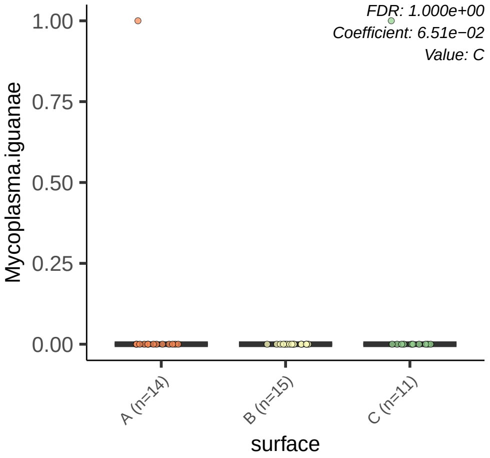
FDR: 1.000e+00
Coefficient: 6.51e−02
Value: C
Mycoplasma.iguanae
1.00
0.75
0.50
0.25
0.00
A (n=14)
B (n=15)
C (n=11)
surface

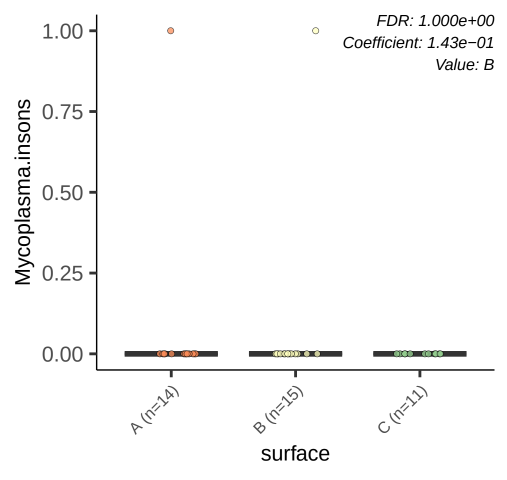
FDR: 1.000e+00
Coefficient: 1.43e−01
Value: B
Mycoplasma.insons
1.00
0.75
0.50
0.25
0.00
A (n=14)
B (n=15)
C (n=11)
surface

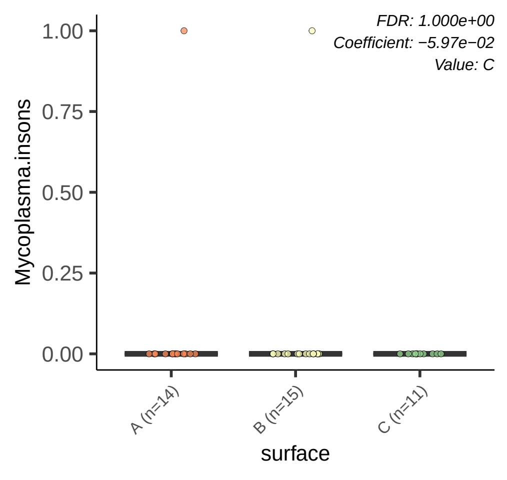
FDR: 1.000e+00
Coefficient: −5.97e−02
Value: C
Mycoplasma.insons
1.00
0.75
0.50
0.25
0.00
A (n=14)
B (n=15)
C (n=11)
surface

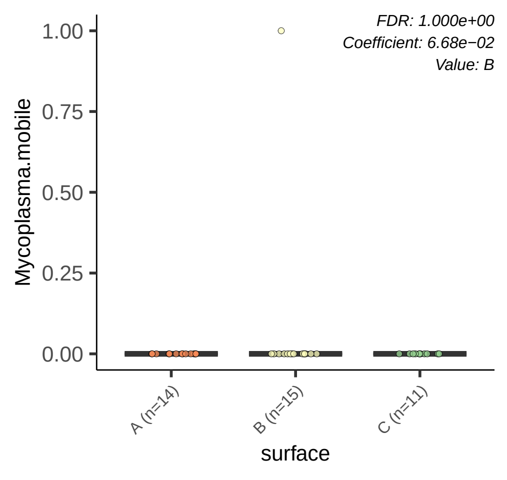
FDR: 1.000e+00
Coefficient: 6.68e−02
Value: B
Mycoplasma.mobile
1.00
0.75
0.50
0.25
0.00
A (n=14)
B (n=15)
C (n=11)
surface

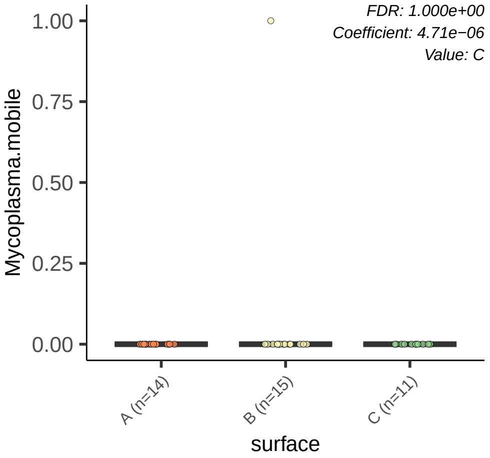
FDR: 1.000e+00
Coefficient: 4.71e−06
Value: C
Mycoplasma.mobile
1.00
0.75
0.50
0.25
0.00
A (n=14)
B (n=15)
C (n=11)
surface

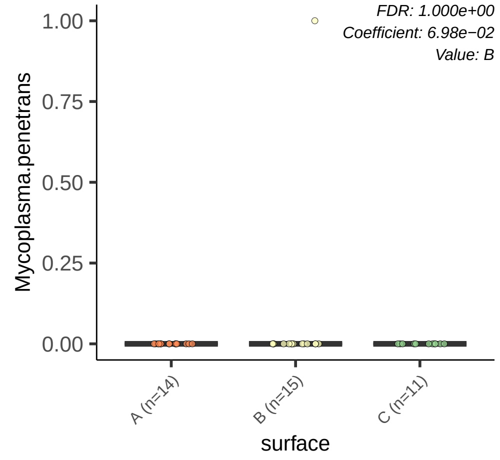
FDR: 1.000e+00
Coefficient: 6.98e−02
Value: B
Mycoplasma.penetrans
1.00
0.75
0.50
0.25
0.00
A (n=14)
B (n=15)
C (n=11)
surface

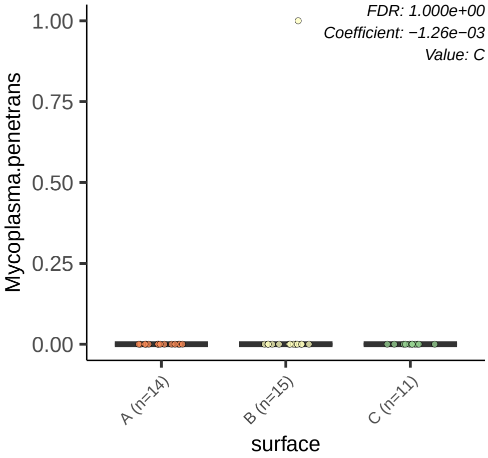

FDR: 1.000e+00
Coefficient: −1.26e−03
Value: C
Mycoplasma.penetrans
1.00
0.75
0.50
0.25
0.00
A (n=14)
B (n=15)
C (n=11)
surface

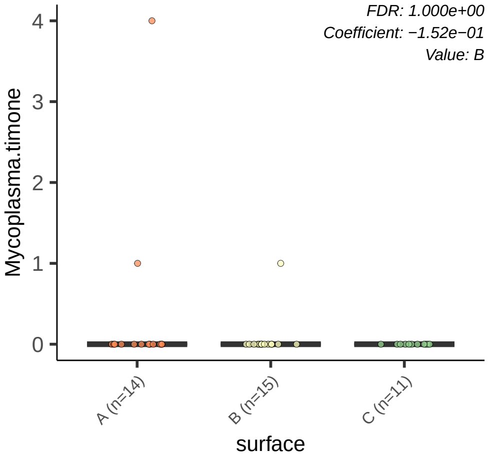
FDR: 1.000e+00
Coefficient: −1.52e−01
Value: B
Mycoplasma.timone
4
3
2
1
0
A (n=14)
B (n=15)
C (n=11)
surface

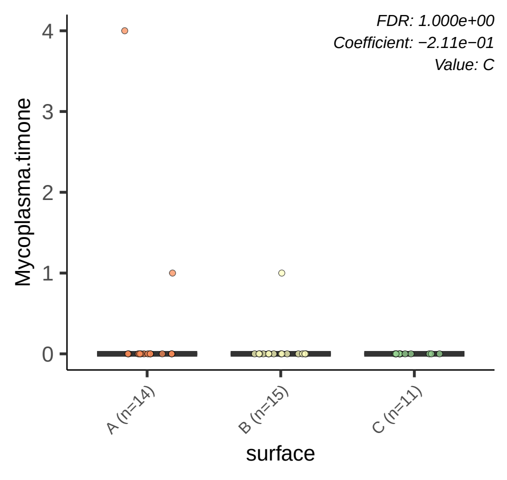

FDR: 1.000e+00
Coefficient: −2.11e−01
Value: C
Mycoplasma.timone
4
3
2
1
0
A (n=14)
B (n=15)
C (n=11)
surface

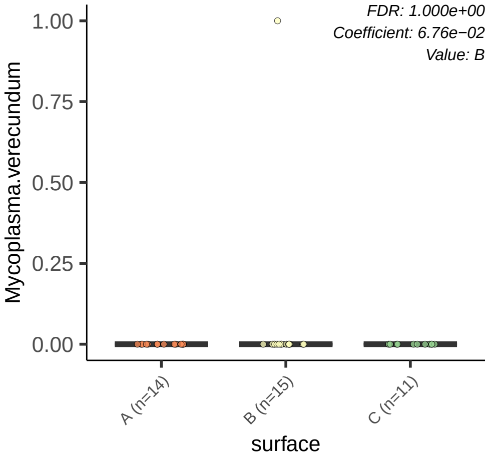
FDR: 1.000e+00
Coefficient: 6.76e−02
Value: B
Mycoplasma.verecundum
1.00
0.75
0.50
0.25
0.00
A (n=14)
B (n=15)
C (n=11)
surface

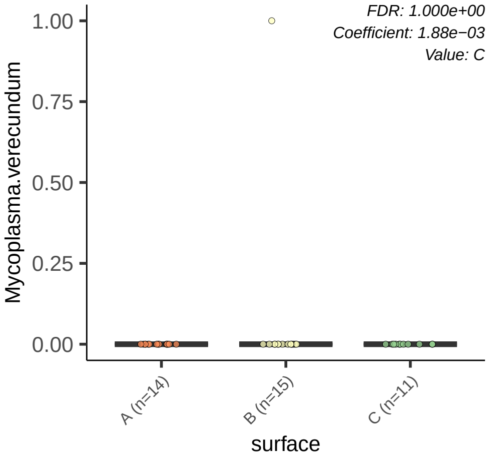

FDR: 1.000e+00
Coefficient: 1.88e−03
Value: C
Mycoplasma.verecundum
1.00
0.75
0.50
0.25
0.00
A (n=14)
B (n=15)
C (n=11)
surface

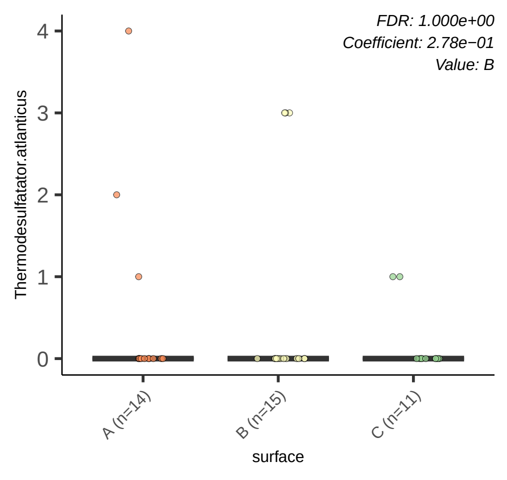

FDR: 1.000e+00
Coefficient: 2.78e−01
Value: B
Thermodesulfatator.atlanticus
0
1
2
3
4
A (n=14)
B (n=15)
C (n=11)
surface

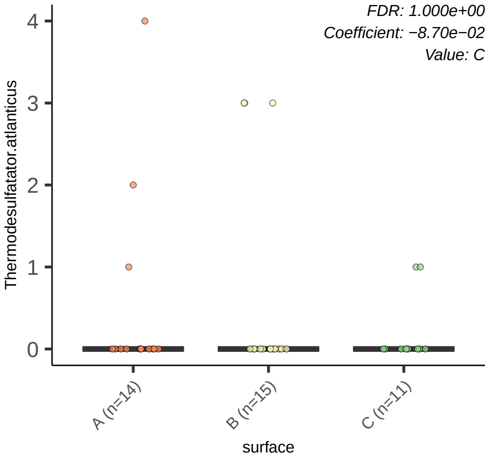

FDR: 1.000e+00
Coefficient: −8.70e−02
Value: C
Thermodesulfatator.atlanticus
0
1
2
3
4
A (n=14)
B (n=15)
C (n=11)
surface

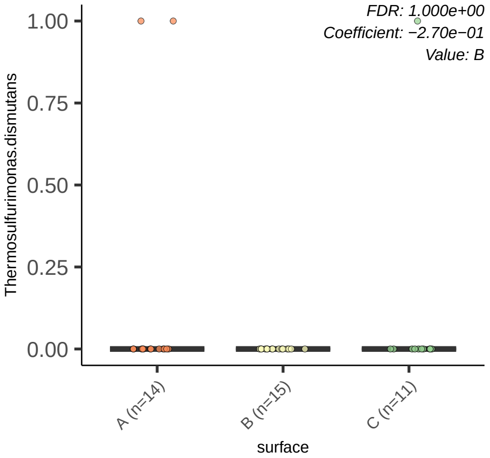

FDR: 1.000e+00
Coefficient: −2.70e−01
Value: B
Thermosulfurimonas.dismutans
1.00
0.75
0.50
0.25
0.00
A (n=14)
B (n=15)
C (n=11)
surface

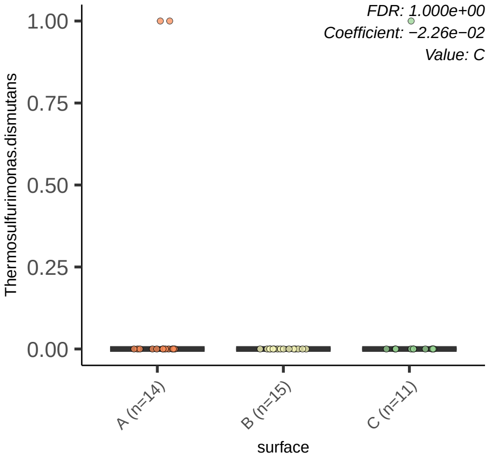

FDR: 1.000e+00
Coefficient: −2.26e−02
Value: C
Thermosulfurimonas.dismutans
1.00
0.75
0.50
0.25
0.00
A (n=14)
B (n=15)
C (n=11)
surface

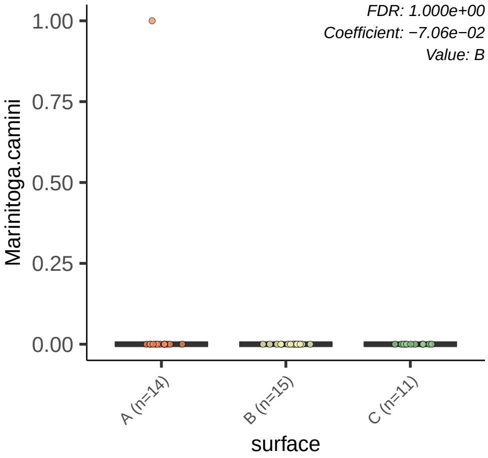

FDR: 1.000e+00
Coefficient: −7.06e−02
Value: B
Marinitoga.camini
1.00
0.75
0.50
0.25
0.00
A (n=14)
B (n=15)
C (n=11)
surface

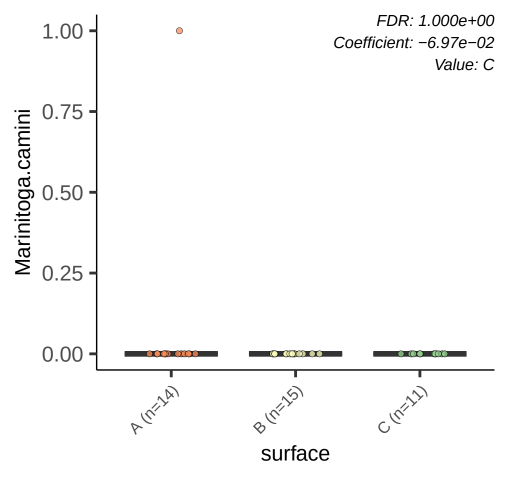
FDR: 1.000e+00
Coefficient: −6.97e−02
Value: C
Marinitoga.camini
1.00
0.75
0.50
0.25
0.00
A (n=14)
B (n=15)
C (n=11)
surface

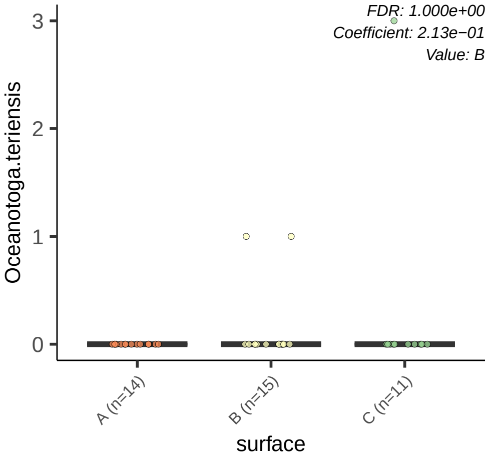
FDR: 1.000e+00
Coefficient: 2.13e−01
Value: B
Oceanotoga.teriensis
3
2
1
0
A (n=14)
B (n=15)
C (n=11)
surface

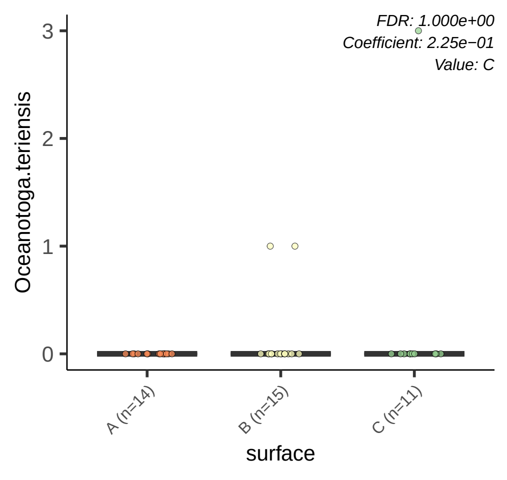
FDR: 1.000e+00
Coefficient: 2.25e−01
Value: C
Oceanotoga.teriensis
0
1
2
3
A (n=14)
B (n=15)
C (n=11)
surface

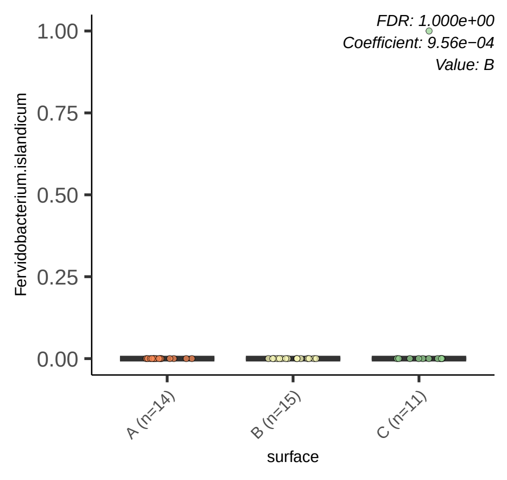

FDR: 1.000e+00
Coefficient: 9.56e−04
Value: B
1.00
0.75
0.50
0.25
0.00
Fervidobacterium.islandicum
A (n=14)
B (n=15)
C (n=11)
surface

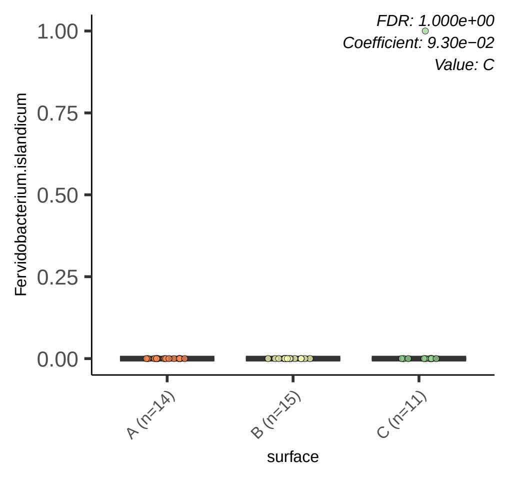
FDR: 1.000e+00
Coefficient: 9.30e−02
Value: C
Fervidobacterium.islandicum
1.00
0.75
0.50
0.25
0.00
A (n=14)
B (n=15)
C (n=11)
surface

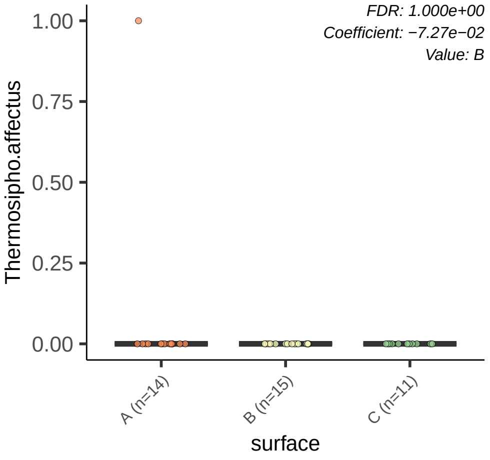
FDR: 1.000e+00
Coefficient: −7.27e−02
Value: B
Thermosipho.affectus
1.00
0.75
0.50
0.25
0.00
A (n=14)
B (n=15)
C (n=11)
surface

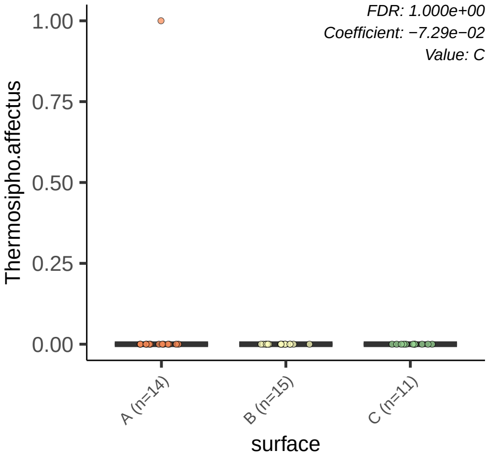
FDR: 1.000e+00
Coefficient: −7.29e−02
Value: C
Thermosipho.affectus
1.00
0.75
0.50
0.25
0.00
A (n=14)
B (n=15)
C (n=11)
surface

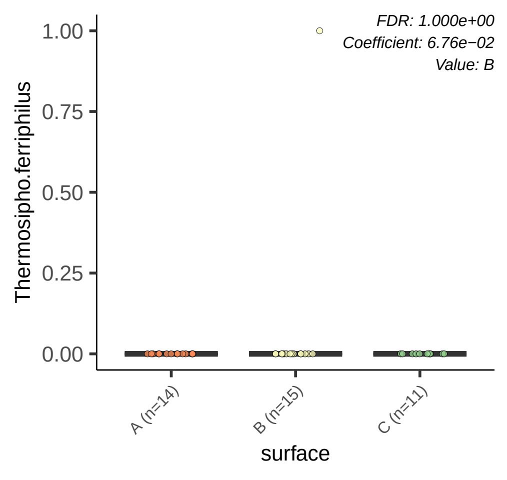

FDR: 1.000e+00
Coefficient: 6.76e−02
Value: B
Thermosipho.ferriphilus
1.00
0.75
0.50
0.25
0.00
A (n=14)
B (n=15)
C (n=11)
surface

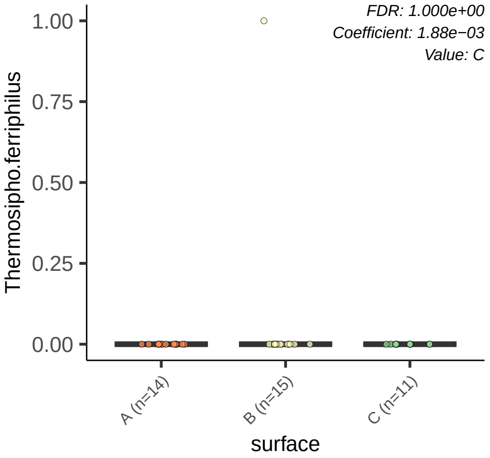

FDR: 1.000e+00
Coefficient: 1.88e−03
Value: C
Thermosipho.ferriphilus
1.00
0.75
0.50
0.25
0.00
A (n=14)
B (n=15)
C (n=11)
surface

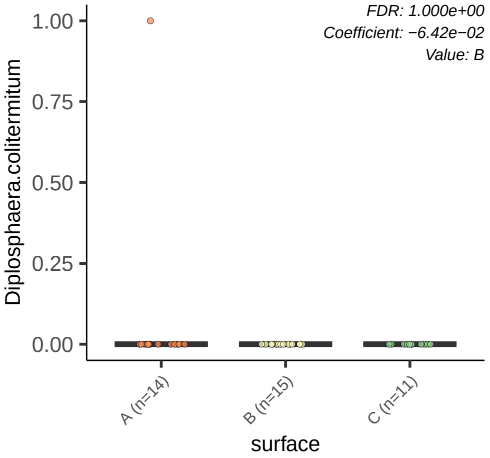
FDR: 1.000e+00
Coefficient: −6.42e−02
Value: B
Diplosphaera.colitermitum
1.00
0.75
0.50
0.25
0.00
A (n=14)
B (n=15)
C (n=11)
surface

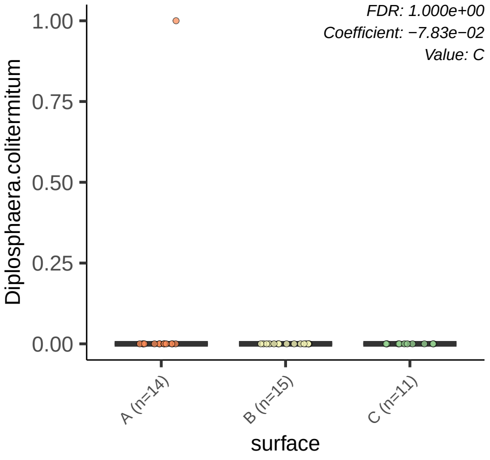
FDR: 1.000e+00
Coefficient: −7.83e−02
Value: C
Diplosphaera.colitermitum
1.00
0.75
0.50
0.25
0.00
A (n=14)
B (n=15)
C (n=11)
surface

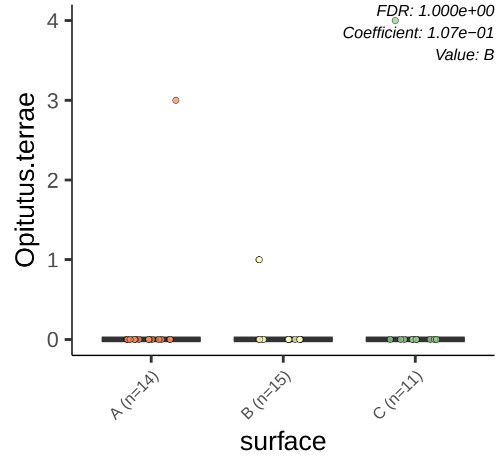
FDR: 1.000e+00
Coefficient: 1.07e−01
Value: B
Opitutus.terrae
4
3
2
1
0
A (n=14)
B (n=15)
C (n=11)
surface

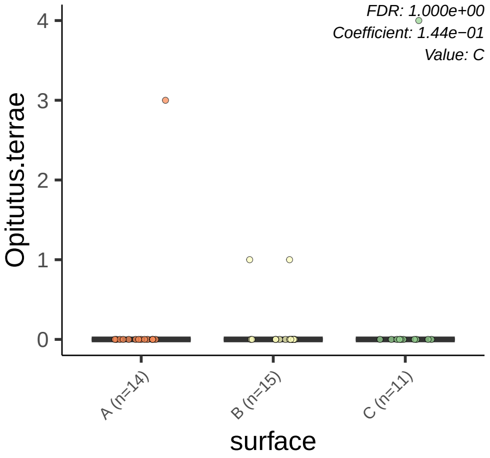
FDR: 1.000e+00
Coefficient: 1.44e−01
Value: C
Opitutus.terrae
0
1
2
3
4
A (n=14)
B (n=15)
C (n=11)
surface

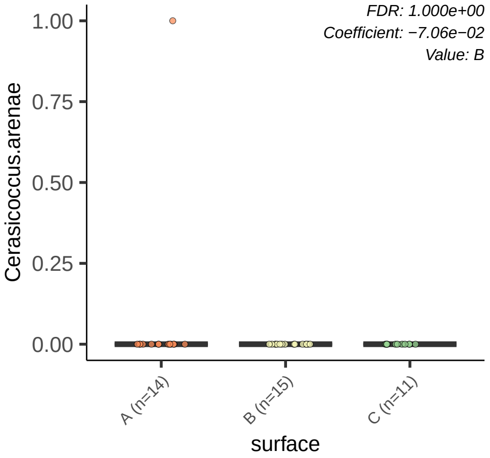
FDR: 1.000e+00
Coefficient: −7.06e−02
Value: B
Cerasicoccus.arenae
1.00
0.75
0.50
0.25
0.00
A (n=14)
B (n=15)
C (n=11)
surface

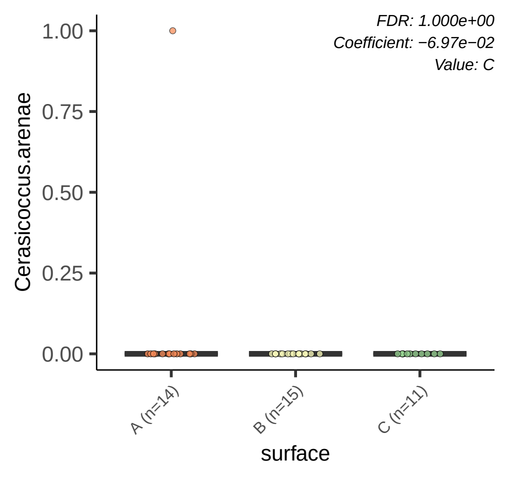

FDR: 1.000e+00
Coefficient: −6.97e−02
Value: C
Cerasicoccus.arenae
1.00
0.75
0.50
0.25
0.00
A (n=14)
B (n=15)
C (n=11)
surface

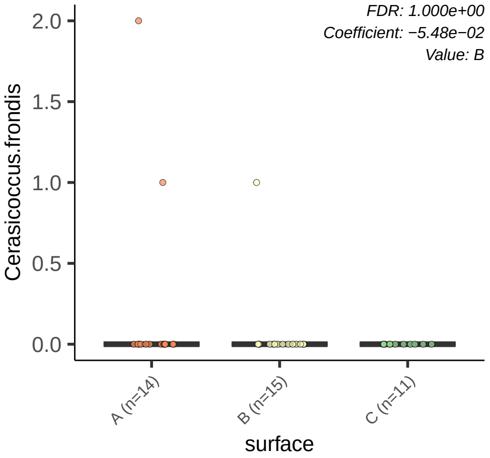
FDR: 1.000e+00
Coefficient: −5.48e−02
Value: B
Cerasicoccus.frondis
2.0
1.5
1.0
0.5
0.0
A (n=14)
B (n=15)
C (n=11)
surface

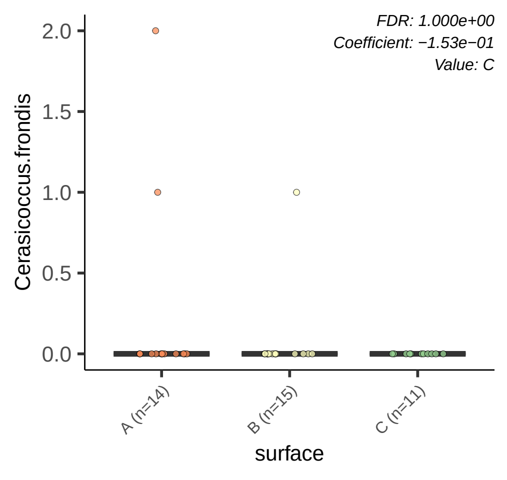
FDR: 1.000e+00
Coefficient: −1.53e−01
Value: C
Cerasicoccus.frondis
2.0
1.5
1.0
0.5
0.0
A (n=14)
B (n=15)
C (n=11)
surface

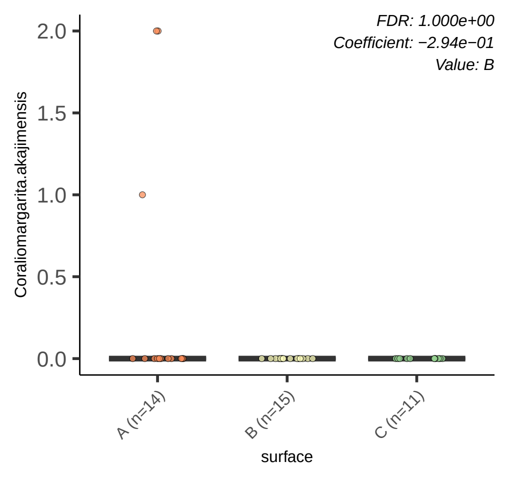
FDR: 1.000e+00
Coefficient: −2.94e−01
Value: B
Coraliomargarita.akajimensis
2.0
1.5
1.0
0.5
0.0
A (n=14)
B (n=15)
C (n=11)
surface

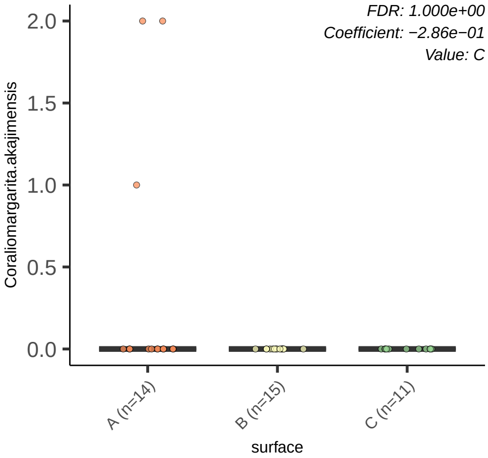

FDR: 1.000e+00
Coefficient: −2.86e−01
Value: C
Coraliomargarita.akajimensis
2.0
1.5
1.0
0.5
0.0
A (n=14)
B (n=15)
C (n=11)
surface

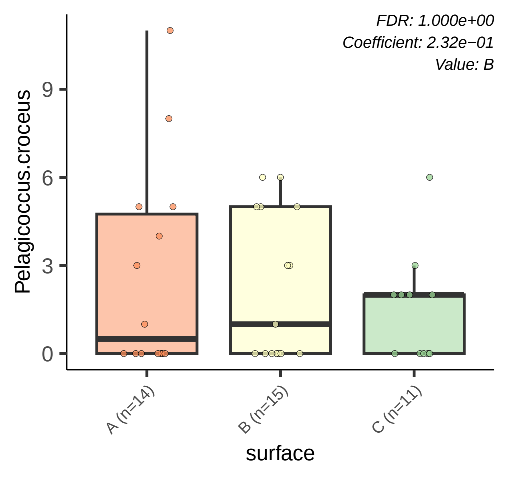
FDR: 1.000e+00
Coefficient: 2.32e−01
Value: B
Pelagicoccus.croceus
0
3
6
9
A (n=14)
B (n=15)
C (n=11)
surface

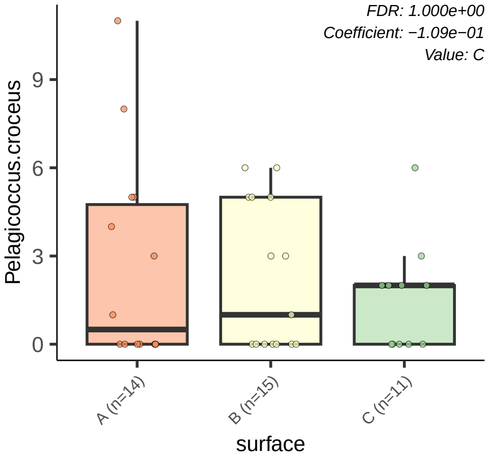

FDR: 1.000e+00
Coefficient: −1.09e−01
Value: C
Pelagicoccus.croceus
0
3
6
9
A (n=14)
B (n=15)
C (n=11)
surface

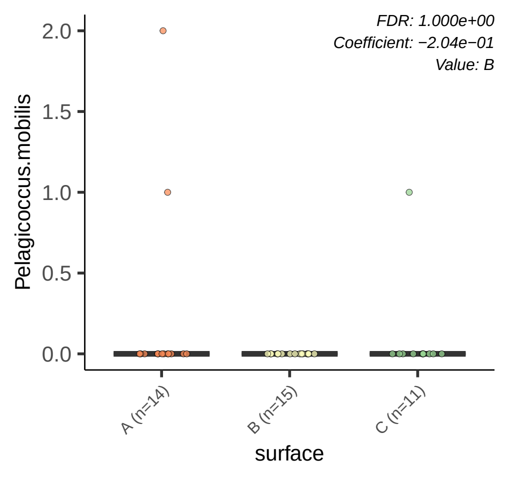
FDR: 1.000e+00
Coefficient: −2.04e−01
Value: B
Pelagicoccus.mobilis
2.0
1.5
1.0
0.5
0.0
A (n=14)
B (n=15)
C (n=11)
surface

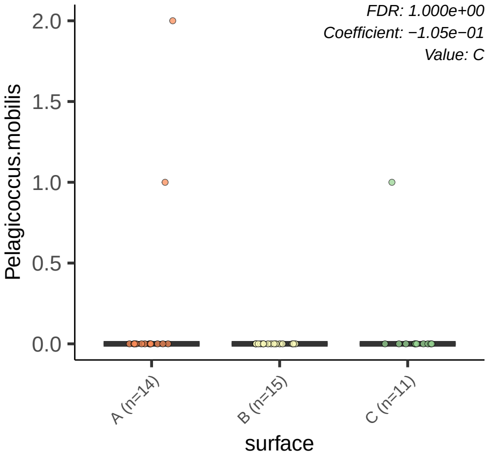
FDR: 1.000e+00
Coefficient: −1.05e−01
Value: C
Pelagicoccus.mobilis
2.0
1.5
1.0
0.5
0.0
A (n=14)
B (n=15)
C (n=11)
surface

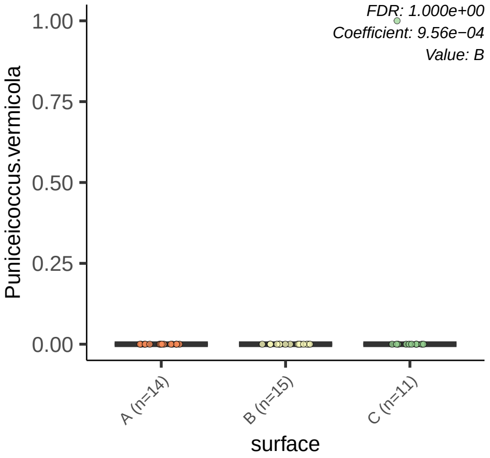
FDR: 1.000e+00
Coefficient: 9.56e−04
Value: B
Puniceicoccus.vermicola
1.00
0.75
0.50
0.25
0.00
A (n=14)
B (n=15)
C (n=11)
surface

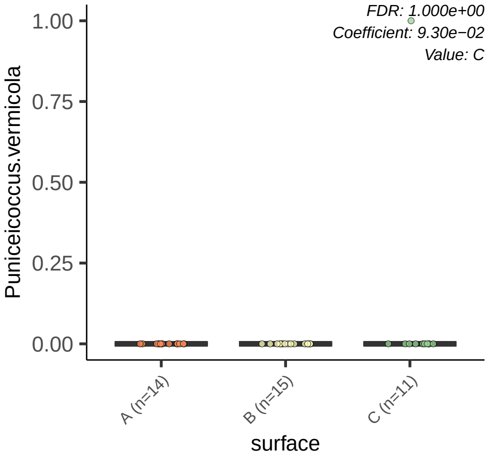
FDR: 1.000e+00
Coefficient: 9.30e−02
Value: C
Puniceicoccus.vermicola
1.00
0.75
0.50
0.25
0.00
A (n=14)
B (n=15)
C (n=11)
surface

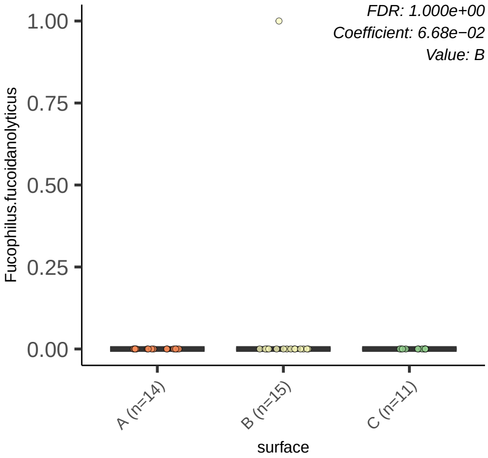
FDR: 1.000e+00
Coefficient: 6.68e−02
Value: B
Fucophilus.fucoidanolyticus
1.00
0.75
0.50
0.25
0.00
A (n=14)
B (n=15)
C (n=11)
surface

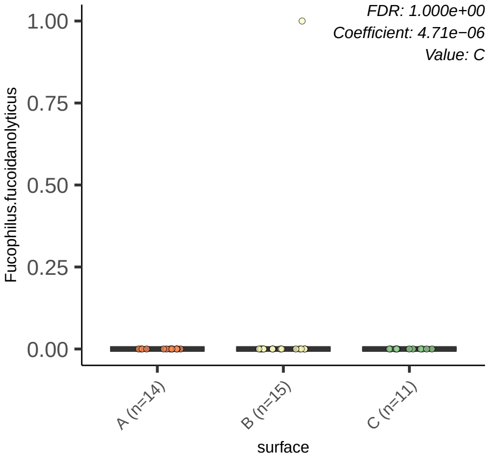
FDR: 1.000e+00
Coefficient: 4.71e−06
Value: C
Fucophilus.fucoidanolyticus
1.00
0.75
0.50
0.25
0.00
A (n=14)
B (n=15)
C (n=11)
surface

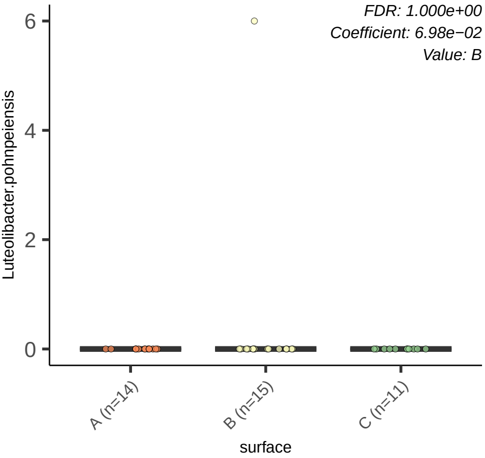
FDR: 1.000e+00
Coefficient: 6.98e−02
Value: B
Luteolibacter.pohnpeiensis
6
4
2
0
A (n=14)
B (n=15)
C (n=11)
surface

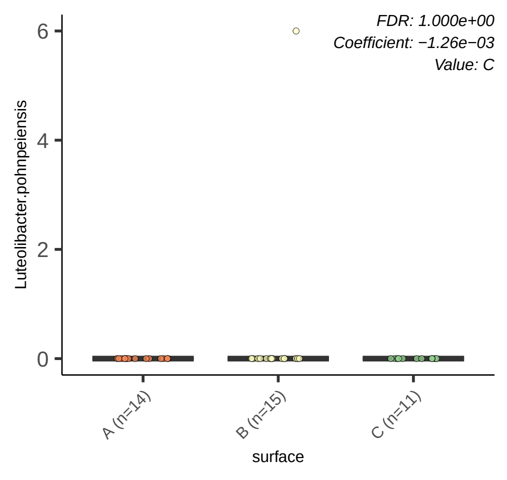

FDR: 1.000e+00
Coefficient: −1.26e−03
Value: C
Luteolibacter.pohnpeiensis
0
2
4
6
A (n=14)
B (n=15)
C (n=11)
surface

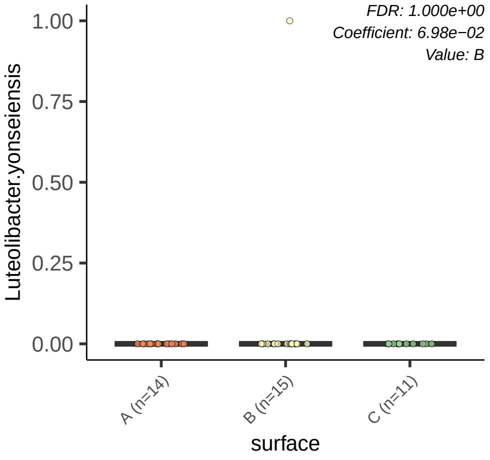

FDR: 1.000e+00
Coefficient: 6.98e−02
Value: B
Luteolibacter.yonseiensis
1.00
0.75
0.50
0.25
0.00
A (n=14)
B (n=15)
C (n=11)
surface

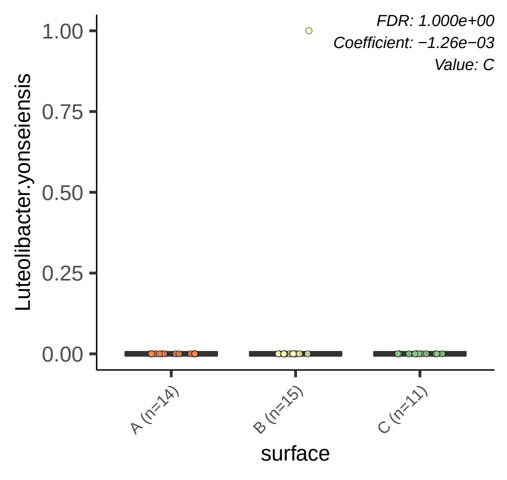
FDR: 1.000e+00
Coefficient: −1.26e−03
Value: C
Luteolibacter.yonseiensis
1.00
0.75
0.50
0.25
0.00
A (n=14)
B (n=15)
C (n=11)
surface

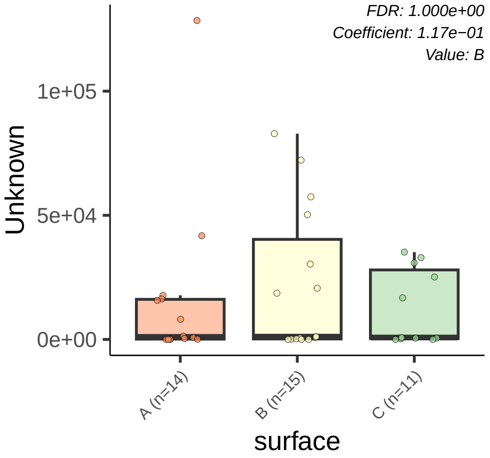

FDR: 1.000e+00
Coefficient: 1.17e−01
Value: B
Unknown
1e+05
5e+04
0e+00
A (n=14)
B (n=15)
C (n=11)
surface

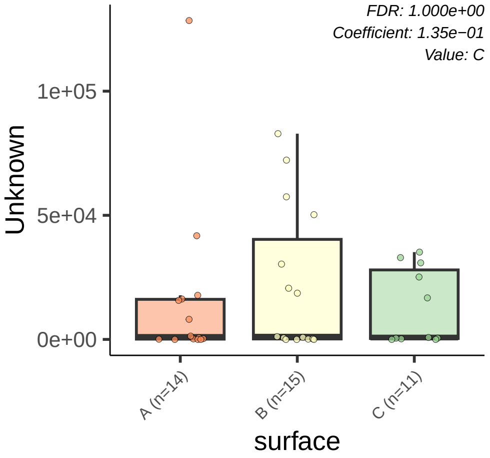

FDR: 1.000e+00
Coefficient: 1.35e−01
Value: C
Unknown
1e+05
5e+04
0e+00
A (n=14)
B (n=15)
C (n=11)
surface

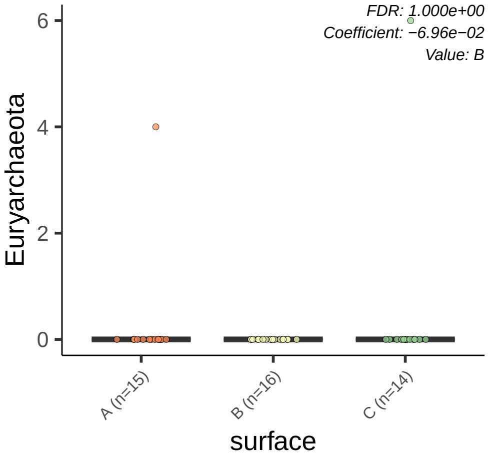
FDR: 1.000e+00
Coefficient: −6.96e−02
Value: B
Euryarchaeota
0
2
4
6
A (n=15)
B (n=16)
C (n=14)
surface

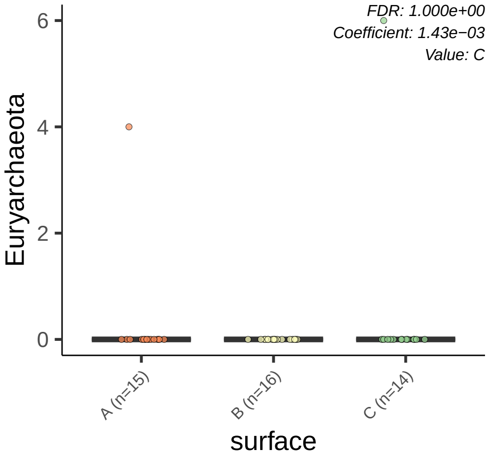

FDR: 1.000e+00
Coefficient: 1.43e−03
Value: C
Euryarchaeota
6
4
2
0
A (n=15)
B (n=16)
C (n=14)
surface

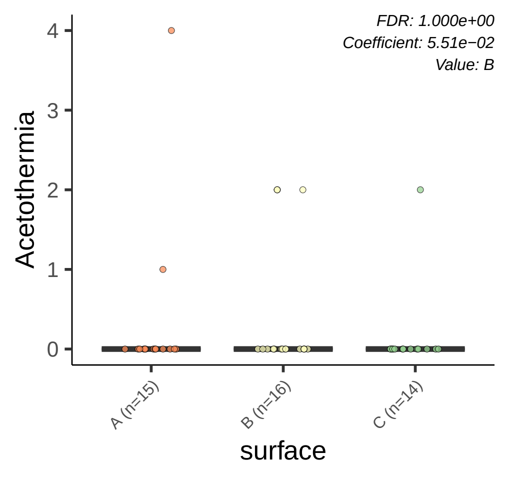

FDR: 1.000e+00
Coefficient: 5.51e−02
Value: B
Acetothermia
0
1
2
3
4
A (n=15)
B (n=16)
C (n=14)
surface

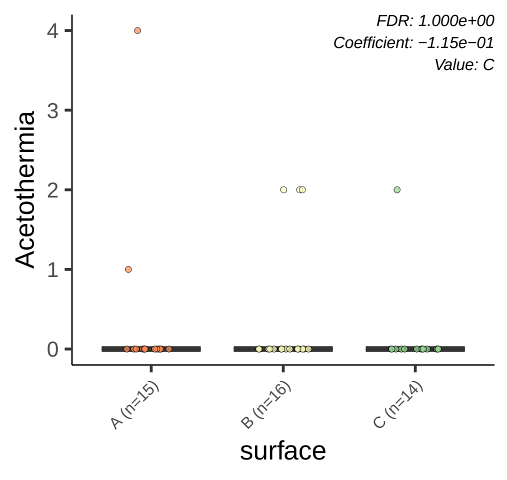

FDR: 1.000e+00
Coefficient: −1.15e−01
Value: C
Acetothermia
0
1
2
3
4
A (n=15)
B (n=16)
C (n=14)
surface

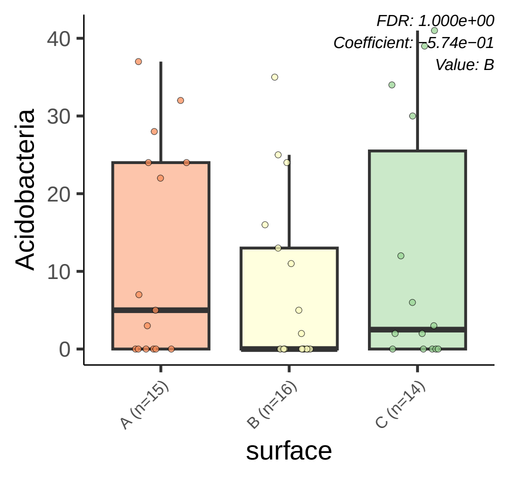
FDR: 1.000e+00
Coefficient: −5.74e−01
Value: B
Acidobacteria
0
10
20
30
40
A (n=15)
B (n=16)
C (n=14)
surface

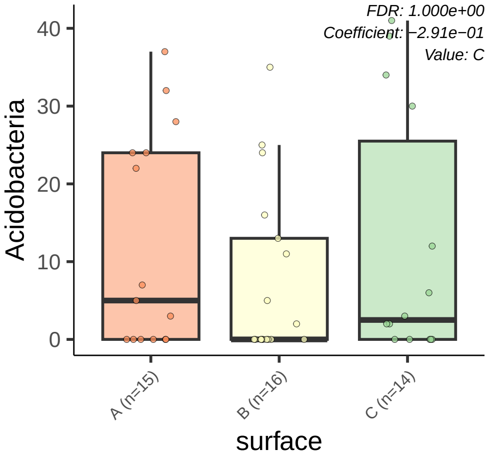

FDR: 1.000e+00
Coefficient: −2.91e−01
Value: C
Acidobacteria
40
30
20
10
0
A (n=15)
B (n=16)
C (n=14)
surface

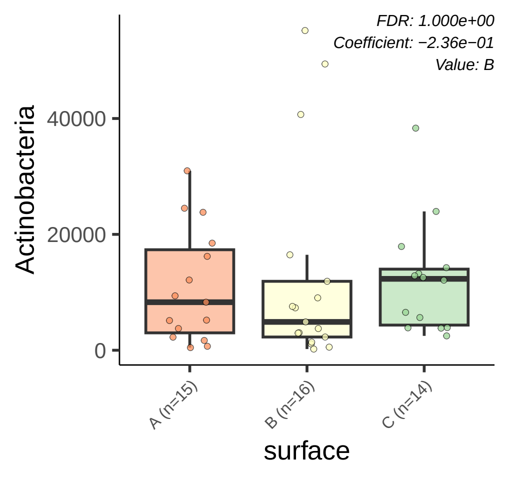

FDR: 1.000e+00
Coefficient: −2.36e−01
Value: B
Actinobacteria
40000
20000
0
A (n=15)
B (n=16)
C (n=14)
surface

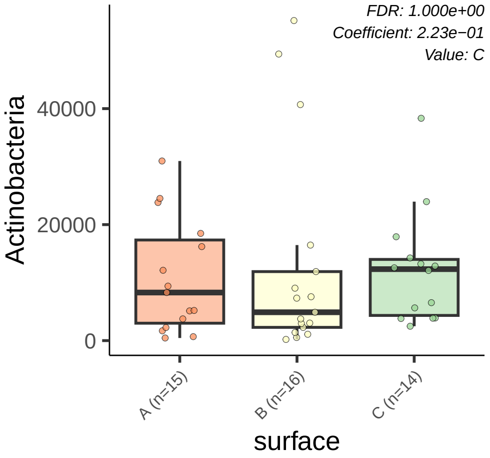
FDR: 1.000e+00
Coefficient: 2.23e−01
Value: C
Actinobacteria
40000
20000
0
A (n=15)
B (n=16)
C (n=14)
surface

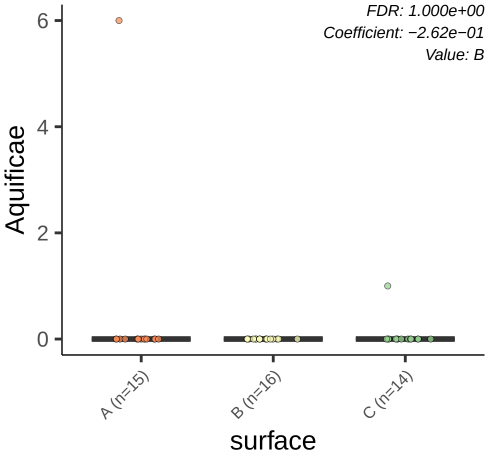

FDR: 1.000e+00
Coefficient: −2.62e−01
Value: B
Aquificae
0
2
4
6
A (n=15)
B (n=16)
C (n=14)
surface

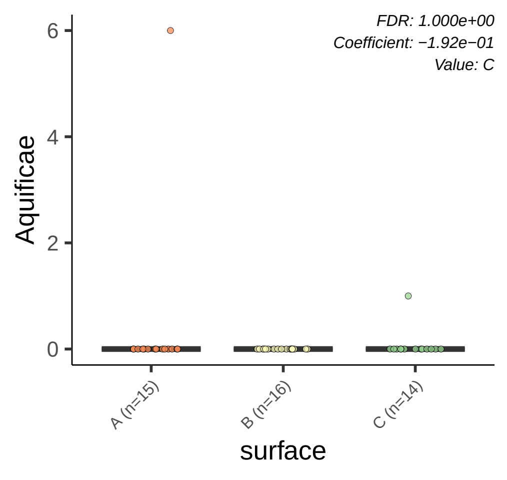
FDR: 1.000e+00
Coefficient: −1.92e−01
Value: C
Aquificae
6
4
2
0
A (n=15)
B (n=16)
C (n=14)
surface

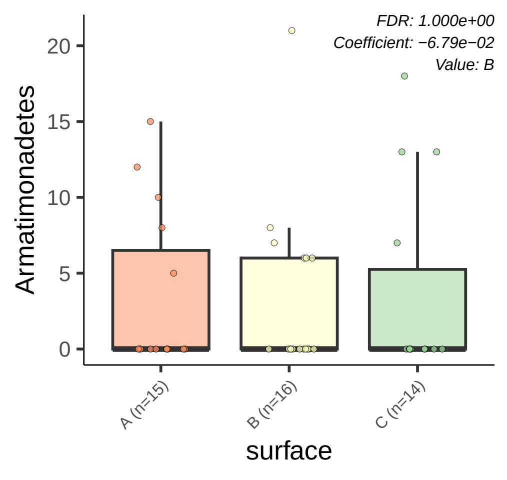
FDR: 1.000e+00
Coefficient: −6.79e−02
Value: B
Armatimonadetes
20
15
10
5
0
A (n=15)
B (n=16)
C (n=14)
surface

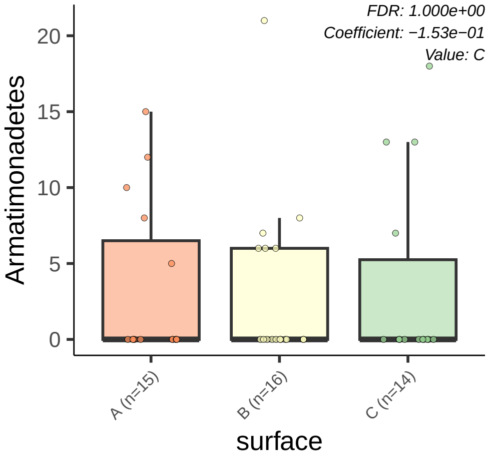
FDR: 1.000e+00
Coefficient: −1.53e−01
Value: C
Armatimonadetes
20
15
10
5
0
A (n=15)
B (n=16)
C (n=14)
surface

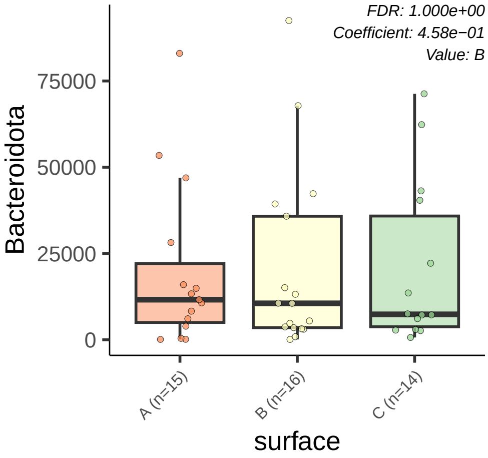
FDR: 1.000e+00
Coefficient: 4.58e−01
Value: B
Bacteroidota
75000
50000
25000
0
A (n=15)
B (n=16)
C (n=14)
surface

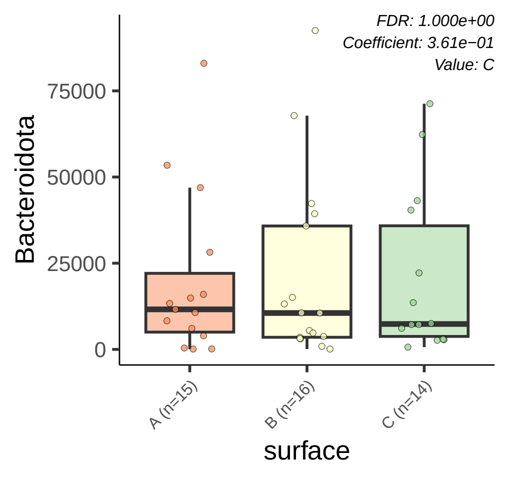
FDR: 1.000e+00
Coefficient: 3.61e−01
Value: C
Bacteroidota
75000
50000
25000
0
A (n=15)
B (n=16)
C (n=14)
surface

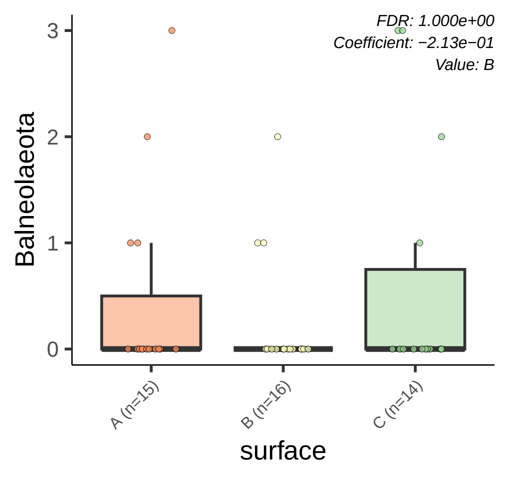
FDR: 1.000e+00
Coefficient: −2.13e−01
Value: B
Balneolaeota
3
2
1
0
A (n=15)
B (n=16)
C (n=14)
surface

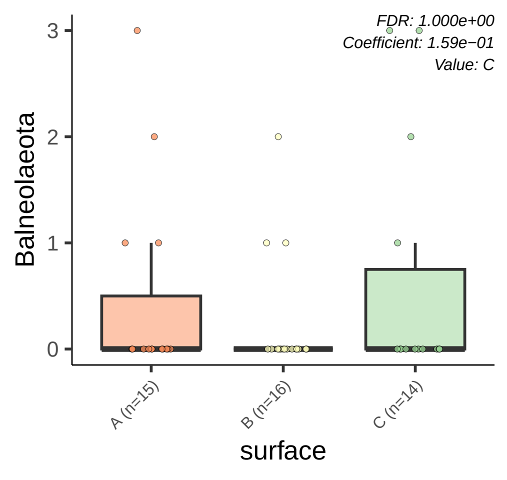

FDR: 1.000e+00
Coefficient: 1.59e−01
Value: C
Balneolaeota
3
2
1
0
A (n=15)
B (n=16)
C (n=14)
surface

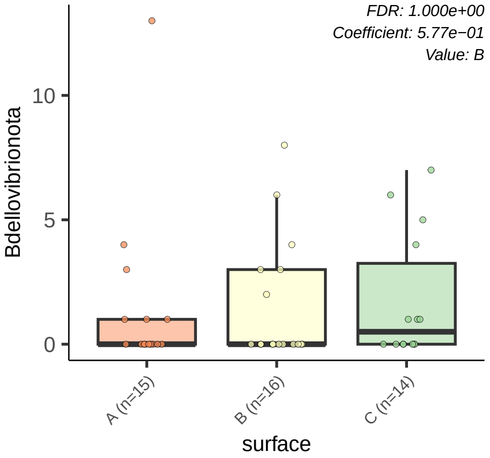
FDR: 1.000e+00
Coefficient: 5.77e−01
Value: B
Bdellovibrionota
10
5
0
A (n=15)
B (n=16)
C (n=14)
surface

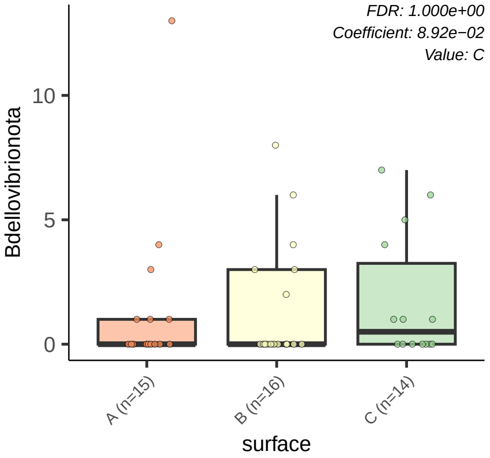

FDR: 1.000e+00
Coefficient: 8.92e−02
Value: C
10
5
0
Bdellovibrionota
A (n=15)
B (n=16)
C (n=14)
surface

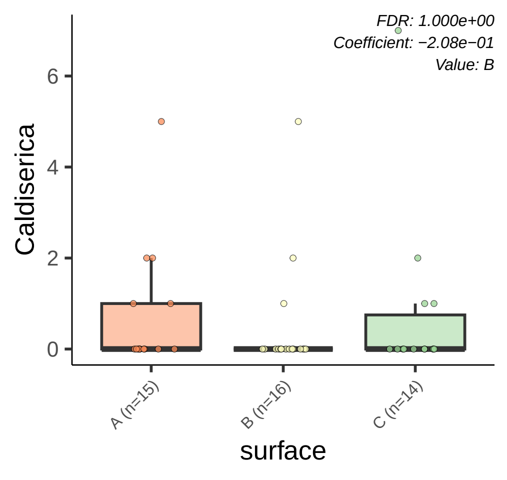
FDR: 1.000e+00
Coefficient: −2.08e−01
Value: B
Caldiserica
0
2
4
6
A (n=15)
B (n=16)
C (n=14)
surface

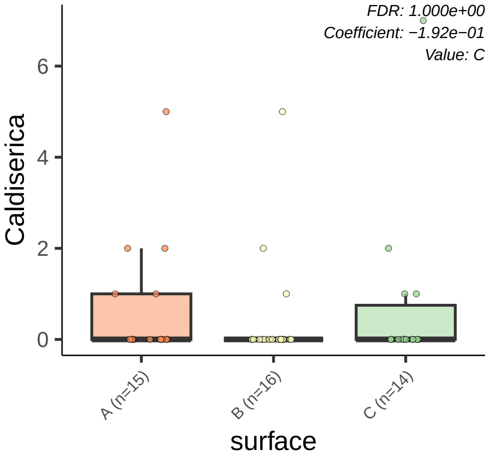
FDR: 1.000e+00
Coefficient: −1.92e−01
Value: C
Caldiserica
0
2
4
6
A (n=15)
B (n=16)
C (n=14)
surface

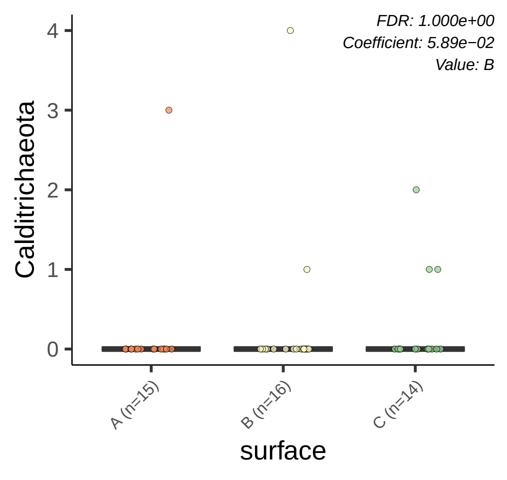
FDR: 1.000e+00
Coefficient: 5.89e−02
Value: B
Calditrichaeota
4
3
2
1
0
A (n=15)
B (n=16)
C (n=14)
surface

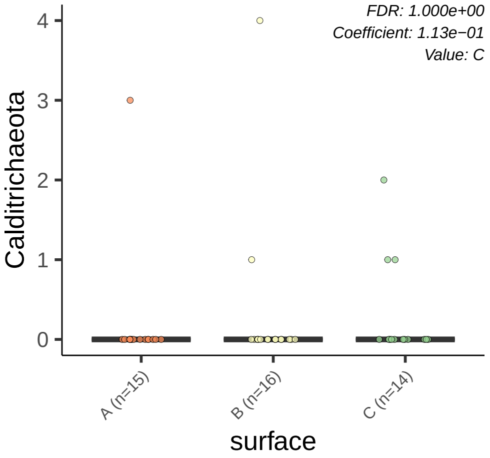

FDR: 1.000e+00
Coefficient: 1.13e−01
Value: C
Calditrichaeota
4
3
2
1
0
A (n=15)
B (n=16)
C (n=14)
surface

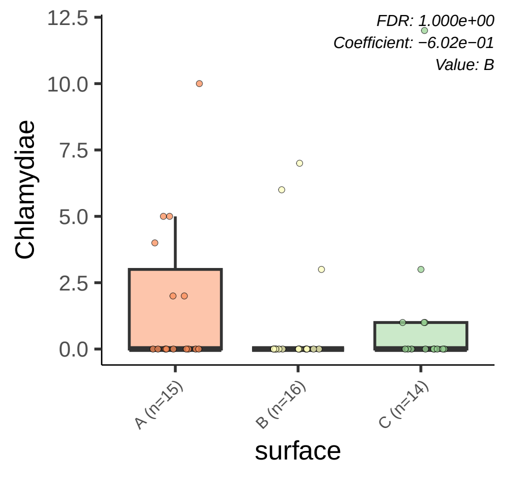
FDR: 1.000e+00
Coefficient: −6.02e−01
Value: B
Chlamydiae
0.0
2.5
5.0
7.5
10.0
12.5
A (n=15)
B (n=16)
C (n=14)
surface

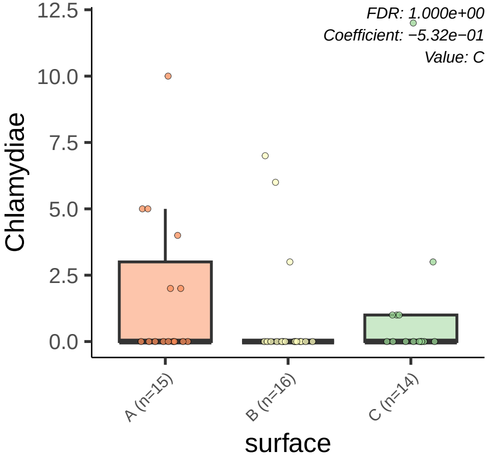

FDR: 1.000e+00
Coefficient: −5.32e−01
Value: C
Chlamydiae
12.5
10.0
7.5
5.0
2.5
0.0
A (n=15)
B (n=16)
C (n=14)
surface

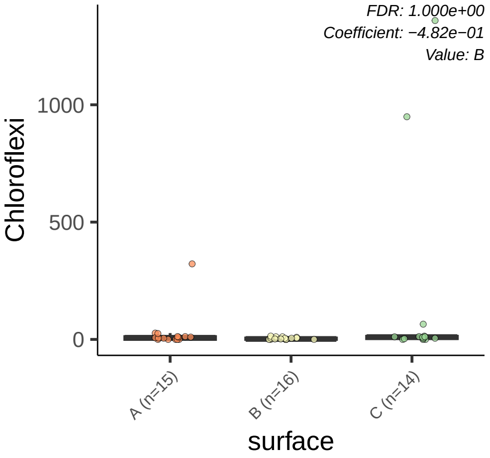

FDR: 1.000e+00
Coefficient: −4.82e−01
Value: B
Chloroflexi
1000
500
0
A (n=15)
B (n=16)
C (n=14)
surface

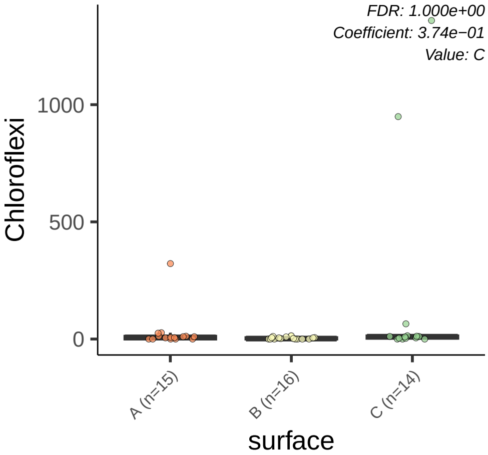

FDR: 1.000e+00
Coefficient: 3.74e−01
Value: C
Chloroflexi
1000
500
0
A (n=15)
B (n=16)
C (n=14)
surface

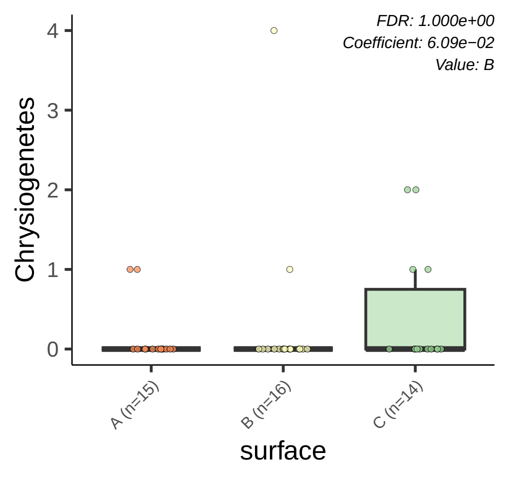
FDR: 1.000e+00
Coefficient: 6.09e−02
Value: B
Chrysiogenetes
0
1
2
3
4
A (n=15)
B (n=16)
C (n=14)
surface

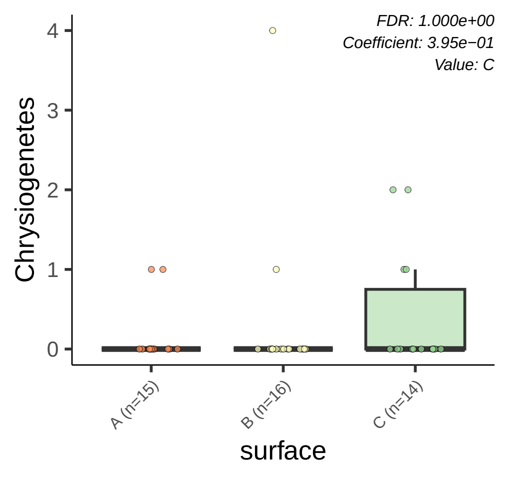

FDR: 1.000e+00
Coefficient: 3.95e−01
Value: C
Chrysiogenetes
0
1
2
3
4
A (n=15)
B (n=16)
C (n=14)
surface

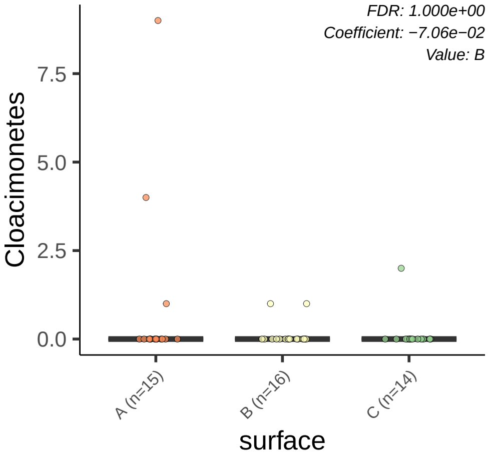

FDR: 1.000e+00
Coefficient: −7.06e−02
Value: B
Cloacimonetes
7.5
5.0
2.5
0.0
A (n=15)
B (n=16)
C (n=14)
surface

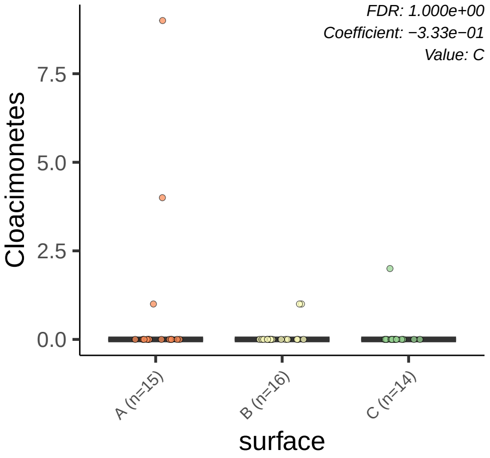

FDR: 1.000e+00
Coefficient: −3.33e−01
Value: C
Cloacimonetes
7.5
5.0
2.5
0.0
A (n=15)
B (n=16)
C (n=14)
surface

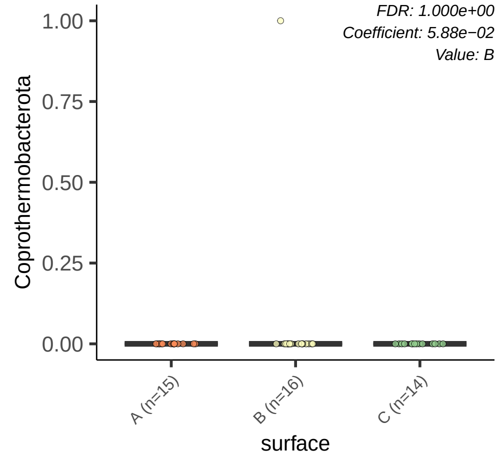

FDR: 1.000e+00
Coefficient: 5.88e−02
Value: B
Coprothermobacterota
1.00
0.75
0.50
0.25
0.00
A (n=15)
B (n=16)
C (n=14)
surface

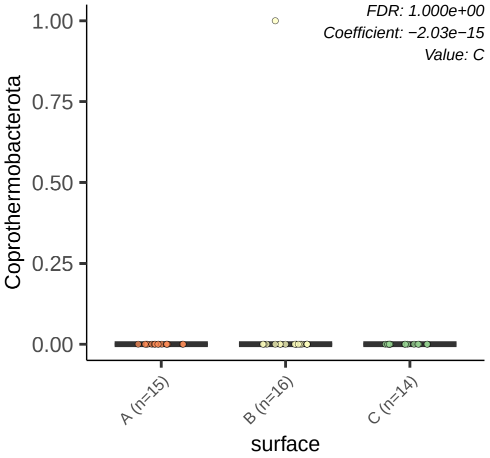
FDR: 1.000e+00
Coefficient: −2.03e−15
Value: C
Coprothermobacterota
1.00
0.75
0.50
0.25
0.00
A (n=15)
B (n=16)
C (n=14)
surface

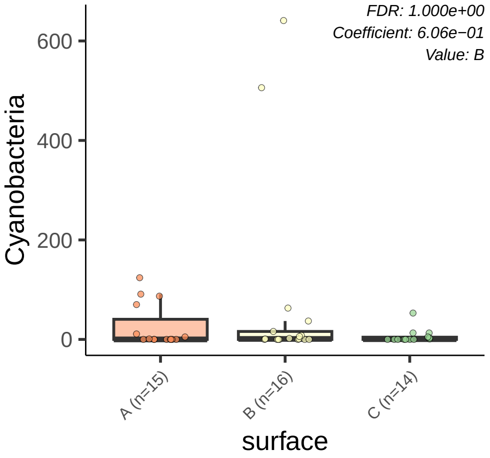
FDR: 1.000e+00
Coefficient: 6.06e−01
Value: B
Cyanobacteria
600
400
200
0
A (n=15)
B (n=16)
C (n=14)
surface

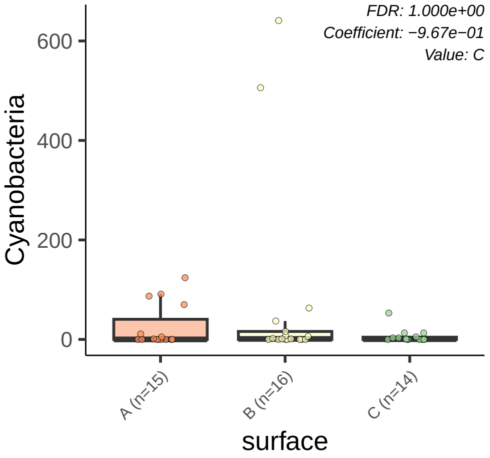

FDR: 1.000e+00
Coefficient: −9.67e−01
Value: C
Cyanobacteria
600
400
200
0
A (n=15)
B (n=16)
C (n=14)
surface

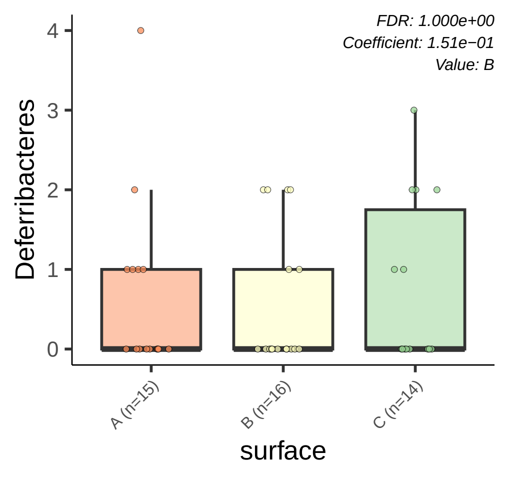

FDR: 1.000e+00
Coefficient: 1.51e−01
Value: B
Deferribacteres
0
1
2
3
4
A (n=15)
B (n=16)
C (n=14)
surface

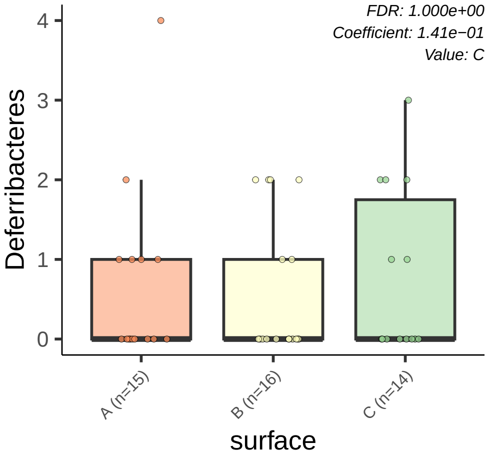
FDR: 1.000e+00
Coefficient: 1.41e−01
Value: C
Deferribacteres
0
1
2
3
4
A (n=15)
B (n=16)
C (n=14)
surface

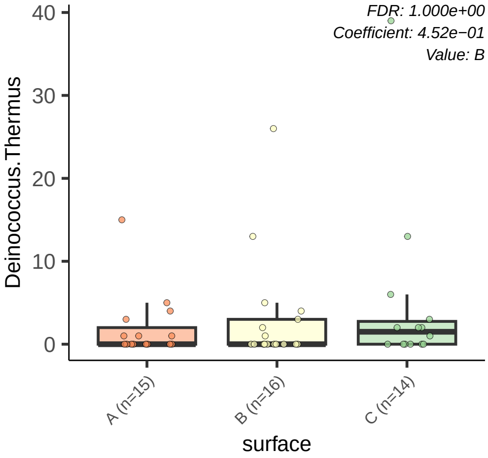
FDR: 1.000e+00
Coefficient: 4.52e−01
Value: B
Deinococcus.Thermus
40
30
20
10
0
A (n=15)
B (n=16)
C (n=14)
surface

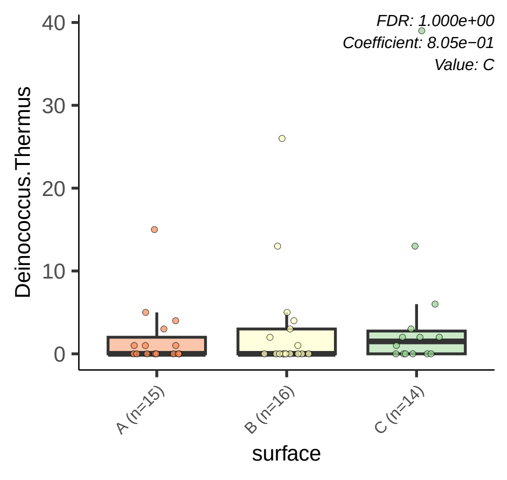

FDR: 1.000e+00
Coefficient: 8.05e−01
Value: C
Deinococcus.Thermus
0
10
20
30
40
A (n=15)
B (n=16)
C (n=14)
surface

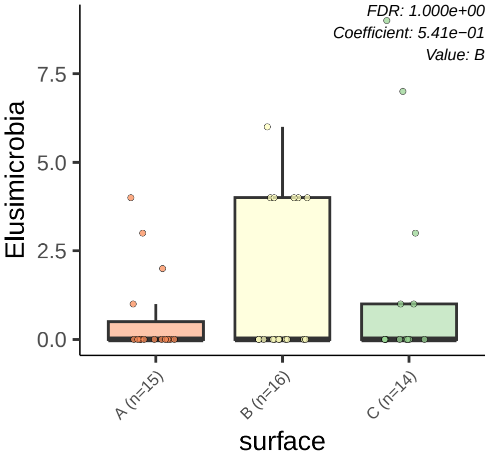

FDR: 1.000e+00
Coefficient: 5.41e−01
Value: B
Elusimicrobia
7.5
5.0
2.5
0.0
A (n=15)
B (n=16)
C (n=14)
surface

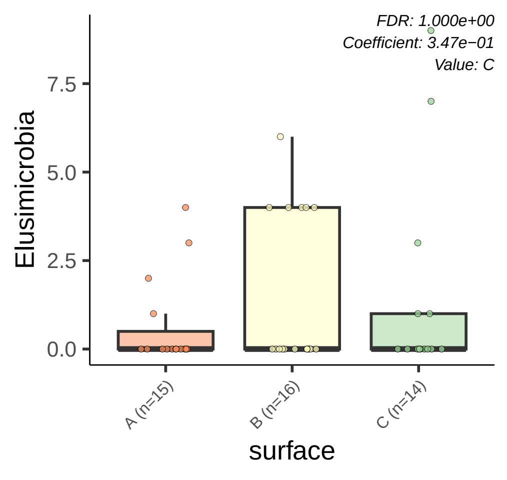
FDR: 1.000e+00
Coefficient: 3.47e−01
Value: C
Elusimicrobia
7.5
5.0
2.5
0.0
A (n=15)
B (n=16)
C (n=14)
surface

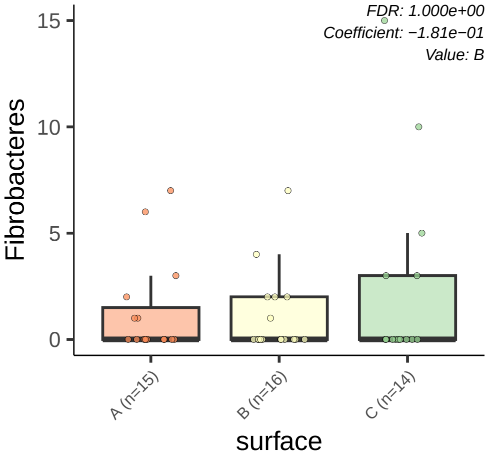
FDR: 1.000e+00
Coefficient: −1.81e−01
Value: B
Fibrobacteres
15
10
5
0
A (n=15)
B (n=16)
C (n=14)
surface

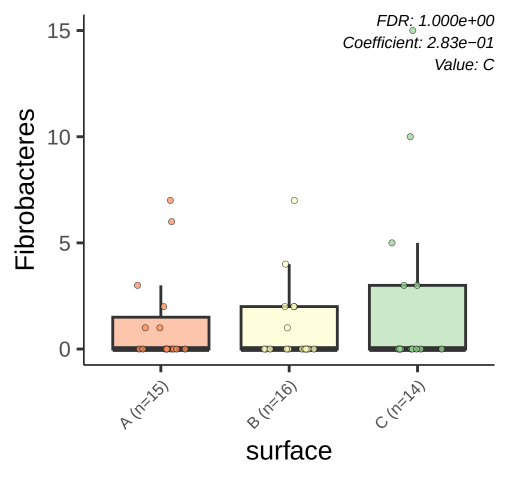

FDR: 1.000e+00
Coefficient: 2.83e−01
Value: C
Fibrobacteres
15
10
5
0
A (n=15)
B (n=16)
C (n=14)
surface

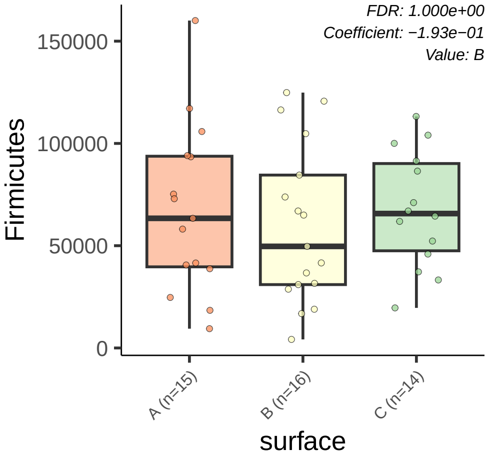

FDR: 1.000e+00
Coefficient: −1.93e−01
Value: B
Firmicutes
150000
100000
50000
0
A (n=15)
B (n=16)
C (n=14)
surface

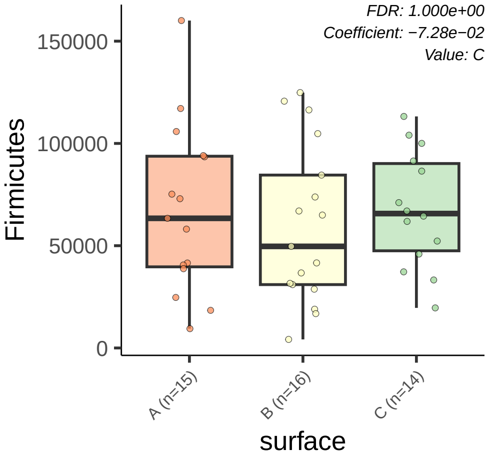
FDR: 1.000e+00
Coefficient: −7.28e−02
Value: C
Firmicutes
150000
100000
50000
0
A (n=15)
B (n=16)
C (n=14)
surface

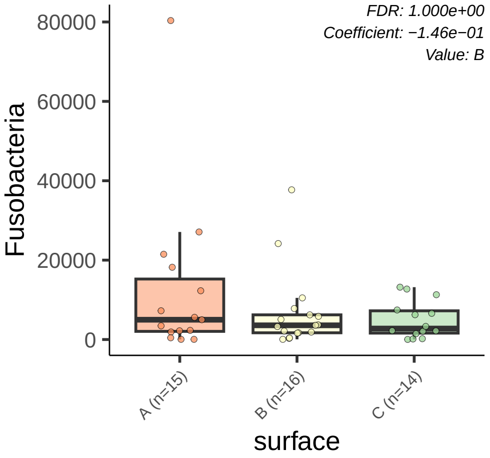
FDR: 1.000e+00
Coefficient: −1.46e−01
Value: B
Fusobacteria
80000
60000
40000
20000
0
A (n=15)
B (n=16)
C (n=14)
surface

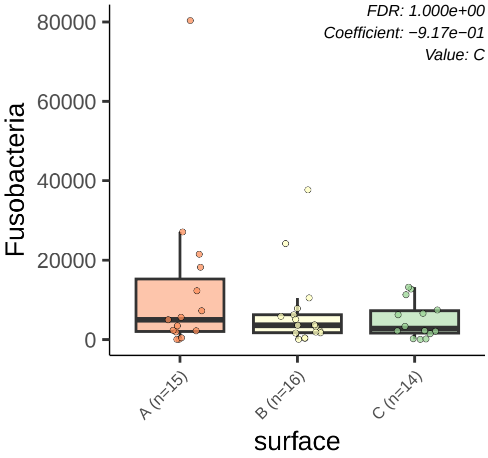

FDR: 1.000e+00
Coefficient: −9.17e−01
Value: C
Fusobacteria
80000
60000
40000
20000
0
A (n=15)
B (n=16)
C (n=14)
surface

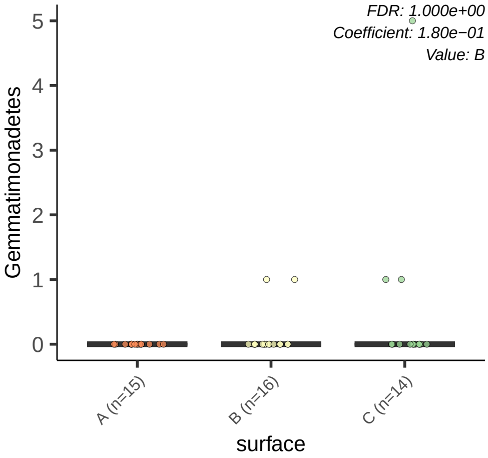

FDR: 1.000e+00
Coefficient: 1.80e−01
Value: B
Gemmatimonadetes
0
1
2
3
4
5
A (n=15)
B (n=16)
C (n=14)
surface

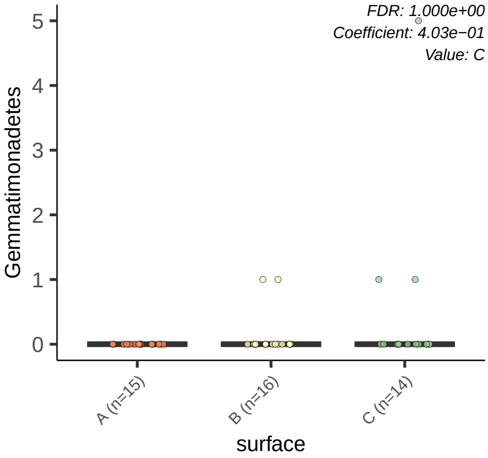
FDR: 1.000e+00
Coefficient: 4.03e−01
Value: C
Gemmatimonadetes
0
1
2
3
4
5
A (n=15)
B (n=16)
C (n=14)
surface

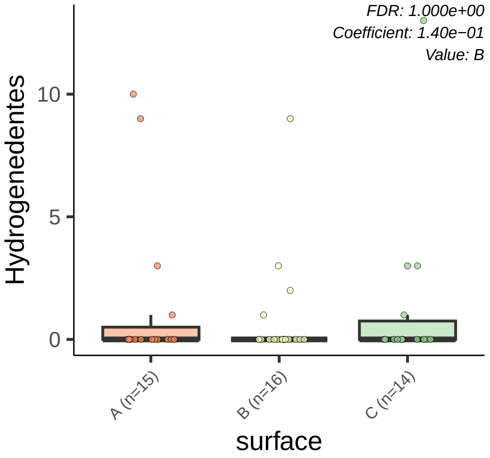
FDR: 1.000e+00
Coefficient: 1.40e−01
Value: B
Hydrogenedentes
10
5
0
A (n=15)
B (n=16)
C (n=14)
surface

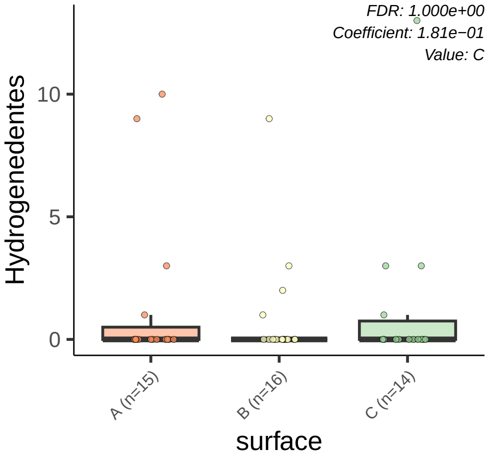
FDR: 1.000e+00
Coefficient: 1.81e−01
Value: C
Hydrogenedentes
10
5
0
A (n=15)
B (n=16)
C (n=14)
surface

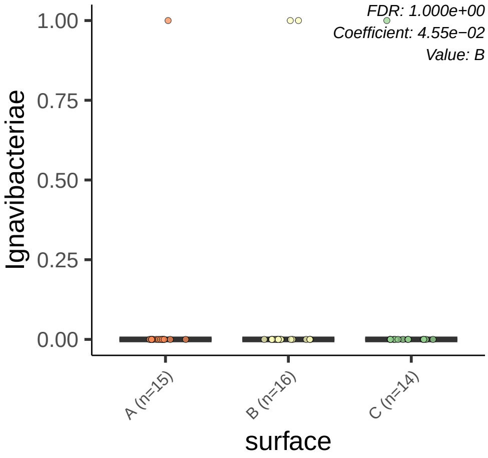

FDR: 1.000e+00
Coefficient: 4.55e−02
Value: B
Ignavibacteriae
1.00
0.75
0.50
0.25
0.00
A (n=15)
B (n=16)
C (n=14)
surface

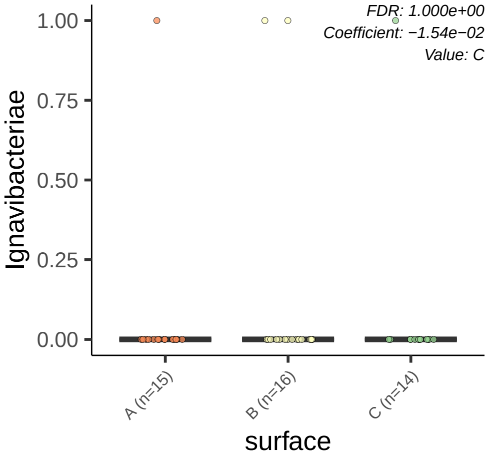
FDR: 1.000e+00
Coefficient: −1.54e−02
Value: C
Ignavibacteriae
1.00
0.75
0.50
0.25
0.00
A (n=15)
B (n=16)
C (n=14)
surface

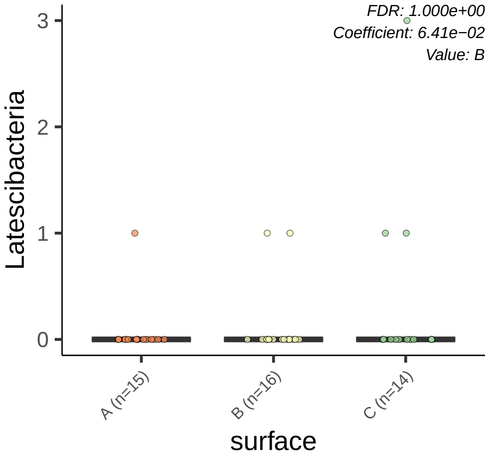
FDR: 1.000e+00
Coefficient: 6.41e−02
Value: B
Latescibacteria
3
2
1
0
A (n=15)
B (n=16)
C (n=14)
surface

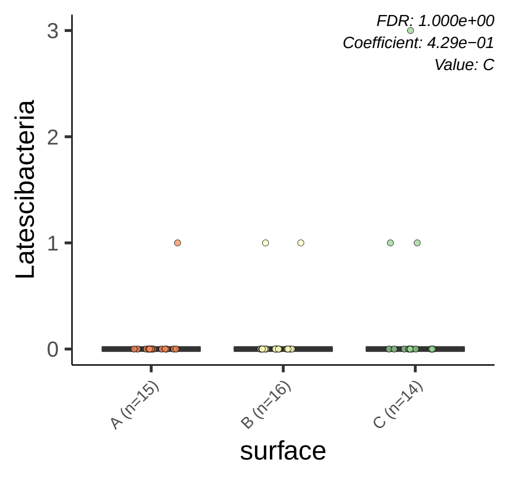
FDR: 1.000e+00
Coefficient: 4.29e−01
Value: C
Latescibacteria
3
2
1
0
A (n=15)
B (n=16)
C (n=14)
surface

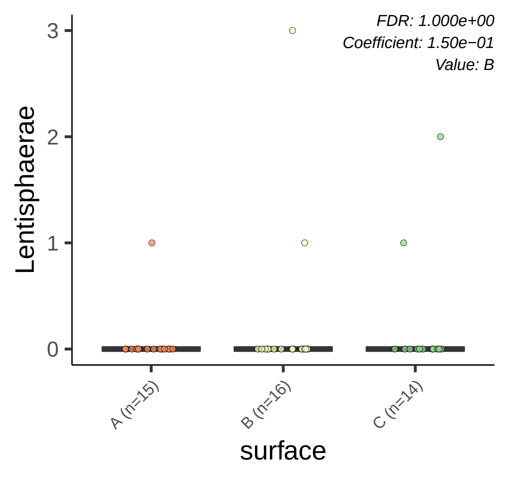
FDR: 1.000e+00
Coefficient: 1.50e−01
Value: B
Lentisphaerae
3
2
1
0
A (n=15)
B (n=16)
C (n=14)
surface

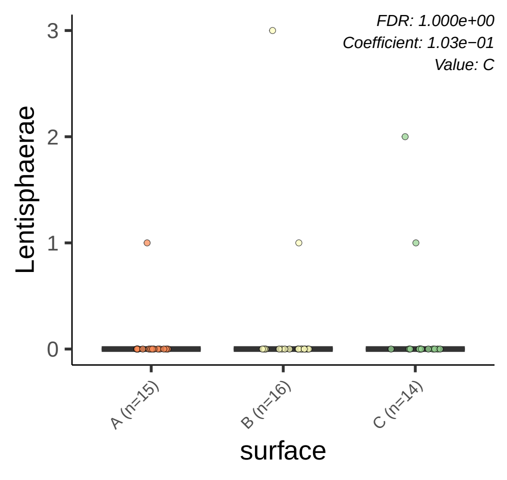

FDR: 1.000e+00
Coefficient: 1.03e−01
Value: C
Lentisphaerae
3
2
1
0
A (n=15)
B (n=16)
C (n=14)
surface

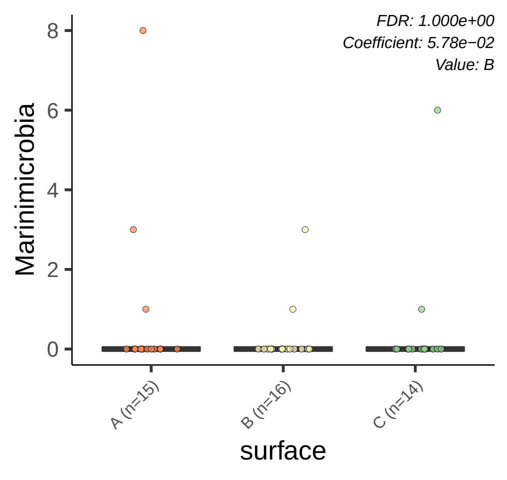

FDR: 1.000e+00
Coefficient: 5.78e−02
Value: B
Marinimicrobia
8
6
4
2
0
A (n=15)
B (n=16)
C (n=14)
surface

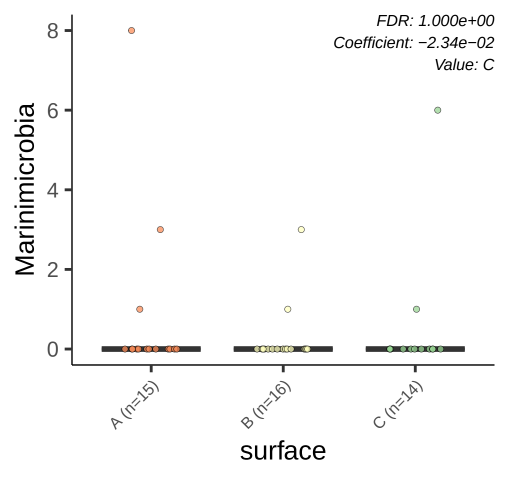
FDR: 1.000e+00
Coefficient: −2.34e−02
Value: C
Marinimicrobia
8
6
4
2
0
A (n=15)
B (n=16)
C (n=14)
surface

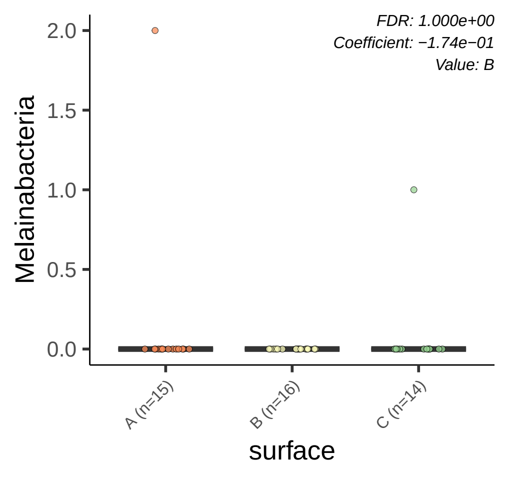
FDR: 1.000e+00
Coefficient: −1.74e−01
Value: B
Melainabacteria
2.0
1.5
1.0
0.5
0.0
A (n=15)
B (n=16)
C (n=14)
surface

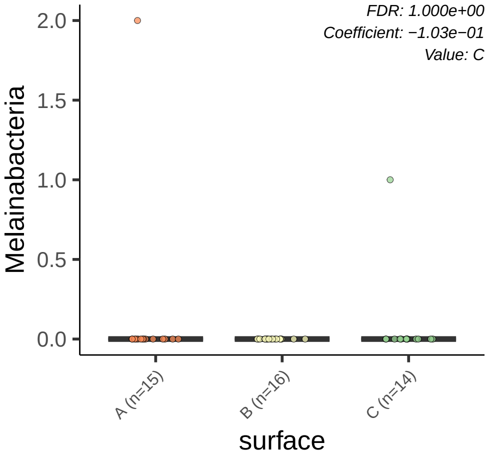
FDR: 1.000e+00
Coefficient: −1.03e−01
Value: C
Melainabacteria
2.0
1.5
1.0
0.5
0.0
A (n=15)
B (n=16)
C (n=14)
surface

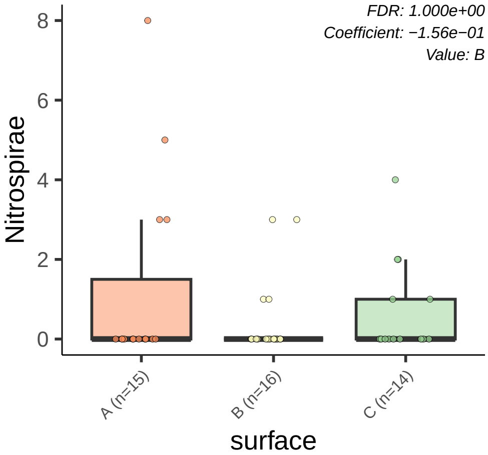

FDR: 1.000e+00
Coefficient: −1.56e−01
Value: B
Nitrospirae
8
6
4
2
0
A (n=15)
B (n=16)
C (n=14)
surface

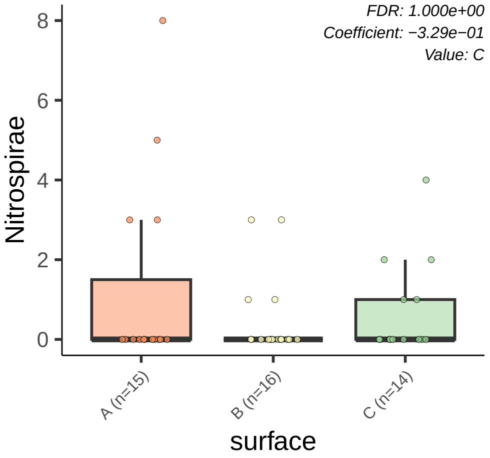

FDR: 1.000e+00
Coefficient: −3.29e−01
Value: C
Nitrospirae
0
2
4
6
8
A (n=15)
B (n=16)
C (n=14)
surface

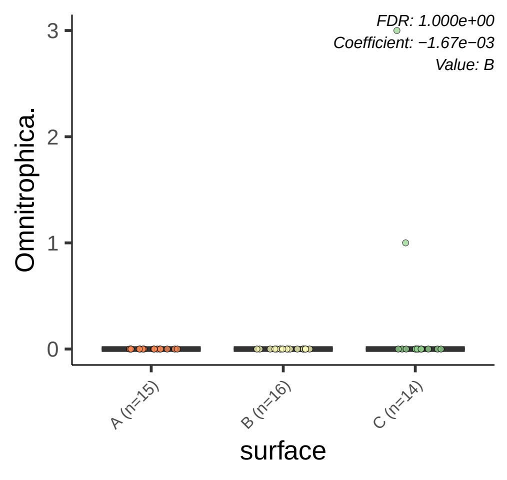

FDR: 1.000e+00
Coefficient: −1.67e−03
Value: B
Omnitrophica.
3
2
1
0
A (n=15)
B (n=16)
C (n=14)
surface

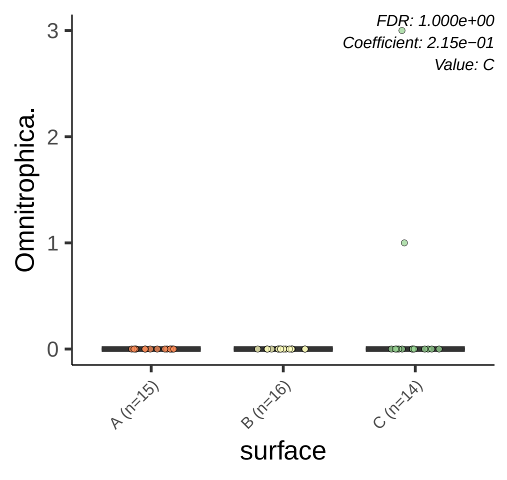
FDR: 1.000e+00
Coefficient: 2.15e−01
Value: C
Omnitrophica.
3
2
1
0
A (n=15)
B (n=16)
C (n=14)
surface

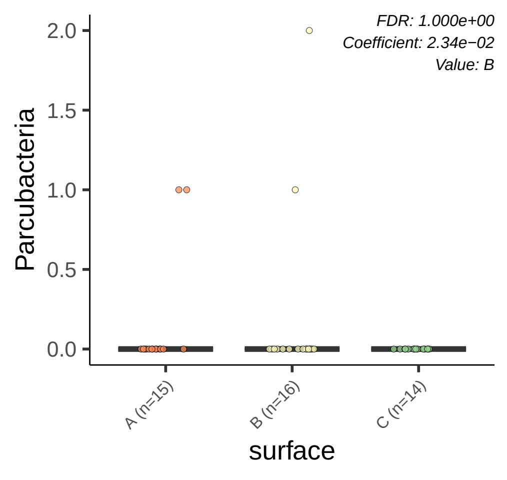

FDR: 1.000e+00
Coefficient: 2.34e−02
Value: B
Parcubacteria
2.0
1.5
1.0
0.5
0.0
A (n=15)
B (n=16)
C (n=14)
surface

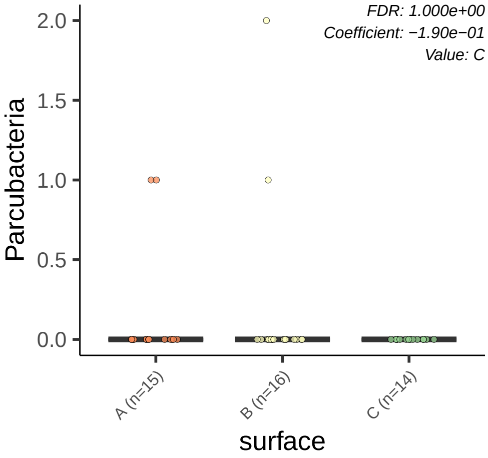

FDR: 1.000e+00
Coefficient: −1.90e−01
Value: C
Parcubacteria
2.0
1.5
1.0
0.5
0.0
A (n=15)
B (n=16)
C (n=14)
surface

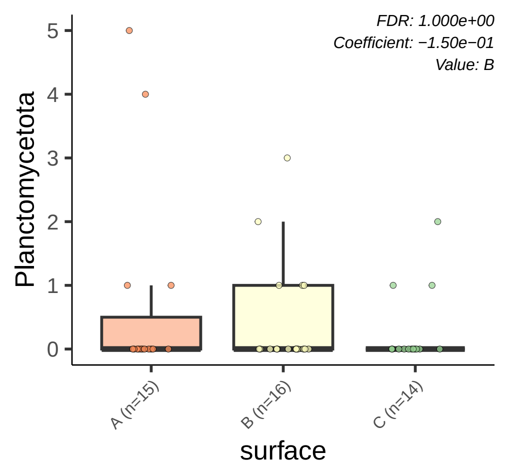
FDR: 1.000e+00
Coefficient: −1.50e−01
Value: B
Planctomycetota
5
4
3
2
1
0
A (n=15)
B (n=16)
C (n=14)
surface

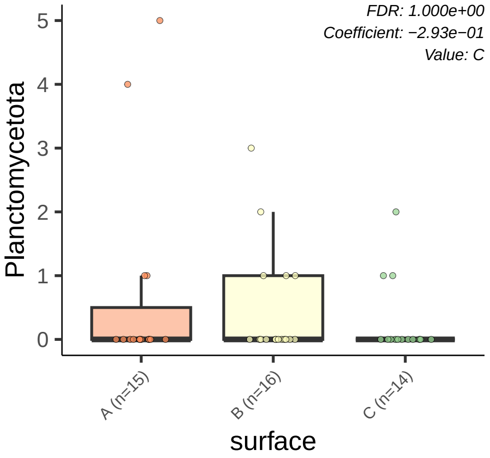

FDR: 1.000e+00
Coefficient: −2.93e−01
Value: C
Planctomycetota
0
1
2
3
4
5
A (n=15)
B (n=16)
C (n=14)
surface

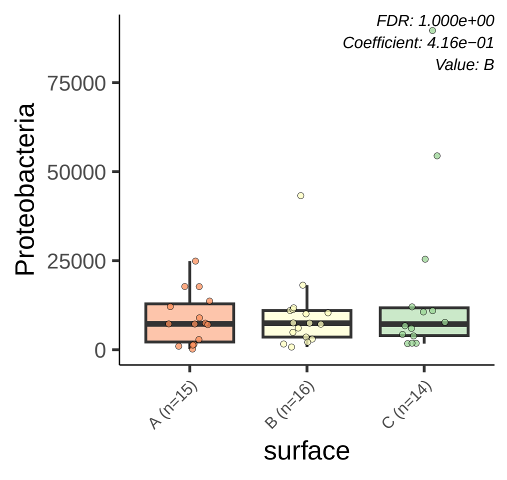

FDR: 1.000e+00
Coefficient: 4.16e−01
Value: B
Proteobacteria
75000
50000
25000
0
A (n=15)
B (n=16)
C (n=14)
surface

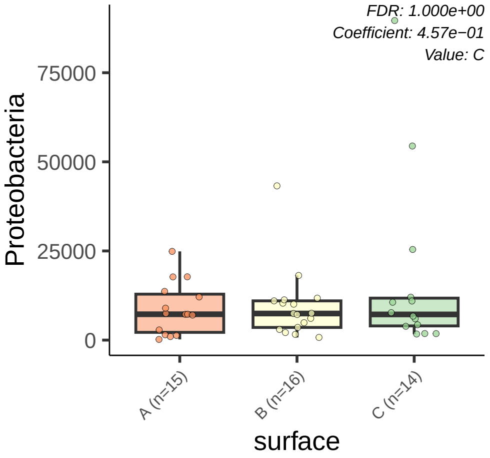

FDR: 1.000e+00
Coefficient: 4.57e−01
Value: C
Proteobacteria
75000
50000
25000
0
A (n=15)
B (n=16)
C (n=14)
surface

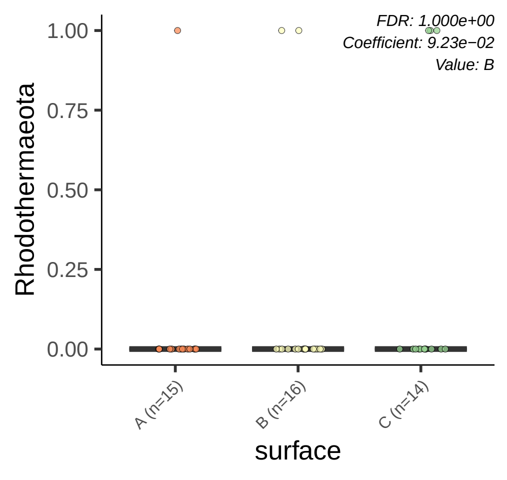
FDR: 1.000e+00
Coefficient: 9.23e−02
Value: B
Rhodothermaeota
1.00
0.75
0.50
0.25
0.00
A (n=15)
B (n=16)
C (n=14)
surface

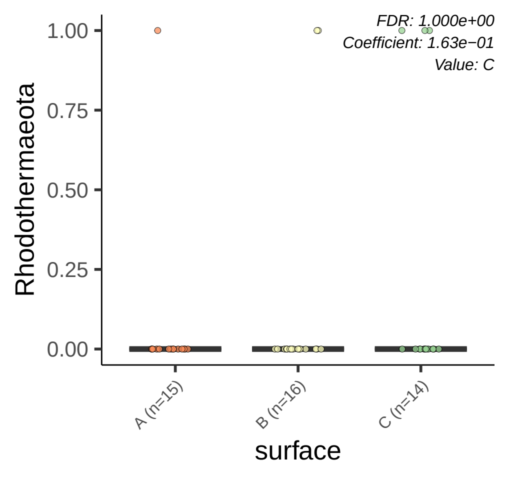

FDR: 1.000e+00
Coefficient: 1.63e−01
Value: C
Rhodothermaeota
1.00
0.75
0.50
0.25
0.00
A (n=15)
B (n=16)
C (n=14)
surface

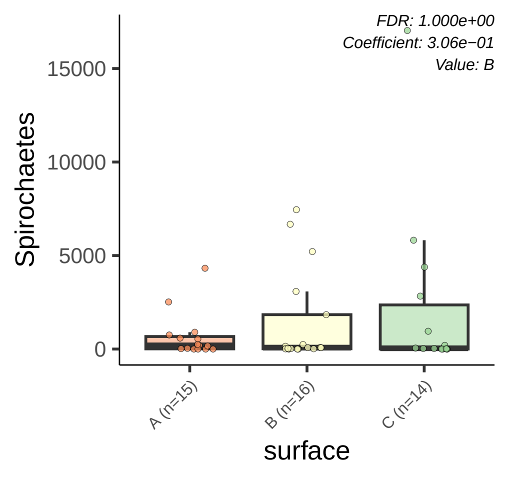
FDR: 1.000e+00
Coefficient: 3.06e−01
Value: B
Spirochaetes
15000
10000
5000
0
A (n=15)
B (n=16)
C (n=14)
surface

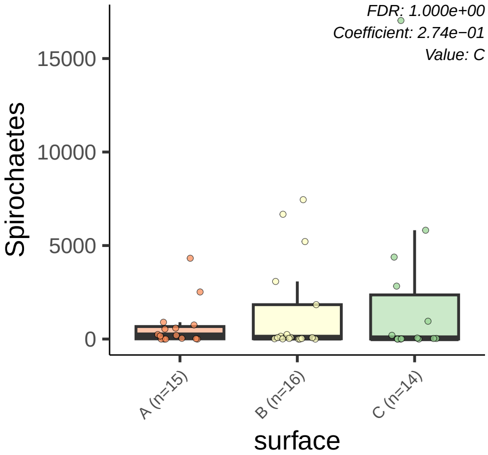
FDR: 1.000e+00
Coefficient: 2.74e−01
Value: C
Spirochaetes
15000
10000
5000
0
A (n=15)
B (n=16)
C (n=14)
surface

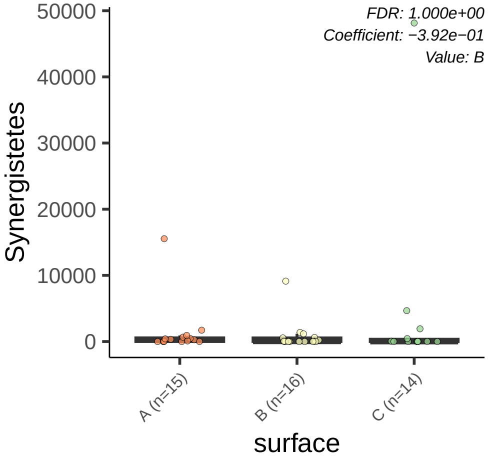

FDR: 1.000e+00
Coefficient: −3.92e−01
Value: B
Synergistetes
50000
40000
30000
20000
10000
0
A (n=15)
B (n=16)
C (n=14)
surface

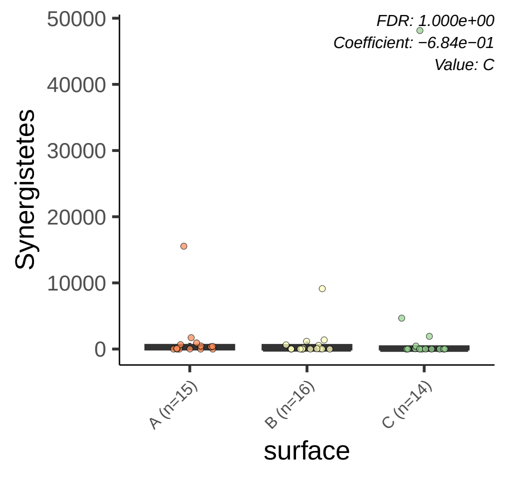
FDR: 1.000e+00
Coefficient: −6.84e−01
Value: C
50000
40000
30000
20000
10000
0
Synergistetes
A (n=15)
B (n=16)
C (n=14)
surface

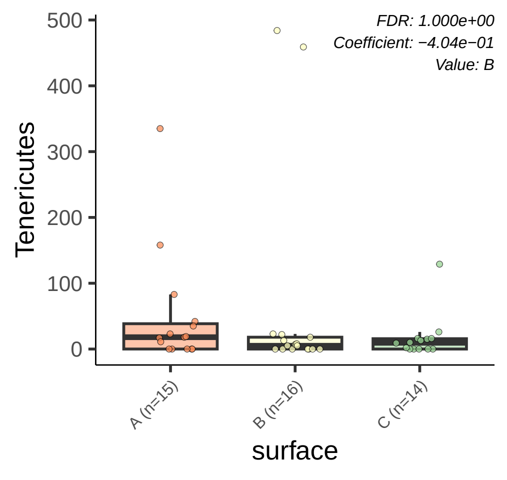
FDR: 1.000e+00
Coefficient: −4.04e−01
Value: B
Tenericutes
0
100
200
300
400
500
A (n=15)
B (n=16)
C (n=14)
surface

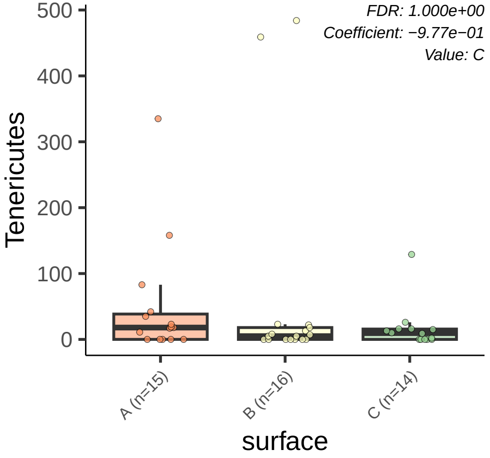
FDR: 1.000e+00
Coefficient: −9.77e−01
Value: C
Tenericutes
0
100
200
300
400
500
A (n=15)
B (n=16)
C (n=14)
surface

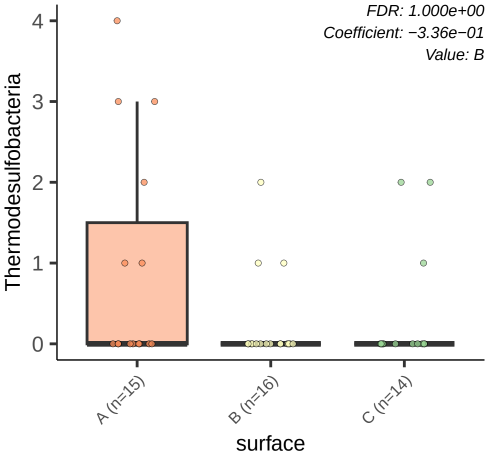
FDR: 1.000e+00
Coefficient: −3.36e−01
Value: B
Thermodesulfobacteria
0
1
2
3
4
A (n=15)
B (n=16)
C (n=14)
surface

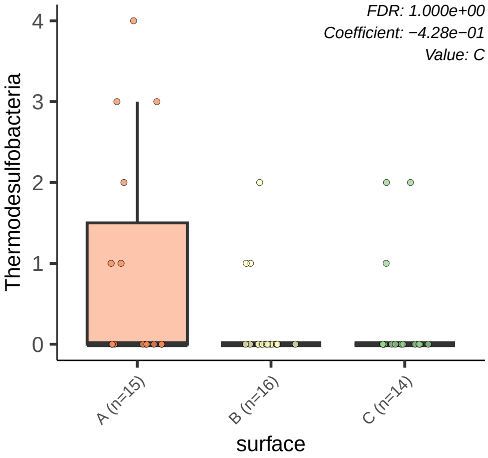
FDR: 1.000e+00
Coefficient: −4.28e−01
Value: C
Thermodesulfobacteria
0
1
2
3
4
A (n=15)
B (n=16)
C (n=14)
surface

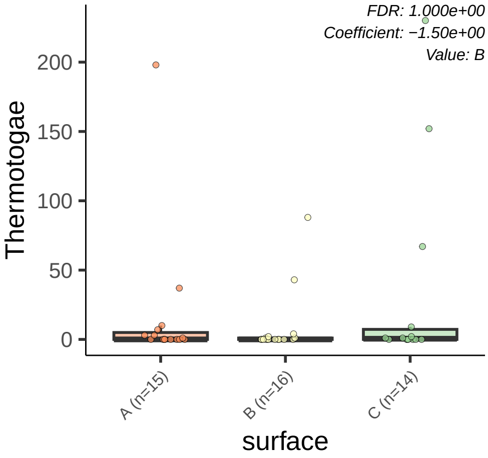
FDR: 1.000e+00
Coefficient: −1.50e+00
Value: B
Thermotogae
200
150
100
50
0
A (n=15)
B (n=16)
C (n=14)
surface

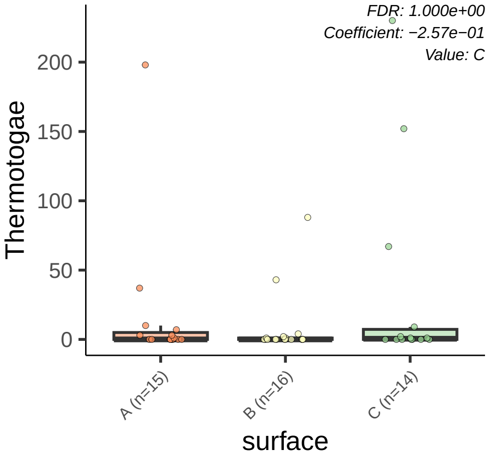

FDR: 1.000e+00
Coefficient: −2.57e−01
Value: C
Thermotogae
200
150
100
50
0
A (n=15)
B (n=16)
C (n=14)
surface

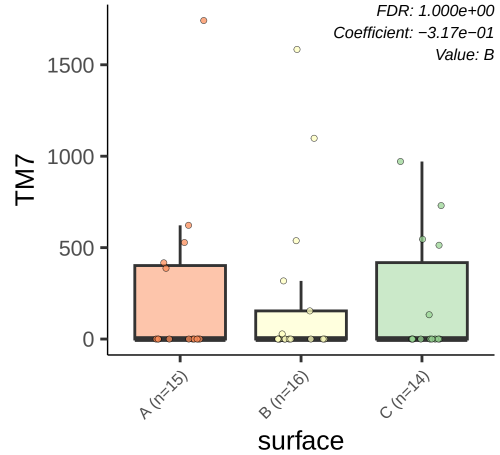

FDR: 1.000e+00
Coefficient: −3.17e−01
Value: B
TM7
1500
1000
500
0
A (n=15)
B (n=16)
C (n=14)
surface

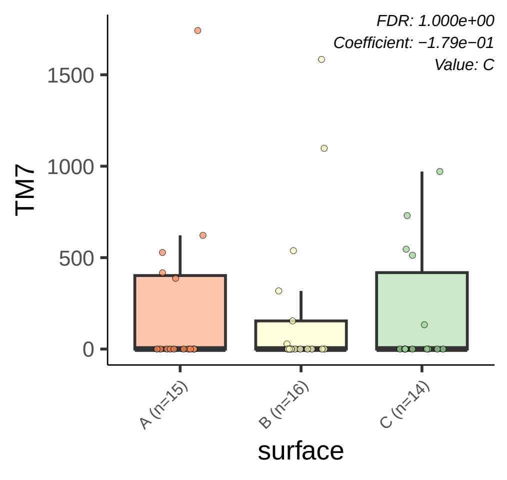
FDR: 1.000e+00
Coefficient: −1.79e−01
Value: C
TM7
1500
1000
500
0
A (n=15)
B (n=16)
C (n=14)
surface

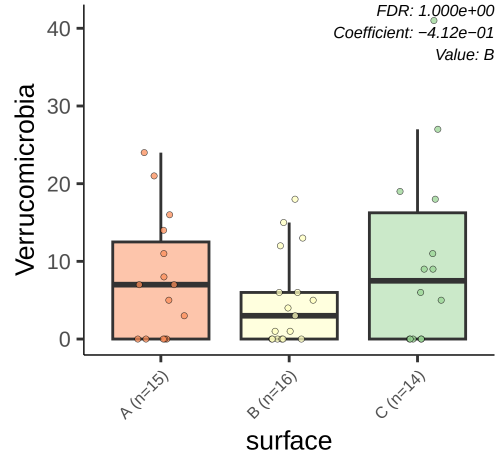

FDR: 1.000e+00
Coefficient: −4.12e−01
Value: B
Verrucomicrobia
40
30
20
10
0
A (n=15)
B (n=16)
C (n=14)
surface

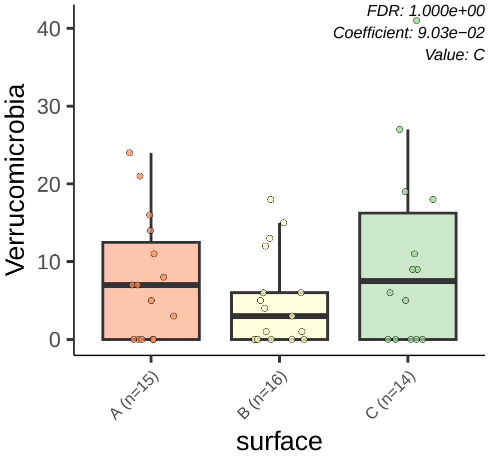
FDR: 1.000e+00
Coefficient: 9.03e−02
Value: C
Verrucomicrobia
40
30
20
10
0
A (n=15)
B (n=16)
C (n=14)
surface

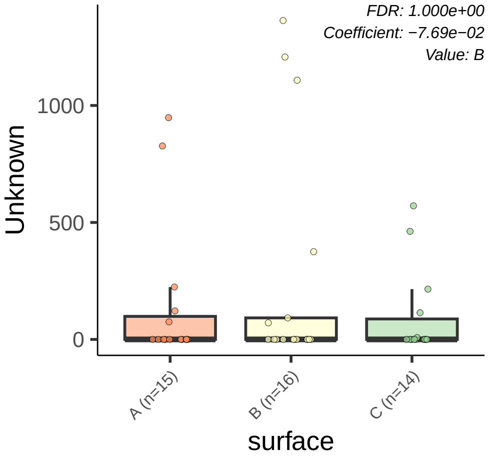
FDR: 1.000e+00
Coefficient: −7.69e−02
Value: B
Unknown
1000
500
0
A (n=15)
B (n=16)
C (n=14)
surface

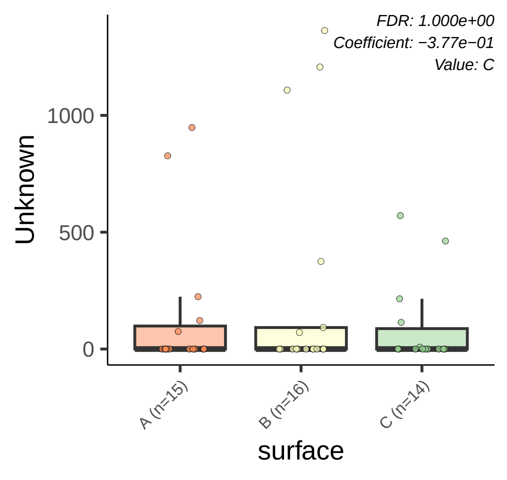
FDR: 1.000e+00
Coefficient: −3.77e−01
Value: C
Unknown
1000
500
0
A (n=15)
B (n=16)
C (n=14)
surface

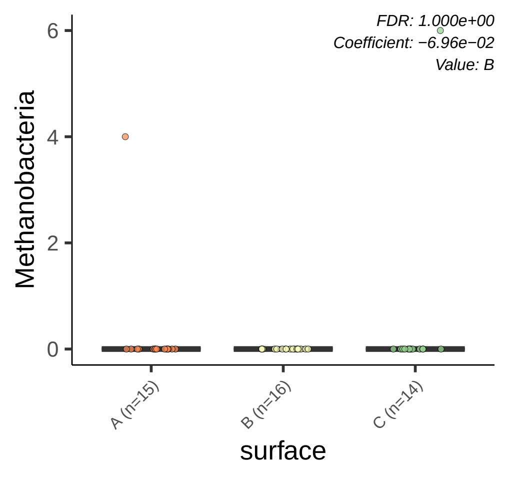

FDR: 1.000e+00
Coefficient: −6.96e−02
Value: B
Methanobacteria
6
4
2
0
A (n=15)
B (n=16)
C (n=14)
surface

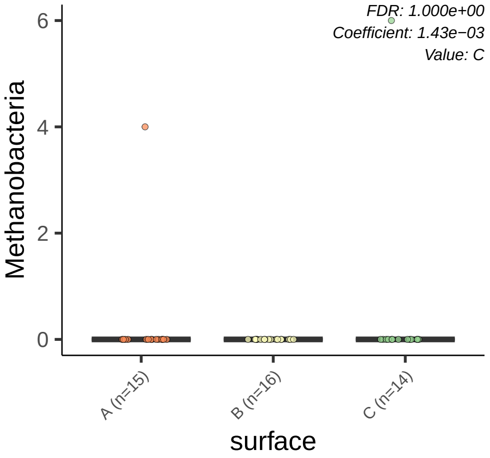
FDR: 1.000e+00
Coefficient: 1.43e−03
Value: C
Methanobacteria
0
2
4
6
A (n=15)
B (n=16)
C (n=14)
surface

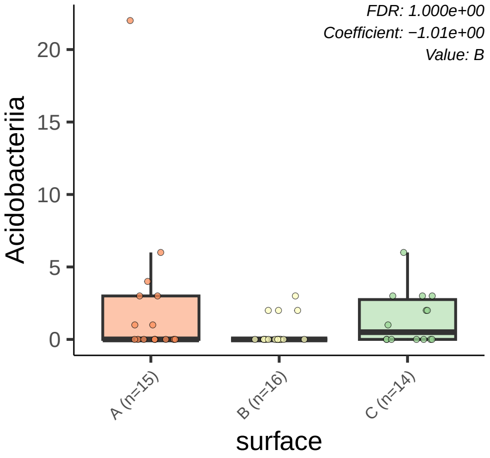
FDR: 1.000e+00
Coefficient: −1.01e+00
Value: B
Acidobacteriia
0
5
10
15
20
A (n=15)
B (n=16)
C (n=14)
surface

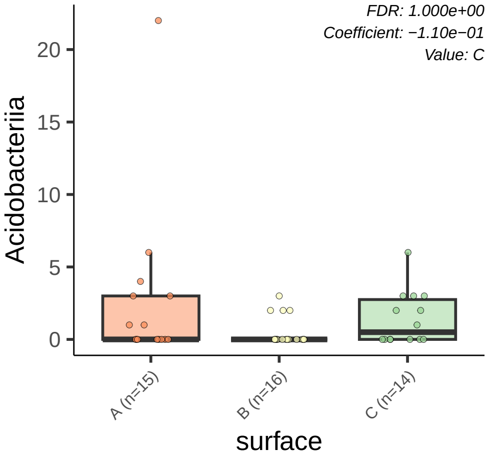
FDR: 1.000e+00
Coefficient: −1.10e−01
Value: C
Acidobacteriia
20
15
10
5
0
A (n=15)
B (n=16)
C (n=14)
surface

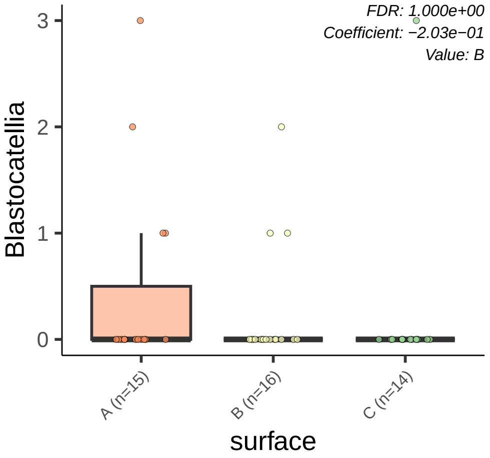

FDR: 1.000e+00
Coefficient: −2.03e−01
Value: B
Blastocatellia
0
1
2
3
A (n=15)
B (n=16)
C (n=14)
surface

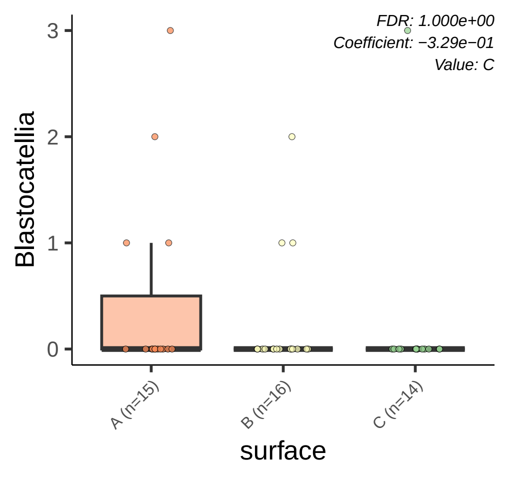
FDR: 1.000e+00
Coefficient: −3.29e−01
Value: C
Blastocatellia
3
2
1
0
A (n=15)
B (n=16)
C (n=14)
surface

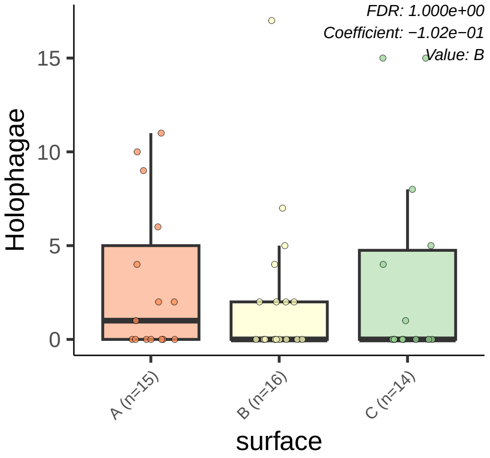

FDR: 1.000e+00
Coefficient: −1.02e−01
Value: B
Holophagae
0
5
10
15
A (n=15)
B (n=16)
C (n=14)
surface

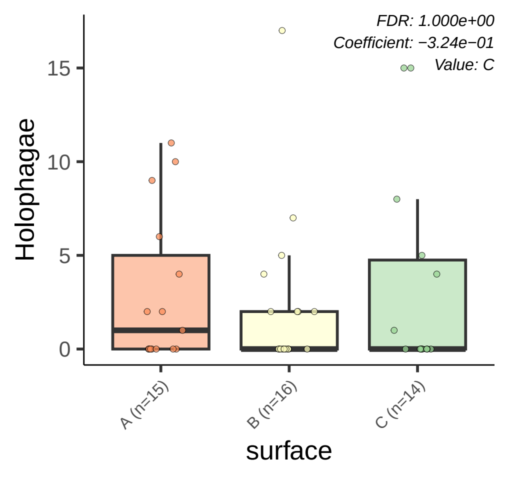

FDR: 1.000e+00
Coefficient: −3.24e−01
Value: C
Holophagae
0
5
10
15
A (n=15)
B (n=16)
C (n=14)
surface

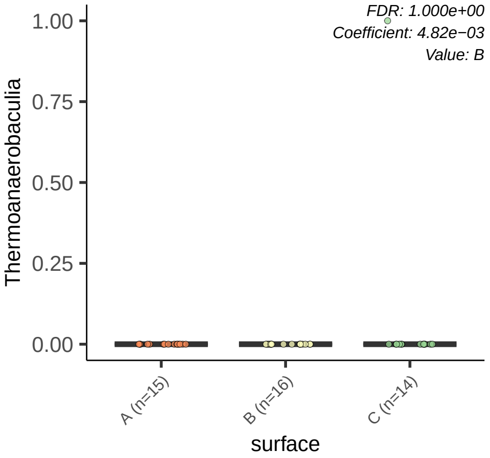

FDR: 1.000e+00
Coefficient: 4.82e−03
Value: B
1.00
0.75
0.50
0.25
0.00
Thermoanaerobaculia
A (n=15)
B (n=16)
C (n=14)
surface

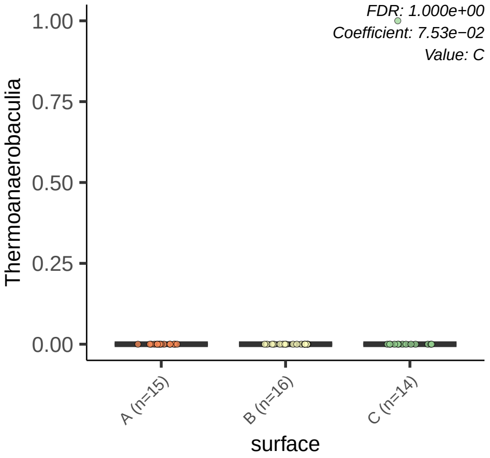
FDR: 1.000e+00
Coefficient: 7.53e−02
Value: C
Thermoanaerobaculia
1.00
0.75
0.50
0.25
0.00
A (n=15)
B (n=16)
C (n=14)
surface

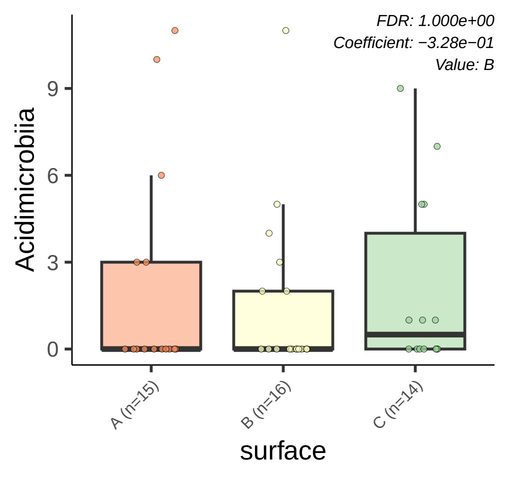

FDR: 1.000e+00
Coefficient: −3.28e−01
Value: B
Acidimicrobiia
0
3
6
9
A (n=15)
B (n=16)
C (n=14)
surface

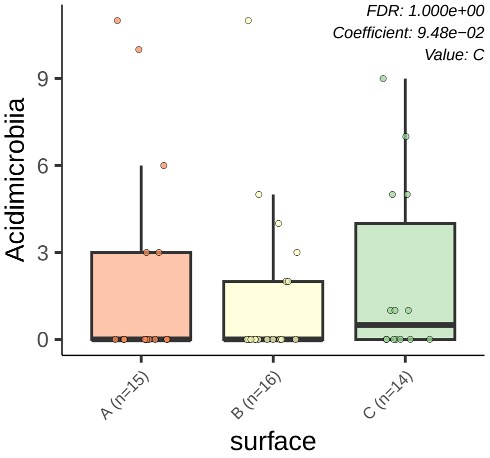
FDR: 1.000e+00
Coefficient: 9.48e−02
Value: C
Acidimicrobiia
0
3
6
9
A (n=15)
B (n=16)
C (n=14)
surface

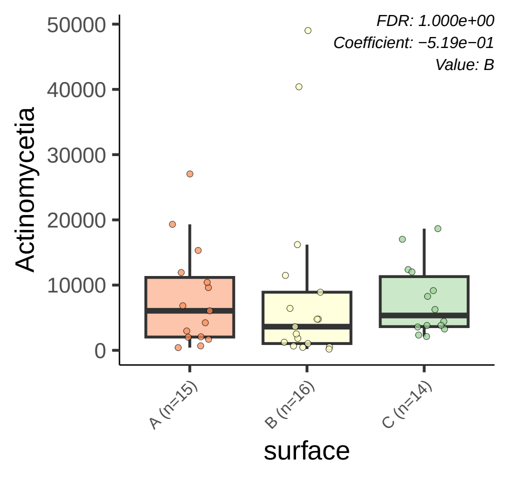

FDR: 1.000e+00
Coefficient: −5.19e−01
Value: B
Actinomycetia
50000
40000
30000
20000
10000
0
A (n=15)
B (n=16)
C (n=14)
surface

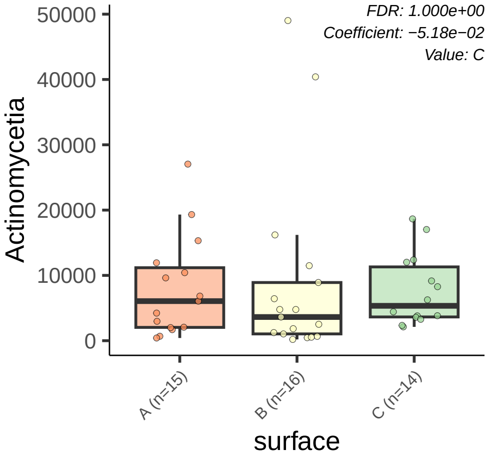
FDR: 1.000e+00
Coefficient: −5.18e−02
Value: C
Actinomycetia
50000
40000
30000
20000
10000
0
A (n=15)
B (n=16)
C (n=14)
surface

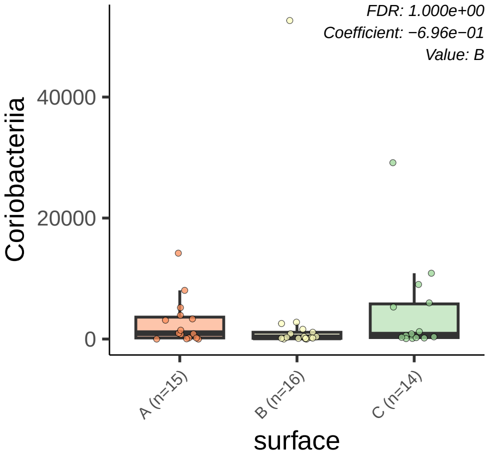
FDR: 1.000e+00
Coefficient: −6.96e−01
Value: B
Coriobacteriia
40000
20000
0
A (n=15)
B (n=16)
C (n=14)
surface

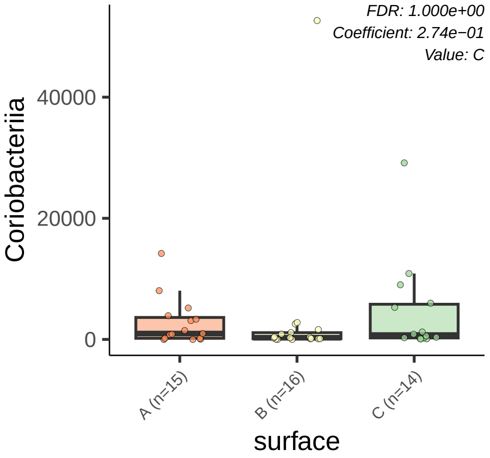

FDR: 1.000e+00
Coefficient: 2.74e−01
Value: C
Coriobacteriia
40000
20000
0
A (n=15)
B (n=16)
C (n=14)
surface

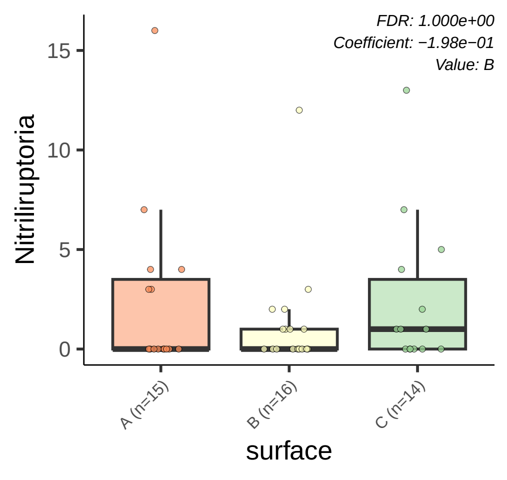
FDR: 1.000e+00
Coefficient: −1.98e−01
Value: B
Nitriliruptoria
0
5
10
15
A (n=15)
B (n=16)
C (n=14)
surface

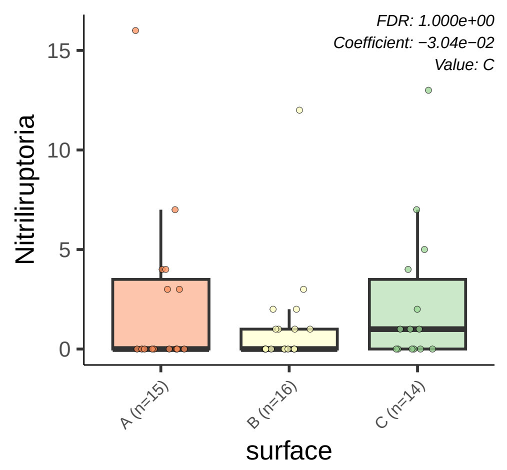

FDR: 1.000e+00
Coefficient: −3.04e−02
Value: C
Nitriliruptoria
15
10
5
0
A (n=15)
B (n=16)
C (n=14)
surface

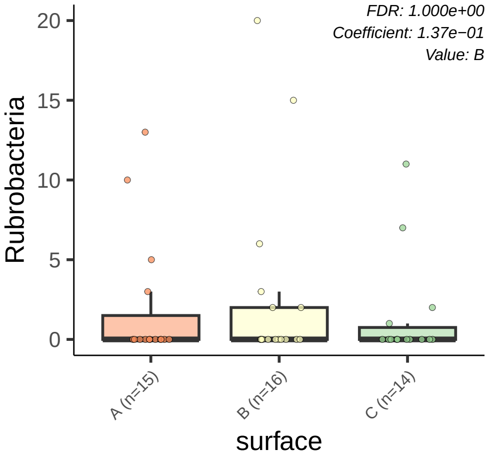
FDR: 1.000e+00
Coefficient: 1.37e−01
Value: B
Rubrobacteria
0
5
10
15
20
A (n=15)
B (n=16)
C (n=14)
surface

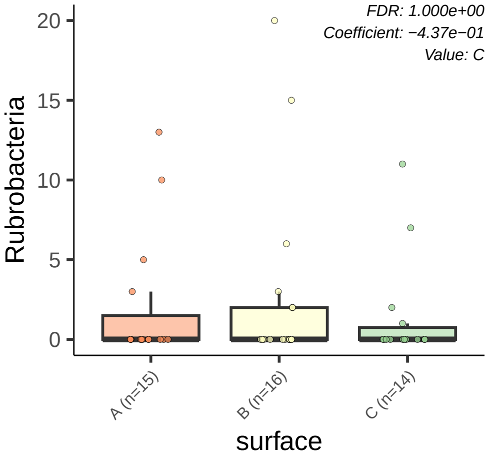

FDR: 1.000e+00
Coefficient: −4.37e−01
Value: C
Rubrobacteria
20
15
10
5
0
A (n=15)
B (n=16)
C (n=14)
surface

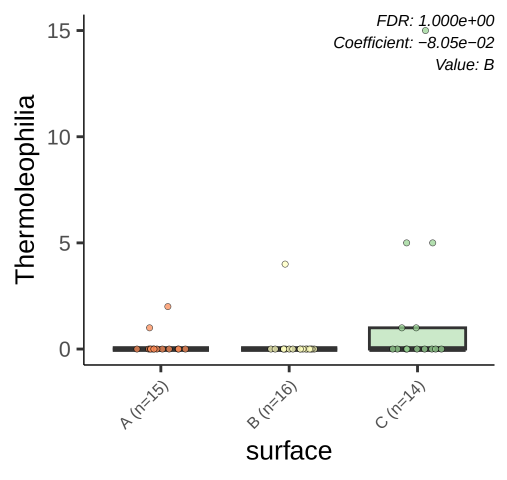
FDR: 1.000e+00
Coefficient: −8.05e−02
Value: B
Thermoleophilia
15
10
5
0
A (n=15)
B (n=16)
C (n=14)
surface

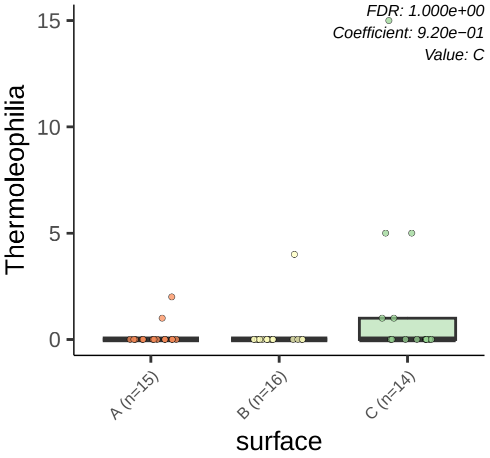
FDR: 1.000e+00
Coefficient: 9.20e−01
Value: C
Thermoleophilia
15
10
5
0
A (n=15)
B (n=16)
C (n=14)
surface

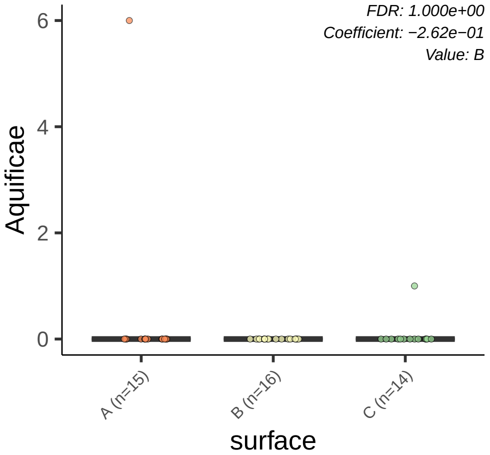
FDR: 1.000e+00
Coefficient: −2.62e−01
Value: B
Aquificae
0
2
4
6
A (n=15)
B (n=16)
C (n=14)
surface

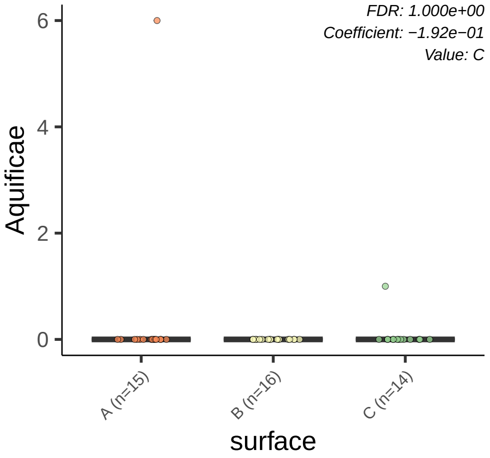
FDR: 1.000e+00
Coefficient: −1.92e−01
Value: C
Aquificae
6
4
2
0
A (n=15)
B (n=16)
C (n=14)
surface

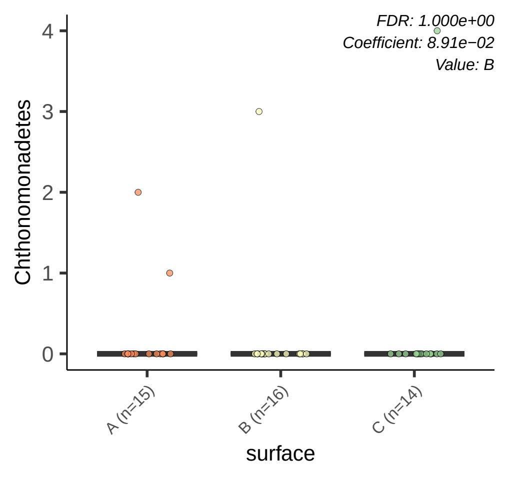
FDR: 1.000e+00
Coefficient: 8.91e−02
Value: B
Chthonomonadetes
0
1
2
3
4
A (n=15)
B (n=16)
C (n=14)
surface

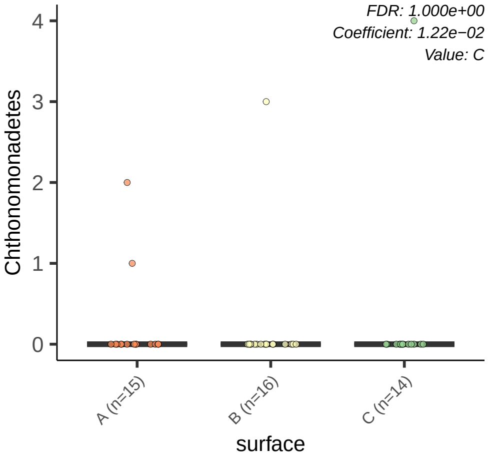
FDR: 1.000e+00
Coefficient: 1.22e−02
Value: C
Chthonomonadetes
4
3
2
1
0
A (n=15)
B (n=16)
C (n=14)
surface

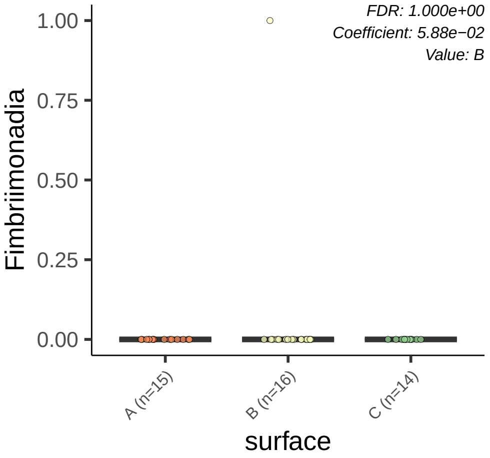
FDR: 1.000e+00
Coefficient: 5.88e−02
Value: B
Fimbriimonadia
1.00
0.75
0.50
0.25
0.00
A (n=15)
B (n=16)
C (n=14)
surface

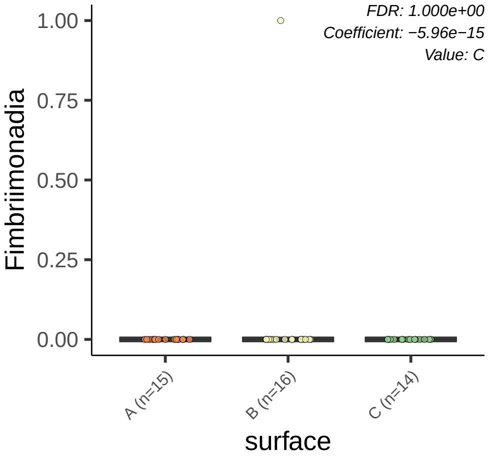
FDR: 1.000e+00
Coefficient: −5.96e−15
Value: C
Fimbriimonadia
1.00
0.75
0.50
0.25
0.00
A (n=15)
B (n=16)
C (n=14)
surface

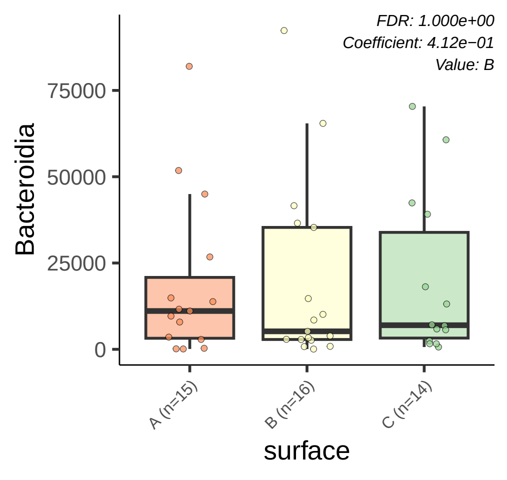

FDR: 1.000e+00
Coefficient: 4.12e−01
Value: B
Bacteroidia
75000
50000
25000
0
A (n=15)
B (n=16)
C (n=14)
surface

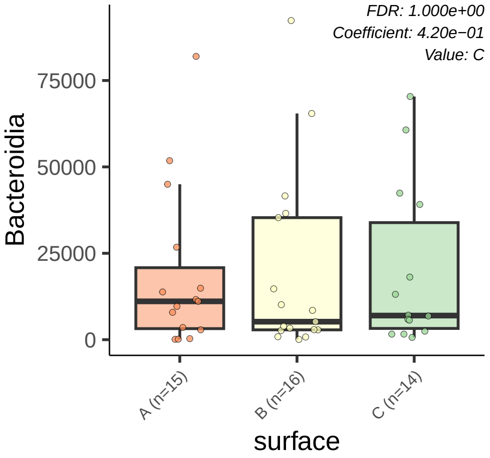
FDR: 1.000e+00
Coefficient: 4.20e−01
Value: C
Bacteroidia
75000
50000
25000
0
A (n=15)
B (n=16)
C (n=14)
surface

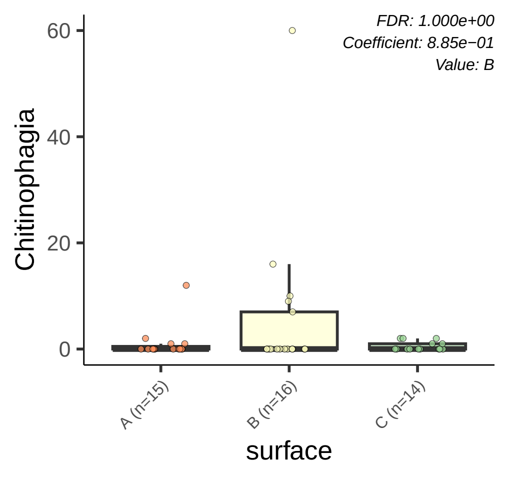

FDR: 1.000e+00
Coefficient: 8.85e−01
Value: B
Chitinophagia
0
20
40
60
A (n=15)
B (n=16)
C (n=14)
surface

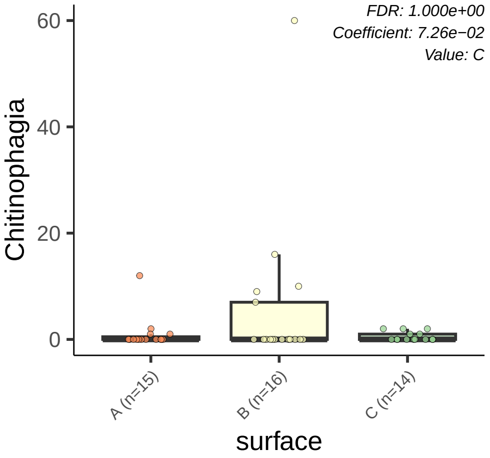
FDR: 1.000e+00
Coefficient: 7.26e−02
Value: C
Chitinophagia
0
20
40
60
A (n=15)
B (n=16)
C (n=14)
surface

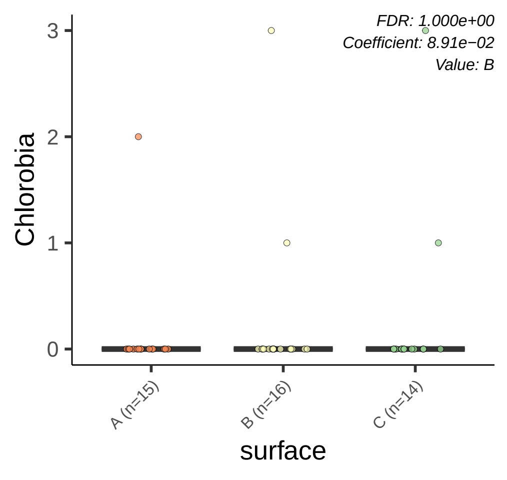

FDR: 1.000e+00
Coefficient: 8.91e−02
Value: B
Chlorobia
0
1
2
3
A (n=15)
B (n=16)
C (n=14)
surface

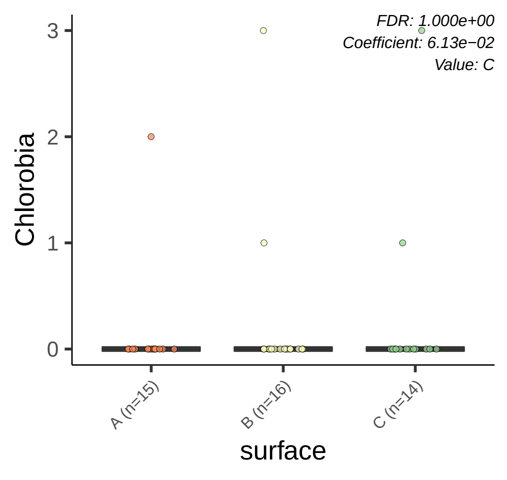

FDR: 1.000e+00
Coefficient: 6.13e−02
Value: C
Chlorobia
3
2
1
0
A (n=15)
B (n=16)
C (n=14)
surface

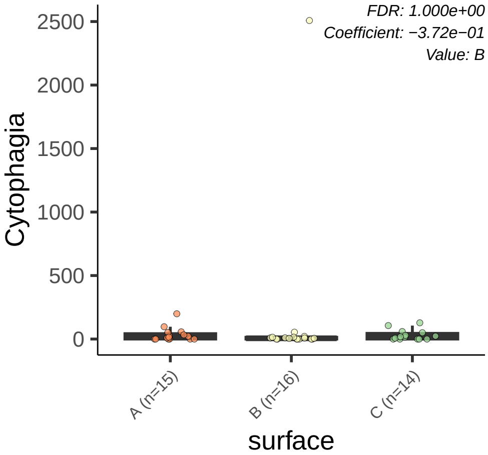

FDR: 1.000e+00
Coefficient: −3.72e−01
Value: B
Cytophagia
2500
2000
1500
1000
500
0
A (n=15)
B (n=16)
C (n=14)
surface

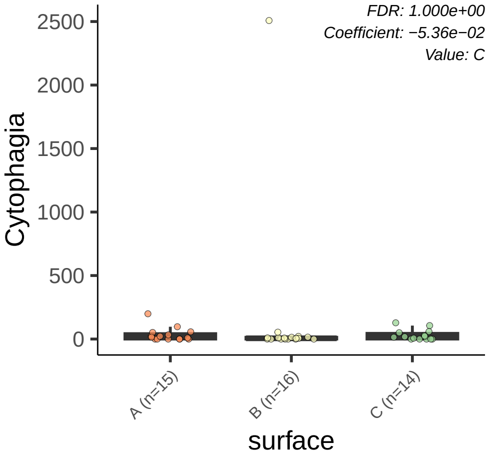
FDR: 1.000e+00
Coefficient: −5.36e−02
Value: C
Cytophagia
2500
2000
1500
1000
500
0
A (n=15)
B (n=16)
C (n=14)
surface

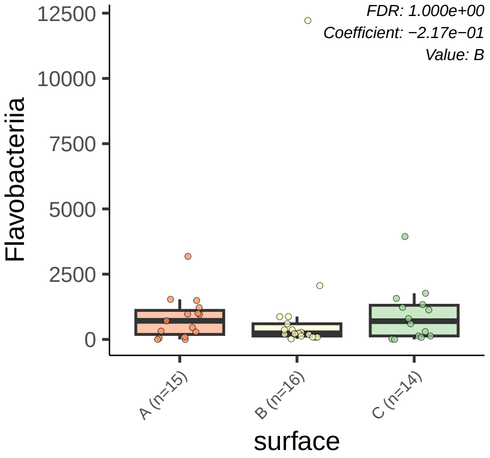

FDR: 1.000e+00
Coefficient: −2.17e−01
Value: B
Flavobacteriia
12500
10000
7500
5000
2500
0
A (n=15)
B (n=16)
C (n=14)
surface

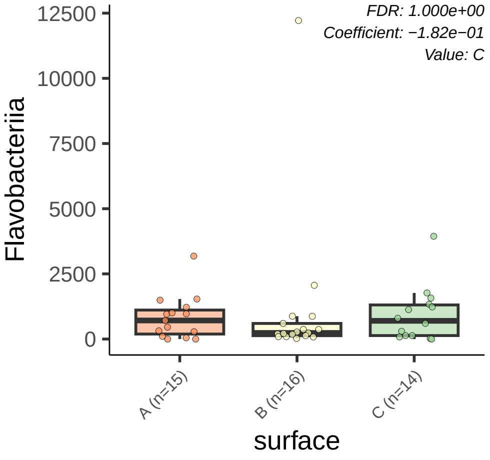
FDR: 1.000e+00
Coefficient: −1.82e−01
Value: C
Flavobacteriia
12500
10000
7500
5000
2500
0
A (n=15)
B (n=16)
C (n=14)
surface

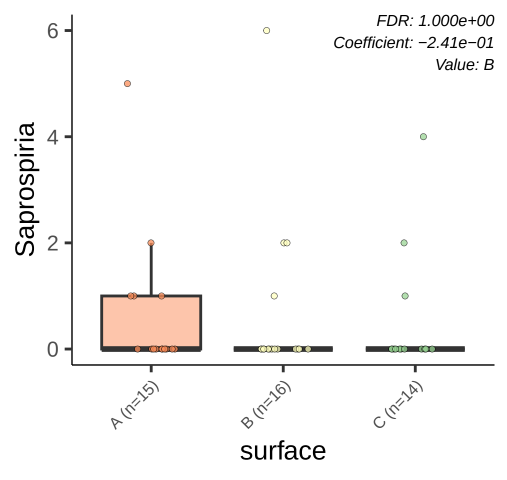
FDR: 1.000e+00
Coefficient: −2.41e−01
Value: B
Saprospiria
0
2
4
6
A (n=15)
B (n=16)
C (n=14)
surface

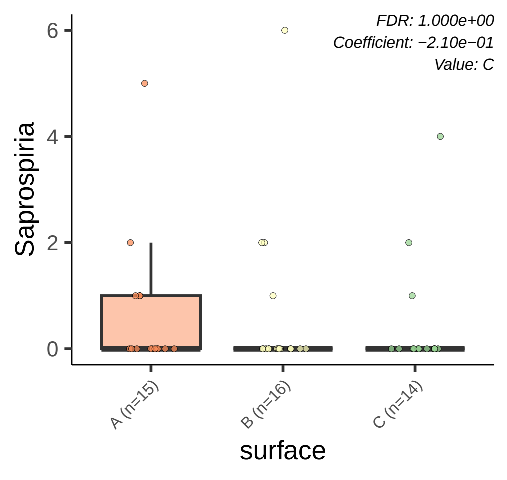
FDR: 1.000e+00
Coefficient: −2.10e−01
Value: C
Saprospiria
0
2
4
6
A (n=15)
B (n=16)
C (n=14)
surface

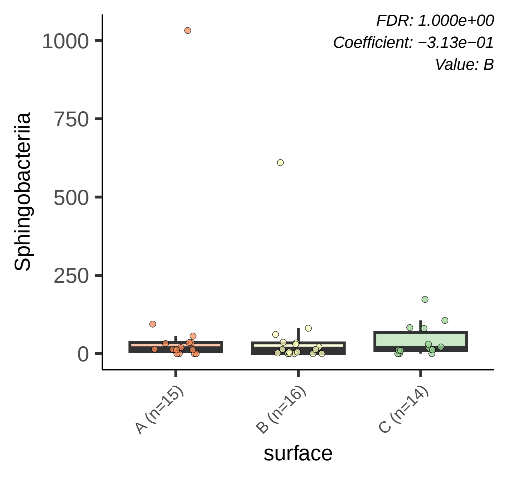

FDR: 1.000e+00
Coefficient: −3.13e−01
Value: B
Sphingobacteriia
1000
750
500
250
0
A (n=15)
B (n=16)
C (n=14)
surface

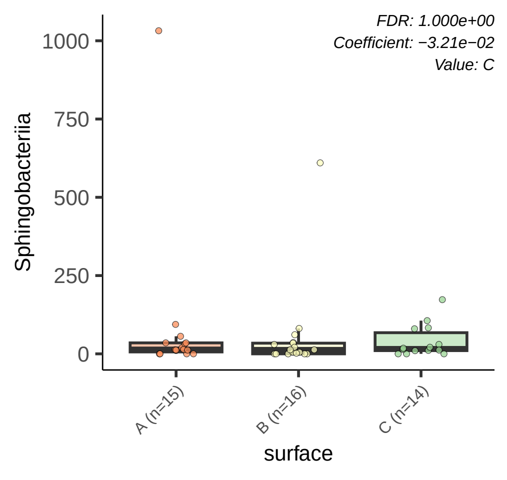

FDR: 1.000e+00
Coefficient: −3.21e−02
Value: C
Sphingobacteriia
1000
750
500
250
0
A (n=15)
B (n=16)
C (n=14)
surface

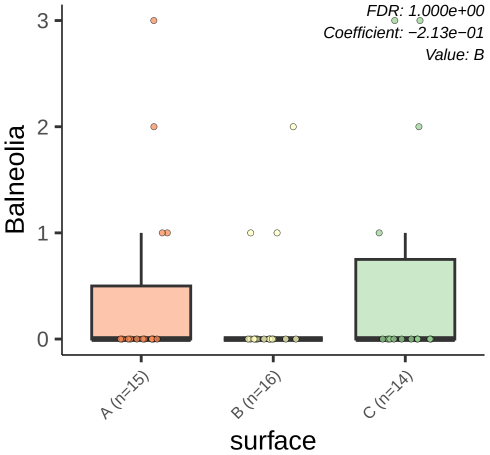
FDR: 1.000e+00
Coefficient: −2.13e−01
Value: B
Balneolia
3
2
1
0
A (n=15)
B (n=16)
C (n=14)
surface

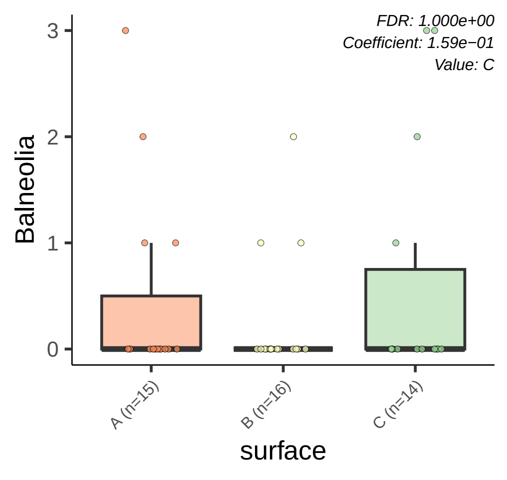

FDR: 1.000e+00
Coefficient: 1.59e−01
Value: C
Balneolia
0
1
2
3
A (n=15)
B (n=16)
C (n=14)
surface

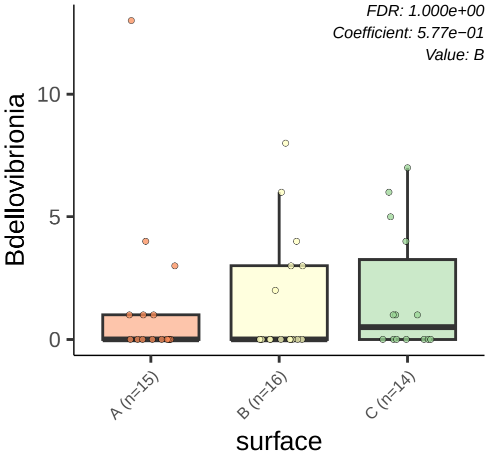

FDR: 1.000e+00
Coefficient: 5.77e−01
Value: B
Bdellovibrionia
0
5
10
A (n=15)
B (n=16)
C (n=14)
surface

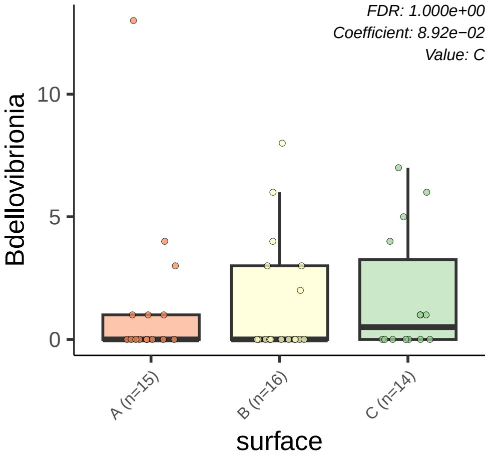
FDR: 1.000e+00
Coefficient: 8.92e−02
Value: C
Bdellovibrionia
10
5
0
A (n=15)
B (n=16)
C (n=14)
surface

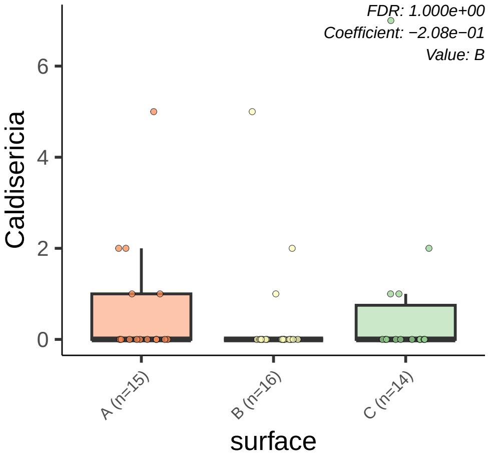

FDR: 1.000e+00
Coefficient: −2.08e−01
Value: B
Caldisericia
0
2
4
6
A (n=15)
B (n=16)
C (n=14)
surface

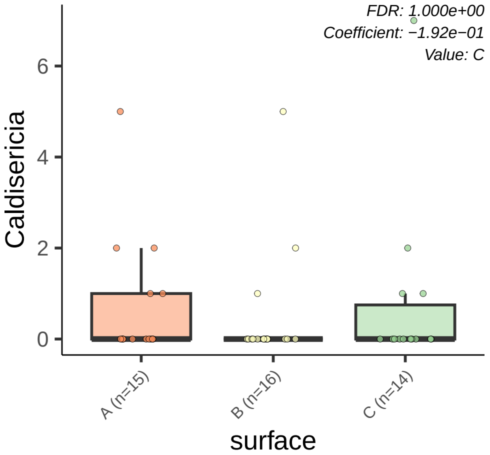

FDR: 1.000e+00
Coefficient: −1.92e−01
Value: C
Caldisericia
0
2
4
6
A (n=15)
B (n=16)
C (n=14)
surface

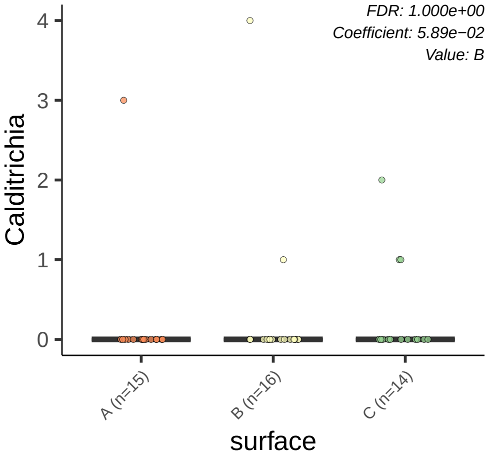

FDR: 1.000e+00
Coefficient: 5.89e−02
Value: B
Calditrichia
0
1
2
3
4
A (n=15)
B (n=16)
C (n=14)
surface

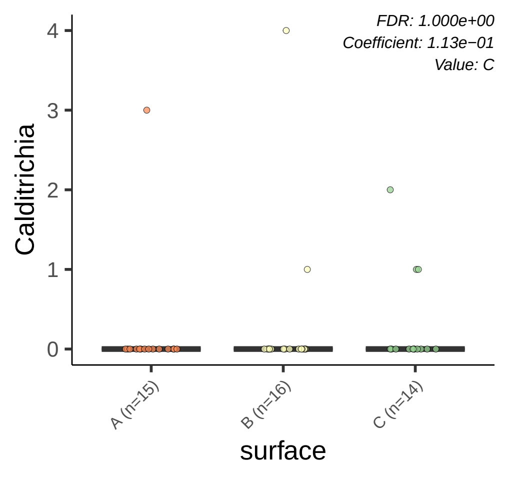

FDR: 1.000e+00
Coefficient: 1.13e−01
Value: C
Calditrichia
4
3
2
1
0
A (n=15)
B (n=16)
C (n=14)
surface

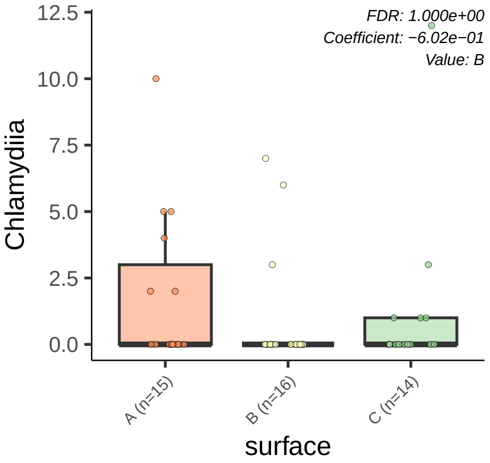

FDR: 1.000e+00
Coefficient: −6.02e−01
Value: B
Chlamydiia
12.5
10.0
7.5
5.0
2.5
0.0
A (n=15)
B (n=16)
C (n=14)
surface

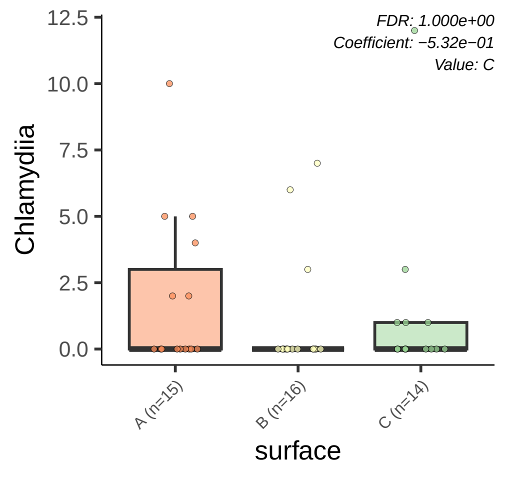
FDR: 1.000e+00
Coefficient: −5.32e−01
Value: C
Chlamydiia
12.5
10.0
7.5
5.0
2.5
0.0
A (n=15)
B (n=16)
C (n=14)
surface

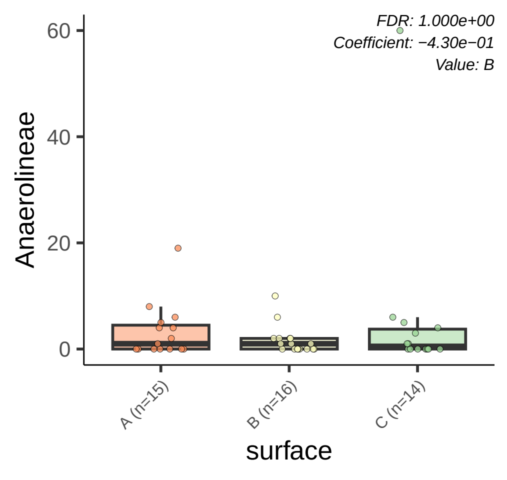
FDR: 1.000e+00
Coefficient: −4.30e−01
Value: B
Anaerolineae
0
20
40
60
A (n=15)
B (n=16)
C (n=14)
surface

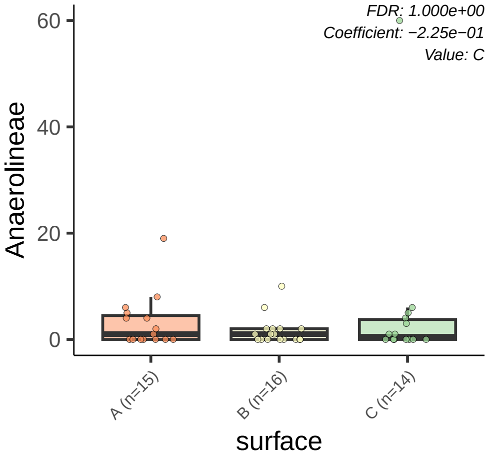

FDR: 1.000e+00
Coefficient: −2.25e−01
Value: C
Anaerolineae
60
40
20
0
A (n=15)
B (n=16)
C (n=14)
surface

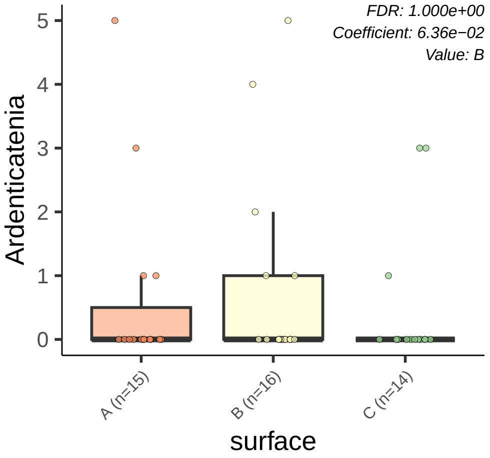

FDR: 1.000e+00
Coefficient: 6.36e−02
Value: B
Ardenticatenia
0
1
2
3
4
5
A (n=15)
B (n=16)
C (n=14)
surface

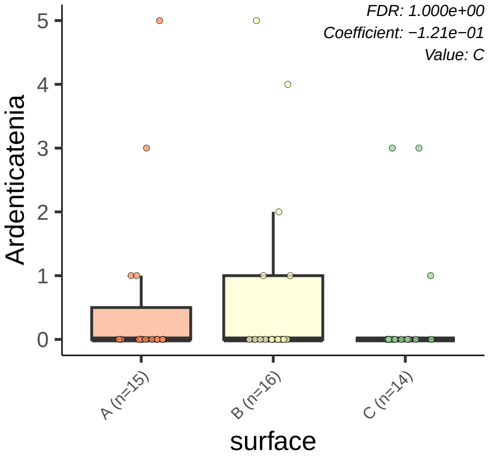
FDR: 1.000e+00
Coefficient: −1.21e−01
Value: C
Ardenticatenia
0
1
2
3
4
5
A (n=15)
B (n=16)
C (n=14)
surface

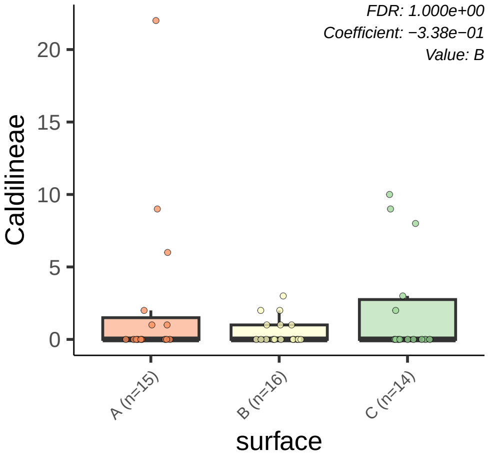
FDR: 1.000e+00
Coefficient: −3.38e−01
Value: B
Caldilineae
0
5
10
15
20
A (n=15)
B (n=16)
C (n=14)
surface

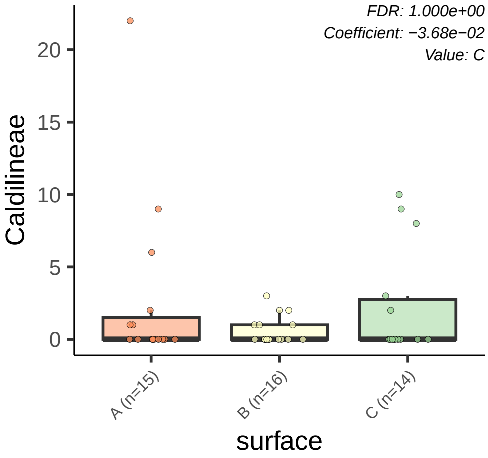

FDR: 1.000e+00
Coefficient: −3.68e−02
Value: C
Caldilineae
20
15
10
5
0
A (n=15)
B (n=16)
C (n=14)
surface

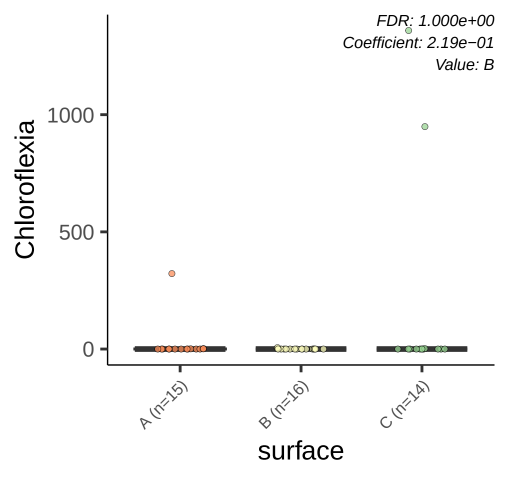
FDR: 1.000e+00
Coefficient: 2.19e−01
Value: B
Chloroflexia
1000
500
0
A (n=15)
B (n=16)
C (n=14)
surface

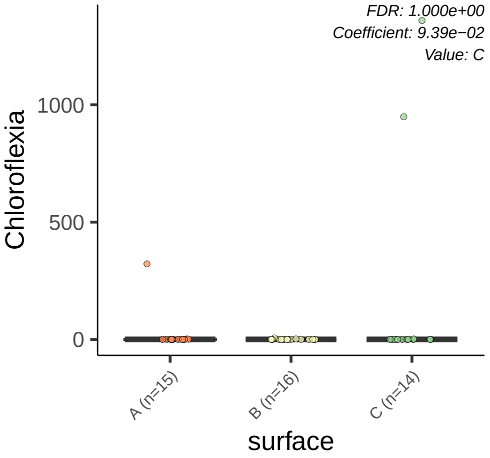

FDR: 1.000e+00
Coefficient: 9.39e−02
Value: C
Chloroflexia
1000
500
0
A (n=15)
B (n=16)
C (n=14)
surface

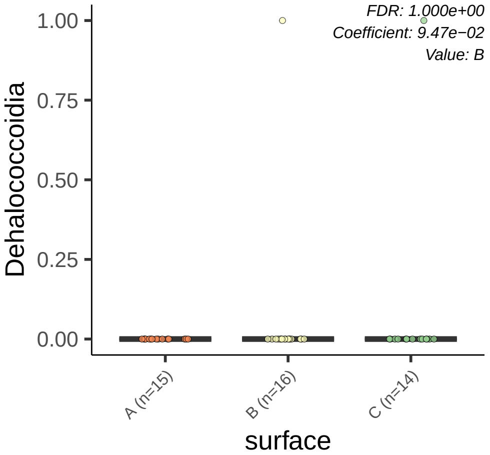
FDR: 1.000e+00
Coefficient: 9.47e−02
Value: B
Dehalococcoidia
1.00
0.75
0.50
0.25
0.00
A (n=15)
B (n=16)
C (n=14)
surface

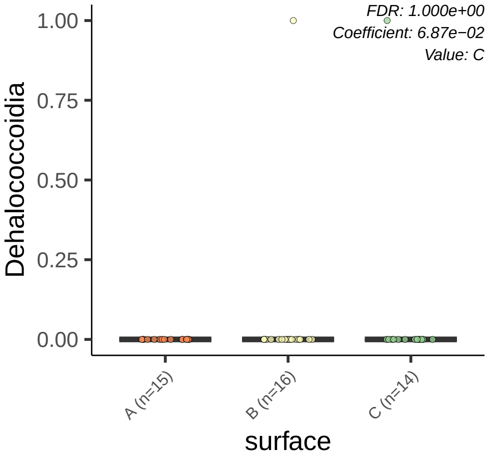
FDR: 1.000e+00
Coefficient: 6.87e−02
Value: C
Dehalococcoidia
1.00
0.75
0.50
0.25
0.00
A (n=15)
B (n=16)
C (n=14)
surface

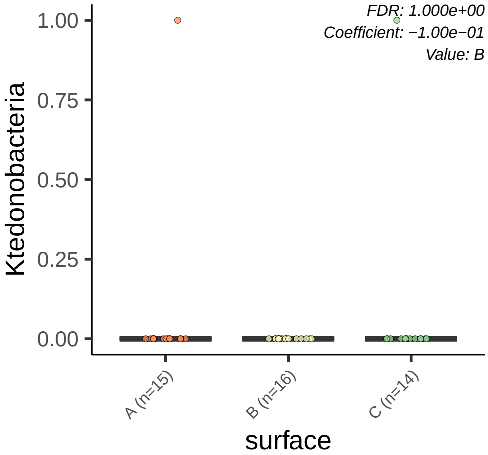
FDR: 1.000e+00
Coefficient: −1.00e−01
Value: B
Ktedonobacteria
1.00
0.75
0.50
0.25
0.00
A (n=15)
B (n=16)
C (n=14)
surface

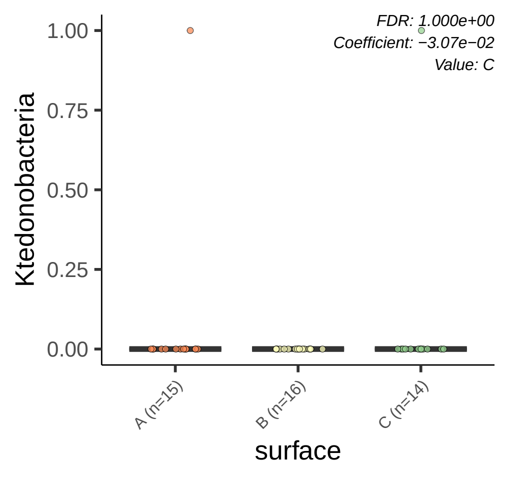
FDR: 1.000e+00
Coefficient: −3.07e−02
Value: C
Ktedonobacteria
1.00
0.75
0.50
0.25
0.00
A (n=15)
B (n=16)
C (n=14)
surface

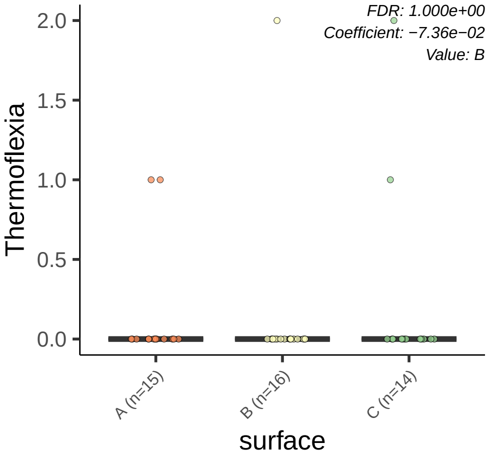
FDR: 1.000e+00
Coefficient: −7.36e−02
Value: B
Thermoflexia
2.0
1.5
1.0
0.5
0.0
A (n=15)
B (n=16)
C (n=14)
surface

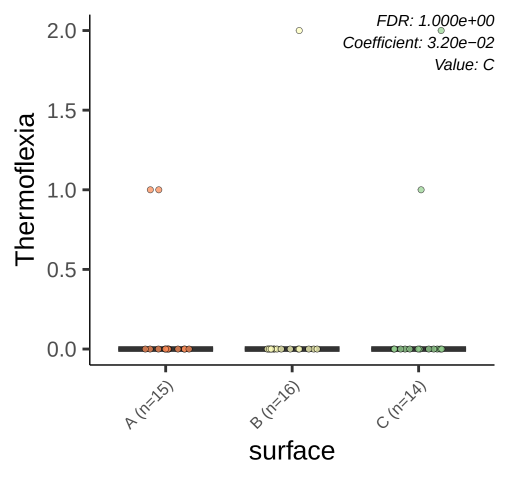

FDR: 1.000e+00
Coefficient: 3.20e−02
Value: C
Thermoflexia
2.0
1.5
1.0
0.5
0.0
A (n=15)
B (n=16)
C (n=14)
surface

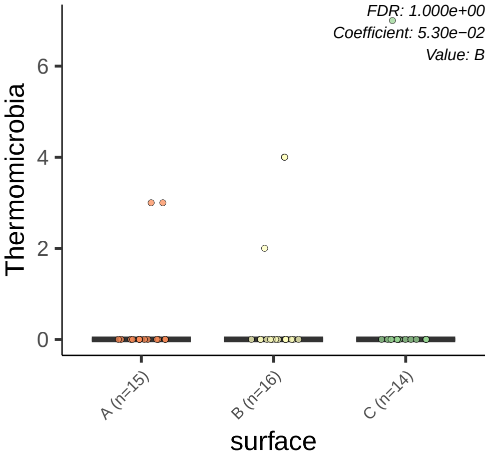
FDR: 1.000e+00
Coefficient: 5.30e−02
Value: B
Thermomicrobia
0
2
4
6
A (n=15)
B (n=16)
C (n=14)
surface

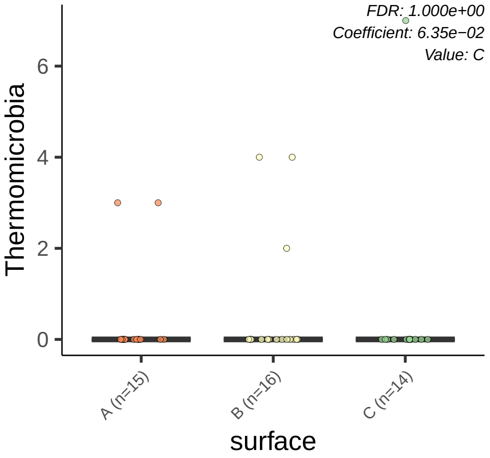
FDR: 1.000e+00
Coefficient: 6.35e−02
Value: C
Thermomicrobia
0
2
4
6
A (n=15)
B (n=16)
C (n=14)
surface

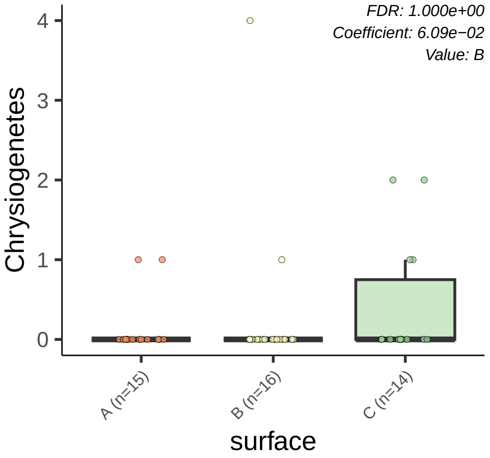

FDR: 1.000e+00
Coefficient: 6.09e−02
Value: B
Chrysiogenetes
0
1
2
3
4
A (n=15)
B (n=16)
C (n=14)
surface

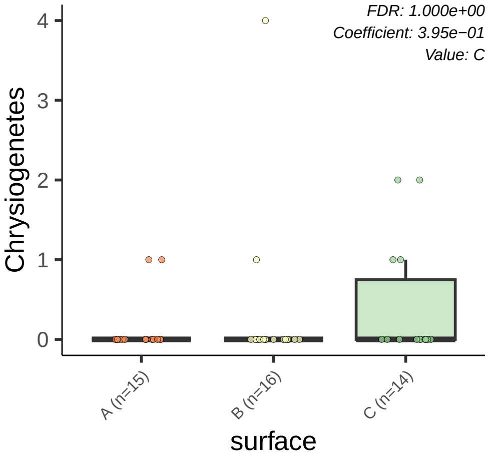
FDR: 1.000e+00
Coefficient: 3.95e−01
Value: C
Chrysiogenetes
0
1
2
3
4
A (n=15)
B (n=16)
C (n=14)
surface

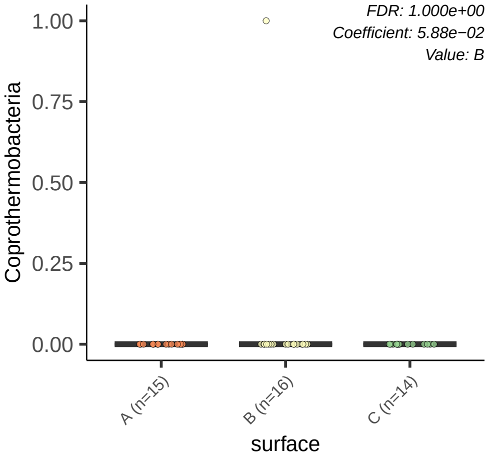
FDR: 1.000e+00
Coefficient: 5.88e−02
Value: B
Coprothermobacteria
1.00
0.75
0.50
0.25
0.00
A (n=15)
B (n=16)
C (n=14)
surface

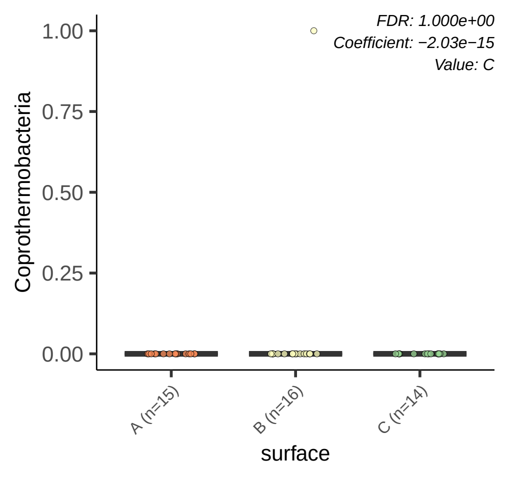
FDR: 1.000e+00
Coefficient: −2.03e−15
Value: C
Coprothermobacteria
1.00
0.75
0.50
0.25
0.00
A (n=15)
B (n=16)
C (n=14)
surface

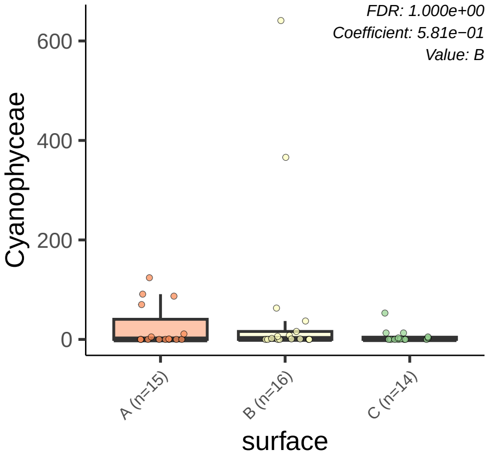
FDR: 1.000e+00
Coefficient: 5.81e−01
Value: B
Cyanophyceae
600
400
200
0
A (n=15)
B (n=16)
C (n=14)
surface

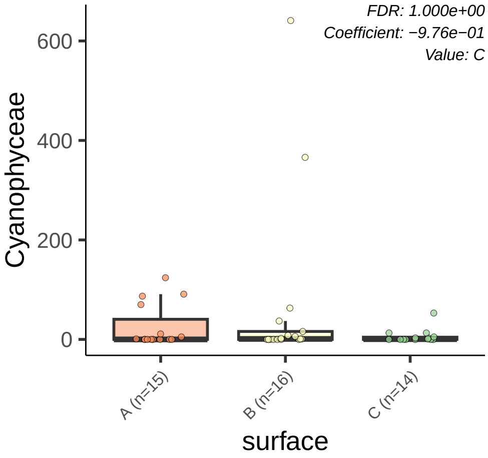

FDR: 1.000e+00
Coefficient: −9.76e−01
Value: C
Cyanophyceae
600
400
200
0
A (n=15)
B (n=16)
C (n=14)
surface

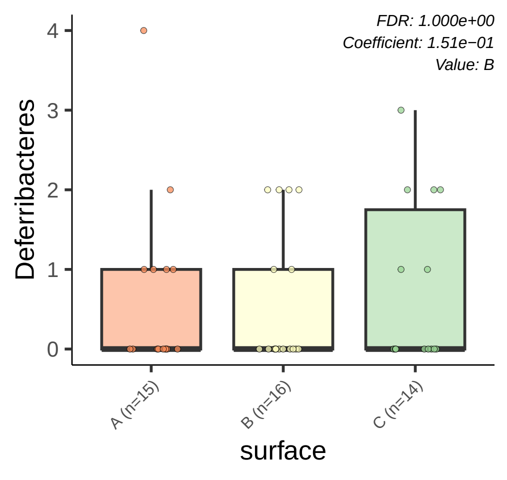

FDR: 1.000e+00
Coefficient: 1.51e−01
Value: B
Deferribacteres
0
1
2
3
4
A (n=15)
B (n=16)
C (n=14)
surface

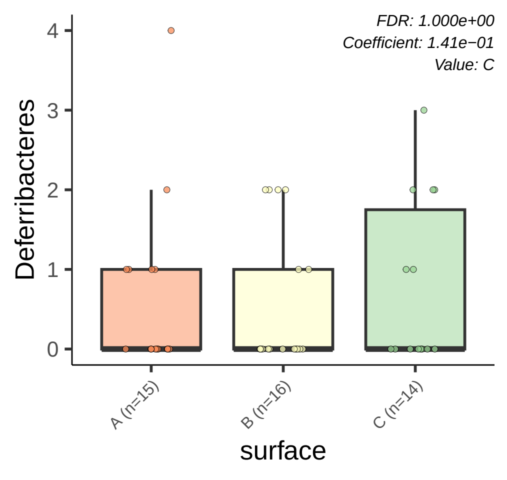

FDR: 1.000e+00
Coefficient: 1.41e−01
Value: C
Deferribacteres
4
3
2
1
0
A (n=15)
B (n=16)
C (n=14)
surface

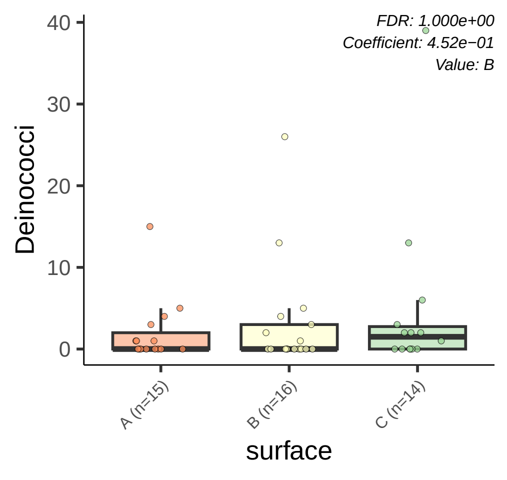
FDR: 1.000e+00
Coefficient: 4.52e−01
Value: B
Deinococci
40
30
20
10
0
A (n=15)
B (n=16)
C (n=14)
surface

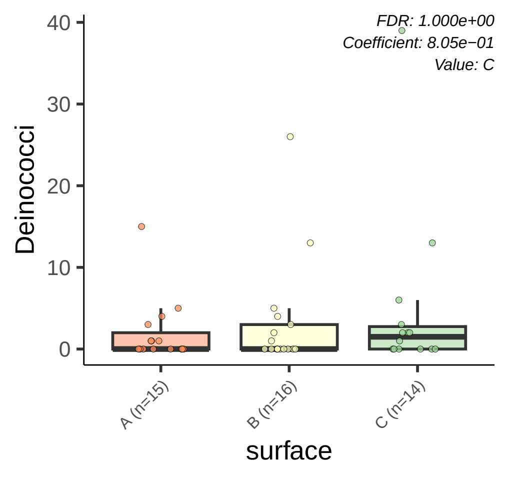
FDR: 1.000e+00
Coefficient: 8.05e−01
Value: C
Deinococci
40
30
20
10
0
A (n=15)
B (n=16)
C (n=14)
surface

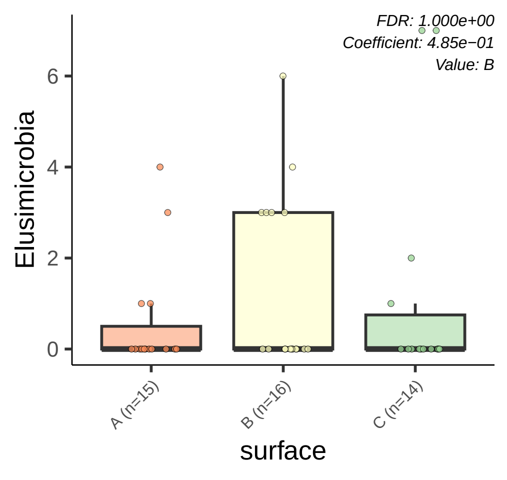
FDR: 1.000e+00
Coefficient: 4.85e−01
Value: B
Elusimicrobia
0
2
4
6
A (n=15)
B (n=16)
C (n=14)
surface

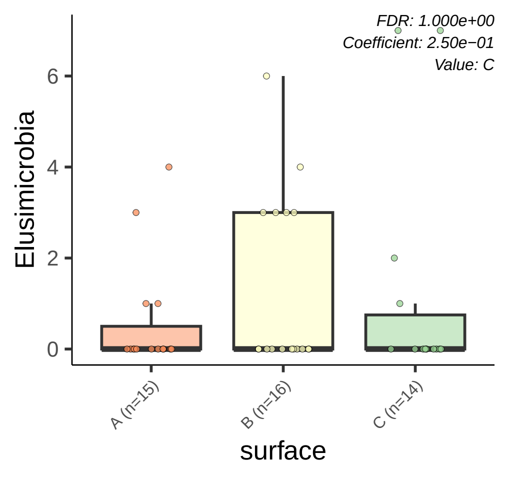
FDR: 1.000e+00
Coefficient: 2.50e−01
Value: C
Elusimicrobia
0
2
4
6
A (n=15)
B (n=16)
C (n=14)
surface

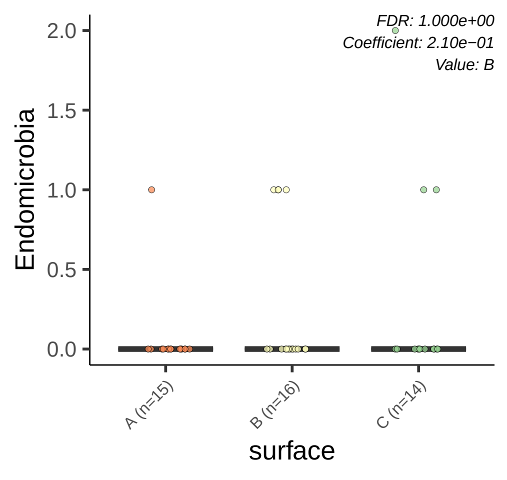

FDR: 1.000e+00
Coefficient: 2.10e−01
Value: B
Endomicrobia
2.0
1.5
1.0
0.5
0.0
A (n=15)
B (n=16)
C (n=14)
surface

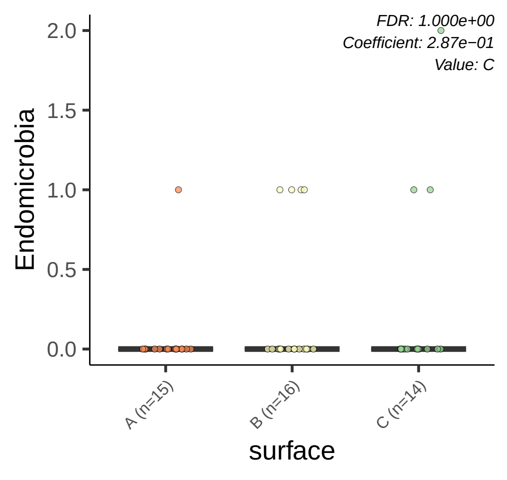

FDR: 1.000e+00
Coefficient: 2.87e−01
Value: C
Endomicrobia
2.0
1.5
1.0
0.5
0.0
A (n=15)
B (n=16)
C (n=14)
surface

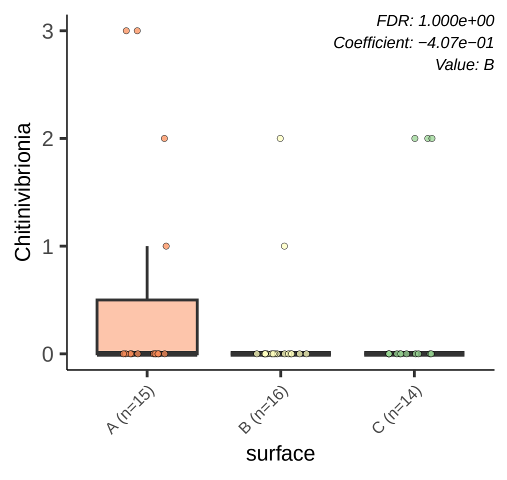

FDR: 1.000e+00
Coefficient: −4.07e−01
Value: B
Chitinivibrionia
3
2
1
0
A (n=15)
B (n=16)
C (n=14)
surface

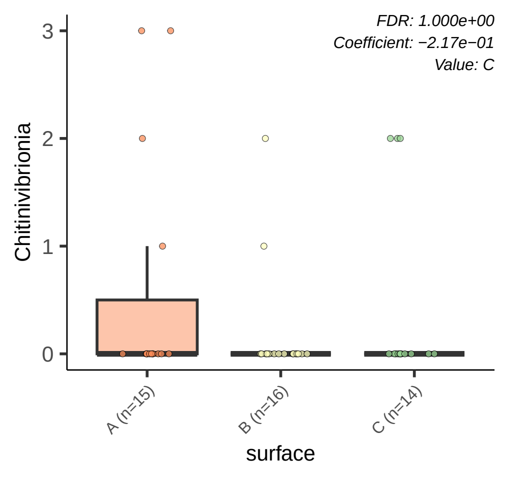

FDR: 1.000e+00
Coefficient: −2.17e−01
Value: C
Chitinivibrionia
3
2
1
0
A (n=15)
B (n=16)
C (n=14)
surface

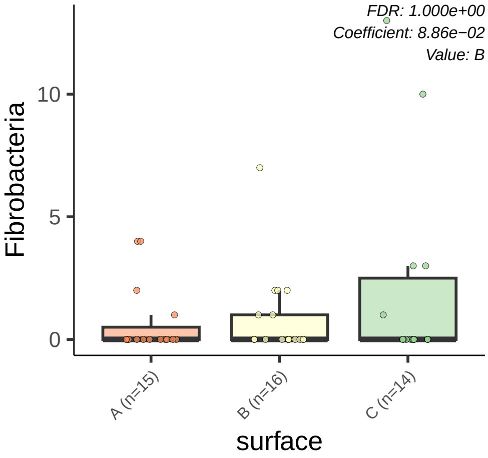
FDR: 1.000e+00
Coefficient: 8.86e−02
Value: B
Fibrobacteria
0
5
10
A (n=15)
B (n=16)
C (n=14)
surface

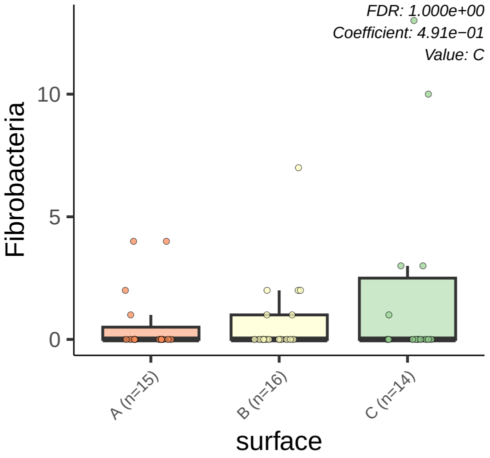

FDR: 1.000e+00
Coefficient: 4.91e−01
Value: C
Fibrobacteria
0
5
10
A (n=15)
B (n=16)
C (n=14)
surface

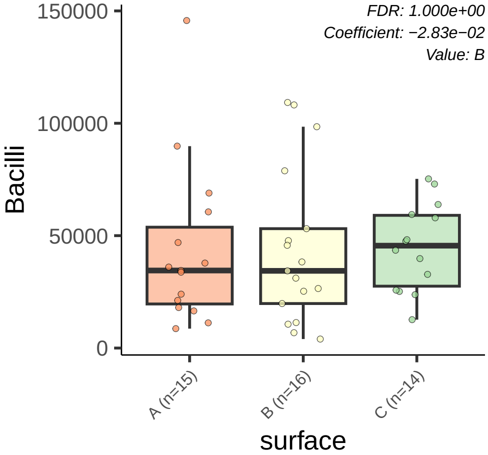
FDR: 1.000e+00
Coefficient: −2.83e−02
Value: B
Bacilli
150000
100000
50000
0
A (n=15)
B (n=16)
C (n=14)
surface

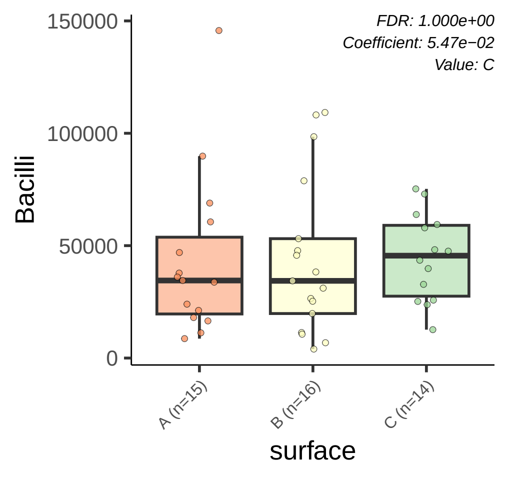
FDR: 1.000e+00
Coefficient: 5.47e−02
Value: C
150000
100000
50000
0
Bacilli
A (n=15)
B (n=16)
C (n=14)
surface

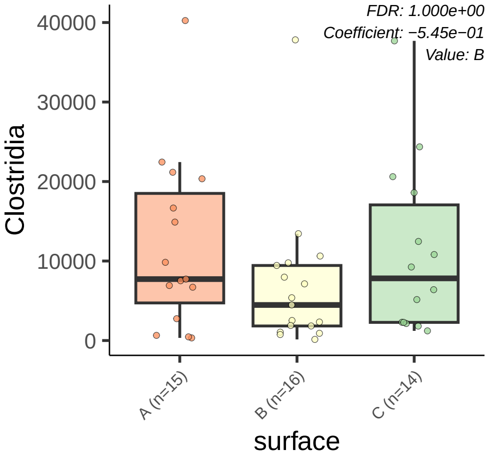

FDR: 1.000e+00
Coefficient: −5.45e−01
Value: B
Clostridia
40000
30000
20000
10000
0
A (n=15)
B (n=16)
C (n=14)
surface

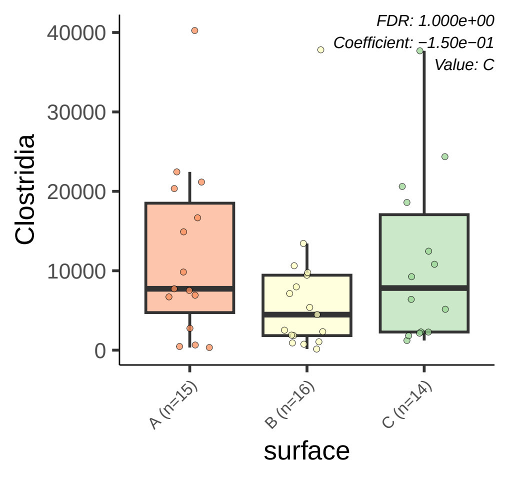

FDR: 1.000e+00
Coefficient: −1.50e−01
Value: C
Clostridia
40000
30000
20000
10000
0
A (n=15)
B (n=16)
C (n=14)
surface

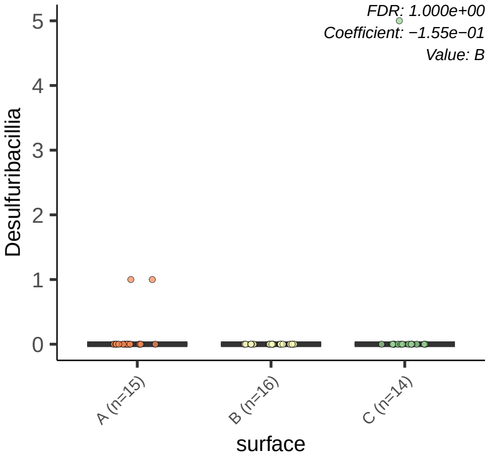

FDR: 1.000e+00
Coefficient: −1.55e−01
Value: B
Desulfuribacillia
0
1
2
3
4
5
A (n=15)
B (n=16)
C (n=14)
surface

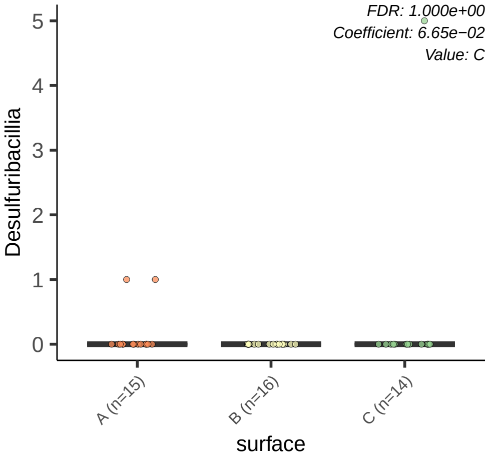

FDR: 1.000e+00
Coefficient: 6.65e−02
Value: C
Desulfuribacillia
0
1
2
3
4
5
A (n=15)
B (n=16)
C (n=14)
surface

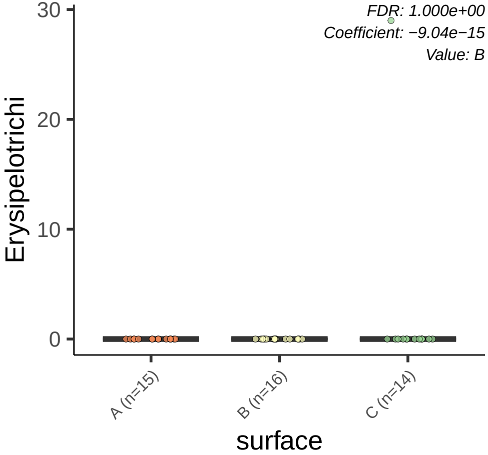
FDR: 1.000e+00
Coefficient: −9.04e−15
Value: B
Erysipelotrichi
30
20
10
0
A (n=15)
B (n=16)
C (n=14)
surface

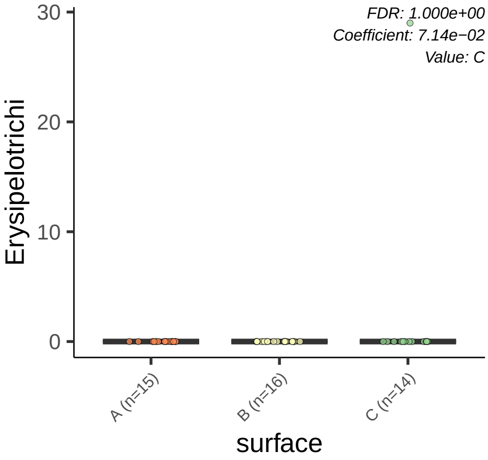

FDR: 1.000e+00
Coefficient: 7.14e−02
Value: C
Erysipelotrichi
30
20
10
0
A (n=15)
B (n=16)
C (n=14)
surface

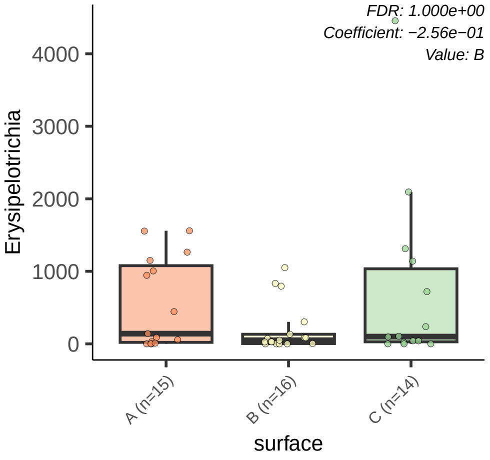
FDR: 1.000e+00
Coefficient: −2.56e−01
Value: B
Erysipelotrichia
4000
3000
2000
1000
0
A (n=15)
B (n=16)
C (n=14)
surface

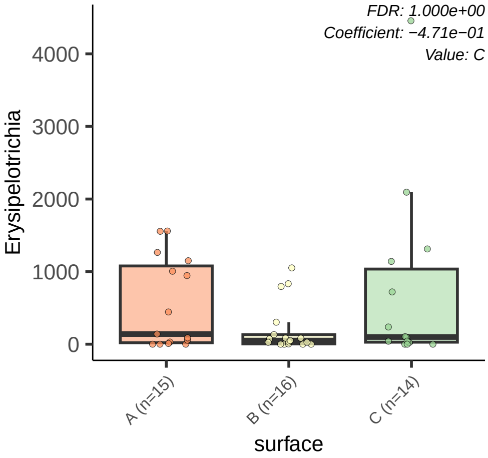

FDR: 1.000e+00
Coefficient: −4.71e−01
Value: C
4000
3000
2000
1000
0
Erysipelotrichia
A (n=15)
B (n=16)
C (n=14)
surface

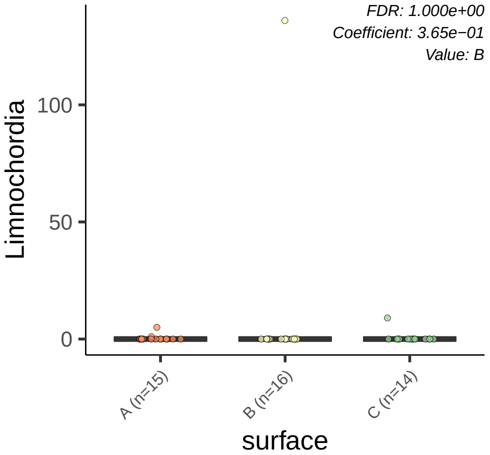
FDR: 1.000e+00
Coefficient: 3.65e−01
Value: B
Limnochordia
100
50
0
A (n=15)
B (n=16)
C (n=14)
surface

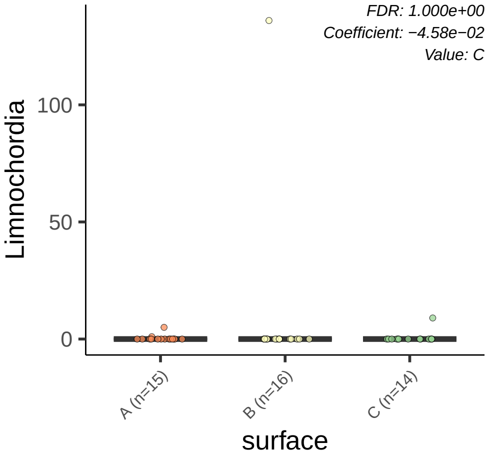

FDR: 1.000e+00
Coefficient: −4.58e−02
Value: C
Limnochordia
100
50
0
A (n=15)
B (n=16)
C (n=14)
surface

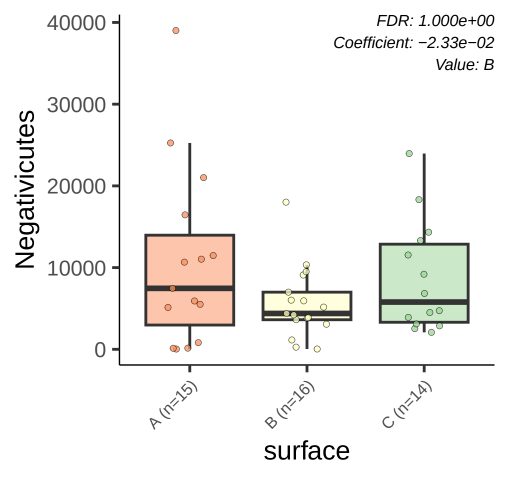

FDR: 1.000e+00
Coefficient: −2.33e−02
Value: B
Negativicutes
40000
30000
20000
10000
0
A (n=15)
B (n=16)
C (n=14)
surface

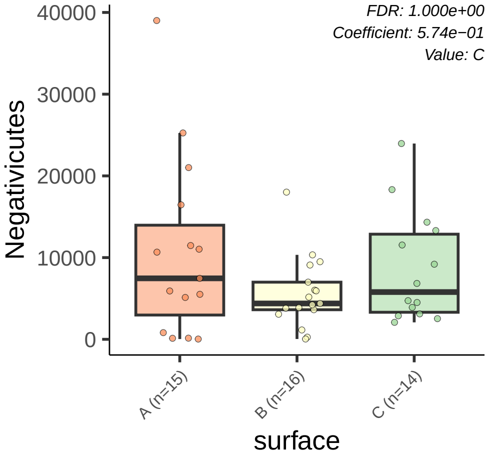
FDR: 1.000e+00
Coefficient: 5.74e−01
Value: C
Negativicutes
40000
30000
20000
10000
0
A (n=15)
B (n=16)
C (n=14)
surface

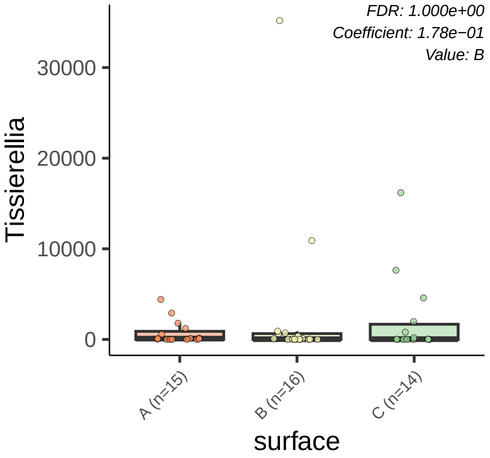
FDR: 1.000e+00
Coefficient: 1.78e−01
Value: B
Tissierellia
30000
20000
10000
0
A (n=15)
B (n=16)
C (n=14)
surface

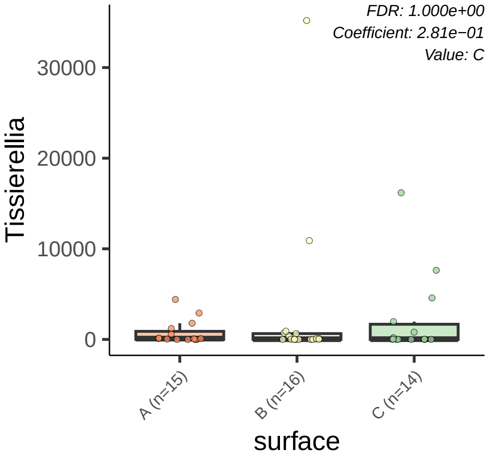
FDR: 1.000e+00
Coefficient: 2.81e−01
Value: C
Tissierellia
30000
20000
10000
0
A (n=15)
B (n=16)
C (n=14)
surface

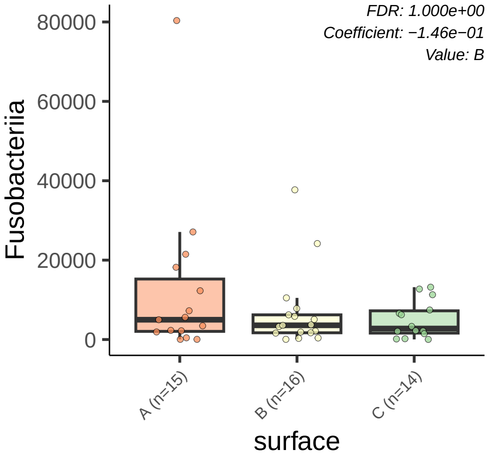

FDR: 1.000e+00
Coefficient: −1.46e−01
Value: B
80000
60000
40000
20000
0
Fusobacteriia
A (n=15)
B (n=16)
C (n=14)
surface

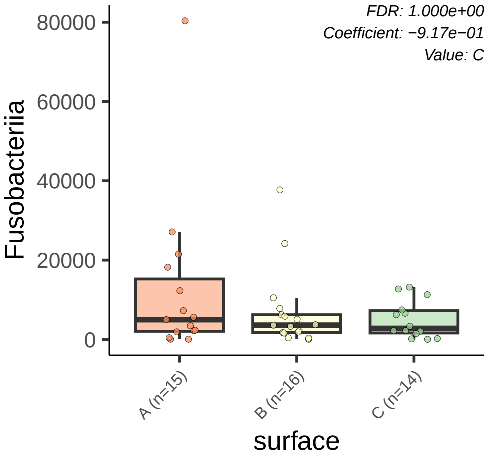

FDR: 1.000e+00
Coefficient: −9.17e−01
Value: C
Fusobacteriia
80000
60000
40000
20000
0
A (n=15)
B (n=16)
C (n=14)
surface

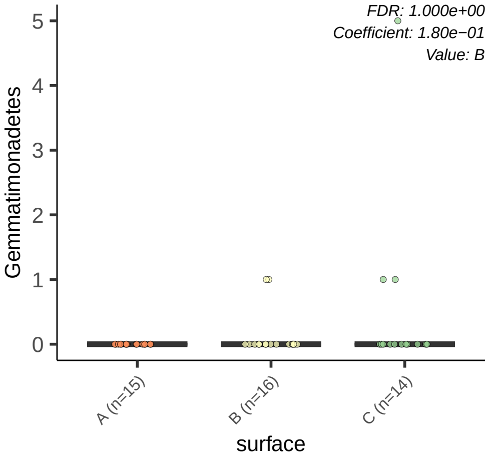
FDR: 1.000e+00
Coefficient: 1.80e−01
Value: B
Gemmatimonadetes
5
4
3
2
1
0
A (n=15)
B (n=16)
C (n=14)
surface

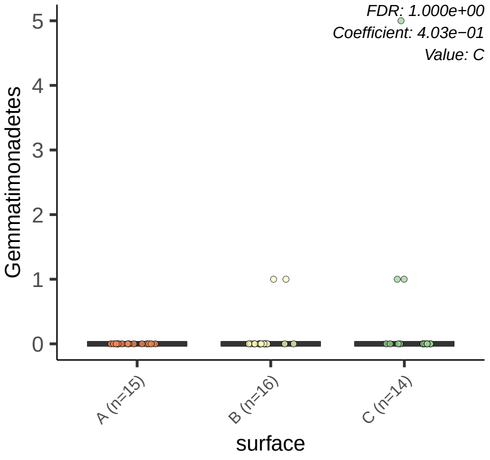

FDR: 1.000e+00
Coefficient: 4.03e−01
Value: C
Gemmatimonadetes
5
4
3
2
1
0
A (n=15)
B (n=16)
C (n=14)
surface

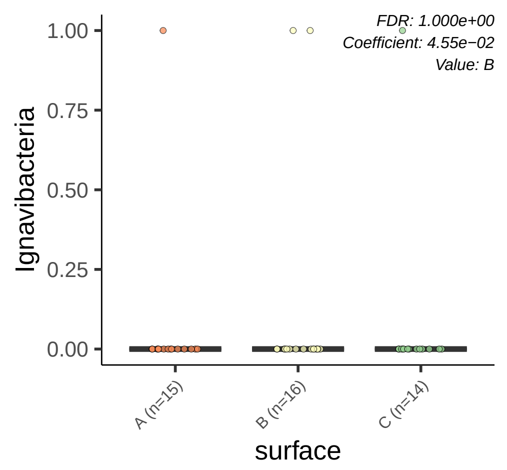

FDR: 1.000e+00
Coefficient: 4.55e−02
Value: B
Ignavibacteria
1.00
0.75
0.50
0.25
0.00
A (n=15)
B (n=16)
C (n=14)
surface

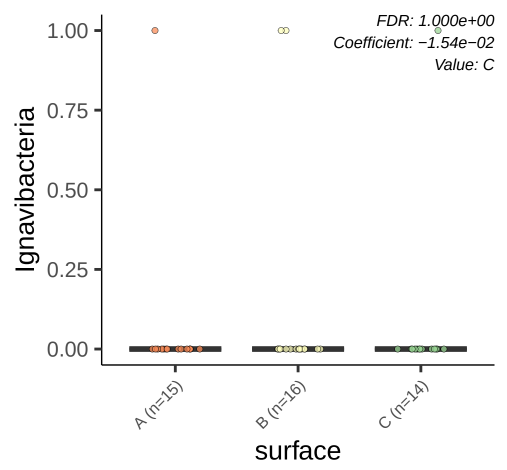
FDR: 1.000e+00
Coefficient: −1.54e−02
Value: C
Ignavibacteria
1.00
0.75
0.50
0.25
0.00
A (n=15)
B (n=16)
C (n=14)
surface

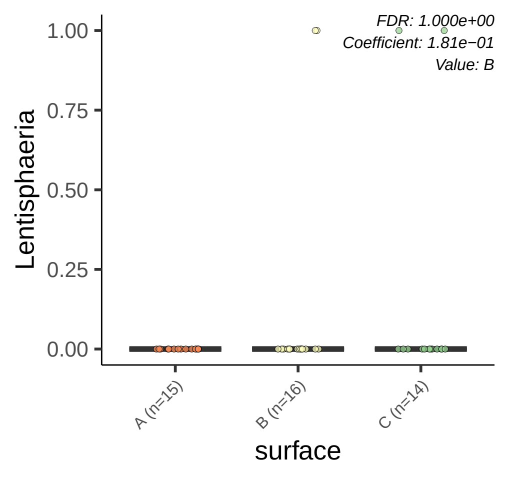
FDR: 1.000e+00
Coefficient: 1.81e−01
Value: B
Lentisphaeria
1.00
0.75
0.50
0.25
0.00
A (n=15)
B (n=16)
C (n=14)
surface

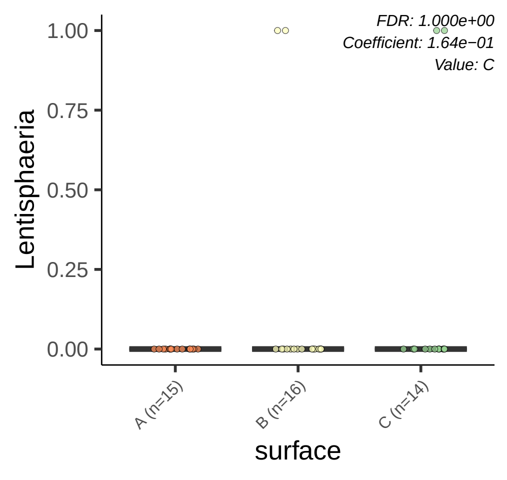
FDR: 1.000e+00
Coefficient: 1.64e−01
Value: C
Lentisphaeria
1.00
0.75
0.50
0.25
0.00
A (n=15)
B (n=16)
C (n=14)
surface

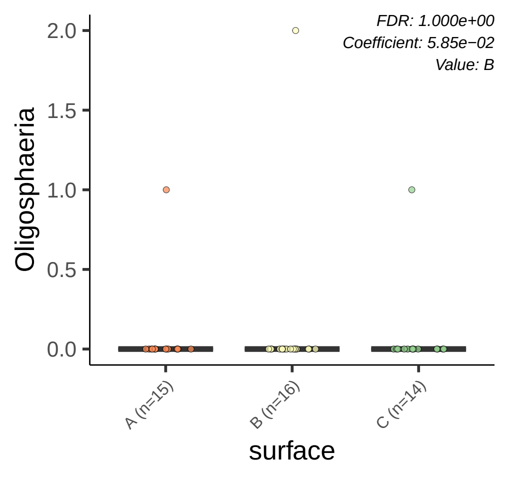
FDR: 1.000e+00
Coefficient: 5.85e−02
Value: B
Oligosphaeria
2.0
1.5
1.0
0.5
0.0
A (n=15)
B (n=16)
C (n=14)
surface

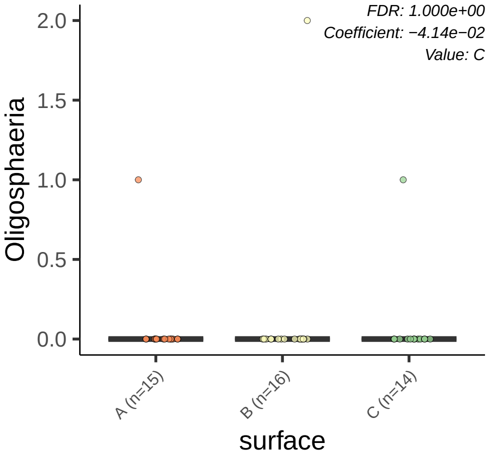

FDR: 1.000e+00
Coefficient: −4.14e−02
Value: C
Oligosphaeria
2.0
1.5
1.0
0.5
0.0
A (n=15)
B (n=16)
C (n=14)
surface

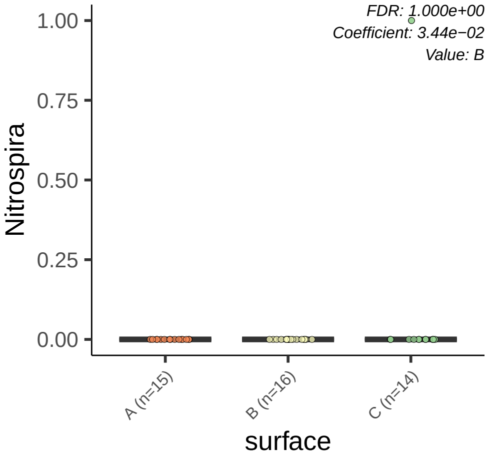
FDR: 1.000e+00
Coefficient: 3.44e−02
Value: B
Nitrospira
1.00
0.75
0.50
0.25
0.00
A (n=15)
B (n=16)
C (n=14)
surface

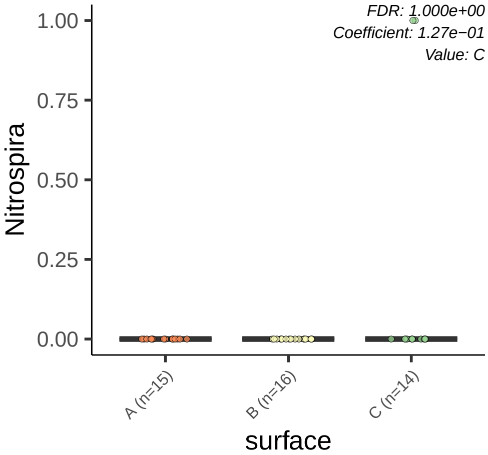
FDR: 1.000e+00
Coefficient: 1.27e−01
Value: C
Nitrospira
1.00
0.75
0.50
0.25
0.00
A (n=15)
B (n=16)
C (n=14)
surface

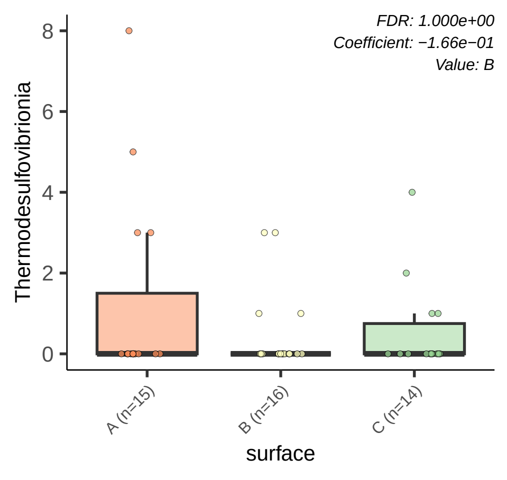

FDR: 1.000e+00
Coefficient: −1.66e−01
Value: B
Thermodesulfovibrionia
8
6
4
2
0
A (n=15)
B (n=16)
C (n=14)
surface

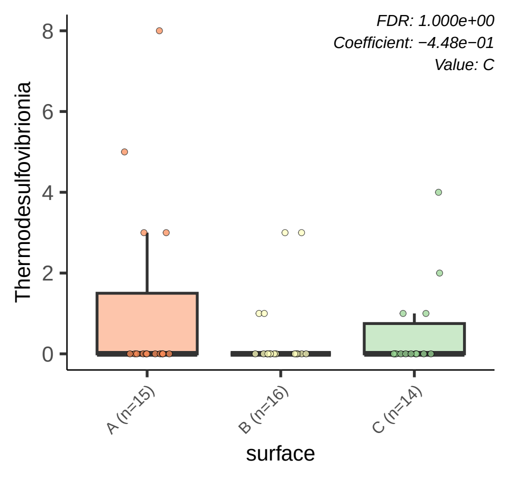
FDR: 1.000e+00
Coefficient: −4.48e−01
Value: C
Thermodesulfovibrionia
8
6
4
2
0
A (n=15)
B (n=16)
C (n=14)
surface

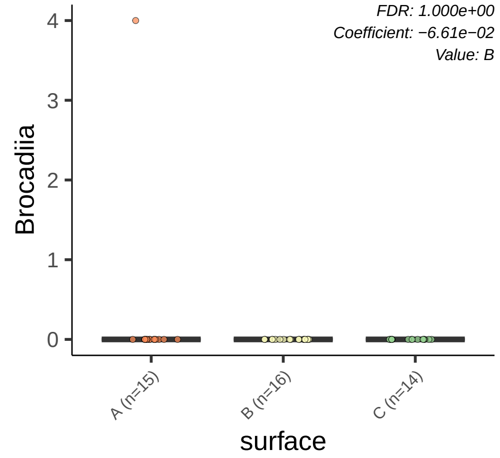
FDR: 1.000e+00
Coefficient: −6.61e−02
Value: B
Brocadiia
0
1
2
3
4
A (n=15)
B (n=16)
C (n=14)
surface

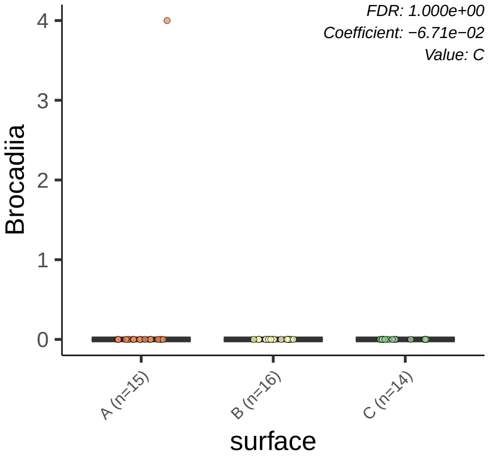

FDR: 1.000e+00
Coefficient: −6.71e−02
Value: C
Brocadiia
4
3
2
1
0
A (n=15)
B (n=16)
C (n=14)
surface

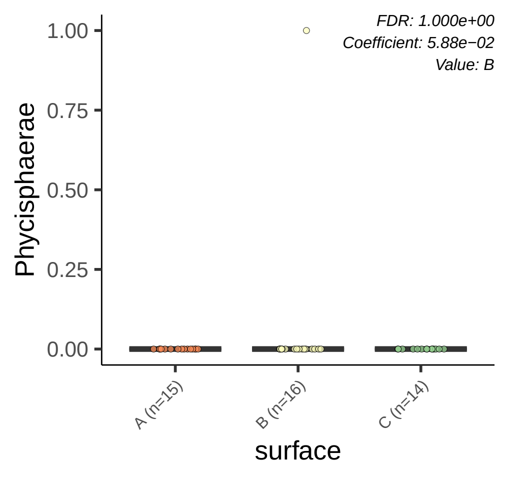

FDR: 1.000e+00
Coefficient: 5.88e−02
Value: B
Phycisphaerae
1.00
0.75
0.50
0.25
0.00
A (n=15)
B (n=16)
C (n=14)
surface

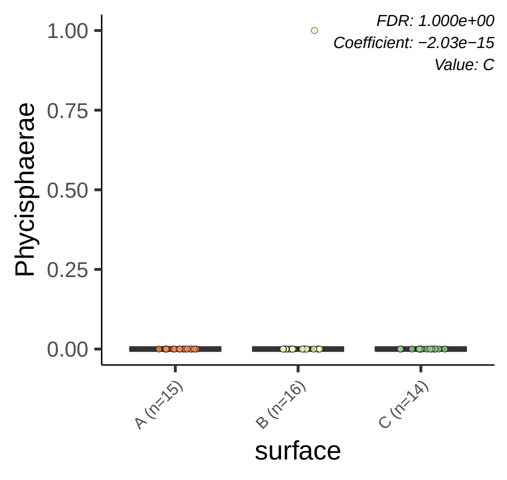
FDR: 1.000e+00
Coefficient: −2.03e−15
Value: C
Phycisphaerae
1.00
0.75
0.50
0.25
0.00
A (n=15)
B (n=16)
C (n=14)
surface

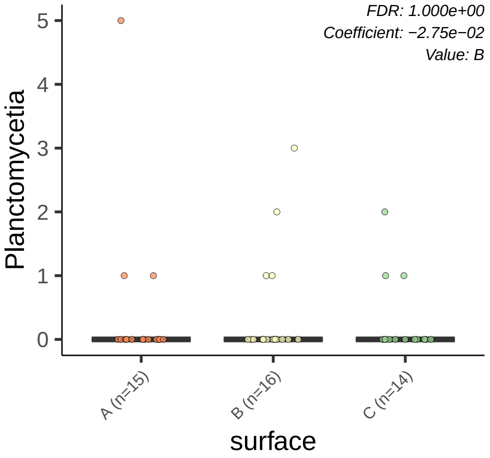
FDR: 1.000e+00
Coefficient: −2.75e−02
Value: B
Planctomycetia
5
4
3
2
1
0
A (n=15)
B (n=16)
C (n=14)
surface

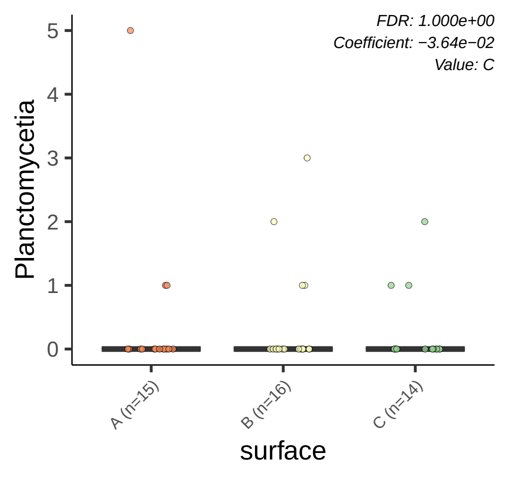
FDR: 1.000e+00
Coefficient: −3.64e−02
Value: C
Planctomycetia
5
4
3
2
1
0
A (n=15)
B (n=16)
C (n=14)
surface

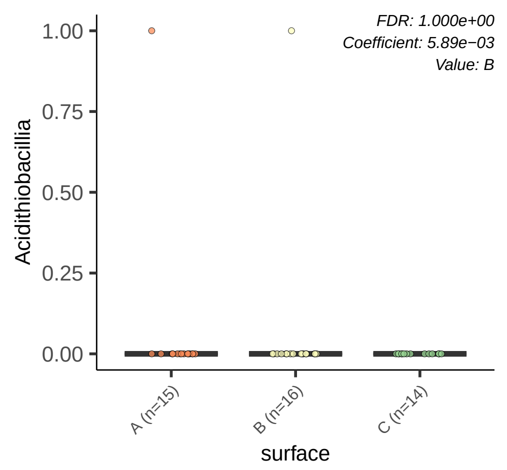

FDR: 1.000e+00
Coefficient: 5.89e−03
Value: B
Acidithiobacillia
1.00
0.75
0.50
0.25
0.00
A (n=15)
B (n=16)
C (n=14)
surface

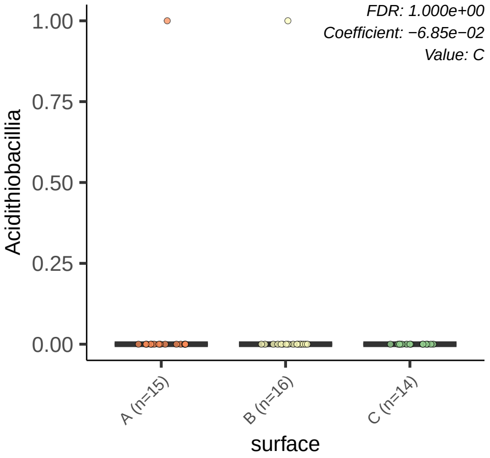
FDR: 1.000e+00
Coefficient: −6.85e−02
Value: C
Acidithiobacillia
1.00
0.75
0.50
0.25
0.00
A (n=15)
B (n=16)
C (n=14)
surface

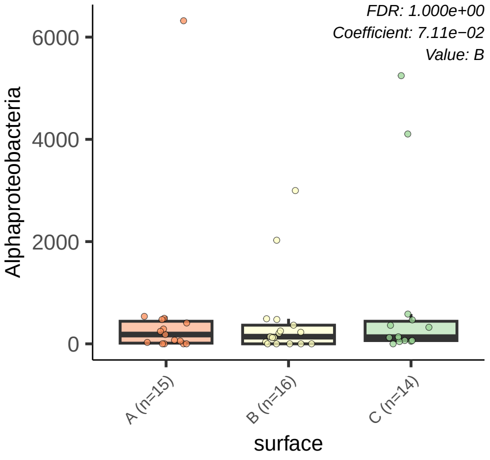

FDR: 1.000e+00
Coefficient: 7.11e−02
Value: B
Alphaproteobacteria
6000
4000
2000
0
A (n=15)
B (n=16)
C (n=14)
surface

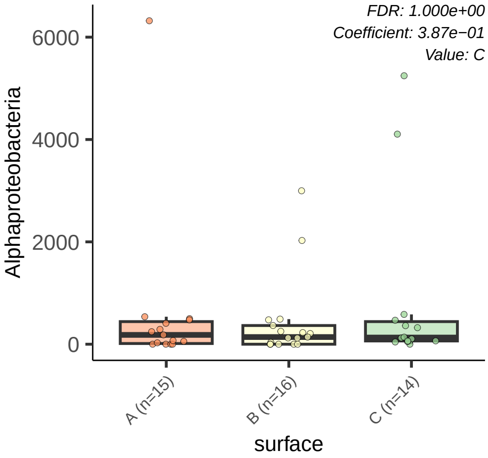

FDR: 1.000e+00
Coefficient: 3.87e−01
Value: C
6000
4000
2000
0
Alphaproteobacteria
A (n=15)
B (n=16)
C (n=14)
surface

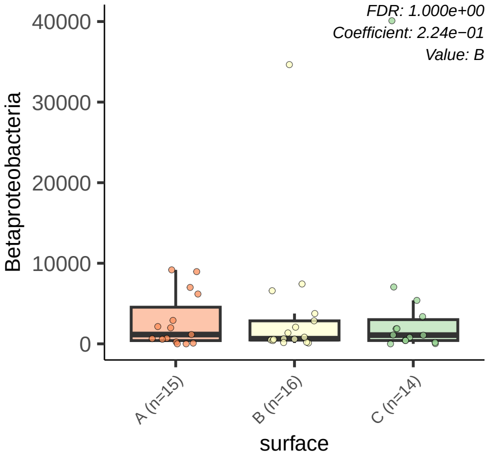
FDR: 1.000e+00
Coefficient: 2.24e−01
Value: B
Betaproteobacteria
40000
30000
20000
10000
0
A (n=15)
B (n=16)
C (n=14)
surface

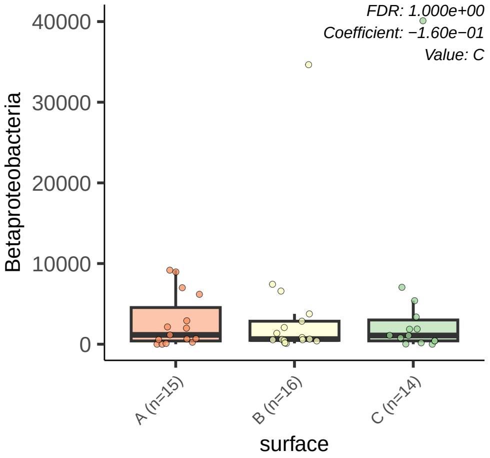
FDR: 1.000e+00
Coefficient: −1.60e−01
Value: C
Betaproteobacteria
40000
30000
20000
10000
0
A (n=15)
B (n=16)
C (n=14)
surface

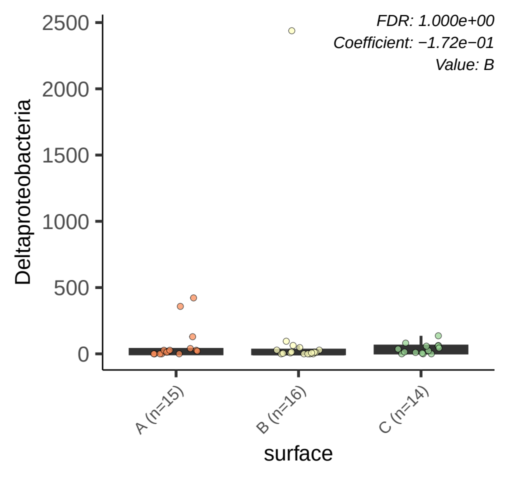

FDR: 1.000e+00
Coefficient: −1.72e−01
Value: B
Deltaproteobacteria
2500
2000
1500
1000
500
0
A (n=15)
B (n=16)
C (n=14)
surface

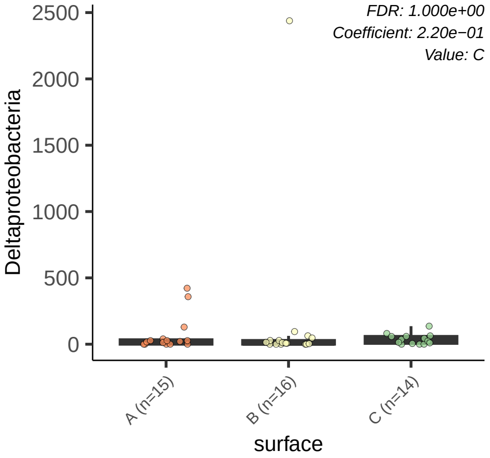

FDR: 1.000e+00
Coefficient: 2.20e−01
Value: C
Deltaproteobacteria
2500
2000
1500
1000
500
0
A (n=15)
B (n=16)
C (n=14)
surface

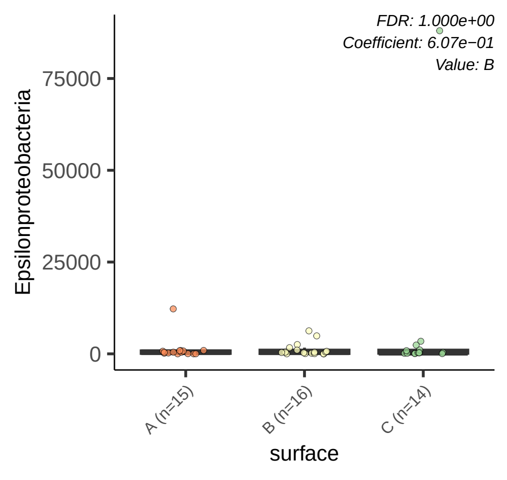
FDR: 1.000e+00
Coefficient: 6.07e−01
Value: B
Epsilonproteobacteria
75000
50000
25000
0
A (n=15)
B (n=16)
C (n=14)
surface

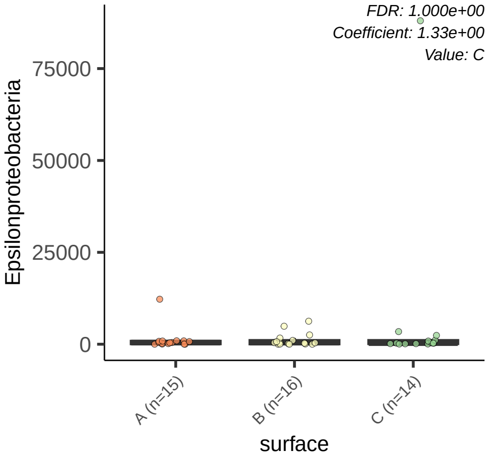

FDR: 1.000e+00
Coefficient: 1.33e+00
Value: C
Epsilonproteobacteria
75000
50000
25000
0
A (n=15)
B (n=16)
C (n=14)
surface

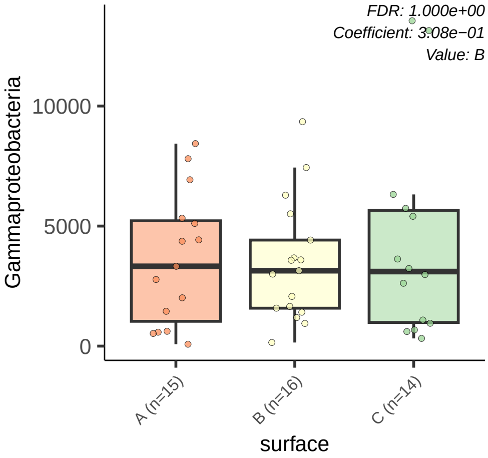

FDR: 1.000e+00
Coefficient: 3.08e−01
Value: B
Gammaproteobacteria
10000
5000
0
A (n=15)
B (n=16)
C (n=14)
surface

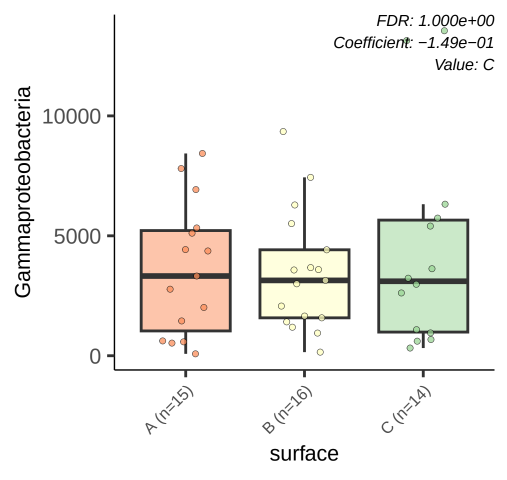
FDR: 1.000e+00
Coefficient: −1.49e−01
Value: C
Gammaproteobacteria
10000
5000
0
A (n=15)
B (n=16)
C (n=14)
surface

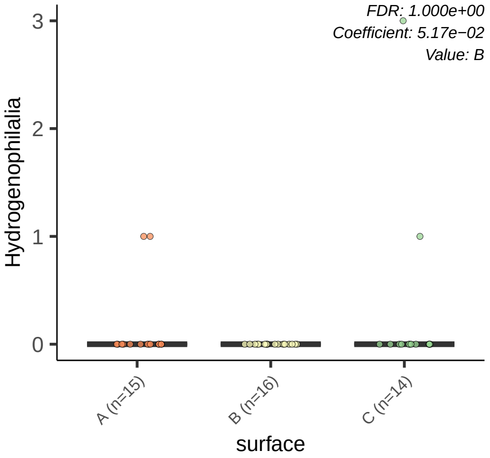

FDR: 1.000e+00
Coefficient: 5.17e−02
Value: B
Hydrogenophilalia
3
2
1
0
A (n=15)
B (n=16)
C (n=14)
surface

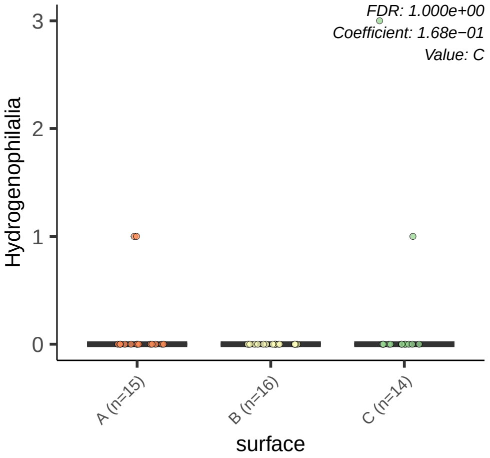
FDR: 1.000e+00
Coefficient: 1.68e−01
Value: C
Hydrogenophilalia
3
2
1
0
A (n=15)
B (n=16)
C (n=14)
surface

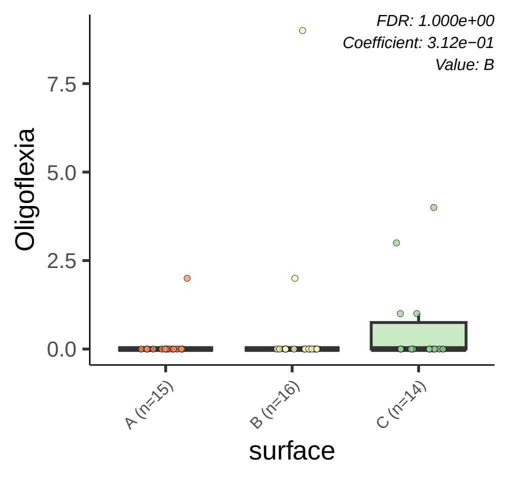
FDR: 1.000e+00
Coefficient: 3.12e−01
Value: B
Oligoflexia
7.5
5.0
2.5
0.0
A (n=15)
B (n=16)
C (n=14)
surface

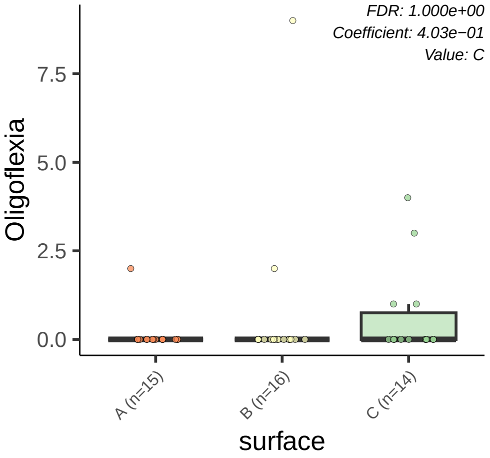

FDR: 1.000e+00
Coefficient: 4.03e−01
Value: C
Oligoflexia
7.5
5.0
2.5
0.0
A (n=15)
B (n=16)
C (n=14)
surface

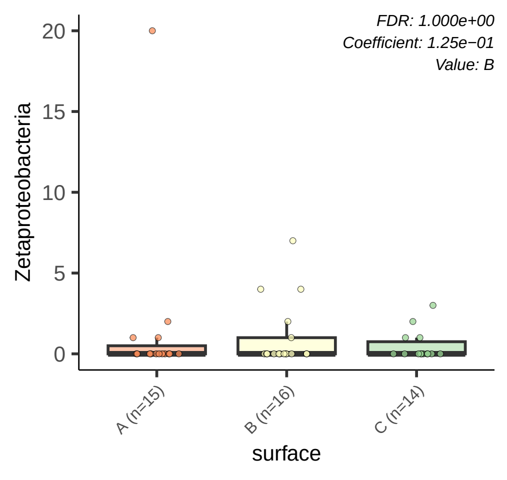

FDR: 1.000e+00
Coefficient: 1.25e−01
Value: B
Zetaproteobacteria
20
15
10
5
0
A (n=15)
B (n=16)
C (n=14)
surface

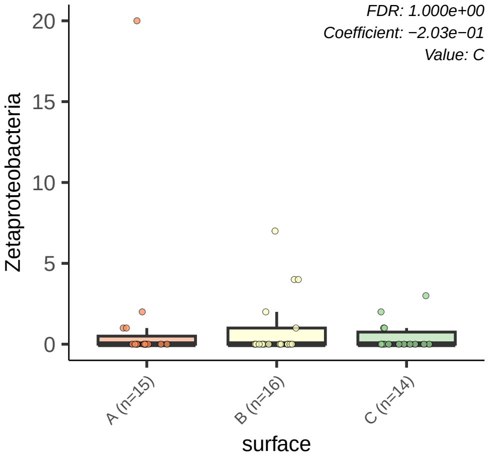
FDR: 1.000e+00
Coefficient: −2.03e−01
Value: C
Zetaproteobacteria
20
15
10
5
0
A (n=15)
B (n=16)
C (n=14)
surface

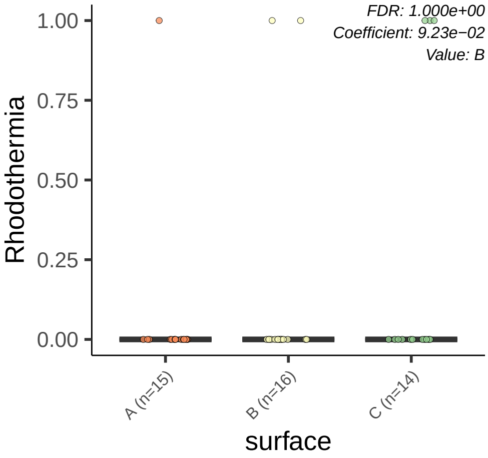
FDR: 1.000e+00
Coefficient: 9.23e−02
Value: B
Rhodothermia
1.00
0.75
0.50
0.25
0.00
A (n=15)
B (n=16)
C (n=14)
surface

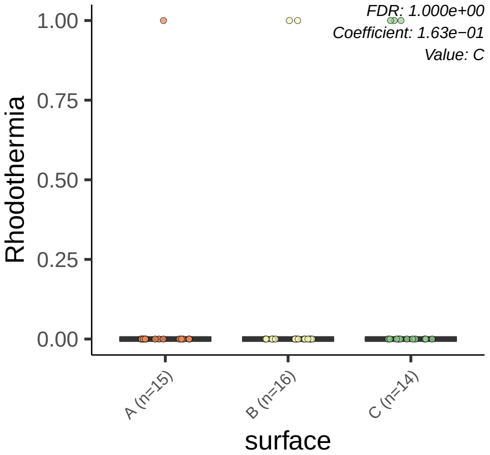

FDR: 1.000e+00
Coefficient: 1.63e−01
Value: C
Rhodothermia
1.00
0.75
0.50
0.25
0.00
A (n=15)
B (n=16)
C (n=14)
surface

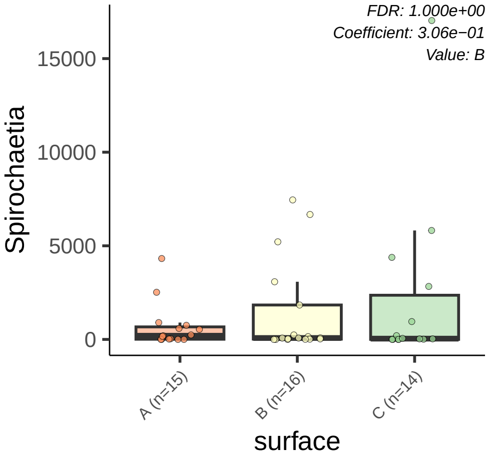

FDR: 1.000e+00
Coefficient: 3.06e−01
Value: B
15000
10000
5000
0
Spirochaetia
A (n=15)
B (n=16)
C (n=14)
surface

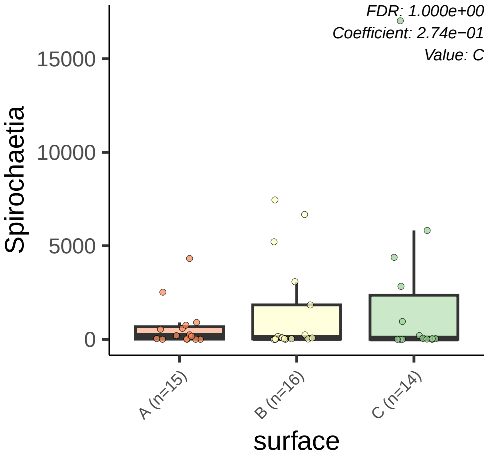

FDR: 1.000e+00
Coefficient: 2.74e−01
Value: C
Spirochaetia
15000
10000
5000
0
A (n=15)
B (n=16)
C (n=14)
surface

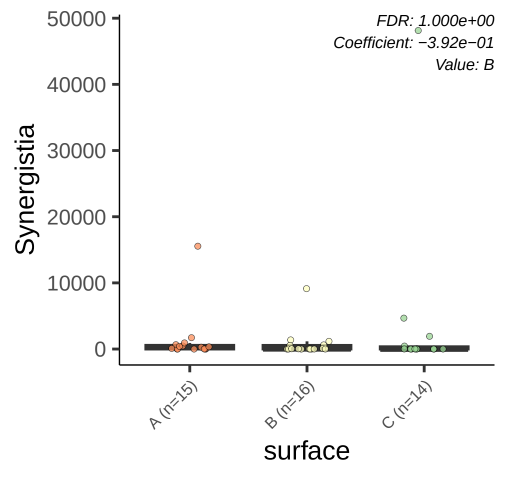
FDR: 1.000e+00
Coefficient: −3.92e−01
Value: B
Synergistia
50000
40000
30000
20000
10000
0
A (n=15)
B (n=16)
C (n=14)
surface

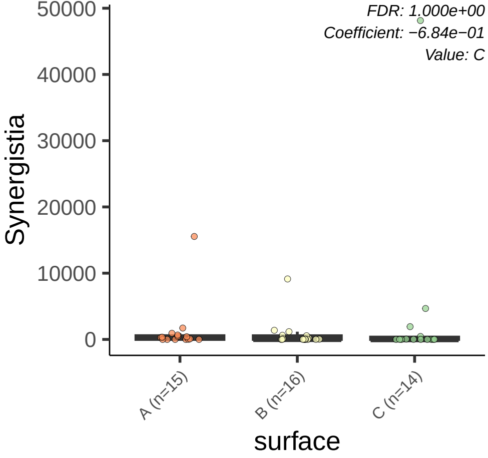

FDR: 1.000e+00
Coefficient: −6.84e−01
Value: C
Synergistia
50000
40000
30000
20000
10000
0
A (n=15)
B (n=16)
C (n=14)
surface

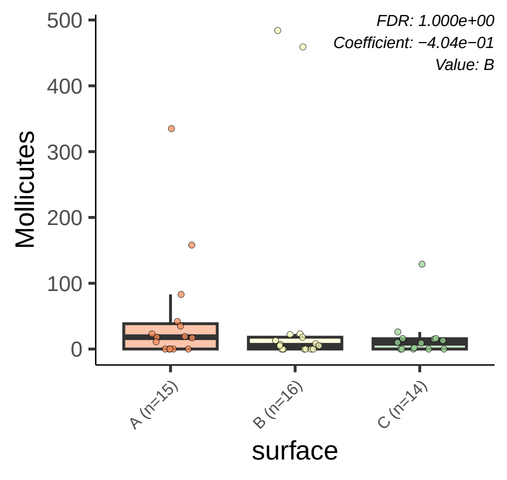

FDR: 1.000e+00
Coefficient: −4.04e−01
Value: B
Mollicutes
500
400
300
200
100
0
A (n=15)
B (n=16)
C (n=14)
surface

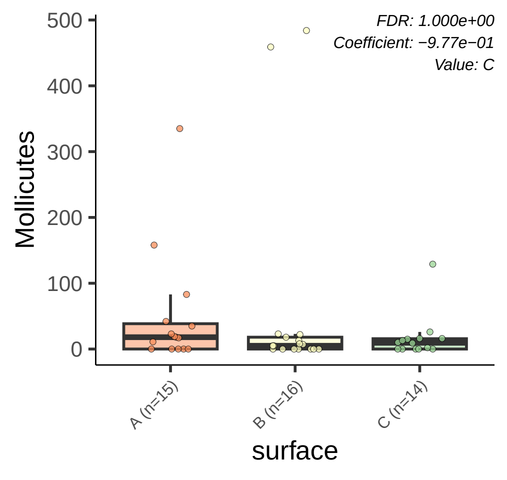

FDR: 1.000e+00
Coefficient: −9.77e−01
Value: C
Mollicutes
0
100
200
300
400
500
A (n=15)
B (n=16)
C (n=14)
surface

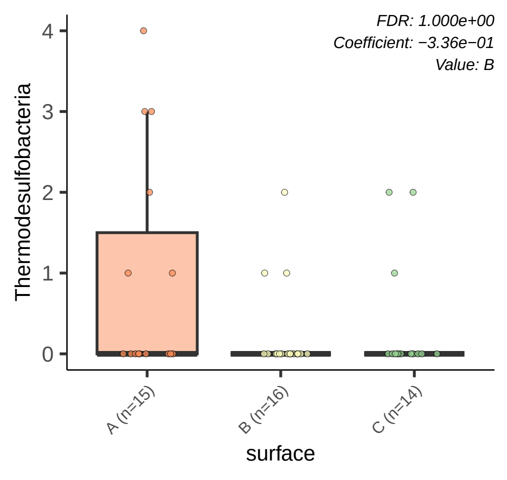

FDR: 1.000e+00
Coefficient: −3.36e−01
Value: B
Thermodesulfobacteria
0
1
2
3
4
A (n=15)
B (n=16)
C (n=14)
surface

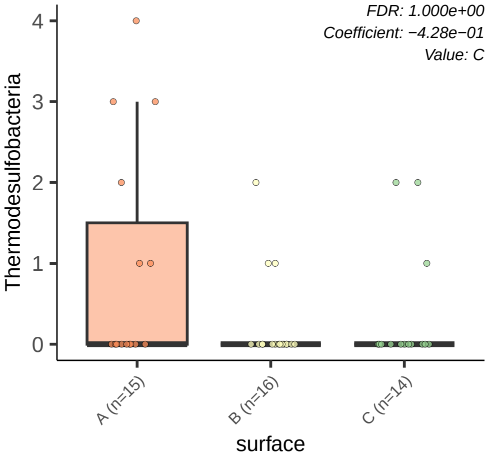
FDR: 1.000e+00
Coefficient: −4.28e−01
Value: C
Thermodesulfobacteria
4
3
2
1
0
A (n=15)
B (n=16)
C (n=14)
surface

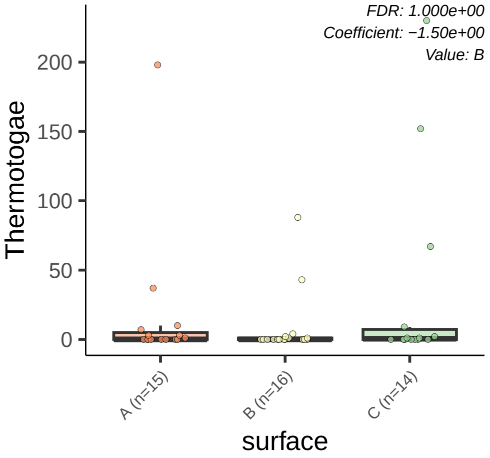

FDR: 1.000e+00
Coefficient: −1.50e+00
Value: B
Thermotogae
200
150
100
50
0
A (n=15)
B (n=16)
C (n=14)
surface

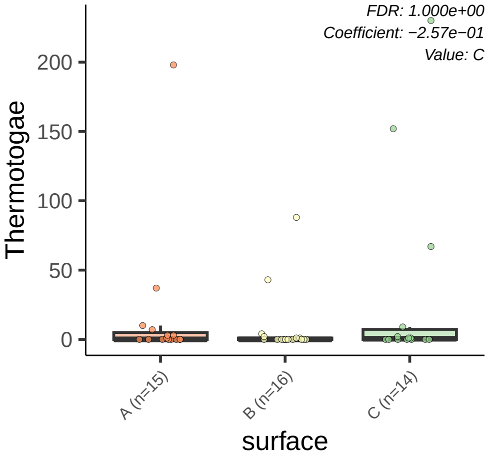

FDR: 1.000e+00
Coefficient: −2.57e−01
Value: C
Thermotogae
200
150
100
50
0
A (n=15)
B (n=16)
C (n=14)
surface

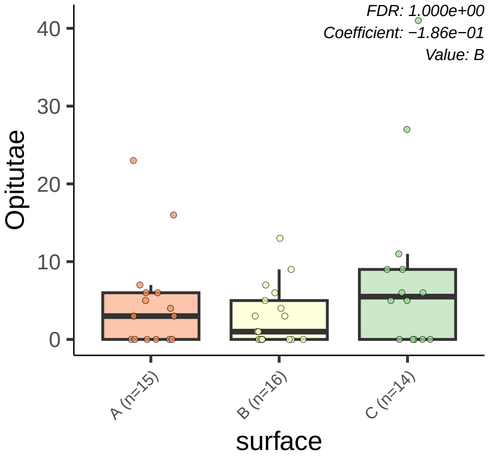
FDR: 1.000e+00
Coefficient: −1.86e−01
Value: B
Opitutae
40
30
20
10
0
A (n=15)
B (n=16)
C (n=14)
surface

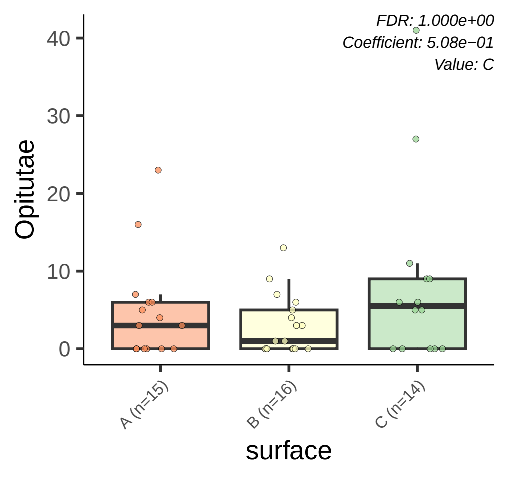

FDR: 1.000e+00
Coefficient: 5.08e−01
Value: C
Opitutae
40
30
20
10
0
A (n=15)
B (n=16)
C (n=14)
surface

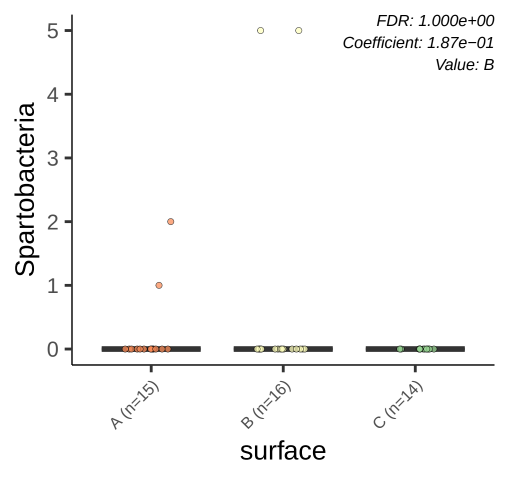

FDR: 1.000e+00
Coefficient: 1.87e−01
Value: B
Spartobacteria
5
4
3
2
1
0
A (n=15)
B (n=16)
C (n=14)
surface

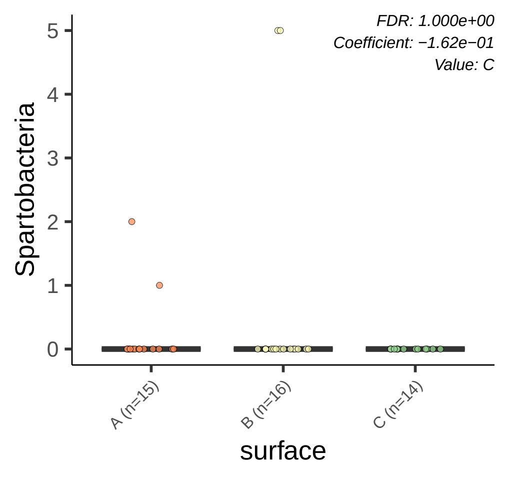
FDR: 1.000e+00
Coefficient: −1.62e−01
Value: C
Spartobacteria
0
1
2
3
4
5
A (n=15)
B (n=16)
C (n=14)
surface

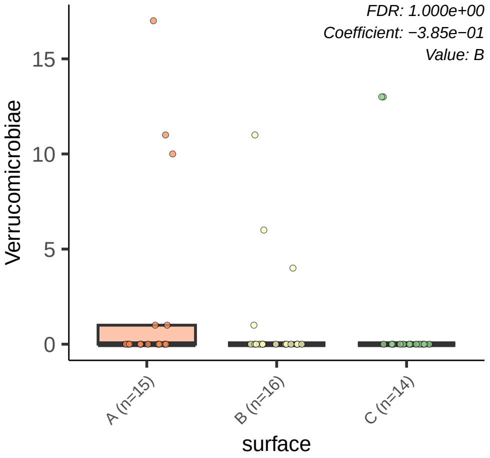
FDR: 1.000e+00
Coefficient: −3.85e−01
Value: B
Verrucomicrobiae
0
5
10
15
A (n=15)
B (n=16)
C (n=14)
surface

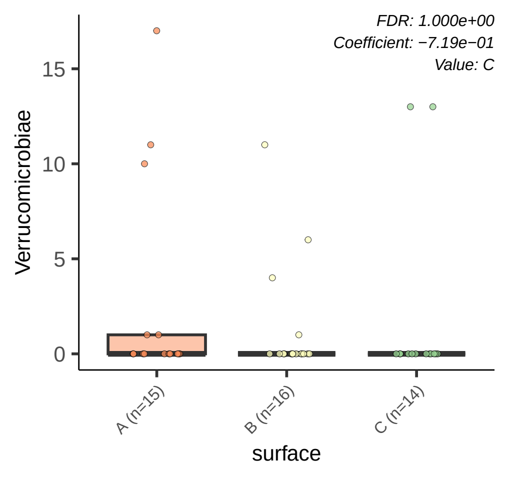

FDR: 1.000e+00
Coefficient: −7.19e−01
Value: C
Verrucomicrobiae
15
10
5
0
A (n=15)
B (n=16)
C (n=14)
surface

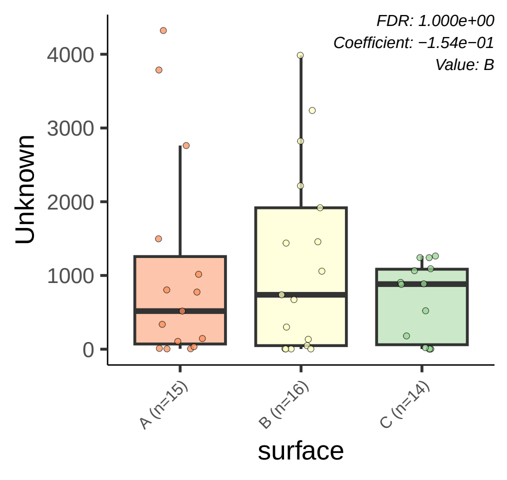
FDR: 1.000e+00
Coefficient: −1.54e−01
Value: B
Unknown
4000
3000
2000
1000
0
A (n=15)
B (n=16)
C (n=14)
surface

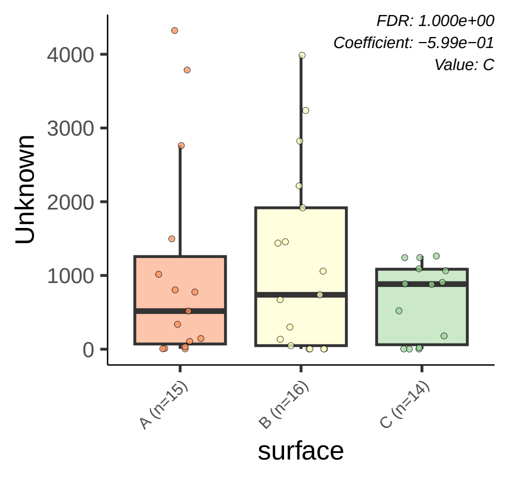
FDR: 1.000e+00
Coefficient: −5.99e−01
Value: C
Unknown
4000
3000
2000
1000
0
A (n=15)
B (n=16)
C (n=14)
surface

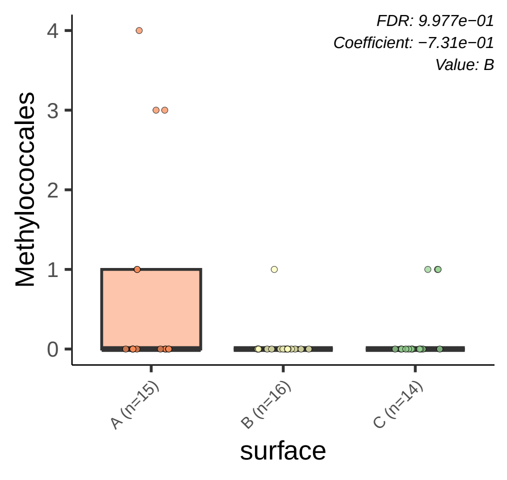

FDR: 9.977e−01
Coefficient: −7.31e−01
Value: B
Methylococcales
0
1
2
3
4
A (n=15)
B (n=16)
C (n=14)
surface

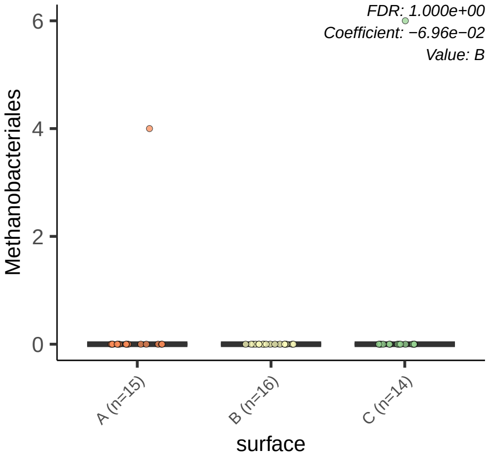

FDR: 1.000e+00
Coefficient: −6.96e−02
Value: B
Methanobacteriales
0
2
4
6
A (n=15)
B (n=16)
C (n=14)
surface

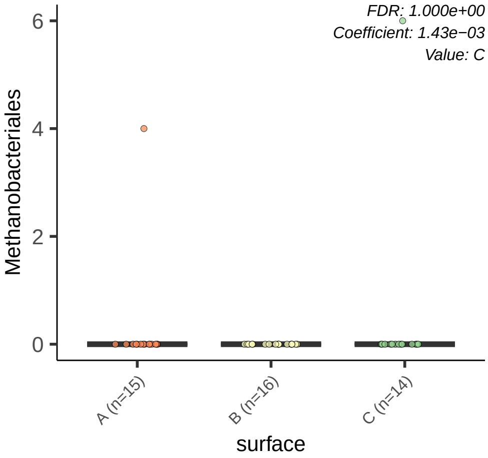

FDR: 1.000e+00
Coefficient: 1.43e−03
Value: C
Methanobacteriales
6
4
2
0
A (n=15)
B (n=16)
C (n=14)
surface

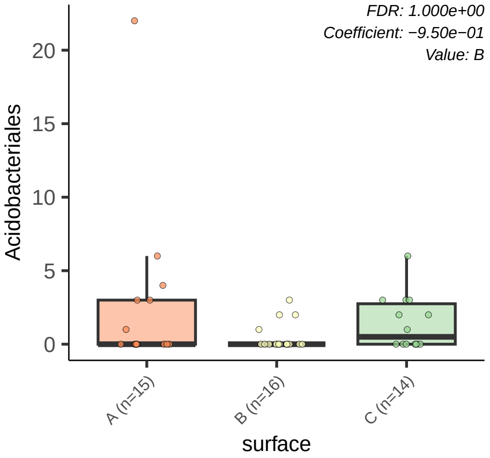
FDR: 1.000e+00
Coefficient: −9.50e−01
Value: B
Acidobacteriales
0
5
10
15
20
A (n=15)
B (n=16)
C (n=14)
surface

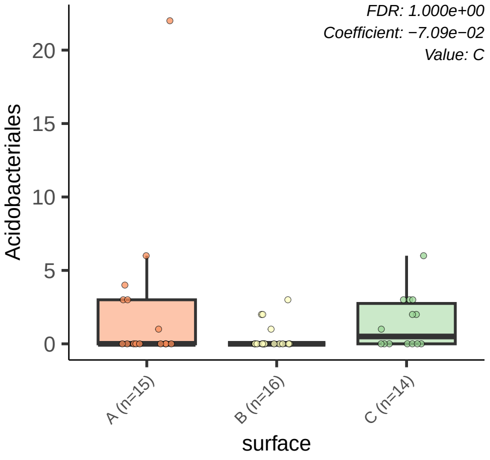
FDR: 1.000e+00
Coefficient: −7.09e−02
Value: C
Acidobacteriales
20
15
10
5
0
A (n=15)
B (n=16)
C (n=14)
surface

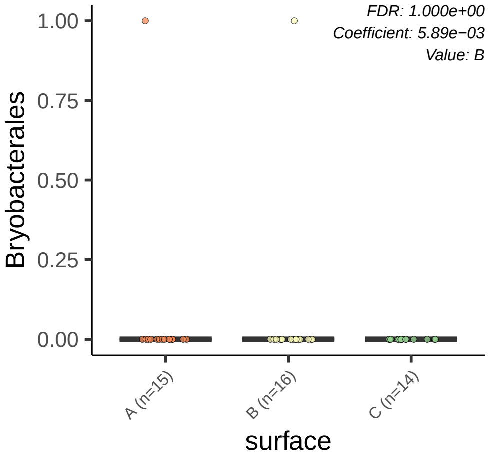
FDR: 1.000e+00
Coefficient: 5.89e−03
Value: B
Bryobacterales
1.00
0.75
0.50
0.25
0.00
A (n=15)
B (n=16)
C (n=14)
surface

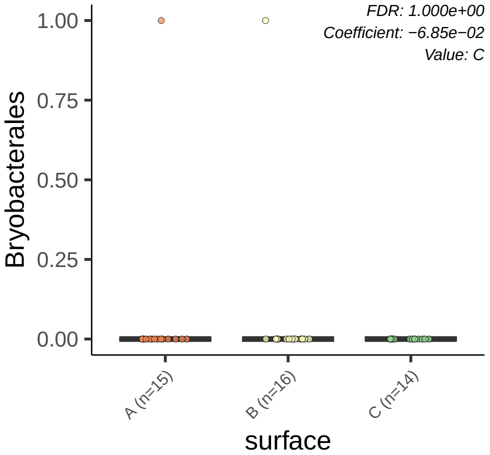
FDR: 1.000e+00
Coefficient: −6.85e−02
Value: C
Bryobacterales
1.00
0.75
0.50
0.25
0.00
A (n=15)
B (n=16)
C (n=14)
surface

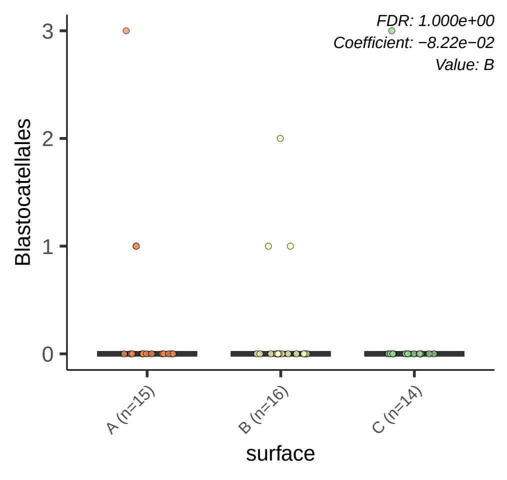

FDR: 1.000e+00
Coefficient: −8.22e−02
Value: B
Blastocatellales
0
1
2
3
A (n=15)
B (n=16)
C (n=14)
surface

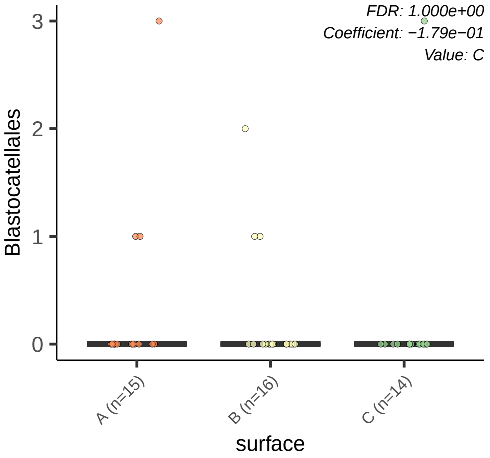

FDR: 1.000e+00
Coefficient: −1.79e−01
Value: C
Blastocatellales
3
2
1
0
A (n=15)
B (n=16)
C (n=14)
surface

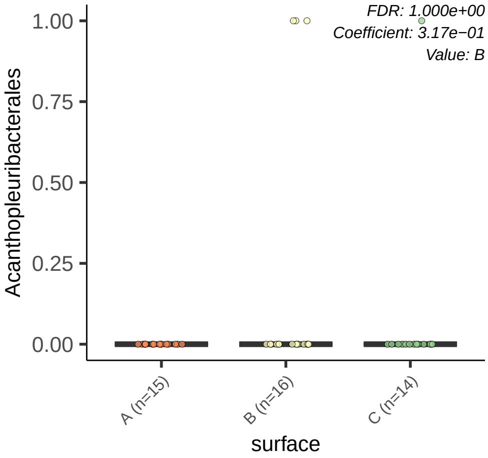
FDR: 1.000e+00
Coefficient: 3.17e−01
Value: B
Acanthopleuribacterales
1.00
0.75
0.50
0.25
0.00
A (n=15)
B (n=16)
C (n=14)
surface

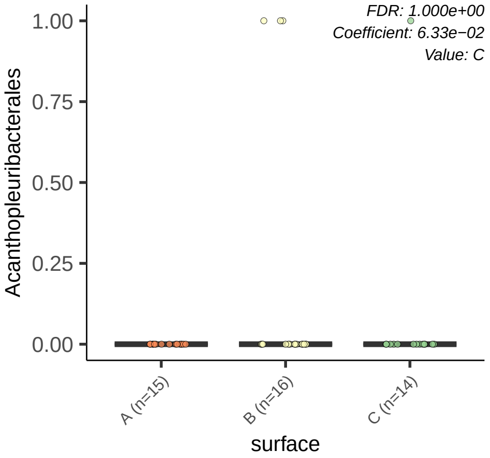

FDR: 1.000e+00
Coefficient: 6.33e−02
Value: C
Acanthopleuribacterales
1.00
0.75
0.50
0.25
0.00
A (n=15)
B (n=16)
C (n=14)
surface

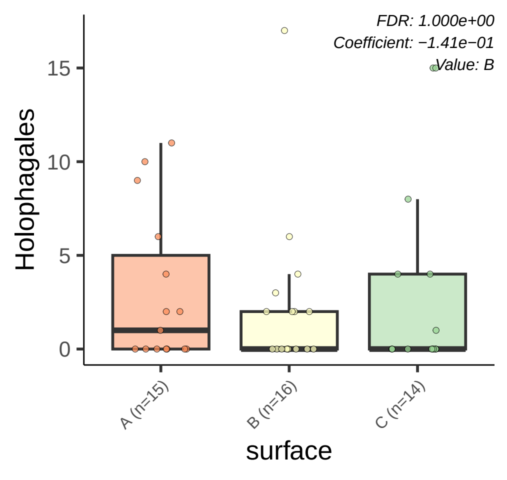
FDR: 1.000e+00
Coefficient: −1.41e−01
Value: B
Holophagales
0
5
10
15
A (n=15)
B (n=16)
C (n=14)
surface

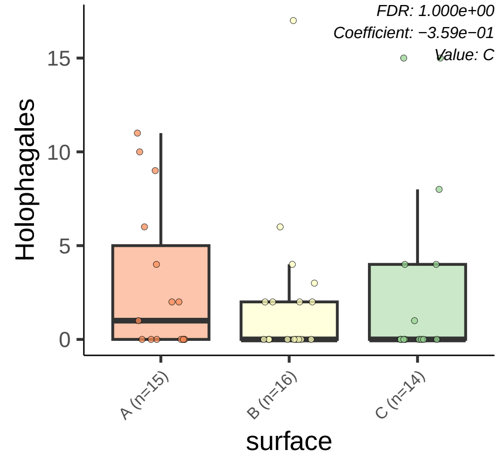
FDR: 1.000e+00
Coefficient: −3.59e−01
Value: C
Holophagales
0
5
10
15
A (n=15)
B (n=16)
C (n=14)
surface

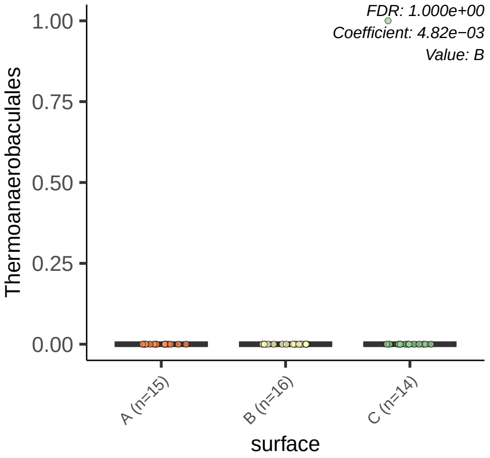
FDR: 1.000e+00
Coefficient: 4.82e−03
Value: B
Thermoanaerobaculales
1.00
0.75
0.50
0.25
0.00
A (n=15)
B (n=16)
C (n=14)
surface

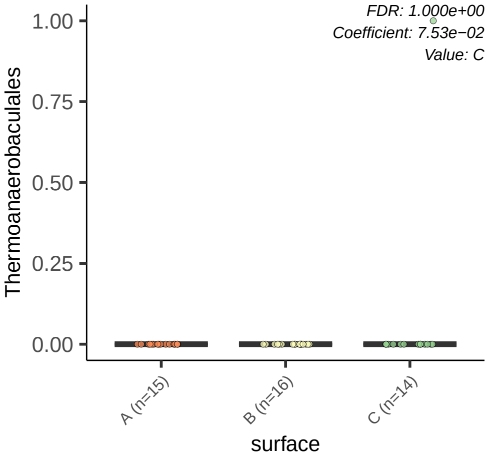
FDR: 1.000e+00
Coefficient: 7.53e−02
Value: C
Thermoanaerobaculales
1.00
0.75
0.50
0.25
0.00
A (n=15)
B (n=16)
C (n=14)
surface

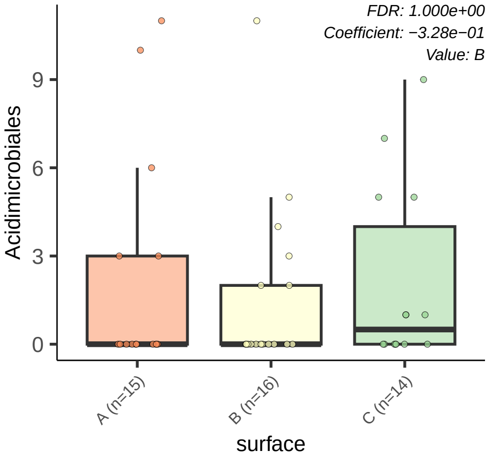

FDR: 1.000e+00
Coefficient: −3.28e−01
Value: B
Acidimicrobiales
0
3
6
9
A (n=15)
B (n=16)
C (n=14)
surface

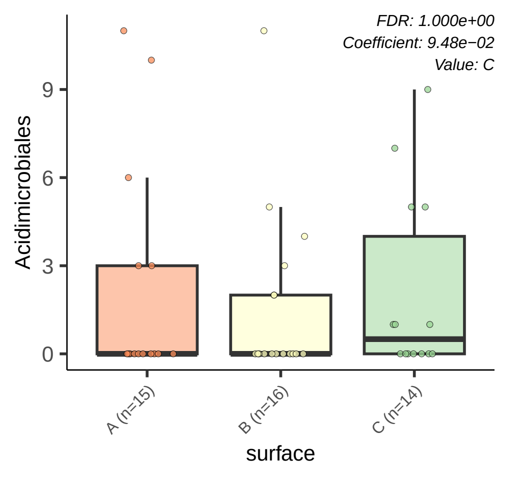
FDR: 1.000e+00
Coefficient: 9.48e−02
Value: C
Acidimicrobiales
0
3
6
9
A (n=15)
B (n=16)
C (n=14)
surface

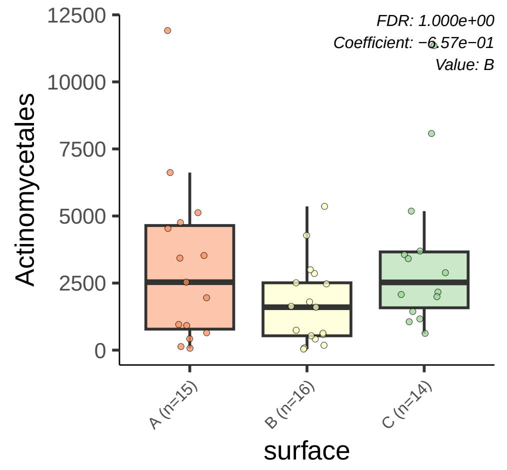
FDR: 1.000e+00
Coefficient: −6.57e−01
Value: B
Actinomycetales
12500
10000
7500
5000
2500
0
A (n=15)
B (n=16)
C (n=14)
surface

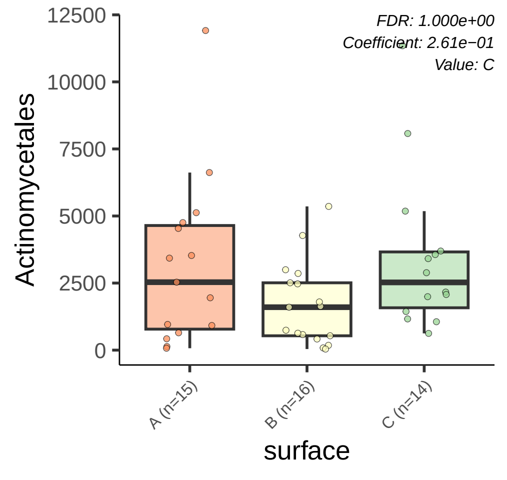

FDR: 1.000e+00
Coefficient: 2.61e−01
Value: C
Actinomycetales
12500
10000
7500
5000
2500
0
A (n=15)
B (n=16)
C (n=14)
surface

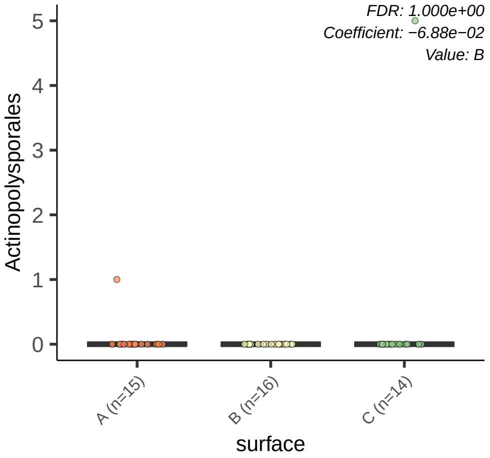
FDR: 1.000e+00
Coefficient: −6.88e−02
Value: B
Actinopolysporales
0
1
2
3
4
5
A (n=15)
B (n=16)
C (n=14)
surface

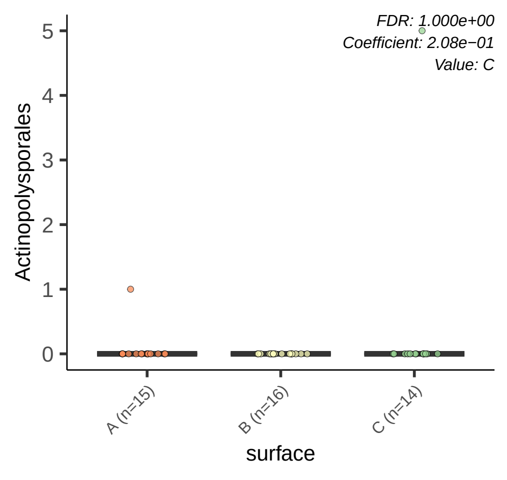
FDR: 1.000e+00
Coefficient: 2.08e−01
Value: C
Actinopolysporales
5
4
3
2
1
0
A (n=15)
B (n=16)
C (n=14)
surface

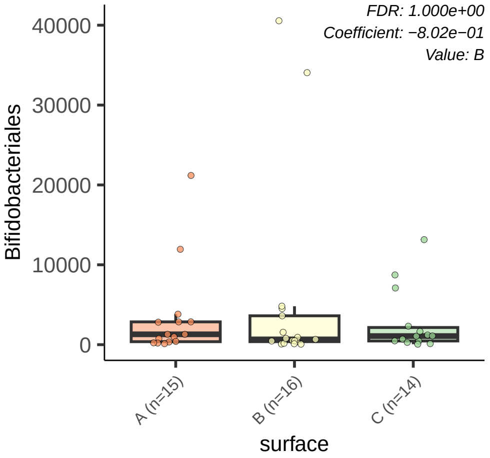
FDR: 1.000e+00
Coefficient: −8.02e−01
Value: B
Bifidobacteriales
40000
30000
20000
10000
0
A (n=15)
B (n=16)
C (n=14)
surface

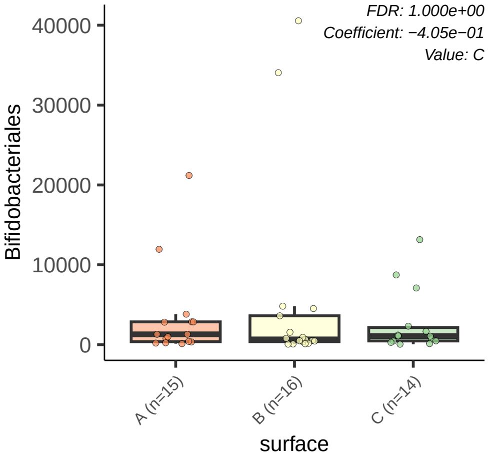
FDR: 1.000e+00
Coefficient: −4.05e−01
Value: C
Bifidobacteriales
40000
30000
20000
10000
0
A (n=15)
B (n=16)
C (n=14)
surface

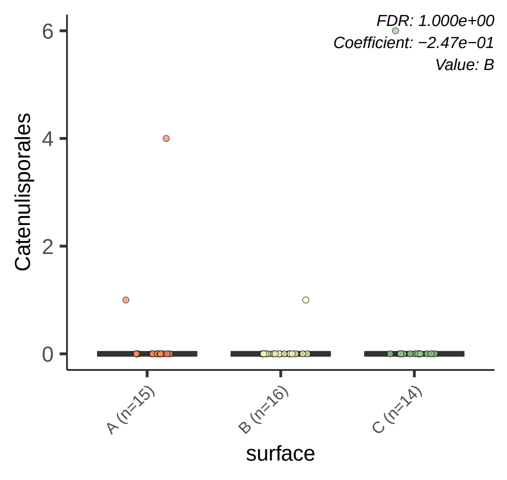
FDR: 1.000e+00
Coefficient: −2.47e−01
Value: B
Catenulisporales
0
2
4
6
A (n=15)
B (n=16)
C (n=14)
surface

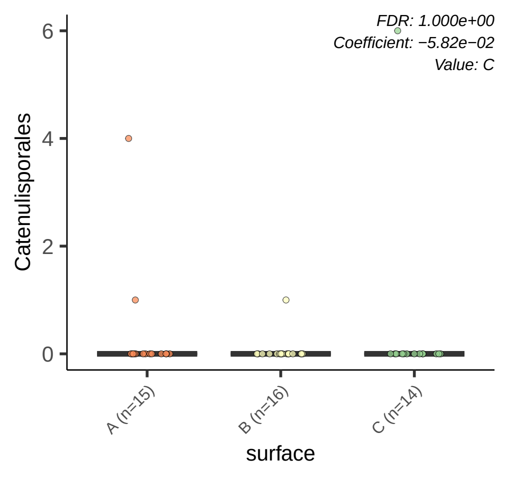

FDR: 1.000e+00
Coefficient: −5.82e−02
Value: C
Catenulisporales
0
2
4
6
A (n=15)
B (n=16)
C (n=14)
surface

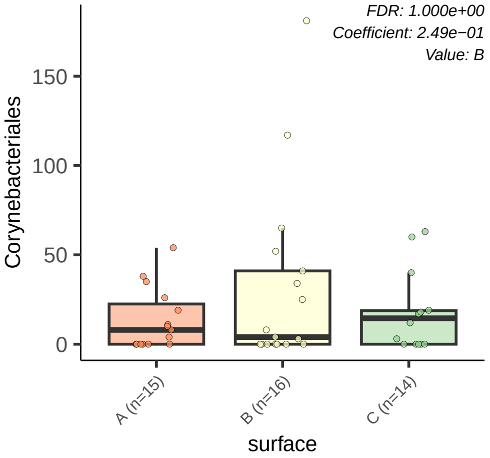
FDR: 1.000e+00
Coefficient: 2.49e−01
Value: B
Corynebacteriales
150
100
50
0
A (n=15)
B (n=16)
C (n=14)
surface

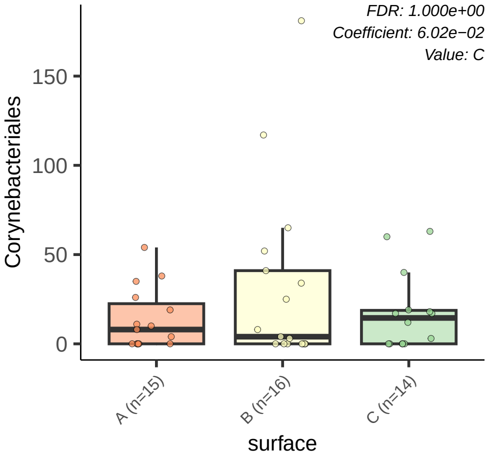

FDR: 1.000e+00
Coefficient: 6.02e−02
Value: C
Corynebacteriales
150
100
50
0
A (n=15)
B (n=16)
C (n=14)
surface

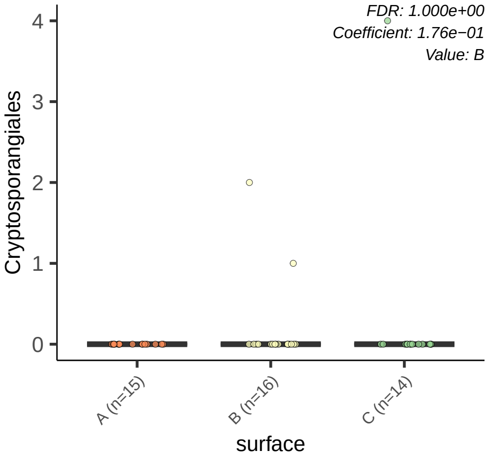
FDR: 1.000e+00
Coefficient: 1.76e−01
Value: B
Cryptosporangiales
0
1
2
3
4
A (n=15)
B (n=16)
C (n=14)
surface

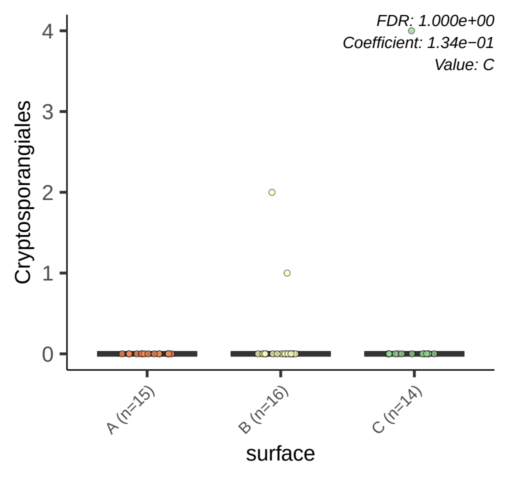

FDR: 1.000e+00
Coefficient: 1.34e−01
Value: C
Cryptosporangiales
0
1
2
3
4
A (n=15)
B (n=16)
C (n=14)
surface

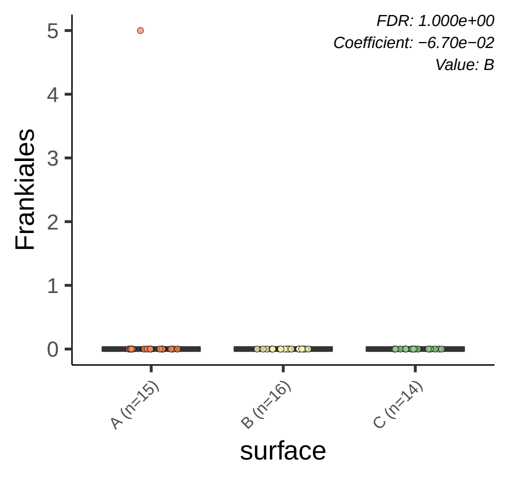

FDR: 1.000e+00
Coefficient: −6.70e−02
Value: B
Frankiales
5
4
3
2
1
0
A (n=15)
B (n=16)
C (n=14)
surface

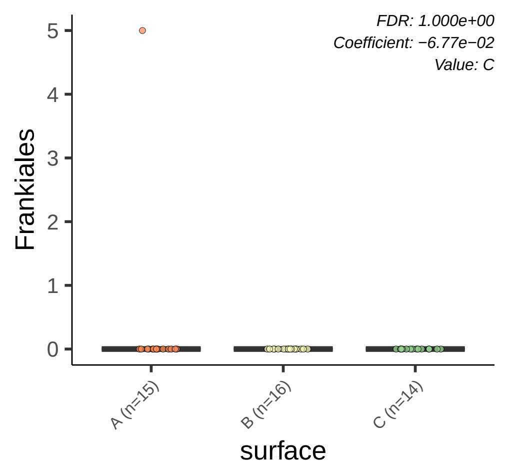

FDR: 1.000e+00
Coefficient: −6.77e−02
Value: C
Frankiales
5
4
3
2
1
0
A (n=15)
B (n=16)
C (n=14)
surface

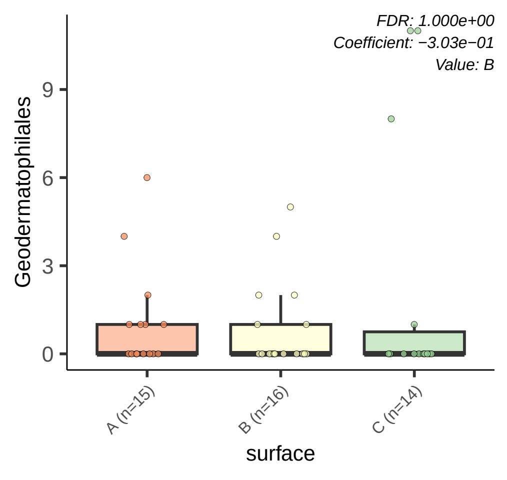
FDR: 1.000e+00
Coefficient: −3.03e−01
Value: B
Geodermatophilales
0
3
6
9
A (n=15)
B (n=16)
C (n=14)
surface

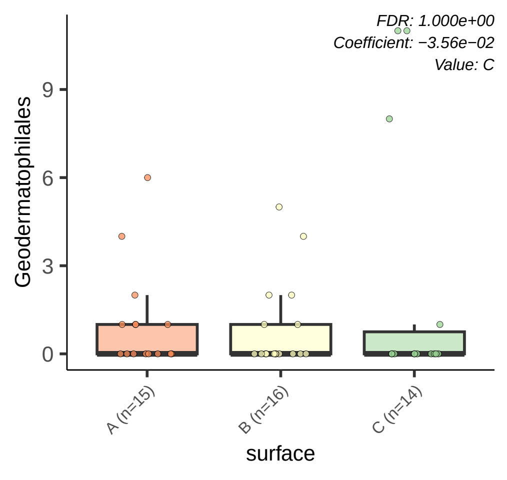
FDR: 1.000e+00
Coefficient: −3.56e−02
Value: C
Geodermatophilales
0
3
6
9
A (n=15)
B (n=16)
C (n=14)
surface

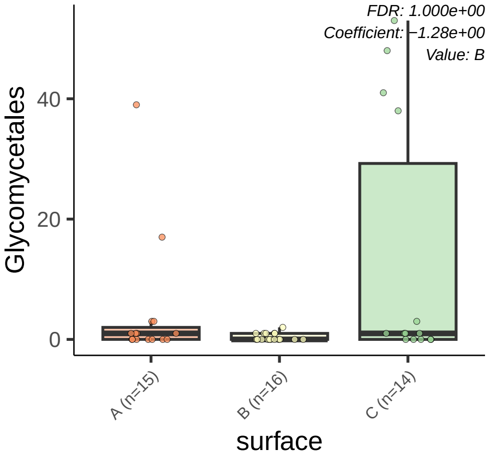

FDR: 1.000e+00
Coefficient: −1.28e+00
Value: B
Glycomycetales
0
20
40
A (n=15)
B (n=16)
C (n=14)
surface

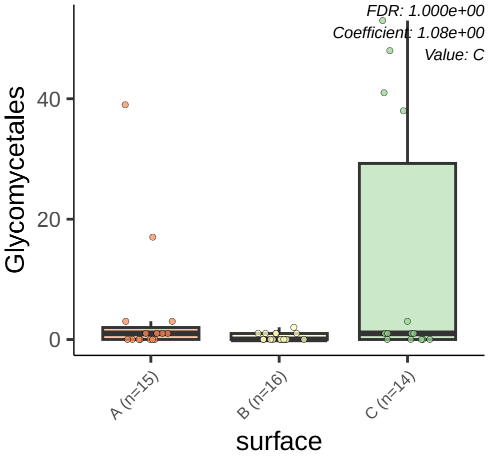

FDR: 1.000e+00
Coefficient: 1.08e+00
Value: C
Glycomycetales
40
20
0
A (n=15)
B (n=16)
C (n=14)
surface

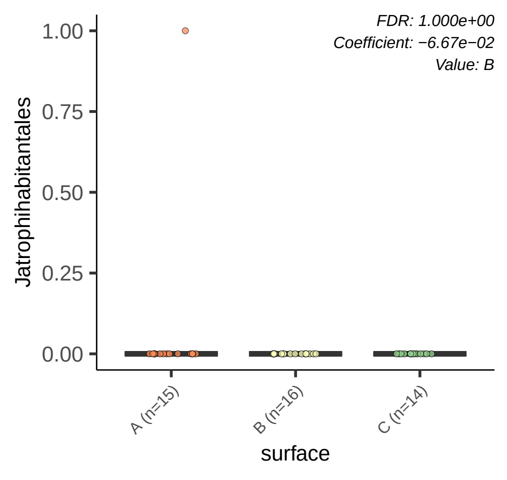
FDR: 1.000e+00
Coefficient: −6.67e−02
Value: B
Jatrophihabitantales
1.00
0.75
0.50
0.25
0.00
A (n=15)
B (n=16)
C (n=14)
surface

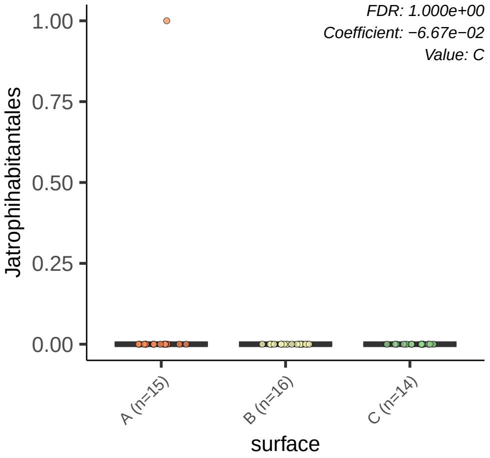

FDR: 1.000e+00
Coefficient: −6.67e−02
Value: C
Jatrophihabitantales
1.00
0.75
0.50
0.25
0.00
A (n=15)
B (n=16)
C (n=14)
surface

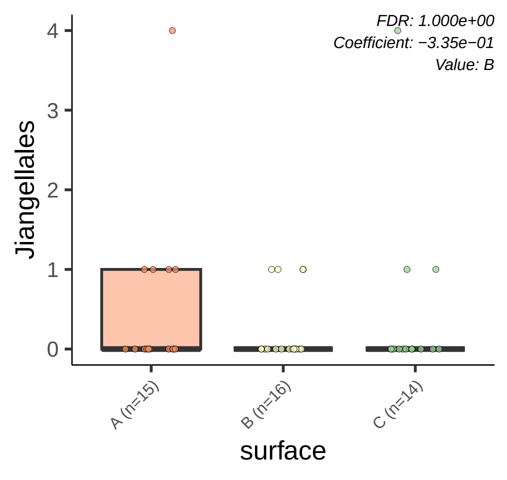
FDR: 1.000e+00
Coefficient: −3.35e−01
Value: B
Jiangellales
4
3
2
1
0
A (n=15)
B (n=16)
C (n=14)
surface

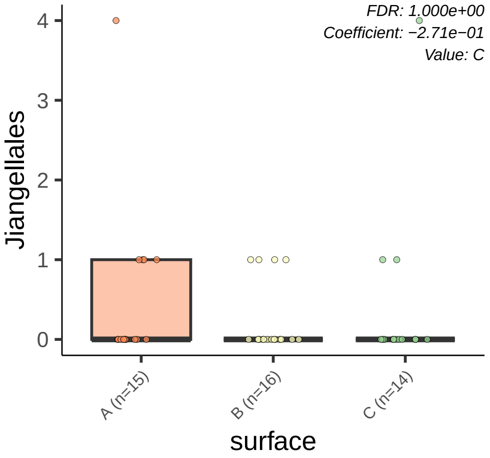

FDR: 1.000e+00
Coefficient: −2.71e−01
Value: C
Jiangellales
4
3
2
1
0
A (n=15)
B (n=16)
C (n=14)
surface

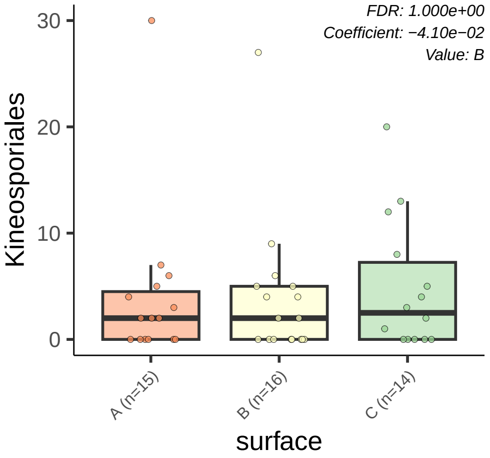
FDR: 1.000e+00
Coefficient: −4.10e−02
Value: B
Kineosporiales
30
20
10
0
A (n=15)
B (n=16)
C (n=14)
surface

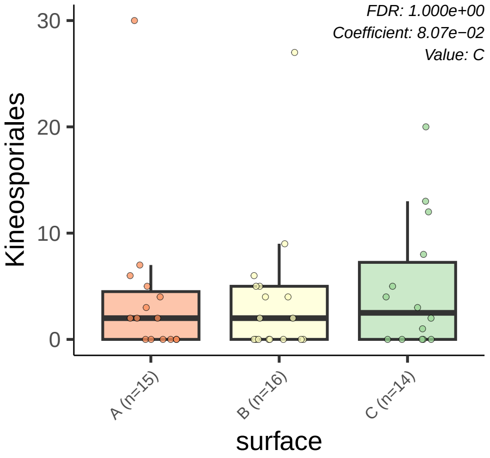
FDR: 1.000e+00
Coefficient: 8.07e−02
Value: C
Kineosporiales
30
20
10
0
A (n=15)
B (n=16)
C (n=14)
surface

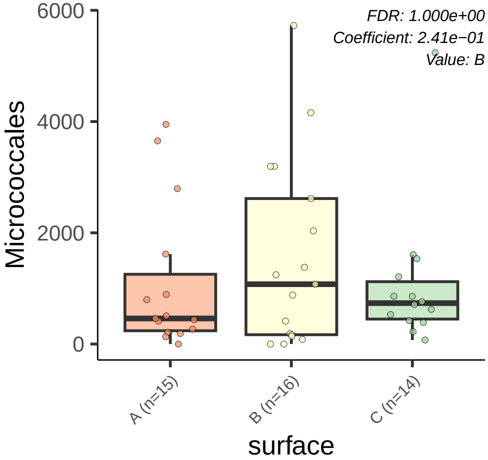
FDR: 1.000e+00
Coefficient: 2.41e−01
Value: B
Micrococcales
6000
4000
2000
0
A (n=15)
B (n=16)
C (n=14)
surface

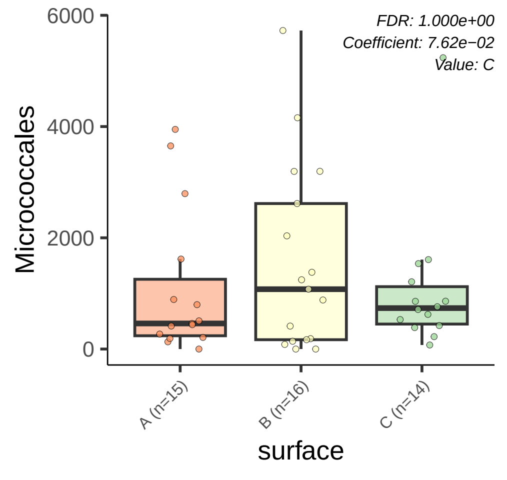

FDR: 1.000e+00
Coefficient: 7.62e−02
Value: C
Micrococcales
6000
4000
2000
0
A (n=15)
B (n=16)
C (n=14)
surface

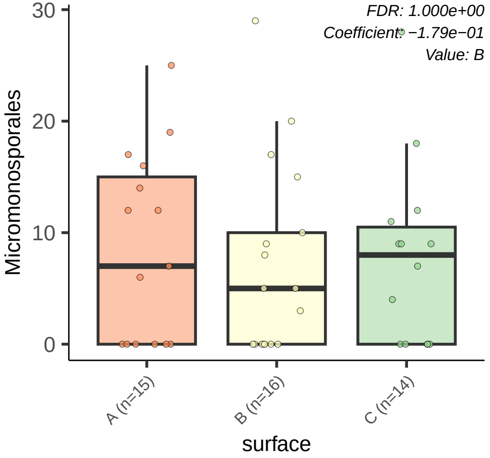

FDR: 1.000e+00
Coefficient: −1.79e−01
Value: B
Micromonosporales
30
20
10
0
A (n=15)
B (n=16)
C (n=14)
surface

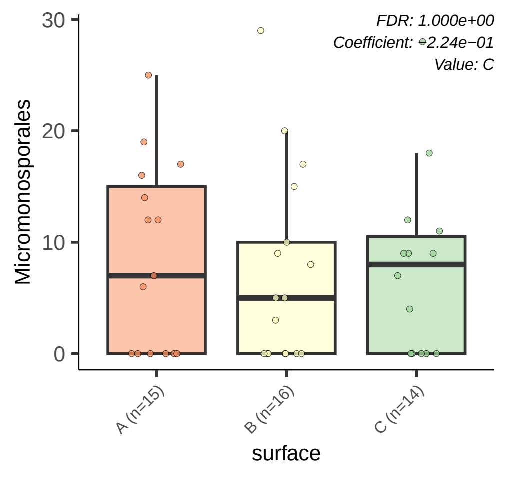
FDR: 1.000e+00
Coefficient: −2.24e−01
Value: C
Micromonosporales
30
20
10
0
A (n=15)
B (n=16)
C (n=14)
surface

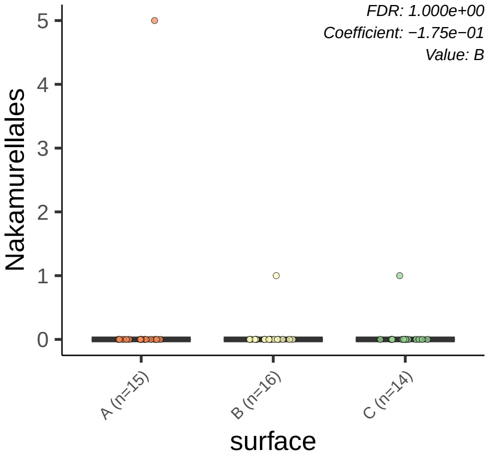
FDR: 1.000e+00
Coefficient: −1.75e−01
Value: B
Nakamurellales
0
1
2
3
4
5
A (n=15)
B (n=16)
C (n=14)
surface

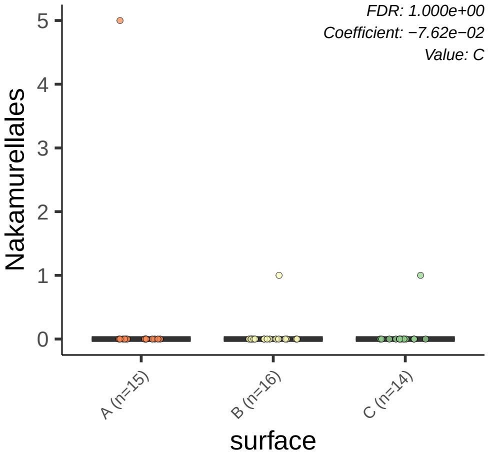
FDR: 1.000e+00
Coefficient: −7.62e−02
Value: C
Nakamurellales
5
4
3
2
1
0
A (n=15)
B (n=16)
C (n=14)
surface

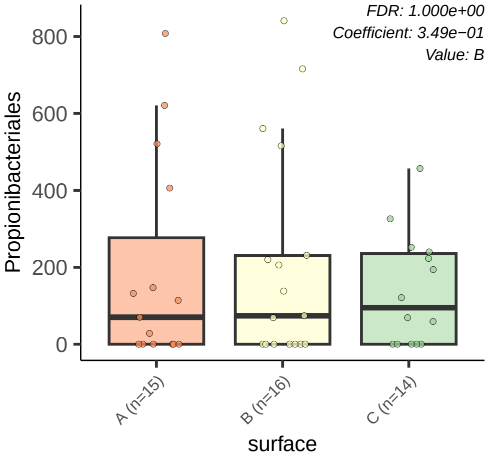

FDR: 1.000e+00
Coefficient: 3.49e−01
Value: B
Propionibacteriales
800
600
400
200
0
A (n=15)
B (n=16)
C (n=14)
surface

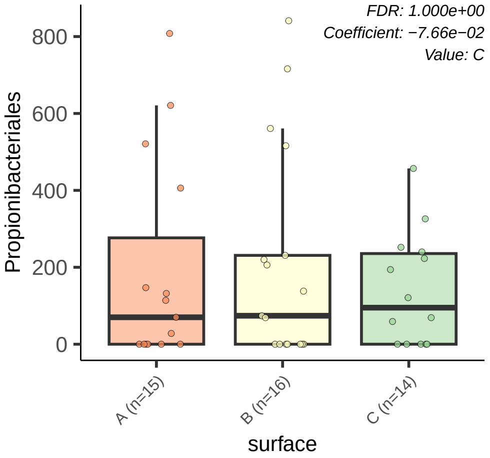
FDR: 1.000e+00
Coefficient: −7.66e−02
Value: C
Propionibacteriales
800
600
400
200
0
A (n=15)
B (n=16)
C (n=14)
surface

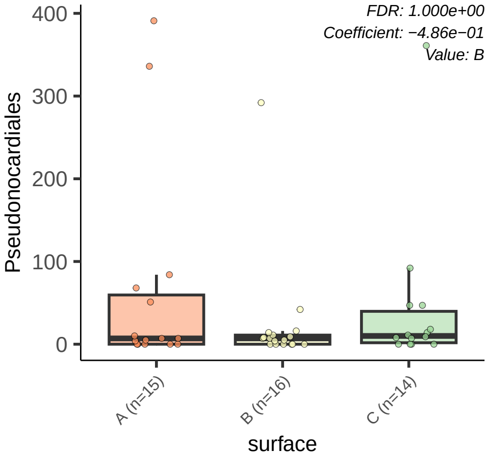
FDR: 1.000e+00
Coefficient: −4.86e−01
Value: B
Pseudonocardiales
400
300
200
100
0
A (n=15)
B (n=16)
C (n=14)
surface

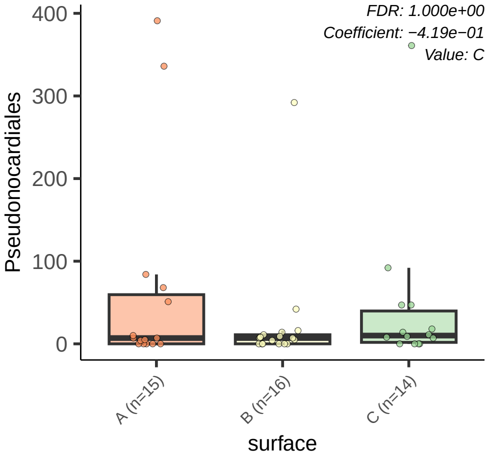

FDR: 1.000e+00
Coefficient: −4.19e−01
Value: C
Pseudonocardiales
400
300
200
100
0
A (n=15)
B (n=16)
C (n=14)
surface

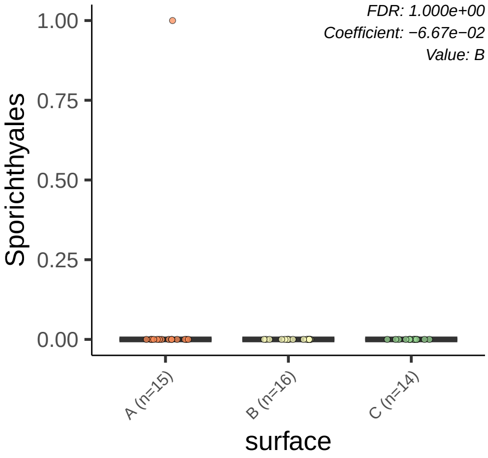

FDR: 1.000e+00
Coefficient: −6.67e−02
Value: B
Sporichthyales
1.00
0.75
0.50
0.25
0.00
A (n=15)
B (n=16)
C (n=14)
surface

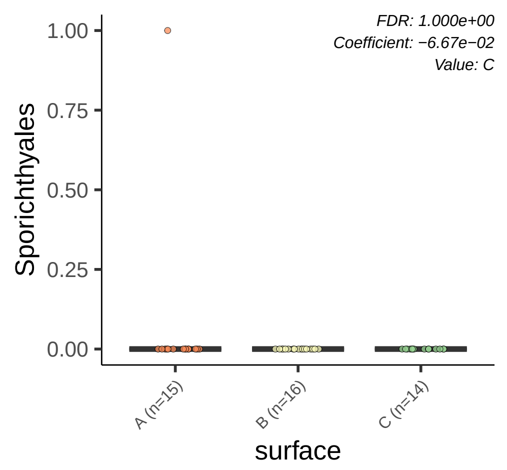
FDR: 1.000e+00
Coefficient: −6.67e−02
Value: C
Sporichthyales
1.00
0.75
0.50
0.25
0.00
A (n=15)
B (n=16)
C (n=14)
surface

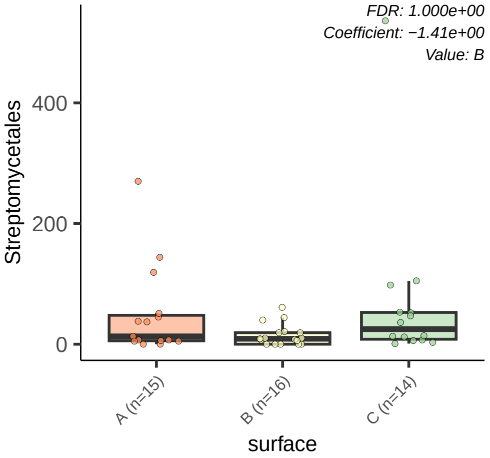
FDR: 1.000e+00
Coefficient: −1.41e+00
Value: B
Streptomycetales
400
200
0
A (n=15)
B (n=16)
C (n=14)
surface

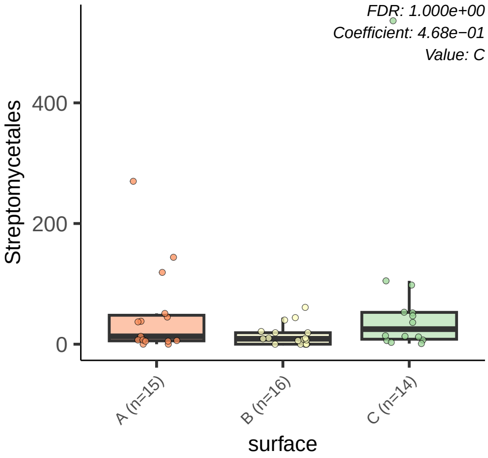

FDR: 1.000e+00
Coefficient: 4.68e−01
Value: C
Streptomycetales
400
200
0
A (n=15)
B (n=16)
C (n=14)
surface

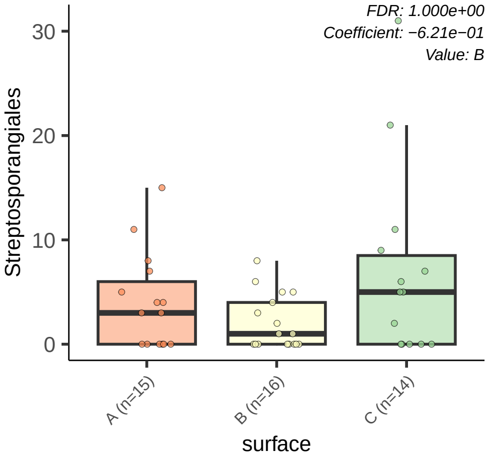
FDR: 1.000e+00
Coefficient: −6.21e−01
Value: B
Streptosporangiales
30
20
10
0
A (n=15)
B (n=16)
C (n=14)
surface

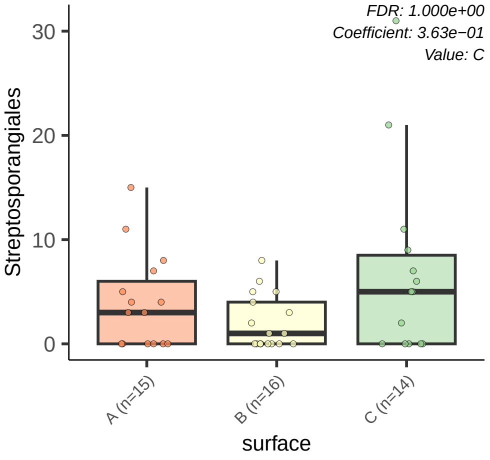

FDR: 1.000e+00
Coefficient: 3.63e−01
Value: C
Streptosporangiales
30
20
10
0
A (n=15)
B (n=16)
C (n=14)
surface

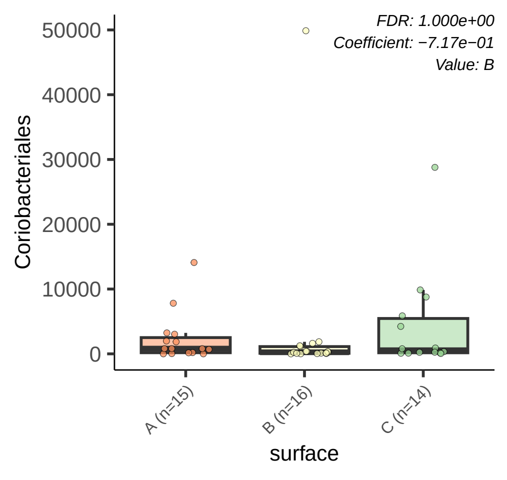
FDR: 1.000e+00
Coefficient: −7.17e−01
Value: B
Coriobacteriales
50000
40000
30000
20000
10000
0
A (n=15)
B (n=16)
C (n=14)
surface

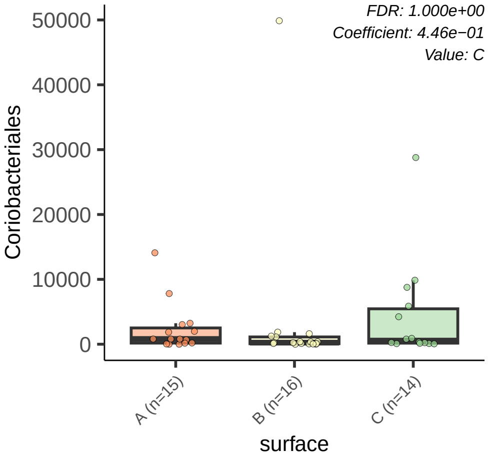
FDR: 1.000e+00
Coefficient: 4.46e−01
Value: C
Coriobacteriales
50000
40000
30000
20000
10000
0
A (n=15)
B (n=16)
C (n=14)
surface

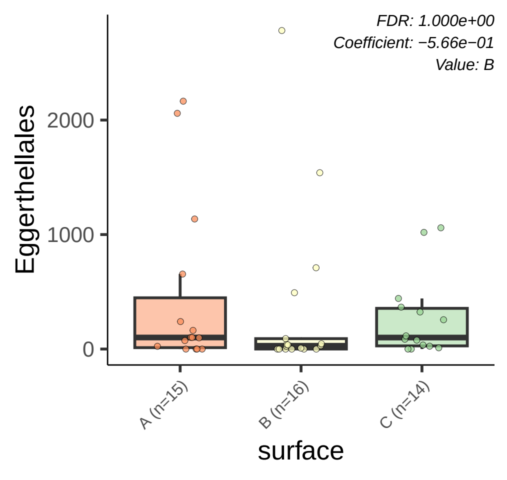
FDR: 1.000e+00
Coefficient: −5.66e−01
Value: B
Eggerthellales
2000
1000
0
A (n=15)
B (n=16)
C (n=14)
surface

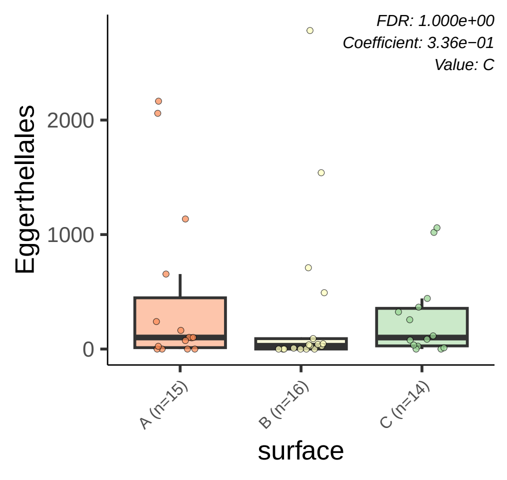
FDR: 1.000e+00
Coefficient: 3.36e−01
Value: C
Eggerthellales
2000
1000
0
A (n=15)
B (n=16)
C (n=14)
surface

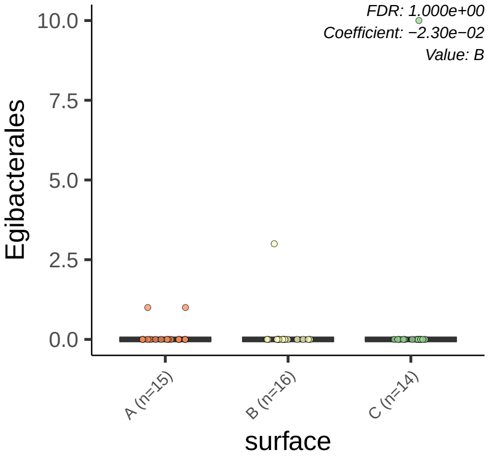

FDR: 1.000e+00
Coefficient: −2.30e−02
Value: B
Egibacterales
10.0
7.5
5.0
2.5
0.0
A (n=15)
B (n=16)
C (n=14)
surface

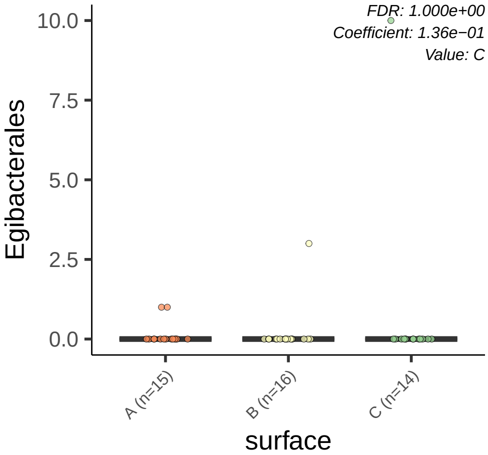
FDR: 1.000e+00
Coefficient: 1.36e−01
Value: C
Egibacterales
10.0
7.5
5.0
2.5
0.0
A (n=15)
B (n=16)
C (n=14)
surface

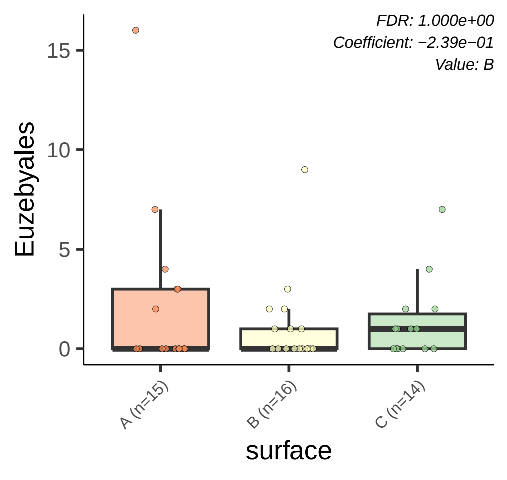

FDR: 1.000e+00
Coefficient: −2.39e−01
Value: B
Euzebyales
15
10
5
0
A (n=15)
B (n=16)
C (n=14)
surface

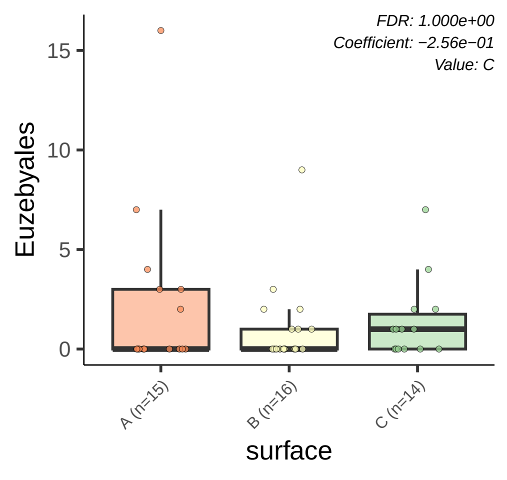
FDR: 1.000e+00
Coefficient: −2.56e−01
Value: C
Euzebyales
15
10
5
0
A (n=15)
B (n=16)
C (n=14)
surface

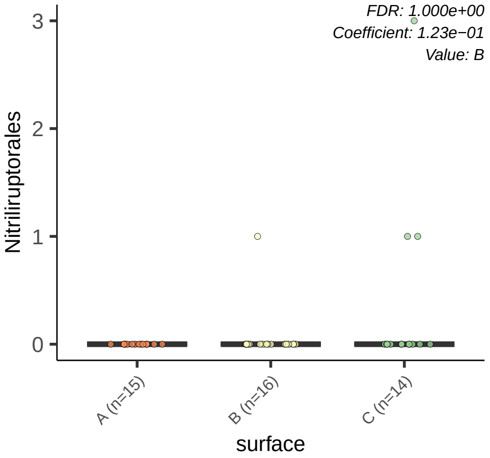
FDR: 1.000e+00
Coefficient: 1.23e−01
Value: B
Nitriliruptorales
3
2
1
0
A (n=15)
B (n=16)
C (n=14)
surface

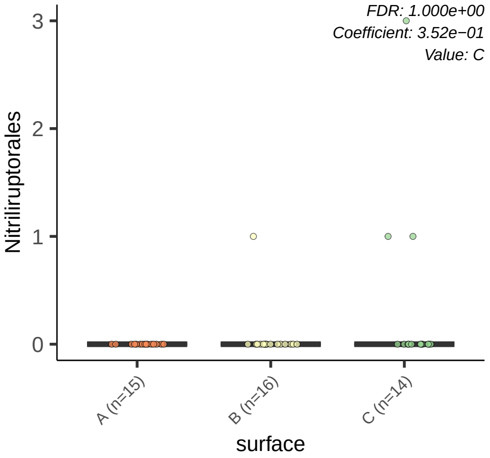
FDR: 1.000e+00
Coefficient: 3.52e−01
Value: C
Nitriliruptorales
0
1
2
3
A (n=15)
B (n=16)
C (n=14)
surface

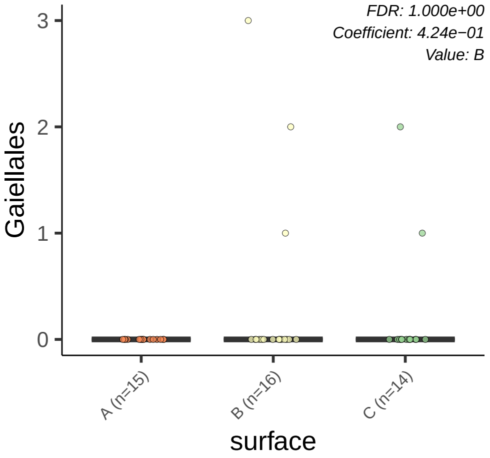
FDR: 1.000e+00
Coefficient: 4.24e−01
Value: B
Gaiellales
3
2
1
0
A (n=15)
B (n=16)
C (n=14)
surface

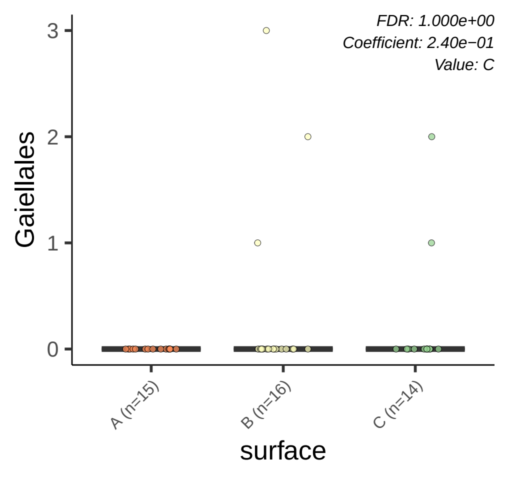

FDR: 1.000e+00
Coefficient: 2.40e−01
Value: C
Gaiellales
3
2
1
0
A (n=15)
B (n=16)
C (n=14)
surface

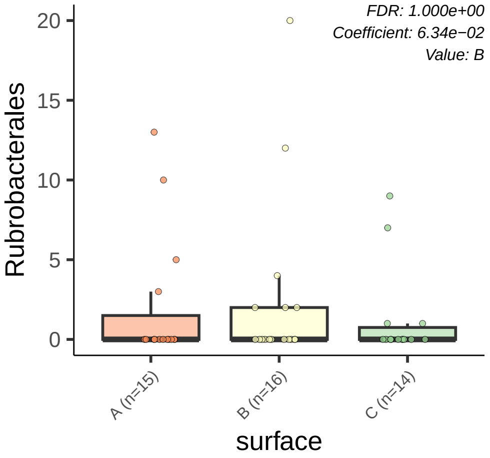
FDR: 1.000e+00
Coefficient: 6.34e−02
Value: B
Rubrobacterales
20
15
10
5
0
A (n=15)
B (n=16)
C (n=14)
surface

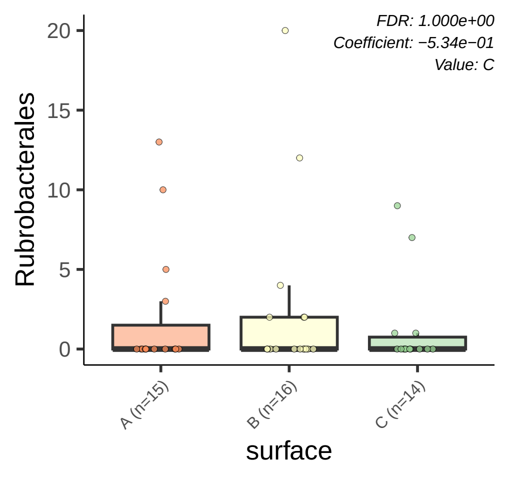
FDR: 1.000e+00
Coefficient: −5.34e−01
Value: C
Rubrobacterales
20
15
10
5
0
A (n=15)
B (n=16)
C (n=14)
surface

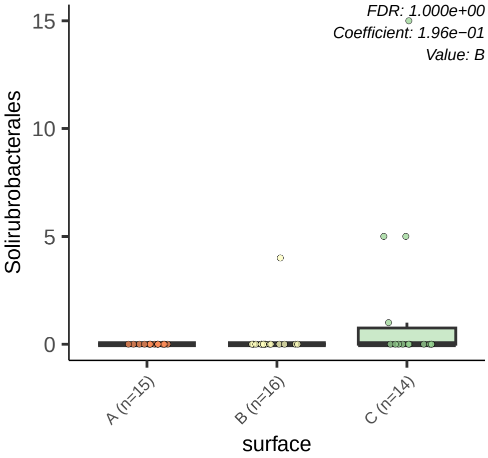
FDR: 1.000e+00
Coefficient: 1.96e−01
Value: B
Solirubrobacterales
15
10
5
0
A (n=15)
B (n=16)
C (n=14)
surface

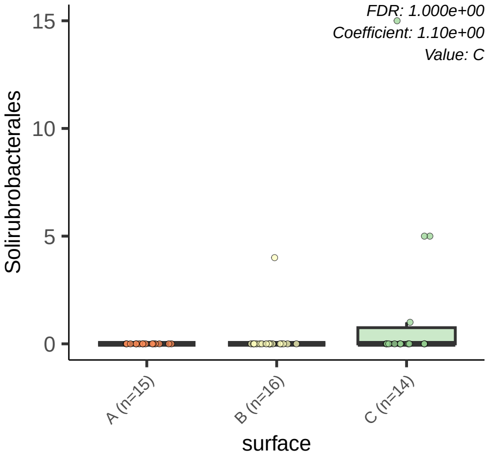

FDR: 1.000e+00
Coefficient: 1.10e+00
Value: C
Solirubrobacterales
15
10
5
0
A (n=15)
B (n=16)
C (n=14)
surface

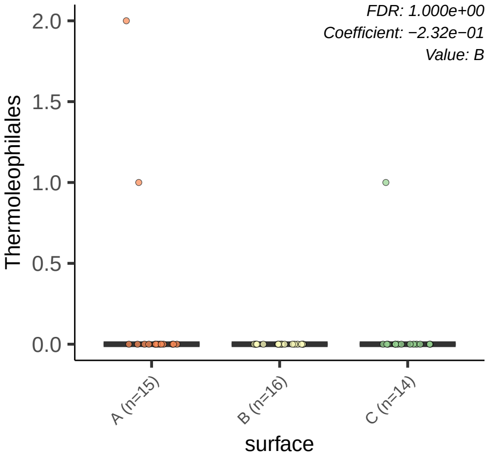

FDR: 1.000e+00
Coefficient: −2.32e−01
Value: B
Thermoleophilales
2.0
1.5
1.0
0.5
0.0
A (n=15)
B (n=16)
C (n=14)
surface

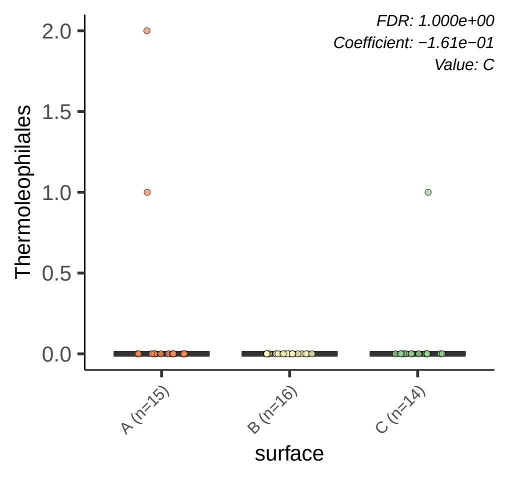

FDR: 1.000e+00
Coefficient: −1.61e−01
Value: C
Thermoleophilales
2.0
1.5
1.0
0.5
0.0
A (n=15)
B (n=16)
C (n=14)
surface

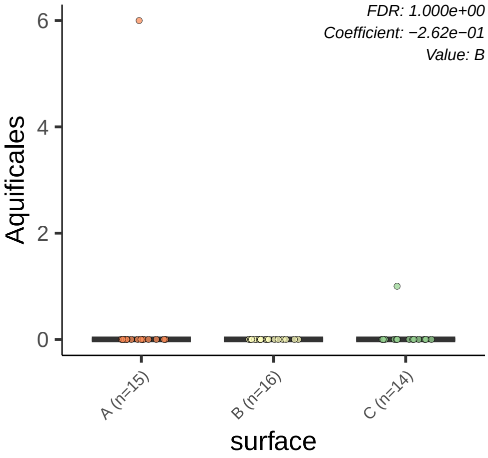
FDR: 1.000e+00
Coefficient: −2.62e−01
Value: B
Aquificales
6
4
2
0
A (n=15)
B (n=16)
C (n=14)
surface

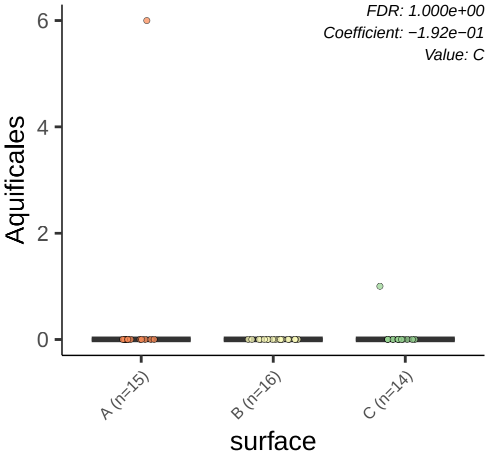

FDR: 1.000e+00
Coefficient: −1.92e−01
Value: C
Aquificales
0
2
4
6
A (n=15)
B (n=16)
C (n=14)
surface

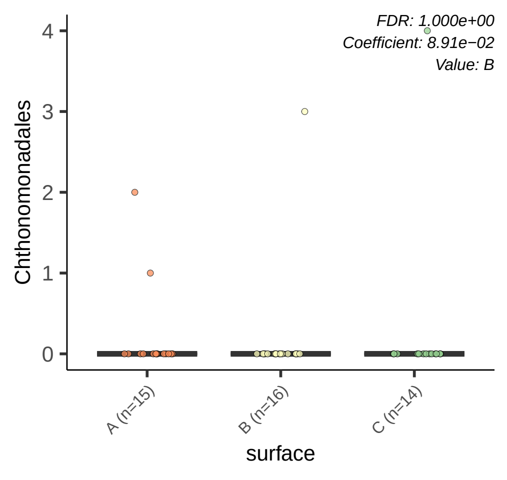
FDR: 1.000e+00
Coefficient: 8.91e−02
Value: B
Chthonomonadales
4
3
2
1
0
A (n=15)
B (n=16)
C (n=14)
surface

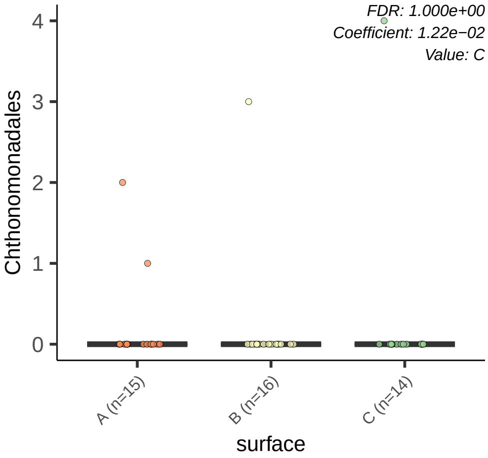
FDR: 1.000e+00
Coefficient: 1.22e−02
Value: C
Chthonomonadales
0
1
2
3
4
A (n=15)
B (n=16)
C (n=14)
surface

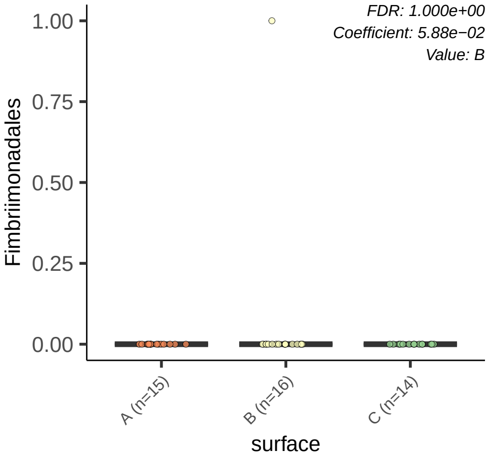
FDR: 1.000e+00
Coefficient: 5.88e−02
Value: B
1.00
0.75
0.50
0.25
0.00
Fimbriimonadales
A (n=15)
B (n=16)
C (n=14)
surface

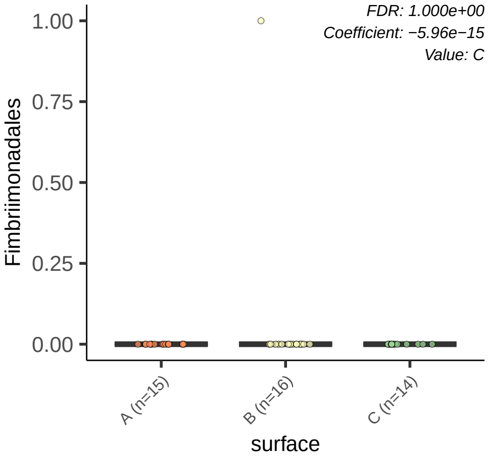

FDR: 1.000e+00
Coefficient: −5.96e−15
Value: C
Fimbriimonadales
1.00
0.75
0.50
0.25
0.00
A (n=15)
B (n=16)
C (n=14)
surface

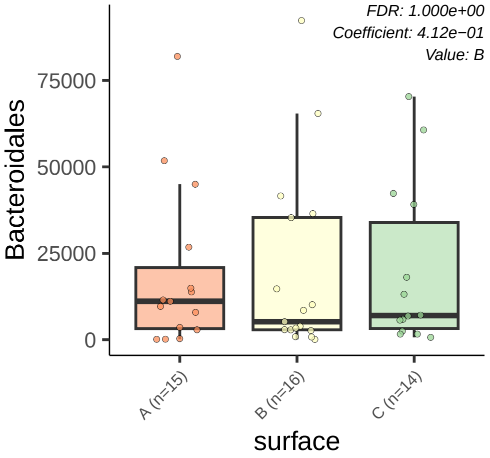
FDR: 1.000e+00
Coefficient: 4.12e−01
Value: B
Bacteroidales
75000
50000
25000
0
A (n=15)
B (n=16)
C (n=14)
surface

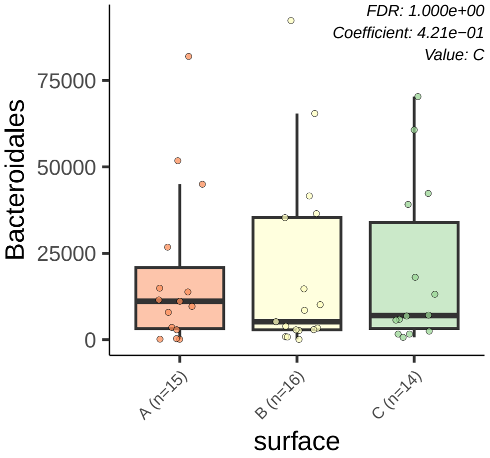
FDR: 1.000e+00
Coefficient: 4.21e−01
Value: C
Bacteroidales
75000
50000
25000
0
A (n=15)
B (n=16)
C (n=14)
surface

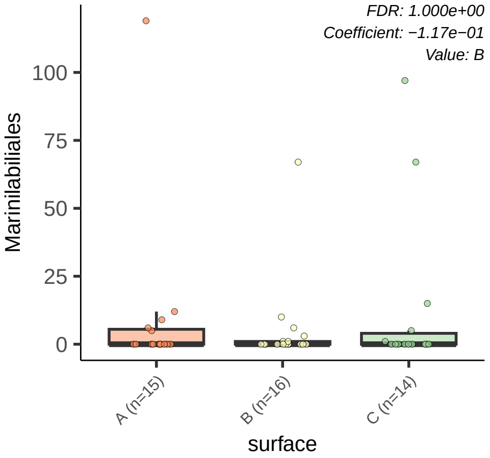

FDR: 1.000e+00
Coefficient: −1.17e−01
Value: B
Marinilabiliales
100
75
50
25
0
A (n=15)
B (n=16)
C (n=14)
surface

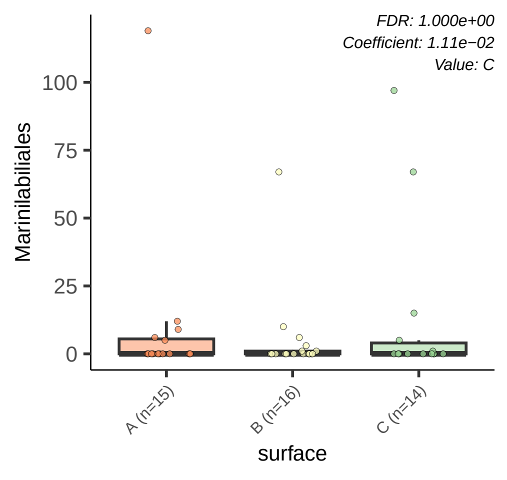
FDR: 1.000e+00
Coefficient: 1.11e−02
Value: C
Marinilabiliales
100
75
50
25
0
A (n=15)
B (n=16)
C (n=14)
surface

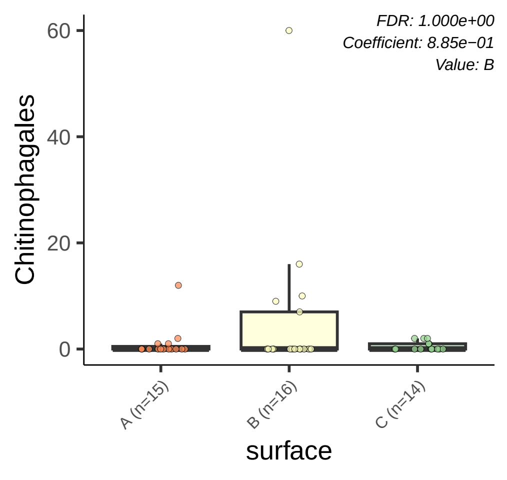

FDR: 1.000e+00
Coefficient: 8.85e−01
Value: B
Chitinophagales
0
20
40
60
A (n=15)
B (n=16)
C (n=14)
surface

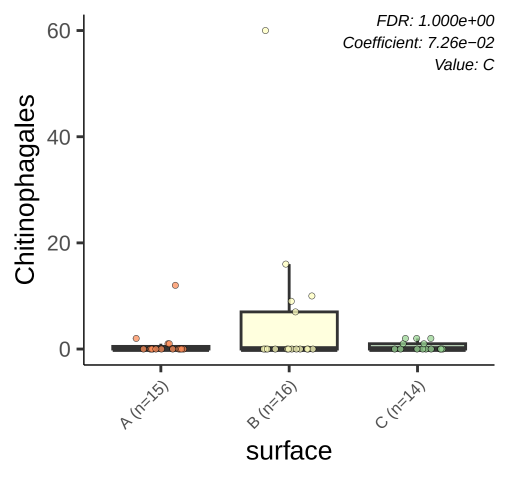
FDR: 1.000e+00
Coefficient: 7.26e−02
Value: C
Chitinophagales
60
40
20
0
A (n=15)
B (n=16)
C (n=14)
surface

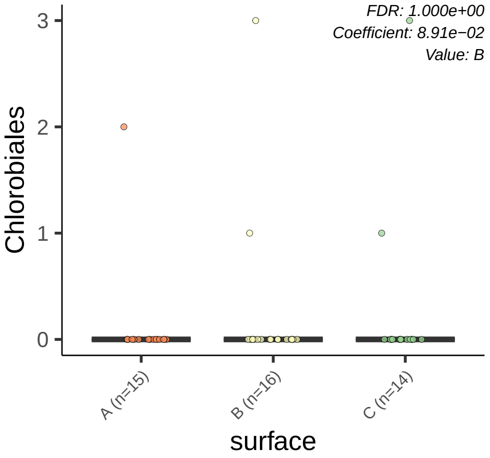

FDR: 1.000e+00
Coefficient: 8.91e−02
Value: B
Chlorobiales
3
2
1
0
A (n=15)
B (n=16)
C (n=14)
surface

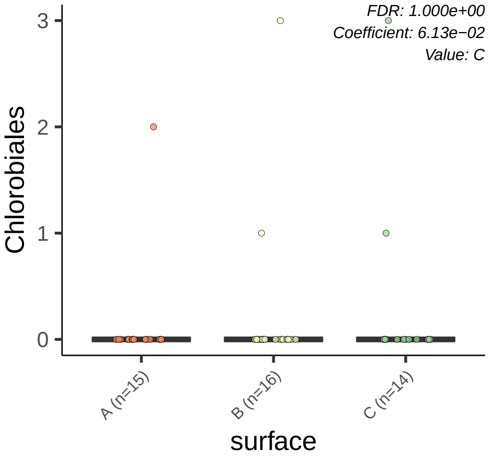
FDR: 1.000e+00
Coefficient: 6.13e−02
Value: C
Chlorobiales
3
2
1
0
A (n=15)
B (n=16)
C (n=14)
surface

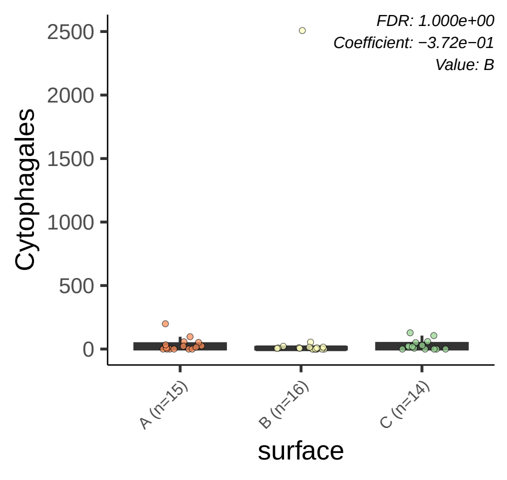

FDR: 1.000e+00
Coefficient: −3.72e−01
Value: B
Cytophagales
2500
2000
1500
1000
500
0
A (n=15)
B (n=16)
C (n=14)
surface

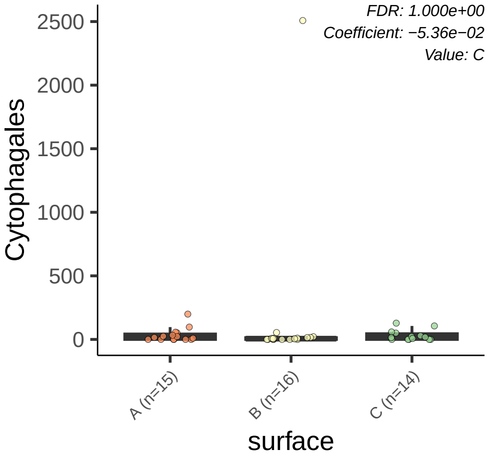
FDR: 1.000e+00
Coefficient: −5.36e−02
Value: C
Cytophagales
2500
2000
1500
1000
500
0
A (n=15)
B (n=16)
C (n=14)
surface

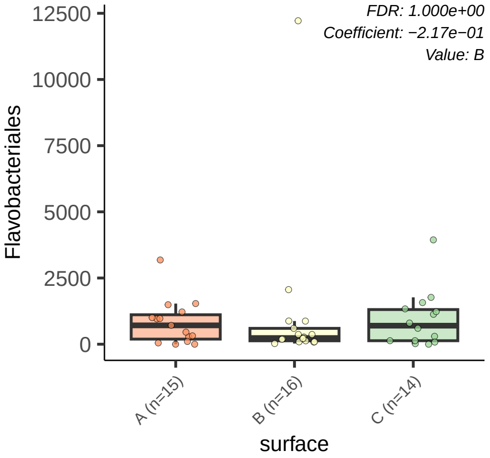

FDR: 1.000e+00
Coefficient: −2.17e−01
Value: B
Flavobacteriales
12500
10000
7500
5000
2500
0
A (n=15)
B (n=16)
C (n=14)
surface

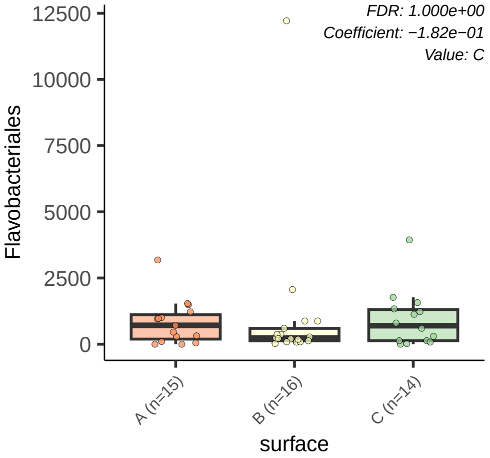
FDR: 1.000e+00
Coefficient: −1.82e−01
Value: C
12500
10000
7500
5000
2500
0
Flavobacteriales
A (n=15)
B (n=16)
C (n=14)
surface

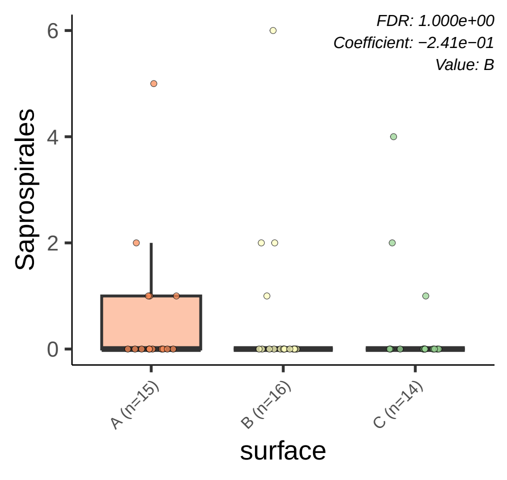

FDR: 1.000e+00
Coefficient: −2.41e−01
Value: B
Saprospirales
6
4
2
0
A (n=15)
B (n=16)
C (n=14)
surface

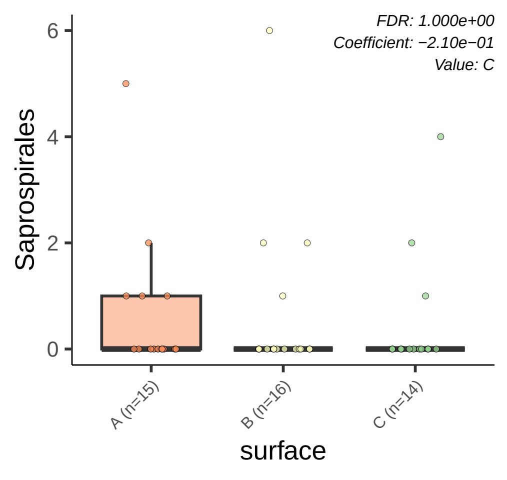
FDR: 1.000e+00
Coefficient: −2.10e−01
Value: C
Saprospirales
0
2
4
6
A (n=15)
B (n=16)
C (n=14)
surface

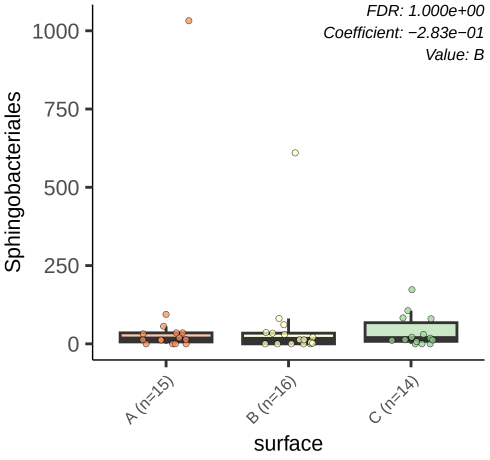
FDR: 1.000e+00
Coefficient: −2.83e−01
Value: B
Sphingobacteriales
1000
750
500
250
0
A (n=15)
B (n=16)
C (n=14)
surface

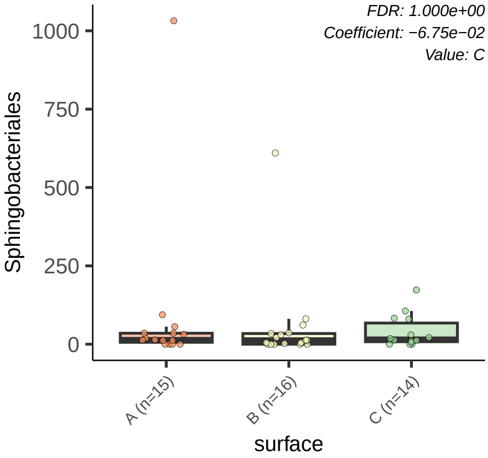
FDR: 1.000e+00
Coefficient: −6.75e−02
Value: C
Sphingobacteriales
1000
750
500
250
0
A (n=15)
B (n=16)
C (n=14)
surface

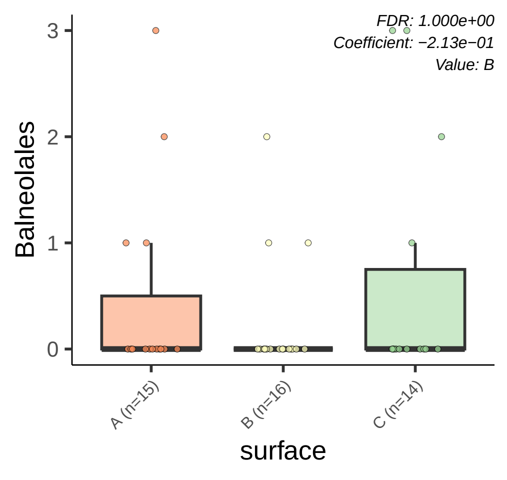

FDR: 1.000e+00
Coefficient: −2.13e−01
Value: B
Balneolales
3
2
1
0
A (n=15)
B (n=16)
C (n=14)
surface

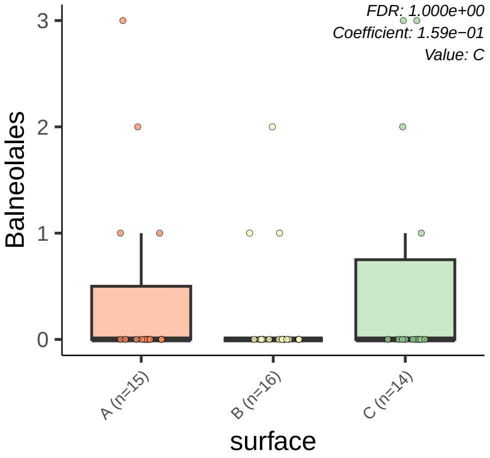

FDR: 1.000e+00
Coefficient: 1.59e−01
Value: C
Balneolales
3
2
1
0
A (n=15)
B (n=16)
C (n=14)
surface

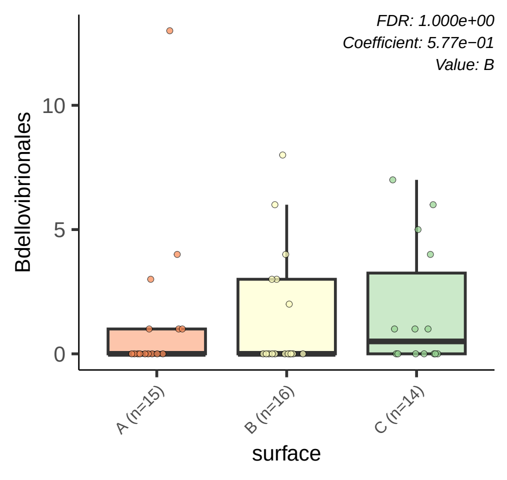

FDR: 1.000e+00
Coefficient: 5.77e−01
Value: B
Bdellovibrionales
10
5
0
A (n=15)
B (n=16)
C (n=14)
surface

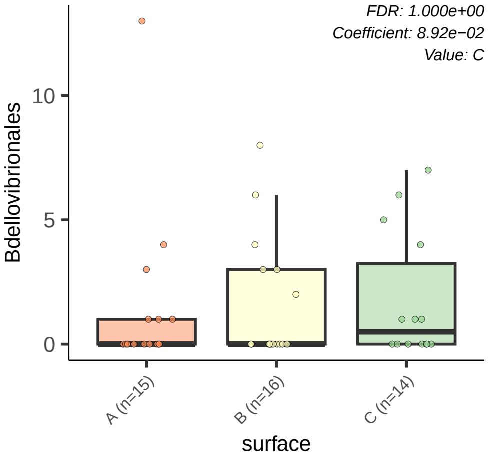
FDR: 1.000e+00
Coefficient: 8.92e−02
Value: C
Bdellovibrionales
10
5
0
A (n=15)
B (n=16)
C (n=14)
surface

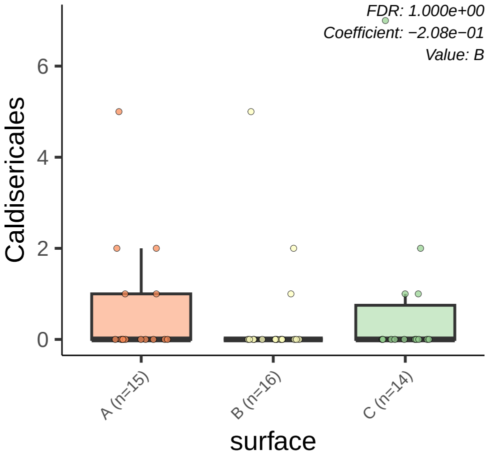

FDR: 1.000e+00
Coefficient: −2.08e−01
Value: B
Caldisericales
0
2
4
6
A (n=15)
B (n=16)
C (n=14)
surface

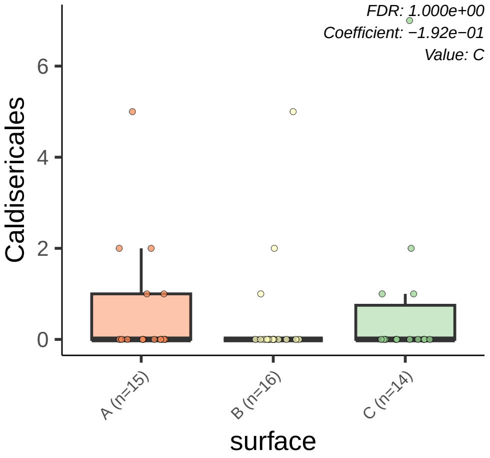
FDR: 1.000e+00
Coefficient: −1.92e−01
Value: C
Caldisericales
0
2
4
6
A (n=15)
B (n=16)
C (n=14)
surface

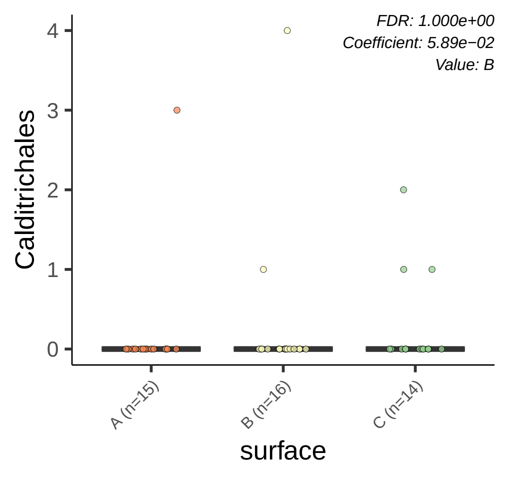
FDR: 1.000e+00
Coefficient: 5.89e−02
Value: B
Calditrichales
0
1
2
3
4
A (n=15)
B (n=16)
C (n=14)
surface

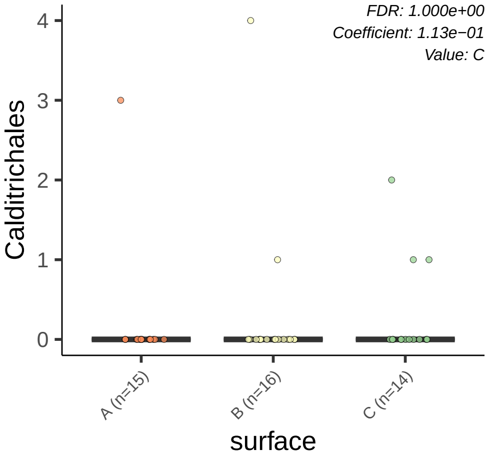
FDR: 1.000e+00
Coefficient: 1.13e−01
Value: C
Calditrichales
4
3
2
1
0
A (n=15)
B (n=16)
C (n=14)
surface

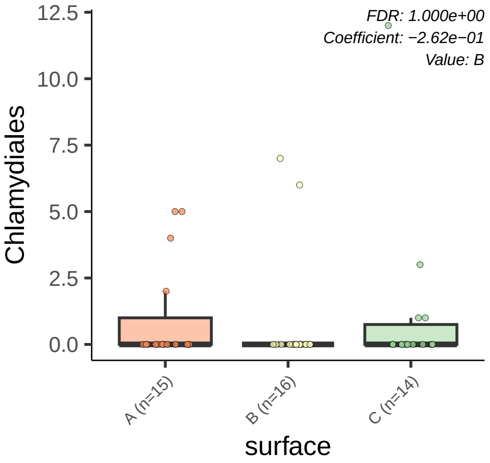
FDR: 1.000e+00
Coefficient: −2.62e−01
Value: B
Chlamydiales
12.5
10.0
7.5
5.0
2.5
0.0
A (n=15)
B (n=16)
C (n=14)
surface

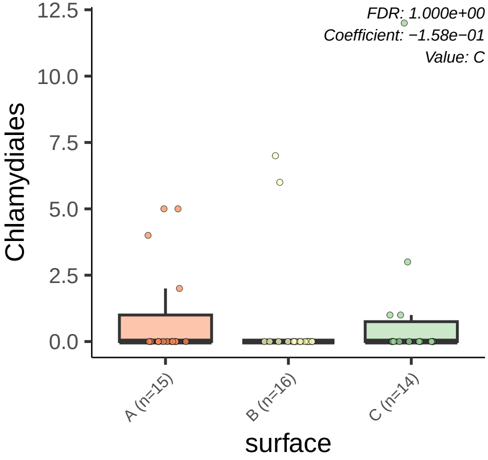

FDR: 1.000e+00
Coefficient: −1.58e−01
Value: C
Chlamydiales
12.5
10.0
7.5
5.0
2.5
0.0
A (n=15)
B (n=16)
C (n=14)
surface

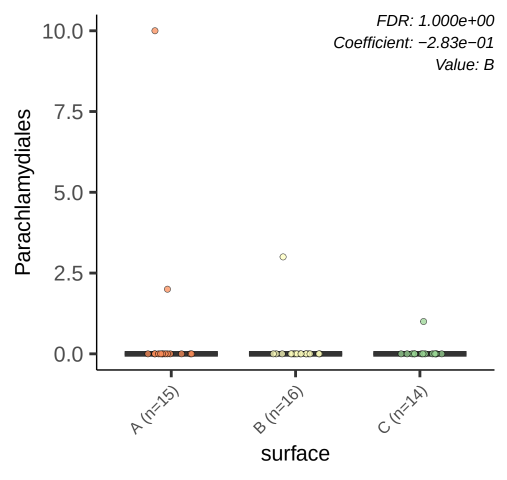
FDR: 1.000e+00
Coefficient: −2.83e−01
Value: B
Parachlamydiales
10.0
7.5
5.0
2.5
0.0
A (n=15)
B (n=16)
C (n=14)
surface

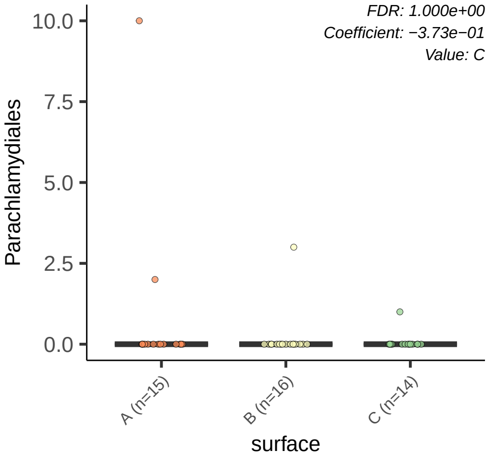
FDR: 1.000e+00
Coefficient: −3.73e−01
Value: C
Parachlamydiales
10.0
7.5
5.0
2.5
0.0
A (n=15)
B (n=16)
C (n=14)
surface

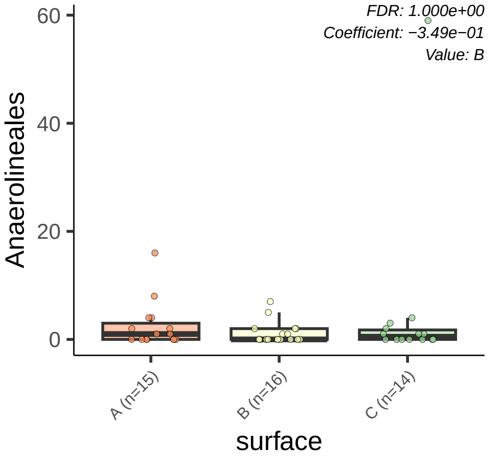

FDR: 1.000e+00
Coefficient: −3.49e−01
Value: B
Anaerolineales
60
40
20
0
A (n=15)
B (n=16)
C (n=14)
surface

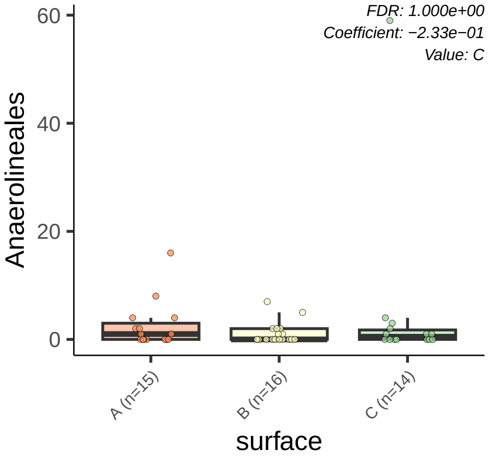
FDR: 1.000e+00
Coefficient: −2.33e−01
Value: C
Anaerolineales
60
40
20
0
A (n=15)
B (n=16)
C (n=14)
surface

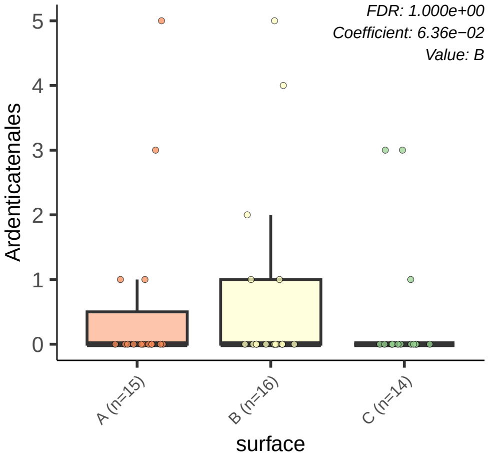

FDR: 1.000e+00
Coefficient: 6.36e−02
Value: B
Ardenticatenales
0
1
2
3
4
5
A (n=15)
B (n=16)
C (n=14)
surface

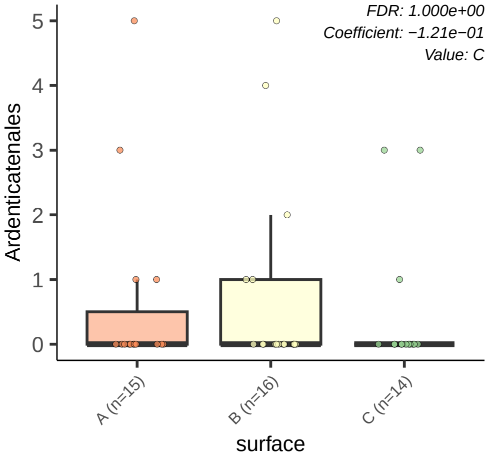

FDR: 1.000e+00
Coefficient: −1.21e−01
Value: C
Ardenticatenales
0
1
2
3
4
5
A (n=15)
B (n=16)
C (n=14)
surface

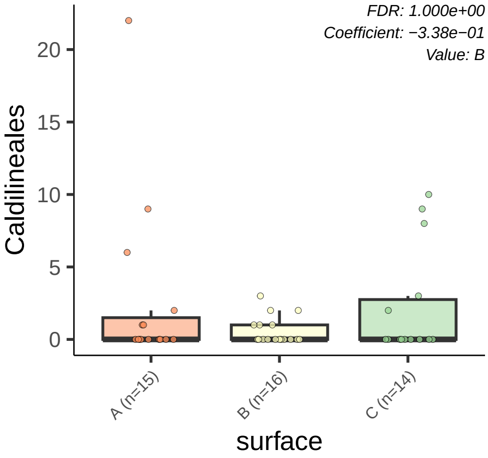

FDR: 1.000e+00
Coefficient: −3.38e−01
Value: B
Caldilineales
0
5
10
15
20
A (n=15)
B (n=16)
C (n=14)
surface

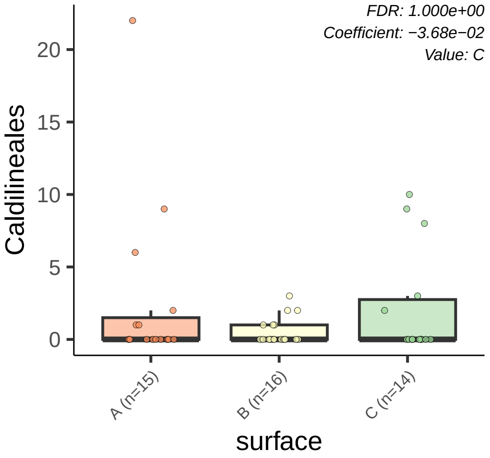
FDR: 1.000e+00
Coefficient: −3.68e−02
Value: C
Caldilineales
20
15
10
5
0
A (n=15)
B (n=16)
C (n=14)
surface

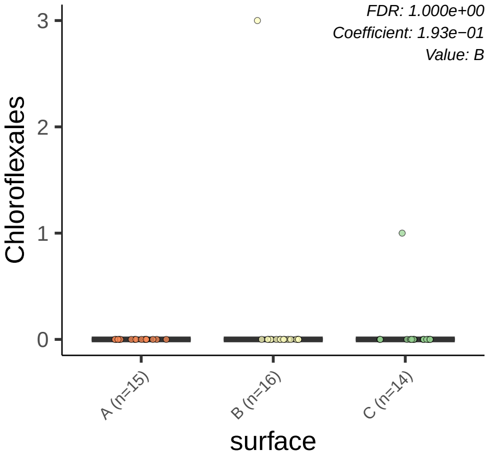
FDR: 1.000e+00
Coefficient: 1.93e−01
Value: B
Chloroflexales
0
1
2
3
A (n=15)
B (n=16)
C (n=14)
surface

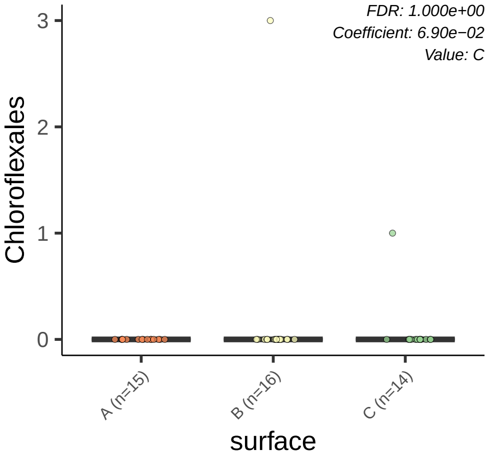
FDR: 1.000e+00
Coefficient: 6.90e−02
Value: C
Chloroflexales
3
2
1
0
A (n=15)
B (n=16)
C (n=14)
surface

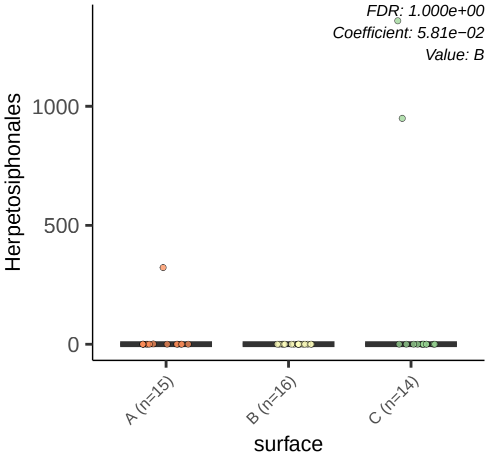
FDR: 1.000e+00
Coefficient: 5.81e−02
Value: B
Herpetosiphonales
1000
500
0
A (n=15)
B (n=16)
C (n=14)
surface

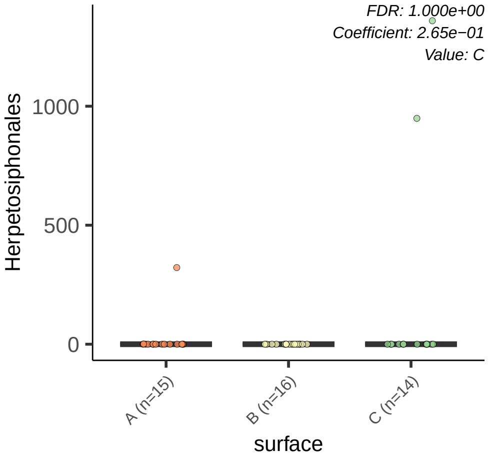

FDR: 1.000e+00
Coefficient: 2.65e−01
Value: C
Herpetosiphonales
1000
500
0
A (n=15)
B (n=16)
C (n=14)
surface

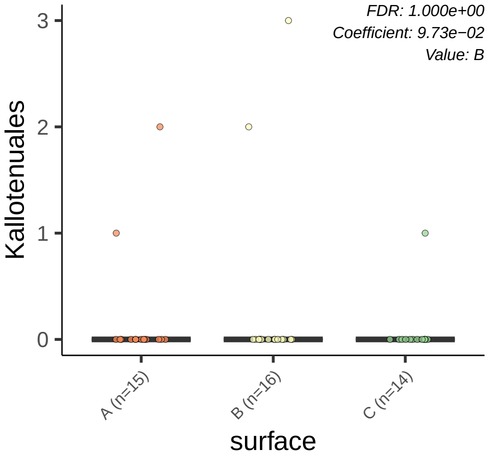
FDR: 1.000e+00
Coefficient: 9.73e−02
Value: B
Kallotenuales
3
2
1
0
A (n=15)
B (n=16)
C (n=14)
surface

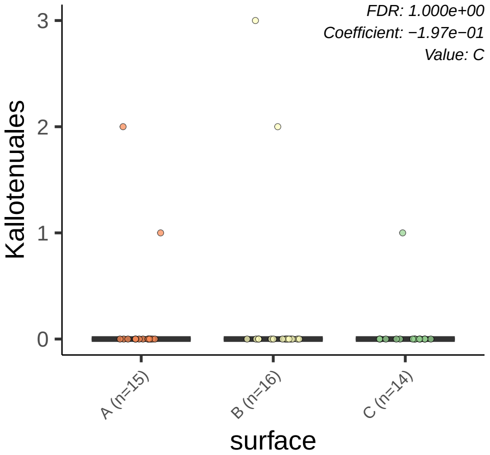
FDR: 1.000e+00
Coefficient: −1.97e−01
Value: C
Kallotenuales
3
2
1
0
A (n=15)
B (n=16)
C (n=14)
surface

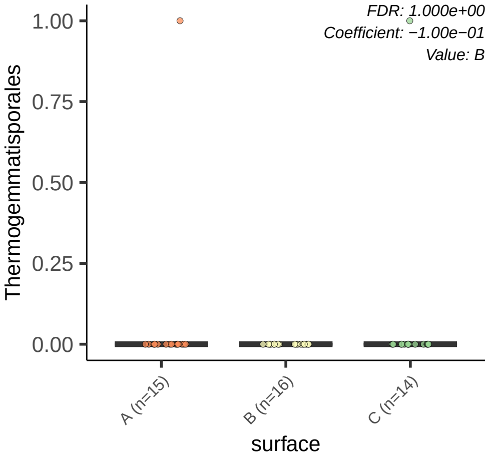
FDR: 1.000e+00
Coefficient: −1.00e−01
Value: B
Thermogemmatisporales
1.00
0.75
0.50
0.25
0.00
A (n=15)
B (n=16)
C (n=14)
surface

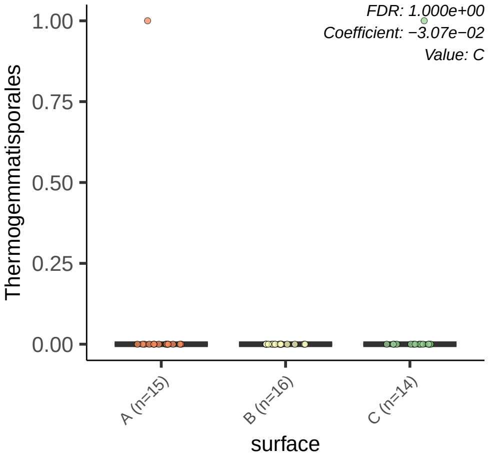

FDR: 1.000e+00
Coefficient: −3.07e−02
Value: C
Thermogemmatisporales
1.00
0.75
0.50
0.25
0.00
A (n=15)
B (n=16)
C (n=14)
surface

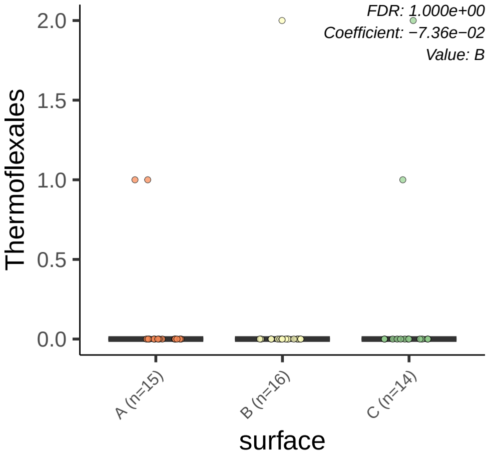

FDR: 1.000e+00
Coefficient: −7.36e−02
Value: B
Thermoflexales
2.0
1.5
1.0
0.5
0.0
A (n=15)
B (n=16)
C (n=14)
surface

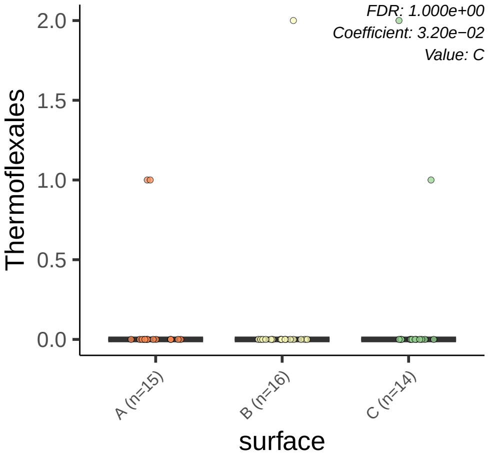

FDR: 1.000e+00
Coefficient: 3.20e−02
Value: C
Thermoflexales
2.0
1.5
1.0
0.5
0.0
A (n=15)
B (n=16)
C (n=14)
surface

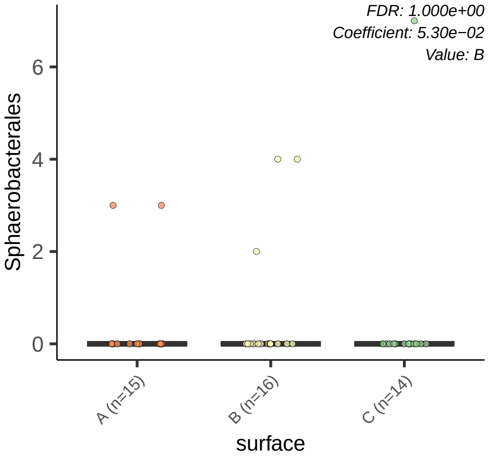

FDR: 1.000e+00
Coefficient: 5.30e−02
Value: B
Sphaerobacterales
0
2
4
6
A (n=15)
B (n=16)
C (n=14)
surface

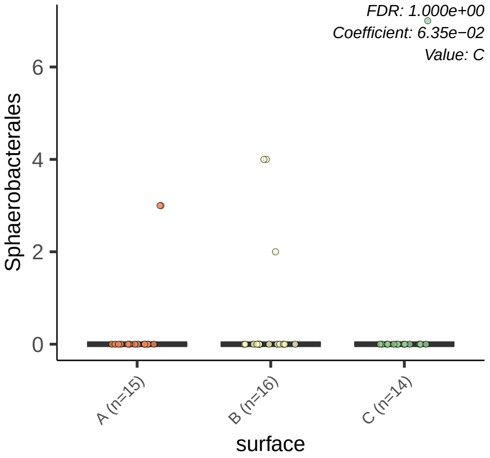

FDR: 1.000e+00
Coefficient: 6.35e−02
Value: C
Sphaerobacterales
0
2
4
6
A (n=15)
B (n=16)
C (n=14)
surface

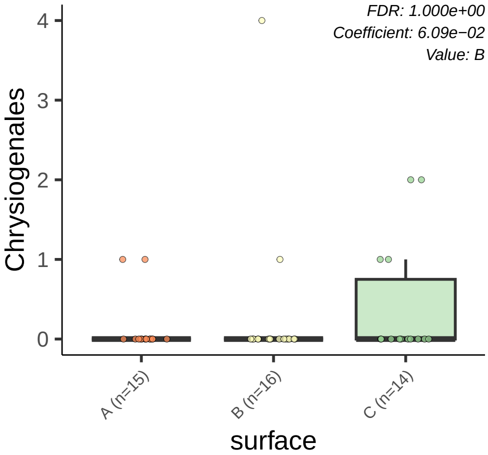
FDR: 1.000e+00
Coefficient: 6.09e−02
Value: B
Chrysiogenales
0
1
2
3
4
A (n=15)
B (n=16)
C (n=14)
surface

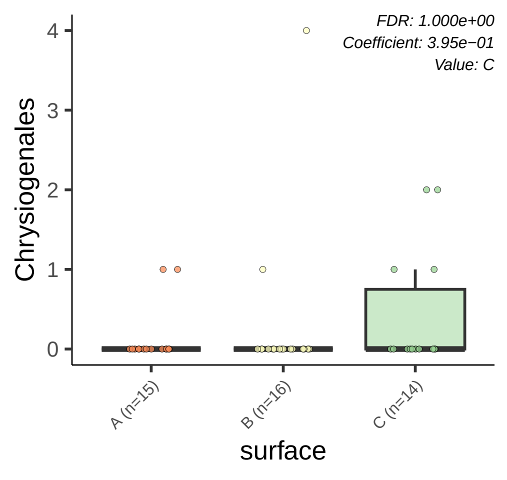
FDR: 1.000e+00
Coefficient: 3.95e−01
Value: C
Chrysiogenales
4
3
2
1
0
A (n=15)
B (n=16)
C (n=14)
surface

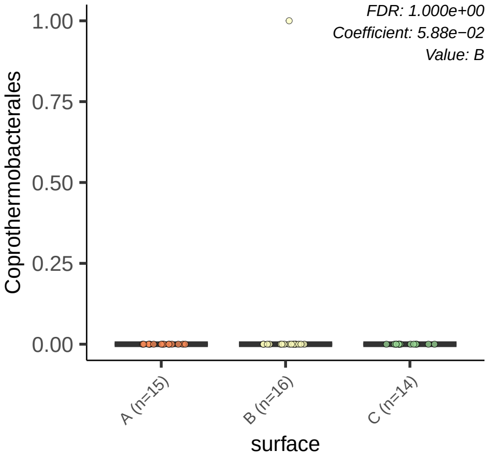
FDR: 1.000e+00
Coefficient: 5.88e−02
Value: B
Coprothermobacterales
1.00
0.75
0.50
0.25
0.00
A (n=15)
B (n=16)
C (n=14)
surface

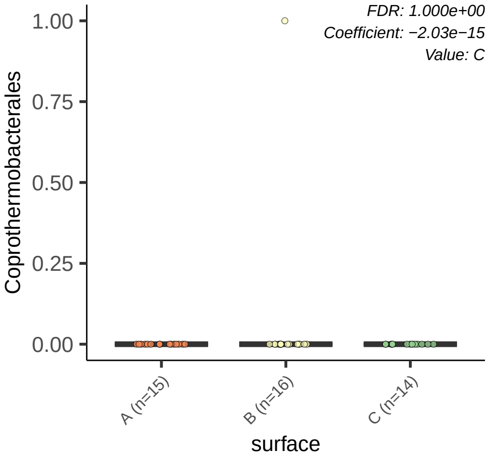

FDR: 1.000e+00
Coefficient: −2.03e−15
Value: C
Coprothermobacterales
1.00
0.75
0.50
0.25
0.00
A (n=15)
B (n=16)
C (n=14)
surface

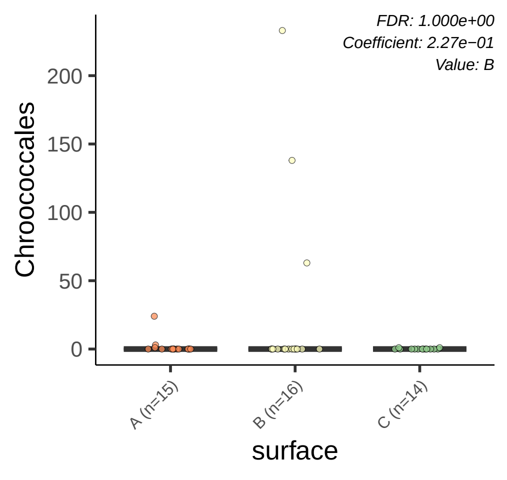

FDR: 1.000e+00
Coefficient: 2.27e−01
Value: B
Chroococcales
0
50
100
150
200
A (n=15)
B (n=16)
C (n=14)
surface

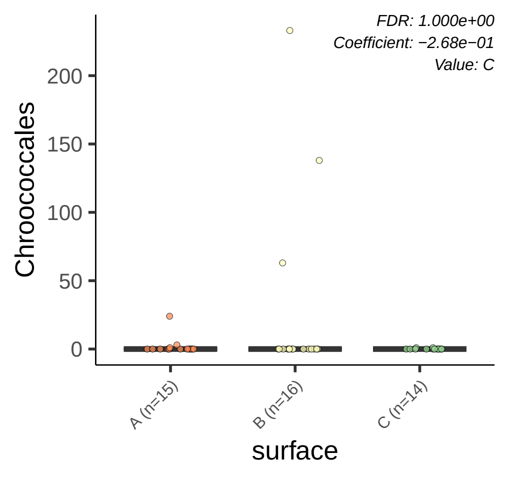

FDR: 1.000e+00
Coefficient: −2.68e−01
Value: C
Chroococcales
200
150
100
50
0
A (n=15)
B (n=16)
C (n=14)
surface

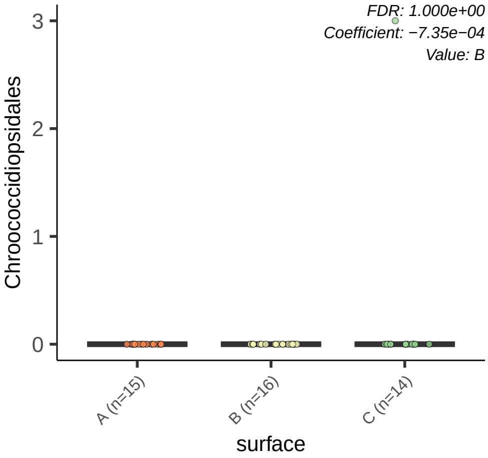

FDR: 1.000e+00
Coefficient: −7.35e−04
Value: B
Chroococcidiopsidales
3
2
1
0
A (n=15)
B (n=16)
C (n=14)
surface

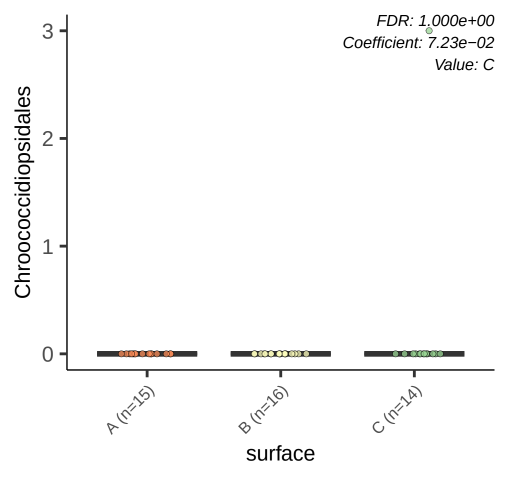
FDR: 1.000e+00
Coefficient: 7.23e−02
Value: C
Chroococcidiopsidales
0
1
2
3
A (n=15)
B (n=16)
C (n=14)
surface

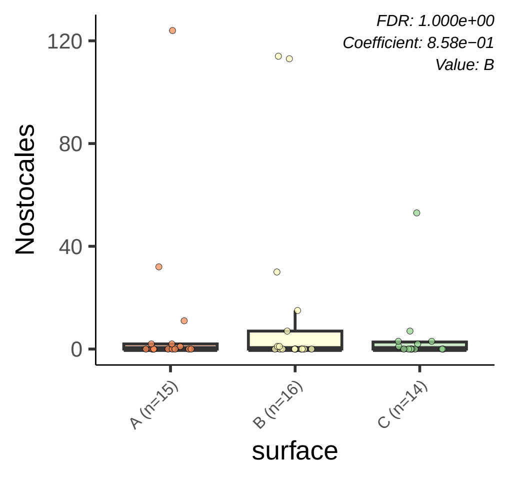

FDR: 1.000e+00
Coefficient: 8.58e−01
Value: B
Nostocales
120
80
40
0
A (n=15)
B (n=16)
C (n=14)
surface

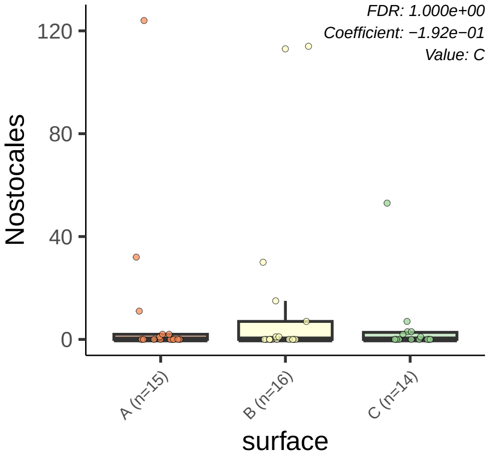

FDR: 1.000e+00
Coefficient: −1.92e−01
Value: C
Nostocales
120
80
40
0
A (n=15)
B (n=16)
C (n=14)
surface

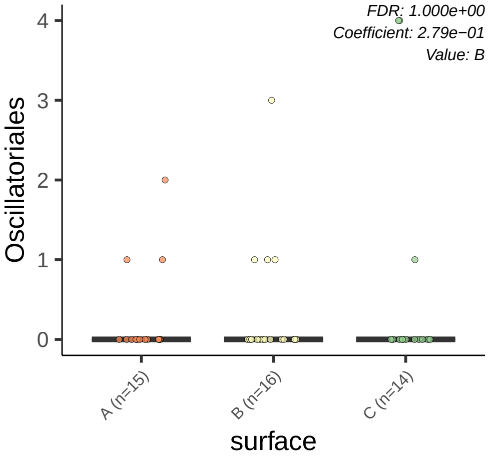
FDR: 1.000e+00
Coefficient: 2.79e−01
Value: B
Oscillatoriales
0
1
2
3
4
A (n=15)
B (n=16)
C (n=14)
surface

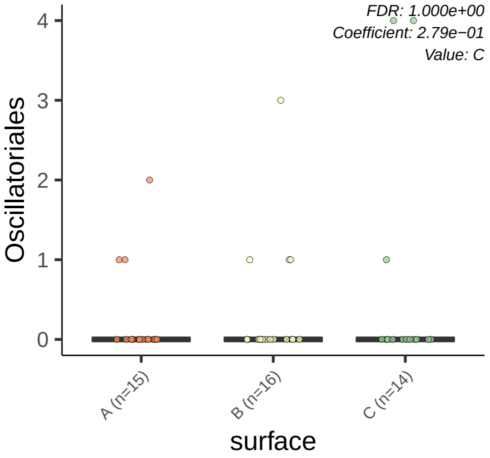
FDR: 1.000e+00
Coefficient: 2.79e−01
Value: C
Oscillatoriales
0
1
2
3
4
A (n=15)
B (n=16)
C (n=14)
surface

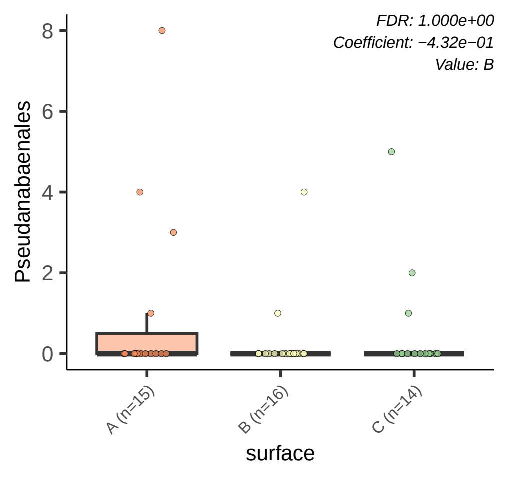

FDR: 1.000e+00
Coefficient: −4.32e−01
Value: B
Pseudanabaenales
8
6
4
2
0
A (n=15)
B (n=16)
C (n=14)
surface

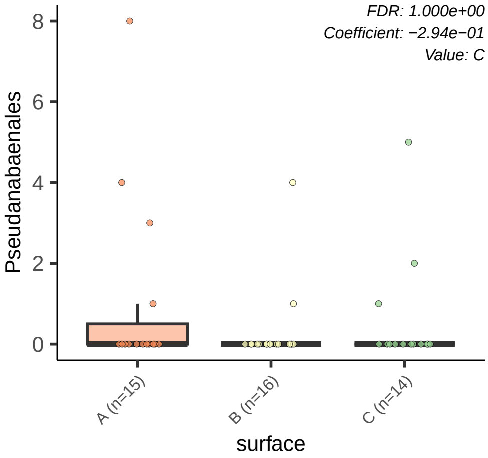

FDR: 1.000e+00
Coefficient: −2.94e−01
Value: C
Pseudanabaenales
8
6
4
2
0
A (n=15)
B (n=16)
C (n=14)
surface

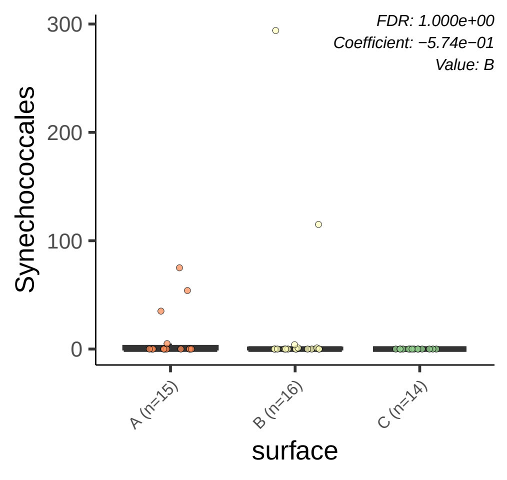
FDR: 1.000e+00
Coefficient: −5.74e−01
Value: B
Synechococcales
300
200
100
0
A (n=15)
B (n=16)
C (n=14)
surface

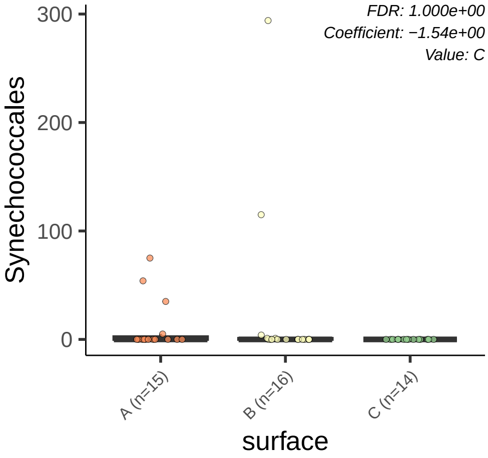

FDR: 1.000e+00
Coefficient: −1.54e+00
Value: C
Synechococcales
300
200
100
0
A (n=15)
B (n=16)
C (n=14)
surface

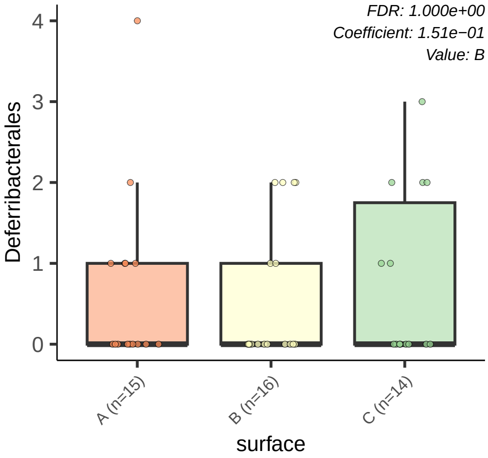

FDR: 1.000e+00
Coefficient: 1.51e−01
Value: B
Deferribacterales
0
1
2
3
4
A (n=15)
B (n=16)
C (n=14)
surface

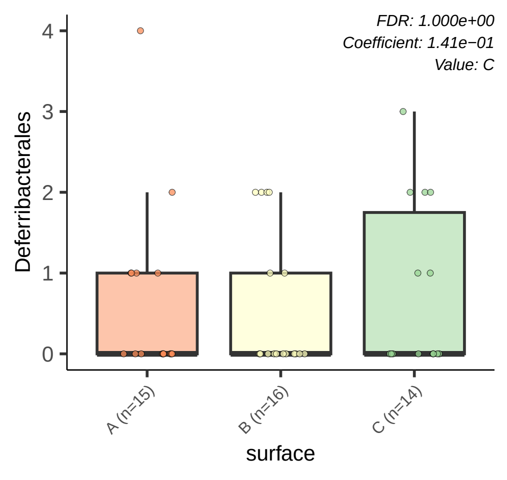
FDR: 1.000e+00
Coefficient: 1.41e−01
Value: C
Deferribacterales
0
1
2
3
4
A (n=15)
B (n=16)
C (n=14)
surface

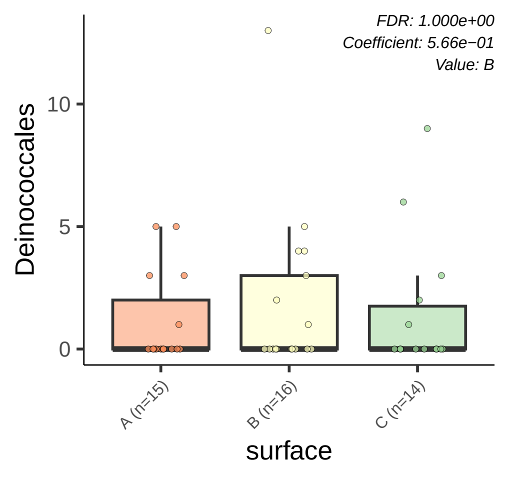
FDR: 1.000e+00
Coefficient: 5.66e−01
Value: B
Deinococcales
0
5
10
A (n=15)
B (n=16)
C (n=14)
surface

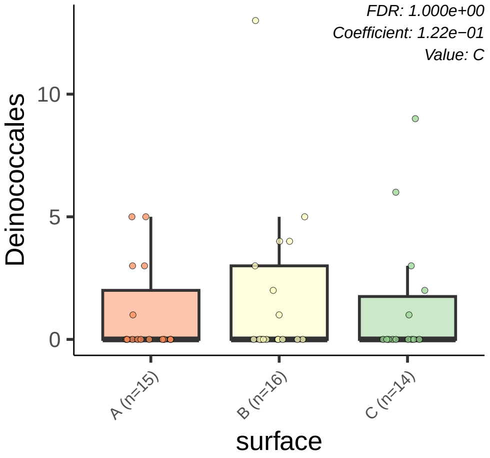
FDR: 1.000e+00
Coefficient: 1.22e−01
Value: C
Deinococcales
10
5
0
A (n=15)
B (n=16)
C (n=14)
surface

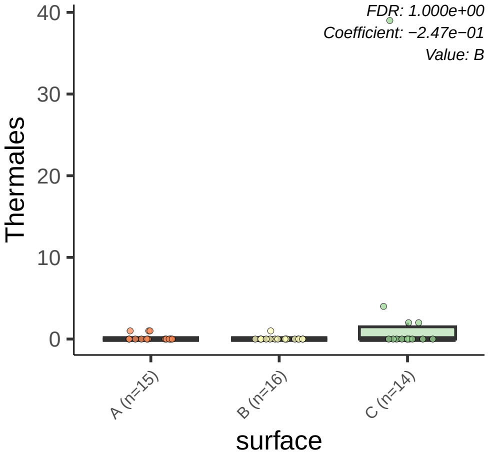
FDR: 1.000e+00
Coefficient: −2.47e−01
Value: B
Thermales
40
30
20
10
0
A (n=15)
B (n=16)
C (n=14)
surface

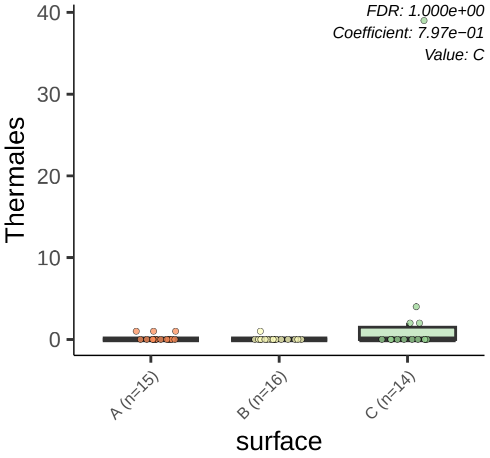

FDR: 1.000e+00
Coefficient: 7.97e−01
Value: C
Thermales
40
30
20
10
0
A (n=15)
B (n=16)
C (n=14)
surface

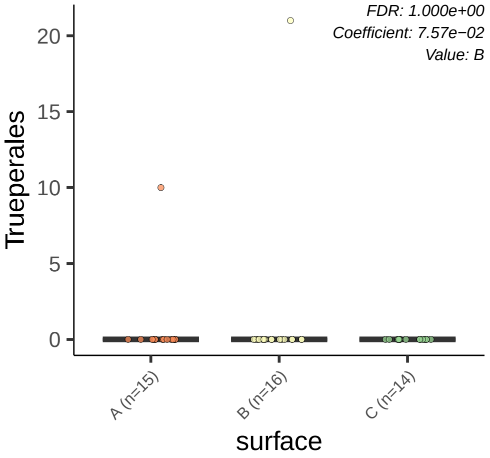
FDR: 1.000e+00
Coefficient: 7.57e−02
Value: B
Trueperales
20
15
10
5
0
A (n=15)
B (n=16)
C (n=14)
surface

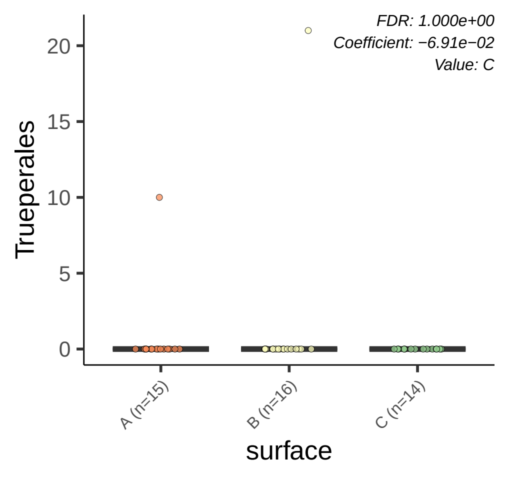

FDR: 1.000e+00
Coefficient: −6.91e−02
Value: C
Trueperales
20
15
10
5
0
A (n=15)
B (n=16)
C (n=14)
surface

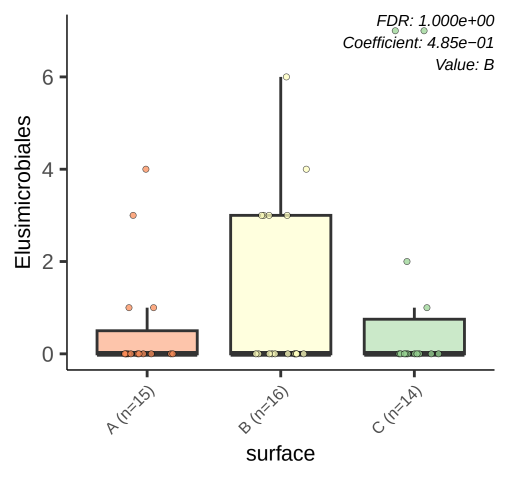
FDR: 1.000e+00
Coefficient: 4.85e−01
Value: B
Elusimicrobiales
0
2
4
6
A (n=15)
B (n=16)
C (n=14)
surface

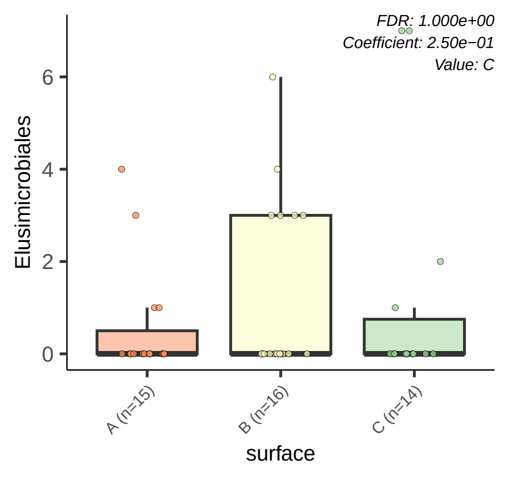

FDR: 1.000e+00
Coefficient: 2.50e−01
Value: C
Elusimicrobiales
0
2
4
6
A (n=15)
B (n=16)
C (n=14)
surface

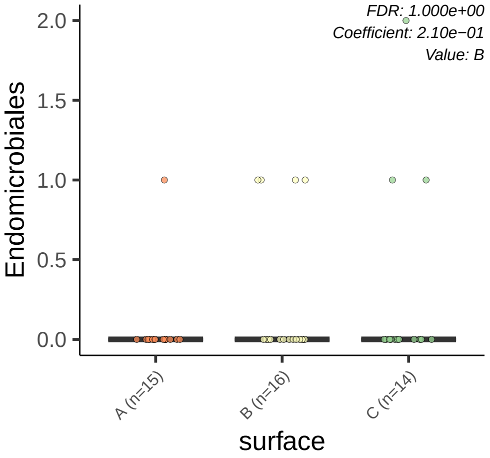

FDR: 1.000e+00
Coefficient: 2.10e−01
Value: B
Endomicrobiales
2.0
1.5
1.0
0.5
0.0
A (n=15)
B (n=16)
C (n=14)
surface

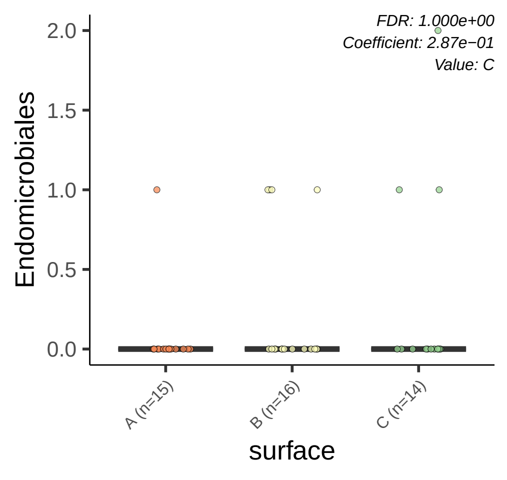

FDR: 1.000e+00
Coefficient: 2.87e−01
Value: C
Endomicrobiales
2.0
1.5
1.0
0.5
0.0
A (n=15)
B (n=16)
C (n=14)
surface

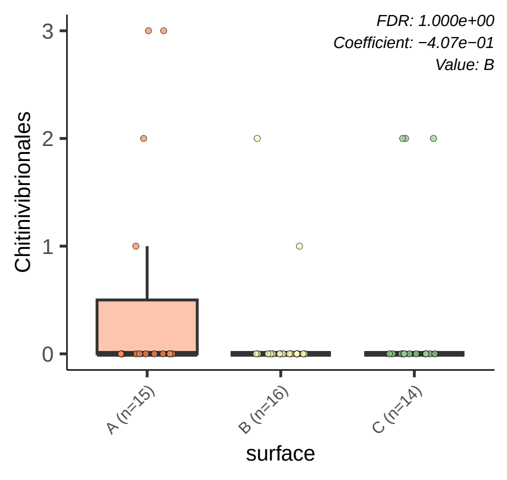
FDR: 1.000e+00
Coefficient: −4.07e−01
Value: B
Chitinivibrionales
3
2
1
0
A (n=15)
B (n=16)
C (n=14)
surface

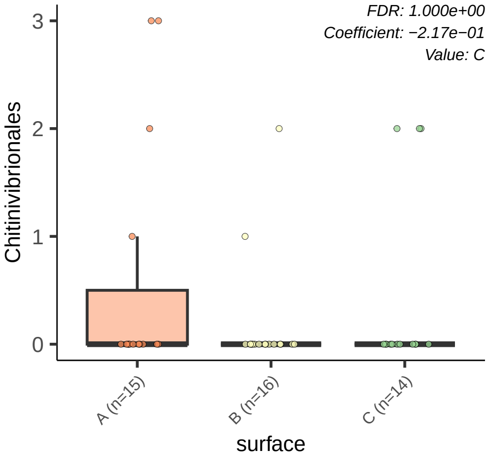
FDR: 1.000e+00
Coefficient: −2.17e−01
Value: C
Chitinivibrionales
0
1
2
3
A (n=15)
B (n=16)
C (n=14)
surface

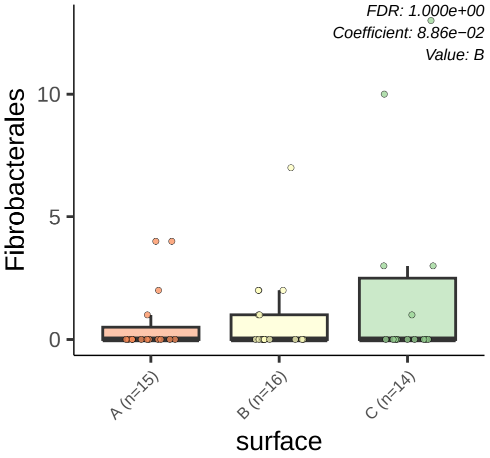

FDR: 1.000e+00
Coefficient: 8.86e−02
Value: B
Fibrobacterales
10
5
0
A (n=15)
B (n=16)
C (n=14)
surface

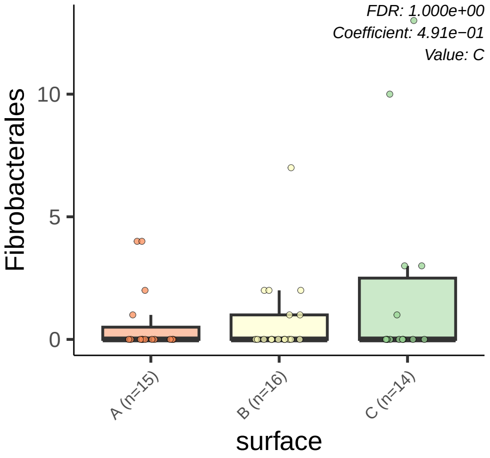
FDR: 1.000e+00
Coefficient: 4.91e−01
Value: C
Fibrobacterales
0
5
10
A (n=15)
B (n=16)
C (n=14)
surface

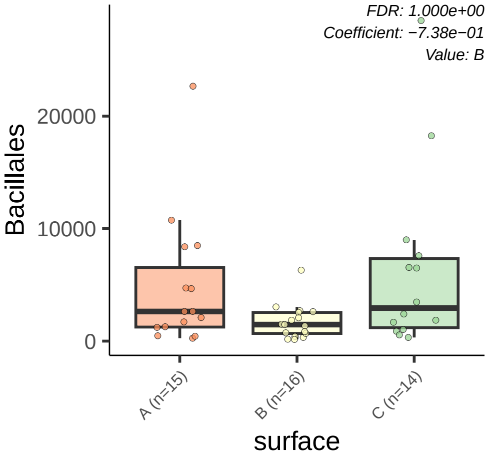

FDR: 1.000e+00
Coefficient: −7.38e−01
Value: B
Bacillales
20000
10000
0
A (n=15)
B (n=16)
C (n=14)
surface

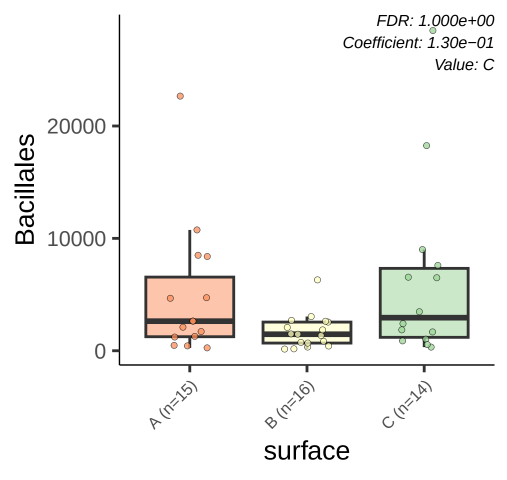

FDR: 1.000e+00
Coefficient: 1.30e−01
Value: C
Bacillales
20000
10000
0
A (n=15)
B (n=16)
C (n=14)
surface

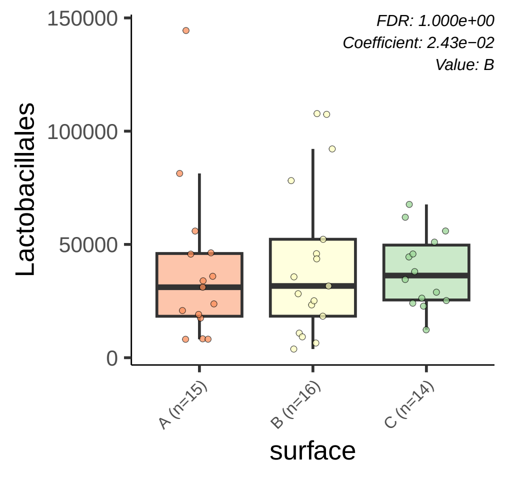
FDR: 1.000e+00
Coefficient: 2.43e−02
Value: B
150000
100000
50000
0
Lactobacillales
A (n=15)
B (n=16)
C (n=14)
surface

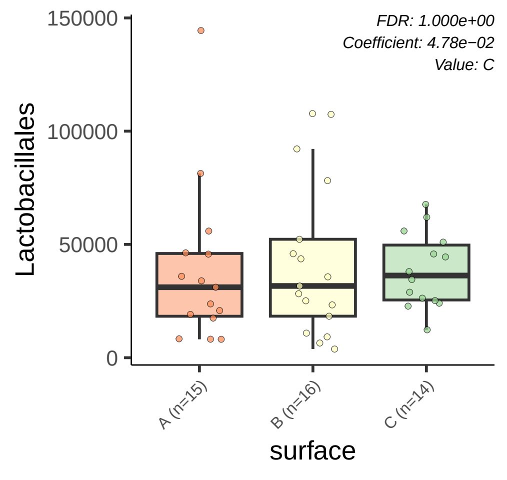

FDR: 1.000e+00
Coefficient: 4.78e−02
Value: C
Lactobacillales
150000
100000
50000
0
A (n=15)
B (n=16)
C (n=14)
surface

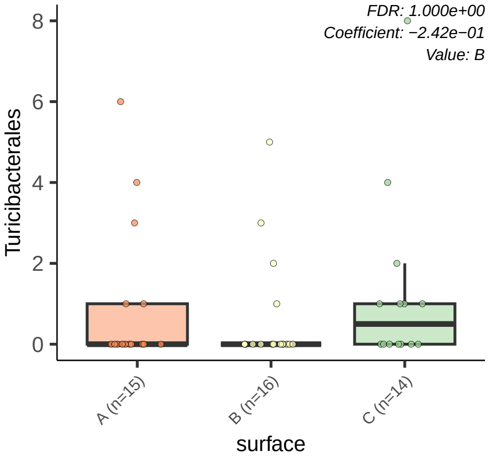

FDR: 1.000e+00
Coefficient: −2.42e−01
Value: B
Turicibacterales
8
6
4
2
0
A (n=15)
B (n=16)
C (n=14)
surface

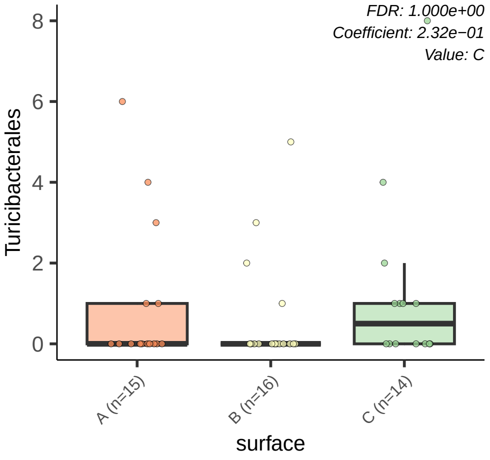

FDR: 1.000e+00
Coefficient: 2.32e−01
Value: C
Turicibacterales
0
2
4
6
8
A (n=15)
B (n=16)
C (n=14)
surface

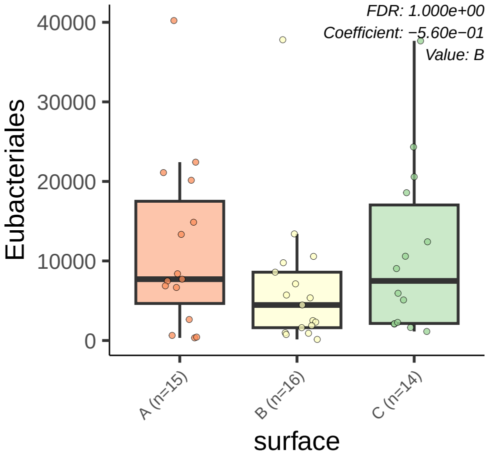
FDR: 1.000e+00
Coefficient: −5.60e−01
Value: B
Eubacteriales
40000
30000
20000
10000
0
A (n=15)
B (n=16)
C (n=14)
surface

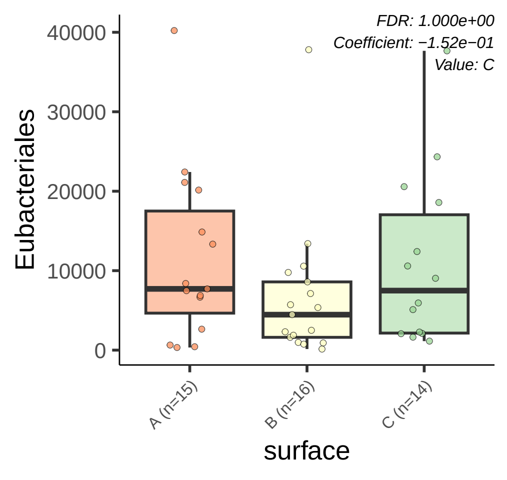
FDR: 1.000e+00
Coefficient: −1.52e−01
Value: C
Eubacteriales
40000
30000
20000
10000
0
A (n=15)
B (n=16)
C (n=14)
surface

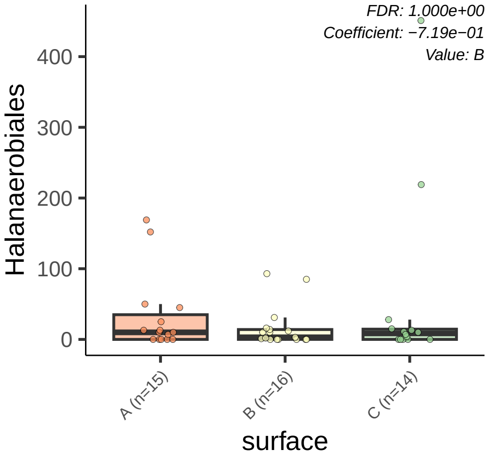

FDR: 1.000e+00
Coefficient: −7.19e−01
Value: B
Halanaerobiales
400
300
200
100
0
A (n=15)
B (n=16)
C (n=14)
surface

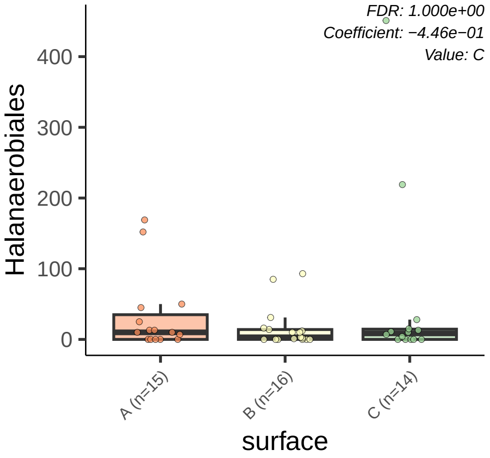

FDR: 1.000e+00
Coefficient: −4.46e−01
Value: C
Halanaerobiales
400
300
200
100
0
A (n=15)
B (n=16)
C (n=14)
surface

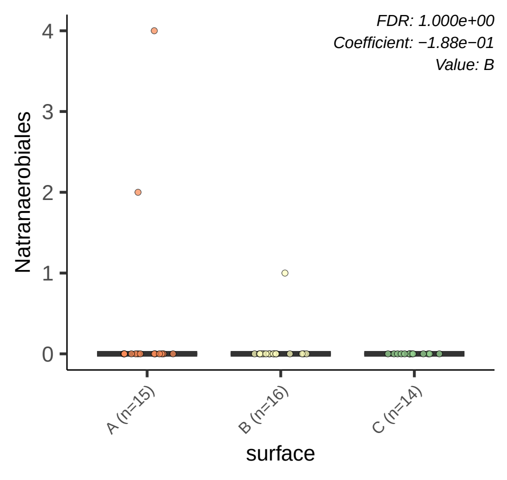
FDR: 1.000e+00
Coefficient: −1.88e−01
Value: B
Natranaerobiales
0
1
2
3
4
A (n=15)
B (n=16)
C (n=14)
surface

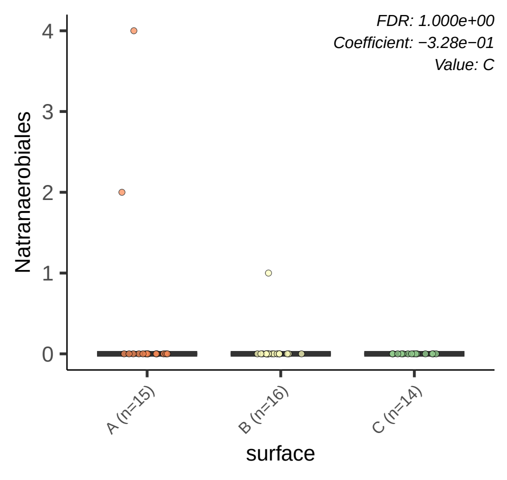

FDR: 1.000e+00
Coefficient: −3.28e−01
Value: C
Natranaerobiales
4
3
2
1
0
A (n=15)
B (n=16)
C (n=14)
surface

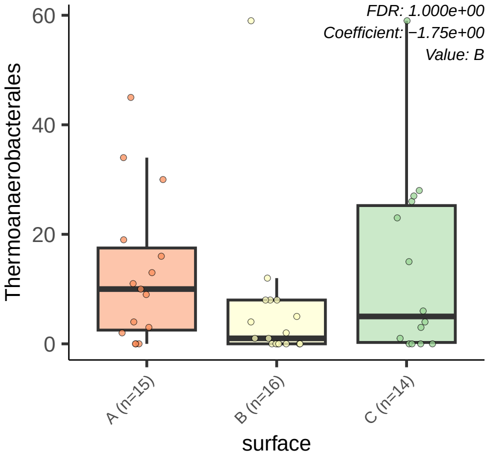

FDR: 1.000e+00
Coefficient: −1.75e+00
Value: B
Thermoanaerobacterales
0
20
40
60
A (n=15)
B (n=16)
C (n=14)
surface

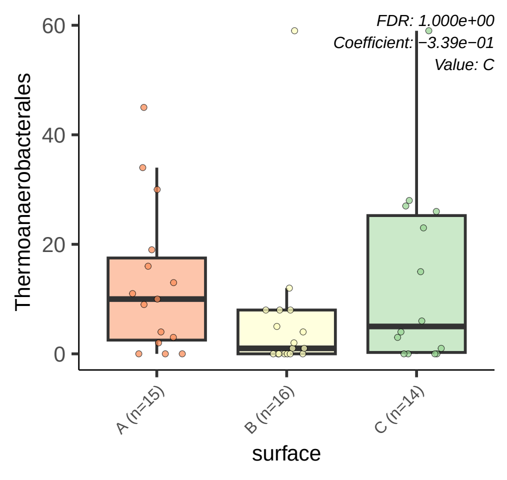

FDR: 1.000e+00
Coefficient: −3.39e−01
Value: C
Thermoanaerobacterales
60
40
20
0
A (n=15)
B (n=16)
C (n=14)
surface

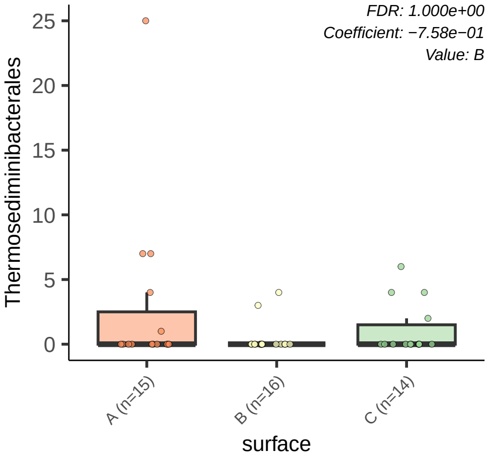

FDR: 1.000e+00
Coefficient: −7.58e−01
Value: B
Thermosediminibacterales
25
20
15
10
5
0
A (n=15)
B (n=16)
C (n=14)
surface

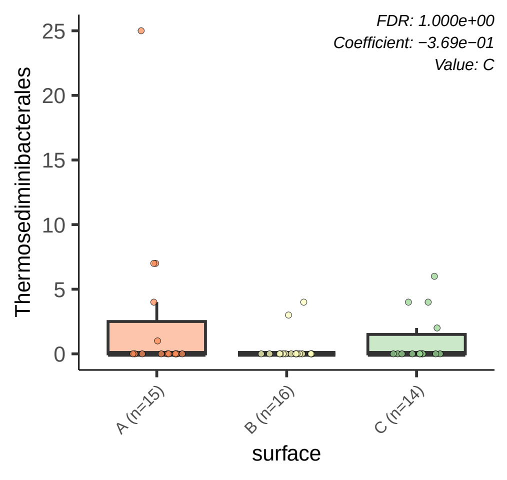

FDR: 1.000e+00
Coefficient: −3.69e−01
Value: C
Thermosediminibacterales
25
20
15
10
5
0
A (n=15)
B (n=16)
C (n=14)
surface

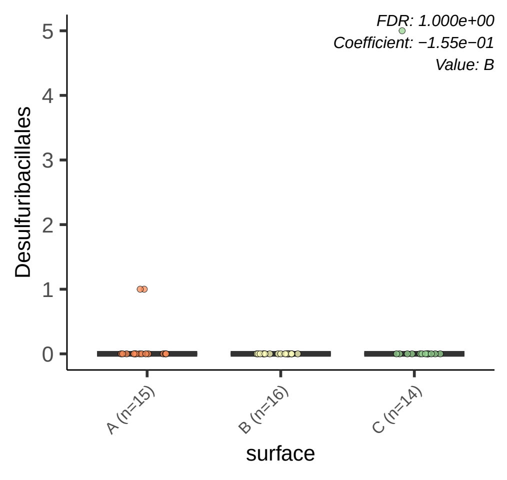
FDR: 1.000e+00
Coefficient: −1.55e−01
Value: B
Desulfuribacillales
5
4
3
2
1
0
A (n=15)
B (n=16)
C (n=14)
surface

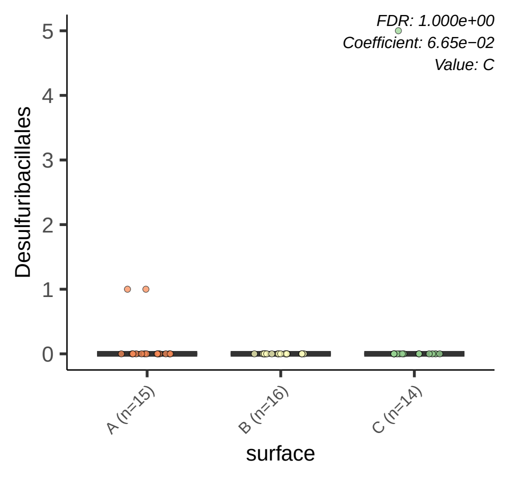

FDR: 1.000e+00
Coefficient: 6.65e−02
Value: C
Desulfuribacillales
0
1
2
3
4
5
A (n=15)
B (n=16)
C (n=14)
surface

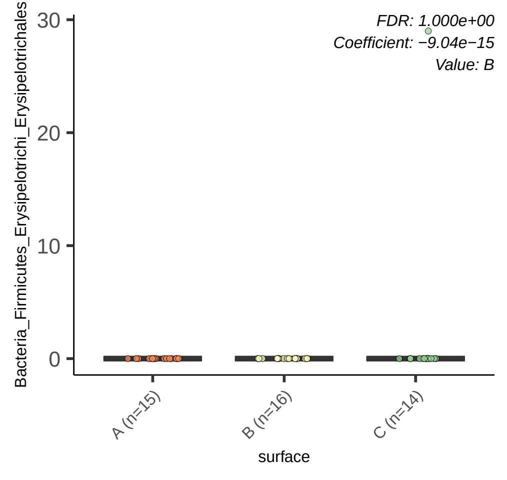

FDR: 1.000e+00
Coefficient: −9.04e−15
Value: B
Bacteria_Firmicutes_Erysipelotrichi_Erysipelotrichales
30
20
10
0
A (n=15)
B (n=16)
C (n=14)
surface

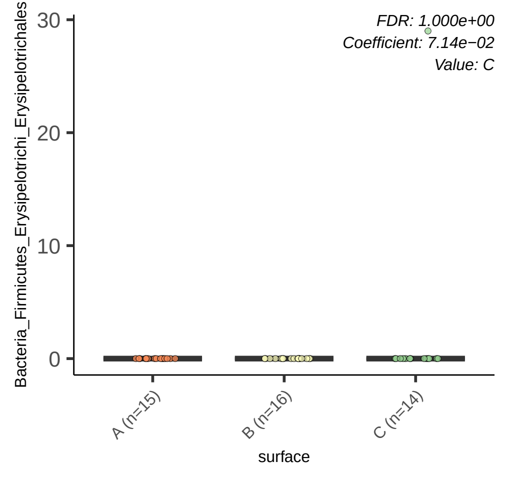

FDR: 1.000e+00
Coefficient: 7.14e−02
Value: C
Bacteria_Firmicutes_Erysipelotrichi_Erysipelotrichales
30
20
10
0
A (n=15)
B (n=16)
C (n=14)
surface

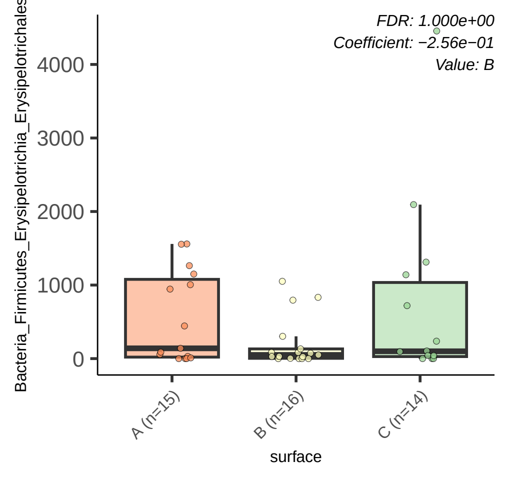

FDR: 1.000e+00
Coefficient: −2.56e−01
Value: B
Bacteria_Firmicutes_Erysipelotrichia_Erysipelotrichales
4000
3000
2000
1000
0
A (n=15)
B (n=16)
C (n=14)
surface

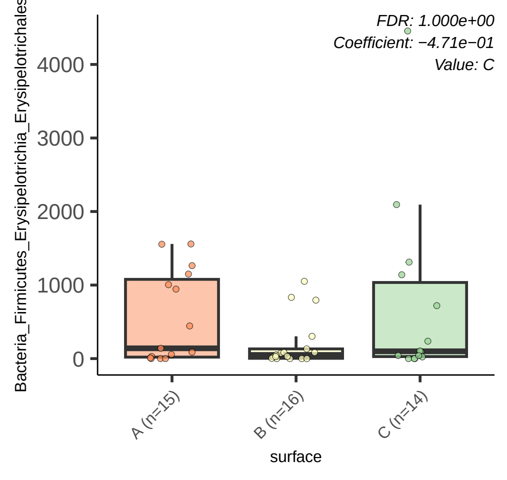

FDR: 1.000e+00
Coefficient: −4.71e−01
Value: C
Bacteria_Firmicutes_Erysipelotrichia_Erysipelotrichales
4000
3000
2000
1000
0
A (n=15)
B (n=16)
C (n=14)
surface

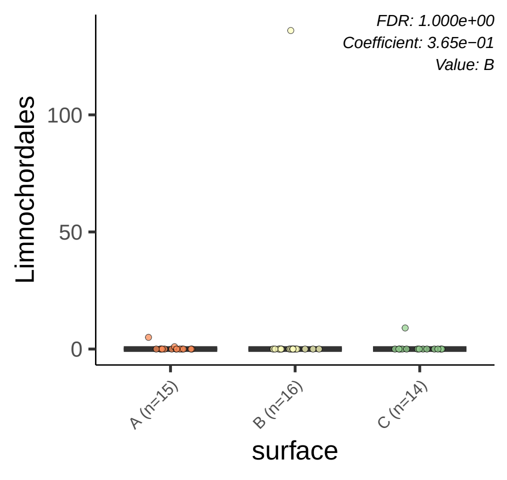
FDR: 1.000e+00
Coefficient: 3.65e−01
Value: B
Limnochordales
100
50
0
A (n=15)
B (n=16)
C (n=14)
surface

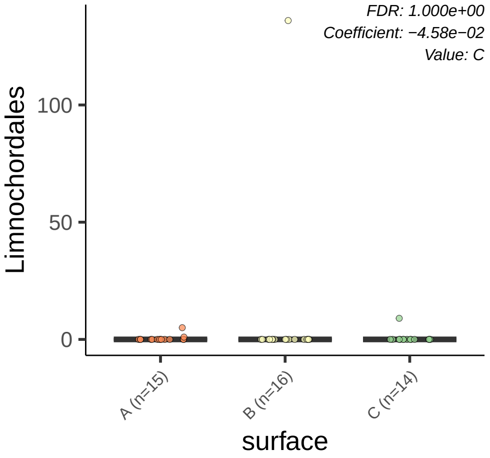
FDR: 1.000e+00
Coefficient: −4.58e−02
Value: C
Limnochordales
100
50
0
A (n=15)
B (n=16)
C (n=14)
surface

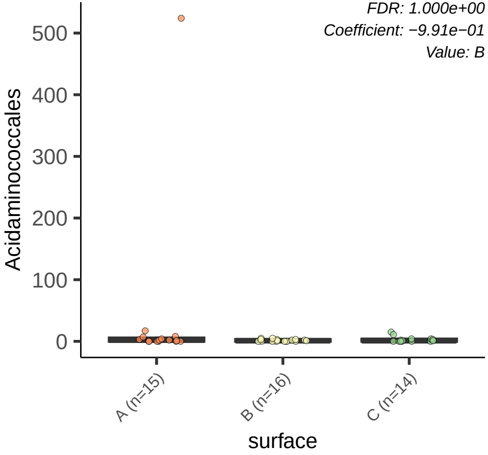
FDR: 1.000e+00
Coefficient: −9.91e−01
Value: B
Acidaminococcales
500
400
300
200
100
0
A (n=15)
B (n=16)
C (n=14)
surface

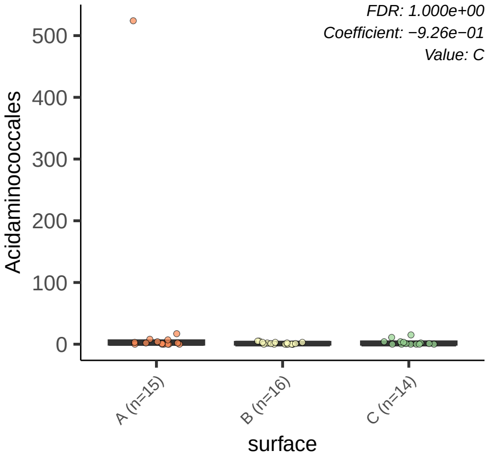
FDR: 1.000e+00
Coefficient: −9.26e−01
Value: C
Acidaminococcales
500
400
300
200
100
0
A (n=15)
B (n=16)
C (n=14)
surface

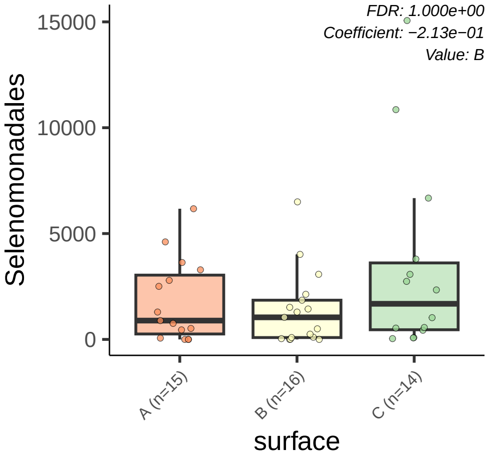

FDR: 1.000e+00
Coefficient: −2.13e−01
Value: B
Selenomonadales
15000
10000
5000
0
A (n=15)
B (n=16)
C (n=14)
surface

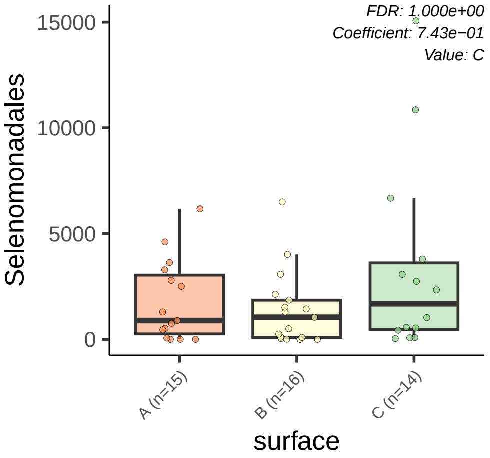
FDR: 1.000e+00
Coefficient: 7.43e−01
Value: C
Selenomonadales
15000
10000
5000
0
A (n=15)
B (n=16)
C (n=14)
surface

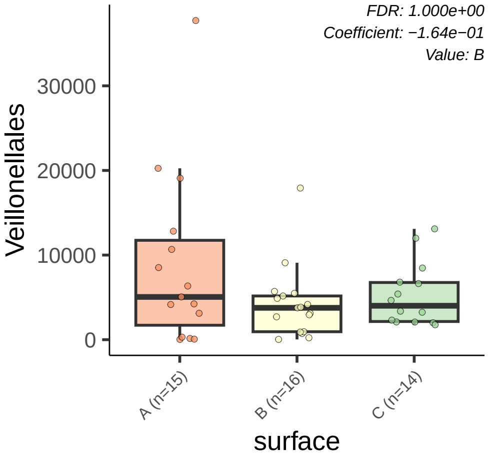
FDR: 1.000e+00
Coefficient: −1.64e−01
Value: B
Veillonellales
30000
20000
10000
0
A (n=15)
B (n=16)
C (n=14)
surface

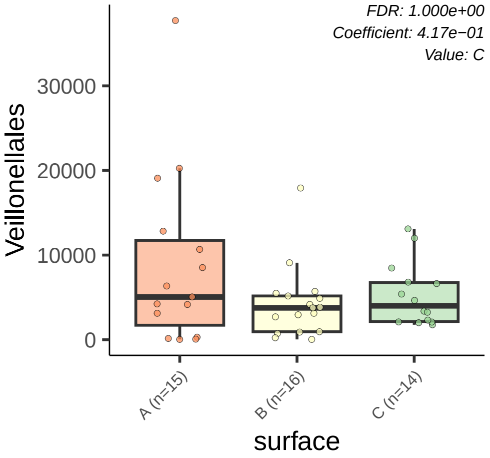
FDR: 1.000e+00
Coefficient: 4.17e−01
Value: C
Veillonellales
30000
20000
10000
0
A (n=15)
B (n=16)
C (n=14)
surface

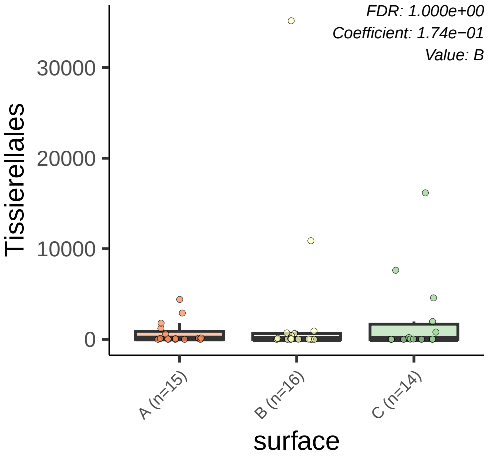

FDR: 1.000e+00
Coefficient: 1.74e−01
Value: B
Tissierellales
30000
20000
10000
0
A (n=15)
B (n=16)
C (n=14)
surface

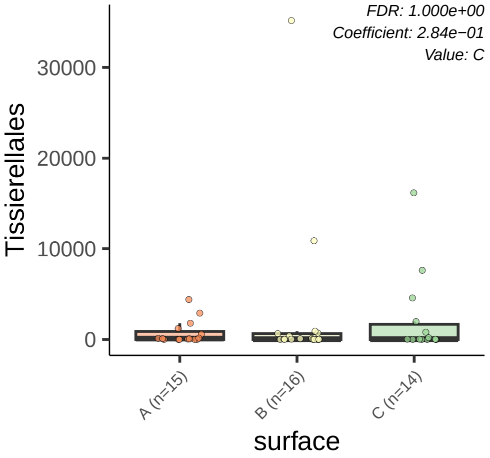
FDR: 1.000e+00
Coefficient: 2.84e−01
Value: C
Tissierellales
30000
20000
10000
0
A (n=15)
B (n=16)
C (n=14)
surface

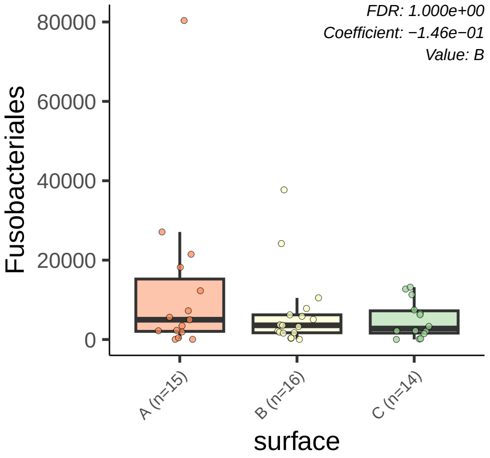
FDR: 1.000e+00
Coefficient: −1.46e−01
Value: B
Fusobacteriales
80000
60000
40000
20000
0
A (n=15)
B (n=16)
C (n=14)
surface

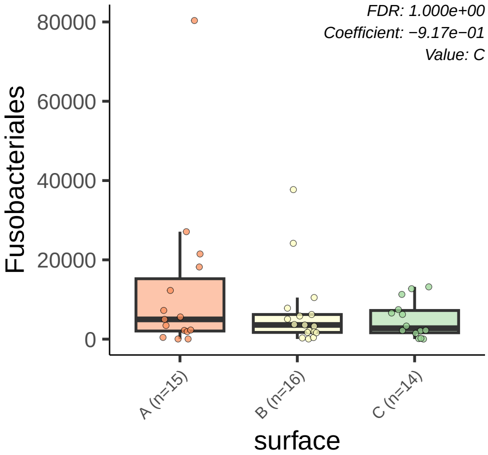
FDR: 1.000e+00
Coefficient: −9.17e−01
Value: C
Fusobacteriales
80000
60000
40000
20000
0
A (n=15)
B (n=16)
C (n=14)
surface

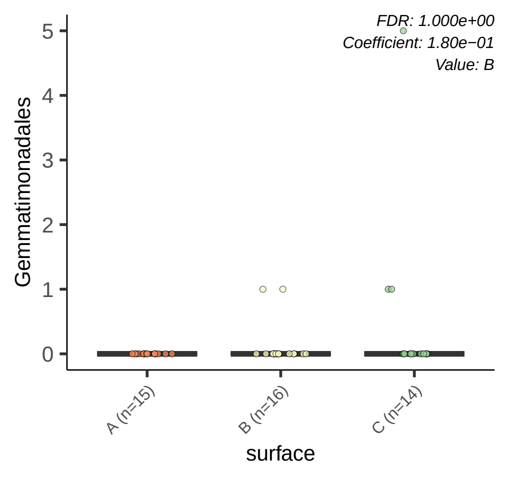
FDR: 1.000e+00
Coefficient: 1.80e−01
Value: B
Gemmatimonadales
0
1
2
3
4
5
A (n=15)
B (n=16)
C (n=14)
surface

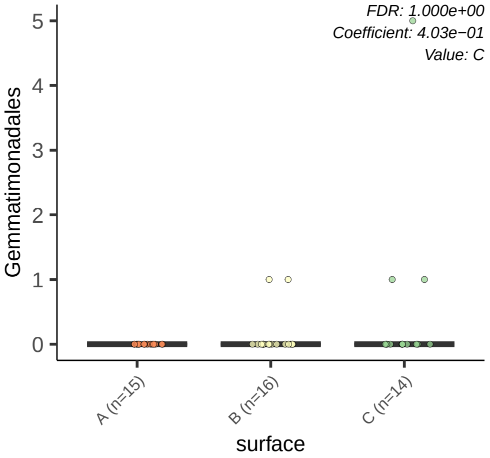
FDR: 1.000e+00
Coefficient: 4.03e−01
Value: C
Gemmatimonadales
0
1
2
3
4
5
A (n=15)
B (n=16)
C (n=14)
surface

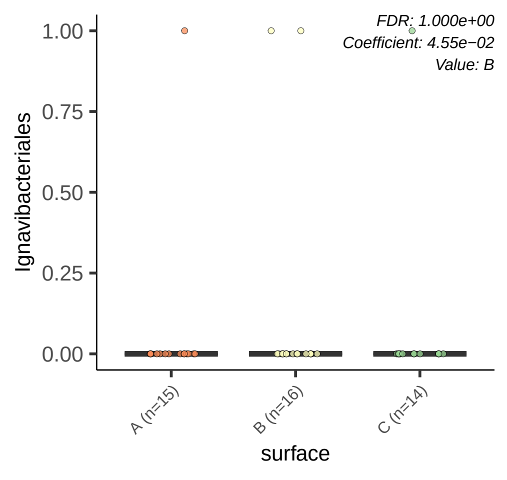
FDR: 1.000e+00
Coefficient: 4.55e−02
Value: B
Ignavibacteriales
1.00
0.75
0.50
0.25
0.00
A (n=15)
B (n=16)
C (n=14)
surface

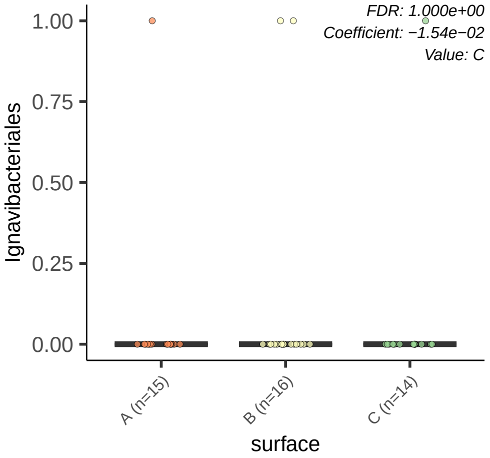
FDR: 1.000e+00
Coefficient: −1.54e−02
Value: C
Ignavibacteriales
1.00
0.75
0.50
0.25
0.00
A (n=15)
B (n=16)
C (n=14)
surface

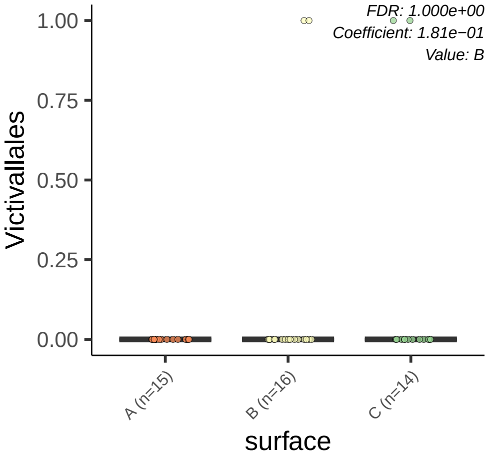
FDR: 1.000e+00
Coefficient: 1.81e−01
Value: B
1.00
0.75
0.50
0.25
0.00
Victivallales
A (n=15)
B (n=16)
C (n=14)
surface

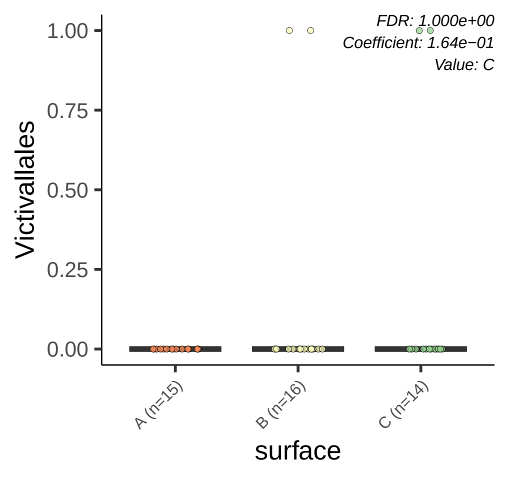
FDR: 1.000e+00
Coefficient: 1.64e−01
Value: C
Victivallales
1.00
0.75
0.50
0.25
0.00
A (n=15)
B (n=16)
C (n=14)
surface

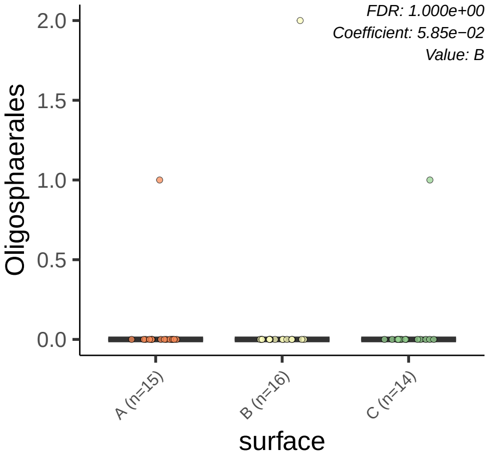
FDR: 1.000e+00
Coefficient: 5.85e−02
Value: B
Oligosphaerales
2.0
1.5
1.0
0.5
0.0
A (n=15)
B (n=16)
C (n=14)
surface

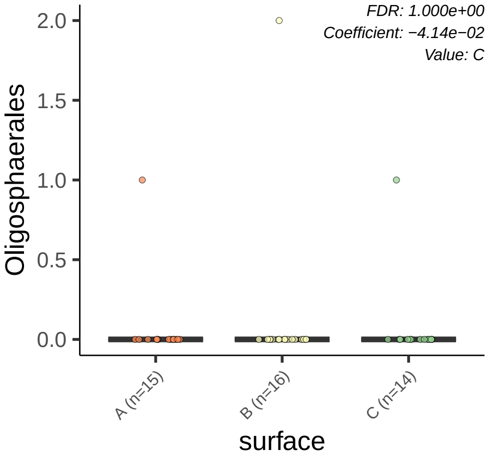
FDR: 1.000e+00
Coefficient: −4.14e−02
Value: C
Oligosphaerales
2.0
1.5
1.0
0.5
0.0
A (n=15)
B (n=16)
C (n=14)
surface

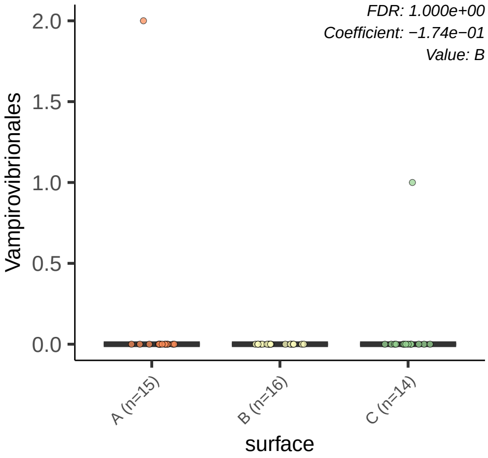

FDR: 1.000e+00
Coefficient: −1.74e−01
Value: B
Vampirovibrionales
2.0
1.5
1.0
0.5
0.0
A (n=15)
B (n=16)
C (n=14)
surface

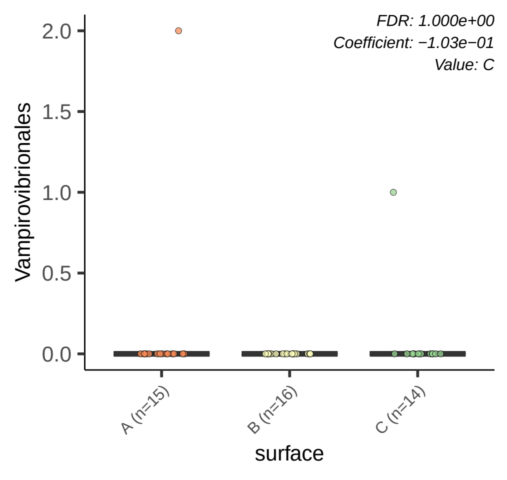
FDR: 1.000e+00
Coefficient: −1.03e−01
Value: C
Vampirovibrionales
2.0
1.5
1.0
0.5
0.0
A (n=15)
B (n=16)
C (n=14)
surface

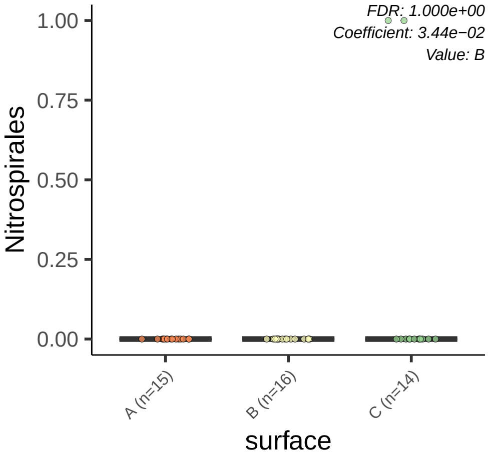
FDR: 1.000e+00
Coefficient: 3.44e−02
Value: B
Nitrospirales
1.00
0.75
0.50
0.25
0.00
A (n=15)
B (n=16)
C (n=14)
surface

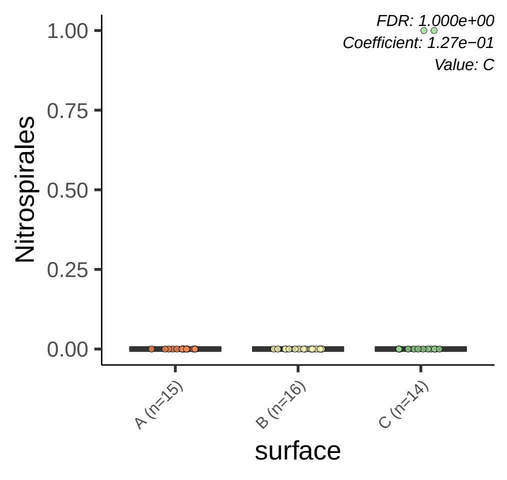
FDR: 1.000e+00
Coefficient: 1.27e−01
Value: C
Nitrospirales
1.00
0.75
0.50
0.25
0.00
A (n=15)
B (n=16)
C (n=14)
surface

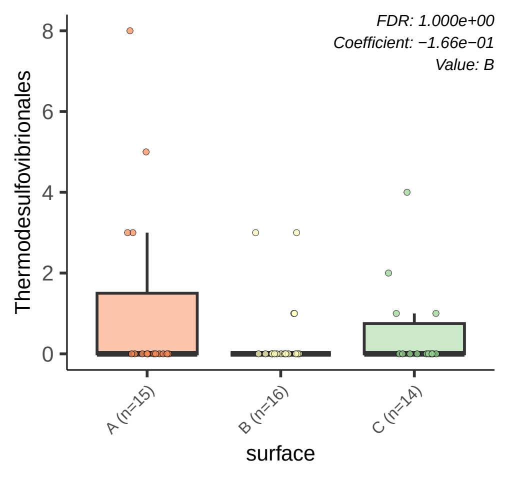

FDR: 1.000e+00
Coefficient: −1.66e−01
Value: B
Thermodesulfovibrionales
0
2
4
6
8
A (n=15)
B (n=16)
C (n=14)
surface

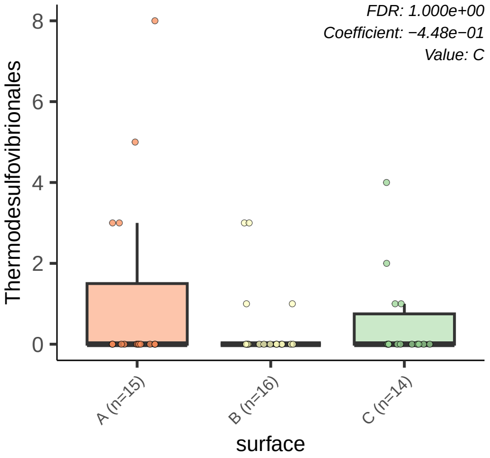

FDR: 1.000e+00
Coefficient: −4.48e−01
Value: C
Thermodesulfovibrionales
8
6
4
2
0
A (n=15)
B (n=16)
C (n=14)
surface

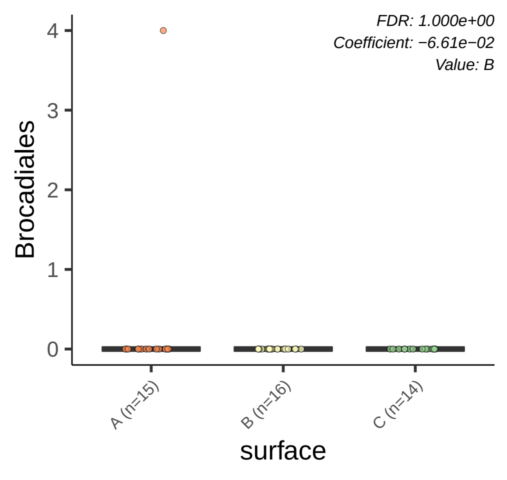
FDR: 1.000e+00
Coefficient: −6.61e−02
Value: B
Brocadiales
4
3
2
1
0
A (n=15)
B (n=16)
C (n=14)
surface

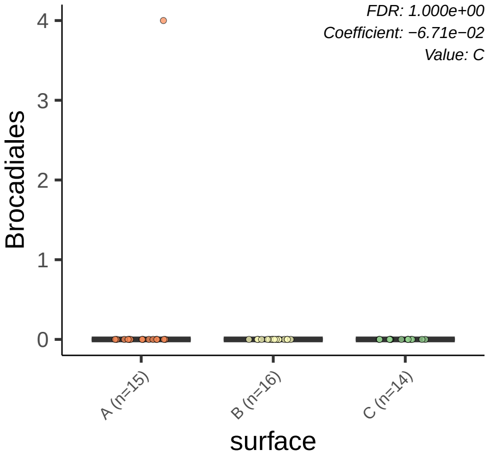
FDR: 1.000e+00
Coefficient: −6.71e−02
Value: C
Brocadiales
4
3
2
1
0
A (n=15)
B (n=16)
C (n=14)
surface

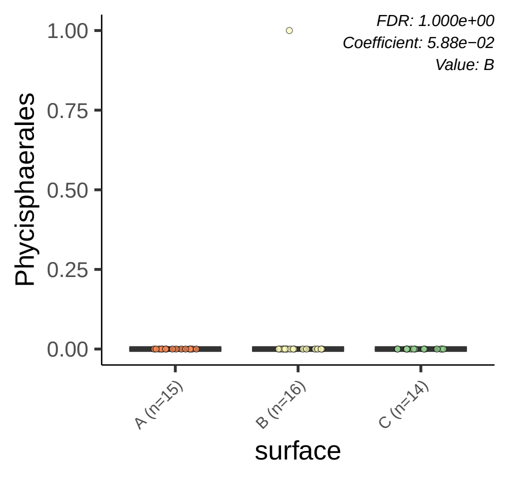
FDR: 1.000e+00
Coefficient: 5.88e−02
Value: B
Phycisphaerales
1.00
0.75
0.50
0.25
0.00
A (n=15)
B (n=16)
C (n=14)
surface

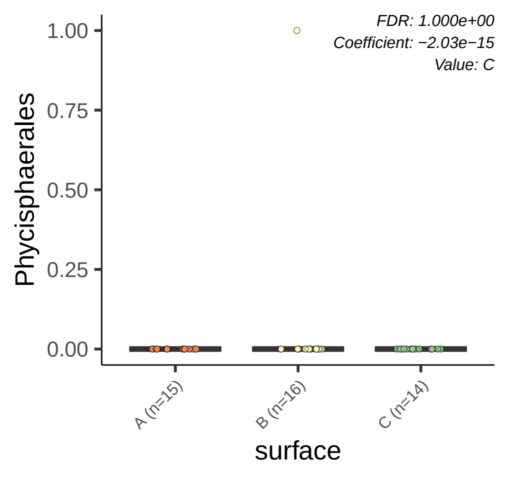

FDR: 1.000e+00
Coefficient: −2.03e−15
Value: C
Phycisphaerales
1.00
0.75
0.50
0.25
0.00
A (n=15)
B (n=16)
C (n=14)
surface

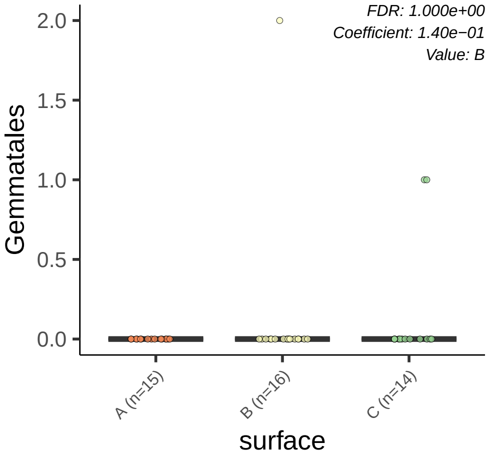
FDR: 1.000e+00
Coefficient: 1.40e−01
Value: B
Gemmatales
2.0
1.5
1.0
0.5
0.0
A (n=15)
B (n=16)
C (n=14)
surface

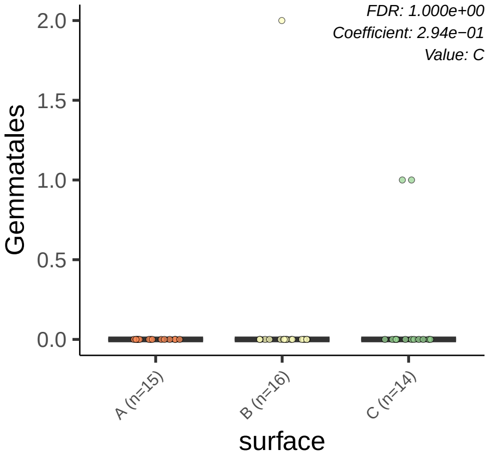
FDR: 1.000e+00
Coefficient: 2.94e−01
Value: C
Gemmatales
2.0
1.5
1.0
0.5
0.0
A (n=15)
B (n=16)
C (n=14)
surface

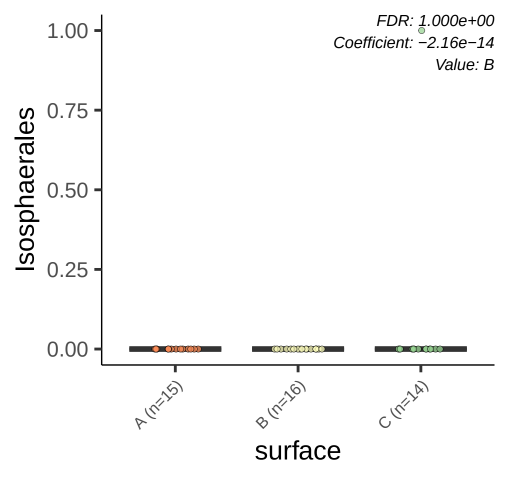

FDR: 1.000e+00
Coefficient: −2.16e−14
Value: B
Isosphaerales
1.00
0.75
0.50
0.25
0.00
A (n=15)
B (n=16)
C (n=14)
surface

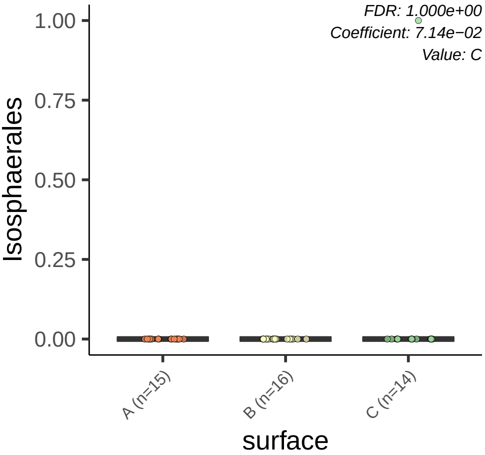
FDR: 1.000e+00
Coefficient: 7.14e−02
Value: C
Isosphaerales
1.00
0.75
0.50
0.25
0.00
A (n=15)
B (n=16)
C (n=14)
surface

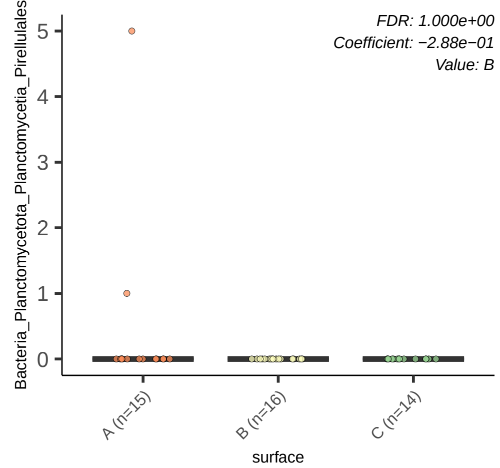

FDR: 1.000e+00
Coefficient: −2.88e−01
Value: B
Bacteria_Planctomycetota_Planctomycetia_Pirellulales
5
4
3
2
1
0
A (n=15)
B (n=16)
C (n=14)
surface

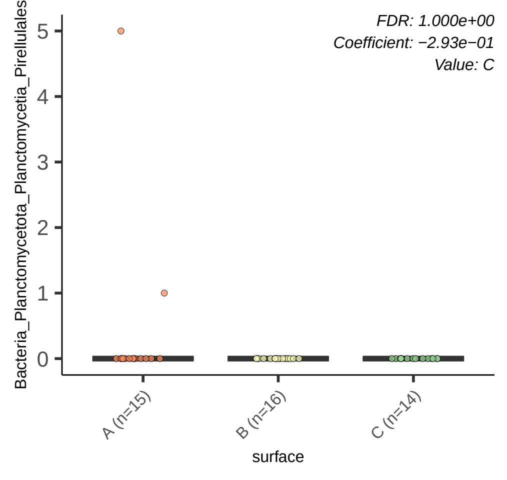
FDR: 1.000e+00
Coefficient: −2.93e−01
Value: C
Bacteria_Planctomycetota_Planctomycetia_Pirellulales
0
1
2
3
4
5
A (n=15)
B (n=16)
C (n=14)
surface

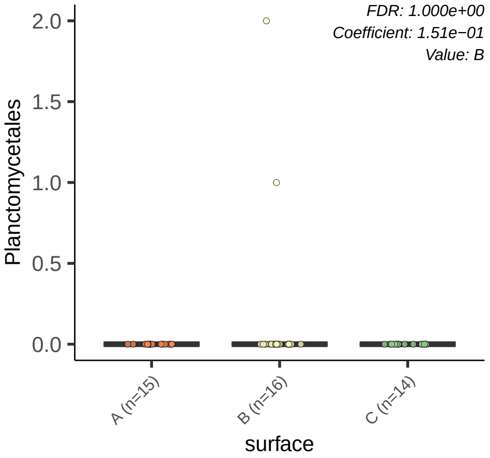

FDR: 1.000e+00
Coefficient: 1.51e−01
Value: B
Planctomycetales
2.0
1.5
1.0
0.5
0.0
A (n=15)
B (n=16)
C (n=14)
surface

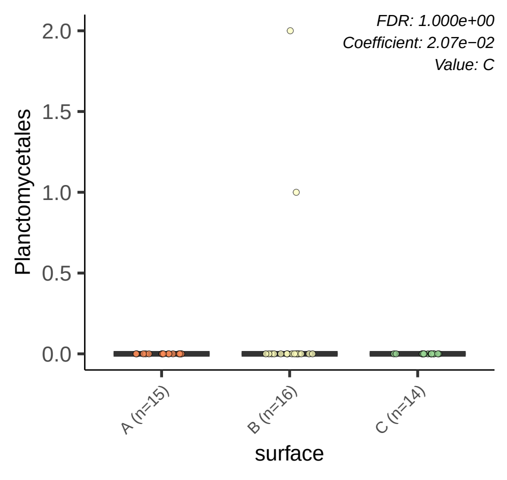
FDR: 1.000e+00
Coefficient: 2.07e−02
Value: C
Planctomycetales
2.0
1.5
1.0
0.5
0.0
A (n=15)
B (n=16)
C (n=14)
surface

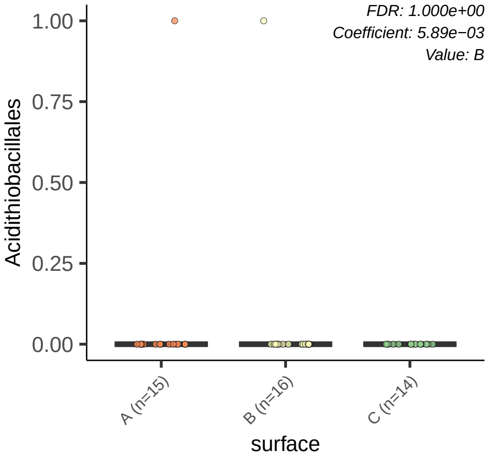

FDR: 1.000e+00
Coefficient: 5.89e−03
Value: B
Acidithiobacillales
1.00
0.75
0.50
0.25
0.00
A (n=15)
B (n=16)
C (n=14)
surface

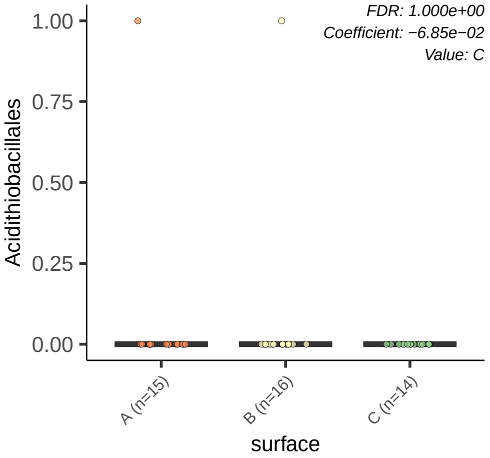
FDR: 1.000e+00
Coefficient: −6.85e−02
Value: C
Acidithiobacillales
1.00
0.75
0.50
0.25
0.00
A (n=15)
B (n=16)
C (n=14)
surface

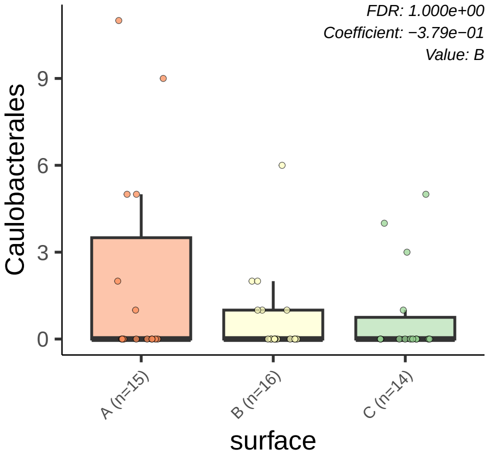
FDR: 1.000e+00
Coefficient: −3.79e−01
Value: B
Caulobacterales
0
3
6
9
A (n=15)
B (n=16)
C (n=14)
surface

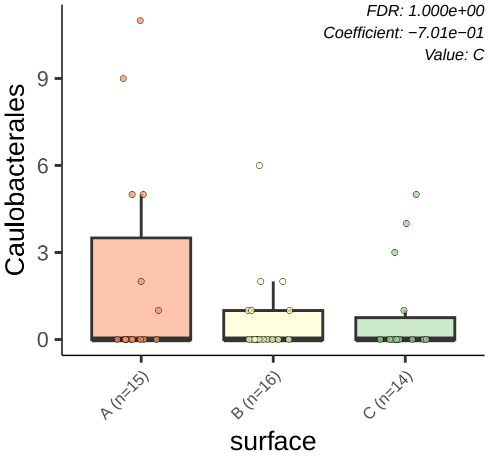
FDR: 1.000e+00
Coefficient: −7.01e−01
Value: C
Caulobacterales
0
3
6
9
A (n=15)
B (n=16)
C (n=14)
surface

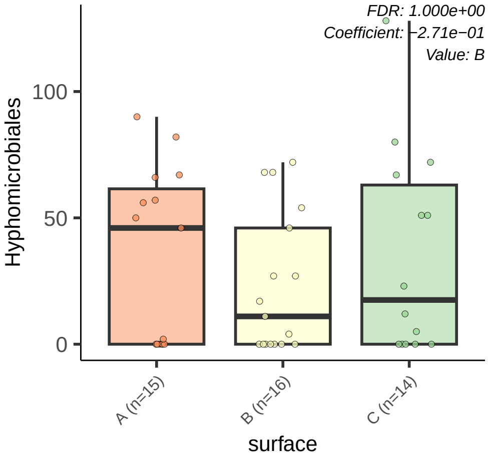
FDR: 1.000e+00
Coefficient: −2.71e−01
Value: B
Hyphomicrobiales
100
50
0
A (n=15)
B (n=16)
C (n=14)
surface

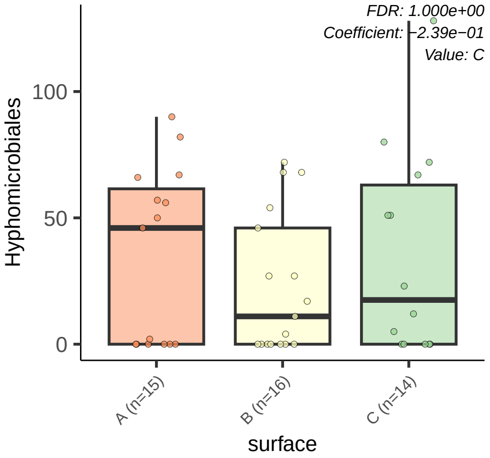

FDR: 1.000e+00
Coefficient: −2.39e−01
Value: C
Hyphomicrobiales
100
50
0
A (n=15)
B (n=16)
C (n=14)
surface

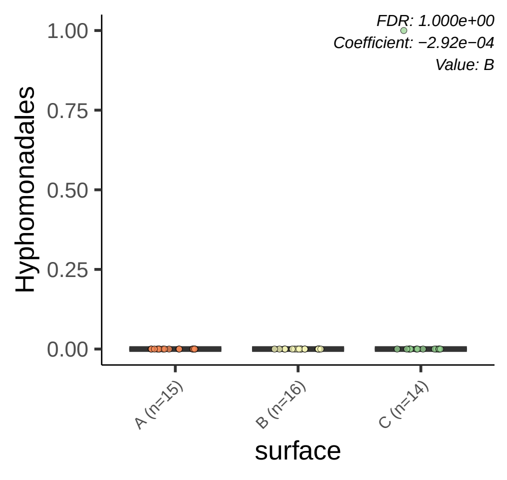

FDR: 1.000e+00
Coefficient: −2.92e−04
Value: B
Hyphomonadales
1.00
0.75
0.50
0.25
0.00
A (n=15)
B (n=16)
C (n=14)
surface

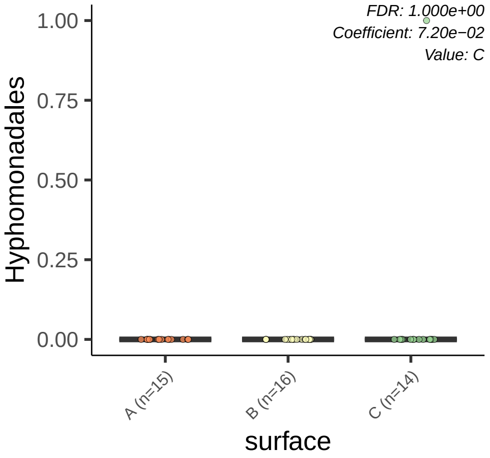
FDR: 1.000e+00
Coefficient: 7.20e−02
Value: C
Hyphomonadales
1.00
0.75
0.50
0.25
0.00
A (n=15)
B (n=16)
C (n=14)
surface

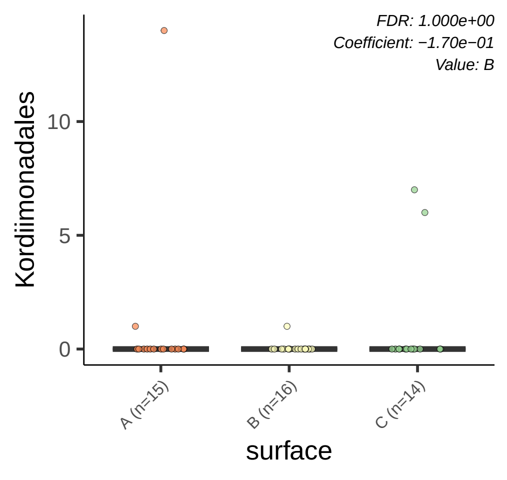

FDR: 1.000e+00
Coefficient: −1.70e−01
Value: B
Kordiimonadales
10
5
0
A (n=15)
B (n=16)
C (n=14)
surface

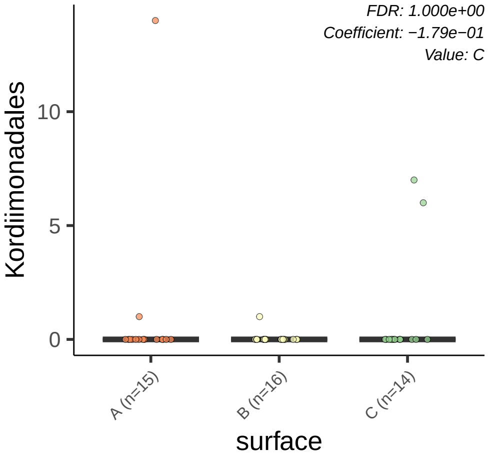
FDR: 1.000e+00
Coefficient: −1.79e−01
Value: C
Kordiimonadales
10
5
0
A (n=15)
B (n=16)
C (n=14)
surface

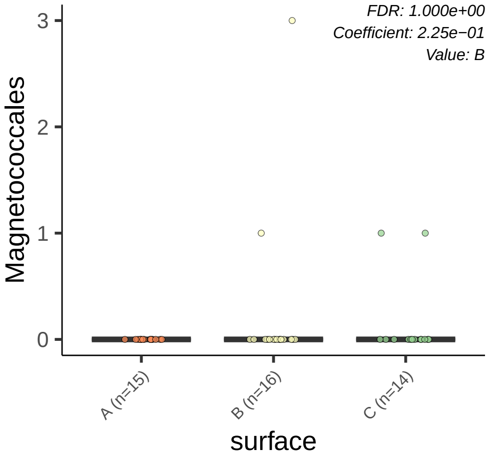

FDR: 1.000e+00
Coefficient: 2.25e−01
Value: B
Magnetococcales
0
1
2
3
A (n=15)
B (n=16)
C (n=14)
surface

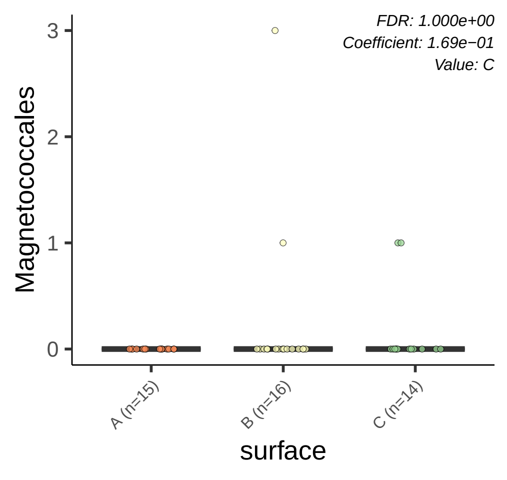
FDR: 1.000e+00
Coefficient: 1.69e−01
Value: C
Magnetococcales
3
2
1
0
A (n=15)
B (n=16)
C (n=14)
surface

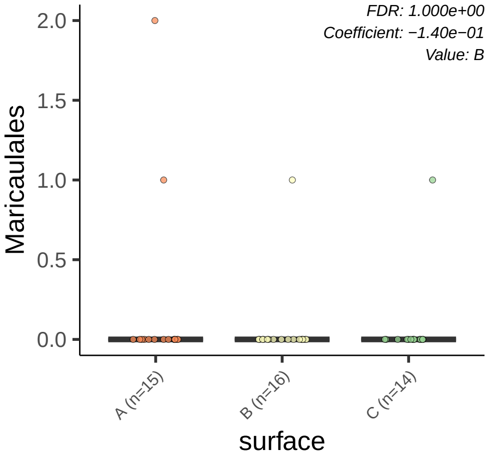
FDR: 1.000e+00
Coefficient: −1.40e−01
Value: B
Maricaulales
2.0
1.5
1.0
0.5
0.0
A (n=15)
B (n=16)
C (n=14)
surface

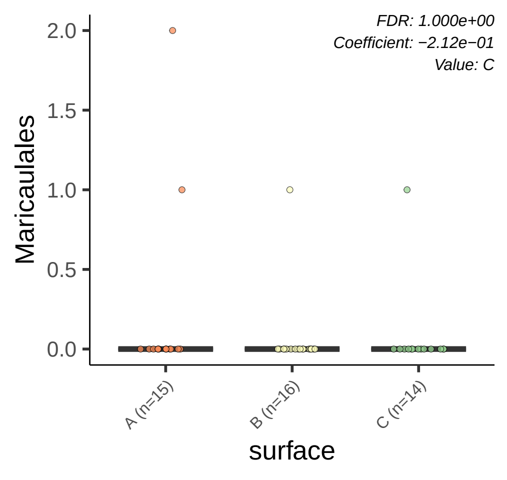
FDR: 1.000e+00
Coefficient: −2.12e−01
Value: C
Maricaulales
2.0
1.5
1.0
0.5
0.0
A (n=15)
B (n=16)
C (n=14)
surface

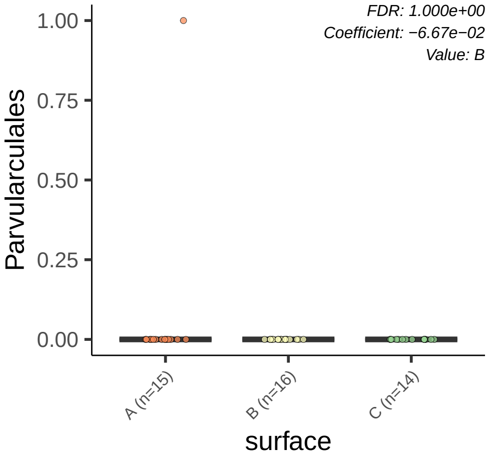
FDR: 1.000e+00
Coefficient: −6.67e−02
Value: B
Parvularculales
1.00
0.75
0.50
0.25
0.00
A (n=15)
B (n=16)
C (n=14)
surface

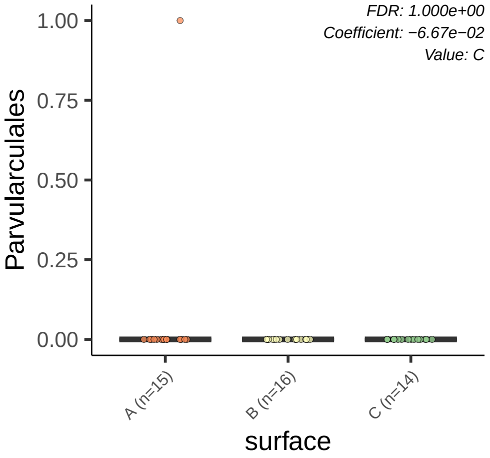
FDR: 1.000e+00
Coefficient: −6.67e−02
Value: C
Parvularculales
1.00
0.75
0.50
0.25
0.00
A (n=15)
B (n=16)
C (n=14)
surface

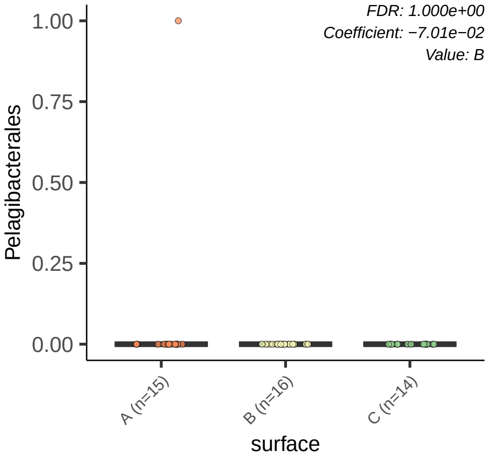
FDR: 1.000e+00
Coefficient: −7.01e−02
Value: B
Pelagibacterales
1.00
0.75
0.50
0.25
0.00
A (n=15)
B (n=16)
C (n=14)
surface

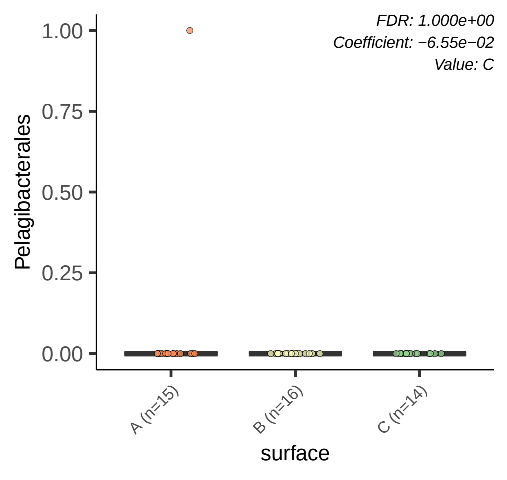
FDR: 1.000e+00
Coefficient: −6.55e−02
Value: C
Pelagibacterales
1.00
0.75
0.50
0.25
0.00
A (n=15)
B (n=16)
C (n=14)
surface

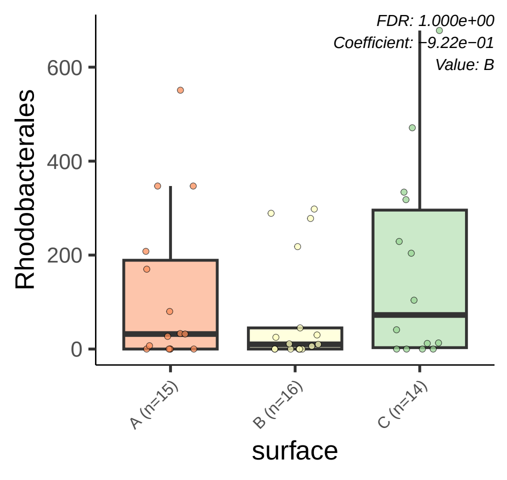

FDR: 1.000e+00
Coefficient: −9.22e−01
Value: B
Rhodobacterales
600
400
200
0
A (n=15)
B (n=16)
C (n=14)
surface

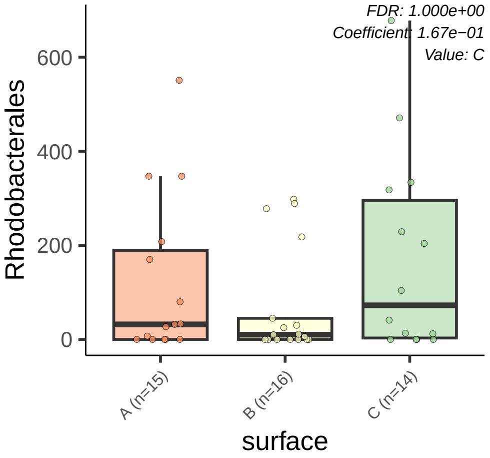
FDR: 1.000e+00
Coefficient: 1.67e−01
Value: C
Rhodobacterales
600
400
200
0
A (n=15)
B (n=16)
C (n=14)
surface

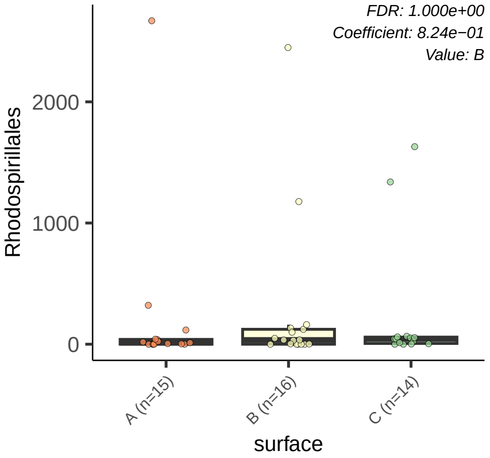
FDR: 1.000e+00
Coefficient: 8.24e−01
Value: B
Rhodospirillales
2000
1000
0
A (n=15)
B (n=16)
C (n=14)
surface

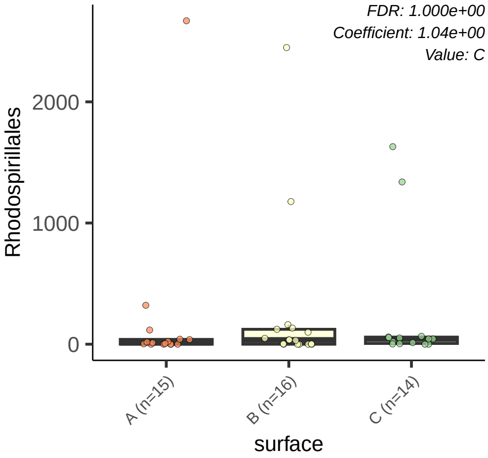

FDR: 1.000e+00
Coefficient: 1.04e+00
Value: C
Rhodospirillales
2000
1000
0
A (n=15)
B (n=16)
C (n=14)
surface

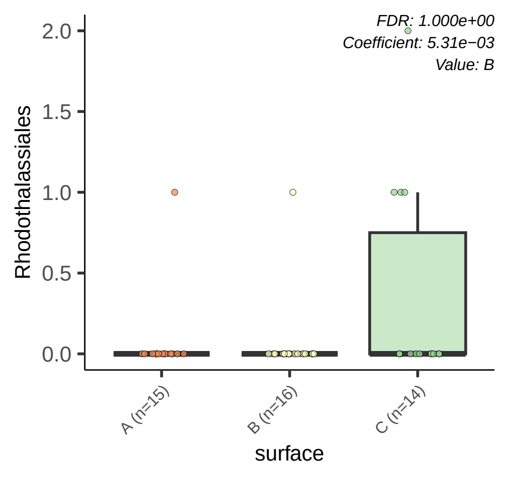
FDR: 1.000e+00
Coefficient: 5.31e−03
Value: B
Rhodothalassiales
2.0
1.5
1.0
0.5
0.0
A (n=15)
B (n=16)
C (n=14)
surface

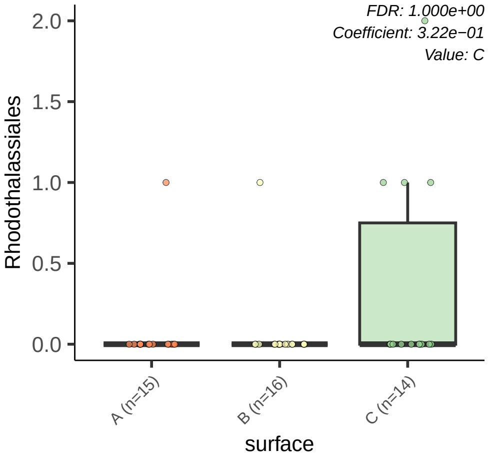
FDR: 1.000e+00
Coefficient: 3.22e−01
Value: C
Rhodothalassiales
2.0
1.5
1.0
0.5
0.0
A (n=15)
B (n=16)
C (n=14)
surface

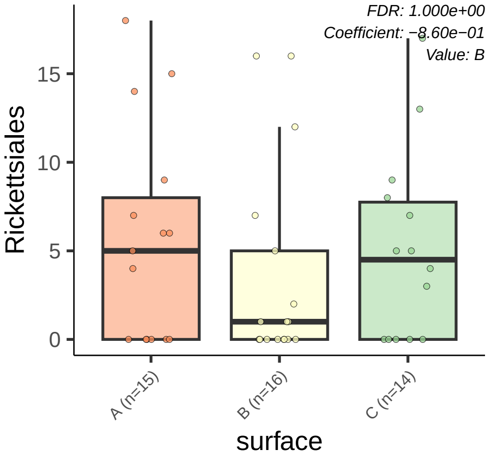

FDR: 1.000e+00
Coefficient: −8.60e−01
Value: B
Rickettsiales
0
5
10
15
A (n=15)
B (n=16)
C (n=14)
surface

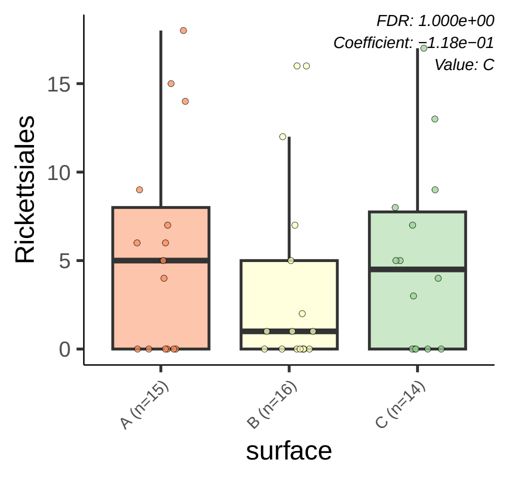
FDR: 1.000e+00
Coefficient: −1.18e−01
Value: C
Rickettsiales
0
5
10
15
A (n=15)
B (n=16)
C (n=14)
surface

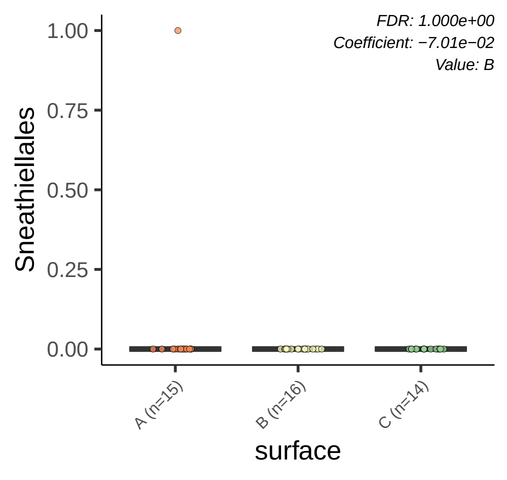
FDR: 1.000e+00
Coefficient: −7.01e−02
Value: B
Sneathiellales
1.00
0.75
0.50
0.25
0.00
A (n=15)
B (n=16)
C (n=14)
surface

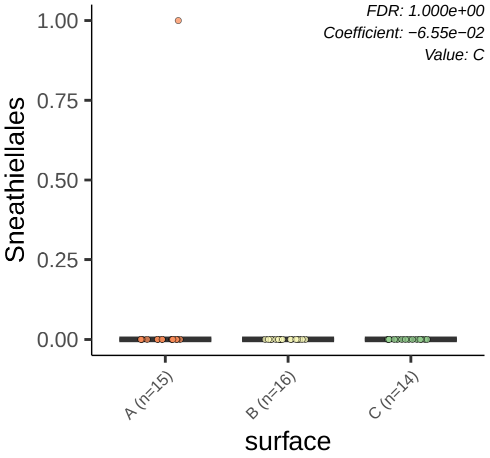
FDR: 1.000e+00
Coefficient: −6.55e−02
Value: C
Sneathiellales
1.00
0.75
0.50
0.25
0.00
A (n=15)
B (n=16)
C (n=14)
surface

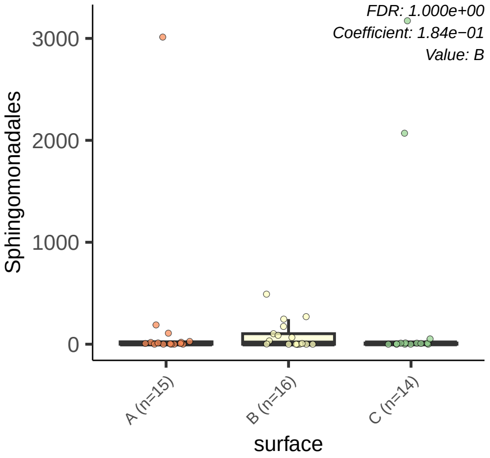

FDR: 1.000e+00
Coefficient: 1.84e−01
Value: B
Sphingomonadales
3000
2000
1000
0
A (n=15)
B (n=16)
C (n=14)
surface

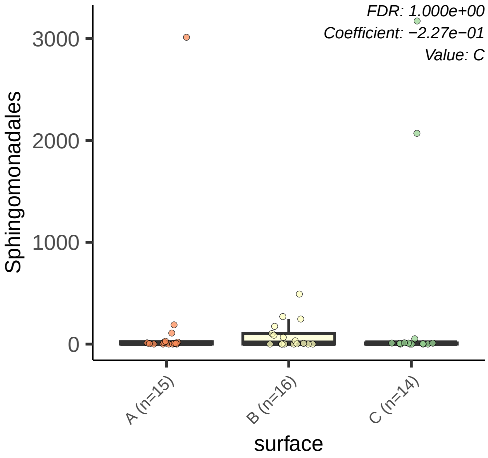

FDR: 1.000e+00
Coefficient: −2.27e−01
Value: C
Sphingomonadales
3000
2000
1000
0
A (n=15)
B (n=16)
C (n=14)
surface

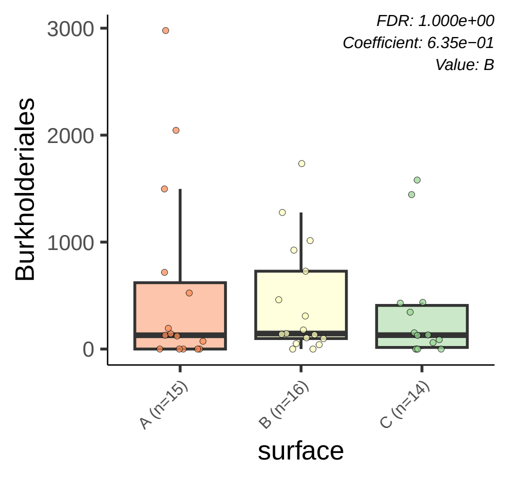
FDR: 1.000e+00
Coefficient: 6.35e−01
Value: B
Burkholderiales
3000
2000
1000
0
A (n=15)
B (n=16)
C (n=14)
surface

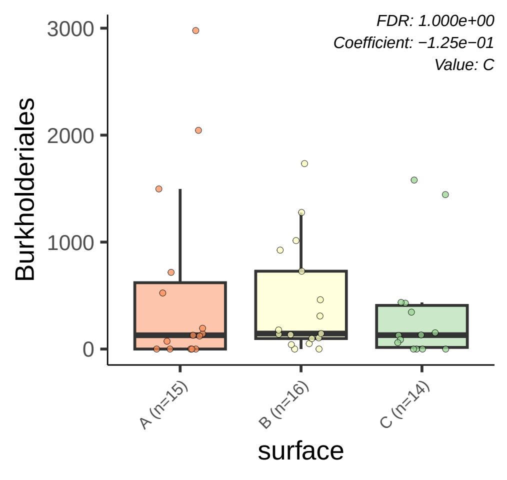

FDR: 1.000e+00
Coefficient: −1.25e−01
Value: C
Burkholderiales
3000
2000
1000
0
A (n=15)
B (n=16)
C (n=14)
surface

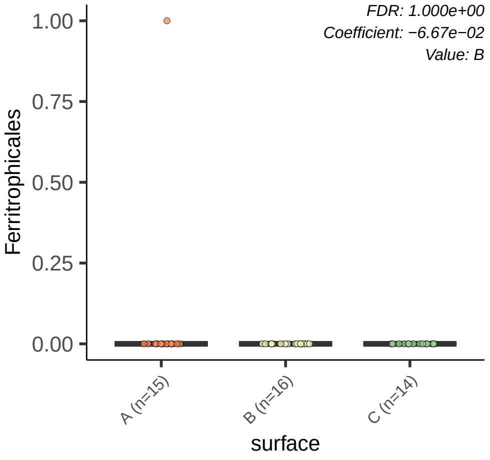
FDR: 1.000e+00
Coefficient: −6.67e−02
Value: B
Ferritrophicales
1.00
0.75
0.50
0.25
0.00
A (n=15)
B (n=16)
C (n=14)
surface

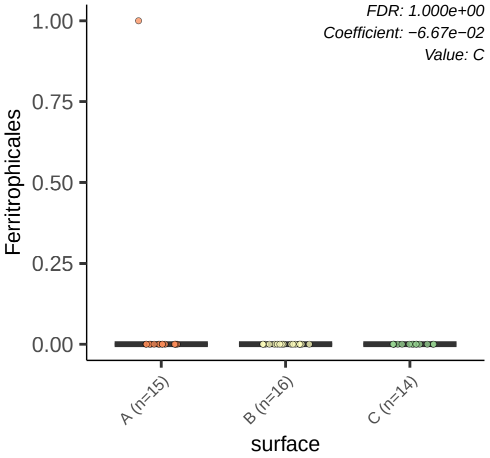
FDR: 1.000e+00
Coefficient: −6.67e−02
Value: C
Ferritrophicales
1.00
0.75
0.50
0.25
0.00
A (n=15)
B (n=16)
C (n=14)
surface

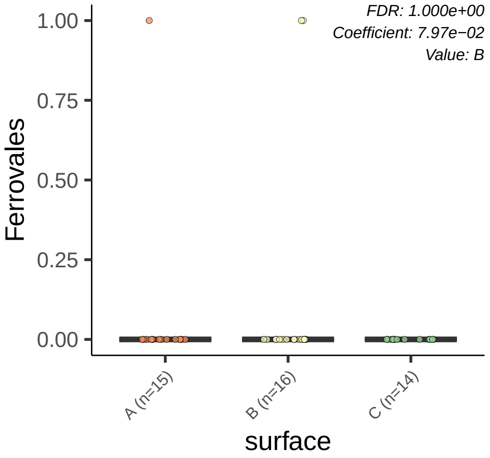
FDR: 1.000e+00
Coefficient: 7.97e−02
Value: B
Ferrovales
1.00
0.75
0.50
0.25
0.00
A (n=15)
B (n=16)
C (n=14)
surface

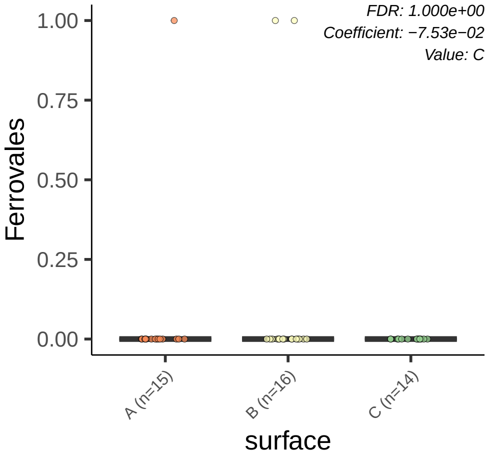
FDR: 1.000e+00
Coefficient: −7.53e−02
Value: C
1.00
0.75
0.50
0.25
0.00
Ferrovales
A (n=15)
B (n=16)
C (n=14)
surface

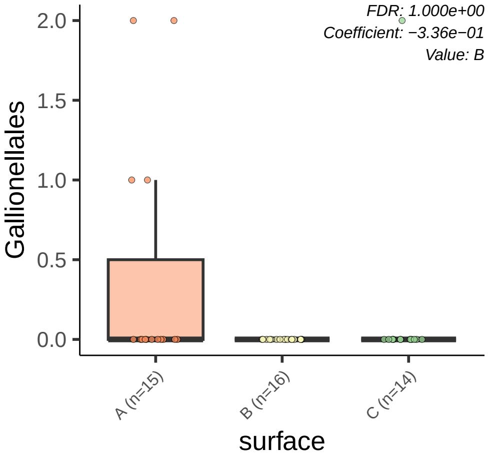

FDR: 1.000e+00
Coefficient: −3.36e−01
Value: B
Gallionellales
2.0
1.5
1.0
0.5
0.0
A (n=15)
B (n=16)
C (n=14)
surface

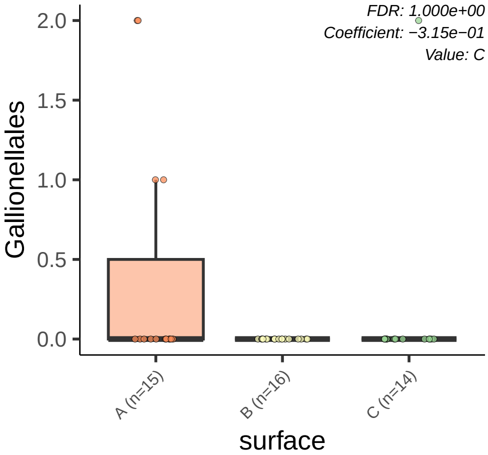
FDR: 1.000e+00
Coefficient: −3.15e−01
Value: C
Gallionellales
2.0
1.5
1.0
0.5
0.0
A (n=15)
B (n=16)
C (n=14)
surface

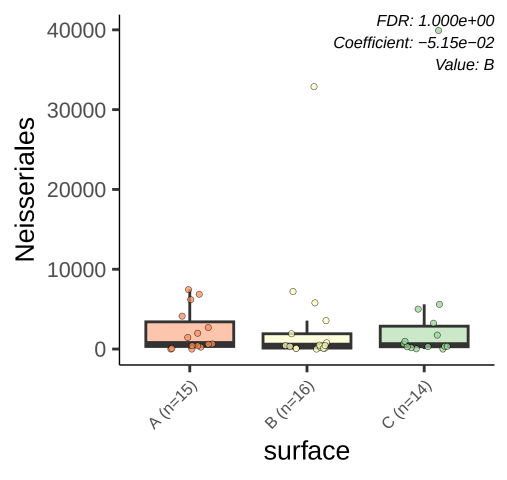
FDR: 1.000e+00
Coefficient: −5.15e−02
Value: B
Neisseriales
40000
30000
20000
10000
0
A (n=15)
B (n=16)
C (n=14)
surface

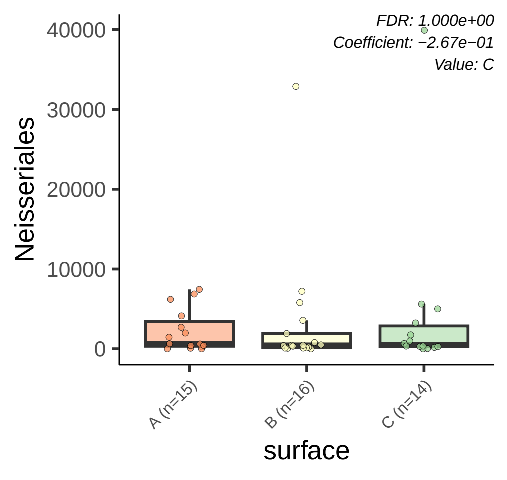
FDR: 1.000e+00
Coefficient: −2.67e−01
Value: C
Neisseriales
40000
30000
20000
10000
0
A (n=15)
B (n=16)
C (n=14)
surface

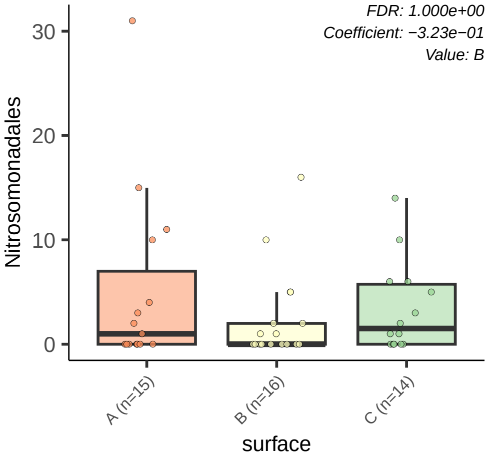

FDR: 1.000e+00
Coefficient: −3.23e−01
Value: B
Nitrosomonadales
30
20
10
0
A (n=15)
B (n=16)
C (n=14)
surface

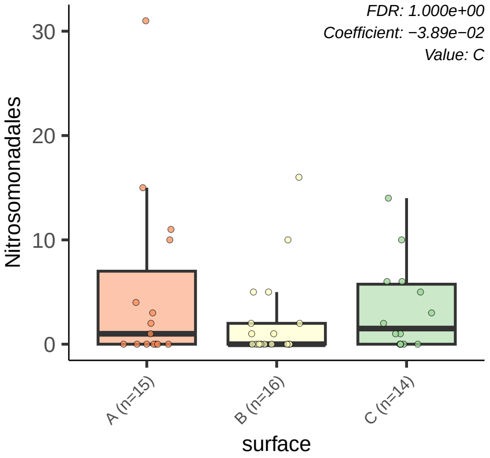
FDR: 1.000e+00
Coefficient: −3.89e−02
Value: C
Nitrosomonadales
30
20
10
0
A (n=15)
B (n=16)
C (n=14)
surface

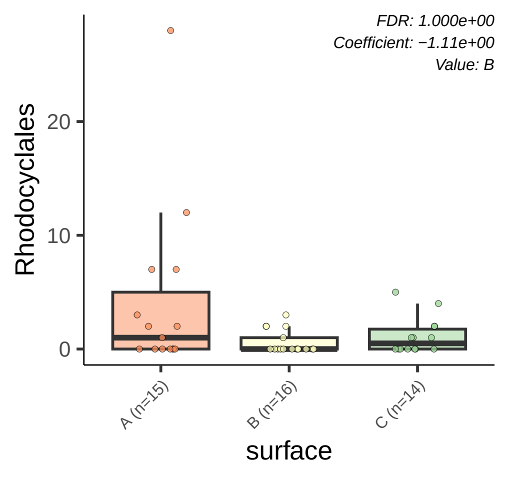

FDR: 1.000e+00
Coefficient: −1.11e+00
Value: B
Rhodocyclales
0
10
20
A (n=15)
B (n=16)
C (n=14)
surface

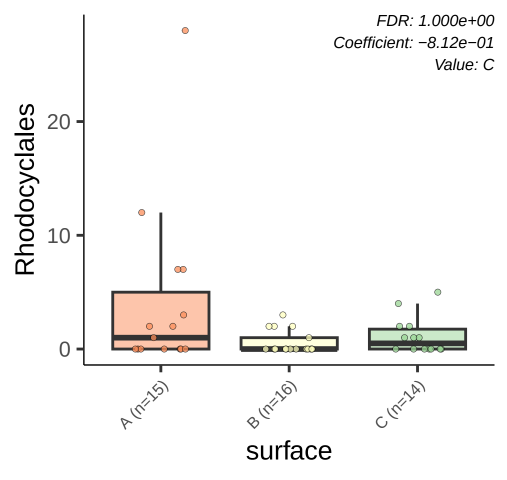
FDR: 1.000e+00
Coefficient: −8.12e−01
Value: C
Rhodocyclales
0
10
20
A (n=15)
B (n=16)
C (n=14)
surface

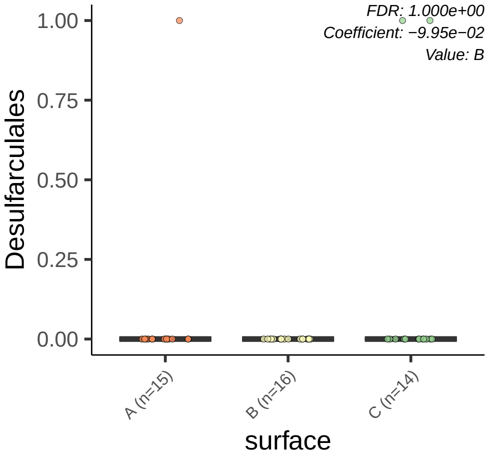
FDR: 1.000e+00
Coefficient: −9.95e−02
Value: B
Desulfarculales
1.00
0.75
0.50
0.25
0.00
A (n=15)
B (n=16)
C (n=14)
surface

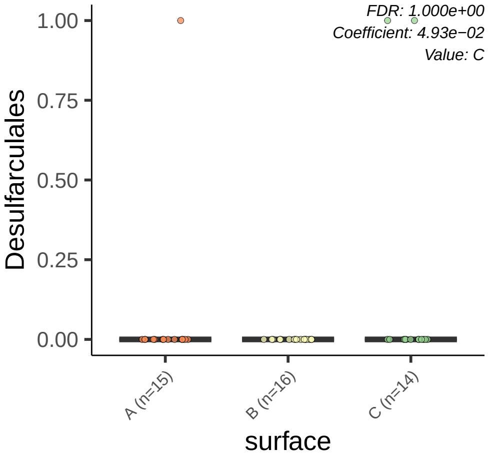

FDR: 1.000e+00
Coefficient: 4.93e−02
Value: C
Desulfarculales
1.00
0.75
0.50
0.25
0.00
A (n=15)
B (n=16)
C (n=14)
surface

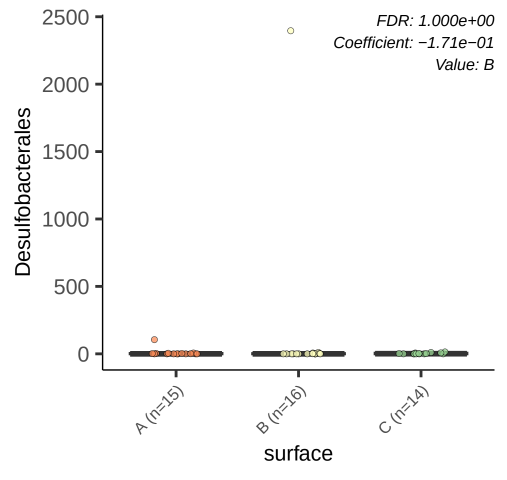

FDR: 1.000e+00
Coefficient: −1.71e−01
Value: B
Desulfobacterales
2500
2000
1500
1000
500
0
A (n=15)
B (n=16)
C (n=14)
surface

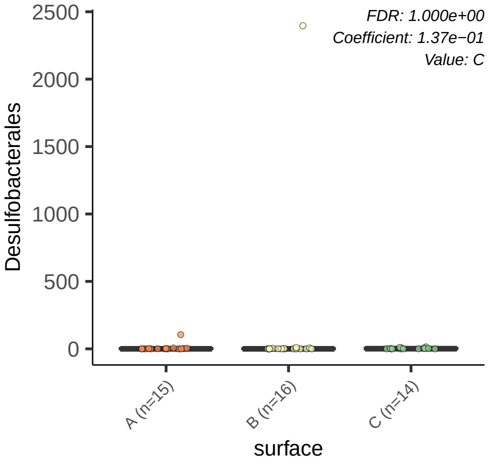
FDR: 1.000e+00
Coefficient: 1.37e−01
Value: C
Desulfobacterales
2500
2000
1500
1000
500
0
A (n=15)
B (n=16)
C (n=14)
surface

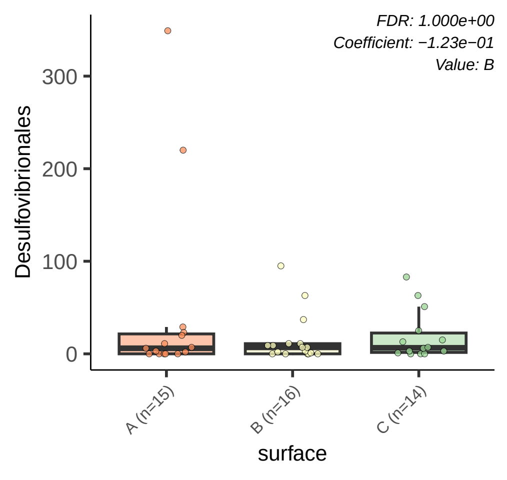

FDR: 1.000e+00
Coefficient: −1.23e−01
Value: B
Desulfovibrionales
300
200
100
0
A (n=15)
B (n=16)
C (n=14)
surface

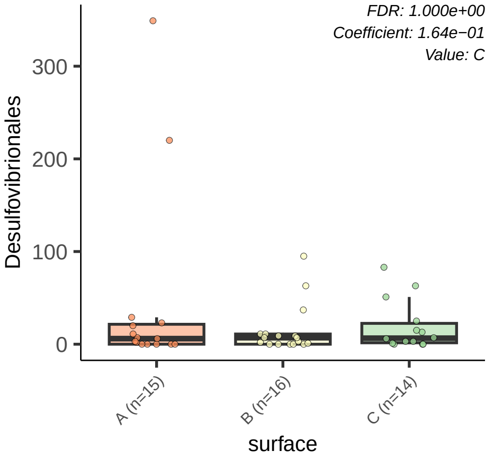

FDR: 1.000e+00
Coefficient: 1.64e−01
Value: C
Desulfovibrionales
300
200
100
0
A (n=15)
B (n=16)
C (n=14)
surface

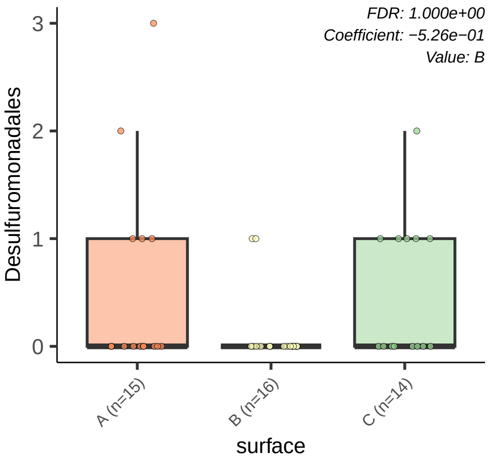

FDR: 1.000e+00
Coefficient: −5.26e−01
Value: B
Desulfuromonadales
3
2
1
0
A (n=15)
B (n=16)
C (n=14)
surface

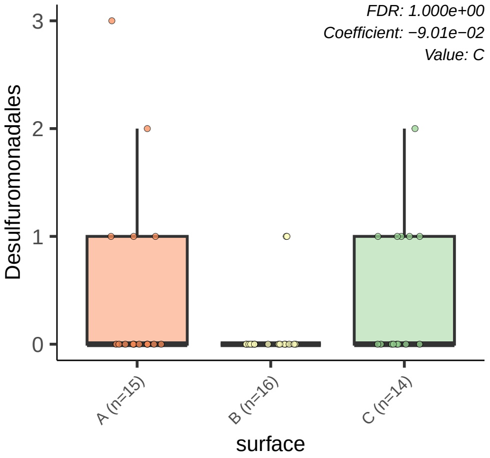

FDR: 1.000e+00
Coefficient: −9.01e−02
Value: C
Desulfuromonadales
3
2
1
0
A (n=15)
B (n=16)
C (n=14)
surface

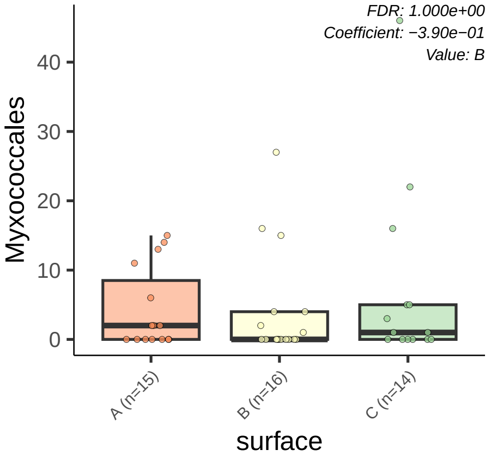
FDR: 1.000e+00
Coefficient: −3.90e−01
Value: B
Myxococcales
0
10
20
30
40
A (n=15)
B (n=16)
C (n=14)
surface

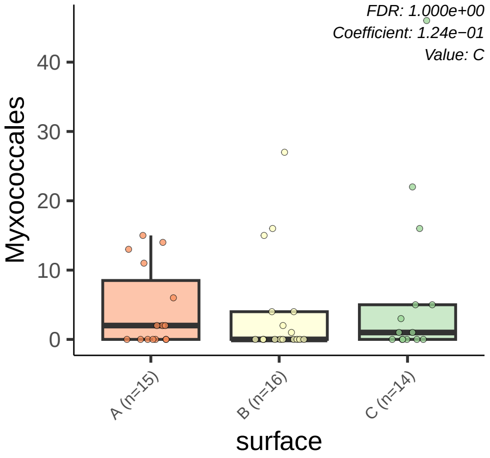

FDR: 1.000e+00
Coefficient: 1.24e−01
Value: C
Myxococcales
40
30
20
10
0
A (n=15)
B (n=16)
C (n=14)
surface

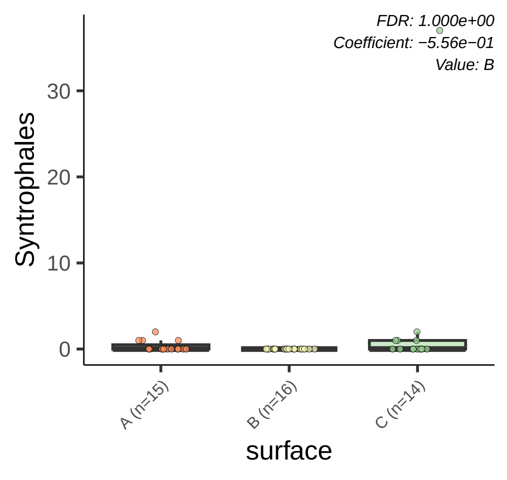
FDR: 1.000e+00
Coefficient: −5.56e−01
Value: B
Syntrophales
30
20
10
0
A (n=15)
B (n=16)
C (n=14)
surface

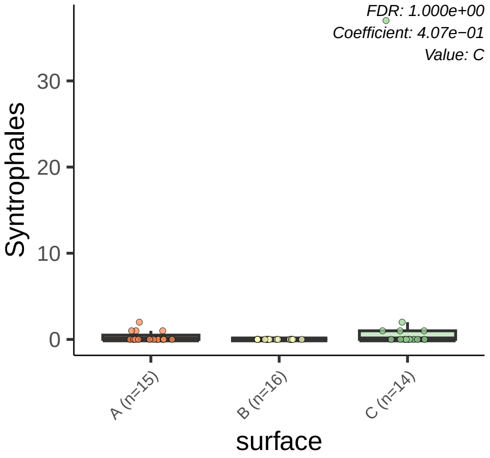
FDR: 1.000e+00
Coefficient: 4.07e−01
Value: C
Syntrophales
30
20
10
0
A (n=15)
B (n=16)
C (n=14)
surface

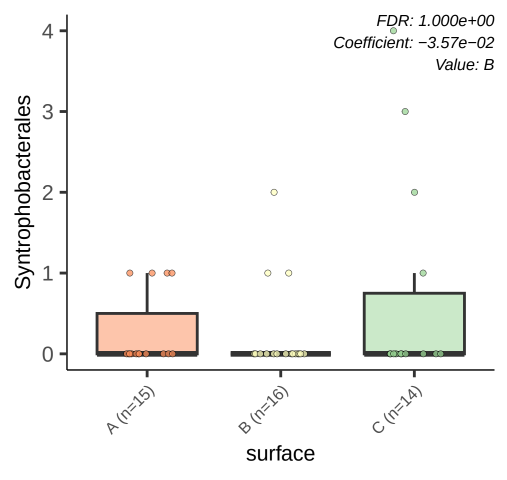

FDR: 1.000e+00
Coefficient: −3.57e−02
Value: B
Syntrophobacterales
0
1
2
3
4
A (n=15)
B (n=16)
C (n=14)
surface

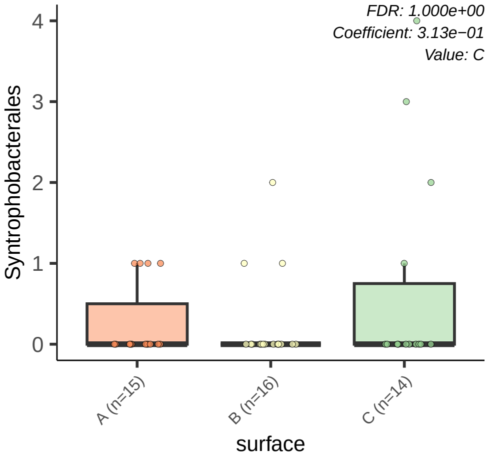

FDR: 1.000e+00
Coefficient: 3.13e−01
Value: C
Syntrophobacterales
0
1
2
3
4
A (n=15)
B (n=16)
C (n=14)
surface

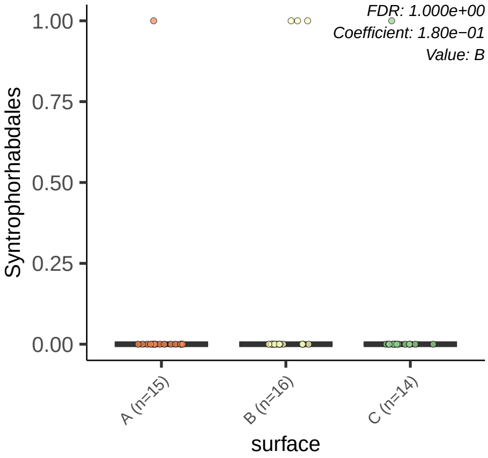

FDR: 1.000e+00
Coefficient: 1.80e−01
Value: B
Syntrophorhabdales
1.00
0.75
0.50
0.25
0.00
A (n=15)
B (n=16)
C (n=14)
surface

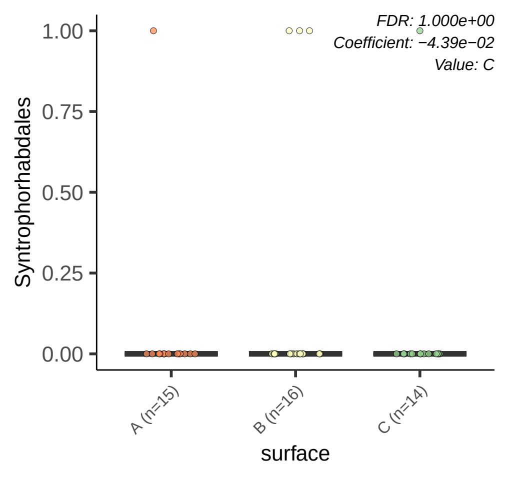
FDR: 1.000e+00
Coefficient: −4.39e−02
Value: C
Syntrophorhabdales
1.00
0.75
0.50
0.25
0.00
A (n=15)
B (n=16)
C (n=14)
surface

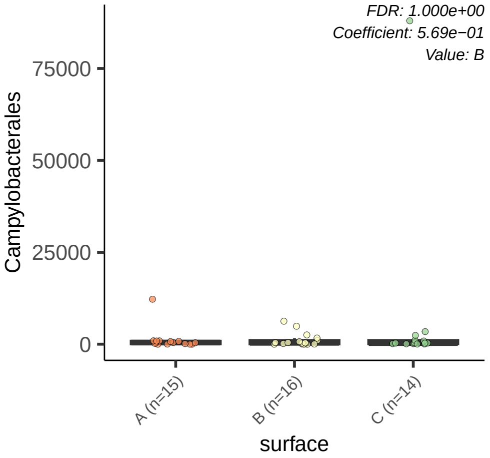

FDR: 1.000e+00
Coefficient: 5.69e−01
Value: B
Campylobacterales
75000
50000
25000
0
A (n=15)
B (n=16)
C (n=14)
surface

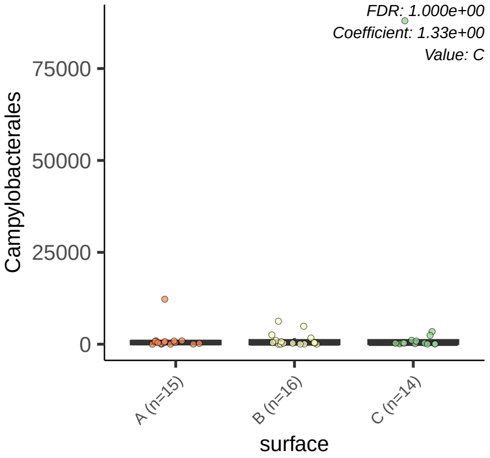
FDR: 1.000e+00
Coefficient: 1.33e+00
Value: C
Campylobacterales
75000
50000
25000
0
A (n=15)
B (n=16)
C (n=14)
surface

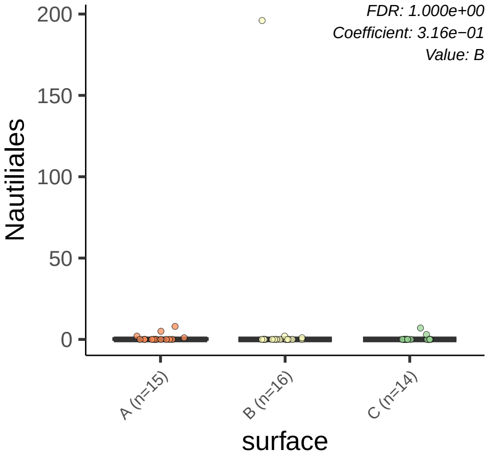

FDR: 1.000e+00
Coefficient: 3.16e−01
Value: B
Nautiliales
200
150
100
50
0
A (n=15)
B (n=16)
C (n=14)
surface

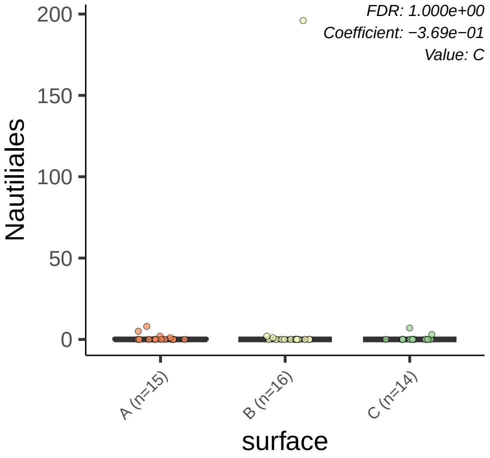

FDR: 1.000e+00
Coefficient: −3.69e−01
Value: C
Nautiliales
200
150
100
50
0
A (n=15)
B (n=16)
C (n=14)
surface

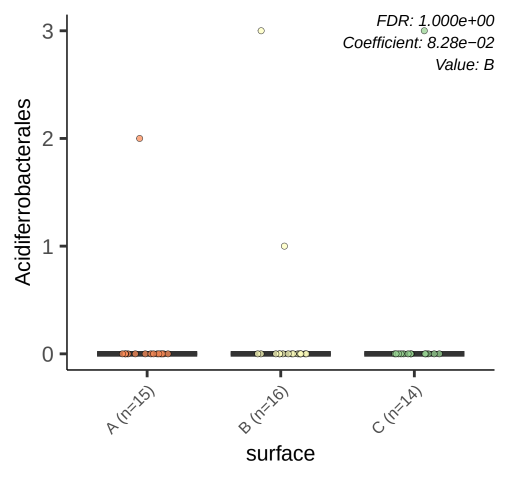

FDR: 1.000e+00
Coefficient: 8.28e−02
Value: B
Acidiferrobacterales
3
2
1
0
A (n=15)
B (n=16)
C (n=14)
surface

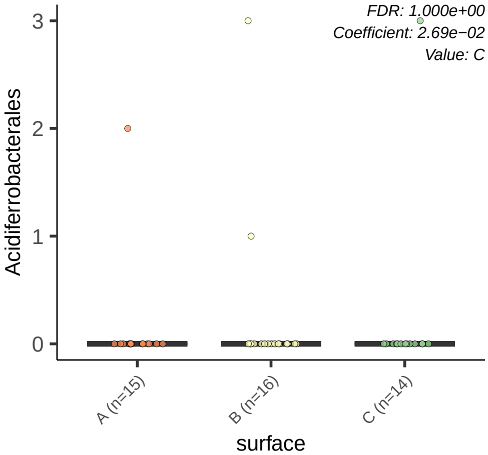

FDR: 1.000e+00
Coefficient: 2.69e−02
Value: C
Acidiferrobacterales
3
2
1
0
A (n=15)
B (n=16)
C (n=14)
surface

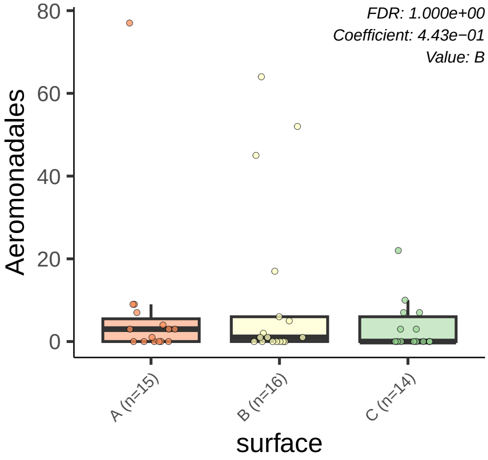

FDR: 1.000e+00
Coefficient: 4.43e−01
Value: B
Aeromonadales
80
60
40
20
0
A (n=15)
B (n=16)
C (n=14)
surface

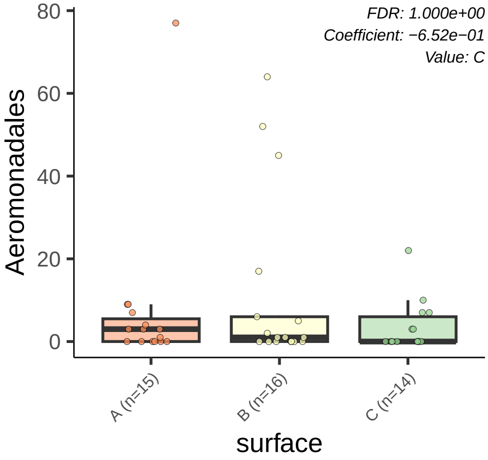
FDR: 1.000e+00
Coefficient: −6.52e−01
Value: C
Aeromonadales
80
60
40
20
0
A (n=15)
B (n=16)
C (n=14)
surface

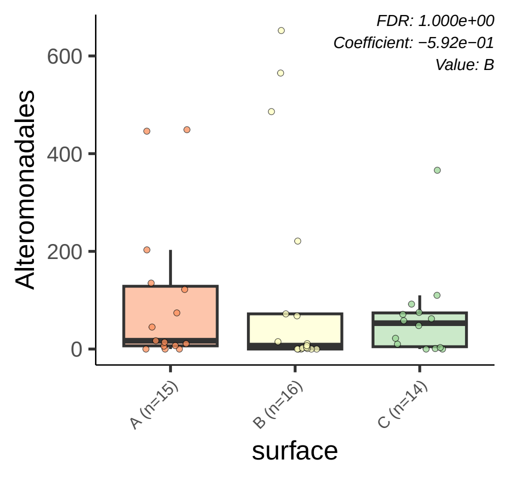
FDR: 1.000e+00
Coefficient: −5.92e−01
Value: B
Alteromonadales
600
400
200
0
A (n=15)
B (n=16)
C (n=14)
surface

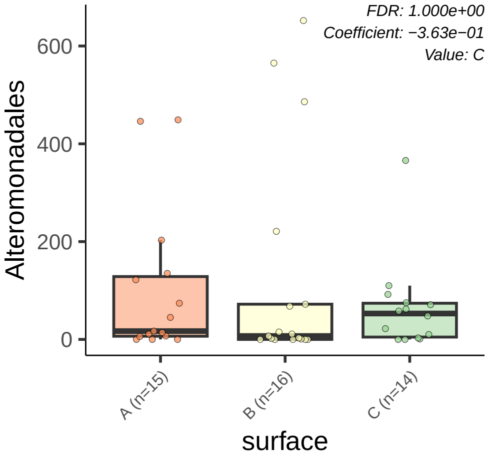

FDR: 1.000e+00
Coefficient: −3.63e−01
Value: C
Alteromonadales
600
400
200
0
A (n=15)
B (n=16)
C (n=14)
surface

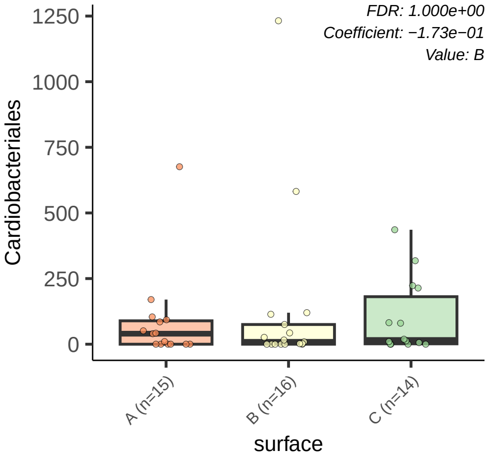
FDR: 1.000e+00
Coefficient: −1.73e−01
Value: B
Cardiobacteriales
1250
1000
750
500
250
0
A (n=15)
B (n=16)
C (n=14)
surface

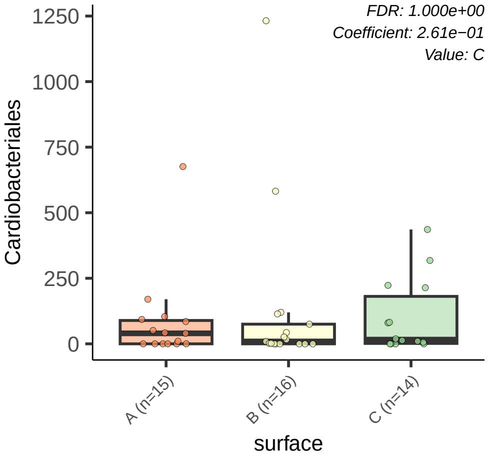
FDR: 1.000e+00
Coefficient: 2.61e−01
Value: C
Cardiobacteriales
1250
1000
750
500
250
0
A (n=15)
B (n=16)
C (n=14)
surface

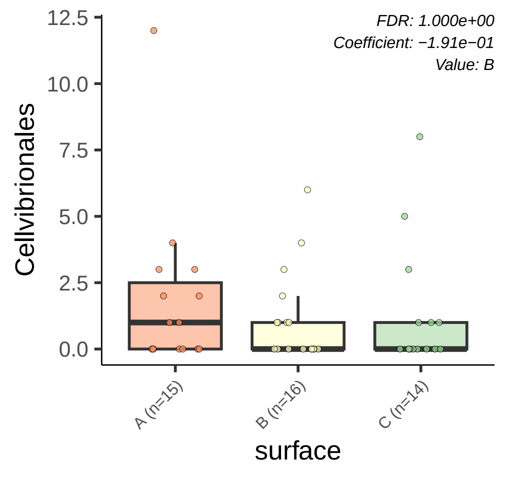
FDR: 1.000e+00
Coefficient: −1.91e−01
Value: B
Cellvibrionales
12.5
10.0
7.5
5.0
2.5
0.0
A (n=15)
B (n=16)
C (n=14)
surface

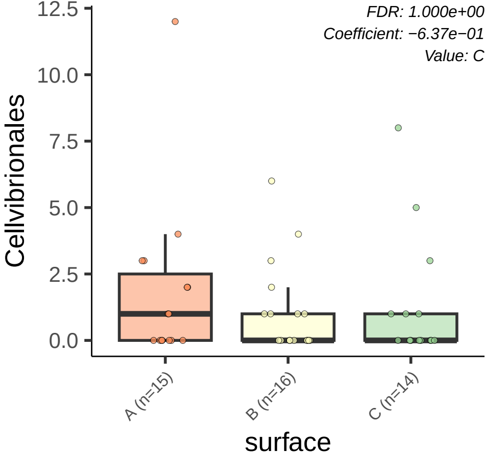

FDR: 1.000e+00
Coefficient: −6.37e−01
Value: C
Cellvibrionales
12.5
10.0
7.5
5.0
2.5
0.0
A (n=15)
B (n=16)
C (n=14)
surface

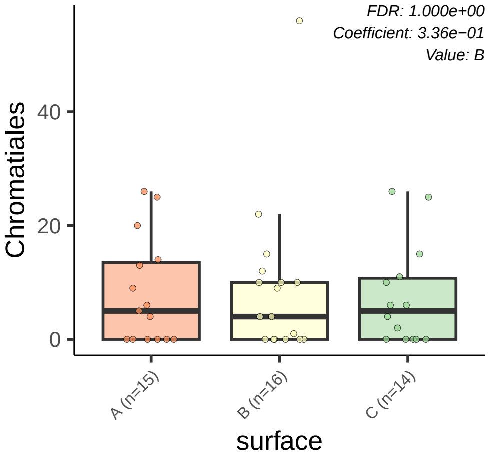
FDR: 1.000e+00
Coefficient: 3.36e−01
Value: B
Chromatiales
0
20
40
A (n=15)
B (n=16)
C (n=14)
surface

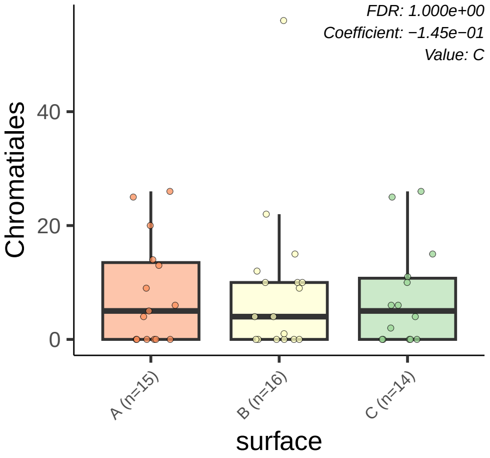

FDR: 1.000e+00
Coefficient: −1.45e−01
Value: C
Chromatiales
40
20
0
A (n=15)
B (n=16)
C (n=14)
surface

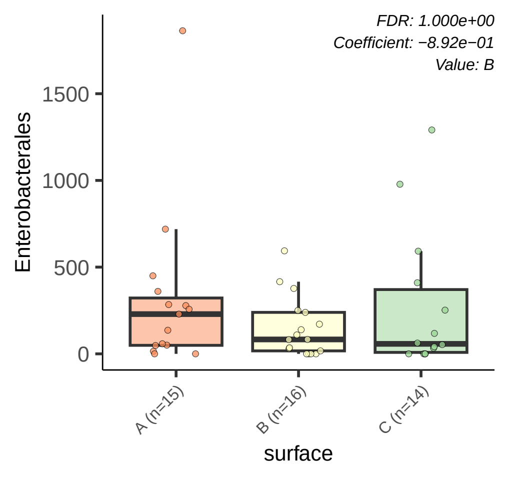

FDR: 1.000e+00
Coefficient: −8.92e−01
Value: B
Enterobacterales
1500
1000
500
0
A (n=15)
B (n=16)
C (n=14)
surface

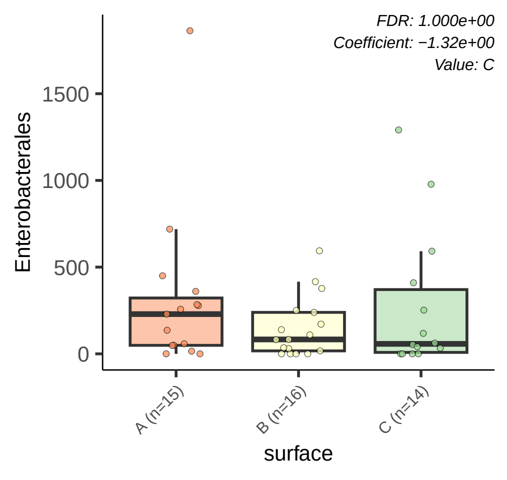
FDR: 1.000e+00
Coefficient: −1.32e+00
Value: C
Enterobacterales
1500
1000
500
0
A (n=15)
B (n=16)
C (n=14)
surface

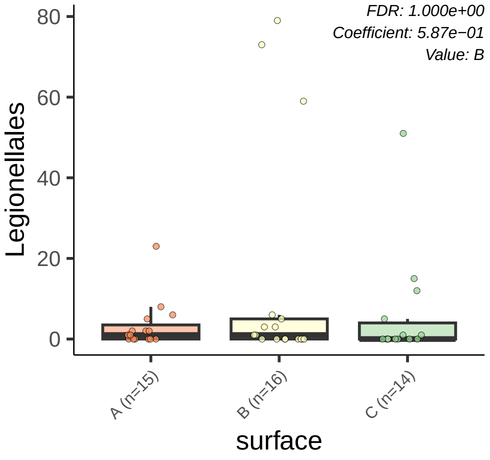
FDR: 1.000e+00
Coefficient: 5.87e−01
Value: B
Legionellales
80
60
40
20
0
A (n=15)
B (n=16)
C (n=14)
surface

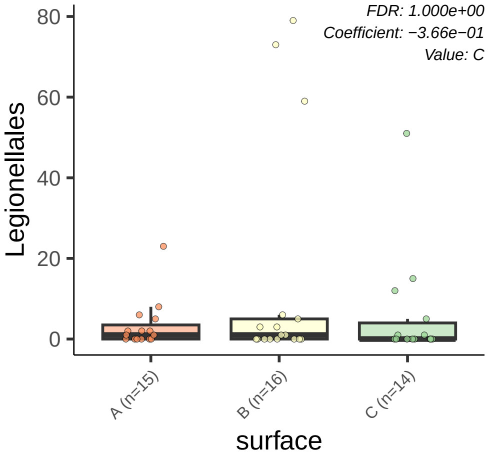
FDR: 1.000e+00
Coefficient: −3.66e−01
Value: C
Legionellales
0
20
40
60
80
A (n=15)
B (n=16)
C (n=14)
surface

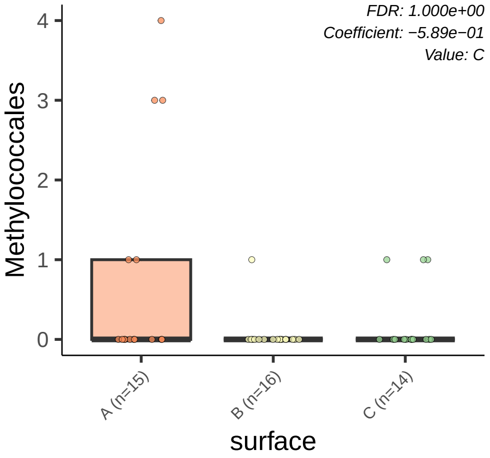
FDR: 1.000e+00
Coefficient: −5.89e−01
Value: C
Methylococcales
0
1
2
3
4
A (n=15)
B (n=16)
C (n=14)
surface

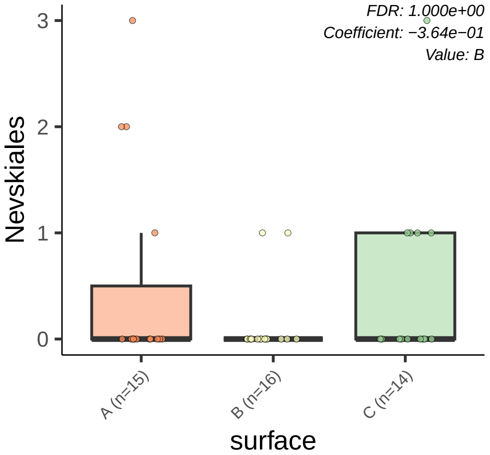
FDR: 1.000e+00
Coefficient: −3.64e−01
Value: B
Nevskiales
3
2
1
0
A (n=15)
B (n=16)
C (n=14)
surface

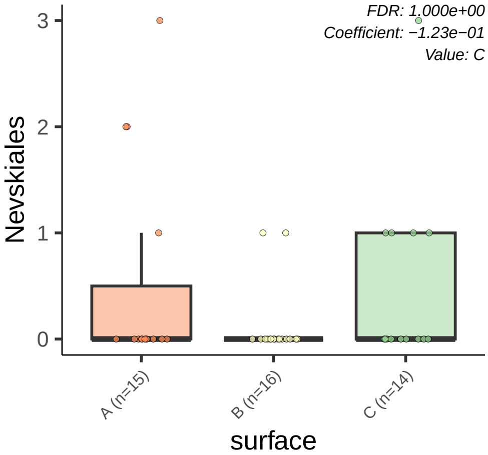
FDR: 1.000e+00
Coefficient: −1.23e−01
Value: C
Nevskiales
0
1
2
3
A (n=15)
B (n=16)
C (n=14)
surface

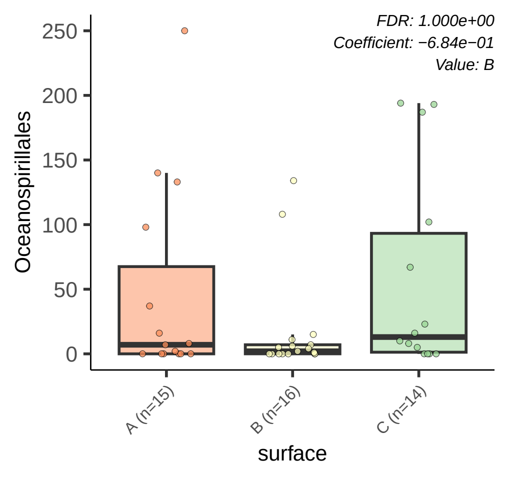
FDR: 1.000e+00
Coefficient: −6.84e−01
Value: B
Oceanospirillales
250
200
150
100
50
0
A (n=15)
B (n=16)
C (n=14)
surface

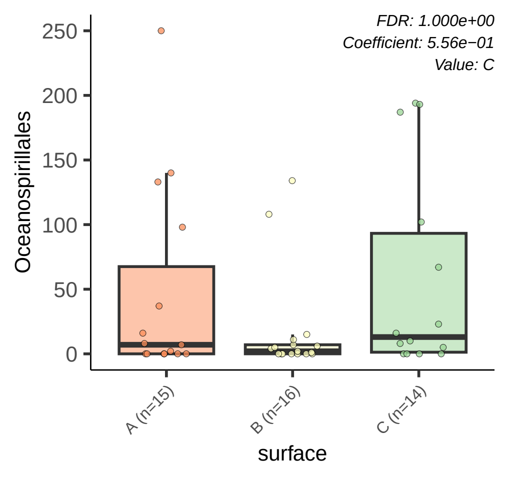
FDR: 1.000e+00
Coefficient: 5.56e−01
Value: C
Oceanospirillales
250
200
150
100
50
0
A (n=15)
B (n=16)
C (n=14)
surface

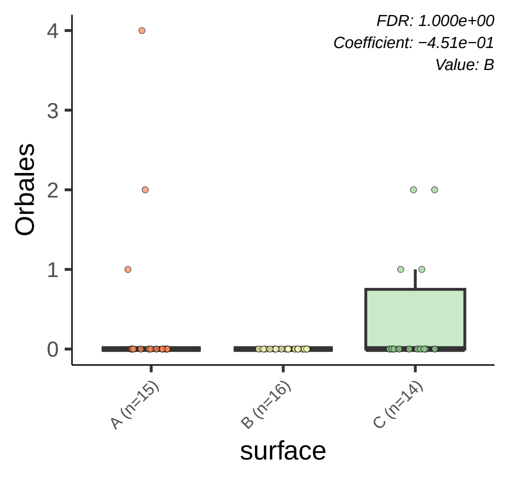
FDR: 1.000e+00
Coefficient: −4.51e−01
Value: B
Orbales
0
1
2
3
4
A (n=15)
B (n=16)
C (n=14)
surface

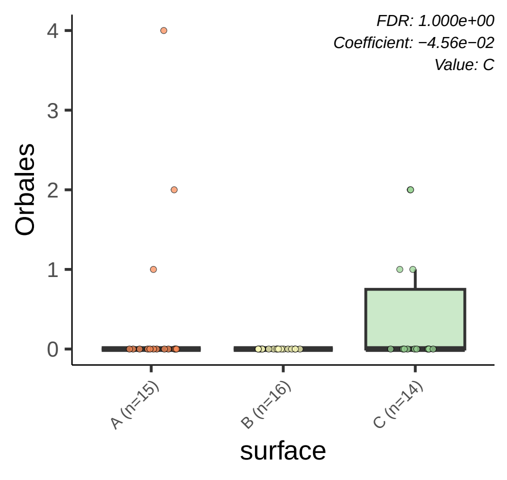
FDR: 1.000e+00
Coefficient: −4.56e−02
Value: C
Orbales
4
3
2
1
0
A (n=15)
B (n=16)
C (n=14)
surface

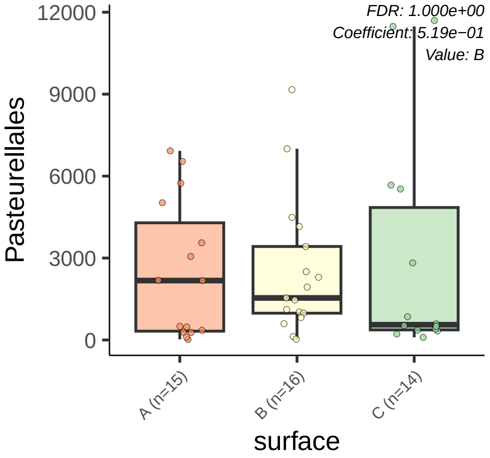

FDR: 1.000e+00
Coefficient: 5.19e−01
Value: B
12000
9000
6000
3000
0
Pasteurellales
A (n=15)
B (n=16)
C (n=14)
surface

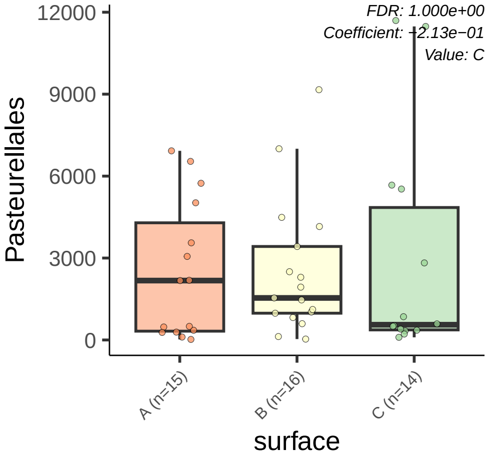

FDR: 1.000e+00
Coefficient: −2.13e−01
Value: C
Pasteurellales
12000
9000
6000
3000
0
A (n=15)
B (n=16)
C (n=14)
surface

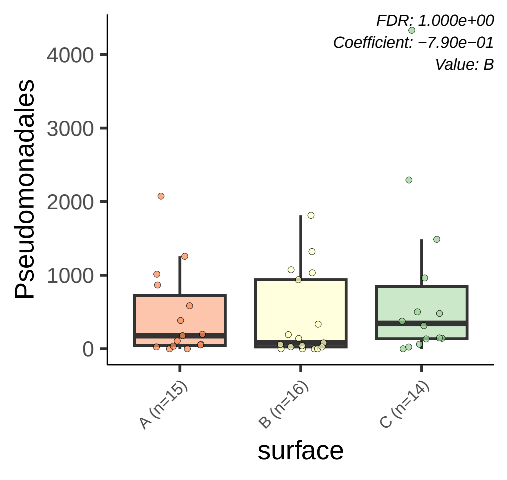
FDR: 1.000e+00
Coefficient: −7.90e−01
Value: B
Pseudomonadales
4000
3000
2000
1000
0
A (n=15)
B (n=16)
C (n=14)
surface

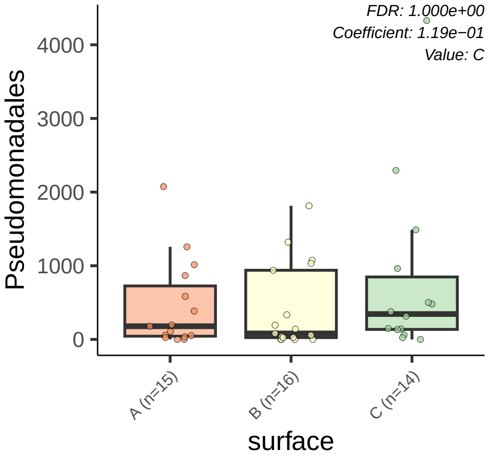
FDR: 1.000e+00
Coefficient: 1.19e−01
Value: C
Pseudomonadales
4000
3000
2000
1000
0
A (n=15)
B (n=16)
C (n=14)
surface

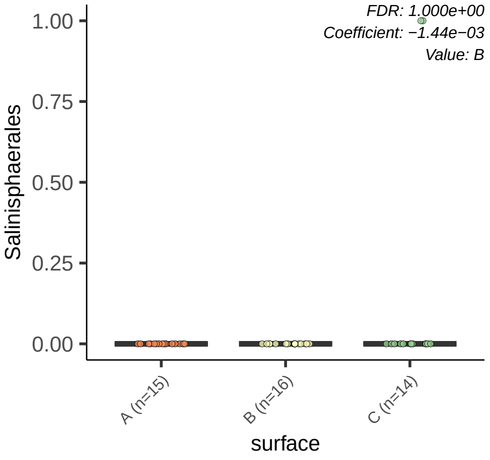

FDR: 1.000e+00
Coefficient: −1.44e−03
Value: B
Salinisphaerales
1.00
0.75
0.50
0.25
0.00
A (n=15)
B (n=16)
C (n=14)
surface

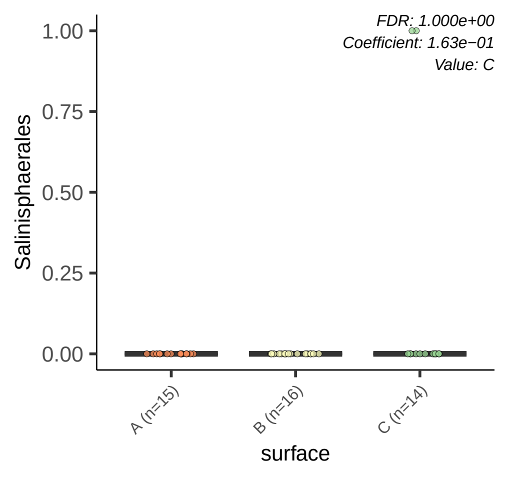

FDR: 1.000e+00
Coefficient: 1.63e−01
Value: C
Salinisphaerales
1.00
0.75
0.50
0.25
0.00
A (n=15)
B (n=16)
C (n=14)
surface

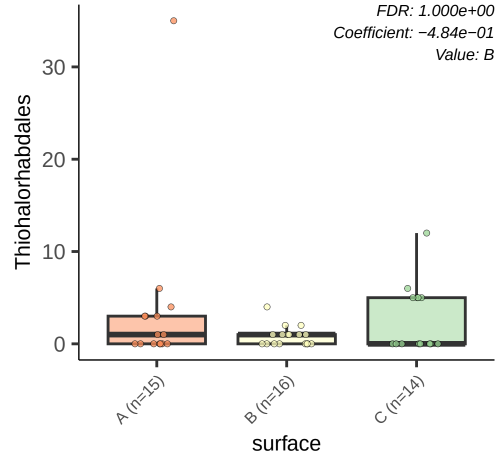

FDR: 1.000e+00
Coefficient: −4.84e−01
Value: B
Thiohalorhabdales
0
10
20
30
A (n=15)
B (n=16)
C (n=14)
surface

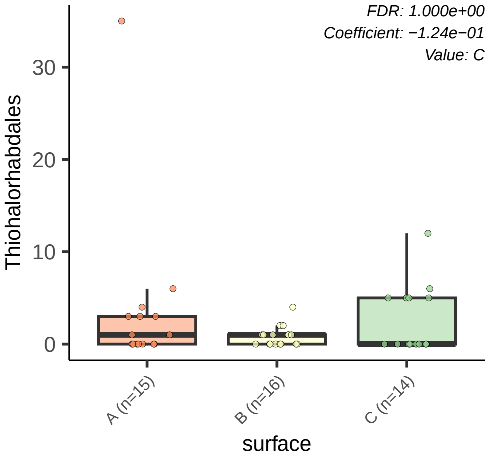

FDR: 1.000e+00
Coefficient: −1.24e−01
Value: C
Thiohalorhabdales
30
20
10
0
A (n=15)
B (n=16)
C (n=14)
surface

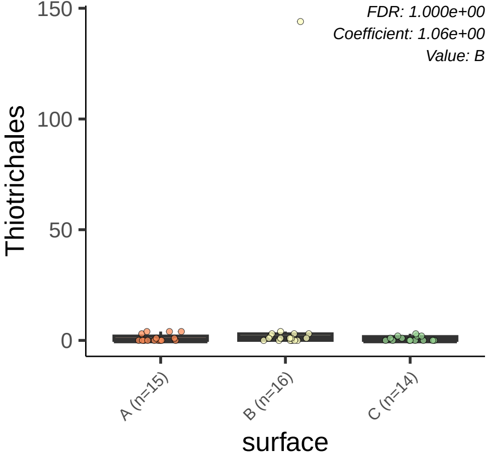
FDR: 1.000e+00
Coefficient: 1.06e+00
Value: B
Thiotrichales
150
100
50
0
A (n=15)
B (n=16)
C (n=14)
surface

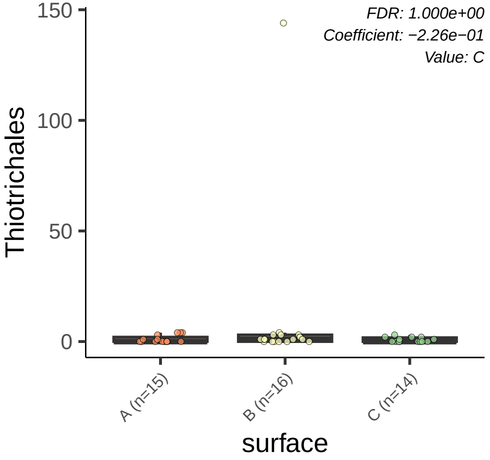

FDR: 1.000e+00
Coefficient: −2.26e−01
Value: C
Thiotrichales
150
100
50
0
A (n=15)
B (n=16)
C (n=14)
surface

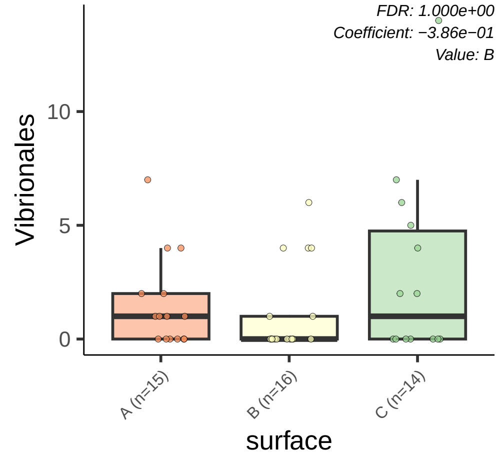
FDR: 1.000e+00
Coefficient: −3.86e−01
Value: B
Vibrionales
10
5
0
A (n=15)
B (n=16)
C (n=14)
surface

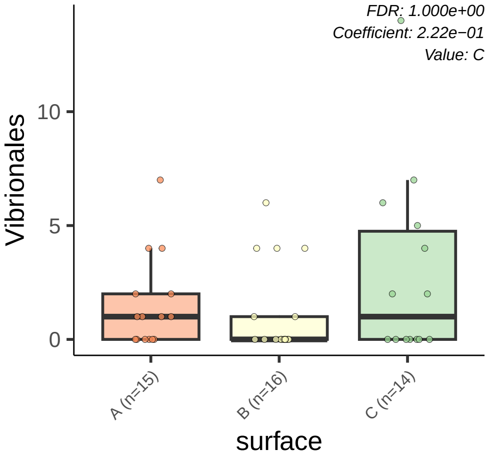

FDR: 1.000e+00
Coefficient: 2.22e−01
Value: C
Vibrionales
10
5
0
A (n=15)
B (n=16)
C (n=14)
surface

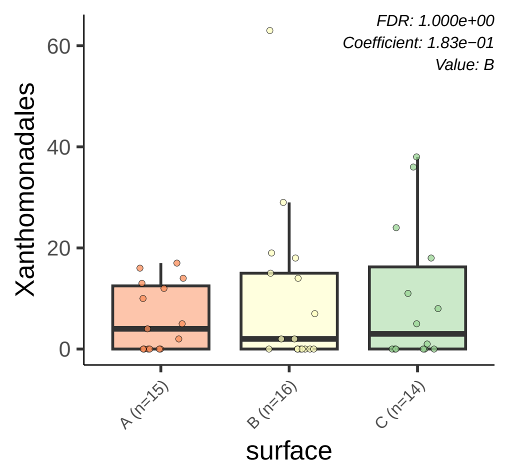

FDR: 1.000e+00
Coefficient: 1.83e−01
Value: B
Xanthomonadales
0
20
40
60
A (n=15)
B (n=16)
C (n=14)
surface

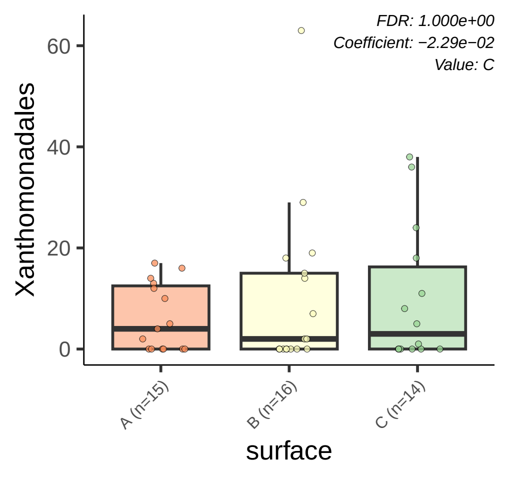

FDR: 1.000e+00
Coefficient: −2.29e−02
Value: C
Xanthomonadales
60
40
20
0
A (n=15)
B (n=16)
C (n=14)
surface

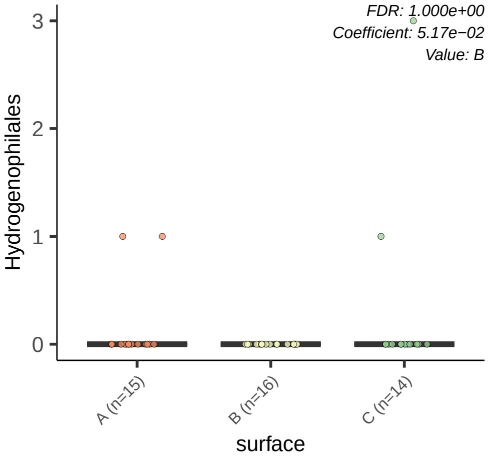

FDR: 1.000e+00
Coefficient: 5.17e−02
Value: B
Hydrogenophilales
3
2
1
0
A (n=15)
B (n=16)
C (n=14)
surface

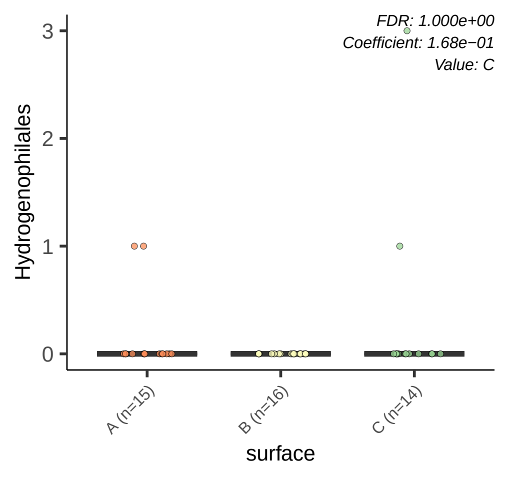
FDR: 1.000e+00
Coefficient: 1.68e−01
Value: C
Hydrogenophilales
3
2
1
0
A (n=15)
B (n=16)
C (n=14)
surface

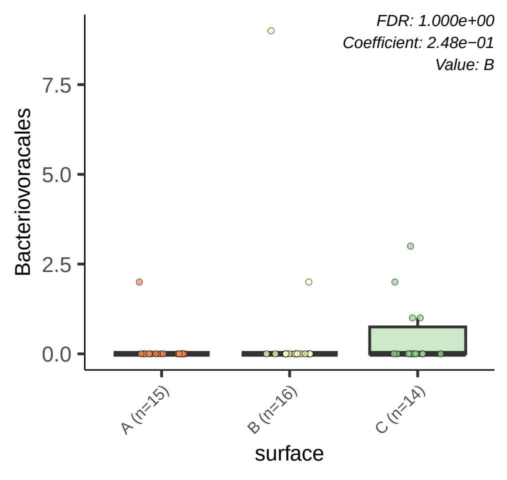
FDR: 1.000e+00
Coefficient: 2.48e−01
Value: B
Bacteriovoracales
7.5
5.0
2.5
0.0
A (n=15)
B (n=16)
C (n=14)
surface

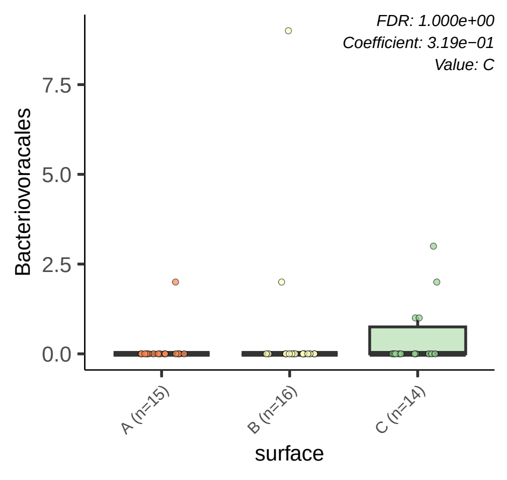
FDR: 1.000e+00
Coefficient: 3.19e−01
Value: C
Bacteriovoracales
7.5
5.0
2.5
0.0
A (n=15)
B (n=16)
C (n=14)
surface

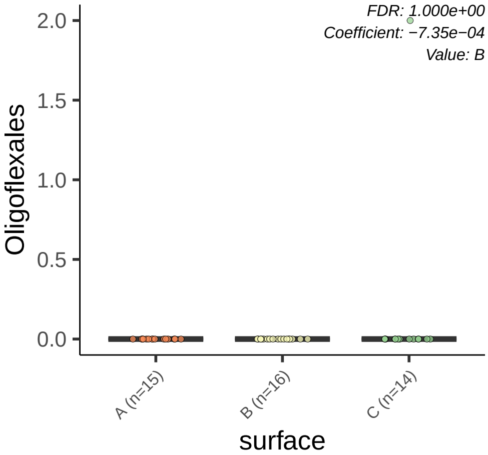
FDR: 1.000e+00
Coefficient: −7.35e−04
Value: B
Oligoflexales
2.0
1.5
1.0
0.5
0.0
A (n=15)
B (n=16)
C (n=14)
surface

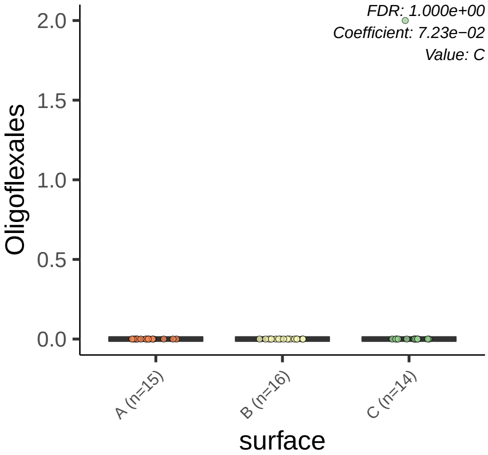

FDR: 1.000e+00
Coefficient: 7.23e−02
Value: C
Oligoflexales
2.0
1.5
1.0
0.5
0.0
A (n=15)
B (n=16)
C (n=14)
surface

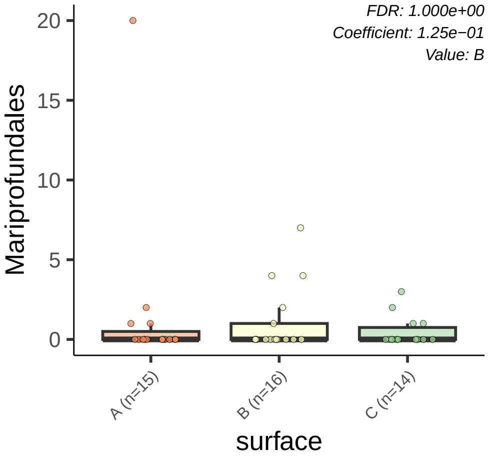
FDR: 1.000e+00
Coefficient: 1.25e−01
Value: B
Mariprofundales
20
15
10
5
0
A (n=15)
B (n=16)
C (n=14)
surface

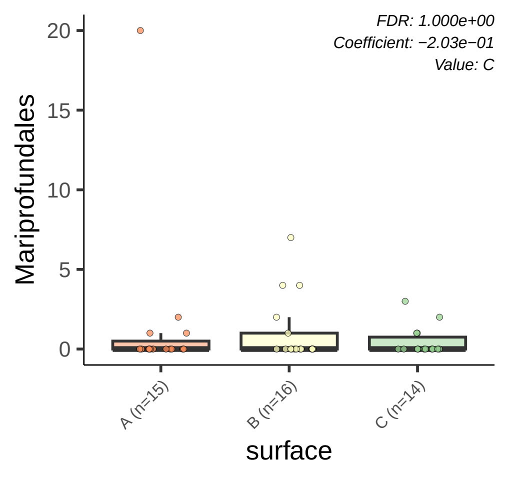
FDR: 1.000e+00
Coefficient: −2.03e−01
Value: C
Mariprofundales
20
15
10
5
0
A (n=15)
B (n=16)
C (n=14)
surface

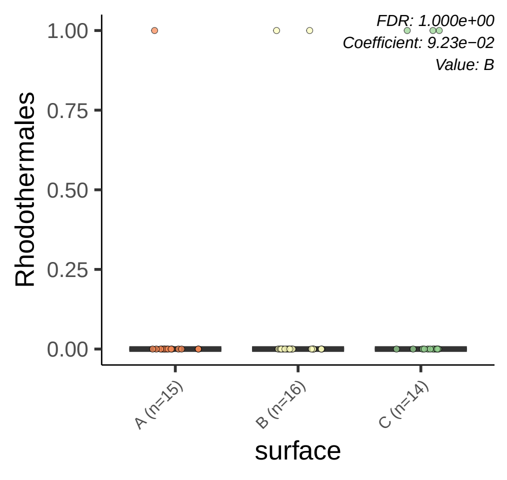

FDR: 1.000e+00
Coefficient: 9.23e−02
Value: B
Rhodothermales
1.00
0.75
0.50
0.25
0.00
A (n=15)
B (n=16)
C (n=14)
surface

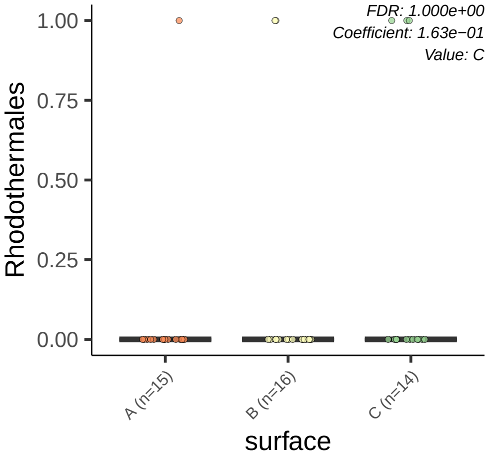
FDR: 1.000e+00
Coefficient: 1.63e−01
Value: C
Rhodothermales
1.00
0.75
0.50
0.25
0.00
A (n=15)
B (n=16)
C (n=14)
surface

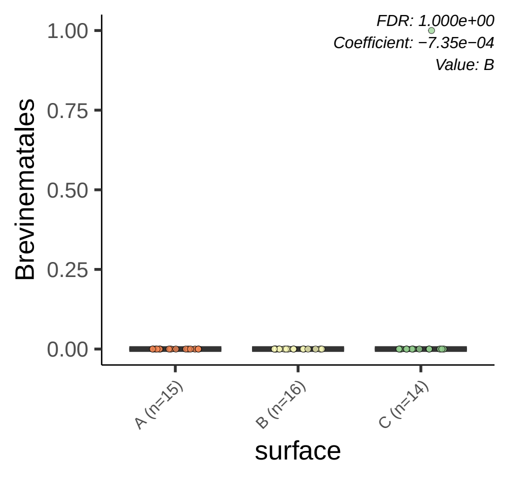

FDR: 1.000e+00
Coefficient: −7.35e−04
Value: B
Brevinematales
1.00
0.75
0.50
0.25
0.00
A (n=15)
B (n=16)
C (n=14)
surface

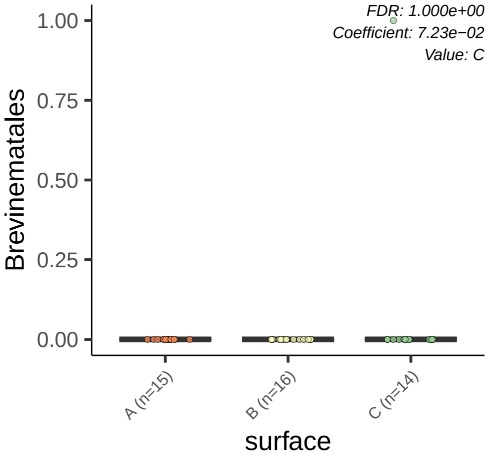

FDR: 1.000e+00
Coefficient: 7.23e−02
Value: C
Brevinematales
1.00
0.75
0.50
0.25
0.00
A (n=15)
B (n=16)
C (n=14)
surface

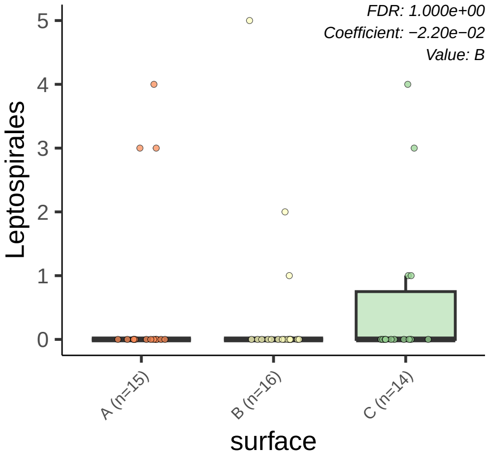
FDR: 1.000e+00
Coefficient: −2.20e−02
Value: B
Leptospirales
5
4
3
2
1
0
A (n=15)
B (n=16)
C (n=14)
surface

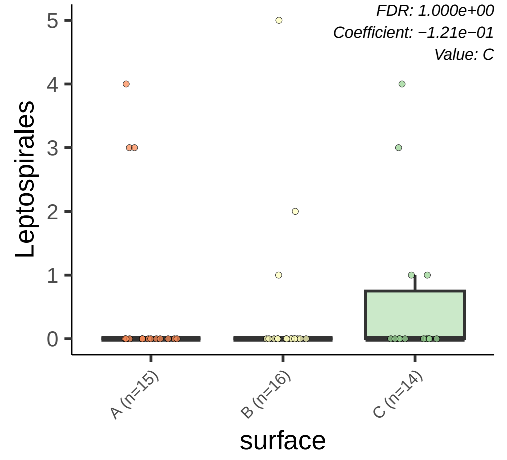

FDR: 1.000e+00
Coefficient: −1.21e−01
Value: C
Leptospirales
0
1
2
3
4
5
A (n=15)
B (n=16)
C (n=14)
surface

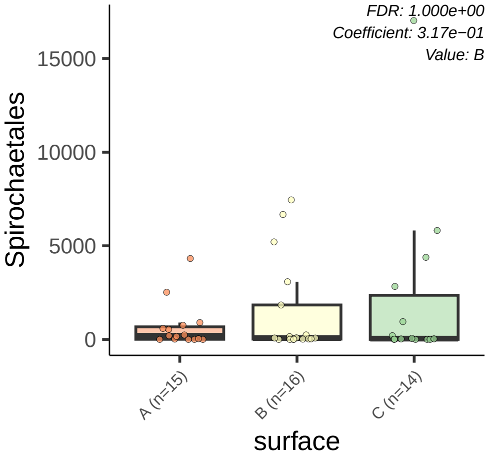
FDR: 1.000e+00
Coefficient: 3.17e−01
Value: B
Spirochaetales
15000
10000
5000
0
A (n=15)
B (n=16)
C (n=14)
surface

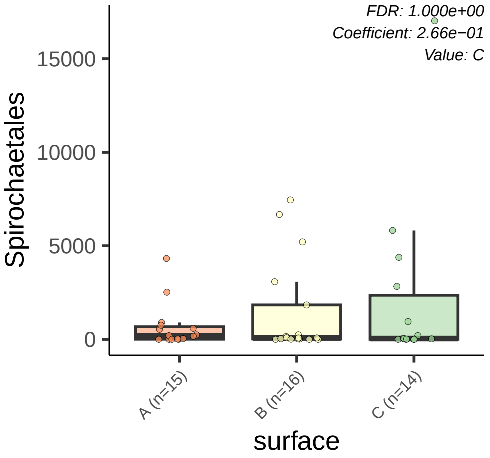

FDR: 1.000e+00
Coefficient: 2.66e−01
Value: C
Spirochaetales
15000
10000
5000
0
A (n=15)
B (n=16)
C (n=14)
surface

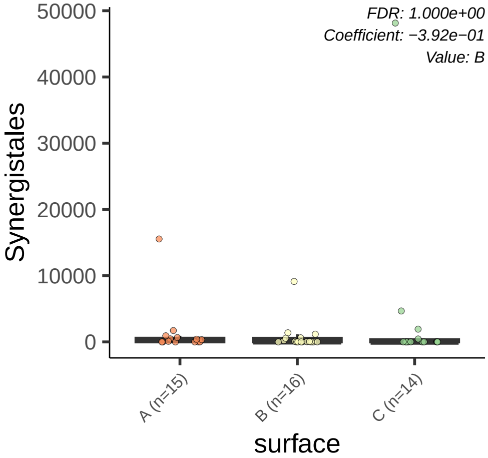

FDR: 1.000e+00
Coefficient: −3.92e−01
Value: B
Synergistales
50000
40000
30000
20000
10000
0
A (n=15)
B (n=16)
C (n=14)
surface

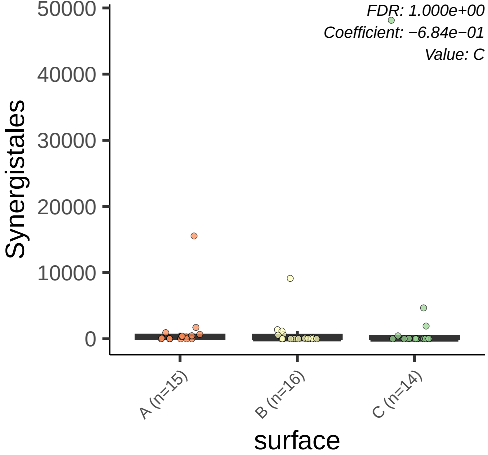

FDR: 1.000e+00
Coefficient: −6.84e−01
Value: C
Synergistales
50000
40000
30000
20000
10000
0
A (n=15)
B (n=16)
C (n=14)
surface

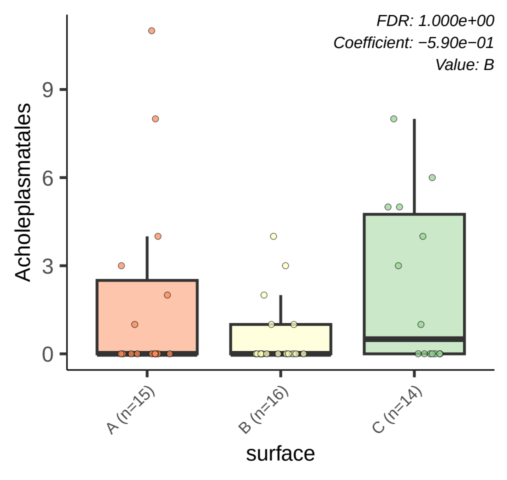

FDR: 1.000e+00
Coefficient: −5.90e−01
Value: B
Acholeplasmatales
0
3
6
9
A (n=15)
B (n=16)
C (n=14)
surface

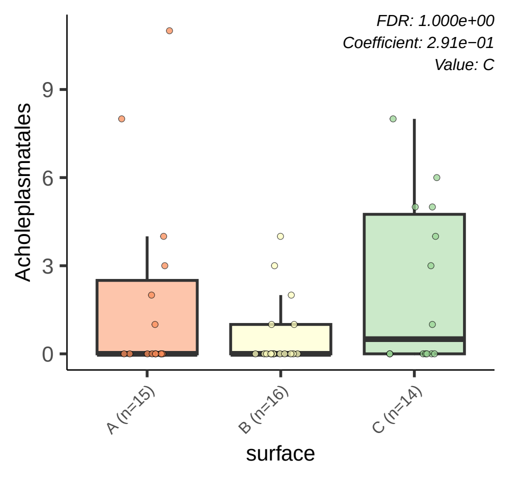
FDR: 1.000e+00
Coefficient: 2.91e−01
Value: C
Acholeplasmatales
0
3
6
9
A (n=15)
B (n=16)
C (n=14)
surface

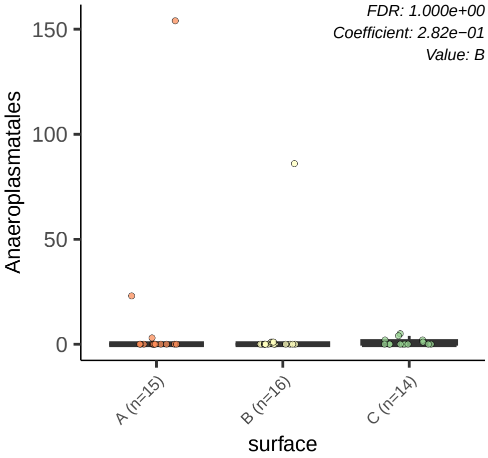
FDR: 1.000e+00
Coefficient: 2.82e−01
Value: B
Anaeroplasmatales
150
100
50
0
A (n=15)
B (n=16)
C (n=14)
surface

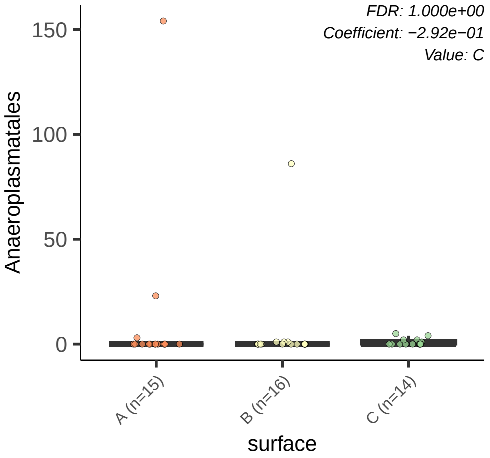
FDR: 1.000e+00
Coefficient: −2.92e−01
Value: C
Anaeroplasmatales
150
100
50
0
A (n=15)
B (n=16)
C (n=14)
surface

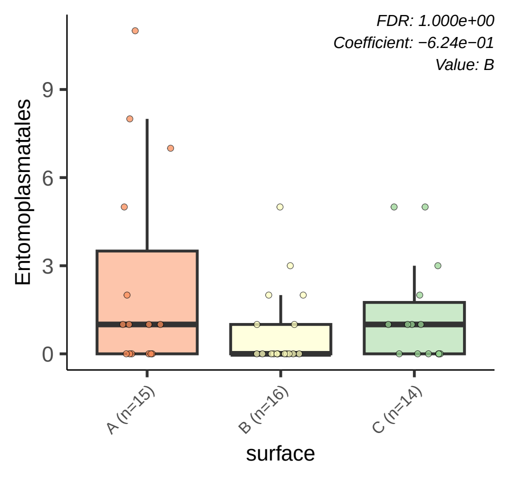
FDR: 1.000e+00
Coefficient: −6.24e−01
Value: B
Entomoplasmatales
0
3
6
9
A (n=15)
B (n=16)
C (n=14)
surface

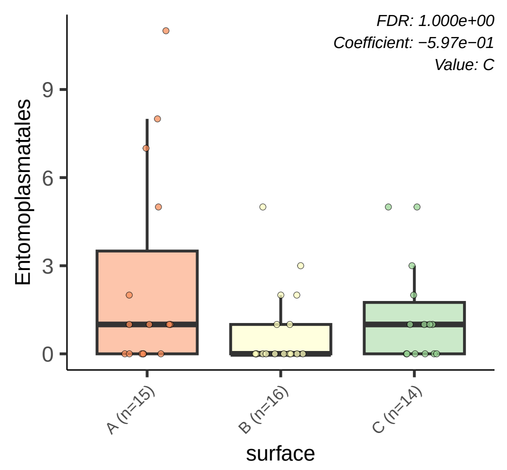

FDR: 1.000e+00
Coefficient: −5.97e−01
Value: C
Entomoplasmatales
0
3
6
9
A (n=15)
B (n=16)
C (n=14)
surface

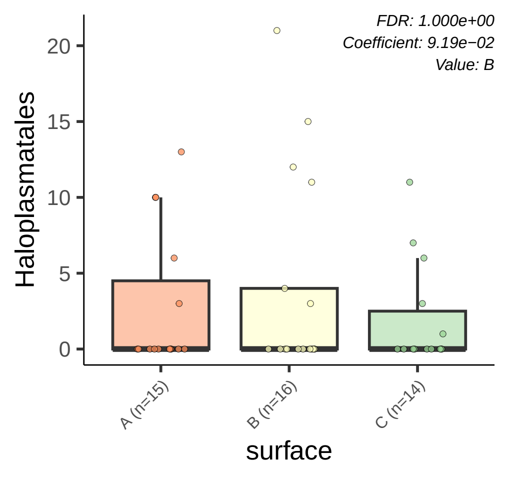
FDR: 1.000e+00
Coefficient: 9.19e−02
Value: B
Haloplasmatales
20
15
10
5
0
A (n=15)
B (n=16)
C (n=14)
surface

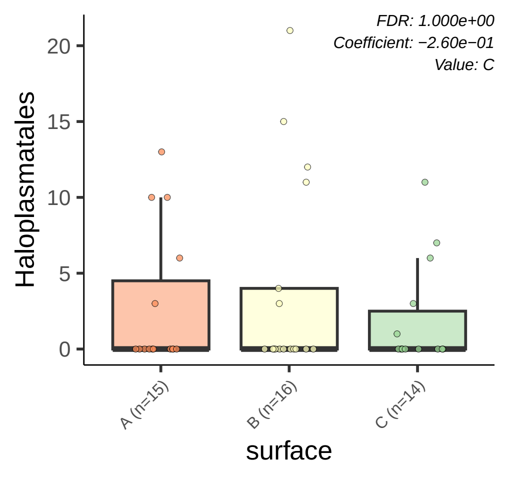
FDR: 1.000e+00
Coefficient: −2.60e−01
Value: C
Haloplasmatales
0
5
10
15
20
A (n=15)
B (n=16)
C (n=14)
surface

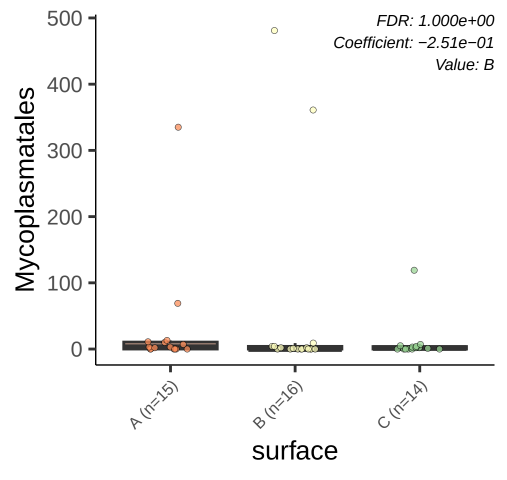
FDR: 1.000e+00
Coefficient: −2.51e−01
Value: B
Mycoplasmatales
500
400
300
200
100
0
A (n=15)
B (n=16)
C (n=14)
surface

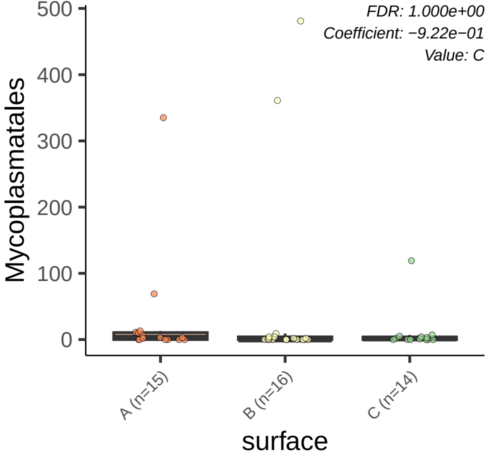
FDR: 1.000e+00
Coefficient: −9.22e−01
Value: C
Mycoplasmatales
500
400
300
200
100
0
A (n=15)
B (n=16)
C (n=14)
surface

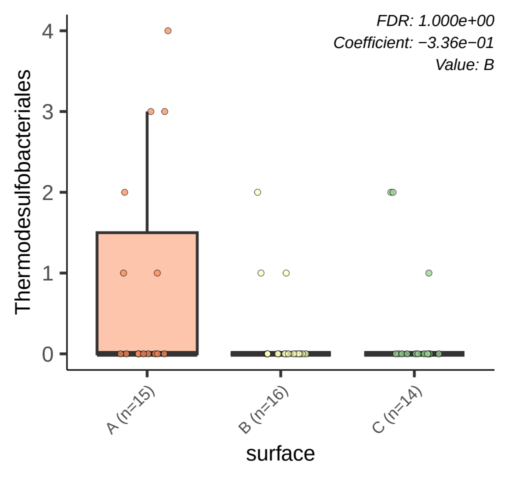

FDR: 1.000e+00
Coefficient: −3.36e−01
Value: B
Thermodesulfobacteriales
0
1
2
3
4
A (n=15)
B (n=16)
C (n=14)
surface

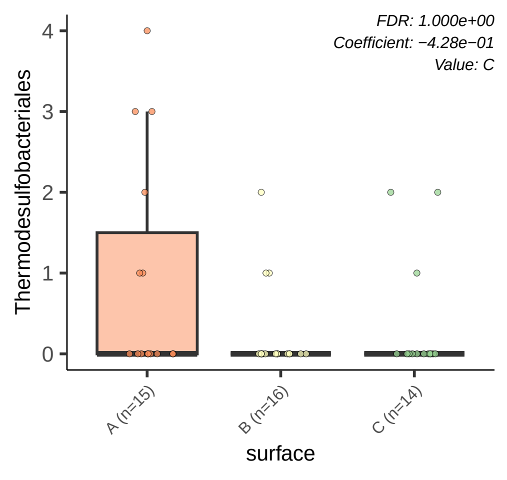

FDR: 1.000e+00
Coefficient: −4.28e−01
Value: C
Thermodesulfobacteriales
0
1
2
3
4
A (n=15)
B (n=16)
C (n=14)
surface

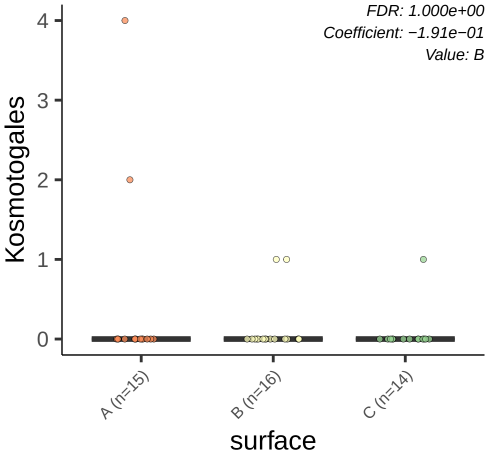
FDR: 1.000e+00
Coefficient: −1.91e−01
Value: B
Kosmotogales
4
3
2
1
0
A (n=15)
B (n=16)
C (n=14)
surface

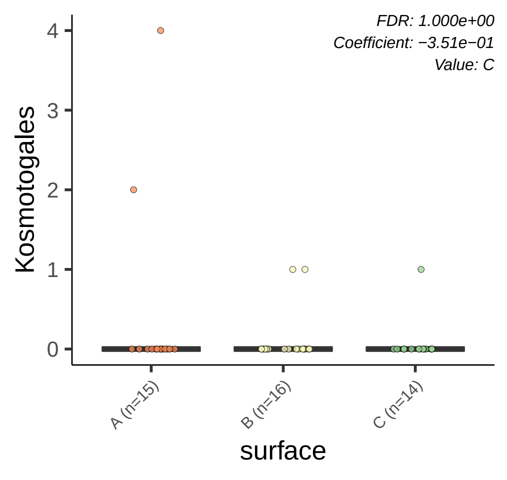

FDR: 1.000e+00
Coefficient: −3.51e−01
Value: C
Kosmotogales
4
3
2
1
0
A (n=15)
B (n=16)
C (n=14)
surface

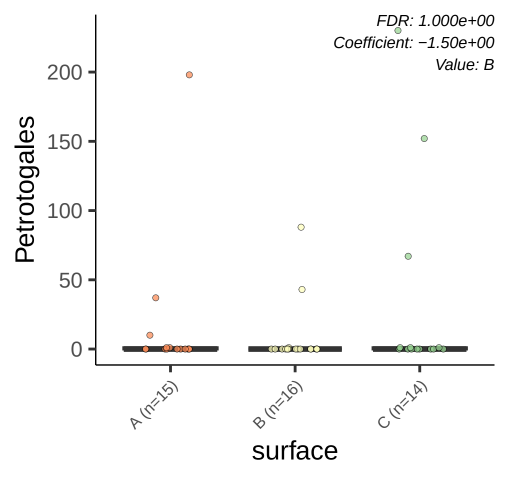

FDR: 1.000e+00
Coefficient: −1.50e+00
Value: B
Petrotogales
200
150
100
50
0
A (n=15)
B (n=16)
C (n=14)
surface

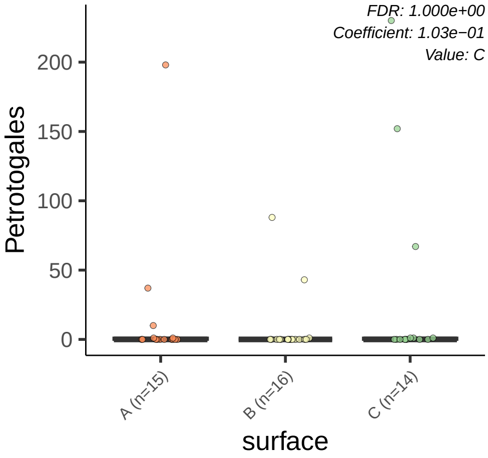

FDR: 1.000e+00
Coefficient: 1.03e−01
Value: C
Petrotogales
200
150
100
50
0
A (n=15)
B (n=16)
C (n=14)
surface

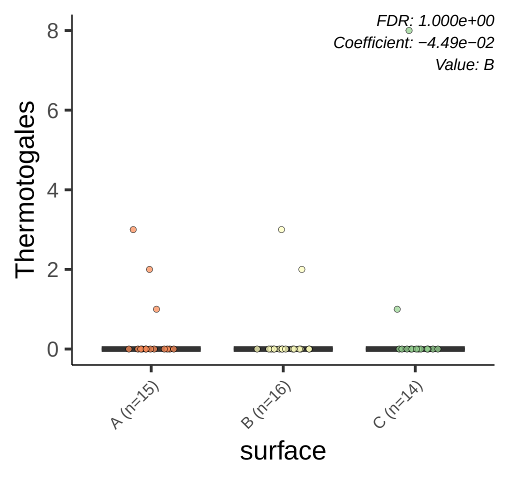
FDR: 1.000e+00
Coefficient: −4.49e−02
Value: B
Thermotogales
8
6
4
2
0
A (n=15)
B (n=16)
C (n=14)
surface

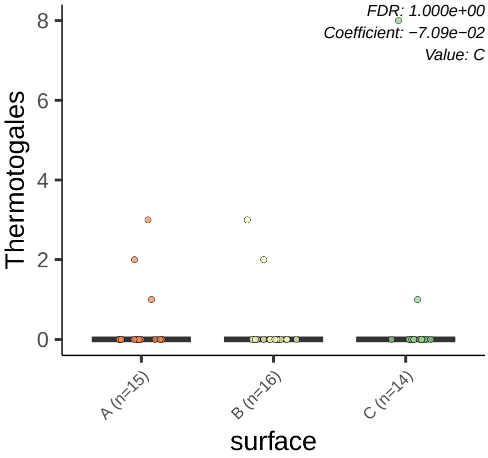
FDR: 1.000e+00
Coefficient: −7.09e−02
Value: C
Thermotogales
0
2
4
6
8
A (n=15)
B (n=16)
C (n=14)
surface

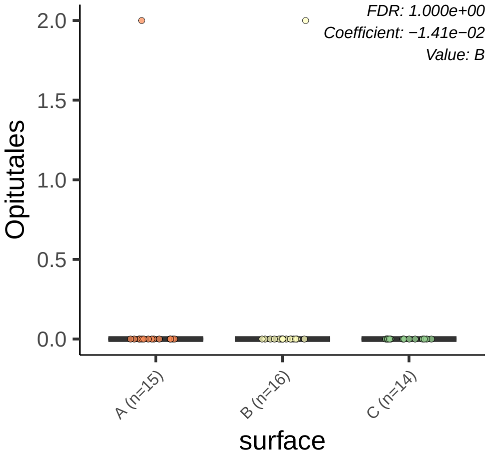
FDR: 1.000e+00
Coefficient: −1.41e−02
Value: B
Opitutales
2.0
1.5
1.0
0.5
0.0
A (n=15)
B (n=16)
C (n=14)
surface

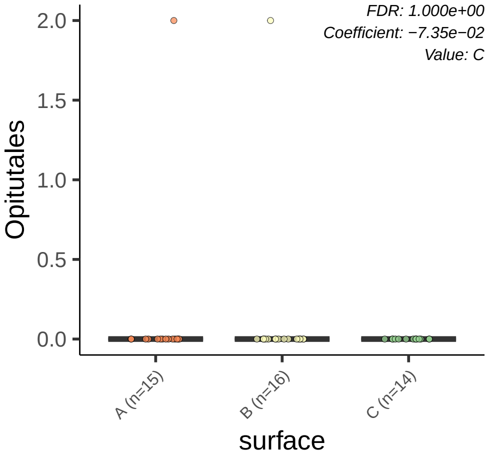
FDR: 1.000e+00
Coefficient: −7.35e−02
Value: C
Opitutales
2.0
1.5
1.0
0.5
0.0
A (n=15)
B (n=16)
C (n=14)
surface

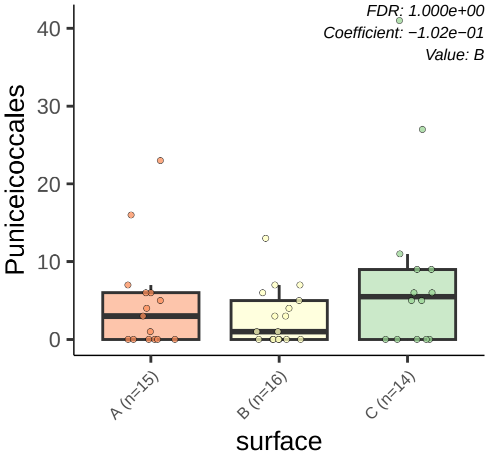

FDR: 1.000e+00
Coefficient: −1.02e−01
Value: B
Puniceicoccales
0
10
20
30
40
A (n=15)
B (n=16)
C (n=14)
surface

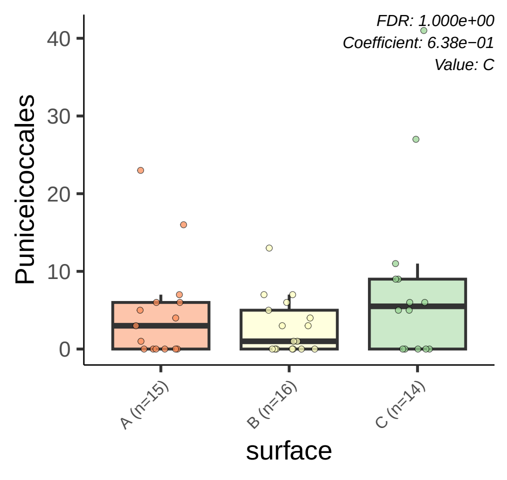

FDR: 1.000e+00
Coefficient: 6.38e−01
Value: C
Puniceicoccales
40
30
20
10
0
A (n=15)
B (n=16)
C (n=14)
surface

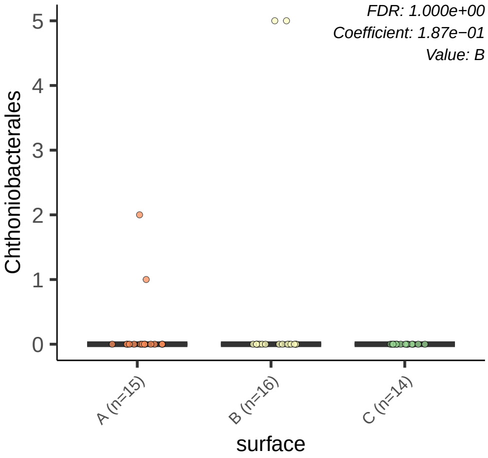

FDR: 1.000e+00
Coefficient: 1.87e−01
Value: B
Chthoniobacterales
0
1
2
3
4
5
A (n=15)
B (n=16)
C (n=14)
surface

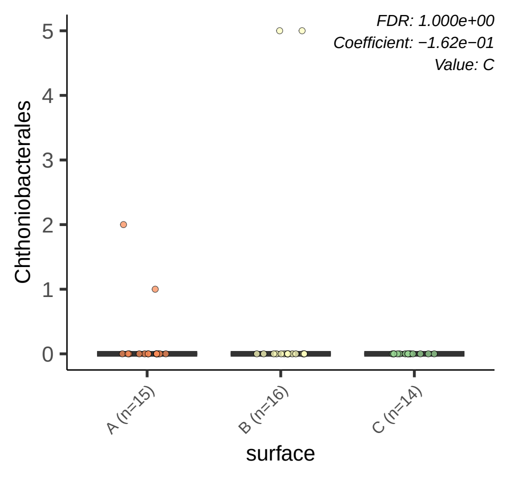

FDR: 1.000e+00
Coefficient: −1.62e−01
Value: C
Chthoniobacterales
0
1
2
3
4
5
A (n=15)
B (n=16)
C (n=14)
surface

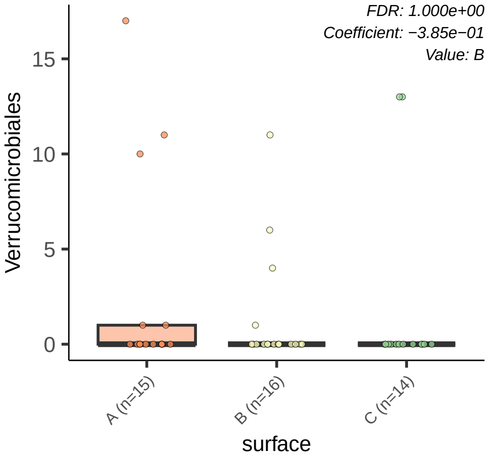

FDR: 1.000e+00
Coefficient: −3.85e−01
Value: B
Verrucomicrobiales
15
10
5
0
A (n=15)
B (n=16)
C (n=14)
surface

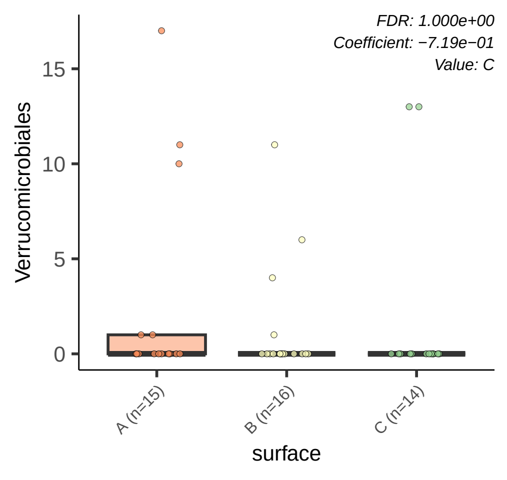

FDR: 1.000e+00
Coefficient: −7.19e−01
Value: C
Verrucomicrobiales
15
10
5
0
A (n=15)
B (n=16)
C (n=14)
surface

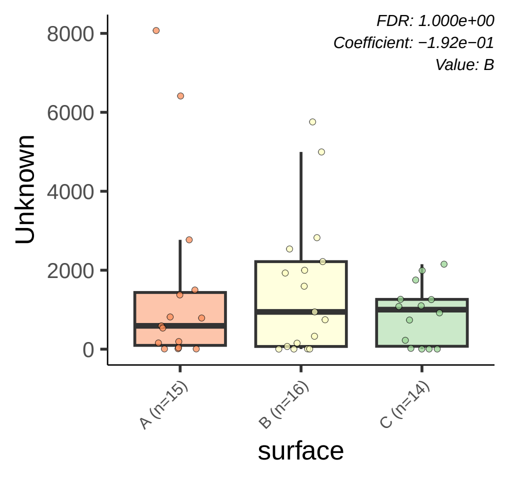

FDR: 1.000e+00
Coefficient: −1.92e−01
Value: B
Unknown
8000
6000
4000
2000
0
A (n=15)
B (n=16)
C (n=14)
surface

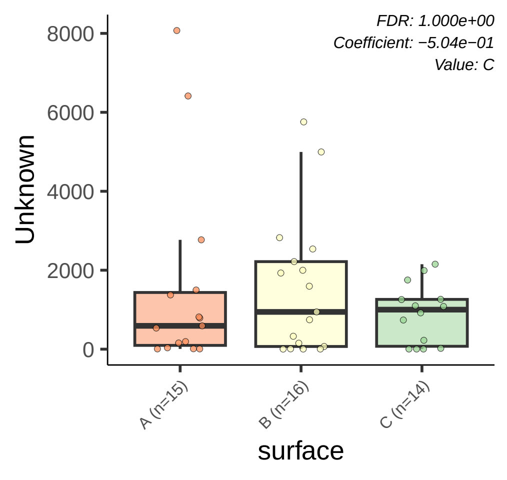

FDR: 1.000e+00
Coefficient: −5.04e−01
Value: C
Unknown
8000
6000
4000
2000
0
A (n=15)
B (n=16)
C (n=14)
surface

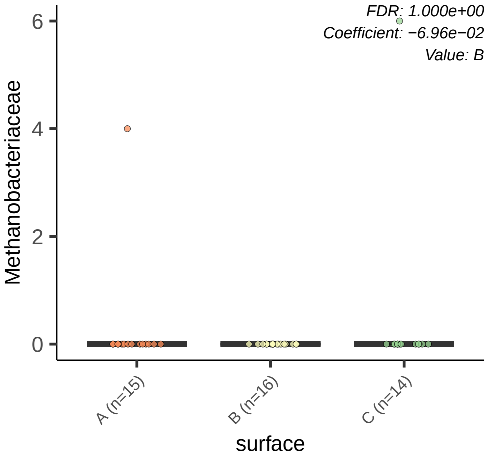
FDR: 1.000e+00
Coefficient: −6.96e−02
Value: B
Methanobacteriaceae
6
4
2
0
A (n=15)
B (n=16)
C (n=14)
surface

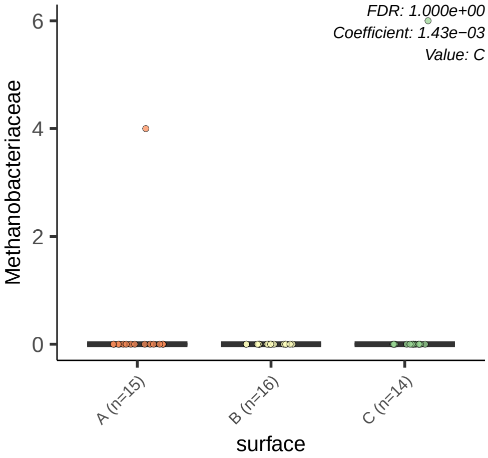

FDR: 1.000e+00
Coefficient: 1.43e−03
Value: C
Methanobacteriaceae
6
4
2
0
A (n=15)
B (n=16)
C (n=14)
surface

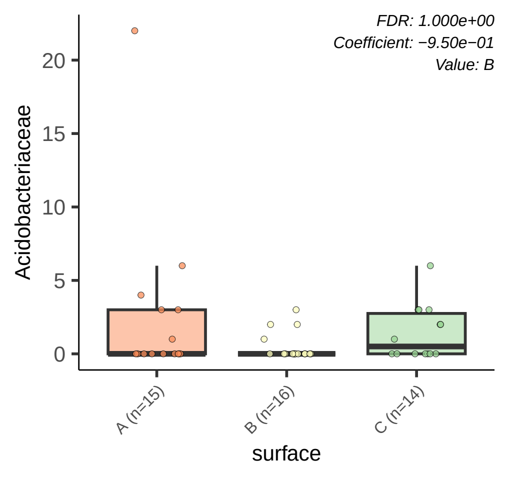
FDR: 1.000e+00
Coefficient: −9.50e−01
Value: B
Acidobacteriaceae
20
15
10
5
0
A (n=15)
B (n=16)
C (n=14)
surface

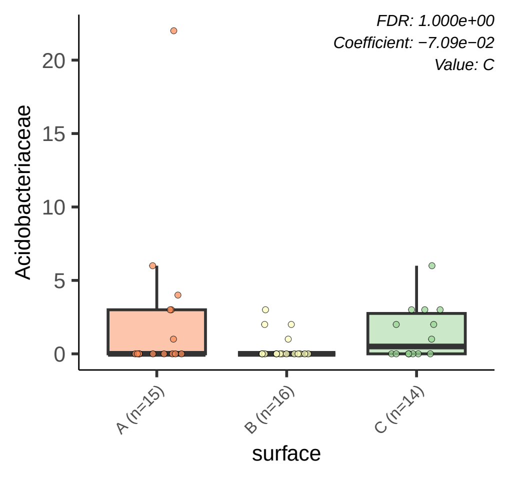
FDR: 1.000e+00
Coefficient: −7.09e−02
Value: C
Acidobacteriaceae
20
15
10
5
0
A (n=15)
B (n=16)
C (n=14)
surface

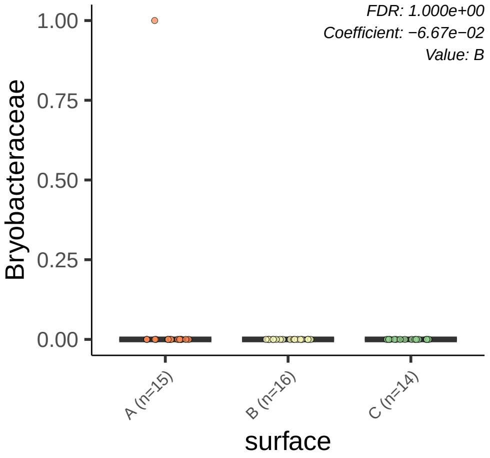

FDR: 1.000e+00
Coefficient: −6.67e−02
Value: B
Bryobacteraceae
1.00
0.75
0.50
0.25
0.00
A (n=15)
B (n=16)
C (n=14)
surface

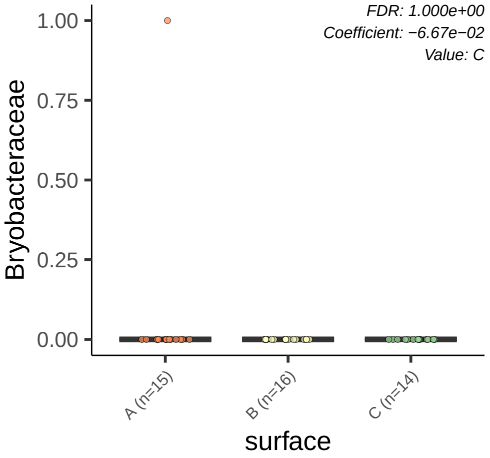
FDR: 1.000e+00
Coefficient: −6.67e−02
Value: C
Bryobacteraceae
1.00
0.75
0.50
0.25
0.00
A (n=15)
B (n=16)
C (n=14)
surface

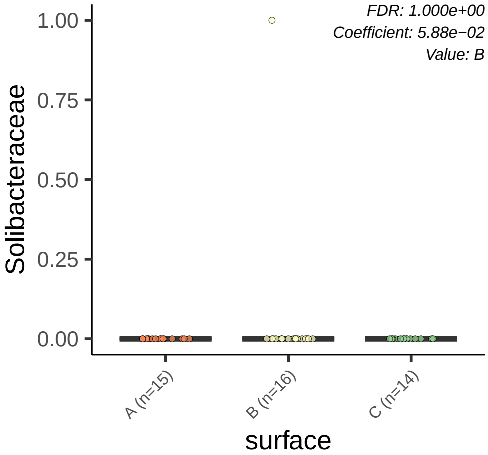
FDR: 1.000e+00
Coefficient: 5.88e−02
Value: B
Solibacteraceae
1.00
0.75
0.50
0.25
0.00
A (n=15)
B (n=16)
C (n=14)
surface

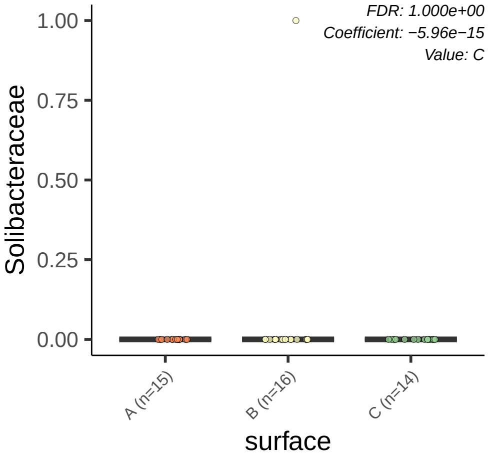
FDR: 1.000e+00
Coefficient: −5.96e−15
Value: C
Solibacteraceae
1.00
0.75
0.50
0.25
0.00
A (n=15)
B (n=16)
C (n=14)
surface

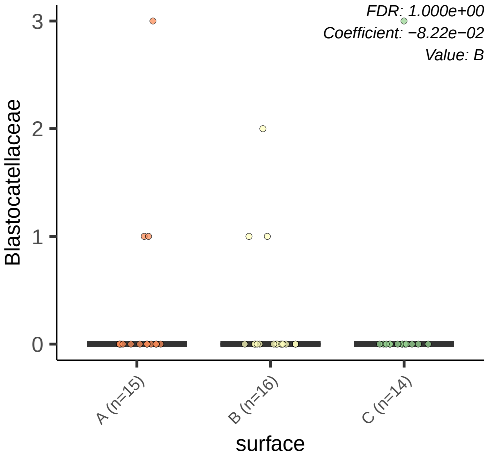
FDR: 1.000e+00
Coefficient: −8.22e−02
Value: B
Blastocatellaceae
3
2
1
0
A (n=15)
B (n=16)
C (n=14)
surface

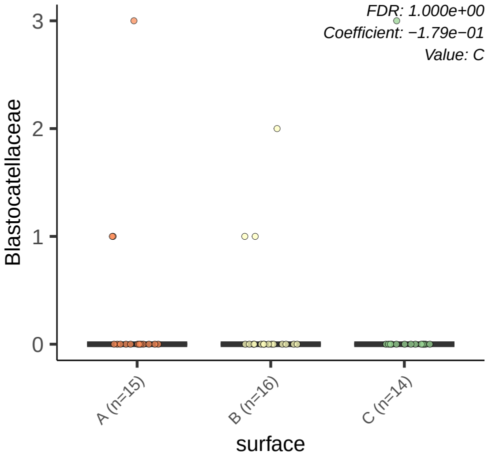
FDR: 1.000e+00
Coefficient: −1.79e−01
Value: C
Blastocatellaceae
3
2
1
0
A (n=15)
B (n=16)
C (n=14)
surface

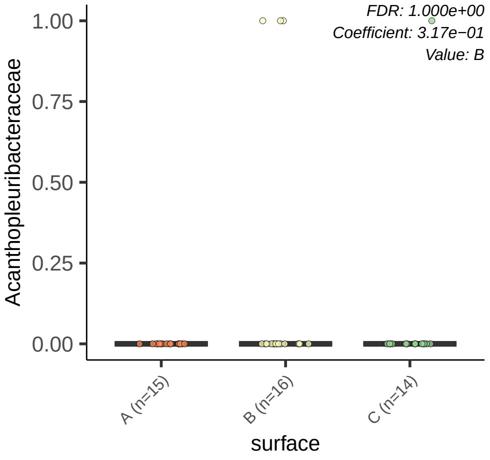
FDR: 1.000e+00
Coefficient: 3.17e−01
Value: B
Acanthopleuribacteraceae
1.00
0.75
0.50
0.25
0.00
A (n=15)
B (n=16)
C (n=14)
surface

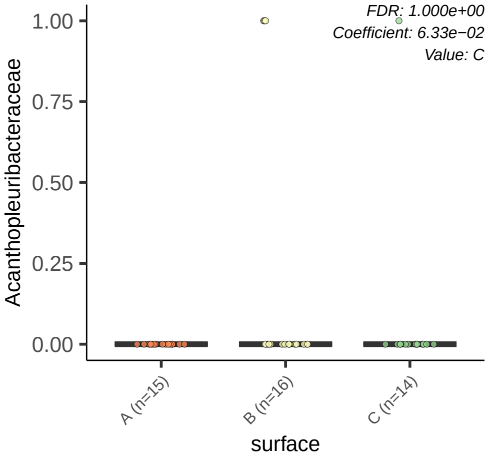
FDR: 1.000e+00
Coefficient: 6.33e−02
Value: C
Acanthopleuribacteraceae
1.00
0.75
0.50
0.25
0.00
A (n=15)
B (n=16)
C (n=14)
surface

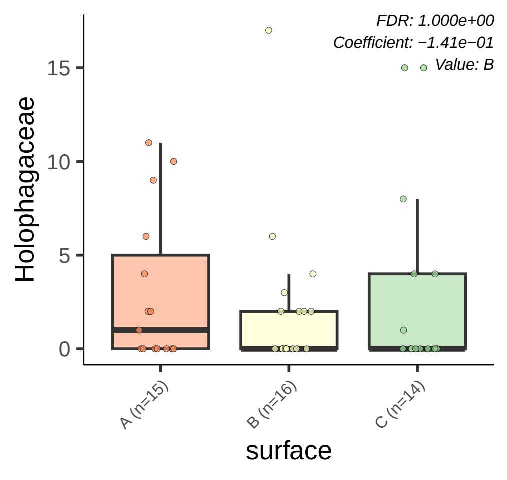
FDR: 1.000e+00
Coefficient: −1.41e−01
Value: B
Holophagaceae
0
5
10
15
A (n=15)
B (n=16)
C (n=14)
surface

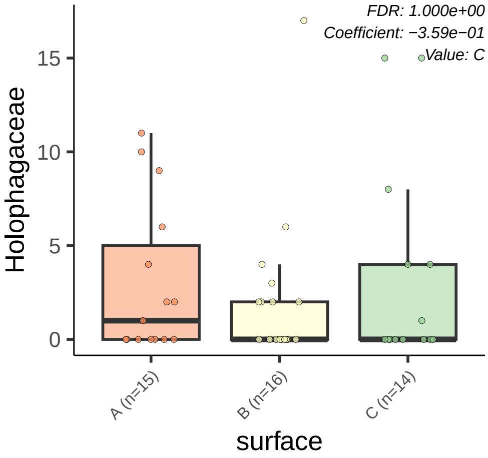

FDR: 1.000e+00
Coefficient: −3.59e−01
Value: C
Holophagaceae
15
10
5
0
A (n=15)
B (n=16)
C (n=14)
surface

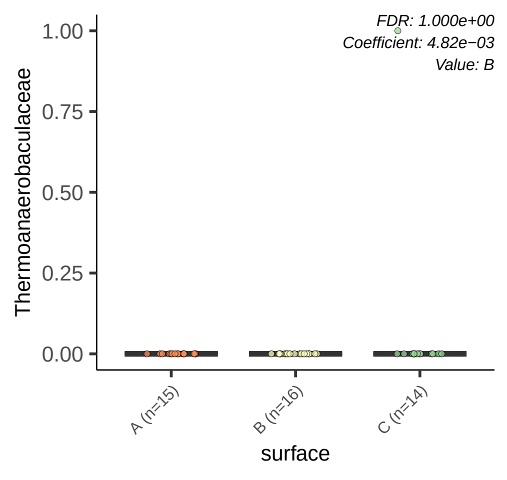
FDR: 1.000e+00
Coefficient: 4.82e−03
Value: B
Thermoanaerobaculaceae
1.00
0.75
0.50
0.25
0.00
A (n=15)
B (n=16)
C (n=14)
surface

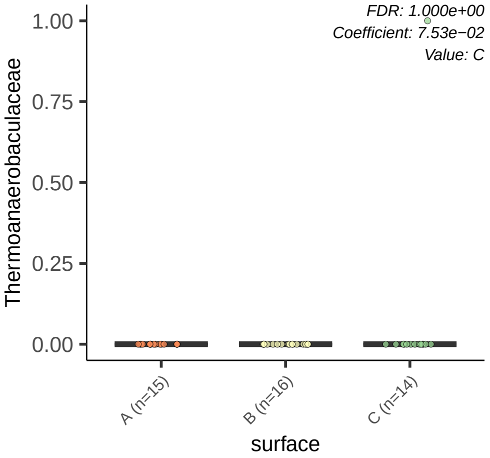

FDR: 1.000e+00
Coefficient: 7.53e−02
Value: C
Thermoanaerobaculaceae
1.00
0.75
0.50
0.25
0.00
A (n=15)
B (n=16)
C (n=14)
surface

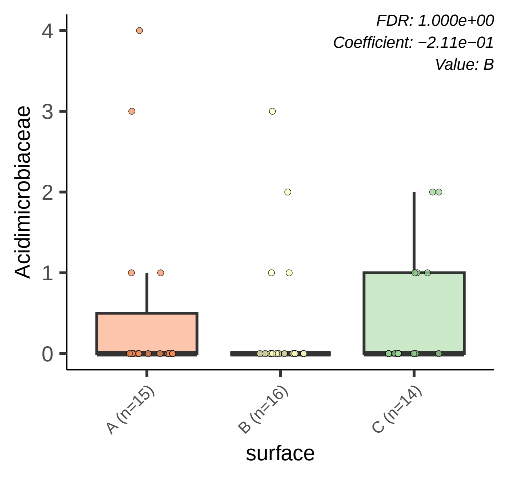
FDR: 1.000e+00
Coefficient: −2.11e−01
Value: B
Acidimicrobiaceae
0
1
2
3
4
A (n=15)
B (n=16)
C (n=14)
surface

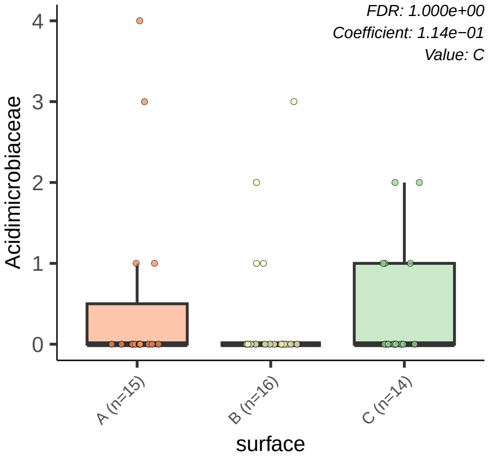
FDR: 1.000e+00
Coefficient: 1.14e−01
Value: C
Acidimicrobiaceae
0
1
2
3
4
A (n=15)
B (n=16)
C (n=14)
surface

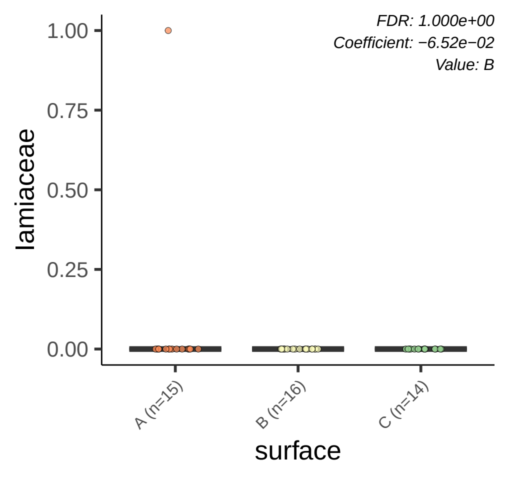

FDR: 1.000e+00
Coefficient: −6.52e−02
Value: B
1.00
0.75
0.50
0.25
0.00
lamiaceae
A (n=15)
B (n=16)
C (n=14)
surface

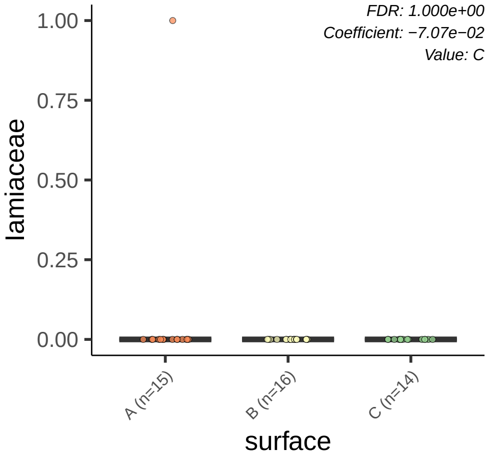

FDR: 1.000e+00
Coefficient: −7.07e−02
Value: C
lamiaceae
1.00
0.75
0.50
0.25
0.00
A (n=15)
B (n=16)
C (n=14)
surface

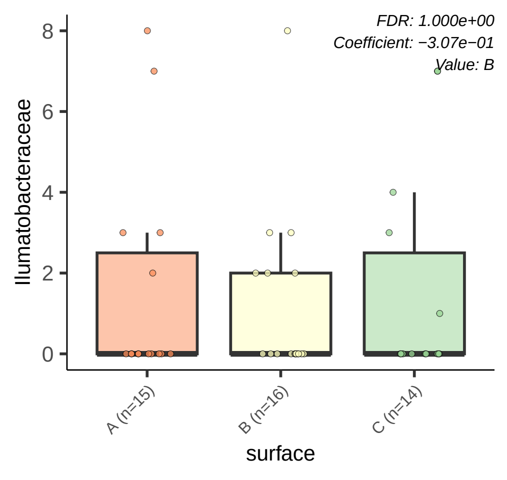
FDR: 1.000e+00
Coefficient: −3.07e−01
Value: B
Ilumatobacteraceae
0
2
4
6
8
A (n=15)
B (n=16)
C (n=14)
surface

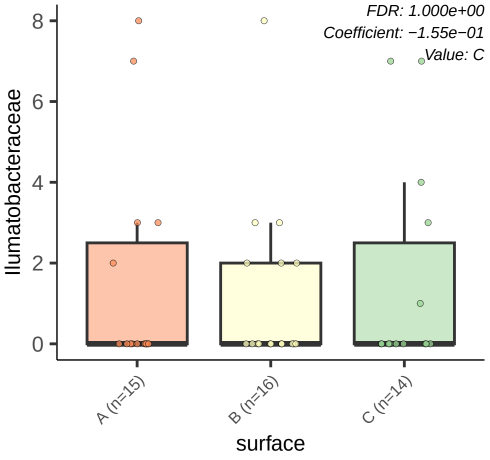
FDR: 1.000e+00
Coefficient: −1.55e−01
Value: C
Ilumatobacteraceae
8
6
4
2
0
A (n=15)
B (n=16)
C (n=14)
surface

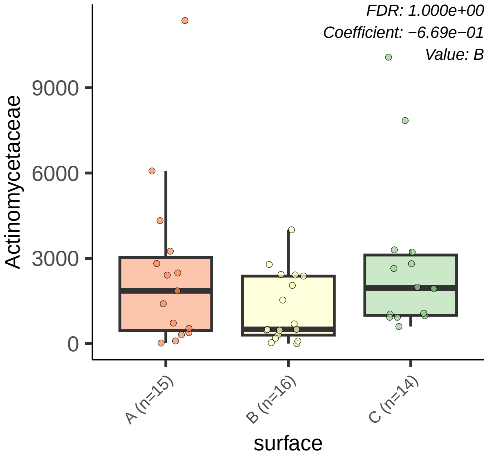

FDR: 1.000e+00
Coefficient: −6.69e−01
Value: B
Actinomycetaceae
9000
6000
3000
0
A (n=15)
B (n=16)
C (n=14)
surface

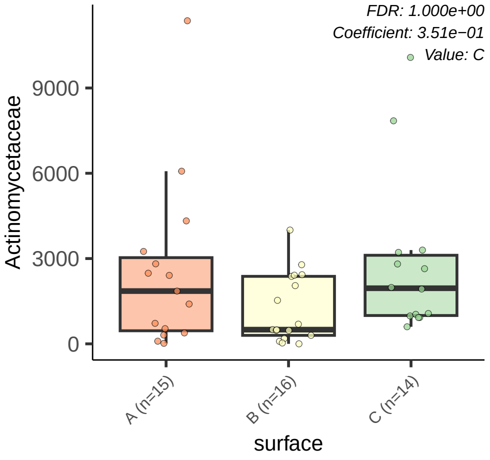
FDR: 1.000e+00
Coefficient: 3.51e−01
Value: C
Actinomycetaceae
9000
6000
3000
0
A (n=15)
B (n=16)
C (n=14)
surface

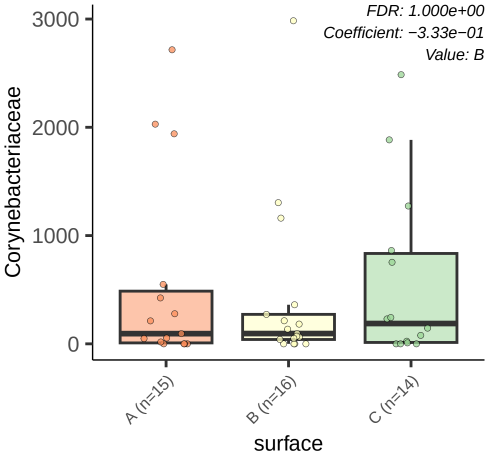
FDR: 1.000e+00
Coefficient: −3.33e−01
Value: B
Corynebacteriaceae
3000
2000
1000
0
A (n=15)
B (n=16)
C (n=14)
surface

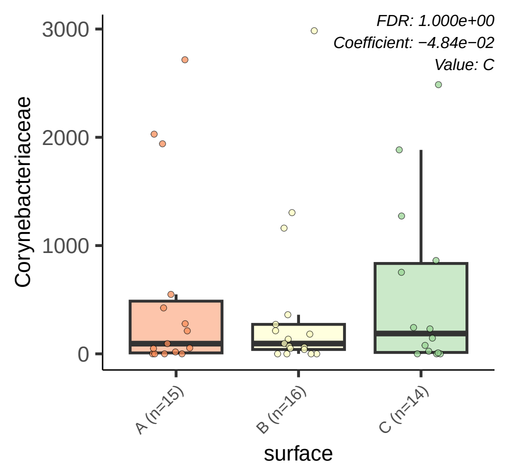
FDR: 1.000e+00
Coefficient: −4.84e−02
Value: C
Corynebacteriaceae
3000
2000
1000
0
A (n=15)
B (n=16)
C (n=14)
surface

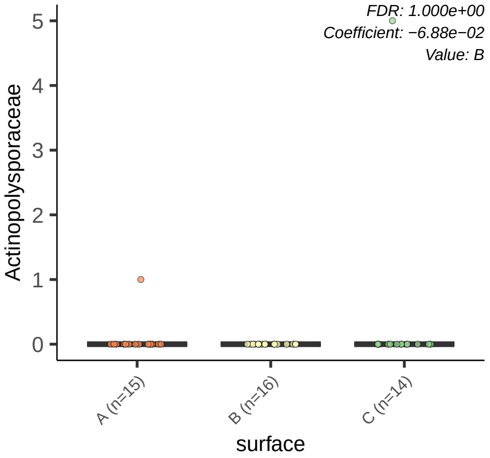
FDR: 1.000e+00
Coefficient: −6.88e−02
Value: B
Actinopolysporaceae
0
1
2
3
4
5
A (n=15)
B (n=16)
C (n=14)
surface

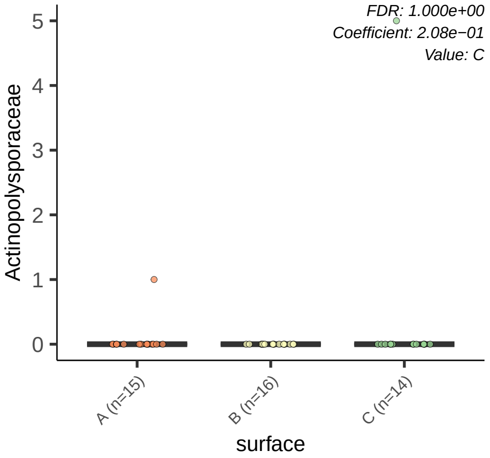
FDR: 1.000e+00
Coefficient: 2.08e−01
Value: C
Actinopolysporaceae
0
1
2
3
4
5
A (n=15)
B (n=16)
C (n=14)
surface

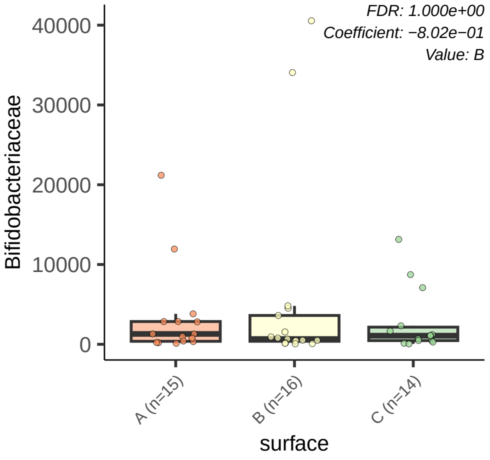

FDR: 1.000e+00
Coefficient: −8.02e−01
Value: B
Bifidobacteriaceae
40000
30000
20000
10000
0
A (n=15)
B (n=16)
C (n=14)
surface

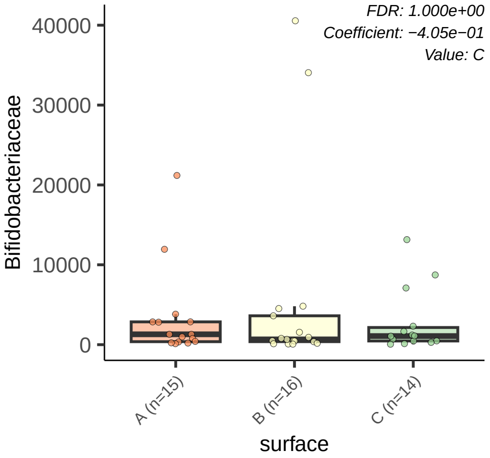
FDR: 1.000e+00
Coefficient: −4.05e−01
Value: C
Bifidobacteriaceae
40000
30000
20000
10000
0
A (n=15)
B (n=16)
C (n=14)
surface

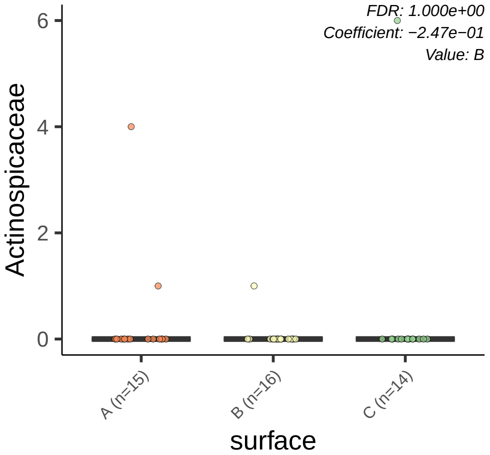
FDR: 1.000e+00
Coefficient: −2.47e−01
Value: B
Actinospicaceae
0
2
4
6
A (n=15)
B (n=16)
C (n=14)
surface

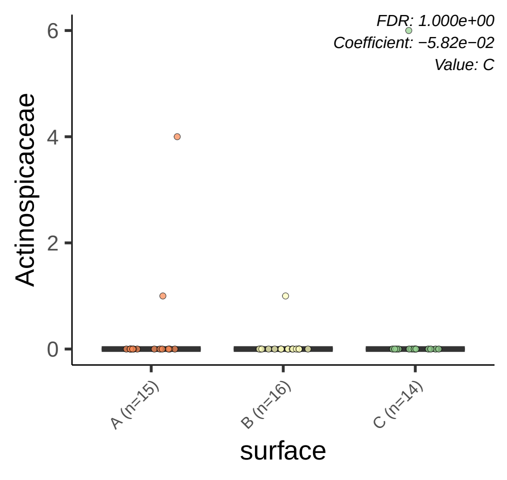
FDR: 1.000e+00
Coefficient: −5.82e−02
Value: C
Actinospicaceae
6
4
2
0
A (n=15)
B (n=16)
C (n=14)
surface

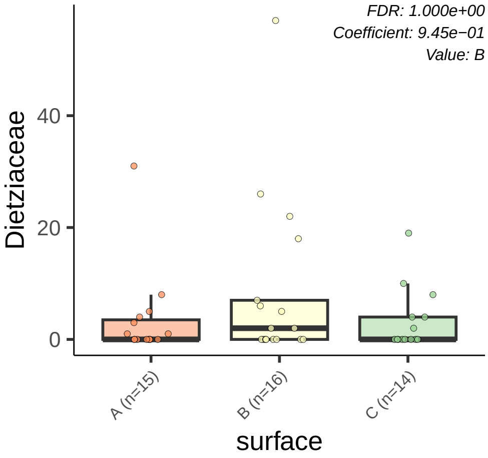
FDR: 1.000e+00
Coefficient: 9.45e−01
Value: B
Dietziaceae
0
20
40
A (n=15)
B (n=16)
C (n=14)
surface

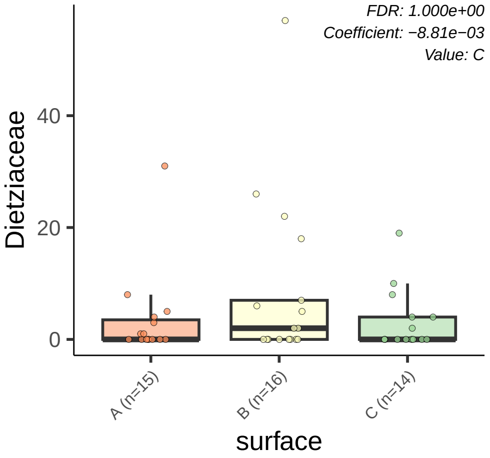
FDR: 1.000e+00
Coefficient: −8.81e−03
Value: C
Dietziaceae
40
20
0
A (n=15)
B (n=16)
C (n=14)
surface

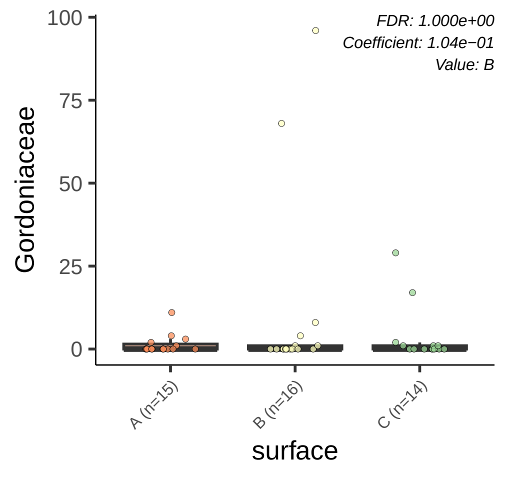
FDR: 1.000e+00
Coefficient: 1.04e−01
Value: B
Gordoniaceae
100
75
50
25
0
A (n=15)
B (n=16)
C (n=14)
surface

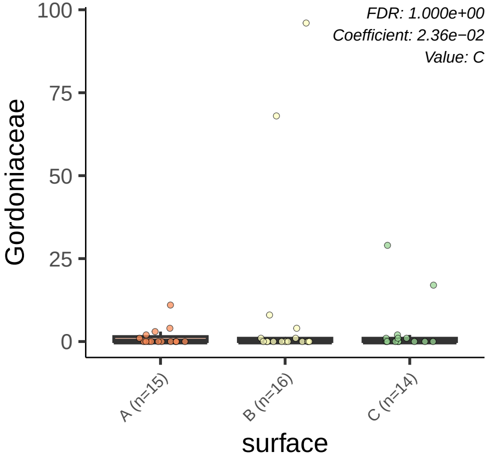
FDR: 1.000e+00
Coefficient: 2.36e−02
Value: C
Gordoniaceae
100
75
50
25
0
A (n=15)
B (n=16)
C (n=14)
surface

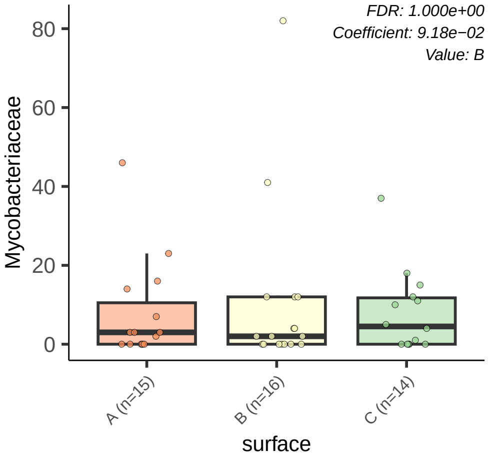

FDR: 1.000e+00
Coefficient: 9.18e−02
Value: B
Mycobacteriaceae
0
20
40
60
80
A (n=15)
B (n=16)
C (n=14)
surface

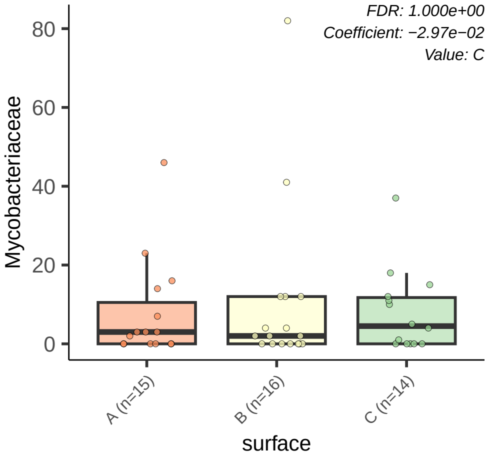
FDR: 1.000e+00
Coefficient: −2.97e−02
Value: C
Mycobacteriaceae
0
20
40
60
80
A (n=15)
B (n=16)
C (n=14)
surface

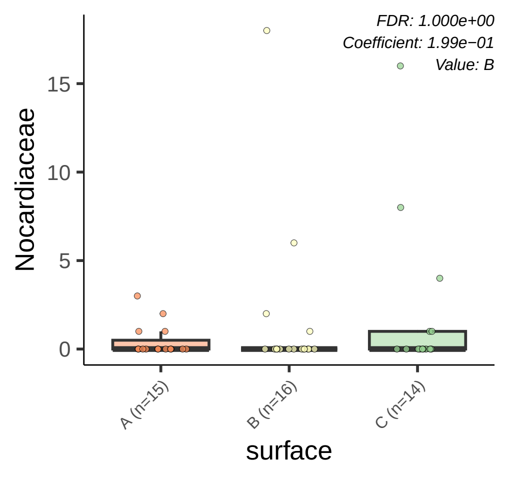
FDR: 1.000e+00
Coefficient: 1.99e−01
Value: B
Nocardiaceae
15
10
5
0
A (n=15)
B (n=16)
C (n=14)
surface

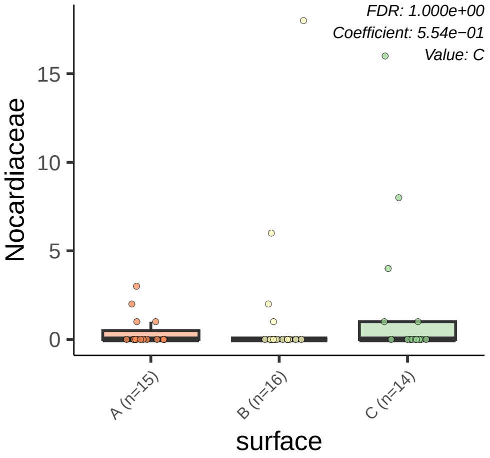
FDR: 1.000e+00
Coefficient: 5.54e−01
Value: C
Nocardiaceae
0
5
10
15
A (n=15)
B (n=16)
C (n=14)
surface

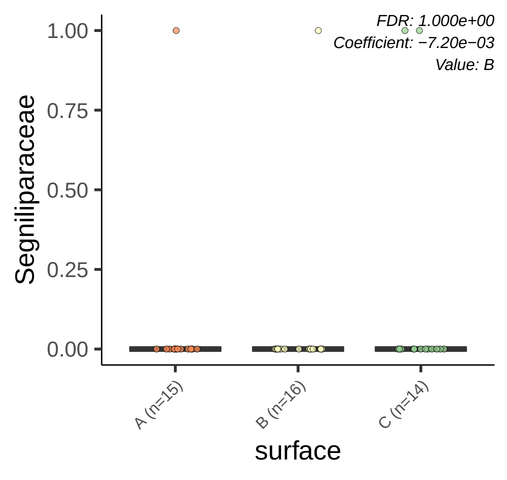
FDR: 1.000e+00
Coefficient: −7.20e−03
Value: B
Segniliparaceae
1.00
0.75
0.50
0.25
0.00
A (n=15)
B (n=16)
C (n=14)
surface

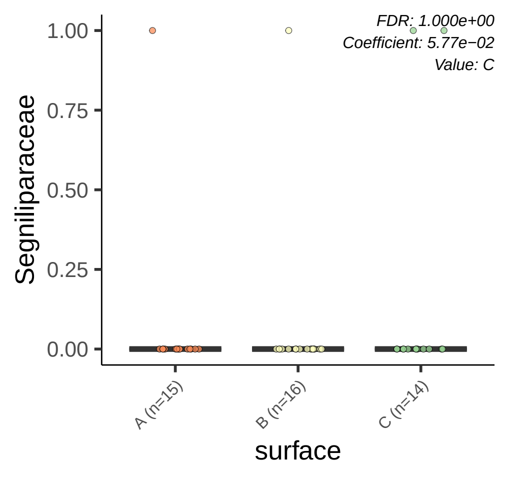

FDR: 1.000e+00
Coefficient: 5.77e−02
Value: C
Segniliparaceae
1.00
0.75
0.50
0.25
0.00
A (n=15)
B (n=16)
C (n=14)
surface

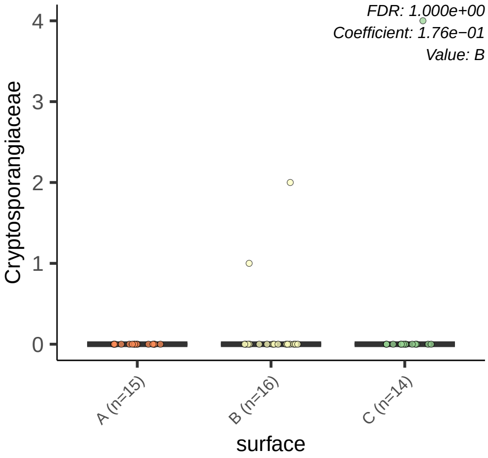
FDR: 1.000e+00
Coefficient: 1.76e−01
Value: B
Cryptosporangiaceae
0
1
2
3
4
A (n=15)
B (n=16)
C (n=14)
surface

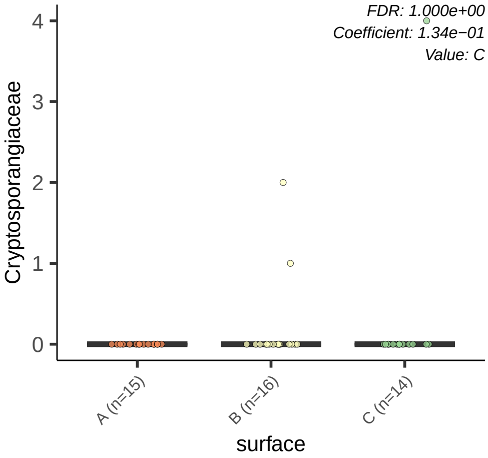

FDR: 1.000e+00
Coefficient: 1.34e−01
Value: C
Cryptosporangiaceae
4
3
2
1
0
A (n=15)
B (n=16)
C (n=14)
surface

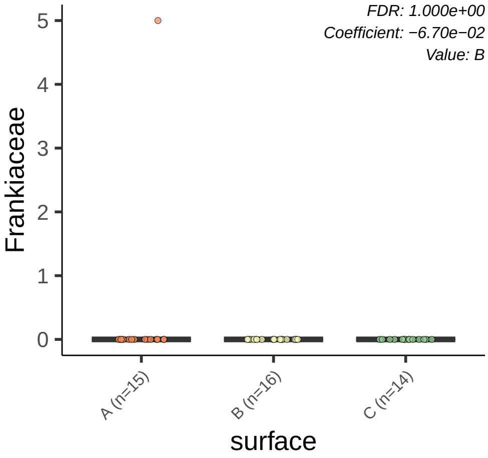

FDR: 1.000e+00
Coefficient: −6.70e−02
Value: B
Frankiaceae
0
1
2
3
4
5
A (n=15)
B (n=16)
C (n=14)
surface

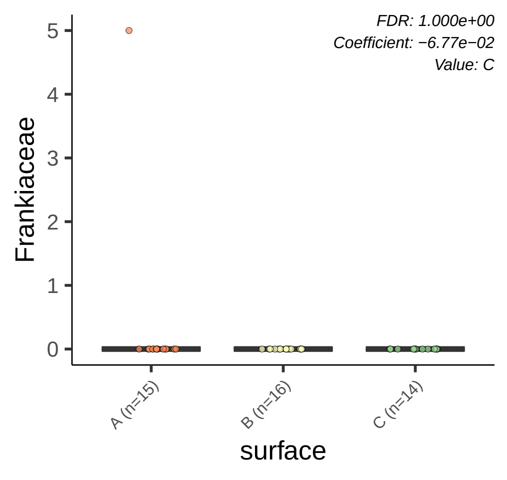
FDR: 1.000e+00
Coefficient: −6.77e−02
Value: C
Frankiaceae
0
1
2
3
4
5
A (n=15)
B (n=16)
C (n=14)
surface

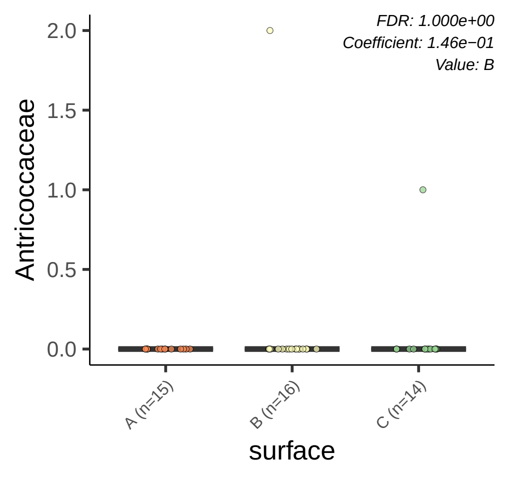

FDR: 1.000e+00
Coefficient: 1.46e−01
Value: B
Antricoccaceae
2.0
1.5
1.0
0.5
0.0
A (n=15)
B (n=16)
C (n=14)
surface

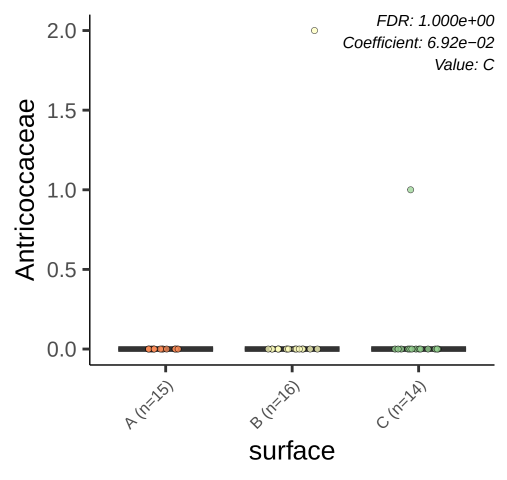

FDR: 1.000e+00
Coefficient: 6.92e−02
Value: C
Antricoccaceae
2.0
1.5
1.0
0.5
0.0
A (n=15)
B (n=16)
C (n=14)
surface

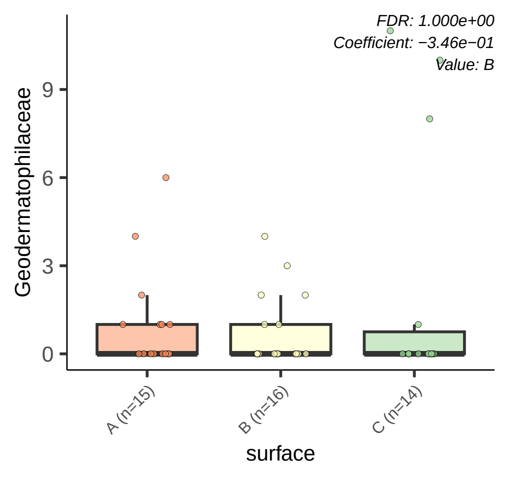
FDR: 1.000e+00
Coefficient: −3.46e−01
Value: B
Geodermatophilaceae
0
3
6
9
A (n=15)
B (n=16)
C (n=14)
surface

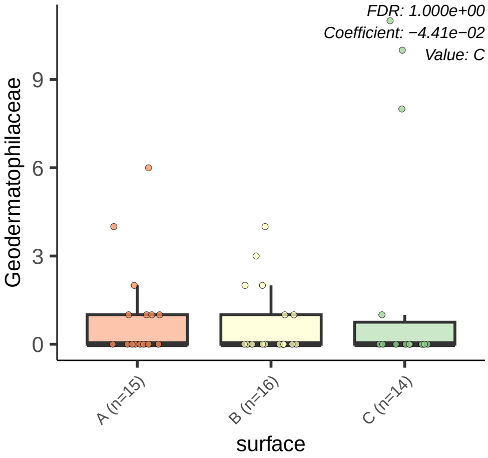
FDR: 1.000e+00
Coefficient: −4.41e−02
Value: C
Geodermatophilaceae
0
3
6
9
A (n=15)
B (n=16)
C (n=14)
surface

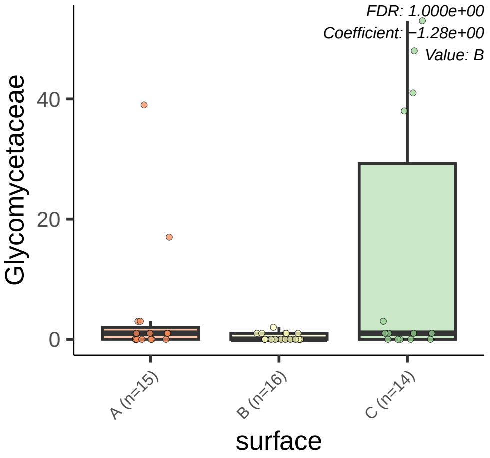

FDR: 1.000e+00
Coefficient: −1.28e+00
Value: B
Glycomycetaceae
40
20
0
A (n=15)
B (n=16)
C (n=14)
surface

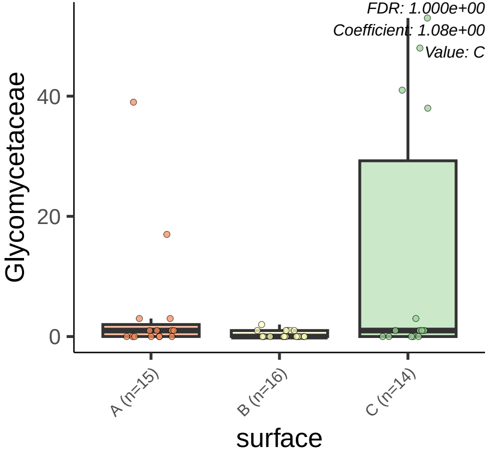
FDR: 1.000e+00
Coefficient: 1.08e+00
Value: C
Glycomycetaceae
40
20
0
A (n=15)
B (n=16)
C (n=14)
surface

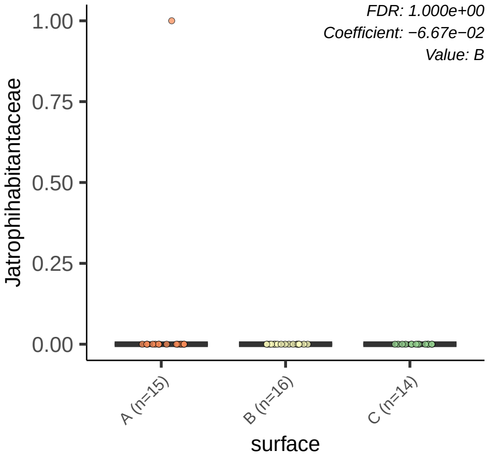

FDR: 1.000e+00
Coefficient: −6.67e−02
Value: B
Jatrophihabitantaceae
1.00
0.75
0.50
0.25
0.00
A (n=15)
B (n=16)
C (n=14)
surface

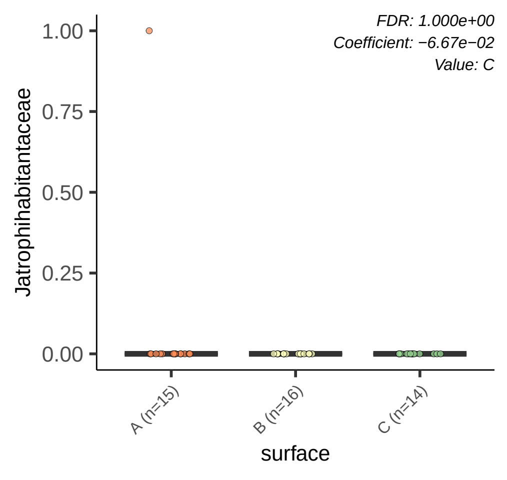
FDR: 1.000e+00
Coefficient: −6.67e−02
Value: C
Jatrophihabitantaceae
1.00
0.75
0.50
0.25
0.00
A (n=15)
B (n=16)
C (n=14)
surface

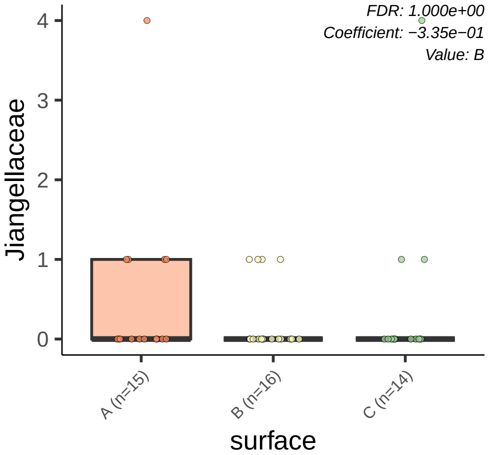

FDR: 1.000e+00
Coefficient: −3.35e−01
Value: B
Jiangellaceae
0
1
2
3
4
A (n=15)
B (n=16)
C (n=14)
surface

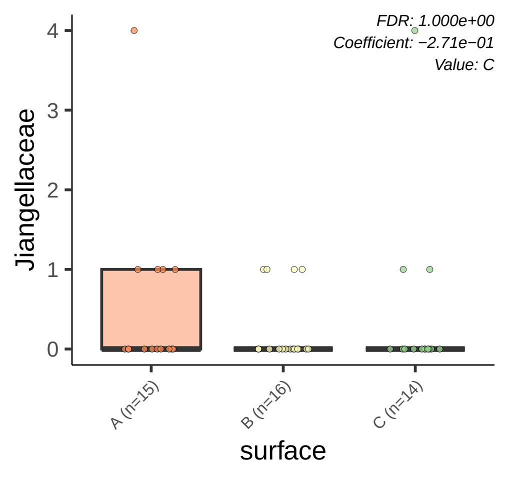

FDR: 1.000e+00
Coefficient: −2.71e−01
Value: C
Jiangellaceae
0
1
2
3
4
A (n=15)
B (n=16)
C (n=14)
surface

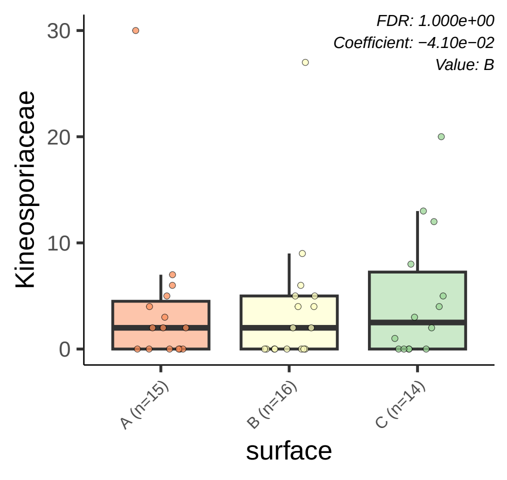

FDR: 1.000e+00
Coefficient: −4.10e−02
Value: B
Kineosporiaceae
0
10
20
30
A (n=15)
B (n=16)
C (n=14)
surface

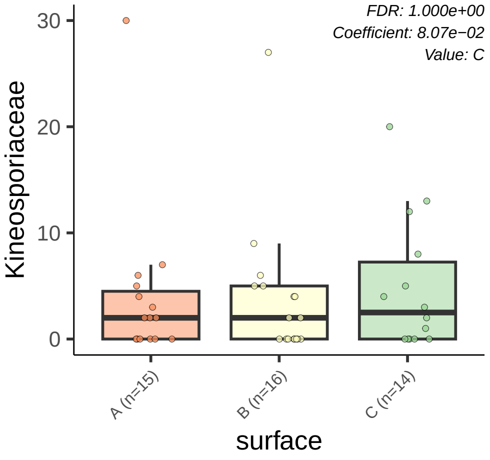

FDR: 1.000e+00
Coefficient: 8.07e−02
Value: C
Kineosporiaceae
30
20
10
0
A (n=15)
B (n=16)
C (n=14)
surface

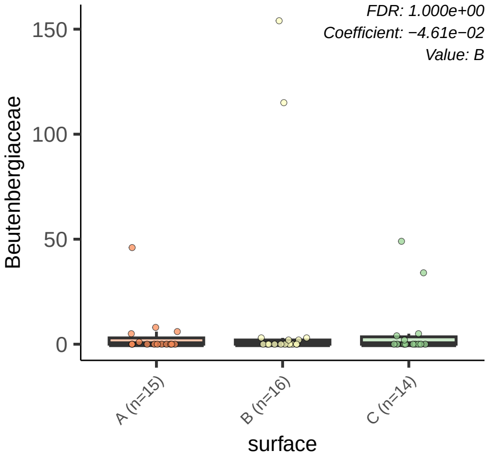
FDR: 1.000e+00
Coefficient: −4.61e−02
Value: B
150
100
50
0
Beutenbergiaceae
A (n=15)
B (n=16)
C (n=14)
surface

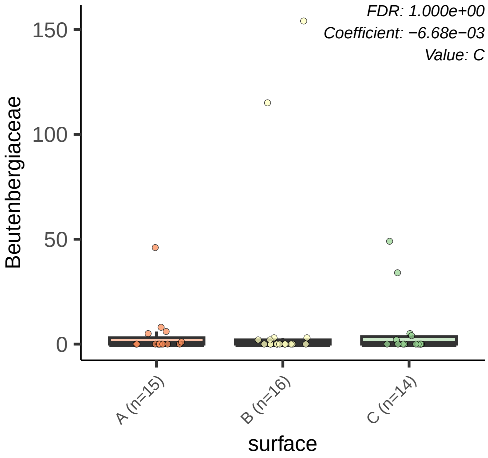

FDR: 1.000e+00
Coefficient: −6.68e−03
Value: C
Beutenbergiaceae
150
100
50
0
A (n=15)
B (n=16)
C (n=14)
surface

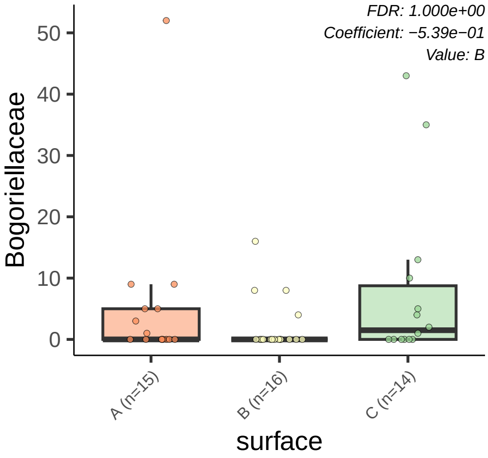

FDR: 1.000e+00
Coefficient: −5.39e−01
Value: B
Bogoriellaceae
0
10
20
30
40
50
A (n=15)
B (n=16)
C (n=14)
surface

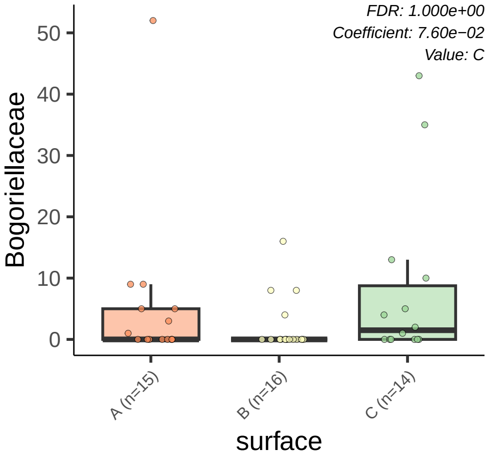
FDR: 1.000e+00
Coefficient: 7.60e−02
Value: C
Bogoriellaceae
50
40
30
20
10
0
A (n=15)
B (n=16)
C (n=14)
surface

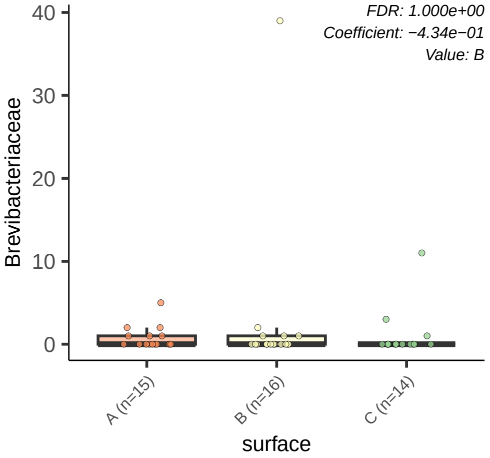
FDR: 1.000e+00
Coefficient: −4.34e−01
Value: B
Brevibacteriaceae
40
30
20
10
0
A (n=15)
B (n=16)
C (n=14)
surface

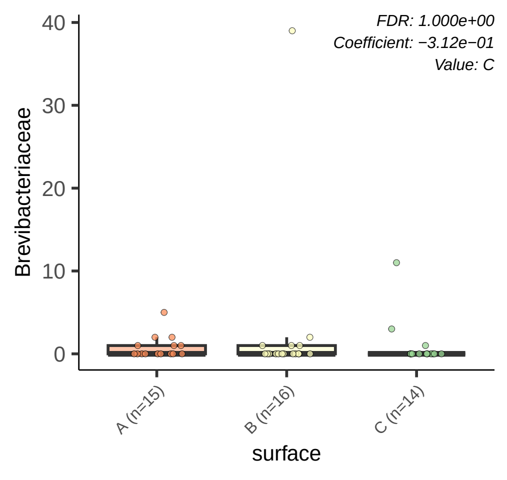

FDR: 1.000e+00
Coefficient: −3.12e−01
Value: C
Brevibacteriaceae
40
30
20
10
0
A (n=15)
B (n=16)
C (n=14)
surface

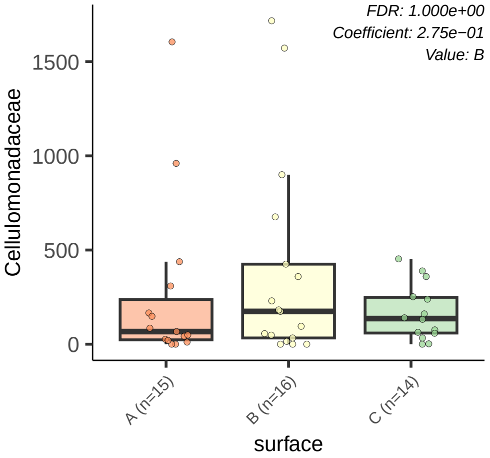

FDR: 1.000e+00
Coefficient: 2.75e−01
Value: B
Cellulomonadaceae
1500
1000
500
0
A (n=15)
B (n=16)
C (n=14)
surface

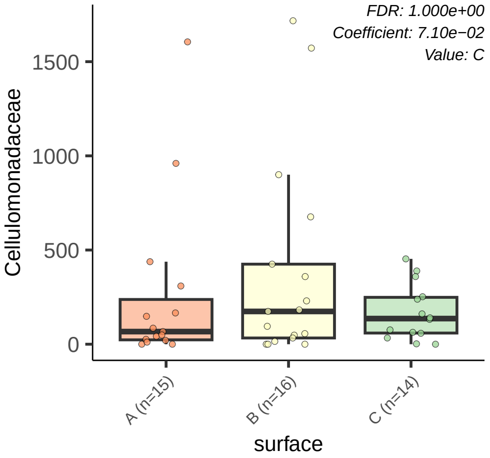
FDR: 1.000e+00
Coefficient: 7.10e−02
Value: C
Cellulomonadaceae
1500
1000
500
0
A (n=15)
B (n=16)
C (n=14)
surface

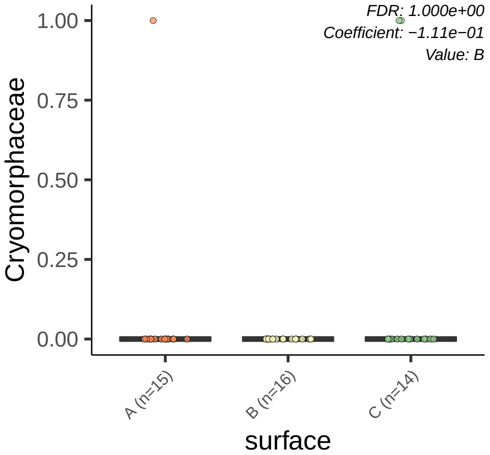

FDR: 1.000e+00
Coefficient: −1.11e−01
Value: B
Cryomorphaceae
1.00
0.75
0.50
0.25
0.00
A (n=15)
B (n=16)
C (n=14)
surface

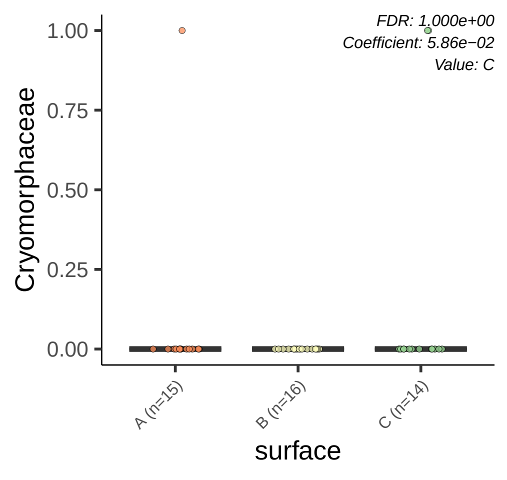
FDR: 1.000e+00
Coefficient: 5.86e−02
Value: C
Cryomorphaceae
1.00
0.75
0.50
0.25
0.00
A (n=15)
B (n=16)
C (n=14)
surface

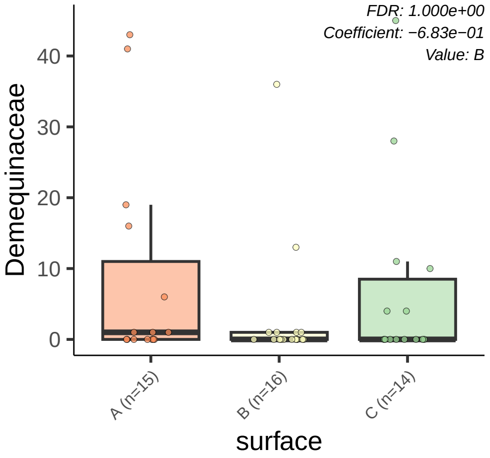
FDR: 1.000e+00
Coefficient: −6.83e−01
Value: B
Demequinaceae
0
10
20
30
40
A (n=15)
B (n=16)
C (n=14)
surface

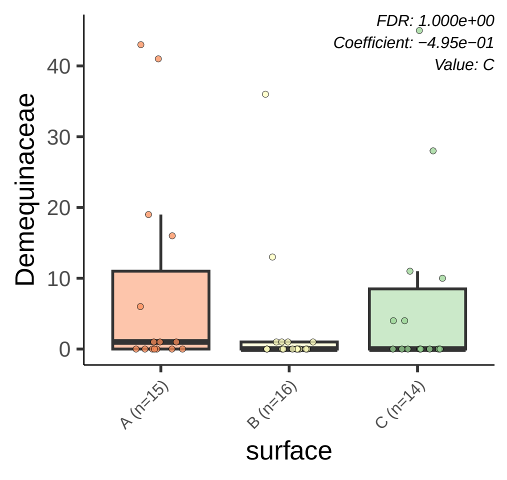
FDR: 1.000e+00
Coefficient: −4.95e−01
Value: C
Demequinaceae
0
10
20
30
40
A (n=15)
B (n=16)
C (n=14)
surface

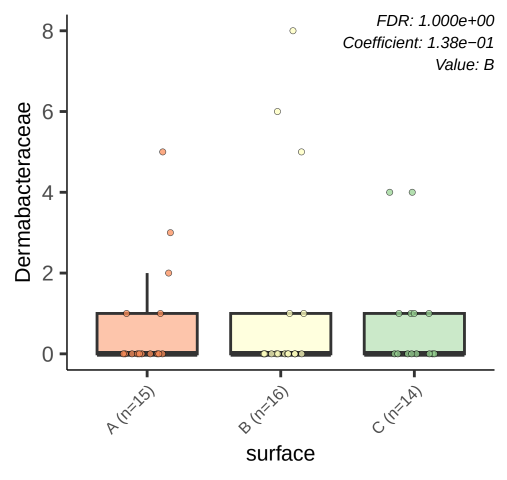
FDR: 1.000e+00
Coefficient: 1.38e−01
Value: B
Dermabacteraceae
0
2
4
6
8
A (n=15)
B (n=16)
C (n=14)
surface

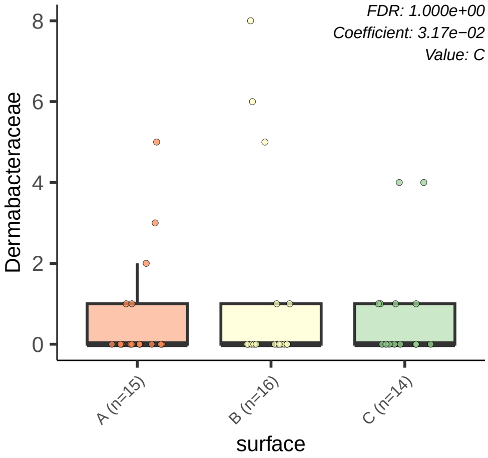

FDR: 1.000e+00
Coefficient: 3.17e−02
Value: C
Dermabacteraceae
0
2
4
6
8
A (n=15)
B (n=16)
C (n=14)
surface

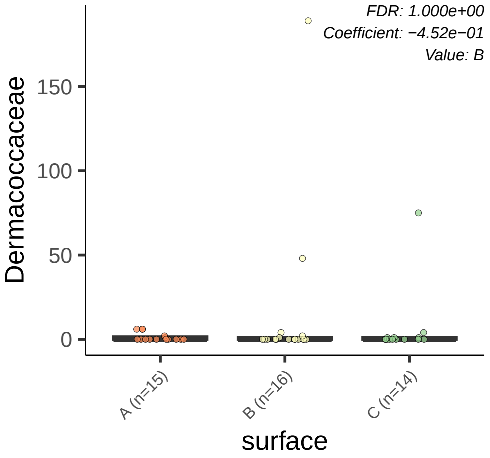
FDR: 1.000e+00
Coefficient: −4.52e−01
Value: B
Dermacoccaceae
150
100
50
0
A (n=15)
B (n=16)
C (n=14)
surface

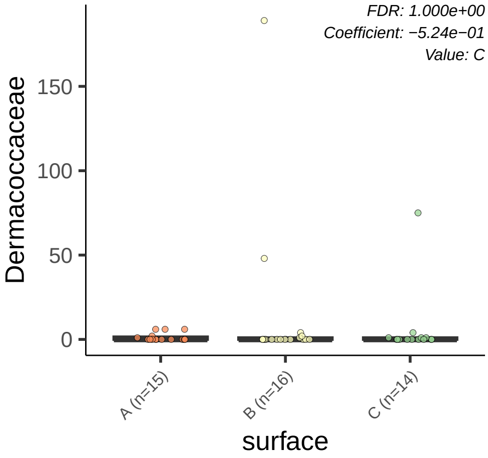
FDR: 1.000e+00
Coefficient: −5.24e−01
Value: C
Dermacoccaceae
150
100
50
0
A (n=15)
B (n=16)
C (n=14)
surface

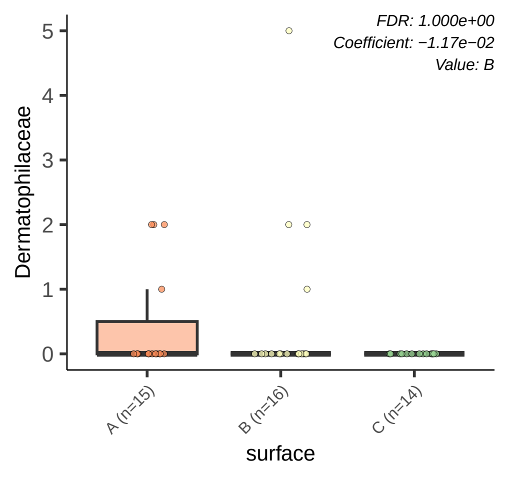

FDR: 1.000e+00
Coefficient: −1.17e−02
Value: B
Dermatophilaceae
0
1
2
3
4
5
A (n=15)
B (n=16)
C (n=14)
surface

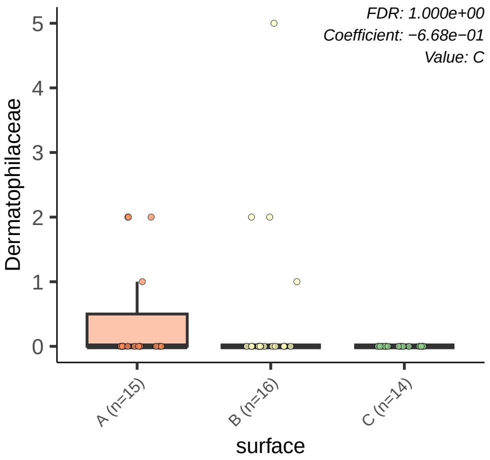
FDR: 1.000e+00
Coefficient: −6.68e−01
Value: C
Dermatophilaceae
5
4
3
2
1
0
A (n=15)
B (n=16)
C (n=14)
surface

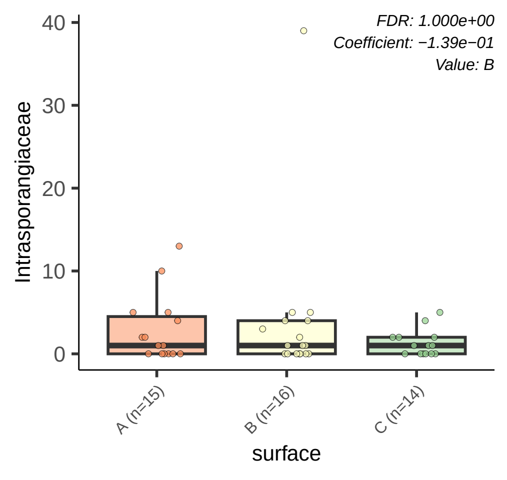

FDR: 1.000e+00
Coefficient: −1.39e−01
Value: B
Intrasporangiaceae
0
10
20
30
40
A (n=15)
B (n=16)
C (n=14)
surface

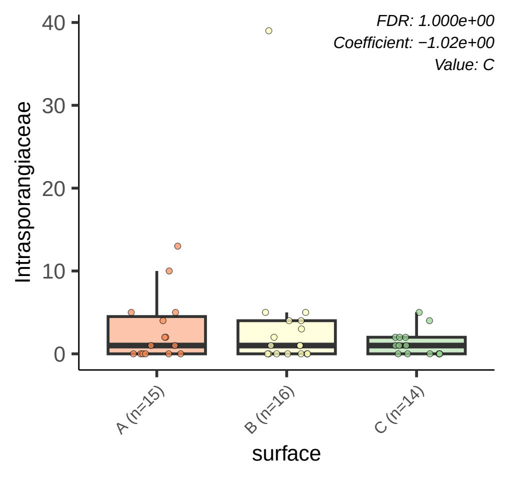

FDR: 1.000e+00
Coefficient: −1.02e+00
Value: C
Intrasporangiaceae
40
30
20
10
0
A (n=15)
B (n=16)
C (n=14)
surface

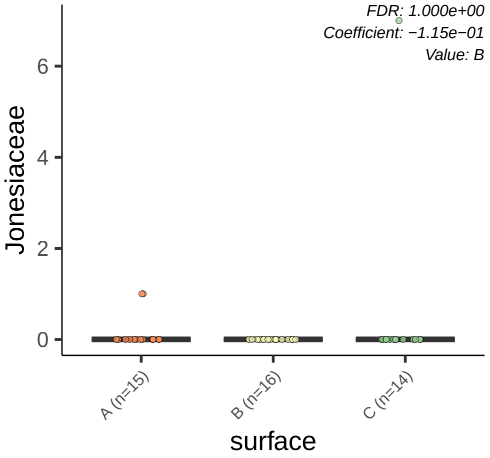
FDR: 1.000e+00
Coefficient: −1.15e−01
Value: B
Jonesiaceae
0
2
4
6
A (n=15)
B (n=16)
C (n=14)
surface

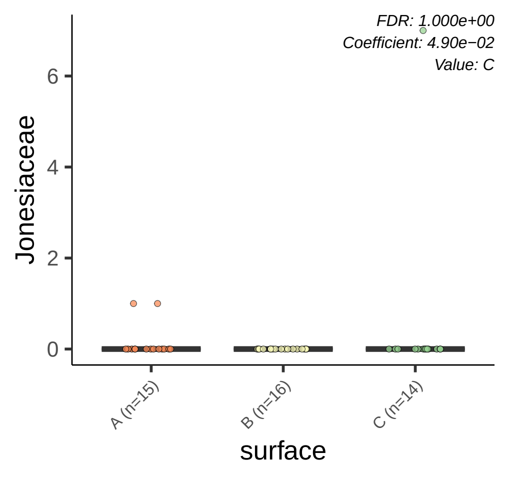
FDR: 1.000e+00
Coefficient: 4.90e−02
Value: C
Jonesiaceae
0
2
4
6
A (n=15)
B (n=16)
C (n=14)
surface

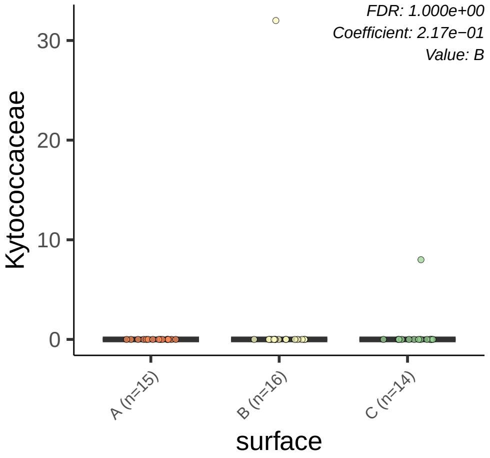
FDR: 1.000e+00
Coefficient: 2.17e−01
Value: B
Kytococcaceae
30
20
10
0
A (n=15)
B (n=16)
C (n=14)
surface

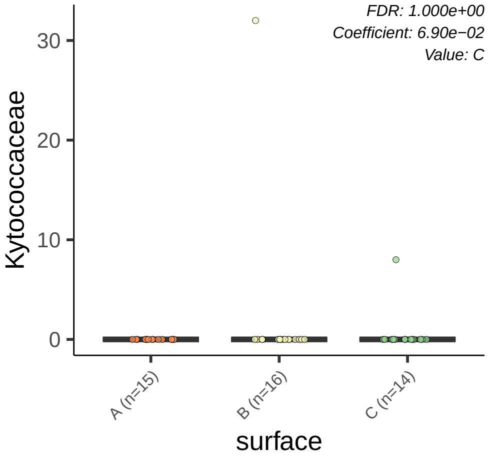
FDR: 1.000e+00
Coefficient: 6.90e−02
Value: C
Kytococcaceae
30
20
10
0
A (n=15)
B (n=16)
C (n=14)
surface

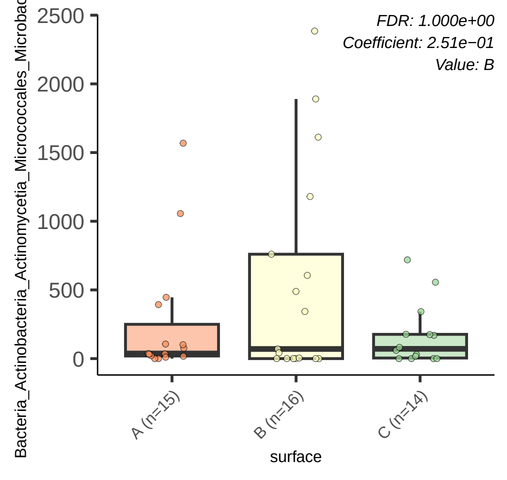

FDR: 1.000e+00
Coefficient: 2.51e−01
Value: B
Bacteria_Actinobacteria_Actinomycetia_Micrococcales_Microbac
2500
2000
1500
1000
500
0
A (n=15)
B (n=16)
C (n=14)
surface

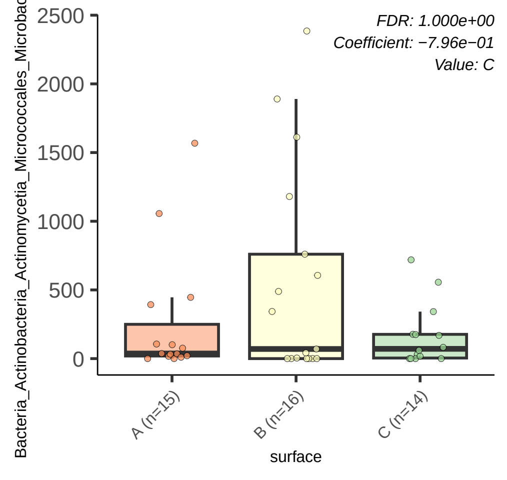
FDR: 1.000e+00
Coefficient: −7.96e−01
Value: C
Bacteria_Actinobacteria_Actinomycetia_Micrococcales_Microbac
2500
2000
1500
1000
500
0
A (n=15)
B (n=16)
C (n=14)
surface

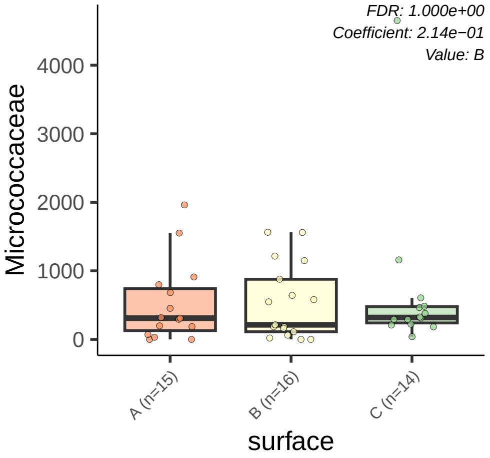

FDR: 1.000e+00
Coefficient: 2.14e−01
Value: B
Micrococcaceae
4000
3000
2000
1000
0
A (n=15)
B (n=16)
C (n=14)
surface

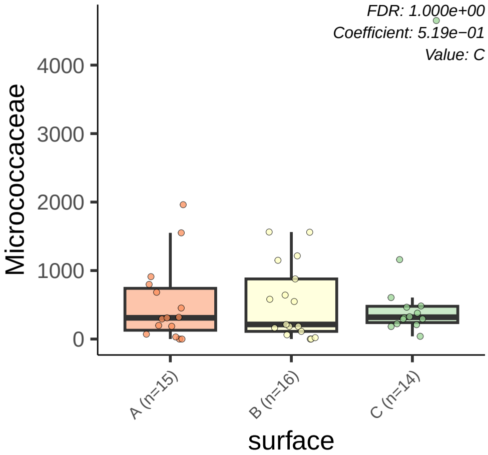
FDR: 1.000e+00
Coefficient: 5.19e−01
Value: C
Micrococcaceae
4000
3000
2000
1000
0
A (n=15)
B (n=16)
C (n=14)
surface

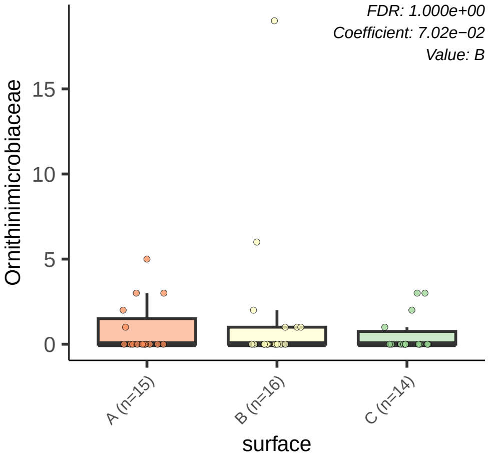

FDR: 1.000e+00
Coefficient: 7.02e−02
Value: B
Ornithinimicrobiaceae
0
5
10
15
A (n=15)
B (n=16)
C (n=14)
surface

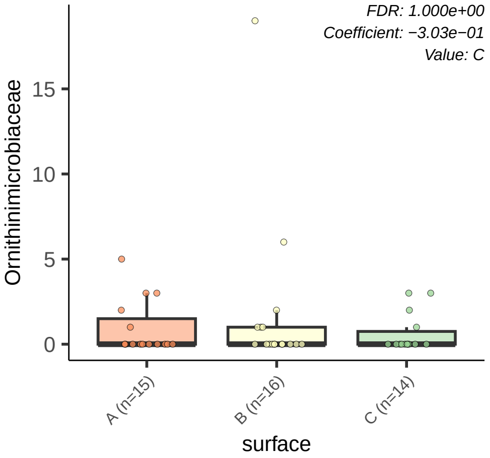
FDR: 1.000e+00
Coefficient: −3.03e−01
Value: C
Ornithinimicrobiaceae
0
5
10
15
A (n=15)
B (n=16)
C (n=14)
surface

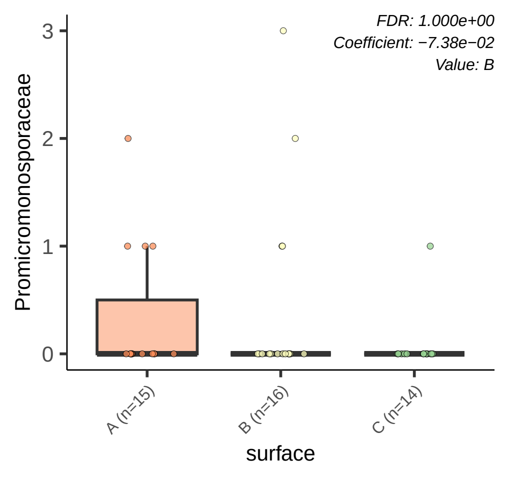

FDR: 1.000e+00
Coefficient: −7.38e−02
Value: B
Promicromonosporaceae
0
1
2
3
A (n=15)
B (n=16)
C (n=14)
surface

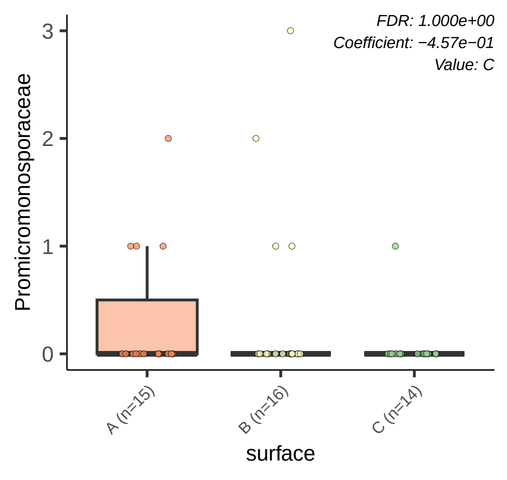
FDR: 1.000e+00
Coefficient: −4.57e−01
Value: C
Promicromonosporaceae
3
2
1
0
A (n=15)
B (n=16)
C (n=14)
surface

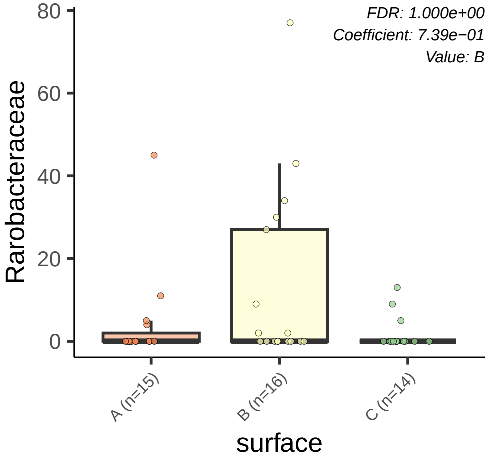

FDR: 1.000e+00
Coefficient: 7.39e−01
Value: B
Rarobacteraceae
0
20
40
60
80
A (n=15)
B (n=16)
C (n=14)
surface

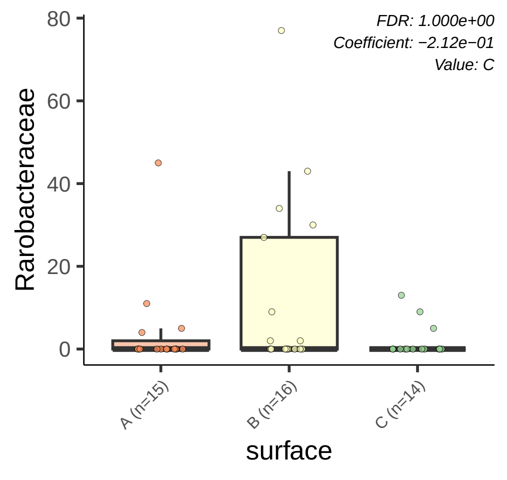
FDR: 1.000e+00
Coefficient: −2.12e−01
Value: C
Rarobacteraceae
80
60
40
20
0
A (n=15)
B (n=16)
C (n=14)
surface

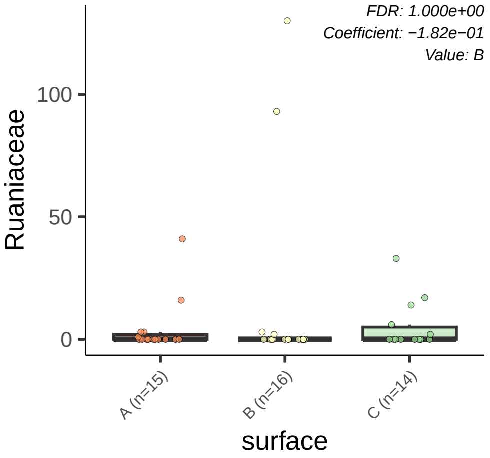

FDR: 1.000e+00
Coefficient: −1.82e−01
Value: B
Ruaniaceae
100
50
0
A (n=15)
B (n=16)
C (n=14)
surface

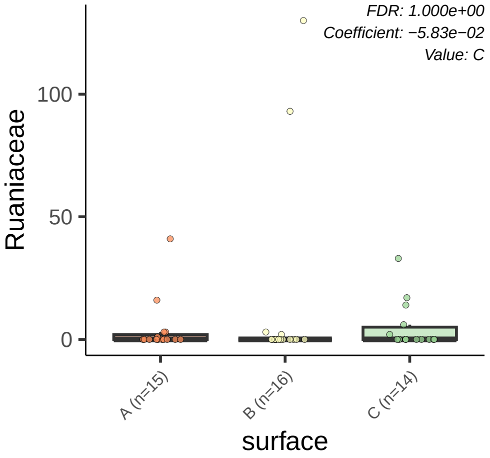
FDR: 1.000e+00
Coefficient: −5.83e−02
Value: C
Ruaniaceae
100
50
0
A (n=15)
B (n=16)
C (n=14)
surface

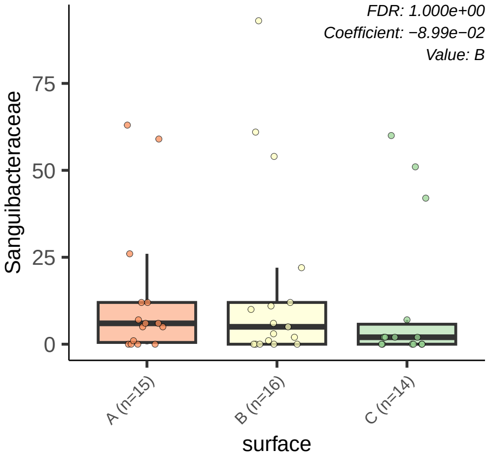
FDR: 1.000e+00
Coefficient: −8.99e−02
Value: B
Sanguibacteraceae
75
50
25
0
A (n=15)
B (n=16)
C (n=14)
surface

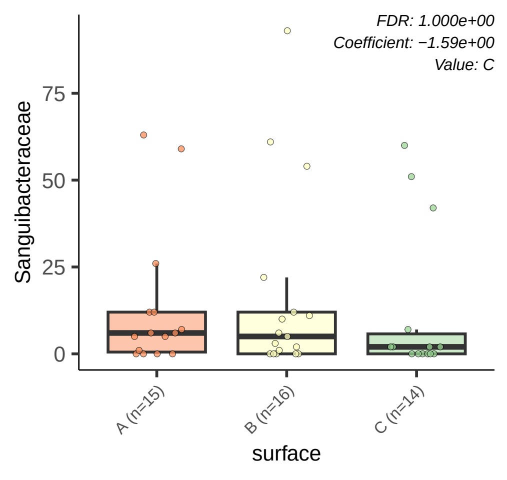
FDR: 1.000e+00
Coefficient: −1.59e+00
Value: C
Sanguibacteraceae
75
50
25
0
A (n=15)
B (n=16)
C (n=14)
surface

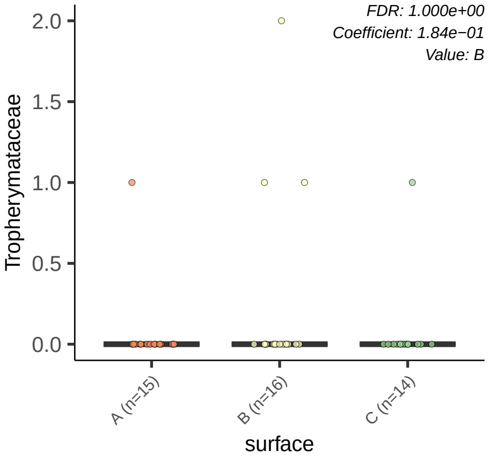

FDR: 1.000e+00
Coefficient: 1.84e−01
Value: B
Tropherymataceae
2.0
1.5
1.0
0.5
0.0
A (n=15)
B (n=16)
C (n=14)
surface

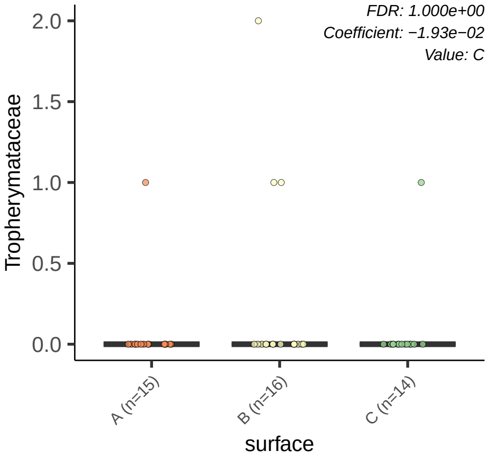
FDR: 1.000e+00
Coefficient: −1.93e−02
Value: C
Tropherymataceae
2.0
1.5
1.0
0.5
0.0
A (n=15)
B (n=16)
C (n=14)
surface

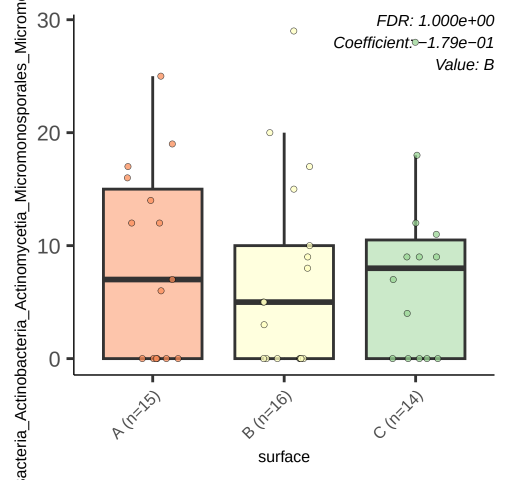
FDR: 1.000e+00
Coefficient: −1.79e−01
Value: B
bacteria_Actinobacteria_Actinomycetia_Micromonosporales_Microm
30
20
10
0
A (n=15)
B (n=16)
C (n=14)
surface

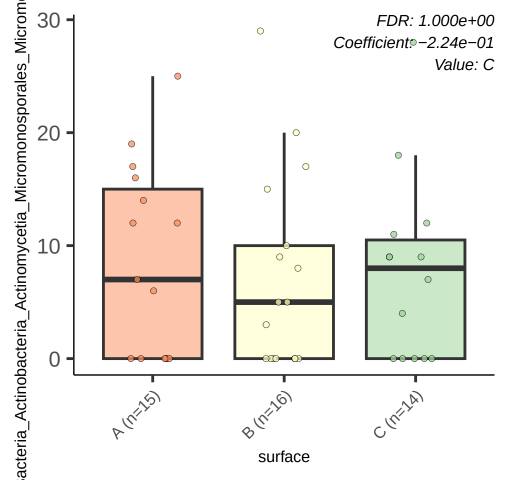
FDR: 1.000e+00
Coefficient: −2.24e−01
Value: C
bacteria_Actinobacteria_Actinomycetia_Micromonosporales_Micromo
0
10
20
30
A (n=15)
B (n=16)
C (n=14)
surface

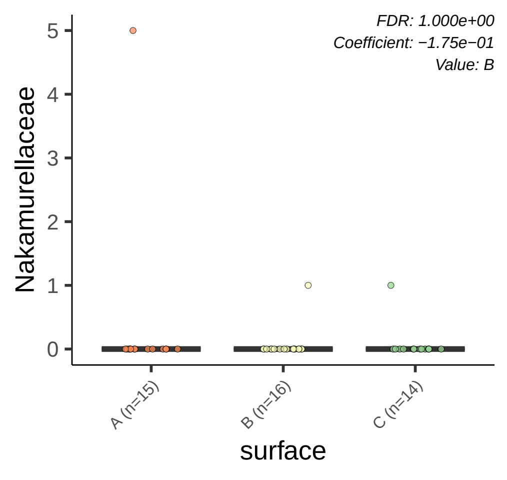

FDR: 1.000e+00
Coefficient: −1.75e−01
Value: B
Nakamurellaceae
0
1
2
3
4
5
A (n=15)
B (n=16)
C (n=14)
surface

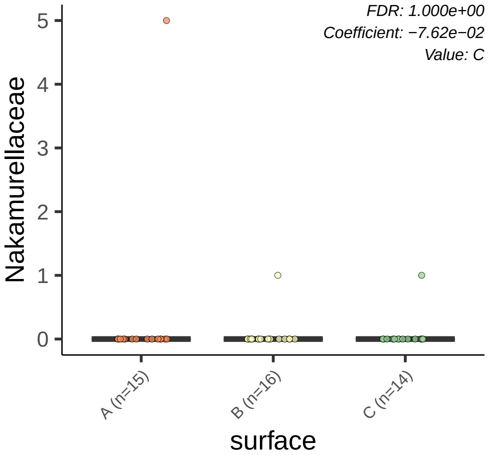
FDR: 1.000e+00
Coefficient: −7.62e−02
Value: C
Nakamurellaceae
5
4
3
2
1
0
A (n=15)
B (n=16)
C (n=14)
surface

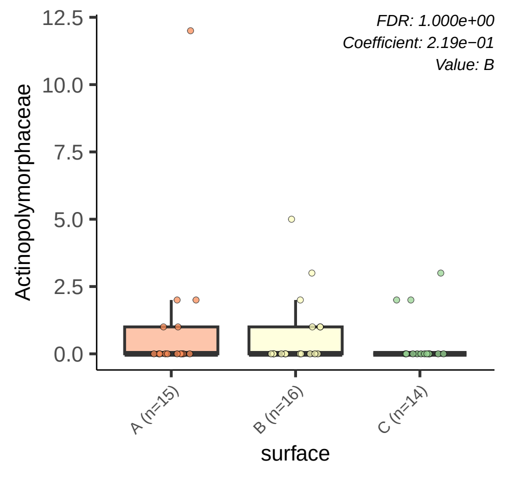

FDR: 1.000e+00
Coefficient: 2.19e−01
Value: B
Actinopolymorphaceae
12.5
10.0
7.5
5.0
2.5
0.0
A (n=15)
B (n=16)
C (n=14)
surface

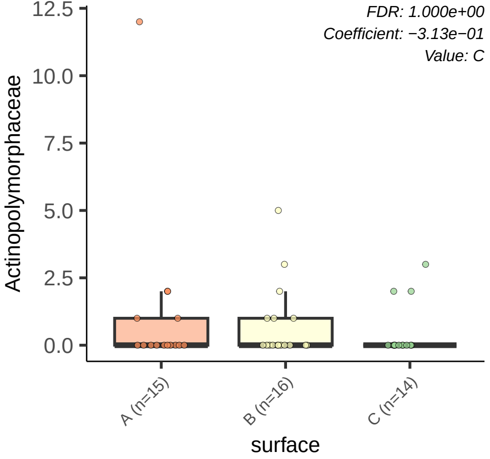

FDR: 1.000e+00
Coefficient: −3.13e−01
Value: C
Actinopolymorphaceae
12.5
10.0
7.5
5.0
2.5
0.0
A (n=15)
B (n=16)
C (n=14)
surface

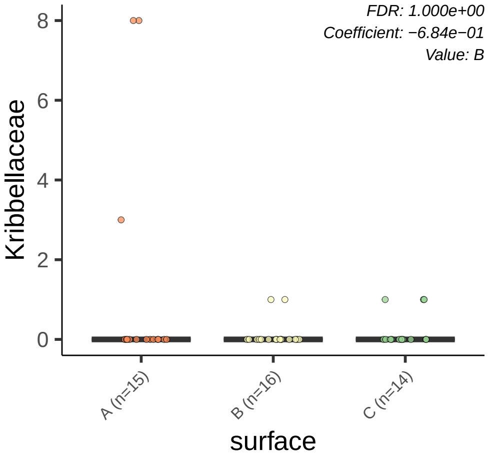

FDR: 1.000e+00
Coefficient: −6.84e−01
Value: B
Kribbellaceae
8
6
4
2
0
A (n=15)
B (n=16)
C (n=14)
surface

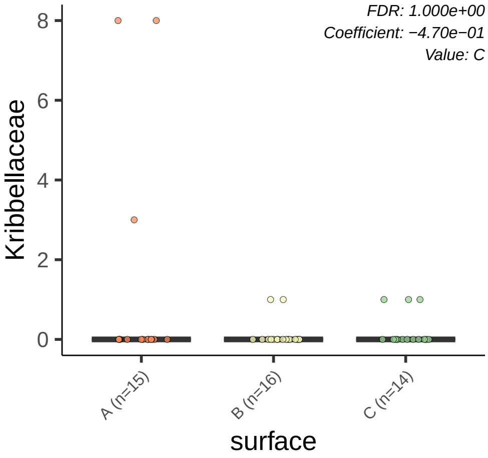
FDR: 1.000e+00
Coefficient: −4.70e−01
Value: C
Kribbellaceae
8
6
4
2
0
A (n=15)
B (n=16)
C (n=14)
surface

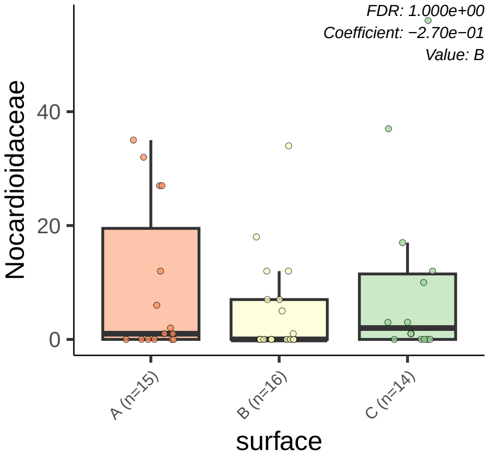

FDR: 1.000e+00
Coefficient: −2.70e−01
Value: B
Nocardioidaceae
40
20
0
A (n=15)
B (n=16)
C (n=14)
surface

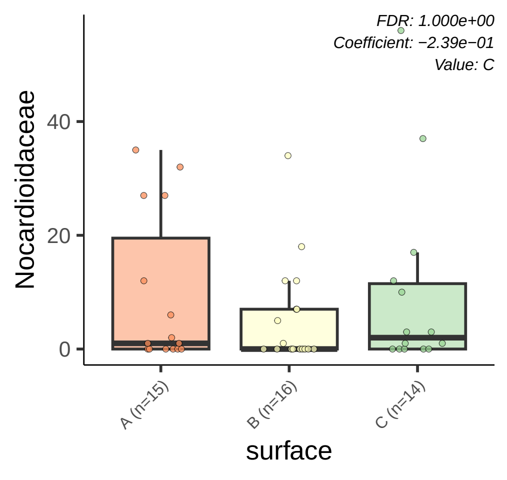

FDR: 1.000e+00
Coefficient: −2.39e−01
Value: C
Nocardioidaceae
0
20
40
A (n=15)
B (n=16)
C (n=14)
surface

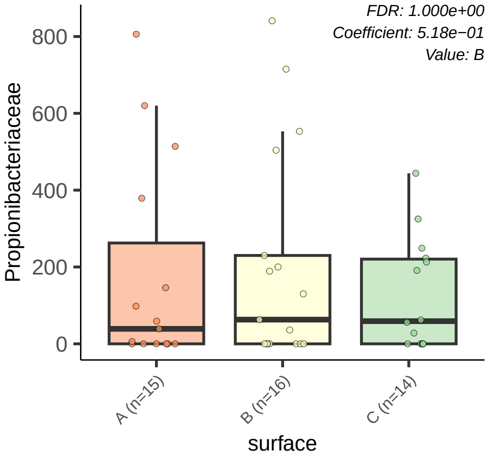

FDR: 1.000e+00
Coefficient: 5.18e−01
Value: B
Propionibacteriaceae
800
600
400
200
0
A (n=15)
B (n=16)
C (n=14)
surface

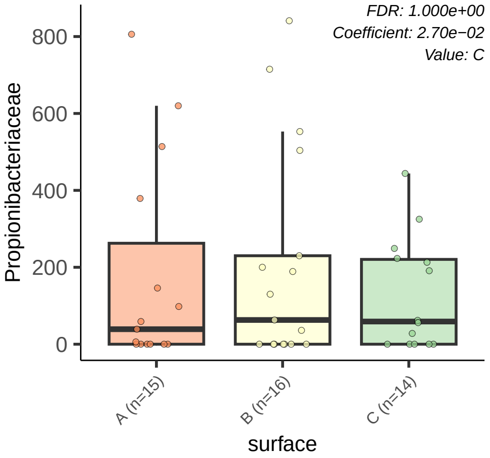
FDR: 1.000e+00
Coefficient: 2.70e−02
Value: C
Propionibacteriaceae
800
600
400
200
0
A (n=15)
B (n=16)
C (n=14)
surface

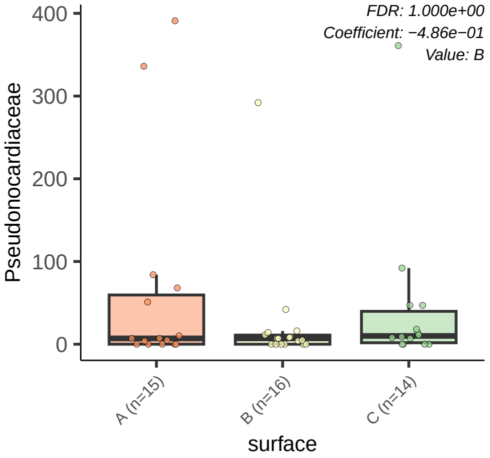
FDR: 1.000e+00
Coefficient: −4.86e−01
Value: B
Pseudonocardiaceae
400
300
200
100
0
A (n=15)
B (n=16)
C (n=14)
surface

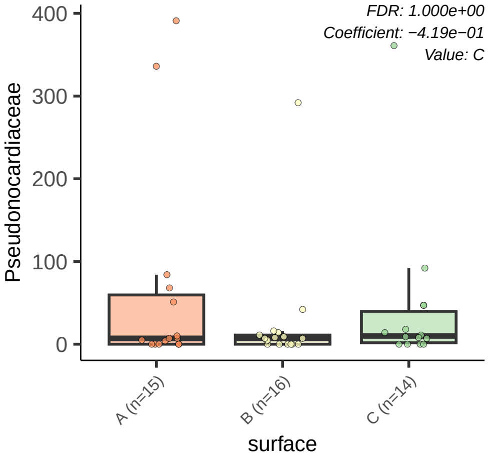
FDR: 1.000e+00
Coefficient: −4.19e−01
Value: C
Pseudonocardiaceae
400
300
200
100
0
A (n=15)
B (n=16)
C (n=14)
surface

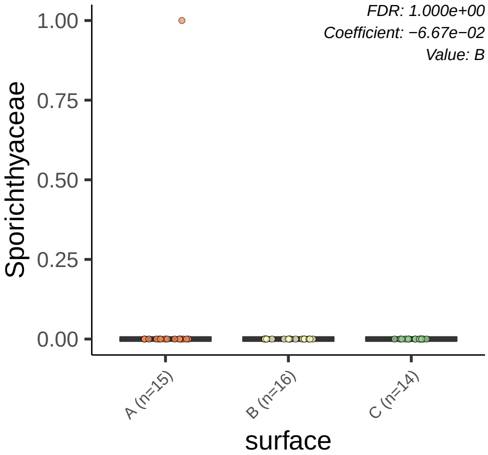

FDR: 1.000e+00
Coefficient: −6.67e−02
Value: B
Sporichthyaceae
1.00
0.75
0.50
0.25
0.00
A (n=15)
B (n=16)
C (n=14)
surface

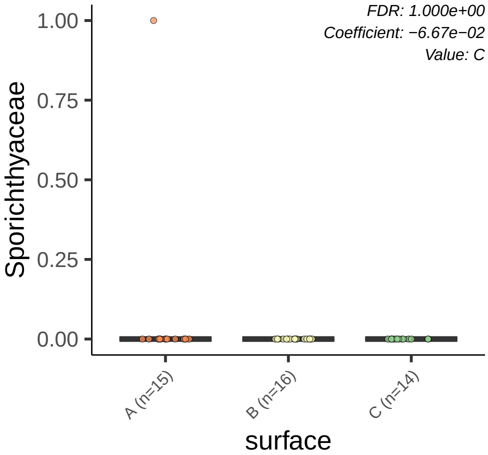
FDR: 1.000e+00
Coefficient: −6.67e−02
Value: C
Sporichthyaceae
1.00
0.75
0.50
0.25
0.00
A (n=15)
B (n=16)
C (n=14)
surface

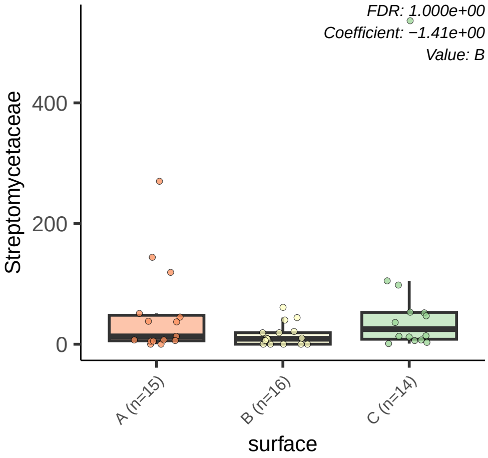
FDR: 1.000e+00
Coefficient: −1.41e+00
Value: B
Streptomycetaceae
400
200
0
A (n=15)
B (n=16)
C (n=14)
surface

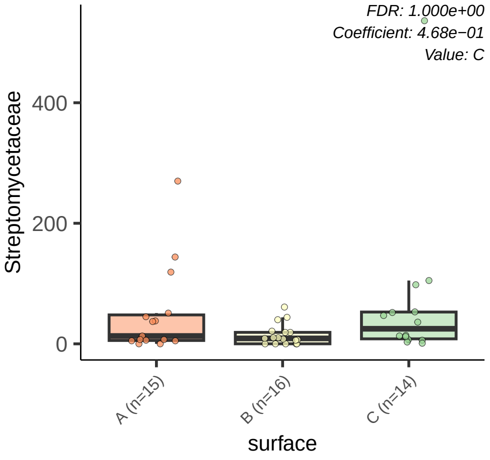
FDR: 1.000e+00
Coefficient: 4.68e−01
Value: C
Streptomycetaceae
400
200
0
A (n=15)
B (n=16)
C (n=14)
surface

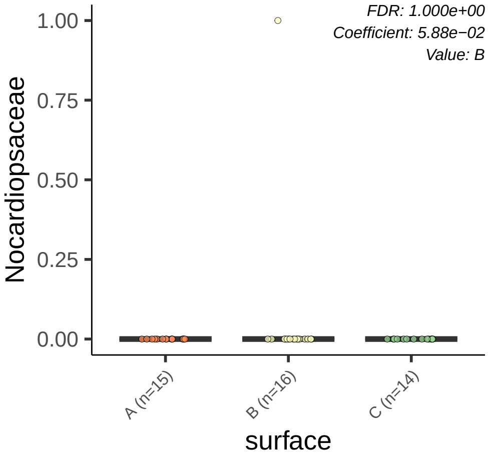

FDR: 1.000e+00
Coefficient: 5.88e−02
Value: B
Nocardiopsaceae
1.00
0.75
0.50
0.25
0.00
A (n=15)
B (n=16)
C (n=14)
surface

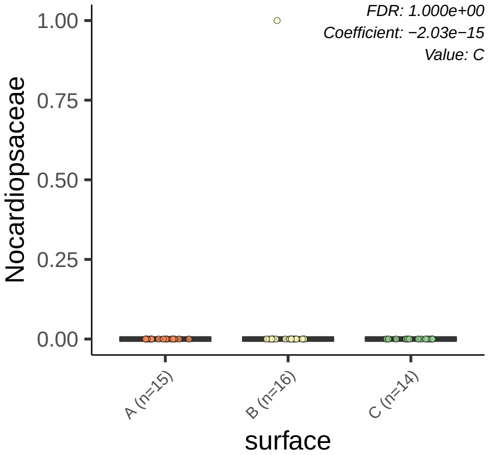
FDR: 1.000e+00
Coefficient: −2.03e−15
Value: C
Nocardiopsaceae
1.00
0.75
0.50
0.25
0.00
A (n=15)
B (n=16)
C (n=14)
surface

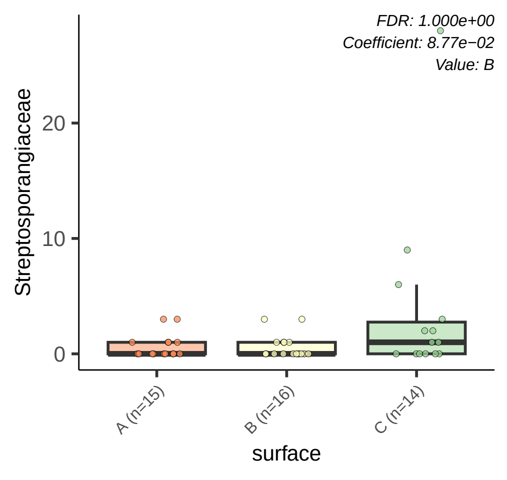

FDR: 1.000e+00
Coefficient: 8.77e−02
Value: B
Streptosporangiaceae
0
10
20
A (n=15)
B (n=16)
C (n=14)
surface

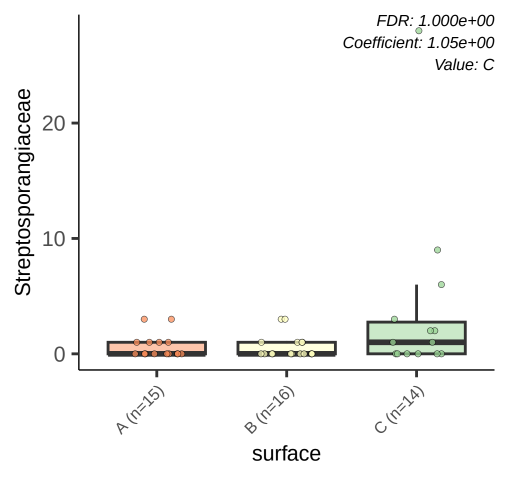
FDR: 1.000e+00
Coefficient: 1.05e+00
Value: C
Streptosporangiaceae
20
10
0
A (n=15)
B (n=16)
C (n=14)
surface

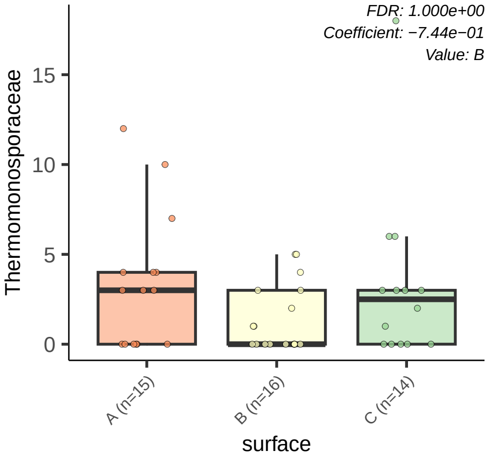

FDR: 1.000e+00
Coefficient: −7.44e−01
Value: B
Thermomonosporaceae
15
10
5
0
A (n=15)
B (n=16)
C (n=14)
surface

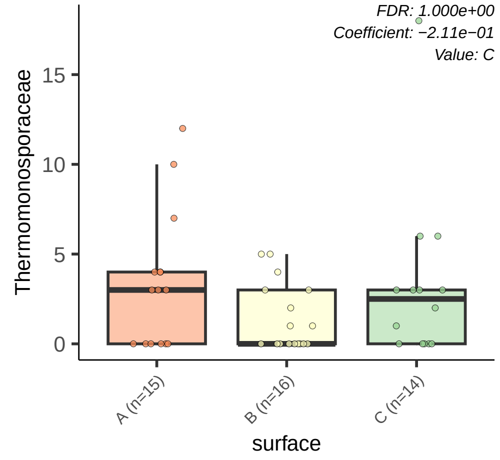

FDR: 1.000e+00
Coefficient: −2.11e−01
Value: C
Thermomonosporaceae
15
10
5
0
A (n=15)
B (n=16)
C (n=14)
surface

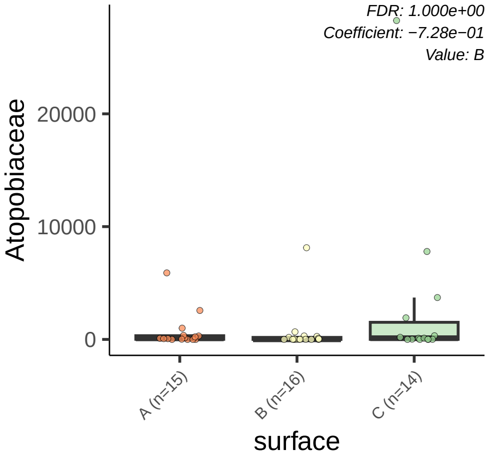
FDR: 1.000e+00
Coefficient: −7.28e−01
Value: B
Atopobiaceae
20000
10000
0
A (n=15)
B (n=16)
C (n=14)
surface

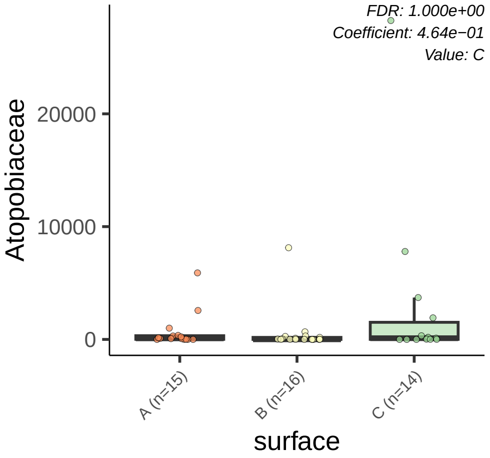
FDR: 1.000e+00
Coefficient: 4.64e−01
Value: C
Atopobiaceae
20000
10000
0
A (n=15)
B (n=16)
C (n=14)
surface

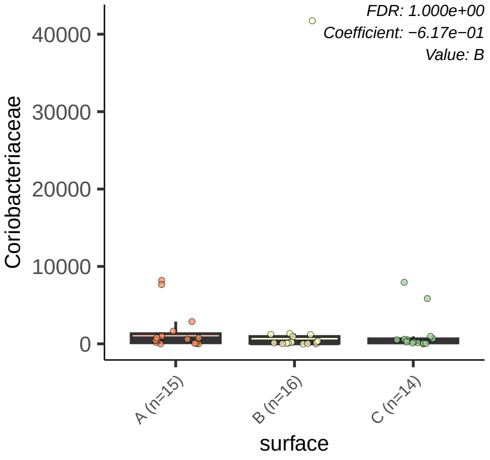

FDR: 1.000e+00
Coefficient: −6.17e−01
Value: B
Coriobacteriaceae
40000
30000
20000
10000
0
A (n=15)
B (n=16)
C (n=14)
surface

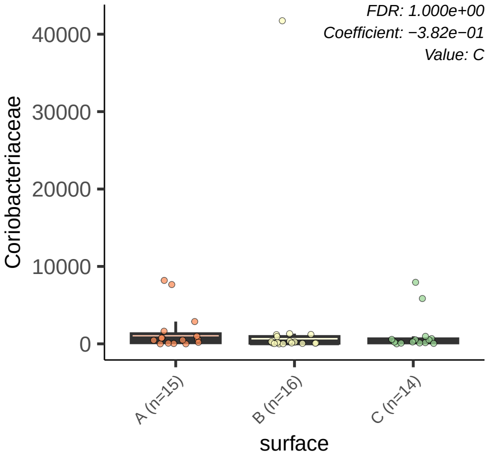
FDR: 1.000e+00
Coefficient: −3.82e−01
Value: C
Coriobacteriaceae
40000
30000
20000
10000
0
A (n=15)
B (n=16)
C (n=14)
surface

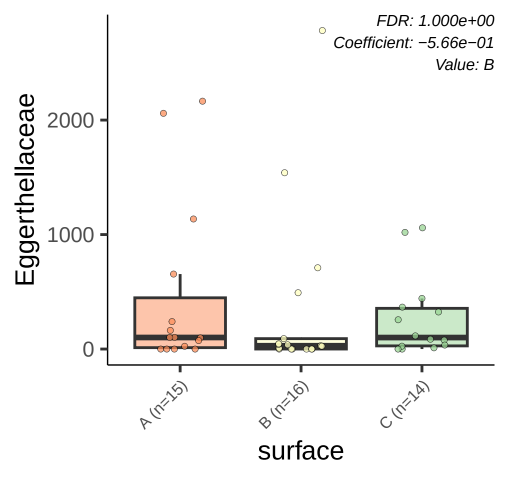

FDR: 1.000e+00
Coefficient: −5.66e−01
Value: B
Eggerthellaceae
2000
1000
0
A (n=15)
B (n=16)
C (n=14)
surface

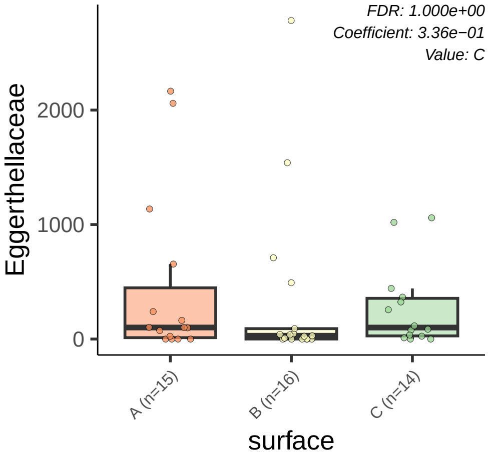
FDR: 1.000e+00
Coefficient: 3.36e−01
Value: C
Eggerthellaceae
2000
1000
0
A (n=15)
B (n=16)
C (n=14)
surface

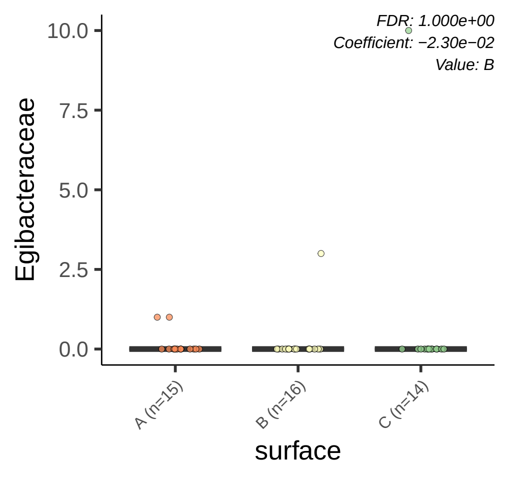

FDR: 1.000e+00
Coefficient: −2.30e−02
Value: B
Egibacteraceae
10.0
7.5
5.0
2.5
0.0
A (n=15)
B (n=16)
C (n=14)
surface

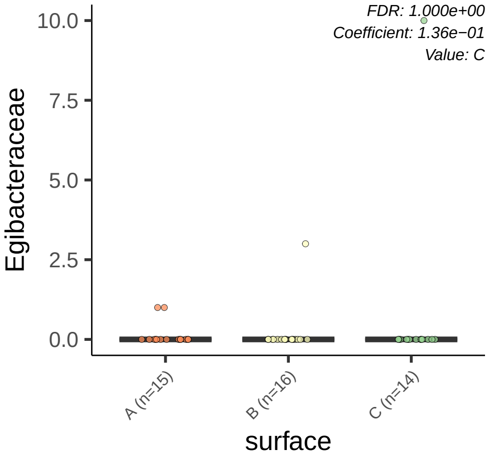

FDR: 1.000e+00
Coefficient: 1.36e−01
Value: C
Egibacteraceae
10.0
7.5
5.0
2.5
0.0
A (n=15)
B (n=16)
C (n=14)
surface

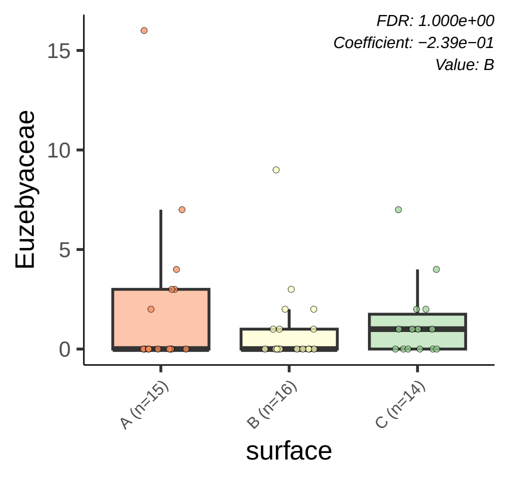
FDR: 1.000e+00
Coefficient: −2.39e−01
Value: B
Euzebyaceae
15
10
5
0
A (n=15)
B (n=16)
C (n=14)
surface

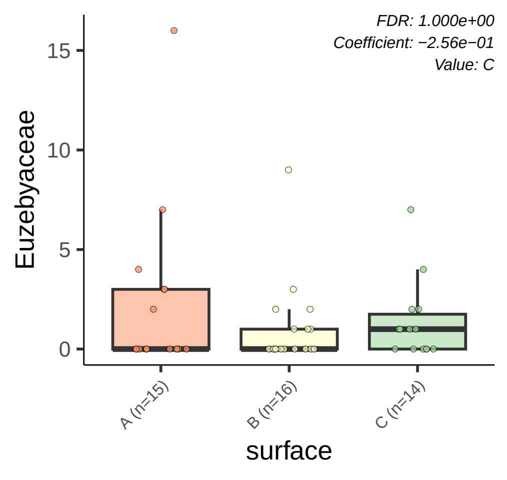
FDR: 1.000e+00
Coefficient: −2.56e−01
Value: C
Euzebyaceae
15
10
5
0
A (n=15)
B (n=16)
C (n=14)
surface

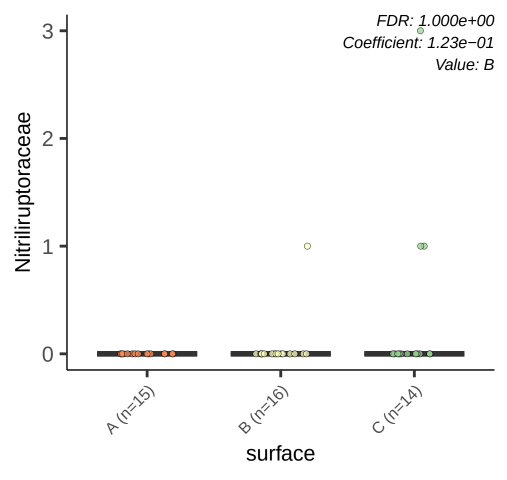

FDR: 1.000e+00
Coefficient: 1.23e−01
Value: B
Nitriliruptoraceae
3
2
1
0
A (n=15)
B (n=16)
C (n=14)
surface

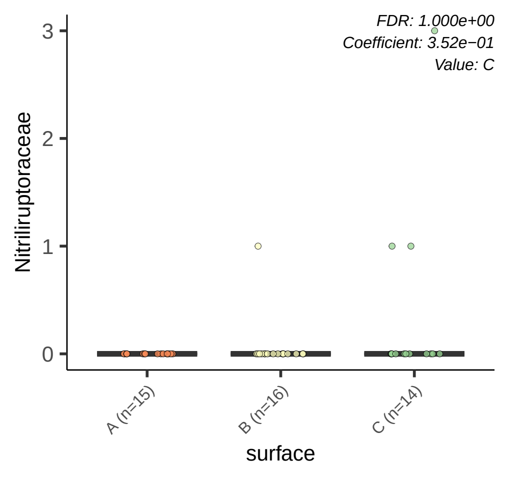
FDR: 1.000e+00
Coefficient: 3.52e−01
Value: C
Nitriliruptoraceae
3
2
1
0
A (n=15)
B (n=16)
C (n=14)
surface

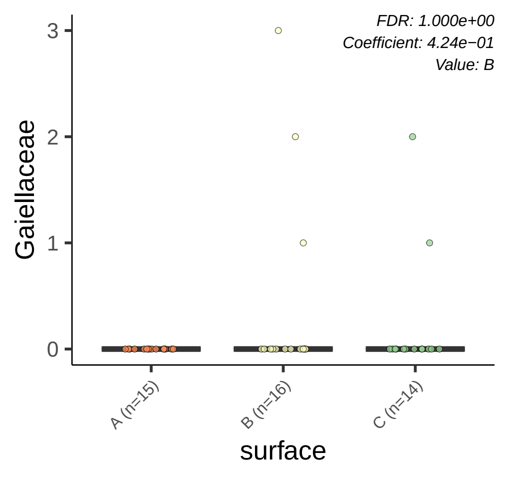
FDR: 1.000e+00
Coefficient: 4.24e−01
Value: B
Gaiellaceae
3
2
1
0
A (n=15)
B (n=16)
C (n=14)
surface

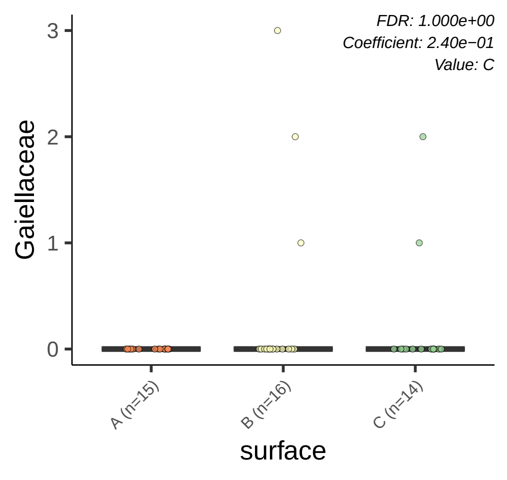

FDR: 1.000e+00
Coefficient: 2.40e−01
Value: C
Gaiellaceae
3
2
1
0
A (n=15)
B (n=16)
C (n=14)
surface

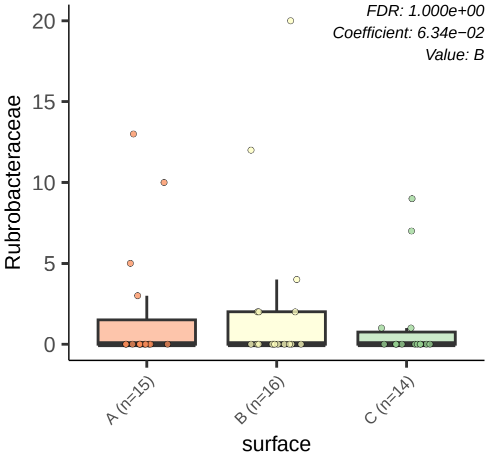

FDR: 1.000e+00
Coefficient: 6.34e−02
Value: B
Rubrobacteraceae
20
15
10
5
0
A (n=15)
B (n=16)
C (n=14)
surface

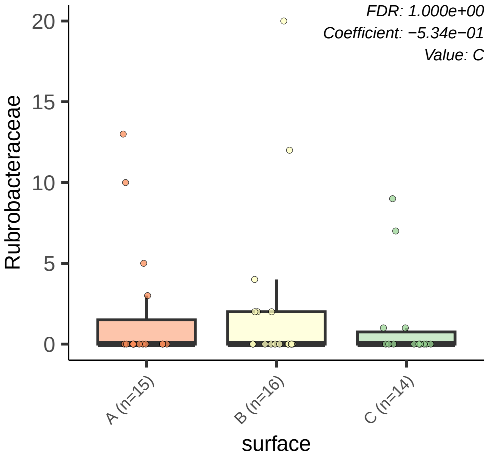
FDR: 1.000e+00
Coefficient: −5.34e−01
Value: C
Rubrobacteraceae
20
15
10
5
0
A (n=15)
B (n=16)
C (n=14)
surface

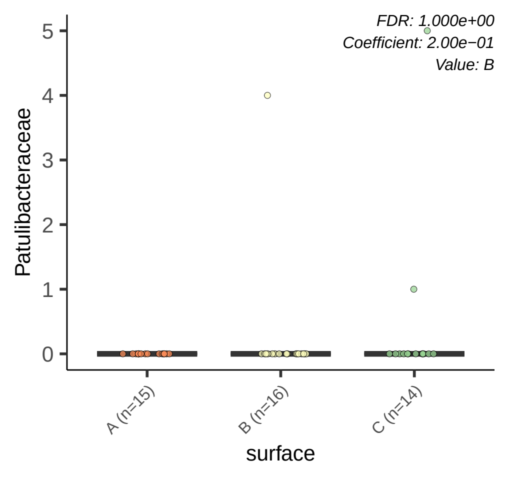
FDR: 1.000e+00
Coefficient: 2.00e−01
Value: B
Patulibacteraceae
0
1
2
3
4
5
A (n=15)
B (n=16)
C (n=14)
surface

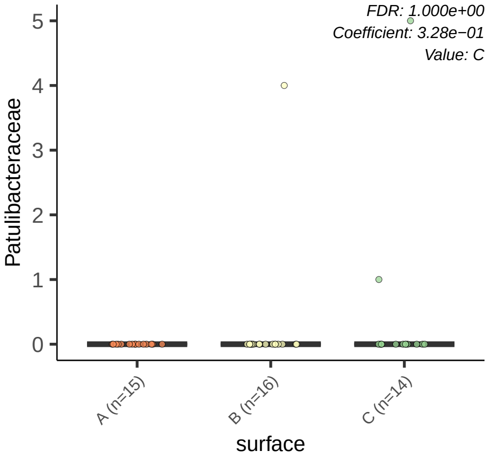
FDR: 1.000e+00
Coefficient: 3.28e−01
Value: C
Patulibacteraceae
0
1
2
3
4
5
A (n=15)
B (n=16)
C (n=14)
surface

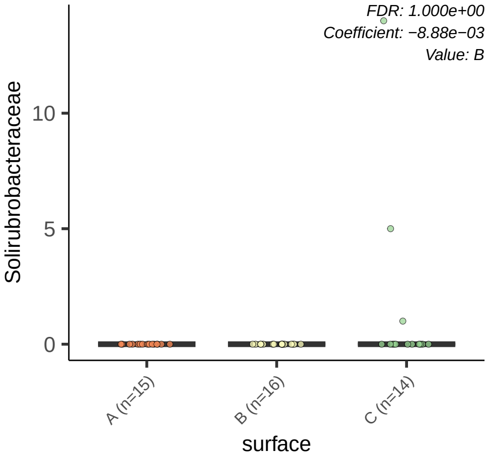

FDR: 1.000e+00
Coefficient: −8.88e−03
Value: B
Solirubrobacteraceae
10
5
0
A (n=15)
B (n=16)
C (n=14)
surface

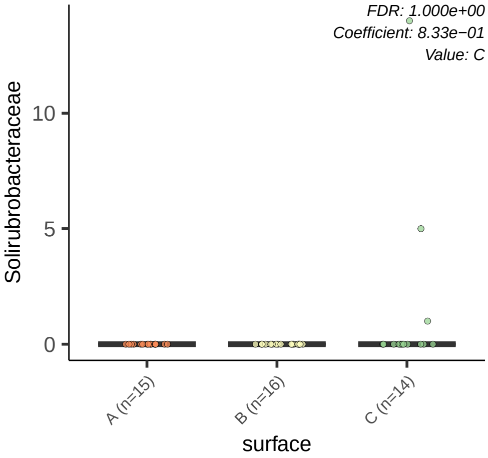
FDR: 1.000e+00
Coefficient: 8.33e−01
Value: C
Solirubrobacteraceae
10
5
0
A (n=15)
B (n=16)
C (n=14)
surface

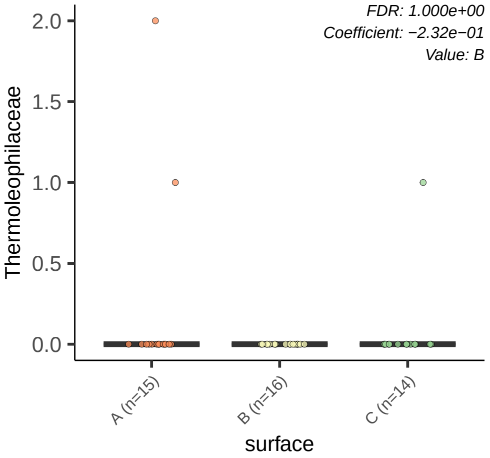
FDR: 1.000e+00
Coefficient: −2.32e−01
Value: B
Thermoleophilaceae
2.0
1.5
1.0
0.5
0.0
A (n=15)
B (n=16)
C (n=14)
surface

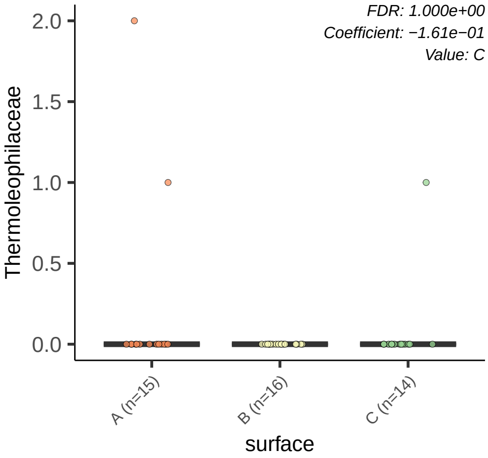

FDR: 1.000e+00
Coefficient: −1.61e−01
Value: C
Thermoleophilaceae
2.0
1.5
1.0
0.5
0.0
A (n=15)
B (n=16)
C (n=14)
surface

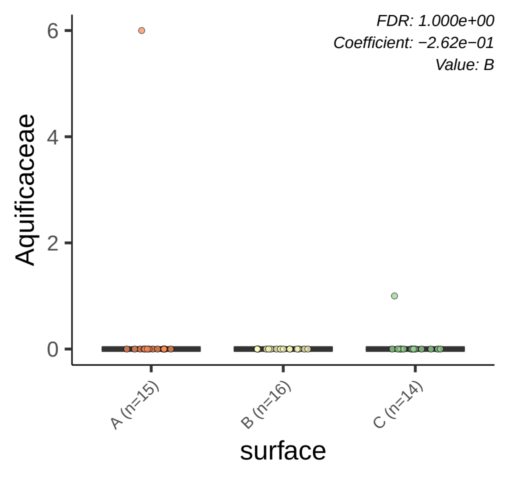

FDR: 1.000e+00
Coefficient: −2.62e−01
Value: B
Aquificaceae
6
4
2
0
A (n=15)
B (n=16)
C (n=14)
surface

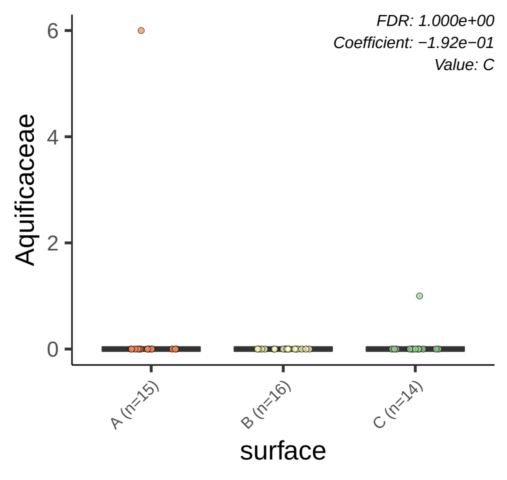
FDR: 1.000e+00
Coefficient: −1.92e−01
Value: C
Aquificaceae
6
4
2
0
A (n=15)
B (n=16)
C (n=14)
surface

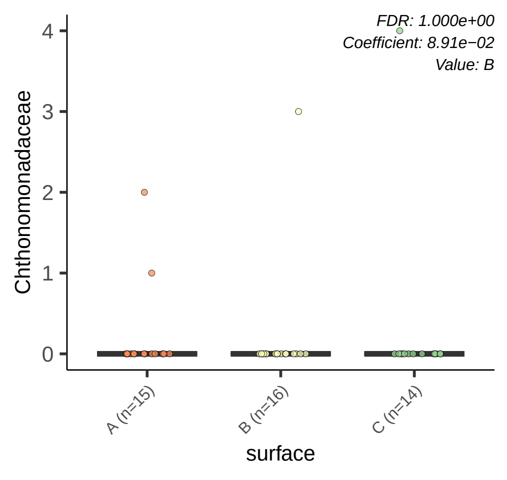

FDR: 1.000e+00
Coefficient: 8.91e−02
Value: B
Chthonomonadaceae
4
3
2
1
0
A (n=15)
B (n=16)
C (n=14)
surface

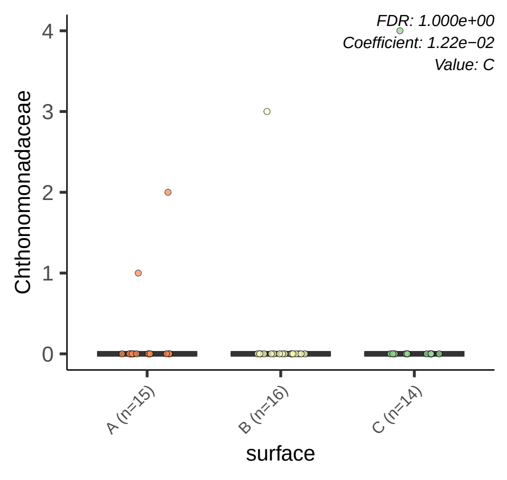

FDR: 1.000e+00
Coefficient: 1.22e−02
Value: C
Chthonomonadaceae
0
1
2
3
4
A (n=15)
B (n=16)
C (n=14)
surface

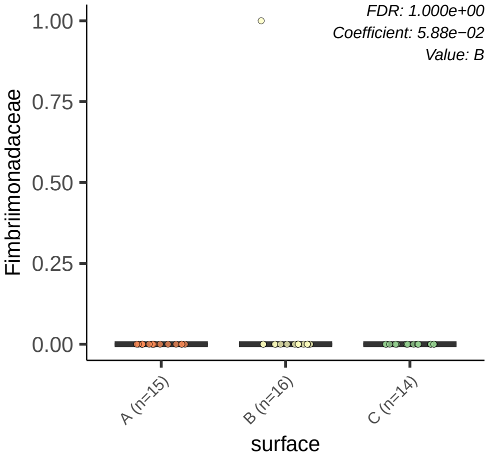

FDR: 1.000e+00
Coefficient: 5.88e−02
Value: B
Fimbriimonadaceae
1.00
0.75
0.50
0.25
0.00
A (n=15)
B (n=16)
C (n=14)
surface

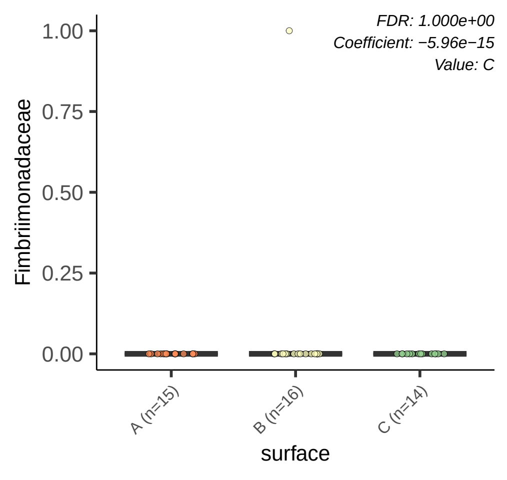
FDR: 1.000e+00
Coefficient: −5.96e−15
Value: C
Fimbriimonadaceae
1.00
0.75
0.50
0.25
0.00
A (n=15)
B (n=16)
C (n=14)
surface

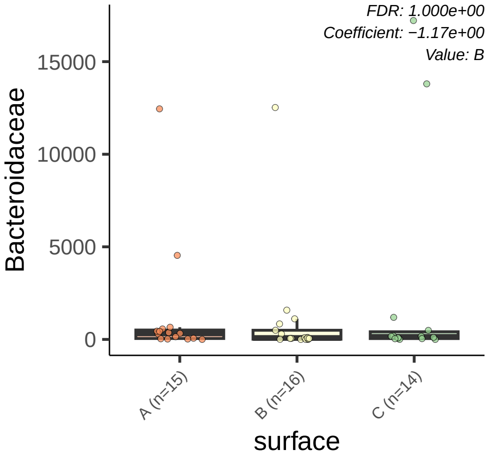
FDR: 1.000e+00
Coefficient: −1.17e+00
Value: B
Bacteroidaceae
15000
10000
5000
0
A (n=15)
B (n=16)
C (n=14)
surface

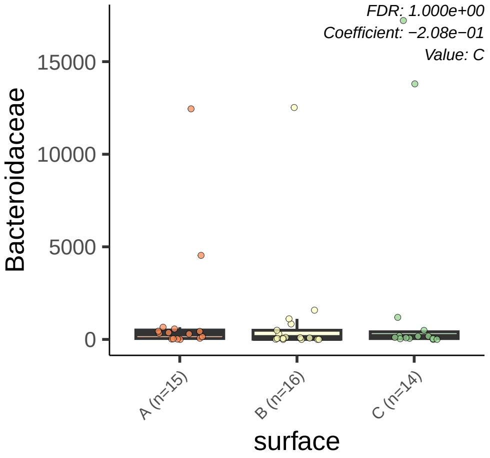
FDR: 1.000e+00
Coefficient: −2.08e−01
Value: C
Bacteroidaceae
15000
10000
5000
0
A (n=15)
B (n=16)
C (n=14)
surface

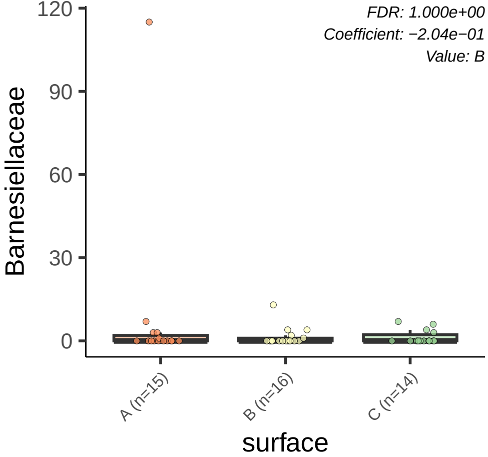
FDR: 1.000e+00
Coefficient: −2.04e−01
Value: B
Barnesiellaceae
120
90
60
30
0
A (n=15)
B (n=16)
C (n=14)
surface

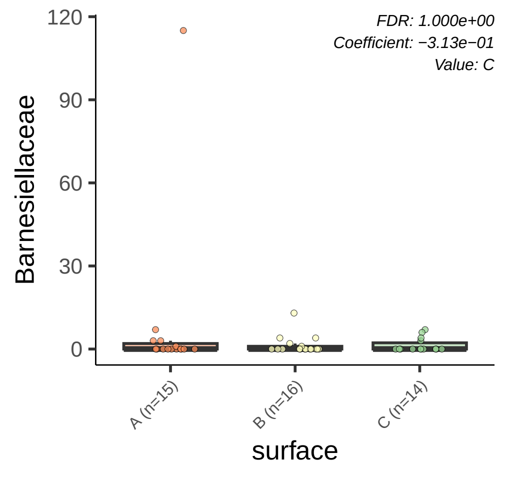
FDR: 1.000e+00
Coefficient: −3.13e−01
Value: C
Barnesiellaceae
120
90
60
30
0
A (n=15)
B (n=16)
C (n=14)
surface

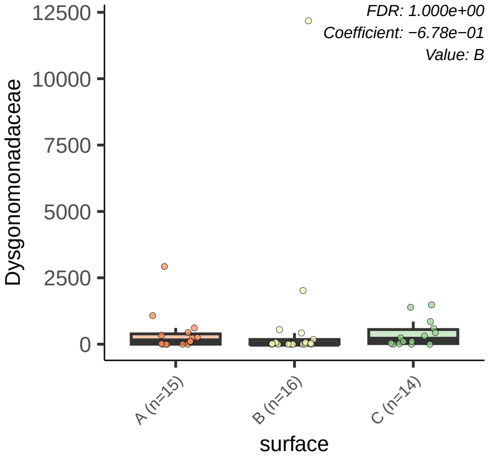
FDR: 1.000e+00
Coefficient: −6.78e−01
Value: B
Dysgonomonadaceae
12500
10000
7500
5000
2500
0
A (n=15)
B (n=16)
C (n=14)
surface

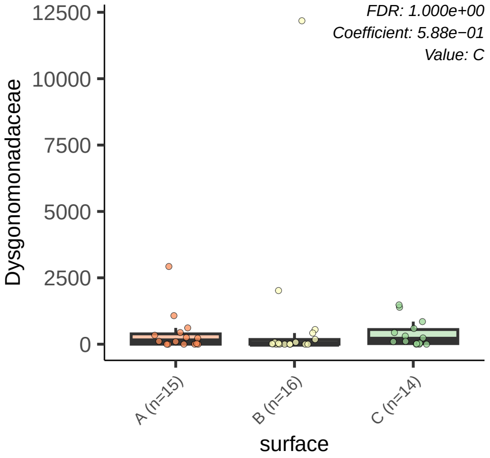
FDR: 1.000e+00
Coefficient: 5.88e−01
Value: C
Dysgonomonadaceae
12500
10000
7500
5000
2500
0
A (n=15)
B (n=16)
C (n=14)
surface

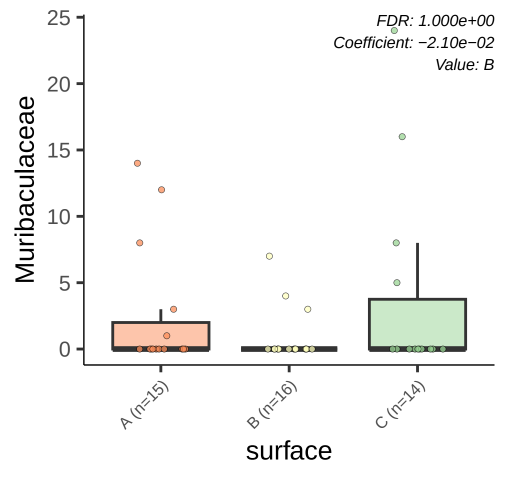

FDR: 1.000e+00
Coefficient: −2.10e−02
Value: B
Muribaculaceae
25
20
15
10
5
0
A (n=15)
B (n=16)
C (n=14)
surface

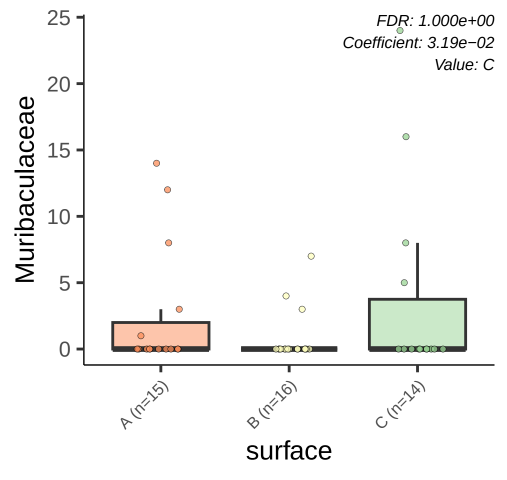

FDR: 1.000e+00
Coefficient: 3.19e−02
Value: C
Muribaculaceae
25
20
15
10
5
0
A (n=15)
B (n=16)
C (n=14)
surface

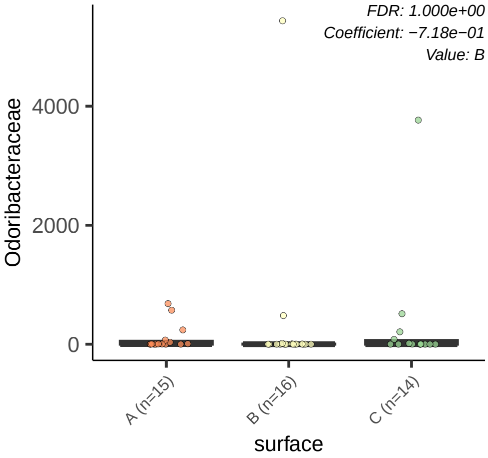
FDR: 1.000e+00
Coefficient: −7.18e−01
Value: B
Odoribacteraceae
4000
2000
0
A (n=15)
B (n=16)
C (n=14)
surface

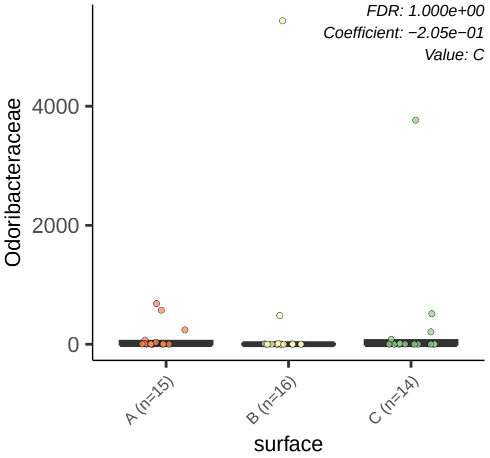

FDR: 1.000e+00
Coefficient: −2.05e−01
Value: C
Odoribacteraceae
4000
2000
0
A (n=15)
B (n=16)
C (n=14)
surface

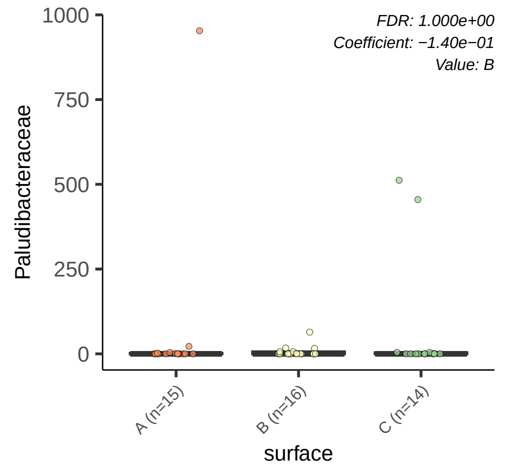

FDR: 1.000e+00
Coefficient: −1.40e−01
Value: B
Paludibacteraceae
1000
750
500
250
0
A (n=15)
B (n=16)
C (n=14)
surface

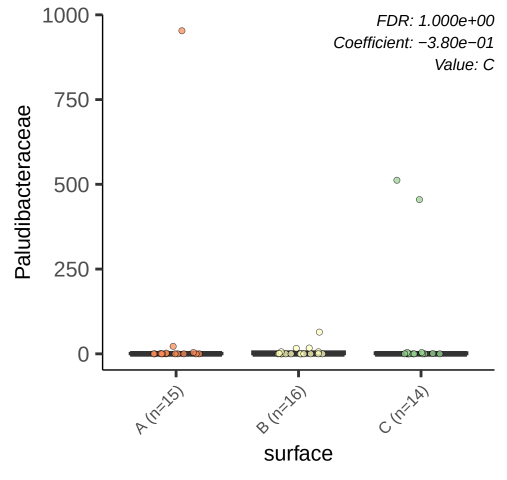

FDR: 1.000e+00
Coefficient: −3.80e−01
Value: C
Paludibacteraceae
1000
750
500
250
0
A (n=15)
B (n=16)
C (n=14)
surface

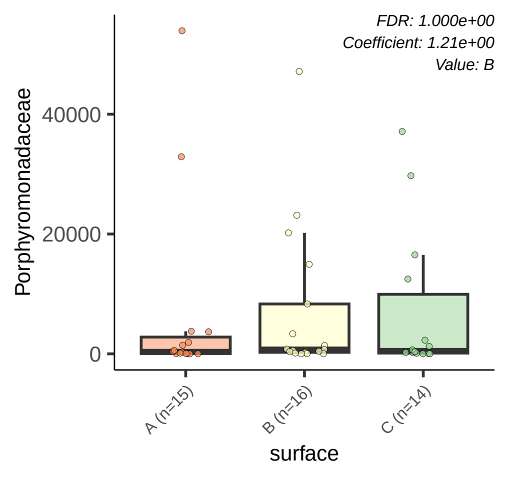
FDR: 1.000e+00
Coefficient: 1.21e+00
Value: B
Porphyromonadaceae
40000
20000
0
A (n=15)
B (n=16)
C (n=14)
surface

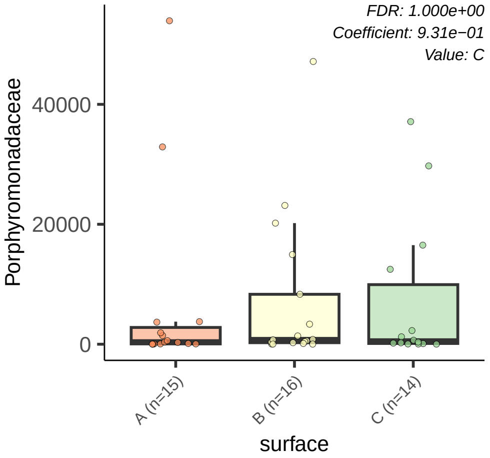
FDR: 1.000e+00
Coefficient: 9.31e−01
Value: C
Porphyromonadaceae
40000
20000
0
A (n=15)
B (n=16)
C (n=14)
surface

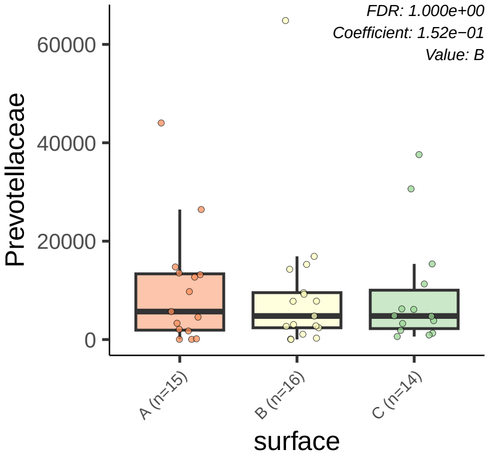
FDR: 1.000e+00
Coefficient: 1.52e−01
Value: B
Prevotellaceae
60000
40000
20000
0
A (n=15)
B (n=16)
C (n=14)
surface

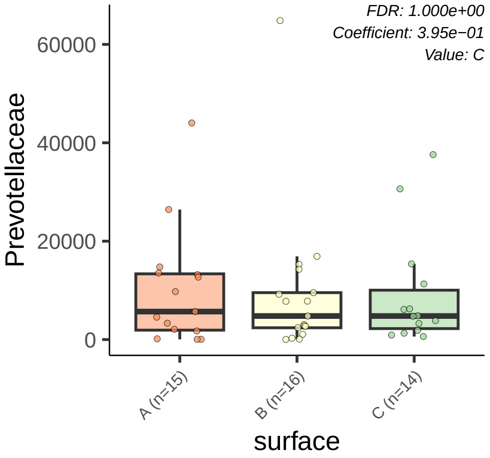

FDR: 1.000e+00
Coefficient: 3.95e−01
Value: C
Prevotellaceae
60000
40000
20000
0
A (n=15)
B (n=16)
C (n=14)
surface

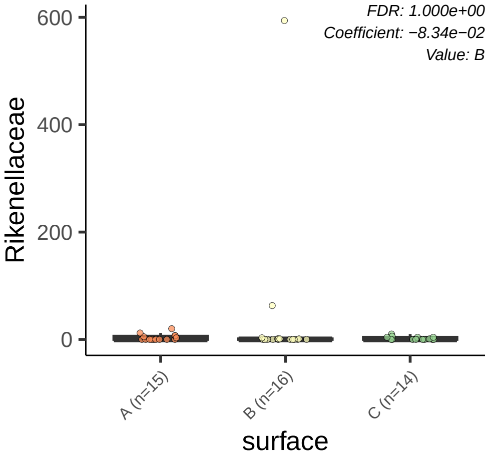

FDR: 1.000e+00
Coefficient: −8.34e−02
Value: B
Rikenellaceae
600
400
200
0
A (n=15)
B (n=16)
C (n=14)
surface

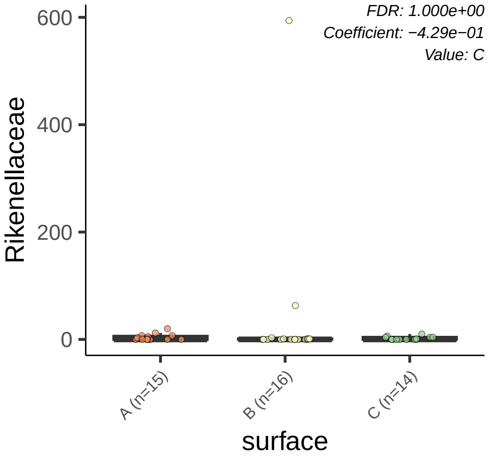

FDR: 1.000e+00
Coefficient: −4.29e−01
Value: C
600
400
200
0
Rikenellaceae
A (n=15)
B (n=16)
C (n=14)
surface

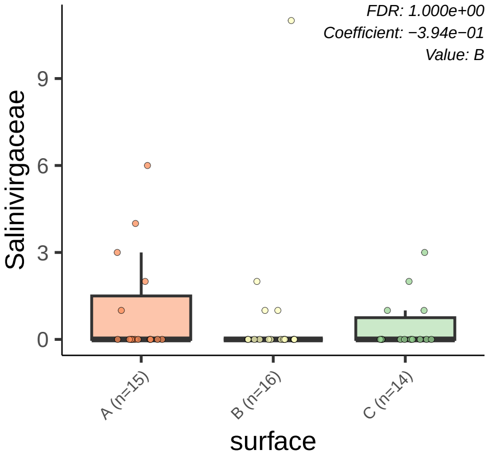
FDR: 1.000e+00
Coefficient: −3.94e−01
Value: B
Salinivirgaceae
0
3
6
9
A (n=15)
B (n=16)
C (n=14)
surface

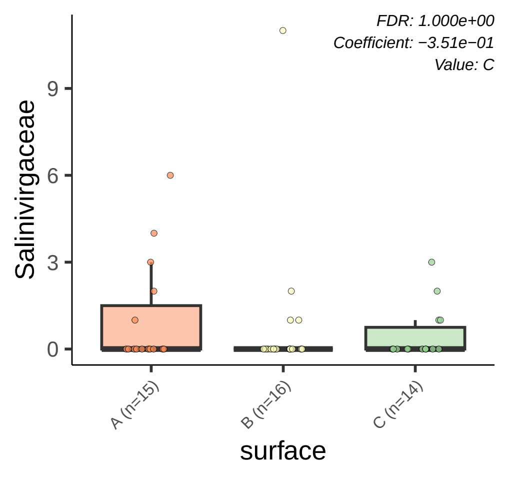
FDR: 1.000e+00
Coefficient: −3.51e−01
Value: C
Salinivirgaceae
0
3
6
9
A (n=15)
B (n=16)
C (n=14)
surface

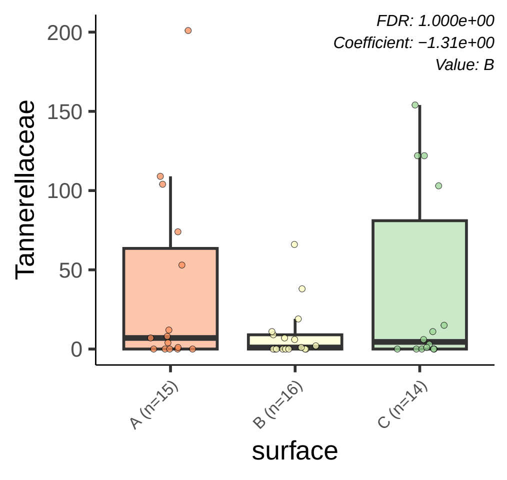
FDR: 1.000e+00
Coefficient: −1.31e+00
Value: B
Tannerellaceae
200
150
100
50
0
A (n=15)
B (n=16)
C (n=14)
surface

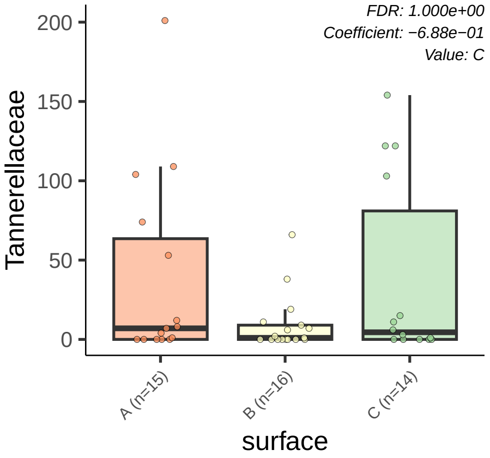
FDR: 1.000e+00
Coefficient: −6.88e−01
Value: C
Tannerellaceae
200
150
100
50
0
A (n=15)
B (n=16)
C (n=14)
surface

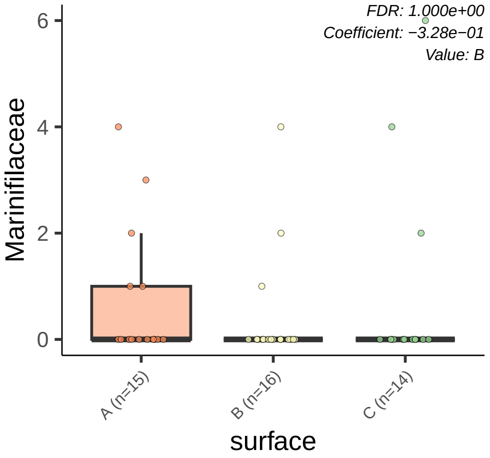

FDR: 1.000e+00
Coefficient: −3.28e−01
Value: B
Marinifilaceae
0
2
4
6
A (n=15)
B (n=16)
C (n=14)
surface

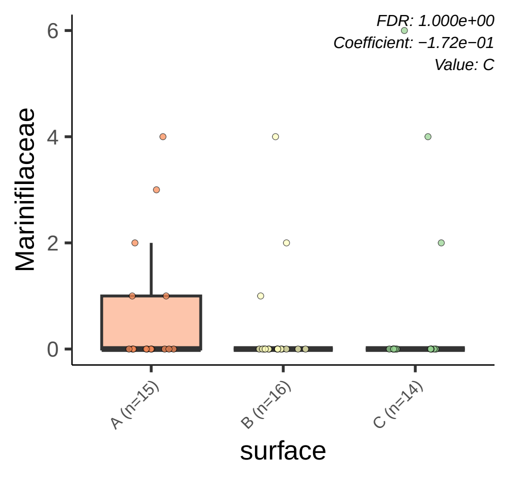
FDR: 1.000e+00
Coefficient: −1.72e−01
Value: C
Marinifilaceae
0
2
4
6
A (n=15)
B (n=16)
C (n=14)
surface

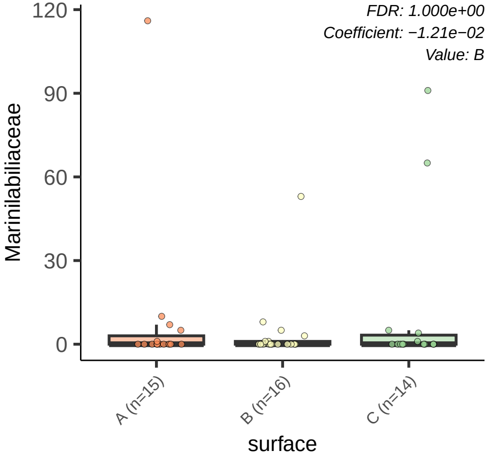

FDR: 1.000e+00
Coefficient: −1.21e−02
Value: B
120
90
60
30
0
Marinilabiliaceae
A (n=15)
B (n=16)
C (n=14)
surface

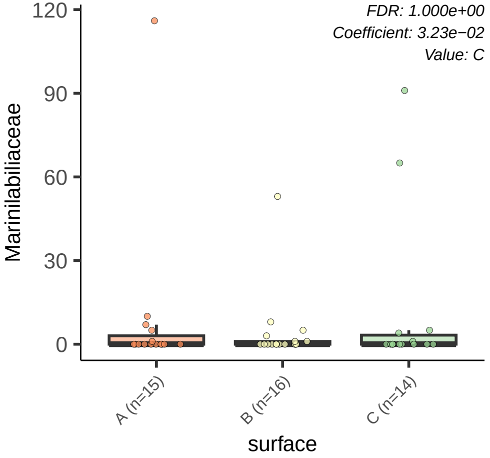

FDR: 1.000e+00
Coefficient: 3.23e−02
Value: C
Marinilabiliaceae
120
90
60
30
0
A (n=15)
B (n=16)
C (n=14)
surface

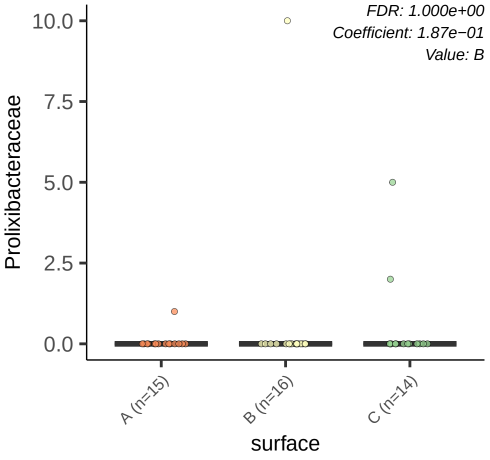
FDR: 1.000e+00
Coefficient: 1.87e−01
Value: B
Prolixibacteraceae
10.0
7.5
5.0
2.5
0.0
A (n=15)
B (n=16)
C (n=14)
surface

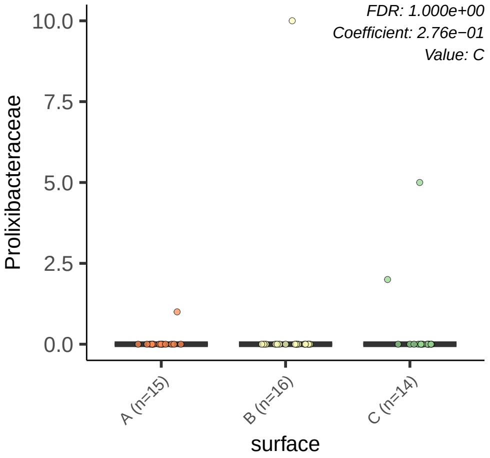

FDR: 1.000e+00
Coefficient: 2.76e−01
Value: C
Prolixibacteraceae
10.0
7.5
5.0
2.5
0.0
A (n=15)
B (n=16)
C (n=14)
surface

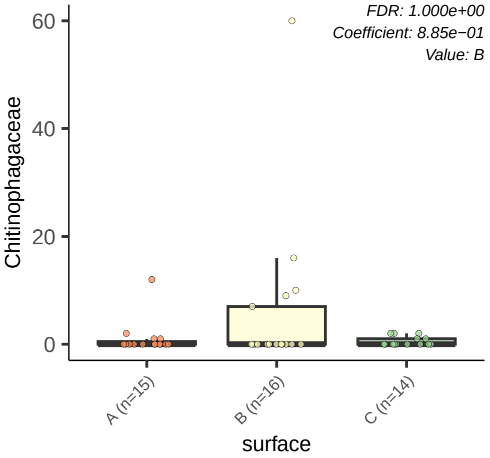

FDR: 1.000e+00
Coefficient: 8.85e−01
Value: B
Chitinophagaceae
60
40
20
0
A (n=15)
B (n=16)
C (n=14)
surface

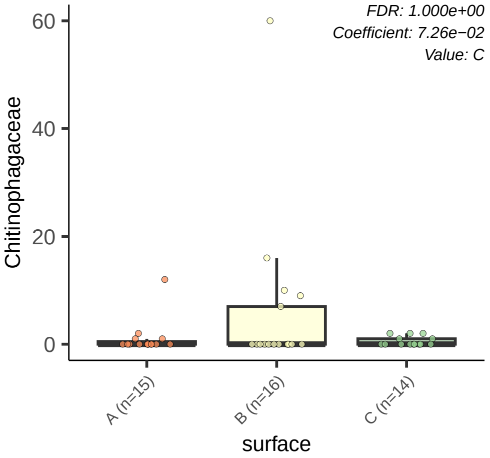
FDR: 1.000e+00
Coefficient: 7.26e−02
Value: C
Chitinophagaceae
0
20
40
60
A (n=15)
B (n=16)
C (n=14)
surface

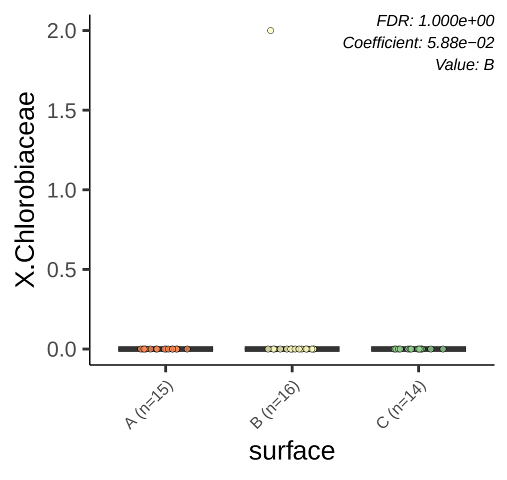
FDR: 1.000e+00
Coefficient: 5.88e−02
Value: B
X.Chlorobiaceae
2.0
1.5
1.0
0.5
0.0
A (n=15)
B (n=16)
C (n=14)
surface

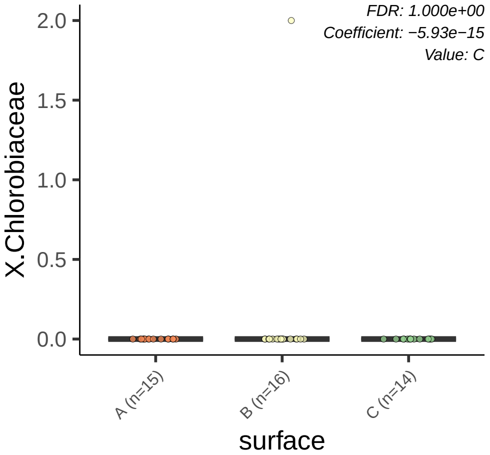

FDR: 1.000e+00
Coefficient: −5.93e−15
Value: C
X.Chlorobiaceae
2.0
1.5
1.0
0.5
0.0
A (n=15)
B (n=16)
C (n=14)
surface

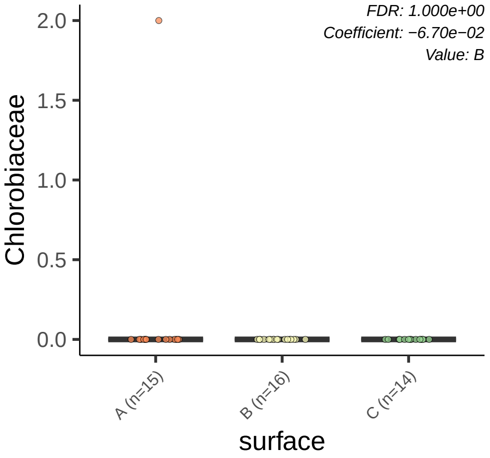

FDR: 1.000e+00
Coefficient: −6.70e−02
Value: B
Chlorobiaceae
2.0
1.5
1.0
0.5
0.0
A (n=15)
B (n=16)
C (n=14)
surface

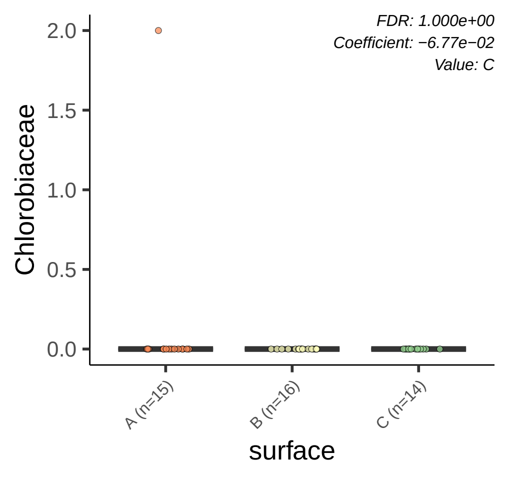
FDR: 1.000e+00
Coefficient: −6.77e−02
Value: C
Chlorobiaceae
2.0
1.5
1.0
0.5
0.0
A (n=15)
B (n=16)
C (n=14)
surface

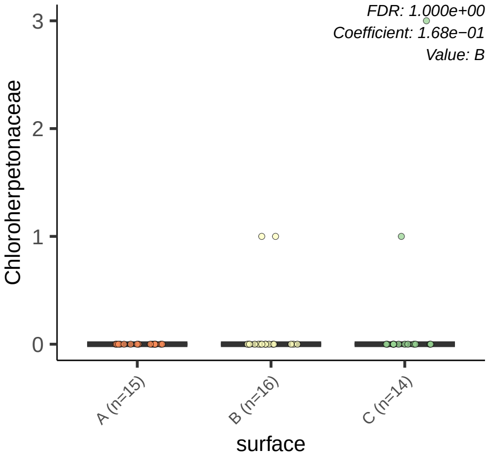

FDR: 1.000e+00
Coefficient: 1.68e−01
Value: B
Chloroherpetonaceae
0
1
2
3
A (n=15)
B (n=16)
C (n=14)
surface

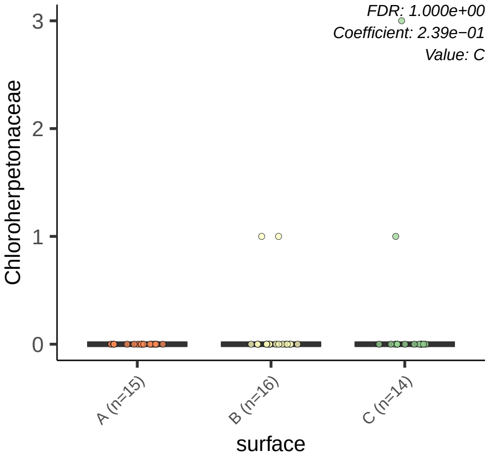
FDR: 1.000e+00
Coefficient: 2.39e−01
Value: C
Chloroherpetonaceae
0
1
2
3
A (n=15)
B (n=16)
C (n=14)
surface

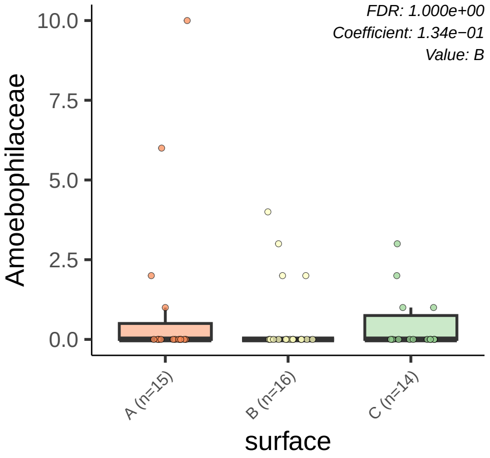

FDR: 1.000e+00
Coefficient: 1.34e−01
Value: B
Amoebophilaceae
10.0
7.5
5.0
2.5
0.0
A (n=15)
B (n=16)
C (n=14)
surface

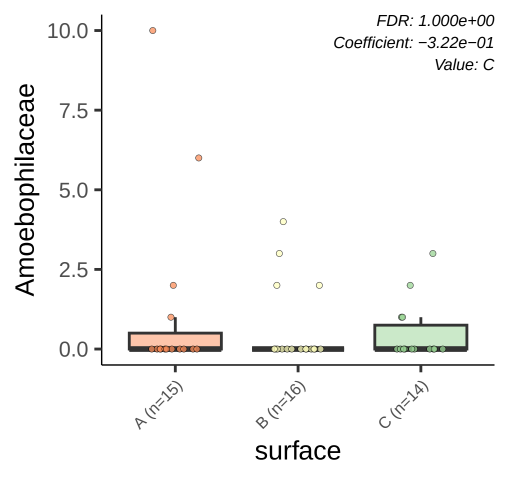
FDR: 1.000e+00
Coefficient: −3.22e−01
Value: C
Amoebophilaceae
10.0
7.5
5.0
2.5
0.0
A (n=15)
B (n=16)
C (n=14)
surface

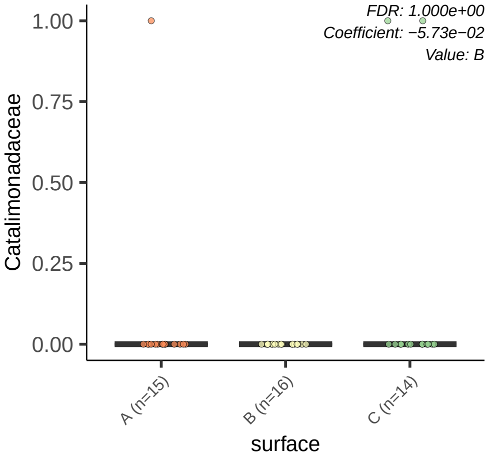

FDR: 1.000e+00
Coefficient: −5.73e−02
Value: B
Catalimonadaceae
1.00
0.75
0.50
0.25
0.00
A (n=15)
B (n=16)
C (n=14)
surface

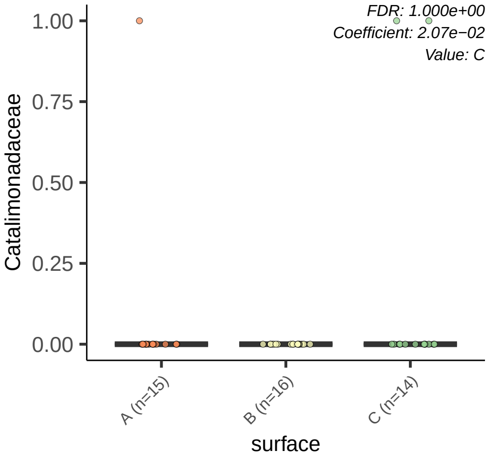
FDR: 1.000e+00
Coefficient: 2.07e−02
Value: C
Catalimonadaceae
1.00
0.75
0.50
0.25
0.00
A (n=15)
B (n=16)
C (n=14)
surface

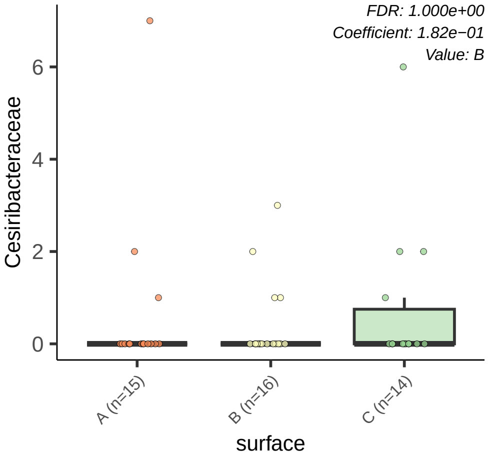
FDR: 1.000e+00
Coefficient: 1.82e−01
Value: B
Cesiribacteraceae
0
2
4
6
A (n=15)
B (n=16)
C (n=14)
surface

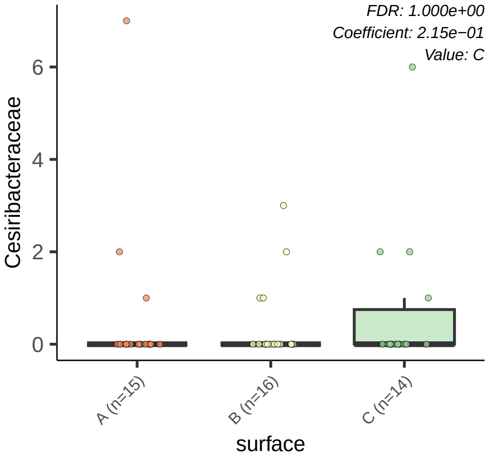

FDR: 1.000e+00
Coefficient: 2.15e−01
Value: C
Cesiribacteraceae
0
2
4
6
A (n=15)
B (n=16)
C (n=14)
surface

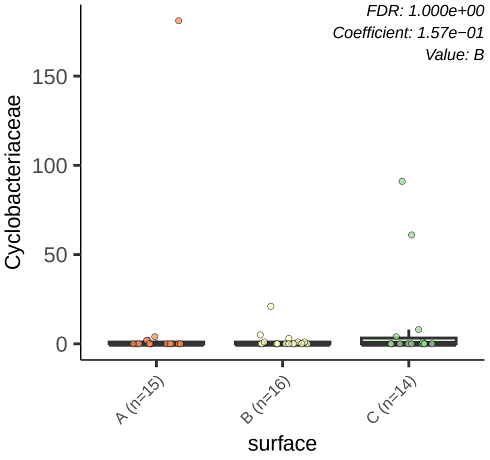
FDR: 1.000e+00
Coefficient: 1.57e−01
Value: B
Cyclobacteriaceae
150
100
50
0
A (n=15)
B (n=16)
C (n=14)
surface

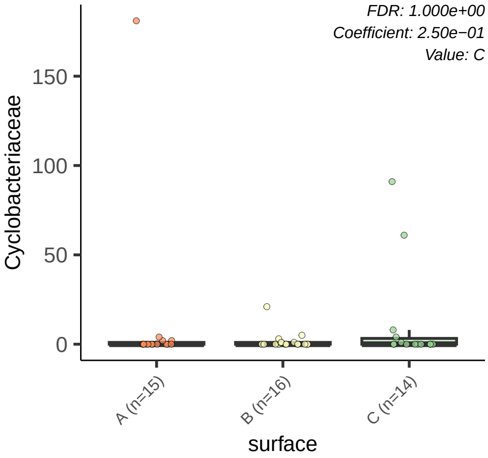

FDR: 1.000e+00
Coefficient: 2.50e−01
Value: C
Cyclobacteriaceae
150
100
50
0
A (n=15)
B (n=16)
C (n=14)
surface

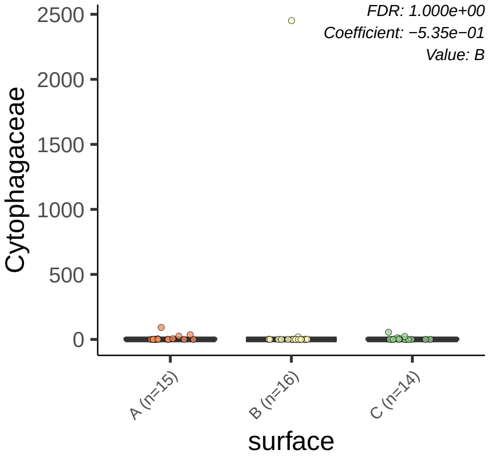

FDR: 1.000e+00
Coefficient: −5.35e−01
Value: B
Cytophagaceae
2500
2000
1500
1000
500
0
A (n=15)
B (n=16)
C (n=14)
surface

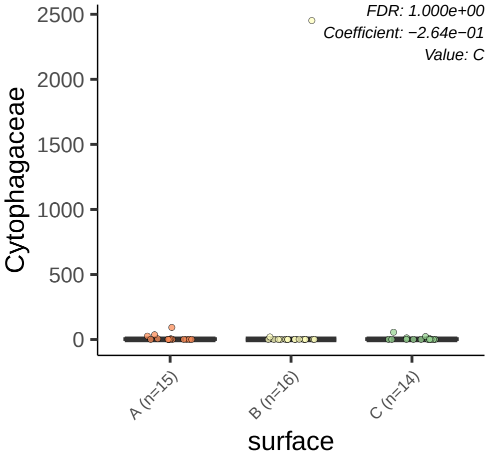

FDR: 1.000e+00
Coefficient: −2.64e−01
Value: C
Cytophagaceae
2500
2000
1500
1000
500
0
A (n=15)
B (n=16)
C (n=14)
surface

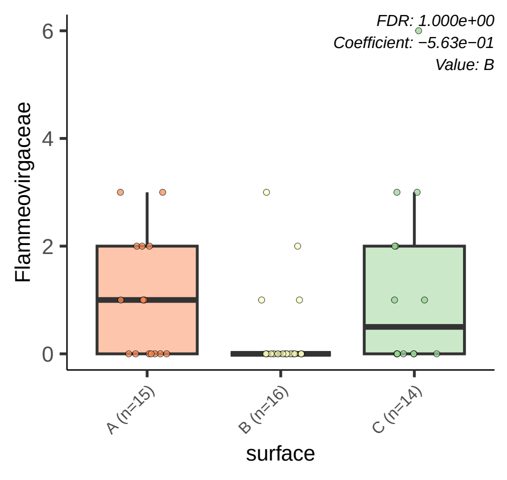

FDR: 1.000e+00
Coefficient: −5.63e−01
Value: B
Flammeovirgaceae
0
2
4
6
A (n=15)
B (n=16)
C (n=14)
surface

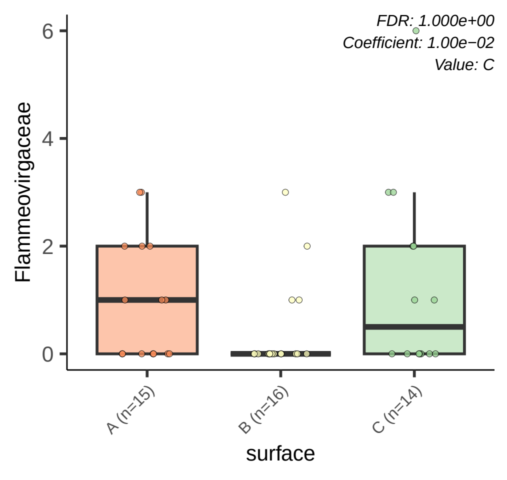

FDR: 1.000e+00
Coefficient: 1.00e−02
Value: C
Flammeovirgaceae
0
2
4
6
A (n=15)
B (n=16)
C (n=14)
surface

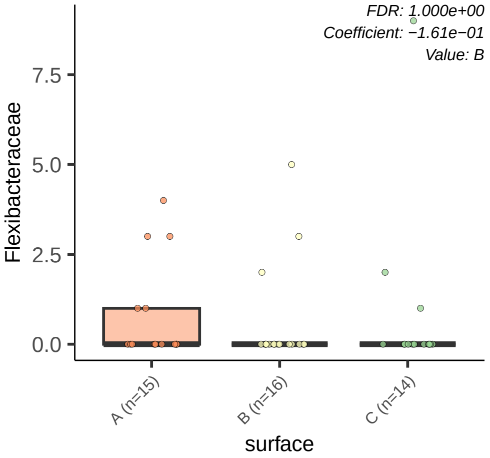

FDR: 1.000e+00
Coefficient: −1.61e−01
Value: B
Flexibacteraceae
7.5
5.0
2.5
0.0
A (n=15)
B (n=16)
C (n=14)
surface

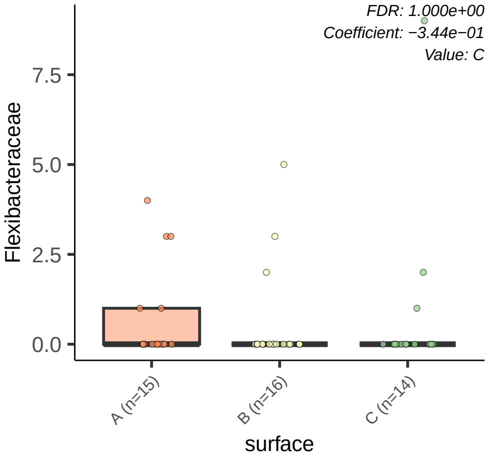
FDR: 1.000e+00
Coefficient: −3.44e−01
Value: C
Flexibacteraceae
7.5
5.0
2.5
0.0
A (n=15)
B (n=16)
C (n=14)
surface

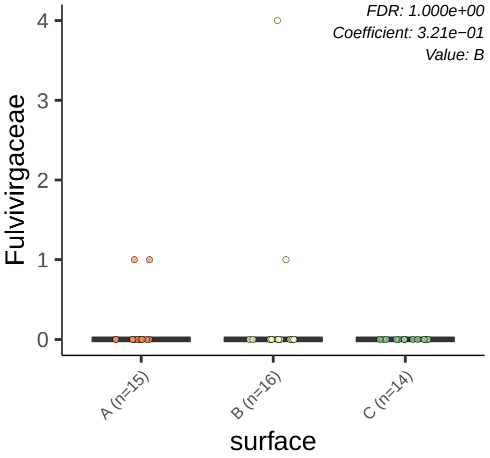

FDR: 1.000e+00
Coefficient: 3.21e−01
Value: B
Fulvivirgaceae
0
1
2
3
4
A (n=15)
B (n=16)
C (n=14)
surface

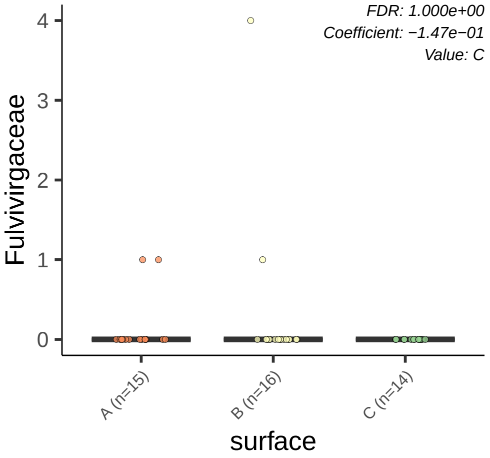
FDR: 1.000e+00
Coefficient: −1.47e−01
Value: C
Fulvivirgaceae
0
1
2
3
4
A (n=15)
B (n=16)
C (n=14)
surface

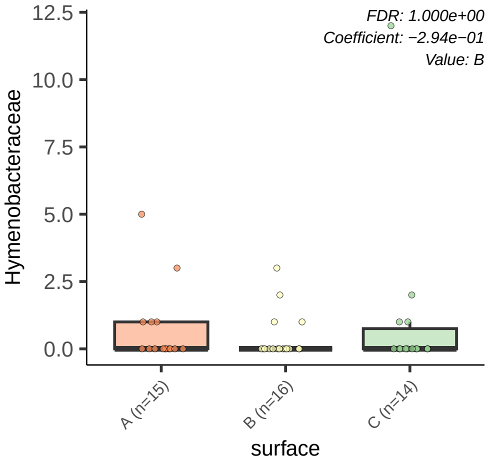
FDR: 1.000e+00
Coefficient: −2.94e−01
Value: B
Hymenobacteraceae
12.5
10.0
7.5
5.0
2.5
0.0
A (n=15)
B (n=16)
C (n=14)
surface

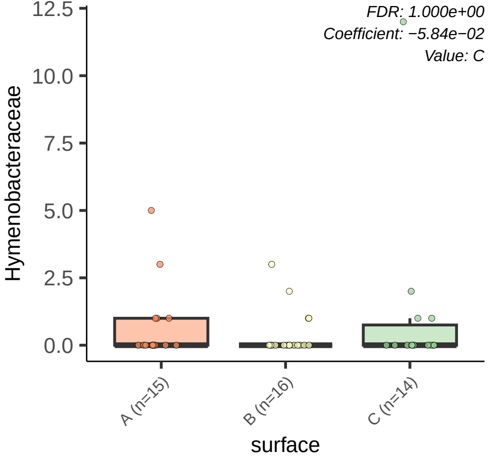

FDR: 1.000e+00
Coefficient: −5.84e−02
Value: C
Hymenobacteraceae
12.5
10.0
7.5
5.0
2.5
0.0
A (n=15)
B (n=16)
C (n=14)
surface

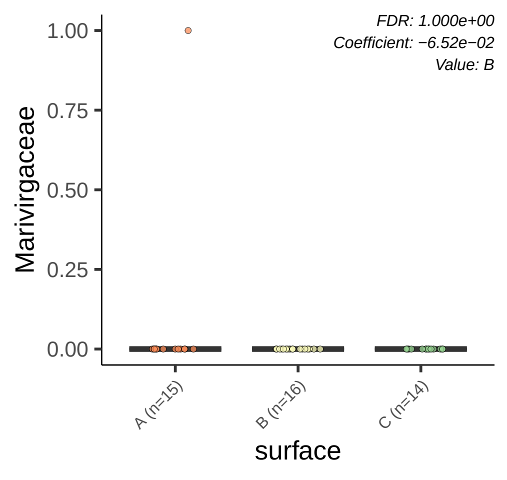
FDR: 1.000e+00
Coefficient: −6.52e−02
Value: B
Marivirgaceae
1.00
0.75
0.50
0.25
0.00
A (n=15)
B (n=16)
C (n=14)
surface

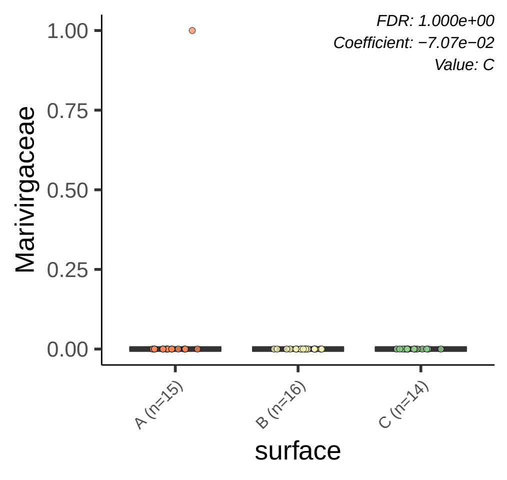

FDR: 1.000e+00
Coefficient: −7.07e−02
Value: C
Marivirgaceae
1.00
0.75
0.50
0.25
0.00
A (n=15)
B (n=16)
C (n=14)
surface

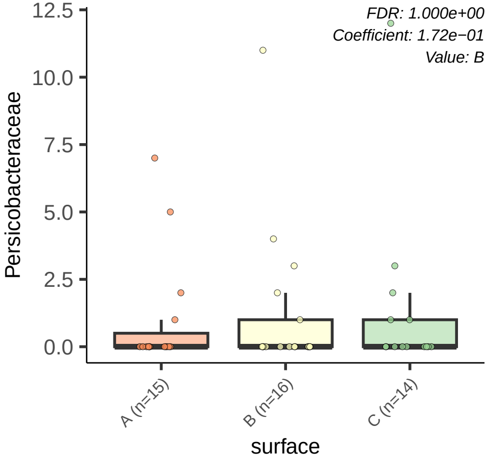

FDR: 1.000e+00
Coefficient: 1.72e−01
Value: B
Persicobacteraceae
12.5
10.0
7.5
5.0
2.5
0.0
A (n=15)
B (n=16)
C (n=14)
surface

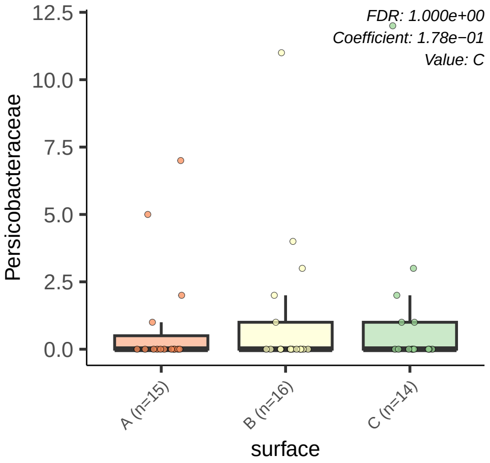

FDR: 1.000e+00
Coefficient: 1.78e−01
Value: C
Persicobacteraceae
12.5
10.0
7.5
5.0
2.5
0.0
A (n=15)
B (n=16)
C (n=14)
surface

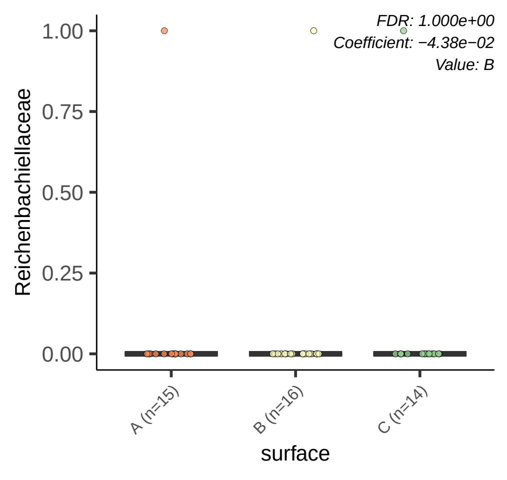

FDR: 1.000e+00
Coefficient: −4.38e−02
Value: B
Reichenbachiellaceae
1.00
0.75
0.50
0.25
0.00
A (n=15)
B (n=16)
C (n=14)
surface

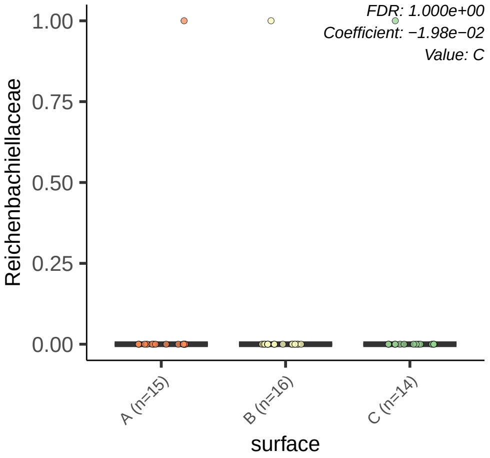
FDR: 1.000e+00
Coefficient: −1.98e−02
Value: C
Reichenbachiellaceae
1.00
0.75
0.50
0.25
0.00
A (n=15)
B (n=16)
C (n=14)
surface

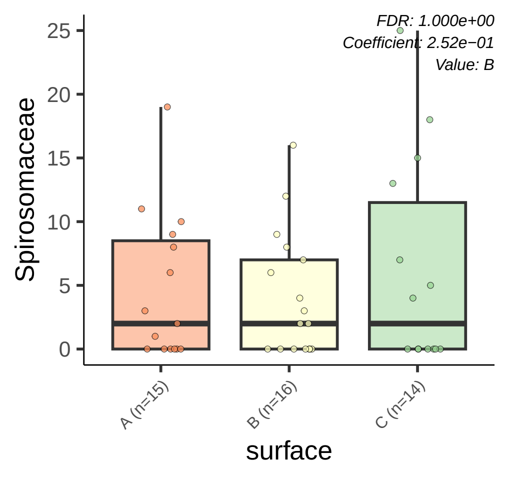

FDR: 1.000e+00
Coefficient: 2.52e−01
Value: B
Spirosomaceae
0
5
10
15
20
25
A (n=15)
B (n=16)
C (n=14)
surface

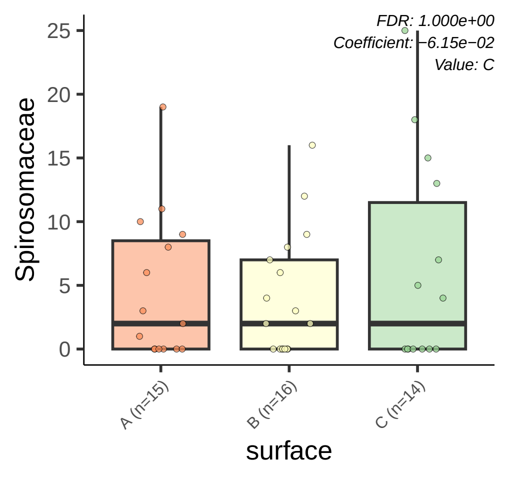
FDR: 1.000e+00
Coefficient: −6.15e−02
Value: C
Spirosomaceae
0
5
10
15
20
25
A (n=15)
B (n=16)
C (n=14)
surface

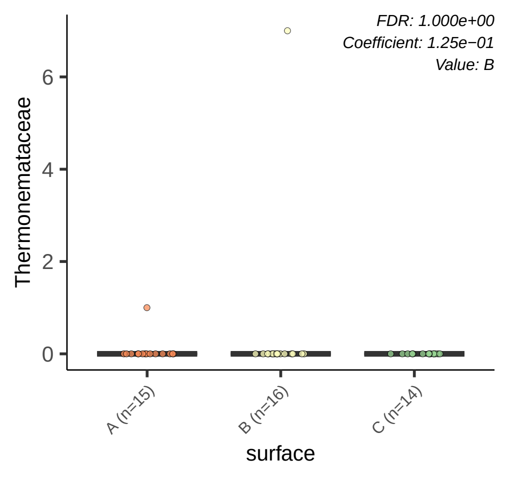
FDR: 1.000e+00
Coefficient: 1.25e−01
Value: B
Thermonemataceae
0
2
4
6
A (n=15)
B (n=16)
C (n=14)
surface

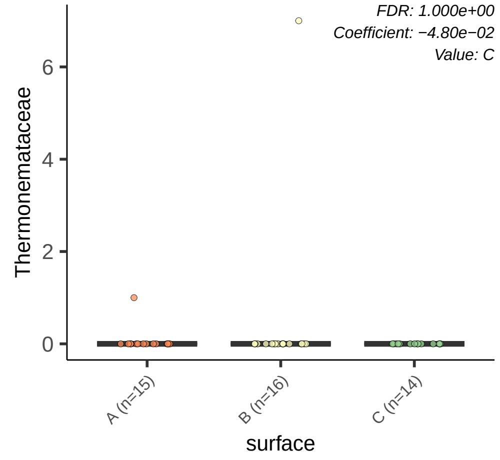
FDR: 1.000e+00
Coefficient: −4.80e−02
Value: C
Thermonemataceae
0
2
4
6
A (n=15)
B (n=16)
C (n=14)
surface

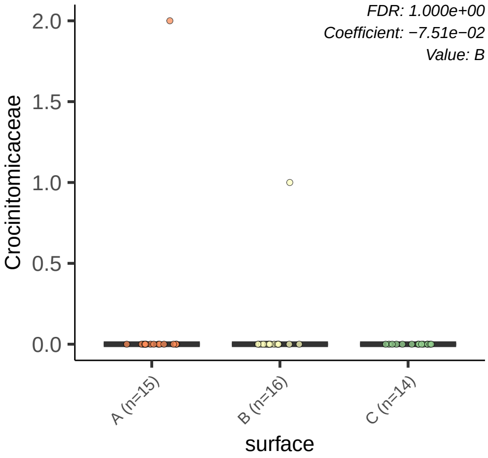
FDR: 1.000e+00
Coefficient: −7.51e−02
Value: B
Crocinitomicaceae
2.0
1.5
1.0
0.5
0.0
A (n=15)
B (n=16)
C (n=14)
surface

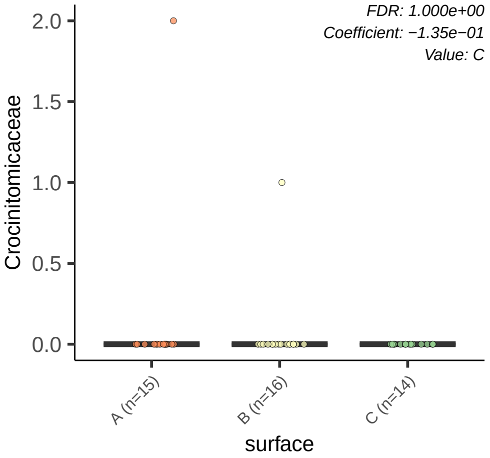

FDR: 1.000e+00
Coefficient: −1.35e−01
Value: C
Crocinitomicaceae
2.0
1.5
1.0
0.5
0.0
A (n=15)
B (n=16)
C (n=14)
surface

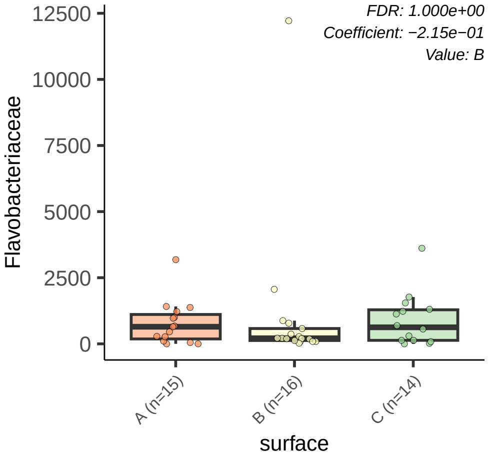
FDR: 1.000e+00
Coefficient: −2.15e−01
Value: B
Flavobacteriaceae
12500
10000
7500
5000
2500
0
A (n=15)
B (n=16)
C (n=14)
surface

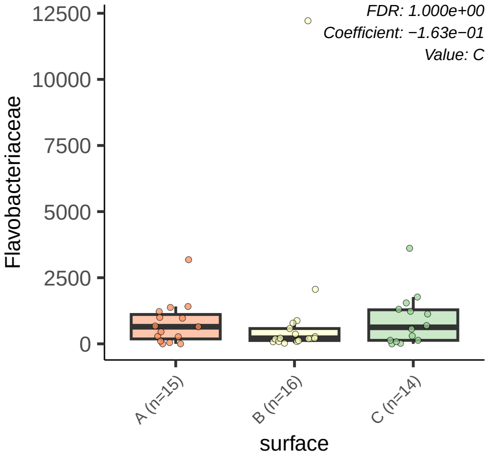

FDR: 1.000e+00
Coefficient: −1.63e−01
Value: C
12500
10000
7500
5000
2500
0
Flavobacteriaceae
A (n=15)
B (n=16)
C (n=14)
surface

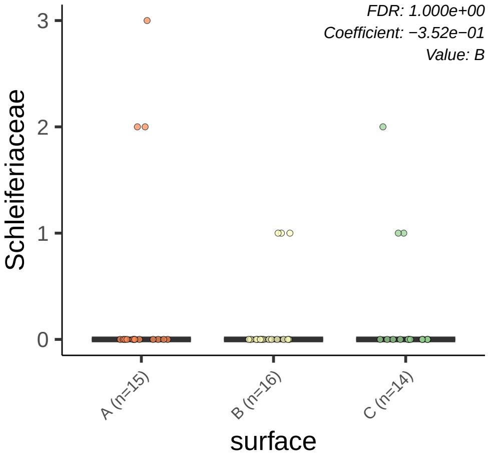
FDR: 1.000e+00
Coefficient: −3.52e−01
Value: B
Schleiferiaceae
3
2
1
0
A (n=15)
B (n=16)
C (n=14)
surface

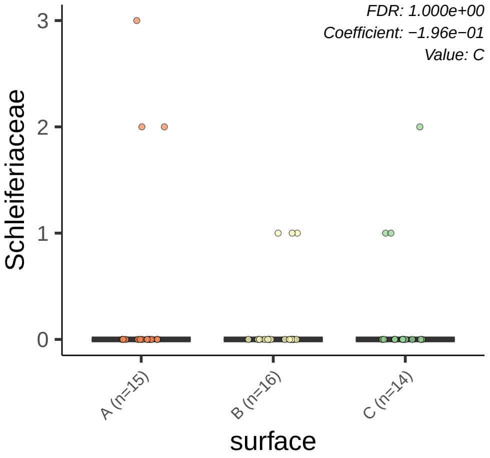

FDR: 1.000e+00
Coefficient: −1.96e−01
Value: C
Schleiferiaceae
3
2
1
0
A (n=15)
B (n=16)
C (n=14)
surface

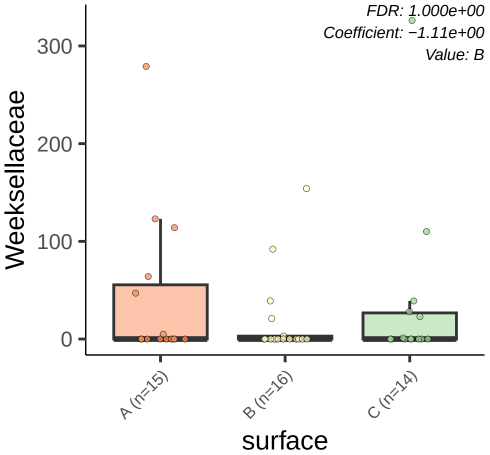

FDR: 1.000e+00
Coefficient: −1.11e+00
Value: B
Weeksellaceae
300
200
100
0
A (n=15)
B (n=16)
C (n=14)
surface

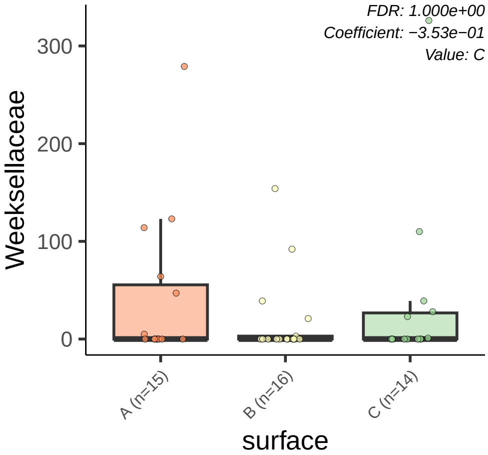

FDR: 1.000e+00
Coefficient: −3.53e−01
Value: C
Weeksellaceae
300
200
100
0
A (n=15)
B (n=16)
C (n=14)
surface

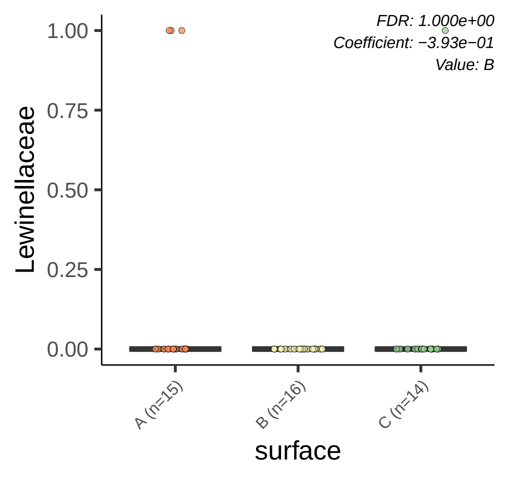
FDR: 1.000e+00
Coefficient: −3.93e−01
Value: B
Lewinellaceae
1.00
0.75
0.50
0.25
0.00
A (n=15)
B (n=16)
C (n=14)
surface

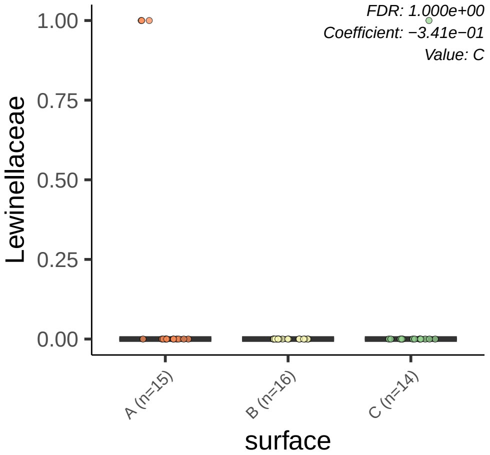
FDR: 1.000e+00
Coefficient: −3.41e−01
Value: C
Lewinellaceae
1.00
0.75
0.50
0.25
0.00
A (n=15)
B (n=16)
C (n=14)
surface

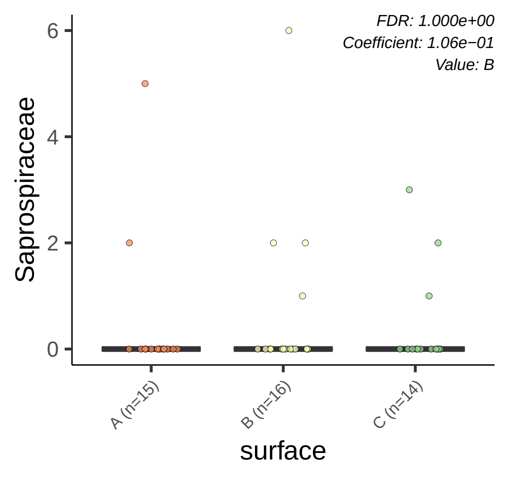

FDR: 1.000e+00
Coefficient: 1.06e−01
Value: B
Saprospiraceae
6
4
2
0
A (n=15)
B (n=16)
C (n=14)
surface

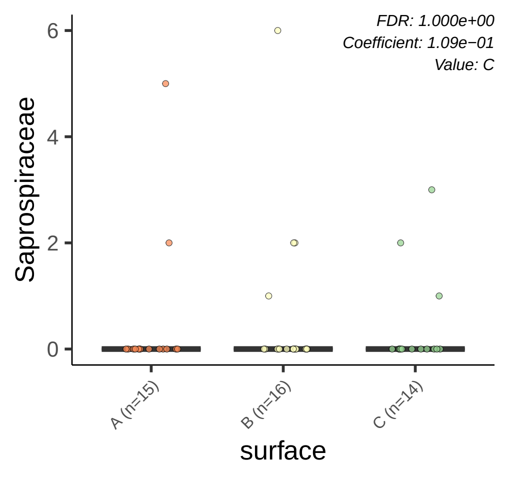

FDR: 1.000e+00
Coefficient: 1.09e−01
Value: C
Saprospiraceae
0
2
4
6
A (n=15)
B (n=16)
C (n=14)
surface

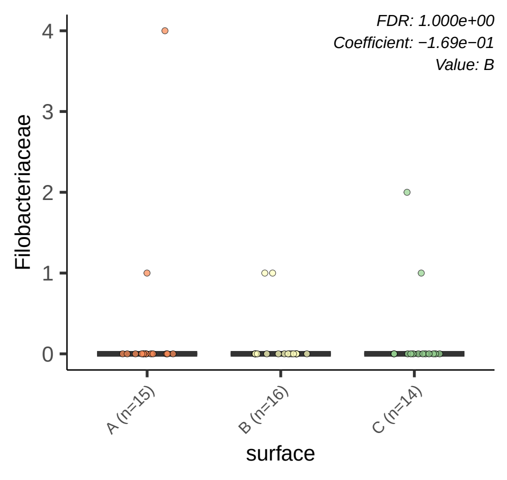
FDR: 1.000e+00
Coefficient: −1.69e−01
Value: B
Filobacteriaceae
0
1
2
3
4
A (n=15)
B (n=16)
C (n=14)
surface

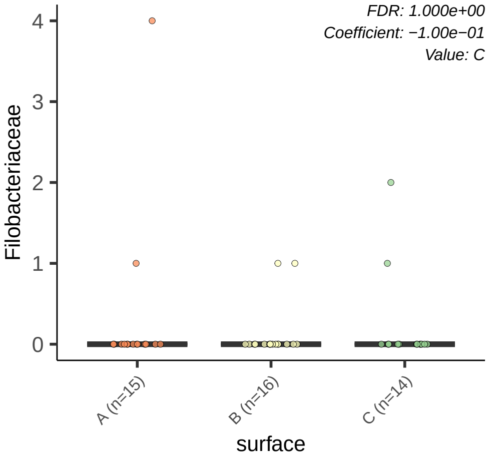

FDR: 1.000e+00
Coefficient: −1.00e−01
Value: C
Filobacteriaceae
4
3
2
1
0
A (n=15)
B (n=16)
C (n=14)
surface

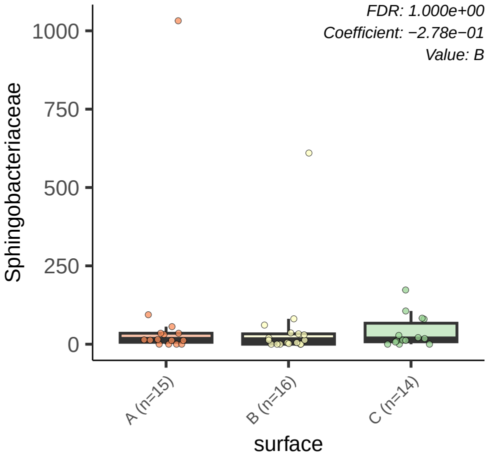

FDR: 1.000e+00
Coefficient: −2.78e−01
Value: B
Sphingobacteriaceae
1000
750
500
250
0
A (n=15)
B (n=16)
C (n=14)
surface

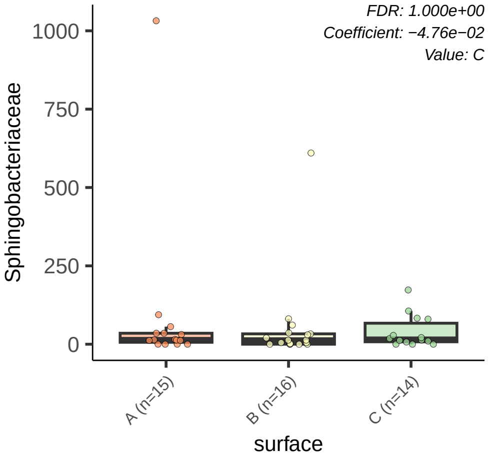
FDR: 1.000e+00
Coefficient: −4.76e−02
Value: C
Sphingobacteriaceae
1000
750
500
250
0
A (n=15)
B (n=16)
C (n=14)
surface

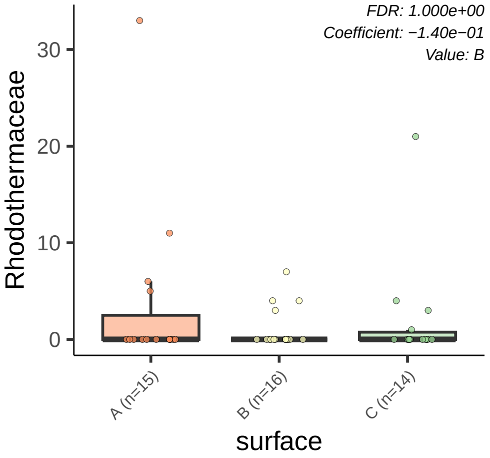

FDR: 1.000e+00
Coefficient: −1.40e−01
Value: B
Rhodothermaceae
30
20
10
0
A (n=15)
B (n=16)
C (n=14)
surface

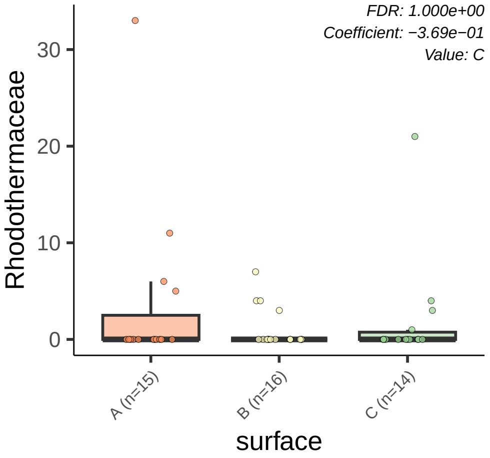

FDR: 1.000e+00
Coefficient: −3.69e−01
Value: C
Rhodothermaceae
0
10
20
30
A (n=15)
B (n=16)
C (n=14)
surface

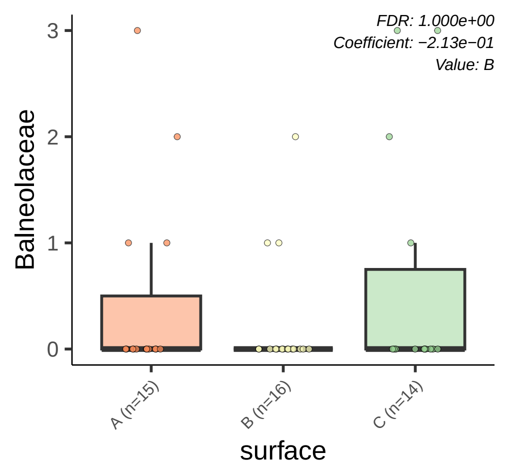

FDR: 1.000e+00
Coefficient: −2.13e−01
Value: B
Balneolaceae
3
2
1
0
A (n=15)
B (n=16)
C (n=14)
surface

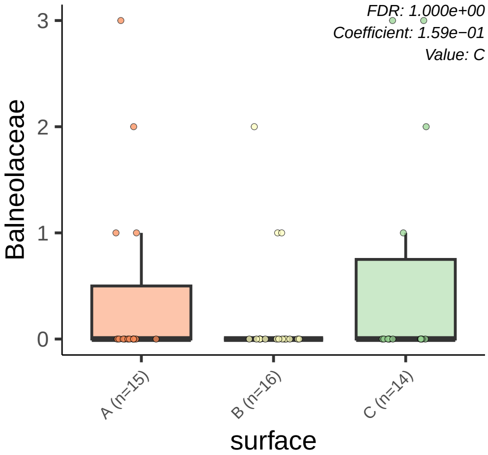

FDR: 1.000e+00
Coefficient: 1.59e−01
Value: C
Balneolaceae
3
2
1
0
A (n=15)
B (n=16)
C (n=14)
surface

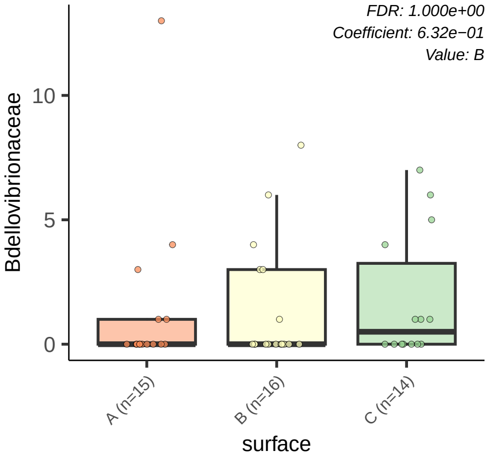

FDR: 1.000e+00
Coefficient: 6.32e−01
Value: B
Bdellovibrionaceae
0
5
10
A (n=15)
B (n=16)
C (n=14)
surface

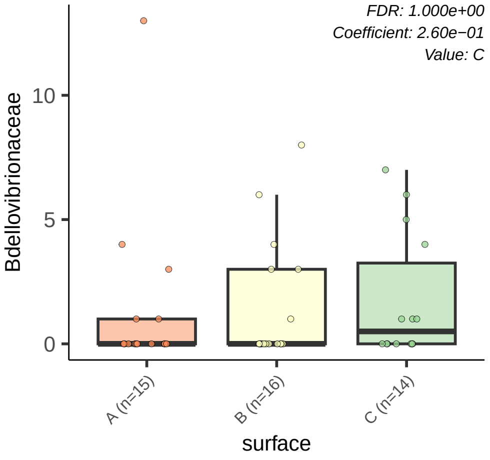

FDR: 1.000e+00
Coefficient: 2.60e−01
Value: C
Bdellovibrionaceae
10
5
0
A (n=15)
B (n=16)
C (n=14)
surface

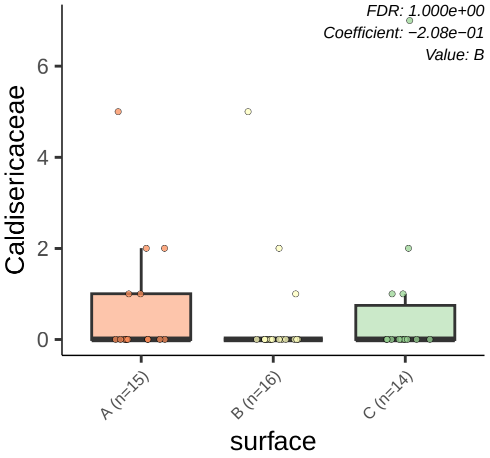

FDR: 1.000e+00
Coefficient: −2.08e−01
Value: B
Caldisericaceae
0
2
4
6
A (n=15)
B (n=16)
C (n=14)
surface

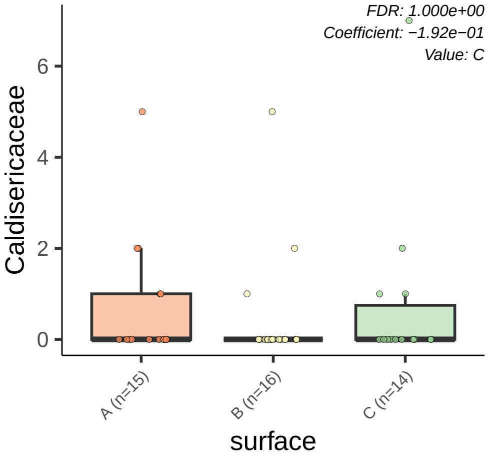

FDR: 1.000e+00
Coefficient: −1.92e−01
Value: C
Caldisericaceae
0
2
4
6
A (n=15)
B (n=16)
C (n=14)
surface

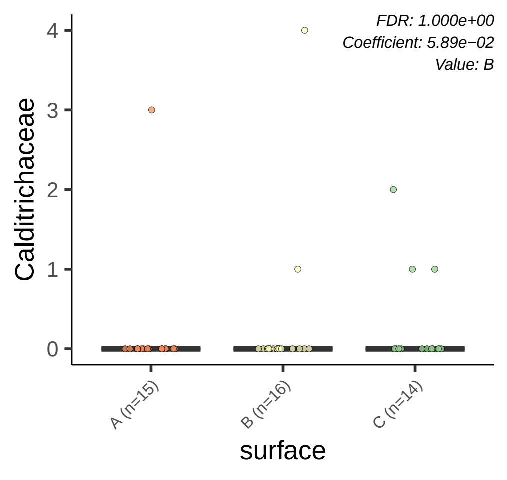
FDR: 1.000e+00
Coefficient: 5.89e−02
Value: B
Calditrichaceae
0
1
2
3
4
A (n=15)
B (n=16)
C (n=14)
surface

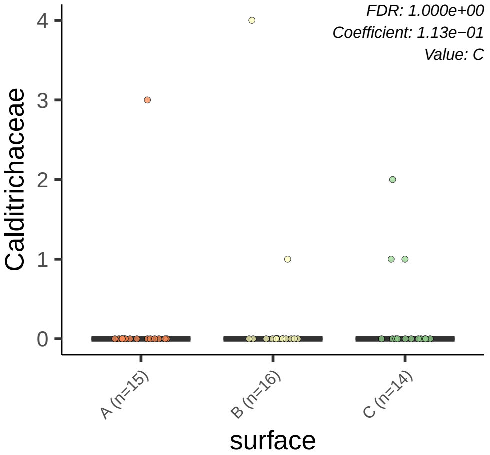
FDR: 1.000e+00
Coefficient: 1.13e−01
Value: C
Calditrichaceae
0
1
2
3
4
A (n=15)
B (n=16)
C (n=14)
surface

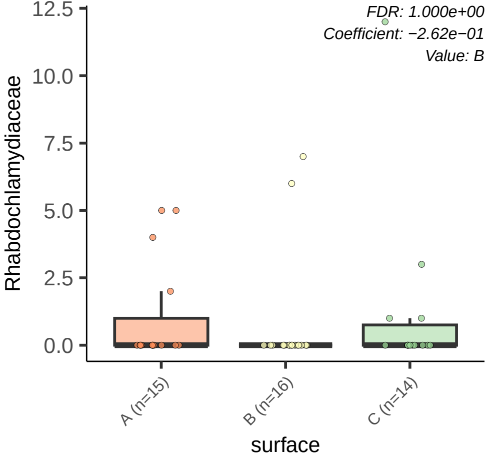

FDR: 1.000e+00
Coefficient: −2.62e−01
Value: B
Rhabdochlamydiaceae
12.5
10.0
7.5
5.0
2.5
0.0
A (n=15)
B (n=16)
C (n=14)
surface

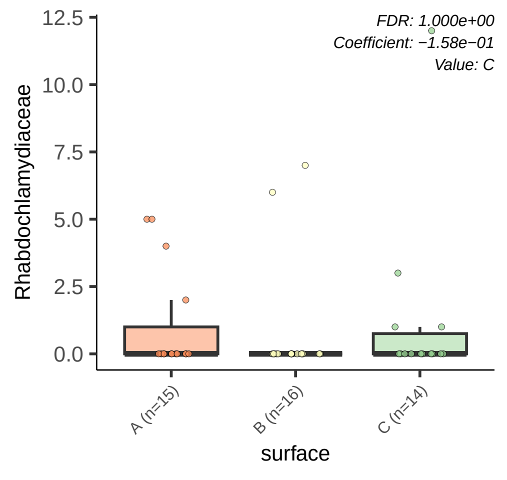
FDR: 1.000e+00
Coefficient: −1.58e−01
Value: C
Rhabdochlamydiaceae
12.5
10.0
7.5
5.0
2.5
0.0
A (n=15)
B (n=16)
C (n=14)
surface

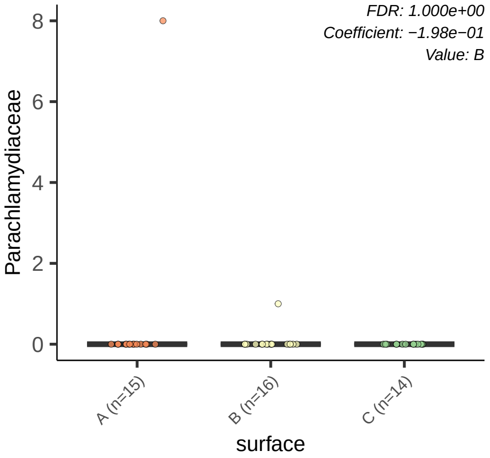

FDR: 1.000e+00
Coefficient: −1.98e−01
Value: B
Parachlamydiaceae
8
6
4
2
0
A (n=15)
B (n=16)
C (n=14)
surface

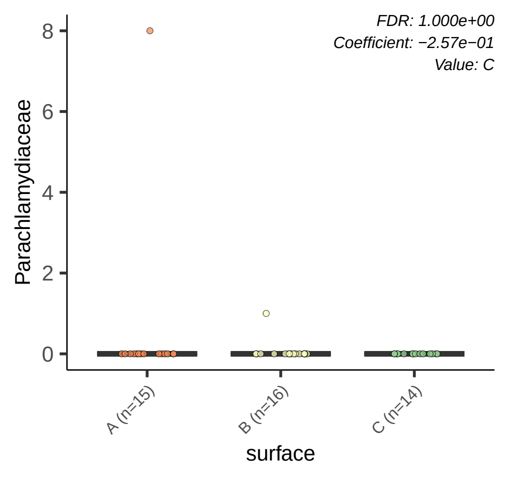
FDR: 1.000e+00
Coefficient: −2.57e−01
Value: C
Parachlamydiaceae
0
2
4
6
8
A (n=15)
B (n=16)
C (n=14)
surface

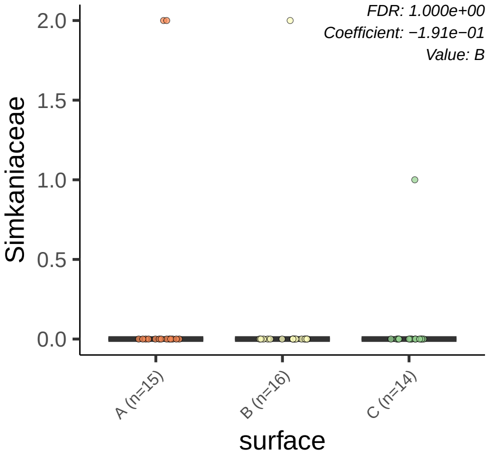
FDR: 1.000e+00
Coefficient: −1.91e−01
Value: B
Simkaniaceae
2.0
1.5
1.0
0.5
0.0
A (n=15)
B (n=16)
C (n=14)
surface

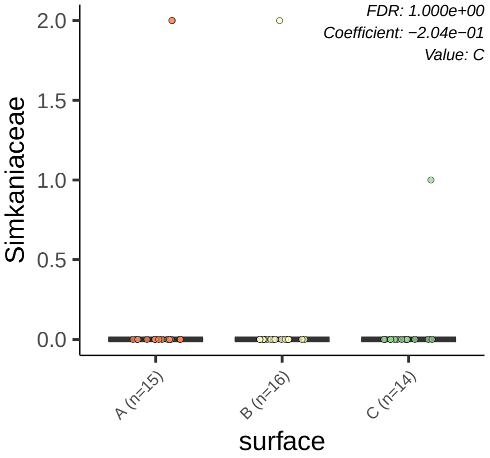

FDR: 1.000e+00
Coefficient: −2.04e−01
Value: C
Simkaniaceae
2.0
1.5
1.0
0.5
0.0
A (n=15)
B (n=16)
C (n=14)
surface

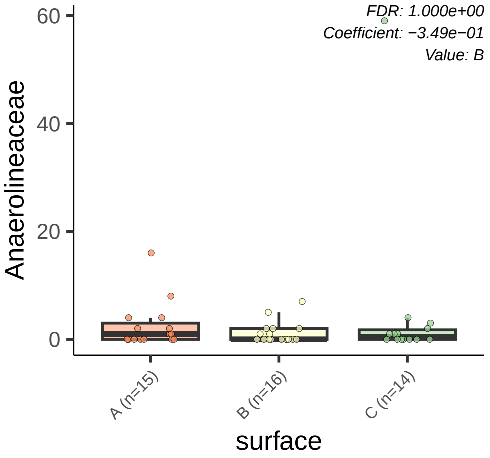

FDR: 1.000e+00
Coefficient: −3.49e−01
Value: B
Anaerolineaceae
60
40
20
0
A (n=15)
B (n=16)
C (n=14)
surface

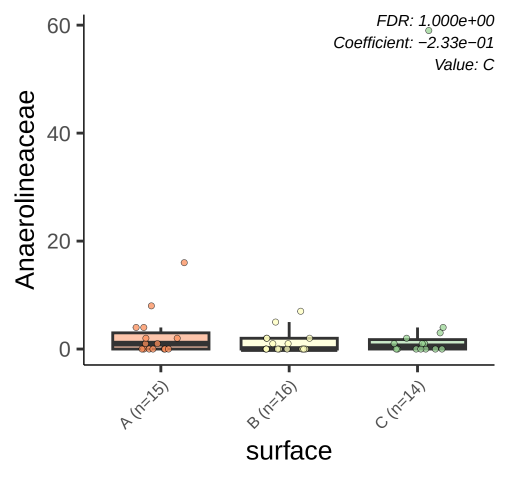
FDR: 1.000e+00
Coefficient: −2.33e−01
Value: C
Anaerolineaceae
60
40
20
0
A (n=15)
B (n=16)
C (n=14)
surface

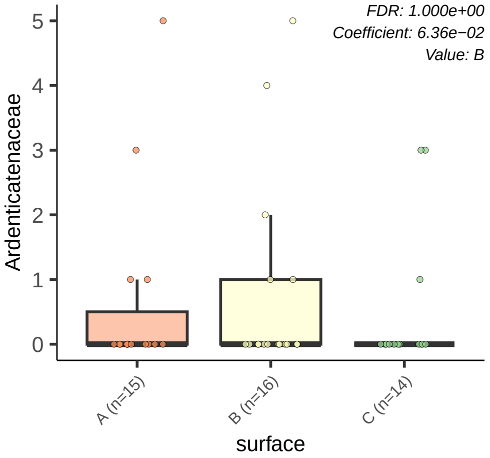
FDR: 1.000e+00
Coefficient: 6.36e−02
Value: B
Ardenticatenaceae
0
1
2
3
4
5
A (n=15)
B (n=16)
C (n=14)
surface

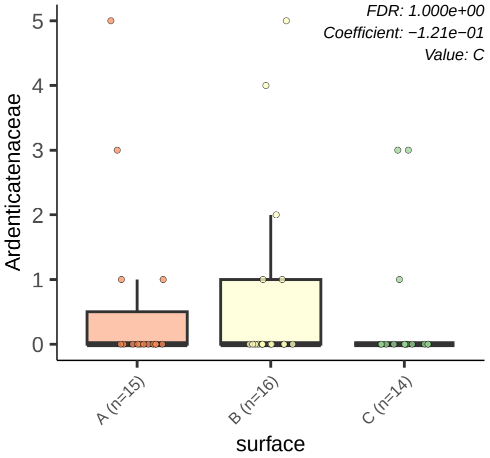

FDR: 1.000e+00
Coefficient: −1.21e−01
Value: C
Ardenticatenaceae
0
1
2
3
4
5
A (n=15)
B (n=16)
C (n=14)
surface

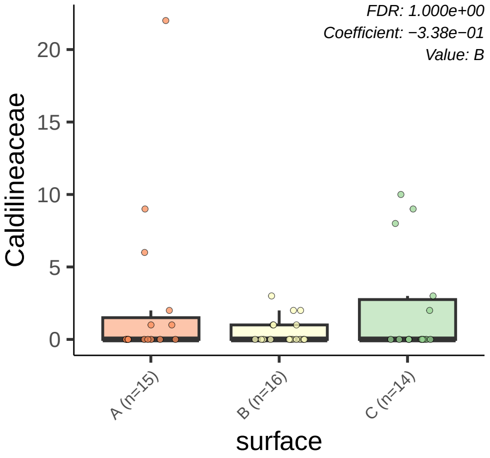

FDR: 1.000e+00
Coefficient: −3.38e−01
Value: B
Caldilineaceae
0
5
10
15
20
A (n=15)
B (n=16)
C (n=14)
surface

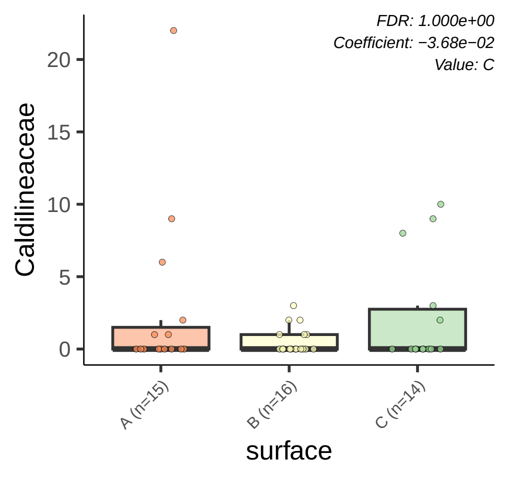
FDR: 1.000e+00
Coefficient: −3.68e−02
Value: C
Caldilineaceae
0
5
10
15
20
A (n=15)
B (n=16)
C (n=14)
surface

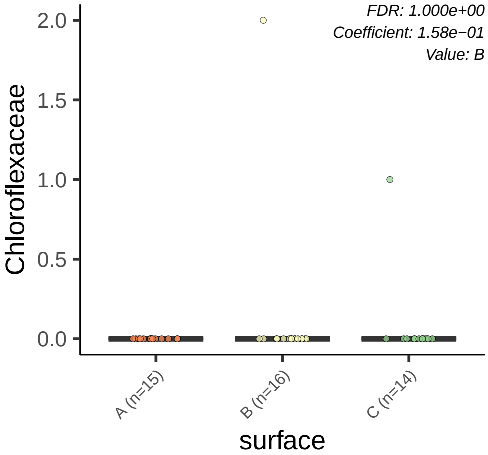

FDR: 1.000e+00
Coefficient: 1.58e−01
Value: B
Chloroflexaceae
2.0
1.5
1.0
0.5
0.0
A (n=15)
B (n=16)
C (n=14)
surface

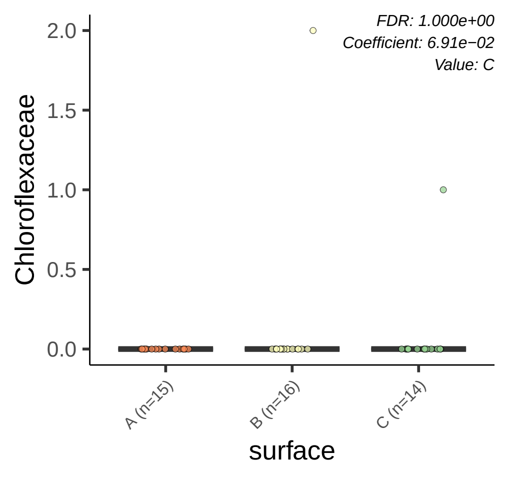
FDR: 1.000e+00
Coefficient: 6.91e−02
Value: C
Chloroflexaceae
2.0
1.5
1.0
0.5
0.0
A (n=15)
B (n=16)
C (n=14)
surface

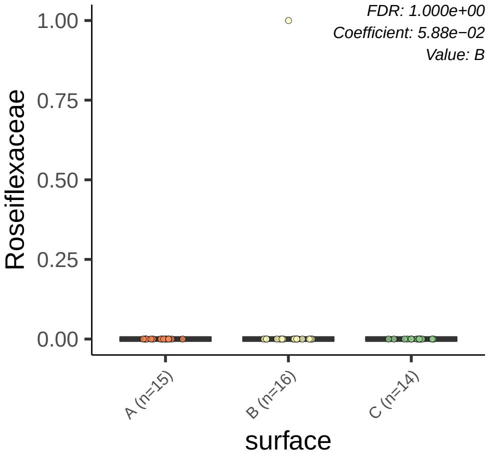

FDR: 1.000e+00
Coefficient: 5.88e−02
Value: B
Roseiflexaceae
1.00
0.75
0.50
0.25
0.00
A (n=15)
B (n=16)
C (n=14)
surface

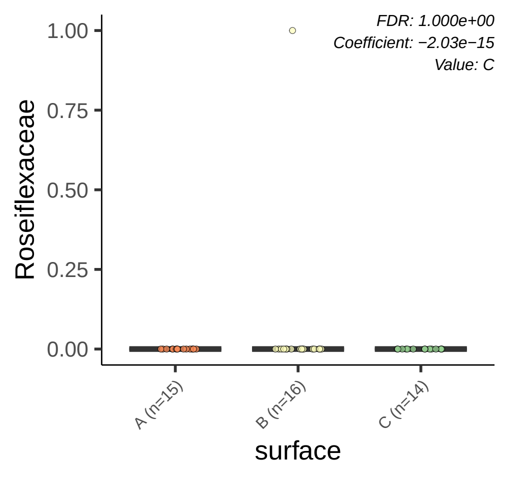
FDR: 1.000e+00
Coefficient: −2.03e−15
Value: C
Roseiflexaceae
1.00
0.75
0.50
0.25
0.00
A (n=15)
B (n=16)
C (n=14)
surface

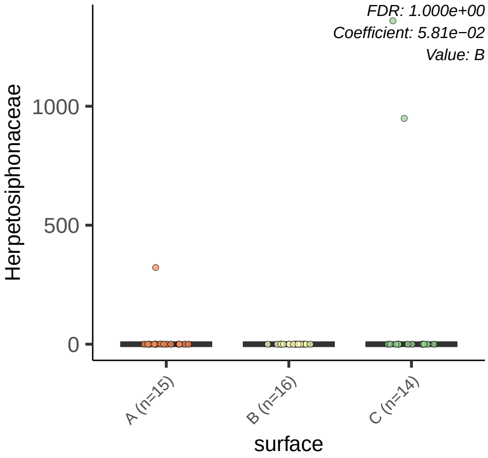
FDR: 1.000e+00
Coefficient: 5.81e−02
Value: B
Herpetosiphonaceae
1000
500
0
A (n=15)
B (n=16)
C (n=14)
surface

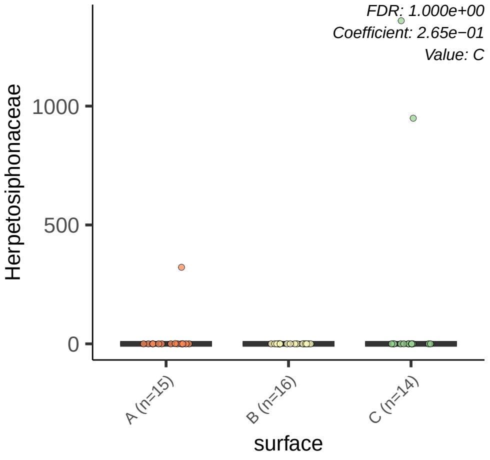
FDR: 1.000e+00
Coefficient: 2.65e−01
Value: C
Herpetosiphonaceae
1000
500
0
A (n=15)
B (n=16)
C (n=14)
surface

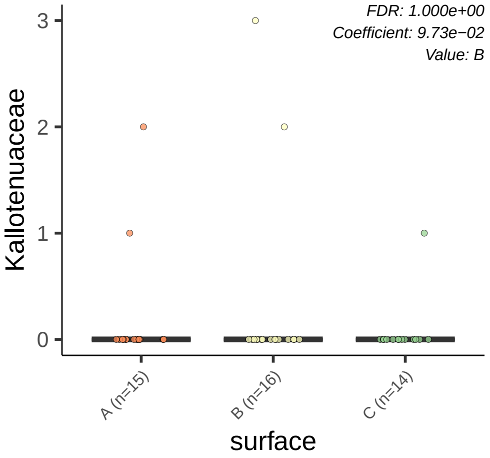

FDR: 1.000e+00
Coefficient: 9.73e−02
Value: B
Kallotenuaceae
3
2
1
0
A (n=15)
B (n=16)
C (n=14)
surface

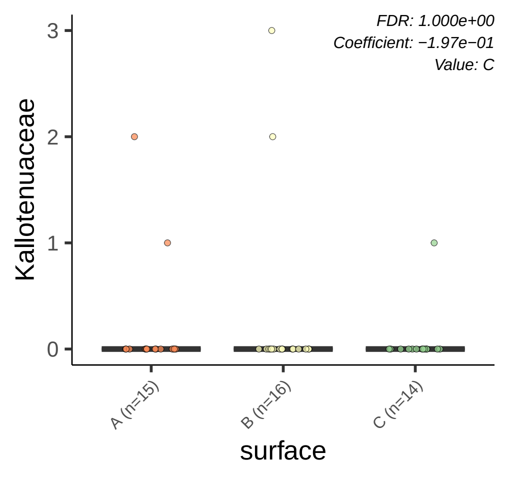
FDR: 1.000e+00
Coefficient: −1.97e−01
Value: C
Kallotenuaceae
3
2
1
0
A (n=15)
B (n=16)
C (n=14)
surface

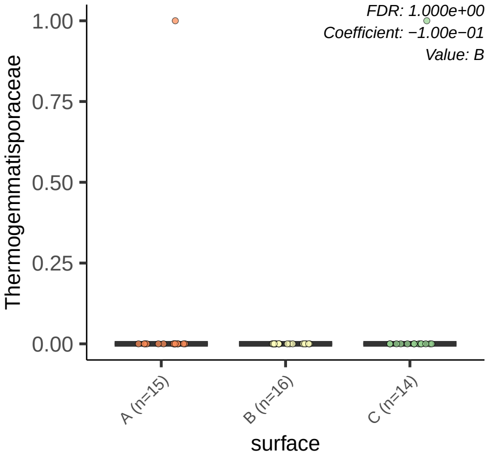
FDR: 1.000e+00
Coefficient: −1.00e−01
Value: B
Thermogemmatisporaceae
1.00
0.75
0.50
0.25
0.00
A (n=15)
B (n=16)
C (n=14)
surface

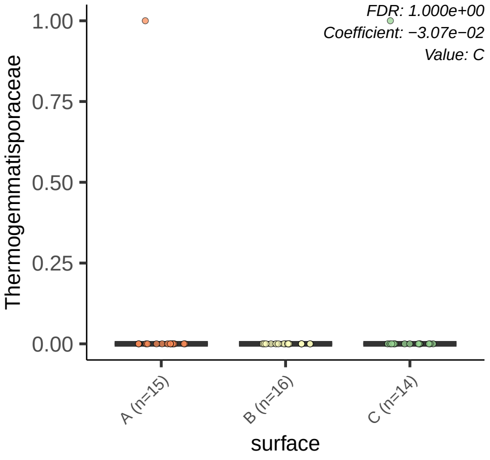

FDR: 1.000e+00
Coefficient: −3.07e−02
Value: C
Thermogemmatisporaceae
1.00
0.75
0.50
0.25
0.00
A (n=15)
B (n=16)
C (n=14)
surface

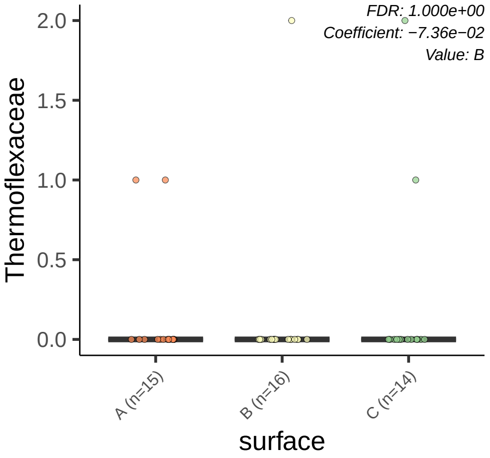
FDR: 1.000e+00
Coefficient: −7.36e−02
Value: B
Thermoflexaceae
2.0
1.5
1.0
0.5
0.0
A (n=15)
B (n=16)
C (n=14)
surface

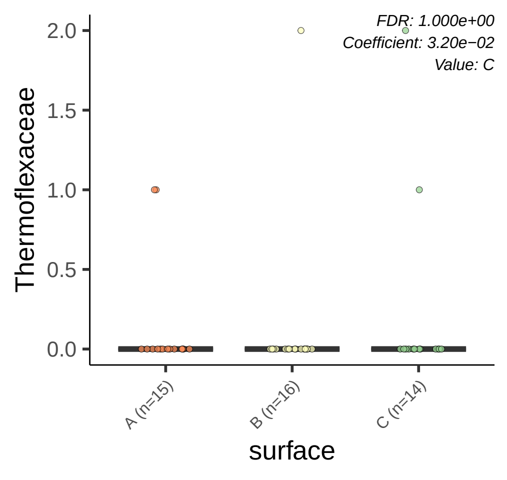
FDR: 1.000e+00
Coefficient: 3.20e−02
Value: C
Thermoflexaceae
2.0
1.5
1.0
0.5
0.0
A (n=15)
B (n=16)
C (n=14)
surface

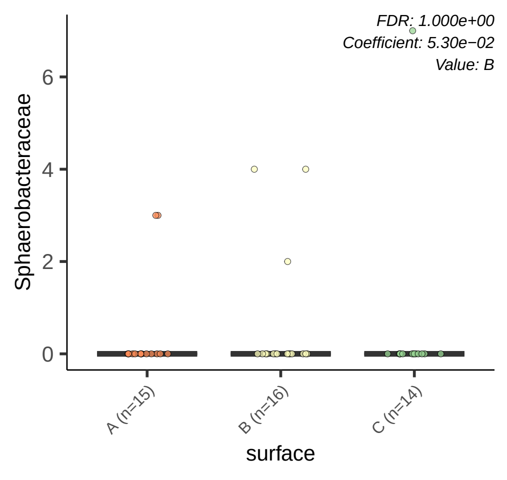
FDR: 1.000e+00
Coefficient: 5.30e−02
Value: B
Sphaerobacteraceae
0
2
4
6
A (n=15)
B (n=16)
C (n=14)
surface

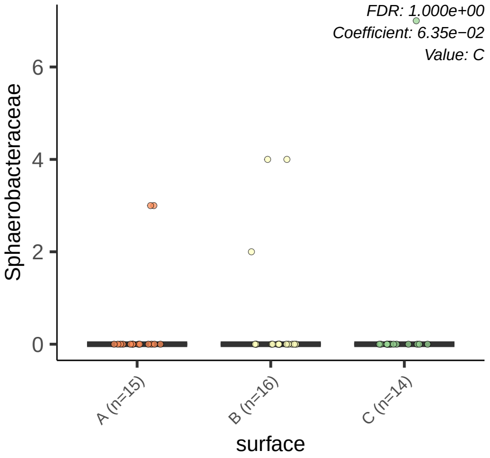
FDR: 1.000e+00
Coefficient: 6.35e−02
Value: C
Sphaerobacteraceae
0
2
4
6
A (n=15)
B (n=16)
C (n=14)
surface

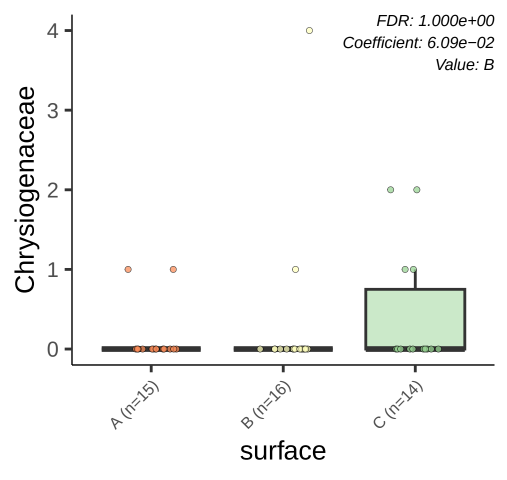

FDR: 1.000e+00
Coefficient: 6.09e−02
Value: B
Chrysiogenaceae
0
1
2
3
4
A (n=15)
B (n=16)
C (n=14)
surface

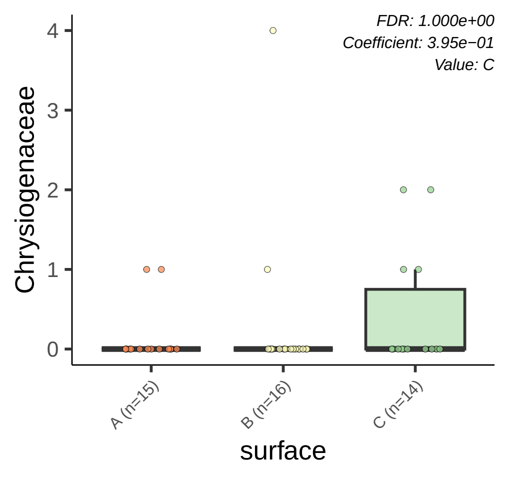
FDR: 1.000e+00
Coefficient: 3.95e−01
Value: C
Chrysiogenaceae
4
3
2
1
0
A (n=15)
B (n=16)
C (n=14)
surface

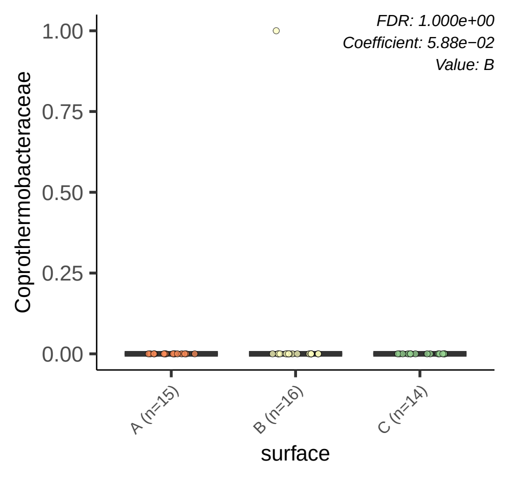
FDR: 1.000e+00
Coefficient: 5.88e−02
Value: B
Coprothermobacteraceae
1.00
0.75
0.50
0.25
0.00
A (n=15)
B (n=16)
C (n=14)
surface

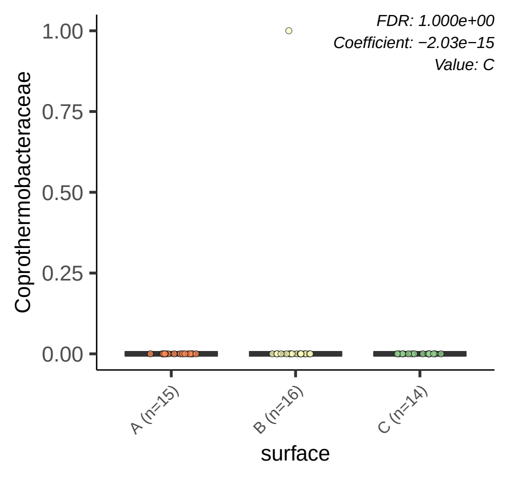
FDR: 1.000e+00
Coefficient: −2.03e−15
Value: C
Coprothermobacteraceae
1.00
0.75
0.50
0.25
0.00
A (n=15)
B (n=16)
C (n=14)
surface

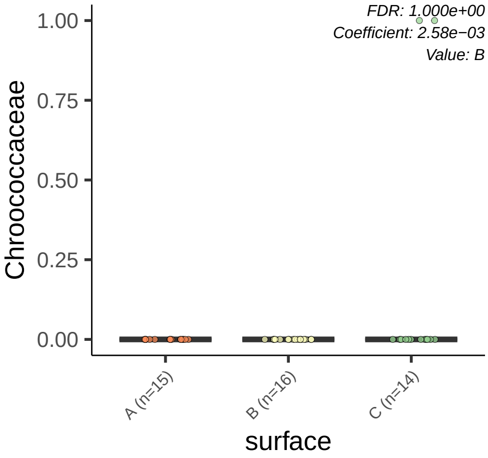
FDR: 1.000e+00
Coefficient: 2.58e−03
Value: B
Chroococcaceae
1.00
0.75
0.50
0.25
0.00
A (n=15)
B (n=16)
C (n=14)
surface

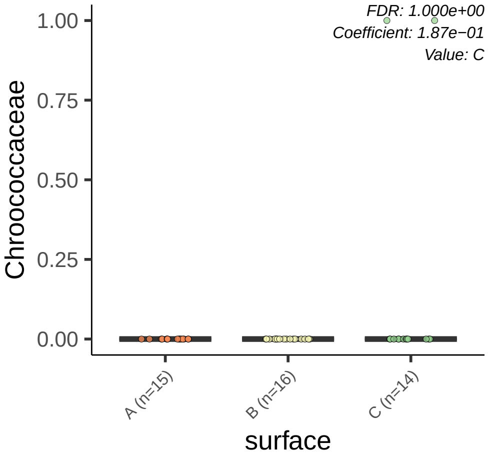
FDR: 1.000e+00
Coefficient: 1.87e−01
Value: C
Chroococcaceae
1.00
0.75
0.50
0.25
0.00
A (n=15)
B (n=16)
C (n=14)
surface

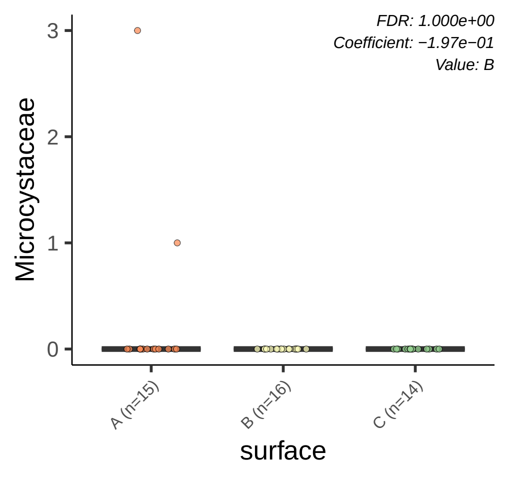
FDR: 1.000e+00
Coefficient: −1.97e−01
Value: B
Microcystaceae
3
2
1
0
A (n=15)
B (n=16)
C (n=14)
surface

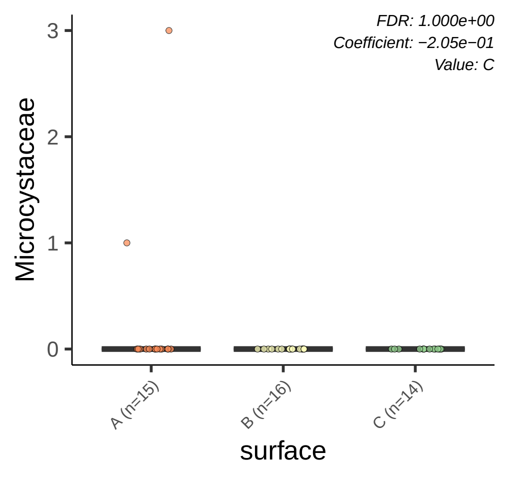
FDR: 1.000e+00
Coefficient: −2.05e−01
Value: C
Microcystaceae
3
2
1
0
A (n=15)
B (n=16)
C (n=14)
surface

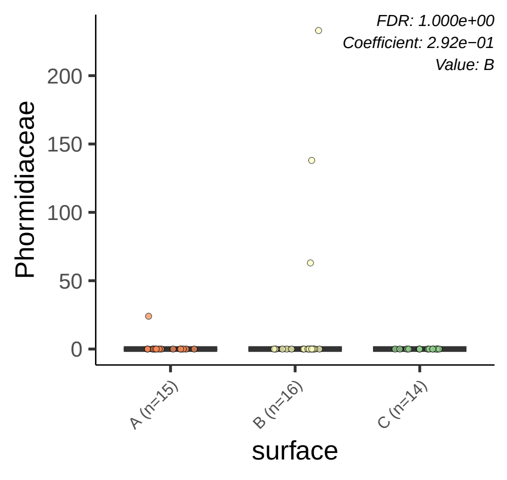
FDR: 1.000e+00
Coefficient: 2.92e−01
Value: B
Phormidiaceae
200
150
100
50
0
A (n=15)
B (n=16)
C (n=14)
surface

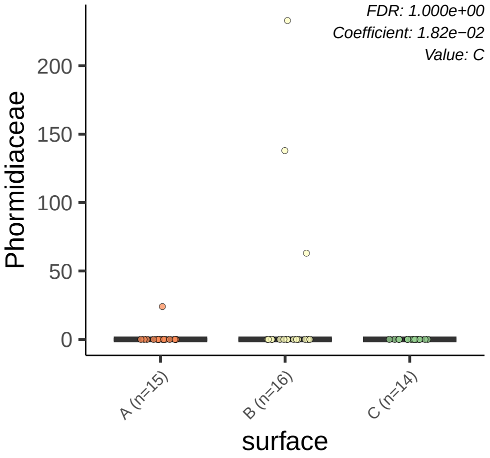
FDR: 1.000e+00
Coefficient: 1.82e−02
Value: C
Phormidiaceae
200
150
100
50
0
A (n=15)
B (n=16)
C (n=14)
surface

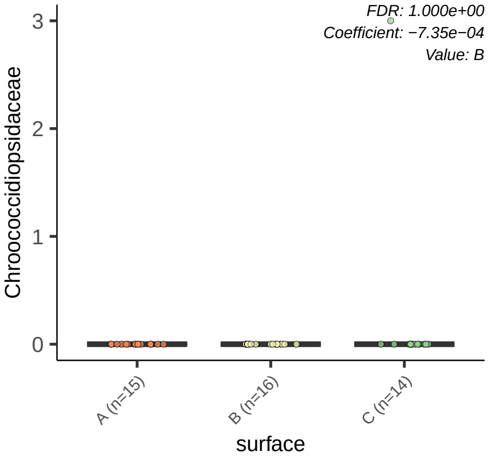
FDR: 1.000e+00
Coefficient: −7.35e−04
Value: B
Chroococcidiopsidaceae
3
2
1
0
A (n=15)
B (n=16)
C (n=14)
surface

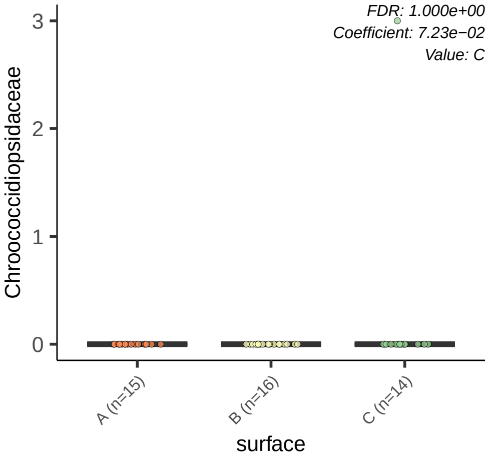
FDR: 1.000e+00
Coefficient: 7.23e−02
Value: C
Chroococcidiopsidaceae
3
2
1
0
A (n=15)
B (n=16)
C (n=14)
surface

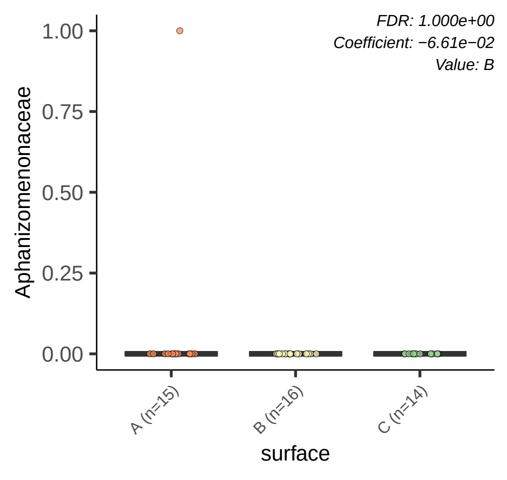
FDR: 1.000e+00
Coefficient: −6.61e−02
Value: B
Aphanizomenonaceae
1.00
0.75
0.50
0.25
0.00
A (n=15)
B (n=16)
C (n=14)
surface

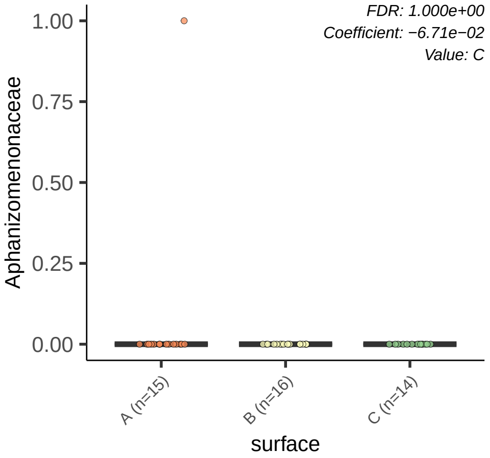
FDR: 1.000e+00
Coefficient: −6.71e−02
Value: C
Aphanizomenonaceae
1.00
0.75
0.50
0.25
0.00
A (n=15)
B (n=16)
C (n=14)
surface

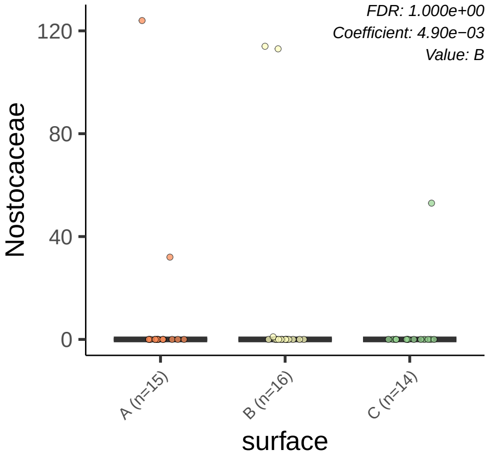
FDR: 1.000e+00
Coefficient: 4.90e−03
Value: B
Nostocaceae
120
80
40
0
A (n=15)
B (n=16)
C (n=14)
surface

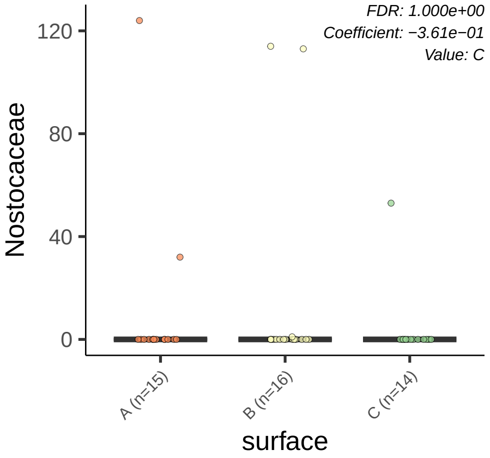
FDR: 1.000e+00
Coefficient: −3.61e−01
Value: C
Nostocaceae
120
80
40
0
A (n=15)
B (n=16)
C (n=14)
surface

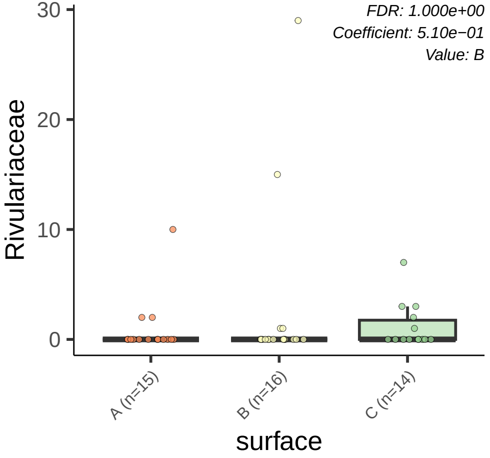

FDR: 1.000e+00
Coefficient: 5.10e−01
Value: B
Rivulariaceae
30
20
10
0
A (n=15)
B (n=16)
C (n=14)
surface

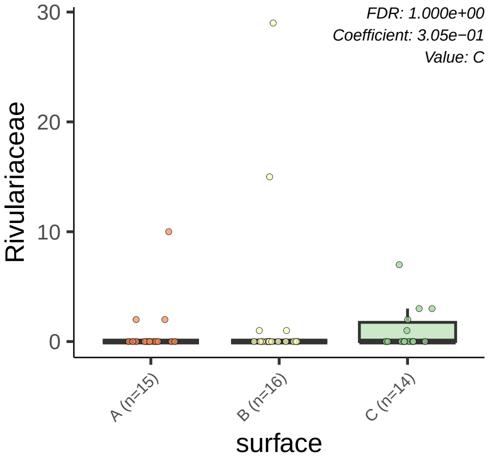

FDR: 1.000e+00
Coefficient: 3.05e−01
Value: C
Rivulariaceae
30
20
10
0
A (n=15)
B (n=16)
C (n=14)
surface

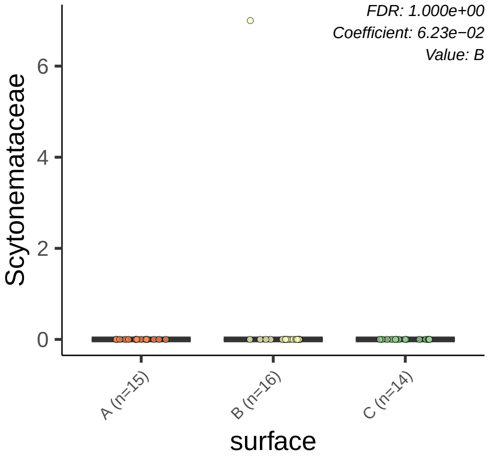

FDR: 1.000e+00
Coefficient: 6.23e−02
Value: B
Scytonemataceae
0
2
4
6
A (n=15)
B (n=16)
C (n=14)
surface

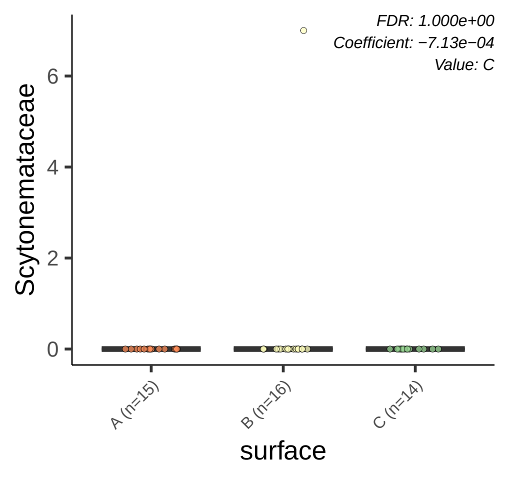
FDR: 1.000e+00
Coefficient: −7.13e−04
Value: C
Scytonemataceae
6
4
2
0
A (n=15)
B (n=16)
C (n=14)
surface

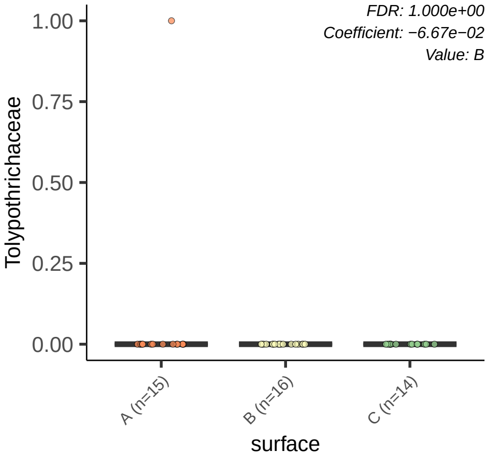
FDR: 1.000e+00
Coefficient: −6.67e−02
Value: B
Tolypothrichaceae
1.00
0.75
0.50
0.25
0.00
A (n=15)
B (n=16)
C (n=14)
surface

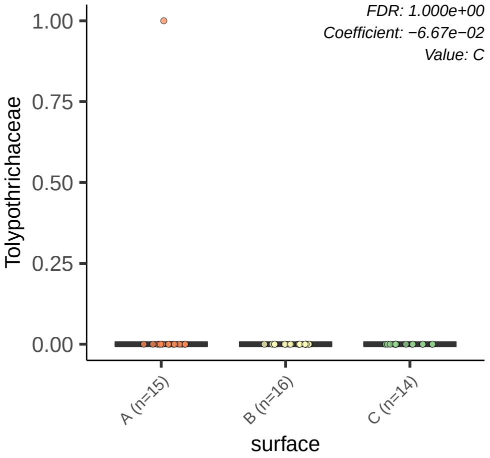
FDR: 1.000e+00
Coefficient: −6.67e−02
Value: C
Tolypothrichaceae
1.00
0.75
0.50
0.25
0.00
A (n=15)
B (n=16)
C (n=14)
surface

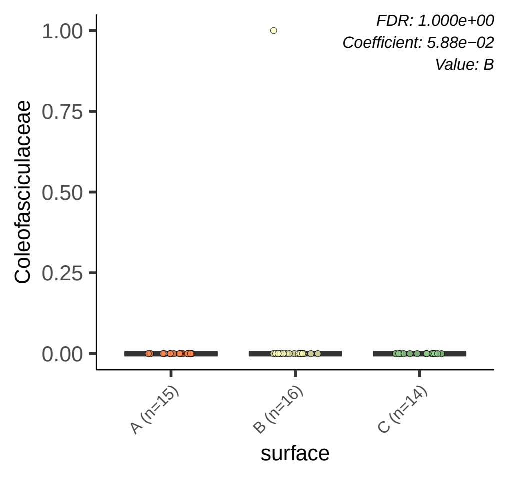

FDR: 1.000e+00
Coefficient: 5.88e−02
Value: B
Coleofasciculaceae
1.00
0.75
0.50
0.25
0.00
A (n=15)
B (n=16)
C (n=14)
surface

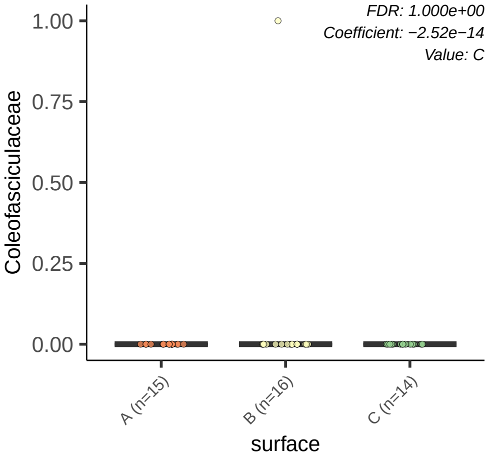
FDR: 1.000e+00
Coefficient: −2.52e−14
Value: C
Coleofasciculaceae
1.00
0.75
0.50
0.25
0.00
A (n=15)
B (n=16)
C (n=14)
surface

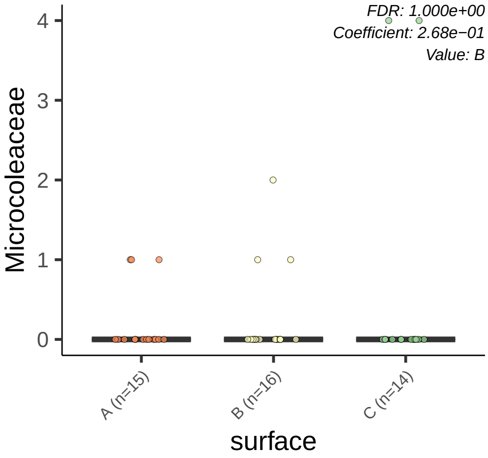
FDR: 1.000e+00
Coefficient: 2.68e−01
Value: B
Microcoleaceae
0
1
2
3
4
A (n=15)
B (n=16)
C (n=14)
surface

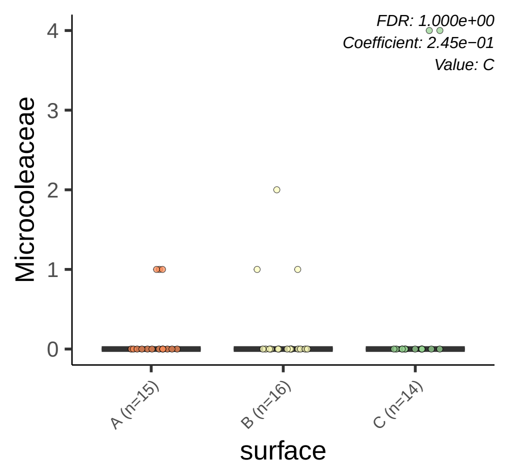

FDR: 1.000e+00
Coefficient: 2.45e−01
Value: C
Microcoleaceae
0
1
2
3
4
A (n=15)
B (n=16)
C (n=14)
surface

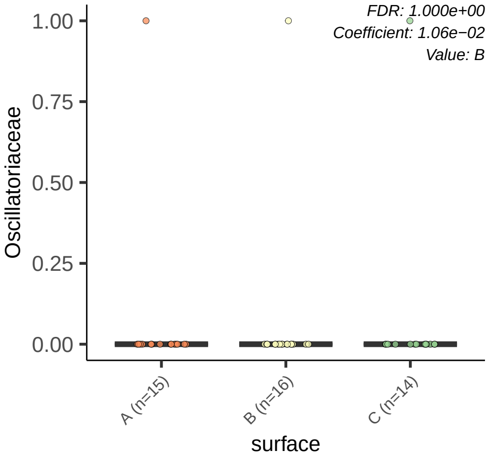
FDR: 1.000e+00
Coefficient: 1.06e−02
Value: B
Oscillatoriaceae
1.00
0.75
0.50
0.25
0.00
A (n=15)
B (n=16)
C (n=14)
surface

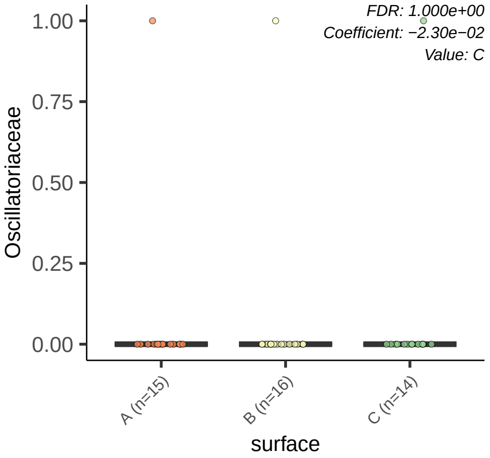

FDR: 1.000e+00
Coefficient: −2.30e−02
Value: C
Oscillatoriaceae
1.00
0.75
0.50
0.25
0.00
A (n=15)
B (n=16)
C (n=14)
surface

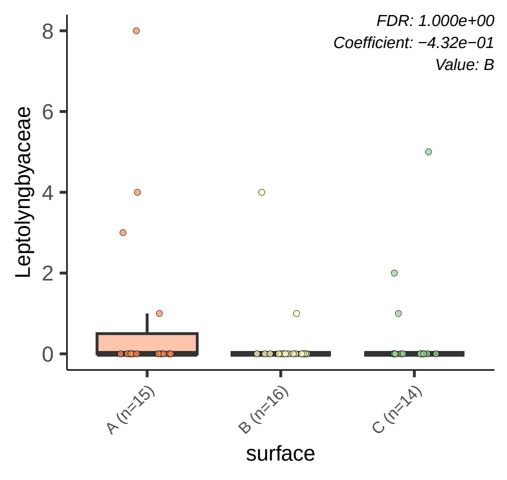

FDR: 1.000e+00
Coefficient: −4.32e−01
Value: B
Leptolyngbyaceae
0
2
4
6
8
A (n=15)
B (n=16)
C (n=14)
surface

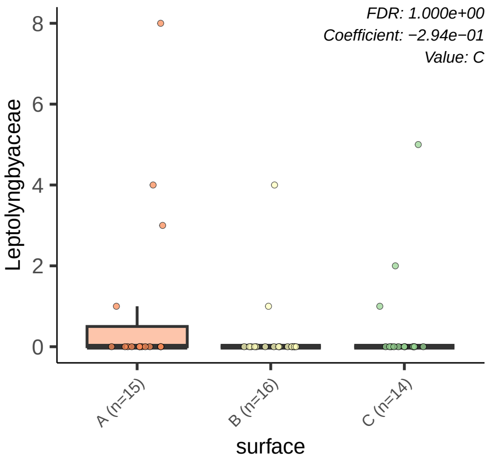

FDR: 1.000e+00
Coefficient: −2.94e−01
Value: C
Leptolyngbyaceae
0
2
4
6
8
A (n=15)
B (n=16)
C (n=14)
surface

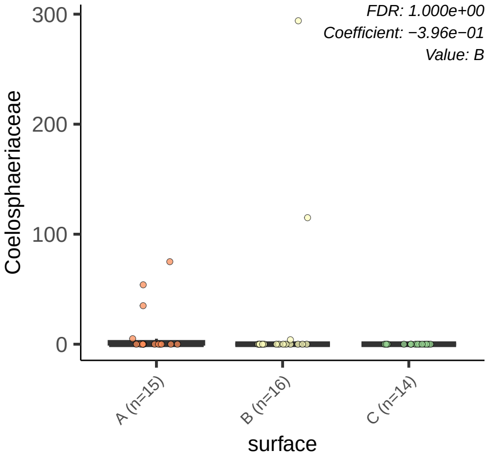

FDR: 1.000e+00
Coefficient: −3.96e−01
Value: B
Coelosphaeriaceae
300
200
100
0
A (n=15)
B (n=16)
C (n=14)
surface

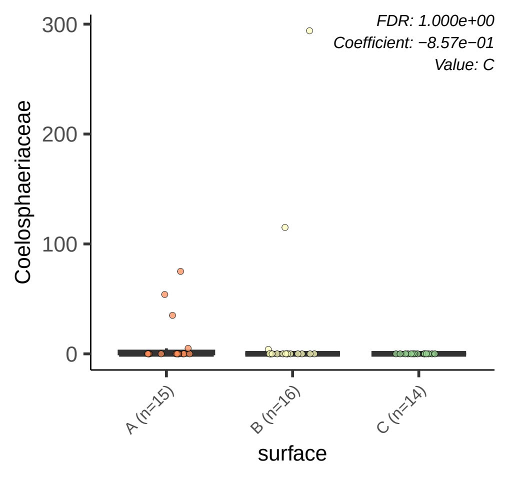
FDR: 1.000e+00
Coefficient: −8.57e−01
Value: C
Coelosphaeriaceae
300
200
100
0
A (n=15)
B (n=16)
C (n=14)
surface

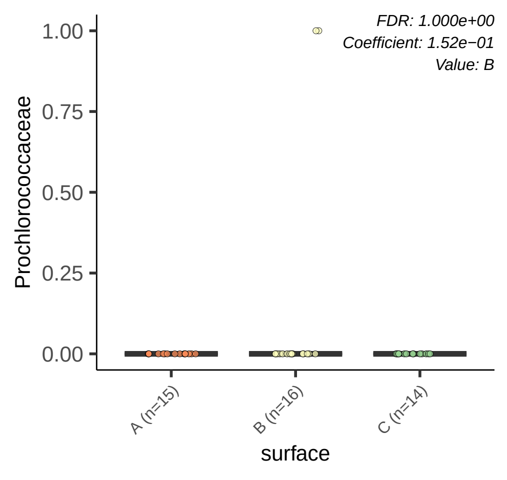
FDR: 1.000e+00
Coefficient: 1.52e−01
Value: B
Prochlorococcaceae
1.00
0.75
0.50
0.25
0.00
A (n=15)
B (n=16)
C (n=14)
surface

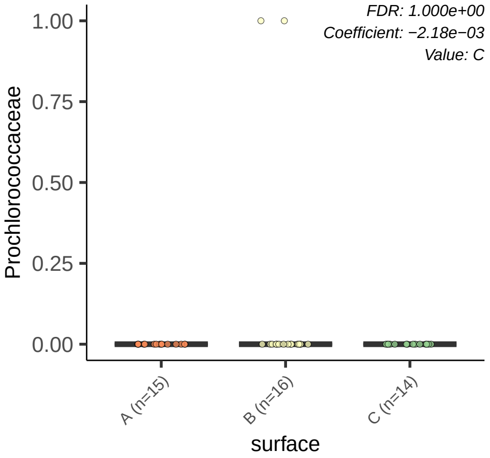
FDR: 1.000e+00
Coefficient: −2.18e−03
Value: C
Prochlorococcaceae
1.00
0.75
0.50
0.25
0.00
A (n=15)
B (n=16)
C (n=14)
surface

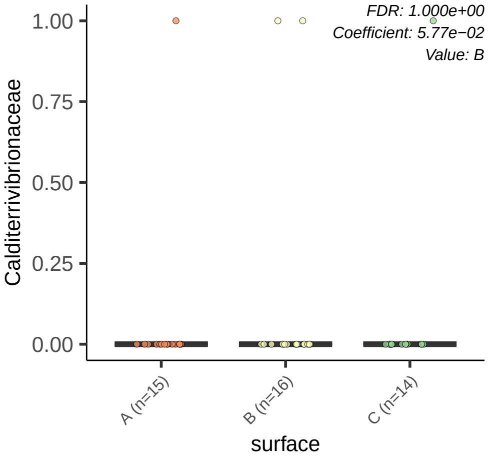

FDR: 1.000e+00
Coefficient: 5.77e−02
Value: B
1.00
0.75
0.50
0.25
0.00
Calditerrivibrionaceae
A (n=15)
B (n=16)
C (n=14)
surface

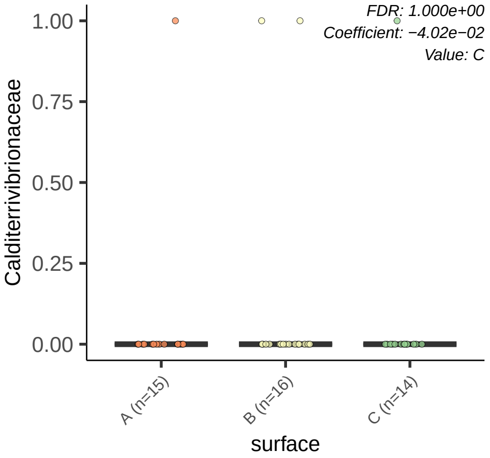

FDR: 1.000e+00
Coefficient: −4.02e−02
Value: C
Calditerrivibrionaceae
1.00
0.75
0.50
0.25
0.00
A (n=15)
B (n=16)
C (n=14)
surface

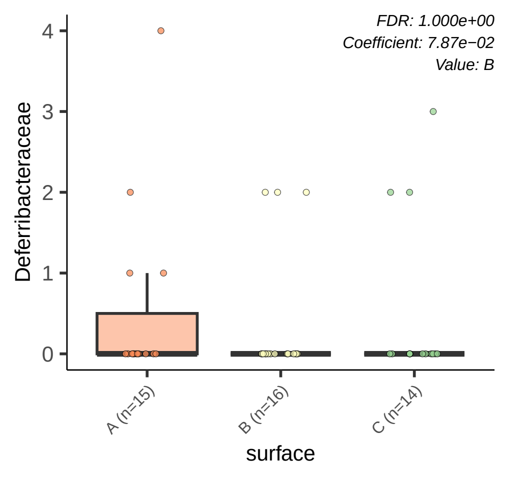

FDR: 1.000e+00
Coefficient: 7.87e−02
Value: B
Deferribacteraceae
4
3
2
1
0
A (n=15)
B (n=16)
C (n=14)
surface

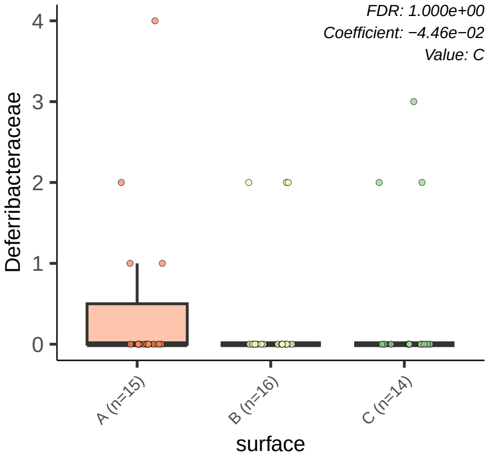
FDR: 1.000e+00
Coefficient: −4.46e−02
Value: C
Deferribacteraceae
0
1
2
3
4
A (n=15)
B (n=16)
C (n=14)
surface

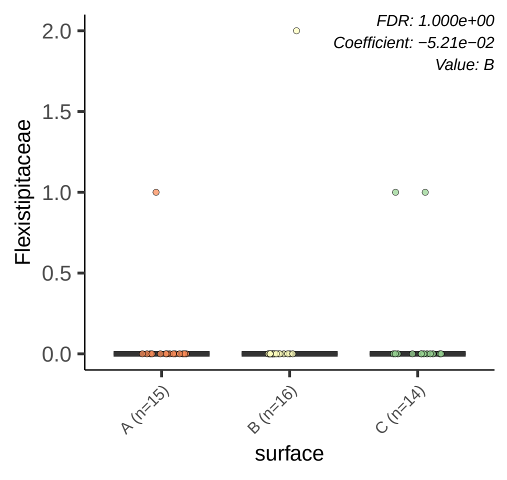
FDR: 1.000e+00
Coefficient: −5.21e−02
Value: B
Flexistipitaceae
2.0
1.5
1.0
0.5
0.0
A (n=15)
B (n=16)
C (n=14)
surface

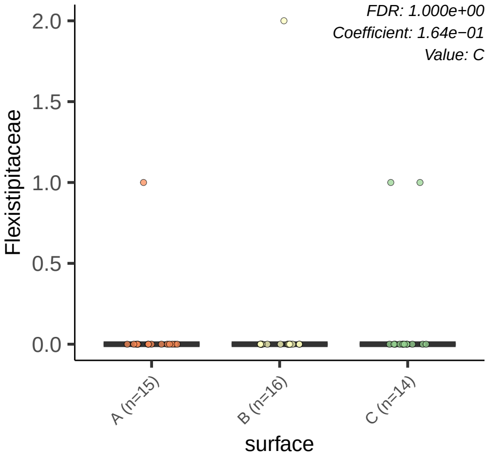
FDR: 1.000e+00
Coefficient: 1.64e−01
Value: C
Flexistipitaceae
2.0
1.5
1.0
0.5
0.0
A (n=15)
B (n=16)
C (n=14)
surface

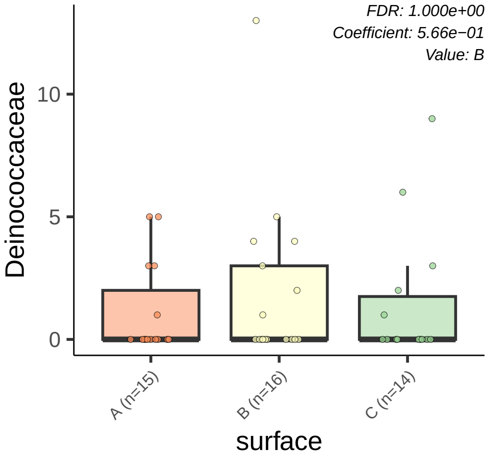
FDR: 1.000e+00
Coefficient: 5.66e−01
Value: B
Deinococcaceae
10
5
0
A (n=15)
B (n=16)
C (n=14)
surface

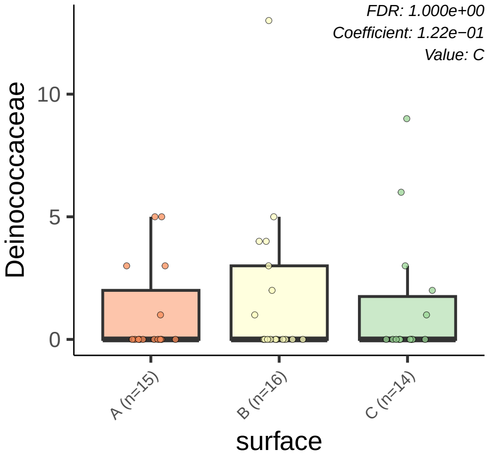
FDR: 1.000e+00
Coefficient: 1.22e−01
Value: C
Deinococcaceae
10
5
0
A (n=15)
B (n=16)
C (n=14)
surface

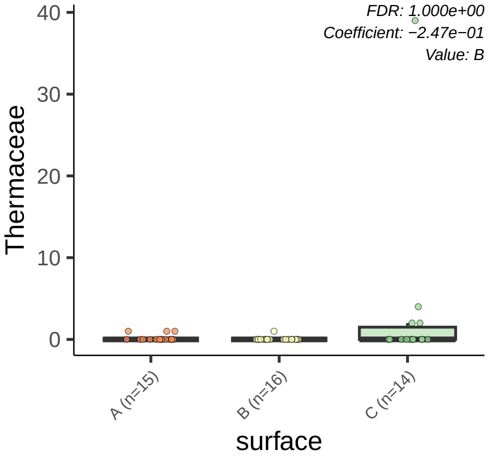
FDR: 1.000e+00
Coefficient: −2.47e−01
Value: B
Thermaceae
40
30
20
10
0
A (n=15)
B (n=16)
C (n=14)
surface

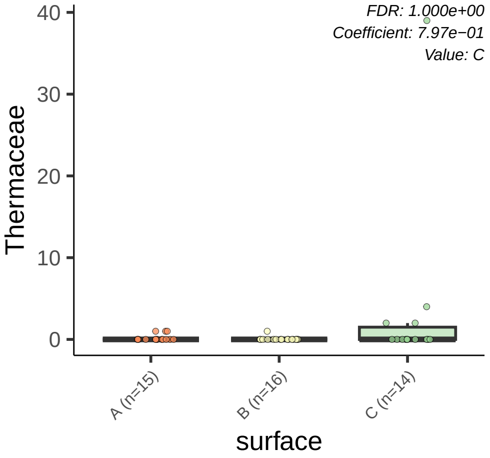
FDR: 1.000e+00
Coefficient: 7.97e−01
Value: C
Thermaceae
40
30
20
10
0
A (n=15)
B (n=16)
C (n=14)
surface

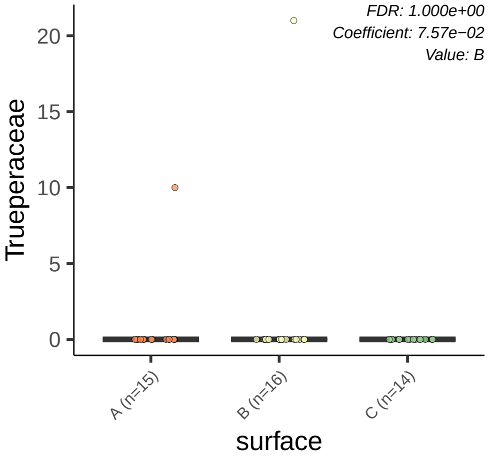
FDR: 1.000e+00
Coefficient: 7.57e−02
Value: B
Trueperaceae
0
5
10
15
20
A (n=15)
B (n=16)
C (n=14)
surface

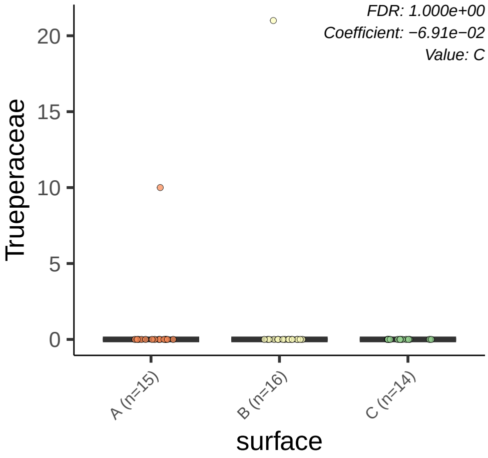

FDR: 1.000e+00
Coefficient: −6.91e−02
Value: C
Trueperaceae
0
5
10
15
20
A (n=15)
B (n=16)
C (n=14)
surface

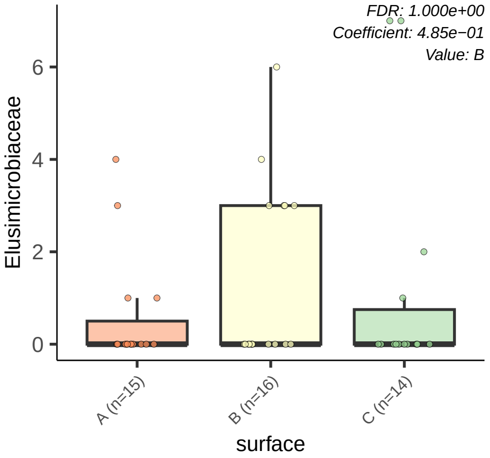

FDR: 1.000e+00
Coefficient: 4.85e−01
Value: B
Elusimicrobiaceae
0
2
4
6
A (n=15)
B (n=16)
C (n=14)
surface

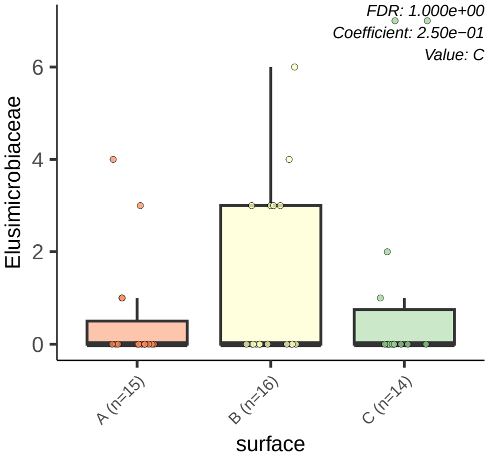

FDR: 1.000e+00
Coefficient: 2.50e−01
Value: C
Elusimicrobiaceae
0
2
4
6
A (n=15)
B (n=16)
C (n=14)
surface

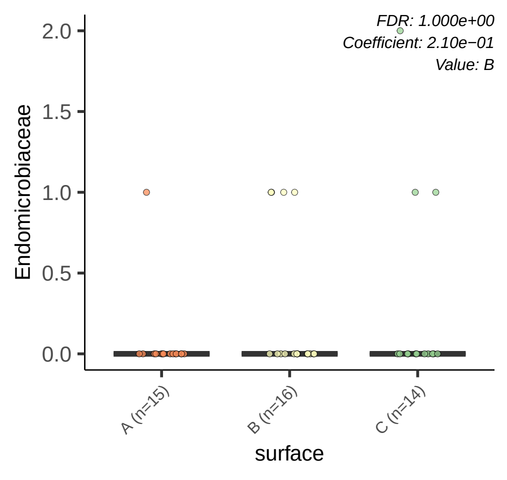

FDR: 1.000e+00
Coefficient: 2.10e−01
Value: B
Endomicrobiaceae
2.0
1.5
1.0
0.5
0.0
A (n=15)
B (n=16)
C (n=14)
surface

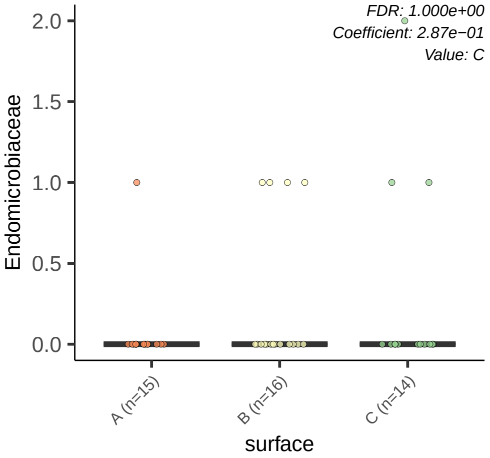

FDR: 1.000e+00
Coefficient: 2.87e−01
Value: C
Endomicrobiaceae
2.0
1.5
1.0
0.5
0.0
A (n=15)
B (n=16)
C (n=14)
surface

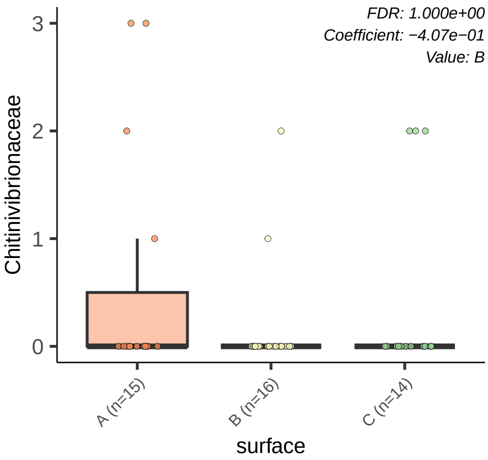

FDR: 1.000e+00
Coefficient: −4.07e−01
Value: B
Chitinivibrionaceae
3
2
1
0
A (n=15)
B (n=16)
C (n=14)
surface

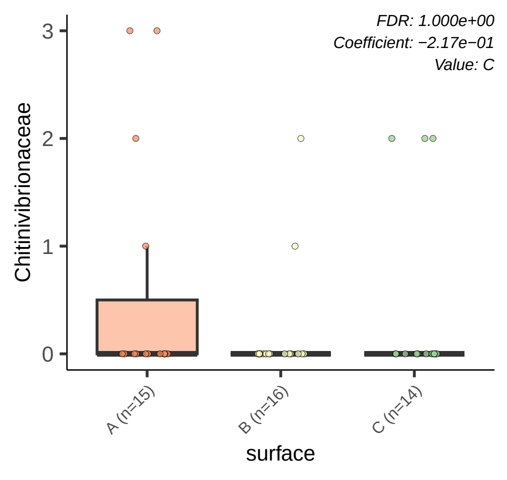
FDR: 1.000e+00
Coefficient: −2.17e−01
Value: C
Chitinivibrionaceae
3
2
1
0
A (n=15)
B (n=16)
C (n=14)
surface

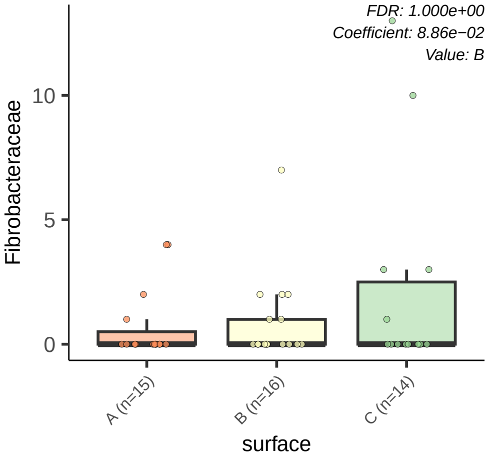

FDR: 1.000e+00
Coefficient: 8.86e−02
Value: B
Fibrobacteraceae
0
5
10
A (n=15)
B (n=16)
C (n=14)
surface

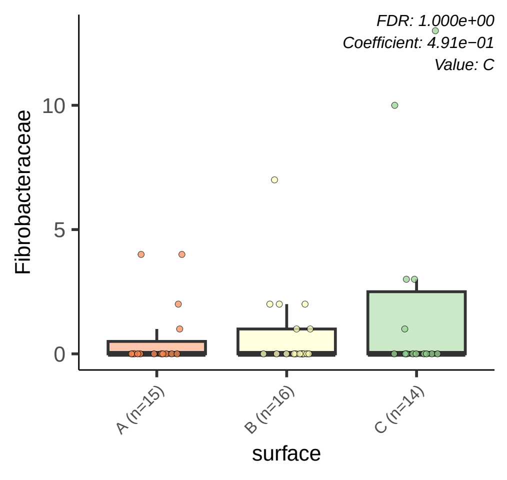

FDR: 1.000e+00
Coefficient: 4.91e−01
Value: C
Fibrobacteraceae
10
5
0
A (n=15)
B (n=16)
C (n=14)
surface

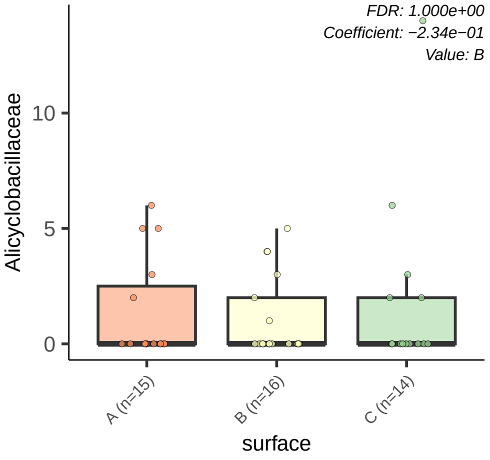
FDR: 1.000e+00
Coefficient: −2.34e−01
Value: B
Alicyclobacillaceae
10
5
0
A (n=15)
B (n=16)
C (n=14)
surface

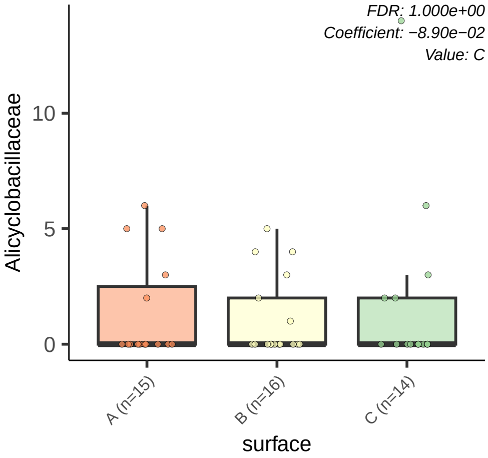

FDR: 1.000e+00
Coefficient: −8.90e−02
Value: C
Alicyclobacillaceae
10
5
0
A (n=15)
B (n=16)
C (n=14)
surface

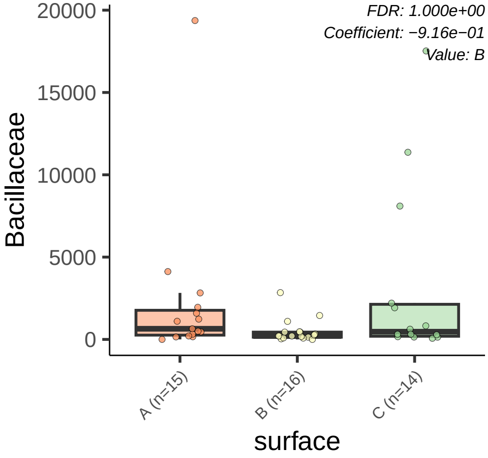

FDR: 1.000e+00
Coefficient: −9.16e−01
Value: B
Bacillaceae
20000
15000
10000
5000
0
A (n=15)
B (n=16)
C (n=14)
surface

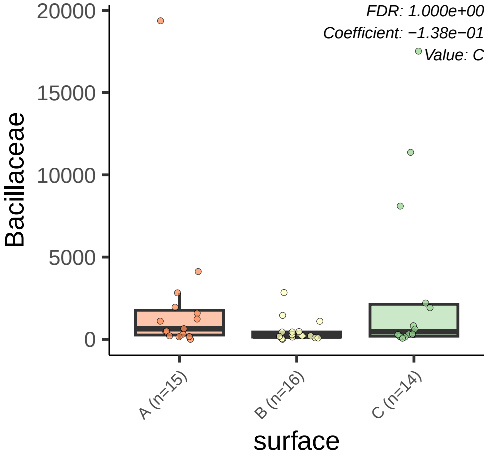

FDR: 1.000e+00
Coefficient: −1.38e−01
Value: C
Bacillaceae
20000
15000
10000
5000
0
A (n=15)
B (n=16)
C (n=14)
surface

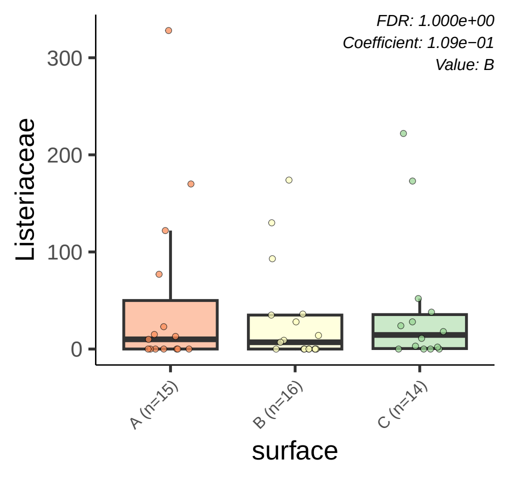
FDR: 1.000e+00
Coefficient: 1.09e−01
Value: B
Listeriaceae
300
200
100
0
A (n=15)
B (n=16)
C (n=14)
surface

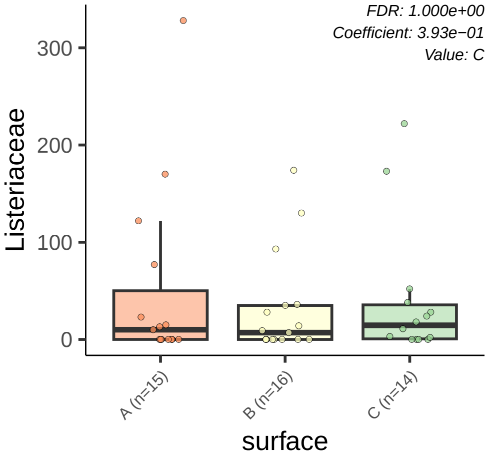

FDR: 1.000e+00
Coefficient: 3.93e−01
Value: C
Listeriaceae
300
200
100
0
A (n=15)
B (n=16)
C (n=14)
surface

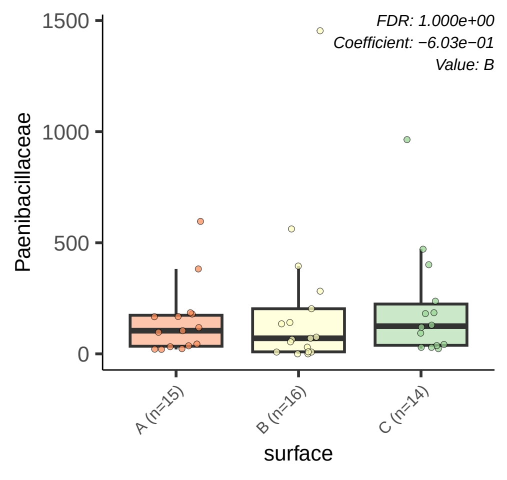
FDR: 1.000e+00
Coefficient: −6.03e−01
Value: B
Paenibacillaceae
1500
1000
500
0
A (n=15)
B (n=16)
C (n=14)
surface

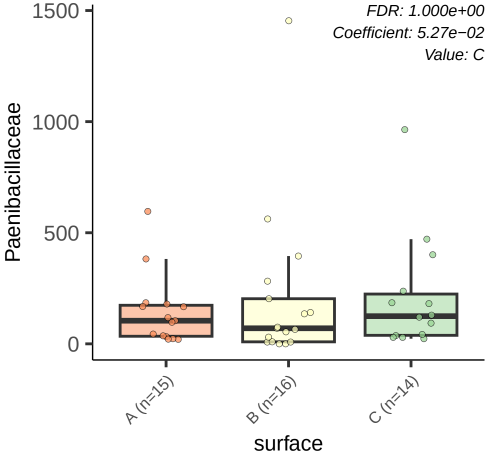

FDR: 1.000e+00
Coefficient: 5.27e−02
Value: C
Paenibacillaceae
1500
1000
500
0
A (n=15)
B (n=16)
C (n=14)
surface

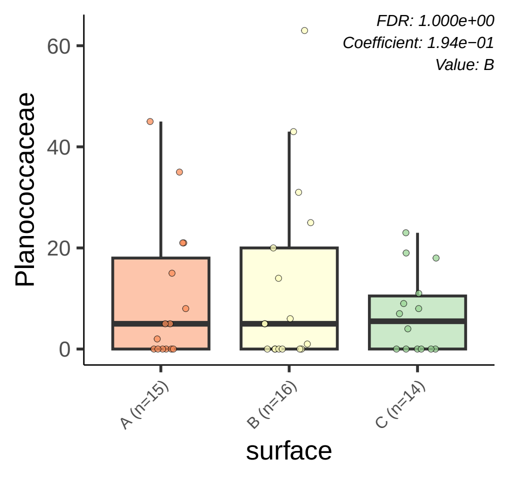
FDR: 1.000e+00
Coefficient: 1.94e−01
Value: B
Planococcaceae
60
40
20
0
A (n=15)
B (n=16)
C (n=14)
surface

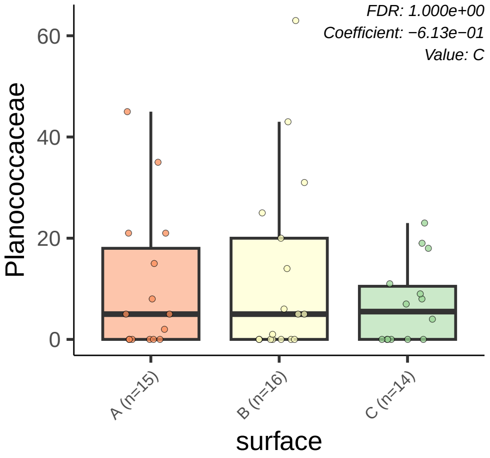

FDR: 1.000e+00
Coefficient: −6.13e−01
Value: C
Planococcaceae
0
20
40
60
A (n=15)
B (n=16)
C (n=14)
surface

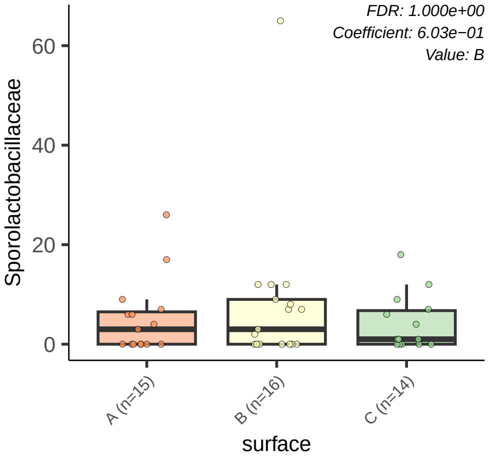

FDR: 1.000e+00
Coefficient: 6.03e−01
Value: B
Sporolactobacillaceae
0
20
40
60
A (n=15)
B (n=16)
C (n=14)
surface

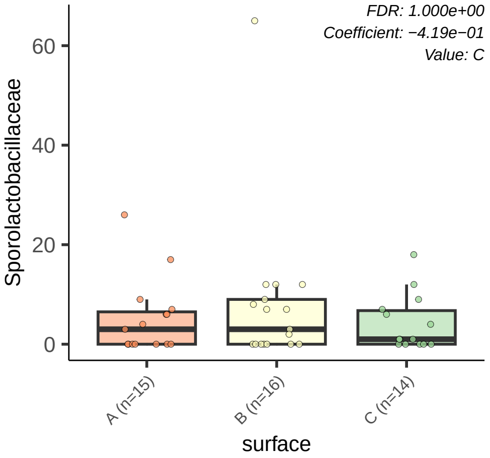
FDR: 1.000e+00
Coefficient: −4.19e−01
Value: C
Sporolactobacillaceae
0
20
40
60
A (n=15)
B (n=16)
C (n=14)
surface

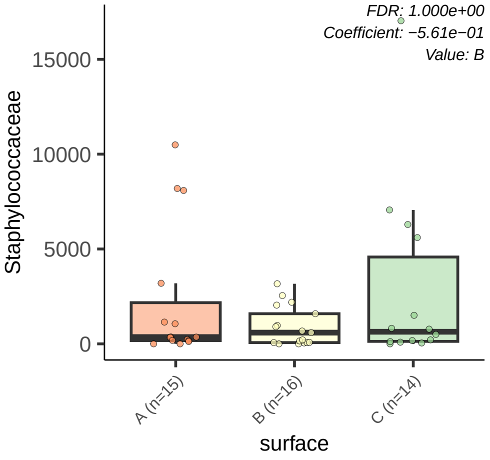
FDR: 1.000e+00
Coefficient: −5.61e−01
Value: B
Staphylococcaceae
15000
10000
5000
0
A (n=15)
B (n=16)
C (n=14)
surface

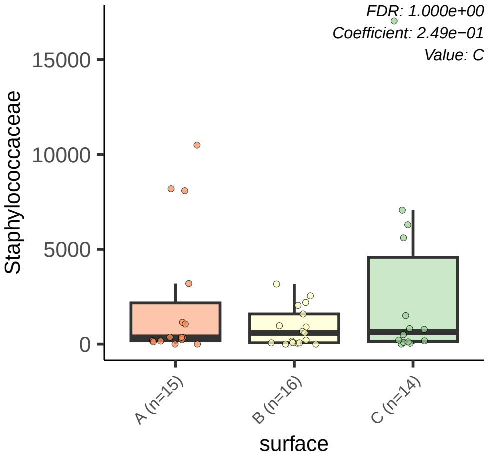

FDR: 1.000e+00
Coefficient: 2.49e−01
Value: C
Staphylococcaceae
15000
10000
5000
0
A (n=15)
B (n=16)
C (n=14)
surface

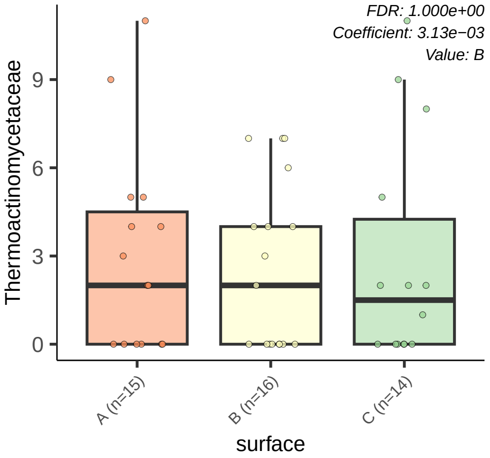

FDR: 1.000e+00
Coefficient: 3.13e−03
Value: B
Thermoactinomycetaceae
0
3
6
9
A (n=15)
B (n=16)
C (n=14)
surface

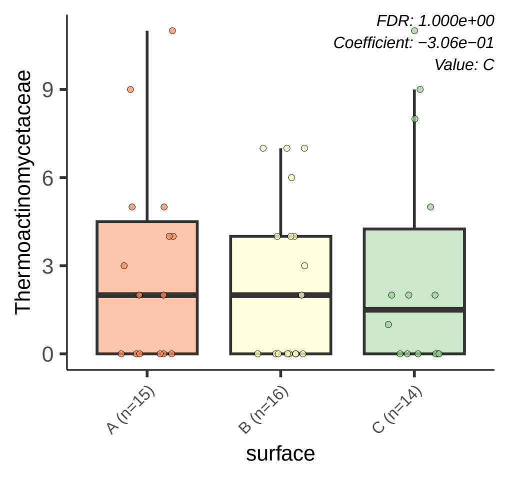

FDR: 1.000e+00
Coefficient: −3.06e−01
Value: C
Thermoactinomycetaceae
0
3
6
9
A (n=15)
B (n=16)
C (n=14)
surface

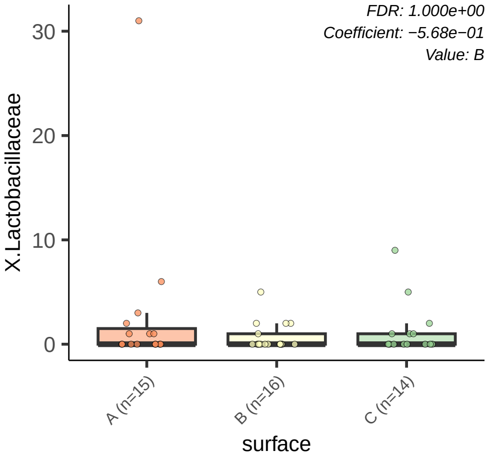

FDR: 1.000e+00
Coefficient: −5.68e−01
Value: B
X.Lactobacillaceae
30
20
10
0
A (n=15)
B (n=16)
C (n=14)
surface

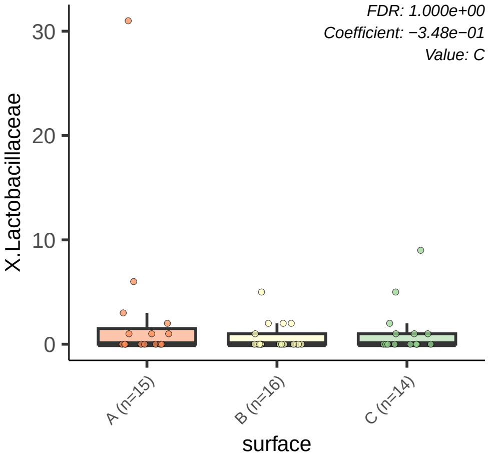

FDR: 1.000e+00
Coefficient: −3.48e−01
Value: C
X.Lactobacillaceae
30
20
10
0
A (n=15)
B (n=16)
C (n=14)
surface

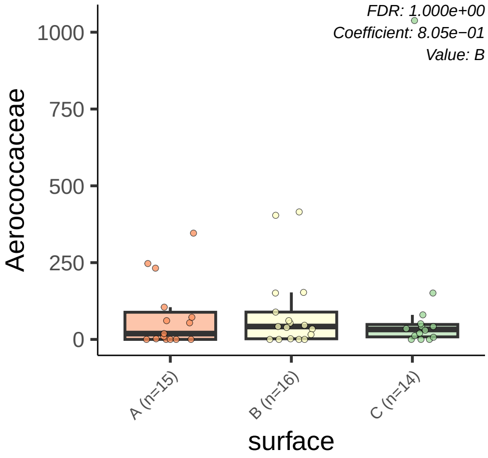
FDR: 1.000e+00
Coefficient: 8.05e−01
Value: B
Aerococcaceae
1000
750
500
250
0
A (n=15)
B (n=16)
C (n=14)
surface

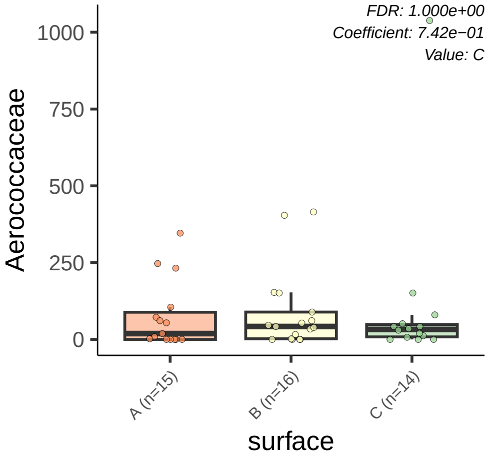
FDR: 1.000e+00
Coefficient: 7.42e−01
Value: C
Aerococcaceae
1000
750
500
250
0
A (n=15)
B (n=16)
C (n=14)
surface

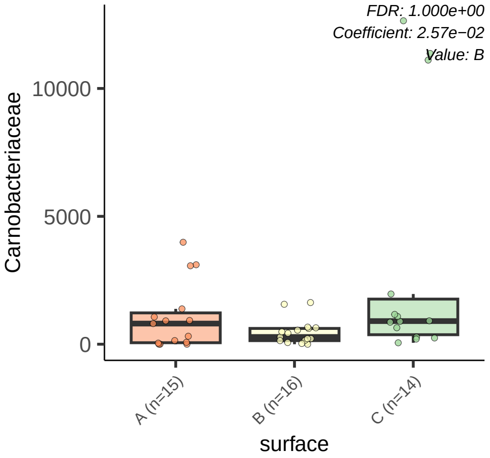
FDR: 1.000e+00
Coefficient: 2.57e−02
Value: B
Carnobacteriaceae
10000
5000
0
A (n=15)
B (n=16)
C (n=14)
surface

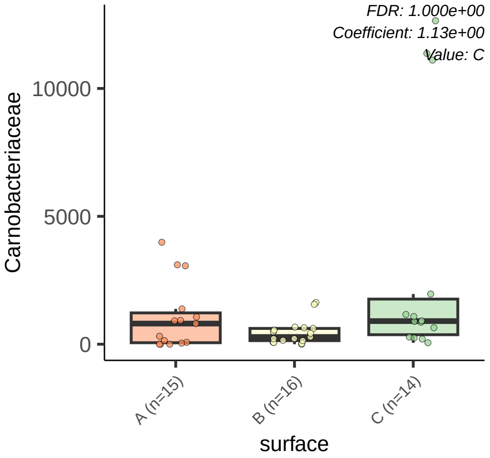

FDR: 1.000e+00
Coefficient: 1.13e+00
Value: C
Carnobacteriaceae
10000
5000
0
A (n=15)
B (n=16)
C (n=14)
surface

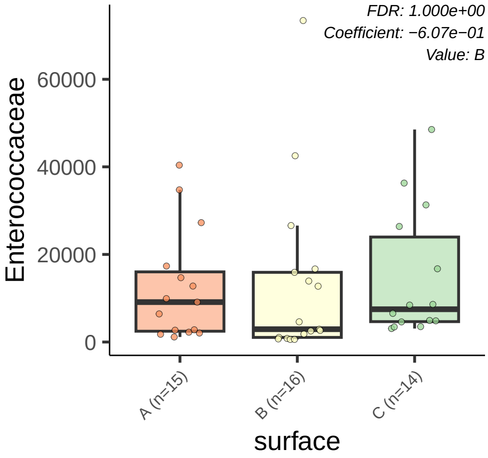

FDR: 1.000e+00
Coefficient: −6.07e−01
Value: B
Enterococcaceae
60000
40000
20000
0
A (n=15)
B (n=16)
C (n=14)
surface

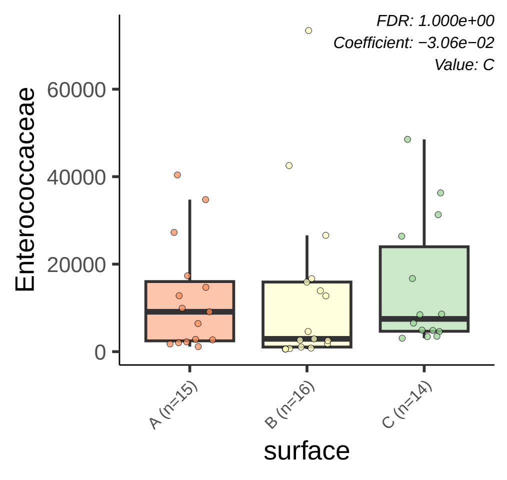
FDR: 1.000e+00
Coefficient: −3.06e−02
Value: C
Enterococcaceae
60000
40000
20000
0
A (n=15)
B (n=16)
C (n=14)
surface

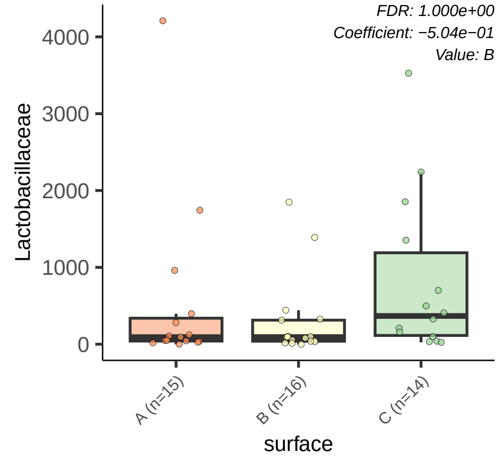

FDR: 1.000e+00
Coefficient: −5.04e−01
Value: B
Lactobacillaceae
4000
3000
2000
1000
0
A (n=15)
B (n=16)
C (n=14)
surface

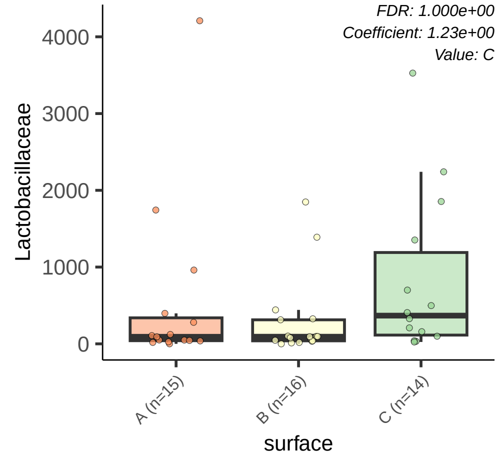

FDR: 1.000e+00
Coefficient: 1.23e+00
Value: C
Lactobacillaceae
4000
3000
2000
1000
0
A (n=15)
B (n=16)
C (n=14)
surface

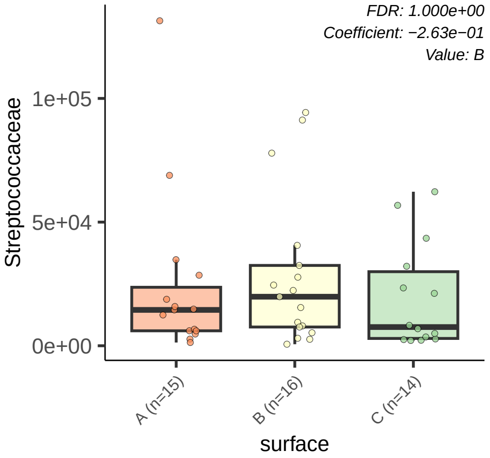

FDR: 1.000e+00
Coefficient: −2.63e−01
Value: B
Streptococcaceae
1e+05
5e+04
0e+00
A (n=15)
B (n=16)
C (n=14)
surface

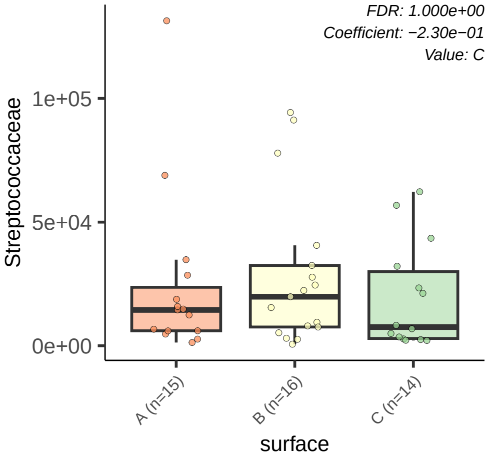

FDR: 1.000e+00
Coefficient: −2.30e−01
Value: C
Streptococcaceae
1e+05
5e+04
0e+00
A (n=15)
B (n=16)
C (n=14)
surface

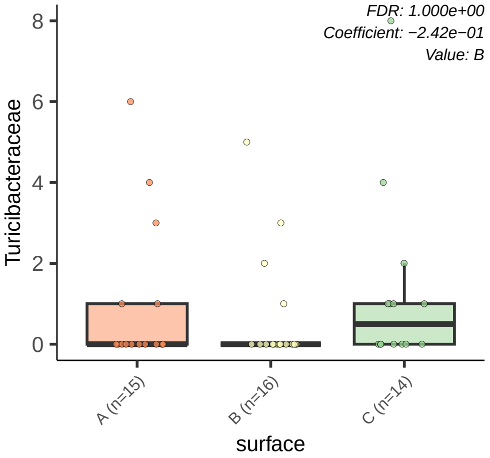

FDR: 1.000e+00
Coefficient: −2.42e−01
Value: B
Turicibacteraceae
0
2
4
6
8
A (n=15)
B (n=16)
C (n=14)
surface

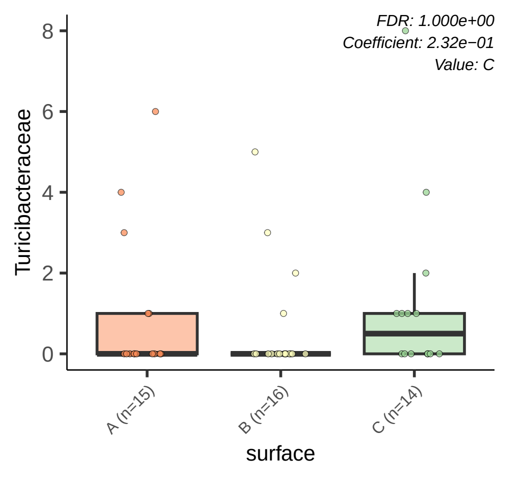
FDR: 1.000e+00
Coefficient: 2.32e−01
Value: C
Turicibacteraceae
0
2
4
6
8
A (n=15)
B (n=16)
C (n=14)
surface

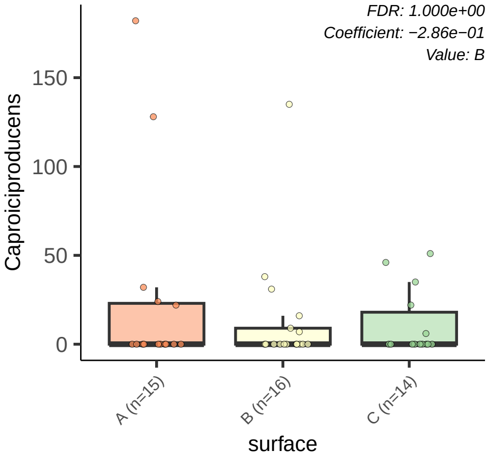
FDR: 1.000e+00
Coefficient: −2.86e−01
Value: B
Caproiciproducens
150
100
50
0
A (n=15)
B (n=16)
C (n=14)
surface

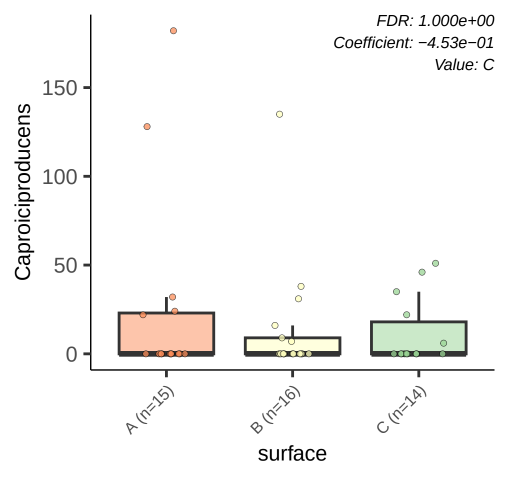
FDR: 1.000e+00
Coefficient: −4.53e−01
Value: C
Caproiciproducens
150
100
50
0
A (n=15)
B (n=16)
C (n=14)
surface

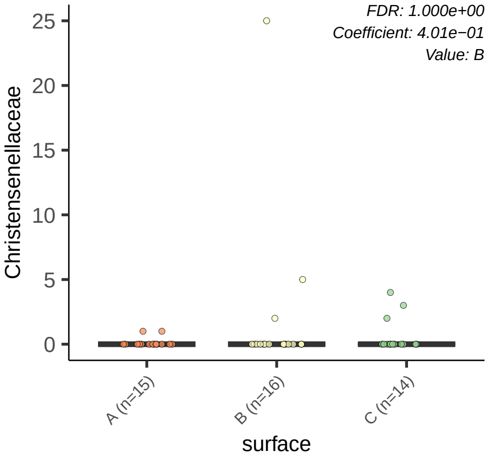

FDR: 1.000e+00
Coefficient: 4.01e−01
Value: B
Christensenellaceae
25
20
15
10
5
0
A (n=15)
B (n=16)
C (n=14)
surface

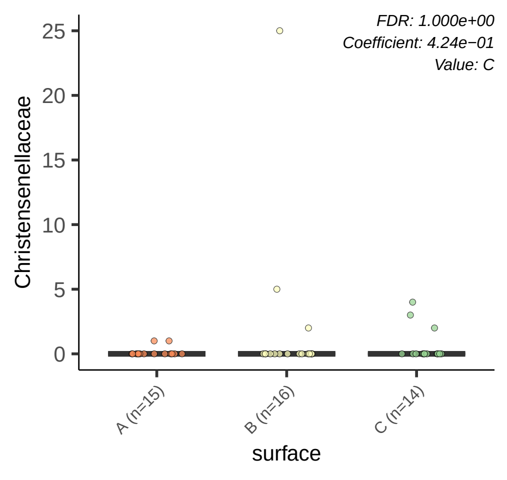
FDR: 1.000e+00
Coefficient: 4.24e−01
Value: C
Christensenellaceae
0
5
10
15
20
25
A (n=15)
B (n=16)
C (n=14)
surface

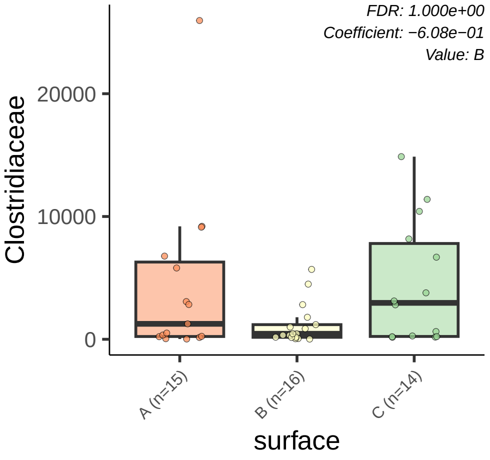
FDR: 1.000e+00
Coefficient: −6.08e−01
Value: B
Clostridiaceae
20000
10000
0
A (n=15)
B (n=16)
C (n=14)
surface

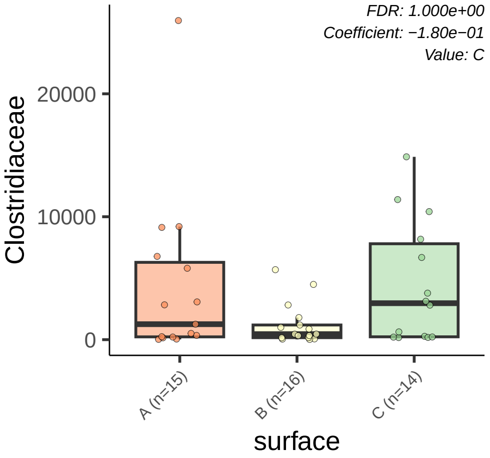
FDR: 1.000e+00
Coefficient: −1.80e−01
Value: C
Clostridiaceae
20000
10000
0
A (n=15)
B (n=16)
C (n=14)
surface

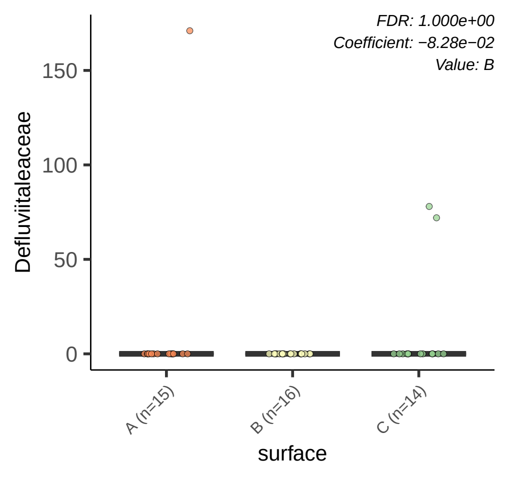
FDR: 1.000e+00
Coefficient: −8.28e−02
Value: B
Defluviitaleaceae
150
100
50
0
A (n=15)
B (n=16)
C (n=14)
surface

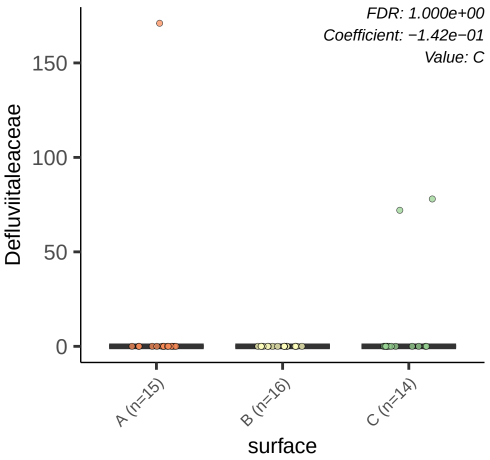
FDR: 1.000e+00
Coefficient: −1.42e−01
Value: C
Defluviitaleaceae
150
100
50
0
A (n=15)
B (n=16)
C (n=14)
surface

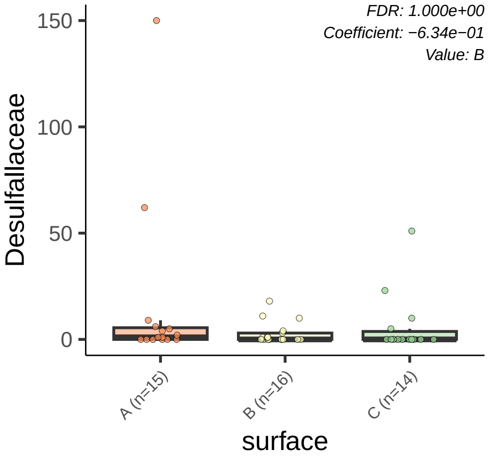
FDR: 1.000e+00
Coefficient: −6.34e−01
Value: B
Desulfallaceae
150
100
50
0
A (n=15)
B (n=16)
C (n=14)
surface

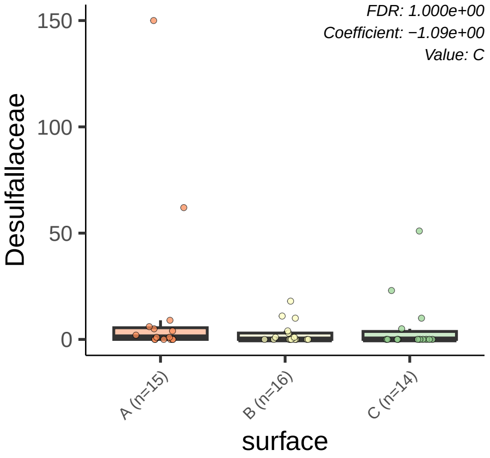
FDR: 1.000e+00
Coefficient: −1.09e+00
Value: C
Desulfallaceae
150
100
50
0
A (n=15)
B (n=16)
C (n=14)
surface

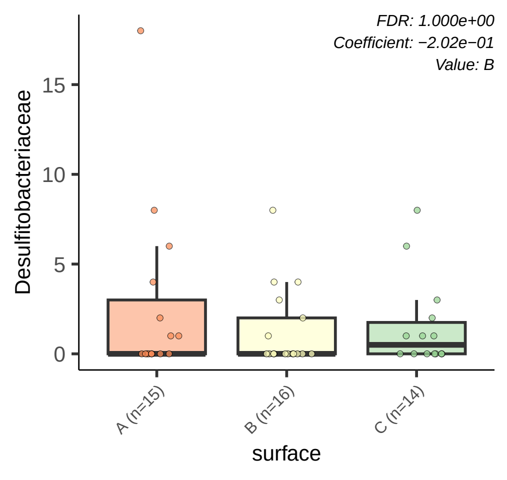

FDR: 1.000e+00
Coefficient: −2.02e−01
Value: B
Desulfitobacteriaceae
0
5
10
15
A (n=15)
B (n=16)
C (n=14)
surface

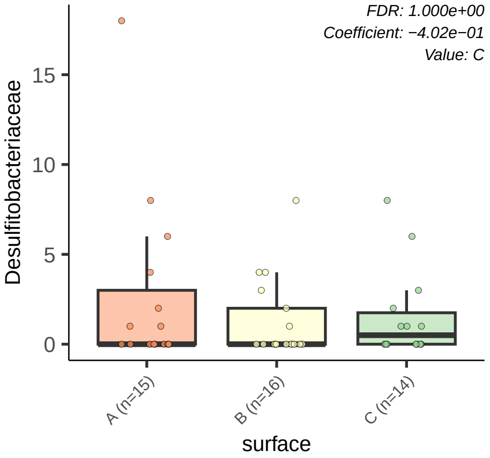
FDR: 1.000e+00
Coefficient: −4.02e−01
Value: C
Desulfitobacteriaceae
0
5
10
15
A (n=15)
B (n=16)
C (n=14)
surface

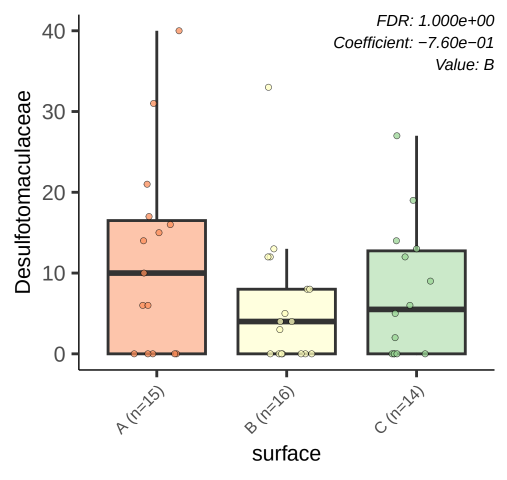
FDR: 1.000e+00
Coefficient: −7.60e−01
Value: B
Desulfotomaculaceae
0
10
20
30
40
A (n=15)
B (n=16)
C (n=14)
surface

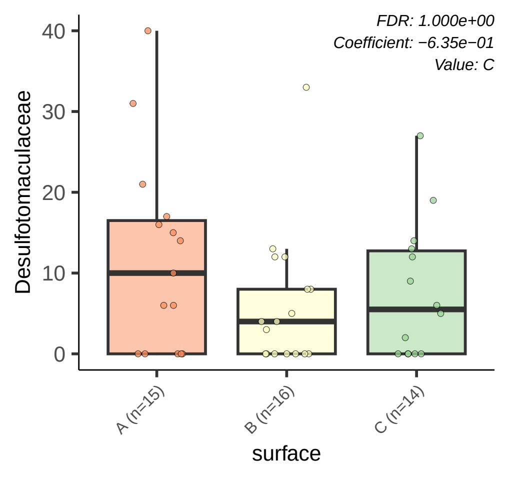

FDR: 1.000e+00
Coefficient: −6.35e−01
Value: C
Desulfotomaculaceae
40
30
20
10
0
A (n=15)
B (n=16)
C (n=14)
surface

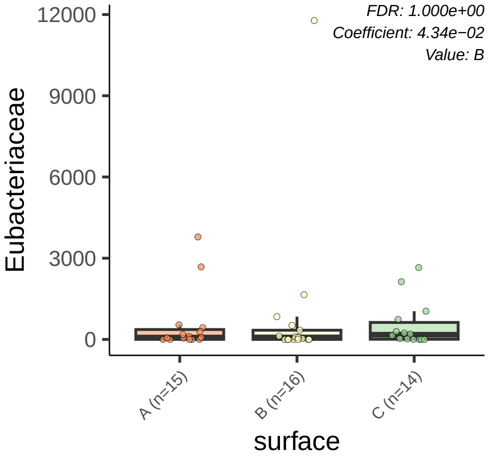
FDR: 1.000e+00
Coefficient: 4.34e−02
Value: B
Eubacteriaceae
12000
9000
6000
3000
0
A (n=15)
B (n=16)
C (n=14)
surface

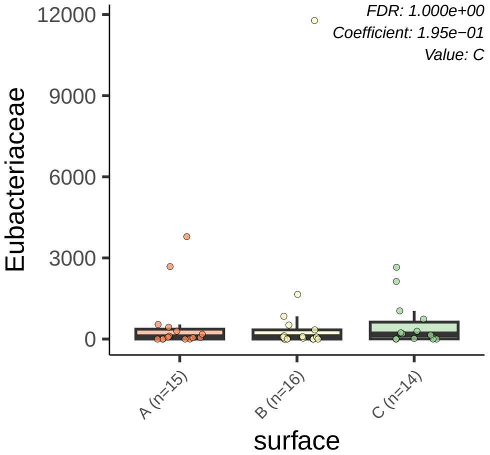
FDR: 1.000e+00
Coefficient: 1.95e−01
Value: C
Eubacteriaceae
12000
9000
6000
3000
0
A (n=15)
B (n=16)
C (n=14)
surface

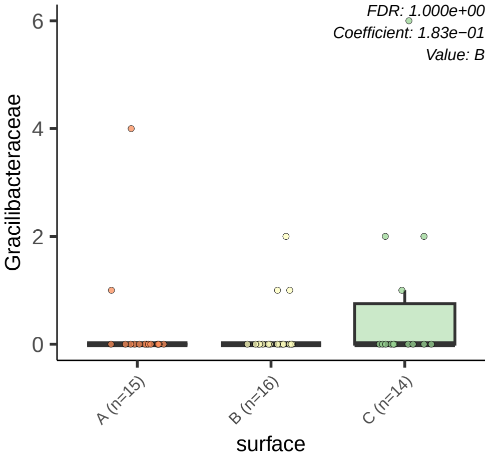
FDR: 1.000e+00
Coefficient: 1.83e−01
Value: B
Gracilibacteraceae
0
2
4
6
A (n=15)
B (n=16)
C (n=14)
surface

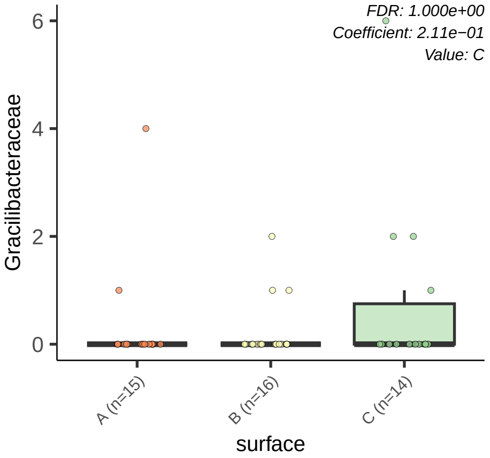

FDR: 1.000e+00
Coefficient: 2.11e−01
Value: C
Gracilibacteraceae
0
2
4
6
A (n=15)
B (n=16)
C (n=14)
surface

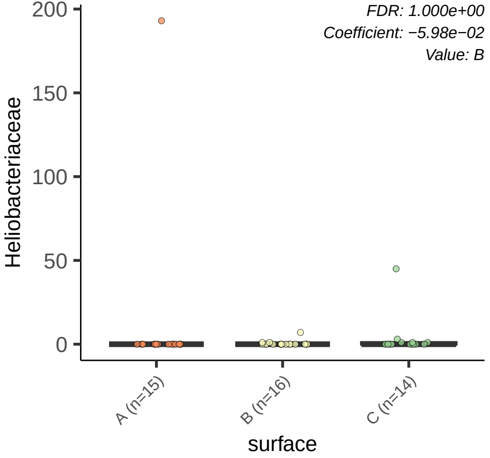

FDR: 1.000e+00
Coefficient: −5.98e−02
Value: B
Heliobacteriaceae
200
150
100
50
0
A (n=15)
B (n=16)
C (n=14)
surface

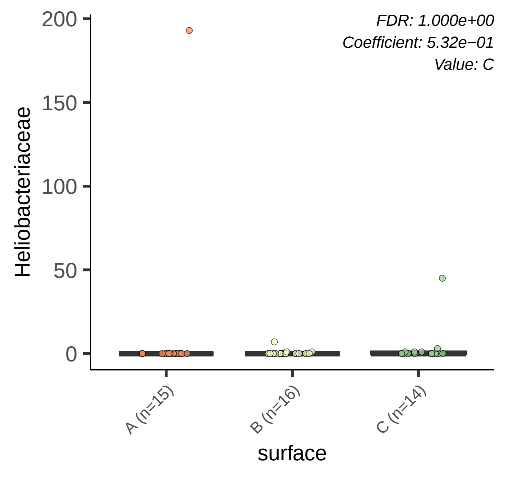
FDR: 1.000e+00
Coefficient: 5.32e−01
Value: C
Heliobacteriaceae
200
150
100
50
0
A (n=15)
B (n=16)
C (n=14)
surface

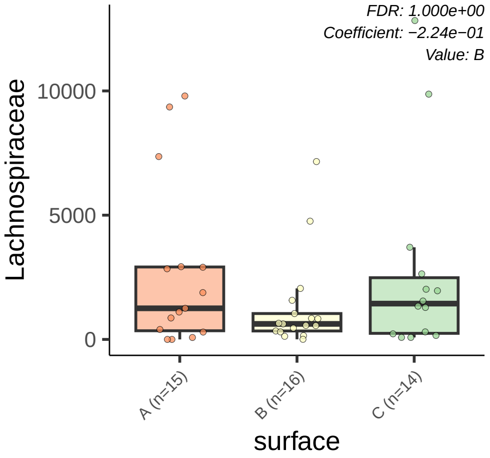
FDR: 1.000e+00
Coefficient: −2.24e−01
Value: B
Lachnospiraceae
10000
5000
0
A (n=15)
B (n=16)
C (n=14)
surface

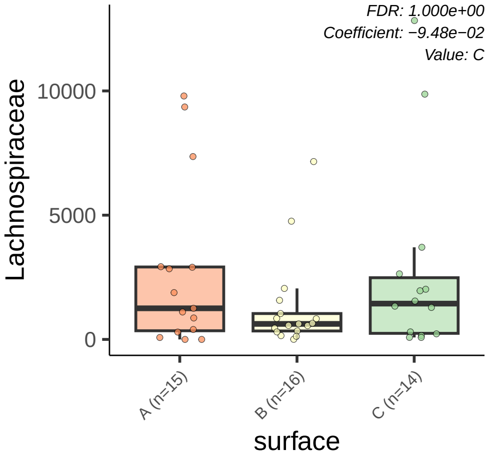

FDR: 1.000e+00
Coefficient: −9.48e−02
Value: C
Lachnospiraceae
10000
5000
0
A (n=15)
B (n=16)
C (n=14)
surface

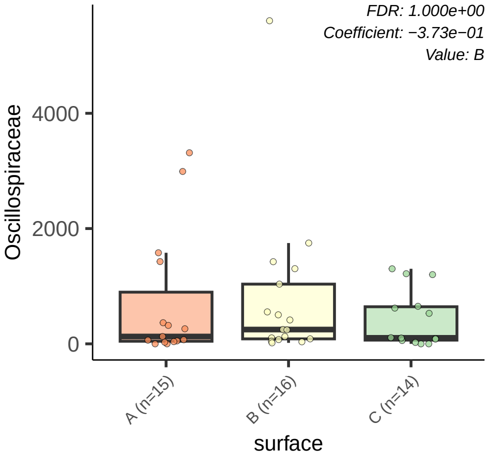
FDR: 1.000e+00
Coefficient: −3.73e−01
Value: B
Oscillospiraceae
4000
2000
0
A (n=15)
B (n=16)
C (n=14)
surface

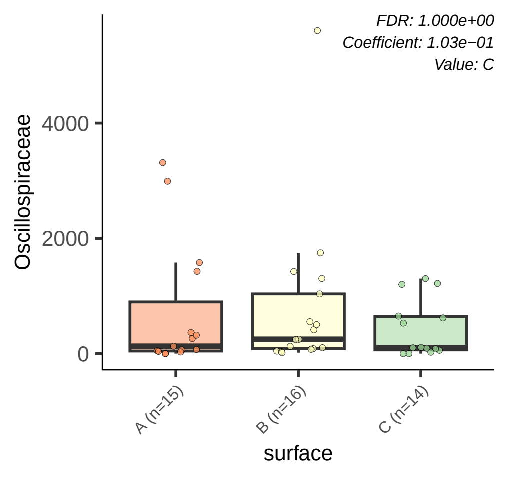
FDR: 1.000e+00
Coefficient: 1.03e−01
Value: C
Oscillospiraceae
4000
2000
0
A (n=15)
B (n=16)
C (n=14)
surface

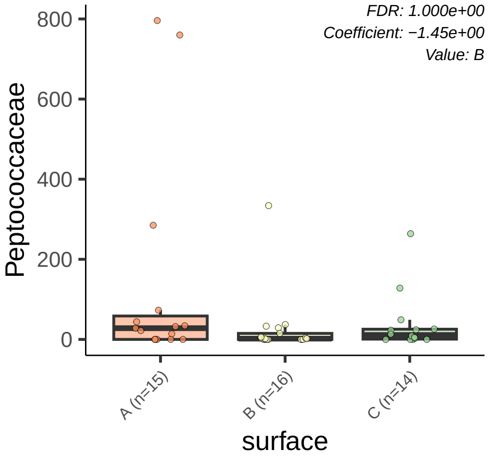

FDR: 1.000e+00
Coefficient: −1.45e+00
Value: B
Peptococcaceae
800
600
400
200
0
A (n=15)
B (n=16)
C (n=14)
surface

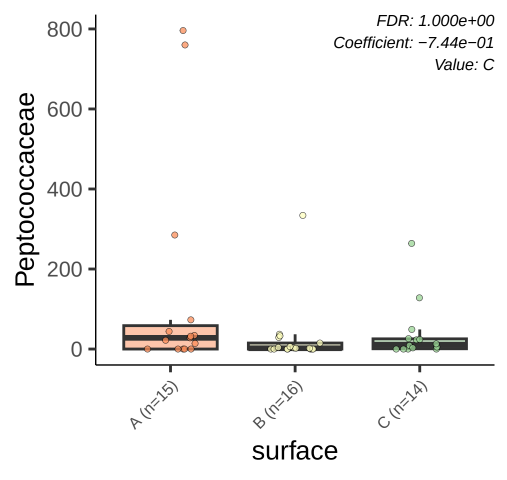
FDR: 1.000e+00
Coefficient: −7.44e−01
Value: C
Peptococcaceae
800
600
400
200
0
A (n=15)
B (n=16)
C (n=14)
surface

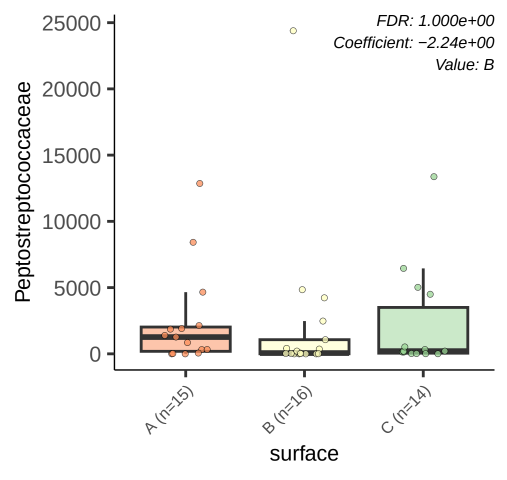

FDR: 1.000e+00
Coefficient: −2.24e+00
Value: B
Peptostreptococcaceae
25000
20000
15000
10000
5000
0
A (n=15)
B (n=16)
C (n=14)
surface

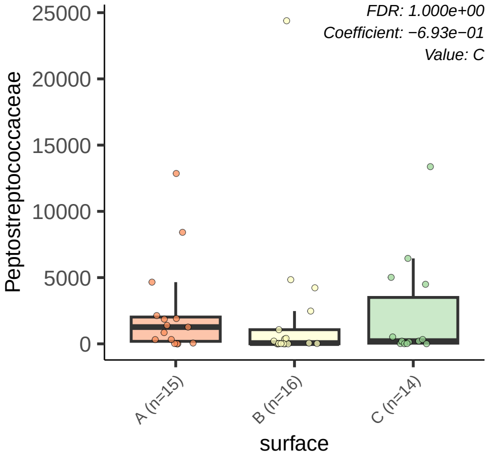
FDR: 1.000e+00
Coefficient: −6.93e−01
Value: C
Peptostreptococcaceae
25000
20000
15000
10000
5000
0
A (n=15)
B (n=16)
C (n=14)
surface

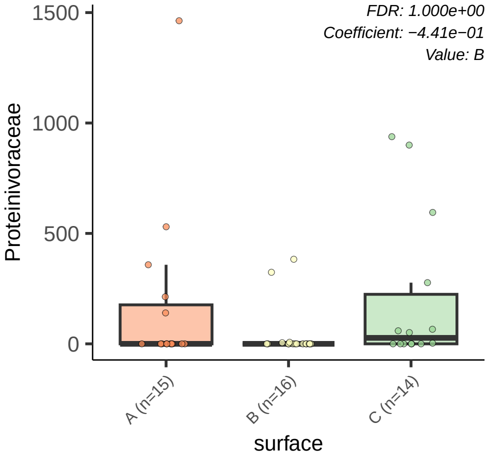
FDR: 1.000e+00
Coefficient: −4.41e−01
Value: B
Proteinivoraceae
1500
1000
500
0
A (n=15)
B (n=16)
C (n=14)
surface

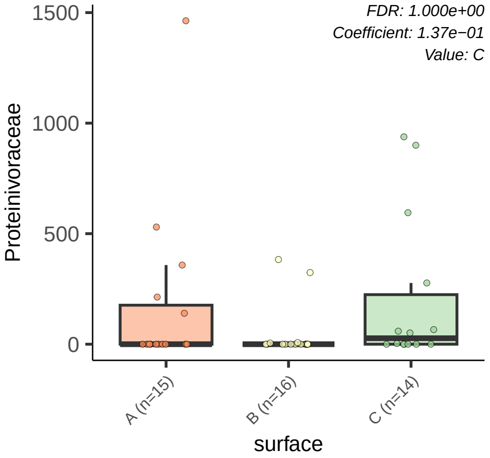

FDR: 1.000e+00
Coefficient: 1.37e−01
Value: C
Proteinivoraceae
1500
1000
500
0
A (n=15)
B (n=16)
C (n=14)
surface

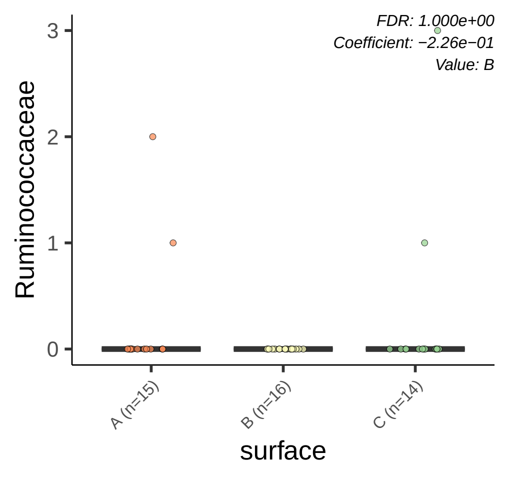

FDR: 1.000e+00
Coefficient: −2.26e−01
Value: B
Ruminococcaceae
0
1
2
3
A (n=15)
B (n=16)
C (n=14)
surface

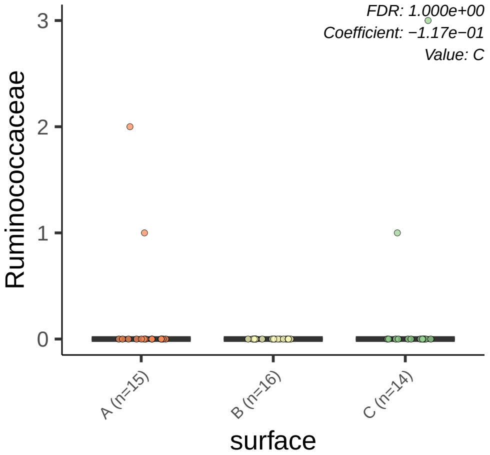

FDR: 1.000e+00
Coefficient: −1.17e−01
Value: C
Ruminococcaceae
3
2
1
0
A (n=15)
B (n=16)
C (n=14)
surface

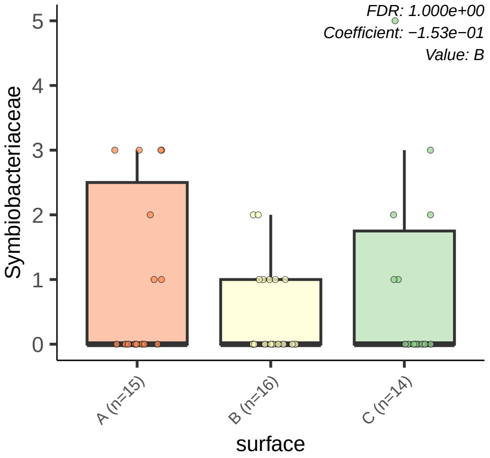
FDR: 1.000e+00
Coefficient: −1.53e−01
Value: B
Symbiobacteriaceae
0
1
2
3
4
5
A (n=15)
B (n=16)
C (n=14)
surface

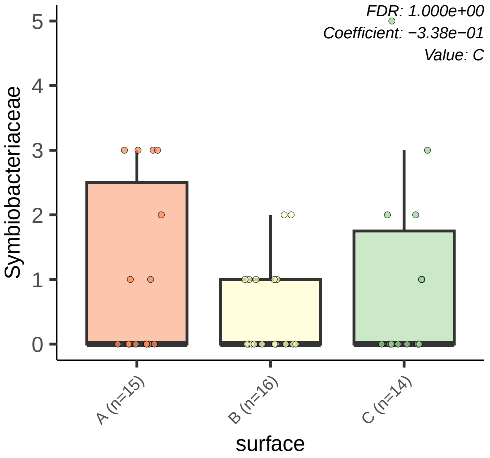

FDR: 1.000e+00
Coefficient: −3.38e−01
Value: C
Symbiobacteriaceae
0
1
2
3
4
5
A (n=15)
B (n=16)
C (n=14)
surface

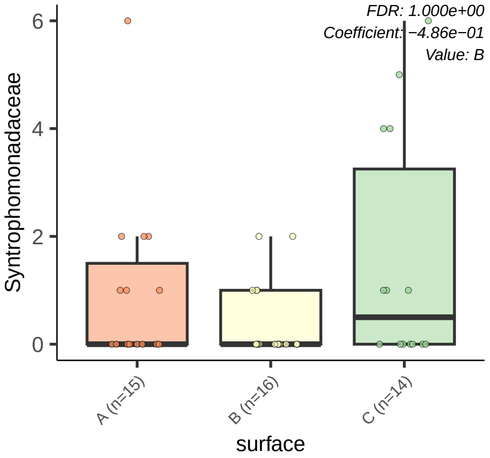
FDR: 1.000e+00
Coefficient: −4.86e−01
Value: B
Syntrophomonadaceae
0
2
4
6
A (n=15)
B (n=16)
C (n=14)
surface

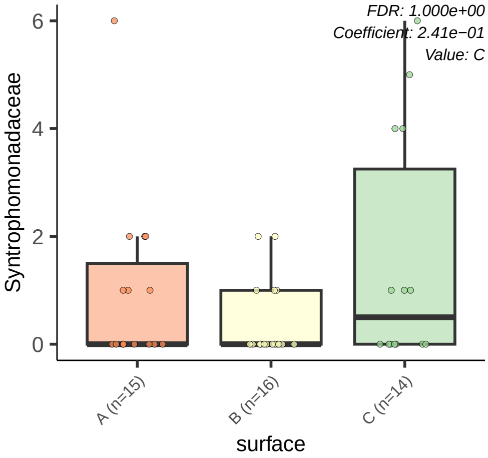
FDR: 1.000e+00
Coefficient: 2.41e−01
Value: C
Syntrophomonadaceae
0
2
4
6
A (n=15)
B (n=16)
C (n=14)
surface

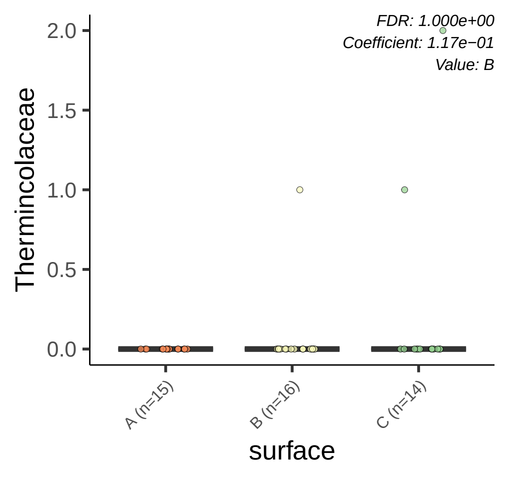
FDR: 1.000e+00
Coefficient: 1.17e−01
Value: B
Thermincolaceae
2.0
1.5
1.0
0.5
0.0
A (n=15)
B (n=16)
C (n=14)
surface

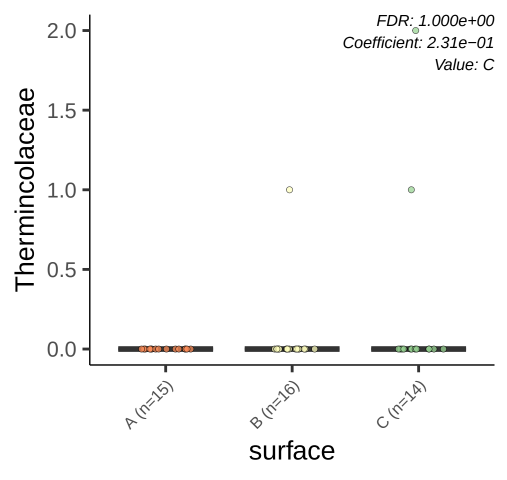

FDR: 1.000e+00
Coefficient: 2.31e−01
Value: C
Thermincolaceae
2.0
1.5
1.0
0.5
0.0
A (n=15)
B (n=16)
C (n=14)
surface

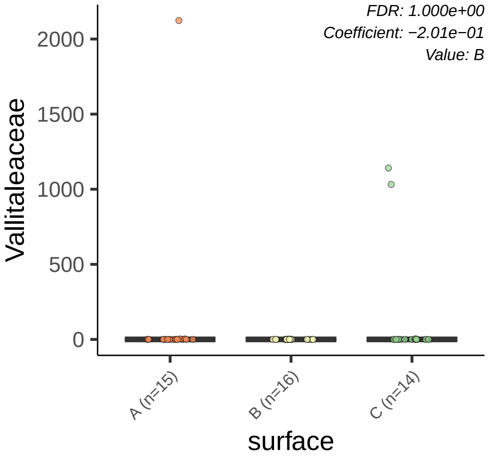

FDR: 1.000e+00
Coefficient: −2.01e−01
Value: B
Vallitaleaceae
2000
1500
1000
500
0
A (n=15)
B (n=16)
C (n=14)
surface

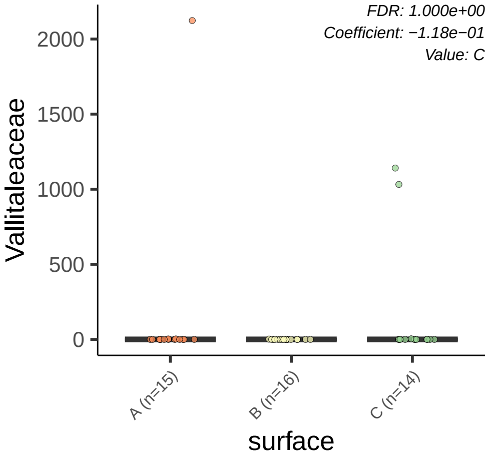
FDR: 1.000e+00
Coefficient: −1.18e−01
Value: C
Vallitaleaceae
2000
1500
1000
500
0
A (n=15)
B (n=16)
C (n=14)
surface

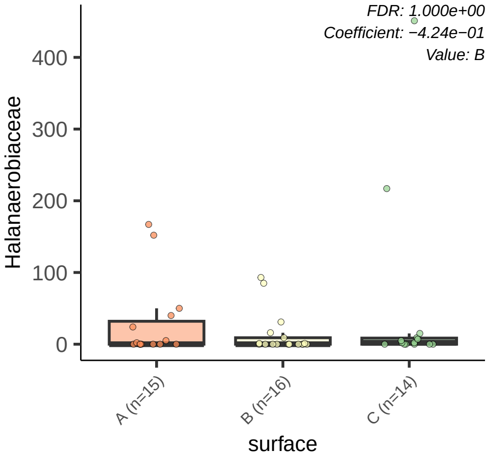
FDR: 1.000e+00
Coefficient: −4.24e−01
Value: B
Halanaerobiaceae
0
100
200
300
400
A (n=15)
B (n=16)
C (n=14)
surface

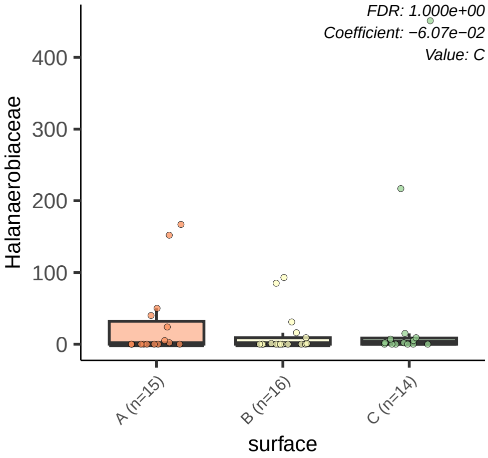

FDR: 1.000e+00
Coefficient: −6.07e−02
Value: C
Halanaerobiaceae
400
300
200
100
0
A (n=15)
B (n=16)
C (n=14)
surface

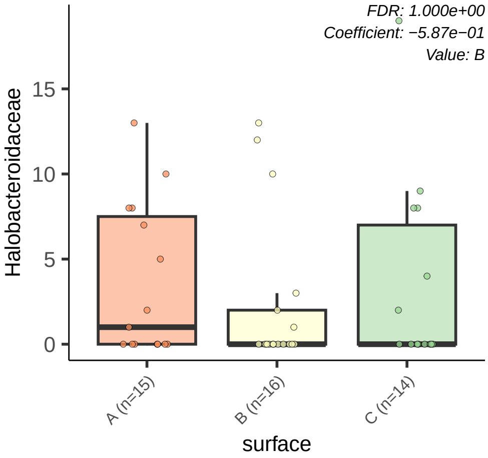

FDR: 1.000e+00
Coefficient: −5.87e−01
Value: B
Halobacteroidaceae
0
5
10
15
A (n=15)
B (n=16)
C (n=14)
surface

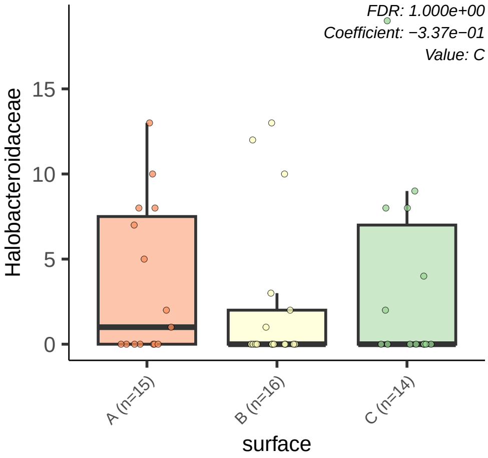

FDR: 1.000e+00
Coefficient: −3.37e−01
Value: C
Halobacteroidaceae
15
10
5
0
A (n=15)
B (n=16)
C (n=14)
surface

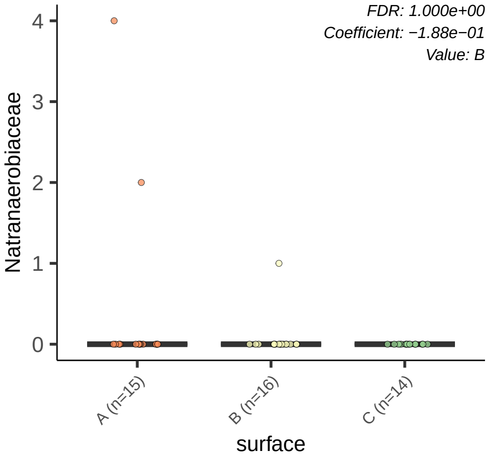
FDR: 1.000e+00
Coefficient: −1.88e−01
Value: B
Natranaerobiaceae
4
3
2
1
0
A (n=15)
B (n=16)
C (n=14)
surface

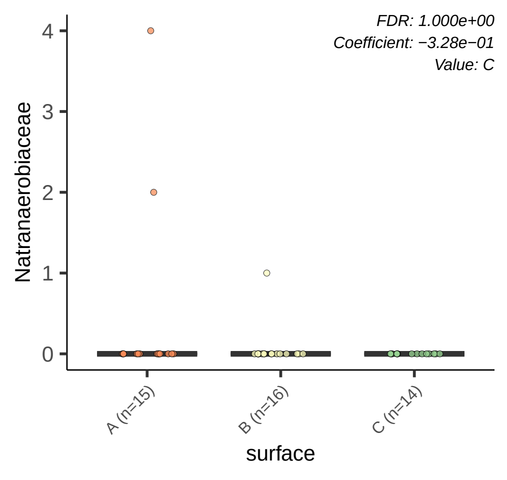

FDR: 1.000e+00
Coefficient: −3.28e−01
Value: C
Natranaerobiaceae
0
1
2
3
4
A (n=15)
B (n=16)
C (n=14)
surface

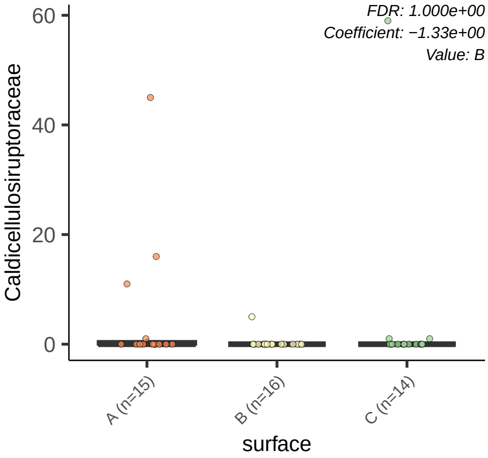

FDR: 1.000e+00
Coefficient: −1.33e+00
Value: B
Caldicellulosiruptoraceae
0
20
40
60
A (n=15)
B (n=16)
C (n=14)
surface

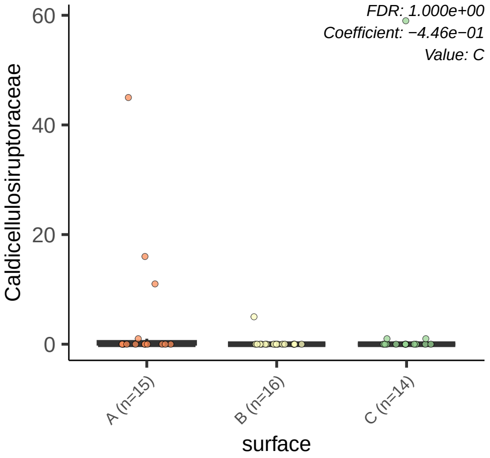

FDR: 1.000e+00
Coefficient: −4.46e−01
Value: C
Caldicellulosiruptoraceae
0
20
40
60
A (n=15)
B (n=16)
C (n=14)
surface

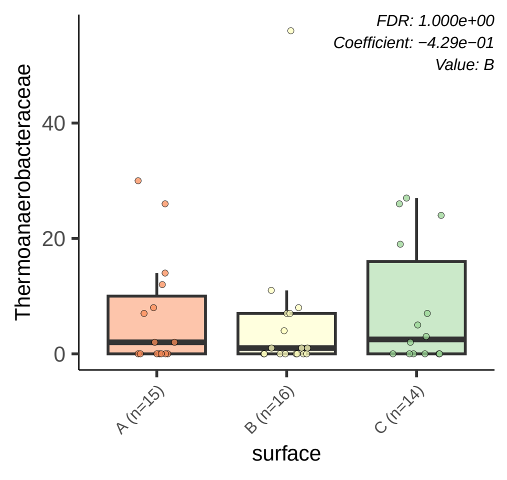

FDR: 1.000e+00
Coefficient: −4.29e−01
Value: B
Thermoanaerobacteraceae
0
20
40
A (n=15)
B (n=16)
C (n=14)
surface

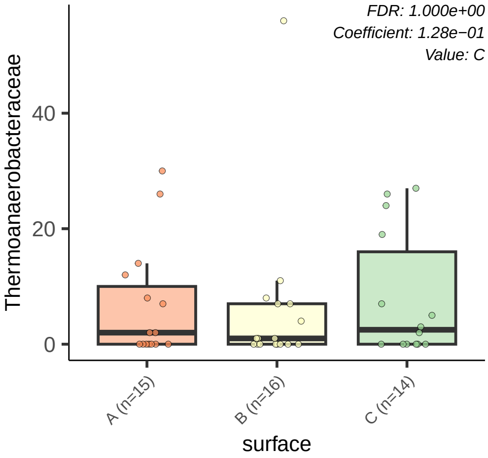

FDR: 1.000e+00
Coefficient: 1.28e−01
Value: C
Thermoanaerobacteraceae
0
20
40
A (n=15)
B (n=16)
C (n=14)
surface

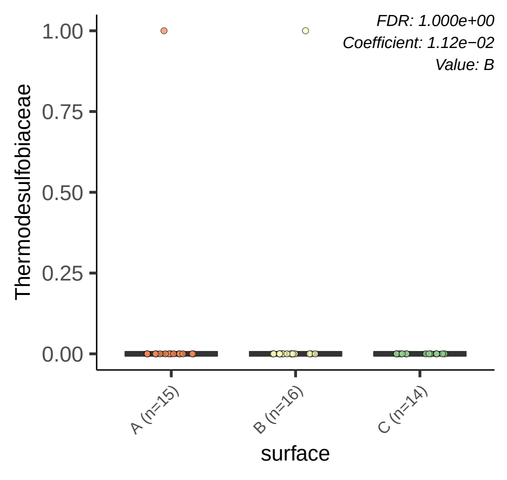
FDR: 1.000e+00
Coefficient: 1.12e−02
Value: B
Thermodesulfobiaceae
1.00
0.75
0.50
0.25
0.00
A (n=15)
B (n=16)
C (n=14)
surface

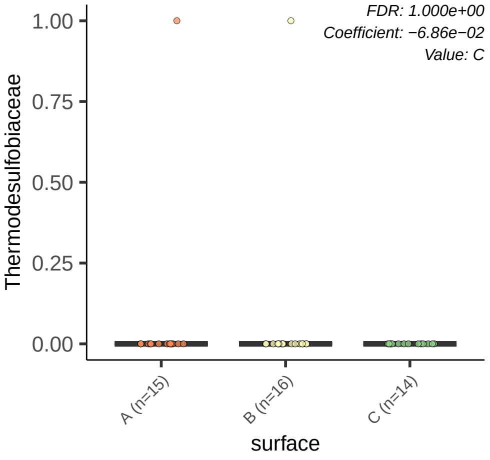
FDR: 1.000e+00
Coefficient: −6.86e−02
Value: C
Thermodesulfobiaceae
1.00
0.75
0.50
0.25
0.00
A (n=15)
B (n=16)
C (n=14)
surface

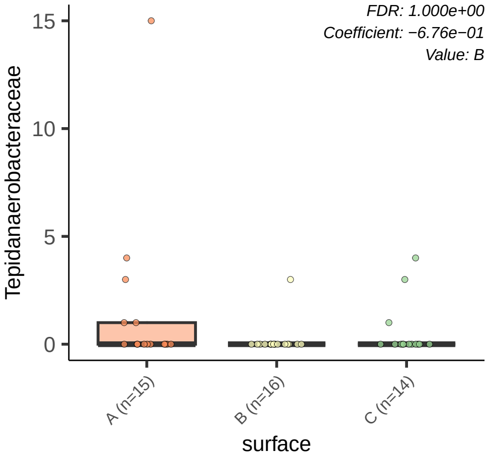
FDR: 1.000e+00
Coefficient: −6.76e−01
Value: B
Tepidanaerobacteraceae
15
10
5
0
A (n=15)
B (n=16)
C (n=14)
surface

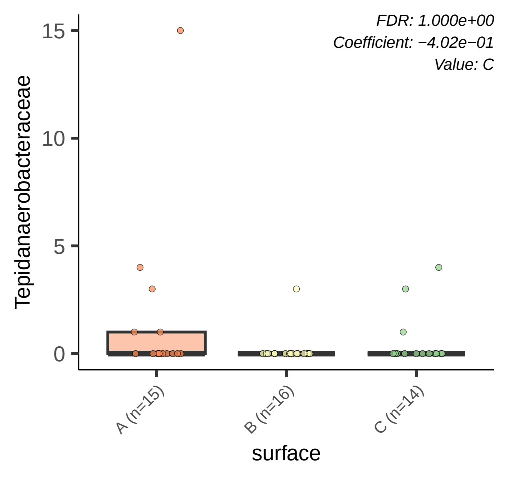
FDR: 1.000e+00
Coefficient: −4.02e−01
Value: C
Tepidanaerobacteraceae
15
10
5
0
A (n=15)
B (n=16)
C (n=14)
surface

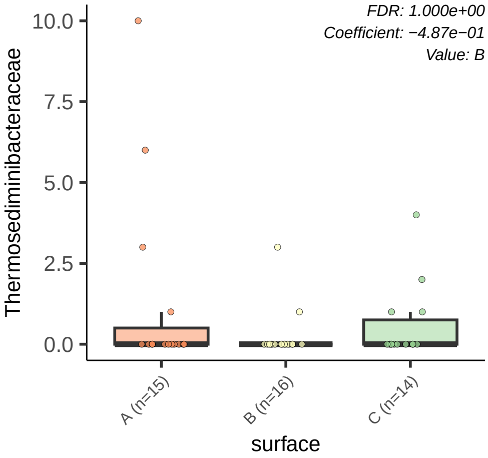

FDR: 1.000e+00
Coefficient: −4.87e−01
Value: B
Thermosediminibacteraceae
10.0
7.5
5.0
2.5
0.0
A (n=15)
B (n=16)
C (n=14)
surface

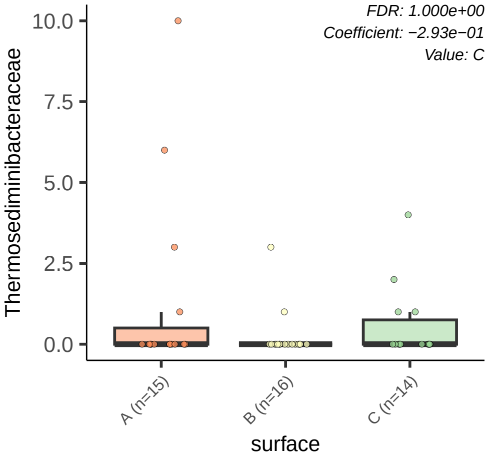

FDR: 1.000e+00
Coefficient: −2.93e−01
Value: C
Thermosediminibacteraceae
10.0
7.5
5.0
2.5
0.0
A (n=15)
B (n=16)
C (n=14)
surface

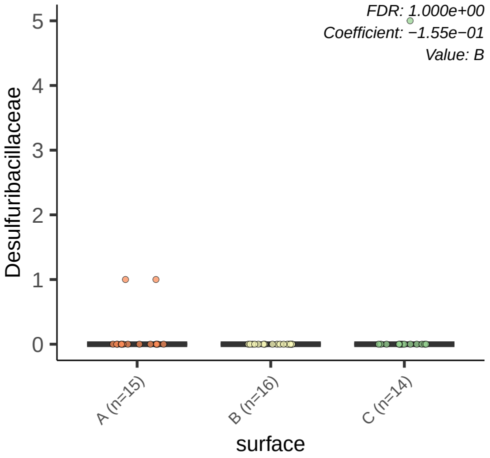

FDR: 1.000e+00
Coefficient: −1.55e−01
Value: B
Desulfuribacillaceae
0
1
2
3
4
5
A (n=15)
B (n=16)
C (n=14)
surface

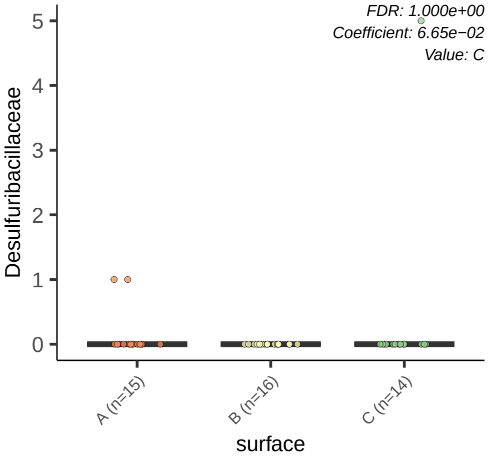

FDR: 1.000e+00
Coefficient: 6.65e−02
Value: C
Desulfuribacillaceae
0
1
2
3
4
5
A (n=15)
B (n=16)
C (n=14)
surface

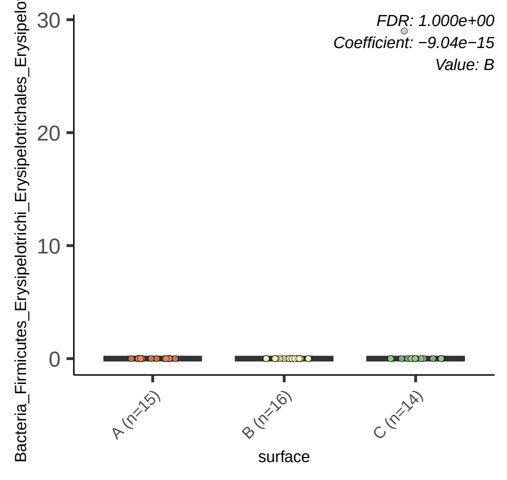

FDR: 1.000e+00
Coefficient: −9.04e−15
Value: B
30
20
10
0
Bacteria_Firmicutes_Erysipelotrichi_Erysipelotrichales_Erysipelo
A (n=15)
B (n=16)
C (n=14)
surface

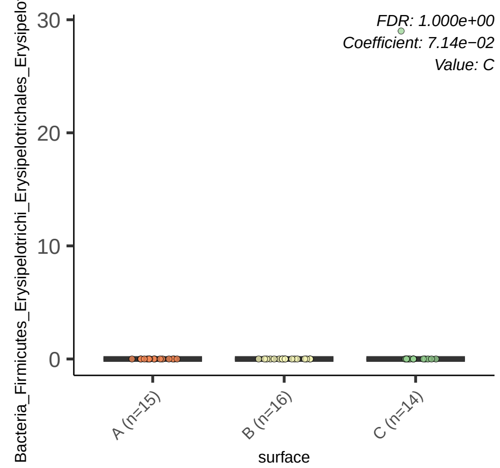
Bacteria_Firmicutes_Erysipelotrichi_Erysipelotrichales_Erysipelo
FDR: 1.000e+00
Coefficient: 7.14e−02
Value: C
30
20
10
0
A (n=15)
B (n=16)
C (n=14)
surface

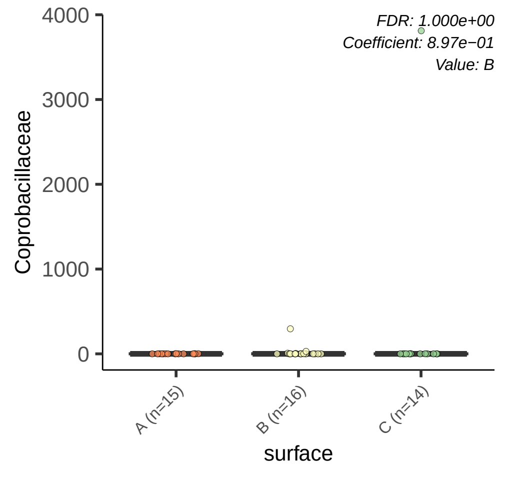
FDR: 1.000e+00
Coefficient: 8.97e−01
Value: B
Coprobacillaceae
4000
3000
2000
1000
0
A (n=15)
B (n=16)
C (n=14)
surface

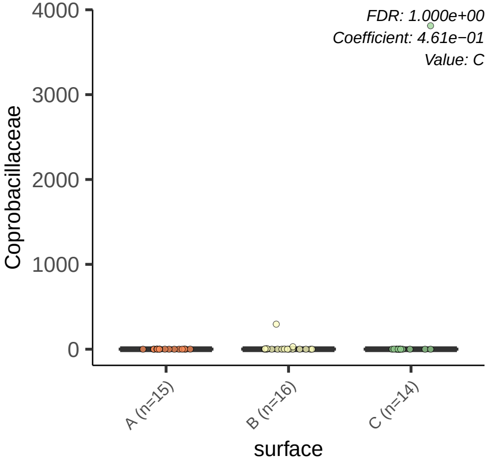

FDR: 1.000e+00
Coefficient: 4.61e−01
Value: C
Coprobacillaceae
4000
3000
2000
1000
0
A (n=15)
B (n=16)
C (n=14)
surface

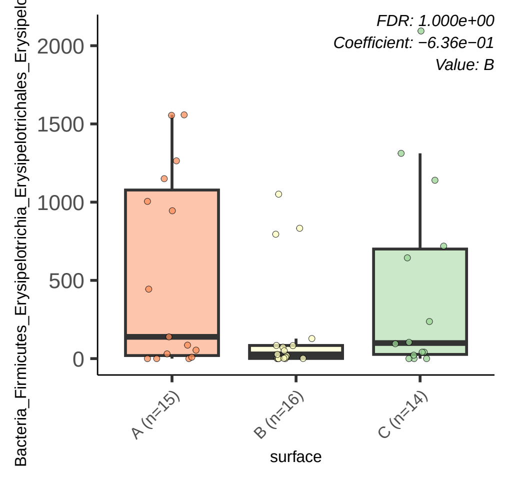
FDR: 1.000e+00
Coefficient: −6.36e−01
Value: B
2000
1500
1000
500
0
Bacteria_Firmicutes_Erysipelotrichia_Erysipelotrichales_Erysipelo
A (n=15)
B (n=16)
C (n=14)
surface

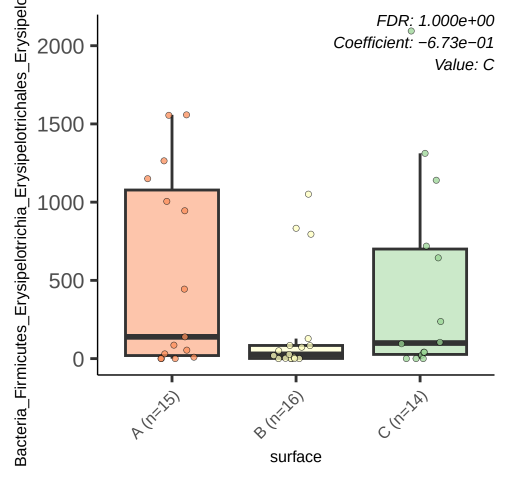
FDR: 1.000e+00
Coefficient: −6.73e−01
Value: C
2000
1500
1000
500
0
Bacteria_Firmicutes_Erysipelotrichia_Erysipelotrichales_Erysipelo
A (n=15)
B (n=16)
C (n=14)
surface

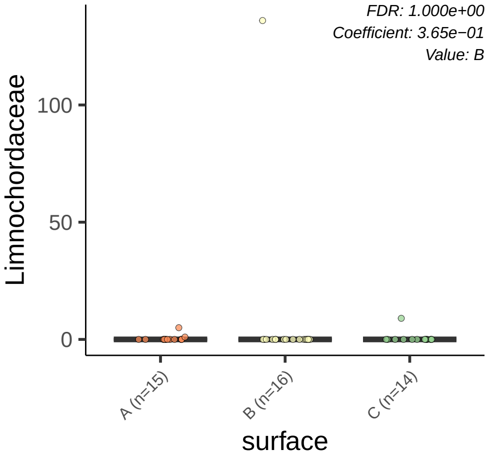
FDR: 1.000e+00
Coefficient: 3.65e−01
Value: B
Limnochordaceae
100
50
0
A (n=15)
B (n=16)
C (n=14)
surface

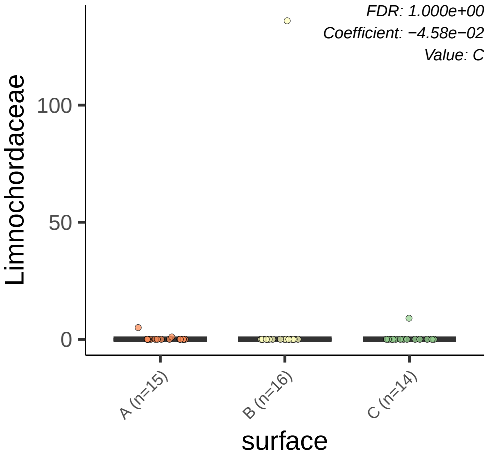

FDR: 1.000e+00
Coefficient: −4.58e−02
Value: C
Limnochordaceae
100
50
0
A (n=15)
B (n=16)
C (n=14)
surface

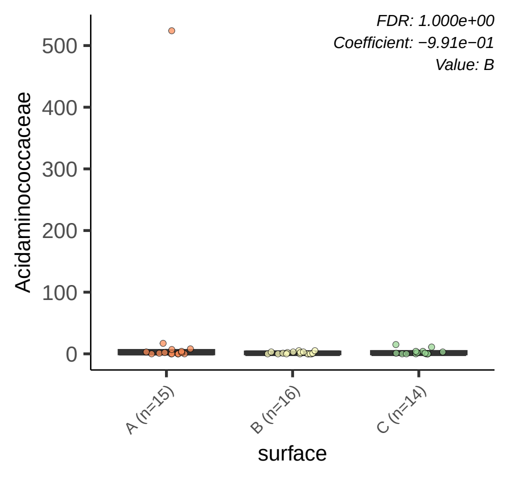
FDR: 1.000e+00
Coefficient: −9.91e−01
Value: B
Acidaminococcaceae
500
400
300
200
100
0
A (n=15)
B (n=16)
C (n=14)
surface

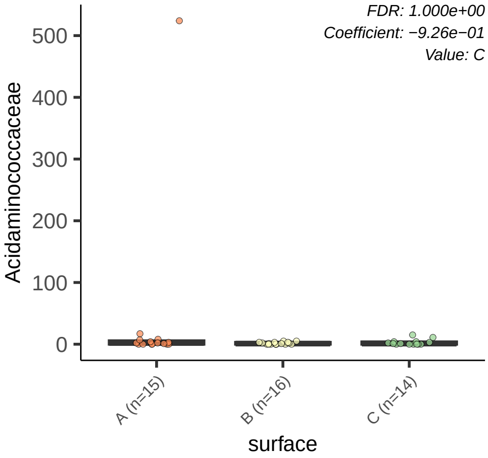

FDR: 1.000e+00
Coefficient: −9.26e−01
Value: C
Acidaminococcaceae
0
100
200
300
400
500
A (n=15)
B (n=16)
C (n=14)
surface

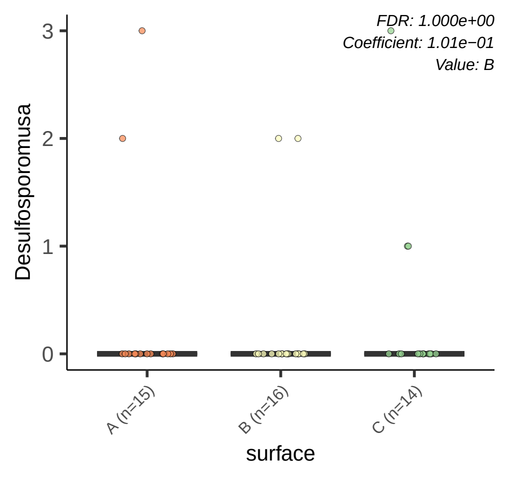
FDR: 1.000e+00
Coefficient: 1.01e−01
Value: B
Desulfosporomusa
3
2
1
0
A (n=15)
B (n=16)
C (n=14)
surface

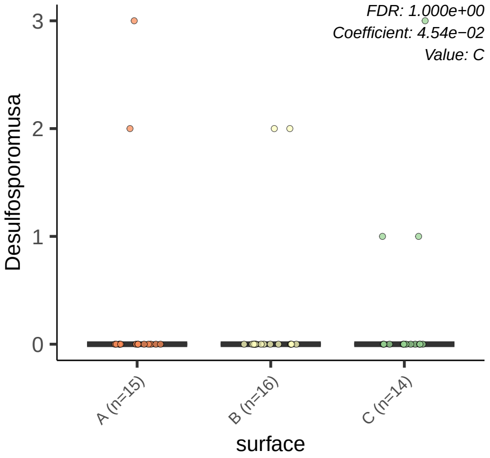

FDR: 1.000e+00
Coefficient: 4.54e−02
Value: C
Desulfosporomusa
3
2
1
0
A (n=15)
B (n=16)
C (n=14)
surface

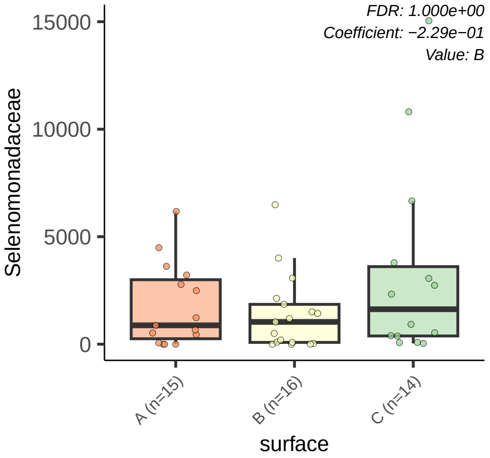

FDR: 1.000e+00
Coefficient: −2.29e−01
Value: B
Selenomonadaceae
15000
10000
5000
0
A (n=15)
B (n=16)
C (n=14)
surface

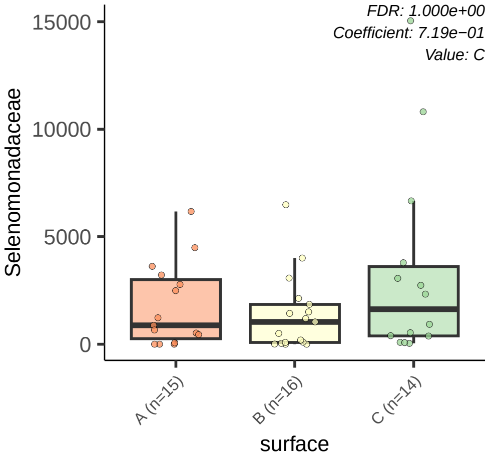

FDR: 1.000e+00
Coefficient: 7.19e−01
Value: C
15000
10000
5000
0
Selenomonadaceae
A (n=15)
B (n=16)
C (n=14)
surface

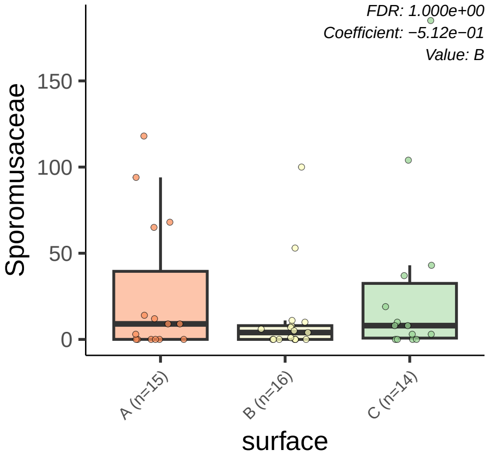

FDR: 1.000e+00
Coefficient: −5.12e−01
Value: B
Sporomusaceae
150
100
50
0
A (n=15)
B (n=16)
C (n=14)
surface

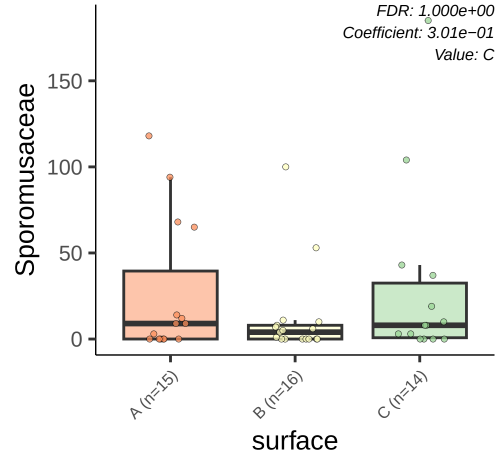
FDR: 1.000e+00
Coefficient: 3.01e−01
Value: C
Sporomusaceae
150
100
50
0
A (n=15)
B (n=16)
C (n=14)
surface

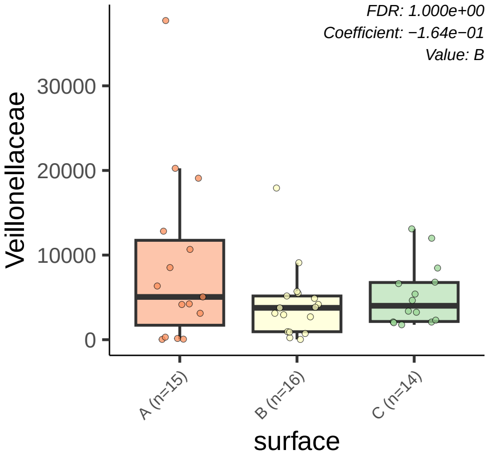
FDR: 1.000e+00
Coefficient: −1.64e−01
Value: B
Veillonellaceae
30000
20000
10000
0
A (n=15)
B (n=16)
C (n=14)
surface

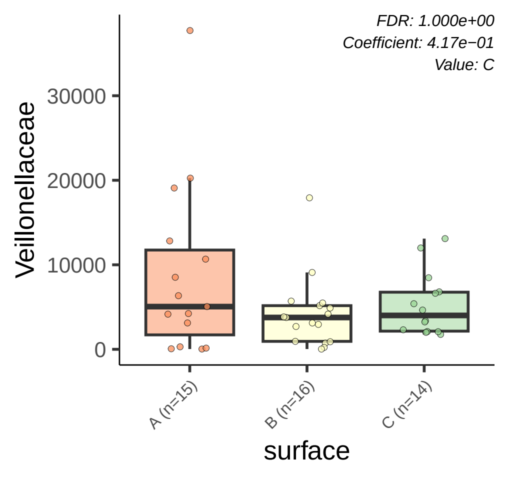

FDR: 1.000e+00
Coefficient: 4.17e−01
Value: C
Veillonellaceae
30000
20000
10000
0
A (n=15)
B (n=16)
C (n=14)
surface

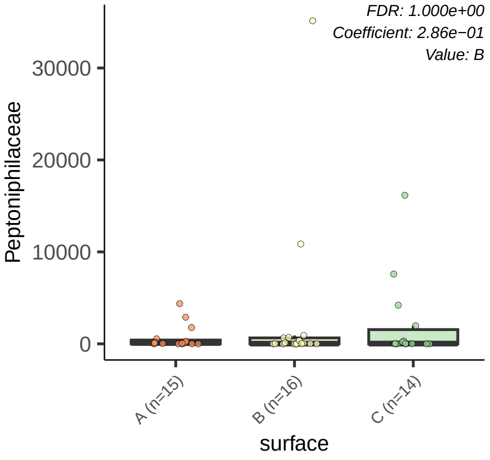
FDR: 1.000e+00
Coefficient: 2.86e−01
Value: B
Peptoniphilaceae
30000
20000
10000
0
A (n=15)
B (n=16)
C (n=14)
surface

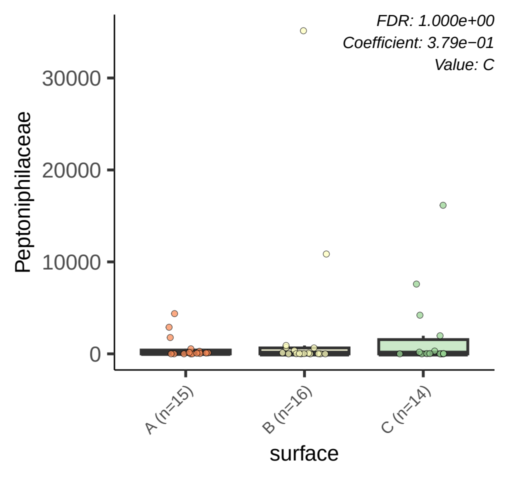

FDR: 1.000e+00
Coefficient: 3.79e−01
Value: C
Peptoniphilaceae
30000
20000
10000
0
A (n=15)
B (n=16)
C (n=14)
surface

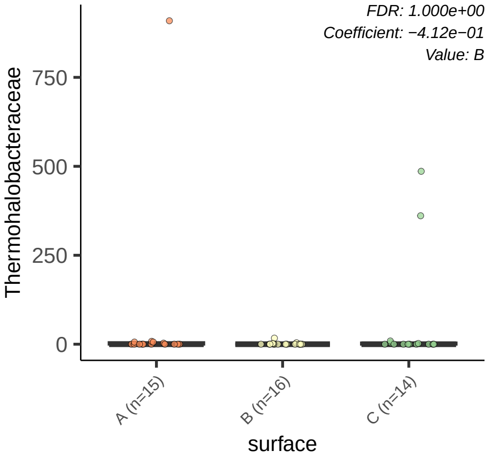

FDR: 1.000e+00
Coefficient: −4.12e−01
Value: B
Thermohalobacteraceae
750
500
250
0
A (n=15)
B (n=16)
C (n=14)
surface

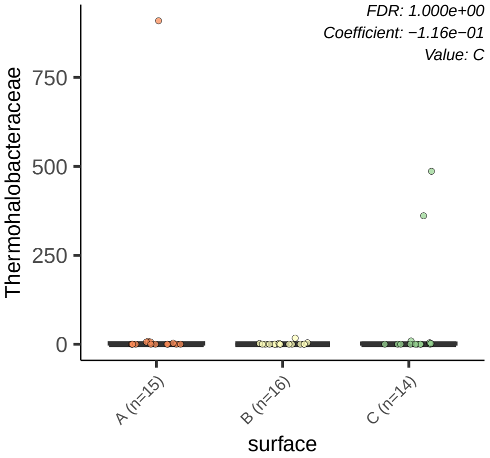

FDR: 1.000e+00
Coefficient: −1.16e−01
Value: C
Thermohalobacteraceae
750
500
250
0
A (n=15)
B (n=16)
C (n=14)
surface

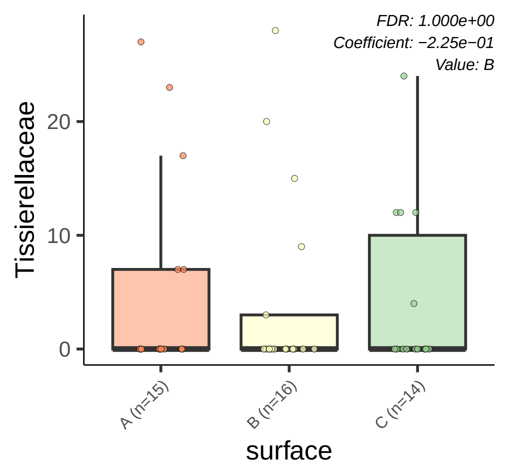
FDR: 1.000e+00
Coefficient: −2.25e−01
Value: B
Tissierellaceae
20
10
0
A (n=15)
B (n=16)
C (n=14)
surface

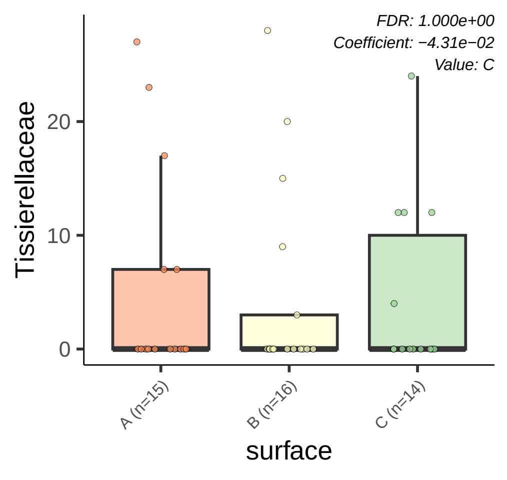

FDR: 1.000e+00
Coefficient: −4.31e−02
Value: C
Tissierellaceae
0
10
20
A (n=15)
B (n=16)
C (n=14)
surface

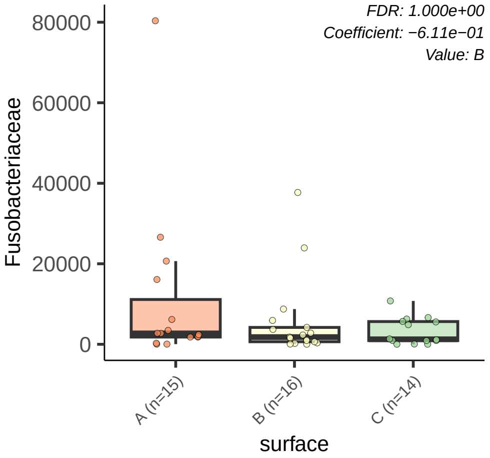
FDR: 1.000e+00
Coefficient: −6.11e−01
Value: B
Fusobacteriaceae
80000
60000
40000
20000
0
A (n=15)
B (n=16)
C (n=14)
surface

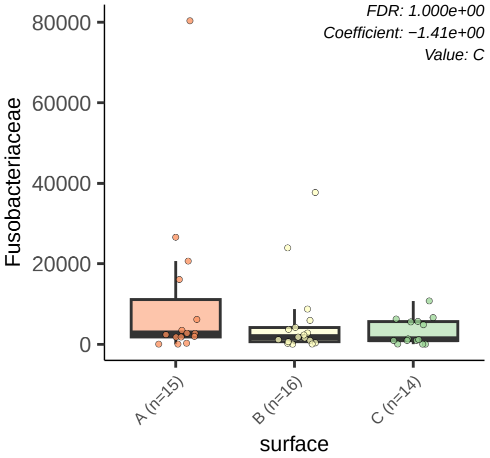
FDR: 1.000e+00
Coefficient: −1.41e+00
Value: C
Fusobacteriaceae
80000
60000
40000
20000
0
A (n=15)
B (n=16)
C (n=14)
surface

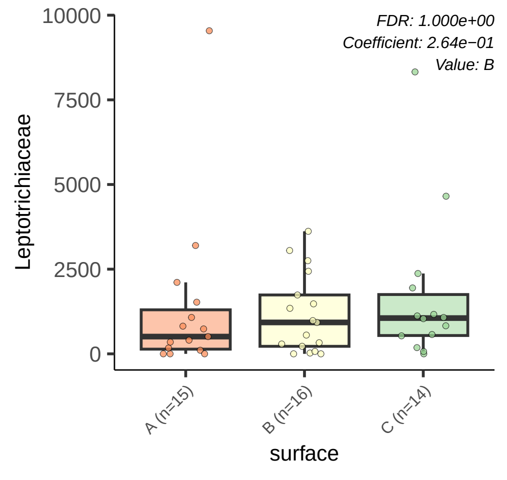
FDR: 1.000e+00
Coefficient: 2.64e−01
Value: B
Leptotrichiaceae
10000
7500
5000
2500
0
A (n=15)
B (n=16)
C (n=14)
surface

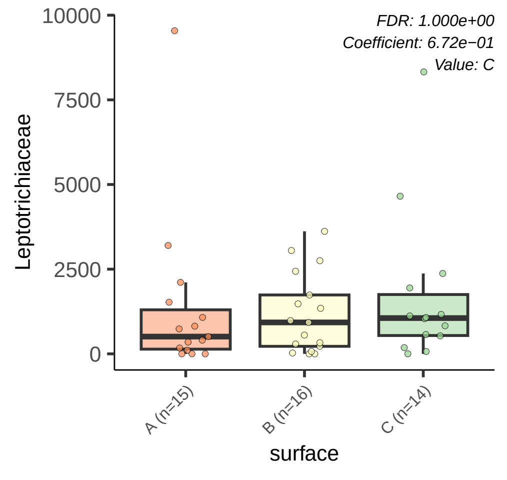
FDR: 1.000e+00
Coefficient: 6.72e−01
Value: C
Leptotrichiaceae
10000
7500
5000
2500
0
A (n=15)
B (n=16)
C (n=14)
surface

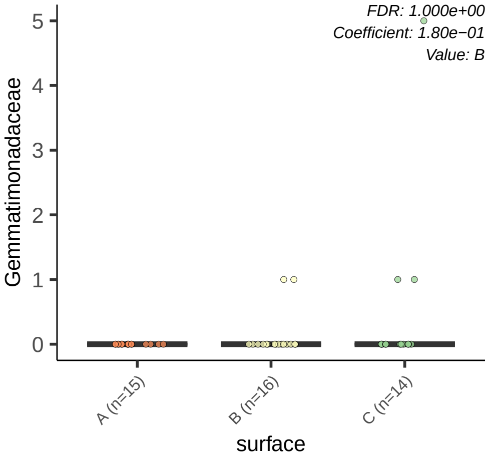
FDR: 1.000e+00
Coefficient: 1.80e−01
Value: B
Gemmatimonadaceae
0
1
2
3
4
5
A (n=15)
B (n=16)
C (n=14)
surface

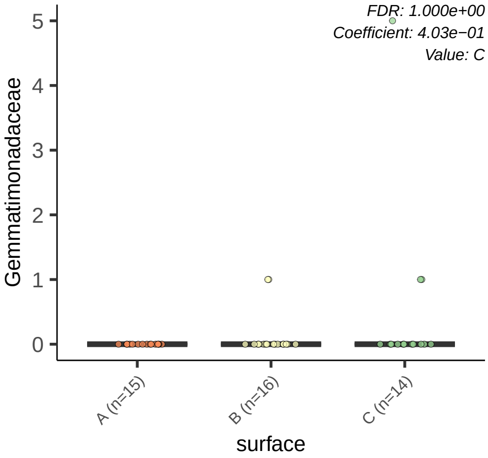

FDR: 1.000e+00
Coefficient: 4.03e−01
Value: C
Gemmatimonadaceae
0
1
2
3
4
5
A (n=15)
B (n=16)
C (n=14)
surface

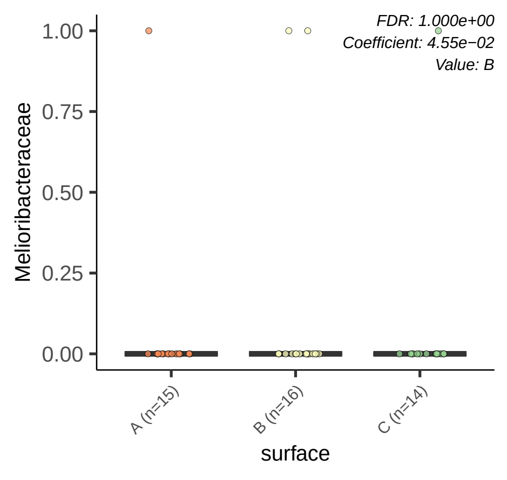
FDR: 1.000e+00
Coefficient: 4.55e−02
Value: B
Melioribacteraceae
1.00
0.75
0.50
0.25
0.00
A (n=15)
B (n=16)
C (n=14)
surface

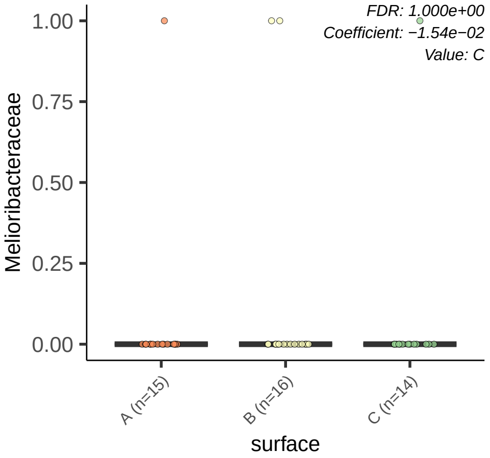
FDR: 1.000e+00
Coefficient: −1.54e−02
Value: C
Melioribacteraceae
1.00
0.75
0.50
0.25
0.00
A (n=15)
B (n=16)
C (n=14)
surface

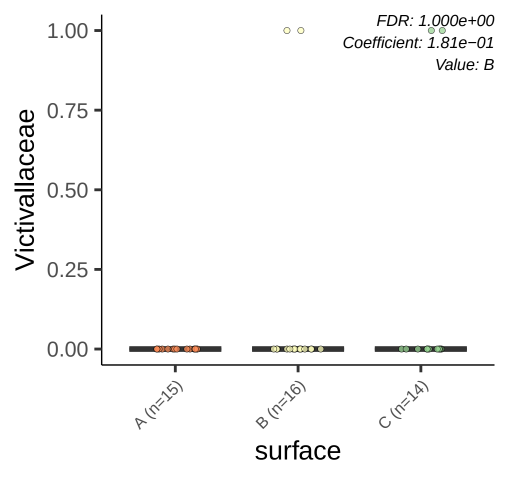

FDR: 1.000e+00
Coefficient: 1.81e−01
Value: B
Victivallaceae
1.00
0.75
0.50
0.25
0.00
A (n=15)
B (n=16)
C (n=14)
surface

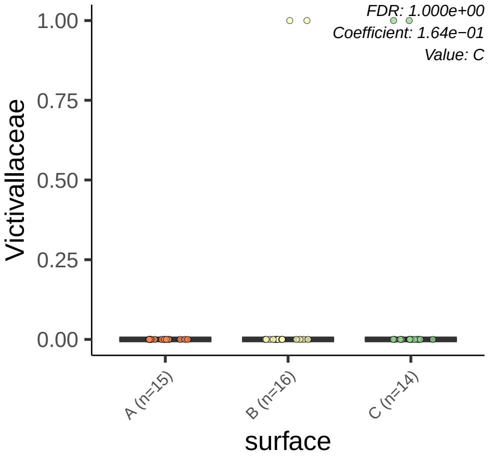

FDR: 1.000e+00
Coefficient: 1.64e−01
Value: C
Victivallaceae
1.00
0.75
0.50
0.25
0.00
A (n=15)
B (n=16)
C (n=14)
surface

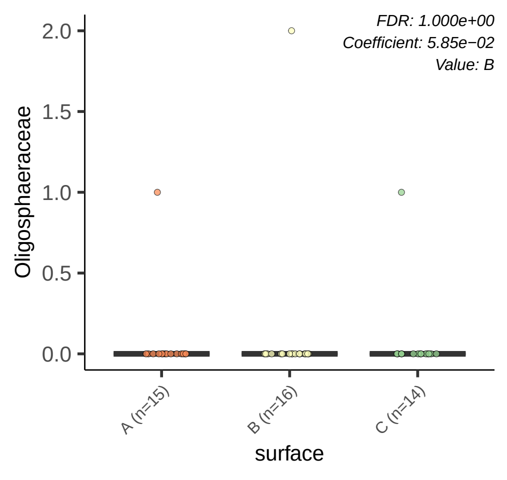
FDR: 1.000e+00
Coefficient: 5.85e−02
Value: B
Oligosphaeraceae
2.0
1.5
1.0
0.5
0.0
A (n=15)
B (n=16)
C (n=14)
surface

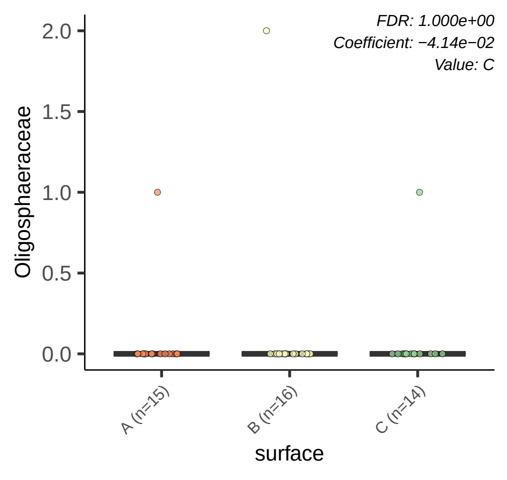
FDR: 1.000e+00
Coefficient: −4.14e−02
Value: C
Oligosphaeraceae
2.0
1.5
1.0
0.5
0.0
A (n=15)
B (n=16)
C (n=14)
surface

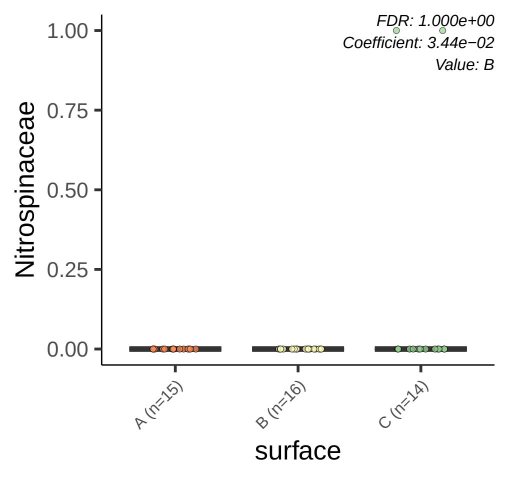
FDR: 1.000e+00
Coefficient: 3.44e−02
Value: B
Nitrospinaceae
1.00
0.75
0.50
0.25
0.00
A (n=15)
B (n=16)
C (n=14)
surface

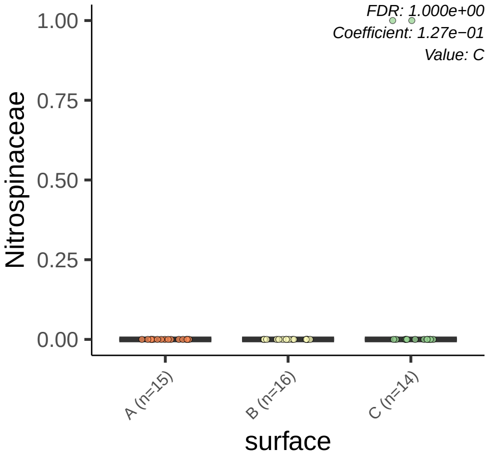

FDR: 1.000e+00
Coefficient: 1.27e−01
Value: C
Nitrospinaceae
1.00
0.75
0.50
0.25
0.00
A (n=15)
B (n=16)
C (n=14)
surface

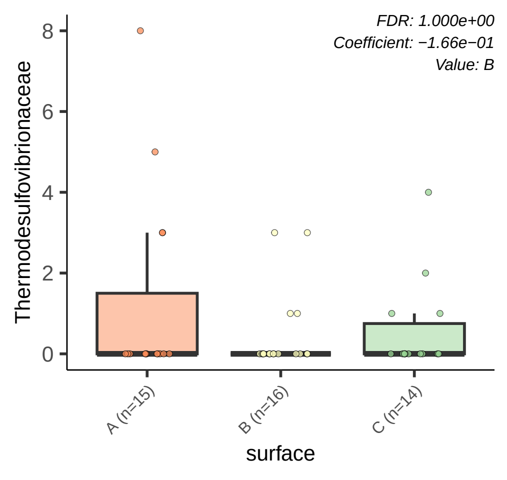

FDR: 1.000e+00
Coefficient: −1.66e−01
Value: B
Thermodesulfovibrionaceae
8
6
4
2
0
A (n=15)
B (n=16)
C (n=14)
surface

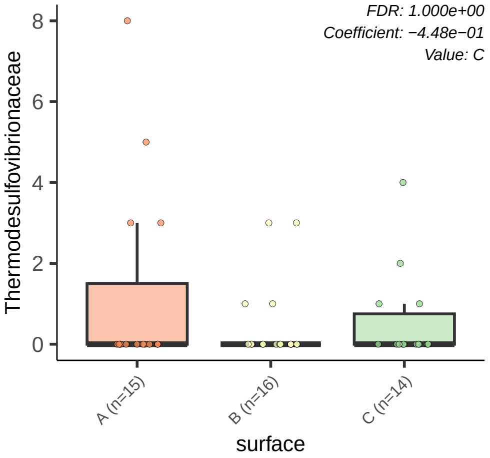
FDR: 1.000e+00
Coefficient: −4.48e−01
Value: C
Thermodesulfovibrionaceae
8
6
4
2
0
A (n=15)
B (n=16)
C (n=14)
surface

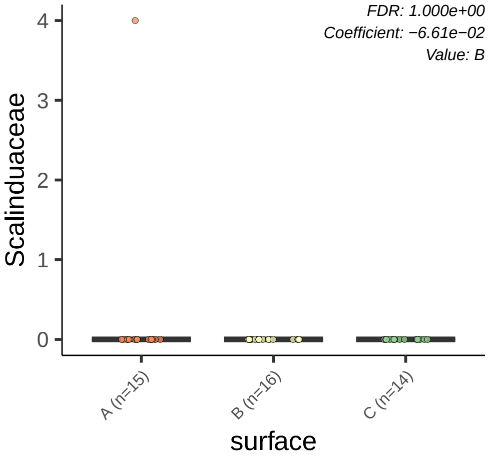
FDR: 1.000e+00
Coefficient: −6.61e−02
Value: B
Scalinduaceae
4
3
2
1
0
A (n=15)
B (n=16)
C (n=14)
surface

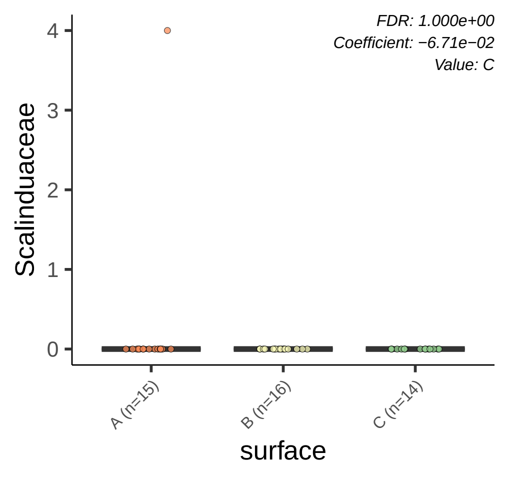
FDR: 1.000e+00
Coefficient: −6.71e−02
Value: C
Scalinduaceae
4
3
2
1
0
A (n=15)
B (n=16)
C (n=14)
surface

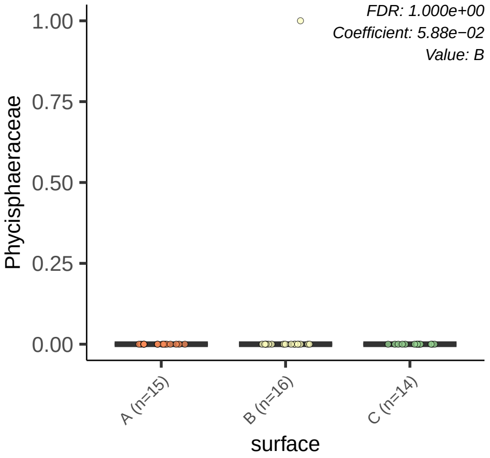
FDR: 1.000e+00
Coefficient: 5.88e−02
Value: B
Phycisphaeraceae
1.00
0.75
0.50
0.25
0.00
A (n=15)
B (n=16)
C (n=14)
surface

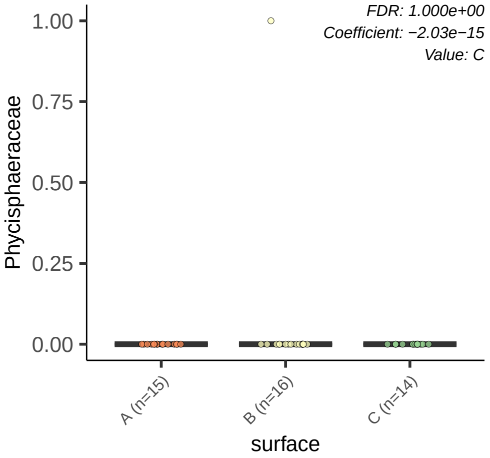
FDR: 1.000e+00
Coefficient: −2.03e−15
Value: C
Phycisphaeraceae
1.00
0.75
0.50
0.25
0.00
A (n=15)
B (n=16)
C (n=14)
surface

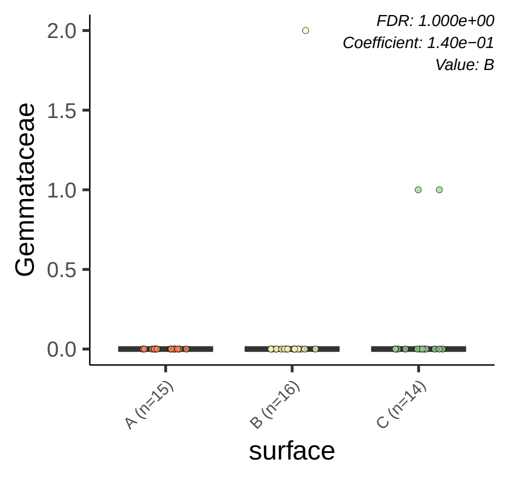
FDR: 1.000e+00
Coefficient: 1.40e−01
Value: B
Gemmataceae
2.0
1.5
1.0
0.5
0.0
A (n=15)
B (n=16)
C (n=14)
surface

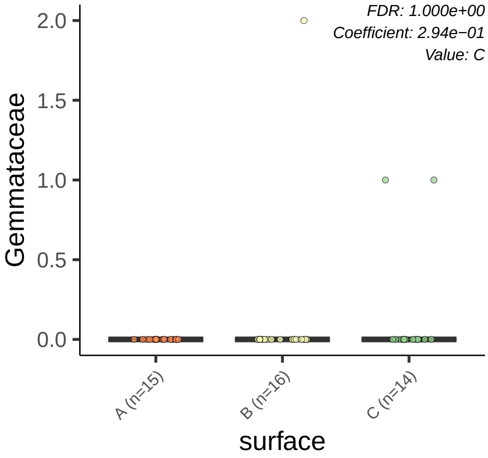

FDR: 1.000e+00
Coefficient: 2.94e−01
Value: C
Gemmataceae
2.0
1.5
1.0
0.5
0.0
A (n=15)
B (n=16)
C (n=14)
surface

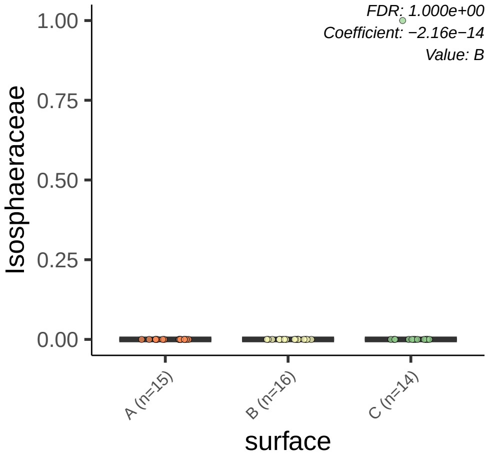
FDR: 1.000e+00
Coefficient: −2.16e−14
Value: B
Isosphaeraceae
1.00
0.75
0.50
0.25
0.00
A (n=15)
B (n=16)
C (n=14)
surface

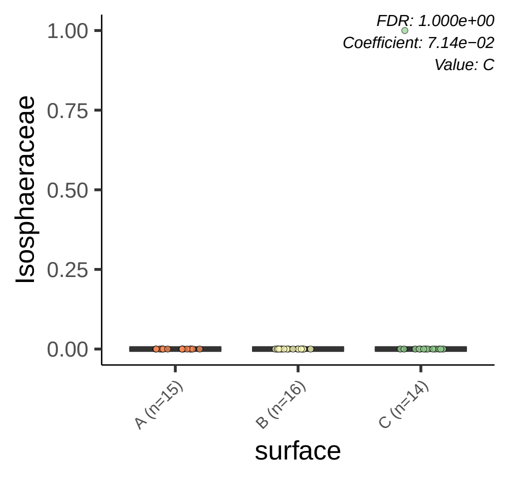

FDR: 1.000e+00
Coefficient: 7.14e−02
Value: C
Isosphaeraceae
1.00
0.75
0.50
0.25
0.00
A (n=15)
B (n=16)
C (n=14)
surface

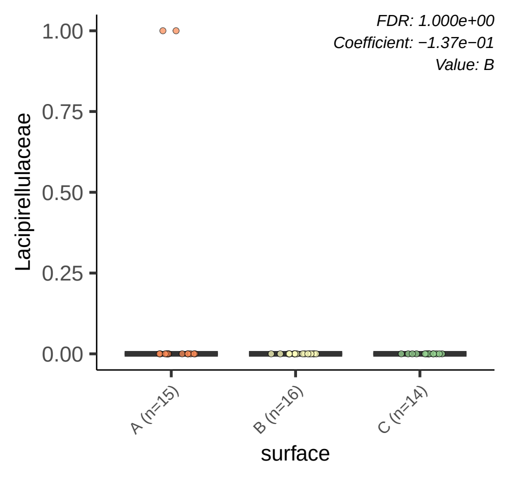
FDR: 1.000e+00
Coefficient: −1.37e−01
Value: B
Lacipirellulaceae
1.00
0.75
0.50
0.25
0.00
A (n=15)
B (n=16)
C (n=14)
surface

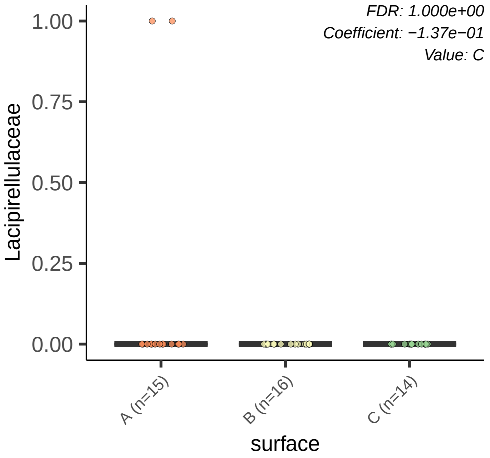
FDR: 1.000e+00
Coefficient: −1.37e−01
Value: C
Lacipirellulaceae
1.00
0.75
0.50
0.25
0.00
A (n=15)
B (n=16)
C (n=14)
surface

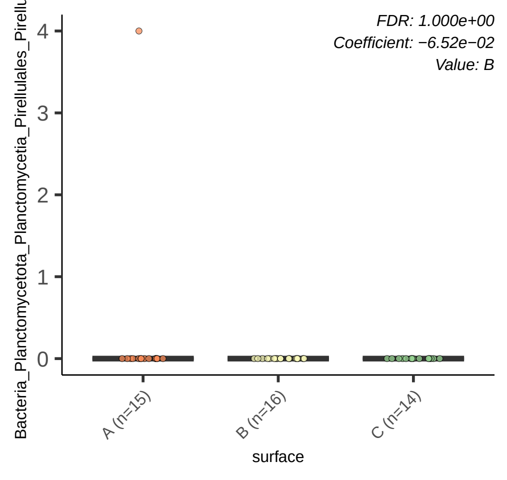

FDR: 1.000e+00
Coefficient: −6.52e−02
Value: B
Bacteria_Planctomycetota_Planctomycetia_Pirellulales_Pirellu
4
3
2
1
0
A (n=15)
B (n=16)
C (n=14)
surface

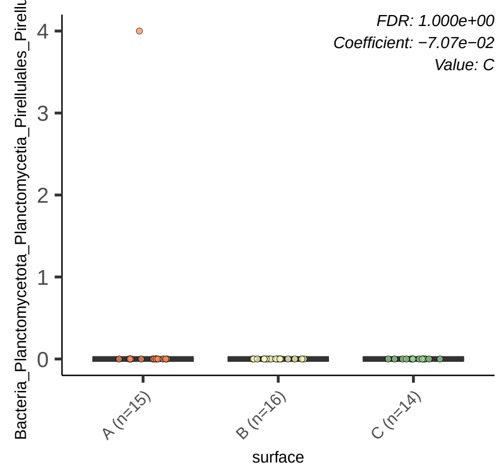
FDR: 1.000e+00
Coefficient: −7.07e−02
Value: C
4
3
2
1
0
Bacteria_Planctomycetota_Planctomycetia_Pirellulales_Pirellu
A (n=15)
B (n=16)
C (n=14)
surface

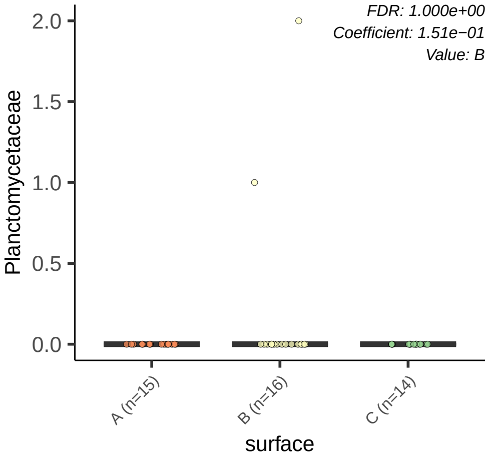

FDR: 1.000e+00
Coefficient: 1.51e−01
Value: B
Planctomycetaceae
2.0
1.5
1.0
0.5
0.0
A (n=15)
B (n=16)
C (n=14)
surface

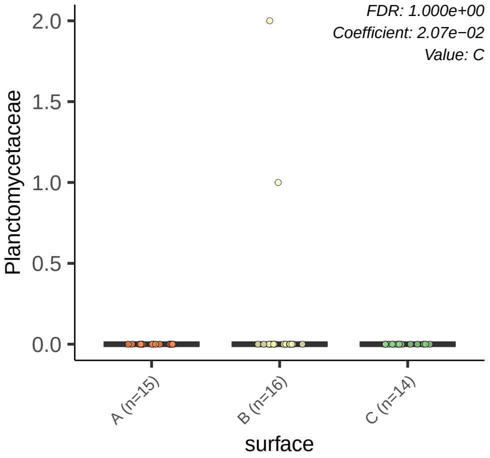

FDR: 1.000e+00
Coefficient: 2.07e−02
Value: C
Planctomycetaceae
2.0
1.5
1.0
0.5
0.0
A (n=15)
B (n=16)
C (n=14)
surface

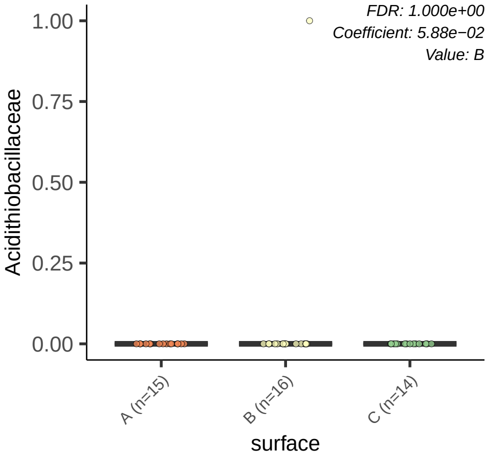

FDR: 1.000e+00
Coefficient: 5.88e−02
Value: B
Acidithiobacillaceae
1.00
0.75
0.50
0.25
0.00
A (n=15)
B (n=16)
C (n=14)
surface

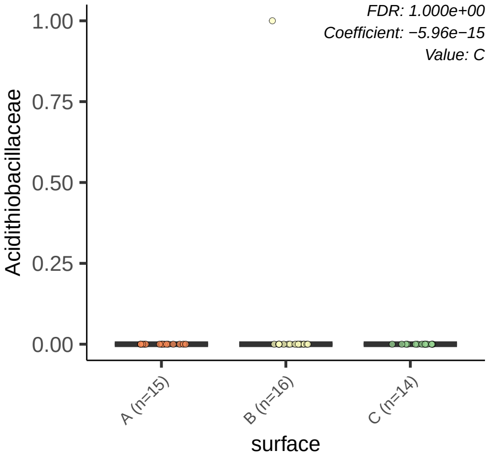
FDR: 1.000e+00
Coefficient: −5.96e−15
Value: C
Acidithiobacillaceae
1.00
0.75
0.50
0.25
0.00
A (n=15)
B (n=16)
C (n=14)
surface

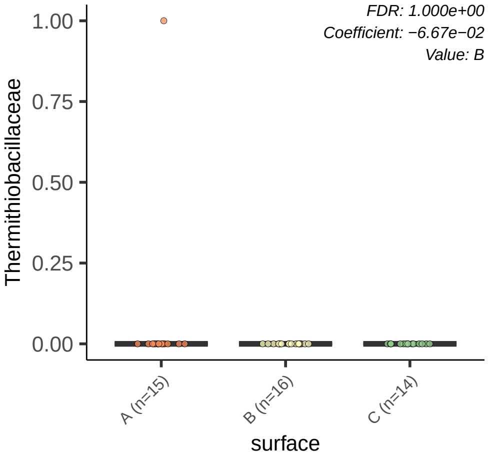

FDR: 1.000e+00
Coefficient: −6.67e−02
Value: B
Thermithiobacillaceae
1.00
0.75
0.50
0.25
0.00
A (n=15)
B (n=16)
C (n=14)
surface

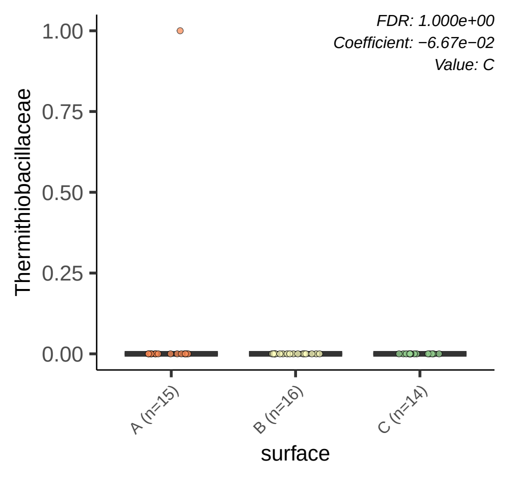
FDR: 1.000e+00
Coefficient: −6.67e−02
Value: C
Thermithiobacillaceae
1.00
0.75
0.50
0.25
0.00
A (n=15)
B (n=16)
C (n=14)
surface

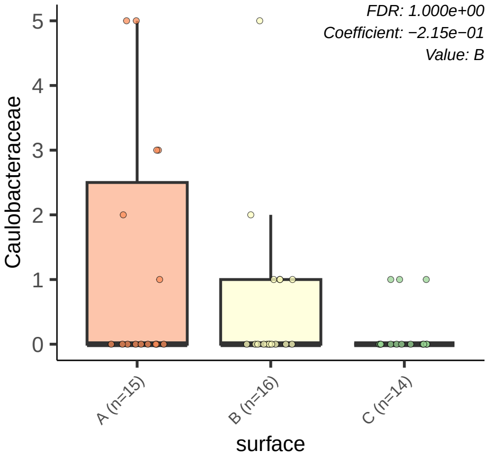

FDR: 1.000e+00
Coefficient: −2.15e−01
Value: B
Caulobacteraceae
0
1
2
3
4
5
A (n=15)
B (n=16)
C (n=14)
surface

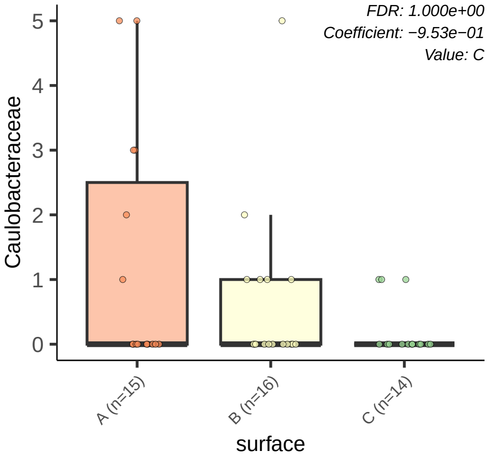

FDR: 1.000e+00
Coefficient: −9.53e−01
Value: C
Caulobacteraceae
0
1
2
3
4
5
A (n=15)
B (n=16)
C (n=14)
surface

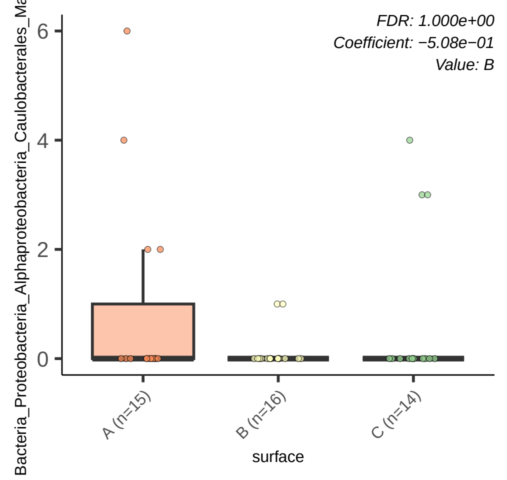
FDR: 1.000e+00
Coefficient: −5.08e−01
Value: B
Bacteria_Proteobacteria_Alphaproteobacteria_Caulobacterales_Ma
6
4
2
0
A (n=15)
B (n=16)
C (n=14)
surface

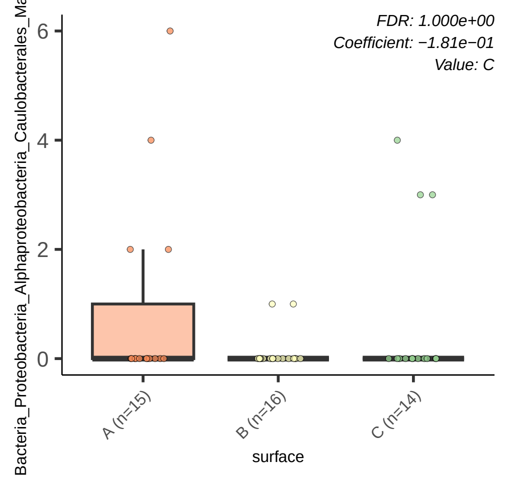
FDR: 1.000e+00
Coefficient: −1.81e−01
Value: C
Bacteria_Proteobacteria_Alphaproteobacteria_Caulobacterales_Ma
6
4
2
0
A (n=15)
B (n=16)
C (n=14)
surface

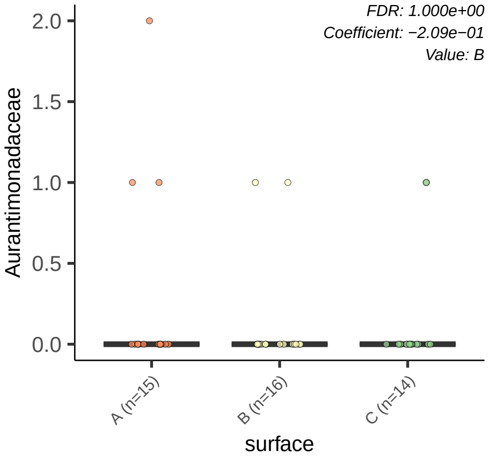
FDR: 1.000e+00
Coefficient: −2.09e−01
Value: B
Aurantimonadaceae
2.0
1.5
1.0
0.5
0.0
A (n=15)
B (n=16)
C (n=14)
surface

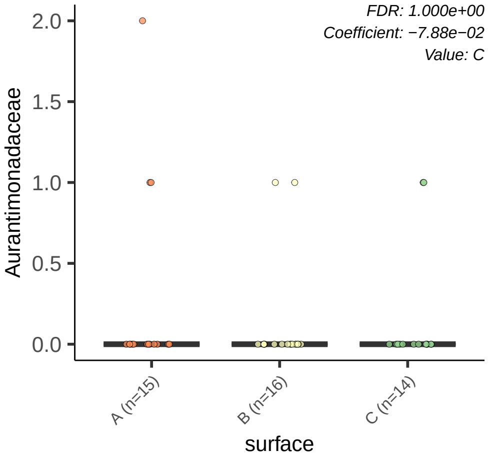

FDR: 1.000e+00
Coefficient: −7.88e−02
Value: C
Aurantimonadaceae
2.0
1.5
1.0
0.5
0.0
A (n=15)
B (n=16)
C (n=14)
surface

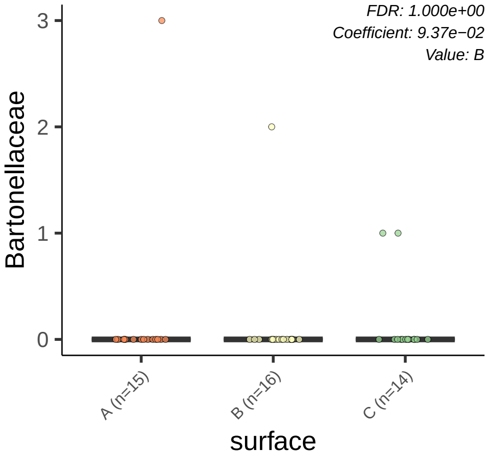
FDR: 1.000e+00
Coefficient: 9.37e−02
Value: B
Bartonellaceae
3
2
1
0
A (n=15)
B (n=16)
C (n=14)
surface

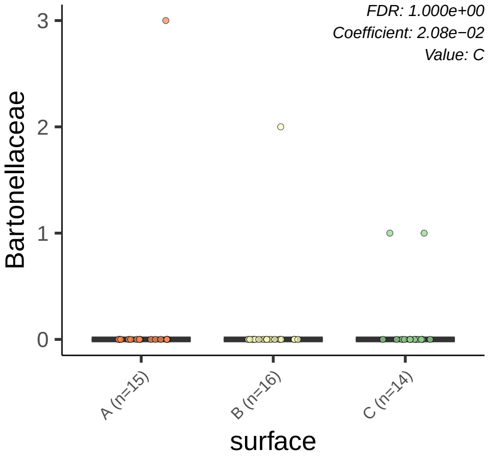
FDR: 1.000e+00
Coefficient: 2.08e−02
Value: C
Bartonellaceae
3
2
1
0
A (n=15)
B (n=16)
C (n=14)
surface

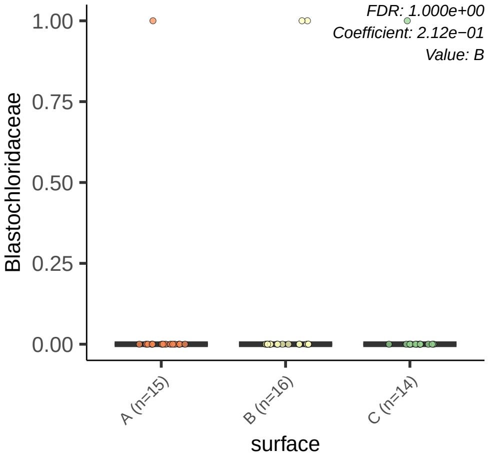
FDR: 1.000e+00
Coefficient: 2.12e−01
Value: B
1.00
0.75
0.50
0.25
0.00
Blastochloridaceae
A (n=15)
B (n=16)
C (n=14)
surface

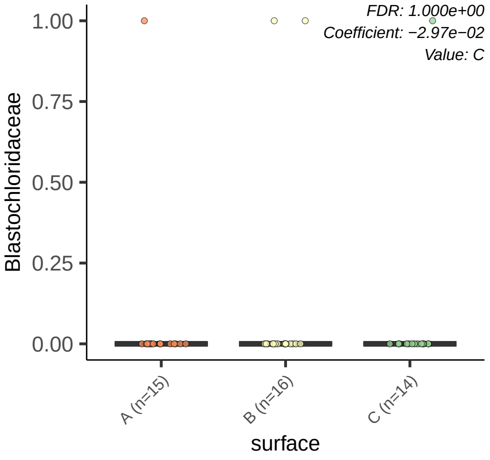

FDR: 1.000e+00
Coefficient: −2.97e−02
Value: C
Blastochloridaceae
1.00
0.75
0.50
0.25
0.00
A (n=15)
B (n=16)
C (n=14)
surface

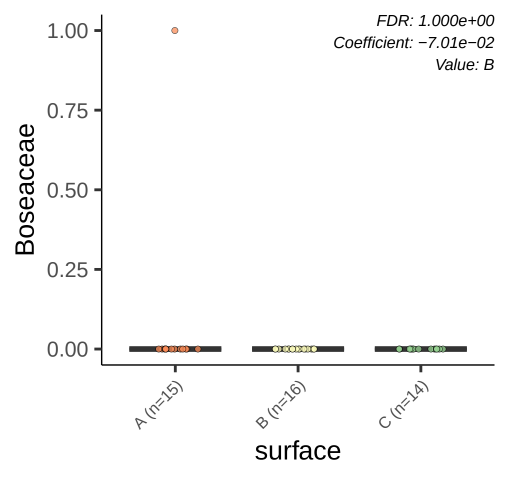

FDR: 1.000e+00
Coefficient: −7.01e−02
Value: B
1.00
0.75
0.50
0.25
0.00
Boseaceae
A (n=15)
B (n=16)
C (n=14)
surface

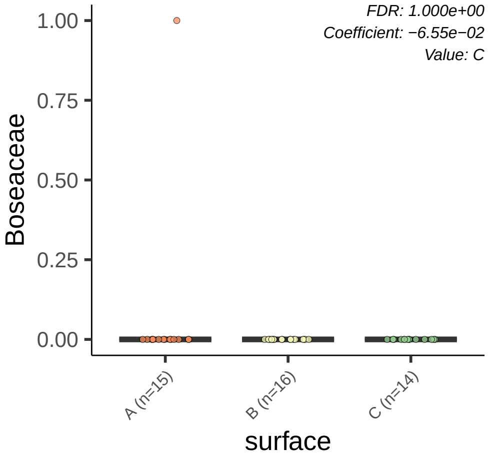

FDR: 1.000e+00
Coefficient: −6.55e−02
Value: C
Boseaceae
1.00
0.75
0.50
0.25
0.00
A (n=15)
B (n=16)
C (n=14)
surface

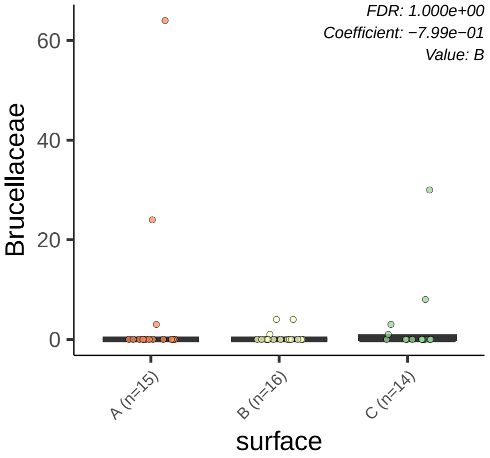
FDR: 1.000e+00
Coefficient: −7.99e−01
Value: B
Brucellaceae
0
20
40
60
A (n=15)
B (n=16)
C (n=14)
surface

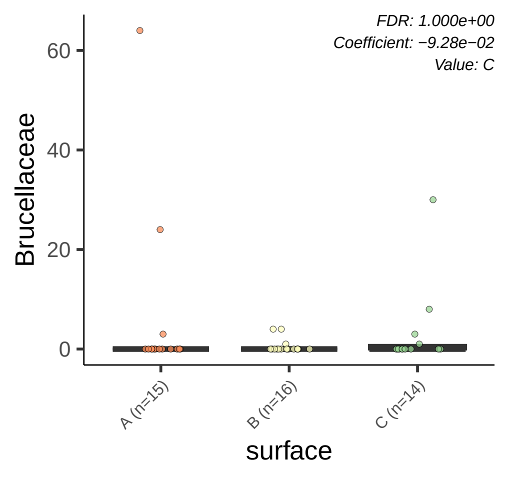
FDR: 1.000e+00
Coefficient: −9.28e−02
Value: C
Brucellaceae
60
40
20
0
A (n=15)
B (n=16)
C (n=14)
surface

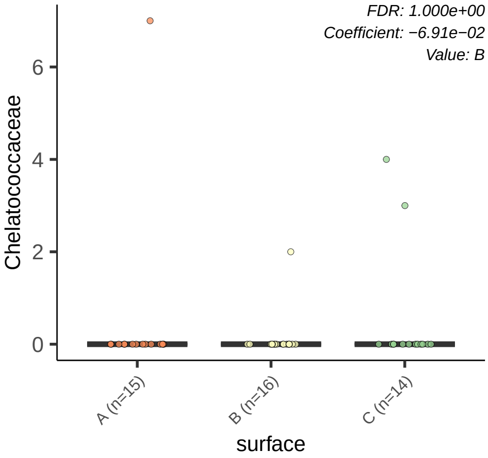
FDR: 1.000e+00
Coefficient: −6.91e−02
Value: B
Chelatococcaceae
0
2
4
6
A (n=15)
B (n=16)
C (n=14)
surface

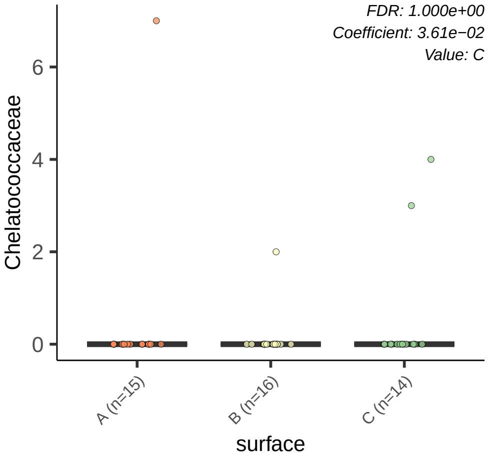
FDR: 1.000e+00
Coefficient: 3.61e−02
Value: C
Chelatococcaceae
0
2
4
6
A (n=15)
B (n=16)
C (n=14)
surface

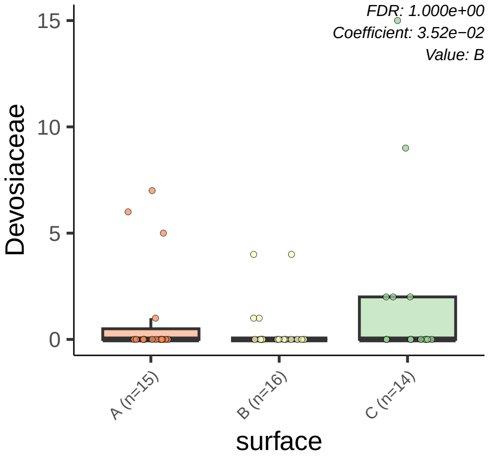
FDR: 1.000e+00
Coefficient: 3.52e−02
Value: B
Devosiaceae
15
10
5
0
A (n=15)
B (n=16)
C (n=14)
surface

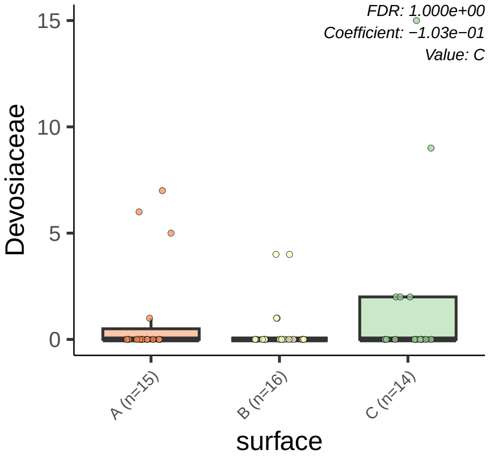
FDR: 1.000e+00
Coefficient: −1.03e−01
Value: C
Devosiaceae
15
10
5
0
A (n=15)
B (n=16)
C (n=14)
surface

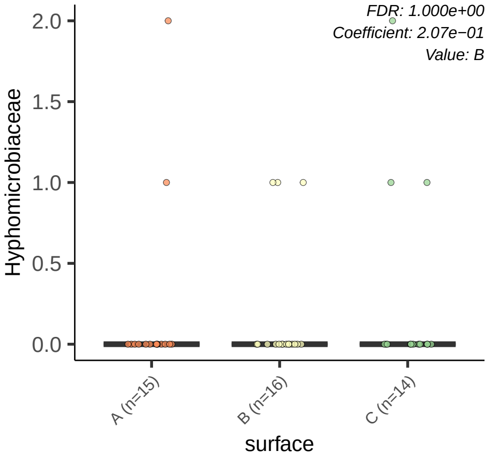
FDR: 1.000e+00
Coefficient: 2.07e−01
Value: B
Hyphomicrobiaceae
2.0
1.5
1.0
0.5
0.0
A (n=15)
B (n=16)
C (n=14)
surface

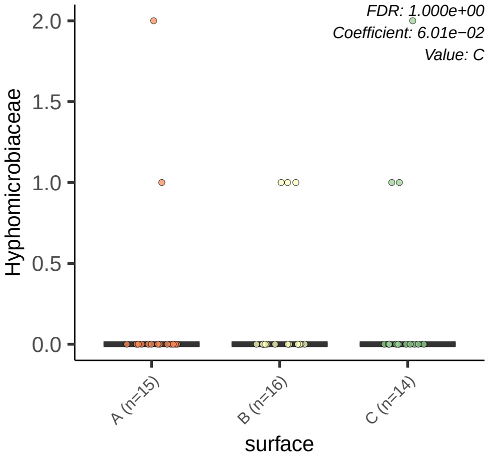

FDR: 1.000e+00
Coefficient: 6.01e−02
Value: C
Hyphomicrobiaceae
2.0
1.5
1.0
0.5
0.0
A (n=15)
B (n=16)
C (n=14)
surface

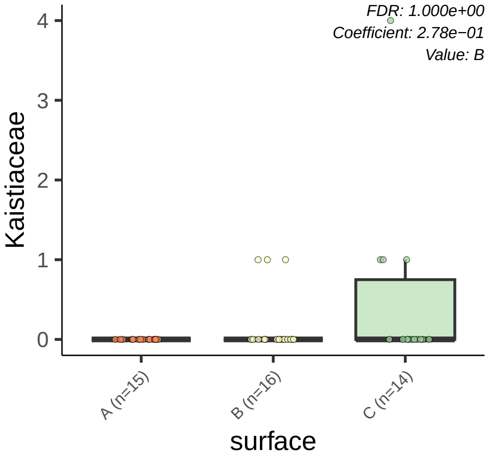

FDR: 1.000e+00
Coefficient: 2.78e−01
Value: B
Kaistiaceae
0
1
2
3
4
A (n=15)
B (n=16)
C (n=14)
surface

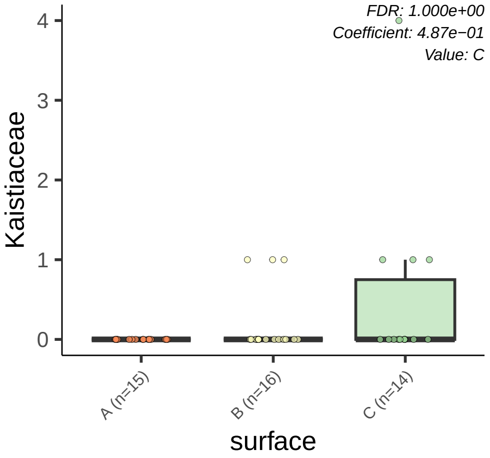
FDR: 1.000e+00
Coefficient: 4.87e−01
Value: C
Kaistiaceae
0
1
2
3
4
A (n=15)
B (n=16)
C (n=14)
surface

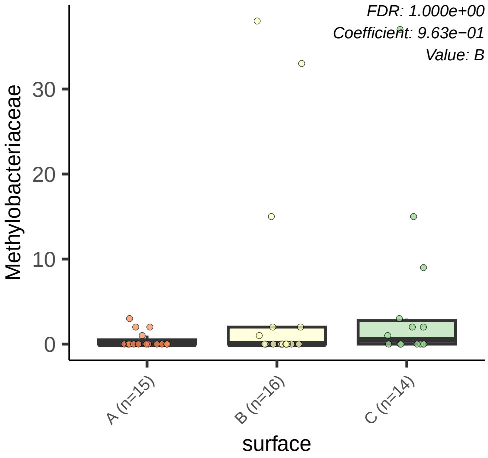

FDR: 1.000e+00
Coefficient: 9.63e−01
Value: B
Methylobacteriaceae
0
10
20
30
A (n=15)
B (n=16)
C (n=14)
surface

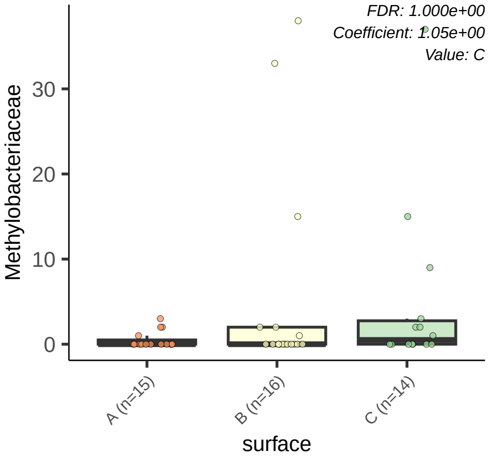
FDR: 1.000e+00
Coefficient: 1.05e+00
Value: C
Methylobacteriaceae
0
10
20
30
A (n=15)
B (n=16)
C (n=14)
surface

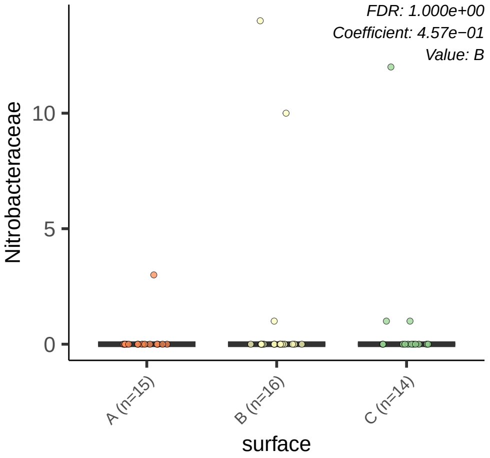
FDR: 1.000e+00
Coefficient: 4.57e−01
Value: B
Nitrobacteraceae
0
5
10
A (n=15)
B (n=16)
C (n=14)
surface

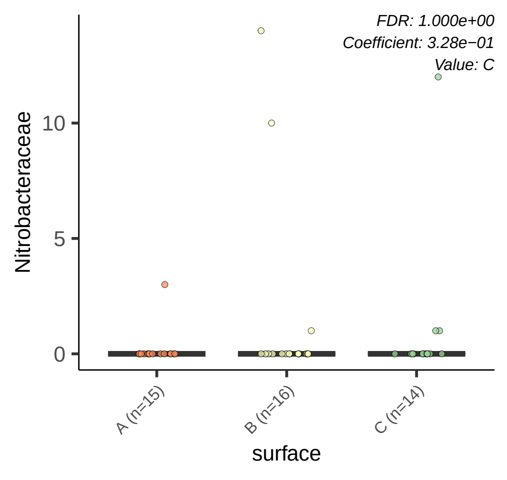
FDR: 1.000e+00
Coefficient: 3.28e−01
Value: C
Nitrobacteraceae
0
5
10
A (n=15)
B (n=16)
C (n=14)
surface

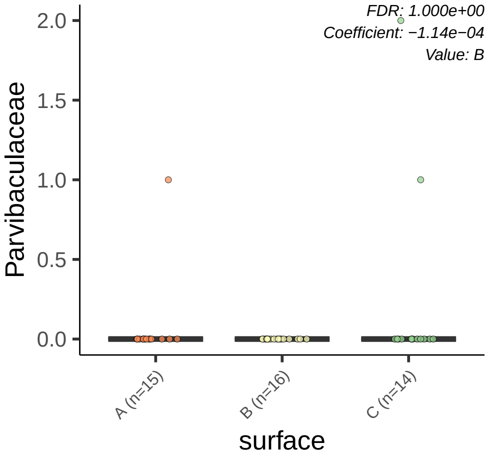

FDR: 1.000e+00
Coefficient: −1.14e−04
Value: B
Parvibaculaceae
2.0
1.5
1.0
0.5
0.0
A (n=15)
B (n=16)
C (n=14)
surface

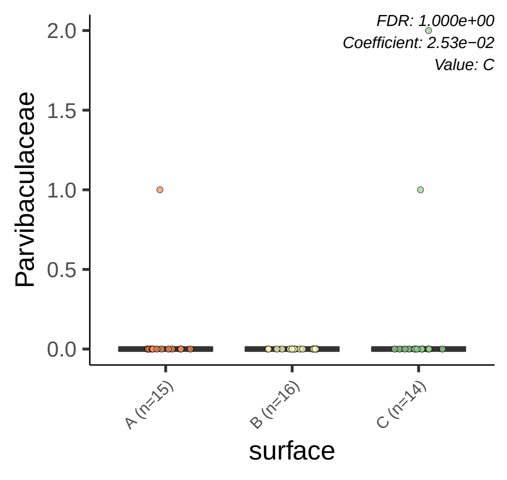
FDR: 1.000e+00
Coefficient: 2.53e−02
Value: C
Parvibaculaceae
2.0
1.5
1.0
0.5
0.0
A (n=15)
B (n=16)
C (n=14)
surface

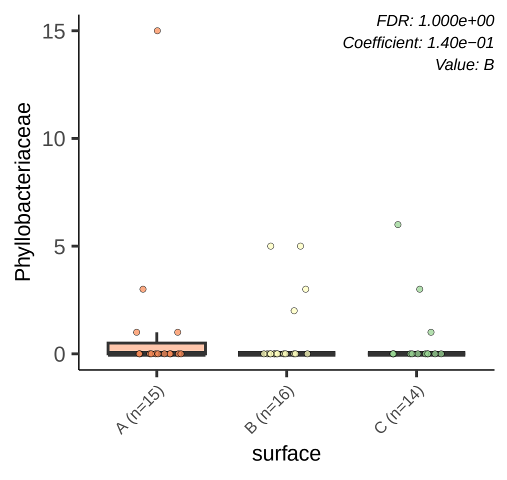
FDR: 1.000e+00
Coefficient: 1.40e−01
Value: B
Phyllobacteriaceae
15
10
5
0
A (n=15)
B (n=16)
C (n=14)
surface

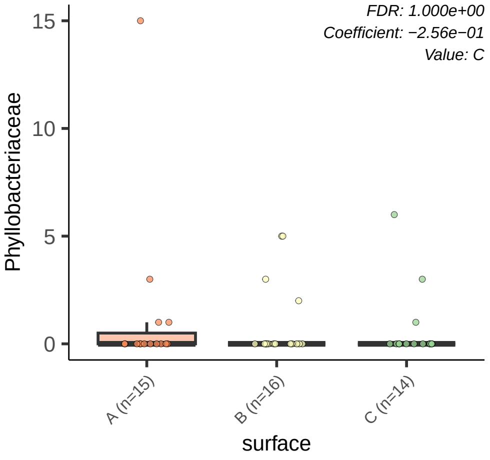
FDR: 1.000e+00
Coefficient: −2.56e−01
Value: C
Phyllobacteriaceae
15
10
5
0
A (n=15)
B (n=16)
C (n=14)
surface

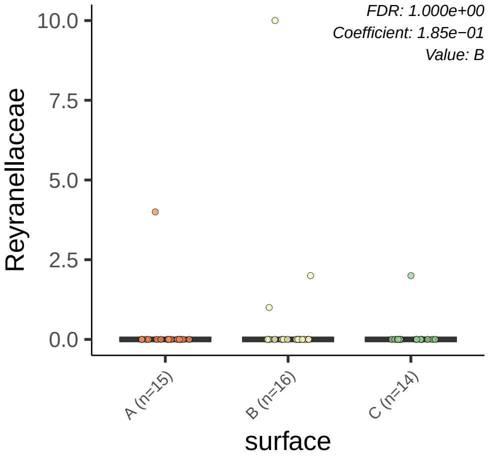

FDR: 1.000e+00
Coefficient: 1.85e−01
Value: B
Reyranellaceae
10.0
7.5
5.0
2.5
0.0
A (n=15)
B (n=16)
C (n=14)
surface

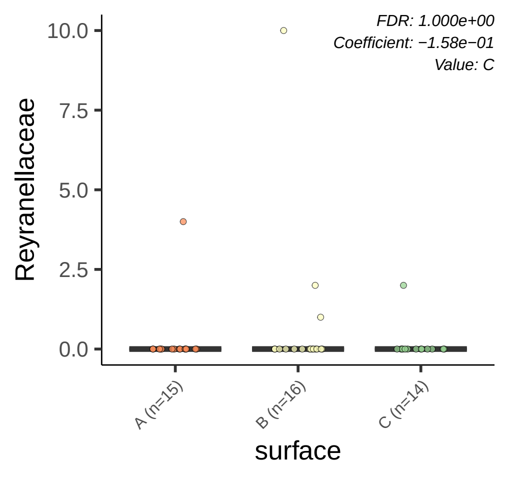
FDR: 1.000e+00
Coefficient: −1.58e−01
Value: C
Reyranellaceae
10.0
7.5
5.0
2.5
0.0
A (n=15)
B (n=16)
C (n=14)
surface

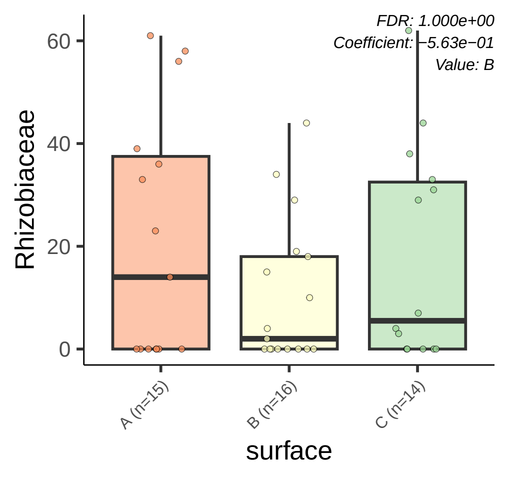

FDR: 1.000e+00
Coefficient: −5.63e−01
Value: B
Rhizobiaceae
60
40
20
0
A (n=15)
B (n=16)
C (n=14)
surface

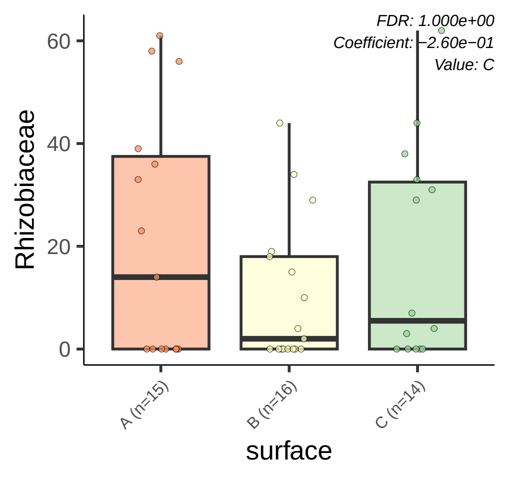
FDR: 1.000e+00
Coefficient: −2.60e−01
Value: C
Rhizobiaceae
0
20
40
60
A (n=15)
B (n=16)
C (n=14)
surface

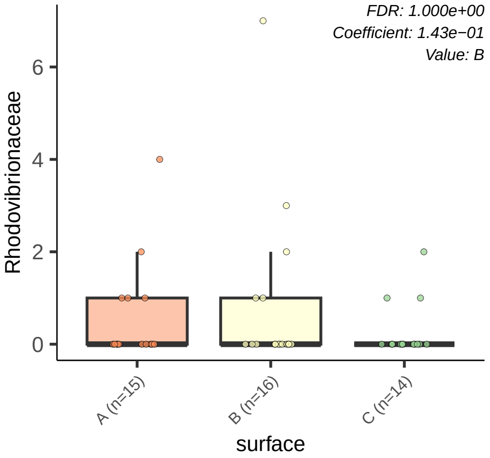
FDR: 1.000e+00
Coefficient: 1.43e−01
Value: B
Rhodovibrionaceae
0
2
4
6
A (n=15)
B (n=16)
C (n=14)
surface

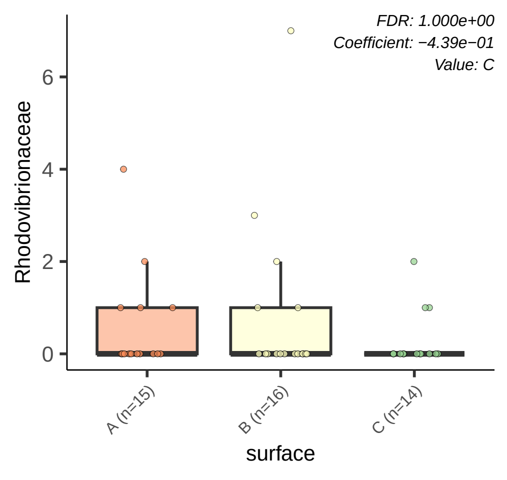

FDR: 1.000e+00
Coefficient: −4.39e−01
Value: C
Rhodovibrionaceae
0
2
4
6
A (n=15)
B (n=16)
C (n=14)
surface

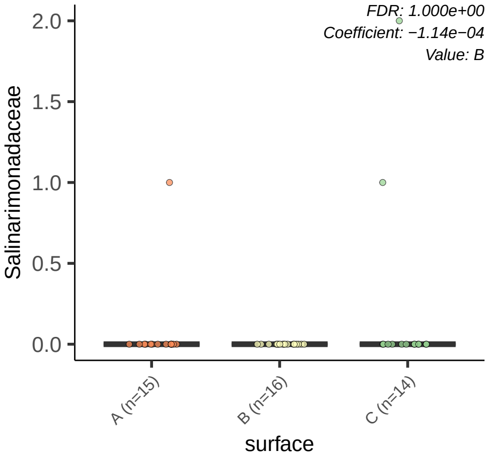

FDR: 1.000e+00
Coefficient: −1.14e−04
Value: B
Salinarimonadaceae
2.0
1.5
1.0
0.5
0.0
A (n=15)
B (n=16)
C (n=14)
surface

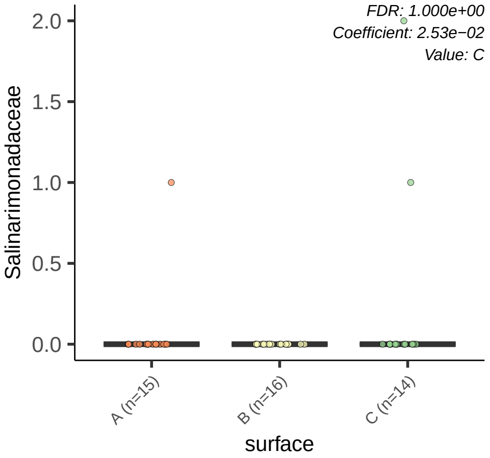
FDR: 1.000e+00
Coefficient: 2.53e−02
Value: C
Salinarimonadaceae
2.0
1.5
1.0
0.5
0.0
A (n=15)
B (n=16)
C (n=14)
surface

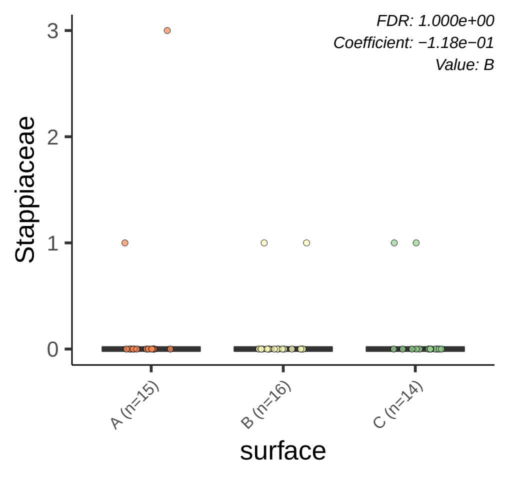

FDR: 1.000e+00
Coefficient: −1.18e−01
Value: B
Stappiaceae
3
2
1
0
A (n=15)
B (n=16)
C (n=14)
surface

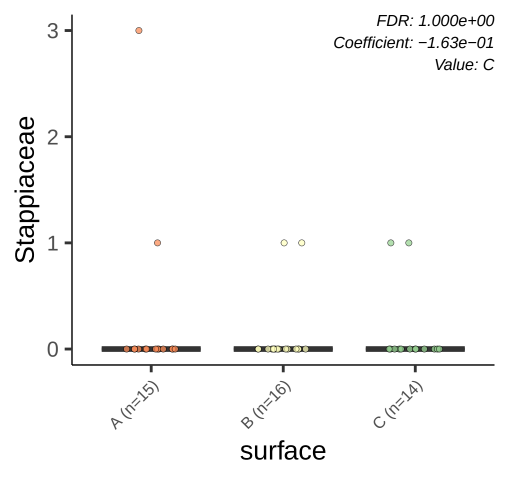
FDR: 1.000e+00
Coefficient: −1.63e−01
Value: C
Stappiaceae
3
2
1
0
A (n=15)
B (n=16)
C (n=14)
surface

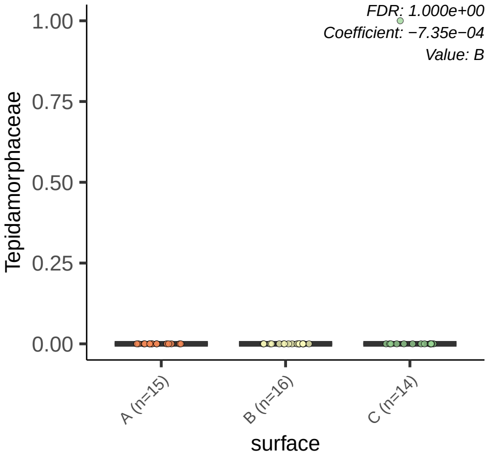
FDR: 1.000e+00
Coefficient: −7.35e−04
Value: B
Tepidamorphaceae
1.00
0.75
0.50
0.25
0.00
A (n=15)
B (n=16)
C (n=14)
surface

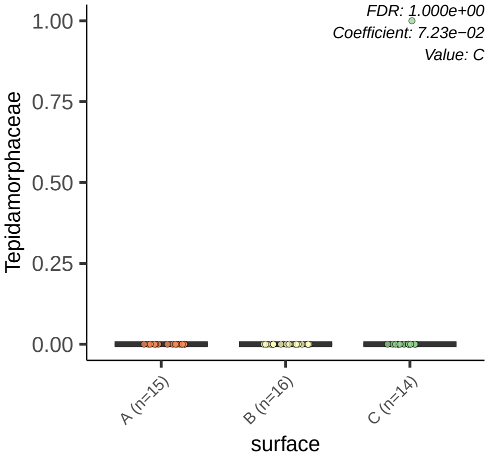

FDR: 1.000e+00
Coefficient: 7.23e−02
Value: C
Tepidamorphaceae
1.00
0.75
0.50
0.25
0.00
A (n=15)
B (n=16)
C (n=14)
surface

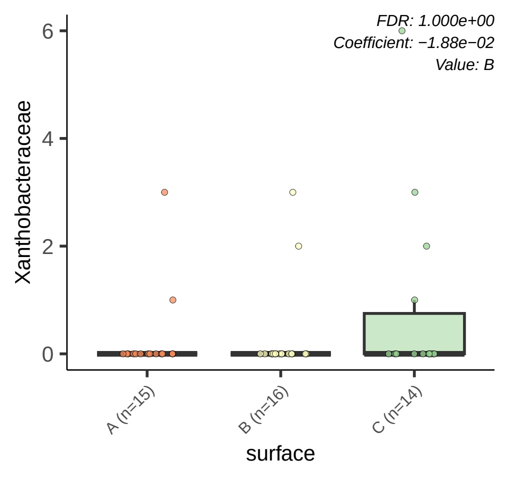

FDR: 1.000e+00
Coefficient: −1.88e−02
Value: B
Xanthobacteraceae
6
4
2
0
A (n=15)
B (n=16)
C (n=14)
surface

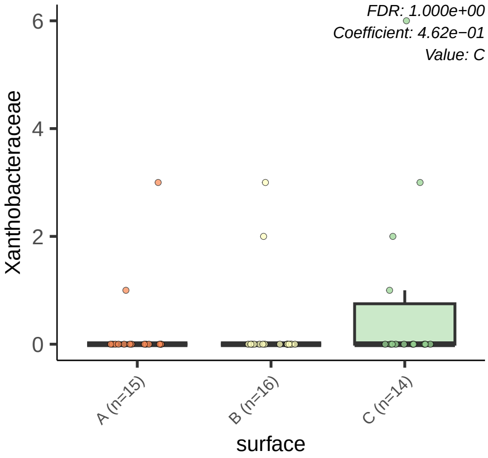

FDR: 1.000e+00
Coefficient: 4.62e−01
Value: C
Xanthobacteraceae
0
2
4
6
A (n=15)
B (n=16)
C (n=14)
surface

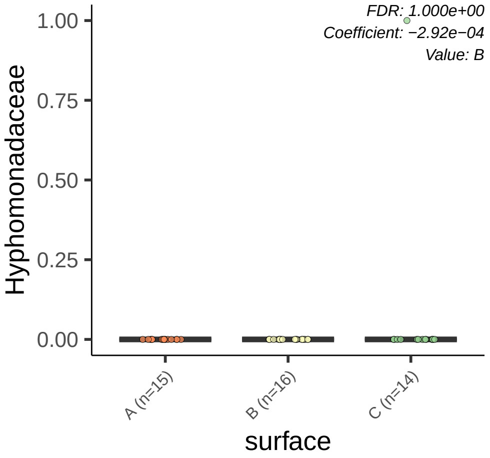

FDR: 1.000e+00
Coefficient: −2.92e−04
Value: B
Hyphomonadaceae
1.00
0.75
0.50
0.25
0.00
A (n=15)
B (n=16)
C (n=14)
surface

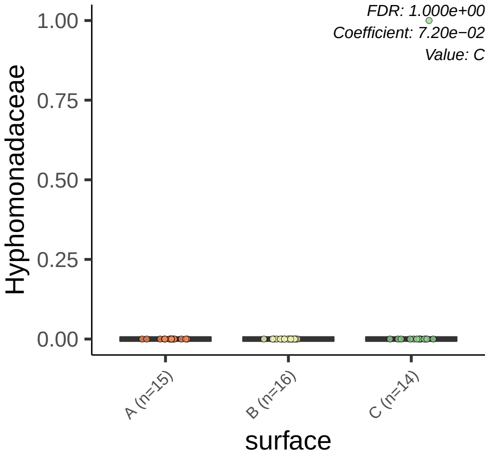
FDR: 1.000e+00
Coefficient: 7.20e−02
Value: C
Hyphomonadaceae
1.00
0.75
0.50
0.25
0.00
A (n=15)
B (n=16)
C (n=14)
surface

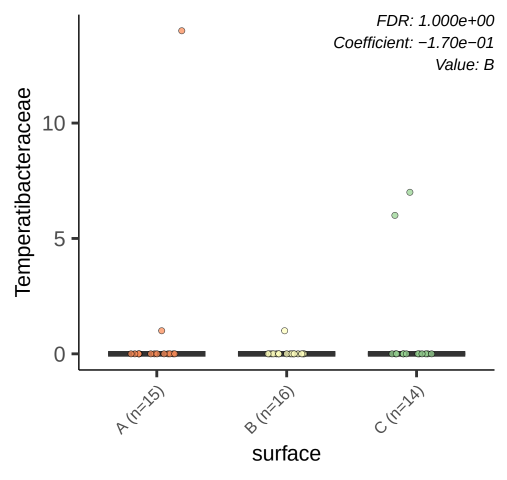

FDR: 1.000e+00
Coefficient: −1.70e−01
Value: B
Temperatibacteraceae
10
5
0
A (n=15)
B (n=16)
C (n=14)
surface

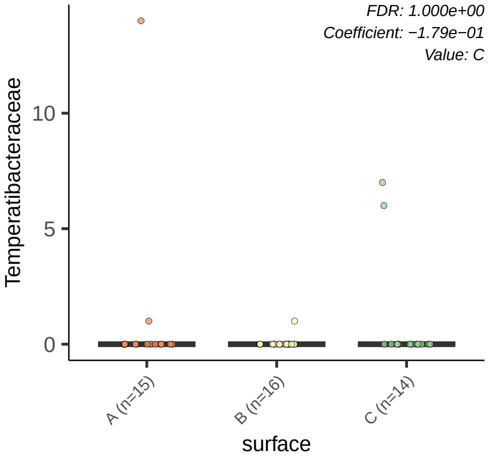
FDR: 1.000e+00
Coefficient: −1.79e−01
Value: C
Temperatibacteraceae
10
5
0
A (n=15)
B (n=16)
C (n=14)
surface

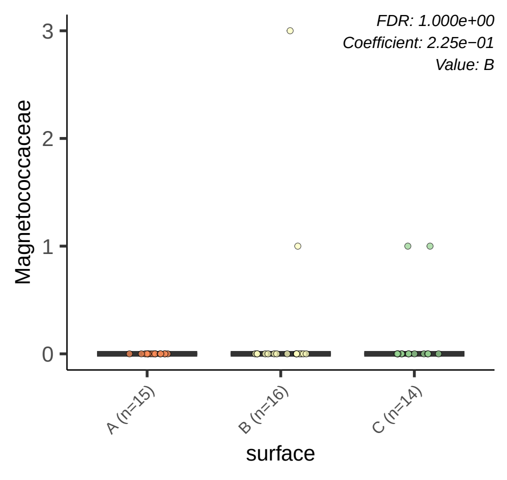
FDR: 1.000e+00
Coefficient: 2.25e−01
Value: B
Magnetococcaceae
0
1
2
3
A (n=15)
B (n=16)
C (n=14)
surface

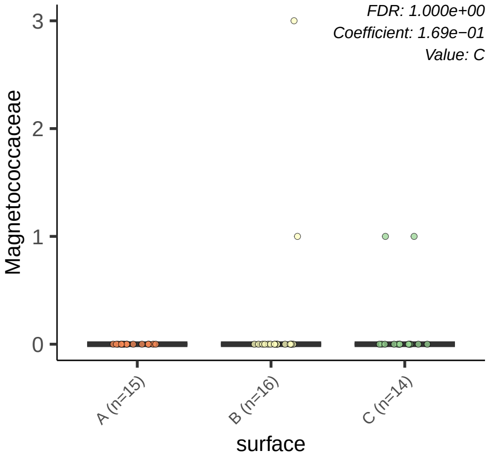
FDR: 1.000e+00
Coefficient: 1.69e−01
Value: C
Magnetococcaceae
3
2
1
0
A (n=15)
B (n=16)
C (n=14)
surface

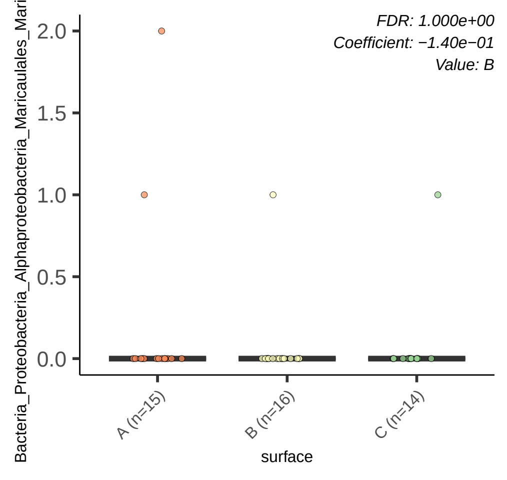

FDR: 1.000e+00
Coefficient: −1.40e−01
Value: B
Bacteria_Proteobacteria_Alphaproteobacteria_Maricaulales_Mari
2.0
1.5
1.0
0.5
0.0
A (n=15)
B (n=16)
C (n=14)
surface

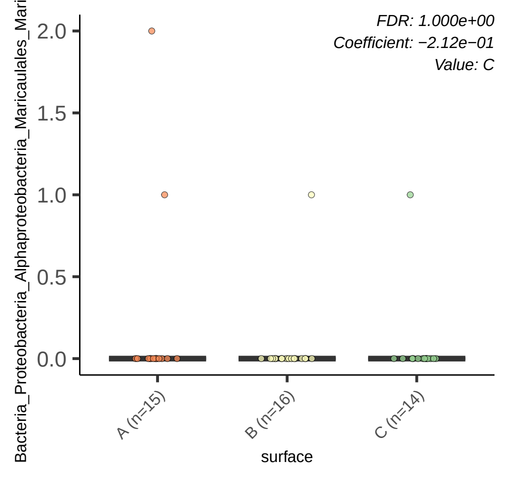

FDR: 1.000e+00
Coefficient: −2.12e−01
Value: C
Bacteria_Proteobacteria_Alphaproteobacteria_Maricaulales_Mari
2.0
1.5
1.0
0.5
0.0
A (n=15)
B (n=16)
C (n=14)
surface

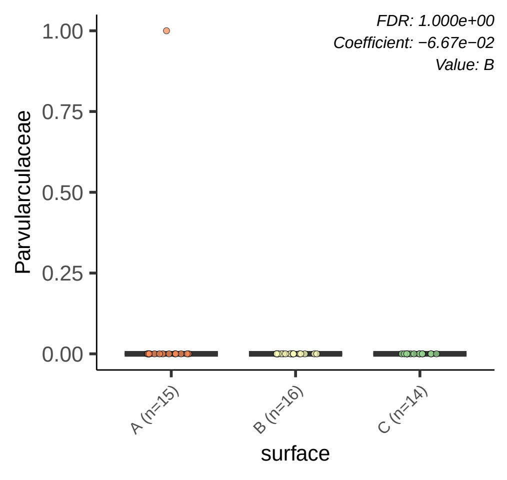
FDR: 1.000e+00
Coefficient: −6.67e−02
Value: B
Parvularculaceae
1.00
0.75
0.50
0.25
0.00
A (n=15)
B (n=16)
C (n=14)
surface

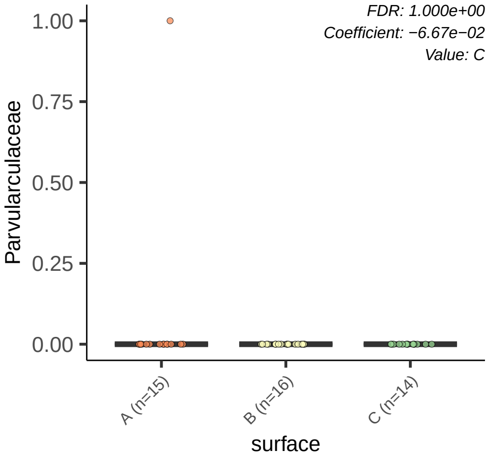

FDR: 1.000e+00
Coefficient: −6.67e−02
Value: C
Parvularculaceae
1.00
0.75
0.50
0.25
0.00
A (n=15)
B (n=16)
C (n=14)
surface

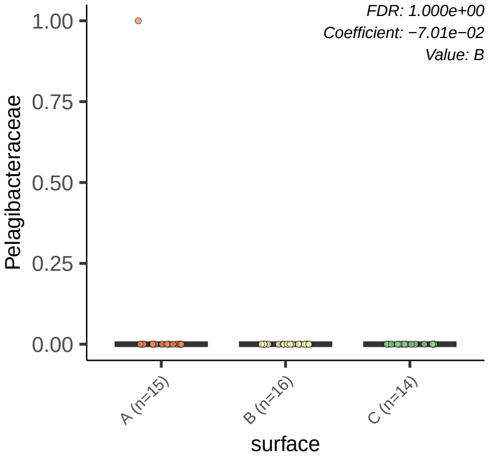
FDR: 1.000e+00
Coefficient: −7.01e−02
Value: B
Pelagibacteraceae
1.00
0.75
0.50
0.25
0.00
A (n=15)
B (n=16)
C (n=14)
surface

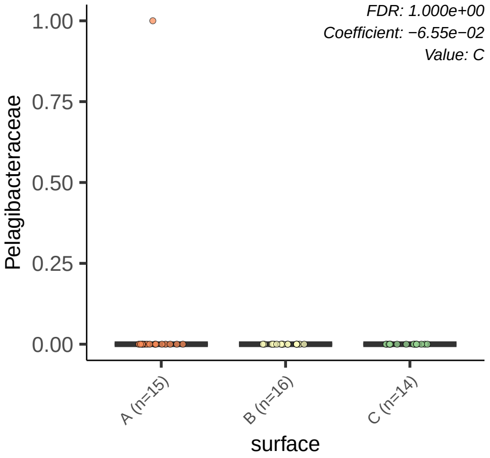
FDR: 1.000e+00
Coefficient: −6.55e−02
Value: C
Pelagibacteraceae
1.00
0.75
0.50
0.25
0.00
A (n=15)
B (n=16)
C (n=14)
surface

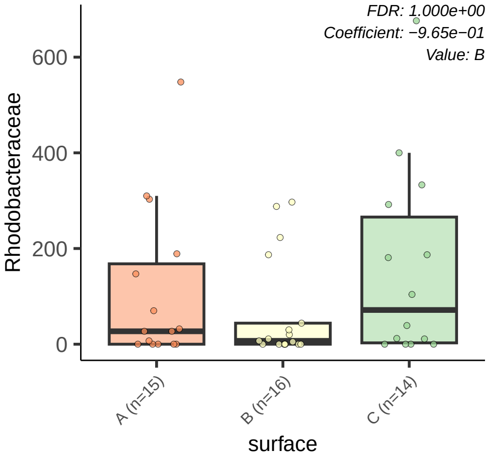
FDR: 1.000e+00
Coefficient: −9.65e−01
Value: B
Rhodobacteraceae
600
400
200
0
A (n=15)
B (n=16)
C (n=14)
surface

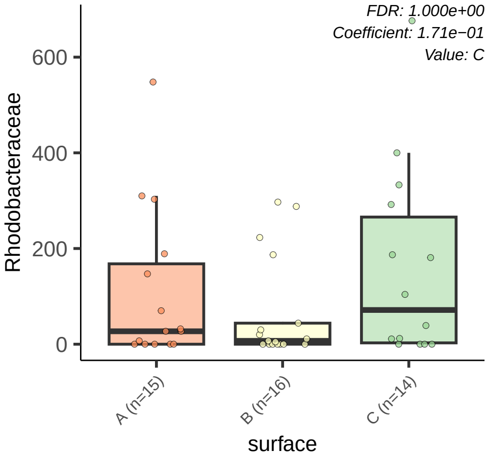
FDR: 1.000e+00
Coefficient: 1.71e−01
Value: C
Rhodobacteraceae
600
400
200
0
A (n=15)
B (n=16)
C (n=14)
surface

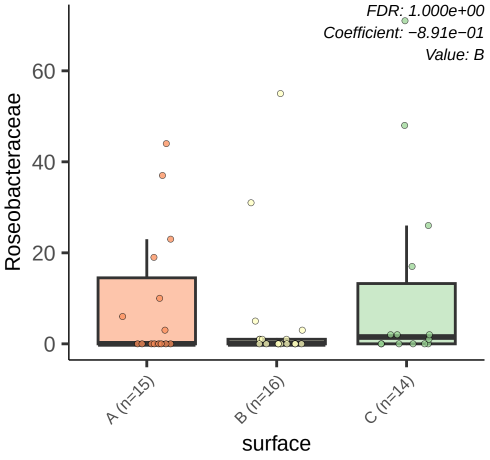

FDR: 1.000e+00
Coefficient: −8.91e−01
Value: B
Roseobacteraceae
60
40
20
0
A (n=15)
B (n=16)
C (n=14)
surface

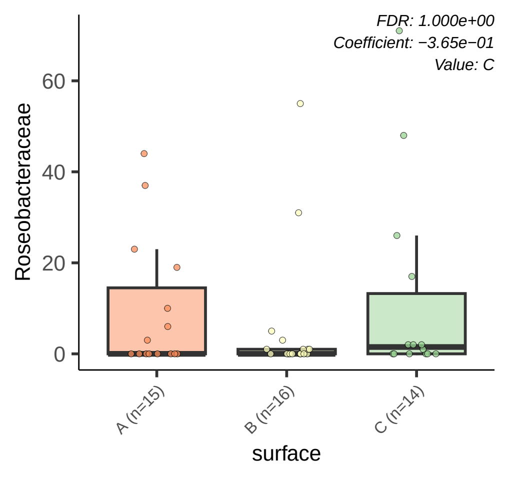
FDR: 1.000e+00
Coefficient: −3.65e−01
Value: C
Roseobacteraceae
60
40
20
0
A (n=15)
B (n=16)
C (n=14)
surface

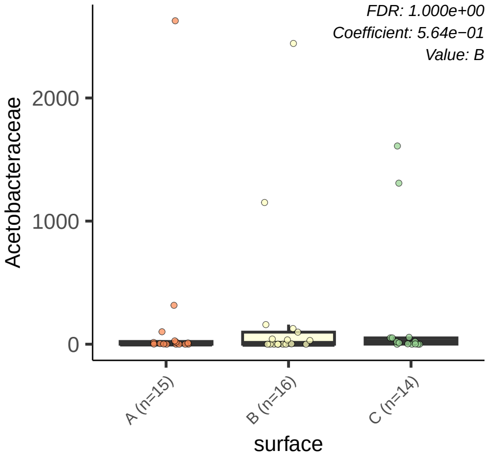

FDR: 1.000e+00
Coefficient: 5.64e−01
Value: B
Acetobacteraceae
2000
1000
0
A (n=15)
B (n=16)
C (n=14)
surface

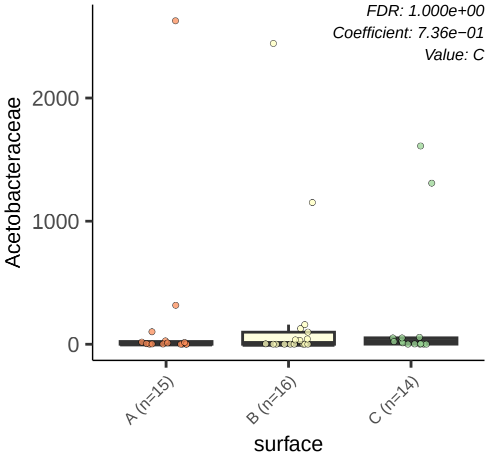
FDR: 1.000e+00
Coefficient: 7.36e−01
Value: C
Acetobacteraceae
2000
1000
0
A (n=15)
B (n=16)
C (n=14)
surface

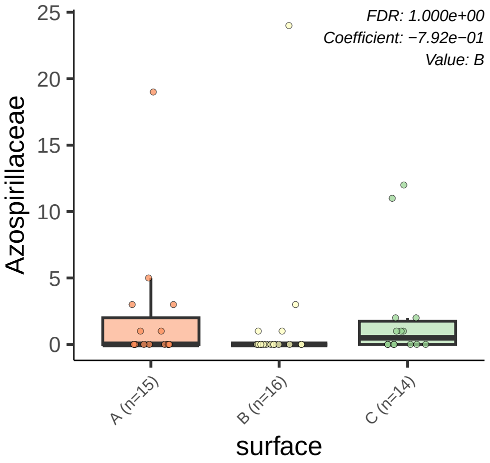

FDR: 1.000e+00
Coefficient: −7.92e−01
Value: B
Azospirillaceae
25
20
15
10
5
0
A (n=15)
B (n=16)
C (n=14)
surface

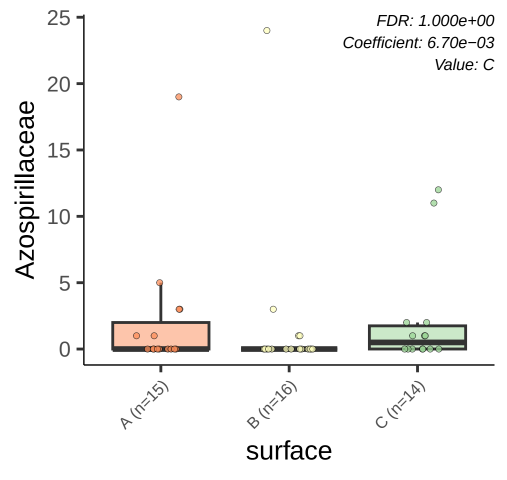

FDR: 1.000e+00
Coefficient: 6.70e−03
Value: C
Azospirillaceae
25
20
15
10
5
0
A (n=15)
B (n=16)
C (n=14)
surface

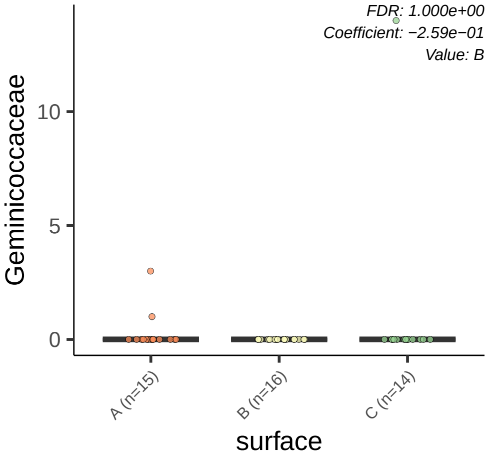
FDR: 1.000e+00
Coefficient: −2.59e−01
Value: B
Geminicoccaceae
10
5
0
A (n=15)
B (n=16)
C (n=14)
surface

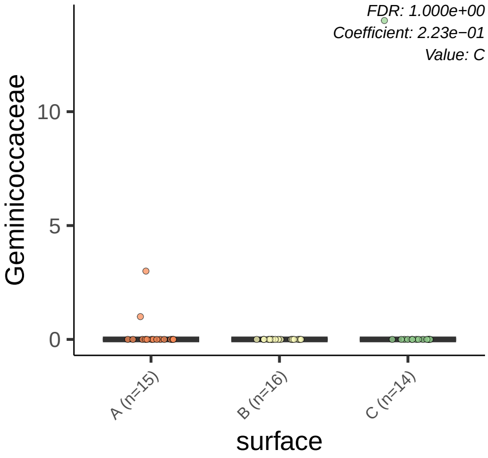
FDR: 1.000e+00
Coefficient: 2.23e−01
Value: C
Geminicoccaceae
10
5
0
A (n=15)
B (n=16)
C (n=14)
surface

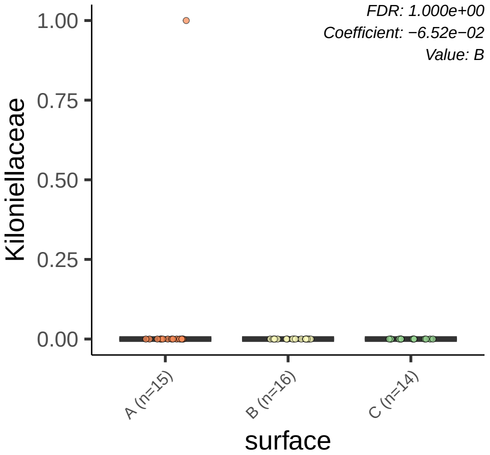
FDR: 1.000e+00
Coefficient: −6.52e−02
Value: B
Kiloniellaceae
1.00
0.75
0.50
0.25
0.00
A (n=15)
B (n=16)
C (n=14)
surface

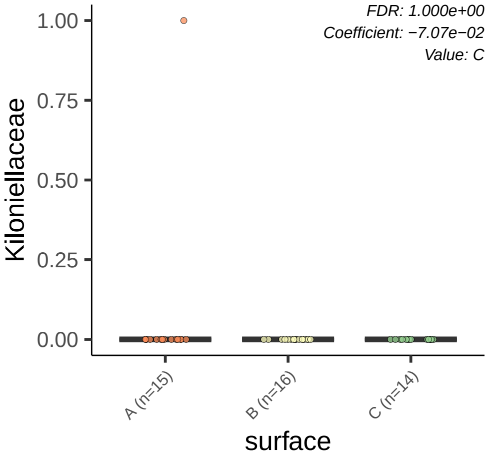
FDR: 1.000e+00
Coefficient: −7.07e−02
Value: C
Kiloniellaceae
1.00
0.75
0.50
0.25
0.00
A (n=15)
B (n=16)
C (n=14)
surface

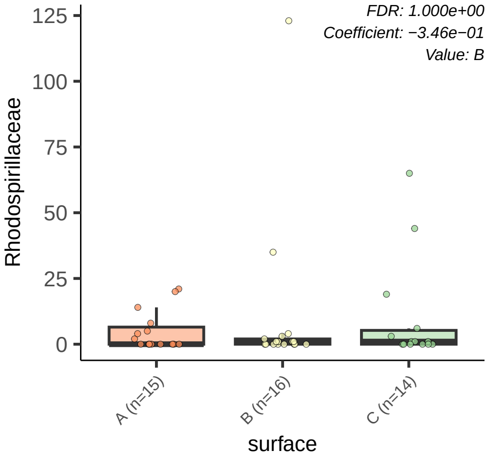
FDR: 1.000e+00
Coefficient: −3.46e−01
Value: B
Rhodospirillaceae
125
100
75
50
25
0
A (n=15)
B (n=16)
C (n=14)
surface

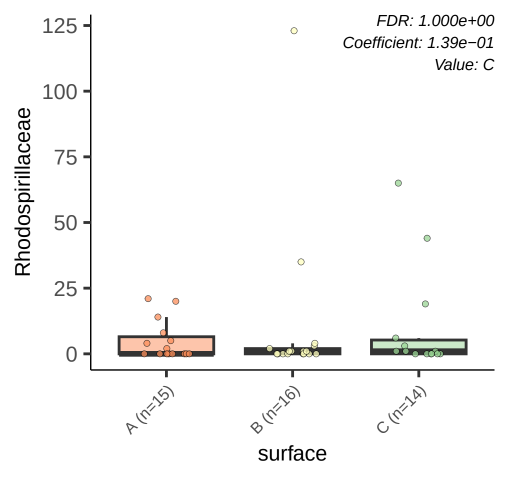

FDR: 1.000e+00
Coefficient: 1.39e−01
Value: C
Rhodospirillaceae
125
100
75
50
25
0
A (n=15)
B (n=16)
C (n=14)
surface

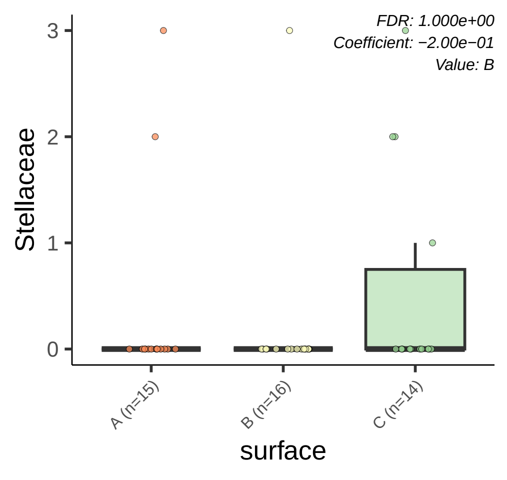

FDR: 1.000e+00
Coefficient: −2.00e−01
Value: B
Stellaceae
0
1
2
3
A (n=15)
B (n=16)
C (n=14)
surface

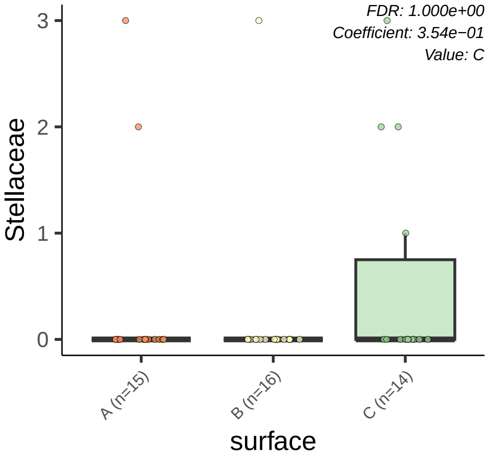
FDR: 1.000e+00
Coefficient: 3.54e−01
Value: C
Stellaceae
3
2
1
0
A (n=15)
B (n=16)
C (n=14)
surface

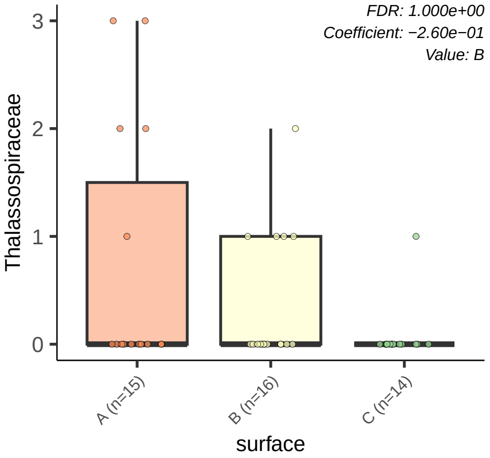
FDR: 1.000e+00
Coefficient: −2.60e−01
Value: B
Thalassospiraceae
0
1
2
3
A (n=15)
B (n=16)
C (n=14)
surface

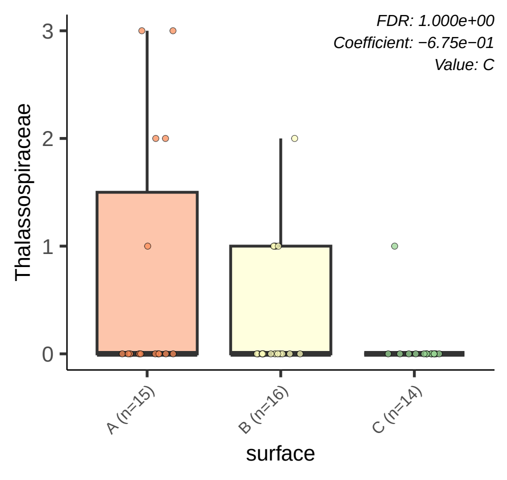
FDR: 1.000e+00
Coefficient: −6.75e−01
Value: C
Thalassospiraceae
0
1
2
3
A (n=15)
B (n=16)
C (n=14)
surface

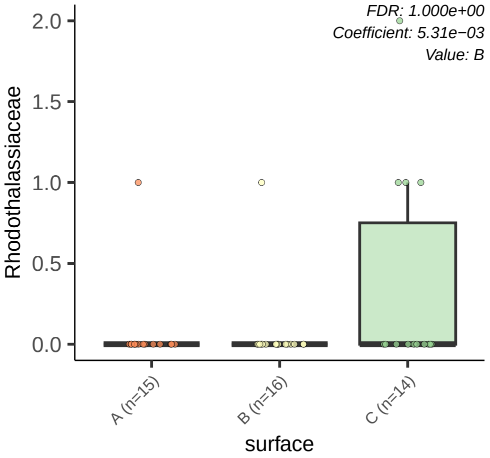

FDR: 1.000e+00
Coefficient: 5.31e−03
Value: B
Rhodothalassiaceae
2.0
1.5
1.0
0.5
0.0
A (n=15)
B (n=16)
C (n=14)
surface

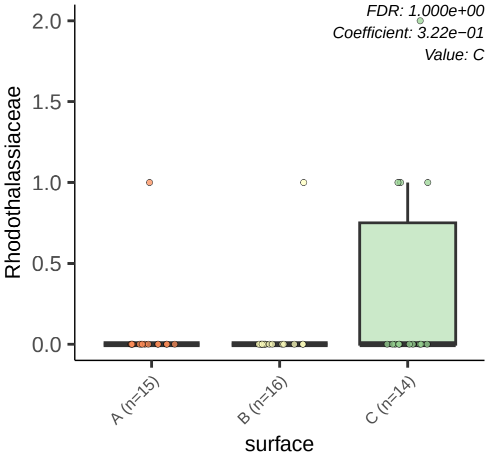
FDR: 1.000e+00
Coefficient: 3.22e−01
Value: C
Rhodothalassiaceae
2.0
1.5
1.0
0.5
0.0
A (n=15)
B (n=16)
C (n=14)
surface

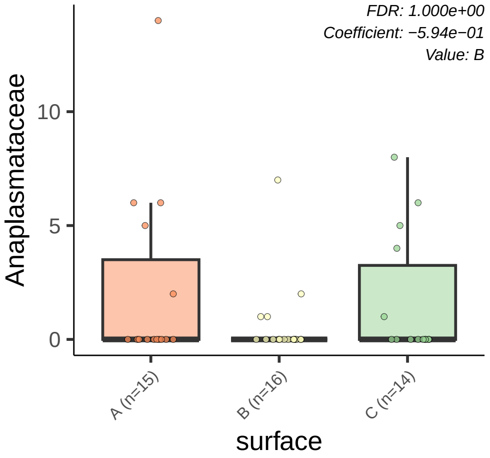
FDR: 1.000e+00
Coefficient: −5.94e−01
Value: B
Anaplasmataceae
10
5
0
A (n=15)
B (n=16)
C (n=14)
surface

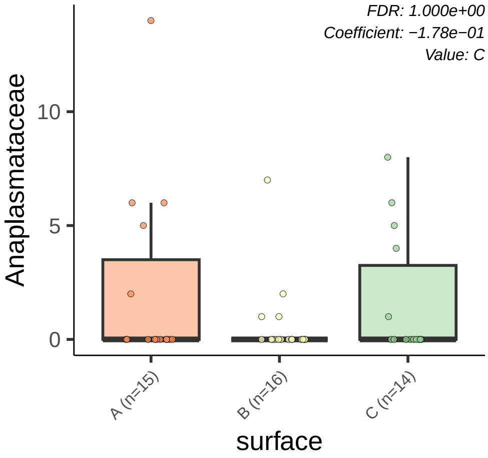

FDR: 1.000e+00
Coefficient: −1.78e−01
Value: C
Anaplasmataceae
0
5
10
A (n=15)
B (n=16)
C (n=14)
surface

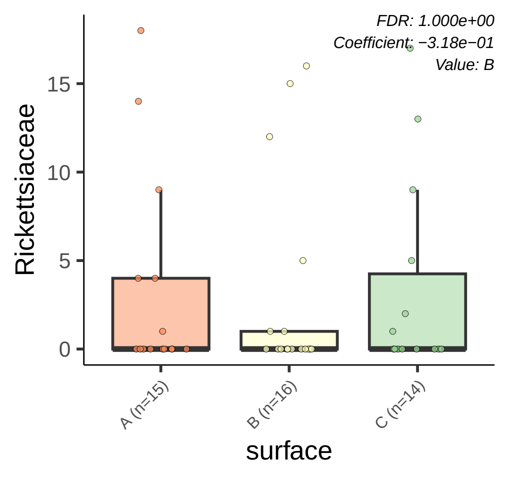
FDR: 1.000e+00
Coefficient: −3.18e−01
Value: B
Rickettsiaceae
0
5
10
15
A (n=15)
B (n=16)
C (n=14)
surface

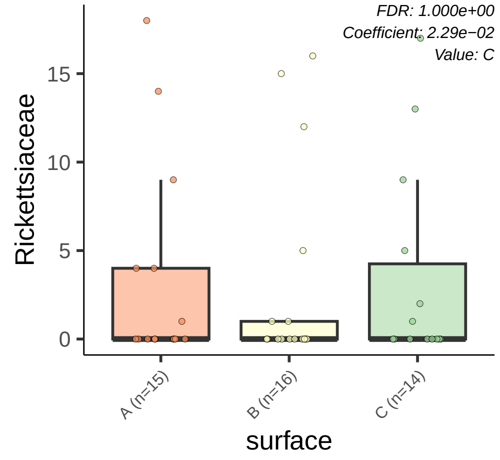

FDR: 1.000e+00
Coefficient: 2.29e−02
Value: C
Rickettsiaceae
0
5
10
15
A (n=15)
B (n=16)
C (n=14)
surface

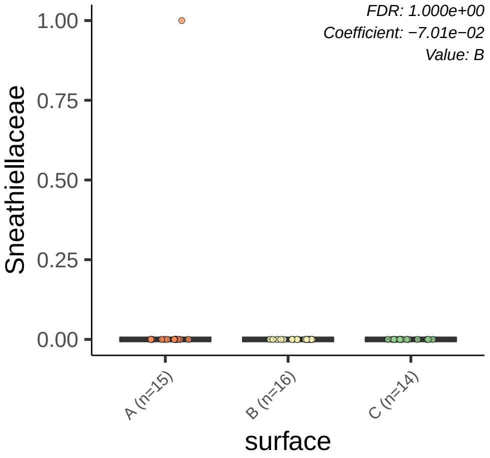
FDR: 1.000e+00
Coefficient: −7.01e−02
Value: B
Sneathiellaceae
1.00
0.75
0.50
0.25
0.00
A (n=15)
B (n=16)
C (n=14)
surface

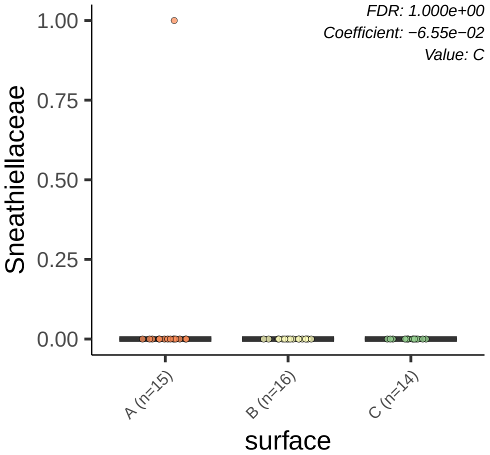
FDR: 1.000e+00
Coefficient: −6.55e−02
Value: C
Sneathiellaceae
1.00
0.75
0.50
0.25
0.00
A (n=15)
B (n=16)
C (n=14)
surface

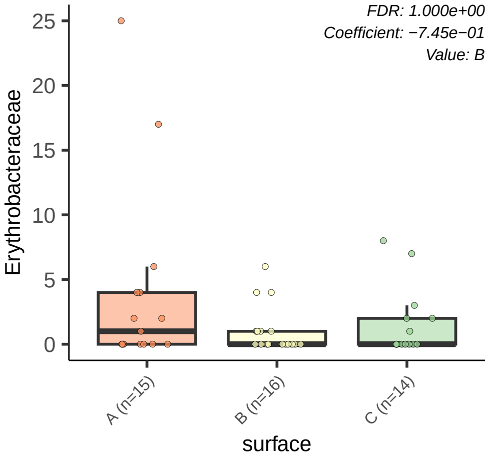

FDR: 1.000e+00
Coefficient: −7.45e−01
Value: B
Erythrobacteraceae
0
5
10
15
20
25
A (n=15)
B (n=16)
C (n=14)
surface

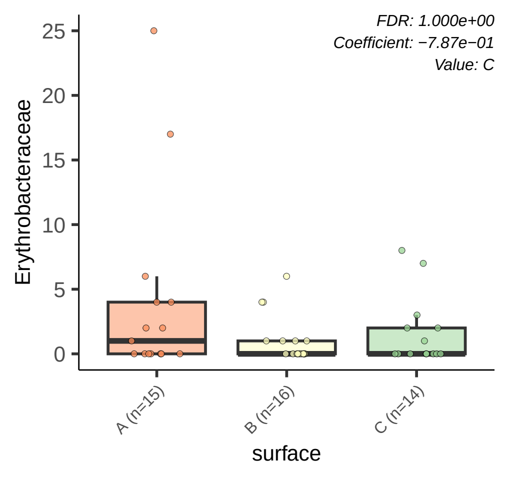

FDR: 1.000e+00
Coefficient: −7.87e−01
Value: C
Erythrobacteraceae
0
5
10
15
20
25
A (n=15)
B (n=16)
C (n=14)
surface

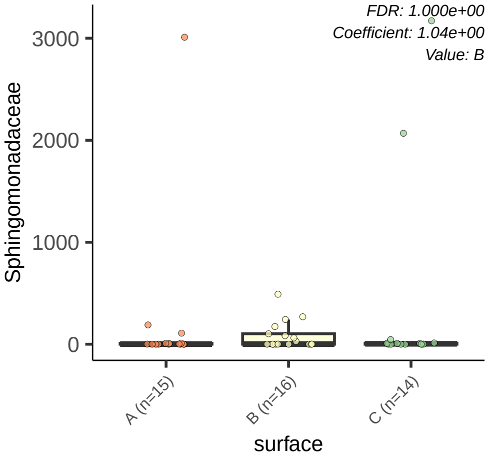

FDR: 1.000e+00
Coefficient: 1.04e+00
Value: B
3000
2000
1000
0
Sphingomonadaceae
A (n=15)
B (n=16)
C (n=14)
surface

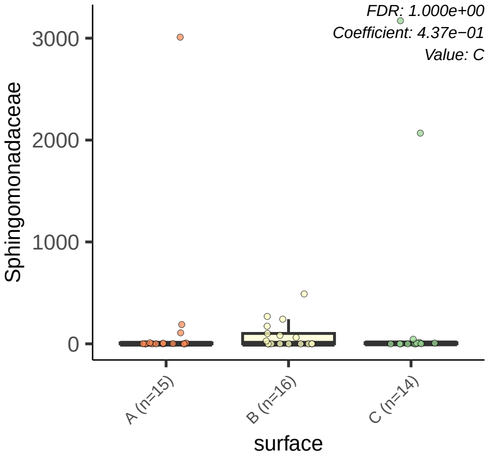

FDR: 1.000e+00
Coefficient: 4.37e−01
Value: C
Sphingomonadaceae
3000
2000
1000
0
A (n=15)
B (n=16)
C (n=14)
surface

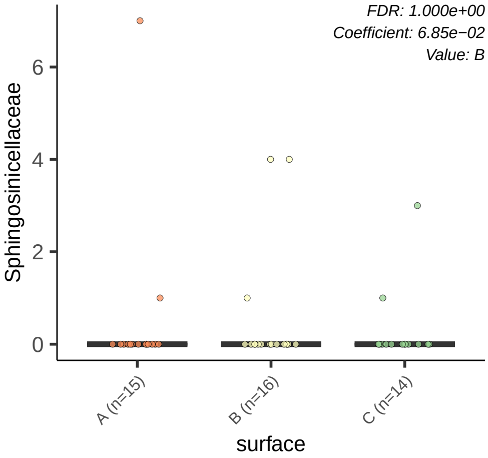
FDR: 1.000e+00
Coefficient: 6.85e−02
Value: B
Sphingosinicellaceae
0
2
4
6
A (n=15)
B (n=16)
C (n=14)
surface

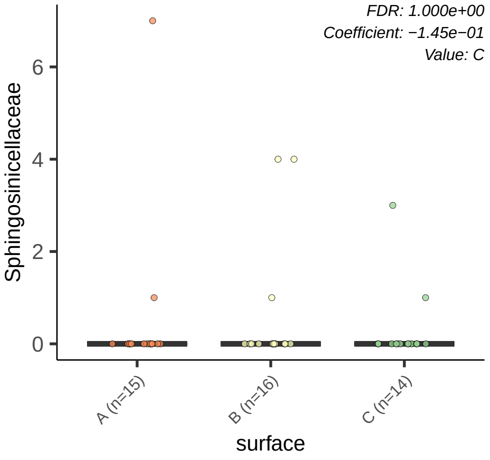
FDR: 1.000e+00
Coefficient: −1.45e−01
Value: C
Sphingosinicellaceae
0
2
4
6
A (n=15)
B (n=16)
C (n=14)
surface

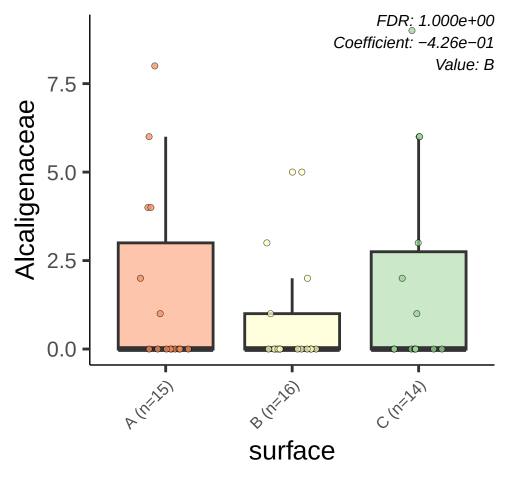
FDR: 1.000e+00
Coefficient: −4.26e−01
Value: B
Alcaligenaceae
7.5
5.0
2.5
0.0
A (n=15)
B (n=16)
C (n=14)
surface

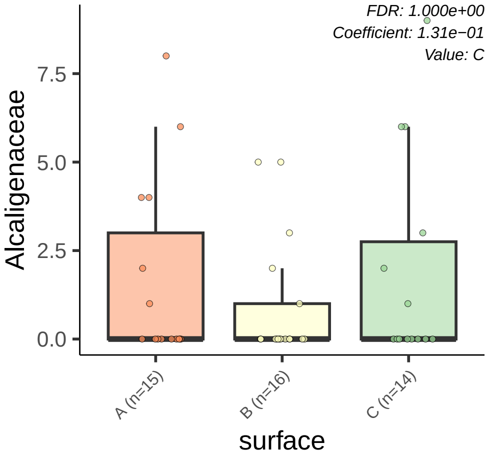
FDR: 1.000e+00
Coefficient: 1.31e−01
Value: C
Alcaligenaceae
7.5
5.0
2.5
0.0
A (n=15)
B (n=16)
C (n=14)
surface

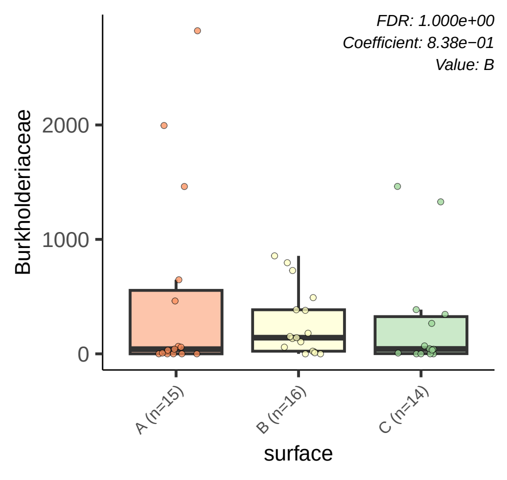
FDR: 1.000e+00
Coefficient: 8.38e−01
Value: B
Burkholderiaceae
2000
1000
0
A (n=15)
B (n=16)
C (n=14)
surface

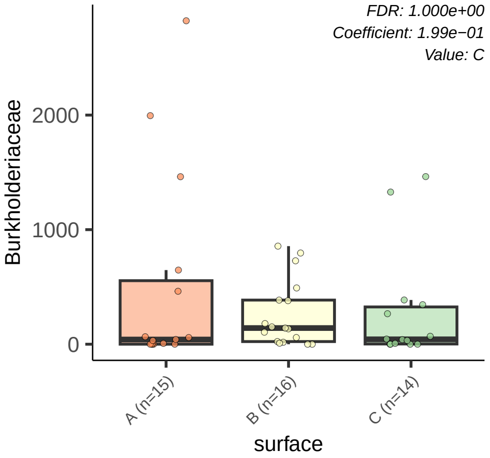
FDR: 1.000e+00
Coefficient: 1.99e−01
Value: C
Burkholderiaceae
2000
1000
0
A (n=15)
B (n=16)
C (n=14)
surface

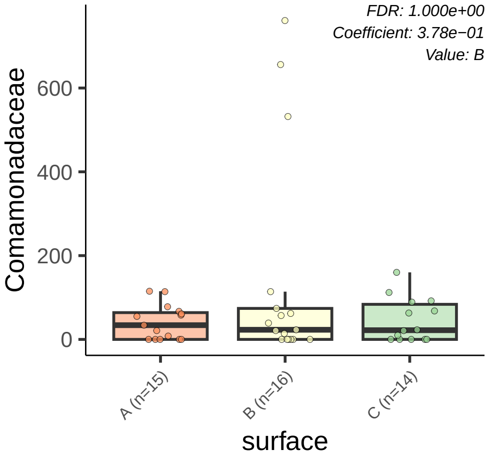

FDR: 1.000e+00
Coefficient: 3.78e−01
Value: B
Comamonadaceae
0
200
400
600
A (n=15)
B (n=16)
C (n=14)
surface

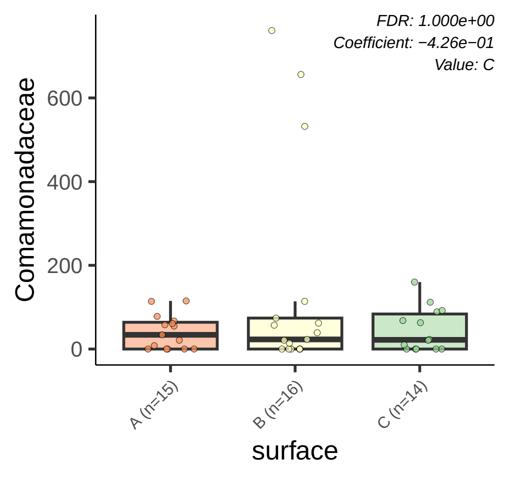

FDR: 1.000e+00
Coefficient: −4.26e−01
Value: C
Comamonadaceae
0
200
400
600
A (n=15)
B (n=16)
C (n=14)
surface

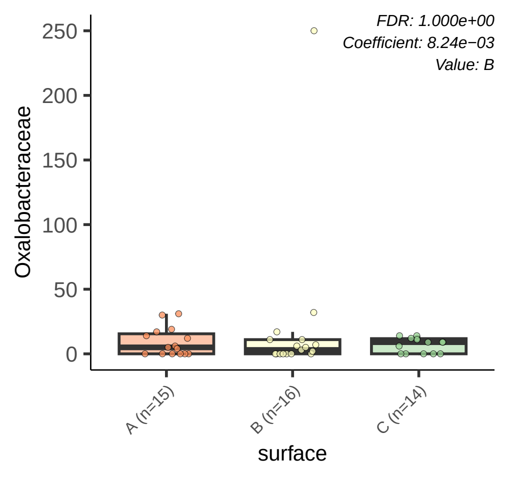
FDR: 1.000e+00
Coefficient: 8.24e−03
Value: B
Oxalobacteraceae
250
200
150
100
50
0
A (n=15)
B (n=16)
C (n=14)
surface

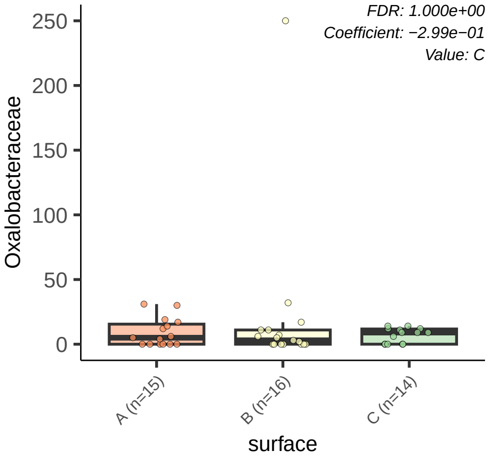

FDR: 1.000e+00
Coefficient: −2.99e−01
Value: C
250
200
150
100
50
0
Oxalobacteraceae
A (n=15)
B (n=16)
C (n=14)
surface

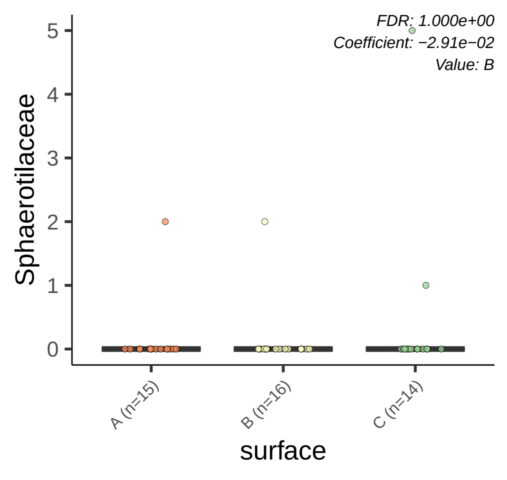

FDR: 1.000e+00
Coefficient: −2.91e−02
Value: B
Sphaerotilaceae
0
1
2
3
4
5
A (n=15)
B (n=16)
C (n=14)
surface

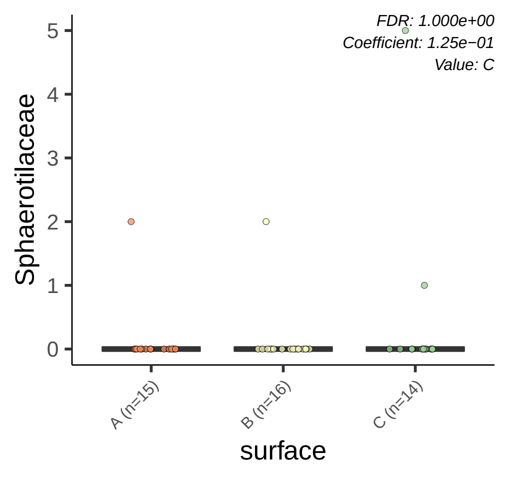
FDR: 1.000e+00
Coefficient: 1.25e−01
Value: C
Sphaerotilaceae
0
1
2
3
4
5
A (n=15)
B (n=16)
C (n=14)
surface

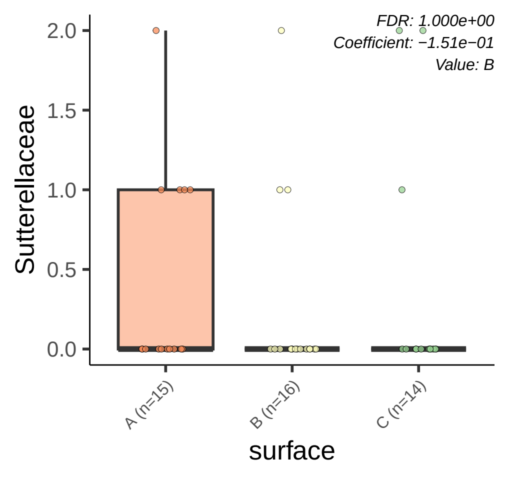
FDR: 1.000e+00
Coefficient: −1.51e−01
Value: B
Sutterellaceae
2.0
1.5
1.0
0.5
0.0
A (n=15)
B (n=16)
C (n=14)
surface

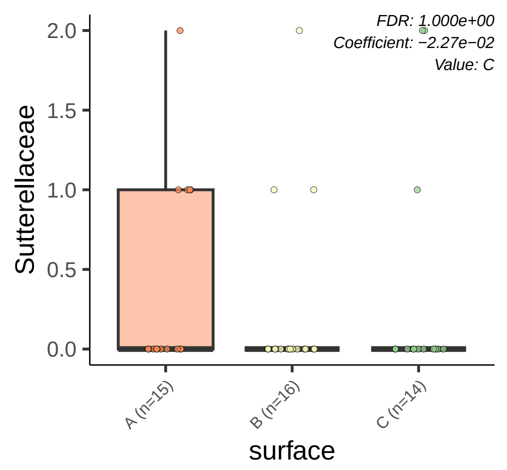
FDR: 1.000e+00
Coefficient: −2.27e−02
Value: C
Sutterellaceae
2.0
1.5
1.0
0.5
0.0
A (n=15)
B (n=16)
C (n=14)
surface

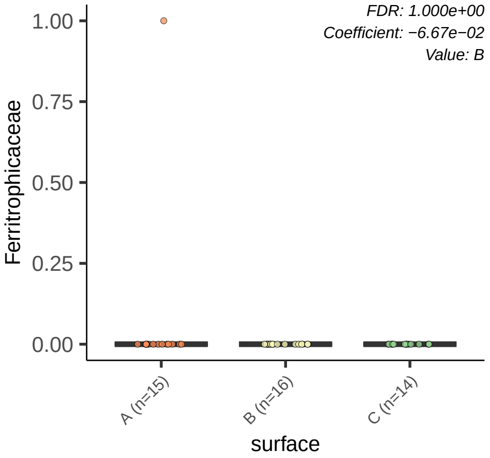

FDR: 1.000e+00
Coefficient: −6.67e−02
Value: B
Ferritrophicaceae
1.00
0.75
0.50
0.25
0.00
A (n=15)
B (n=16)
C (n=14)
surface

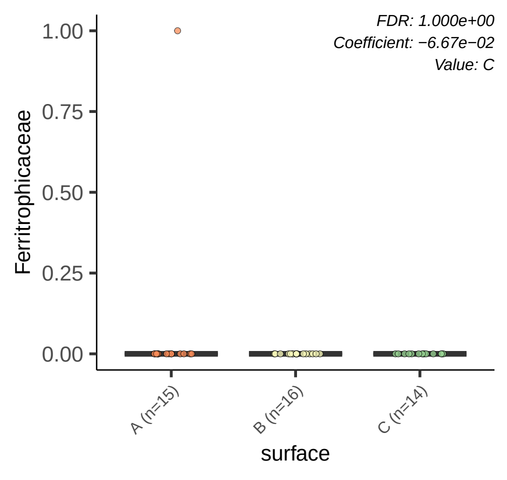

FDR: 1.000e+00
Coefficient: −6.67e−02
Value: C
Ferritrophicaceae
1.00
0.75
0.50
0.25
0.00
A (n=15)
B (n=16)
C (n=14)
surface

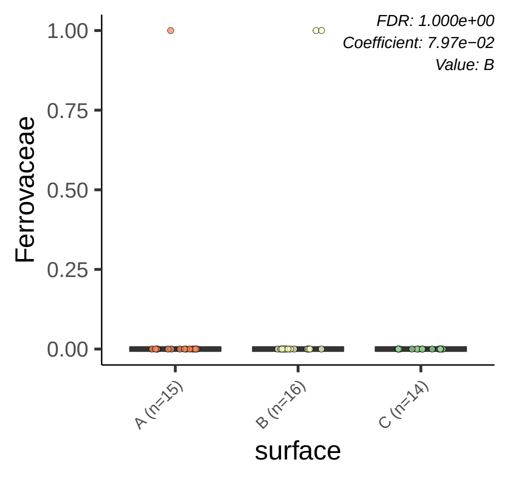

FDR: 1.000e+00
Coefficient: 7.97e−02
Value: B
Ferrovaceae
1.00
0.75
0.50
0.25
0.00
A (n=15)
B (n=16)
C (n=14)
surface

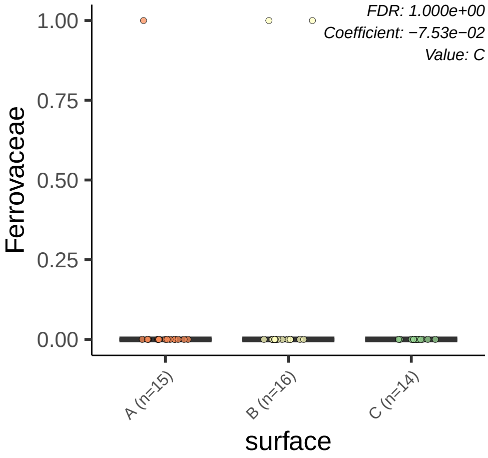
FDR: 1.000e+00
Coefficient: −7.53e−02
Value: C
Ferrovaceae
1.00
0.75
0.50
0.25
0.00
A (n=15)
B (n=16)
C (n=14)
surface

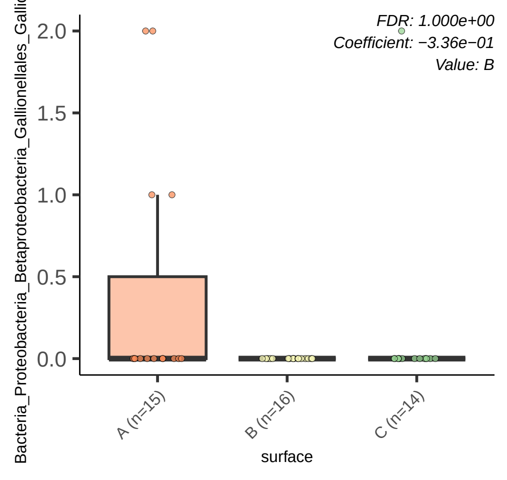

FDR: 1.000e+00
Coefficient: −3.36e−01
Value: B
Bacteria_Proteobacteria_Betaproteobacteria_Gallionellales_Gallio
2.0
1.5
1.0
0.5
0.0
A (n=15)
B (n=16)
C (n=14)
surface

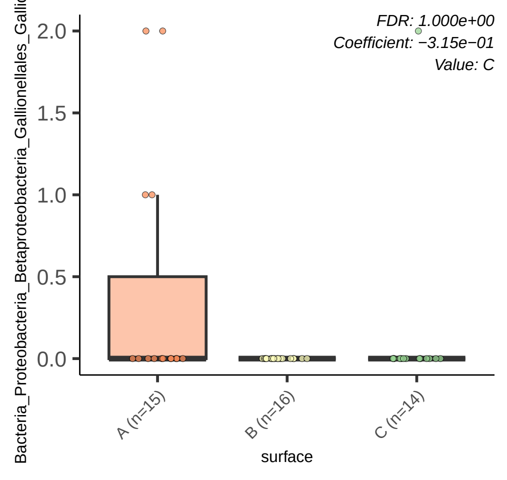
FDR: 1.000e+00
Coefficient: −3.15e−01
Value: C
Bacteria_Proteobacteria_Betaproteobacteria_Gallionellales_Gallio
2.0
1.5
1.0
0.5
0.0
A (n=15)
B (n=16)
C (n=14)
surface

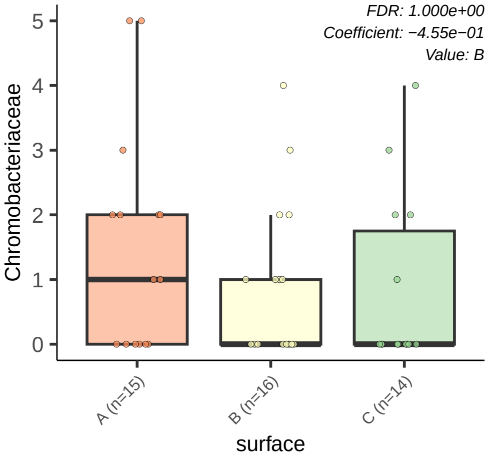
FDR: 1.000e+00
Coefficient: −4.55e−01
Value: B
Chromobacteriaceae
0
1
2
3
4
5
A (n=15)
B (n=16)
C (n=14)
surface

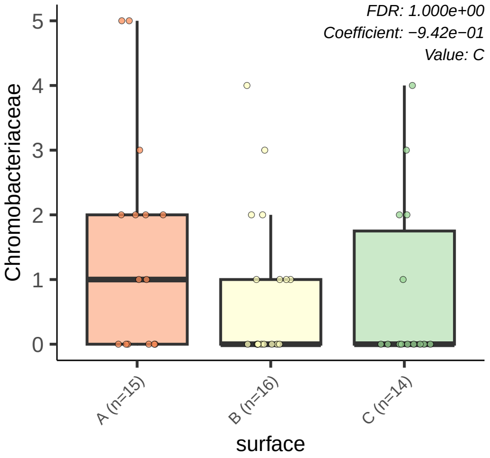

FDR: 1.000e+00
Coefficient: −9.42e−01
Value: C
Chromobacteriaceae
0
1
2
3
4
5
A (n=15)
B (n=16)
C (n=14)
surface

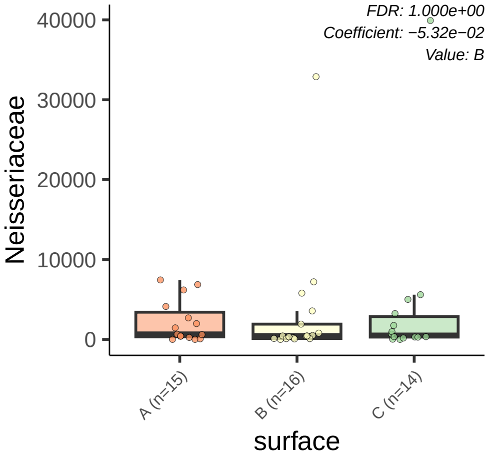
FDR: 1.000e+00
Coefficient: −5.32e−02
Value: B
40000
30000
20000
10000
0
Neisseriaceae
A (n=15)
B (n=16)
C (n=14)
surface

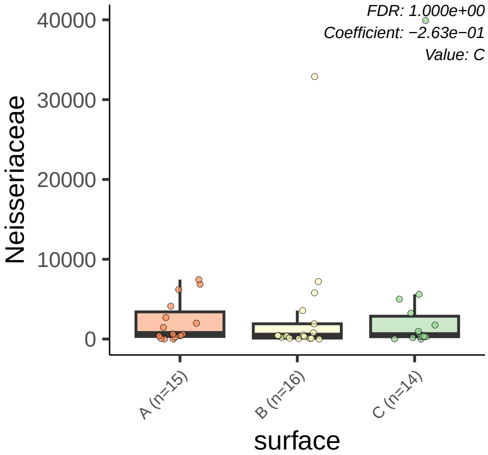

FDR: 1.000e+00
Coefficient: −2.63e−01
Value: C
Neisseriaceae
40000
30000
20000
10000
0
A (n=15)
B (n=16)
C (n=14)
surface

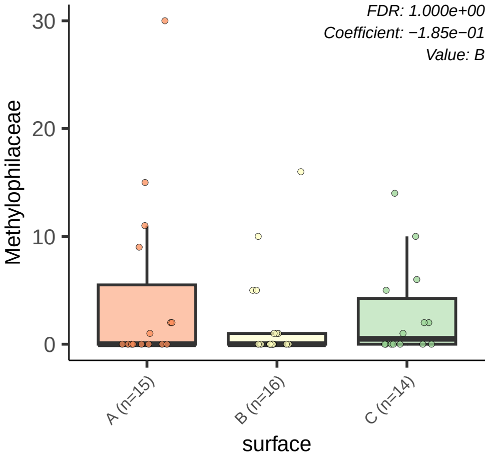
FDR: 1.000e+00
Coefficient: −1.85e−01
Value: B
Methylophilaceae
30
20
10
0
A (n=15)
B (n=16)
C (n=14)
surface

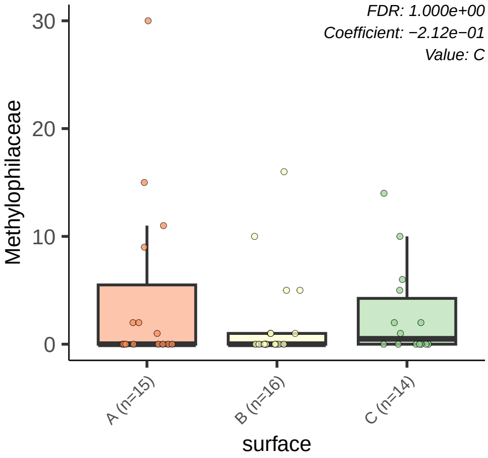
FDR: 1.000e+00
Coefficient: −2.12e−01
Value: C
Methylophilaceae
30
20
10
0
A (n=15)
B (n=16)
C (n=14)
surface

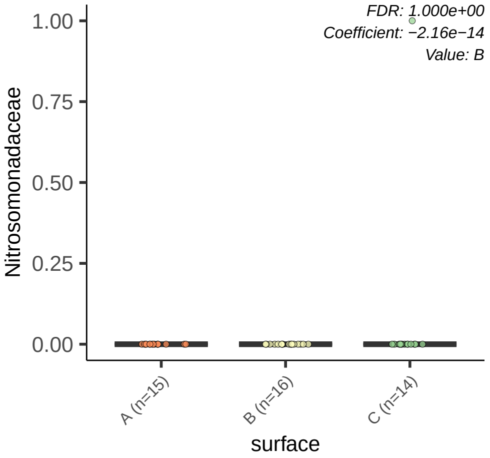
FDR: 1.000e+00
Coefficient: −2.16e−14
Value: B
Nitrosomonadaceae
1.00
0.75
0.50
0.25
0.00
A (n=15)
B (n=16)
C (n=14)
surface

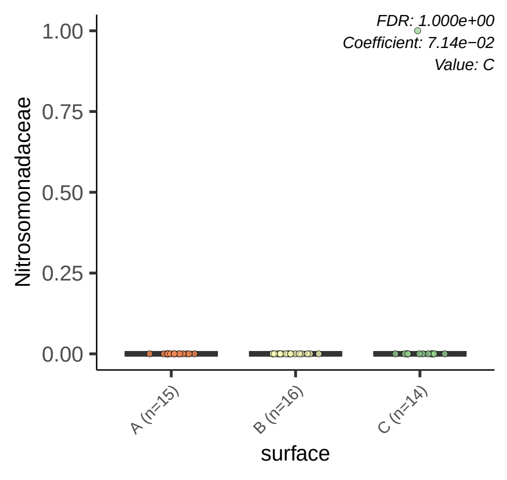
FDR: 1.000e+00
Coefficient: 7.14e−02
Value: C
1.00
0.75
0.50
0.25
0.00
Nitrosomonadaceae
A (n=15)
B (n=16)
C (n=14)
surface

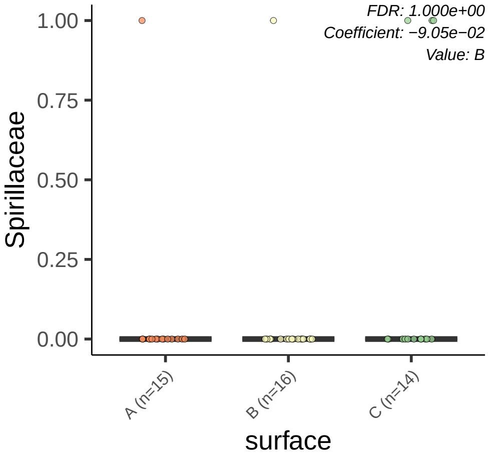

FDR: 1.000e+00
Coefficient: −9.05e−02
Value: B
Spirillaceae
1.00
0.75
0.50
0.25
0.00
A (n=15)
B (n=16)
C (n=14)
surface

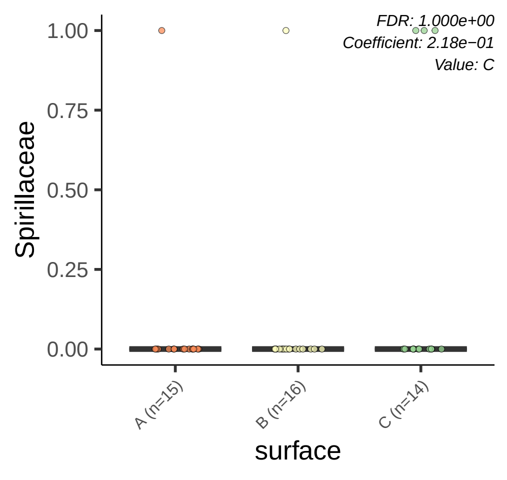

FDR: 1.000e+00
Coefficient: 2.18e−01
Value: C
Spirillaceae
1.00
0.75
0.50
0.25
0.00
A (n=15)
B (n=16)
C (n=14)
surface

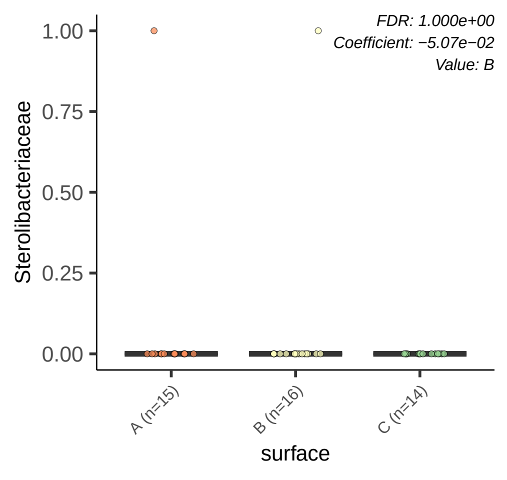

FDR: 1.000e+00
Coefficient: −5.07e−02
Value: B
Sterolibacteriaceae
1.00
0.75
0.50
0.25
0.00
A (n=15)
B (n=16)
C (n=14)
surface

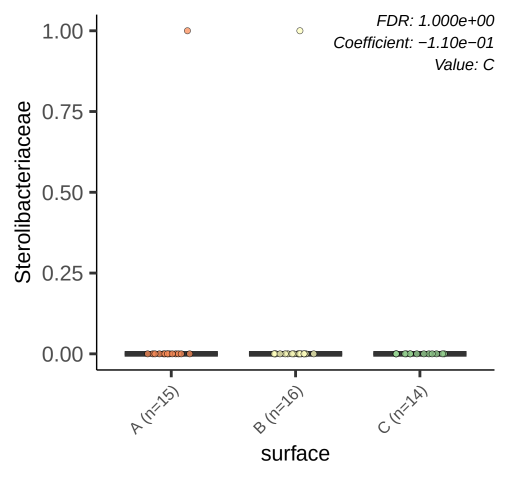
FDR: 1.000e+00
Coefficient: −1.10e−01
Value: C
Sterolibacteriaceae
1.00
0.75
0.50
0.25
0.00
A (n=15)
B (n=16)
C (n=14)
surface

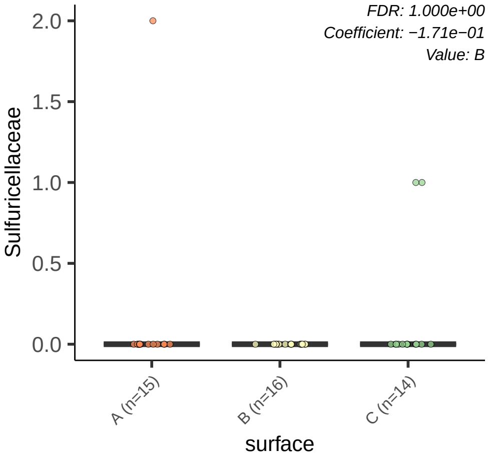

FDR: 1.000e+00
Coefficient: −1.71e−01
Value: B
Sulfuricellaceae
2.0
1.5
1.0
0.5
0.0
A (n=15)
B (n=16)
C (n=14)
surface

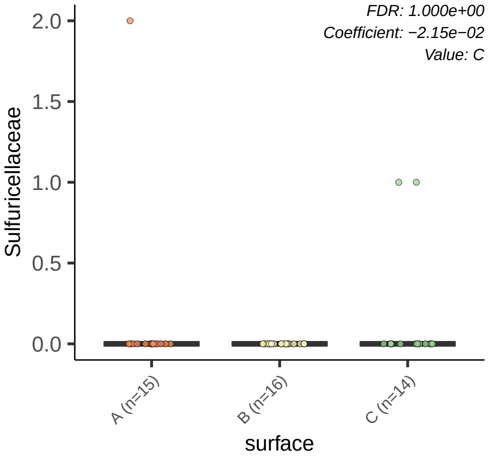

FDR: 1.000e+00
Coefficient: −2.15e−02
Value: C
Sulfuricellaceae
2.0
1.5
1.0
0.5
0.0
A (n=15)
B (n=16)
C (n=14)
surface

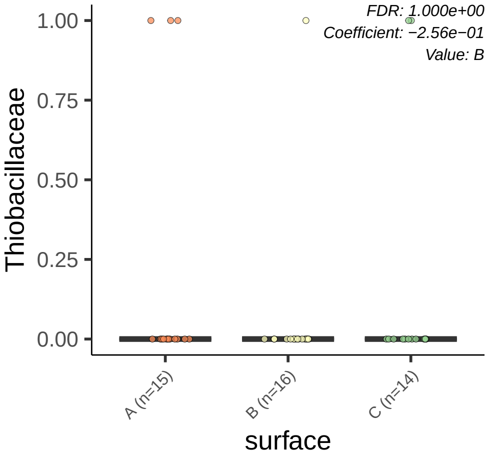
FDR: 1.000e+00
Coefficient: −2.56e−01
Value: B
Thiobacillaceae
1.00
0.75
0.50
0.25
0.00
A (n=15)
B (n=16)
C (n=14)
surface

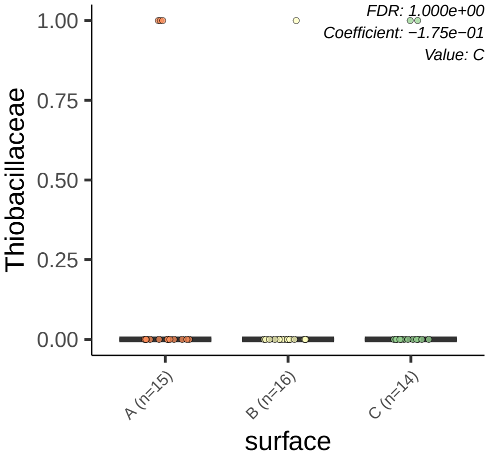

FDR: 1.000e+00
Coefficient: −1.75e−01
Value: C
Thiobacillaceae
1.00
0.75
0.50
0.25
0.00
A (n=15)
B (n=16)
C (n=14)
surface

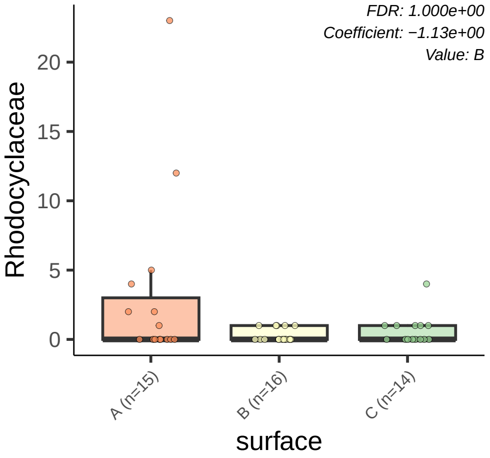
FDR: 1.000e+00
Coefficient: −1.13e+00
Value: B
Rhodocyclaceae
20
15
10
5
0
A (n=15)
B (n=16)
C (n=14)
surface

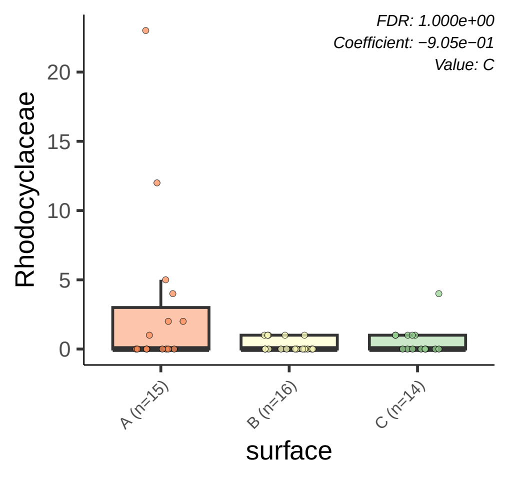

FDR: 1.000e+00
Coefficient: −9.05e−01
Value: C
Rhodocyclaceae
0
5
10
15
20
A (n=15)
B (n=16)
C (n=14)
surface

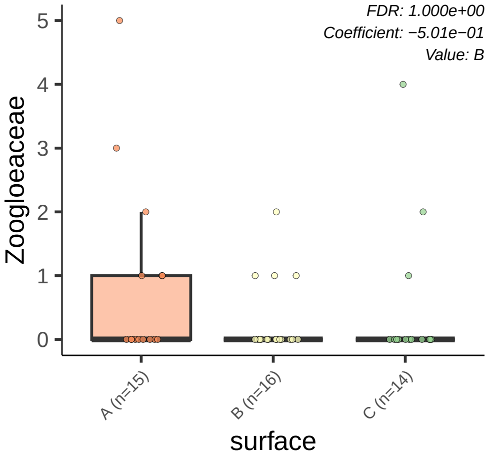

FDR: 1.000e+00
Coefficient: −5.01e−01
Value: B
Zoogloeaceae
0
1
2
3
4
5
A (n=15)
B (n=16)
C (n=14)
surface

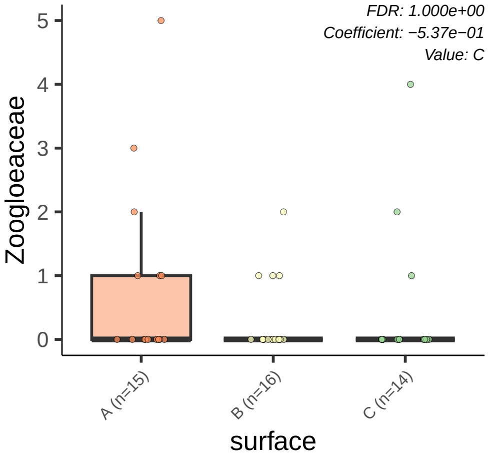
FDR: 1.000e+00
Coefficient: −5.37e−01
Value: C
Zoogloeaceae
0
1
2
3
4
5
A (n=15)
B (n=16)
C (n=14)
surface

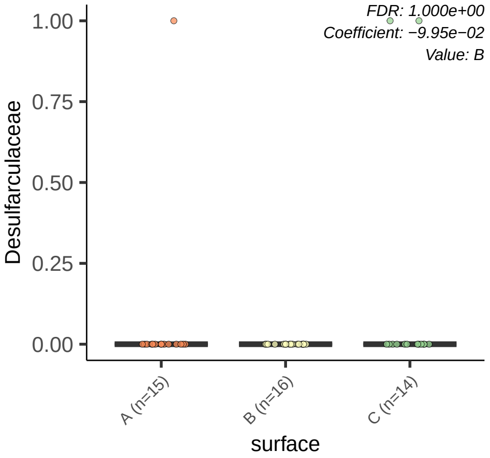

FDR: 1.000e+00
Coefficient: −9.95e−02
Value: B
Desulfarculaceae
1.00
0.75
0.50
0.25
0.00
A (n=15)
B (n=16)
C (n=14)
surface

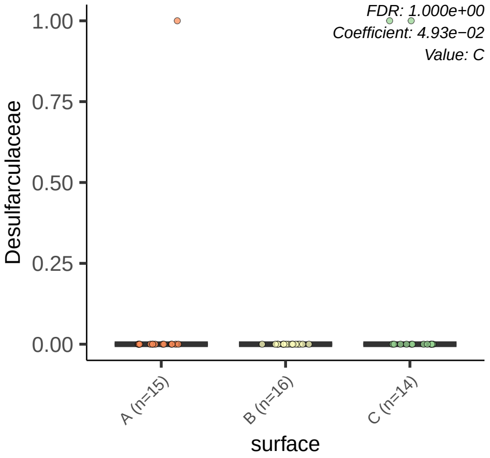

FDR: 1.000e+00
Coefficient: 4.93e−02
Value: C
Desulfarculaceae
1.00
0.75
0.50
0.25
0.00
A (n=15)
B (n=16)
C (n=14)
surface

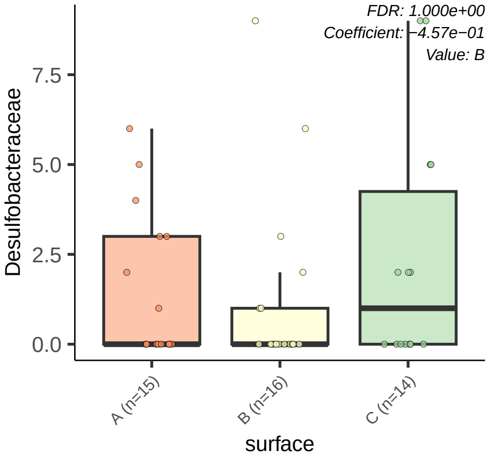

FDR: 1.000e+00
Coefficient: −4.57e−01
Value: B
Desulfobacteraceae
7.5
5.0
2.5
0.0
A (n=15)
B (n=16)
C (n=14)
surface

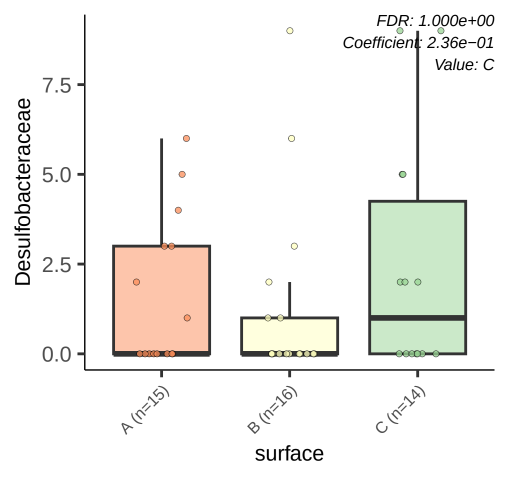
FDR: 1.000e+00
Coefficient: 2.36e−01
Value: C
Desulfobacteraceae
0.0
2.5
5.0
7.5
A (n=15)
B (n=16)
C (n=14)
surface

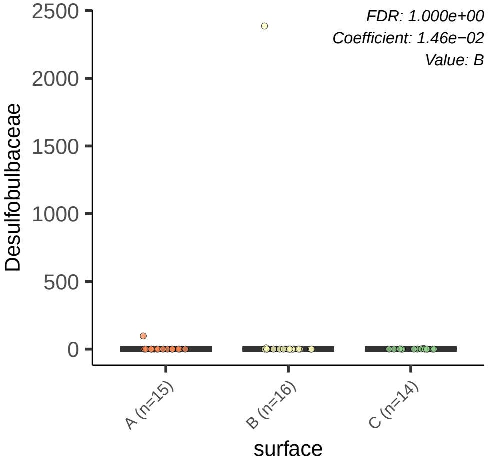
FDR: 1.000e+00
Coefficient: 1.46e−02
Value: B
Desulfobulbaceae
2500
2000
1500
1000
500
0
A (n=15)
B (n=16)
C (n=14)
surface

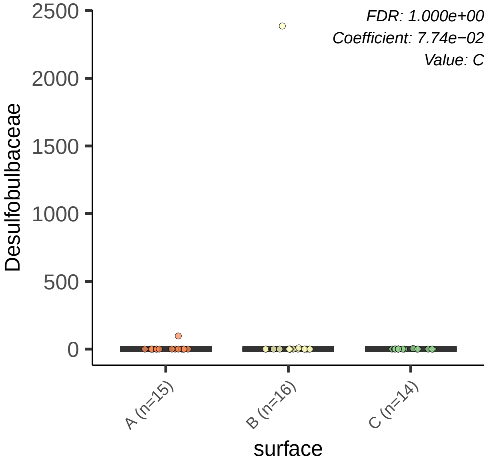
FDR: 1.000e+00
Coefficient: 7.74e−02
Value: C
Desulfobulbaceae
2500
2000
1500
1000
500
0
A (n=15)
B (n=16)
C (n=14)
surface

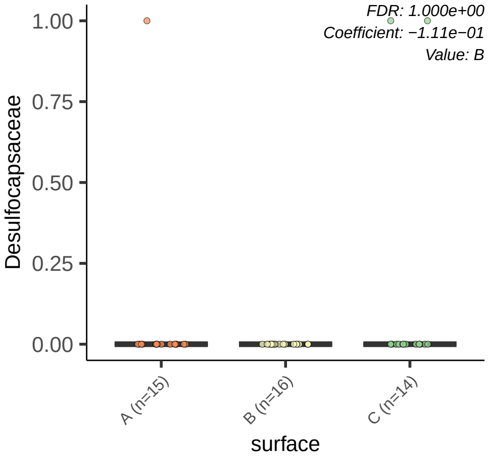
FDR: 1.000e+00
Coefficient: −1.11e−01
Value: B
Desulfocapsaceae
1.00
0.75
0.50
0.25
0.00
A (n=15)
B (n=16)
C (n=14)
surface

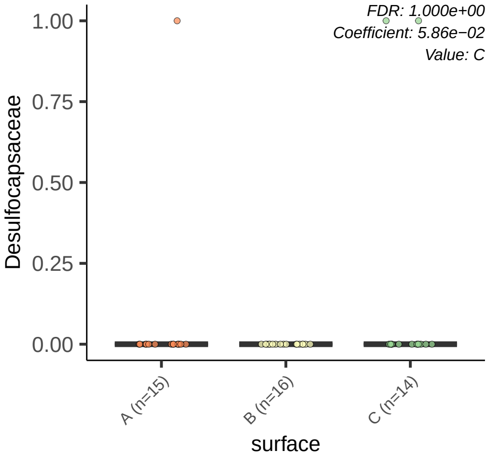
FDR: 1.000e+00
Coefficient: 5.86e−02
Value: C
1.00
0.75
0.50
0.25
0.00
Desulfocapsaceae
A (n=15)
B (n=16)
C (n=14)
surface

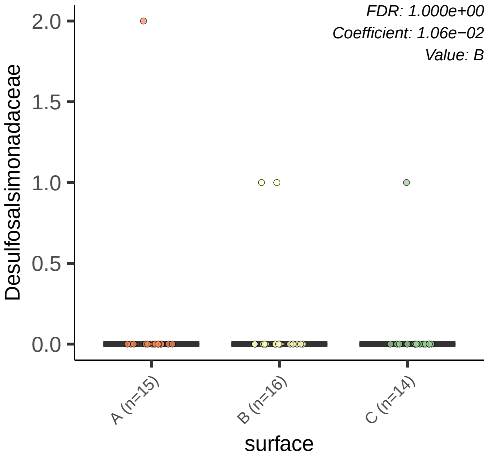

FDR: 1.000e+00
Coefficient: 1.06e−02
Value: B
Desulfosalsimonadaceae
2.0
1.5
1.0
0.5
0.0
A (n=15)
B (n=16)
C (n=14)
surface

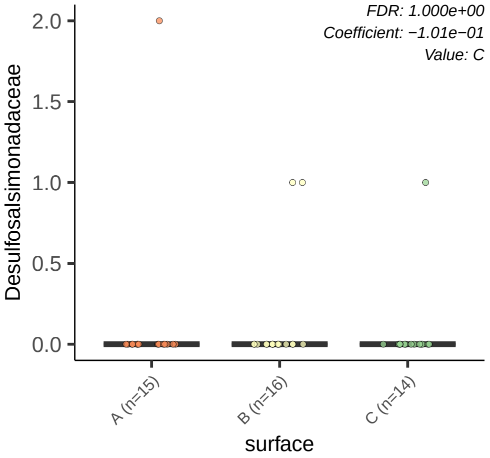

FDR: 1.000e+00
Coefficient: −1.01e−01
Value: C
Desulfosalsimonadaceae
2.0
1.5
1.0
0.5
0.0
A (n=15)
B (n=16)
C (n=14)
surface

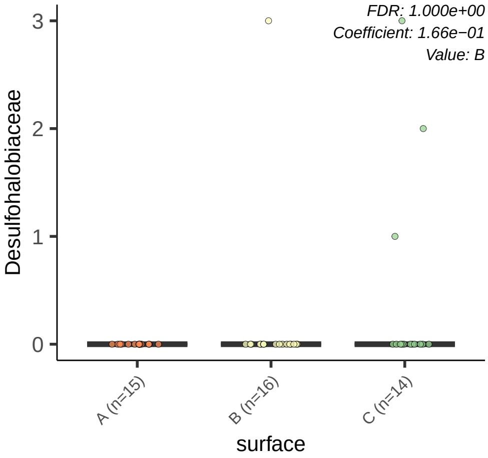
FDR: 1.000e+00
Coefficient: 1.66e−01
Value: B
Desulfohalobiaceae
3
2
1
0
A (n=15)
B (n=16)
C (n=14)
surface

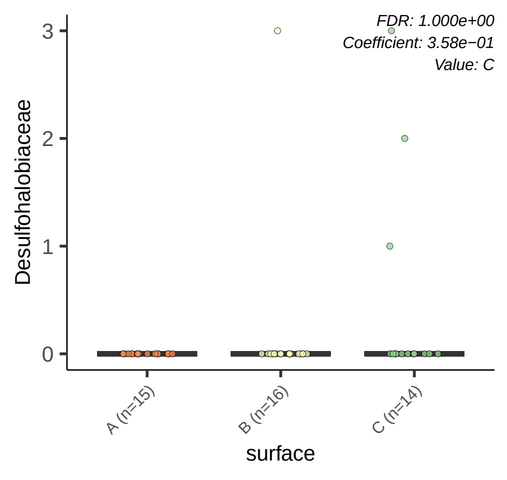

FDR: 1.000e+00
Coefficient: 3.58e−01
Value: C
Desulfohalobiaceae
3
2
1
0
A (n=15)
B (n=16)
C (n=14)
surface

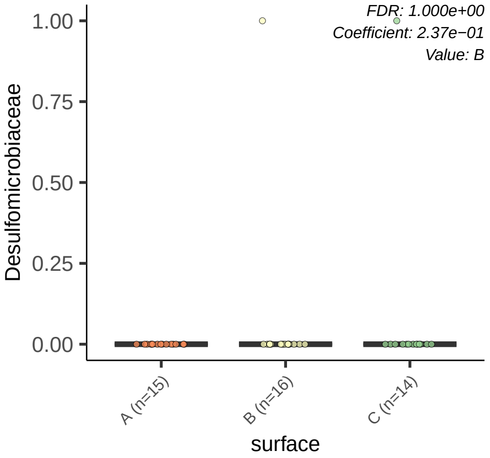
FDR: 1.000e+00
Coefficient: 2.37e−01
Value: B
Desulfomicrobiaceae
1.00
0.75
0.50
0.25
0.00
A (n=15)
B (n=16)
C (n=14)
surface

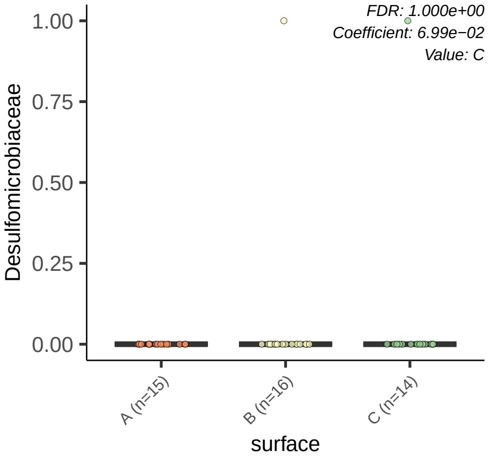
FDR: 1.000e+00
Coefficient: 6.99e−02
Value: C
Desulfomicrobiaceae
1.00
0.75
0.50
0.25
0.00
A (n=15)
B (n=16)
C (n=14)
surface

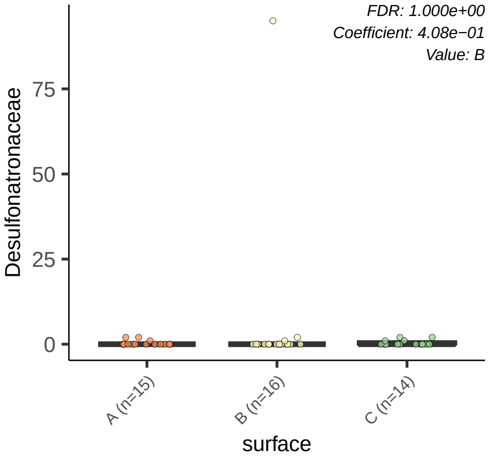

FDR: 1.000e+00
Coefficient: 4.08e−01
Value: B
Desulfonatronaceae
75
50
25
0
A (n=15)
B (n=16)
C (n=14)
surface

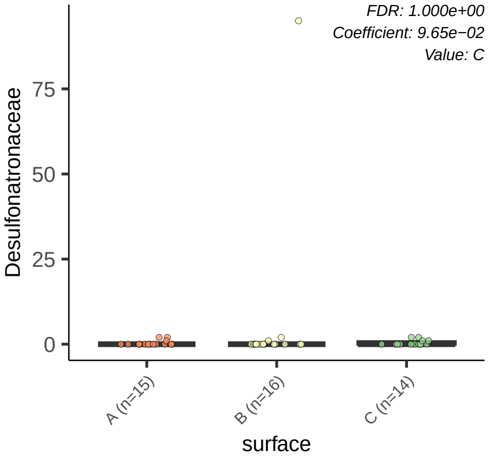

FDR: 1.000e+00
Coefficient: 9.65e−02
Value: C
Desulfonatronaceae
0
25
50
75
A (n=15)
B (n=16)
C (n=14)
surface

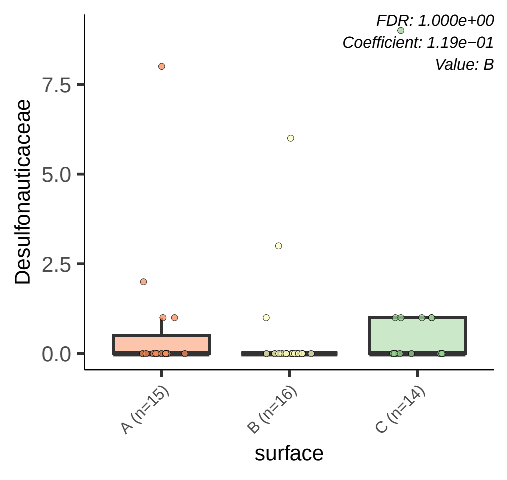
FDR: 1.000e+00
Coefficient: 1.19e−01
Value: B
Desulfonauticaceae
7.5
5.0
2.5
0.0
A (n=15)
B (n=16)
C (n=14)
surface

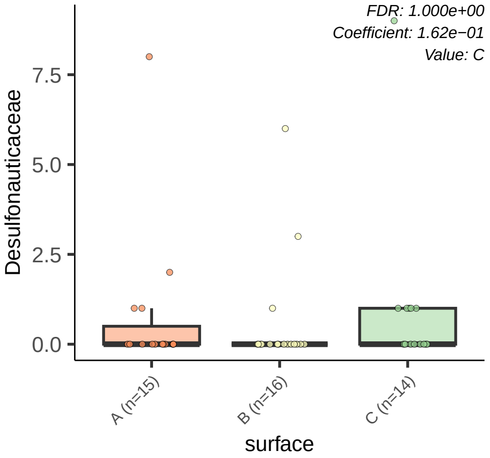
FDR: 1.000e+00
Coefficient: 1.62e−01
Value: C
Desulfonauticaceae
7.5
5.0
2.5
0.0
A (n=15)
B (n=16)
C (n=14)
surface

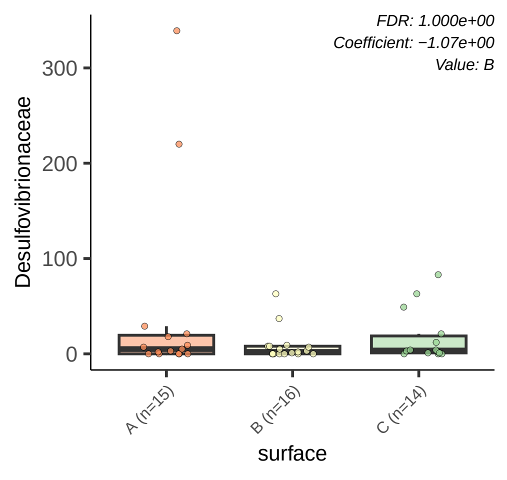
FDR: 1.000e+00
Coefficient: −1.07e+00
Value: B
Desulfovibrionaceae
300
200
100
0
A (n=15)
B (n=16)
C (n=14)
surface

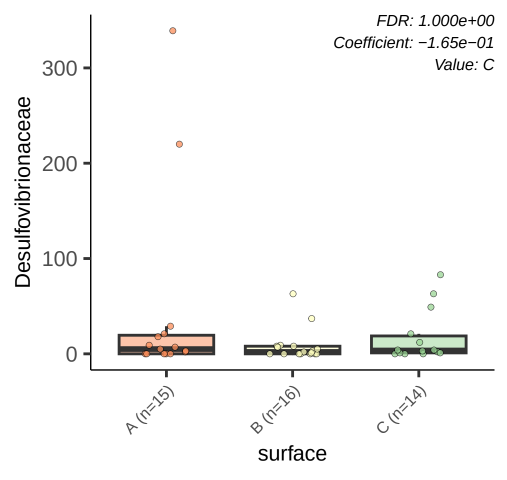
FDR: 1.000e+00
Coefficient: −1.65e−01
Value: C
Desulfovibrionaceae
300
200
100
0
A (n=15)
B (n=16)
C (n=14)
surface

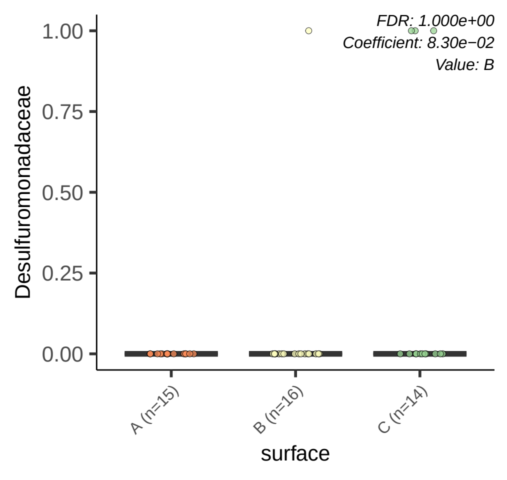
FDR: 1.000e+00
Coefficient: 8.30e−02
Value: B
Desulfuromonadaceae
1.00
0.75
0.50
0.25
0.00
A (n=15)
B (n=16)
C (n=14)
surface

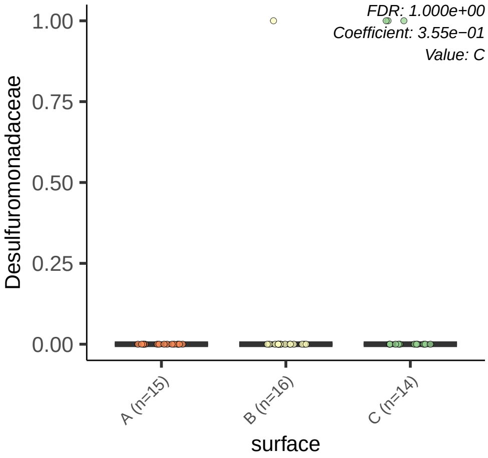

FDR: 1.000e+00
Coefficient: 3.55e−01
Value: C
Desulfuromonadaceae
1.00
0.75
0.50
0.25
0.00
A (n=15)
B (n=16)
C (n=14)
surface

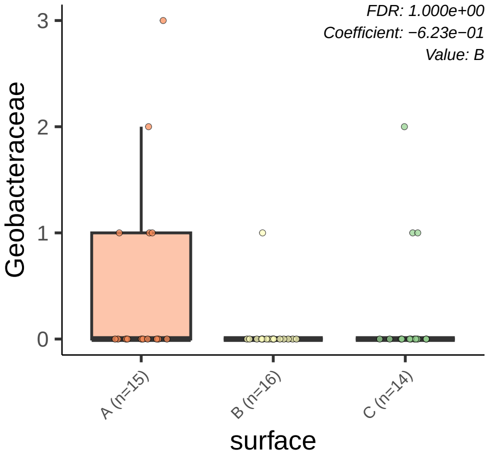
FDR: 1.000e+00
Coefficient: −6.23e−01
Value: B
Geobacteraceae
3
2
1
0
A (n=15)
B (n=16)
C (n=14)
surface

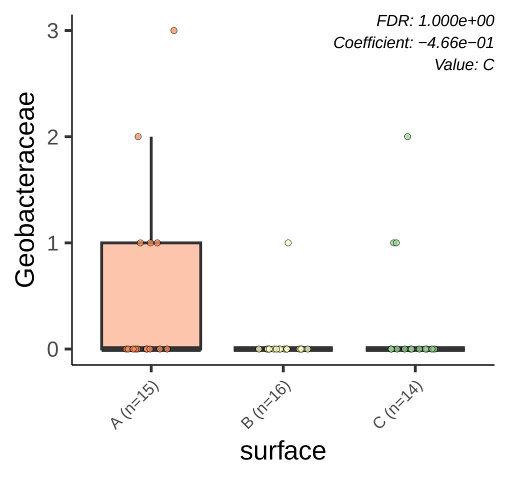
FDR: 1.000e+00
Coefficient: −4.66e−01
Value: C
Geobacteraceae
0
1
2
3
A (n=15)
B (n=16)
C (n=14)
surface

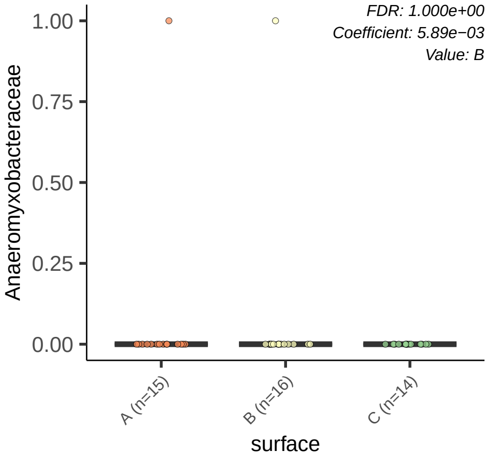

FDR: 1.000e+00
Coefficient: 5.89e−03
Value: B
Anaeromyxobacteraceae
1.00
0.75
0.50
0.25
0.00
A (n=15)
B (n=16)
C (n=14)
surface

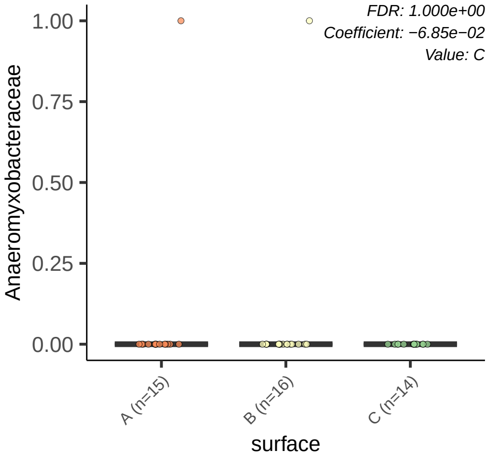
FDR: 1.000e+00
Coefficient: −6.85e−02
Value: C
Anaeromyxobacteraceae
1.00
0.75
0.50
0.25
0.00
A (n=15)
B (n=16)
C (n=14)
surface

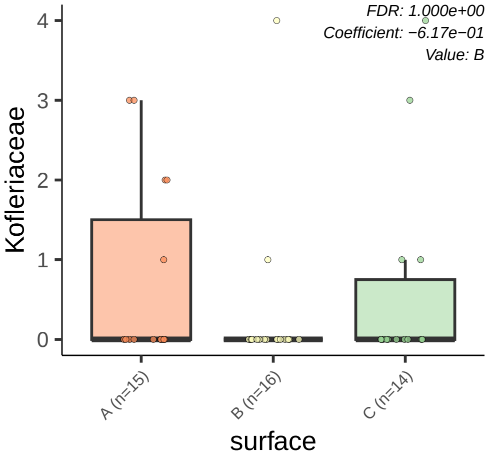
FDR: 1.000e+00
Coefficient: −6.17e−01
Value: B
Kofleriaceae
0
1
2
3
4
A (n=15)
B (n=16)
C (n=14)
surface

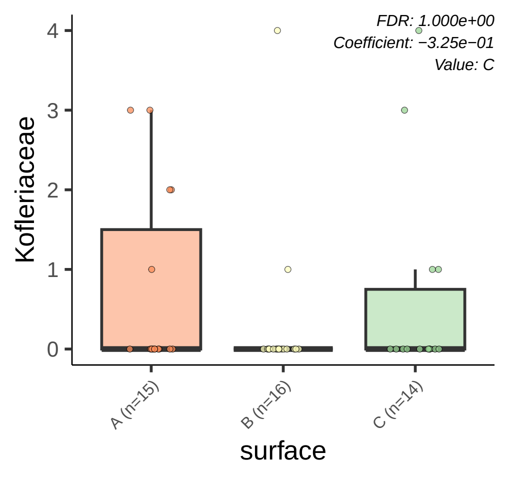
FDR: 1.000e+00
Coefficient: −3.25e−01
Value: C
Kofleriaceae
0
1
2
3
4
A (n=15)
B (n=16)
C (n=14)
surface

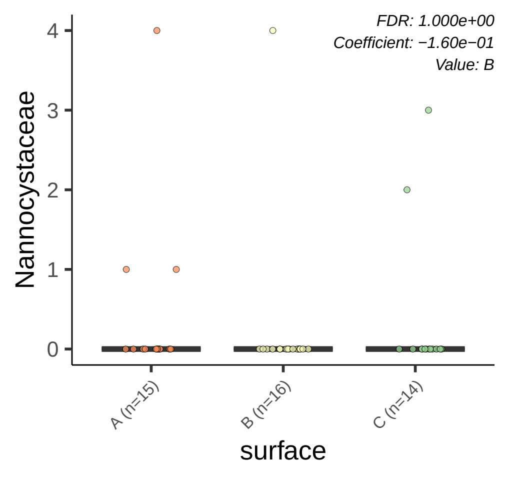
FDR: 1.000e+00
Coefficient: −1.60e−01
Value: B
Nannocystaceae
0
1
2
3
4
A (n=15)
B (n=16)
C (n=14)
surface

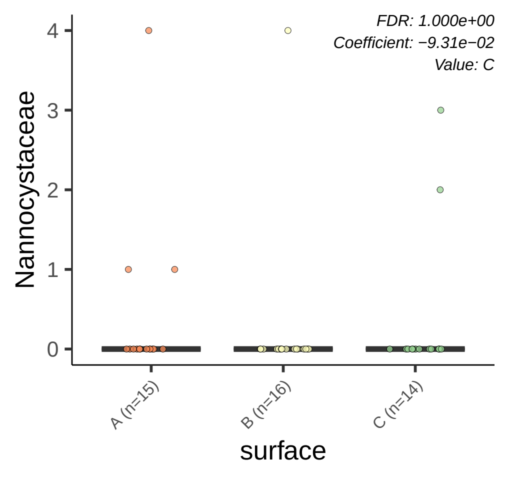
FDR: 1.000e+00
Coefficient: −9.31e−02
Value: C
Nannocystaceae
0
1
2
3
4
A (n=15)
B (n=16)
C (n=14)
surface

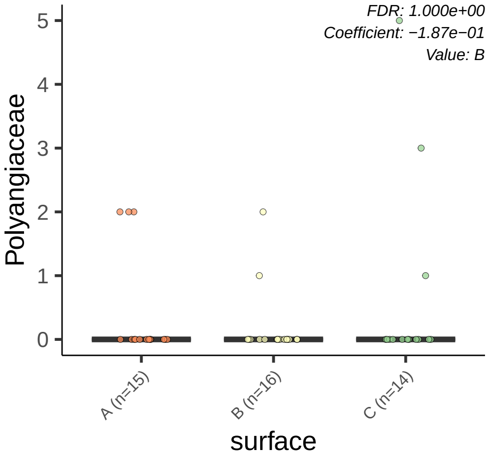
FDR: 1.000e+00
Coefficient: −1.87e−01
Value: B
Polyangiaceae
0
1
2
3
4
5
A (n=15)
B (n=16)
C (n=14)
surface

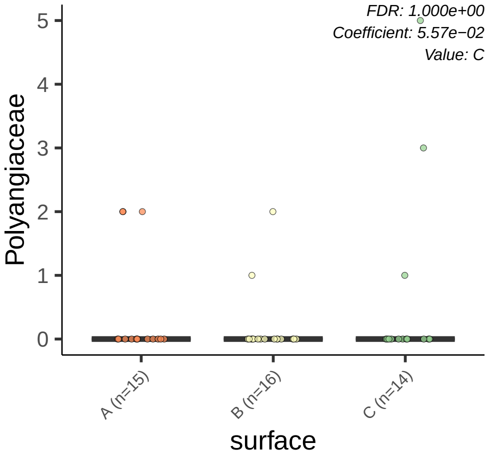
FDR: 1.000e+00
Coefficient: 5.57e−02
Value: C
Polyangiaceae
0
1
2
3
4
5
A (n=15)
B (n=16)
C (n=14)
surface

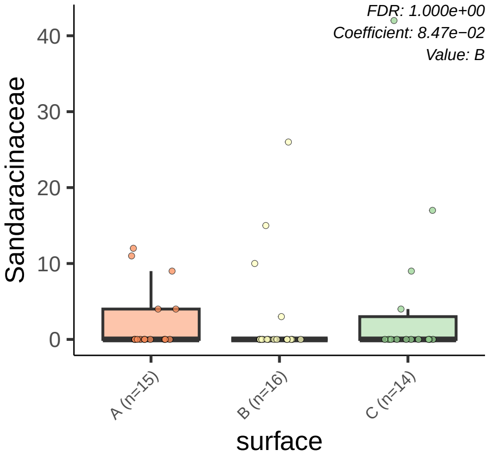
FDR: 1.000e+00
Coefficient: 8.47e−02
Value: B
Sandaracinaceae
40
30
20
10
0
A (n=15)
B (n=16)
C (n=14)
surface

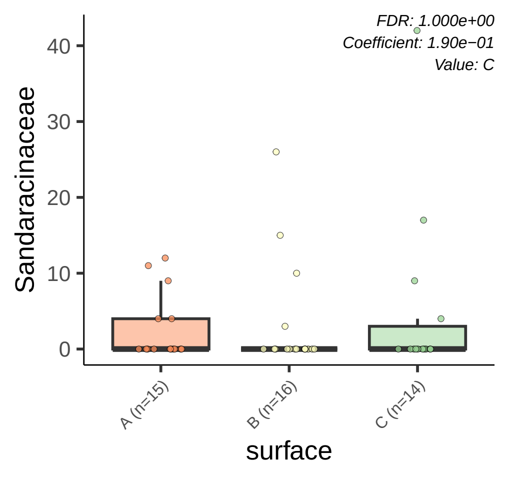

FDR: 1.000e+00
Coefficient: 1.90e−01
Value: C
Sandaracinaceae
0
10
20
30
40
A (n=15)
B (n=16)
C (n=14)
surface

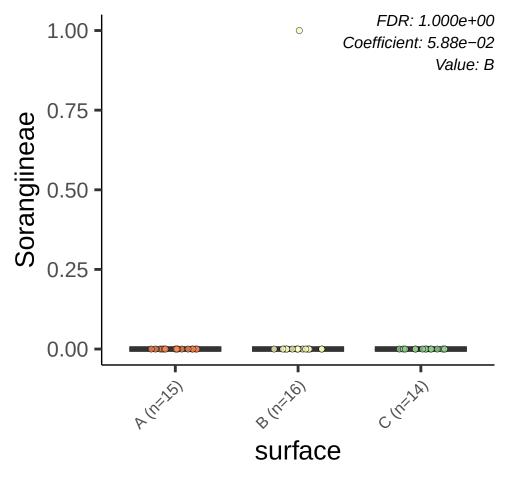
FDR: 1.000e+00
Coefficient: 5.88e−02
Value: B
Sorangineae
1.00
0.75
0.50
0.25
0.00
A (n=15)
B (n=16)
C (n=14)
surface

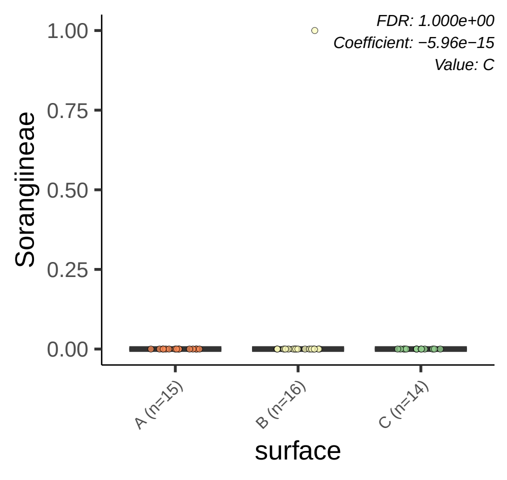

FDR: 1.000e+00
Coefficient: −5.96e−15
Value: C
Sorangineae
1.00
0.75
0.50
0.25
0.00
A (n=15)
B (n=16)
C (n=14)
surface

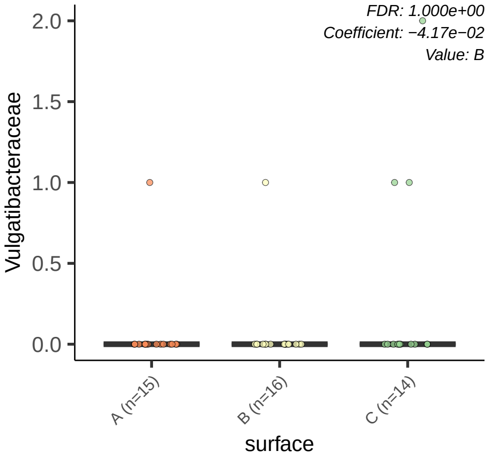
FDR: 1.000e+00
Coefficient: −4.17e−02
Value: B
Vulgatibacteraceae
2.0
1.5
1.0
0.5
0.0
A (n=15)
B (n=16)
C (n=14)
surface

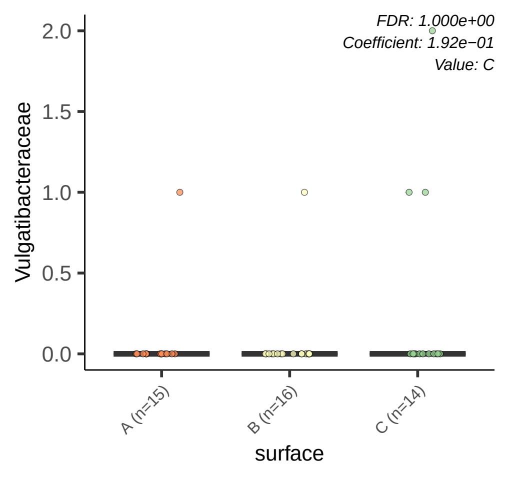
FDR: 1.000e+00
Coefficient: 1.92e−01
Value: C
Vulgatibacteraceae
2.0
1.5
1.0
0.5
0.0
A (n=15)
B (n=16)
C (n=14)
surface

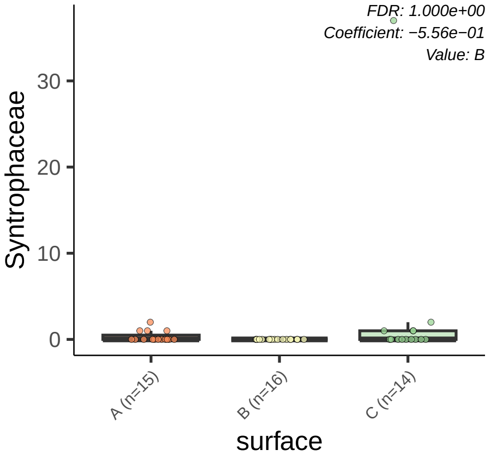
FDR: 1.000e+00
Coefficient: −5.56e−01
Value: B
Syntrophaceae
0
10
20
30
A (n=15)
B (n=16)
C (n=14)
surface

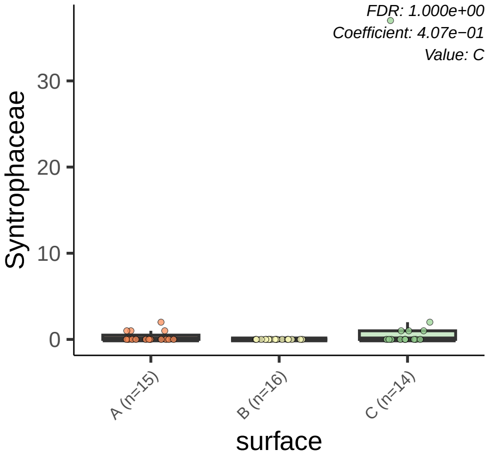

FDR: 1.000e+00
Coefficient: 4.07e−01
Value: C
Syntrophaceae
30
20
10
0
A (n=15)
B (n=16)
C (n=14)
surface

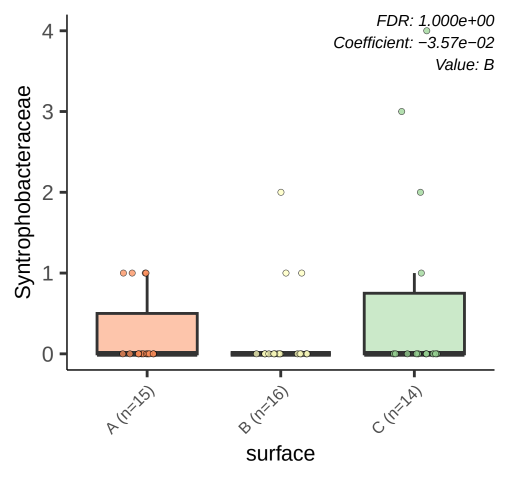

FDR: 1.000e+00
Coefficient: −3.57e−02
Value: B
Syntrophobacteraceae
0
1
2
3
4
A (n=15)
B (n=16)
C (n=14)
surface

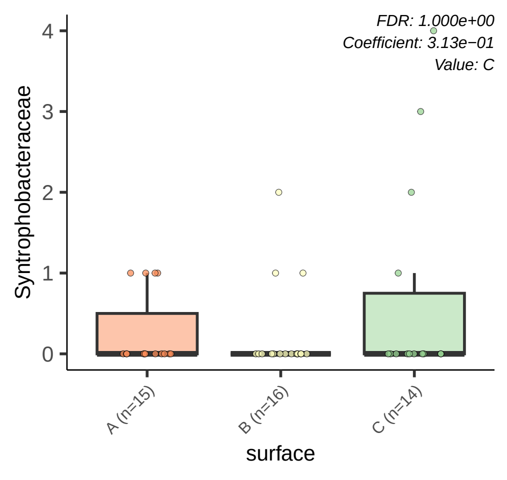
FDR: 1.000e+00
Coefficient: 3.13e−01
Value: C
Syntrophobacteraceae
4
3
2
1
0
A (n=15)
B (n=16)
C (n=14)
surface

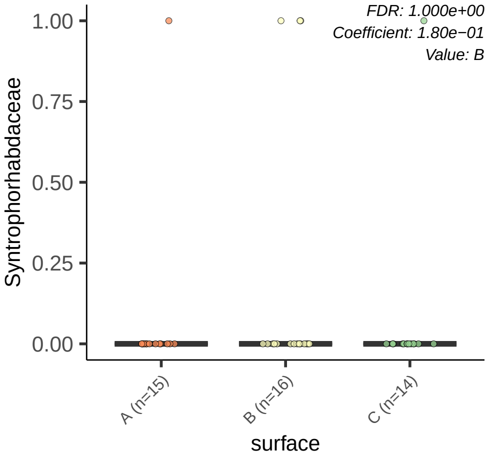

FDR: 1.000e+00
Coefficient: 1.80e−01
Value: B
Syntrophorhabdaceae
1.00
0.75
0.50
0.25
0.00
A (n=15)
B (n=16)
C (n=14)
surface

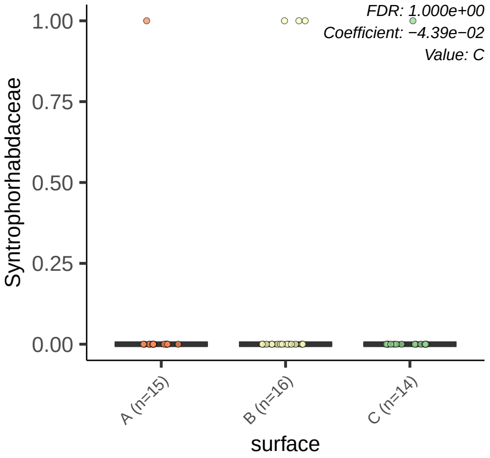
FDR: 1.000e+00
Coefficient: −4.39e−02
Value: C
Syntrophorhabdaceae
1.00
0.75
0.50
0.25
0.00
A (n=15)
B (n=16)
C (n=14)
surface

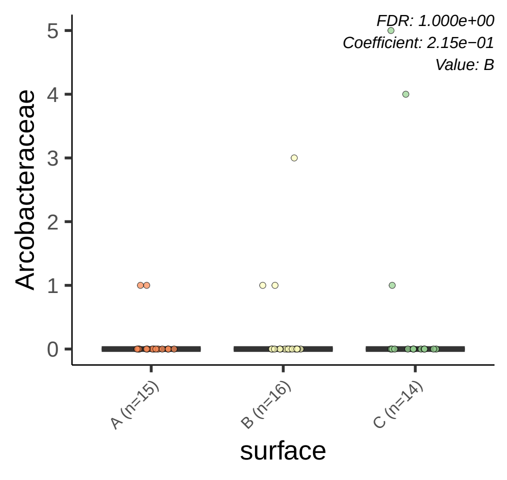
FDR: 1.000e+00
Coefficient: 2.15e−01
Value: B
Arcobacteraceae
0
1
2
3
4
5
A (n=15)
B (n=16)
C (n=14)
surface

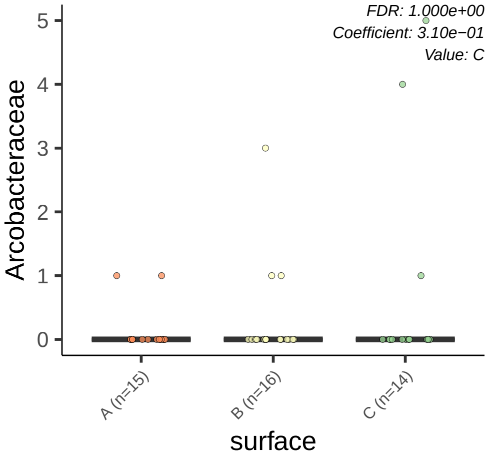
FDR: 1.000e+00
Coefficient: 3.10e−01
Value: C
Arcobacteraceae
0
1
2
3
4
5
A (n=15)
B (n=16)
C (n=14)
surface

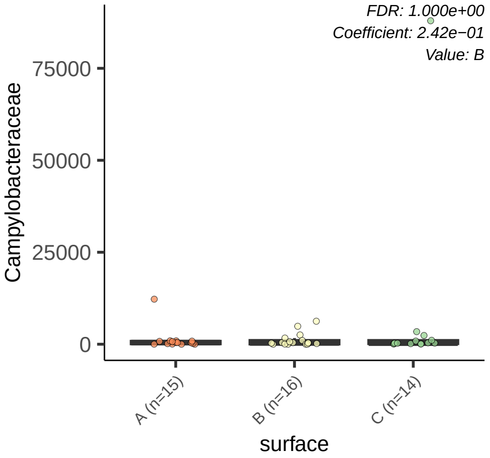
FDR: 1.000e+00
Coefficient: 2.42e−01
Value: B
Campylobacteraceae
75000
50000
25000
0
A (n=15)
B (n=16)
C (n=14)
surface

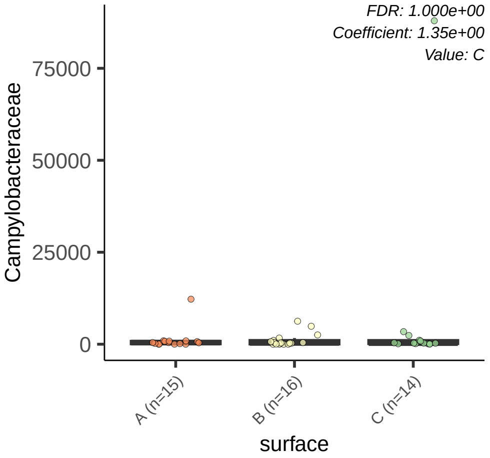
FDR: 1.000e+00
Coefficient: 1.35e+00
Value: C
Campylobacteraceae
75000
50000
25000
0
A (n=15)
B (n=16)
C (n=14)
surface

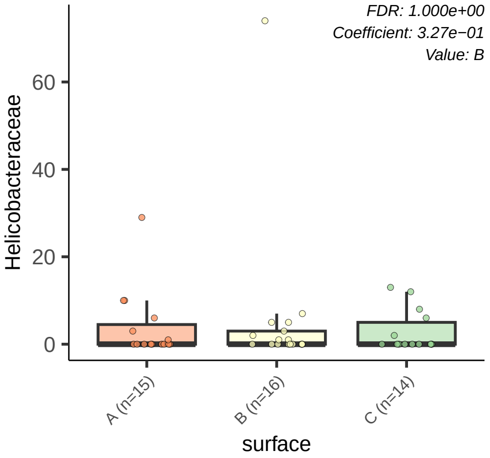
FDR: 1.000e+00
Coefficient: 3.27e−01
Value: B
Helicobacteraceae
0
20
40
60
A (n=15)
B (n=16)
C (n=14)
surface

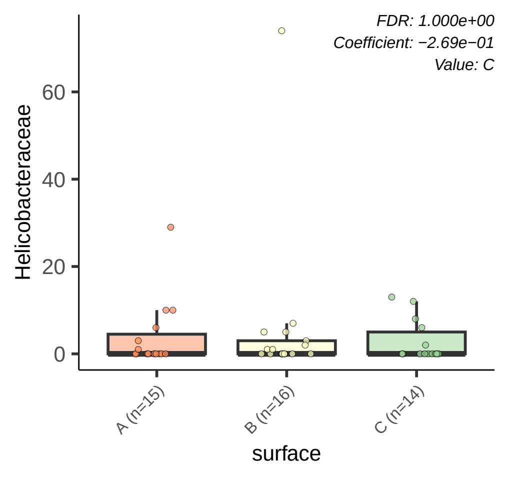

FDR: 1.000e+00
Coefficient: −2.69e−01
Value: C
Helicobacteraceae
0
20
40
60
A (n=15)
B (n=16)
C (n=14)
surface

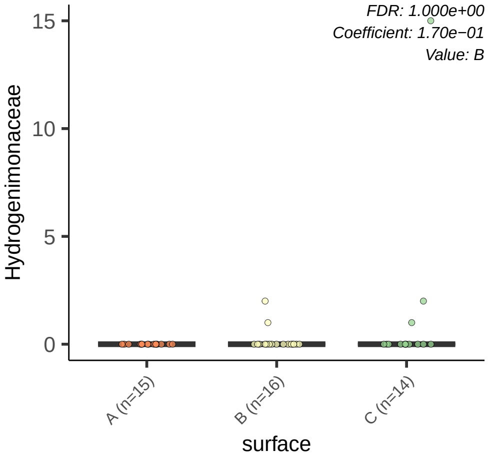
FDR: 1.000e+00
Coefficient: 1.70e−01
Value: B
Hydrogenimonaceae
15
10
5
0
A (n=15)
B (n=16)
C (n=14)
surface

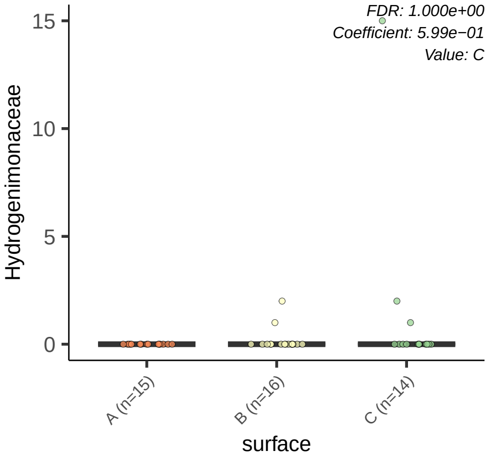

FDR: 1.000e+00
Coefficient: 5.99e−01
Value: C
Hydrogenimonaceae
15
10
5
0
A (n=15)
B (n=16)
C (n=14)
surface

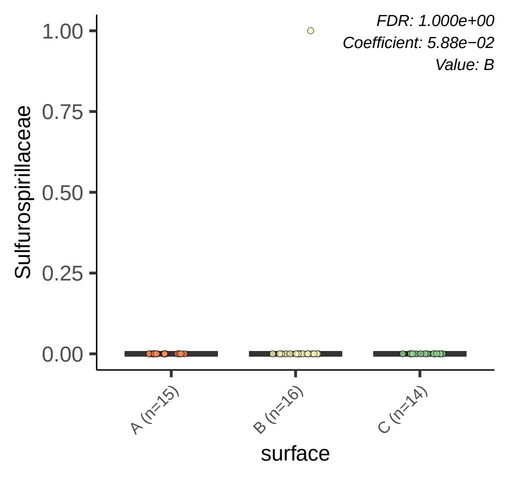

FDR: 1.000e+00
Coefficient: 5.88e−02
Value: B
Sulfurospirillaceae
1.00
0.75
0.50
0.25
0.00
A (n=15)
B (n=16)
C (n=14)
surface

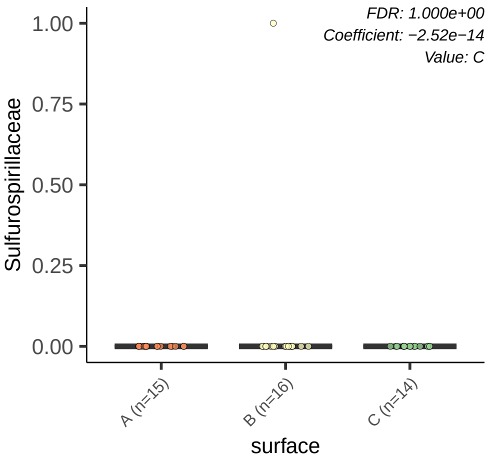
FDR: 1.000e+00
Coefficient: −2.52e−14
Value: C
Sulfurospirillaceae
1.00
0.75
0.50
0.25
0.00
A (n=15)
B (n=16)
C (n=14)
surface

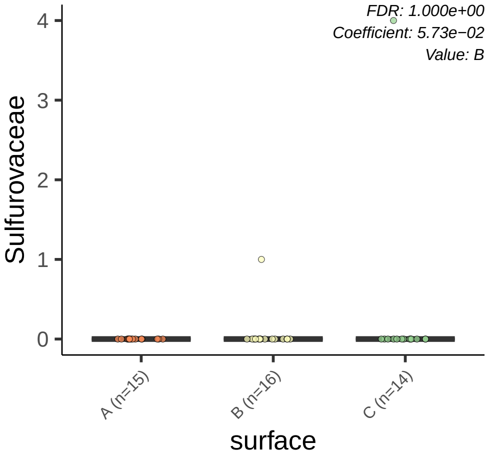
FDR: 1.000e+00
Coefficient: 5.73e−02
Value: B
Sulfurovaceae
0
1
2
3
4
A (n=15)
B (n=16)
C (n=14)
surface

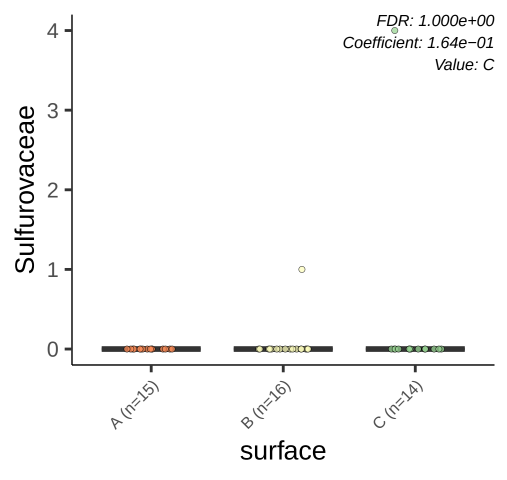
FDR: 1.000e+00
Coefficient: 1.64e−01
Value: C
Sulfurovaceae
0
1
2
3
4
A (n=15)
B (n=16)
C (n=14)
surface

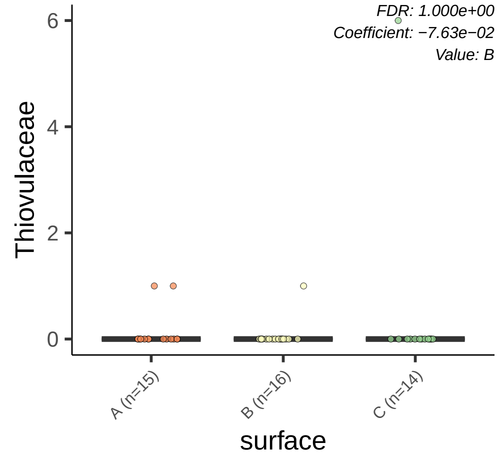

FDR: 1.000e+00
Coefficient: −7.63e−02
Value: B
Thiovulaceae
0
2
4
6
A (n=15)
B (n=16)
C (n=14)
surface

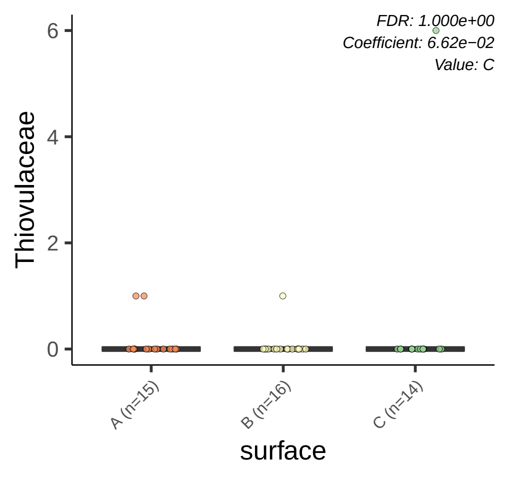
FDR: 1.000e+00
Coefficient: 6.62e−02
Value: C
Thiovulaceae
0
2
4
6
A (n=15)
B (n=16)
C (n=14)
surface

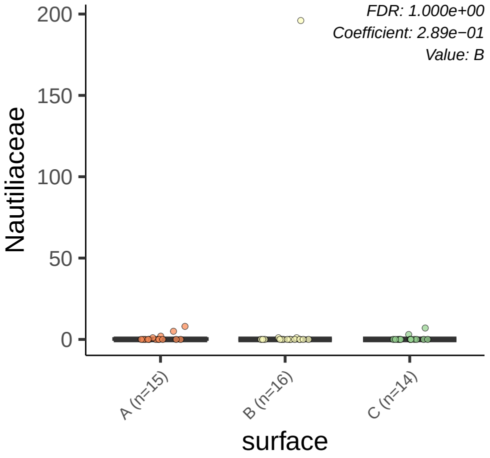

FDR: 1.000e+00
Coefficient: 2.89e−01
Value: B
Nautiliaceae
200
150
100
50
0
A (n=15)
B (n=16)
C (n=14)
surface

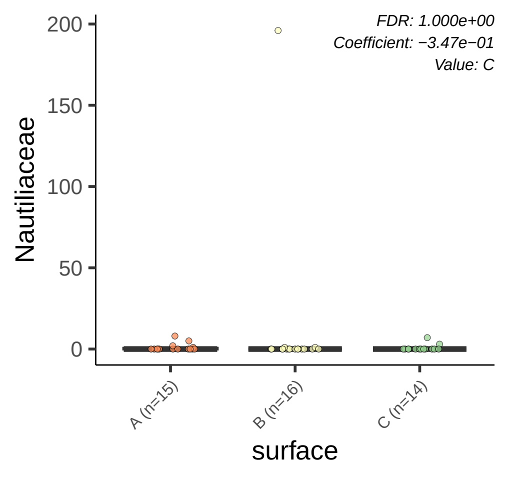
FDR: 1.000e+00
Coefficient: −3.47e−01
Value: C
Nautiliaceae
200
150
100
50
0
A (n=15)
B (n=16)
C (n=14)
surface

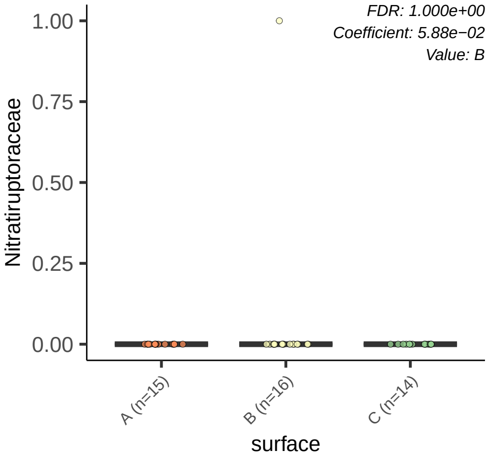

FDR: 1.000e+00
Coefficient: 5.88e−02
Value: B
Nitratiruptoraceae
1.00
0.75
0.50
0.25
0.00
A (n=15)
B (n=16)
C (n=14)
surface

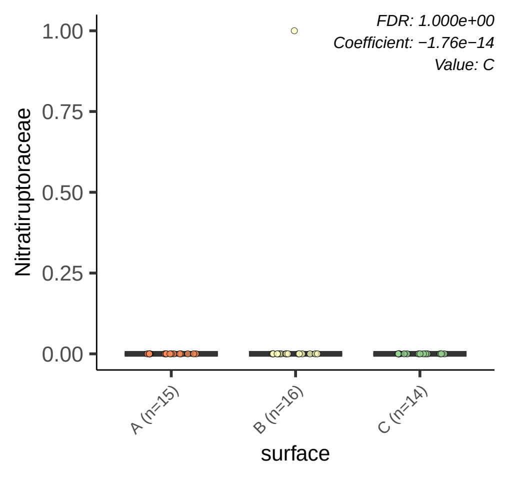
FDR: 1.000e+00
Coefficient: −1.76e−14
Value: C
Nitratiruptoraceae
1.00
0.75
0.50
0.25
0.00
A (n=15)
B (n=16)
C (n=14)
surface

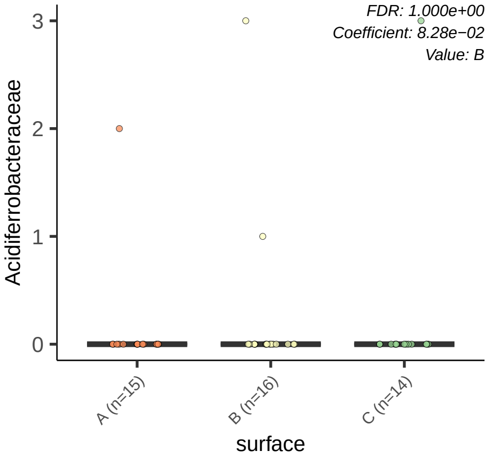
FDR: 1.000e+00
Coefficient: 8.28e−02
Value: B
Acidiferrobacteraceae
3
2
1
0
A (n=15)
B (n=16)
C (n=14)
surface

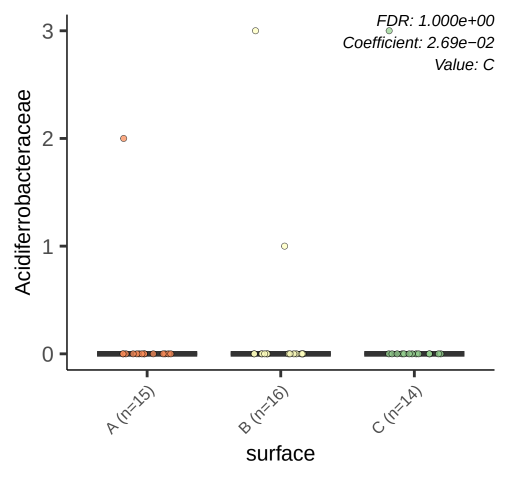

FDR: 1.000e+00
Coefficient: 2.69e−02
Value: C
Acidiferrobacteraceae
0
1
2
3
A (n=15)
B (n=16)
C (n=14)
surface

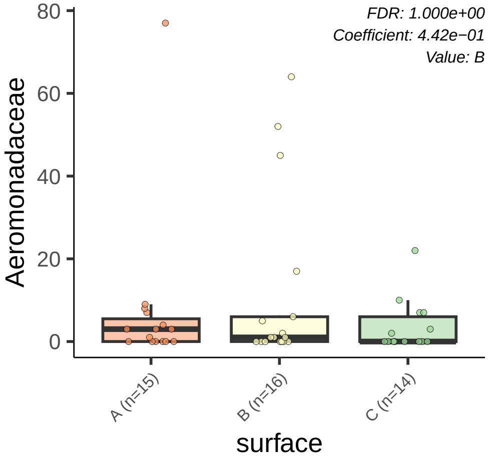
FDR: 1.000e+00
Coefficient: 4.42e−01
Value: B
Aeromonadaceae
80
60
40
20
0
A (n=15)
B (n=16)
C (n=14)
surface

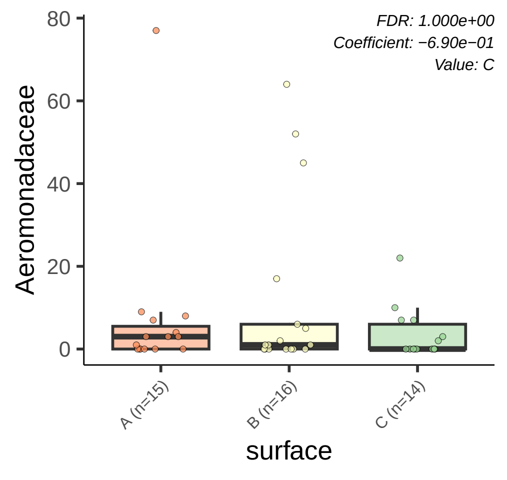

FDR: 1.000e+00
Coefficient: −6.90e−01
Value: C
Aeromonadaceae
80
60
40
20
0
A (n=15)
B (n=16)
C (n=14)
surface

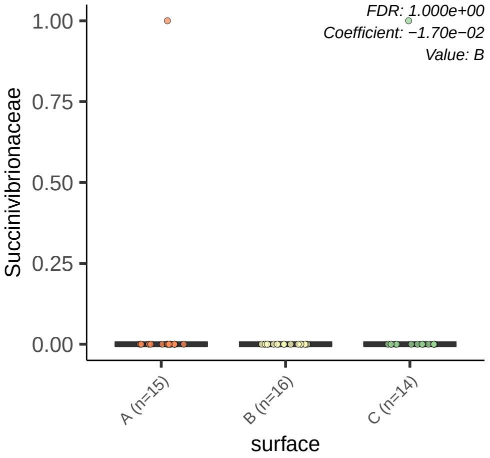

FDR: 1.000e+00
Coefficient: −1.70e−02
Value: B
Succinivibrionaceae
1.00
0.75
0.50
0.25
0.00
A (n=15)
B (n=16)
C (n=14)
surface

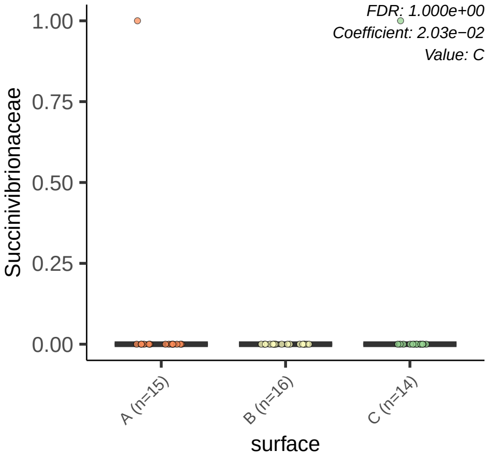
FDR: 1.000e+00
Coefficient: 2.03e−02
Value: C
Succinivibrionaceae
1.00
0.75
0.50
0.25
0.00
A (n=15)
B (n=16)
C (n=14)
surface

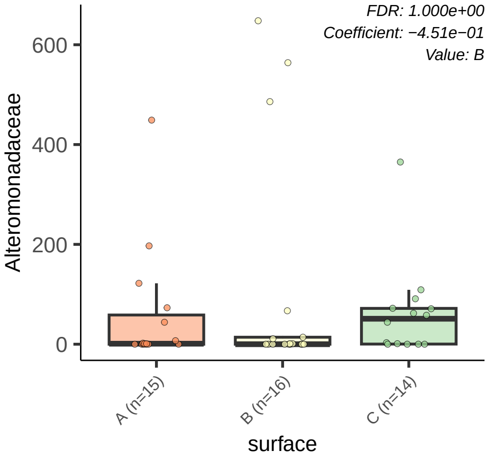

FDR: 1.000e+00
Coefficient: −4.51e−01
Value: B
Alteromonadaceae
0
200
400
600
A (n=15)
B (n=16)
C (n=14)
surface

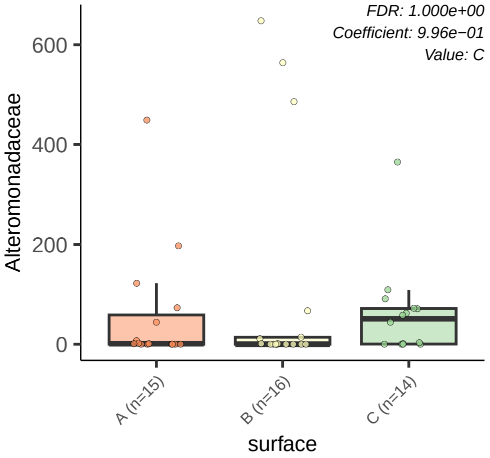
FDR: 1.000e+00
Coefficient: 9.96e−01
Value: C
600
400
200
0
Alteromonadaceae
A (n=15)
B (n=16)
C (n=14)
surface

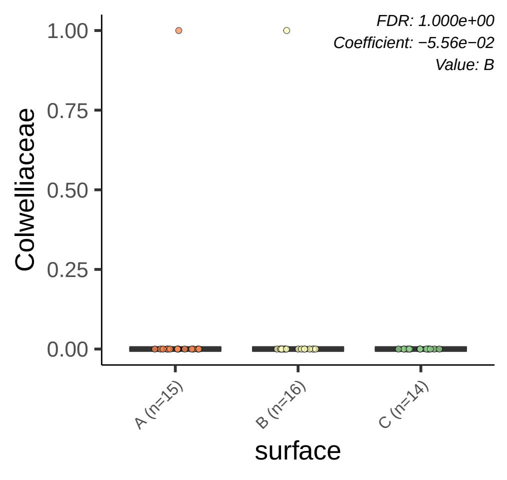
FDR: 1.000e+00
Coefficient: −5.56e−02
Value: B
Colwelliaceae
1.00
0.75
0.50
0.25
0.00
A (n=15)
B (n=16)
C (n=14)
surface

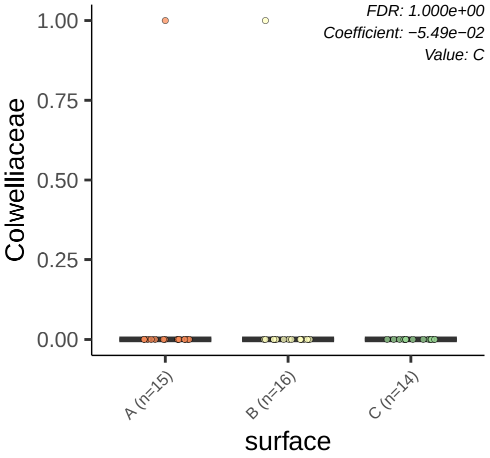

FDR: 1.000e+00
Coefficient: −5.49e−02
Value: C
Colwelliaceae
1.00
0.75
0.50
0.25
0.00
A (n=15)
B (n=16)
C (n=14)
surface

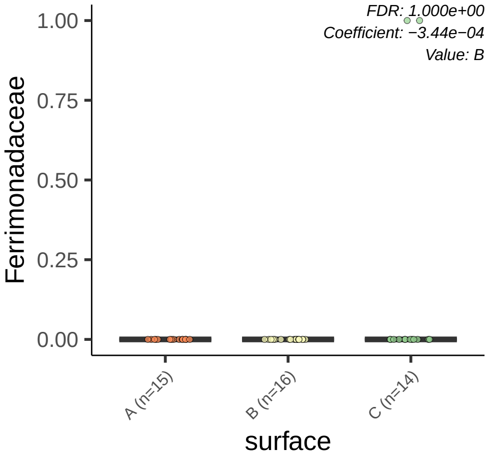
FDR: 1.000e+00
Coefficient: −3.44e−04
Value: B
Ferrimonadaceae
1.00
0.75
0.50
0.25
0.00
A (n=15)
B (n=16)
C (n=14)
surface

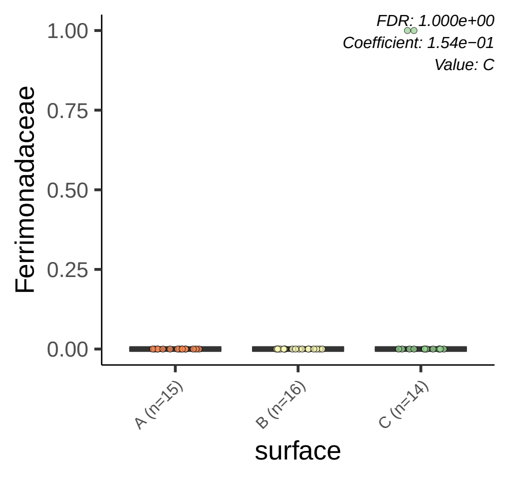
FDR: 1.000e+00
Coefficient: 1.54e−01
Value: C
Ferrimonadaceae
1.00
0.75
0.50
0.25
0.00
A (n=15)
B (n=16)
C (n=14)
surface

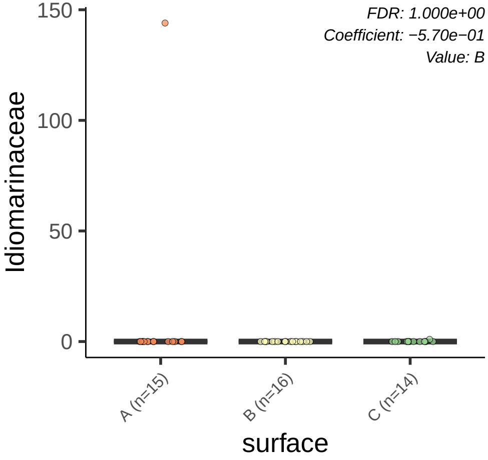

FDR: 1.000e+00
Coefficient: −5.70e−01
Value: B
Idiomarinaceae
150
100
50
0
A (n=15)
B (n=16)
C (n=14)
surface

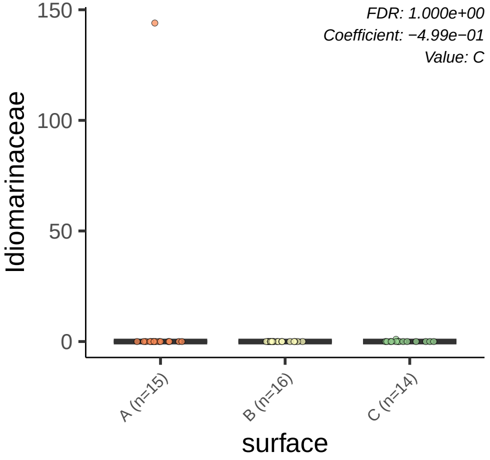

FDR: 1.000e+00
Coefficient: −4.99e−01
Value: C
Idiomarinaceae
150
100
50
0
A (n=15)
B (n=16)
C (n=14)
surface

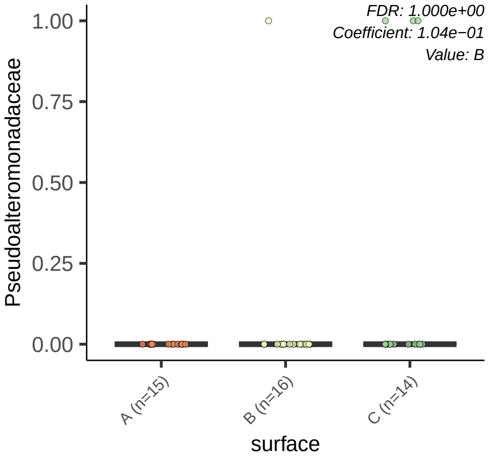
FDR: 1.000e+00
Coefficient: 1.04e−01
Value: B
Pseudoalteromonadaceae
1.00
0.75
0.50
0.25
0.00
A (n=15)
B (n=16)
C (n=14)
surface

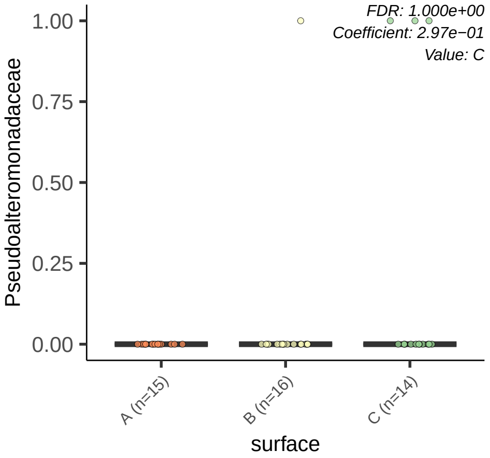

FDR: 1.000e+00
Coefficient: 2.97e−01
Value: C
Pseudoalteromonadaceae
1.00
0.75
0.50
0.25
0.00
A (n=15)
B (n=16)
C (n=14)
surface

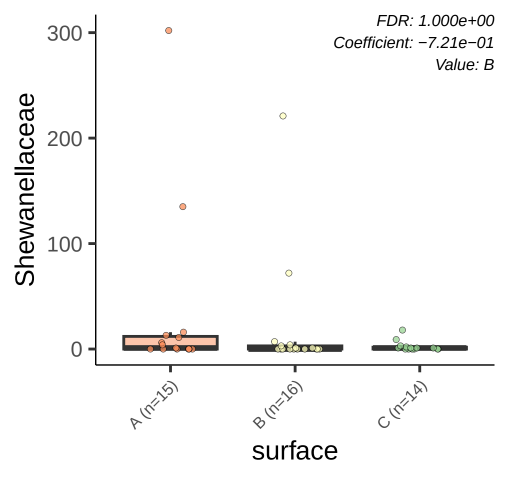
FDR: 1.000e+00
Coefficient: −7.21e−01
Value: B
Shewanellaceae
300
200
100
0
A (n=15)
B (n=16)
C (n=14)
surface

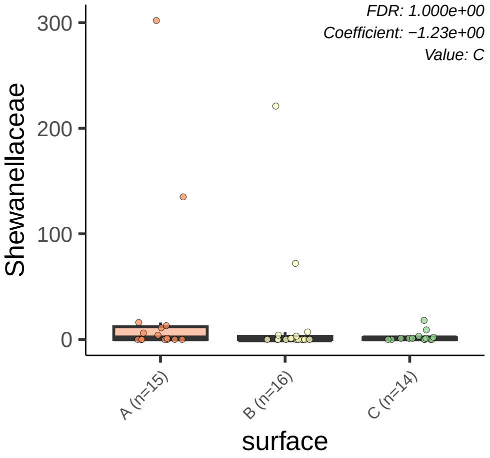
FDR: 1.000e+00
Coefficient: −1.23e+00
Value: C
Shewanellaceae
300
200
100
0
A (n=15)
B (n=16)
C (n=14)
surface

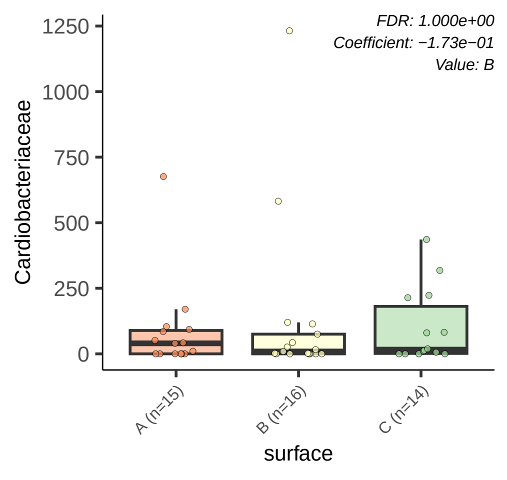
FDR: 1.000e+00
Coefficient: −1.73e−01
Value: B
Cardiobacteriaceae
1250
1000
750
500
250
0
A (n=15)
B (n=16)
C (n=14)
surface

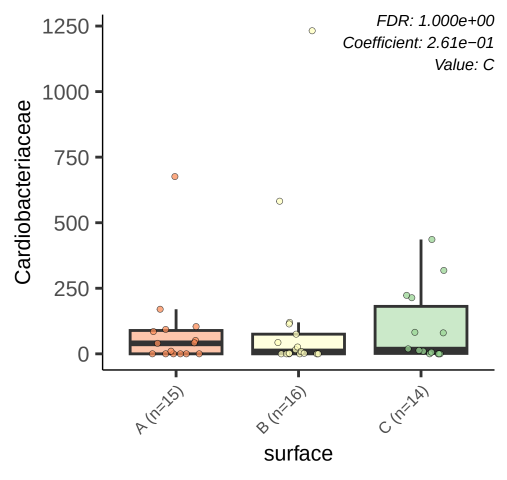

FDR: 1.000e+00
Coefficient: 2.61e−01
Value: C
Cardiobacteriaceae
1250
1000
750
500
250
0
A (n=15)
B (n=16)
C (n=14)
surface

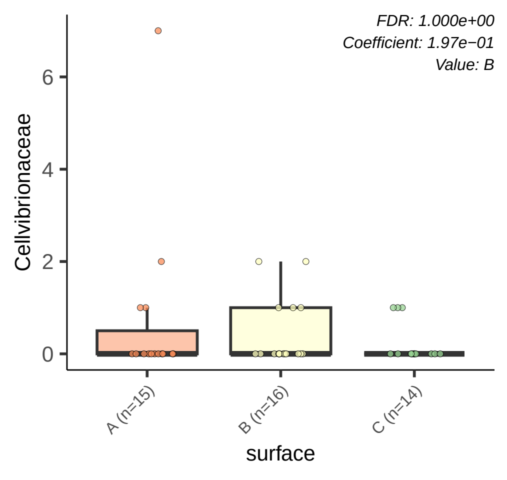
FDR: 1.000e+00
Coefficient: 1.97e−01
Value: B
Cellvibrionaceae
0
2
4
6
A (n=15)
B (n=16)
C (n=14)
surface

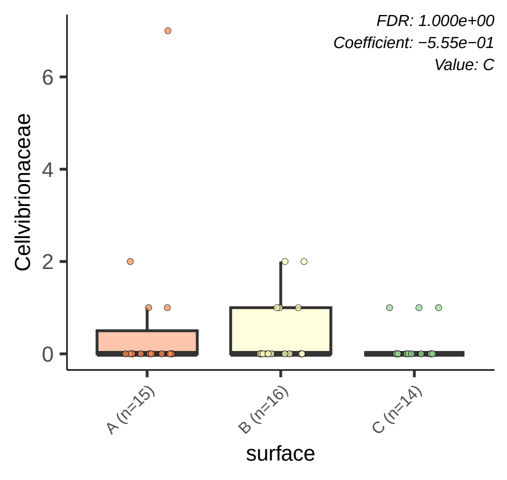
FDR: 1.000e+00
Coefficient: −5.55e−01
Value: C
Cellvibrionaceae
0
2
4
6
A (n=15)
B (n=16)
C (n=14)
surface

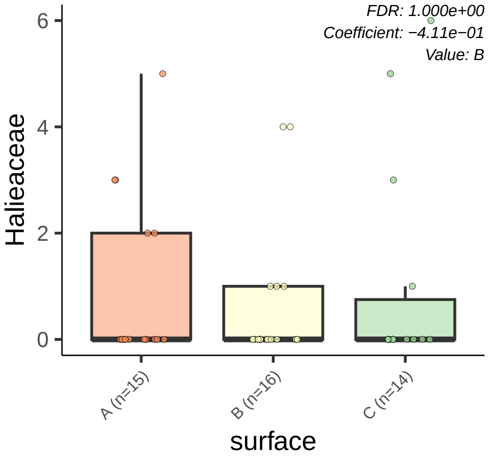

FDR: 1.000e+00
Coefficient: −4.11e−01
Value: B
Halieaceae
0
2
4
6
A (n=15)
B (n=16)
C (n=14)
surface

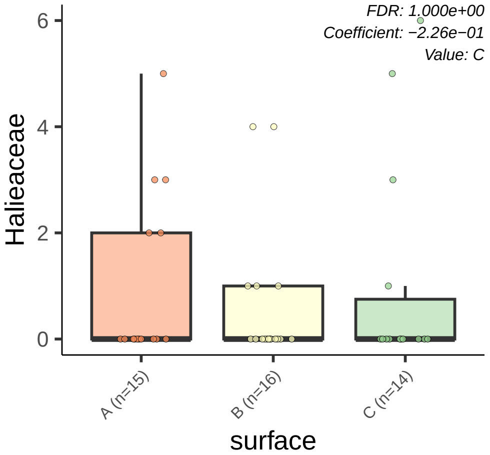
FDR: 1.000e+00
Coefficient: −2.26e−01
Value: C
Halieaceae
0
2
4
6
A (n=15)
B (n=16)
C (n=14)
surface

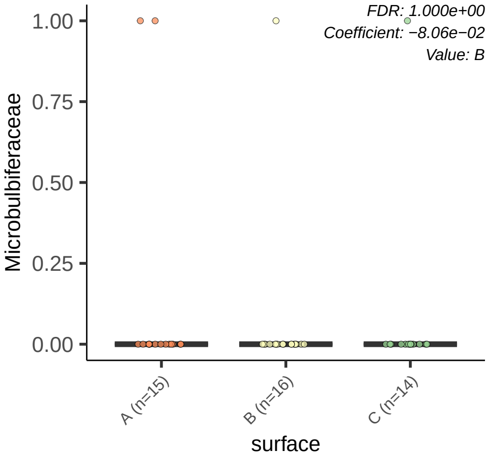
FDR: 1.000e+00
Coefficient: −8.06e−02
Value: B
Microbulbiferaceae
1.00
0.75
0.50
0.25
0.00
A (n=15)
B (n=16)
C (n=14)
surface

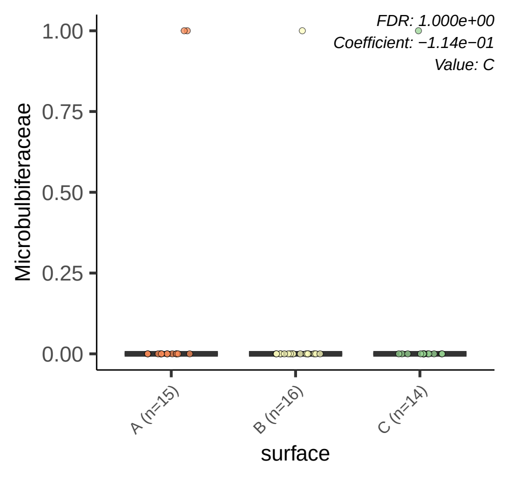
FDR: 1.000e+00
Coefficient: −1.14e−01
Value: C
Microbulbiferaceae
1.00
0.75
0.50
0.25
0.00
A (n=15)
B (n=16)
C (n=14)
surface

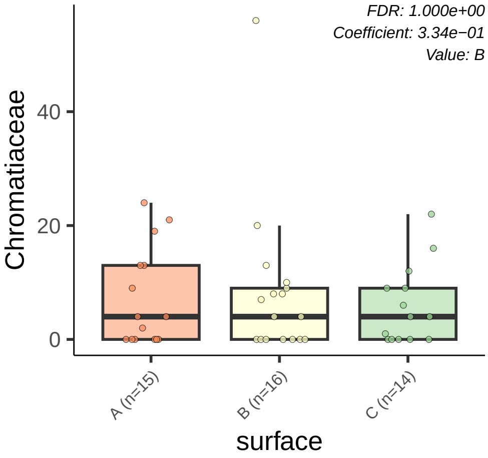

FDR: 1.000e+00
Coefficient: 3.34e−01
Value: B
Chromatiaceae
0
20
40
A (n=15)
B (n=16)
C (n=14)
surface

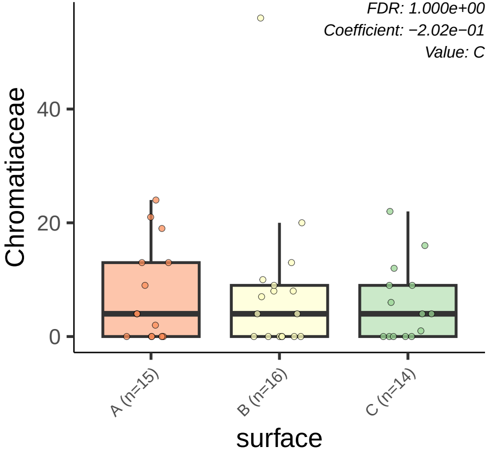
FDR: 1.000e+00
Coefficient: −2.02e−01
Value: C
Chromatiaceae
0
20
40
A (n=15)
B (n=16)
C (n=14)
surface

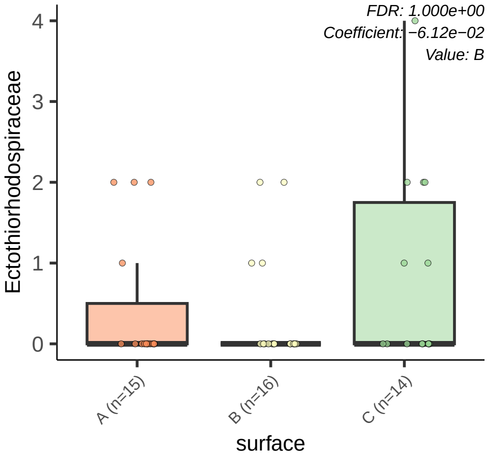
FDR: 1.000e+00
Coefficient: −6.12e−02
Value: B
Ectothiorhodospiraceae
4
3
2
1
0
A (n=15)
B (n=16)
C (n=14)
surface

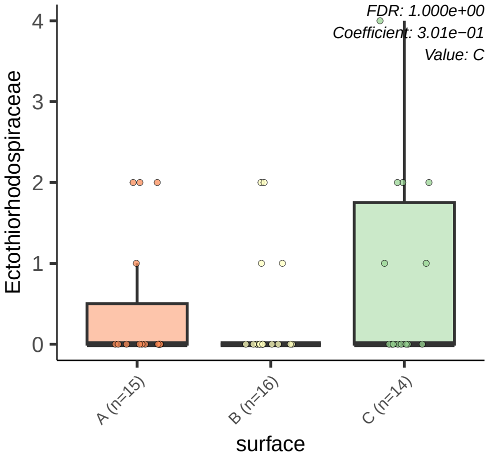

FDR: 1.000e+00
Coefficient: 3.01e−01
Value: C
Ectothiorhodospiraceae
4
3
2
1
0
A (n=15)
B (n=16)
C (n=14)
surface

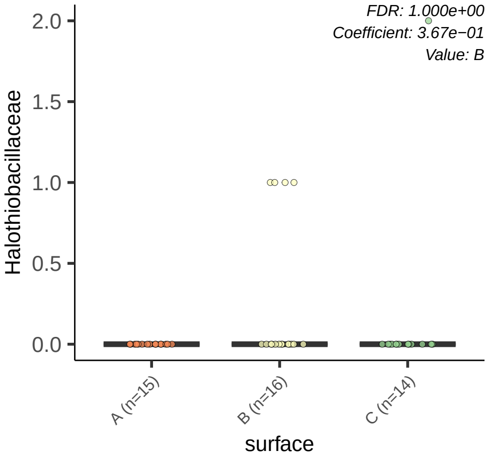
FDR: 1.000e+00
Coefficient: 3.67e−01
Value: B
Halothiobacillaceae
2.0
1.5
1.0
0.5
0.0
A (n=15)
B (n=16)
C (n=14)
surface

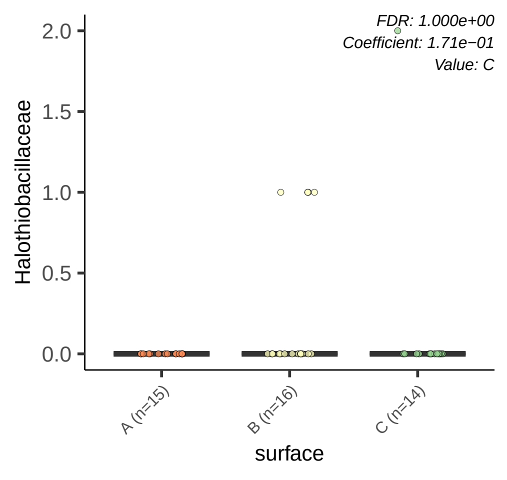
FDR: 1.000e+00
Coefficient: 1.71e−01
Value: C
Halothiobacillaceae
2.0
1.5
1.0
0.5
0.0
A (n=15)
B (n=16)
C (n=14)
surface

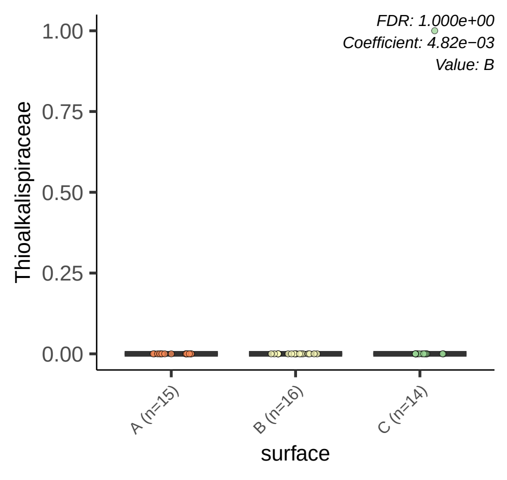

FDR: 1.000e+00
Coefficient: 4.82e−03
Value: B
Thioalkalispiraceae
1.00
0.75
0.50
0.25
0.00
A (n=15)
B (n=16)
C (n=14)
surface

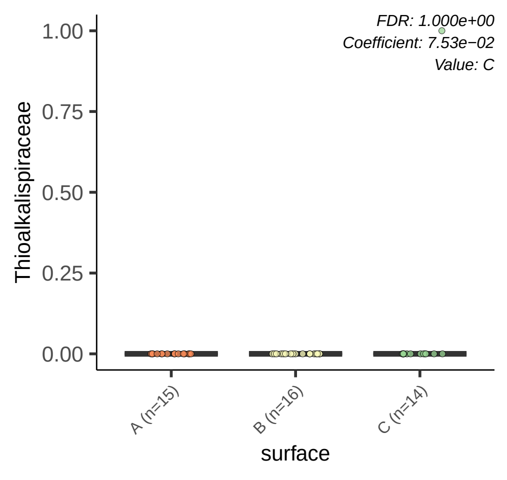
FDR: 1.000e+00
Coefficient: 7.53e−02
Value: C
Thioalkalispiraceae
1.00
0.75
0.50
0.25
0.00
A (n=15)
B (n=16)
C (n=14)
surface

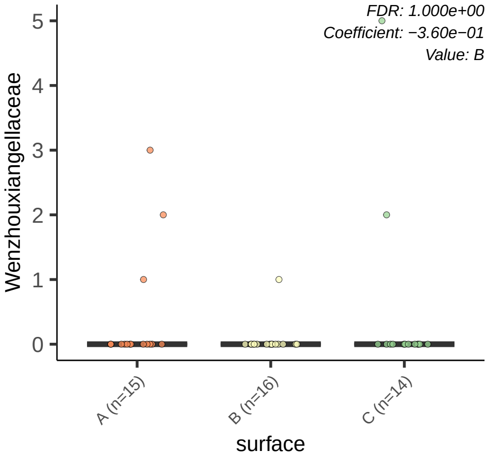

FDR: 1.000e+00
Coefficient: −3.60e−01
Value: B
Wenzhouxiangellaceae
0
1
2
3
4
5
A (n=15)
B (n=16)
C (n=14)
surface

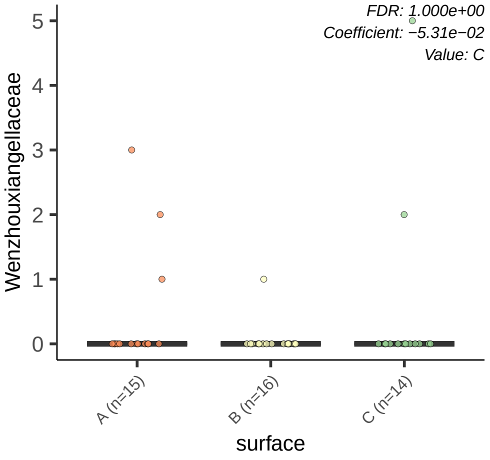

FDR: 1.000e+00
Coefficient: −5.31e−02
Value: C
Wenzhouxiangellaceae
0
1
2
3
4
5
A (n=15)
B (n=16)
C (n=14)
surface

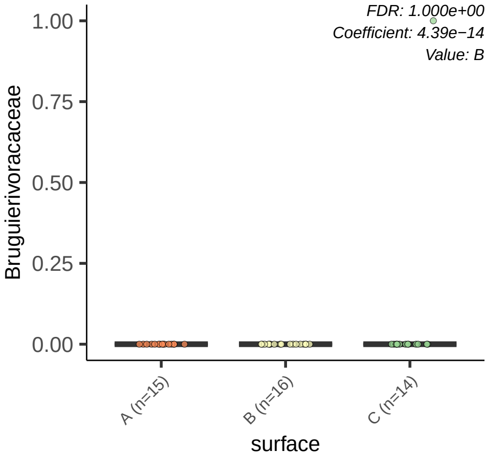
FDR: 1.000e+00
Coefficient: 4.39e−14
Value: B
Bruguierivoracaceae
1.00
0.75
0.50
0.25
0.00
A (n=15)
B (n=16)
C (n=14)
surface

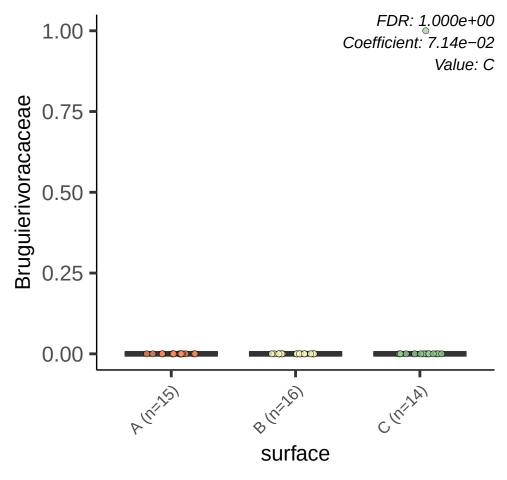
FDR: 1.000e+00
Coefficient: 7.14e−02
Value: C
Bruguierivoracaceae
1.00
0.75
0.50
0.25
0.00
A (n=15)
B (n=16)
C (n=14)
surface

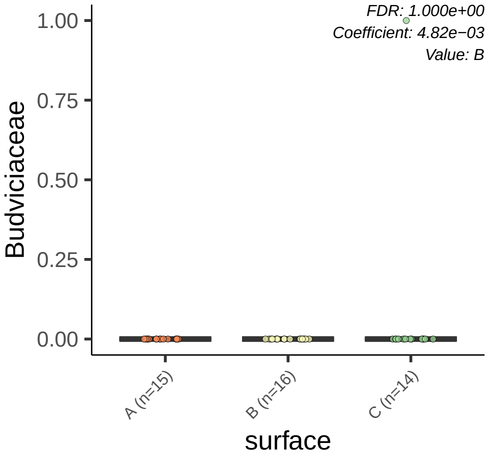
FDR: 1.000e+00
Coefficient: 4.82e−03
Value: B
Budviciaceae
1.00
0.75
0.50
0.25
0.00
A (n=15)
B (n=16)
C (n=14)
surface

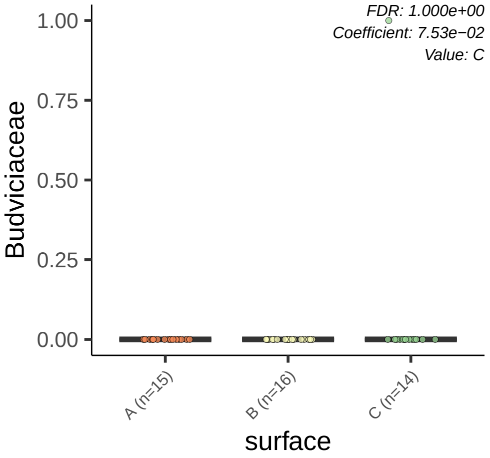
FDR: 1.000e+00
Coefficient: 7.53e−02
Value: C
Budviciaceae
1.00
0.75
0.50
0.25
0.00
A (n=15)
B (n=16)
C (n=14)
surface

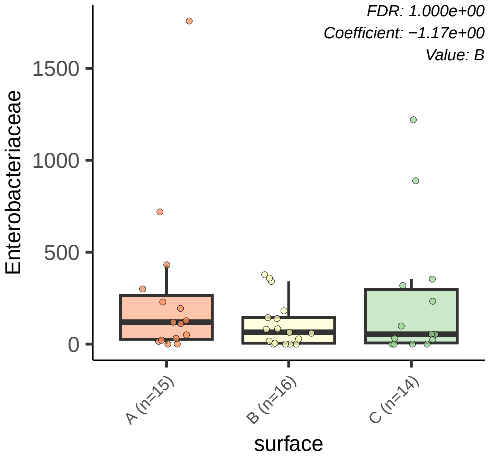
FDR: 1.000e+00
Coefficient: −1.17e+00
Value: B
Enterobacteriaceae
1500
1000
500
0
A (n=15)
B (n=16)
C (n=14)
surface

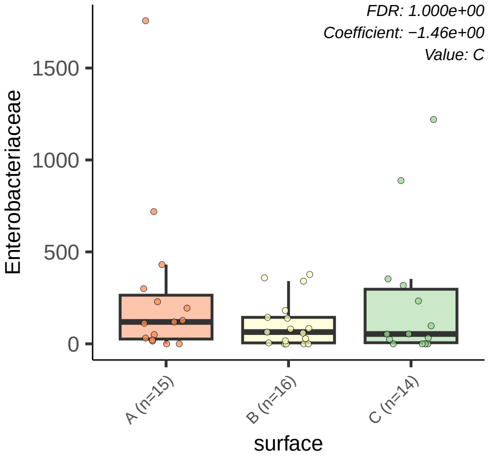
FDR: 1.000e+00
Coefficient: −1.46e+00
Value: C
Enterobacteriaceae
1500
1000
500
0
A (n=15)
B (n=16)
C (n=14)
surface

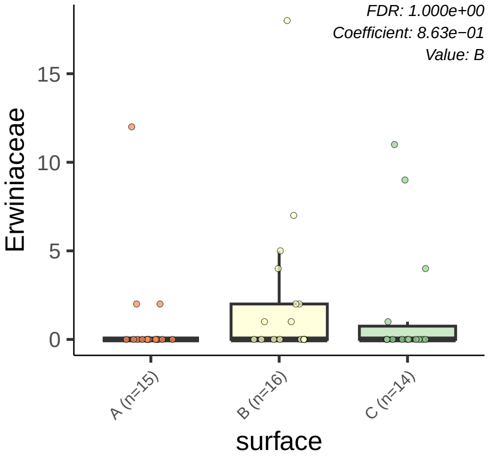

FDR: 1.000e+00
Coefficient: 8.63e−01
Value: B
Erwiniaceae
0
5
10
15
A (n=15)
B (n=16)
C (n=14)
surface

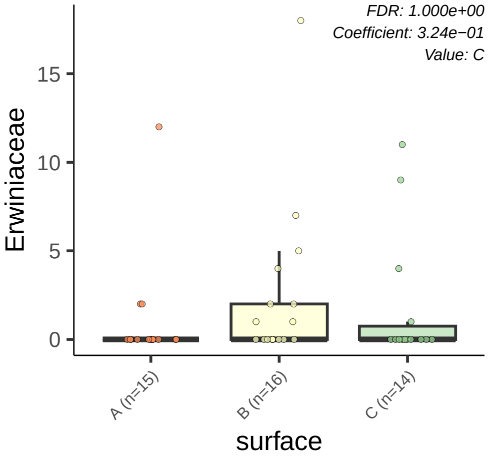

FDR: 1.000e+00
Coefficient: 3.24e−01
Value: C
Erwiniaceae
0
5
10
15
A (n=15)
B (n=16)
C (n=14)
surface

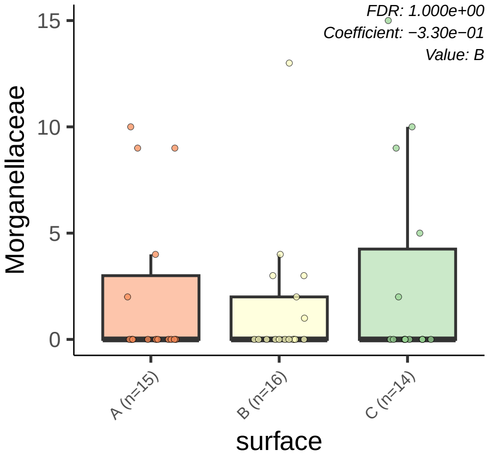

FDR: 1.000e+00
Coefficient: −3.30e−01
Value: B
Morganellaceae
15
10
5
0
A (n=15)
B (n=16)
C (n=14)
surface

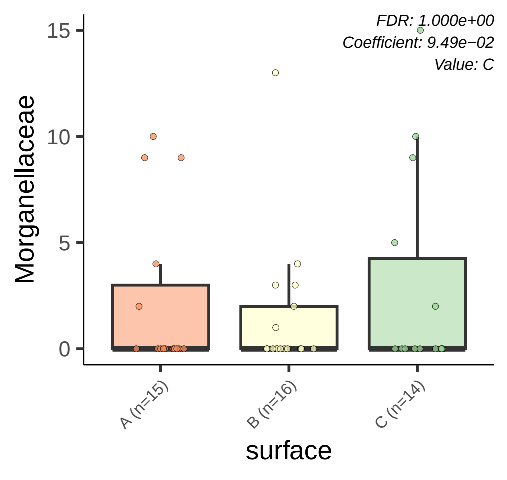
FDR: 1.000e+00
Coefficient: 9.49e−02
Value: C
Morganellaceae
0
5
10
15
A (n=15)
B (n=16)
C (n=14)
surface

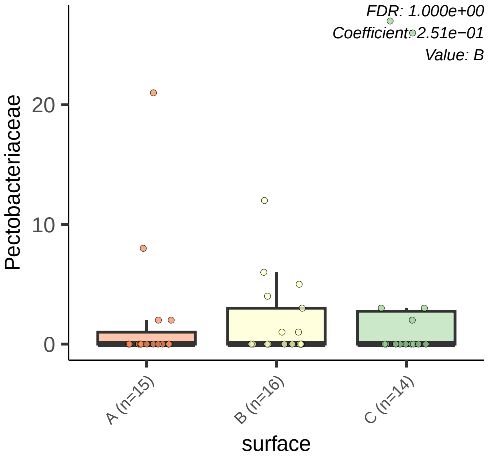
FDR: 1.000e+00
Coefficient: 2.51e−01
Value: B
Pectobacteriaceae
0
10
20
A (n=15)
B (n=16)
C (n=14)
surface

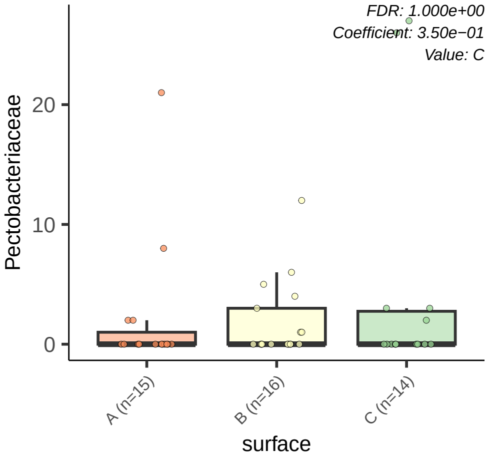

FDR: 1.000e+00
Coefficient: 3.50e−01
Value: C
Pectobacteriaceae
0
10
20
A (n=15)
B (n=16)
C (n=14)
surface

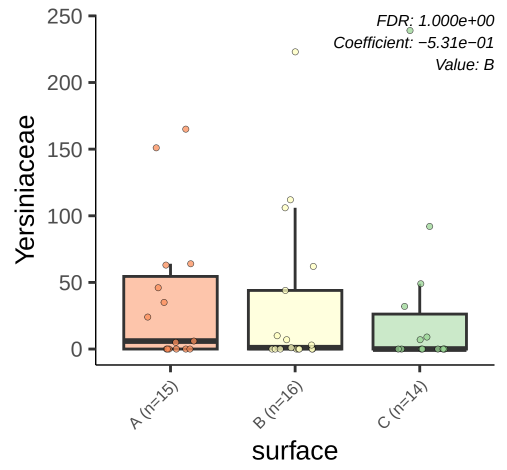

FDR: 1.000e+00
Coefficient: −5.31e−01
Value: B
Yersiniaceae
250
200
150
100
50
0
A (n=15)
B (n=16)
C (n=14)
surface

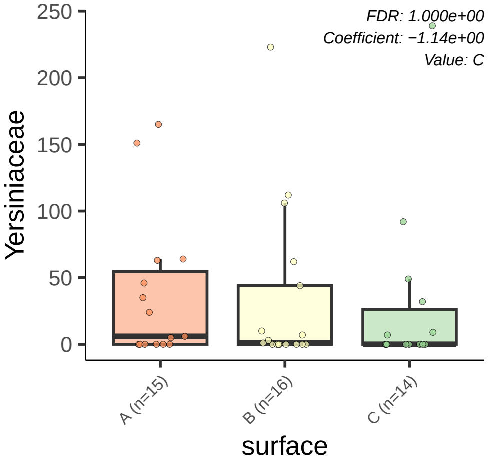
FDR: 1.000e+00
Coefficient: −1.14e+00
Value: C
Yersiniaceae
250
200
150
100
50
0
A (n=15)
B (n=16)
C (n=14)
surface

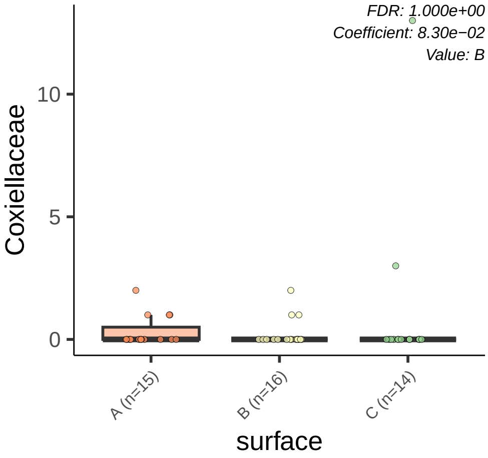
FDR: 1.000e+00
Coefficient: 8.30e−02
Value: B
Coxiellaceae
10
5
0
A (n=15)
B (n=16)
C (n=14)
surface

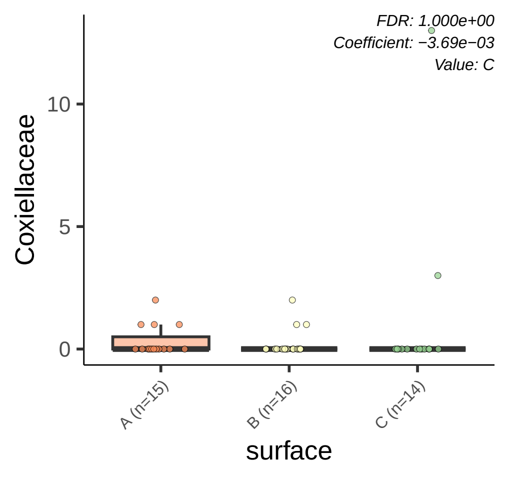
FDR: 1.000e+00
Coefficient: −3.69e−03
Value: C
Coxiellaceae
10
5
0
A (n=15)
B (n=16)
C (n=14)
surface

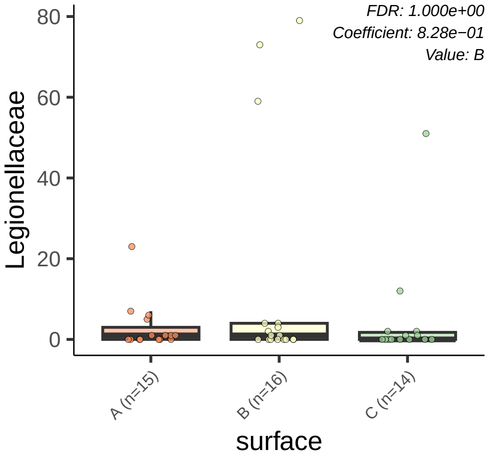

FDR: 1.000e+00
Coefficient: 8.28e−01
Value: B
Legionellaceae
80
60
40
20
0
A (n=15)
B (n=16)
C (n=14)
surface

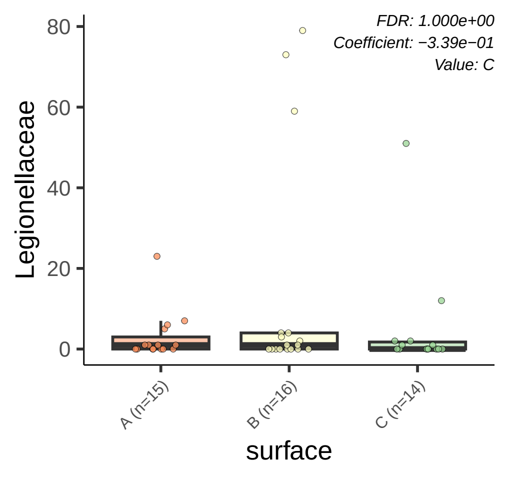
FDR: 1.000e+00
Coefficient: −3.39e−01
Value: C
Legionellaceae
0
20
40
60
80
A (n=15)
B (n=16)
C (n=14)
surface

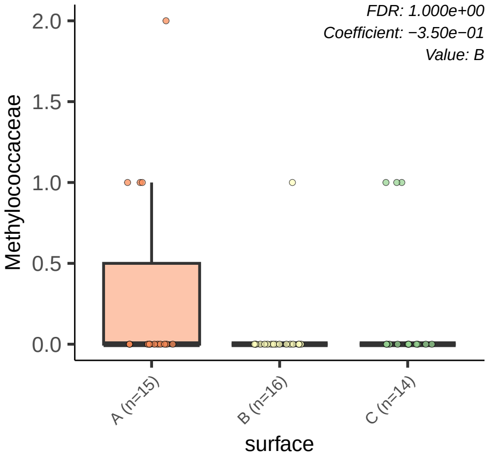

FDR: 1.000e+00
Coefficient: −3.50e−01
Value: B
Methylococcaceae
2.0
1.5
1.0
0.5
0.0
A (n=15)
B (n=16)
C (n=14)
surface

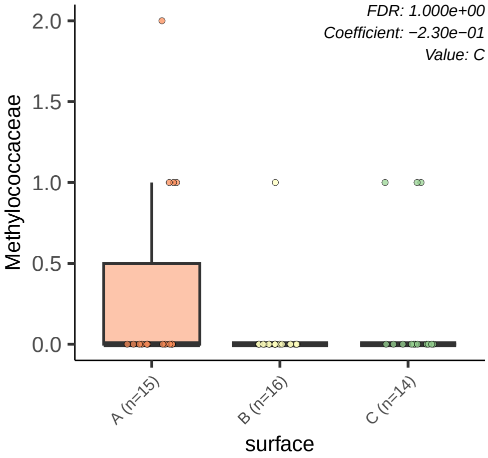

FDR: 1.000e+00
Coefficient: −2.30e−01
Value: C
Methylococcaceae
2.0
1.5
1.0
0.5
0.0
A (n=15)
B (n=16)
C (n=14)
surface

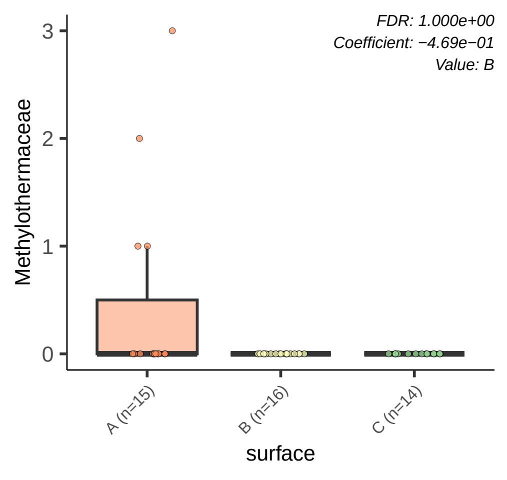

FDR: 1.000e+00
Coefficient: −4.69e−01
Value: B
Methylothermaceae
3
2
1
0
A (n=15)
B (n=16)
C (n=14)
surface

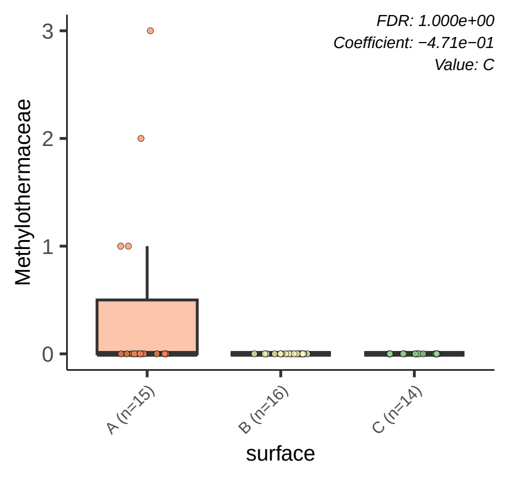
FDR: 1.000e+00
Coefficient: −4.71e−01
Value: C
Methylothermaceae
3
2
1
0
A (n=15)
B (n=16)
C (n=14)
surface

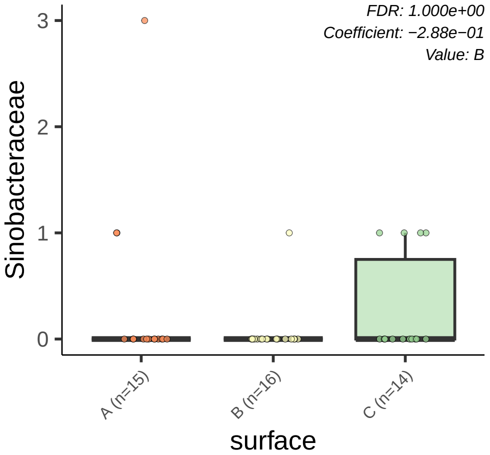

FDR: 1.000e+00
Coefficient: −2.88e−01
Value: B
Sinobacteraceae
3
2
1
0
A (n=15)
B (n=16)
C (n=14)
surface

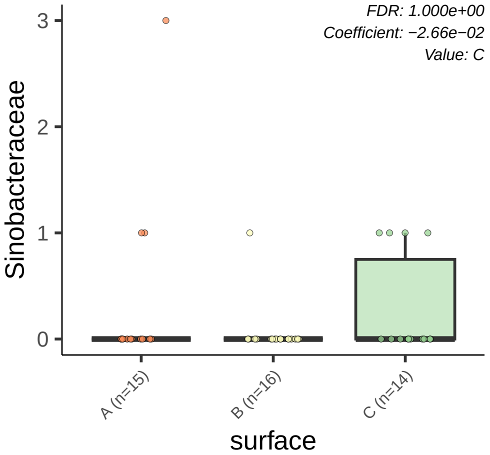
FDR: 1.000e+00
Coefficient: −2.66e−02
Value: C
Sinobacteraceae
3
2
1
0
A (n=15)
B (n=16)
C (n=14)
surface

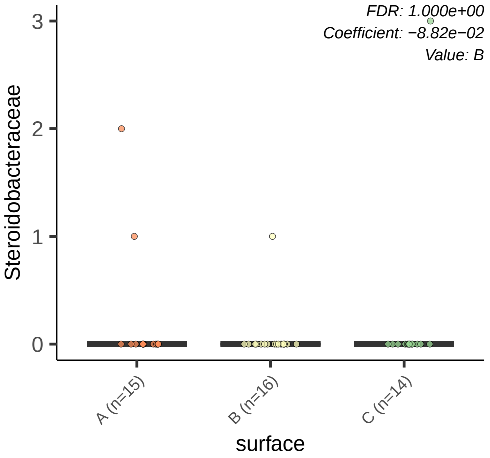

FDR: 1.000e+00
Coefficient: −8.82e−02
Value: B
Steroidobacteraceae
3
2
1
0
A (n=15)
B (n=16)
C (n=14)
surface

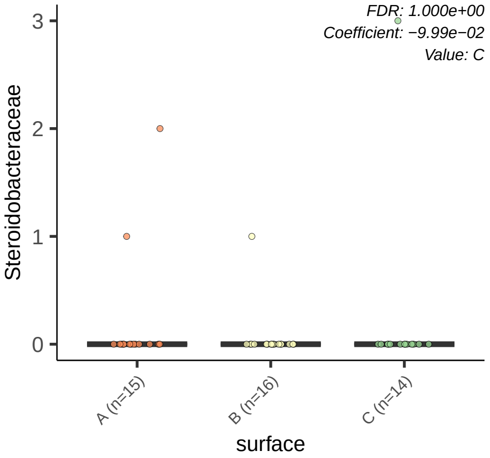

FDR: 1.000e+00
Coefficient: −9.99e−02
Value: C
Steroidobacteraceae
0
1
2
3
A (n=15)
B (n=16)
C (n=14)
surface

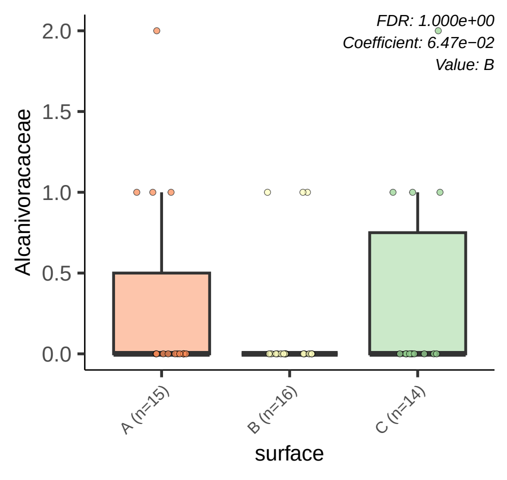
FDR: 1.000e+00
Coefficient: 6.47e−02
Value: B
Alcanivoracaceae
2.0
1.5
1.0
0.5
0.0
A (n=15)
B (n=16)
C (n=14)
surface

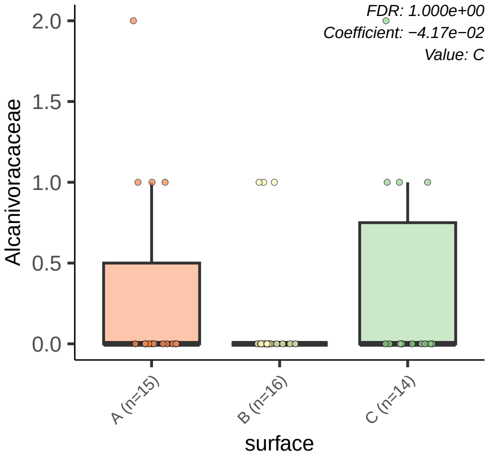
FDR: 1.000e+00
Coefficient: −4.17e−02
Value: C
Alcanivoracaceae
2.0
1.5
1.0
0.5
0.0
A (n=15)
B (n=16)
C (n=14)
surface

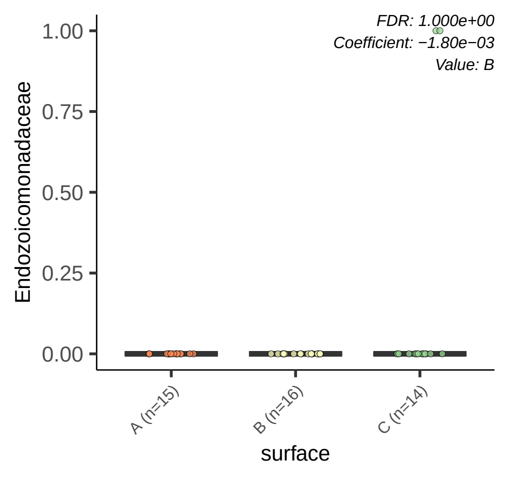
FDR: 1.000e+00
Coefficient: −1.80e−03
Value: B
Endozoicomonadaceae
1.00
0.75
0.50
0.25
0.00
A (n=15)
B (n=16)
C (n=14)
surface

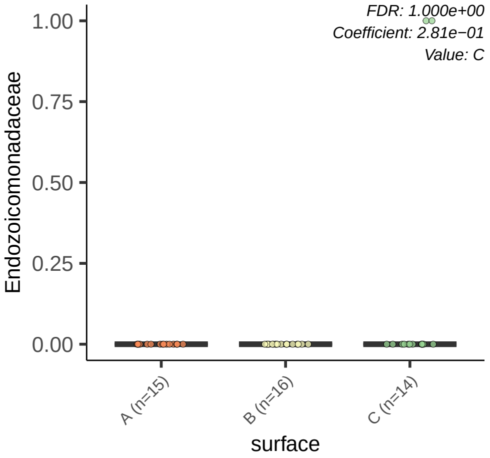

FDR: 1.000e+00
Coefficient: 2.81e−01
Value: C
Endozoicomonadaceae
1.00
0.75
0.50
0.25
0.00
A (n=15)
B (n=16)
C (n=14)
surface

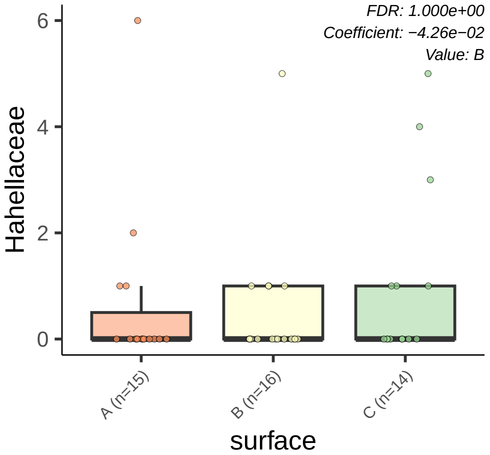

FDR: 1.000e+00
Coefficient: −4.26e−02
Value: B
Hahellaceae
6
4
2
0
A (n=15)
B (n=16)
C (n=14)
surface

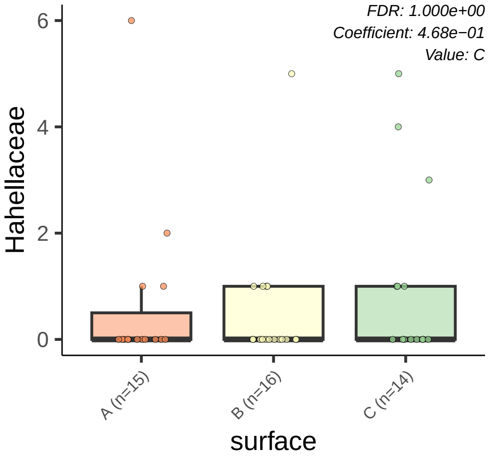
FDR: 1.000e+00
Coefficient: 4.68e−01
Value: C
Hahellaceae
0
2
4
6
A (n=15)
B (n=16)
C (n=14)
surface

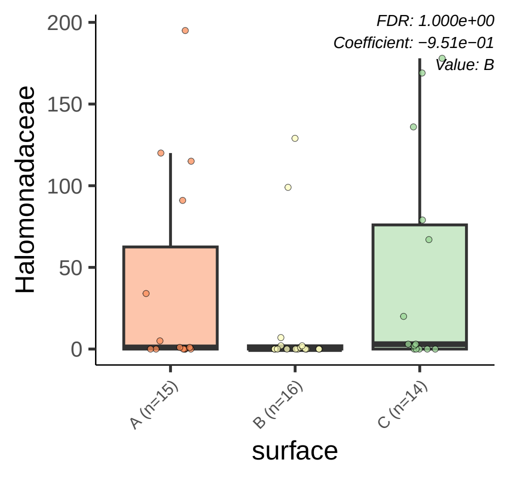
FDR: 1.000e+00
Coefficient: −9.51e−01
Value: B
Halomonadaceae
200
150
100
50
0
A (n=15)
B (n=16)
C (n=14)
surface

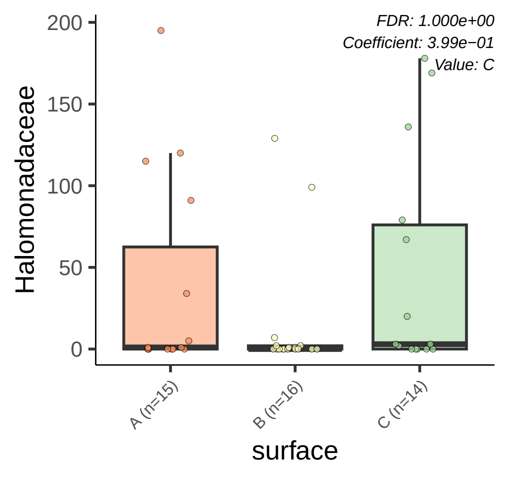

FDR: 1.000e+00
Coefficient: 3.99e−01
Value: C
Halomonadaceae
200
150
100
50
0
A (n=15)
B (n=16)
C (n=14)
surface

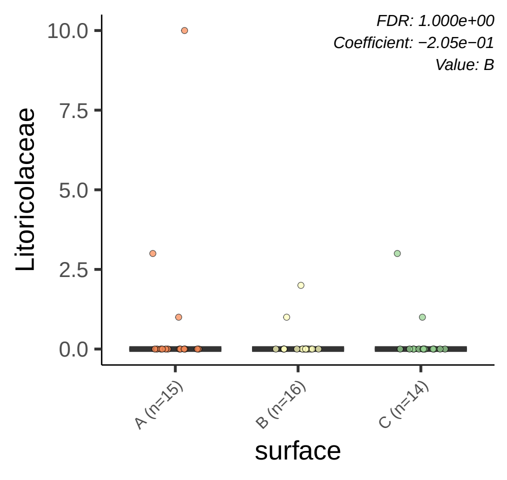
FDR: 1.000e+00
Coefficient: −2.05e−01
Value: B
Litoricolaceae
10.0
7.5
5.0
2.5
0.0
A (n=15)
B (n=16)
C (n=14)
surface

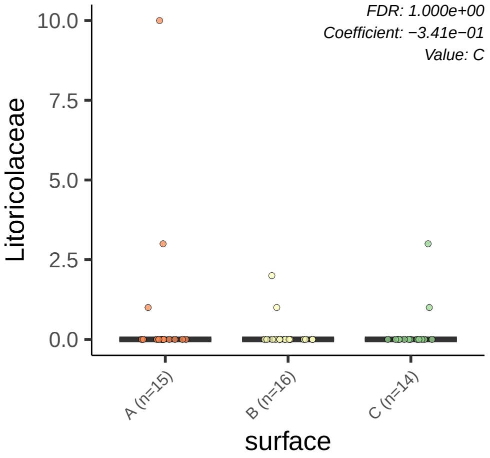

FDR: 1.000e+00
Coefficient: −3.41e−01
Value: C
Litoricolaceae
10.0
7.5
5.0
2.5
0.0
A (n=15)
B (n=16)
C (n=14)
surface

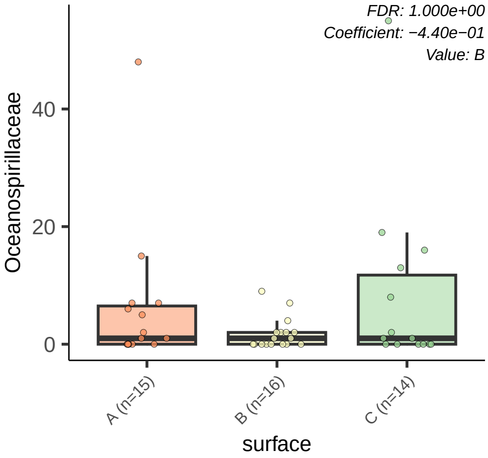

FDR: 1.000e+00
Coefficient: −4.40e−01
Value: B
Oceanospirillaceae
0
20
40
A (n=15)
B (n=16)
C (n=14)
surface

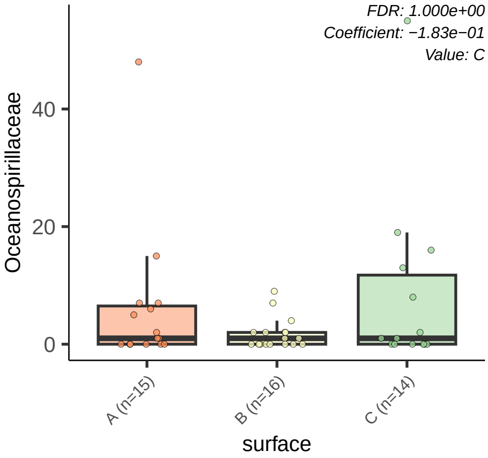
FDR: 1.000e+00
Coefficient: −1.83e−01
Value: C
Oceanospirillaceae
0
20
40
A (n=15)
B (n=16)
C (n=14)
surface

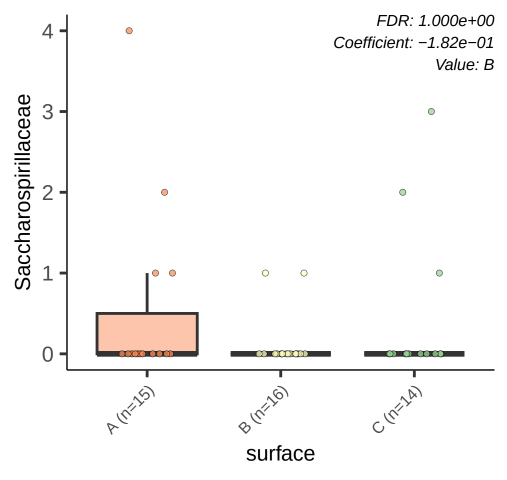

FDR: 1.000e+00
Coefficient: −1.82e−01
Value: B
Saccharospirillaceae
0
1
2
3
4
A (n=15)
B (n=16)
C (n=14)
surface

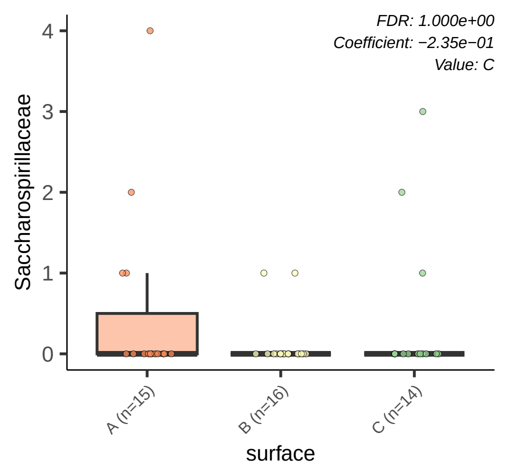

FDR: 1.000e+00
Coefficient: −2.35e−01
Value: C
Saccharospirillaceae
0
1
2
3
4
A (n=15)
B (n=16)
C (n=14)
surface

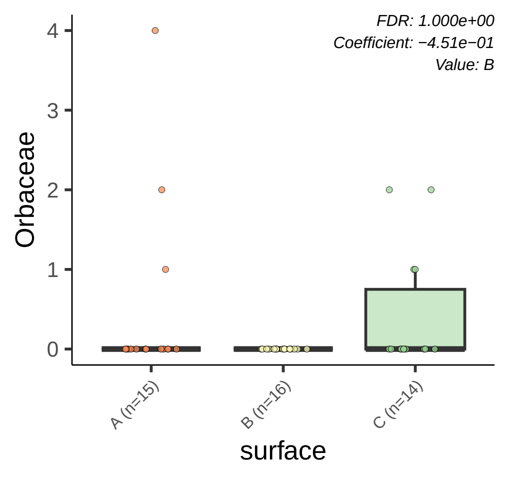
FDR: 1.000e+00
Coefficient: −4.51e−01
Value: B
Orbaceae
4
3
2
1
0
A (n=15)
B (n=16)
C (n=14)
surface

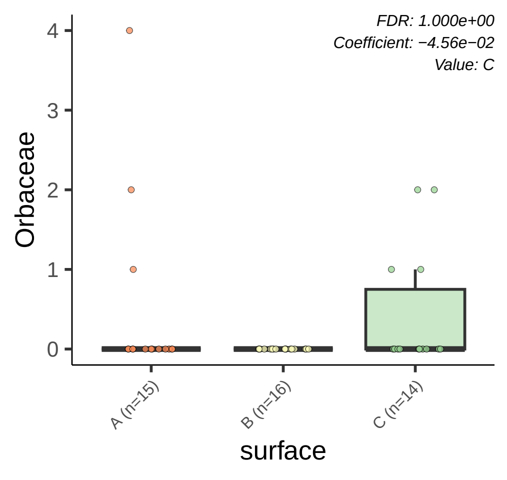
FDR: 1.000e+00
Coefficient: −4.56e−02
Value: C
Orbaceae
0
1
2
3
4
A (n=15)
B (n=16)
C (n=14)
surface

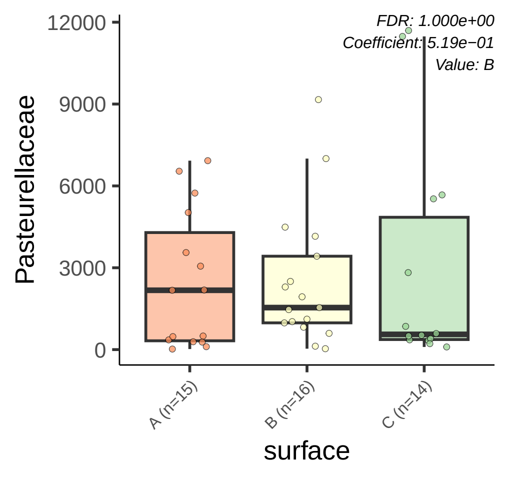
FDR: 1.000e+00
Coefficient: 5.19e−01
Value: B
Pasteurellaceae
12000
9000
6000
3000
0
A (n=15)
B (n=16)
C (n=14)
surface

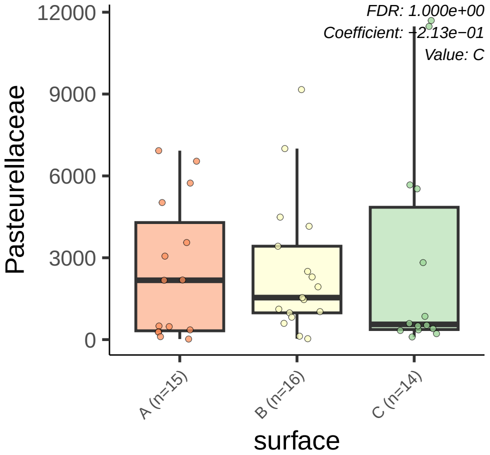
FDR: 1.000e+00
Coefficient: −2.13e−01
Value: C
Pasteurellaceae
12000
9000
6000
3000
0
A (n=15)
B (n=16)
C (n=14)
surface

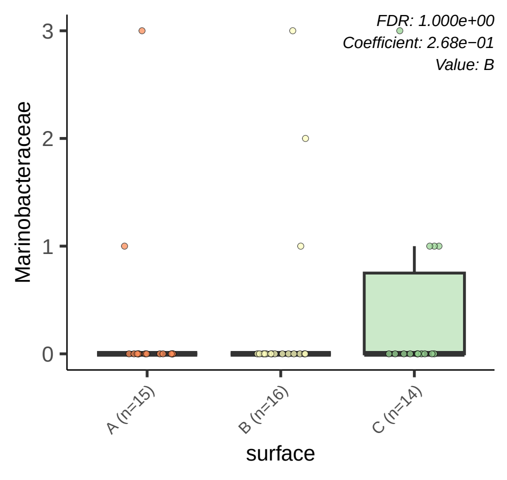
FDR: 1.000e+00
Coefficient: 2.68e−01
Value: B
Marinobacteraceae
3
2
1
0
A (n=15)
B (n=16)
C (n=14)
surface

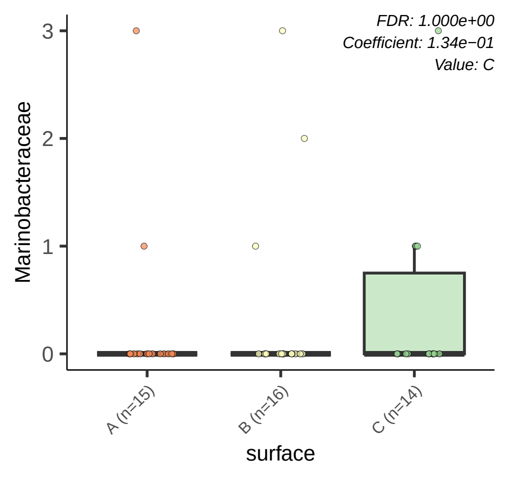

FDR: 1.000e+00
Coefficient: 1.34e−01
Value: C
Marinobacteraceae
0
1
2
3
A (n=15)
B (n=16)
C (n=14)
surface

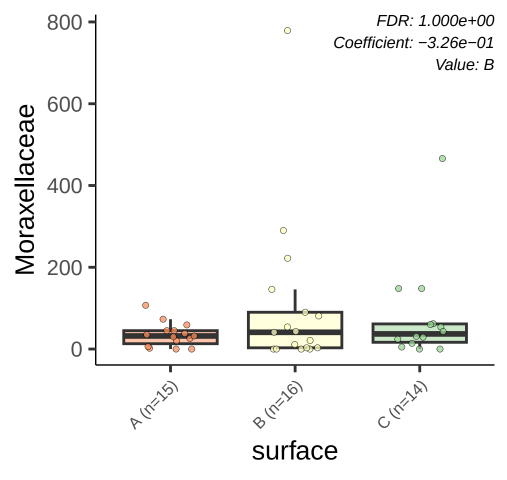
FDR: 1.000e+00
Coefficient: −3.26e−01
Value: B
Moraxellaceae
800
600
400
200
0
A (n=15)
B (n=16)
C (n=14)
surface

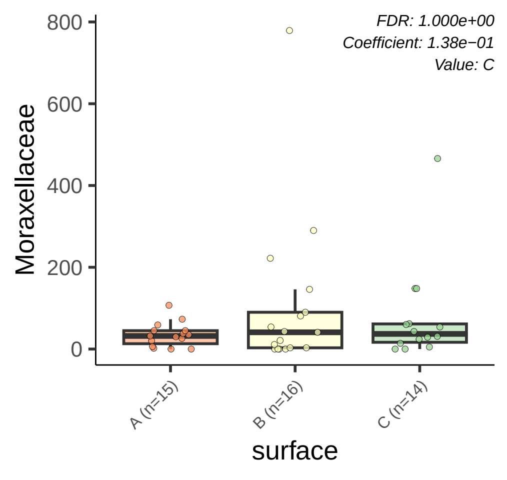

FDR: 1.000e+00
Coefficient: 1.38e−01
Value: C
Moraxellaceae
800
600
400
200
0
A (n=15)
B (n=16)
C (n=14)
surface

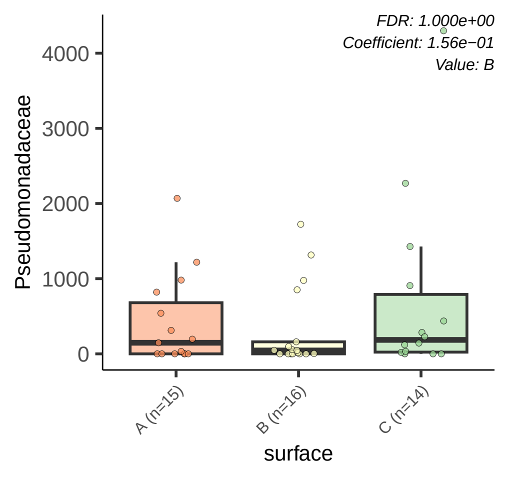

FDR: 1.000e+00
Coefficient: 1.56e−01
Value: B
Pseudomonadaceae
4000
3000
2000
1000
0
A (n=15)
B (n=16)
C (n=14)
surface

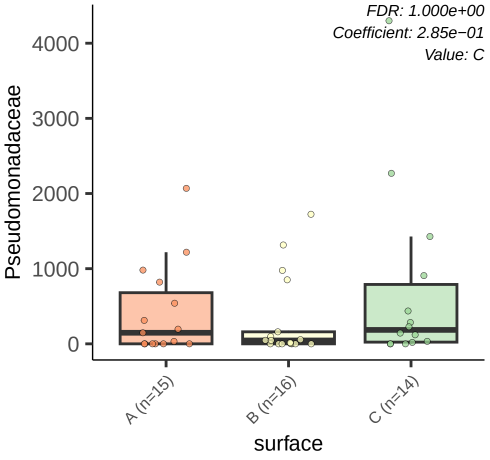
FDR: 1.000e+00
Coefficient: 2.85e−01
Value: C
Pseudomonadaceae
4000
3000
2000
1000
0
A (n=15)
B (n=16)
C (n=14)
surface

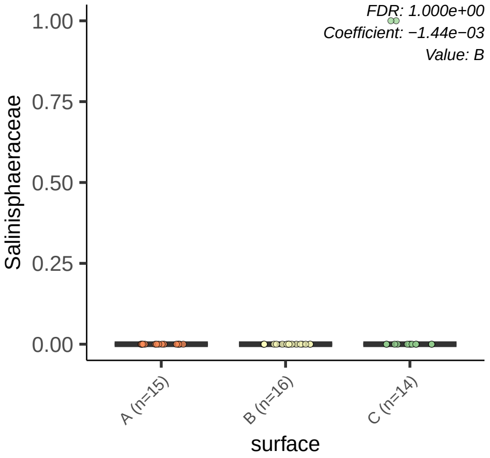
FDR: 1.000e+00
Coefficient: −1.44e−03
Value: B
1.00
0.75
0.50
0.25
0.00
Salinisphaeraceae
A (n=15)
B (n=16)
C (n=14)
surface

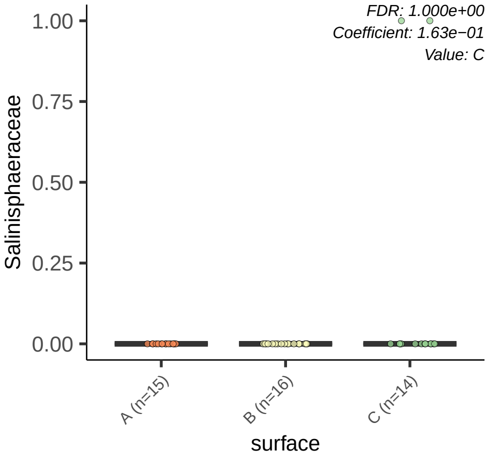
FDR: 1.000e+00
Coefficient: 1.63e−01
Value: C
Salinisphaeraceae
1.00
0.75
0.50
0.25
0.00
A (n=15)
B (n=16)
C (n=14)
surface

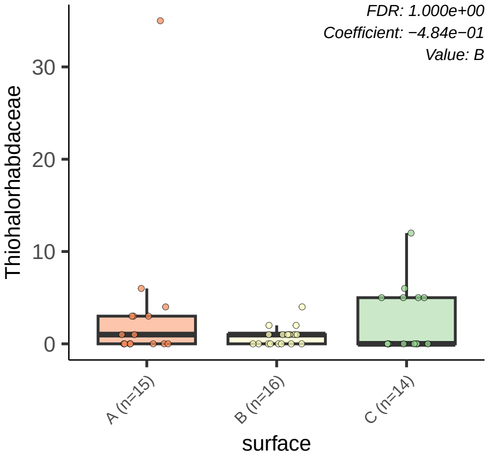

FDR: 1.000e+00
Coefficient: −4.84e−01
Value: B
Thiohalorhabdaceae
0
10
20
30
A (n=15)
B (n=16)
C (n=14)
surface

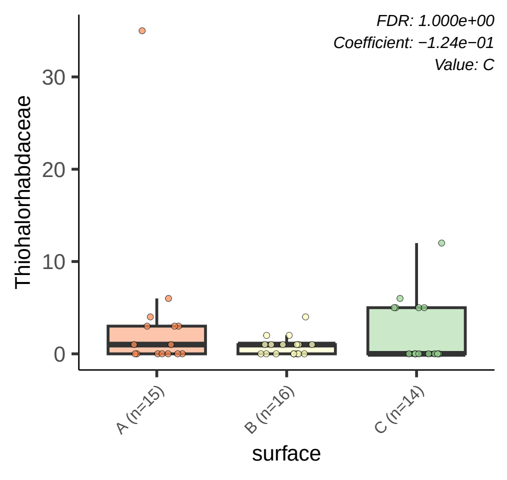

FDR: 1.000e+00
Coefficient: −1.24e−01
Value: C
Thiohalorhabdaceae
30
20
10
0
A (n=15)
B (n=16)
C (n=14)
surface

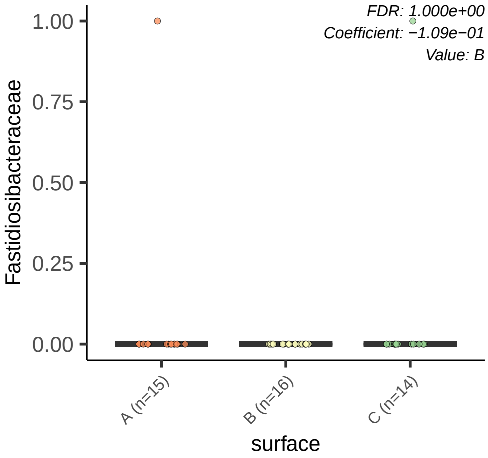
FDR: 1.000e+00
Coefficient: −1.09e−01
Value: B
Fastidiosibacteraceae
1.00
0.75
0.50
0.25
0.00
A (n=15)
B (n=16)
C (n=14)
surface

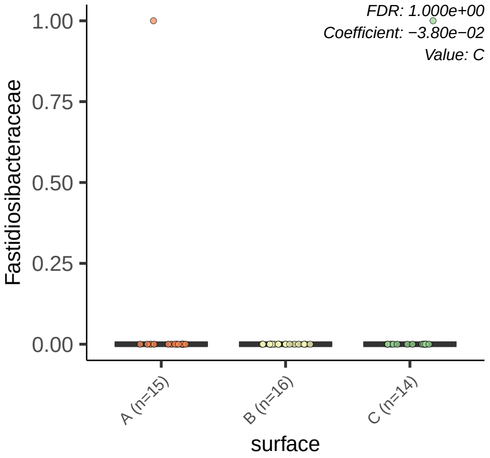
FDR: 1.000e+00
Coefficient: −3.80e−02
Value: C
Fastidiosibacteraceae
1.00
0.75
0.50
0.25
0.00
A (n=15)
B (n=16)
C (n=14)
surface

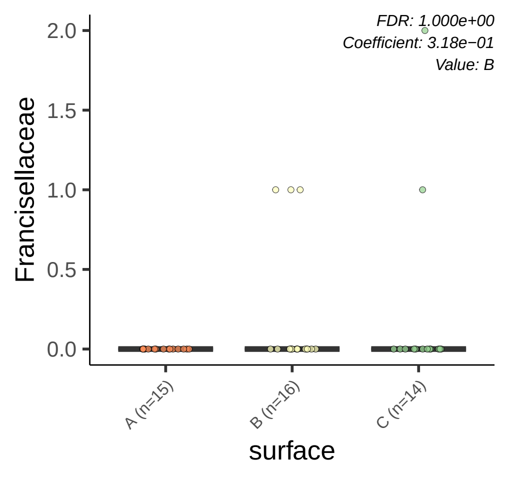

FDR: 1.000e+00
Coefficient: 3.18e−01
Value: B
Francisellaceae
2.0
1.5
1.0
0.5
0.0
A (n=15)
B (n=16)
C (n=14)
surface

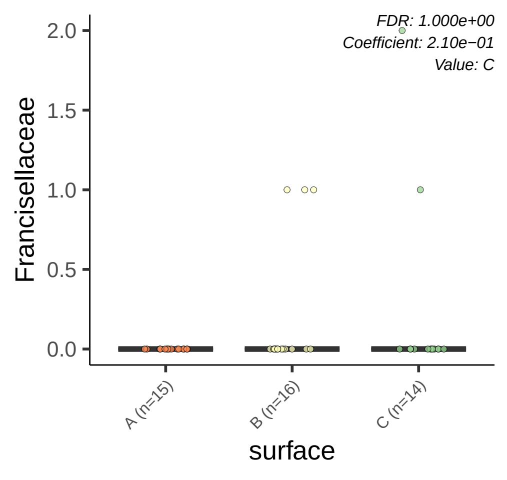
FDR: 1.000e+00
Coefficient: 2.10e−01
Value: C
Francisellaceae
2.0
1.5
1.0
0.5
0.0
A (n=15)
B (n=16)
C (n=14)
surface

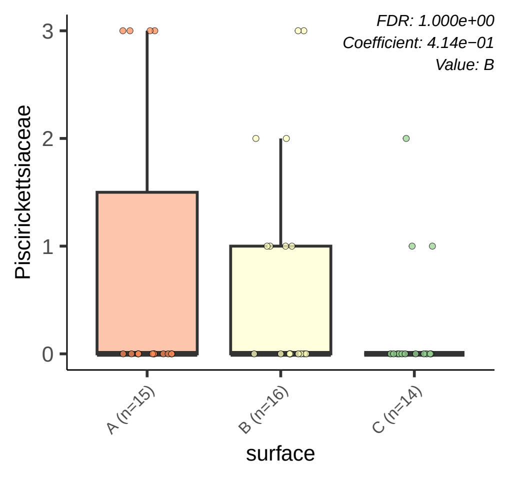

FDR: 1.000e+00
Coefficient: 4.14e−01
Value: B
Piscirickettsiaceae
0
1
2
3
A (n=15)
B (n=16)
C (n=14)
surface

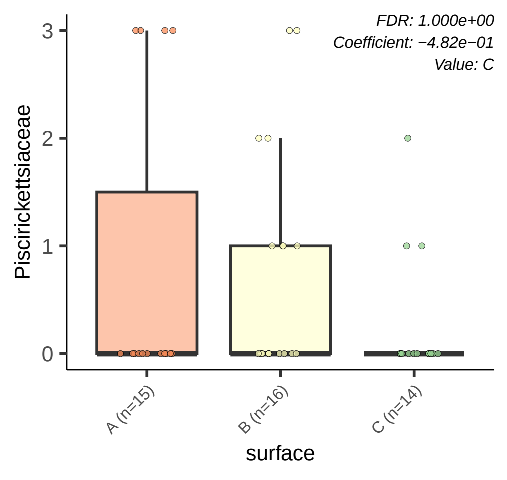

FDR: 1.000e+00
Coefficient: −4.82e−01
Value: C
Piscirickettsiaceae
0
1
2
3
A (n=15)
B (n=16)
C (n=14)
surface

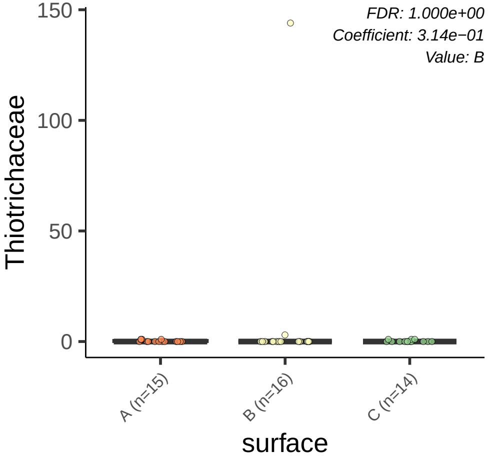
FDR: 1.000e+00
Coefficient: 3.14e−01
Value: B
Thiotrichaceae
150
100
50
0
A (n=15)
B (n=16)
C (n=14)
surface

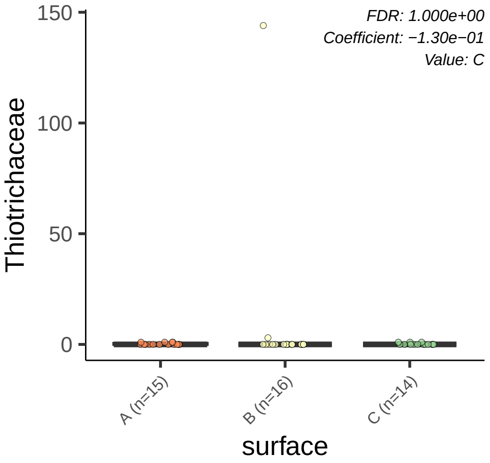

FDR: 1.000e+00
Coefficient: −1.30e−01
Value: C
Thiotrichaceae
150
100
50
0
A (n=15)
B (n=16)
C (n=14)
surface

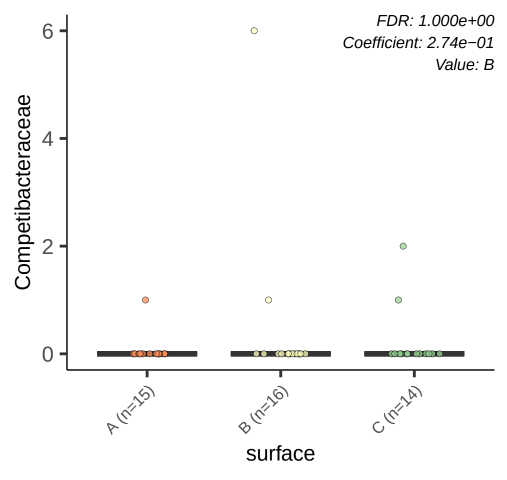
FDR: 1.000e+00
Coefficient: 2.74e−01
Value: B
Competibacteraceae
6
4
2
0
A (n=15)
B (n=16)
C (n=14)
surface

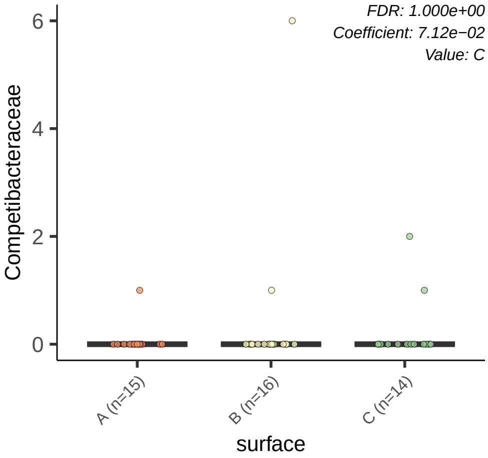

FDR: 1.000e+00
Coefficient: 7.12e−02
Value: C
Competibacteraceae
0
2
4
6
A (n=15)
B (n=16)
C (n=14)
surface

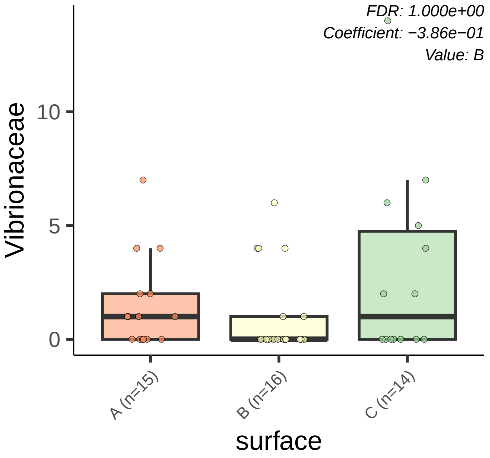
FDR: 1.000e+00
Coefficient: −3.86e−01
Value: B
Vibrionaceae
10
5
0
A (n=15)
B (n=16)
C (n=14)
surface

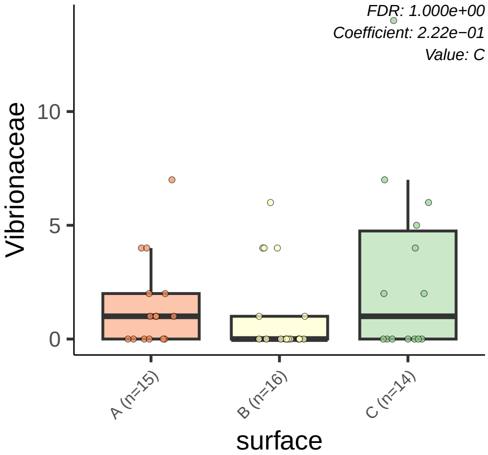
FDR: 1.000e+00
Coefficient: 2.22e−01
Value: C
Vibrionaceae
10
5
0
A (n=15)
B (n=16)
C (n=14)
surface

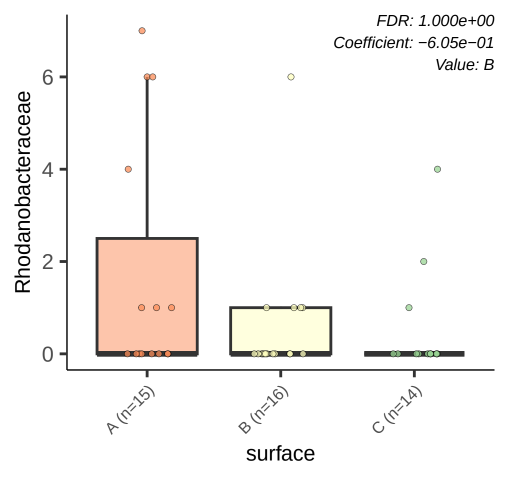

FDR: 1.000e+00
Coefficient: −6.05e−01
Value: B
Rhodanobacteraceae
0
2
4
6
A (n=15)
B (n=16)
C (n=14)
surface

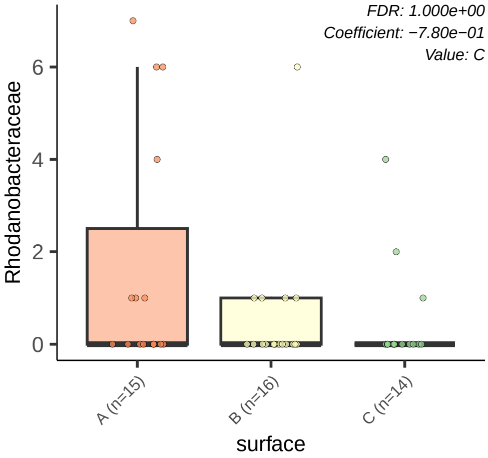

FDR: 1.000e+00
Coefficient: −7.80e−01
Value: C
Rhodanobacteraceae
0
2
4
6
A (n=15)
B (n=16)
C (n=14)
surface

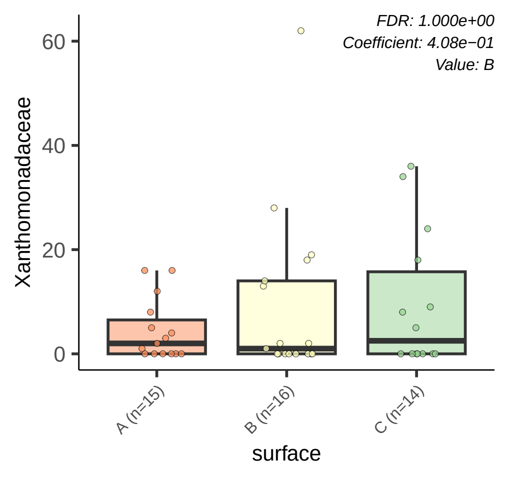
FDR: 1.000e+00
Coefficient: 4.08e−01
Value: B
Xanthomonadaceae
60
40
20
0
A (n=15)
B (n=16)
C (n=14)
surface

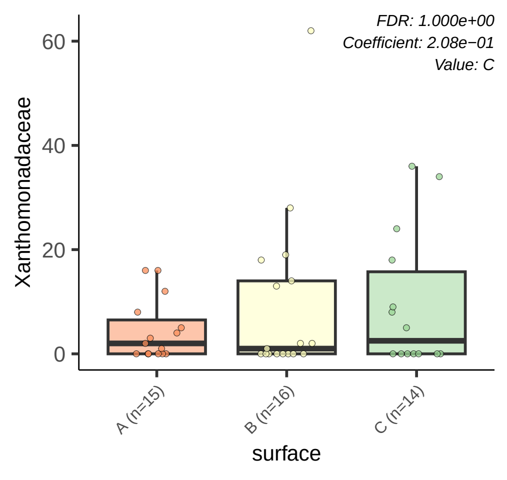
FDR: 1.000e+00
Coefficient: 2.08e−01
Value: C
Xanthomonadaceae
60
40
20
0
A (n=15)
B (n=16)
C (n=14)
surface

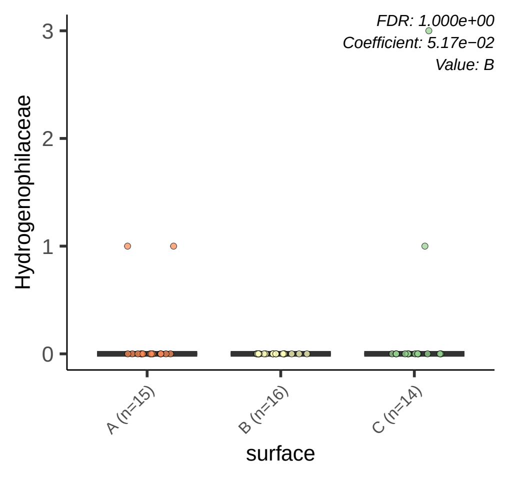
FDR: 1.000e+00
Coefficient: 5.17e−02
Value: B
Hydrogenophilaceae
3
2
1
0
A (n=15)
B (n=16)
C (n=14)
surface

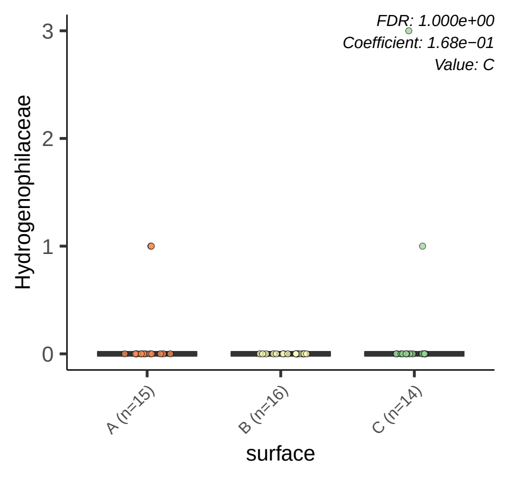

FDR: 1.000e+00
Coefficient: 1.68e−01
Value: C
Hydrogenophilaceae
3
2
1
0
A (n=15)
B (n=16)
C (n=14)
surface

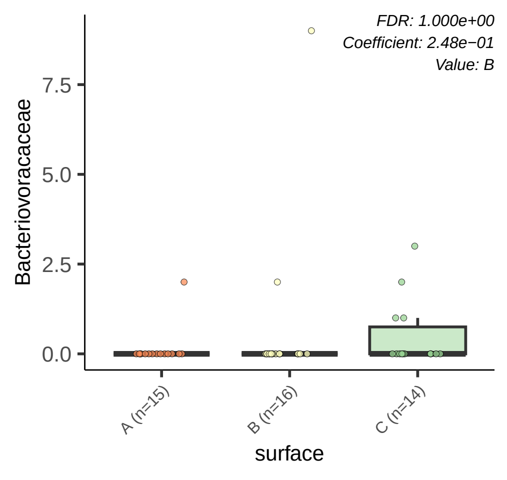

FDR: 1.000e+00
Coefficient: 2.48e−01
Value: B
Bacteriovoracaceae
7.5
5.0
2.5
0.0
A (n=15)
B (n=16)
C (n=14)
surface

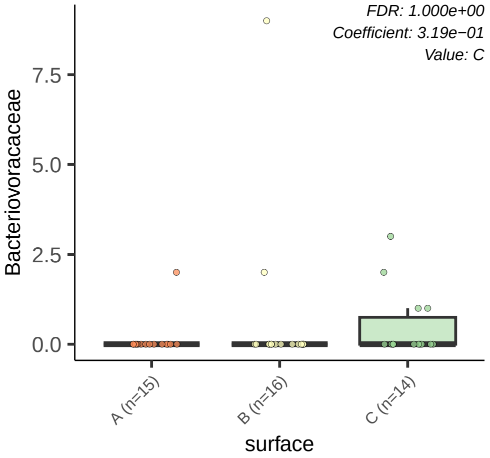
FDR: 1.000e+00
Coefficient: 3.19e−01
Value: C
Bacteriovoracaceae
7.5
5.0
2.5
0.0
A (n=15)
B (n=16)
C (n=14)
surface

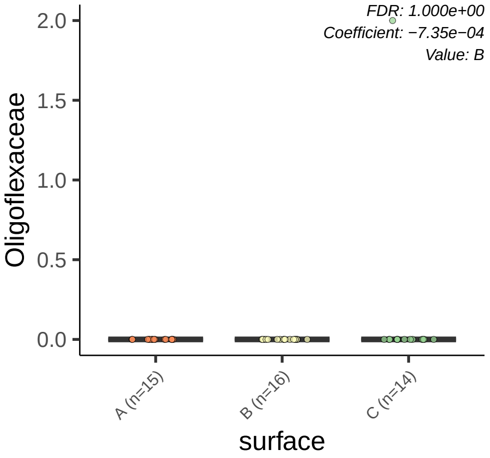

FDR: 1.000e+00
Coefficient: −7.35e−04
Value: B
Oligoflexaceae
2.0
1.5
1.0
0.5
0.0
A (n=15)
B (n=16)
C (n=14)
surface

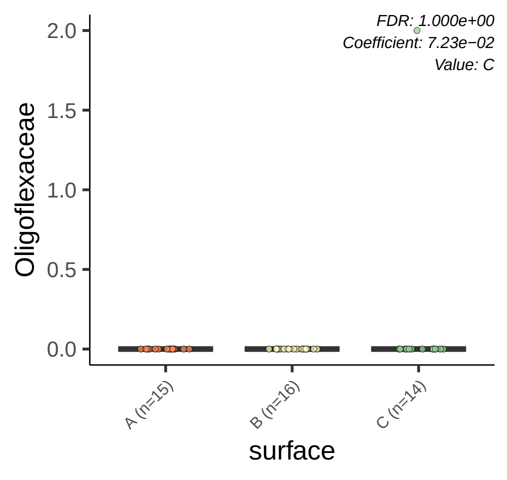
FDR: 1.000e+00
Coefficient: 7.23e−02
Value: C
Oligoflexaceae
2.0
1.5
1.0
0.5
0.0
A (n=15)
B (n=16)
C (n=14)
surface

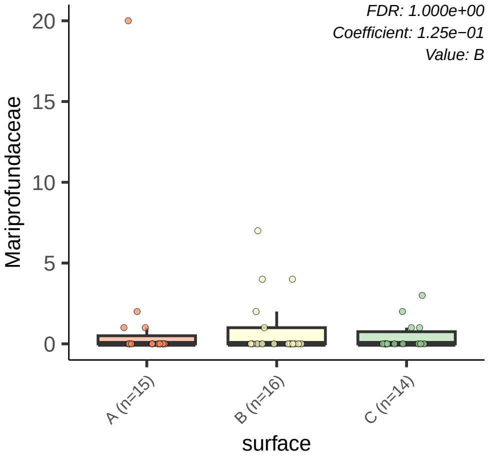
FDR: 1.000e+00
Coefficient: 1.25e−01
Value: B
Mariprofundaceae
20
15
10
5
0
A (n=15)
B (n=16)
C (n=14)
surface

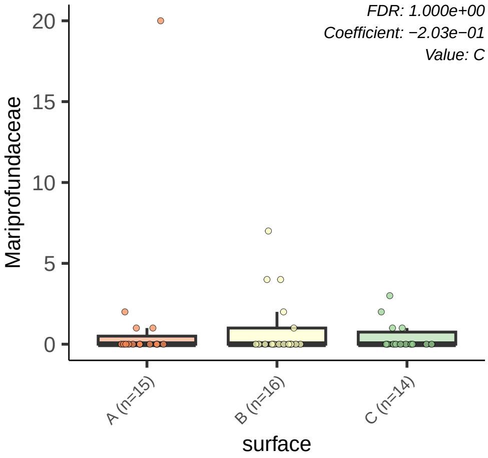
FDR: 1.000e+00
Coefficient: −2.03e−01
Value: C
Mariprofundaceae
0
5
10
15
20
A (n=15)
B (n=16)
C (n=14)
surface

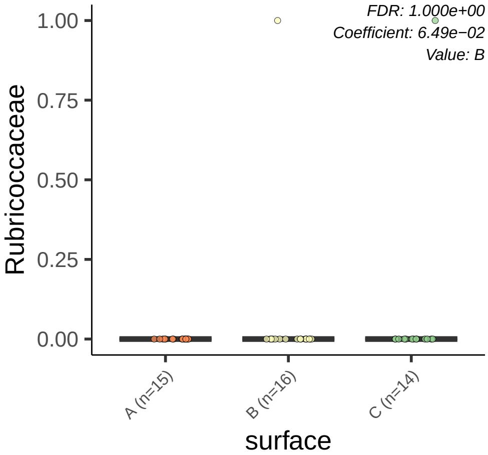

FDR: 1.000e+00
Coefficient: 6.49e−02
Value: B
Rubricoccaceae
1.00
0.75
0.50
0.25
0.00
A (n=15)
B (n=16)
C (n=14)
surface

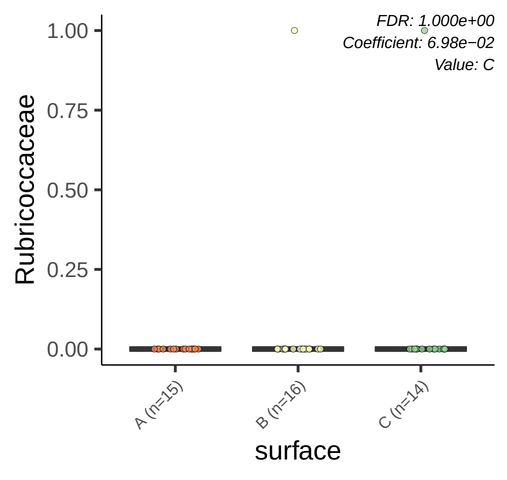
FDR: 1.000e+00
Coefficient: 6.98e−02
Value: C
Rubricoccaceae
1.00
0.75
0.50
0.25
0.00
A (n=15)
B (n=16)
C (n=14)
surface

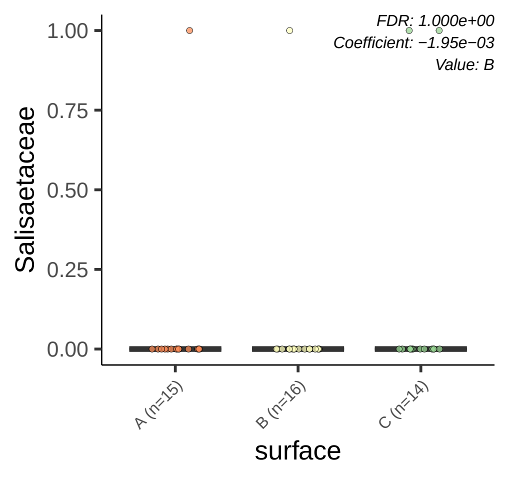

FDR: 1.000e+00
Coefficient: −1.95e−03
Value: B
Salisaetaceae
1.00
0.75
0.50
0.25
0.00
A (n=15)
B (n=16)
C (n=14)
surface

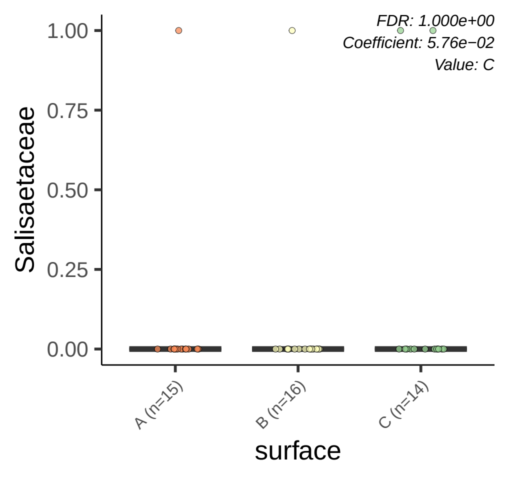

FDR: 1.000e+00
Coefficient: 5.76e−02
Value: C
Salisaetaceae
1.00
0.75
0.50
0.25
0.00
A (n=15)
B (n=16)
C (n=14)
surface

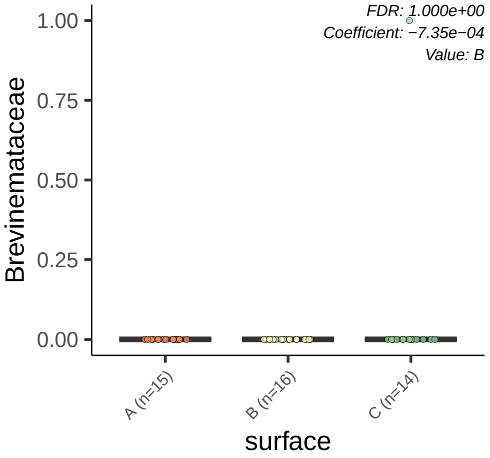
FDR: 1.000e+00
Coefficient: −7.35e−04
Value: B
Brevinemataceae
1.00
0.75
0.50
0.25
0.00
A (n=15)
B (n=16)
C (n=14)
surface

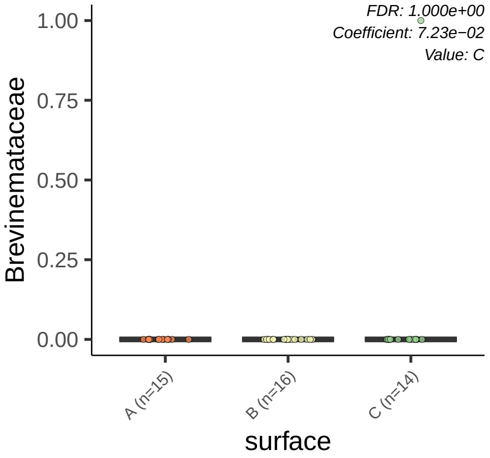

FDR: 1.000e+00
Coefficient: 7.23e−02
Value: C
1.00
0.75
0.50
0.25
0.00
Brevinemataceae
A (n=15)
B (n=16)
C (n=14)
surface

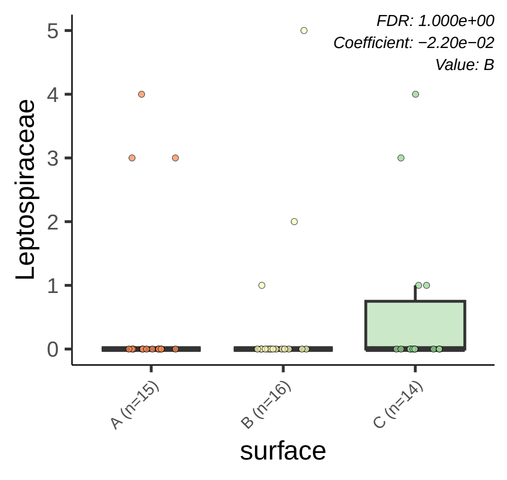
FDR: 1.000e+00
Coefficient: −2.20e−02
Value: B
Leptospiraceae
0
1
2
3
4
5
A (n=15)
B (n=16)
C (n=14)
surface

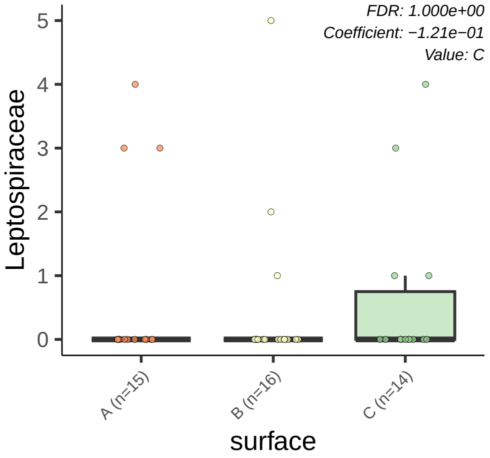

FDR: 1.000e+00
Coefficient: −1.21e−01
Value: C
Leptospiraceae
0
1
2
3
4
5
A (n=15)
B (n=16)
C (n=14)
surface

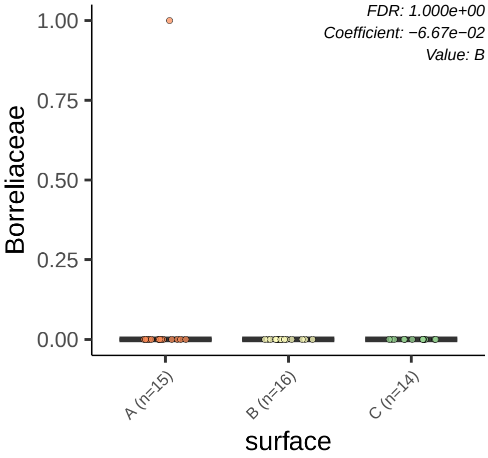
FDR: 1.000e+00
Coefficient: −6.67e−02
Value: B
Borreliaceae
1.00
0.75
0.50
0.25
0.00
A (n=15)
B (n=16)
C (n=14)
surface

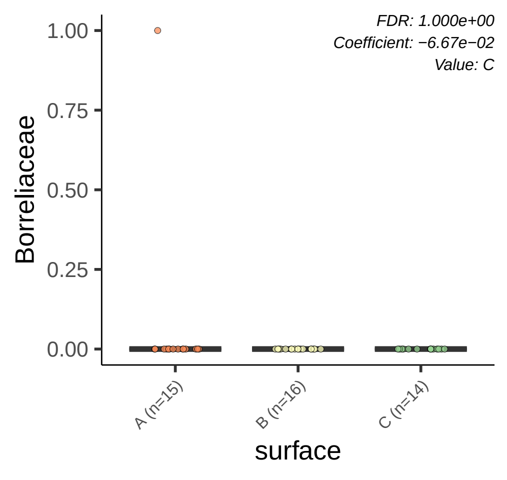
FDR: 1.000e+00
Coefficient: −6.67e−02
Value: C
Borreliaceae
1.00
0.75
0.50
0.25
0.00
A (n=15)
B (n=16)
C (n=14)
surface

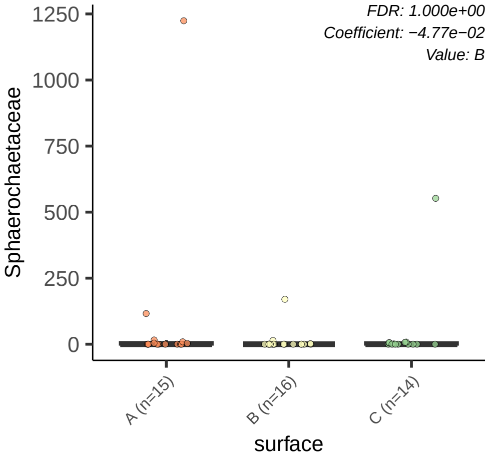
FDR: 1.000e+00
Coefficient: −4.77e−02
Value: B
Sphaerochaetaceae
1250
1000
750
500
250
0
A (n=15)
B (n=16)
C (n=14)
surface

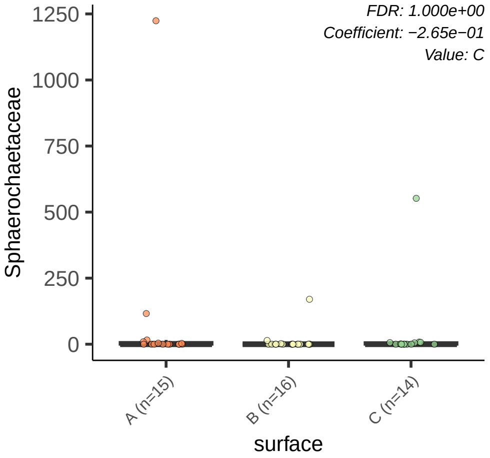

FDR: 1.000e+00
Coefficient: −2.65e−01
Value: C
Sphaerochaetaceae
1250
1000
750
500
250
0
A (n=15)
B (n=16)
C (n=14)
surface

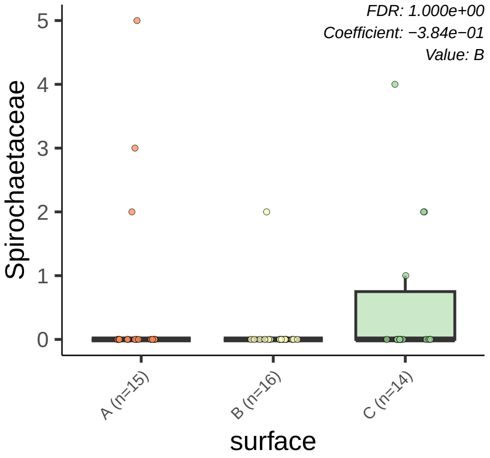
FDR: 1.000e+00
Coefficient: −3.84e−01
Value: B
Spirochaetaceae
5
4
3
2
1
0
A (n=15)
B (n=16)
C (n=14)
surface

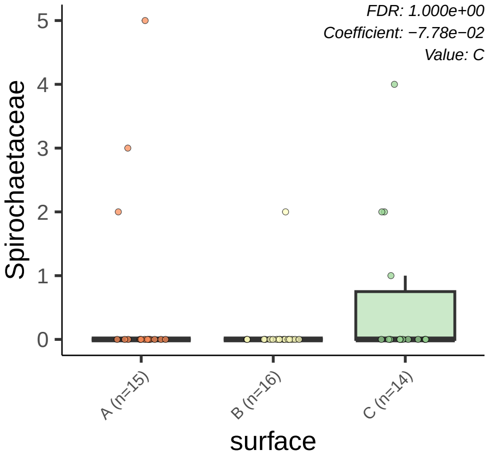

FDR: 1.000e+00
Coefficient: −7.78e−02
Value: C
Spirochaetaceae
0
1
2
3
4
5
A (n=15)
B (n=16)
C (n=14)
surface

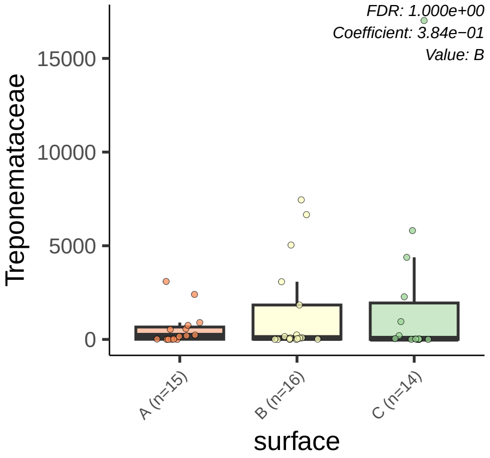
FDR: 1.000e+00
Coefficient: 3.84e−01
Value: B
Treponemataceae
15000
10000
5000
0
A (n=15)
B (n=16)
C (n=14)
surface

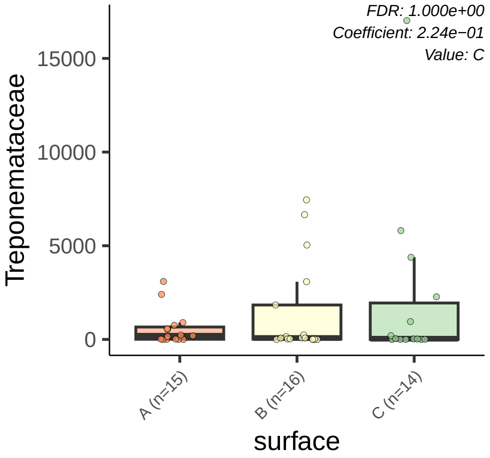

FDR: 1.000e+00
Coefficient: 2.24e−01
Value: C
Treponemataceae
15000
10000
5000
0
A (n=15)
B (n=16)
C (n=14)
surface

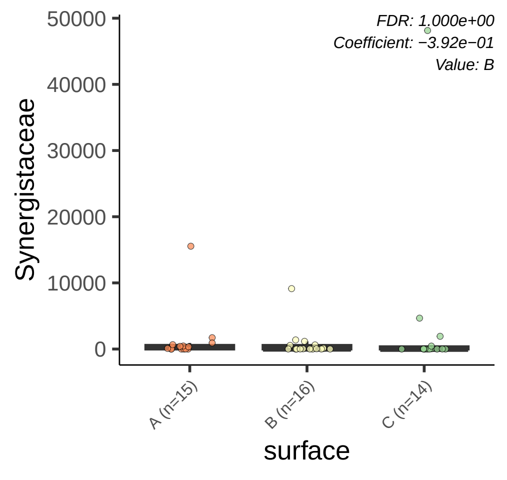

FDR: 1.000e+00
Coefficient: −3.92e−01
Value: B
Synergistaceae
50000
40000
30000
20000
10000
0
A (n=15)
B (n=16)
C (n=14)
surface

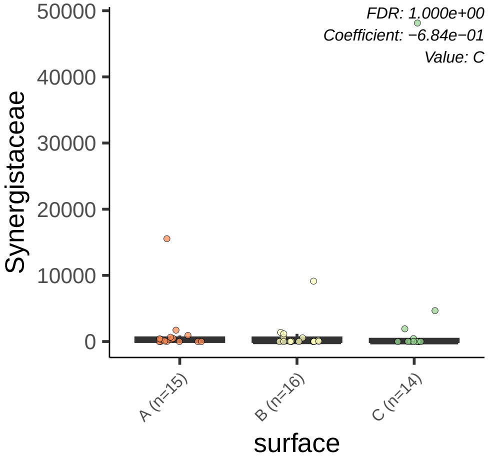
FDR: 1.000e+00
Coefficient: −6.84e−01
Value: C
Synergistaceae
50000
40000
30000
20000
10000
0
A (n=15)
B (n=16)
C (n=14)
surface

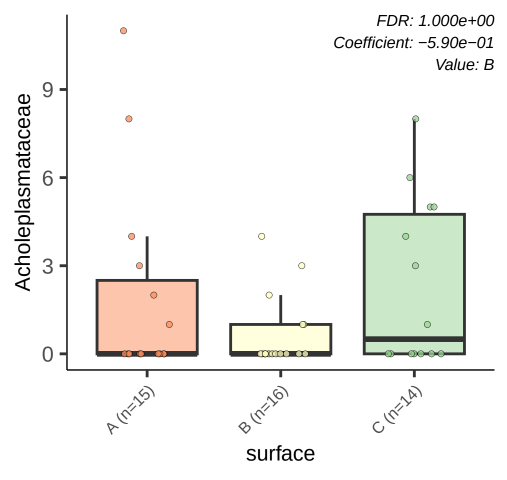

FDR: 1.000e+00
Coefficient: −5.90e−01
Value: B
Acholeplasmataceae
0
3
6
9
A (n=15)
B (n=16)
C (n=14)
surface

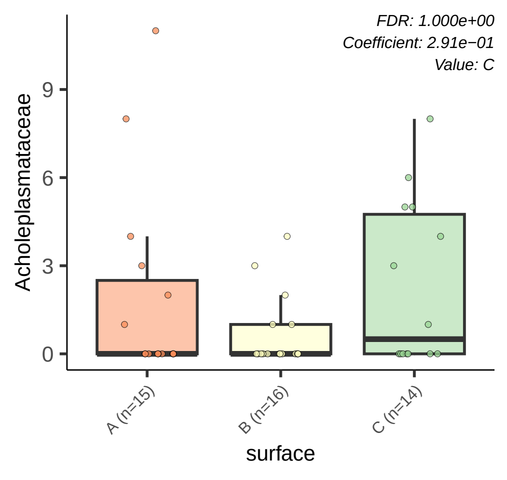

FDR: 1.000e+00
Coefficient: 2.91e−01
Value: C
Acholeplasmataceae
0
3
6
9
A (n=15)
B (n=16)
C (n=14)
surface

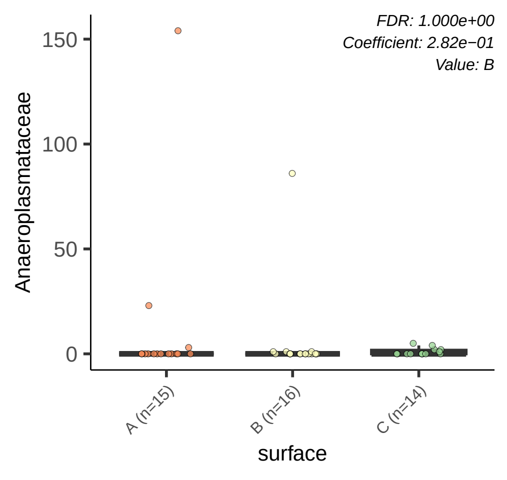
FDR: 1.000e+00
Coefficient: 2.82e−01
Value: B
Anaeroplasmataceae
150
100
50
0
A (n=15)
B (n=16)
C (n=14)
surface

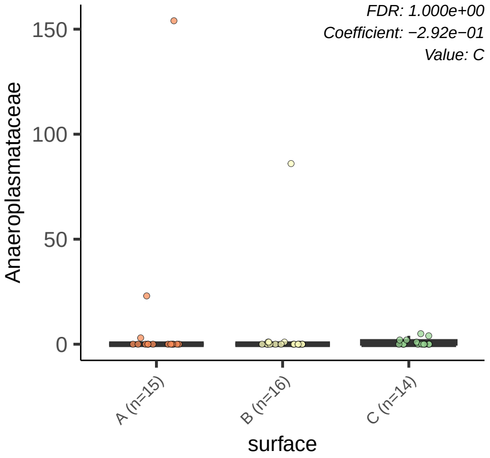

FDR: 1.000e+00
Coefficient: −2.92e−01
Value: C
Anaeroplasmataceae
150
100
50
0
A (n=15)
B (n=16)
C (n=14)
surface

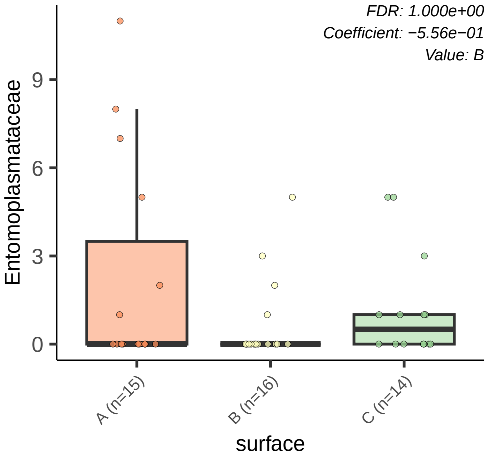

FDR: 1.000e+00
Coefficient: −5.56e−01
Value: B
Entomoplasmataceae
9
6
3
0
A (n=15)
B (n=16)
C (n=14)
surface

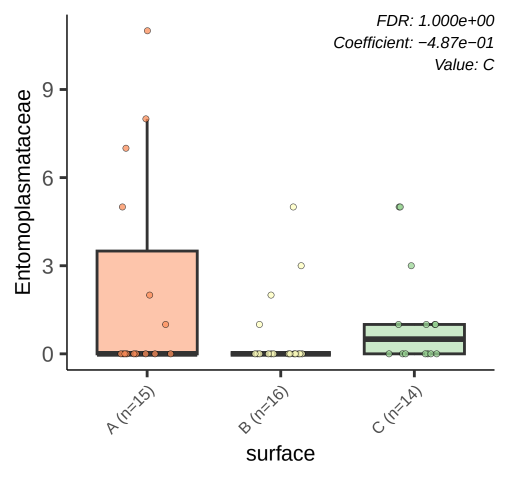
FDR: 1.000e+00
Coefficient: −4.87e−01
Value: C
Entomoplasmataceae
0
3
6
9
A (n=15)
B (n=16)
C (n=14)
surface

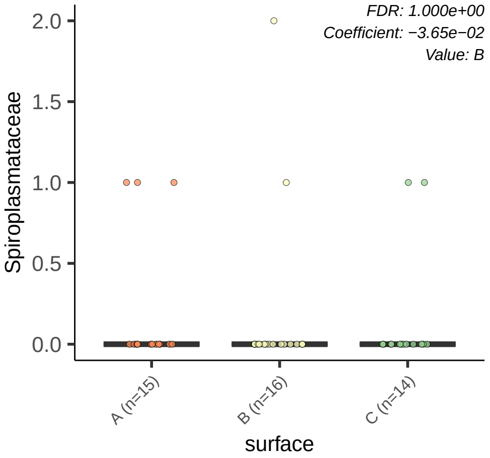
FDR: 1.000e+00
Coefficient: −3.65e−02
Value: B
Spiroplasmataceae
2.0
1.5
1.0
0.5
0.0
A (n=15)
B (n=16)
C (n=14)
surface

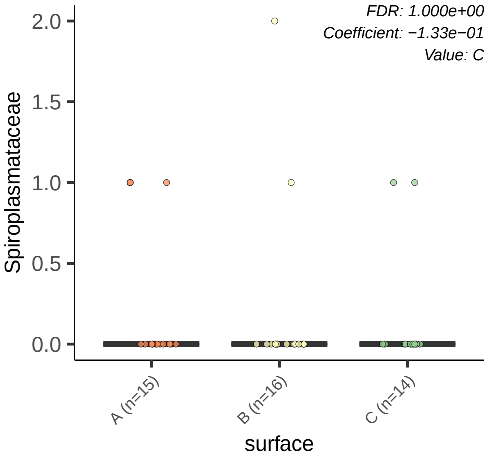
FDR: 1.000e+00
Coefficient: −1.33e−01
Value: C
2.0
1.5
1.0
0.5
0.0
Spiroplasmataceae
A (n=15)
B (n=16)
C (n=14)
surface

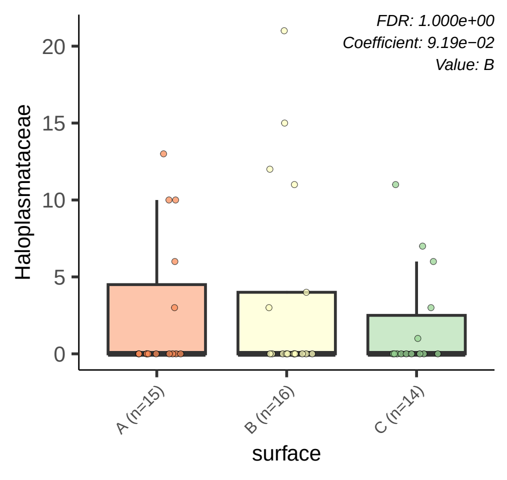

FDR: 1.000e+00
Coefficient: 9.19e−02
Value: B
Haloplasmataceae
0
5
10
15
20
A (n=15)
B (n=16)
C (n=14)
surface

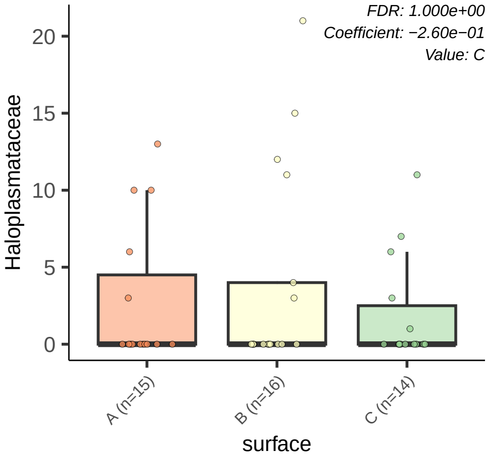
FDR: 1.000e+00
Coefficient: −2.60e−01
Value: C
Haloplasmataceae
0
5
10
15
20
A (n=15)
B (n=16)
C (n=14)
surface

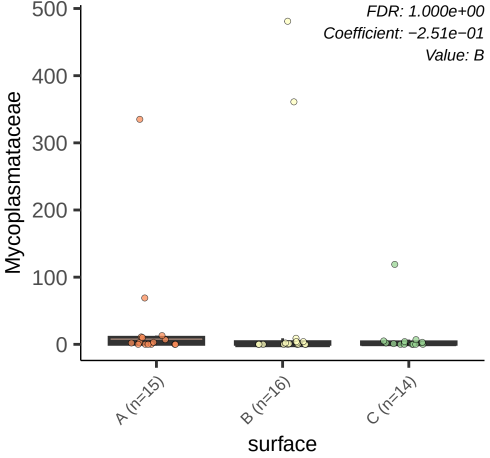

FDR: 1.000e+00
Coefficient: −2.51e−01
Value: B
Mycoplasmataceae
500
400
300
200
100
0
A (n=15)
B (n=16)
C (n=14)
surface

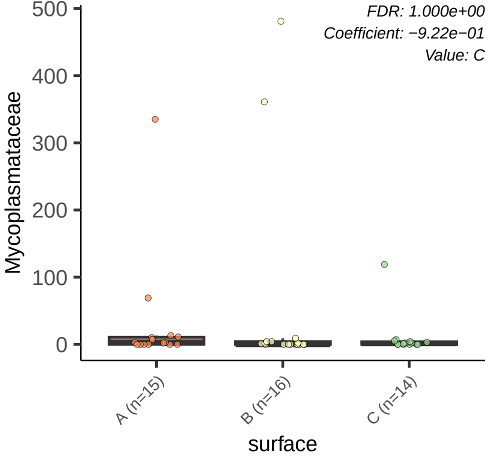
FDR: 1.000e+00
Coefficient: −9.22e−01
Value: C
Mycoplasmataceae
500
400
300
200
100
0
A (n=15)
B (n=16)
C (n=14)
surface

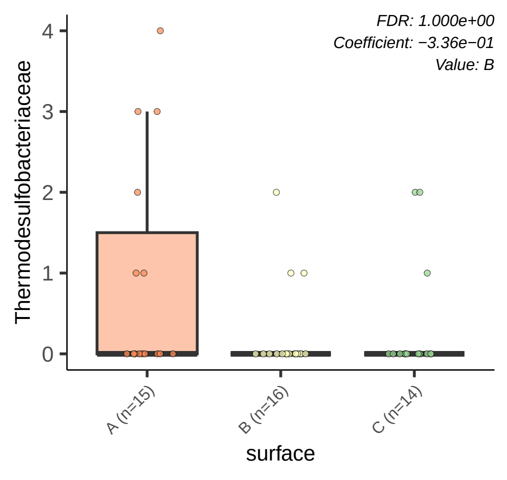
FDR: 1.000e+00
Coefficient: −3.36e−01
Value: B
Thermodesulfobacteriaceae
0
1
2
3
4
A (n=15)
B (n=16)
C (n=14)
surface

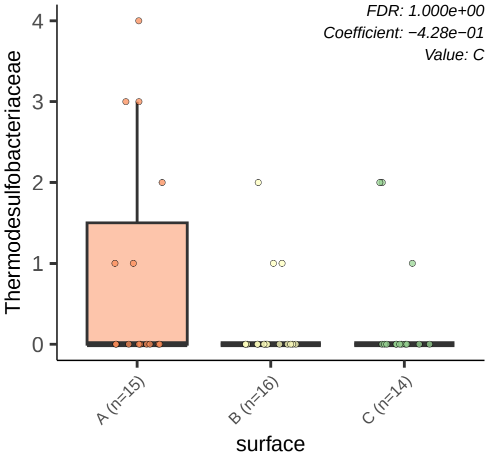
FDR: 1.000e+00
Coefficient: −4.28e−01
Value: C
Thermodesulfobacteriaceae
0
1
2
3
4
A (n=15)
B (n=16)
C (n=14)
surface

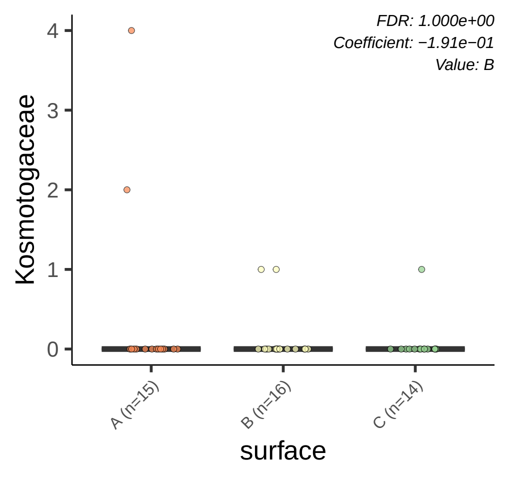
FDR: 1.000e+00
Coefficient: −1.91e−01
Value: B
Kosmotogaceae
4
3
2
1
0
A (n=15)
B (n=16)
C (n=14)
surface

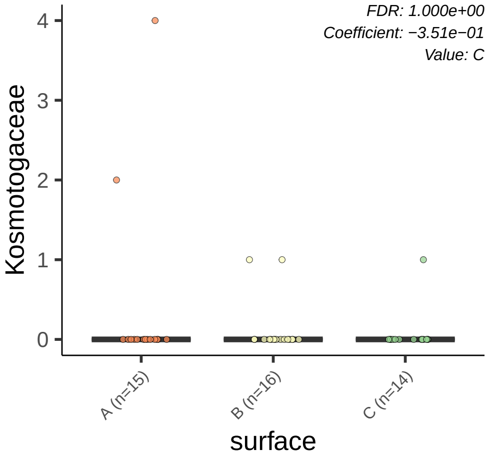
FDR: 1.000e+00
Coefficient: −3.51e−01
Value: C
Kosmotogaceae
0
1
2
3
4
A (n=15)
B (n=16)
C (n=14)
surface

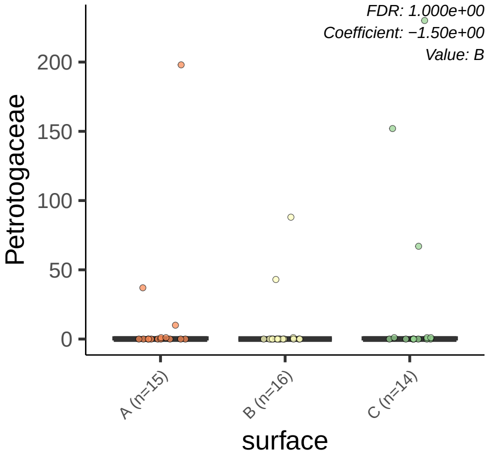

FDR: 1.000e+00
Coefficient: −1.50e+00
Value: B
Petrotogaceae
200
150
100
50
0
A (n=15)
B (n=16)
C (n=14)
surface

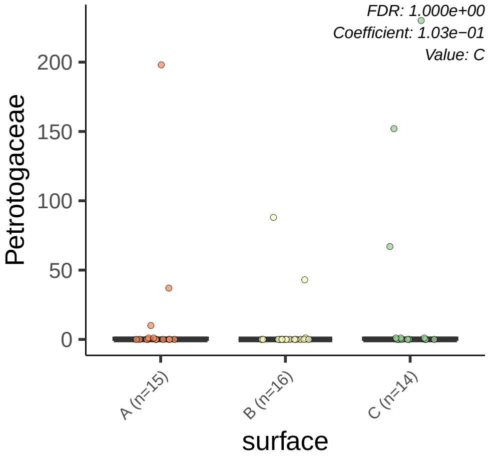
FDR: 1.000e+00
Coefficient: 1.03e−01
Value: C
Petrotogaceae
200
150
100
50
0
A (n=15)
B (n=16)
C (n=14)
surface

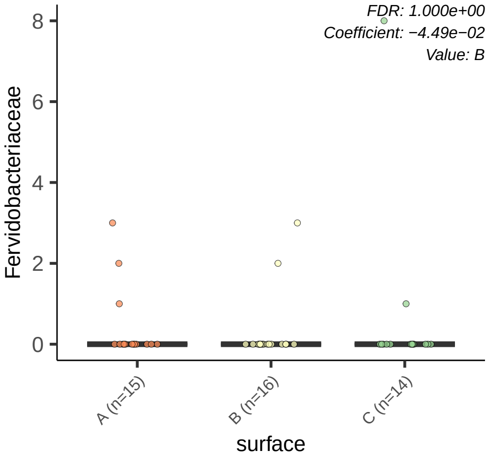

FDR: 1.000e+00
Coefficient: −4.49e−02
Value: B
Fervidobacteriaceae
8
6
4
2
0
A (n=15)
B (n=16)
C (n=14)
surface

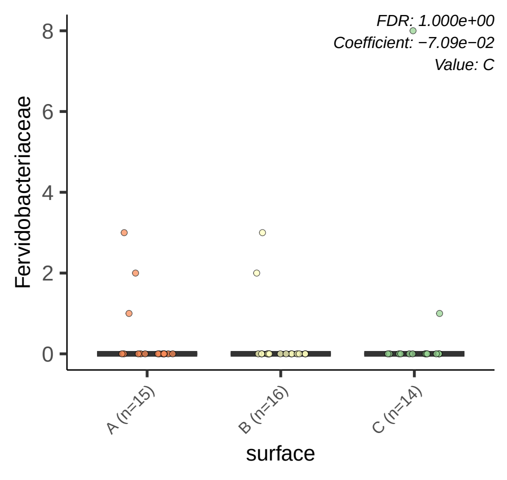
FDR: 1.000e+00
Coefficient: −7.09e−02
Value: C
Fervidobacteriaceae
0
2
4
6
8
A (n=15)
B (n=16)
C (n=14)
surface

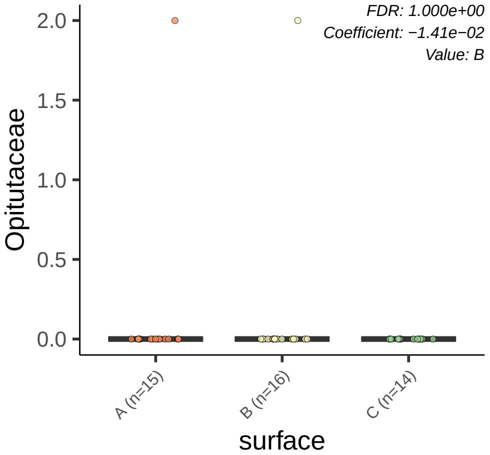
FDR: 1.000e+00
Coefficient: −1.41e−02
Value: B
Opitutaceae
2.0
1.5
1.0
0.5
0.0
A (n=15)
B (n=16)
C (n=14)
surface

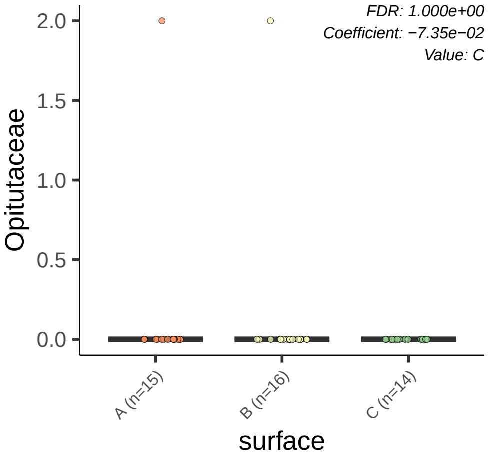
FDR: 1.000e+00
Coefficient: −7.35e−02
Value: C
Opitutaceae
2.0
1.5
1.0
0.5
0.0
A (n=15)
B (n=16)
C (n=14)
surface

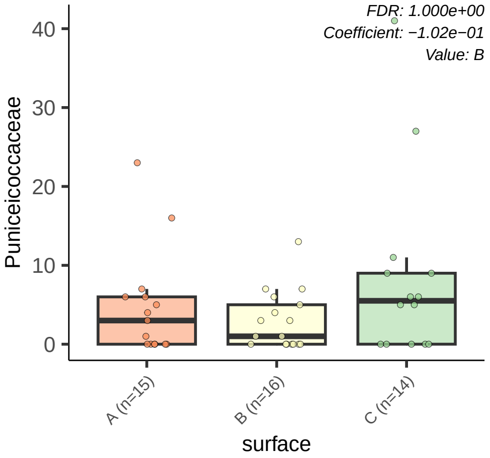
FDR: 1.000e+00
Coefficient: −1.02e−01
Value: B
Puniceicoccaceae
0
10
20
30
40
A (n=15)
B (n=16)
C (n=14)
surface

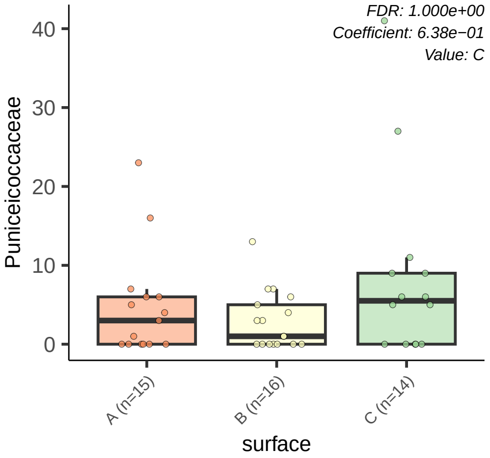
FDR: 1.000e+00
Coefficient: 6.38e−01
Value: C
Puniceicoccaceae
40
30
20
10
0
A (n=15)
B (n=16)
C (n=14)
surface

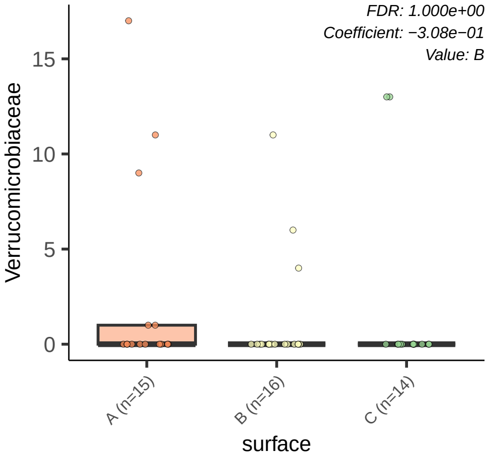
FDR: 1.000e+00
Coefficient: −3.08e−01
Value: B
Verrucomicrobiaceae
15
10
5
0
A (n=15)
B (n=16)
C (n=14)
surface

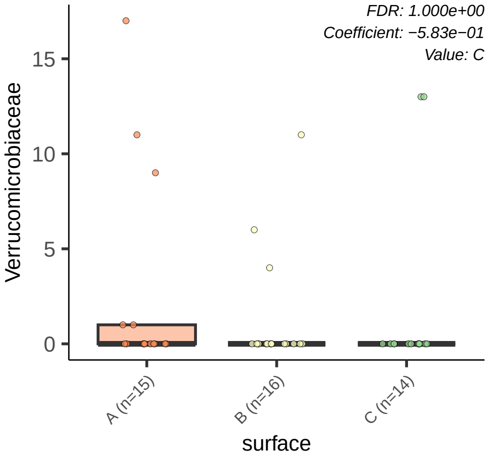
FDR: 1.000e+00
Coefficient: −5.83e−01
Value: C
Verrucomicrobiaceae
15
10
5
0
A (n=15)
B (n=16)
C (n=14)
surface

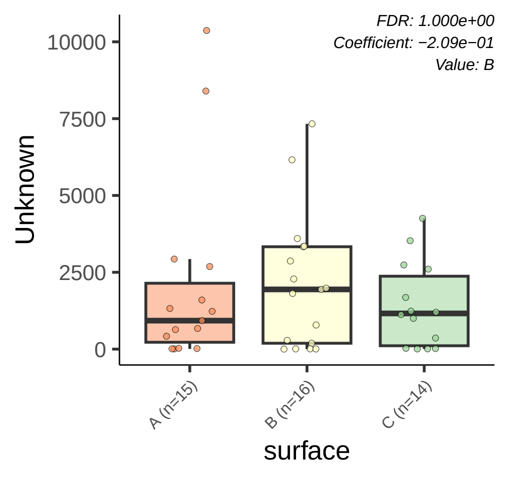
FDR: 1.000e+00
Coefficient: −2.09e−01
Value: B
Unknown
10000
7500
5000
2500
0
A (n=15)
B (n=16)
C (n=14)
surface

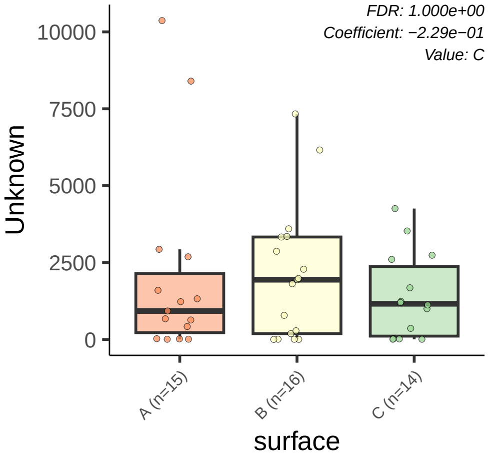
FDR: 1.000e+00
Coefficient: −2.29e−01
Value: C
Unknown
10000
7500
5000
2500
0
A (n=15)
B (n=16)
C (n=14)
surface

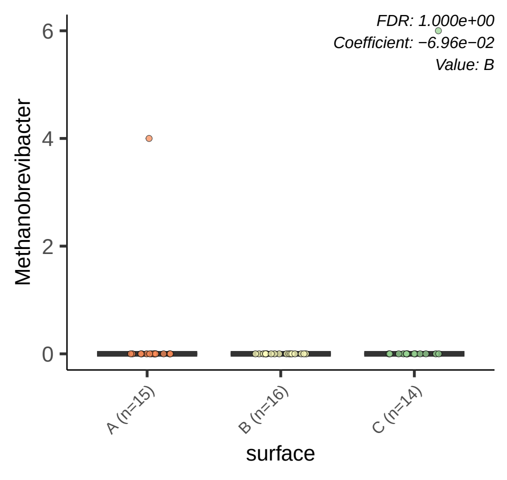

FDR: 1.000e+00
Coefficient: −6.96e−02
Value: B
Methanobrevibacter
6
4
2
0
A (n=15)
B (n=16)
C (n=14)
surface

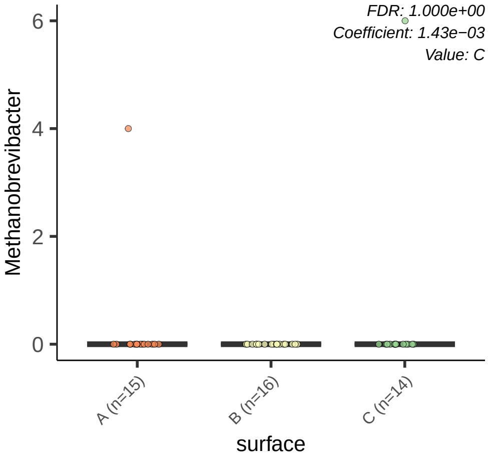
FDR: 1.000e+00
Coefficient: 1.43e−03
Value: C
Methanobrevibacter
6
4
2
0
A (n=15)
B (n=16)
C (n=14)
surface

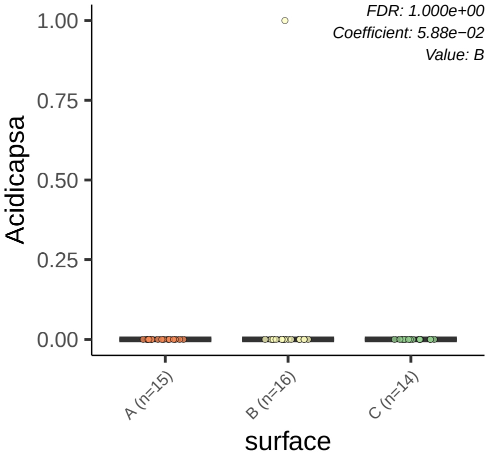
FDR: 1.000e+00
Coefficient: 5.88e−02
Value: B
1.00
0.75
0.50
0.25
0.00
Acidicapsa
A (n=15)
B (n=16)
C (n=14)
surface

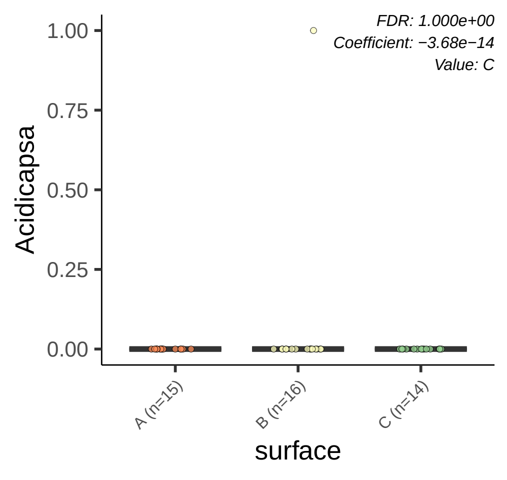
FDR: 1.000e+00
Coefficient: −3.68e−14
Value: C
Acidicapsa
1.00
0.75
0.50
0.25
0.00
A (n=15)
B (n=16)
C (n=14)
surface

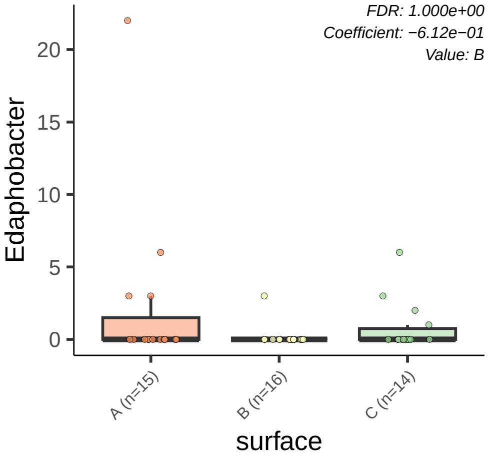

FDR: 1.000e+00
Coefficient: −6.12e−01
Value: B
Edaphobacter
20
15
10
5
0
A (n=15)
B (n=16)
C (n=14)
surface

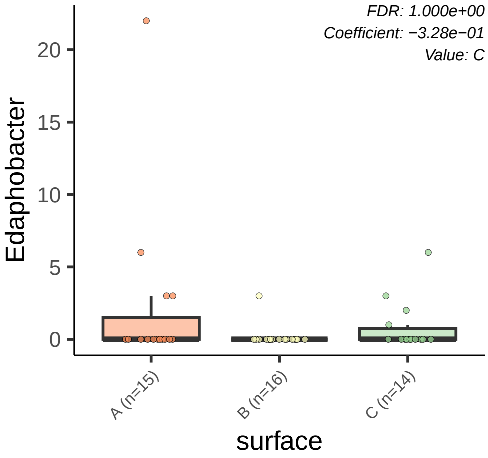
FDR: 1.000e+00
Coefficient: −3.28e−01
Value: C
Edaphobacter
20
15
10
5
0
A (n=15)
B (n=16)
C (n=14)
surface

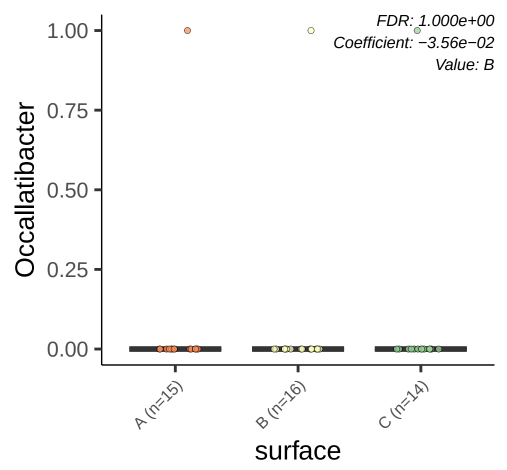
FDR: 1.000e+00
Coefficient: −3.56e−02
Value: B
Occallatibacter
1.00
0.75
0.50
0.25
0.00
A (n=15)
B (n=16)
C (n=14)
surface

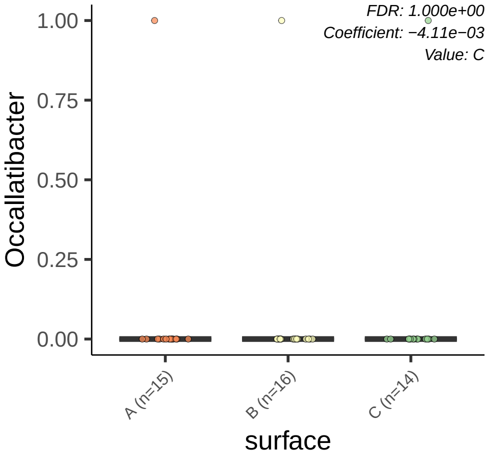
FDR: 1.000e+00
Coefficient: −4.11e−03
Value: C
Occallatibacter
1.00
0.75
0.50
0.25
0.00
A (n=15)
B (n=16)
C (n=14)
surface

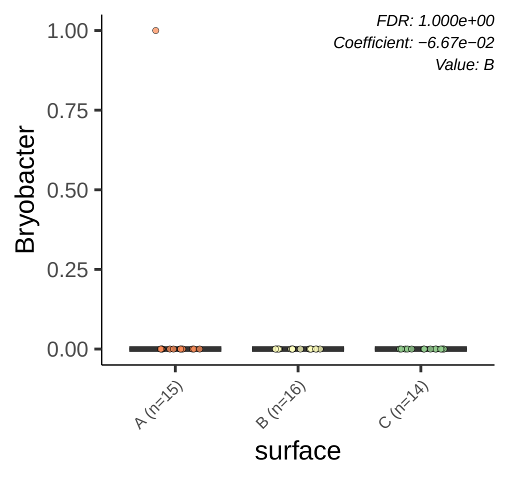

FDR: 1.000e+00
Coefficient: −6.67e−02
Value: B
Bryobacter
1.00
0.75
0.50
0.25
0.00
A (n=15)
B (n=16)
C (n=14)
surface

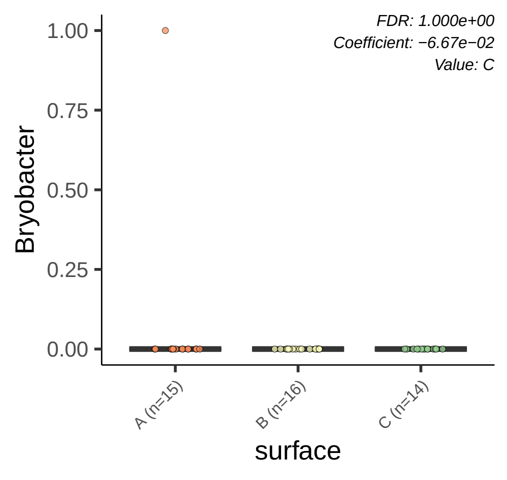

FDR: 1.000e+00
Coefficient: −6.67e−02
Value: C
Bryobacter
1.00
0.75
0.50
0.25
0.00
A (n=15)
B (n=16)
C (n=14)
surface

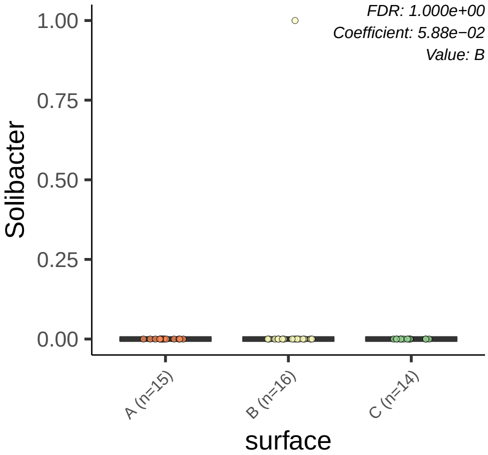
FDR: 1.000e+00
Coefficient: 5.88e−02
Value: B
Solibacter
1.00
0.75
0.50
0.25
0.00
A (n=15)
B (n=16)
C (n=14)
surface

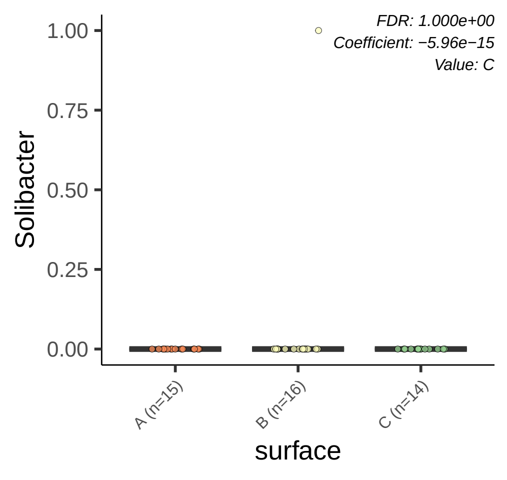
FDR: 1.000e+00
Coefficient: −5.96e−15
Value: C
Solibacter
1.00
0.75
0.50
0.25
0.00
A (n=15)
B (n=16)
C (n=14)
surface

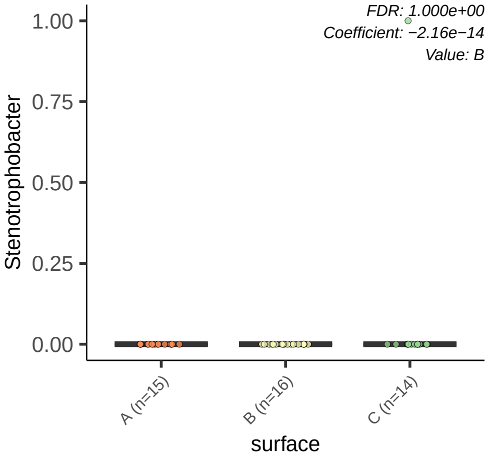

FDR: 1.000e+00
Coefficient: −2.16e−14
Value: B
Stenotrophobacter
1.00
0.75
0.50
0.25
0.00
A (n=15)
B (n=16)
C (n=14)
surface

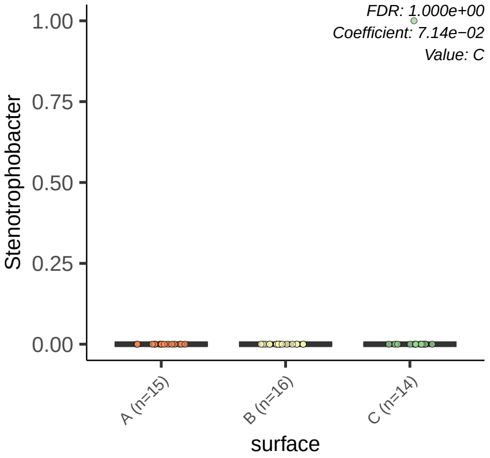
FDR: 1.000e+00
Coefficient: 7.14e−02
Value: C
Stenotrophobacter
1.00
0.75
0.50
0.25
0.00
A (n=15)
B (n=16)
C (n=14)
surface

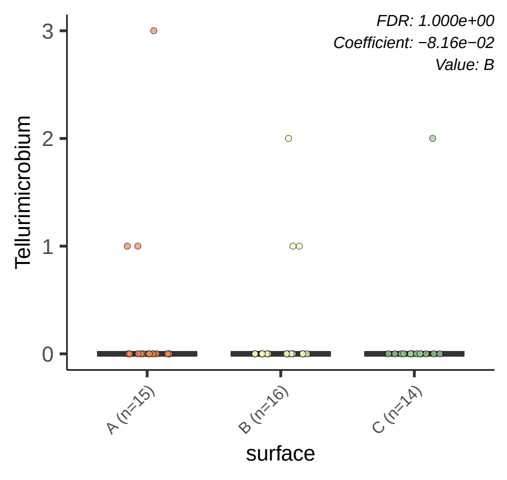
FDR: 1.000e+00
Coefficient: −8.16e−02
Value: B
Tellurimicrobium
3
2
1
0
A (n=15)
B (n=16)
C (n=14)
surface

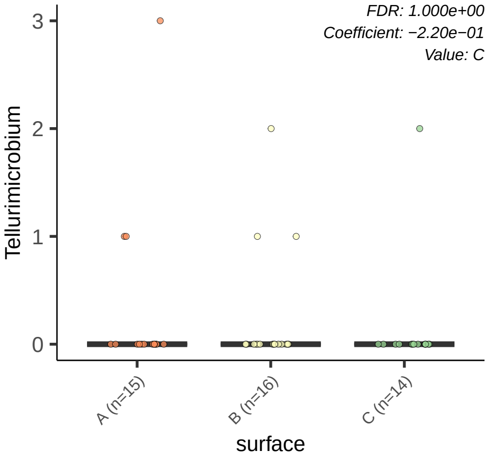

FDR: 1.000e+00
Coefficient: −2.20e−01
Value: C
Tellurimicrobium
3
2
1
0
A (n=15)
B (n=16)
C (n=14)
surface

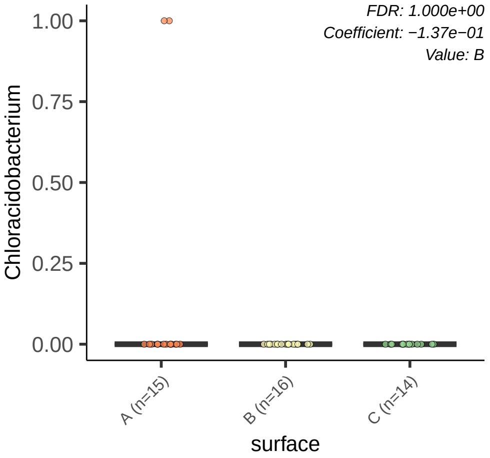
FDR: 1.000e+00
Coefficient: −1.37e−01
Value: B
Chloracidobacterium
1.00
0.75
0.50
0.25
0.00
A (n=15)
B (n=16)
C (n=14)
surface

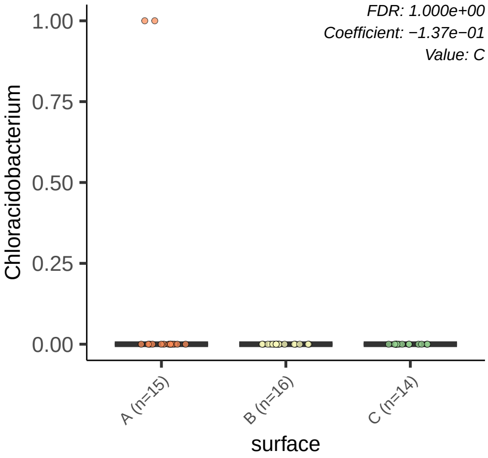

FDR: 1.000e+00
Coefficient: −1.37e−01
Value: C
Chloracidobacterium
1.00
0.75
0.50
0.25
0.00
A (n=15)
B (n=16)
C (n=14)
surface

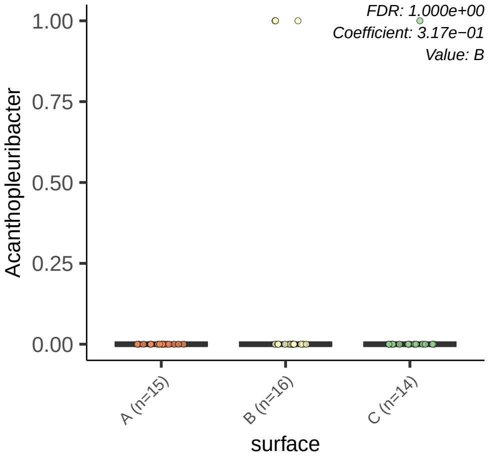
FDR: 1.000e+00
Coefficient: 3.17e−01
Value: B
Acanthopleuribacter
1.00
0.75
0.50
0.25
0.00
A (n=15)
B (n=16)
C (n=14)
surface

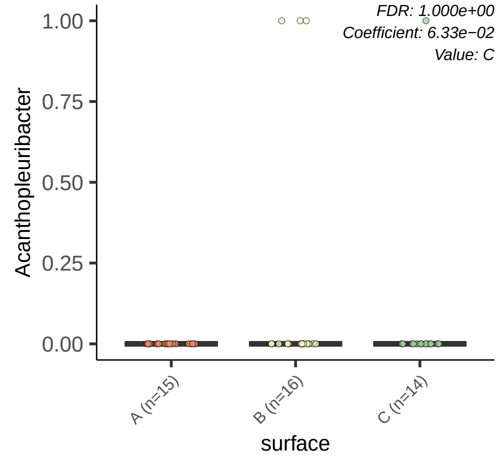
FDR: 1.000e+00
Coefficient: 6.33e−02
Value: C
Acanthopleuribacter
1.00
0.75
0.50
0.25
0.00
A (n=15)
B (n=16)
C (n=14)
surface

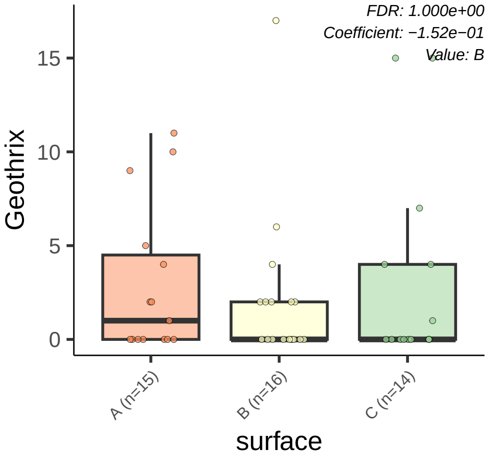

FDR: 1.000e+00
Coefficient: −1.52e−01
Value: B
Geothrix
0
5
10
15
A (n=15)
B (n=16)
C (n=14)
surface

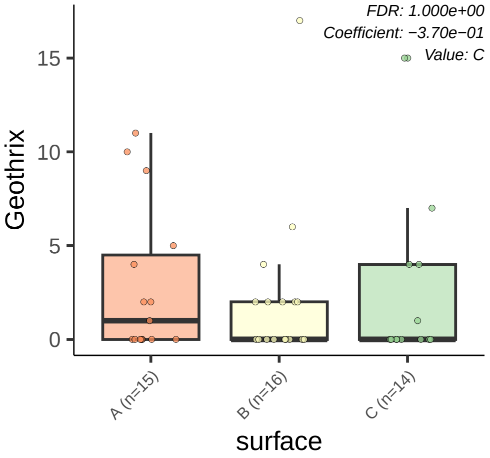

FDR: 1.000e+00
Coefficient: −3.70e−01
Value: C
Geothrix
0
5
10
15
A (n=15)
B (n=16)
C (n=14)
surface

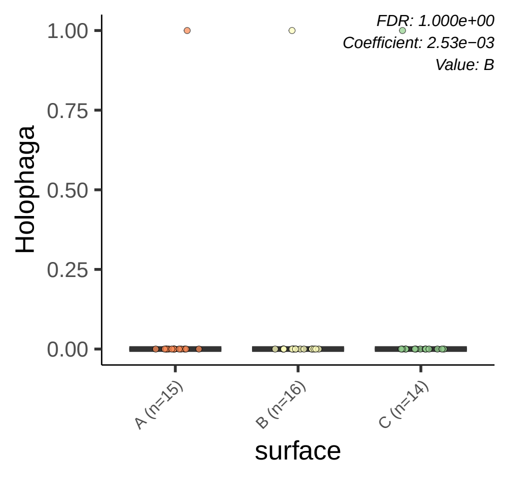
FDR: 1.000e+00
Coefficient: 2.53e−03
Value: B
1.00
0.75
0.50
0.25
0.00
Holophaga
A (n=15)
B (n=16)
C (n=14)
surface

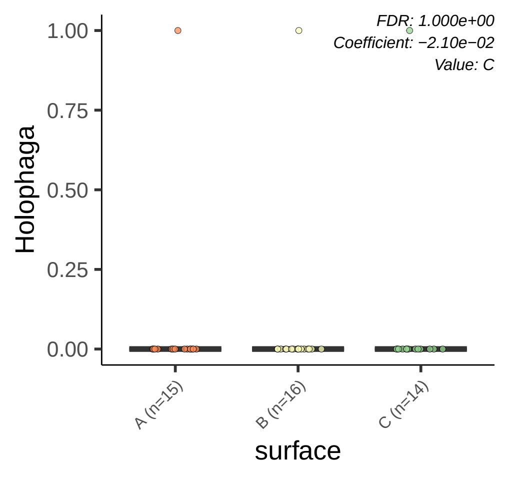
FDR: 1.000e+00
Coefficient: −2.10e−02
Value: C
Holophaga
1.00
0.75
0.50
0.25
0.00
A (n=15)
B (n=16)
C (n=14)
surface

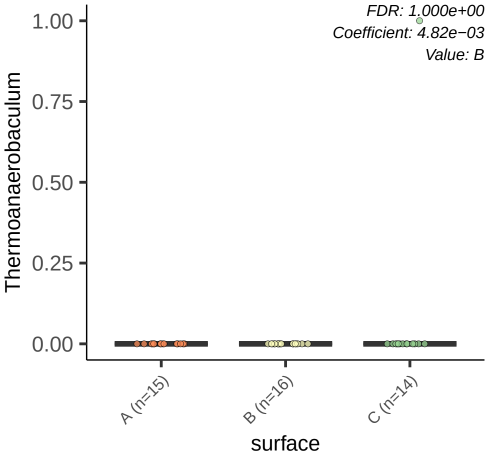
FDR: 1.000e+00
Coefficient: 4.82e−03
Value: B
Thermoanaerobaculum
1.00
0.75
0.50
0.25
0.00
A (n=15)
B (n=16)
C (n=14)
surface

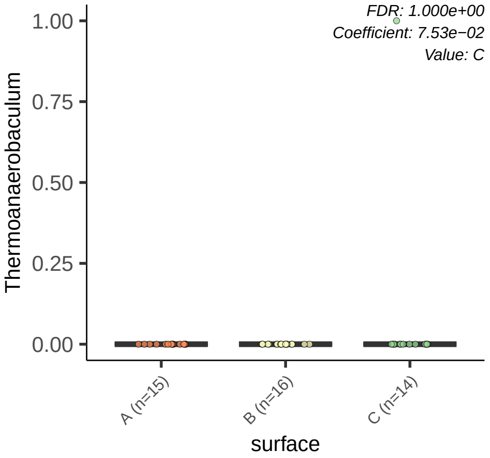
FDR: 1.000e+00
Coefficient: 7.53e−02
Value: C
1.00
0.75
0.50
0.25
0.00
Thermoanaerobaculum
A (n=15)
B (n=16)
C (n=14)
surface

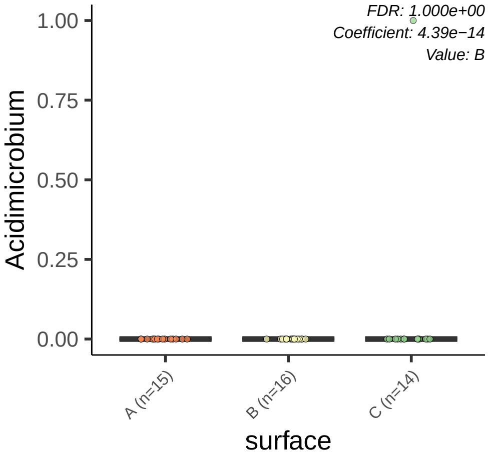
FDR: 1.000e+00
Coefficient: 4.39e−14
Value: B
Acidimicrobium
1.00
0.75
0.50
0.25
0.00
A (n=15)
B (n=16)
C (n=14)
surface

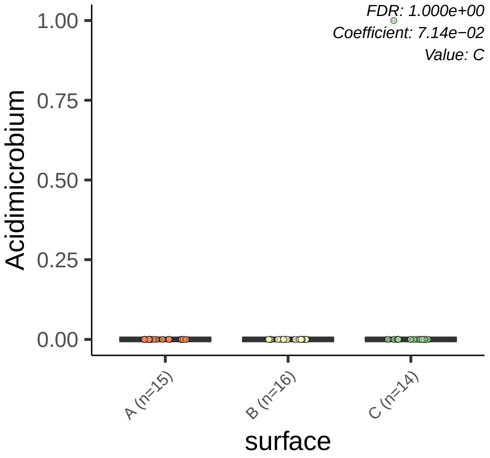

FDR: 1.000e+00
Coefficient: 7.14e−02
Value: C
Acidimicrobium
1.00
0.75
0.50
0.25
0.00
A (n=15)
B (n=16)
C (n=14)
surface

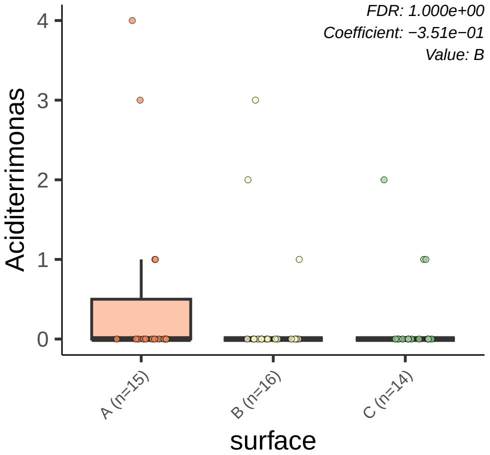

FDR: 1.000e+00
Coefficient: −3.51e−01
Value: B
Aciditerrimonas
4
3
2
1
0
A (n=15)
B (n=16)
C (n=14)
surface

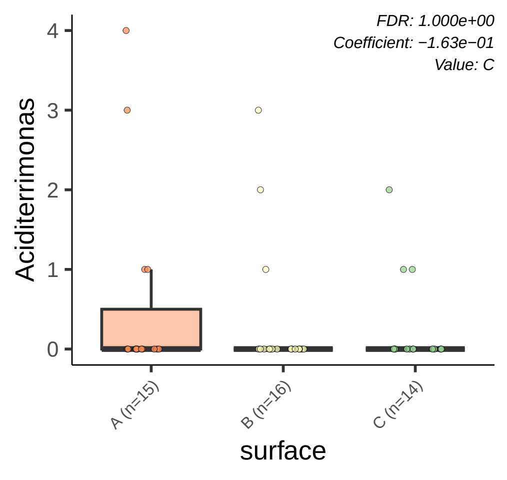

FDR: 1.000e+00
Coefficient: −1.63e−01
Value: C
Aciditerrimonas
0
1
2
3
4
A (n=15)
B (n=16)
C (n=14)
surface

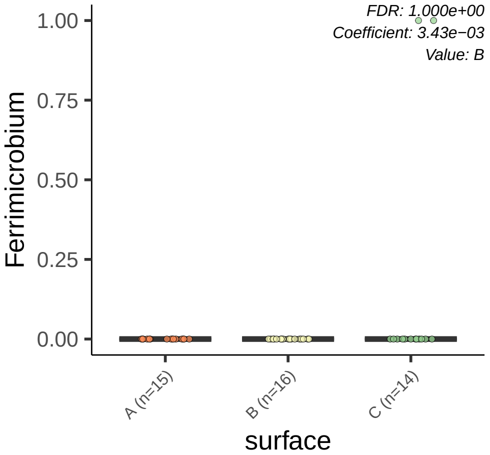
FDR: 1.000e+00
Coefficient: 3.43e−03
Value: B
Ferrimicrobium
1.00
0.75
0.50
0.25
0.00
A (n=15)
B (n=16)
C (n=14)
surface

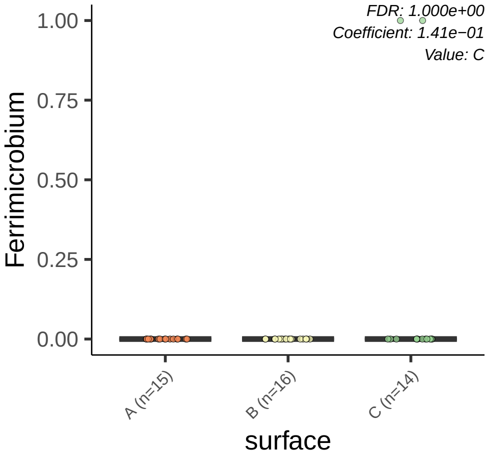
FDR: 1.000e+00
Coefficient: 1.41e−01
Value: C
1.00
0.75
0.50
0.25
0.00
Ferrimicrobium
A (n=15)
B (n=16)
C (n=14)
surface

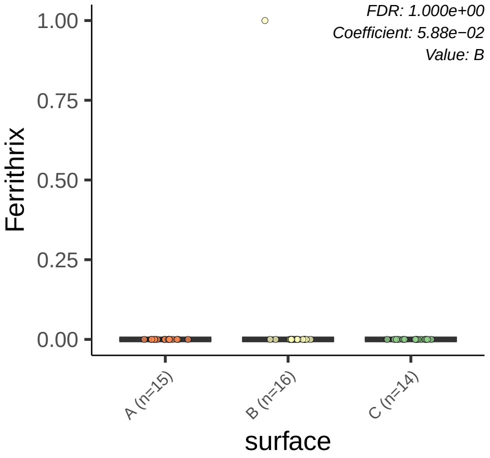
FDR: 1.000e+00
Coefficient: 5.88e−02
Value: B
Ferrithrix
1.00
0.75
0.50
0.25
0.00
A (n=15)
B (n=16)
C (n=14)
surface

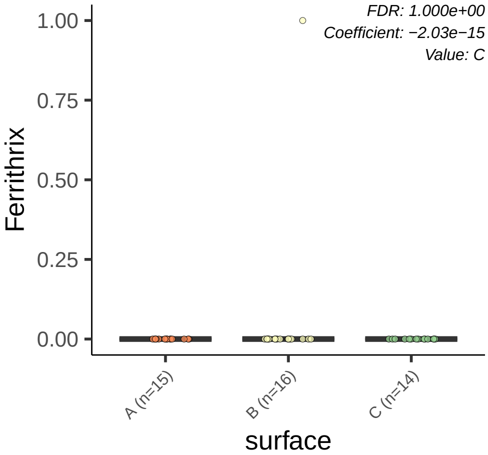
FDR: 1.000e+00
Coefficient: −2.03e−15
Value: C
1.00
0.75
0.50
0.25
0.00
Ferrithrix
A (n=15)
B (n=16)
C (n=14)
surface

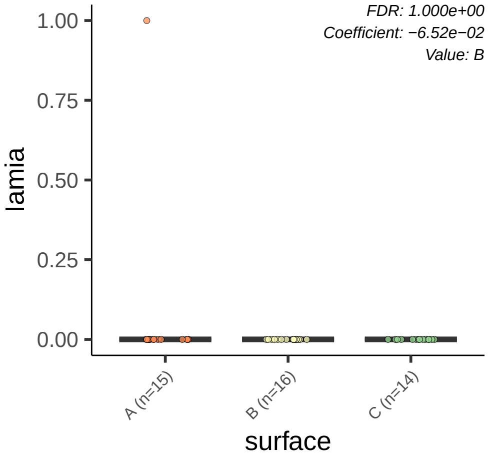

FDR: 1.000e+00
Coefficient: −6.52e−02
Value: B
lamia
1.00
0.75
0.50
0.25
0.00
A (n=15)
B (n=16)
C (n=14)
surface

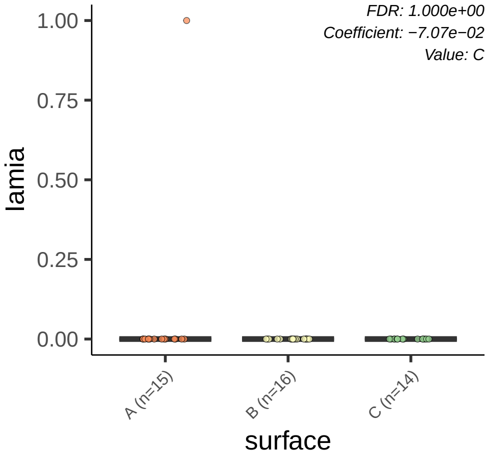
FDR: 1.000e+00
Coefficient: −7.07e−02
Value: C
lamia
1.00
0.75
0.50
0.25
0.00
A (n=15)
B (n=16)
C (n=14)
surface

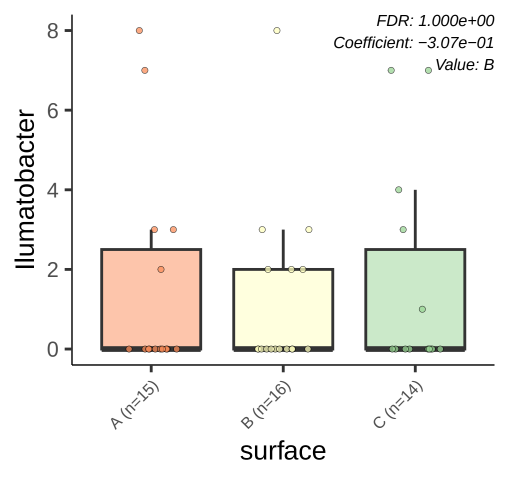
FDR: 1.000e+00
Coefficient: −3.07e−01
Value: B
Ilumatobacter
0
2
4
6
8
A (n=15)
B (n=16)
C (n=14)
surface

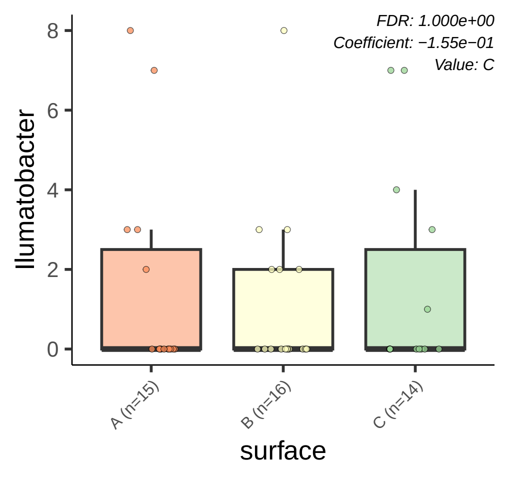
FDR: 1.000e+00
Coefficient: −1.55e−01
Value: C
Ilumatobacter
0
2
4
6
8
A (n=15)
B (n=16)
C (n=14)
surface

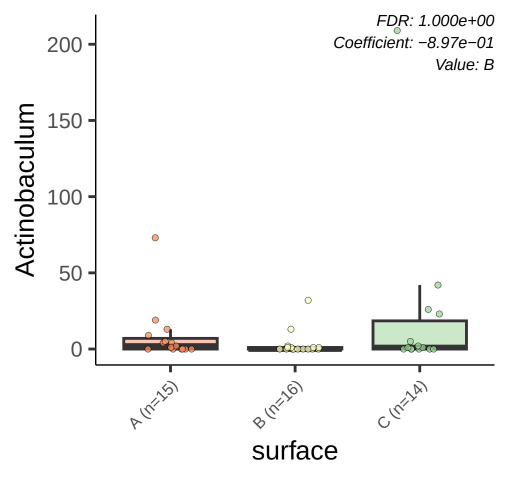
FDR: 1.000e+00
Coefficient: −8.97e−01
Value: B
Actinobaculum
200
150
100
50
0
A (n=15)
B (n=16)
C (n=14)
surface

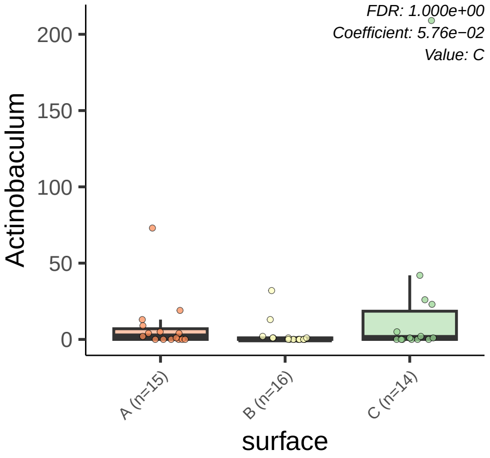
FDR: 1.000e+00
Coefficient: 5.76e−02
Value: C
Actinobaculum
0
50
100
150
200
A (n=15)
B (n=16)
C (n=14)
surface

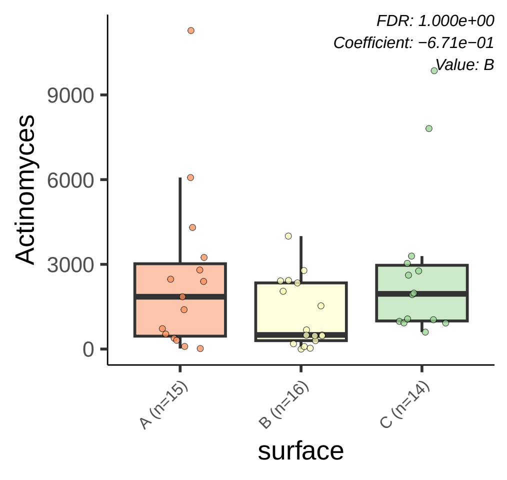
FDR: 1.000e+00
Coefficient: −6.71e−01
Value: B
Actinomyces
9000
6000
3000
0
A (n=15)
B (n=16)
C (n=14)
surface

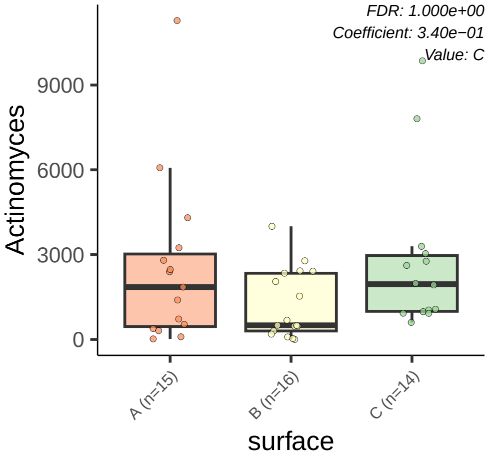
FDR: 1.000e+00
Coefficient: 3.40e−01
Value: C
9000
6000
3000
0
Actinomyces
A (n=15)
B (n=16)
C (n=14)
surface

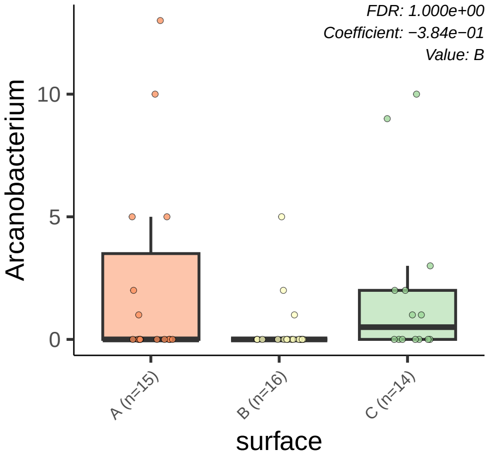
FDR: 1.000e+00
Coefficient: −3.84e−01
Value: B
Arcanobacterium
10
5
0
A (n=15)
B (n=16)
C (n=14)
surface

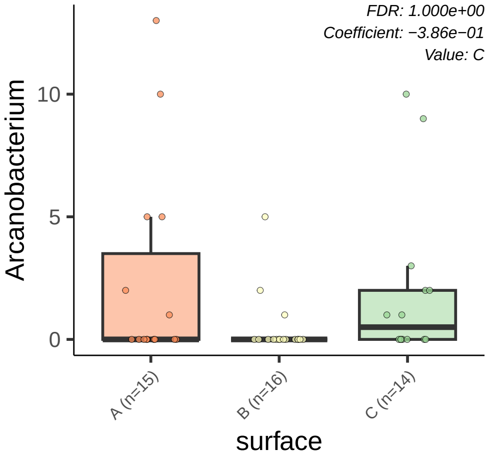

FDR: 1.000e+00
Coefficient: −3.86e−01
Value: C
Arcanobacterium
10
5
0
A (n=15)
B (n=16)
C (n=14)
surface

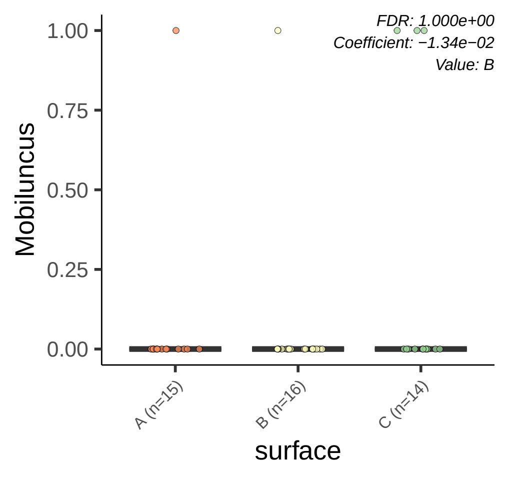
FDR: 1.000e+00
Coefficient: −1.34e−02
Value: B
Mobiluncus
1.00
0.75
0.50
0.25
0.00
A (n=15)
B (n=16)
C (n=14)
surface

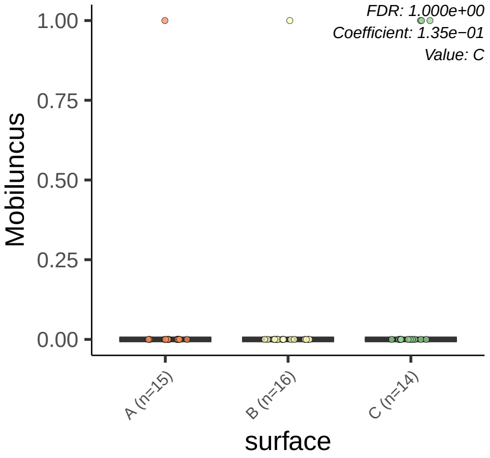
FDR: 1.000e+00
Coefficient: 1.35e−01
Value: C
Mobiluncus
1.00
0.75
0.50
0.25
0.00
A (n=15)
B (n=16)
C (n=14)
surface

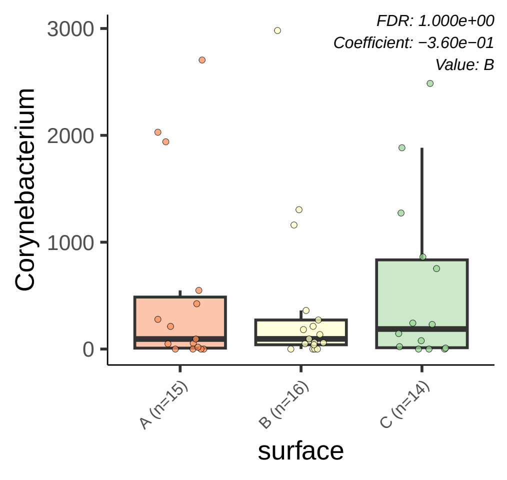
FDR: 1.000e+00
Coefficient: −3.60e−01
Value: B
Corynebacterium
3000
2000
1000
0
A (n=15)
B (n=16)
C (n=14)
surface

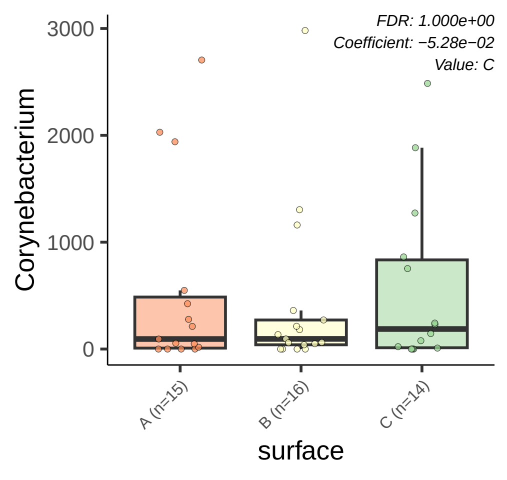
FDR: 1.000e+00
Coefficient: −5.28e−02
Value: C
Corynebacterium
3000
2000
1000
0
A (n=15)
B (n=16)
C (n=14)
surface

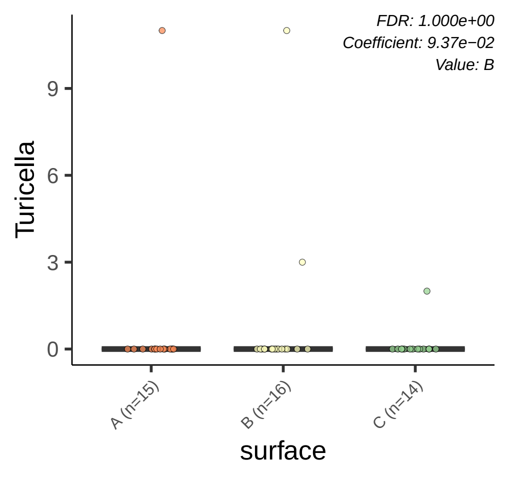

FDR: 1.000e+00
Coefficient: 9.37e−02
Value: B
Turicella
9
6
3
0
A (n=15)
B (n=16)
C (n=14)
surface

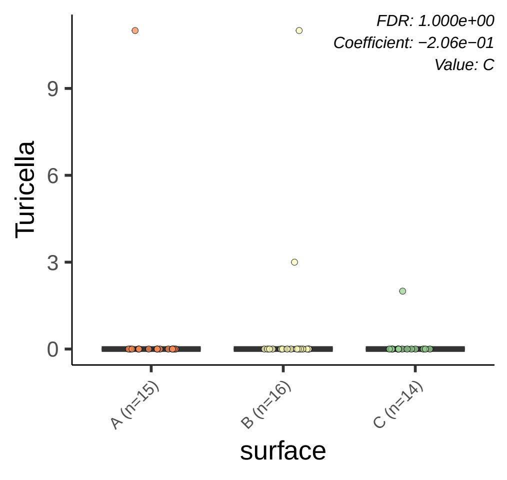
FDR: 1.000e+00
Coefficient: −2.06e−01
Value: C
Turicella
9
6
3
0
A (n=15)
B (n=16)
C (n=14)
surface

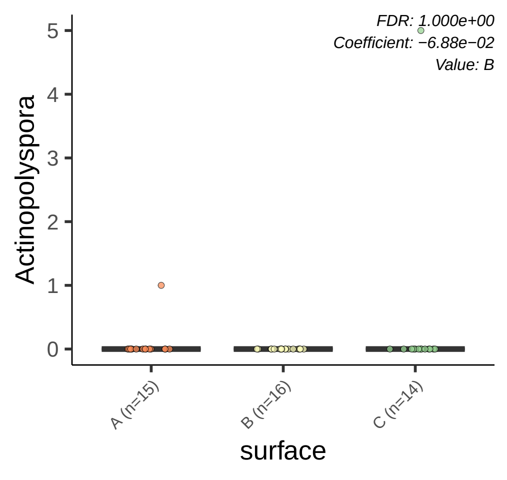

FDR: 1.000e+00
Coefficient: −6.88e−02
Value: B
Actinopolyspora
5
4
3
2
1
0
A (n=15)
B (n=16)
C (n=14)
surface

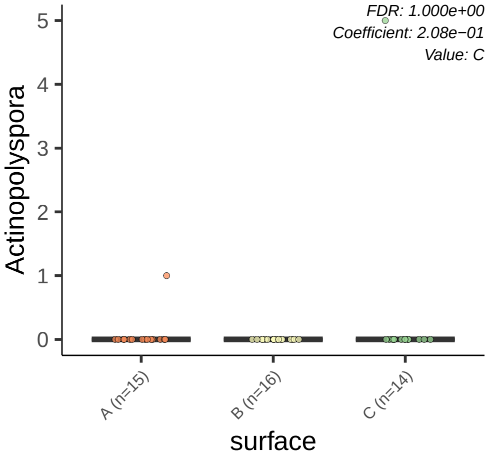
FDR: 1.000e+00
Coefficient: 2.08e−01
Value: C
Actinopolyspora
0
1
2
3
4
5
A (n=15)
B (n=16)
C (n=14)
surface

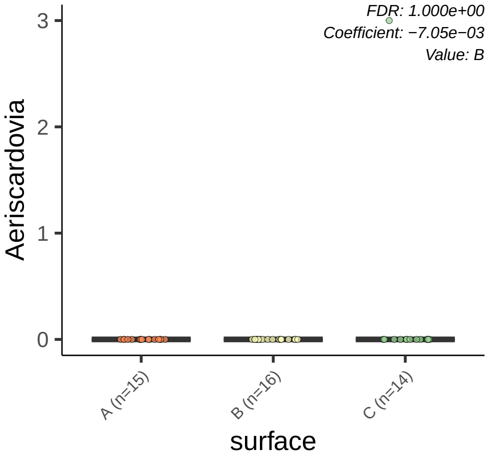
FDR: 1.000e+00
Coefficient: −7.05e−03
Value: B
Aeriscardovia
3
2
1
0
A (n=15)
B (n=16)
C (n=14)
surface

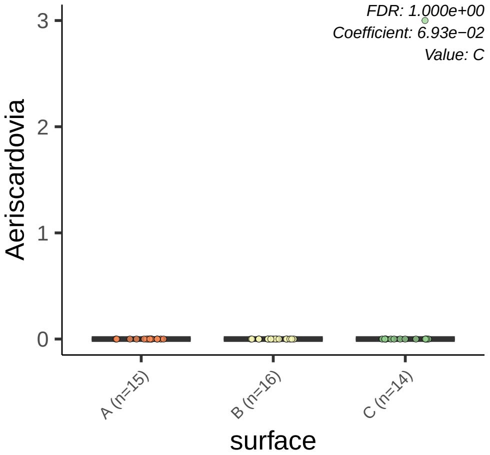
FDR: 1.000e+00
Coefficient: 6.93e−02
Value: C
Aeriscardovia
3
2
1
0
A (n=15)
B (n=16)
C (n=14)
surface

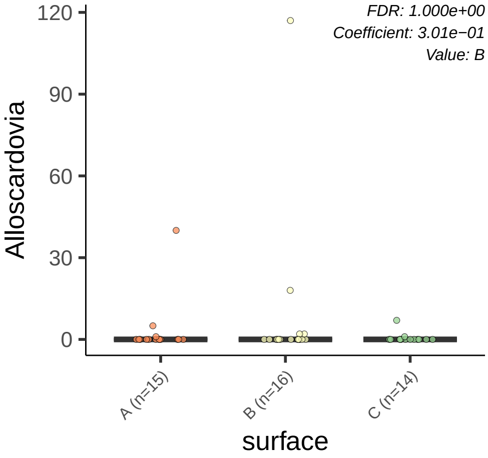

FDR: 1.000e+00
Coefficient: 3.01e−01
Value: B
Alloscardovia
120
90
60
30
0
A (n=15)
B (n=16)
C (n=14)
surface

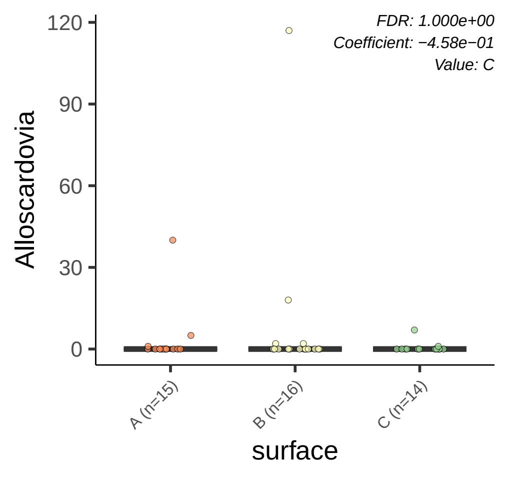
FDR: 1.000e+00
Coefficient: −4.58e−01
Value: C
Alloscardovia
120
90
60
30
0
A (n=15)
B (n=16)
C (n=14)
surface

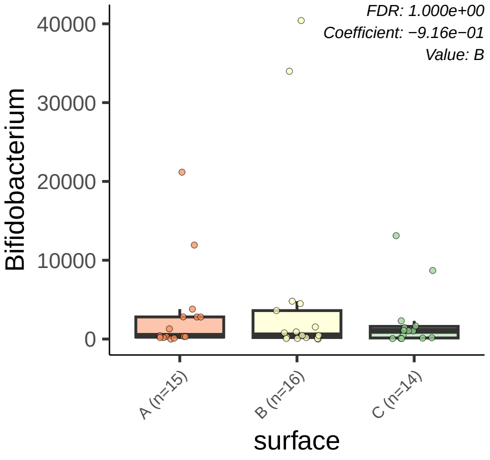

FDR: 1.000e+00
Coefficient: −9.16e−01
Value: B
40000
30000
20000
10000
0
Bifidobacterium
A (n=15)
B (n=16)
C (n=14)
surface

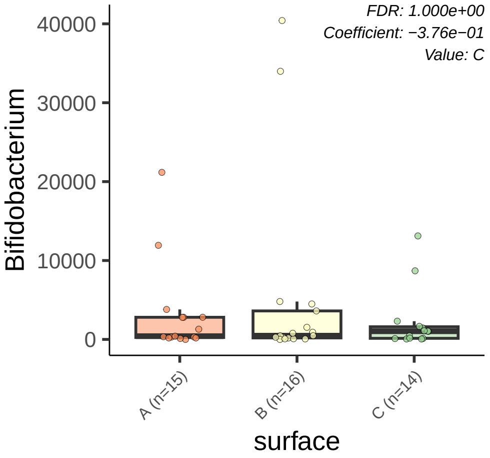

FDR: 1.000e+00
Coefficient: −3.76e−01
Value: C
Bifidobacterium
40000
30000
20000
10000
0
A (n=15)
B (n=16)
C (n=14)
surface

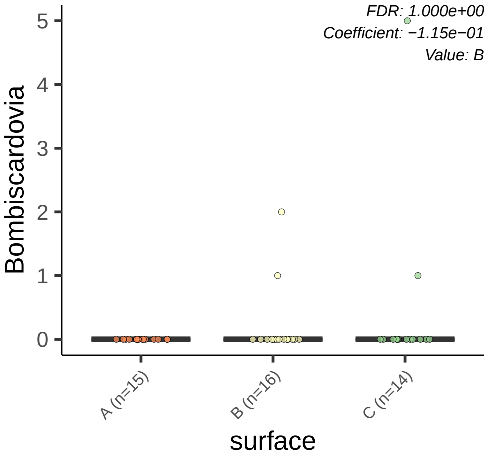

FDR: 1.000e+00
Coefficient: −1.15e−01
Value: B
Bombiscardovia
0
1
2
3
4
5
A (n=15)
B (n=16)
C (n=14)
surface

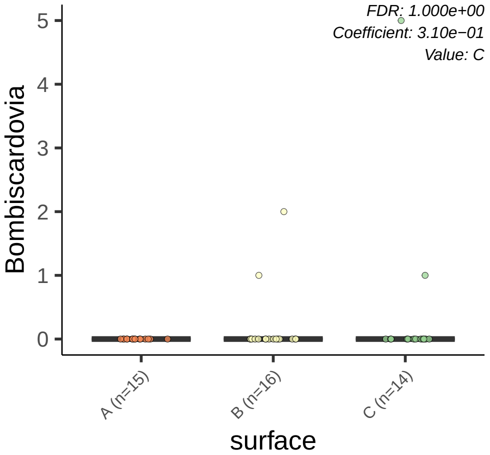
FDR: 1.000e+00
Coefficient: 3.10e−01
Value: C
Bombiscardovia
0
1
2
3
4
5
A (n=15)
B (n=16)
C (n=14)
surface

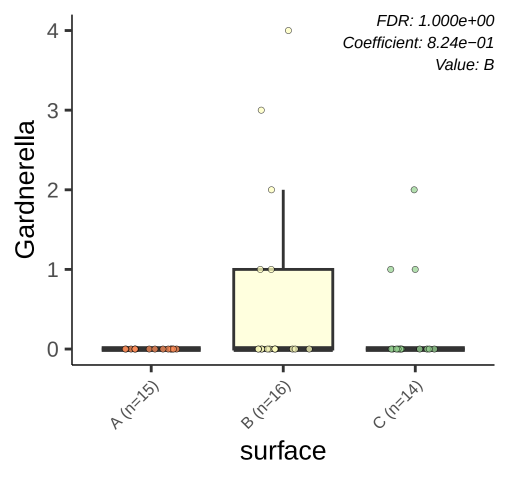

FDR: 1.000e+00
Coefficient: 8.24e−01
Value: B
Gardnerella
0
1
2
3
4
A (n=15)
B (n=16)
C (n=14)
surface

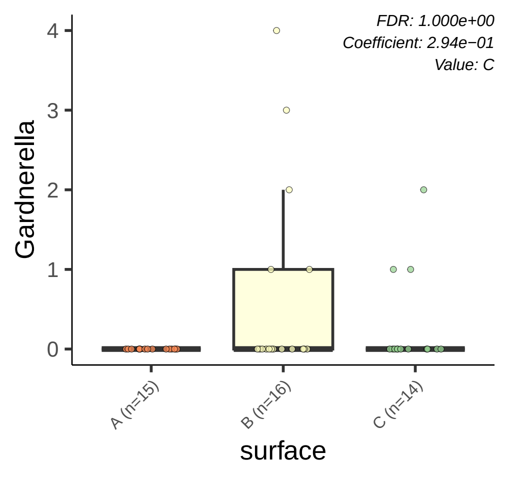
FDR: 1.000e+00
Coefficient: 2.94e−01
Value: C
Gardnerella
0
1
2
3
4
A (n=15)
B (n=16)
C (n=14)
surface

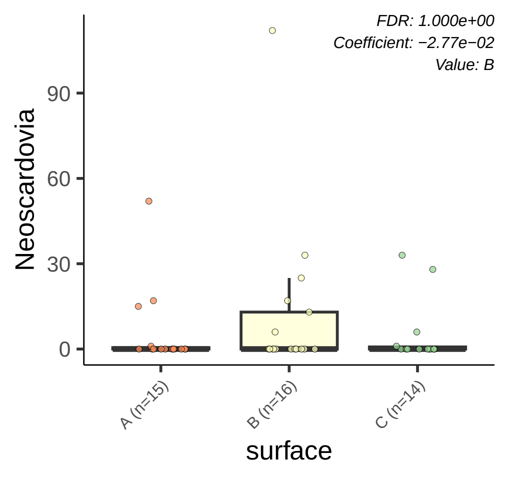

FDR: 1.000e+00
Coefficient: −2.77e−02
Value: B
Neoscardovia
90
60
30
0
A (n=15)
B (n=16)
C (n=14)
surface

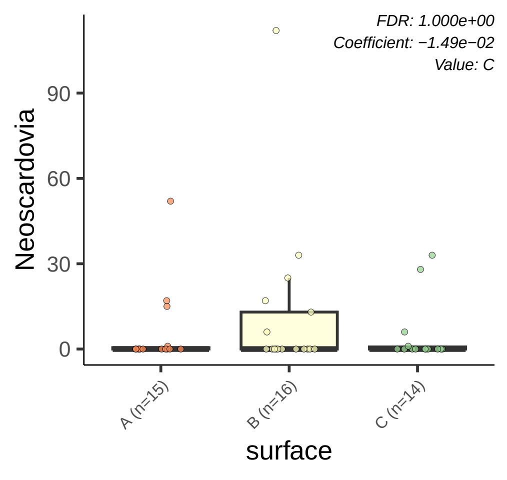

FDR: 1.000e+00
Coefficient: −1.49e−02
Value: C
Neoscardovia
90
60
30
0
A (n=15)
B (n=16)
C (n=14)
surface

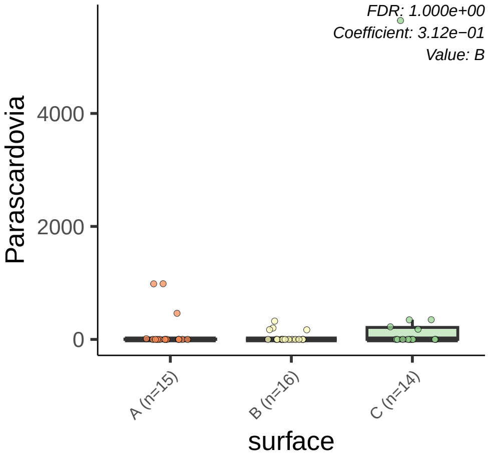

FDR: 1.000e+00
Coefficient: 3.12e−01
Value: B
Parascardovia
4000
2000
0
A (n=15)
B (n=16)
C (n=14)
surface

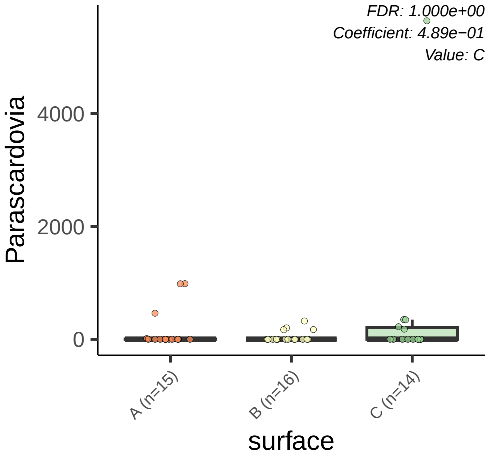
FDR: 1.000e+00
Coefficient: 4.89e−01
Value: C
Parascardovia
4000
2000
0
A (n=15)
B (n=16)
C (n=14)
surface

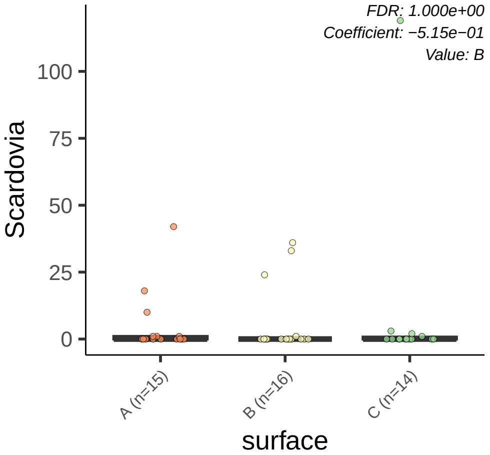
FDR: 1.000e+00
Coefficient: −5.15e−01
Value: B
Scardovia
100
75
50
25
0
A (n=15)
B (n=16)
C (n=14)
surface

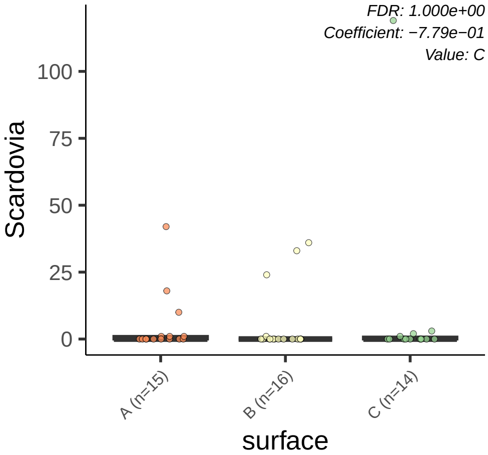
FDR: 1.000e+00
Coefficient: −7.79e−01
Value: C
Scardovia
100
75
50
25
0
A (n=15)
B (n=16)
C (n=14)
surface

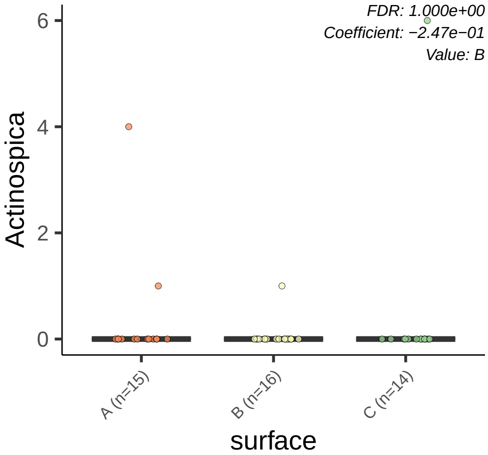

FDR: 1.000e+00
Coefficient: −2.47e−01
Value: B
Actinospica
0
2
4
6
A (n=15)
B (n=16)
C (n=14)
surface

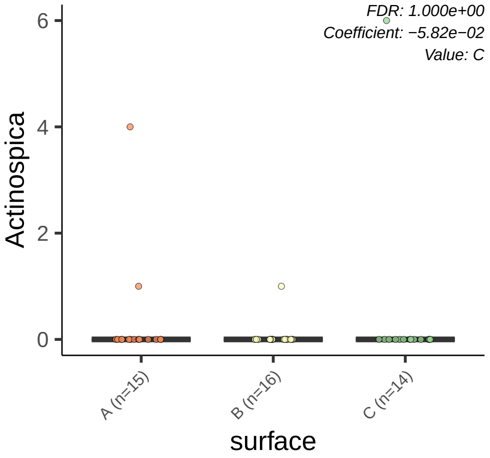
FDR: 1.000e+00
Coefficient: −5.82e−02
Value: C
Actinospica
6
4
2
0
A (n=15)
B (n=16)
C (n=14)
surface

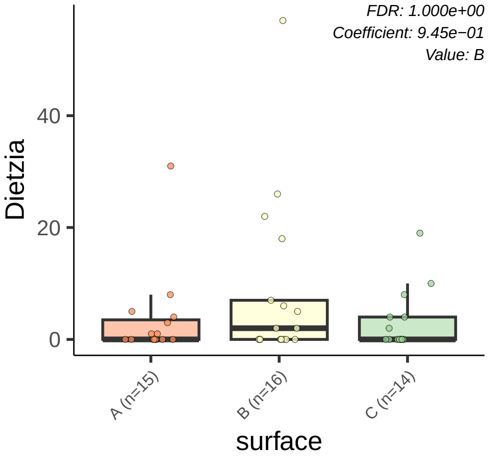
FDR: 1.000e+00
Coefficient: 9.45e−01
Value: B
Dietzia
0
20
40
A (n=15)
B (n=16)
C (n=14)
surface

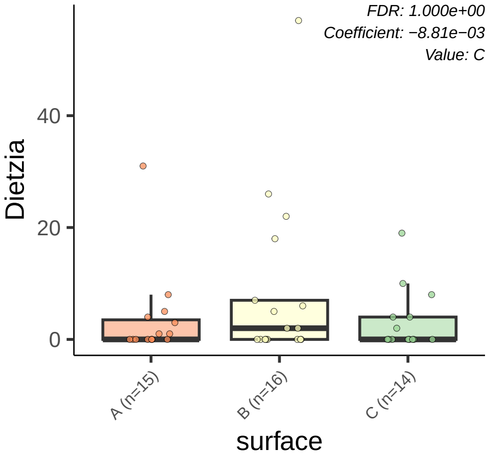

FDR: 1.000e+00
Coefficient: −8.81e−03
Value: C
Dietzia
40
20
0
A (n=15)
B (n=16)
C (n=14)
surface

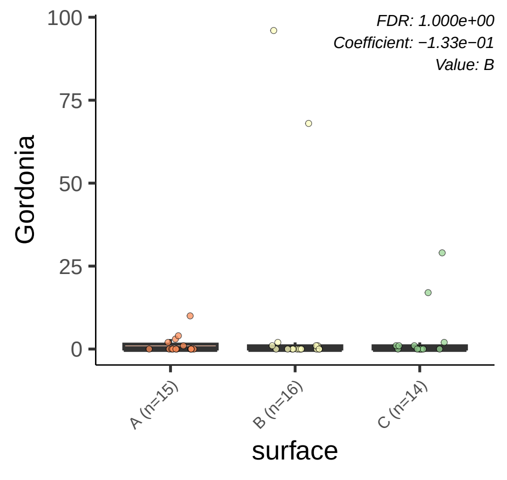
FDR: 1.000e+00
Coefficient: −1.33e−01
Value: B
Gordonia
100
75
50
25
0
A (n=15)
B (n=16)
C (n=14)
surface

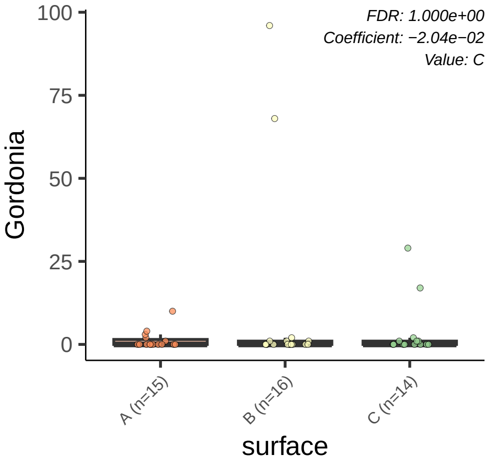
FDR: 1.000e+00
Coefficient: −2.04e−02
Value: C
100
75
50
25
0
Gordonia
A (n=15)
B (n=16)
C (n=14)
surface

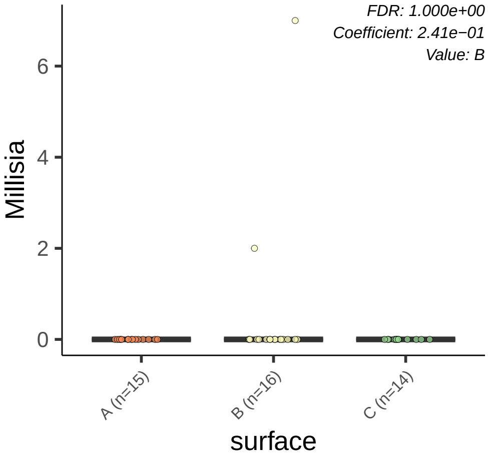

FDR: 1.000e+00
Coefficient: 2.41e−01
Value: B
Millisia
0
2
4
6
A (n=15)
B (n=16)
C (n=14)
surface

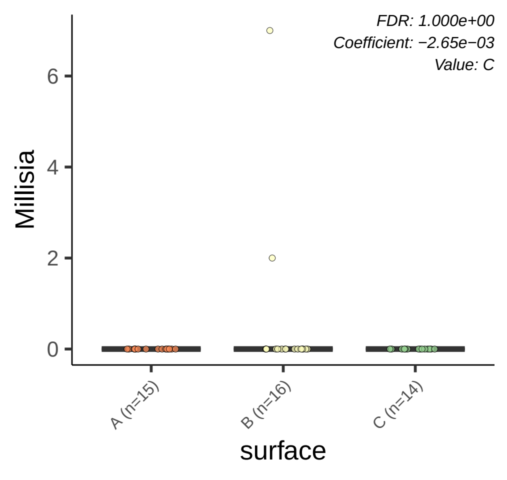
FDR: 1.000e+00
Coefficient: −2.65e−03
Value: C
Millisia
0
2
4
6
A (n=15)
B (n=16)
C (n=14)
surface

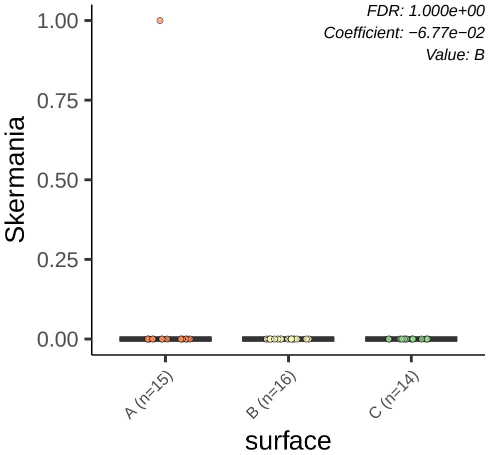

FDR: 1.000e+00
Coefficient: −6.77e−02
Value: B
Skermania
1.00
0.75
0.50
0.25
0.00
A (n=15)
B (n=16)
C (n=14)
surface

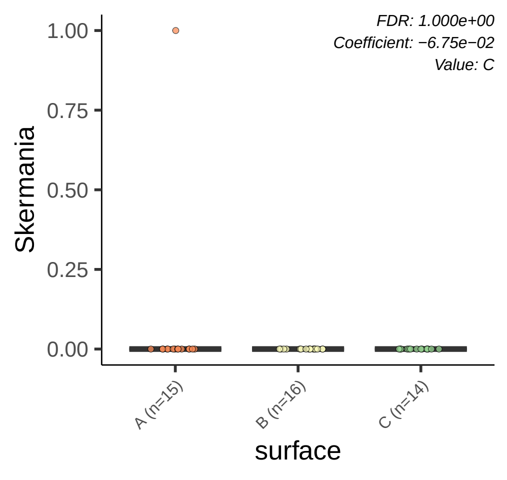
FDR: 1.000e+00
Coefficient: −6.75e−02
Value: C
Skermania
1.00
0.75
0.50
0.25
0.00
A (n=15)
B (n=16)
C (n=14)
surface

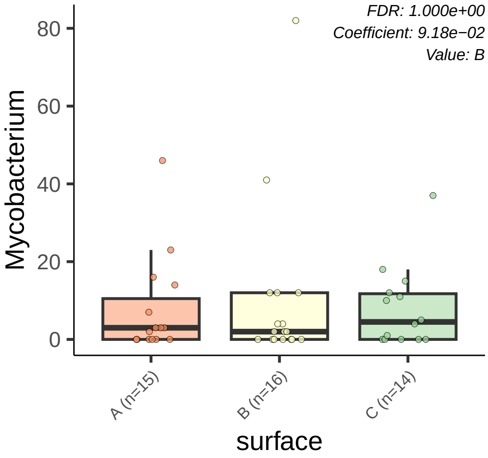

FDR: 1.000e+00
Coefficient: 9.18e−02
Value: B
Mycobacterium
0
20
40
60
80
A (n=15)
B (n=16)
C (n=14)
surface

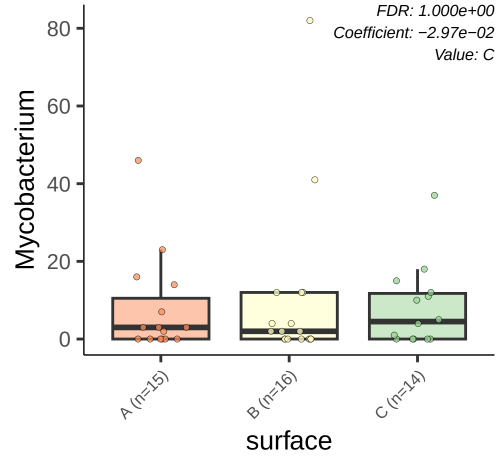
FDR: 1.000e+00
Coefficient: −2.97e−02
Value: C
Mycobacterium
0
20
40
60
80
A (n=15)
B (n=16)
C (n=14)
surface

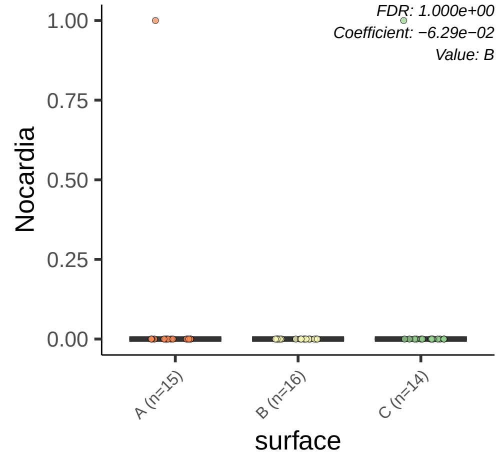

FDR: 1.000e+00
Coefficient: −6.29e−02
Value: B
Nocardia
1.00
0.75
0.50
0.25
0.00
A (n=15)
B (n=16)
C (n=14)
surface

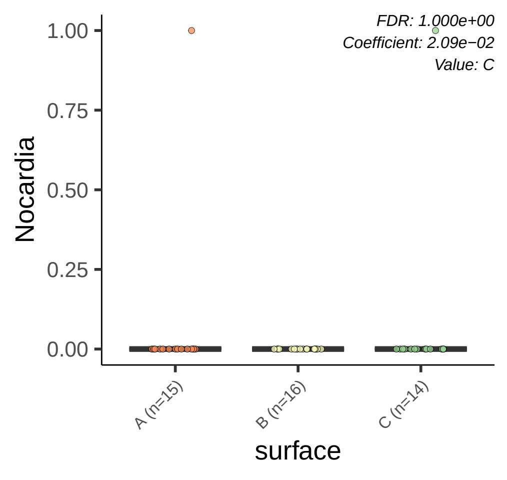
FDR: 1.000e+00
Coefficient: 2.09e−02
Value: C
Nocardia
1.00
0.75
0.50
0.25
0.00
A (n=15)
B (n=16)
C (n=14)
surface

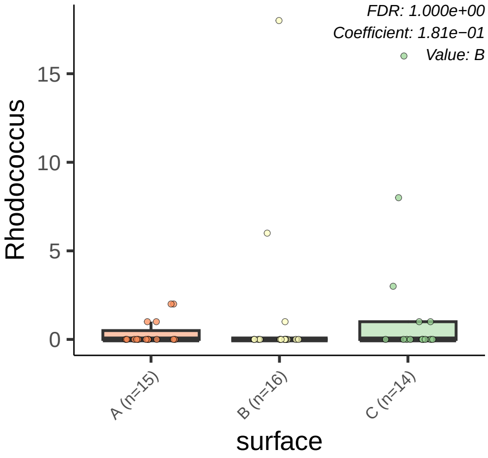
FDR: 1.000e+00
Coefficient: 1.81e−01
Value: B
Rhodococcus
15
10
5
0
A (n=15)
B (n=16)
C (n=14)
surface

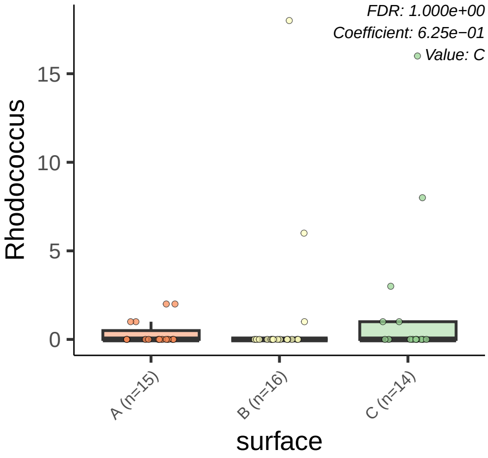

FDR: 1.000e+00
Coefficient: 6.25e−01
Value: C
Rhodococcus
15
10
5
0
A (n=15)
B (n=16)
C (n=14)
surface

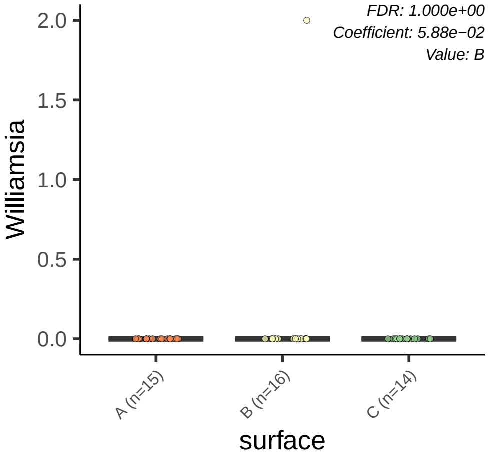

FDR: 1.000e+00
Coefficient: 5.88e−02
Value: B
Williamsia
2.0
1.5
1.0
0.5
0.0
A (n=15)
B (n=16)
C (n=14)
surface

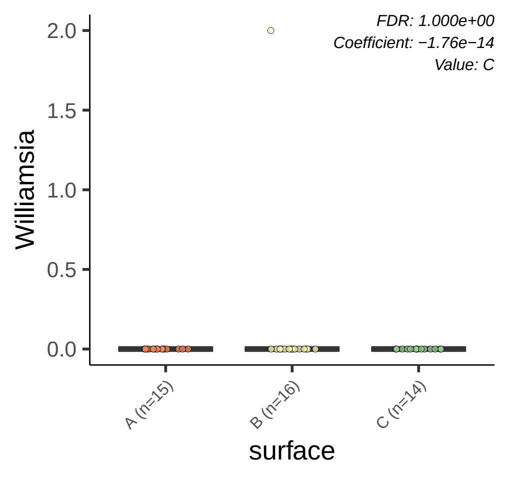
FDR: 1.000e+00
Coefficient: −1.76e−14
Value: C
Williamsia
2.0
1.5
1.0
0.5
0.0
A (n=15)
B (n=16)
C (n=14)
surface

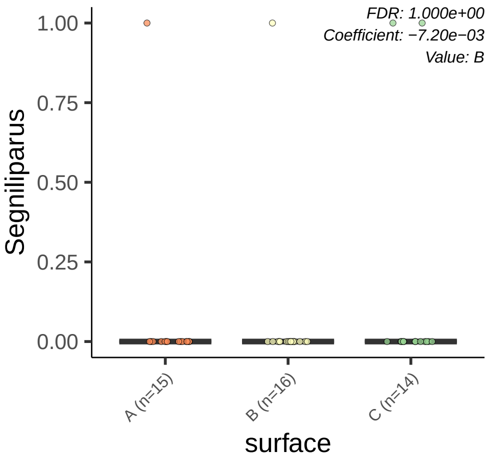

FDR: 1.000e+00
Coefficient: −7.20e−03
Value: B
Segniliparus
1.00
0.75
0.50
0.25
0.00
A (n=15)
B (n=16)
C (n=14)
surface

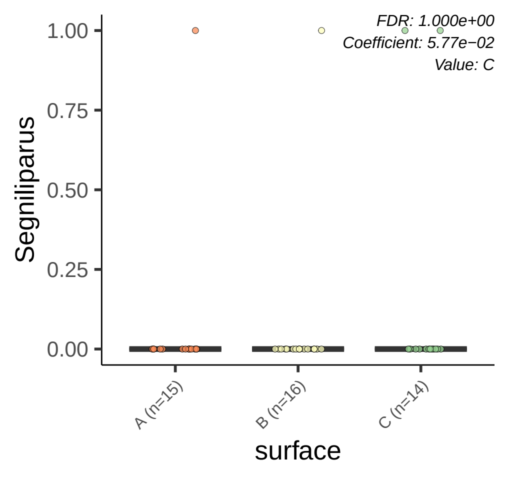

FDR: 1.000e+00
Coefficient: 5.77e−02
Value: C
Segniliparus
1.00
0.75
0.50
0.25
0.00
A (n=15)
B (n=16)
C (n=14)
surface

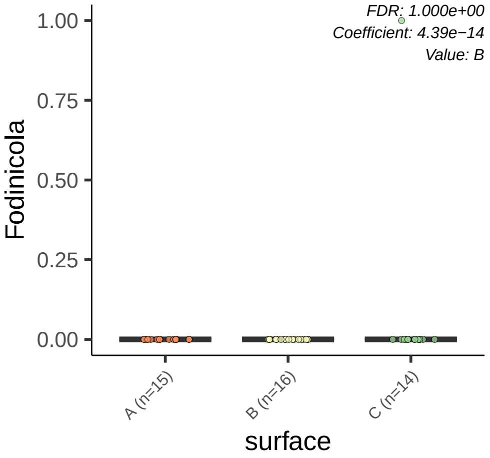
FDR: 1.000e+00
Coefficient: 4.39e−14
Value: B
Fodinicola
1.00
0.75
0.50
0.25
0.00
A (n=15)
B (n=16)
C (n=14)
surface

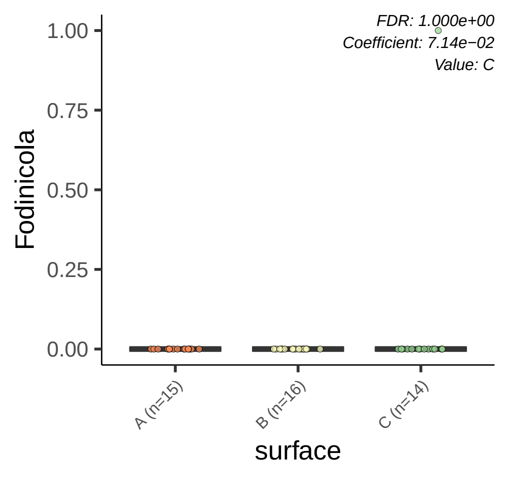
FDR: 1.000e+00
Coefficient: 7.14e−02
Value: C
Fodinicola
1.00
0.75
0.50
0.25
0.00
A (n=15)
B (n=16)
C (n=14)
surface

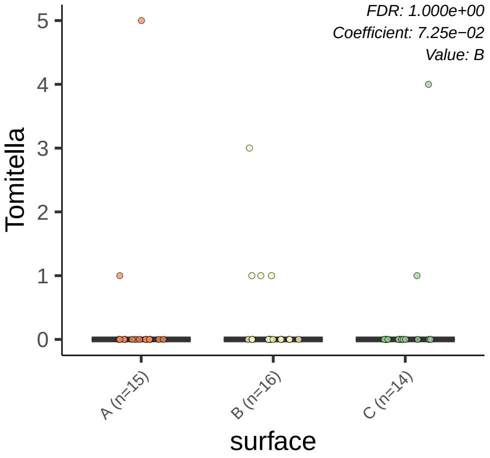

FDR: 1.000e+00
Coefficient: 7.25e−02
Value: B
Tomitella
5
4
3
2
1
0
A (n=15)
B (n=16)
C (n=14)
surface

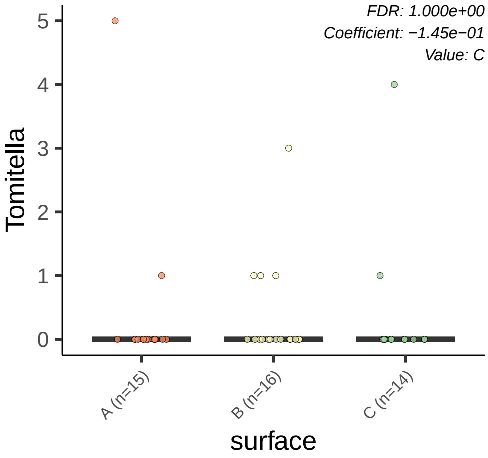

FDR: 1.000e+00
Coefficient: −1.45e−01
Value: C
Tomitella
0
1
2
3
4
5
A (n=15)
B (n=16)
C (n=14)
surface

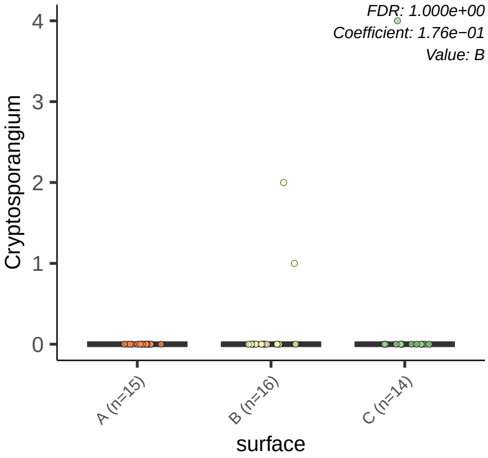

FDR: 1.000e+00
Coefficient: 1.76e−01
Value: B
Cryptosporangium
4
3
2
1
0
A (n=15)
B (n=16)
C (n=14)
surface

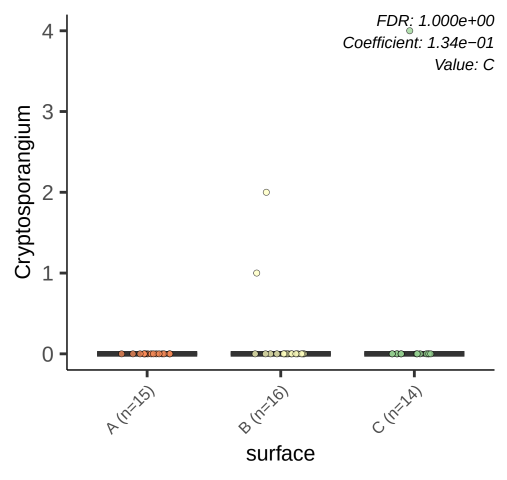
FDR: 1.000e+00
Coefficient: 1.34e−01
Value: C
Cryptosporangium
0
1
2
3
4
A (n=15)
B (n=16)
C (n=14)
surface

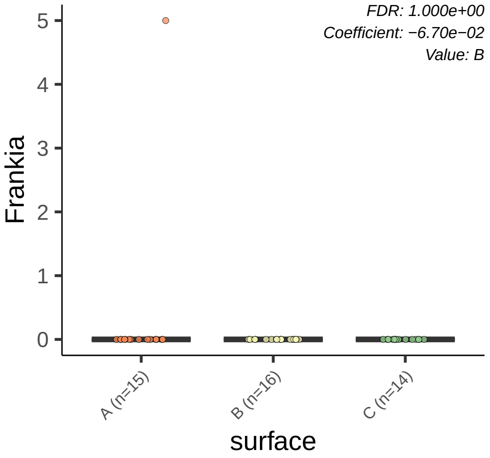

FDR: 1.000e+00
Coefficient: −6.70e−02
Value: B
Frankia
5
4
3
2
1
0
A (n=15)
B (n=16)
C (n=14)
surface

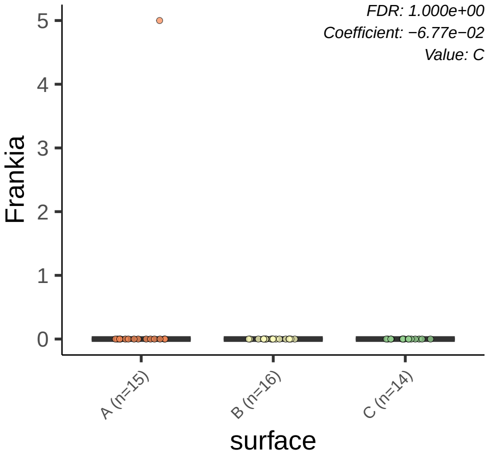
FDR: 1.000e+00
Coefficient: −6.77e−02
Value: C
Frankia
0
1
2
3
4
5
A (n=15)
B (n=16)
C (n=14)
surface

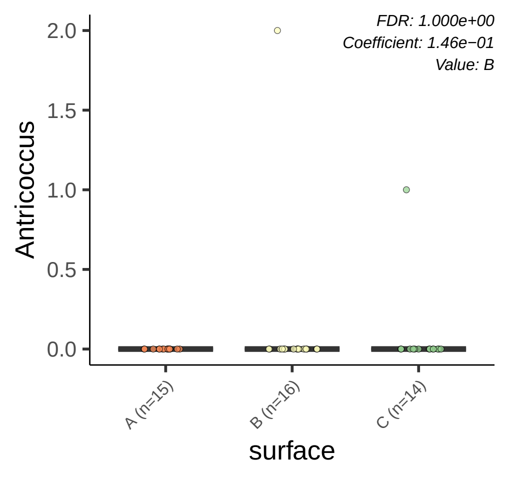
FDR: 1.000e+00
Coefficient: 1.46e−01
Value: B
Antricoccus
2.0
1.5
1.0
0.5
0.0
A (n=15)
B (n=16)
C (n=14)
surface

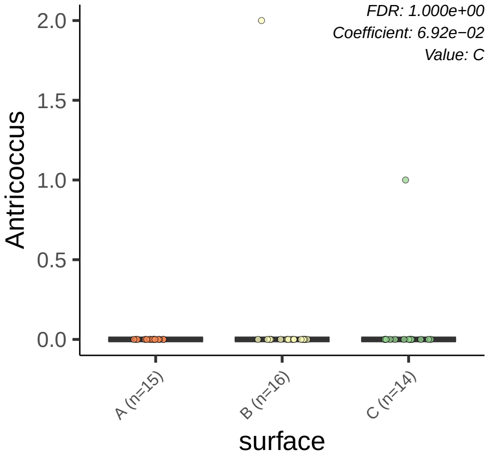
FDR: 1.000e+00
Coefficient: 6.92e−02
Value: C
Antricoccus
2.0
1.5
1.0
0.5
0.0
A (n=15)
B (n=16)
C (n=14)
surface

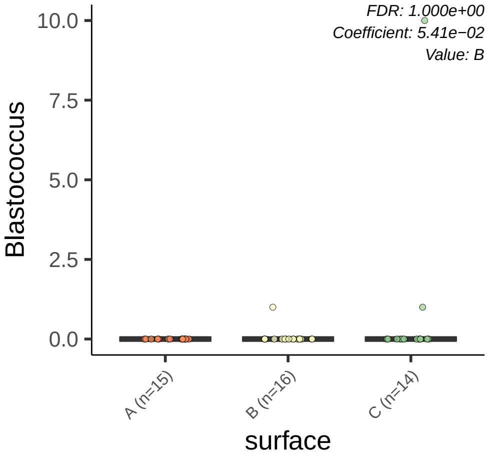
FDR: 1.000e+00
Coefficient: 5.41e−02
Value: B
Blastococcus
10.0
7.5
5.0
2.5
0.0
A (n=15)
B (n=16)
C (n=14)
surface

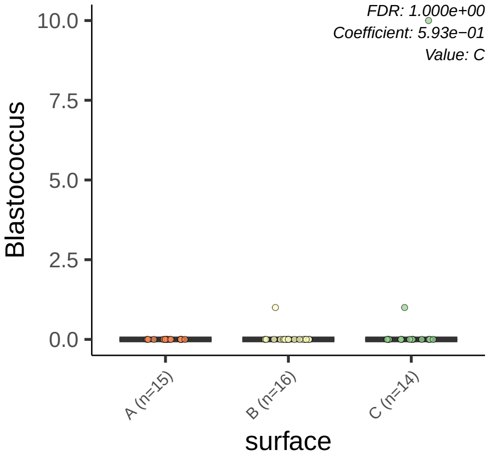
FDR: 1.000e+00
Coefficient: 5.93e−01
Value: C
Blastococcus
10.0
7.5
5.0
2.5
0.0
A (n=15)
B (n=16)
C (n=14)
surface

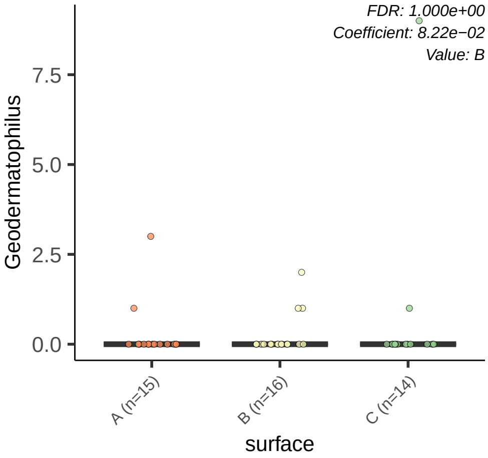
FDR: 1.000e+00
Coefficient: 8.22e−02
Value: B
Geodermatophilus
7.5
5.0
2.5
0.0
A (n=15)
B (n=16)
C (n=14)
surface

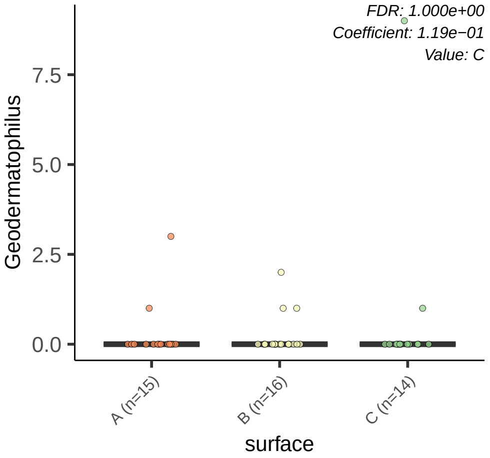
FDR: 1.000e+00
Coefficient: 1.19e−01
Value: C
Geodermatophilus
7.5
5.0
2.5
0.0
A (n=15)
B (n=16)
C (n=14)
surface

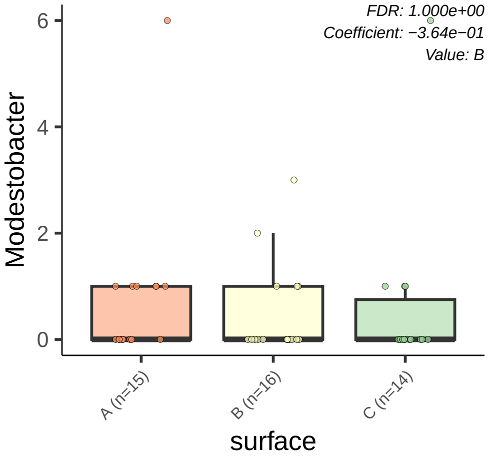

FDR: 1.000e+00
Coefficient: −3.64e−01
Value: B
Modestobacter
6
4
2
0
A (n=15)
B (n=16)
C (n=14)
surface

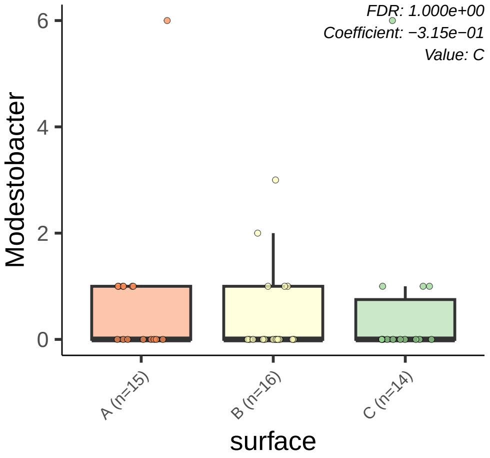

FDR: 1.000e+00
Coefficient: −3.15e−01
Value: C
Modestobacter
0
2
4
6
A (n=15)
B (n=16)
C (n=14)
surface

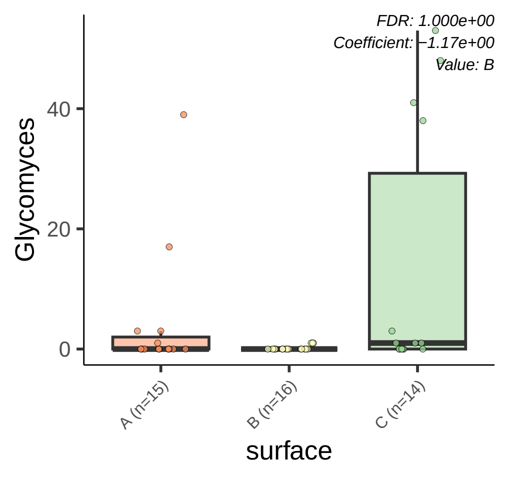
FDR: 1.000e+00
Coefficient: −1.17e+00
Value: B
Glycomyces
0
20
40
A (n=15)
B (n=16)
C (n=14)
surface

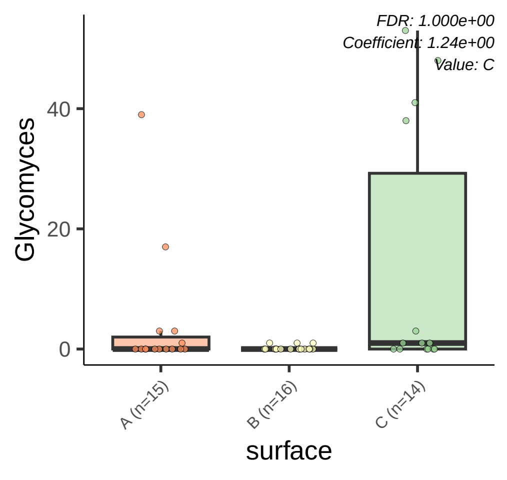

FDR: 1.000e+00
Coefficient: 1.24e+00
Value: C
Glycomyces
40
20
0
A (n=15)
B (n=16)
C (n=14)
surface

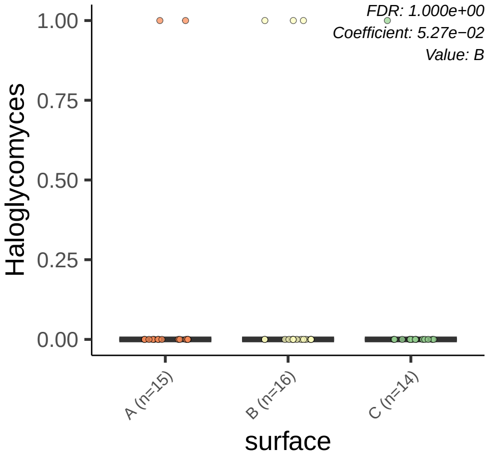
FDR: 1.000e+00
Coefficient: 5.27e−02
Value: B
Haloglycomyces
1.00
0.75
0.50
0.25
0.00
A (n=15)
B (n=16)
C (n=14)
surface

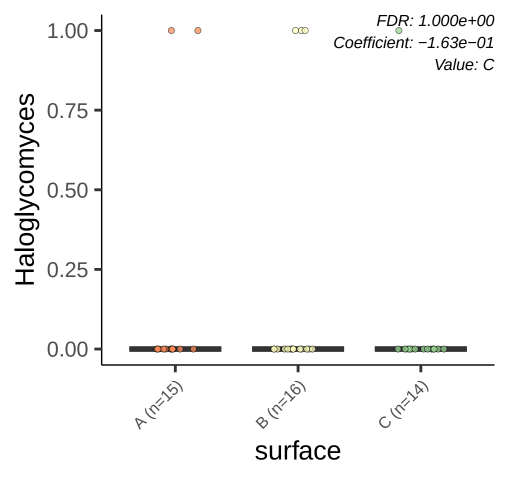
FDR: 1.000e+00
Coefficient: −1.63e−01
Value: C
Haloglycomyces
1.00
0.75
0.50
0.25
0.00
A (n=15)
B (n=16)
C (n=14)
surface

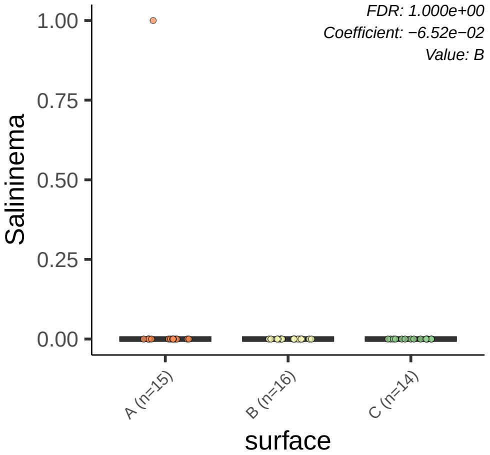
FDR: 1.000e+00
Coefficient: −6.52e−02
Value: B
Salininema
1.00
0.75
0.50
0.25
0.00
A (n=15)
B (n=16)
C (n=14)
surface

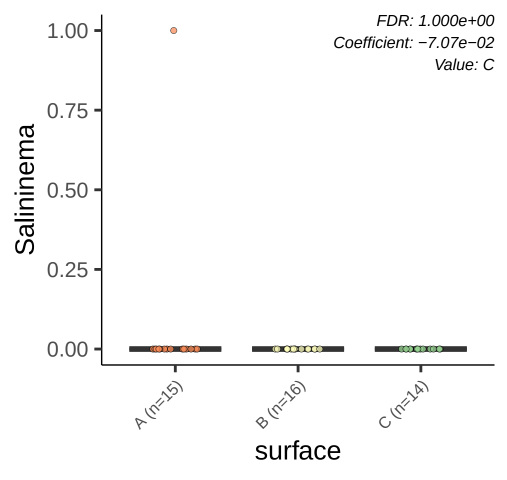
FDR: 1.000e+00
Coefficient: −7.07e−02
Value: C
Salininema
1.00
0.75
0.50
0.25
0.00
A (n=15)
B (n=16)
C (n=14)
surface

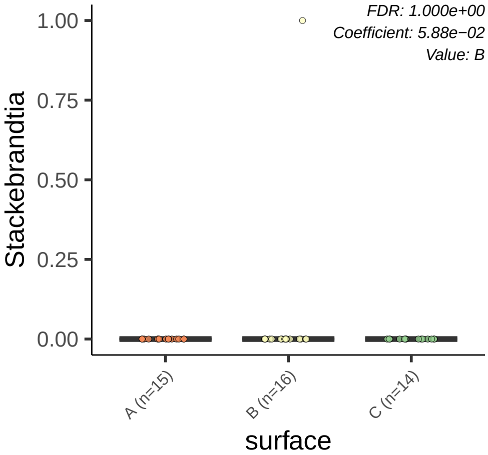

FDR: 1.000e+00
Coefficient: 5.88e−02
Value: B
Stackebrandtia
1.00
0.75
0.50
0.25
0.00
A (n=15)
B (n=16)
C (n=14)
surface

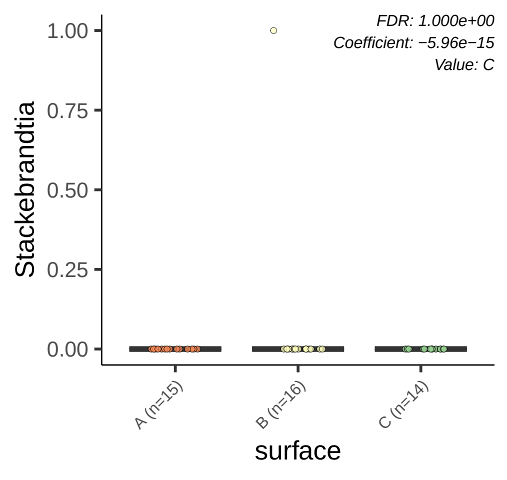

FDR: 1.000e+00
Coefficient: −5.96e−15
Value: C
Stackebrandtia
1.00
0.75
0.50
0.25
0.00
A (n=15)
B (n=16)
C (n=14)
surface

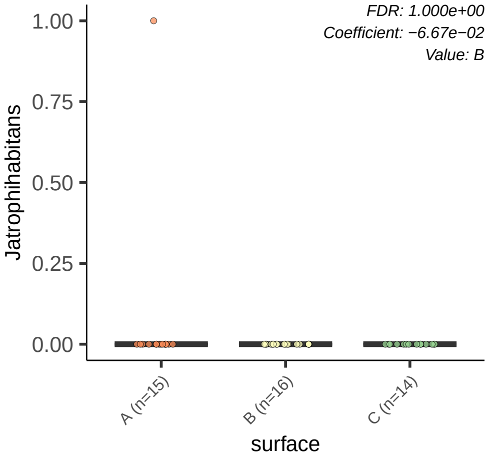

FDR: 1.000e+00
Coefficient: −6.67e−02
Value: B
Jatrophihabitans
1.00
0.75
0.50
0.25
0.00
A (n=15)
B (n=16)
C (n=14)
surface

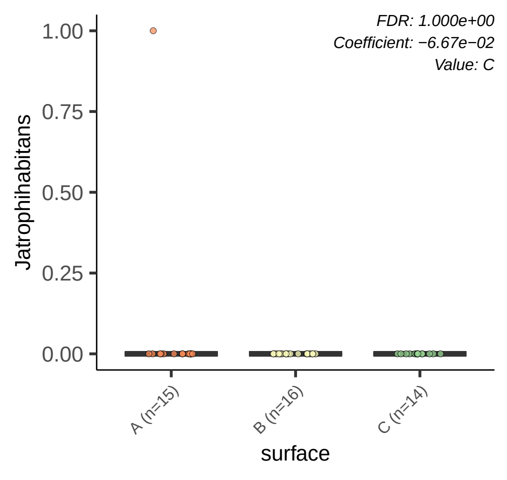
FDR: 1.000e+00
Coefficient: −6.67e−02
Value: C
Jatrophihabitans
1.00
0.75
0.50
0.25
0.00
A (n=15)
B (n=16)
C (n=14)
surface

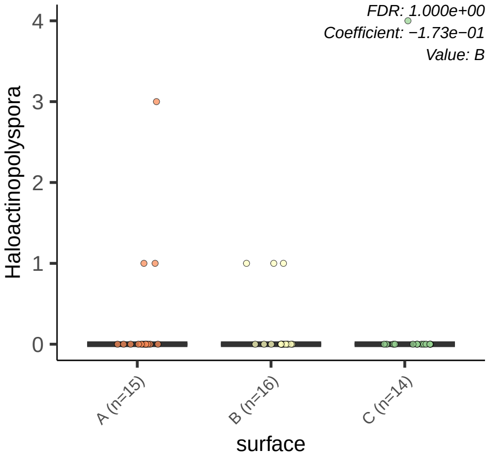
FDR: 1.000e+00
Coefficient: −1.73e−01
Value: B
Haloactinopolyspora
0
1
2
3
4
A (n=15)
B (n=16)
C (n=14)
surface

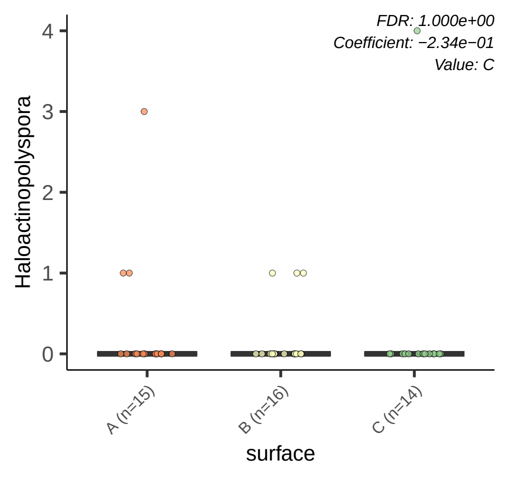

FDR: 1.000e+00
Coefficient: −2.34e−01
Value: C
Haloactinopolyspora
0
1
2
3
4
A (n=15)
B (n=16)
C (n=14)
surface

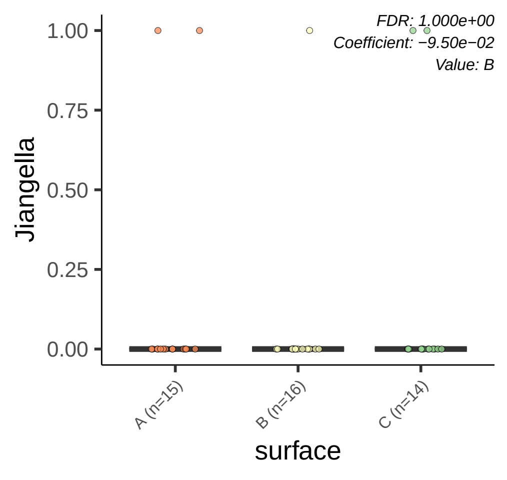

FDR: 1.000e+00
Coefficient: −9.50e−02
Value: B
1.00
0.75
0.50
0.25
0.00
Jiangella
A (n=15)
B (n=16)
C (n=14)
surface

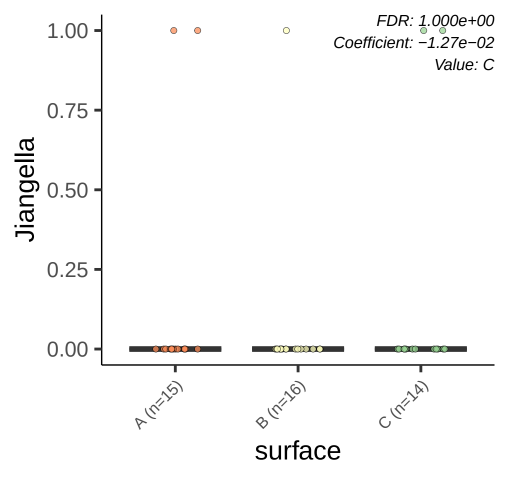
FDR: 1.000e+00
Coefficient: −1.27e−02
Value: C
Jiangella
1.00
0.75
0.50
0.25
0.00
A (n=15)
B (n=16)
C (n=14)
surface

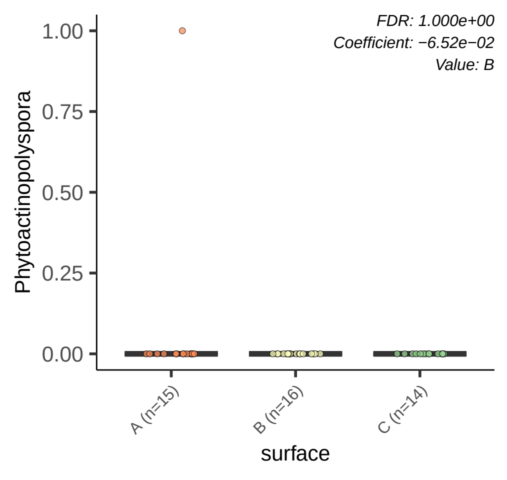

FDR: 1.000e+00
Coefficient: −6.52e−02
Value: B
Phytoactinopolyspora
1.00
0.75
0.50
0.25
0.00
A (n=15)
B (n=16)
C (n=14)
surface

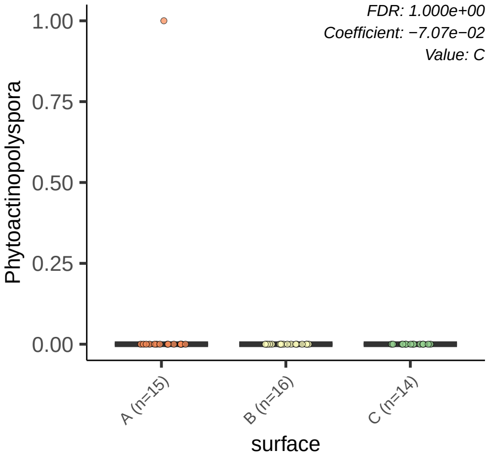

FDR: 1.000e+00
Coefficient: −7.07e−02
Value: C
Phytoactinopolyspora
1.00
0.75
0.50
0.25
0.00
A (n=15)
B (n=16)
C (n=14)
surface

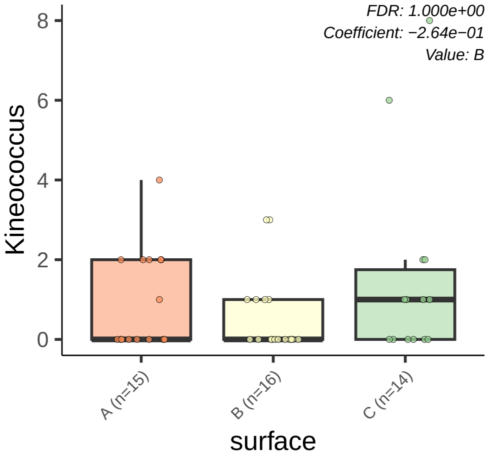
FDR: 1.000e+00
Coefficient: −2.64e−01
Value: B
Kineococcus
8
6
4
2
0
A (n=15)
B (n=16)
C (n=14)
surface

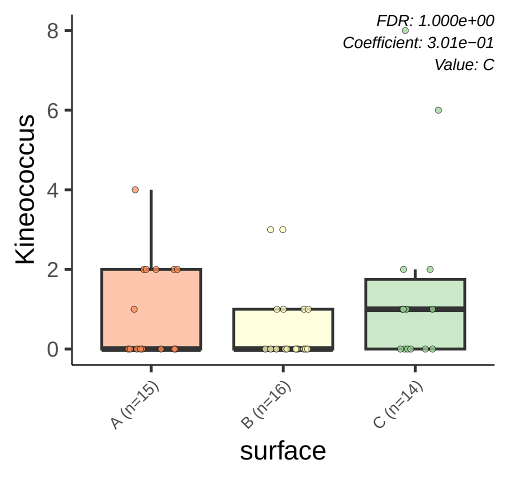

FDR: 1.000e+00
Coefficient: 3.01e−01
Value: C
Kineococcus
0
2
4
6
8
A (n=15)
B (n=16)
C (n=14)
surface

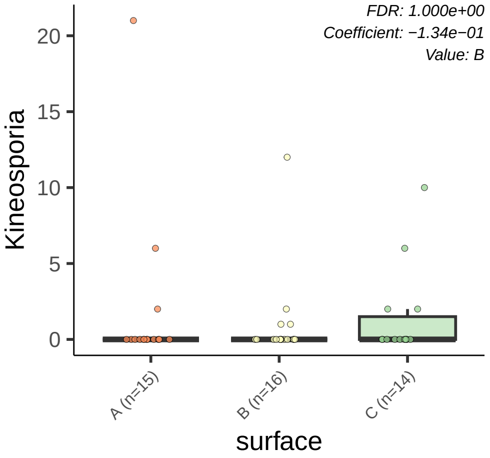
FDR: 1.000e+00
Coefficient: −1.34e−01
Value: B
Kineosporia
20
15
10
5
0
A (n=15)
B (n=16)
C (n=14)
surface

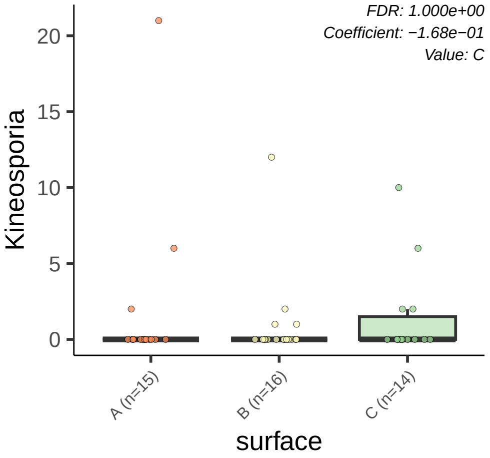
FDR: 1.000e+00
Coefficient: −1.68e−01
Value: C
Kineosporia
20
15
10
5
0
A (n=15)
B (n=16)
C (n=14)
surface

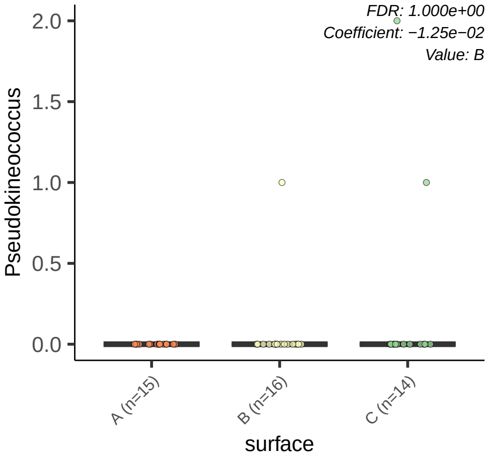
FDR: 1.000e+00
Coefficient: −1.25e−02
Value: B
Pseudokineococcus
2.0
1.5
1.0
0.5
0.0
A (n=15)
B (n=16)
C (n=14)
surface

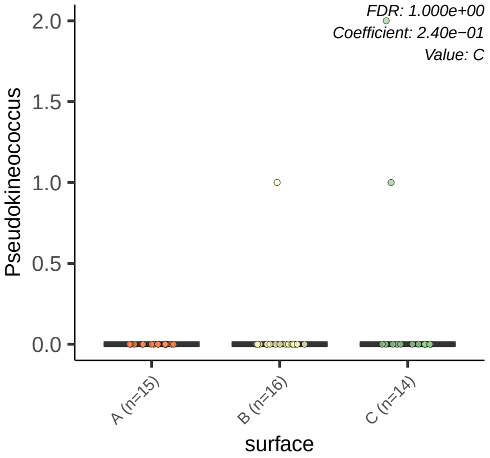
FDR: 1.000e+00
Coefficient: 2.40e−01
Value: C
Pseudokineococcus
2.0
1.5
1.0
0.5
0.0
A (n=15)
B (n=16)
C (n=14)
surface

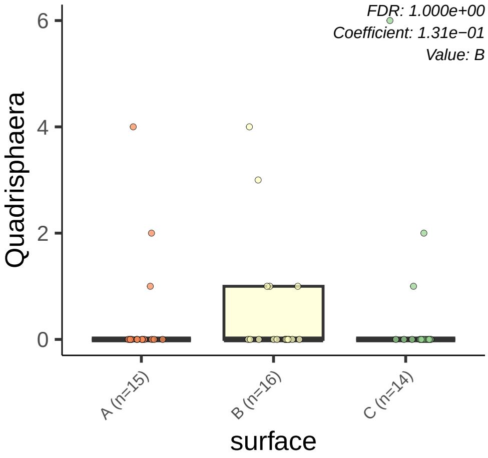

FDR: 1.000e+00
Coefficient: 1.31e−01
Value: B
Quadrisphaera
0
2
4
6
A (n=15)
B (n=16)
C (n=14)
surface

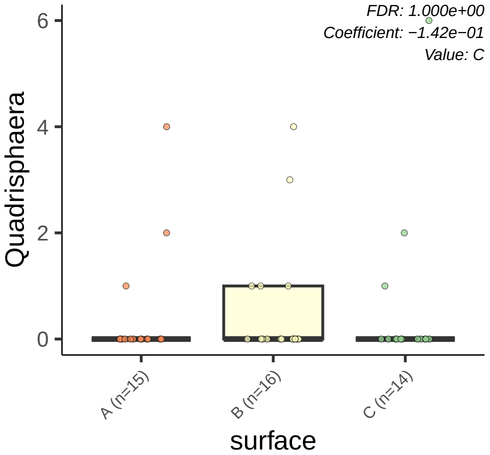
FDR: 1.000e+00
Coefficient: −1.42e−01
Value: C
Quadrisphaera
0
2
4
6
A (n=15)
B (n=16)
C (n=14)
surface

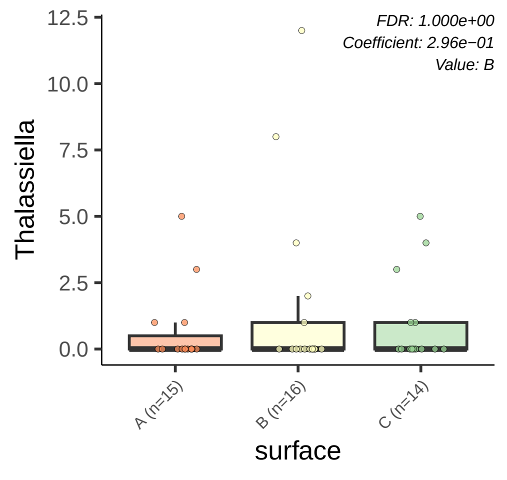

FDR: 1.000e+00
Coefficient: 2.96e−01
Value: B
Thalassiella
12.5
10.0
7.5
5.0
2.5
0.0
A (n=15)
B (n=16)
C (n=14)
surface

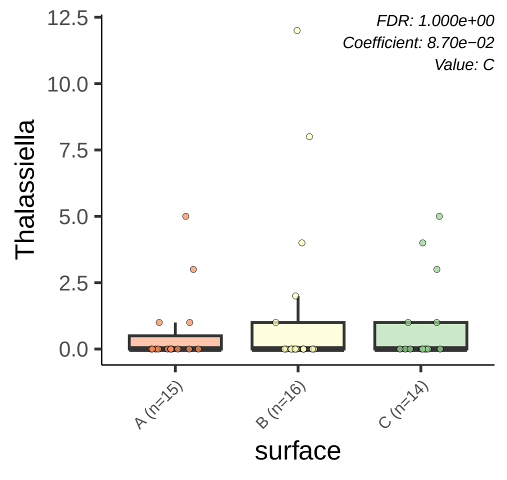
FDR: 1.000e+00
Coefficient: 8.70e−02
Value: C
Thalassiella
12.5
10.0
7.5
5.0
2.5
0.0
A (n=15)
B (n=16)
C (n=14)
surface

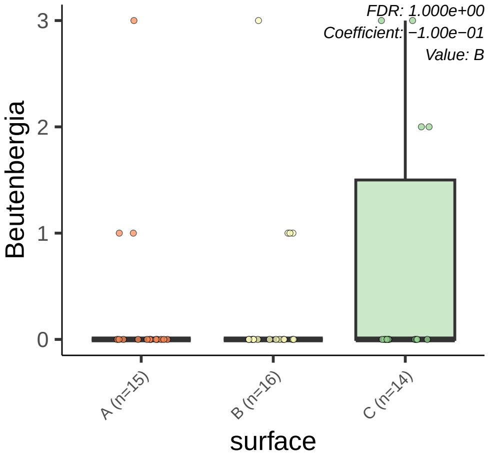
FDR: 1.000e+00
Coefficient: −1.00e−01
Value: B
Beutenbergia
3
2
1
0
A (n=15)
B (n=16)
C (n=14)
surface

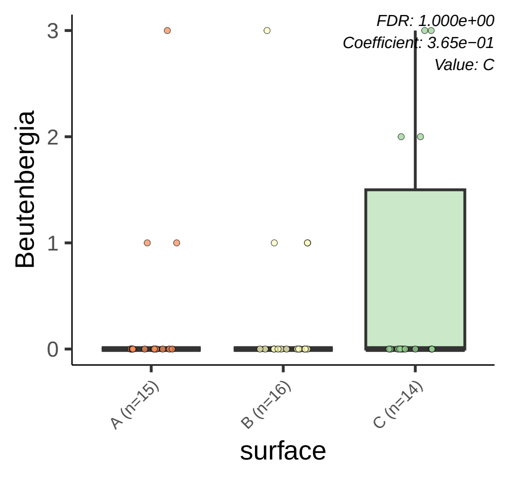

FDR: 1.000e+00
Coefficient: 3.65e−01
Value: C
Beutenbergia
0
1
2
3
A (n=15)
B (n=16)
C (n=14)
surface

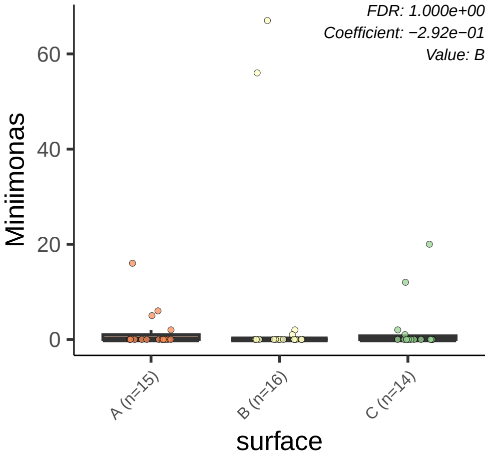

FDR: 1.000e+00
Coefficient: −2.92e−01
Value: B
Miniimonas
0
20
40
60
A (n=15)
B (n=16)
C (n=14)
surface

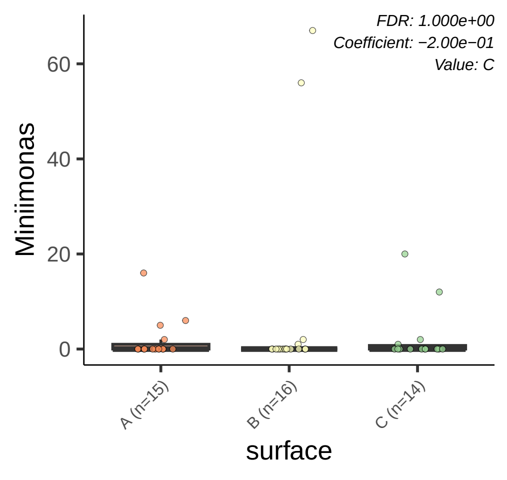
FDR: 1.000e+00
Coefficient: −2.00e−01
Value: C
Miniimonas
60
40
20
0
A (n=15)
B (n=16)
C (n=14)
surface

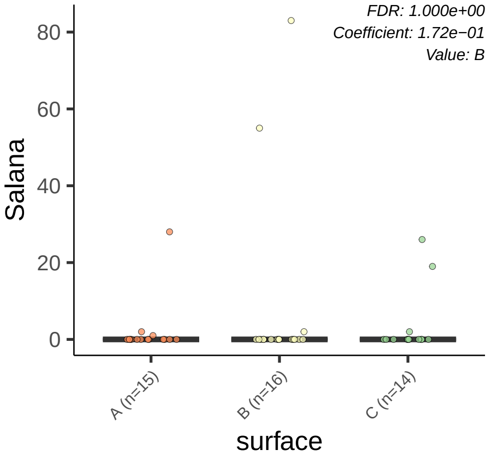

FDR: 1.000e+00
Coefficient: 1.72e−01
Value: B
Salana
0
20
40
60
80
A (n=15)
B (n=16)
C (n=14)
surface

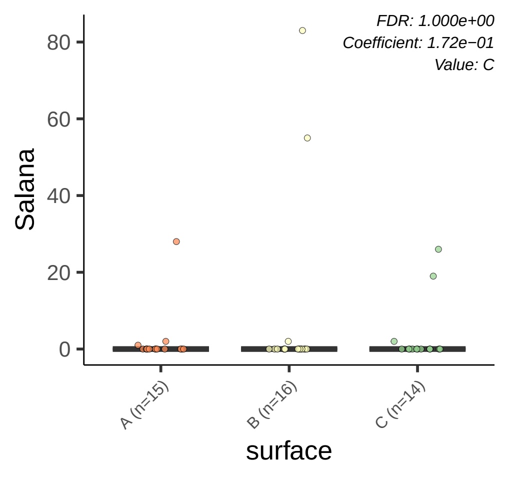
FDR: 1.000e+00
Coefficient: 1.72e−01
Value: C
Salana
0
20
40
60
80
A (n=15)
B (n=16)
C (n=14)
surface

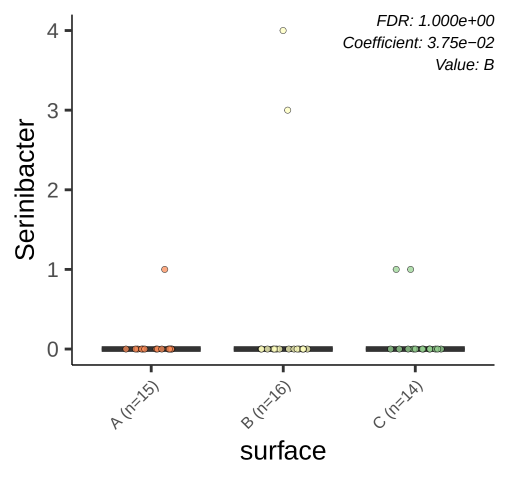
FDR: 1.000e+00
Coefficient: 3.75e−02
Value: B
Serinibacter
4
3
2
1
0
A (n=15)
B (n=16)
C (n=14)
surface

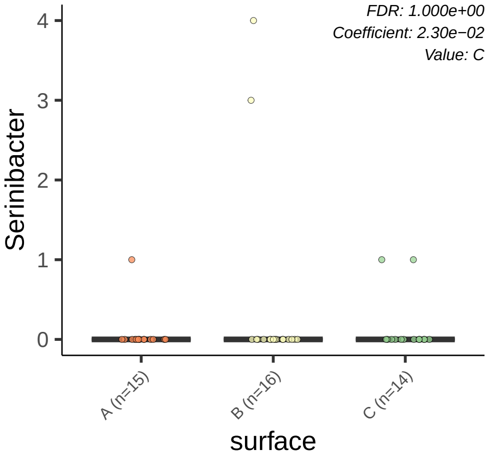

FDR: 1.000e+00
Coefficient: 2.30e−02
Value: C
Serinibacter
0
1
2
3
4
A (n=15)
B (n=16)
C (n=14)
surface

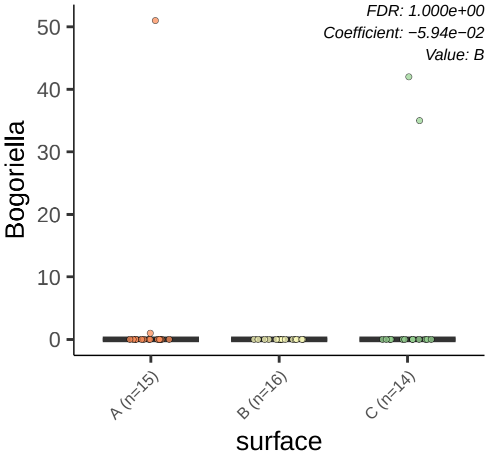

FDR: 1.000e+00
Coefficient: −5.94e−02
Value: B
Bogoriella
50
40
30
20
10
0
A (n=15)
B (n=16)
C (n=14)
surface

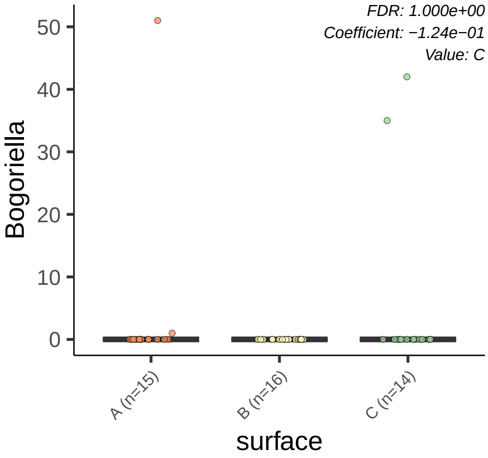
FDR: 1.000e+00
Coefficient: −1.24e−01
Value: C
Bogoriella
50
40
30
20
10
0
A (n=15)
B (n=16)
C (n=14)
surface

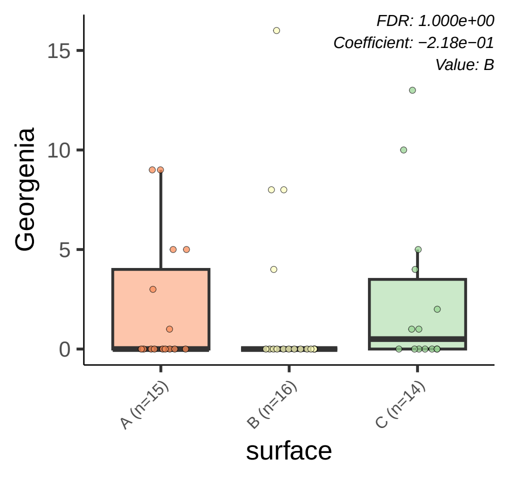
FDR: 1.000e+00
Coefficient: −2.18e−01
Value: B
Georgenia
0
5
10
15
A (n=15)
B (n=16)
C (n=14)
surface

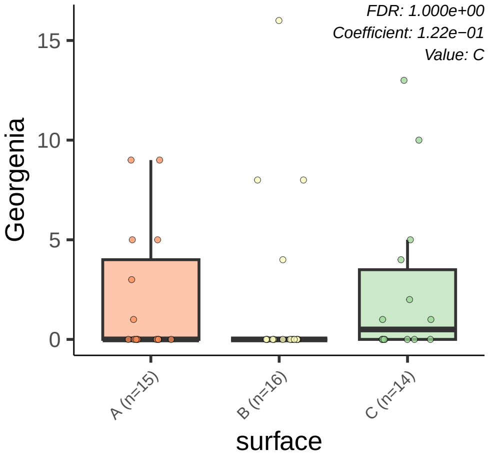

FDR: 1.000e+00
Coefficient: 1.22e−01
Value: C
Georgenia
0
5
10
15
A (n=15)
B (n=16)
C (n=14)
surface

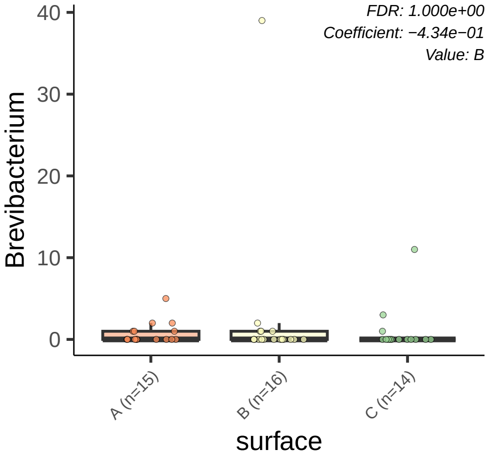

FDR: 1.000e+00
Coefficient: −4.34e−01
Value: B
Brevibacterium
40
30
20
10
0
A (n=15)
B (n=16)
C (n=14)
surface

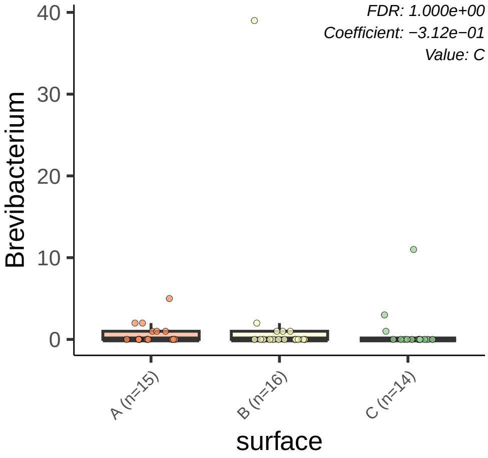
FDR: 1.000e+00
Coefficient: −3.12e−01
Value: C
Brevibacterium
40
30
20
10
0
A (n=15)
B (n=16)
C (n=14)
surface

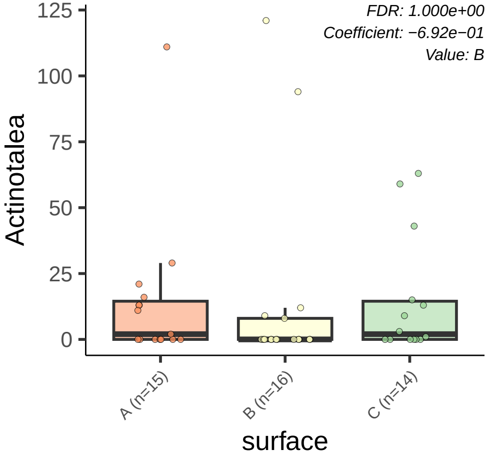

FDR: 1.000e+00
Coefficient: −6.92e−01
Value: B
Actinotalea
125
100
75
50
25
0
A (n=15)
B (n=16)
C (n=14)
surface

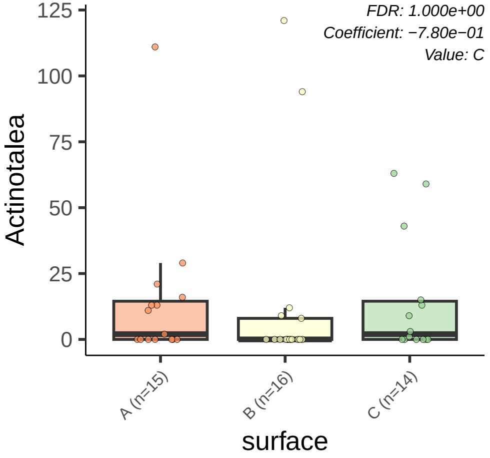

FDR: 1.000e+00
Coefficient: −7.80e−01
Value: C
Actinotalea
125
100
75
50
25
0
A (n=15)
B (n=16)
C (n=14)
surface

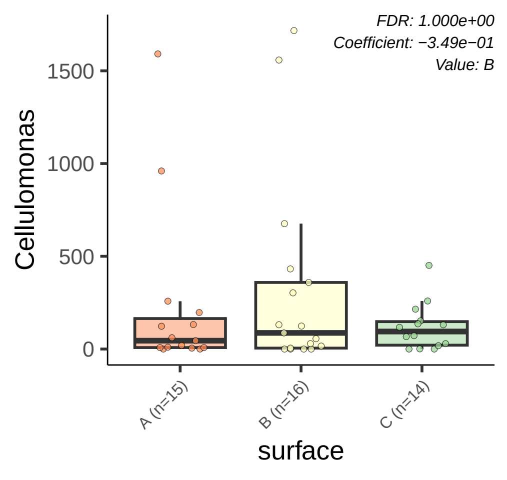

FDR: 1.000e+00
Coefficient: −3.49e−01
Value: B
Cellulomonas
1500
1000
500
0
A (n=15)
B (n=16)
C (n=14)
surface

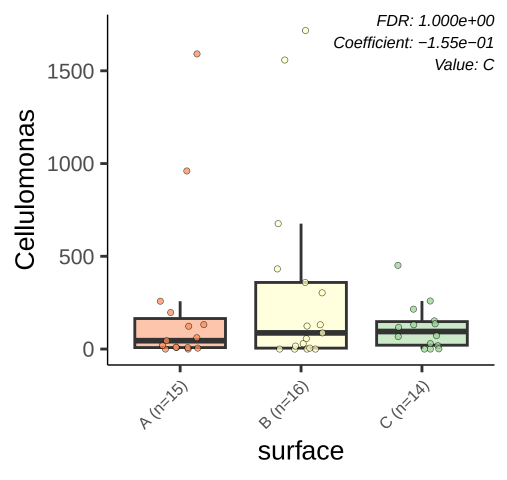

FDR: 1.000e+00
Coefficient: −1.55e−01
Value: C
Cellulomonas
1500
1000
500
0
A (n=15)
B (n=16)
C (n=14)
surface

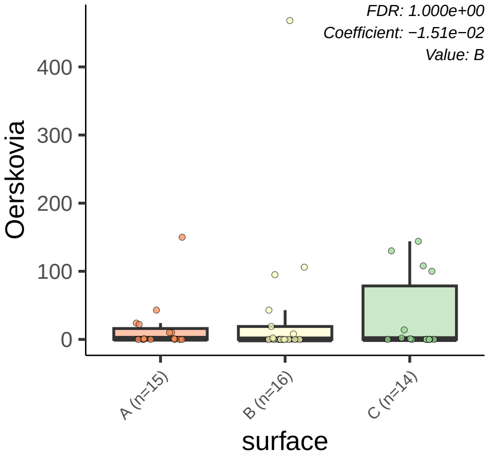

FDR: 1.000e+00
Coefficient: −1.51e−02
Value: B
Oerskovia
0
100
200
300
400
A (n=15)
B (n=16)
C (n=14)
surface

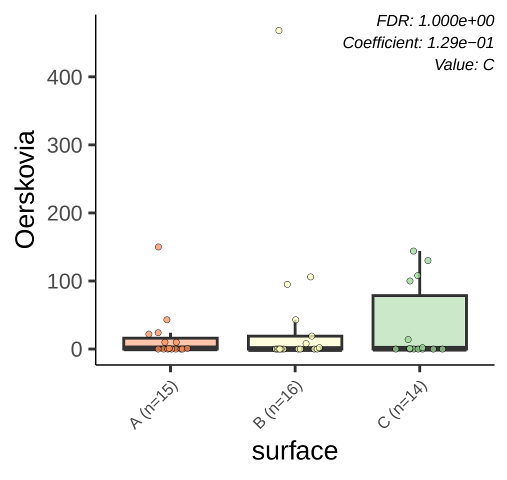

FDR: 1.000e+00
Coefficient: 1.29e−01
Value: C
Oerskovia
400
300
200
100
0
A (n=15)
B (n=16)
C (n=14)
surface

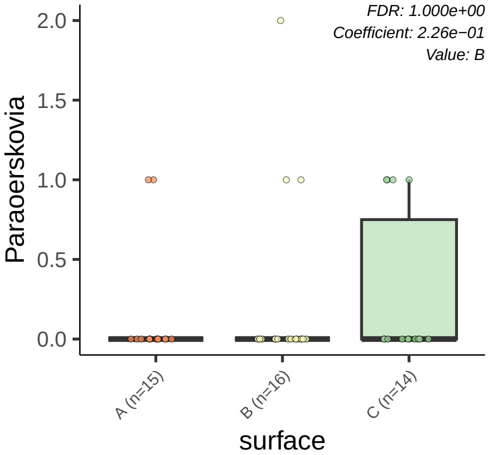
FDR: 1.000e+00
Coefficient: 2.26e−01
Value: B
Paraoerskovia
2.0
1.5
1.0
0.5
0.0
A (n=15)
B (n=16)
C (n=14)
surface

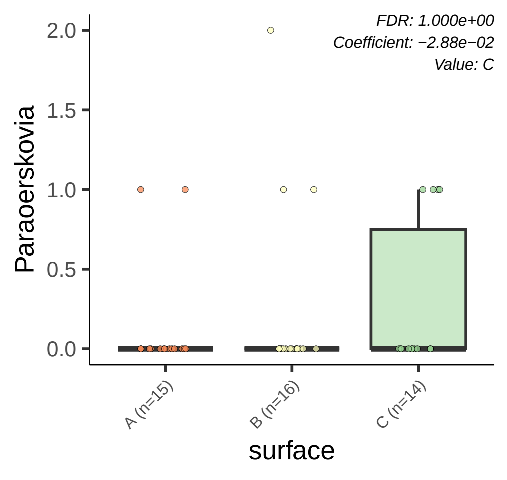
FDR: 1.000e+00
Coefficient: −2.88e−02
Value: C
Paraoerskovia
2.0
1.5
1.0
0.5
0.0
A (n=15)
B (n=16)
C (n=14)
surface

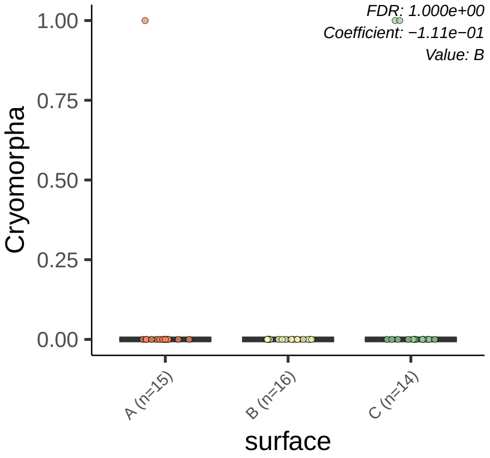
FDR: 1.000e+00
Coefficient: −1.11e−01
Value: B
Cryomorpha
1.00
0.75
0.50
0.25
0.00
A (n=15)
B (n=16)
C (n=14)
surface

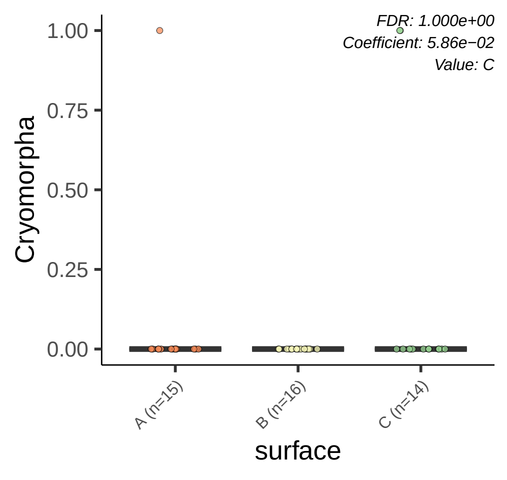
FDR: 1.000e+00
Coefficient: 5.86e−02
Value: C
Cryomorpha
1.00
0.75
0.50
0.25
0.00
A (n=15)
B (n=16)
C (n=14)
surface

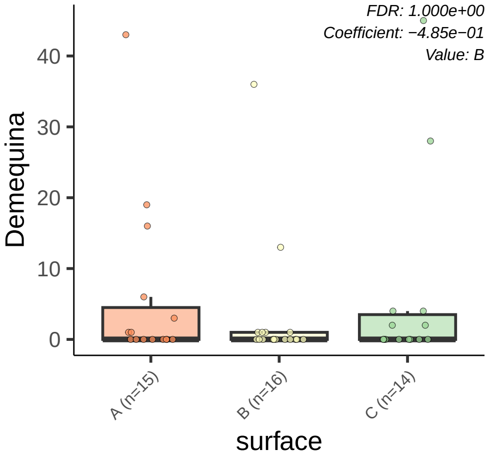

FDR: 1.000e+00
Coefficient: −4.85e−01
Value: B
Demequina
40
30
20
10
0
A (n=15)
B (n=16)
C (n=14)
surface

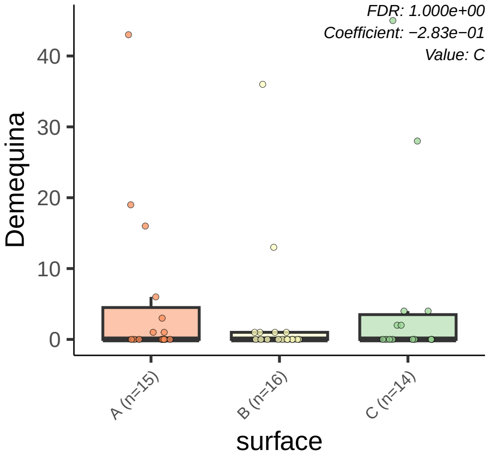

FDR: 1.000e+00
Coefficient: −2.83e−01
Value: C
Demequina
0
10
20
30
40
A (n=15)
B (n=16)
C (n=14)
surface

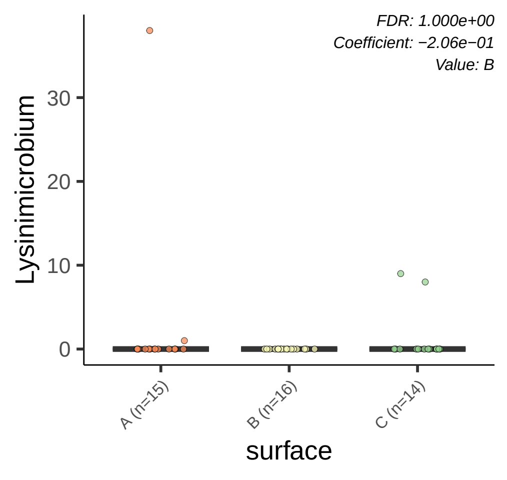
FDR: 1.000e+00
Coefficient: −2.06e−01
Value: B
Lysinimicrobium
30
20
10
0
A (n=15)
B (n=16)
C (n=14)
surface

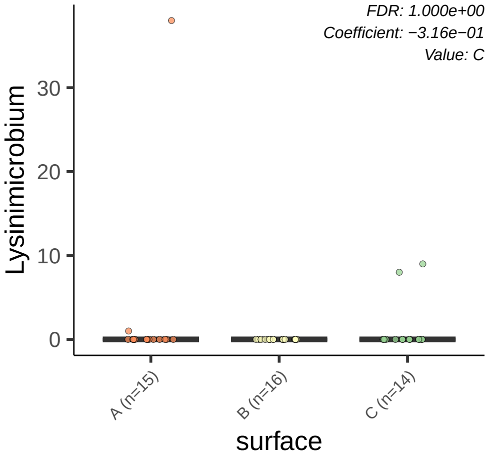
FDR: 1.000e+00
Coefficient: −3.16e−01
Value: C
Lysinimicrobium
30
20
10
0
A (n=15)
B (n=16)
C (n=14)
surface

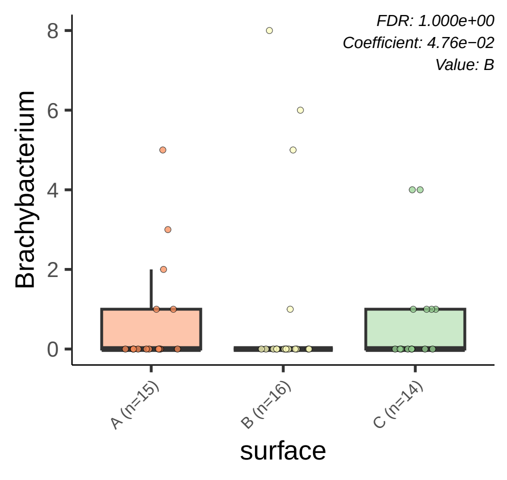

FDR: 1.000e+00
Coefficient: 4.76e−02
Value: B
Brachybacterium
8
6
4
2
0
A (n=15)
B (n=16)
C (n=14)
surface

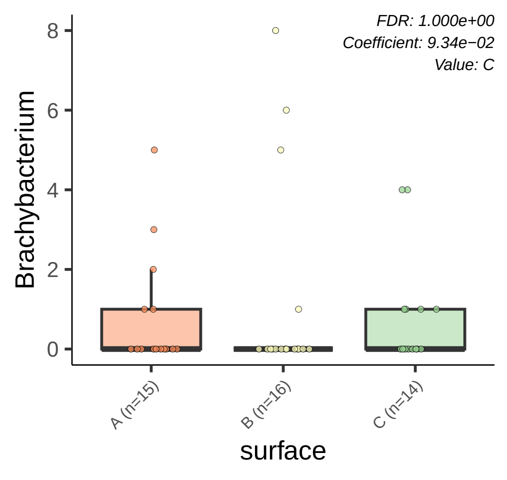

FDR: 1.000e+00
Coefficient: 9.34e−02
Value: C
Brachybacterium
8
6
4
2
0
A (n=15)
B (n=16)
C (n=14)
surface

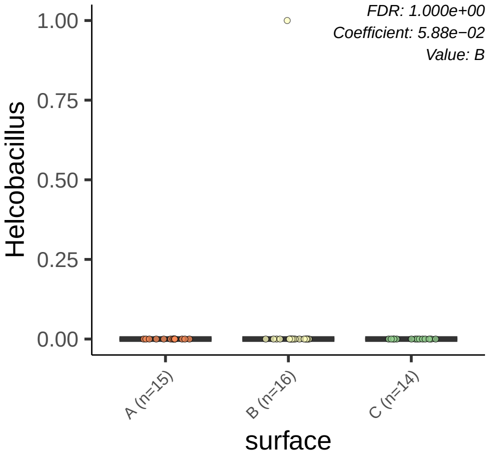
FDR: 1.000e+00
Coefficient: 5.88e−02
Value: B
Helcobacillus
1.00
0.75
0.50
0.25
0.00
A (n=15)
B (n=16)
C (n=14)
surface

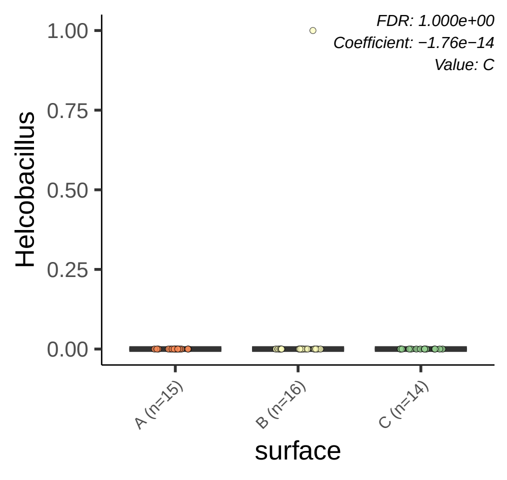

FDR: 1.000e+00
Coefficient: −1.76e−14
Value: C
Helcobacillus
1.00
0.75
0.50
0.25
0.00
A (n=15)
B (n=16)
C (n=14)
surface

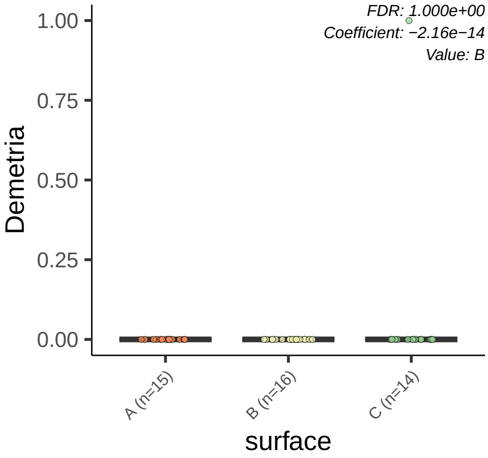
FDR: 1.000e+00
Coefficient: −2.16e−14
Value: B
1.00
0.75
0.50
0.25
0.00
Demetria
A (n=15)
B (n=16)
C (n=14)
surface

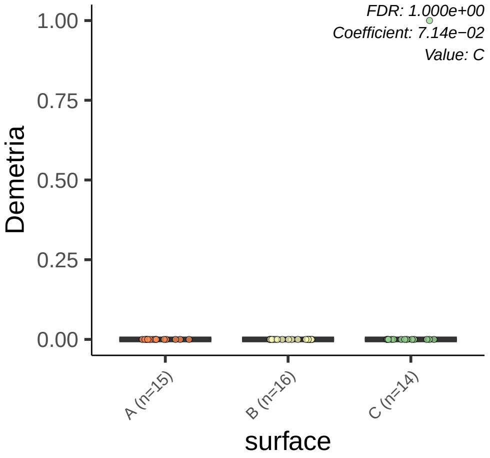
FDR: 1.000e+00
Coefficient: 7.14e−02
Value: C
1.00
0.75
0.50
0.25
0.00
Demetria
A (n=15)
B (n=16)
C (n=14)
surface

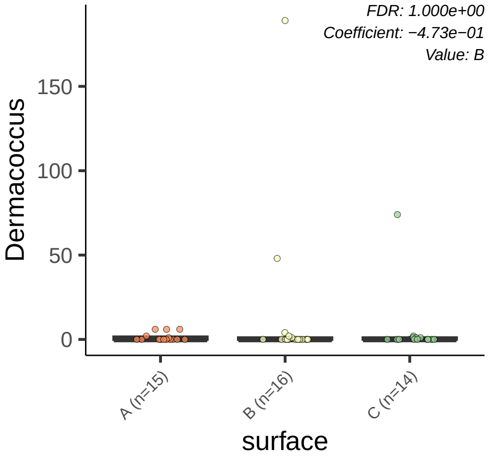

FDR: 1.000e+00
Coefficient: −4.73e−01
Value: B
Dermacoccus
150
100
50
0
A (n=15)
B (n=16)
C (n=14)
surface

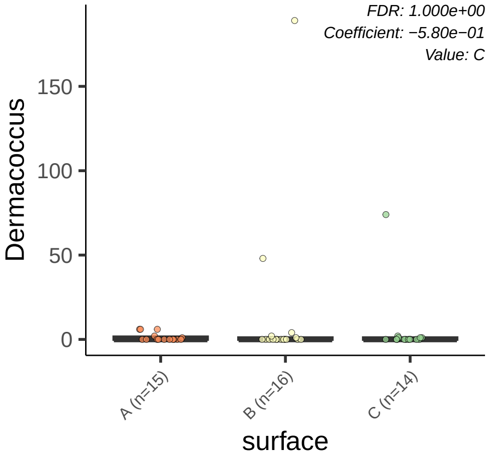

FDR: 1.000e+00
Coefficient: −5.80e−01
Value: C
Dermacoccus
150
100
50
0
A (n=15)
B (n=16)
C (n=14)
surface

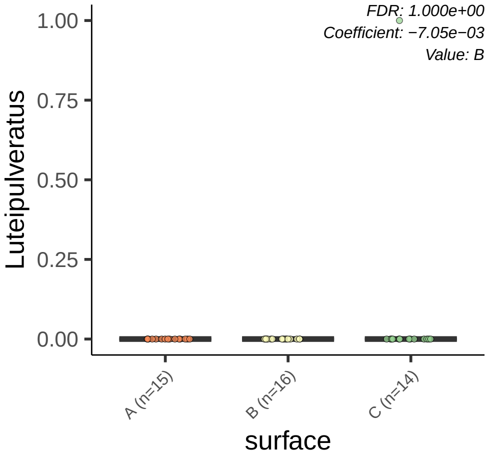
FDR: 1.000e+00
Coefficient: −7.05e−03
Value: B
Luteipulveratus
1.00
0.75
0.50
0.25
0.00
A (n=15)
B (n=16)
C (n=14)
surface

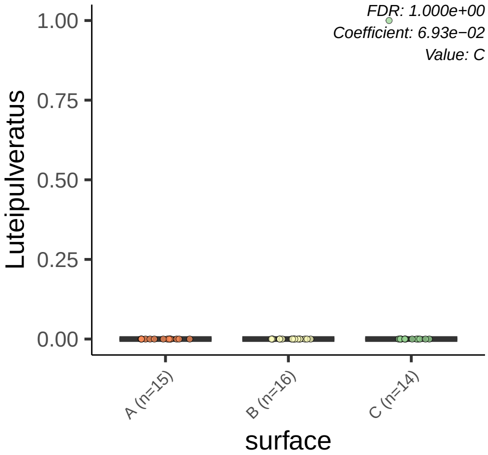

FDR: 1.000e+00
Coefficient: 6.93e−02
Value: C
Luteipulveratus
1.00
0.75
0.50
0.25
0.00
A (n=15)
B (n=16)
C (n=14)
surface

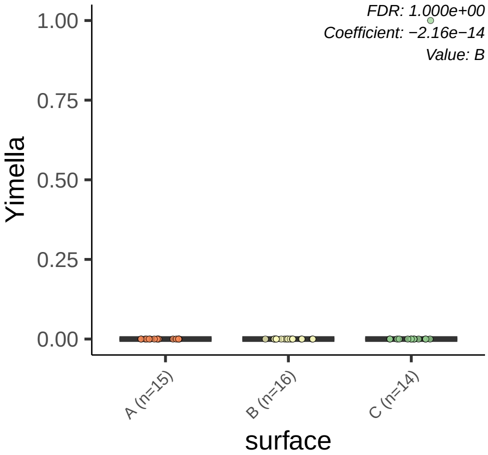
FDR: 1.000e+00
Coefficient: −2.16e−14
Value: B
Yimella
1.00
0.75
0.50
0.25
0.00
A (n=15)
B (n=16)
C (n=14)
surface

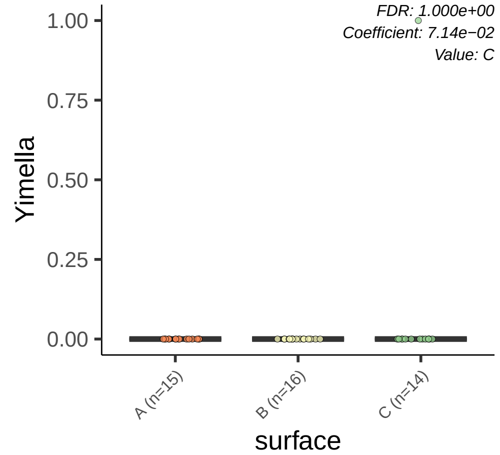

FDR: 1.000e+00
Coefficient: 7.14e−02
Value: C
1.00
0.75
0.50
0.25
0.00
Yimella
A (n=15)
B (n=16)
C (n=14)
surface

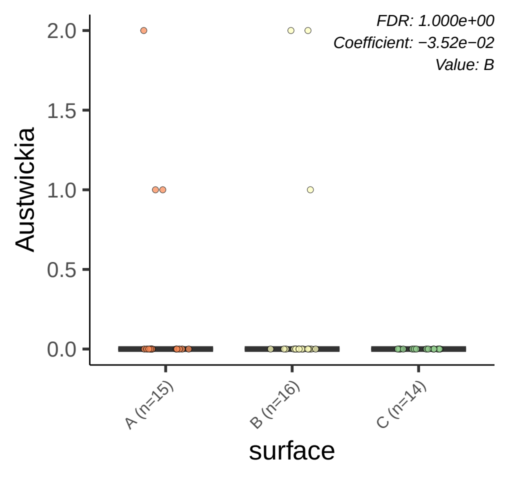

FDR: 1.000e+00
Coefficient: −3.52e−02
Value: B
Austwickia
2.0
1.5
1.0
0.5
0.0
A (n=15)
B (n=16)
C (n=14)
surface

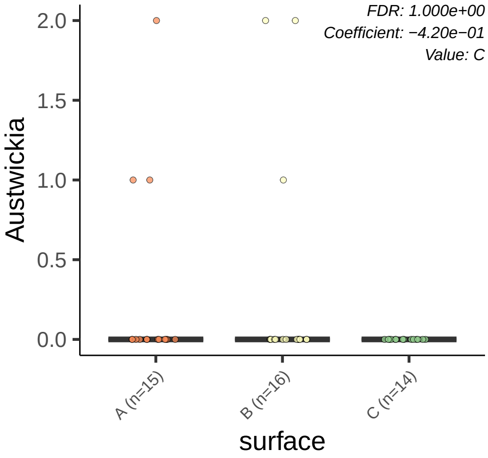

FDR: 1.000e+00
Coefficient: −4.20e−01
Value: C
Austwickia
2.0
1.5
1.0
0.5
0.0
A (n=15)
B (n=16)
C (n=14)
surface

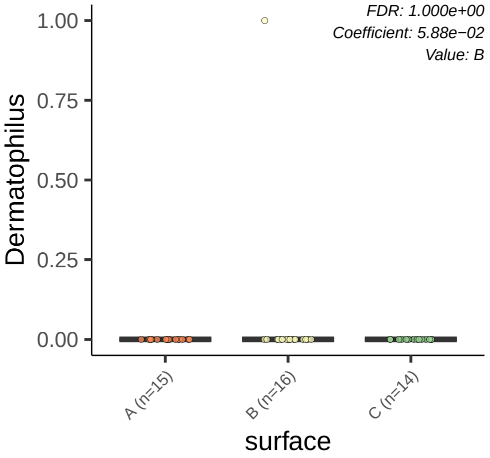

FDR: 1.000e+00
Coefficient: 5.88e−02
Value: B
1.00
0.75
0.50
0.25
0.00
Dermatophilus
A (n=15)
B (n=16)
C (n=14)
surface

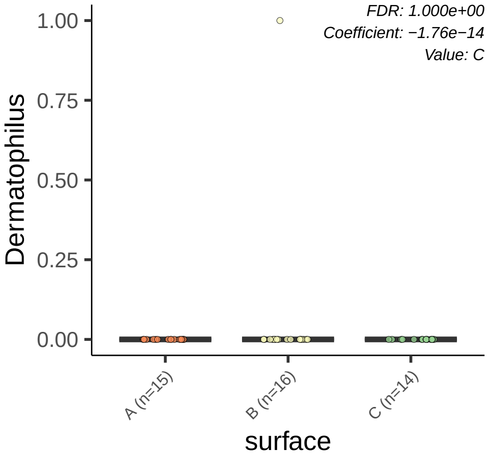
FDR: 1.000e+00
Coefficient: −1.76e−14
Value: C
Dermatophilus
1.00
0.75
0.50
0.25
0.00
A (n=15)
B (n=16)
C (n=14)
surface

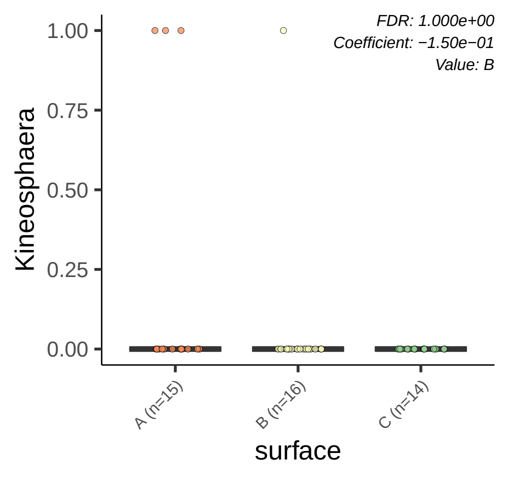
FDR: 1.000e+00
Coefficient: −1.50e−01
Value: B
Kineosphaera
1.00
0.75
0.50
0.25
0.00
A (n=15)
B (n=16)
C (n=14)
surface

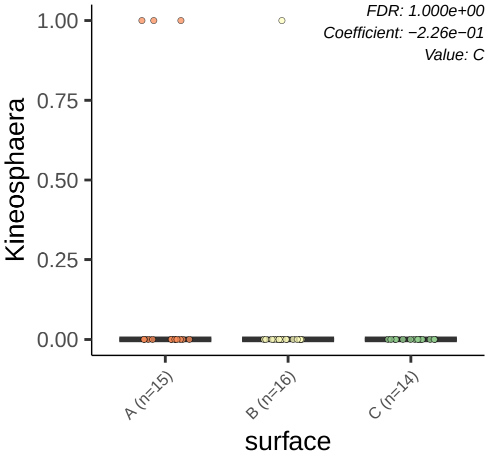
FDR: 1.000e+00
Coefficient: −2.26e−01
Value: C
Kineosphaera
1.00
0.75
0.50
0.25
0.00
A (n=15)
B (n=16)
C (n=14)
surface

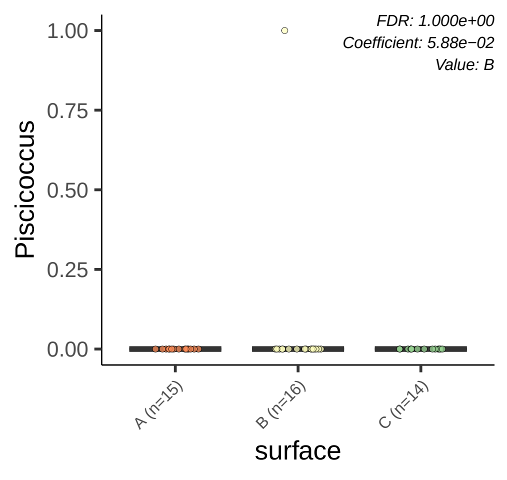
FDR: 1.000e+00
Coefficient: 5.88e−02
Value: B
Piscicoccus
1.00
0.75
0.50
0.25
0.00
A (n=15)
B (n=16)
C (n=14)
surface

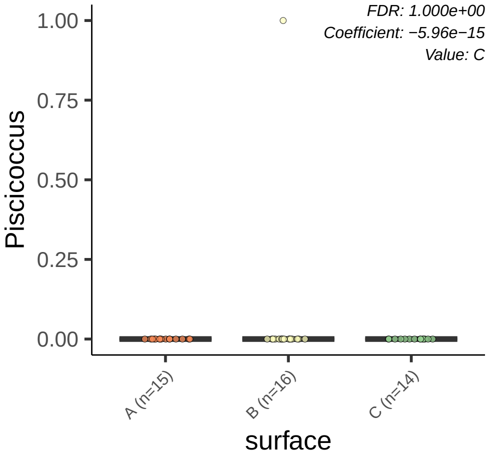

FDR: 1.000e+00
Coefficient: −5.96e−15
Value: C
Piscicoccus
1.00
0.75
0.50
0.25
0.00
A (n=15)
B (n=16)
C (n=14)
surface

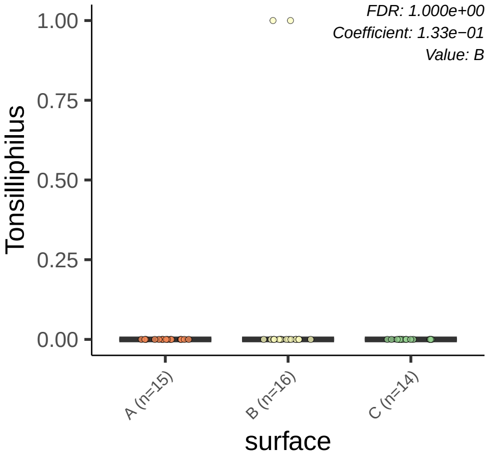
FDR: 1.000e+00
Coefficient: 1.33e−01
Value: B
Tonsilliphilus
1.00
0.75
0.50
0.25
0.00
A (n=15)
B (n=16)
C (n=14)
surface

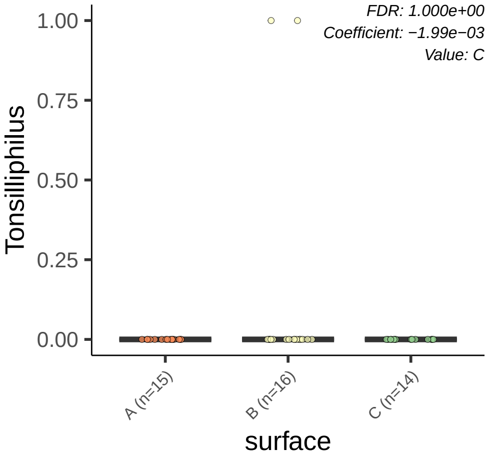

FDR: 1.000e+00
Coefficient: −1.99e−03
Value: C
1.00
0.75
0.50
0.25
0.00
Tonsilliphilus
A (n=15)
B (n=16)
C (n=14)
surface

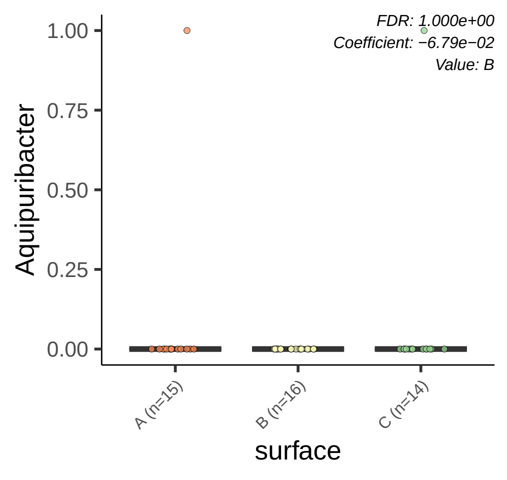
FDR: 1.000e+00
Coefficient: −6.79e−02
Value: B
Aquipuribacter
1.00
0.75
0.50
0.25
0.00
A (n=15)
B (n=16)
C (n=14)
surface

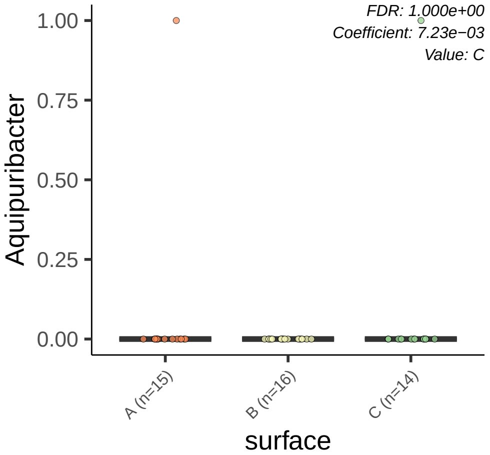

FDR: 1.000e+00
Coefficient: 7.23e−03
Value: C
Aquipuribacter
1.00
0.75
0.50
0.25
0.00
A (n=15)
B (n=16)
C (n=14)
surface

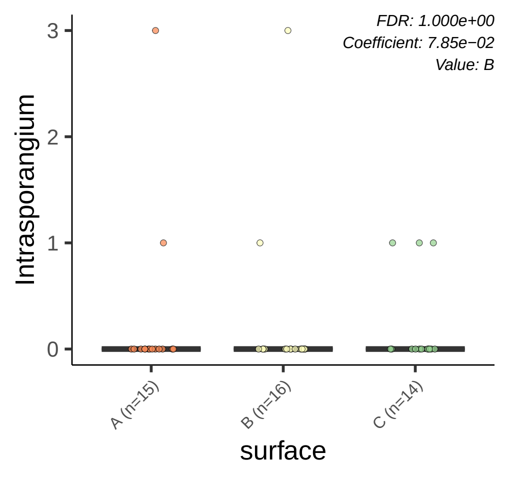

FDR: 1.000e+00
Coefficient: 7.85e−02
Value: B
Intrasporangium
0
1
2
3
A (n=15)
B (n=16)
C (n=14)
surface

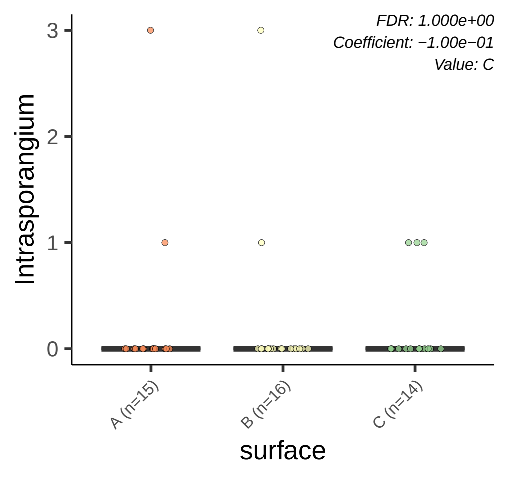
FDR: 1.000e+00
Coefficient: −1.00e−01
Value: C
Intrasporangium
0
1
2
3
A (n=15)
B (n=16)
C (n=14)
surface

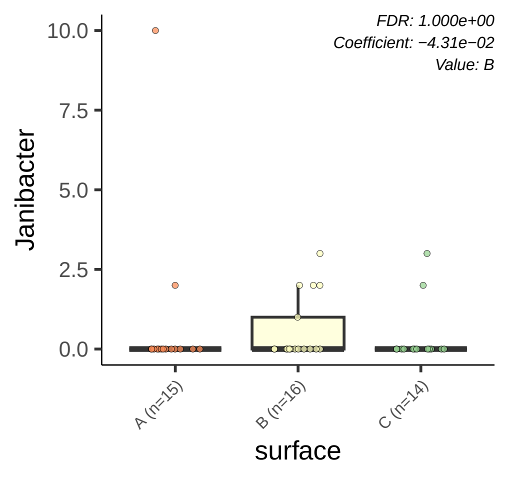
FDR: 1.000e+00
Coefficient: −4.31e−02
Value: B
Janibacter
10.0
7.5
5.0
2.5
0.0
A (n=15)
B (n=16)
C (n=14)
surface

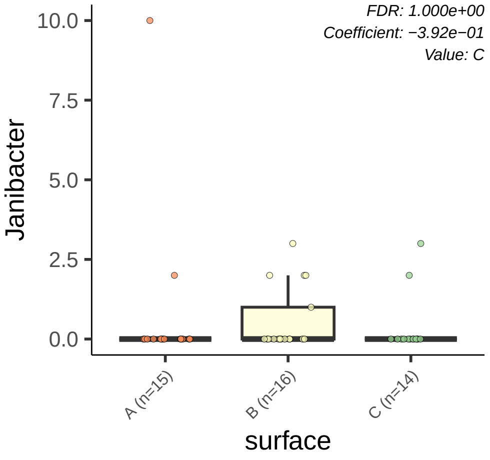
FDR: 1.000e+00
Coefficient: −3.92e−01
Value: C
Janibacter
10.0
7.5
5.0
2.5
0.0
A (n=15)
B (n=16)
C (n=14)
surface

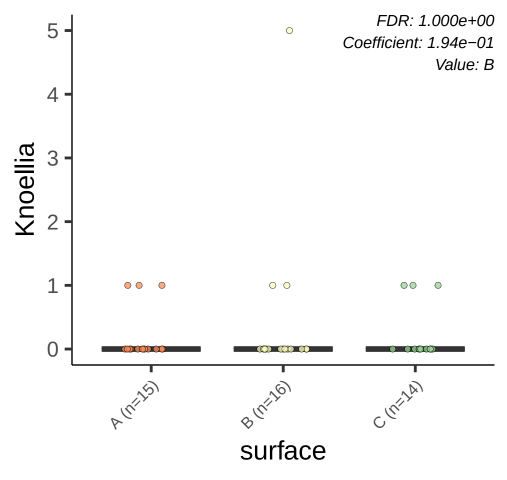
FDR: 1.000e+00
Coefficient: 1.94e−01
Value: B
Knoellia
5
4
3
2
1
0
A (n=15)
B (n=16)
C (n=14)
surface

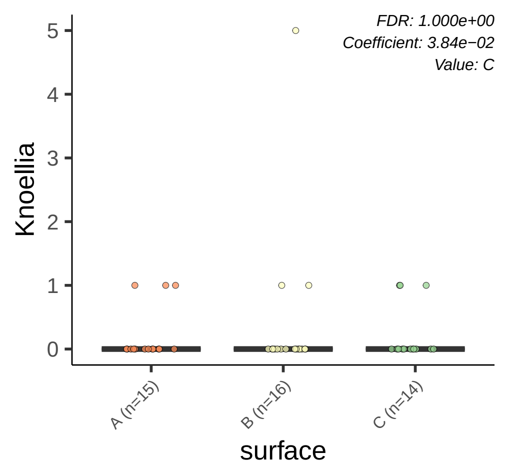
FDR: 1.000e+00
Coefficient: 3.84e−02
Value: C
Knoellia
0
1
2
3
4
5
A (n=15)
B (n=16)
C (n=14)
surface

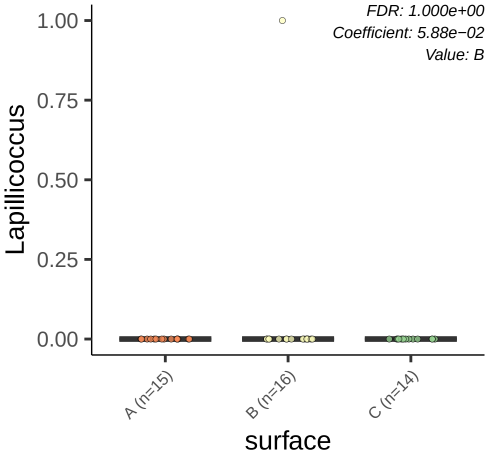

FDR: 1.000e+00
Coefficient: 5.88e−02
Value: B
Lapillicoccus
1.00
0.75
0.50
0.25
0.00
A (n=15)
B (n=16)
C (n=14)
surface

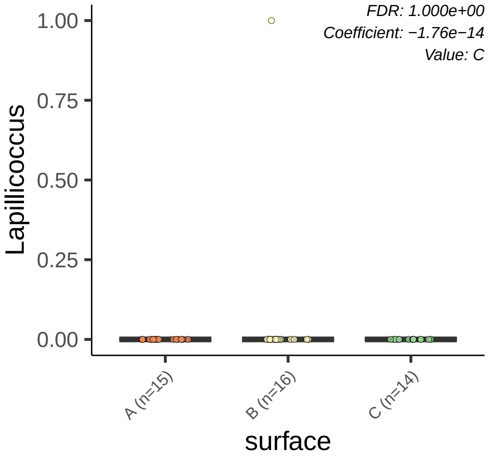
FDR: 1.000e+00
Coefficient: −1.76e−14
Value: C
Lapillicoccus
1.00
0.75
0.50
0.25
0.00
A (n=15)
B (n=16)
C (n=14)
surface

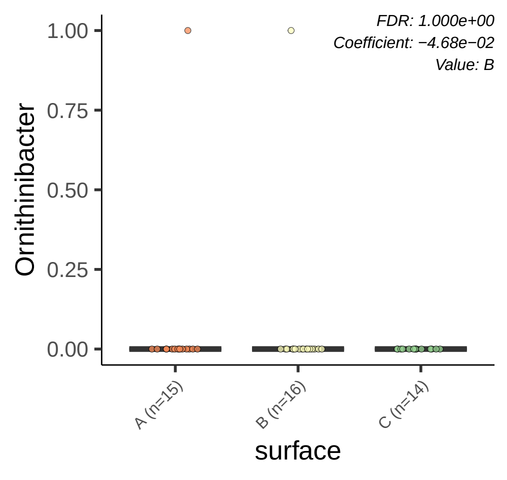

FDR: 1.000e+00
Coefficient: −4.68e−02
Value: B
Ornithinibacter
1.00
0.75
0.50
0.25
0.00
A (n=15)
B (n=16)
C (n=14)
surface

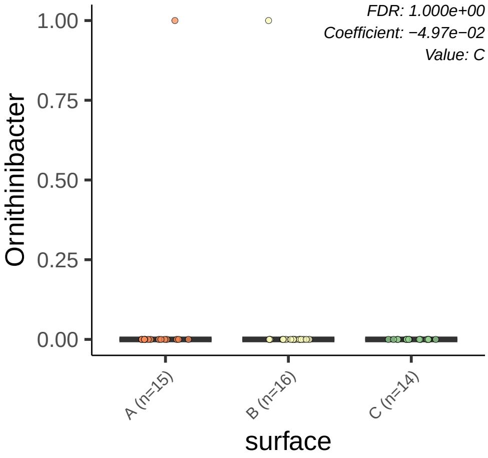

FDR: 1.000e+00
Coefficient: −4.97e−02
Value: C
Ornithinibacter
1.00
0.75
0.50
0.25
0.00
A (n=15)
B (n=16)
C (n=14)
surface

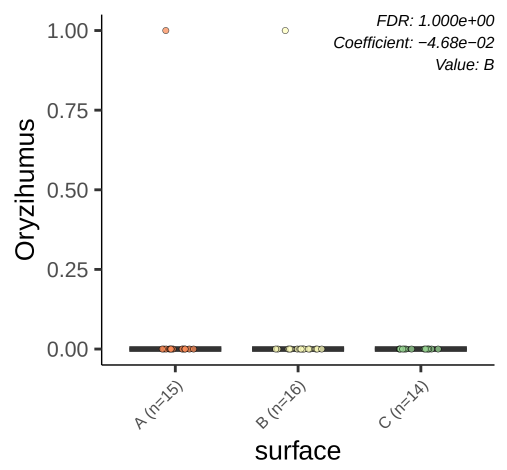

FDR: 1.000e+00
Coefficient: −4.68e−02
Value: B
Oryzihumus
1.00
0.75
0.50
0.25
0.00
A (n=15)
B (n=16)
C (n=14)
surface

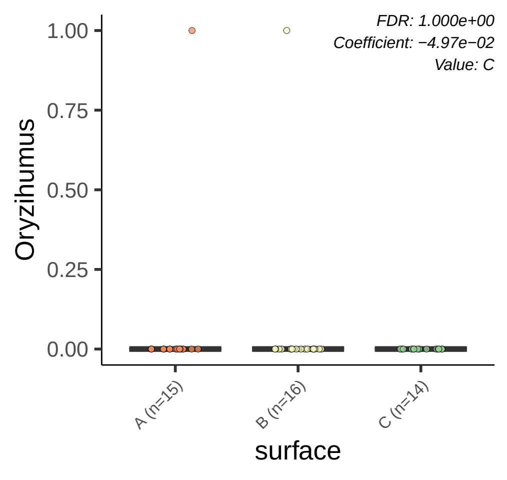

FDR: 1.000e+00
Coefficient: −4.97e−02
Value: C
Oryzihumus
1.00
0.75
0.50
0.25
0.00
A (n=15)
B (n=16)
C (n=14)
surface

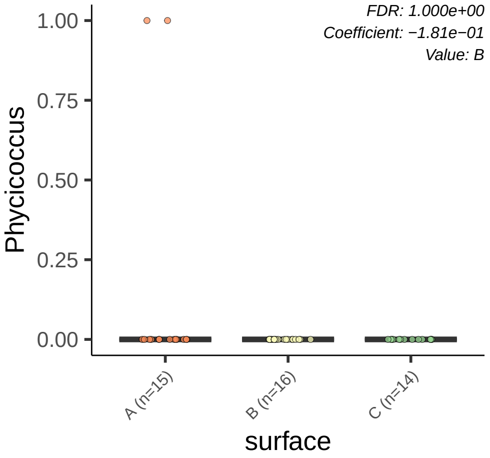

FDR: 1.000e+00
Coefficient: −1.81e−01
Value: B
Phycicoccus
1.00
0.75
0.50
0.25
0.00
A (n=15)
B (n=16)
C (n=14)
surface

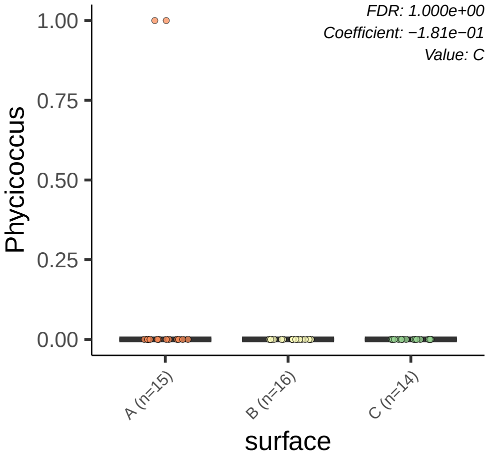

FDR: 1.000e+00
Coefficient: −1.81e−01
Value: C
Phycicoccus
1.00
0.75
0.50
0.25
0.00
A (n=15)
B (n=16)
C (n=14)
surface

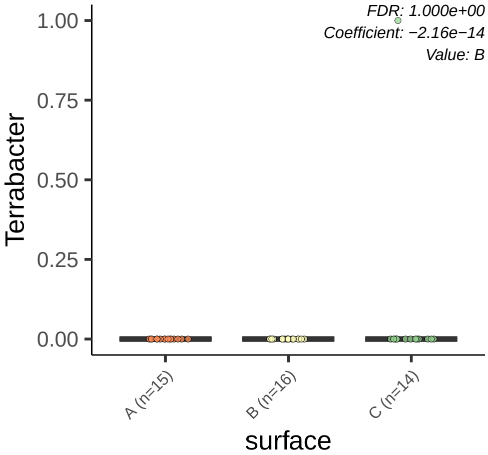
FDR: 1.000e+00
Coefficient: −2.16e−14
Value: B
Terrabacter
1.00
0.75
0.50
0.25
0.00
A (n=15)
B (n=16)
C (n=14)
surface

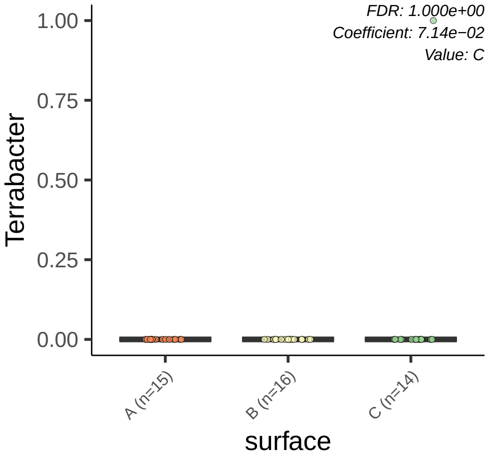
FDR: 1.000e+00
Coefficient: 7.14e−02
Value: C
Terrabacter
1.00
0.75
0.50
0.25
0.00
A (n=15)
B (n=16)
C (n=14)
surface

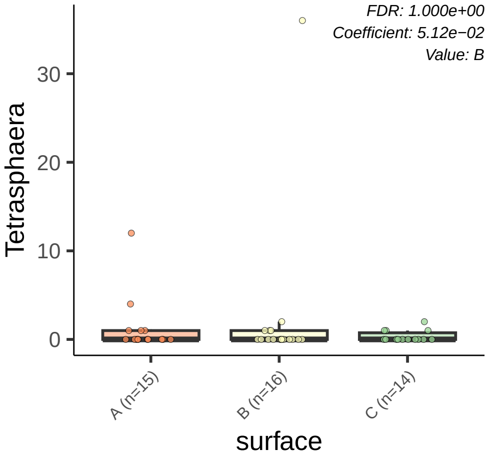
FDR: 1.000e+00
Coefficient: 5.12e−02
Value: B
Tetrasphaera
0
10
20
30
A (n=15)
B (n=16)
C (n=14)
surface

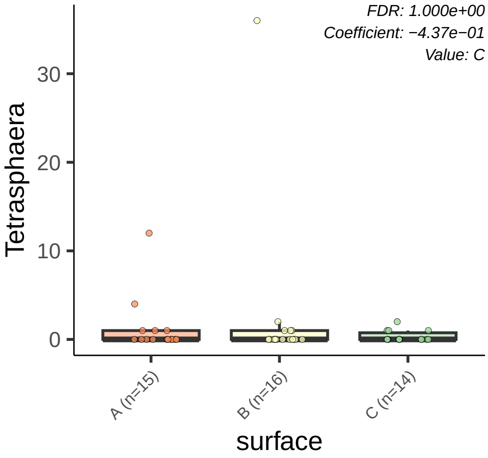

FDR: 1.000e+00
Coefficient: −4.37e−01
Value: C
Tetrasphaera
30
20
10
0
A (n=15)
B (n=16)
C (n=14)
surface

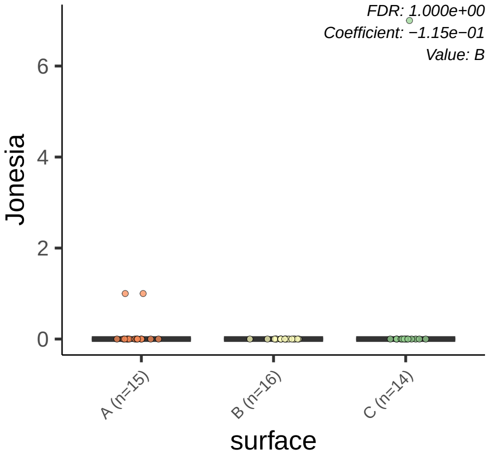
FDR: 1.000e+00
Coefficient: −1.15e−01
Value: B
Jonesia
6
4
2
0
A (n=15)
B (n=16)
C (n=14)
surface

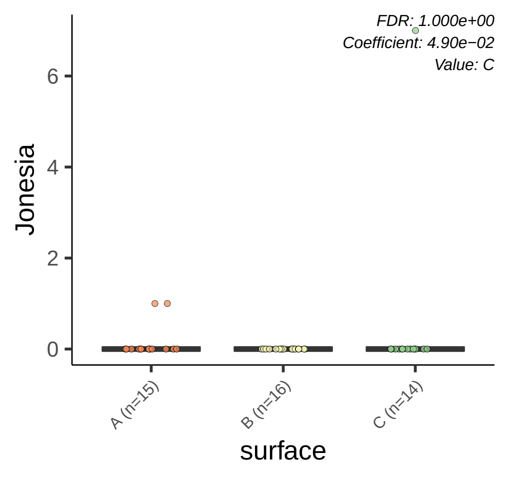
FDR: 1.000e+00
Coefficient: 4.90e−02
Value: C
Jonesia
6
4
2
0
A (n=15)
B (n=16)
C (n=14)
surface

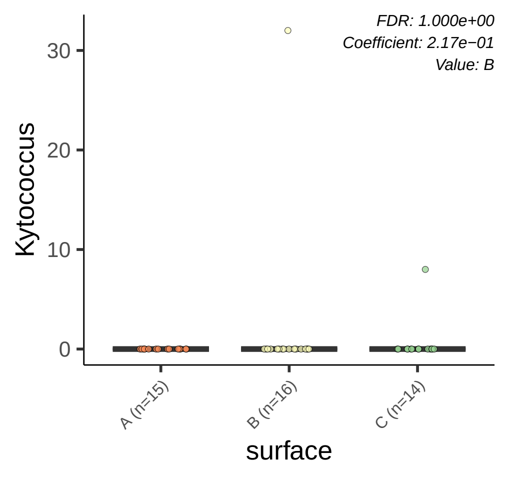
FDR: 1.000e+00
Coefficient: 2.17e−01
Value: B
Kytococcus
30
20
10
0
A (n=15)
B (n=16)
C (n=14)
surface

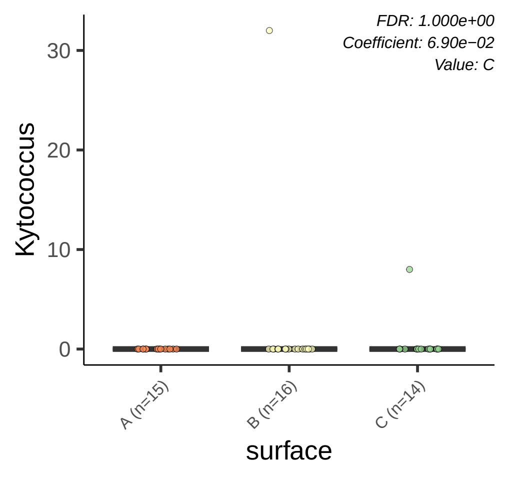
FDR: 1.000e+00
Coefficient: 6.90e−02
Value: C
Kytococcus
30
20
10
0
A (n=15)
B (n=16)
C (n=14)
surface

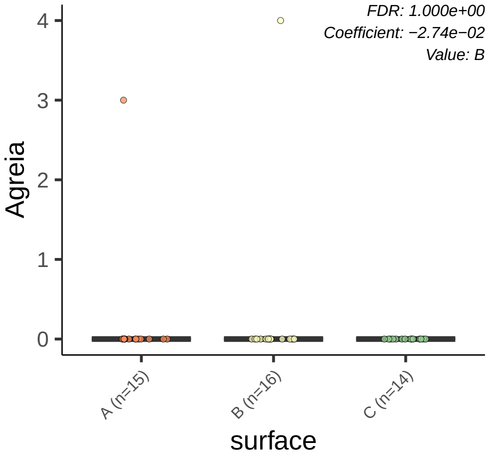

FDR: 1.000e+00
Coefficient: −2.74e−02
Value: B
Agreia
4
3
2
1
0
A (n=15)
B (n=16)
C (n=14)
surface

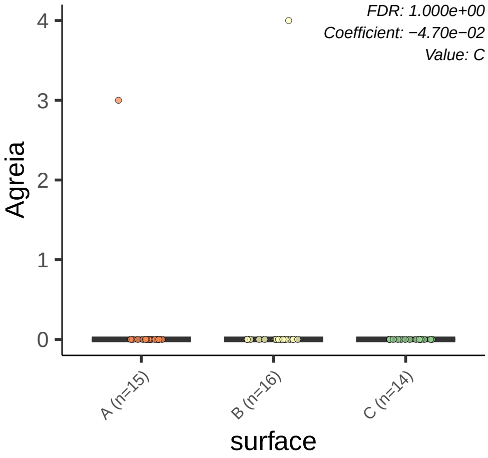

FDR: 1.000e+00
Coefficient: −4.70e−02
Value: C
Agreia
0
1
2
3
4
A (n=15)
B (n=16)
C (n=14)
surface

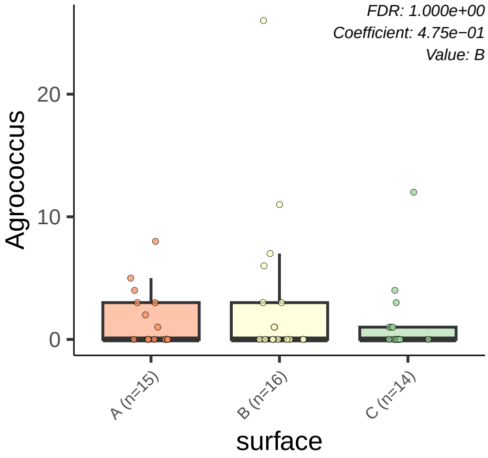

FDR: 1.000e+00
Coefficient: 4.75e−01
Value: B
Agrococcus
0
10
20
A (n=15)
B (n=16)
C (n=14)
surface

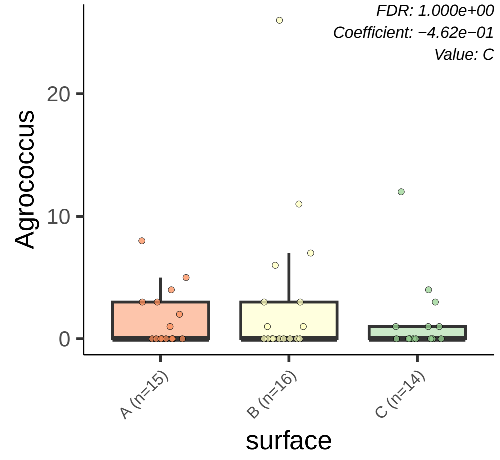

FDR: 1.000e+00
Coefficient: −4.62e−01
Value: C
Agrococcus
20
10
0
A (n=15)
B (n=16)
C (n=14)
surface

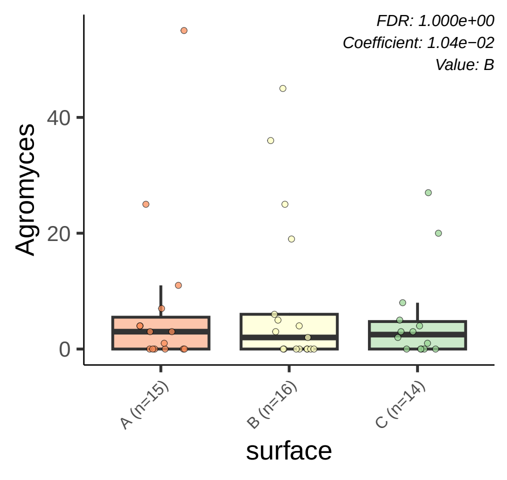
FDR: 1.000e+00
Coefficient: 1.04e−02
Value: B
Agromyces
0
20
40
A (n=15)
B (n=16)
C (n=14)
surface

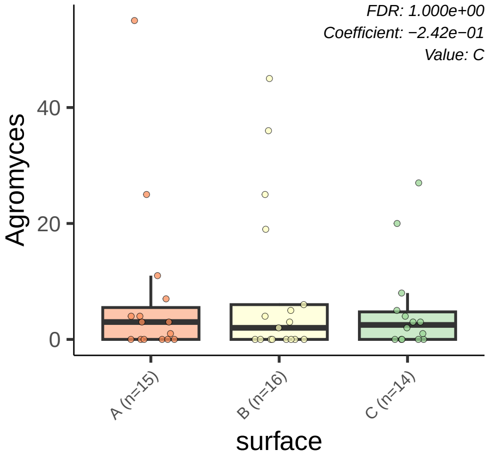
FDR: 1.000e+00
Coefficient: −2.42e−01
Value: C
Agromyces
0
20
40
A (n=15)
B (n=16)
C (n=14)
surface

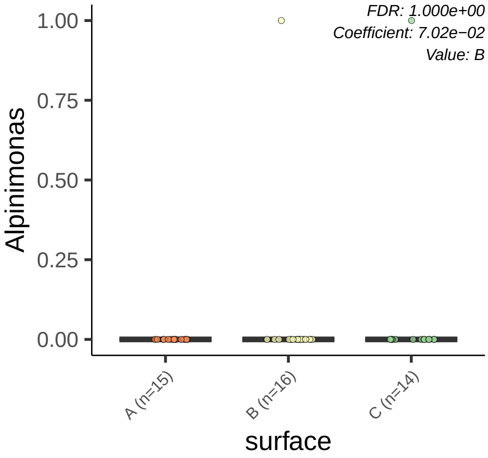

FDR: 1.000e+00
Coefficient: 7.02e−02
Value: B
Alpinimonas
1.00
0.75
0.50
0.25
0.00
A (n=15)
B (n=16)
C (n=14)
surface

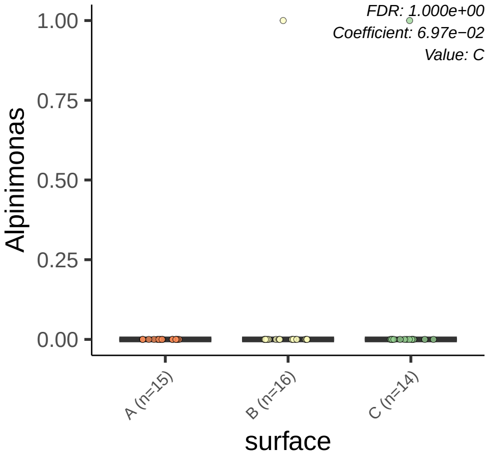
FDR: 1.000e+00
Coefficient: 6.97e−02
Value: C
Alpinimonas
1.00
0.75
0.50
0.25
0.00
A (n=15)
B (n=16)
C (n=14)
surface

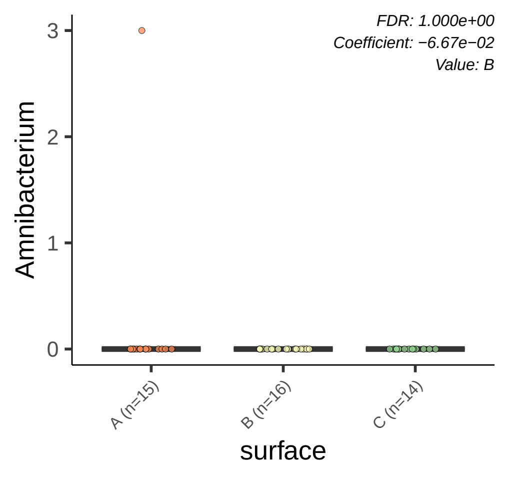
FDR: 1.000e+00
Coefficient: −6.67e−02
Value: B
Amnibacterium
3
2
1
0
A (n=15)
B (n=16)
C (n=14)
surface

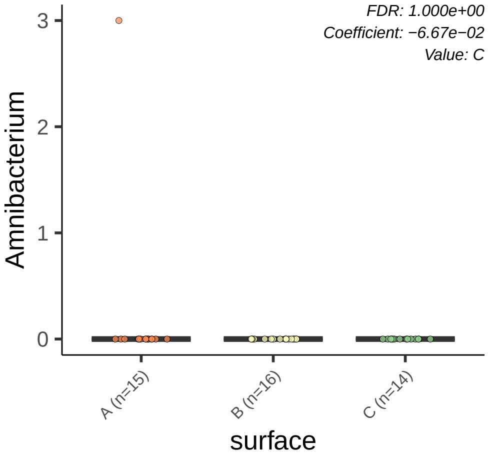

FDR: 1.000e+00
Coefficient: −6.67e−02
Value: C
Amnibacterium
3
2
1
0
A (n=15)
B (n=16)
C (n=14)
surface

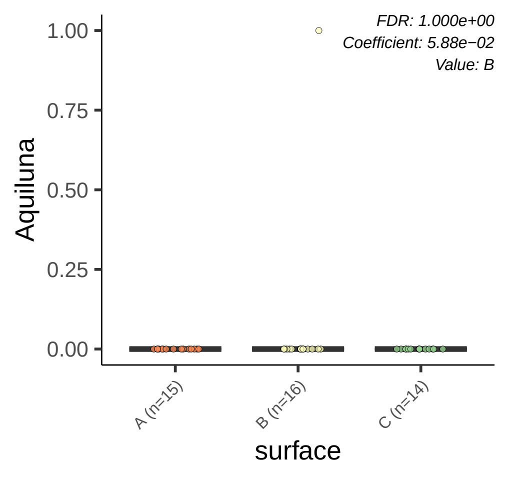
FDR: 1.000e+00
Coefficient: 5.88e−02
Value: B
1.00
0.75
0.50
0.25
0.00
Aquiluna
A (n=15)
B (n=16)
C (n=14)
surface

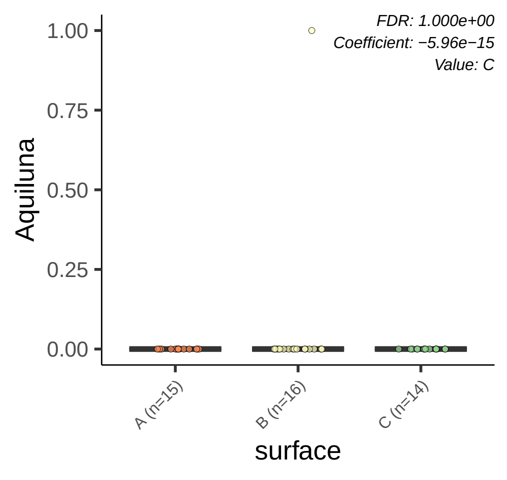

FDR: 1.000e+00
Coefficient: −5.96e−15
Value: C
Aquiluna
1.00
0.75
0.50
0.25
0.00
A (n=15)
B (n=16)
C (n=14)
surface

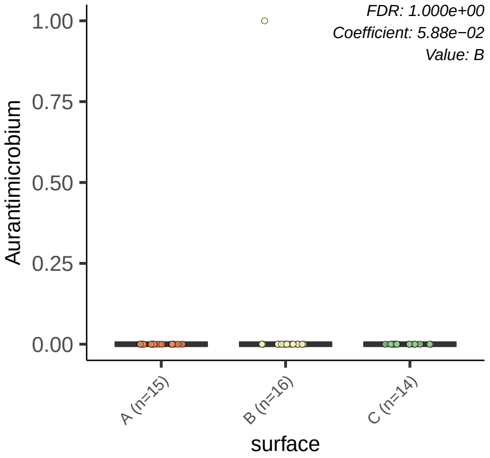
FDR: 1.000e+00
Coefficient: 5.88e−02
Value: B
1.00
0.75
0.50
0.25
0.00
Aurantimicrobium
A (n=15)
B (n=16)
C (n=14)
surface

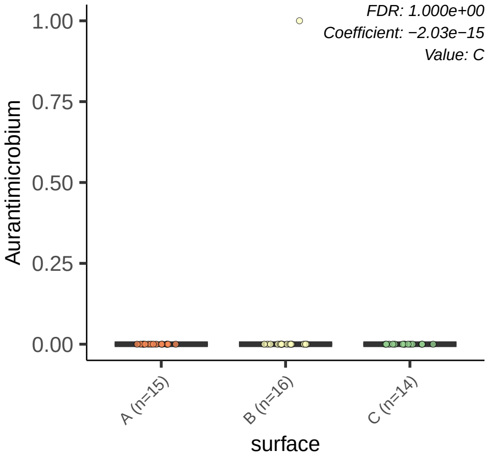
FDR: 1.000e+00
Coefficient: −2.03e−15
Value: C
Aurantimicrobium
1.00
0.75
0.50
0.25
0.00
A (n=15)
B (n=16)
C (n=14)
surface

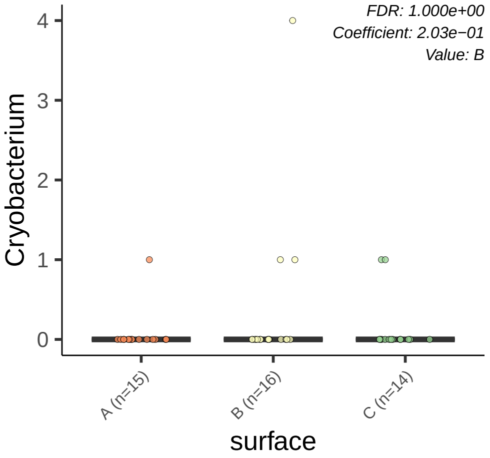

FDR: 1.000e+00
Coefficient: 2.03e−01
Value: B
Cryobacterium
4
3
2
1
0
A (n=15)
B (n=16)
C (n=14)
surface

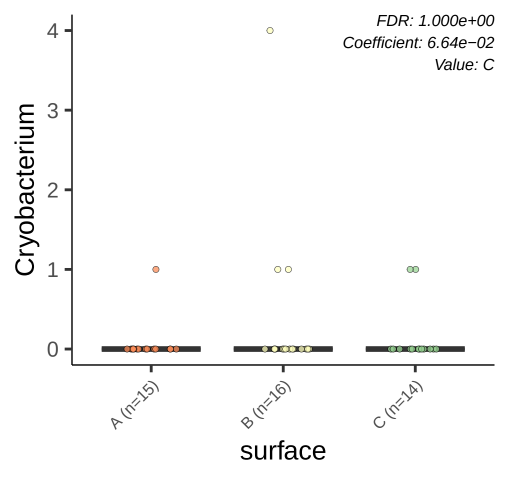
FDR: 1.000e+00
Coefficient: 6.64e−02
Value: C
Cryobacterium
4
3
2
1
0
A (n=15)
B (n=16)
C (n=14)
surface

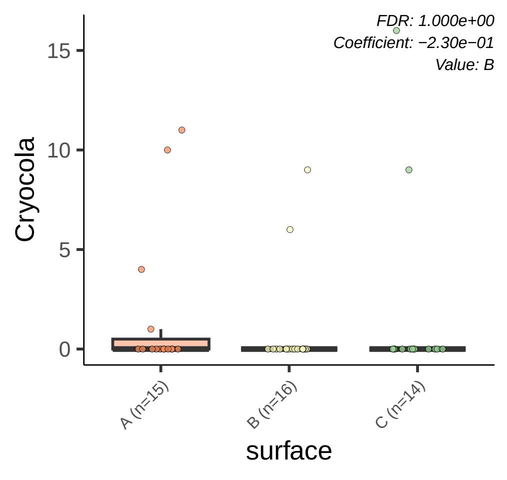

FDR: 1.000e+00
Coefficient: −2.30e−01
Value: B
Cryocola
15
10
5
0
A (n=15)
B (n=16)
C (n=14)
surface

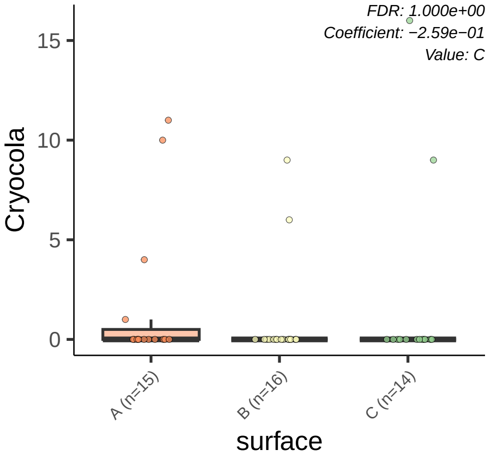
FDR: 1.000e+00
Coefficient: −2.59e−01
Value: C
Cryocola
15
10
5
0
A (n=15)
B (n=16)
C (n=14)
surface

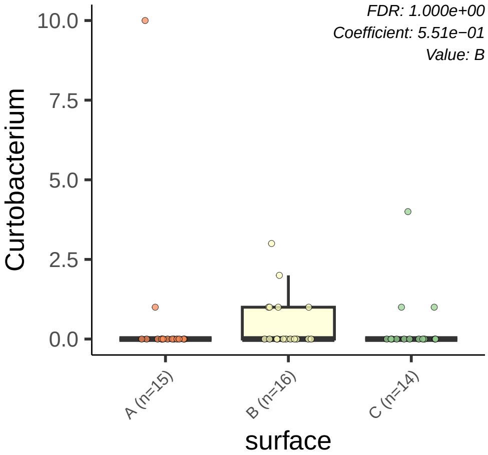

FDR: 1.000e+00
Coefficient: 5.51e−01
Value: B
Curtobacterium
10.0
7.5
5.0
2.5
0.0
A (n=15)
B (n=16)
C (n=14)
surface

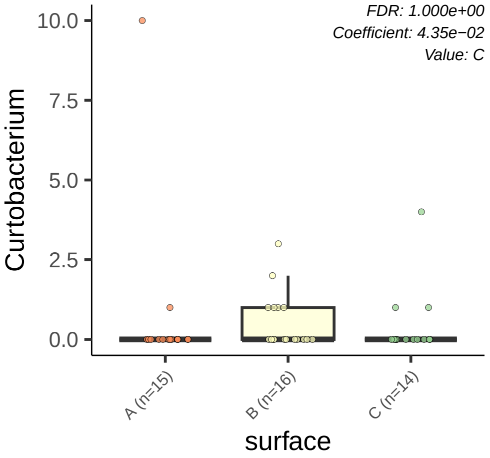
FDR: 1.000e+00
Coefficient: 4.35e−02
Value: C
Curtobacterium
10.0
7.5
5.0
2.5
0.0
A (n=15)
B (n=16)
C (n=14)
surface

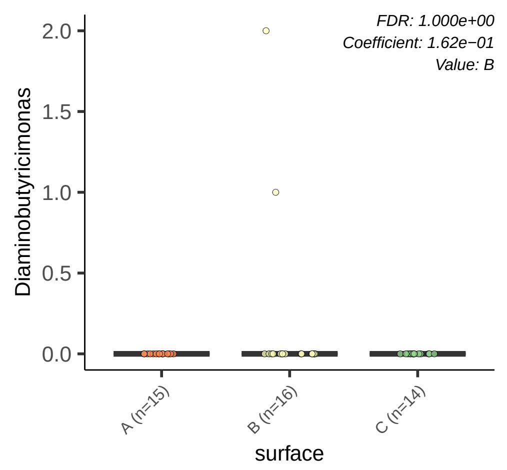

FDR: 1.000e+00
Coefficient: 1.62e−01
Value: B
Diaminobutyricimonas
2.0
1.5
1.0
0.5
0.0
A (n=15)
B (n=16)
C (n=14)
surface

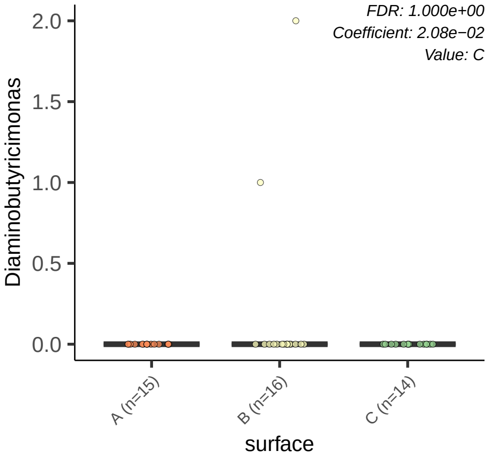

FDR: 1.000e+00
Coefficient: 2.08e−02
Value: C
Diaminobutyricimonas
2.0
1.5
1.0
0.5
0.0
A (n=15)
B (n=16)
C (n=14)
surface

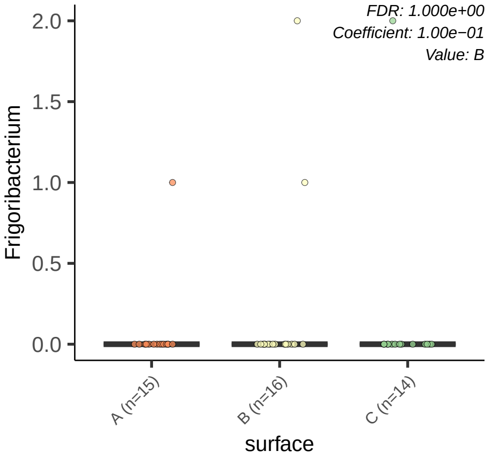
FDR: 1.000e+00
Coefficient: 1.00e−01
Value: B
Frigoribacterium
2.0
1.5
1.0
0.5
0.0
A (n=15)
B (n=16)
C (n=14)
surface

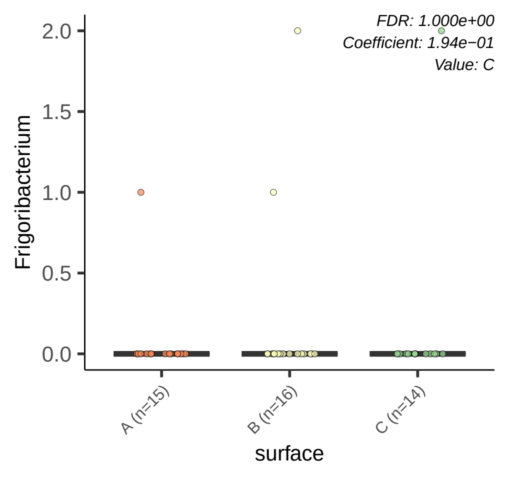
FDR: 1.000e+00
Coefficient: 1.94e−01
Value: C
Frigoribacterium
2.0
1.5
1.0
0.5
0.0
A (n=15)
B (n=16)
C (n=14)
surface

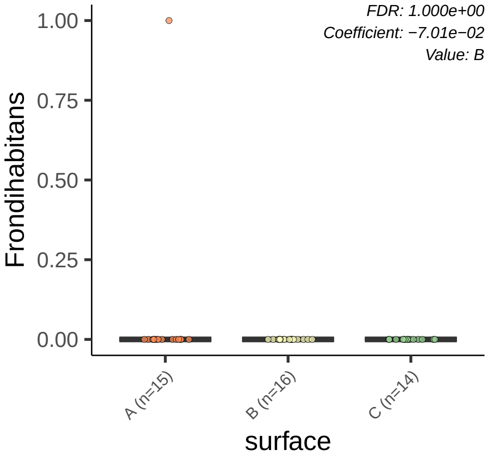

FDR: 1.000e+00
Coefficient: −7.01e−02
Value: B
Frondihabitans
1.00
0.75
0.50
0.25
0.00
A (n=15)
B (n=16)
C (n=14)
surface

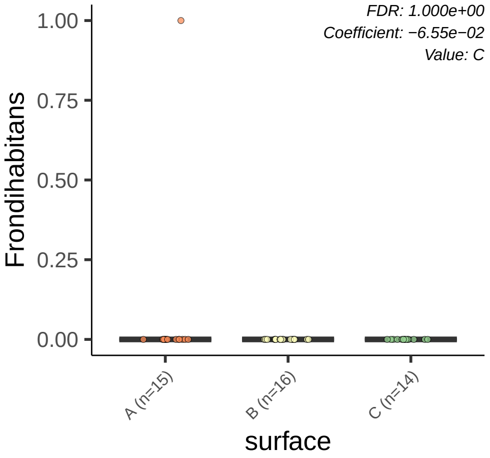
FDR: 1.000e+00
Coefficient: −6.55e−02
Value: C
Frondihabitans
1.00
0.75
0.50
0.25
0.00
A (n=15)
B (n=16)
C (n=14)
surface

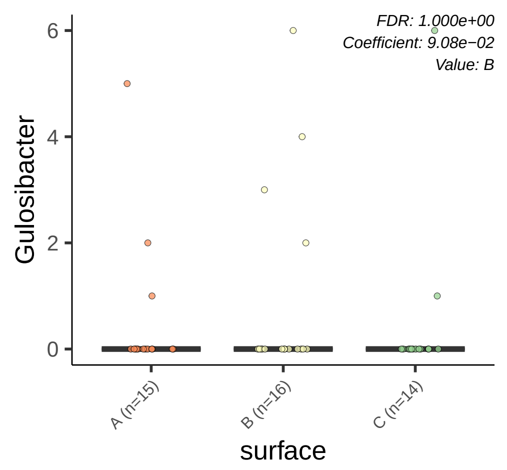

FDR: 1.000e+00
Coefficient: 9.08e−02
Value: B
Gulosibacter
6
4
2
0
A (n=15)
B (n=16)
C (n=14)
surface

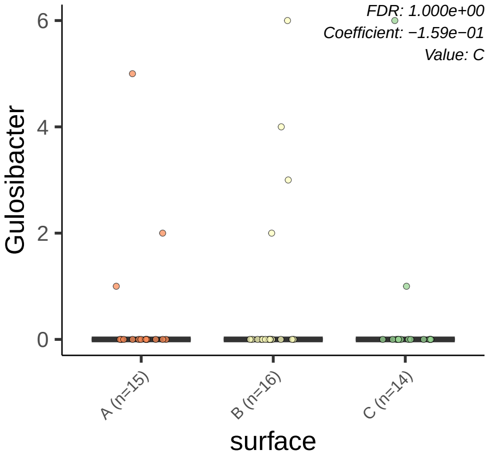

FDR: 1.000e+00
Coefficient: −1.59e−01
Value: C
Gulosibacter
0
2
4
6
A (n=15)
B (n=16)
C (n=14)
surface

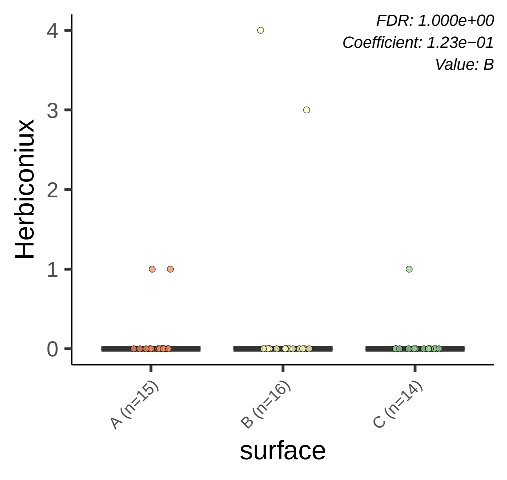

FDR: 1.000e+00
Coefficient: 1.23e−01
Value: B
Herbiconiux
4
3
2
1
0
A (n=15)
B (n=16)
C (n=14)
surface

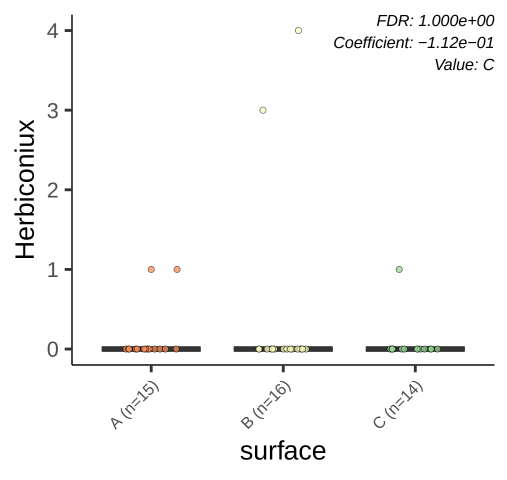
FDR: 1.000e+00
Coefficient: −1.12e−01
Value: C
Herbiconiux
0
1
2
3
4
A (n=15)
B (n=16)
C (n=14)
surface

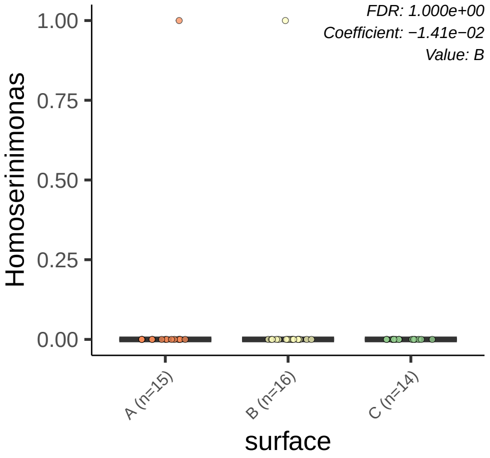

FDR: 1.000e+00
Coefficient: −1.41e−02
Value: B
Homoserinimonas
1.00
0.75
0.50
0.25
0.00
A (n=15)
B (n=16)
C (n=14)
surface

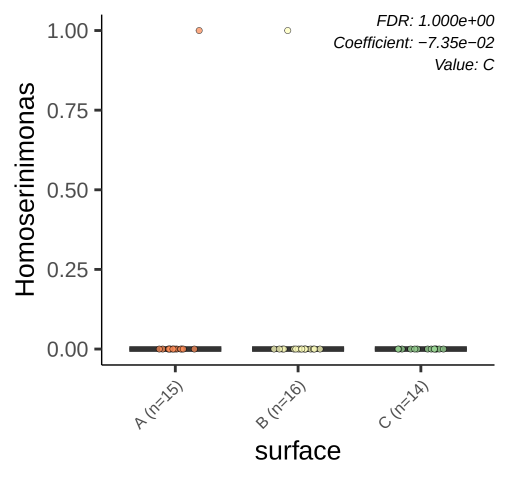
FDR: 1.000e+00
Coefficient: −7.35e−02
Value: C
Homoserinimonas
1.00
0.75
0.50
0.25
0.00
A (n=15)
B (n=16)
C (n=14)
surface

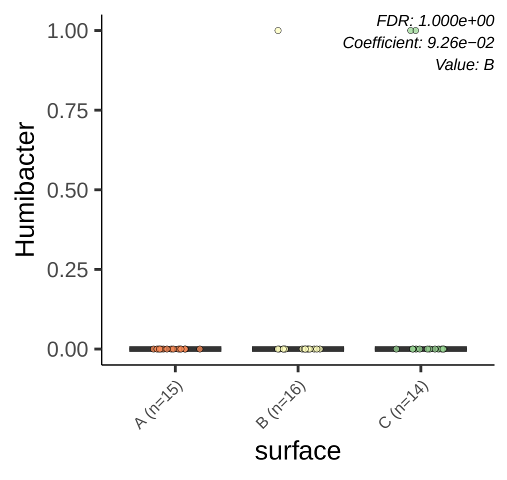
FDR: 1.000e+00
Coefficient: 9.26e−02
Value: B
Humibacter
1.00
0.75
0.50
0.25
0.00
A (n=15)
B (n=16)
C (n=14)
surface

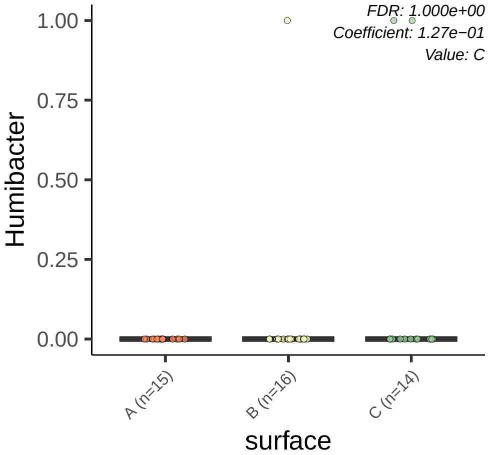

FDR: 1.000e+00
Coefficient: 1.27e−01
Value: C
Humibacter
1.00
0.75
0.50
0.25
0.00
A (n=15)
B (n=16)
C (n=14)
surface

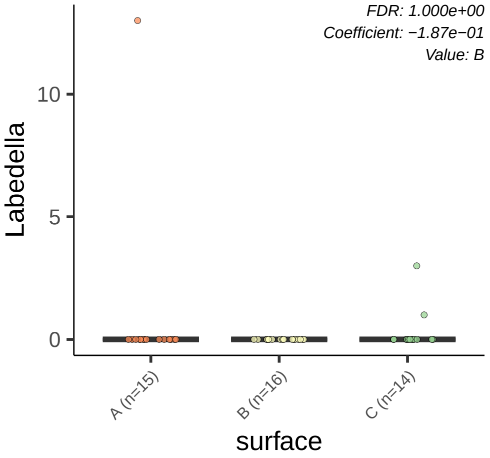
FDR: 1.000e+00
Coefficient: −1.87e−01
Value: B
Labedella
0
5
10
A (n=15)
B (n=16)
C (n=14)
surface

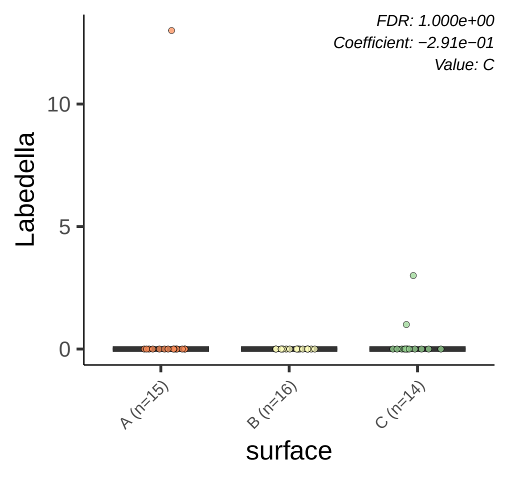
FDR: 1.000e+00
Coefficient: −2.91e−01
Value: C
Labedella
10
5
0
A (n=15)
B (n=16)
C (n=14)
surface

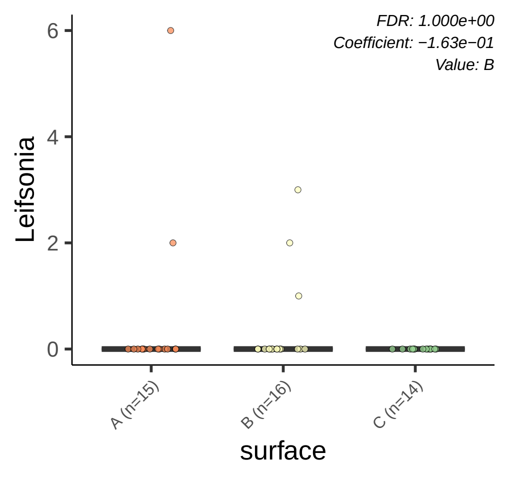
FDR: 1.000e+00
Coefficient: −1.63e−01
Value: B
Leifsonia
6
4
2
0
A (n=15)
B (n=16)
C (n=14)
surface

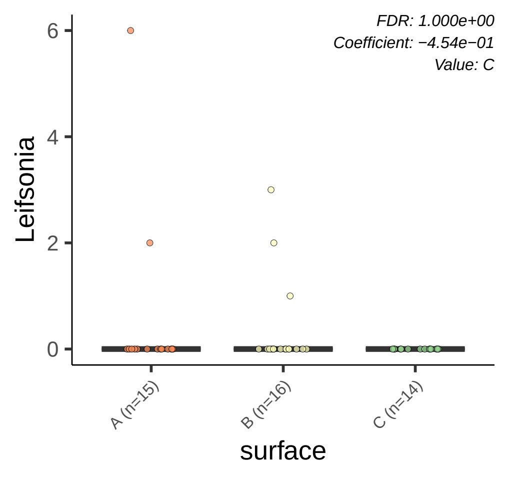
FDR: 1.000e+00
Coefficient: −4.54e−01
Value: C
Leifsonia
6
4
2
0
A (n=15)
B (n=16)
C (n=14)
surface

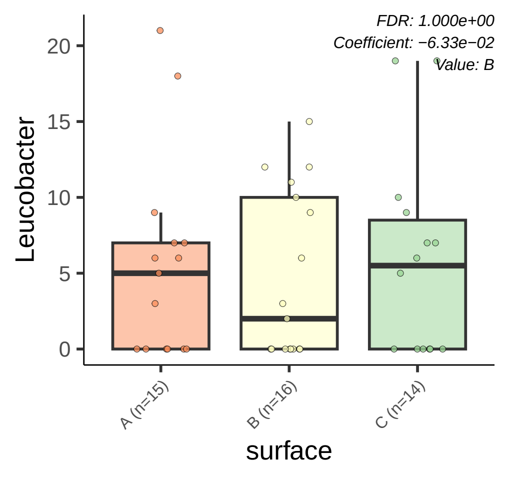

FDR: 1.000e+00
Coefficient: −6.33e−02
Value: B
Leucobacter
0
5
10
15
20
A (n=15)
B (n=16)
C (n=14)
surface

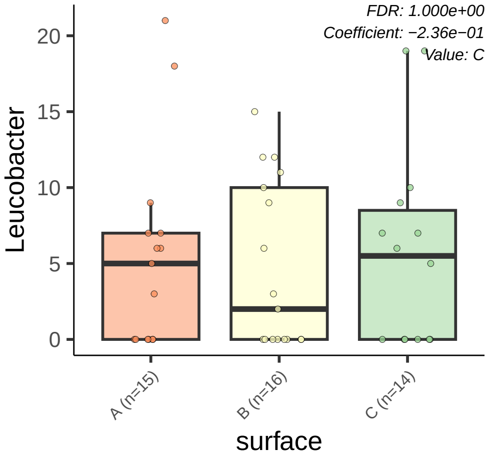
FDR: 1.000e+00
Coefficient: −2.36e−01
Value: C
Leucobacter
20
15
10
5
0
A (n=15)
B (n=16)
C (n=14)
surface

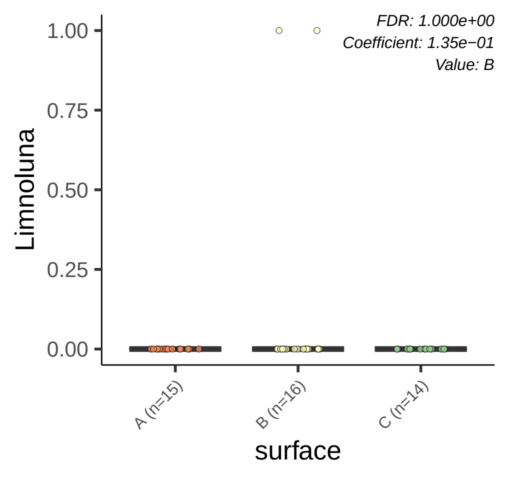
FDR: 1.000e+00
Coefficient: 1.35e−01
Value: B
Limnoluna
1.00
0.75
0.50
0.25
0.00
A (n=15)
B (n=16)
C (n=14)
surface

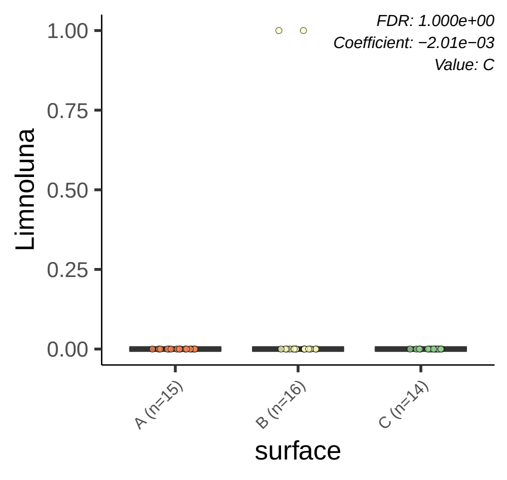
FDR: 1.000e+00
Coefficient: −2.01e−03
Value: C
Limnoluna
1.00
0.75
0.50
0.25
0.00
A (n=15)
B (n=16)
C (n=14)
surface

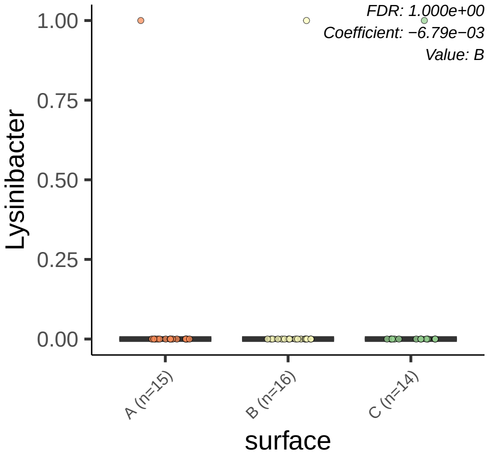

FDR: 1.000e+00
Coefficient: −6.79e−03
Value: B
Lysinibacter
1.00
0.75
0.50
0.25
0.00
A (n=15)
B (n=16)
C (n=14)
surface

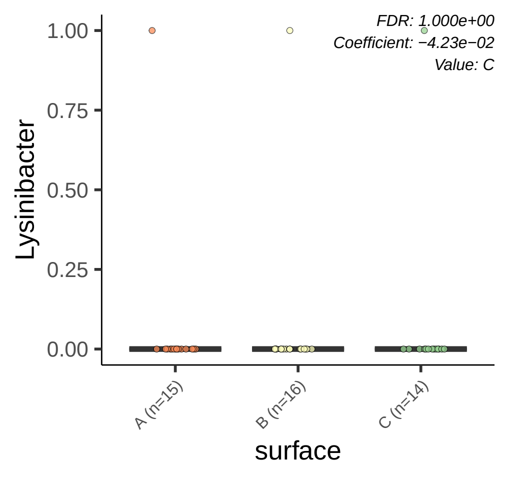

FDR: 1.000e+00
Coefficient: −4.23e−02
Value: C
Lysinibacter
1.00
0.75
0.50
0.25
0.00
A (n=15)
B (n=16)
C (n=14)
surface

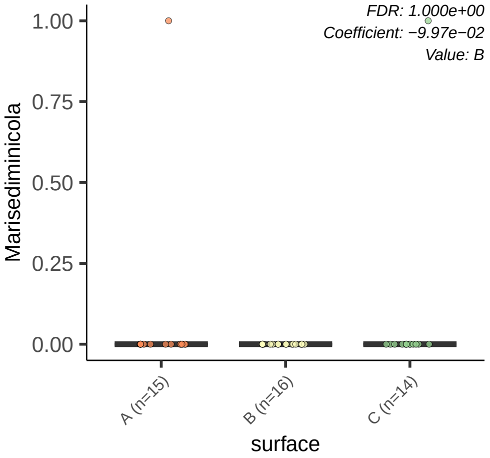
FDR: 1.000e+00
Coefficient: −9.97e−02
Value: B
Marisediminicola
1.00
0.75
0.50
0.25
0.00
A (n=15)
B (n=16)
C (n=14)
surface

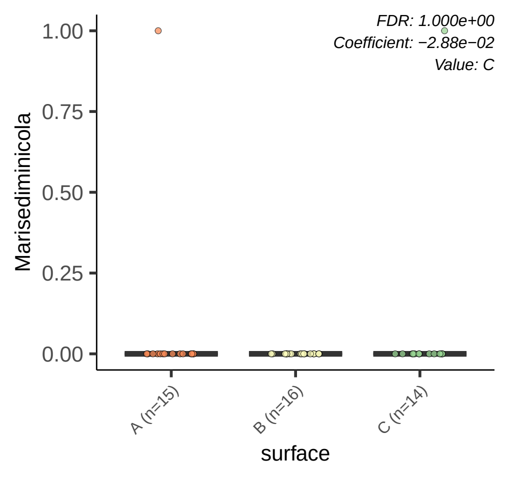
FDR: 1.000e+00
Coefficient: −2.88e−02
Value: C
Marisediminicola
1.00
0.75
0.50
0.25
0.00
A (n=15)
B (n=16)
C (n=14)
surface

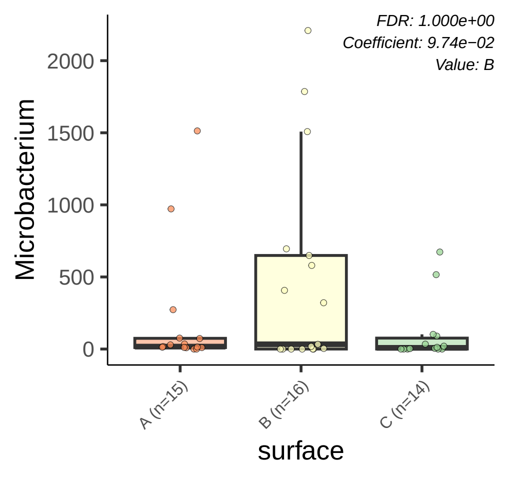
FDR: 1.000e+00
Coefficient: 9.74e−02
Value: B
Microbacterium
2000
1500
1000
500
0
A (n=15)
B (n=16)
C (n=14)
surface

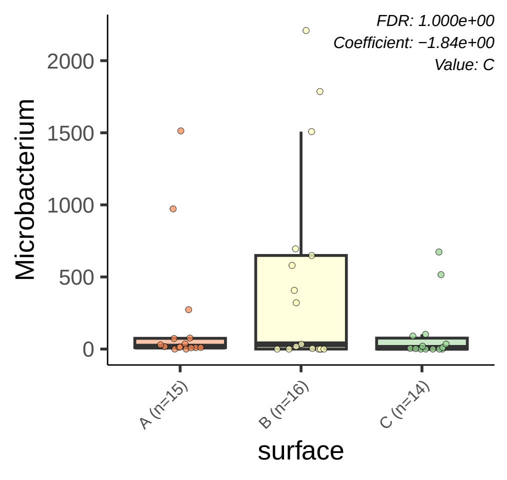
FDR: 1.000e+00
Coefficient: −1.84e+00
Value: C
Microbacterium
2000
1500
1000
500
0
A (n=15)
B (n=16)
C (n=14)
surface

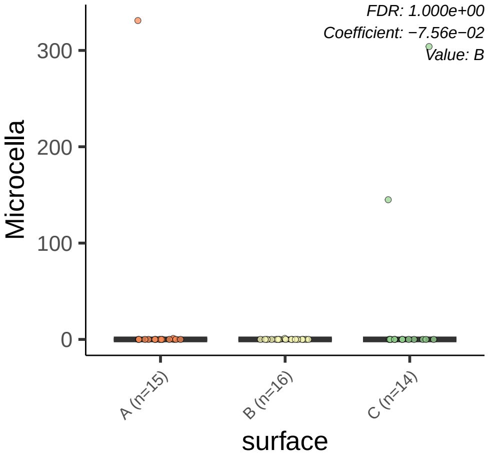
FDR: 1.000e+00
Coefficient: −7.56e−02
Value: B
Microcella
300
200
100
0
A (n=15)
B (n=16)
C (n=14)
surface

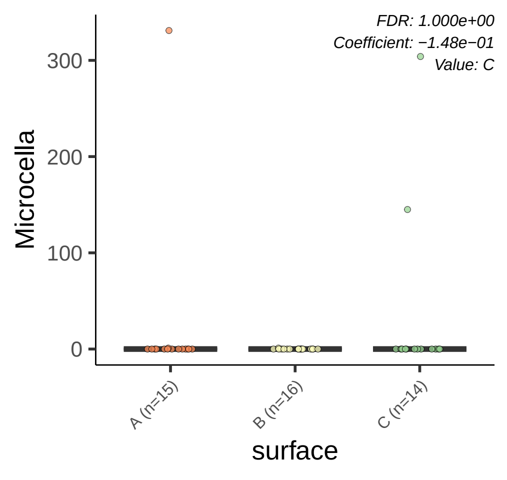

FDR: 1.000e+00
Coefficient: −1.48e−01
Value: C
Microcella
300
200
100
0
A (n=15)
B (n=16)
C (n=14)
surface

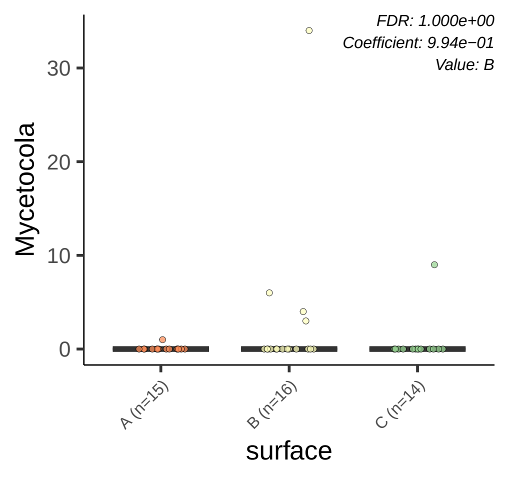

FDR: 1.000e+00
Coefficient: 9.94e−01
Value: B
Mycetocola
0
10
20
30
A (n=15)
B (n=16)
C (n=14)
surface

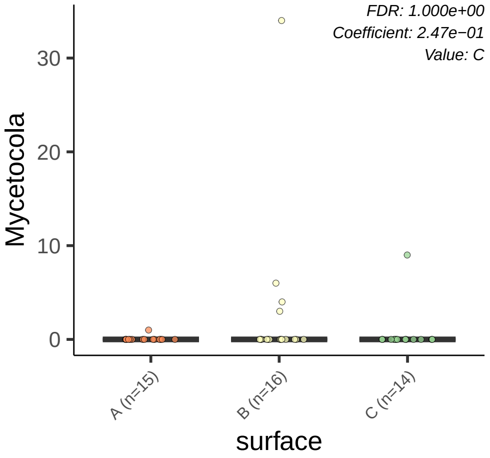

FDR: 1.000e+00
Coefficient: 2.47e−01
Value: C
Mycetocola
30
20
10
0
A (n=15)
B (n=16)
C (n=14)
surface

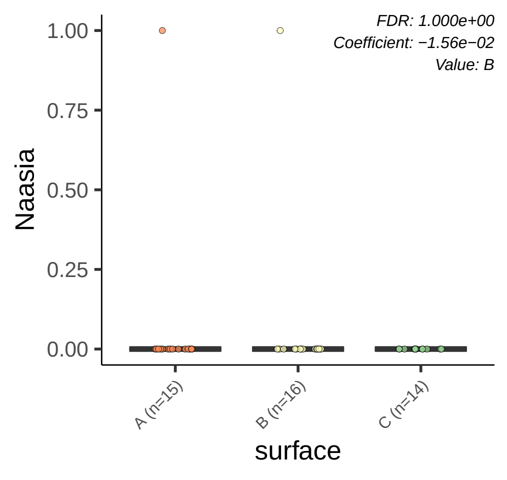
FDR: 1.000e+00
Coefficient: −1.56e−02
Value: B
1.00
0.75
0.50
0.25
0.00
Naasia
A (n=15)
B (n=16)
C (n=14)
surface

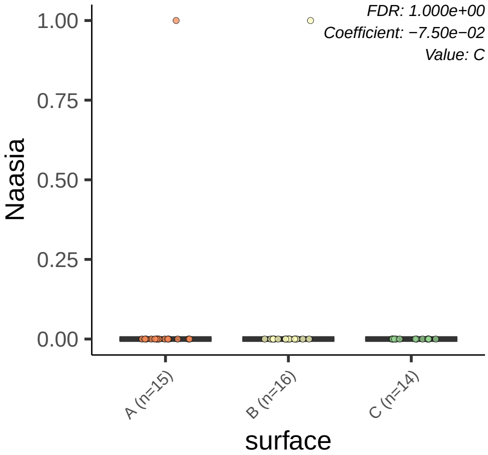
FDR: 1.000e+00
Coefficient: −7.50e−02
Value: C
Naasia
1.00
0.75
0.50
0.25
0.00
A (n=15)
B (n=16)
C (n=14)
surface

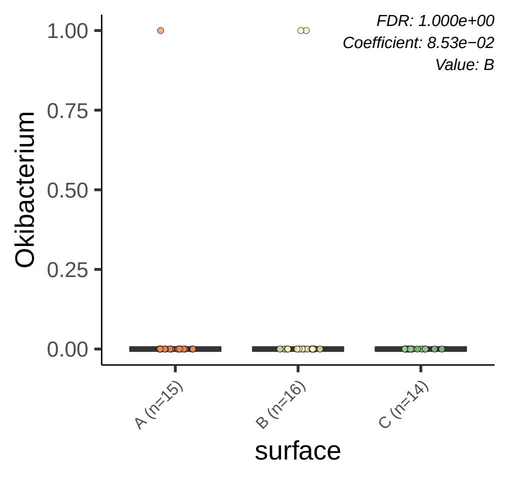
FDR: 1.000e+00
Coefficient: 8.53e−02
Value: B
Okibacterium
1.00
0.75
0.50
0.25
0.00
A (n=15)
B (n=16)
C (n=14)
surface

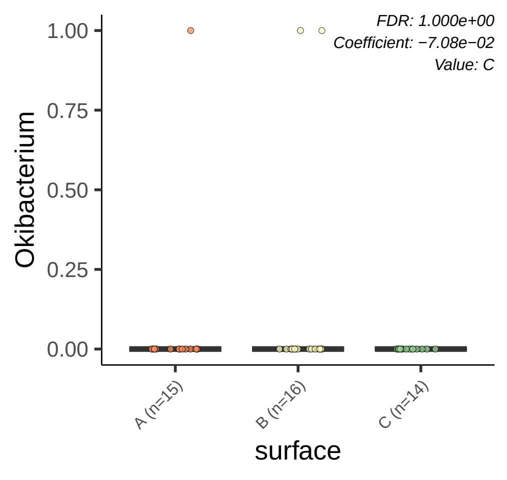
FDR: 1.000e+00
Coefficient: −7.08e−02
Value: C
Okibacterium
1.00
0.75
0.50
0.25
0.00
A (n=15)
B (n=16)
C (n=14)
surface

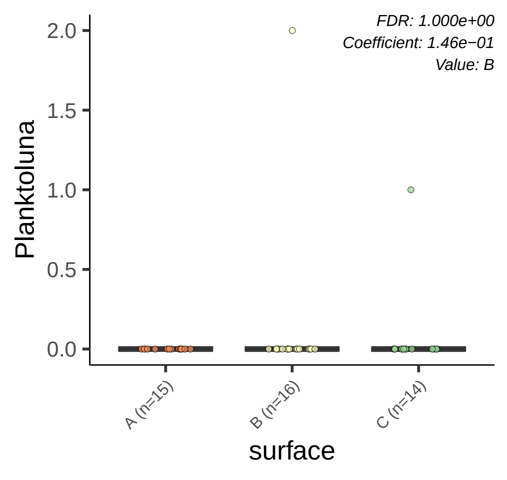

FDR: 1.000e+00
Coefficient: 1.46e−01
Value: B
Planktoluna
2.0
1.5
1.0
0.5
0.0
A (n=15)
B (n=16)
C (n=14)
surface

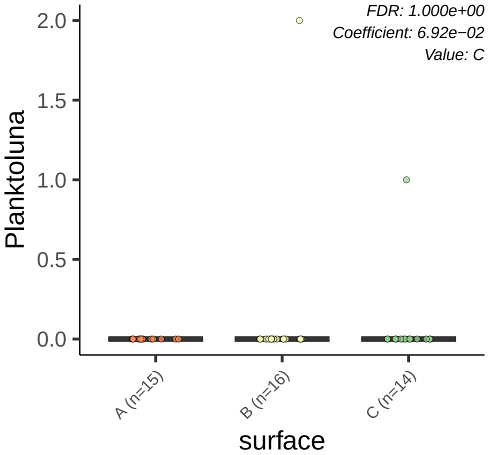

FDR: 1.000e+00
Coefficient: 6.92e−02
Value: C
Planktoluna
2.0
1.5
1.0
0.5
0.0
A (n=15)
B (n=16)
C (n=14)
surface

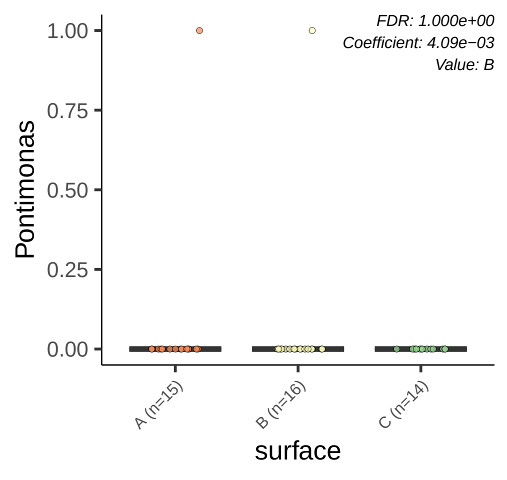
FDR: 1.000e+00
Coefficient: 4.09e−03
Value: B
Pontimonas
1.00
0.75
0.50
0.25
0.00
A (n=15)
B (n=16)
C (n=14)
surface

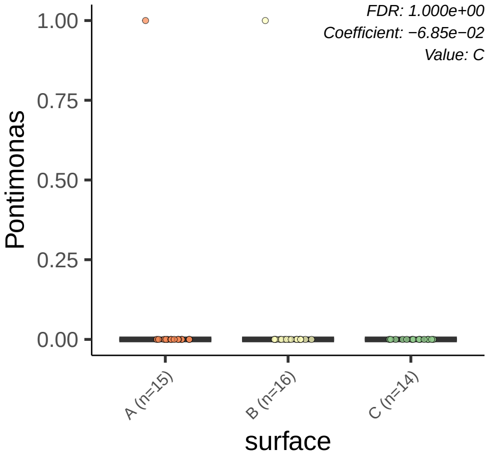
FDR: 1.000e+00
Coefficient: −6.85e−02
Value: C
Pontimonas
1.00
0.75
0.50
0.25
0.00
A (n=15)
B (n=16)
C (n=14)
surface

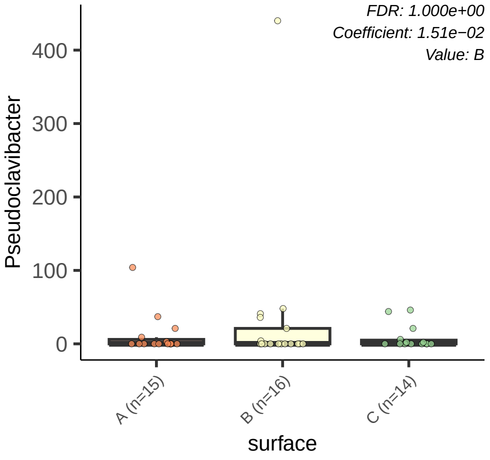

FDR: 1.000e+00
Coefficient: 1.51e−02
Value: B
Pseudoclavibacter
400
300
200
100
0
A (n=15)
B (n=16)
C (n=14)
surface

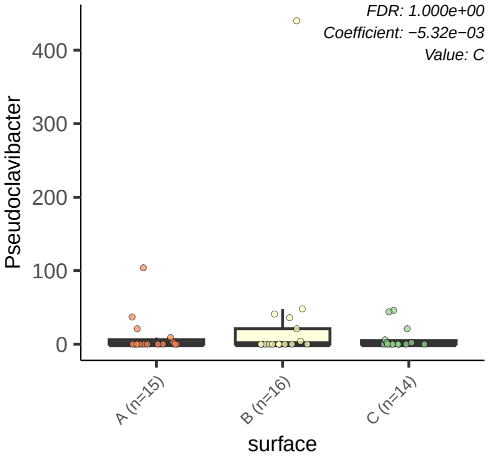

FDR: 1.000e+00
Coefficient: −5.32e−03
Value: C
Pseudoclavibacter
400
300
200
100
0
A (n=15)
B (n=16)
C (n=14)
surface

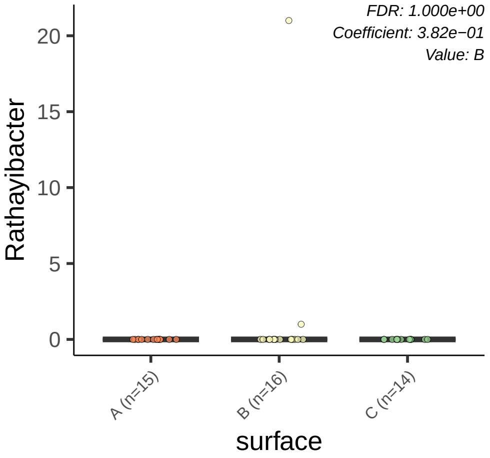
FDR: 1.000e+00
Coefficient: 3.82e−01
Value: B
Rathayibacter
0
5
10
15
20
A (n=15)
B (n=16)
C (n=14)
surface

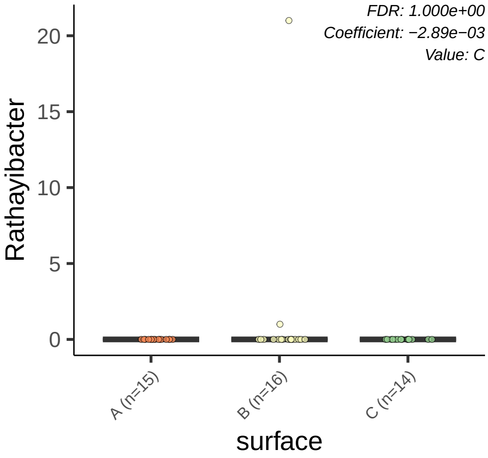
FDR: 1.000e+00
Coefficient: −2.89e−03
Value: C
Rathayibacter
20
15
10
5
0
A (n=15)
B (n=16)
C (n=14)
surface

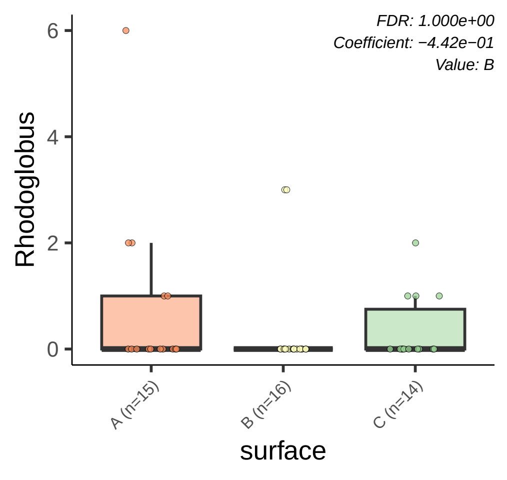

FDR: 1.000e+00
Coefficient: −4.42e−01
Value: B
Rhodoglobus
0
2
4
6
A (n=15)
B (n=16)
C (n=14)
surface

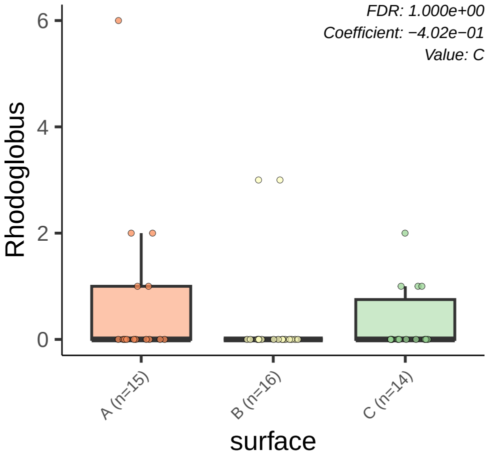

FDR: 1.000e+00
Coefficient: −4.02e−01
Value: C
Rhodoglobus
0
2
4
6
A (n=15)
B (n=16)
C (n=14)
surface

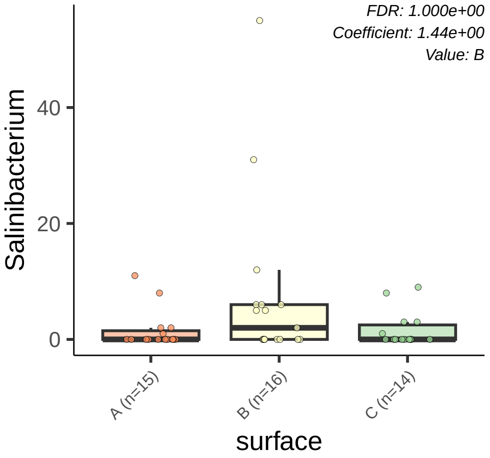

FDR: 1.000e+00
Coefficient: 1.44e+00
Value: B
Salinibacterium
40
20
0
A (n=15)
B (n=16)
C (n=14)
surface

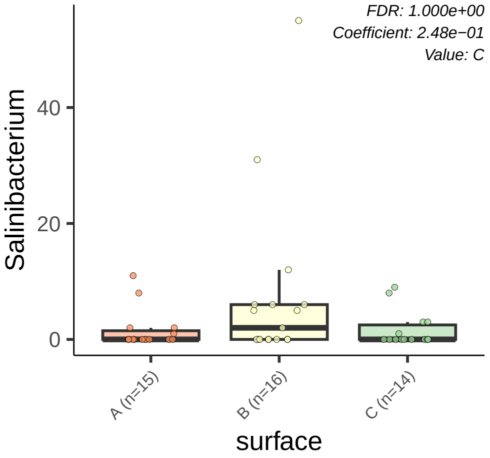
FDR: 1.000e+00
Coefficient: 2.48e−01
Value: C
Salinibacterium
40
20
0
A (n=15)
B (n=16)
C (n=14)
surface

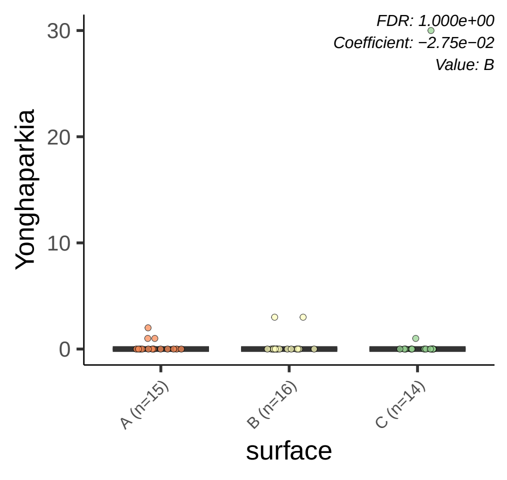
FDR: 1.000e+00
Coefficient: −2.75e−02
Value: B
Yonghaparkia
30
20
10
0
A (n=15)
B (n=16)
C (n=14)
surface

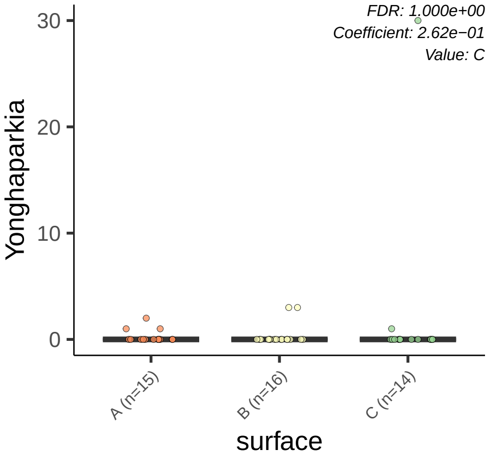

FDR: 1.000e+00
Coefficient: 2.62e−01
Value: C
Yonghaparkia
30
20
10
0
A (n=15)
B (n=16)
C (n=14)
surface

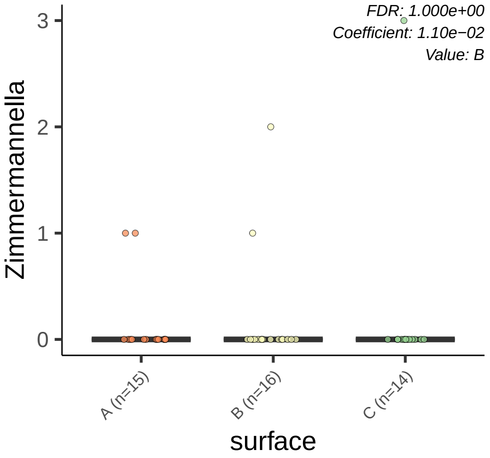

FDR: 1.000e+00
Coefficient: 1.10e−02
Value: B
Zimmermannella
3
2
1
0
A (n=15)
B (n=16)
C (n=14)
surface

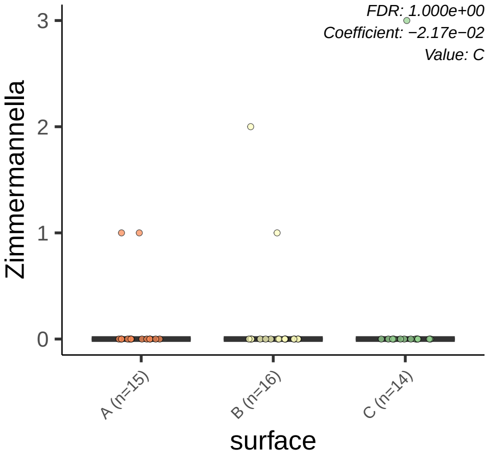

FDR: 1.000e+00
Coefficient: −2.17e−02
Value: C
Zimmermannella
3
2
1
0
A (n=15)
B (n=16)
C (n=14)
surface

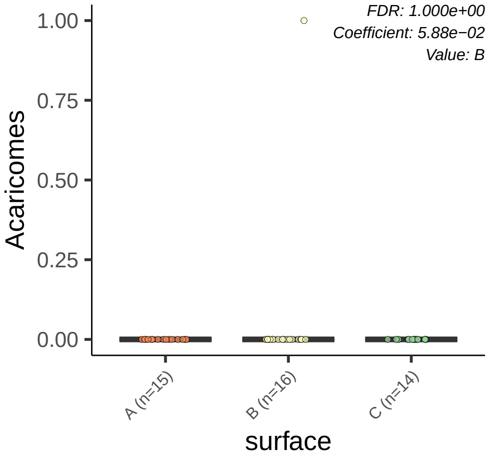
FDR: 1.000e+00
Coefficient: 5.88e−02
Value: B
Acaricomes
1.00
0.75
0.50
0.25
0.00
A (n=15)
B (n=16)
C (n=14)
surface

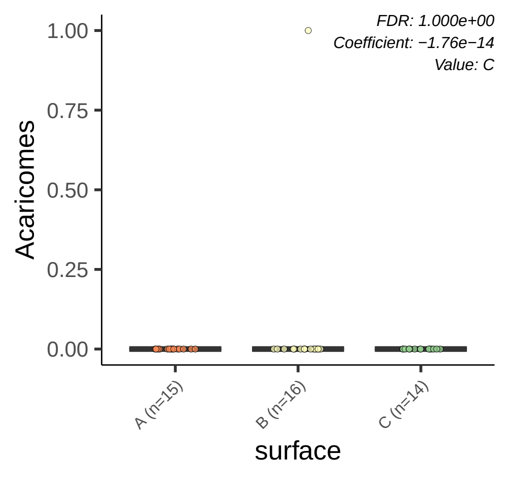

FDR: 1.000e+00
Coefficient: −1.76e−14
Value: C
Acaricomes
1.00
0.75
0.50
0.25
0.00
A (n=15)
B (n=16)
C (n=14)
surface

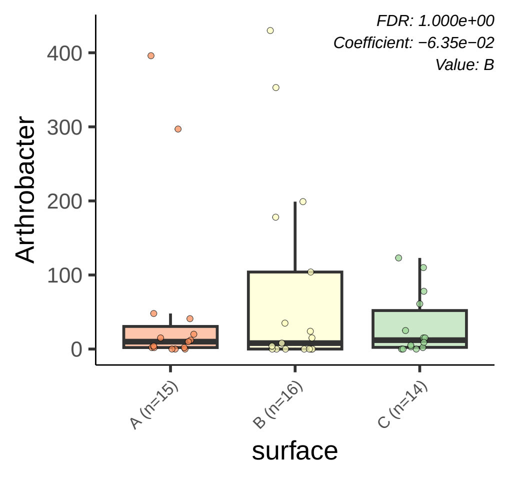
FDR: 1.000e+00
Coefficient: −6.35e−02
Value: B
Arthrobacter
400
300
200
100
0
A (n=15)
B (n=16)
C (n=14)
surface

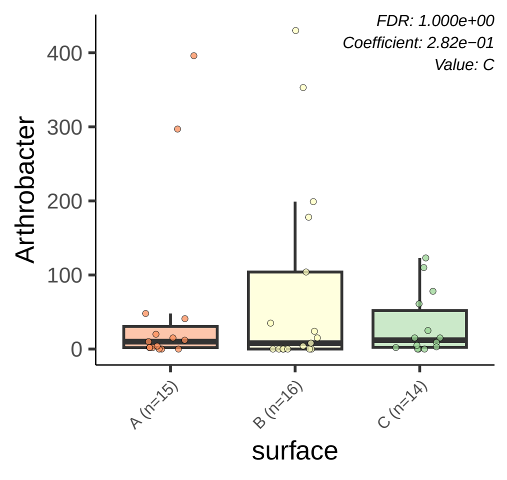
FDR: 1.000e+00
Coefficient: 2.82e−01
Value: C
Arthrobacter
0
100
200
300
400
A (n=15)
B (n=16)
C (n=14)
surface

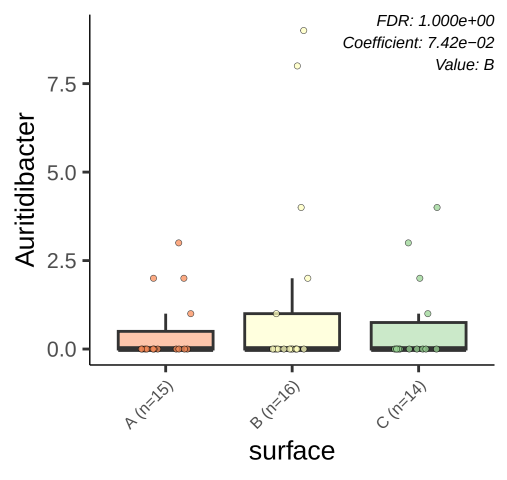

FDR: 1.000e+00
Coefficient: 7.42e−02
Value: B
Auritidibacter
7.5
5.0
2.5
0.0
A (n=15)
B (n=16)
C (n=14)
surface

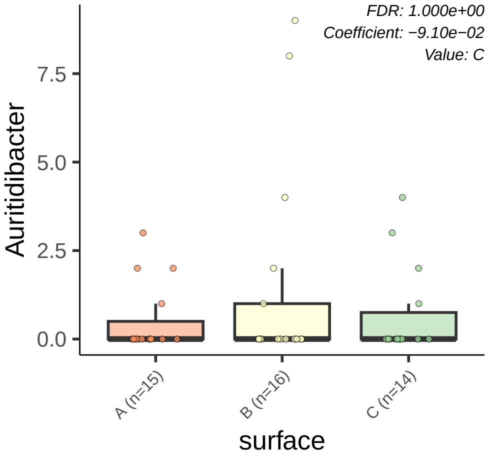
FDR: 1.000e+00
Coefficient: −9.10e−02
Value: C
Auritidibacter
7.5
5.0
2.5
0.0
A (n=15)
B (n=16)
C (n=14)
surface

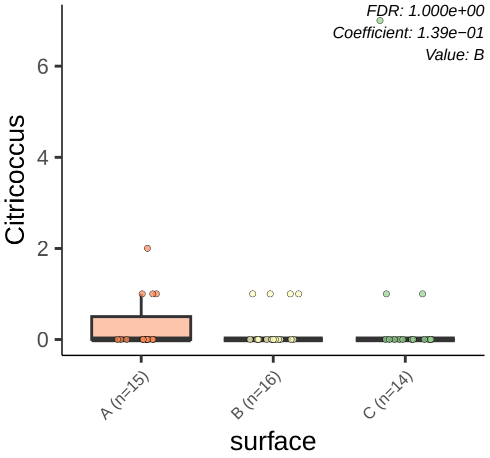

FDR: 1.000e+00
Coefficient: 1.39e−01
Value: B
Citricoccus
0
2
4
6
A (n=15)
B (n=16)
C (n=14)
surface

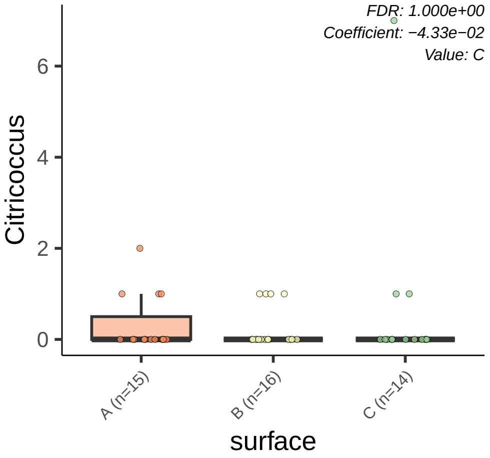
FDR: 1.000e+00
Coefficient: −4.33e−02
Value: C
Citricoccus
6
4
2
0
A (n=15)
B (n=16)
C (n=14)
surface

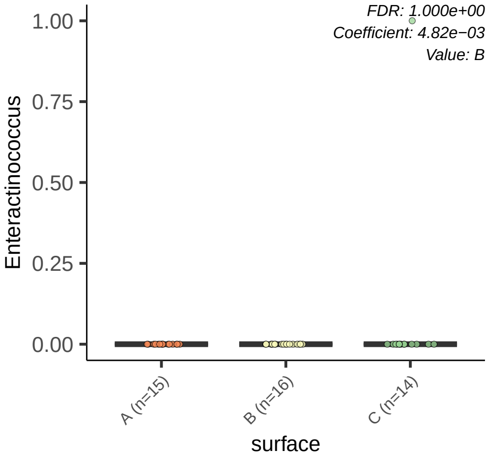
FDR: 1.000e+00
Coefficient: 4.82e−03
Value: B
Enteractinococcus
1.00
0.75
0.50
0.25
0.00
A (n=15)
B (n=16)
C (n=14)
surface

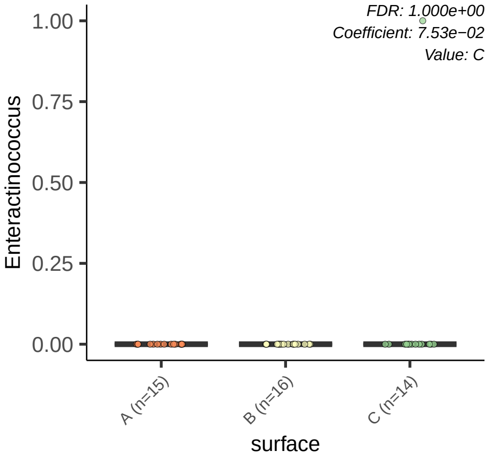

FDR: 1.000e+00
Coefficient: 7.53e−02
Value: C
1.00
0.75
0.50
0.25
0.00
Enteractinococcus
A (n=15)
B (n=16)
C (n=14)
surface

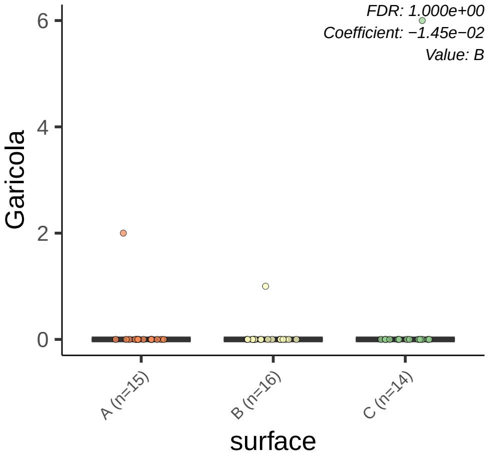
FDR: 1.000e+00
Coefficient: −1.45e−02
Value: B
Garicola
6
4
2
0
A (n=15)
B (n=16)
C (n=14)
surface

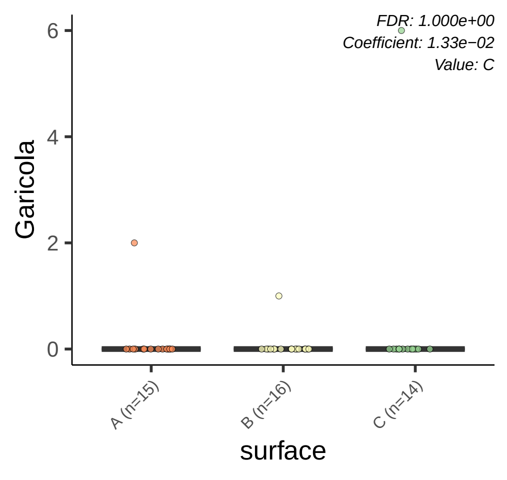

FDR: 1.000e+00
Coefficient: 1.33e−02
Value: C
Garicola
6
4
2
0
A (n=15)
B (n=16)
C (n=14)
surface

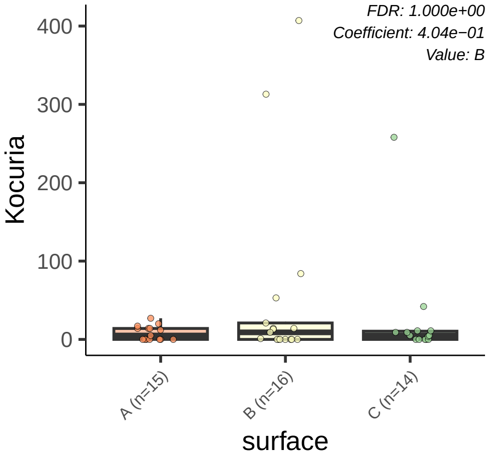
FDR: 1.000e+00
Coefficient: 4.04e−01
Value: B
Kocuria
400
300
200
100
0
A (n=15)
B (n=16)
C (n=14)
surface

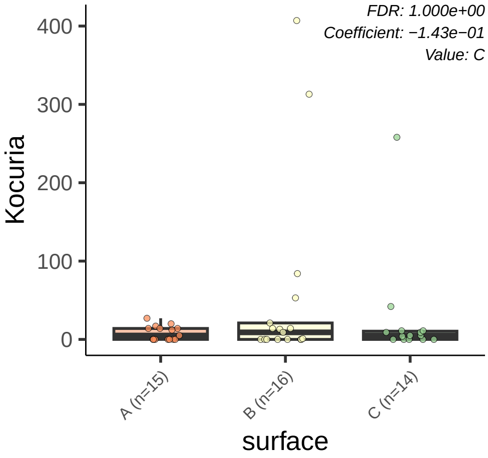
FDR: 1.000e+00
Coefficient: −1.43e−01
Value: C
Kocuria
400
300
200
100
0
A (n=15)
B (n=16)
C (n=14)
surface

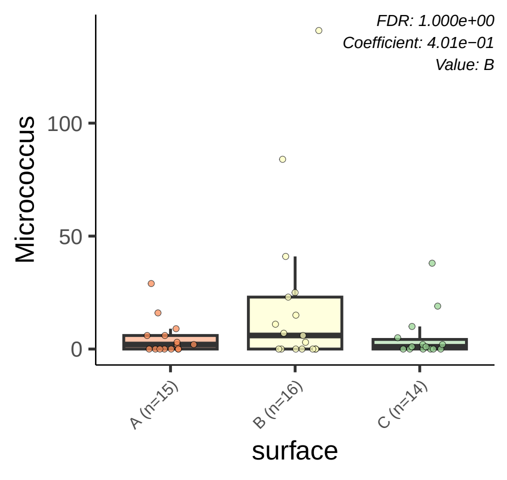

FDR: 1.000e+00
Coefficient: 4.01e−01
Value: B
Micrococcus
100
50
0
A (n=15)
B (n=16)
C (n=14)
surface

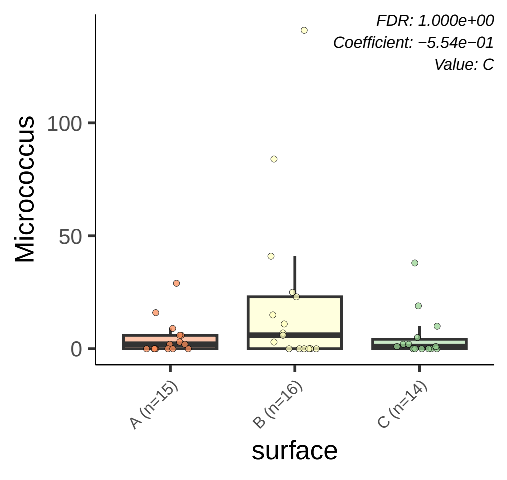
FDR: 1.000e+00
Coefficient: −5.54e−01
Value: C
Micrococcus
100
50
0
A (n=15)
B (n=16)
C (n=14)
surface

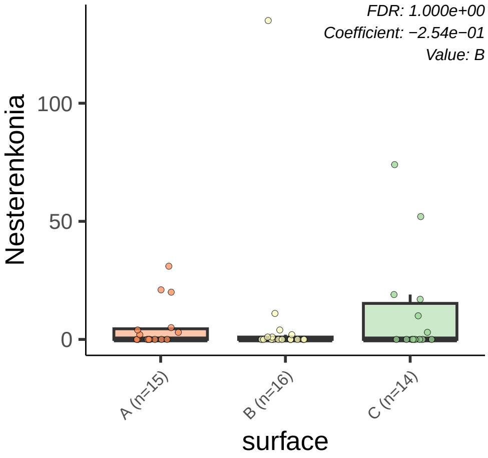

FDR: 1.000e+00
Coefficient: −2.54e−01
Value: B
Nesterenkonia
100
50
0
A (n=15)
B (n=16)
C (n=14)
surface

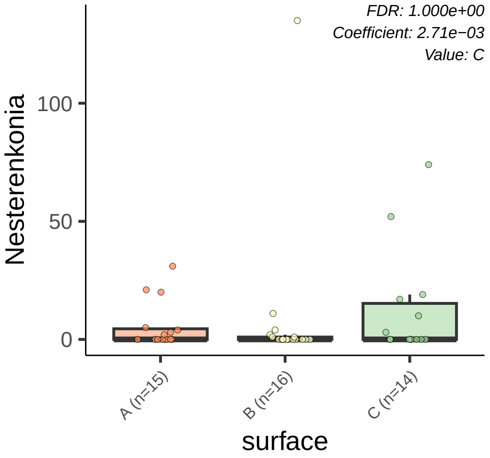
FDR: 1.000e+00
Coefficient: 2.71e−03
Value: C
Nesterenkonia
100
50
0
A (n=15)
B (n=16)
C (n=14)
surface

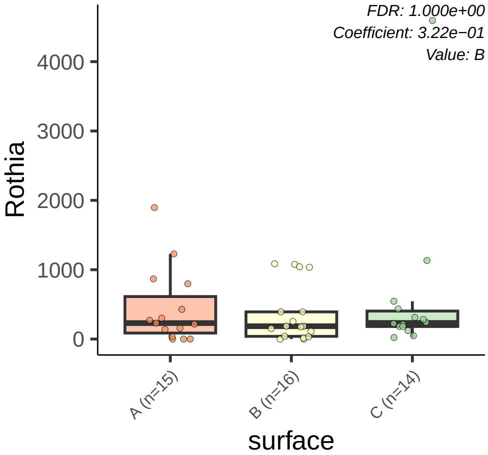
FDR: 1.000e+00
Coefficient: 3.22e−01
Value: B
Rothia
4000
3000
2000
1000
0
A (n=15)
B (n=16)
C (n=14)
surface

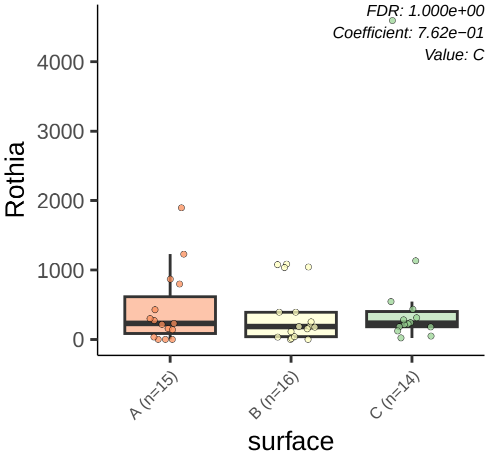

FDR: 1.000e+00
Coefficient: 7.62e−01
Value: C
Rothia
4000
3000
2000
1000
0
A (n=15)
B (n=16)
C (n=14)
surface

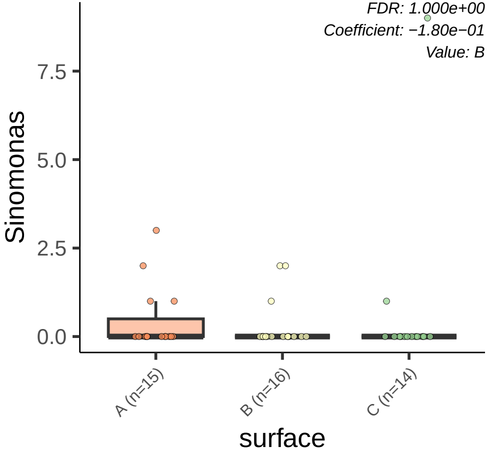
FDR: 1.000e+00
Coefficient: −1.80e−01
Value: B
Sinomonas
7.5
5.0
2.5
0.0
A (n=15)
B (n=16)
C (n=14)
surface

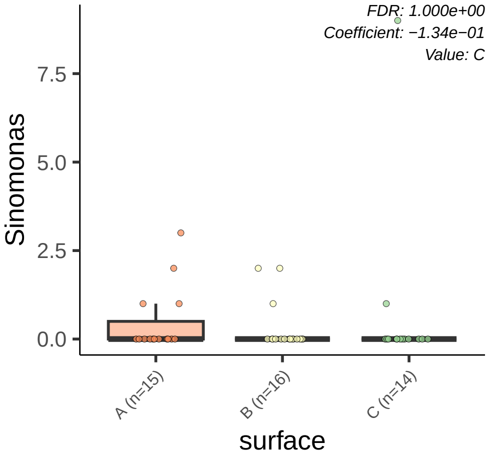
FDR: 1.000e+00
Coefficient: −1.34e−01
Value: C
Sinomonas
7.5
5.0
2.5
0.0
A (n=15)
B (n=16)
C (n=14)
surface

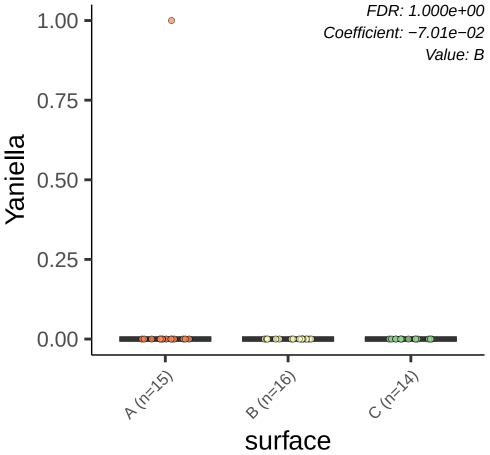

FDR: 1.000e+00
Coefficient: −7.01e−02
Value: B
Yaniella
1.00
0.75
0.50
0.25
0.00
A (n=15)
B (n=16)
C (n=14)
surface

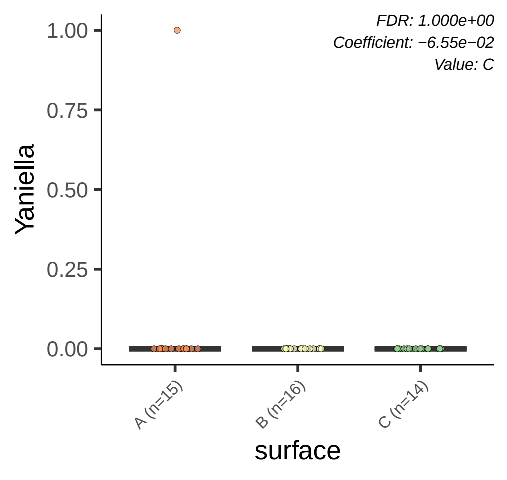
FDR: 1.000e+00
Coefficient: −6.55e−02
Value: C
Yaniella
1.00
0.75
0.50
0.25
0.00
A (n=15)
B (n=16)
C (n=14)
surface

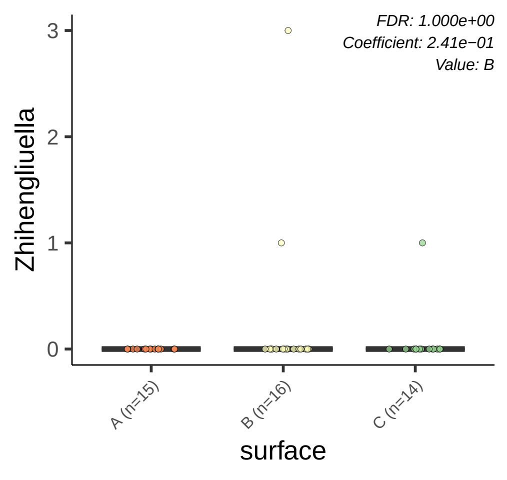

FDR: 1.000e+00
Coefficient: 2.41e−01
Value: B
Zhihengliuella
3
2
1
0
A (n=15)
B (n=16)
C (n=14)
surface

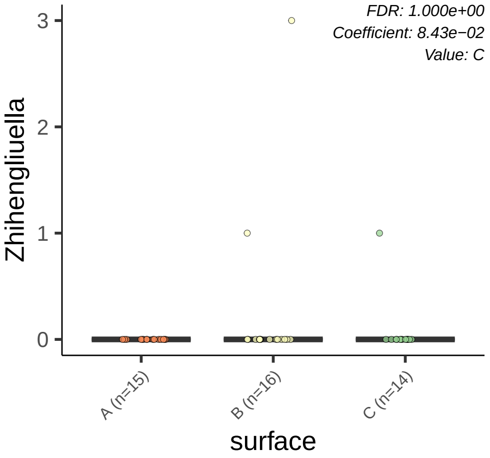

FDR: 1.000e+00
Coefficient: 8.43e−02
Value: C
Zhihengliuella
3
2
1
0
A (n=15)
B (n=16)
C (n=14)
surface

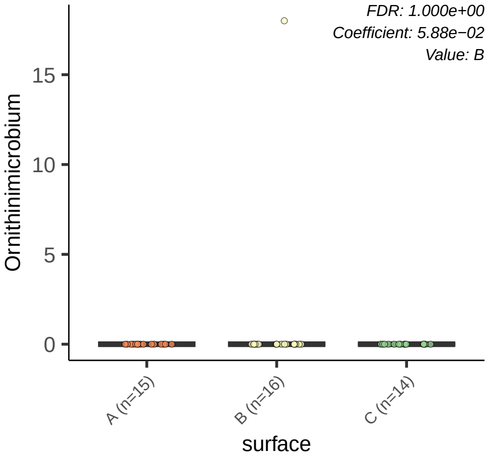

FDR: 1.000e+00
Coefficient: 5.88e−02
Value: B
Ornithinimicrobium
0
5
10
15
A (n=15)
B (n=16)
C (n=14)
surface

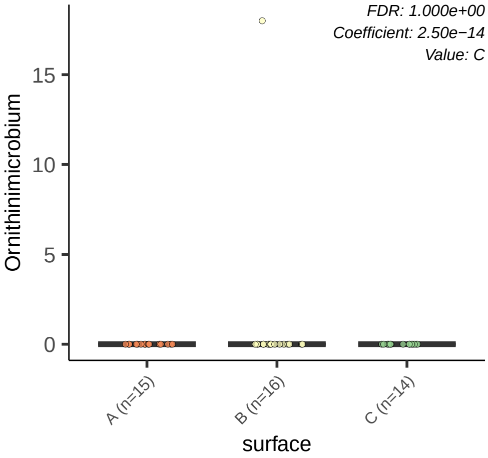
FDR: 1.000e+00
Coefficient: 2.50e−14
Value: C
Ornithinimicrobium
0
5
10
15
A (n=15)
B (n=16)
C (n=14)
surface

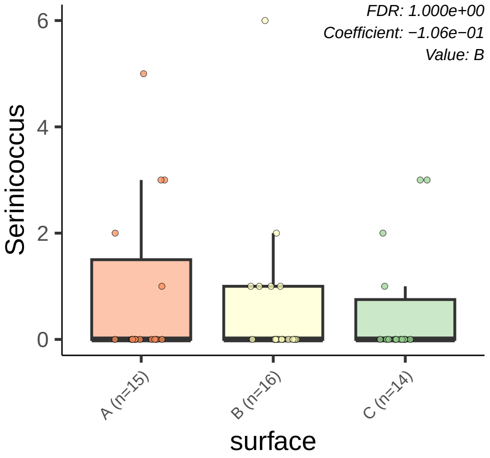

FDR: 1.000e+00
Coefficient: −1.06e−01
Value: B
Serinicoccus
0
2
4
6
A (n=15)
B (n=16)
C (n=14)
surface

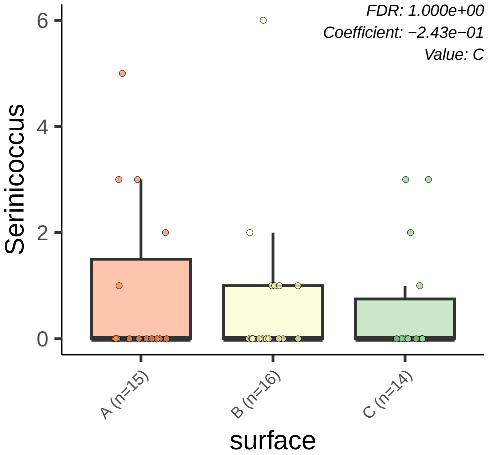

FDR: 1.000e+00
Coefficient: −2.43e−01
Value: C
Serinicoccus
0
2
4
6
A (n=15)
B (n=16)
C (n=14)
surface

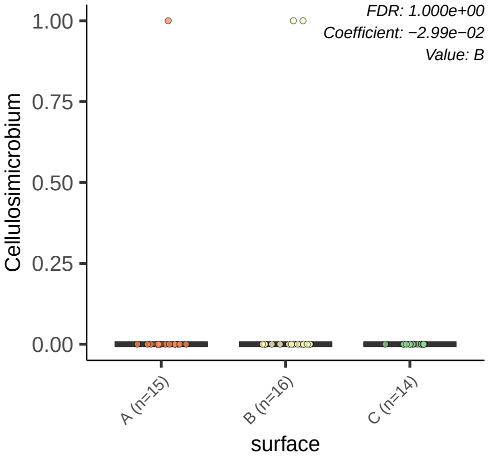

FDR: 1.000e+00
Coefficient: −2.99e−02
Value: B
Cellulosimicrobium
1.00
0.75
0.50
0.25
0.00
A (n=15)
B (n=16)
C (n=14)
surface

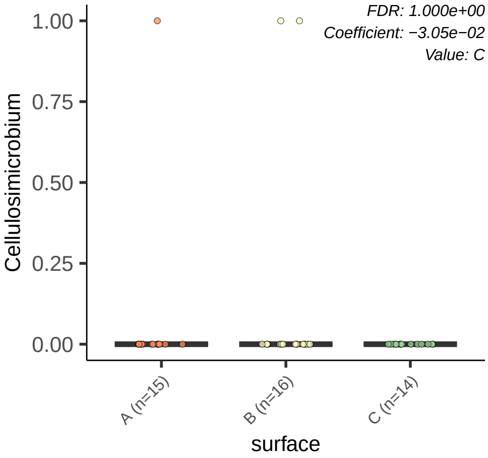

FDR: 1.000e+00
Coefficient: −3.05e−02
Value: C
Cellulosimicrobium
1.00
0.75
0.50
0.25
0.00
A (n=15)
B (n=16)
C (n=14)
surface

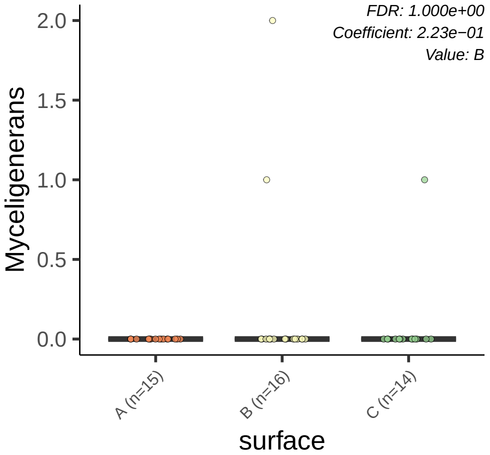

FDR: 1.000e+00
Coefficient: 2.23e−01
Value: B
Myceligenerans
2.0
1.5
1.0
0.5
0.0
A (n=15)
B (n=16)
C (n=14)
surface

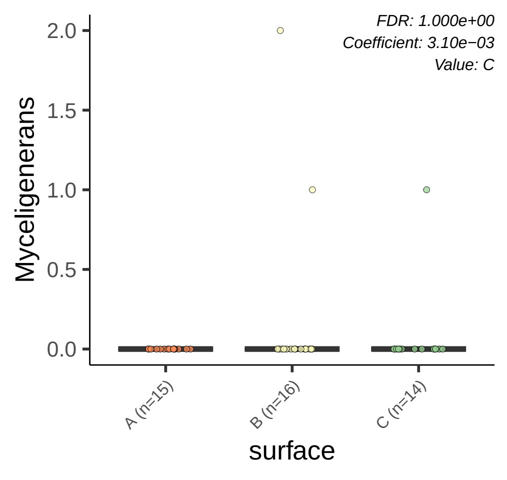

FDR: 1.000e+00
Coefficient: 3.10e−03
Value: C
Myceligenerans
2.0
1.5
1.0
0.5
0.0
A (n=15)
B (n=16)
C (n=14)
surface

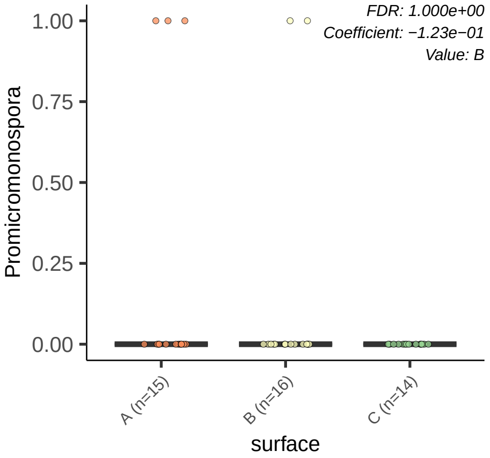
FDR: 1.000e+00
Coefficient: −1.23e−01
Value: B
Promicromonospora
1.00
0.75
0.50
0.25
0.00
A (n=15)
B (n=16)
C (n=14)
surface

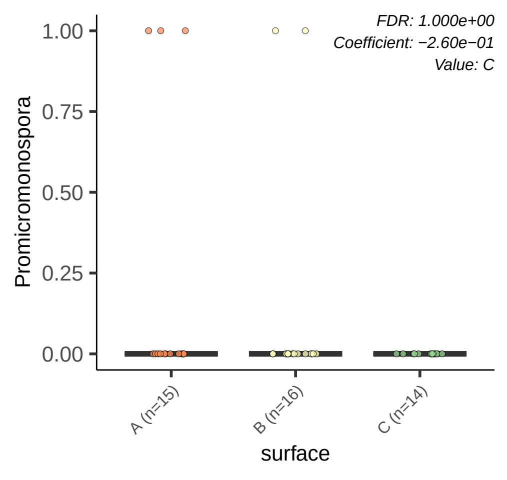

FDR: 1.000e+00
Coefficient: −2.60e−01
Value: C
Promicromonospora
1.00
0.75
0.50
0.25
0.00
A (n=15)
B (n=16)
C (n=14)
surface

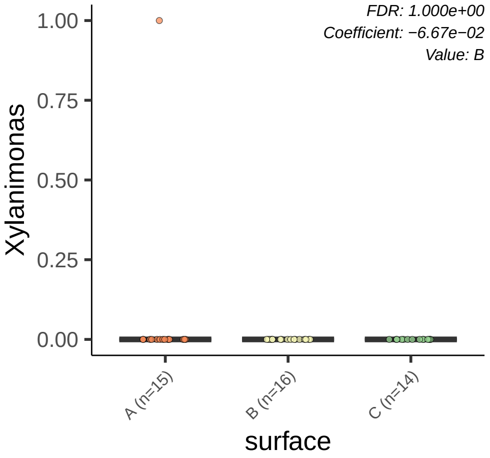
FDR: 1.000e+00
Coefficient: −6.67e−02
Value: B
Xylanimonas
1.00
0.75
0.50
0.25
0.00
A (n=15)
B (n=16)
C (n=14)
surface

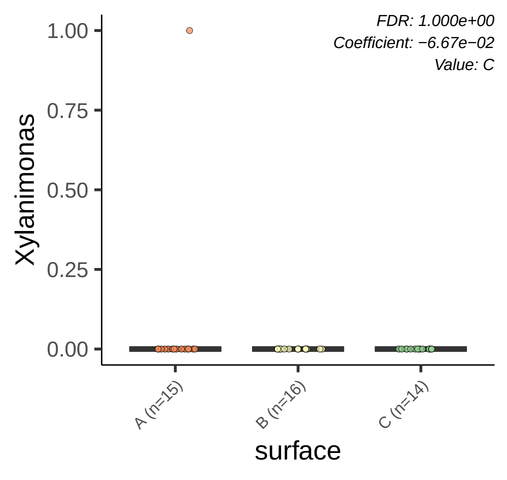
FDR: 1.000e+00
Coefficient: −6.67e−02
Value: C
Xylanimonas
1.00
0.75
0.50
0.25
0.00
A (n=15)
B (n=16)
C (n=14)
surface

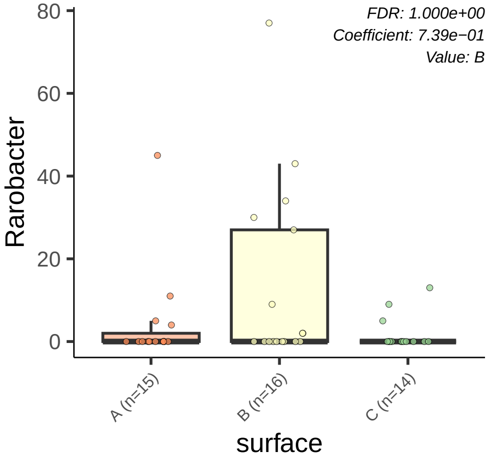
FDR: 1.000e+00
Coefficient: 7.39e−01
Value: B
Rarobacter
0
20
40
60
80
A (n=15)
B (n=16)
C (n=14)
surface

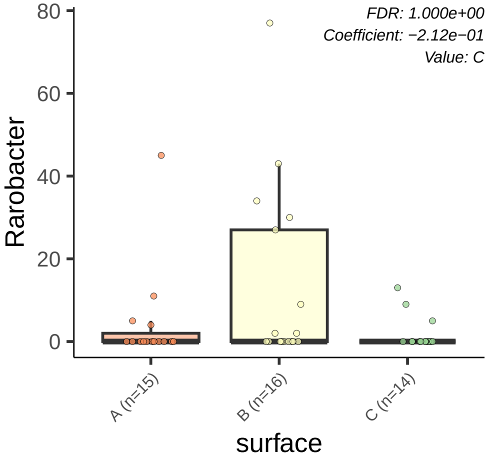
FDR: 1.000e+00
Coefficient: −2.12e−01
Value: C
Rarobacter
0
20
40
60
80
A (n=15)
B (n=16)
C (n=14)
surface

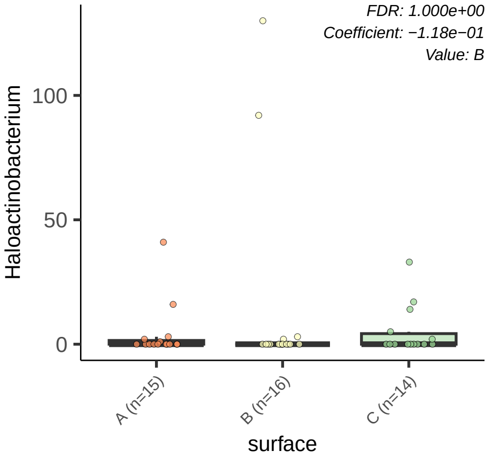
FDR: 1.000e+00
Coefficient: −1.18e−01
Value: B
Haloactinobacterium
100
50
0
A (n=15)
B (n=16)
C (n=14)
surface

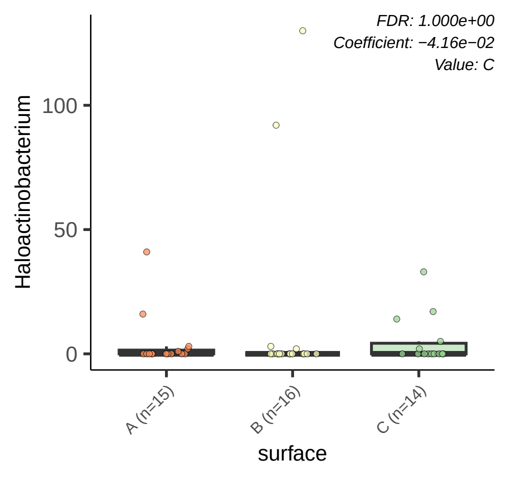

FDR: 1.000e+00
Coefficient: −4.16e−02
Value: C
Haloactinobacterium
100
50
0
A (n=15)
B (n=16)
C (n=14)
surface

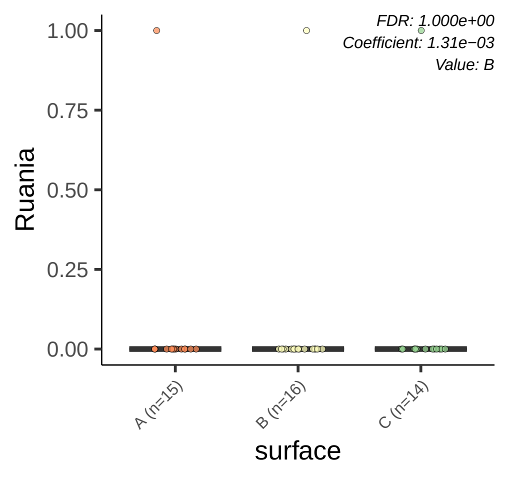

FDR: 1.000e+00
Coefficient: 1.31e−03
Value: B
Ruania
1.00
0.75
0.50
0.25
0.00
A (n=15)
B (n=16)
C (n=14)
surface

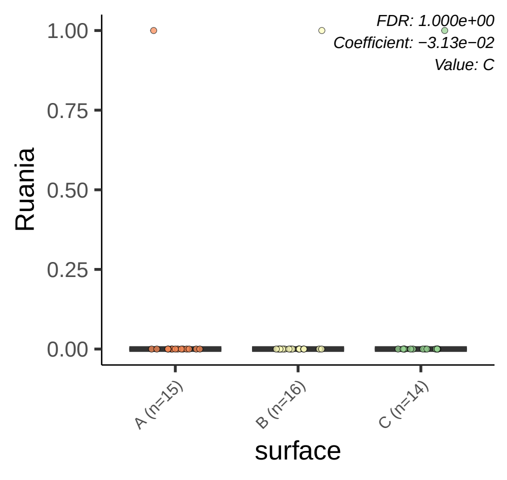
FDR: 1.000e+00
Coefficient: −3.13e−02
Value: C
Ruania
1.00
0.75
0.50
0.25
0.00
A (n=15)
B (n=16)
C (n=14)
surface

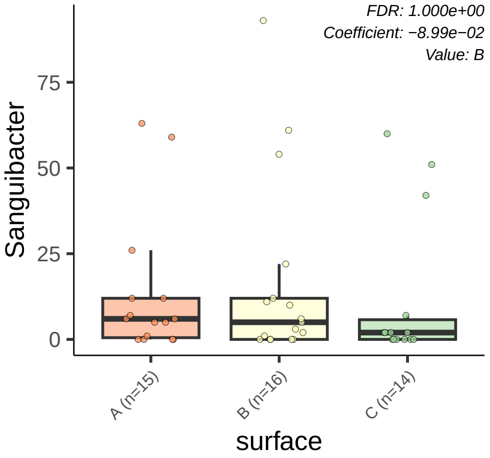
FDR: 1.000e+00
Coefficient: −8.99e−02
Value: B
Sanguibacter
75
50
25
0
A (n=15)
B (n=16)
C (n=14)
surface

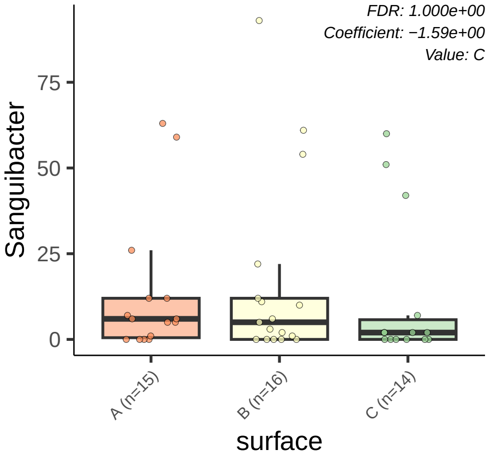
FDR: 1.000e+00
Coefficient: −1.59e+00
Value: C
Sanguibacter
75
50
25
0
A (n=15)
B (n=16)
C (n=14)
surface

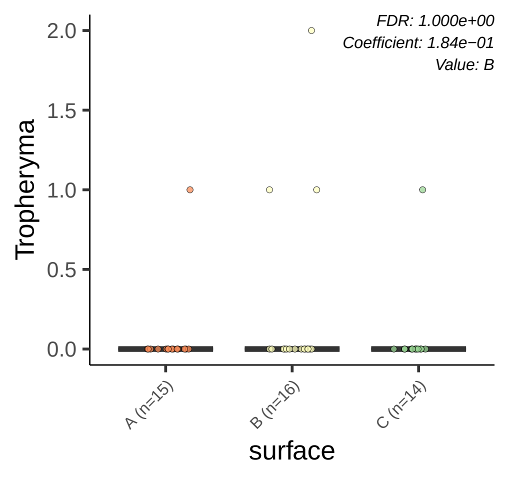

FDR: 1.000e+00
Coefficient: 1.84e−01
Value: B
Tropheryma
2.0
1.5
1.0
0.5
0.0
A (n=15)
B (n=16)
C (n=14)
surface

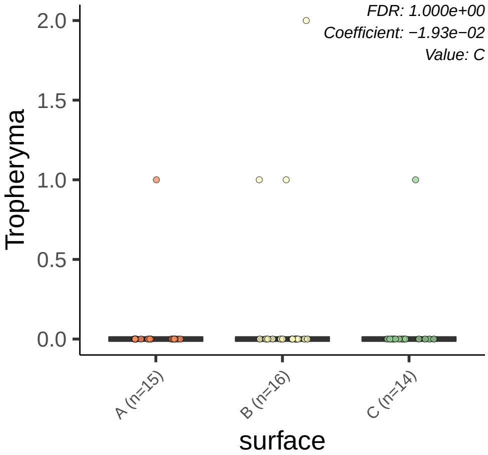
FDR: 1.000e+00
Coefficient: −1.93e−02
Value: C
Tropheryma
2.0
1.5
1.0
0.5
0.0
A (n=15)
B (n=16)
C (n=14)
surface

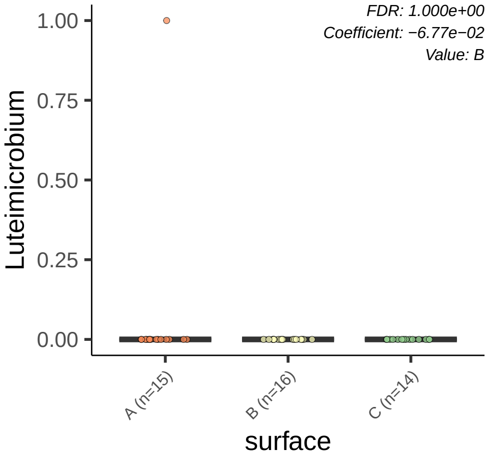
FDR: 1.000e+00
Coefficient: −6.77e−02
Value: B
Luteimicrobium
1.00
0.75
0.50
0.25
0.00
A (n=15)
B (n=16)
C (n=14)
surface

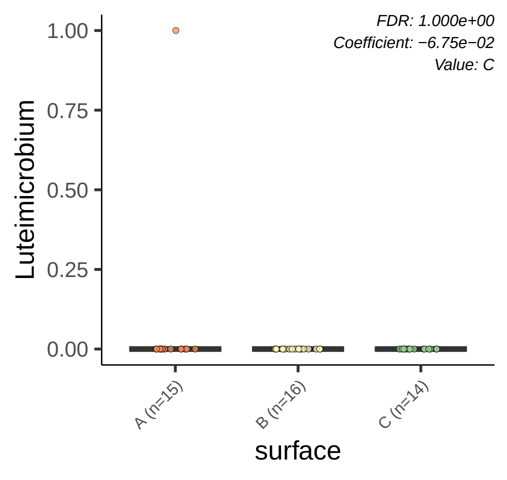
FDR: 1.000e+00
Coefficient: −6.75e−02
Value: C
Luteimicrobium
1.00
0.75
0.50
0.25
0.00
A (n=15)
B (n=16)
C (n=14)
surface

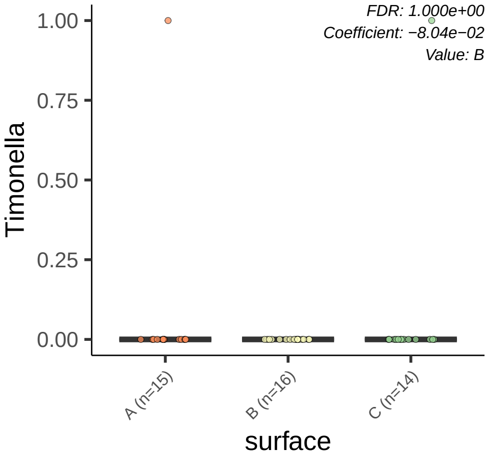

FDR: 1.000e+00
Coefficient: −8.04e−02
Value: B
Timonella
1.00
0.75
0.50
0.25
0.00
A (n=15)
B (n=16)
C (n=14)
surface

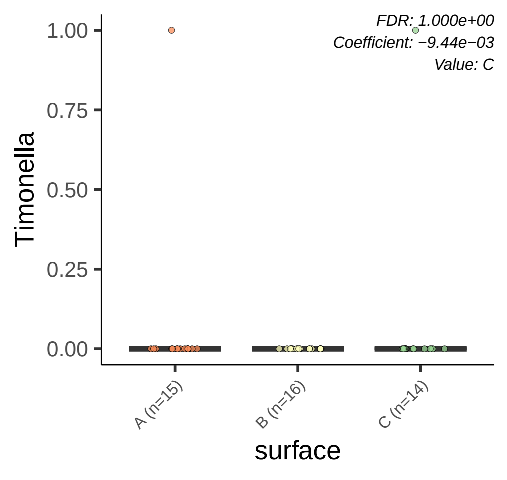
FDR: 1.000e+00
Coefficient: −9.44e−03
Value: C
Timonella
1.00
0.75
0.50
0.25
0.00
A (n=15)
B (n=16)
C (n=14)
surface

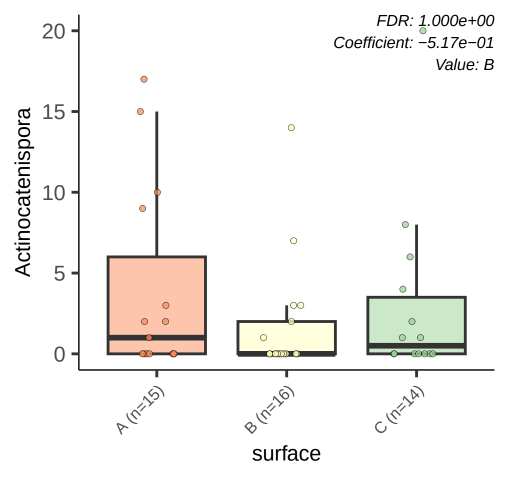
FDR: 1.000e+00
Coefficient: −5.17e−01
Value: B
Actinocatenispora
20
15
10
5
0
A (n=15)
B (n=16)
C (n=14)
surface

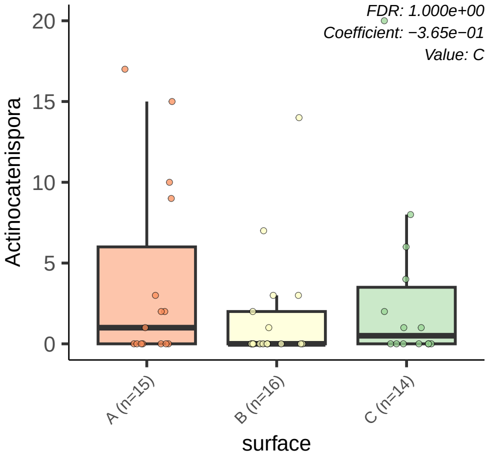

FDR: 1.000e+00
Coefficient: −3.65e−01
Value: C
Actinocatenispora
20
15
10
5
0
A (n=15)
B (n=16)
C (n=14)
surface

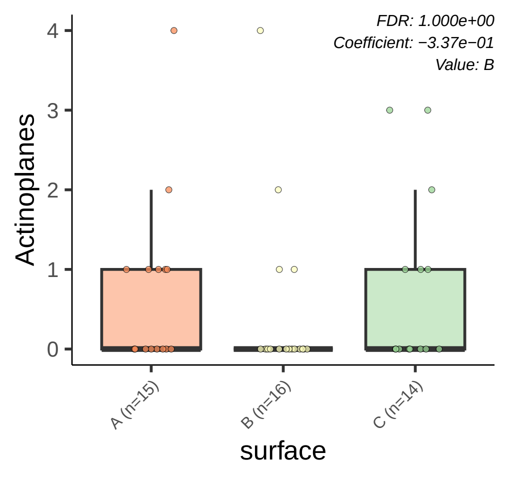
FDR: 1.000e+00
Coefficient: −3.37e−01
Value: B
Actinoplanes
4
3
2
1
0
A (n=15)
B (n=16)
C (n=14)
surface

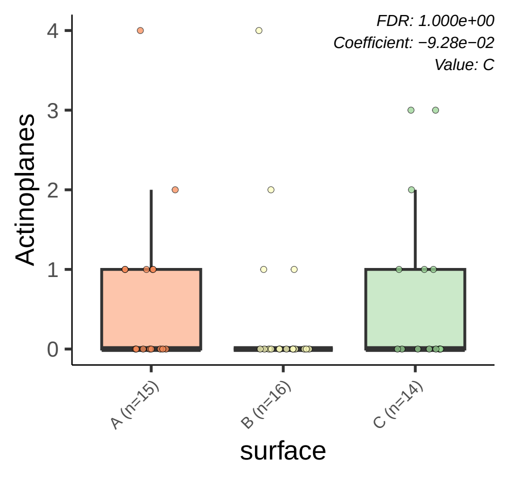

FDR: 1.000e+00
Coefficient: −9.28e−02
Value: C
Actinoplanes
4
3
2
1
0
A (n=15)
B (n=16)
C (n=14)
surface

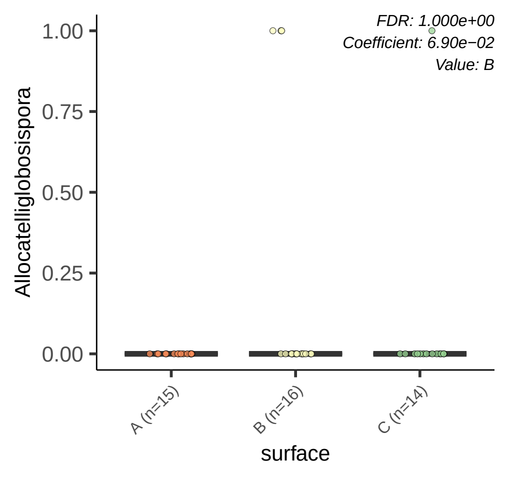
FDR: 1.000e+00
Coefficient: 6.90e−02
Value: B
Allocatelliglobosispora
1.00
0.75
0.50
0.25
0.00
A (n=15)
B (n=16)
C (n=14)
surface

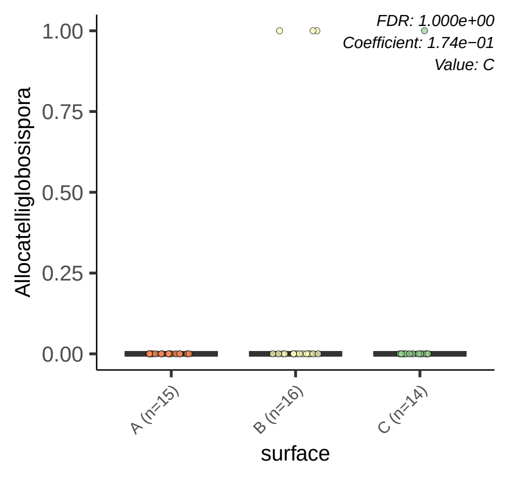

FDR: 1.000e+00
Coefficient: 1.74e−01
Value: C
Allocatelliglobosispora
1.00
0.75
0.50
0.25
0.00
A (n=15)
B (n=16)
C (n=14)
surface

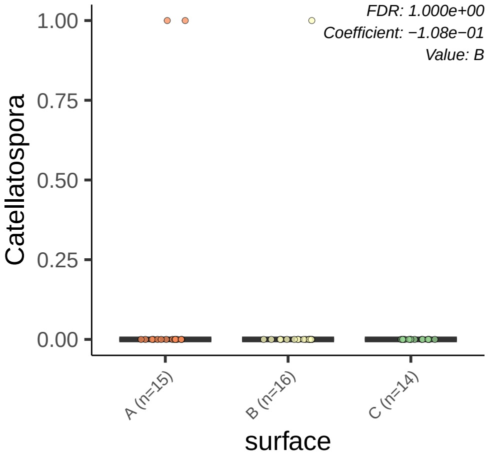

FDR: 1.000e+00
Coefficient: −1.08e−01
Value: B
Catellatospora
1.00
0.75
0.50
0.25
0.00
A (n=15)
B (n=16)
C (n=14)
surface

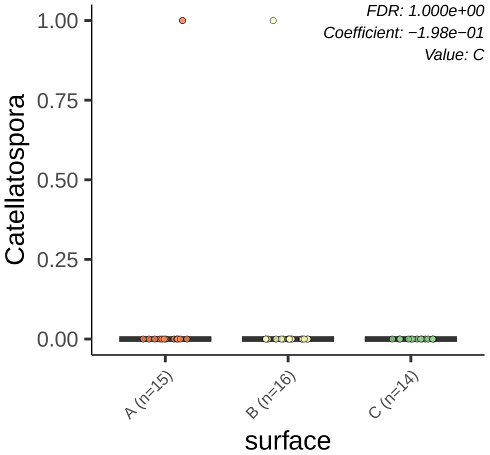
FDR: 1.000e+00
Coefficient: −1.98e−01
Value: C
1.00
0.75
0.50
0.25
0.00
Catellatospora
A (n=15)
B (n=16)
C (n=14)
surface

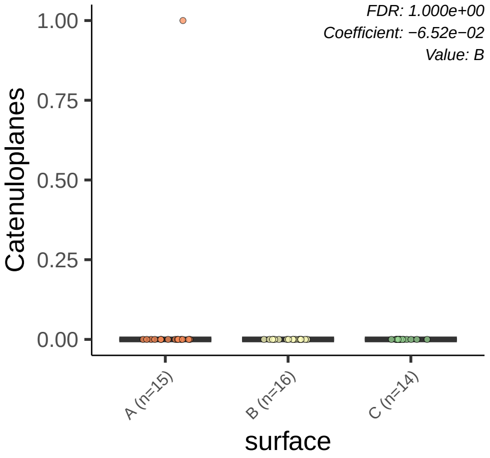

FDR: 1.000e+00
Coefficient: −6.52e−02
Value: B
Catenuloplanes
1.00
0.75
0.50
0.25
0.00
A (n=15)
B (n=16)
C (n=14)
surface

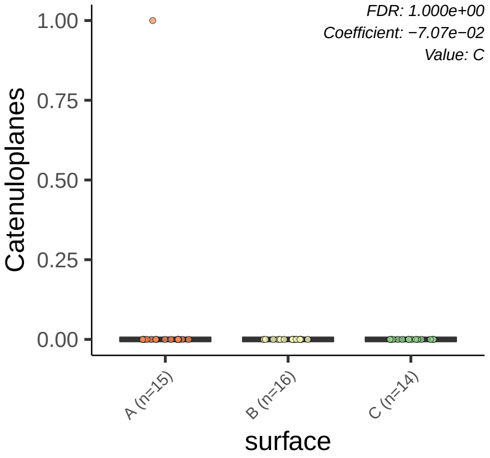

FDR: 1.000e+00
Coefficient: −7.07e−02
Value: C
Catenuloplanes
1.00
0.75
0.50
0.25
0.00
A (n=15)
B (n=16)
C (n=14)
surface

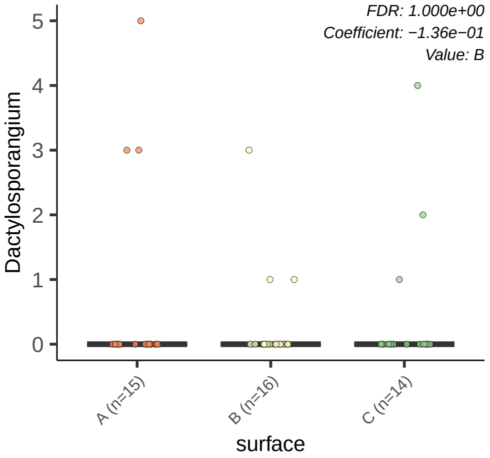
FDR: 1.000e+00
Coefficient: −1.36e−01
Value: B
Dactylosporangium
0
1
2
3
4
5
A (n=15)
B (n=16)
C (n=14)
surface

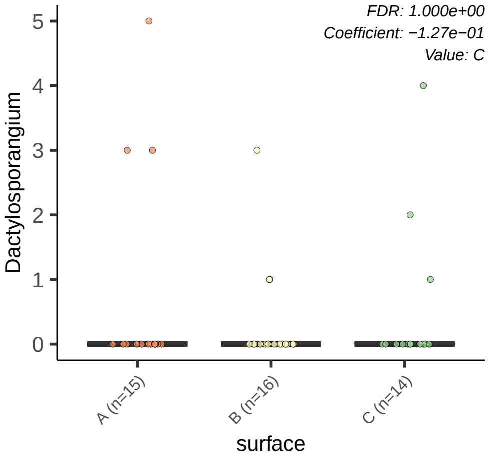

FDR: 1.000e+00
Coefficient: −1.27e−01
Value: C
Dactylosporangium
0
1
2
3
4
5
A (n=15)
B (n=16)
C (n=14)
surface

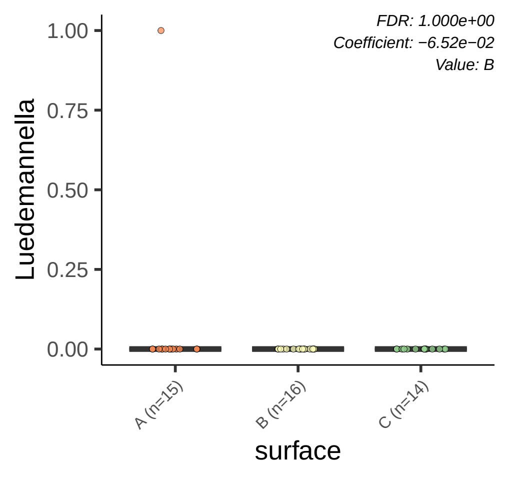
FDR: 1.000e+00
Coefficient: −6.52e−02
Value: B
Luedemannella
1.00
0.75
0.50
0.25
0.00
A (n=15)
B (n=16)
C (n=14)
surface

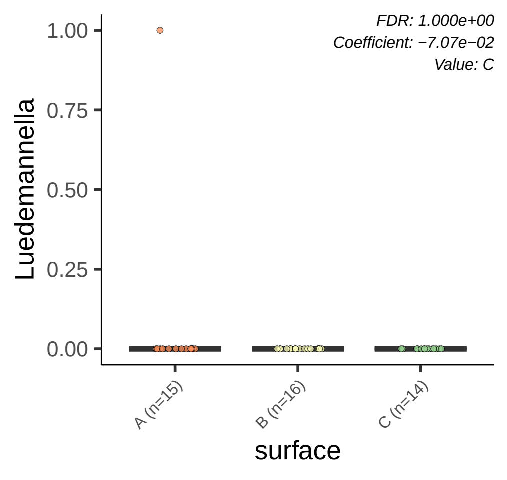
FDR: 1.000e+00
Coefficient: −7.07e−02
Value: C
Luedemannella
1.00
0.75
0.50
0.25
0.00
A (n=15)
B (n=16)
C (n=14)
surface

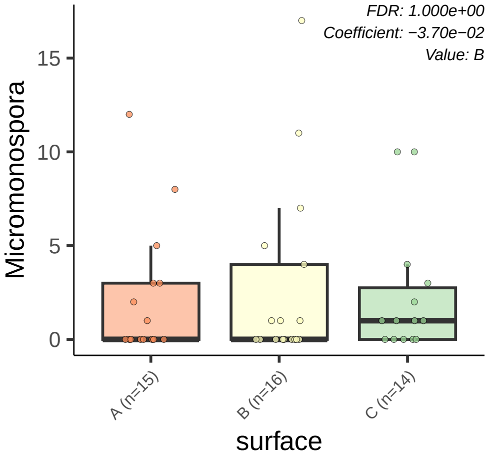
FDR: 1.000e+00
Coefficient: −3.70e−02
Value: B
Micromonospora
0
5
10
15
A (n=15)
B (n=16)
C (n=14)
surface

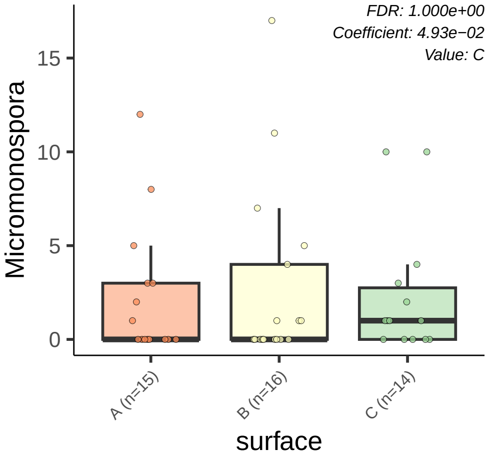

FDR: 1.000e+00
Coefficient: 4.93e−02
Value: C
Micromonospora
0
5
10
15
A (n=15)
B (n=16)
C (n=14)
surface

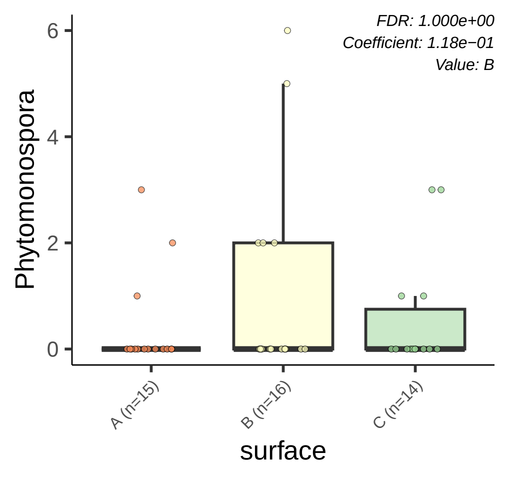

FDR: 1.000e+00
Coefficient: 1.18e−01
Value: B
Phytomonospora
0
2
4
6
A (n=15)
B (n=16)
C (n=14)
surface

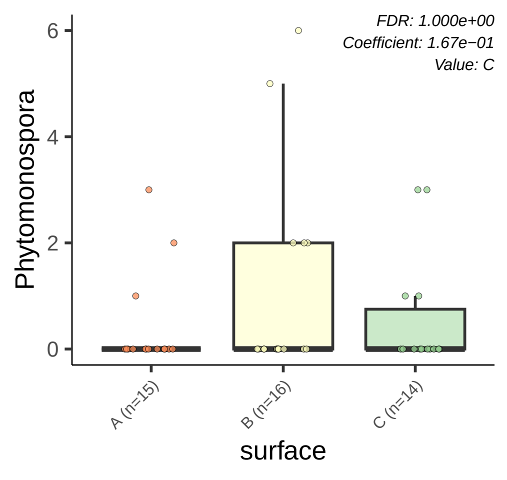
FDR: 1.000e+00
Coefficient: 1.67e−01
Value: C
Phytomonospora
6
4
2
0
A (n=15)
B (n=16)
C (n=14)
surface

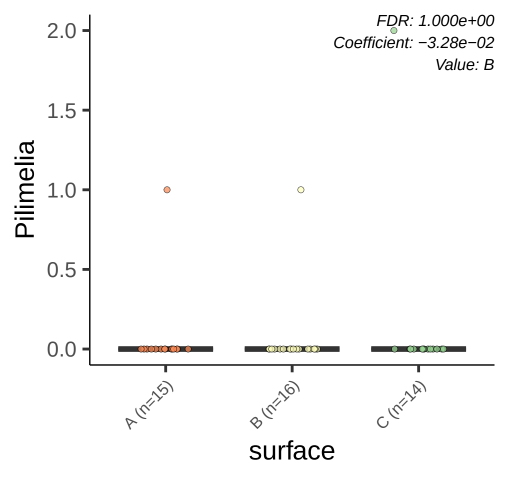
FDR: 1.000e+00
Coefficient: −3.28e−02
Value: B
Pilimelia
2.0
1.5
1.0
0.5
0.0
A (n=15)
B (n=16)
C (n=14)
surface

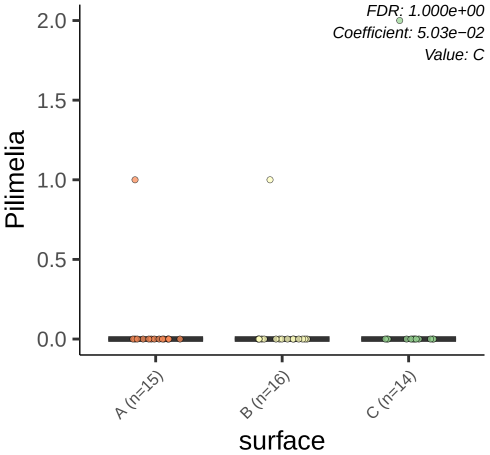

FDR: 1.000e+00
Coefficient: 5.03e−02
Value: C
Pilimelia
2.0
1.5
1.0
0.5
0.0
A (n=15)
B (n=16)
C (n=14)
surface

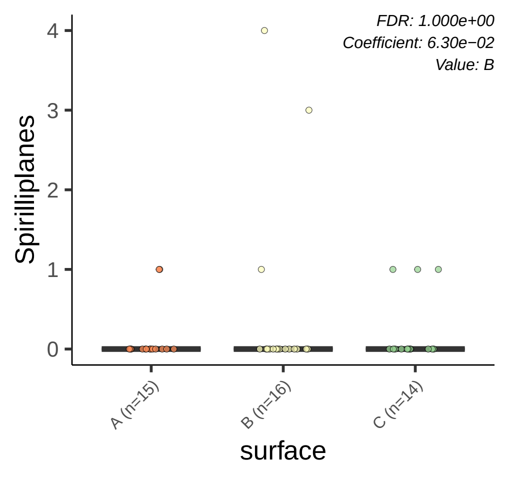
FDR: 1.000e+00
Coefficient: 6.30e−02
Value: B
Spirilliplanes
0
1
2
3
4
A (n=15)
B (n=16)
C (n=14)
surface

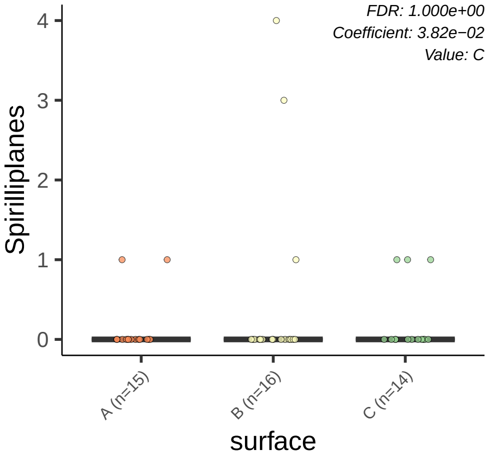

FDR: 1.000e+00
Coefficient: 3.82e−02
Value: C
Spirilliplanes
0
1
2
3
4
A (n=15)
B (n=16)
C (n=14)
surface

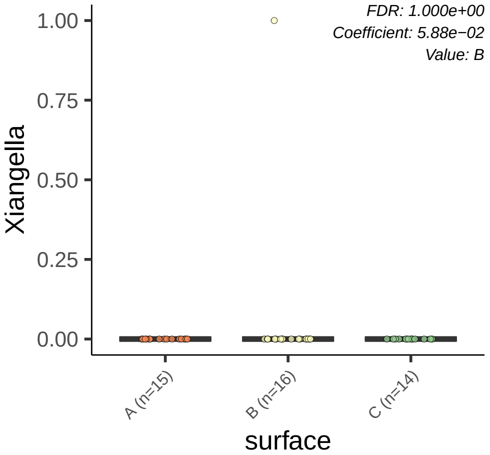

FDR: 1.000e+00
Coefficient: 5.88e−02
Value: B
1.00
0.75
0.50
0.25
0.00
Xiangella
A (n=15)
B (n=16)
C (n=14)
surface

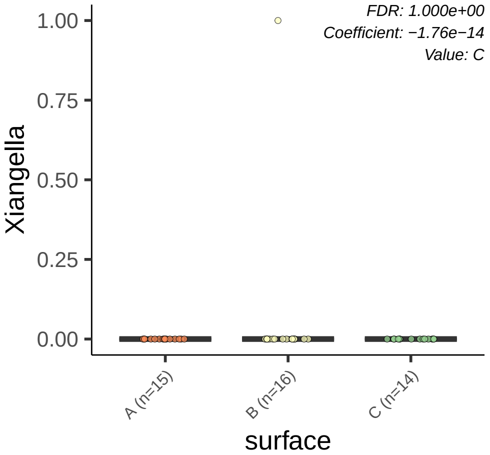
FDR: 1.000e+00
Coefficient: −1.76e−14
Value: C
1.00
0.75
0.50
0.25
0.00
Xiangella
A (n=15)
B (n=16)
C (n=14)
surface

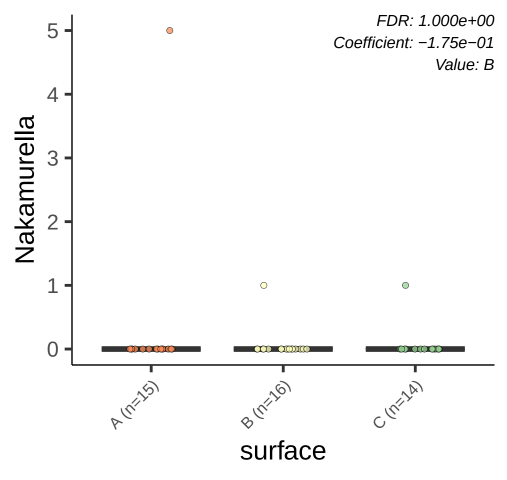
FDR: 1.000e+00
Coefficient: −1.75e−01
Value: B
Nakamurella
5
4
3
2
1
0
A (n=15)
B (n=16)
C (n=14)
surface

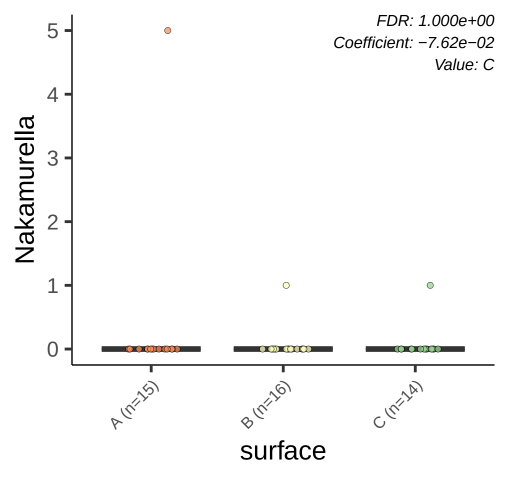
FDR: 1.000e+00
Coefficient: −7.62e−02
Value: C
Nakamurella
0
1
2
3
4
5
A (n=15)
B (n=16)
C (n=14)
surface

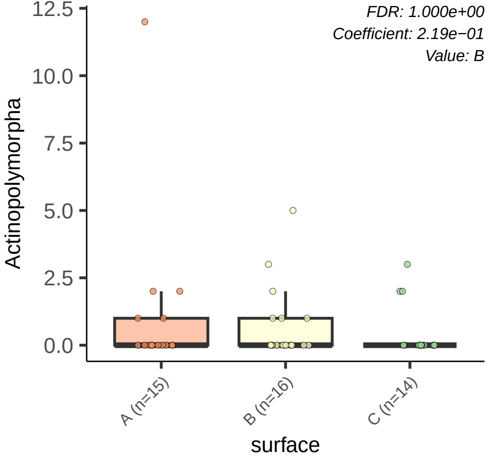

FDR: 1.000e+00
Coefficient: 2.19e−01
Value: B
Actinopolymorpha
12.5
10.0
7.5
5.0
2.5
0.0
A (n=15)
B (n=16)
C (n=14)
surface

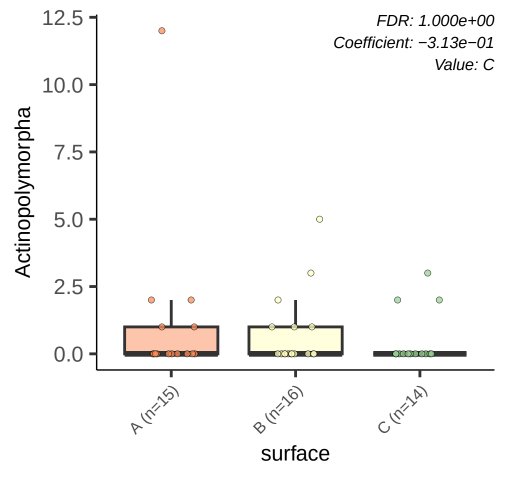
FDR: 1.000e+00
Coefficient: −3.13e−01
Value: C
Actinopolymorpha
12.5
10.0
7.5
5.0
2.5
0.0
A (n=15)
B (n=16)
C (n=14)
surface

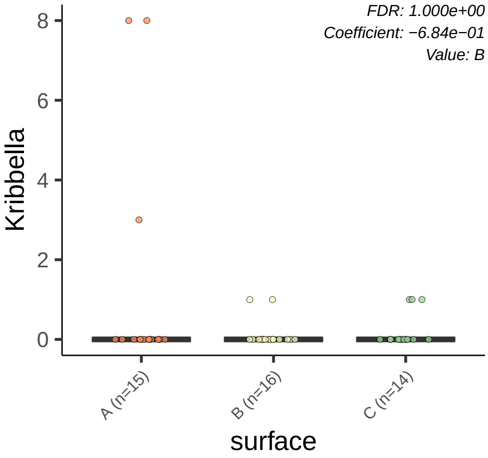

FDR: 1.000e+00
Coefficient: −6.84e−01
Value: B
Kribbella
0
2
4
6
8
A (n=15)
B (n=16)
C (n=14)
surface

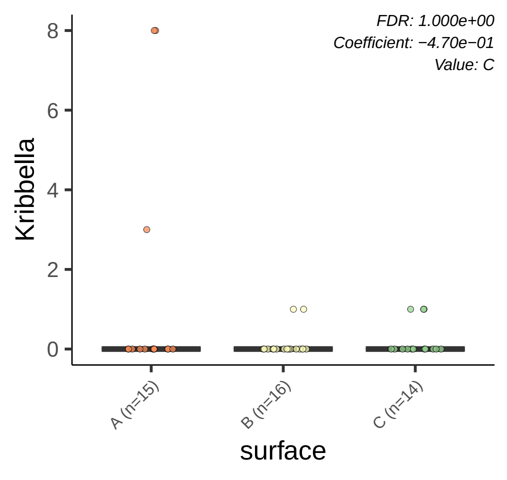
FDR: 1.000e+00
Coefficient: −4.70e−01
Value: C
Kribbella
0
2
4
6
8
A (n=15)
B (n=16)
C (n=14)
surface

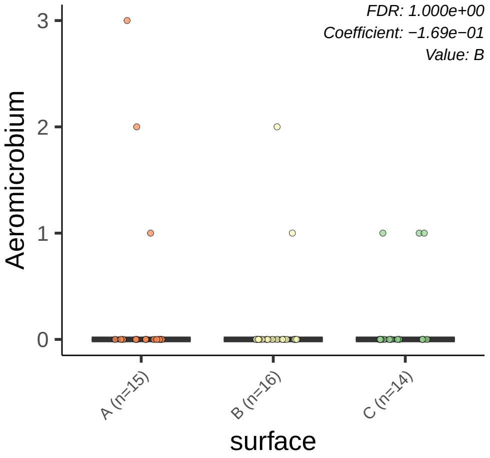

FDR: 1.000e+00
Coefficient: −1.69e−01
Value: B
Aeromicrobium
0
1
2
3
A (n=15)
B (n=16)
C (n=14)
surface

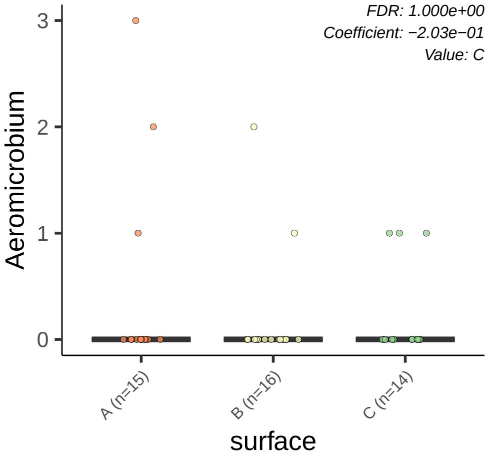
FDR: 1.000e+00
Coefficient: −2.03e−01
Value: C
Aeromicrobium
3
2
1
0
A (n=15)
B (n=16)
C (n=14)
surface

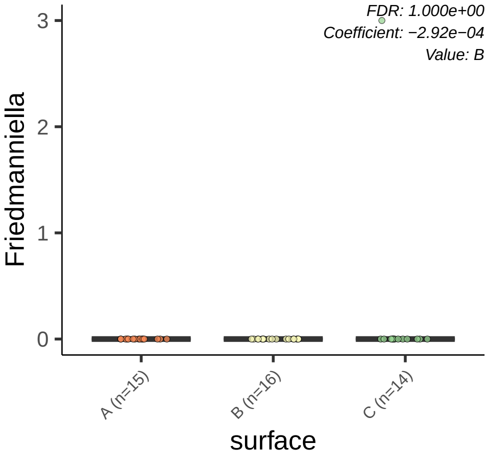
FDR: 1.000e+00
Coefficient: −2.92e−04
Value: B
Friedmanniella
3
2
1
0
A (n=15)
B (n=16)
C (n=14)
surface

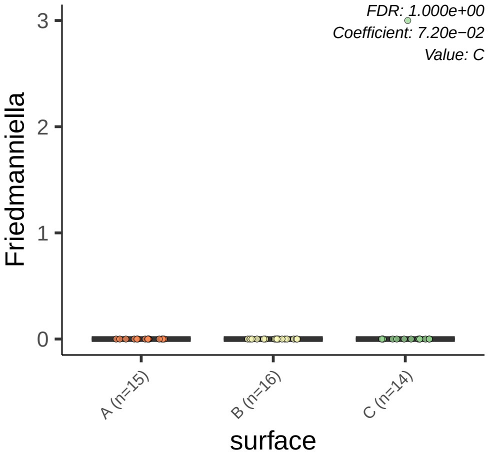
FDR: 1.000e+00
Coefficient: 7.20e−02
Value: C
Friedmanniella
3
2
1
0
A (n=15)
B (n=16)
C (n=14)
surface

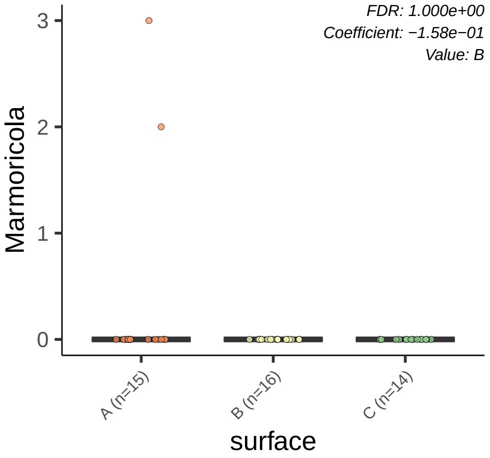

FDR: 1.000e+00
Coefficient: −1.58e−01
Value: B
Marmoricola
3
2
1
0
A (n=15)
B (n=16)
C (n=14)
surface

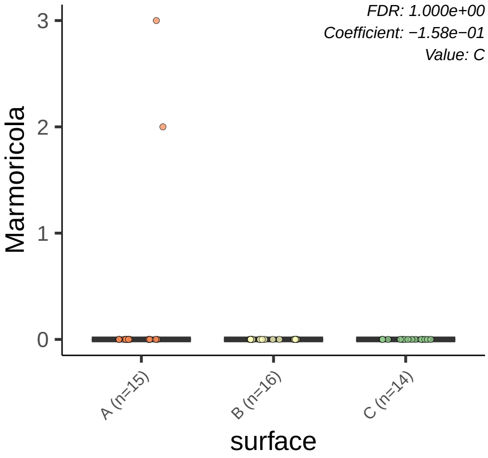
FDR: 1.000e+00
Coefficient: −1.58e−01
Value: C
Marmoricola
3
2
1
0
A (n=15)
B (n=16)
C (n=14)
surface

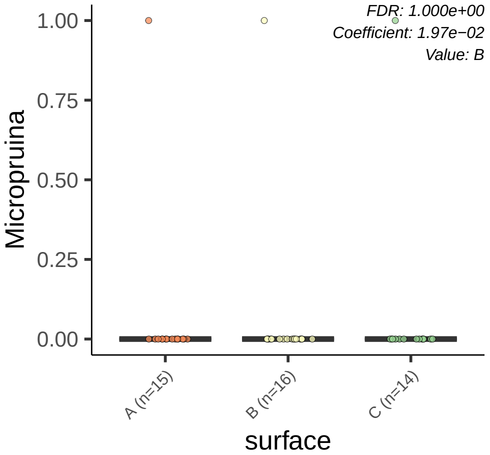
FDR: 1.000e+00
Coefficient: 1.97e−02
Value: B
Micropruina
1.00
0.75
0.50
0.25
0.00
A (n=15)
B (n=16)
C (n=14)
surface

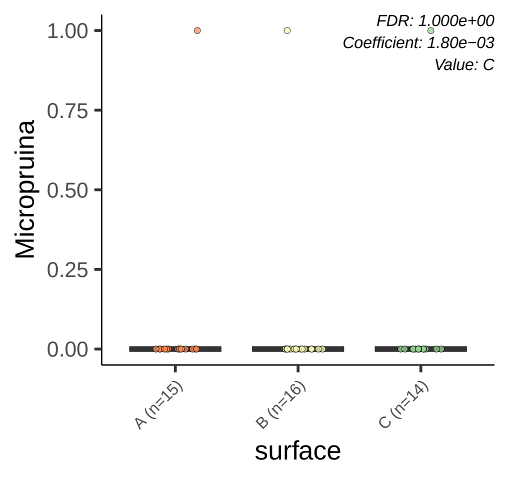
FDR: 1.000e+00
Coefficient: 1.80e−03
Value: C
Micropruina
1.00
0.75
0.50
0.25
0.00
A (n=15)
B (n=16)
C (n=14)
surface

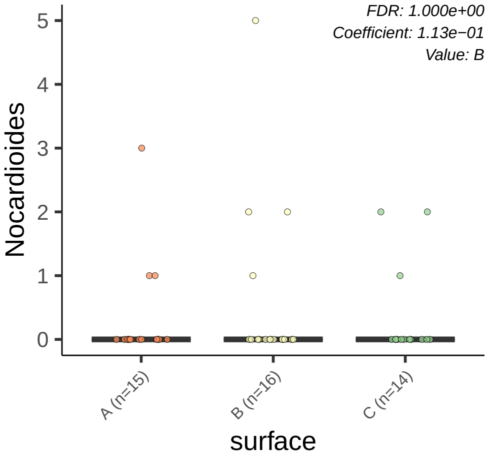
FDR: 1.000e+00
Coefficient: 1.13e−01
Value: B
Nocardioides
0
1
2
3
4
5
A (n=15)
B (n=16)
C (n=14)
surface

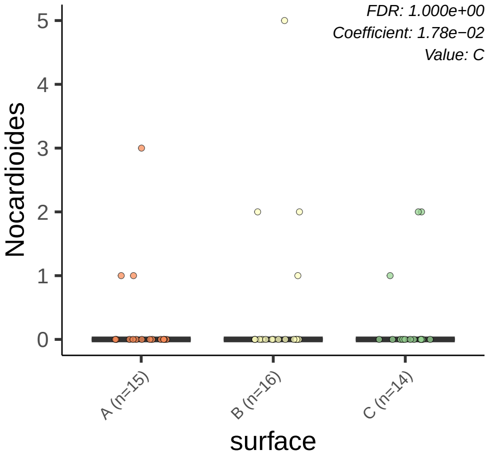
FDR: 1.000e+00
Coefficient: 1.78e−02
Value: C
Nocardioides
0
1
2
3
4
5
A (n=15)
B (n=16)
C (n=14)
surface

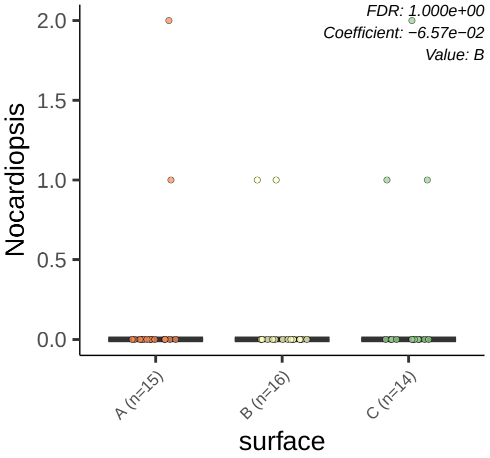
FDR: 1.000e+00
Coefficient: −6.57e−02
Value: B
Nocardiopsis
2.0
1.5
1.0
0.5
0.0
A (n=15)
B (n=16)
C (n=14)
surface

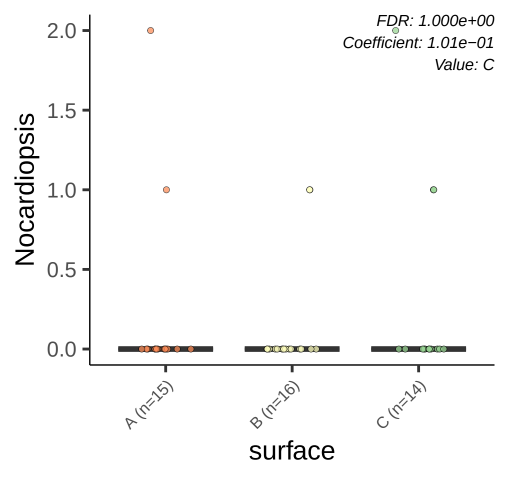
FDR: 1.000e+00
Coefficient: 1.01e−01
Value: C
Nocardiopsis
2.0
1.5
1.0
0.5
0.0
A (n=15)
B (n=16)
C (n=14)
surface

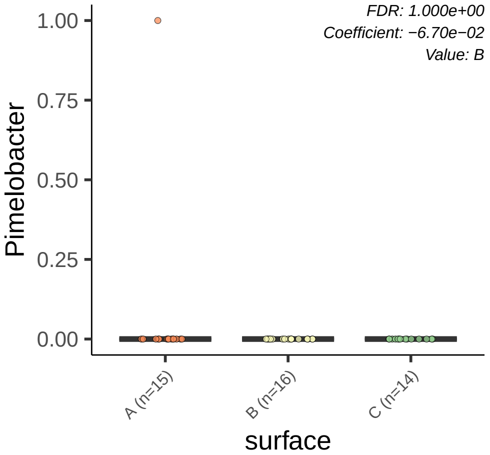
FDR: 1.000e+00
Coefficient: −6.70e−02
Value: B
Pimelobacter
1.00
0.75
0.50
0.25
0.00
A (n=15)
B (n=16)
C (n=14)
surface

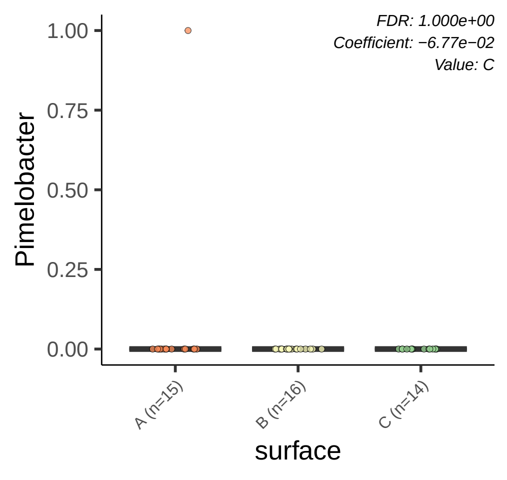

FDR: 1.000e+00
Coefficient: −6.77e−02
Value: C
Pimelobacter
1.00
0.75
0.50
0.25
0.00
A (n=15)
B (n=16)
C (n=14)
surface

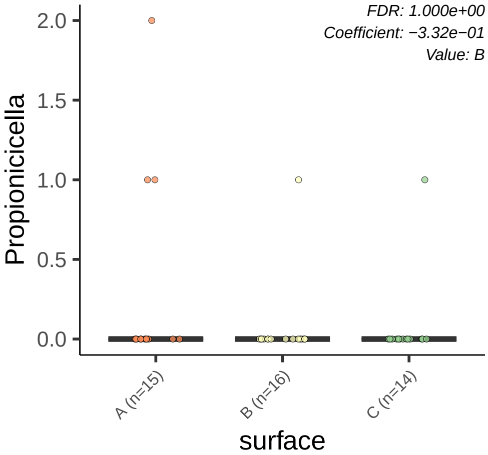

FDR: 1.000e+00
Coefficient: −3.32e−01
Value: B
Propionicicella
2.0
1.5
1.0
0.5
0.0
A (n=15)
B (n=16)
C (n=14)
surface

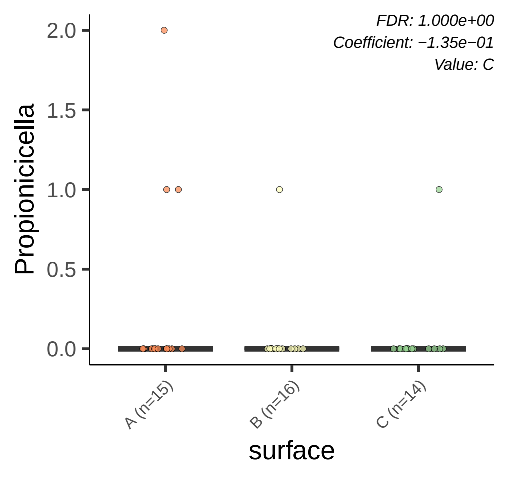
FDR: 1.000e+00
Coefficient: −1.35e−01
Value: C
Propionicicella
2.0
1.5
1.0
0.5
0.0
A (n=15)
B (n=16)
C (n=14)
surface

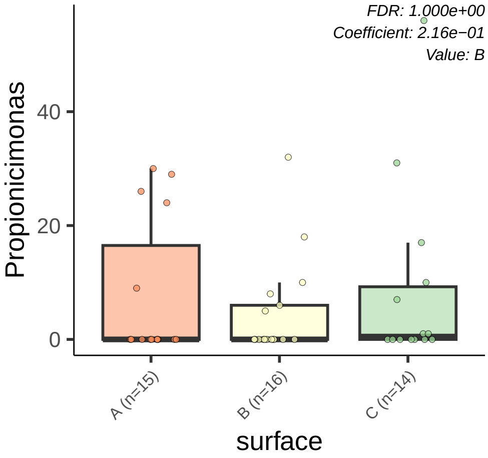
FDR: 1.000e+00
Coefficient: 2.16e−01
Value: B
Propionicimonas
40
20
0
A (n=15)
B (n=16)
C (n=14)
surface

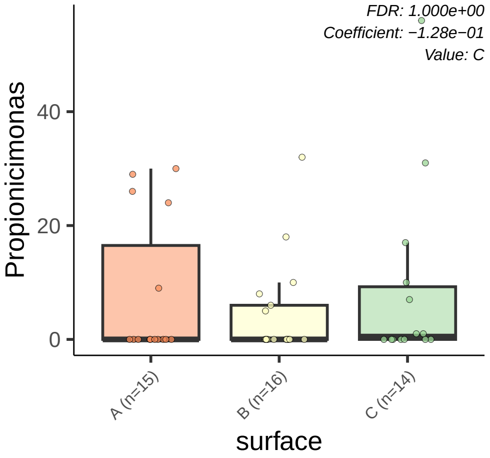
FDR: 1.000e+00
Coefficient: −1.28e−01
Value: C
Propionicimonas
40
20
0
A (n=15)
B (n=16)
C (n=14)
surface

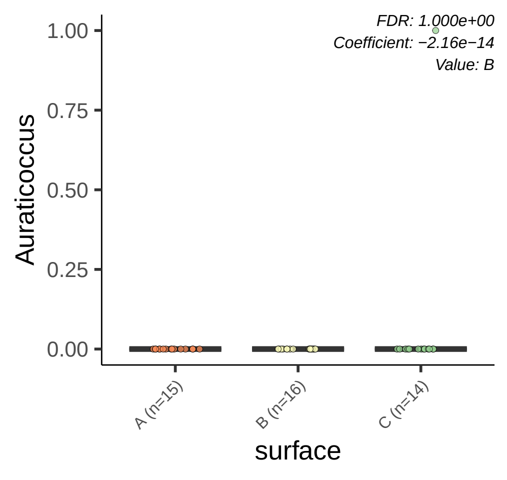
FDR: 1.000e+00
Coefficient: −2.16e−14
Value: B
Auraticoccus
1.00
0.75
0.50
0.25
0.00
A (n=15)
B (n=16)
C (n=14)
surface

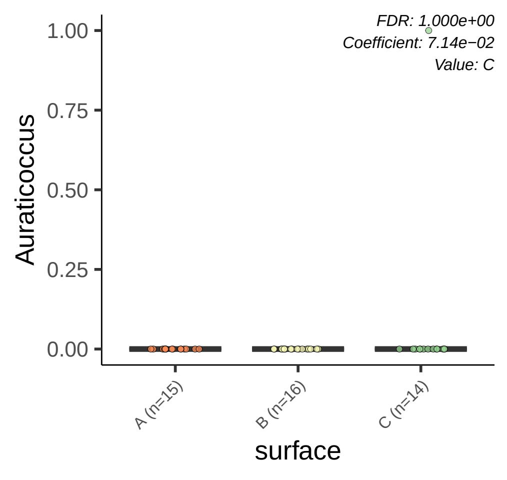
FDR: 1.000e+00
Coefficient: 7.14e−02
Value: C
Auraticoccus
1.00
0.75
0.50
0.25
0.00
A (n=15)
B (n=16)
C (n=14)
surface

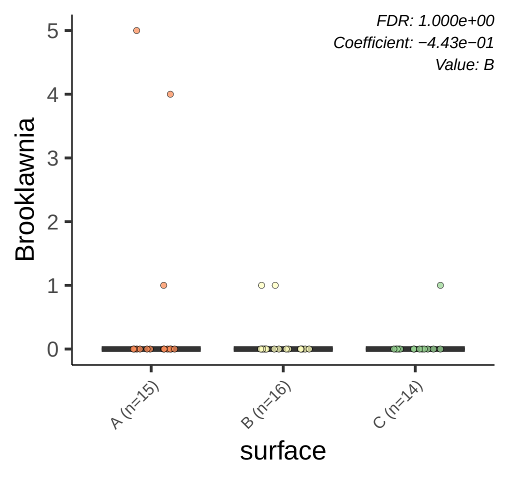
FDR: 1.000e+00
Coefficient: −4.43e−01
Value: B
Brooklawnia
5
4
3
2
1
0
A (n=15)
B (n=16)
C (n=14)
surface

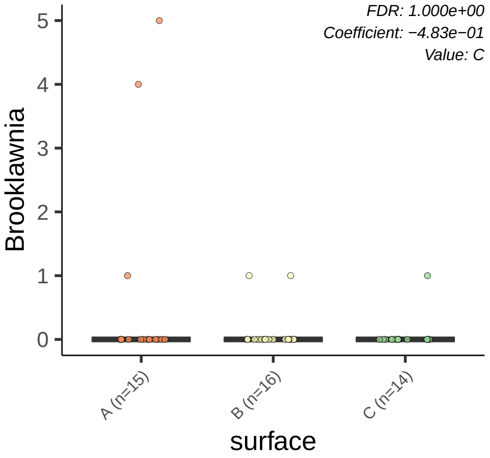
FDR: 1.000e+00
Coefficient: −4.83e−01
Value: C
Brooklawnia
5
4
3
2
1
0
A (n=15)
B (n=16)
C (n=14)
surface

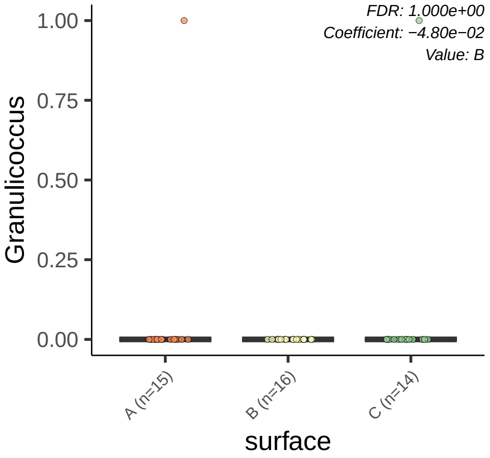

FDR: 1.000e+00
Coefficient: −4.80e−02
Value: B
Granulicoccus
1.00
0.75
0.50
0.25
0.00
A (n=15)
B (n=16)
C (n=14)
surface

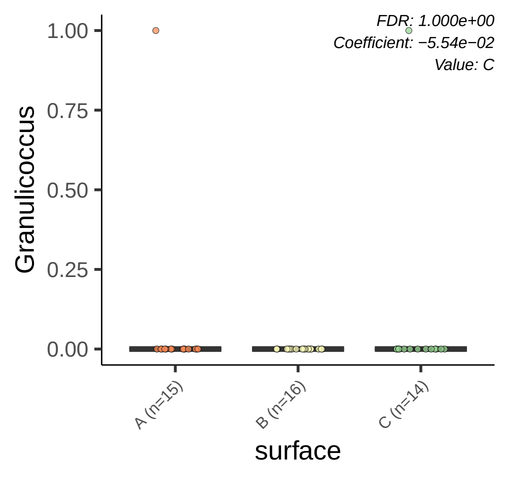
FDR: 1.000e+00
Coefficient: −5.54e−02
Value: C
Granulicoccus
1.00
0.75
0.50
0.25
0.00
A (n=15)
B (n=16)
C (n=14)
surface

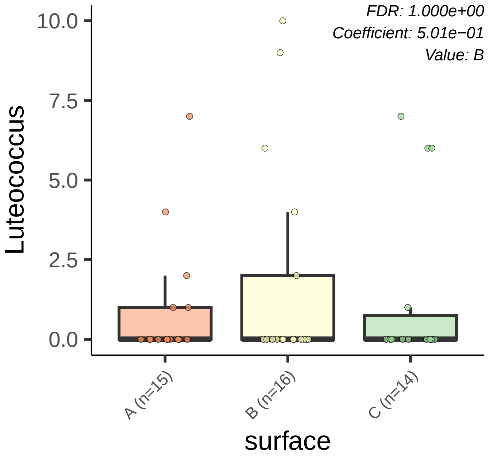

FDR: 1.000e+00
Coefficient: 5.01e−01
Value: B
Luteococcus
10.0
7.5
5.0
2.5
0.0
A (n=15)
B (n=16)
C (n=14)
surface

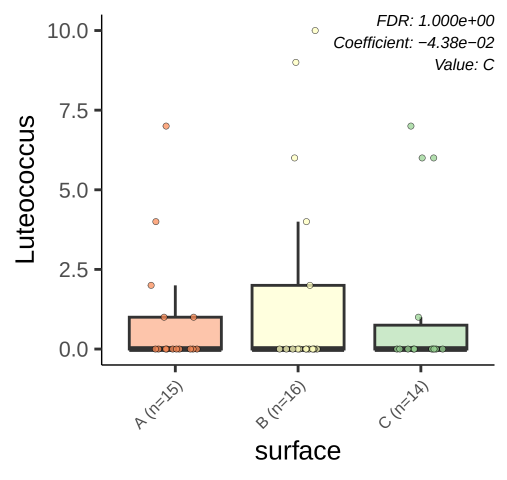
FDR: 1.000e+00
Coefficient: −4.38e−02
Value: C
Luteococcus
10.0
7.5
5.0
2.5
0.0
A (n=15)
B (n=16)
C (n=14)
surface

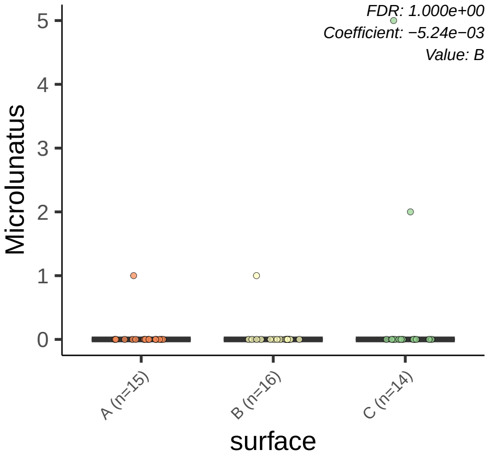
FDR: 1.000e+00
Coefficient: −5.24e−03
Value: B
Microlunatus
5
4
3
2
1
0
A (n=15)
B (n=16)
C (n=14)
surface

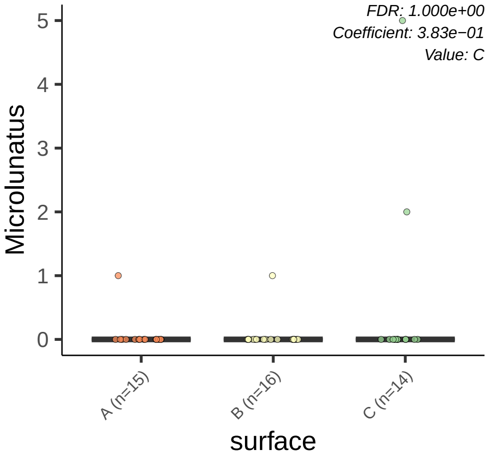
FDR: 1.000e+00
Coefficient: 3.83e−01
Value: C
Microlunatus
5
4
3
2
1
0
A (n=15)
B (n=16)
C (n=14)
surface

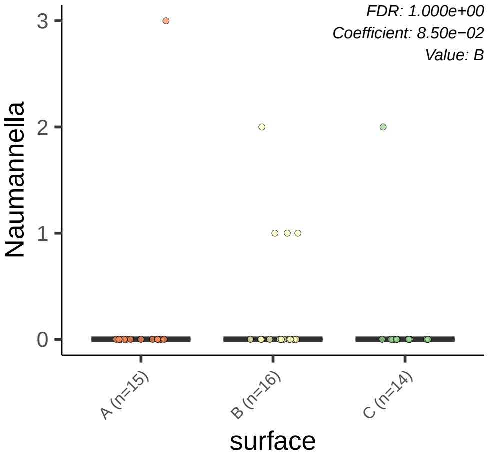
FDR: 1.000e+00
Coefficient: 8.50e−02
Value: B
Naumannella
3
2
1
0
A (n=15)
B (n=16)
C (n=14)
surface

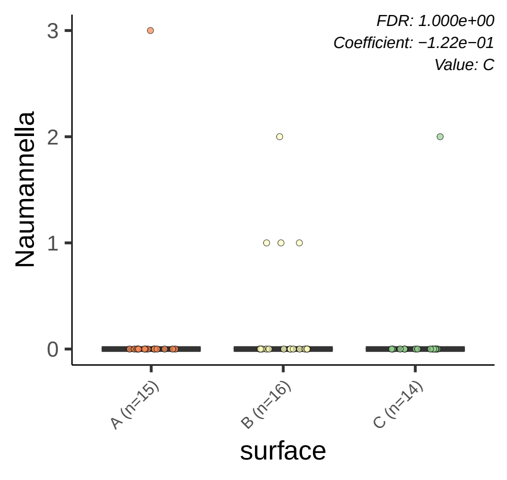

FDR: 1.000e+00
Coefficient: −1.22e−01
Value: C
Naumannella
3
2
1
0
A (n=15)
B (n=16)
C (n=14)
surface

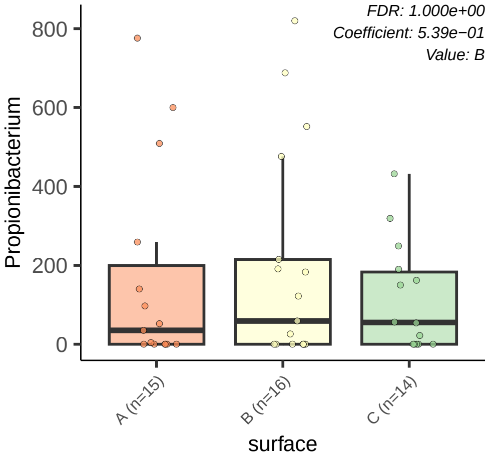
FDR: 1.000e+00
Coefficient: 5.39e−01
Value: B
Propionibacterium
800
600
400
200
0
A (n=15)
B (n=16)
C (n=14)
surface

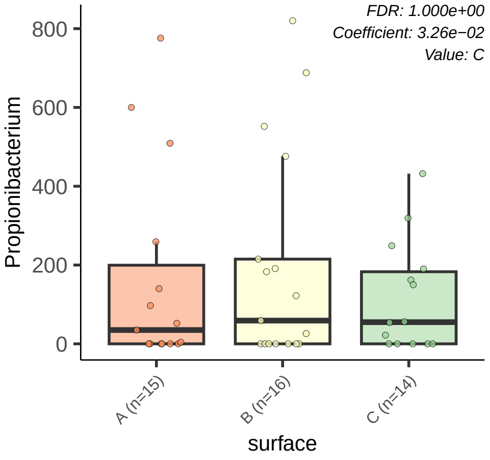

FDR: 1.000e+00
Coefficient: 3.26e−02
Value: C
Propionibacterium
0
200
400
600
800
A (n=15)
B (n=16)
C (n=14)
surface

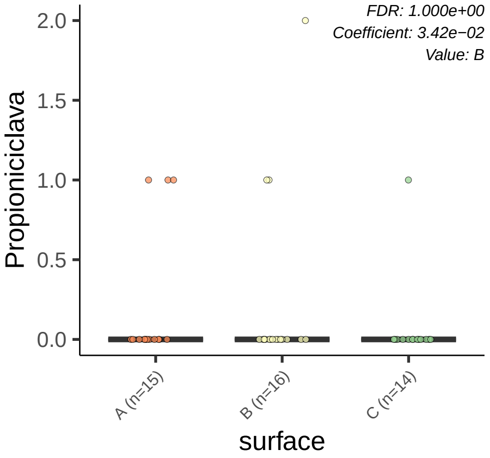
FDR: 1.000e+00
Coefficient: 3.42e−02
Value: B
Propioniciclava
2.0
1.5
1.0
0.5
0.0
A (n=15)
B (n=16)
C (n=14)
surface

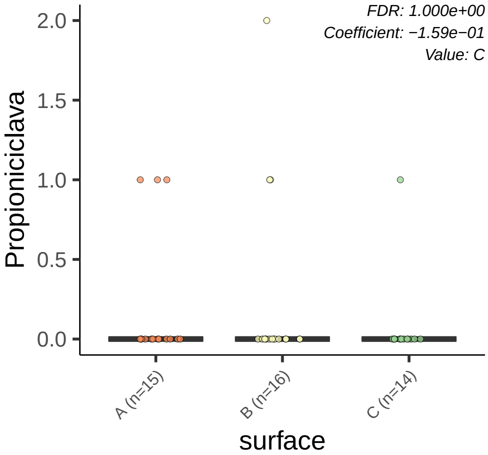
FDR: 1.000e+00
Coefficient: −1.59e−01
Value: C
Propioniciclava
2.0
1.5
1.0
0.5
0.0
A (n=15)
B (n=16)
C (n=14)
surface

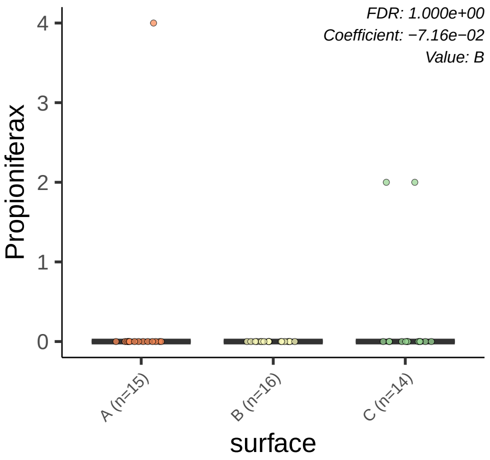
FDR: 1.000e+00
Coefficient: −7.16e−02
Value: B
Propioniferax
4
3
2
1
0
A (n=15)
B (n=16)
C (n=14)
surface

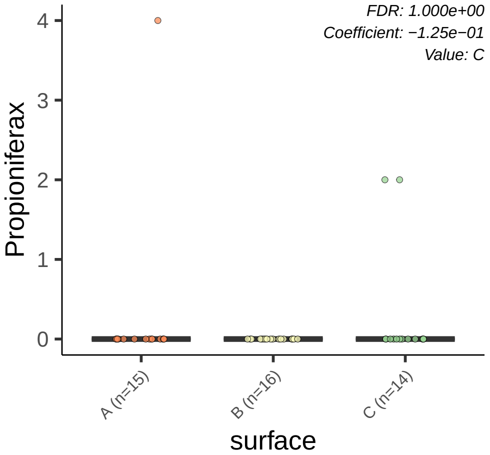
FDR: 1.000e+00
Coefficient: −1.25e−01
Value: C
Propioniferax
4
3
2
1
0
A (n=15)
B (n=16)
C (n=14)
surface

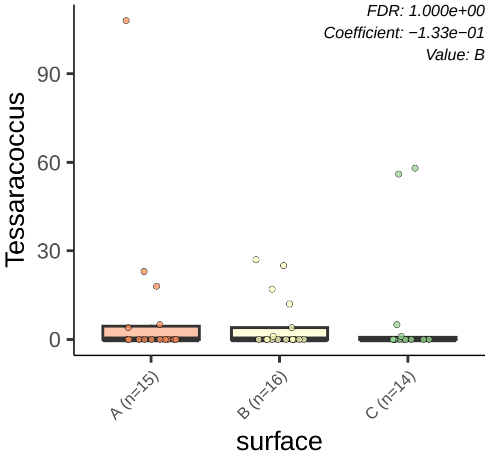
FDR: 1.000e+00
Coefficient: −1.33e−01
Value: B
Tessaracoccus
90
60
30
0
A (n=15)
B (n=16)
C (n=14)
surface

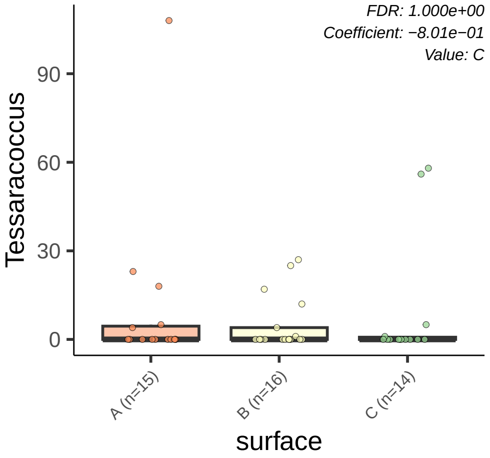
FDR: 1.000e+00
Coefficient: −8.01e−01
Value: C
Tessaracoccus
90
60
30
0
A (n=15)
B (n=16)
C (n=14)
surface

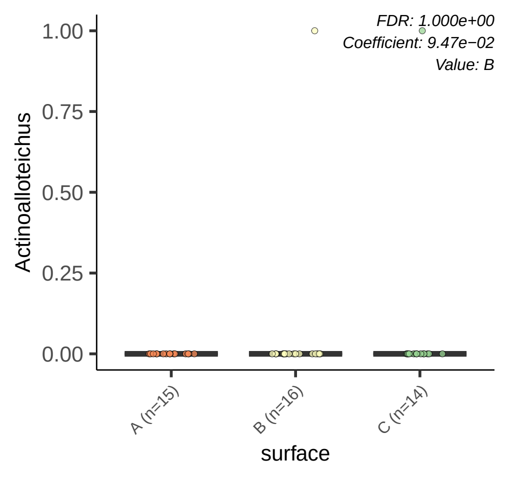

FDR: 1.000e+00
Coefficient: 9.47e−02
Value: B
Actinoalloteichus
1.00
0.75
0.50
0.25
0.00
A (n=15)
B (n=16)
C (n=14)
surface

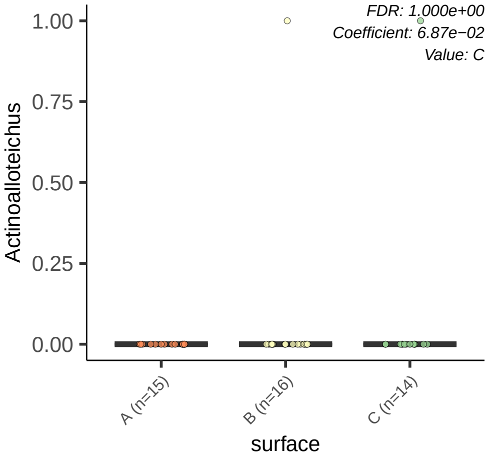

FDR: 1.000e+00
Coefficient: 6.87e−02
Value: C
1.00
0.75
0.50
0.25
0.00
Actinoalloteichus
A (n=15)
B (n=16)
C (n=14)
surface

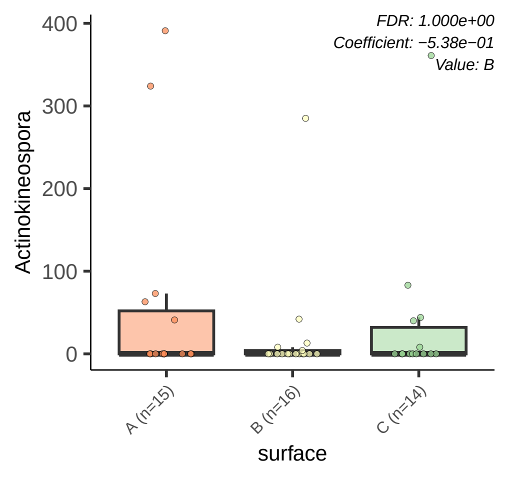
FDR: 1.000e+00
Coefficient: −5.38e−01
Value: B
Actinokineospora
400
300
200
100
0
A (n=15)
B (n=16)
C (n=14)
surface

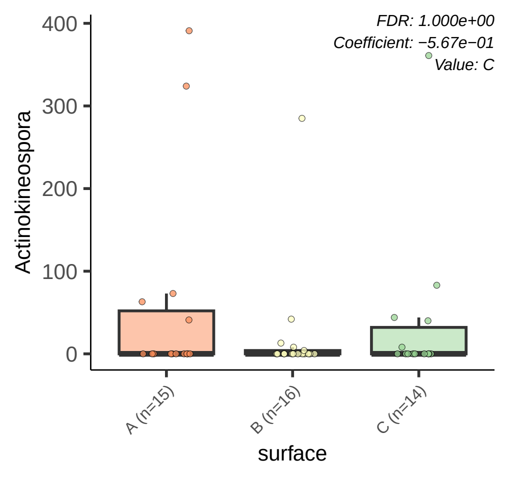
FDR: 1.000e+00
Coefficient: −5.67e−01
Value: C
Actinokineospora
400
300
200
100
0
A (n=15)
B (n=16)
C (n=14)
surface

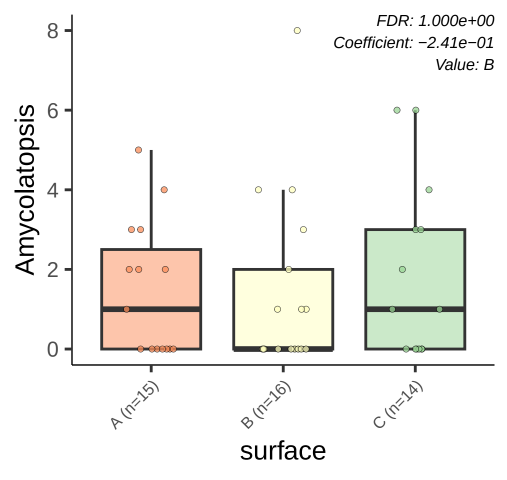

FDR: 1.000e+00
Coefficient: −2.41e−01
Value: B
Amycolatopsis
0
2
4
6
8
A (n=15)
B (n=16)
C (n=14)
surface

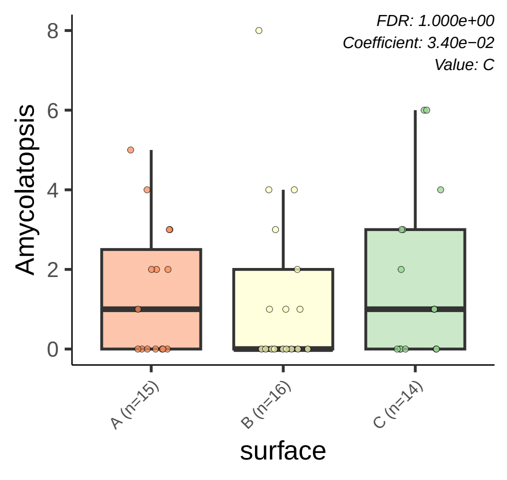
FDR: 1.000e+00
Coefficient: 3.40e−02
Value: C
Amycolatopsis
0
2
4
6
8
A (n=15)
B (n=16)
C (n=14)
surface

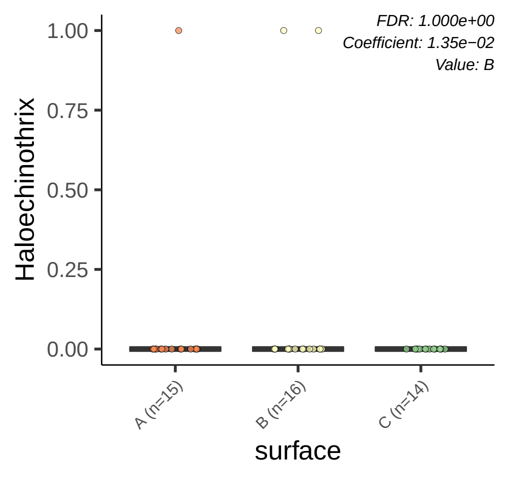
FDR: 1.000e+00
Coefficient: 1.35e−02
Value: B
Haloechinothrix
1.00
0.75
0.50
0.25
0.00
A (n=15)
B (n=16)
C (n=14)
surface

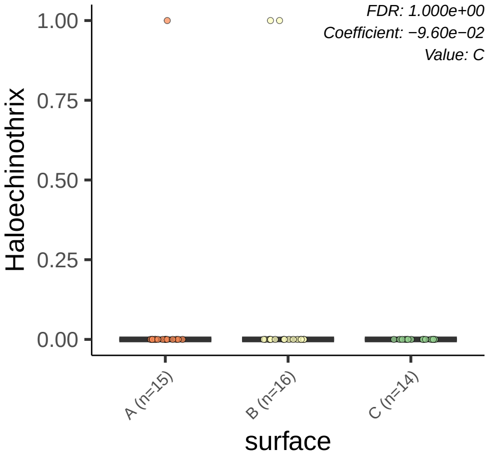

FDR: 1.000e+00
Coefficient: −9.60e−02
Value: C
Haloechinothrix
1.00
0.75
0.50
0.25
0.00
A (n=15)
B (n=16)
C (n=14)
surface

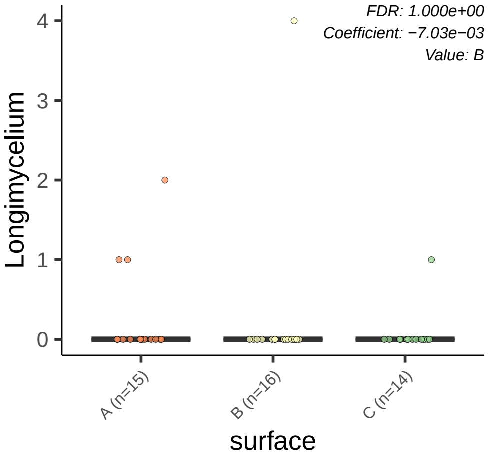
FDR: 1.000e+00
Coefficient: −7.03e−03
Value: B
Longimycelium
0
1
2
3
4
A (n=15)
B (n=16)
C (n=14)
surface

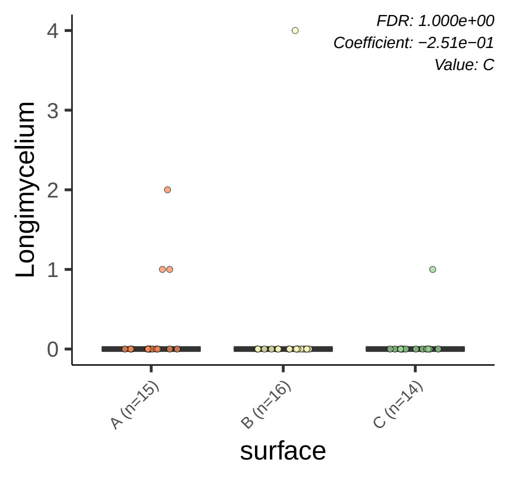

FDR: 1.000e+00
Coefficient: −2.51e−01
Value: C
Longimycelium
0
1
2
3
4
A (n=15)
B (n=16)
C (n=14)
surface

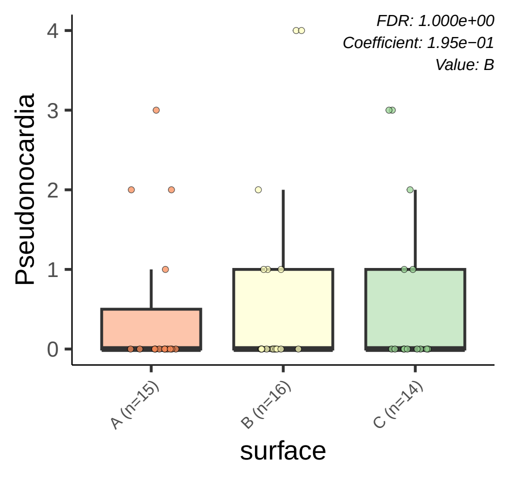
FDR: 1.000e+00
Coefficient: 1.95e−01
Value: B
Pseudonocardia
0
1
2
3
4
A (n=15)
B (n=16)
C (n=14)
surface

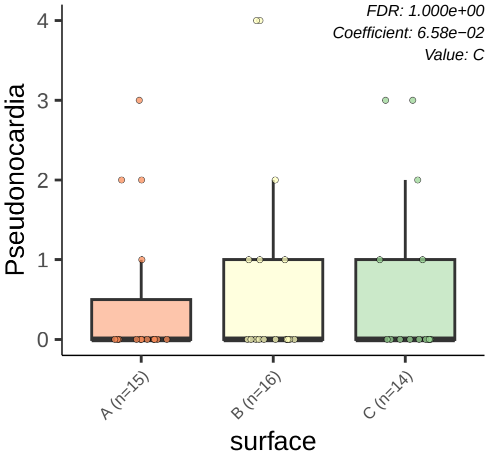
FDR: 1.000e+00
Coefficient: 6.58e−02
Value: C
Pseudonocardia
0
1
2
3
4
A (n=15)
B (n=16)
C (n=14)
surface

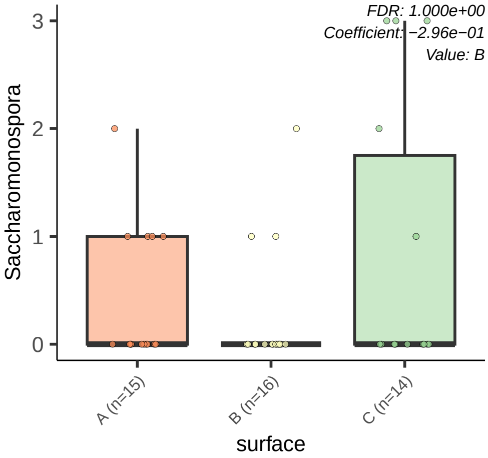
FDR: 1.000e+00
Coefficient: −2.96e−01
Value: B
Saccharomonospora
3
2
1
0
A (n=15)
B (n=16)
C (n=14)
surface

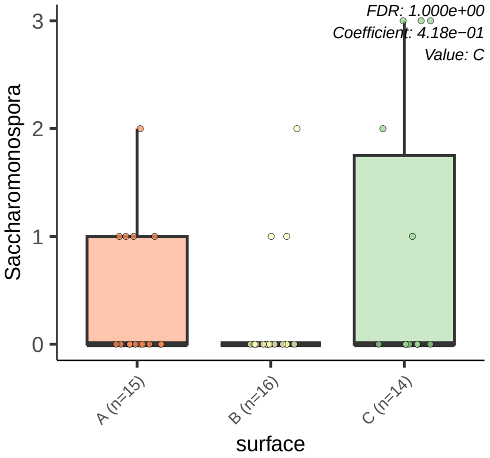
FDR: 1.000e+00
Coefficient: 4.18e−01
Value: C
Saccharomonospora
3
2
1
0
A (n=15)
B (n=16)
C (n=14)
surface

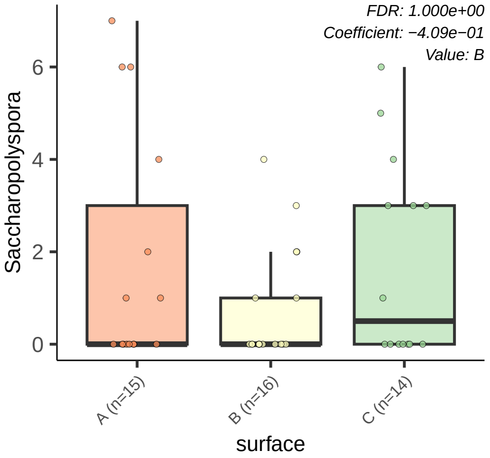

FDR: 1.000e+00
Coefficient: −4.09e−01
Value: B
Saccharopolyspora
0
2
4
6
A (n=15)
B (n=16)
C (n=14)
surface

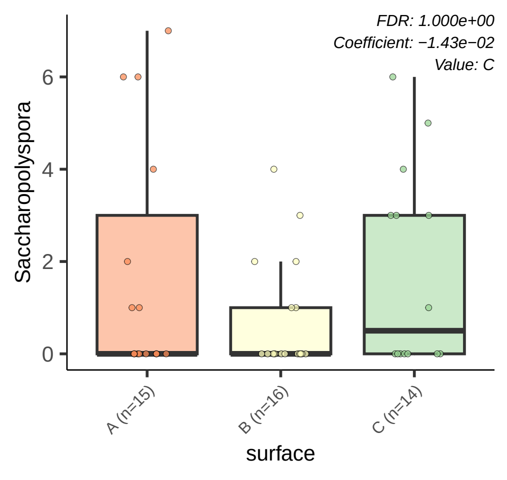

FDR: 1.000e+00
Coefficient: −1.43e−02
Value: C
Saccharopolyspora
0
2
4
6
A (n=15)
B (n=16)
C (n=14)
surface

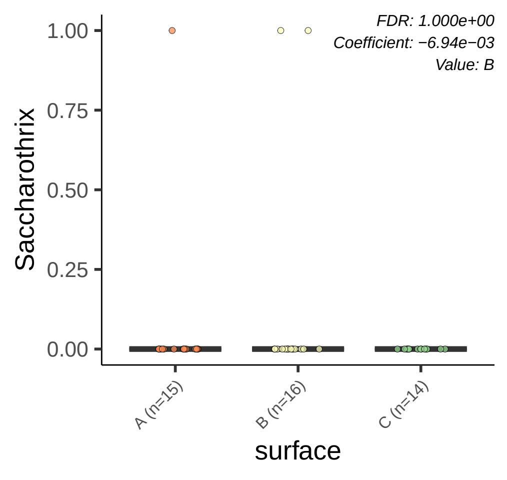
FDR: 1.000e+00
Coefficient: −6.94e−03
Value: B
Saccharothrix
1.00
0.75
0.50
0.25
0.00
A (n=15)
B (n=16)
C (n=14)
surface

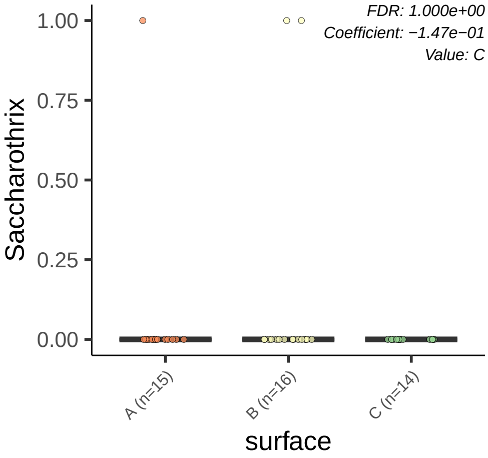

FDR: 1.000e+00
Coefficient: −1.47e−01
Value: C
Saccharothrix
1.00
0.75
0.50
0.25
0.00
A (n=15)
B (n=16)
C (n=14)
surface

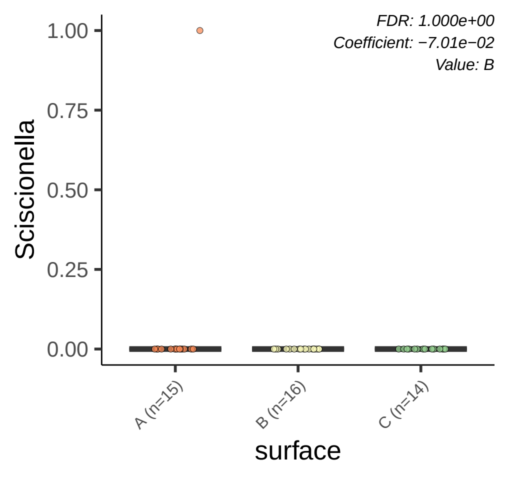
FDR: 1.000e+00
Coefficient: −7.01e−02
Value: B
Sciscionella
1.00
0.75
0.50
0.25
0.00
A (n=15)
B (n=16)
C (n=14)
surface

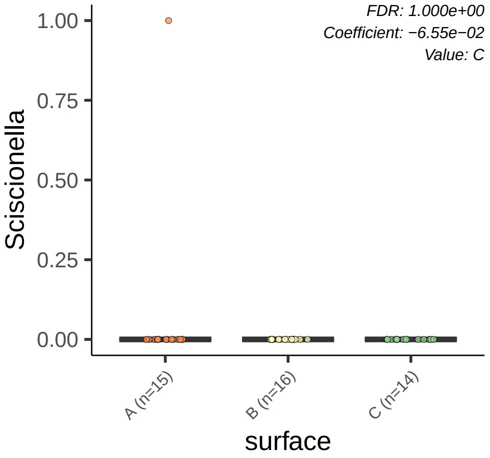
FDR: 1.000e+00
Coefficient: −6.55e−02
Value: C
Sciscionella
1.00
0.75
0.50
0.25
0.00
A (n=15)
B (n=16)
C (n=14)
surface

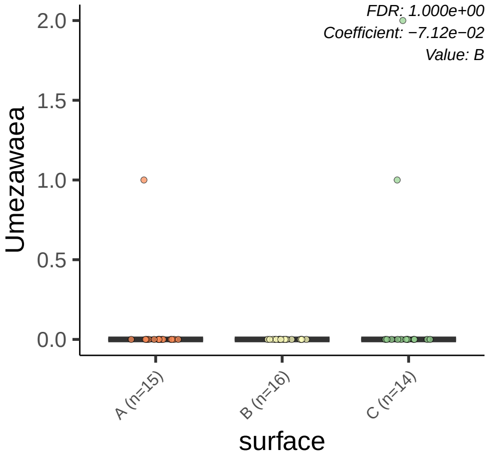
FDR: 1.000e+00
Coefficient: −7.12e−02
Value: B
Umezawaea
2.0
1.5
1.0
0.5
0.0
A (n=15)
B (n=16)
C (n=14)
surface

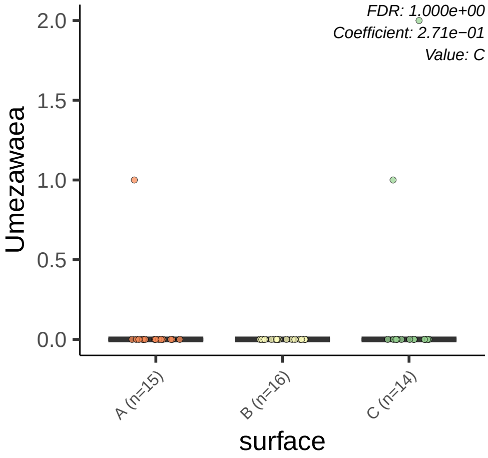

FDR: 1.000e+00
Coefficient: 2.71e−01
Value: C
Umezawaea
2.0
1.5
1.0
0.5
0.0
A (n=15)
B (n=16)
C (n=14)
surface

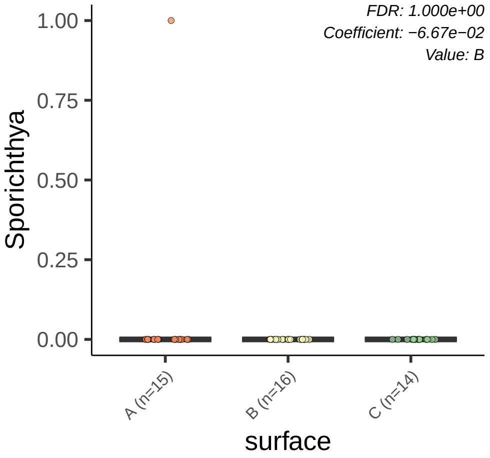
FDR: 1.000e+00
Coefficient: −6.67e−02
Value: B
Sporichthya
1.00
0.75
0.50
0.25
0.00
A (n=15)
B (n=16)
C (n=14)
surface

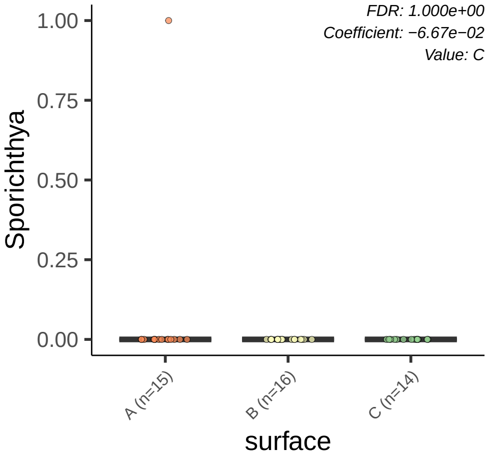
FDR: 1.000e+00
Coefficient: −6.67e−02
Value: C
Sporichthya
1.00
0.75
0.50
0.25
0.00
A (n=15)
B (n=16)
C (n=14)
surface

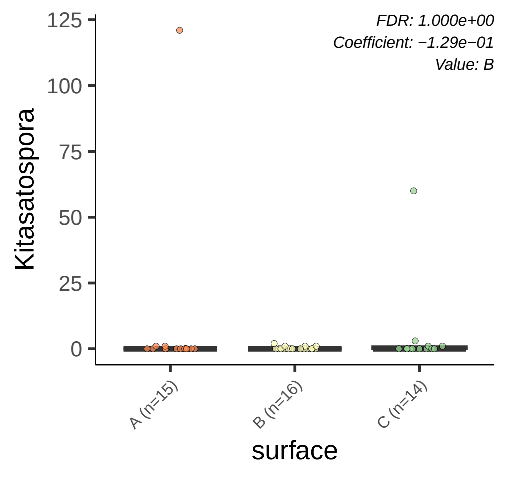
FDR: 1.000e+00
Coefficient: −1.29e−01
Value: B
Kitasatospora
125
100
75
50
25
0
A (n=15)
B (n=16)
C (n=14)
surface

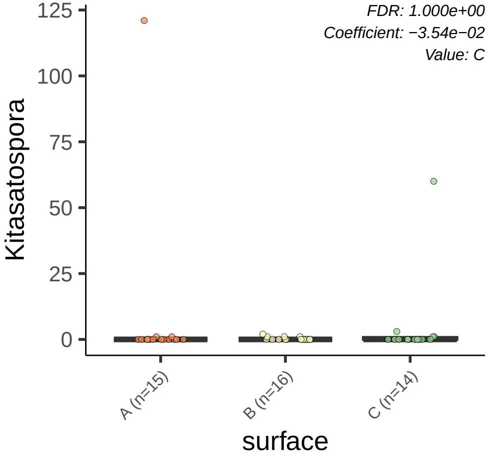

FDR: 1.000e+00
Coefficient: −3.54e−02
Value: C
Kitasatospora
125
100
75
50
25
0
A (n=15)
B (n=16)
C (n=14)
surface

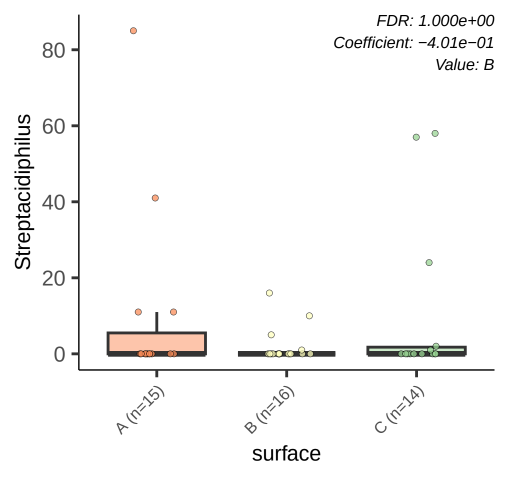

FDR: 1.000e+00
Coefficient: −4.01e−01
Value: B
Streptacidiphilus
0
20
40
60
80
A (n=15)
B (n=16)
C (n=14)
surface

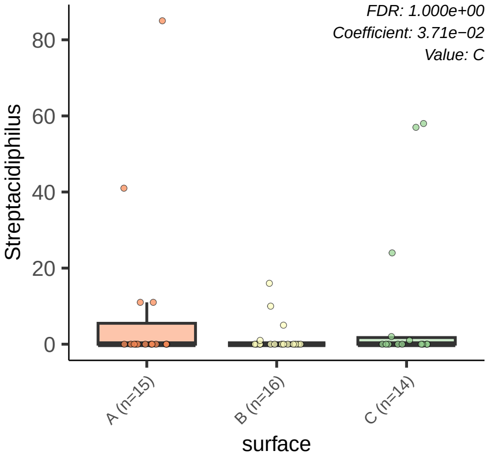

FDR: 1.000e+00
Coefficient: 3.71e−02
Value: C
Streptacidiphilus
0
20
40
60
80
A (n=15)
B (n=16)
C (n=14)
surface

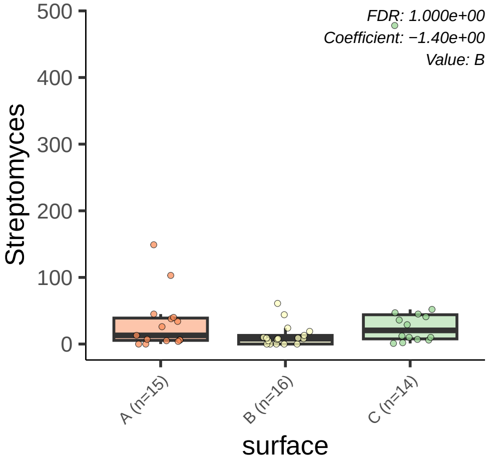

FDR: 1.000e+00
Coefficient: −1.40e+00
Value: B
Streptomyces
0
100
200
300
400
500
A (n=15)
B (n=16)
C (n=14)
surface

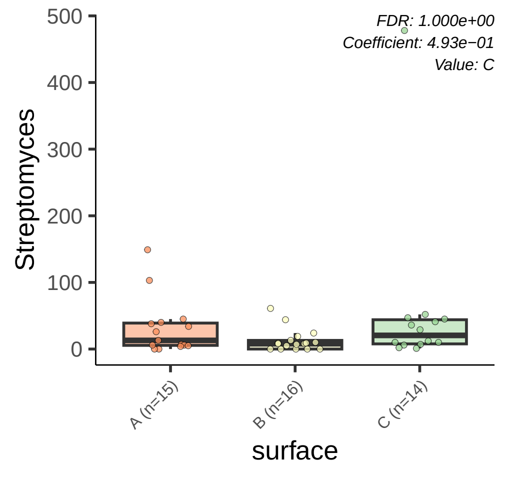
FDR: 1.000e+00
Coefficient: 4.93e−01
Value: C
Streptomyces
500
400
300
200
100
0
A (n=15)
B (n=16)
C (n=14)
surface

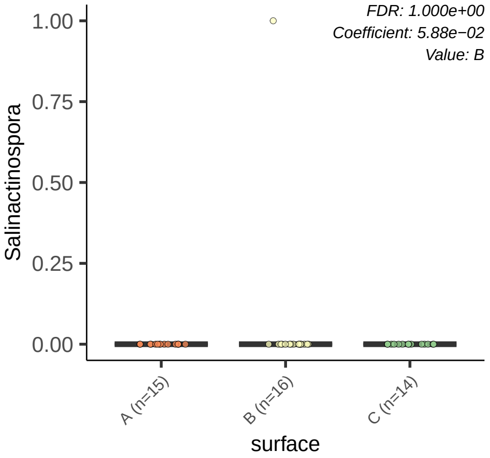
FDR: 1.000e+00
Coefficient: 5.88e−02
Value: B
Salinactinospora
1.00
0.75
0.50
0.25
0.00
A (n=15)
B (n=16)
C (n=14)
surface

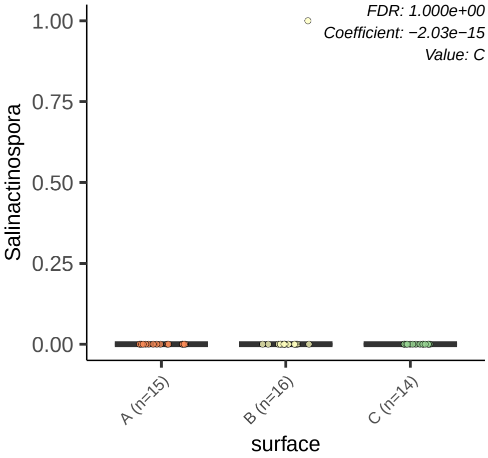

FDR: 1.000e+00
Coefficient: −2.03e−15
Value: C
Salinactinospora
1.00
0.75
0.50
0.25
0.00
A (n=15)
B (n=16)
C (n=14)
surface

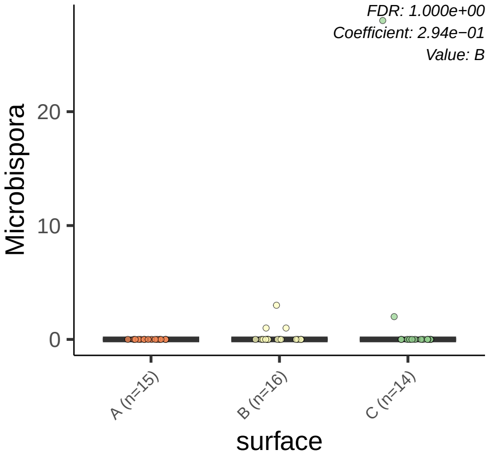

FDR: 1.000e+00
Coefficient: 2.94e−01
Value: B
Microbispora
20
10
0
A (n=15)
B (n=16)
C (n=14)
surface

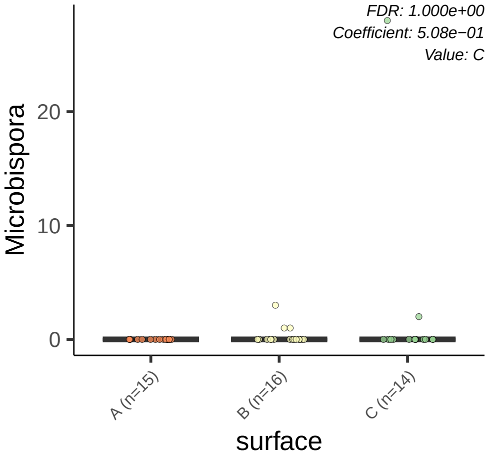

FDR: 1.000e+00
Coefficient: 5.08e−01
Value: C
Microbispora
20
10
0
A (n=15)
B (n=16)
C (n=14)
surface

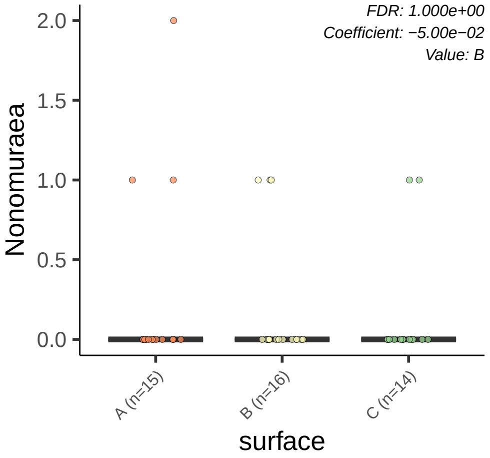

FDR: 1.000e+00
Coefficient: −5.00e−02
Value: B
Nonomuraea
2.0
1.5
1.0
0.5
0.0
A (n=15)
B (n=16)
C (n=14)
surface

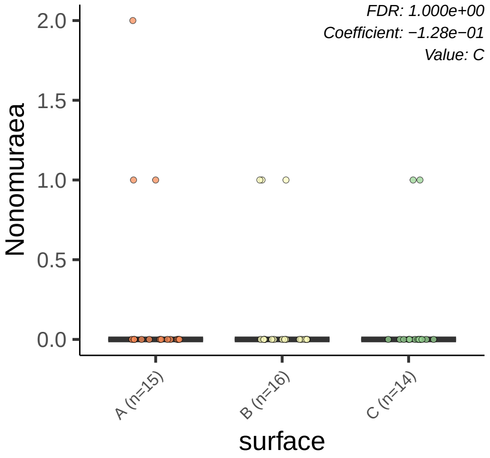

FDR: 1.000e+00
Coefficient: −1.28e−01
Value: C
Nonomuraea
2.0
1.5
1.0
0.5
0.0
A (n=15)
B (n=16)
C (n=14)
surface

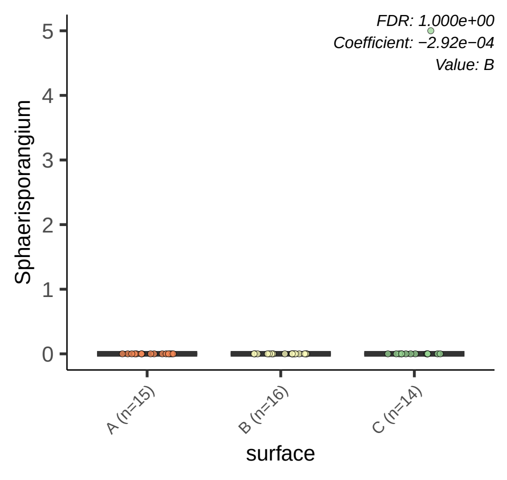

FDR: 1.000e+00
Coefficient: −2.92e−04
Value: B
Sphaerisporangium
5
4
3
2
1
0
A (n=15)
B (n=16)
C (n=14)
surface

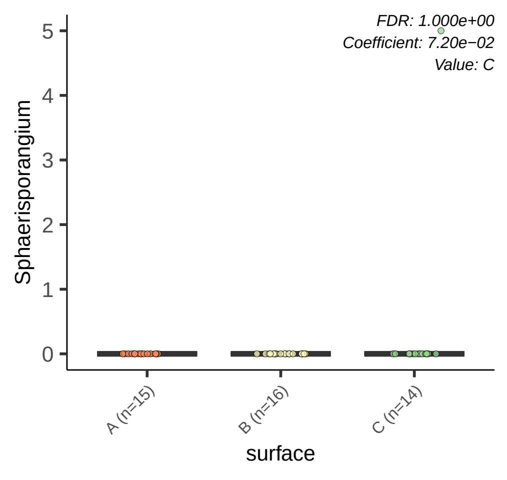

FDR: 1.000e+00
Coefficient: 7.20e−02
Value: C
Sphaerisporangium
0
1
2
3
4
5
A (n=15)
B (n=16)
C (n=14)
surface

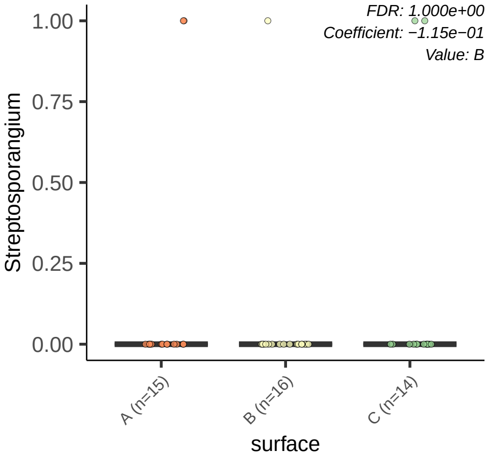
FDR: 1.000e+00
Coefficient: −1.15e−01
Value: B
Streptosporangium
1.00
0.75
0.50
0.25
0.00
A (n=15)
B (n=16)
C (n=14)
surface

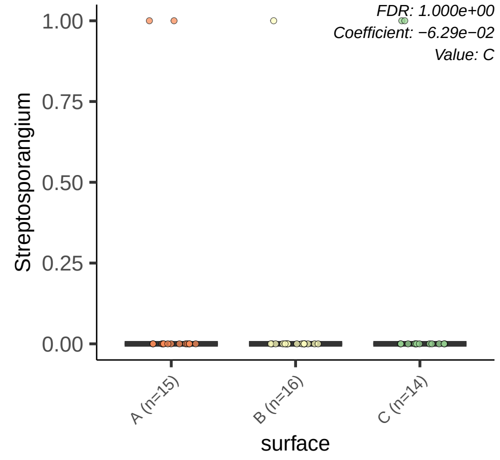
FDR: 1.000e+00
Coefficient: −6.29e−02
Value: C
Streptosporangium
1.00
0.75
0.50
0.25
0.00
A (n=15)
B (n=16)
C (n=14)
surface

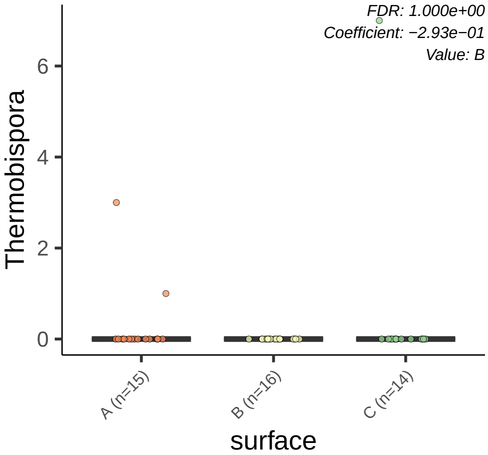

FDR: 1.000e+00
Coefficient: −2.93e−01
Value: B
Thermobispora
0
2
4
6
A (n=15)
B (n=16)
C (n=14)
surface

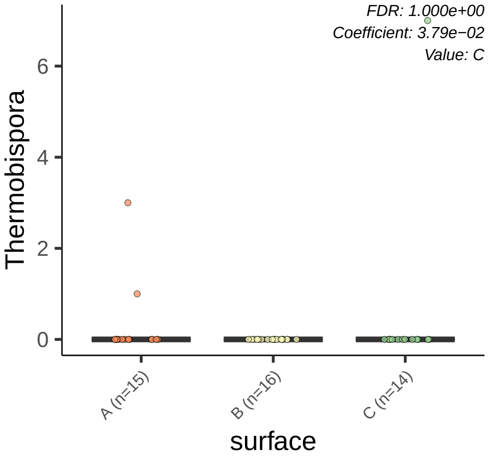

FDR: 1.000e+00
Coefficient: 3.79e−02
Value: C
Thermobispora
0
2
4
6
A (n=15)
B (n=16)
C (n=14)
surface

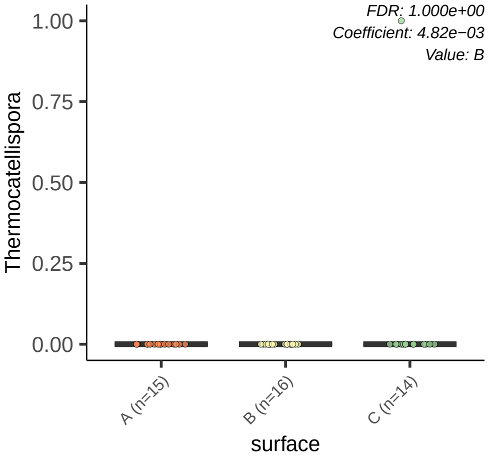
FDR: 1.000e+00
Coefficient: 4.82e−03
Value: B
Thermocatellispora
1.00
0.75
0.50
0.25
0.00
A (n=15)
B (n=16)
C (n=14)
surface

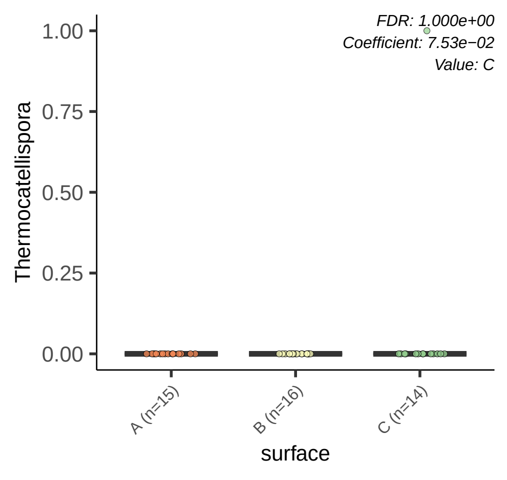

FDR: 1.000e+00
Coefficient: 7.53e−02
Value: C
Thermocatellispora
1.00
0.75
0.50
0.25
0.00
A (n=15)
B (n=16)
C (n=14)
surface

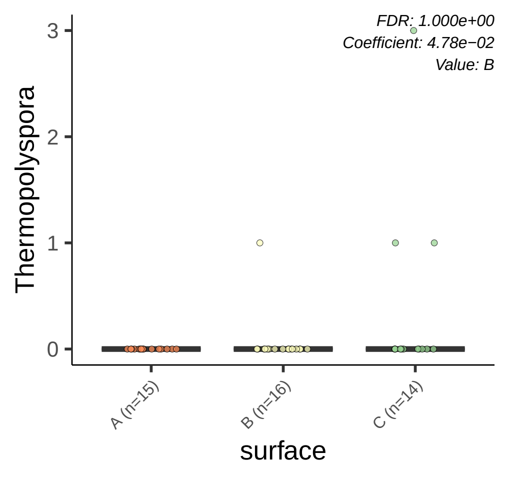

FDR: 1.000e+00
Coefficient: 4.78e−02
Value: B
Thermopolyspora
3
2
1
0
A (n=15)
B (n=16)
C (n=14)
surface

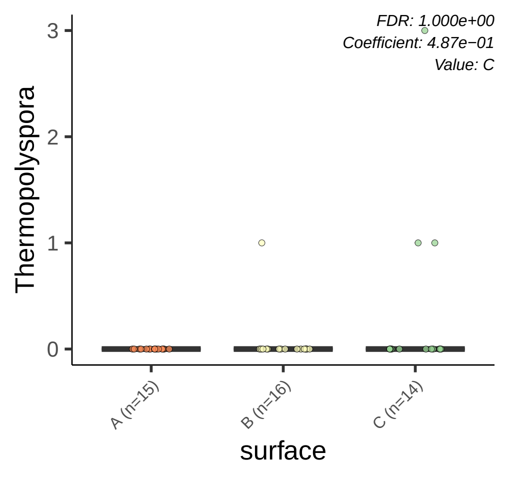

FDR: 1.000e+00
Coefficient: 4.87e−01
Value: C
Thermopolyspora
0
1
2
3
A (n=15)
B (n=16)
C (n=14)
surface

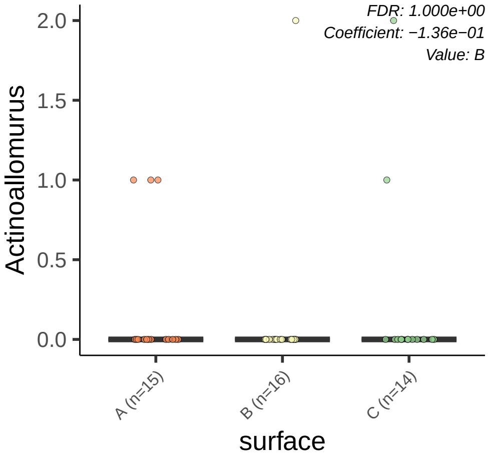
FDR: 1.000e+00
Coefficient: −1.36e−01
Value: B
Actinoallomurus
2.0
1.5
1.0
0.5
0.0
A (n=15)
B (n=16)
C (n=14)
surface

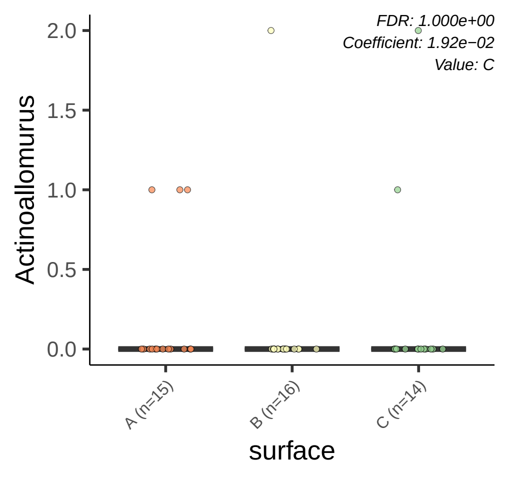
FDR: 1.000e+00
Coefficient: 1.92e−02
Value: C
Actinoallomurus
2.0
1.5
1.0
0.5
0.0
A (n=15)
B (n=16)
C (n=14)
surface

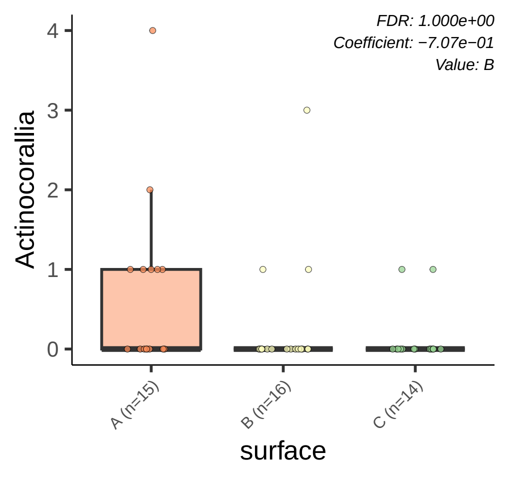
FDR: 1.000e+00
Coefficient: −7.07e−01
Value: B
Actinocorallia
4
3
2
1
0
A (n=15)
B (n=16)
C (n=14)
surface

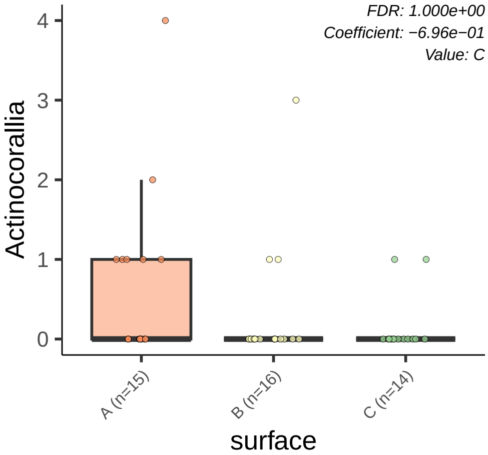
FDR: 1.000e+00
Coefficient: −6.96e−01
Value: C
Actinocorallia
0
1
2
3
4
A (n=15)
B (n=16)
C (n=14)
surface

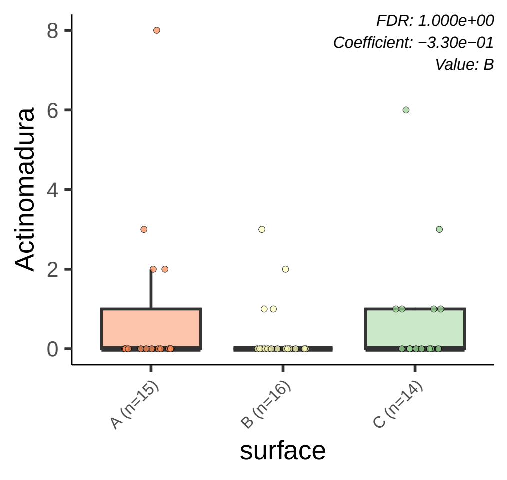
FDR: 1.000e+00
Coefficient: −3.30e−01
Value: B
Actinomadura
0
2
4
6
8
A (n=15)
B (n=16)
C (n=14)
surface

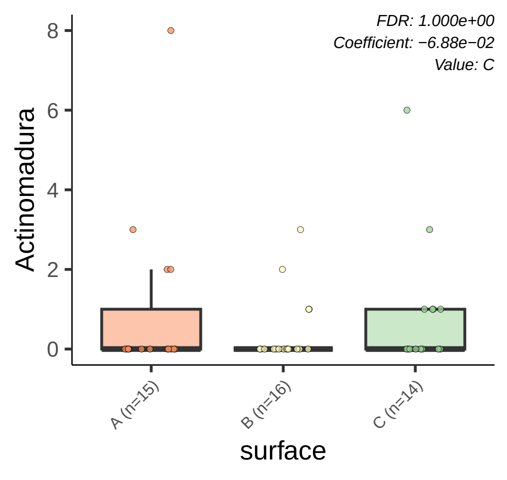
FDR: 1.000e+00
Coefficient: −6.88e−02
Value: C
Actinomadura
8
6
4
2
0
A (n=15)
B (n=16)
C (n=14)
surface

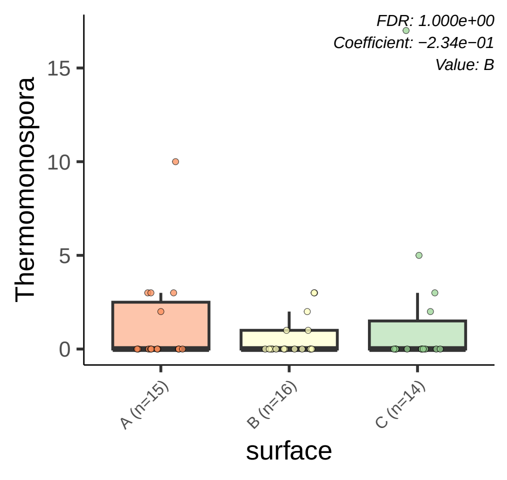
FDR: 1.000e+00
Coefficient: −2.34e−01
Value: B
Thermomonospora
15
10
5
0
A (n=15)
B (n=16)
C (n=14)
surface

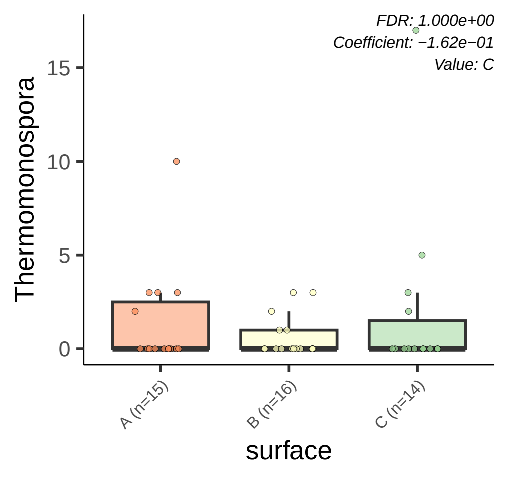
FDR: 1.000e+00
Coefficient: −1.62e−01
Value: C
Thermomonospora
15
10
5
0
A (n=15)
B (n=16)
C (n=14)
surface

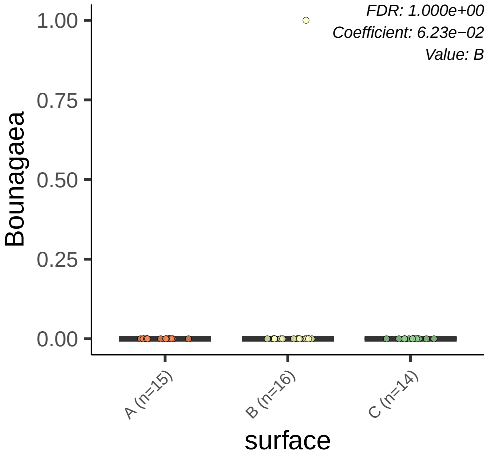

FDR: 1.000e+00
Coefficient: 6.23e−02
Value: B
Bounagaea
1.00
0.75
0.50
0.25
0.00
A (n=15)
B (n=16)
C (n=14)
surface

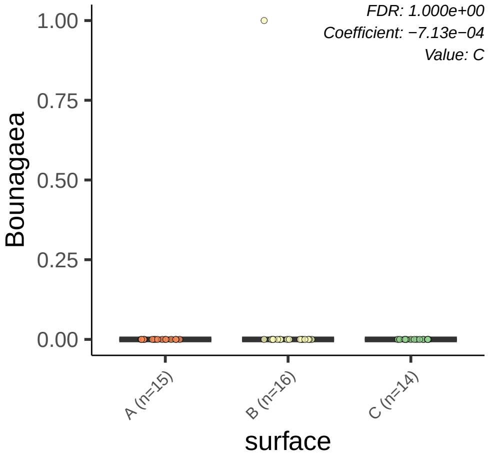
FDR: 1.000e+00
Coefficient: −7.13e−04
Value: C
1.00
0.75
0.50
0.25
0.00
Bounagaea
A (n=15)
B (n=16)
C (n=14)
surface

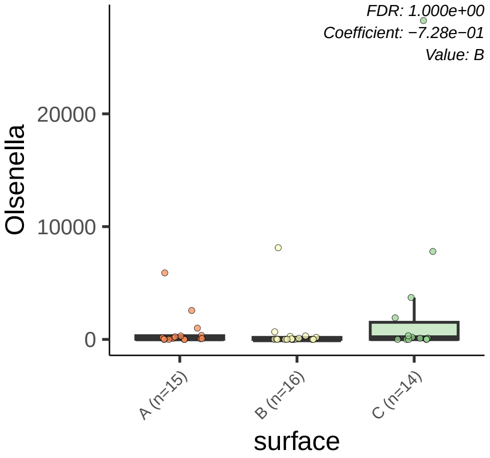

FDR: 1.000e+00
Coefficient: −7.28e−01
Value: B
Olsenella
20000
10000
0
A (n=15)
B (n=16)
C (n=14)
surface

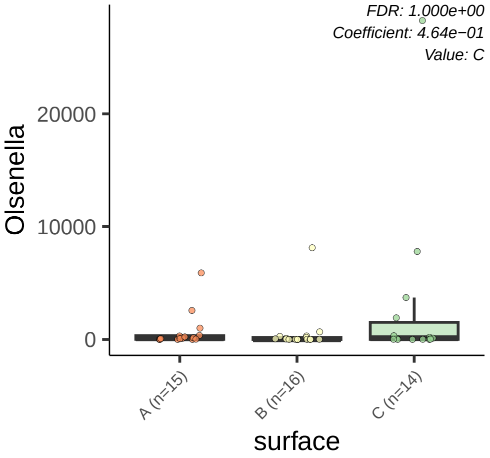
FDR: 1.000e+00
Coefficient: 4.64e−01
Value: C
Olsenella
20000
10000
0
A (n=15)
B (n=16)
C (n=14)
surface

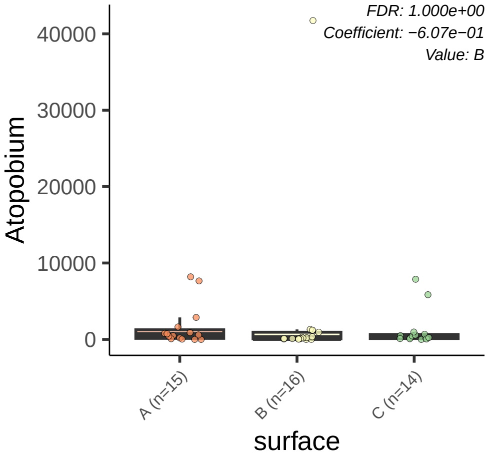

FDR: 1.000e+00
Coefficient: −6.07e−01
Value: B
Atopobium
40000
30000
20000
10000
0
A (n=15)
B (n=16)
C (n=14)
surface

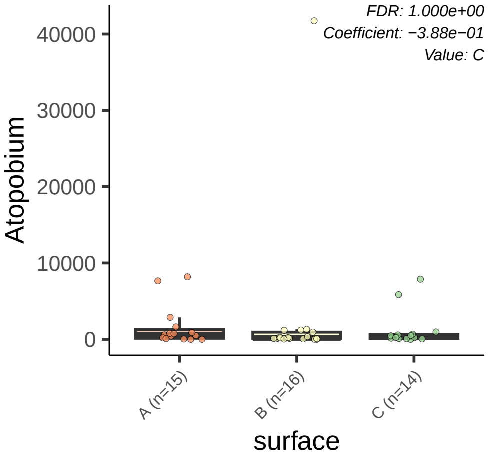

FDR: 1.000e+00
Coefficient: −3.88e−01
Value: C
Atopobium
40000
30000
20000
10000
0
A (n=15)
B (n=16)
C (n=14)
surface

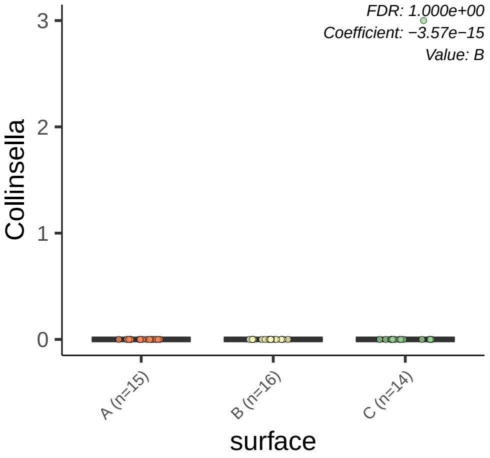

FDR: 1.000e+00
Coefficient: −3.57e−15
Value: B
Collinsella
3
2
1
0
A (n=15)
B (n=16)
C (n=14)
surface

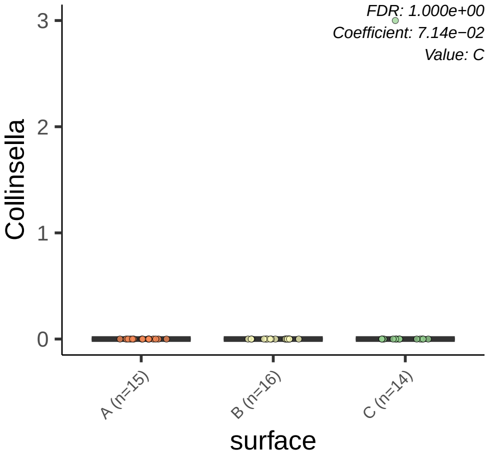
FDR: 1.000e+00
Coefficient: 7.14e−02
Value: C
Collinsella
3
2
1
0
A (n=15)
B (n=16)
C (n=14)
surface

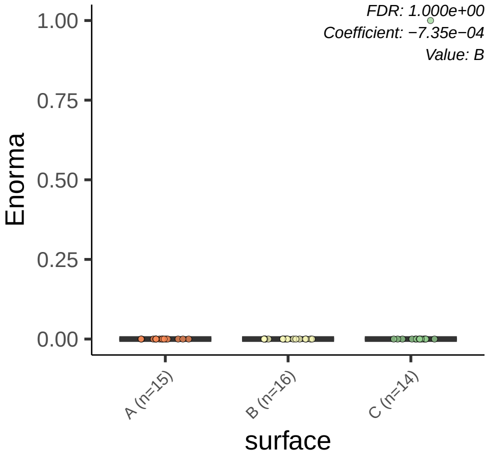
FDR: 1.000e+00
Coefficient: −7.35e−04
Value: B
Enorma
1.00
0.75
0.50
0.25
0.00
A (n=15)
B (n=16)
C (n=14)
surface

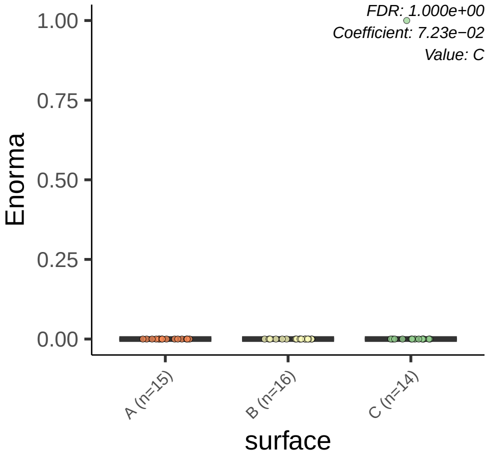
FDR: 1.000e+00
Coefficient: 7.23e−02
Value: C
Enorma
1.00
0.75
0.50
0.25
0.00
A (n=15)
B (n=16)
C (n=14)
surface

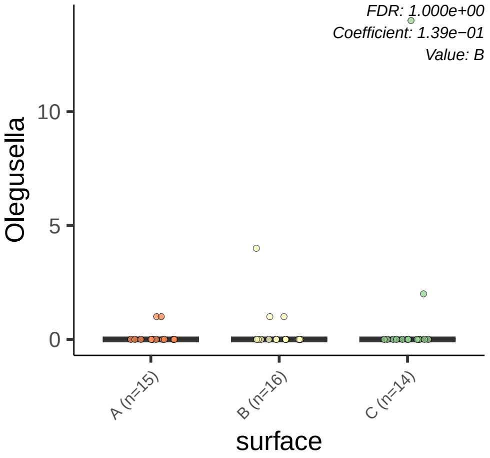
FDR: 1.000e+00
Coefficient: 1.39e−01
Value: B
Olegusella
0
5
10
A (n=15)
B (n=16)
C (n=14)
surface

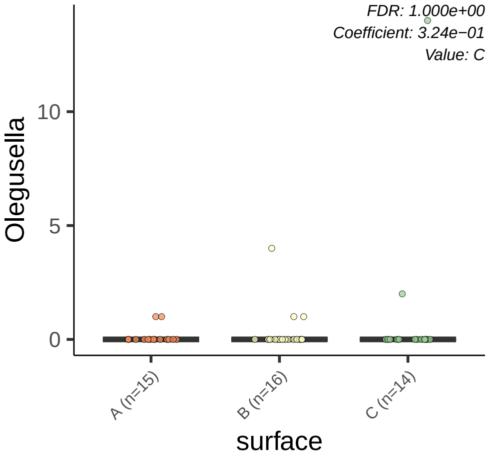
FDR: 1.000e+00
Coefficient: 3.24e−01
Value: C
Olegusella
10
5
0
A (n=15)
B (n=16)
C (n=14)
surface

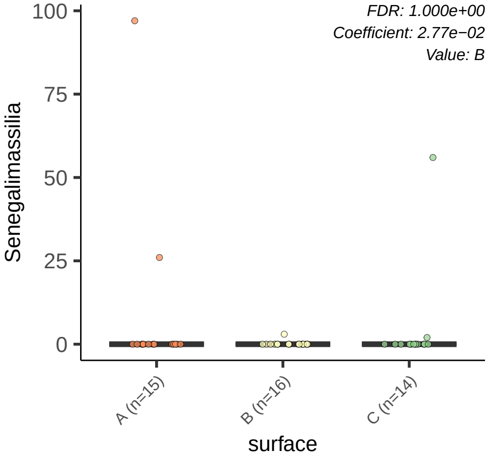

FDR: 1.000e+00
Coefficient: 2.77e−02
Value: B
Senegalimassilia
100
75
50
25
0
A (n=15)
B (n=16)
C (n=14)
surface

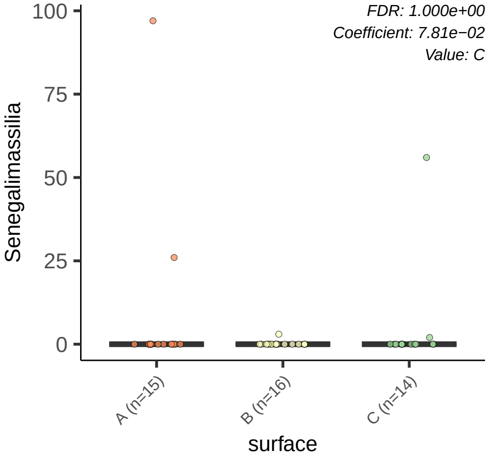

FDR: 1.000e+00
Coefficient: 7.81e−02
Value: C
100
75
50
25
0
Senegalimassilia
A (n=15)
B (n=16)
C (n=14)
surface

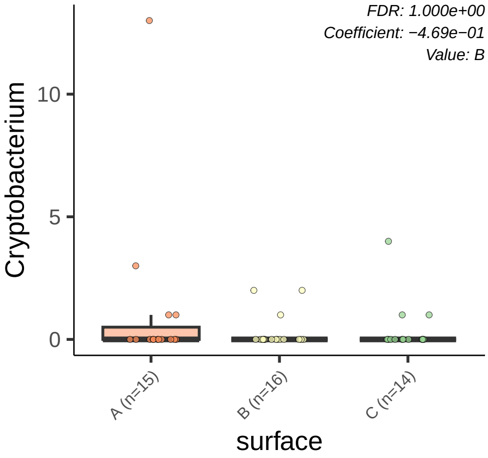

FDR: 1.000e+00
Coefficient: −4.69e−01
Value: B
Cryptobacterium
10
5
0
A (n=15)
B (n=16)
C (n=14)
surface

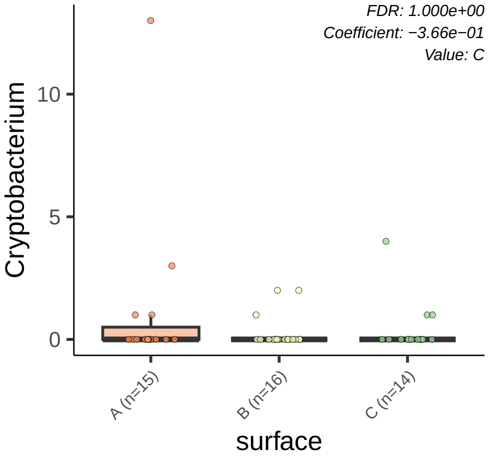
FDR: 1.000e+00
Coefficient: −3.66e−01
Value: C
Cryptobacterium
10
5
0
A (n=15)
B (n=16)
C (n=14)
surface

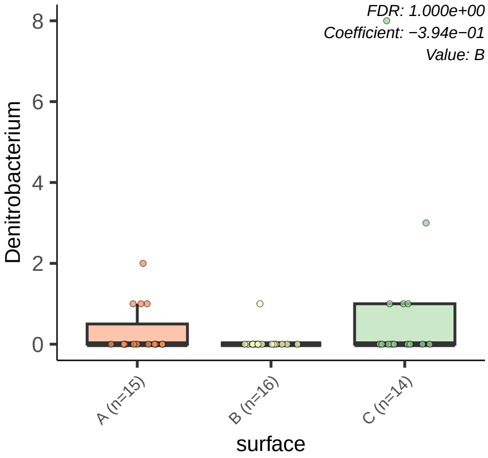
FDR: 1.000e+00
Coefficient: −3.94e−01
Value: B
Denitrobacterium
0
2
4
6
8
A (n=15)
B (n=16)
C (n=14)
surface

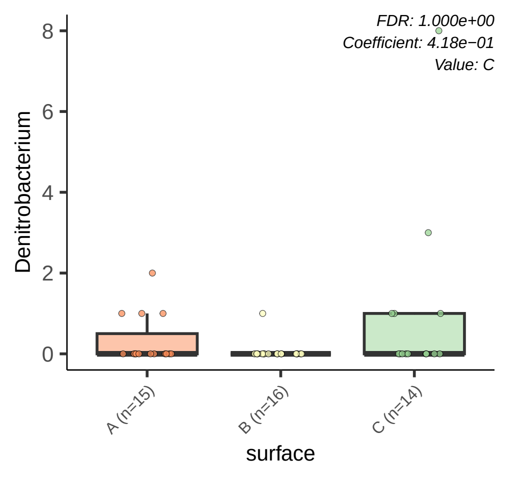
FDR: 1.000e+00
Coefficient: 4.18e−01
Value: C
Denitrobacterium
0
2
4
6
8
A (n=15)
B (n=16)
C (n=14)
surface

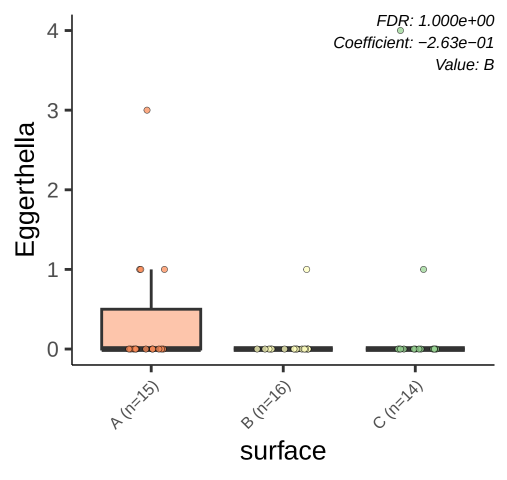

FDR: 1.000e+00
Coefficient: −2.63e−01
Value: B
Eggerthella
0
1
2
3
4
A (n=15)
B (n=16)
C (n=14)
surface

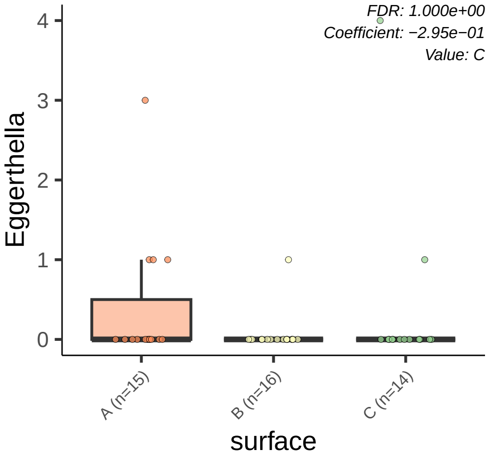
FDR: 1.000e+00
Coefficient: −2.95e−01
Value: C
Eggerthella
0
1
2
3
4
A (n=15)
B (n=16)
C (n=14)
surface

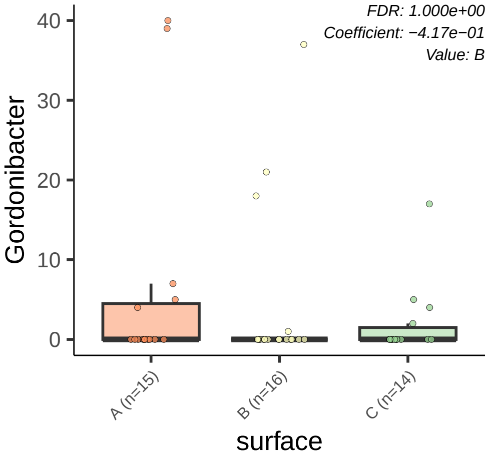

FDR: 1.000e+00
Coefficient: −4.17e−01
Value: B
Gordonibacter
40
30
20
10
0
A (n=15)
B (n=16)
C (n=14)
surface

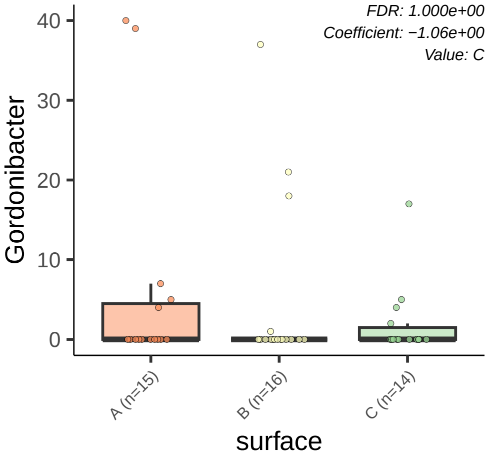
FDR: 1.000e+00
Coefficient: −1.06e+00
Value: C
Gordonibacter
40
30
20
10
0
A (n=15)
B (n=16)
C (n=14)
surface

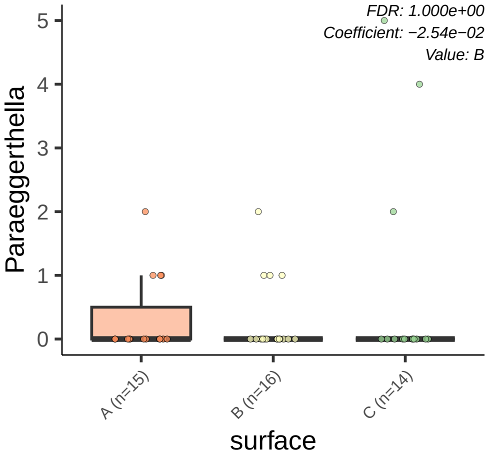

FDR: 1.000e+00
Coefficient: −2.54e−02
Value: B
Paraeggerthella
0
1
2
3
4
5
A (n=15)
B (n=16)
C (n=14)
surface

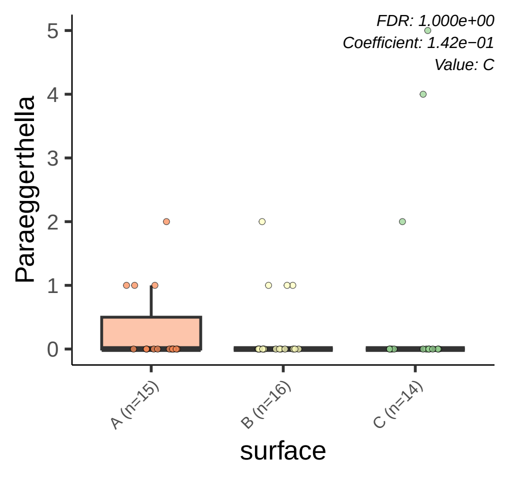
FDR: 1.000e+00
Coefficient: 1.42e−01
Value: C
Paraeggerthella
0
1
2
3
4
5
A (n=15)
B (n=16)
C (n=14)
surface

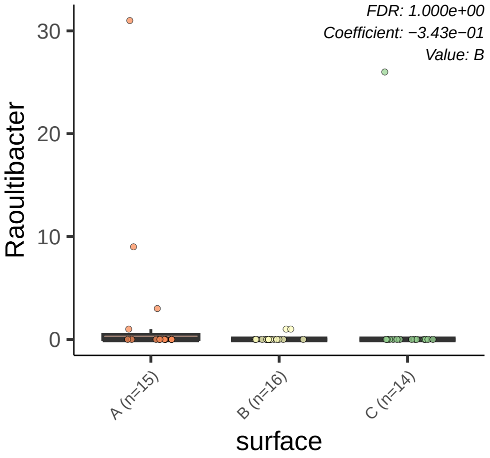

FDR: 1.000e+00
Coefficient: −3.43e−01
Value: B
Raoultibacter
30
20
10
0
A (n=15)
B (n=16)
C (n=14)
surface

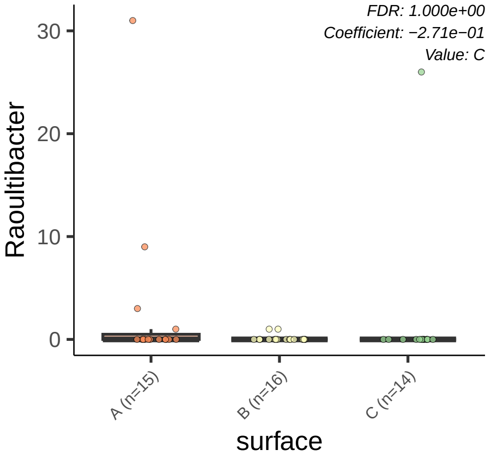

FDR: 1.000e+00
Coefficient: −2.71e−01
Value: C
Raoultibacter
30
20
10
0
A (n=15)
B (n=16)
C (n=14)
surface

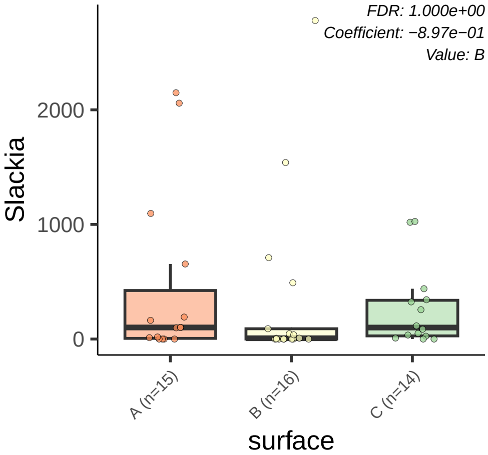

FDR: 1.000e+00
Coefficient: −8.97e−01
Value: B
Slackia
2000
1000
0
A (n=15)
B (n=16)
C (n=14)
surface

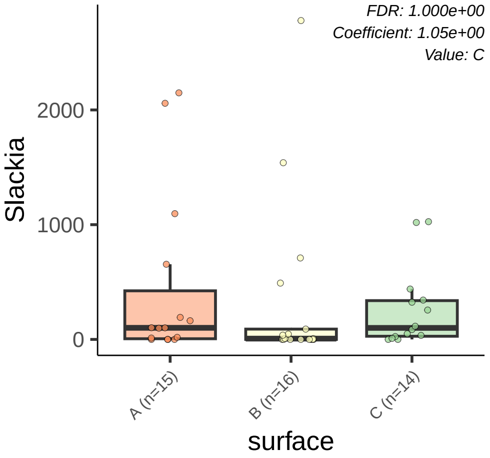
FDR: 1.000e+00
Coefficient: 1.05e+00
Value: C
Slackia
2000
1000
0
A (n=15)
B (n=16)
C (n=14)
surface

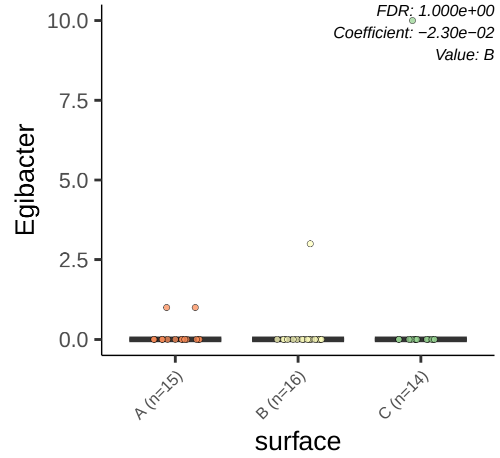
FDR: 1.000e+00
Coefficient: −2.30e−02
Value: B
Egibacter
10.0
7.5
5.0
2.5
0.0
A (n=15)
B (n=16)
C (n=14)
surface

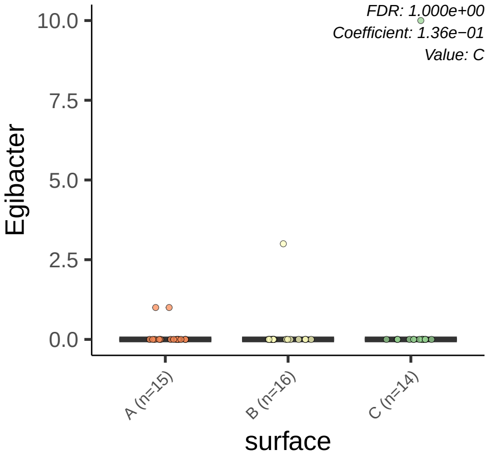

FDR: 1.000e+00
Coefficient: 1.36e−01
Value: C
Egibacter
10.0
7.5
5.0
2.5
0.0
A (n=15)
B (n=16)
C (n=14)
surface

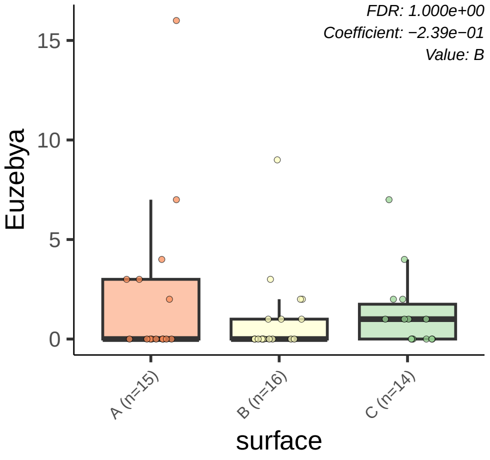
FDR: 1.000e+00
Coefficient: −2.39e−01
Value: B
Euzebya
0
5
10
15
A (n=15)
B (n=16)
C (n=14)
surface

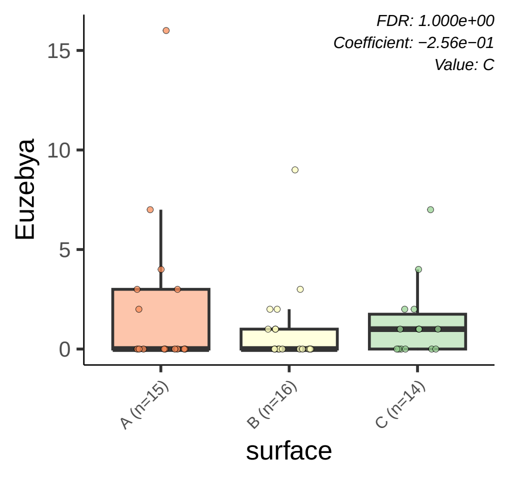
FDR: 1.000e+00
Coefficient: −2.56e−01
Value: C
Euzebya
15
10
5
0
A (n=15)
B (n=16)
C (n=14)
surface

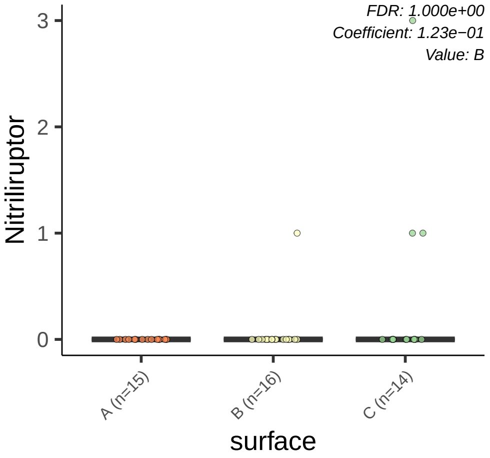

FDR: 1.000e+00
Coefficient: 1.23e−01
Value: B
Nitriliruptor
0
1
2
3
A (n=15)
B (n=16)
C (n=14)
surface

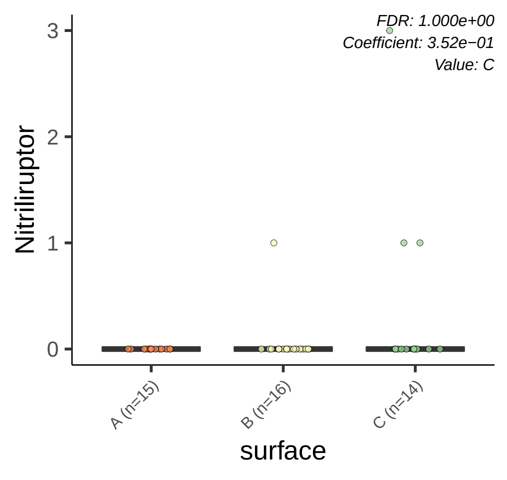

FDR: 1.000e+00
Coefficient: 3.52e−01
Value: C
Nitriliruptor
3
2
1
0
A (n=15)
B (n=16)
C (n=14)
surface

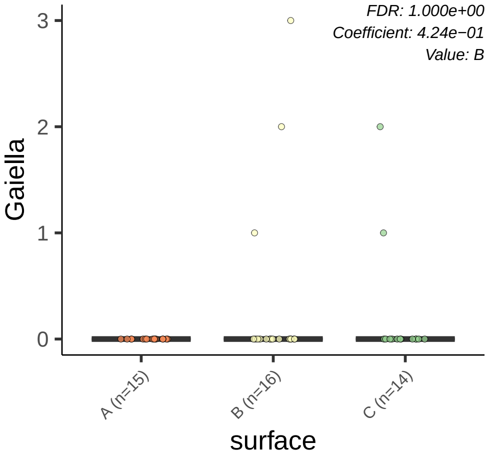
FDR: 1.000e+00
Coefficient: 4.24e−01
Value: B
Gaiella
3
2
1
0
A (n=15)
B (n=16)
C (n=14)
surface

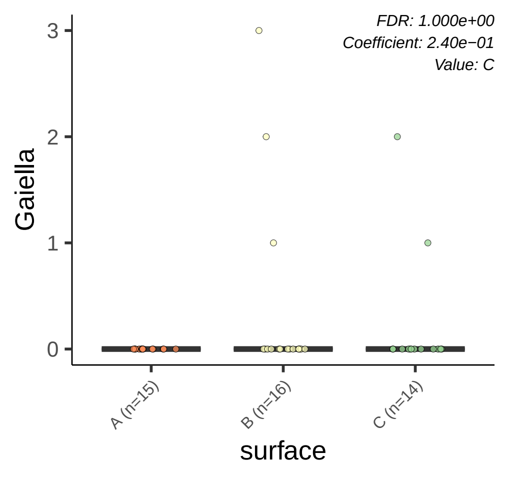

FDR: 1.000e+00
Coefficient: 2.40e−01
Value: C
Gaiella
3
2
1
0
A (n=15)
B (n=16)
C (n=14)
surface

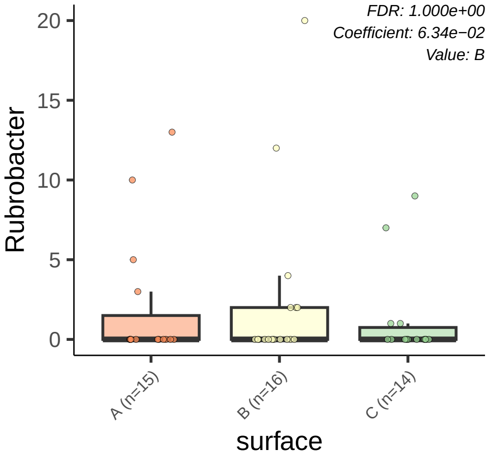
FDR: 1.000e+00
Coefficient: 6.34e−02
Value: B
Rubrobacter
20
15
10
5
0
A (n=15)
B (n=16)
C (n=14)
surface

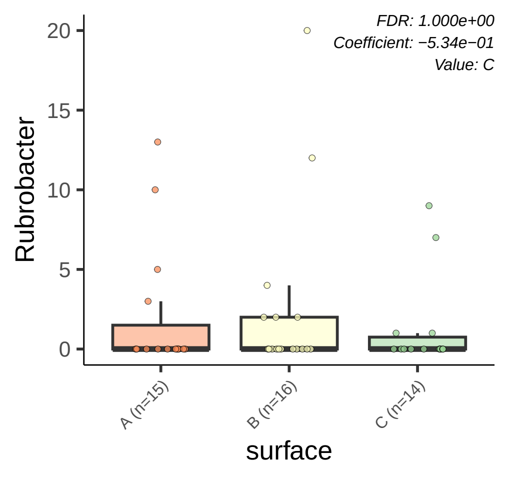
FDR: 1.000e+00
Coefficient: −5.34e−01
Value: C
Rubrobacter
0
5
10
15
20
A (n=15)
B (n=16)
C (n=14)
surface

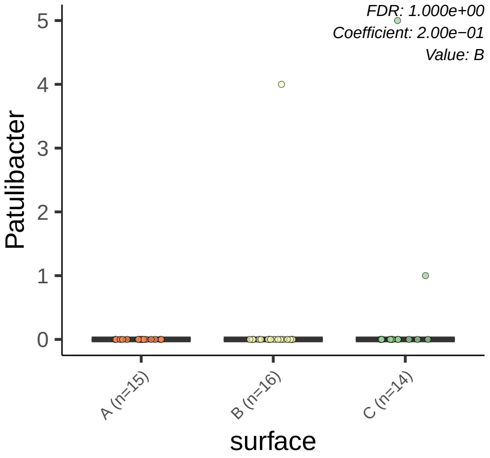
FDR: 1.000e+00
Coefficient: 2.00e−01
Value: B
Patulibacter
5
4
3
2
1
0
A (n=15)
B (n=16)
C (n=14)
surface

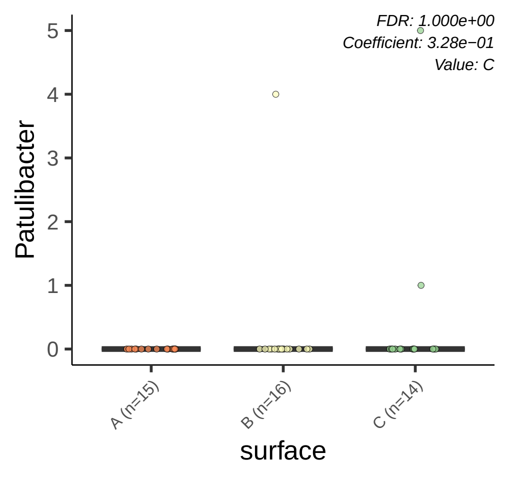
FDR: 1.000e+00
Coefficient: 3.28e−01
Value: C
Patulibacter
0
1
2
3
4
5
A (n=15)
B (n=16)
C (n=14)
surface

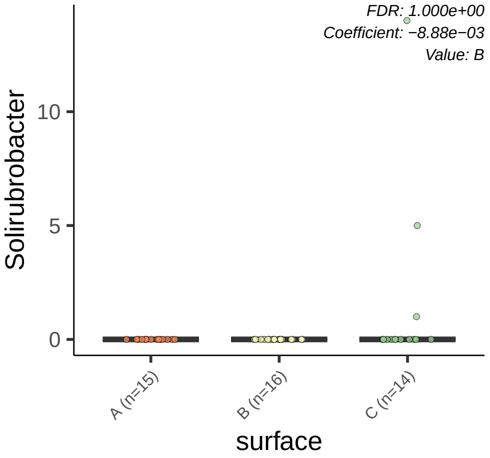

FDR: 1.000e+00
Coefficient: −8.88e−03
Value: B
Solirubrobacter
10
5
0
A (n=15)
B (n=16)
C (n=14)
surface

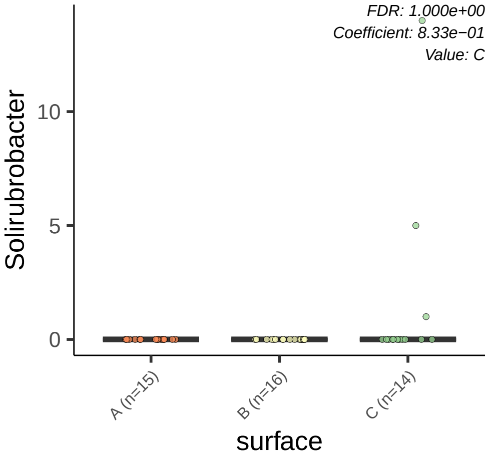
FDR: 1.000e+00
Coefficient: 8.33e−01
Value: C
Solirubrobacter
10
5
0
A (n=15)
B (n=16)
C (n=14)
surface

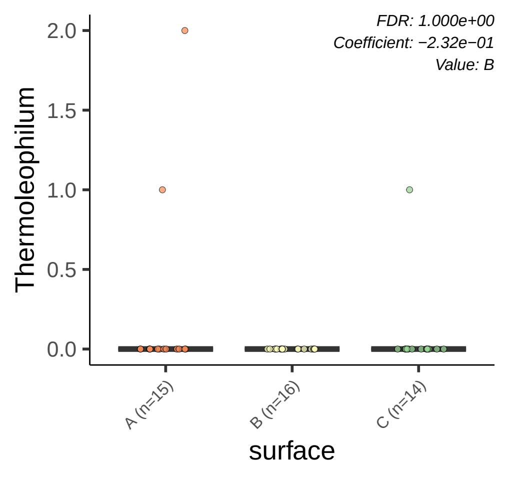

FDR: 1.000e+00
Coefficient: −2.32e−01
Value: B
Thermoleophilum
2.0
1.5
1.0
0.5
0.0
A (n=15)
B (n=16)
C (n=14)
surface

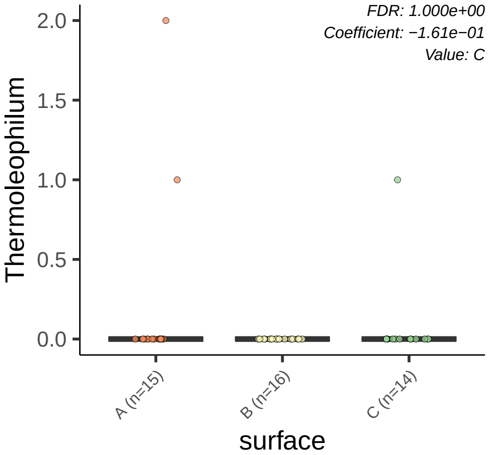
FDR: 1.000e+00
Coefficient: −1.61e−01
Value: C
Thermoleophilum
2.0
1.5
1.0
0.5
0.0
A (n=15)
B (n=16)
C (n=14)
surface

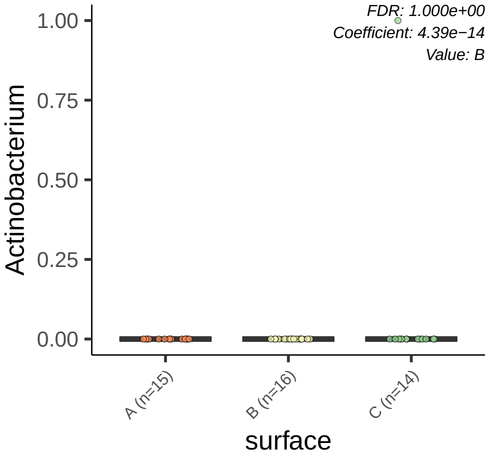
FDR: 1.000e+00
Coefficient: 4.39e−14
Value: B
1.00
0.75
0.50
0.25
0.00
Actinobacterium
A (n=15)
B (n=16)
C (n=14)
surface

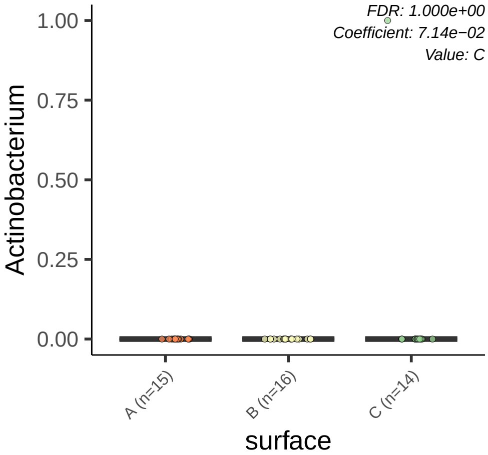

FDR: 1.000e+00
Coefficient: 7.14e−02
Value: C
1.00
0.75
0.50
0.25
0.00
Actinobacterium
A (n=15)
B (n=16)
C (n=14)
surface

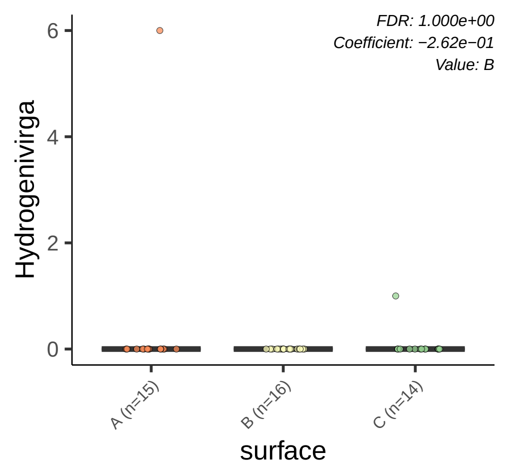

FDR: 1.000e+00
Coefficient: −2.62e−01
Value: B
Hydrogenivirga
6
4
2
0
A (n=15)
B (n=16)
C (n=14)
surface

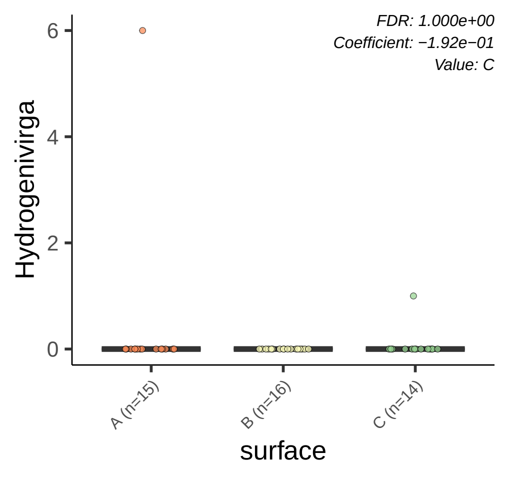
FDR: 1.000e+00
Coefficient: −1.92e−01
Value: C
Hydrogenivirga
0
2
4
6
A (n=15)
B (n=16)
C (n=14)
surface

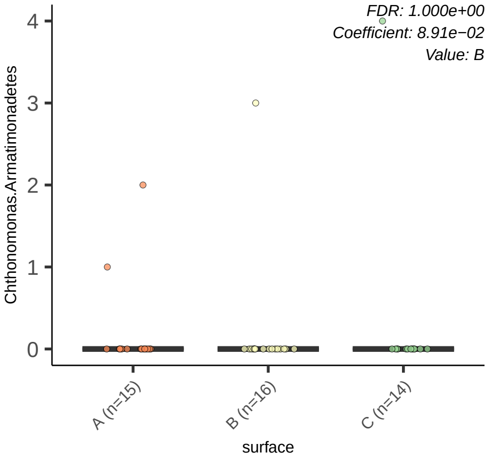

FDR: 1.000e+00
Coefficient: 8.91e−02
Value: B
Chthonomonas.Armatimonadetes
4
3
2
1
0
A (n=15)
B (n=16)
C (n=14)
surface

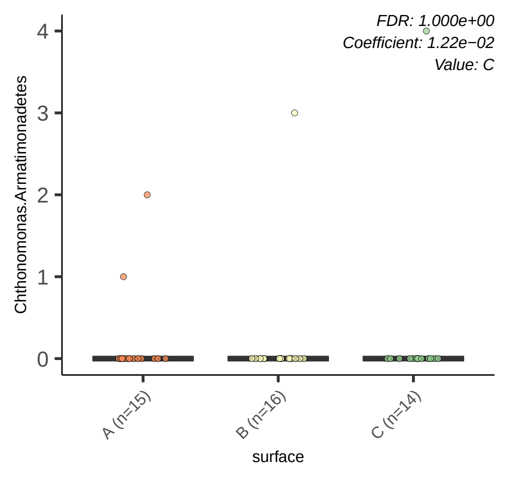

FDR: 1.000e+00
Coefficient: 1.22e−02
Value: C
Chthonomonas.Armatimonadetes
4
3
2
1
0
A (n=15)
B (n=16)
C (n=14)
surface

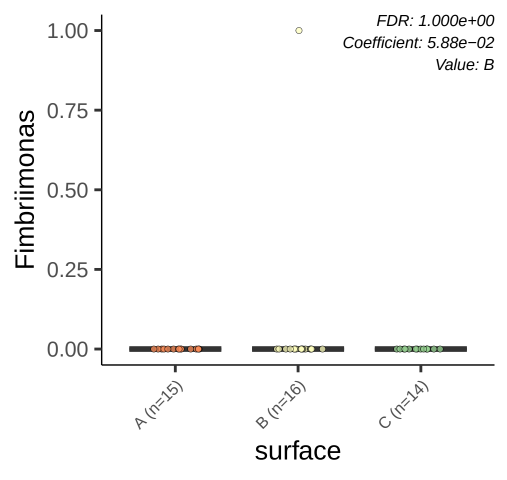

FDR: 1.000e+00
Coefficient: 5.88e−02
Value: B
1.00
0.75
0.50
0.25
0.00
Fimbriimonas
A (n=15)
B (n=16)
C (n=14)
surface

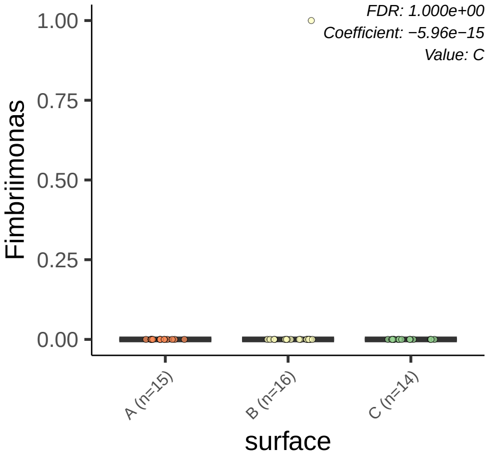
FDR: 1.000e+00
Coefficient: −5.96e−15
Value: C
1.00
0.75
0.50
0.25
0.00
Fimbriimonas
A (n=15)
B (n=16)
C (n=14)
surface

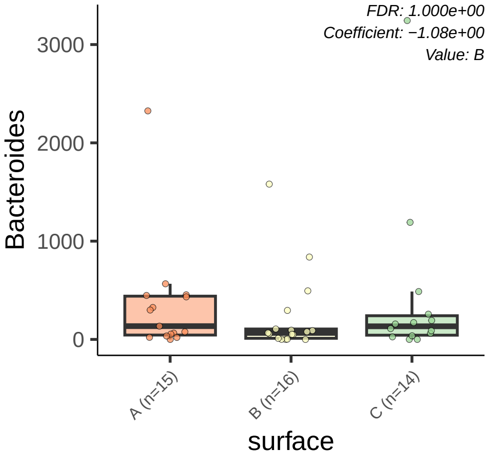

FDR: 1.000e+00
Coefficient: −1.08e+00
Value: B
Bacteroides
3000
2000
1000
0
A (n=15)
B (n=16)
C (n=14)
surface

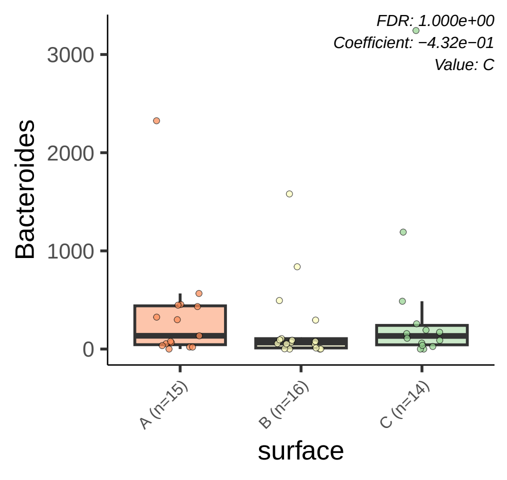
FDR: 1.000e+00
Coefficient: −4.32e−01
Value: C
Bacteroides
3000
2000
1000
0
A (n=15)
B (n=16)
C (n=14)
surface

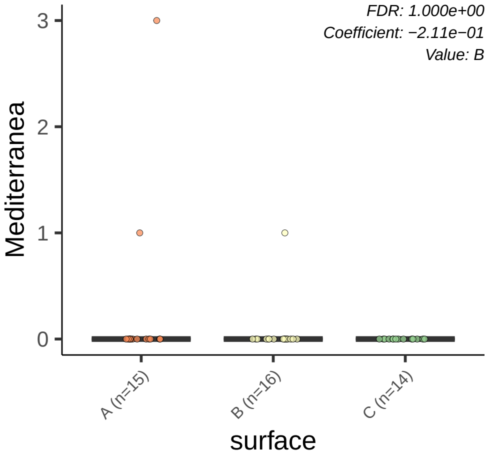
FDR: 1.000e+00
Coefficient: −2.11e−01
Value: B
Mediterranea
3
2
1
0
A (n=15)
B (n=16)
C (n=14)
surface

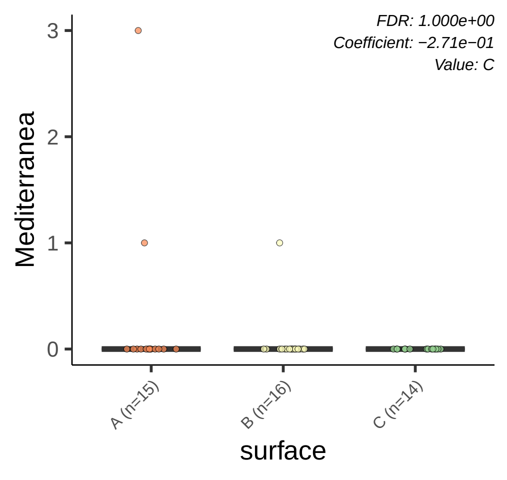
FDR: 1.000e+00
Coefficient: −2.71e−01
Value: C
Mediterranea
3
2
1
0
A (n=15)
B (n=16)
C (n=14)
surface

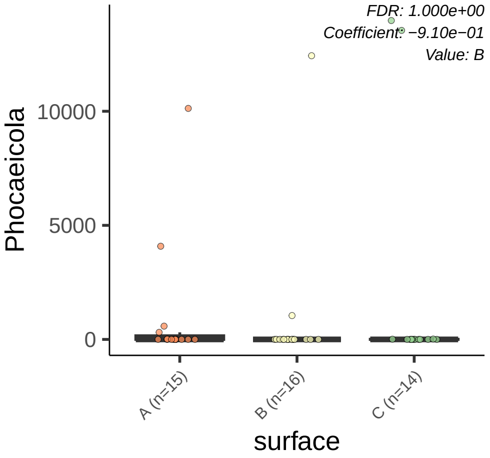

FDR: 1.000e+00
Coefficient: −9.10e−01
Value: B
Phocaeicola
10000
5000
0
A (n=15)
B (n=16)
C (n=14)
surface

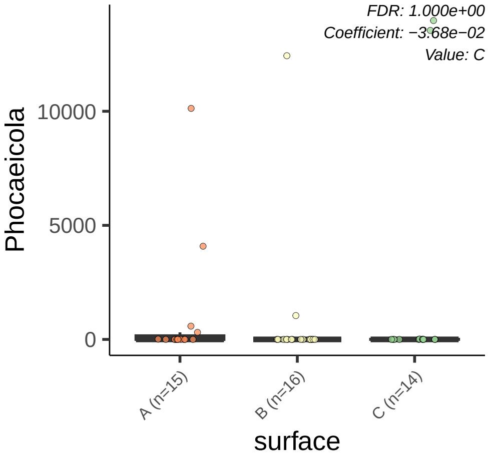
FDR: 1.000e+00
Coefficient: −3.68e−02
Value: C
Phocaeicola
10000
5000
0
A (n=15)
B (n=16)
C (n=14)
surface

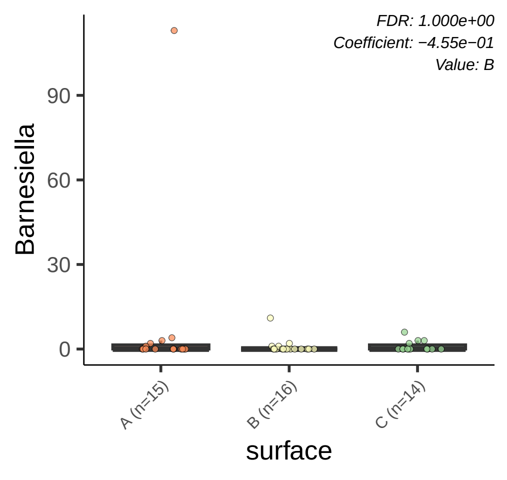
FDR: 1.000e+00
Coefficient: −4.55e−01
Value: B
Barnesiella
90
60
30
0
A (n=15)
B (n=16)
C (n=14)
surface

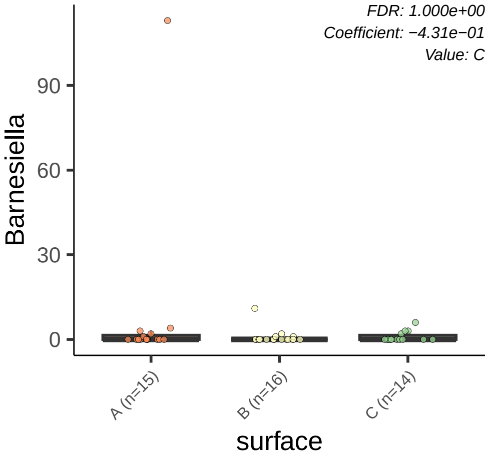
FDR: 1.000e+00
Coefficient: −4.31e−01
Value: C
Barnesiella
90
60
30
0
A (n=15)
B (n=16)
C (n=14)
surface

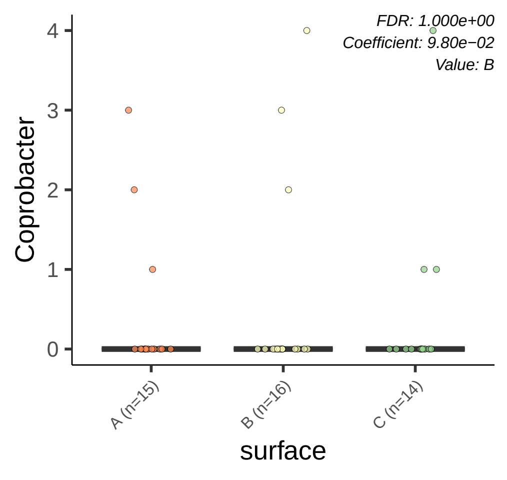
FDR: 1.000e+00
Coefficient: 9.80e−02
Value: B
Coprobacter
0
1
2
3
4
A (n=15)
B (n=16)
C (n=14)
surface

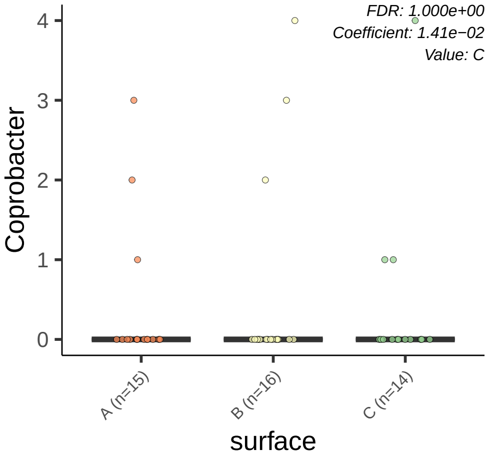

FDR: 1.000e+00
Coefficient: 1.41e−02
Value: C
Coprobacter
0
1
2
3
4
A (n=15)
B (n=16)
C (n=14)
surface

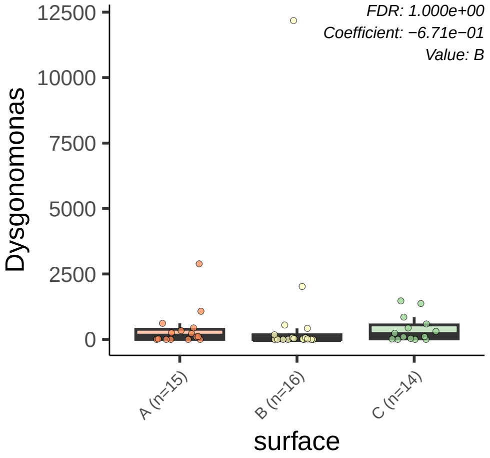
FDR: 1.000e+00
Coefficient: −6.71e−01
Value: B
Dysgonomonas
12500
10000
7500
5000
2500
0
A (n=15)
B (n=16)
C (n=14)
surface

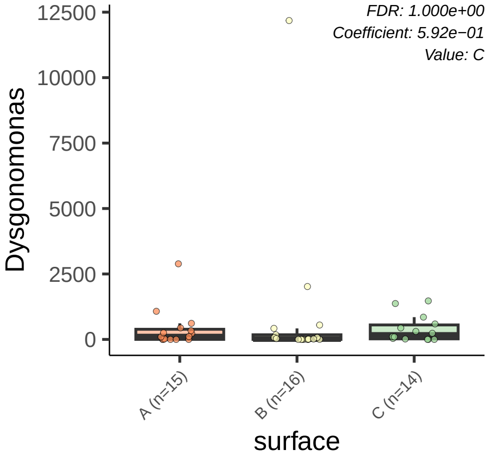

FDR: 1.000e+00
Coefficient: 5.92e−01
Value: C
Dysgonomonas
12500
10000
7500
5000
2500
0
A (n=15)
B (n=16)
C (n=14)
surface

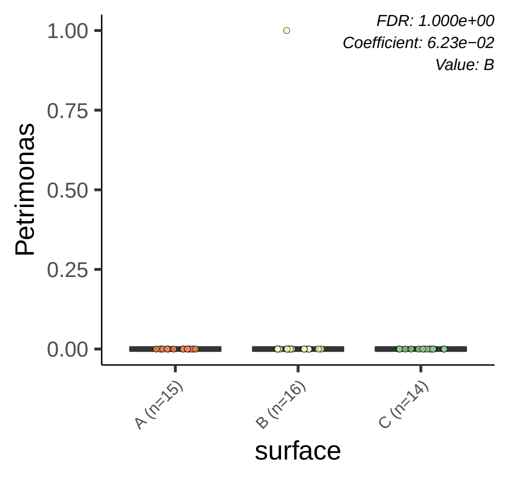
FDR: 1.000e+00
Coefficient: 6.23e−02
Value: B
Petrimonas
1.00
0.75
0.50
0.25
0.00
A (n=15)
B (n=16)
C (n=14)
surface

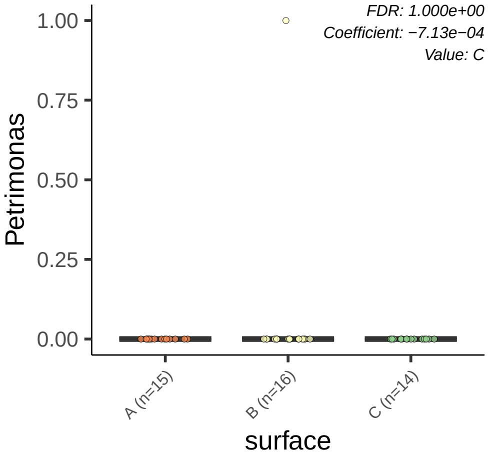
FDR: 1.000e+00
Coefficient: −7.13e−04
Value: C
Petrimonas
1.00
0.75
0.50
0.25
0.00
A (n=15)
B (n=16)
C (n=14)
surface

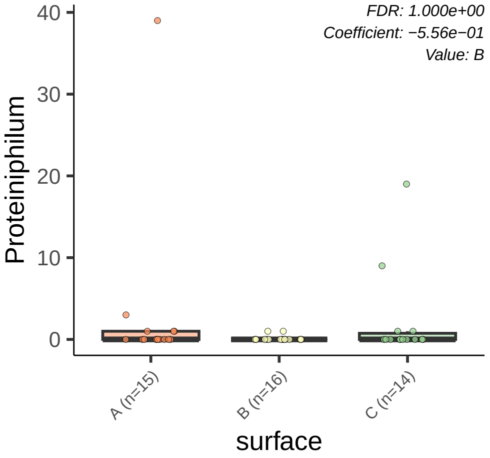

FDR: 1.000e+00
Coefficient: −5.56e−01
Value: B
Proteiniphilum
0
10
20
30
40
A (n=15)
B (n=16)
C (n=14)
surface

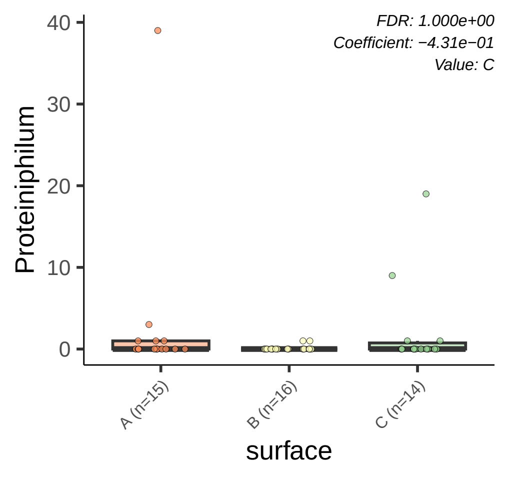
FDR: 1.000e+00
Coefficient: −4.31e−01
Value: C
Proteiniphilum
0
10
20
30
40
A (n=15)
B (n=16)
C (n=14)
surface

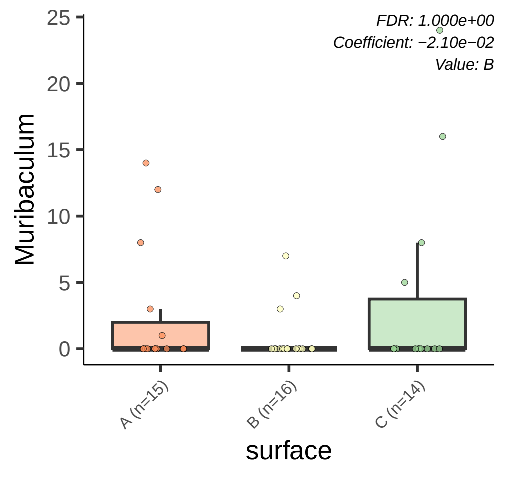

FDR: 1.000e+00
Coefficient: −2.10e−02
Value: B
Muribaculum
0
5
10
15
20
25
A (n=15)
B (n=16)
C (n=14)
surface

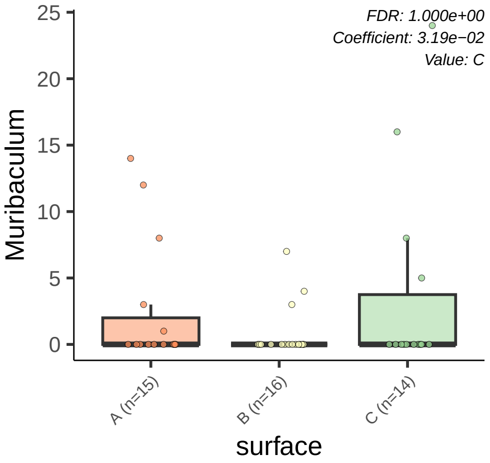

FDR: 1.000e+00
Coefficient: 3.19e−02
Value: C
Muribaculum
0
5
10
15
20
25
A (n=15)
B (n=16)
C (n=14)
surface

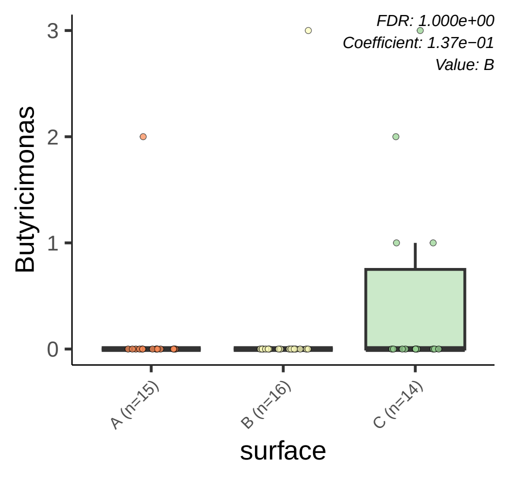

FDR: 1.000e+00
Coefficient: 1.37e−01
Value: B
Butyricimonas
3
2
1
0
A (n=15)
B (n=16)
C (n=14)
surface

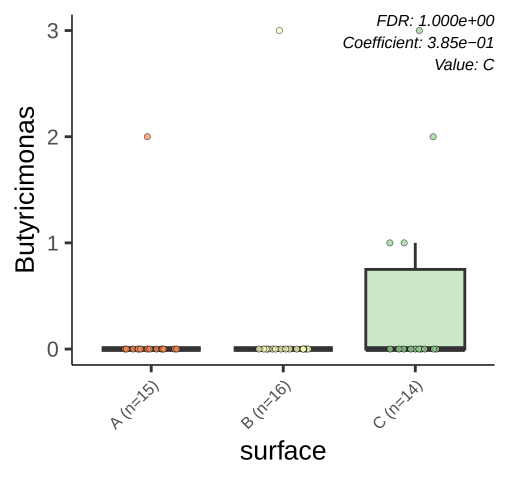

FDR: 1.000e+00
Coefficient: 3.85e−01
Value: C
Butyricimonas
0
1
2
3
A (n=15)
B (n=16)
C (n=14)
surface

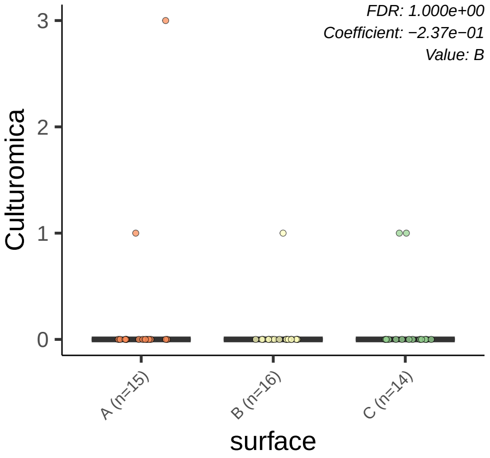
FDR: 1.000e+00
Coefficient: −2.37e−01
Value: B
Culturomica
3
2
1
0
A (n=15)
B (n=16)
C (n=14)
surface

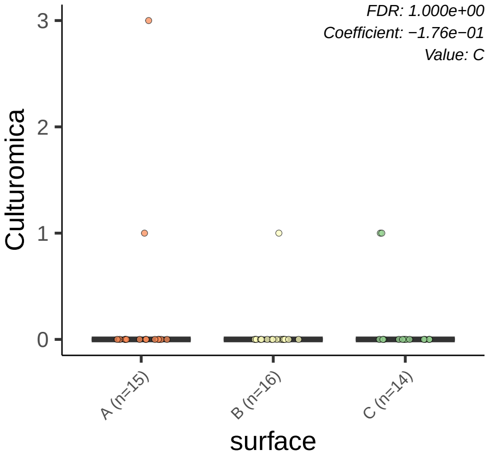
FDR: 1.000e+00
Coefficient: −1.76e−01
Value: C
Culturomica
3
2
1
0
A (n=15)
B (n=16)
C (n=14)
surface

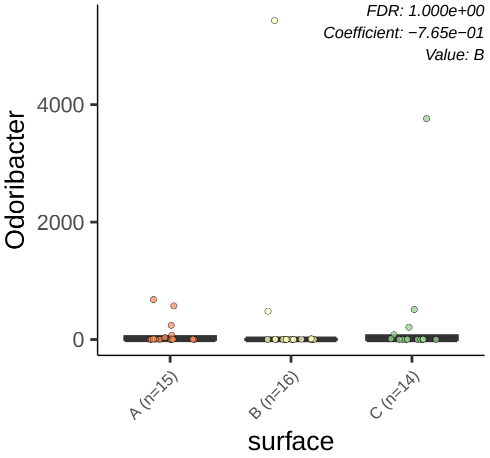
FDR: 1.000e+00
Coefficient: −7.65e−01
Value: B
Odoribacter
4000
2000
0
A (n=15)
B (n=16)
C (n=14)
surface

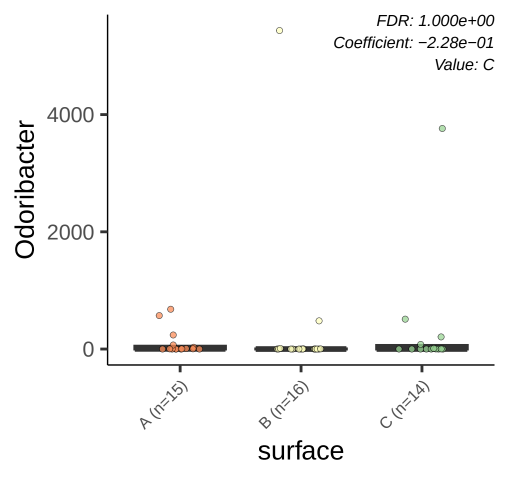

FDR: 1.000e+00
Coefficient: −2.28e−01
Value: C
Odoribacter
0
2000
4000
A (n=15)
B (n=16)
C (n=14)
surface

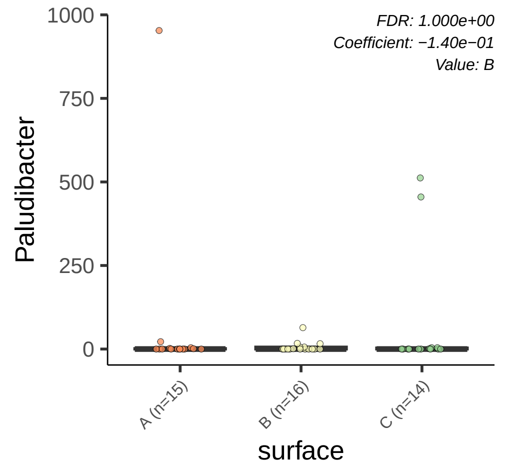

FDR: 1.000e+00
Coefficient: −1.40e−01
Value: B
Paludibacter
1000
750
500
250
0
A (n=15)
B (n=16)
C (n=14)
surface

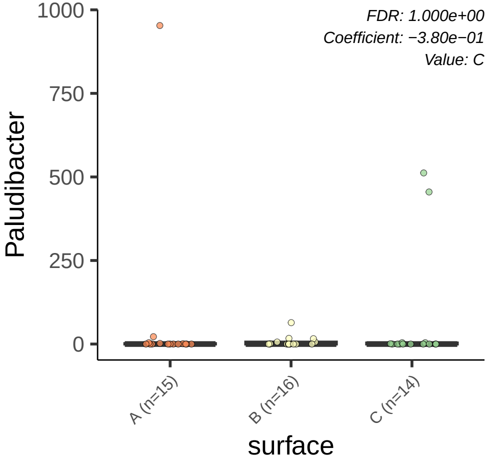

FDR: 1.000e+00
Coefficient: −3.80e−01
Value: C
Paludibacter
1000
750
500
250
0
A (n=15)
B (n=16)
C (n=14)
surface

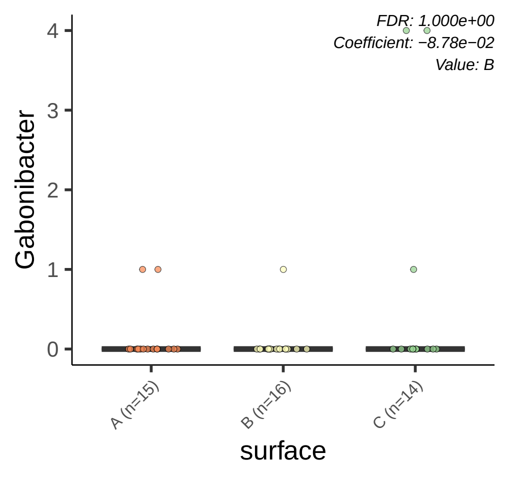

FDR: 1.000e+00
Coefficient: −8.78e−02
Value: B
Gabonibacter
4
3
2
1
0
A (n=15)
B (n=16)
C (n=14)
surface

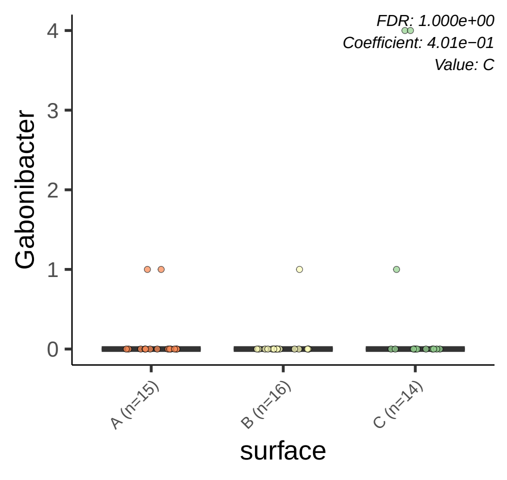

FDR: 1.000e+00
Coefficient: 4.01e−01
Value: C
Gabonibacter
4
3
2
1
0
A (n=15)
B (n=16)
C (n=14)
surface

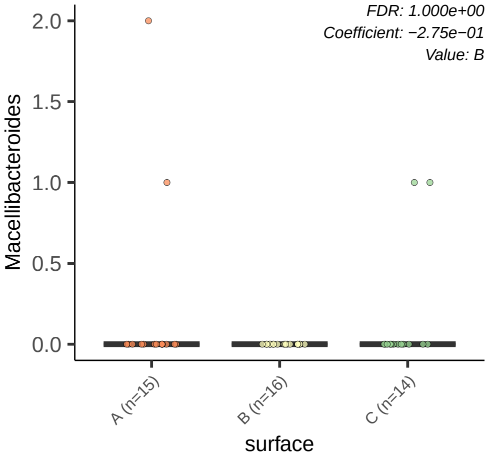
FDR: 1.000e+00
Coefficient: −2.75e−01
Value: B
Macellibacteroides
2.0
1.5
1.0
0.5
0.0
A (n=15)
B (n=16)
C (n=14)
surface

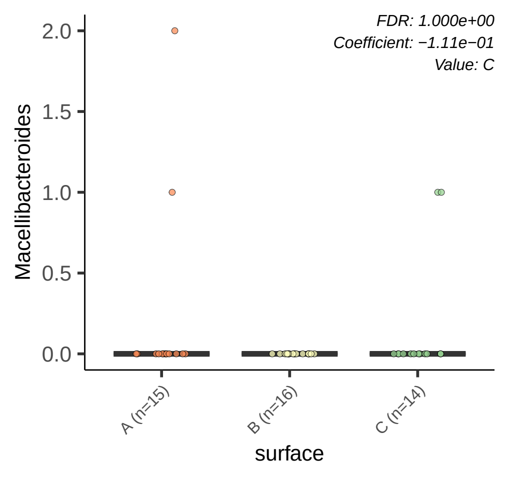
FDR: 1.000e+00
Coefficient: −1.11e−01
Value: C
Macellibacteroides
2.0
1.5
1.0
0.5
0.0
A (n=15)
B (n=16)
C (n=14)
surface

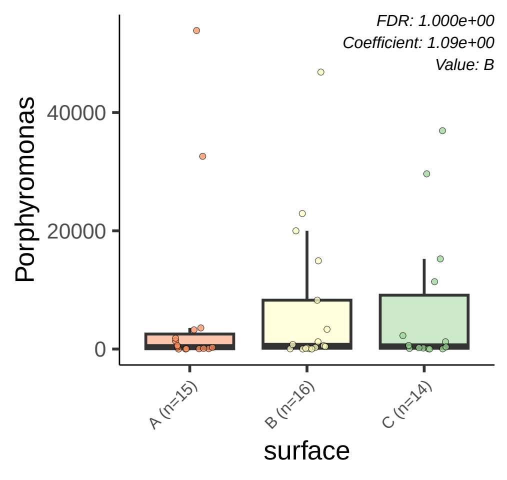

FDR: 1.000e+00
Coefficient: 1.09e+00
Value: B
Porphyromonas
40000
20000
0
A (n=15)
B (n=16)
C (n=14)
surface

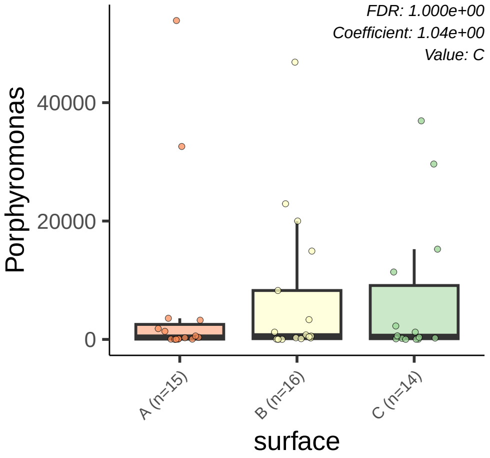
FDR: 1.000e+00
Coefficient: 1.04e+00
Value: C
Porphyromonas
40000
20000
0
A (n=15)
B (n=16)
C (n=14)
surface

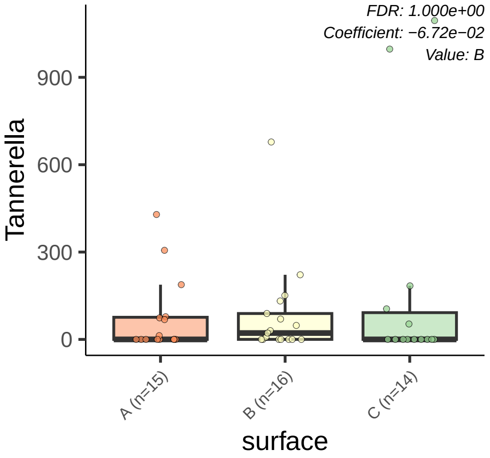
FDR: 1.000e+00
Coefficient: −6.72e−02
Value: B
Tannerella
900
600
300
0
A (n=15)
B (n=16)
C (n=14)
surface

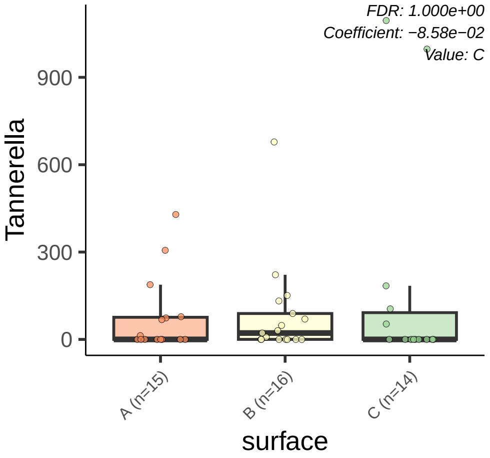
FDR: 1.000e+00
Coefficient: −8.58e−02
Value: C
Tannerella
900
600
300
0
A (n=15)
B (n=16)
C (n=14)
surface

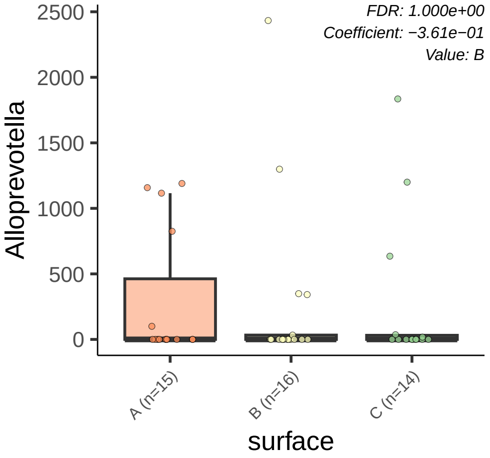
FDR: 1.000e+00
Coefficient: −3.61e−01
Value: B
Alloprevotella
2500
2000
1500
1000
500
0
A (n=15)
B (n=16)
C (n=14)
surface

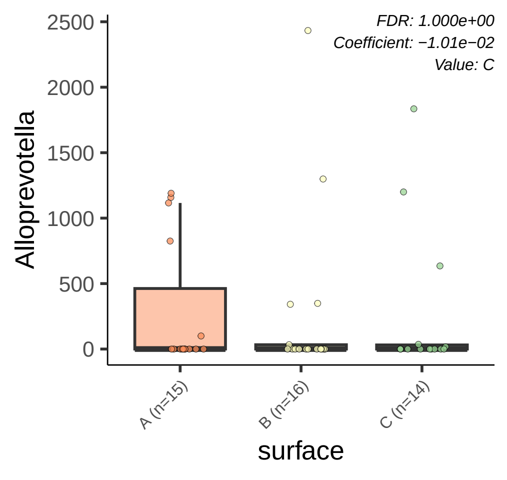
FDR: 1.000e+00
Coefficient: −1.01e−02
Value: C
Alloprevotella
2500
2000
1500
1000
500
0
A (n=15)
B (n=16)
C (n=14)
surface

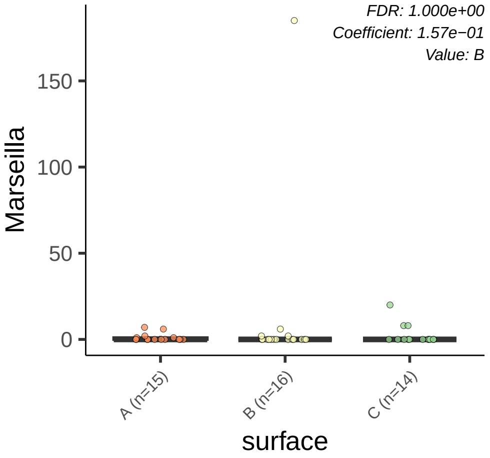

FDR: 1.000e+00
Coefficient: 1.57e−01
Value: B
Marseilla
150
100
50
0
A (n=15)
B (n=16)
C (n=14)
surface

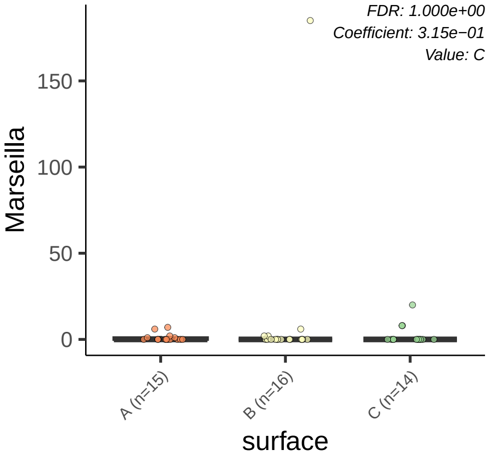

FDR: 1.000e+00
Coefficient: 3.15e−01
Value: C
Marseilla
150
100
50
0
A (n=15)
B (n=16)
C (n=14)
surface

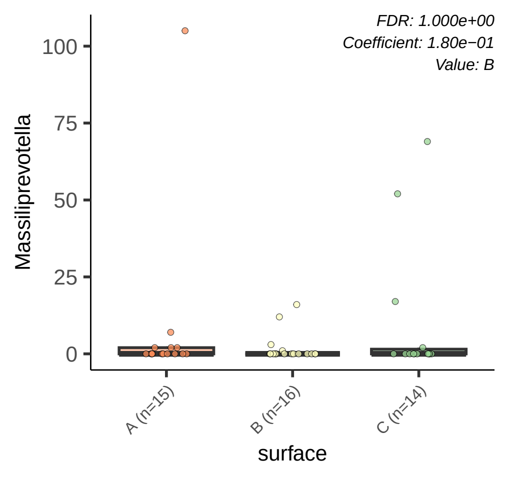

FDR: 1.000e+00
Coefficient: 1.80e−01
Value: B
Massiliprevotella
100
75
50
25
0
A (n=15)
B (n=16)
C (n=14)
surface

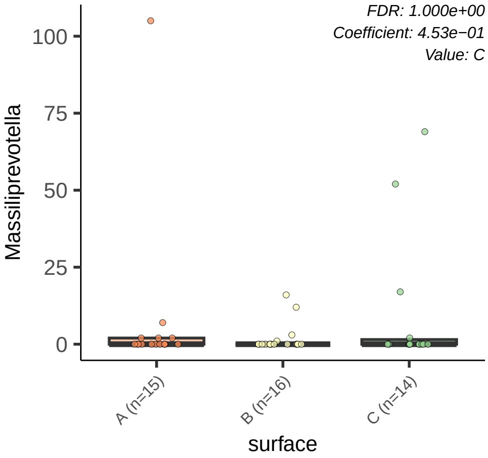
FDR: 1.000e+00
Coefficient: 4.53e−01
Value: C
Massiliprevotella
100
75
50
25
0
A (n=15)
B (n=16)
C (n=14)
surface

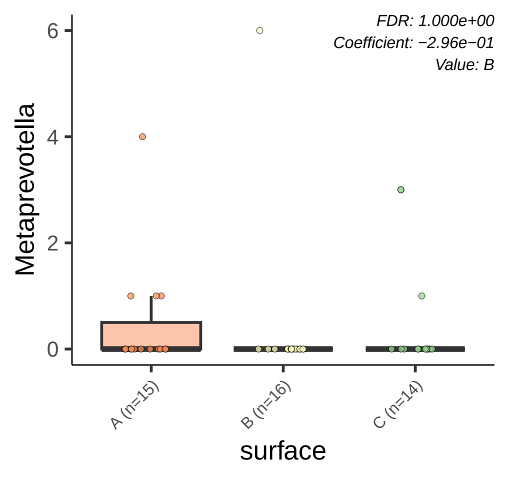
FDR: 1.000e+00
Coefficient: −2.96e−01
Value: B
Metaprevotella
6
4
2
0
A (n=15)
B (n=16)
C (n=14)
surface

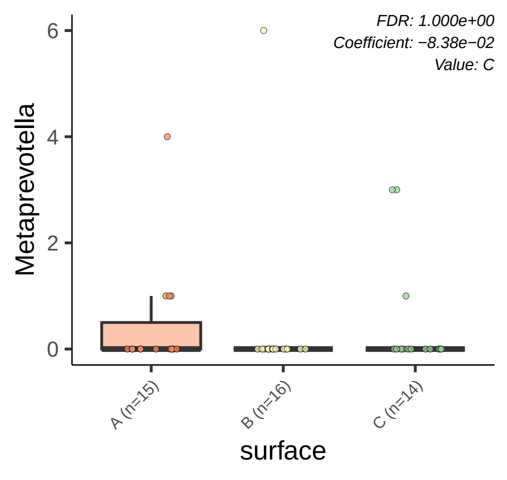

FDR: 1.000e+00
Coefficient: −8.38e−02
Value: C
Metaprevotella
6
4
2
0
A (n=15)
B (n=16)
C (n=14)
surface

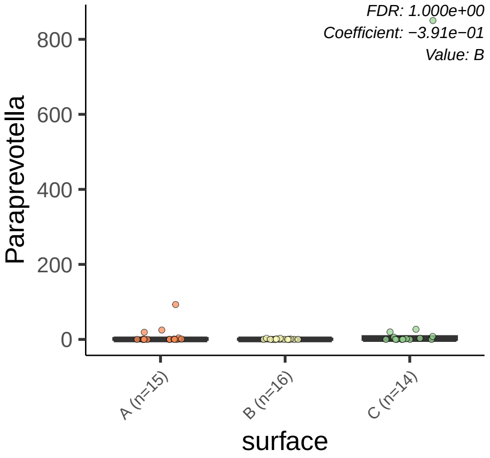
FDR: 1.000e+00
Coefficient: −3.91e−01
Value: B
Paraprevotella
800
600
400
200
0
A (n=15)
B (n=16)
C (n=14)
surface

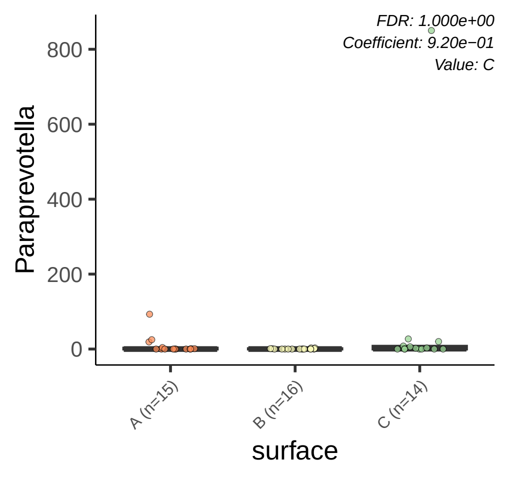
FDR: 1.000e+00
Coefficient: 9.20e−01
Value: C
Paraprevotella
800
600
400
200
0
A (n=15)
B (n=16)
C (n=14)
surface

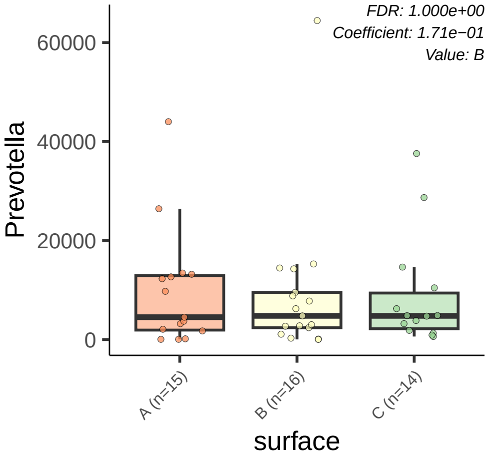

FDR: 1.000e+00
Coefficient: 1.71e−01
Value: B
60000
40000
20000
0
Prevotella
A (n=15)
B (n=16)
C (n=14)
surface

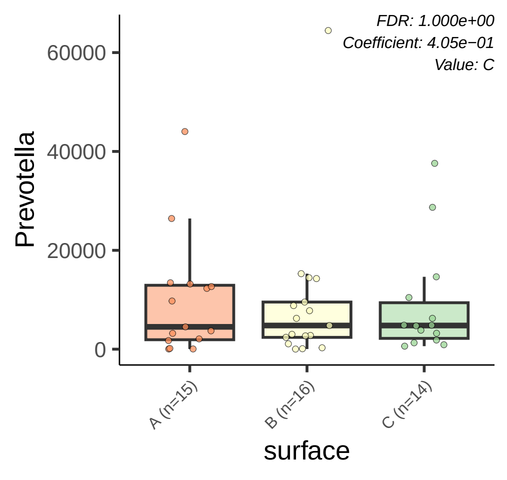

FDR: 1.000e+00
Coefficient: 4.05e−01
Value: C
Prevotella
60000
40000
20000
0
A (n=15)
B (n=16)
C (n=14)
surface

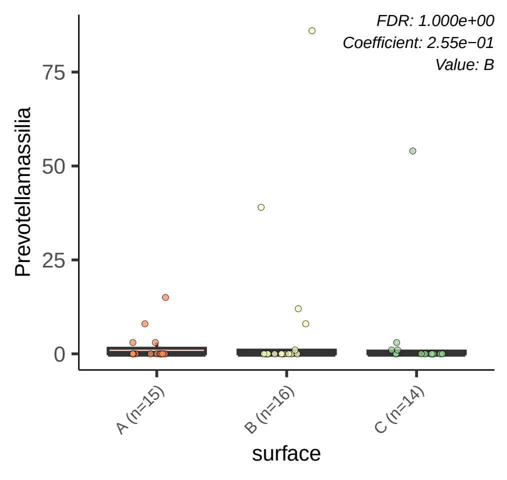
FDR: 1.000e+00
Coefficient: 2.55e−01
Value: B
Prevotellamassilia
0
25
50
75
A (n=15)
B (n=16)
C (n=14)
surface

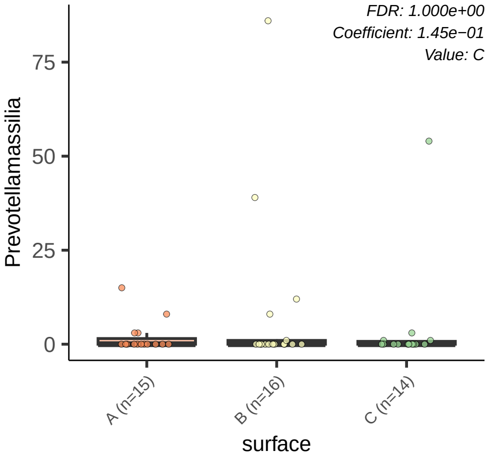
FDR: 1.000e+00
Coefficient: 1.45e−01
Value: C
Prevotellamassilia
0
25
50
75
A (n=15)
B (n=16)
C (n=14)
surface

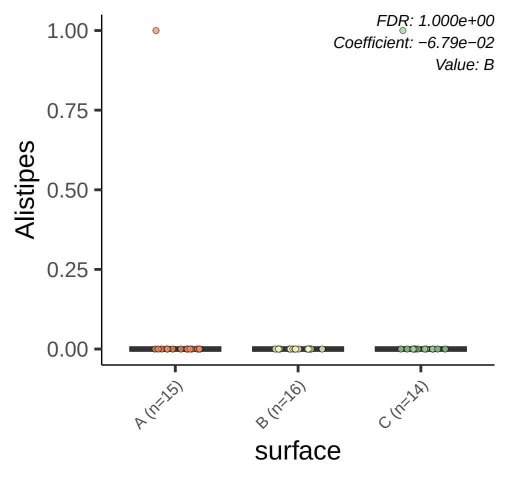

FDR: 1.000e+00
Coefficient: −6.79e−02
Value: B
Alistipes
1.00
0.75
0.50
0.25
0.00
A (n=15)
B (n=16)
C (n=14)
surface

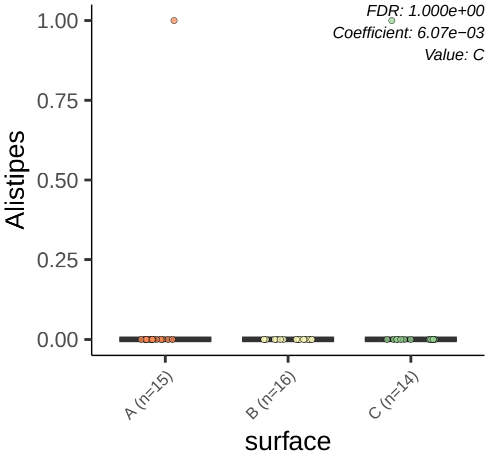
FDR: 1.000e+00
Coefficient: 6.07e−03
Value: C
Alistipes
1.00
0.75
0.50
0.25
0.00
A (n=15)
B (n=16)
C (n=14)
surface

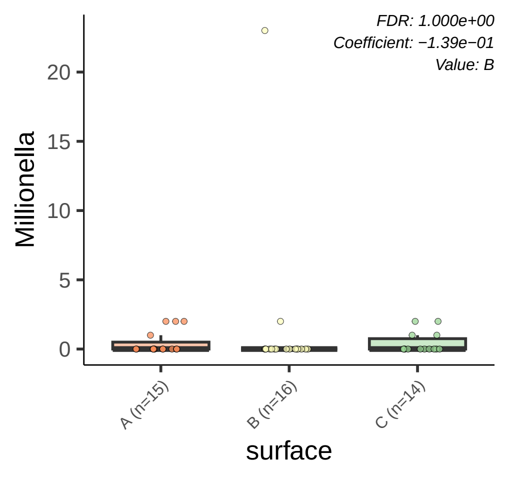

FDR: 1.000e+00
Coefficient: −1.39e−01
Value: B
Millionella
0
5
10
15
20
A (n=15)
B (n=16)
C (n=14)
surface

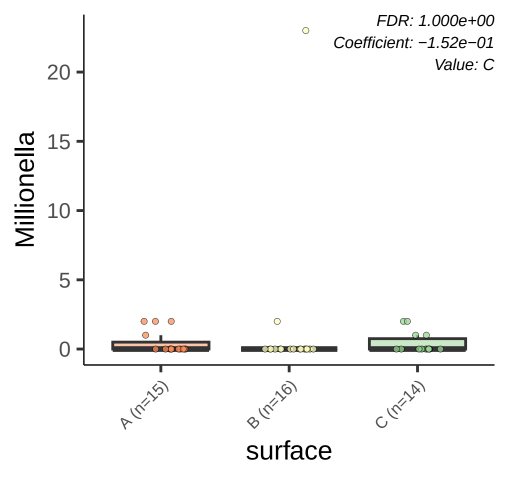
FDR: 1.000e+00
Coefficient: −1.52e−01
Value: C
Millionella
0
5
10
15
20
A (n=15)
B (n=16)
C (n=14)
surface

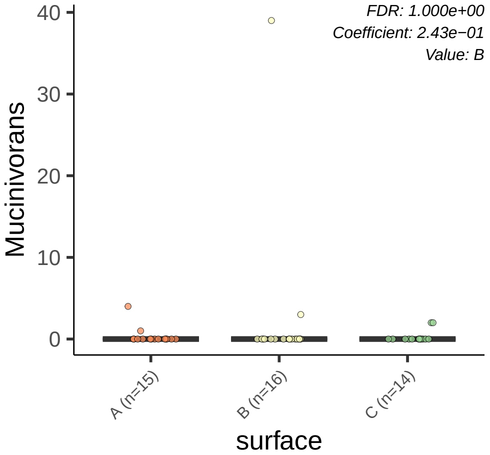
FDR: 1.000e+00
Coefficient: 2.43e−01
Value: B
Mucinivorans
40
30
20
10
0
A (n=15)
B (n=16)
C (n=14)
surface

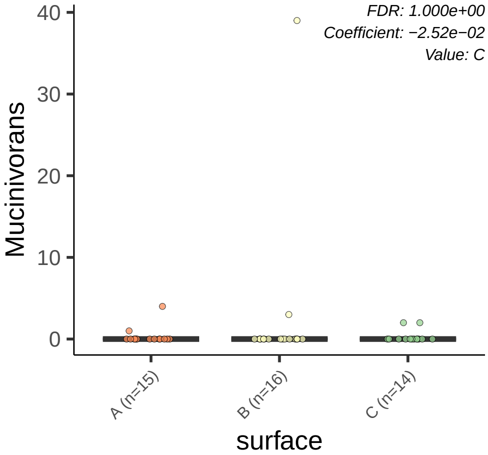
FDR: 1.000e+00
Coefficient: −2.52e−02
Value: C
Mucinivorans
40
30
20
10
0
A (n=15)
B (n=16)
C (n=14)
surface

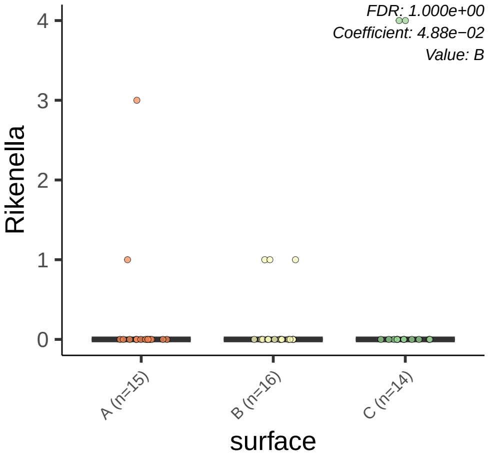

FDR: 1.000e+00
Coefficient: 4.88e−02
Value: B
Rikenella
4
3
2
1
0
A (n=15)
B (n=16)
C (n=14)
surface

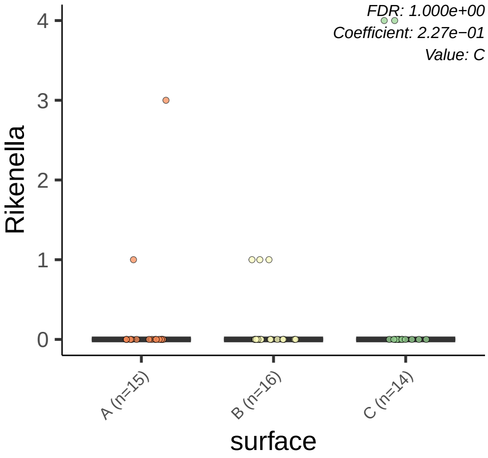

FDR: 1.000e+00
Coefficient: 2.27e−01
Value: C
Rikenella
0
1
2
3
4
A (n=15)
B (n=16)
C (n=14)
surface

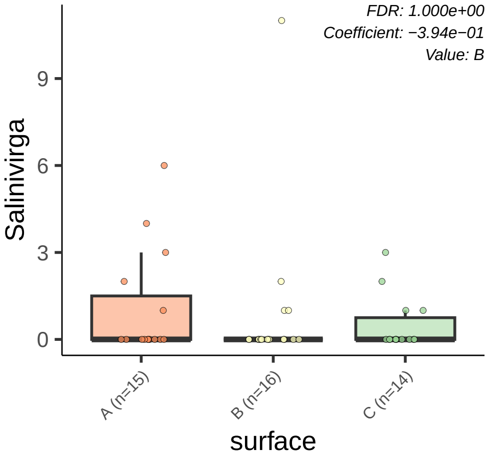

FDR: 1.000e+00
Coefficient: −3.94e−01
Value: B
Salinivirga
0
3
6
9
A (n=15)
B (n=16)
C (n=14)
surface

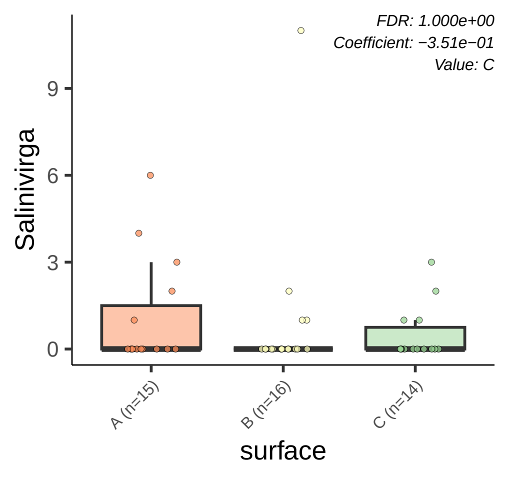
FDR: 1.000e+00
Coefficient: −3.51e−01
Value: C
Salinivirga
9
6
3
0
A (n=15)
B (n=16)
C (n=14)
surface

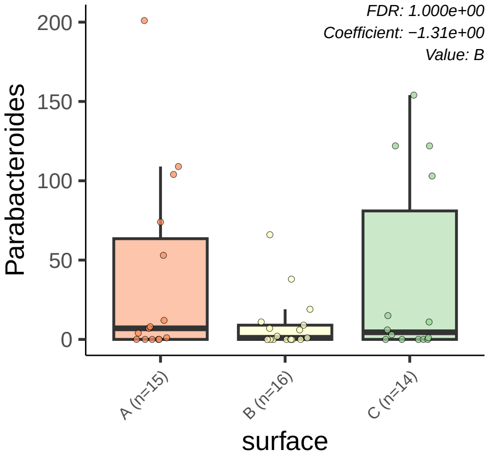

FDR: 1.000e+00
Coefficient: −1.31e+00
Value: B
Parabacteroides
200
150
100
50
0
A (n=15)
B (n=16)
C (n=14)
surface

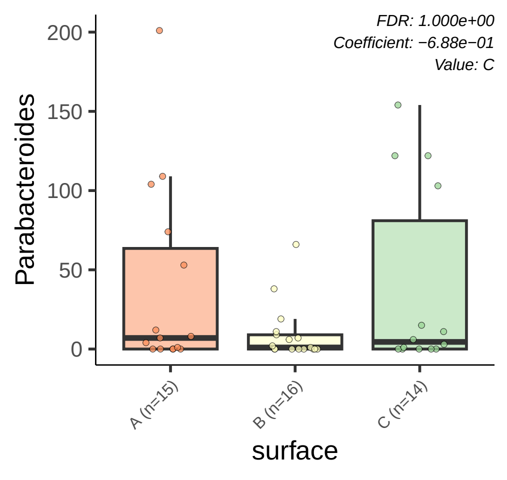
FDR: 1.000e+00
Coefficient: −6.88e−01
Value: C
Parabacteroides
200
150
100
50
0
A (n=15)
B (n=16)
C (n=14)
surface

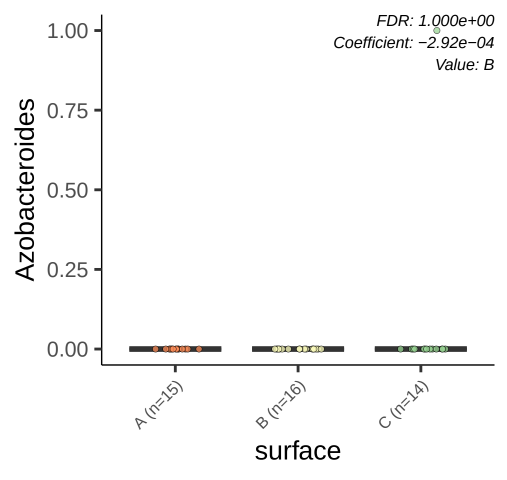
FDR: 1.000e+00
Coefficient: −2.92e−04
Value: B
Azobacteroides
1.00
0.75
0.50
0.25
0.00
A (n=15)
B (n=16)
C (n=14)
surface

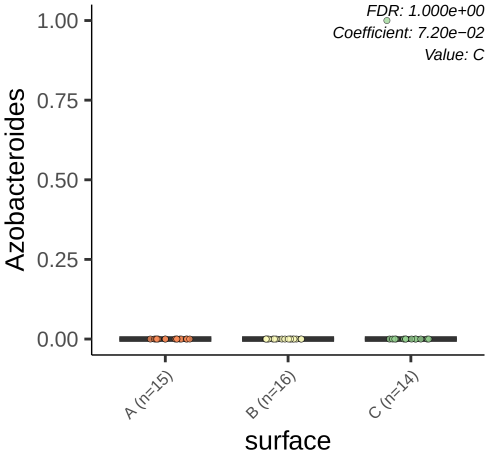
FDR: 1.000e+00
Coefficient: 7.20e−02
Value: C
Azobacteroides
1.00
0.75
0.50
0.25
0.00
A (n=15)
B (n=16)
C (n=14)
surface

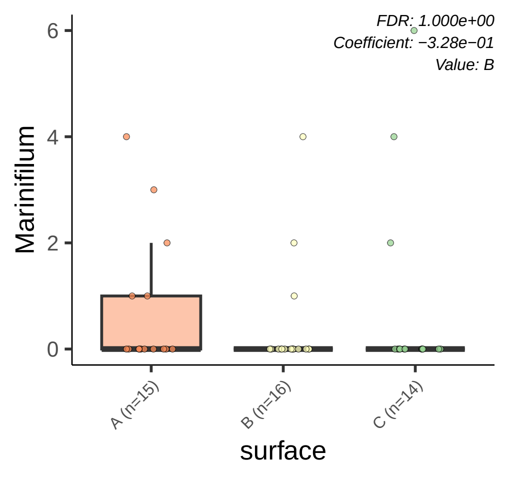
FDR: 1.000e+00
Coefficient: −3.28e−01
Value: B
Marinifilum
0
2
4
6
A (n=15)
B (n=16)
C (n=14)
surface

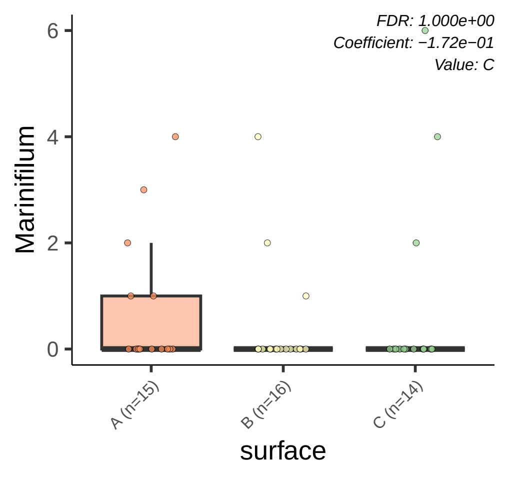
FDR: 1.000e+00
Coefficient: −1.72e−01
Value: C
Marinifilum
surface
A (n=15)
B (n=16)
C (n=14)
0
2
4
6

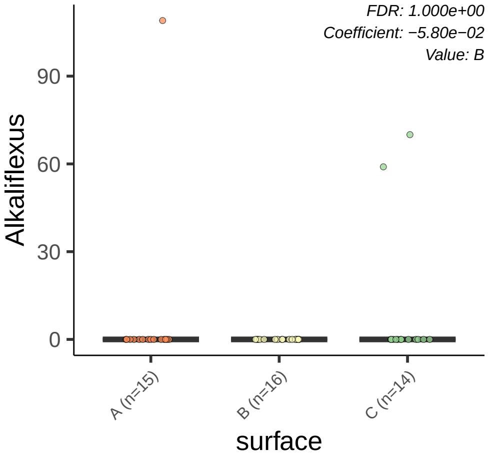

FDR: 1.000e+00
Coefficient: −5.80e−02
Value: B
Alkaliflexus
90
60
30
0
A (n=15)
B (n=16)
C (n=14)
surface

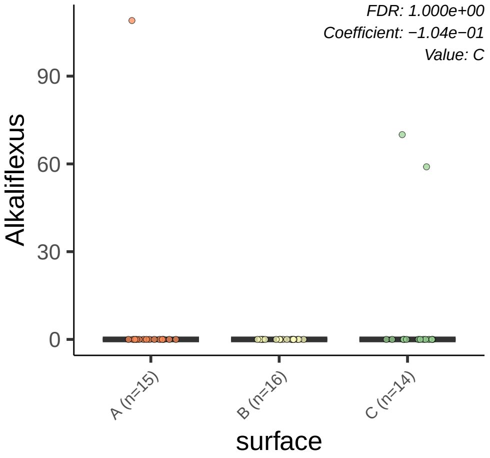

FDR: 1.000e+00
Coefficient: −1.04e−01
Value: C
Alkaliflexus
90
60
30
0
A (n=15)
B (n=16)
C (n=14)
surface

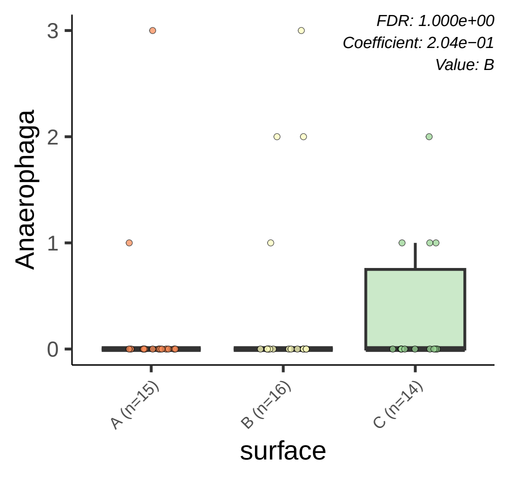

FDR: 1.000e+00
Coefficient: 2.04e−01
Value: B
Anaerophaga
3
2
1
0
A (n=15)
B (n=16)
C (n=14)
surface

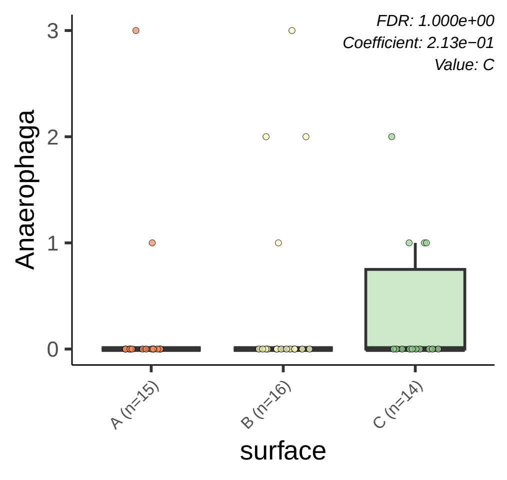
FDR: 1.000e+00
Coefficient: 2.13e−01
Value: C
Anaerophaga
0
1
2
3
A (n=15)
B (n=16)
C (n=14)
surface

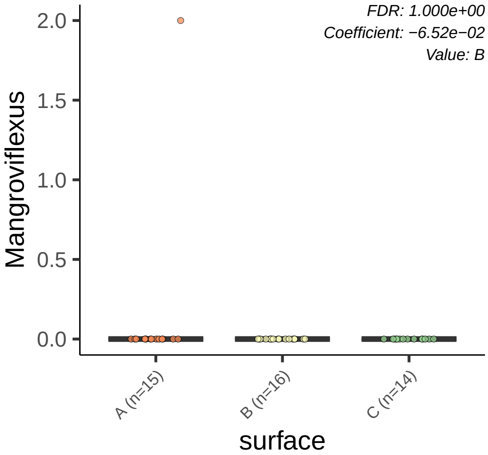

FDR: 1.000e+00
Coefficient: −6.52e−02
Value: B
Mangroviflexus
2.0
1.5
1.0
0.5
0.0
A (n=15)
B (n=16)
C (n=14)
surface

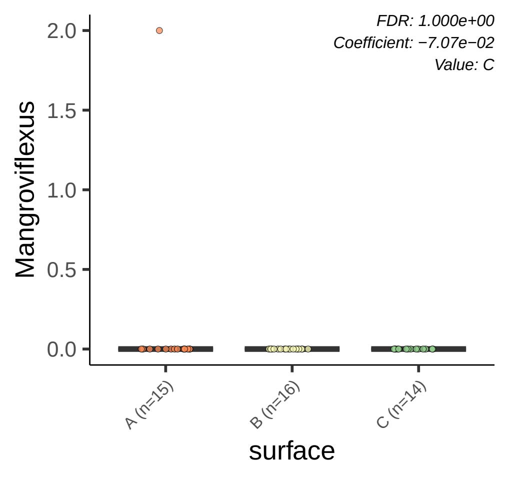

FDR: 1.000e+00
Coefficient: −7.07e−02
Value: C
Mangroviflexus
2.0
1.5
1.0
0.5
0.0
A (n=15)
B (n=16)
C (n=14)
surface

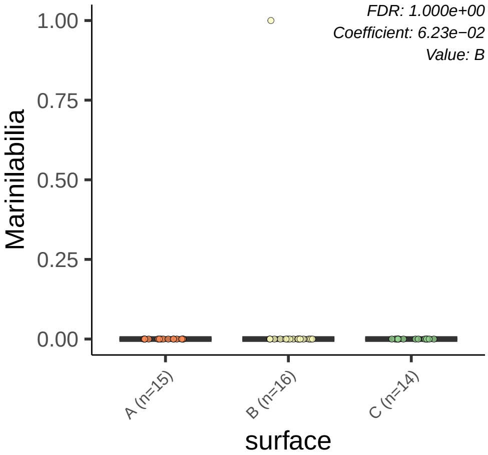
FDR: 1.000e+00
Coefficient: 6.23e−02
Value: B
Marinilabilia
1.00
0.75
0.50
0.25
0.00
A (n=15)
B (n=16)
C (n=14)
surface

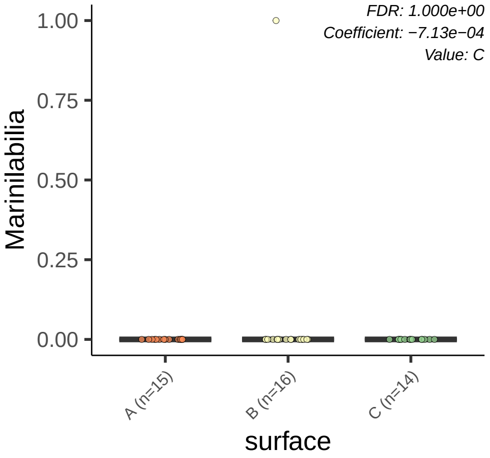

FDR: 1.000e+00
Coefficient: −7.13e−04
Value: C
Marinilabilia
1.00
0.75
0.50
0.25
0.00
A (n=15)
B (n=16)
C (n=14)
surface

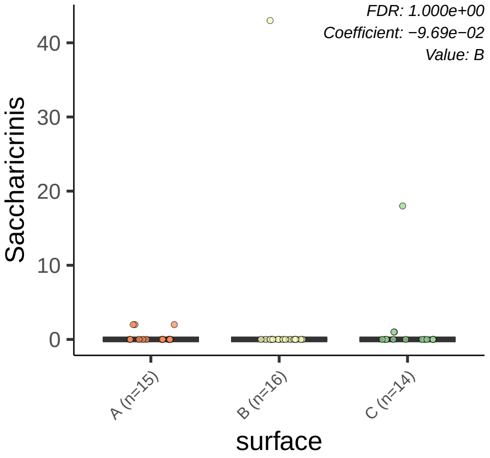

FDR: 1.000e+00
Coefficient: −9.69e−02
Value: B
Saccharicrinis
0
10
20
30
40
A (n=15)
B (n=16)
C (n=14)
surface

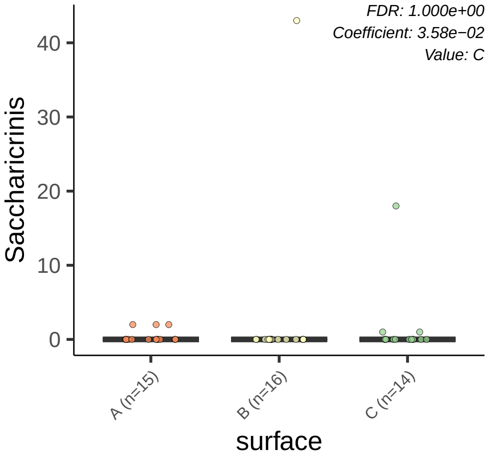

FDR: 1.000e+00
Coefficient: 3.58e−02
Value: C
Saccharicrinis
40
30
20
10
0
A (n=15)
B (n=16)
C (n=14)
surface

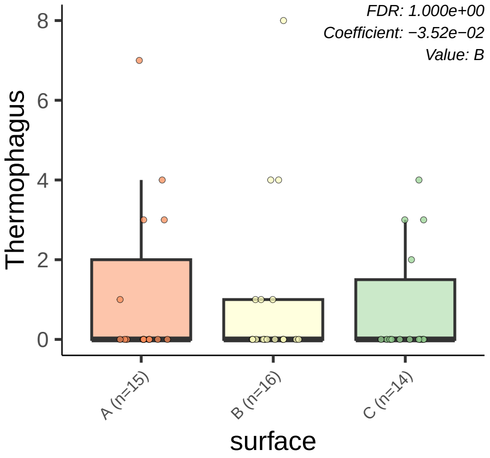

FDR: 1.000e+00
Coefficient: −3.52e−02
Value: B
Thermophagus
0
2
4
6
8
A (n=15)
B (n=16)
C (n=14)
surface

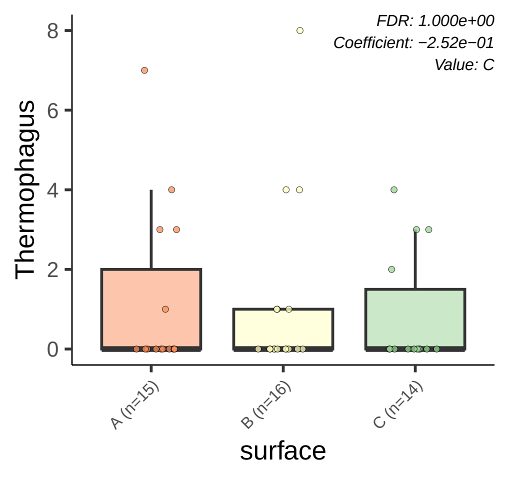
FDR: 1.000e+00
Coefficient: −2.52e−01
Value: C
Thermophagus
0
2
4
6
8
A (n=15)
B (n=16)
C (n=14)
surface

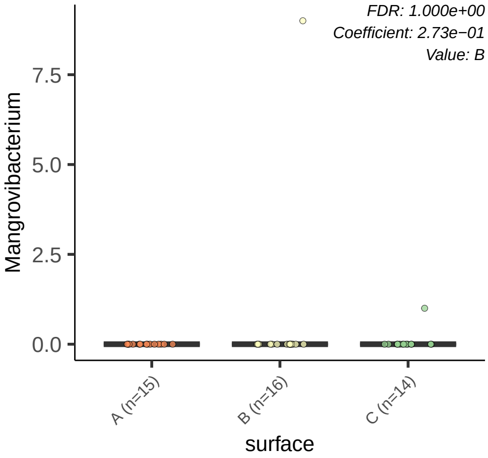
FDR: 1.000e+00
Coefficient: 2.73e−01
Value: B
Mangrovibacterium
7.5
5.0
2.5
0.0
A (n=15)
B (n=16)
C (n=14)
surface

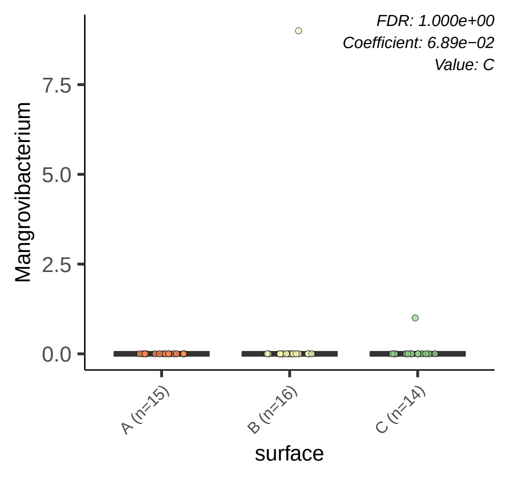
FDR: 1.000e+00
Coefficient: 6.89e−02
Value: C
Mangrovibacterium
7.5
5.0
2.5
0.0
A (n=15)
B (n=16)
C (n=14)
surface

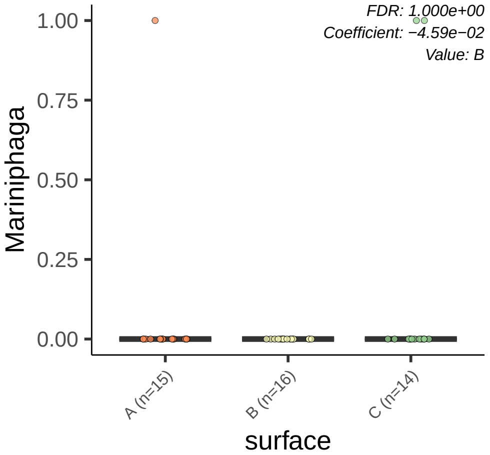

FDR: 1.000e+00
Coefficient: −4.59e−02
Value: B
Mariniphaga
1.00
0.75
0.50
0.25
0.00
A (n=15)
B (n=16)
C (n=14)
surface

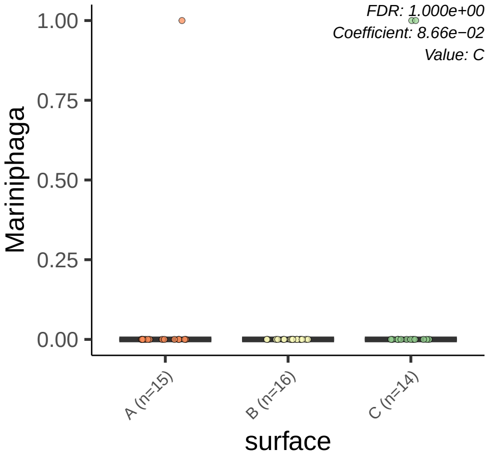

FDR: 1.000e+00
Coefficient: 8.66e−02
Value: C
Mariniphaga
1.00
0.75
0.50
0.25
0.00
A (n=15)
B (n=16)
C (n=14)
surface

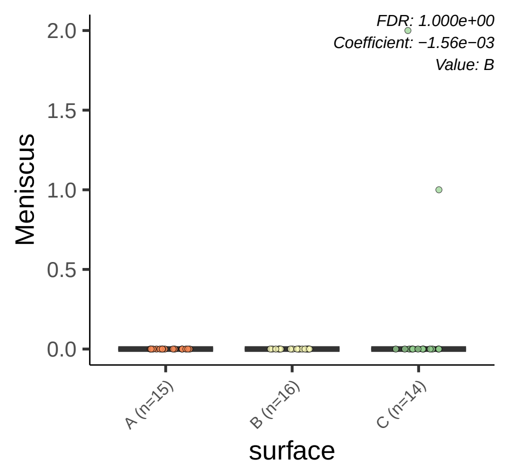
FDR: 1.000e+00
Coefficient: −1.56e−03
Value: B
Meniscus
2.0
1.5
1.0
0.5
0.0
A (n=15)
B (n=16)
C (n=14)
surface

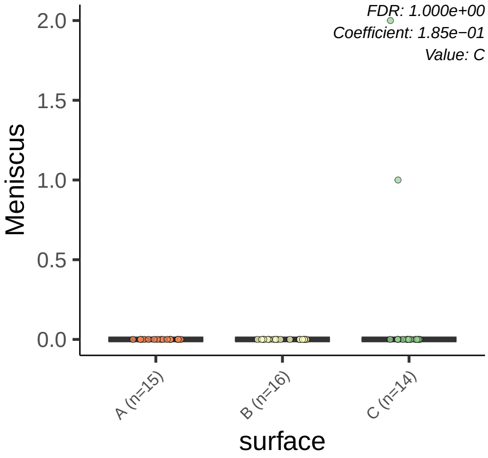
FDR: 1.000e+00
Coefficient: 1.85e−01
Value: C
Meniscus
2.0
1.5
1.0
0.5
0.0
A (n=15)
B (n=16)
C (n=14)
surface

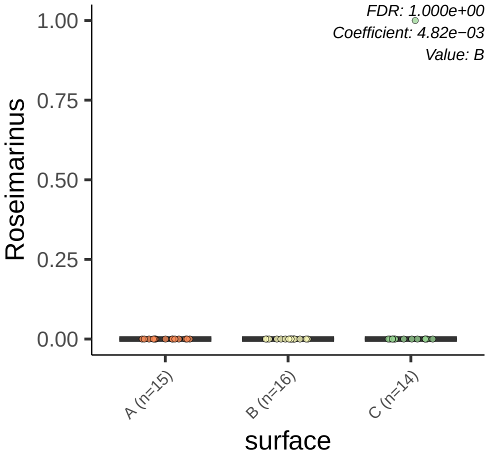

FDR: 1.000e+00
Coefficient: 4.82e−03
Value: B
Roseimarinus
1.00
0.75
0.50
0.25
0.00
A (n=15)
B (n=16)
C (n=14)
surface

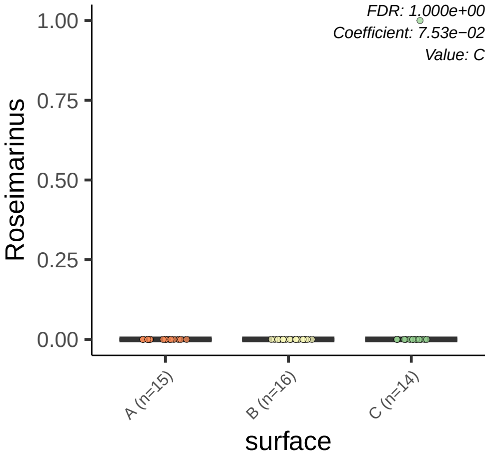
FDR: 1.000e+00
Coefficient: 7.53e−02
Value: C
Roseimarinus
1.00
0.75
0.50
0.25
0.00
A (n=15)
B (n=16)
C (n=14)
surface

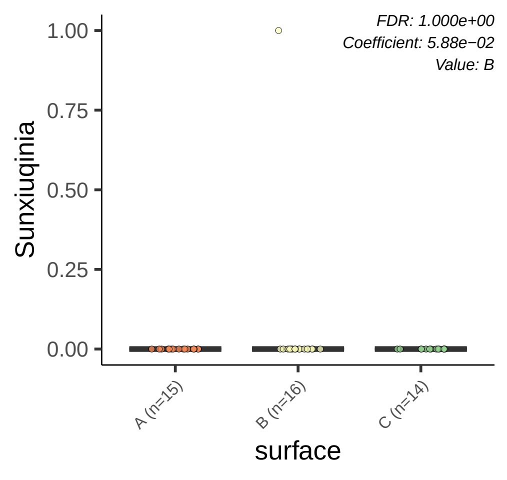

FDR: 1.000e+00
Coefficient: 5.88e−02
Value: B
1.00
0.75
0.50
0.25
0.00
Sunxiuqinia
A (n=15)
B (n=16)
C (n=14)
surface

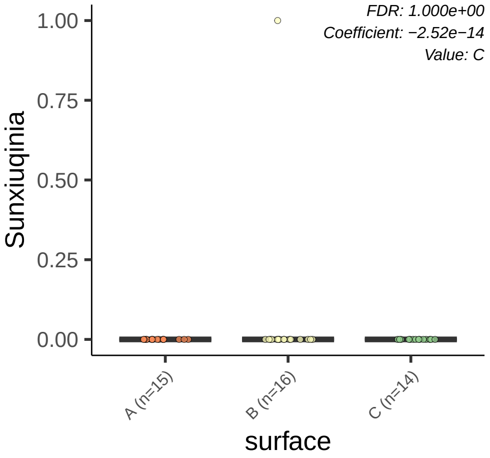

FDR: 1.000e+00
Coefficient: −2.52e−14
Value: C
Sunxiuqinia
1.00
0.75
0.50
0.25
0.00
A (n=15)
B (n=16)
C (n=14)
surface

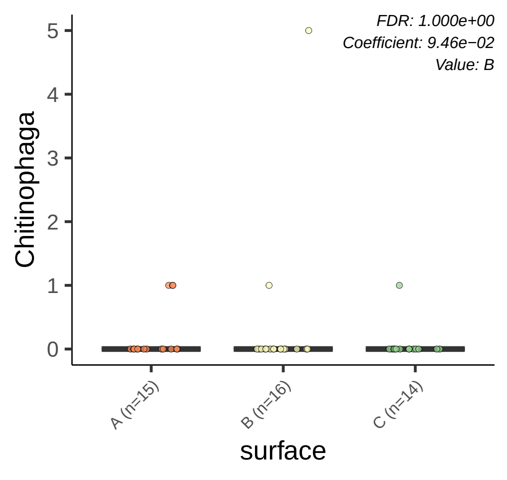
FDR: 1.000e+00
Coefficient: 9.46e−02
Value: B
Chitinophaga
5
4
3
2
1
0
A (n=15)
B (n=16)
C (n=14)
surface

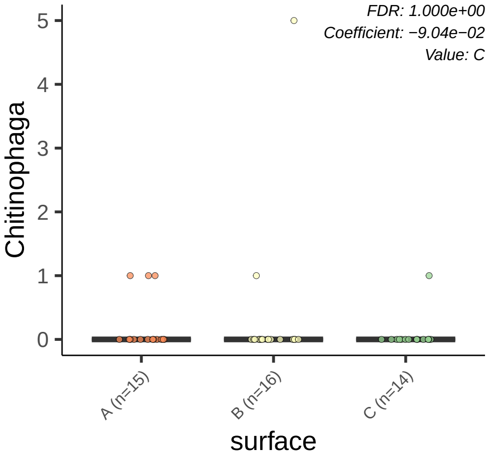

FDR: 1.000e+00
Coefficient: −9.04e−02
Value: C
Chitinophaga
0
1
2
3
4
5
A (n=15)
B (n=16)
C (n=14)
surface

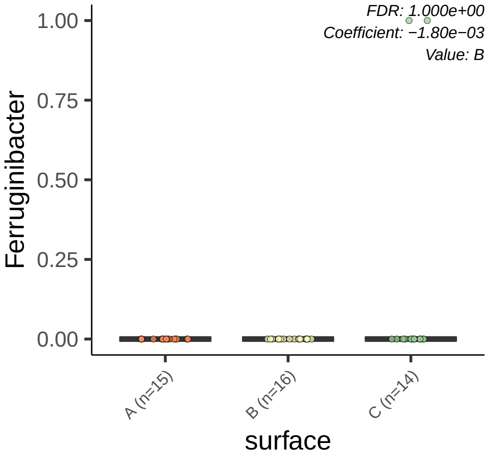
FDR: 1.000e+00
Coefficient: −1.80e−03
Value: B
Ferruginibacter
1.00
0.75
0.50
0.25
0.00
A (n=15)
B (n=16)
C (n=14)
surface

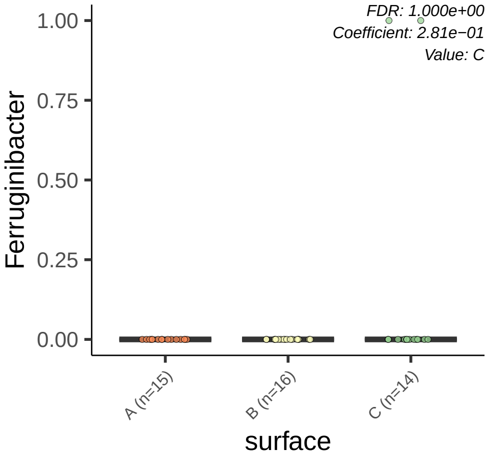
FDR: 1.000e+00
Coefficient: 2.81e−01
Value: C
Ferruginibacter
1.00
0.75
0.50
0.25
0.00
A (n=15)
B (n=16)
C (n=14)
surface

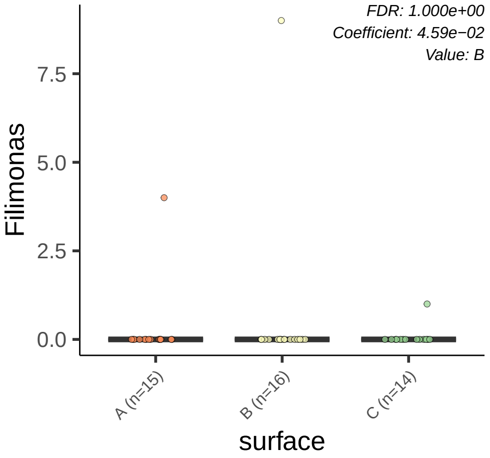

FDR: 1.000e+00
Coefficient: 4.59e−02
Value: B
Filimonas
7.5
5.0
2.5
0.0
A (n=15)
B (n=16)
C (n=14)
surface

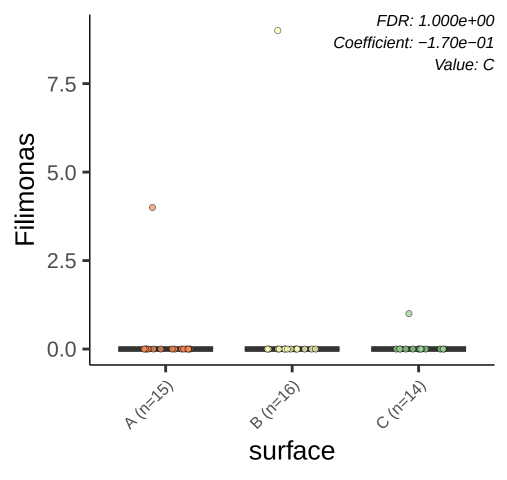
FDR: 1.000e+00
Coefficient: −1.70e−01
Value: C
Filimonas
7.5
5.0
2.5
0.0
A (n=15)
B (n=16)
C (n=14)
surface

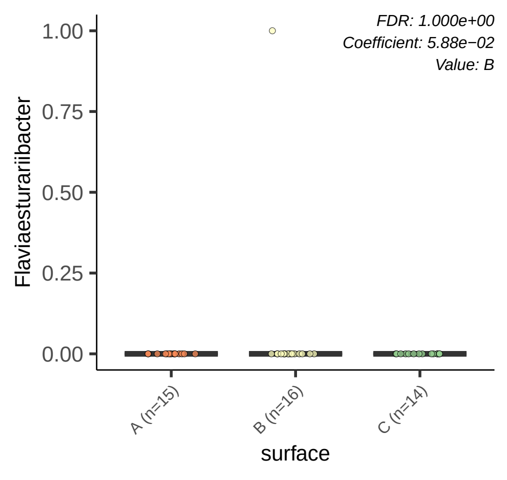
FDR: 1.000e+00
Coefficient: 5.88e−02
Value: B
Flaviaesturariibacter
1.00
0.75
0.50
0.25
0.00
A (n=15)
B (n=16)
C (n=14)
surface

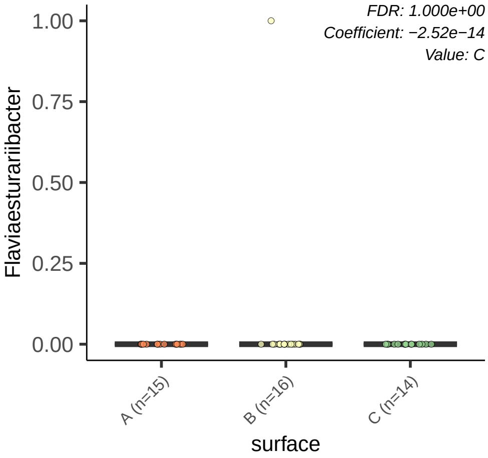
FDR: 1.000e+00
Coefficient: −2.52e−14
Value: C
Flaviaesturariibacter
1.00
0.75
0.50
0.25
0.00
A (n=15)
B (n=16)
C (n=14)
surface

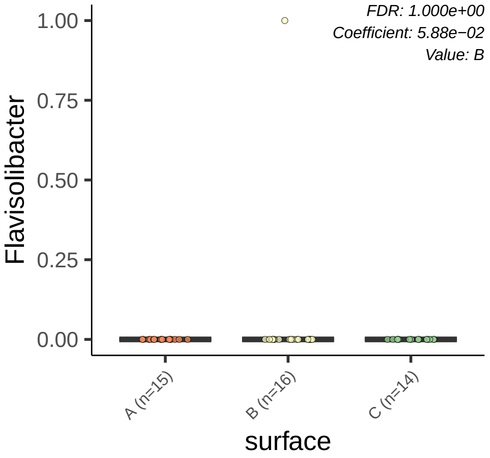
FDR: 1.000e+00
Coefficient: 5.88e−02
Value: B
Flavisolibacter
1.00
0.75
0.50
0.25
0.00
A (n=15)
B (n=16)
C (n=14)
surface

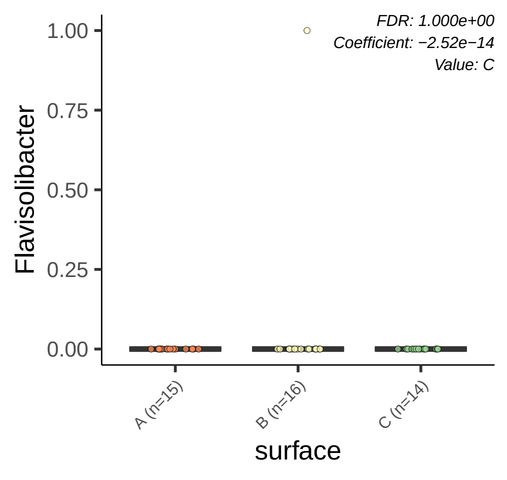
FDR: 1.000e+00
Coefficient: −2.52e−14
Value: C
Flavisolibacter
1.00
0.75
0.50
0.25
0.00
A (n=15)
B (n=16)
C (n=14)
surface

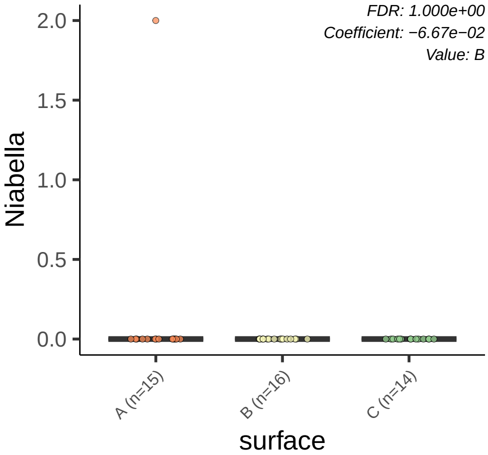
FDR: 1.000e+00
Coefficient: −6.67e−02
Value: B
Niabella
2.0
1.5
1.0
0.5
0.0
A (n=15)
B (n=16)
C (n=14)
surface

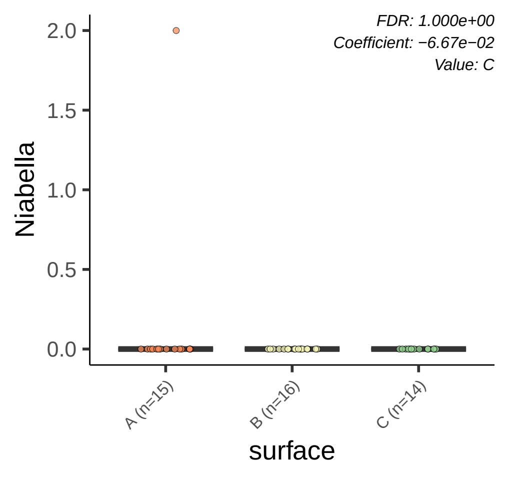
FDR: 1.000e+00
Coefficient: −6.67e−02
Value: C
Niabella
2.0
1.5
1.0
0.5
0.0
A (n=15)
B (n=16)
C (n=14)
surface

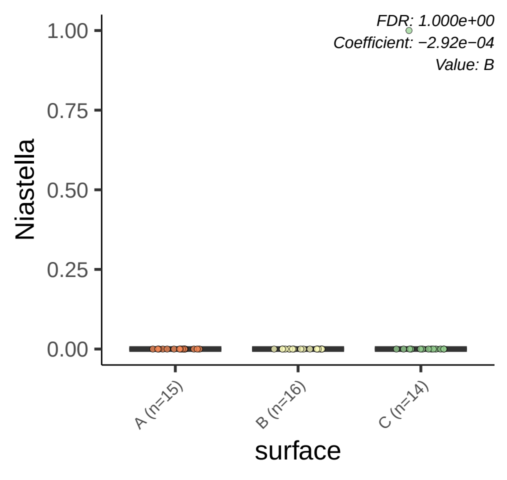
FDR: 1.000e+00
Coefficient: −2.92e−04
Value: B
Niastella
1.00
0.75
0.50
0.25
0.00
A (n=15)
B (n=16)
C (n=14)
surface

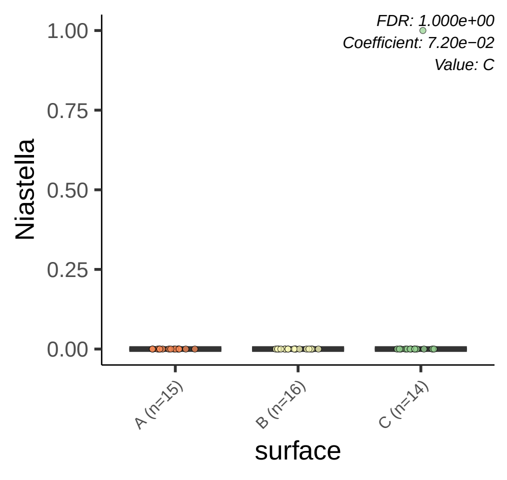
FDR: 1.000e+00
Coefficient: 7.20e−02
Value: C
Niastella
1.00
0.75
0.50
0.25
0.00
A (n=15)
B (n=16)
C (n=14)
surface

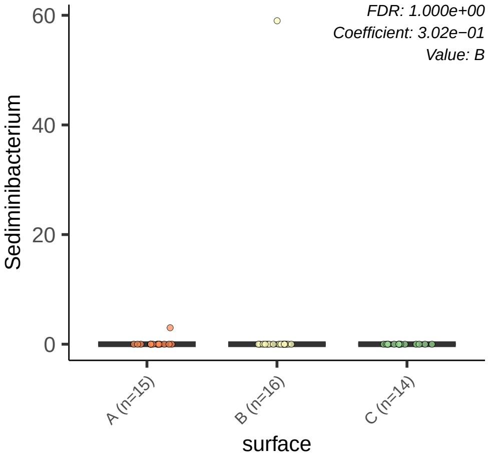
FDR: 1.000e+00
Coefficient: 3.02e−01
Value: B
Sediminibacterium
60
40
20
0
A (n=15)
B (n=16)
C (n=14)
surface

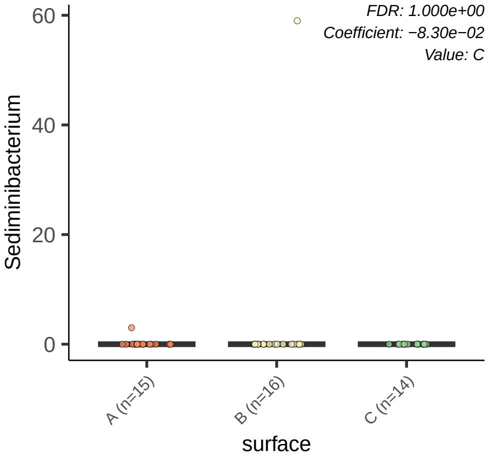
FDR: 1.000e+00
Coefficient: −8.30e−02
Value: C
Sediminibacterium
60
40
20
0
A (n=15)
B (n=16)
C (n=14)
surface

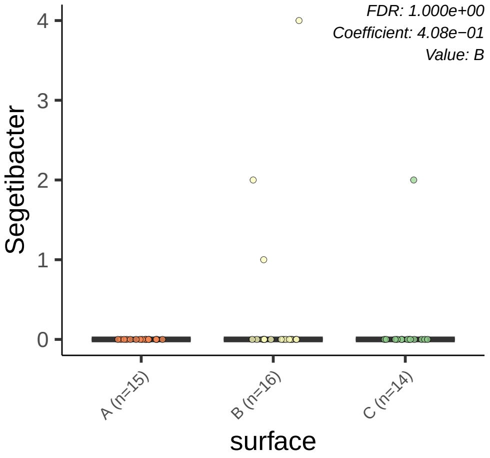

FDR: 1.000e+00
Coefficient: 4.08e−01
Value: B
Segetibacter
0
1
2
3
4
A (n=15)
B (n=16)
C (n=14)
surface

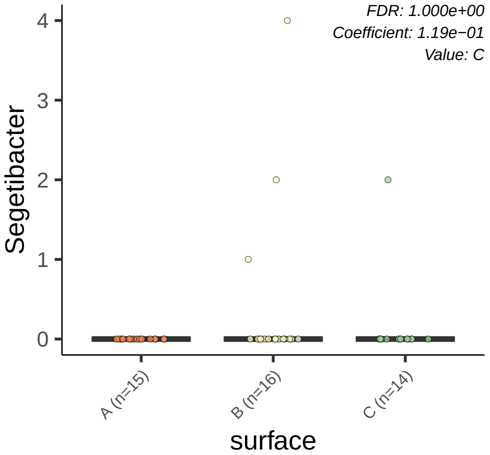

FDR: 1.000e+00
Coefficient: 1.19e−01
Value: C
Segetibacter
4
3
2
1
0
A (n=15)
B (n=16)
C (n=14)
surface

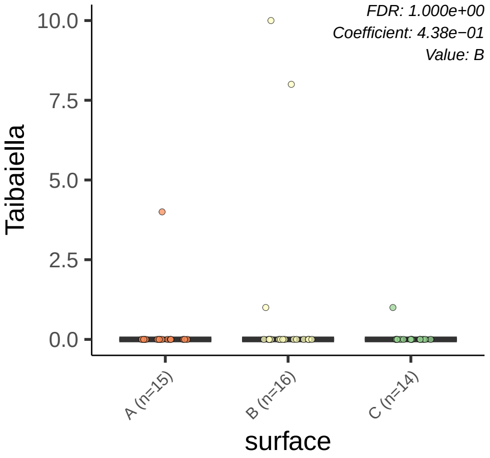
FDR: 1.000e+00
Coefficient: 4.38e−01
Value: B
Taibaiella
10.0
7.5
5.0
2.5
0.0
A (n=15)
B (n=16)
C (n=14)
surface

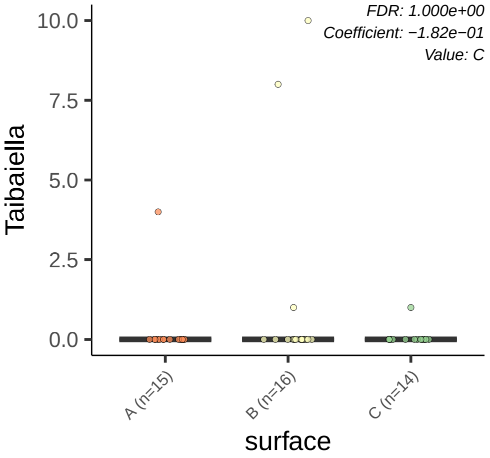
FDR: 1.000e+00
Coefficient: −1.82e−01
Value: C
Taibaiella
10.0
7.5
5.0
2.5
0.0
A (n=15)
B (n=16)
C (n=14)
surface

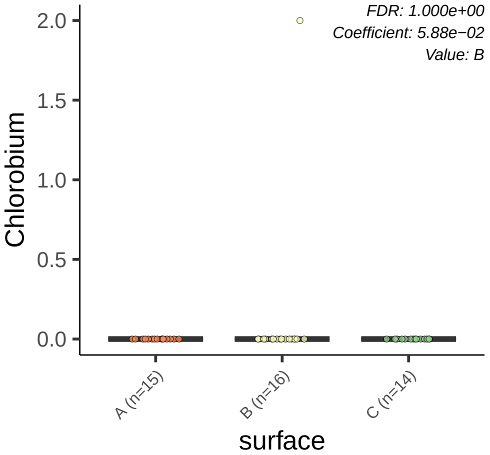
FDR: 1.000e+00
Coefficient: 5.88e−02
Value: B
Chlorobium
2.0
1.5
1.0
0.5
0.0
A (n=15)
B (n=16)
C (n=14)
surface

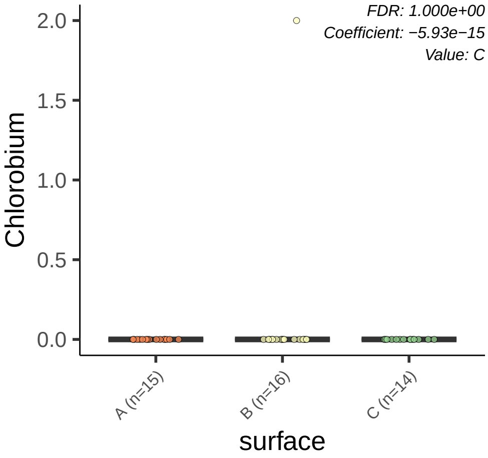

FDR: 1.000e+00
Coefficient: −5.93e−15
Value: C
Chlorobium
2.0
1.5
1.0
0.5
0.0
A (n=15)
B (n=16)
C (n=14)
surface

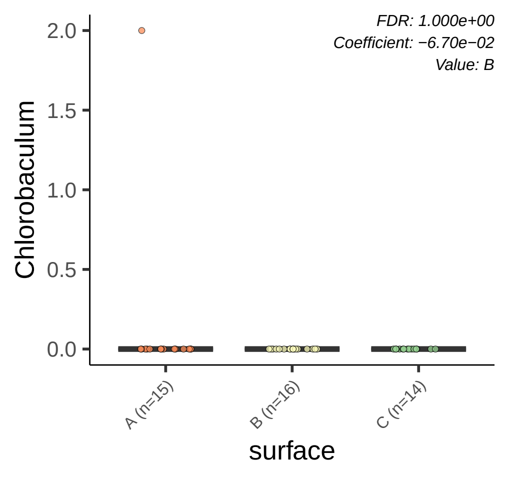

FDR: 1.000e+00
Coefficient: −6.70e−02
Value: B
Chlorobaculum
2.0
1.5
1.0
0.5
0.0
A (n=15)
B (n=16)
C (n=14)
surface

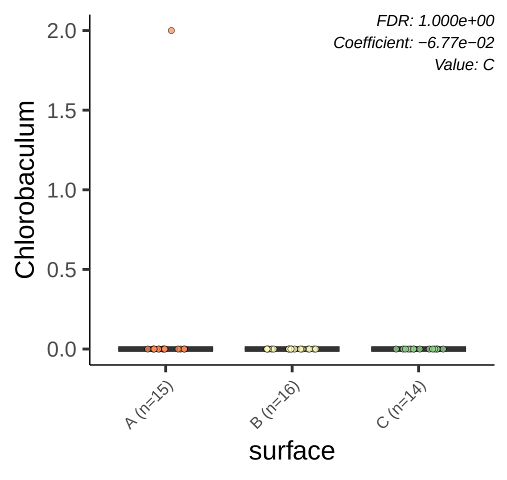
FDR: 1.000e+00
Coefficient: −6.77e−02
Value: C
Chlorobaculum
2.0
1.5
1.0
0.5
0.0
A (n=15)
B (n=16)
C (n=14)
surface

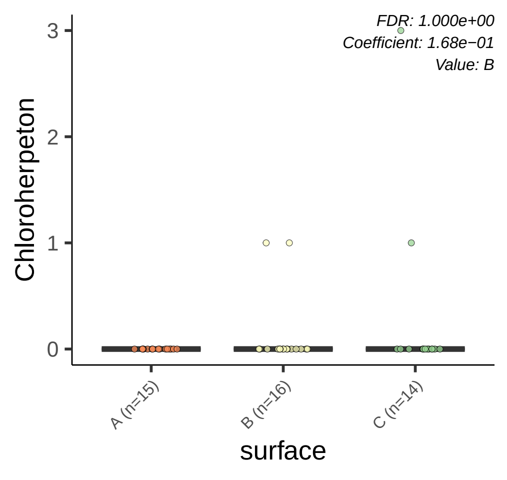
FDR: 1.000e+00
Coefficient: 1.68e−01
Value: B
Chloroherpeton
3
2
1
0
A (n=15)
B (n=16)
C (n=14)
surface

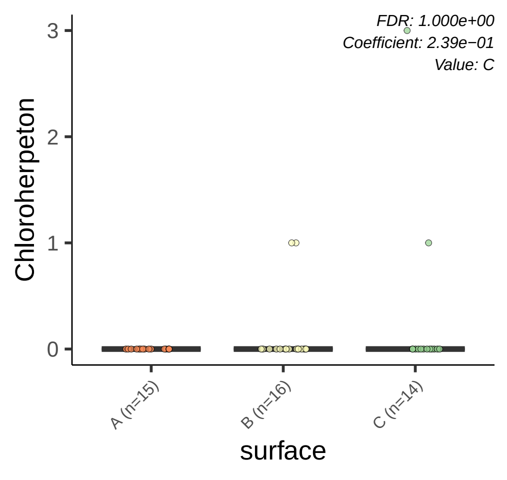

FDR: 1.000e+00
Coefficient: 2.39e−01
Value: C
Chloroherpeton
3
2
1
0
A (n=15)
B (n=16)
C (n=14)
surface

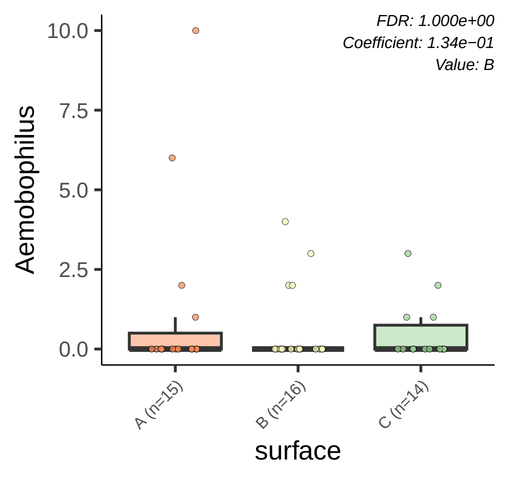
FDR: 1.000e+00
Coefficient: 1.34e−01
Value: B
Aemobophilus
10.0
7.5
5.0
2.5
0.0
A (n=15)
B (n=16)
C (n=14)
surface

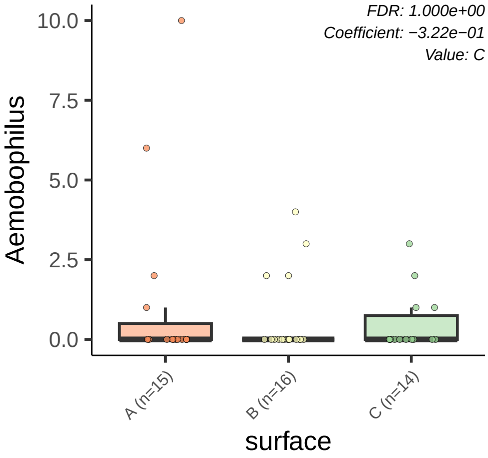

FDR: 1.000e+00
Coefficient: −3.22e−01
Value: C
Aemobophilus
10.0
7.5
5.0
2.5
0.0
A (n=15)
B (n=16)
C (n=14)
surface

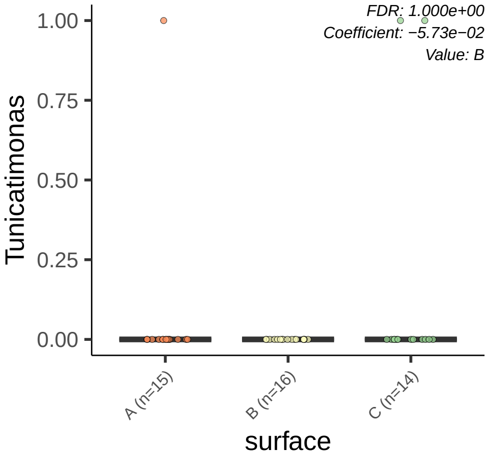
FDR: 1.000e+00
Coefficient: −5.73e−02
Value: B
Tunicatimonas
1.00
0.75
0.50
0.25
0.00
A (n=15)
B (n=16)
C (n=14)
surface

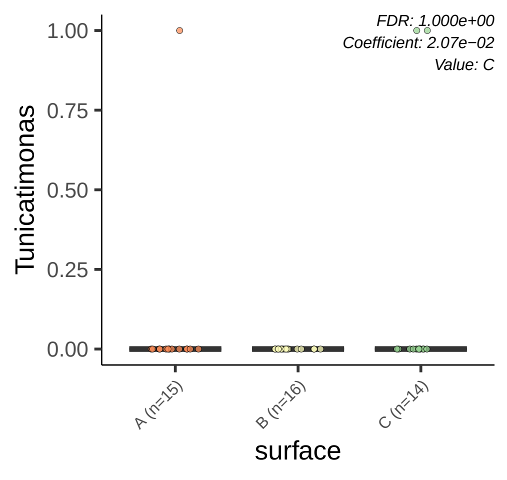
FDR: 1.000e+00
Coefficient: 2.07e−02
Value: C
Tunicatimonas
1.00
0.75
0.50
0.25
0.00
A (n=15)
B (n=16)
C (n=14)
surface

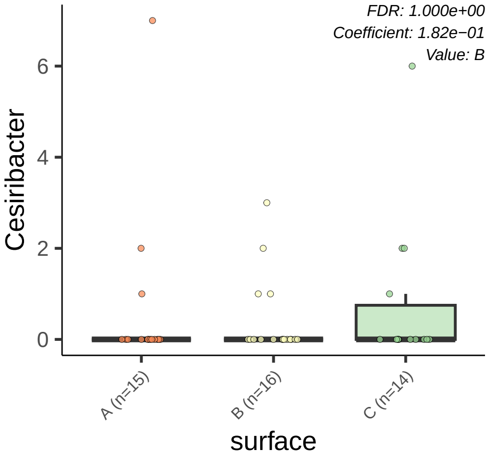

FDR: 1.000e+00
Coefficient: 1.82e−01
Value: B
Cesiribacter
0
2
4
6
A (n=15)
B (n=16)
C (n=14)
surface

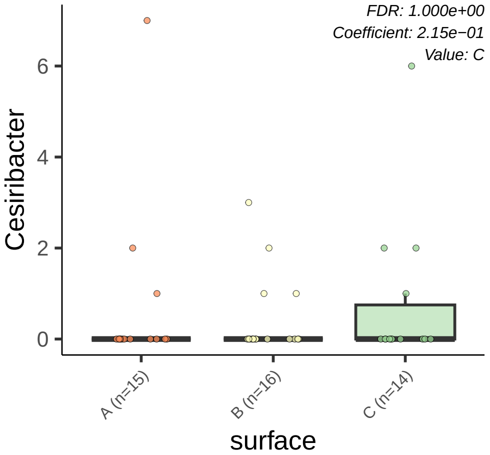

FDR: 1.000e+00
Coefficient: 2.15e−01
Value: C
Cesiribacter
0
2
4
6
A (n=15)
B (n=16)
C (n=14)
surface

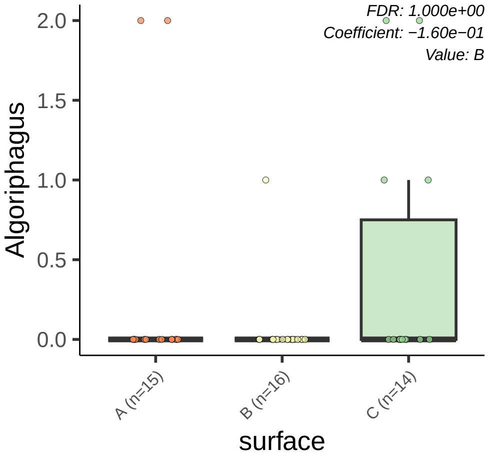
FDR: 1.000e+00
Coefficient: −1.60e−01
Value: B
Algoriphagus
2.0
1.5
1.0
0.5
0.0
A (n=15)
B (n=16)
C (n=14)
surface

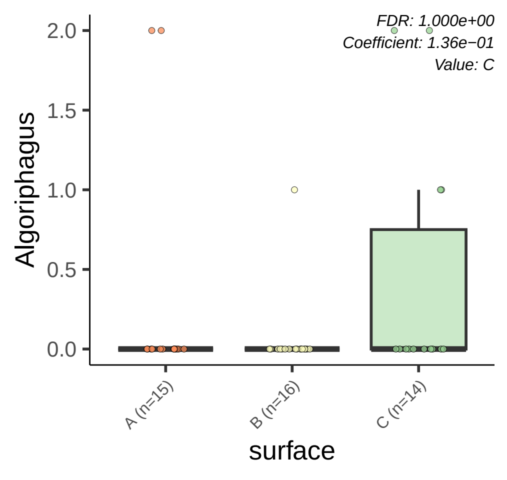

FDR: 1.000e+00
Coefficient: 1.36e−01
Value: C
Algoriphagus
2.0
1.5
1.0
0.5
0.0
A (n=15)
B (n=16)
C (n=14)
surface

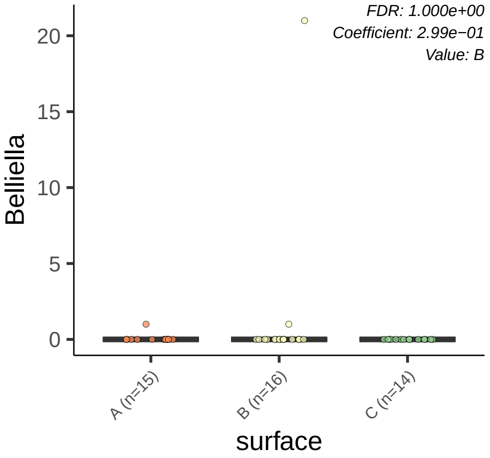
FDR: 1.000e+00
Coefficient: 2.99e−01
Value: B
Belliella
20
15
10
5
0
A (n=15)
B (n=16)
C (n=14)
surface

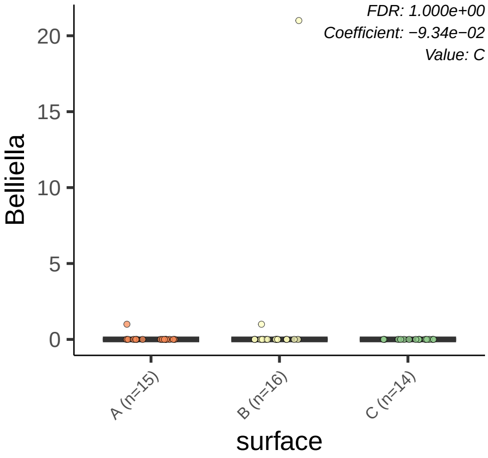
FDR: 1.000e+00
Coefficient: −9.34e−02
Value: C
Belliella
20
15
10
5
0
A (n=15)
B (n=16)
C (n=14)
surface

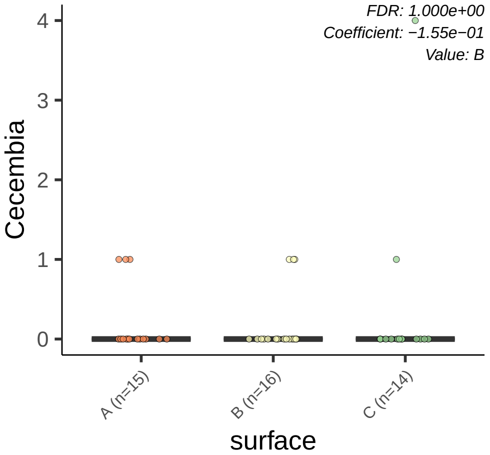

FDR: 1.000e+00
Coefficient: −1.55e−01
Value: B
Cecembia
0
1
2
3
4
A (n=15)
B (n=16)
C (n=14)
surface

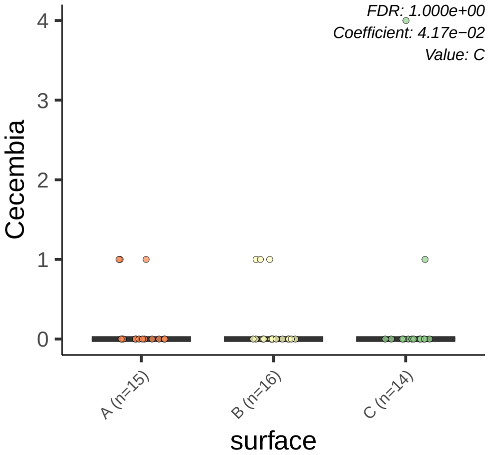
FDR: 1.000e+00
Coefficient: 4.17e−02
Value: C
Cecembia
0
1
2
3
4
A (n=15)
B (n=16)
C (n=14)
surface

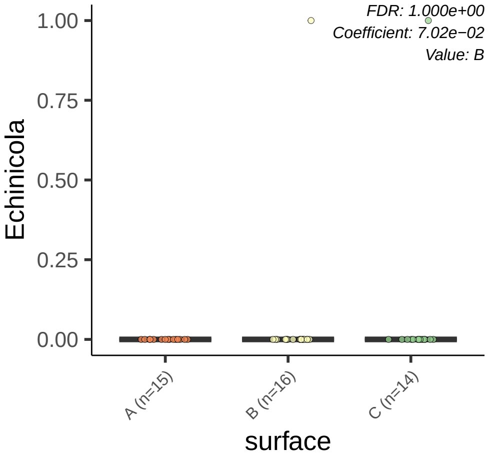
FDR: 1.000e+00
Coefficient: 7.02e−02
Value: B
1.00
0.75
0.50
0.25
0.00
Echinicola
A (n=15)
B (n=16)
C (n=14)
surface

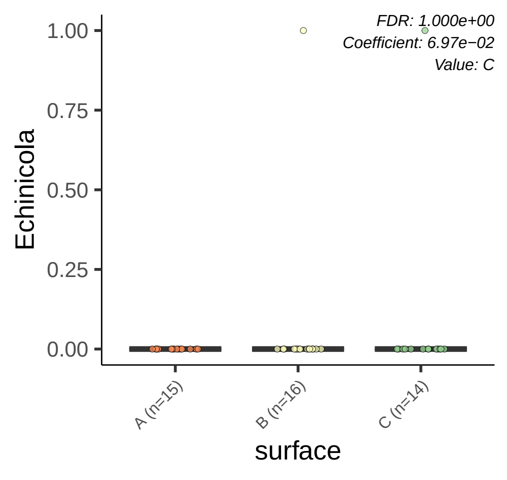
FDR: 1.000e+00
Coefficient: 6.97e−02
Value: C
Echinicola
1.00
0.75
0.50
0.25
0.00
A (n=15)
B (n=16)
C (n=14)
surface

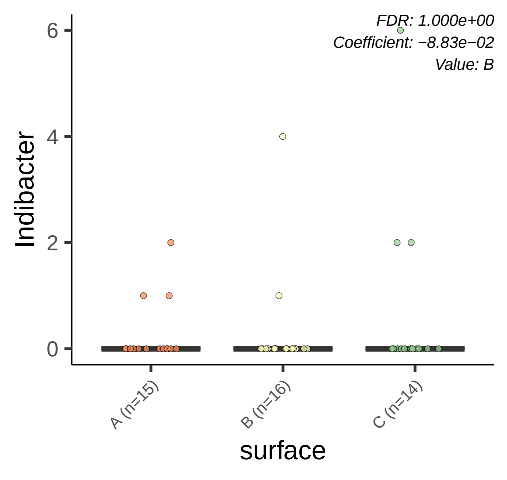
FDR: 1.000e+00
Coefficient: −8.83e−02
Value: B
Indibacter
0
2
4
6
A (n=15)
B (n=16)
C (n=14)
surface

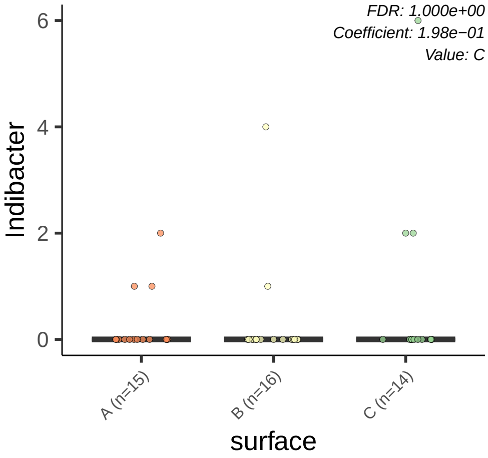

FDR: 1.000e+00
Coefficient: 1.98e−01
Value: C
Indibacter
0
2
4
6
A (n=15)
B (n=16)
C (n=14)
surface

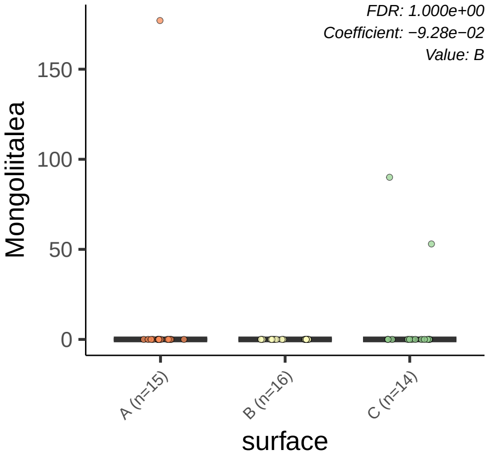
FDR: 1.000e+00
Coefficient: −9.28e−02
Value: B
Mongoliitalea
150
100
50
0
A (n=15)
B (n=16)
C (n=14)
surface

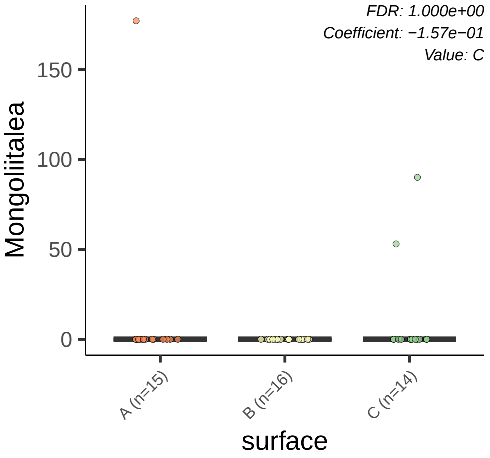

FDR: 1.000e+00
Coefficient: −1.57e−01
Value: C
Mongoliitalea
150
100
50
0
A (n=15)
B (n=16)
C (n=14)
surface

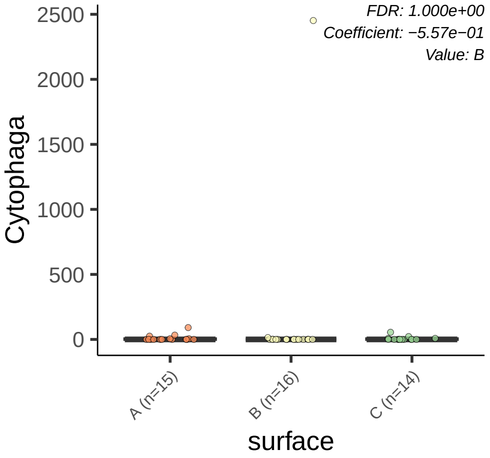
FDR: 1.000e+00
Coefficient: −5.57e−01
Value: B
Cytophaga
2500
2000
1500
1000
500
0
A (n=15)
B (n=16)
C (n=14)
surface

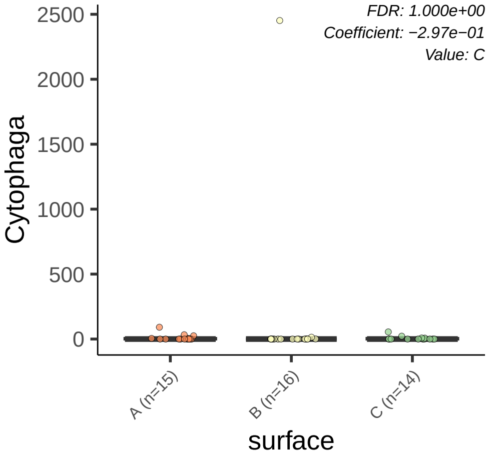
FDR: 1.000e+00
Coefficient: −2.97e−01
Value: C
Cytophaga
2500
2000
1500
1000
500
0
A (n=15)
B (n=16)
C (n=14)
surface

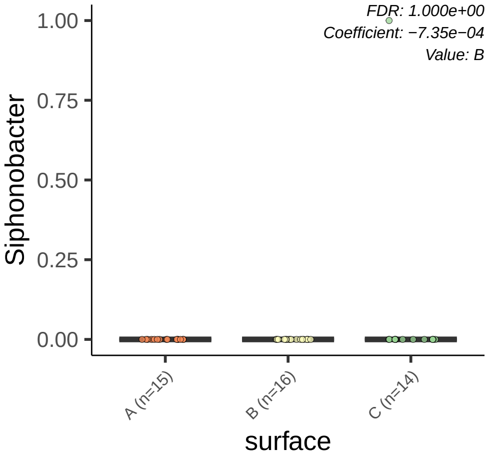
FDR: 1.000e+00
Coefficient: −7.35e−04
Value: B
Siphonobacter
1.00
0.75
0.50
0.25
0.00
A (n=15)
B (n=16)
C (n=14)
surface

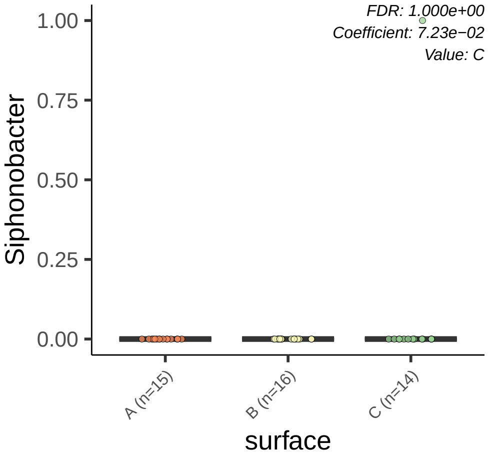

FDR: 1.000e+00
Coefficient: 7.23e−02
Value: C
Siphonobacter
1.00
0.75
0.50
0.25
0.00
A (n=15)
B (n=16)
C (n=14)
surface

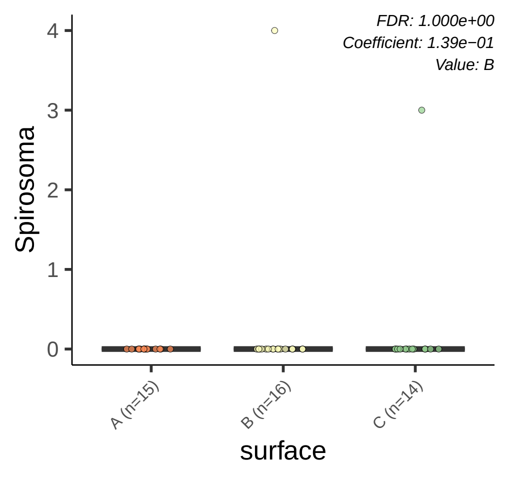
FDR: 1.000e+00
Coefficient: 1.39e−01
Value: B
Spirosoma
0
1
2
3
4
A (n=15)
B (n=16)
C (n=14)
surface

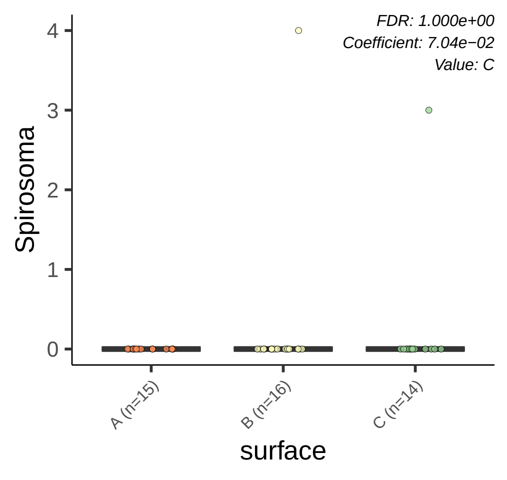

FDR: 1.000e+00
Coefficient: 7.04e−02
Value: C
Spirosoma
0
1
2
3
4
A (n=15)
B (n=16)
C (n=14)
surface

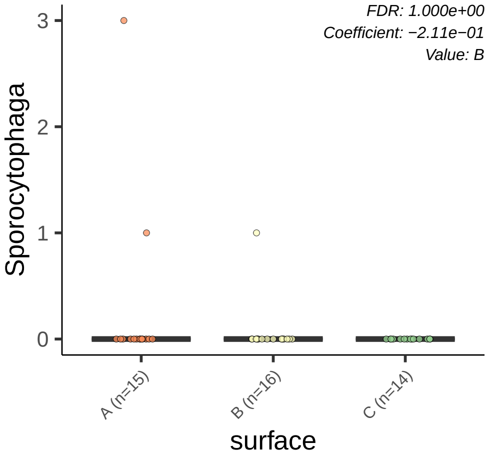
FDR: 1.000e+00
Coefficient: −2.11e−01
Value: B
Sporocytophaga
3
2
1
0
A (n=15)
B (n=16)
C (n=14)
surface

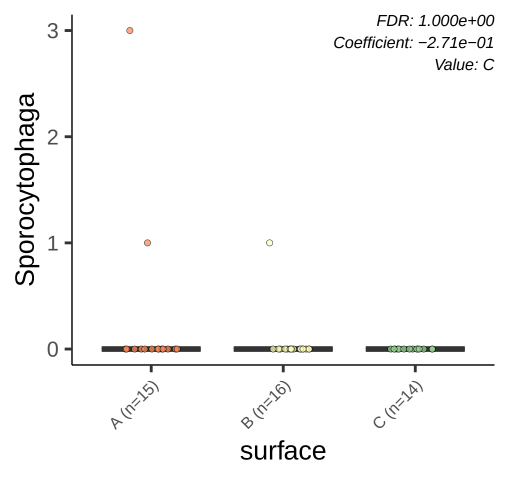
FDR: 1.000e+00
Coefficient: −2.71e−01
Value: C
Sporocytophaga
3
2
1
0
A (n=15)
B (n=16)
C (n=14)
surface

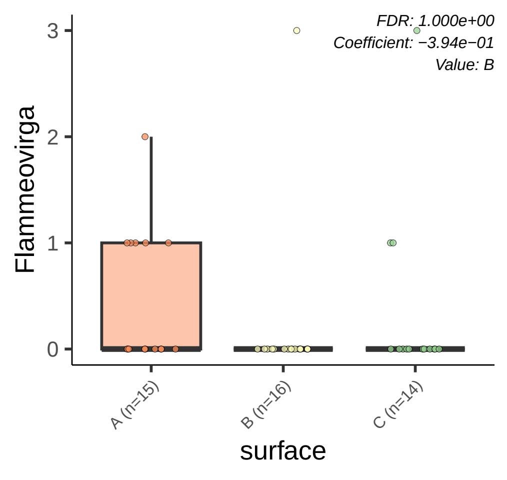

FDR: 1.000e+00
Coefficient: −3.94e−01
Value: B
Flammeovirga
3
2
1
0
A (n=15)
B (n=16)
C (n=14)
surface

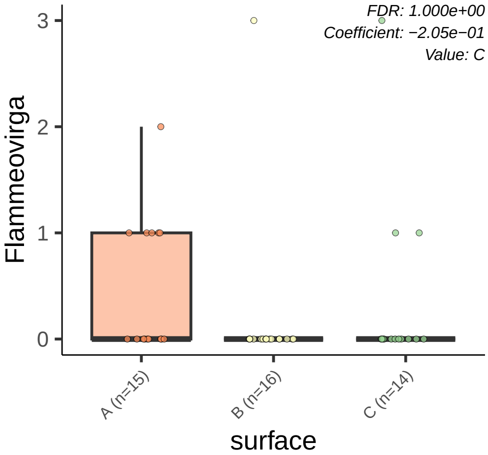
FDR: 1.000e+00
Coefficient: −2.05e−01
Value: C
Flammeovirga
3
2
1
0
A (n=15)
B (n=16)
C (n=14)
surface

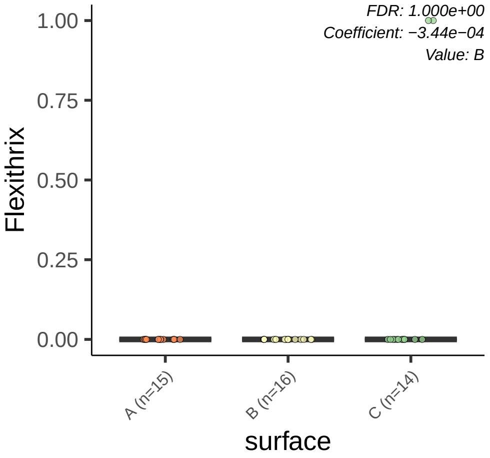

FDR: 1.000e+00
Coefficient: −3.44e−04
Value: B
1.00
0.75
0.50
0.25
0.00
Flexithrix
A (n=15)
B (n=16)
C (n=14)
surface

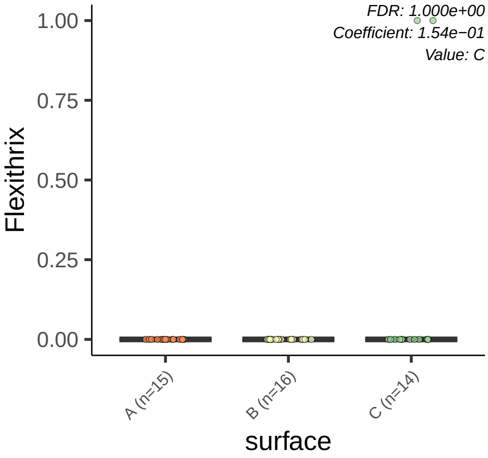
FDR: 1.000e+00
Coefficient: 1.54e−01
Value: C
Flexithrix
1.00
0.75
0.50
0.25
0.00
A (n=15)
B (n=16)
C (n=14)
surface

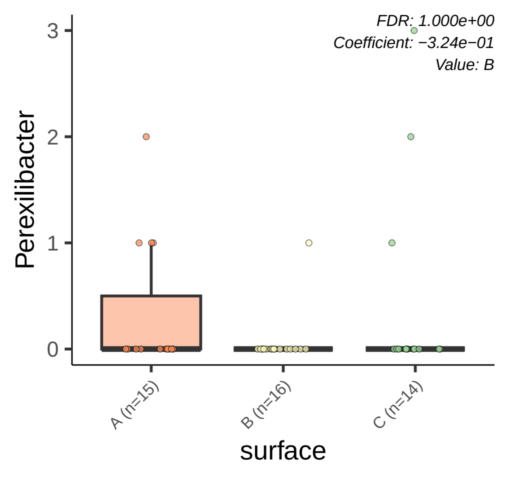

FDR: 1.000e+00
Coefficient: −3.24e−01
Value: B
Perexilibacter
0
1
2
3
A (n=15)
B (n=16)
C (n=14)
surface

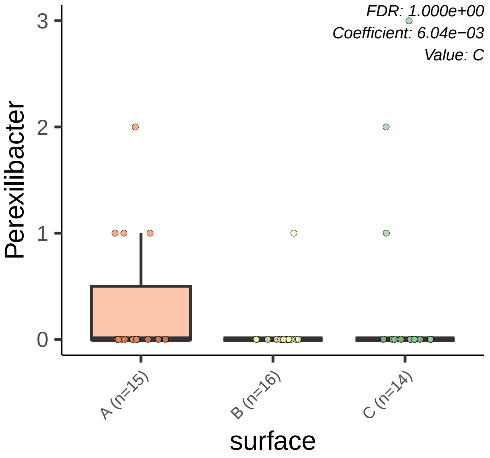

FDR: 1.000e+00
Coefficient: 6.04e−03
Value: C
Perexilibacter
3
2
1
0
A (n=15)
B (n=16)
C (n=14)
surface

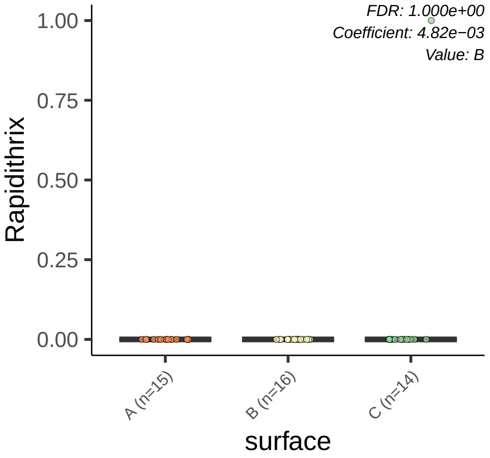
FDR: 1.000e+00
Coefficient: 4.82e−03
Value: B
Rapidithrix
1.00
0.75
0.50
0.25
0.00
A (n=15)
B (n=16)
C (n=14)
surface

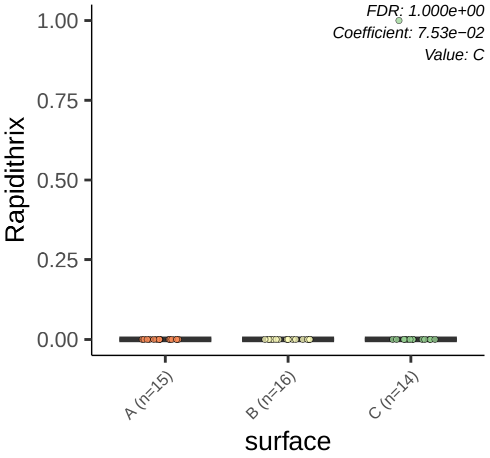
FDR: 1.000e+00
Coefficient: 7.53e−02
Value: C
1.00
0.75
0.50
0.25
0.00
Rapidithrix
A (n=15)
B (n=16)
C (n=14)
surface

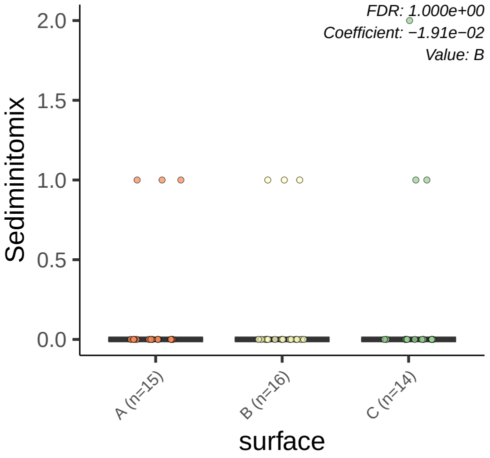
FDR: 1.000e+00
Coefficient: −1.91e−02
Value: B
Sediminitomix
2.0
1.5
1.0
0.5
0.0
A (n=15)
B (n=16)
C (n=14)
surface

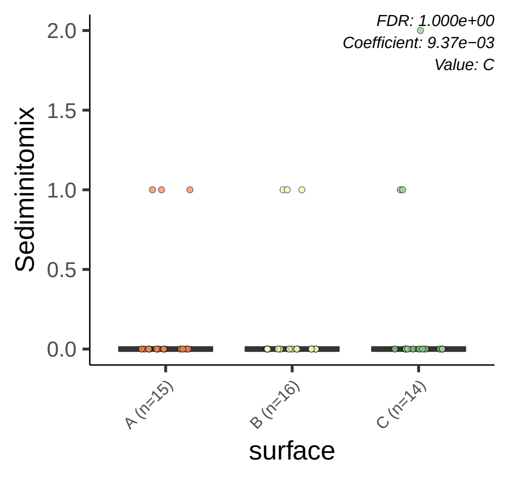
FDR: 1.000e+00
Coefficient: 9.37e−03
Value: C
Sedimintomix
2.0
1.5
1.0
0.5
0.0
A (n=15)
B (n=16)
C (n=14)
surface

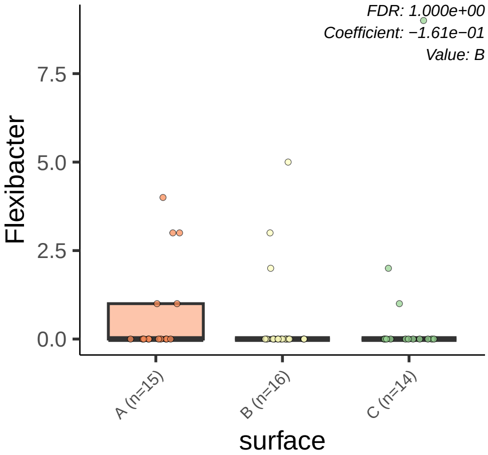

FDR: 1.000e+00
Coefficient: −1.61e−01
Value: B
Flexibacter
7.5
5.0
2.5
0.0
A (n=15)
B (n=16)
C (n=14)
surface

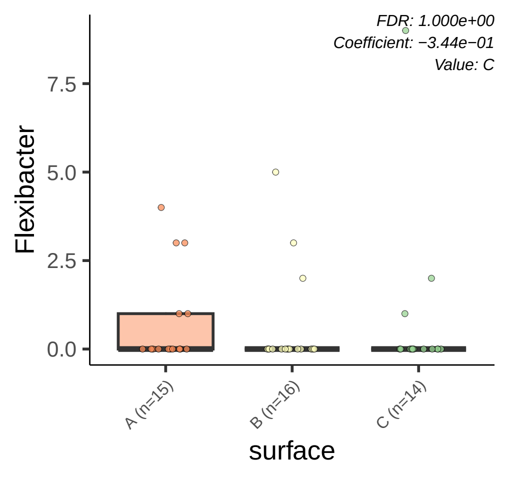
FDR: 1.000e+00
Coefficient: −3.44e−01
Value: C
Flexibacter
7.5
5.0
2.5
0.0
A (n=15)
B (n=16)
C (n=14)
surface

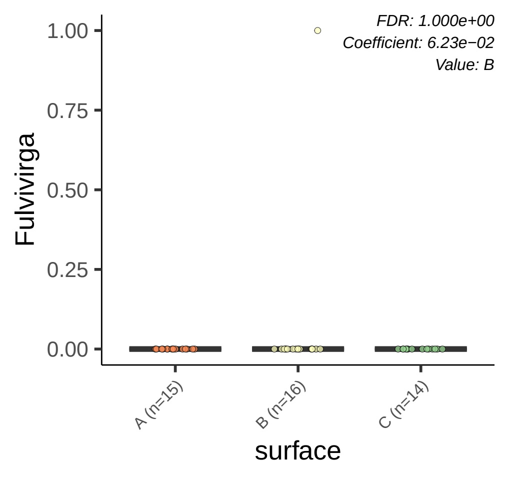
FDR: 1.000e+00
Coefficient: 6.23e−02
Value: B
Fulvivirga
1.00
0.75
0.50
0.25
0.00
A (n=15)
B (n=16)
C (n=14)
surface

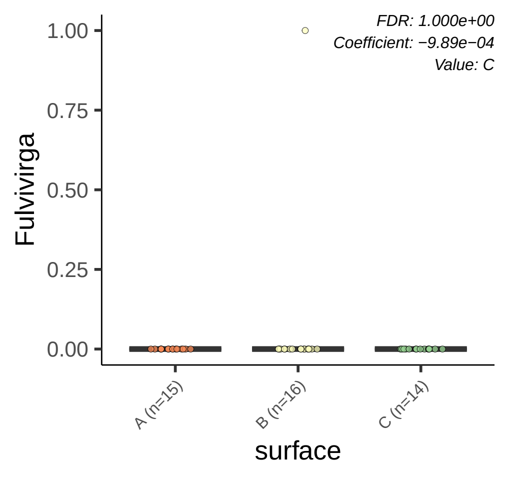

FDR: 1.000e+00
Coefficient: −9.89e−04
Value: C
Fulvivirga
1.00
0.75
0.50
0.25
0.00
A (n=15)
B (n=16)
C (n=14)
surface

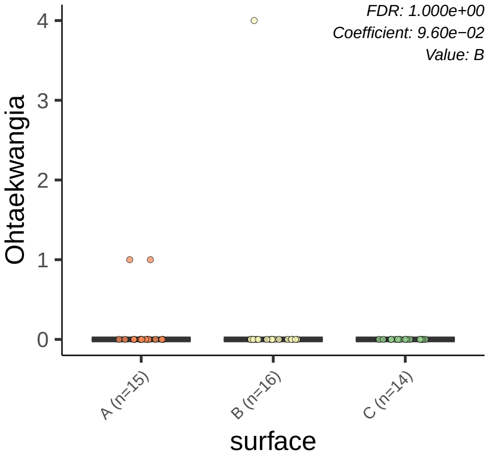
FDR: 1.000e+00
Coefficient: 9.60e−02
Value: B
Ohtaekwangia
0
1
2
3
4
A (n=15)
B (n=16)
C (n=14)
surface

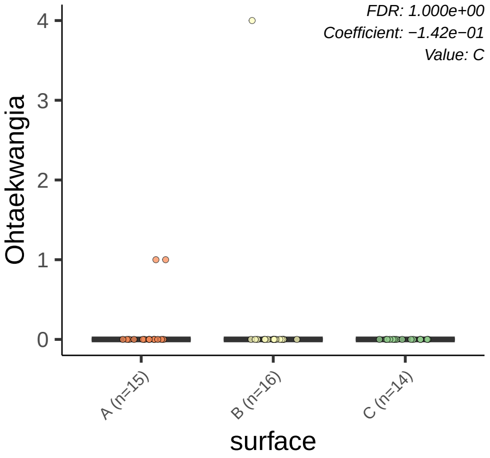
FDR: 1.000e+00
Coefficient: −1.42e−01
Value: C
Ohtaekwangia
4
3
2
1
0
A (n=15)
B (n=16)
C (n=14)
surface

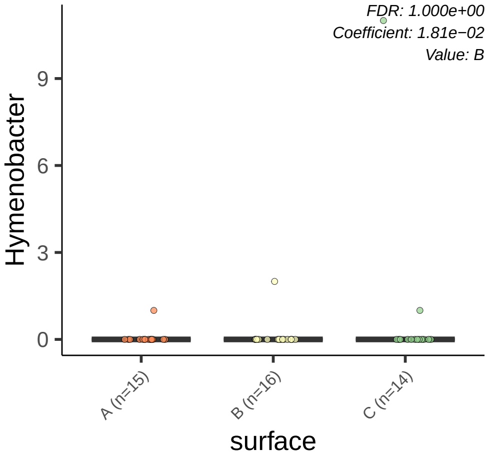
FDR: 1.000e+00
Coefficient: 1.81e−02
Value: B
Hymenobacter
9
6
3
0
A (n=15)
B (n=16)
C (n=14)
surface

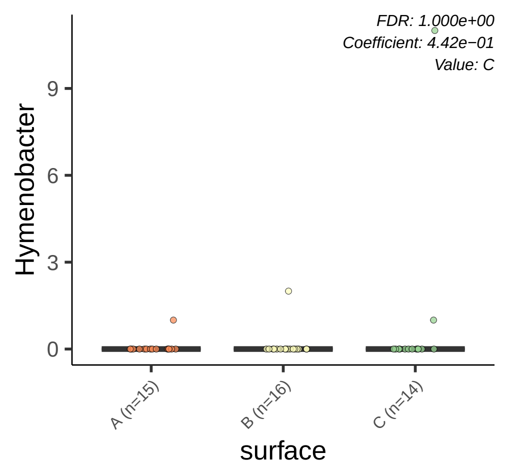
FDR: 1.000e+00
Coefficient: 4.42e−01
Value: C
Hymenobacter
0
3
6
9
A (n=15)
B (n=16)
C (n=14)
surface

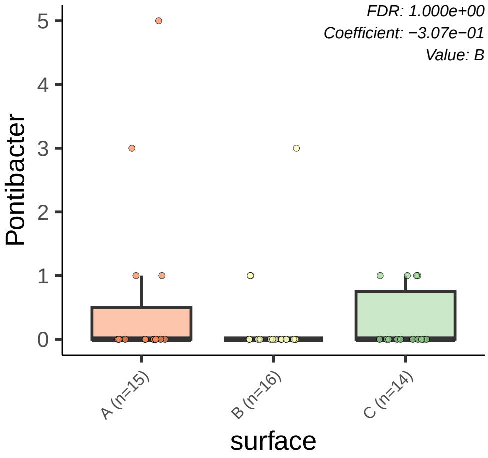

FDR: 1.000e+00
Coefficient: −3.07e−01
Value: B
Pontibacter
0
1
2
3
4
5
A (n=15)
B (n=16)
C (n=14)
surface

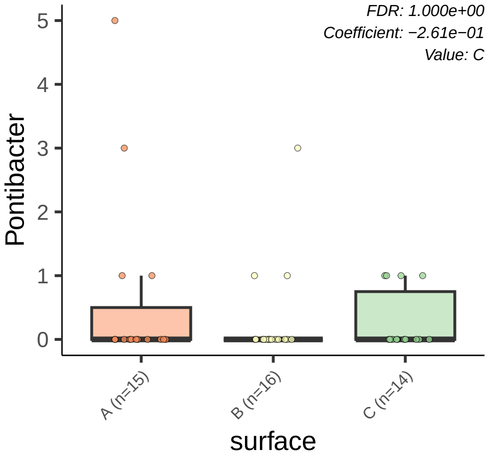
FDR: 1.000e+00
Coefficient: −2.61e−01
Value: C
Pontibacter
0
1
2
3
4
5
A (n=15)
B (n=16)
C (n=14)
surface

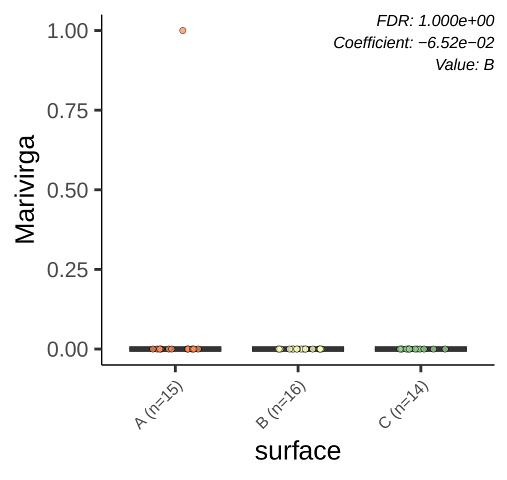
FDR: 1.000e+00
Coefficient: −6.52e−02
Value: B
Marivirga
1.00
0.75
0.50
0.25
0.00
A (n=15)
B (n=16)
C (n=14)
surface

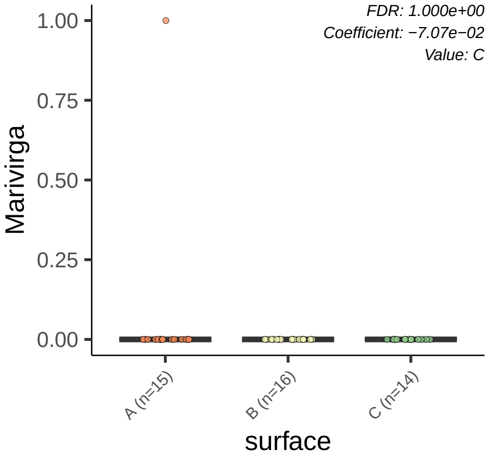

FDR: 1.000e+00
Coefficient: −7.07e−02
Value: C
Marivirga
1.00
0.75
0.50
0.25
0.00
A (n=15)
B (n=16)
C (n=14)
surface

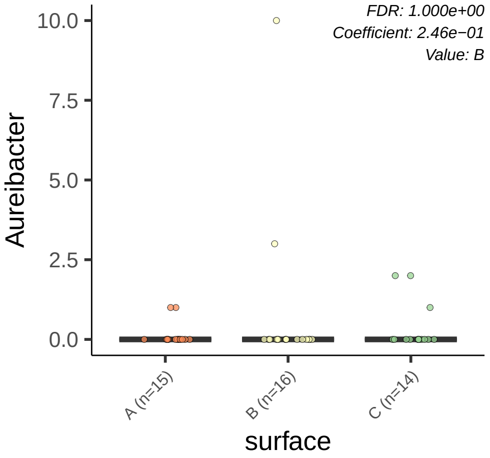
FDR: 1.000e+00
Coefficient: 2.46e−01
Value: B
10.0
7.5
5.0
2.5
0.0
Aureibacter
A (n=15)
B (n=16)
C (n=14)
surface

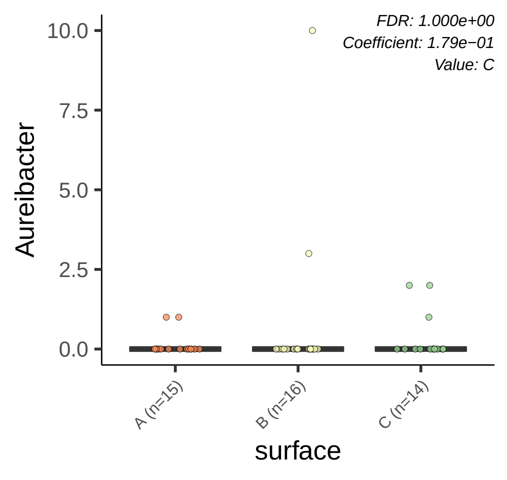

FDR: 1.000e+00
Coefficient: 1.79e−01
Value: C
Aureibacter
10.0
7.5
5.0
2.5
0.0
A (n=15)
B (n=16)
C (n=14)
surface

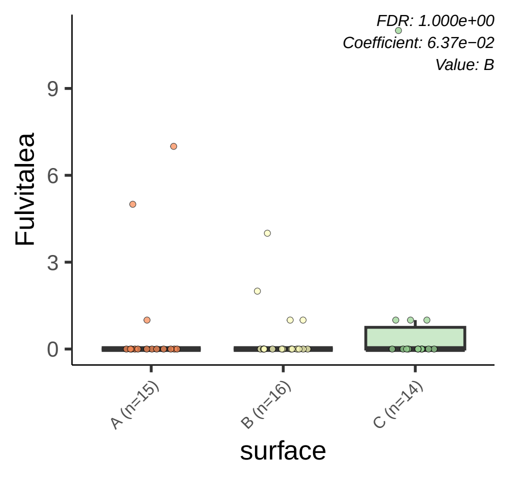

FDR: 1.000e+00
Coefficient: 6.37e−02
Value: B
Fulvitalea
0
3
6
9
A (n=15)
B (n=16)
C (n=14)
surface

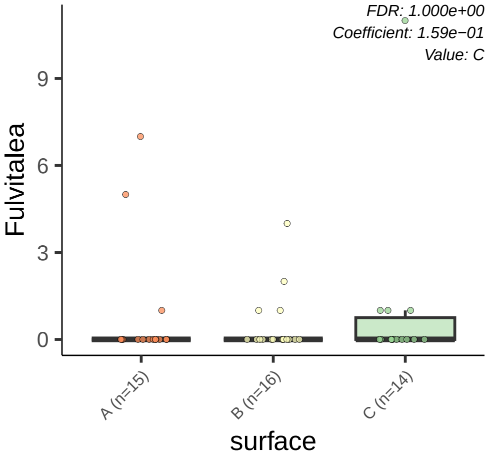

FDR: 1.000e+00
Coefficient: 1.59e−01
Value: C
Fulvitalea
0
3
6
9
A (n=15)
B (n=16)
C (n=14)
surface

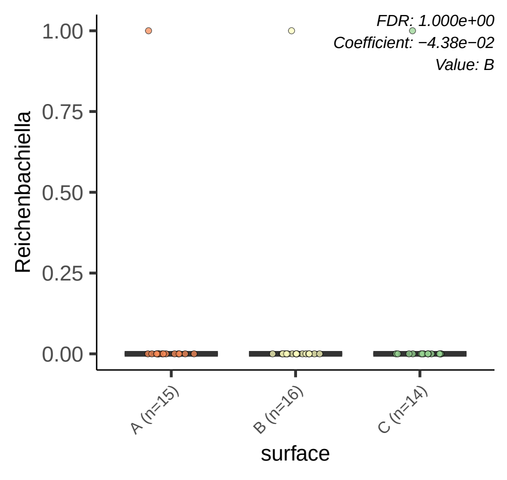

FDR: 1.000e+00
Coefficient: −4.38e−02
Value: B
Reichenbachiella
1.00
0.75
0.50
0.25
0.00
A (n=15)
B (n=16)
C (n=14)
surface

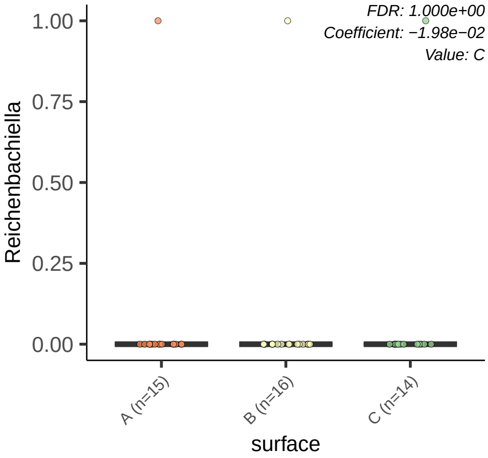
FDR: 1.000e+00
Coefficient: −1.98e−02
Value: C
Reichenbachiella
1.00
0.75
0.50
0.25
0.00
A (n=15)
B (n=16)
C (n=14)
surface

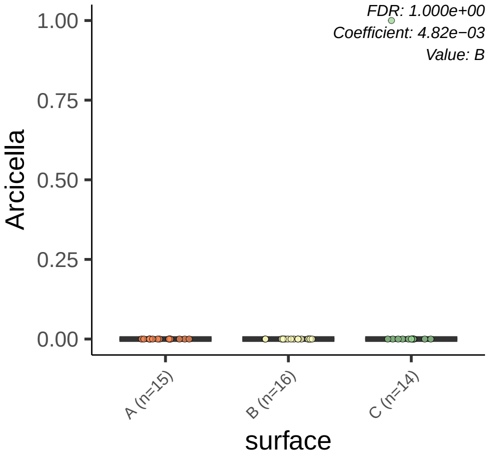
FDR: 1.000e+00
Coefficient: 4.82e−03
Value: B
Arcicella
1.00
0.75
0.50
0.25
0.00
A (n=15)
B (n=16)
C (n=14)
surface

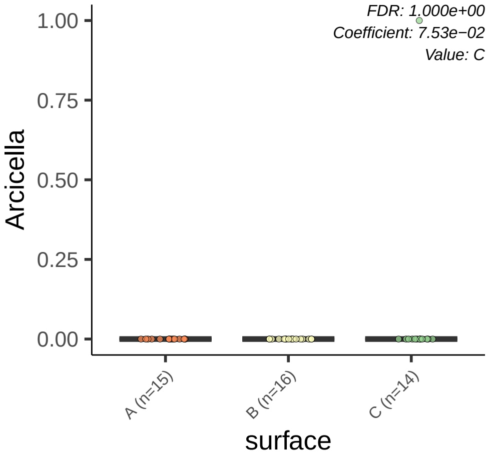
FDR: 1.000e+00
Coefficient: 7.53e−02
Value: C
1.00
0.75
0.50
0.25
0.00
Arcicella
A (n=15)
B (n=16)
C (n=14)
surface

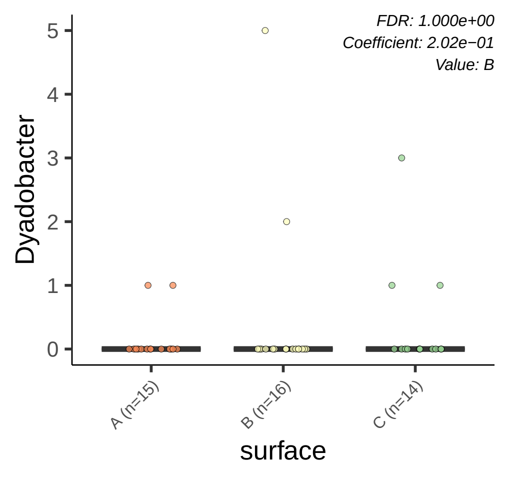
FDR: 1.000e+00
Coefficient: 2.02e−01
Value: B
Dyadobacter
0
1
2
3
4
5
A (n=15)
B (n=16)
C (n=14)
surface

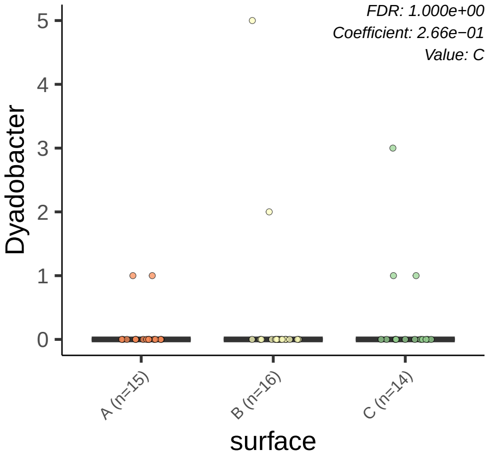
FDR: 1.000e+00
Coefficient: 2.66e−01
Value: C
Dyadobacter
0
1
2
3
4
5
A (n=15)
B (n=16)
C (n=14)
surface

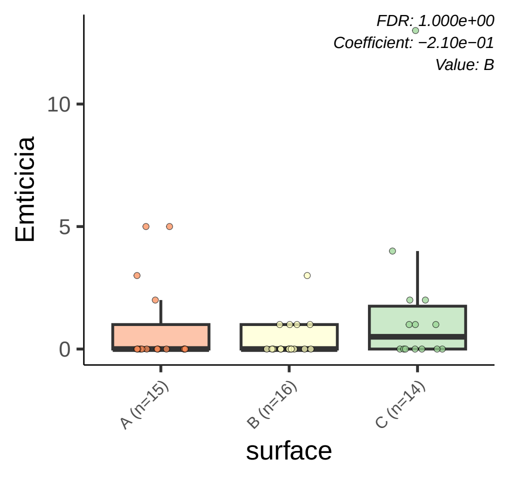

FDR: 1.000e+00
Coefficient: −2.10e−01
Value: B
Emticicia
10
5
0
A (n=15)
B (n=16)
C (n=14)
surface

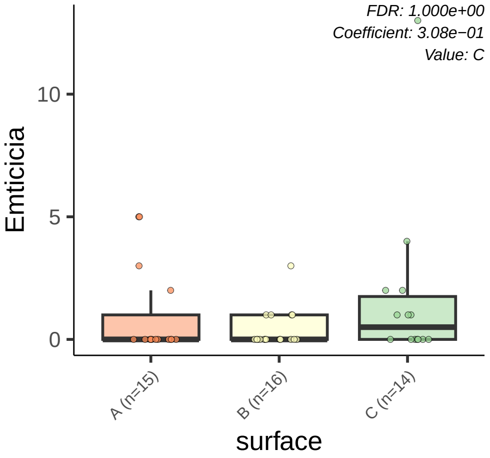

FDR: 1.000e+00
Coefficient: 3.08e−01
Value: C
Emticicia
10
5
0
A (n=15)
B (n=16)
C (n=14)
surface

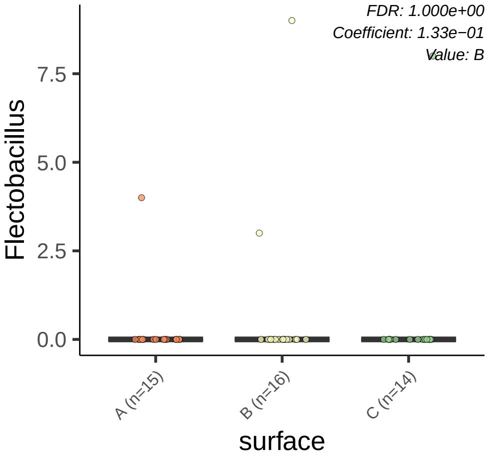
FDR: 1.000e+00
Coefficient: 1.33e−01
Value: B
Flectobacillus
7.5
5.0
2.5
0.0
A (n=15)
B (n=16)
C (n=14)
surface

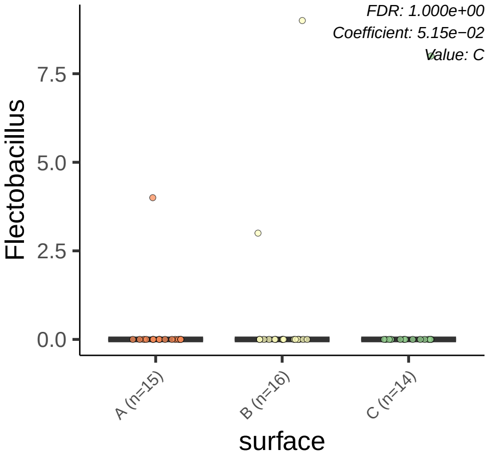
FDR: 1.000e+00
Coefficient: 5.15e−02
Value: C
Flectobacillus
7.5
5.0
2.5
0.0
A (n=15)
B (n=16)
C (n=14)
surface

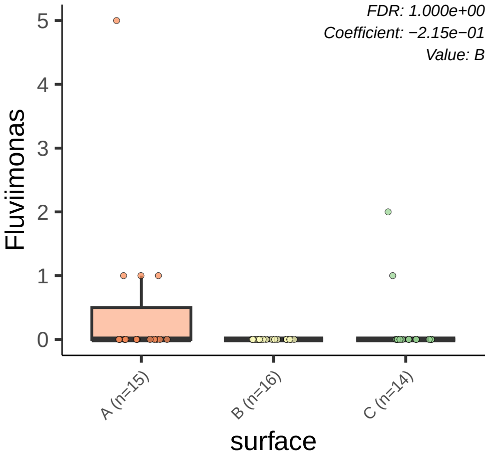

FDR: 1.000e+00
Coefficient: −2.15e−01
Value: B
Fluviimonas
5
4
3
2
1
0
A (n=15)
B (n=16)
C (n=14)
surface

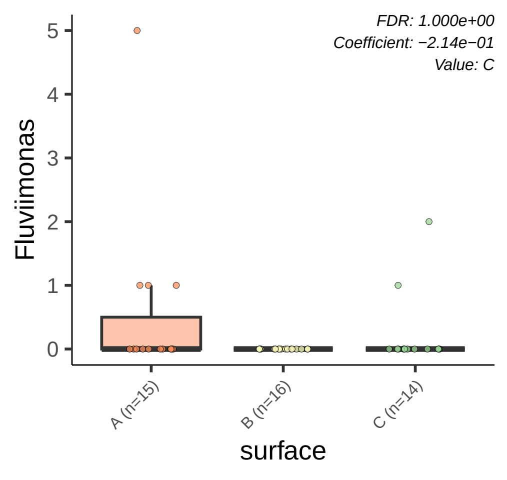
FDR: 1.000e+00
Coefficient: −2.14e−01
Value: C
Fluviimonas
5
4
3
2
1
0
A (n=15)
B (n=16)
C (n=14)
surface

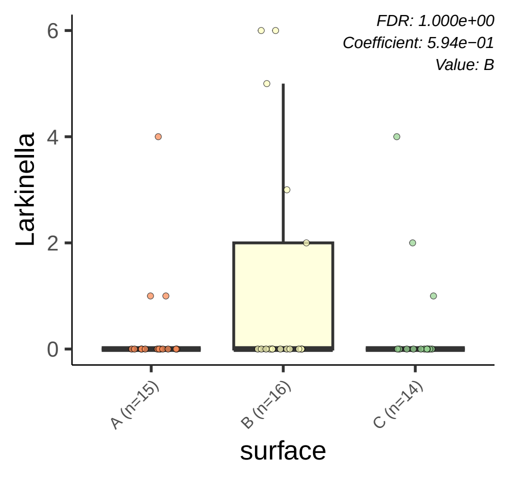
FDR: 1.000e+00
Coefficient: 5.94e−01
Value: B
Larkinella
0
2
4
6
A (n=15)
B (n=16)
C (n=14)
surface

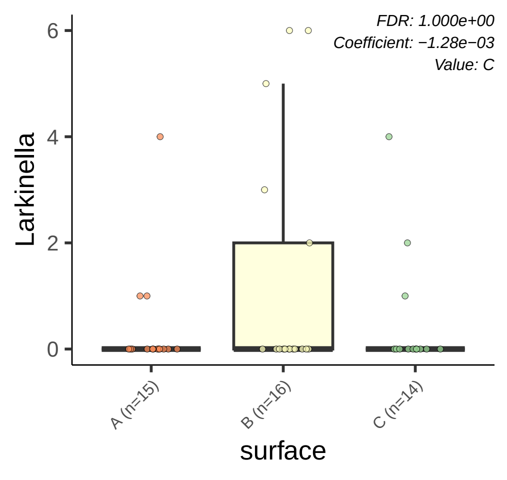
FDR: 1.000e+00
Coefficient: −1.28e−03
Value: C
Larkinella
0
2
4
6
A (n=15)
B (n=16)
C (n=14)
surface

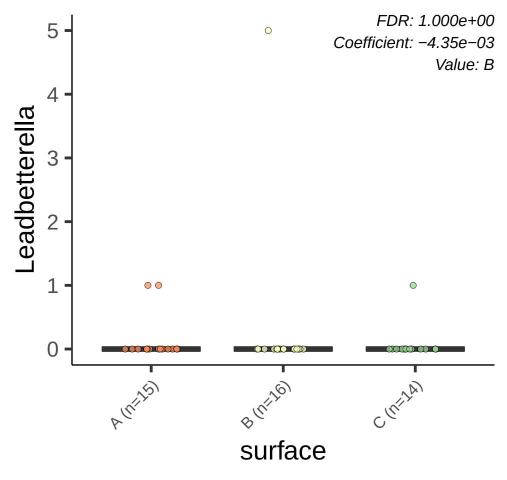
FDR: 1.000e+00
Coefficient: −4.35e−03
Value: B
Leadbetterella
0
1
2
3
4
5
A (n=15)
B (n=16)
C (n=14)
surface

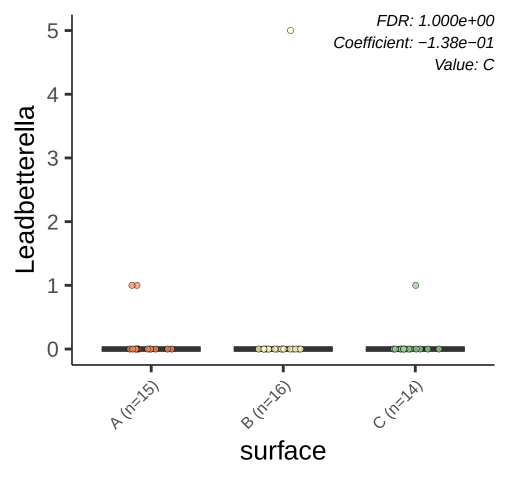
FDR: 1.000e+00
Coefficient: −1.38e−01
Value: C
Leadbetterella
0
1
2
3
4
5
A (n=15)
B (n=16)
C (n=14)
surface

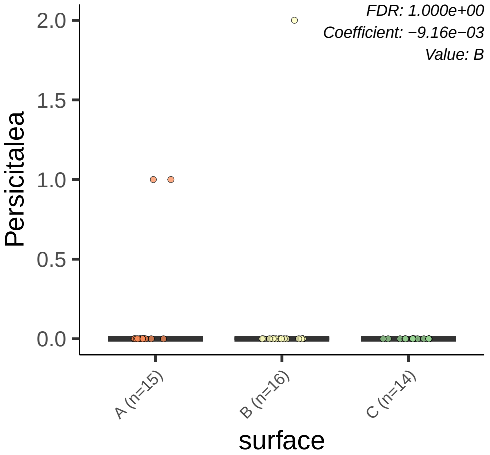

FDR: 1.000e+00
Coefficient: −9.16e−03
Value: B
Persicitalea
2.0
1.5
1.0
0.5
0.0
A (n=15)
B (n=16)
C (n=14)
surface

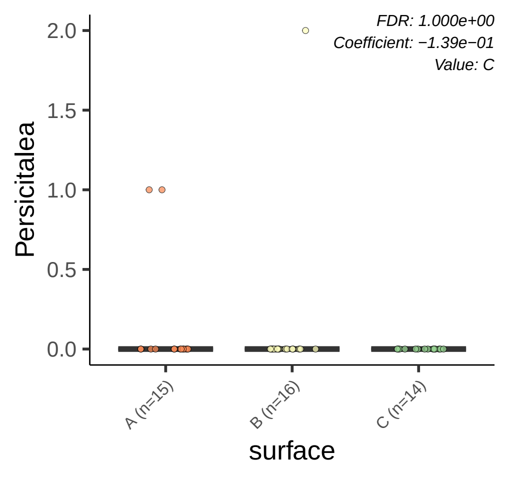
FDR: 1.000e+00
Coefficient: −1.39e−01
Value: C
Persicitalea
2.0
1.5
1.0
0.5
0.0
A (n=15)
B (n=16)
C (n=14)
surface

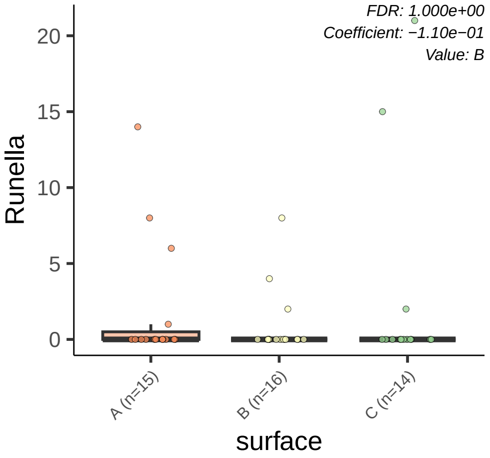

FDR: 1.000e+00
Coefficient: −1.10e−01
Value: B
Runella
20
15
10
5
0
A (n=15)
B (n=16)
C (n=14)
surface

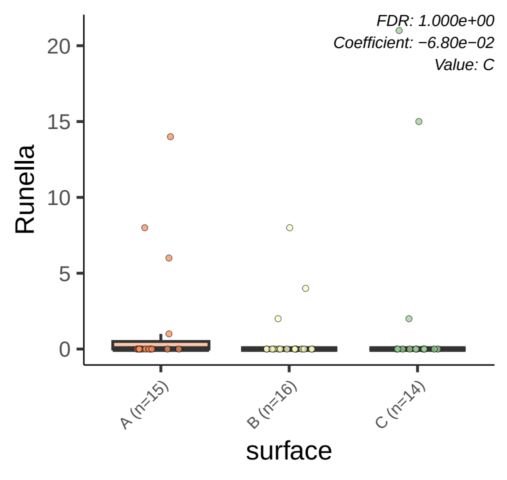
FDR: 1.000e+00
Coefficient: −6.80e−02
Value: C
Runella
20
15
10
5
0
A (n=15)
B (n=16)
C (n=14)
surface

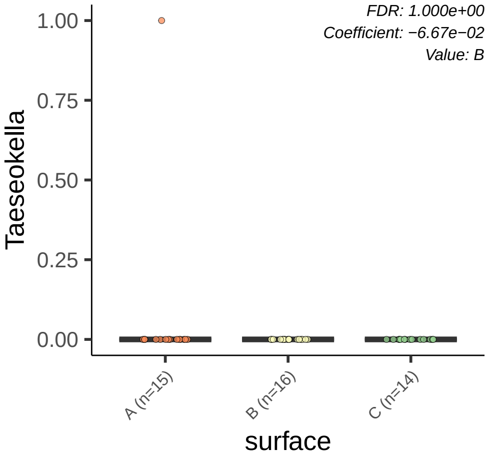
FDR: 1.000e+00
Coefficient: −6.67e−02
Value: B
Taeseokella
1.00
0.75
0.50
0.25
0.00
A (n=15)
B (n=16)
C (n=14)
surface

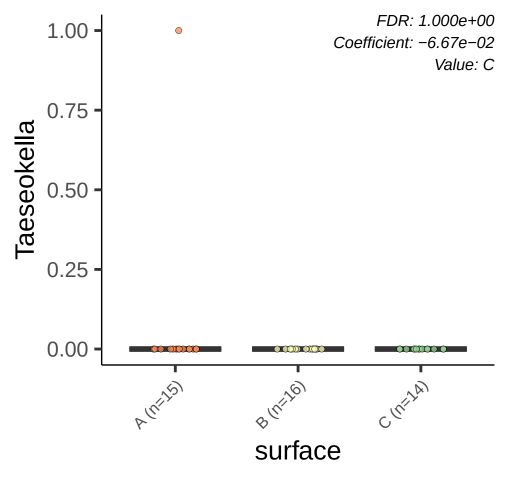
FDR: 1.000e+00
Coefficient: −6.67e−02
Value: C
Taeseokella
1.00
0.75
0.50
0.25
0.00
A (n=15)
B (n=16)
C (n=14)
surface

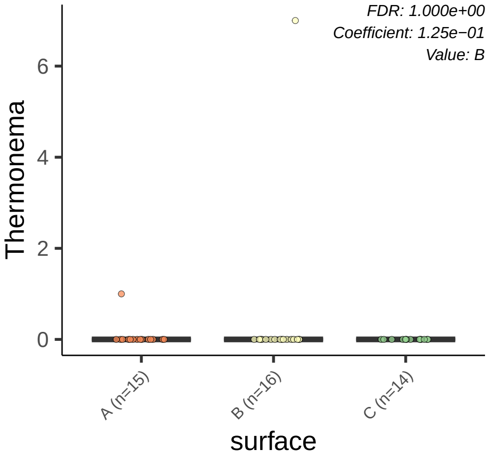

FDR: 1.000e+00
Coefficient: 1.25e−01
Value: B
Thermonema
0
2
4
6
A (n=15)
B (n=16)
C (n=14)
surface

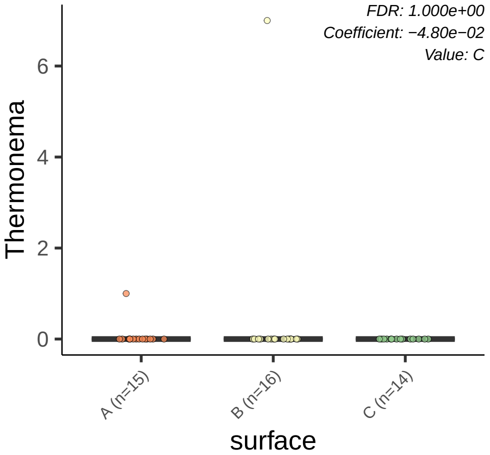

FDR: 1.000e+00
Coefficient: −4.80e−02
Value: C
Thermonema
6
4
2
0
A (n=15)
B (n=16)
C (n=14)
surface

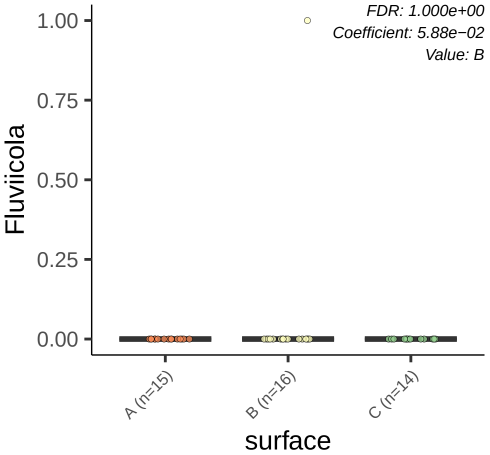
FDR: 1.000e+00
Coefficient: 5.88e−02
Value: B
Fluviicola
1.00
0.75
0.50
0.25
0.00
A (n=15)
B (n=16)
C (n=14)
surface

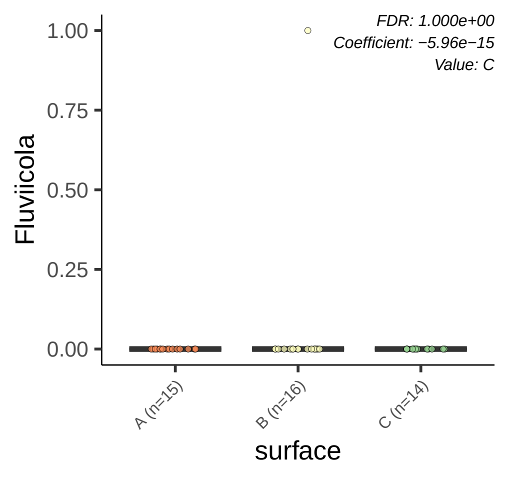
FDR: 1.000e+00
Coefficient: −5.96e−15
Value: C
Fluviicola
1.00
0.75
0.50
0.25
0.00
A (n=15)
B (n=16)
C (n=14)
surface

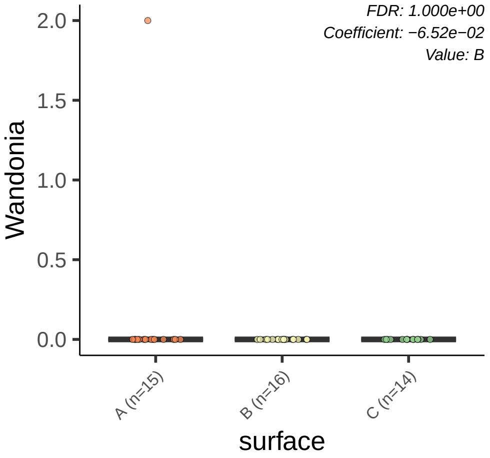

FDR: 1.000e+00
Coefficient: −6.52e−02
Value: B
Wandonia
2.0
1.5
1.0
0.5
0.0
A (n=15)
B (n=16)
C (n=14)
surface

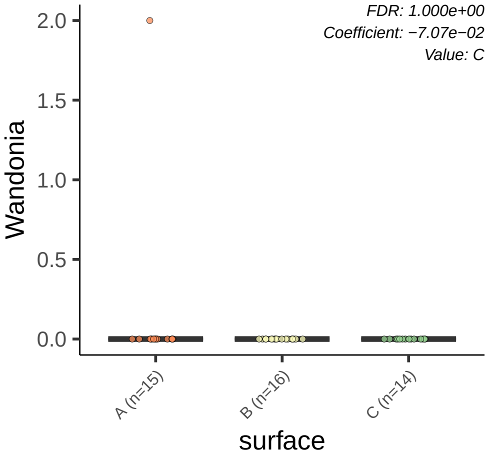

FDR: 1.000e+00
Coefficient: −7.07e−02
Value: C
Wandonia
2.0
1.5
1.0
0.5
0.0
A (n=15)
B (n=16)
C (n=14)
surface

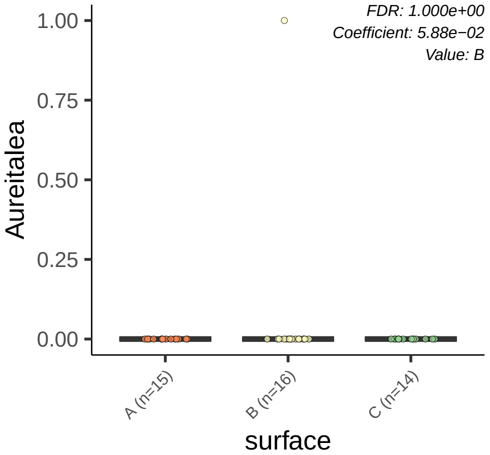
FDR: 1.000e+00
Coefficient: 5.88e−02
Value: B
Aureitalea
1.00
0.75
0.50
0.25
0.00
A (n=15)
B (n=16)
C (n=14)
surface

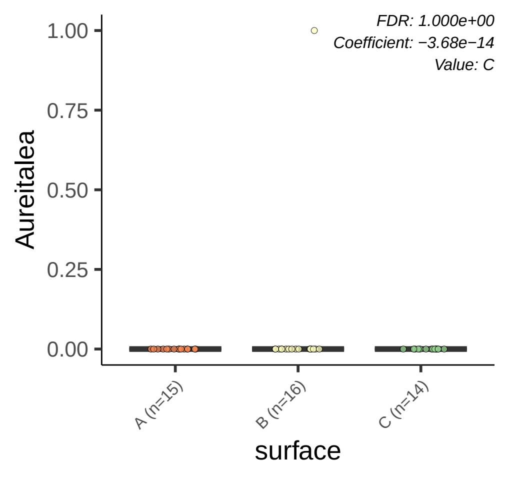
FDR: 1.000e+00
Coefficient: −3.68e−14
Value: C
1.00
0.75
0.50
0.25
0.00
Aureitalea
A (n=15)
B (n=16)
C (n=14)
surface

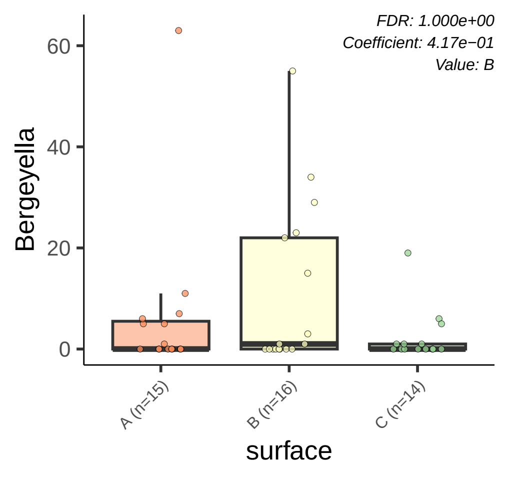
FDR: 1.000e+00
Coefficient: 4.17e−01
Value: B
Bergeyella
60
40
20
0
A (n=15)
B (n=16)
C (n=14)
surface

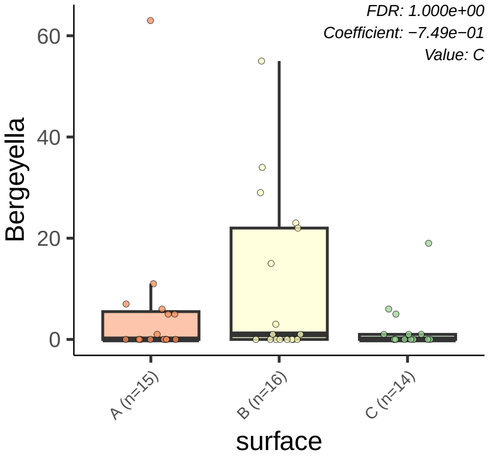

FDR: 1.000e+00
Coefficient: −7.49e−01
Value: C
Bergeyella
60
40
20
0
A (n=15)
B (n=16)
C (n=14)
surface

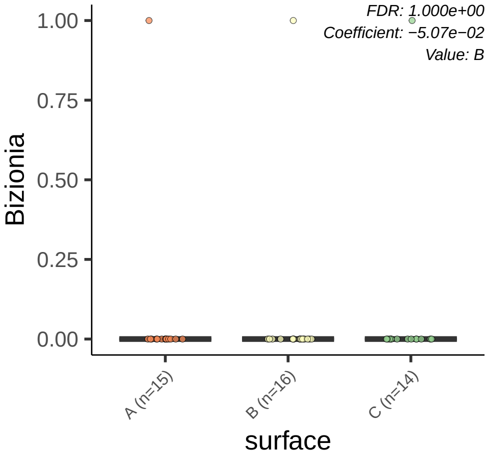
FDR: 1.000e+00
Coefficient: −5.07e−02
Value: B
Bizionia
1.00
0.75
0.50
0.25
0.00
A (n=15)
B (n=16)
C (n=14)
surface

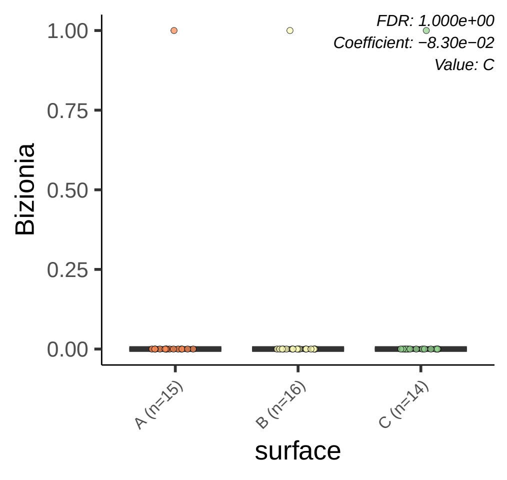
FDR: 1.000e+00
Coefficient: −8.30e−02
Value: C
Bizionia
1.00
0.75
0.50
0.25
0.00
A (n=15)
B (n=16)
C (n=14)
surface

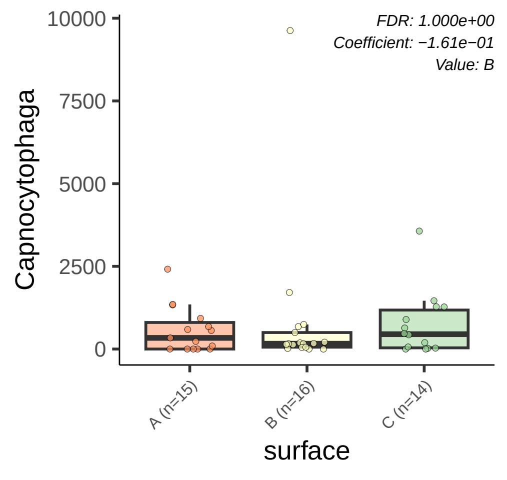
FDR: 1.000e+00
Coefficient: −1.61e−01
Value: B
Capnocytophaga
10000
7500
5000
2500
0
A (n=15)
B (n=16)
C (n=14)
surface

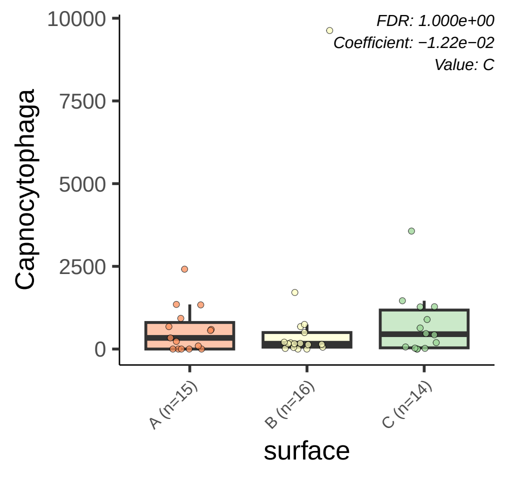
FDR: 1.000e+00
Coefficient: −1.22e−02
Value: C
Capnocytophaga
10000
7500
5000
2500
0
A (n=15)
B (n=16)
C (n=14)
surface

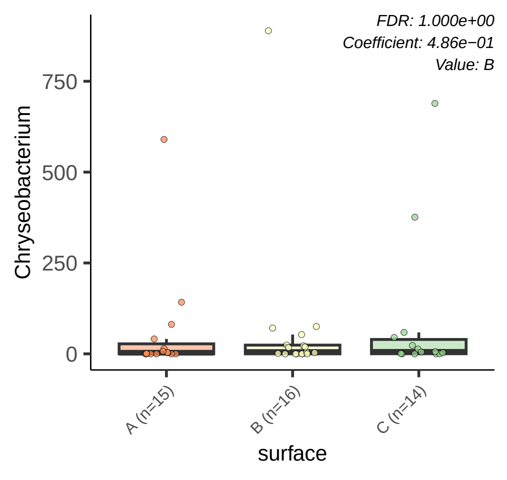

FDR: 1.000e+00
Coefficient: 4.86e−01
Value: B
Chryseobacterium
750
500
250
0
A (n=15)
B (n=16)
C (n=14)
surface

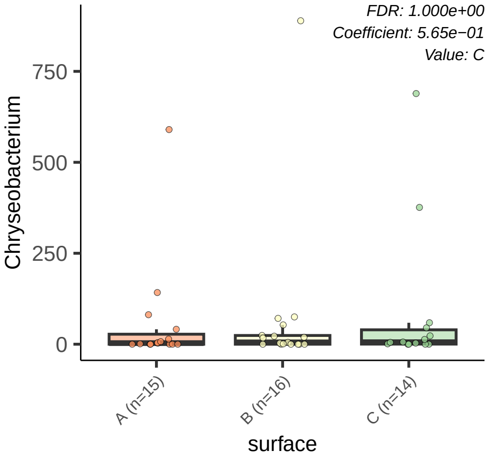
FDR: 1.000e+00
Coefficient: 5.65e−01
Value: C
Chryseobacterium
750
500
250
0
A (n=15)
B (n=16)
C (n=14)
surface

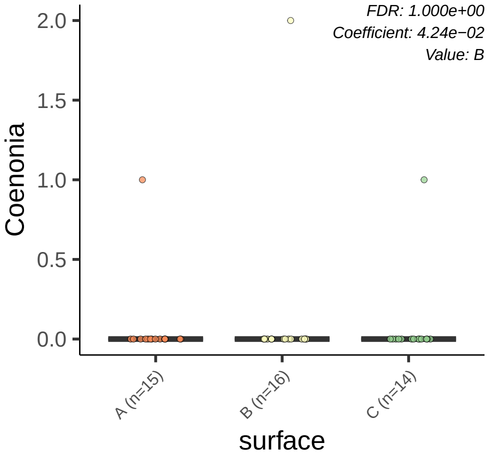

FDR: 1.000e+00
Coefficient: 4.24e−02
Value: B
2.0
1.5
1.0
0.5
0.0
Coenonia
A (n=15)
B (n=16)
C (n=14)
surface

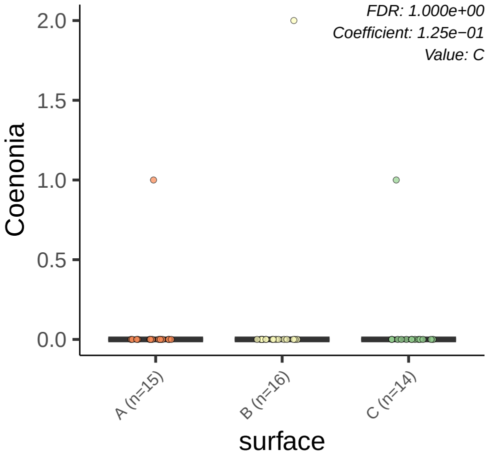
FDR: 1.000e+00
Coefficient: 1.25e−01
Value: C
Coenonia
2.0
1.5
1.0
0.5
0.0
A (n=15)
B (n=16)
C (n=14)
surface

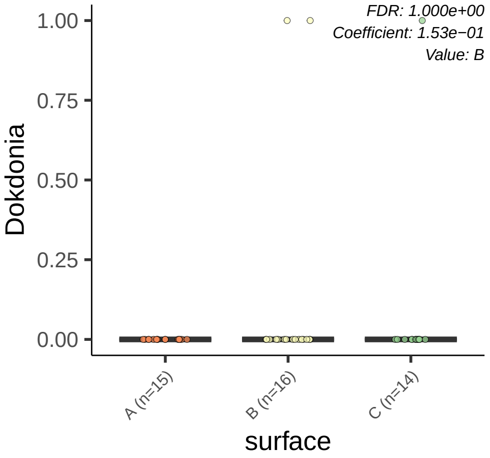
FDR: 1.000e+00
Coefficient: 1.53e−01
Value: B
1.00
0.75
0.50
0.25
0.00
Dokdonia
A (n=15)
B (n=16)
C (n=14)
surface

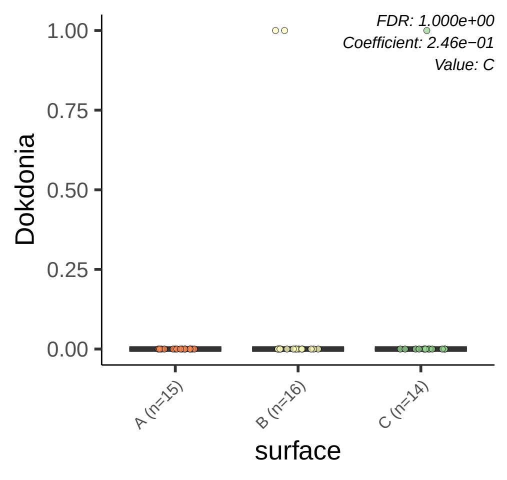
FDR: 1.000e+00
Coefficient: 2.46e−01
Value: C
Dokdonia
1.00
0.75
0.50
0.25
0.00
A (n=15)
B (n=16)
C (n=14)
surface

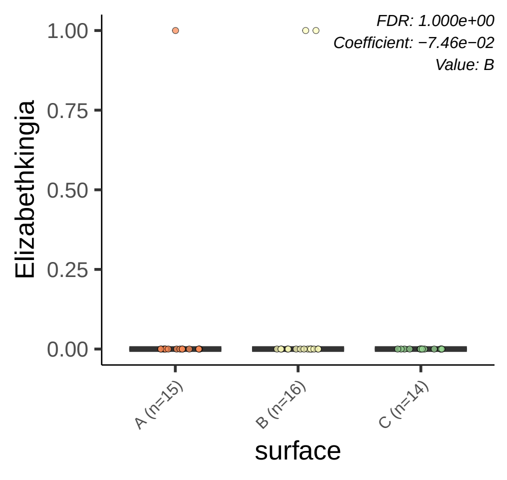

FDR: 1.000e+00
Coefficient: −7.46e−02
Value: B
Elizabethkingia
1.00
0.75
0.50
0.25
0.00
A (n=15)
B (n=16)
C (n=14)
surface

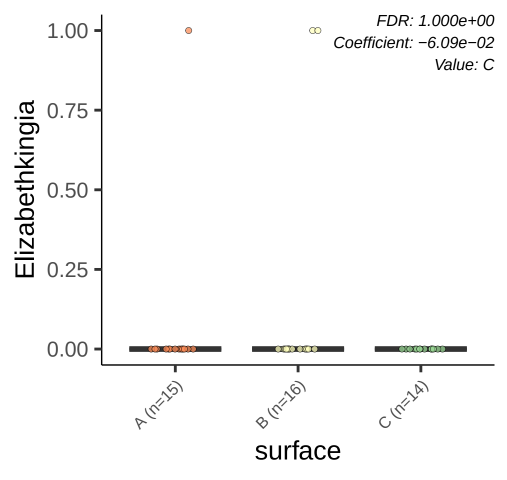

FDR: 1.000e+00
Coefficient: −6.09e−02
Value: C
Elizabethkingia
1.00
0.75
0.50
0.25
0.00
A (n=15)
B (n=16)
C (n=14)
surface

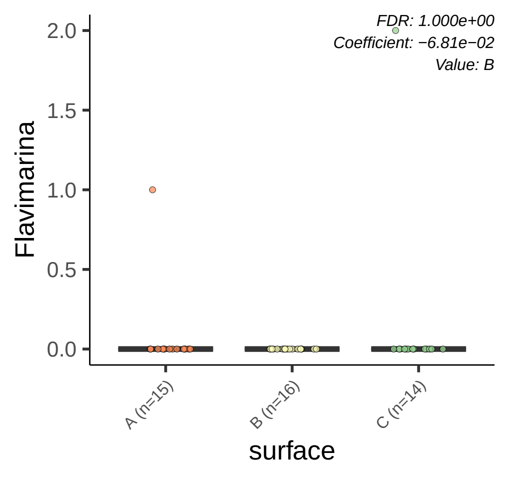
FDR: 1.000e+00
Coefficient: −6.81e−02
Value: B
Flavimarina
2.0
1.5
1.0
0.5
0.0
A (n=15)
B (n=16)
C (n=14)
surface

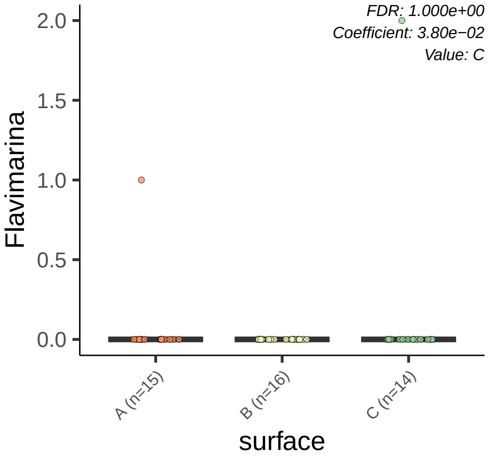
FDR: 1.000e+00
Coefficient: 3.80e−02
Value: C
Flavimarina
2.0
1.5
1.0
0.5
0.0
A (n=15)
B (n=16)
C (n=14)
surface

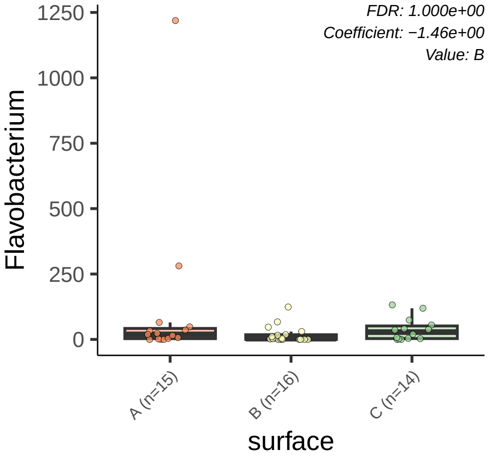

FDR: 1.000e+00
Coefficient: −1.46e+00
Value: B
Flavobacterium
1250
1000
750
500
250
0
A (n=15)
B (n=16)
C (n=14)
surface

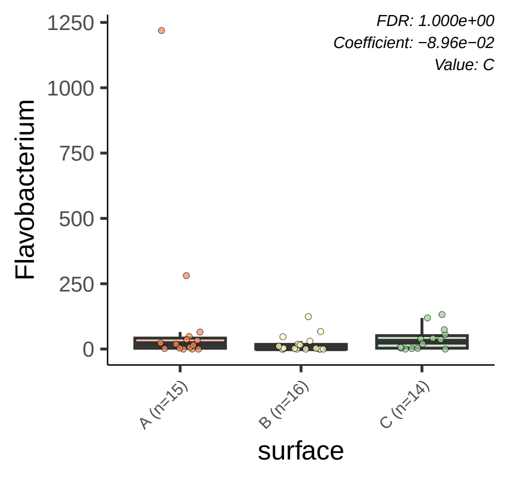

FDR: 1.000e+00
Coefficient: −8.96e−02
Value: C
Flavobacterium
1250
1000
750
500
250
0
A (n=15)
B (n=16)
C (n=14)
surface

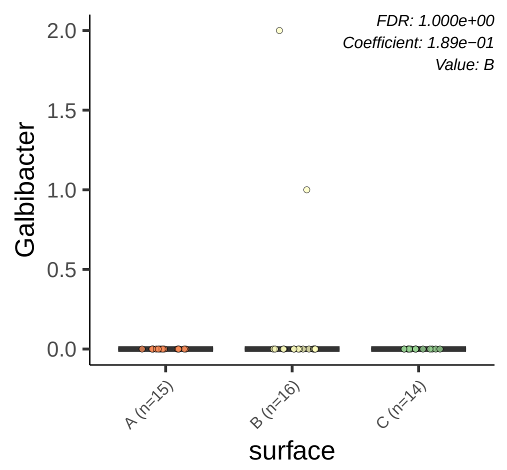
FDR: 1.000e+00
Coefficient: 1.89e−01
Value: B
Galbibacter
2.0
1.5
1.0
0.5
0.0
A (n=15)
B (n=16)
C (n=14)
surface

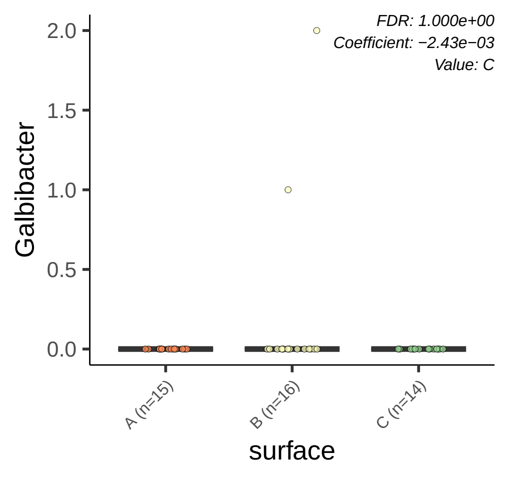
FDR: 1.000e+00
Coefficient: −2.43e−03
Value: C
Galbibacter
2.0
1.5
1.0
0.5
0.0
A (n=15)
B (n=16)
C (n=14)
surface

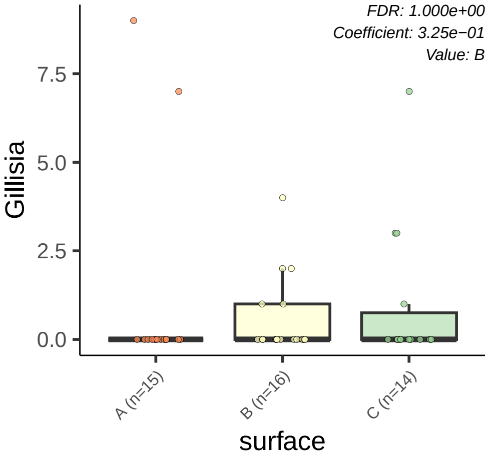

FDR: 1.000e+00
Coefficient: 3.25e−01
Value: B
Gillisia
7.5
5.0
2.5
0.0
A (n=15)
B (n=16)
C (n=14)
surface

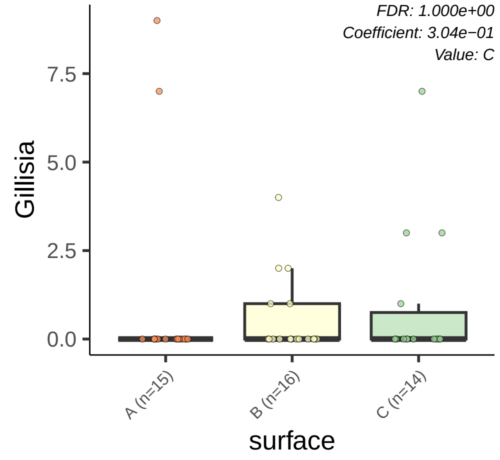
FDR: 1.000e+00
Coefficient: 3.04e−01
Value: C
Gillisia
7.5
5.0
2.5
0.0
A (n=15)
B (n=16)
C (n=14)
surface

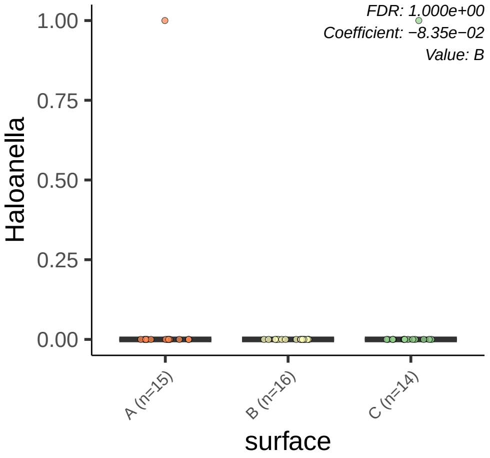
FDR: 1.000e+00
Coefficient: −8.35e−02
Value: B
Haloanella
1.00
0.75
0.50
0.25
0.00
A (n=15)
B (n=16)
C (n=14)
surface

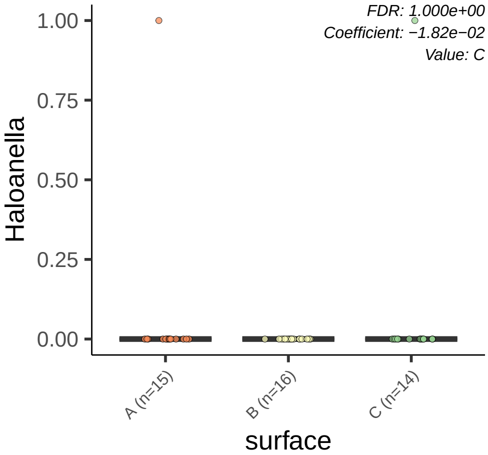
FDR: 1.000e+00
Coefficient: −1.82e−02
Value: C
Haloanella
1.00
0.75
0.50
0.25
0.00
A (n=15)
B (n=16)
C (n=14)
surface

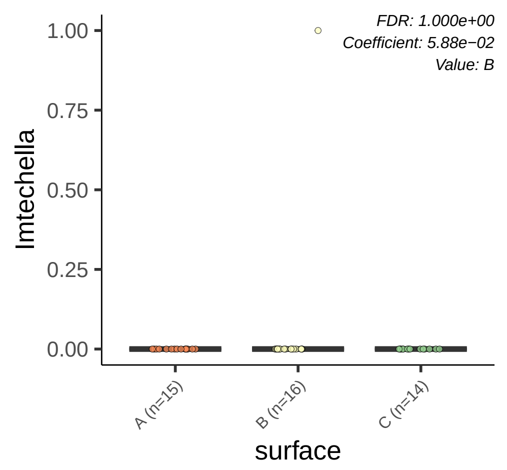

FDR: 1.000e+00
Coefficient: 5.88e−02
Value: B
Imtechella
1.00
0.75
0.50
0.25
0.00
A (n=15)
B (n=16)
C (n=14)
surface

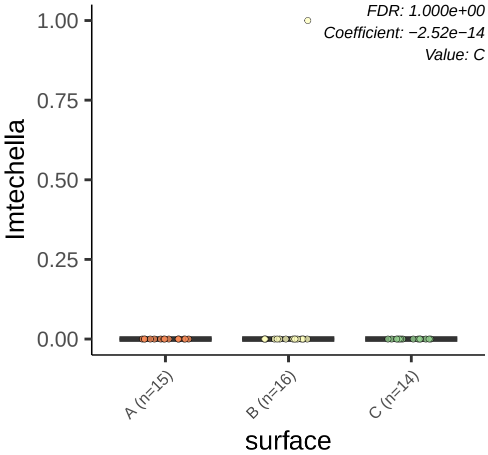

FDR: 1.000e+00
Coefficient: −2.52e−14
Value: C
Imtechella
1.00
0.75
0.50
0.25
0.00
A (n=15)
B (n=16)
C (n=14)
surface

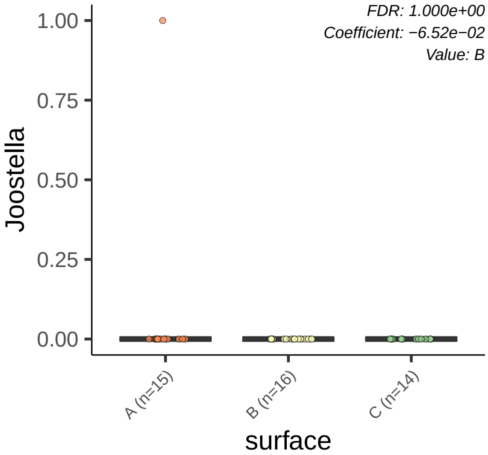
FDR: 1.000e+00
Coefficient: −6.52e−02
Value: B
Joostella
1.00
0.75
0.50
0.25
0.00
A (n=15)
B (n=16)
C (n=14)
surface

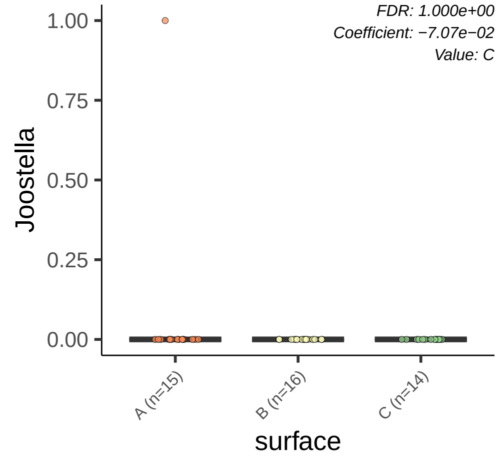

FDR: 1.000e+00
Coefficient: −7.07e−02
Value: C
Joostella
1.00
0.75
0.50
0.25
0.00
A (n=15)
B (n=16)
C (n=14)
surface

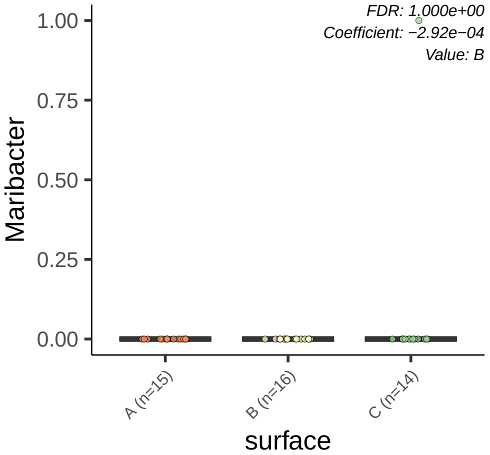

FDR: 1.000e+00
Coefficient: −2.92e−04
Value: B
1.00
0.75
0.50
0.25
0.00
Maribacter
A (n=15)
B (n=16)
C (n=14)
surface

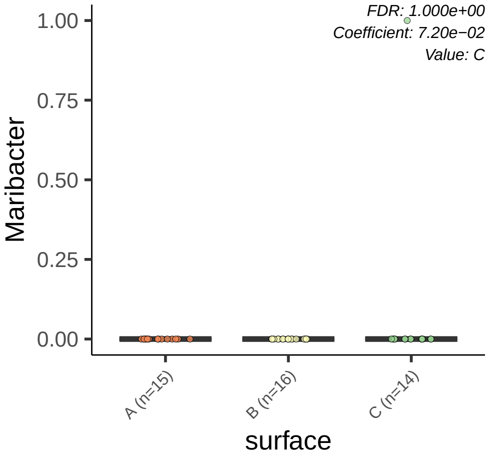

FDR: 1.000e+00
Coefficient: 7.20e−02
Value: C
Maribacter
1.00
0.75
0.50
0.25
0.00
A (n=15)
B (n=16)
C (n=14)
surface

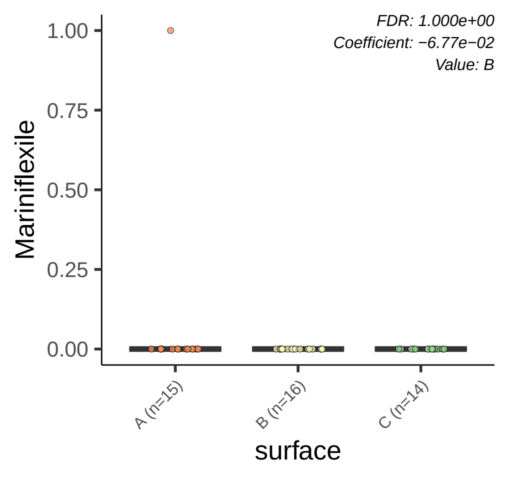
FDR: 1.000e+00
Coefficient: −6.77e−02
Value: B
Mariniflexile
1.00
0.75
0.50
0.25
0.00
A (n=15)
B (n=16)
C (n=14)
surface

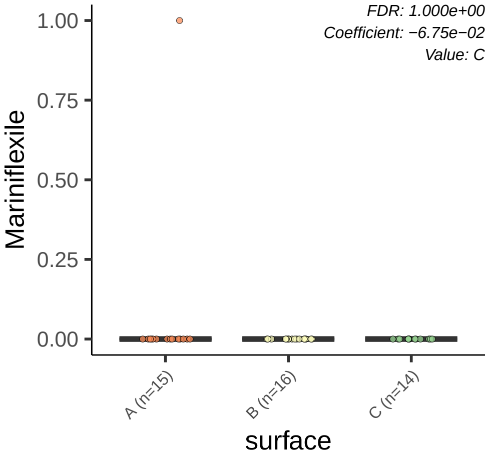
FDR: 1.000e+00
Coefficient: −6.75e−02
Value: C
Mariniflexile
1.00
0.75
0.50
0.25
0.00
A (n=15)
B (n=16)
C (n=14)
surface

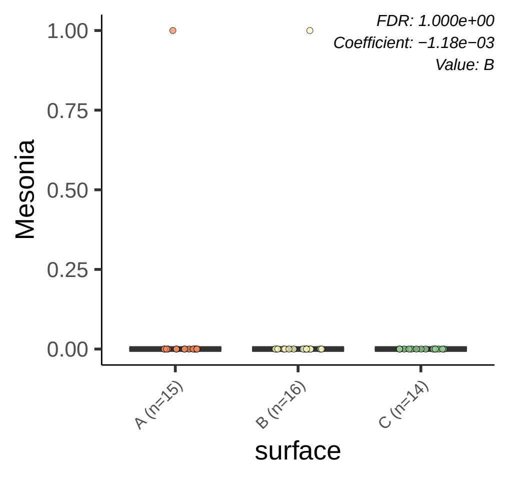
FDR: 1.000e+00
Coefficient: −1.18e−03
Value: B
Mesonia
1.00
0.75
0.50
0.25
0.00
A (n=15)
B (n=16)
C (n=14)
surface

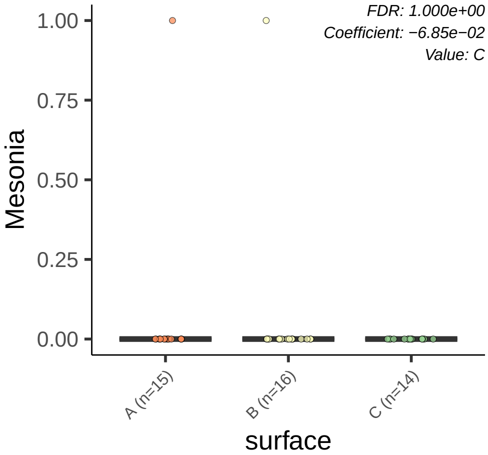

FDR: 1.000e+00
Coefficient: −6.85e−02
Value: C
Mesonia
1.00
0.75
0.50
0.25
0.00
A (n=15)
B (n=16)
C (n=14)
surface

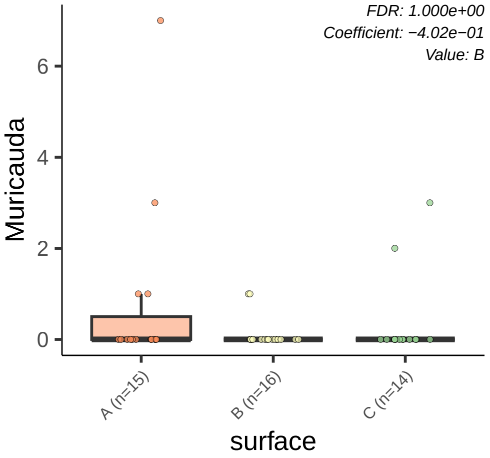
FDR: 1.000e+00
Coefficient: −4.02e−01
Value: B
Muricauda
0
2
4
6
A (n=15)
B (n=16)
C (n=14)
surface

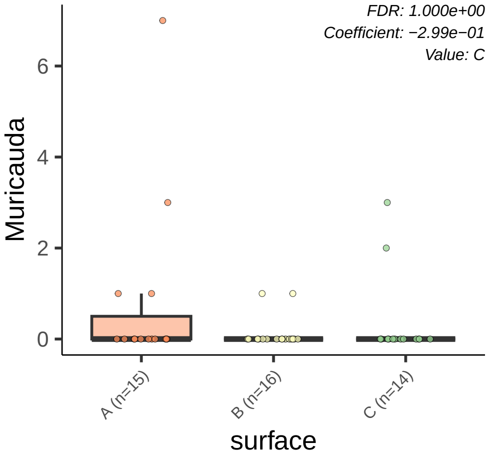

FDR: 1.000e+00
Coefficient: −2.99e−01
Value: C
Muricauda
0
2
4
6
A (n=15)
B (n=16)
C (n=14)
surface

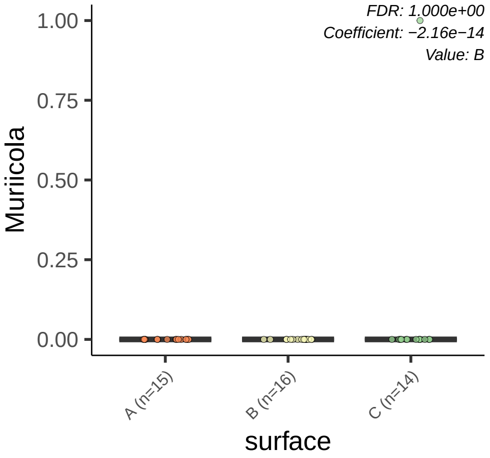

FDR: 1.000e+00
Coefficient: −2.16e−14
Value: B
Muriicola
1.00
0.75
0.50
0.25
0.00
A (n=15)
B (n=16)
C (n=14)
surface

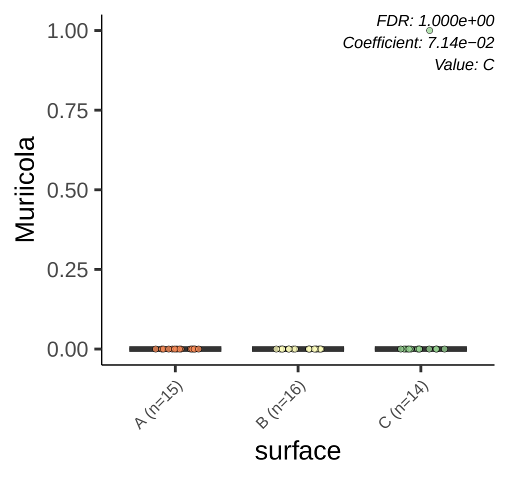
FDR: 1.000e+00
Coefficient: 7.14e−02
Value: C
Muriicola
1.00
0.75
0.50
0.25
0.00
A (n=15)
B (n=16)
C (n=14)
surface

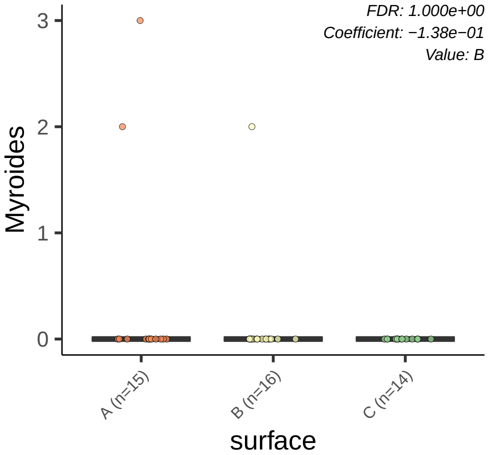

FDR: 1.000e+00
Coefficient: −1.38e−01
Value: B
Myroides
3
2
1
0
A (n=15)
B (n=16)
C (n=14)
surface

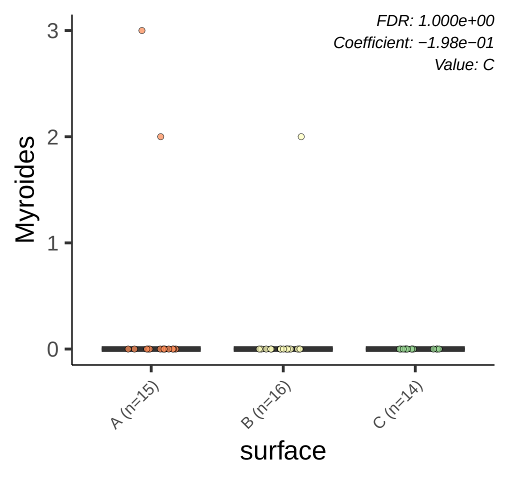
FDR: 1.000e+00
Coefficient: −1.98e−01
Value: C
Myroides
0
1
2
3
A (n=15)
B (n=16)
C (n=14)
surface

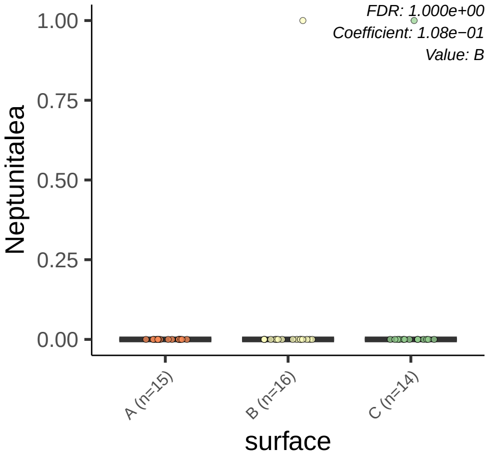

FDR: 1.000e+00
Coefficient: 1.08e−01
Value: B
1.00
0.75
0.50
0.25
0.00
Neptunitalea
A (n=15)
B (n=16)
C (n=14)
surface

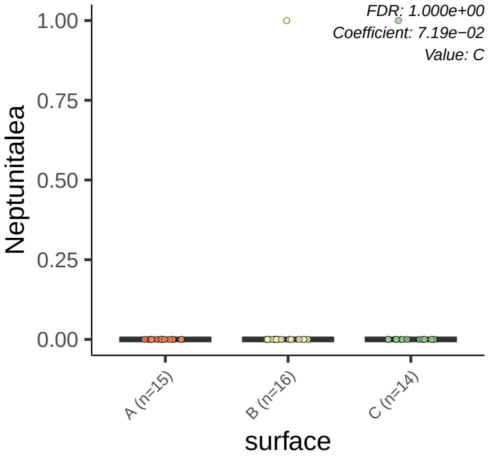
FDR: 1.000e+00
Coefficient: 7.19e−02
Value: C
Neptunitalea
1.00
0.75
0.50
0.25
0.00
A (n=15)
B (n=16)
C (n=14)
surface

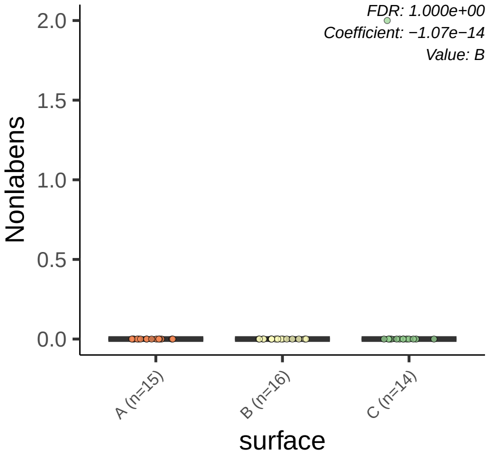
FDR: 1.000e+00
Coefficient: −1.07e−14
Value: B
Nonlabens
2.0
1.5
1.0
0.5
0.0
A (n=15)
B (n=16)
C (n=14)
surface

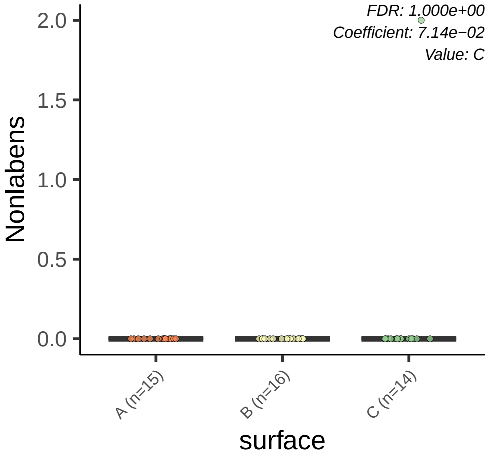

FDR: 1.000e+00
Coefficient: 7.14e−02
Value: C
Nonlabens
2.0
1.5
1.0
0.5
0.0
A (n=15)
B (n=16)
C (n=14)
surface

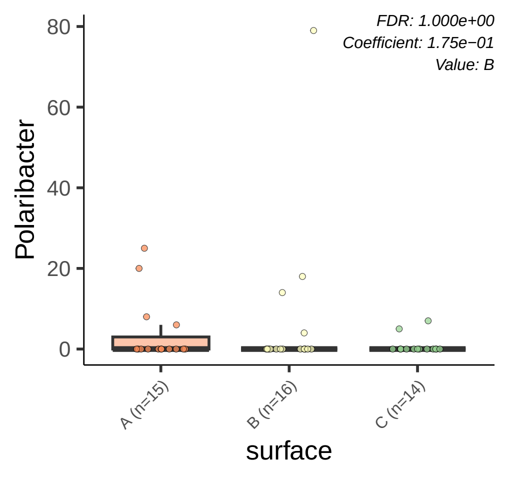
FDR: 1.000e+00
Coefficient: 1.75e−01
Value: B
Polaribacter
80
60
40
20
0
A (n=15)
B (n=16)
C (n=14)
surface

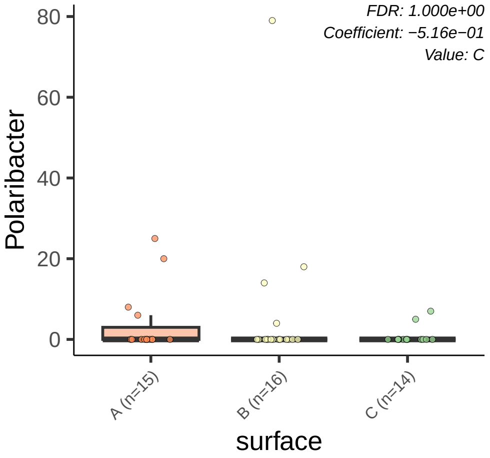

FDR: 1.000e+00
Coefficient: −5.16e−01
Value: C
Polaribacter
0
20
40
60
80
A (n=15)
B (n=16)
C (n=14)
surface

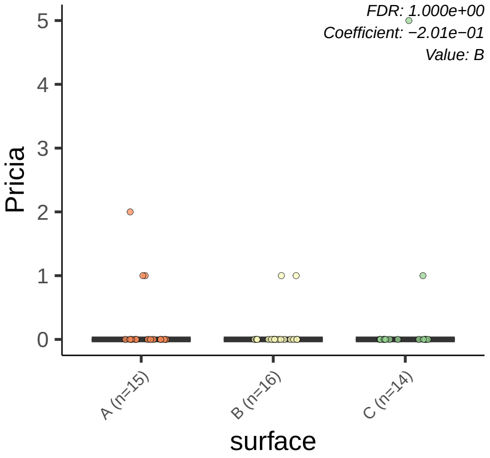
FDR: 1.000e+00
Coefficient: −2.01e−01
Value: B
Pricia
0
1
2
3
4
5
A (n=15)
B (n=16)
C (n=14)
surface

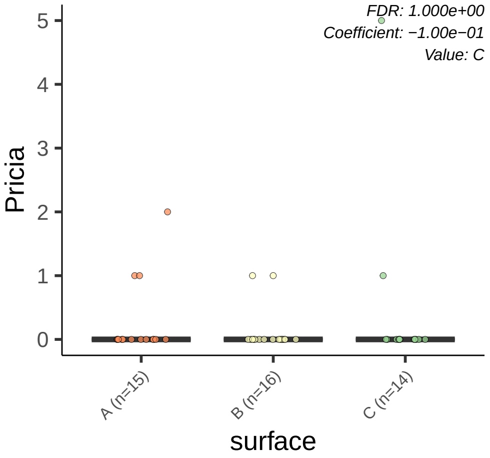

FDR: 1.000e+00
Coefficient: −1.00e−01
Value: C
Pricia
5
4
3
2
1
0
A (n=15)
B (n=16)
C (n=14)
surface

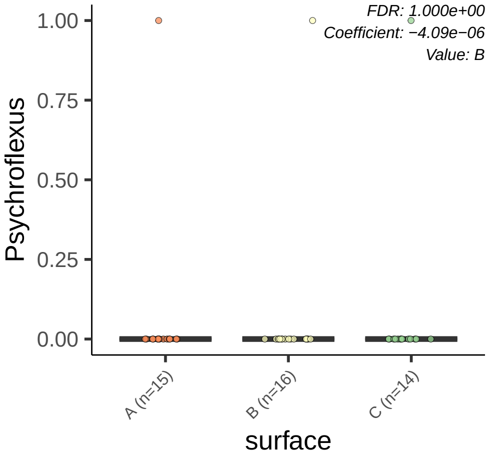

FDR: 1.000e+00
Coefficient: −4.09e−06
Value: B
Psychroflexus
1.00
0.75
0.50
0.25
0.00
A (n=15)
B (n=16)
C (n=14)
surface

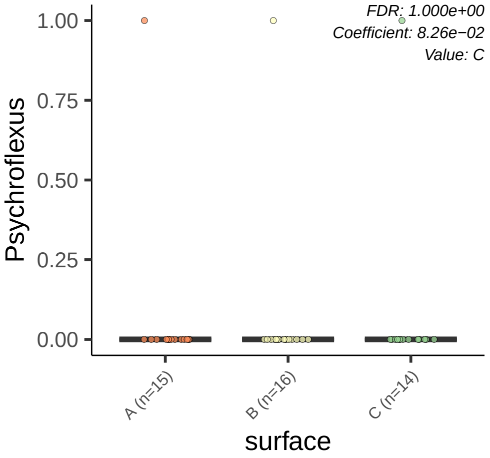
FDR: 1.000e+00
Coefficient: 8.26e−02
Value: C
Psychroflexus
1.00
0.75
0.50
0.25
0.00
A (n=15)
B (n=16)
C (n=14)
surface

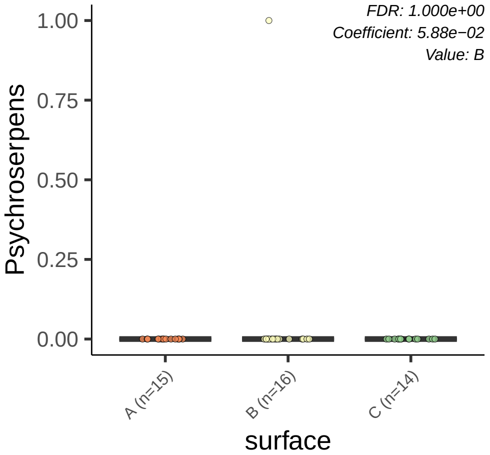
FDR: 1.000e+00
Coefficient: 5.88e−02
Value: B
Psychroserpens
1.00
0.75
0.50
0.25
0.00
A (n=15)
B (n=16)
C (n=14)
surface

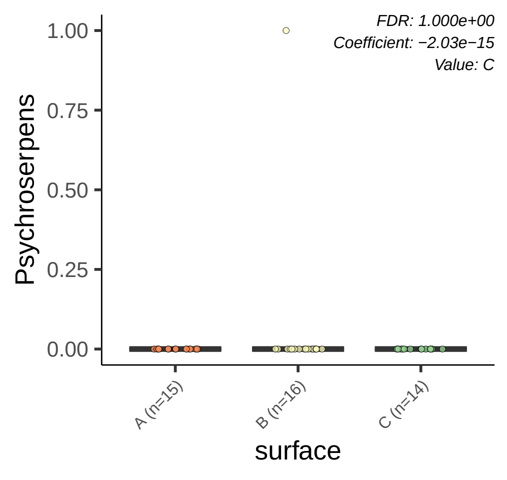
FDR: 1.000e+00
Coefficient: −2.03e−15
Value: C
Psychroserpens
1.00
0.75
0.50
0.25
0.00
A (n=15)
B (n=16)
C (n=14)
surface

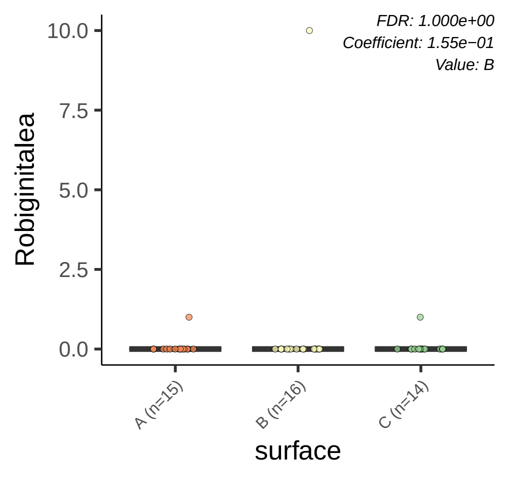

FDR: 1.000e+00
Coefficient: 1.55e−01
Value: B
Robiginitalea
10.0
7.5
5.0
2.5
0.0
A (n=15)
B (n=16)
C (n=14)
surface

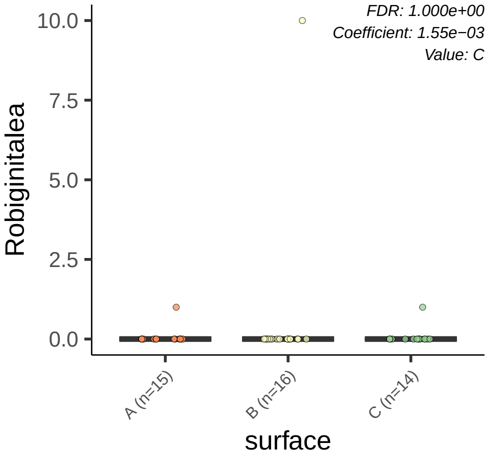

FDR: 1.000e+00
Coefficient: 1.55e−03
Value: C
10.0
7.5
5.0
2.5
0.0
Robiginitalea
A (n=15)
B (n=16)
C (n=14)
surface

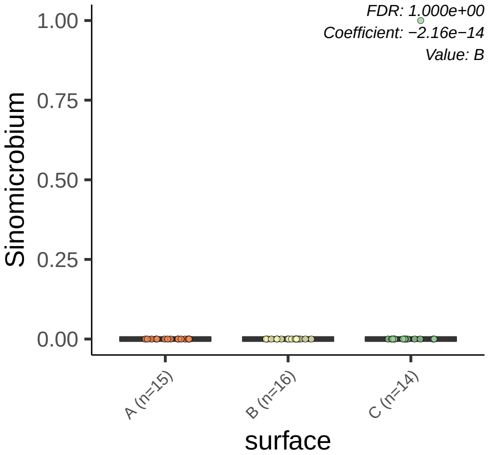
FDR: 1.000e+00
Coefficient: −2.16e−14
Value: B
Sinomicrobium
1.00
0.75
0.50
0.25
0.00
A (n=15)
B (n=16)
C (n=14)
surface

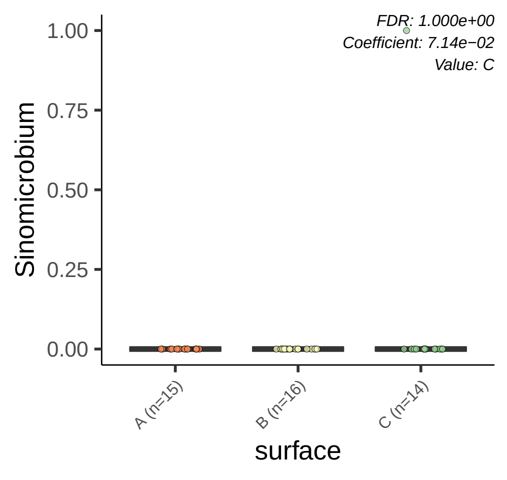

FDR: 1.000e+00
Coefficient: 7.14e−02
Value: C
Sinomicrobium
1.00
0.75
0.50
0.25
0.00
A (n=15)
B (n=16)
C (n=14)
surface

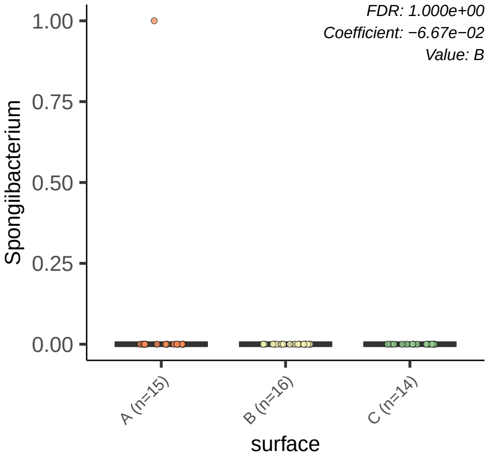
FDR: 1.000e+00
Coefficient: −6.67e−02
Value: B
Spongiibacterium
1.00
0.75
0.50
0.25
0.00
A (n=15)
B (n=16)
C (n=14)
surface

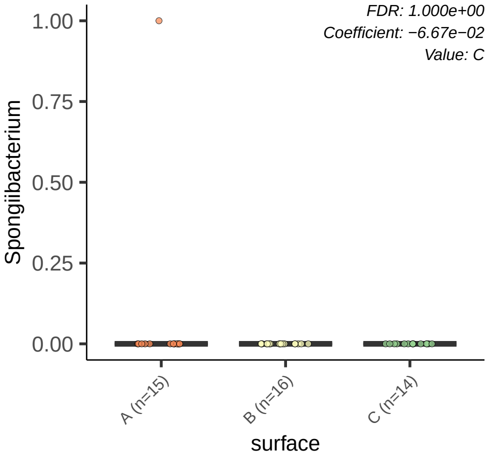

FDR: 1.000e+00
Coefficient: −6.67e−02
Value: C
Spongiibacterium
1.00
0.75
0.50
0.25
0.00
A (n=15)
B (n=16)
C (n=14)
surface

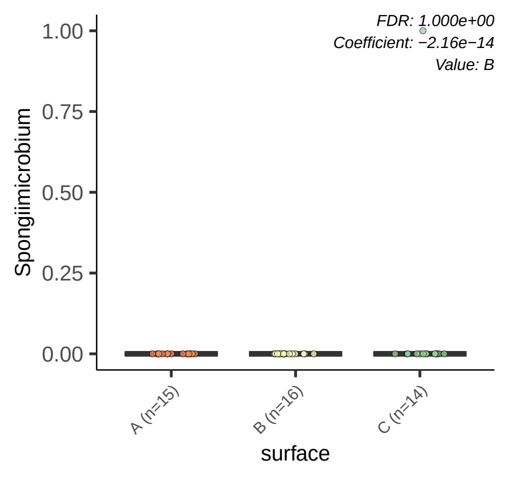
FDR: 1.000e+00
Coefficient: −2.16e−14
Value: B
Spongiimicrobium
1.00
0.75
0.50
0.25
0.00
A (n=15)
B (n=16)
C (n=14)
surface

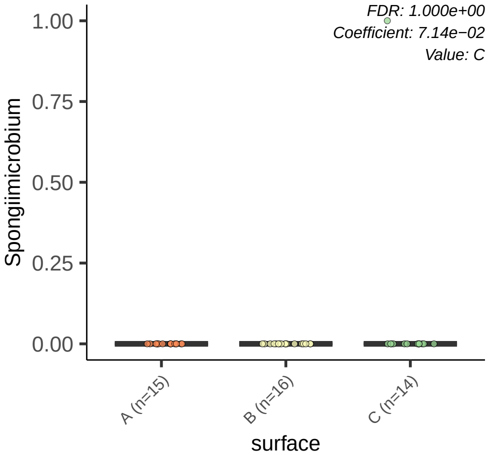
FDR: 1.000e+00
Coefficient: 7.14e−02
Value: C
1.00
0.75
0.50
0.25
0.00
Spongiimicrobium
A (n=15)
B (n=16)
C (n=14)
surface

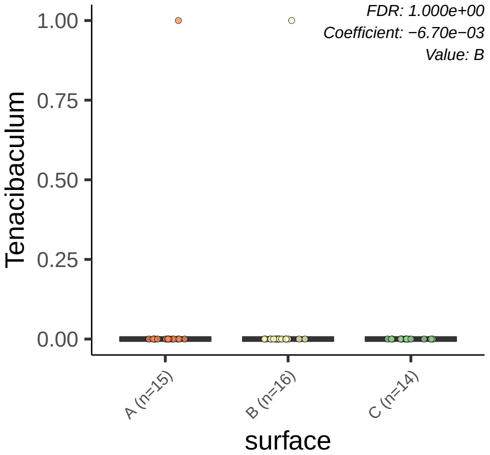
FDR: 1.000e+00
Coefficient: −6.70e−03
Value: B
Tenacibaculum
1.00
0.75
0.50
0.25
0.00
A (n=15)
B (n=16)
C (n=14)
surface

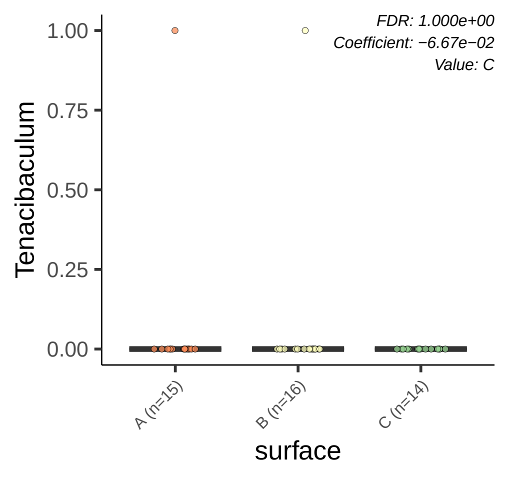
FDR: 1.000e+00
Coefficient: −6.67e−02
Value: C
Tenacibaculum
1.00
0.75
0.50
0.25
0.00
A (n=15)
B (n=16)
C (n=14)
surface

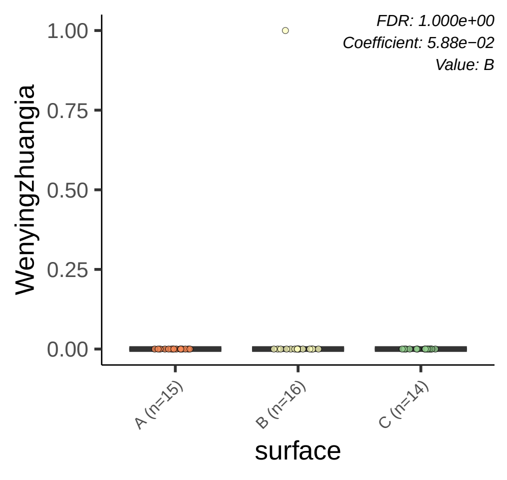
FDR: 1.000e+00
Coefficient: 5.88e−02
Value: B
Wenyingzhuangia
1.00
0.75
0.50
0.25
0.00
A (n=15)
B (n=16)
C (n=14)
surface

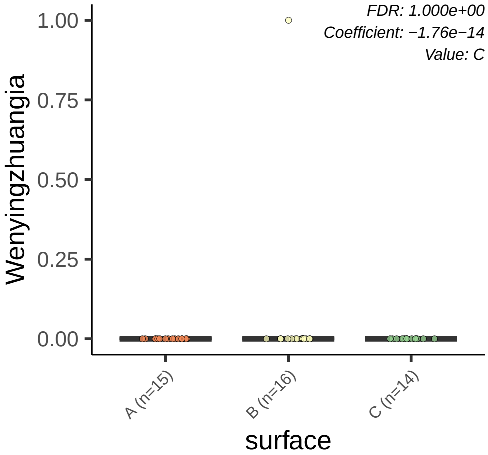

FDR: 1.000e+00
Coefficient: −1.76e−14
Value: C
Wenyingzhuangia
1.00
0.75
0.50
0.25
0.00
A (n=15)
B (n=16)
C (n=14)
surface

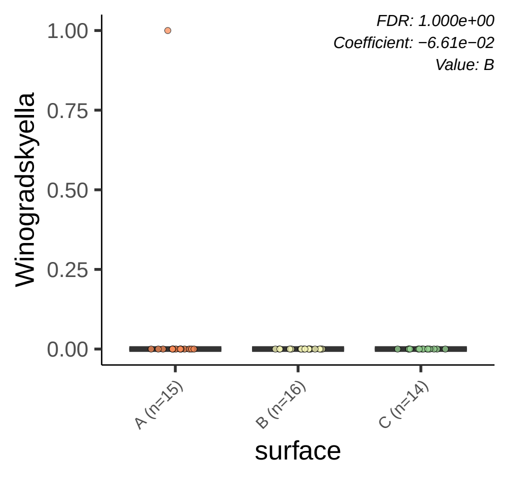
FDR: 1.000e+00
Coefficient: −6.61e−02
Value: B
Winogradskyella
1.00
0.75
0.50
0.25
0.00
A (n=15)
B (n=16)
C (n=14)
surface

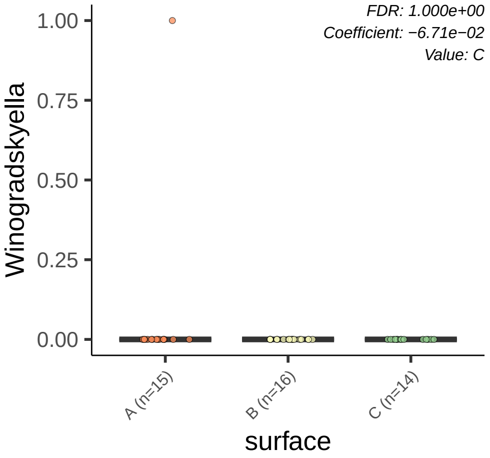
FDR: 1.000e+00
Coefficient: −6.71e−02
Value: C
Winogradskyella
1.00
0.75
0.50
0.25
0.00
A (n=15)
B (n=16)
C (n=14)
surface

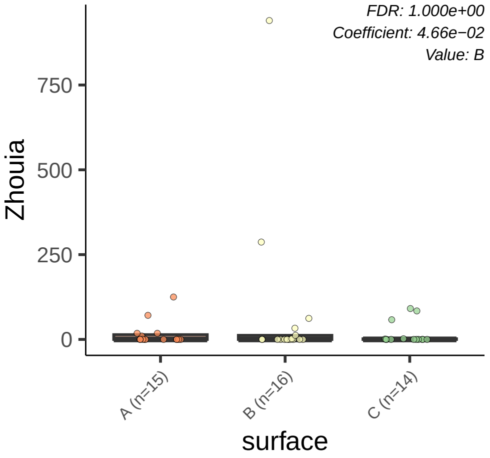

FDR: 1.000e+00
Coefficient: 4.66e−02
Value: B
Zhouia
750
500
250
0
A (n=15)
B (n=16)
C (n=14)
surface

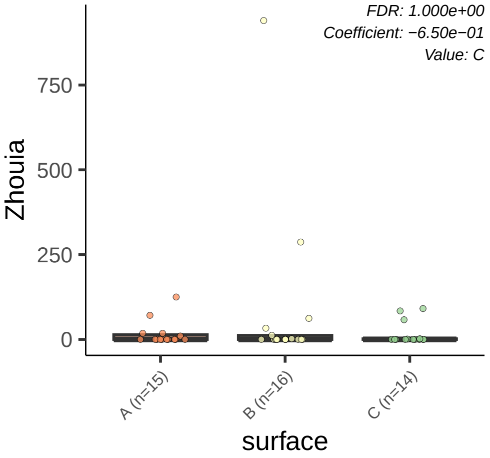

FDR: 1.000e+00
Coefficient: −6.50e−01
Value: C
Zhouia
750
500
250
0
A (n=15)
B (n=16)
C (n=14)
surface

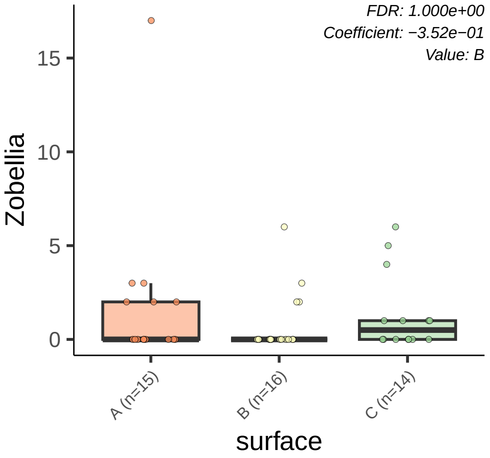
FDR: 1.000e+00
Coefficient: −3.52e−01
Value: B
Zobellia
15
10
5
0
A (n=15)
B (n=16)
C (n=14)
surface

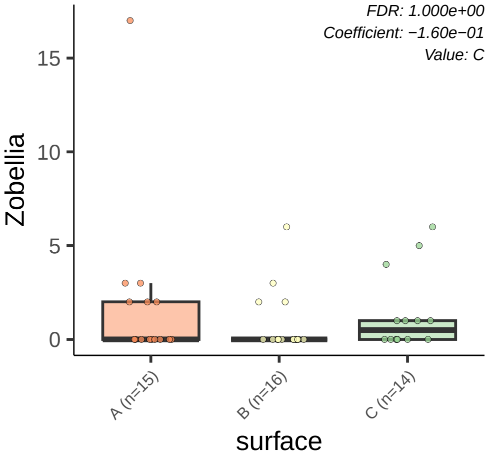
FDR: 1.000e+00
Coefficient: −1.60e−01
Value: C
Zobellia
15
10
5
0
A (n=15)
B (n=16)
C (n=14)
surface

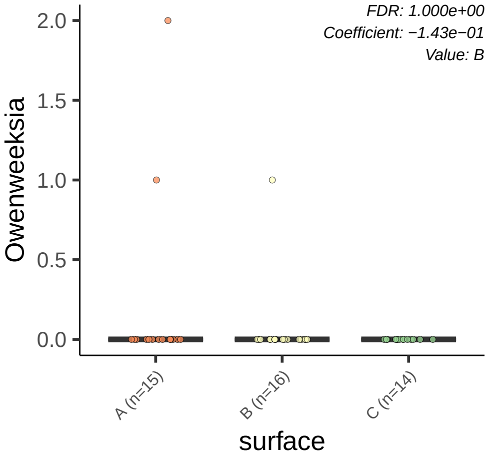
FDR: 1.000e+00
Coefficient: −1.43e−01
Value: B
Owenweeksia
2.0
1.5
1.0
0.5
0.0
A (n=15)
B (n=16)
C (n=14)
surface

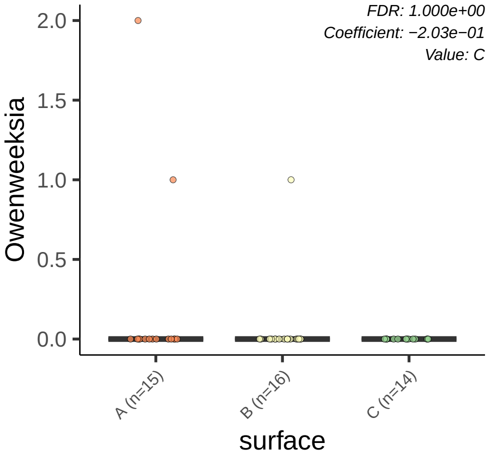
FDR: 1.000e+00
Coefficient: −2.03e−01
Value: C
Owenweeksia
2.0
1.5
1.0
0.5
0.0
A (n=15)
B (n=16)
C (n=14)
surface

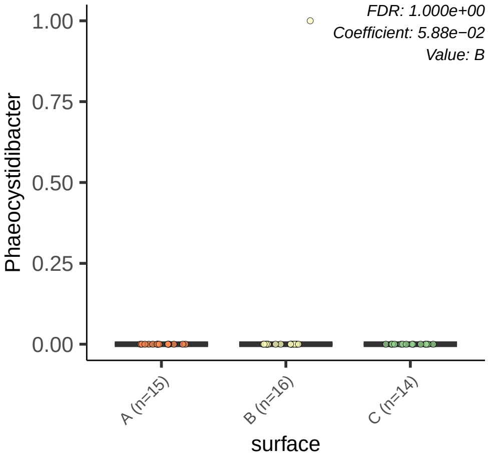

FDR: 1.000e+00
Coefficient: 5.88e−02
Value: B
Phaeocystidibacter
1.00
0.75
0.50
0.25
0.00
A (n=15)
B (n=16)
C (n=14)
surface

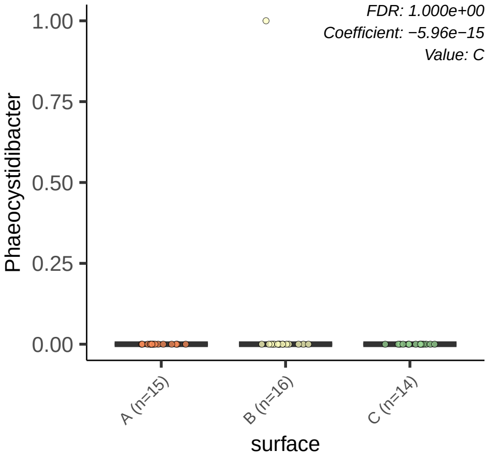
FDR: 1.000e+00
Coefficient: −5.96e−15
Value: C
Phaeocystidibacter
1.00
0.75
0.50
0.25
0.00
A (n=15)
B (n=16)
C (n=14)
surface

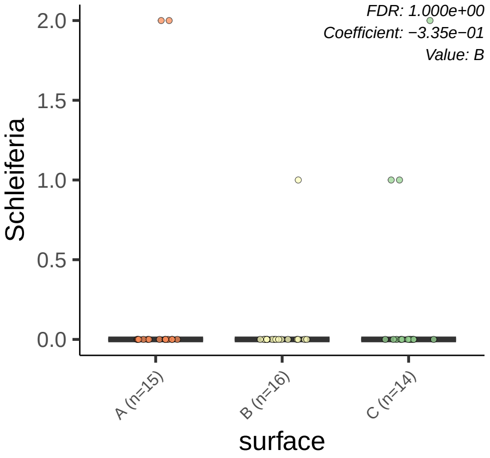
FDR: 1.000e+00
Coefficient: −3.35e−01
Value: B
Schleiferia
2.0
1.5
1.0
0.5
0.0
A (n=15)
B (n=16)
C (n=14)
surface

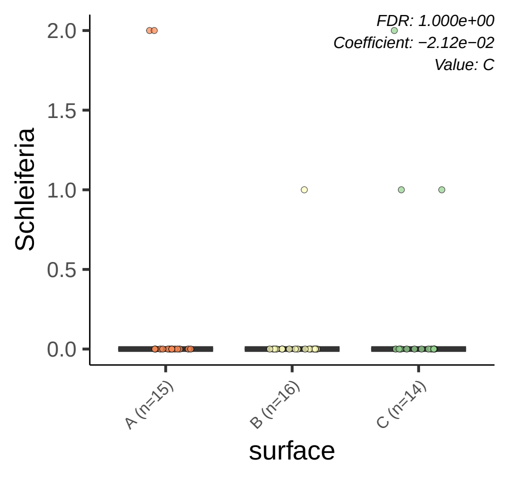
FDR: 1.000e+00
Coefficient: −2.12e−02
Value: C
Schleiferia
2.0
1.5
1.0
0.5
0.0
A (n=15)
B (n=16)
C (n=14)
surface

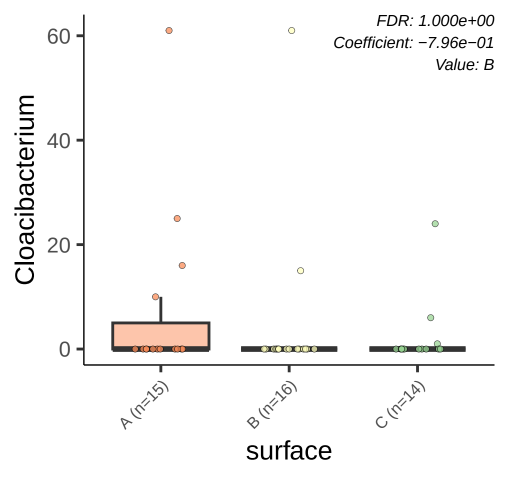
FDR: 1.000e+00
Coefficient: −7.96e−01
Value: B
Cloacibacterium
60
40
20
0
A (n=15)
B (n=16)
C (n=14)
surface

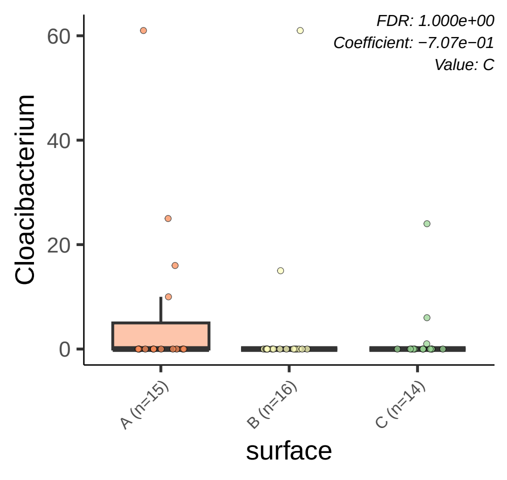
FDR: 1.000e+00
Coefficient: −7.07e−01
Value: C
Cloacibacterium
0
20
40
60
A (n=15)
B (n=16)
C (n=14)
surface

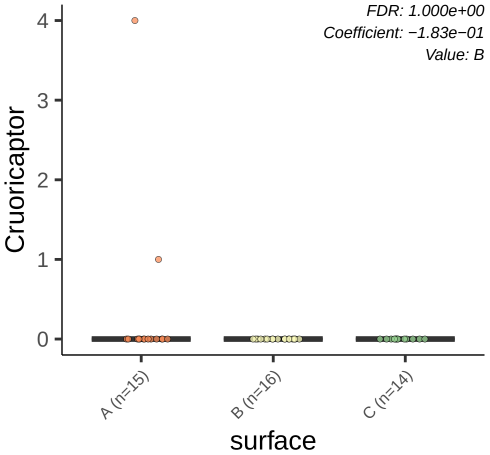

FDR: 1.000e+00
Coefficient: −1.83e−01
Value: B
Cruoricaptor
4
3
2
1
0
A (n=15)
B (n=16)
C (n=14)
surface

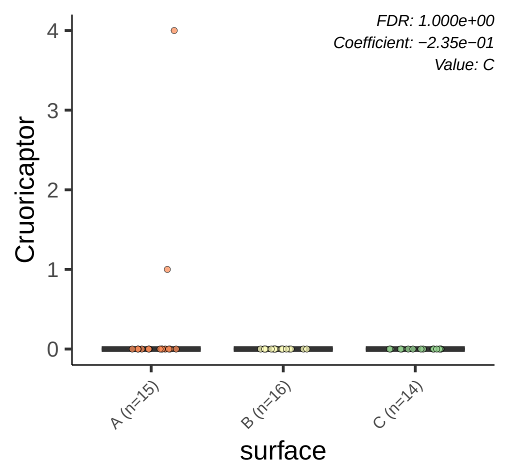

FDR: 1.000e+00
Coefficient: −2.35e−01
Value: C
Cruoricaptor
0
1
2
3
4
A (n=15)
B (n=16)
C (n=14)
surface

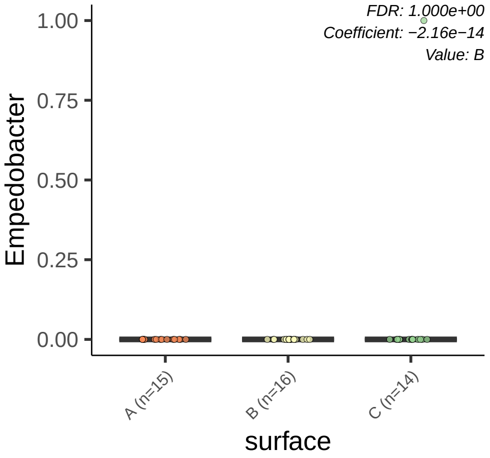
FDR: 1.000e+00
Coefficient: −2.16e−14
Value: B
Empedobacter
1.00
0.75
0.50
0.25
0.00
A (n=15)
B (n=16)
C (n=14)
surface

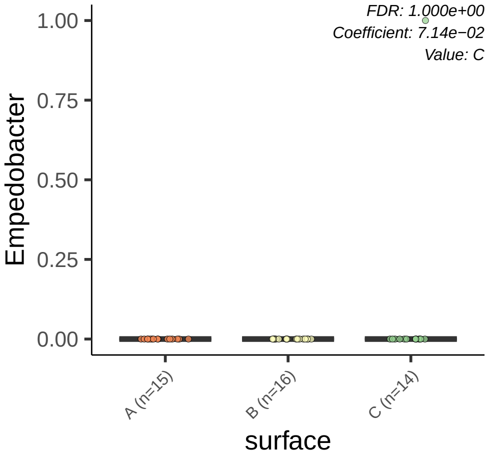
FDR: 1.000e+00
Coefficient: 7.14e−02
Value: C
Empedobacter
1.00
0.75
0.50
0.25
0.00
A (n=15)
B (n=16)
C (n=14)
surface

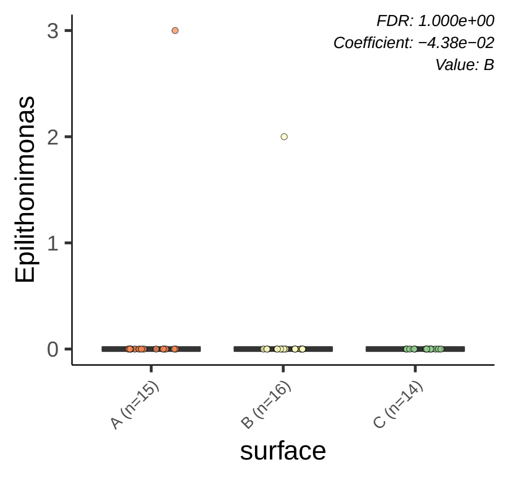
FDR: 1.000e+00
Coefficient: −4.38e−02
Value: B
Epilithonimonas
0
1
2
3
surface
A (n=15)
B (n=16)
C (n=14)

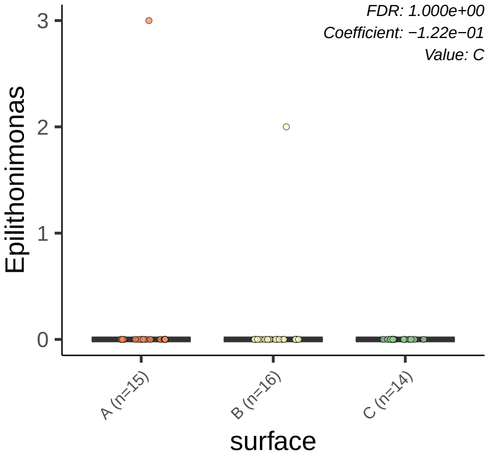

FDR: 1.000e+00
Coefficient: −1.22e−01
Value: C
Epilithonimonas
3
2
1
0
A (n=15)
B (n=16)
C (n=14)
surface

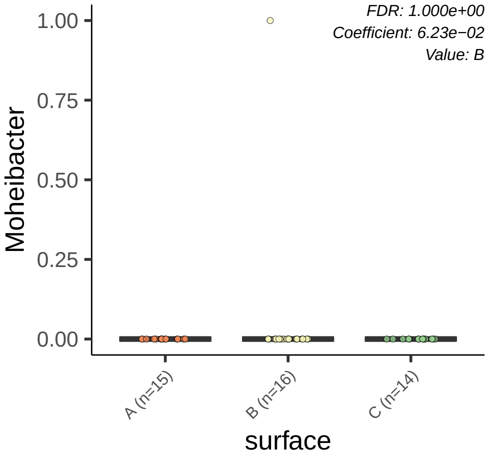
FDR: 1.000e+00
Coefficient: 6.23e−02
Value: B
Moheibacter
1.00
0.75
0.50
0.25
0.00
A (n=15)
B (n=16)
C (n=14)
surface

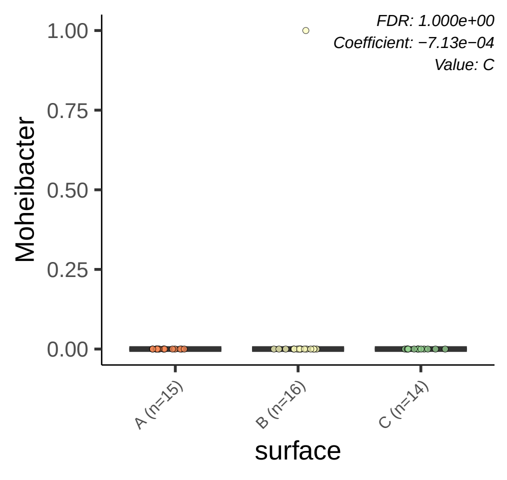

FDR: 1.000e+00
Coefficient: −7.13e−04
Value: C
Moheibacter
1.00
0.75
0.50
0.25
0.00
A (n=15)
B (n=16)
C (n=14)
surface

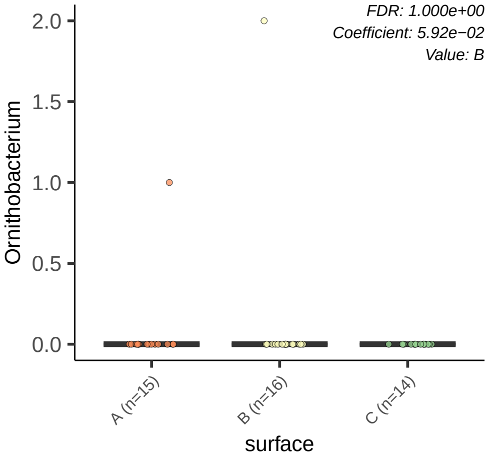

FDR: 1.000e+00
Coefficient: 5.92e−02
Value: B
Ornithobacterium
2.0
1.5
1.0
0.5
0.0
A (n=15)
B (n=16)
C (n=14)
surface

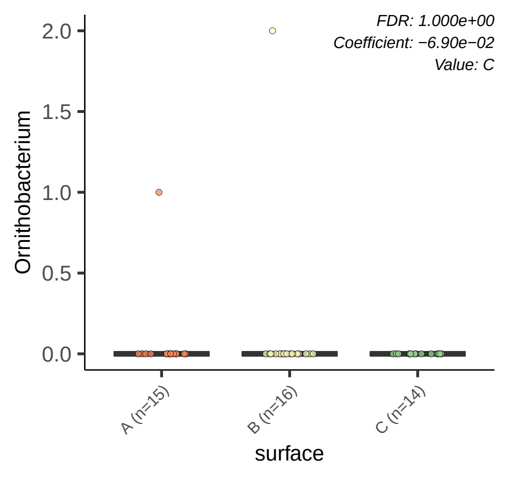
FDR: 1.000e+00
Coefficient: −6.90e−02
Value: C
Ornithobacterium
2.0
1.5
1.0
0.5
0.0
A (n=15)
B (n=16)
C (n=14)
surface

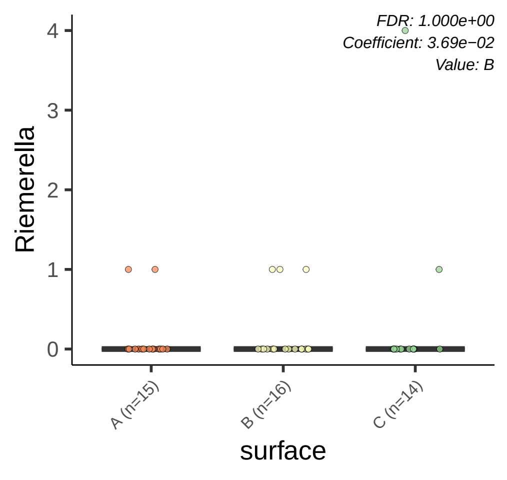
FDR: 1.000e+00
Coefficient: 3.69e−02
Value: B
Riemerella
0
1
2
3
4
A (n=15)
B (n=16)
C (n=14)
surface

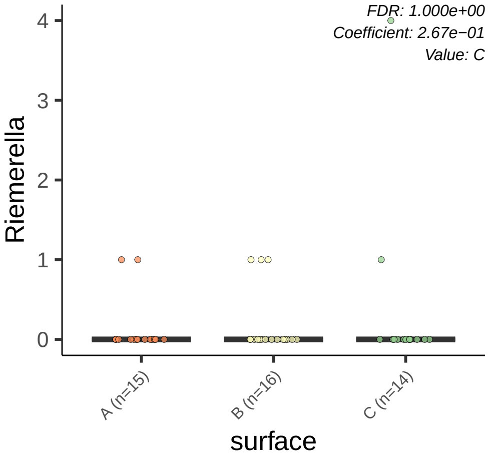
FDR: 1.000e+00
Coefficient: 2.67e−01
Value: C
Riemerella
0
1
2
3
4
A (n=15)
B (n=16)
C (n=14)
surface

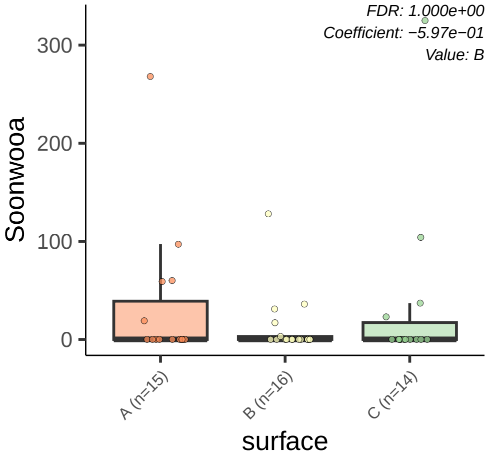

FDR: 1.000e+00
Coefficient: −5.97e−01
Value: B
Soonwooa
300
200
100
0
A (n=15)
B (n=16)
C (n=14)
surface

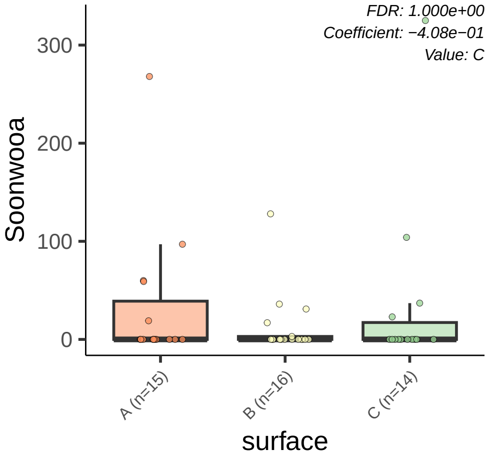
FDR: 1.000e+00
Coefficient: −4.08e−01
Value: C
Soonwooa
300
200
100
0
A (n=15)
B (n=16)
C (n=14)
surface

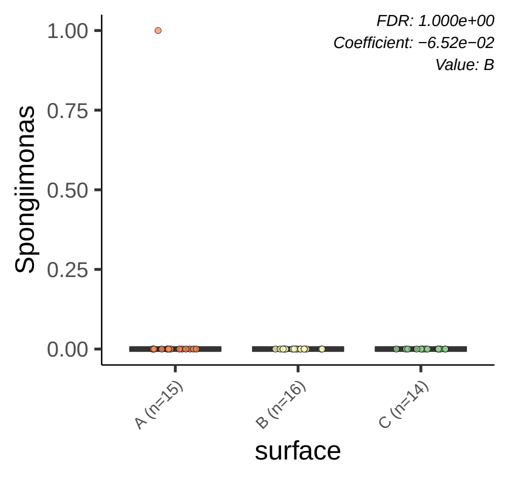

FDR: 1.000e+00
Coefficient: −6.52e−02
Value: B
Spongiimonas
1.00
0.75
0.50
0.25
0.00
A (n=15)
B (n=16)
C (n=14)
surface

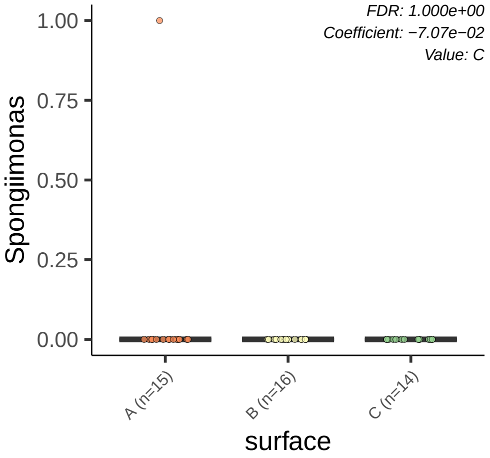
FDR: 1.000e+00
Coefficient: −7.07e−02
Value: C
Spongiimonas
1.00
0.75
0.50
0.25
0.00
A (n=15)
B (n=16)
C (n=14)
surface

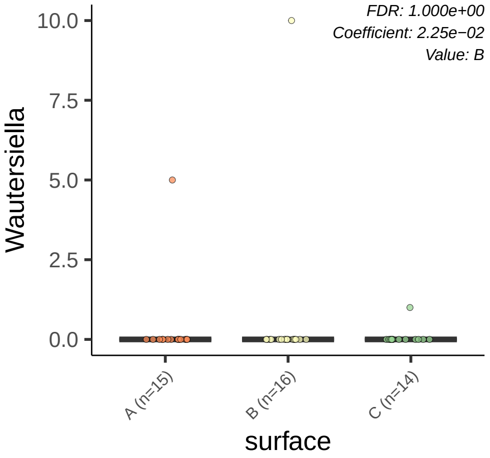
FDR: 1.000e+00
Coefficient: 2.25e−02
Value: B
Wautersiella
10.0
7.5
5.0
2.5
0.0
A (n=15)
B (n=16)
C (n=14)
surface

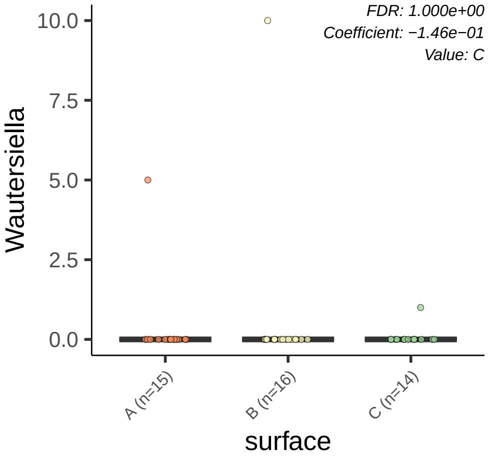
FDR: 1.000e+00
Coefficient: −1.46e−01
Value: C
Wautersiella
10.0
7.5
5.0
2.5
0.0
A (n=15)
B (n=16)
C (n=14)
surface

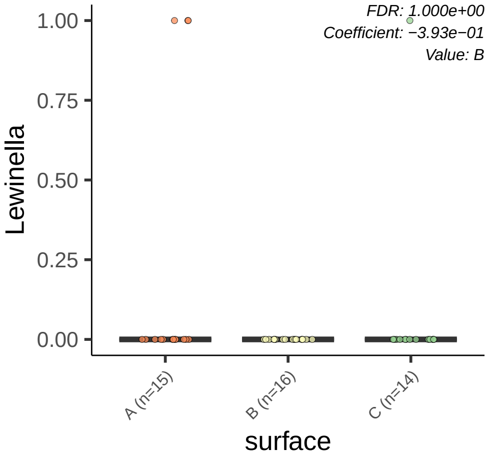
FDR: 1.000e+00
Coefficient: −3.93e−01
Value: B
Lewinella
1.00
0.75
0.50
0.25
0.00
A (n=15)
B (n=16)
C (n=14)
surface

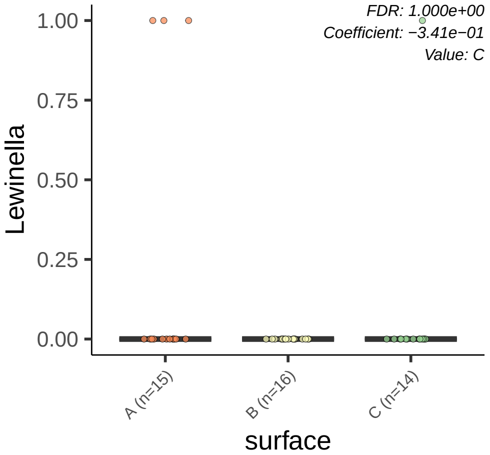

FDR: 1.000e+00
Coefficient: −3.41e−01
Value: C
1.00
0.75
0.50
0.25
0.00
Lewinella
A (n=15)
B (n=16)
C (n=14)
surface

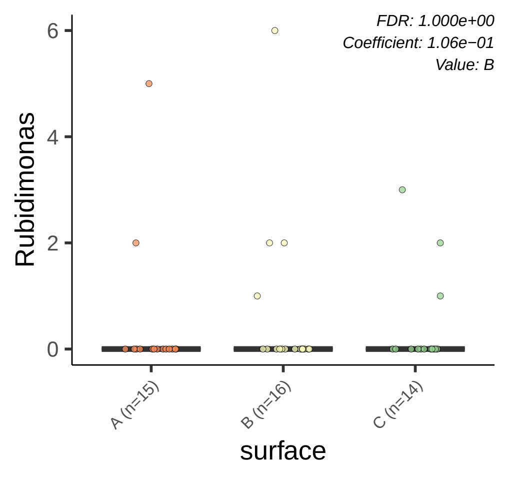

FDR: 1.000e+00
Coefficient: 1.06e−01
Value: B
Rubidimonas
0
2
4
6
A (n=15)
B (n=16)
C (n=14)
surface

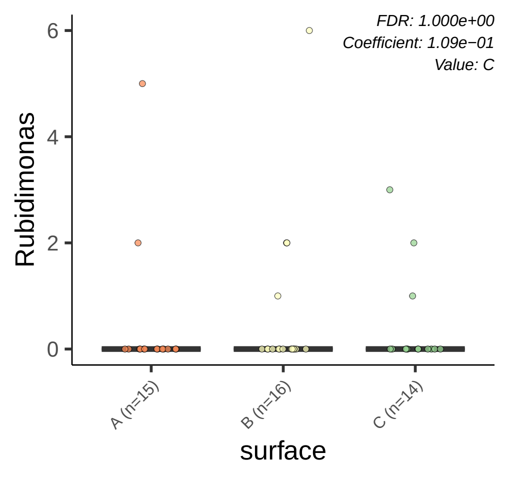

FDR: 1.000e+00
Coefficient: 1.09e−01
Value: C
Rubidimonas
0
2
4
6
A (n=15)
B (n=16)
C (n=14)
surface

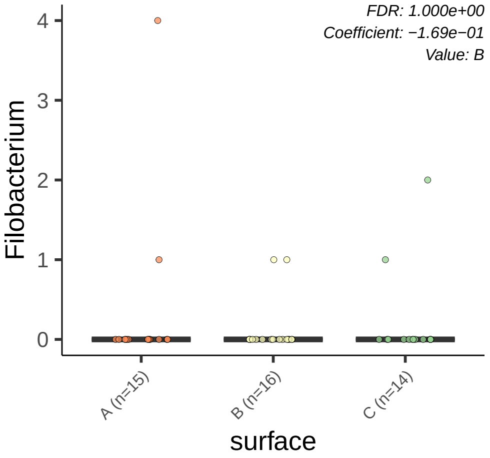

FDR: 1.000e+00
Coefficient: −1.69e−01
Value: B
Filobacterium
0
1
2
3
4
A (n=15)
B (n=16)
C (n=14)
surface

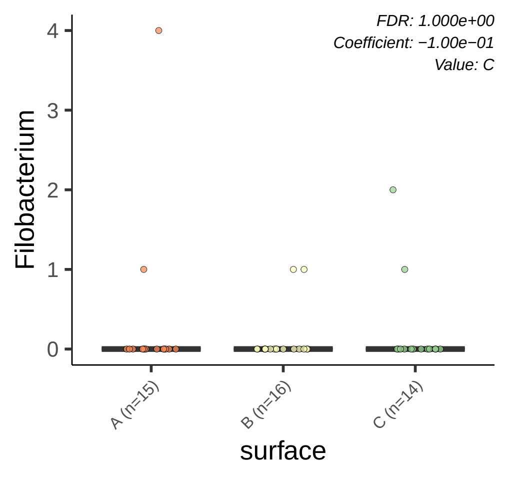

FDR: 1.000e+00
Coefficient: −1.00e−01
Value: C
Filobacterium
0
1
2
3
4
A (n=15)
B (n=16)
C (n=14)
surface

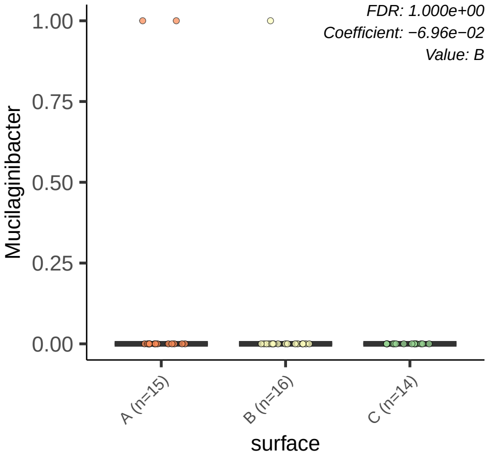
FDR: 1.000e+00
Coefficient: −6.96e−02
Value: B
Mucilaginibacter
1.00
0.75
0.50
0.25
0.00
A (n=15)
B (n=16)
C (n=14)
surface

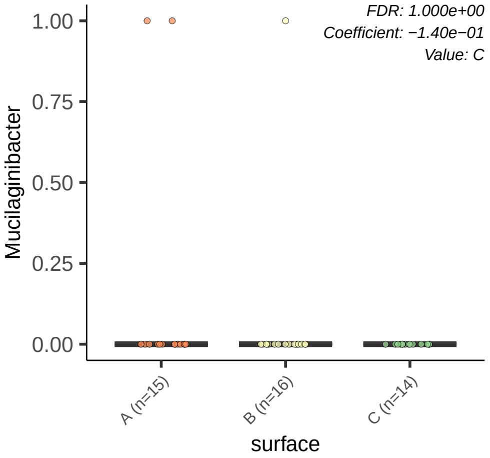

FDR: 1.000e+00
Coefficient: −1.40e−01
Value: C
Mucilaginibacter
1.00
0.75
0.50
0.25
0.00
A (n=15)
B (n=16)
C (n=14)
surface

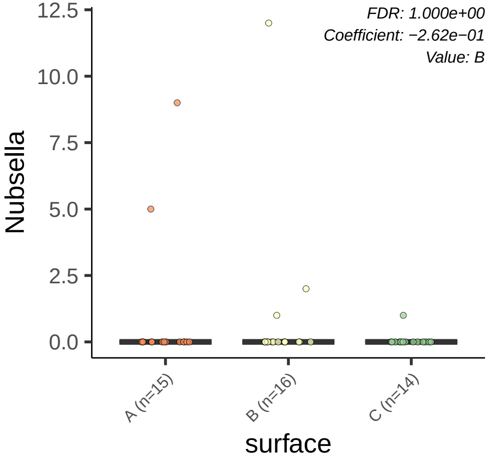

FDR: 1.000e+00
Coefficient: −2.62e−01
Value: B
Nubsella
12.5
10.0
7.5
5.0
2.5
0.0
A (n=15)
B (n=16)
C (n=14)
surface

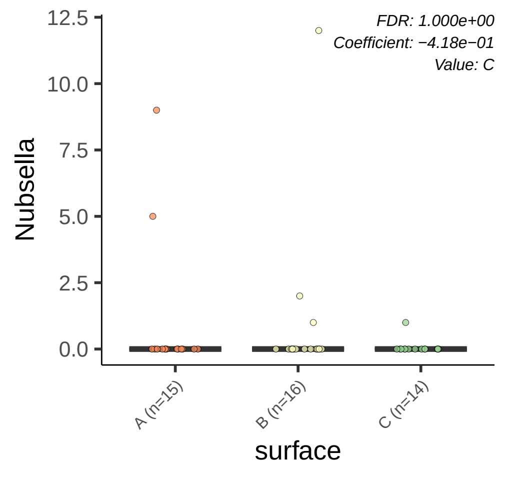

FDR: 1.000e+00
Coefficient: −4.18e−01
Value: C
Nubsella
12.5
10.0
7.5
5.0
2.5
0.0
A (n=15)
B (n=16)
C (n=14)
surface

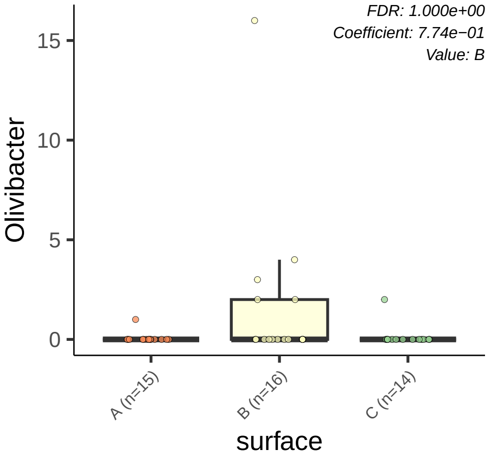

FDR: 1.000e+00
Coefficient: 7.74e−01
Value: B
Olivibacter
15
10
5
0
A (n=15)
B (n=16)
C (n=14)
surface

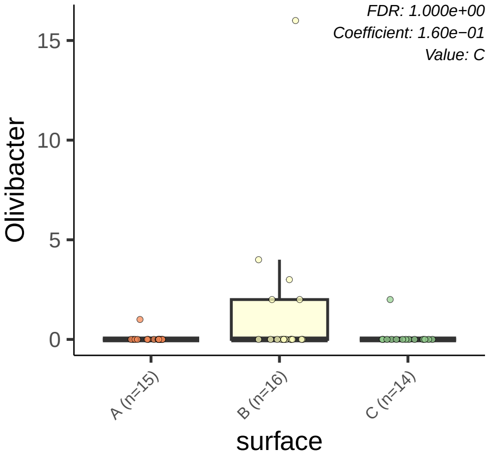

FDR: 1.000e+00
Coefficient: 1.60e−01
Value: C
Olivibacter
15
10
5
0
A (n=15)
B (n=16)
C (n=14)
surface

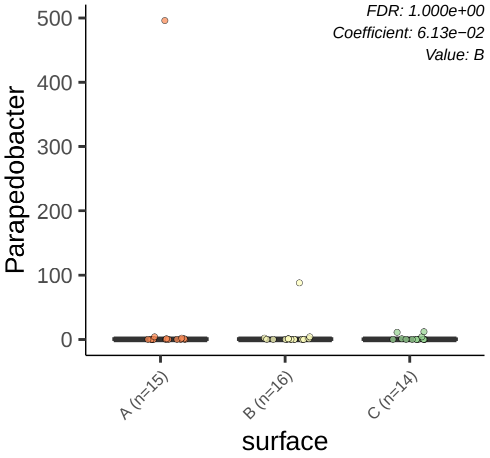
FDR: 1.000e+00
Coefficient: 6.13e−02
Value: B
Parapedobacter
500
400
300
200
100
0
A (n=15)
B (n=16)
C (n=14)
surface

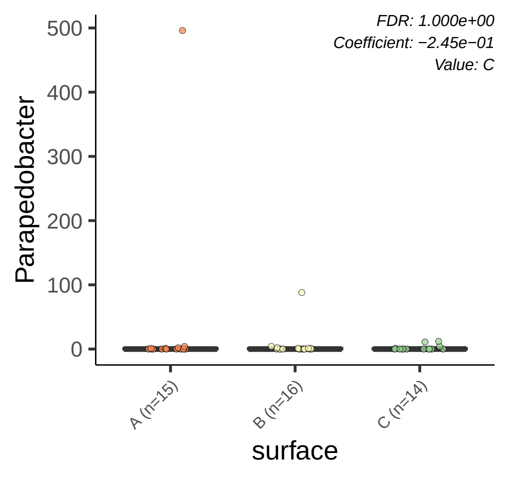
FDR: 1.000e+00
Coefficient: −2.45e−01
Value: C
Parapedobacter
500
400
300
200
100
0
A (n=15)
B (n=16)
C (n=14)
surface

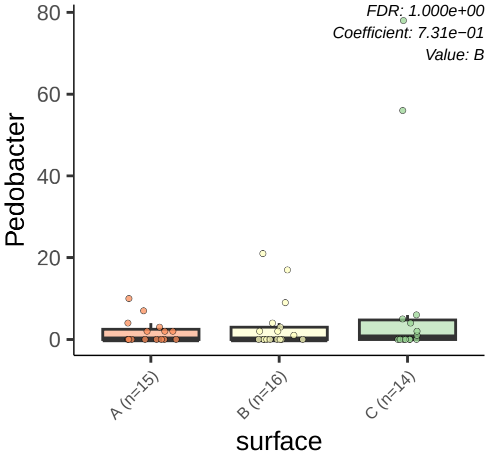
FDR: 1.000e+00
Coefficient: 7.31e−01
Value: B
Pedobacter
80
60
40
20
0
A (n=15)
B (n=16)
C (n=14)
surface

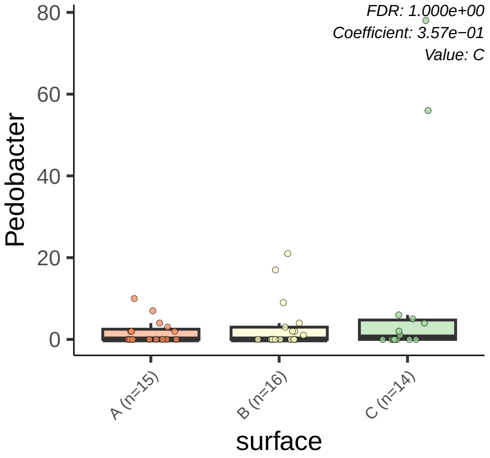
FDR: 1.000e+00
Coefficient: 3.57e−01
Value: C
Pedobacter
80
60
40
20
0
A (n=15)
B (n=16)
C (n=14)
surface

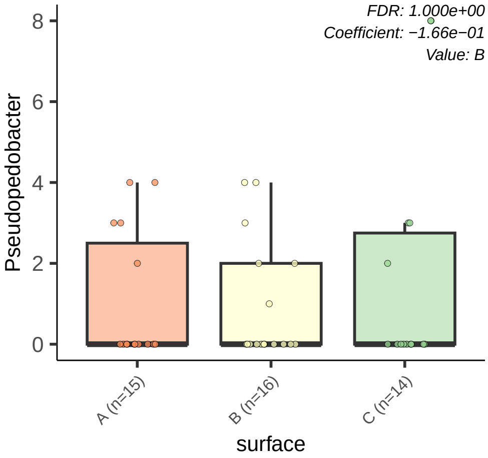
FDR: 1.000e+00
Coefficient: −1.66e−01
Value: B
Pseudopedobacter
8
6
4
2
0
A (n=15)
B (n=16)
C (n=14)
surface

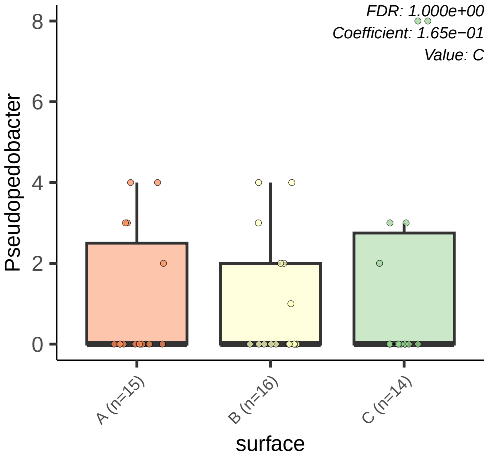

FDR: 1.000e+00
Coefficient: 1.65e−01
Value: C
Pseudopedobacter
0
2
4
6
8
A (n=15)
B (n=16)
C (n=14)
surface

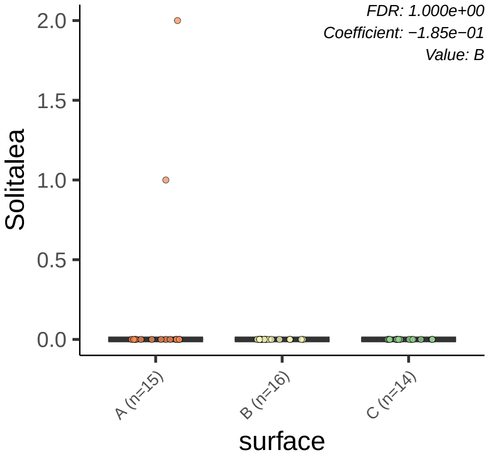

FDR: 1.000e+00
Coefficient: −1.85e−01
Value: B
Solitalea
2.0
1.5
1.0
0.5
0.0
A (n=15)
B (n=16)
C (n=14)
surface

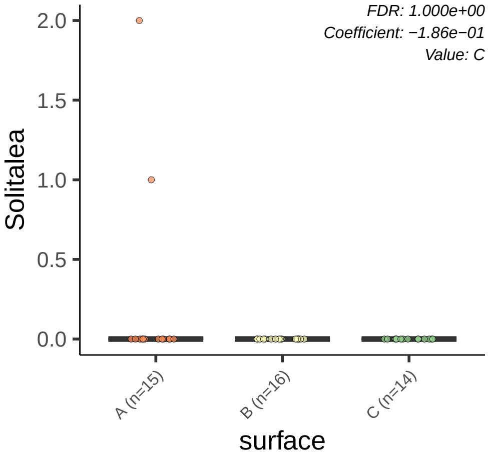
FDR: 1.000e+00
Coefficient: −1.86e−01
Value: C
Solitalea
2.0
1.5
1.0
0.5
0.0
A (n=15)
B (n=16)
C (n=14)
surface

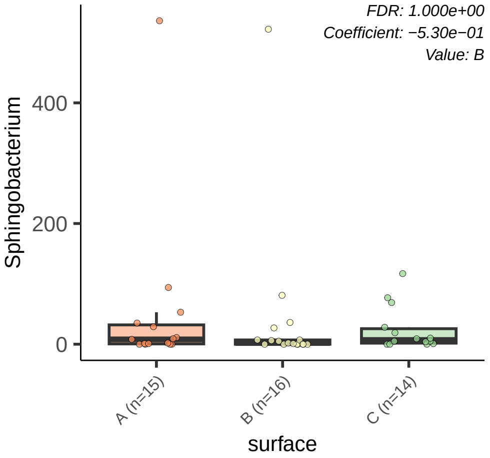
FDR: 1.000e+00
Coefficient: −5.30e−01
Value: B
Sphingobacterium
400
200
0
A (n=15)
B (n=16)
C (n=14)
surface

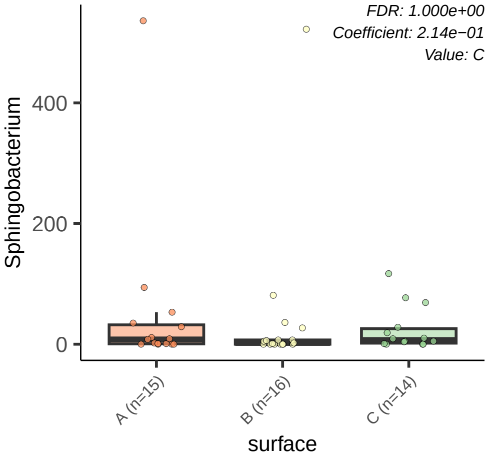

FDR: 1.000e+00
Coefficient: 2.14e−01
Value: C
Sphingobacterium
400
200
0
A (n=15)
B (n=16)
C (n=14)
surface

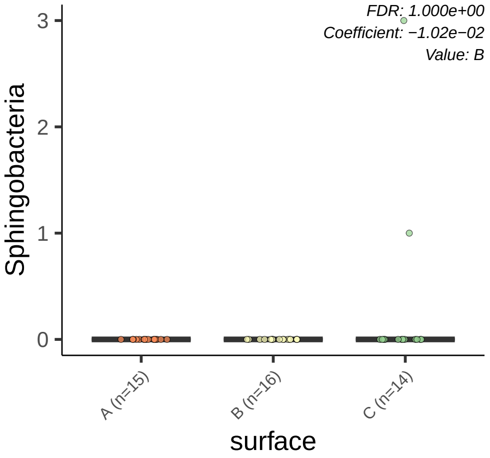
FDR: 1.000e+00
Coefficient: −1.02e−02
Value: B
Sphingobacteria
3
2
1
0
A (n=15)
B (n=16)
C (n=14)
surface

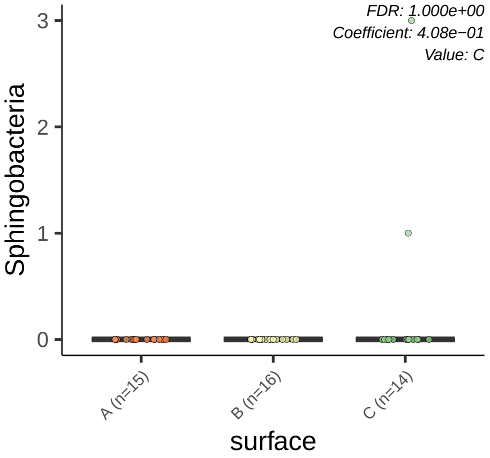

FDR: 1.000e+00
Coefficient: 4.08e−01
Value: C
Sphingobacteria
3
2
1
0
A (n=15)
B (n=16)
C (n=14)
surface

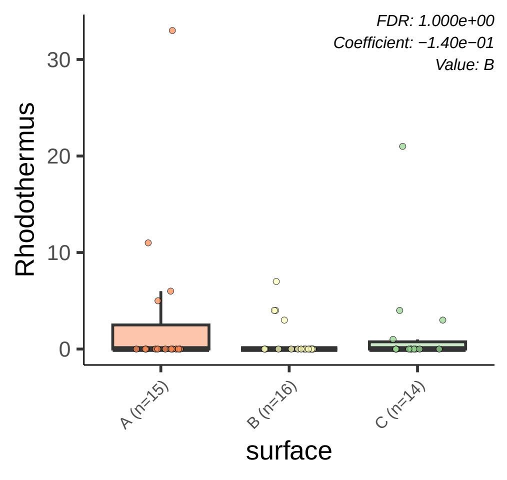
FDR: 1.000e+00
Coefficient: −1.40e−01
Value: B
Rhodothermus
30
20
10
0
A (n=15)
B (n=16)
C (n=14)
surface

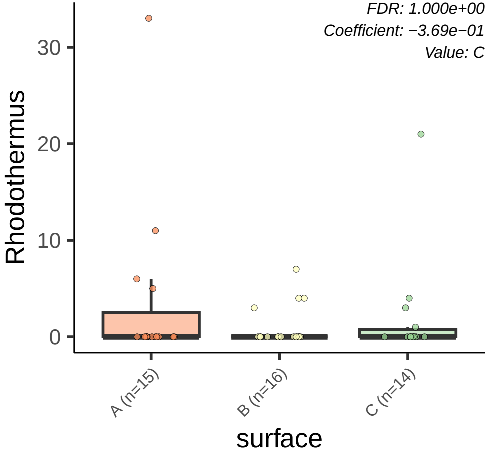

FDR: 1.000e+00
Coefficient: −3.69e−01
Value: C
Rhodothermus
30
20
10
0
A (n=15)
B (n=16)
C (n=14)
surface

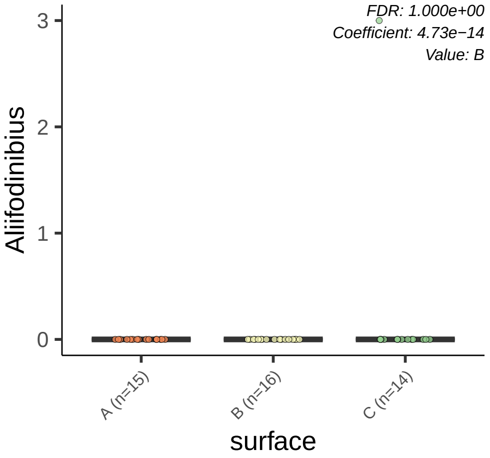

FDR: 1.000e+00
Coefficient: 4.73e−14
Value: B
Aliifodinibius
3
2
1
0
A (n=15)
B (n=16)
C (n=14)
surface

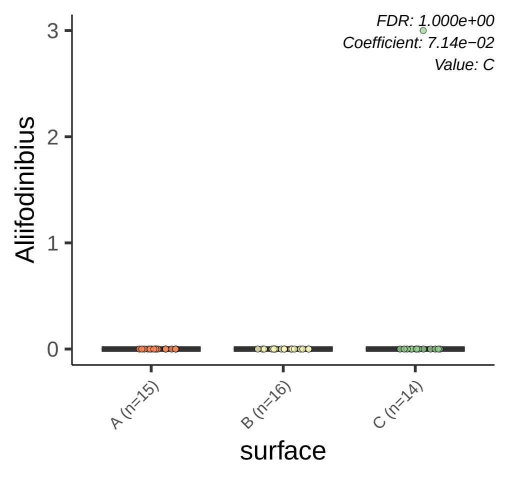
FDR: 1.000e+00
Coefficient: 7.14e−02
Value: C
Aliifodinibius
0
1
2
3
A (n=15)
B (n=16)
C (n=14)
surface

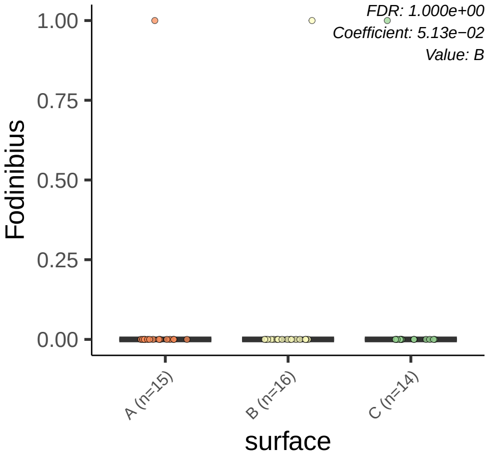

FDR: 1.000e+00
Coefficient: 5.13e−02
Value: B
Fodinibius
1.00
0.75
0.50
0.25
0.00
A (n=15)
B (n=16)
C (n=14)
surface

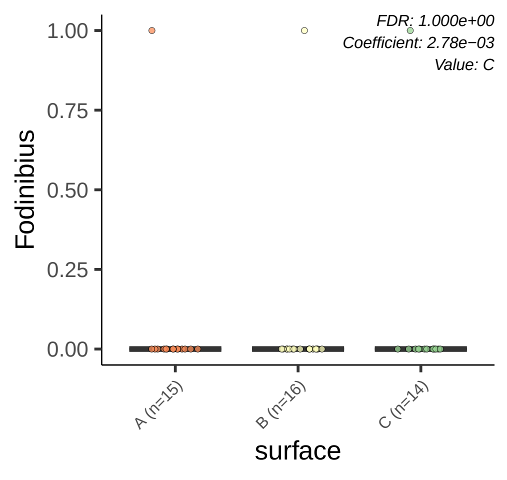

FDR: 1.000e+00
Coefficient: 2.78e−03
Value: C
Fodinibius
1.00
0.75
0.50
0.25
0.00
A (n=15)
B (n=16)
C (n=14)
surface

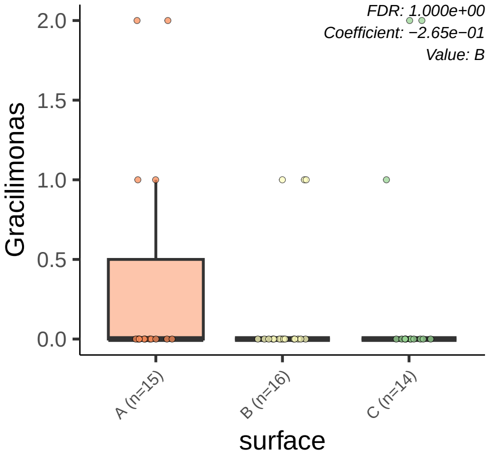
FDR: 1.000e+00
Coefficient: −2.65e−01
Value: B
Gracilimonas
2.0
1.5
1.0
0.5
0.0
A (n=15)
B (n=16)
C (n=14)
surface

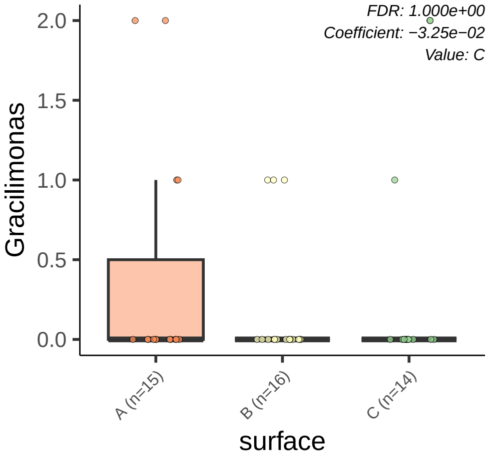
FDR: 1.000e+00
Coefficient: −3.25e−02
Value: C
Gracilimonas
2.0
1.5
1.0
0.5
0.0
A (n=15)
B (n=16)
C (n=14)
surface

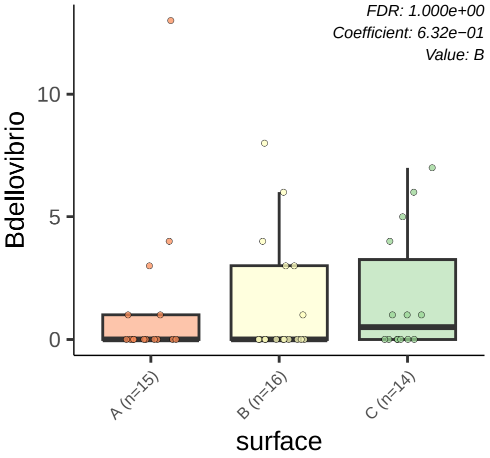
FDR: 1.000e+00
Coefficient: 6.32e−01
Value: B
Bdellovibrio
10
5
0
A (n=15)
B (n=16)
C (n=14)
surface

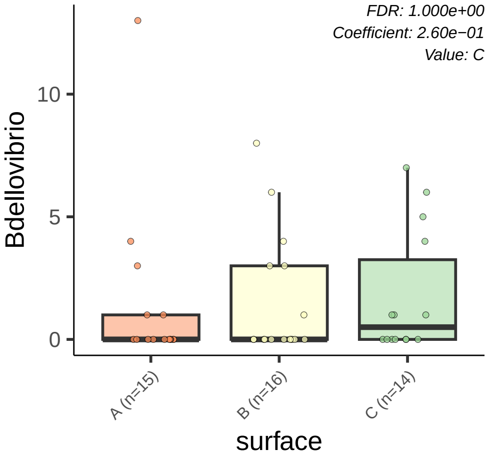
FDR: 1.000e+00
Coefficient: 2.60e−01
Value: C
Bdellovibrio
10
5
0
A (n=15)
B (n=16)
C (n=14)
surface

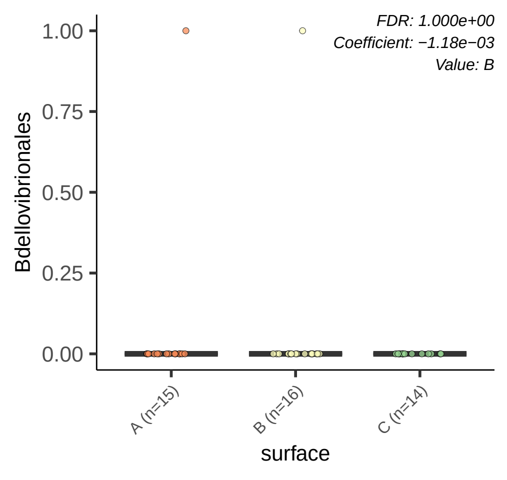

FDR: 1.000e+00
Coefficient: −1.18e−03
Value: B
Bdellovibrionales
1.00
0.75
0.50
0.25
0.00
A (n=15)
B (n=16)
C (n=14)
surface

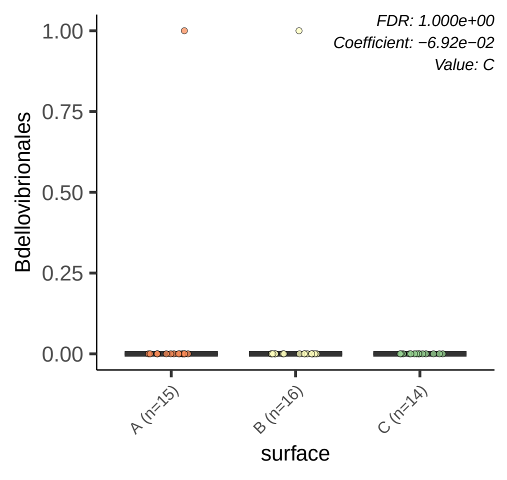
FDR: 1.000e+00
Coefficient: −6.92e−02
Value: C
Bdellovibrionales
1.00
0.75
0.50
0.25
0.00
A (n=15)
B (n=16)
C (n=14)
surface

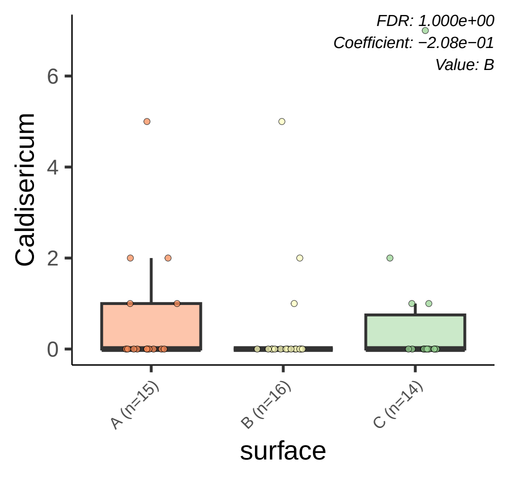

FDR: 1.000e+00
Coefficient: −2.08e−01
Value: B
Caldisericum
0
2
4
6
A (n=15)
B (n=16)
C (n=14)
surface

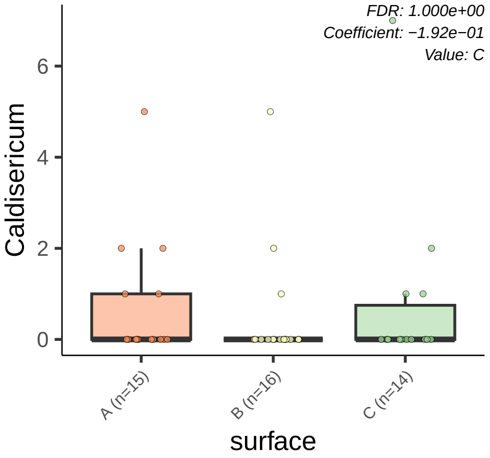
FDR: 1.000e+00
Coefficient: −1.92e−01
Value: C
Caldisericum
0
2
4
6
A (n=15)
B (n=16)
C (n=14)
surface

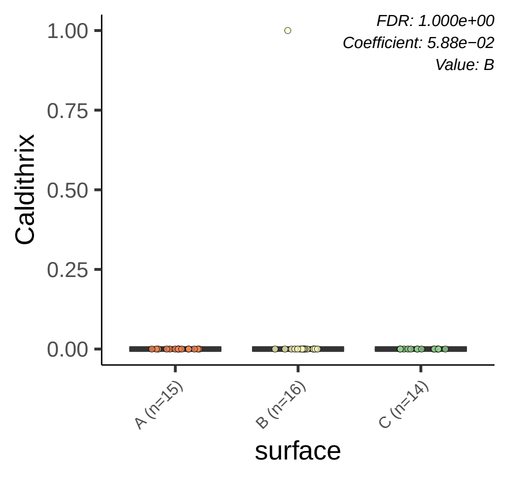

FDR: 1.000e+00
Coefficient: 5.88e−02
Value: B
Caldithrix
1.00
0.75
0.50
0.25
0.00
A (n=15)
B (n=16)
C (n=14)
surface

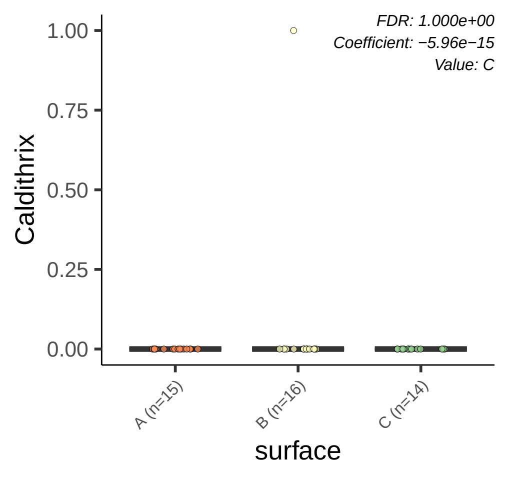
FDR: 1.000e+00
Coefficient: −5.96e−15
Value: C
Caldithrix
1.00
0.75
0.50
0.25
0.00
A (n=15)
B (n=16)
C (n=14)
surface

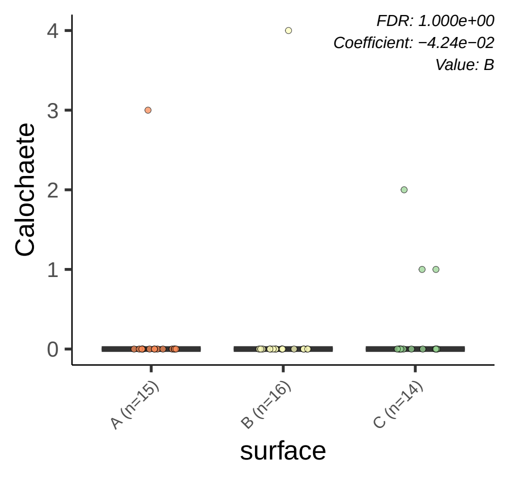
FDR: 1.000e+00
Coefficient: −4.24e−02
Value: B
Calochaete
4
3
2
1
0
A (n=15)
B (n=16)
C (n=14)
surface

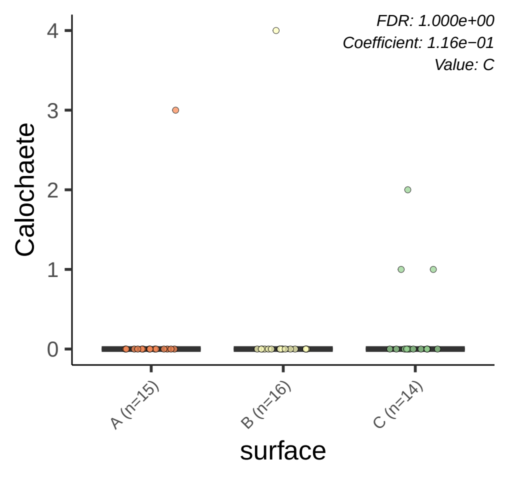
FDR: 1.000e+00
Coefficient: 1.16e−01
Value: C
Calochaete
4
3
2
1
0
A (n=15)
B (n=16)
C (n=14)
surface

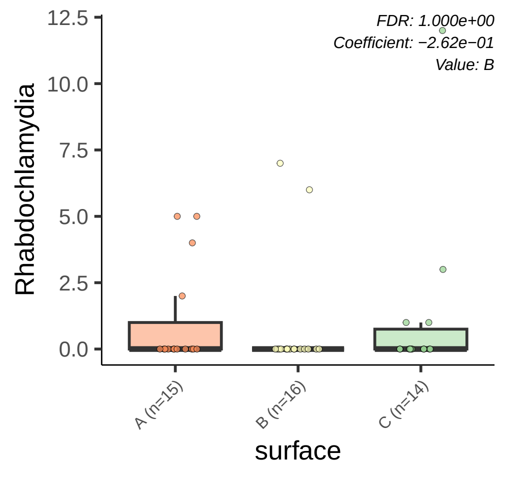
FDR: 1.000e+00
Coefficient: −2.62e−01
Value: B
Rhabdochlamydia
12.5
10.0
7.5
5.0
2.5
0.0
A (n=15)
B (n=16)
C (n=14)
surface

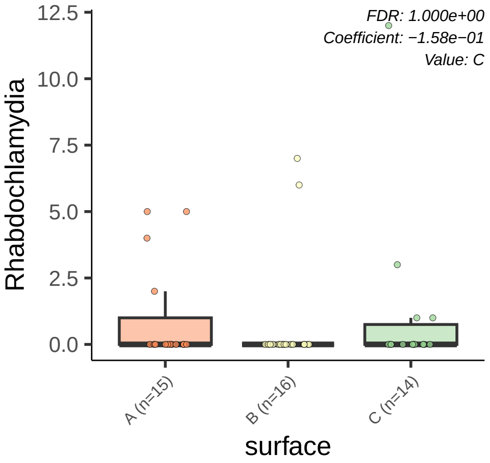

FDR: 1.000e+00
Coefficient: −1.58e−01
Value: C
Rhabdochlamydia
12.5
10.0
7.5
5.0
2.5
0.0
A (n=15)
B (n=16)
C (n=14)
surface

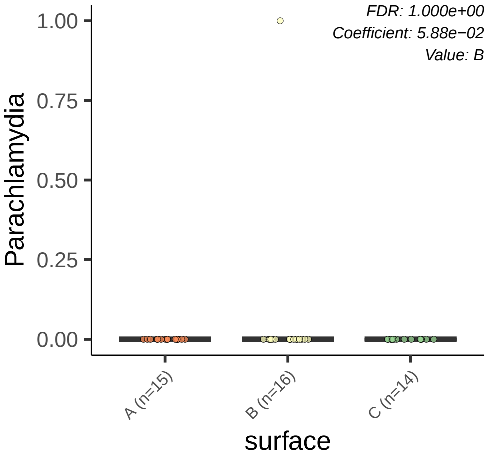

FDR: 1.000e+00
Coefficient: 5.88e−02
Value: B
Parachlamydia
1.00
0.75
0.50
0.25
0.00
A (n=15)
B (n=16)
C (n=14)
surface

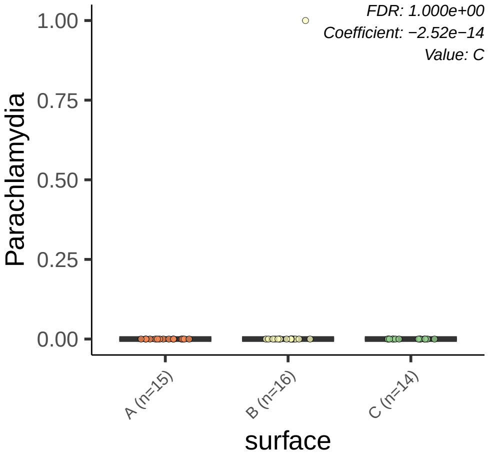
FDR: 1.000e+00
Coefficient: −2.52e−14
Value: C
Parachlamydia
1.00
0.75
0.50
0.25
0.00
A (n=15)
B (n=16)
C (n=14)
surface

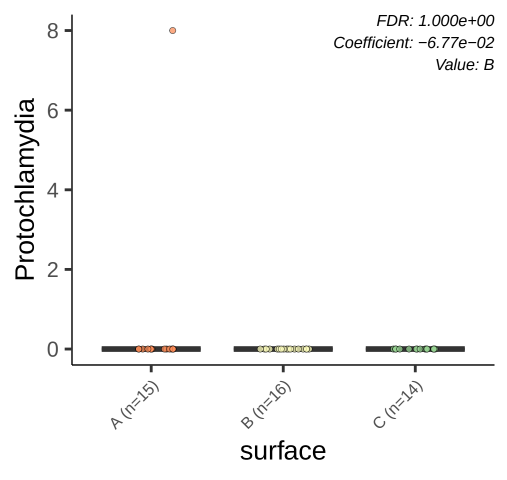
FDR: 1.000e+00
Coefficient: −6.77e−02
Value: B
Protochlamydia
8
6
4
2
0
A (n=15)
B (n=16)
C (n=14)
surface

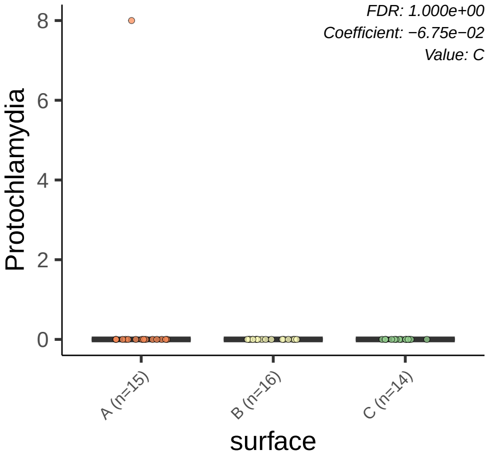

FDR: 1.000e+00
Coefficient: −6.75e−02
Value: C
Protochlamydia
8
6
4
2
0
A (n=15)
B (n=16)
C (n=14)
surface

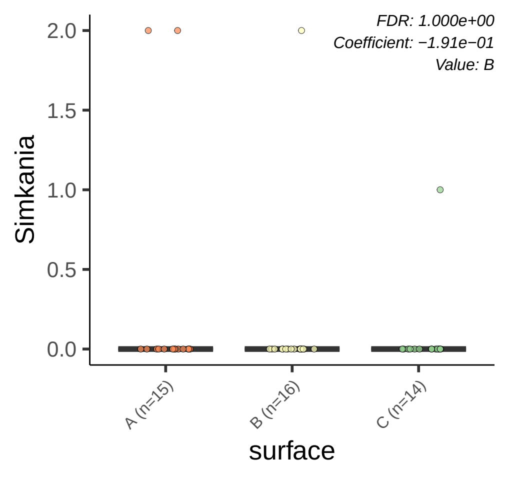

FDR: 1.000e+00
Coefficient: −1.91e−01
Value: B
Simkania
2.0
1.5
1.0
0.5
0.0
A (n=15)
B (n=16)
C (n=14)
surface

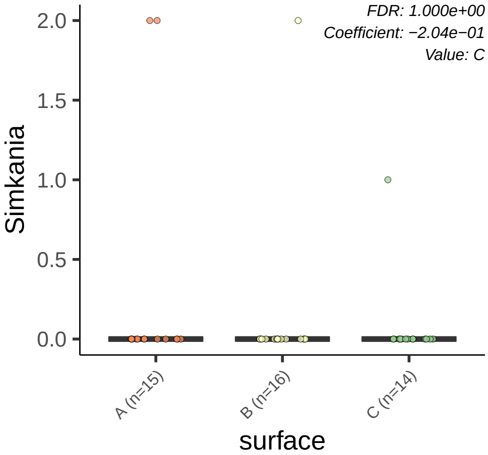

FDR: 1.000e+00
Coefficient: −2.04e−01
Value: C
2.0
1.5
1.0
0.5
0.0
Simkania
A (n=15)
B (n=16)
C (n=14)
surface

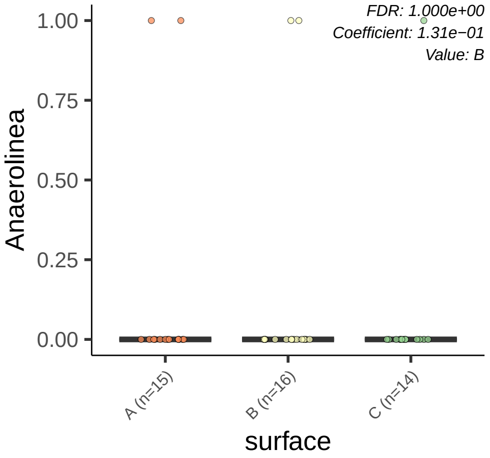

FDR: 1.000e+00
Coefficient: 1.31e−01
Value: B
Anaerolinea
1.00
0.75
0.50
0.25
0.00
A (n=15)
B (n=16)
C (n=14)
surface

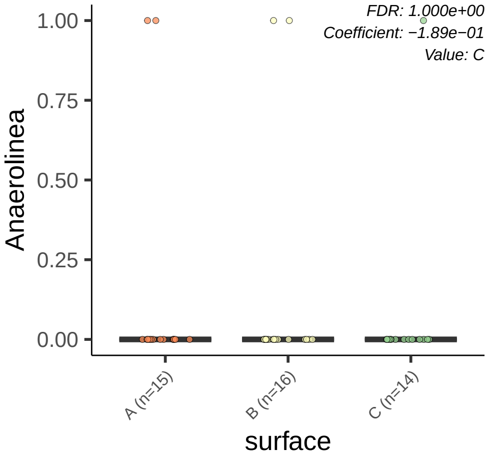

FDR: 1.000e+00
Coefficient: −1.89e−01
Value: C
Anaerolinea
1.00
0.75
0.50
0.25
0.00
A (n=15)
B (n=16)
C (n=14)
surface

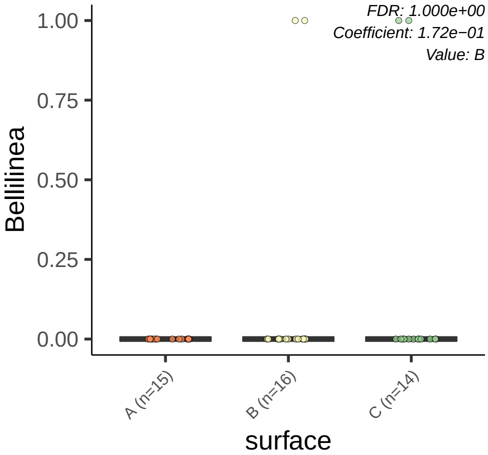

FDR: 1.000e+00
Coefficient: 1.72e−01
Value: B
Bellilinea
1.00
0.75
0.50
0.25
0.00
A (n=15)
B (n=16)
C (n=14)
surface

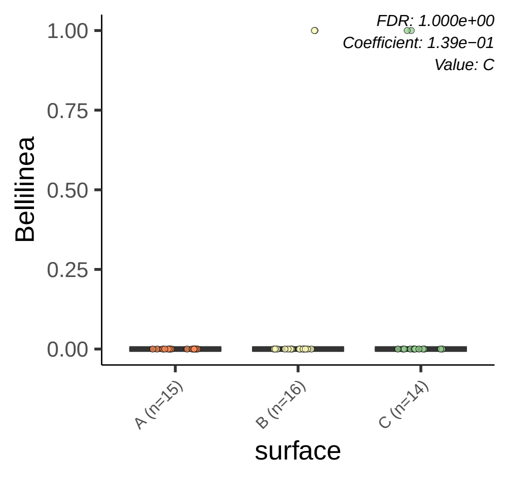
FDR: 1.000e+00
Coefficient: 1.39e−01
Value: C
Bellilinea
1.00
0.75
0.50
0.25
0.00
A (n=15)
B (n=16)
C (n=14)
surface

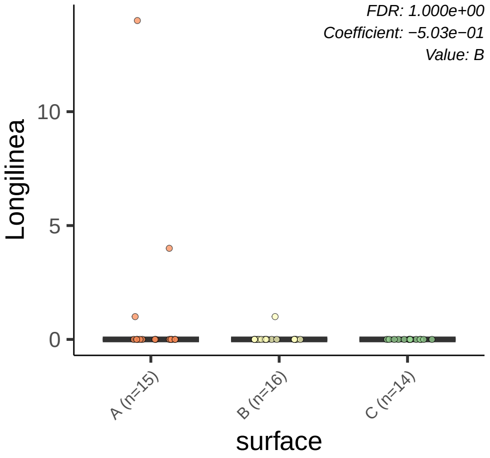
FDR: 1.000e+00
Coefficient: −5.03e−01
Value: B
Longilinea
10
5
0
A (n=15)
B (n=16)
C (n=14)
surface

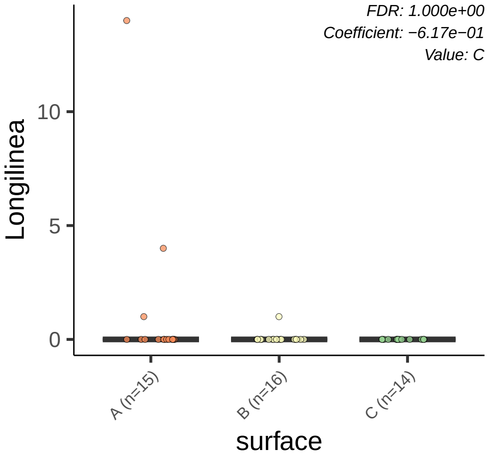

FDR: 1.000e+00
Coefficient: −6.17e−01
Value: C
Longilinea
10
5
0
A (n=15)
B (n=16)
C (n=14)
surface

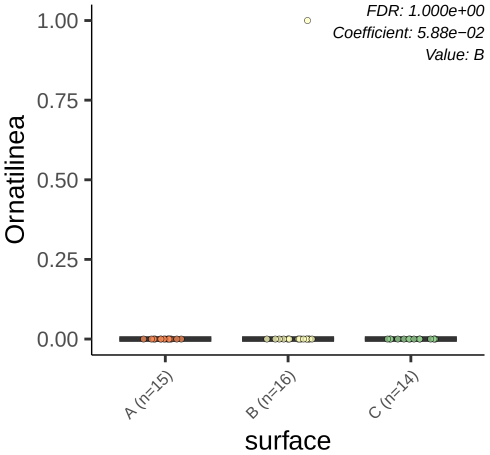
FDR: 1.000e+00
Coefficient: 5.88e−02
Value: B
Ornatilinea
1.00
0.75
0.50
0.25
0.00
A (n=15)
B (n=16)
C (n=14)
surface

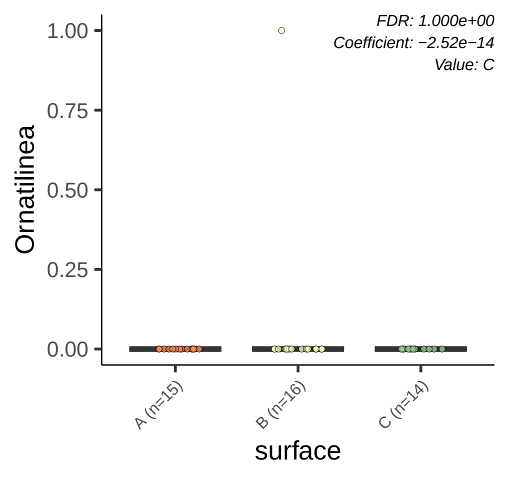

FDR: 1.000e+00
Coefficient: −2.52e−14
Value: C
Ornatilinea
1.00
0.75
0.50
0.25
0.00
A (n=15)
B (n=16)
C (n=14)
surface

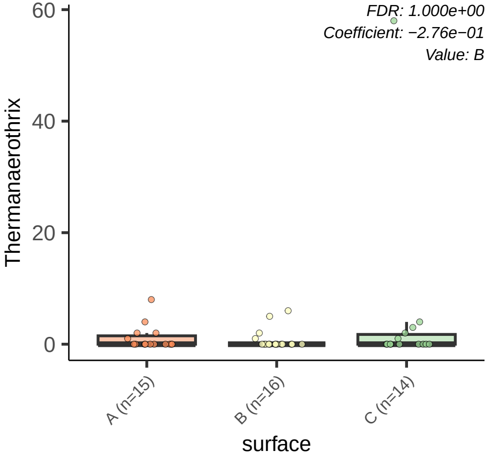

FDR: 1.000e+00
Coefficient: −2.76e−01
Value: B
Thermanaerothrix
60
40
20
0
A (n=15)
B (n=16)
C (n=14)
surface

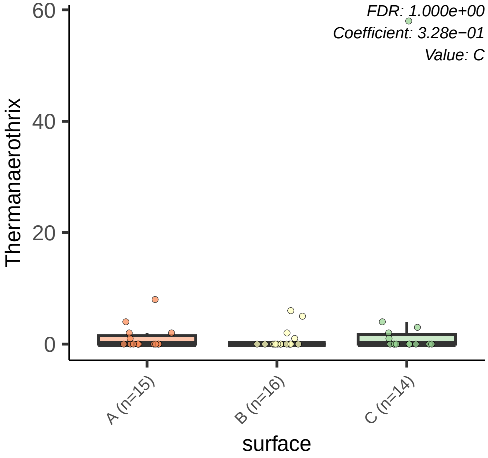

FDR: 1.000e+00
Coefficient: 3.28e−01
Value: C
Thermanaerothrix
60
40
20
0
A (n=15)
B (n=16)
C (n=14)
surface

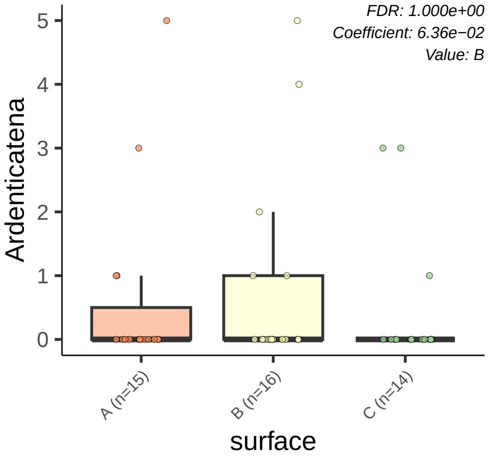
FDR: 1.000e+00
Coefficient: 6.36e−02
Value: B
Ardenticatena
0
1
2
3
4
5
A (n=15)
B (n=16)
C (n=14)
surface

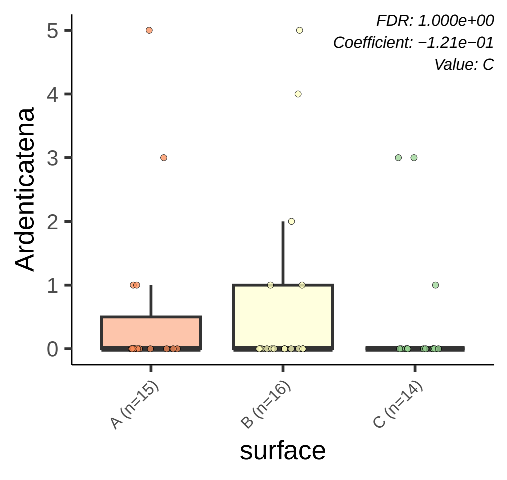
FDR: 1.000e+00
Coefficient: −1.21e−01
Value: C
Ardenticatena
0
1
2
3
4
5
A (n=15)
B (n=16)
C (n=14)
surface

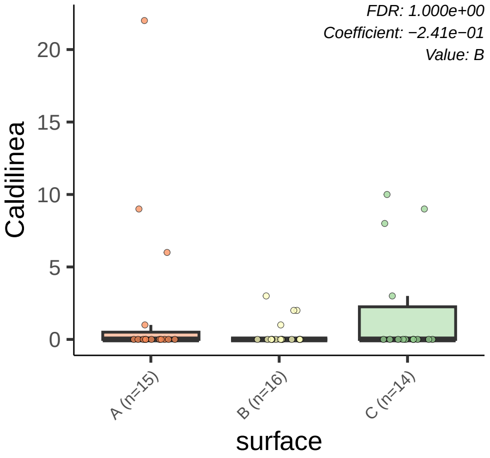

FDR: 1.000e+00
Coefficient: −2.41e−01
Value: B
Caldilinea
20
15
10
5
0
A (n=15)
B (n=16)
C (n=14)
surface

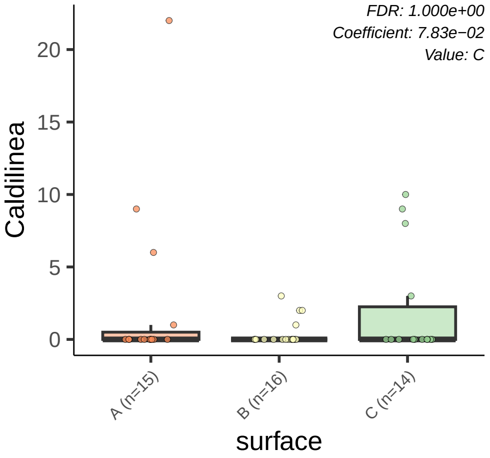
FDR: 1.000e+00
Coefficient: 7.83e−02
Value: C
Caldilinea
20
15
10
5
0
A (n=15)
B (n=16)
C (n=14)
surface

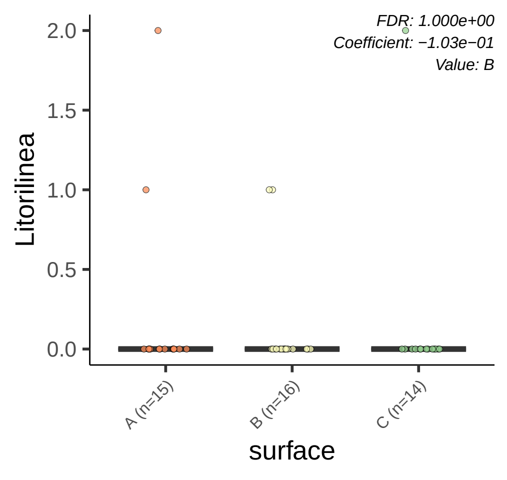
FDR: 1.000e+00
Coefficient: −1.03e−01
Value: B
Litorilinea
2.0
1.5
1.0
0.5
0.0
A (n=15)
B (n=16)
C (n=14)
surface

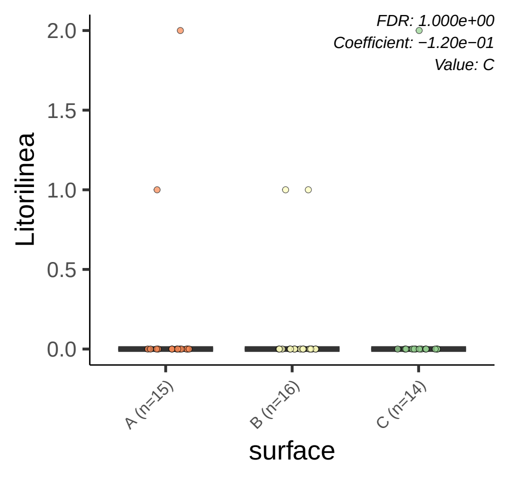

FDR: 1.000e+00
Coefficient: −1.20e−01
Value: C
Litorilinea
2.0
1.5
1.0
0.5
0.0
A (n=15)
B (n=16)
C (n=14)
surface

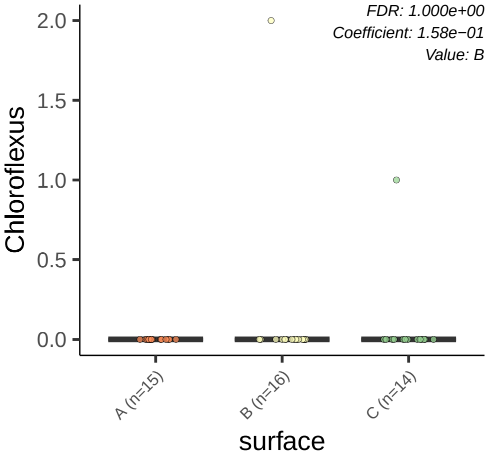
FDR: 1.000e+00
Coefficient: 1.58e−01
Value: B
Chloroflexus
2.0
1.5
1.0
0.5
0.0
A (n=15)
B (n=16)
C (n=14)
surface

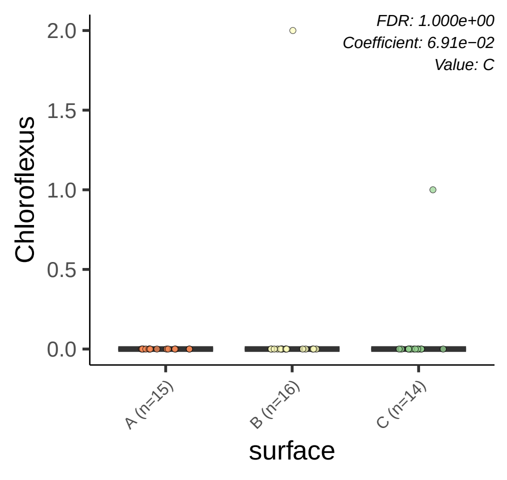

FDR: 1.000e+00
Coefficient: 6.91e−02
Value: C
Chloroflexus
2.0
1.5
1.0
0.5
0.0
A (n=15)
B (n=16)
C (n=14)
surface

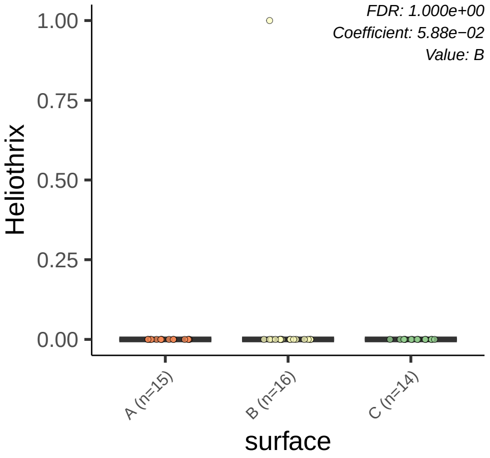
FDR: 1.000e+00
Coefficient: 5.88e−02
Value: B
Heliothrix
1.00
0.75
0.50
0.25
0.00
A (n=15)
B (n=16)
C (n=14)
surface

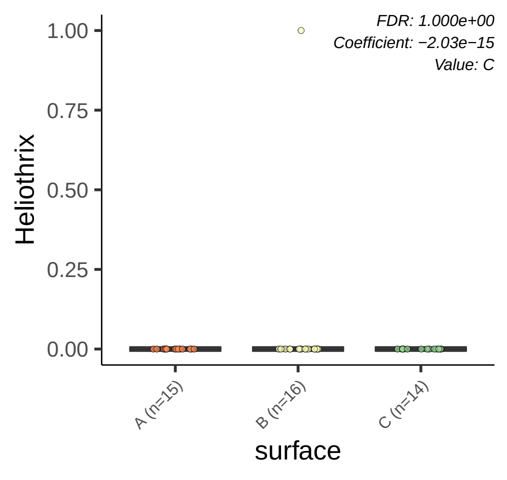
FDR: 1.000e+00
Coefficient: −2.03e−15
Value: C
Heliothrix
1.00
0.75
0.50
0.25
0.00
A (n=15)
B (n=16)
C (n=14)
surface

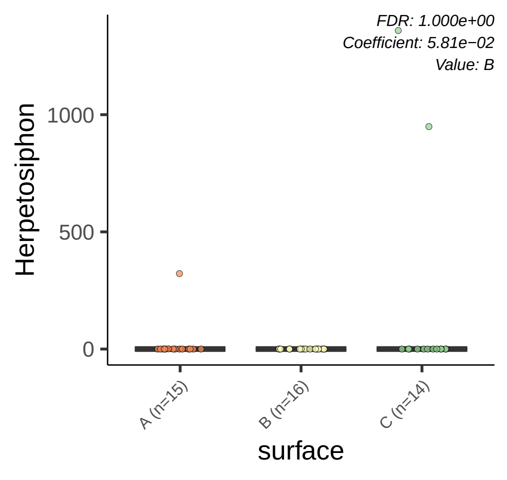
FDR: 1.000e+00
Coefficient: 5.81e−02
Value: B
Herpetosiphon
1000
500
0
A (n=15)
B (n=16)
C (n=14)
surface

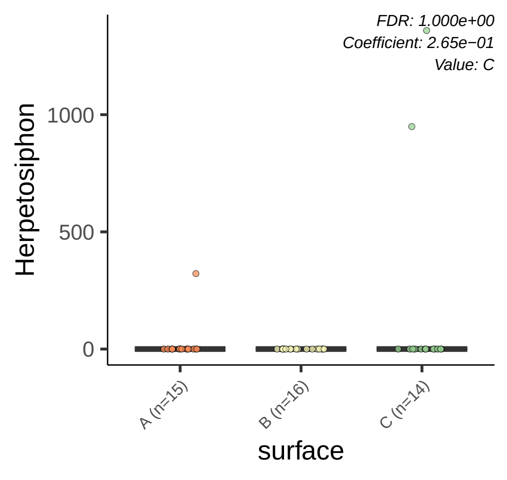
FDR: 1.000e+00
Coefficient: 2.65e−01
Value: C
Herpetosiphon
1000
500
0
A (n=15)
B (n=16)
C (n=14)
surface

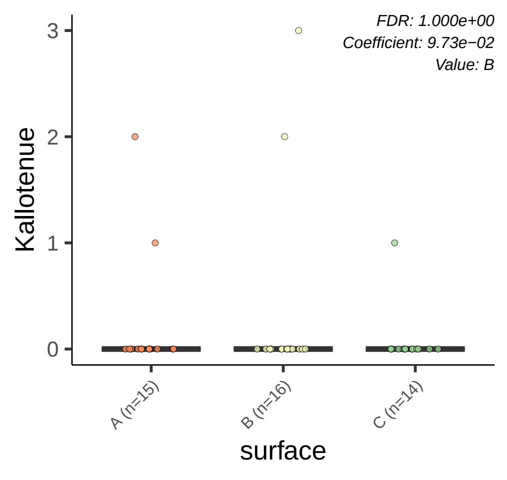

FDR: 1.000e+00
Coefficient: 9.73e−02
Value: B
Kallotenue
3
2
1
0
A (n=15)
B (n=16)
C (n=14)
surface

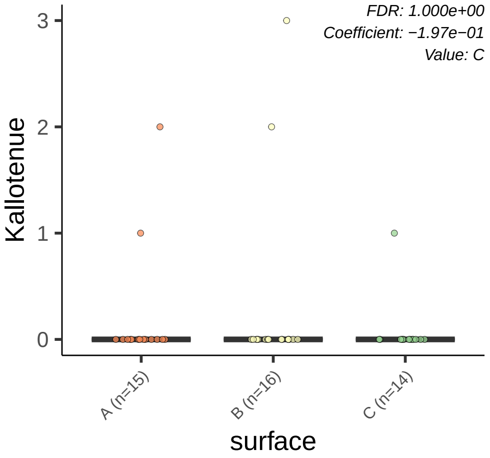

FDR: 1.000e+00
Coefficient: −1.97e−01
Value: C
Kallotenue
3
2
1
0
A (n=15)
B (n=16)
C (n=14)
surface

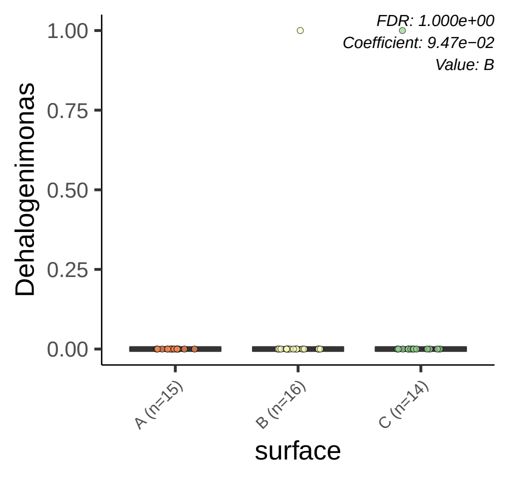
FDR: 1.000e+00
Coefficient: 9.47e−02
Value: B
Dehalogenimonas
1.00
0.75
0.50
0.25
0.00
A (n=15)
B (n=16)
C (n=14)
surface

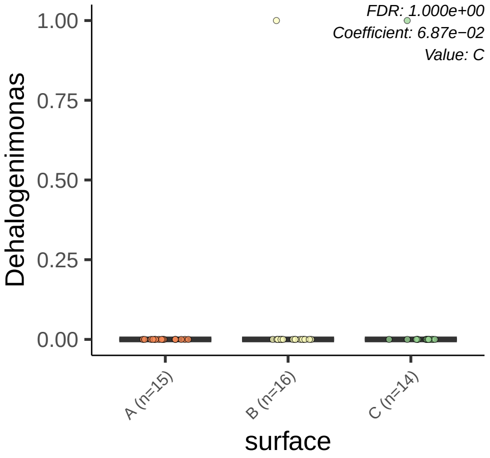

FDR: 1.000e+00
Coefficient: 6.87e−02
Value: C
Dehalogenimonas
1.00
0.75
0.50
0.25
0.00
A (n=15)
B (n=16)
C (n=14)
surface

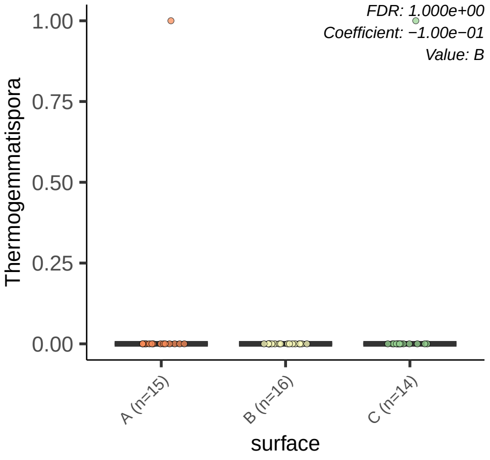

FDR: 1.000e+00
Coefficient: −1.00e−01
Value: B
Thermogemmatispora
1.00
0.75
0.50
0.25
0.00
A (n=15)
B (n=16)
C (n=14)
surface

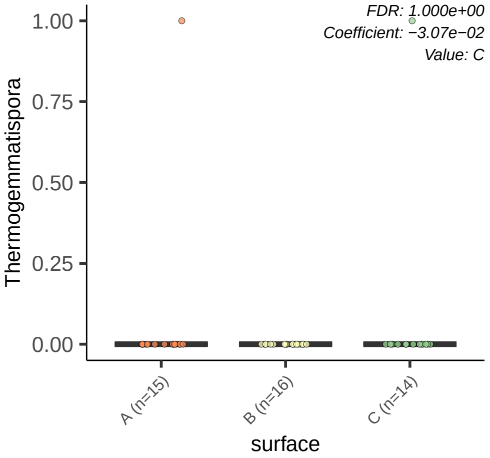

FDR: 1.000e+00
Coefficient: −3.07e−02
Value: C
Thermogemmatispora
1.00
0.75
0.50
0.25
0.00
A (n=15)
B (n=16)
C (n=14)
surface

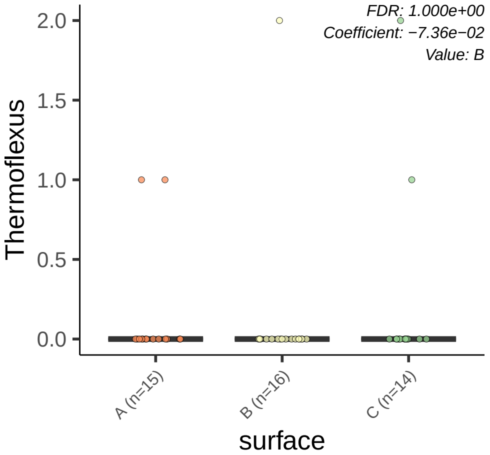

FDR: 1.000e+00
Coefficient: −7.36e−02
Value: B
Thermoflexus
2.0
1.5
1.0
0.5
0.0
A (n=15)
B (n=16)
C (n=14)
surface

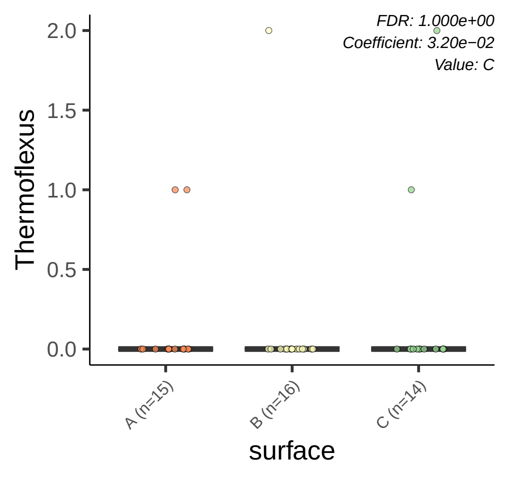
FDR: 1.000e+00
Coefficient: 3.20e−02
Value: C
Thermoflexus
2.0
1.5
1.0
0.5
0.0
A (n=15)
B (n=16)
C (n=14)
surface

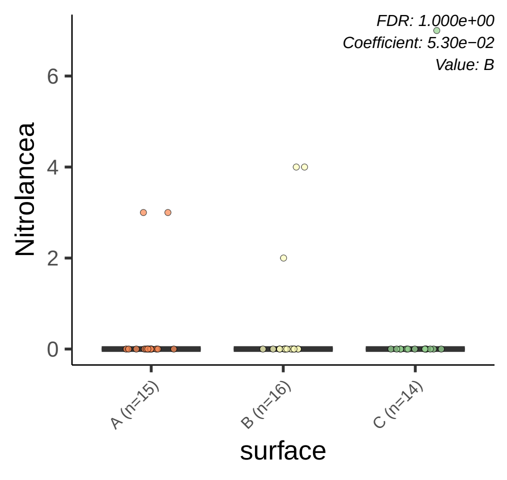

FDR: 1.000e+00
Coefficient: 5.30e−02
Value: B
Nitrolancea
0
2
4
6
A (n=15)
B (n=16)
C (n=14)
surface

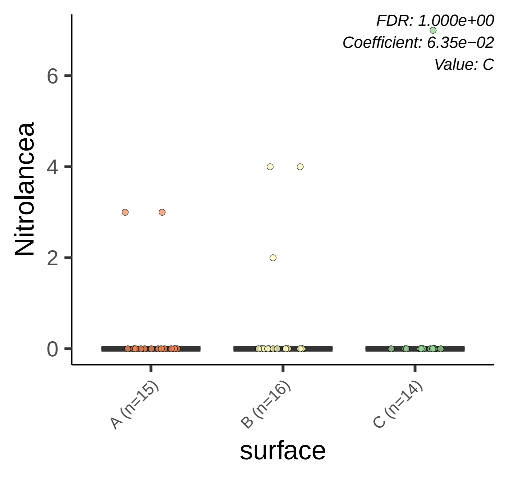
FDR: 1.000e+00
Coefficient: 6.35e−02
Value: C
Nitrolancea
0
2
4
6
A (n=15)
B (n=16)
C (n=14)
surface

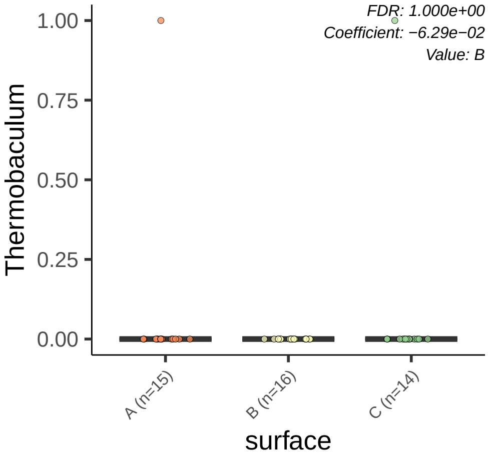

FDR: 1.000e+00
Coefficient: −6.29e−02
Value: B
Thermobaculum
1.00
0.75
0.50
0.25
0.00
A (n=15)
B (n=16)
C (n=14)
surface

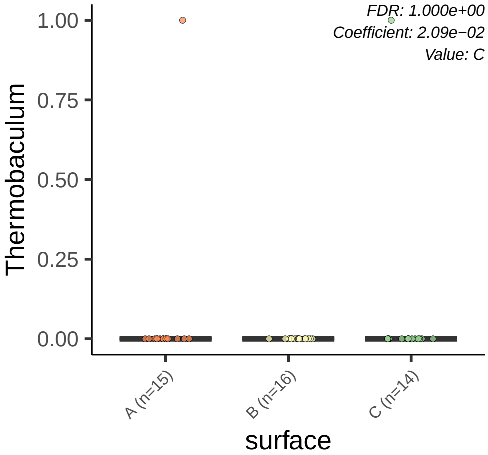
FDR: 1.000e+00
Coefficient: 2.09e−02
Value: C
Thermobaculum
1.00
0.75
0.50
0.25
0.00
A (n=15)
B (n=16)
C (n=14)
surface

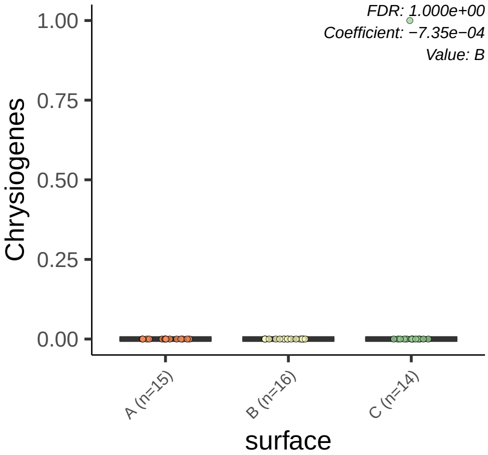

FDR: 1.000e+00
Coefficient: −7.35e−04
Value: B
Chrysiogenes
1.00
0.75
0.50
0.25
0.00
A (n=15)
B (n=16)
C (n=14)
surface

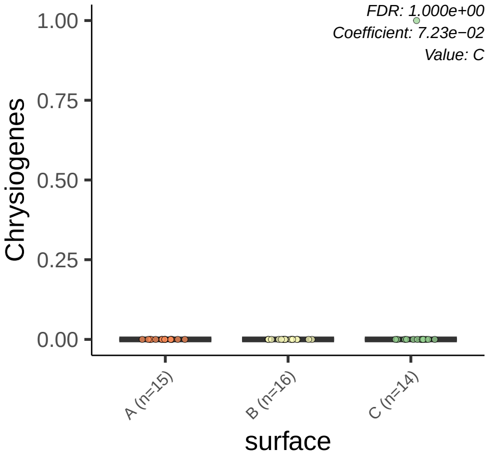
FDR: 1.000e+00
Coefficient: 7.23e−02
Value: C
Chrysiogenes
1.00
0.75
0.50
0.25
0.00
A (n=15)
B (n=16)
C (n=14)
surface

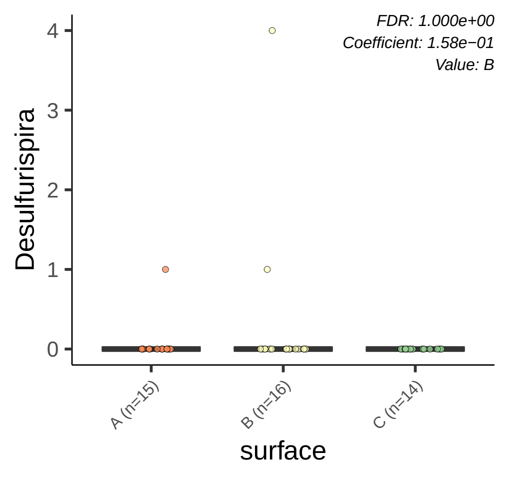
FDR: 1.000e+00
Coefficient: 1.58e−01
Value: B
Desulfurispira
0
1
2
3
4
A (n=15)
B (n=16)
C (n=14)
surface

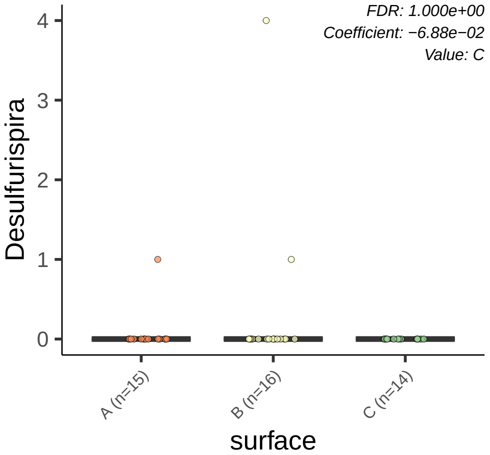
FDR: 1.000e+00
Coefficient: −6.88e−02
Value: C
Desulfurispira
0
1
2
3
4
A (n=15)
B (n=16)
C (n=14)
surface

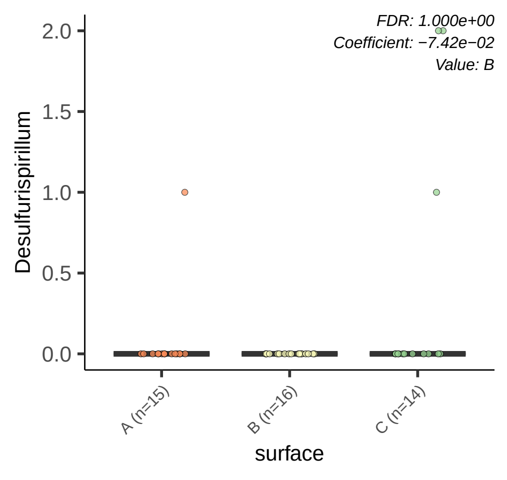
FDR: 1.000e+00
Coefficient: −7.42e−02
Value: B
Desulfurispirillum
2.0
1.5
1.0
0.5
0.0
A (n=15)
B (n=16)
C (n=14)
surface

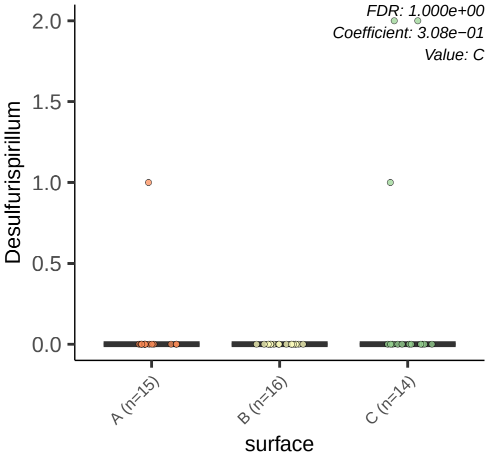

FDR: 1.000e+00
Coefficient: 3.08e−01
Value: C
Desulfurispirillum
2.0
1.5
1.0
0.5
0.0
A (n=15)
B (n=16)
C (n=14)
surface

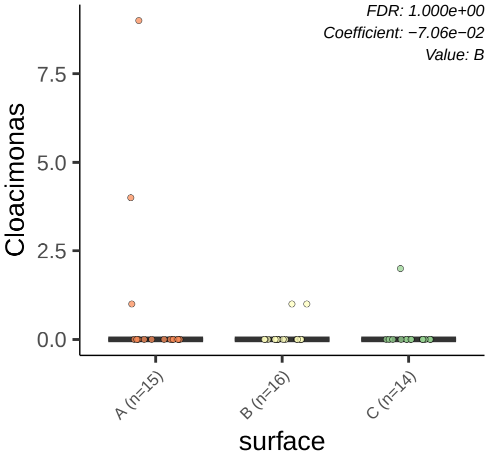
FDR: 1.000e+00
Coefficient: −7.06e−02
Value: B
Cloacimonas
7.5
5.0
2.5
0.0
A (n=15)
B (n=16)
C (n=14)
surface

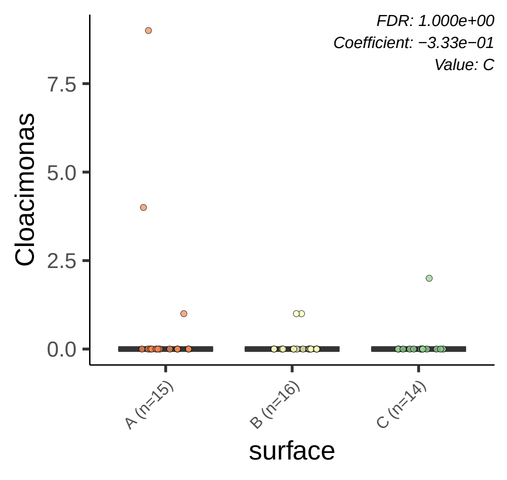

FDR: 1.000e+00
Coefficient: −3.33e−01
Value: C
Cloacimonas
7.5
5.0
2.5
0.0
A (n=15)
B (n=16)
C (n=14)
surface

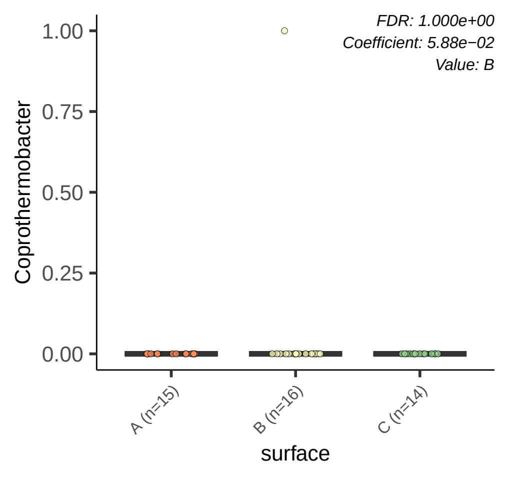

FDR: 1.000e+00
Coefficient: 5.88e−02
Value: B
Coprothermobacter
1.00
0.75
0.50
0.25
0.00
A (n=15)
B (n=16)
C (n=14)
surface

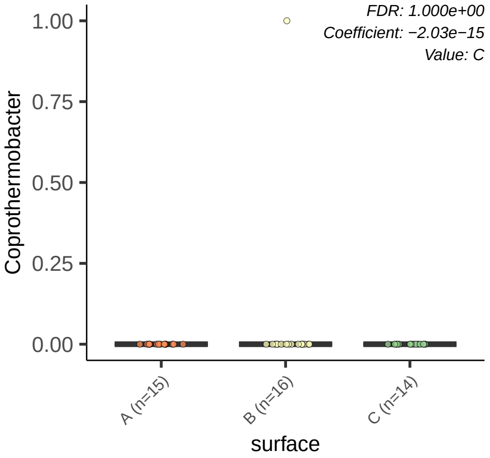
FDR: 1.000e+00
Coefficient: −2.03e−15
Value: C
Coprothermobacter
1.00
0.75
0.50
0.25
0.00
A (n=15)
B (n=16)
C (n=14)
surface

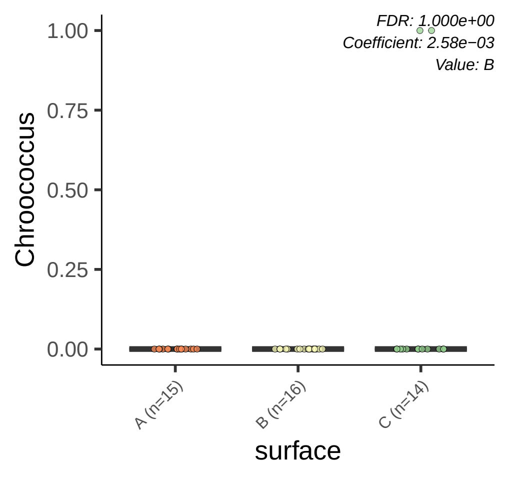

FDR: 1.000e+00
Coefficient: 2.58e−03
Value: B
1.00
0.75
0.50
0.25
0.00
Chroococcus
A (n=15)
B (n=16)
C (n=14)
surface

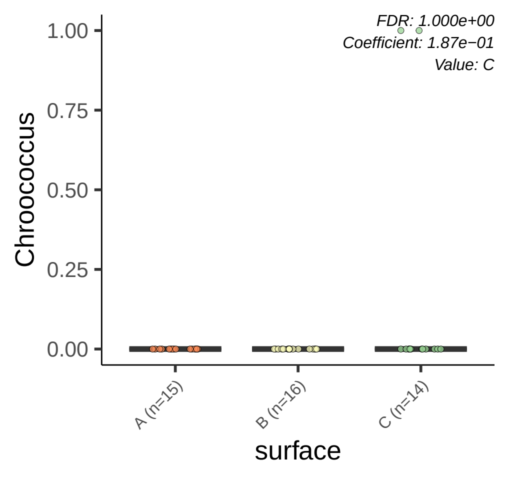
FDR: 1.000e+00
Coefficient: 1.87e−01
Value: C
Chroococcus
1.00
0.75
0.50
0.25
0.00
A (n=15)
B (n=16)
C (n=14)
surface

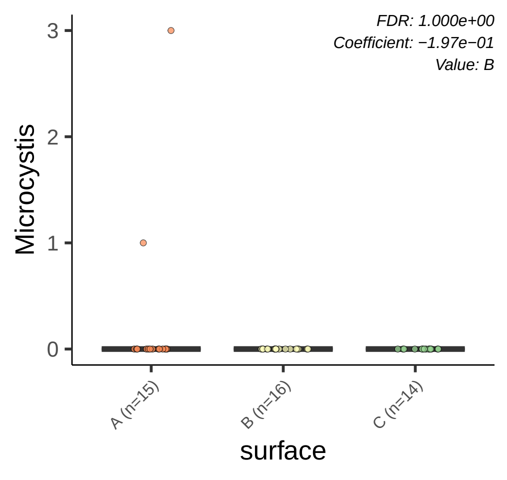
FDR: 1.000e+00
Coefficient: −1.97e−01
Value: B
Microcystis
3
2
1
0
A (n=15)
B (n=16)
C (n=14)
surface

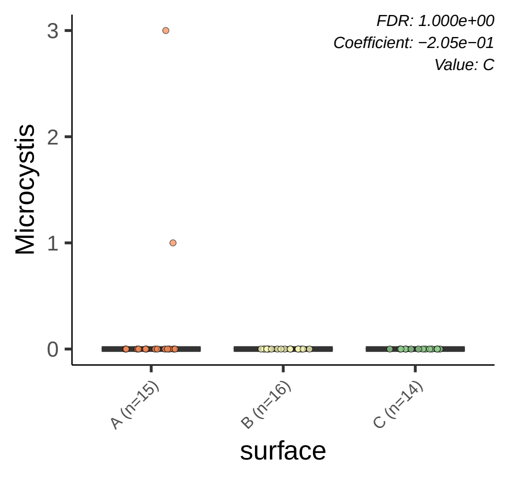
FDR: 1.000e+00
Coefficient: −2.05e−01
Value: C
Microcystis
3
2
1
0
A (n=15)
B (n=16)
C (n=14)
surface

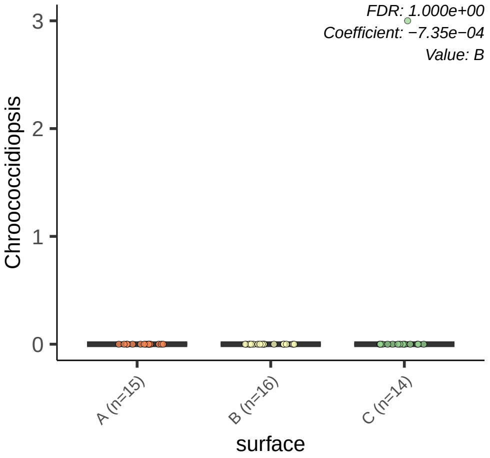

FDR: 1.000e+00
Coefficient: −7.35e−04
Value: B
Chroococcidiopsis
3
2
1
0
A (n=15)
B (n=16)
C (n=14)
surface

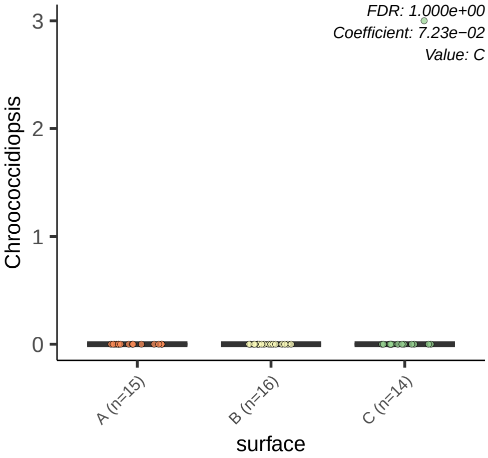

FDR: 1.000e+00
Coefficient: 7.23e−02
Value: C
Chroococcidiopsis
3
2
1
0
A (n=15)
B (n=16)
C (n=14)
surface

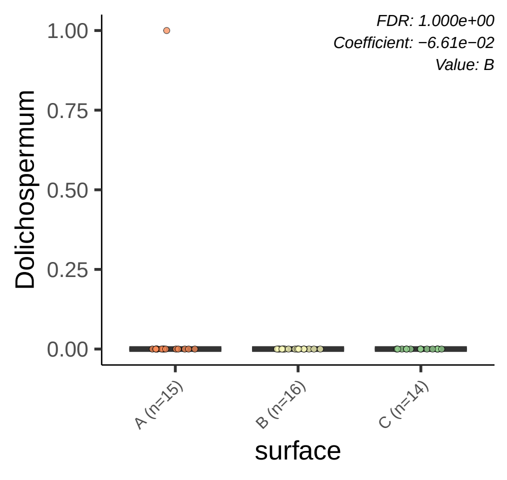
FDR: 1.000e+00
Coefficient: −6.61e−02
Value: B
Dolichospermum
1.00
0.75
0.50
0.25
0.00
A (n=15)
B (n=16)
C (n=14)
surface

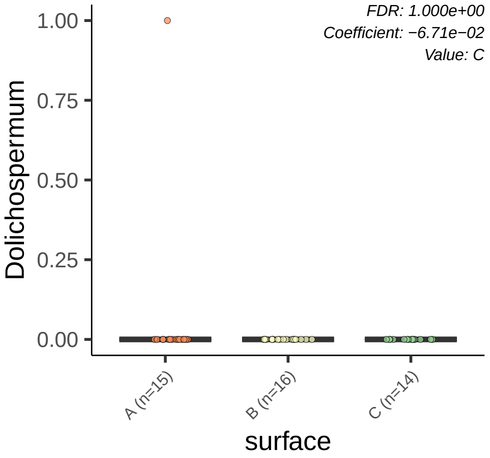
FDR: 1.000e+00
Coefficient: −6.71e−02
Value: C
Dolichospermum
1.00
0.75
0.50
0.25
0.00
A (n=15)
B (n=16)
C (n=14)
surface

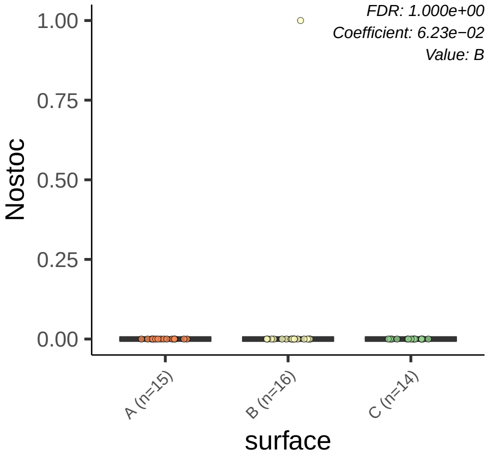
FDR: 1.000e+00
Coefficient: 6.23e−02
Value: B
1.00
0.75
0.50
0.25
0.00
Nostoc
A (n=15)
B (n=16)
C (n=14)
surface

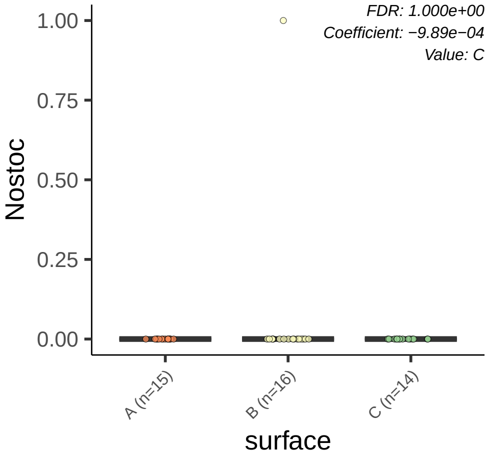
FDR: 1.000e+00
Coefficient: −9.89e−04
Value: C
Nostoc
1.00
0.75
0.50
0.25
0.00
A (n=15)
B (n=16)
C (n=14)
surface

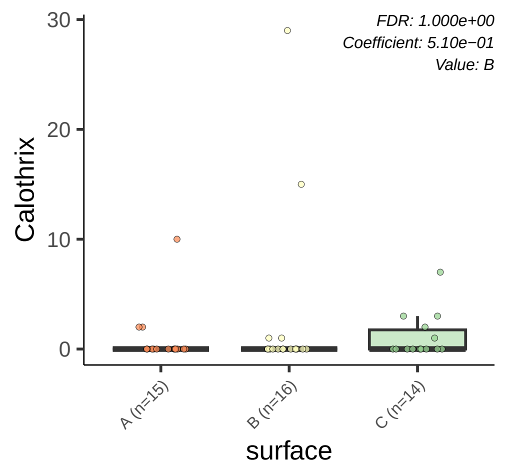
FDR: 1.000e+00
Coefficient: 5.10e−01
Value: B
Calothrix
0
10
20
30
A (n=15)
B (n=16)
C (n=14)
surface

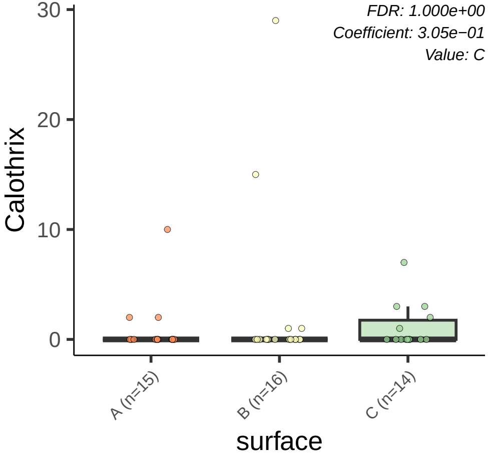

FDR: 1.000e+00
Coefficient: 3.05e−01
Value: C
Calothrix
0
10
20
30
A (n=15)
B (n=16)
C (n=14)
surface

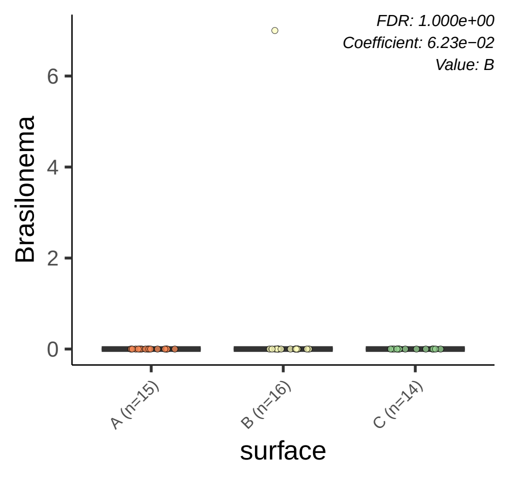

FDR: 1.000e+00
Coefficient: 6.23e−02
Value: B
Brasilonema
0
2
4
6
A (n=15)
B (n=16)
C (n=14)
surface

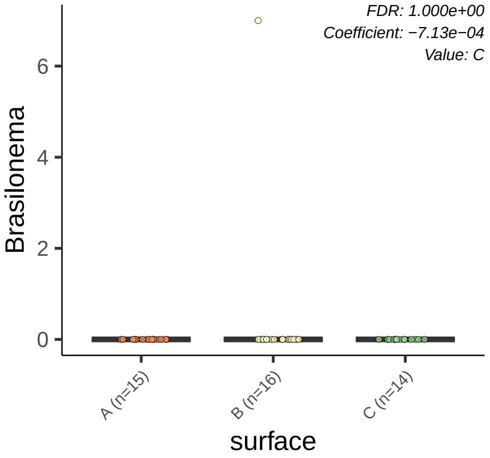
FDR: 1.000e+00
Coefficient: −7.13e−04
Value: C
Brasilonema
0
2
4
6
A (n=15)
B (n=16)
C (n=14)
surface

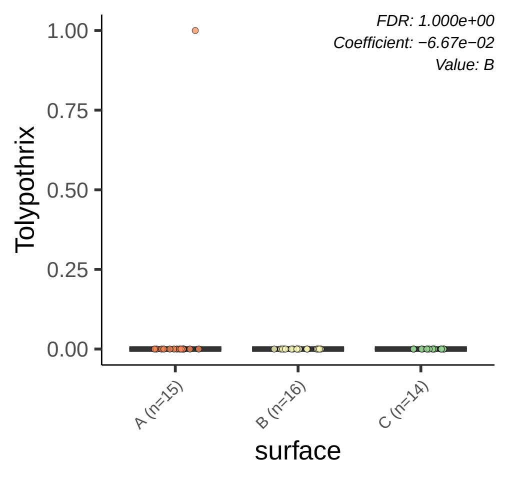

FDR: 1.000e+00
Coefficient: −6.67e−02
Value: B
Tolypothrix
1.00
0.75
0.50
0.25
0.00
A (n=15)
B (n=16)
C (n=14)
surface

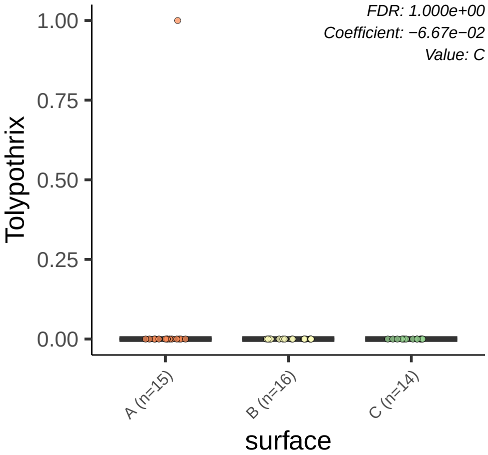
FDR: 1.000e+00
Coefficient: −6.67e−02
Value: C
Tolypothrix
1.00
0.75
0.50
0.25
0.00
A (n=15)
B (n=16)
C (n=14)
surface

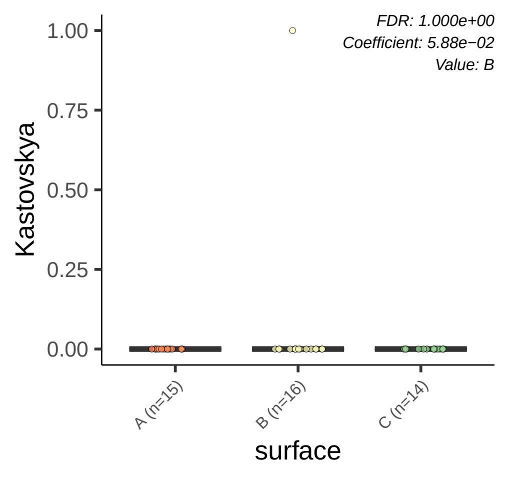
FDR: 1.000e+00
Coefficient: 5.88e−02
Value: B
Kastovskya
1.00
0.75
0.50
0.25
0.00
A (n=15)
B (n=16)
C (n=14)
surface

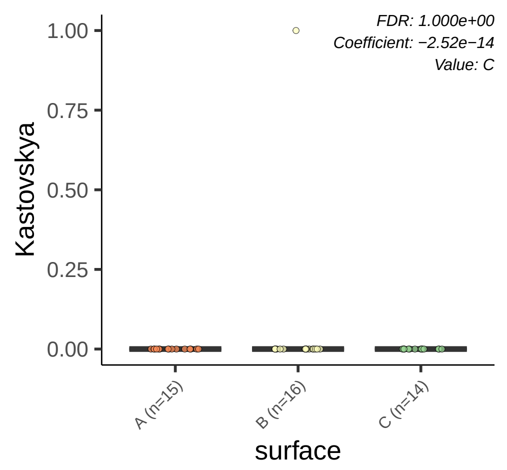
FDR: 1.000e+00
Coefficient: −2.52e−14
Value: C
1.00
0.75
0.50
0.25
0.00
Kastovskya
A (n=15)
B (n=16)
C (n=14)
surface

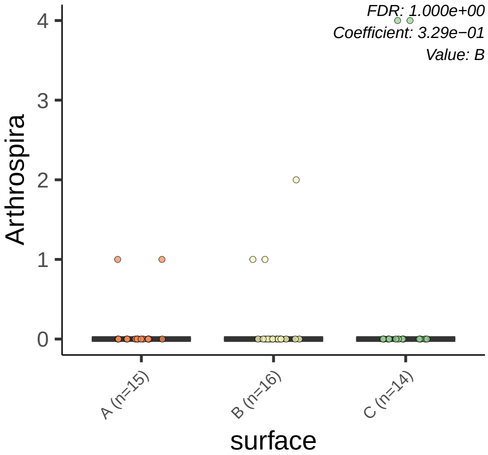

FDR: 1.000e+00
Coefficient: 3.29e−01
Value: B
Arthrospira
4
3
2
1
0
A (n=15)
B (n=16)
C (n=14)
surface

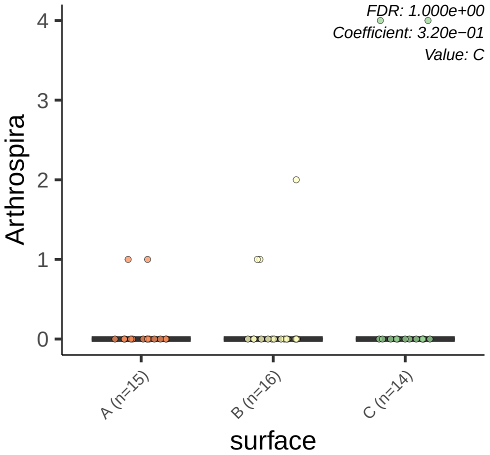

FDR: 1.000e+00
Coefficient: 3.20e−01
Value: C
Arthrospira
0
1
2
3
4
A (n=15)
B (n=16)
C (n=14)
surface

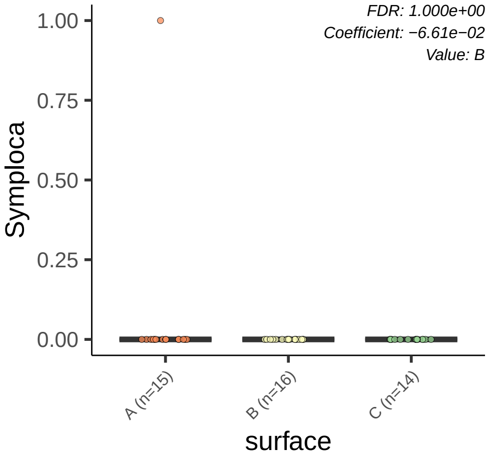

FDR: 1.000e+00
Coefficient: −6.61e−02
Value: B
Symploca
1.00
0.75
0.50
0.25
0.00
A (n=15)
B (n=16)
C (n=14)
surface

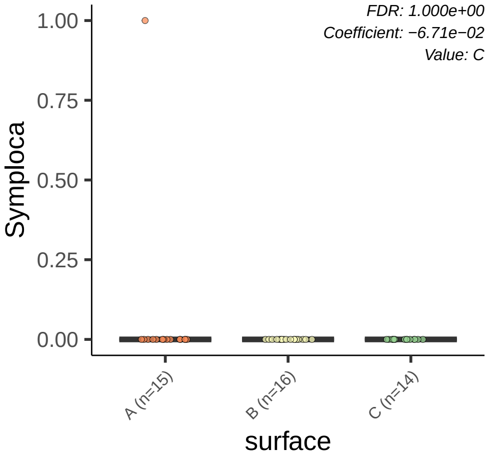

FDR: 1.000e+00
Coefficient: −6.71e−02
Value: C
Symploca
1.00
0.75
0.50
0.25
0.00
A (n=15)
B (n=16)
C (n=14)
surface

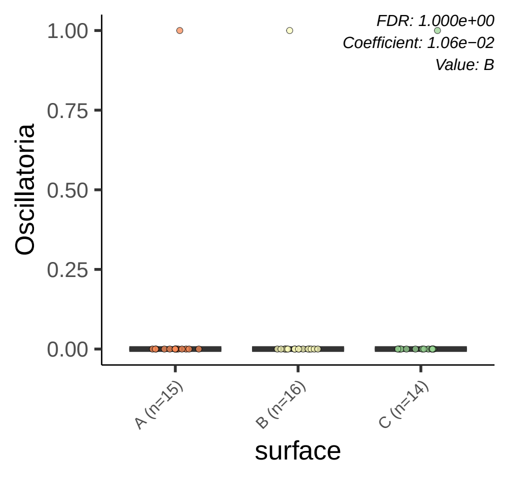
FDR: 1.000e+00
Coefficient: 1.06e−02
Value: B
Oscillatoria
1.00
0.75
0.50
0.25
0.00
A (n=15)
B (n=16)
C (n=14)
surface

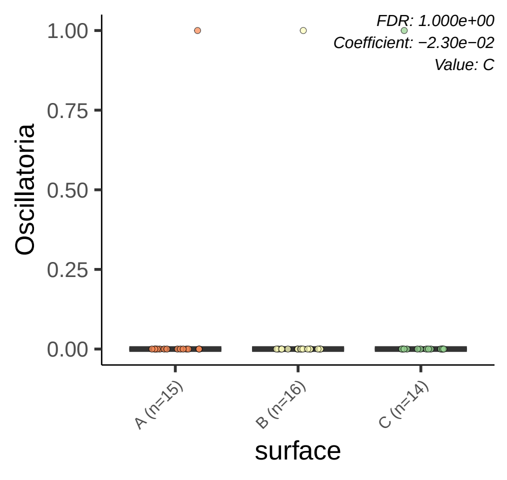
FDR: 1.000e+00
Coefficient: −2.30e−02
Value: C
Oscillatoria
1.00
0.75
0.50
0.25
0.00
A (n=15)
B (n=16)
C (n=14)
surface

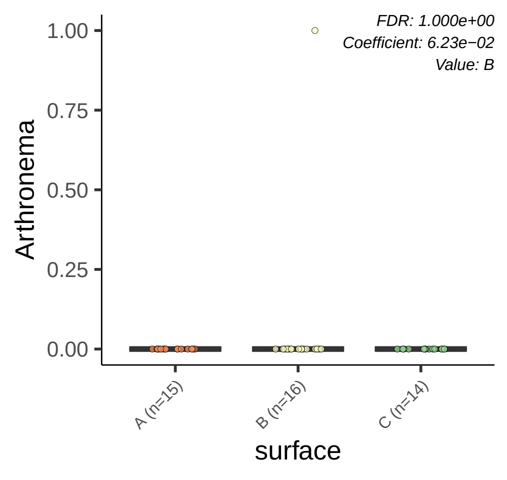
FDR: 1.000e+00
Coefficient: 6.23e−02
Value: B
1.00
0.75
0.50
0.25
0.00
Arthronema
A (n=15)
B (n=16)
C (n=14)
surface

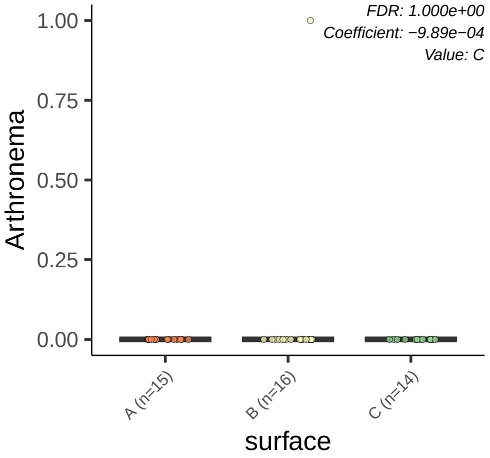
FDR: 1.000e+00
Coefficient: −9.89e−04
Value: C
Arthronema
1.00
0.75
0.50
0.25
0.00
A (n=15)
B (n=16)
C (n=14)
surface

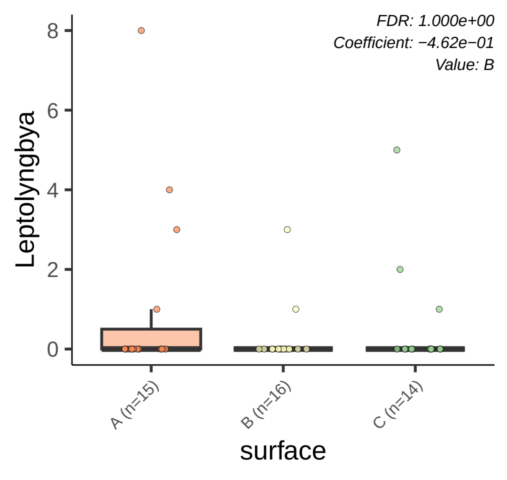
FDR: 1.000e+00
Coefficient: −4.62e−01
Value: B
Leptolyngbya
8
6
4
2
0
A (n=15)
B (n=16)
C (n=14)
surface

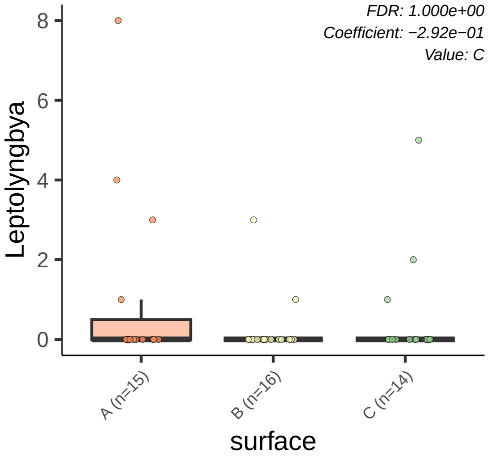
FDR: 1.000e+00
Coefficient: −2.92e−01
Value: C
Leptolyngbya
0
2
4
6
8
A (n=15)
B (n=16)
C (n=14)
surface

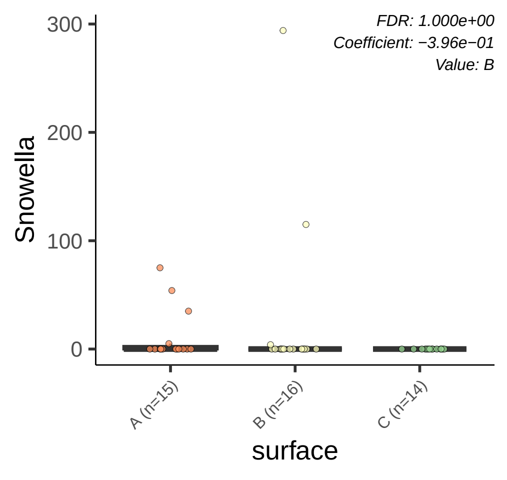

FDR: 1.000e+00
Coefficient: −3.96e−01
Value: B
Snowella
300
200
100
0
A (n=15)
B (n=16)
C (n=14)
surface

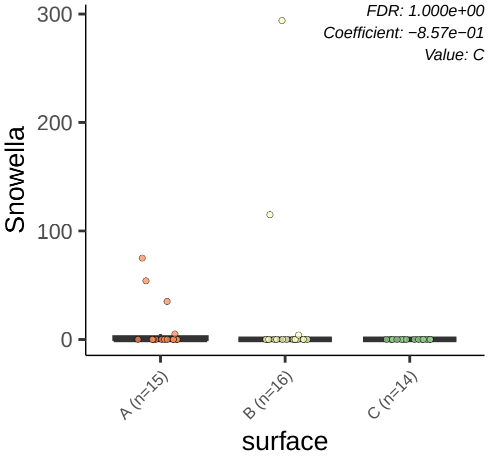
FDR: 1.000e+00
Coefficient: −8.57e−01
Value: C
Snowella
300
200
100
0
A (n=15)
B (n=16)
C (n=14)
surface

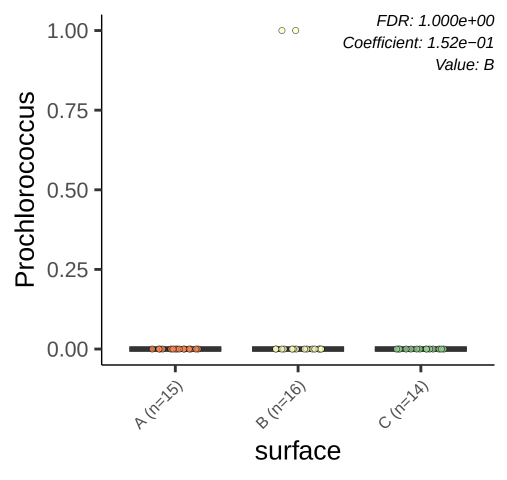
FDR: 1.000e+00
Coefficient: 1.52e−01
Value: B
Prochlorococcus
1.00
0.75
0.50
0.25
0.00
A (n=15)
B (n=16)
C (n=14)
surface

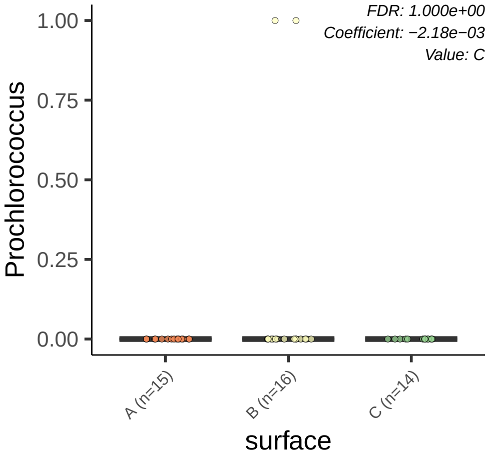
FDR: 1.000e+00
Coefficient: −2.18e−03
Value: C
Prochlorococcus
1.00
0.75
0.50
0.25
0.00
A (n=15)
B (n=16)
C (n=14)
surface

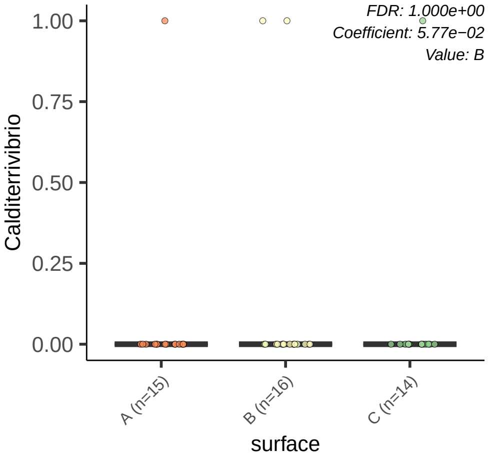

FDR: 1.000e+00
Coefficient: 5.77e−02
Value: B
Calditerrivibrio
1.00
0.75
0.50
0.25
0.00
A (n=15)
B (n=16)
C (n=14)
surface

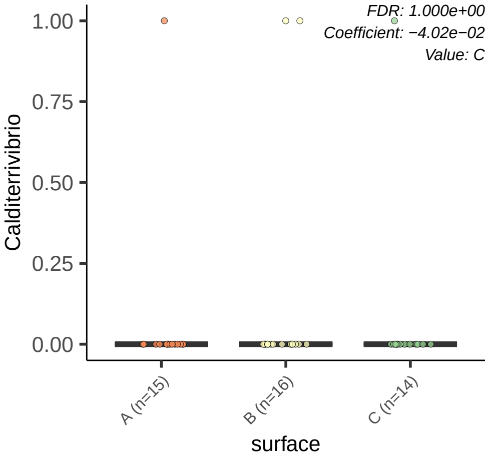

FDR: 1.000e+00
Coefficient: −4.02e−02
Value: C
Calditerrivibrio
1.00
0.75
0.50
0.25
0.00
A (n=15)
B (n=16)
C (n=14)
surface

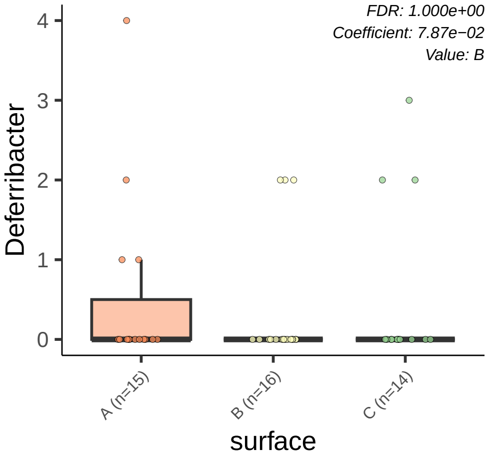

FDR: 1.000e+00
Coefficient: 7.87e−02
Value: B
Deferribacter
0
1
2
3
4
A (n=15)
B (n=16)
C (n=14)
surface

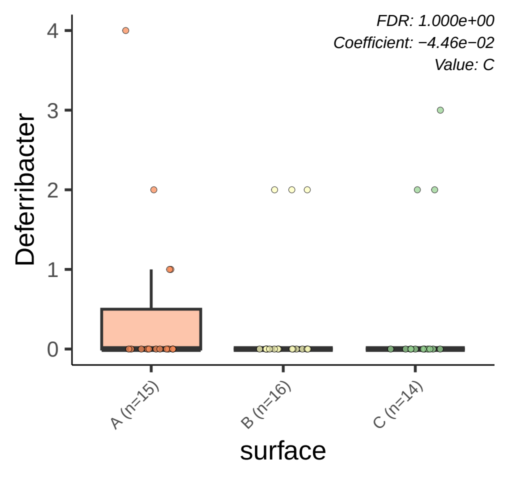

FDR: 1.000e+00
Coefficient: −4.46e−02
Value: C
Deferribacter
0
1
2
3
4
A (n=15)
B (n=16)
C (n=14)
surface

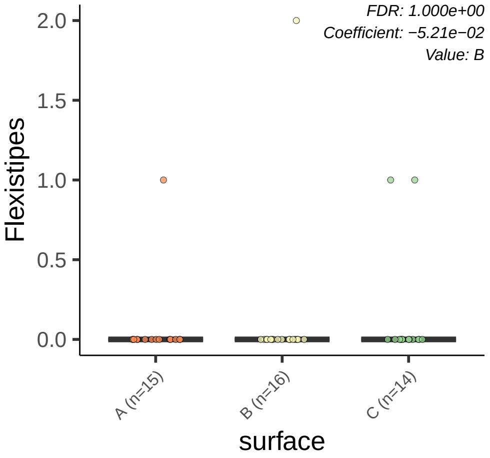

FDR: 1.000e+00
Coefficient: −5.21e−02
Value: B
Flexistipes
2.0
1.5
1.0
0.5
0.0
A (n=15)
B (n=16)
C (n=14)
surface

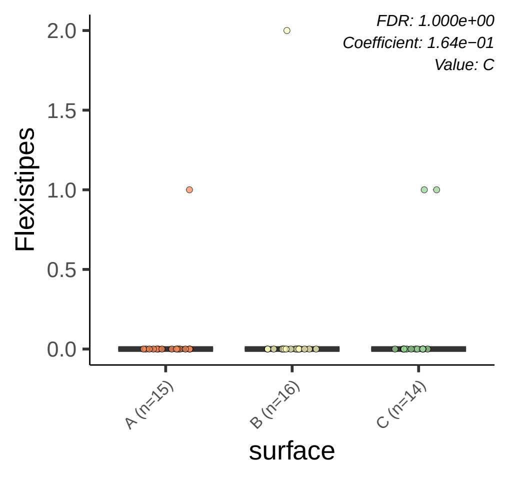

FDR: 1.000e+00
Coefficient: 1.64e−01
Value: C
Flexistipes
2.0
1.5
1.0
0.5
0.0
A (n=15)
B (n=16)
C (n=14)
surface

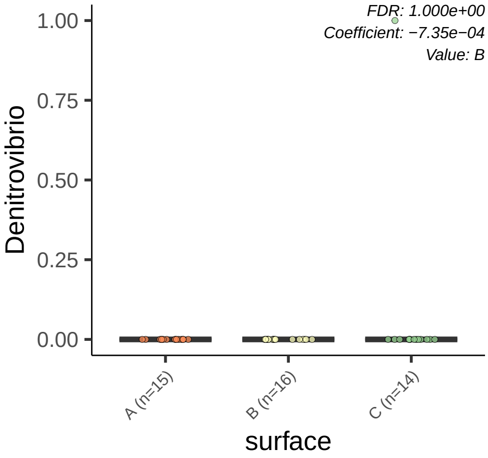

FDR: 1.000e+00
Coefficient: −7.35e−04
Value: B
Denitrovibrio
1.00
0.75
0.50
0.25
0.00
A (n=15)
B (n=16)
C (n=14)
surface

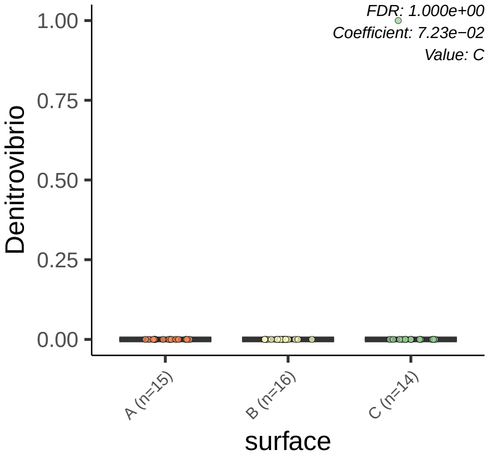

FDR: 1.000e+00
Coefficient: 7.23e−02
Value: C
Denitrovibrio
1.00
0.75
0.50
0.25
0.00
A (n=15)
B (n=16)
C (n=14)
surface

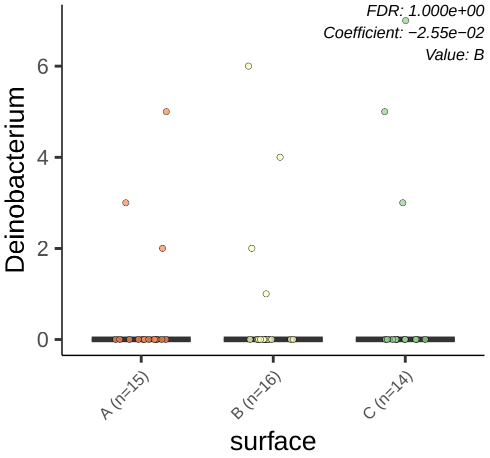

FDR: 1.000e+00
Coefficient: −2.55e−02
Value: B
Deinobacterium
0
2
4
6
A (n=15)
B (n=16)
C (n=14)
surface

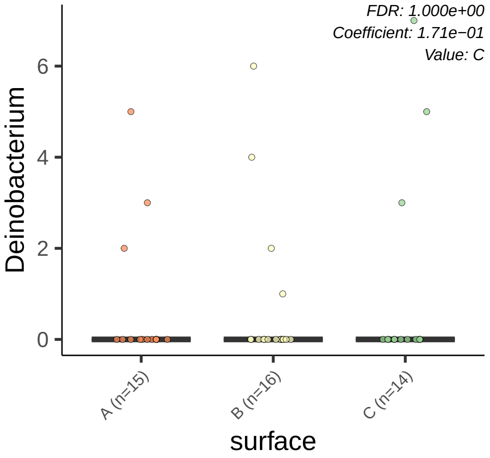
FDR: 1.000e+00
Coefficient: 1.71e−01
Value: C
Deinobacterium
0
2
4
6
A (n=15)
B (n=16)
C (n=14)
surface

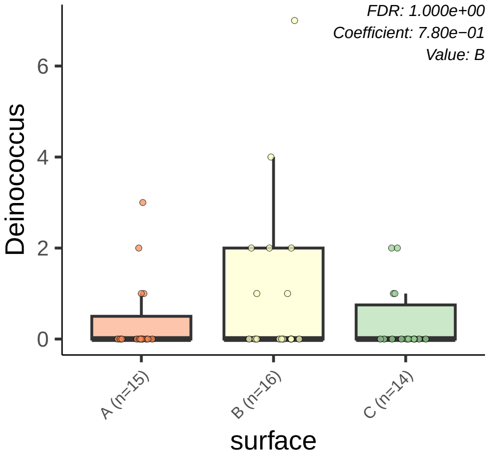

FDR: 1.000e+00
Coefficient: 7.80e−01
Value: B
Deinococcus
0
2
4
6
A (n=15)
B (n=16)
C (n=14)
surface

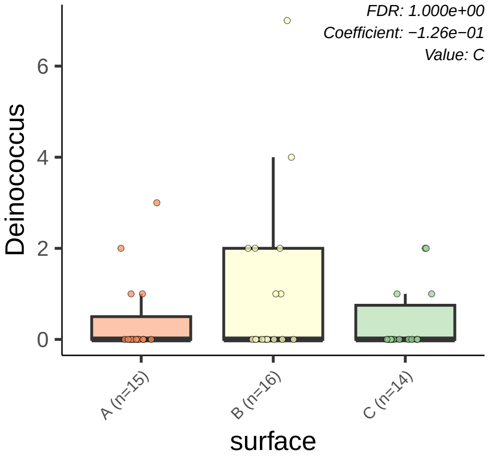

FDR: 1.000e+00
Coefficient: −1.26e−01
Value: C
Deinococcus
0
2
4
6
A (n=15)
B (n=16)
C (n=14)
surface

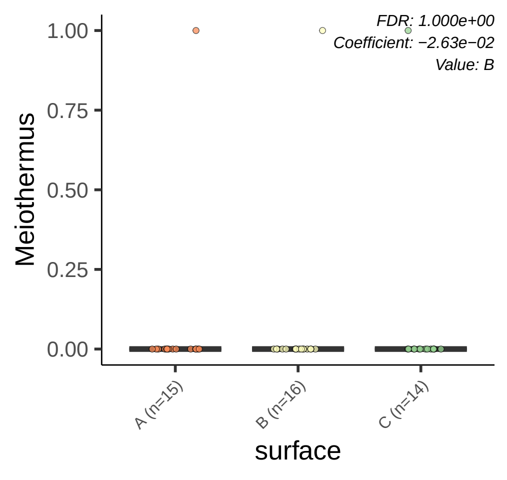
FDR: 1.000e+00
Coefficient: −2.63e−02
Value: B
Meiothermus
1.00
0.75
0.50
0.25
0.00
A (n=15)
B (n=16)
C (n=14)
surface

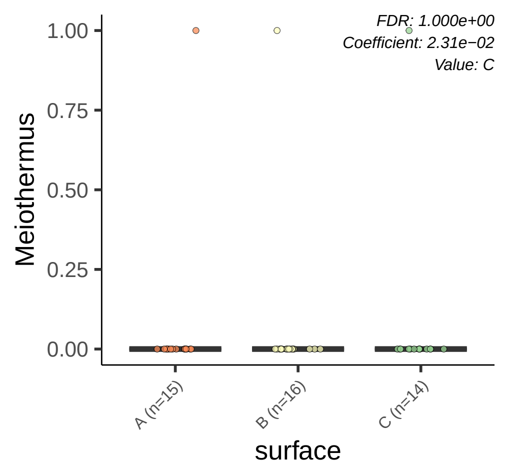

FDR: 1.000e+00
Coefficient: 2.31e−02
Value: C
Meiothermus
1.00
0.75
0.50
0.25
0.00
A (n=15)
B (n=16)
C (n=14)
surface

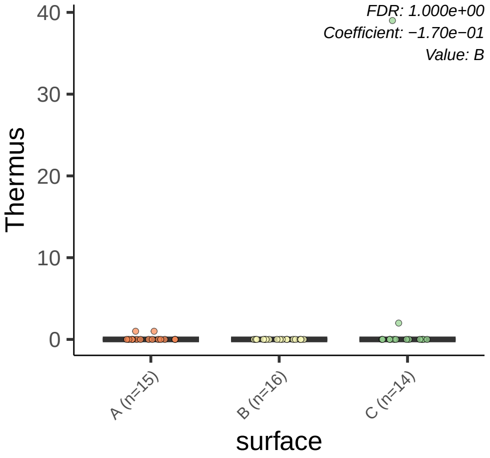
FDR: 1.000e+00
Coefficient: −1.70e−01
Value: B
Thermus
40
30
20
10
0
A (n=15)
B (n=16)
C (n=14)
surface

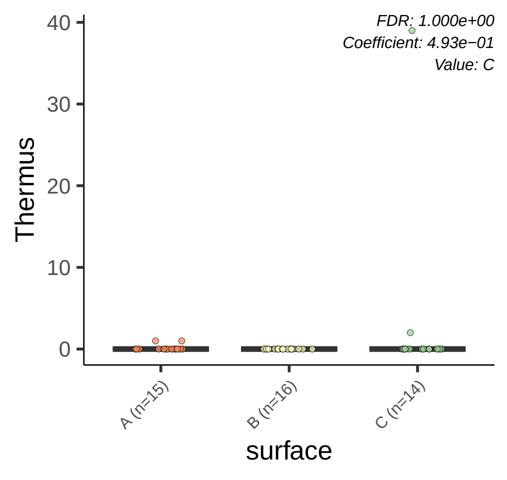
FDR: 1.000e+00
Coefficient: 4.93e−01
Value: C
Thermus
0
10
20
30
40
A (n=15)
B (n=16)
C (n=14)
surface

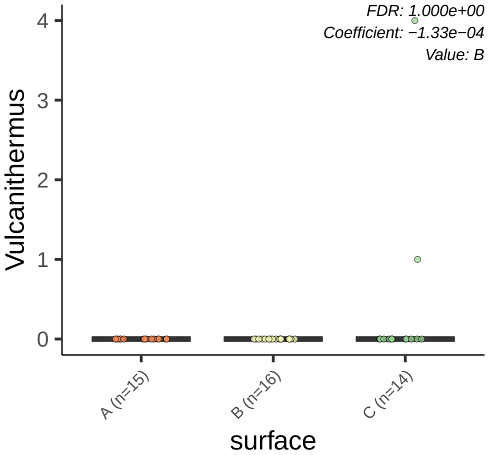

FDR: 1.000e+00
Coefficient: −1.33e−04
Value: B
Vulcanithermus
0
1
2
3
4
A (n=15)
B (n=16)
C (n=14)
surface

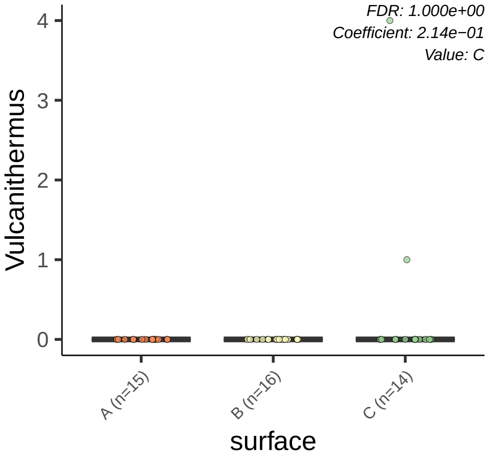
FDR: 1.000e+00
Coefficient: 2.14e−01
Value: C
Vulcanithermus
0
1
2
3
4
A (n=15)
B (n=16)
C (n=14)
surface

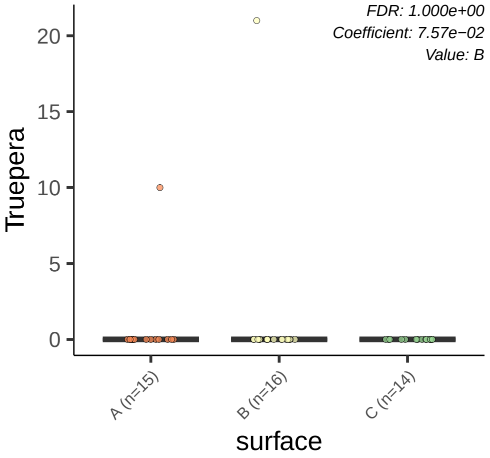

FDR: 1.000e+00
Coefficient: 7.57e−02
Value: B
Truepera
20
15
10
5
0
A (n=15)
B (n=16)
C (n=14)
surface

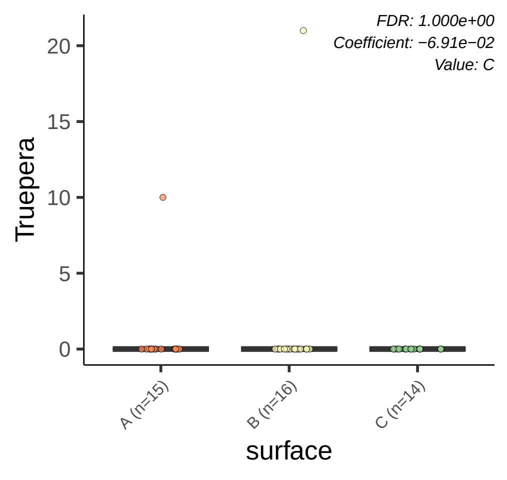

FDR: 1.000e+00
Coefficient: −6.91e−02
Value: C
Truepera
20
15
10
5
0
A (n=15)
B (n=16)
C (n=14)
surface

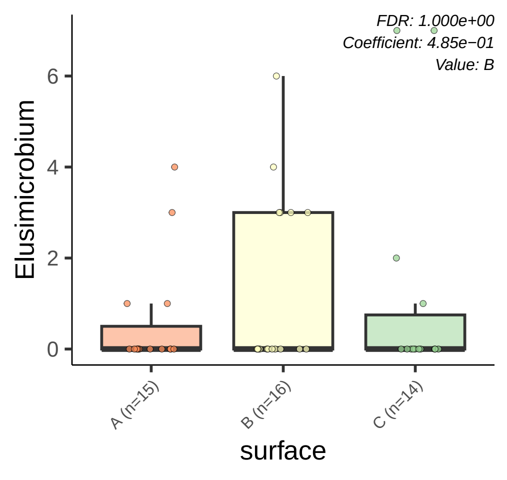

FDR: 1.000e+00
Coefficient: 4.85e−01
Value: B
Elusimicrobium
0
2
4
6
A (n=15)
B (n=16)
C (n=14)
surface

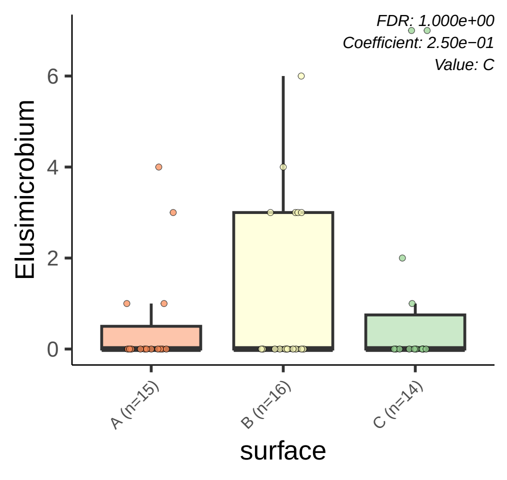

FDR: 1.000e+00
Coefficient: 2.50e−01
Value: C
Elusimicrobium
0
2
4
6
A (n=15)
B (n=16)
C (n=14)
surface

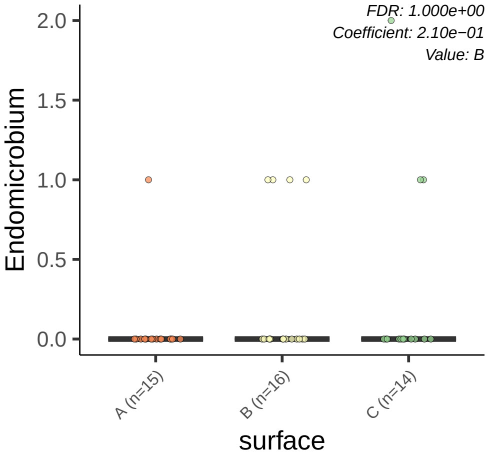
FDR: 1.000e+00
Coefficient: 2.10e−01
Value: B
Endomicrobium
2.0
1.5
1.0
0.5
0.0
A (n=15)
B (n=16)
C (n=14)
surface

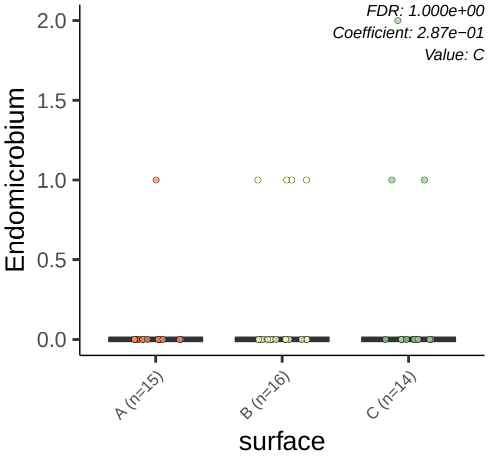

FDR: 1.000e+00
Coefficient: 2.87e−01
Value: C
Endomicrobium
2.0
1.5
1.0
0.5
0.0
A (n=15)
B (n=16)
C (n=14)
surface

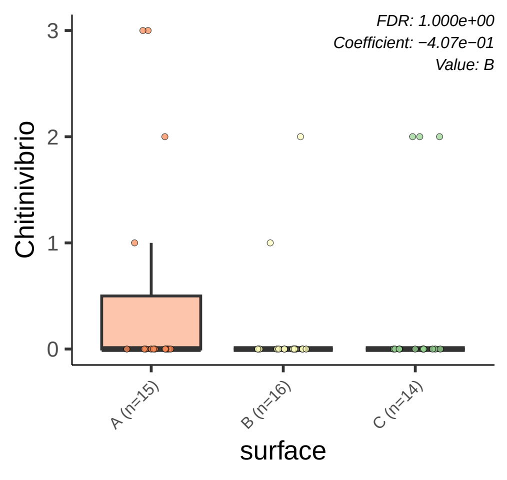
FDR: 1.000e+00
Coefficient: −4.07e−01
Value: B
Chitinivibrio
3
2
1
0
A (n=15)
B (n=16)
C (n=14)
surface

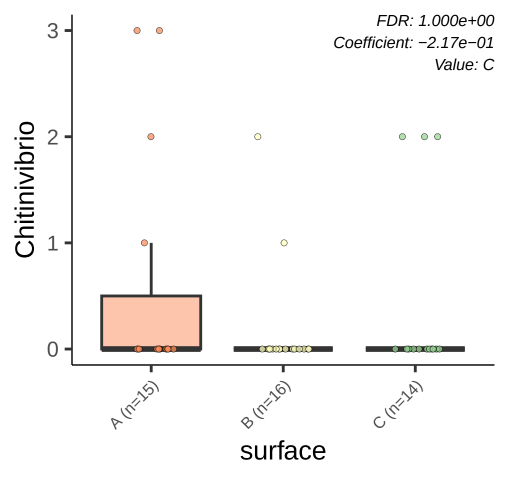
FDR: 1.000e+00
Coefficient: −2.17e−01
Value: C
Chitinivibrio
3
2
1
0
A (n=15)
B (n=16)
C (n=14)
surface

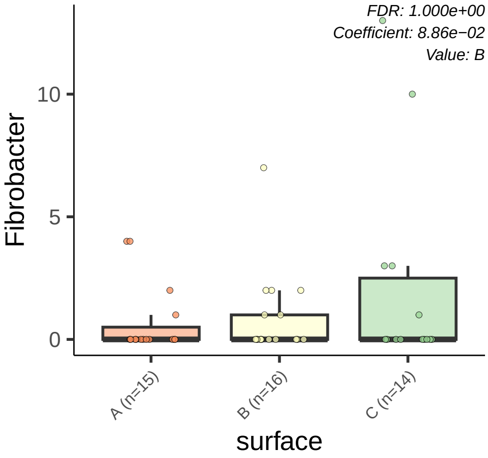

FDR: 1.000e+00
Coefficient: 8.86e−02
Value: B
Fibrobacter
10
5
0
A (n=15)
B (n=16)
C (n=14)
surface

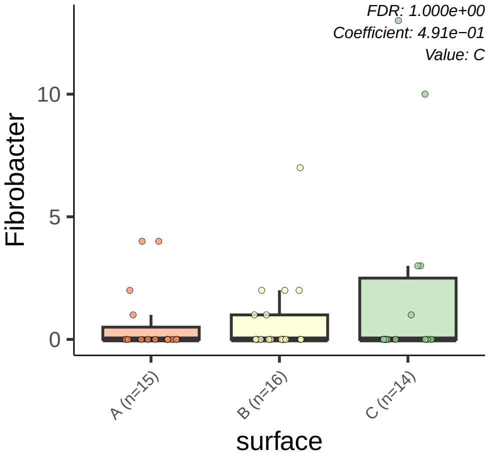

FDR: 1.000e+00
Coefficient: 4.91e−01
Value: C
Fibrobacter
0
5
10
A (n=15)
B (n=16)
C (n=14)
surface

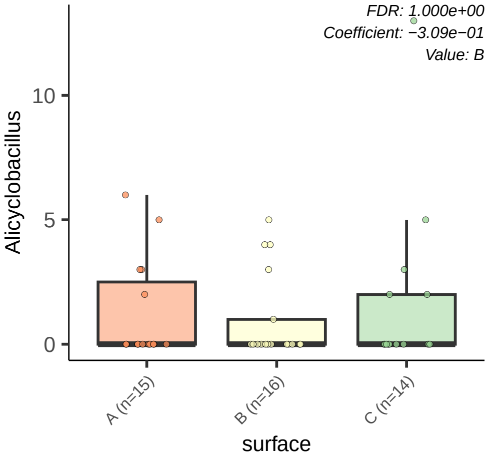
FDR: 1.000e+00
Coefficient: −3.09e−01
Value: B
Alicyclobacillus
10
5
0
A (n=15)
B (n=16)
C (n=14)
surface

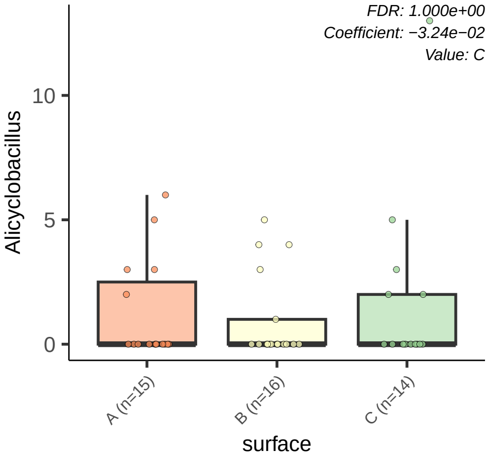

FDR: 1.000e+00
Coefficient: −3.24e−02
Value: C
Alicyclobacillus
10
5
0
A (n=15)
B (n=16)
C (n=14)
surface

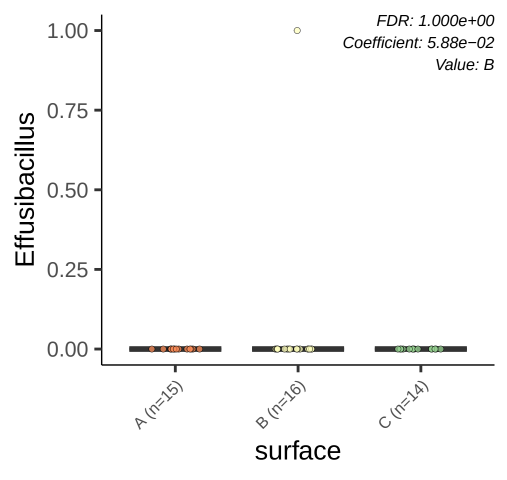

FDR: 1.000e+00
Coefficient: 5.88e−02
Value: B
Effusibacillus
1.00
0.75
0.50
0.25
0.00
A (n=15)
B (n=16)
C (n=14)
surface

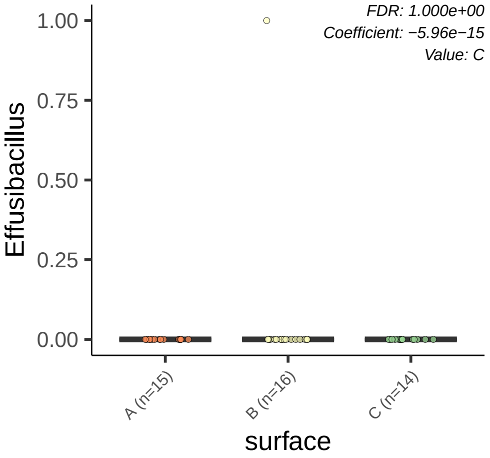
FDR: 1.000e+00
Coefficient: −5.96e−15
Value: C
Effusibacillus
1.00
0.75
0.50
0.25
0.00
A (n=15)
B (n=16)
C (n=14)
surface

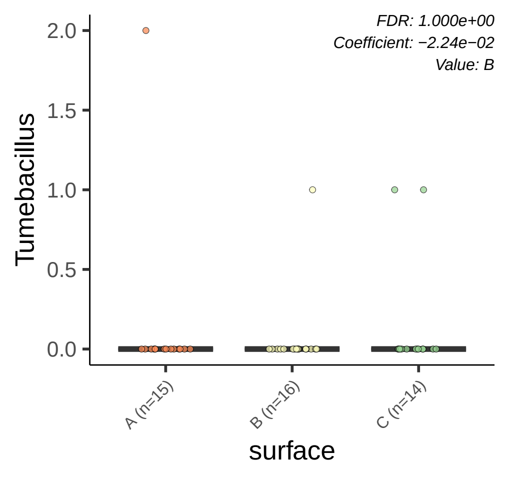
FDR: 1.000e+00
Coefficient: −2.24e−02
Value: B
Tumebacillus
2.0
1.5
1.0
0.5
0.0
A (n=15)
B (n=16)
C (n=14)
surface

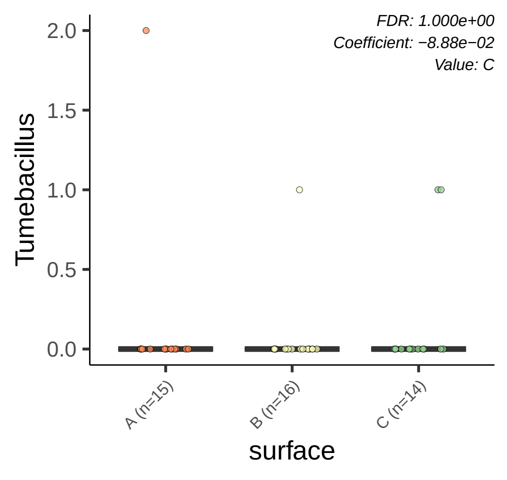

FDR: 1.000e+00
Coefficient: −8.88e−02
Value: C
Tumebacillus
2.0
1.5
1.0
0.5
0.0
A (n=15)
B (n=16)
C (n=14)
surface

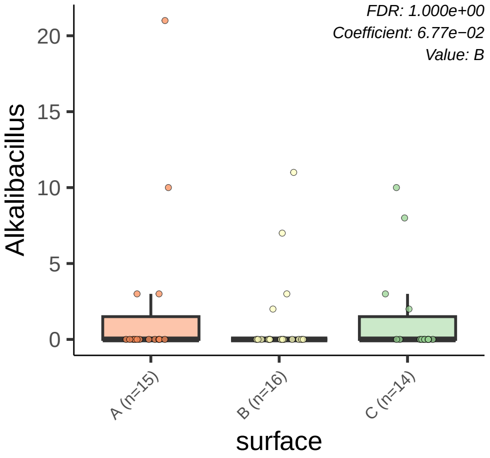

FDR: 1.000e+00
Coefficient: 6.77e−02
Value: B
Alkalibacillus
0
5
10
15
20
A (n=15)
B (n=16)
C (n=14)
surface

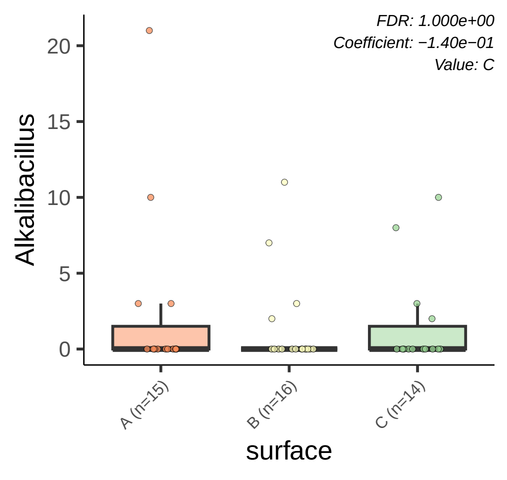

FDR: 1.000e+00
Coefficient: −1.40e−01
Value: C
Alkalibacillus
20
15
10
5
0
A (n=15)
B (n=16)
C (n=14)
surface

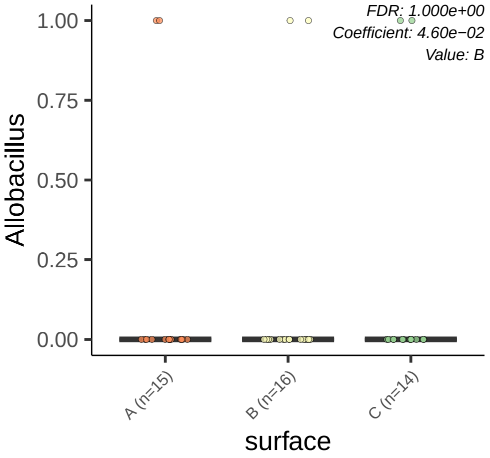
FDR: 1.000e+00
Coefficient: 4.60e−02
Value: B
1.00
0.75
0.50
0.25
0.00
Allobacillus
A (n=15)
B (n=16)
C (n=14)
surface

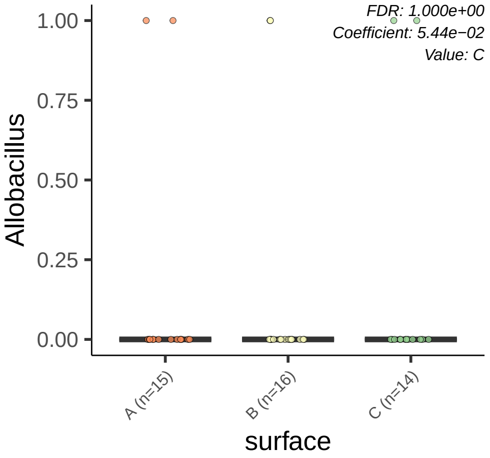

FDR: 1.000e+00
Coefficient: 5.44e−02
Value: C
Allobacillus
1.00
0.75
0.50
0.25
0.00
A (n=15)
B (n=16)
C (n=14)
surface

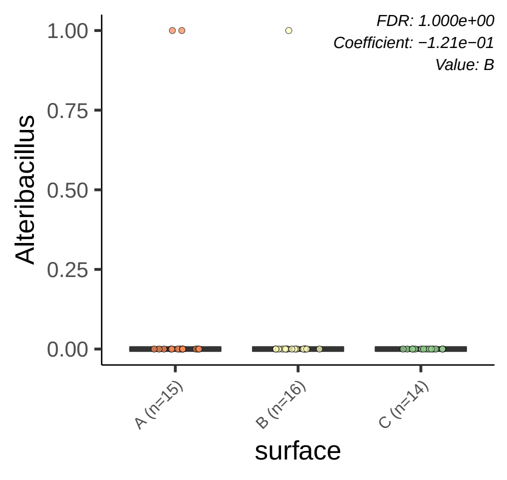

FDR: 1.000e+00
Coefficient: −1.21e−01
Value: B
Alteribacillus
1.00
0.75
0.50
0.25
0.00
A (n=15)
B (n=16)
C (n=14)
surface

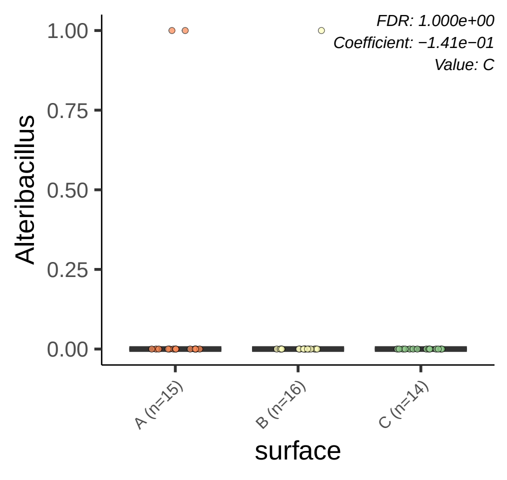
FDR: 1.000e+00
Coefficient: −1.41e−01
Value: C
Alteribacillus
1.00
0.75
0.50
0.25
0.00
A (n=15)
B (n=16)
C (n=14)
surface

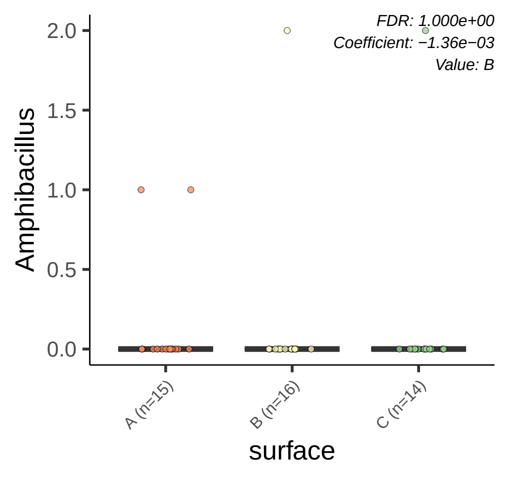
FDR: 1.000e+00
Coefficient: −1.36e−03
Value: B
Amphibacillus
2.0
1.5
1.0
0.5
0.0
A (n=15)
B (n=16)
C (n=14)
surface

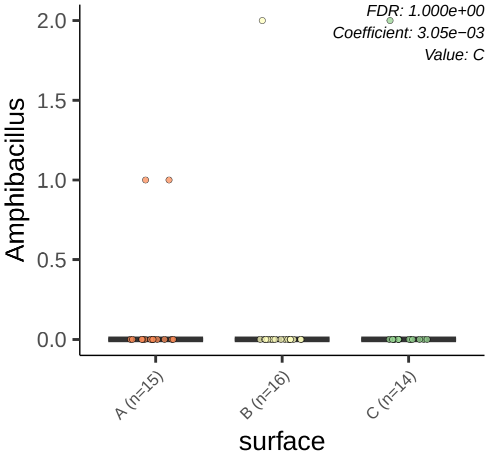
FDR: 1.000e+00
Coefficient: 3.05e−03
Value: C
Amphibacillus
2.0
1.5
1.0
0.5
0.0
A (n=15)
B (n=16)
C (n=14)
surface

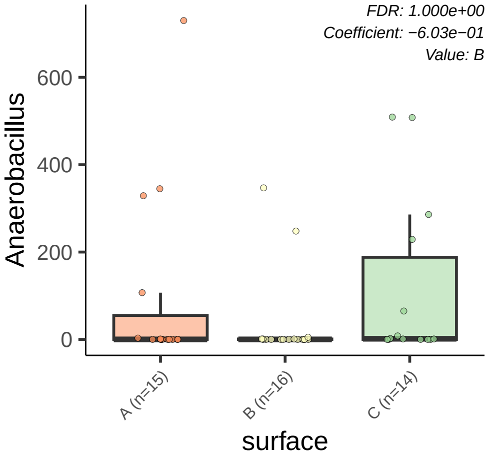

FDR: 1.000e+00
Coefficient: −6.03e−01
Value: B
Anaerobacillus
600
400
200
0
A (n=15)
B (n=16)
C (n=14)
surface

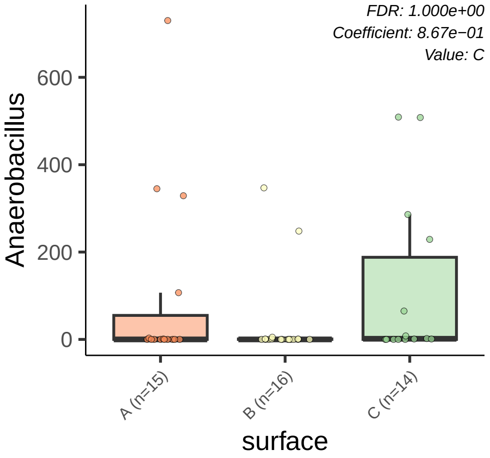

FDR: 1.000e+00
Coefficient: 8.67e−01
Value: C
Anaerobacillus
0
200
400
600
A (n=15)
B (n=16)
C (n=14)
surface

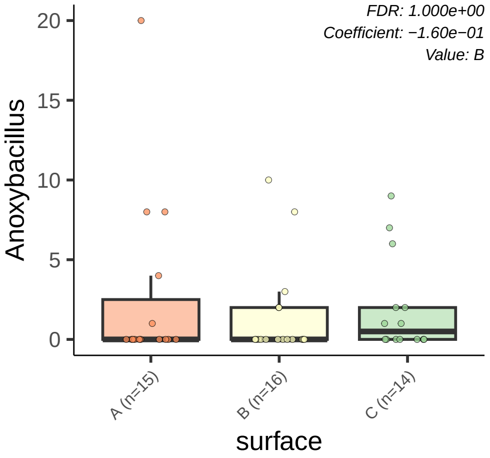
FDR: 1.000e+00
Coefficient: −1.60e−01
Value: B
Anoxybacillus
20
15
10
5
0
A (n=15)
B (n=16)
C (n=14)
surface

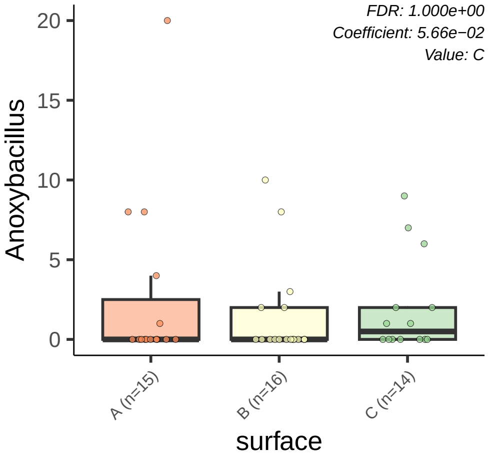
FDR: 1.000e+00
Coefficient: 5.66e−02
Value: C
Anoxybacillus
20
15
10
5
0
A (n=15)
B (n=16)
C (n=14)
surface

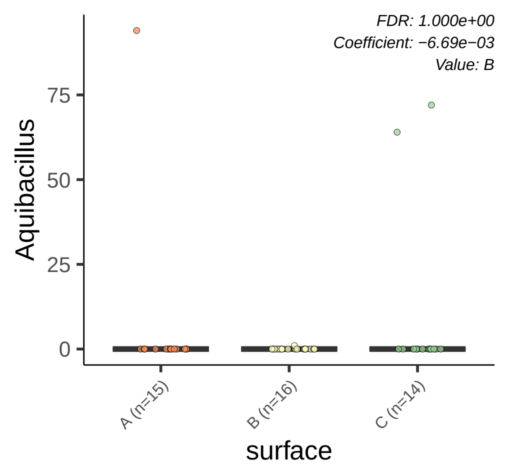
FDR: 1.000e+00
Coefficient: −6.69e−03
Value: B
Aquibacillus
75
50
25
0
A (n=15)
B (n=16)
C (n=14)
surface

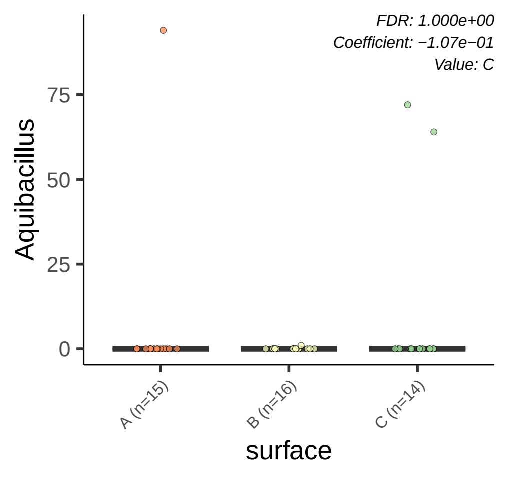
FDR: 1.000e+00
Coefficient: −1.07e−01
Value: C
Aquibacillus
75
50
25
0
A (n=15)
B (n=16)
C (n=14)
surface

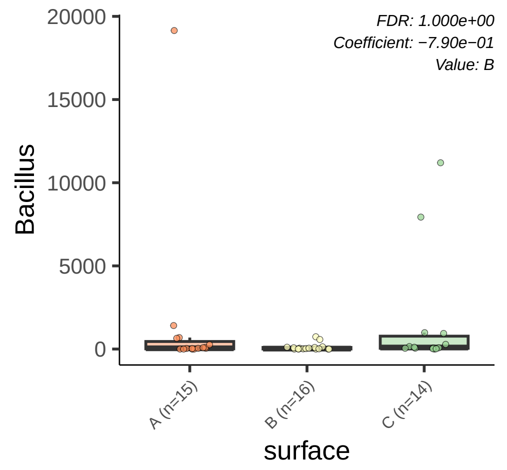

FDR: 1.000e+00
Coefficient: −7.90e−01
Value: B
Bacillus
20000
15000
10000
5000
0
A (n=15)
B (n=16)
C (n=14)
surface

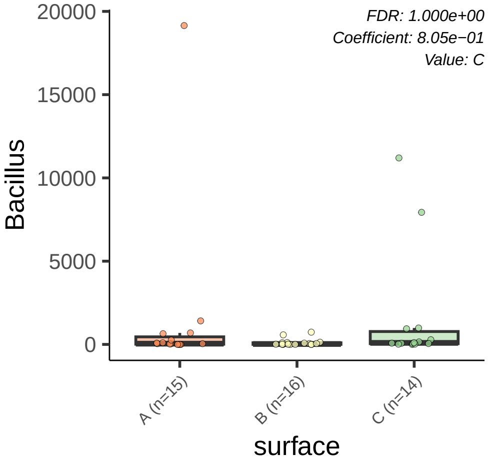

FDR: 1.000e+00
Coefficient: 8.05e−01
Value: C
Bacillus
20000
15000
10000
5000
0
A (n=15)
B (n=16)
C (n=14)
surface

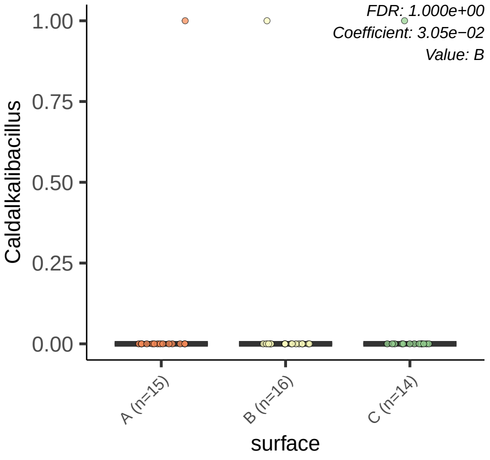

FDR: 1.000e+00
Coefficient: 3.05e−02
Value: B
Caldalkalibacillus
1.00
0.75
0.50
0.25
0.00
A (n=15)
B (n=16)
C (n=14)
surface

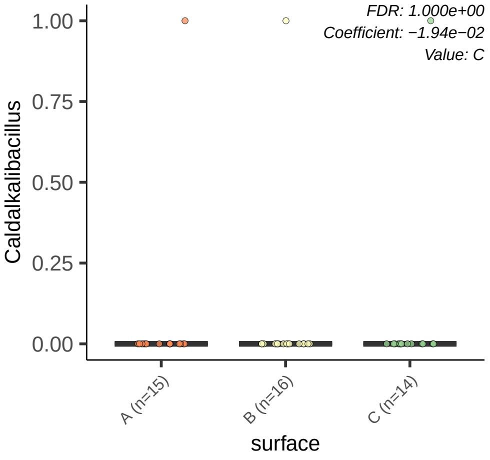

FDR: 1.000e+00
Coefficient: −1.94e−02
Value: C
Caldalkalibacillus
1.00
0.75
0.50
0.25
0.00
A (n=15)
B (n=16)
C (n=14)
surface

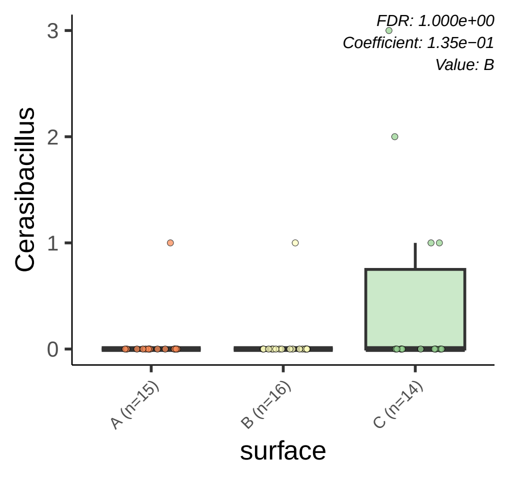

FDR: 1.000e+00
Coefficient: 1.35e−01
Value: B
Cerasibacillus
0
1
2
3
A (n=15)
B (n=16)
C (n=14)
surface

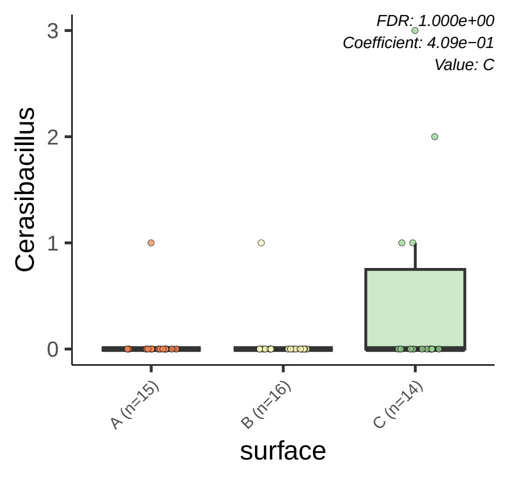
FDR: 1.000e+00
Coefficient: 4.09e−01
Value: C
Cerasibacillus
3
2
1
0
A (n=15)
B (n=16)
C (n=14)
surface

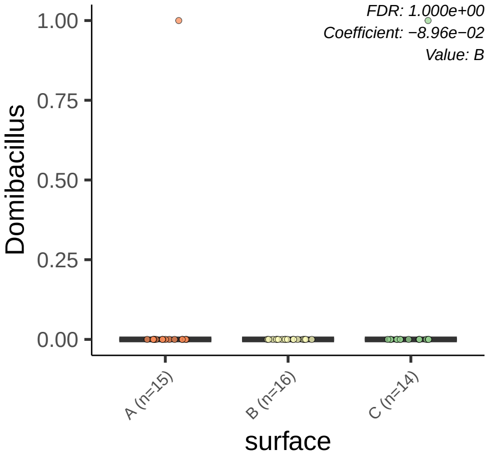
FDR: 1.000e+00
Coefficient: −8.96e−02
Value: B
Domibacillus
1.00
0.75
0.50
0.25
0.00
A (n=15)
B (n=16)
C (n=14)
surface

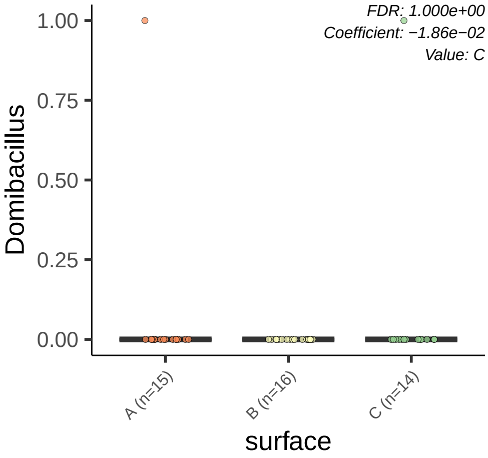

FDR: 1.000e+00
Coefficient: −1.86e−02
Value: C
Dombacillus
1.00
0.75
0.50
0.25
0.00
A (n=15)
B (n=16)
C (n=14)
surface

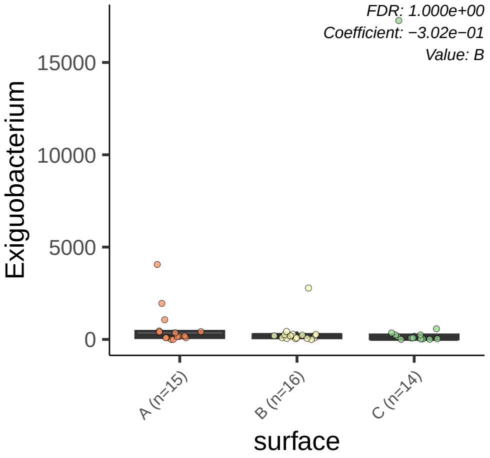
FDR: 1.000e+00
Coefficient: −3.02e−01
Value: B
15000
10000
5000
0
Exiguobacterium
A (n=15)
B (n=16)
C (n=14)
surface

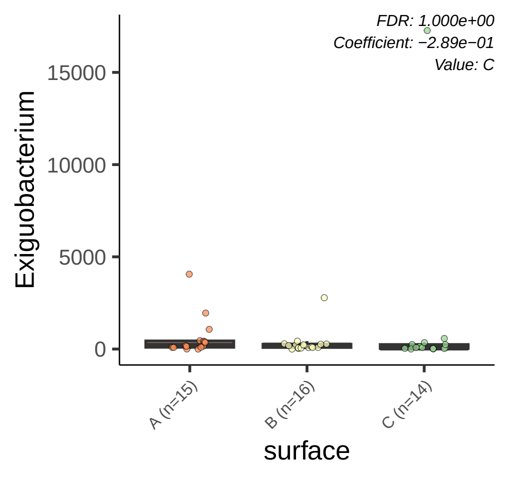
FDR: 1.000e+00
Coefficient: −2.89e−01
Value: C
Exiguobacterium
15000
10000
5000
0
A (n=15)
B (n=16)
C (n=14)
surface

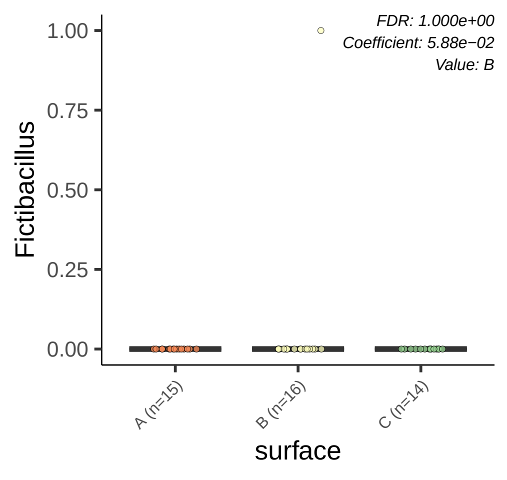
FDR: 1.000e+00
Coefficient: 5.88e−02
Value: B
Fictibacillus
1.00
0.75
0.50
0.25
0.00
A (n=15)
B (n=16)
C (n=14)
surface

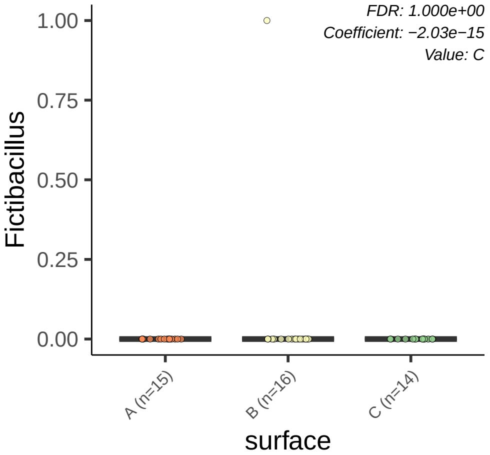
FDR: 1.000e+00
Coefficient: −2.03e−15
Value: C
Fictibacillus
1.00
0.75
0.50
0.25
0.00
A (n=15)
B (n=16)
C (n=14)
surface

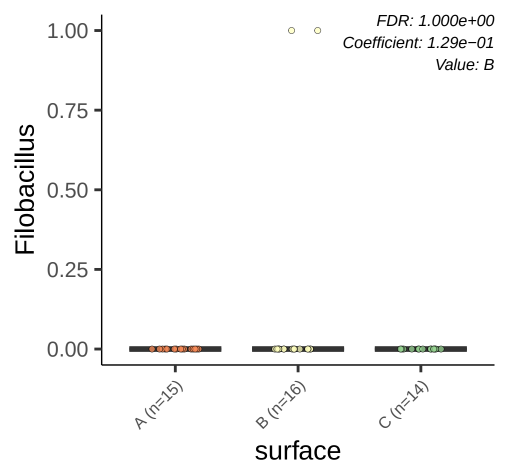

FDR: 1.000e+00
Coefficient: 1.29e−01
Value: B
Filobacillus
1.00
0.75
0.50
0.25
0.00
A (n=15)
B (n=16)
C (n=14)
surface

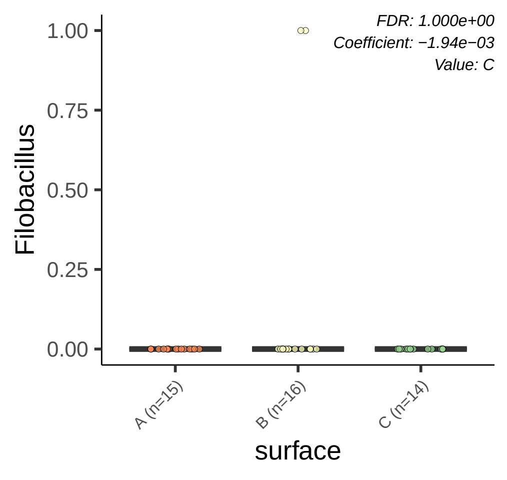
FDR: 1.000e+00
Coefficient: −1.94e−03
Value: C
1.00
0.75
0.50
0.25
0.00
Filobacillus
A (n=15)
B (n=16)
C (n=14)
surface

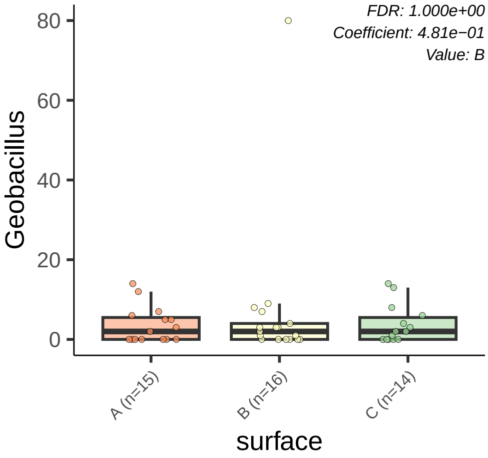
FDR: 1.000e+00
Coefficient: 4.81e−01
Value: B
Geobacillus
0
20
40
60
80
A (n=15)
B (n=16)
C (n=14)
surface

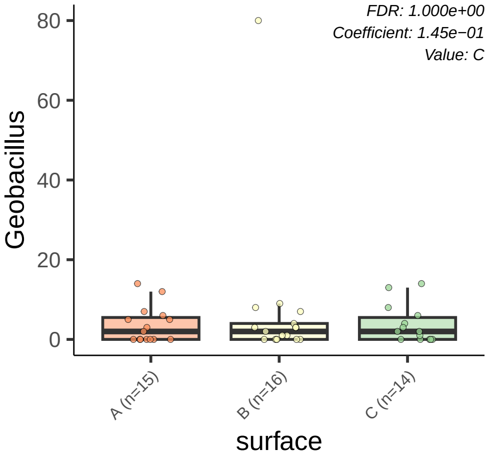

FDR: 1.000e+00
Coefficient: 1.45e−01
Value: C
Geobacillus
0
20
40
60
80
A (n=15)
B (n=16)
C (n=14)
surface

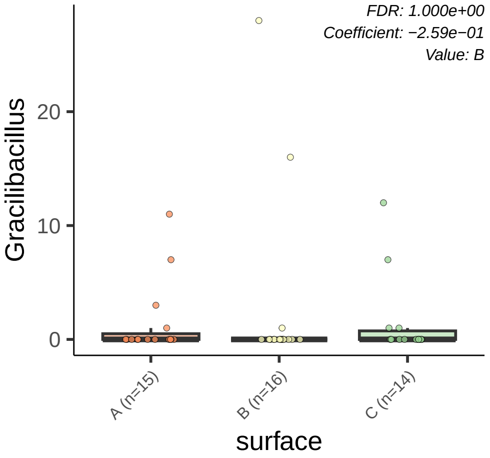
FDR: 1.000e+00
Coefficient: −2.59e−01
Value: B
Gracilibacillus
20
10
0
A (n=15)
B (n=16)
C (n=14)
surface

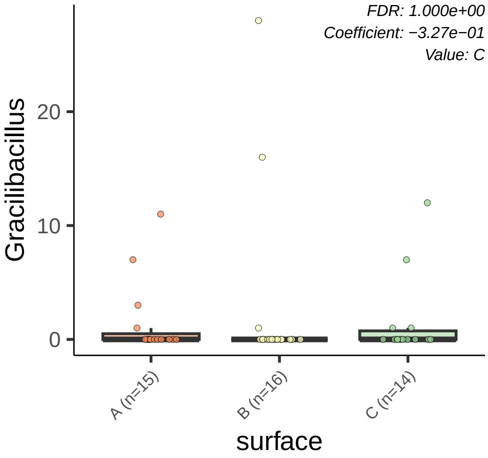
FDR: 1.000e+00
Coefficient: −3.27e−01
Value: C
Gracilibacillus
20
10
0
A (n=15)
B (n=16)
C (n=14)
surface

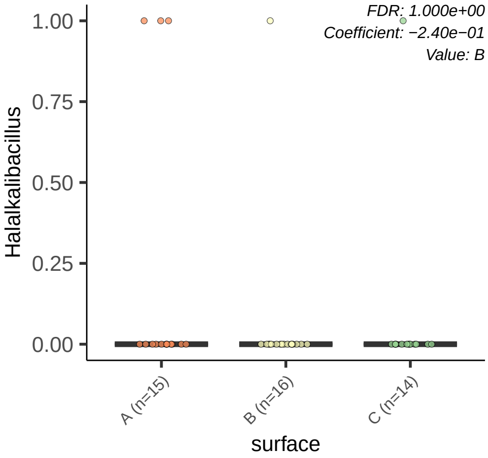

FDR: 1.000e+00
Coefficient: −2.40e−01
Value: B
Halalkalibacillus
1.00
0.75
0.50
0.25
0.00
A (n=15)
B (n=16)
C (n=14)
surface

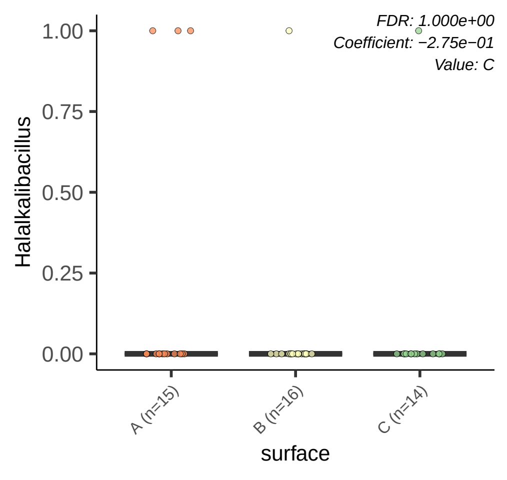
FDR: 1.000e+00
Coefficient: −2.75e−01
Value: C
Halalkalibacillus
1.00
0.75
0.50
0.25
0.00
A (n=15)
B (n=16)
C (n=14)
surface

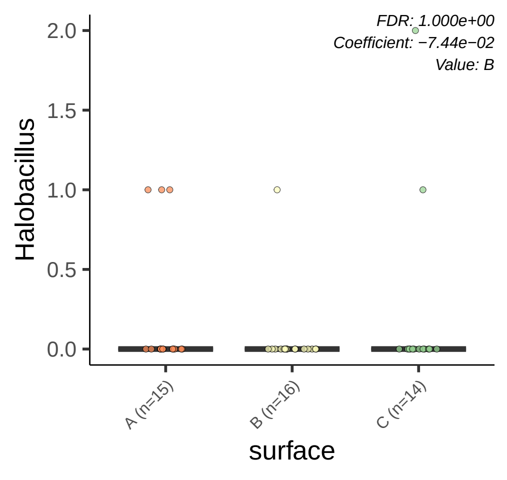
FDR: 1.000e+00
Coefficient: −7.44e−02
Value: B
Halobacillus
2.0
1.5
1.0
0.5
0.0
A (n=15)
B (n=16)
C (n=14)
surface

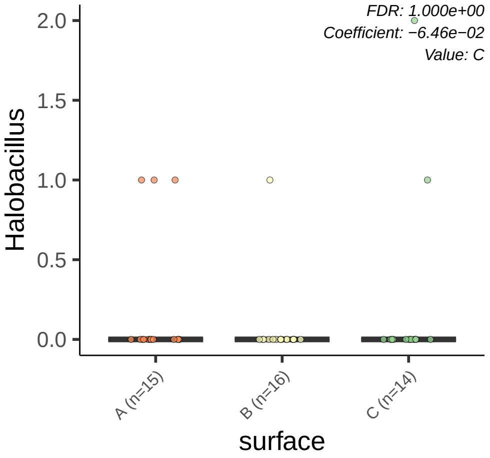
FDR: 1.000e+00
Coefficient: −6.46e−02
Value: C
Halobacillus
2.0
1.5
1.0
0.5
0.0
A (n=15)
B (n=16)
C (n=14)
surface

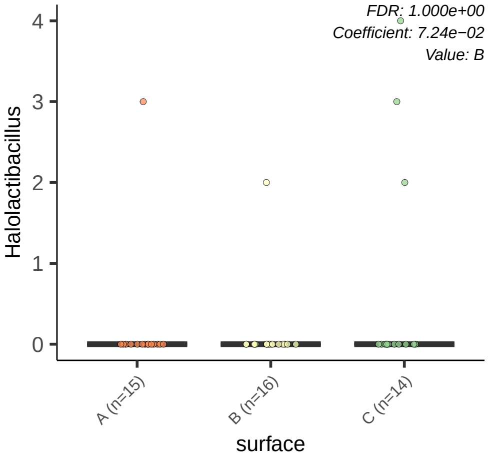
FDR: 1.000e+00
Coefficient: 7.24e−02
Value: B
Halolactibacillus
0
1
2
3
4
A (n=15)
B (n=16)
C (n=14)
surface

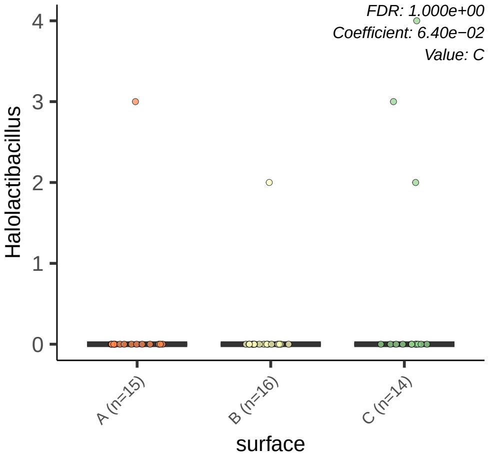

FDR: 1.000e+00
Coefficient: 6.40e−02
Value: C
Halolactibacillus
0
1
2
3
4
A (n=15)
B (n=16)
C (n=14)
surface

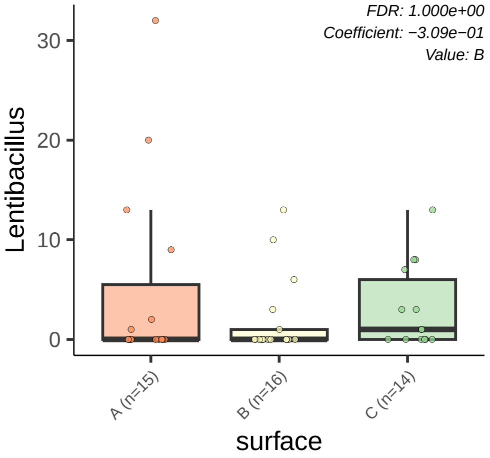

FDR: 1.000e+00
Coefficient: −3.09e−01
Value: B
Lentibacillus
30
20
10
0
A (n=15)
B (n=16)
C (n=14)
surface

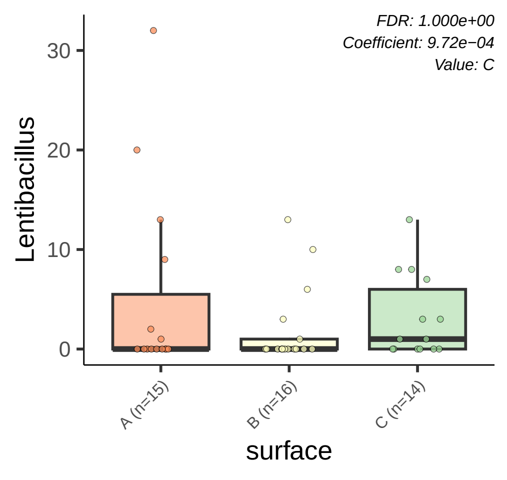

FDR: 1.000e+00
Coefficient: 9.72e−04
Value: C
Lentibacillus
0
10
20
30
A (n=15)
B (n=16)
C (n=14)
surface

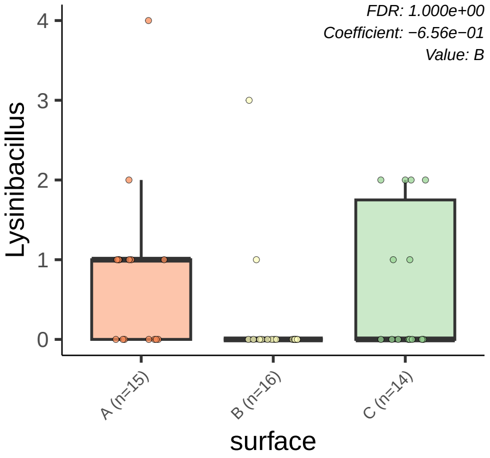
FDR: 1.000e+00
Coefficient: −6.56e−01
Value: B
Lysinibacillus
0
1
2
3
4
A (n=15)
B (n=16)
C (n=14)
surface

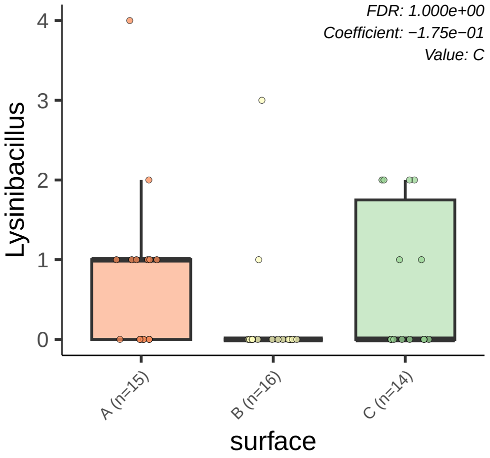
FDR: 1.000e+00
Coefficient: −1.75e−01
Value: C
Lysinibacillus
0
1
2
3
4
A (n=15)
B (n=16)
C (n=14)
surface

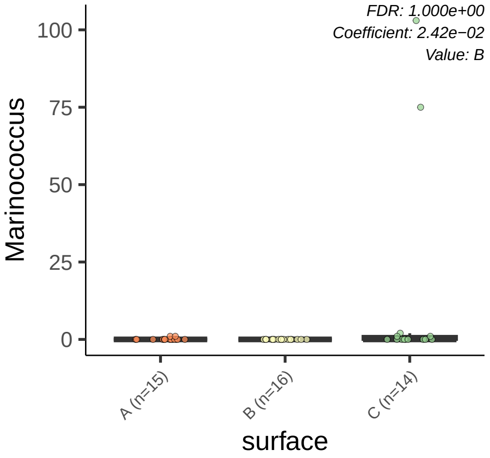
FDR: 1.000e+00
Coefficient: 2.42e−02
Value: B
Marinococcus
100
75
50
25
0
A (n=15)
B (n=16)
C (n=14)
surface

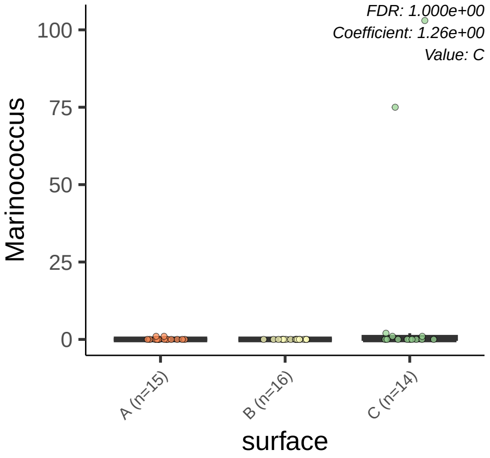
FDR: 1.000e+00
Coefficient: 1.26e+00
Value: C
Marinococcus
100
75
50
25
0
A (n=15)
B (n=16)
C (n=14)
surface

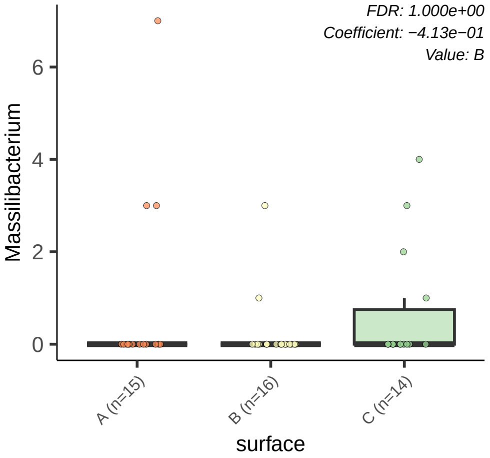

FDR: 1.000e+00
Coefficient: −4.13e−01
Value: B
Massilibacterium
0
2
4
6
A (n=15)
B (n=16)
C (n=14)
surface

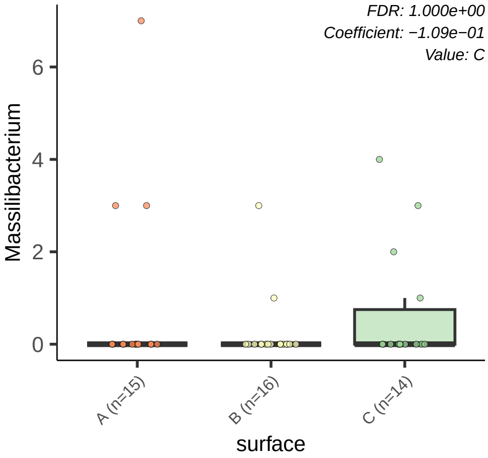
FDR: 1.000e+00
Coefficient: −1.09e−01
Value: C
Massilibacterium
0
2
4
6
A (n=15)
B (n=16)
C (n=14)
surface

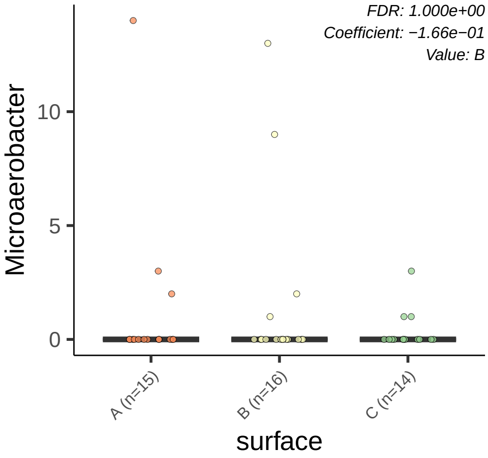

FDR: 1.000e+00
Coefficient: −1.66e−01
Value: B
Microaerobacter
10
5
0
A (n=15)
B (n=16)
C (n=14)
surface

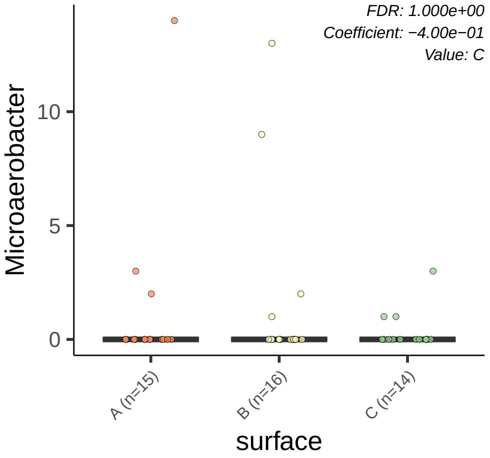
FDR: 1.000e+00
Coefficient: −4.00e−01
Value: C
Microaerobacter
10
5
0
A (n=15)
B (n=16)
C (n=14)
surface

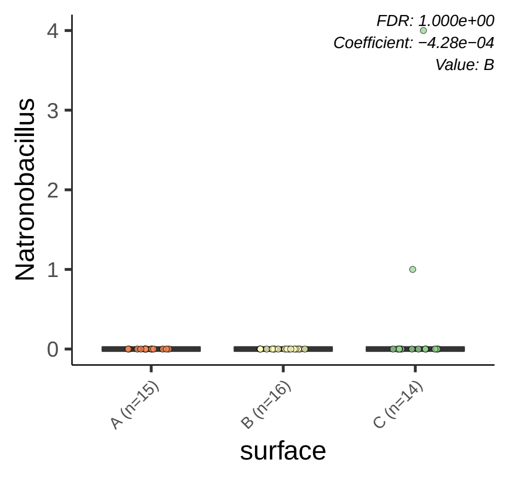
FDR: 1.000e+00
Coefficient: −4.28e−04
Value: B
Natronobacillus
4
3
2
1
0
A (n=15)
B (n=16)
C (n=14)
surface

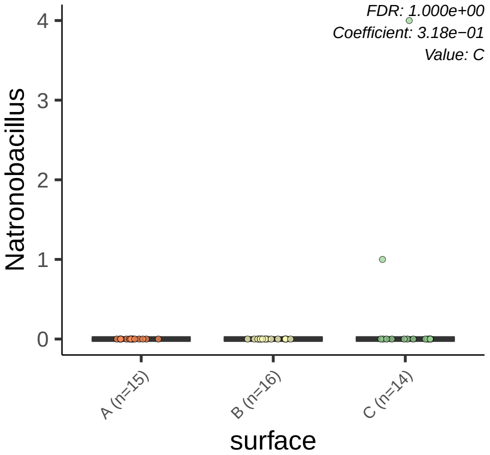

FDR: 1.000e+00
Coefficient: 3.18e−01
Value: C
Natronobacillus
4
3
2
1
0
A (n=15)
B (n=16)
C (n=14)
surface

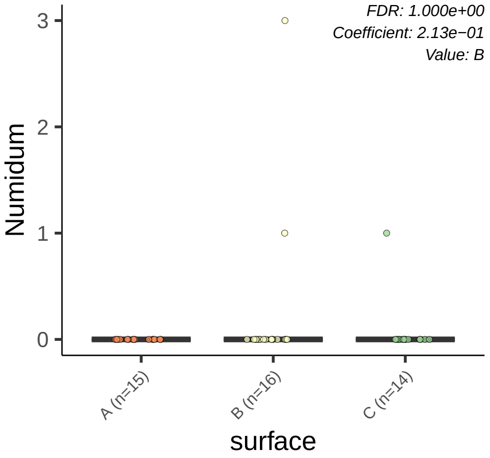

FDR: 1.000e+00
Coefficient: 2.13e−01
Value: B
Numidum
3
2
1
0
A (n=15)
B (n=16)
C (n=14)
surface

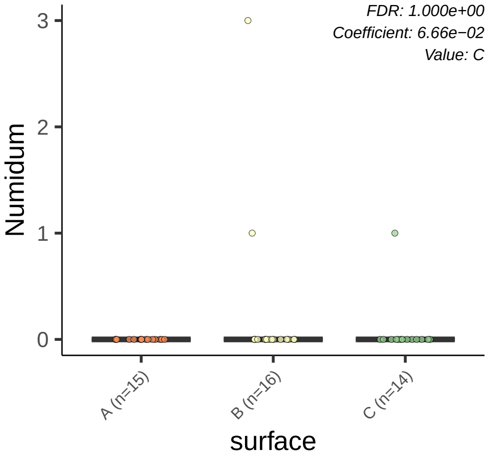
FDR: 1.000e+00
Coefficient: 6.66e−02
Value: C
Numidum
3
2
1
0
A (n=15)
B (n=16)
C (n=14)
surface

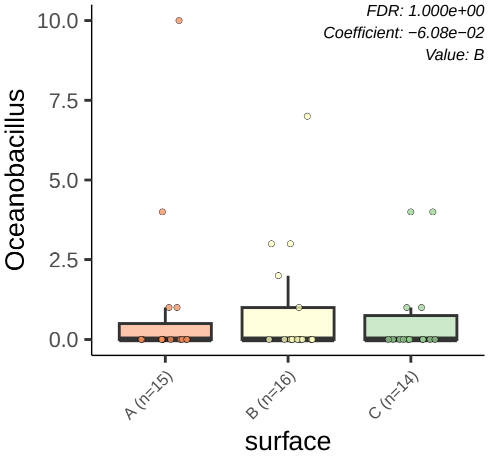
FDR: 1.000e+00
Coefficient: −6.08e−02
Value: B
Oceanobacillus
10.0
7.5
5.0
2.5
0.0
A (n=15)
B (n=16)
C (n=14)
surface

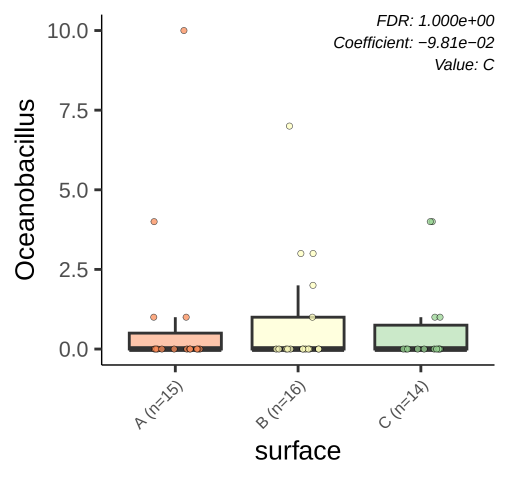
FDR: 1.000e+00
Coefficient: −9.81e−02
Value: C
Oceanobacillus
10.0
7.5
5.0
2.5
0.0
A (n=15)
B (n=16)
C (n=14)
surface

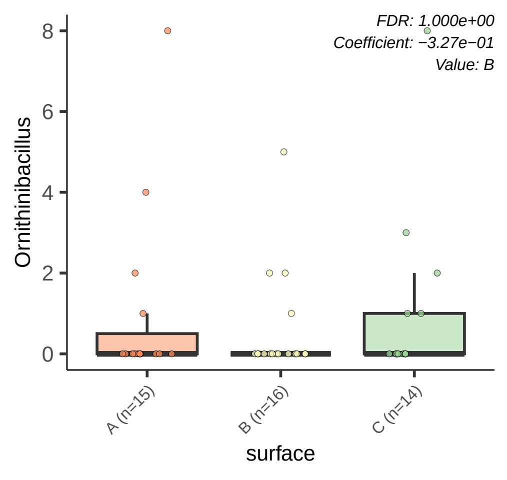

FDR: 1.000e+00
Coefficient: −3.27e−01
Value: B
Ornithinibacillus
0
2
4
6
8
A (n=15)
B (n=16)
C (n=14)
surface

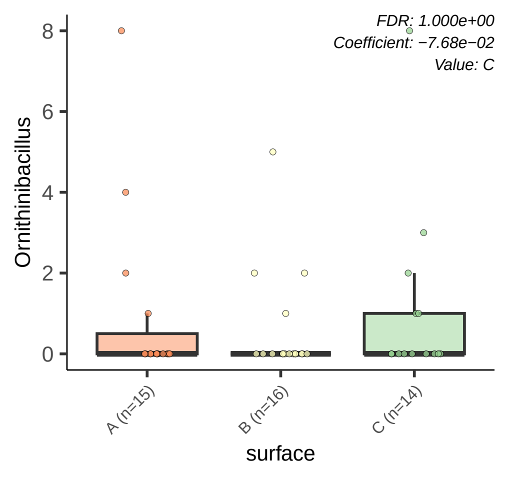
FDR: 1.000e+00
Coefficient: −7.68e−02
Value: C
Ornithinibacillus
0
2
4
6
8
A (n=15)
B (n=16)
C (n=14)
surface

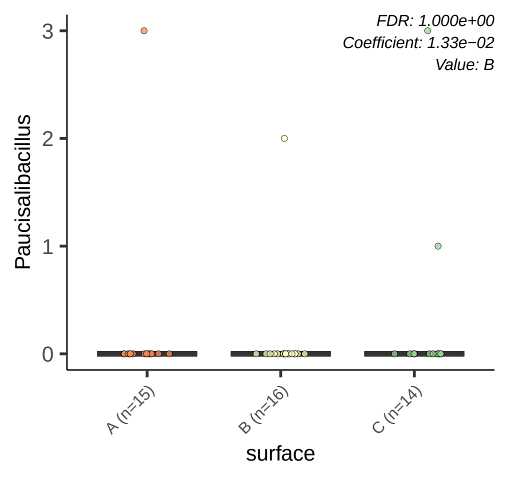

FDR: 1.000e+00
Coefficient: 1.33e−02
Value: B
Paucisalibacillus
3
2
1
0
A (n=15)
B (n=16)
C (n=14)
surface

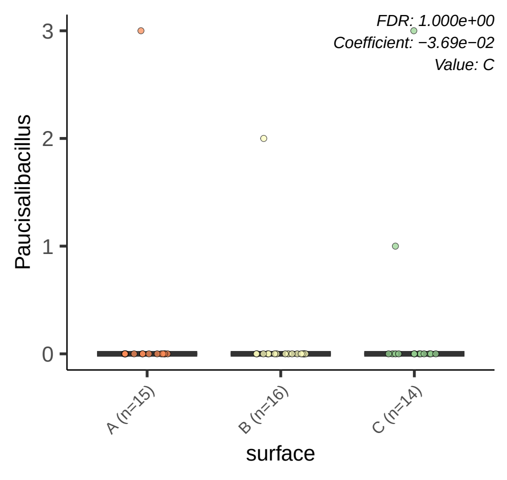

FDR: 1.000e+00
Coefficient: −3.69e−02
Value: C
Paucisalibacillus
3
2
1
0
A (n=15)
B (n=16)
C (n=14)
surface

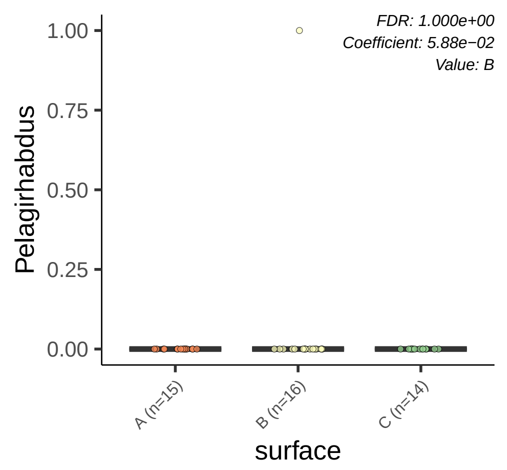
FDR: 1.000e+00
Coefficient: 5.88e−02
Value: B
Pelagirhabdus
1.00
0.75
0.50
0.25
0.00
A (n=15)
B (n=16)
C (n=14)
surface

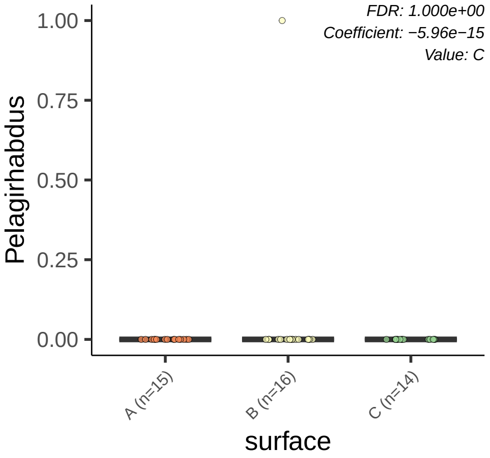

FDR: 1.000e+00
Coefficient: −5.96e−15
Value: C
Pelagirhabdus
1.00
0.75
0.50
0.25
0.00
A (n=15)
B (n=16)
C (n=14)
surface

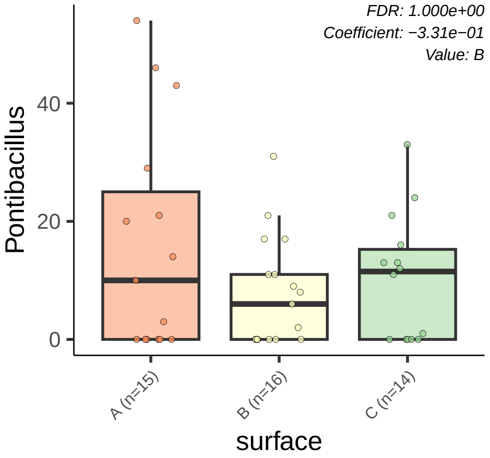
FDR: 1.000e+00
Coefficient: −3.31e−01
Value: B
Pontibacillus
40
20
0
A (n=15)
B (n=16)
C (n=14)
surface

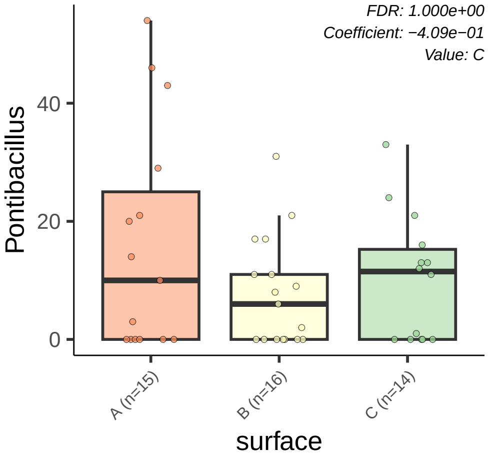

FDR: 1.000e+00
Coefficient: −4.09e−01
Value: C
Pontibacillus
0
20
40
A (n=15)
B (n=16)
C (n=14)
surface

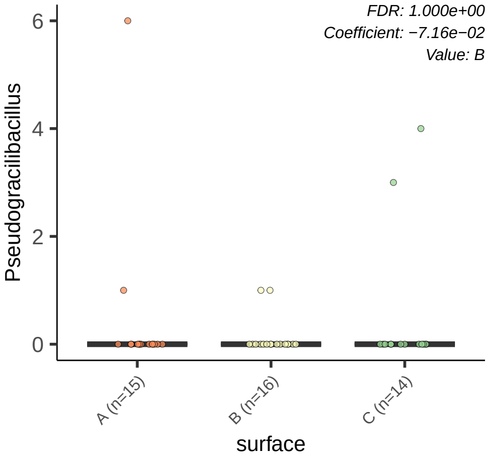

FDR: 1.000e+00
Coefficient: −7.16e−02
Value: B
Pseudogracilibacillus
0
2
4
6
A (n=15)
B (n=16)
C (n=14)
surface

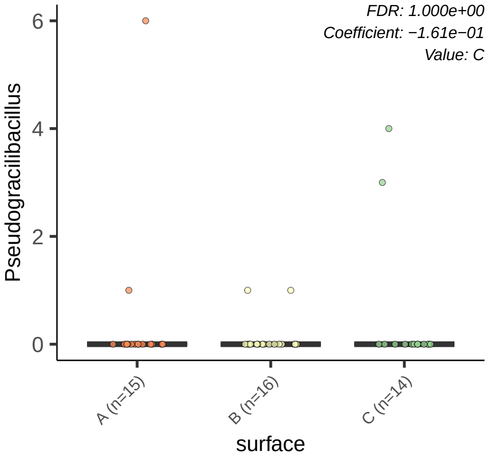
FDR: 1.000e+00
Coefficient: −1.61e−01
Value: C
Pseudogracilibacillus
0
2
4
6
A (n=15)
B (n=16)
C (n=14)
surface

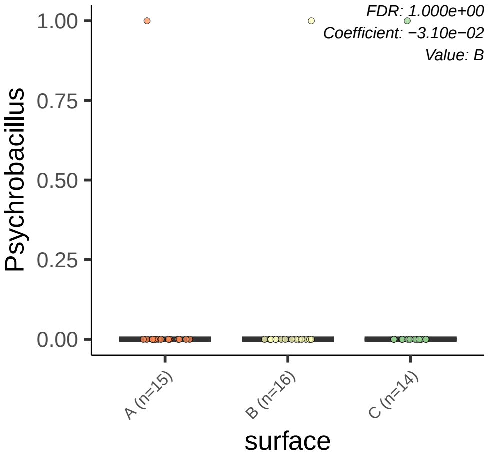
FDR: 1.000e+00
Coefficient: −3.10e−02
Value: B
Psychrobacillus
1.00
0.75
0.50
0.25
0.00
A (n=15)
B (n=16)
C (n=14)
surface

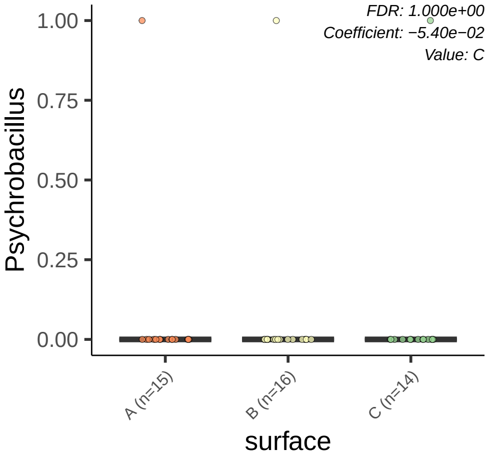
FDR: 1.000e+00
Coefficient: −5.40e−02
Value: C
Psychrobacillus
1.00
0.75
0.50
0.25
0.00
A (n=15)
B (n=16)
C (n=14)
surface

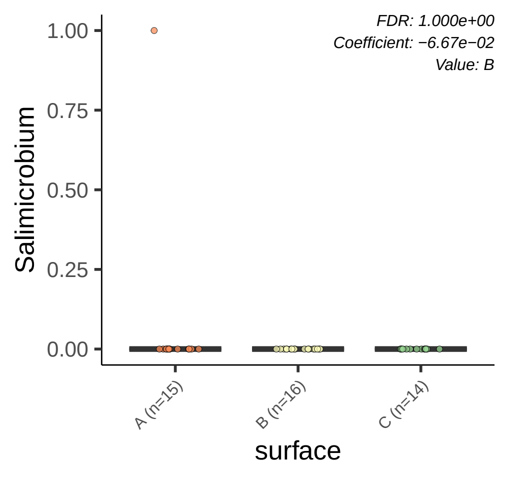
FDR: 1.000e+00
Coefficient: −6.67e−02
Value: B
Salimicrobium
1.00
0.75
0.50
0.25
0.00
A (n=15)
B (n=16)
C (n=14)
surface

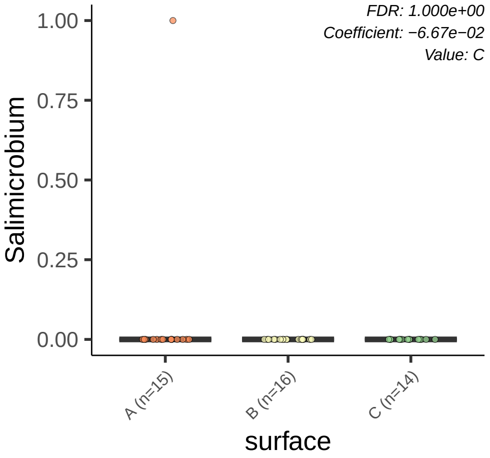
FDR: 1.000e+00
Coefficient: −6.67e−02
Value: C
Salimicrobium
1.00
0.75
0.50
0.25
0.00
A (n=15)
B (n=16)
C (n=14)
surface

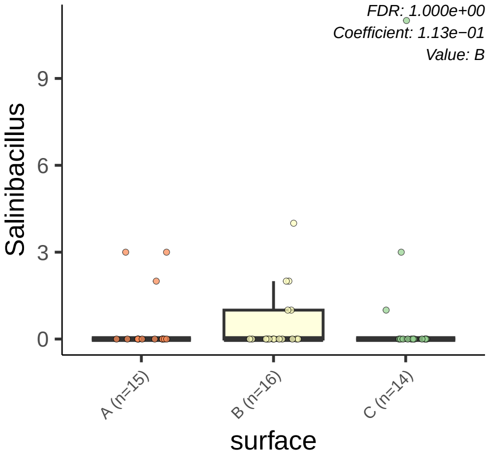
FDR: 1.000e+00
Coefficient: 1.13e−01
Value: B
Salinibacillus
0
3
6
9
A (n=15)
B (n=16)
C (n=14)
surface

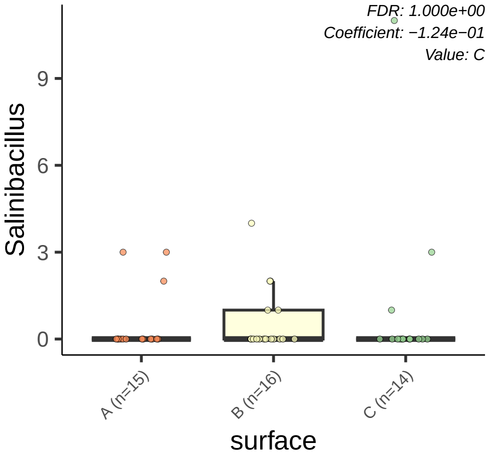

FDR: 1.000e+00
Coefficient: −1.24e−01
Value: C
Salinibacillus
9
6
3
0
A (n=15)
B (n=16)
C (n=14)
surface

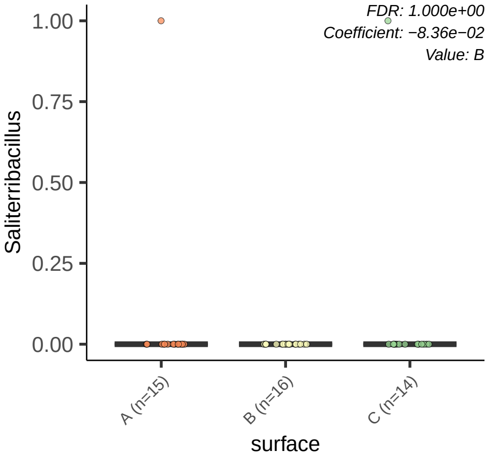
FDR: 1.000e+00
Coefficient: −8.36e−02
Value: B
Saliterribacillus
1.00
0.75
0.50
0.25
0.00
A (n=15)
B (n=16)
C (n=14)
surface

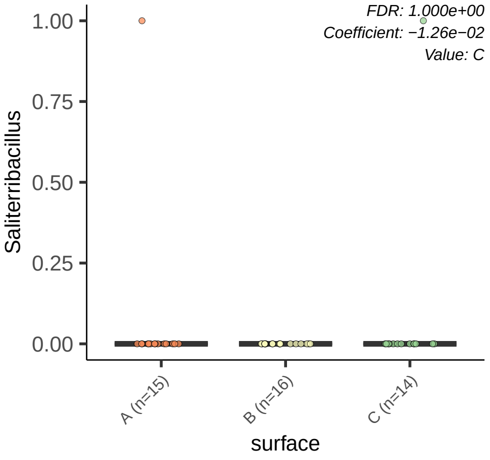
FDR: 1.000e+00
Coefficient: −1.26e−02
Value: C
Saliterribacillus
1.00
0.75
0.50
0.25
0.00
A (n=15)
B (n=16)
C (n=14)
surface

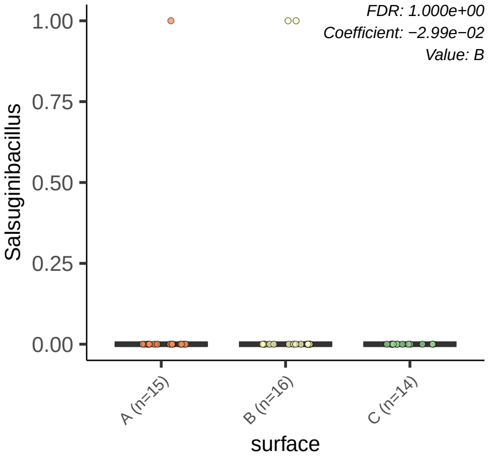

FDR: 1.000e+00
Coefficient: −2.99e−02
Value: B
Salsuginibacillus
1.00
0.75
0.50
0.25
0.00
A (n=15)
B (n=16)
C (n=14)
surface

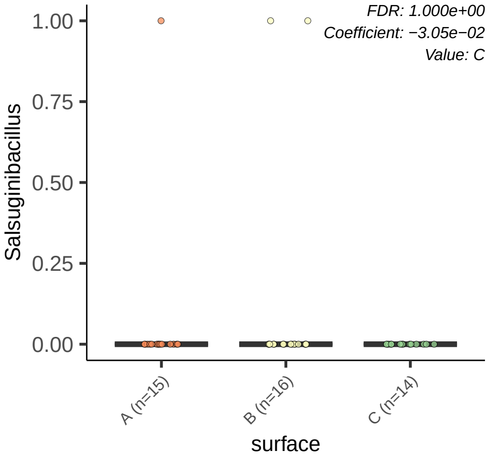
FDR: 1.000e+00
Coefficient: −3.05e−02
Value: C
Salsuginibacillus
1.00
0.75
0.50
0.25
0.00
A (n=15)
B (n=16)
C (n=14)
surface

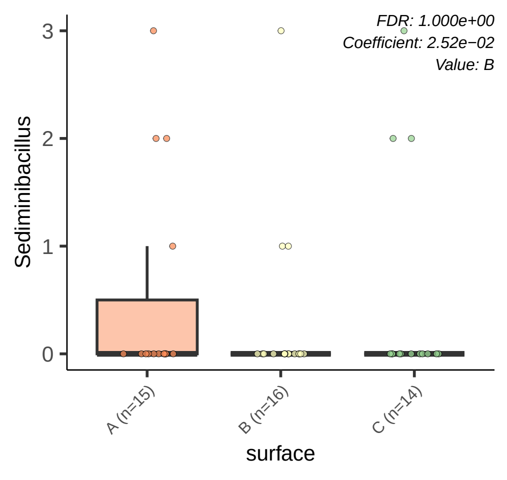

FDR: 1.000e+00
Coefficient: 2.52e−02
Value: B
Sediminibacillus
0
1
2
3
A (n=15)
B (n=16)
C (n=14)
surface

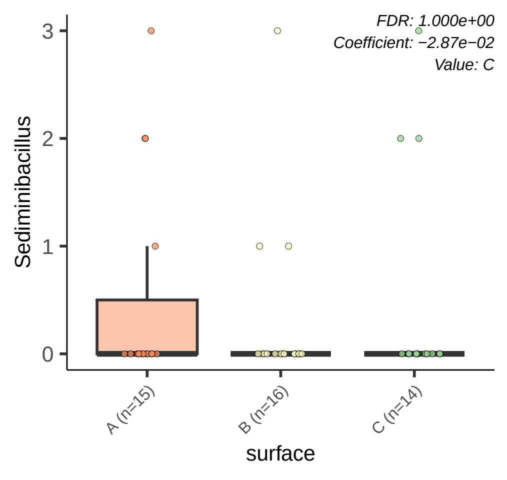
FDR: 1.000e+00
Coefficient: −2.87e−02
Value: C
Sediminibacillus
0
1
2
3
A (n=15)
B (n=16)
C (n=14)
surface

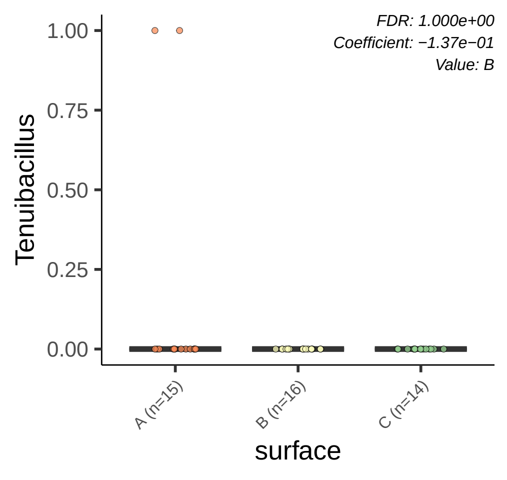
FDR: 1.000e+00
Coefficient: −1.37e−01
Value: B
Tenuibacillus
1.00
0.75
0.50
0.25
0.00
A (n=15)
B (n=16)
C (n=14)
surface

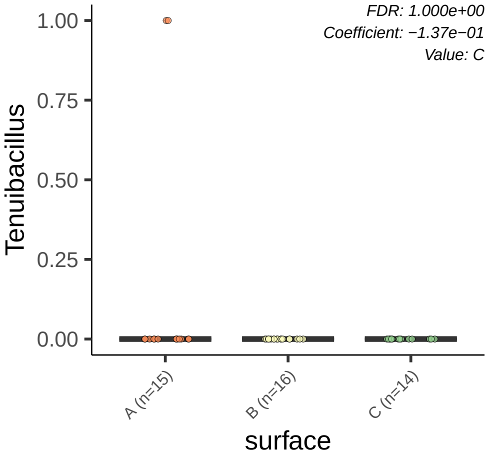
FDR: 1.000e+00
Coefficient: −1.37e−01
Value: C
Tenuibacillus
1.00
0.75
0.50
0.25
0.00
A (n=15)
B (n=16)
C (n=14)
surface

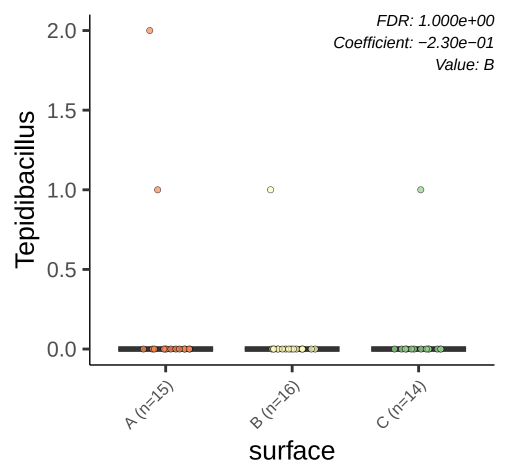
FDR: 1.000e+00
Coefficient: −2.30e−01
Value: B
Tepidibacillus
2.0
1.5
1.0
0.5
0.0
A (n=15)
B (n=16)
C (n=14)
surface

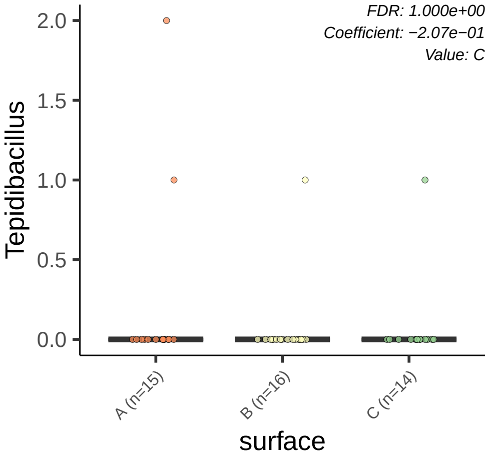
FDR: 1.000e+00
Coefficient: −2.07e−01
Value: C
Tepidibacillus
2.0
1.5
1.0
0.5
0.0
A (n=15)
B (n=16)
C (n=14)
surface

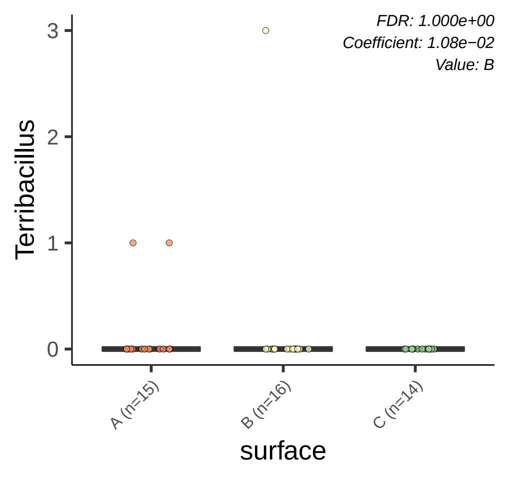

FDR: 1.000e+00
Coefficient: 1.08e−02
Value: B
Terribacillus
3
2
1
0
A (n=15)
B (n=16)
C (n=14)
surface

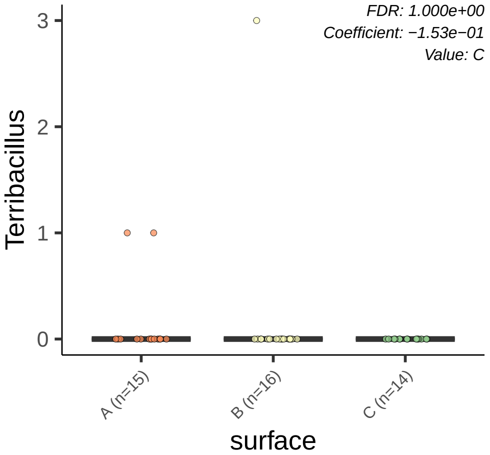
FDR: 1.000e+00
Coefficient: −1.53e−01
Value: C
Terribacillus
3
2
1
0
A (n=15)
B (n=16)
C (n=14)
surface

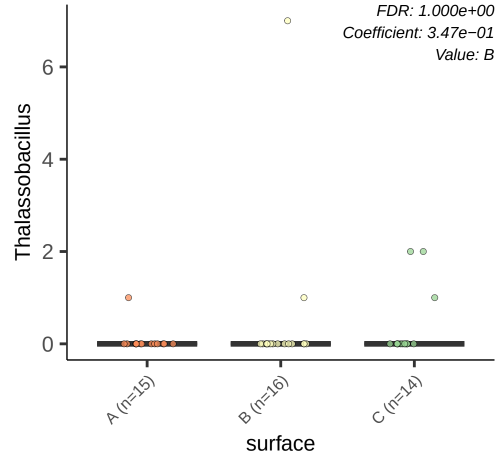
FDR: 1.000e+00
Coefficient: 3.47e−01
Value: B
Thalassobacillus
6
4
2
0
A (n=15)
B (n=16)
C (n=14)
surface

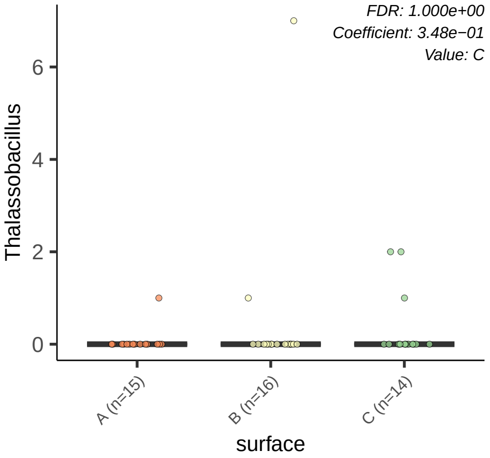

FDR: 1.000e+00
Coefficient: 3.48e−01
Value: C
Thalassobacillus
0
2
4
6
A (n=15)
B (n=16)
C (n=14)
surface

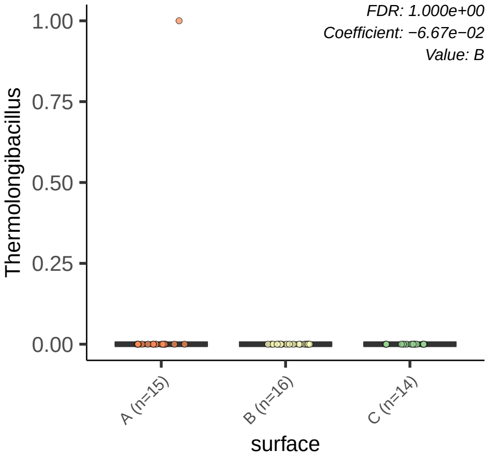
FDR: 1.000e+00
Coefficient: −6.67e−02
Value: B
Thermolongibacillus
1.00
0.75
0.50
0.25
0.00
A (n=15)
B (n=16)
C (n=14)
surface

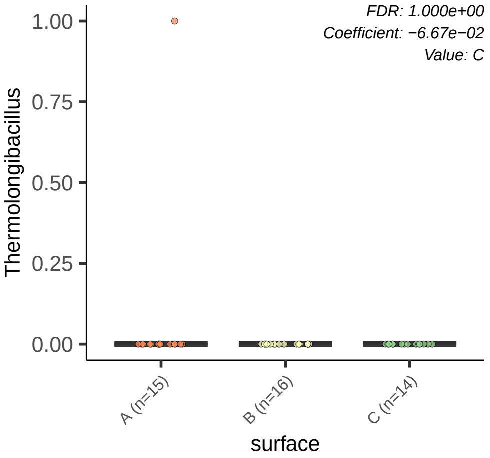

FDR: 1.000e+00
Coefficient: −6.67e−02
Value: C
Thermolongibacillus
1.00
0.75
0.50
0.25
0.00
A (n=15)
B (n=16)
C (n=14)
surface

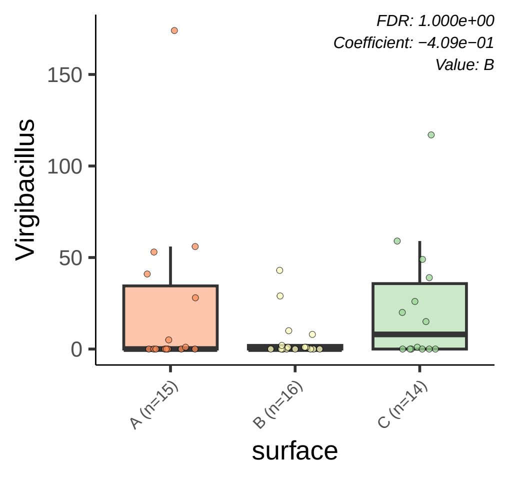
FDR: 1.000e+00
Coefficient: −4.09e−01
Value: B
Virgibacillus
150
100
50
0
A (n=15)
B (n=16)
C (n=14)
surface

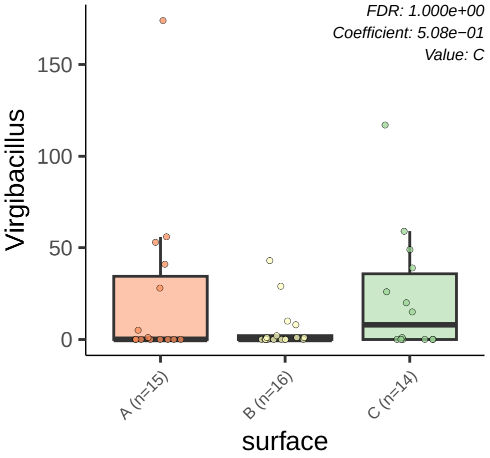

FDR: 1.000e+00
Coefficient: 5.08e−01
Value: C
Virgibacillus
150
100
50
0
A (n=15)
B (n=16)
C (n=14)
surface

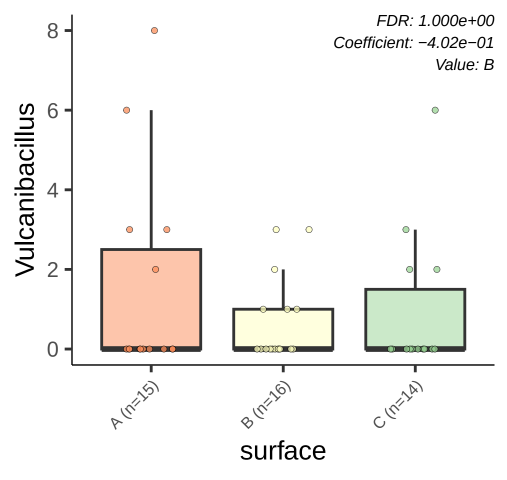
FDR: 1.000e+00
Coefficient: −4.02e−01
Value: B
Vulcanibacillus
0
2
4
6
8
A (n=15)
B (n=16)
C (n=14)
surface

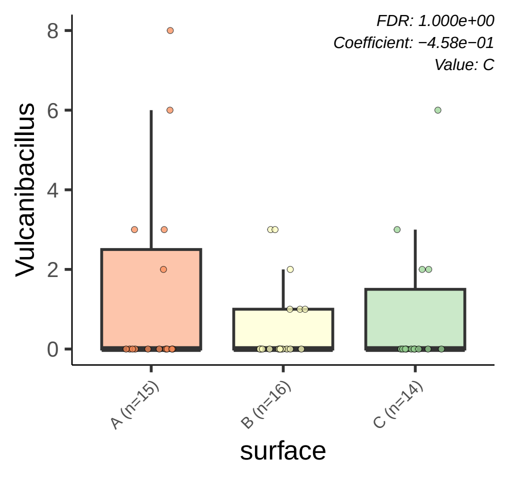

FDR: 1.000e+00
Coefficient: −4.58e−01
Value: C
Vulcanibacillus
0
2
4
6
8
A (n=15)
B (n=16)
C (n=14)
surface

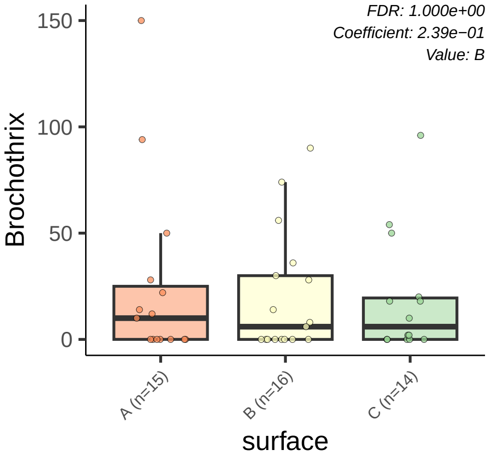
FDR: 1.000e+00
Coefficient: 2.39e−01
Value: B
Brochothrix
150
100
50
0
A (n=15)
B (n=16)
C (n=14)
surface

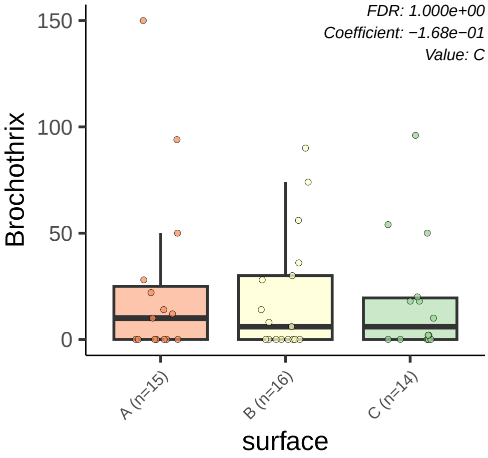
FDR: 1.000e+00
Coefficient: −1.68e−01
Value: C
Brochothrix
150
100
50
0
A (n=15)
B (n=16)
C (n=14)
surface

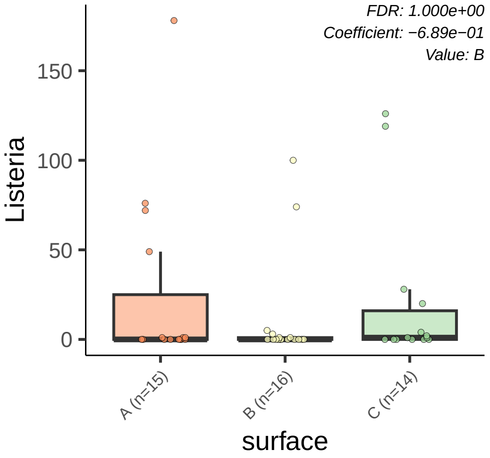
FDR: 1.000e+00
Coefficient: −6.89e−01
Value: B
Listeria
150
100
50
0
A (n=15)
B (n=16)
C (n=14)
surface

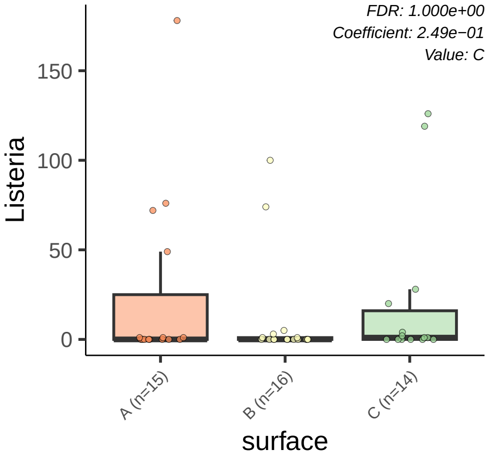
FDR: 1.000e+00
Coefficient: 2.49e−01
Value: C
Listeria
150
100
50
0
A (n=15)
B (n=16)
C (n=14)
surface

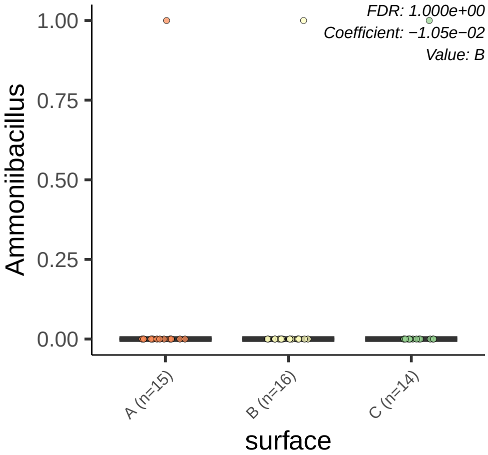

FDR: 1.000e+00
Coefficient: −1.05e−02
Value: B
Ammoniibacillus
1.00
0.75
0.50
0.25
0.00
A (n=15)
B (n=16)
C (n=14)
surface

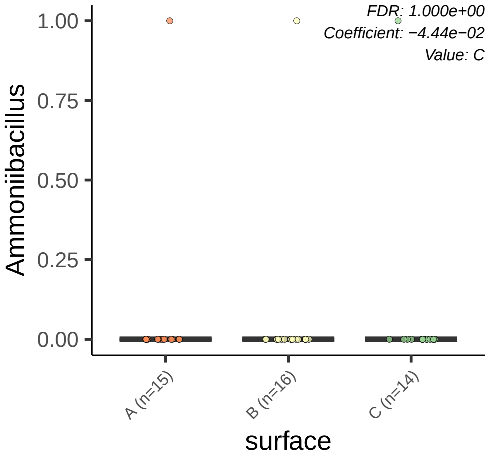
FDR: 1.000e+00
Coefficient: −4.44e−02
Value: C
Ammoniibacillus
1.00
0.75
0.50
0.25
0.00
A (n=15)
B (n=16)
C (n=14)
surface

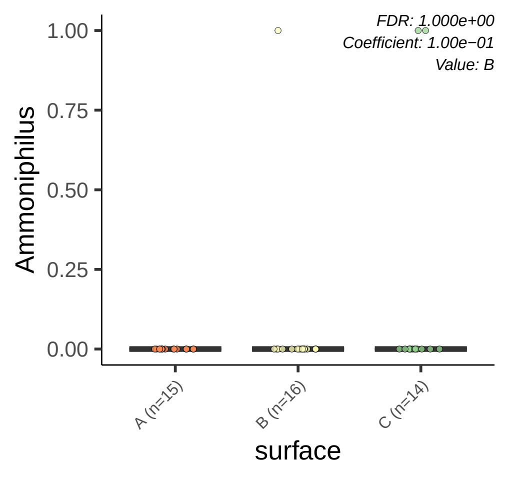
FDR: 1.000e+00
Coefficient: 1.00e−01
Value: B
Ammoniphilus
1.00
0.75
0.50
0.25
0.00
A (n=15)
B (n=16)
C (n=14)
surface

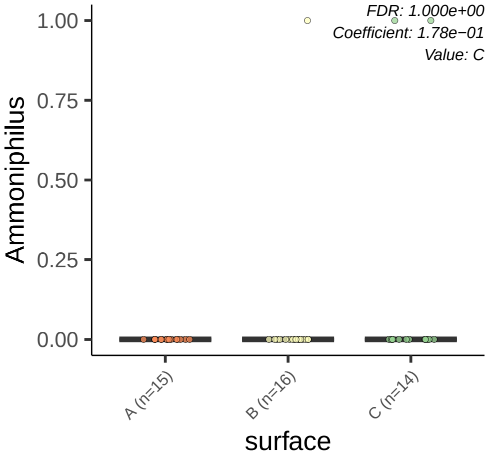
FDR: 1.000e+00
Coefficient: 1.78e−01
Value: C
Ammoniphilus
1.00
0.75
0.50
0.25
0.00
A (n=15)
B (n=16)
C (n=14)
surface

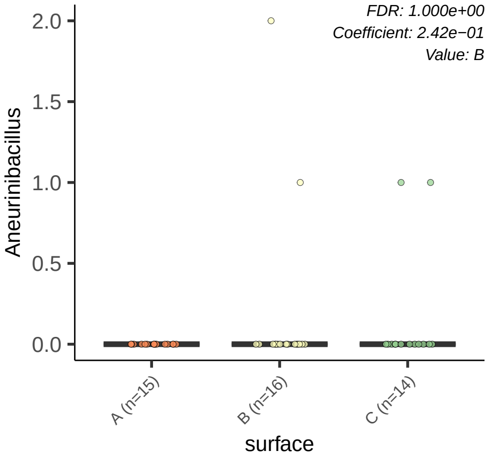

FDR: 1.000e+00
Coefficient: 2.42e−01
Value: B
Aneurinibacillus
2.0
1.5
1.0
0.5
0.0
A (n=15)
B (n=16)
C (n=14)
surface

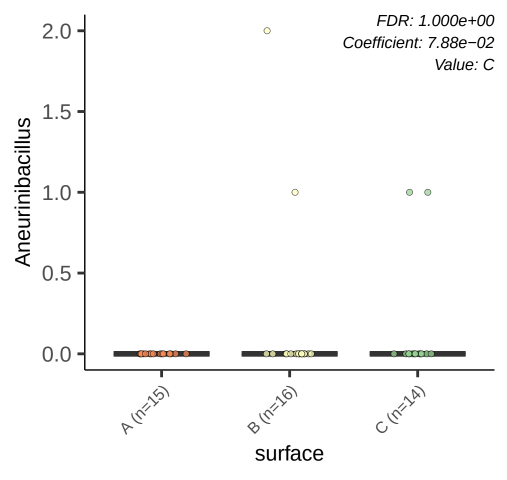
FDR: 1.000e+00
Coefficient: 7.88e−02
Value: C
Aneurinibacillus
2.0
1.5
1.0
0.5
0.0
A (n=15)
B (n=16)
C (n=14)
surface

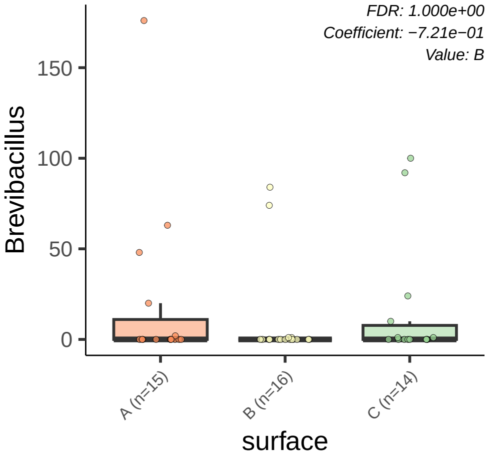
FDR: 1.000e+00
Coefficient: −7.21e−01
Value: B
Brevibacillus
0
50
100
150
A (n=15)
B (n=16)
C (n=14)
surface

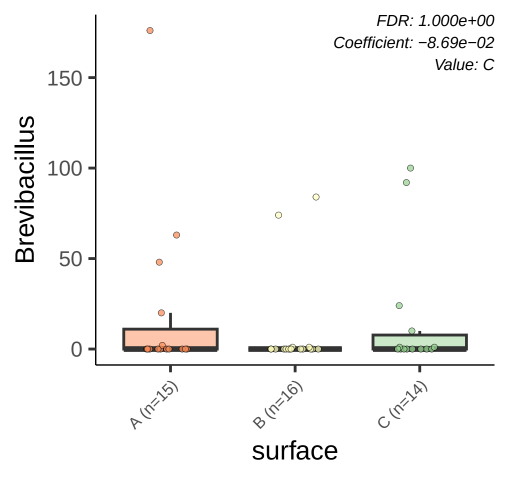

FDR: 1.000e+00
Coefficient: −8.69e−02
Value: C
Brevibacillus
150
100
50
0
A (n=15)
B (n=16)
C (n=14)
surface

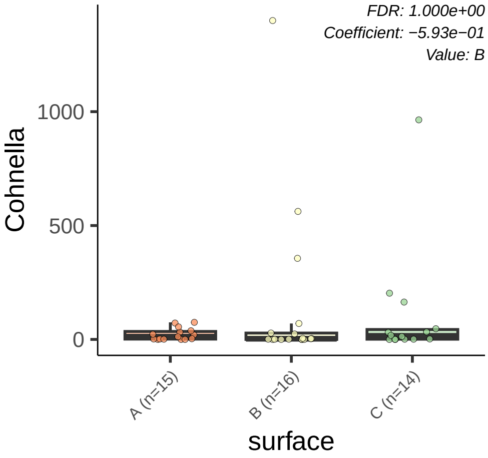

FDR: 1.000e+00
Coefficient: −5.93e−01
Value: B
Cohnella
1000
500
0
A (n=15)
B (n=16)
C (n=14)
surface

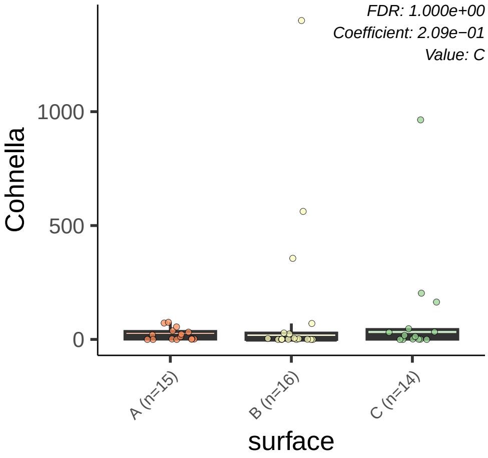

FDR: 1.000e+00
Coefficient: 2.09e−01
Value: C
Cohnella
1000
500
0
A (n=15)
B (n=16)
C (n=14)
surface

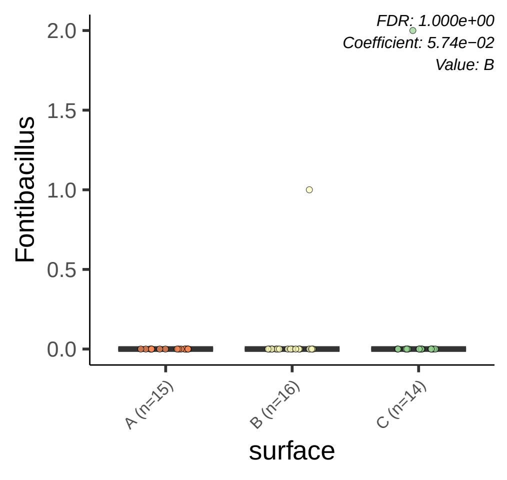

FDR: 1.000e+00
Coefficient: 5.74e−02
Value: B
Fontibacillus
2.0
1.5
1.0
0.5
0.0
A (n=15)
B (n=16)
C (n=14)
surface

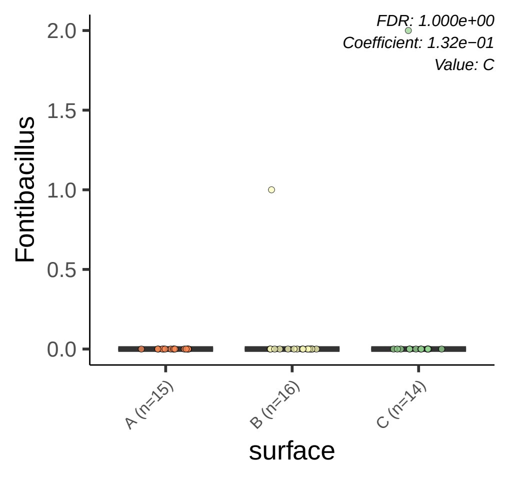

FDR: 1.000e+00
Coefficient: 1.32e−01
Value: C
Fontibacillus
2.0
1.5
1.0
0.5
0.0
A (n=15)
B (n=16)
C (n=14)
surface

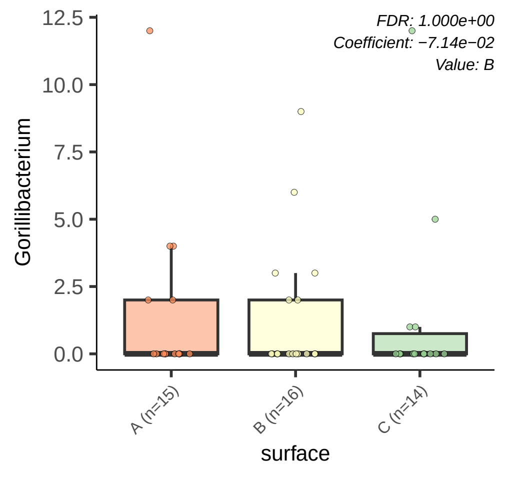
FDR: 1.000e+00
Coefficient: −7.14e−02
Value: B
Gorillibacterium
12.5
10.0
7.5
5.0
2.5
0.0
A (n=15)
B (n=16)
C (n=14)
surface

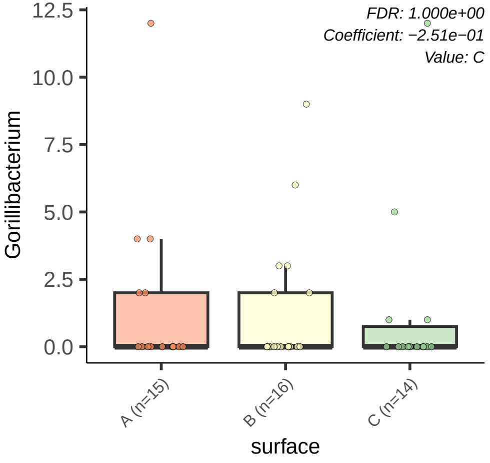

FDR: 1.000e+00
Coefficient: −2.51e−01
Value: C
Gorillibacterium
12.5
10.0
7.5
5.0
2.5
0.0
A (n=15)
B (n=16)
C (n=14)
surface

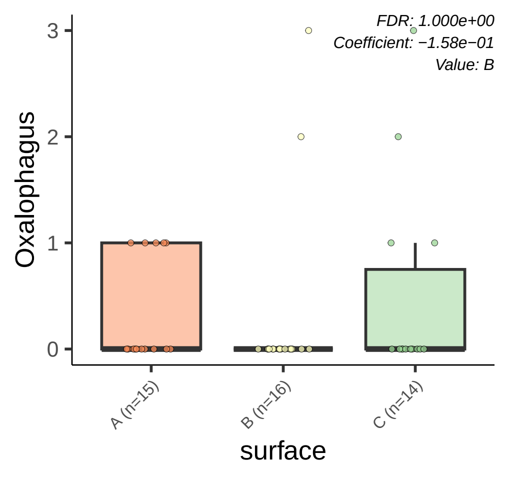

FDR: 1.000e+00
Coefficient: −1.58e−01
Value: B
Oxalophagus
0
1
2
3
A (n=15)
B (n=16)
C (n=14)
surface

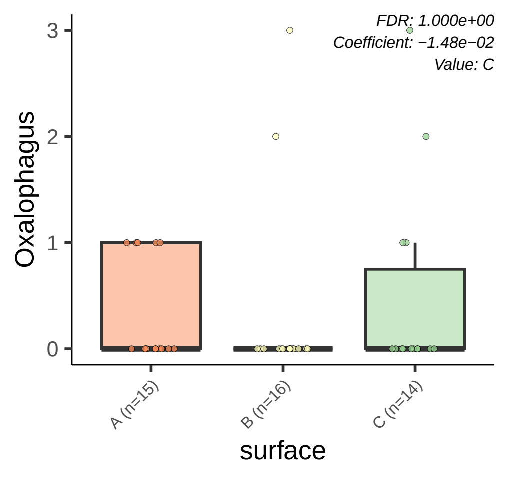

FDR: 1.000e+00
Coefficient: −1.48e−02
Value: C
Oxalophagus
3
2
1
0
A (n=15)
B (n=16)
C (n=14)
surface

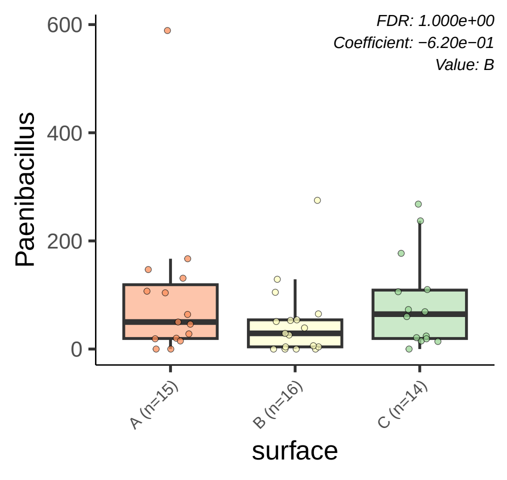
FDR: 1.000e+00
Coefficient: −6.20e−01
Value: B
Paenibacillus
600
400
200
0
A (n=15)
B (n=16)
C (n=14)
surface

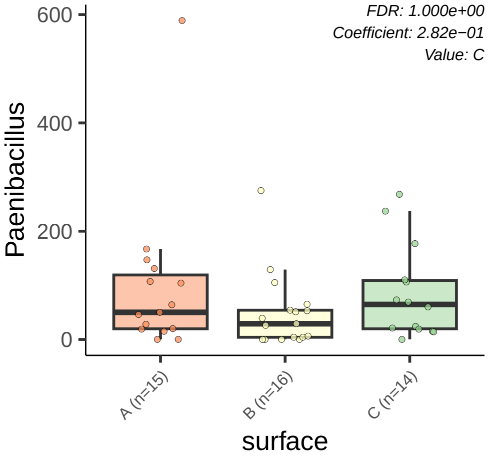
FDR: 1.000e+00
Coefficient: 2.82e−01
Value: C
Paenibacillus
600
400
200
0
A (n=15)
B (n=16)
C (n=14)
surface

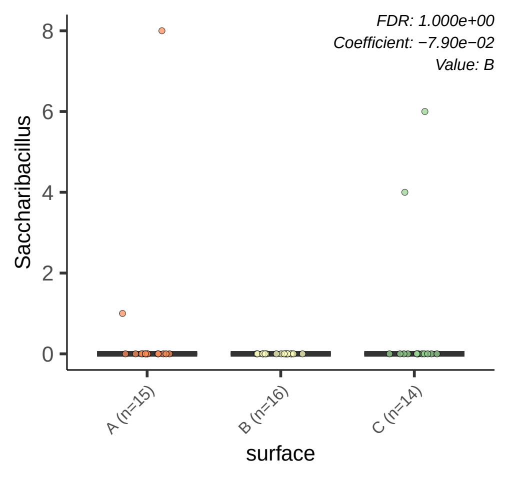

FDR: 1.000e+00
Coefficient: −7.90e−02
Value: B
Saccharibacillus
0
2
4
6
8
A (n=15)
B (n=16)
C (n=14)
surface

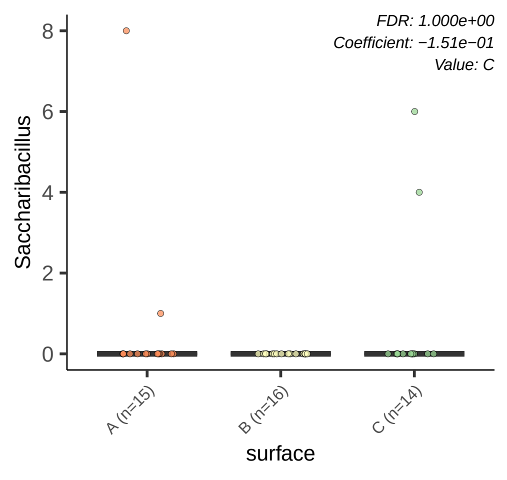

FDR: 1.000e+00
Coefficient: −1.51e−01
Value: C
Saccharibacillus
8
6
4
2
0
A (n=15)
B (n=16)
C (n=14)
surface

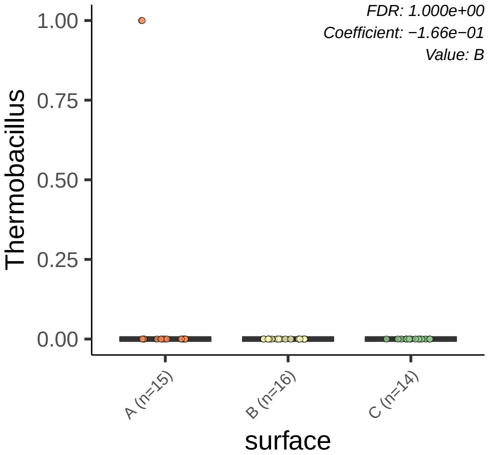
FDR: 1.000e+00
Coefficient: −1.66e−01
Value: B
Thermobacillus
1.00
0.75
0.50
0.25
0.00
A (n=15)
B (n=16)
C (n=14)
surface

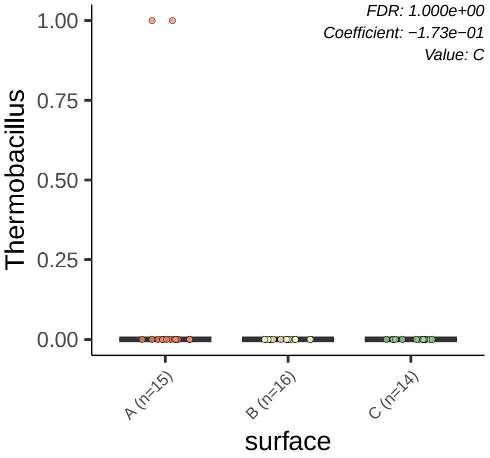

FDR: 1.000e+00
Coefficient: −1.73e−01
Value: C
Thermobacillus
1.00
0.75
0.50
0.25
0.00
A (n=15)
B (n=16)
C (n=14)
surface

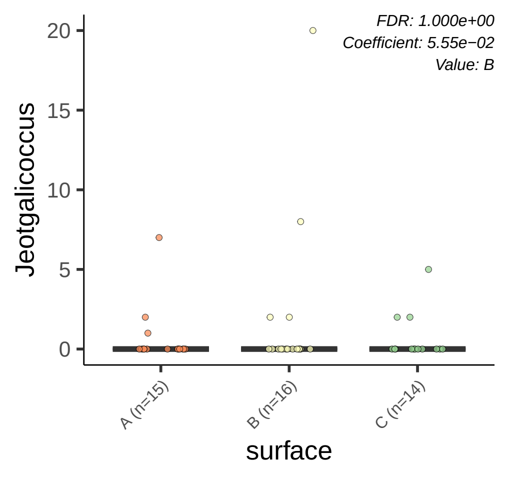

FDR: 1.000e+00
Coefficient: 5.55e−02
Value: B
Jeotgalicoccus
20
15
10
5
0
A (n=15)
B (n=16)
C (n=14)
surface

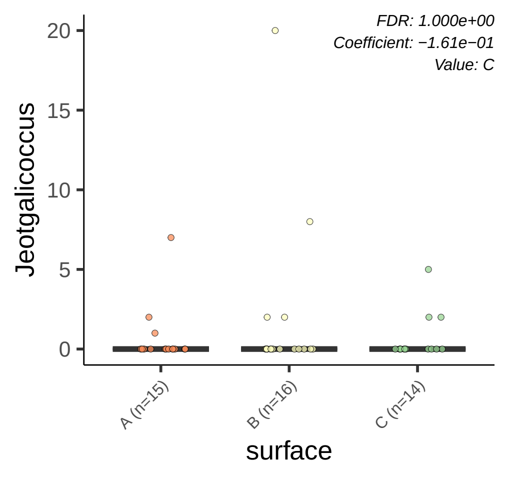

FDR: 1.000e+00
Coefficient: −1.61e−01
Value: C
Jeotgalicoccus
20
15
10
5
0
A (n=15)
B (n=16)
C (n=14)
surface

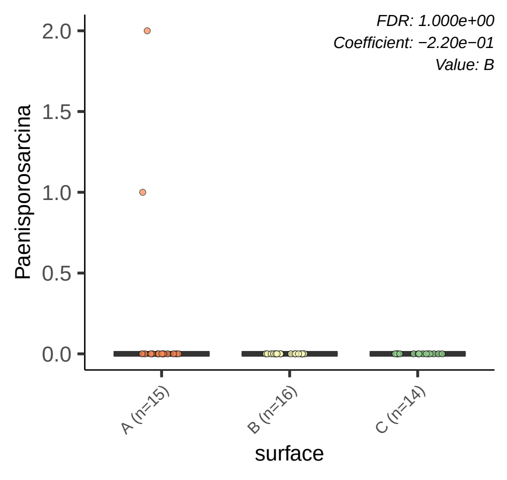

FDR: 1.000e+00
Coefficient: −2.20e−01
Value: B
Paenisporosarcina
2.0
1.5
1.0
0.5
0.0
A (n=15)
B (n=16)
C (n=14)
surface

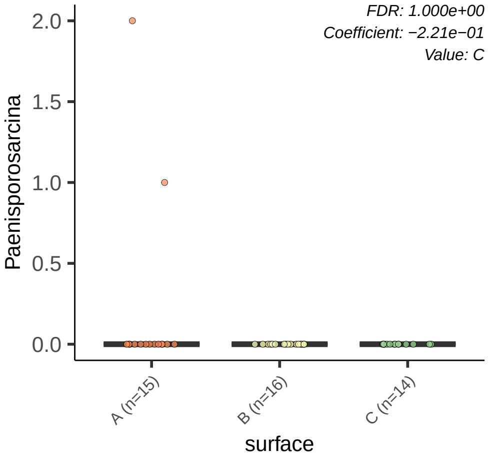
FDR: 1.000e+00
Coefficient: −2.21e−01
Value: C
Paenisporosarcina
2.0
1.5
1.0
0.5
0.0
A (n=15)
B (n=16)
C (n=14)
surface

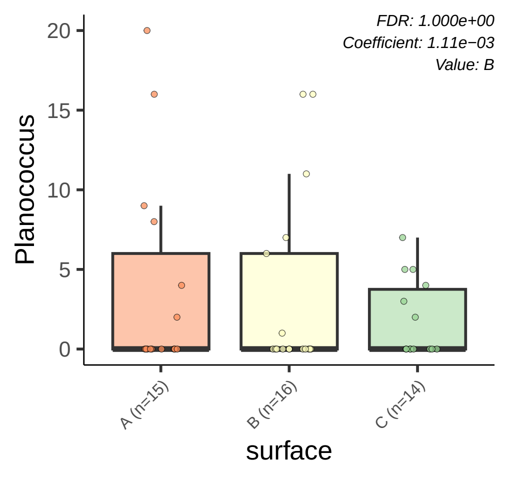

FDR: 1.000e+00
Coefficient: 1.11e−03
Value: B
Planococcus
0
5
10
15
20
A (n=15)
B (n=16)
C (n=14)
surface

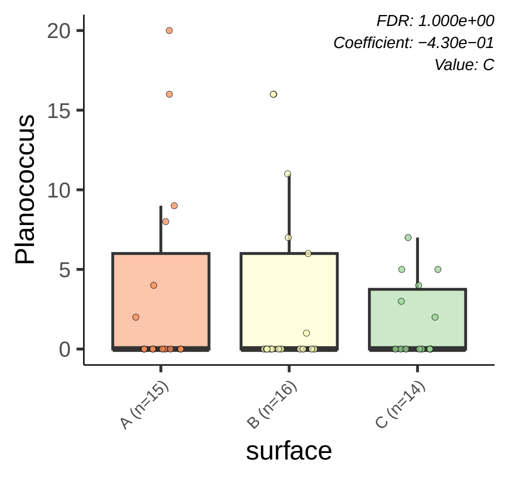
FDR: 1.000e+00
Coefficient: −4.30e−01
Value: C
Planococcus
20
15
10
5
0
A (n=15)
B (n=16)
C (n=14)
surface

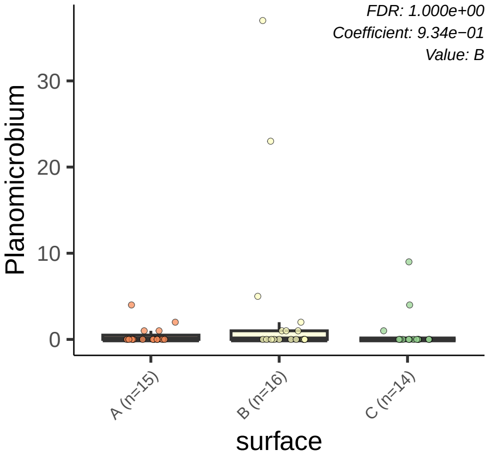

FDR: 1.000e+00
Coefficient: 9.34e−01
Value: B
Planomicrobium
30
20
10
0
A (n=15)
B (n=16)
C (n=14)
surface

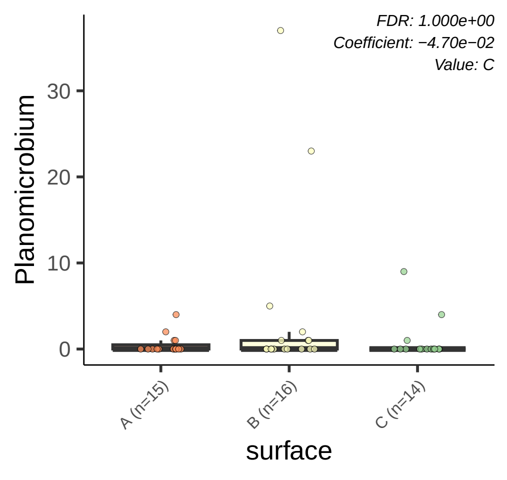
FDR: 1.000e+00
Coefficient: −4.70e−02
Value: C
Planomicrobium
0
10
20
30
A (n=15)
B (n=16)
C (n=14)
surface

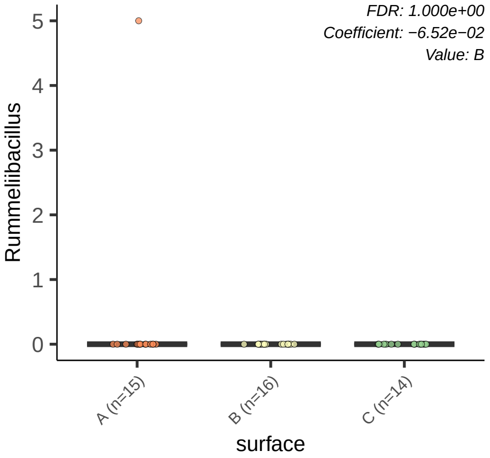
FDR: 1.000e+00
Coefficient: −6.52e−02
Value: B
Rummeliibacillus
0
1
2
3
4
5
A (n=15)
B (n=16)
C (n=14)
surface

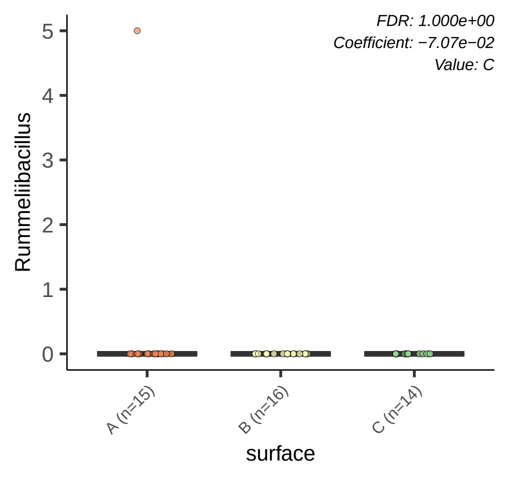
FDR: 1.000e+00
Coefficient: −7.07e−02
Value: C
Rummeliibacillus
0
1
2
3
4
5
A (n=15)
B (n=16)
C (n=14)
surface

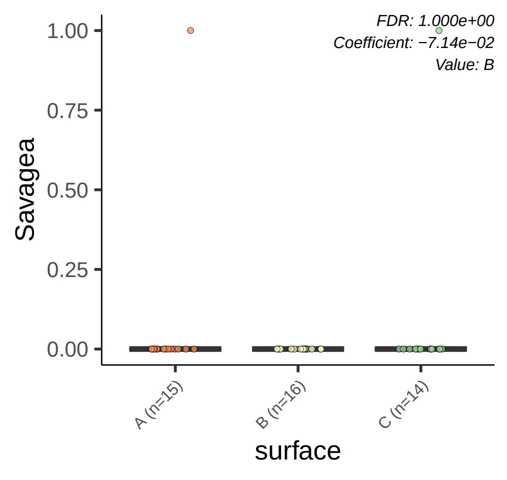
FDR: 1.000e+00
Coefficient: −7.14e−02
Value: B
Savagea
1.00
0.75
0.50
0.25
0.00
A (n=15)
B (n=16)
C (n=14)
surface

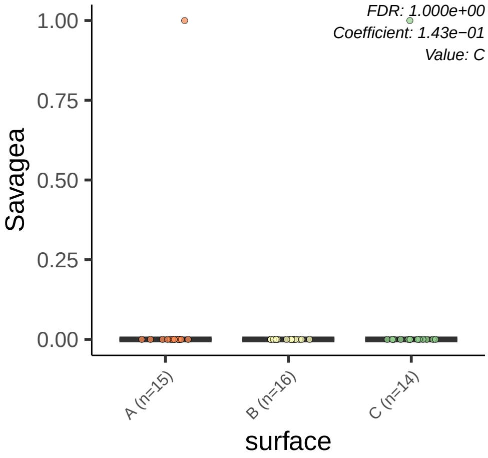
FDR: 1.000e+00
Coefficient: 1.43e−01
Value: C
Savagea
1.00
0.75
0.50
0.25
0.00
A (n=15)
B (n=16)
C (n=14)
surface

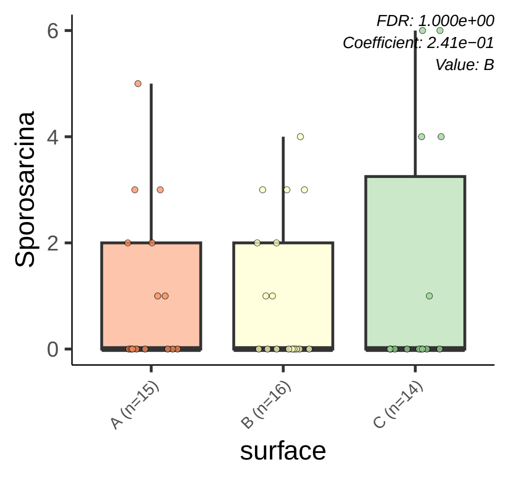
FDR: 1.000e+00
Coefficient: 2.41e−01
Value: B
Sporosarcina
0
2
4
6
A (n=15)
B (n=16)
C (n=14)
surface

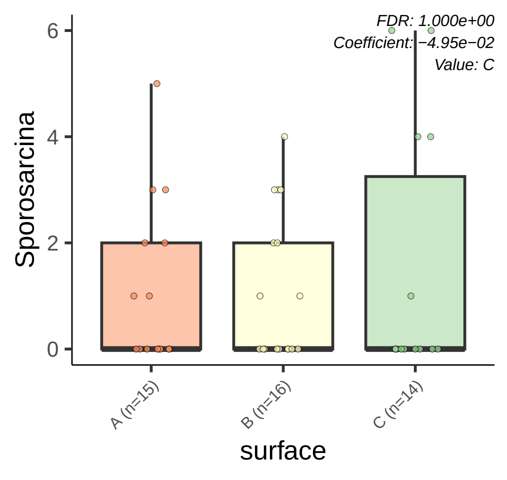

FDR: 1.000e+00
Coefficient: −4.95e−02
Value: C
Sporosarcina
0
2
4
6
A (n=15)
B (n=16)
C (n=14)
surface

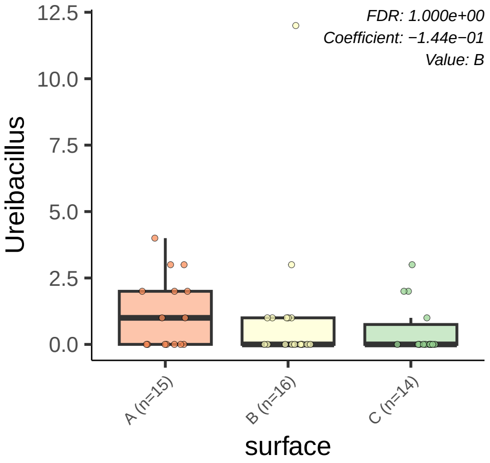

FDR: 1.000e+00
Coefficient: −1.44e−01
Value: B
Ureibacillus
12.5
10.0
7.5
5.0
2.5
0.0
A (n=15)
B (n=16)
C (n=14)
surface

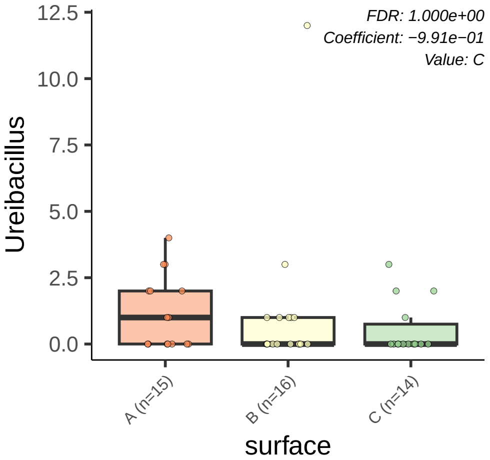
FDR: 1.000e+00
Coefficient: −9.91e−01
Value: C
Ureibacillus
12.5
10.0
7.5
5.0
2.5
0.0
A (n=15)
B (n=16)
C (n=14)
surface

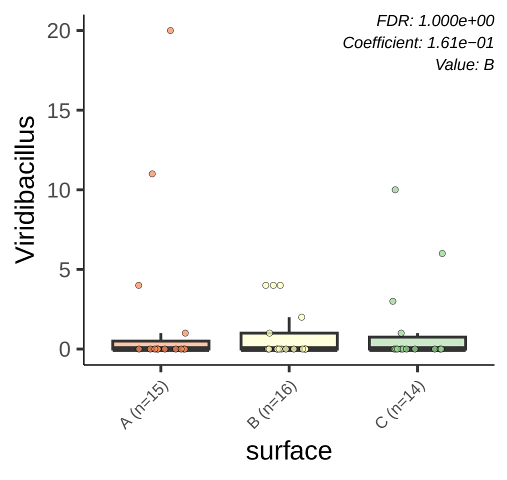

FDR: 1.000e+00
Coefficient: 1.61e−01
Value: B
Viridibacillus
20
15
10
5
0
A (n=15)
B (n=16)
C (n=14)
surface

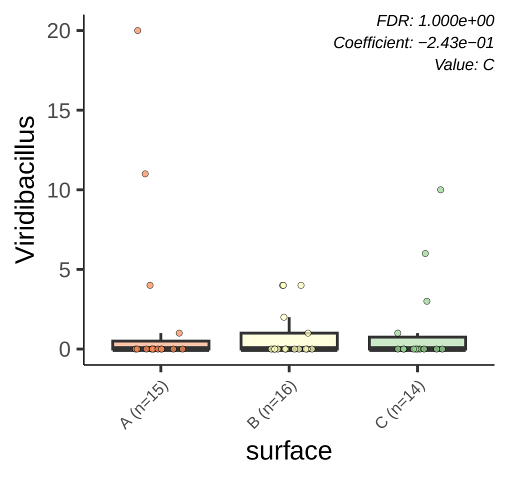
FDR: 1.000e+00
Coefficient: −2.43e−01
Value: C
Viridibacillus
20
15
10
5
0
A (n=15)
B (n=16)
C (n=14)
surface

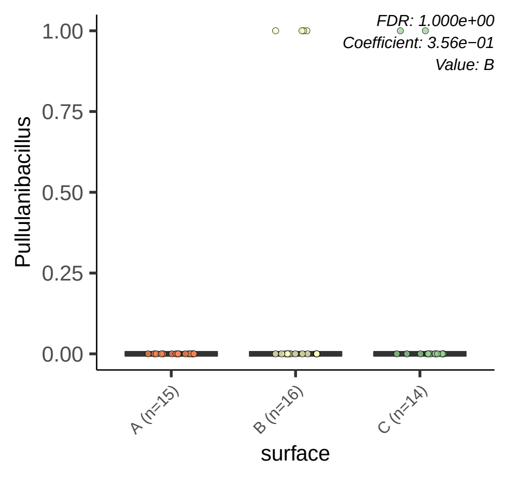
FDR: 1.000e+00
Coefficient: 3.56e−01
Value: B
Pullulanibacillus
1.00
0.75
0.50
0.25
0.00
A (n=15)
B (n=16)
C (n=14)
surface

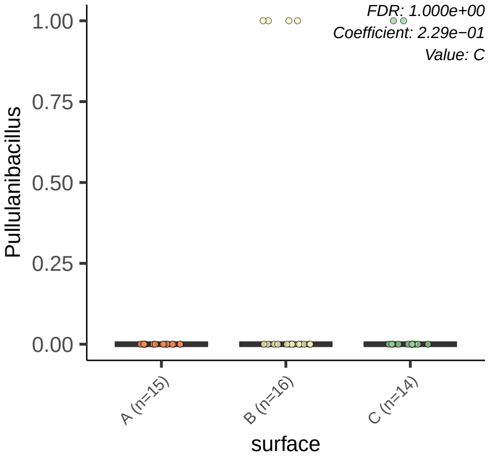
FDR: 1.000e+00
Coefficient: 2.29e−01
Value: C
1.00
0.75
0.50
0.25
0.00
Pullulanibacillus
A (n=15)
B (n=16)
C (n=14)
surface

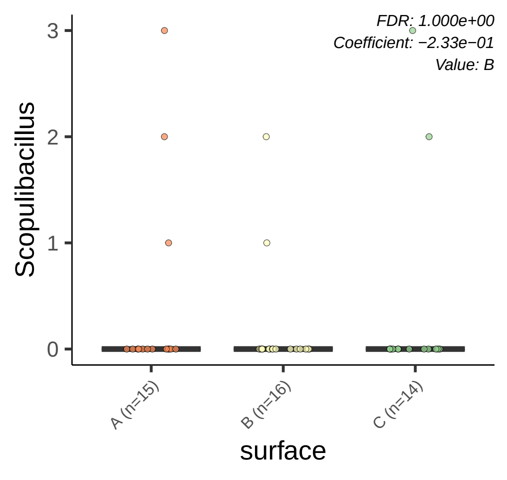
FDR: 1.000e+00
Coefficient: −2.33e−01
Value: B
Scopulibacillus
3
2
1
0
A (n=15)
B (n=16)
C (n=14)
surface

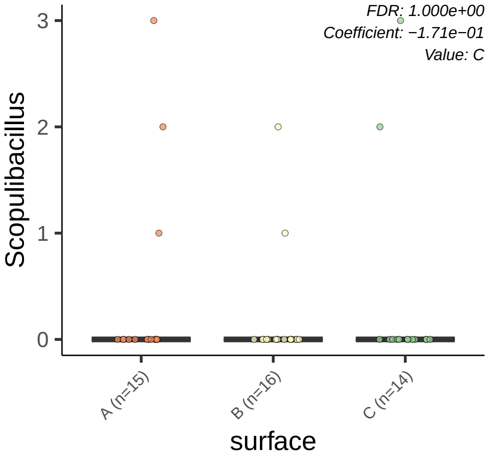
FDR: 1.000e+00
Coefficient: −1.71e−01
Value: C
Scopulibacillus
0
1
2
3
A (n=15)
B (n=16)
C (n=14)
surface

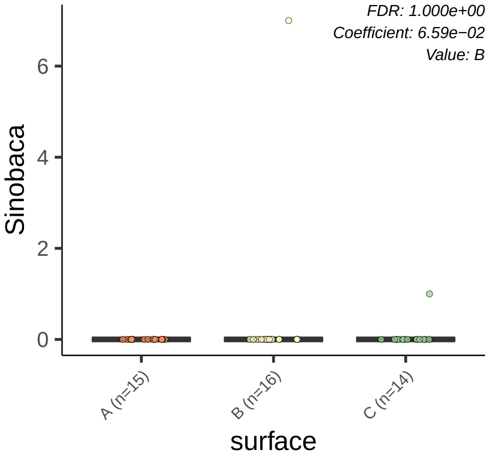
FDR: 1.000e+00
Coefficient: 6.59e−02
Value: B
Sinobaca
0
2
4
6
A (n=15)
B (n=16)
C (n=14)
surface

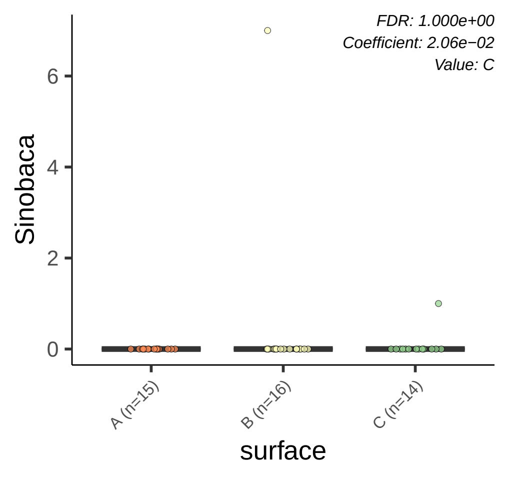
FDR: 1.000e+00
Coefficient: 2.06e−02
Value: C
Sinobaca
0
2
4
6
A (n=15)
B (n=16)
C (n=14)
surface

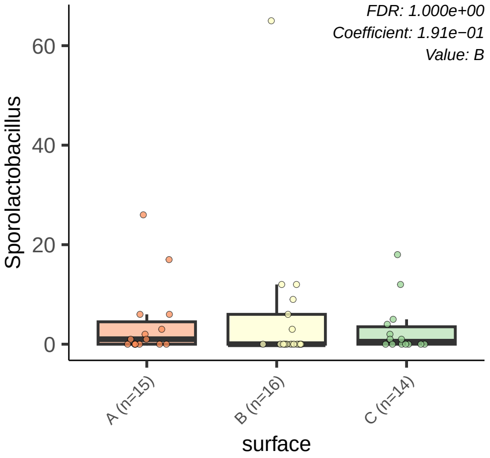
FDR: 1.000e+00
Coefficient: 1.91e−01
Value: B
Sporolactobacillus
0
20
40
60
A (n=15)
B (n=16)
C (n=14)
surface

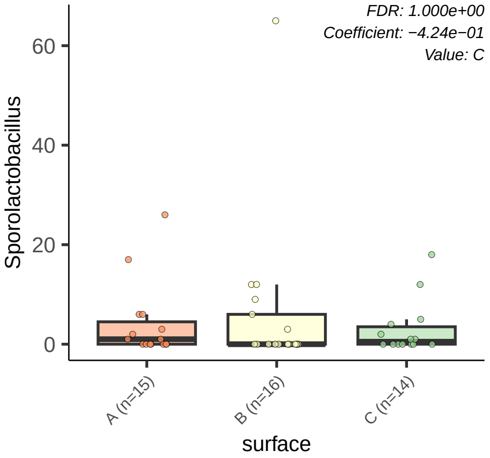

FDR: 1.000e+00
Coefficient: −4.24e−01
Value: C
Sporolactobacillus
0
20
40
60
A (n=15)
B (n=16)
C (n=14)
surface

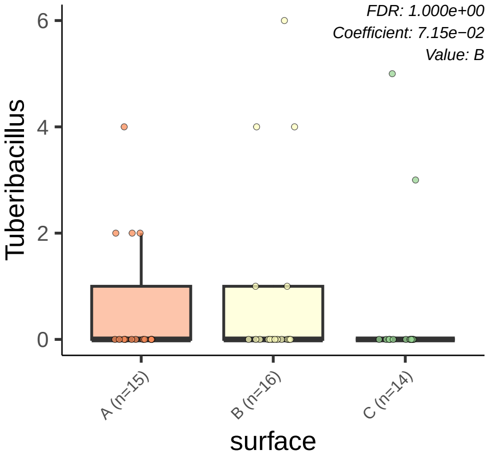

FDR: 1.000e+00
Coefficient: 7.15e−02
Value: B
Tuberibacillus
6
4
2
0
A (n=15)
B (n=16)
C (n=14)
surface

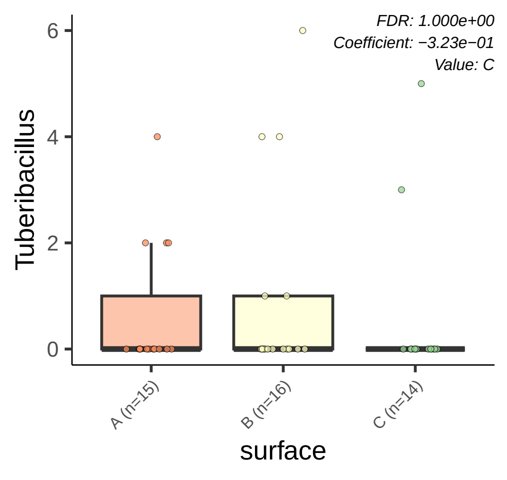

FDR: 1.000e+00
Coefficient: −3.23e−01
Value: C
Tuberibacillus
6
4
2
0
A (n=15)
B (n=16)
C (n=14)
surface

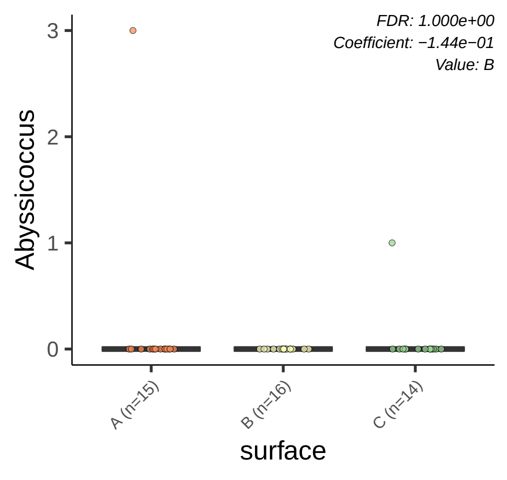
FDR: 1.000e+00
Coefficient: −1.44e−01
Value: B
Abyssicoccus
3
2
1
0
A (n=15)
B (n=16)
C (n=14)
surface

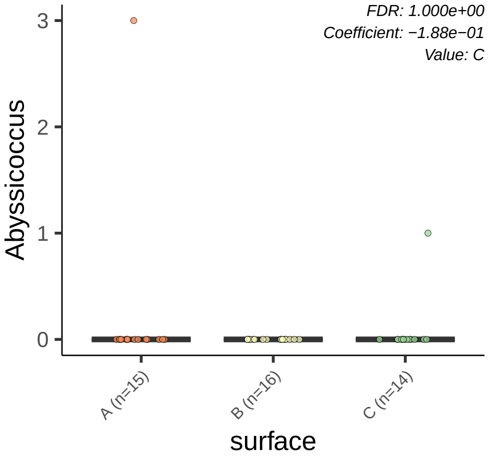
FDR: 1.000e+00
Coefficient: −1.88e−01
Value: C
Abyssicoccus
3
2
1
0
A (n=15)
B (n=16)
C (n=14)
surface

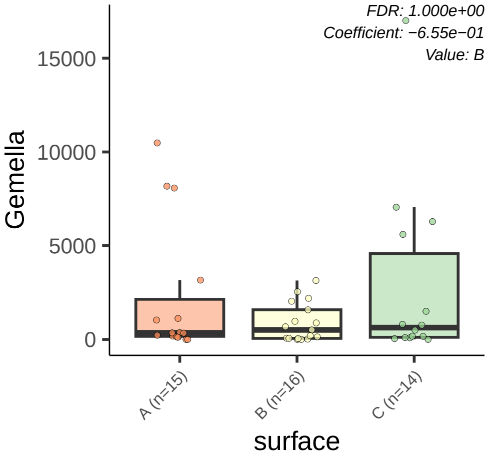
FDR: 1.000e+00
Coefficient: −6.55e−01
Value: B
Gemella
15000
10000
5000
0
A (n=15)
B (n=16)
C (n=14)
surface

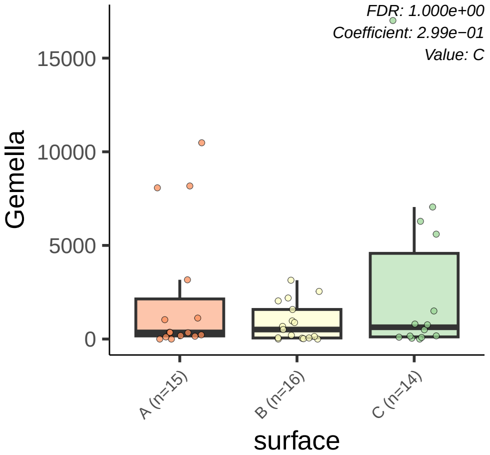
FDR: 1.000e+00
Coefficient: 2.99e−01
Value: C
Gemella
15000
10000
5000
0
A (n=15)
B (n=16)
C (n=14)
surface

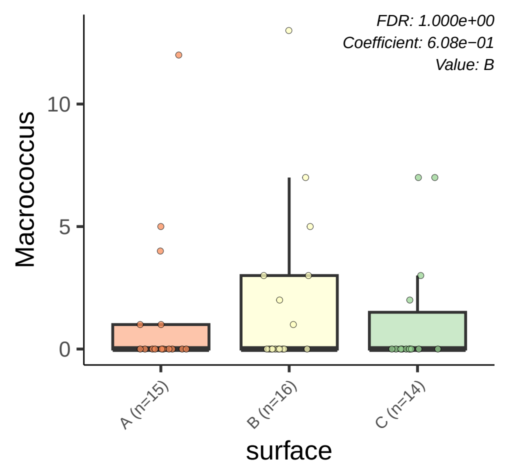
FDR: 1.000e+00
Coefficient: 6.08e−01
Value: B
Macrococcus
10
5
0
A (n=15)
B (n=16)
C (n=14)
surface

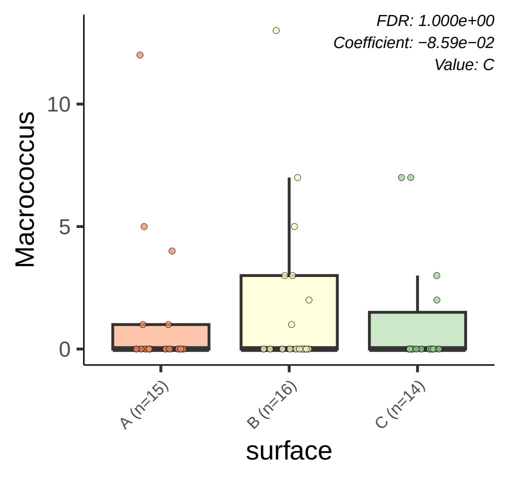

FDR: 1.000e+00
Coefficient: −8.59e−02
Value: C
Macrococcus
10
5
0
A (n=15)
B (n=16)
C (n=14)
surface

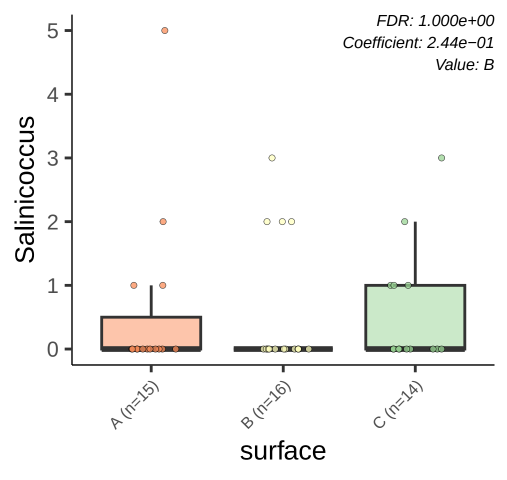
FDR: 1.000e+00
Coefficient: 2.44e−01
Value: B
Salinicoccus
0
1
2
3
4
5
A (n=15)
B (n=16)
C (n=14)
surface

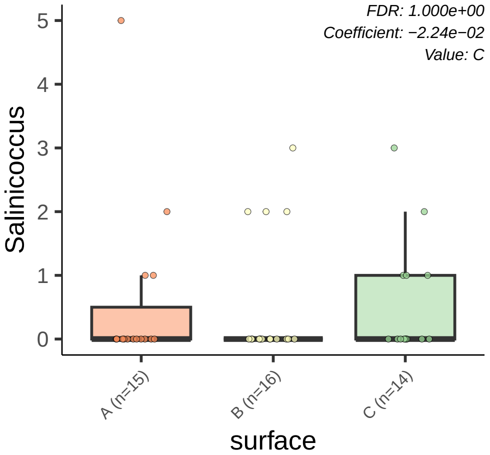

FDR: 1.000e+00
Coefficient: −2.24e−02
Value: C
Salinicoccus
0
1
2
3
4
5
A (n=15)
B (n=16)
C (n=14)
surface

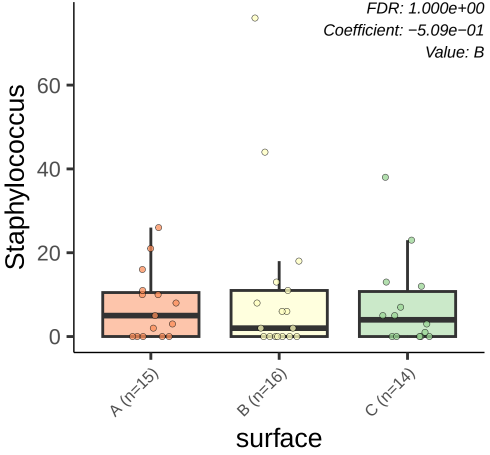
FDR: 1.000e+00
Coefficient: −5.09e−01
Value: B
Staphylococcus
0
20
40
60
A (n=15)
B (n=16)
C (n=14)
surface

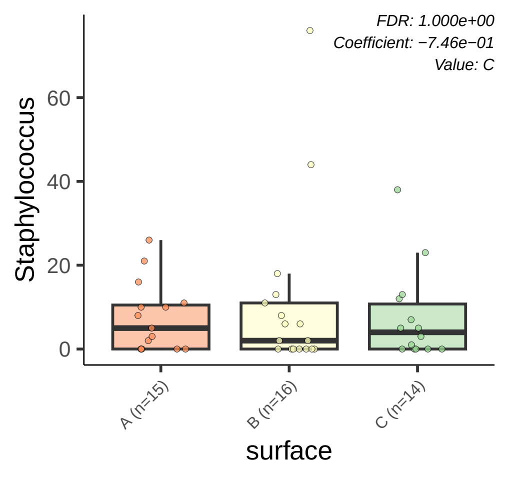
FDR: 1.000e+00
Coefficient: −7.46e−01
Value: C
Staphylococcus
0
20
40
60
A (n=15)
B (n=16)
C (n=14)
surface

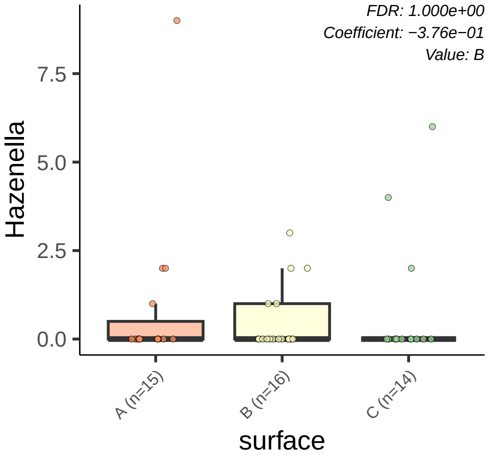

FDR: 1.000e+00
Coefficient: −3.76e−01
Value: B
Hazenella
7.5
5.0
2.5
0.0
A (n=15)
B (n=16)
C (n=14)
surface

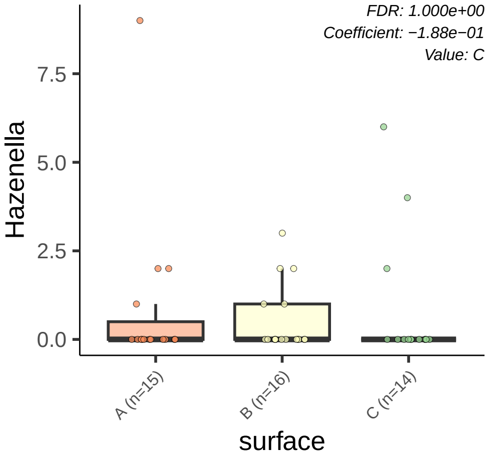
FDR: 1.000e+00
Coefficient: −1.88e−01
Value: C
Hazenella
7.5
5.0
2.5
0.0
A (n=15)
B (n=16)
C (n=14)
surface

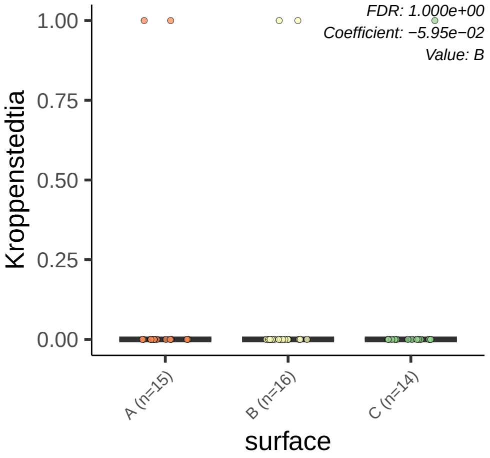
FDR: 1.000e+00
Coefficient: −5.95e−02
Value: B
Kroppenstedtia
1.00
0.75
0.50
0.25
0.00
A (n=15)
B (n=16)
C (n=14)
surface

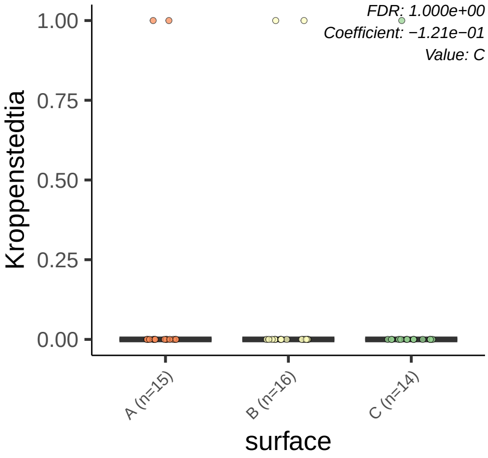
FDR: 1.000e+00
Coefficient: −1.21e−01
Value: C
Kroppenstedtia
1.00
0.75
0.50
0.25
0.00
A (n=15)
B (n=16)
C (n=14)
surface

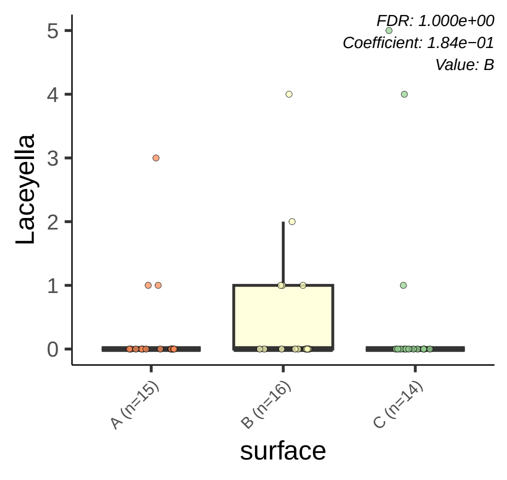

FDR: 1.000e+00
Coefficient: 1.84e−01
Value: B
Laceyella
0
1
2
3
4
5
A (n=15)
B (n=16)
C (n=14)
surface

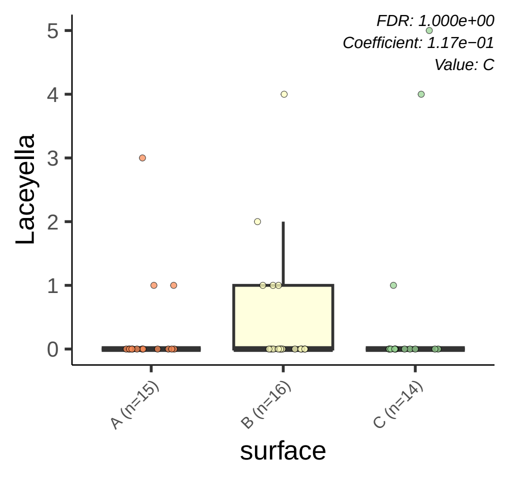
FDR: 1.000e+00
Coefficient: 1.17e−01
Value: C
Laceyella
0
1
2
3
4
5
A (n=15)
B (n=16)
C (n=14)
surface

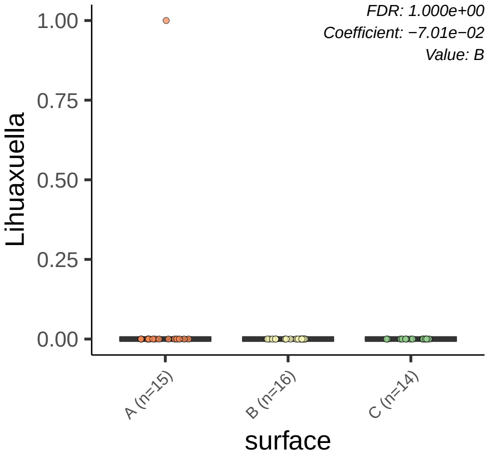
FDR: 1.000e+00
Coefficient: −7.01e−02
Value: B
Lihuaxuella
1.00
0.75
0.50
0.25
0.00
A (n=15)
B (n=16)
C (n=14)
surface

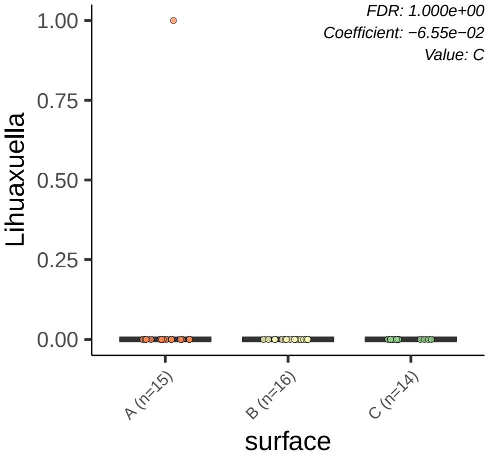
FDR: 1.000e+00
Coefficient: −6.55e−02
Value: C
Lihuaxuella
1.00
0.75
0.50
0.25
0.00
A (n=15)
B (n=16)
C (n=14)
surface

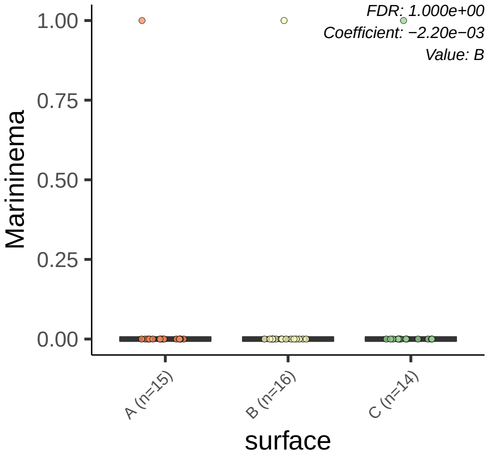
FDR: 1.000e+00
Coefficient: −2.20e−03
Value: B
Marininema
1.00
0.75
0.50
0.25
0.00
A (n=15)
B (n=16)
C (n=14)
surface

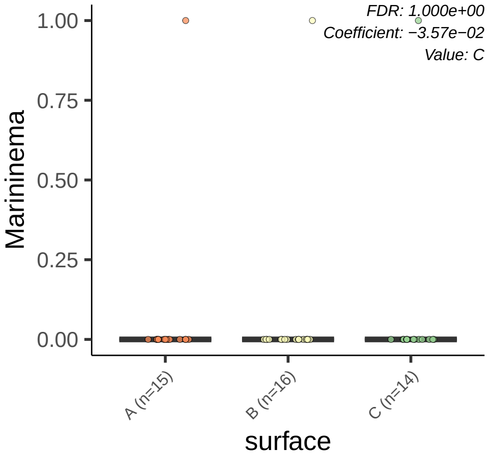

FDR: 1.000e+00
Coefficient: −3.57e−02
Value: C
Marininema
1.00
0.75
0.50
0.25
0.00
A (n=15)
B (n=16)
C (n=14)
surface

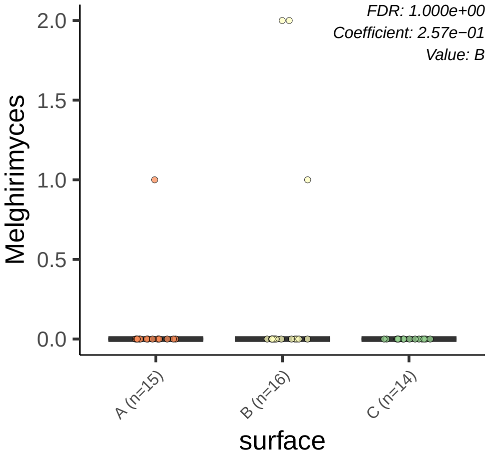
FDR: 1.000e+00
Coefficient: 2.57e−01
Value: B
Melghirimyces
2.0
1.5
1.0
0.5
0.0
A (n=15)
B (n=16)
C (n=14)
surface

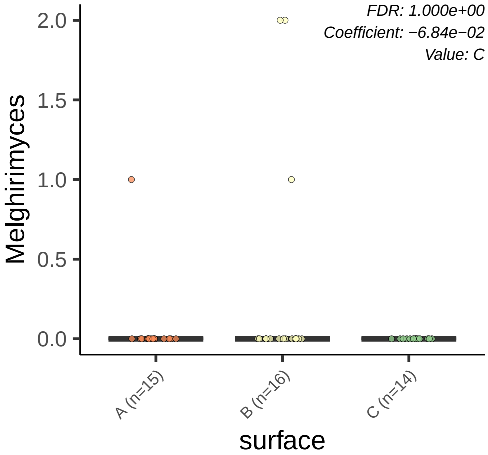

FDR: 1.000e+00
Coefficient: −6.84e−02
Value: C
Melghirimyces
2.0
1.5
1.0
0.5
0.0
A (n=15)
B (n=16)
C (n=14)
surface

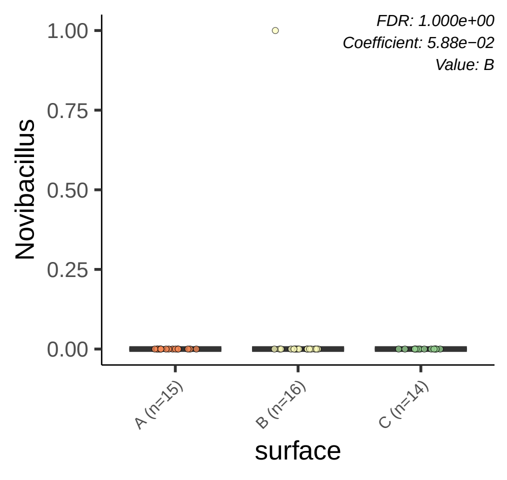

FDR: 1.000e+00
Coefficient: 5.88e−02
Value: B
Novibacillus
1.00
0.75
0.50
0.25
0.00
A (n=15)
B (n=16)
C (n=14)
surface

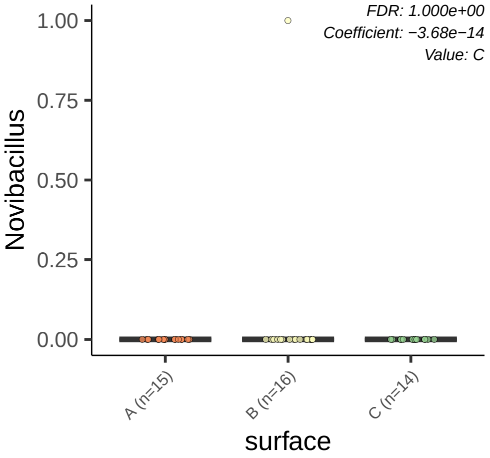

FDR: 1.000e+00
Coefficient: −3.68e−14
Value: C
Novibacillus
1.00
0.75
0.50
0.25
0.00
A (n=15)
B (n=16)
C (n=14)
surface

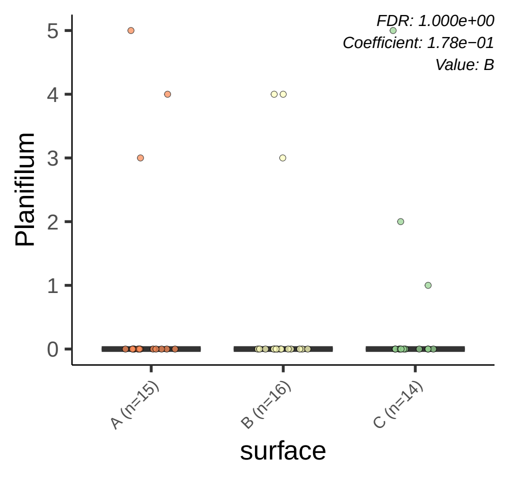

FDR: 1.000e+00
Coefficient: 1.78e−01
Value: B
Planifilum
5
4
3
2
1
0
A (n=15)
B (n=16)
C (n=14)
surface

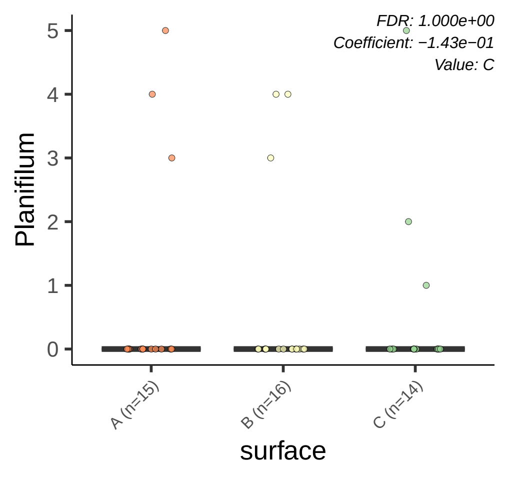

FDR: 1.000e+00
Coefficient: −1.43e−01
Value: C
Planifilum
5
4
3
2
1
0
A (n=15)
B (n=16)
C (n=14)
surface

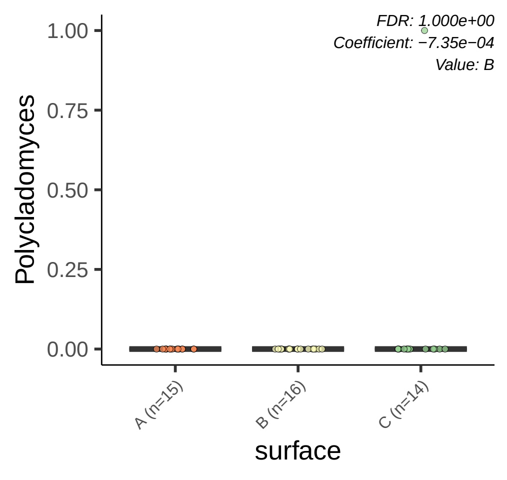

FDR: 1.000e+00
Coefficient: −7.35e−04
Value: B
Polycladomyces
1.00
0.75
0.50
0.25
0.00
A (n=15)
B (n=16)
C (n=14)
surface

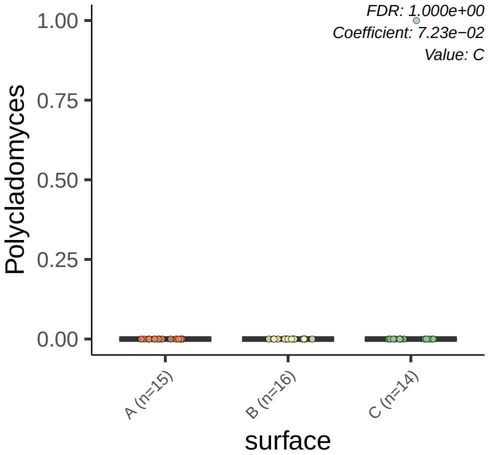
FDR: 1.000e+00
Coefficient: 7.23e−02
Value: C
Polycladomyces
1.00
0.75
0.50
0.25
0.00
A (n=15)
B (n=16)
C (n=14)
surface

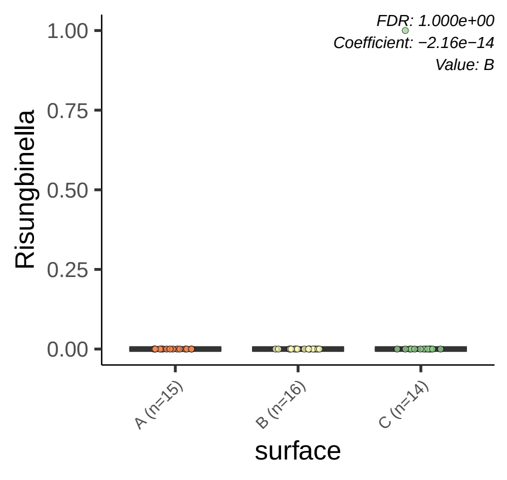
FDR: 1.000e+00
Coefficient: −2.16e−14
Value: B
1.00
0.75
0.50
0.25
0.00
Risungbinella
A (n=15)
B (n=16)
C (n=14)
surface

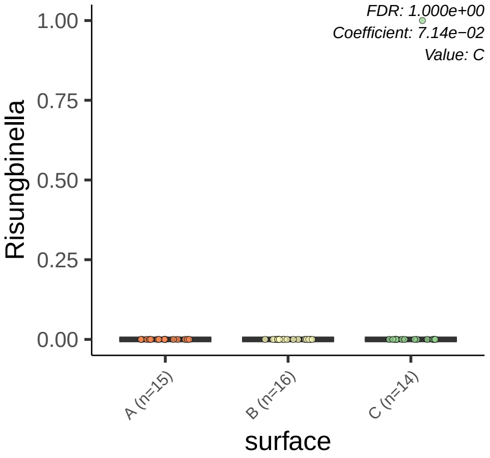
FDR: 1.000e+00
Coefficient: 7.14e−02
Value: C
Risungbinella
1.00
0.75
0.50
0.25
0.00
A (n=15)
B (n=16)
C (n=14)
surface

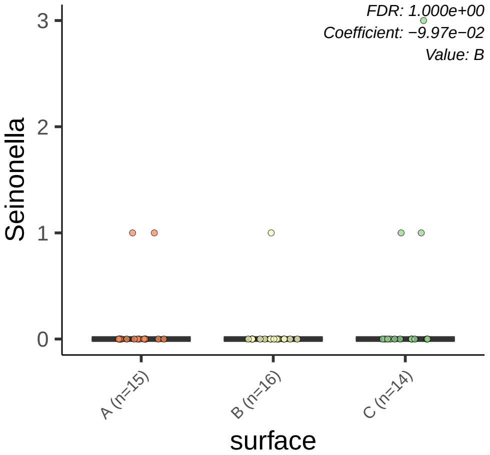

FDR: 1.000e+00
Coefficient: −9.97e−02
Value: B
Seinonella
3
2
1
0
A (n=15)
B (n=16)
C (n=14)
surface

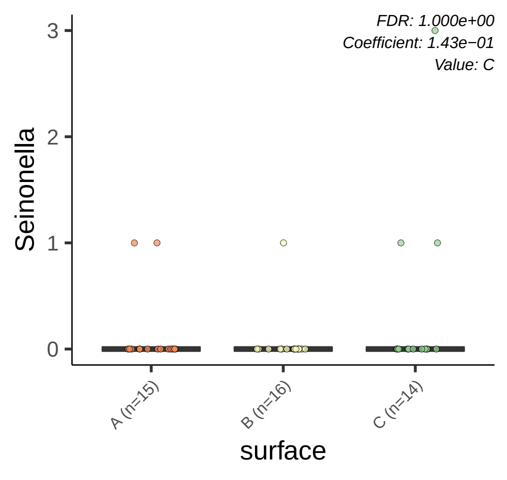
FDR: 1.000e+00
Coefficient: 1.43e−01
Value: C
Seinonella
0
1
2
3
A (n=15)
B (n=16)
C (n=14)
surface

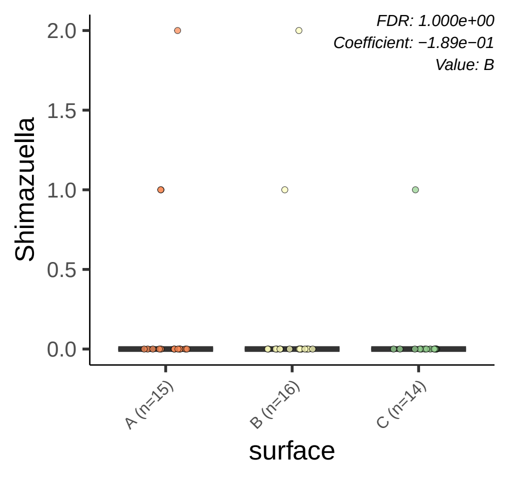
FDR: 1.000e+00
Coefficient: −1.89e−01
Value: B
Shimazuella
2.0
1.5
1.0
0.5
0.0
A (n=15)
B (n=16)
C (n=14)
surface

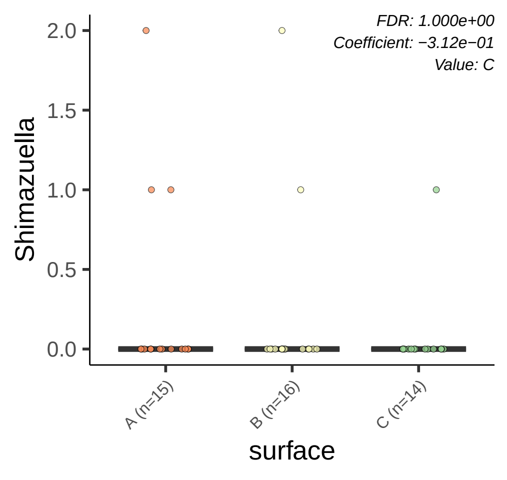
FDR: 1.000e+00
Coefficient: −3.12e−01
Value: C
Shimazuella
2.0
1.5
1.0
0.5
0.0
A (n=15)
B (n=16)
C (n=14)
surface

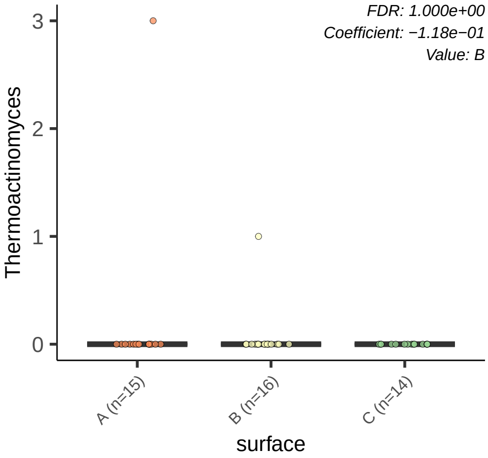
FDR: 1.000e+00
Coefficient: −1.18e−01
Value: B
Thermoactinomyces
3
2
1
0
A (n=15)
B (n=16)
C (n=14)
surface

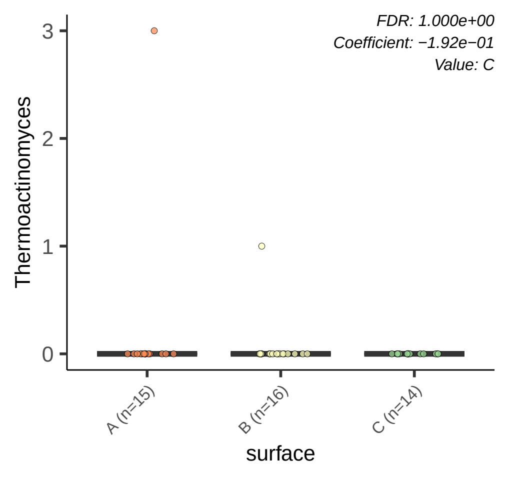

FDR: 1.000e+00
Coefficient: −1.92e−01
Value: C
Thermoactinomyces
3
2
1
0
A (n=15)
B (n=16)
C (n=14)
surface

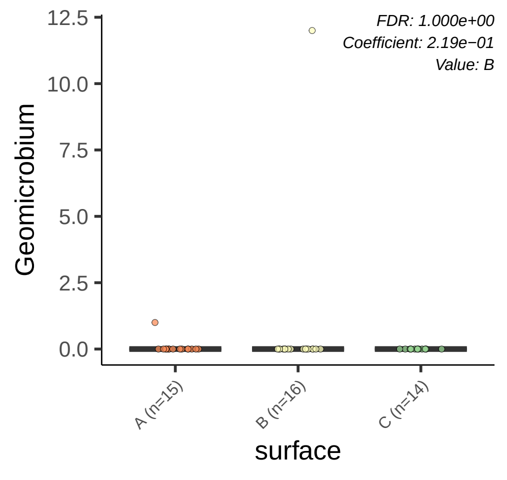
FDR: 1.000e+00
Coefficient: 2.19e−01
Value: B
Geomicrobium
12.5
10.0
7.5
5.0
2.5
0.0
A (n=15)
B (n=16)
C (n=14)
surface

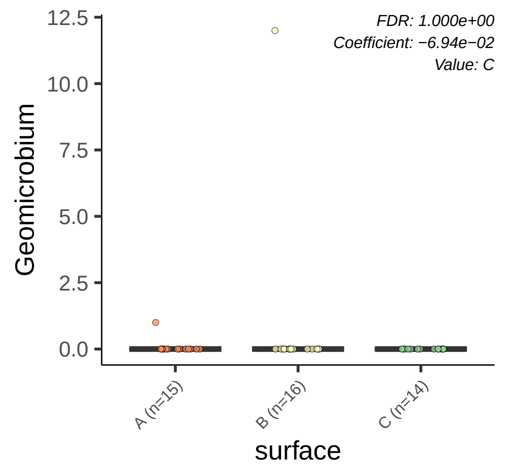
FDR: 1.000e+00
Coefficient: −6.94e−02
Value: C
Geomicrobium
12.5
10.0
7.5
5.0
2.5
0.0
A (n=15)
B (n=16)
C (n=14)
surface

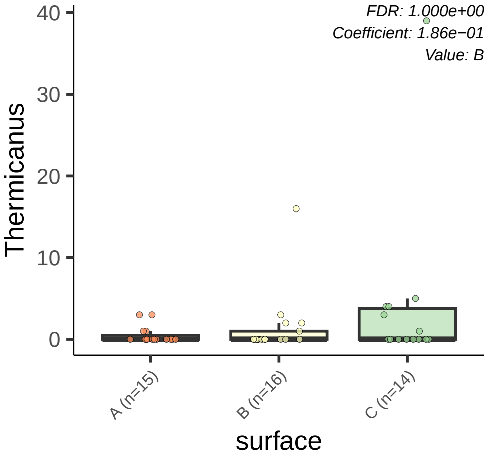

FDR: 1.000e+00
Coefficient: 1.86e−01
Value: B
Thermicanus
40
30
20
10
0
A (n=15)
B (n=16)
C (n=14)
surface

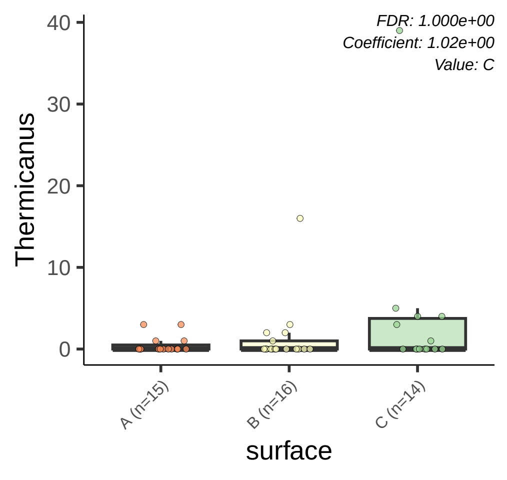
FDR: 1.000e+00
Coefficient: 1.02e+00
Value: C
Thermicanus
0
10
20
30
40
A (n=15)
B (n=16)
C (n=14)
surface

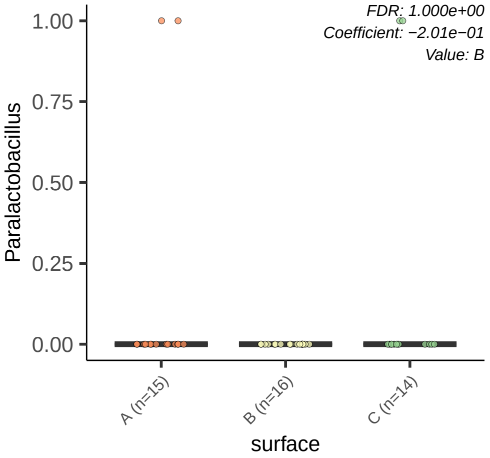
FDR: 1.000e+00
Coefficient: −2.01e−01
Value: B
Paralactobacillus
1.00
0.75
0.50
0.25
0.00
A (n=15)
B (n=16)
C (n=14)
surface

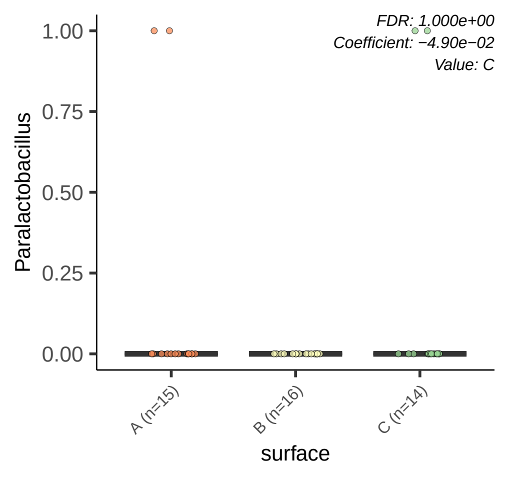
FDR: 1.000e+00
Coefficient: −4.90e−02
Value: C
Paralactobacillus
1.00
0.75
0.50
0.25
0.00
A (n=15)
B (n=16)
C (n=14)
surface

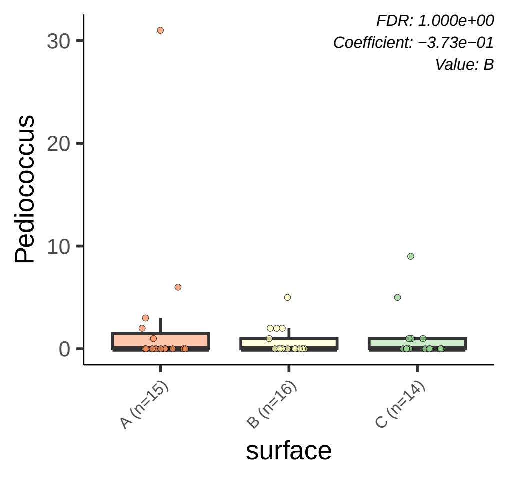
FDR: 1.000e+00
Coefficient: −3.73e−01
Value: B
Pediococcus
30
20
10
0
A (n=15)
B (n=16)
C (n=14)
surface

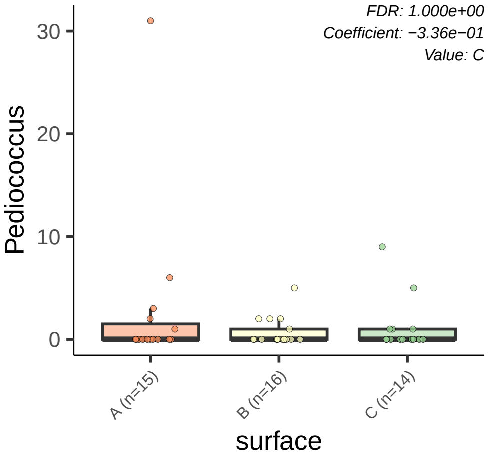

FDR: 1.000e+00
Coefficient: −3.36e−01
Value: C
Pediococcus
30
20
10
0
A (n=15)
B (n=16)
C (n=14)
surface

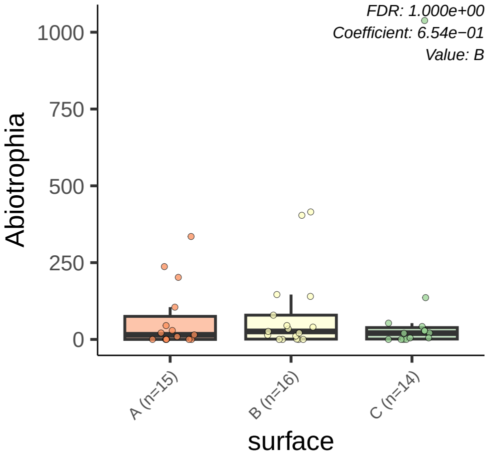
FDR: 1.000e+00
Coefficient: 6.54e−01
Value: B
Abiotrophia
1000
750
500
250
0
A (n=15)
B (n=16)
C (n=14)
surface

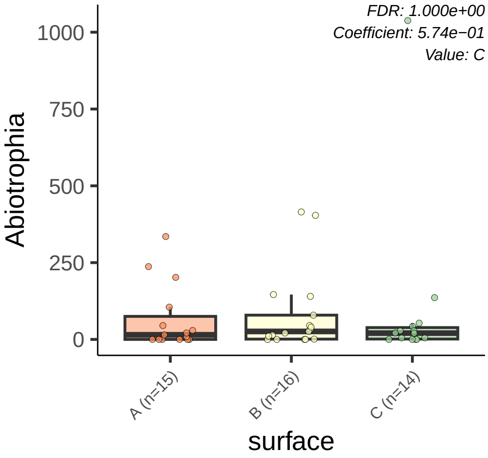

FDR: 1.000e+00
Coefficient: 5.74e−01
Value: C
Abiotrophia
1000
750
500
250
0
A (n=15)
B (n=16)
C (n=14)
surface

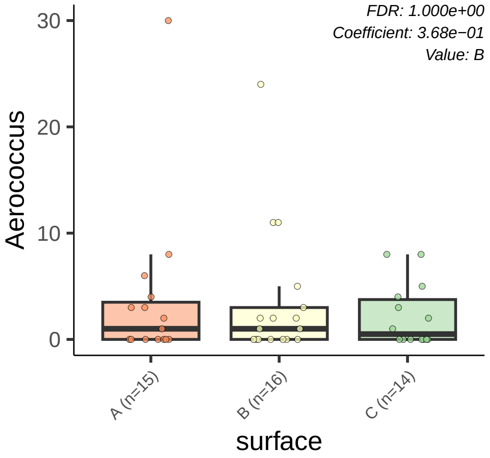

FDR: 1.000e+00
Coefficient: 3.68e−01
Value: B
Aerococcus
30
20
10
0
A (n=15)
B (n=16)
C (n=14)
surface

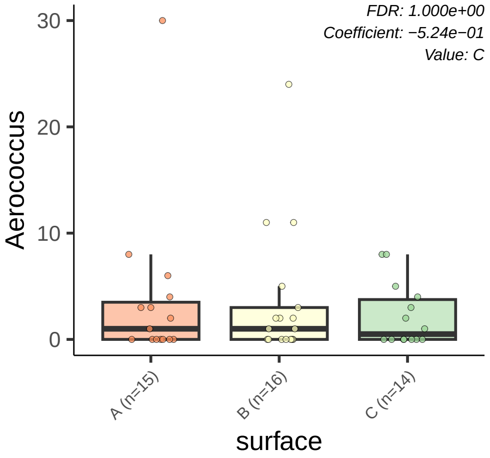
FDR: 1.000e+00
Coefficient: −5.24e−01
Value: C
Aerococcus
30
20
10
0
A (n=15)
B (n=16)
C (n=14)
surface

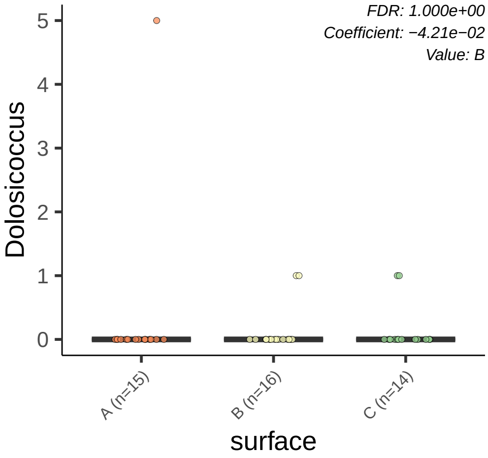

FDR: 1.000e+00
Coefficient: −4.21e−02
Value: B
Dolosicoccus
5
4
3
2
1
0
A (n=15)
B (n=16)
C (n=14)
surface

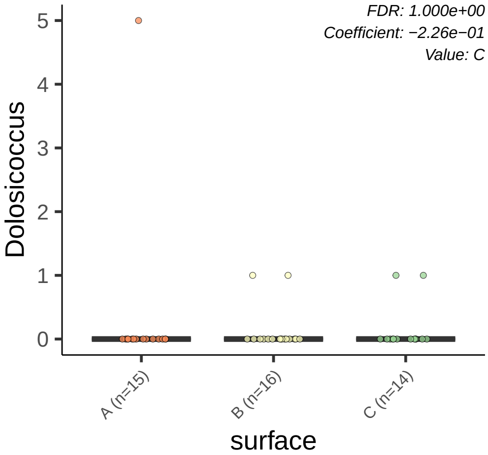
FDR: 1.000e+00
Coefficient: −2.26e−01
Value: C
Dolosicoccus
0
1
2
3
4
5
A (n=15)
B (n=16)
C (n=14)
surface

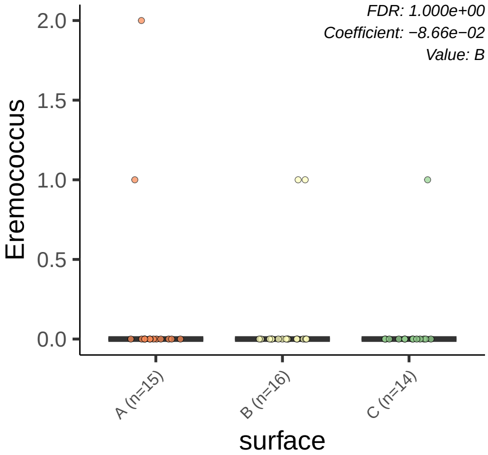
FDR: 1.000e+00
Coefficient: −8.66e−02
Value: B
Eremococcus
2.0
1.5
1.0
0.5
0.0
A (n=15)
B (n=16)
C (n=14)
surface

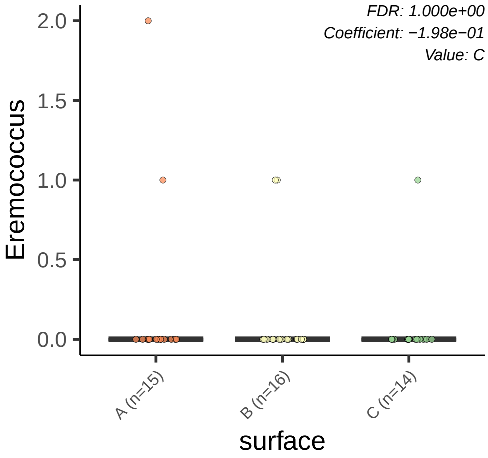

FDR: 1.000e+00
Coefficient: −1.98e−01
Value: C
Eremococcus
2.0
1.5
1.0
0.5
0.0
A (n=15)
B (n=16)
C (n=14)
surface

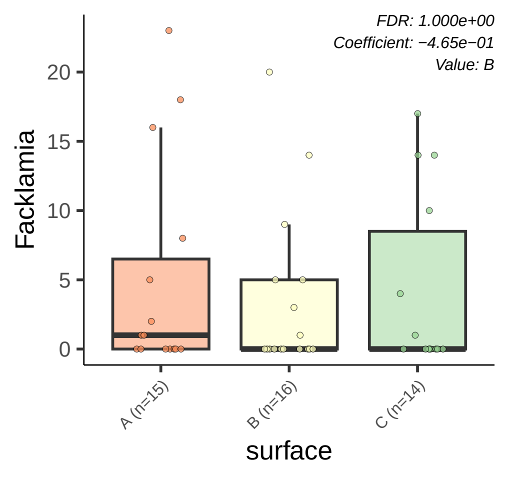

FDR: 1.000e+00
Coefficient: −4.65e−01
Value: B
Facklamia
0
5
10
15
20
A (n=15)
B (n=16)
C (n=14)
surface

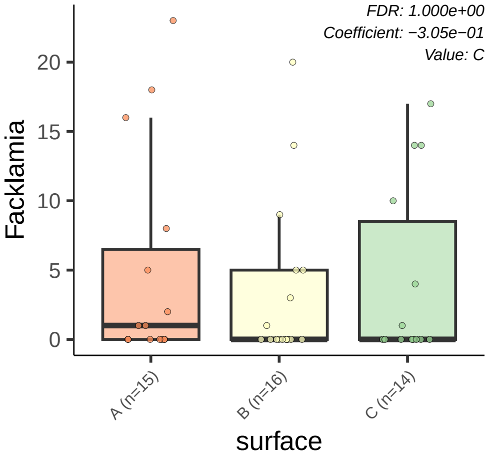
FDR: 1.000e+00
Coefficient: −3.05e−01
Value: C
Facklamia
0
5
10
15
20
A (n=15)
B (n=16)
C (n=14)
surface

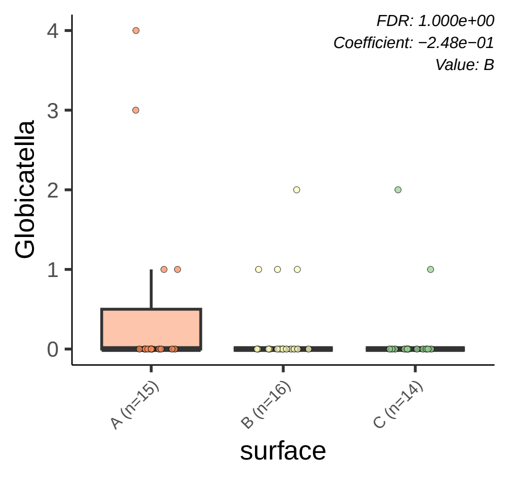
FDR: 1.000e+00
Coefficient: −2.48e−01
Value: B
Globicatella
4
3
2
1
0
A (n=15)
B (n=16)
C (n=14)
surface

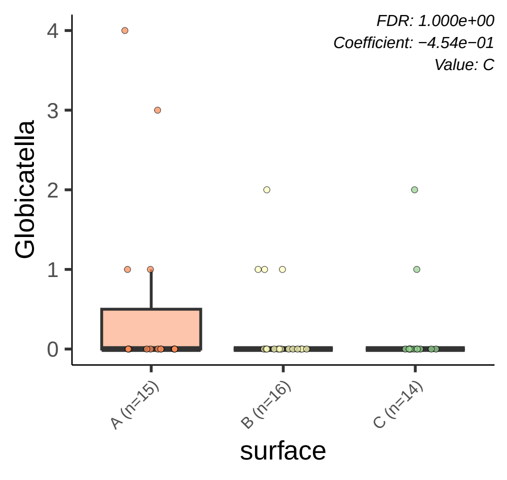
FDR: 1.000e+00
Coefficient: −4.54e−01
Value: C
Globicatella
0
1
2
3
4
A (n=15)
B (n=16)
C (n=14)
surface

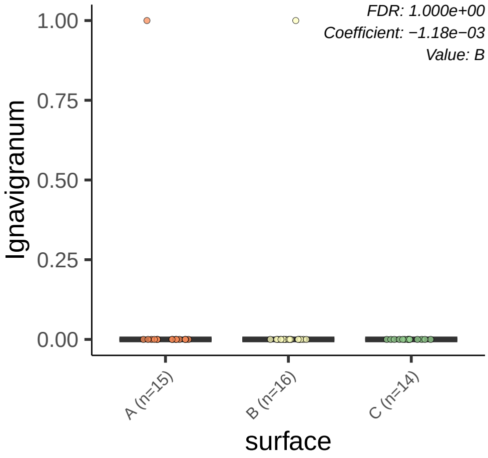
FDR: 1.000e+00
Coefficient: −1.18e−03
Value: B
Ignavigranum
1.00
0.75
0.50
0.25
0.00
A (n=15)
B (n=16)
C (n=14)
surface

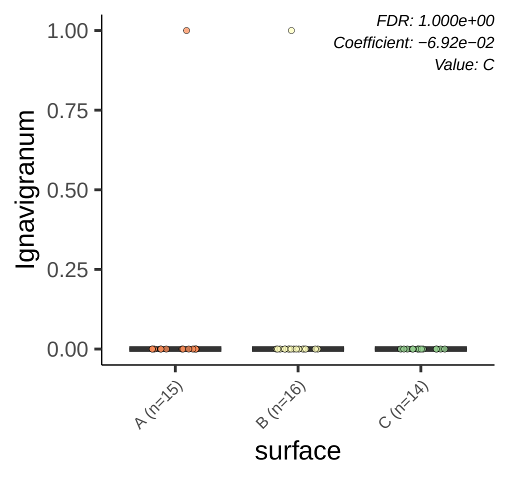
FDR: 1.000e+00
Coefficient: −6.92e−02
Value: C
Ignavigranum
1.00
0.75
0.50
0.25
0.00
A (n=15)
B (n=16)
C (n=14)
surface

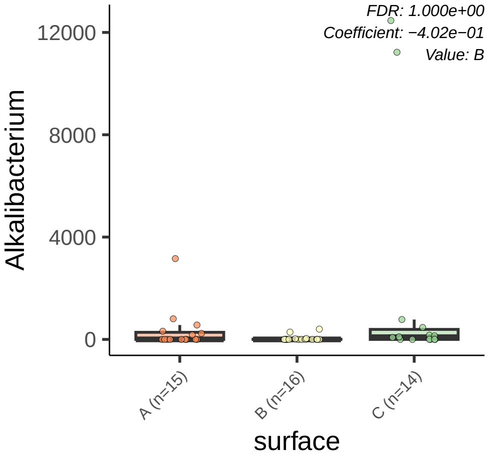
FDR: 1.000e+00
Coefficient: −4.02e−01
Value: B
12000
8000
4000
0
Alkalibacterium
A (n=15)
B (n=16)
C (n=14)
surface

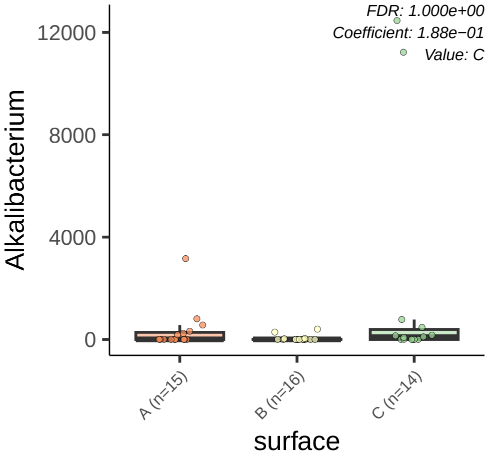
FDR: 1.000e+00
Coefficient: 1.88e−01
Value: C
12000
8000
4000
0
Alkalibacterium
A (n=15)
B (n=16)
C (n=14)
surface

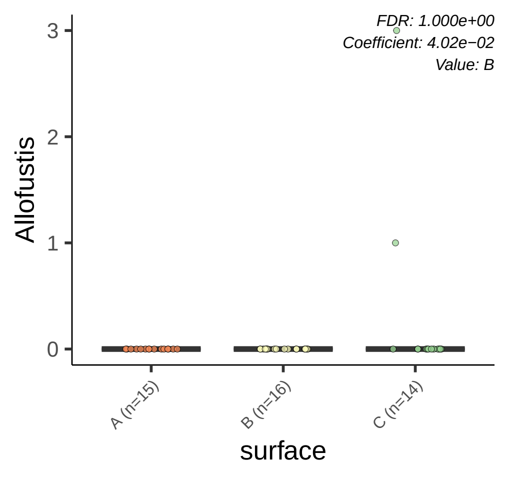
FDR: 1.000e+00
Coefficient: 4.02e−02
Value: B
Allofustis
3
2
1
0
A (n=15)
B (n=16)
C (n=14)
surface

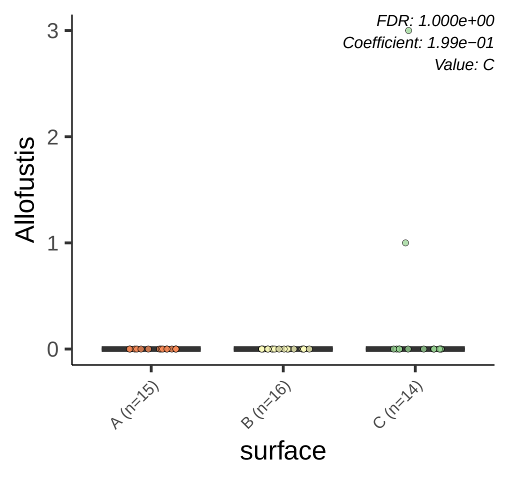
FDR: 1.000e+00
Coefficient: 1.99e−01
Value: C
Allofustis
3
2
1
0
A (n=15)
B (n=16)
C (n=14)
surface

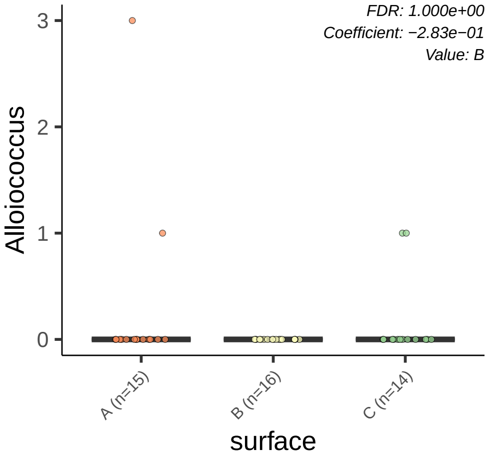
FDR: 1.000e+00
Coefficient: −2.83e−01
Value: B
Alloiococcus
3
2
1
0
A (n=15)
B (n=16)
C (n=14)
surface

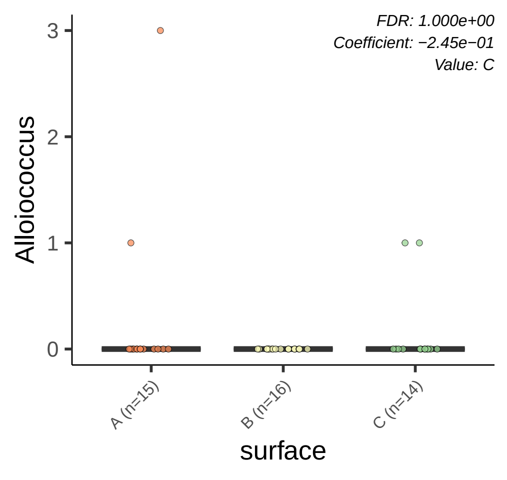
FDR: 1.000e+00
Coefficient: −2.45e−01
Value: C
Alloiococcus
3
2
1
0
A (n=15)
B (n=16)
C (n=14)
surface

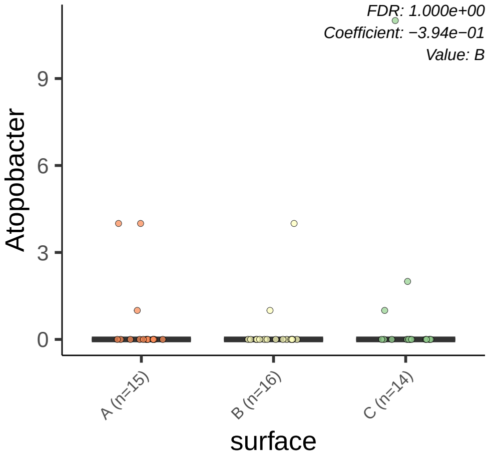
FDR: 1.000e+00
Coefficient: −3.94e−01
Value: B
Atopobacter
0
3
6
9
A (n=15)
B (n=16)
C (n=14)
surface

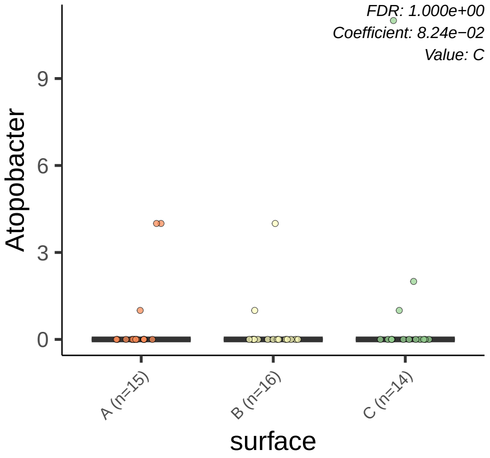

FDR: 1.000e+00
Coefficient: 8.24e−02
Value: C
Atopobacter
9
6
3
0
A (n=15)
B (n=16)
C (n=14)
surface

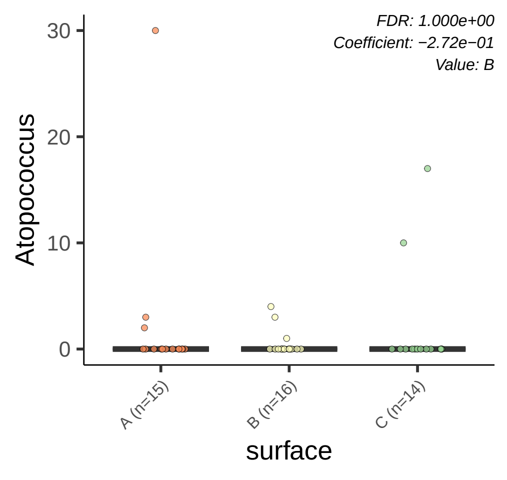

FDR: 1.000e+00
Coefficient: −2.72e−01
Value: B
Atopococcus
30
20
10
0
A (n=15)
B (n=16)
C (n=14)
surface

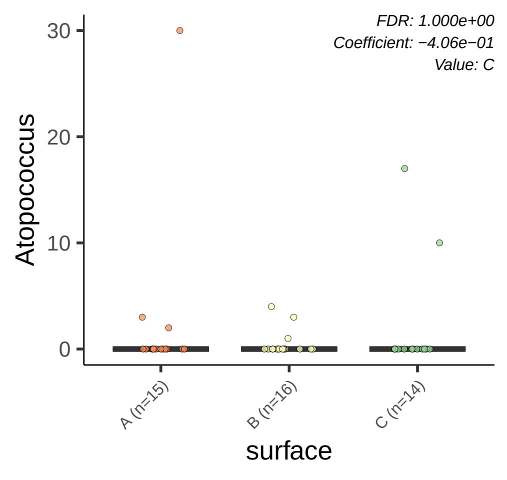
FDR: 1.000e+00
Coefficient: −4.06e−01
Value: C
Atopococcus
30
20
10
0
A (n=15)
B (n=16)
C (n=14)
surface

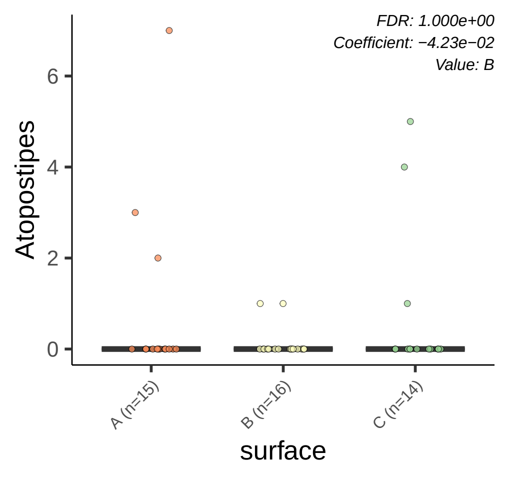
FDR: 1.000e+00
Coefficient: −4.23e−02
Value: B
Atopostipes
0
2
4
6
A (n=15)
B (n=16)
C (n=14)
surface

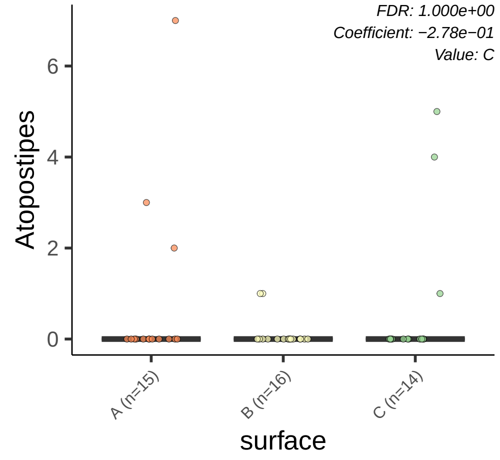
FDR: 1.000e+00
Coefficient: −2.78e−01
Value: C
Atopostipes
6
4
2
0
A (n=15)
B (n=16)
C (n=14)
surface

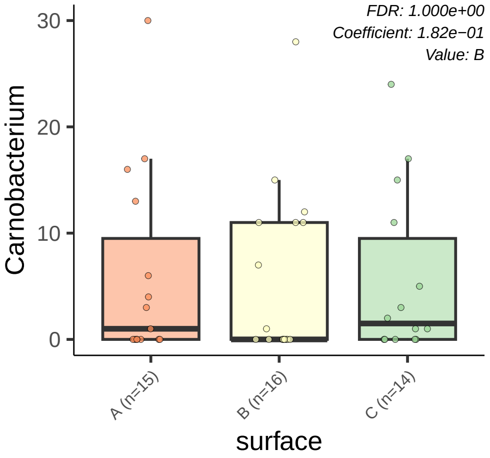

FDR: 1.000e+00
Coefficient: 1.82e−01
Value: B
Carnobacterium
30
20
10
0
A (n=15)
B (n=16)
C (n=14)
surface

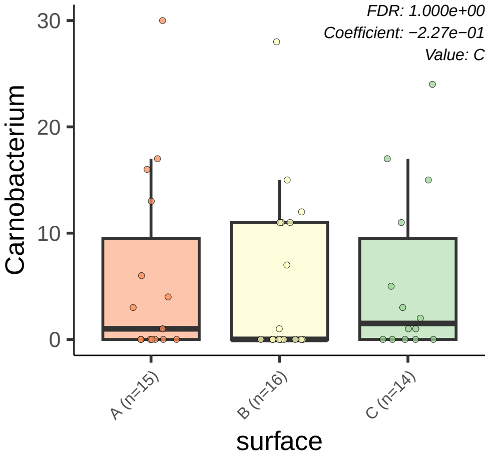
FDR: 1.000e+00
Coefficient: −2.27e−01
Value: C
Carnobacterium
30
20
10
0
A (n=15)
B (n=16)
C (n=14)
surface

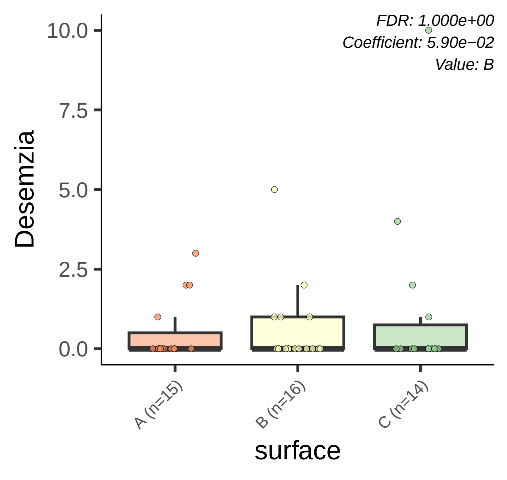
FDR: 1.000e+00
Coefficient: 5.90e−02
Value: B
Desemzia
10.0
7.5
5.0
2.5
0.0
A (n=15)
B (n=16)
C (n=14)
surface

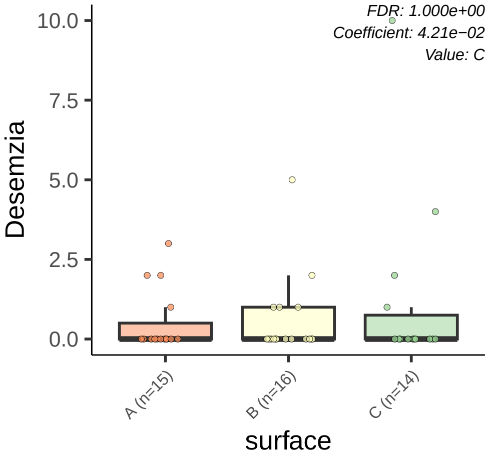
FDR: 1.000e+00
Coefficient: 4.21e−02
Value: C
Desemzia
10.0
7.5
5.0
2.5
0.0
A (n=15)
B (n=16)
C (n=14)
surface

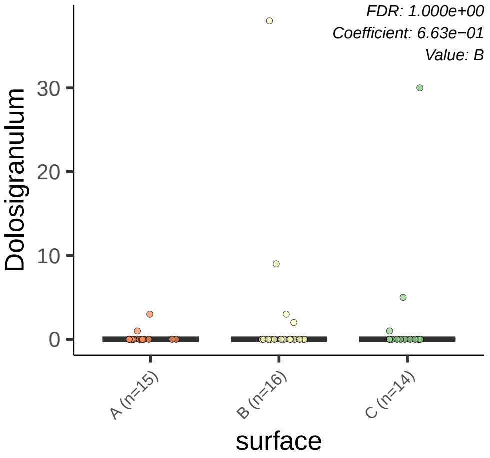

FDR: 1.000e+00
Coefficient: 6.63e−01
Value: B
Dolosigranulum
0
10
20
30
A (n=15)
B (n=16)
C (n=14)
surface

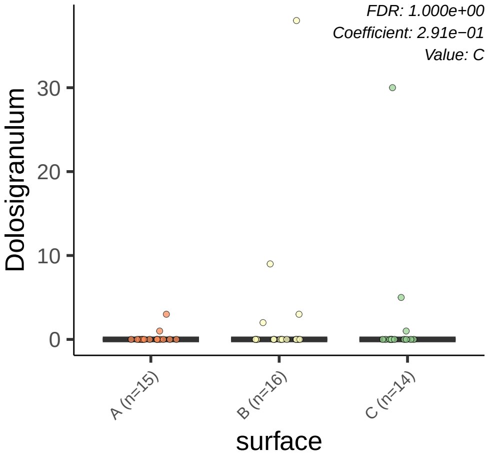

FDR: 1.000e+00
Coefficient: 2.91e−01
Value: C
Dolosigranulum
30
20
10
0
A (n=15)
B (n=16)
C (n=14)
surface

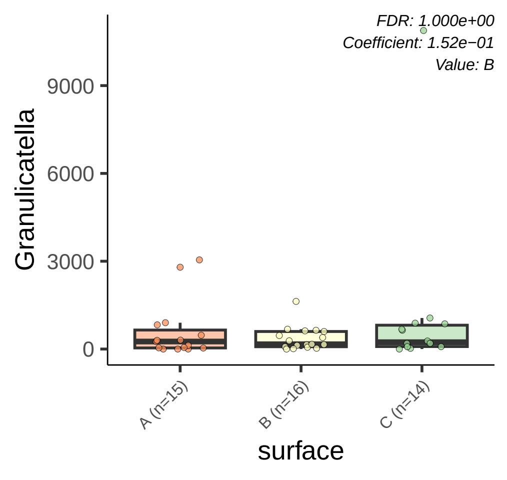

FDR: 1.000e+00
Coefficient: 1.52e−01
Value: B
Granulicatella
9000
6000
3000
0
A (n=15)
B (n=16)
C (n=14)
surface

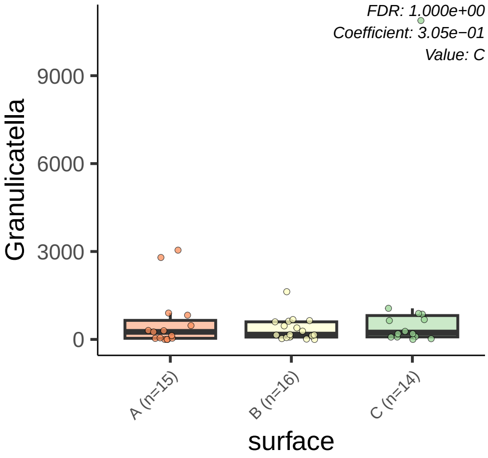
FDR: 1.000e+00
Coefficient: 3.05e−01
Value: C
Granulicatella
9000
6000
3000
0
A (n=15)
B (n=16)
C (n=14)
surface

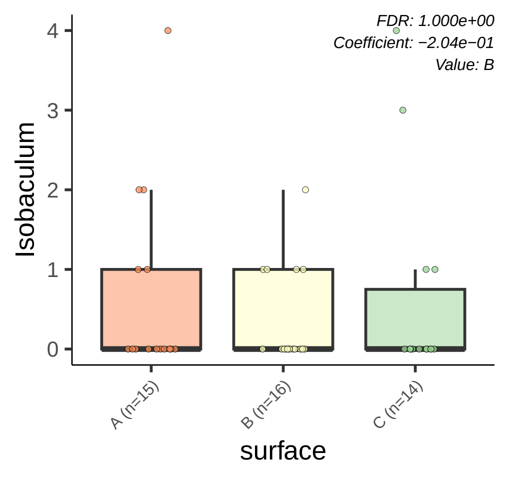
FDR: 1.000e+00
Coefficient: −2.04e−01
Value: B
Isobaculum
4
3
2
1
0
A (n=15)
B (n=16)
C (n=14)
surface

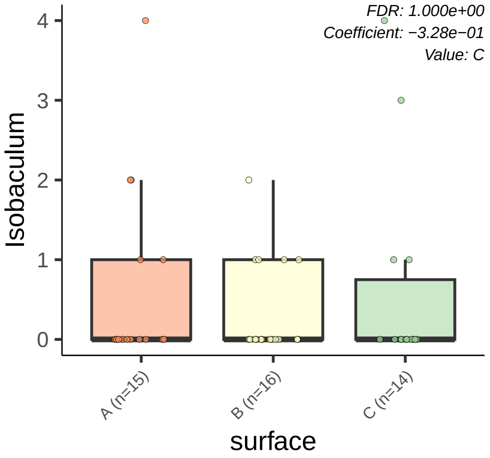
FDR: 1.000e+00
Coefficient: −3.28e−01
Value: C
Isobaculum
0
1
2
3
4
A (n=15)
B (n=16)
C (n=14)
surface

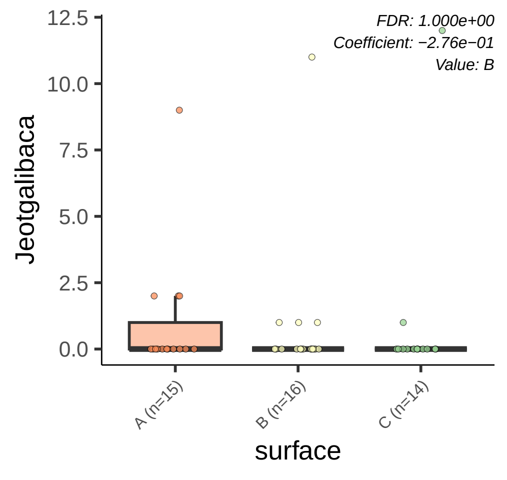
FDR: 1.000e+00
Coefficient: −2.76e−01
Value: B
Jeotgalibaca
12.5
10.0
7.5
5.0
2.5
0.0
A (n=15)
B (n=16)
C (n=14)
surface

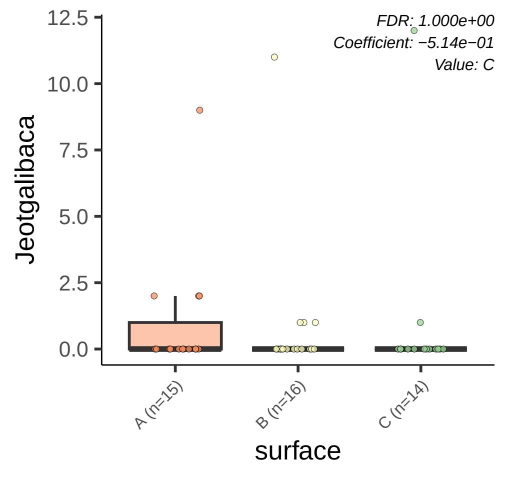

FDR: 1.000e+00
Coefficient: −5.14e−01
Value: C
Jeotgalibaca
12.5
10.0
7.5
5.0
2.5
0.0
A (n=15)
B (n=16)
C (n=14)
surface

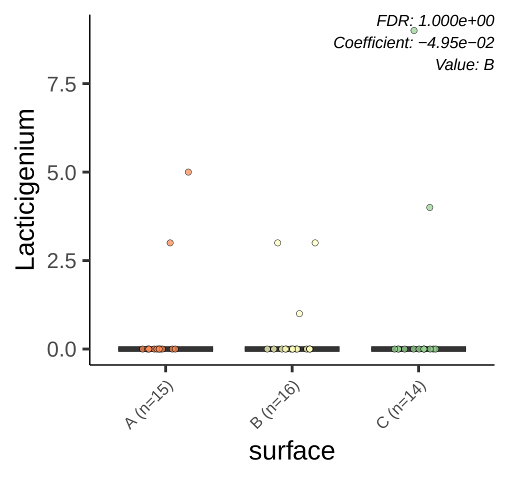

FDR: 1.000e+00
Coefficient: −4.95e−02
Value: B
Lacticigenium
7.5
5.0
2.5
0.0
A (n=15)
B (n=16)
C (n=14)
surface

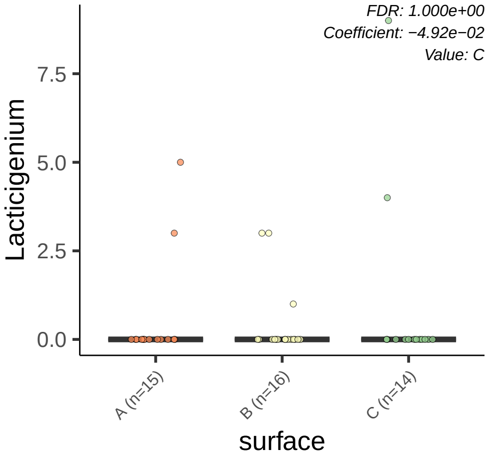

FDR: 1.000e+00
Coefficient: −4.92e−02
Value: C
Lacticigenium
7.5
5.0
2.5
0.0
A (n=15)
B (n=16)
C (n=14)
surface

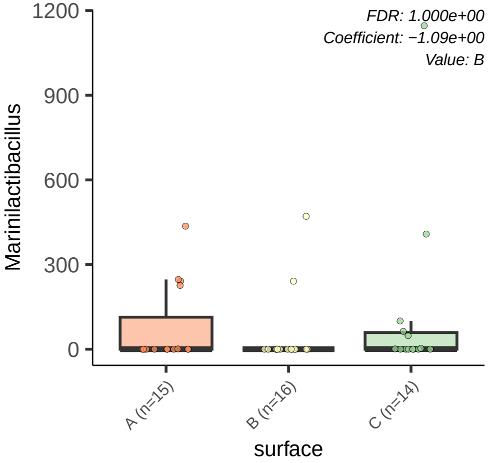

FDR: 1.000e+00
Coefficient: −1.09e+00
Value: B
1200
900
600
300
0
Marinilactibacillus
A (n=15)
B (n=16)
C (n=14)
surface

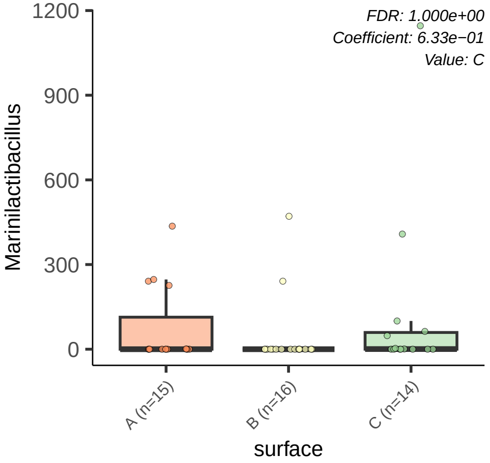
FDR: 1.000e+00
Coefficient: 6.33e−01
Value: C
Marinilactibacillus
1200
900
600
300
0
A (n=15)
B (n=16)
C (n=14)
surface

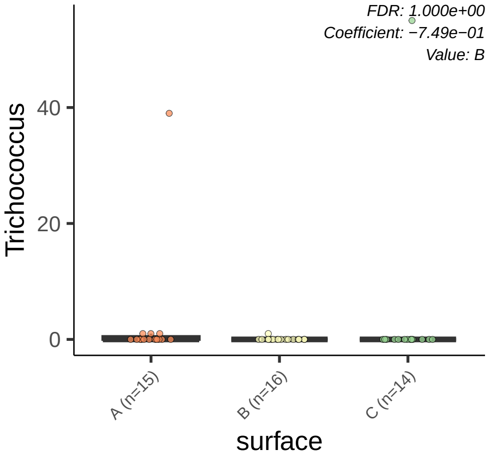

FDR: 1.000e+00
Coefficient: −7.49e−01
Value: B
Trichococcus
40
20
0
A (n=15)
B (n=16)
C (n=14)
surface

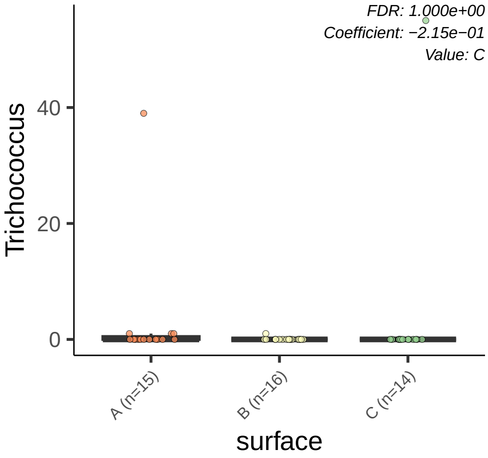

FDR: 1.000e+00
Coefficient: −2.15e−01
Value: C
Trichococcus
40
20
0
A (n=15)
B (n=16)
C (n=14)
surface

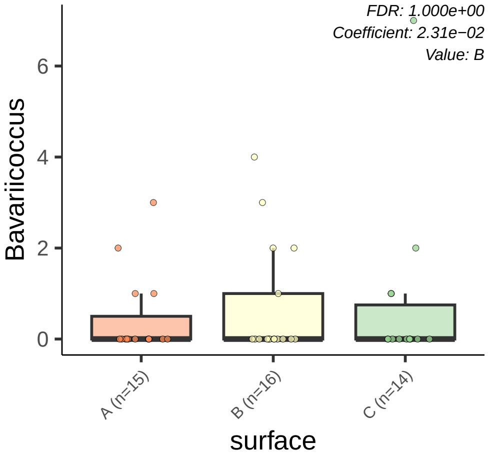
FDR: 1.000e+00
Coefficient: 2.31e−02
Value: B
Bavariicoccus
0
2
4
6
A (n=15)
B (n=16)
C (n=14)
surface

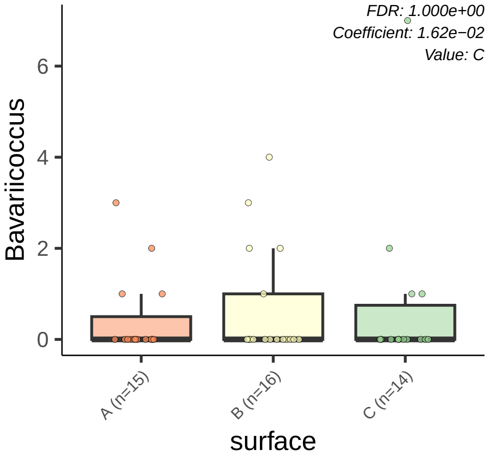
FDR: 1.000e+00
Coefficient: 1.62e−02
Value: C
Bavariicoccus
0
2
4
6
A (n=15)
B (n=16)
C (n=14)
surface

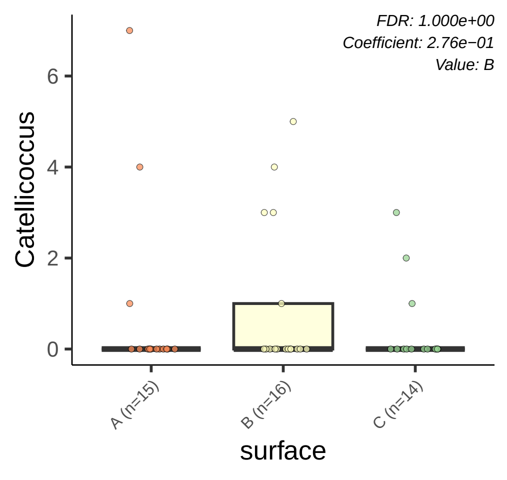
FDR: 1.000e+00
Coefficient: 2.76e−01
Value: B
Catellicoccus
0
2
4
6
A (n=15)
B (n=16)
C (n=14)
surface

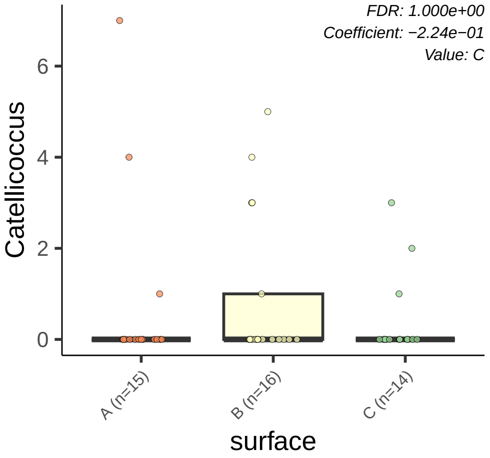
FDR: 1.000e+00
Coefficient: −2.24e−01
Value: C
Catellicoccus
0
2
4
6
A (n=15)
B (n=16)
C (n=14)
surface

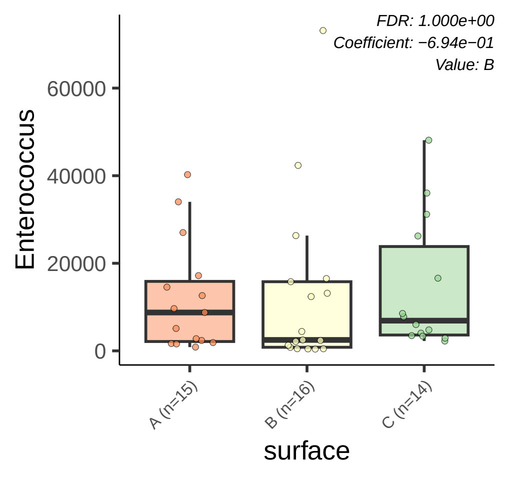
FDR: 1.000e+00
Coefficient: −6.94e−01
Value: B
Enterococcus
60000
40000
20000
0
A (n=15)
B (n=16)
C (n=14)
surface

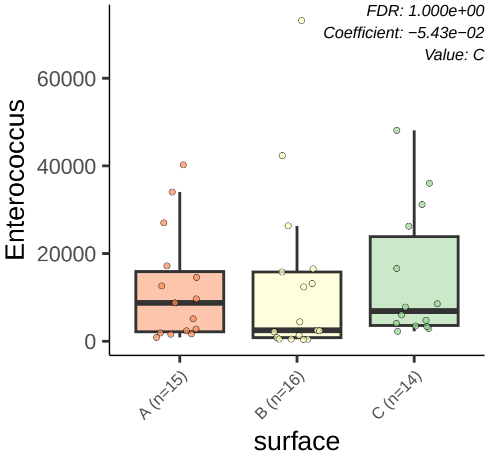
FDR: 1.000e+00
Coefficient: −5.43e−02
Value: C
Enterococcus
60000
40000
20000
0
A (n=15)
B (n=16)
C (n=14)
surface

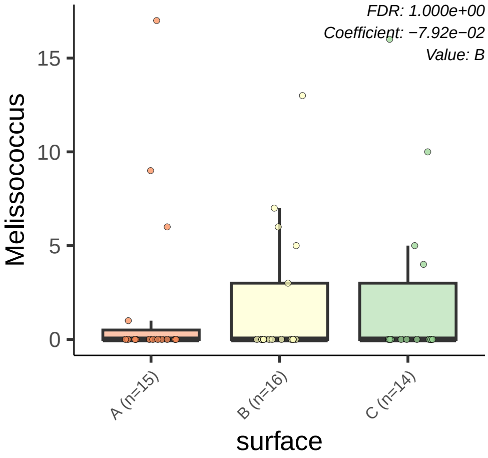
FDR: 1.000e+00
Coefficient: −7.92e−02
Value: B
Melissococcus
0
5
10
15
A (n=15)
B (n=16)
C (n=14)
surface

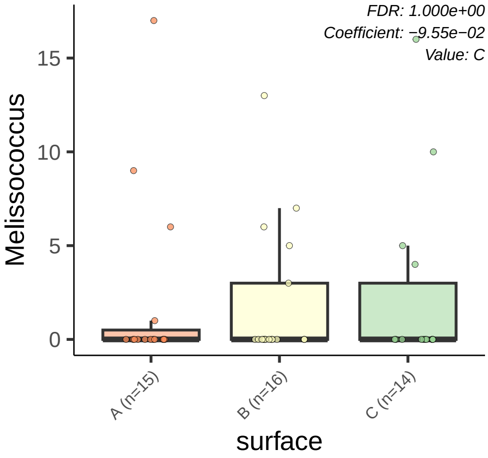

FDR: 1.000e+00
Coefficient: −9.55e−02
Value: C
Melissococcus
0
5
10
15
A (n=15)
B (n=16)
C (n=14)
surface

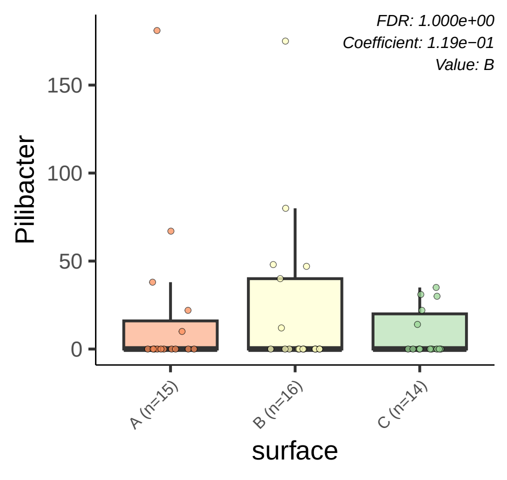
FDR: 1.000e+00
Coefficient: 1.19e−01
Value: B
Pilibacter
150
100
50
0
A (n=15)
B (n=16)
C (n=14)
surface

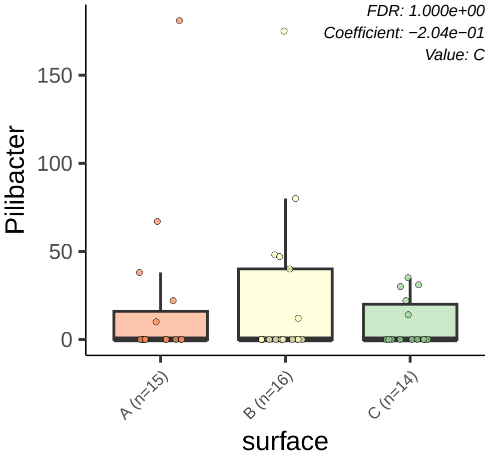
FDR: 1.000e+00
Coefficient: −2.04e−01
Value: C
Pilibacter
150
100
50
0
A (n=15)
B (n=16)
C (n=14)
surface

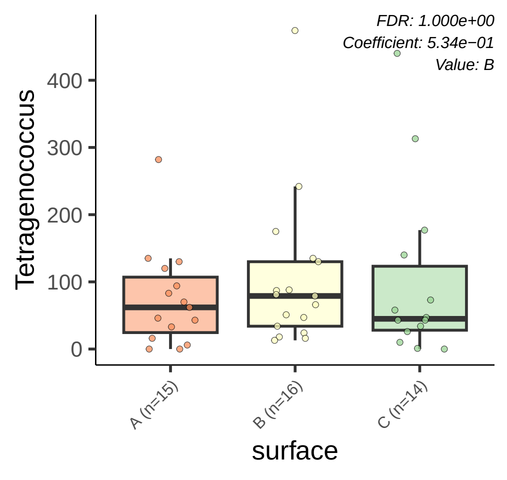
FDR: 1.000e+00
Coefficient: 5.34e−01
Value: B
Tetragenococcus
400
300
200
100
0
A (n=15)
B (n=16)
C (n=14)
surface

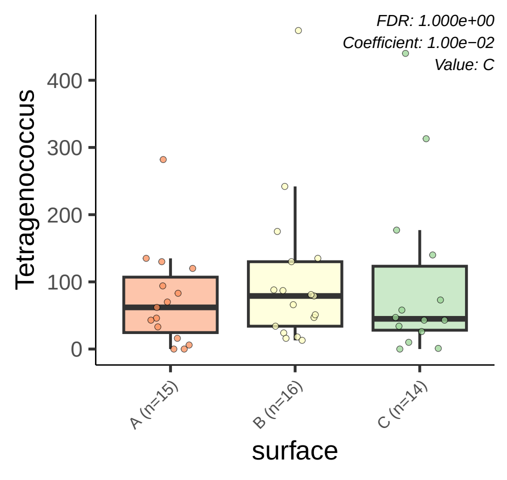
FDR: 1.000e+00
Coefficient: 1.00e−02
Value: C
Tetragenococcus
0
100
200
300
400
A (n=15)
B (n=16)
C (n=14)
surface

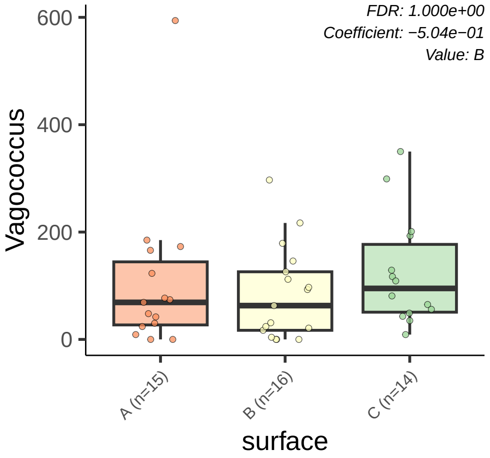
FDR: 1.000e+00
Coefficient: −5.04e−01
Value: B
Vagococcus
600
400
200
0
A (n=15)
B (n=16)
C (n=14)
surface

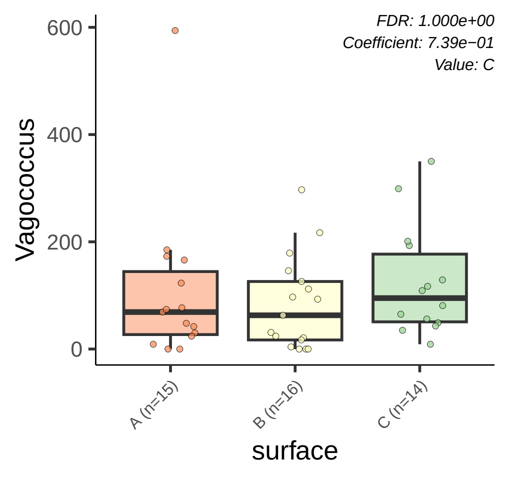

FDR: 1.000e+00
Coefficient: 7.39e−01
Value: C
Vagococcus
600
400
200
0
A (n=15)
B (n=16)
C (n=14)
surface

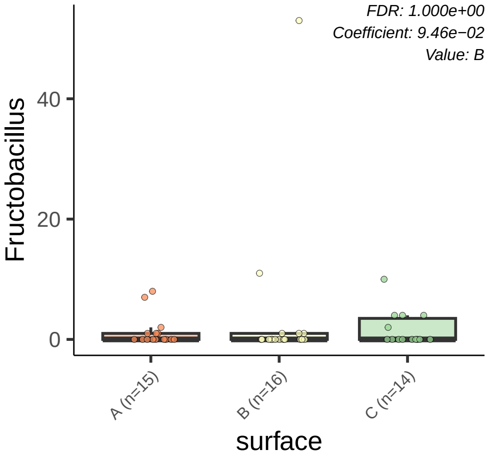
FDR: 1.000e+00
Coefficient: 9.46e−02
Value: B
Fructobacillus
40
20
0
A (n=15)
B (n=16)
C (n=14)
surface

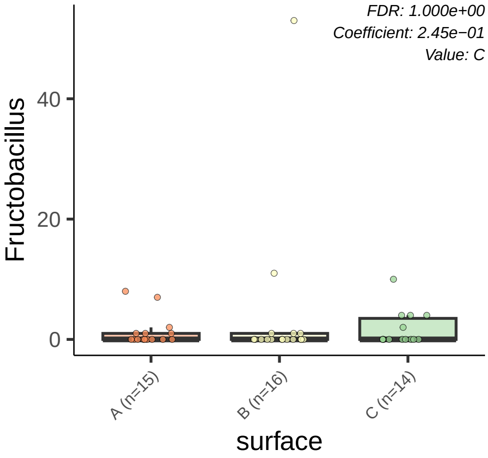

FDR: 1.000e+00
Coefficient: 2.45e−01
Value: C
Fructobacillus
40
20
0
A (n=15)
B (n=16)
C (n=14)
surface

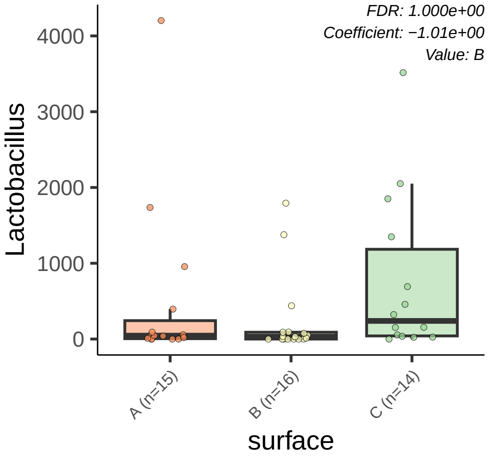
FDR: 1.000e+00
Coefficient: −1.01e+00
Value: B
Lactobacillus
4000
3000
2000
1000
0
A (n=15)
B (n=16)
C (n=14)
surface

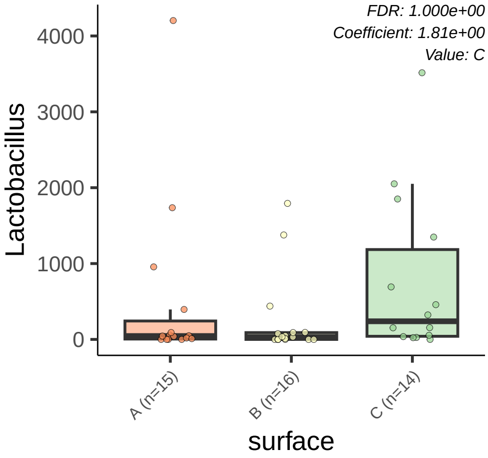

FDR: 1.000e+00
Coefficient: 1.81e+00
Value: C
Lactobacillus
4000
3000
2000
1000
0
A (n=15)
B (n=16)
C (n=14)
surface

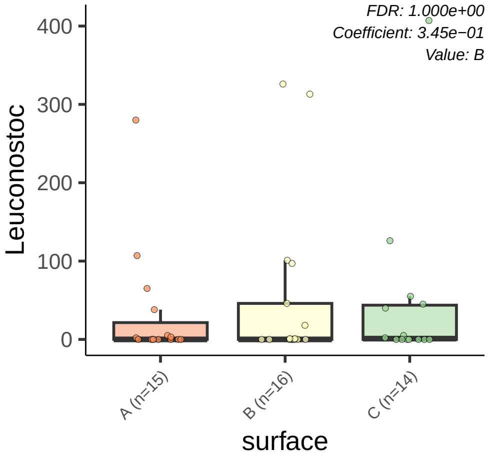

FDR: 1.000e+00
Coefficient: 3.45e−01
Value: B
Leuconostoc
400
300
200
100
0
A (n=15)
B (n=16)
C (n=14)
surface

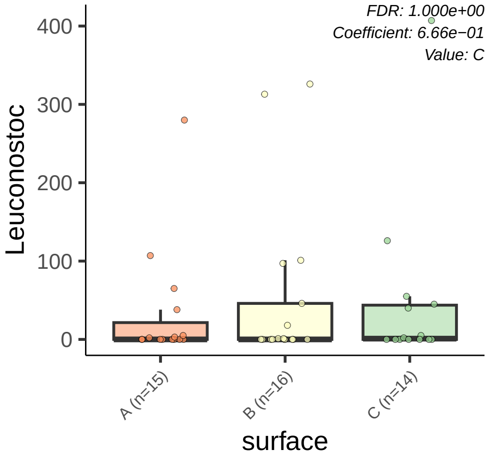

FDR: 1.000e+00
Coefficient: 6.66e−01
Value: C
Leuconostoc
400
300
200
100
0
A (n=15)
B (n=16)
C (n=14)
surface

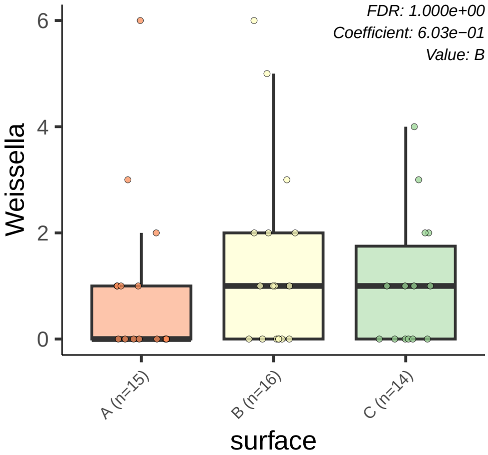
FDR: 1.000e+00
Coefficient: 6.03e−01
Value: B
Weissella
6
4
2
0
A (n=15)
B (n=16)
C (n=14)
surface

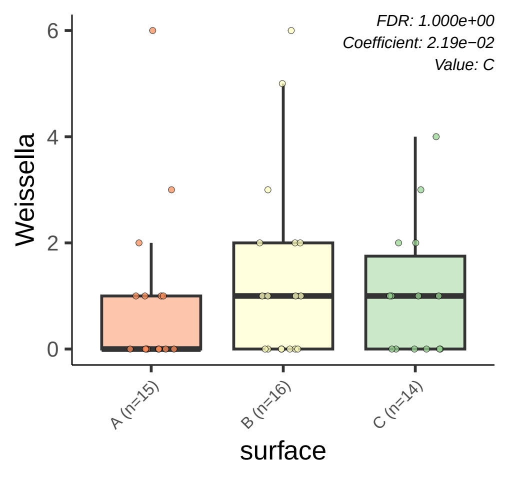

FDR: 1.000e+00
Coefficient: 2.19e−02
Value: C
Weissella
6
4
2
0
A (n=15)
B (n=16)
C (n=14)
surface

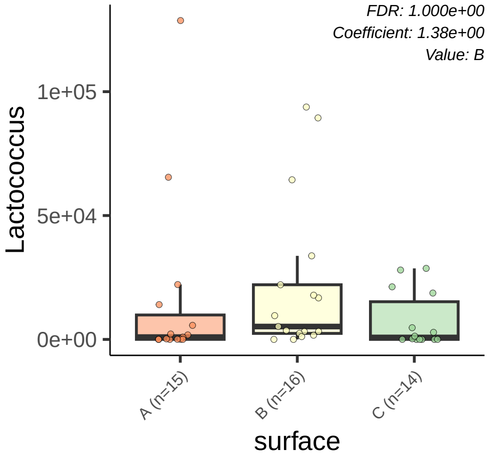
FDR: 1.000e+00
Coefficient: 1.38e+00
Value: B
Lactococcus
1e+05
5e+04
0e+00
A (n=15)
B (n=16)
C (n=14)
surface

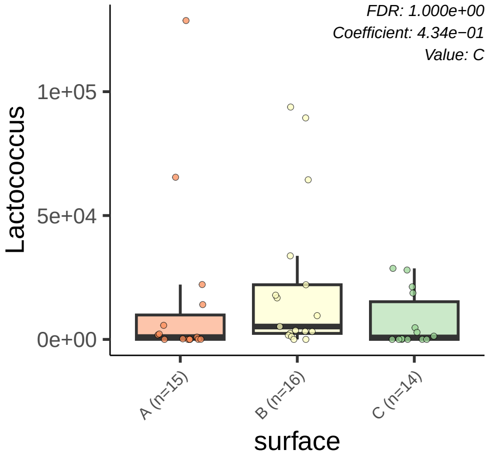

FDR: 1.000e+00
Coefficient: 4.34e−01
Value: C
Lactococcus
1e+05
5e+04
0e+00
A (n=15)
B (n=16)
C (n=14)
surface

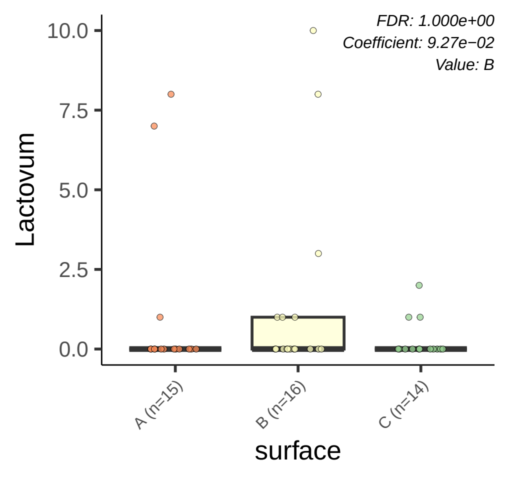
FDR: 1.000e+00
Coefficient: 9.27e−02
Value: B
Lactovum
10.0
7.5
5.0
2.5
0.0
A (n=15)
B (n=16)
C (n=14)
surface

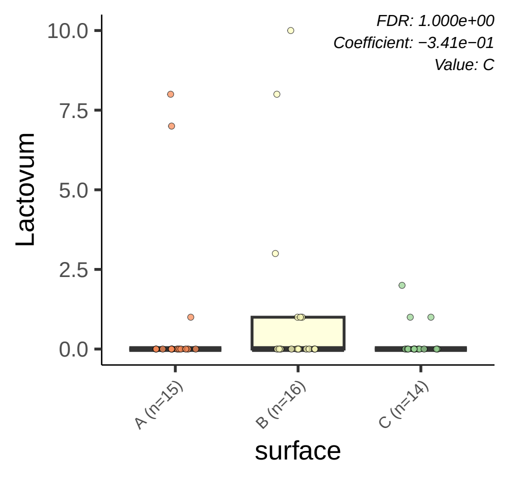

FDR: 1.000e+00
Coefficient: −3.41e−01
Value: C
Lactovum
10.0
7.5
5.0
2.5
0.0
A (n=15)
B (n=16)
C (n=14)
surface

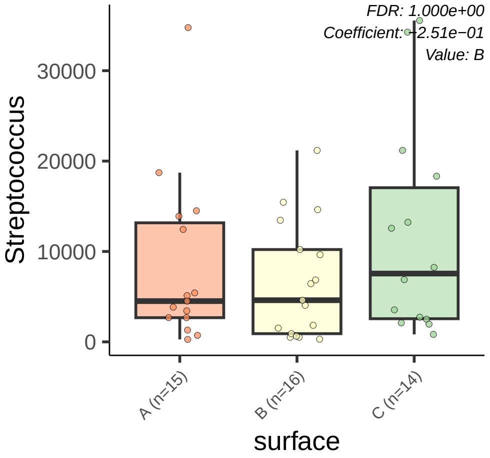
FDR: 1.000e+00
Coefficient: −2.51e−01
Value: B
Streptococcus
30000
20000
10000
0
A (n=15)
B (n=16)
C (n=14)
surface

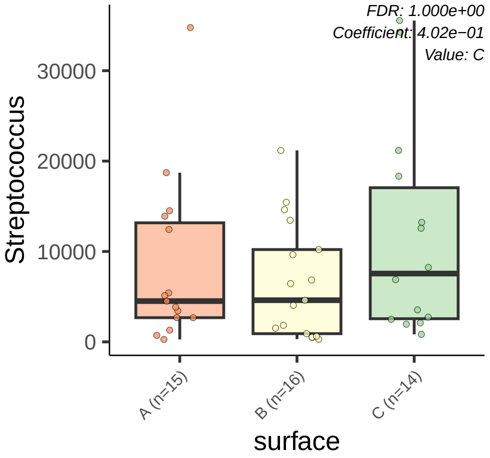
FDR: 1.000e+00
Coefficient: 4.02e−01
Value: C
Streptococcus
30000
20000
10000
0
A (n=15)
B (n=16)
C (n=14)
surface

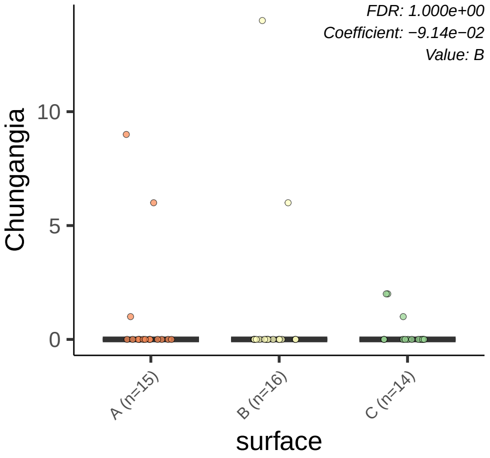

FDR: 1.000e+00
Coefficient: −9.14e−02
Value: B
Chungangia
0
5
10
A (n=15)
B (n=16)
C (n=14)
surface

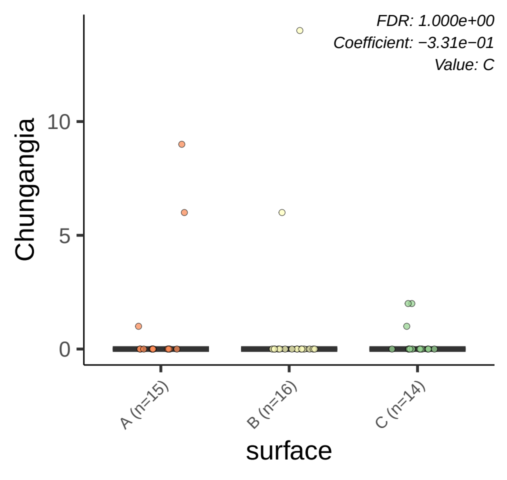
FDR: 1.000e+00
Coefficient: −3.31e−01
Value: C
Chungangia
10
5
0
A (n=15)
B (n=16)
C (n=14)
surface

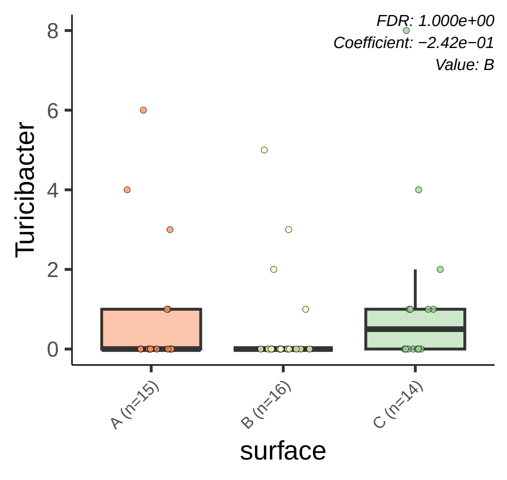
FDR: 1.000e+00
Coefficient: −2.42e−01
Value: B
Turicibacter
0
2
4
6
8
A (n=15)
B (n=16)
C (n=14)
surface

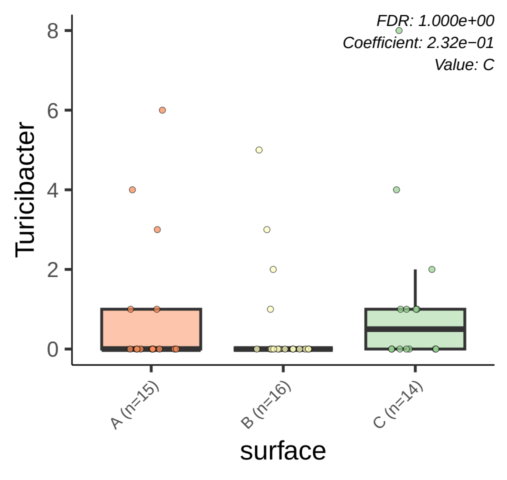

FDR: 1.000e+00
Coefficient: 2.32e−01
Value: C
Turicibacter
0
2
4
6
8
A (n=15)
B (n=16)
C (n=14)
surface

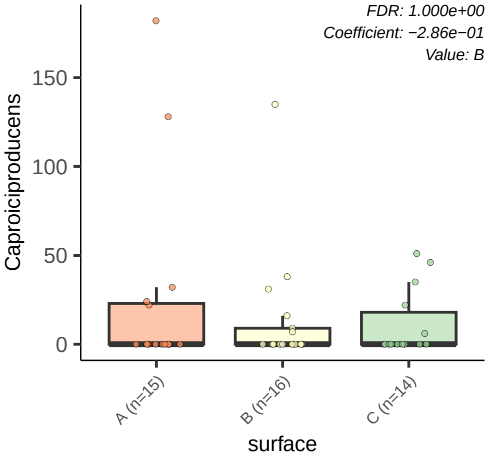

FDR: 1.000e+00
Coefficient: −2.86e−01
Value: B
Caproiciproducens
150
100
50
0
A (n=15)
B (n=16)
C (n=14)
surface

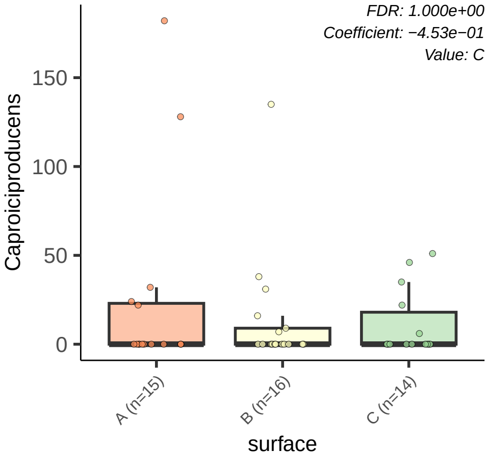
FDR: 1.000e+00
Coefficient: −4.53e−01
Value: C
Caproiciproducens
150
100
50
0
A (n=15)
B (n=16)
C (n=14)
surface

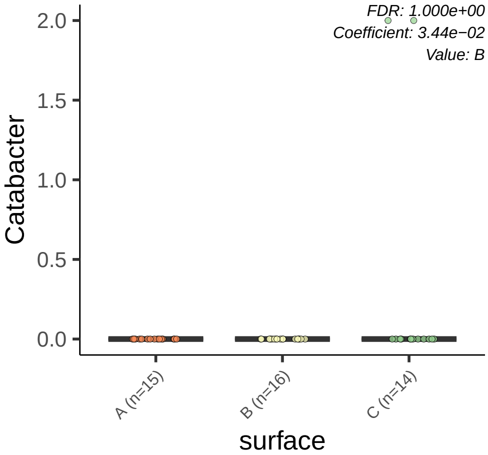
FDR: 1.000e+00
Coefficient: 3.44e−02
Value: B
Catabacter
2.0
1.5
1.0
0.5
0.0
A (n=15)
B (n=16)
C (n=14)
surface

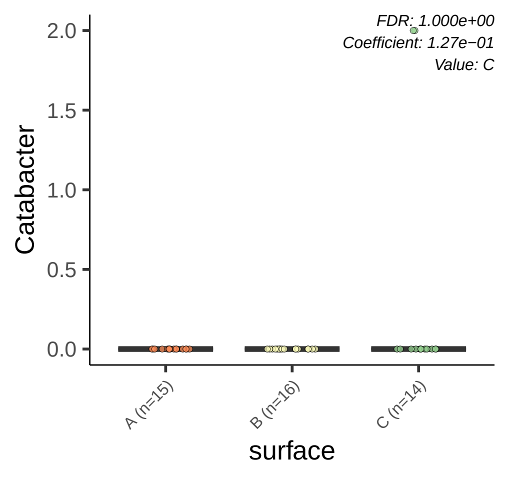

FDR: 1.000e+00
Coefficient: 1.27e−01
Value: C
Catabacter
2.0
1.5
1.0
0.5
0.0
A (n=15)
B (n=16)
C (n=14)
surface

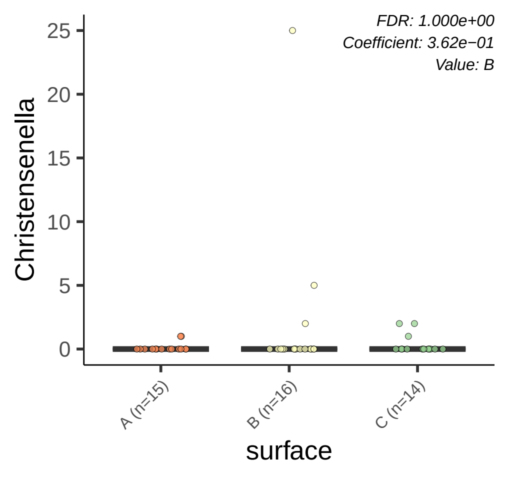
FDR: 1.000e+00
Coefficient: 3.62e−01
Value: B
Christensenella
25
20
15
10
5
0
A (n=15)
B (n=16)
C (n=14)
surface

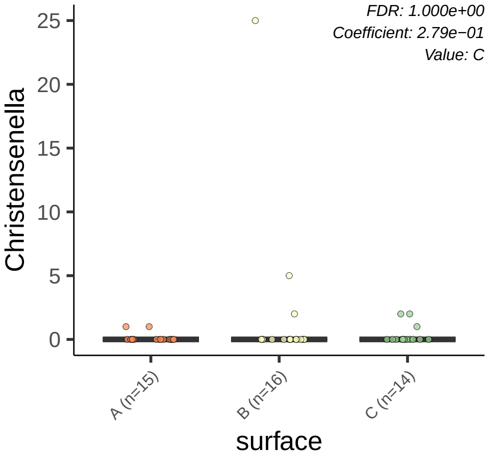

FDR: 1.000e+00
Coefficient: 2.79e−01
Value: C
Christensenella
25
20
15
10
5
0
A (n=15)
B (n=16)
C (n=14)
surface

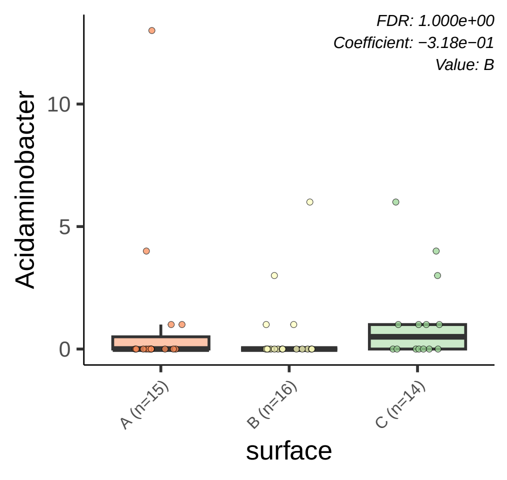

FDR: 1.000e+00
Coefficient: −3.18e−01
Value: B
Acidaminobacter
10
5
0
A (n=15)
B (n=16)
C (n=14)
surface

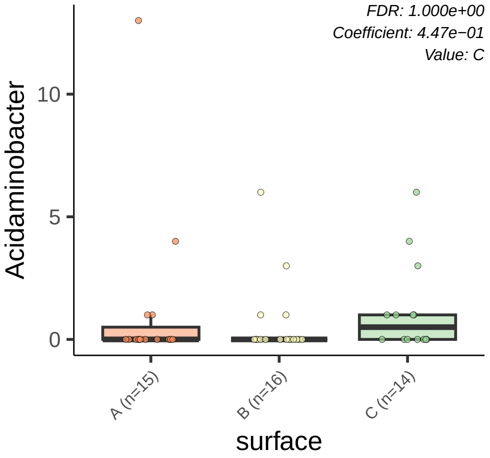
FDR: 1.000e+00
Coefficient: 4.47e−01
Value: C
Acidaminobacter
10
5
0
A (n=15)
B (n=16)
C (n=14)
surface

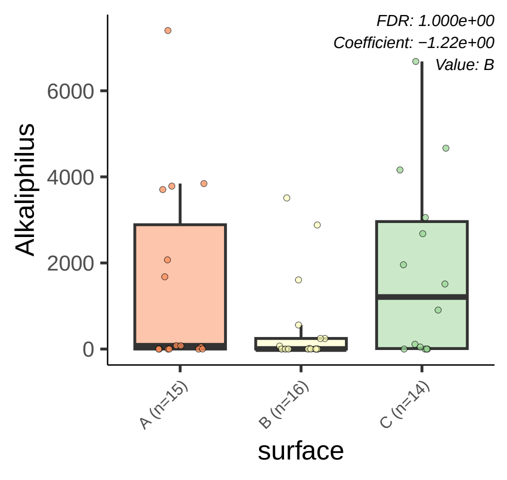

FDR: 1.000e+00
Coefficient: −1.22e+00
Value: B
Alkaliphilus
6000
4000
2000
0
A (n=15)
B (n=16)
C (n=14)
surface

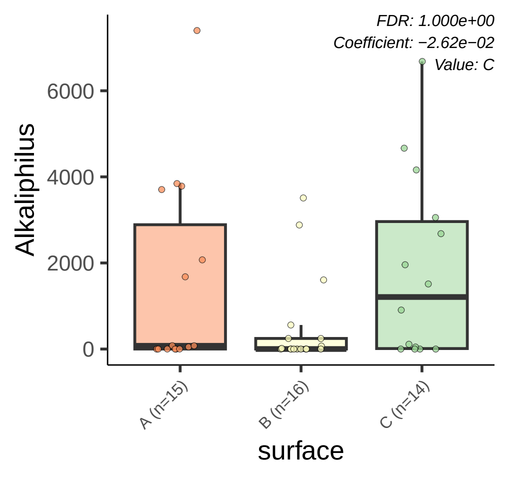
FDR: 1.000e+00
Coefficient: −2.62e−02
Value: C
Alkaliphilus
6000
4000
2000
0
A (n=15)
B (n=16)
C (n=14)
surface

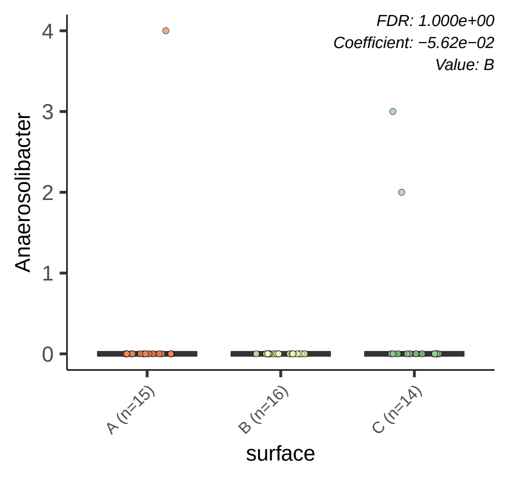

FDR: 1.000e+00
Coefficient: −5.62e−02
Value: B
Anaerosolibacter
4
3
2
1
0
A (n=15)
B (n=16)
C (n=14)
surface

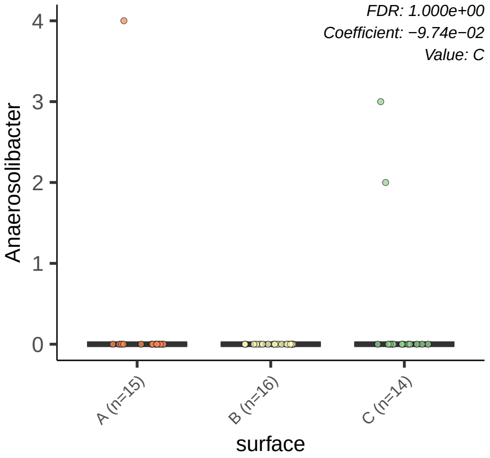
FDR: 1.000e+00
Coefficient: −9.74e−02
Value: C
Anaerosolibacter
4
3
2
1
0
A (n=15)
B (n=16)
C (n=14)
surface

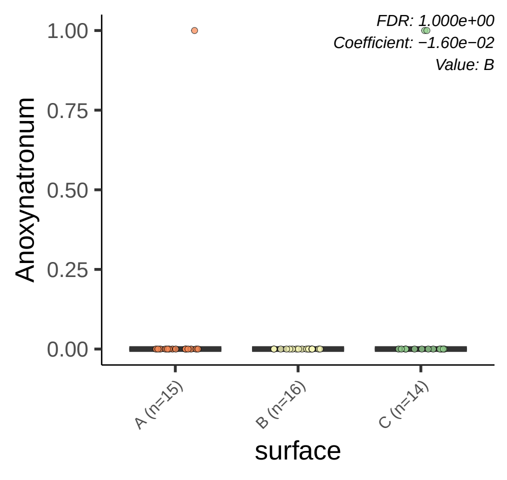
FDR: 1.000e+00
Coefficient: −1.60e−02
Value: B
Anoxynatronum
1.00
0.75
0.50
0.25
0.00
A (n=15)
B (n=16)
C (n=14)
surface

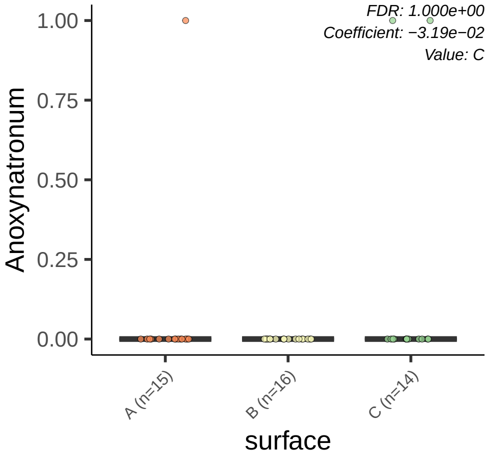
FDR: 1.000e+00
Coefficient: −3.19e−02
Value: C
Anoxynatronum
1.00
0.75
0.50
0.25
0.00
A (n=15)
B (n=16)
C (n=14)
surface

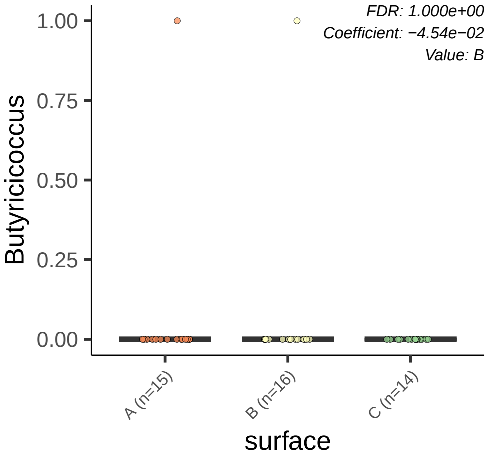

FDR: 1.000e+00
Coefficient: −4.54e−02
Value: B
Butyricicoccus
1.00
0.75
0.50
0.25
0.00
A (n=15)
B (n=16)
C (n=14)
surface

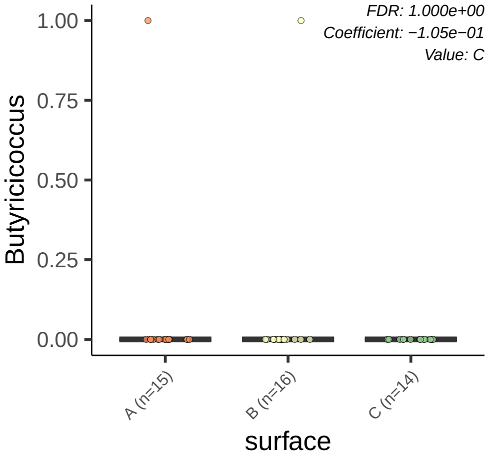
FDR: 1.000e+00
Coefficient: −1.05e−01
Value: C
Butyricicoccus
1.00
0.75
0.50
0.25
0.00
A (n=15)
B (n=16)
C (n=14)
surface

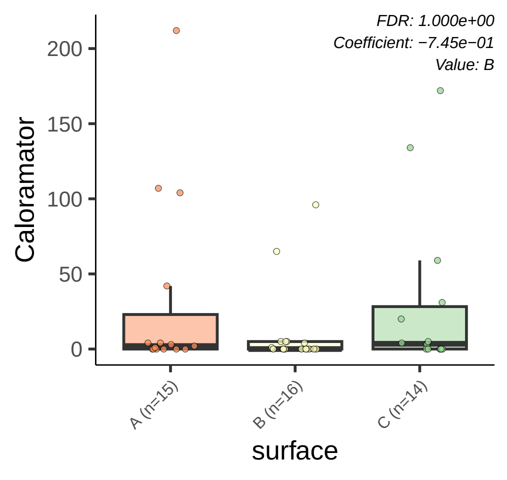
FDR: 1.000e+00
Coefficient: −7.45e−01
Value: B
Caloramator
200
150
100
50
0
A (n=15)
B (n=16)
C (n=14)
surface

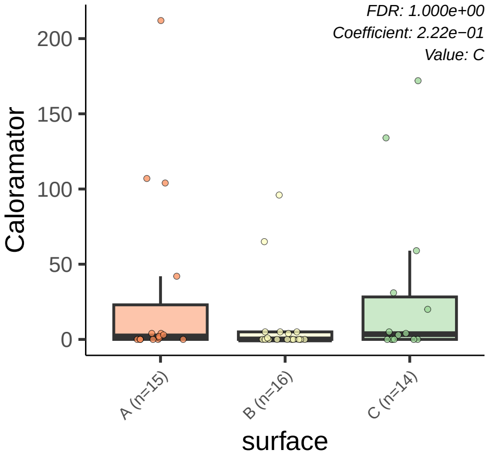

FDR: 1.000e+00
Coefficient: 2.22e−01
Value: C
Caloramator
200
150
100
50
0
A (n=15)
B (n=16)
C (n=14)
surface

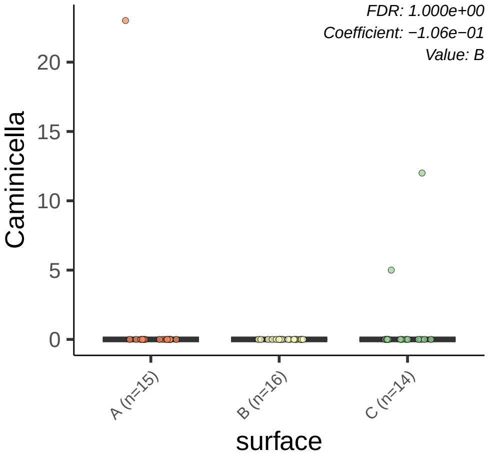

FDR: 1.000e+00
Coefficient: −1.06e−01
Value: B
Caminicella
20
15
10
5
0
A (n=15)
B (n=16)
C (n=14)
surface

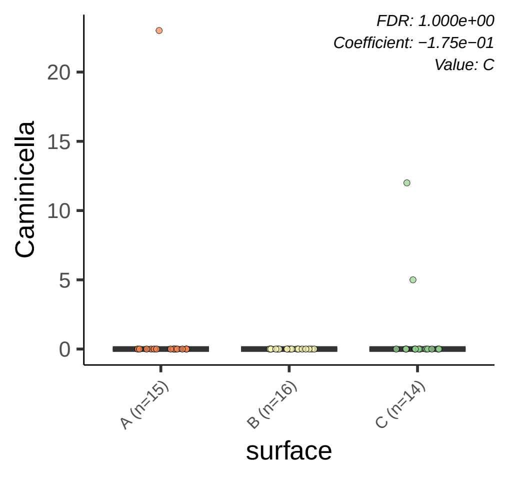

FDR: 1.000e+00
Coefficient: −1.75e−01
Value: C
Caminicella
0
5
10
15
20
A (n=15)
B (n=16)
C (n=14)
surface

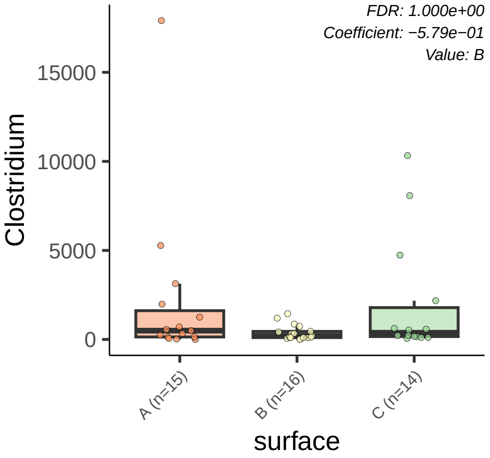

FDR: 1.000e+00
Coefficient: −5.79e−01
Value: B
Clostridium
15000
10000
5000
0
A (n=15)
B (n=16)
C (n=14)
surface

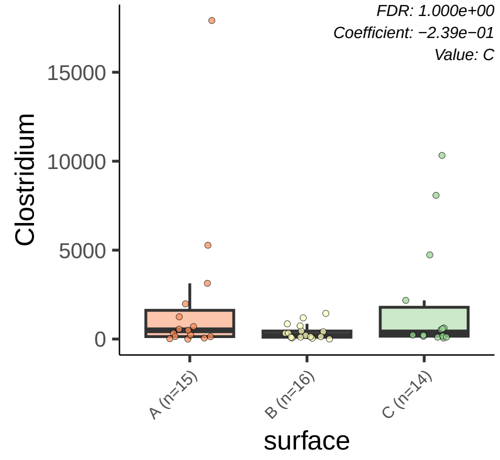
FDR: 1.000e+00
Coefficient: −2.39e−01
Value: C
Clostridium
15000
10000
5000
0
A (n=15)
B (n=16)
C (n=14)
surface

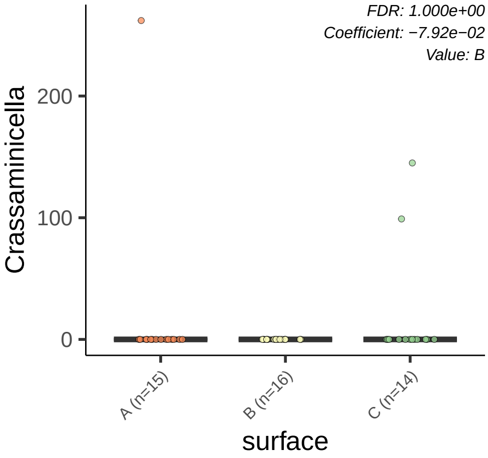

FDR: 1.000e+00
Coefficient: −7.92e−02
Value: B
Crassaminicella
200
100
0
A (n=15)
B (n=16)
C (n=14)
surface

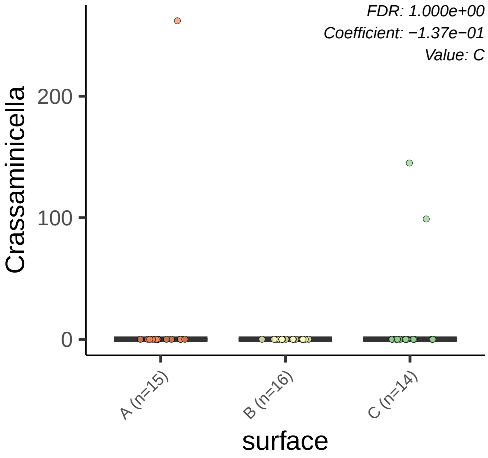
FDR: 1.000e+00
Coefficient: −1.37e−01
Value: C
Crassaminicella
200
100
0
A (n=15)
B (n=16)
C (n=14)
surface

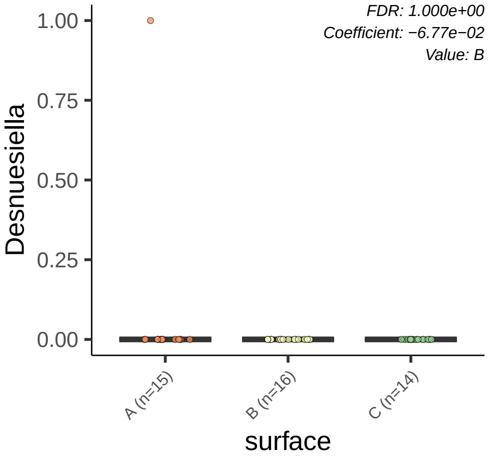

FDR: 1.000e+00
Coefficient: −6.77e−02
Value: B
1.00
0.75
0.50
0.25
0.00
Desnuesiella
A (n=15)
B (n=16)
C (n=14)
surface

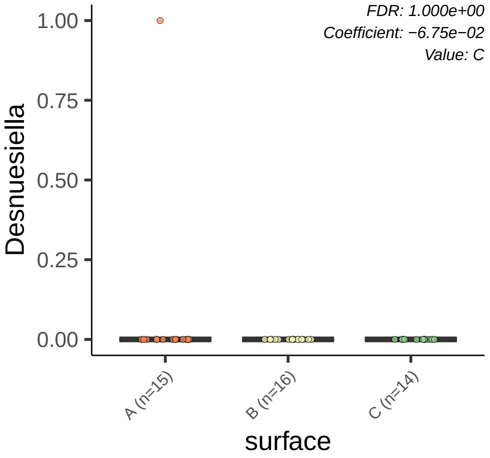

FDR: 1.000e+00
Coefficient: −6.75e−02
Value: C
Desnuesiella
1.00
0.75
0.50
0.25
0.00
A (n=15)
B (n=16)
C (n=14)
surface

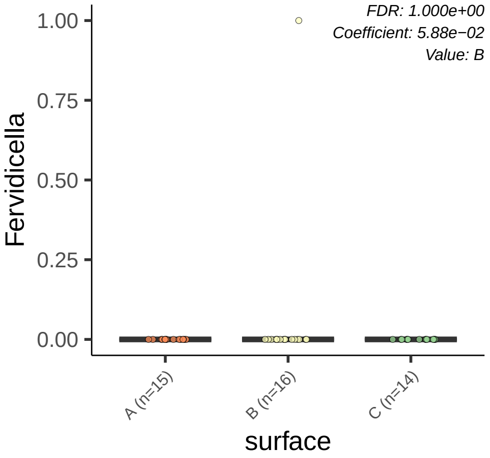

FDR: 1.000e+00
Coefficient: 5.88e−02
Value: B
Fervidicella
1.00
0.75
0.50
0.25
0.00
A (n=15)
B (n=16)
C (n=14)
surface

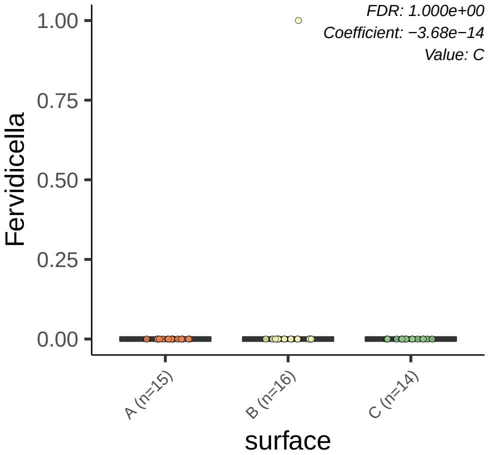

FDR: 1.000e+00
Coefficient: −3.68e−14
Value: C
Fervidicella
1.00
0.75
0.50
0.25
0.00
A (n=15)
B (n=16)
C (n=14)
surface

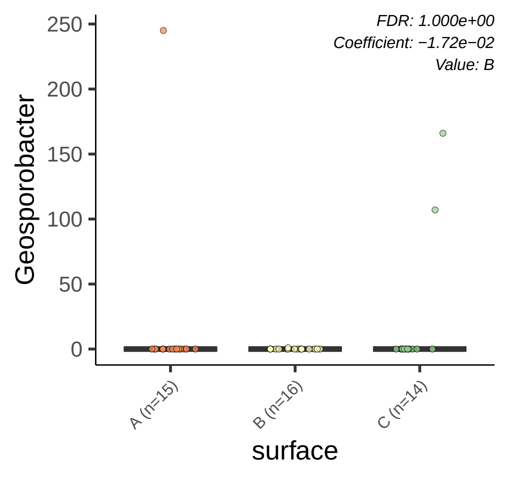

FDR: 1.000e+00
Coefficient: −1.72e−02
Value: B
Geosporobacter
250
200
150
100
50
0
A (n=15)
B (n=16)
C (n=14)
surface

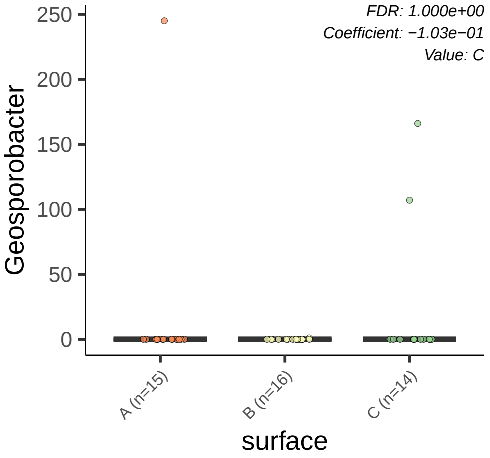

FDR: 1.000e+00
Coefficient: −1.03e−01
Value: C
Geosporobacter
250
200
150
100
50
0
A (n=15)
B (n=16)
C (n=14)
surface

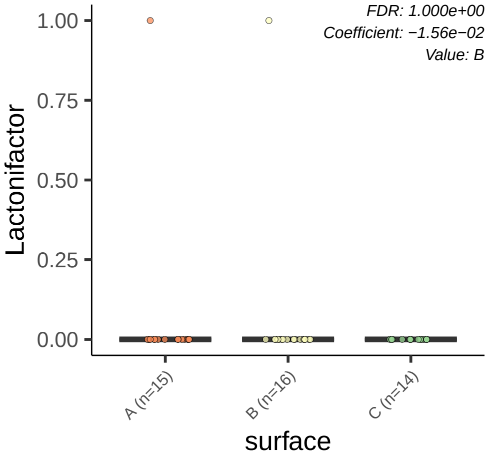
FDR: 1.000e+00
Coefficient: −1.56e−02
Value: B
Lactonifactor
1.00
0.75
0.50
0.25
0.00
A (n=15)
B (n=16)
C (n=14)
surface

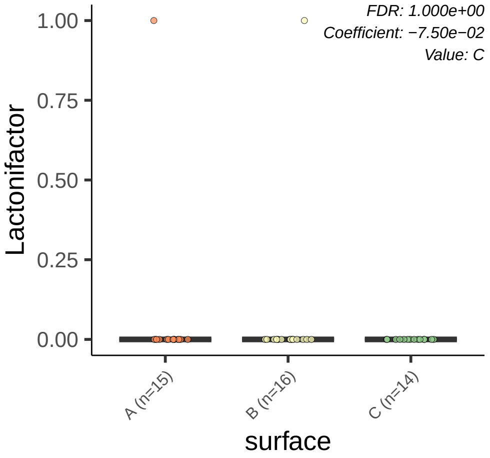
FDR: 1.000e+00
Coefficient: −7.50e−02
Value: C
Lactonifactor
1.00
0.75
0.50
0.25
0.00
A (n=15)
B (n=16)
C (n=14)
surface

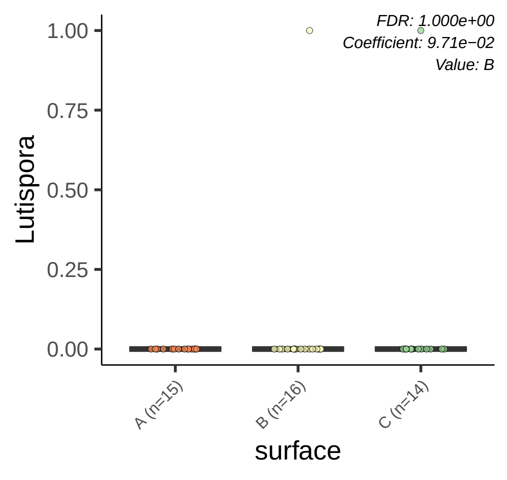
FDR: 1.000e+00
Coefficient: 9.71e−02
Value: B
Lutispora
1.00
0.75
0.50
0.25
0.00
A (n=15)
B (n=16)
C (n=14)
surface

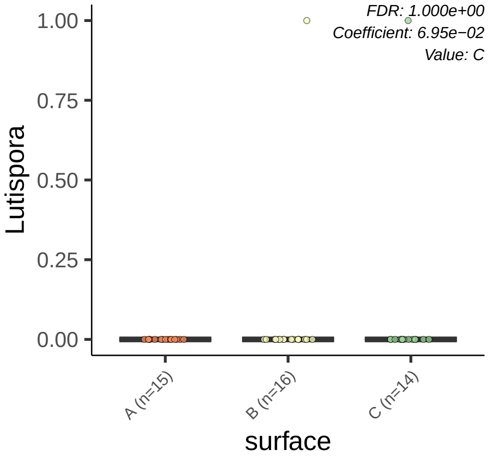

FDR: 1.000e+00
Coefficient: 6.95e−02
Value: C
1.00
0.75
0.50
0.25
0.00
Lutispora
A (n=15)
B (n=16)
C (n=14)
surface

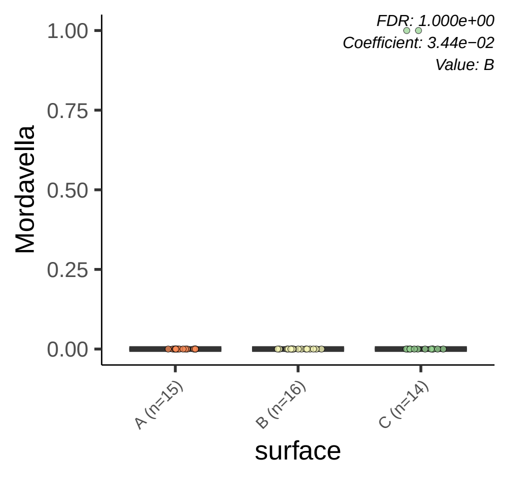
FDR: 1.000e+00
Coefficient: 3.44e−02
Value: B
Mordavella
1.00
0.75
0.50
0.25
0.00
A (n=15)
B (n=16)
C (n=14)
surface

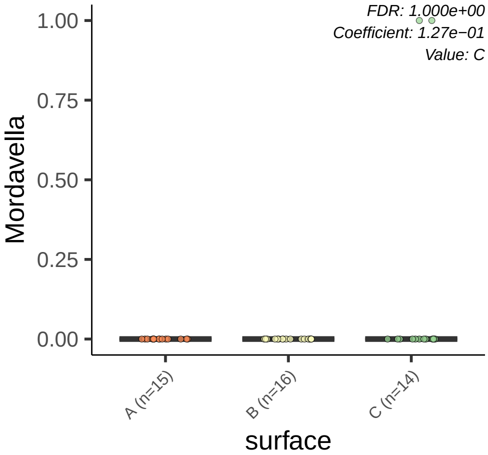
FDR: 1.000e+00
Coefficient: 1.27e−01
Value: C
Mordavella
1.00
0.75
0.50
0.25
0.00
A (n=15)
B (n=16)
C (n=14)
surface

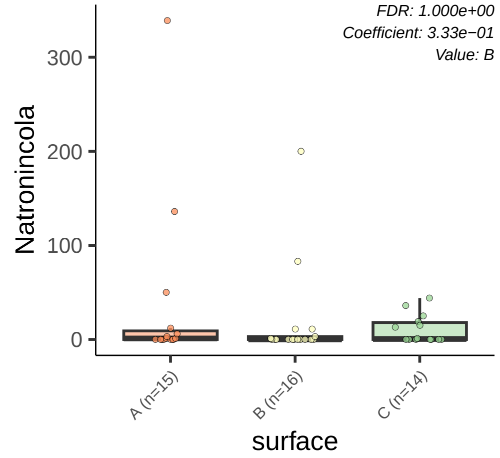

FDR: 1.000e+00
Coefficient: 3.33e−01
Value: B
Natronincola
300
200
100
0
A (n=15)
B (n=16)
C (n=14)
surface

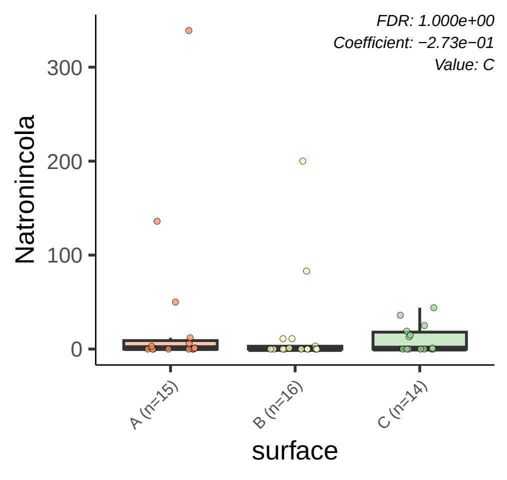

FDR: 1.000e+00
Coefficient: −2.73e−01
Value: C
Natronincola
300
200
100
0
A (n=15)
B (n=16)
C (n=14)
surface

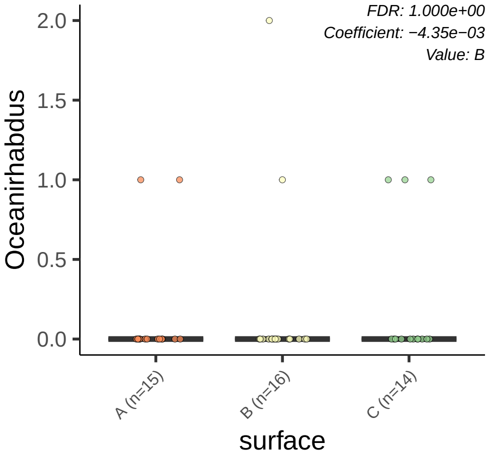

FDR: 1.000e+00
Coefficient: −4.35e−03
Value: B
Oceanirhabdus
2.0
1.5
1.0
0.5
0.0
A (n=15)
B (n=16)
C (n=14)
surface

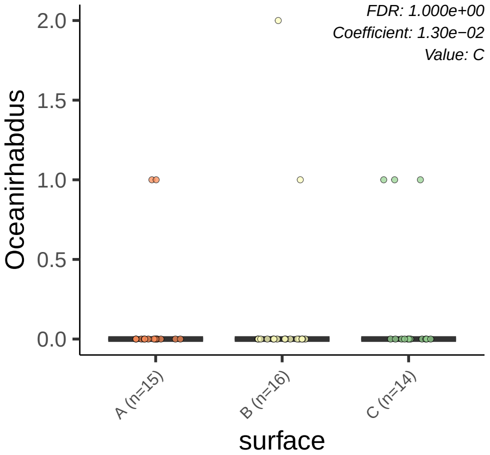
FDR: 1.000e+00
Coefficient: 1.30e−02
Value: C
Oceanirhabdus
2.0
1.5
1.0
0.5
0.0
A (n=15)
B (n=16)
C (n=14)
surface

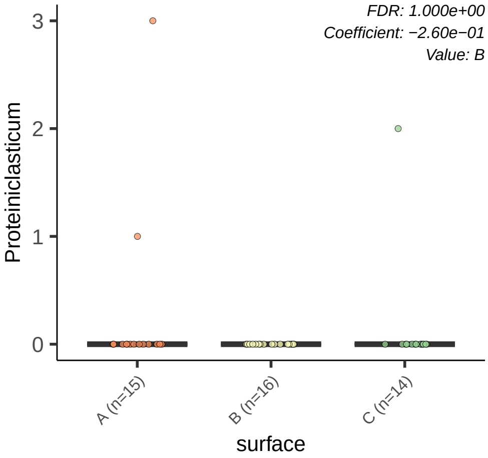
FDR: 1.000e+00
Coefficient: −2.60e−01
Value: B
Proteiniclasticum
3
2
1
0
A (n=15)
B (n=16)
C (n=14)
surface

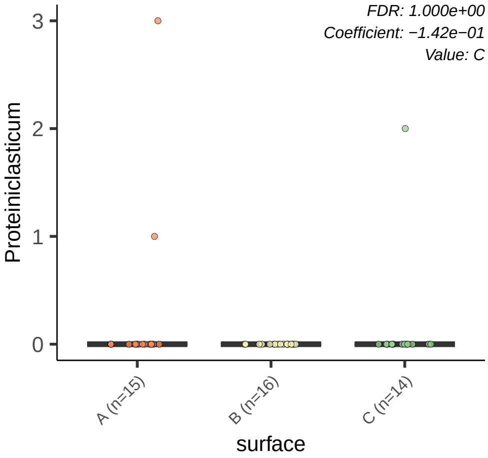
FDR: 1.000e+00
Coefficient: −1.42e−01
Value: C
Proteiniclasticum
3
2
1
0
A (n=15)
B (n=16)
C (n=14)
surface

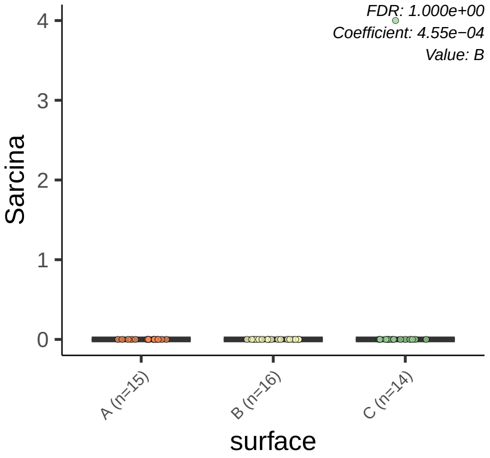

FDR: 1.000e+00
Coefficient: 4.55e−04
Value: B
Sarcina
4
3
2
1
0
A (n=15)
B (n=16)
C (n=14)
surface

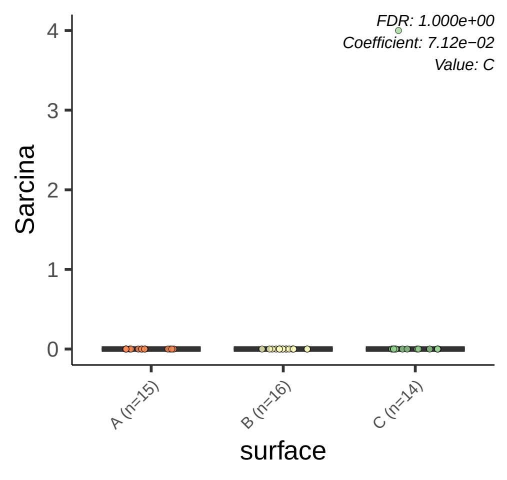

FDR: 1.000e+00
Coefficient: 7.12e−02
Value: C
Sarcina
4
3
2
1
0
A (n=15)
B (n=16)
C (n=14)
surface

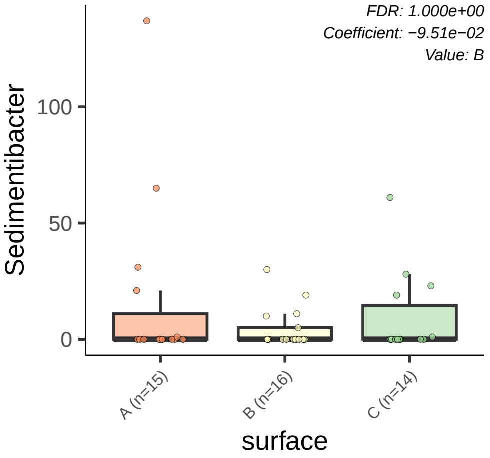
FDR: 1.000e+00
Coefficient: −9.51e−02
Value: B
Sedimentibacter
100
50
0
A (n=15)
B (n=16)
C (n=14)
surface

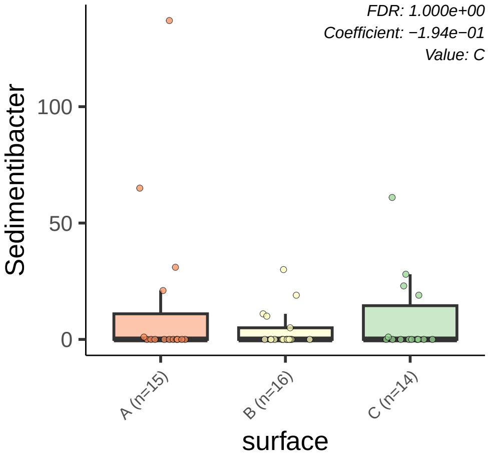

FDR: 1.000e+00
Coefficient: −1.94e−01
Value: C
Sedimentibacter
100
50
0
A (n=15)
B (n=16)
C (n=14)
surface

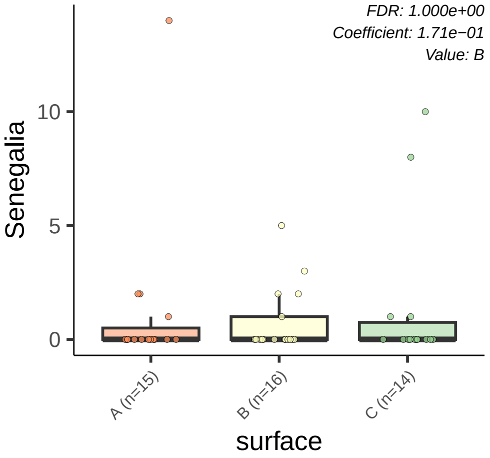
FDR: 1.000e+00
Coefficient: 1.71e−01
Value: B
Senegalia
10
5
0
A (n=15)
B (n=16)
C (n=14)
surface

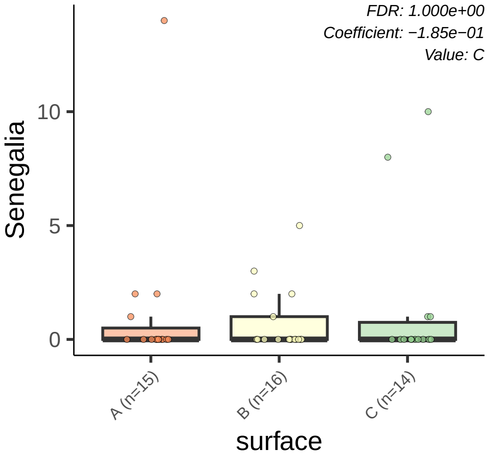

FDR: 1.000e+00
Coefficient: −1.85e−01
Value: C
Senegalia
0
5
10
A (n=15)
B (n=16)
C (n=14)
surface

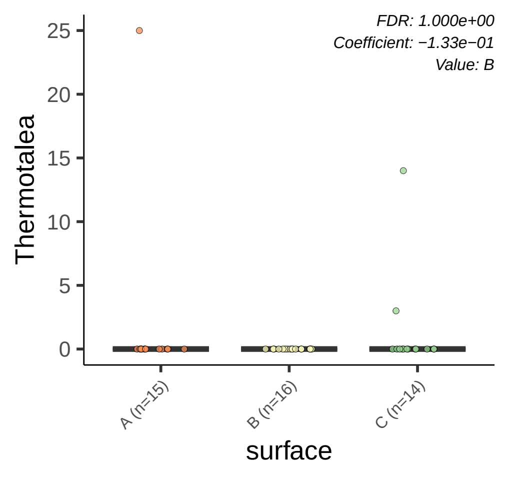

FDR: 1.000e+00
Coefficient: −1.33e−01
Value: B
Thermotalea
25
20
15
10
5
0
A (n=15)
B (n=16)
C (n=14)
surface

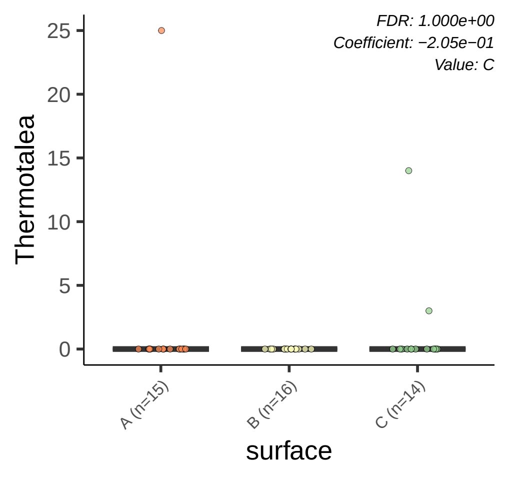
FDR: 1.000e+00
Coefficient: −2.05e−01
Value: C
Thermotalea
25
20
15
10
5
0
A (n=15)
B (n=16)
C (n=14)
surface

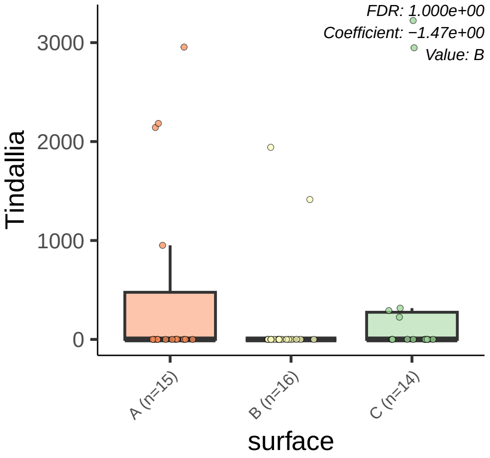
FDR: 1.000e+00
Coefficient: −1.47e+00
Value: B
Tindallia
3000
2000
1000
0
A (n=15)
B (n=16)
C (n=14)
surface

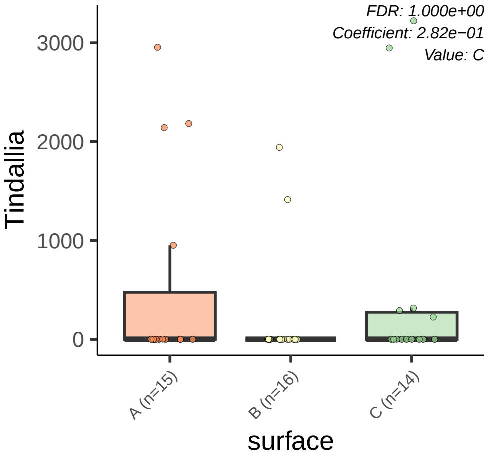

FDR: 1.000e+00
Coefficient: 2.82e−01
Value: C
Tindallia
3000
2000
1000
0
A (n=15)
B (n=16)
C (n=14)
surface

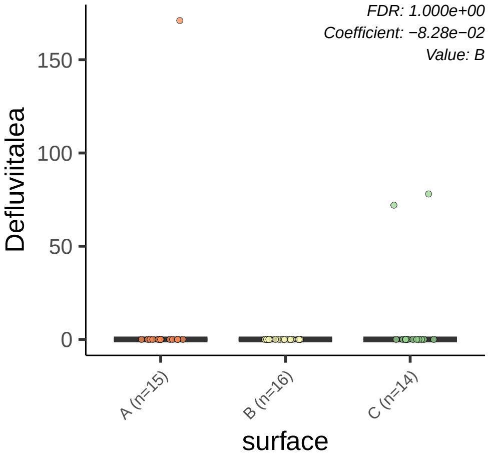

FDR: 1.000e+00
Coefficient: −8.28e−02
Value: B
Defluviitalea
150
100
50
0
A (n=15)
B (n=16)
C (n=14)
surface

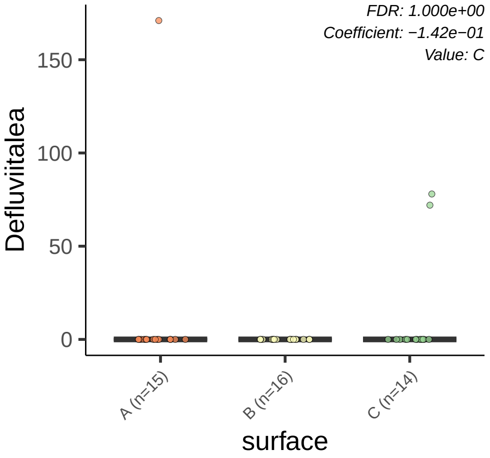
FDR: 1.000e+00
Coefficient: −1.42e−01
Value: C
Defluviitalea
150
100
50
0
A (n=15)
B (n=16)
C (n=14)
surface

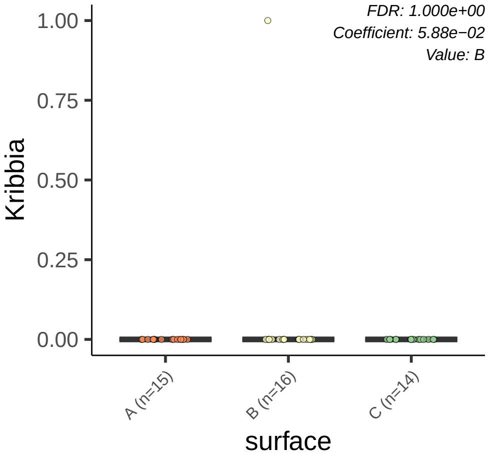
FDR: 1.000e+00
Coefficient: 5.88e−02
Value: B
Kribbia
1.00
0.75
0.50
0.25
0.00
A (n=15)
B (n=16)
C (n=14)
surface

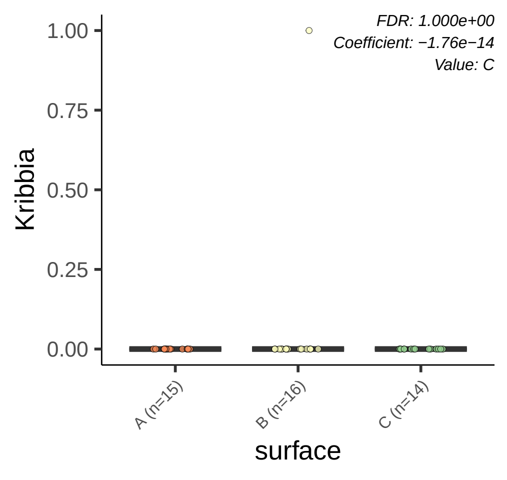

FDR: 1.000e+00
Coefficient: −1.76e−14
Value: C
Kribbia
1.00
0.75
0.50
0.25
0.00
A (n=15)
B (n=16)
C (n=14)
surface

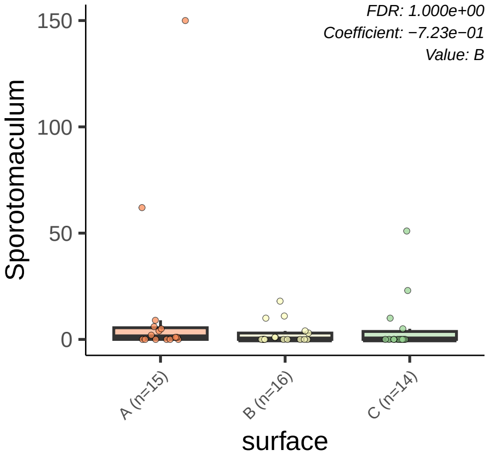

FDR: 1.000e+00
Coefficient: −7.23e−01
Value: B
Sporotomaculum
150
100
50
0
A (n=15)
B (n=16)
C (n=14)
surface

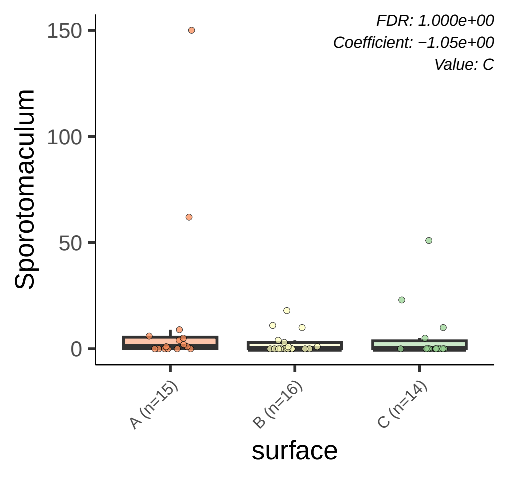
FDR: 1.000e+00
Coefficient: −1.05e+00
Value: C
Sporotomaculum
150
100
50
0
A (n=15)
B (n=16)
C (n=14)
surface

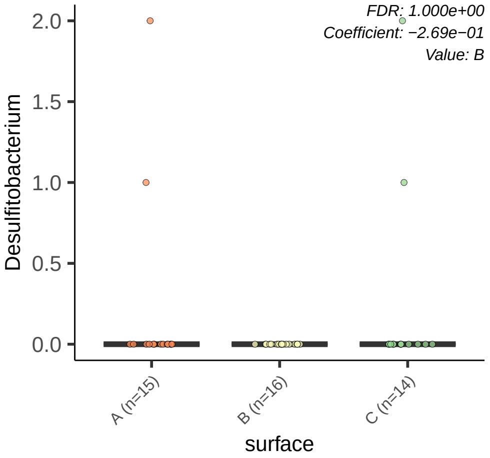
FDR: 1.000e+00
Coefficient: −2.69e−01
Value: B
Desulfitobacterium
2.0
1.5
1.0
0.5
0.0
A (n=15)
B (n=16)
C (n=14)
surface

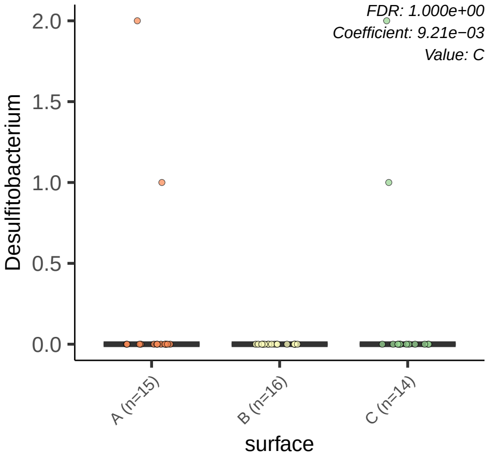

FDR: 1.000e+00
Coefficient: 9.21e−03
Value: C
Desulfitobacterium
2.0
1.5
1.0
0.5
0.0
A (n=15)
B (n=16)
C (n=14)
surface

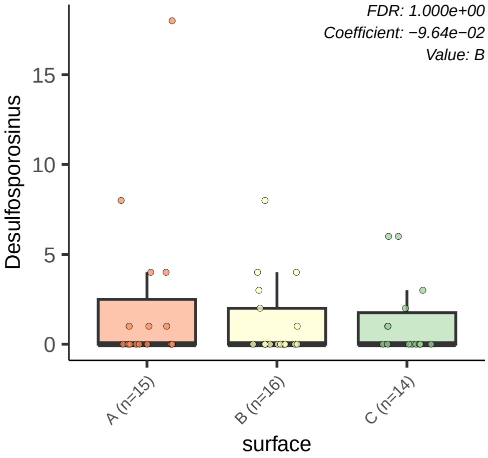

FDR: 1.000e+00
Coefficient: −9.64e−02
Value: B
Desulfosporosinus
0
5
10
15
A (n=15)
B (n=16)
C (n=14)
surface

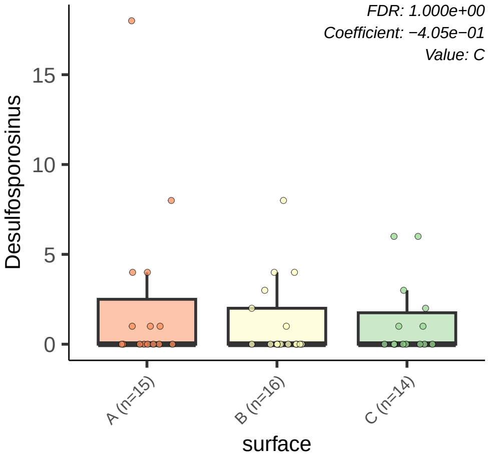

FDR: 1.000e+00
Coefficient: −4.05e−01
Value: C
Desulfosporosinus
15
10
5
0
A (n=15)
B (n=16)
C (n=14)
surface

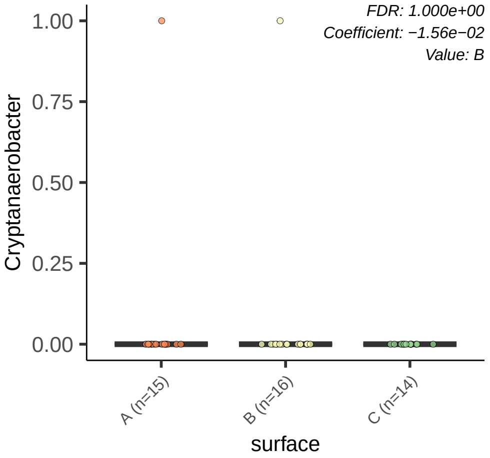
FDR: 1.000e+00
Coefficient: −1.56e−02
Value: B
Cryptanaerobacter
1.00
0.75
0.50
0.25
0.00
A (n=15)
B (n=16)
C (n=14)
surface

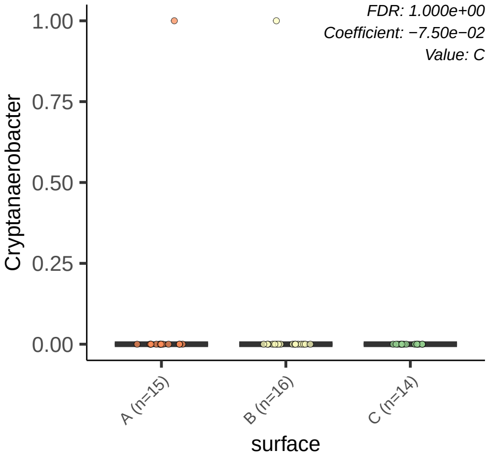
FDR: 1.000e+00
Coefficient: −7.50e−02
Value: C
Cryptanaerobacter
1.00
0.75
0.50
0.25
0.00
A (n=15)
B (n=16)
C (n=14)
surface

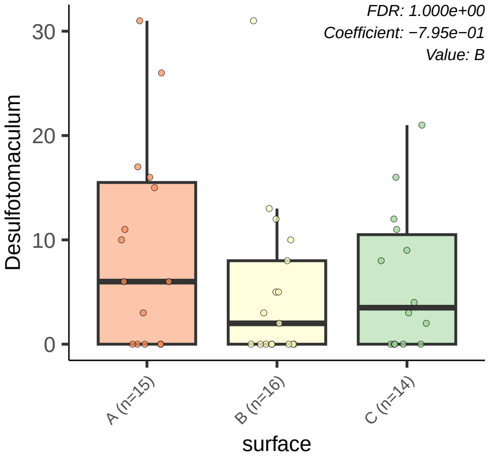
FDR: 1.000e+00
Coefficient: −7.95e−01
Value: B
Desulfotomaculum
30
20
10
0
A (n=15)
B (n=16)
C (n=14)
surface

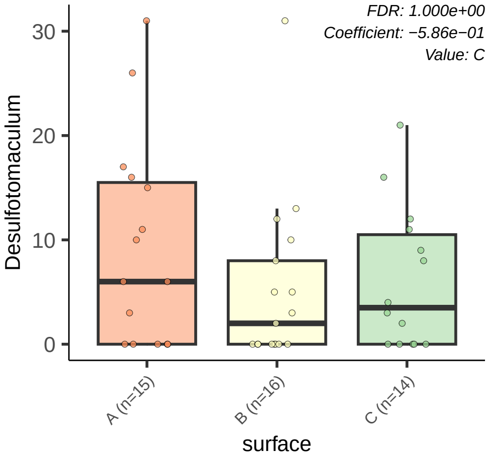
FDR: 1.000e+00
Coefficient: −5.86e−01
Value: C
Desulfotomaculum
0
10
20
30
A (n=15)
B (n=16)
C (n=14)
surface

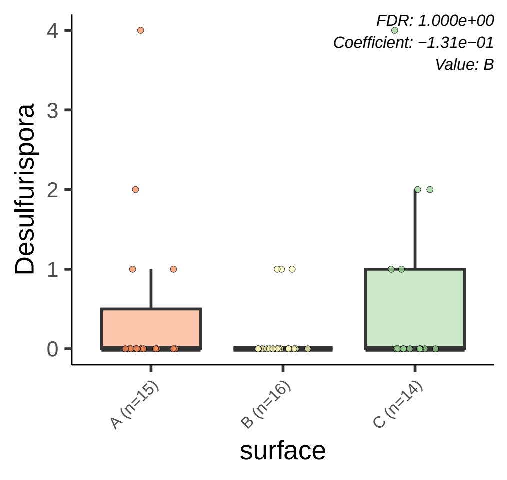
FDR: 1.000e+00
Coefficient: −1.31e−01
Value: B
Desulfurispora
0
1
2
3
4
A (n=15)
B (n=16)
C (n=14)
surface

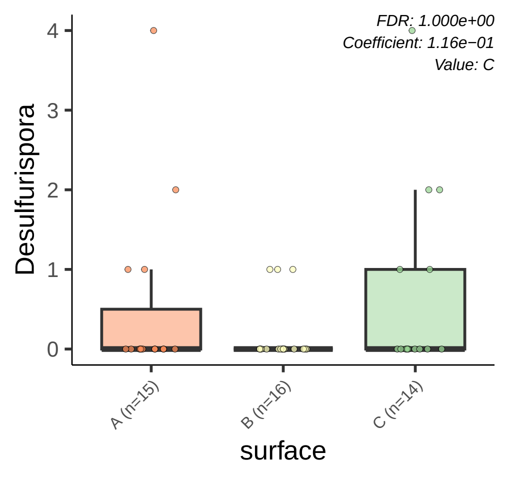

FDR: 1.000e+00
Coefficient: 1.16e−01
Value: C
Desulfurispora
0
1
2
3
4
A (n=15)
B (n=16)
C (n=14)
surface

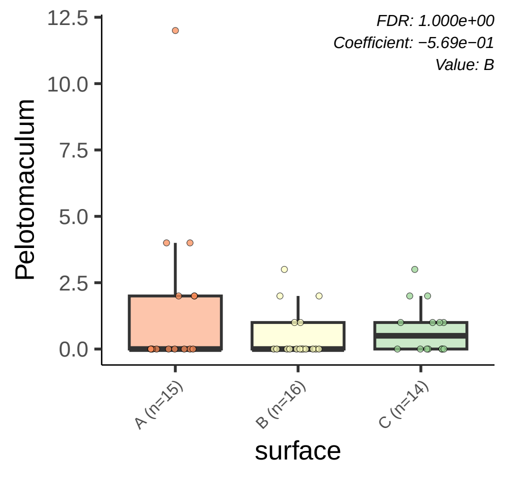

FDR: 1.000e+00
Coefficient: −5.69e−01
Value: B
Pelotomaculum
12.5
10.0
7.5
5.0
2.5
0.0
A (n=15)
B (n=16)
C (n=14)
surface

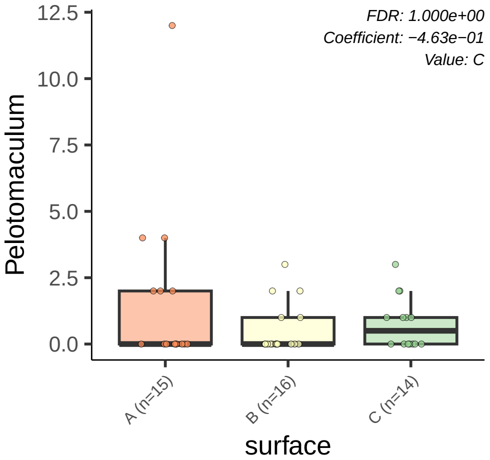

FDR: 1.000e+00
Coefficient: −4.63e−01
Value: C
Pelotomaculum
12.5
10.0
7.5
5.0
2.5
0.0
A (n=15)
B (n=16)
C (n=14)
surface

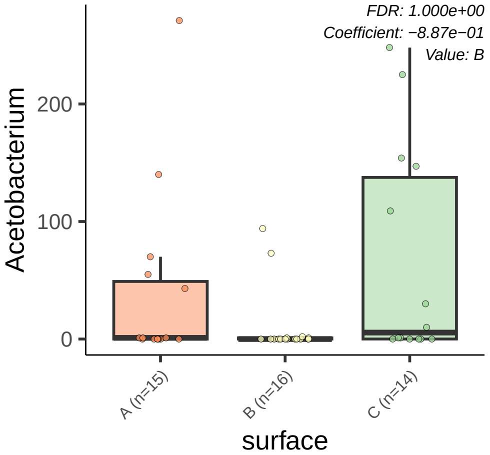
FDR: 1.000e+00
Coefficient: −8.87e−01
Value: B
Acetobacterium
200
100
0
A (n=15)
B (n=16)
C (n=14)
surface

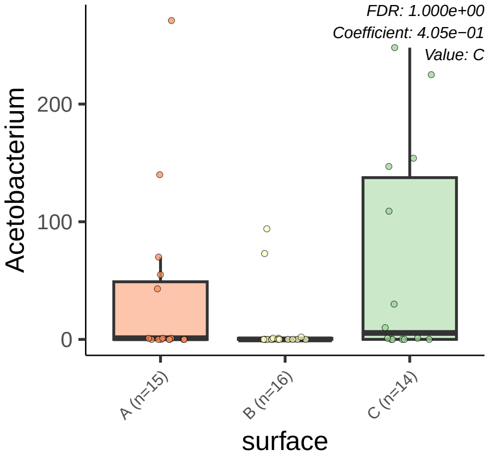
FDR: 1.000e+00
Coefficient: 4.05e−01
Value: C
Acetobacterium
200
100
0
A (n=15)
B (n=16)
C (n=14)
surface

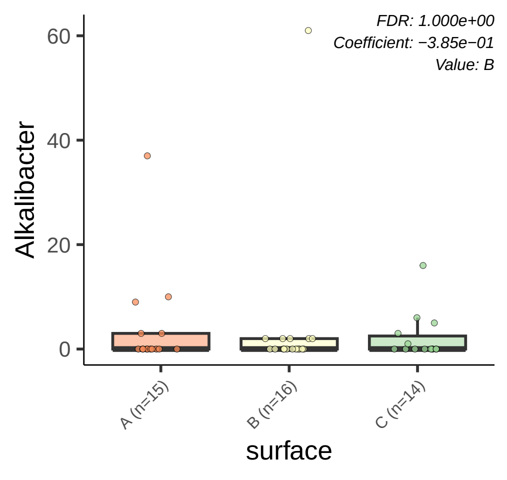
FDR: 1.000e+00
Coefficient: −3.85e−01
Value: B
Alkalibacter
60
40
20
0
A (n=15)
B (n=16)
C (n=14)
surface

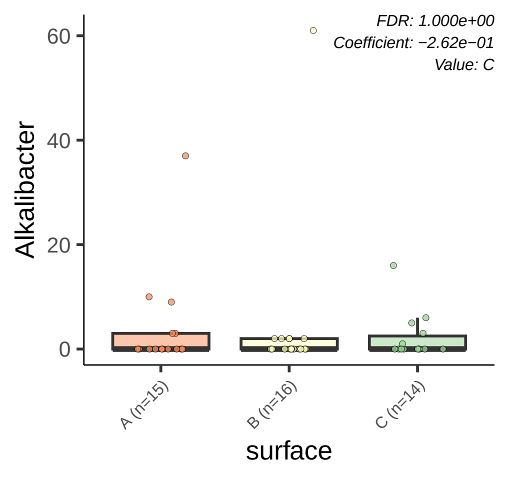
FDR: 1.000e+00
Coefficient: −2.62e−01
Value: C
Alkalibacter
60
40
20
0
A (n=15)
B (n=16)
C (n=14)
surface

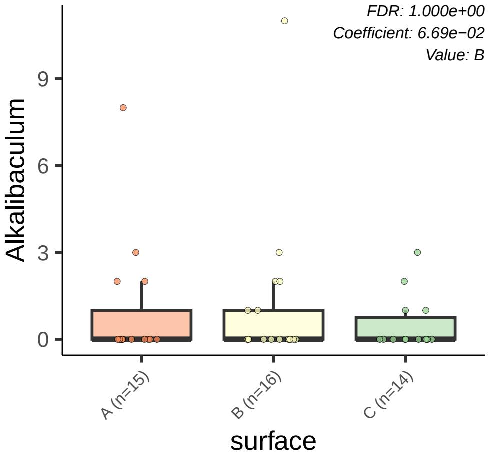
FDR: 1.000e+00
Coefficient: 6.69e−02
Value: B
Alkalibaculum
0
3
6
9
A (n=15)
B (n=16)
C (n=14)
surface

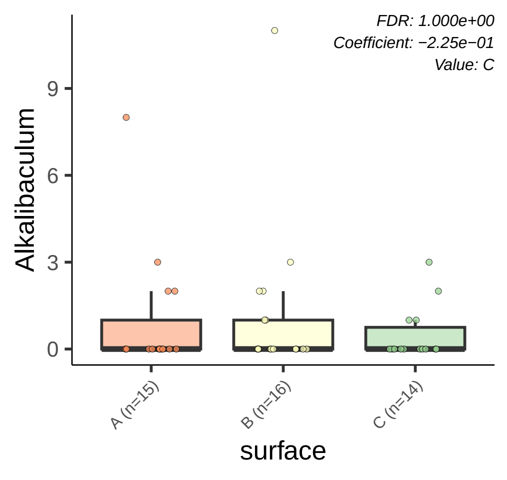
FDR: 1.000e+00
Coefficient: −2.25e−01
Value: C
Alkalibaculum
0
3
6
9
A (n=15)
B (n=16)
C (n=14)
surface

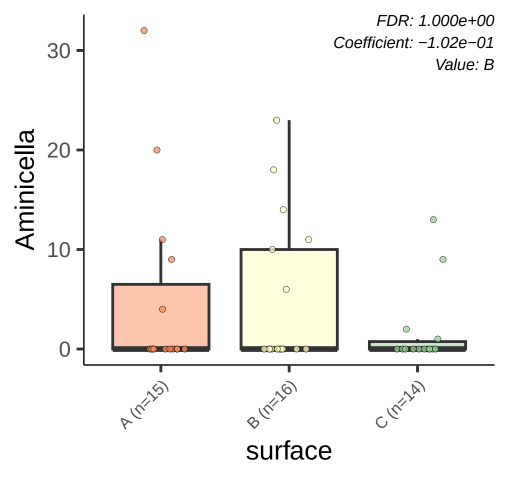
FDR: 1.000e+00
Coefficient: −1.02e−01
Value: B
Aminicella
30
20
10
0
A (n=15)
B (n=16)
C (n=14)
surface

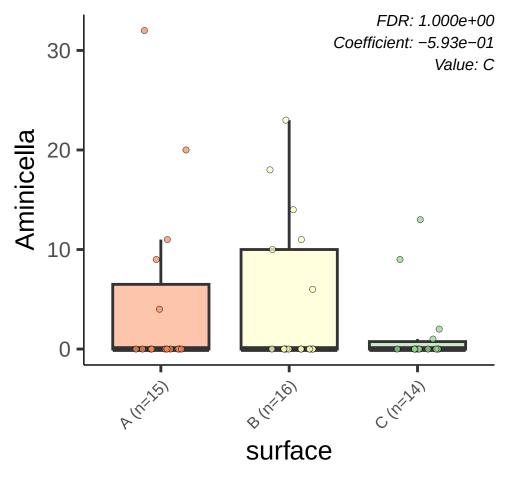
FDR: 1.000e+00
Coefficient: −5.93e−01
Value: C
Aminicella
30
20
10
0
A (n=15)
B (n=16)
C (n=14)
surface

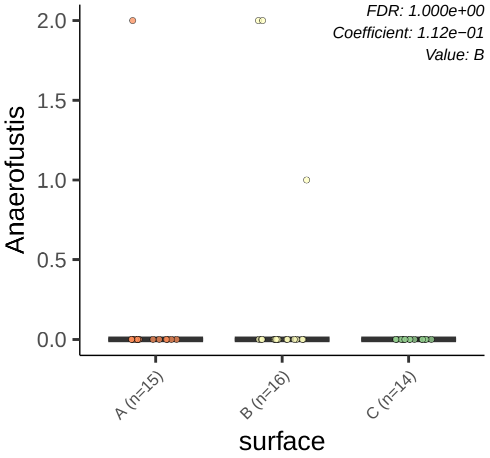
FDR: 1.000e+00
Coefficient: 1.12e−01
Value: B
Anaerofustis
2.0
1.5
1.0
0.5
0.0
A (n=15)
B (n=16)
C (n=14)
surface

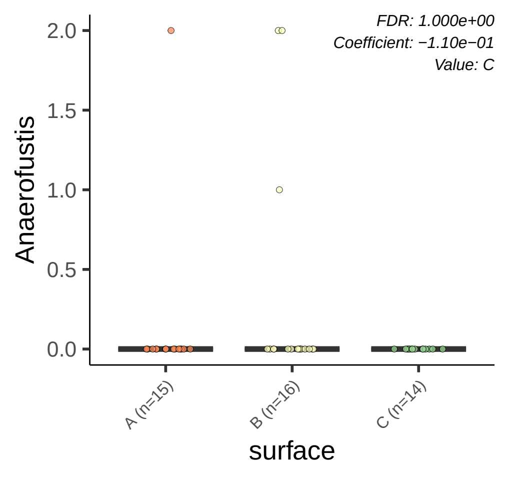
FDR: 1.000e+00
Coefficient: −1.10e−01
Value: C
Anaerofustis
2.0
1.5
1.0
0.5
0.0
A (n=15)
B (n=16)
C (n=14)
surface

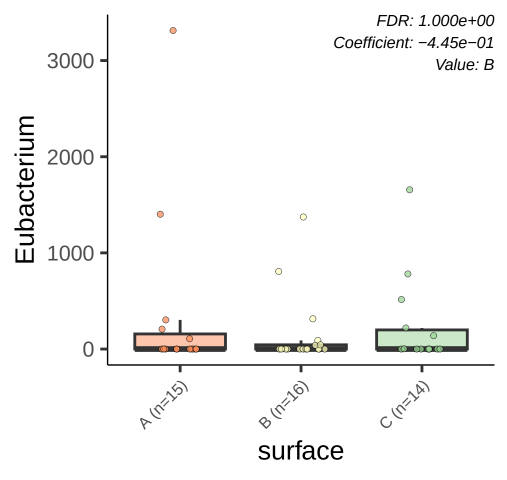
FDR: 1.000e+00
Coefficient: −4.45e−01
Value: B
Eubacterium
3000
2000
1000
0
A (n=15)
B (n=16)
C (n=14)
surface

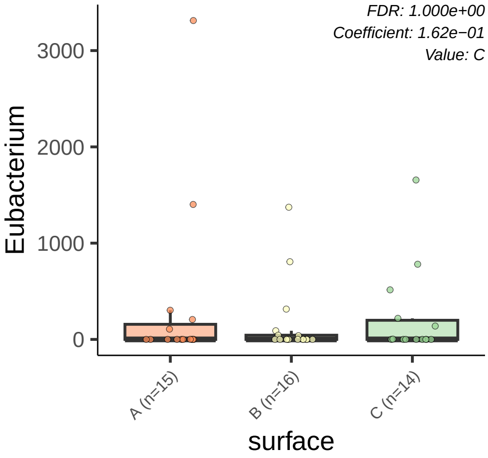

FDR: 1.000e+00
Coefficient: 1.62e−01
Value: C
Eubacterium
3000
2000
1000
0
A (n=15)
B (n=16)
C (n=14)
surface

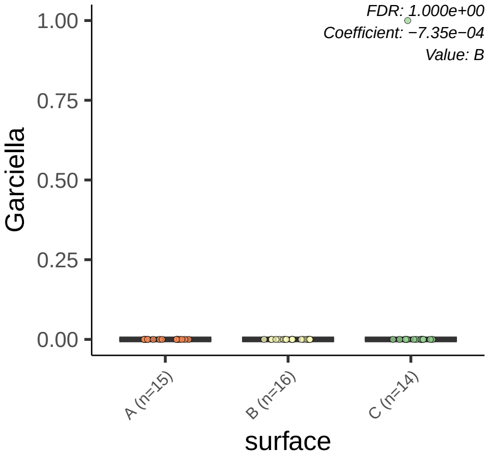
FDR: 1.000e+00
Coefficient: −7.35e−04
Value: B
Garciella
1.00
0.75
0.50
0.25
0.00
A (n=15)
B (n=16)
C (n=14)
surface

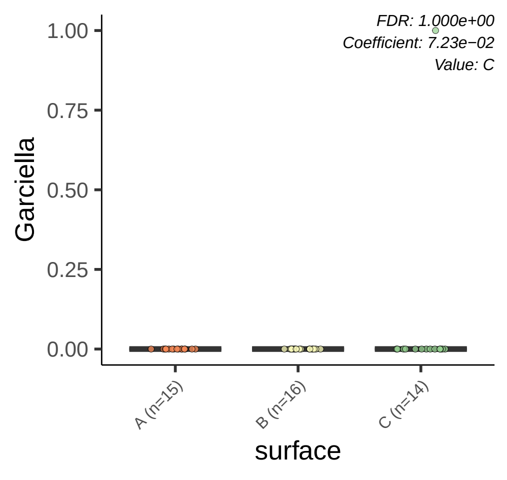
FDR: 1.000e+00
Coefficient: 7.23e−02
Value: C
Garciella
1.00
0.75
0.50
0.25
0.00
A (n=15)
B (n=16)
C (n=14)
surface

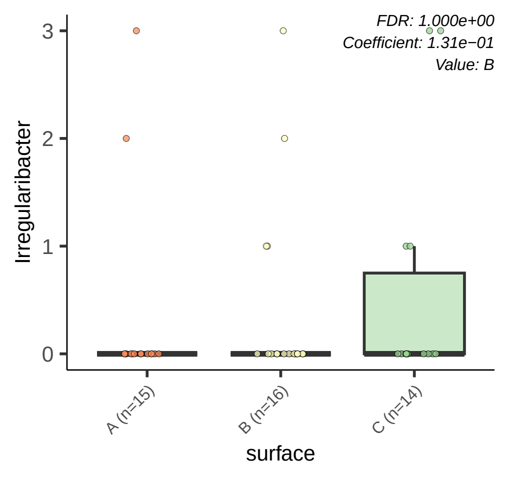
FDR: 1.000e+00
Coefficient: 1.31e−01
Value: B
Irregularibacter
3
2
1
0
A (n=15)
B (n=16)
C (n=14)
surface

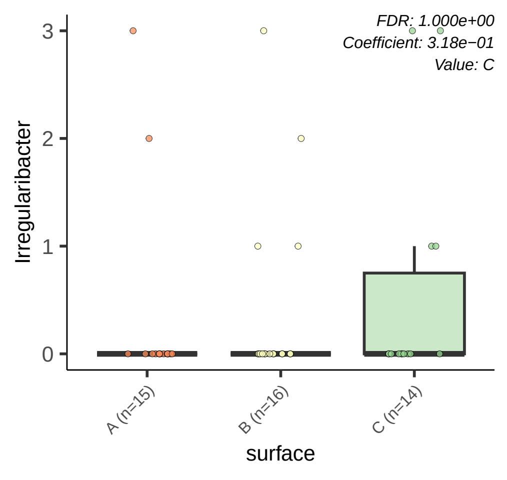

FDR: 1.000e+00
Coefficient: 3.18e−01
Value: C
Irregularibacter
3
2
1
0
A (n=15)
B (n=16)
C (n=14)
surface

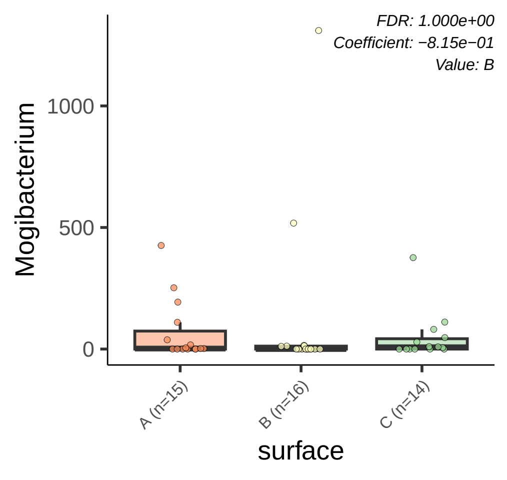
FDR: 1.000e+00
Coefficient: −8.15e−01
Value: B
Mogibacterium
1000
500
0
A (n=15)
B (n=16)
C (n=14)
surface

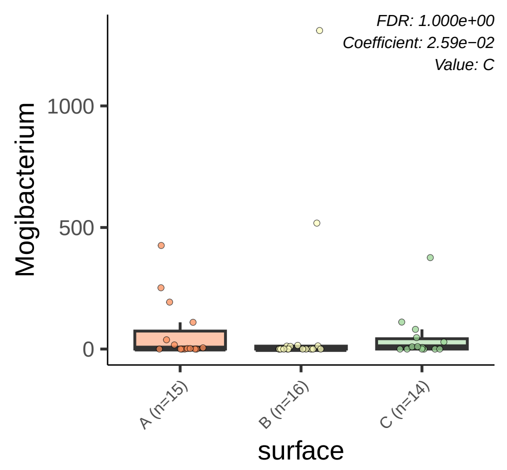
FDR: 1.000e+00
Coefficient: 2.59e−02
Value: C
Mogibacterium
1000
500
0
A (n=15)
B (n=16)
C (n=14)
surface

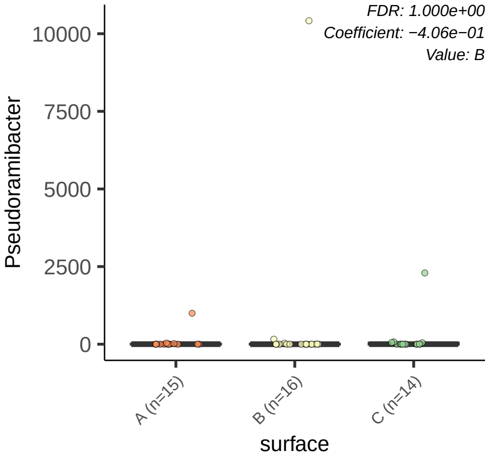

FDR: 1.000e+00
Coefficient: −4.06e−01
Value: B
Pseudoramibacter
10000
7500
5000
2500
0
A (n=15)
B (n=16)
C (n=14)
surface

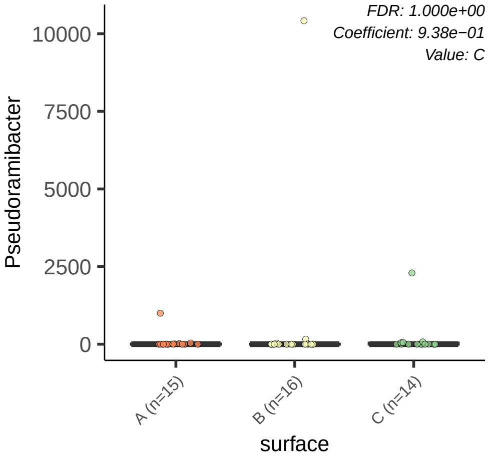

FDR: 1.000e+00
Coefficient: 9.38e−01
Value: C
Pseudoramibacter
10000
7500
5000
2500
0
A (n=15)
B (n=16)
C (n=14)
surface

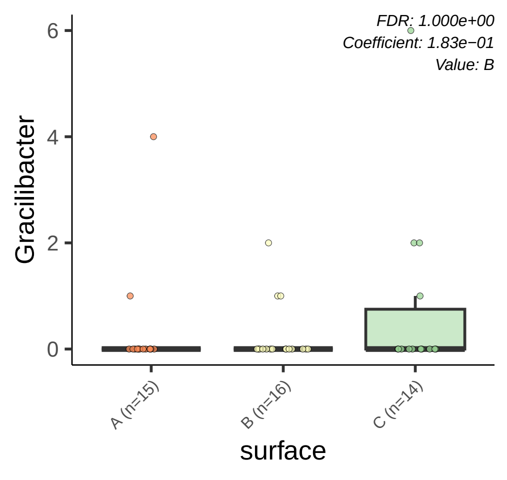
FDR: 1.000e+00
Coefficient: 1.83e−01
Value: B
Gracilibacter
0
2
4
6
A (n=15)
B (n=16)
C (n=14)
surface

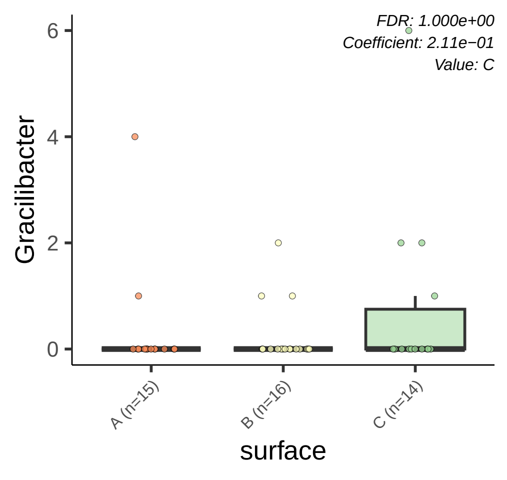
FDR: 1.000e+00
Coefficient: 2.11e−01
Value: C
Gracilibacter
6
4
2
0
A (n=15)
B (n=16)
C (n=14)
surface

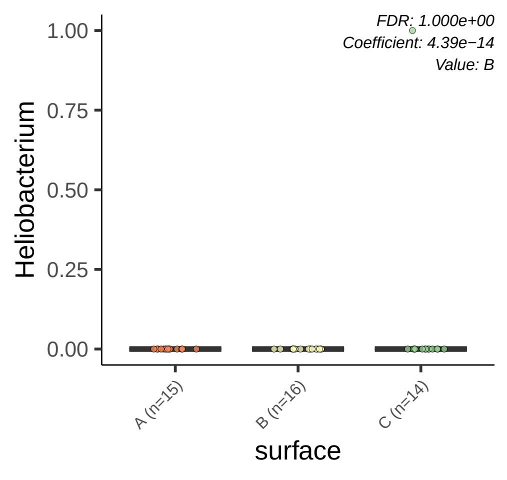
FDR: 1.000e+00
Coefficient: 4.39e−14
Value: B
Heliobacterium
1.00
0.75
0.50
0.25
0.00
A (n=15)
B (n=16)
C (n=14)
surface

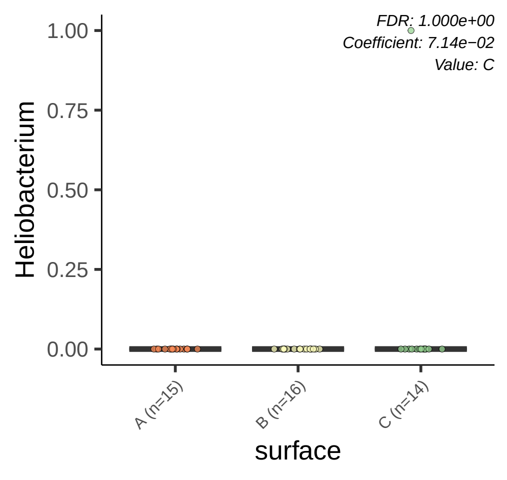
FDR: 1.000e+00
Coefficient: 7.14e−02
Value: C
Heliobacterium
1.00
0.75
0.50
0.25
0.00
A (n=15)
B (n=16)
C (n=14)
surface

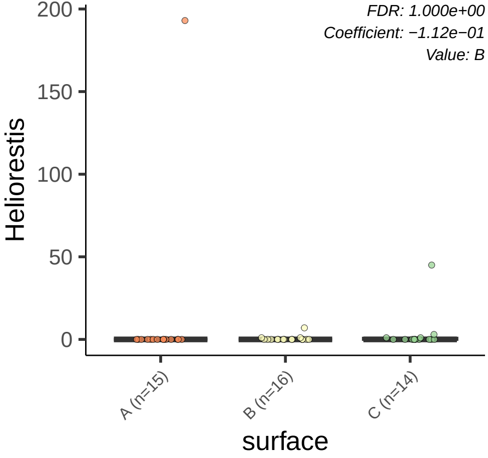

FDR: 1.000e+00
Coefficient: −1.12e−01
Value: B
Heliorestis
200
150
100
50
0
A (n=15)
B (n=16)
C (n=14)
surface

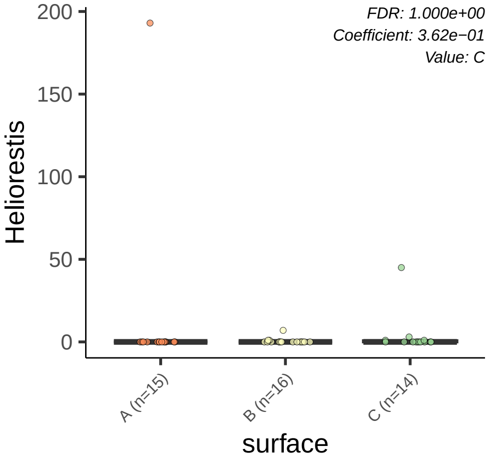

FDR: 1.000e+00
Coefficient: 3.62e−01
Value: C
Heliorestis
200
150
100
50
0
A (n=15)
B (n=16)
C (n=14)
surface

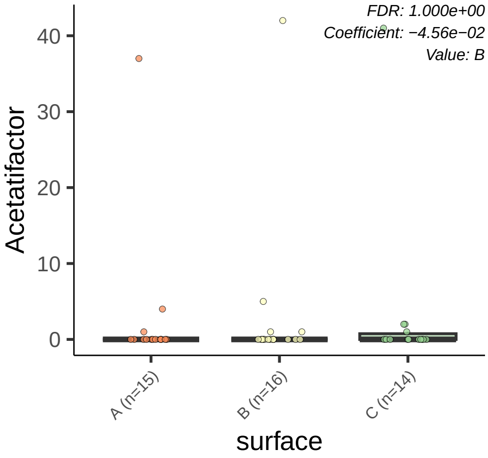

FDR: 1.000e+00
Coefficient: −4.56e−02
Value: B
Acetatifactor
0
10
20
30
40
A (n=15)
B (n=16)
C (n=14)
surface

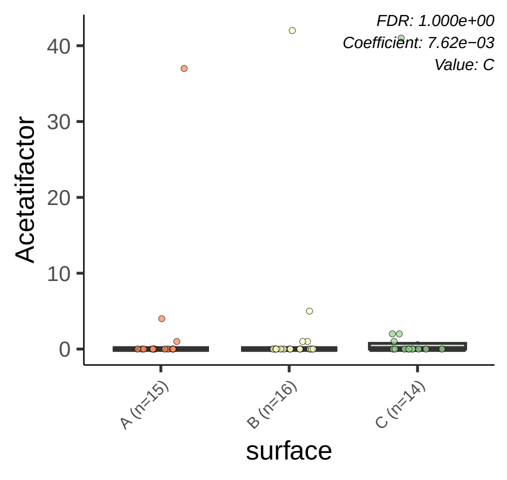

FDR: 1.000e+00
Coefficient: 7.62e−03
Value: C
Acetatifactor
40
30
20
10
0
A (n=15)
B (n=16)
C (n=14)
surface

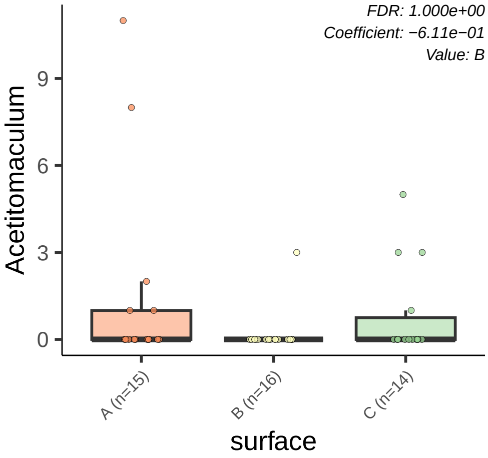
FDR: 1.000e+00
Coefficient: −6.11e−01
Value: B
Acetitomaculum
0
3
6
9
A (n=15)
B (n=16)
C (n=14)
surface

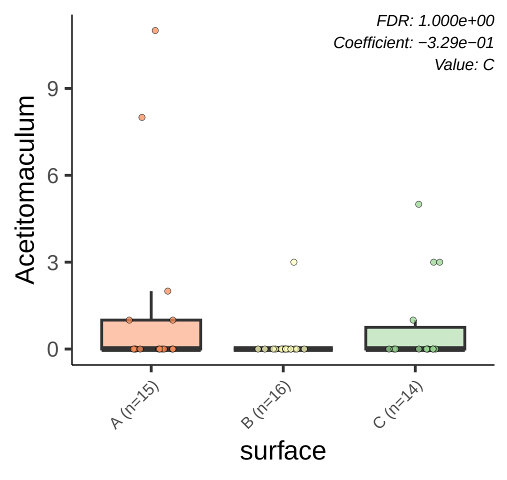
FDR: 1.000e+00
Coefficient: −3.29e−01
Value: C
Acetitomaculum
0
3
6
9
A (n=15)
B (n=16)
C (n=14)
surface

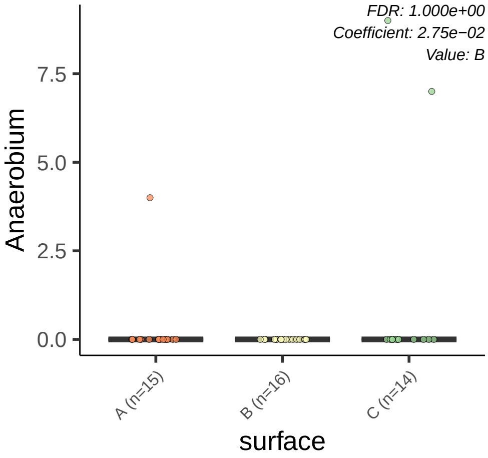

FDR: 1.000e+00
Coefficient: 2.75e−02
Value: B
Anaerobium
7.5
5.0
2.5
0.0
A (n=15)
B (n=16)
C (n=14)
surface

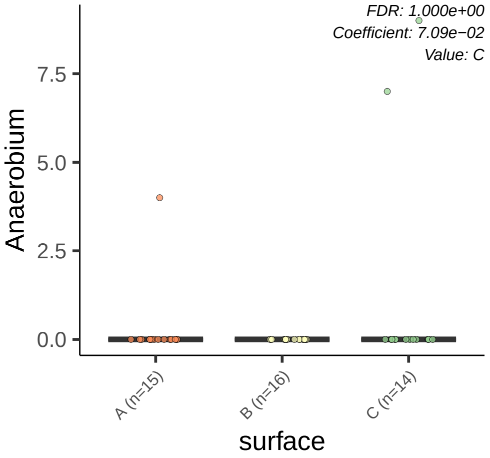
FDR: 1.000e+00
Coefficient: 7.09e−02
Value: C
Anaerobium
7.5
5.0
2.5
0.0
A (n=15)
B (n=16)
C (n=14)
surface

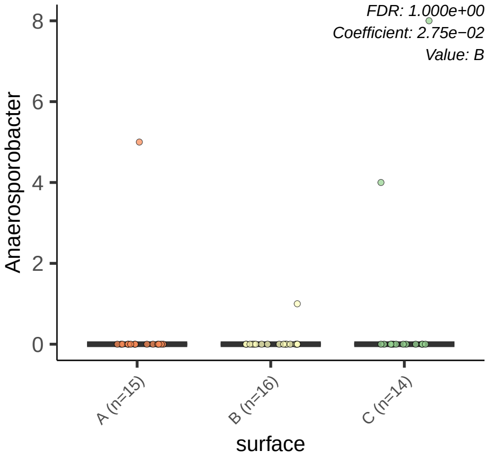

FDR: 1.000e+00
Coefficient: 2.75e−02
Value: B
Anaerosporobacter
0
2
4
6
8
A (n=15)
B (n=16)
C (n=14)
surface

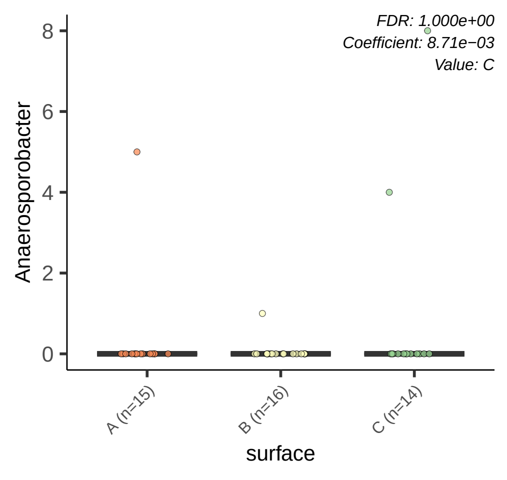

FDR: 1.000e+00
Coefficient: 8.71e−03
Value: C
Anaerosporobacter
0
2
4
6
8
A (n=15)
B (n=16)
C (n=14)
surface

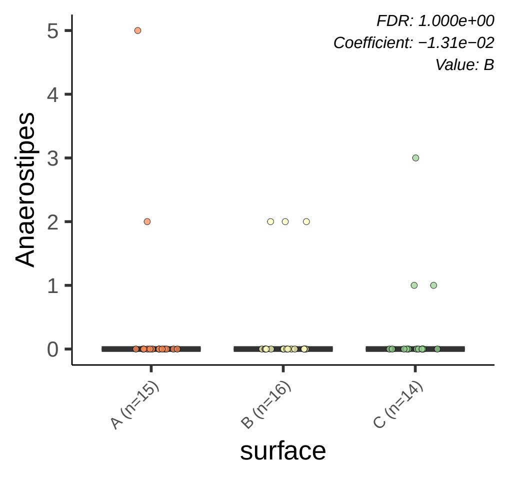

FDR: 1.000e+00
Coefficient: −1.31e−02
Value: B
Anaerostipes
0
1
2
3
4
5
A (n=15)
B (n=16)
C (n=14)
surface

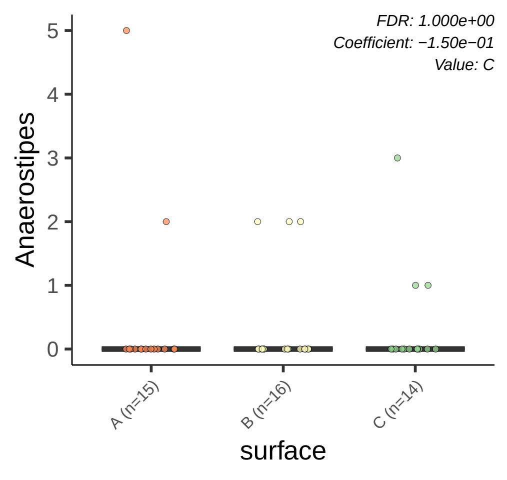
FDR: 1.000e+00
Coefficient: −1.50e−01
Value: C
Anaerostipes
0
1
2
3
4
5
A (n=15)
B (n=16)
C (n=14)
surface

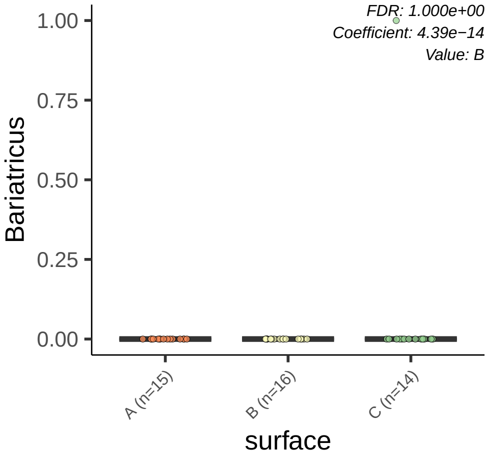
FDR: 1.000e+00
Coefficient: 4.39e−14
Value: B
Bariatricus
1.00
0.75
0.50
0.25
0.00
A (n=15)
B (n=16)
C (n=14)
surface

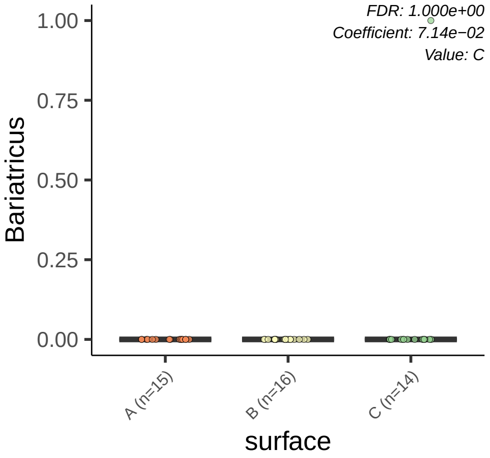
FDR: 1.000e+00
Coefficient: 7.14e−02
Value: C
1.00
0.75
0.50
0.25
0.00
Bariatricus
A (n=15)
B (n=16)
C (n=14)
surface

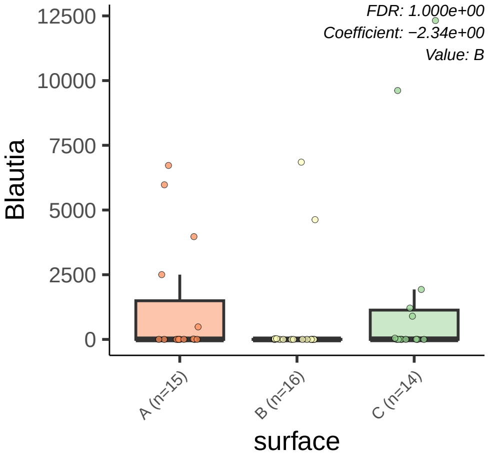

FDR: 1.000e+00
Coefficient: −2.34e+00
Value: B
Blautia
12500
10000
7500
5000
2500
0
A (n=15)
B (n=16)
C (n=14)
surface

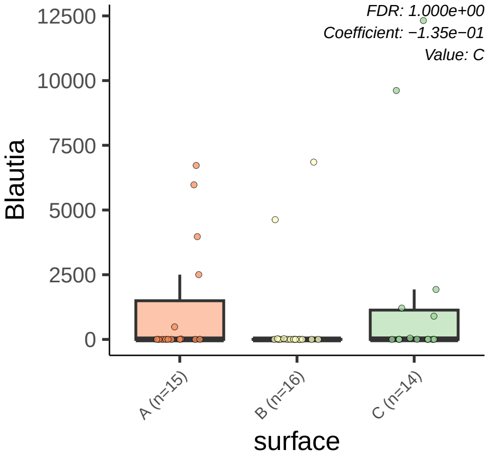
FDR: 1.000e+00
Coefficient: −1.35e−01
Value: C
Blautia
12500
10000
7500
5000
2500
0
A (n=15)
B (n=16)
C (n=14)
surface

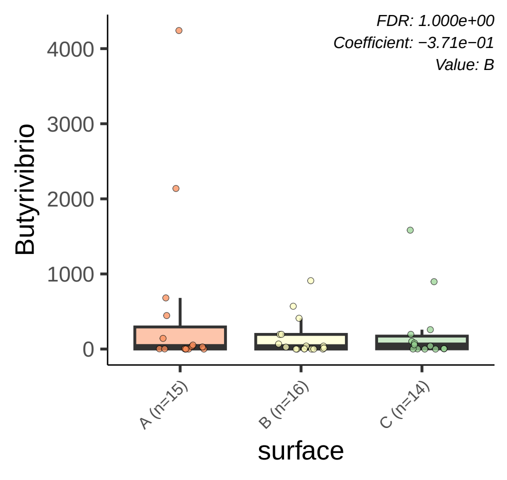
FDR: 1.000e+00
Coefficient: −3.71e−01
Value: B
Butyrivibrio
4000
3000
2000
1000
0
A (n=15)
B (n=16)
C (n=14)
surface

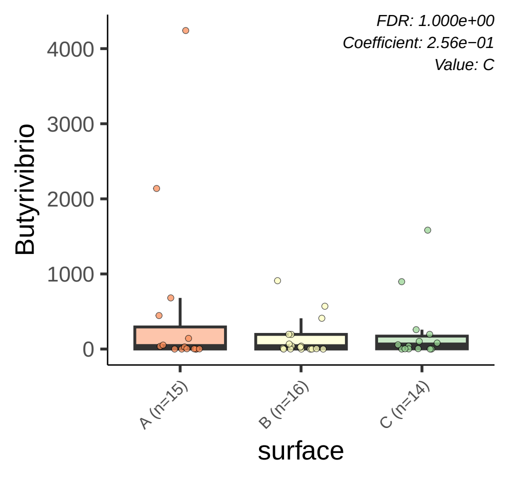

FDR: 1.000e+00
Coefficient: 2.56e−01
Value: C
Butyrivibrio
4000
3000
2000
1000
0
A (n=15)
B (n=16)
C (n=14)
surface

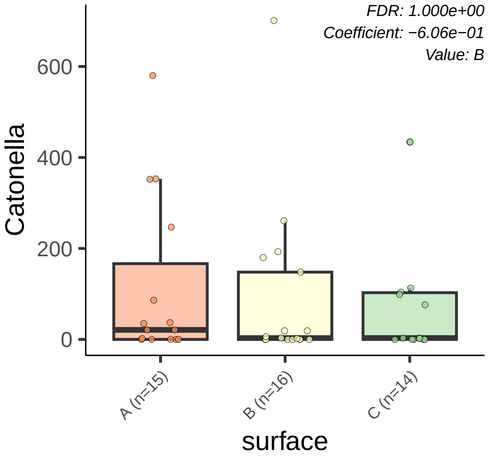

FDR: 1.000e+00
Coefficient: −6.06e−01
Value: B
Catonella
0
200
400
600
A (n=15)
B (n=16)
C (n=14)
surface

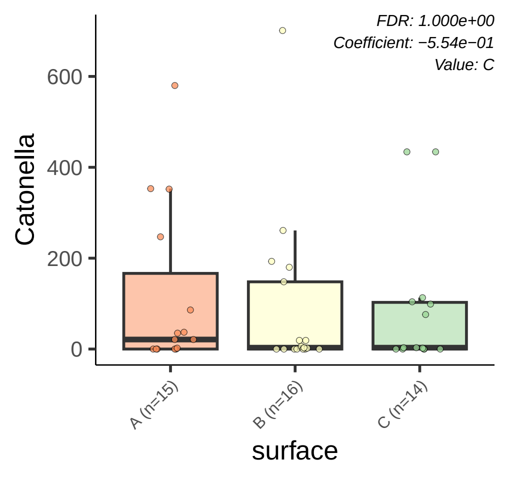
FDR: 1.000e+00
Coefficient: −5.54e−01
Value: C
Catonella
600
400
200
0
A (n=15)
B (n=16)
C (n=14)
surface

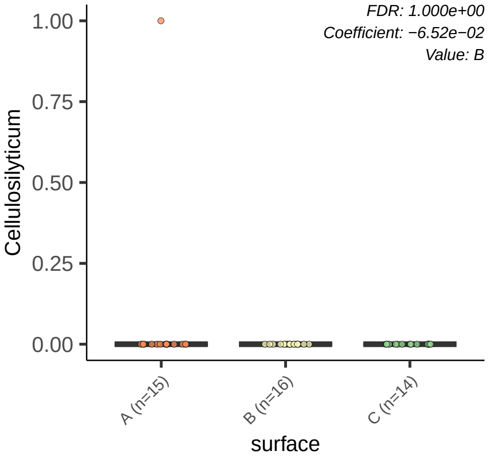
FDR: 1.000e+00
Coefficient: −6.52e−02
Value: B
Cellulosilyticum
1.00
0.75
0.50
0.25
0.00
A (n=15)
B (n=16)
C (n=14)
surface

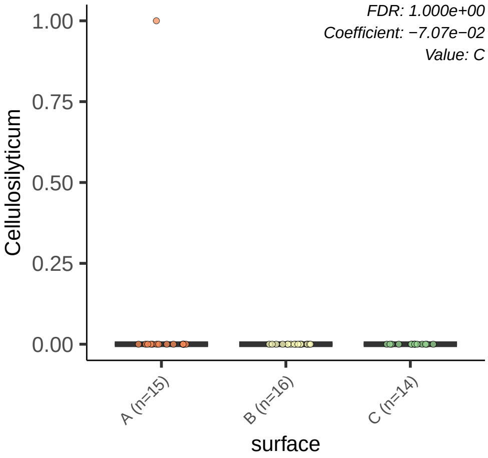
FDR: 1.000e+00
Coefficient: −7.07e−02
Value: C
Cellulosilyticum
1.00
0.75
0.50
0.25
0.00
A (n=15)
B (n=16)
C (n=14)
surface

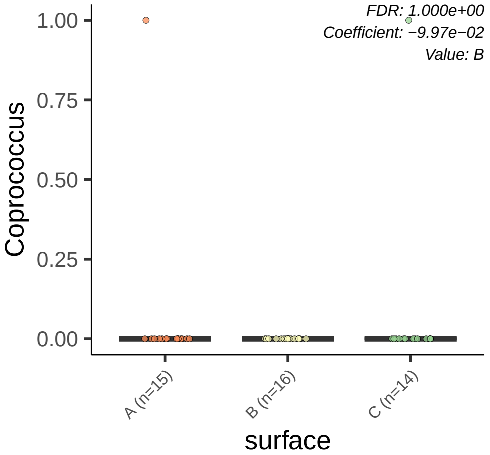

FDR: 1.000e+00
Coefficient: −9.97e−02
Value: B
Coprococcus
1.00
0.75
0.50
0.25
0.00
A (n=15)
B (n=16)
C (n=14)
surface

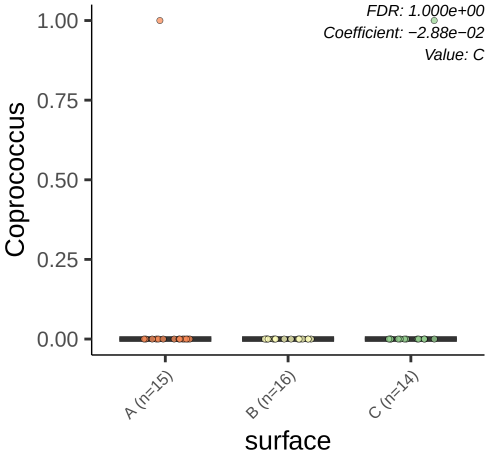
FDR: 1.000e+00
Coefficient: −2.88e−02
Value: C
Coprococcus
1.00
0.75
0.50
0.25
0.00
A (n=15)
B (n=16)
C (n=14)
surface

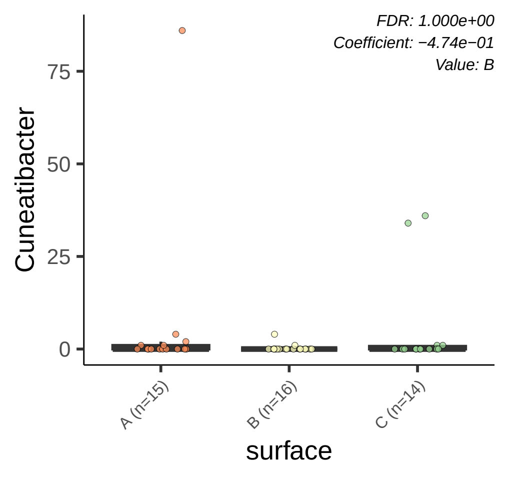
FDR: 1.000e+00
Coefficient: −4.74e−01
Value: B
Cuneatibacter
75
50
25
0
A (n=15)
B (n=16)
C (n=14)
surface

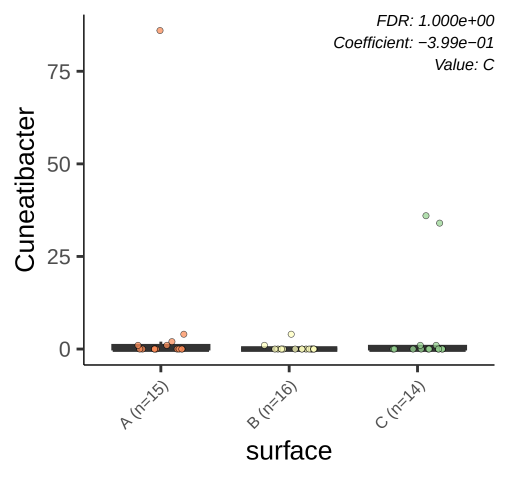

FDR: 1.000e+00
Coefficient: −3.99e−01
Value: C
Cuneatibacter
75
50
25
0
A (n=15)
B (n=16)
C (n=14)
surface

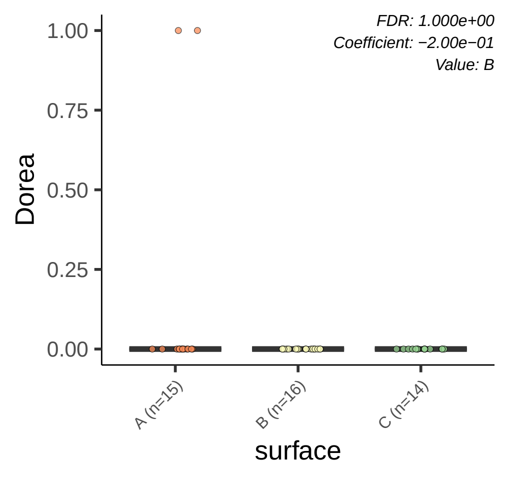
FDR: 1.000e+00
Coefficient: −2.00e−01
Value: B
Dorea
1.00
0.75
0.50
0.25
0.00
A (n=15)
B (n=16)
C (n=14)
surface

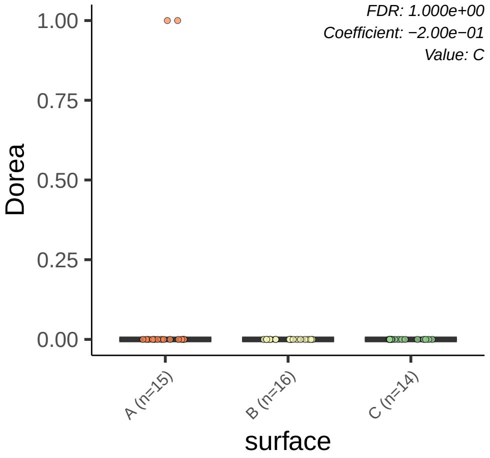

FDR: 1.000e+00
Coefficient: −2.00e−01
Value: C
1.00
0.75
0.50
0.25
0.00
Dorea
A (n=15)
B (n=16)
C (n=14)
surface

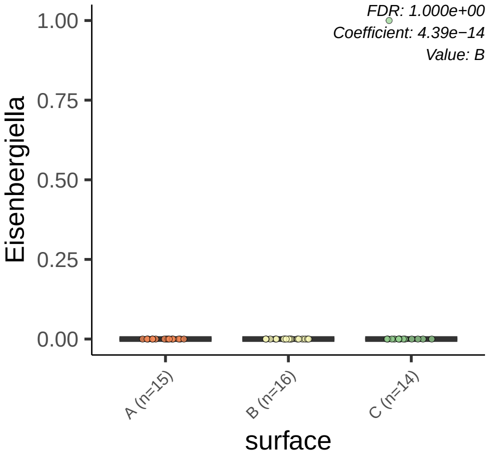

FDR: 1.000e+00
Coefficient: 4.39e−14
Value: B
Eisenbergiella
1.00
0.75
0.50
0.25
0.00
A (n=15)
B (n=16)
C (n=14)
surface

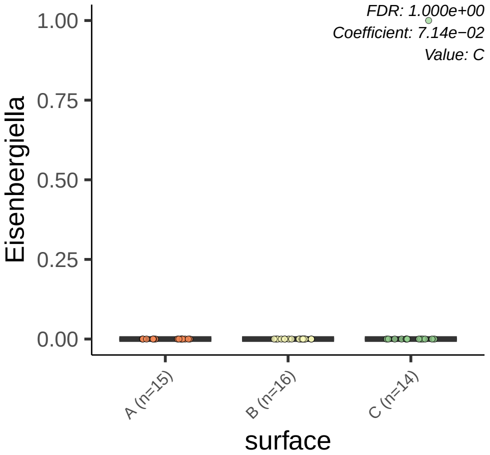

FDR: 1.000e+00
Coefficient: 7.14e−02
Value: C
Eisenbergiella
1.00
0.75
0.50
0.25
0.00
A (n=15)
B (n=16)
C (n=14)
surface

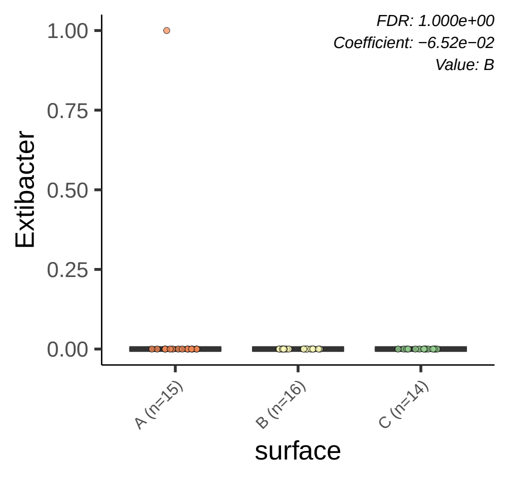
FDR: 1.000e+00
Coefficient: −6.52e−02
Value: B
1.00
0.75
0.50
0.25
0.00
Extibacter
A (n=15)
B (n=16)
C (n=14)
surface

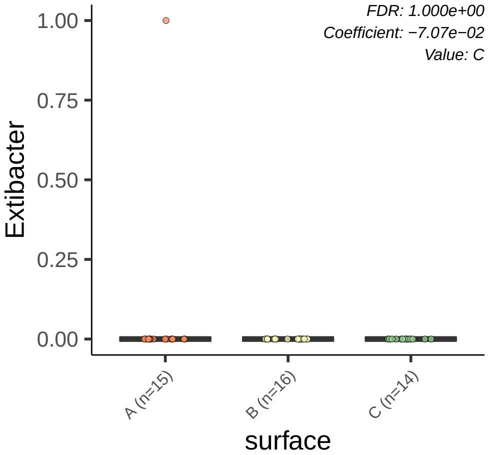
FDR: 1.000e+00
Coefficient: −7.07e−02
Value: C
Extibacter
1.00
0.75
0.50
0.25
0.00
A (n=15)
B (n=16)
C (n=14)
surface

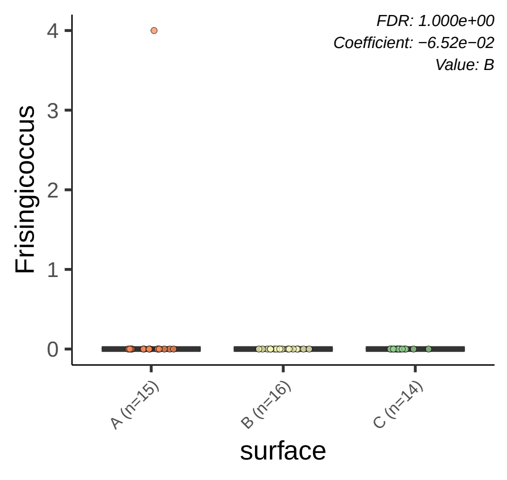

FDR: 1.000e+00
Coefficient: −6.52e−02
Value: B
Frisingicoccus
4
3
2
1
0
A (n=15)
B (n=16)
C (n=14)
surface

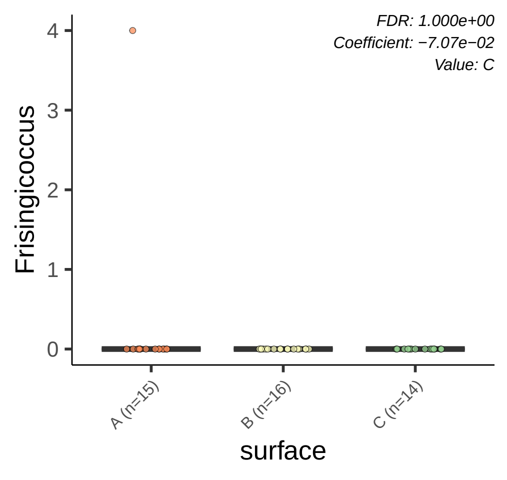
FDR: 1.000e+00
Coefficient: −7.07e−02
Value: C
Frisingicoccus
4
3
2
1
0
A (n=15)
B (n=16)
C (n=14)
surface

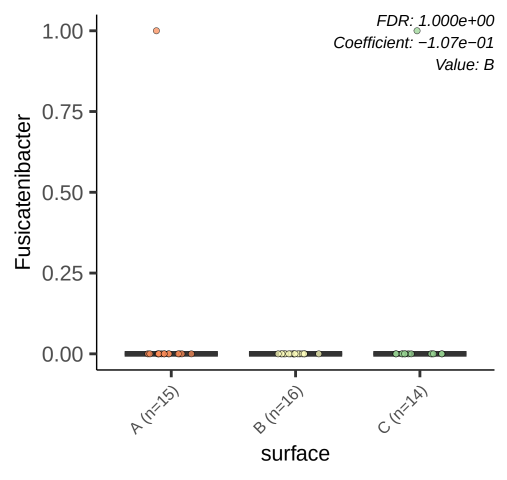

FDR: 1.000e+00
Coefficient: −1.07e−01
Value: B
Fusicatenibacter
1.00
0.75
0.50
0.25
0.00
A (n=15)
B (n=16)
C (n=14)
surface

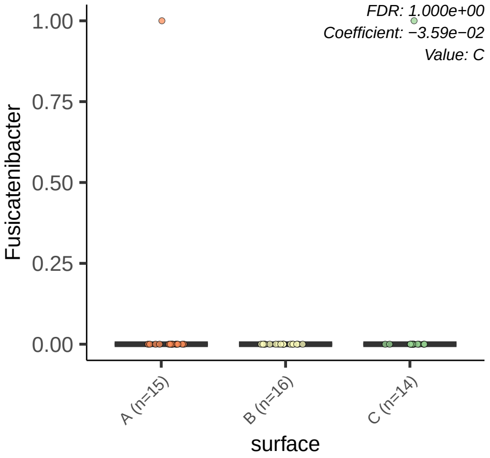

FDR: 1.000e+00
Coefficient: −3.59e−02
Value: C
Fusicatenibacter
1.00
0.75
0.50
0.25
0.00
A (n=15)
B (n=16)
C (n=14)
surface

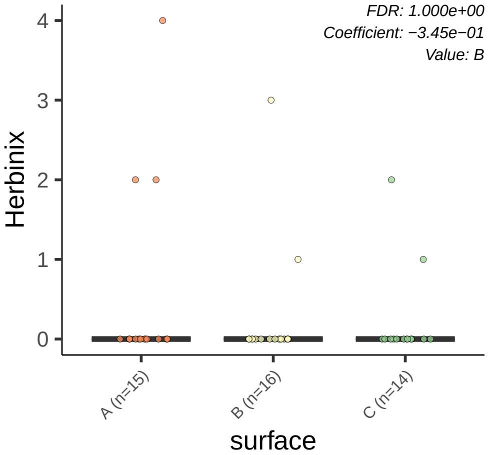
FDR: 1.000e+00
Coefficient: −3.45e−01
Value: B
Herbinix
4
3
2
1
0
A (n=15)
B (n=16)
C (n=14)
surface

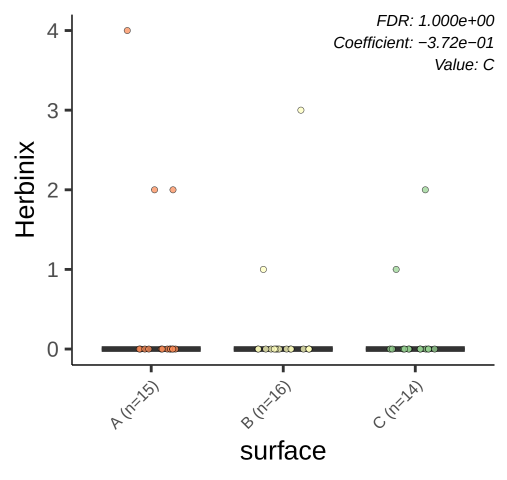
FDR: 1.000e+00
Coefficient: −3.72e−01
Value: C
Herbinix
4
3
2
1
0
A (n=15)
B (n=16)
C (n=14)
surface

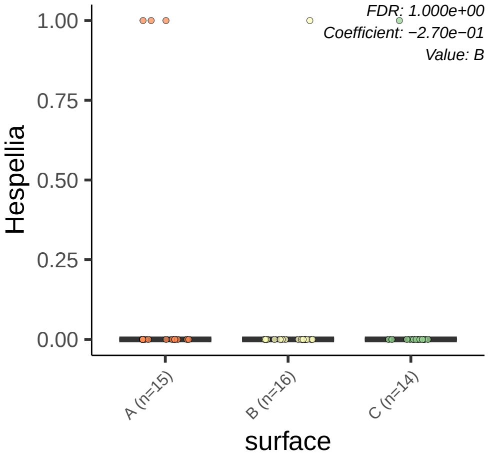

FDR: 1.000e+00
Coefficient: −2.70e−01
Value: B
1.00
0.75
0.50
0.25
0.00
Hespellia
A (n=15)
B (n=16)
C (n=14)
surface

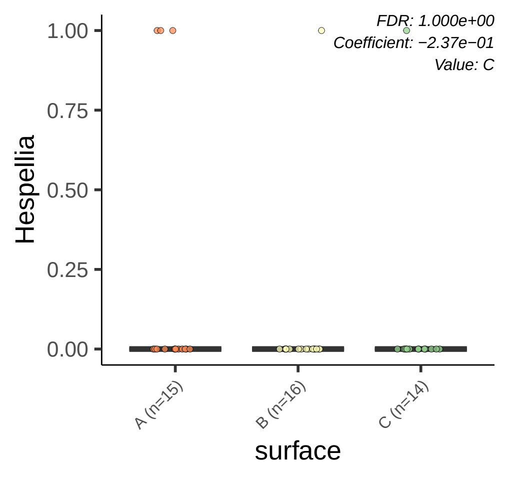
FDR: 1.000e+00
Coefficient: −2.37e−01
Value: C
Hespellia
1.00
0.75
0.50
0.25
0.00
A (n=15)
B (n=16)
C (n=14)
surface

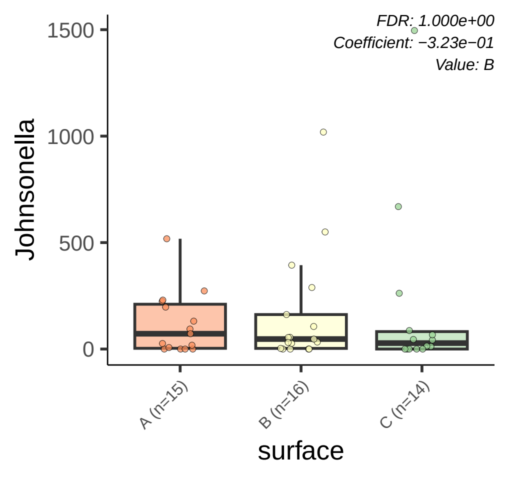
FDR: 1.000e+00
Coefficient: −3.23e−01
Value: B
Johnsonella
1500
1000
500
0
A (n=15)
B (n=16)
C (n=14)
surface

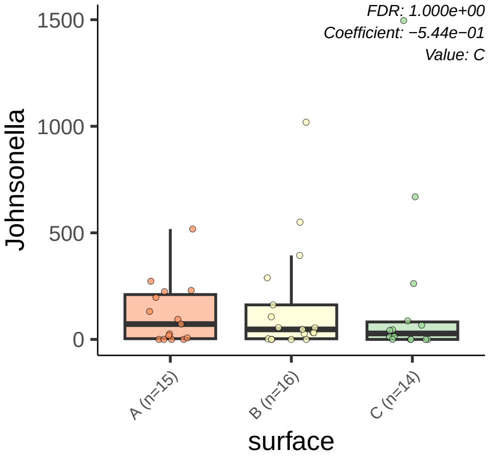

FDR: 1.000e+00
Coefficient: −5.44e−01
Value: C
Johnsonella
1500
1000
500
0
A (n=15)
B (n=16)
C (n=14)
surface

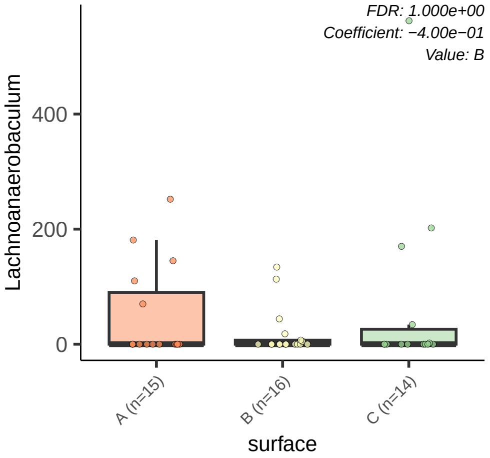
FDR: 1.000e+00
Coefficient: −4.00e−01
Value: B
Lachnoanaerobaculum
400
200
0
A (n=15)
B (n=16)
C (n=14)
surface

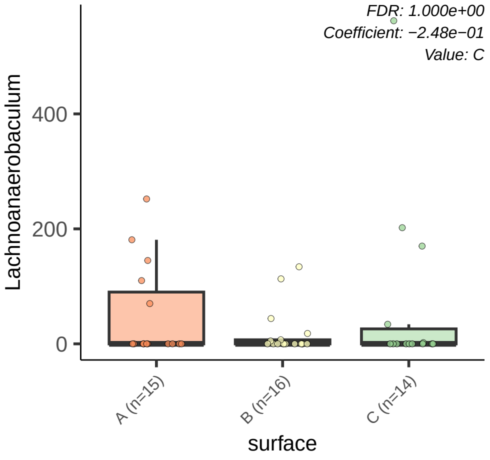

FDR: 1.000e+00
Coefficient: −2.48e−01
Value: C
Lachnoanaerobaculum
400
200
0
A (n=15)
B (n=16)
C (n=14)
surface

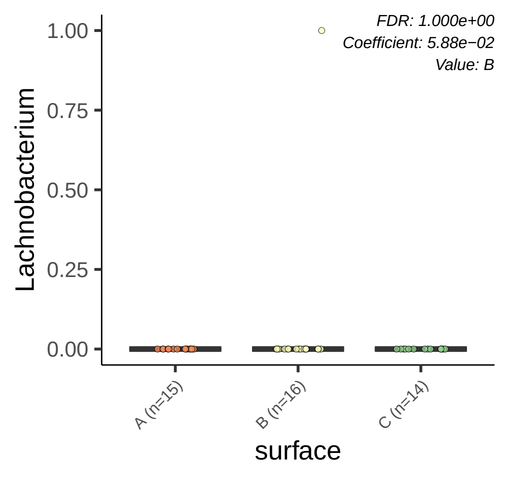
FDR: 1.000e+00
Coefficient: 5.88e−02
Value: B
Lachnobacterium
1.00
0.75
0.50
0.25
0.00
A (n=15)
B (n=16)
C (n=14)
surface

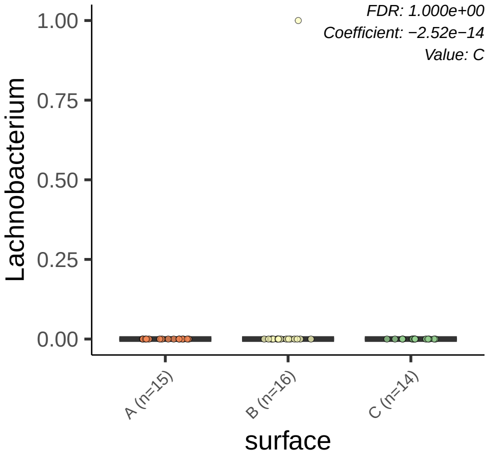
FDR: 1.000e+00
Coefficient: −2.52e−14
Value: C
Lachnobacterium
1.00
0.75
0.50
0.25
0.00
A (n=15)
B (n=16)
C (n=14)
surface

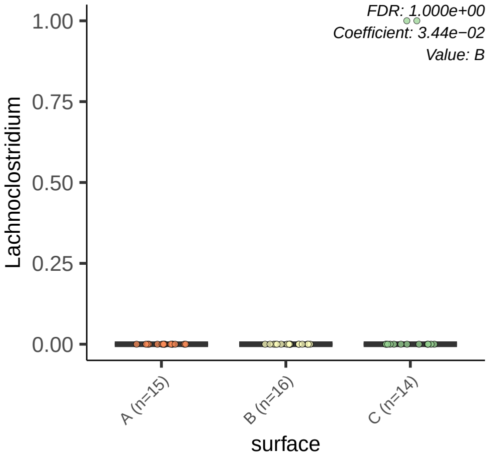

FDR: 1.000e+00
Coefficient: 3.44e−02
Value: B
Lachnoclostridium
1.00
0.75
0.50
0.25
0.00
A (n=15)
B (n=16)
C (n=14)
surface

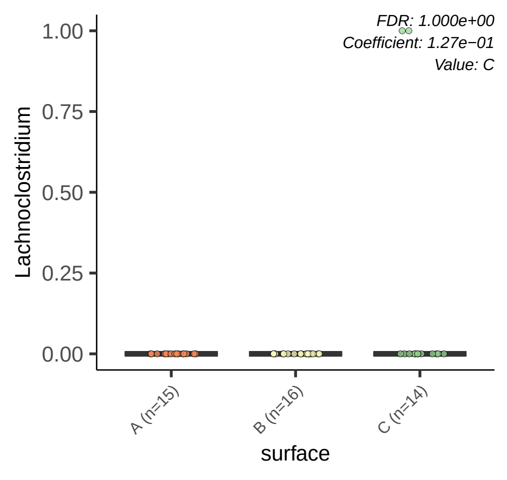
FDR: 1.000e+00
Coefficient: 1.27e−01
Value: C
Lachnoclostridium
1.00
0.75
0.50
0.25
0.00
A (n=15)
B (n=16)
C (n=14)
surface

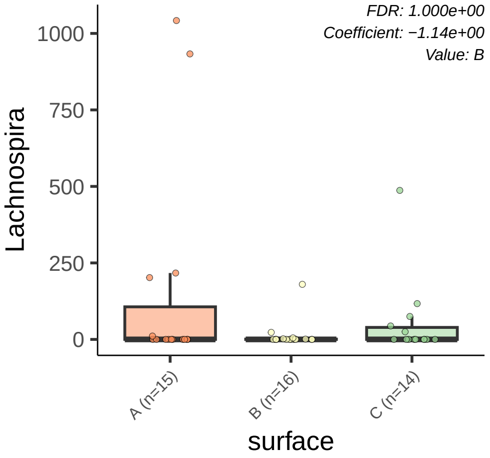

FDR: 1.000e+00
Coefficient: −1.14e+00
Value: B
Lachnospira
1000
750
500
250
0
A (n=15)
B (n=16)
C (n=14)
surface

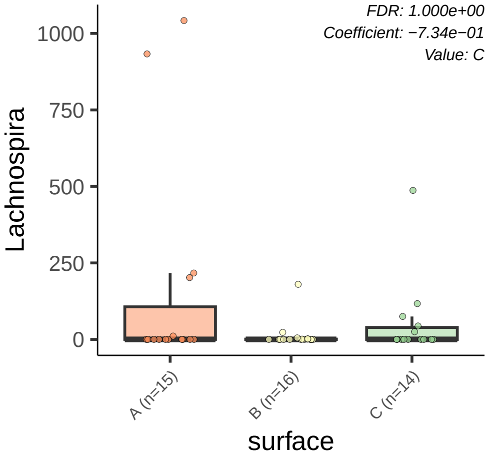
FDR: 1.000e+00
Coefficient: −7.34e−01
Value: C
Lachnospira
1000
750
500
250
0
A (n=15)
B (n=16)
C (n=14)
surface

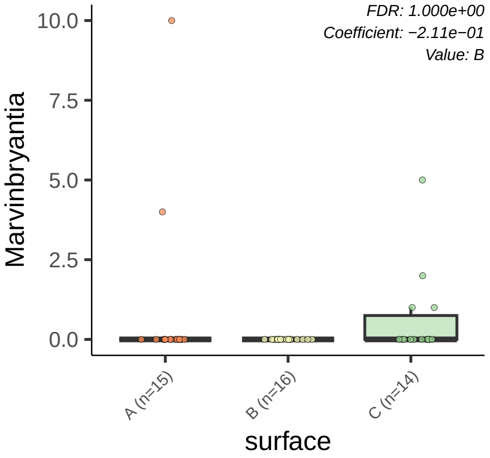
FDR: 1.000e+00
Coefficient: −2.11e−01
Value: B
Marvinbryantia
10.0
7.5
5.0
2.5
0.0
A (n=15)
B (n=16)
C (n=14)
surface

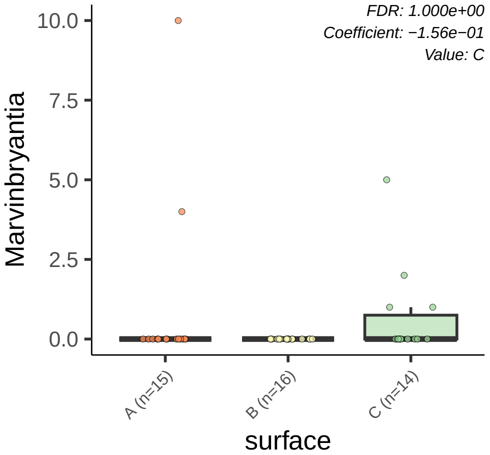
FDR: 1.000e+00
Coefficient: −1.56e−01
Value: C
Marvinbryantia
10.0
7.5
5.0
2.5
0.0
A (n=15)
B (n=16)
C (n=14)
surface

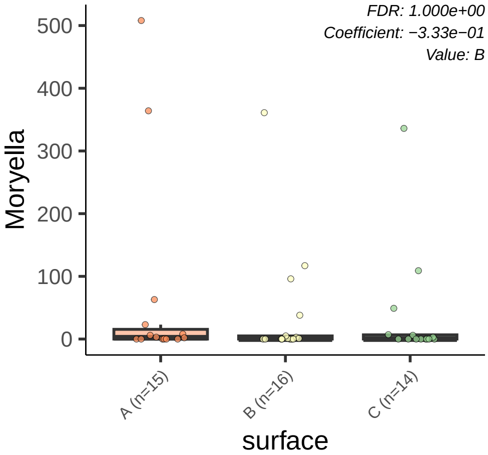
FDR: 1.000e+00
Coefficient: −3.33e−01
Value: B
Moryella
500
400
300
200
100
0
A (n=15)
B (n=16)
C (n=14)
surface

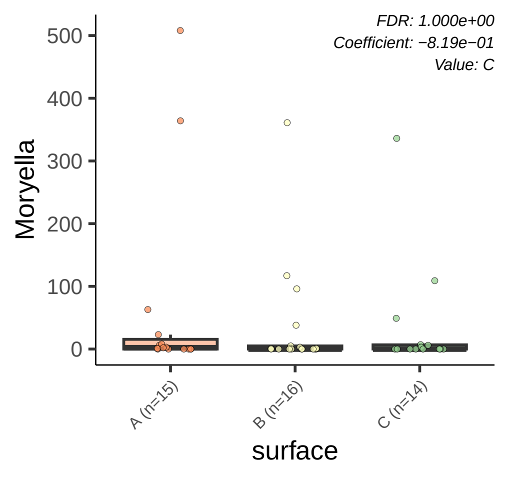
FDR: 1.000e+00
Coefficient: −8.19e−01
Value: C
Moryella
500
400
300
200
100
0
A (n=15)
B (n=16)
C (n=14)
surface

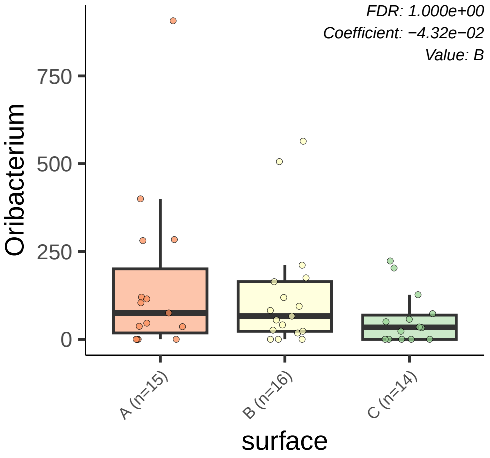
FDR: 1.000e+00
Coefficient: −4.32e−02
Value: B
Oribacterium
750
500
250
0
A (n=15)
B (n=16)
C (n=14)
surface

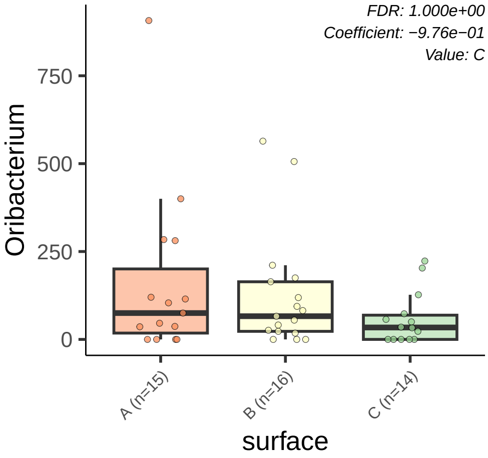

FDR: 1.000e+00
Coefficient: −9.76e−01
Value: C
Oribacterium
750
500
250
0
A (n=15)
B (n=16)
C (n=14)
surface

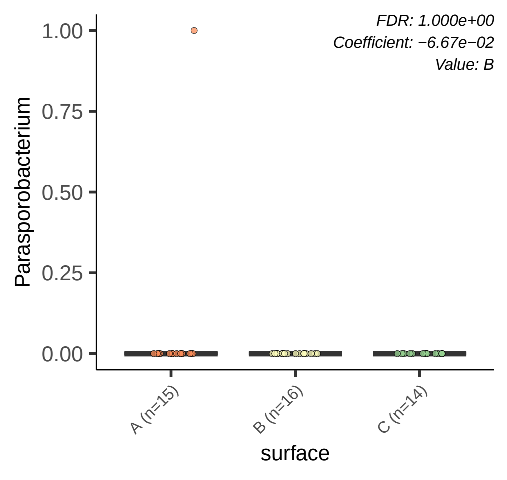
FDR: 1.000e+00
Coefficient: −6.67e−02
Value: B
Parasporobacterium
1.00
0.75
0.50
0.25
0.00
A (n=15)
B (n=16)
C (n=14)
surface

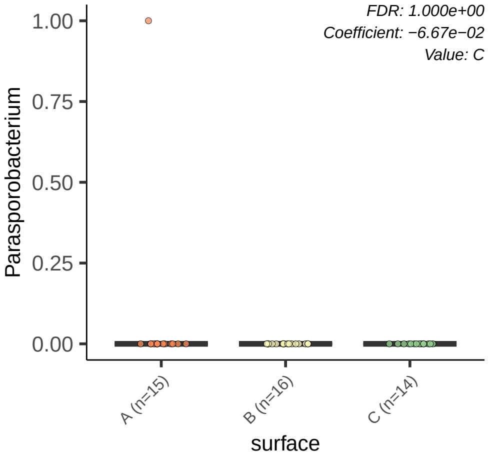
FDR: 1.000e+00
Coefficient: −6.67e−02
Value: C
Parasporobacterium
1.00
0.75
0.50
0.25
0.00
A (n=15)
B (n=16)
C (n=14)
surface

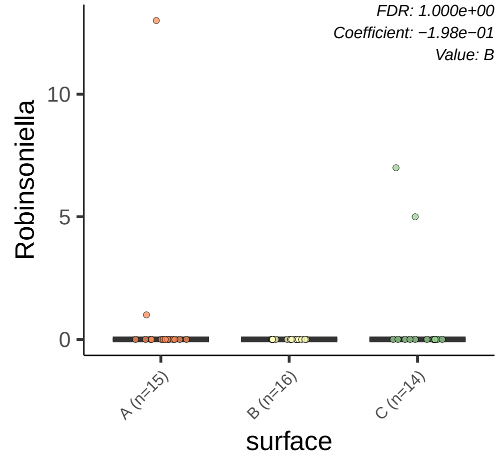
FDR: 1.000e+00
Coefficient: −1.98e−01
Value: B
Robinsoniella
10
5
0
A (n=15)
B (n=16)
C (n=14)
surface

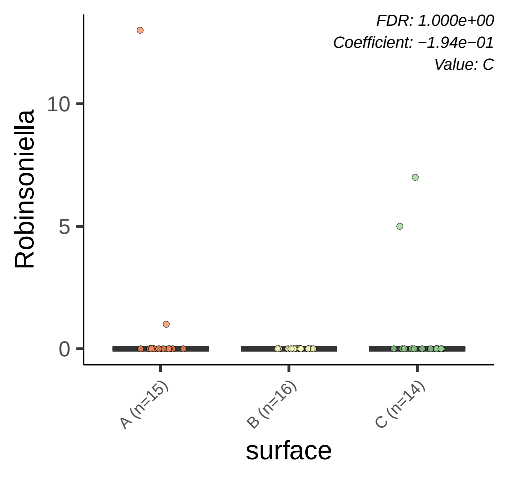
FDR: 1.000e+00
Coefficient: −1.94e−01
Value: C
Robinsoniella
10
5
0
A (n=15)
B (n=16)
C (n=14)
surface

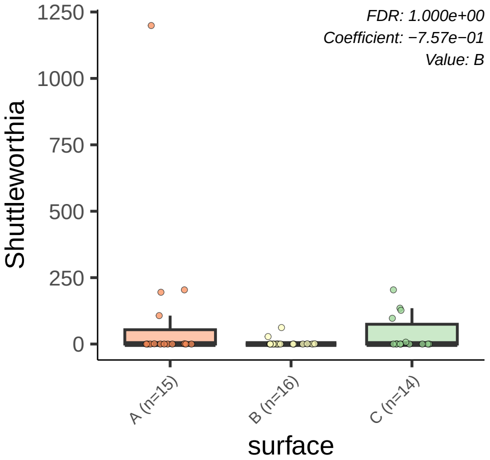

FDR: 1.000e+00
Coefficient: −7.57e−01
Value: B
Shuttleworthia
1250
1000
750
500
250
0
A (n=15)
B (n=16)
C (n=14)
surface

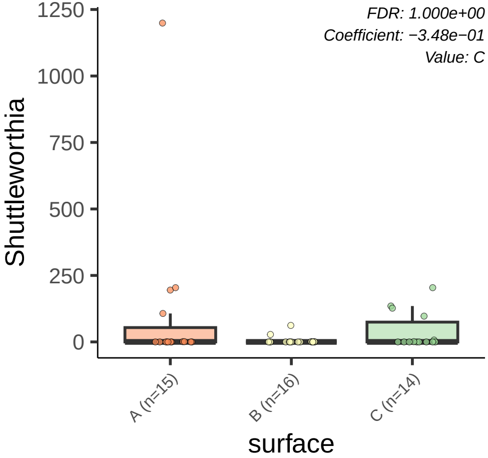

FDR: 1.000e+00
Coefficient: −3.48e−01
Value: C
Shuttleworthia
1250
1000
750
500
250
0
A (n=15)
B (n=16)
C (n=14)
surface

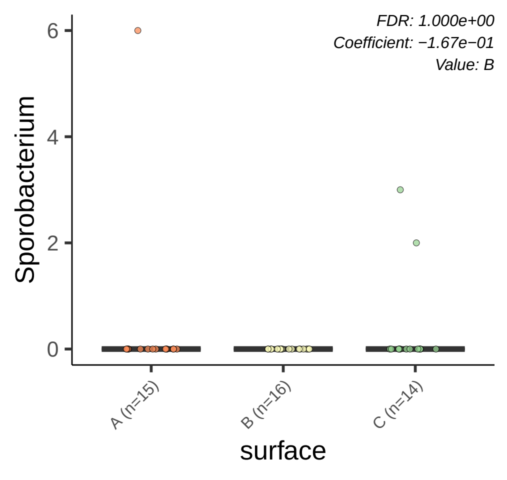

FDR: 1.000e+00
Coefficient: −1.67e−01
Value: B
Sporobacterium
0
2
4
6
A (n=15)
B (n=16)
C (n=14)
surface

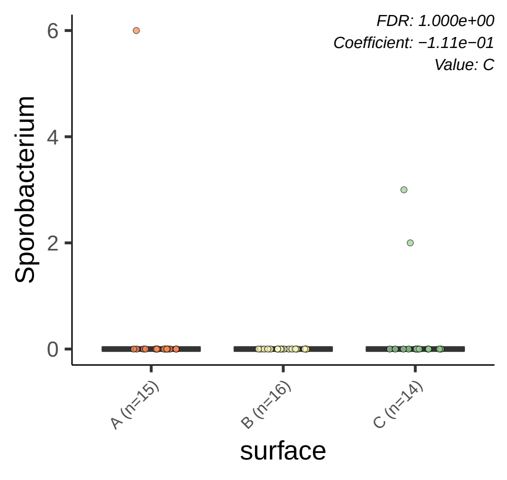

FDR: 1.000e+00
Coefficient: −1.11e−01
Value: C
Sporobacterium
6
4
2
0
A (n=15)
B (n=16)
C (n=14)
surface

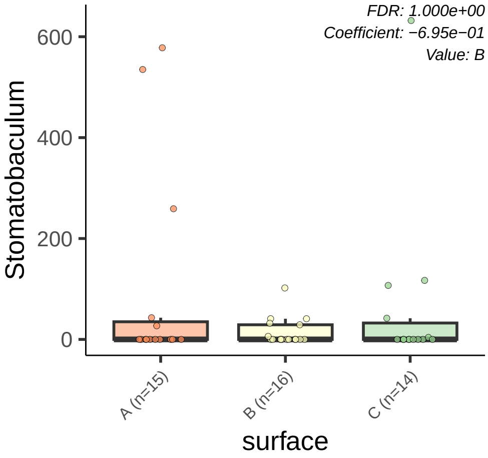
FDR: 1.000e+00
Coefficient: −6.95e−01
Value: B
600
400
200
0
Stomatobaculum
A (n=15)
B (n=16)
C (n=14)
surface

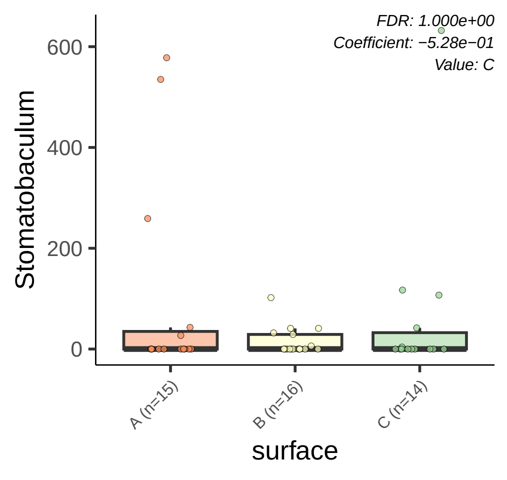

FDR: 1.000e+00
Coefficient: −5.28e−01
Value: C
Stomatobaculum
600
400
200
0
A (n=15)
B (n=16)
C (n=14)
surface

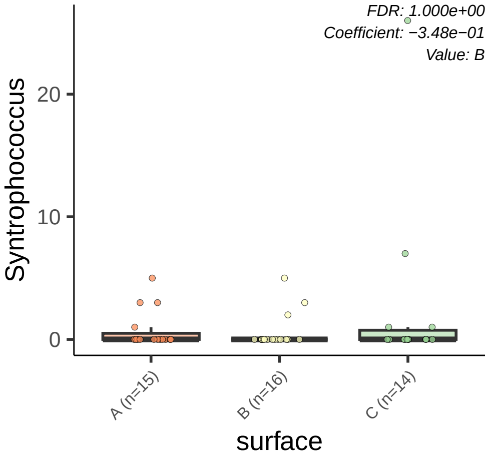
FDR: 1.000e+00
Coefficient: −3.48e−01
Value: B
Syntrophococcus
20
10
0
A (n=15)
B (n=16)
C (n=14)
surface

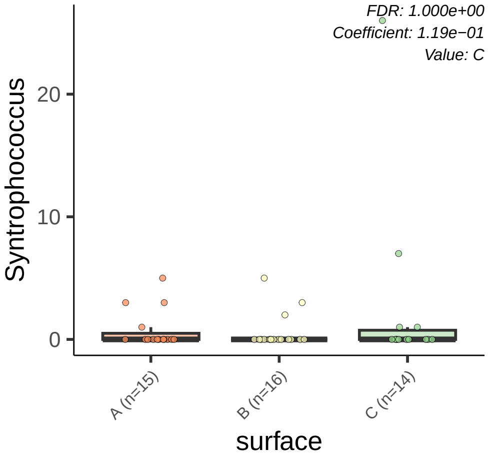

FDR: 1.000e+00
Coefficient: 1.19e−01
Value: C
Syntrophococcus
20
10
0
A (n=15)
B (n=16)
C (n=14)
surface

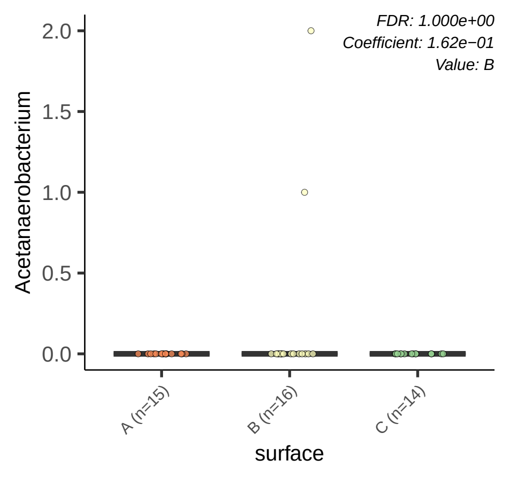

FDR: 1.000e+00
Coefficient: 1.62e−01
Value: B
Acetanaerobacterium
2.0
1.5
1.0
0.5
0.0
A (n=15)
B (n=16)
C (n=14)
surface

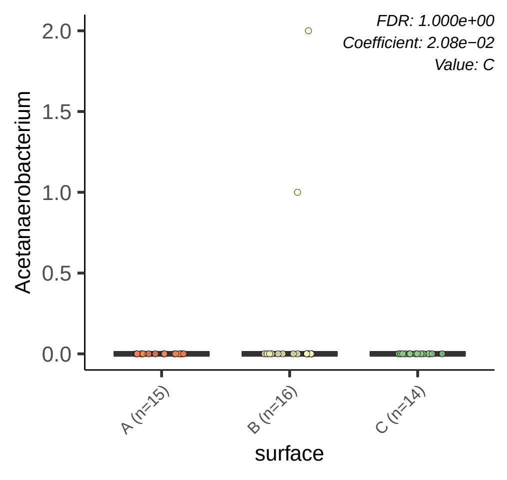

FDR: 1.000e+00
Coefficient: 2.08e−02
Value: C
Acetanaerobacterium
2.0
1.5
1.0
0.5
0.0
A (n=15)
B (n=16)
C (n=14)
surface

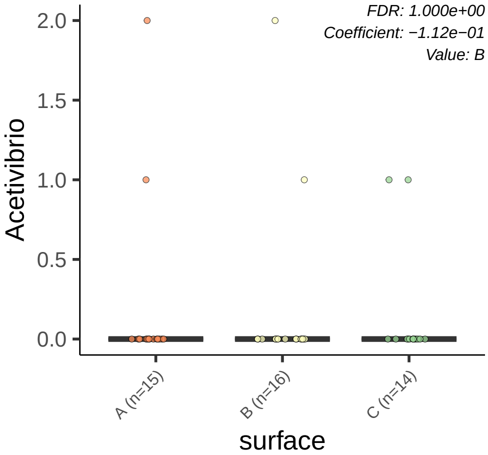

FDR: 1.000e+00
Coefficient: −1.12e−01
Value: B
Acetivibrio
2.0
1.5
1.0
0.5
0.0
A (n=15)
B (n=16)
C (n=14)
surface

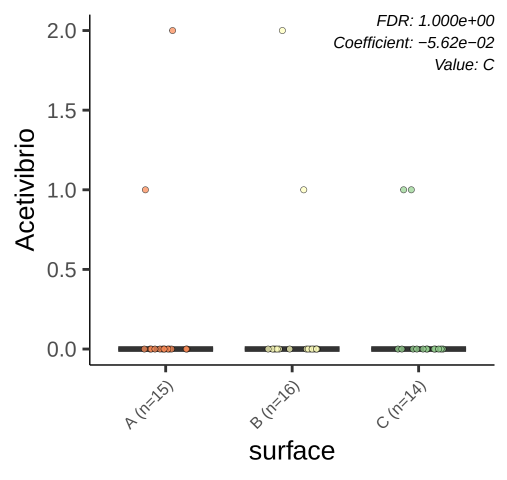
FDR: 1.000e+00
Coefficient: −5.62e−02
Value: C
Acetivibrio
2.0
1.5
1.0
0.5
0.0
A (n=15)
B (n=16)
C (n=14)
surface

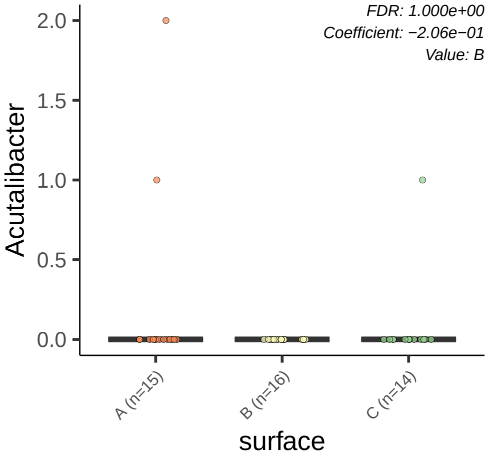
FDR: 1.000e+00
Coefficient: −2.06e−01
Value: B
Acutalibacter
2.0
1.5
1.0
0.5
0.0
A (n=15)
B (n=16)
C (n=14)
surface

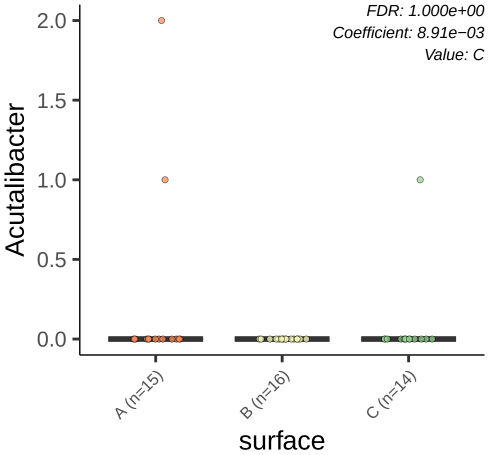
FDR: 1.000e+00
Coefficient: 8.91e−03
Value: C
Acutalibacter
2.0
1.5
1.0
0.5
0.0
A (n=15)
B (n=16)
C (n=14)
surface

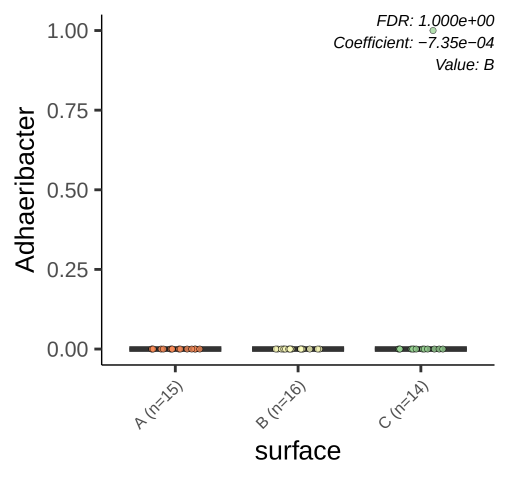
FDR: 1.000e+00
Coefficient: −7.35e−04
Value: B
1.00
0.75
0.50
0.25
0.00
Adhaeribacter
A (n=15)
B (n=16)
C (n=14)
surface

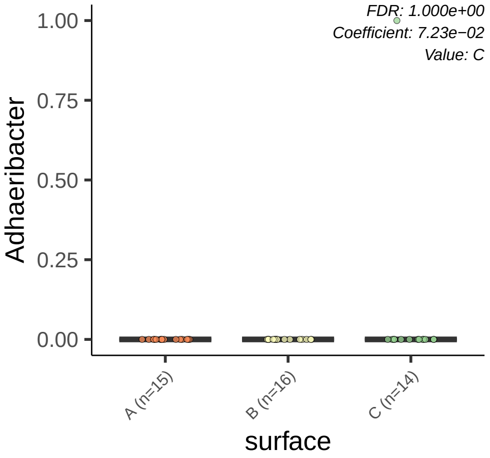

FDR: 1.000e+00
Coefficient: 7.23e−02
Value: C
Adhaeribacter
1.00
0.75
0.50
0.25
0.00
A (n=15)
B (n=16)
C (n=14)
surface

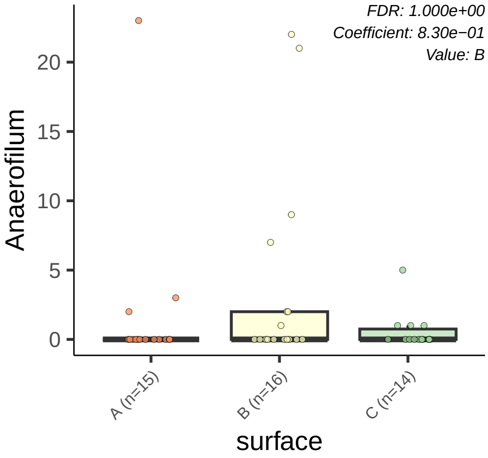

FDR: 1.000e+00
Coefficient: 8.30e−01
Value: B
Anaerofilum
0
5
10
15
20
A (n=15)
B (n=16)
C (n=14)
surface

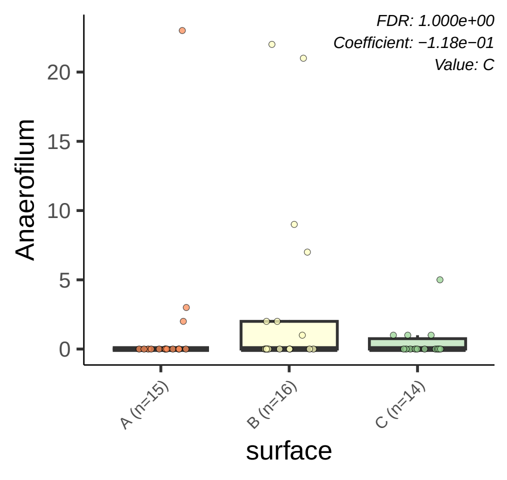
FDR: 1.000e+00
Coefficient: −1.18e−01
Value: C
Anaerofilum
0
5
10
15
20
A (n=15)
B (n=16)
C (n=14)
surface

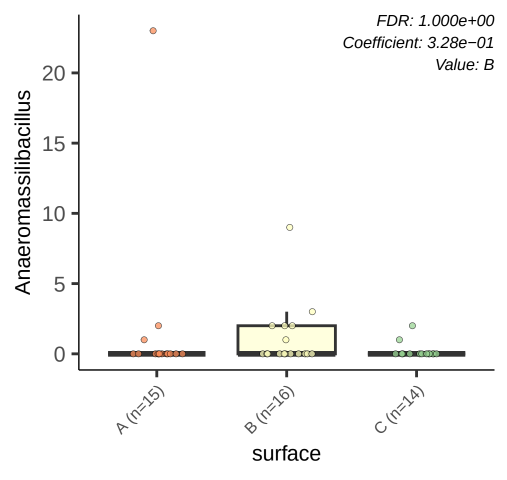
FDR: 1.000e+00
Coefficient: 3.28e−01
Value: B
Anaeromassilibacillus
0
5
10
15
20
A (n=15)
B (n=16)
C (n=14)
surface

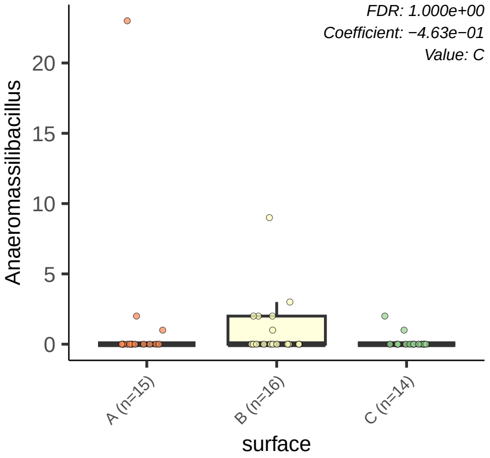
FDR: 1.000e+00
Coefficient: −4.63e−01
Value: C
Anaeromassilibacillus
0
5
10
15
20
A (n=15)
B (n=16)
C (n=14)
surface

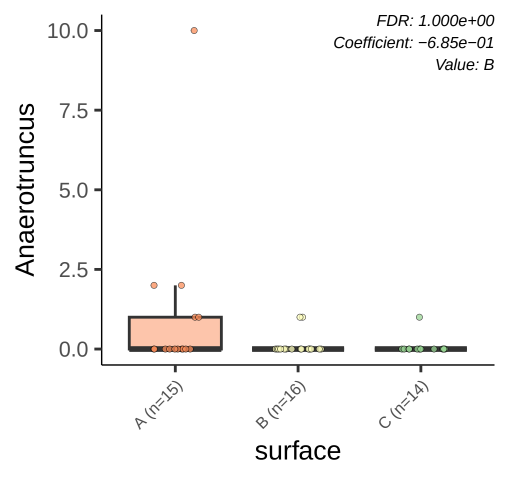

FDR: 1.000e+00
Coefficient: −6.85e−01
Value: B
10.0
7.5
5.0
2.5
0.0
Anaerotruncus
A (n=15)
B (n=16)
C (n=14)
surface

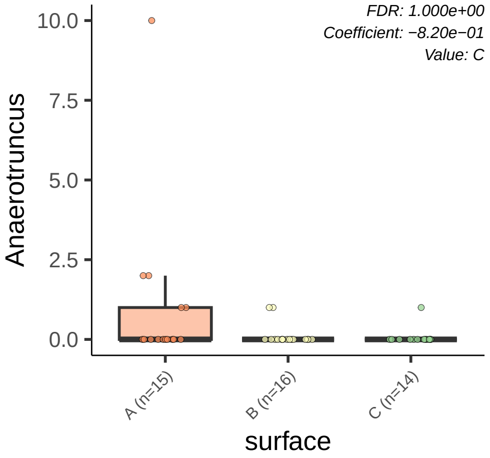
FDR: 1.000e+00
Coefficient: −8.20e−01
Value: C
Anaerotruncus
10.0
7.5
5.0
2.5
0.0
A (n=15)
B (n=16)
C (n=14)
surface

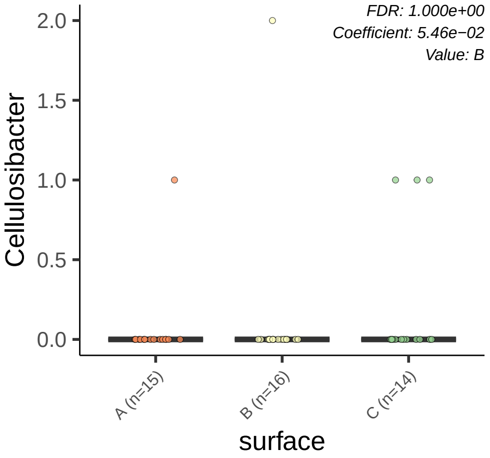

FDR: 1.000e+00
Coefficient: 5.46e−02
Value: B
Cellulosibacter
2.0
1.5
1.0
0.5
0.0
A (n=15)
B (n=16)
C (n=14)
surface

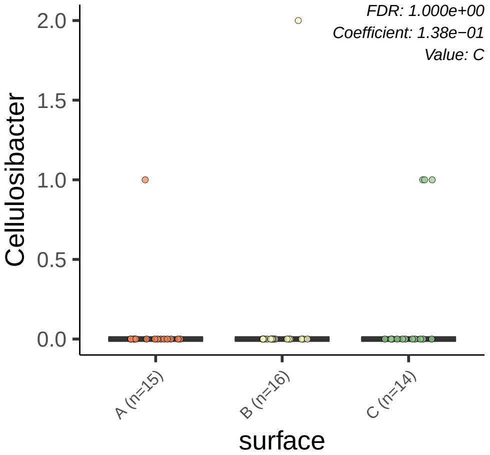
FDR: 1.000e+00
Coefficient: 1.38e−01
Value: C
Cellulosibacter
2.0
1.5
1.0
0.5
0.0
A (n=15)
B (n=16)
C (n=14)
surface

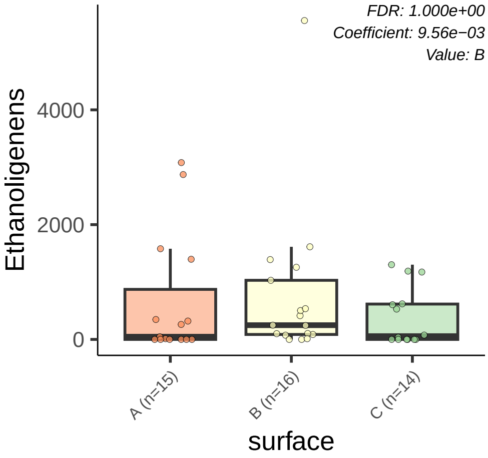
FDR: 1.000e+00
Coefficient: 9.56e−03
Value: B
Ethanoligenens
0
2000
4000
A (n=15)
B (n=16)
C (n=14)
surface

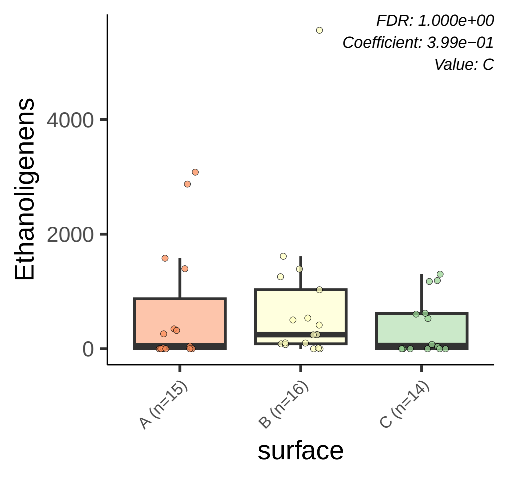

FDR: 1.000e+00
Coefficient: 3.99e−01
Value: C
Ethanoligenens
4000
2000
0
A (n=15)
B (n=16)
C (n=14)
surface

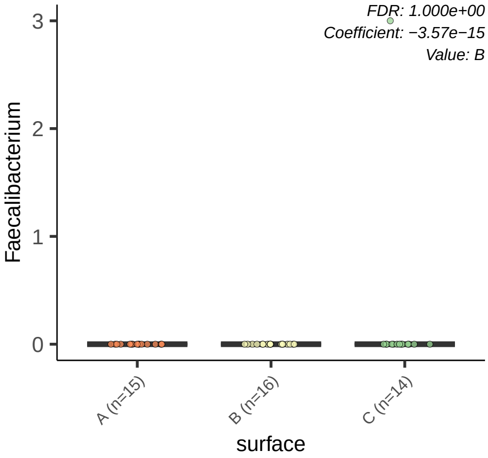
FDR: 1.000e+00
Coefficient: −3.57e−15
Value: B
Faecalibacterium
3
2
1
0
A (n=15)
B (n=16)
C (n=14)
surface

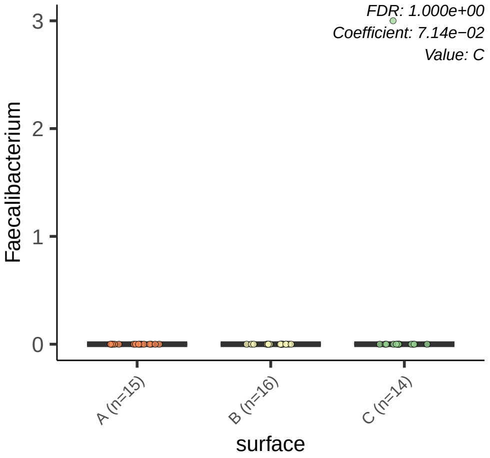
FDR: 1.000e+00
Coefficient: 7.14e−02
Value: C
Faecalibacterium
0
1
2
3
A (n=15)
B (n=16)
C (n=14)
surface

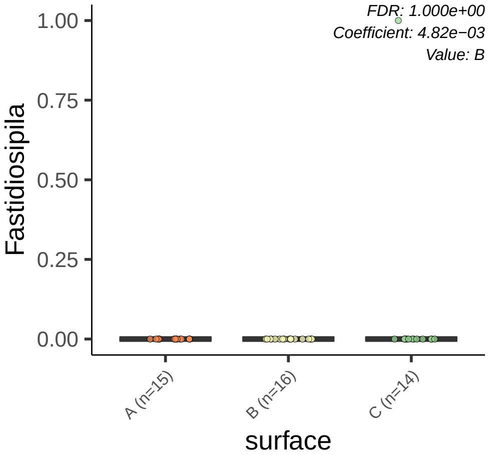

FDR: 1.000e+00
Coefficient: 4.82e−03
Value: B
Fastidiosipila
1.00
0.75
0.50
0.25
0.00
A (n=15)
B (n=16)
C (n=14)
surface

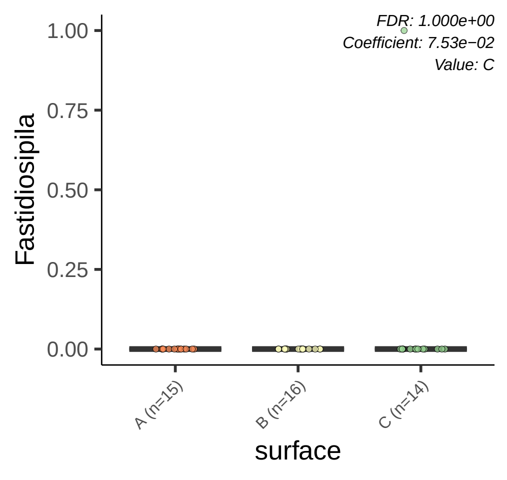
FDR: 1.000e+00
Coefficient: 7.53e−02
Value: C
1.00
0.75
0.50
0.25
0.00
Fastidiosipila
A (n=15)
B (n=16)
C (n=14)
surface

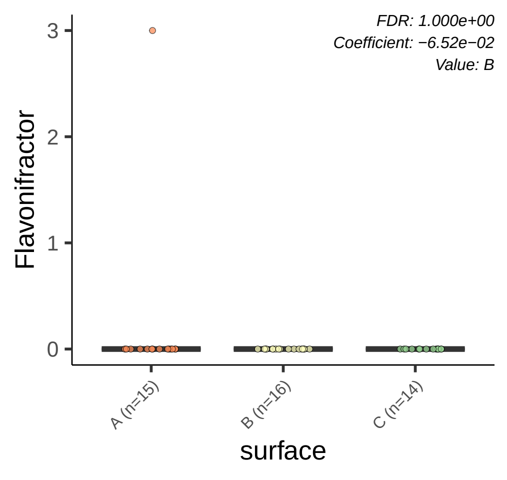
FDR: 1.000e+00
Coefficient: −6.52e−02
Value: B
Flavonifractor
3
2
1
0
A (n=15)
B (n=16)
C (n=14)
surface

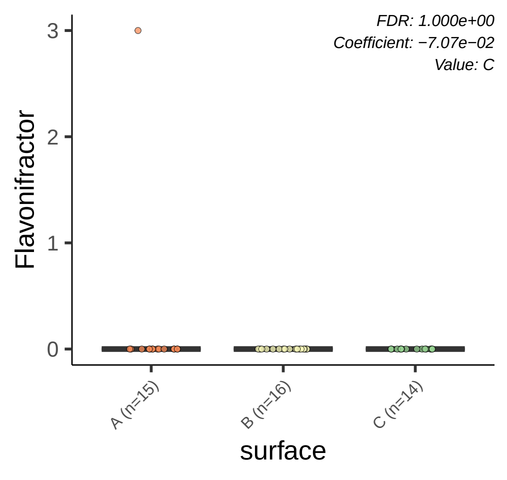
FDR: 1.000e+00
Coefficient: −7.07e−02
Value: C
Flavonifractor
3
2
1
0
A (n=15)
B (n=16)
C (n=14)
surface

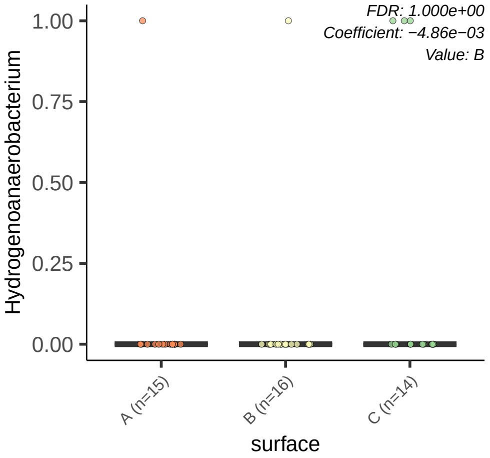

FDR: 1.000e+00
Coefficient: −4.86e−03
Value: B
Hydrogenoanaerobacterium
1.00
0.75
0.50
0.25
0.00
A (n=15)
B (n=16)
C (n=14)
surface

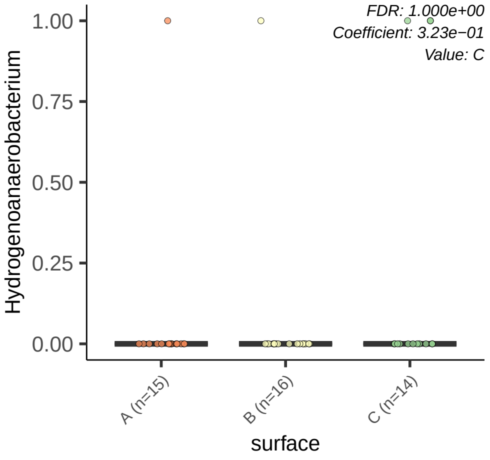

FDR: 1.000e+00
Coefficient: 3.23e−01
Value: C
Hydrogenoanaerobacterium
1.00
0.75
0.50
0.25
0.00
A (n=15)
B (n=16)
C (n=14)
surface

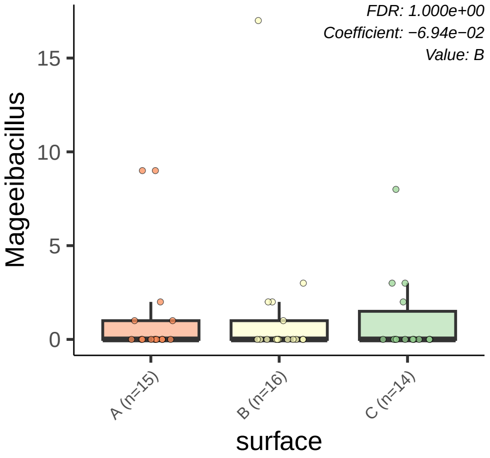
FDR: 1.000e+00
Coefficient: −6.94e−02
Value: B
Mageeibacillus
15
10
5
0
A (n=15)
B (n=16)
C (n=14)
surface

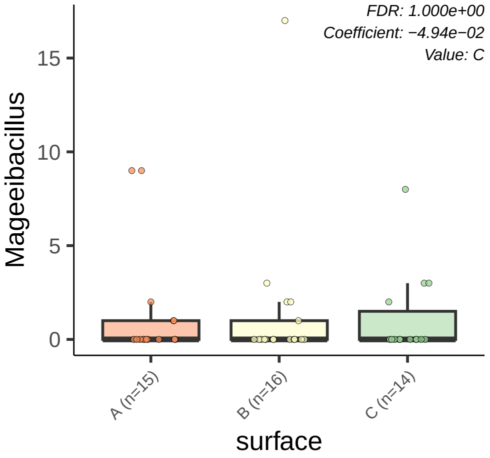

FDR: 1.000e+00
Coefficient: −4.94e−02
Value: C
Mageeibacillus
15
10
5
0
A (n=15)
B (n=16)
C (n=14)
surface

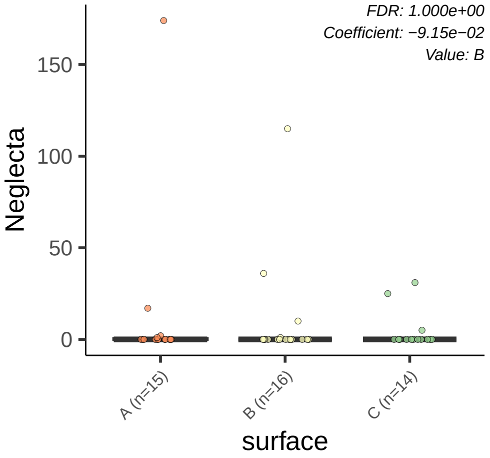
FDR: 1.000e+00
Coefficient: −9.15e−02
Value: B
Neglecta
150
100
50
0
A (n=15)
B (n=16)
C (n=14)
surface

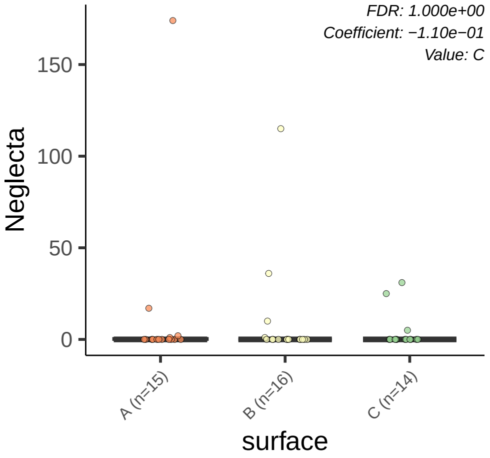
FDR: 1.000e+00
Coefficient: −1.10e−01
Value: C
Neglecta
150
100
50
0
A (n=15)
B (n=16)
C (n=14)
surface

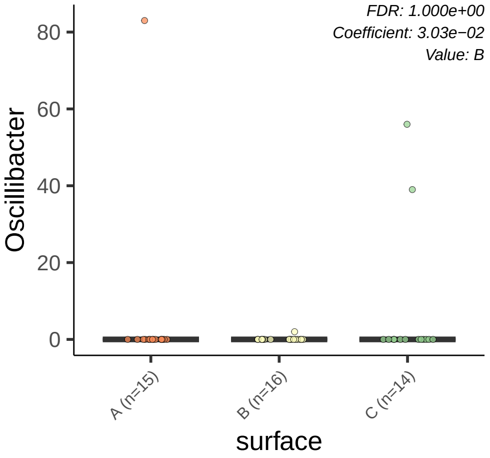

FDR: 1.000e+00
Coefficient: 3.03e−02
Value: B
Oscillibacter
80
60
40
20
0
A (n=15)
B (n=16)
C (n=14)
surface

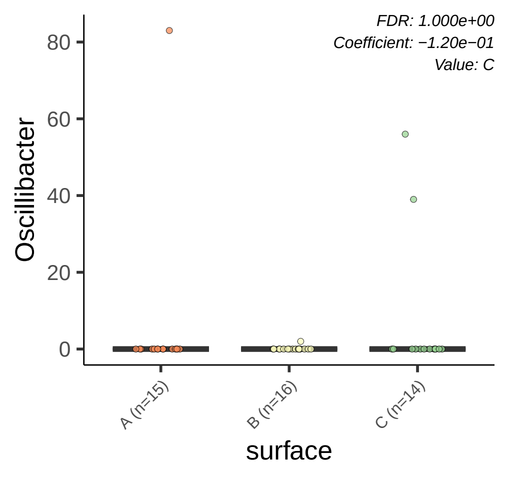

FDR: 1.000e+00
Coefficient: −1.20e−01
Value: C
Oscillibacter
80
60
40
20
0
A (n=15)
B (n=16)
C (n=14)
surface

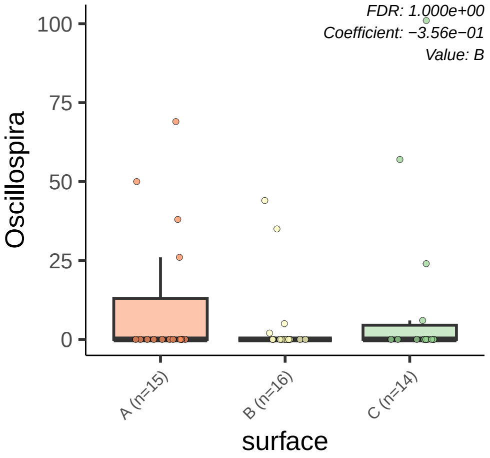

FDR: 1.000e+00
Coefficient: −3.56e−01
Value: B
Oscillospira
100
75
50
25
0
A (n=15)
B (n=16)
C (n=14)
surface

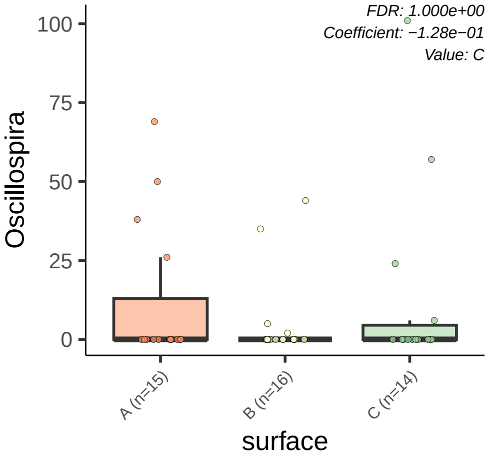

FDR: 1.000e+00
Coefficient: −1.28e−01
Value: C
Oscillospira
100
75
50
25
0
A (n=15)
B (n=16)
C (n=14)
surface

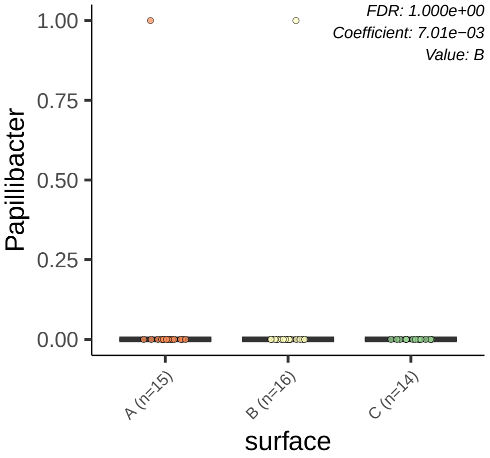

FDR: 1.000e+00
Coefficient: 7.01e−03
Value: B
Papillibacter
1.00
0.75
0.50
0.25
0.00
A (n=15)
B (n=16)
C (n=14)
surface

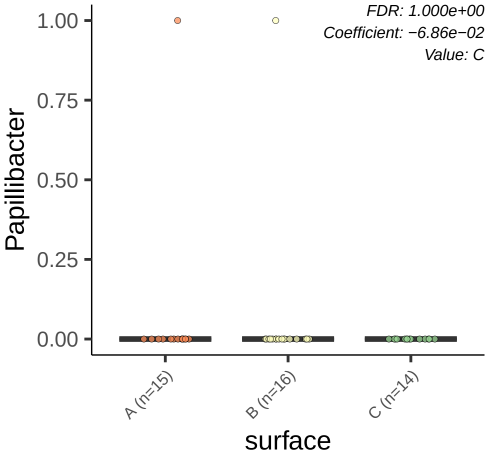

FDR: 1.000e+00
Coefficient: −6.86e−02
Value: C
Papillibacter
1.00
0.75
0.50
0.25
0.00
A (n=15)
B (n=16)
C (n=14)
surface

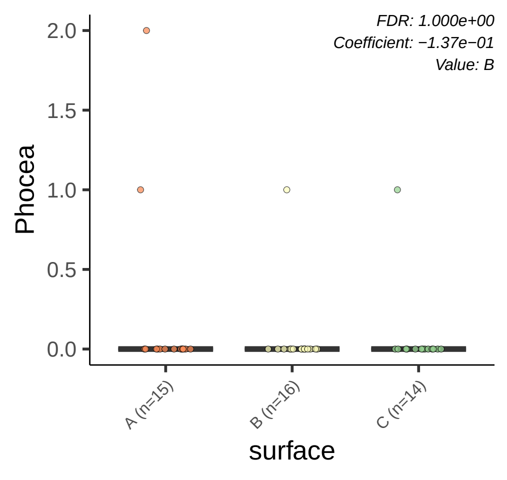

FDR: 1.000e+00
Coefficient: −1.37e−01
Value: B
Phocea
2.0
1.5
1.0
0.5
0.0
A (n=15)
B (n=16)
C (n=14)
surface

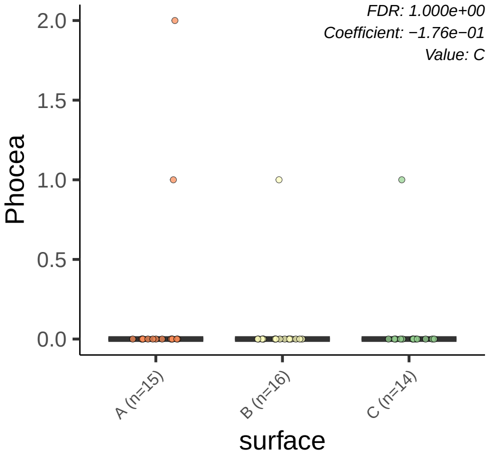
FDR: 1.000e+00
Coefficient: −1.76e−01
Value: C
Phocea
2.0
1.5
1.0
0.5
0.0
A (n=15)
B (n=16)
C (n=14)
surface

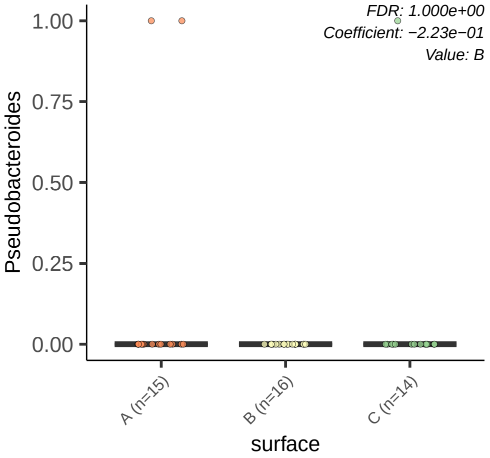
FDR: 1.000e+00
Coefficient: −2.23e−01
Value: B
Pseudobacteroides
1.00
0.75
0.50
0.25
0.00
A (n=15)
B (n=16)
C (n=14)
surface

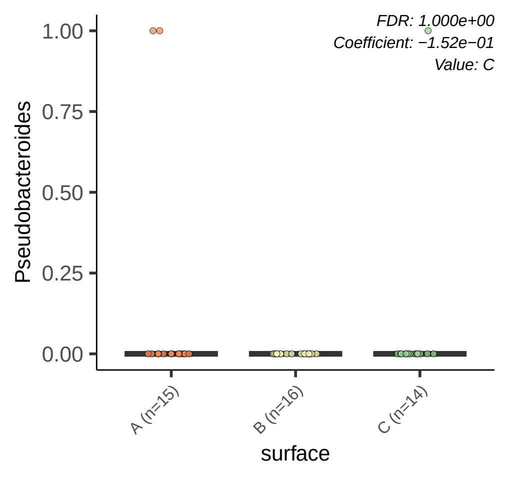

FDR: 1.000e+00
Coefficient: −1.52e−01
Value: C
Pseudobacteroides
1.00
0.75
0.50
0.25
0.00
A (n=15)
B (n=16)
C (n=14)
surface

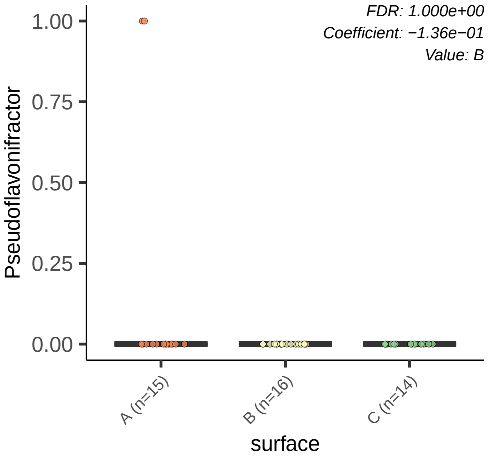
FDR: 1.000e+00
Coefficient: −1.36e−01
Value: B
Pseudoflavonifractor
1.00
0.75
0.50
0.25
0.00
A (n=15)
B (n=16)
C (n=14)
surface

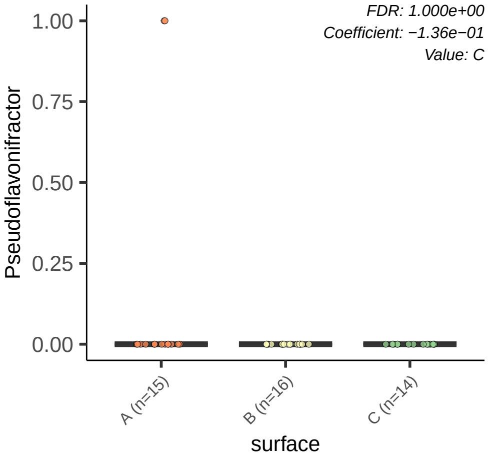

FDR: 1.000e+00
Coefficient: −1.36e−01
Value: C
Pseudoflavonifractor
1.00
0.75
0.50
0.25
0.00
A (n=15)
B (n=16)
C (n=14)
surface

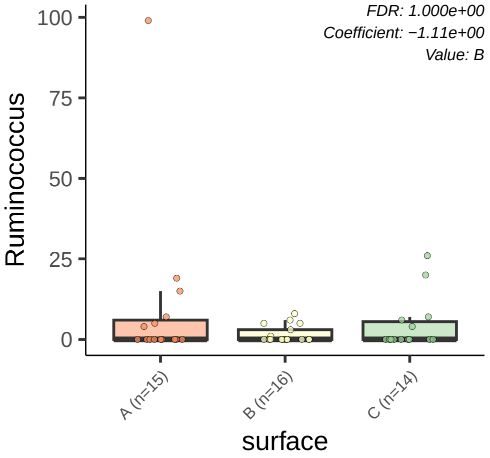

FDR: 1.000e+00
Coefficient: −1.11e+00
Value: B
Ruminococcus
100
75
50
25
0
A (n=15)
B (n=16)
C (n=14)
surface

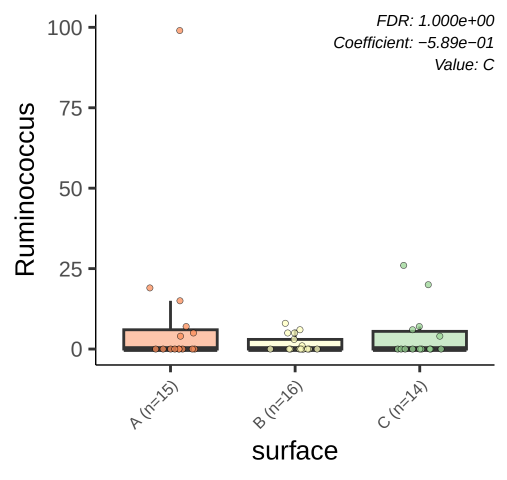
FDR: 1.000e+00
Coefficient: −5.89e−01
Value: C
Ruminococcus
100
75
50
25
0
A (n=15)
B (n=16)
C (n=14)
surface

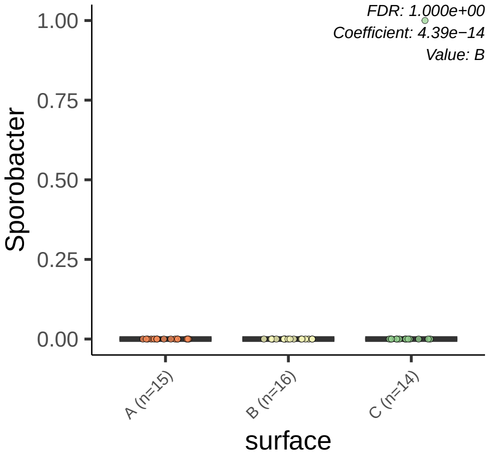

FDR: 1.000e+00
Coefficient: 4.39e−14
Value: B
Sporobacter
1.00
0.75
0.50
0.25
0.00
A (n=15)
B (n=16)
C (n=14)
surface

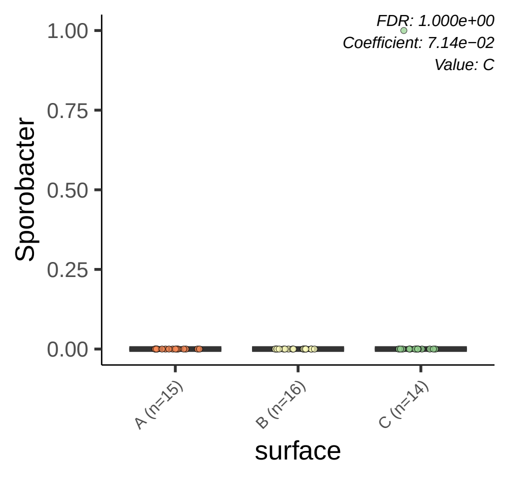
FDR: 1.000e+00
Coefficient: 7.14e−02
Value: C
Sporobacter
1.00
0.75
0.50
0.25
0.00
A (n=15)
B (n=16)
C (n=14)
surface

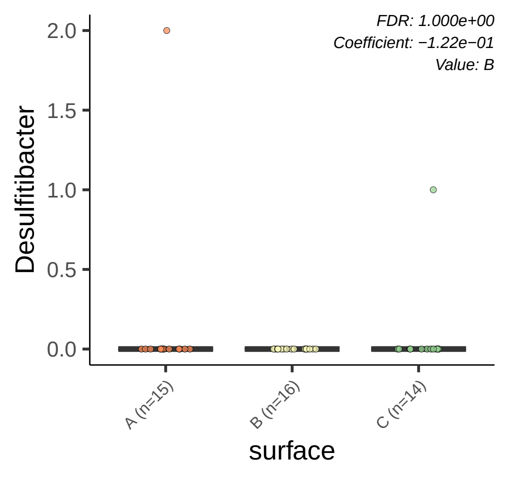

FDR: 1.000e+00
Coefficient: −1.22e−01
Value: B
Desulfitibacter
2.0
1.5
1.0
0.5
0.0
A (n=15)
B (n=16)
C (n=14)
surface

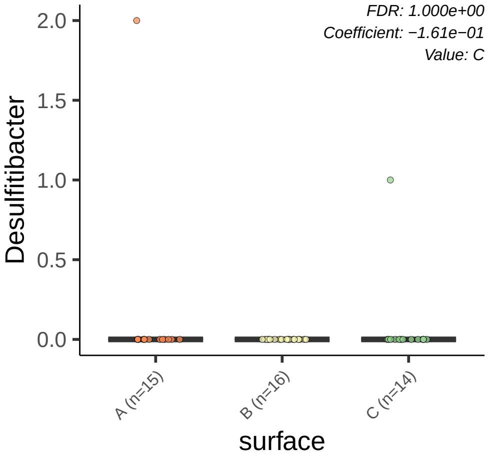

FDR: 1.000e+00
Coefficient: −1.61e−01
Value: C
Desulfitibacter
2.0
1.5
1.0
0.5
0.0
A (n=15)
B (n=16)
C (n=14)
surface

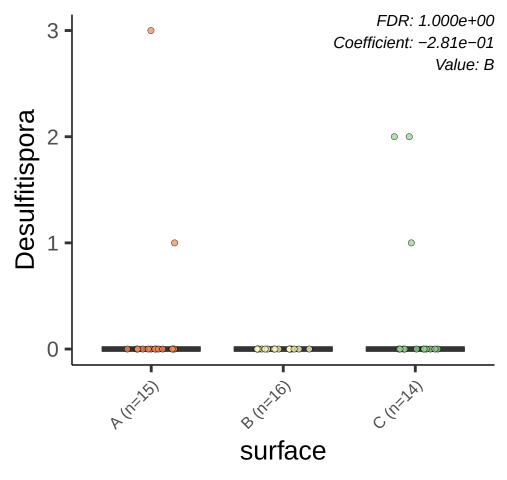

FDR: 1.000e+00
Coefficient: −2.81e−01
Value: B
Desulfitispora
3
2
1
0
A (n=15)
B (n=16)
C (n=14)
surface

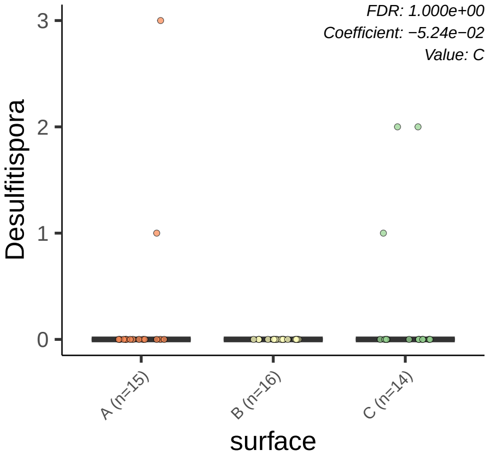
FDR: 1.000e+00
Coefficient: −5.24e−02
Value: C
Desulfitispora
3
2
1
0
A (n=15)
B (n=16)
C (n=14)
surface

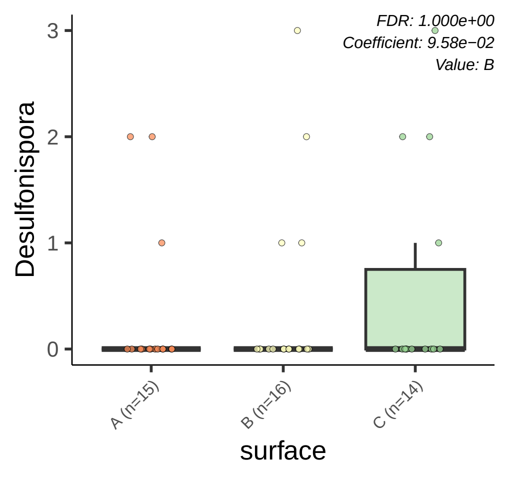

FDR: 1.000e+00
Coefficient: 9.58e−02
Value: B
Desulfonispora
0
1
2
3
A (n=15)
B (n=16)
C (n=14)
surface

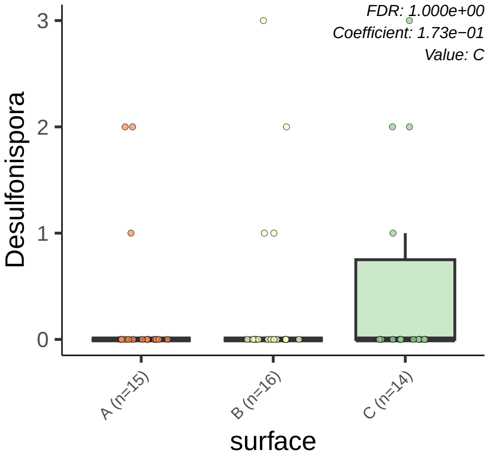
FDR: 1.000e+00
Coefficient: 1.73e−01
Value: C
Desulfonispora
3
2
1
0
A (n=15)
B (n=16)
C (n=14)
surface

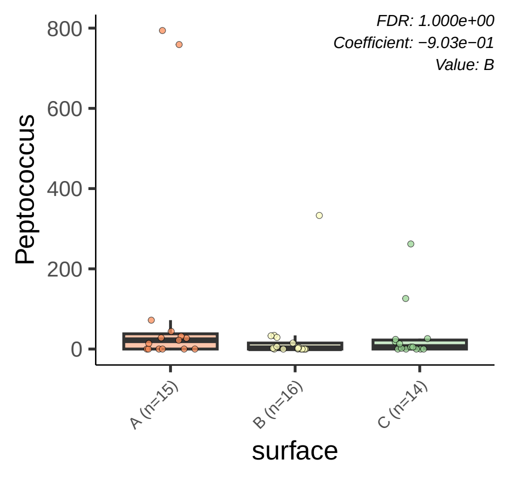

FDR: 1.000e+00
Coefficient: −9.03e−01
Value: B
Peptococcus
800
600
400
200
0
A (n=15)
B (n=16)
C (n=14)
surface

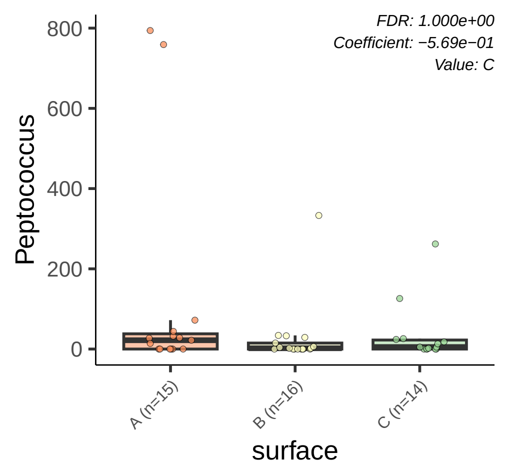

FDR: 1.000e+00
Coefficient: −5.69e−01
Value: C
Peptococcus
800
600
400
200
0
A (n=15)
B (n=16)
C (n=14)
surface

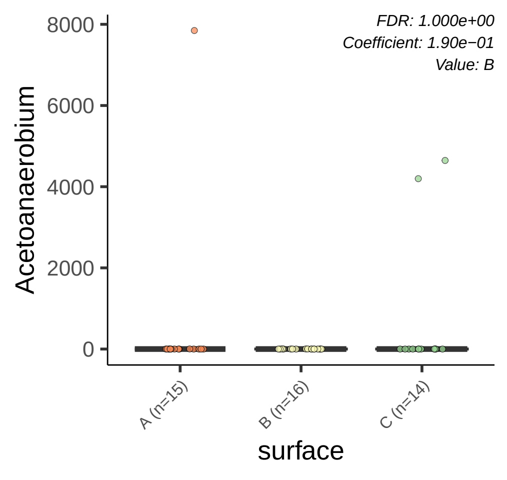

FDR: 1.000e+00
Coefficient: 1.90e−01
Value: B
8000
6000
4000
2000
0
Acetoanaerobium
A (n=15)
B (n=16)
C (n=14)
surface

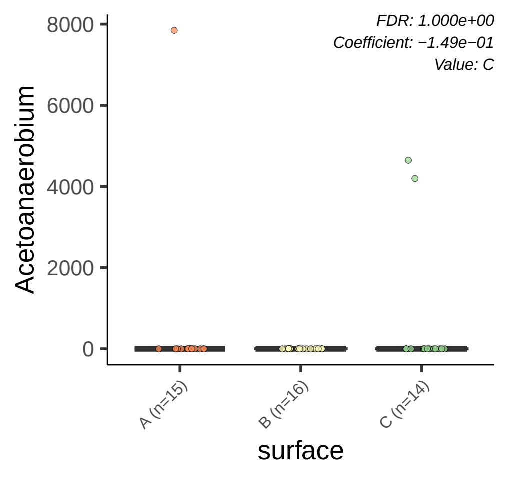
FDR: 1.000e+00
Coefficient: −1.49e−01
Value: C
Acetoanaerobium
8000
6000
4000
2000
0
A (n=15)
B (n=16)
C (n=14)
surface

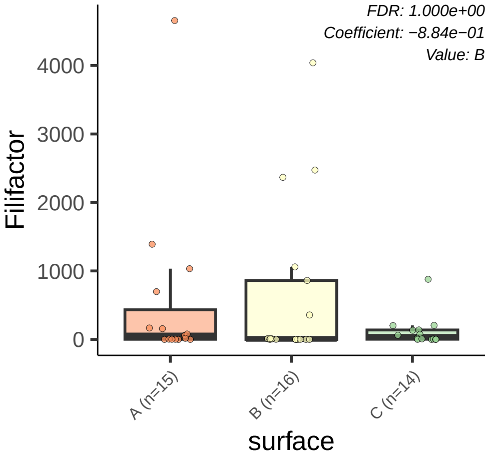
FDR: 1.000e+00
Coefficient: −8.84e−01
Value: B
Filifactor
4000
3000
2000
1000
0
A (n=15)
B (n=16)
C (n=14)
surface

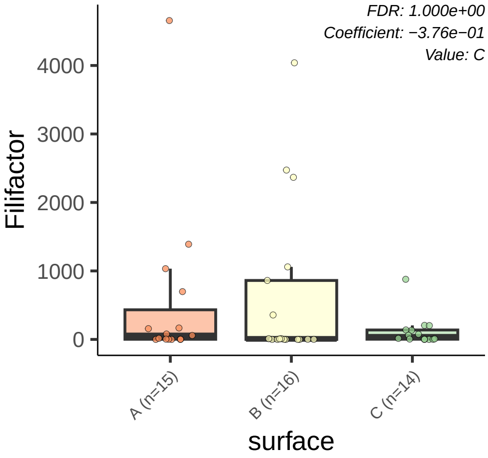
FDR: 1.000e+00
Coefficient: −3.76e−01
Value: C
Filifactor
4000
3000
2000
1000
0
A (n=15)
B (n=16)
C (n=14)
surface

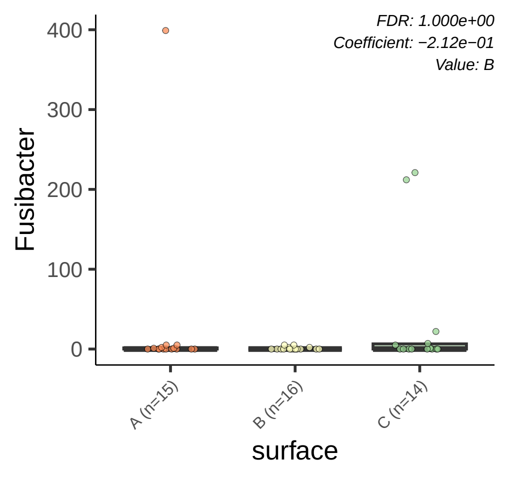
FDR: 1.000e+00
Coefficient: −2.12e−01
Value: B
Fusibacter
400
300
200
100
0
A (n=15)
B (n=16)
C (n=14)
surface

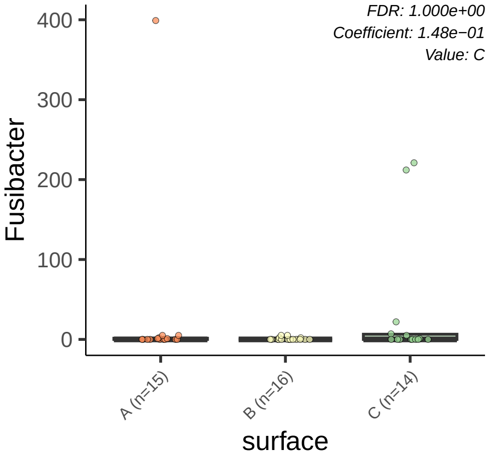
FDR: 1.000e+00
Coefficient: 1.48e−01
Value: C
Fusibacter
400
300
200
100
0
A (n=15)
B (n=16)
C (n=14)
surface

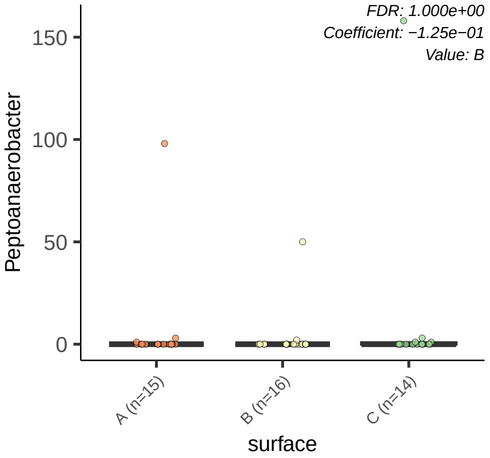

FDR: 1.000e+00
Coefficient: −1.25e−01
Value: B
Peptoanaerobacter
150
100
50
0
A (n=15)
B (n=16)
C (n=14)
surface

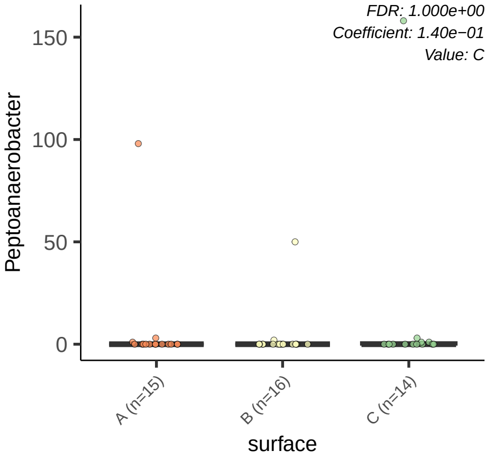

FDR: 1.000e+00
Coefficient: 1.40e−01
Value: C
Peptoanaerobacter
150
100
50
0
A (n=15)
B (n=16)
C (n=14)
surface

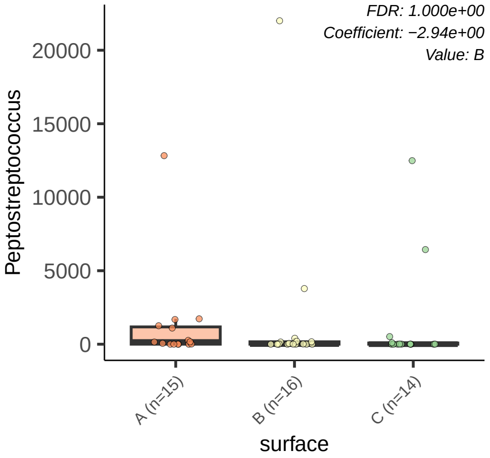

FDR: 1.000e+00
Coefficient: −2.94e+00
Value: B
Peptostreptococcus
20000
15000
10000
5000
0
A (n=15)
B (n=16)
C (n=14)
surface

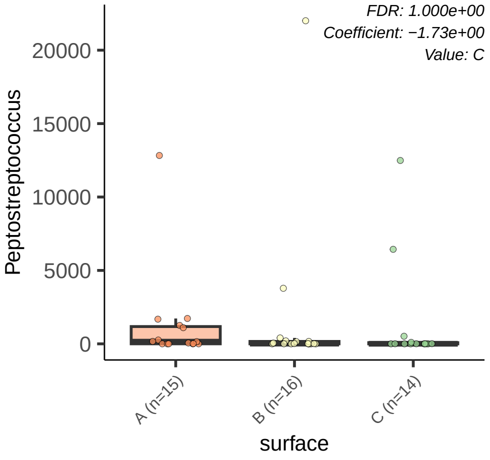

FDR: 1.000e+00
Coefficient: −1.73e+00
Value: C
Peptostreptococcus
20000
15000
10000
5000
0
A (n=15)
B (n=16)
C (n=14)
surface

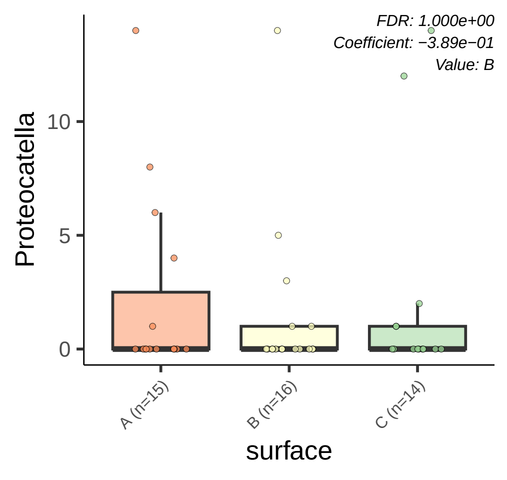

FDR: 1.000e+00
Coefficient: −3.89e−01
Value: B
Proteocatella
0
5
10
A (n=15)
B (n=16)
C (n=14)
surface

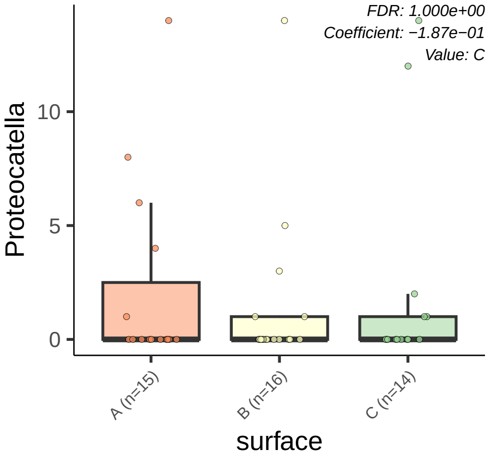

FDR: 1.000e+00
Coefficient: −1.87e−01
Value: C
Proteocatella
0
5
10
A (n=15)
B (n=16)
C (n=14)
surface

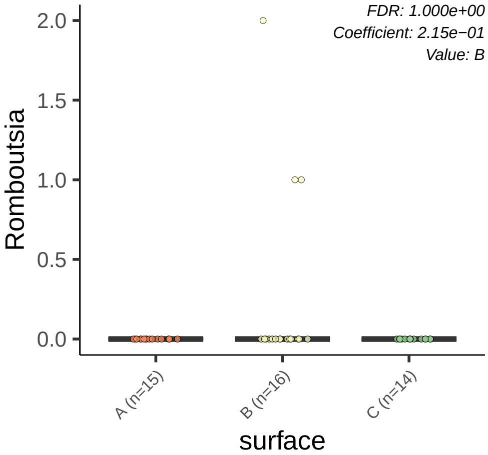
FDR: 1.000e+00
Coefficient: 2.15e−01
Value: B
Romboutsia
2.0
1.5
1.0
0.5
0.0
A (n=15)
B (n=16)
C (n=14)
surface

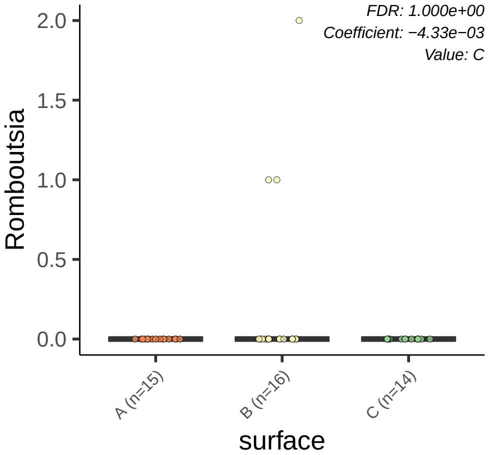

FDR: 1.000e+00
Coefficient: −4.33e−03
Value: C
Rombousia
2.0
1.5
1.0
0.5
0.0
A (n=15)
B (n=16)
C (n=14)
surface

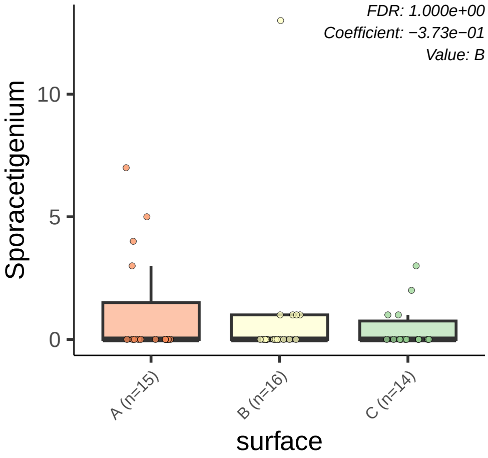
FDR: 1.000e+00
Coefficient: −3.73e−01
Value: B
Sporacetigenium
10
5
0
A (n=15)
B (n=16)
C (n=14)
surface

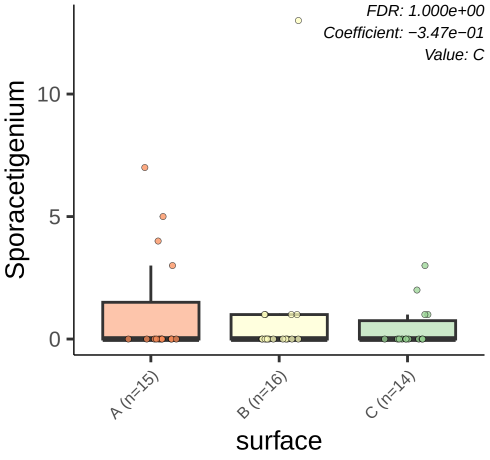
FDR: 1.000e+00
Coefficient: −3.47e−01
Value: C
Sporacetigenium
10
5
0
A (n=15)
B (n=16)
C (n=14)
surface

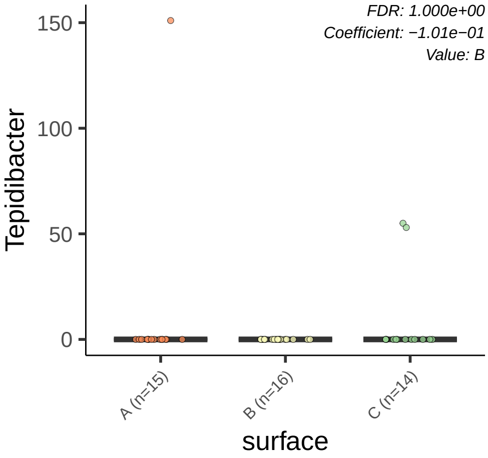
FDR: 1.000e+00
Coefficient: −1.01e−01
Value: B
Tepidibacter
150
100
50
0
A (n=15)
B (n=16)
C (n=14)
surface

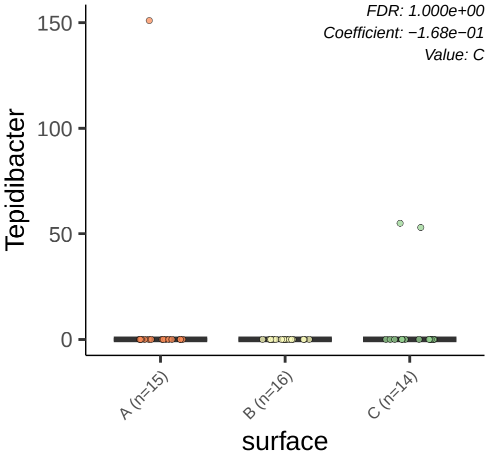
FDR: 1.000e+00
Coefficient: −1.68e−01
Value: C
Tepidibacter
150
100
50
0
A (n=15)
B (n=16)
C (n=14)
surface

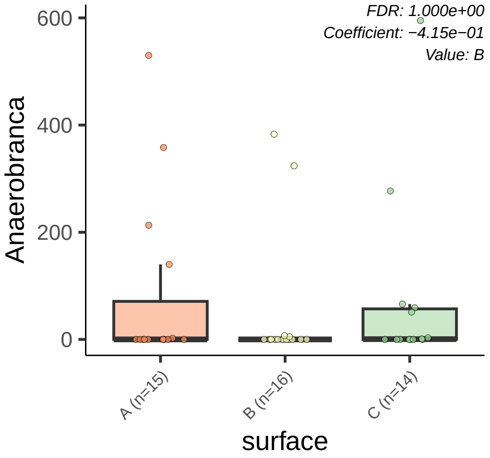

FDR: 1.000e+00
Coefficient: −4.15e−01
Value: B
Anaerobranca
600
400
200
0
A (n=15)
B (n=16)
C (n=14)
surface

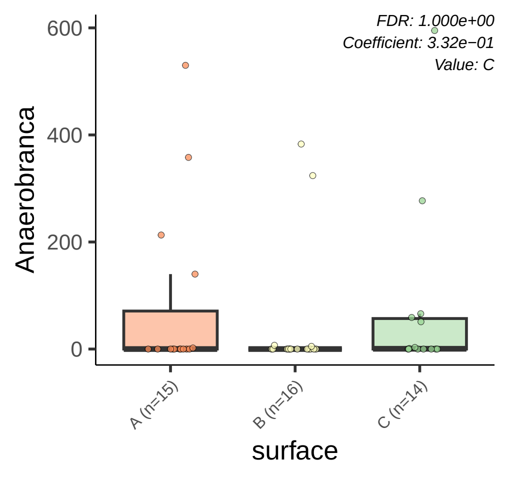
FDR: 1.000e+00
Coefficient: 3.32e−01
Value: C
Anaerobranca
600
400
200
0
A (n=15)
B (n=16)
C (n=14)
surface

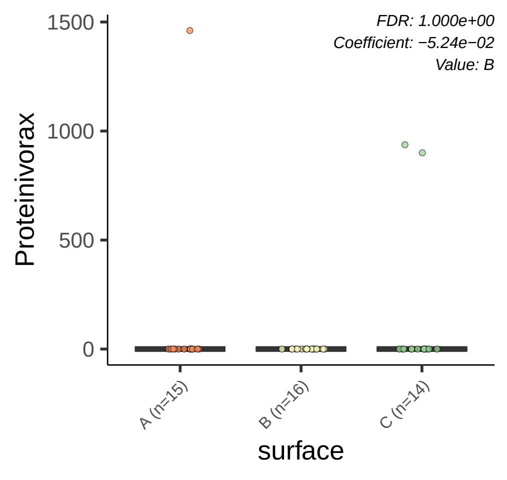
FDR: 1.000e+00
Coefficient: −5.24e−02
Value: B
Proteinivorax
1500
1000
500
0
A (n=15)
B (n=16)
C (n=14)
surface

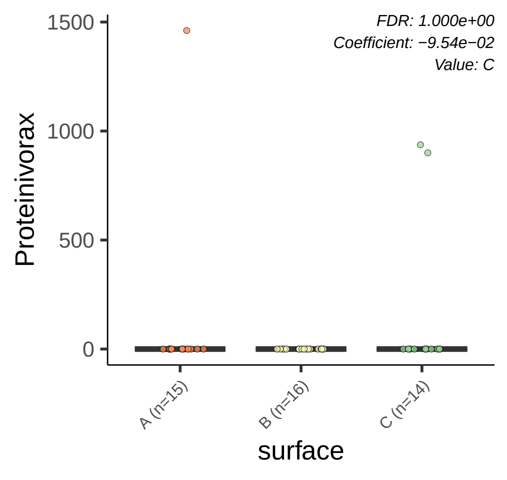

FDR: 1.000e+00
Coefficient: −9.54e−02
Value: C
Proteinivorax
1500
1000
500
0
A (n=15)
B (n=16)
C (n=14)
surface

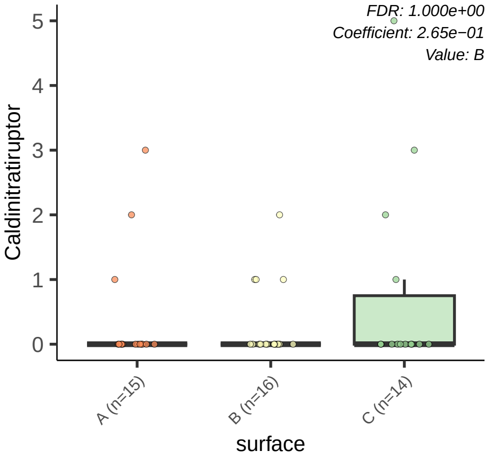

FDR: 1.000e+00
Coefficient: 2.65e−01
Value: B
Caldinitratiruptor
0
1
2
3
4
5
A (n=15)
B (n=16)
C (n=14)
surface

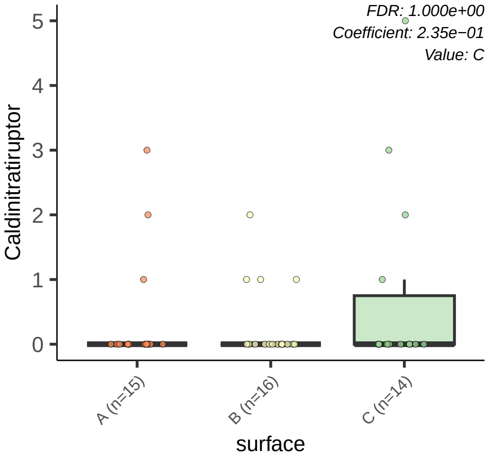

FDR: 1.000e+00
Coefficient: 2.35e−01
Value: C
Caldinitratiruptor
0
1
2
3
4
5
A (n=15)
B (n=16)
C (n=14)
surface

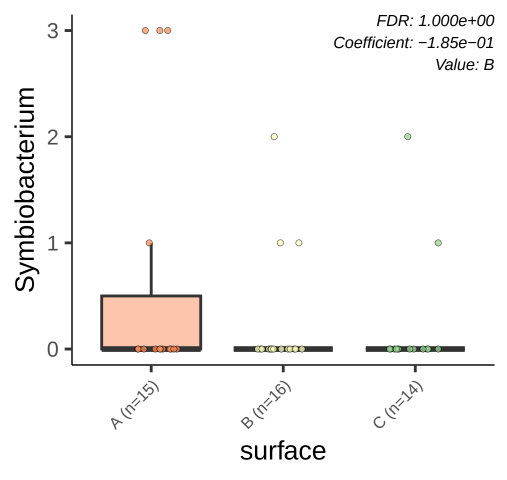
FDR: 1.000e+00
Coefficient: −1.85e−01
Value: B
Symbiobacterium
3
2
1
0
A (n=15)
B (n=16)
C (n=14)
surface

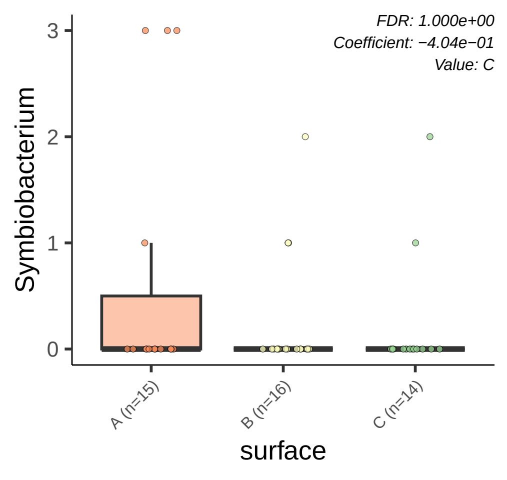
FDR: 1.000e+00
Coefficient: −4.04e−01
Value: C
Symbiobacterium
0
1
2
3
A (n=15)
B (n=16)
C (n=14)
surface

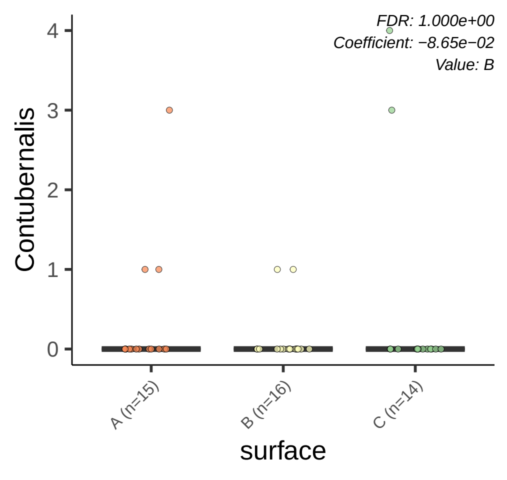

FDR: 1.000e+00
Coefficient: −8.65e−02
Value: B
Contubernalis
0
1
2
3
4
A (n=15)
B (n=16)
C (n=14)
surface

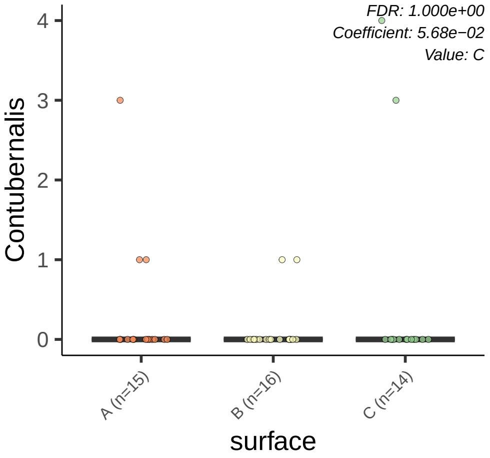
FDR: 1.000e+00
Coefficient: 5.68e−02
Value: C
Contubernalis
0
1
2
3
4
A (n=15)
B (n=16)
C (n=14)
surface

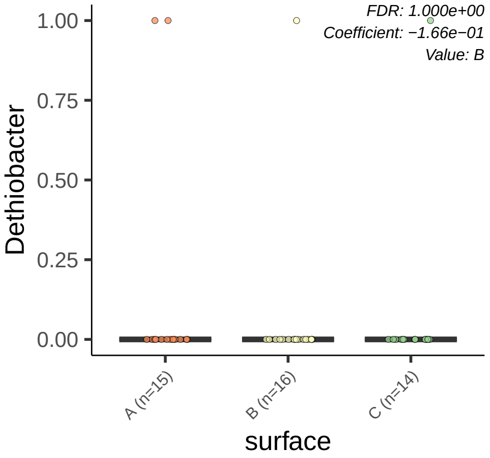
FDR: 1.000e+00
Coefficient: −1.66e−01
Value: B
Dethiobacter
1.00
0.75
0.50
0.25
0.00
A (n=15)
B (n=16)
C (n=14)
surface

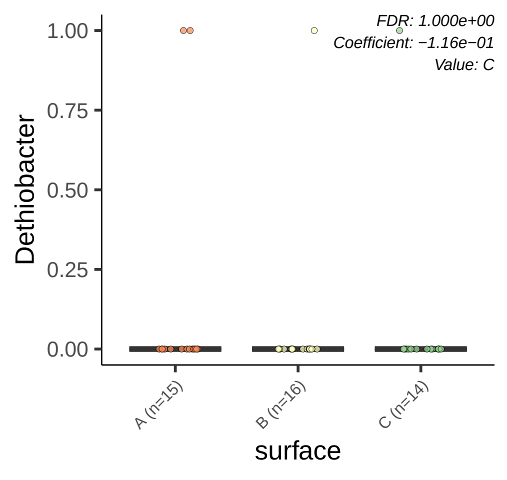
FDR: 1.000e+00
Coefficient: −1.16e−01
Value: C
Dethiobacter
1.00
0.75
0.50
0.25
0.00
A (n=15)
B (n=16)
C (n=14)
surface

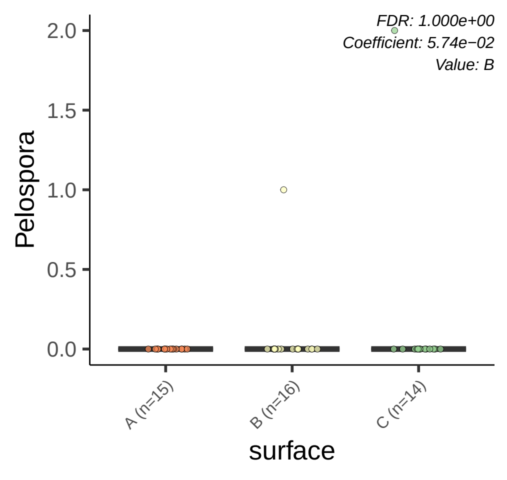
FDR: 1.000e+00
Coefficient: 5.74e−02
Value: B
Pelospora
2.0
1.5
1.0
0.5
0.0
A (n=15)
B (n=16)
C (n=14)
surface

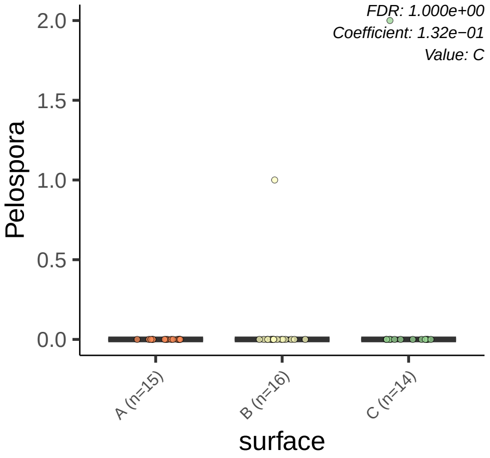

FDR: 1.000e+00
Coefficient: 1.32e−01
Value: C
Pelospora
2.0
1.5
1.0
0.5
0.0
A (n=15)
B (n=16)
C (n=14)
surface

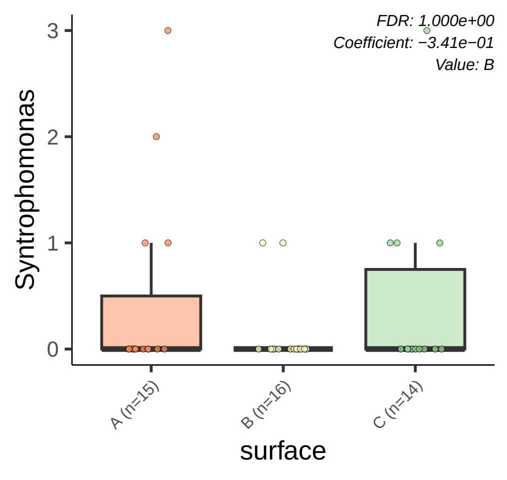
FDR: 1.000e+00
Coefficient: −3.41e−01
Value: B
Syntrophomonas
3
2
1
0
A (n=15)
B (n=16)
C (n=14)
surface

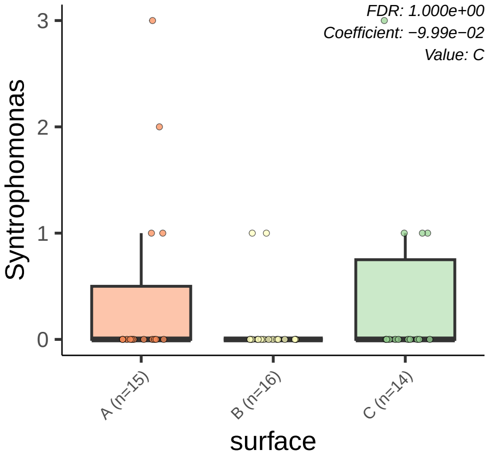

FDR: 1.000e+00
Coefficient: −9.99e−02
Value: C
Syntrophomonas
3
2
1
0
A (n=15)
B (n=16)
C (n=14)
surface

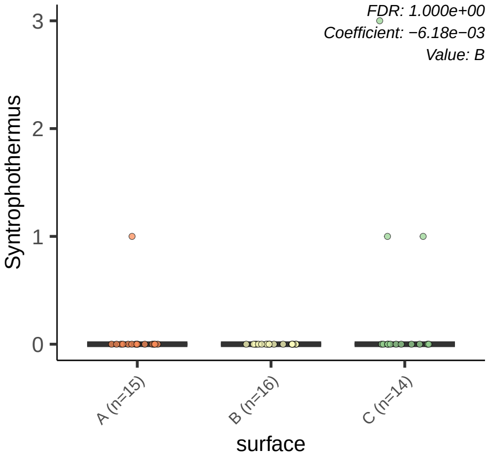
FDR: 1.000e+00
Coefficient: −6.18e−03
Value: B
Syntrophothermus
3
2
1
0
A (n=15)
B (n=16)
C (n=14)
surface

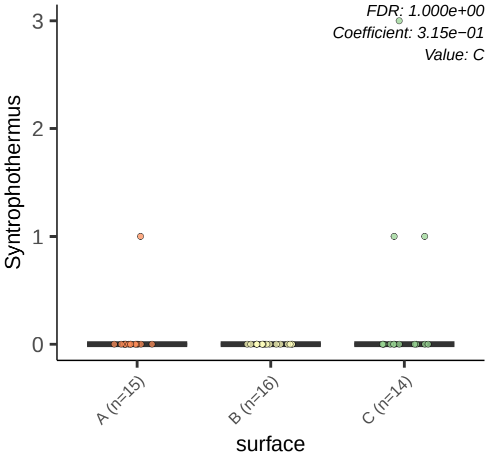
FDR: 1.000e+00
Coefficient: 3.15e−01
Value: C
Syntrophothermus
3
2
1
0
A (n=15)
B (n=16)
C (n=14)
surface

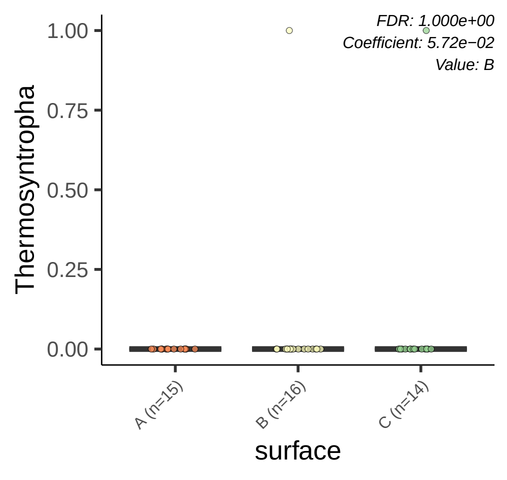

FDR: 1.000e+00
Coefficient: 5.72e−02
Value: B
Thermosyntropha
1.00
0.75
0.50
0.25
0.00
A (n=15)
B (n=16)
C (n=14)
surface

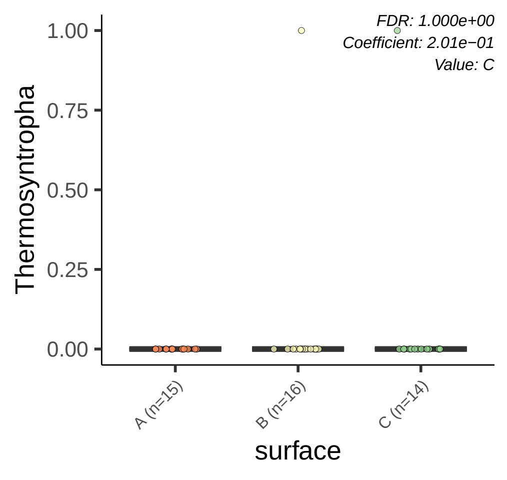
FDR: 1.000e+00
Coefficient: 2.01e−01
Value: C
Thermosyntropha
1.00
0.75
0.50
0.25
0.00
A (n=15)
B (n=16)
C (n=14)
surface

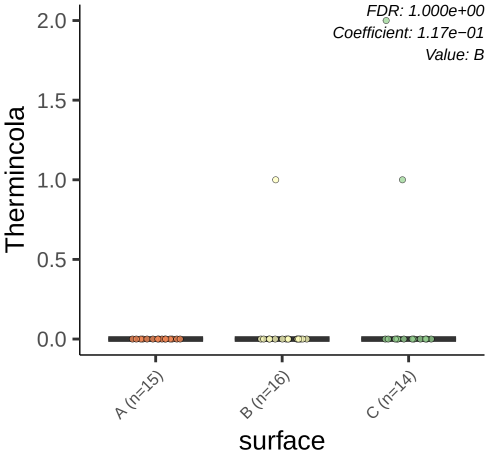

FDR: 1.000e+00
Coefficient: 1.17e−01
Value: B
Thermincola
2.0
1.5
1.0
0.5
0.0
A (n=15)
B (n=16)
C (n=14)
surface

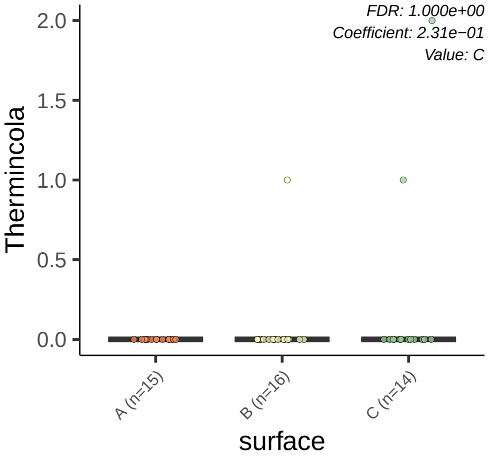
FDR: 1.000e+00
Coefficient: 2.31e−01
Value: C
Thermincola
2.0
1.5
1.0
0.5
0.0
A (n=15)
B (n=16)
C (n=14)
surface

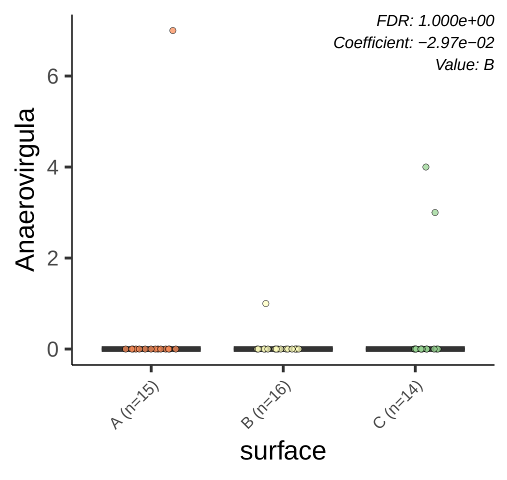

FDR: 1.000e+00
Coefficient: −2.97e−02
Value: B
Anaerovirgula
0
2
4
6
A (n=15)
B (n=16)
C (n=14)
surface

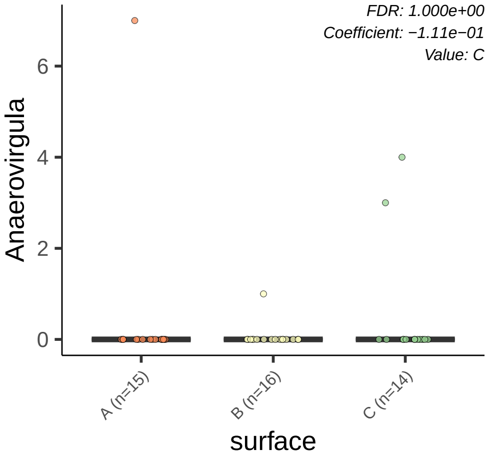
FDR: 1.000e+00
Coefficient: −1.11e−01
Value: C
Anaerovirgula
0
2
4
6
A (n=15)
B (n=16)
C (n=14)
surface

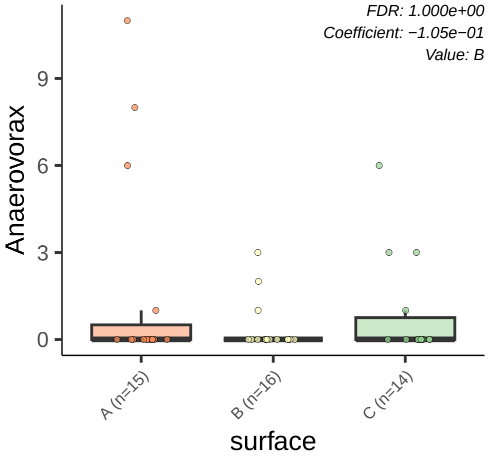
FDR: 1.000e+00
Coefficient: −1.05e−01
Value: B
Anaerovorax
9
6
3
0
A (n=15)
B (n=16)
C (n=14)
surface

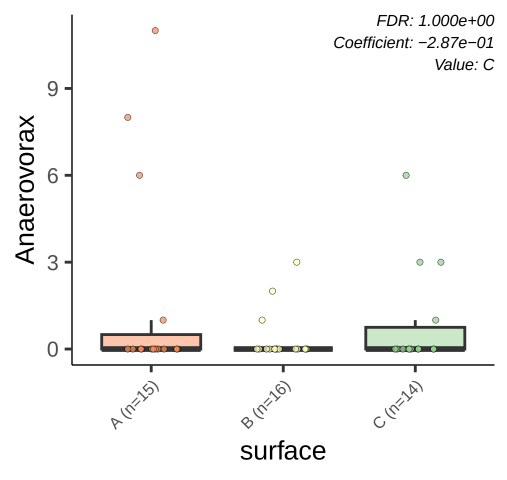

FDR: 1.000e+00
Coefficient: −2.87e−01
Value: C
Anaerovorax
9
6
3
0
A (n=15)
B (n=16)
C (n=14)
surface

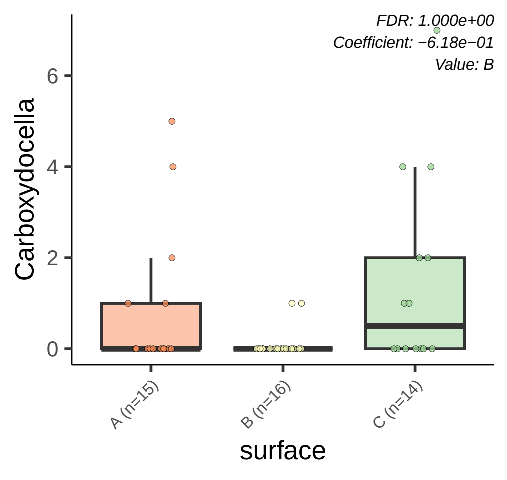
FDR: 1.000e+00
Coefficient: −6.18e−01
Value: B
Carboxydocella
0
2
4
6
A (n=15)
B (n=16)
C (n=14)
surface

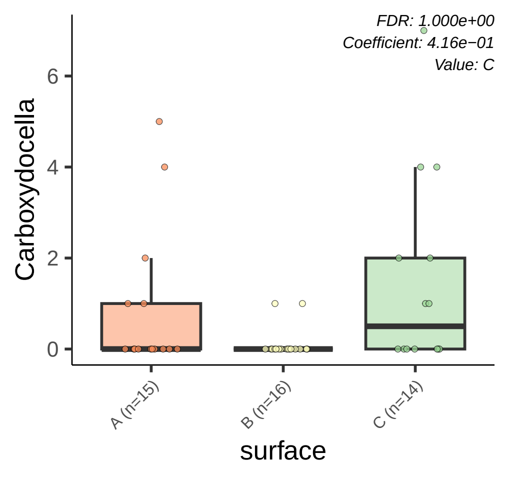
FDR: 1.000e+00
Coefficient: 4.16e−01
Value: C
Carboxydocella
0
2
4
6
A (n=15)
B (n=16)
C (n=14)
surface

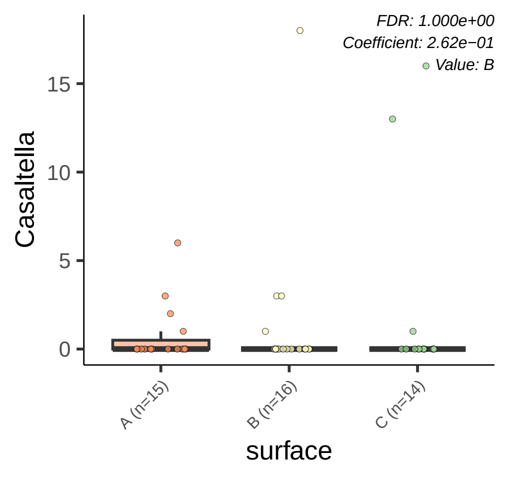
FDR: 1.000e+00
Coefficient: 2.62e−01
Value: B
Casaltella
0
5
10
15
A (n=15)
B (n=16)
C (n=14)
surface

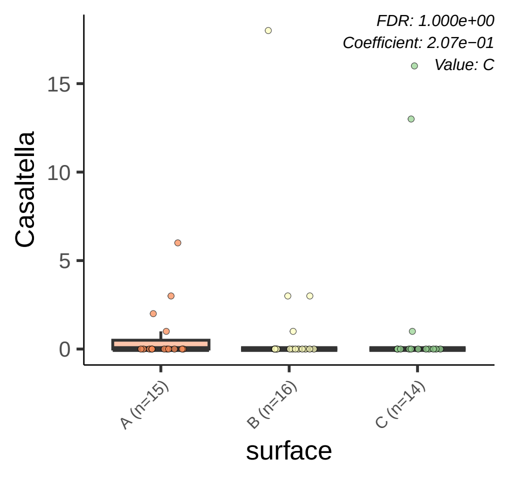

FDR: 1.000e+00
Coefficient: 2.07e−01
Value: C
Casaltella
15
10
5
0
A (n=15)
B (n=16)
C (n=14)
surface

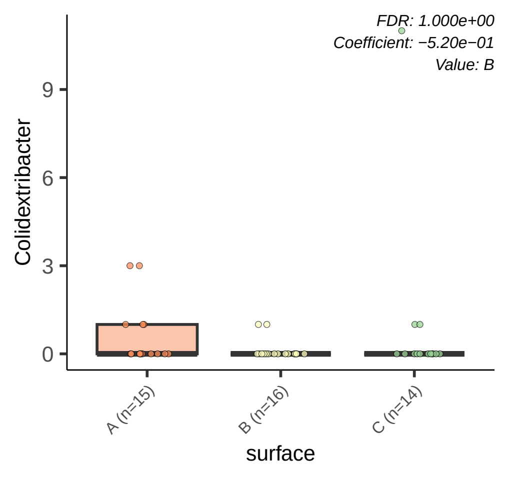

FDR: 1.000e+00
Coefficient: −5.20e−01
Value: B
Colidextribacter
0
3
6
9
A (n=15)
B (n=16)
C (n=14)
surface

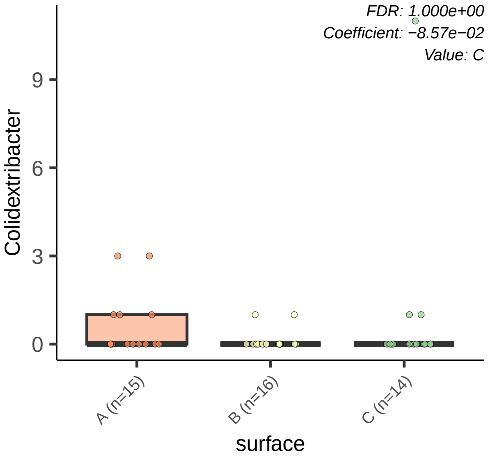

FDR: 1.000e+00
Coefficient: −8.57e−02
Value: C
Colidextribacter
9
6
3
0
A (n=15)
B (n=16)
C (n=14)
surface

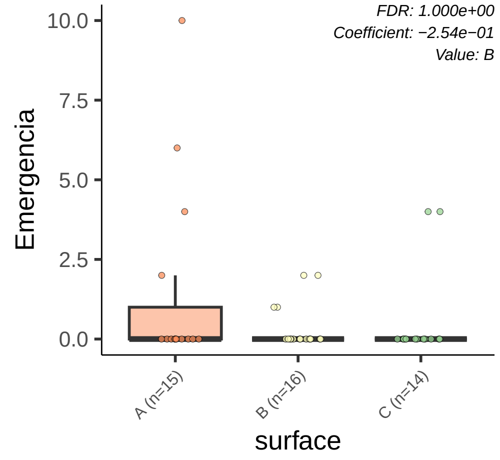

FDR: 1.000e+00
Coefficient: −2.54e−01
Value: B
Emergencia
10.0
7.5
5.0
2.5
0.0
A (n=15)
B (n=16)
C (n=14)
surface

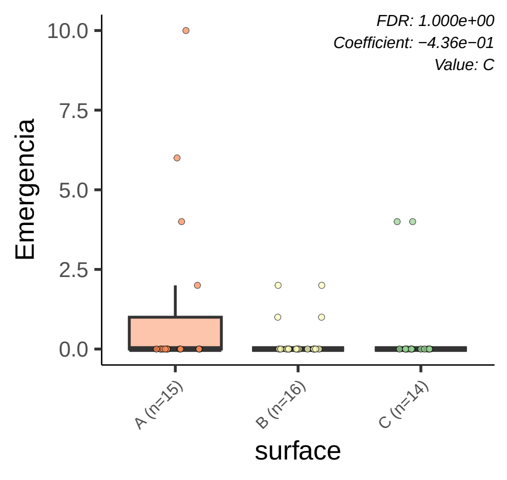

FDR: 1.000e+00
Coefficient: −4.36e−01
Value: C
Emergencia
10.0
7.5
5.0
2.5
0.0
A (n=15)
B (n=16)
C (n=14)
surface

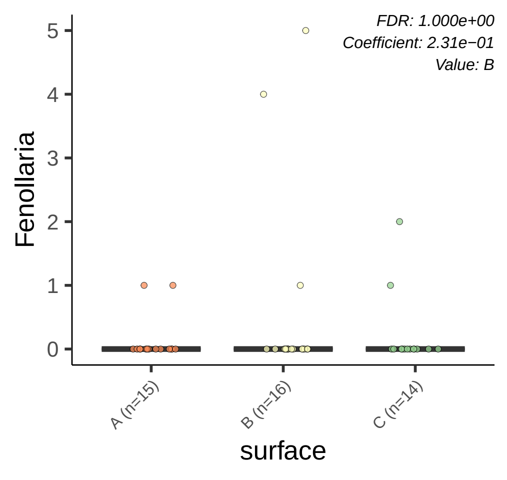
FDR: 1.000e+00
Coefficient: 2.31e−01
Value: B
Fenollaria
0
1
2
3
4
5
A (n=15)
B (n=16)
C (n=14)
surface

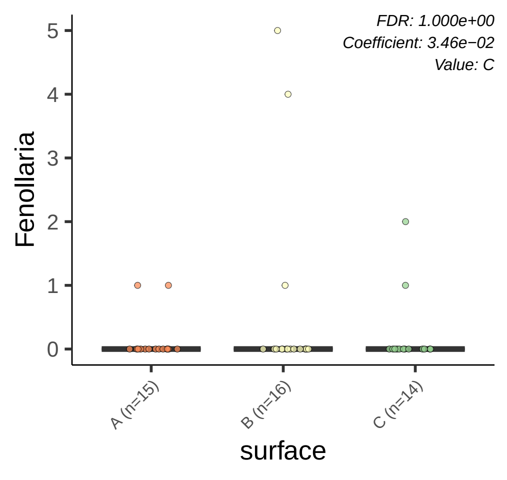

FDR: 1.000e+00
Coefficient: 3.46e−02
Value: C
Fenollaria
0
1
2
3
4
5
A (n=15)
B (n=16)
C (n=14)
surface

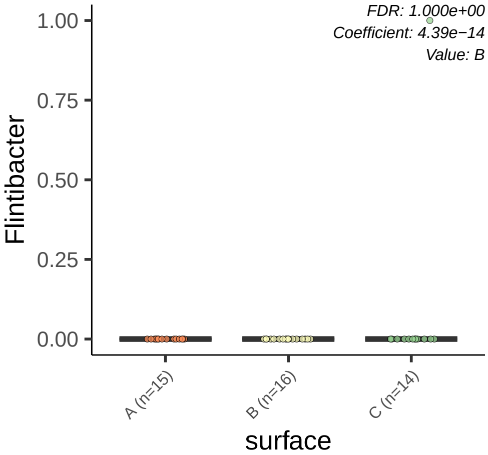

FDR: 1.000e+00
Coefficient: 4.39e−14
Value: B
1.00
0.75
0.50
0.25
0.00
Flintibacter
A (n=15)
B (n=16)
C (n=14)
surface

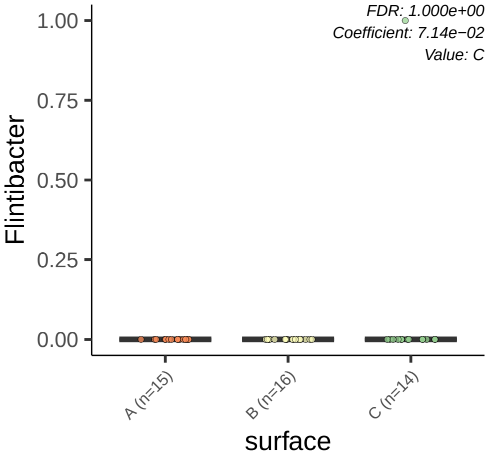
FDR: 1.000e+00
Coefficient: 7.14e−02
Value: C
Flintibacter
1.00
0.75
0.50
0.25
0.00
A (n=15)
B (n=16)
C (n=14)
surface

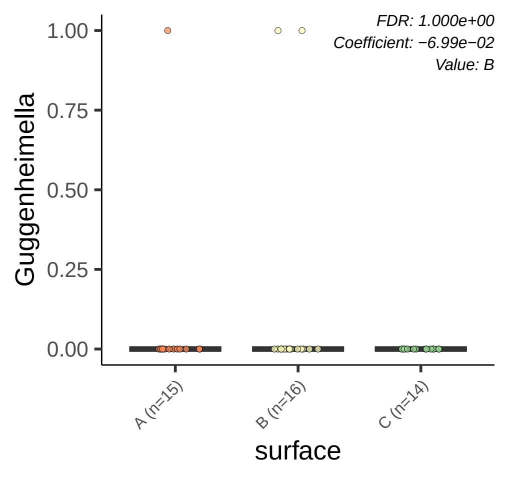
FDR: 1.000e+00
Coefficient: −6.99e−02
Value: B
Guggenheimella
1.00
0.75
0.50
0.25
0.00
A (n=15)
B (n=16)
C (n=14)
surface

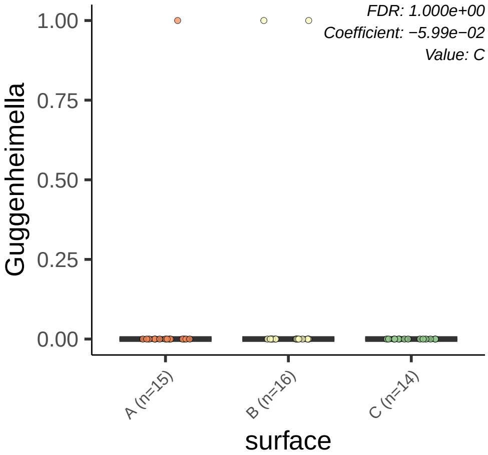

FDR: 1.000e+00
Coefficient: −5.99e−02
Value: C
Guggenheimella
1.00
0.75
0.50
0.25
0.00
A (n=15)
B (n=16)
C (n=14)
surface

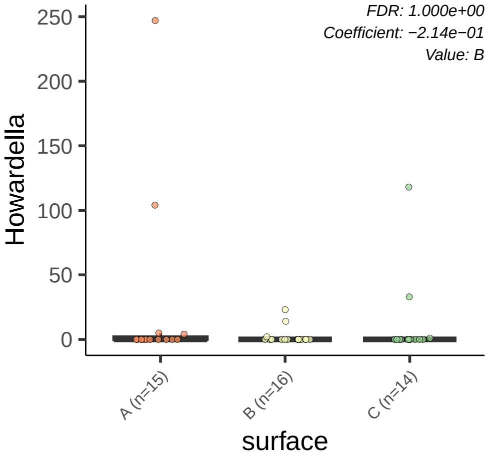

FDR: 1.000e+00
Coefficient: −2.14e−01
Value: B
Howardella
250
200
150
100
50
0
A (n=15)
B (n=16)
C (n=14)
surface

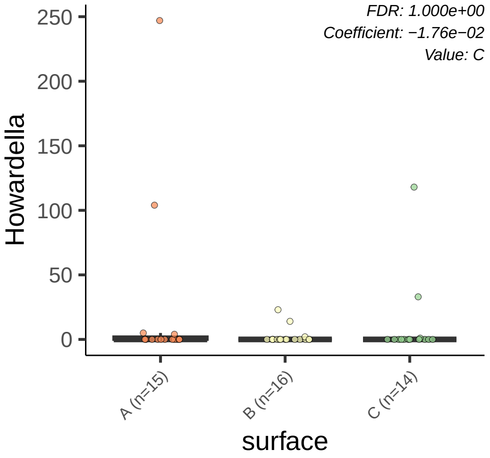

FDR: 1.000e+00
Coefficient: −1.76e−02
Value: C
Howardella
250
200
150
100
50
0
A (n=15)
B (n=16)
C (n=14)
surface

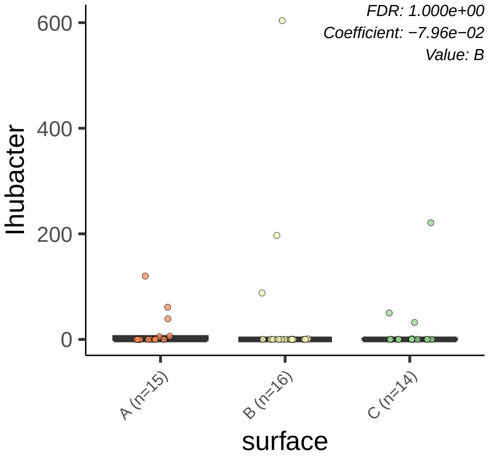
FDR: 1.000e+00
Coefficient: −7.96e−02
Value: B
Ihubacter
600
400
200
0
A (n=15)
B (n=16)
C (n=14)
surface

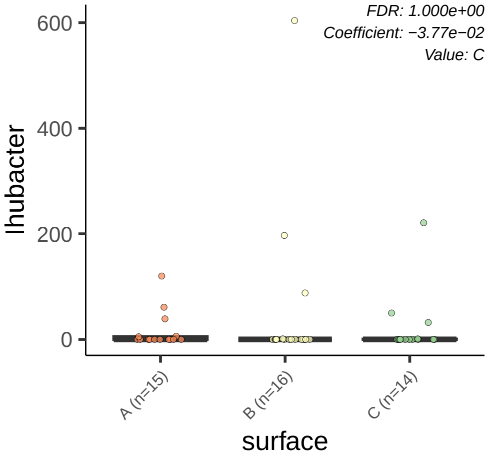
FDR: 1.000e+00
Coefficient: −3.77e−02
Value: C
Ihubacter
600
400
200
0
A (n=15)
B (n=16)
C (n=14)
surface

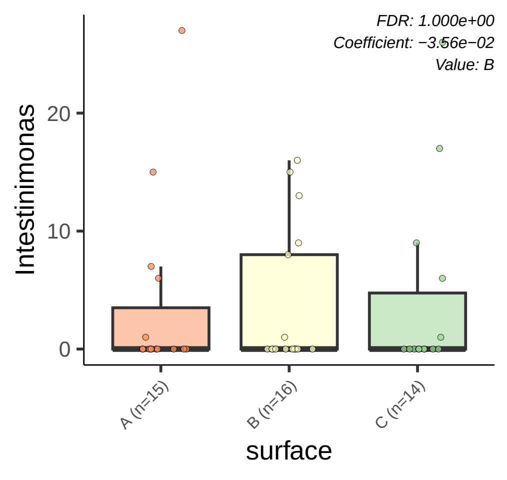
FDR: 1.000e+00
Coefficient: −3.56e−02
Value: B
Intestinimonas
20
10
0
A (n=15)
B (n=16)
C (n=14)
surface

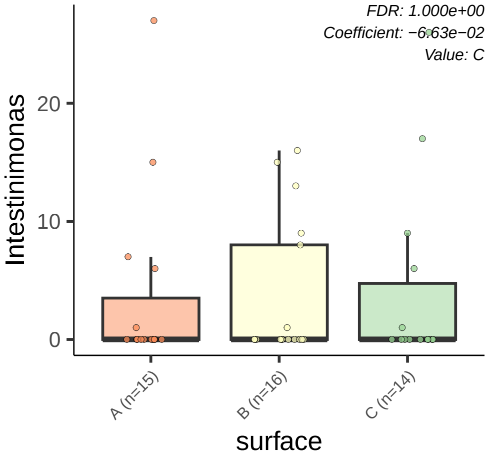
FDR: 1.000e+00
Coefficient: −6.63e−02
Value: C
Intestinimonas
20
10
0
A (n=15)
B (n=16)
C (n=14)
surface

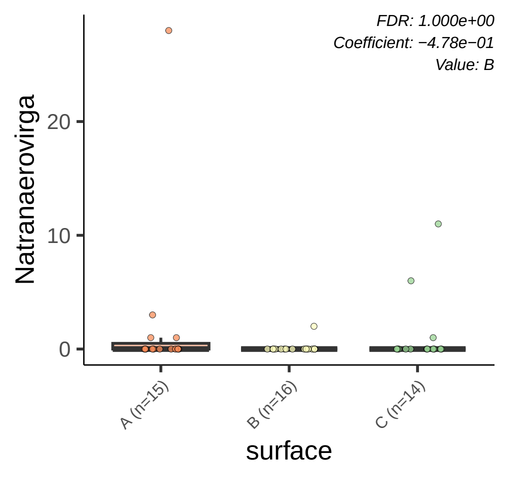

FDR: 1.000e+00
Coefficient: −4.78e−01
Value: B
Natranaerovirga
20
10
0
A (n=15)
B (n=16)
C (n=14)
surface

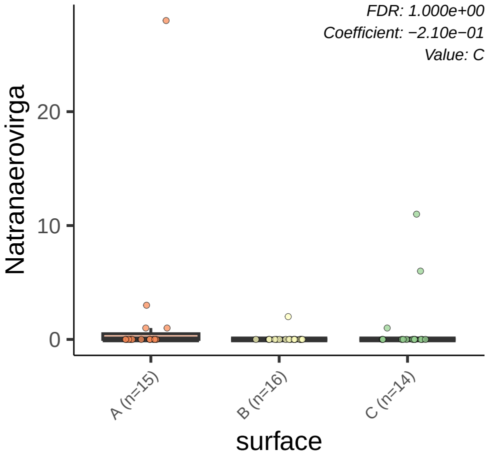

FDR: 1.000e+00
Coefficient: −2.10e−01
Value: C
Natranaerovirga
20
10
0
A (n=15)
B (n=16)
C (n=14)
surface

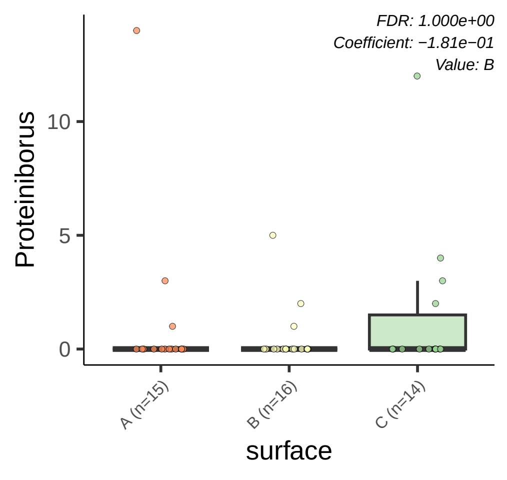
FDR: 1.000e+00
Coefficient: −1.81e−01
Value: B
Proteiniborus
0
5
10
A (n=15)
B (n=16)
C (n=14)
surface

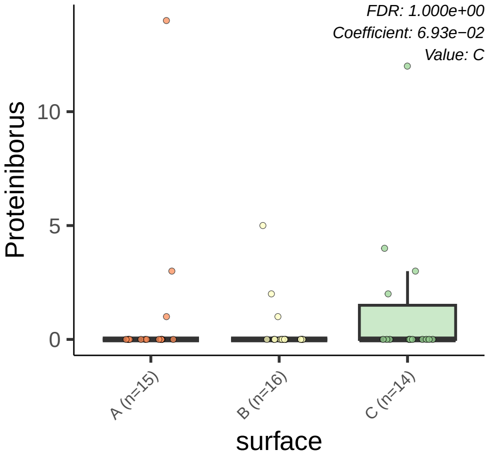
FDR: 1.000e+00
Coefficient: 6.93e−02
Value: C
Proteiniborus
10
5
0
A (n=15)
B (n=16)
C (n=14)
surface

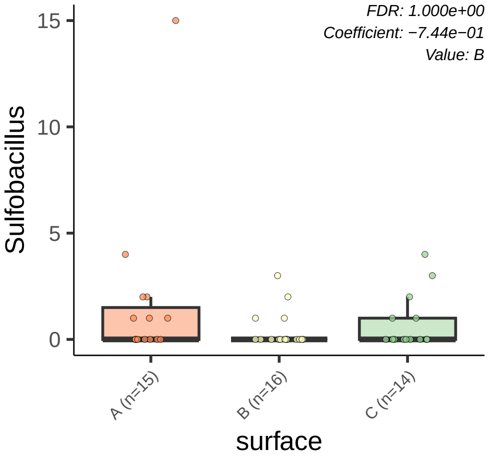
FDR: 1.000e+00
Coefficient: −7.44e−01
Value: B
Sulfobacillus
15
10
5
0
A (n=15)
B (n=16)
C (n=14)
surface

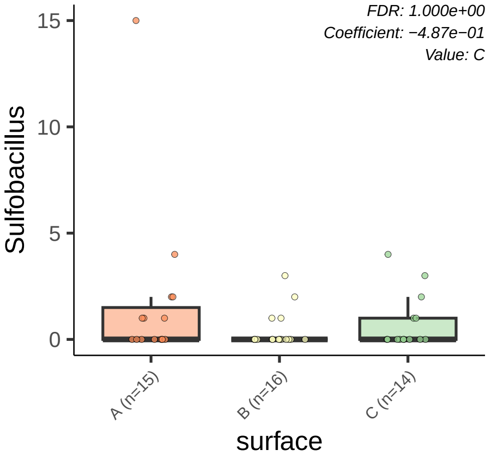

FDR: 1.000e+00
Coefficient: −4.87e−01
Value: C
Sulfobacillus
15
10
5
0
A (n=15)
B (n=16)
C (n=14)
surface

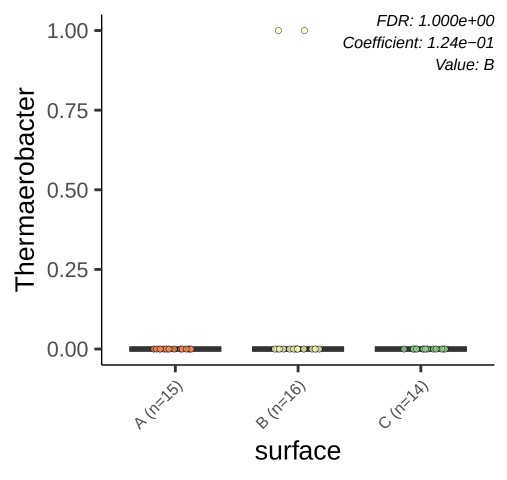

FDR: 1.000e+00
Coefficient: 1.24e−01
Value: B
Thermaerobacter
1.00
0.75
0.50
0.25
0.00
A (n=15)
B (n=16)
C (n=14)
surface

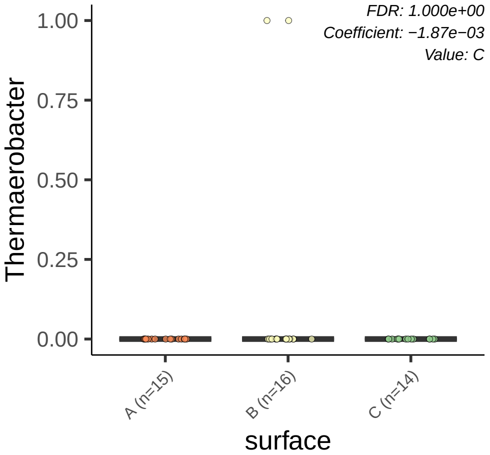

FDR: 1.000e+00
Coefficient: −1.87e−03
Value: C
Thermaerobacter
1.00
0.75
0.50
0.25
0.00
A (n=15)
B (n=16)
C (n=14)
surface

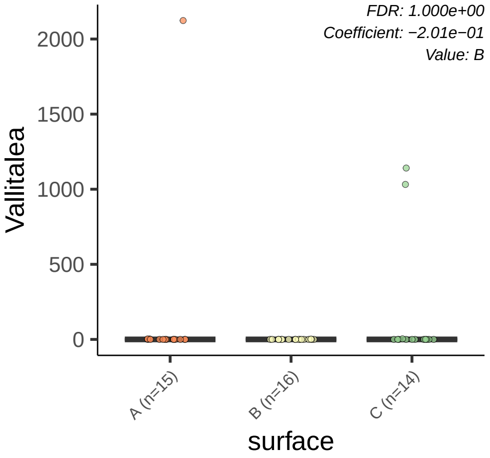

FDR: 1.000e+00
Coefficient: −2.01e−01
Value: B
Vallitalea
2000
1500
1000
500
0
A (n=15)
B (n=16)
C (n=14)
surface

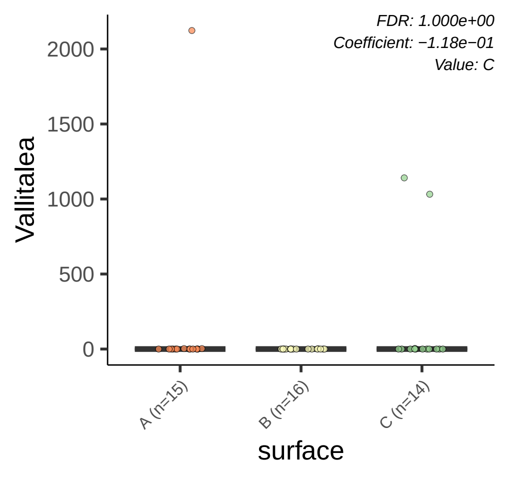

FDR: 1.000e+00
Coefficient: −1.18e−01
Value: C
2000
1500
1000
500
0
Vallitalea
A (n=15)
B (n=16)
C (n=14)
surface

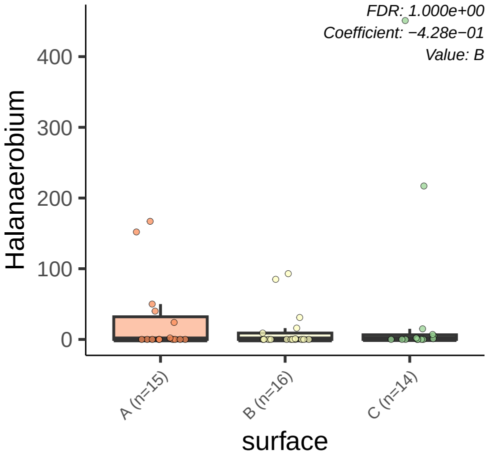
FDR: 1.000e+00
Coefficient: −4.28e−01
Value: B
Halanaerobium
400
300
200
100
0
A (n=15)
B (n=16)
C (n=14)
surface

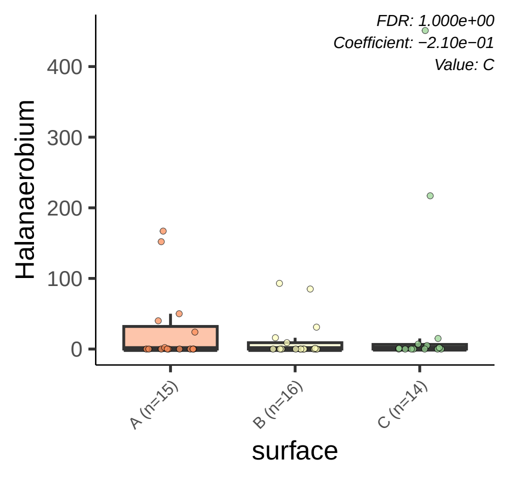
FDR: 1.000e+00
Coefficient: −2.10e−01
Value: C
400
300
200
100
0
Halanaerobium
A (n=15)
B (n=16)
C (n=14)
surface

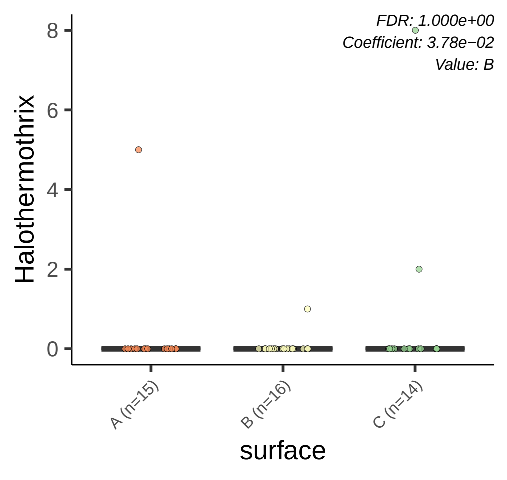
FDR: 1.000e+00
Coefficient: 3.78e−02
Value: B
Halothermothrix
8
6
4
2
0
A (n=15)
B (n=16)
C (n=14)
surface

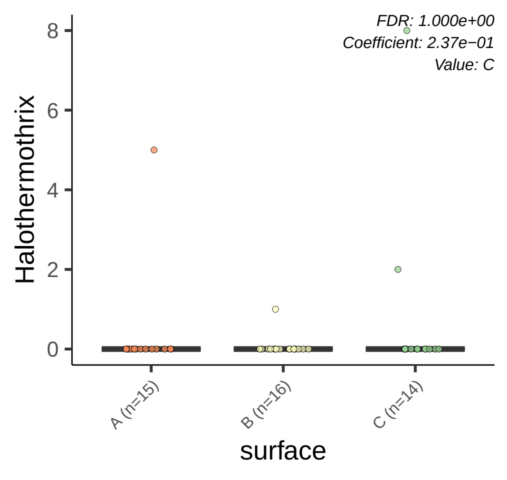

FDR: 1.000e+00
Coefficient: 2.37e−01
Value: C
Halothermothrix
8
6
4
2
0
A (n=15)
B (n=16)
C (n=14)
surface

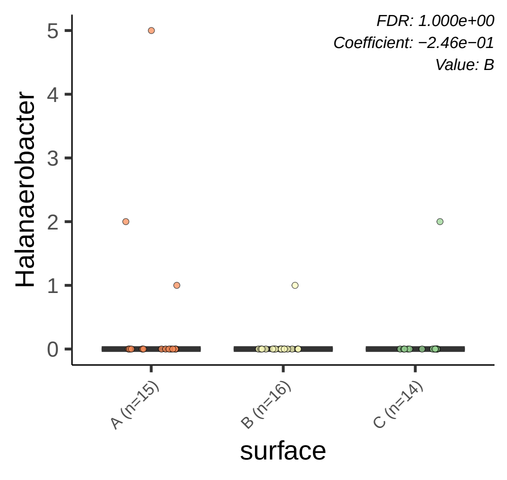
FDR: 1.000e+00
Coefficient: −2.46e−01
Value: B
Halanaerobacter
0
1
2
3
4
5
A (n=15)
B (n=16)
C (n=14)
surface

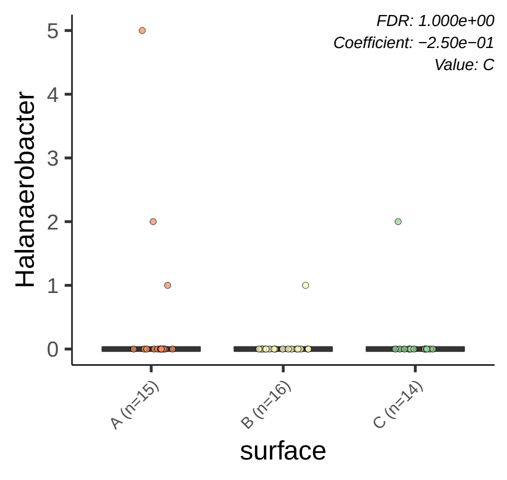

FDR: 1.000e+00
Coefficient: −2.50e−01
Value: C
Halanaerobacter
0
1
2
3
4
5
A (n=15)
B (n=16)
C (n=14)
surface

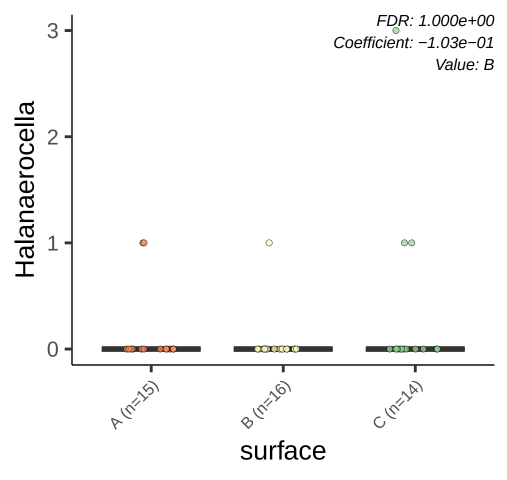

FDR: 1.000e+00
Coefficient: −1.03e−01
Value: B
Halanaerocella
3
2
1
0
A (n=15)
B (n=16)
C (n=14)
surface

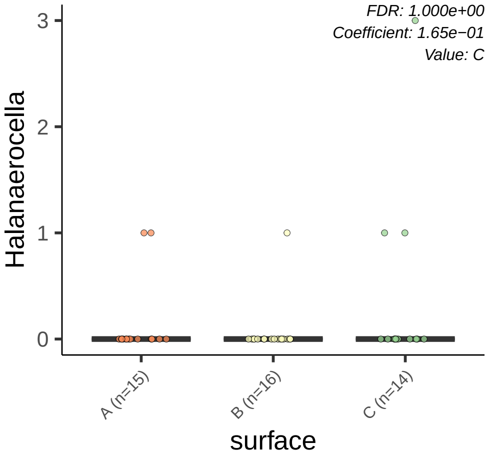
FDR: 1.000e+00
Coefficient: 1.65e−01
Value: C
Halanaerocella
3
2
1
0
A (n=15)
B (n=16)
C (n=14)
surface

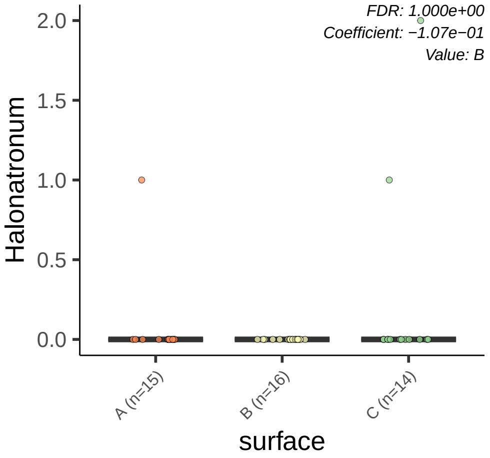
FDR: 1.000e+00
Coefficient: −1.07e−01
Value: B
Halonatronum
2.0
1.5
1.0
0.5
0.0
A (n=15)
B (n=16)
C (n=14)
surface

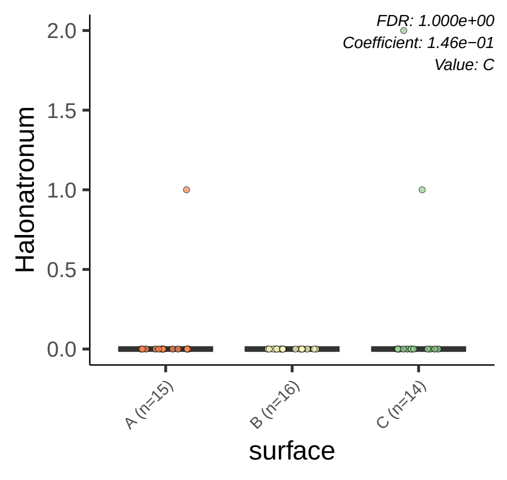
FDR: 1.000e+00
Coefficient: 1.46e−01
Value: C
Halonatronum
2.0
1.5
1.0
0.5
0.0
A (n=15)
B (n=16)
C (n=14)
surface

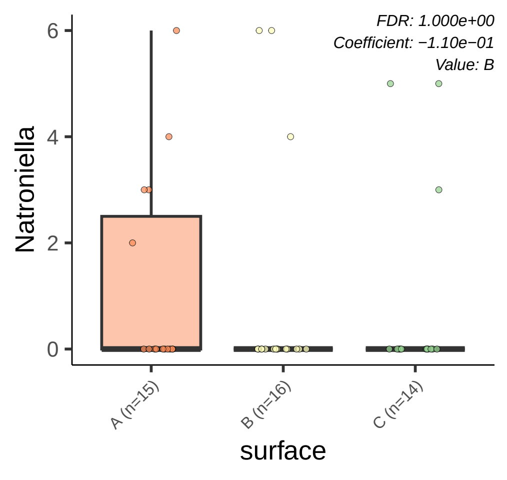

FDR: 1.000e+00
Coefficient: −1.10e−01
Value: B
Natroniella
6
4
2
0
A (n=15)
B (n=16)
C (n=14)
surface

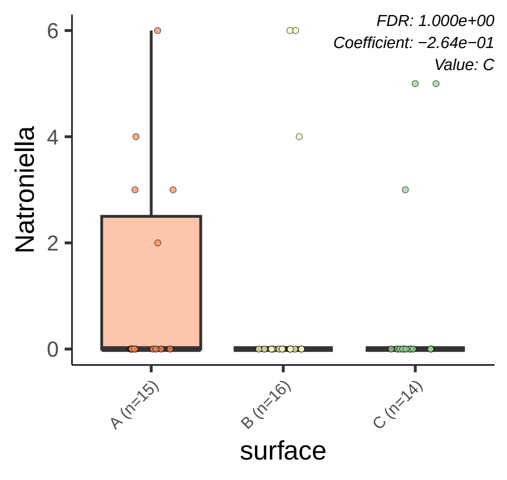

FDR: 1.000e+00
Coefficient: −2.64e−01
Value: C
Natroniella
0
2
4
6
A (n=15)
B (n=16)
C (n=14)
surface

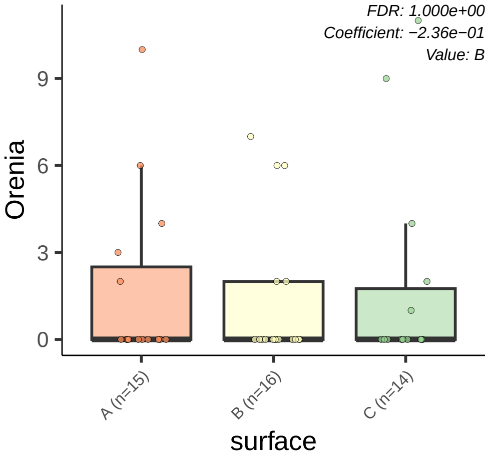
FDR: 1.000e+00
Coefficient: −2.36e−01
Value: B
Orenia
9
6
3
0
A (n=15)
B (n=16)
C (n=14)
surface

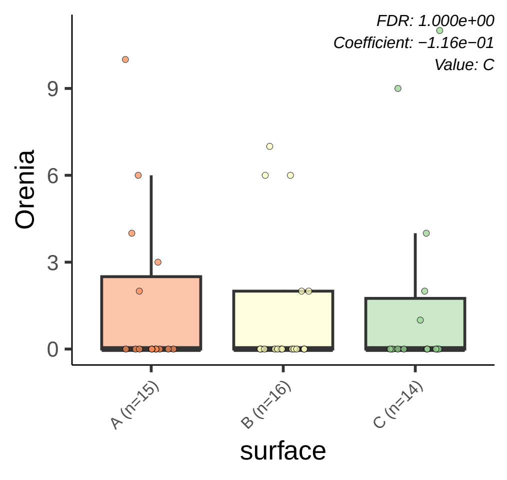
FDR: 1.000e+00
Coefficient: −1.16e−01
Value: C
Orenia
9
6
3
0
A (n=15)
B (n=16)
C (n=14)
surface

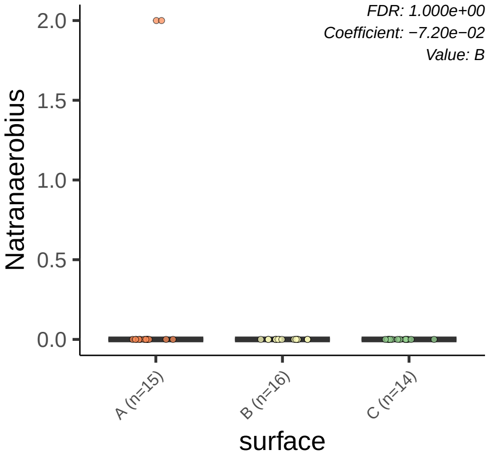

FDR: 1.000e+00
Coefficient: −7.20e−02
Value: B
Natranaerobius
2.0
1.5
1.0
0.5
0.0
A (n=15)
B (n=16)
C (n=14)
surface

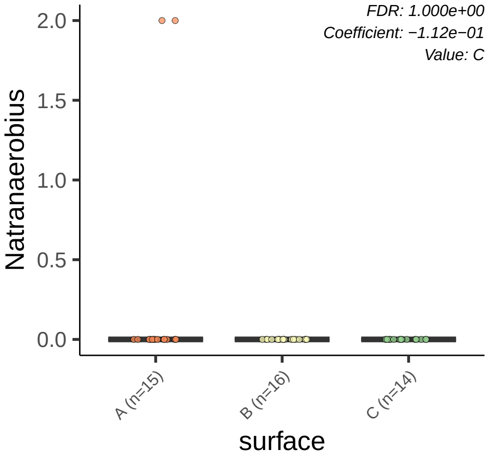
FDR: 1.000e+00
Coefficient: −1.12e−01
Value: C
Natranaerobius
2.0
1.5
1.0
0.5
0.0
A (n=15)
B (n=16)
C (n=14)
surface

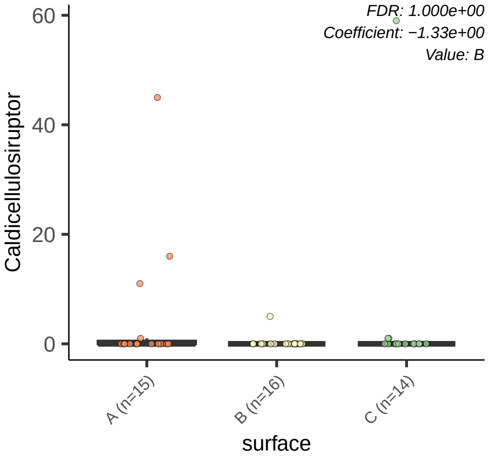
FDR: 1.000e+00
Coefficient: −1.33e+00
Value: B
Caldicellulosiruptor
0
20
40
60
A (n=15)
B (n=16)
C (n=14)
surface

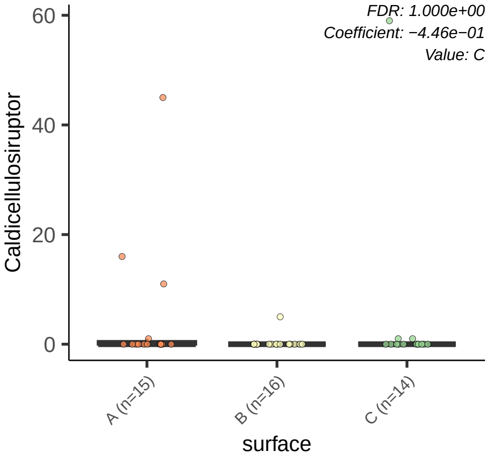
FDR: 1.000e+00
Coefficient: −4.46e−01
Value: C
Caldicellulosiruptor
0
20
40
60
A (n=15)
B (n=16)
C (n=14)
surface

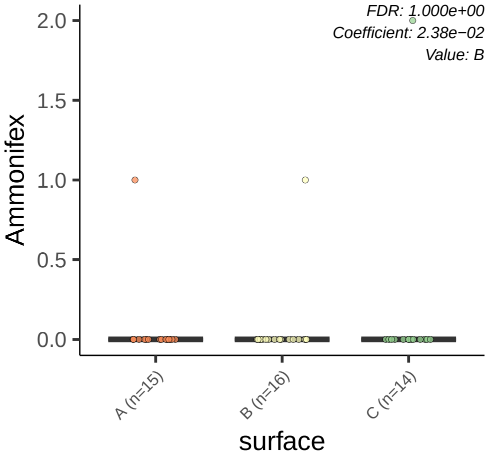
FDR: 1.000e+00
Coefficient: 2.38e−02
Value: B
Ammonifex
2.0
1.5
1.0
0.5
0.0
A (n=15)
B (n=16)
C (n=14)
surface

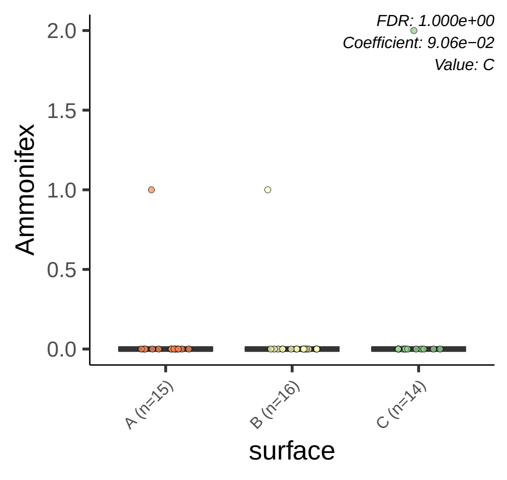
FDR: 1.000e+00
Coefficient: 9.06e−02
Value: C
Ammonifex
2.0
1.5
1.0
0.5
0.0
A (n=15)
B (n=16)
C (n=14)
surface

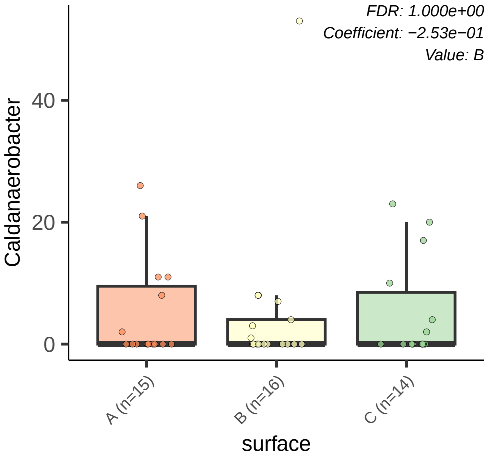
FDR: 1.000e+00
Coefficient: −2.53e−01
Value: B
Caldanaerobacter
0
20
40
A (n=15)
B (n=16)
C (n=14)
surface

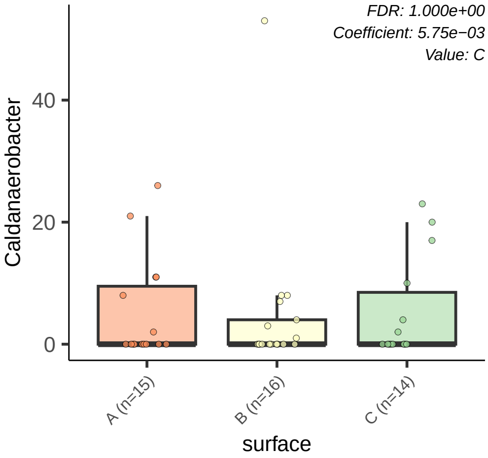
FDR: 1.000e+00
Coefficient: 5.75e−03
Value: C
Caldanaerobacter
0
20
40
A (n=15)
B (n=16)
C (n=14)
surface

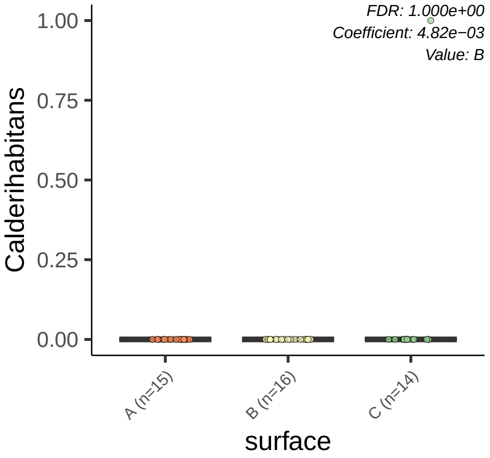

FDR: 1.000e+00
Coefficient: 4.82e−03
Value: B
Calderihabitans
1.00
0.75
0.50
0.25
0.00
A (n=15)
B (n=16)
C (n=14)
surface

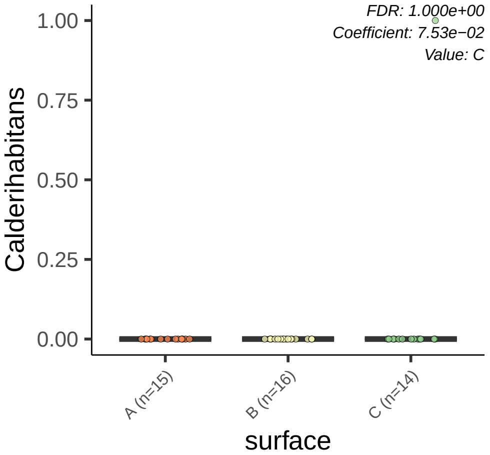

FDR: 1.000e+00
Coefficient: 7.53e−02
Value: C
Calderihabitans
1.00
0.75
0.50
0.25
0.00
A (n=15)
B (n=16)
C (n=14)
surface

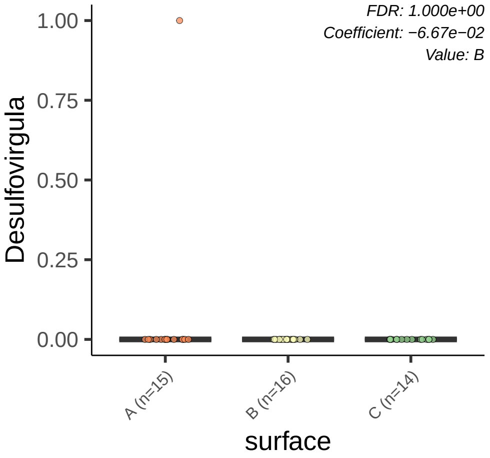
FDR: 1.000e+00
Coefficient: −6.67e−02
Value: B
Desulfovirgula
1.00
0.75
0.50
0.25
0.00
A (n=15)
B (n=16)
C (n=14)
surface

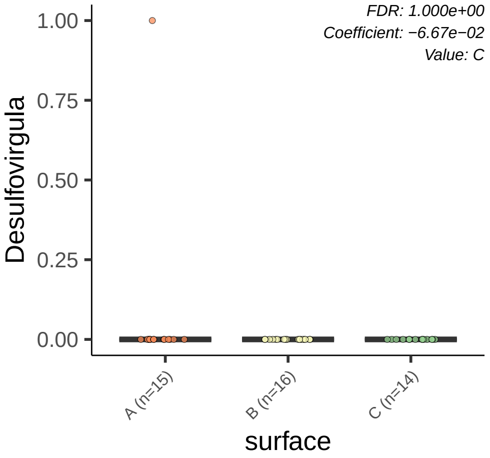
FDR: 1.000e+00
Coefficient: −6.67e−02
Value: C
Desulfovirgula
1.00
0.75
0.50
0.25
0.00
A (n=15)
B (n=16)
C (n=14)
surface

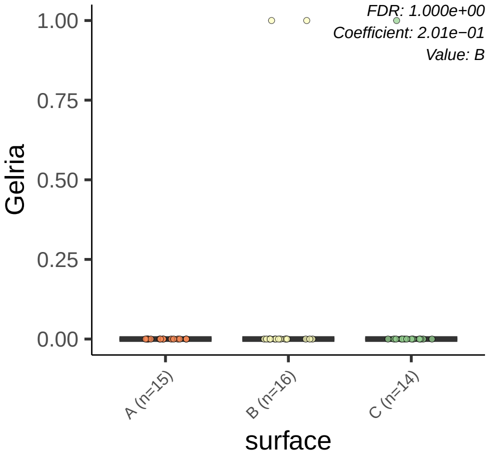

FDR: 1.000e+00
Coefficient: 2.01e−01
Value: B
Gelria
1.00
0.75
0.50
0.25
0.00
A (n=15)
B (n=16)
C (n=14)
surface

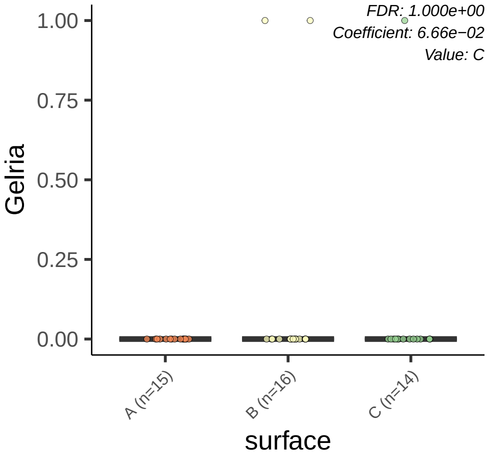
FDR: 1.000e+00
Coefficient: 6.66e−02
Value: C
Gelria
1.00
0.75
0.50
0.25
0.00
A (n=15)
B (n=16)
C (n=14)
surface

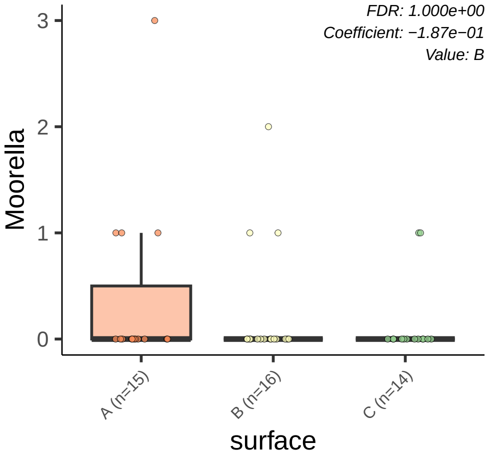

FDR: 1.000e+00
Coefficient: −1.87e−01
Value: B
Moorella
0
1
2
3
A (n=15)
B (n=16)
C (n=14)
surface

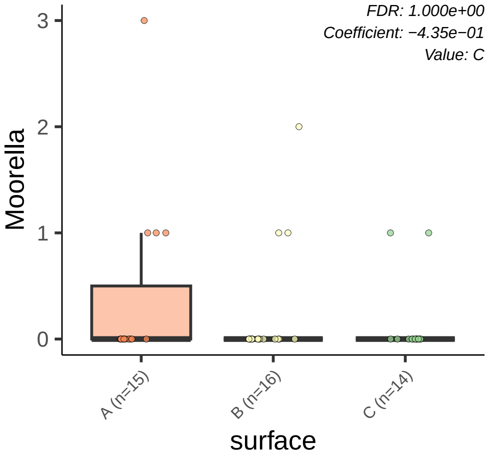
FDR: 1.000e+00
Coefficient: −4.35e−01
Value: C
Moorella
3
2
1
0
A (n=15)
B (n=16)
C (n=14)
surface

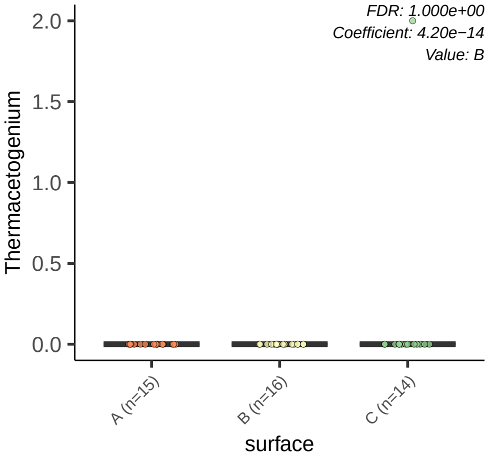

FDR: 1.000e+00
Coefficient: 4.20e−14
Value: B
Thermacetogenium
2.0
1.5
1.0
0.5
0.0
A (n=15)
B (n=16)
C (n=14)
surface

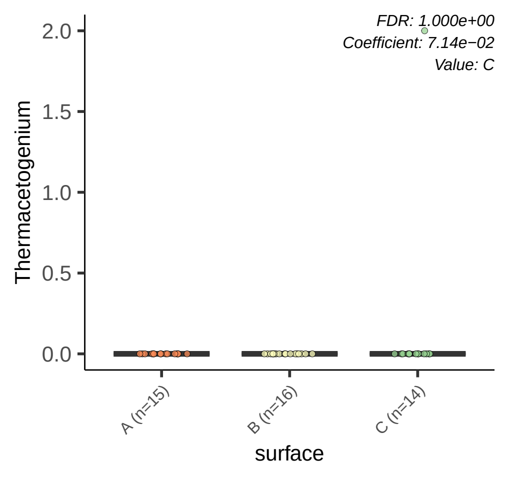

FDR: 1.000e+00
Coefficient: 7.14e−02
Value: C
Thermacetogenium
2.0
1.5
1.0
0.5
0.0
A (n=15)
B (n=16)
C (n=14)
surface

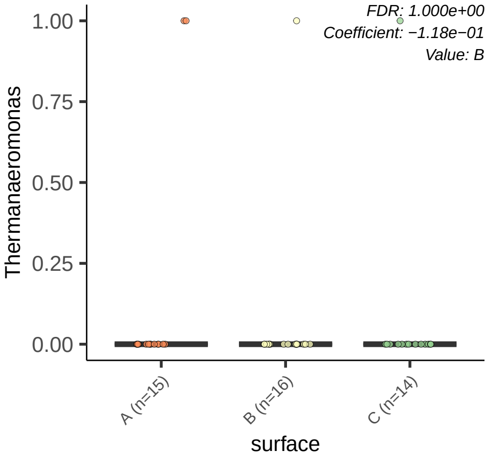
FDR: 1.000e+00
Coefficient: −1.18e−01
Value: B
Thermanaeromonas
1.00
0.75
0.50
0.25
0.00
A (n=15)
B (n=16)
C (n=14)
surface

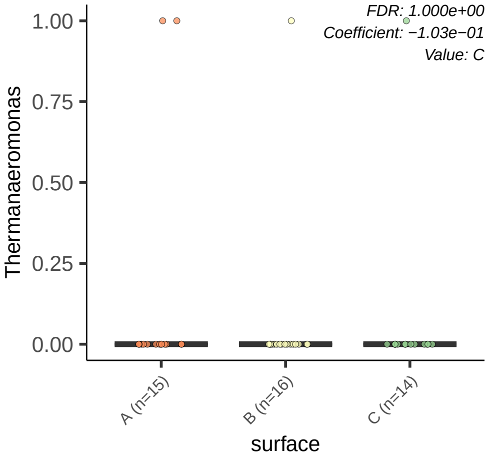
FDR: 1.000e+00
Coefficient: −1.03e−01
Value: C
Thermanaeromonas
1.00
0.75
0.50
0.25
0.00
A (n=15)
B (n=16)
C (n=14)
surface

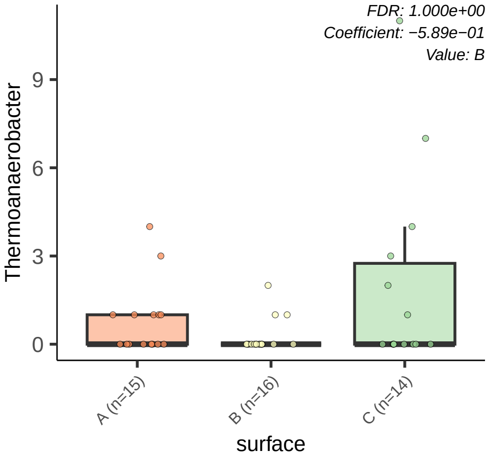

FDR: 1.000e+00
Coefficient: −5.89e−01
Value: B
Thermoanaerobacter
0
3
6
9
A (n=15)
B (n=16)
C (n=14)
surface

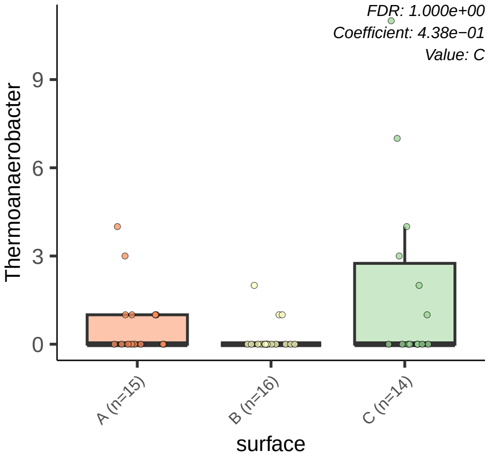

FDR: 1.000e+00
Coefficient: 4.38e−01
Value: C
Thermoanaerobacter
0
3
6
9
A (n=15)
B (n=16)
C (n=14)
surface

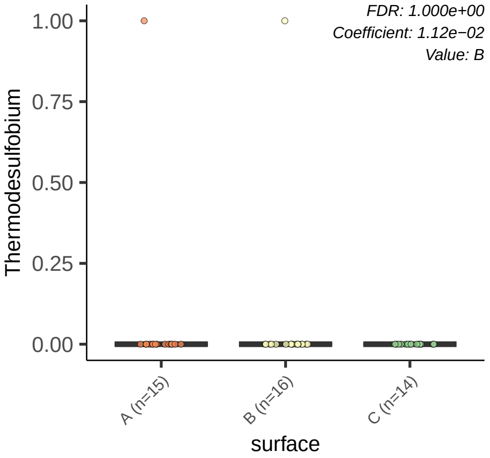
FDR: 1.000e+00
Coefficient: 1.12e−02
Value: B
Thermodesulfobium
1.00
0.75
0.50
0.25
0.00
A (n=15)
B (n=16)
C (n=14)
surface

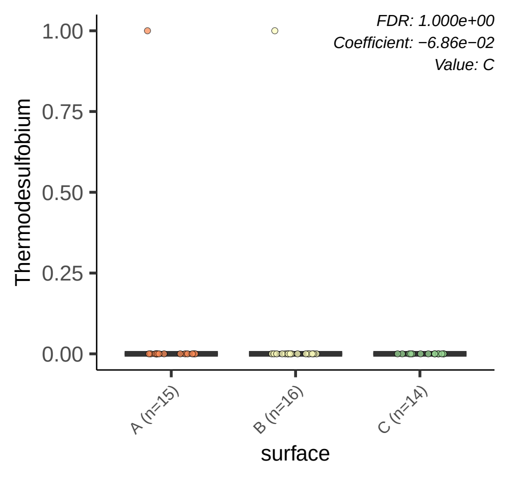
FDR: 1.000e+00
Coefficient: −6.86e−02
Value: C
Thermodesulfobium
1.00
0.75
0.50
0.25
0.00
A (n=15)
B (n=16)
C (n=14)
surface

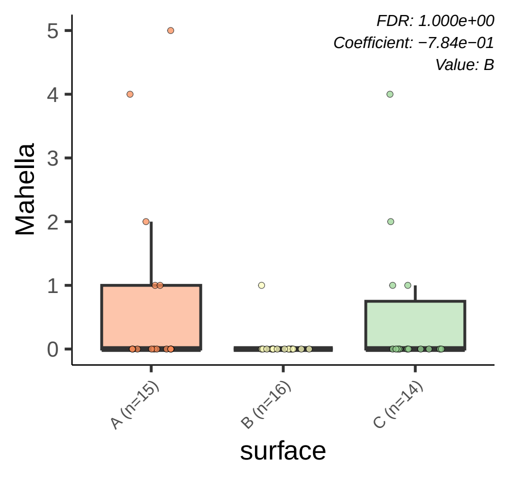
FDR: 1.000e+00
Coefficient: −7.84e−01
Value: B
Mahella
0
1
2
3
4
5
A (n=15)
B (n=16)
C (n=14)
surface

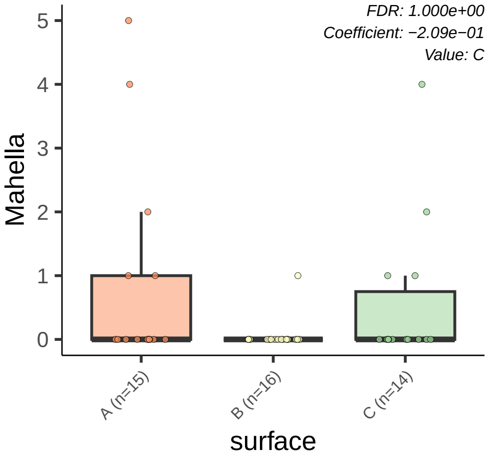
FDR: 1.000e+00
Coefficient: −2.09e−01
Value: C
Mahella
5
4
3
2
1
0
A (n=15)
B (n=16)
C (n=14)
surface

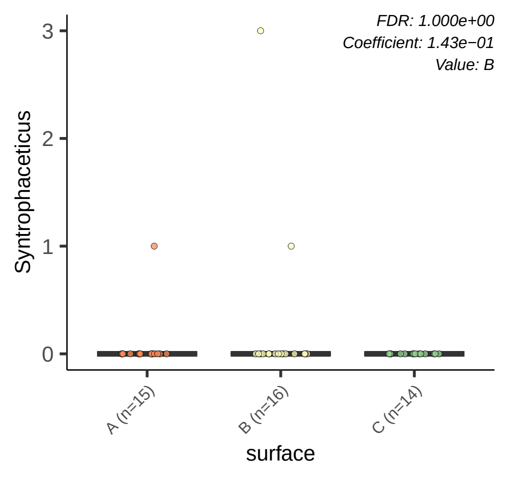

FDR: 1.000e+00
Coefficient: 1.43e−01
Value: B
Syntrophaceticus
0
1
2
3
A (n=15)
B (n=16)
C (n=14)
surface

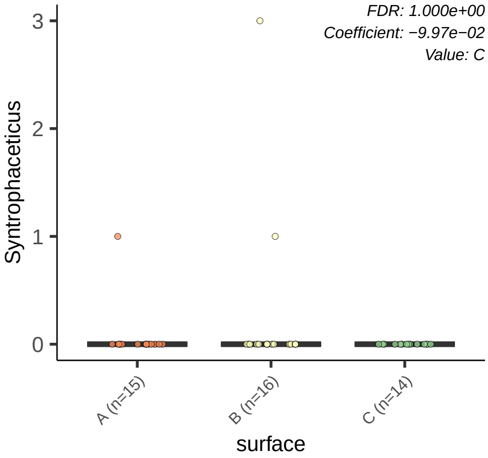
FDR: 1.000e+00
Coefficient: −9.97e−02
Value: C
Syntrophaceticus
3
2
1
0
A (n=15)
B (n=16)
C (n=14)
surface

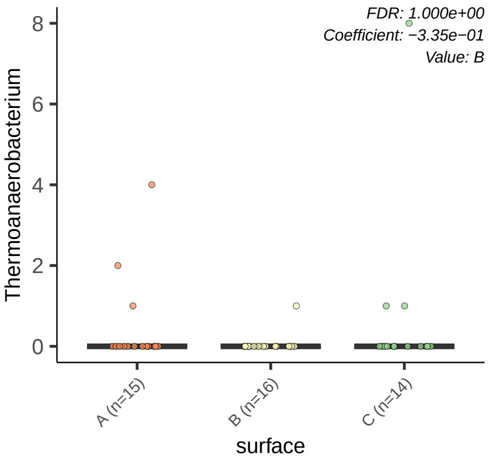
FDR: 1.000e+00
Coefficient: −3.35e−01
Value: B
Thermoanaerobacterium
8
6
4
2
0
A (n=15)
B (n=16)
C (n=14)
surface

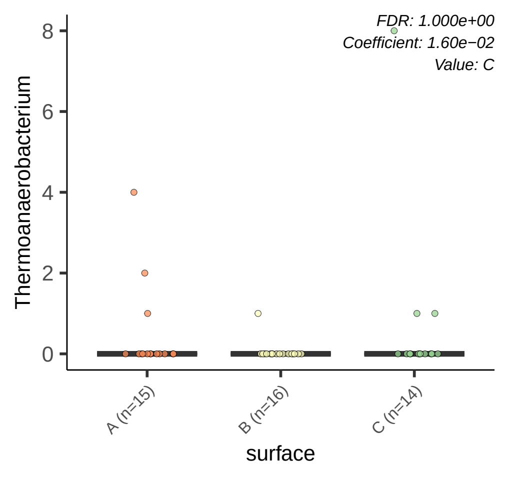
FDR: 1.000e+00
Coefficient: 1.60e−02
Value: C
Thermoanaerobacterium
0
2
4
6
8
A (n=15)
B (n=16)
C (n=14)
surface

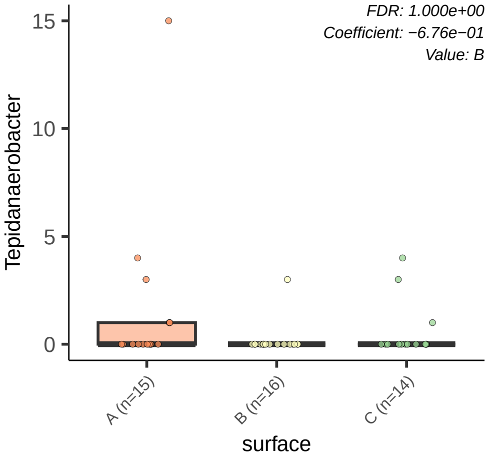
FDR: 1.000e+00
Coefficient: −6.76e−01
Value: B
Tepidanaerobacter
15
10
5
0
A (n=15)
B (n=16)
C (n=14)
surface

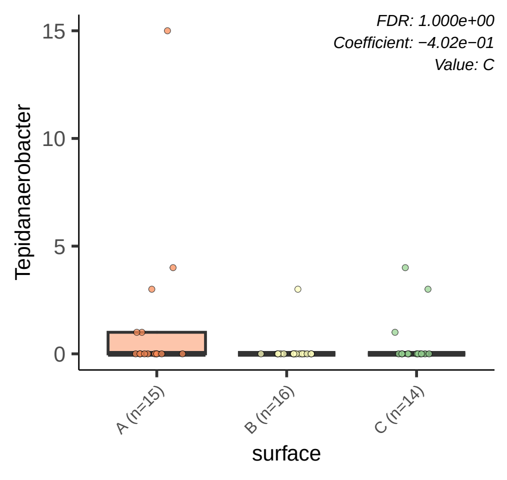

FDR: 1.000e+00
Coefficient: −4.02e−01
Value: C
15
10
5
0
Tepidanaerobacter
A (n=15)
B (n=16)
C (n=14)
surface

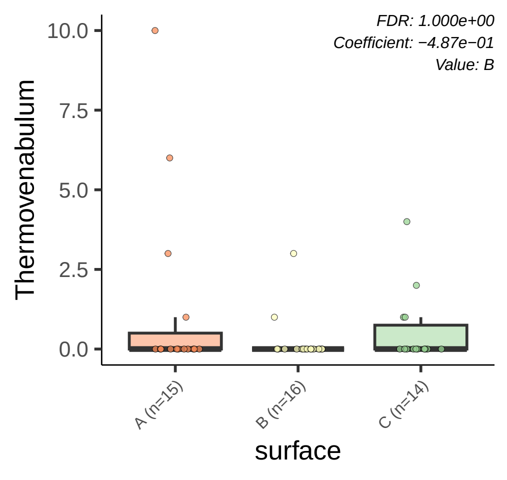
FDR: 1.000e+00
Coefficient: −4.87e−01
Value: B
Thermovenabulum
10.0
7.5
5.0
2.5
0.0
A (n=15)
B (n=16)
C (n=14)
surface

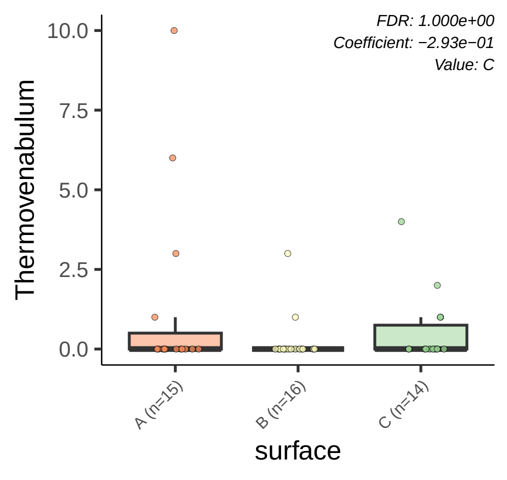

FDR: 1.000e+00
Coefficient: −2.93e−01
Value: C
Thermovenabulum
10.0
7.5
5.0
2.5
0.0
A (n=15)
B (n=16)
C (n=14)
surface

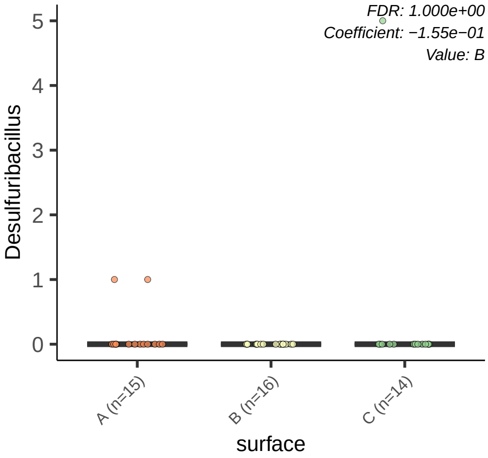

FDR: 1.000e+00
Coefficient: −1.55e−01
Value: B
Desulfuribacillus
5
4
3
2
1
0
A (n=15)
B (n=16)
C (n=14)
surface

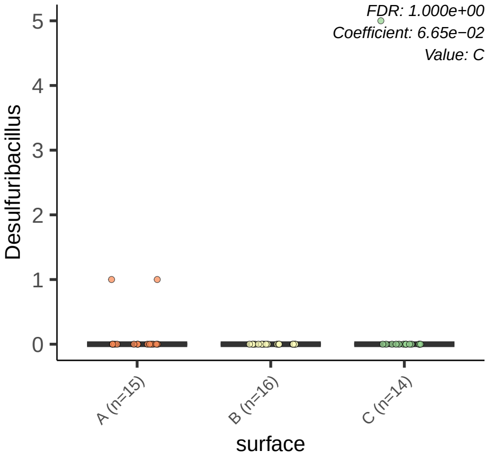

FDR: 1.000e+00
Coefficient: 6.65e−02
Value: C
Desulfuribacillus
0
1
2
3
4
5
A (n=15)
B (n=16)
C (n=14)
surface

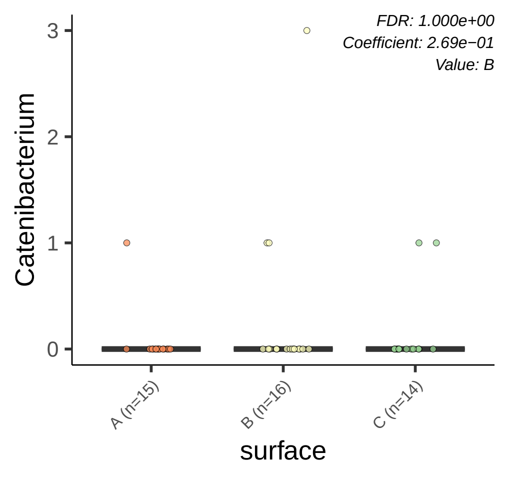
FDR: 1.000e+00
Coefficient: 2.69e−01
Value: B
Catenibacterium
3
2
1
0
A (n=15)
B (n=16)
C (n=14)
surface

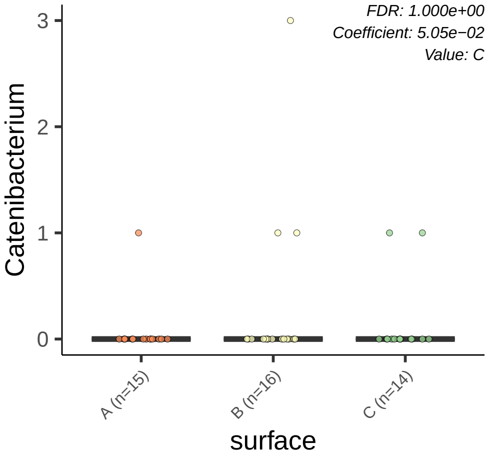
FDR: 1.000e+00
Coefficient: 5.05e−02
Value: C
Catenibacterium
0
1
2
3
A (n=15)
B (n=16)
C (n=14)
surface

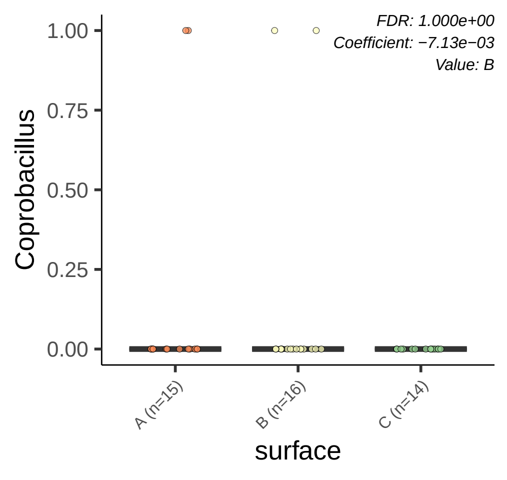

FDR: 1.000e+00
Coefficient: −7.13e−03
Value: B
Coprobacillus
1.00
0.75
0.50
0.25
0.00
A (n=15)
B (n=16)
C (n=14)
surface

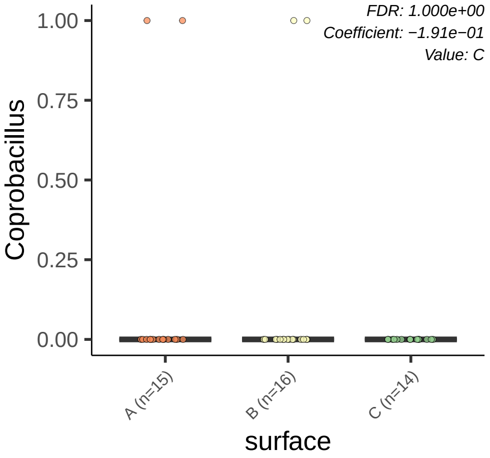
FDR: 1.000e+00
Coefficient: −1.91e−01
Value: C
Coprobacillus
1.00
0.75
0.50
0.25
0.00
A (n=15)
B (n=16)
C (n=14)
surface

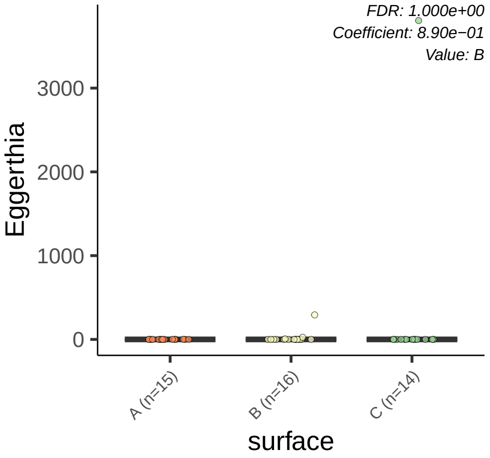
FDR: 1.000e+00
Coefficient: 8.90e−01
Value: B
Eggerthia
3000
2000
1000
0
A (n=15)
B (n=16)
C (n=14)
surface

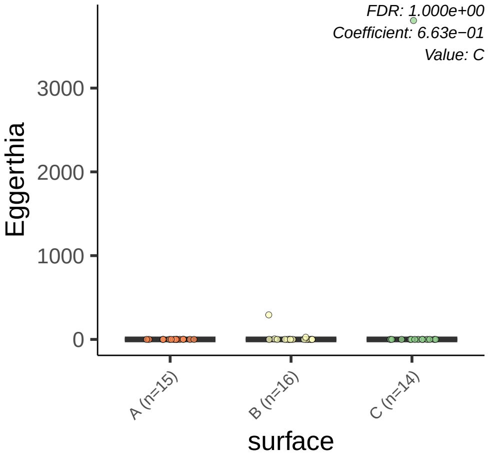
FDR: 1.000e+00
Coefficient: 6.63e−01
Value: C
Eggerthia
3000
2000
1000
0
A (n=15)
B (n=16)
C (n=14)
surface

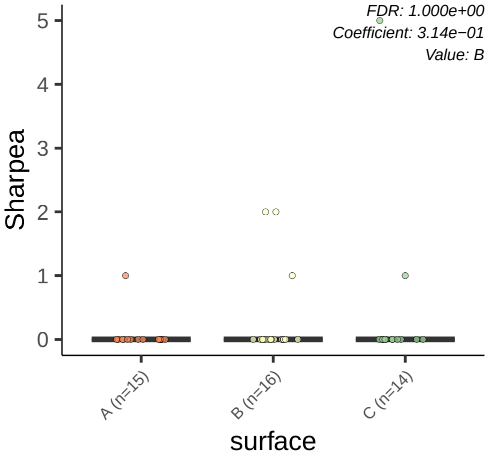
FDR: 1.000e+00
Coefficient: 3.14e−01
Value: B
Sharpea
5
4
3
2
1
0
A (n=15)
B (n=16)
C (n=14)
surface

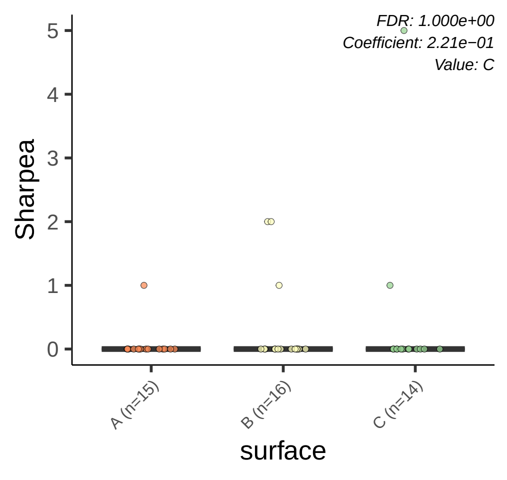

FDR: 1.000e+00
Coefficient: 2.21e−01
Value: C
Sharpea
5
4
3
2
1
0
A (n=15)
B (n=16)
C (n=14)
surface

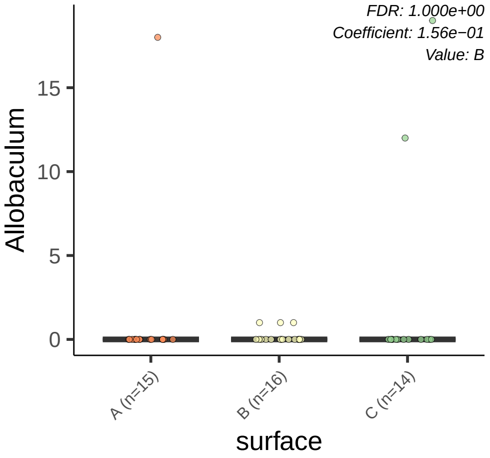
FDR: 1.000e+00
Coefficient: 1.56e−01
Value: B
Allobaculum
15
10
5
0
A (n=15)
B (n=16)
C (n=14)
surface

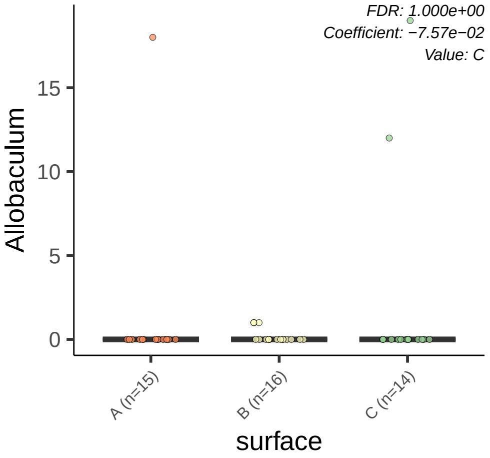

FDR: 1.000e+00
Coefficient: −7.57e−02
Value: C
Allobaculum
15
10
5
0
A (n=15)
B (n=16)
C (n=14)
surface

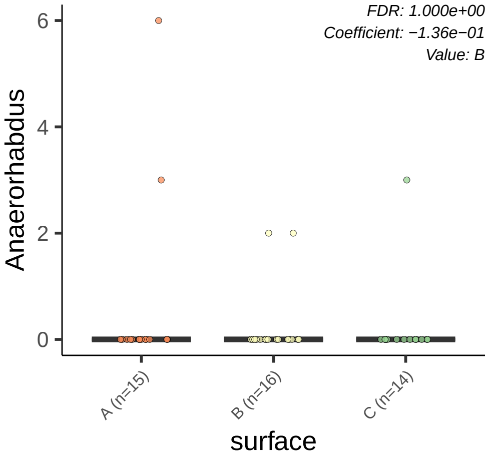
FDR: 1.000e+00
Coefficient: −1.36e−01
Value: B
Anaerorhabdus
6
4
2
0
A (n=15)
B (n=16)
C (n=14)
surface

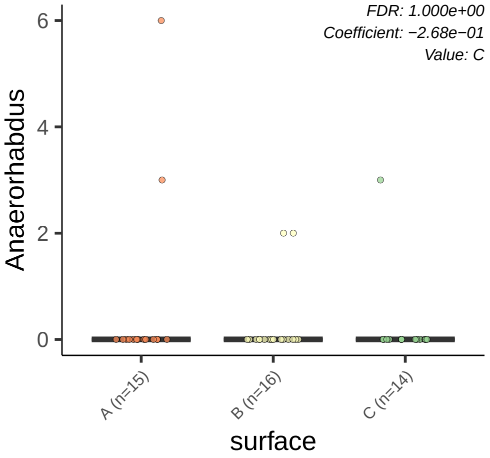

FDR: 1.000e+00
Coefficient: −2.68e−01
Value: C
Anaerorhabdus
6
4
2
0
A (n=15)
B (n=16)
C (n=14)
surface

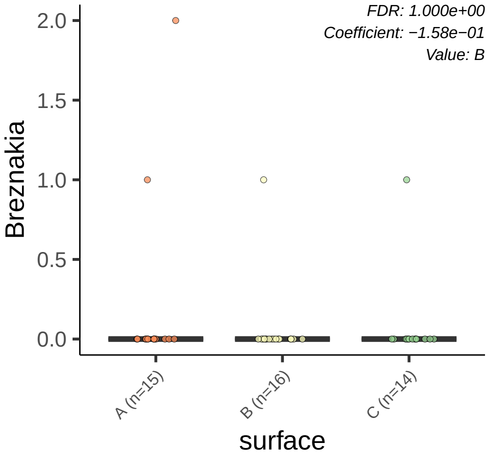
FDR: 1.000e+00
Coefficient: −1.58e−01
Value: B
Breznakia
2.0
1.5
1.0
0.5
0.0
A (n=15)
B (n=16)
C (n=14)
surface

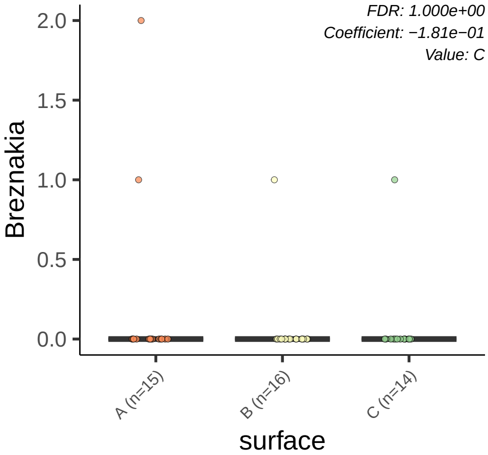
FDR: 1.000e+00
Coefficient: −1.81e−01
Value: C
Breznakia
2.0
1.5
1.0
0.5
0.0
A (n=15)
B (n=16)
C (n=14)
surface

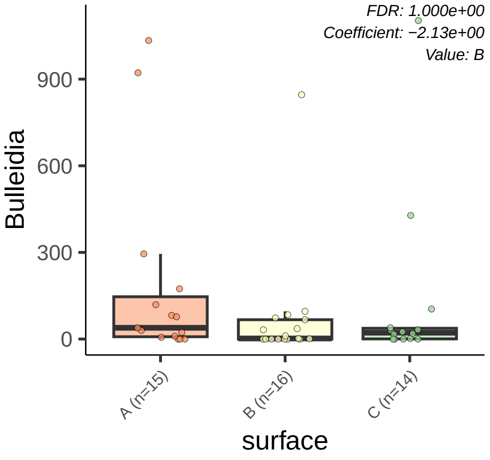

FDR: 1.000e+00
Coefficient: −2.13e+00
Value: B
Bulleidia
900
600
300
0
A (n=15)
B (n=16)
C (n=14)
surface

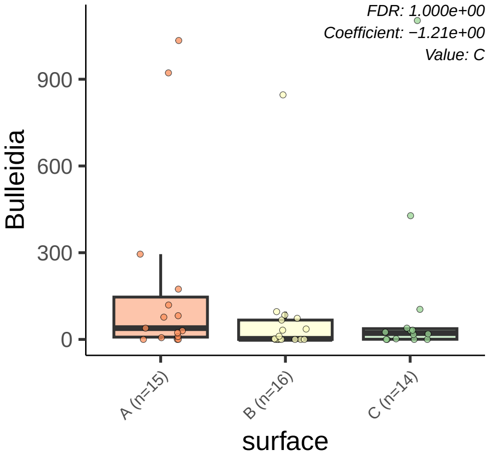

FDR: 1.000e+00
Coefficient: −1.21e+00
Value: C
Bulleidia
900
600
300
0
A (n=15)
B (n=16)
C (n=14)
surface

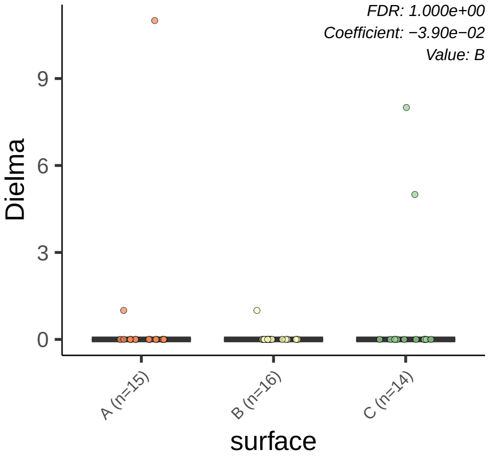

FDR: 1.000e+00
Coefficient: −3.90e−02
Value: B
Dielma
0
3
6
9
A (n=15)
B (n=16)
C (n=14)
surface

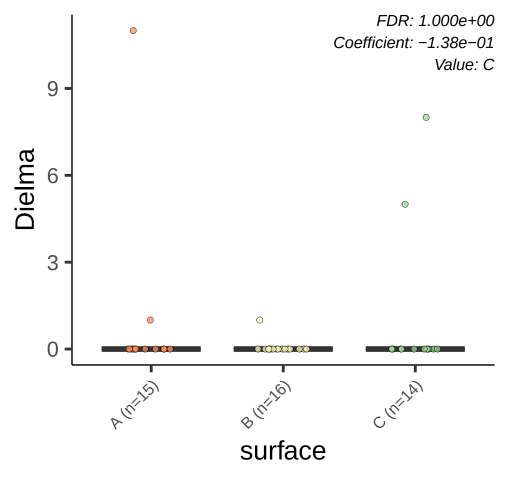
FDR: 1.000e+00
Coefficient: −1.38e−01
Value: C
Dielma
9
6
3
0
A (n=15)
B (n=16)
C (n=14)
surface

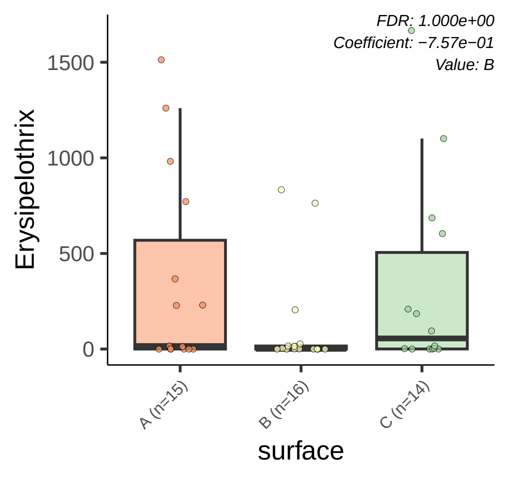

FDR: 1.000e+00
Coefficient: −7.57e−01
Value: B
Erysipelothrix
1500
1000
500
0
A (n=15)
B (n=16)
C (n=14)
surface

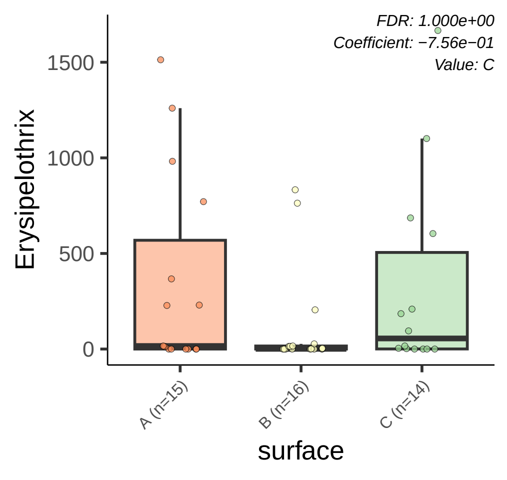

FDR: 1.000e+00
Coefficient: −7.56e−01
Value: C
Erysipelothrix
1500
1000
500
0
A (n=15)
B (n=16)
C (n=14)
surface

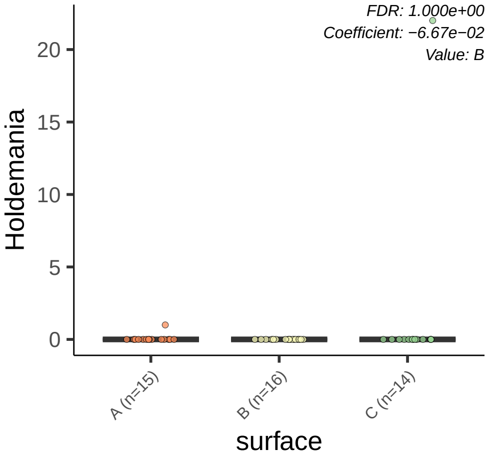
FDR: 1.000e+00
Coefficient: −6.67e−02
Value: B
Holdemania
0
5
10
15
20
A (n=15)
B (n=16)
C (n=14)
surface

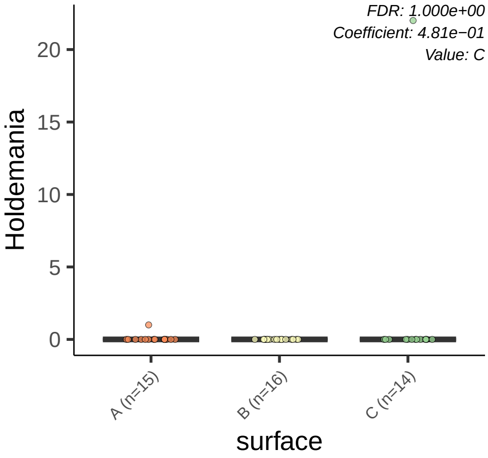
FDR: 1.000e+00
Coefficient: 4.81e−01
Value: C
Holdemania
20
15
10
5
0
A (n=15)
B (n=16)
C (n=14)
surface

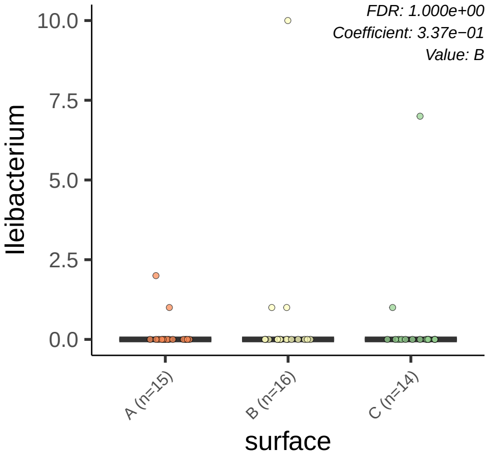

FDR: 1.000e+00
Coefficient: 3.37e−01
Value: B
Ileibacterium
10.0
7.5
5.0
2.5
0.0
A (n=15)
B (n=16)
C (n=14)
surface

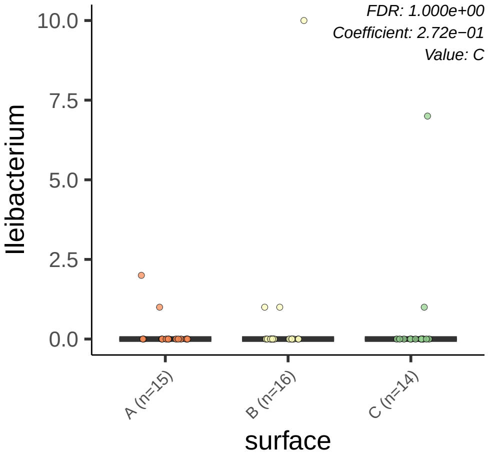
FDR: 1.000e+00
Coefficient: 2.72e−01
Value: C
Ileibacterium
10.0
7.5
5.0
2.5
0.0
A (n=15)
B (n=16)
C (n=14)
surface

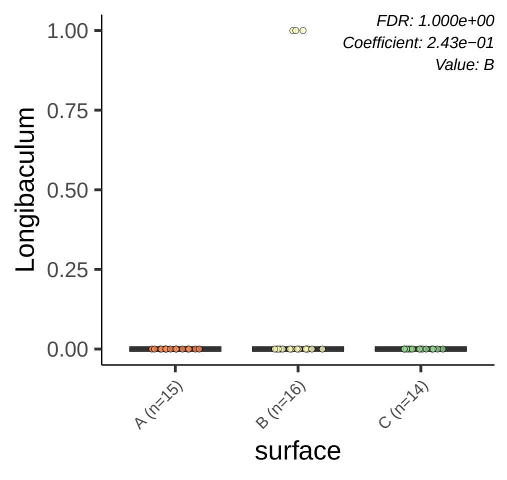
FDR: 1.000e+00
Coefficient: 2.43e−01
Value: B
Longibaculum
1.00
0.75
0.50
0.25
0.00
A (n=15)
B (n=16)
C (n=14)
surface

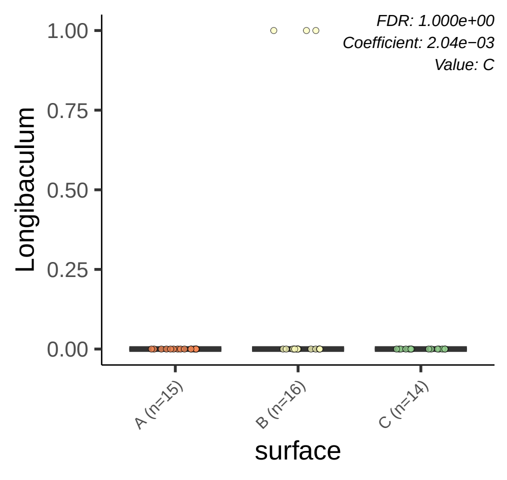
FDR: 1.000e+00
Coefficient: 2.04e−03
Value: C
Longibaculum
1.00
0.75
0.50
0.25
0.00
A (n=15)
B (n=16)
C (n=14)
surface

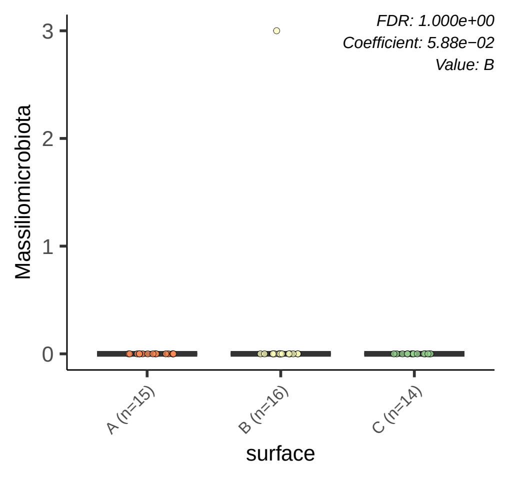
FDR: 1.000e+00
Coefficient: 5.88e−02
Value: B
Massiliomicrobiota
3
2
1
0
A (n=15)
B (n=16)
C (n=14)
surface

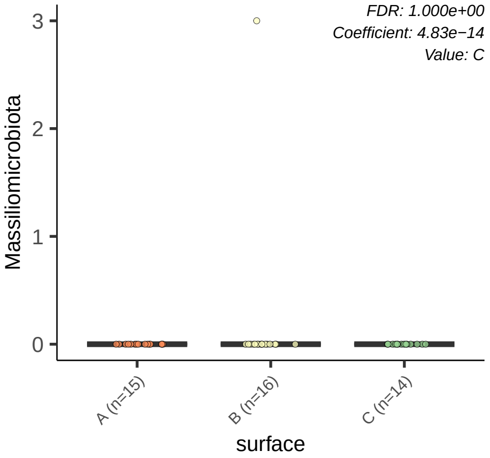

FDR: 1.000e+00
Coefficient: 4.83e−14
Value: C
3
2
1
0
Massiliomicrobiota
A (n=15)
B (n=16)
C (n=14)
surface

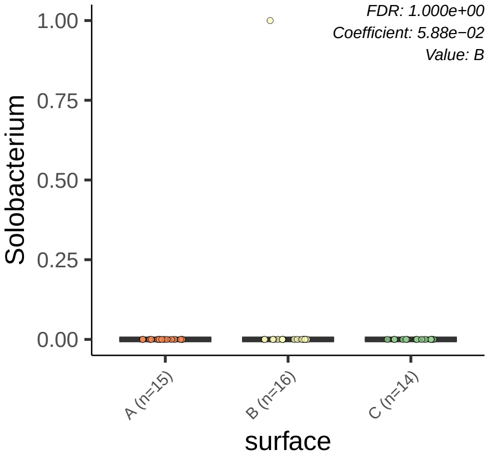

FDR: 1.000e+00
Coefficient: 5.88e−02
Value: B
Solobacterium
1.00
0.75
0.50
0.25
0.00
A (n=15)
B (n=16)
C (n=14)
surface

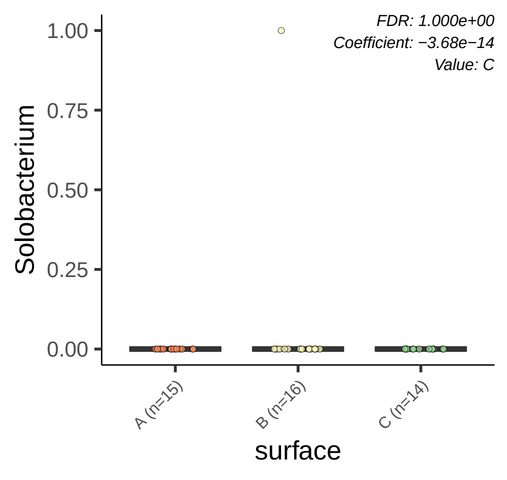
FDR: 1.000e+00
Coefficient: −3.68e−14
Value: C
Solobacterium
1.00
0.75
0.50
0.25
0.00
A (n=15)
B (n=16)
C (n=14)
surface

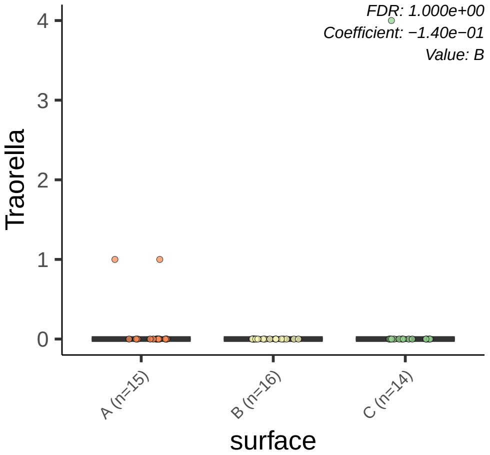
FDR: 1.000e+00
Coefficient: −1.40e−01
Value: B
Traorella
0
1
2
3
4
A (n=15)
B (n=16)
C (n=14)
surface

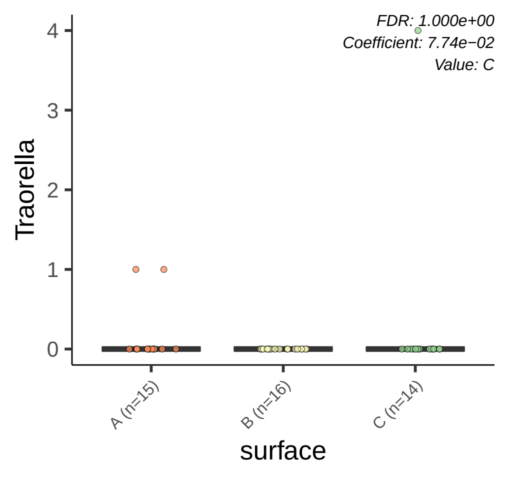

FDR: 1.000e+00
Coefficient: 7.74e−02
Value: C
Traorella
4
3
2
1
0
A (n=15)
B (n=16)
C (n=14)
surface

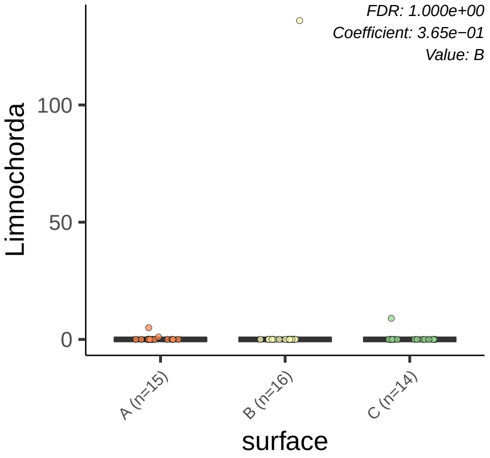
FDR: 1.000e+00
Coefficient: 3.65e−01
Value: B
Limnochorda
100
50
0
A (n=15)
B (n=16)
C (n=14)
surface

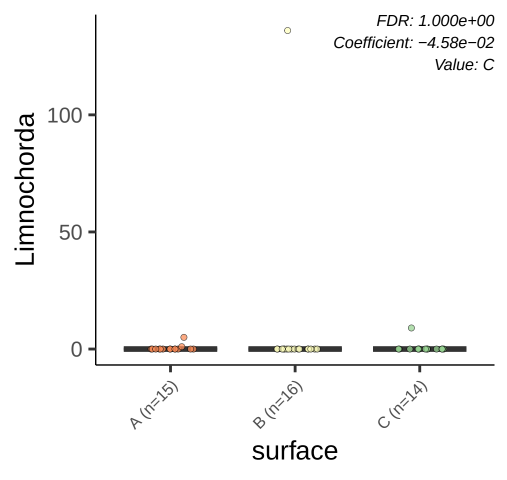
FDR: 1.000e+00
Coefficient: −4.58e−02
Value: C
Limnochorda
100
50
0
A (n=15)
B (n=16)
C (n=14)
surface

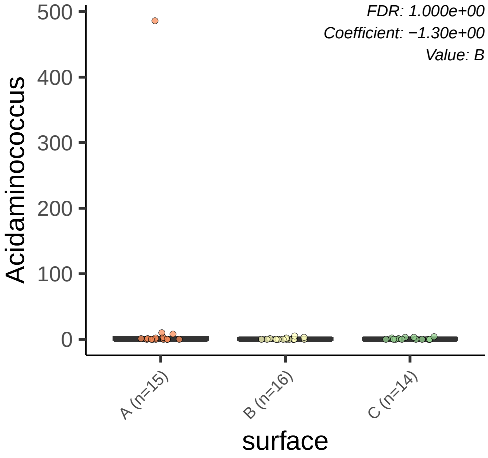

FDR: 1.000e+00
Coefficient: −1.30e+00
Value: B
500
400
300
200
100
0
Acidaminococcus
A (n=15)
B (n=16)
C (n=14)
surface

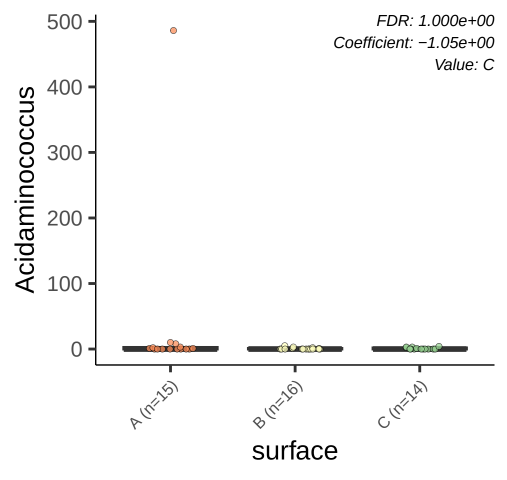

FDR: 1.000e+00
Coefficient: −1.05e+00
Value: C
Acidaminococcus
500
400
300
200
100
0
A (n=15)
B (n=16)
C (n=14)
surface

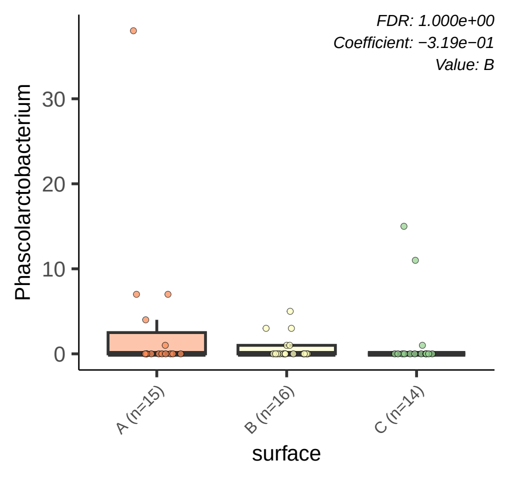

FDR: 1.000e+00
Coefficient: −3.19e−01
Value: B
Phascolarctobacterium
30
20
10
0
A (n=15)
B (n=16)
C (n=14)
surface

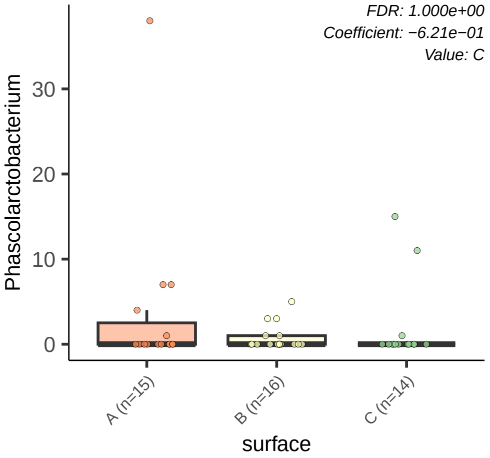
FDR: 1.000e+00
Coefficient: −6.21e−01
Value: C
Phascolarctobacterium
0
10
20
30
A (n=15)
B (n=16)
C (n=14)
surface

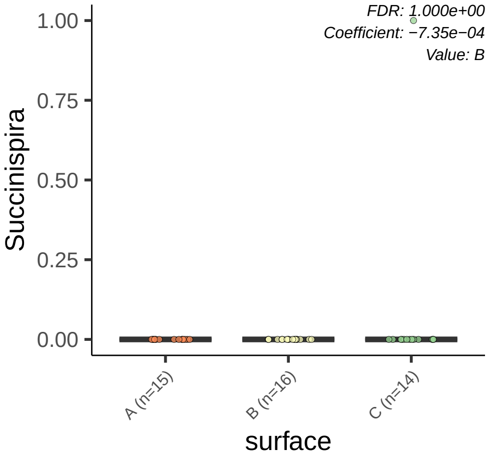
FDR: 1.000e+00
Coefficient: −7.35e−04
Value: B
Succinispira
1.00
0.75
0.50
0.25
0.00
A (n=15)
B (n=16)
C (n=14)
surface

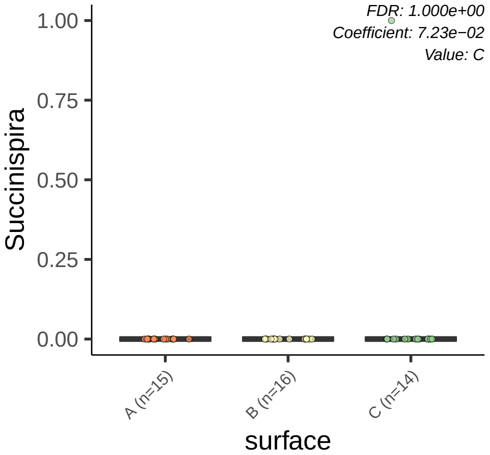
FDR: 1.000e+00
Coefficient: 7.23e−02
Value: C
Succinispira
1.00
0.75
0.50
0.25
0.00
A (n=15)
B (n=16)
C (n=14)
surface

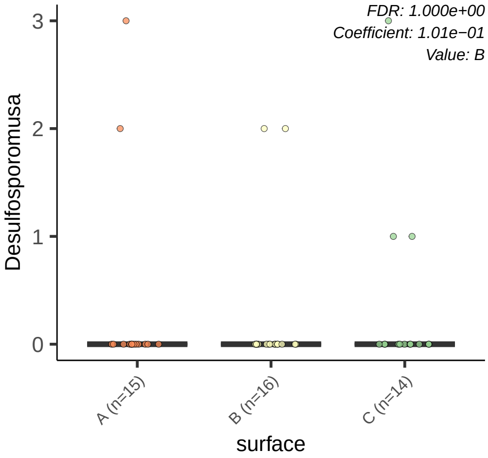
FDR: 1.000e+00
Coefficient: 1.01e−01
Value: B
Desulfosporomusa
3
2
1
0
A (n=15)
B (n=16)
C (n=14)
surface

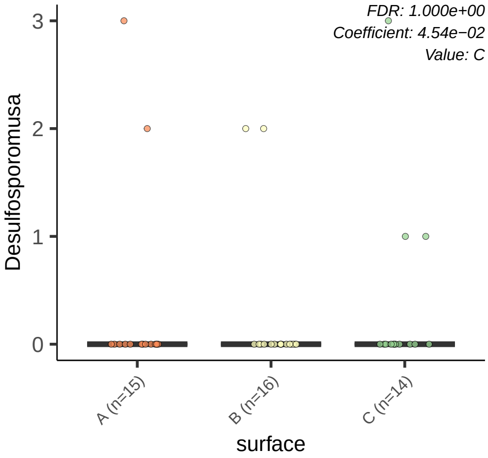

FDR: 1.000e+00
Coefficient: 4.54e−02
Value: C
Desulfosporomusa
3
2
1
0
A (n=15)
B (n=16)
C (n=14)
surface

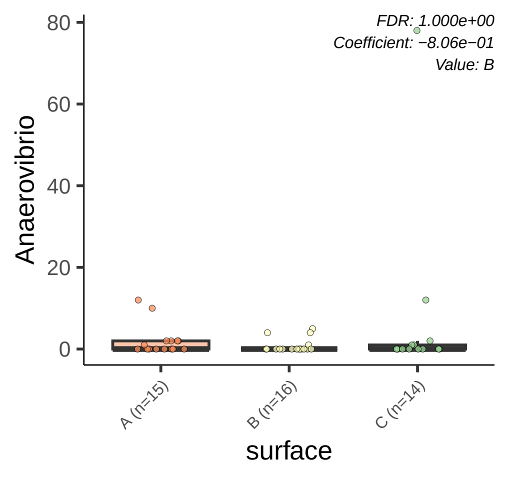
FDR: 1.000e+00
Coefficient: −8.06e−01
Value: B
Anaerovibrio
80
60
40
20
0
A (n=15)
B (n=16)
C (n=14)
surface

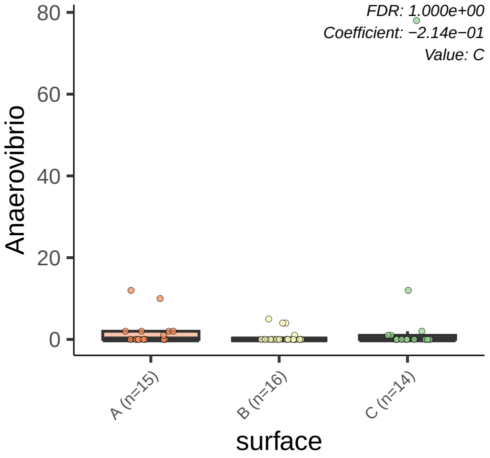

FDR: 1.000e+00
Coefficient: −2.14e−01
Value: C
Anaerovibrio
80
60
40
20
0
A (n=15)
B (n=16)
C (n=14)
surface

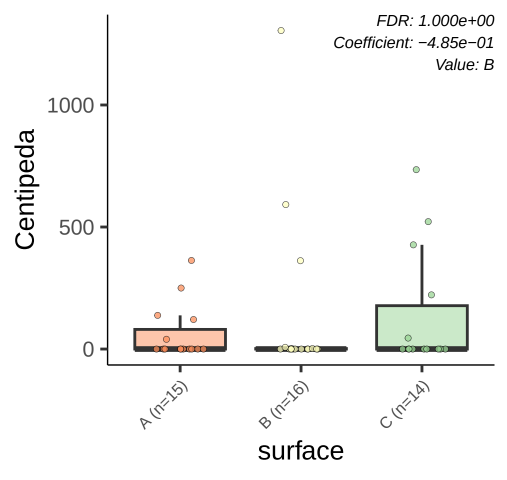

FDR: 1.000e+00
Coefficient: −4.85e−01
Value: B
Centipeda
1000
500
0
A (n=15)
B (n=16)
C (n=14)
surface

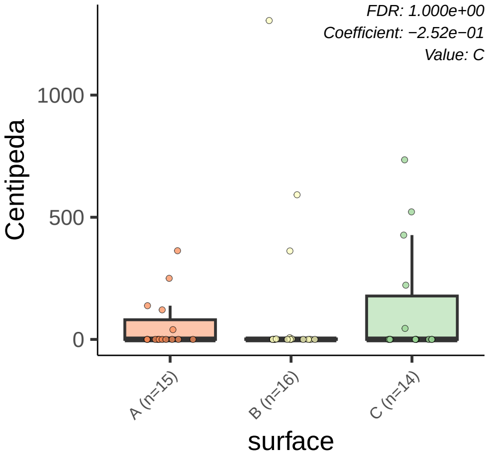
FDR: 1.000e+00
Coefficient: −2.52e−01
Value: C
1000
500
0
Centipeda
A (n=15)
B (n=16)
C (n=14)
surface

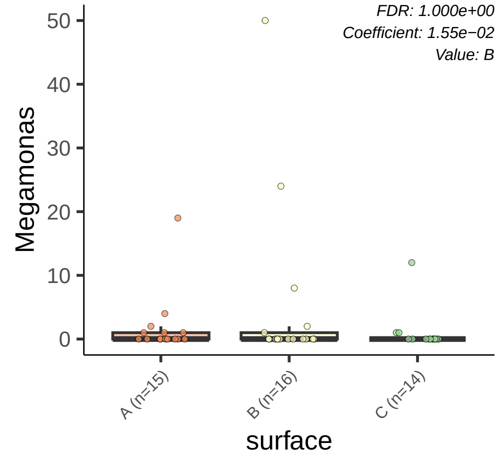
FDR: 1.000e+00
Coefficient: 1.55e−02
Value: B
Megamonas
50
40
30
20
10
0
A (n=15)
B (n=16)
C (n=14)
surface

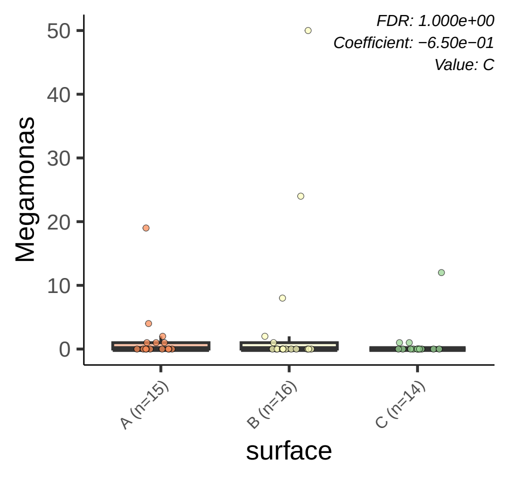
FDR: 1.000e+00
Coefficient: −6.50e−01
Value: C
Megamonas
0
10
20
30
40
50
A (n=15)
B (n=16)
C (n=14)
surface

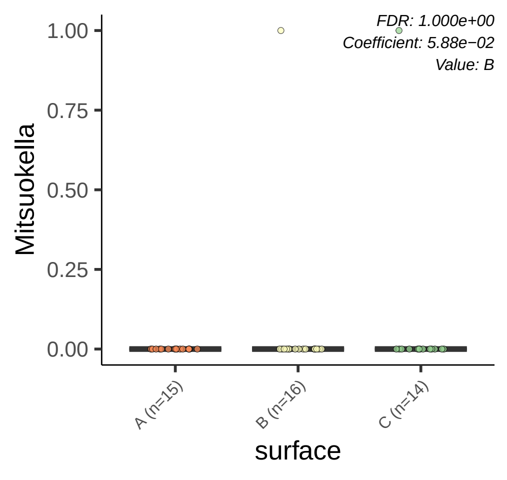
FDR: 1.000e+00
Coefficient: 5.88e−02
Value: B
Mitsuokella
1.00
0.75
0.50
0.25
0.00
A (n=15)
B (n=16)
C (n=14)
surface

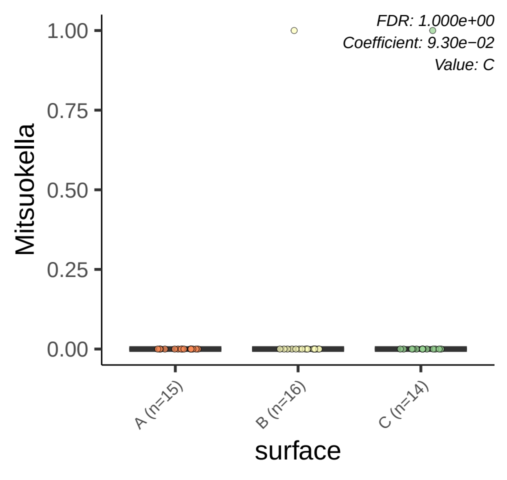
FDR: 1.000e+00
Coefficient: 9.30e−02
Value: C
Mitsuokella
1.00
0.75
0.50
0.25
0.00
A (n=15)
B (n=16)
C (n=14)
surface

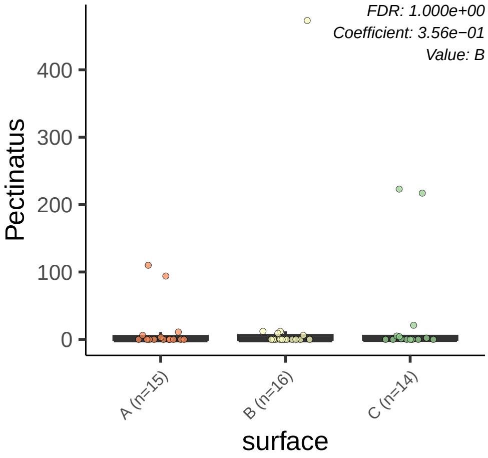
FDR: 1.000e+00
Coefficient: 3.56e−01
Value: B
Pectinatus
400
300
200
100
0
A (n=15)
B (n=16)
C (n=14)
surface

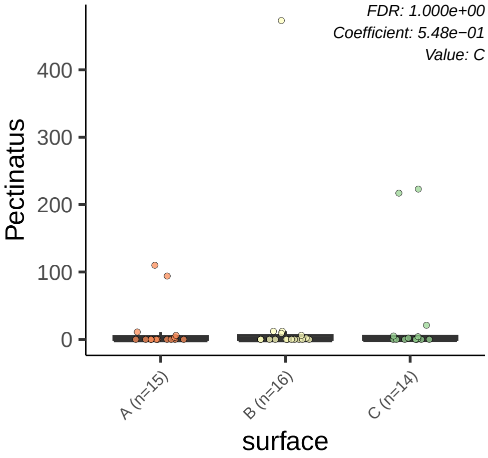
FDR: 1.000e+00
Coefficient: 5.48e−01
Value: C
Pectinatus
400
300
200
100
0
A (n=15)
B (n=16)
C (n=14)
surface

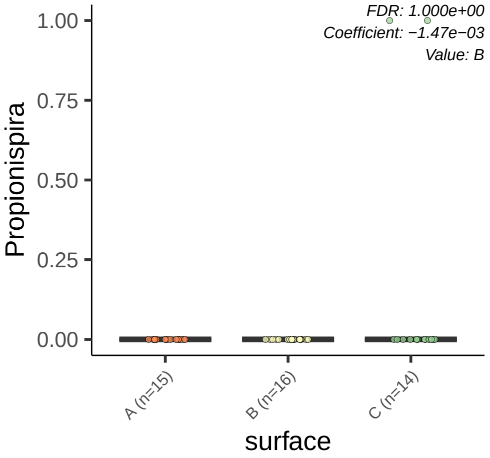

FDR: 1.000e+00
Coefficient: −1.47e−03
Value: B
Propionispira
1.00
0.75
0.50
0.25
0.00
A (n=15)
B (n=16)
C (n=14)
surface

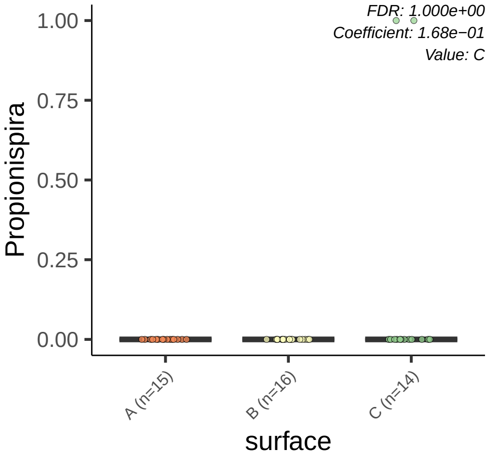

FDR: 1.000e+00
Coefficient: 1.68e−01
Value: C
Propionispira
1.00
0.75
0.50
0.25
0.00
A (n=15)
B (n=16)
C (n=14)
surface

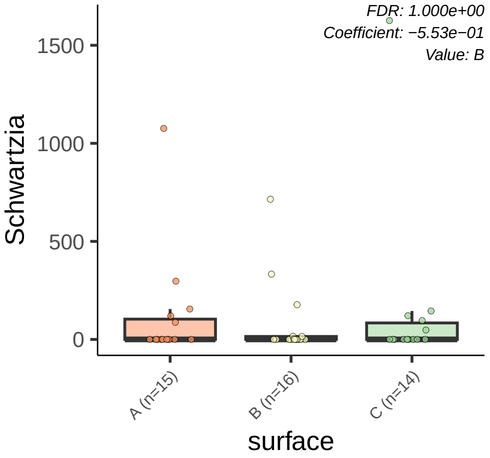
FDR: 1.000e+00
Coefficient: −5.53e−01
Value: B
Schwartzia
1500
1000
500
0
A (n=15)
B (n=16)
C (n=14)
surface

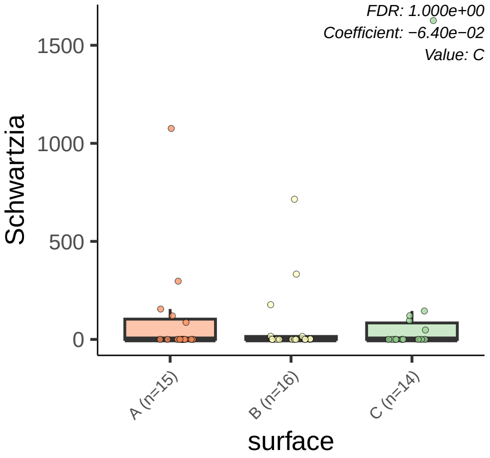
FDR: 1.000e+00
Coefficient: −6.40e−02
Value: C
Schwartzia
1500
1000
500
0
A (n=15)
B (n=16)
C (n=14)
surface

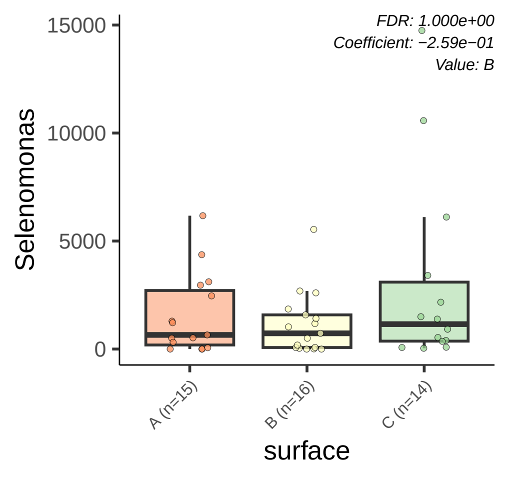
FDR: 1.000e+00
Coefficient: −2.59e−01
Value: B
Selenomonas
15000
10000
5000
0
A (n=15)
B (n=16)
C (n=14)
surface

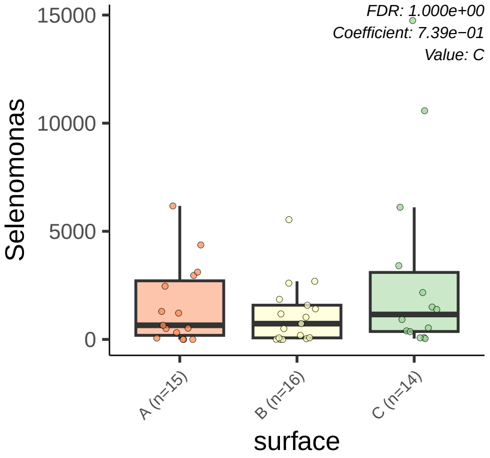

FDR: 1.000e+00
Coefficient: 7.39e−01
Value: C
Selenomonas
15000
10000
5000
0
A (n=15)
B (n=16)
C (n=14)
surface

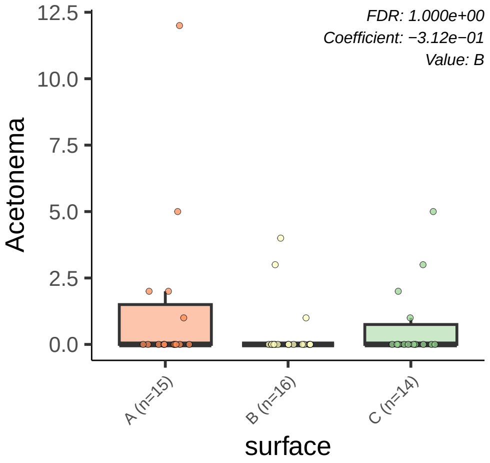

FDR: 1.000e+00
Coefficient: −3.12e−01
Value: B
Acetonema
12.5
10.0
7.5
5.0
2.5
0.0
A (n=15)
B (n=16)
C (n=14)
surface

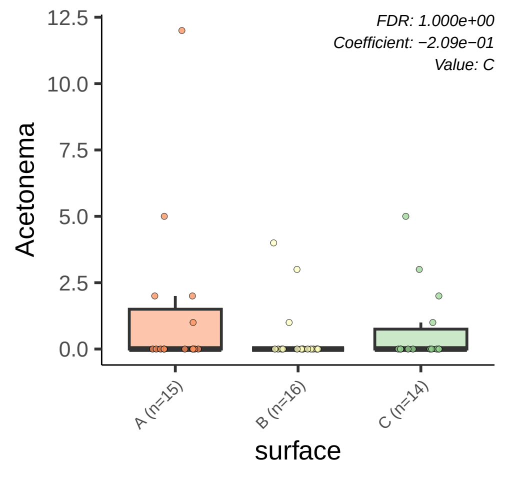
FDR: 1.000e+00
Coefficient: −2.09e−01
Value: C
Acetonema
12.5
10.0
7.5
5.0
2.5
0.0
A (n=15)
B (n=16)
C (n=14)
surface

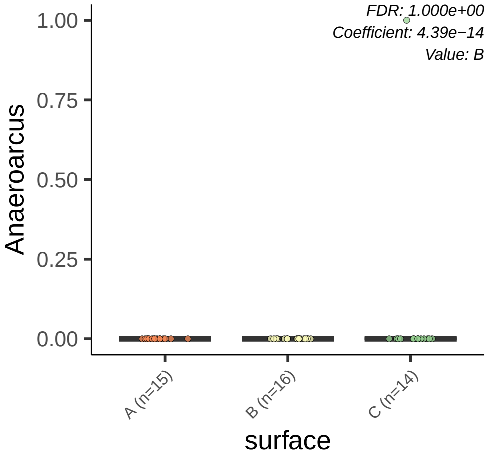
FDR: 1.000e+00
Coefficient: 4.39e−14
Value: B
Anaeroarcus
1.00
0.75
0.50
0.25
0.00
A (n=15)
B (n=16)
C (n=14)
surface

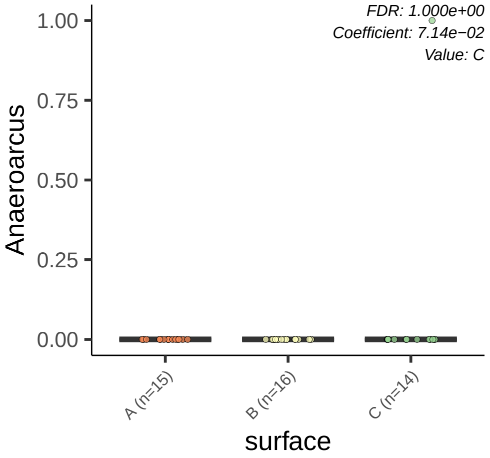
FDR: 1.000e+00
Coefficient: 7.14e−02
Value: C
1.00
0.75
0.50
0.25
0.00
Anaeroarcus
A (n=15)
B (n=16)
C (n=14)
surface

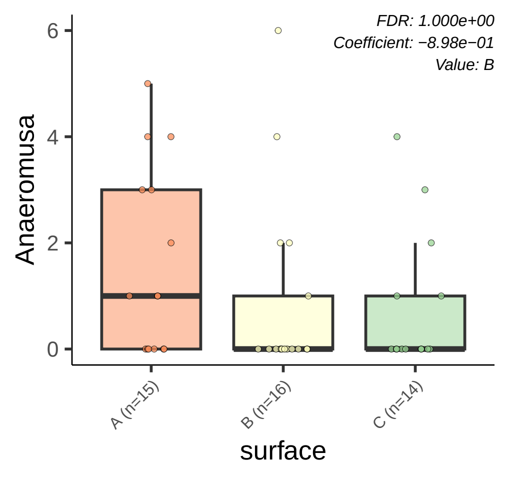

FDR: 1.000e+00
Coefficient: −8.98e−01
Value: B
Anaeromusa
6
4
2
0
A (n=15)
B (n=16)
C (n=14)
surface

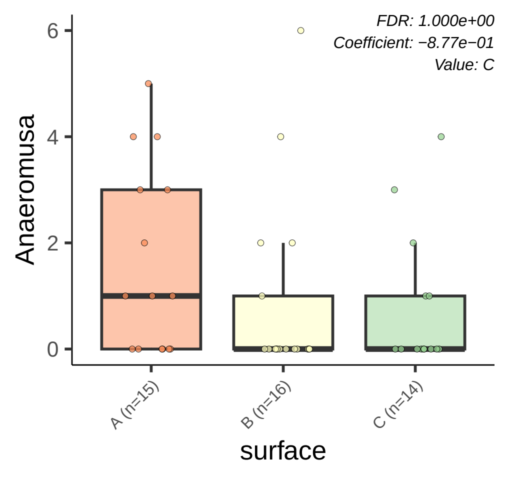
FDR: 1.000e+00
Coefficient: −8.77e−01
Value: C
Anaeromusa
0
2
4
6
A (n=15)
B (n=16)
C (n=14)
surface

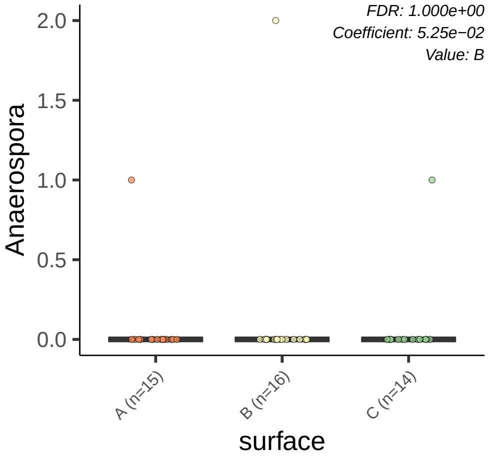

FDR: 1.000e+00
Coefficient: 5.25e−02
Value: B
Anaerospora
2.0
1.5
1.0
0.5
0.0
A (n=15)
B (n=16)
C (n=14)
surface

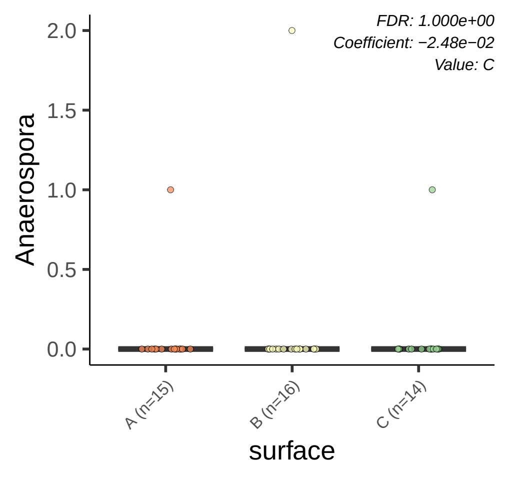
FDR: 1.000e+00
Coefficient: −2.48e−02
Value: C
Anaerospora
2.0
1.5
1.0
0.5
0.0
A (n=15)
B (n=16)
C (n=14)
surface

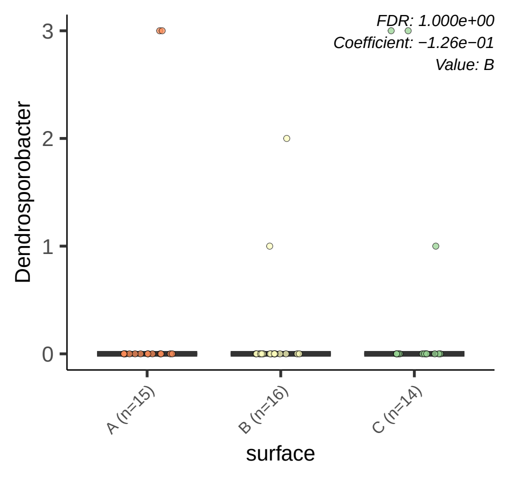

FDR: 1.000e+00
Coefficient: −1.26e−01
Value: B
Dendrosporobacter
3
2
1
0
A (n=15)
B (n=16)
C (n=14)
surface

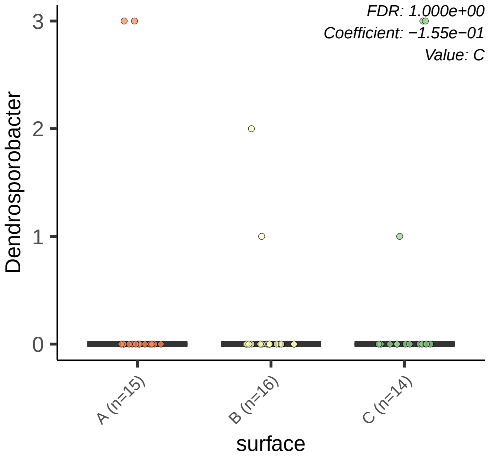
FDR: 1.000e+00
Coefficient: −1.55e−01
Value: C
Dendrosporobacter
3
2
1
0
A (n=15)
B (n=16)
C (n=14)
surface

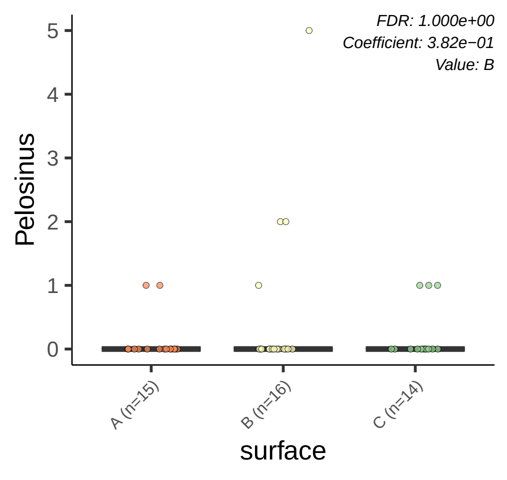
FDR: 1.000e+00
Coefficient: 3.82e−01
Value: B
Pelosinus
5
4
3
2
1
0
A (n=15)
B (n=16)
C (n=14)
surface

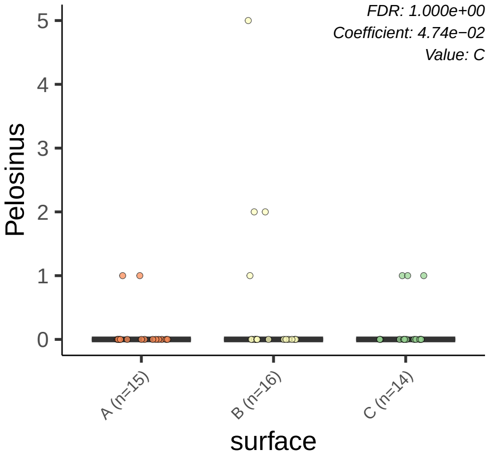

FDR: 1.000e+00
Coefficient: 4.74e−02
Value: C
Pelosinus
5
4
3
2
1
0
A (n=15)
B (n=16)
C (n=14)
surface

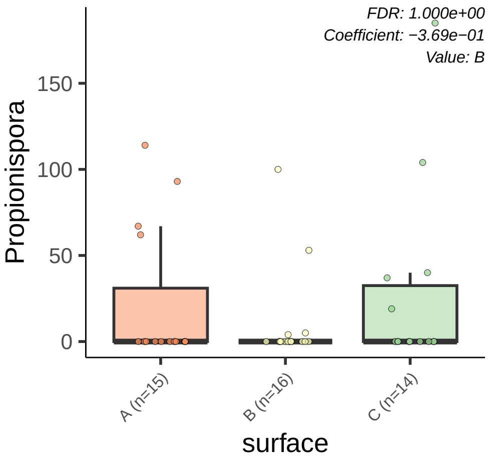

FDR: 1.000e+00
Coefficient: −3.69e−01
Value: B
Propionispora
150
100
50
0
A (n=15)
B (n=16)
C (n=14)
surface

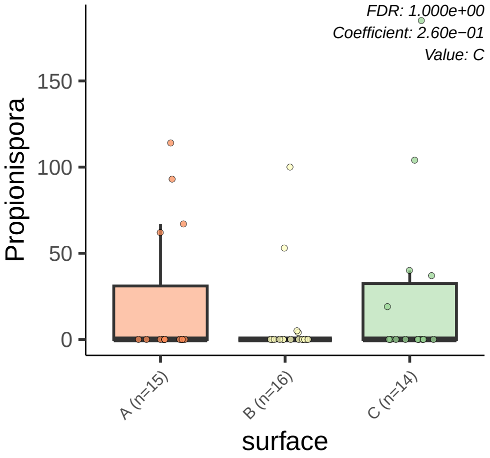
FDR: 1.000e+00
Coefficient: 2.60e−01
Value: C
Propionispora
150
100
50
0
A (n=15)
B (n=16)
C (n=14)
surface

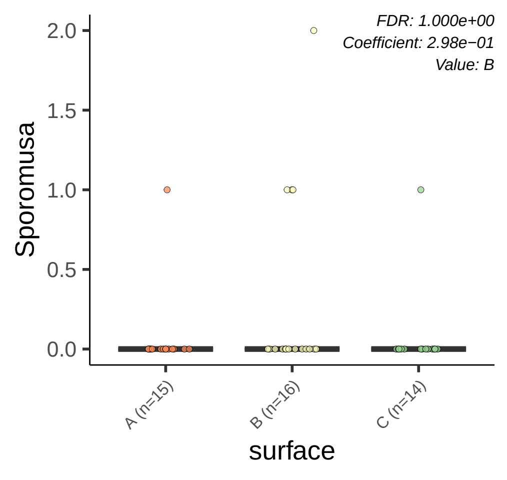
FDR: 1.000e+00
Coefficient: 2.98e−01
Value: B
Sporomusa
2.0
1.5
1.0
0.5
0.0
A (n=15)
B (n=16)
C (n=14)
surface

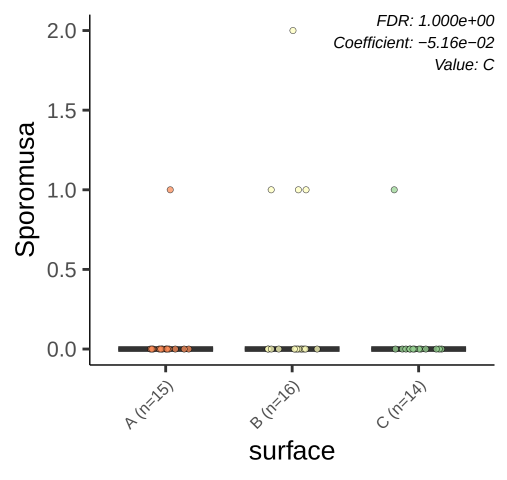
FDR: 1.000e+00
Coefficient: −5.16e−02
Value: C
Sporomusa
2.0
1.5
1.0
0.5
0.0
A (n=15)
B (n=16)
C (n=14)
surface

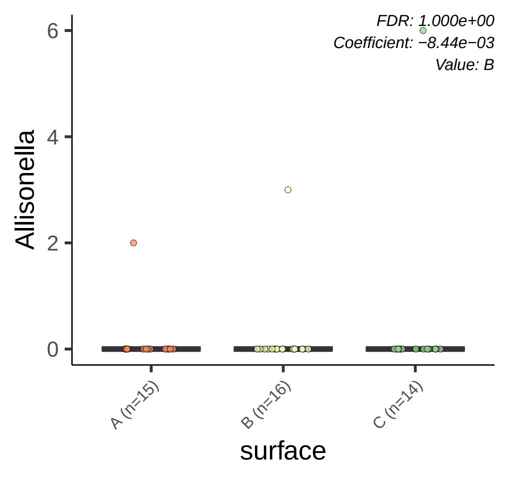

FDR: 1.000e+00
Coefficient: −8.44e−03
Value: B
Allisonella
6
4
2
0
A (n=15)
B (n=16)
C (n=14)
surface

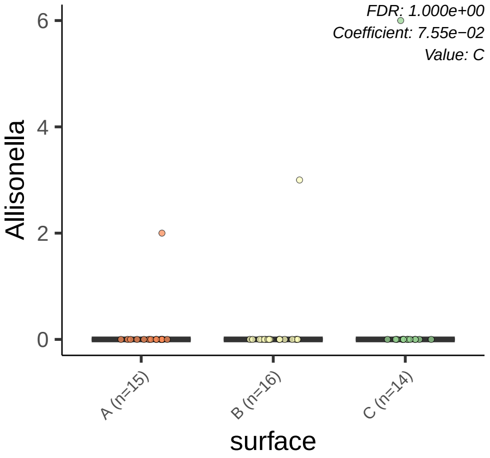

FDR: 1.000e+00
Coefficient: 7.55e−02
Value: C
Allisonella
0
2
4
6
A (n=15)
B (n=16)
C (n=14)
surface

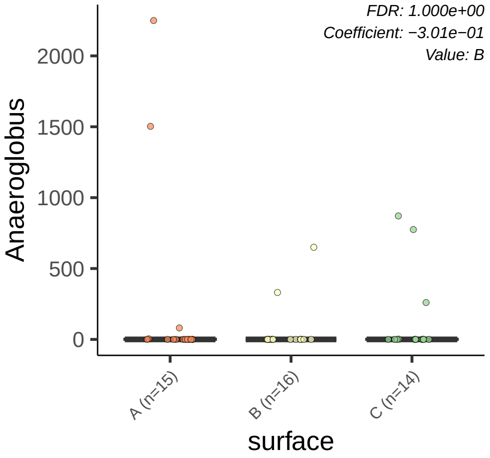
FDR: 1.000e+00
Coefficient: −3.01e−01
Value: B
Anaeroglobus
2000
1500
1000
500
0
A (n=15)
B (n=16)
C (n=14)
surface

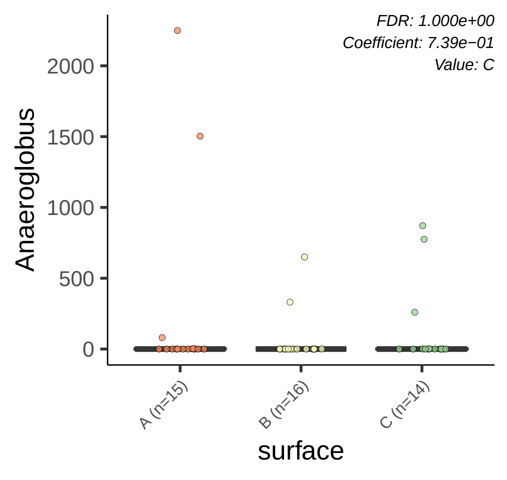
FDR: 1.000e+00
Coefficient: 7.39e−01
Value: C
Anaeroglobus
2000
1500
1000
500
0
A (n=15)
B (n=16)
C (n=14)
surface

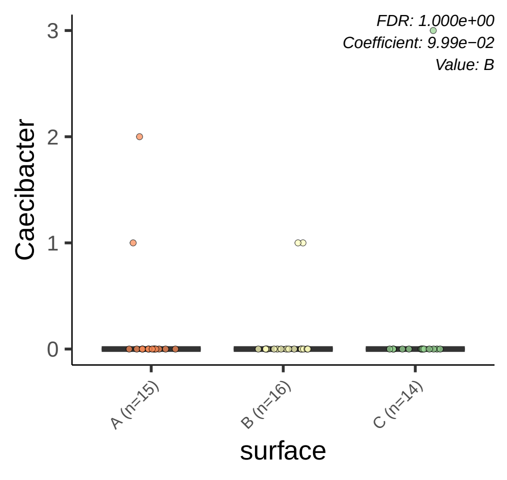

FDR: 1.000e+00
Coefficient: 9.99e−02
Value: B
Caecibacter
0
1
2
3
A (n=15)
B (n=16)
C (n=14)
surface

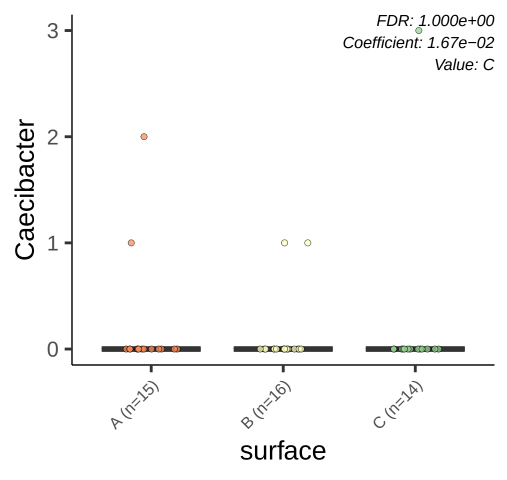
FDR: 1.000e+00
Coefficient: 1.67e−02
Value: C
Caecibacter
0
1
2
3
A (n=15)
B (n=16)
C (n=14)
surface

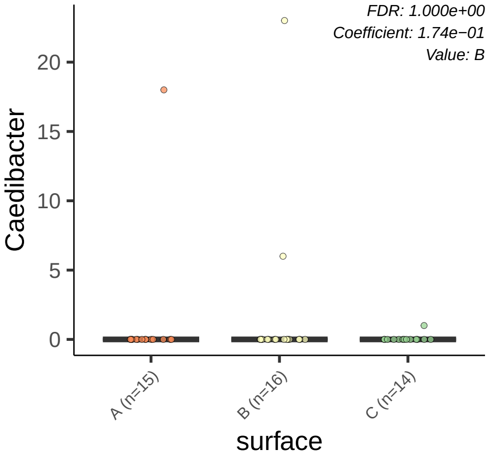

FDR: 1.000e+00
Coefficient: 1.74e−01
Value: B
Caedibacter
20
15
10
5
0
A (n=15)
B (n=16)
C (n=14)
surface

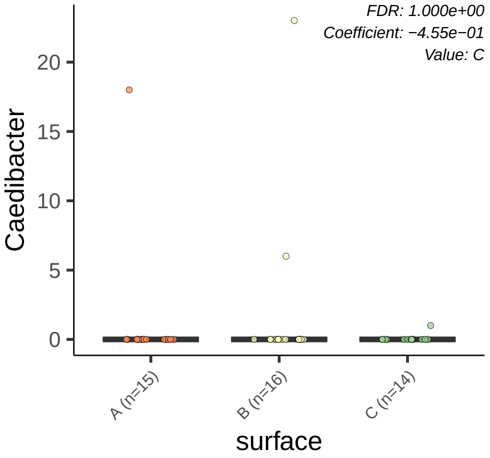
FDR: 1.000e+00
Coefficient: −4.55e−01
Value: C
Caedibacter
0
5
10
15
20
A (n=15)
B (n=16)
C (n=14)
surface

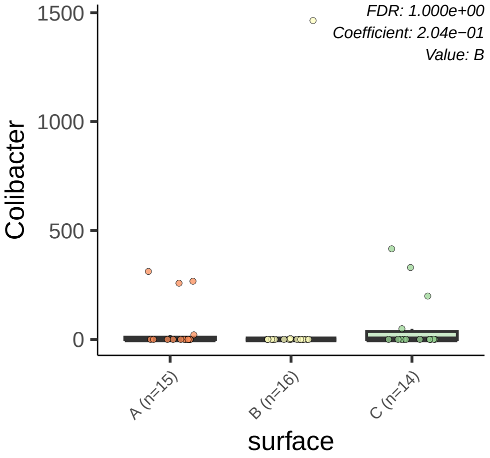

FDR: 1.000e+00
Coefficient: 2.04e−01
Value: B
Colibacter
1500
1000
500
0
A (n=15)
B (n=16)
C (n=14)
surface

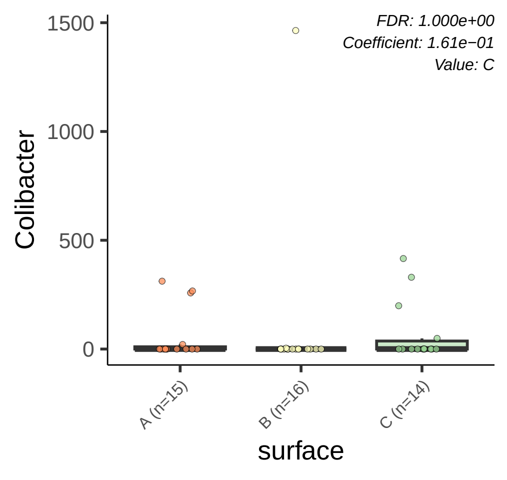

FDR: 1.000e+00
Coefficient: 1.61e−01
Value: C
Colibacter
1500
1000
500
0
A (n=15)
B (n=16)
C (n=14)
surface

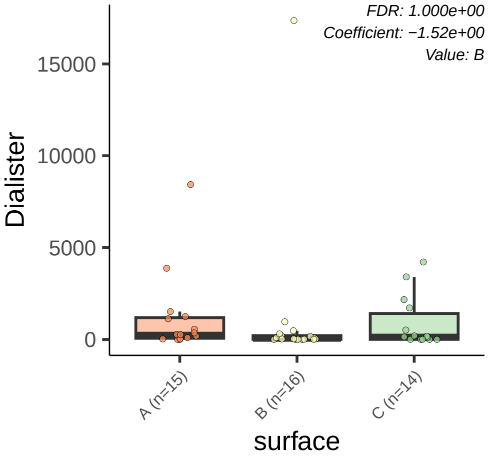

FDR: 1.000e+00
Coefficient: −1.52e+00
Value: B
Dialister
15000
10000
5000
0
A (n=15)
B (n=16)
C (n=14)
surface

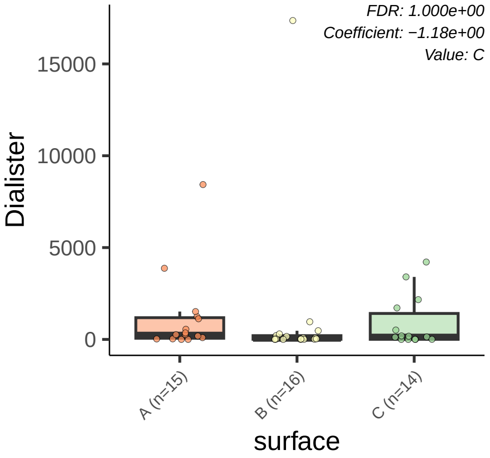
FDR: 1.000e+00
Coefficient: −1.18e+00
Value: C
Dialister
15000
10000
5000
0
A (n=15)
B (n=16)
C (n=14)
surface

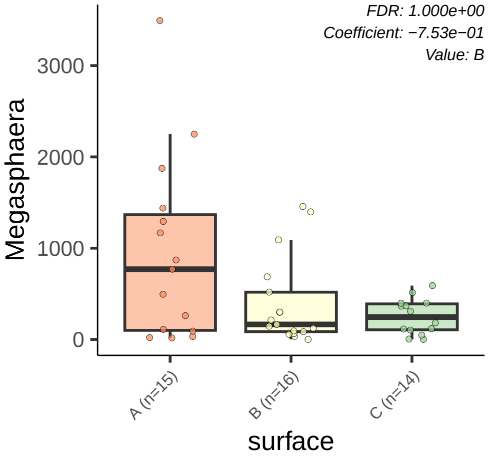

FDR: 1.000e+00
Coefficient: −7.53e−01
Value: B
Megasphaera
3000
2000
1000
0
A (n=15)
B (n=16)
C (n=14)
surface

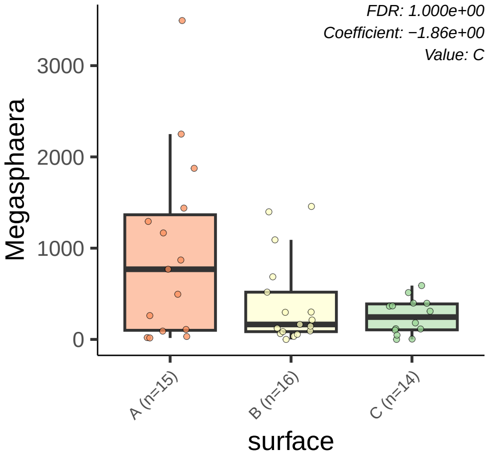

FDR: 1.000e+00
Coefficient: −1.86e+00
Value: C
Megasphaera
3000
2000
1000
0
A (n=15)
B (n=16)
C (n=14)
surface

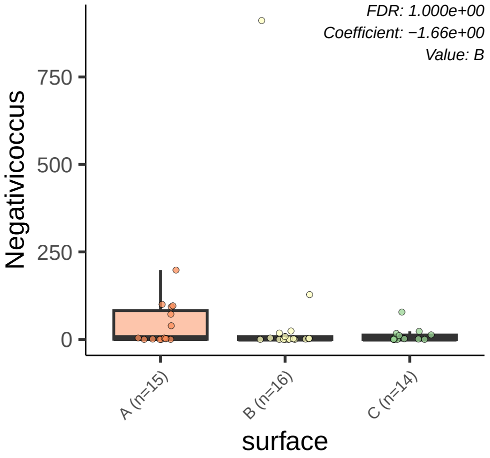

FDR: 1.000e+00
Coefficient: −1.66e+00
Value: B
Negativicoccus
750
500
250
0
A (n=15)
B (n=16)
C (n=14)
surface

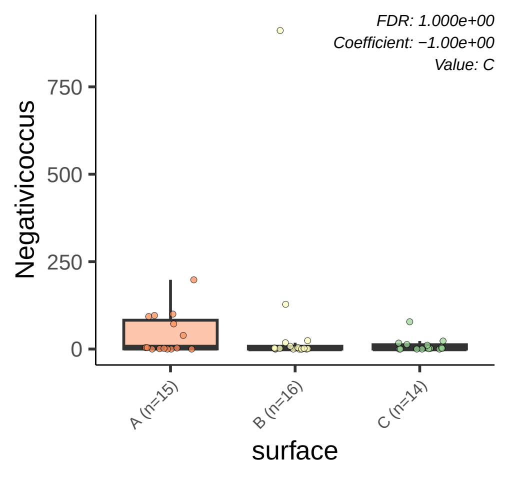
FDR: 1.000e+00
Coefficient: −1.00e+00
Value: C
Negativicoccus
750
500
250
0
A (n=15)
B (n=16)
C (n=14)
surface

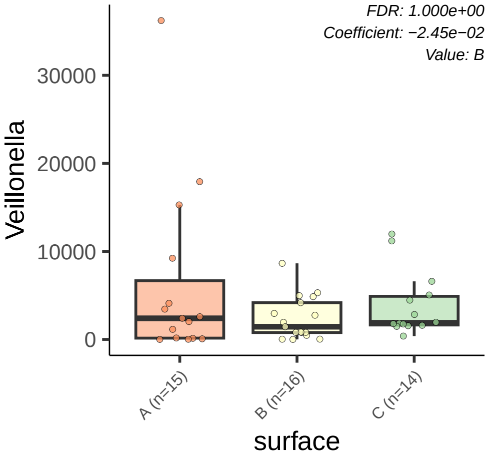

FDR: 1.000e+00
Coefficient: −2.45e−02
Value: B
Veillonella
30000
20000
10000
0
A (n=15)
B (n=16)
C (n=14)
surface

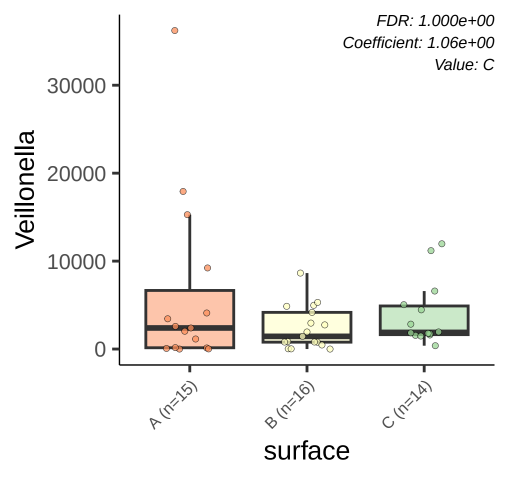

FDR: 1.000e+00
Coefficient: 1.06e+00
Value: C
Veillonella
30000
20000
10000
0
A (n=15)
B (n=16)
C (n=14)
surface

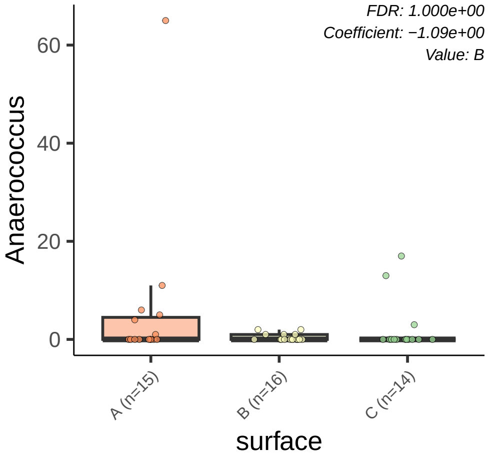
FDR: 1.000e+00
Coefficient: −1.09e+00
Value: B
Anaerococcus
60
40
20
0
A (n=15)
B (n=16)
C (n=14)
surface

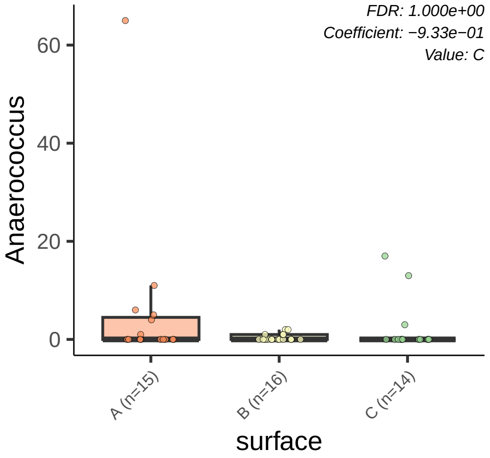
FDR: 1.000e+00
Coefficient: −9.33e−01
Value: C
Anaerococcus
0
20
40
60
A (n=15)
B (n=16)
C (n=14)
surface

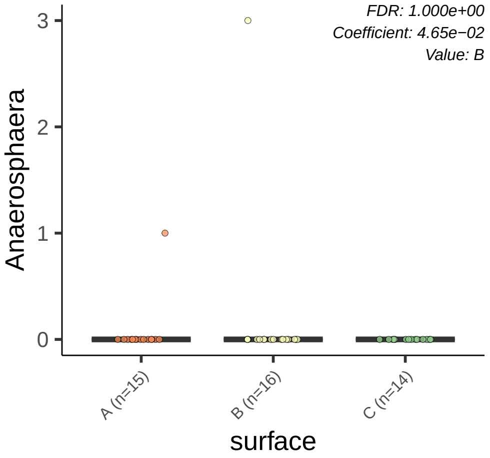

FDR: 1.000e+00
Coefficient: 4.65e−02
Value: B
Anaerosphaera
3
2
1
0
A (n=15)
B (n=16)
C (n=14)
surface

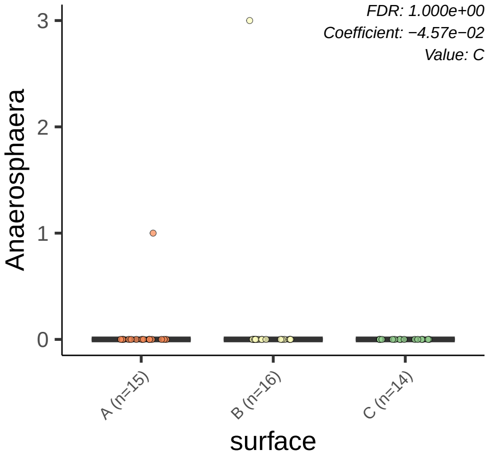
FDR: 1.000e+00
Coefficient: −4.57e−02
Value: C
Anaerosphaera
3
2
1
0
A (n=15)
B (n=16)
C (n=14)
surface

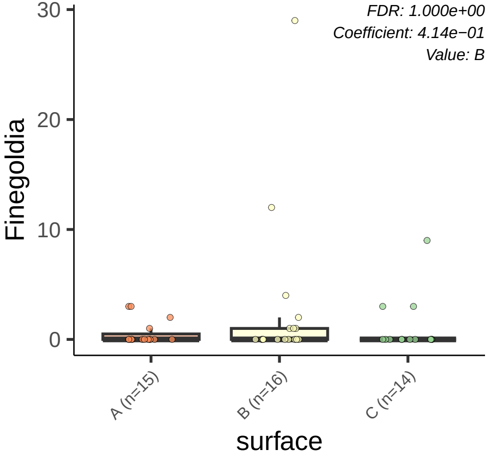

FDR: 1.000e+00
Coefficient: 4.14e−01
Value: B
30
20
10
0
Finegoldia
A (n=15)
B (n=16)
C (n=14)
surface

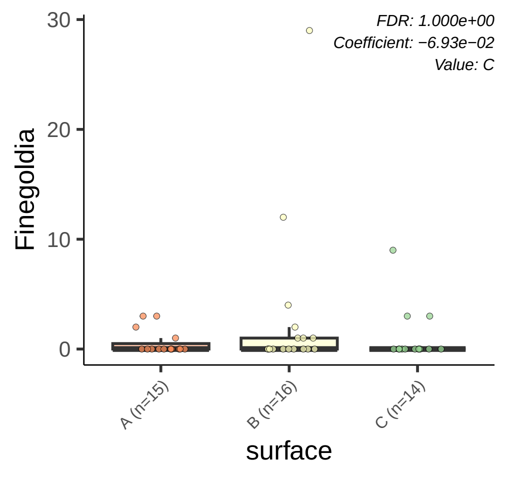
FDR: 1.000e+00
Coefficient: −6.93e−02
Value: C
Finegoldia
30
20
10
0
A (n=15)
B (n=16)
C (n=14)
surface

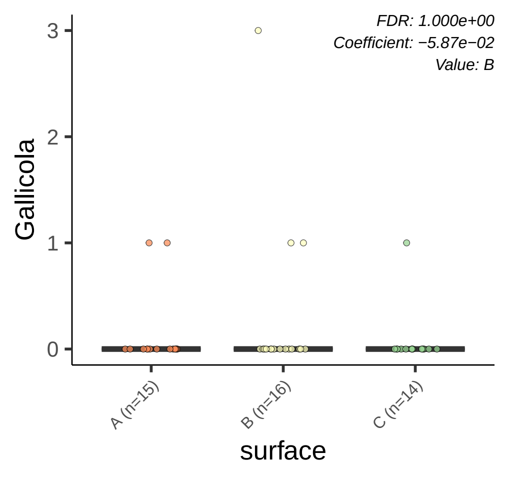
FDR: 1.000e+00
Coefficient: −5.87e−02
Value: B
3
2
1
0
Gallicola
A (n=15)
B (n=16)
C (n=14)
surface

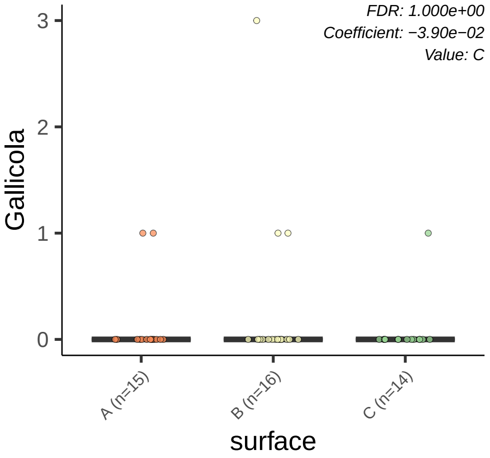

FDR: 1.000e+00
Coefficient: −3.90e−02
Value: C
Gallicola
0
1
2
3
A (n=15)
B (n=16)
C (n=14)
surface

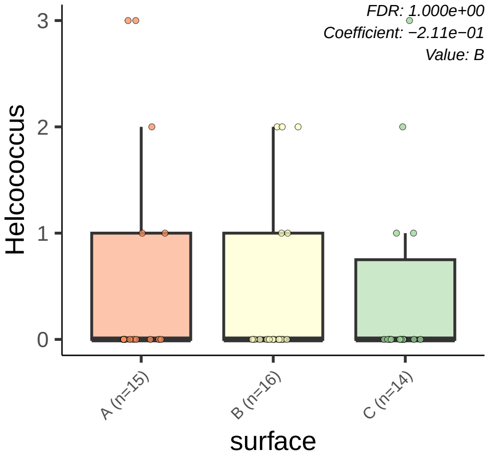
FDR: 1.000e+00
Coefficient: −2.11e−01
Value: B
Helcococcus
3
2
1
0
A (n=15)
B (n=16)
C (n=14)
surface

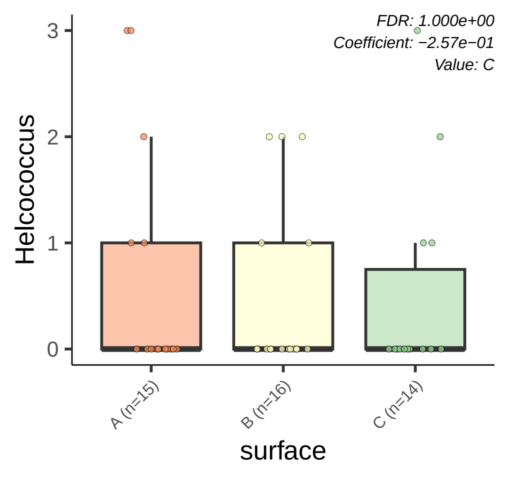

FDR: 1.000e+00
Coefficient: −2.57e−01
Value: C
Helcococcus
3
2
1
0
A (n=15)
B (n=16)
C (n=14)
surface

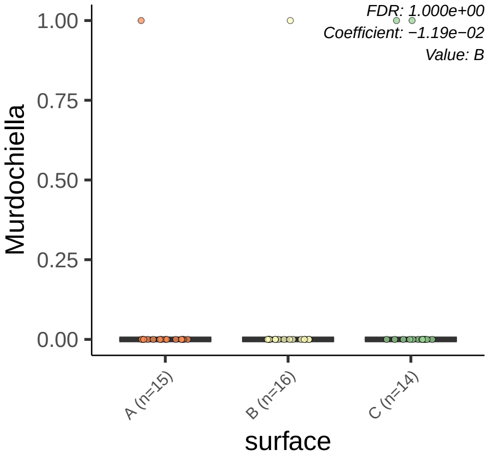
FDR: 1.000e+00
Coefficient: −1.19e−02
Value: B
Murdochiella
1.00
0.75
0.50
0.25
0.00
A (n=15)
B (n=16)
C (n=14)
surface

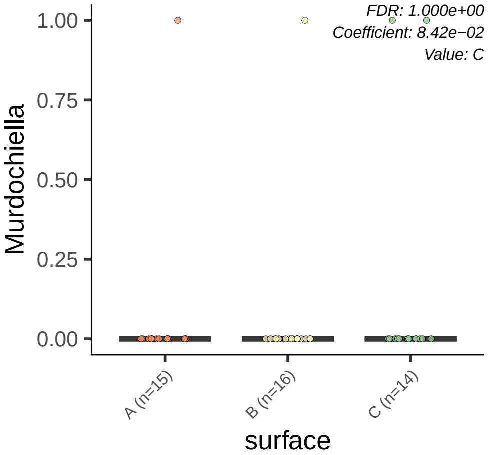
FDR: 1.000e+00
Coefficient: 8.42e−02
Value: C
Murdochiella
1.00
0.75
0.50
0.25
0.00
A (n=15)
B (n=16)
C (n=14)
surface

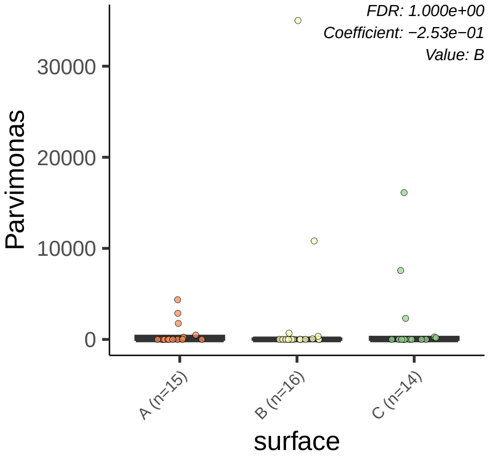
FDR: 1.000e+00
Coefficient: −2.53e−01
Value: B
Parvimonas
30000
20000
10000
0
A (n=15)
B (n=16)
C (n=14)
surface

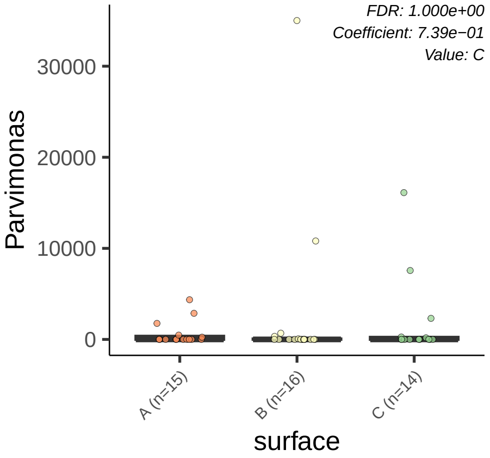
FDR: 1.000e+00
Coefficient: 7.39e−01
Value: C
Parvimonas
30000
20000
10000
0
A (n=15)
B (n=16)
C (n=14)
surface

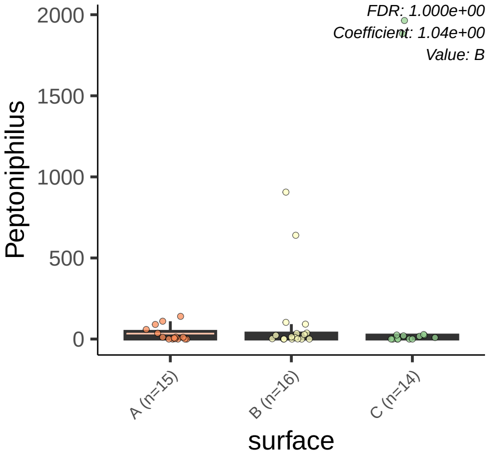
FDR: 1.000e+00
Coefficient: 1.04e+00
Value: B
Peptoniphilus
2000
1500
1000
500
0
A (n=15)
B (n=16)
C (n=14)
surface

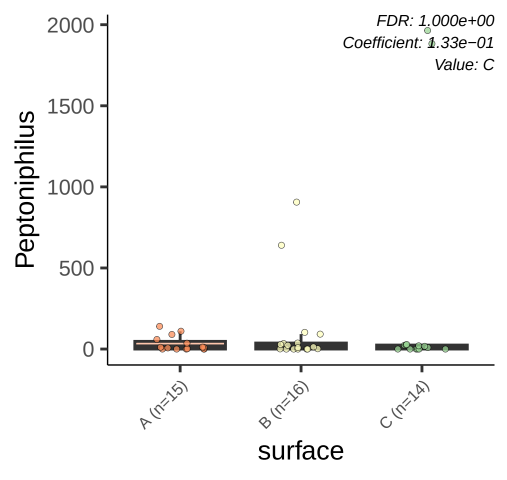
FDR: 1.000e+00
Coefficient: 1.33e−01
Value: C
Peptoniphilus
2000
1500
1000
500
0
A (n=15)
B (n=16)
C (n=14)
surface

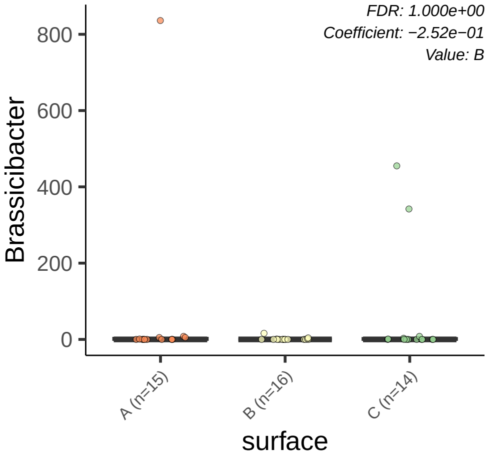

FDR: 1.000e+00
Coefficient: −2.52e−01
Value: B
Brassicibacter
800
600
400
200
0
A (n=15)
B (n=16)
C (n=14)
surface

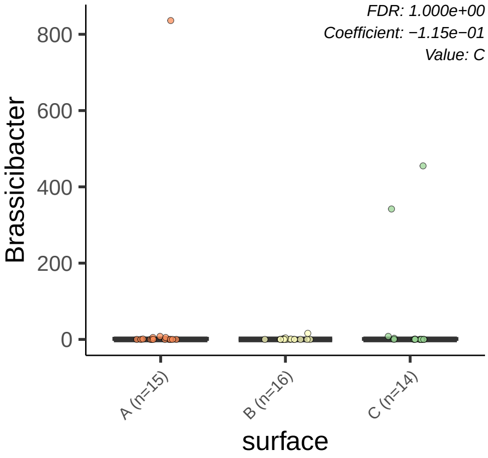
FDR: 1.000e+00
Coefficient: −1.15e−01
Value: C
Brassicibacter
800
600
400
200
0
A (n=15)
B (n=16)
C (n=14)
surface

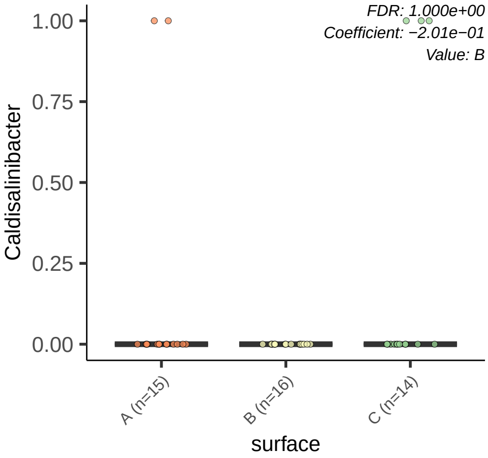

FDR: 1.000e+00
Coefficient: −2.01e−01
Value: B
Caldisalinibacter
1.00
0.75
0.50
0.25
0.00
A (n=15)
B (n=16)
C (n=14)
surface

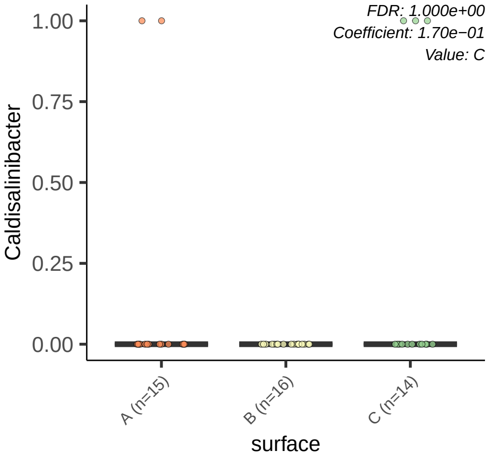
FDR: 1.000e+00
Coefficient: 1.70e−01
Value: C
Caldisalinibacter
1.00
0.75
0.50
0.25
0.00
A (n=15)
B (n=16)
C (n=14)
surface

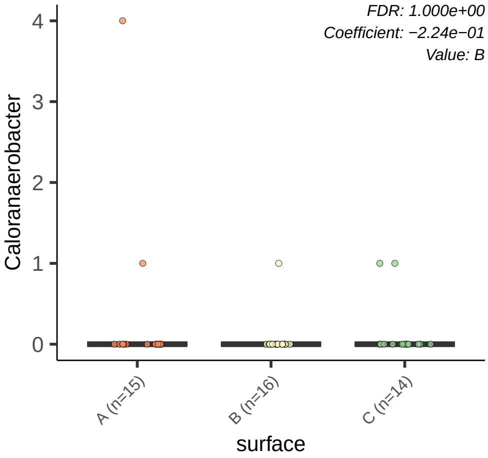
FDR: 1.000e+00
Coefficient: −2.24e−01
Value: B
Caloranaerobacter
4
3
2
1
0
A (n=15)
B (n=16)
C (n=14)
surface

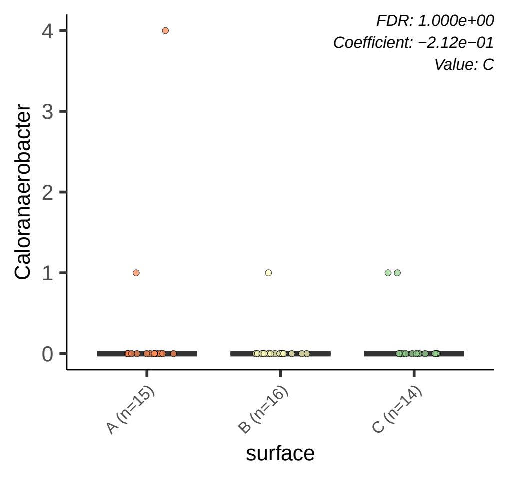

FDR: 1.000e+00
Coefficient: −2.12e−01
Value: C
Caloranaerobacter
4
3
2
1
0
A (n=15)
B (n=16)
C (n=14)
surface

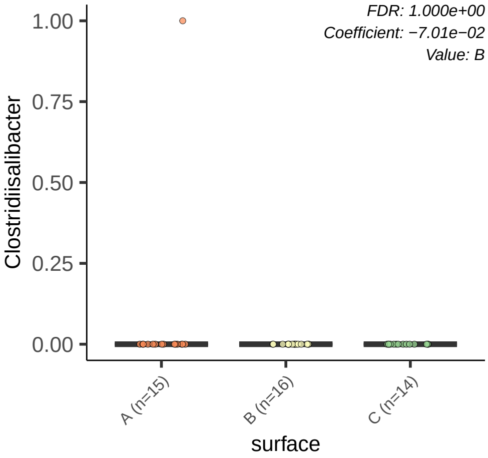

FDR: 1.000e+00
Coefficient: −7.01e−02
Value: B
Clostridiisalibacter
1.00
0.75
0.50
0.25
0.00
A (n=15)
B (n=16)
C (n=14)
surface

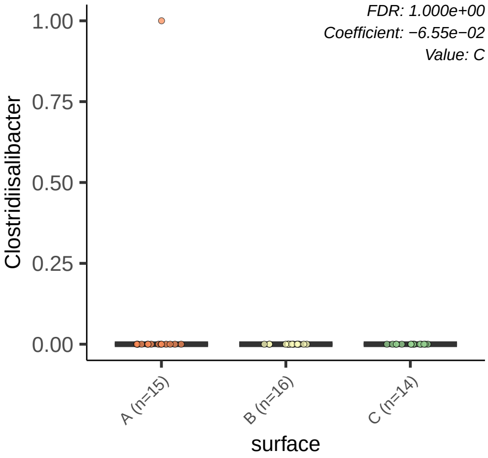

FDR: 1.000e+00
Coefficient: −6.55e−02
Value: C
Clostridiisalibacter
1.00
0.75
0.50
0.25
0.00
A (n=15)
B (n=16)
C (n=14)
surface

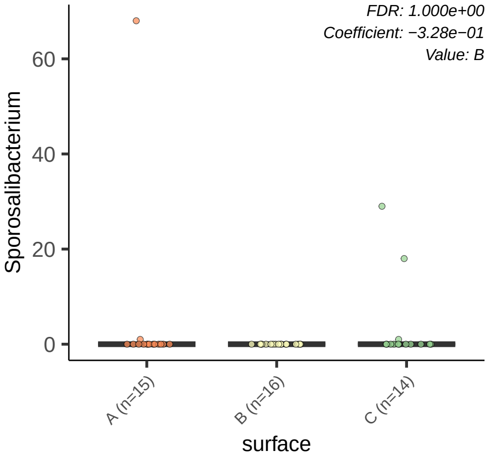

FDR: 1.000e+00
Coefficient: −3.28e−01
Value: B
Sporosalibacterium
0
20
40
60
A (n=15)
B (n=16)
C (n=14)
surface

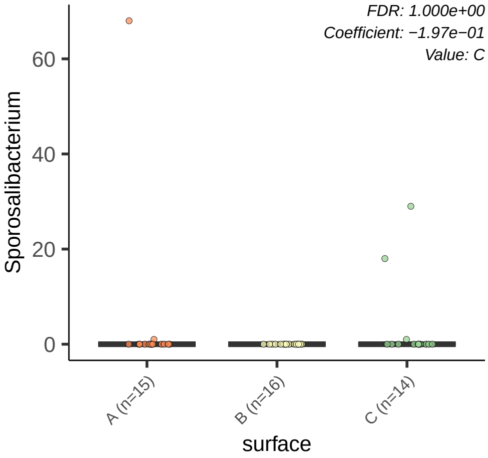

FDR: 1.000e+00
Coefficient: −1.97e−01
Value: C
Sporosalibacterium
60
40
20
0
A (n=15)
B (n=16)
C (n=14)
surface

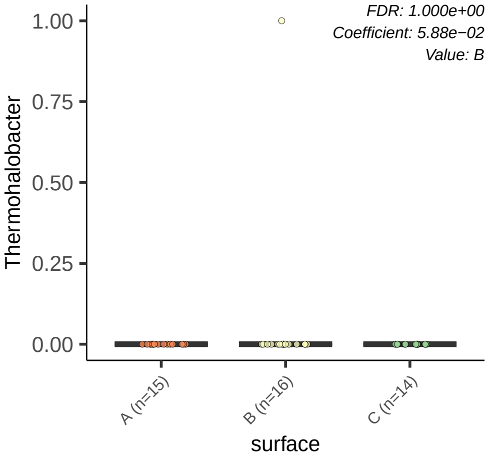
FDR: 1.000e+00
Coefficient: 5.88e−02
Value: B
Thermohalobacter
1.00
0.75
0.50
0.25
0.00
A (n=15)
B (n=16)
C (n=14)
surface

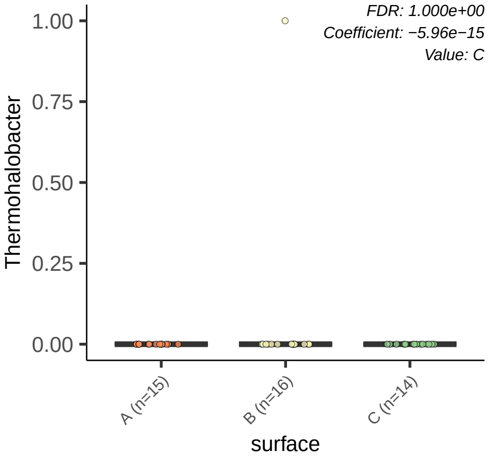

FDR: 1.000e+00
Coefficient: −5.96e−15
Value: C
1.00
0.75
0.50
0.25
0.00
Thermohalobacter
A (n=15)
B (n=16)
C (n=14)
surface

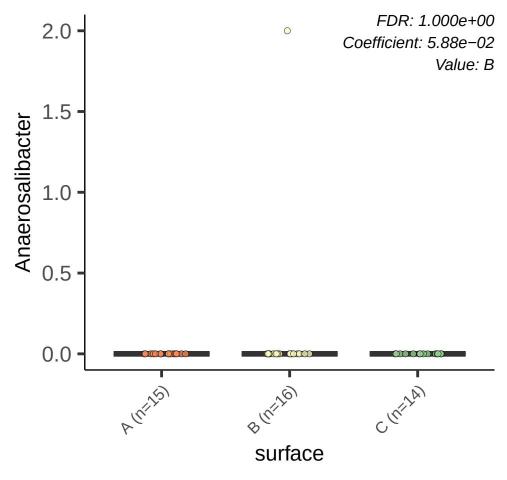
FDR: 1.000e+00
Coefficient: 5.88e−02
Value: B
Anaerosalibacter
2.0
1.5
1.0
0.5
0.0
A (n=15)
B (n=16)
C (n=14)
surface

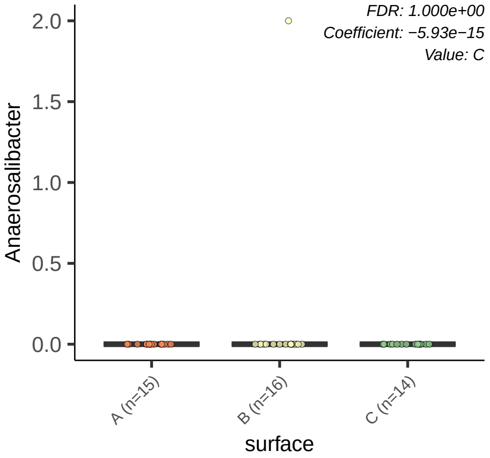
FDR: 1.000e+00
Coefficient: −5.93e−15
Value: C
Anaerosalibacter
2.0
1.5
1.0
0.5
0.0
A (n=15)
B (n=16)
C (n=14)
surface

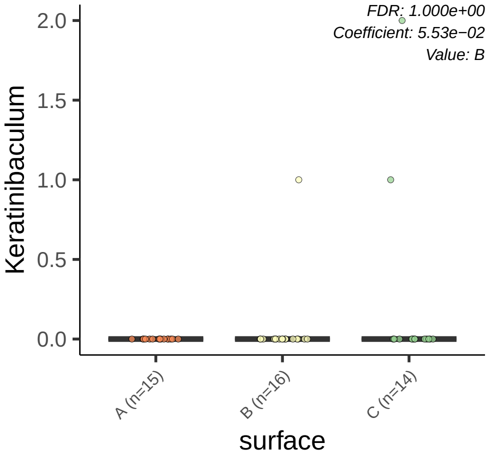
FDR: 1.000e+00
Coefficient: 5.53e−02
Value: B
Keratinibaculum
2.0
1.5
1.0
0.5
0.0
A (n=15)
B (n=16)
C (n=14)
surface

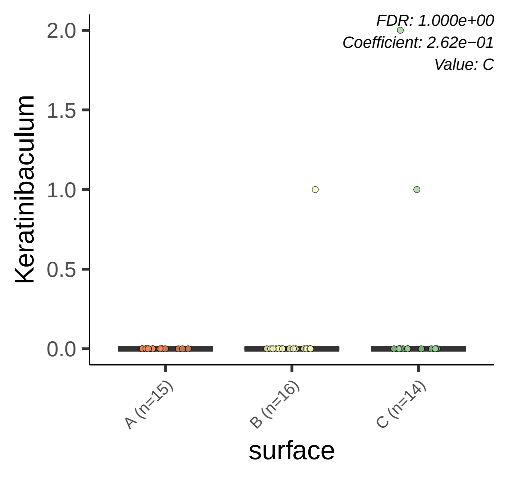
FDR: 1.000e+00
Coefficient: 2.62e−01
Value: C
Keratinibaculum
2.0
1.5
1.0
0.5
0.0
A (n=15)
B (n=16)
C (n=14)
surface

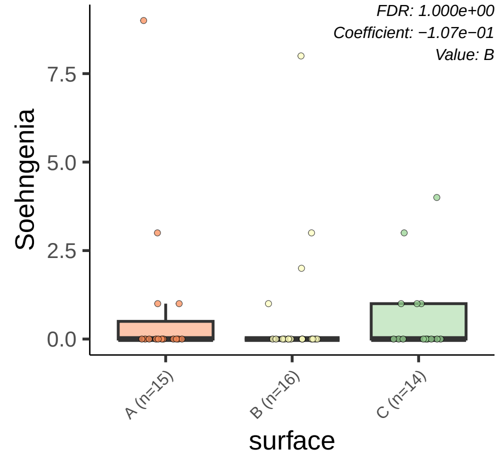
FDR: 1.000e+00
Coefficient: −1.07e−01
Value: B
Soehngenia
7.5
5.0
2.5
0.0
A (n=15)
B (n=16)
C (n=14)
surface

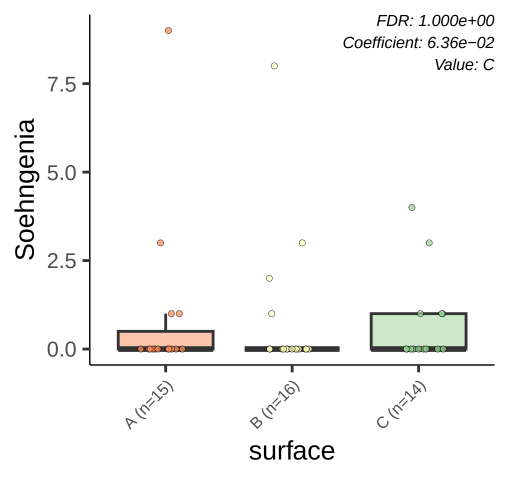

FDR: 1.000e+00
Coefficient: 6.36e−02
Value: C
Soehngenia
7.5
5.0
2.5
0.0
A (n=15)
B (n=16)
C (n=14)
surface

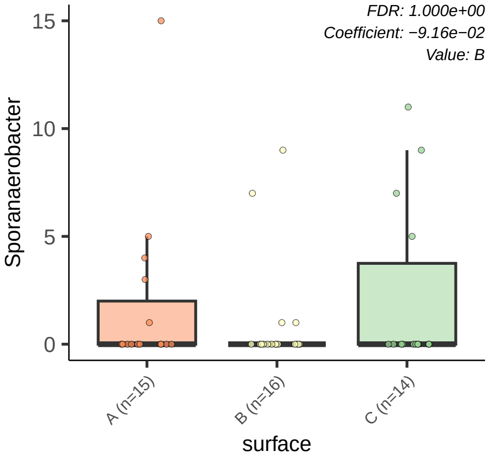

FDR: 1.000e+00
Coefficient: −9.16e−02
Value: B
Sporanaerobacter
15
10
5
0
A (n=15)
B (n=16)
C (n=14)
surface

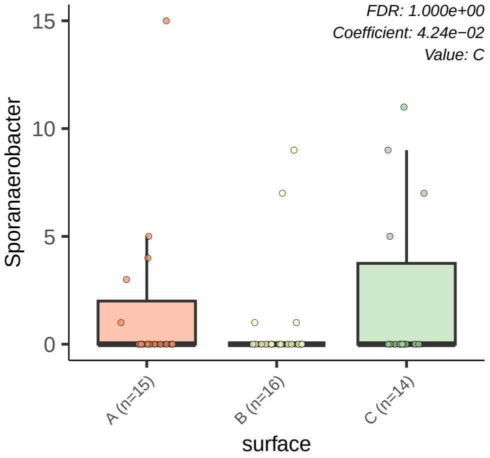
FDR: 1.000e+00
Coefficient: 4.24e−02
Value: C
Sporanaerobacter
15
10
5
0
A (n=15)
B (n=16)
C (n=14)
surface

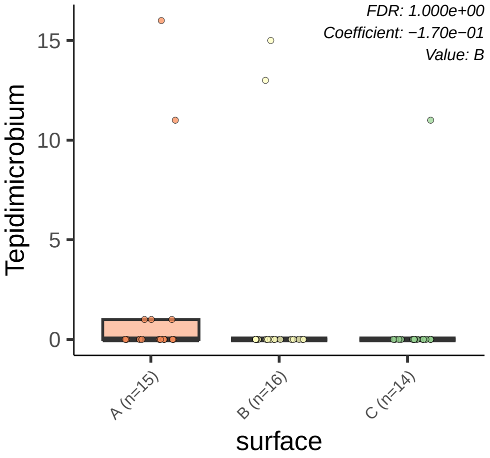
FDR: 1.000e+00
Coefficient: −1.70e−01
Value: B
Tepidimicrobium
15
10
5
0
A (n=15)
B (n=16)
C (n=14)
surface

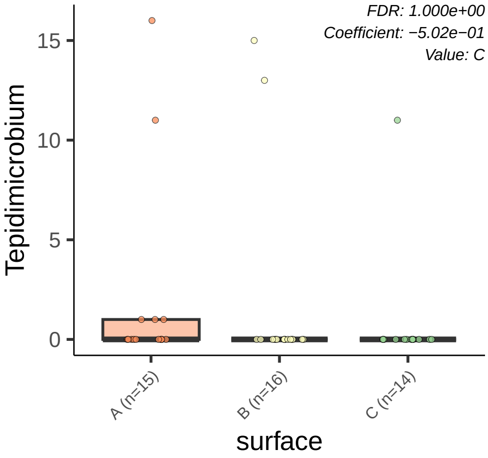

FDR: 1.000e+00
Coefficient: −5.02e−01
Value: C
Tepidimicrobium
15
10
5
0
A (n=15)
B (n=16)
C (n=14)
surface

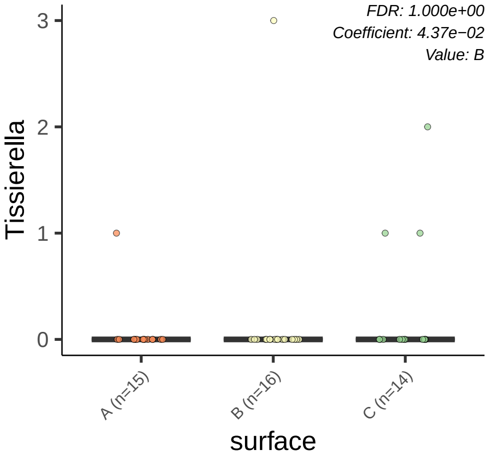
FDR: 1.000e+00
Coefficient: 4.37e−02
Value: B
Tissierella
3
2
1
0
A (n=15)
B (n=16)
C (n=14)
surface

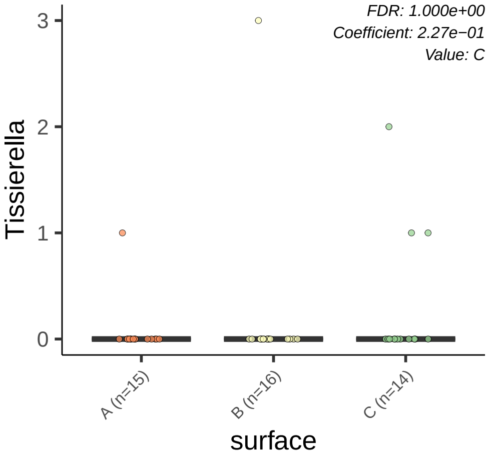
FDR: 1.000e+00
Coefficient: 2.27e−01
Value: C
Tissierella
0
1
2
3
A (n=15)
B (n=16)
C (n=14)
surface

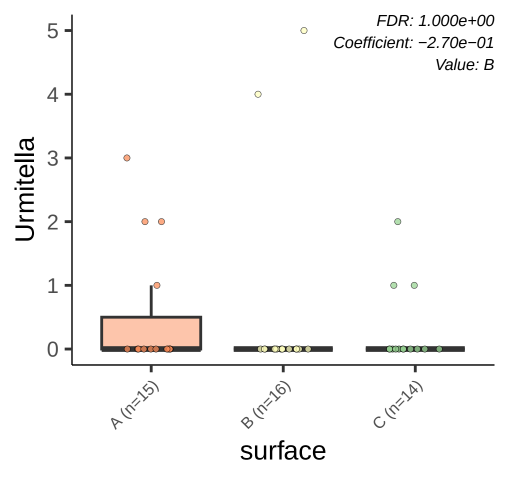

FDR: 1.000e+00
Coefficient: −2.70e−01
Value: B
Urmitella
0
1
2
3
4
5
A (n=15)
B (n=16)
C (n=14)
surface

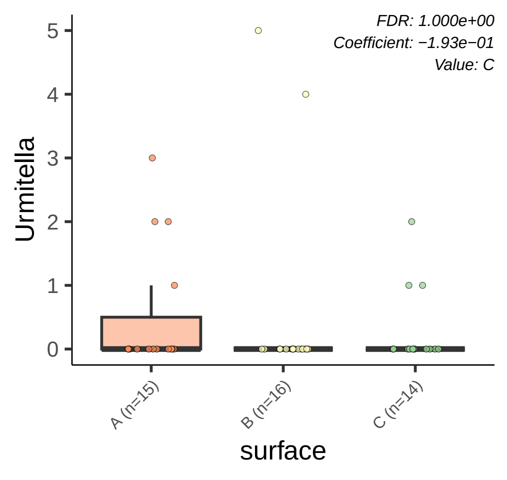
FDR: 1.000e+00
Coefficient: −1.93e−01
Value: C
Urmitella
0
1
2
3
4
5
A (n=15)
B (n=16)
C (n=14)
surface

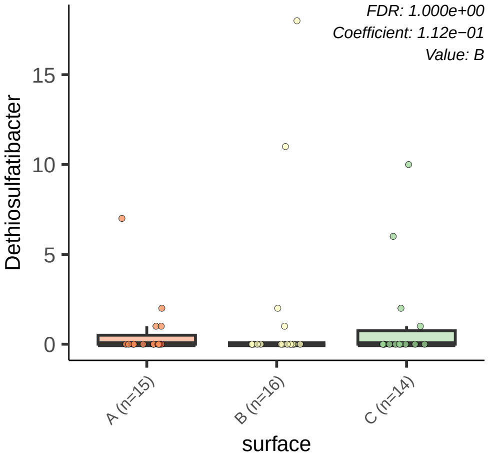

FDR: 1.000e+00
Coefficient: 1.12e−01
Value: B
Dethiosulfatibacter
0
5
10
15
A (n=15)
B (n=16)
C (n=14)
surface

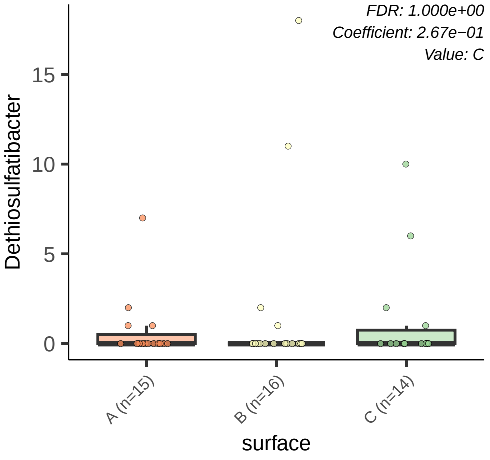
FDR: 1.000e+00
Coefficient: 2.67e−01
Value: C
Dethiosulfatibacter
0
5
10
15
A (n=15)
B (n=16)
C (n=14)
surface

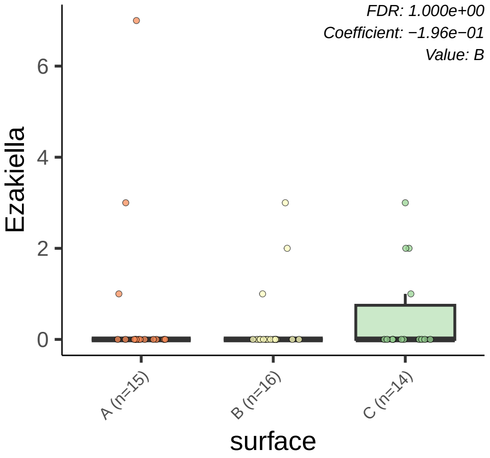
FDR: 1.000e+00
Coefficient: −1.96e−01
Value: B
Ezakiella
0
2
4
6
A (n=15)
B (n=16)
C (n=14)
surface

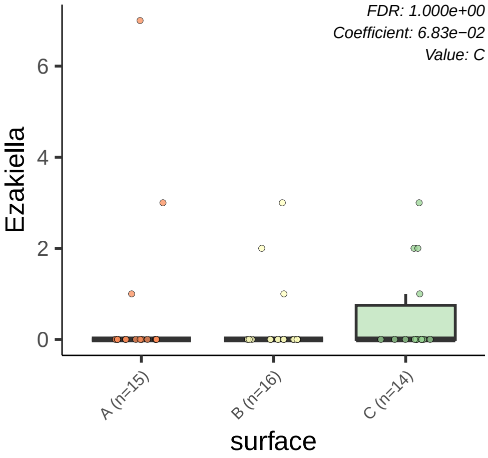
FDR: 1.000e+00
Coefficient: 6.83e−02
Value: C
Ezakiella
0
2
4
6
A (n=15)
B (n=16)
C (n=14)
surface

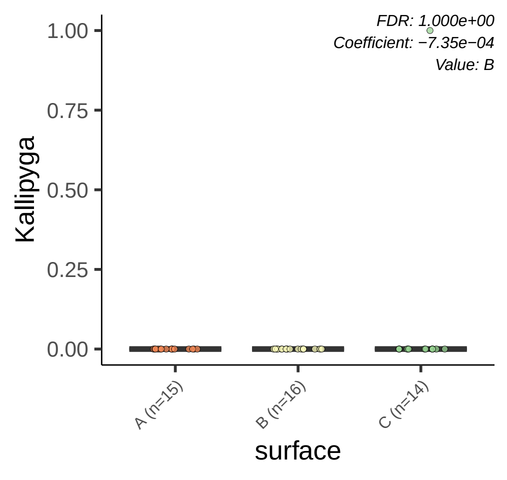
FDR: 1.000e+00
Coefficient: −7.35e−04
Value: B
1.00
0.75
0.50
0.25
0.00
Kallipyga
A (n=15)
B (n=16)
C (n=14)
surface

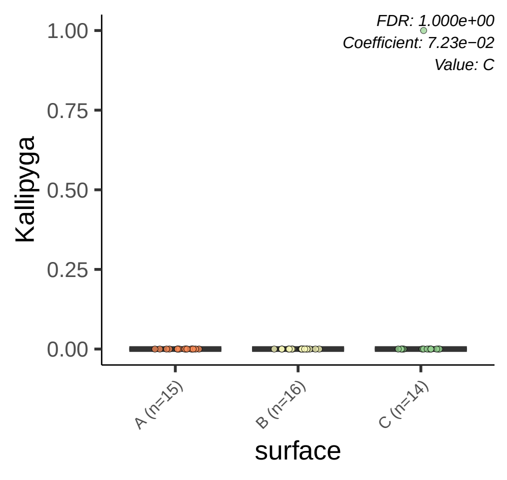
FDR: 1.000e+00
Coefficient: 7.23e−02
Value: C
Kallipyga
1.00
0.75
0.50
0.25
0.00
A (n=15)
B (n=16)
C (n=14)
surface

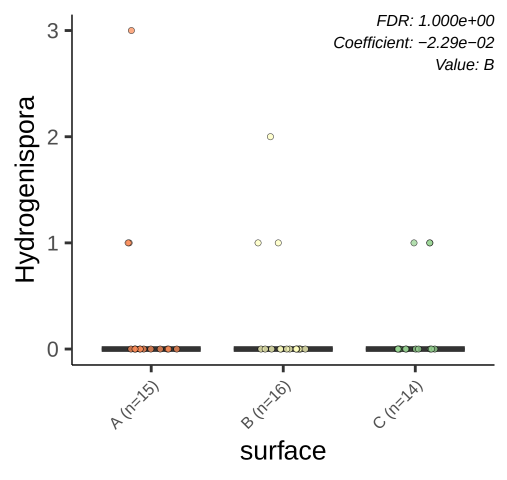
FDR: 1.000e+00
Coefficient: −2.29e−02
Value: B
Hydrogenispora
3
2
1
0
A (n=15)
B (n=16)
C (n=14)
surface

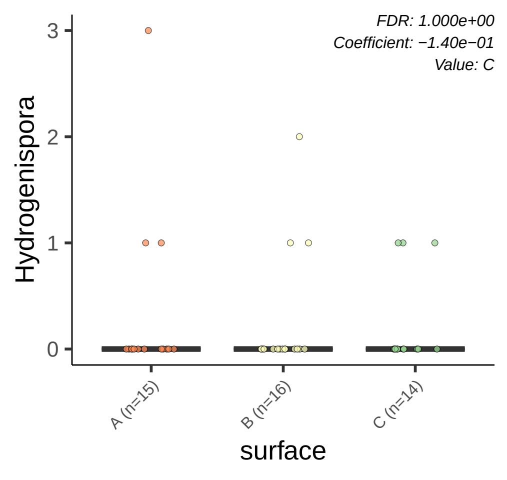
FDR: 1.000e+00
Coefficient: −1.40e−01
Value: C
Hydrogenispora
3
2
1
0
A (n=15)
B (n=16)
C (n=14)
surface

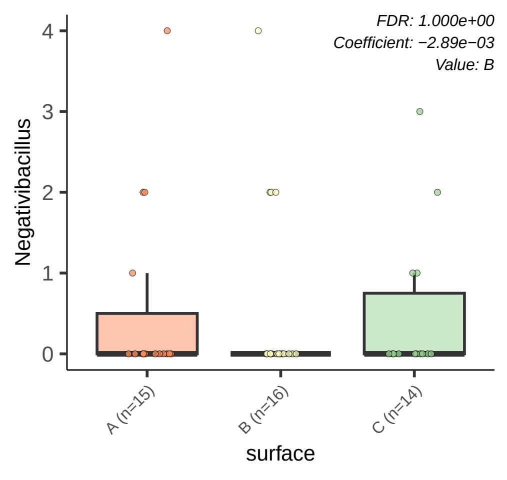

FDR: 1.000e+00
Coefficient: −2.89e−03
Value: B
Negativibacillus
0
1
2
3
4
A (n=15)
B (n=16)
C (n=14)
surface

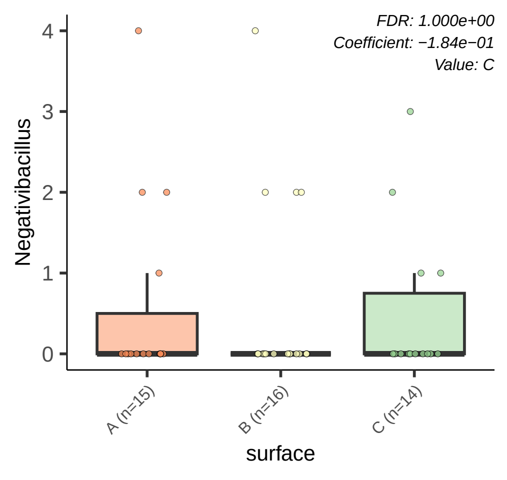
FDR: 1.000e+00
Coefficient: −1.84e−01
Value: C
Negativibacillus
0
1
2
3
4
A (n=15)
B (n=16)
C (n=14)
surface

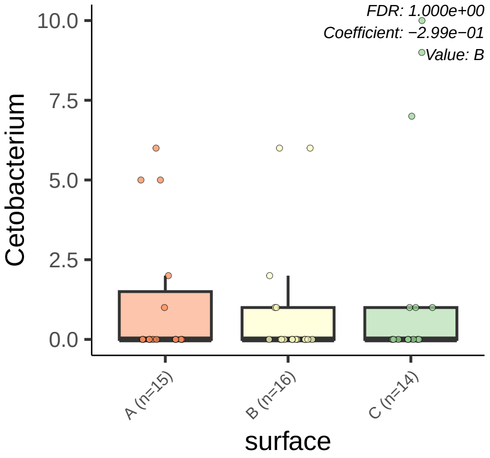

FDR: 1.000e+00
Coefficient: −2.99e−01
Value: B
Cetobacterium
10.0
7.5
5.0
2.5
0.0
A (n=15)
B (n=16)
C (n=14)
surface

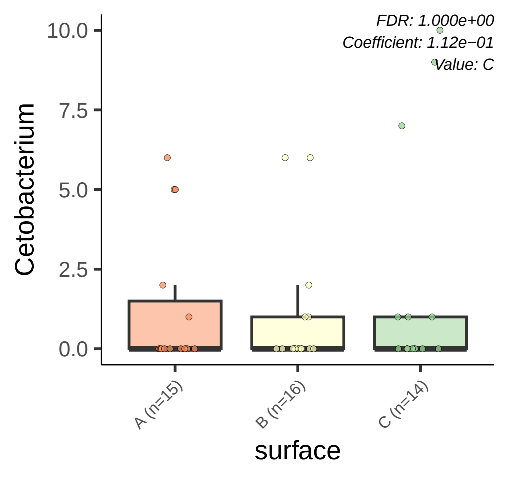
FDR: 1.000e+00
Coefficient: 1.12e−01
Value: C
Cetobacterium
10.0
7.5
5.0
2.5
0.0
A (n=15)
B (n=16)
C (n=14)
surface

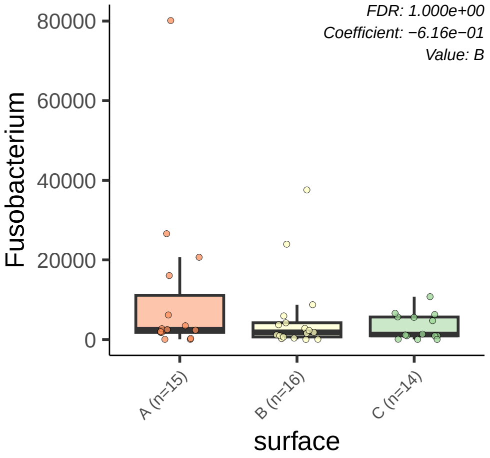

FDR: 1.000e+00
Coefficient: −6.16e−01
Value: B
80000
60000
40000
20000
0
Fusobacterium
A (n=15)
B (n=16)
C (n=14)
surface

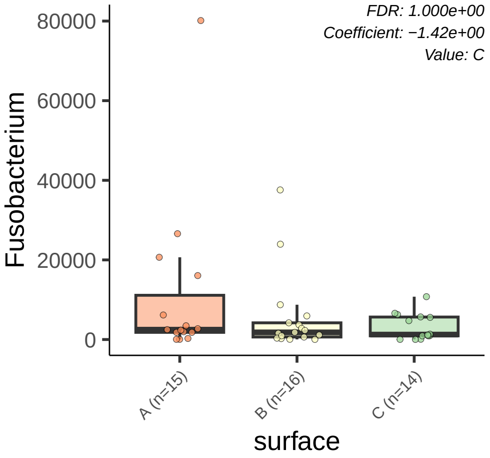
FDR: 1.000e+00
Coefficient: −1.42e+00
Value: C
Fusobacterium
80000
60000
40000
20000
0
A (n=15)
B (n=16)
C (n=14)
surface

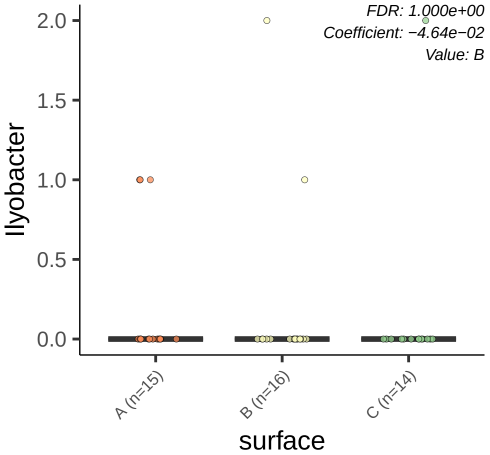

FDR: 1.000e+00
Coefficient: −4.64e−02
Value: B
2.0
1.5
1.0
0.5
0.0
Ilyobacter
A (n=15)
B (n=16)
C (n=14)
surface

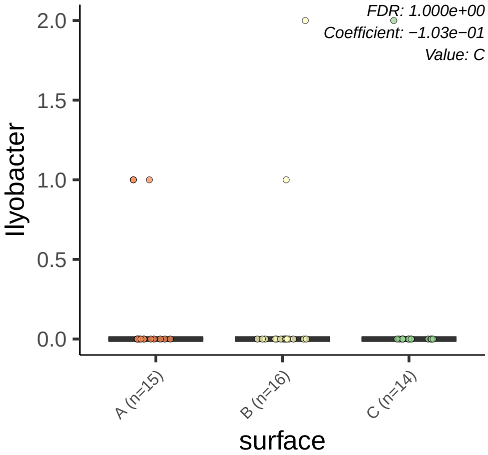

FDR: 1.000e+00
Coefficient: −1.03e−01
Value: C
Ilyobacter
2.0
1.5
1.0
0.5
0.0
A (n=15)
B (n=16)
C (n=14)
surface

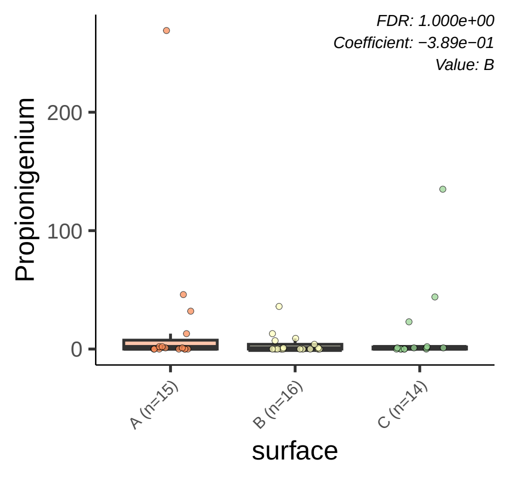
FDR: 1.000e+00
Coefficient: −3.89e−01
Value: B
Propionigenium
200
100
0
A (n=15)
B (n=16)
C (n=14)
surface

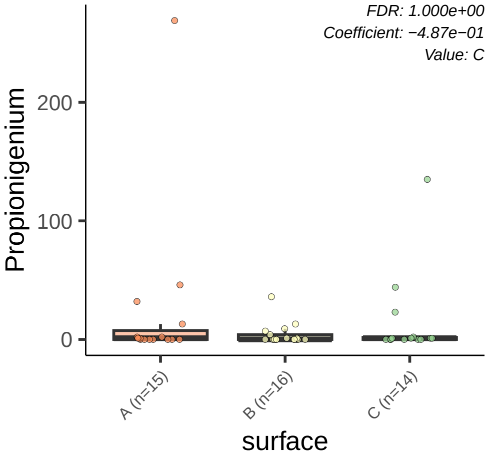

FDR: 1.000e+00
Coefficient: −4.87e−01
Value: C
Propionigenium
200
100
0
A (n=15)
B (n=16)
C (n=14)
surface

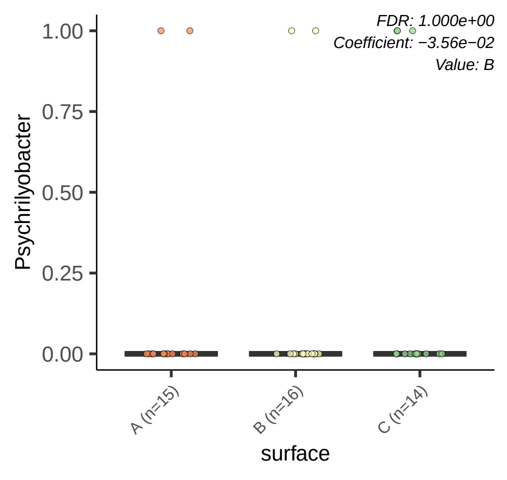
FDR: 1.000e+00
Coefficient: −3.56e−02
Value: B
Psychrilyobacter
1.00
0.75
0.50
0.25
0.00
A (n=15)
B (n=16)
C (n=14)
surface

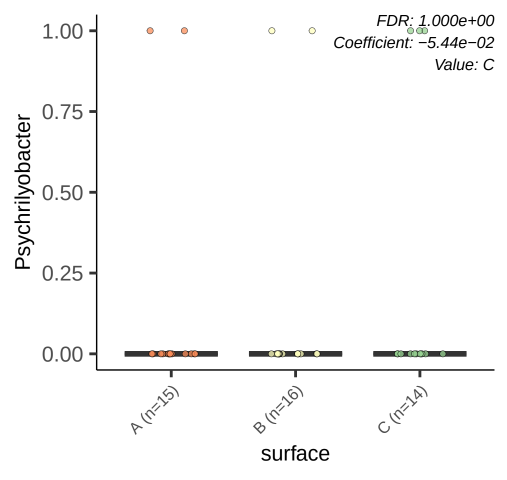
FDR: 1.000e+00
Coefficient: −5.44e−02
Value: C
Psychrilyobacter
1.00
0.75
0.50
0.25
0.00
A (n=15)
B (n=16)
C (n=14)
surface

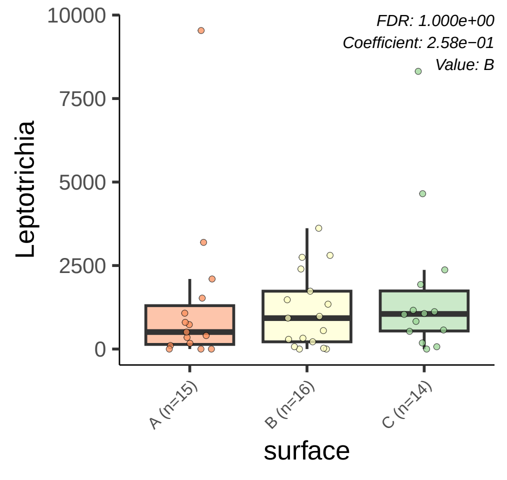

FDR: 1.000e+00
Coefficient: 2.58e−01
Value: B
10000
7500
5000
2500
0
Leptotrichia
A (n=15)
B (n=16)
C (n=14)
surface

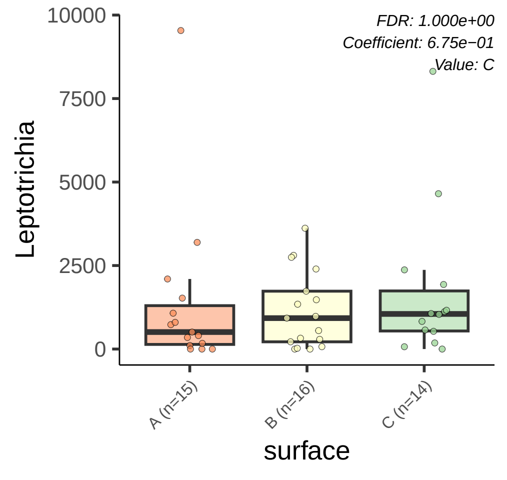

FDR: 1.000e+00
Coefficient: 6.75e−01
Value: C
10000
7500
5000
2500
0
Leptotrichia
A (n=15)
B (n=16)
C (n=14)
surface

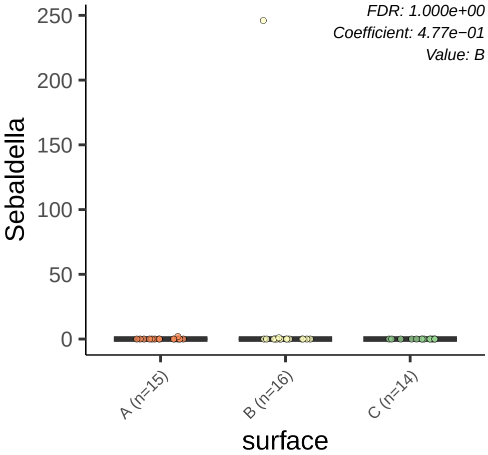
FDR: 1.000e+00
Coefficient: 4.77e−01
Value: B
Sebaldella
250
200
150
100
50
0
A (n=15)
B (n=16)
C (n=14)
surface

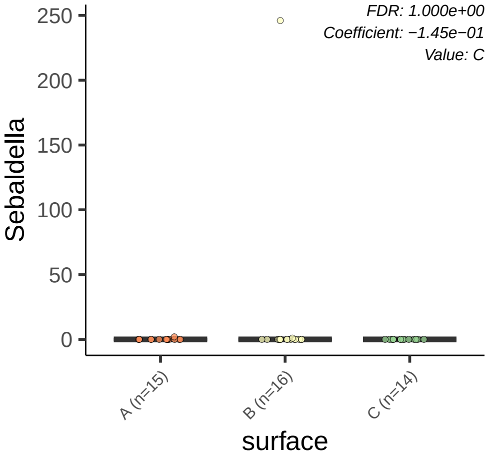
FDR: 1.000e+00
Coefficient: −1.45e−01
Value: C
Sebaldella
250
200
150
100
50
0
A (n=15)
B (n=16)
C (n=14)
surface

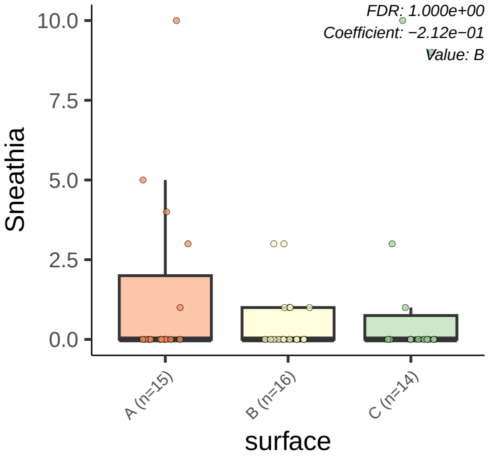
FDR: 1.000e+00
Coefficient: −2.12e−01
Value: B
Sneathia
10.0
7.5
5.0
2.5
0.0
A (n=15)
B (n=16)
C (n=14)
surface

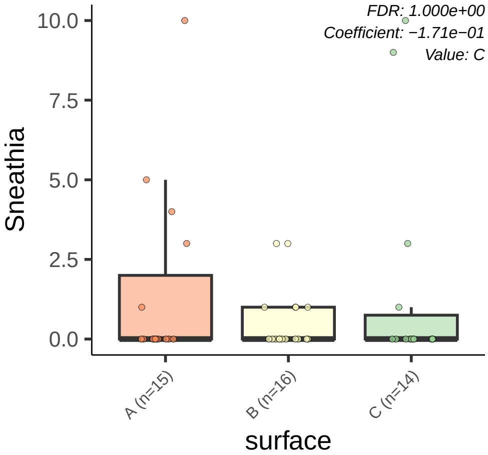
FDR: 1.000e+00
Coefficient: −1.71e−01
Value: C
Sneathia
10.0
7.5
5.0
2.5
0.0
A (n=15)
B (n=16)
C (n=14)
surface

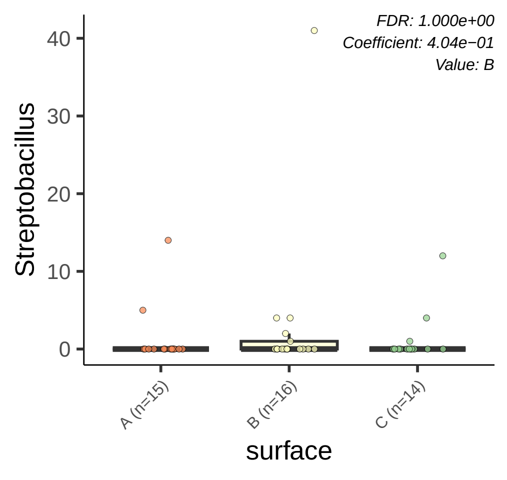
FDR: 1.000e+00
Coefficient: 4.04e−01
Value: B
Streptobacillus
0
10
20
30
40
A (n=15)
B (n=16)
C (n=14)
surface

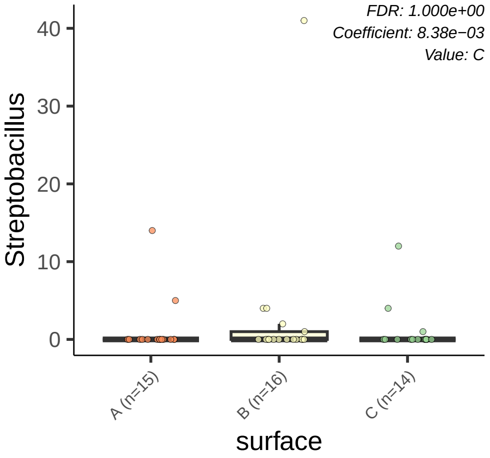

FDR: 1.000e+00
Coefficient: 8.38e−03
Value: C
Streptobacillus
0
10
20
30
40
A (n=15)
B (n=16)
C (n=14)
surface

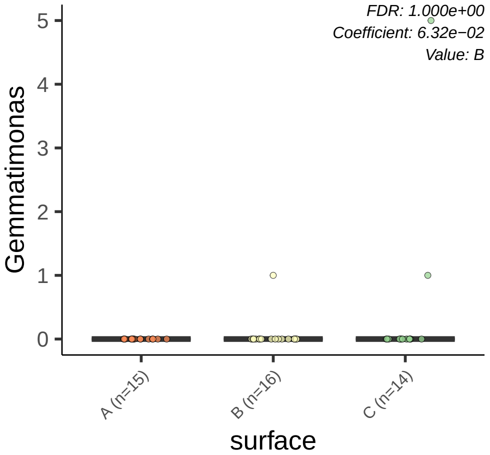
FDR: 1.000e+00
Coefficient: 6.32e−02
Value: B
Gemmatimonas
5
4
3
2
1
0
A (n=15)
B (n=16)
C (n=14)
surface

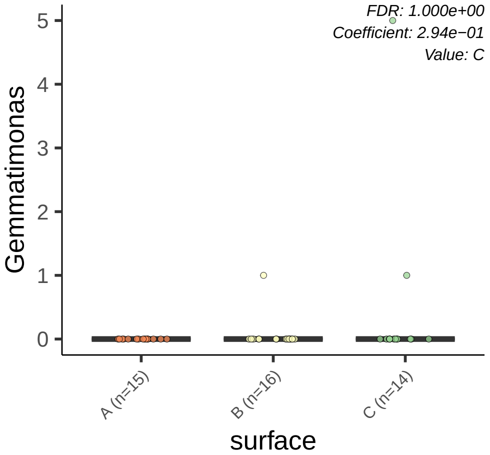
FDR: 1.000e+00
Coefficient: 2.94e−01
Value: C
Gemmatimonas
5
4
3
2
1
0
A (n=15)
B (n=16)
C (n=14)
surface

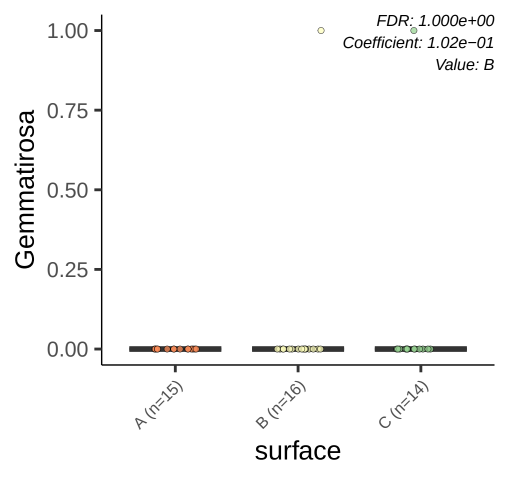
FDR: 1.000e+00
Coefficient: 1.02e−01
Value: B
Gemmatirosa
1.00
0.75
0.50
0.25
0.00
A (n=15)
B (n=16)
C (n=14)
surface

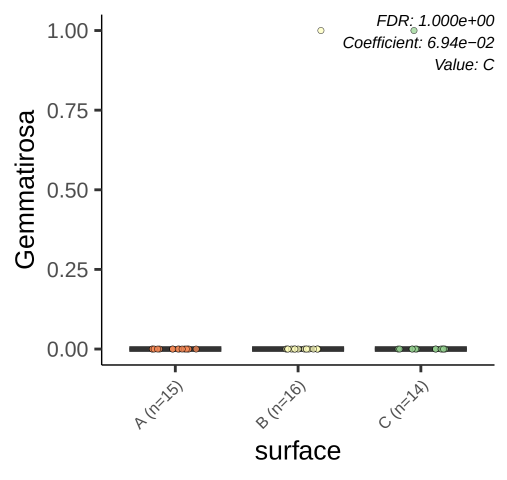
FDR: 1.000e+00
Coefficient: 6.94e−02
Value: C
Gemmatirosa
1.00
0.75
0.50
0.25
0.00
A (n=15)
B (n=16)
C (n=14)
surface

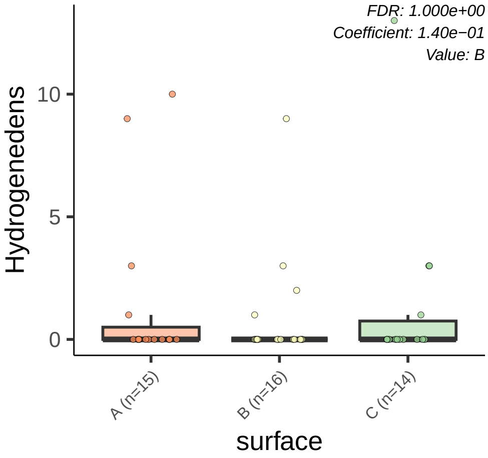
FDR: 1.000e+00
Coefficient: 1.40e−01
Value: B
Hydrogenedens
10
5
0
A (n=15)
B (n=16)
C (n=14)
surface

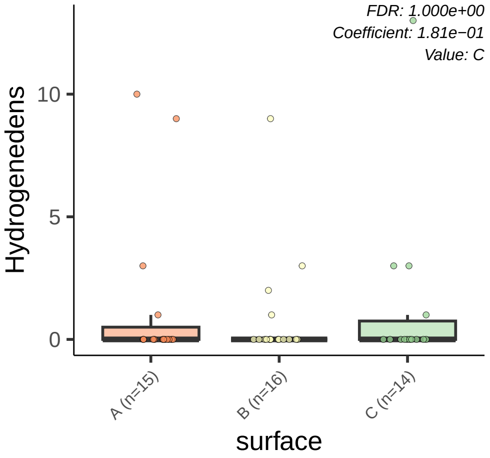
FDR: 1.000e+00
Coefficient: 1.81e−01
Value: C
Hydrogenedens
10
5
0
A (n=15)
B (n=16)
C (n=14)
surface

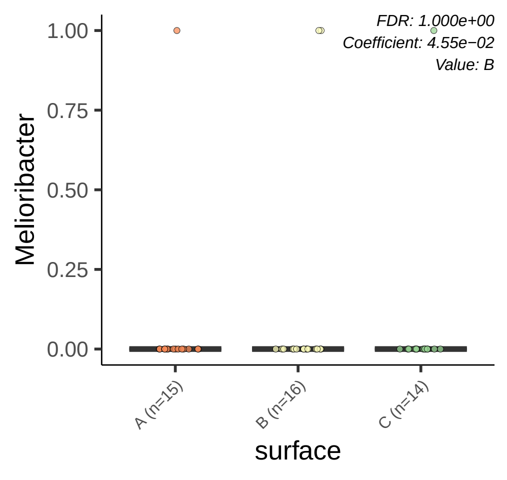
FDR: 1.000e+00
Coefficient: 4.55e−02
Value: B
Melioribacter
1.00
0.75
0.50
0.25
0.00
A (n=15)
B (n=16)
C (n=14)
surface

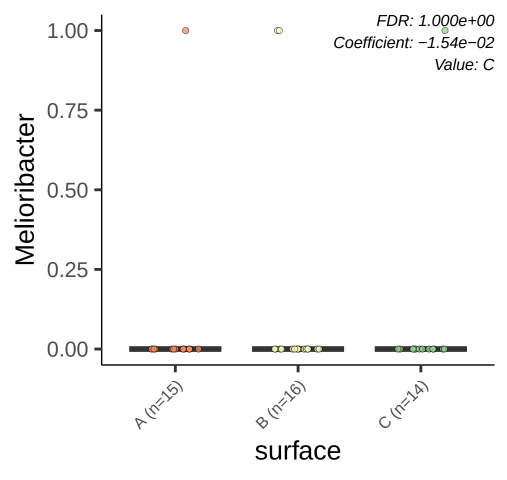

FDR: 1.000e+00
Coefficient: −1.54e−02
Value: C
Melioribacter
1.00
0.75
0.50
0.25
0.00
A (n=15)
B (n=16)
C (n=14)
surface

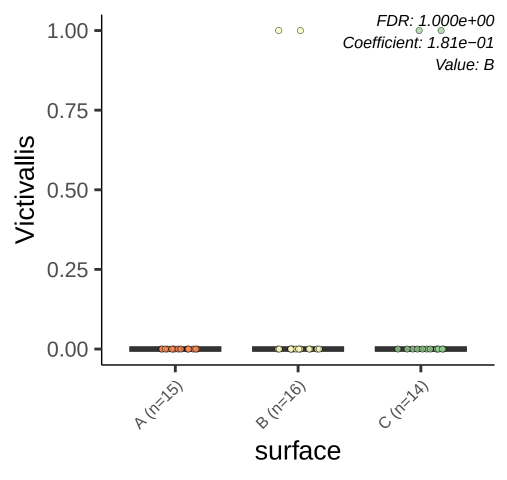
FDR: 1.000e+00
Coefficient: 1.81e−01
Value: B
Victivallis
1.00
0.75
0.50
0.25
0.00
A (n=15)
B (n=16)
C (n=14)
surface

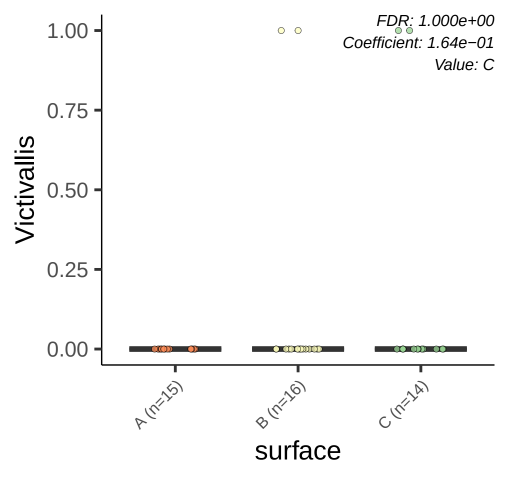
FDR: 1.000e+00
Coefficient: 1.64e−01
Value: C
Victivallis
1.00
0.75
0.50
0.25
0.00
A (n=15)
B (n=16)
C (n=14)
surface

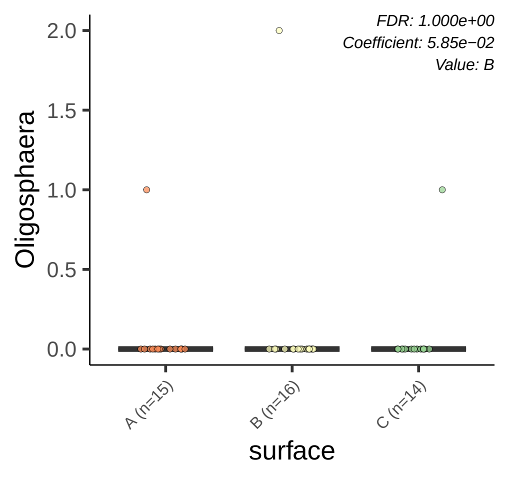

FDR: 1.000e+00
Coefficient: 5.85e−02
Value: B
Oligosphaera
2.0
1.5
1.0
0.5
0.0
A (n=15)
B (n=16)
C (n=14)
surface

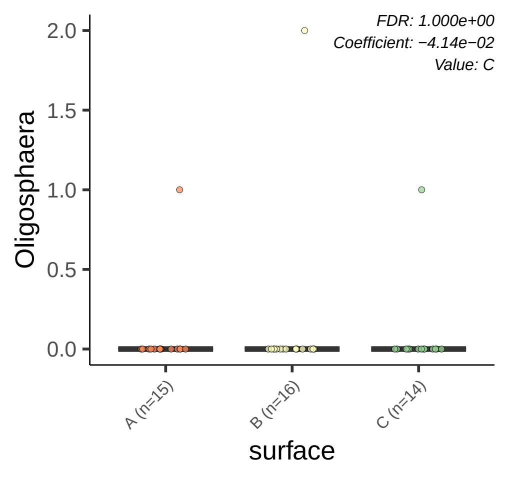

FDR: 1.000e+00
Coefficient: −4.14e−02
Value: C
Oligosphaera
2.0
1.5
1.0
0.5
0.0
A (n=15)
B (n=16)
C (n=14)
surface

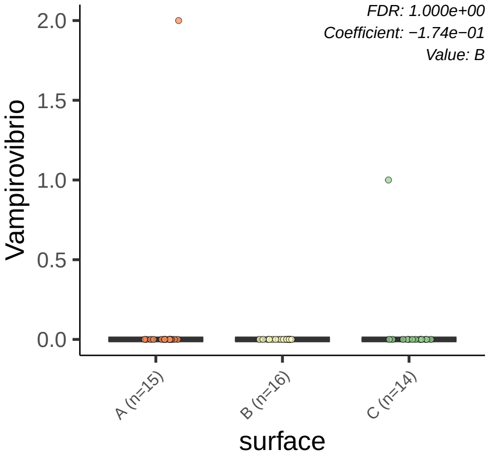

FDR: 1.000e+00
Coefficient: −1.74e−01
Value: B
Vampirovibrio
2.0
1.5
1.0
0.5
0.0
A (n=15)
B (n=16)
C (n=14)
surface

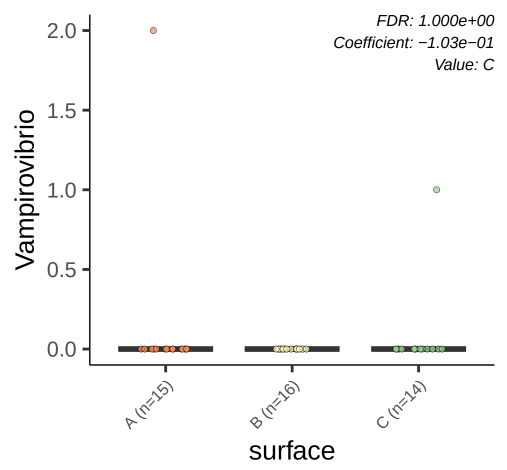
FDR: 1.000e+00
Coefficient: −1.03e−01
Value: C
Vampirovibrio
2.0
1.5
1.0
0.5
0.0
A (n=15)
B (n=16)
C (n=14)
surface

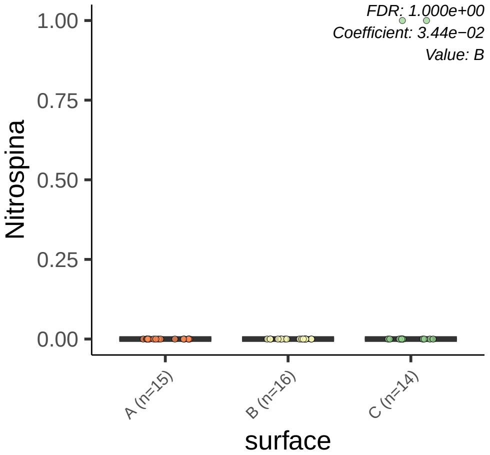
FDR: 1.000e+00
Coefficient: 3.44e−02
Value: B
Nitrospina
1.00
0.75
0.50
0.25
0.00
A (n=15)
B (n=16)
C (n=14)
surface

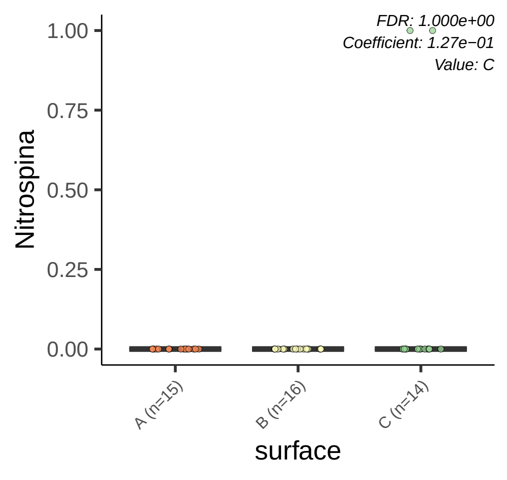

FDR: 1.000e+00
Coefficient: 1.27e−01
Value: C
1.00
0.75
0.50
0.25
0.00
Nitrospina
A (n=15)
B (n=16)
C (n=14)
surface

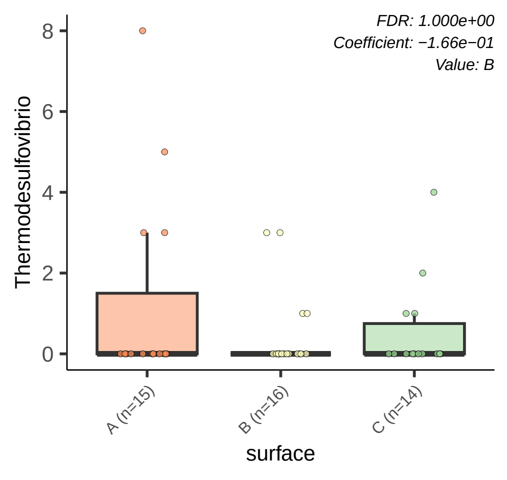

FDR: 1.000e+00
Coefficient: −1.66e−01
Value: B
Thermodesulfovibrio
0
2
4
6
8
A (n=15)
B (n=16)
C (n=14)
surface

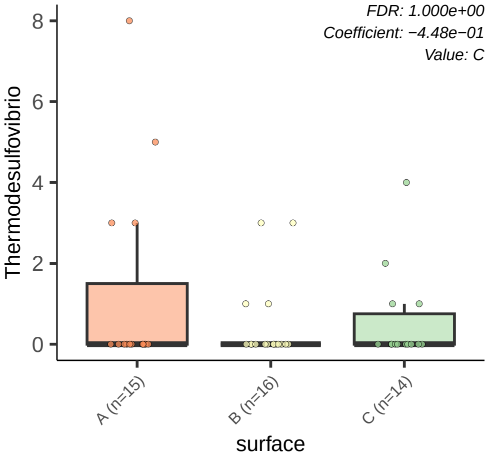

FDR: 1.000e+00
Coefficient: −4.48e−01
Value: C
Thermodesulfovibrio
0
2
4
6
8
A (n=15)
B (n=16)
C (n=14)
surface

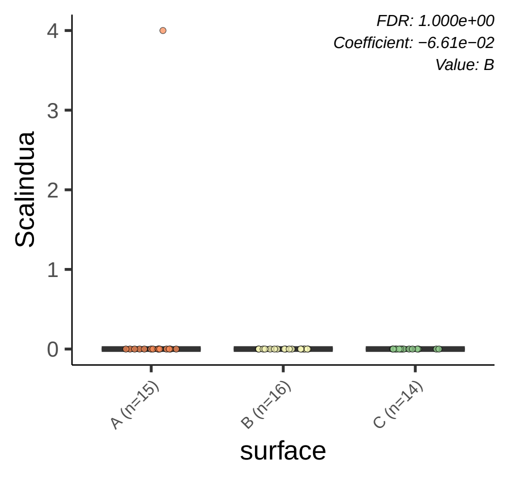
FDR: 1.000e+00
Coefficient: −6.61e−02
Value: B
Scalindua
4
3
2
1
0
A (n=15)
B (n=16)
C (n=14)
surface

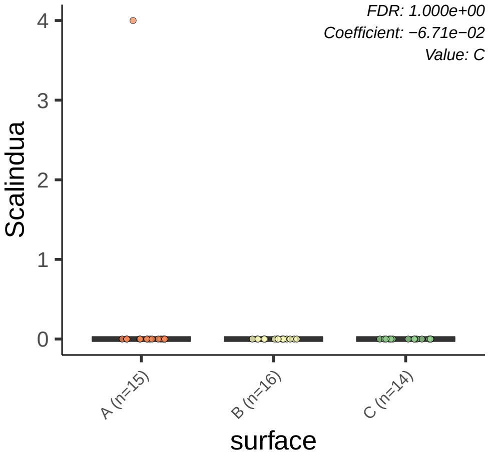
FDR: 1.000e+00
Coefficient: −6.71e−02
Value: C
Scalindua
4
3
2
1
0
A (n=15)
B (n=16)
C (n=14)
surface

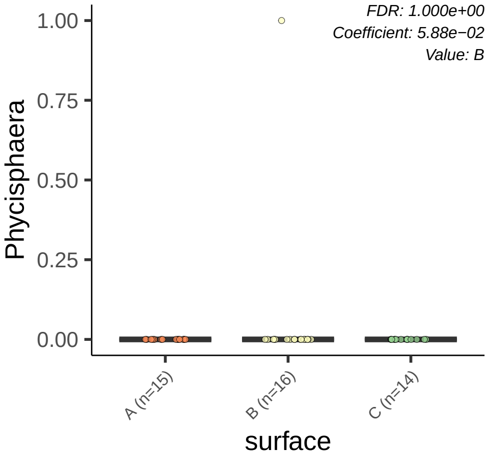
FDR: 1.000e+00
Coefficient: 5.88e−02
Value: B
Phycisphaera
1.00
0.75
0.50
0.25
0.00
A (n=15)
B (n=16)
C (n=14)
surface

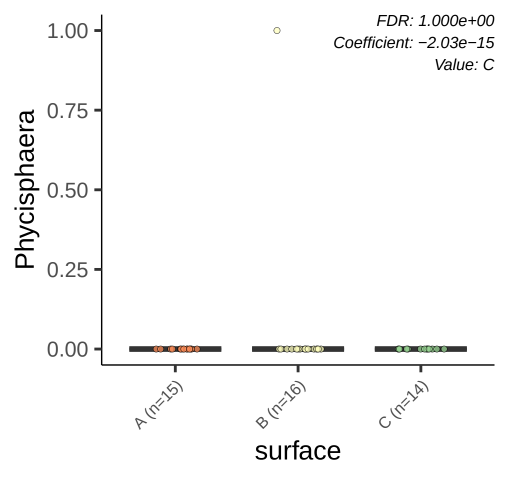
FDR: 1.000e+00
Coefficient: −2.03e−15
Value: C
Phycisphaera
1.00
0.75
0.50
0.25
0.00
A (n=15)
B (n=16)
C (n=14)
surface

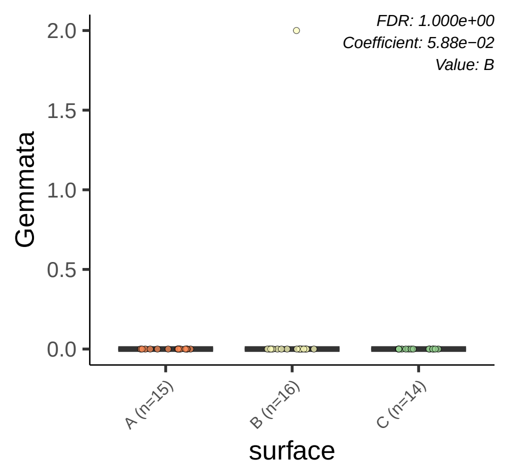
FDR: 1.000e+00
Coefficient: 5.88e−02
Value: B
Gemmata
2.0
1.5
1.0
0.5
0.0
A (n=15)
B (n=16)
C (n=14)
surface

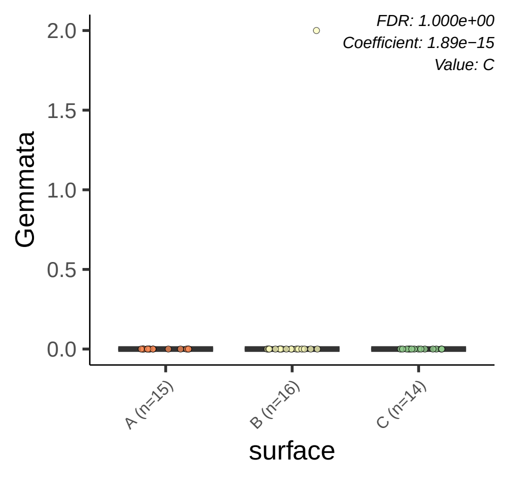
FDR: 1.000e+00
Coefficient: 1.89e−15
Value: C
Gemmata
2.0
1.5
1.0
0.5
0.0
A (n=15)
B (n=16)
C (n=14)
surface

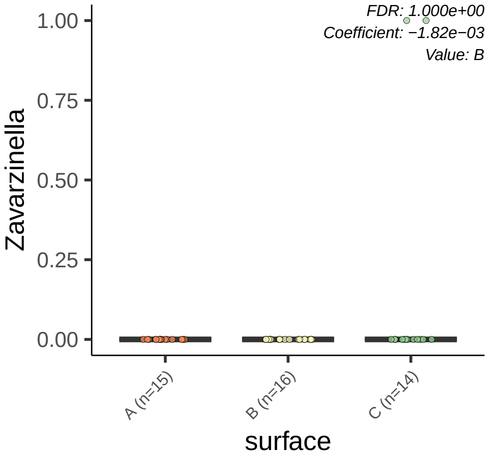
FDR: 1.000e+00
Coefficient: −1.82e−03
Value: B
Zavarzinella
1.00
0.75
0.50
0.25
0.00
A (n=15)
B (n=16)
C (n=14)
surface

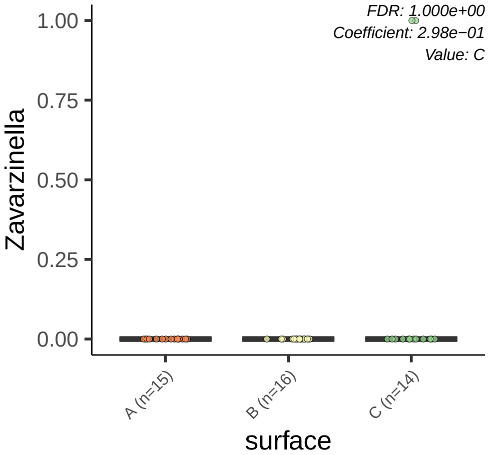
FDR: 1.000e+00
Coefficient: 2.98e−01
Value: C
1.00
0.75
0.50
0.25
0.00
Zavarzinella
A (n=15)
B (n=16)
C (n=14)
surface

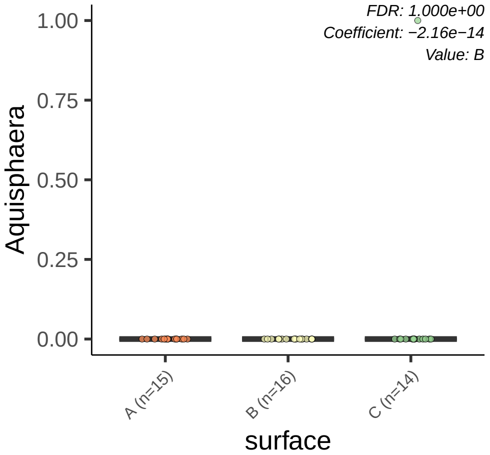

FDR: 1.000e+00
Coefficient: −2.16e−14
Value: B
1.00
0.75
0.50
0.25
0.00
Aquisphaera
A (n=15)
B (n=16)
C (n=14)
surface

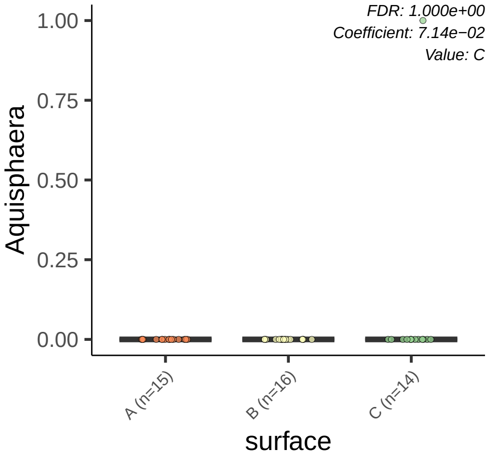
FDR: 1.000e+00
Coefficient: 7.14e−02
Value: C
Aquisphaera
1.00
0.75
0.50
0.25
0.00
A (n=15)
B (n=16)
C (n=14)
surface

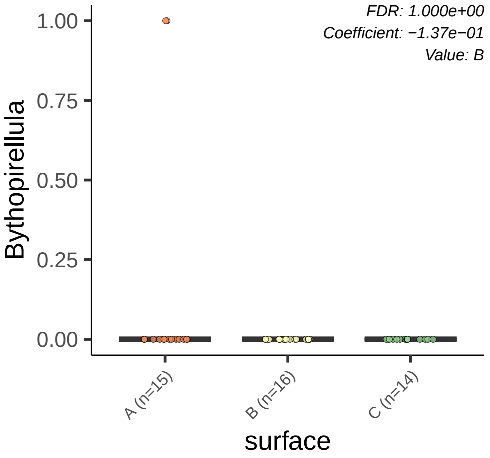
FDR: 1.000e+00
Coefficient: −1.37e−01
Value: B
Bythopirellula
1.00
0.75
0.50
0.25
0.00
A (n=15)
B (n=16)
C (n=14)
surface

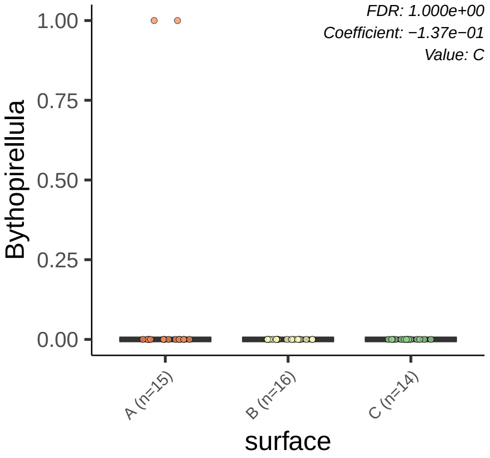
FDR: 1.000e+00
Coefficient: −1.37e−01
Value: C
Bythopirellula
1.00
0.75
0.50
0.25
0.00
A (n=15)
B (n=16)
C (n=14)
surface

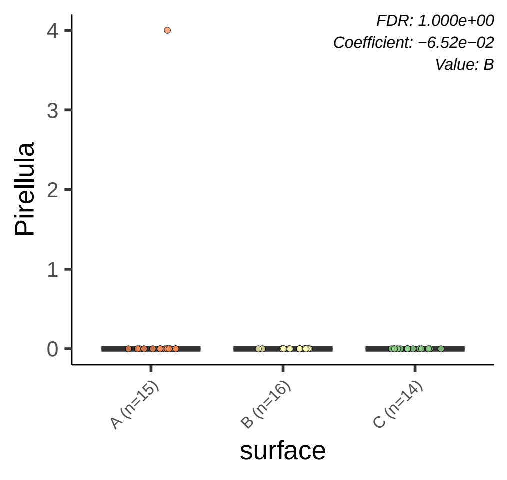
FDR: 1.000e+00
Coefficient: −6.52e−02
Value: B
Pirellula
4
3
2
1
0
A (n=15)
B (n=16)
C (n=14)
surface

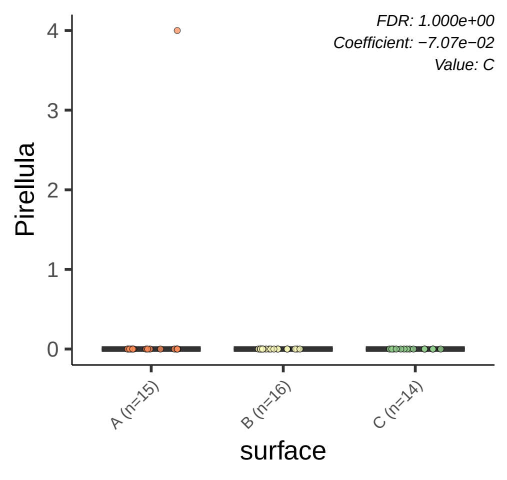

FDR: 1.000e+00
Coefficient: −7.07e−02
Value: C
Pirellula
0
1
2
3
4
A (n=15)
B (n=16)
C (n=14)
surface

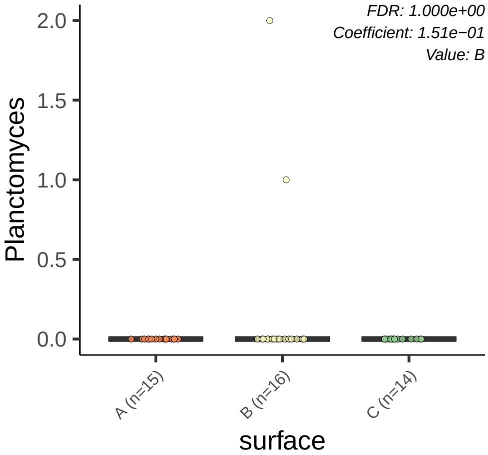
FDR: 1.000e+00
Coefficient: 1.51e−01
Value: B
Planctomyces
2.0
1.5
1.0
0.5
0.0
A (n=15)
B (n=16)
C (n=14)
surface

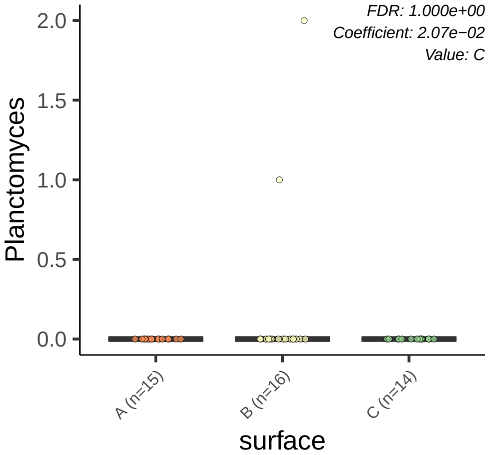

FDR: 1.000e+00
Coefficient: 2.07e−02
Value: C
Planctomyces
2.0
1.5
1.0
0.5
0.0
A (n=15)
B (n=16)
C (n=14)
surface

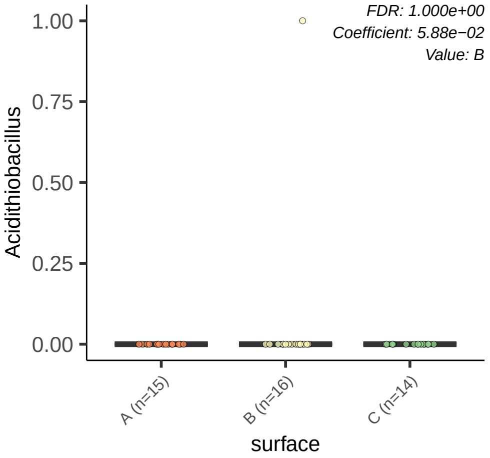

FDR: 1.000e+00
Coefficient: 5.88e−02
Value: B
Acidithiobacillus
1.00
0.75
0.50
0.25
0.00
A (n=15)
B (n=16)
C (n=14)
surface

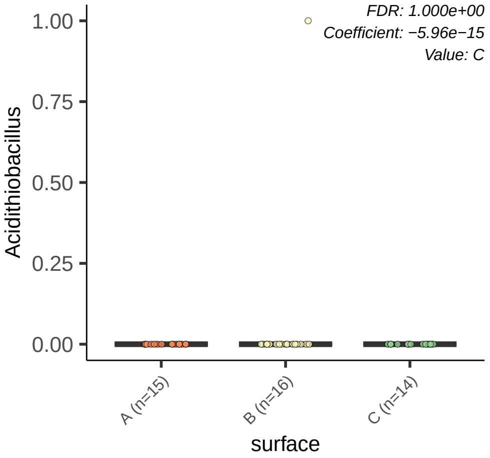
FDR: 1.000e+00
Coefficient: −5.96e−15
Value: C
Acidithiobacillus
1.00
0.75
0.50
0.25
0.00
A (n=15)
B (n=16)
C (n=14)
surface

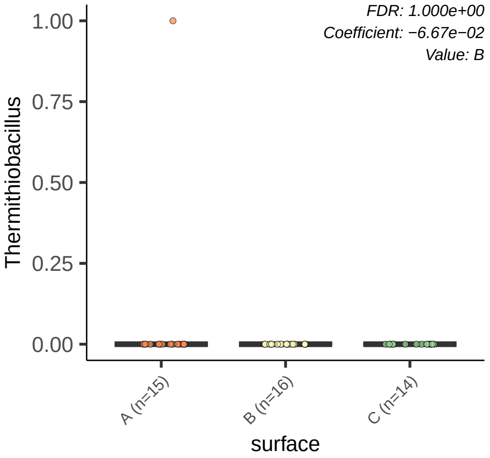

FDR: 1.000e+00
Coefficient: −6.67e−02
Value: B
Thermithiobacillus
1.00
0.75
0.50
0.25
0.00
A (n=15)
B (n=16)
C (n=14)
surface

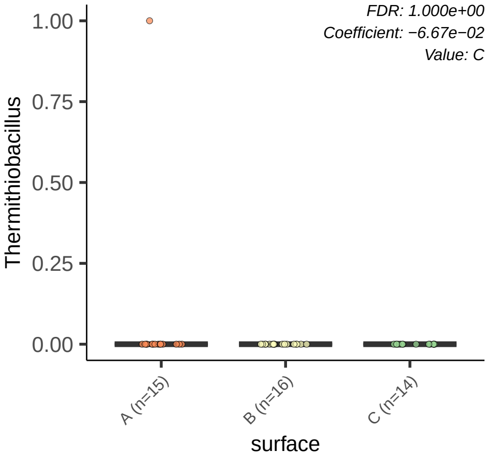
FDR: 1.000e+00
Coefficient: −6.67e−02
Value: C
Thermithiobacillus
1.00
0.75
0.50
0.25
0.00
A (n=15)
B (n=16)
C (n=14)
surface

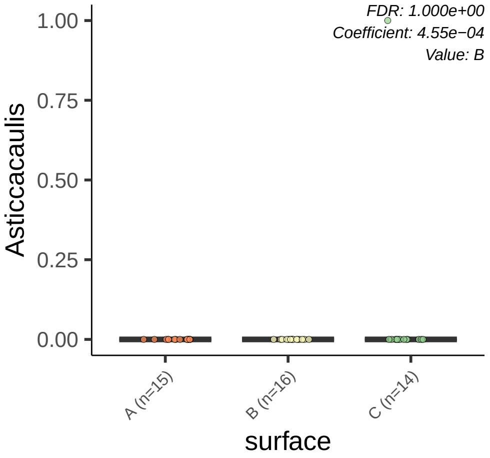

FDR: 1.000e+00
Coefficient: 4.55e−04
Value: B
Asticcacaulis
1.00
0.75
0.50
0.25
0.00
A (n=15)
B (n=16)
C (n=14)
surface

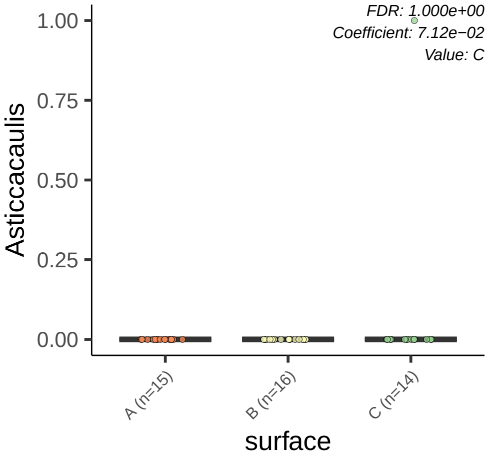

FDR: 1.000e+00
Coefficient: 7.12e−02
Value: C
1.00
0.75
0.50
0.25
0.00
Asticcacaulis
A (n=15)
B (n=16)
C (n=14)
surface

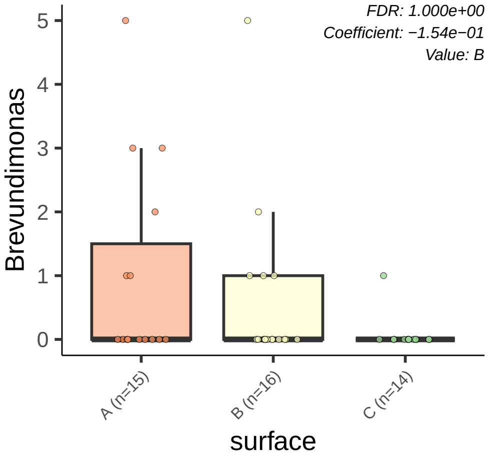
FDR: 1.000e+00
Coefficient: −1.54e−01
Value: B
Brevundimonas
0
1
2
3
4
5
A (n=15)
B (n=16)
C (n=14)
surface

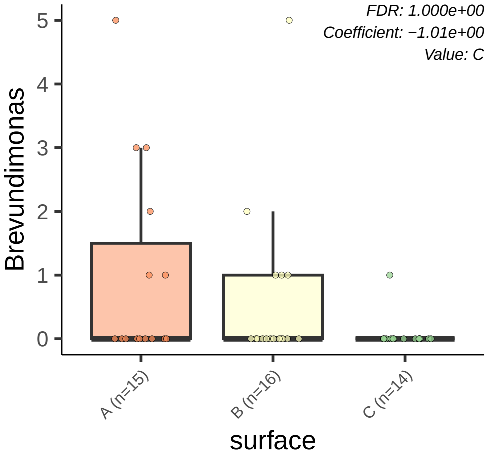

FDR: 1.000e+00
Coefficient: −1.01e+00
Value: C
Brevundimonas
0
1
2
3
4
5
A (n=15)
B (n=16)
C (n=14)
surface

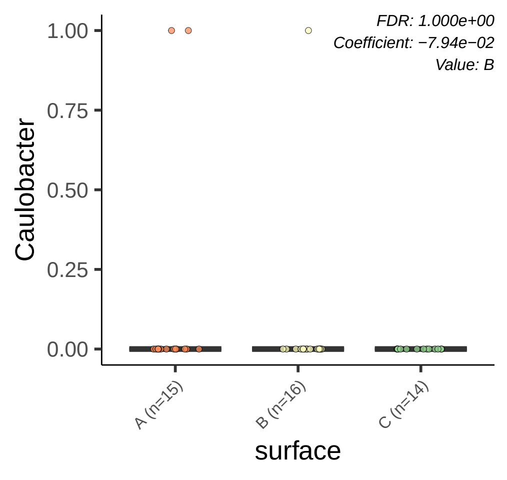
FDR: 1.000e+00
Coefficient: −7.94e−02
Value: B
1.00
0.75
0.50
0.25
0.00
Caulobacter
A (n=15)
B (n=16)
C (n=14)
surface

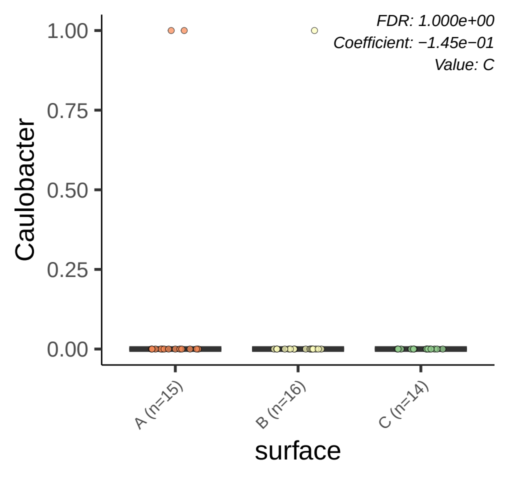

FDR: 1.000e+00
Coefficient: −1.45e−01
Value: C
Caulobacter
1.00
0.75
0.50
0.25
0.00
A (n=15)
B (n=16)
C (n=14)
surface

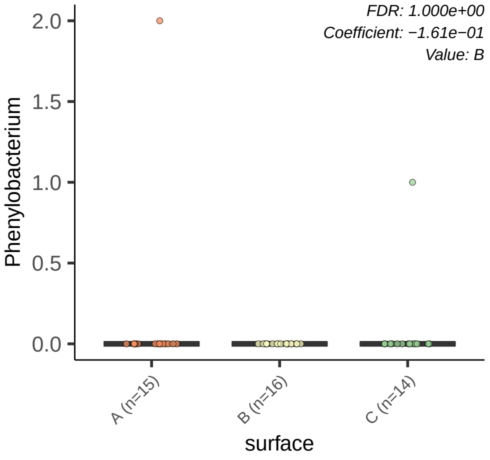
FDR: 1.000e+00
Coefficient: −1.61e−01
Value: B
Phenylobacterium
2.0
1.5
1.0
0.5
0.0
A (n=15)
B (n=16)
C (n=14)
surface

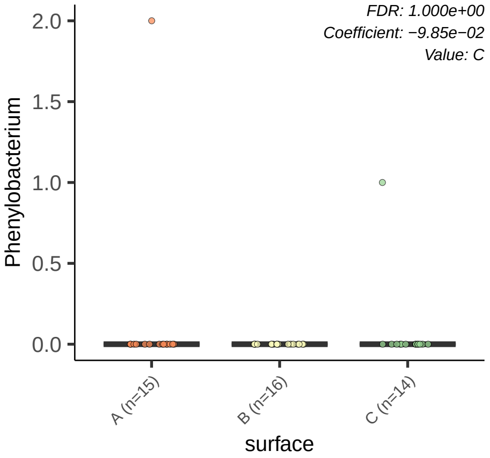
FDR: 1.000e+00
Coefficient: −9.85e−02
Value: C
Phenylobacterium
2.0
1.5
1.0
0.5
0.0
A (n=15)
B (n=16)
C (n=14)
surface

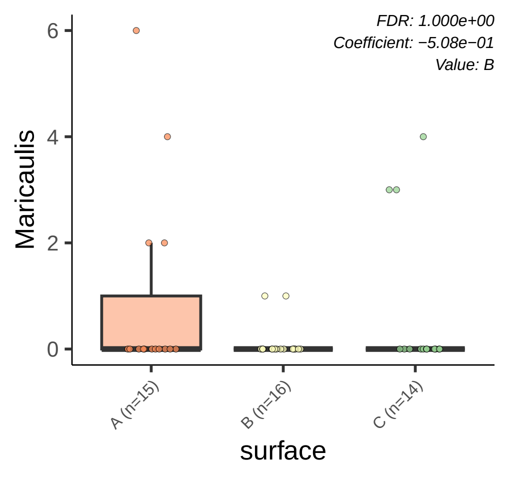
FDR: 1.000e+00
Coefficient: −5.08e−01
Value: B
Maricaulis
6
4
2
0
A (n=15)
B (n=16)
C (n=14)
surface

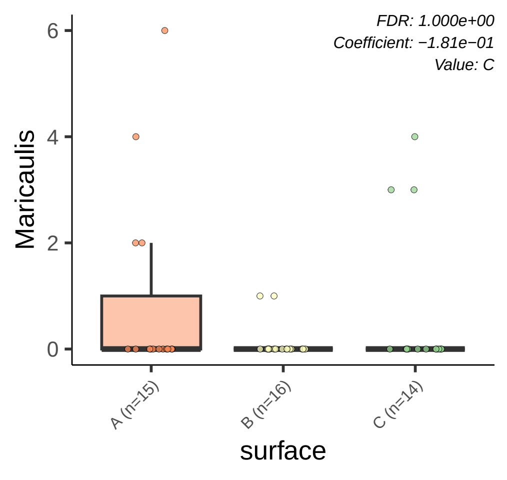

FDR: 1.000e+00
Coefficient: −1.81e−01
Value: C
Maricaulis
6
4
2
0
A (n=15)
B (n=16)
C (n=14)
surface

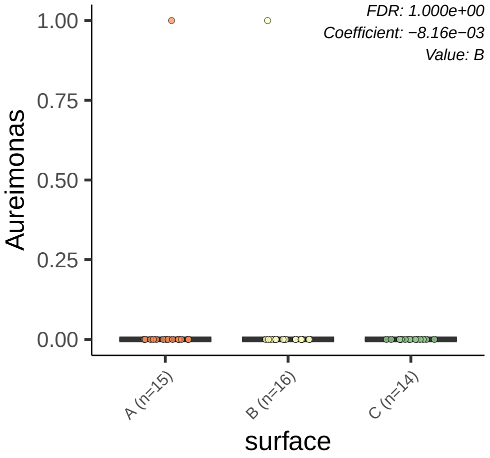

FDR: 1.000e+00
Coefficient: −8.16e−03
Value: B
Aureimonas
1.00
0.75
0.50
0.25
0.00
A (n=15)
B (n=16)
C (n=14)
surface

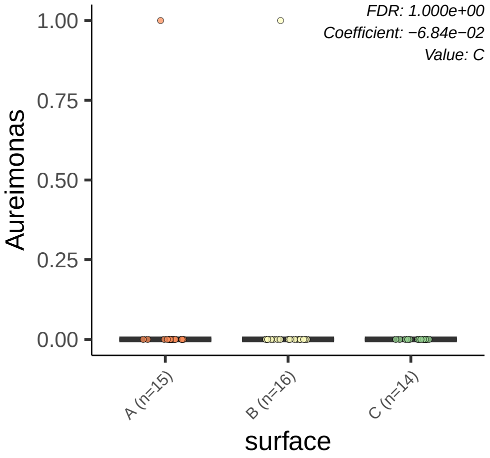

FDR: 1.000e+00
Coefficient: −6.84e−02
Value: C
Aureimonas
1.00
0.75
0.50
0.25
0.00
A (n=15)
B (n=16)
C (n=14)
surface

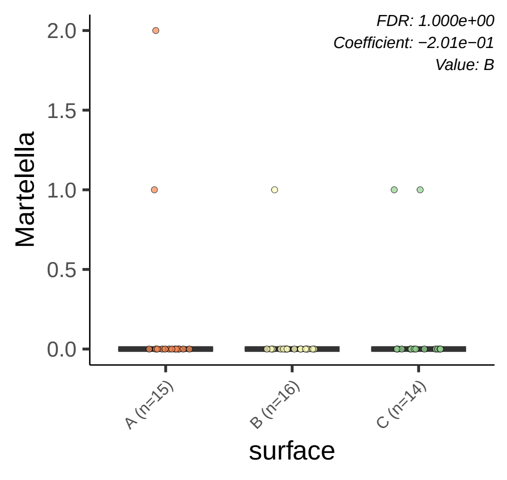
FDR: 1.000e+00
Coefficient: −2.01e−01
Value: B
Martelella
2.0
1.5
1.0
0.5
0.0
A (n=15)
B (n=16)
C (n=14)
surface

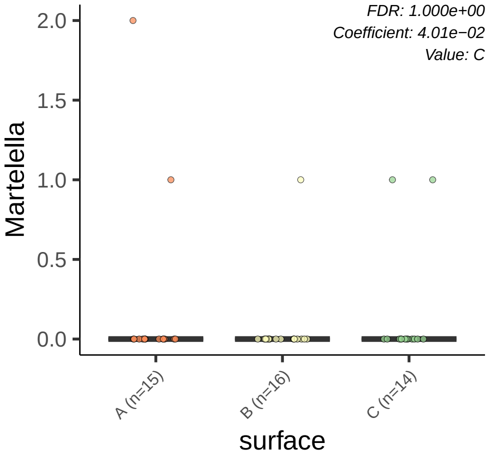

FDR: 1.000e+00
Coefficient: 4.01e−02
Value: C
Martelella
2.0
1.5
1.0
0.5
0.0
A (n=15)
B (n=16)
C (n=14)
surface

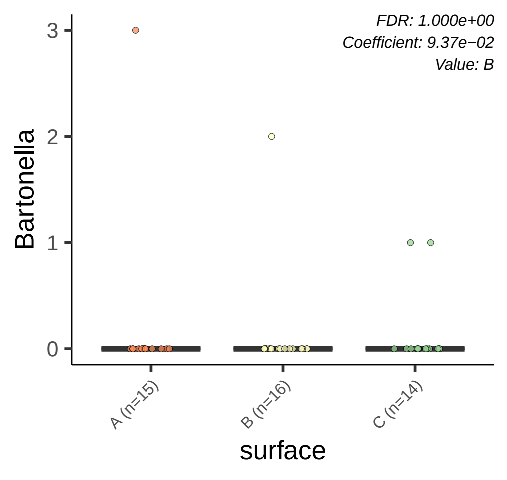

FDR: 1.000e+00
Coefficient: 9.37e−02
Value: B
Bartonella
3
2
1
0
A (n=15)
B (n=16)
C (n=14)
surface

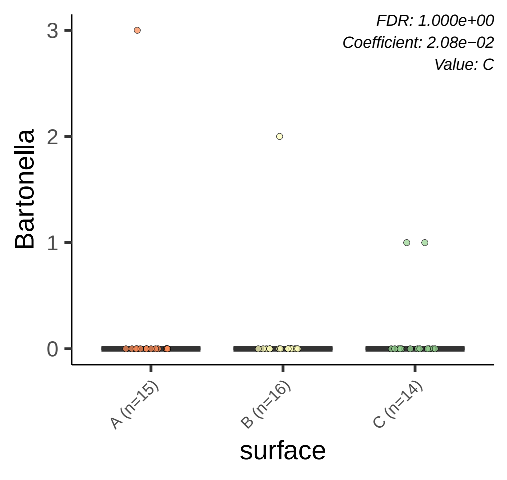
FDR: 1.000e+00
Coefficient: 2.08e−02
Value: C
Bartonella
3
2
1
0
A (n=15)
B (n=16)
C (n=14)
surface

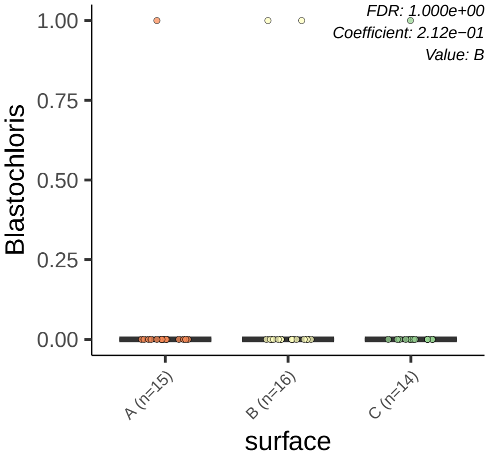
FDR: 1.000e+00
Coefficient: 2.12e−01
Value: B
Blastochloris
1.00
0.75
0.50
0.25
0.00
A (n=15)
B (n=16)
C (n=14)
surface

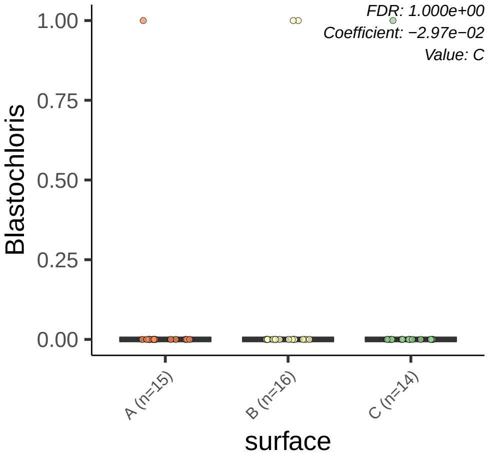

FDR: 1.000e+00
Coefficient: −2.97e−02
Value: C
Blastochloris
1.00
0.75
0.50
0.25
0.00
A (n=15)
B (n=16)
C (n=14)
surface

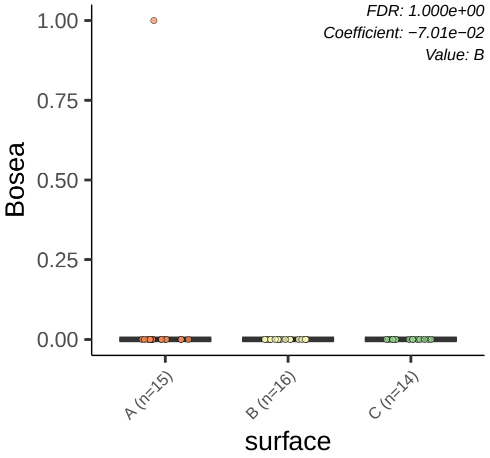

FDR: 1.000e+00
Coefficient: −7.01e−02
Value: B
Bosea
1.00
0.75
0.50
0.25
0.00
A (n=15)
B (n=16)
C (n=14)
surface

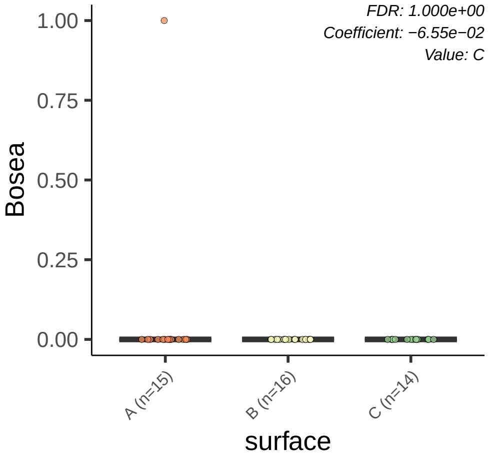

FDR: 1.000e+00
Coefficient: −6.55e−02
Value: C
Bosea
1.00
0.75
0.50
0.25
0.00
A (n=15)
B (n=16)
C (n=14)
surface

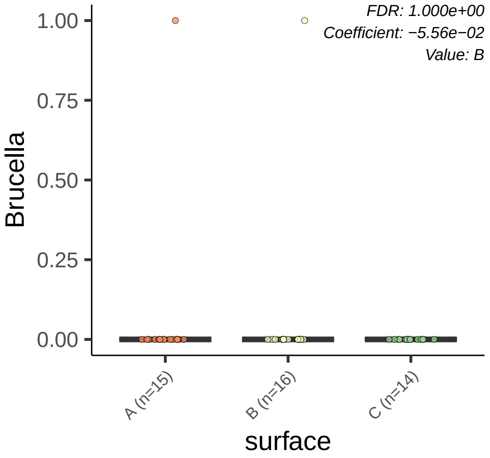
FDR: 1.000e+00
Coefficient: −5.56e−02
Value: B
Brucella
1.00
0.75
0.50
0.25
0.00
A (n=15)
B (n=16)
C (n=14)
surface

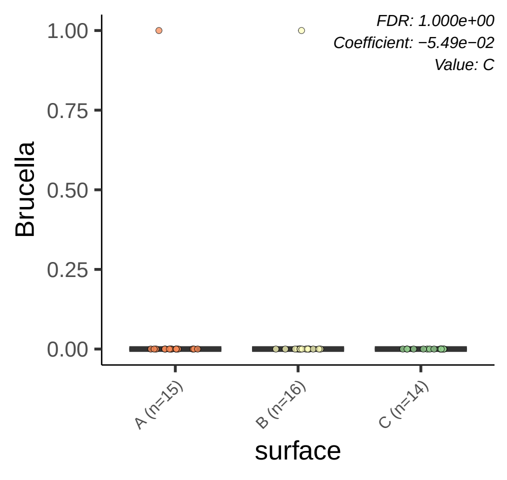
FDR: 1.000e+00
Coefficient: −5.49e−02
Value: C
Brucella
1.00
0.75
0.50
0.25
0.00
A (n=15)
B (n=16)
C (n=14)
surface

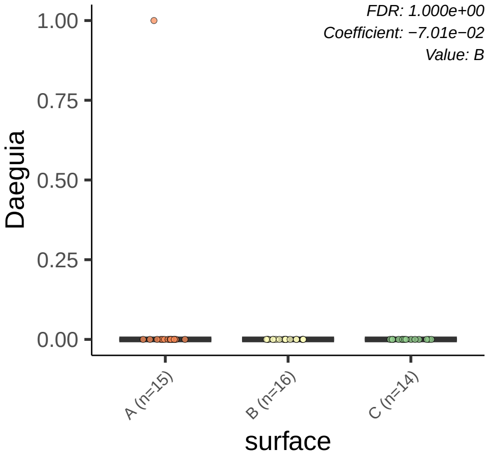
FDR: 1.000e+00
Coefficient: −7.01e−02
Value: B
Daeguia
1.00
0.75
0.50
0.25
0.00
A (n=15)
B (n=16)
C (n=14)
surface

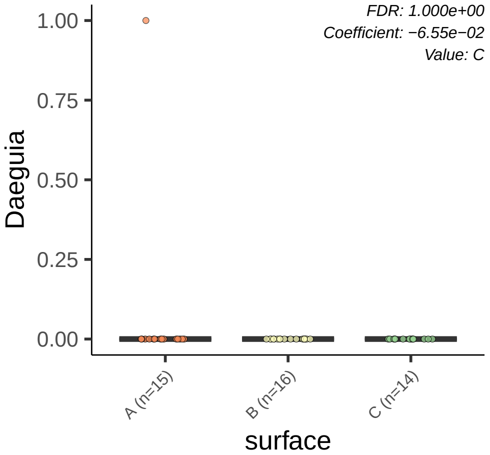
FDR: 1.000e+00
Coefficient: −6.55e−02
Value: C
Daeguia
1.00
0.75
0.50
0.25
0.00
A (n=15)
B (n=16)
C (n=14)
surface

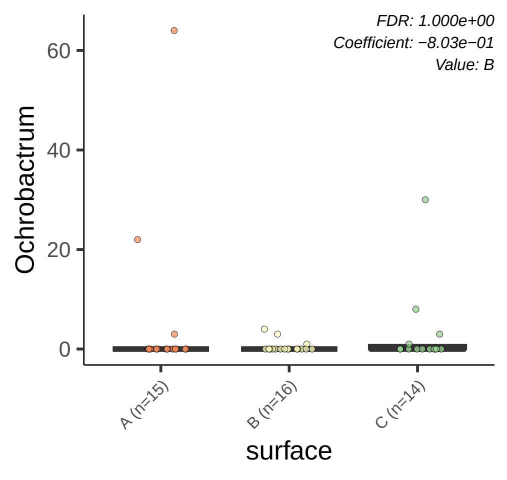

FDR: 1.000e+00
Coefficient: −8.03e−01
Value: B
Ochrobactrum
60
40
20
0
A (n=15)
B (n=16)
C (n=14)
surface

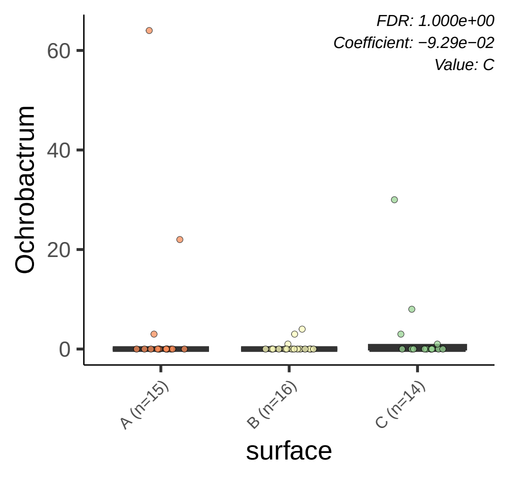
FDR: 1.000e+00
Coefficient: −9.29e−02
Value: C
Ochrobactrum
60
40
20
0
A (n=15)
B (n=16)
C (n=14)
surface

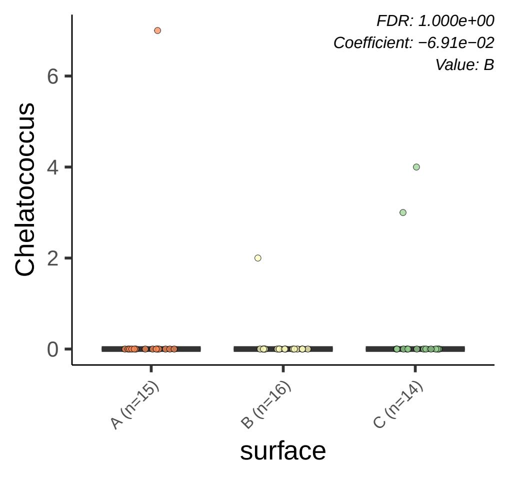
FDR: 1.000e+00
Coefficient: −6.91e−02
Value: B
Chelatococcus
0
2
4
6
A (n=15)
B (n=16)
C (n=14)
surface

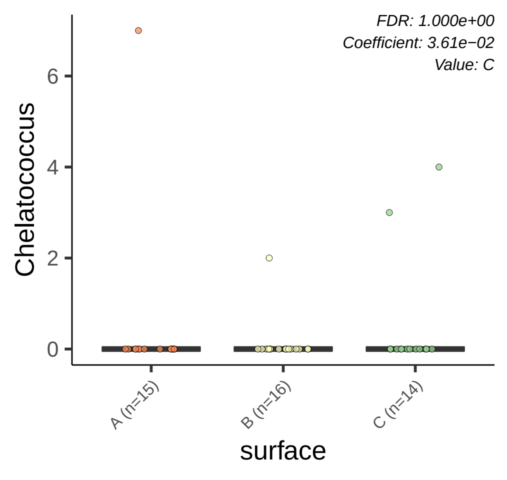

FDR: 1.000e+00
Coefficient: 3.61e−02
Value: C
Chelatococcus
0
2
4
6
A (n=15)
B (n=16)
C (n=14)
surface

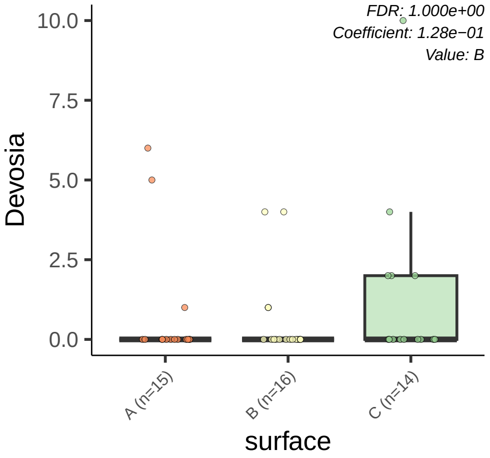
FDR: 1.000e+00
Coefficient: 1.28e−01
Value: B
Devosia
10.0
7.5
5.0
2.5
0.0
A (n=15)
B (n=16)
C (n=14)
surface

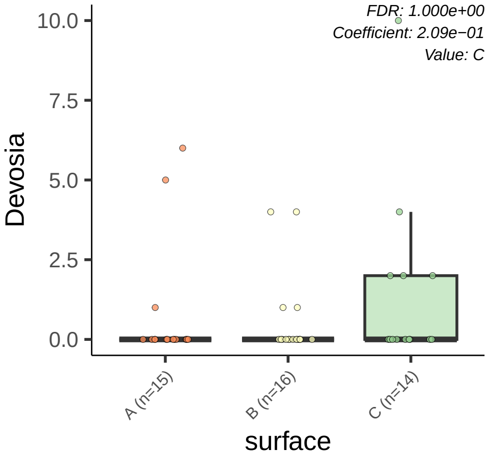
FDR: 1.000e+00
Coefficient: 2.09e−01
Value: C
Devosia
10.0
7.5
5.0
2.5
0.0
A (n=15)
B (n=16)
C (n=14)
surface

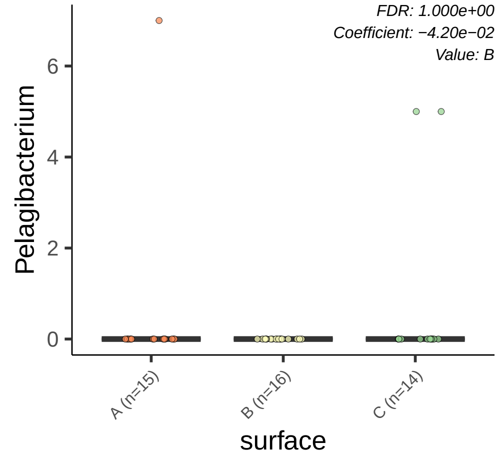
FDR: 1.000e+00
Coefficient: −4.20e−02
Value: B
Pelagibacterium
0
2
4
6
A (n=15)
B (n=16)
C (n=14)
surface

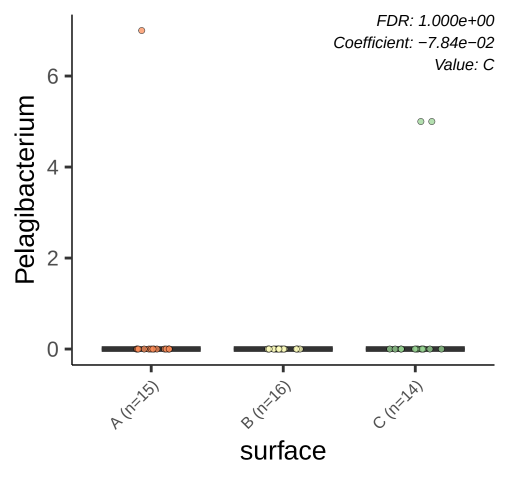

FDR: 1.000e+00
Coefficient: −7.84e−02
Value: C
Pelagibacterium
0
2
4
6
A (n=15)
B (n=16)
C (n=14)
surface

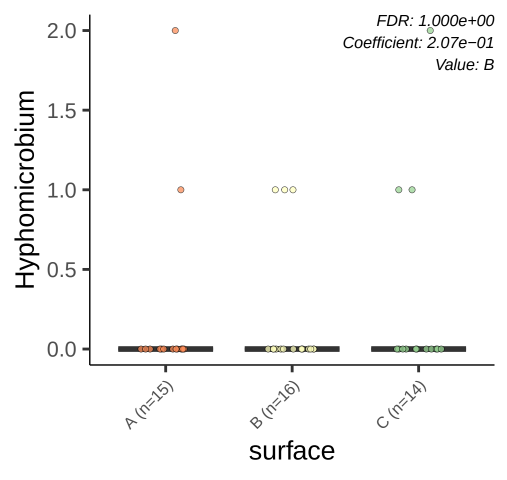
FDR: 1.000e+00
Coefficient: 2.07e−01
Value: B
Hyphomicrobium
2.0
1.5
1.0
0.5
0.0
A (n=15)
B (n=16)
C (n=14)
surface

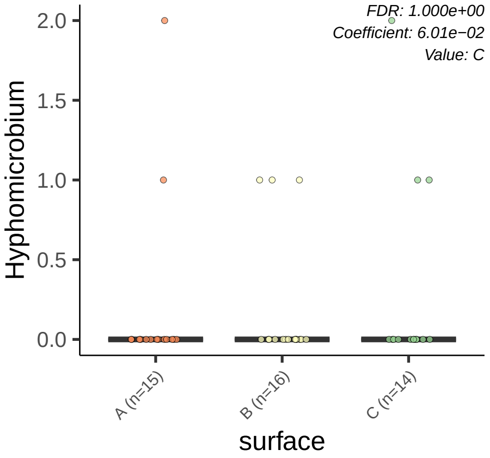

FDR: 1.000e+00
Coefficient: 6.01e−02
Value: C
Hyphomicrobium
2.0
1.5
1.0
0.5
0.0
A (n=15)
B (n=16)
C (n=14)
surface

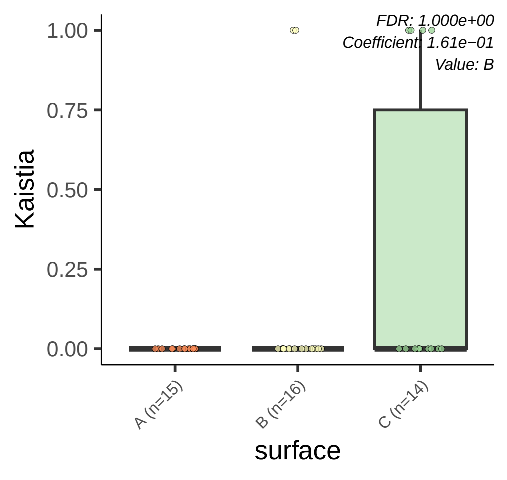

FDR: 1.000e+00
Coefficient: 1.61e−01
Value: B
Kaistia
1.00
0.75
0.50
0.25
0.00
A (n=15)
B (n=16)
C (n=14)
surface

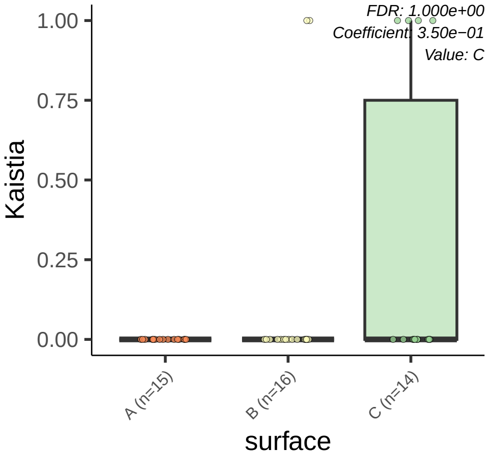
FDR: 1.000e+00
Coefficient: 3.50e−01
Value: C
Kaistia
1.00
0.75
0.50
0.25
0.00
A (n=15)
B (n=16)
C (n=14)
surface

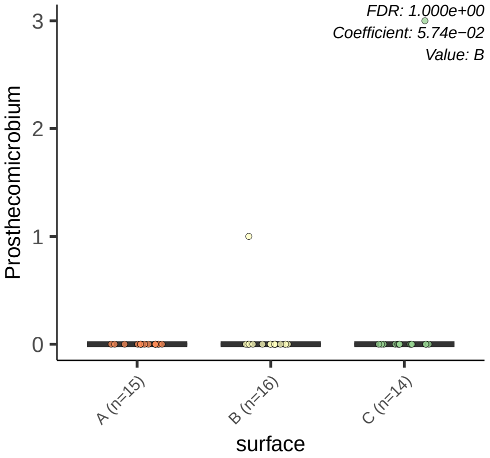

FDR: 1.000e+00
Coefficient: 5.74e−02
Value: B
Prosthecomicrobium
3
2
1
0
A (n=15)
B (n=16)
C (n=14)
surface

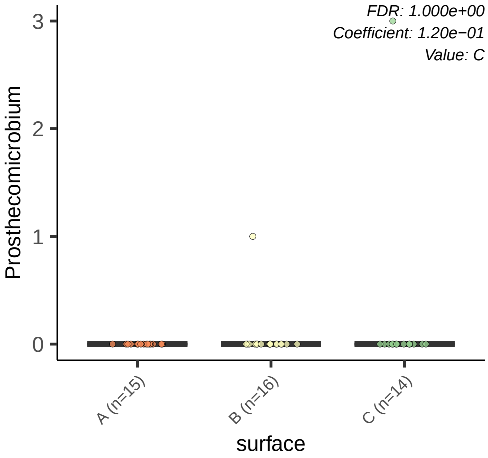
FDR: 1.000e+00
Coefficient: 1.20e−01
Value: C
Prosthecomicrobium
3
2
1
0
A (n=15)
B (n=16)
C (n=14)
surface

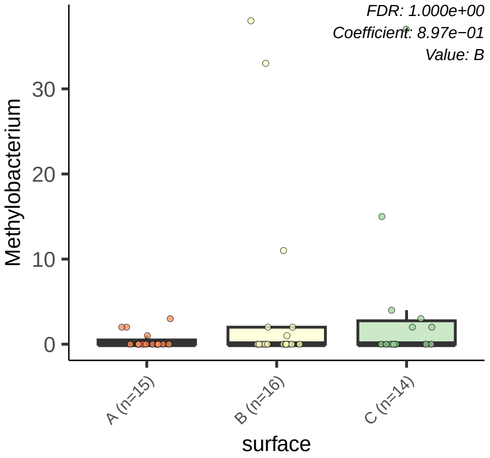

FDR: 1.000e+00
Coefficient: 8.97e−01
Value: B
Methylobacterium
0
10
20
30
A (n=15)
B (n=16)
C (n=14)
surface

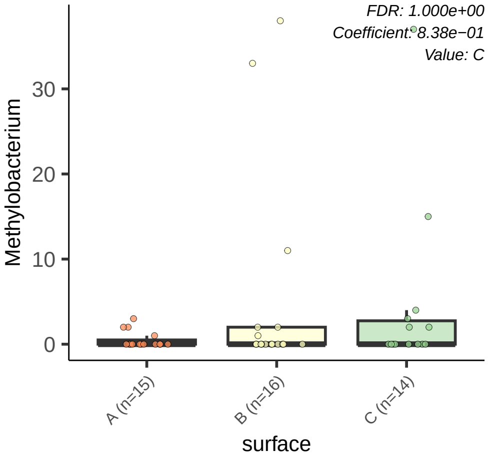

FDR: 1.000e+00
Coefficient: 8.38e−01
Value: C
Methylobacterium
0
10
20
30
A (n=15)
B (n=16)
C (n=14)
surface

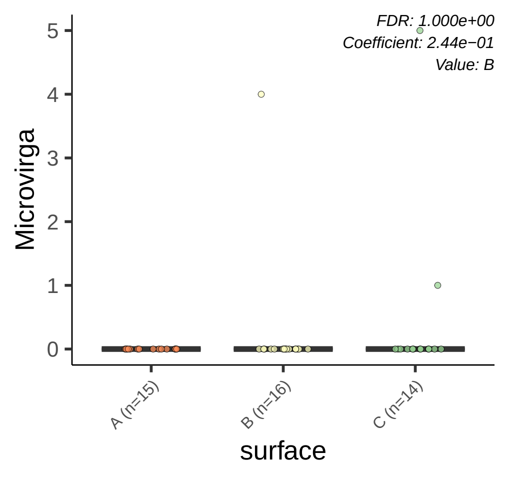
FDR: 1.000e+00
Coefficient: 2.44e−01
Value: B
Microvirga
5
4
3
2
1
0
A (n=15)
B (n=16)
C (n=14)
surface

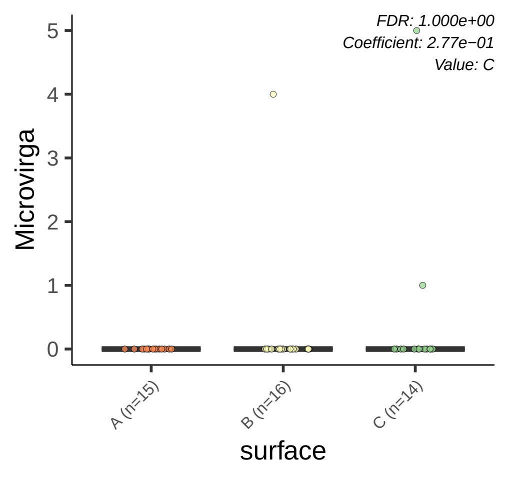

FDR: 1.000e+00
Coefficient: 2.77e−01
Value: C
Microvirga
5
4
3
2
1
0
A (n=15)
B (n=16)
C (n=14)
surface

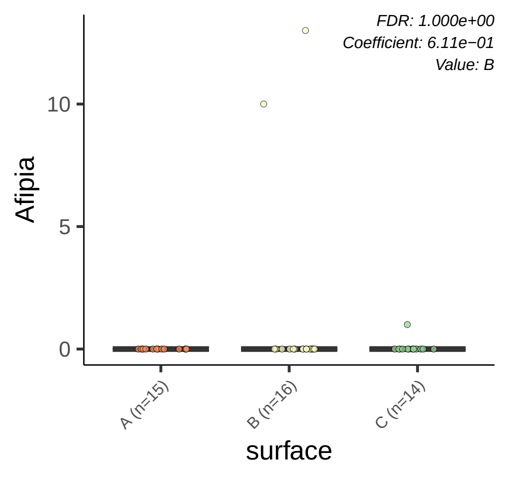
FDR: 1.000e+00
Coefficient: 6.11e−01
Value: B
Afipia
10
5
0
A (n=15)
B (n=16)
C (n=14)
surface

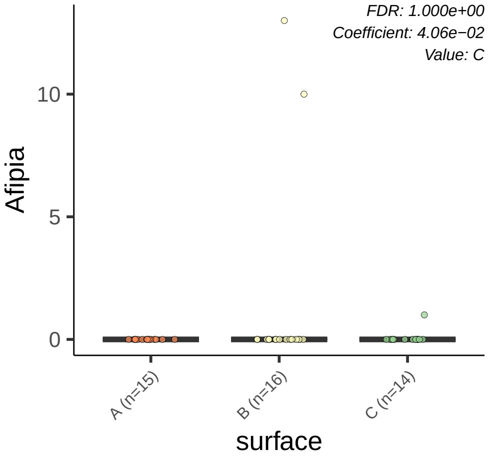

FDR: 1.000e+00
Coefficient: 4.06e−02
Value: C
Afipia
10
5
0
A (n=15)
B (n=16)
C (n=14)
surface

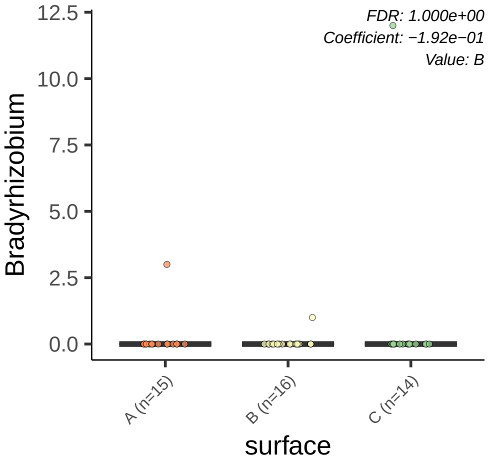
FDR: 1.000e+00
Coefficient: −1.92e−01
Value: B
Bradyrhizobium
12.5
10.0
7.5
5.0
2.5
0.0
A (n=15)
B (n=16)
C (n=14)
surface

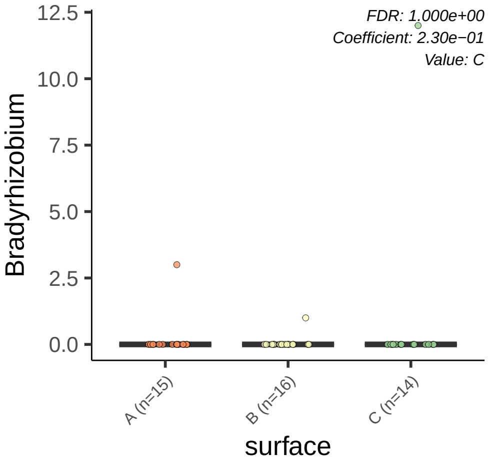

FDR: 1.000e+00
Coefficient: 2.30e−01
Value: C
Bradyrhizobium
12.5
10.0
7.5
5.0
2.5
0.0
A (n=15)
B (n=16)
C (n=14)
surface

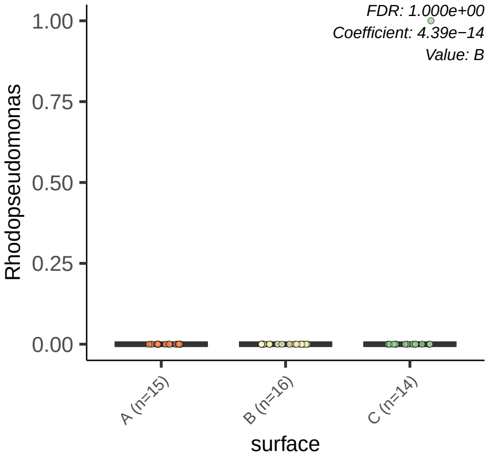
FDR: 1.000e+00
Coefficient: 4.39e−14
Value: B
Rhodopseudomonas
1.00
0.75
0.50
0.25
0.00
A (n=15)
B (n=16)
C (n=14)
surface

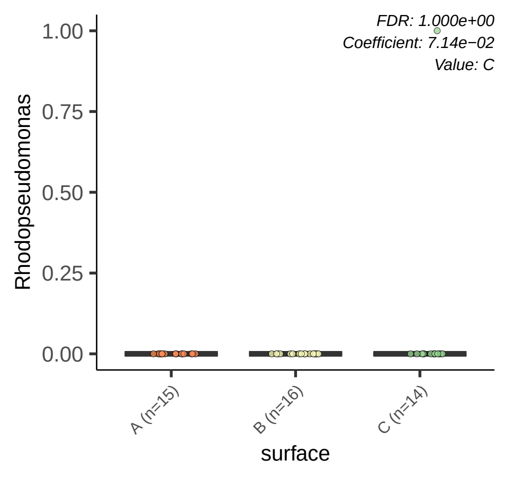
FDR: 1.000e+00
Coefficient: 7.14e−02
Value: C
Rhodopseudomonas
1.00
0.75
0.50
0.25
0.00
A (n=15)
B (n=16)
C (n=14)
surface

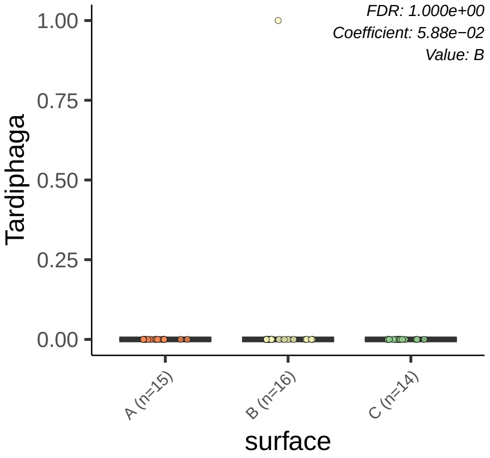

FDR: 1.000e+00
Coefficient: 5.88e−02
Value: B
Tardiphaga
1.00
0.75
0.50
0.25
0.00
A (n=15)
B (n=16)
C (n=14)
surface

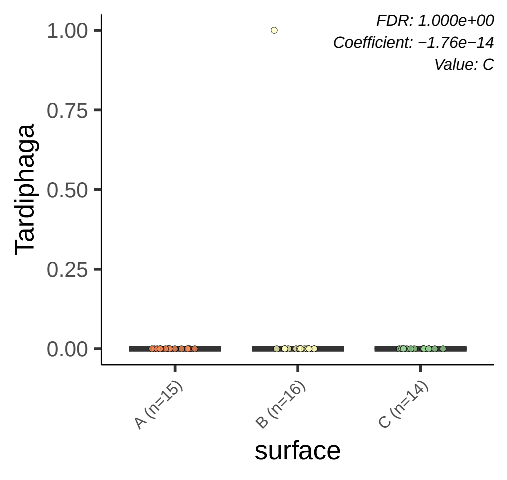
FDR: 1.000e+00
Coefficient: −1.76e−14
Value: C
Tardiphaga
1.00
0.75
0.50
0.25
0.00
A (n=15)
B (n=16)
C (n=14)
surface

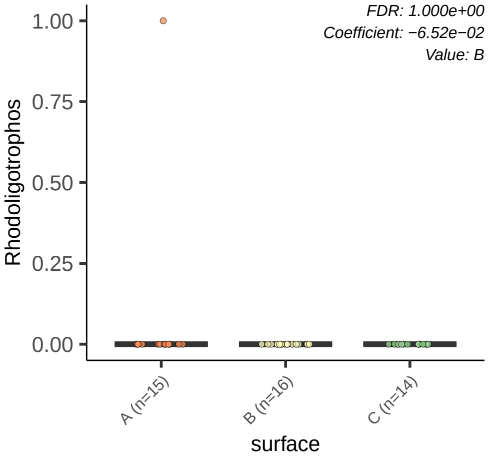

FDR: 1.000e+00
Coefficient: −6.52e−02
Value: B
Rhodoligotrophos
1.00
0.75
0.50
0.25
0.00
A (n=15)
B (n=16)
C (n=14)
surface

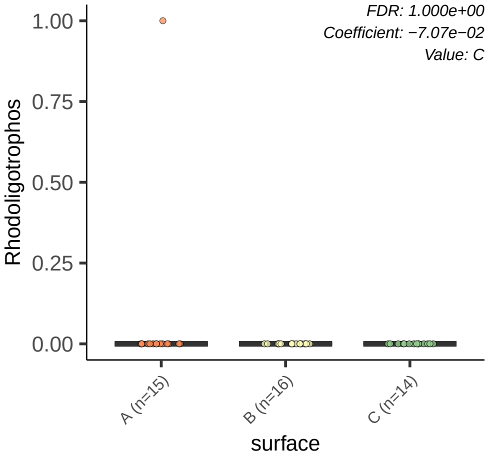
FDR: 1.000e+00
Coefficient: −7.07e−02
Value: C
Rhodoligotrophos
1.00
0.75
0.50
0.25
0.00
A (n=15)
B (n=16)
C (n=14)
surface

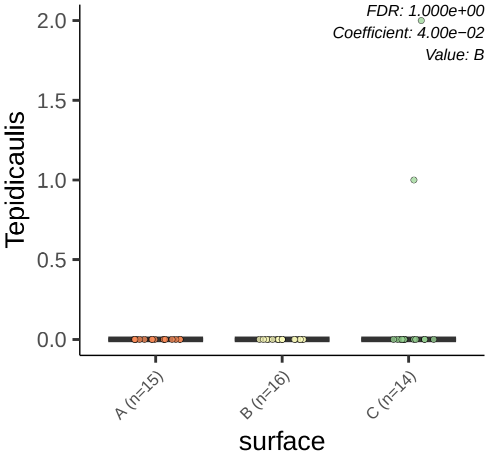
FDR: 1.000e+00
Coefficient: 4.00e−02
Value: B
Tepidicaulis
2.0
1.5
1.0
0.5
0.0
A (n=15)
B (n=16)
C (n=14)
surface

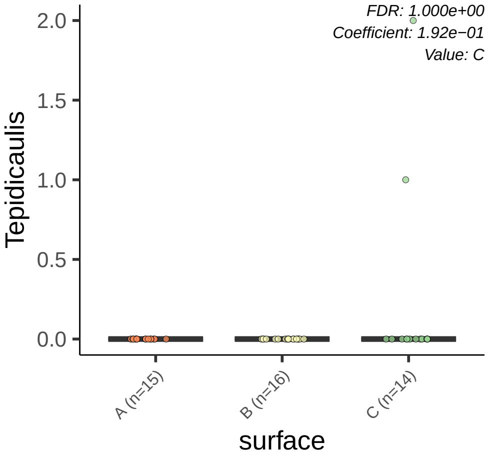
FDR: 1.000e+00
Coefficient: 1.92e−01
Value: C
Tepidicaulis
2.0
1.5
1.0
0.5
0.0
A (n=15)
B (n=16)
C (n=14)
surface

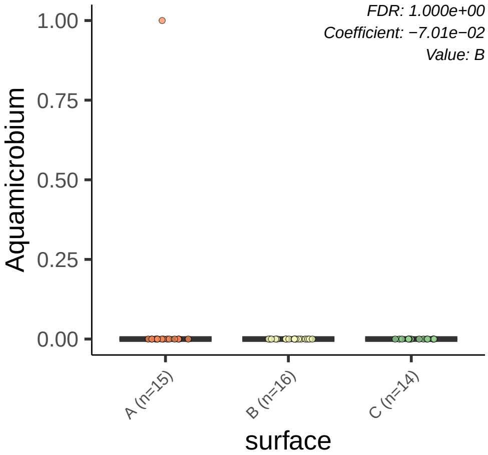
FDR: 1.000e+00
Coefficient: −7.01e−02
Value: B
Aquamicrobium
1.00
0.75
0.50
0.25
0.00
A (n=15)
B (n=16)
C (n=14)
surface

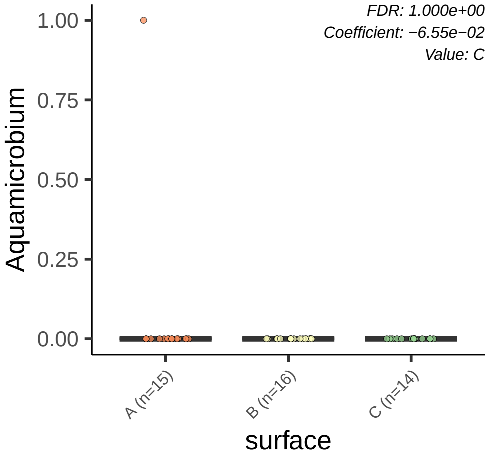

FDR: 1.000e+00
Coefficient: −6.55e−02
Value: C
Aquamicrobium
1.00
0.75
0.50
0.25
0.00
A (n=15)
B (n=16)
C (n=14)
surface

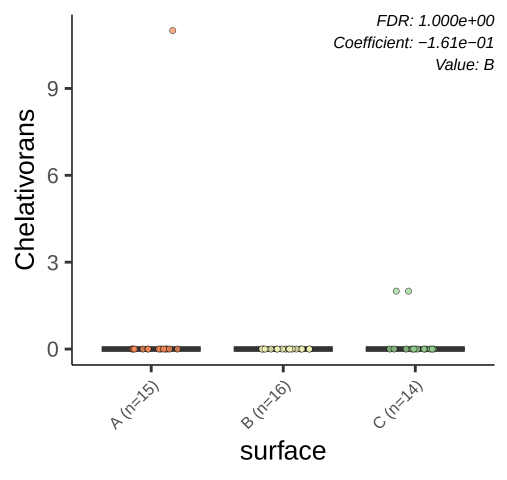

FDR: 1.000e+00
Coefficient: −1.61e−01
Value: B
Chelativorans
9
6
3
0
A (n=15)
B (n=16)
C (n=14)
surface

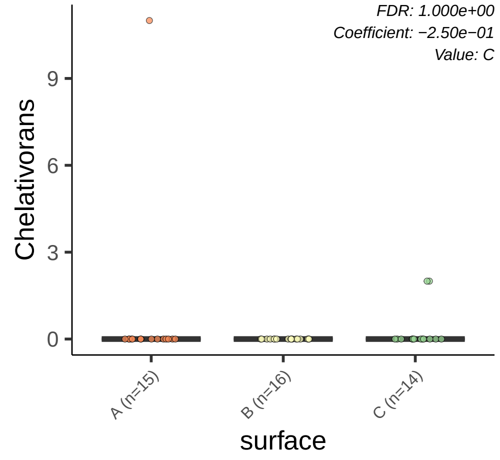

FDR: 1.000e+00
Coefficient: −2.50e−01
Value: C
Chelativorans
9
6
3
0
A (n=15)
B (n=16)
C (n=14)
surface

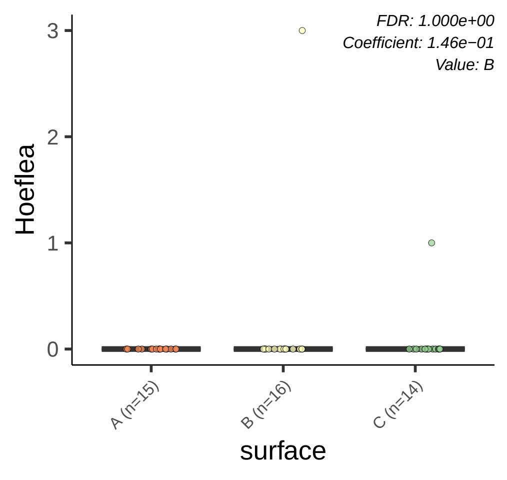

FDR: 1.000e+00
Coefficient: 1.46e−01
Value: B
Hoeflea
3
2
1
0
A (n=15)
B (n=16)
C (n=14)
surface

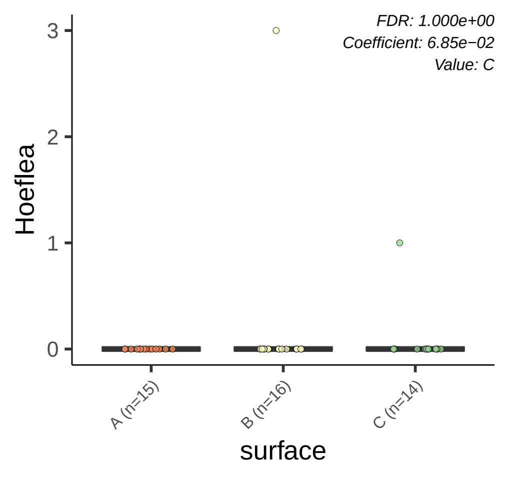
FDR: 1.000e+00
Coefficient: 6.85e−02
Value: C
Hoeflea
0
1
2
3
A (n=15)
B (n=16)
C (n=14)
surface

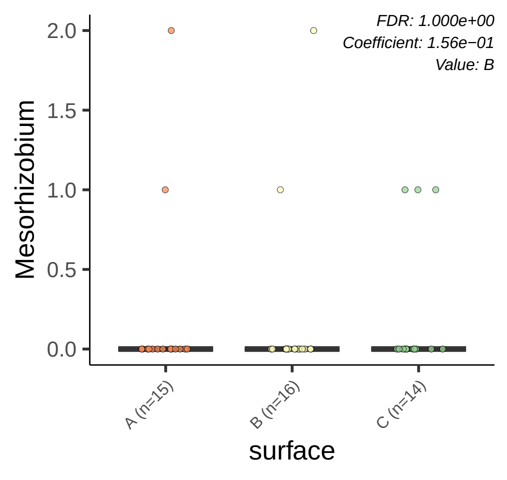

FDR: 1.000e+00
Coefficient: 1.56e−01
Value: B
Mesorhizobium
2.0
1.5
1.0
0.5
0.0
A (n=15)
B (n=16)
C (n=14)
surface

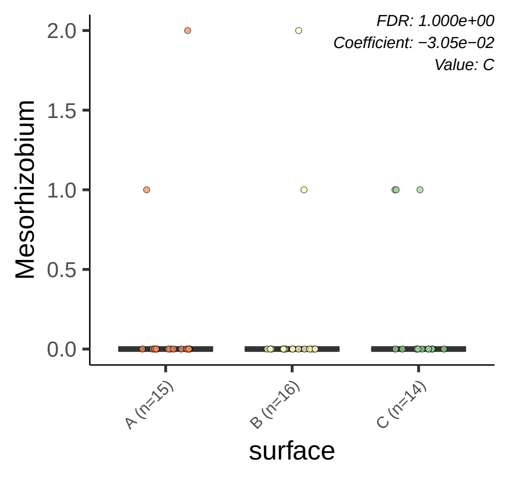

FDR: 1.000e+00
Coefficient: −3.05e−02
Value: C
Mesorhizobium
2.0
1.5
1.0
0.5
0.0
A (n=15)
B (n=16)
C (n=14)
surface

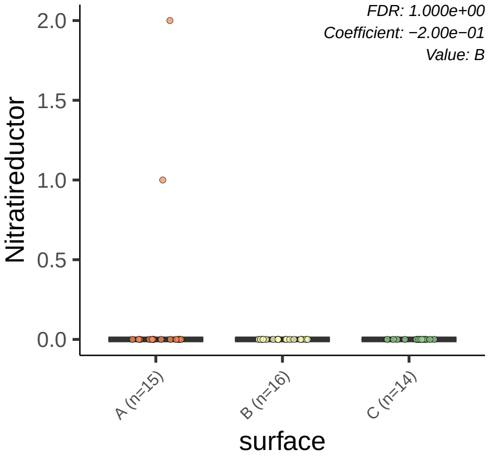

FDR: 1.000e+00
Coefficient: −2.00e−01
Value: B
Nitratireductor
2.0
1.5
1.0
0.5
0.0
A (n=15)
B (n=16)
C (n=14)
surface

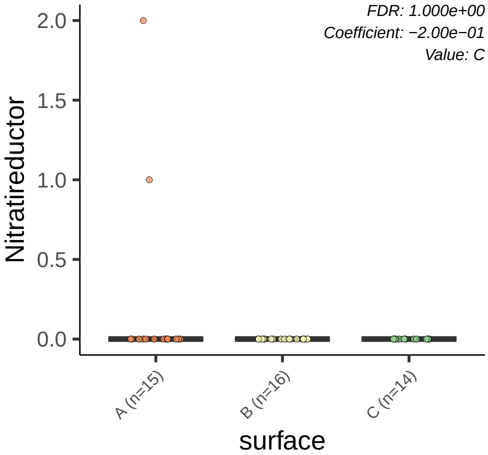

FDR: 1.000e+00
Coefficient: −2.00e−01
Value: C
Nitratireductor
2.0
1.5
1.0
0.5
0.0
A (n=15)
B (n=16)
C (n=14)
surface

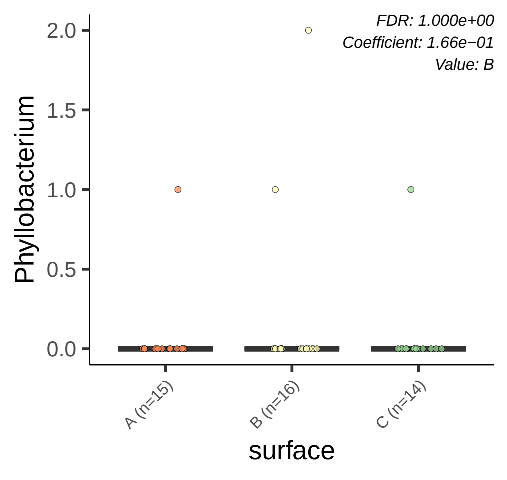
FDR: 1.000e+00
Coefficient: 1.66e−01
Value: B
Phyllobacterium
2.0
1.5
1.0
0.5
0.0
A (n=15)
B (n=16)
C (n=14)
surface

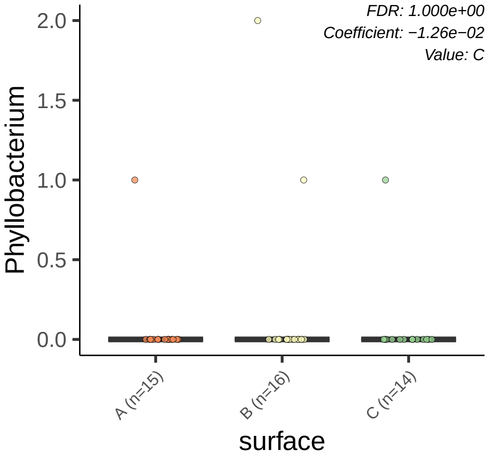
FDR: 1.000e+00
Coefficient: −1.26e−02
Value: C
Phyllobacterium
2.0
1.5
1.0
0.5
0.0
A (n=15)
B (n=16)
C (n=14)
surface

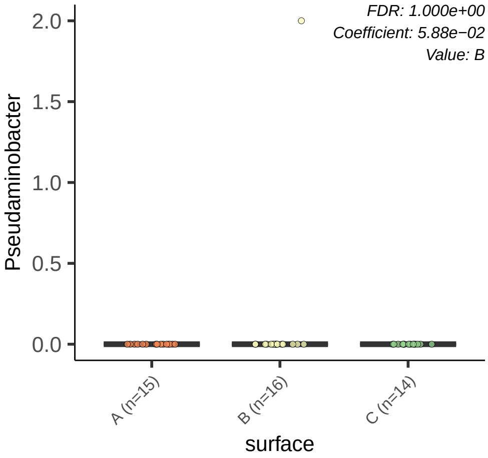

FDR: 1.000e+00
Coefficient: 5.88e−02
Value: B
Pseudaminobacter
2.0
1.5
1.0
0.5
0.0
A (n=15)
B (n=16)
C (n=14)
surface

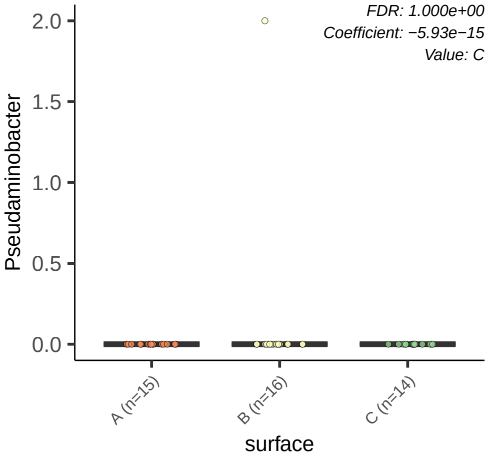

FDR: 1.000e+00
Coefficient: −5.93e−15
Value: C
Pseudaminobacter
2.0
1.5
1.0
0.5
0.0
A (n=15)
B (n=16)
C (n=14)
surface

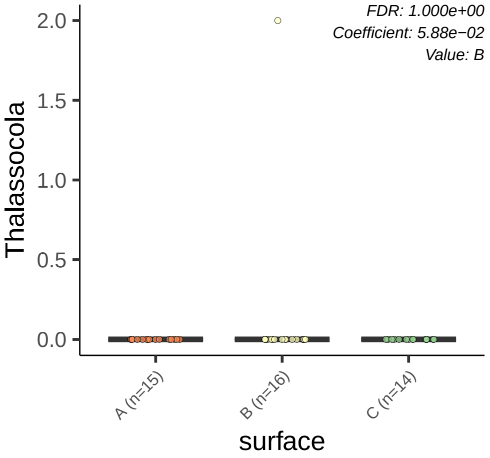

FDR: 1.000e+00
Coefficient: 5.88e−02
Value: B
Thalassocola
2.0
1.5
1.0
0.5
0.0
A (n=15)
B (n=16)
C (n=14)
surface

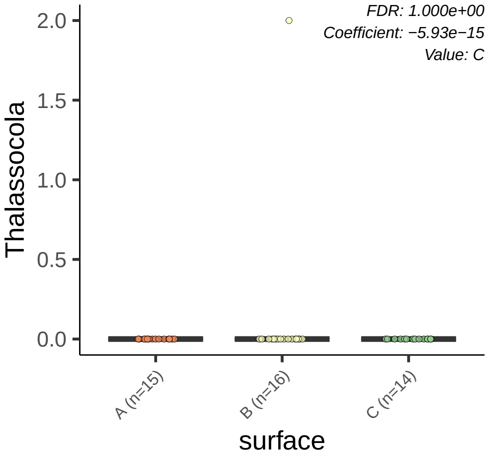
FDR: 1.000e+00
Coefficient: −5.93e−15
Value: C
Thalassocola
2.0
1.5
1.0
0.5
0.0
A (n=15)
B (n=16)
C (n=14)
surface

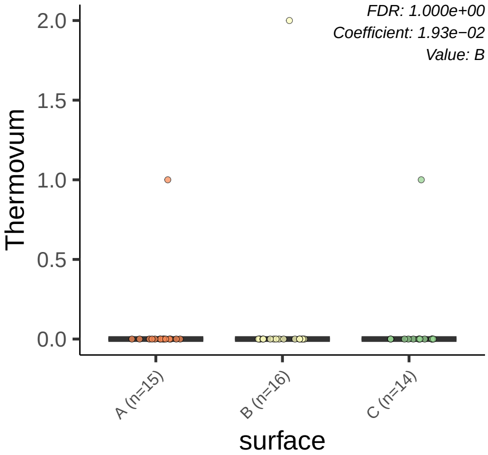
FDR: 1.000e+00
Coefficient: 1.93e−02
Value: B
Thermovum
2.0
1.5
1.0
0.5
0.0
A (n=15)
B (n=16)
C (n=14)
surface

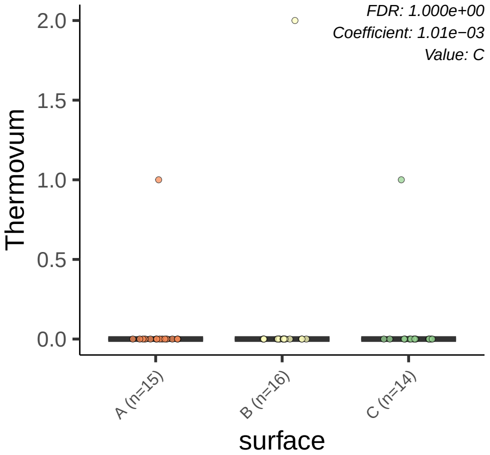

FDR: 1.000e+00
Coefficient: 1.01e−03
Value: C
Thermovum
2.0
1.5
1.0
0.5
0.0
A (n=15)
B (n=16)
C (n=14)
surface

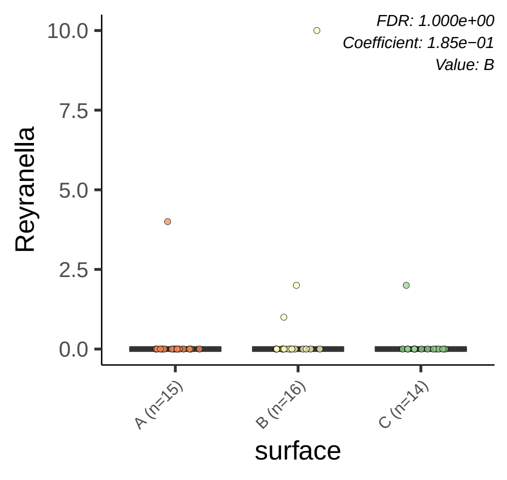
FDR: 1.000e+00
Coefficient: 1.85e−01
Value: B
Reyranella
10.0
7.5
5.0
2.5
0.0
A (n=15)
B (n=16)
C (n=14)
surface

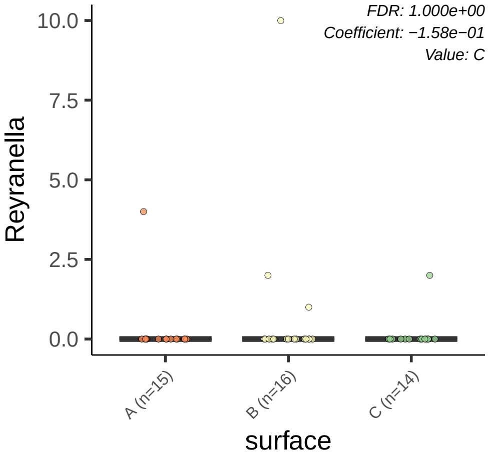
FDR: 1.000e+00
Coefficient: −1.58e−01
Value: C
Reyranella
10.0
7.5
5.0
2.5
0.0
A (n=15)
B (n=16)
C (n=14)
surface

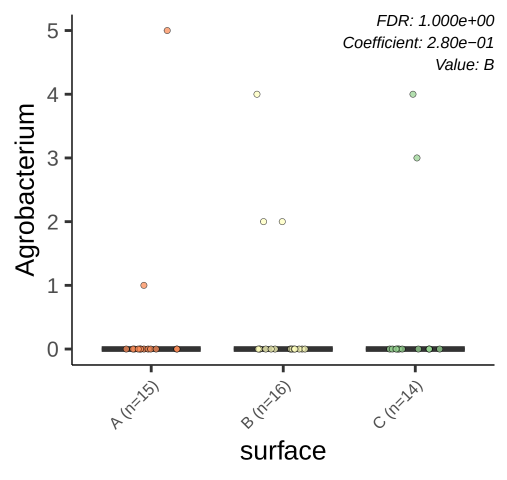
FDR: 1.000e+00
Coefficient: 2.80e−01
Value: B
Agrobacterium
0
1
2
3
4
5
A (n=15)
B (n=16)
C (n=14)
surface

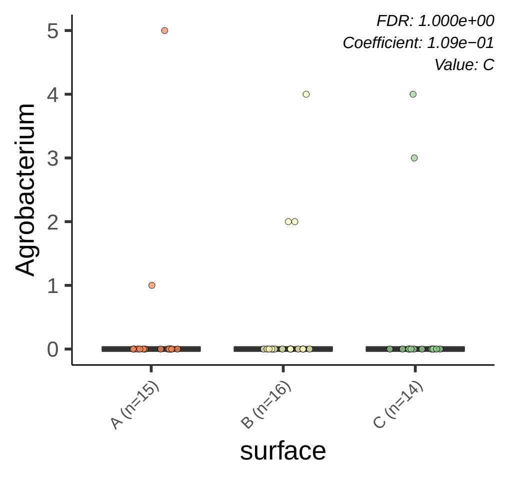

FDR: 1.000e+00
Coefficient: 1.09e−01
Value: C
Agrobacterium
0
1
2
3
4
5
A (n=15)
B (n=16)
C (n=14)
surface

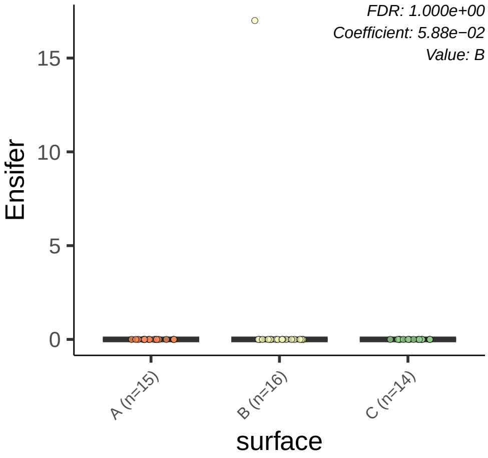
FDR: 1.000e+00
Coefficient: 5.88e−02
Value: B
Ensifer
15
10
5
0
A (n=15)
B (n=16)
C (n=14)
surface

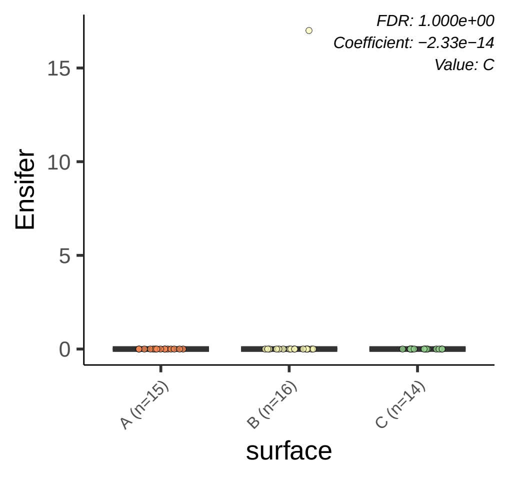
FDR: 1.000e+00
Coefficient: −2.33e−14
Value: C
Ensifer
0
5
10
15
A (n=15)
B (n=16)
C (n=14)
surface

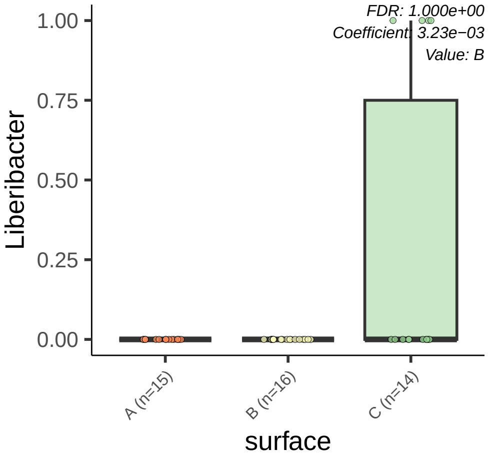
FDR: 1.000e+00
Coefficient: 3.23e−03
Value: B
Liberibacter
1.00
0.75
0.50
0.25
0.00
A (n=15)
B (n=16)
C (n=14)
surface

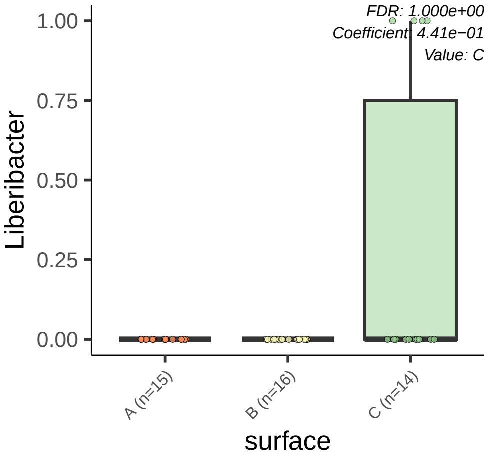
FDR: 1.000e+00
Coefficient: 4.41e−01
Value: C
Liberibacter
1.00
0.75
0.50
0.25
0.00
A (n=15)
B (n=16)
C (n=14)
surface

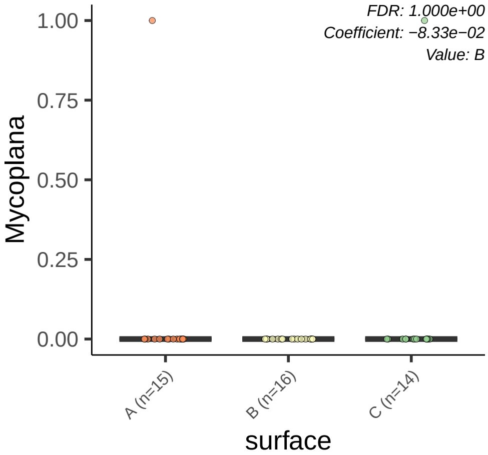
FDR: 1.000e+00
Coefficient: −8.33e−02
Value: B
Mycoplana
1.00
0.75
0.50
0.25
0.00
A (n=15)
B (n=16)
C (n=14)
surface

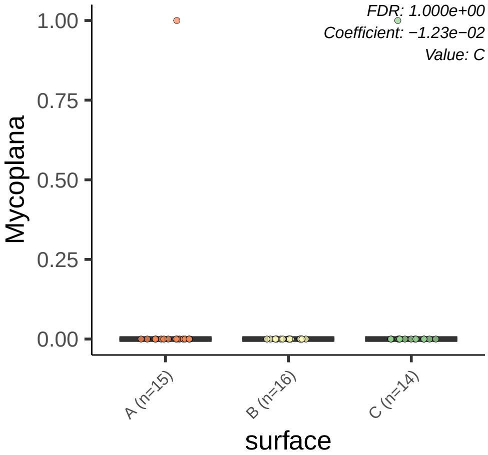

FDR: 1.000e+00
Coefficient: −1.23e−02
Value: C
Mycoplana
1.00
0.75
0.50
0.25
0.00
A (n=15)
B (n=16)
C (n=14)
surface

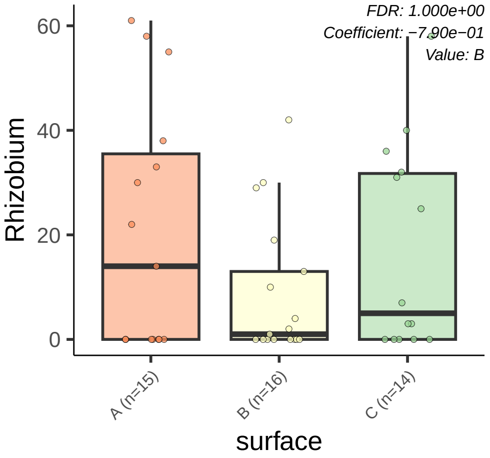
FDR: 1.000e+00
Coefficient: −7.90e−01
Value: B
Rhizobium
60
40
20
0
A (n=15)
B (n=16)
C (n=14)
surface

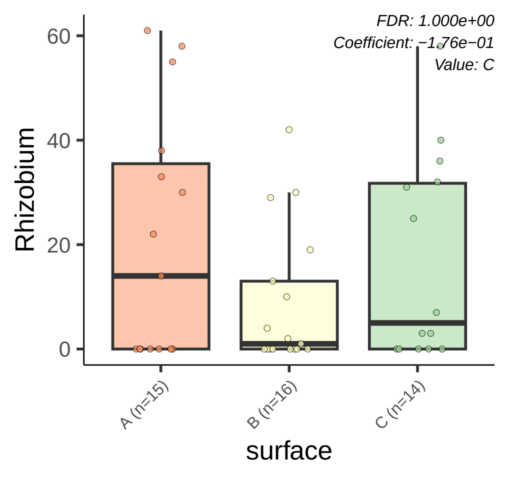

FDR: 1.000e+00
Coefficient: −1.76e−01
Value: C
Rhizobium
60
40
20
0
A (n=15)
B (n=16)
C (n=14)
surface

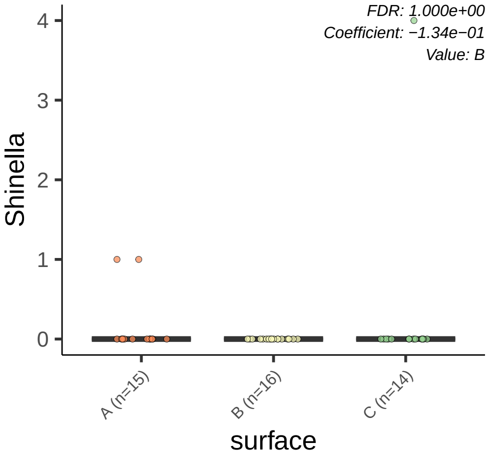

FDR: 1.000e+00
Coefficient: −1.34e−01
Value: B
Shinella
4
3
2
1
0
A (n=15)
B (n=16)
C (n=14)
surface

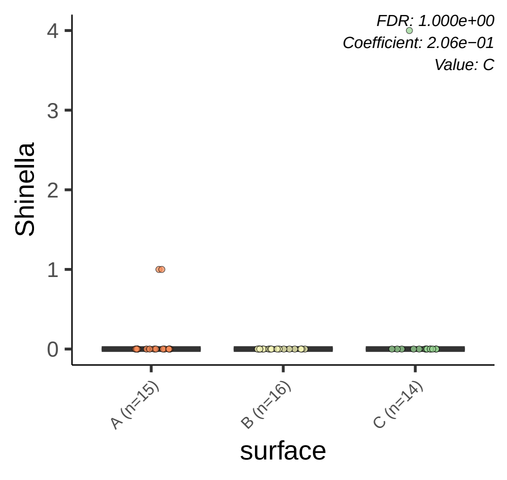
FDR: 1.000e+00
Coefficient: 2.06e−01
Value: C
Shinella
4
3
2
1
0
A (n=15)
B (n=16)
C (n=14)
surface

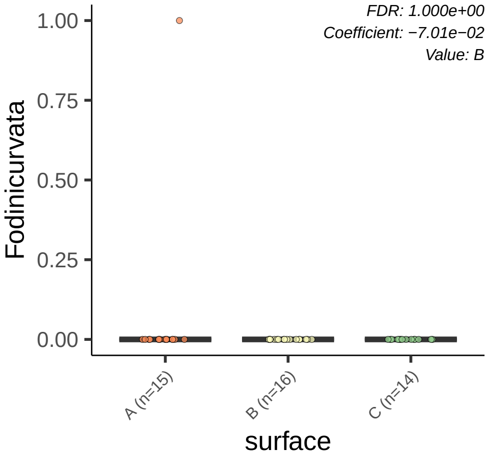
FDR: 1.000e+00
Coefficient: −7.01e−02
Value: B
Fodinicurvata
1.00
0.75
0.50
0.25
0.00
A (n=15)
B (n=16)
C (n=14)
surface

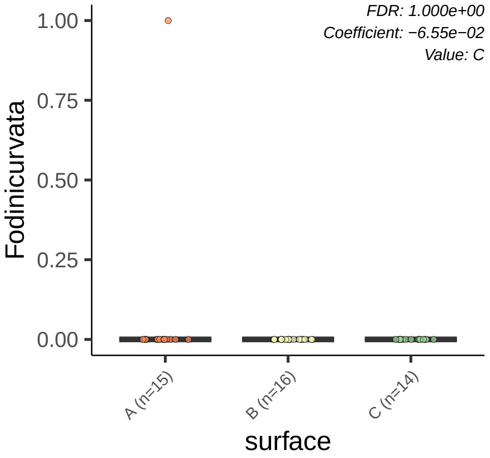
FDR: 1.000e+00
Coefficient: −6.55e−02
Value: C
Fodinicurvata
1.00
0.75
0.50
0.25
0.00
A (n=15)
B (n=16)
C (n=14)
surface

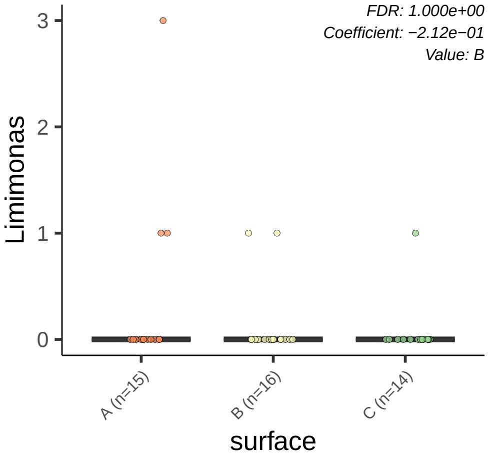

FDR: 1.000e+00
Coefficient: −2.12e−01
Value: B
Limimonas
3
2
1
0
A (n=15)
B (n=16)
C (n=14)
surface

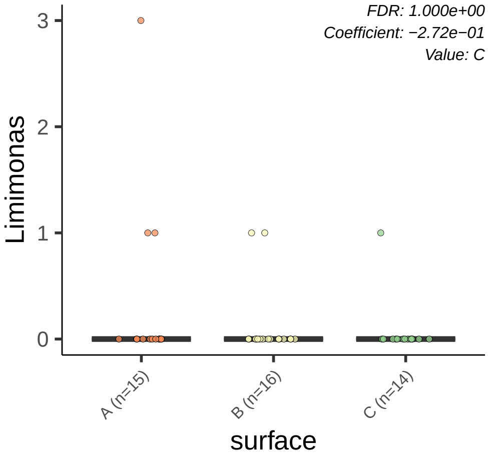
FDR: 1.000e+00
Coefficient: −2.72e−01
Value: C
Limimonas
3
2
1
0
A (n=15)
B (n=16)
C (n=14)
surface

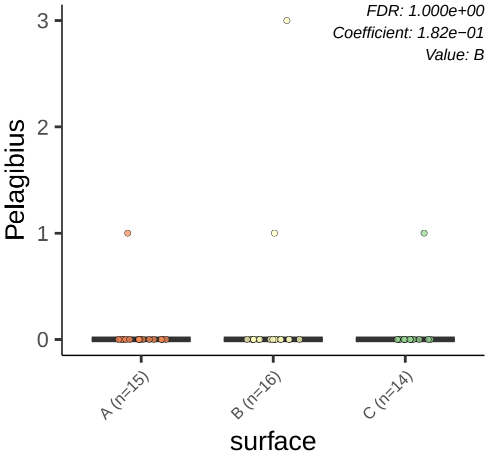
FDR: 1.000e+00
Coefficient: 1.82e−01
Value: B
Pelagibius
0
1
2
3
A (n=15)
B (n=16)
C (n=14)
surface

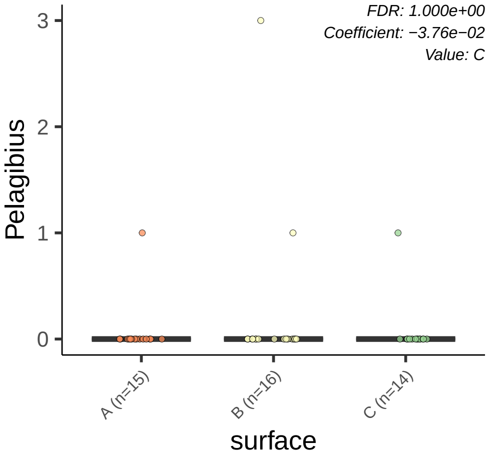
FDR: 1.000e+00
Coefficient: −3.76e−02
Value: C
Pelagibius
3
2
1
0
A (n=15)
B (n=16)
C (n=14)
surface

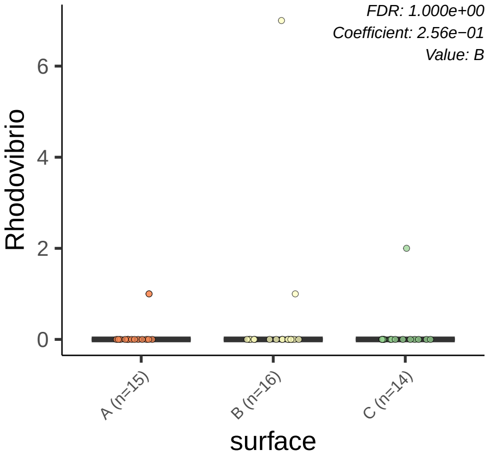
FDR: 1.000e+00
Coefficient: 2.56e−01
Value: B
Rhodovibrio
0
2
4
6
A (n=15)
B (n=16)
C (n=14)
surface

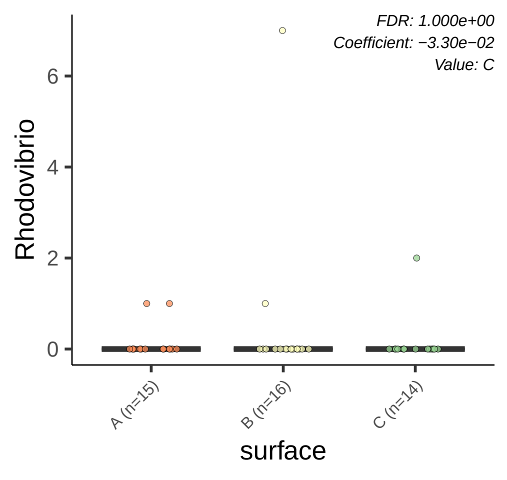

FDR: 1.000e+00
Coefficient: −3.30e−02
Value: C
Rhodovibrio
0
2
4
6
A (n=15)
B (n=16)
C (n=14)
surface

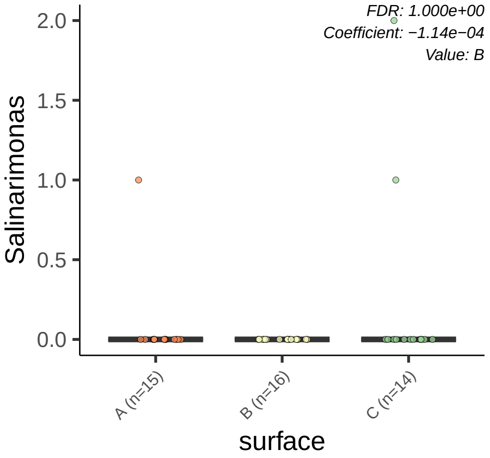
FDR: 1.000e+00
Coefficient: −1.14e−04
Value: B
Salinarimonas
2.0
1.5
1.0
0.5
0.0
A (n=15)
B (n=16)
C (n=14)
surface

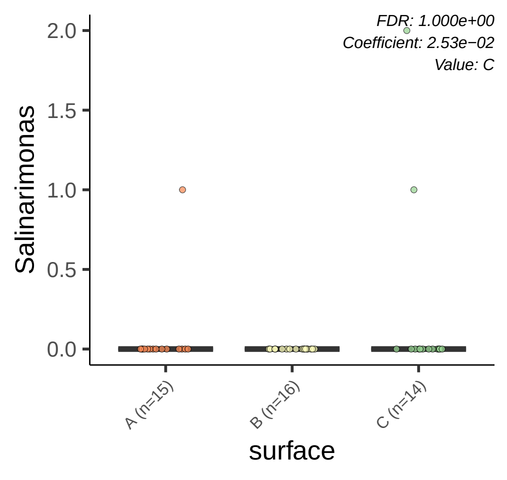
FDR: 1.000e+00
Coefficient: 2.53e−02
Value: C
Salinarimonas
2.0
1.5
1.0
0.5
0.0
A (n=15)
B (n=16)
C (n=14)
surface

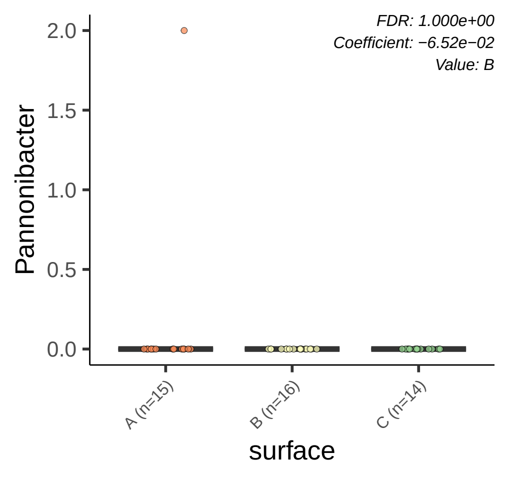

FDR: 1.000e+00
Coefficient: −6.52e−02
Value: B
Pannonibacter
2.0
1.5
1.0
0.5
0.0
A (n=15)
B (n=16)
C (n=14)
surface

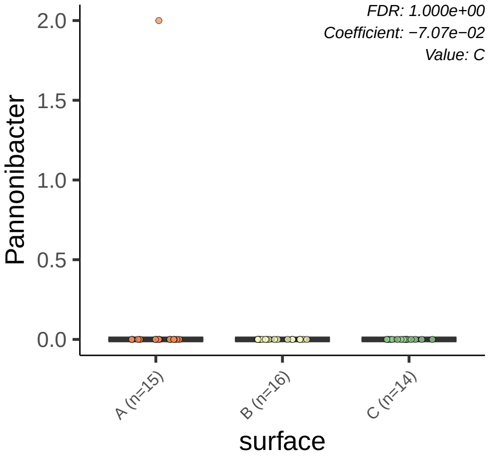
FDR: 1.000e+00
Coefficient: −7.07e−02
Value: C
Pannonibacter
2.0
1.5
1.0
0.5
0.0
A (n=15)
B (n=16)
C (n=14)
surface

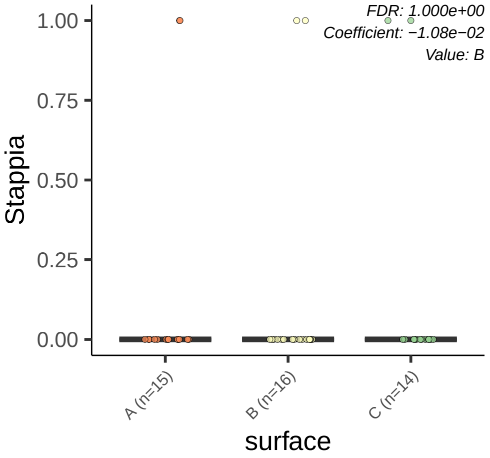

FDR: 1.000e+00
Coefficient: −1.08e−02
Value: B
1.00
0.75
0.50
0.25
0.00
Stappia
A (n=15)
B (n=16)
C (n=14)
surface

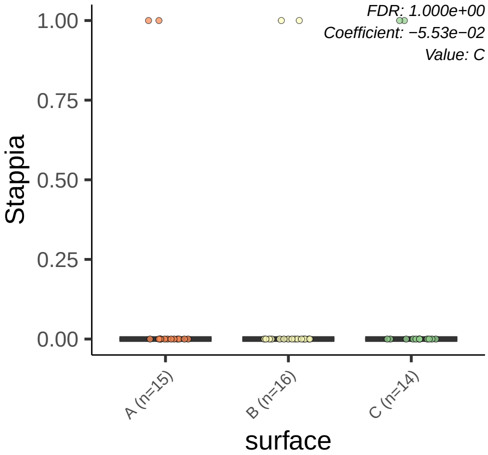

FDR: 1.000e+00
Coefficient: −5.53e−02
Value: C
Stappia
1.00
0.75
0.50
0.25
0.00
A (n=15)
B (n=16)
C (n=14)
surface

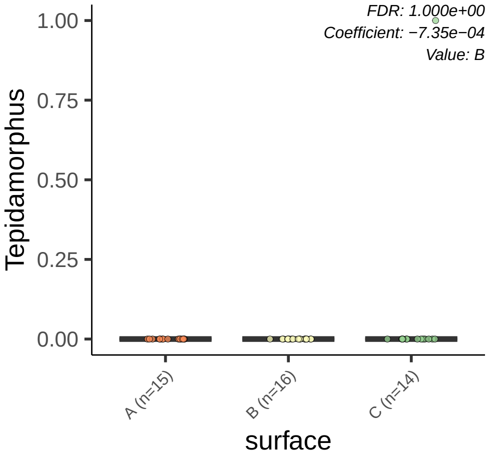
FDR: 1.000e+00
Coefficient: −7.35e−04
Value: B
Tepidamorphus
1.00
0.75
0.50
0.25
0.00
A (n=15)
B (n=16)
C (n=14)
surface

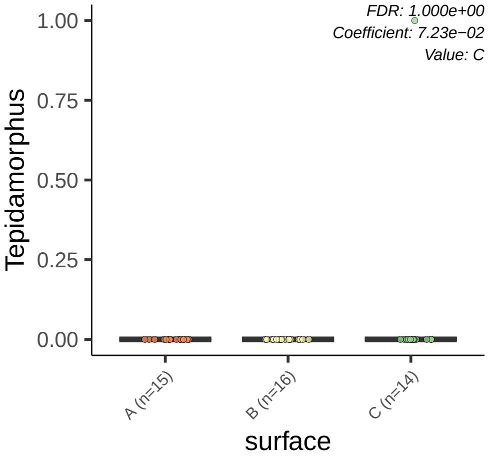
FDR: 1.000e+00
Coefficient: 7.23e−02
Value: C
Tepidamorphus
1.00
0.75
0.50
0.25
0.00
A (n=15)
B (n=16)
C (n=14)
surface

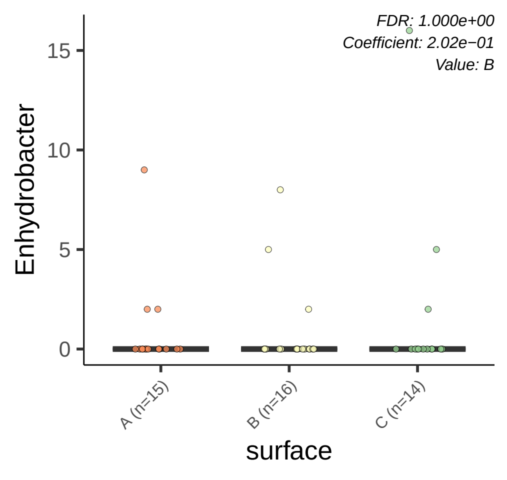

FDR: 1.000e+00
Coefficient: 2.02e−01
Value: B
Enhydrobacter
15
10
5
0
A (n=15)
B (n=16)
C (n=14)
surface

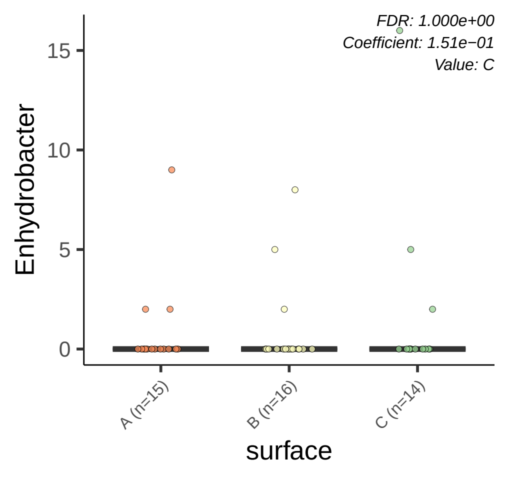

FDR: 1.000e+00
Coefficient: 1.51e−01
Value: C
Enhydrobacter
15
10
5
0
A (n=15)
B (n=16)
C (n=14)
surface

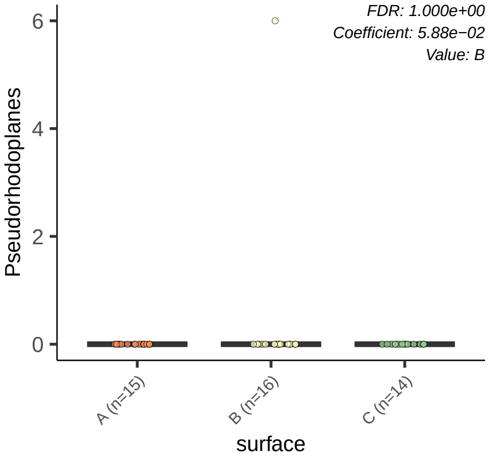
FDR: 1.000e+00
Coefficient: 5.88e−02
Value: B
Pseudorhodoplanes
6
4
2
0
A (n=15)
B (n=16)
C (n=14)
surface

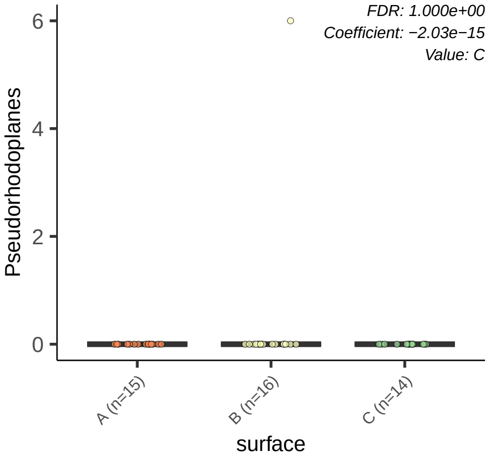

FDR: 1.000e+00
Coefficient: −2.03e−15
Value: C
Pseudorhodoplanes
6
4
2
0
A (n=15)
B (n=16)
C (n=14)
surface

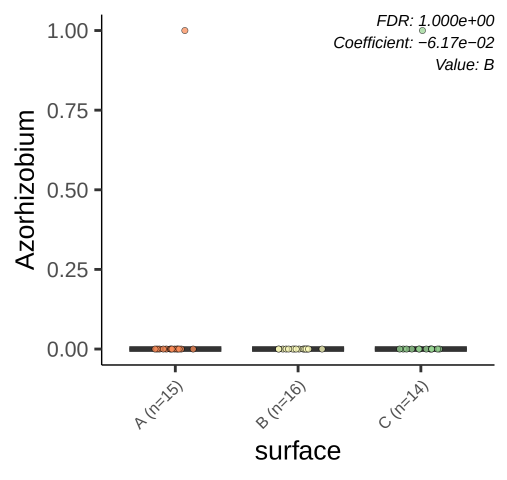
FDR: 1.000e+00
Coefficient: −6.17e−02
Value: B
Azorhizobium
1.00
0.75
0.50
0.25
0.00
A (n=15)
B (n=16)
C (n=14)
surface

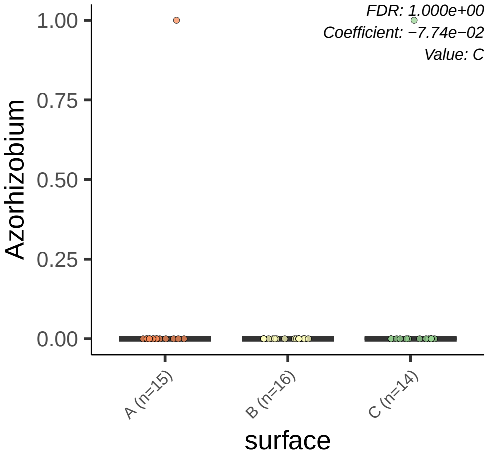

FDR: 1.000e+00
Coefficient: −7.74e−02
Value: C
Azorhizobium
1.00
0.75
0.50
0.25
0.00
A (n=15)
B (n=16)
C (n=14)
surface

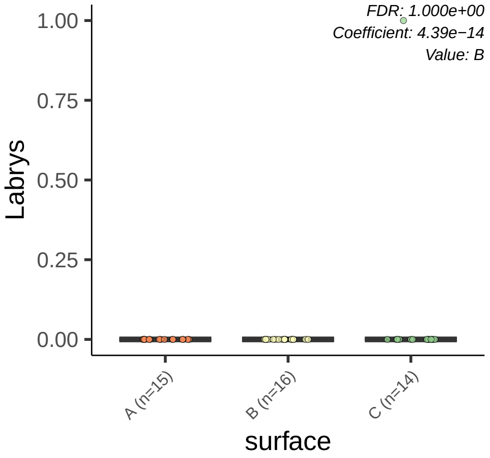
FDR: 1.000e+00
Coefficient: 4.39e−14
Value: B
Labrys
1.00
0.75
0.50
0.25
0.00
A (n=15)
B (n=16)
C (n=14)
surface

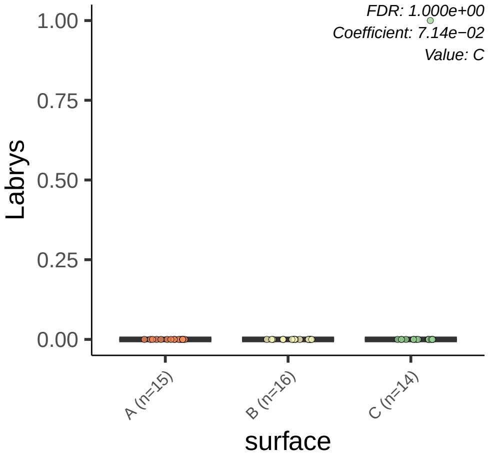
FDR: 1.000e+00
Coefficient: 7.14e−02
Value: C
Labrys
1.00
0.75
0.50
0.25
0.00
A (n=15)
B (n=16)
C (n=14)
surface

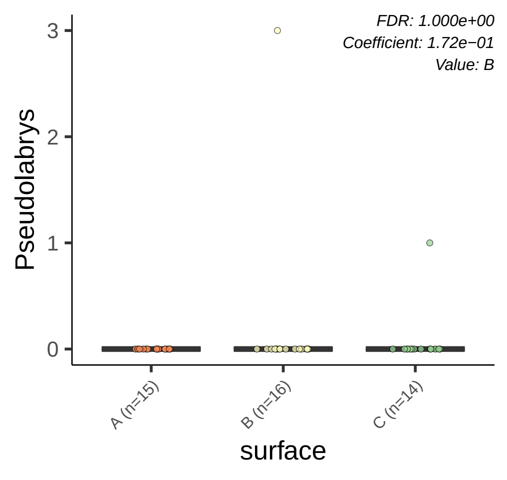

FDR: 1.000e+00
Coefficient: 1.72e−01
Value: B
Pseudolabrys
3
2
1
0
A (n=15)
B (n=16)
C (n=14)
surface

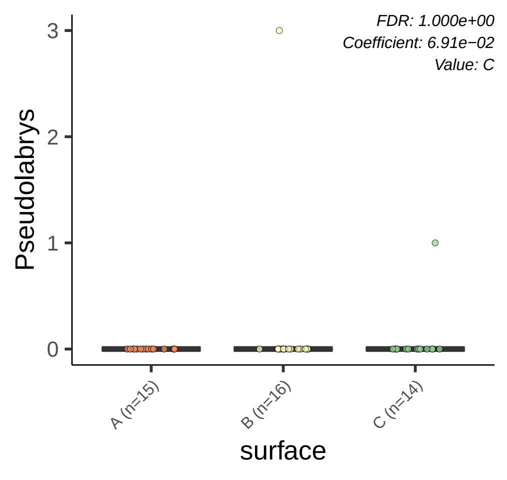
FDR: 1.000e+00
Coefficient: 6.91e−02
Value: C
Pseudolabrys
3
2
1
0
A (n=15)
B (n=16)
C (n=14)
surface

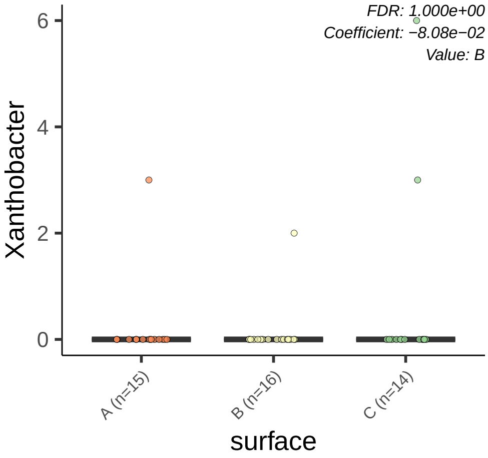

FDR: 1.000e+00
Coefficient: −8.08e−02
Value: B
Xanthobacter
6
4
2
0
A (n=15)
B (n=16)
C (n=14)
surface

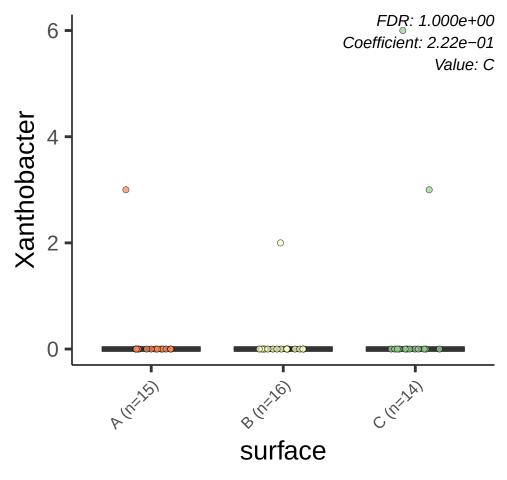

FDR: 1.000e+00
Coefficient: 2.22e−01
Value: C
Xanthobacter
0
2
4
6
A (n=15)
B (n=16)
C (n=14)
surface

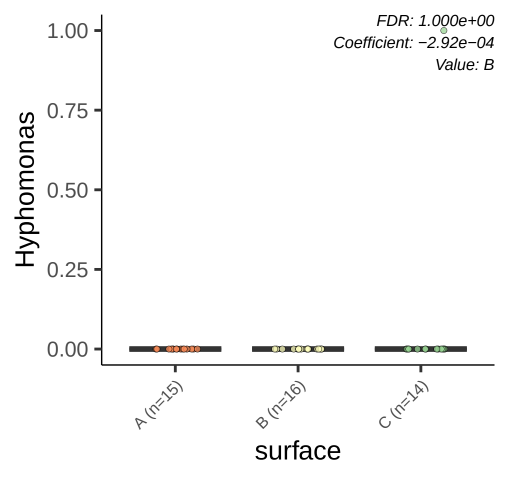

FDR: 1.000e+00
Coefficient: −2.92e−04
Value: B
Hyphomonas
1.00
0.75
0.50
0.25
0.00
A (n=15)
B (n=16)
C (n=14)
surface

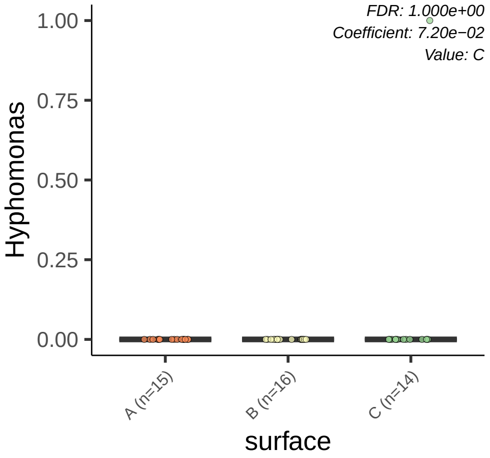

FDR: 1.000e+00
Coefficient: 7.20e−02
Value: C
Hyphomonas
1.00
0.75
0.50
0.25
0.00
A (n=15)
B (n=16)
C (n=14)
surface

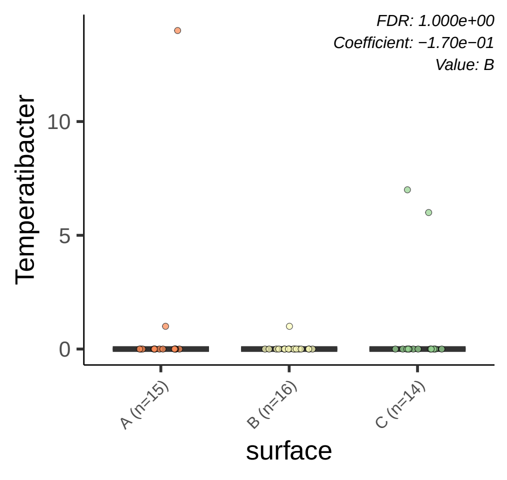

FDR: 1.000e+00
Coefficient: −1.70e−01
Value: B
Temperatibacter
10
5
0
A (n=15)
B (n=16)
C (n=14)
surface

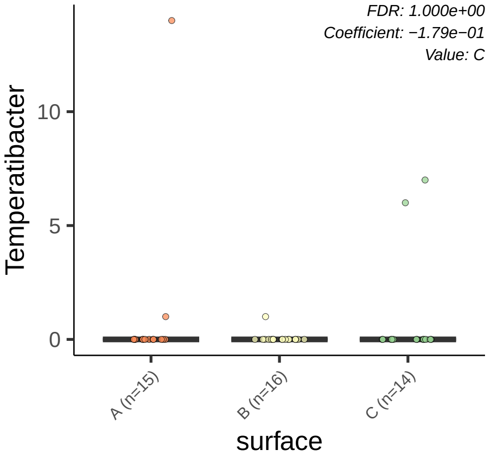
FDR: 1.000e+00
Coefficient: −1.79e−01
Value: C
Temperatibacter
10
5
0
A (n=15)
B (n=16)
C (n=14)
surface

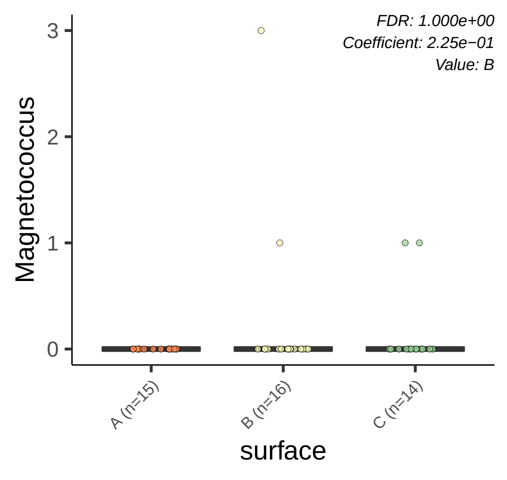
FDR: 1.000e+00
Coefficient: 2.25e−01
Value: B
Magnetococcus
3
2
1
0
A (n=15)
B (n=16)
C (n=14)
surface

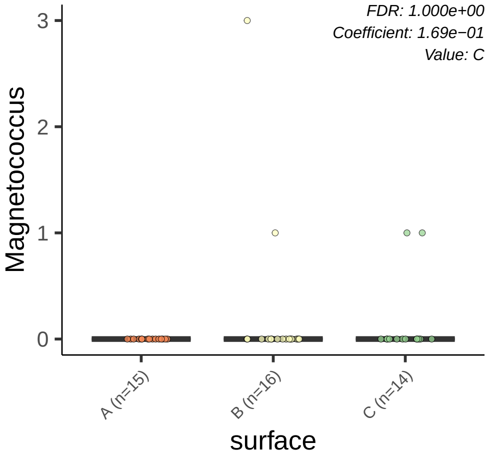

FDR: 1.000e+00
Coefficient: 1.69e−01
Value: C
Magnetococcus
0
1
2
3
A (n=15)
B (n=16)
C (n=14)
surface

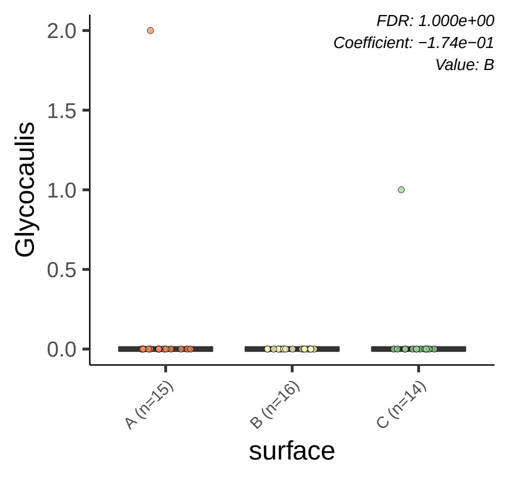
FDR: 1.000e+00
Coefficient: −1.74e−01
Value: B
Glycocaulis
2.0
1.5
1.0
0.5
0.0
A (n=15)
B (n=16)
C (n=14)
surface

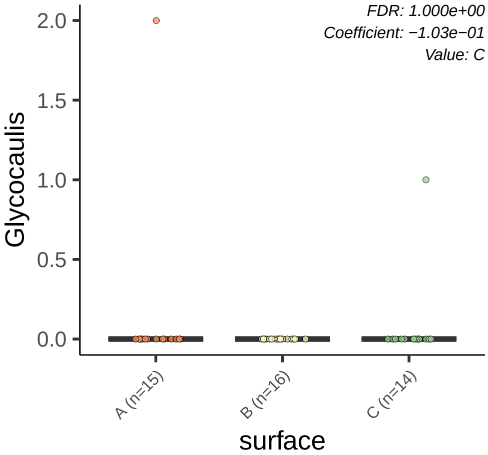
FDR: 1.000e+00
Coefficient: −1.03e−01
Value: C
Glycocaulis
2.0
1.5
1.0
0.5
0.0
A (n=15)
B (n=16)
C (n=14)
surface

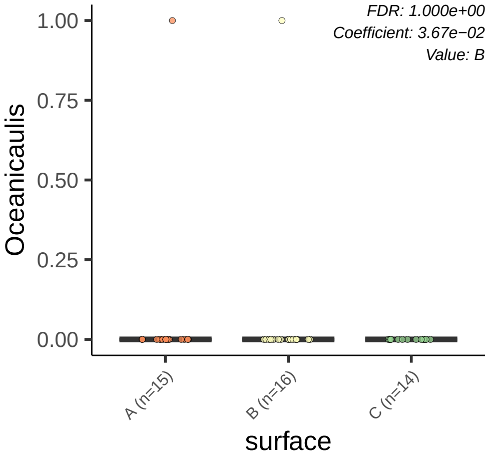
FDR: 1.000e+00
Coefficient: 3.67e−02
Value: B
Oceanicaulis
1.00
0.75
0.50
0.25
0.00
A (n=15)
B (n=16)
C (n=14)
surface

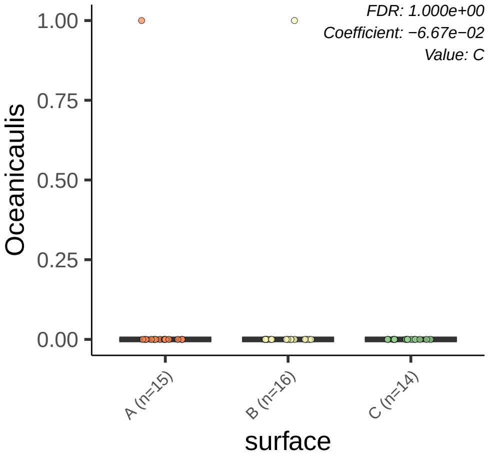

FDR: 1.000e+00
Coefficient: −6.67e−02
Value: C
Oceanicaulis
1.00
0.75
0.50
0.25
0.00
A (n=15)
B (n=16)
C (n=14)
surface

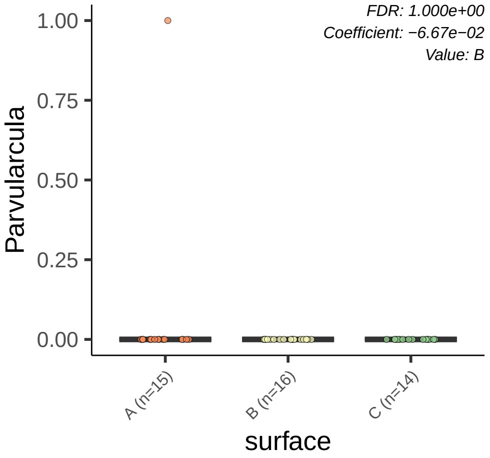

FDR: 1.000e+00
Coefficient: −6.67e−02
Value: B
1.00
0.75
0.50
0.25
0.00
Parvularcula
A (n=15)
B (n=16)
C (n=14)
surface

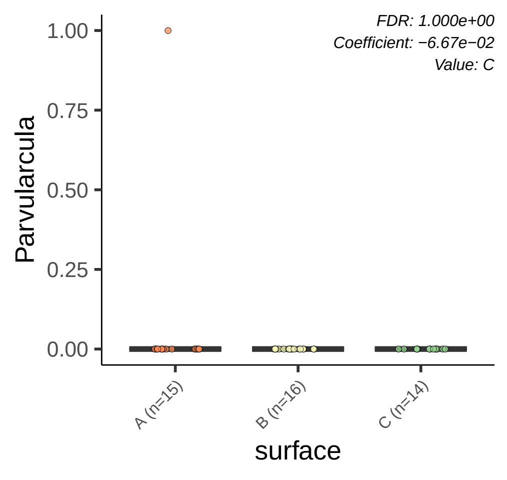
FDR: 1.000e+00
Coefficient: −6.67e−02
Value: C
Parvularcula
1.00
0.75
0.50
0.25
0.00
A (n=15)
B (n=16)
C (n=14)
surface

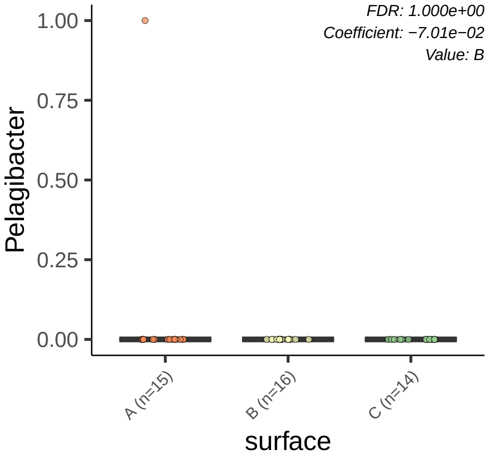
FDR: 1.000e+00
Coefficient: −7.01e−02
Value: B
Pelagibacter
1.00
0.75
0.50
0.25
0.00
A (n=15)
B (n=16)
C (n=14)
surface

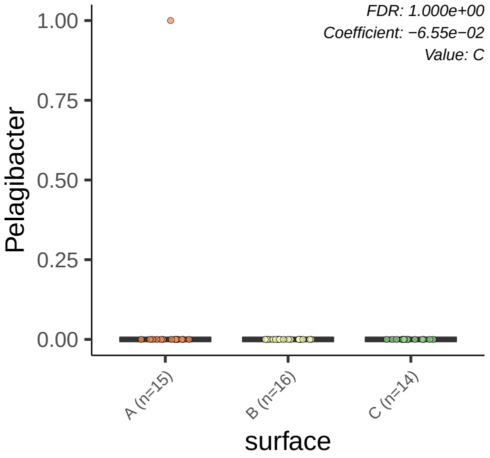
FDR: 1.000e+00
Coefficient: −6.55e−02
Value: C
Pelagibacter
1.00
0.75
0.50
0.25
0.00
A (n=15)
B (n=16)
C (n=14)
surface

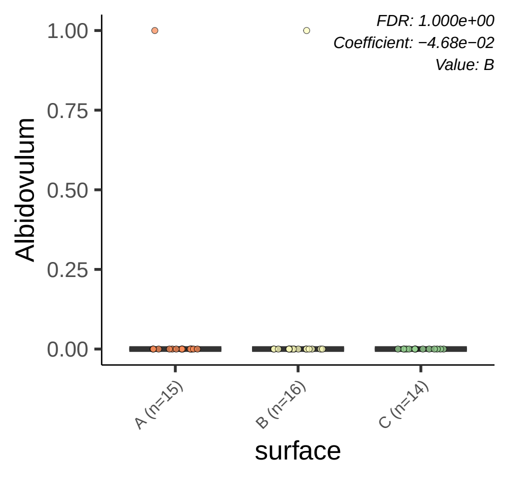
FDR: 1.000e+00
Coefficient: −4.68e−02
Value: B
Albidovulum
1.00
0.75
0.50
0.25
0.00
A (n=15)
B (n=16)
C (n=14)
surface

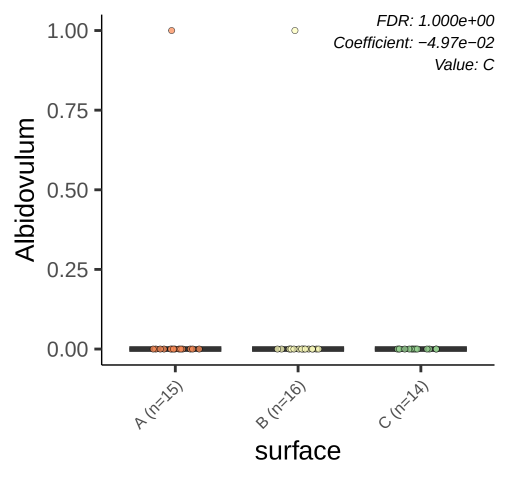
FDR: 1.000e+00
Coefficient: −4.97e−02
Value: C
Albidovulum
1.00
0.75
0.50
0.25
0.00
A (n=15)
B (n=16)
C (n=14)
surface

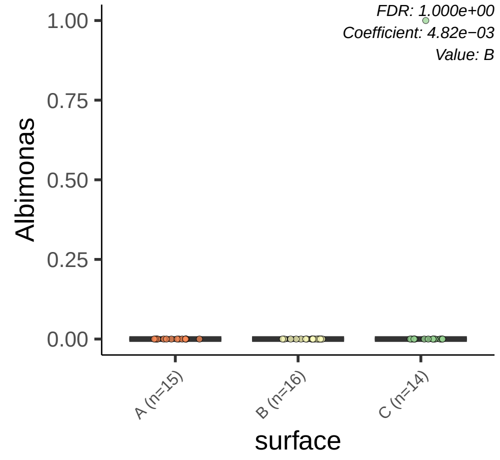

FDR: 1.000e+00
Coefficient: 4.82e−03
Value: B
Albimonas
1.00
0.75
0.50
0.25
0.00
A (n=15)
B (n=16)
C (n=14)
surface

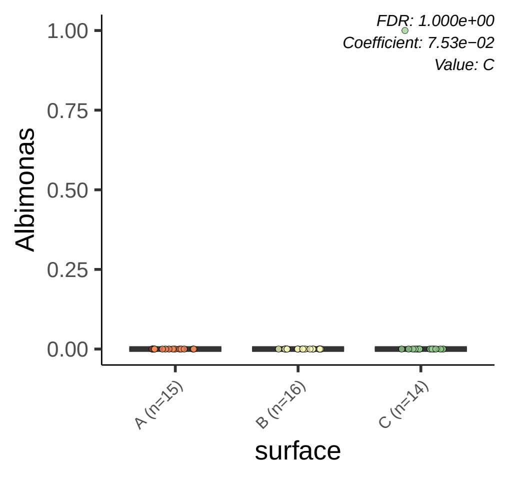
FDR: 1.000e+00
Coefficient: 7.53e−02
Value: C
1.00
0.75
0.50
0.25
0.00
Albimonas
A (n=15)
B (n=16)
C (n=14)
surface

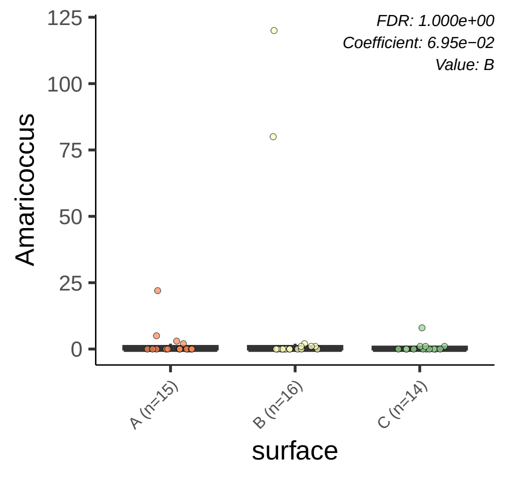

FDR: 1.000e+00
Coefficient: 6.95e−02
Value: B
Amaricoccus
125
100
75
50
25
0
A (n=15)
B (n=16)
C (n=14)
surface

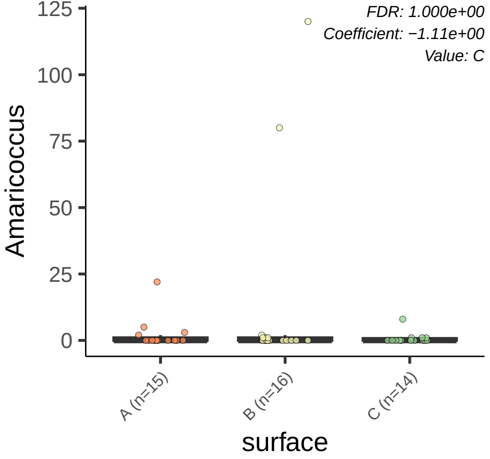
FDR: 1.000e+00
Coefficient: −1.11e+00
Value: C
Amaricoccus
125
100
75
50
25
0
A (n=15)
B (n=16)
C (n=14)
surface

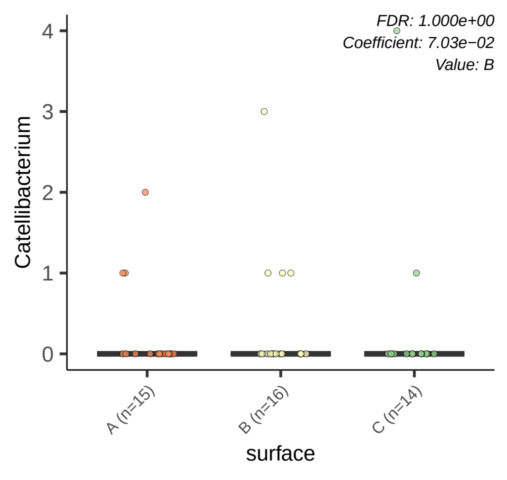

FDR: 1.000e+00
Coefficient: 7.03e−02
Value: B
Catellibacterium
0
1
2
3
4
A (n=15)
B (n=16)
C (n=14)
surface

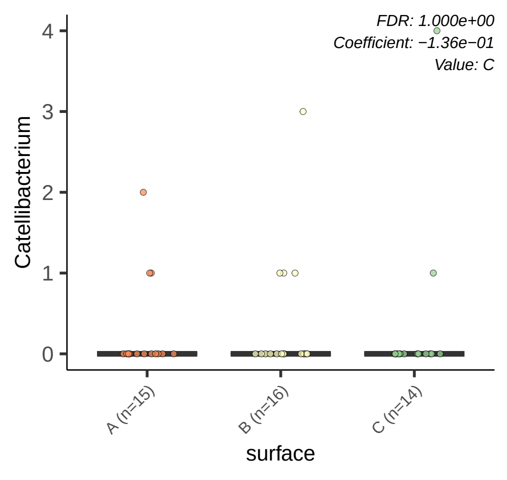

FDR: 1.000e+00
Coefficient: −1.36e−01
Value: C
Catellibacterium
0
1
2
3
4
A (n=15)
B (n=16)
C (n=14)
surface

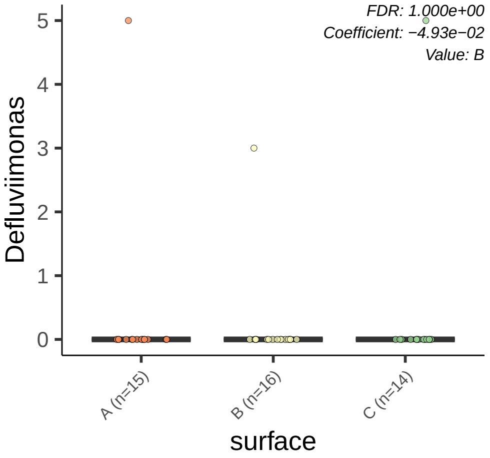
FDR: 1.000e+00
Coefficient: −4.93e−02
Value: B
Defluviimonas
0
1
2
3
4
5
A (n=15)
B (n=16)
C (n=14)
surface

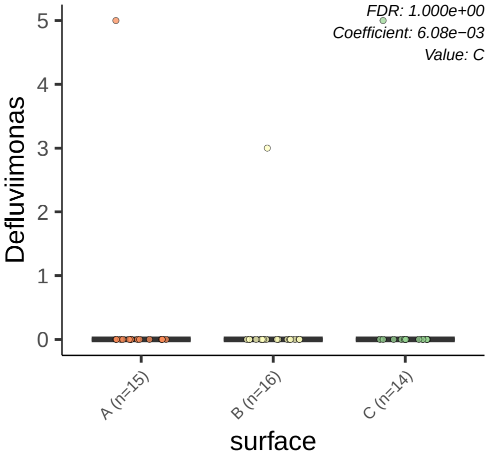
FDR: 1.000e+00
Coefficient: 6.08e−03
Value: C
Defluviimonas
0
1
2
3
4
5
A (n=15)
B (n=16)
C (n=14)
surface

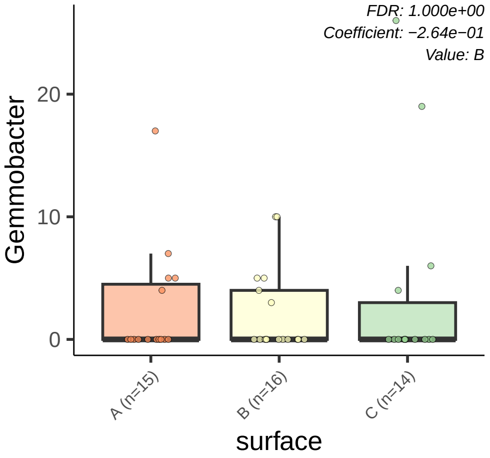

FDR: 1.000e+00
Coefficient: −2.64e−01
Value: B
Gemmobacter
20
10
0
A (n=15)
B (n=16)
C (n=14)
surface

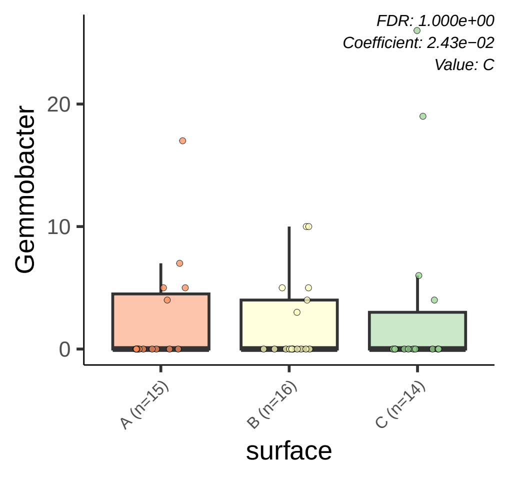
FDR: 1.000e+00
Coefficient: 2.43e−02
Value: C
Gemmobacter
0
10
20
A (n=15)
B (n=16)
C (n=14)
surface

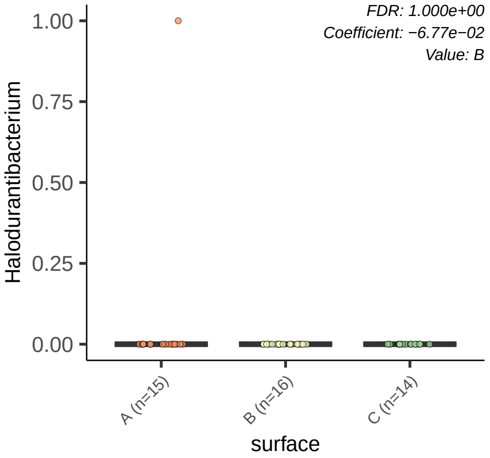
FDR: 1.000e+00
Coefficient: −6.77e−02
Value: B
Halodurantibacterium
1.00
0.75
0.50
0.25
0.00
A (n=15)
B (n=16)
C (n=14)
surface

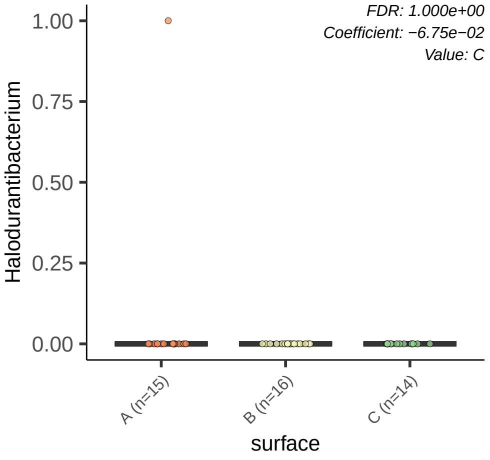

FDR: 1.000e+00
Coefficient: −6.75e−02
Value: C
Halodurantibacterium
1.00
0.75
0.50
0.25
0.00
A (n=15)
B (n=16)
C (n=14)
surface

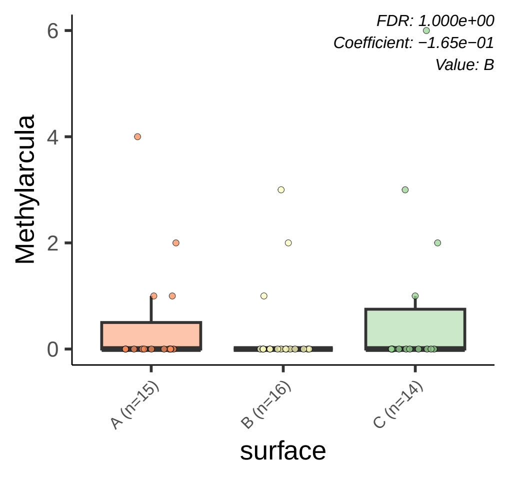
FDR: 1.000e+00
Coefficient: −1.65e−01
Value: B
Methylarcula
0
2
4
6
A (n=15)
B (n=16)
C (n=14)
surface

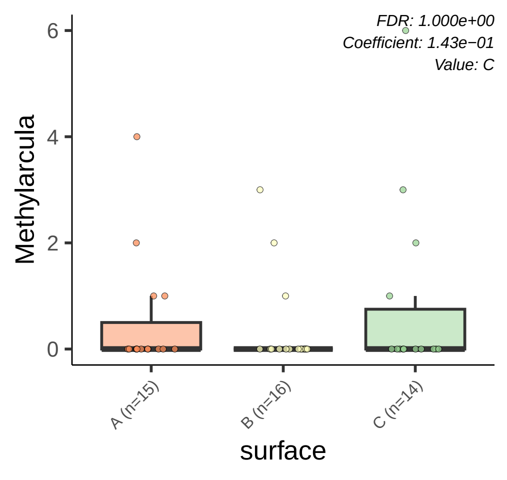

FDR: 1.000e+00
Coefficient: 1.43e−01
Value: C
Methylarcula
0
2
4
6
A (n=15)
B (n=16)
C (n=14)
surface

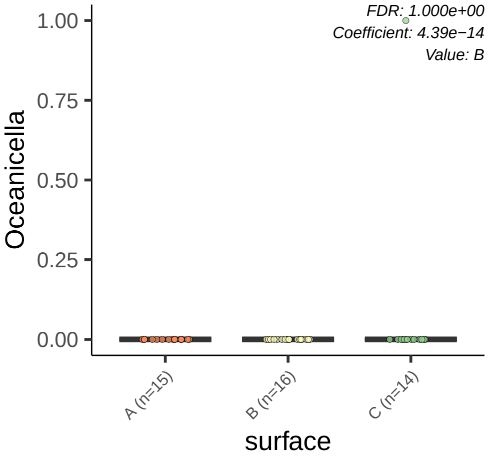
FDR: 1.000e+00
Coefficient: 4.39e−14
Value: B
Oceanicella
1.00
0.75
0.50
0.25
0.00
A (n=15)
B (n=16)
C (n=14)
surface

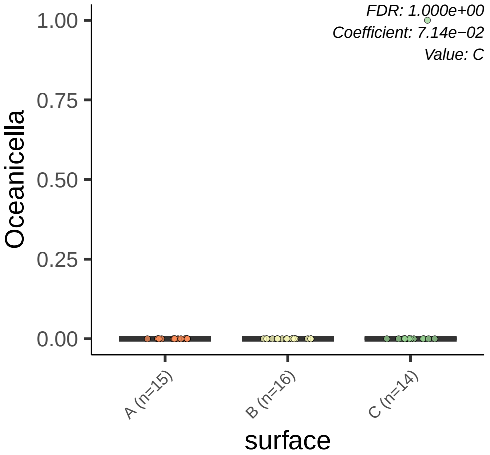

FDR: 1.000e+00
Coefficient: 7.14e−02
Value: C
Oceanicella
1.00
0.75
0.50
0.25
0.00
A (n=15)
B (n=16)
C (n=14)
surface

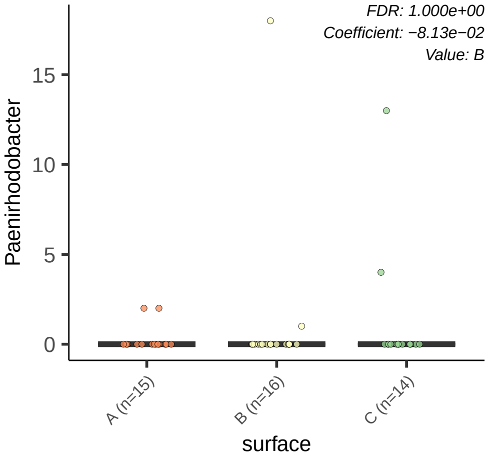

FDR: 1.000e+00
Coefficient: −8.13e−02
Value: B
Paenirhodobacter
15
10
5
0
A (n=15)
B (n=16)
C (n=14)
surface

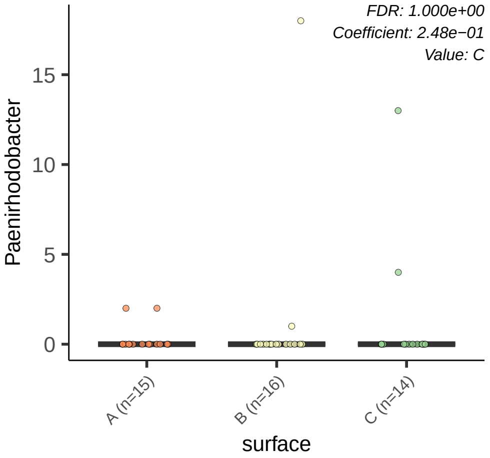
FDR: 1.000e+00
Coefficient: 2.48e−01
Value: C
Paenirhodobacter
0
5
10
15
A (n=15)
B (n=16)
C (n=14)
surface

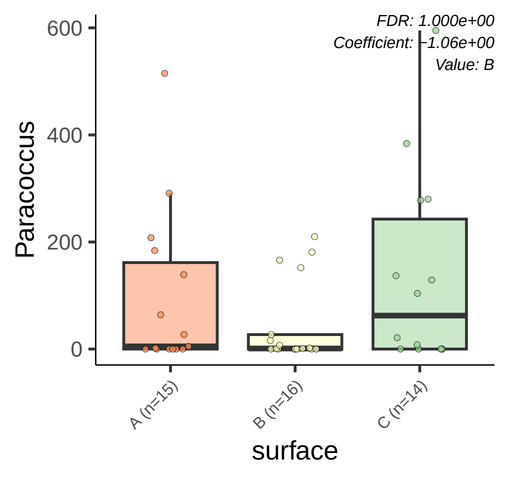

FDR: 1.000e+00
Coefficient: -1.06e+00
Value: B
Paracoccus
600
400
200
0
A (n=15)
B (n=16)
C (n=14)
surface

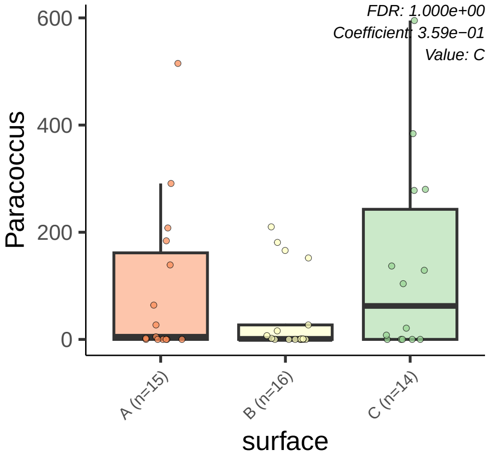
FDR: 1.000e+00
Coefficient: 3.59e−01
Value: C
Paracoccus
600
400
200
0
A (n=15)
B (n=16)
C (n=14)
surface

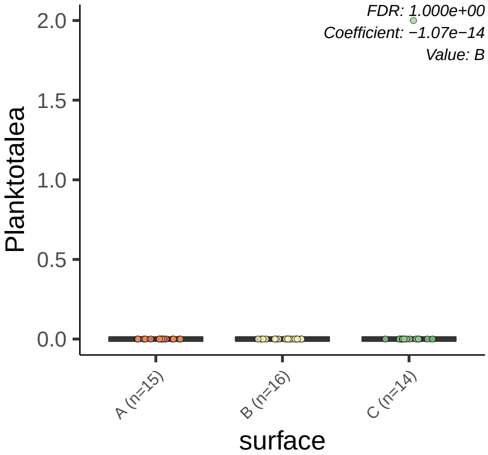
FDR: 1.000e+00
Coefficient: −1.07e−14
Value: B
2.0
1.5
1.0
0.5
0.0
Planktotalea
A (n=15)
B (n=16)
C (n=14)
surface

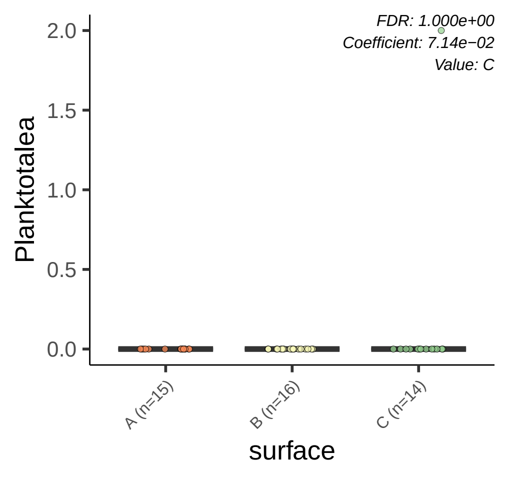
FDR: 1.000e+00
Coefficient: 7.14e−02
Value: C
Planktotalea
2.0
1.5
1.0
0.5
0.0
A (n=15)
B (n=16)
C (n=14)
surface

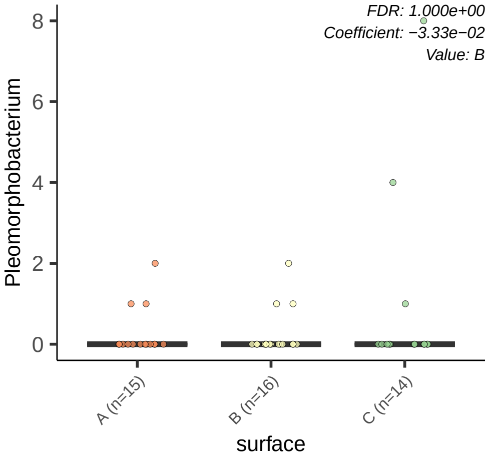
FDR: 1.000e+00
Coefficient: −3.33e−02
Value: B
Pleomorphobacterium
8
6
4
2
0
A (n=15)
B (n=16)
C (n=14)
surface

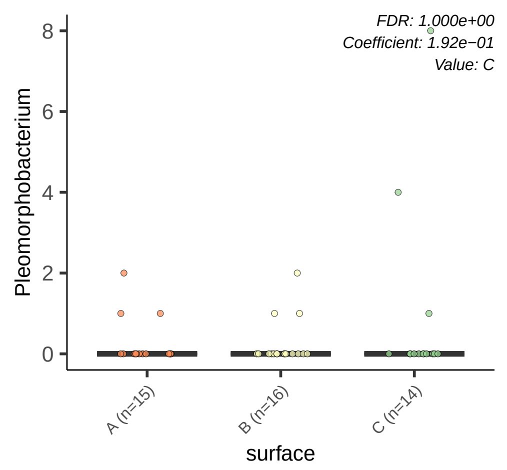

FDR: 1.000e+00
Coefficient: 1.92e−01
Value: C
Pleomorphobacterium
8
6
4
2
0
A (n=15)
B (n=16)
C (n=14)
surface

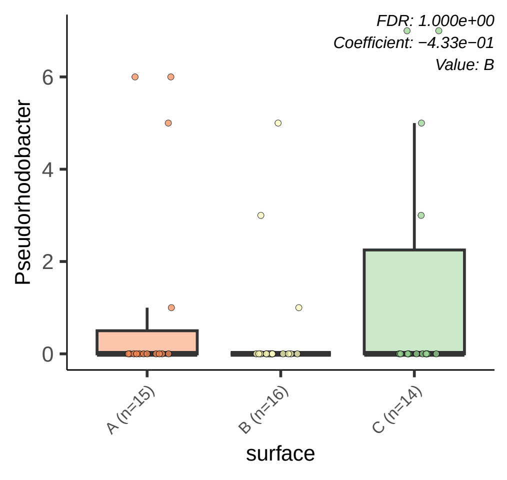
FDR: 1.000e+00
Coefficient: −4.33e−01
Value: B
Pseudorhodobacter
0
2
4
6
A (n=15)
B (n=16)
C (n=14)
surface

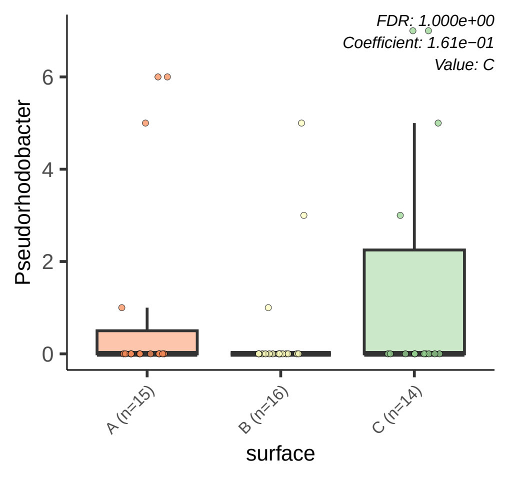
FDR: 1.000e+00
Coefficient: 1.61e−01
Value: C
Pseudorhodobacter
0
2
4
6
A (n=15)
B (n=16)
C (n=14)
surface

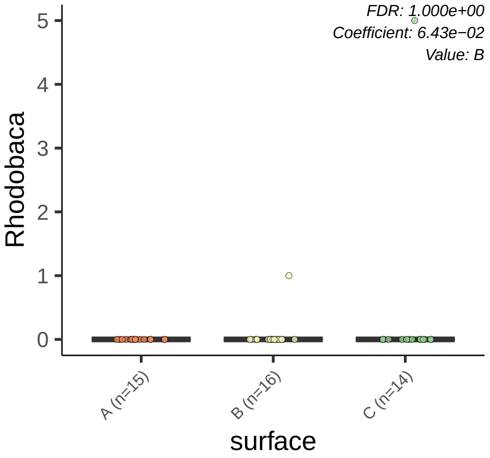
FDR: 1.000e+00
Coefficient: 6.43e−02
Value: B
Rhodobaca
5
4
3
2
1
0
A (n=15)
B (n=16)
C (n=14)
surface

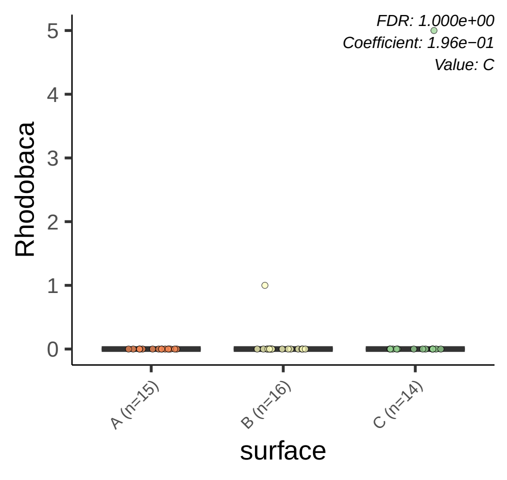

FDR: 1.000e+00
Coefficient: 1.96e−01
Value: C
Rhodobaca
5
4
3
2
1
0
A (n=15)
B (n=16)
C (n=14)
surface

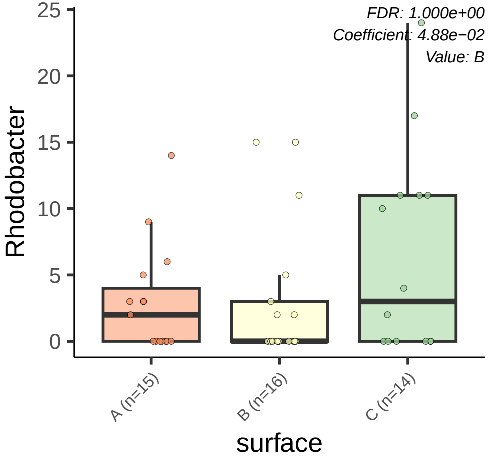

FDR: 1.000e+00
Coefficient: 4.88e−02
Value: B
Rhodobacter
0
5
10
15
20
25
A (n=15)
B (n=16)
C (n=14)
surface

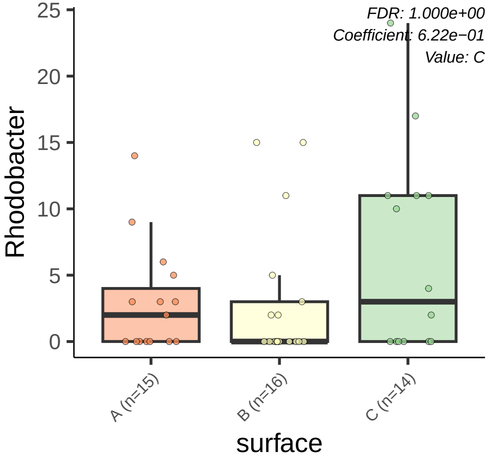

FDR: 1.000e+00
Coefficient: 6.22e−01
Value: C
Rhodobacter
0
5
10
15
20
25
A (n=15)
B (n=16)
C (n=14)
surface

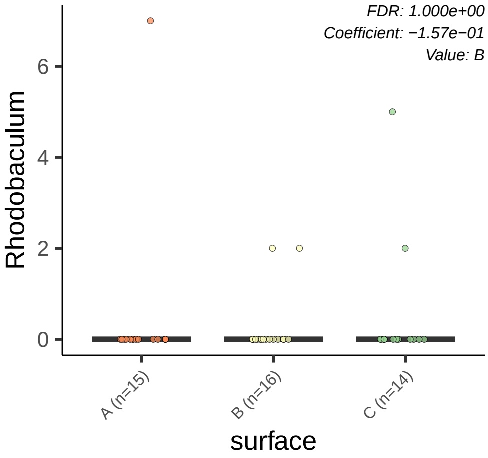

FDR: 1.000e+00
Coefficient: −1.57e−01
Value: B
Rhodobaculum
0
2
4
6
A (n=15)
B (n=16)
C (n=14)
surface

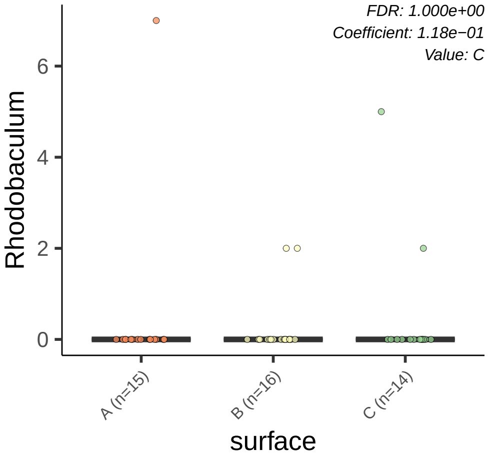
FDR: 1.000e+00
Coefficient: 1.18e−01
Value: C
Rhodobaculum
0
2
4
6
A (n=15)
B (n=16)
C (n=14)
surface

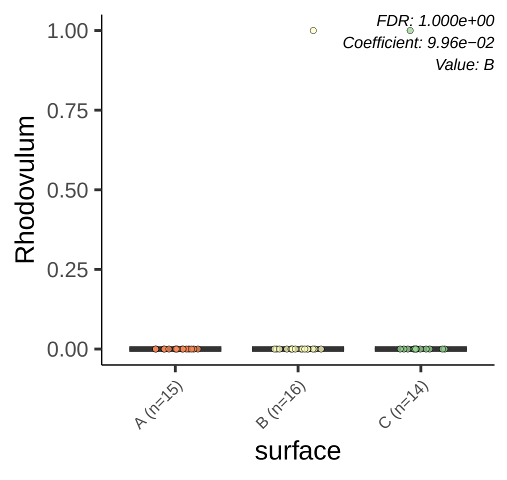

FDR: 1.000e+00
Coefficient: 9.96e−02
Value: B
1.00
0.75
0.50
0.25
0.00
Rhodovulum
A (n=15)
B (n=16)
C (n=14)
surface

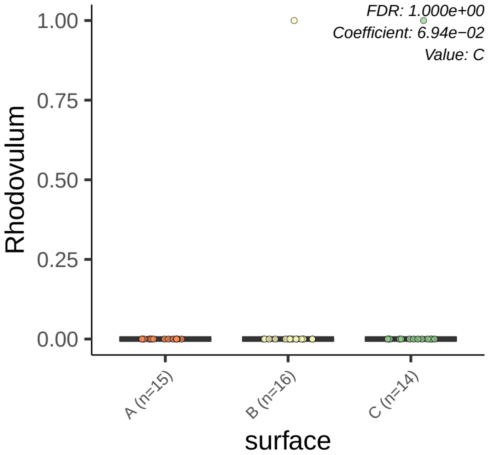

FDR: 1.000e+00
Coefficient: 6.94e−02
Value: C
Rhodovulum
1.00
0.75
0.50
0.25
0.00
A (n=15)
B (n=16)
C (n=14)
surface

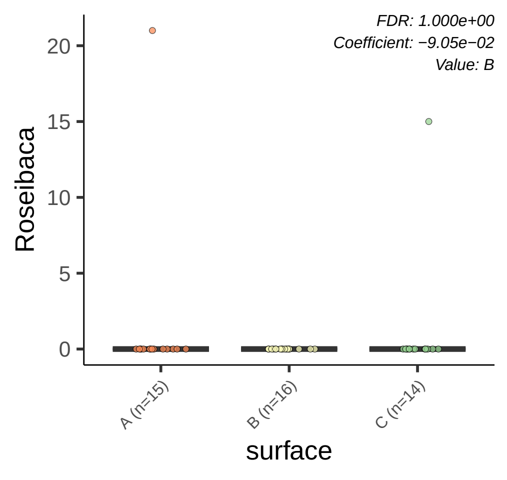
FDR: 1.000e+00
Coefficient: −9.05e−02
Value: B
Roseibaca
20
15
10
5
0
A (n=15)
B (n=16)
C (n=14)
surface

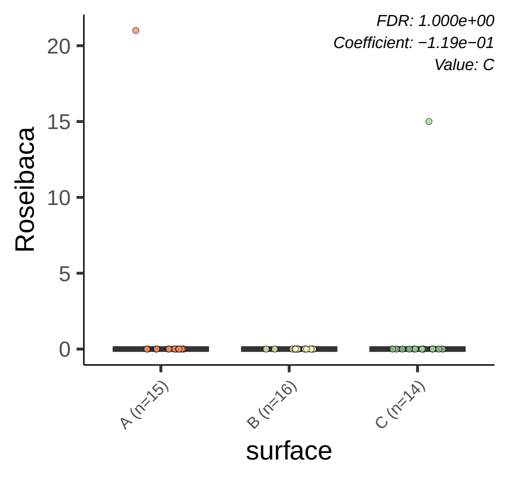

FDR: 1.000e+00
Coefficient: −1.19e−01
Value: C
Roseibaca
0
5
10
15
20
A (n=15)
B (n=16)
C (n=14)
surface

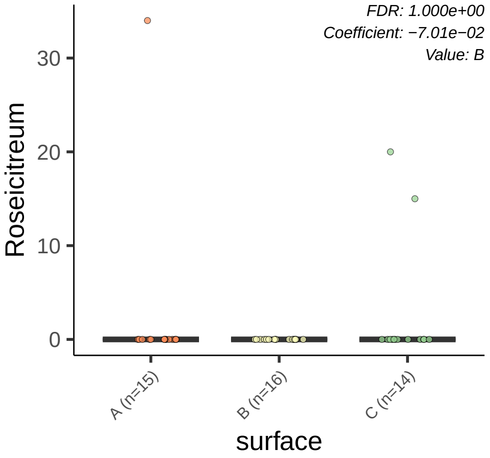
FDR: 1.000e+00
Coefficient: −7.01e−02
Value: B
Roseicitreum
30
20
10
0
A (n=15)
B (n=16)
C (n=14)
surface

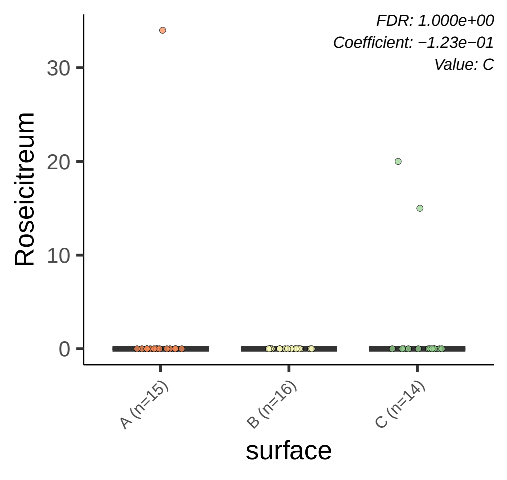

FDR: 1.000e+00
Coefficient: −1.23e−01
Value: C
Roseicitreum
30
20
10
0
A (n=15)
B (n=16)
C (n=14)
surface

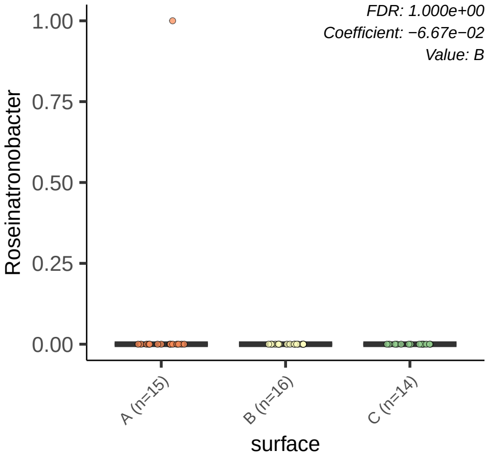

FDR: 1.000e+00
Coefficient: −6.67e−02
Value: B
Roseinatronobacter
1.00
0.75
0.50
0.25
0.00
A (n=15)
B (n=16)
C (n=14)
surface

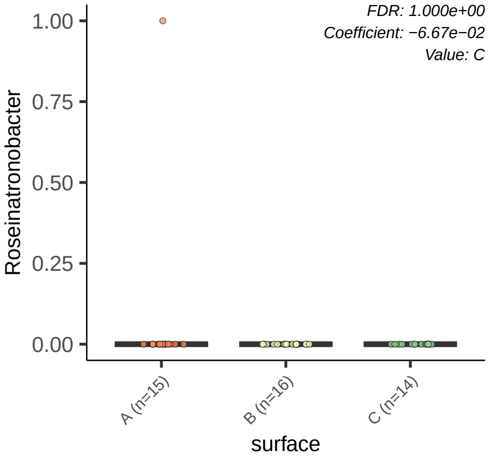
FDR: 1.000e+00
Coefficient: −6.67e−02
Value: C
Roseinatronobacter
1.00
0.75
0.50
0.25
0.00
A (n=15)
B (n=16)
C (n=14)
surface

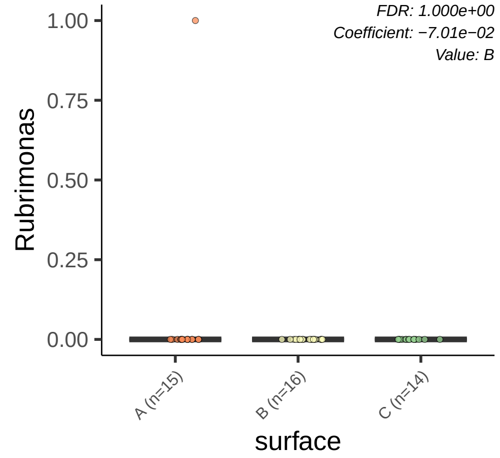

FDR: 1.000e+00
Coefficient: −7.01e−02
Value: B
Rubrimonas
1.00
0.75
0.50
0.25
0.00
A (n=15)
B (n=16)
C (n=14)
surface

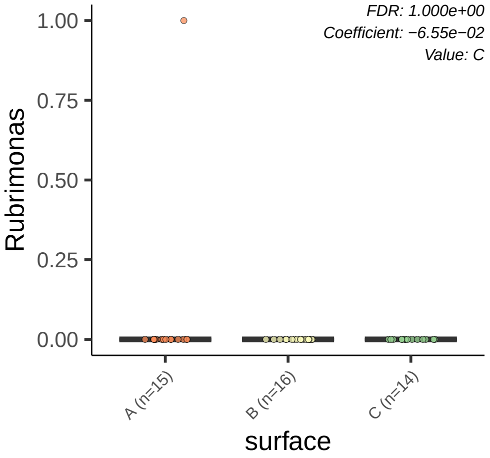
FDR: 1.000e+00
Coefficient: −6.55e−02
Value: C
1.00
0.75
0.50
0.25
0.00
Rubrimonas
A (n=15)
B (n=16)
C (n=14)
surface

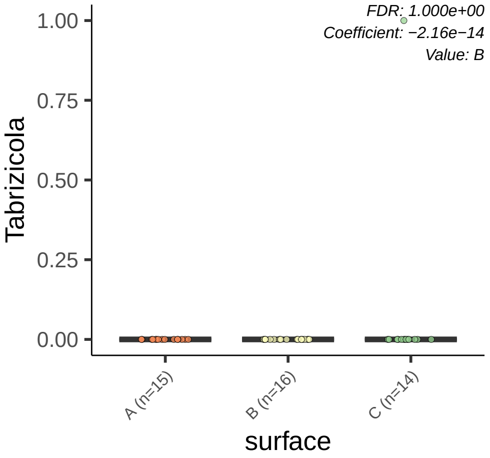

FDR: 1.000e+00
Coefficient: −2.16e−14
Value: B
1.00
0.75
0.50
0.25
0.00
Tabrizicola
A (n=15)
B (n=16)
C (n=14)
surface

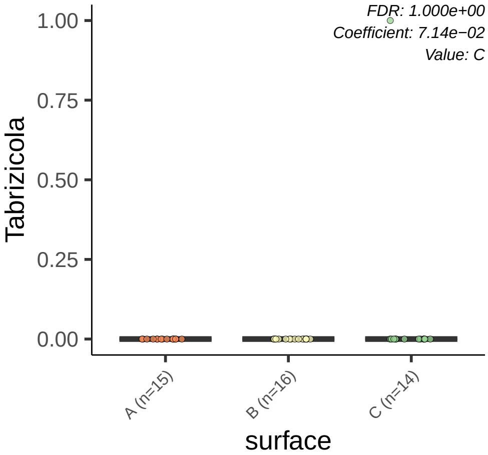

FDR: 1.000e+00
Coefficient: 7.14e−02
Value: C
Tabrizicola
1.00
0.75
0.50
0.25
0.00
A (n=15)
B (n=16)
C (n=14)
surface

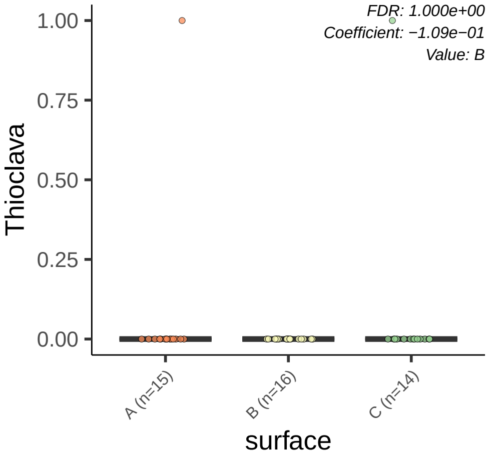
FDR: 1.000e+00
Coefficient: −1.09e−01
Value: B
1.00
0.75
0.50
0.25
0.00
Thioclava
A (n=15)
B (n=16)
C (n=14)
surface

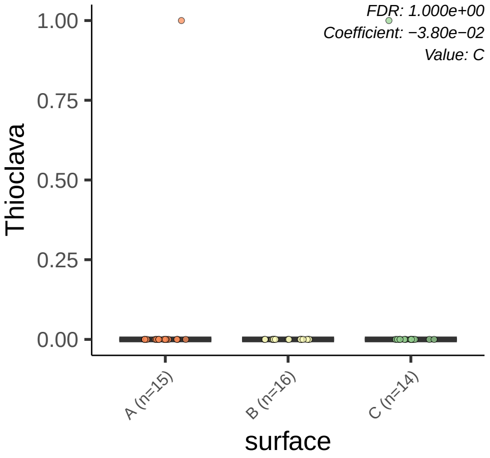
FDR: 1.000e+00
Coefficient: −3.80e−02
Value: C
Thioclava
1.00
0.75
0.50
0.25
0.00
A (n=15)
B (n=16)
C (n=14)
surface

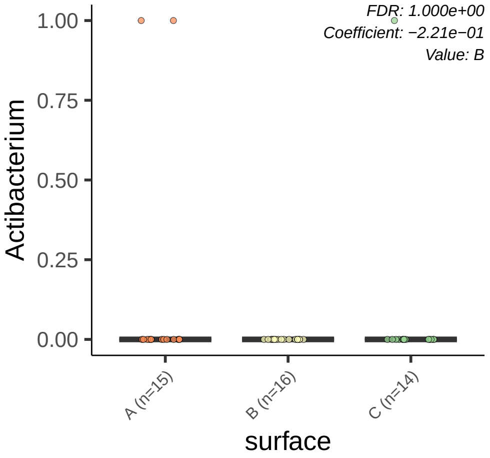
FDR: 1.000e+00
Coefficient: −2.21e−01
Value: B
1.00
0.75
0.50
0.25
0.00
Actibacterium
A (n=15)
B (n=16)
C (n=14)
surface

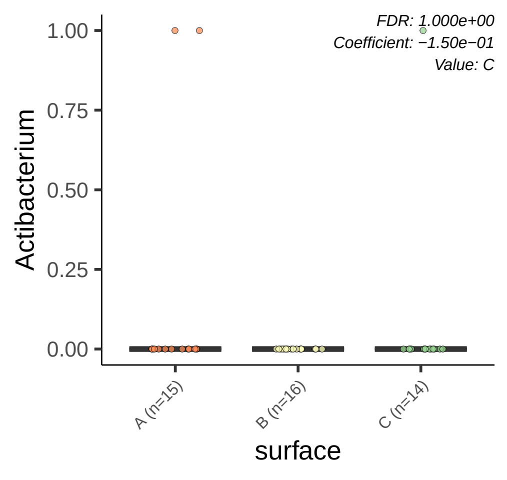
FDR: 1.000e+00
Coefficient: −1.50e−01
Value: C
Actibacterium
1.00
0.75
0.50
0.25
0.00
A (n=15)
B (n=16)
C (n=14)
surface

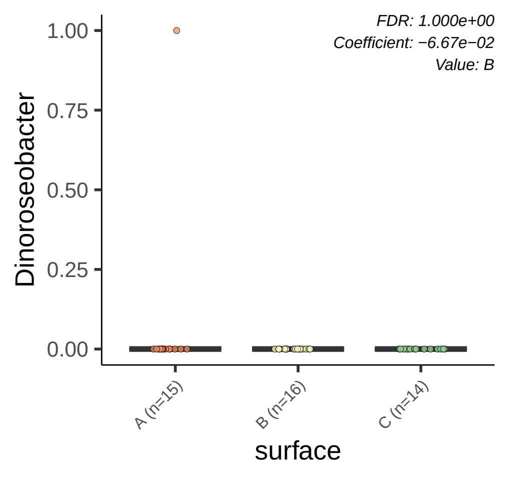
FDR: 1.000e+00
Coefficient: −6.67e−02
Value: B
Dinoroseobacter
1.00
0.75
0.50
0.25
0.00
A (n=15)
B (n=16)
C (n=14)
surface

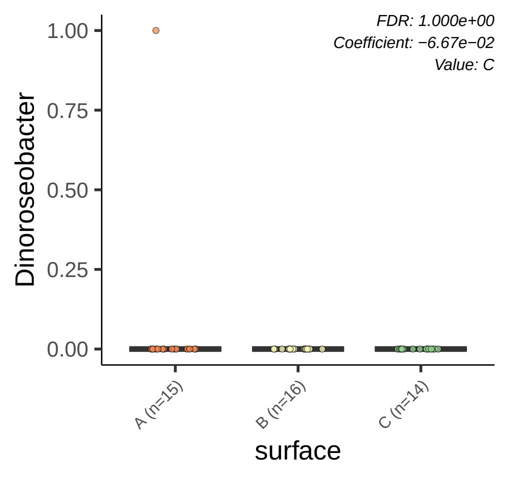
FDR: 1.000e+00
Coefficient: −6.67e−02
Value: C
Dinoroseobacter
1.00
0.75
0.50
0.25
0.00
A (n=15)
B (n=16)
C (n=14)
surface

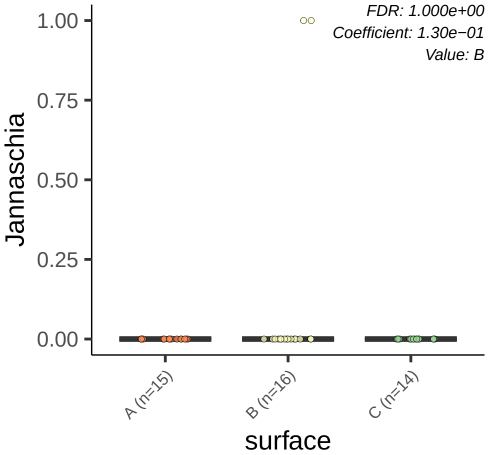
FDR: 1.000e+00
Coefficient: 1.30e−01
Value: B
1.00
0.75
0.50
0.25
0.00
Jannaschia
A (n=15)
B (n=16)
C (n=14)
surface

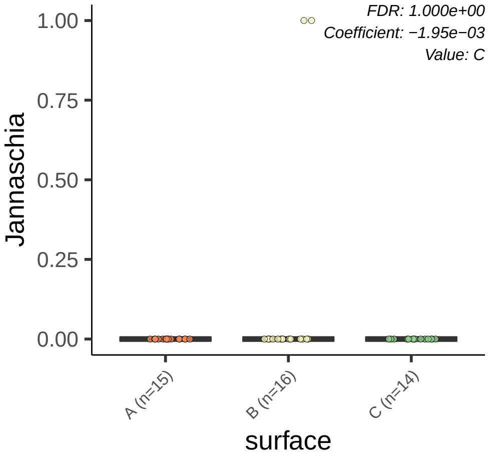
FDR: 1.000e+00
Coefficient: −1.95e−03
Value: C
Jannaschia
1.00
0.75
0.50
0.25
0.00
A (n=15)
B (n=16)
C (n=14)
surface

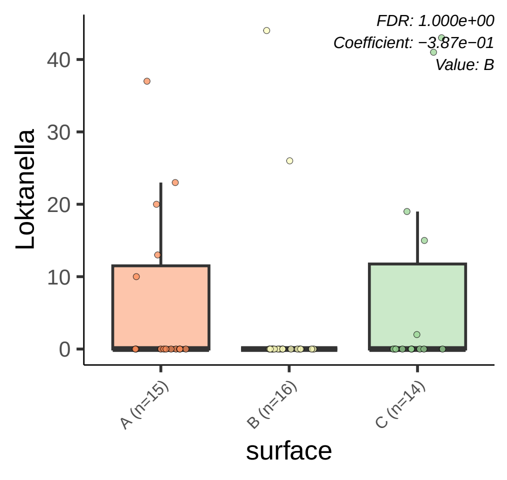
FDR: 1.000e+00
Coefficient: −3.87e−01
Value: B
Loktanella
40
30
20
10
0
A (n=15)
B (n=16)
C (n=14)
surface

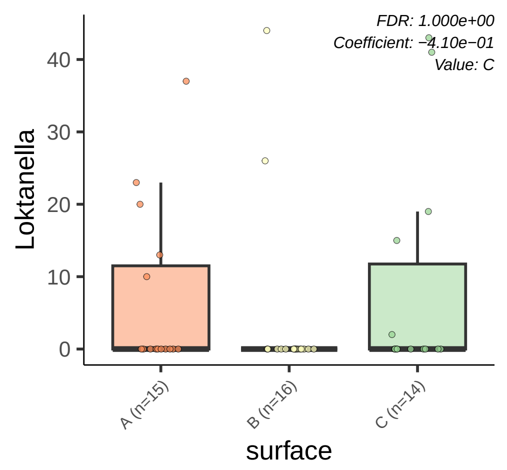

FDR: 1.000e+00
Coefficient: −4.10e−01
Value: C
Loktanella
0
10
20
30
40
A (n=15)
B (n=16)
C (n=14)
surface

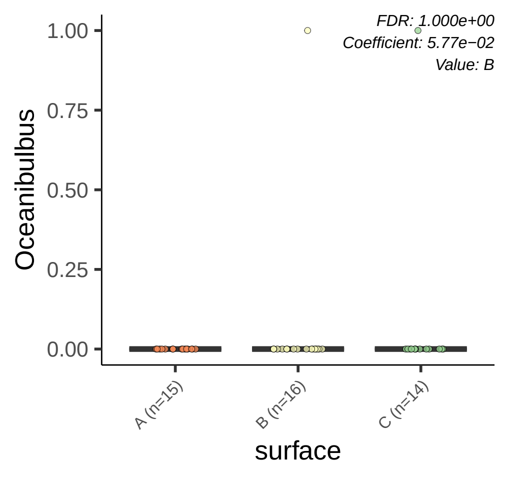
FDR: 1.000e+00
Coefficient: 5.77e−02
Value: B
1.00
0.75
0.50
0.25
0.00
Oceanibulbus
A (n=15)
B (n=16)
C (n=14)
surface

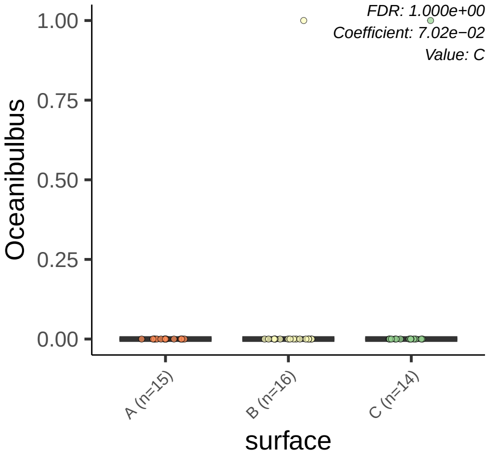
FDR: 1.000e+00
Coefficient: 7.02e−02
Value: C
Oceanibulbus
1.00
0.75
0.50
0.25
0.00
A (n=15)
B (n=16)
C (n=14)
surface

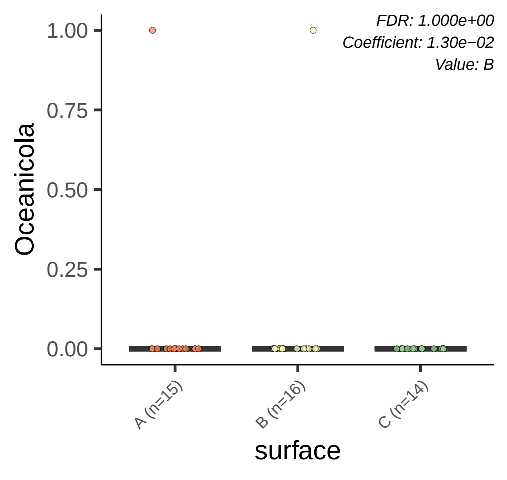
FDR: 1.000e+00
Coefficient: 1.30e−02
Value: B
Oceanicola
1.00
0.75
0.50
0.25
0.00
A (n=15)
B (n=16)
C (n=14)
surface

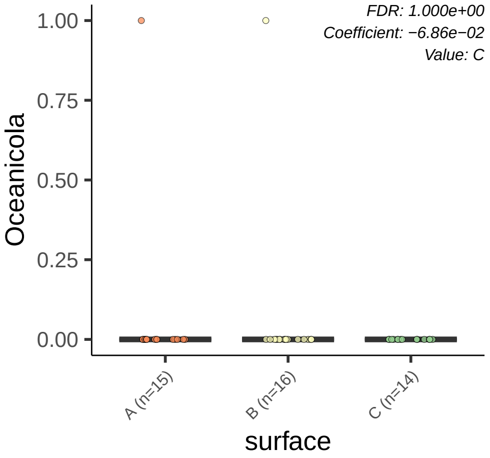
FDR: 1.000e+00
Coefficient: −6.86e−02
Value: C
Oceanicola
1.00
0.75
0.50
0.25
0.00
A (n=15)
B (n=16)
C (n=14)
surface

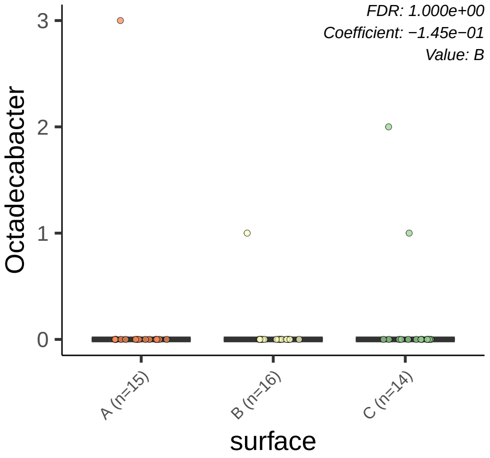

FDR: 1.000e+00
Coefficient: −1.45e−01
Value: B
Octadecabacter
3
2
1
0
A (n=15)
B (n=16)
C (n=14)
surface

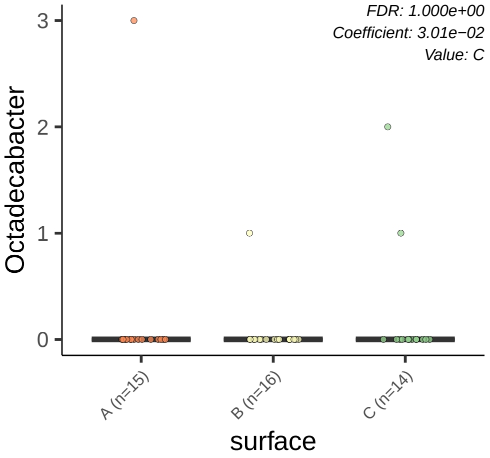
FDR: 1.000e+00
Coefficient: 3.01e−02
Value: C
Octadecabacter
0
1
2
3
A (n=15)
B (n=16)
C (n=14)
surface

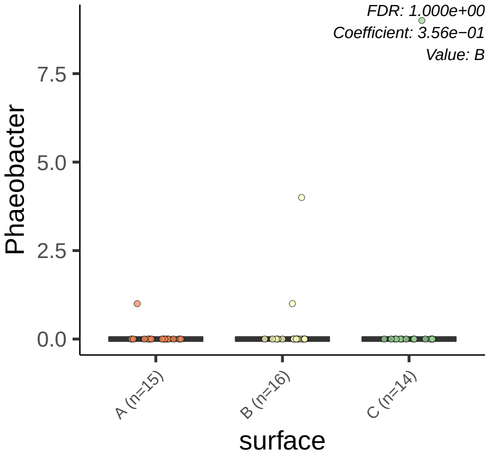

FDR: 1.000e+00
Coefficient: 3.56e−01
Value: B
Phaeobacter
7.5
5.0
2.5
0.0
A (n=15)
B (n=16)
C (n=14)
surface

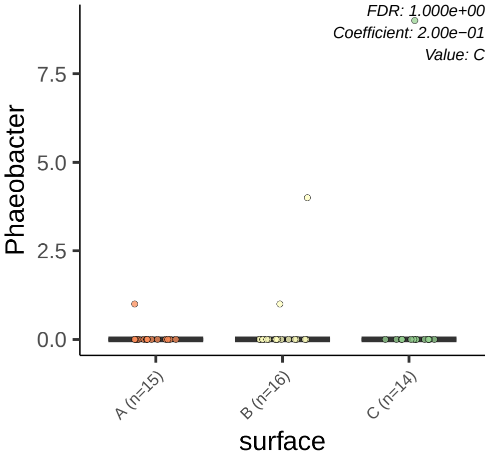
FDR: 1.000e+00
Coefficient: 2.00e−01
Value: C
Phaeobacter
7.5
5.0
2.5
0.0
A (n=15)
B (n=16)
C (n=14)
surface

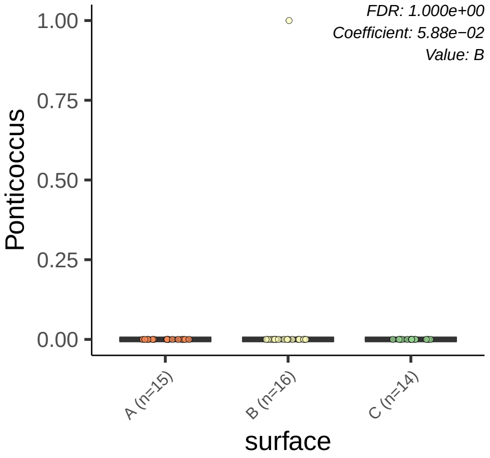
FDR: 1.000e+00
Coefficient: 5.88e−02
Value: B
Ponticoccus
1.00
0.75
0.50
0.25
0.00
A (n=15)
B (n=16)
C (n=14)
surface

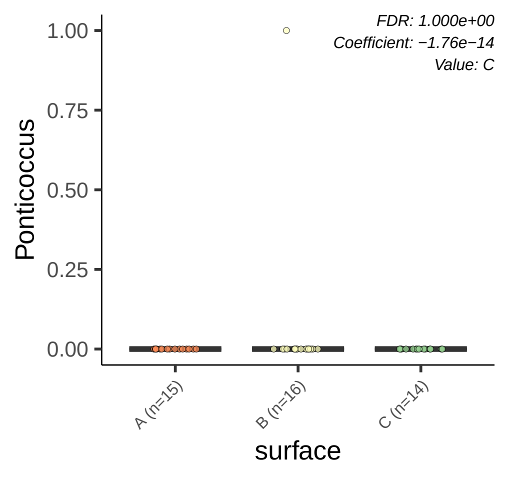
FDR: 1.000e+00
Coefficient: −1.76e−14
Value: C
Ponticoccus
1.00
0.75
0.50
0.25
0.00
A (n=15)
B (n=16)
C (n=14)
surface

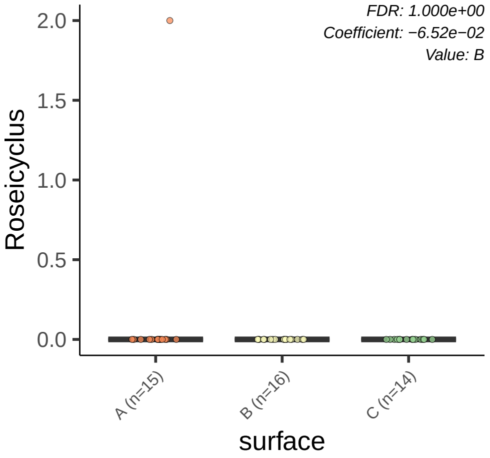

FDR: 1.000e+00
Coefficient: −6.52e−02
Value: B
Roseicyclus
2.0
1.5
1.0
0.5
0.0
A (n=15)
B (n=16)
C (n=14)
surface

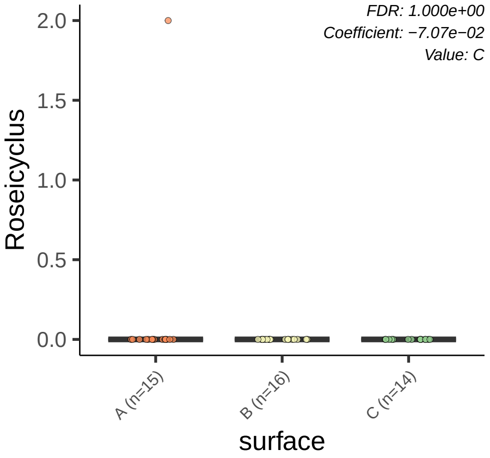

FDR: 1.000e+00
Coefficient: −7.07e−02
Value: C
Roseicyclus
2.0
1.5
1.0
0.5
0.0
A (n=15)
B (n=16)
C (n=14)
surface

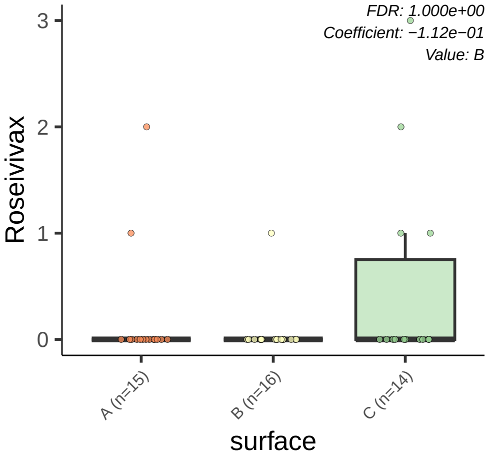

FDR: 1.000e+00
Coefficient: −1.12e−01
Value: B
Roseivivax
3
2
1
0
A (n=15)
B (n=16)
C (n=14)
surface

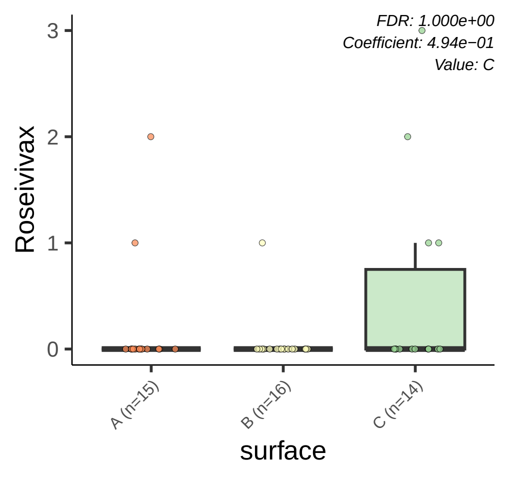
FDR: 1.000e+00
Coefficient: 4.94e−01
Value: C
Roseivivax
0
1
2
3
A (n=15)
B (n=16)
C (n=14)
surface

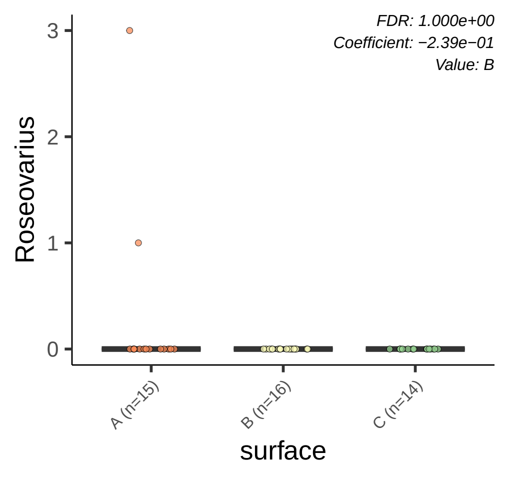
FDR: 1.000e+00
Coefficient: −2.39e−01
Value: B
Roseovarius
3
2
1
0
A (n=15)
B (n=16)
C (n=14)
surface

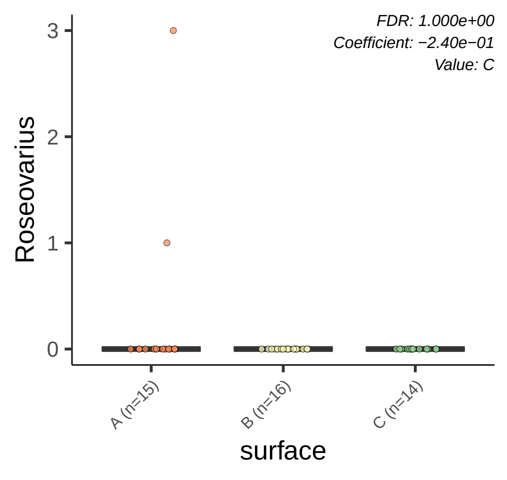
FDR: 1.000e+00
Coefficient: −2.40e−01
Value: C
Roseovarius
3
2
1
0
A (n=15)
B (n=16)
C (n=14)
surface

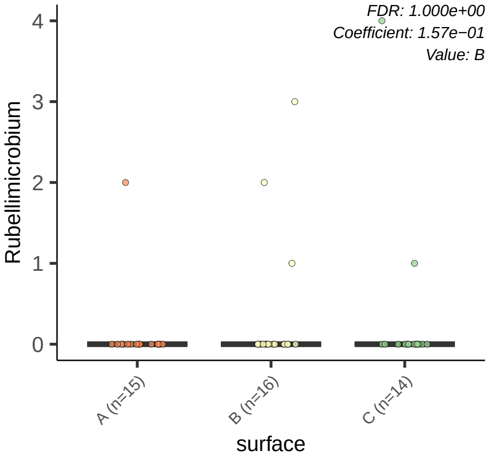
FDR: 1.000e+00
Coefficient: 1.57e−01
Value: B
Rubellimicrobium
0
1
2
3
4
A (n=15)
B (n=16)
C (n=14)
surface

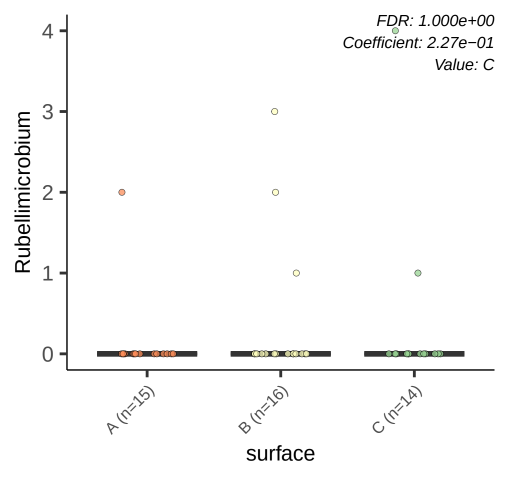
FDR: 1.000e+00
Coefficient: 2.27e−01
Value: C
Rubellimicrobium
0
1
2
3
4
A (n=15)
B (n=16)
C (n=14)
surface

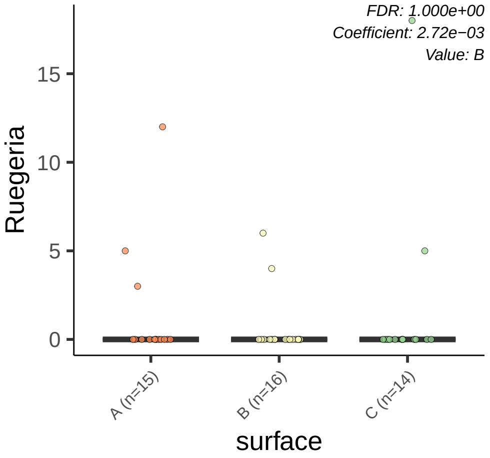

FDR: 1.000e+00
Coefficient: 2.72e−03
Value: B
Ruegeria
15
10
5
0
A (n=15)
B (n=16)
C (n=14)
surface

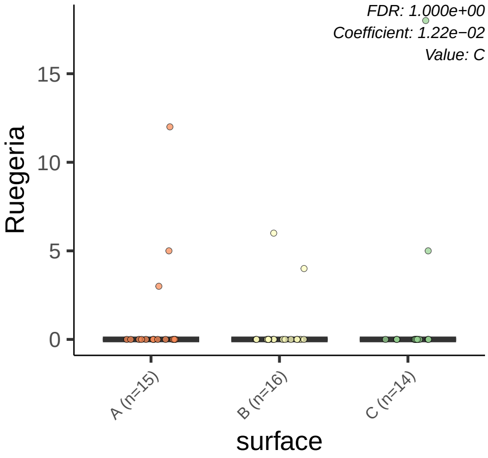

FDR: 1.000e+00
Coefficient: 1.22e−02
Value: C
Ruegeria
15
10
5
0
A (n=15)
B (n=16)
C (n=14)
surface

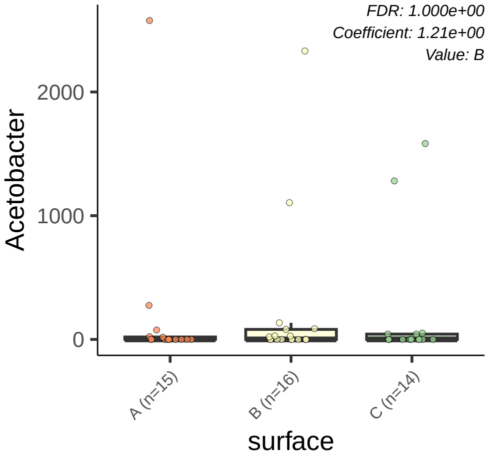
FDR: 1.000e+00
Coefficient: 1.21e+00
Value: B
Acetobacter
2000
1000
0
A (n=15)
B (n=16)
C (n=14)
surface

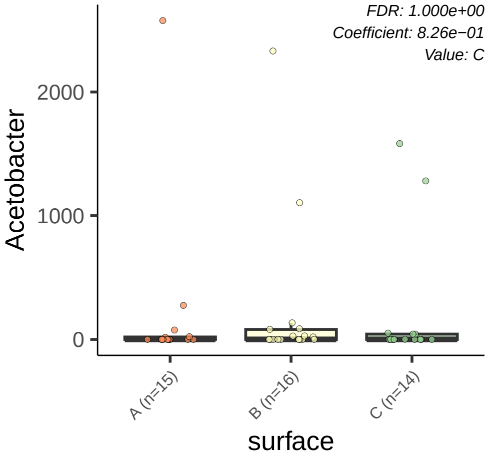
FDR: 1.000e+00
Coefficient: 8.26e−01
Value: C
Acetobacter
2000
1000
0
A (n=15)
B (n=16)
C (n=14)
surface

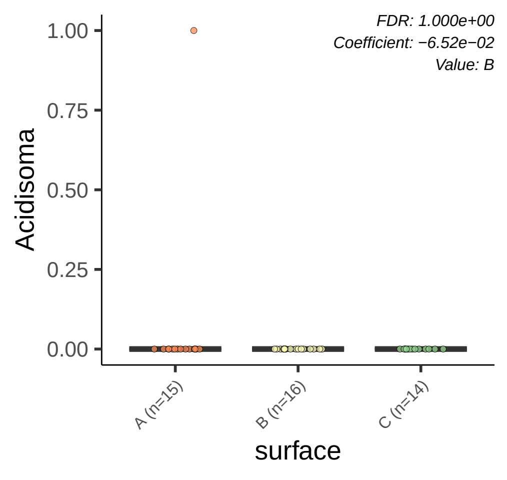
FDR: 1.000e+00
Coefficient: −6.52e−02
Value: B
Acidisoma
1.00
0.75
0.50
0.25
0.00
A (n=15)
B (n=16)
C (n=14)
surface

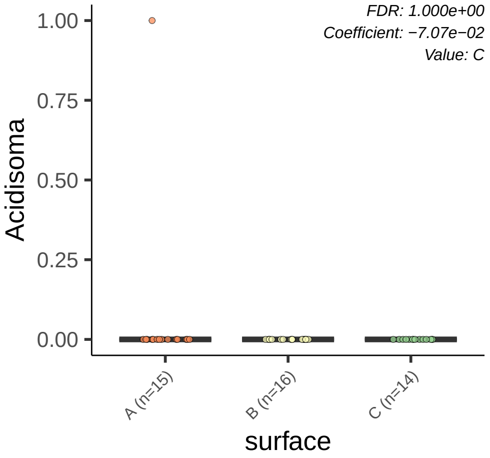
FDR: 1.000e+00
Coefficient: −7.07e−02
Value: C
Acidisoma
1.00
0.75
0.50
0.25
0.00
A (n=15)
B (n=16)
C (n=14)
surface

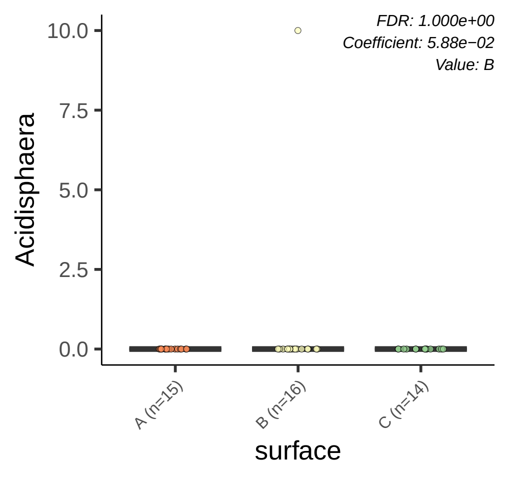

FDR: 1.000e+00
Coefficient: 5.88e−02
Value: B
Acidisphaera
10.0
7.5
5.0
2.5
0.0
A (n=15)
B (n=16)
C (n=14)
surface

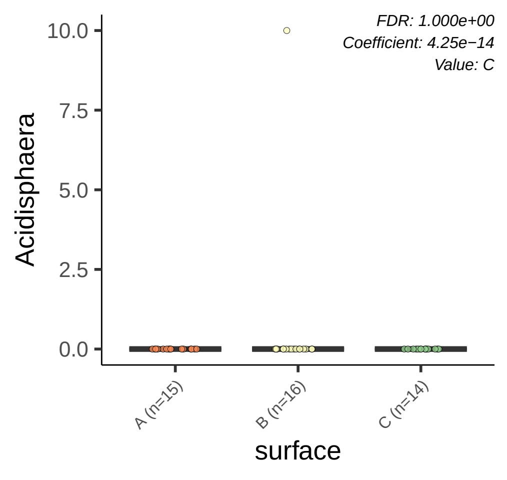
FDR: 1.000e+00
Coefficient: 4.25e−14
Value: C
Acidisphaera
10.0
7.5
5.0
2.5
0.0
A (n=15)
B (n=16)
C (n=14)
surface

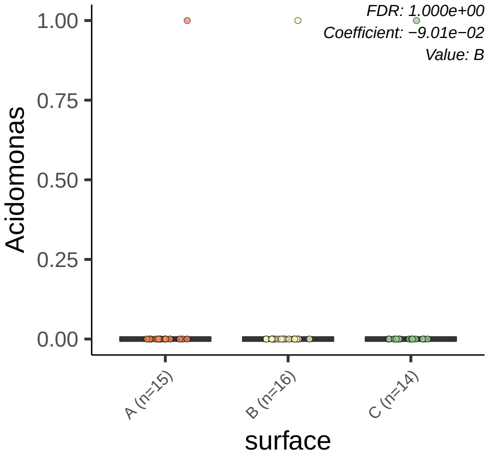
FDR: 1.000e+00
Coefficient: −9.01e−02
Value: B
Acidomonas
1.00
0.75
0.50
0.25
0.00
A (n=15)
B (n=16)
C (n=14)
surface

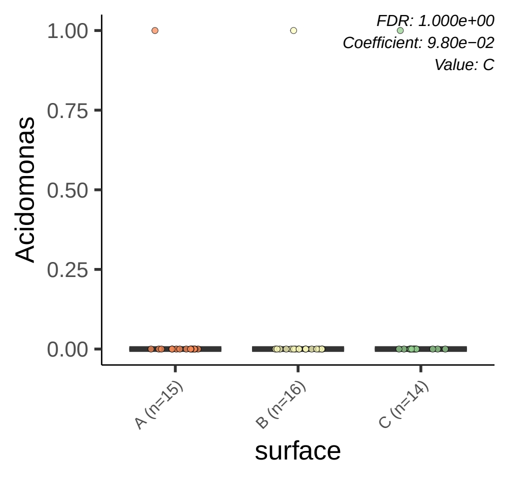

FDR: 1.000e+00
Coefficient: 9.80e−02
Value: C
Acidomonas
1.00
0.75
0.50
0.25
0.00
A (n=15)
B (n=16)
C (n=14)
surface

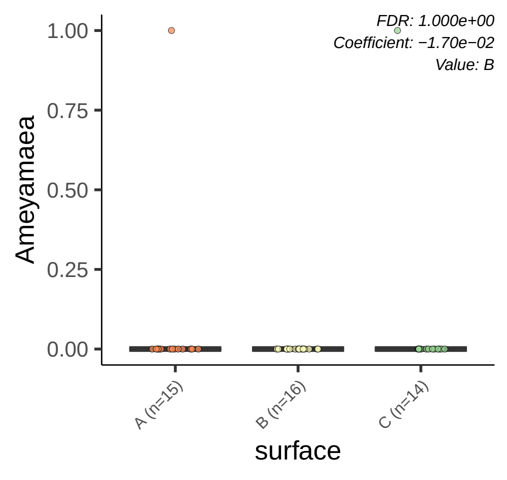
FDR: 1.000e+00
Coefficient: −1.70e−02
Value: B
Ameyamaea
1.00
0.75
0.50
0.25
0.00
A (n=15)
B (n=16)
C (n=14)
surface

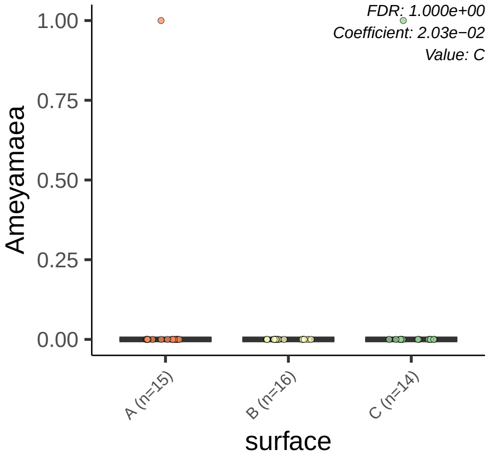

FDR: 1.000e+00
Coefficient: 2.03e−02
Value: C
Ameyamaea
1.00
0.75
0.50
0.25
0.00
A (n=15)
B (n=16)
C (n=14)
surface

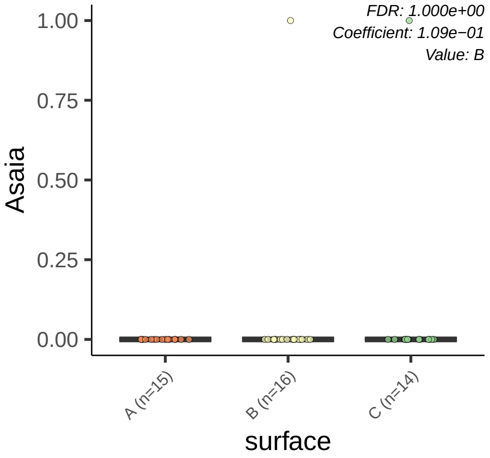
FDR: 1.000e+00
Coefficient: 1.09e−01
Value: B
Asaia
1.00
0.75
0.50
0.25
0.00
A (n=15)
B (n=16)
C (n=14)
surface

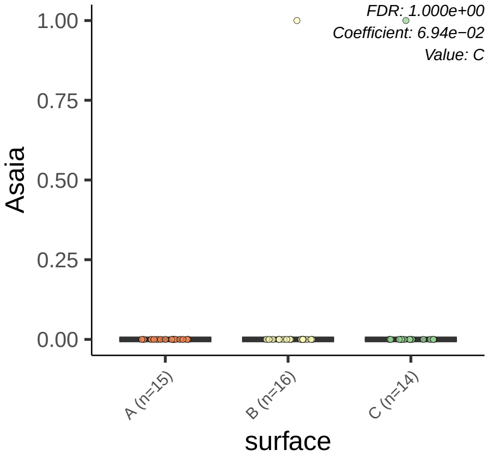

FDR: 1.000e+00
Coefficient: 6.94e−02
Value: C
Asaia
1.00
0.75
0.50
0.25
0.00
A (n=15)
B (n=16)
C (n=14)
surface

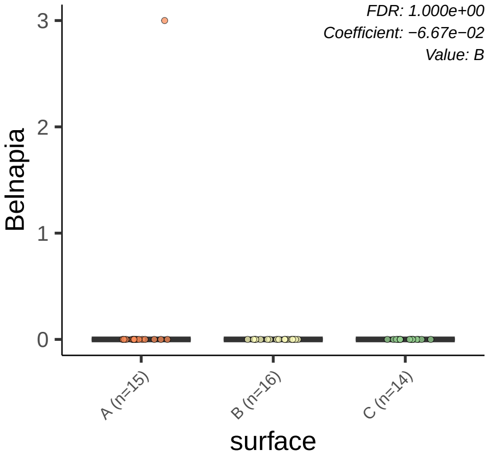

FDR: 1.000e+00
Coefficient: −6.67e−02
Value: B
Belnapia
3
2
1
0
A (n=15)
B (n=16)
C (n=14)
surface

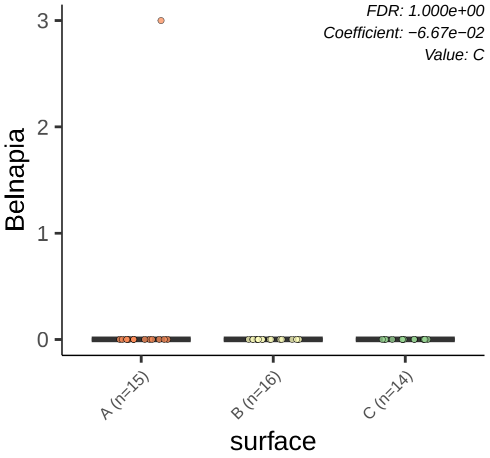
FDR: 1.000e+00
Coefficient: −6.67e−02
Value: C
Belnapia
3
2
1
0
A (n=15)
B (n=16)
C (n=14)
surface

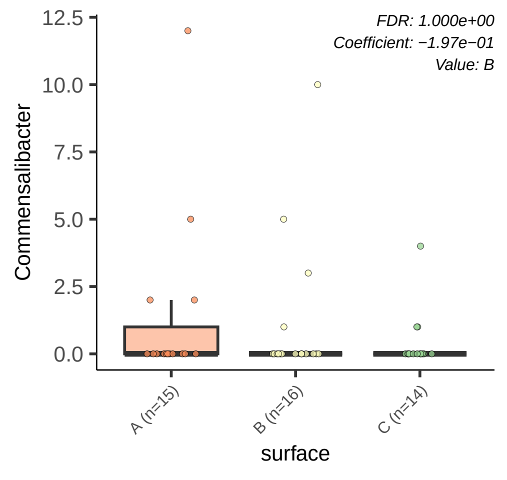
FDR: 1.000e+00
Coefficient: −1.97e−01
Value: B
Commensalibacter
12.5
10.0
7.5
5.0
2.5
0.0
A (n=15)
B (n=16)
C (n=14)
surface

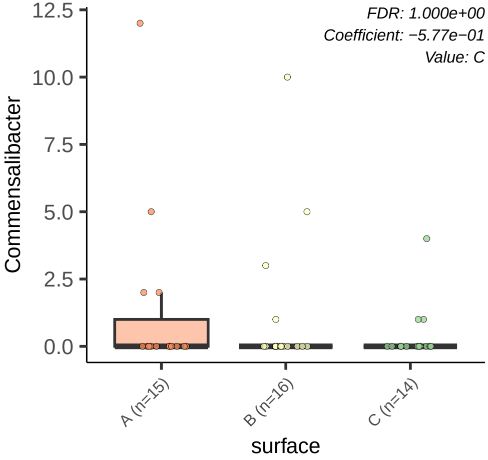

FDR: 1.000e+00
Coefficient: −5.77e−01
Value: C
Commensalibacter
12.5
10.0
7.5
5.0
2.5
0.0
A (n=15)
B (n=16)
C (n=14)
surface

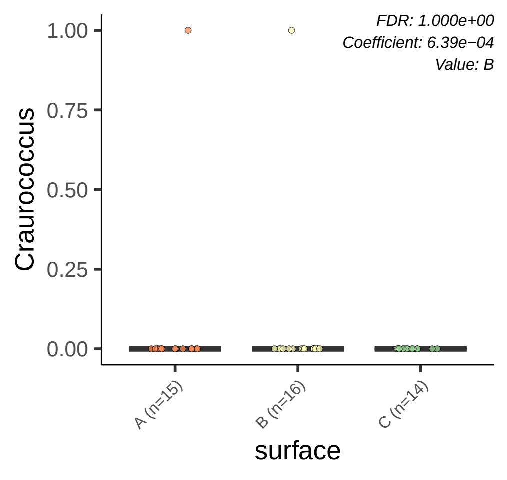

FDR: 1.000e+00
Coefficient: 6.39e−04
Value: B
1.00
0.75
0.50
0.25
0.00
Craurococcus
A (n=15)
B (n=16)
C (n=14)
surface

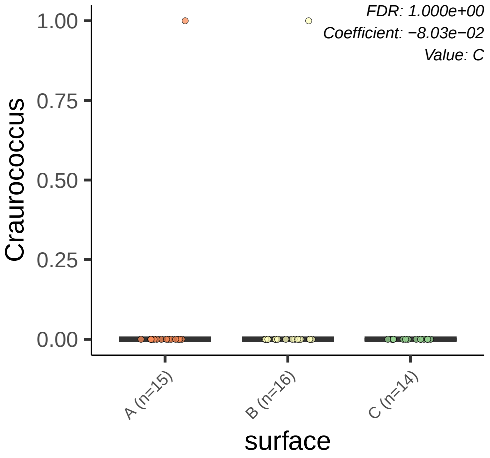
FDR: 1.000e+00
Coefficient: −8.03e−02
Value: C
Craurococcus
1.00
0.75
0.50
0.25
0.00
A (n=15)
B (n=16)
C (n=14)
surface

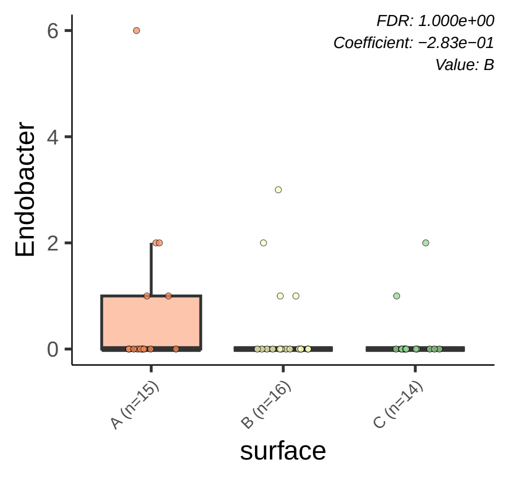
FDR: 1.000e+00
Coefficient: −2.83e−01
Value: B
Endobacter
0
2
4
6
A (n=15)
B (n=16)
C (n=14)
surface

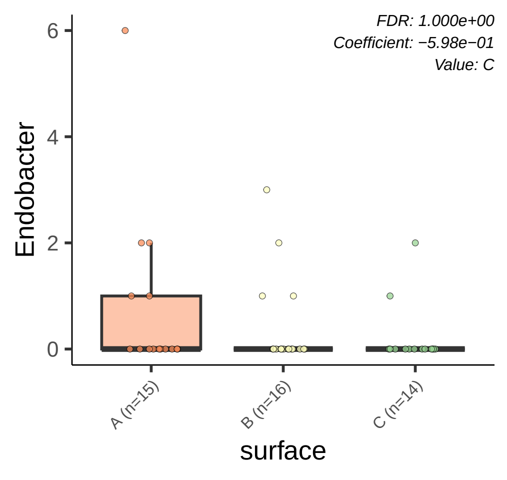
FDR: 1.000e+00
Coefficient: −5.98e−01
Value: C
Endobacter
0
2
4
6
A (n=15)
B (n=16)
C (n=14)
surface

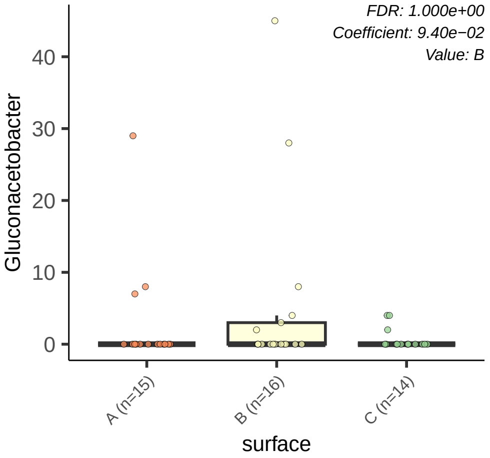

FDR: 1.000e+00
Coefficient: 9.40e−02
Value: B
Gluconacetobacter
0
10
20
30
40
A (n=15)
B (n=16)
C (n=14)
surface

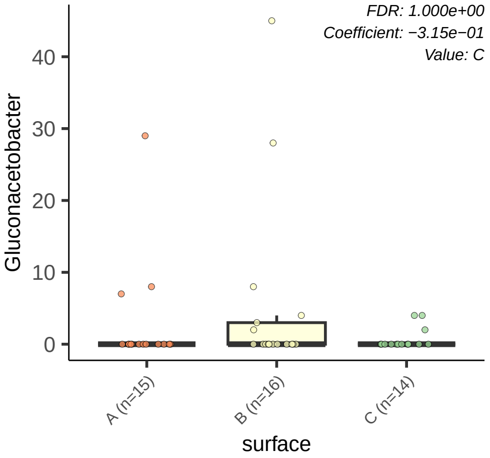
FDR: 1.000e+00
Coefficient: −3.15e−01
Value: C
Gluconacetobacter
40
30
20
10
0
A (n=15)
B (n=16)
C (n=14)
surface

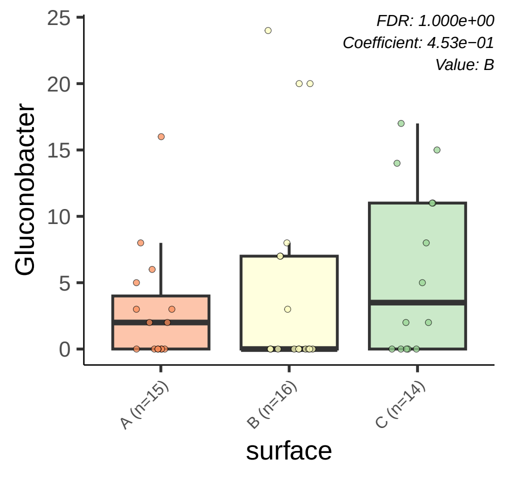

FDR: 1.000e+00
Coefficient: 4.53e−01
Value: B
Gluconobacter
25
20
15
10
5
0
A (n=15)
B (n=16)
C (n=14)
surface

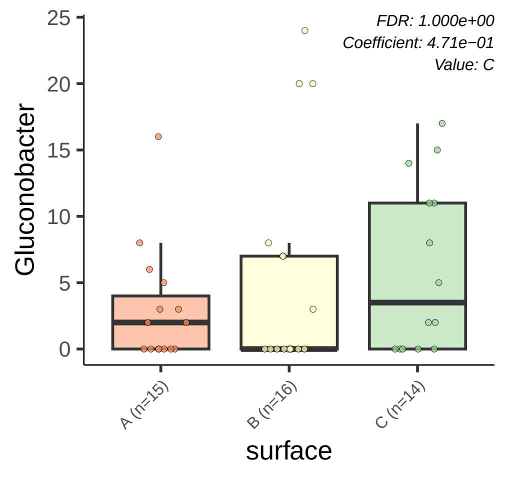
FDR: 1.000e+00
Coefficient: 4.71e−01
Value: C
Gluconobacter
25
20
15
10
5
0
A (n=15)
B (n=16)
C (n=14)
surface

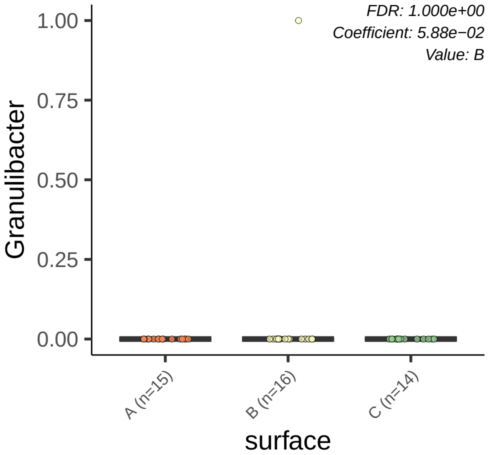
FDR: 1.000e+00
Coefficient: 5.88e−02
Value: B
Granulibacter
1.00
0.75
0.50
0.25
0.00
A (n=15)
B (n=16)
C (n=14)
surface

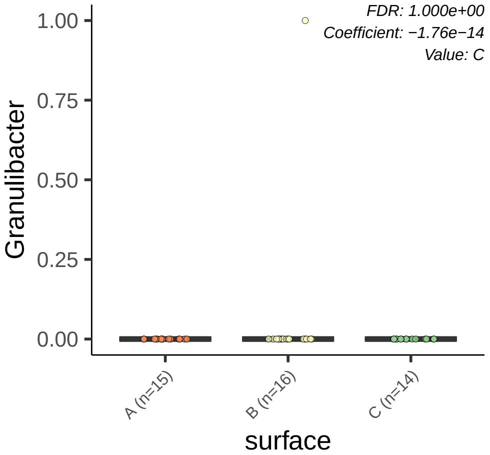
FDR: 1.000e+00
Coefficient: −1.76e−14
Value: C
Granulibacter
1.00
0.75
0.50
0.25
0.00
A (n=15)
B (n=16)
C (n=14)
surface

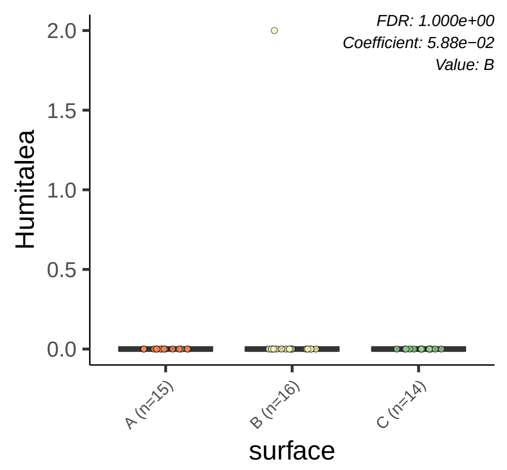
FDR: 1.000e+00
Coefficient: 5.88e−02
Value: B
Humitalea
2.0
1.5
1.0
0.5
0.0
A (n=15)
B (n=16)
C (n=14)
surface

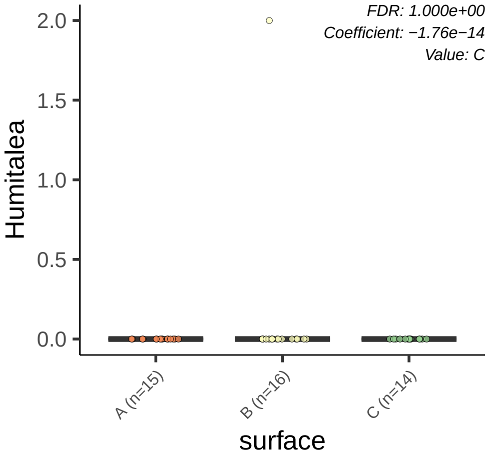

FDR: 1.000e+00
Coefficient: −1.76e−14
Value: C
Humitalea
2.0
1.5
1.0
0.5
0.0
A (n=15)
B (n=16)
C (n=14)
surface

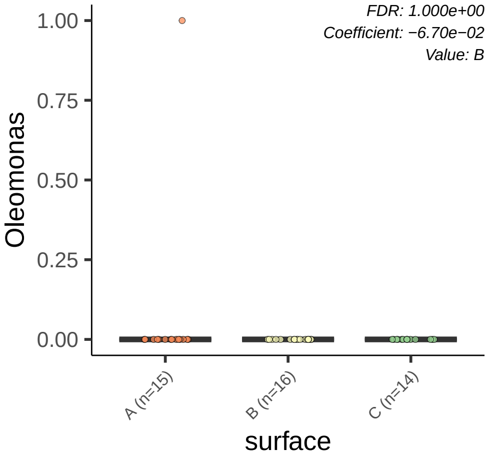

FDR: 1.000e+00
Coefficient: −6.70e−02
Value: B
Oleomonas
1.00
0.75
0.50
0.25
0.00
A (n=15)
B (n=16)
C (n=14)
surface

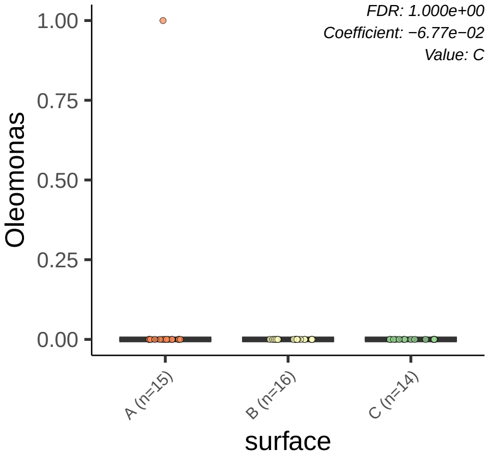

FDR: 1.000e+00
Coefficient: −6.77e−02
Value: C
Oleomonas
1.00
0.75
0.50
0.25
0.00
A (n=15)
B (n=16)
C (n=14)
surface

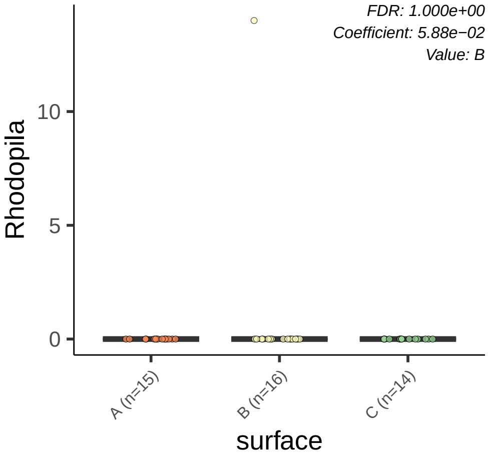
FDR: 1.000e+00
Coefficient: 5.88e−02
Value: B
Rhodopila
10
5
0
A (n=15)
B (n=16)
C (n=14)
surface

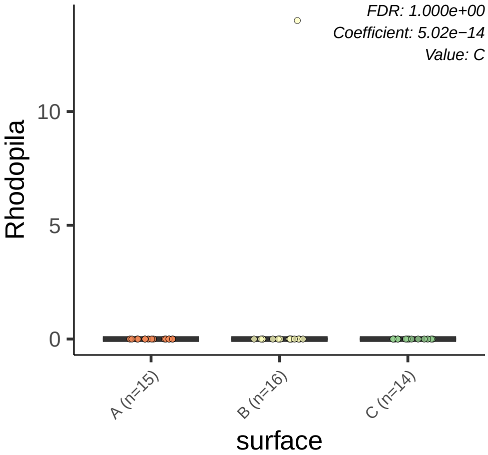
FDR: 1.000e+00
Coefficient: 5.02e−14
Value: C
Rhodopila
10
5
0
A (n=15)
B (n=16)
C (n=14)
surface

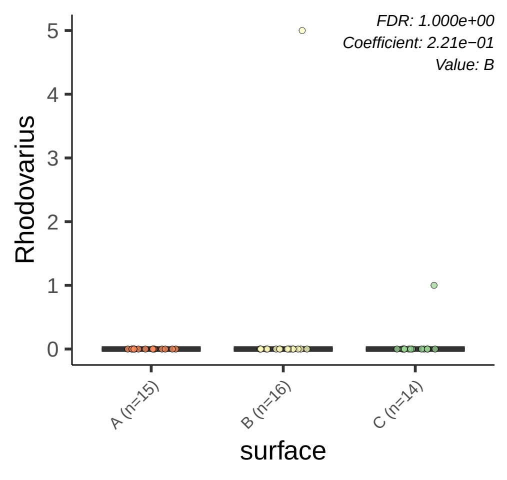

FDR: 1.000e+00
Coefficient: 2.21e−01
Value: B
Rhodovarius
0
1
2
3
4
5
A (n=15)
B (n=16)
C (n=14)
surface

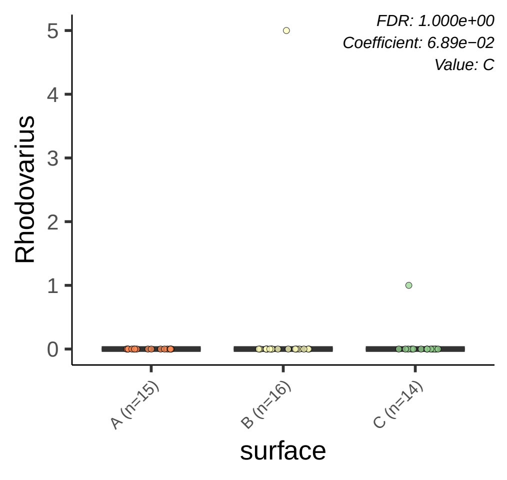

FDR: 1.000e+00
Coefficient: 6.89e−02
Value: C
Rhodovarius
0
1
2
3
4
5
A (n=15)
B (n=16)
C (n=14)
surface

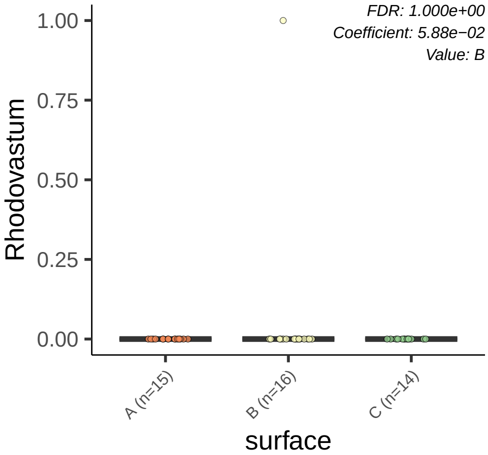

FDR: 1.000e+00
Coefficient: 5.88e−02
Value: B
Rhodovastum
1.00
0.75
0.50
0.25
0.00
A (n=15)
B (n=16)
C (n=14)
surface

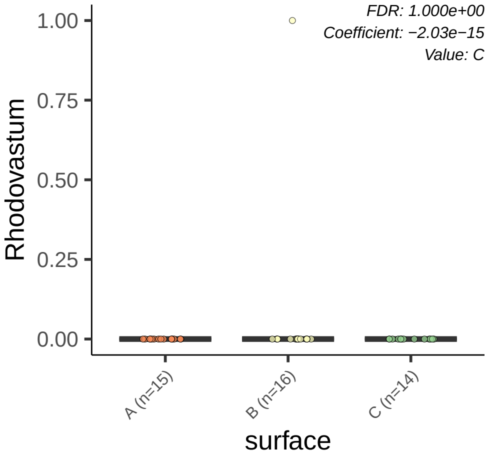
FDR: 1.000e+00
Coefficient: −2.03e−15
Value: C
Rhodovastum
1.00
0.75
0.50
0.25
0.00
A (n=15)
B (n=16)
C (n=14)
surface

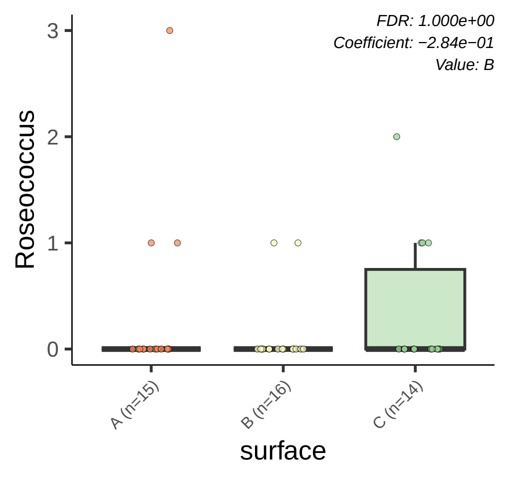

FDR: 1.000e+00
Coefficient: −2.84e−01
Value: B
Roseococcus
3
2
1
0
A (n=15)
B (n=16)
C (n=14)
surface

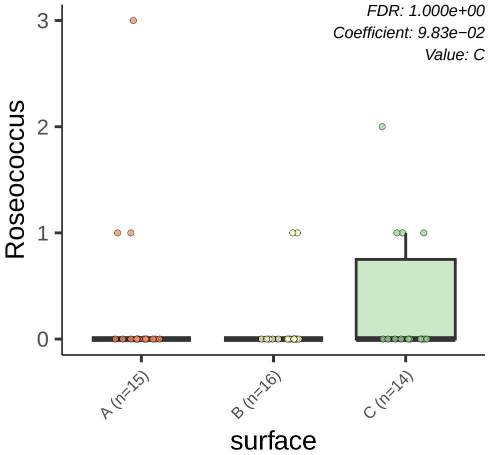

FDR: 1.000e+00
Coefficient: 9.83e−02
Value: C
Roseococcus
3
2
1
0
A (n=15)
B (n=16)
C (n=14)
surface

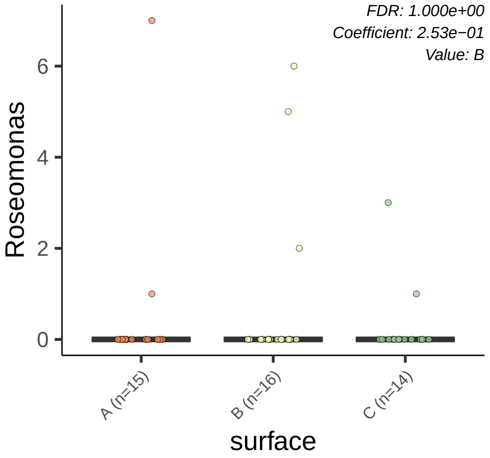
FDR: 1.000e+00
Coefficient: 2.53e−01
Value: B
Roseomonas
0
2
4
6
A (n=15)
B (n=16)
C (n=14)
surface

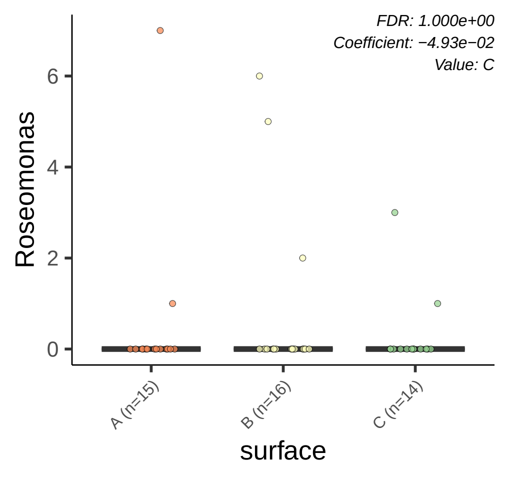
FDR: 1.000e+00
Coefficient: −4.93e−02
Value: C
Roseomonas
0
2
4
6
A (n=15)
B (n=16)
C (n=14)
surface

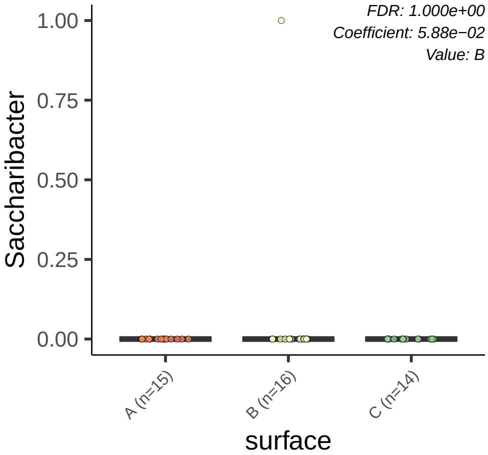

FDR: 1.000e+00
Coefficient: 5.88e−02
Value: B
Saccharibacter
1.00
0.75
0.50
0.25
0.00
A (n=15)
B (n=16)
C (n=14)
surface

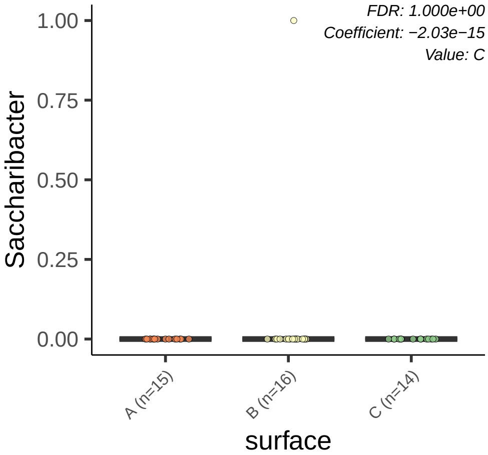
FDR: 1.000e+00
Coefficient: −2.03e−15
Value: C
Saccharibacter
1.00
0.75
0.50
0.25
0.00
A (n=15)
B (n=16)
C (n=14)
surface

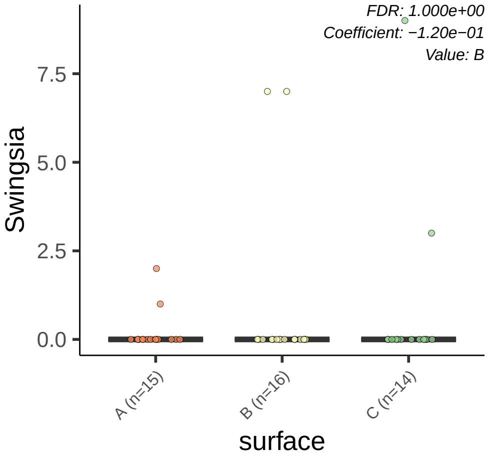
FDR: 1.000e+00
Coefficient: −1.20e−01
Value: B
Swingsia
7.5
5.0
2.5
0.0
A (n=15)
B (n=16)
C (n=14)
surface

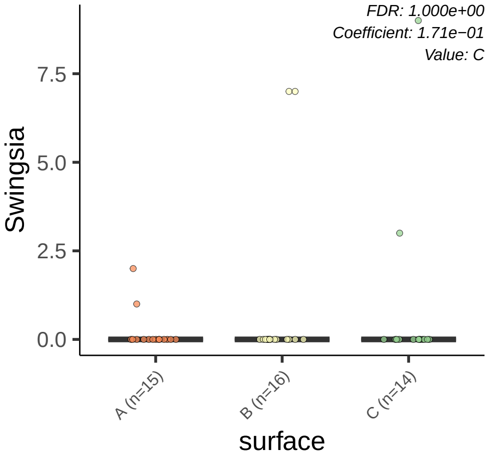

FDR: 1.000e+00
Coefficient: 1.71e−01
Value: C
Swingsia
7.5
5.0
2.5
0.0
A (n=15)
B (n=16)
C (n=14)
surface

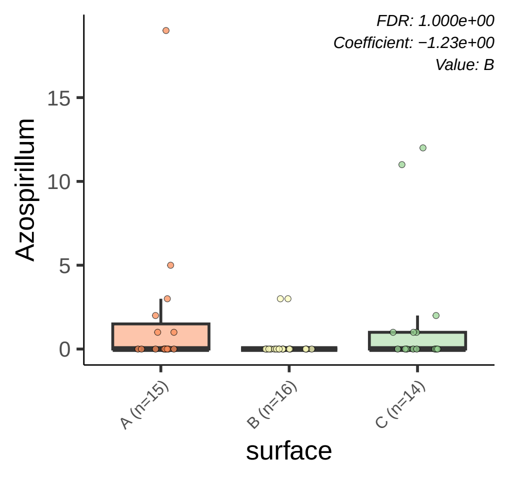

FDR: 1.000e+00
Coefficient: −1.23e+00
Value: B
Azospirillum
15
10
5
0
A (n=15)
B (n=16)
C (n=14)
surface

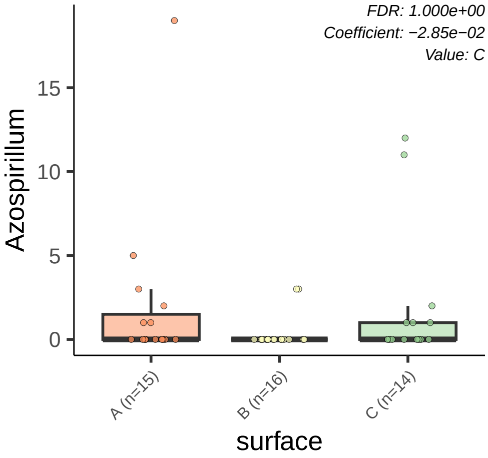

FDR: 1.000e+00
Coefficient: −2.85e−02
Value: C
Azospirillum
0
5
10
15
A (n=15)
B (n=16)
C (n=14)
surface

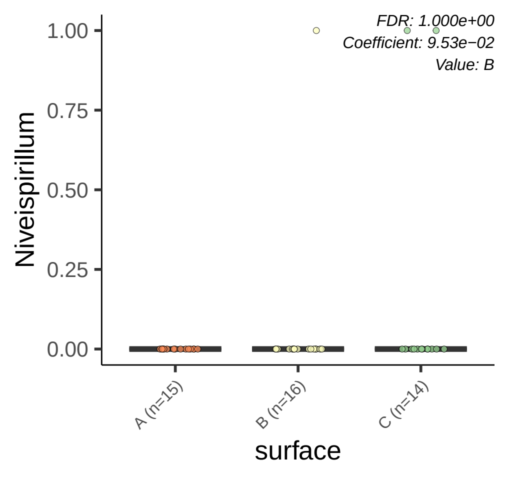

FDR: 1.000e+00
Coefficient: 9.53e−02
Value: B
Niveispirillum
1.00
0.75
0.50
0.25
0.00
A (n=15)
B (n=16)
C (n=14)
surface

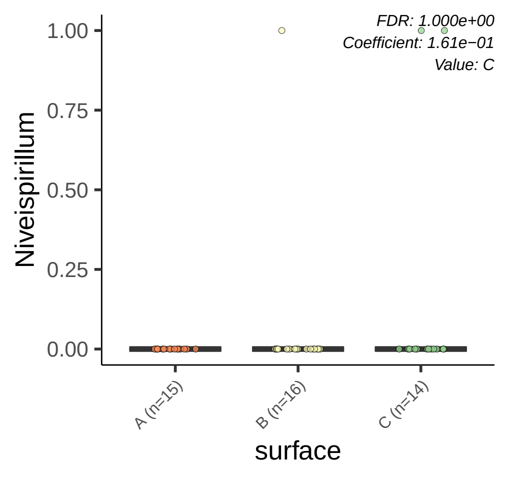
FDR: 1.000e+00
Coefficient: 1.61e−01
Value: C
Niveispirillum
1.00
0.75
0.50
0.25
0.00
A (n=15)
B (n=16)
C (n=14)
surface

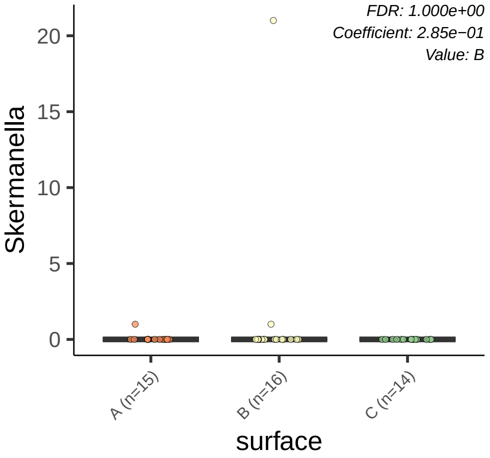
FDR: 1.000e+00
Coefficient: 2.85e−01
Value: B
Skermanella
20
15
10
5
0
A (n=15)
B (n=16)
C (n=14)
surface

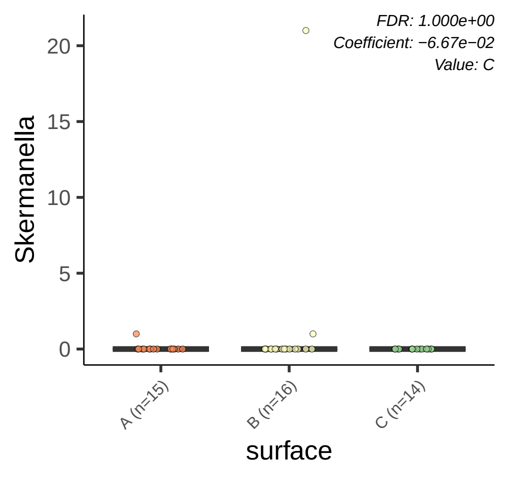
FDR: 1.000e+00
Coefficient: −6.67e−02
Value: C
Skermanella
20
15
10
5
0
A (n=15)
B (n=16)
C (n=14)
surface

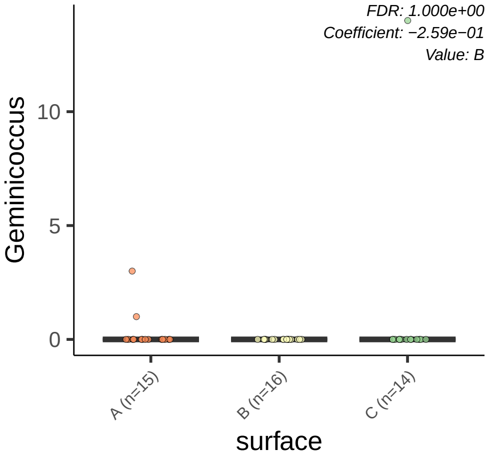

FDR: 1.000e+00
Coefficient: −2.59e−01
Value: B
Geminicoccus
10
5
0
A (n=15)
B (n=16)
C (n=14)
surface

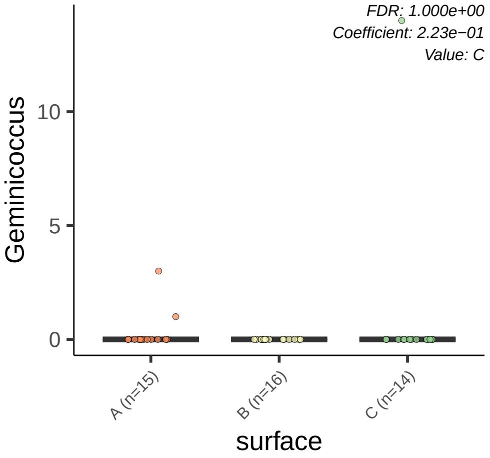
FDR: 1.000e+00
Coefficient: 2.23e−01
Value: C
Geminicoccus
10
5
0
A (n=15)
B (n=16)
C (n=14)
surface

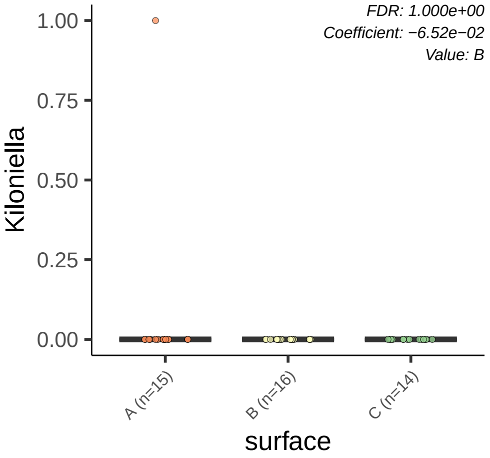

FDR: 1.000e+00
Coefficient: −6.52e−02
Value: B
Kiloniella
1.00
0.75
0.50
0.25
0.00
A (n=15)
B (n=16)
C (n=14)
surface

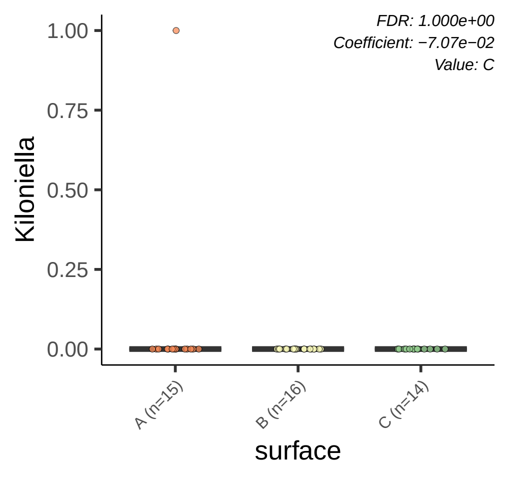
FDR: 1.000e+00
Coefficient: −7.07e−02
Value: C
Kiloniella
1.00
0.75
0.50
0.25
0.00
A (n=15)
B (n=16)
C (n=14)
surface

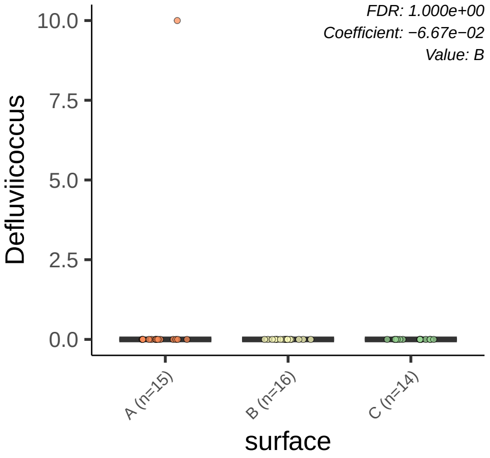
FDR: 1.000e+00
Coefficient: −6.67e−02
Value: B
Defluviicoccus
10.0
7.5
5.0
2.5
0.0
A (n=15)
B (n=16)
C (n=14)
surface

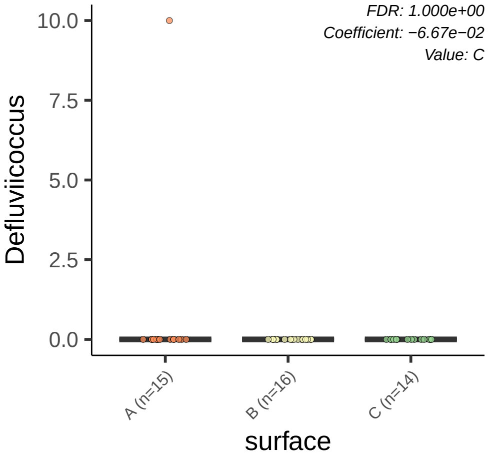
FDR: 1.000e+00
Coefficient: −6.67e−02
Value: C
Defluviicoccus
10.0
7.5
5.0
2.5
0.0
A (n=15)
B (n=16)
C (n=14)
surface

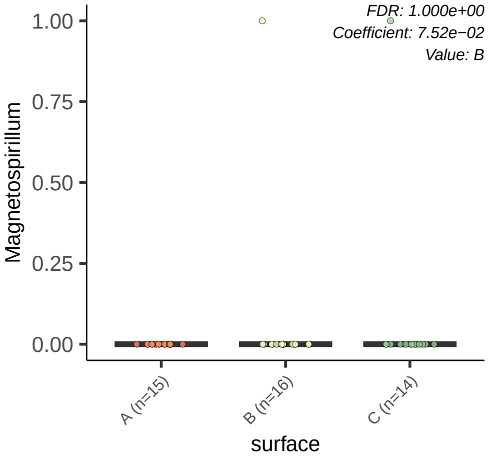

FDR: 1.000e+00
Coefficient: 7.52e−02
Value: B
Magnetospirillum
1.00
0.75
0.50
0.25
0.00
A (n=15)
B (n=16)
C (n=14)
surface

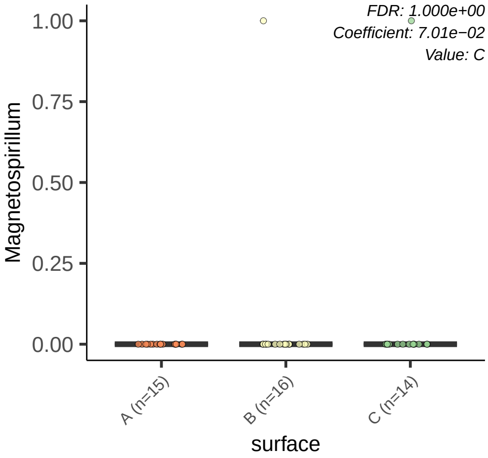
FDR: 1.000e+00
Coefficient: 7.01e−02
Value: C
Magnetospirillum
1.00
0.75
0.50
0.25
0.00
A (n=15)
B (n=16)
C (n=14)
surface

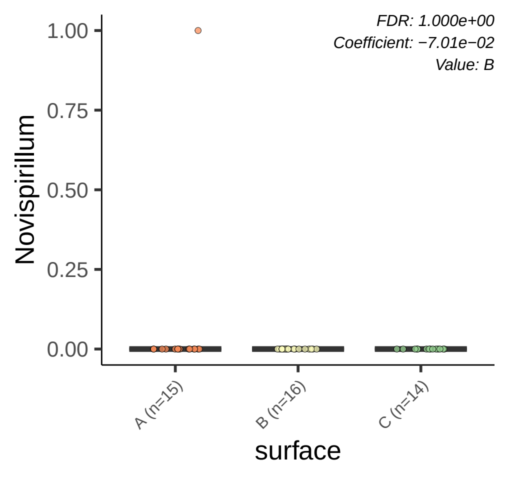
FDR: 1.000e+00
Coefficient: −7.01e−02
Value: B
Novispirillum
1.00
0.75
0.50
0.25
0.00
A (n=15)
B (n=16)
C (n=14)
surface

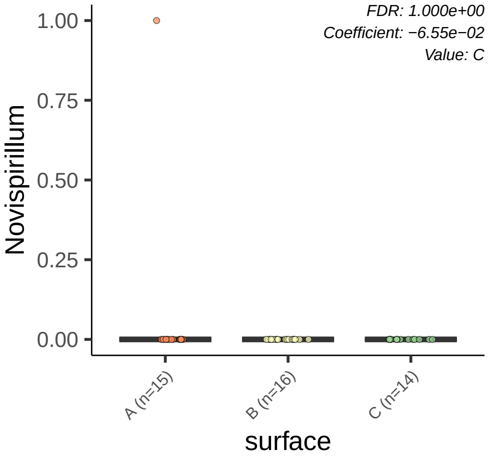

FDR: 1.000e+00
Coefficient: −6.55e−02
Value: C
Novispirillum
1.00
0.75
0.50
0.25
0.00
A (n=15)
B (n=16)
C (n=14)
surface

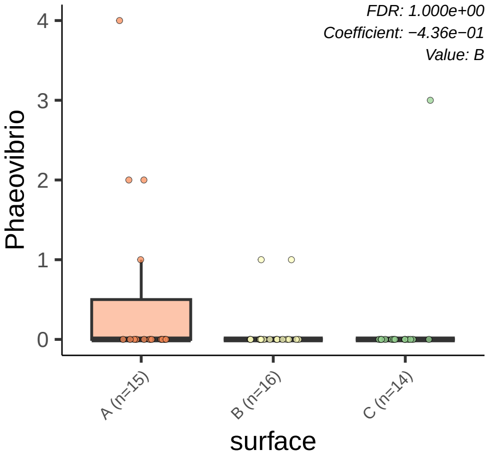

FDR: 1.000e+00
Coefficient: −4.36e−01
Value: B
Phaeovibrio
4
3
2
1
0
A (n=15)
B (n=16)
C (n=14)
surface

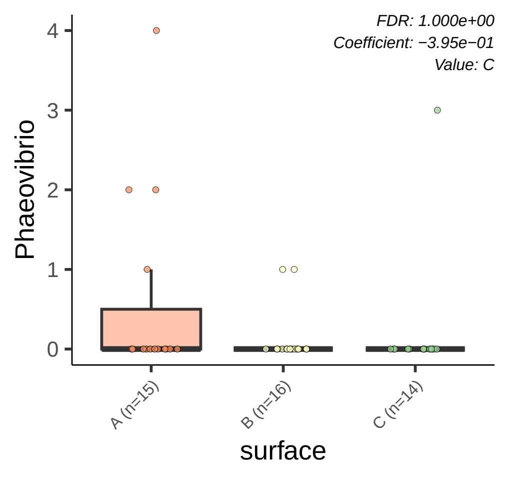
FDR: 1.000e+00
Coefficient: −3.95e−01
Value: C
Phaeovibrio
4
3
2
1
0
A (n=15)
B (n=16)
C (n=14)
surface

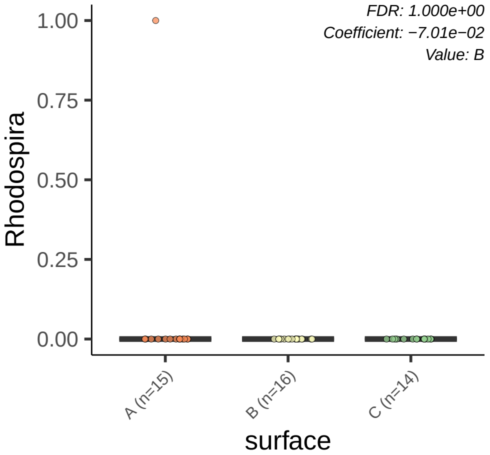

FDR: 1.000e+00
Coefficient: −7.01e−02
Value: B
Rhodospira
1.00
0.75
0.50
0.25
0.00
A (n=15)
B (n=16)
C (n=14)
surface

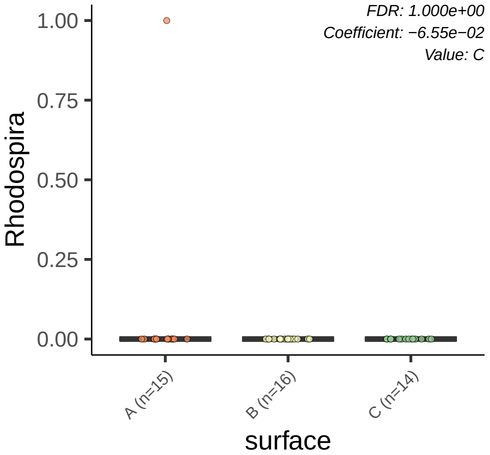

FDR: 1.000e+00
Coefficient: −6.55e−02
Value: C
Rhodospira
1.00
0.75
0.50
0.25
0.00
A (n=15)
B (n=16)
C (n=14)
surface

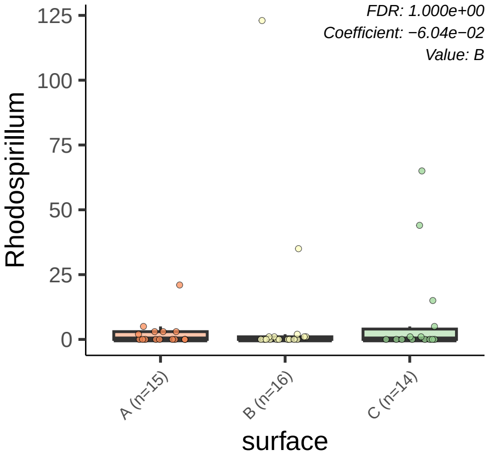

FDR: 1.000e+00
Coefficient: −6.04e−02
Value: B
Rhodospirillum
125
100
75
50
25
0
A (n=15)
B (n=16)
C (n=14)
surface

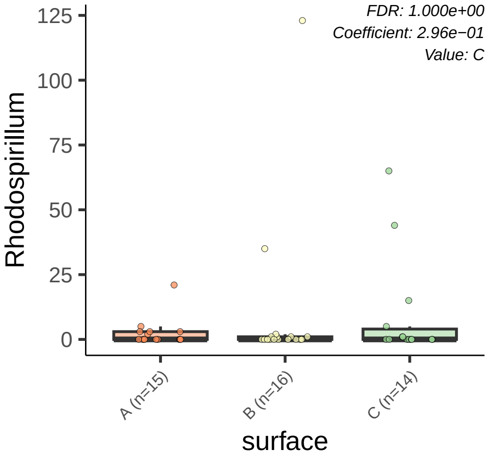
FDR: 1.000e+00
Coefficient: 2.96e−01
Value: C
Rhodospirillum
125
100
75
50
25
0
A (n=15)
B (n=16)
C (n=14)
surface

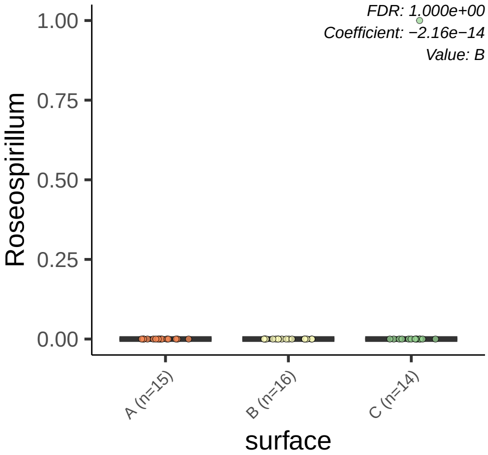
FDR: 1.000e+00
Coefficient: −2.16e−14
Value: B
Roseospirillum
1.00
0.75
0.50
0.25
0.00
A (n=15)
B (n=16)
C (n=14)
surface

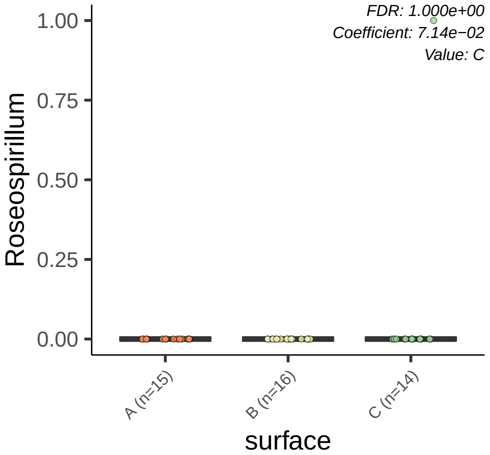
FDR: 1.000e+00
Coefficient: 7.14e−02
Value: C
Roseospirillum
1.00
0.75
0.50
0.25
0.00
A (n=15)
B (n=16)
C (n=14)
surface

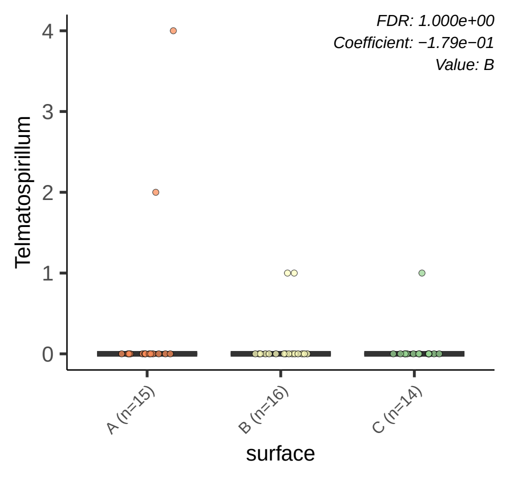

FDR: 1.000e+00
Coefficient: −1.79e−01
Value: B
Telmatospirillum
0
1
2
3
4
A (n=15)
B (n=16)
C (n=14)
surface

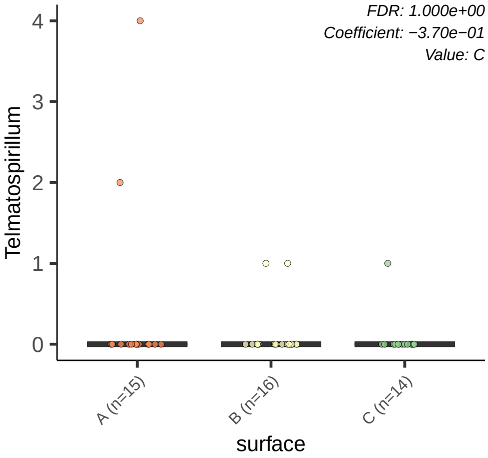
FDR: 1.000e+00
Coefficient: −3.70e−01
Value: C
Telmatospirillum
0
1
2
3
4
A (n=15)
B (n=16)
C (n=14)
surface

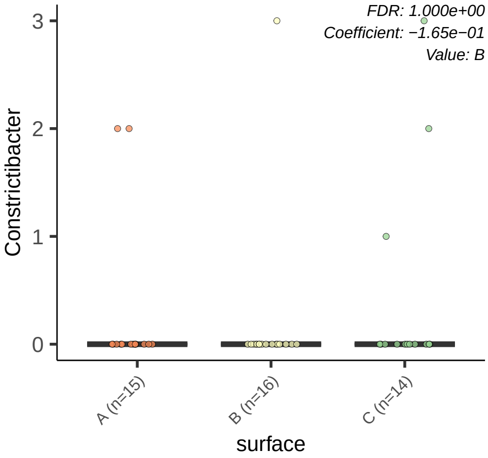
FDR: 1.000e+00
Coefficient: −1.65e−01
Value: B
Constrictibacter
0
1
2
3
A (n=15)
B (n=16)
C (n=14)
surface

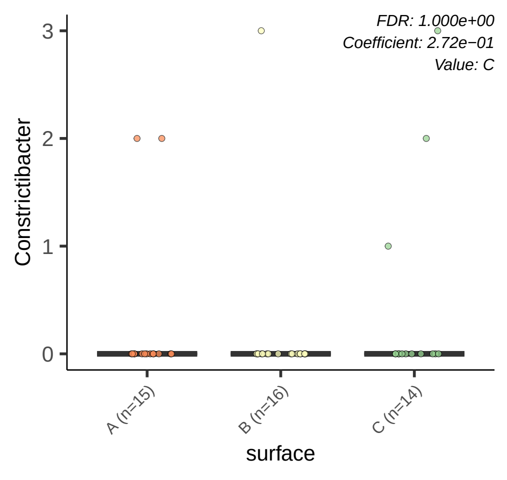

FDR: 1.000e+00
Coefficient: 2.72e−01
Value: C
Constrictibacter
3
2
1
0
A (n=15)
B (n=16)
C (n=14)
surface

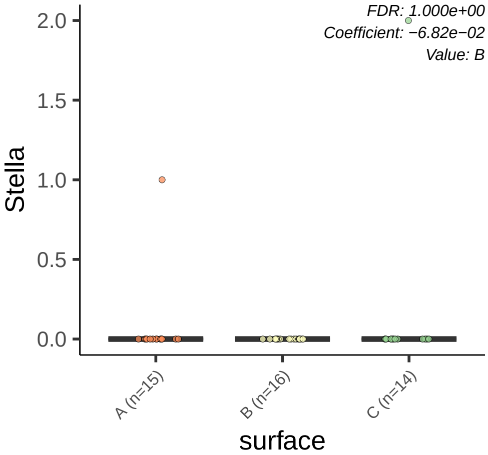
FDR: 1.000e+00
Coefficient: −6.82e−02
Value: B
Stella
2.0
1.5
1.0
0.5
0.0
A (n=15)
B (n=16)
C (n=14)
surface

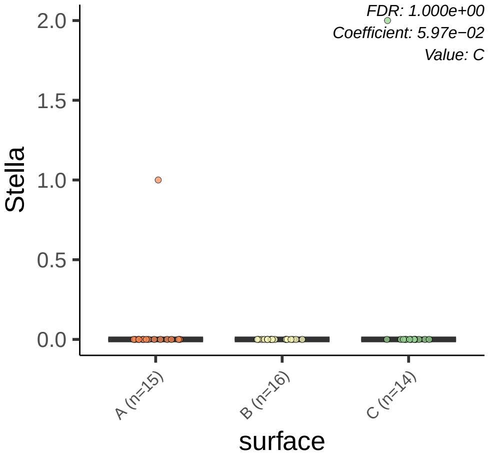
FDR: 1.000e+00
Coefficient: 5.97e−02
Value: C
Stella
2.0
1.5
1.0
0.5
0.0
A (n=15)
B (n=16)
C (n=14)
surface

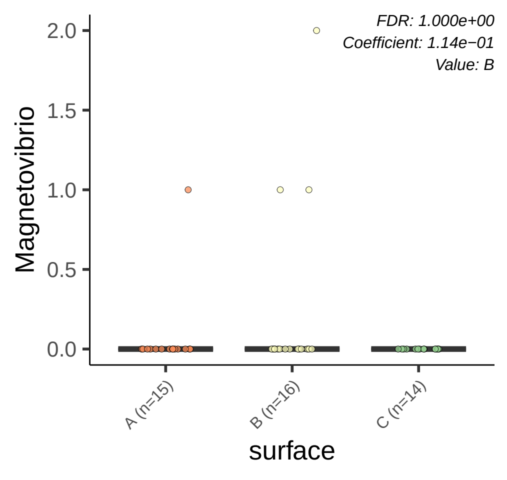
FDR: 1.000e+00
Coefficient: 1.14e−01
Value: B
Magnetovibrio
2.0
1.5
1.0
0.5
0.0
A (n=15)
B (n=16)
C (n=14)
surface

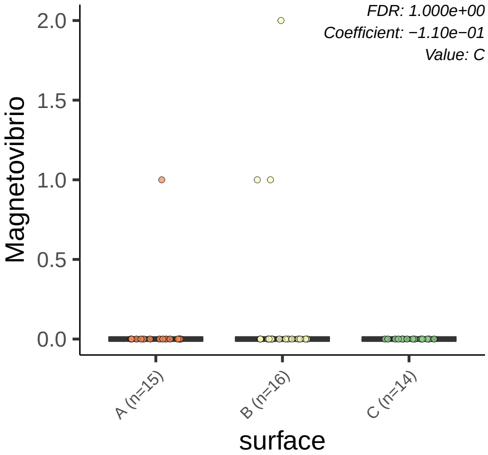

FDR: 1.000e+00
Coefficient: −1.10e−01
Value: C
Magnetovibrio
2.0
1.5
1.0
0.5
0.0
A (n=15)
B (n=16)
C (n=14)
surface

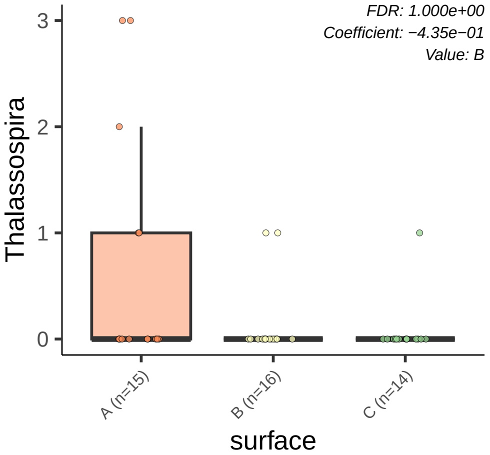
FDR: 1.000e+00
Coefficient: −4.35e−01
Value: B
Thalassospira
0
1
2
3
A (n=15)
B (n=16)
C (n=14)
surface

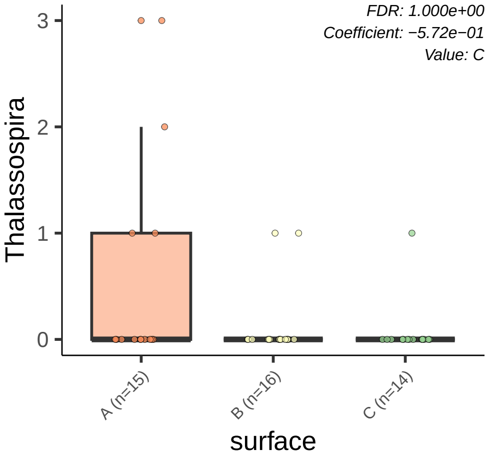
FDR: 1.000e+00
Coefficient: −5.72e−01
Value: C
Thalassospira
3
2
1
0
A (n=15)
B (n=16)
C (n=14)
surface

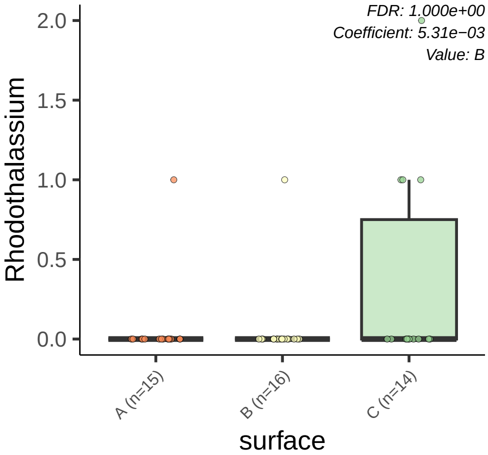

FDR: 1.000e+00
Coefficient: 5.31e−03
Value: B
Rhodothalassium
2.0
1.5
1.0
0.5
0.0
A (n=15)
B (n=16)
C (n=14)
surface

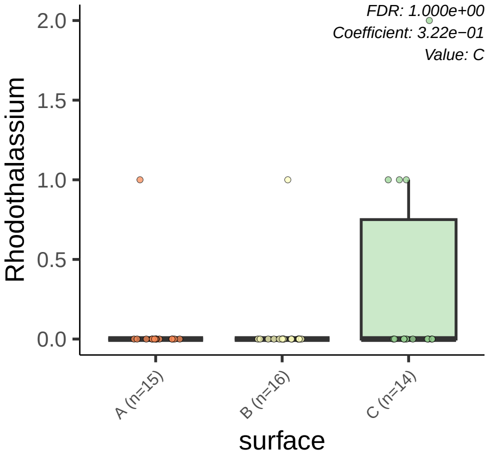
FDR: 1.000e+00
Coefficient: 3.22e−01
Value: C
Rhodothalassium
2.0
1.5
1.0
0.5
0.0
A (n=15)
B (n=16)
C (n=14)
surface

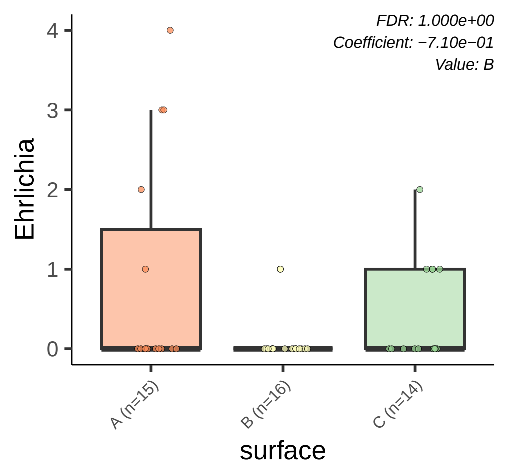

FDR: 1.000e+00
Coefficient: −7.10e−01
Value: B
Ehrlichia
0
1
2
3
4
A (n=15)
B (n=16)
C (n=14)
surface

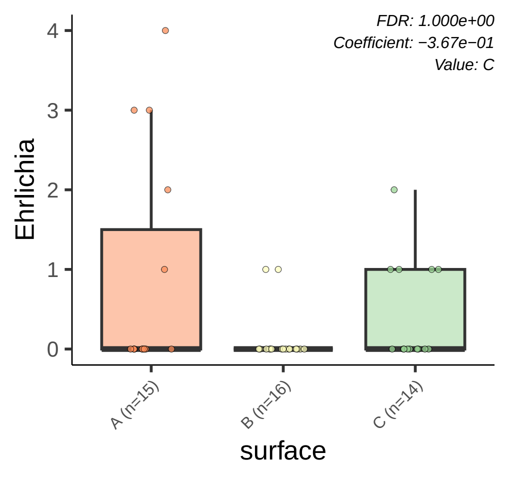
FDR: 1.000e+00
Coefficient: −3.67e−01
Value: C
Ehrlichia
0
1
2
3
4
A (n=15)
B (n=16)
C (n=14)
surface

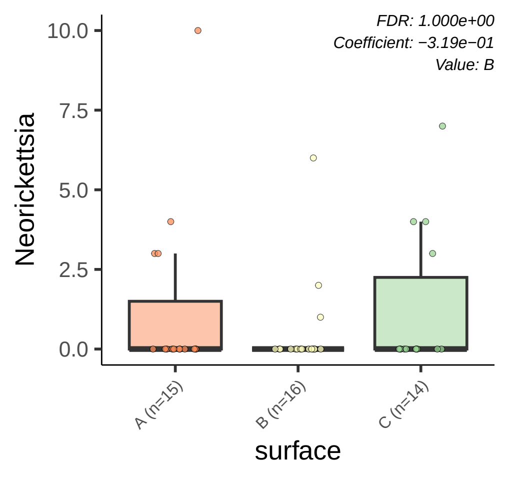
FDR: 1.000e+00
Coefficient: −3.19e−01
Value: B
Neorickettsia
10.0
7.5
5.0
2.5
0.0
A (n=15)
B (n=16)
C (n=14)
surface

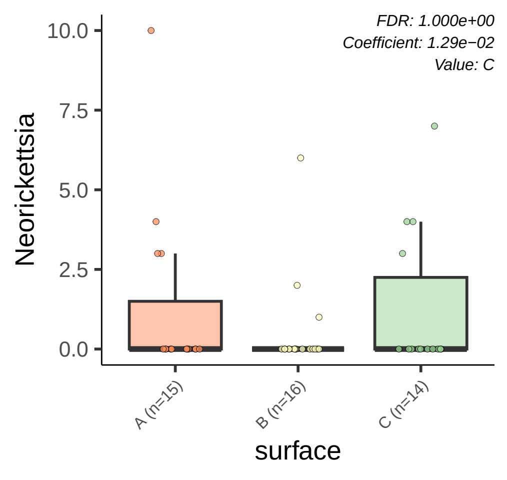

FDR: 1.000e+00
Coefficient: 1.29e−02
Value: C
10.0
7.5
5.0
2.5
0.0
Neorickettsia
A (n=15)
B (n=16)
C (n=14)
surface

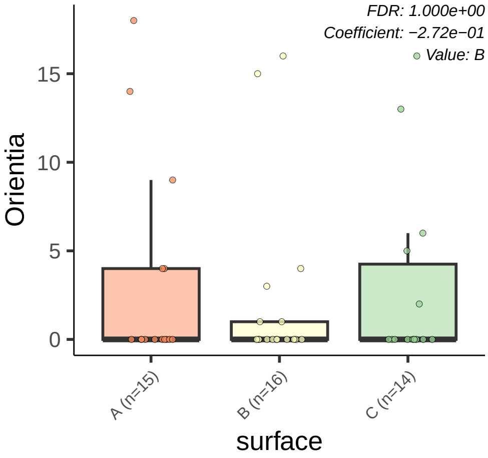
FDR: 1.000e+00
Coefficient: −2.72e−01
Value: B
Orientia
15
10
5
0
A (n=15)
B (n=16)
C (n=14)
surface

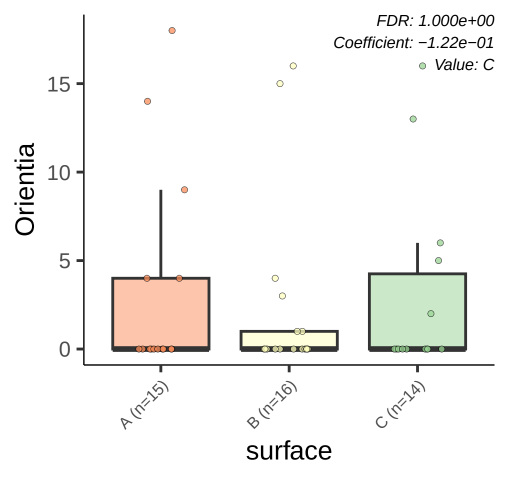
FDR: 1.000e+00
Coefficient: −1.22e−01
Value: C
Orientia
15
10
5
0
A (n=15)
B (n=16)
C (n=14)
surface

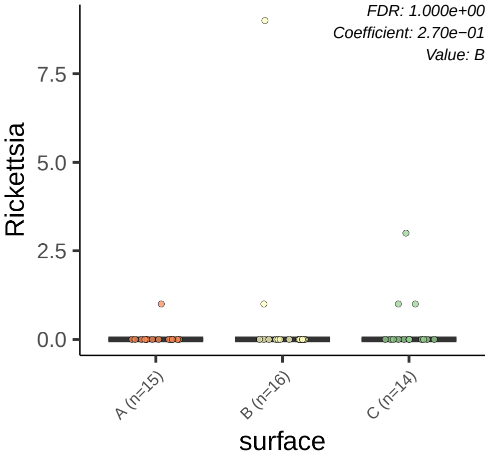

FDR: 1.000e+00
Coefficient: 2.70e−01
Value: B
Rickettsia
7.5
5.0
2.5
0.0
A (n=15)
B (n=16)
C (n=14)
surface

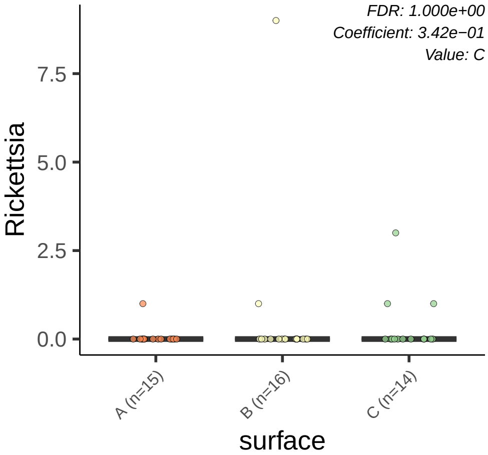
FDR: 1.000e+00
Coefficient: 3.42e−01
Value: C
Rickettsia
7.5
5.0
2.5
0.0
A (n=15)
B (n=16)
C (n=14)
surface

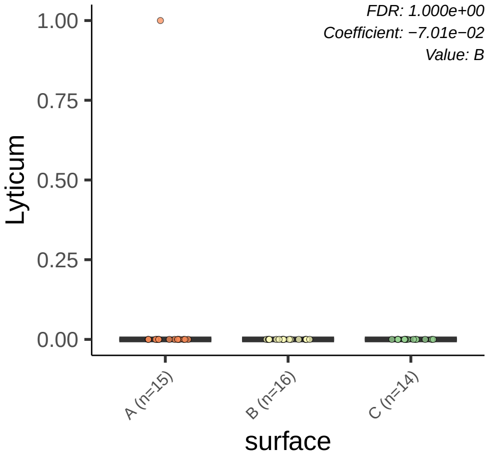
FDR: 1.000e+00
Coefficient: −7.01e−02
Value: B
Lyticum
1.00
0.75
0.50
0.25
0.00
A (n=15)
B (n=16)
C (n=14)
surface

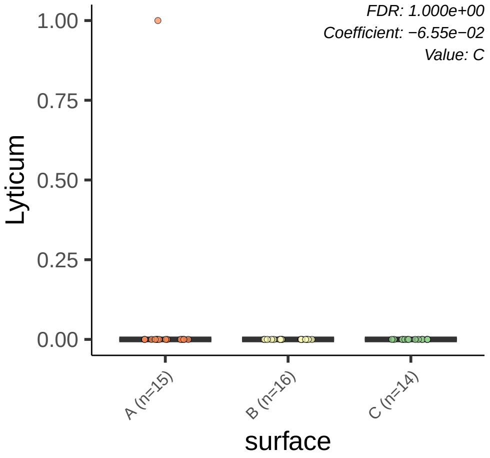
FDR: 1.000e+00
Coefficient: −6.55e−02
Value: C
1.00
0.75
0.50
0.25
0.00
Lyticum
A (n=15)
B (n=16)
C (n=14)
surface

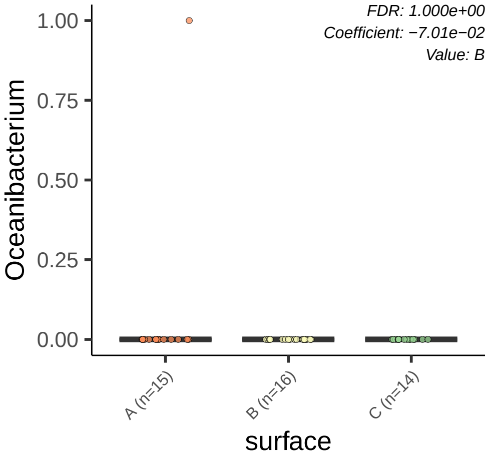

FDR: 1.000e+00
Coefficient: −7.01e−02
Value: B
Oceanibacterium
1.00
0.75
0.50
0.25
0.00
A (n=15)
B (n=16)
C (n=14)
surface

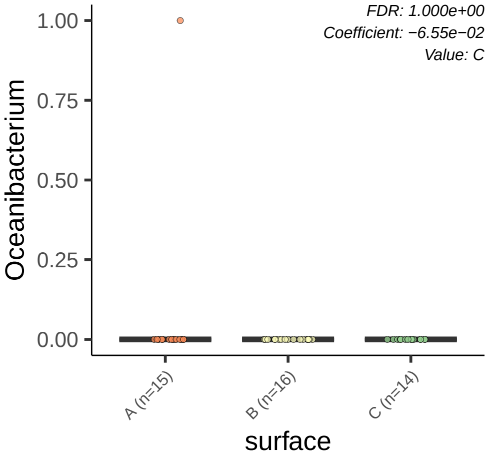
FDR: 1.000e+00
Coefficient: −6.55e−02
Value: C
Oceanibacterium
1.00
0.75
0.50
0.25
0.00
A (n=15)
B (n=16)
C (n=14)
surface

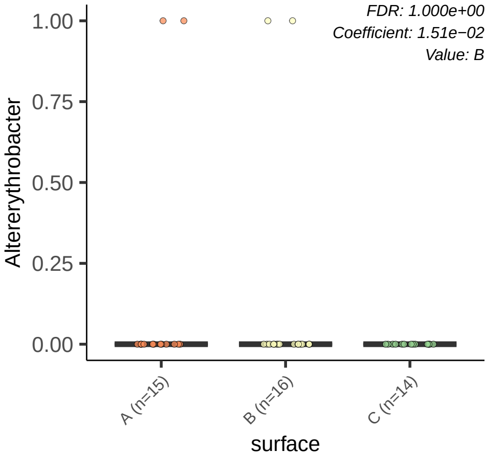

FDR: 1.000e+00
Coefficient: 1.51e−02
Value: B
Altererythrobacter
1.00
0.75
0.50
0.25
0.00
A (n=15)
B (n=16)
C (n=14)
surface

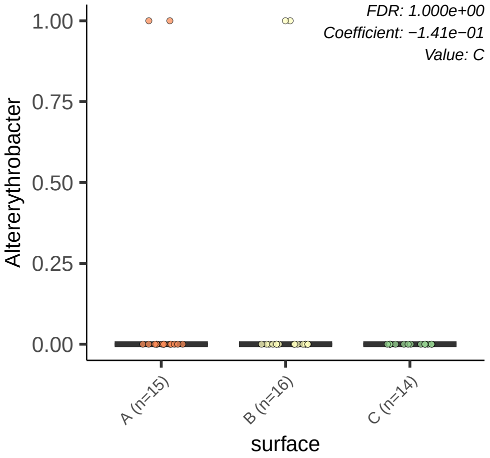
FDR: 1.000e+00
Coefficient: −1.41e−01
Value: C
Altererythrobacter
1.00
0.75
0.50
0.25
0.00
A (n=15)
B (n=16)
C (n=14)
surface

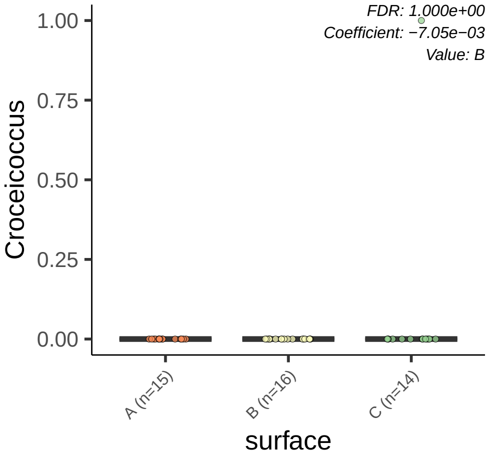

FDR: 1.000e+00
Coefficient: −7.05e−03
Value: B
Croceicoccus
1.00
0.75
0.50
0.25
0.00
A (n=15)
B (n=16)
C (n=14)
surface

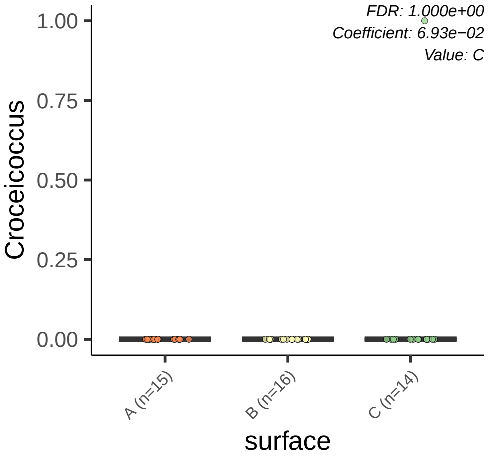
FDR: 1.000e+00
Coefficient: 6.93e−02
Value: C
Croceicoccus
1.00
0.75
0.50
0.25
0.00
A (n=15)
B (n=16)
C (n=14)
surface

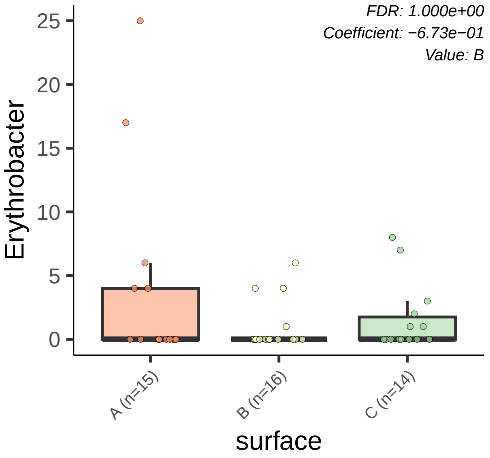

FDR: 1.000e+00
Coefficient: −6.73e−01
Value: B
Erythrobacter
25
20
15
10
5
0
A (n=15)
B (n=16)
C (n=14)
surface

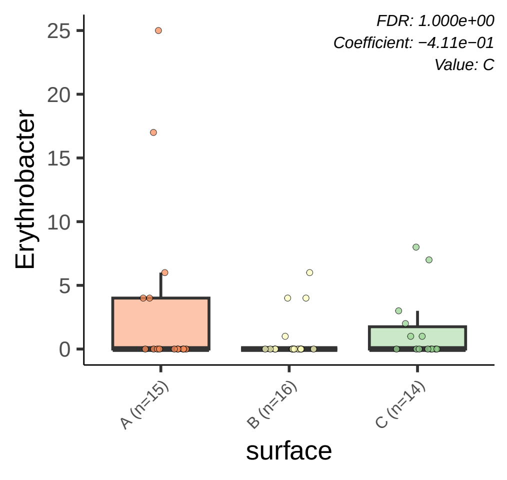
FDR: 1.000e+00
Coefficient: −4.11e−01
Value: C
Erythrobacter
0
5
10
15
20
25
A (n=15)
B (n=16)
C (n=14)
surface

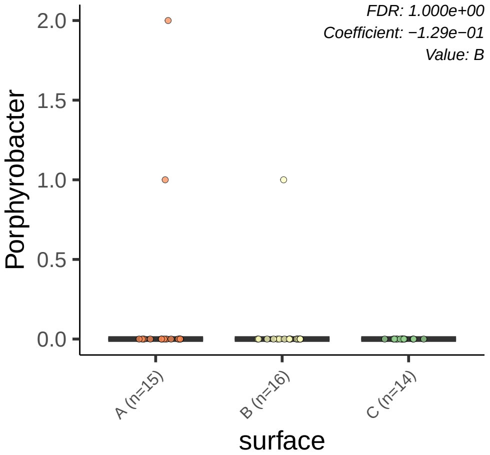
FDR: 1.000e+00
Coefficient: −1.29e−01
Value: B
Porphyrobacter
2.0
1.5
1.0
0.5
0.0
A (n=15)
B (n=16)
C (n=14)
surface

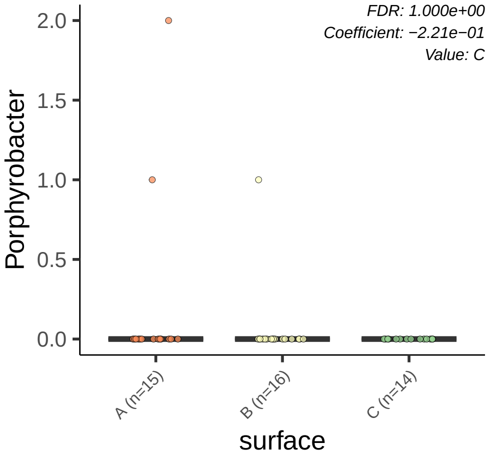
FDR: 1.000e+00
Coefficient: −2.21e−01
Value: C
Porphyrobacter
2.0
1.5
1.0
0.5
0.0
A (n=15)
B (n=16)
C (n=14)
surface

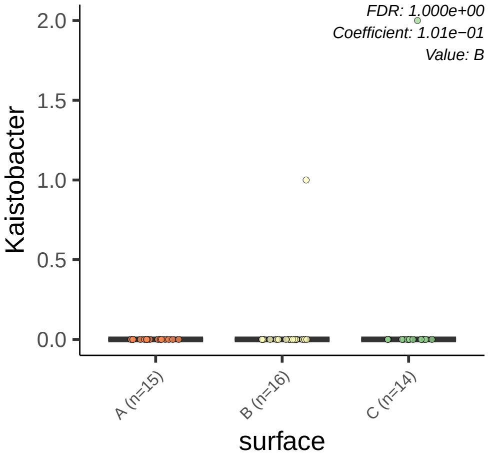

FDR: 1.000e+00
Coefficient: 1.01e−01
Value: B
Kaistobacter
2.0
1.5
1.0
0.5
0.0
A (n=15)
B (n=16)
C (n=14)
surface

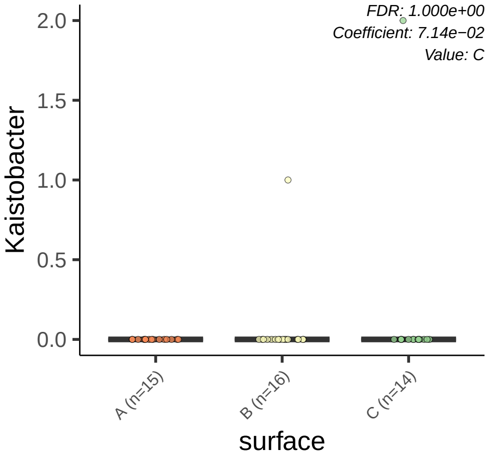
FDR: 1.000e+00
Coefficient: 7.14e−02
Value: C
Kaistobacter
2.0
1.5
1.0
0.5
0.0
A (n=15)
B (n=16)
C (n=14)
surface

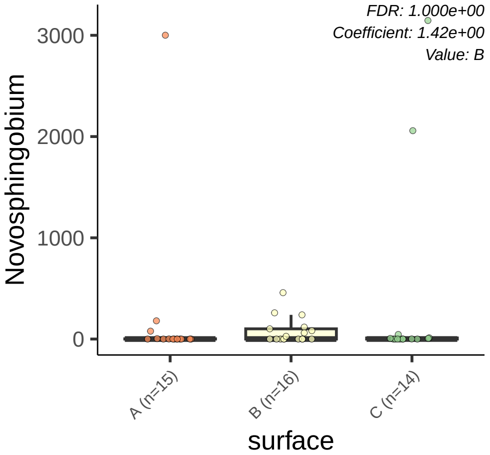

FDR: 1.000e+00
Coefficient: 1.42e+00
Value: B
Novosphingobium
3000
2000
1000
0
A (n=15)
B (n=16)
C (n=14)
surface

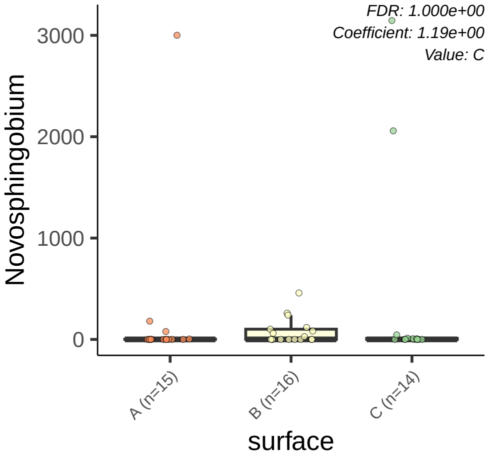
FDR: 1.000e+00
Coefficient: 1.19e+00
Value: C
Novosphingobium
3000
2000
1000
0
A (n=15)
B (n=16)
C (n=14)
surface

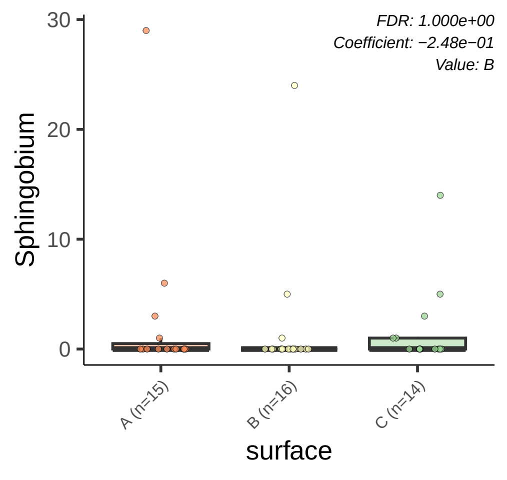

FDR: 1.000e+00
Coefficient: −2.48e−01
Value: B
Sphingobium
30
20
10
0
A (n=15)
B (n=16)
C (n=14)
surface

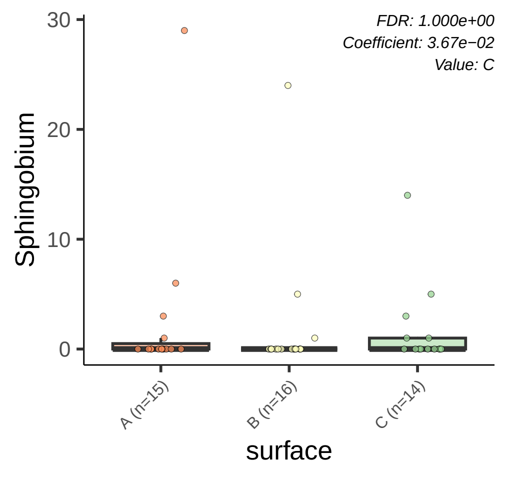

FDR: 1.000e+00
Coefficient: 3.67e−02
Value: C
Sphingobium
30
20
10
0
A (n=15)
B (n=16)
C (n=14)
surface

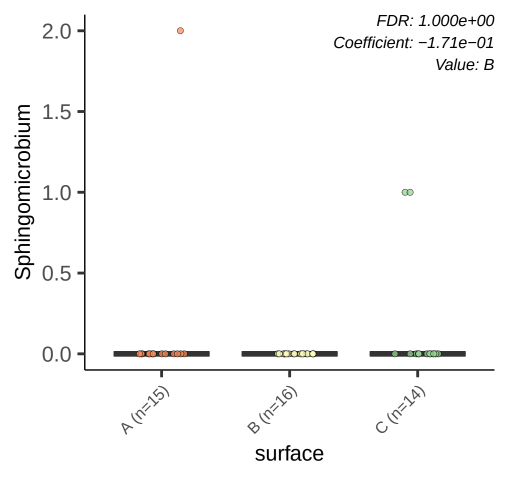

FDR: 1.000e+00
Coefficient: −1.71e−01
Value: B
Sphingomicrobium
2.0
1.5
1.0
0.5
0.0
A (n=15)
B (n=16)
C (n=14)
surface

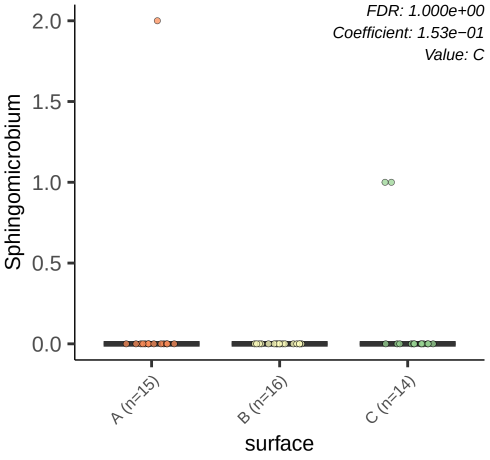
FDR: 1.000e+00
Coefficient: 1.53e−01
Value: C
Sphingomicrobium
2.0
1.5
1.0
0.5
0.0
A (n=15)
B (n=16)
C (n=14)
surface

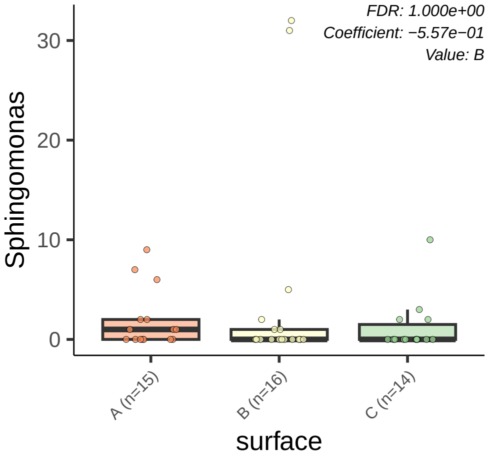

FDR: 1.000e+00
Coefficient: −5.57e−01
Value: B
Sphingomonas
30
20
10
0
A (n=15)
B (n=16)
C (n=14)
surface

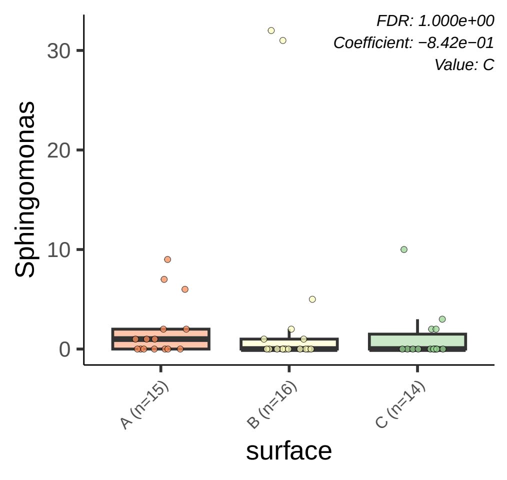
FDR: 1.000e+00
Coefficient: −8.42e−01
Value: C
Sphingomonas
30
20
10
0
A (n=15)
B (n=16)
C (n=14)
surface

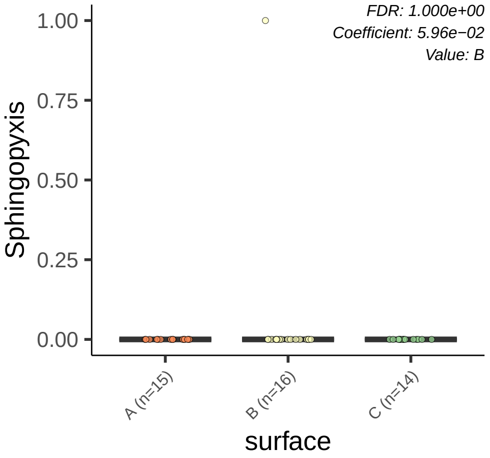
FDR: 1.000e+00
Coefficient: 5.96e−02
Value: B
Sphingopyxis
1.00
0.75
0.50
0.25
0.00
A (n=15)
B (n=16)
C (n=14)
surface

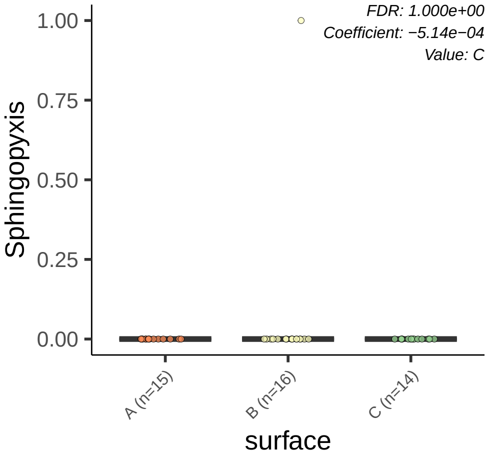

FDR: 1.000e+00
Coefficient: −5.14e−04
Value: C
Sphingopyxis
1.00
0.75
0.50
0.25
0.00
A (n=15)
B (n=16)
C (n=14)
surface

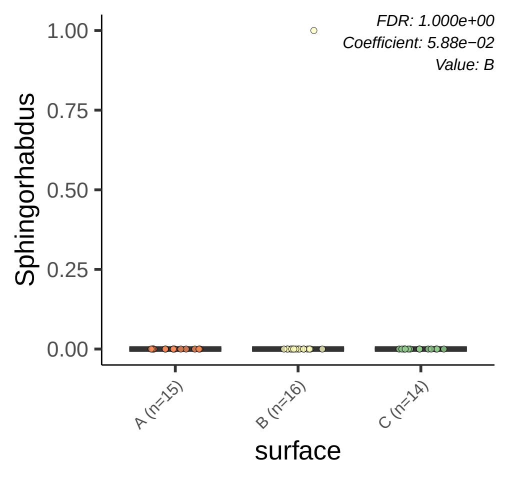
FDR: 1.000e+00
Coefficient: 5.88e−02
Value: B
Sphingorhabdus
1.00
0.75
0.50
0.25
0.00
A (n=15)
B (n=16)
C (n=14)
surface

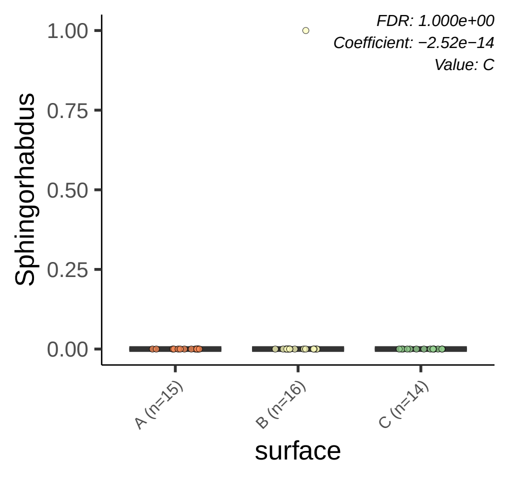
FDR: 1.000e+00
Coefficient: −2.52e−14
Value: C
Sphingorhabdus
1.00
0.75
0.50
0.25
0.00
A (n=15)
B (n=16)
C (n=14)
surface

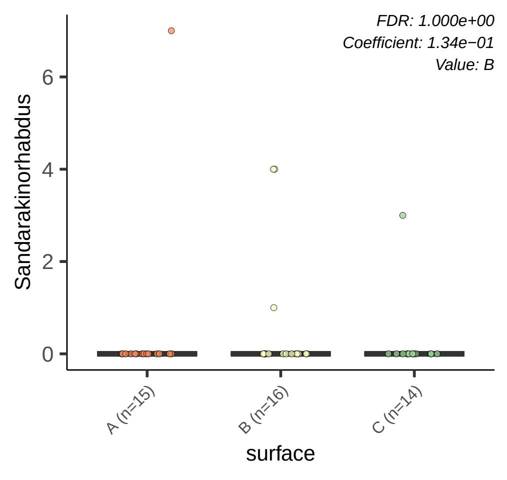
FDR: 1.000e+00
Coefficient: 1.34e−01
Value: B
Sandarakinorhabdus
0
2
4
6
A (n=15)
B (n=16)
C (n=14)
surface

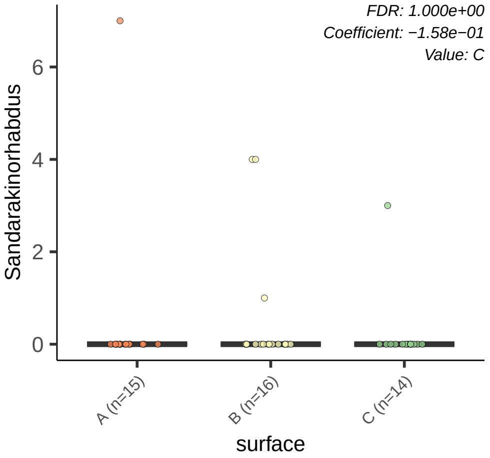

FDR: 1.000e+00
Coefficient: −1.58e−01
Value: C
Sandarakinorhabdus
0
2
4
6
A (n=15)
B (n=16)
C (n=14)
surface

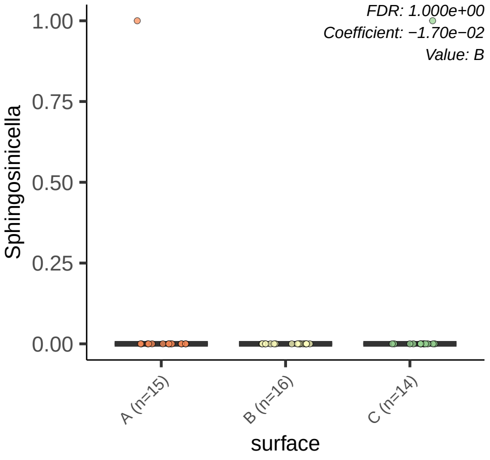
FDR: 1.000e+00
Coefficient: −1.70e−02
Value: B
Sphingosinicella
1.00
0.75
0.50
0.25
0.00
A (n=15)
B (n=16)
C (n=14)
surface

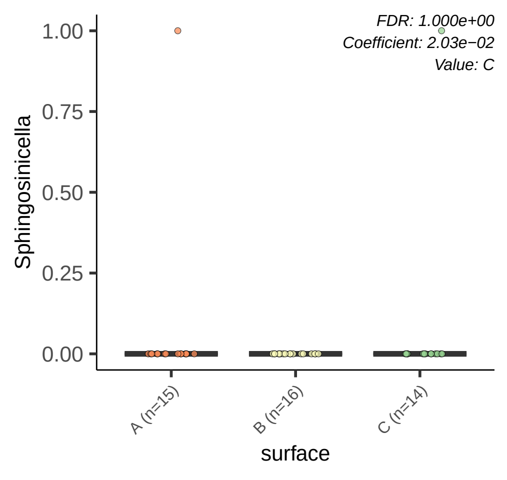
FDR: 1.000e+00
Coefficient: 2.03e−02
Value: C
Sphingosinicella
1.00
0.75
0.50
0.25
0.00
A (n=15)
B (n=16)
C (n=14)
surface

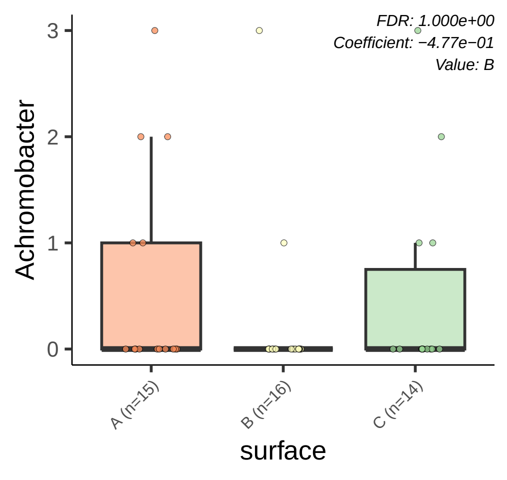
FDR: 1.000e+00
Coefficient: −4.77e−01
Value: B
Achromobacter
3
2
1
0
A (n=15)
B (n=16)
C (n=14)
surface

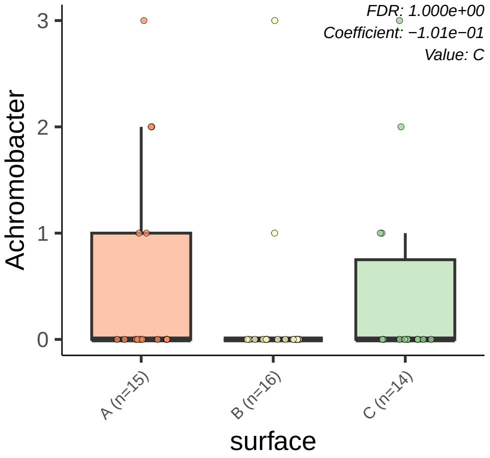
FDR: 1.000e+00
Coefficient: −1.01e−01
Value: C
Achromobacter
3
2
1
0
A (n=15)
B (n=16)
C (n=14)
surface

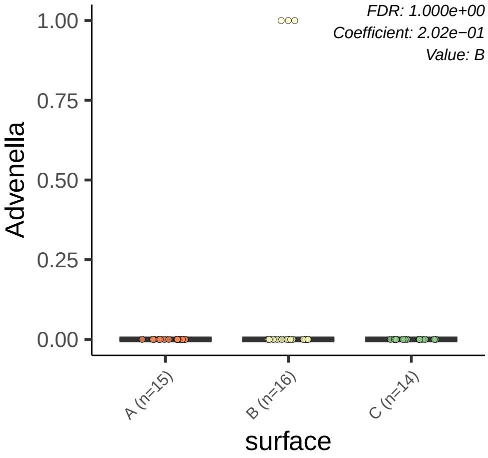

FDR: 1.000e+00
Coefficient: 2.02e−01
Value: B
Advenella
1.00
0.75
0.50
0.25
0.00
A (n=15)
B (n=16)
C (n=14)
surface

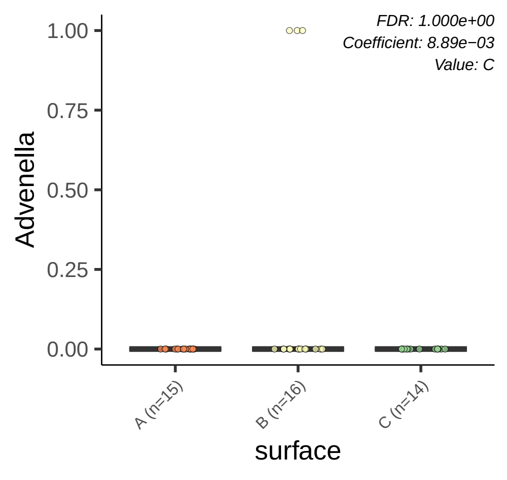
FDR: 1.000e+00
Coefficient: 8.89e−03
Value: C
Advenella
1.00
0.75
0.50
0.25
0.00
A (n=15)
B (n=16)
C (n=14)
surface

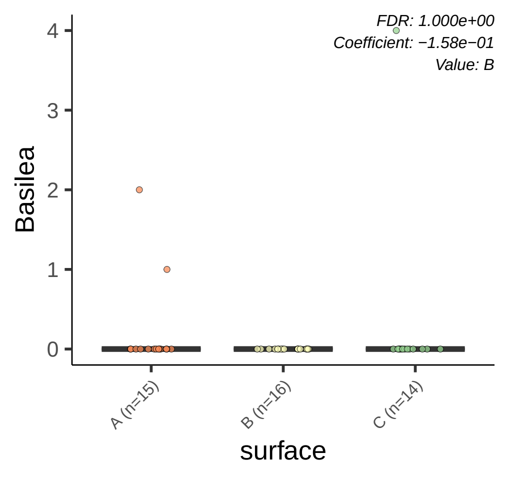

FDR: 1.000e+00
Coefficient: −1.58e−01
Value: B
Basilea
4
3
2
1
0
A (n=15)
B (n=16)
C (n=14)
surface

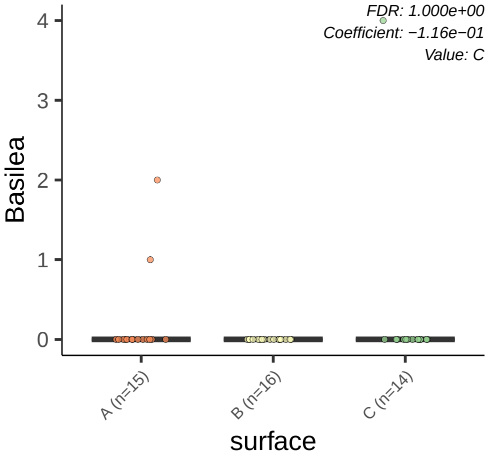
FDR: 1.000e+00
Coefficient: −1.16e−01
Value: C
Basilea
0
1
2
3
4
A (n=15)
B (n=16)
C (n=14)
surface

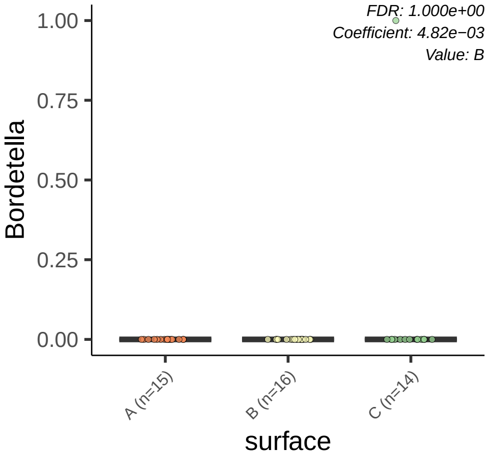
FDR: 1.000e+00
Coefficient: 4.82e−03
Value: B
Bordetella
1.00
0.75
0.50
0.25
0.00
A (n=15)
B (n=16)
C (n=14)
surface

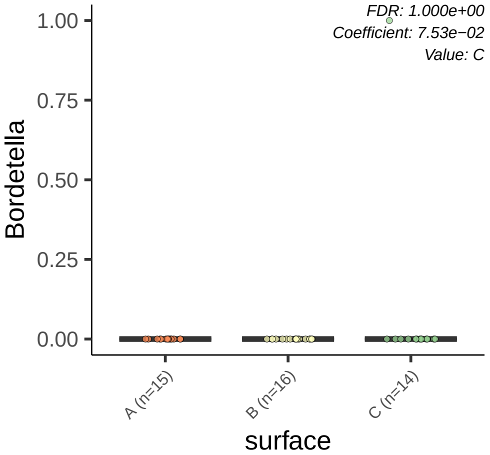
FDR: 1.000e+00
Coefficient: 7.53e−02
Value: C
Bordetella
1.00
0.75
0.50
0.25
0.00
A (n=15)
B (n=16)
C (n=14)
surface

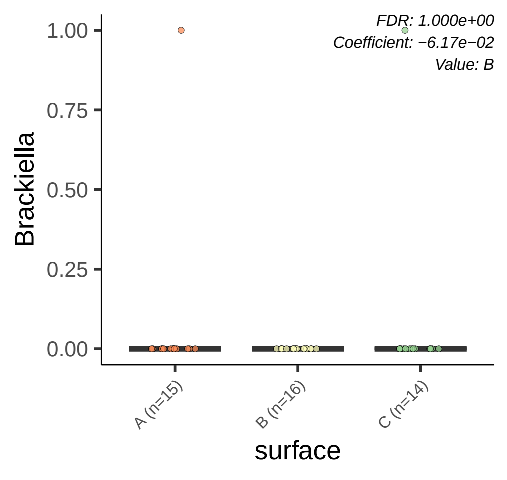
FDR: 1.000e+00
Coefficient: −6.17e−02
Value: B
Brackiella
1.00
0.75
0.50
0.25
0.00
A (n=15)
B (n=16)
C (n=14)
surface

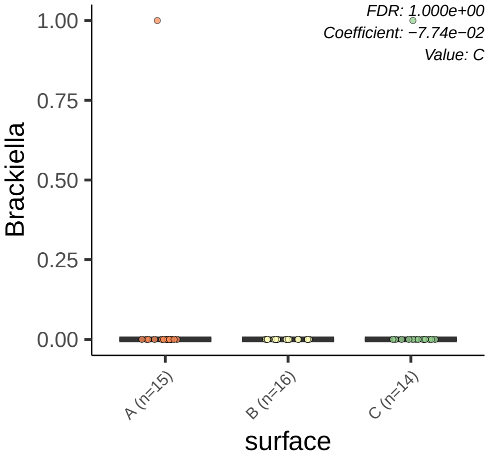
FDR: 1.000e+00
Coefficient: −7.74e−02
Value: C
Brackiella
1.00
0.75
0.50
0.25
0.00
A (n=15)
B (n=16)
C (n=14)
surface

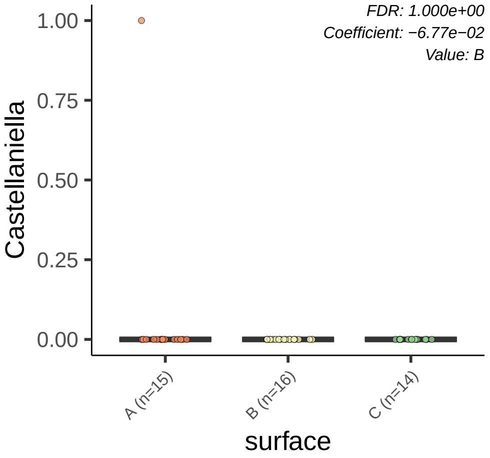
FDR: 1.000e+00
Coefficient: −6.77e−02
Value: B
Castellaniella
1.00
0.75
0.50
0.25
0.00
A (n=15)
B (n=16)
C (n=14)
surface

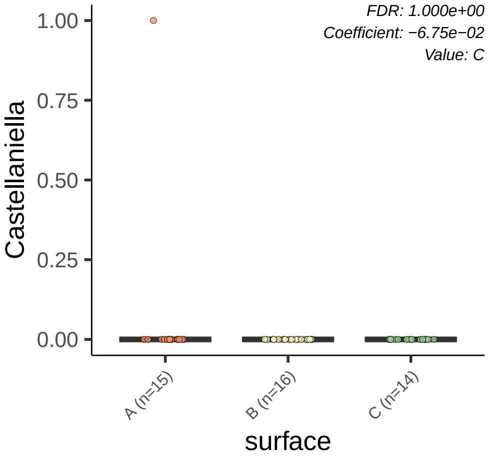

FDR: 1.000e+00
Coefficient: −6.75e−02
Value: C
Castellaniella
1.00
0.75
0.50
0.25
0.00
A (n=15)
B (n=16)
C (n=14)
surface

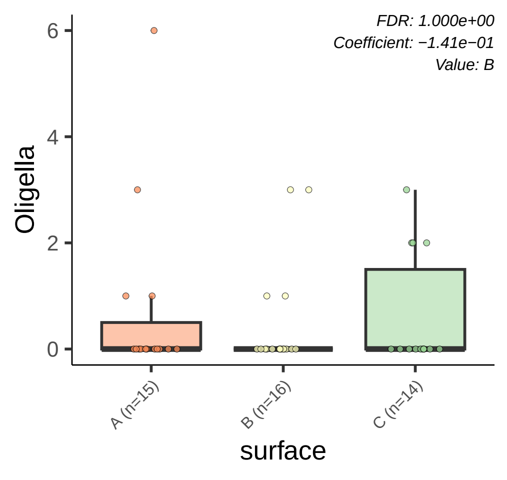

FDR: 1.000e+00
Coefficient: −1.41e−01
Value: B
Oligella
6
4
2
0
A (n=15)
B (n=16)
C (n=14)
surface

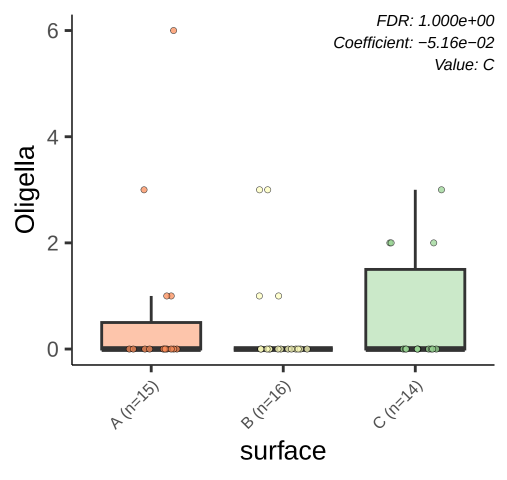
FDR: 1.000e+00
Coefficient: −5.16e−02
Value: C
Oligella
6
4
2
0
A (n=15)
B (n=16)
C (n=14)
surface

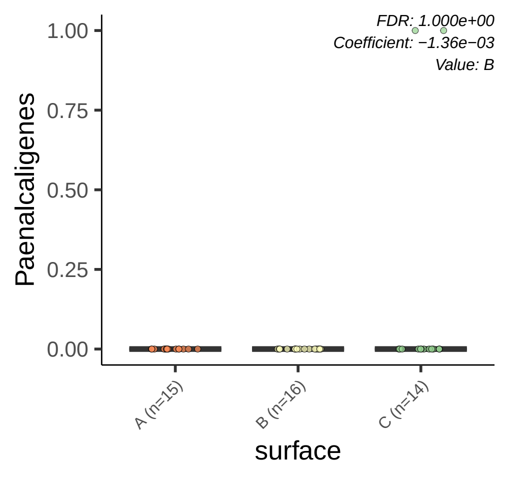
FDR: 1.000e+00
Coefficient: −1.36e−03
Value: B
Paenalcaligenes
1.00
0.75
0.50
0.25
0.00
A (n=15)
B (n=16)
C (n=14)
surface

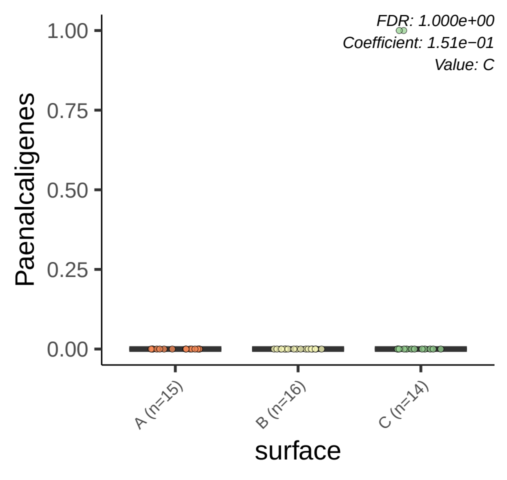
FDR: 1.000e+00
Coefficient: 1.51e−01
Value: C
1.00
0.75
0.50
0.25
0.00
Paenalcaligenes
A (n=15)
B (n=16)
C (n=14)
surface

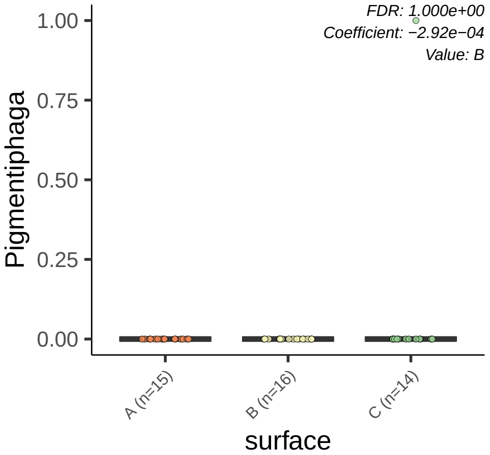
FDR: 1.000e+00
Coefficient: −2.92e−04
Value: B
Pigmentiphaga
1.00
0.75
0.50
0.25
0.00
A (n=15)
B (n=16)
C (n=14)
surface

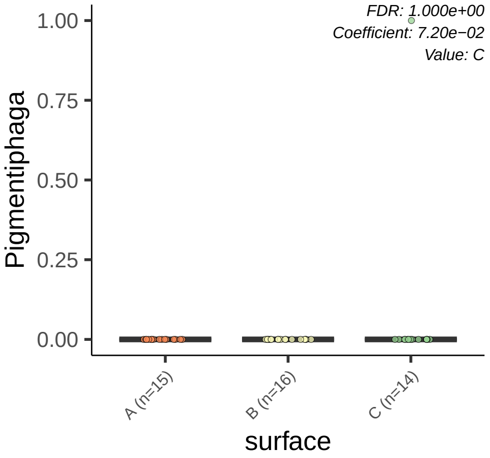

FDR: 1.000e+00
Coefficient: 7.20e−02
Value: C
Pigmentiphaga
1.00
0.75
0.50
0.25
0.00
A (n=15)
B (n=16)
C (n=14)
surface

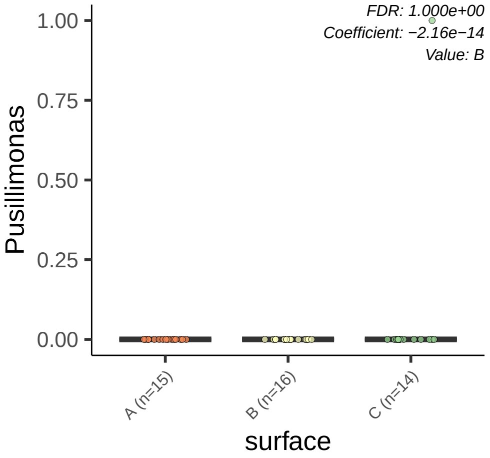
FDR: 1.000e+00
Coefficient: −2.16e−14
Value: B
Pusillimonas
1.00
0.75
0.50
0.25
0.00
A (n=15)
B (n=16)
C (n=14)
surface

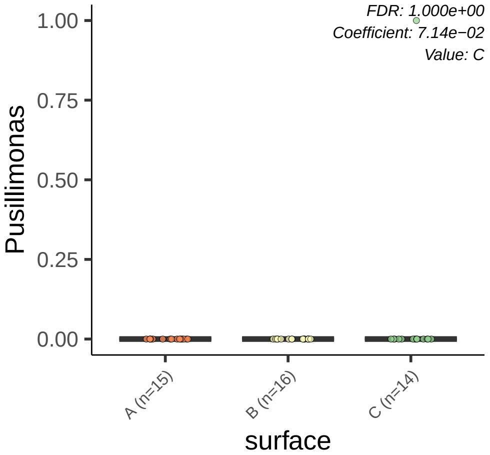
FDR: 1.000e+00
Coefficient: 7.14e−02
Value: C
Pusillimonas
1.00
0.75
0.50
0.25
0.00
A (n=15)
B (n=16)
C (n=14)
surface

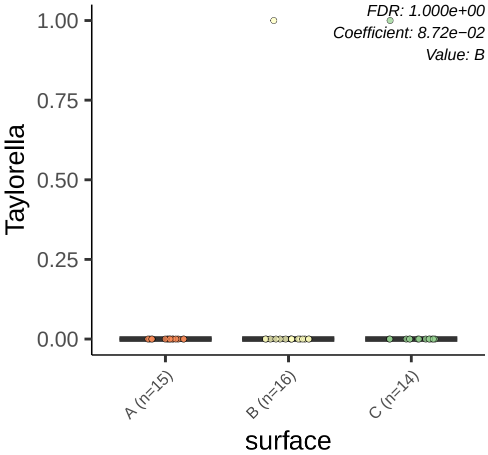
FDR: 1.000e+00
Coefficient: 8.72e−02
Value: B
Taylorella
1.00
0.75
0.50
0.25
0.00
A (n=15)
B (n=16)
C (n=14)
surface

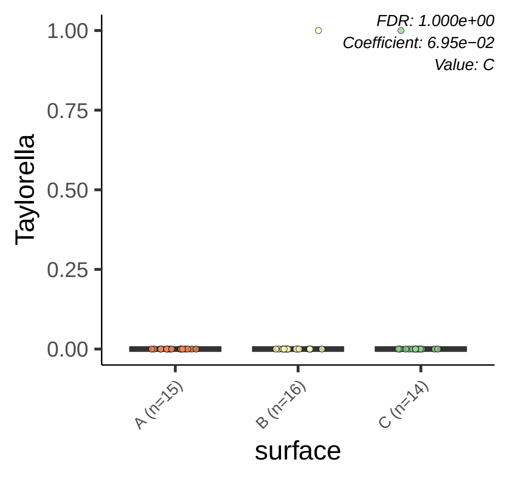
FDR: 1.000e+00
Coefficient: 6.95e−02
Value: C
Taylorella
1.00
0.75
0.50
0.25
0.00
A (n=15)
B (n=16)
C (n=14)
surface

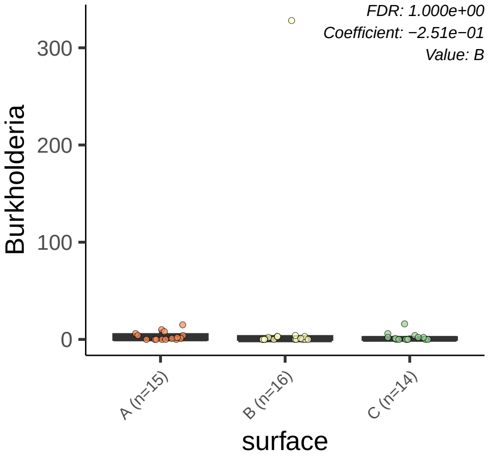
FDR: 1.000e+00
Coefficient: −2.51e−01
Value: B
300
200
100
0
Burkholderia
A (n=15)
B (n=16)
C (n=14)
surface

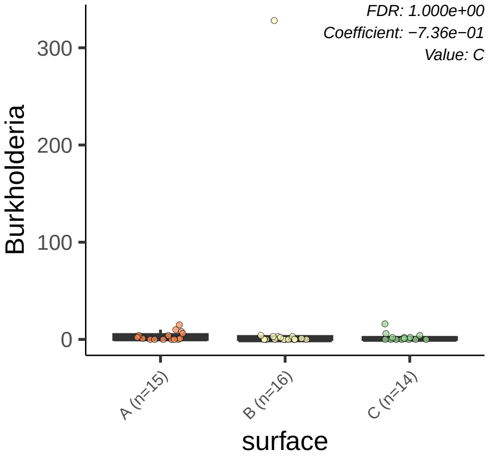
FDR: 1.000e+00
Coefficient: −7.36e−01
Value: C
Burkholderia
300
200
100
0
A (n=15)
B (n=16)
C (n=14)
surface

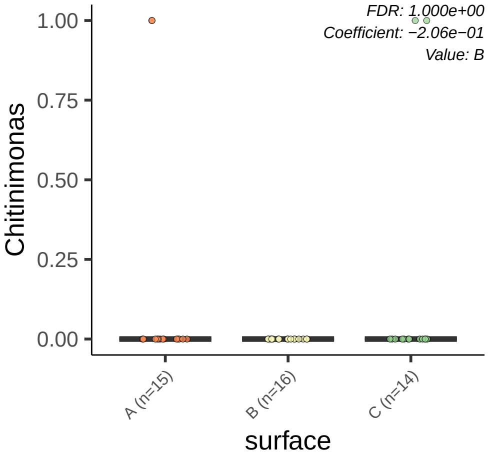
FDR: 1.000e+00
Coefficient: −2.06e−01
Value: B
Chitinimonas
1.00
0.75
0.50
0.25
0.00
A (n=15)
B (n=16)
C (n=14)
surface

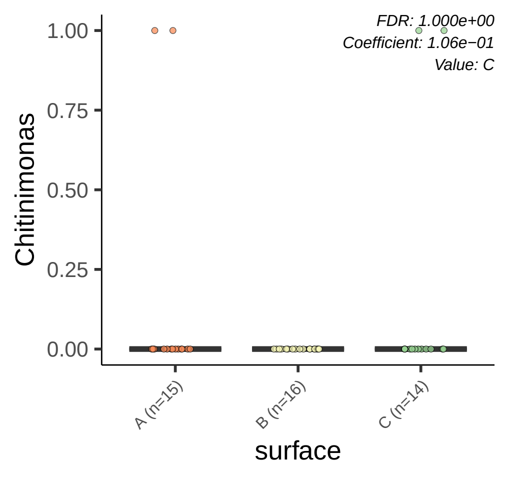
FDR: 1.000e+00
Coefficient: 1.06e−01
Value: C
Chitinimonas
1.00
0.75
0.50
0.25
0.00
A (n=15)
B (n=16)
C (n=14)
surface

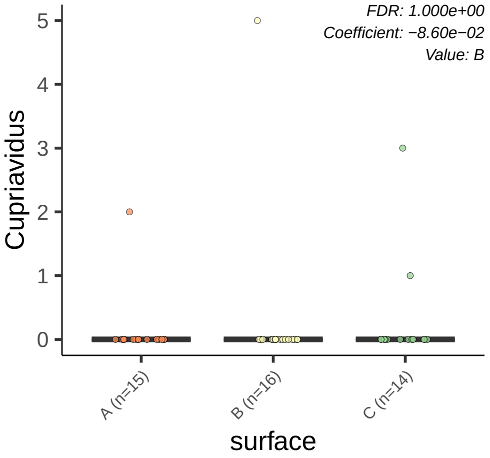
FDR: 1.000e+00
Coefficient: −8.60e−02
Value: B
Cupriavidus
5
4
3
2
1
0
A (n=15)
B (n=16)
C (n=14)
surface

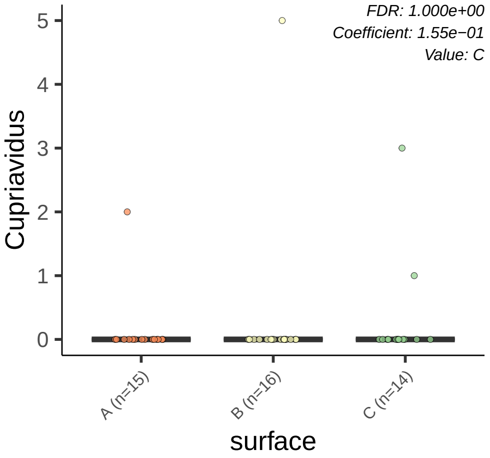

FDR: 1.000e+00
Coefficient: 1.55e−01
Value: C
Cupriavidus
5
4
3
2
1
0
A (n=15)
B (n=16)
C (n=14)
surface

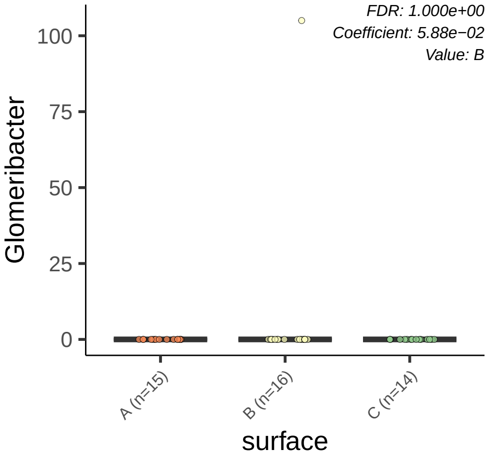

FDR: 1.000e+00
Coefficient: 5.88e−02
Value: B
Glomeribacter
100
75
50
25
0
A (n=15)
B (n=16)
C (n=14)
surface

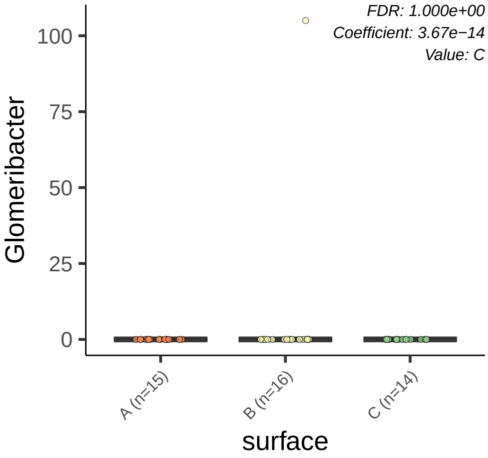
FDR: 1.000e+00
Coefficient: 3.67e−14
Value: C
Glomeribacter
100
75
50
25
0
A (n=15)
B (n=16)
C (n=14)
surface

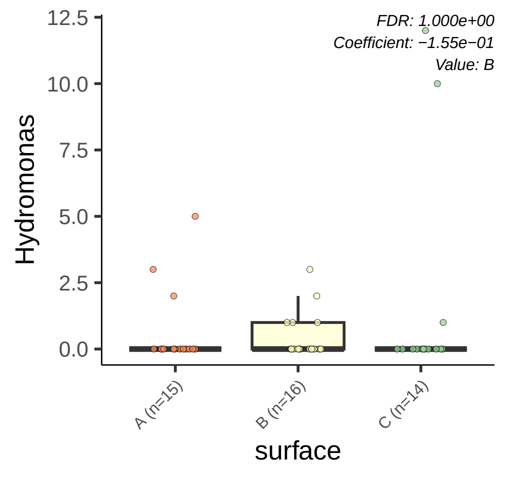

FDR: 1.000e+00
Coefficient: −1.55e−01
Value: B
Hydromonas
12.5
10.0
7.5
5.0
2.5
0.0
A (n=15)
B (n=16)
C (n=14)
surface

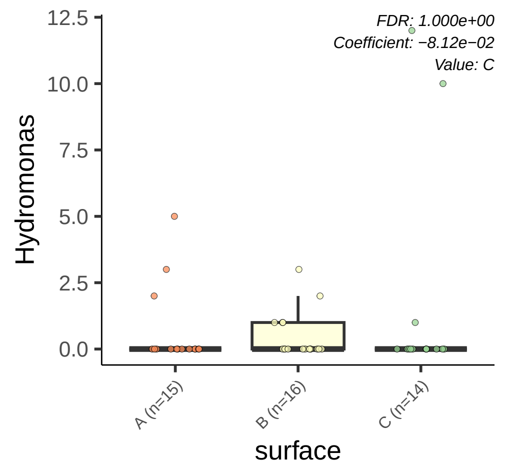
FDR: 1.000e+00
Coefficient: −8.12e−02
Value: C
Hydromonas
12.5
10.0
7.5
5.0
2.5
0.0
A (n=15)
B (n=16)
C (n=14)
surface

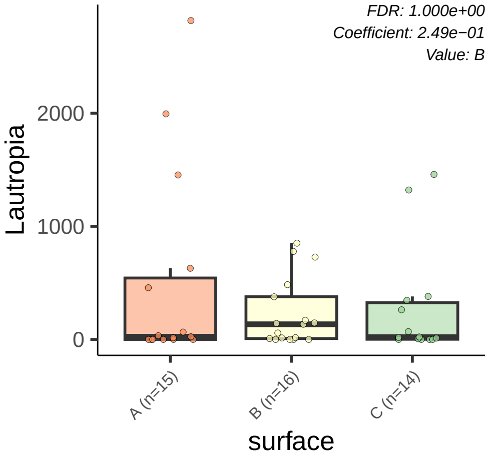
FDR: 1.000e+00
Coefficient: 2.49e−01
Value: B
Lautropia
2000
1000
0
A (n=15)
B (n=16)
C (n=14)
surface

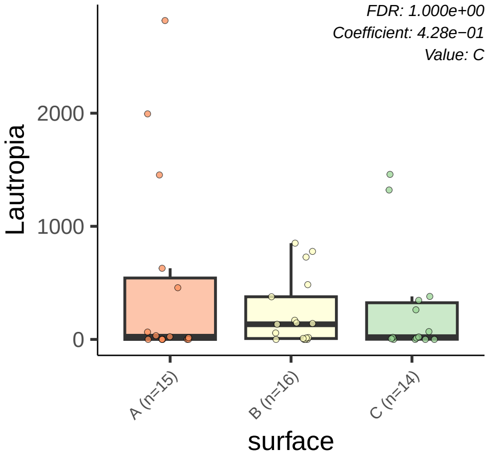
FDR: 1.000e+00
Coefficient: 4.28e−01
Value: C
Lautropia
2000
1000
0
A (n=15)
B (n=16)
C (n=14)
surface

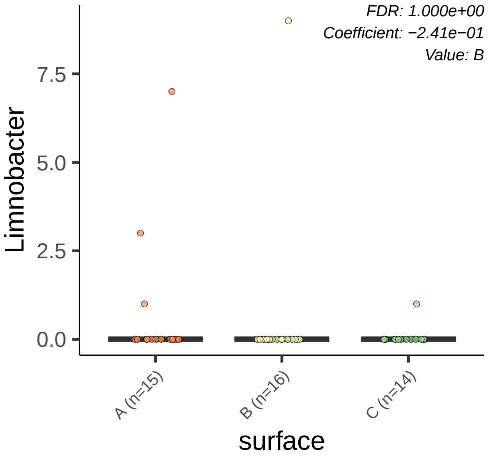
FDR: 1.000e+00
Coefficient: −2.41e−01
Value: B
Limnobacter
7.5
5.0
2.5
0.0
A (n=15)
B (n=16)
C (n=14)
surface

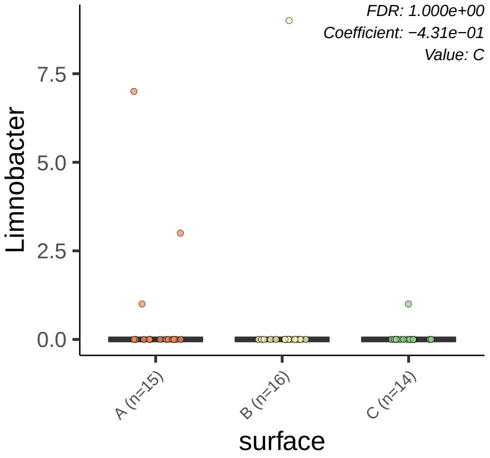
FDR: 1.000e+00
Coefficient: −4.31e−01
Value: C
Limnobacter
7.5
5.0
2.5
0.0
A (n=15)
B (n=16)
C (n=14)
surface

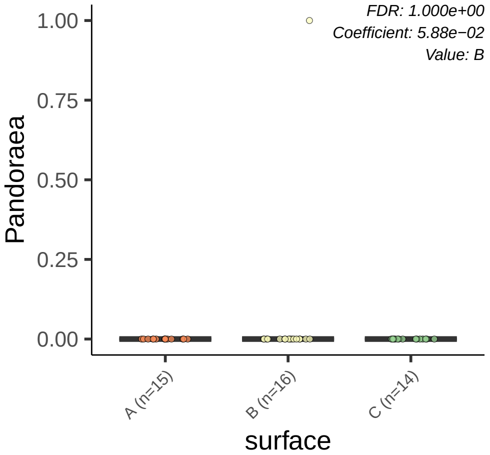
FDR: 1.000e+00
Coefficient: 5.88e−02
Value: B
Pandoraea
1.00
0.75
0.50
0.25
0.00
A (n=15)
B (n=16)
C (n=14)
surface

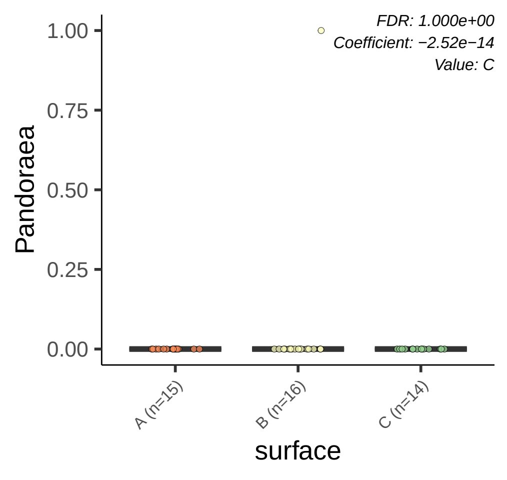
FDR: 1.000e+00
Coefficient: −2.52e−14
Value: C
Pandoraea
1.00
0.75
0.50
0.25
0.00
A (n=15)
B (n=16)
C (n=14)
surface

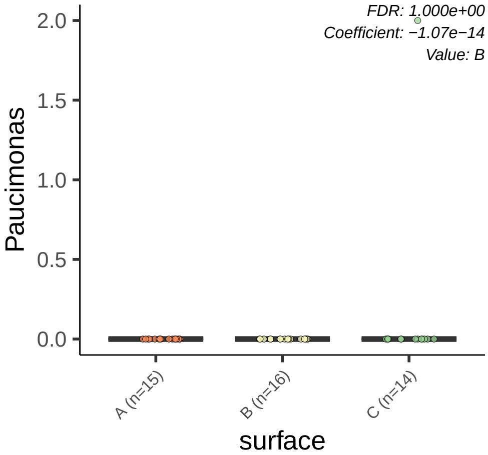

FDR: 1.000e+00
Coefficient: −1.07e−14
Value: B
Paucimonas
2.0
1.5
1.0
0.5
0.0
A (n=15)
B (n=16)
C (n=14)
surface

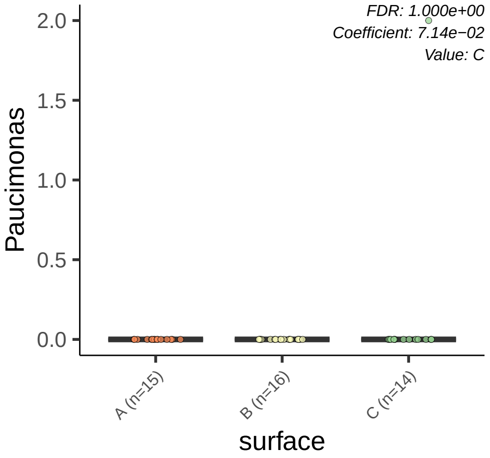

FDR: 1.000e+00
Coefficient: 7.14e−02
Value: C
Paucimonas
2.0
1.5
1.0
0.5
0.0
A (n=15)
B (n=16)
C (n=14)
surface

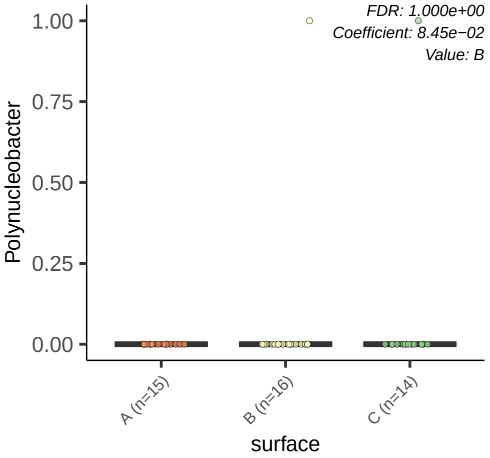
FDR: 1.000e+00
Coefficient: 8.45e−02
Value: B
Polynucleobacter
1.00
0.75
0.50
0.25
0.00
A (n=15)
B (n=16)
C (n=14)
surface

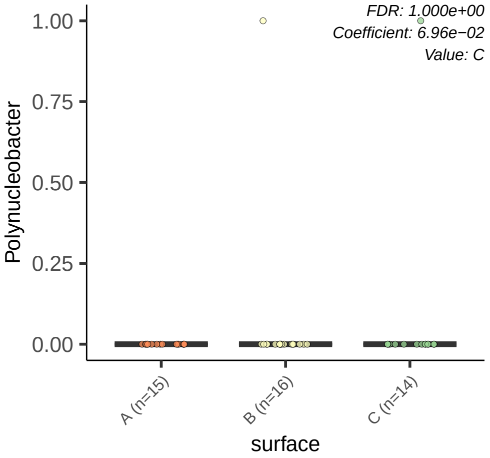

FDR: 1.000e+00
Coefficient: 6.96e−02
Value: C
Polynucleobacter
1.00
0.75
0.50
0.25
0.00
A (n=15)
B (n=16)
C (n=14)
surface

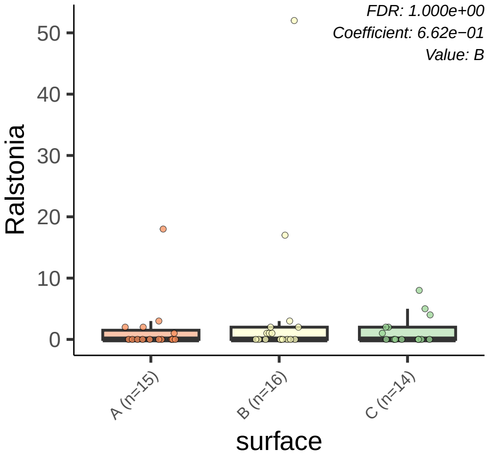
FDR: 1.000e+00
Coefficient: 6.62e−01
Value: B
Ralstonia
0
10
20
30
40
50
A (n=15)
B (n=16)
C (n=14)
surface

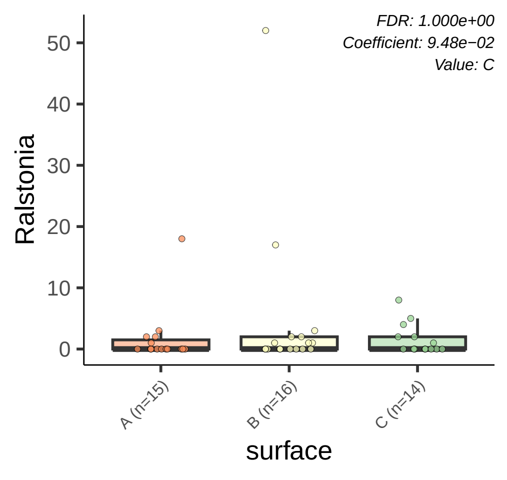

FDR: 1.000e+00
Coefficient: 9.48e−02
Value: C
Ralstonia
0
10
20
30
40
50
A (n=15)
B (n=16)
C (n=14)
surface

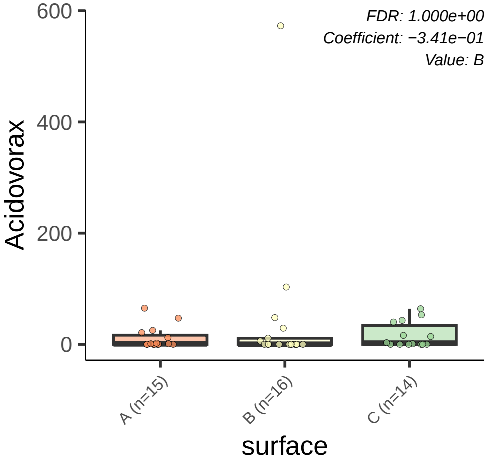

FDR: 1.000e+00
Coefficient: −3.41e−01
Value: B
Acidovorax
600
400
200
0
A (n=15)
B (n=16)
C (n=14)
surface

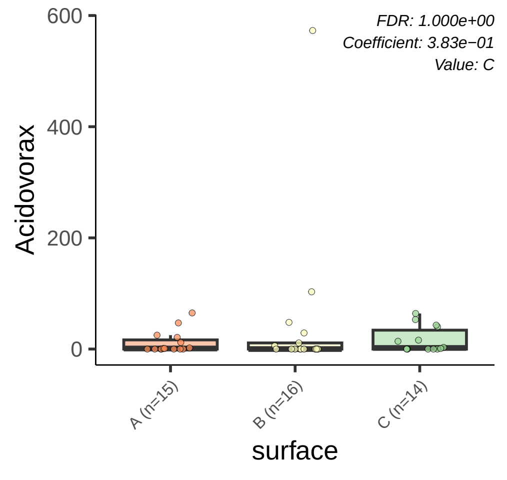
FDR: 1.000e+00
Coefficient: 3.83e−01
Value: C
Acidovorax
600
400
200
0
A (n=15)
B (n=16)
C (n=14)
surface

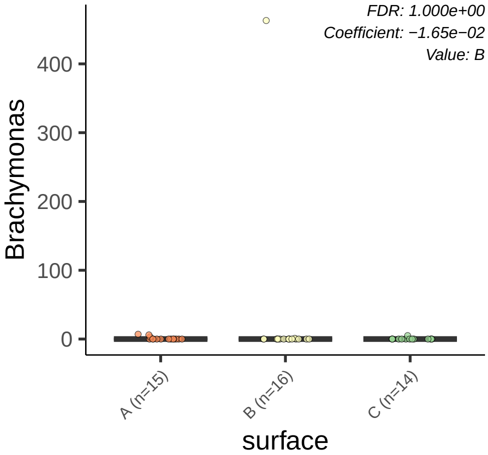
FDR: 1.000e+00
Coefficient: −1.65e−02
Value: B
Brachymonas
400
300
200
100
0
A (n=15)
B (n=16)
C (n=14)
surface

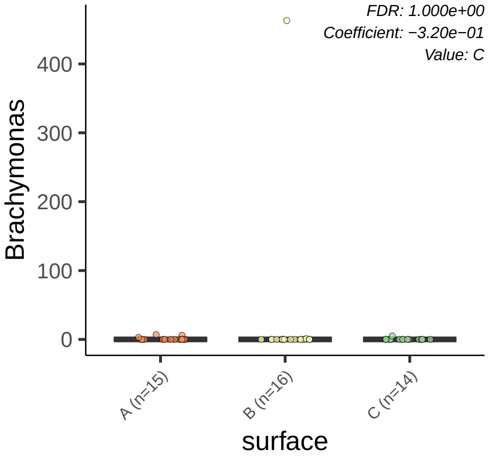
FDR: 1.000e+00
Coefficient: −3.20e−01
Value: C
Brachymonas
400
300
200
100
0
A (n=15)
B (n=16)
C (n=14)
surface

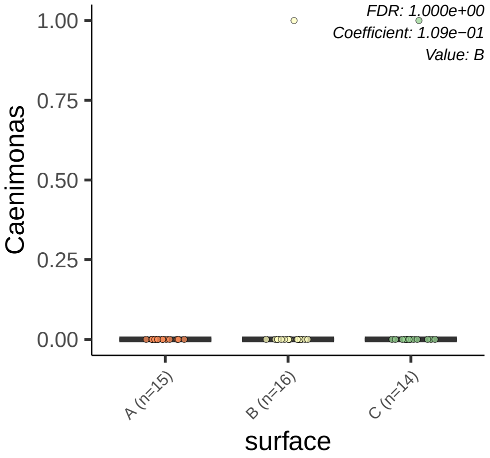
FDR: 1.000e+00
Coefficient: 1.09e−01
Value: B
Caenimonas
1.00
0.75
0.50
0.25
0.00
A (n=15)
B (n=16)
C (n=14)
surface

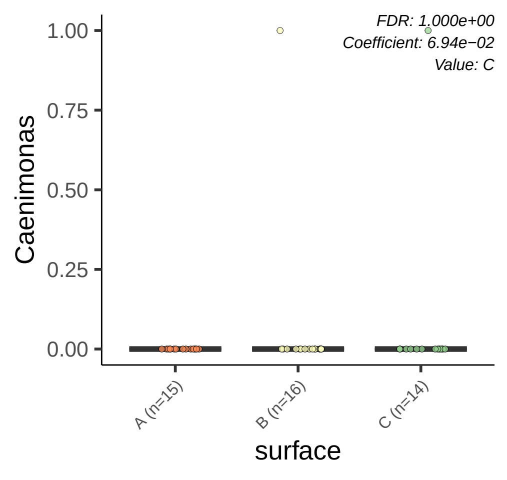
FDR: 1.000e+00
Coefficient: 6.94e−02
Value: C
Caenimonas
1.00
0.75
0.50
0.25
0.00
A (n=15)
B (n=16)
C (n=14)
surface

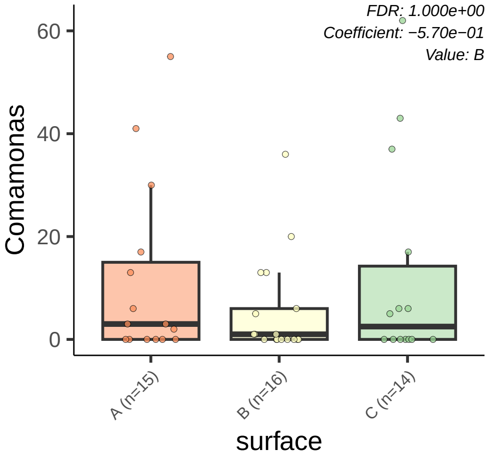

FDR: 1.000e+00
Coefficient: −5.70e−01
Value: B
Comamonas
0
20
40
60
A (n=15)
B (n=16)
C (n=14)
surface

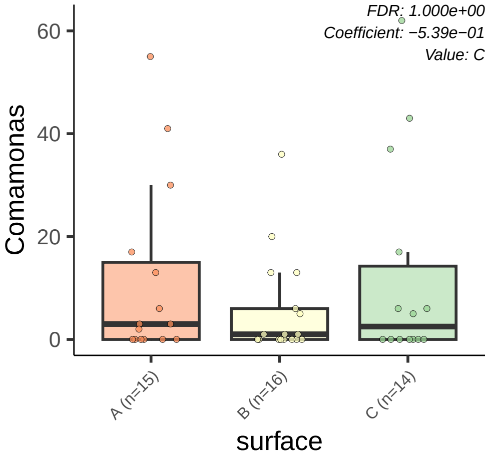
FDR: 1.000e+00
Coefficient: −5.39e−01
Value: C
Comamonas
60
40
20
0
A (n=15)
B (n=16)
C (n=14)
surface

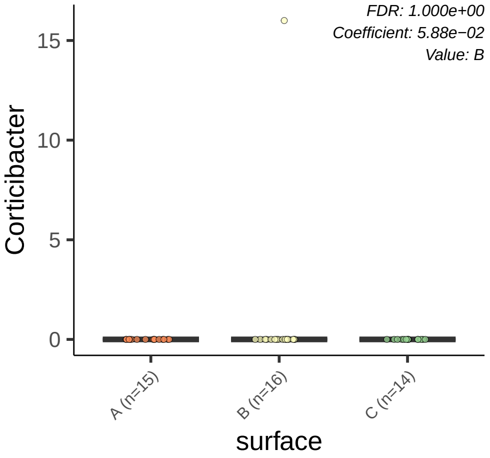
FDR: 1.000e+00
Coefficient: 5.88e−02
Value: B
Corticibacter
15
10
5
0
A (n=15)
B (n=16)
C (n=14)
surface

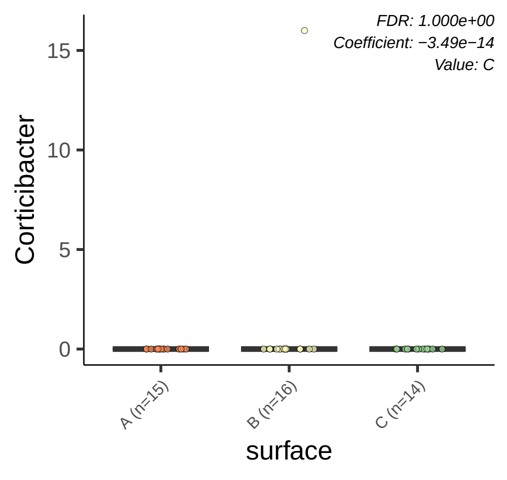
FDR: 1.000e+00
Coefficient: −3.49e−14
Value: C
Corticibacter
15
10
5
0
A (n=15)
B (n=16)
C (n=14)
surface

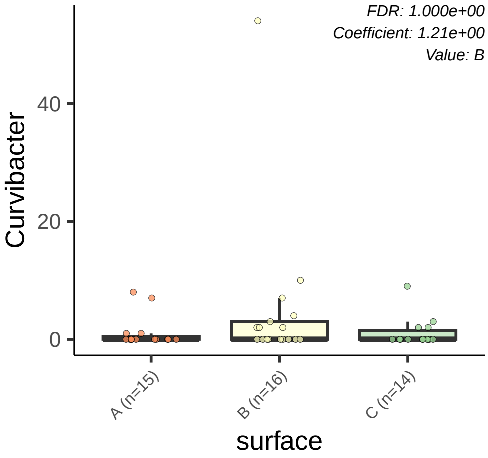

FDR: 1.000e+00
Coefficient: 1.21e+00
Value: B
Curvibacter
0
20
40
A (n=15)
B (n=16)
C (n=14)
surface

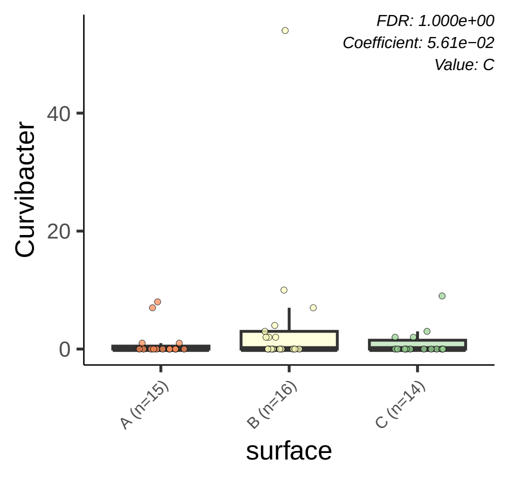

FDR: 1.000e+00
Coefficient: 5.61e−02
Value: C
Curvibacter
40
20
0
A (n=15)
B (n=16)
C (n=14)
surface

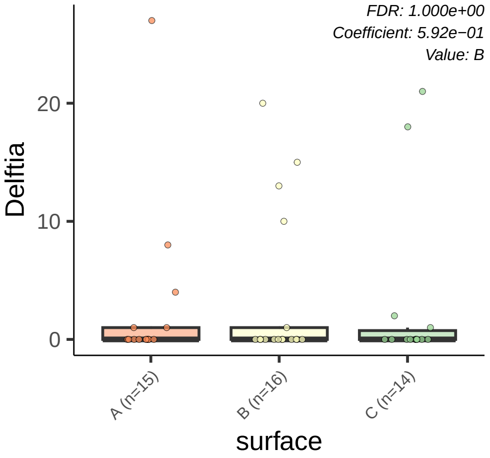
FDR: 1.000e+00
Coefficient: 5.92e−01
Value: B
Delftia
20
10
0
A (n=15)
B (n=16)
C (n=14)
surface

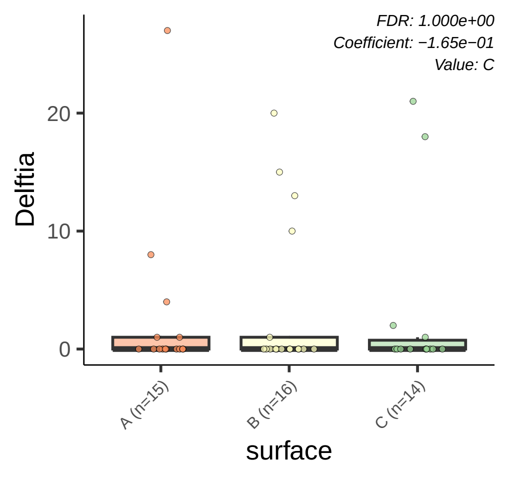
FDR: 1.000e+00
Coefficient: −1.65e−01
Value: C
Delftia
0
10
20
A (n=15)
B (n=16)
C (n=14)
surface

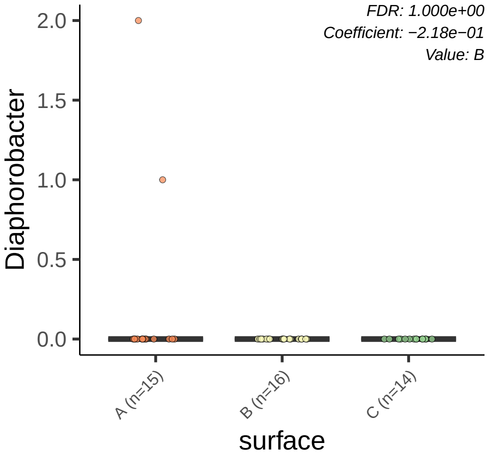
FDR: 1.000e+00
Coefficient: −2.18e−01
Value: B
Diaphorobacter
2.0
1.5
1.0
0.5
0.0
A (n=15)
B (n=16)
C (n=14)
surface

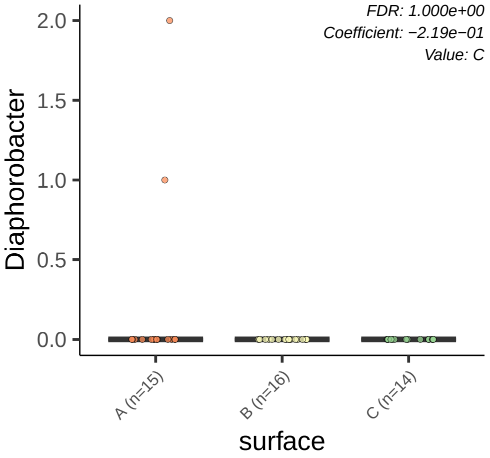
FDR: 1.000e+00
Coefficient: −2.19e−01
Value: C
Diaphorobacter
2.0
1.5
1.0
0.5
0.0
A (n=15)
B (n=16)
C (n=14)
surface

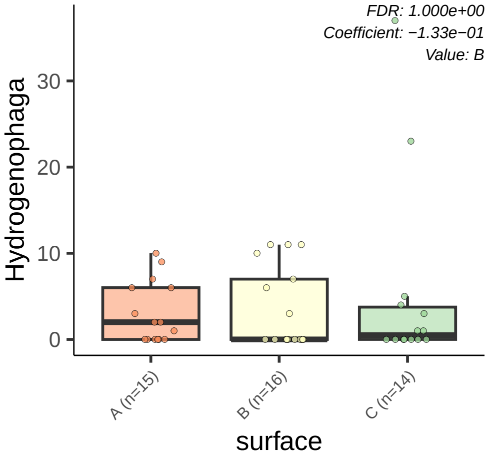

FDR: 1.000e+00
Coefficient: −1.33e−01
Value: B
Hydrogenophaga
0
10
20
30
A (n=15)
B (n=16)
C (n=14)
surface

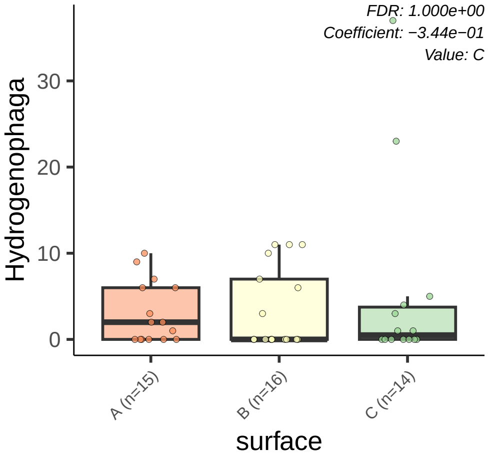

FDR: 1.000e+00
Coefficient: −3.44e−01
Value: C
Hydrogenophaga
0
10
20
30
A (n=15)
B (n=16)
C (n=14)
surface

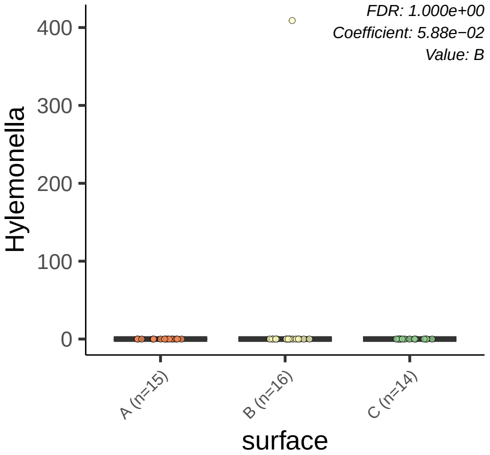

FDR: 1.000e+00
Coefficient: 5.88e−02
Value: B
Hylemonella
400
300
200
100
0
A (n=15)
B (n=16)
C (n=14)
surface

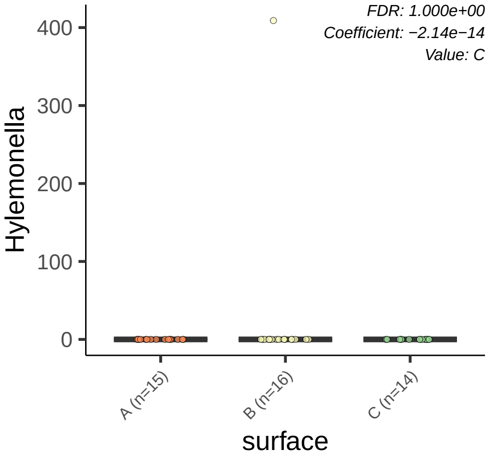
FDR: 1.000e+00
Coefficient: −2.14e−14
Value: C
Hylemonella
400
300
200
100
0
A (n=15)
B (n=16)
C (n=14)
surface

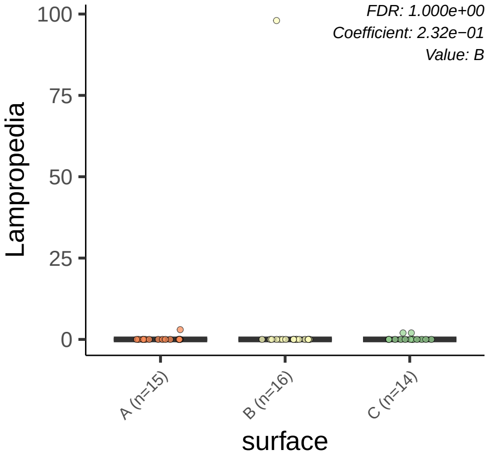
FDR: 1.000e+00
Coefficient: 2.32e−01
Value: B
Lampropedia
100
75
50
25
0
A (n=15)
B (n=16)
C (n=14)
surface

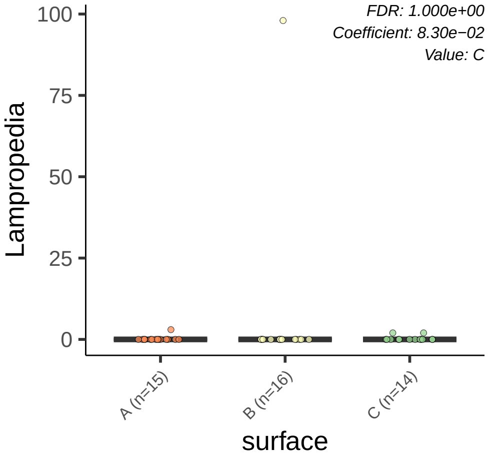

FDR: 1.000e+00
Coefficient: 8.30e−02
Value: C
Lampropedia
100
75
50
25
0
A (n=15)
B (n=16)
C (n=14)
surface

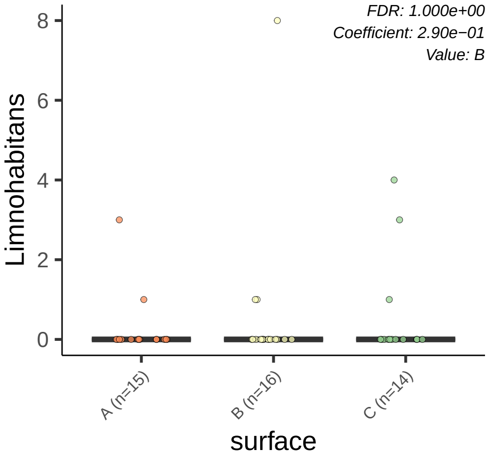
FDR: 1.000e+00
Coefficient: 2.90e−01
Value: B
Limnohabitans
0
2
4
6
8
A (n=15)
B (n=16)
C (n=14)
surface

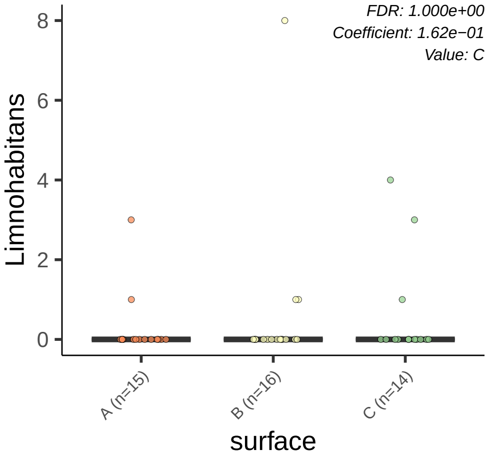

FDR: 1.000e+00
Coefficient: 1.62e−01
Value: C
Limnohabitans
0
2
4
6
8
A (n=15)
B (n=16)
C (n=14)
surface

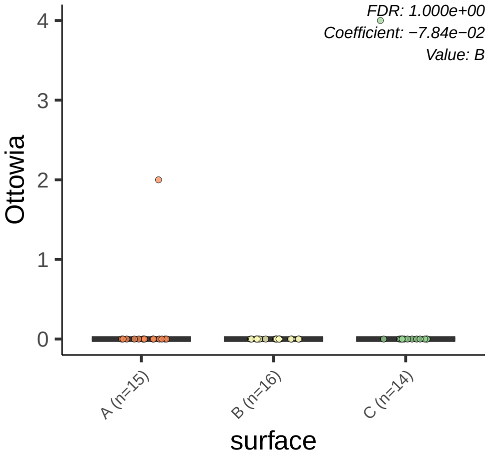

FDR: 1.000e+00
Coefficient: −7.84e−02
Value: B
Ottowia
0
1
2
3
4
A (n=15)
B (n=16)
C (n=14)
surface

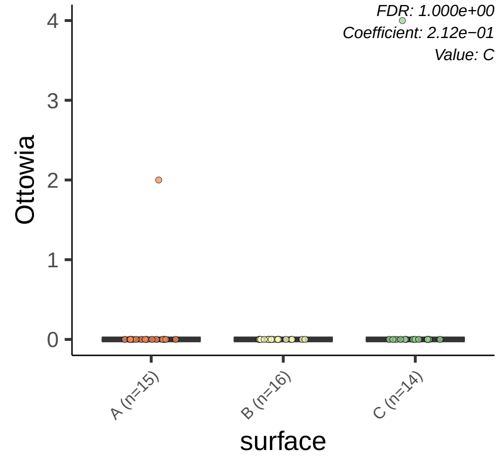
FDR: 1.000e+00
Coefficient: 2.12e−01
Value: C
Ottowia
0
1
2
3
4
A (n=15)
B (n=16)
C (n=14)
surface

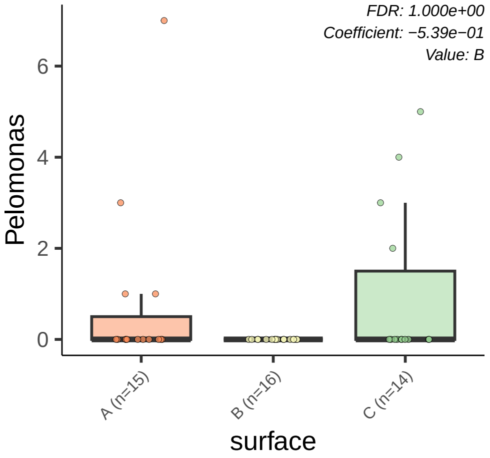
FDR: 1.000e+00
Coefficient: −5.39e−01
Value: B
Pelomonas
0
2
4
6
A (n=15)
B (n=16)
C (n=14)
surface

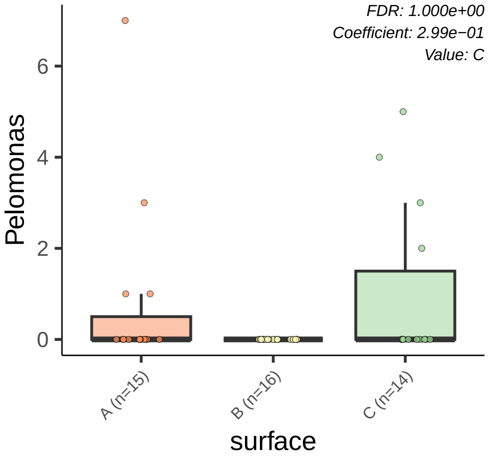

FDR: 1.000e+00
Coefficient: 2.99e−01
Value: C
Pelomonas
0
2
4
6
A (n=15)
B (n=16)
C (n=14)
surface

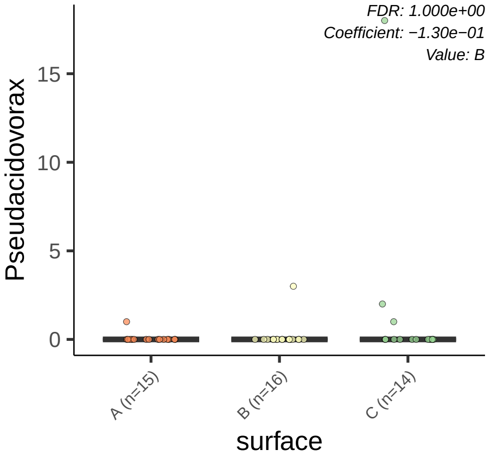
FDR: 1.000e+00
Coefficient: −1.30e−01
Value: B
Pseudacidovorax
15
10
5
0
A (n=15)
B (n=16)
C (n=14)
surface

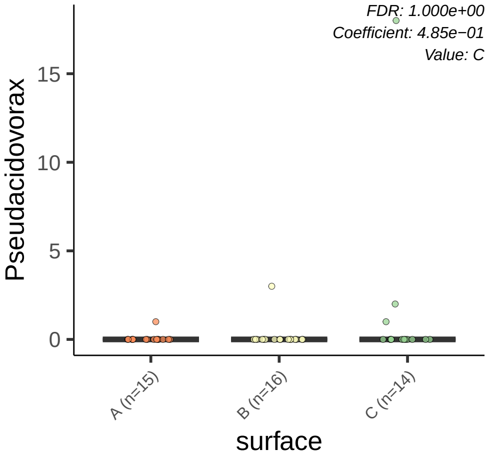

FDR: 1.000e+00
Coefficient: 4.85e−01
Value: C
Pseudacidovorax
15
10
5
0
A (n=15)
B (n=16)
C (n=14)
surface

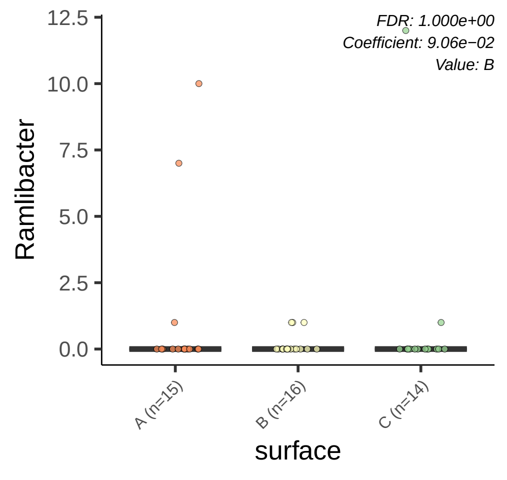
FDR: 1.000e+00
Coefficient: 9.06e−02
Value: B
Ramlibacter
12.5
10.0
7.5
5.0
2.5
0.0
A (n=15)
B (n=16)
C (n=14)
surface

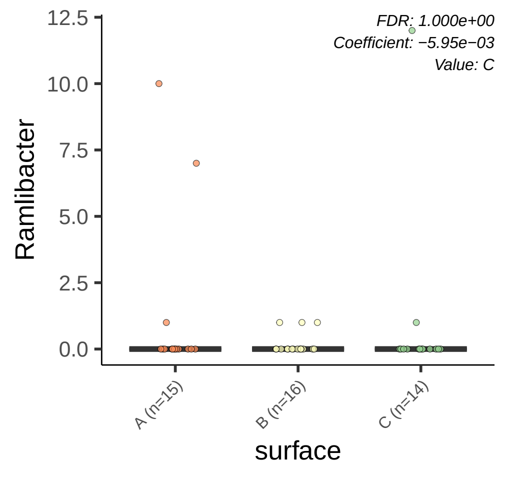
FDR: 1.000e+00
Coefficient: −5.95e−03
Value: C
Ramlibacter
12.5
10.0
7.5
5.0
2.5
0.0
A (n=15)
B (n=16)
C (n=14)
surface

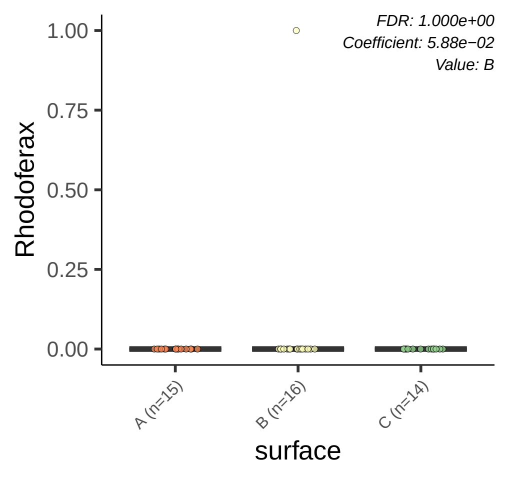

FDR: 1.000e+00
Coefficient: 5.88e−02
Value: B
Rhodoferax
1.00
0.75
0.50
0.25
0.00
A (n=15)
B (n=16)
C (n=14)
surface

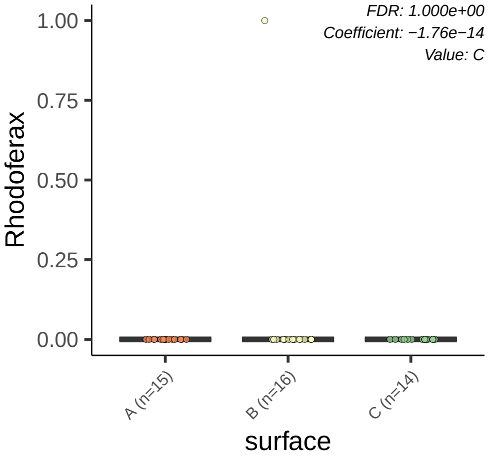
FDR: 1.000e+00
Coefficient: −1.76e−14
Value: C
Rhodoferax
1.00
0.75
0.50
0.25
0.00
A (n=15)
B (n=16)
C (n=14)
surface

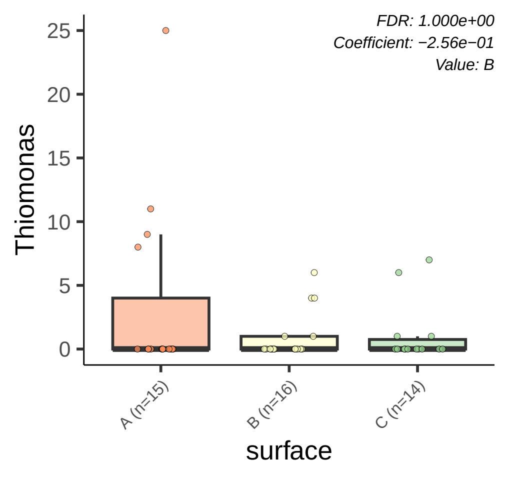
FDR: 1.000e+00
Coefficient: −2.56e−01
Value: B
Thiomonas
0
5
10
15
20
25
A (n=15)
B (n=16)
C (n=14)
surface

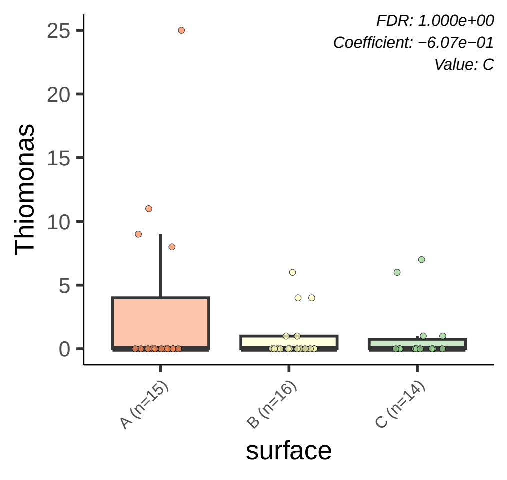
FDR: 1.000e+00
Coefficient: −6.07e−01
Value: C
Thiomonas
25
20
15
10
5
0
A (n=15)
B (n=16)
C (n=14)
surface

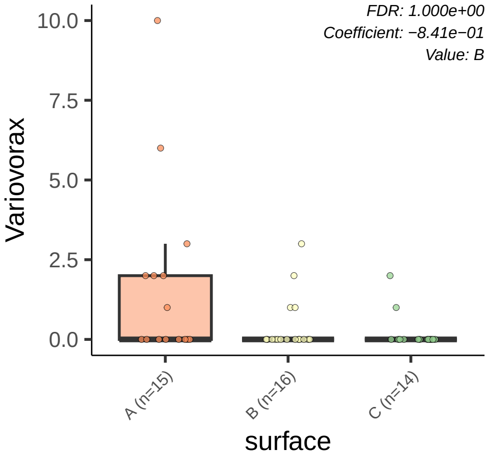

FDR: 1.000e+00
Coefficient: −8.41e−01
Value: B
Variovorax
10.0
7.5
5.0
2.5
0.0
A (n=15)
B (n=16)
C (n=14)
surface

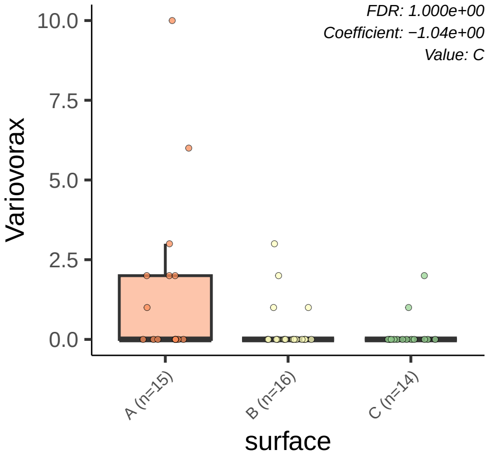

FDR: 1.000e+00
Coefficient: −1.04e+00
Value: C
Variovorax
10.0
7.5
5.0
2.5
0.0
A (n=15)
B (n=16)
C (n=14)
surface

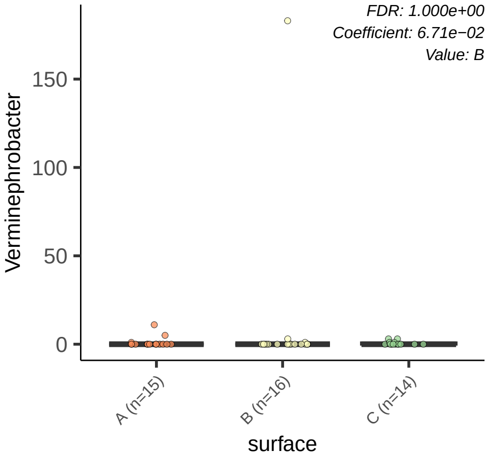

FDR: 1.000e+00
Coefficient: 6.71e−02
Value: B
Verminephrobacter
150
100
50
0
A (n=15)
B (n=16)
C (n=14)
surface

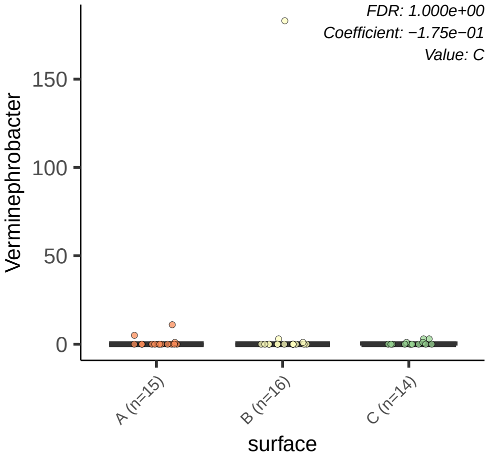
FDR: 1.000e+00
Coefficient: −1.75e−01
Value: C
Verminephrobacter
150
100
50
0
A (n=15)
B (n=16)
C (n=14)
surface

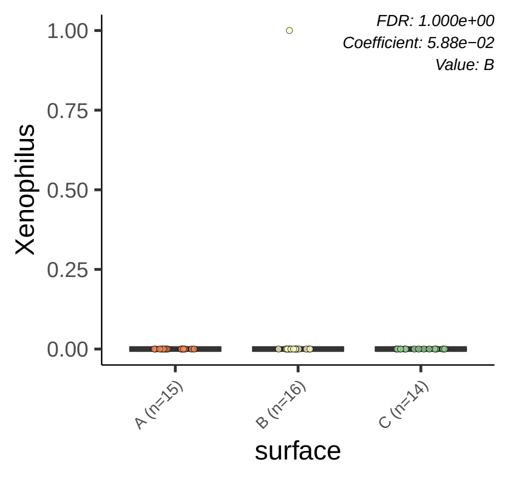

FDR: 1.000e+00
Coefficient: 5.88e−02
Value: B
Xenophilus
1.00
0.75
0.50
0.25
0.00
A (n=15)
B (n=16)
C (n=14)
surface

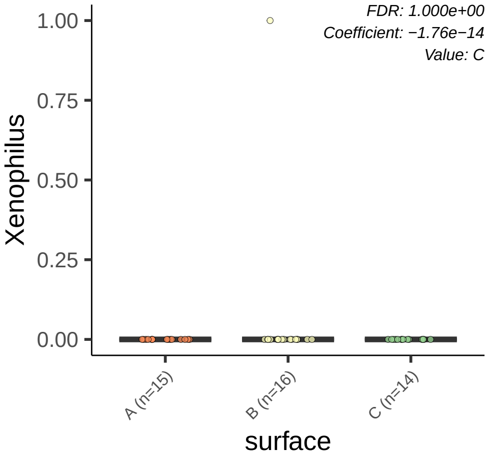
FDR: 1.000e+00
Coefficient: −1.76e−14
Value: C
Xenophilus
1.00
0.75
0.50
0.25
0.00
A (n=15)
B (n=16)
C (n=14)
surface

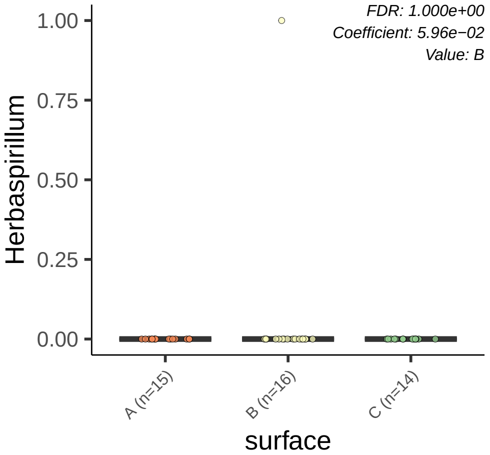
FDR: 1.000e+00
Coefficient: 5.96e−02
Value: B
Herbaspirillum
1.00
0.75
0.50
0.25
0.00
A (n=15)
B (n=16)
C (n=14)
surface

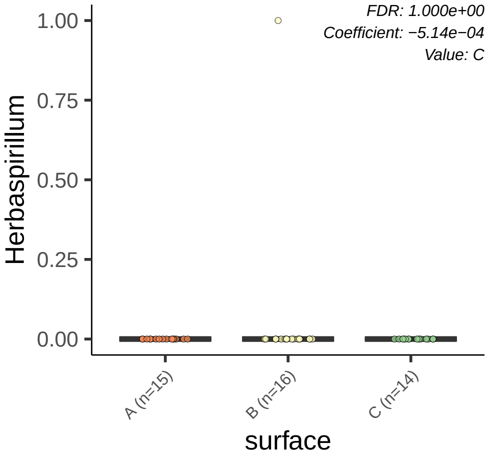

FDR: 1.000e+00
Coefficient: −5.14e−04
Value: C
Herbaspirillum
1.00
0.75
0.50
0.25
0.00
A (n=15)
B (n=16)
C (n=14)
surface

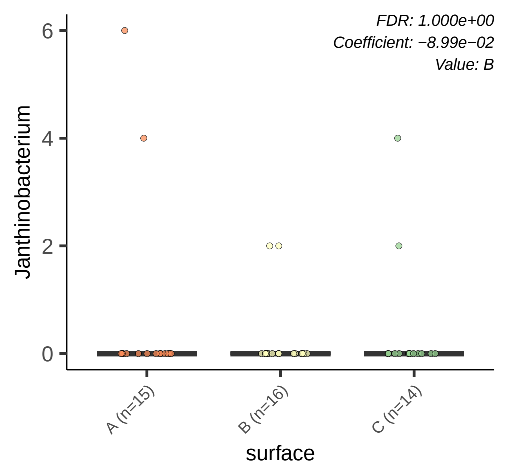

FDR: 1.000e+00
Coefficient: −8.99e−02
Value: B
Janthinobacterium
6
4
2
0
A (n=15)
B (n=16)
C (n=14)
surface

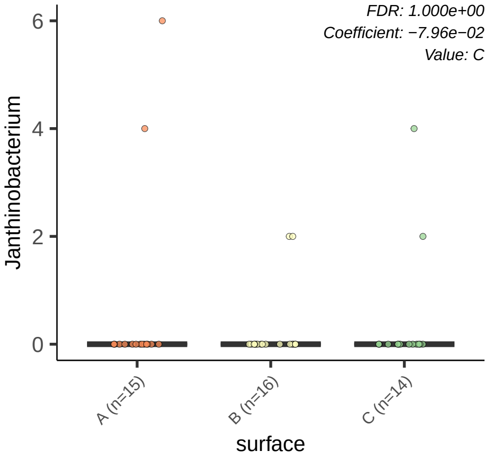
FDR: 1.000e+00
Coefficient: −7.96e−02
Value: C
Janthinobacterium
6
4
2
0
A (n=15)
B (n=16)
C (n=14)
surface

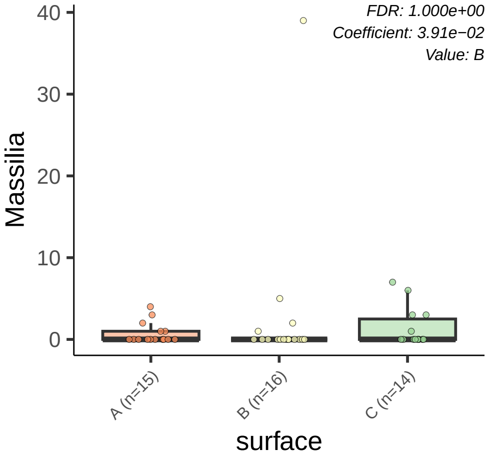

FDR: 1.000e+00
Coefficient: 3.91e−02
Value: B
Massilia
40
30
20
10
0
A (n=15)
B (n=16)
C (n=14)
surface

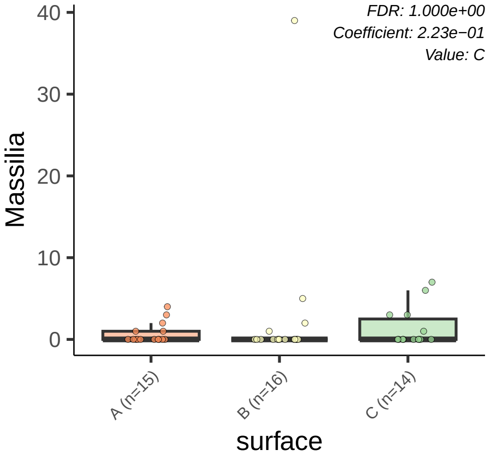

FDR: 1.000e+00
Coefficient: 2.23e−01
Value: C
Massilia
40
30
20
10
0
A (n=15)
B (n=16)
C (n=14)
surface

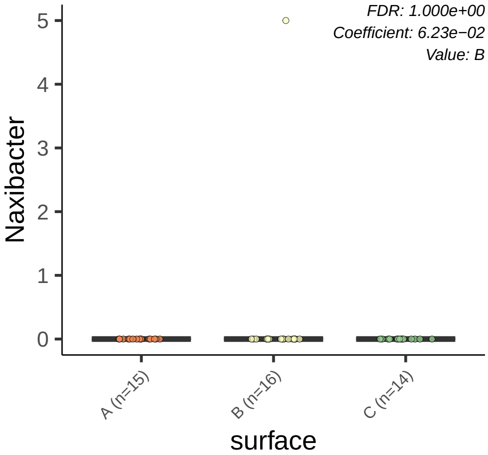

FDR: 1.000e+00
Coefficient: 6.23e−02
Value: B
Naxibacter
0
1
2
3
4
5
A (n=15)
B (n=16)
C (n=14)
surface

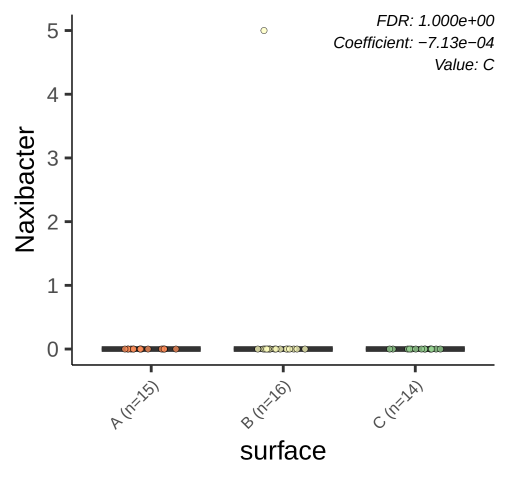

FDR: 1.000e+00
Coefficient: −7.13e−04
Value: C
Naxibacter
5
4
3
2
1
0
A (n=15)
B (n=16)
C (n=14)
surface

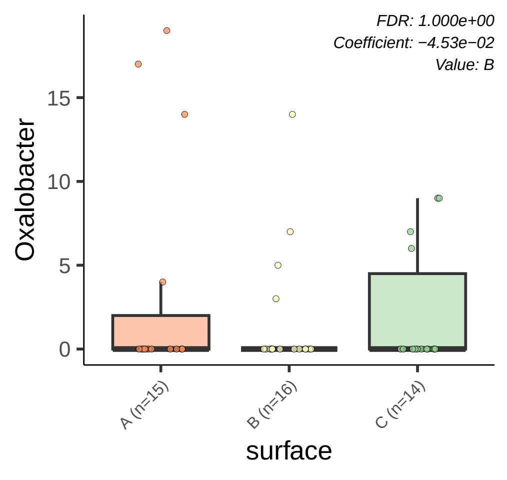

FDR: 1.000e+00
Coefficient: −4.53e−02
Value: B
Oxalobacter
0
5
10
15
A (n=15)
B (n=16)
C (n=14)
surface

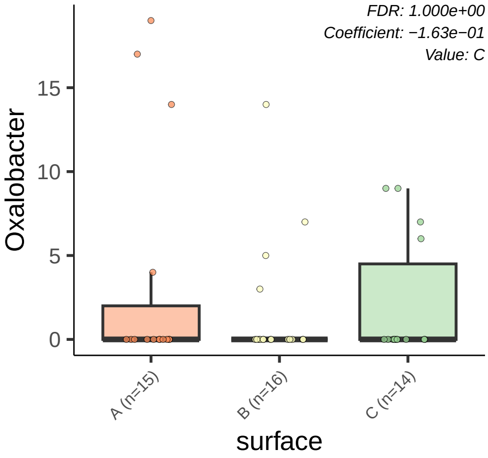

FDR: 1.000e+00
Coefficient: −1.63e−01
Value: C
Oxalobacter
0
5
10
15
A (n=15)
B (n=16)
C (n=14)
surface

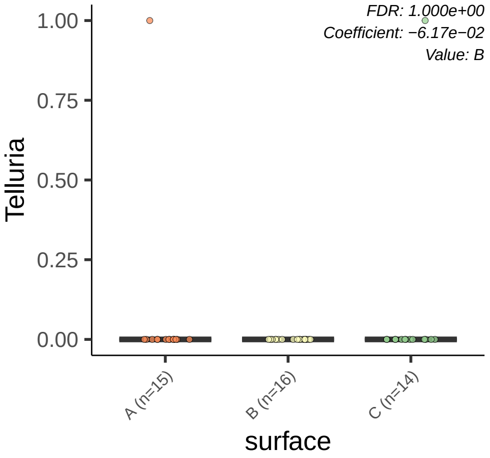
FDR: 1.000e+00
Coefficient: −6.17e−02
Value: B
Telluria
1.00
0.75
0.50
0.25
0.00
A (n=15)
B (n=16)
C (n=14)
surface

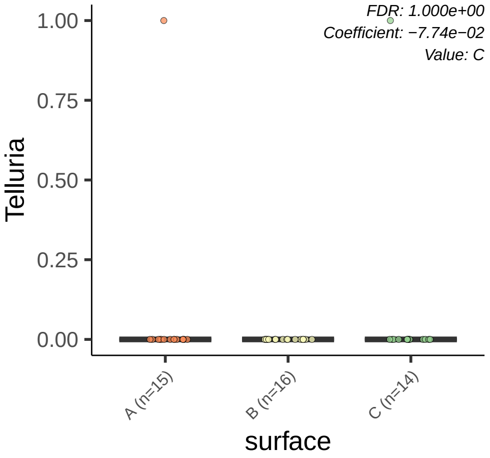
FDR: 1.000e+00
Coefficient: −7.74e−02
Value: C
Telluria
1.00
0.75
0.50
0.25
0.00
A (n=15)
B (n=16)
C (n=14)
surface

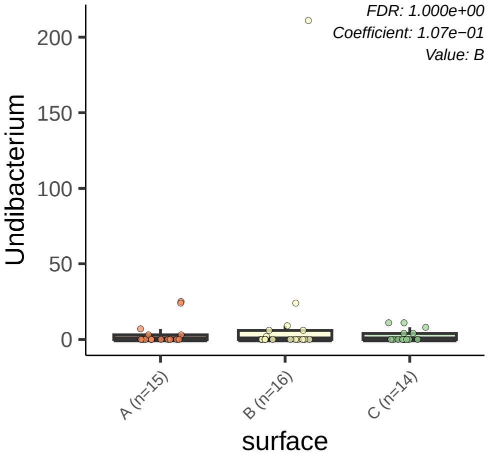

FDR: 1.000e+00
Coefficient: 1.07e−01
Value: B
Undibacterium
200
150
100
50
0
A (n=15)
B (n=16)
C (n=14)
surface

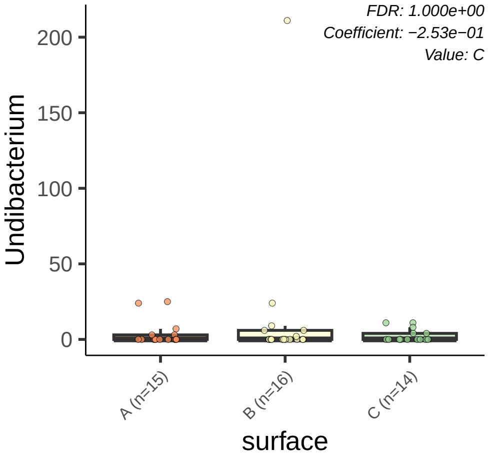

FDR: 1.000e+00
Coefficient: −2.53e−01
Value: C
Undibacterium
200
150
100
50
0
A (n=15)
B (n=16)
C (n=14)
surface

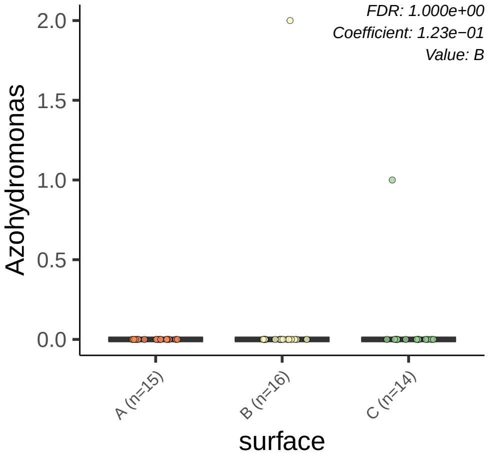
FDR: 1.000e+00
Coefficient: 1.23e−01
Value: B
Azohydromonas
2.0
1.5
1.0
0.5
0.0
A (n=15)
B (n=16)
C (n=14)
surface

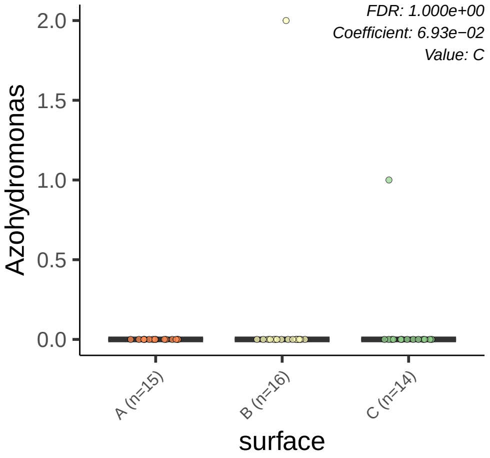
FDR: 1.000e+00
Coefficient: 6.93e−02
Value: C
Azohydromonas
2.0
1.5
1.0
0.5
0.0
A (n=15)
B (n=16)
C (n=14)
surface

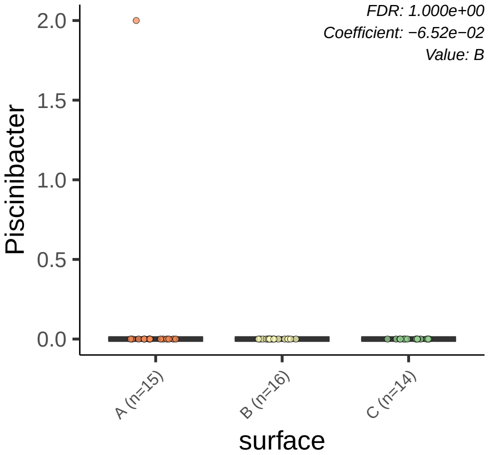

FDR: 1.000e+00
Coefficient: −6.52e−02
Value: B
Piscinibacter
2.0
1.5
1.0
0.5
0.0
A (n=15)
B (n=16)
C (n=14)
surface

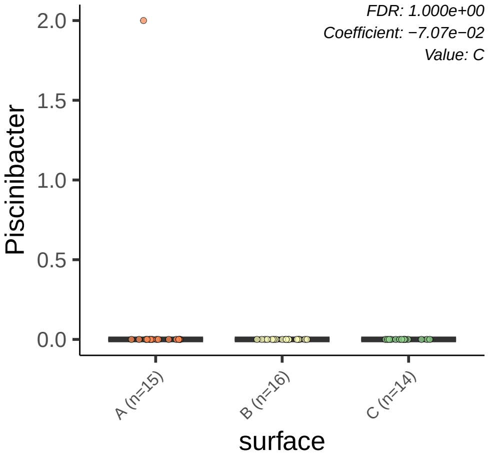
FDR: 1.000e+00
Coefficient: −7.07e−02
Value: C
Piscinibacter
2.0
1.5
1.0
0.5
0.0
A (n=15)
B (n=16)
C (n=14)
surface

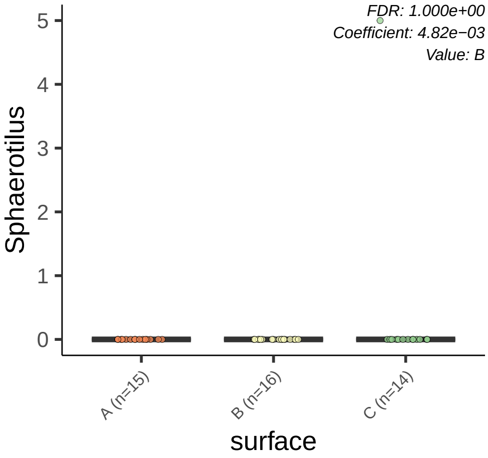
FDR: 1.000e+00
Coefficient: 4.82e−03
Value: B
Sphaerotilus
5
4
3
2
1
0
A (n=15)
B (n=16)
C (n=14)
surface

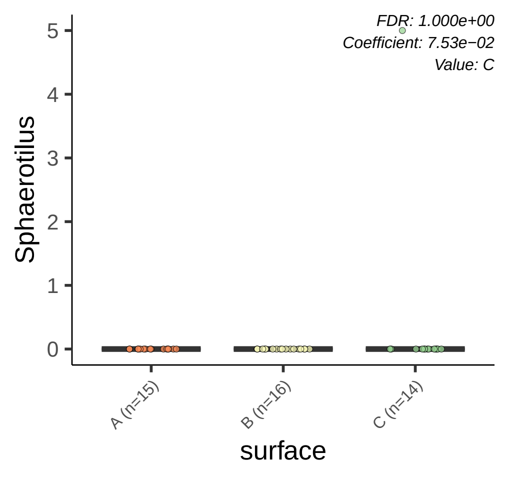
FDR: 1.000e+00
Coefficient: 7.53e−02
Value: C
Sphaerotilus
0
1
2
3
4
5
A (n=15)
B (n=16)
C (n=14)
surface

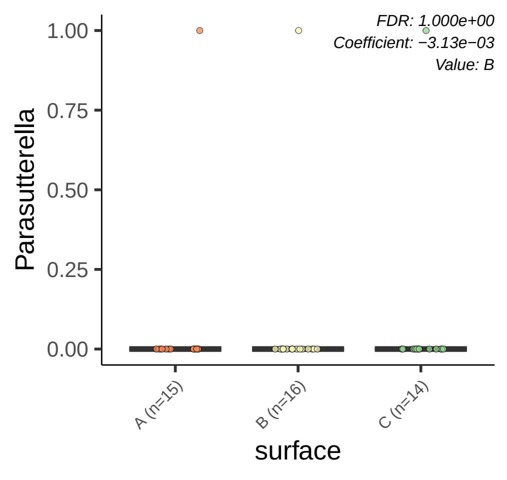
FDR: 1.000e+00
Coefficient: −3.13e−03
Value: B
Parasutterella
1.00
0.75
0.50
0.25
0.00
A (n=15)
B (n=16)
C (n=14)
surface

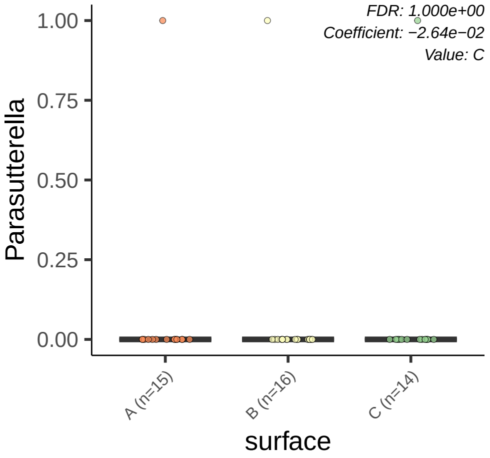

FDR: 1.000e+00
Coefficient: −2.64e−02
Value: C
Parasutterella
1.00
0.75
0.50
0.25
0.00
A (n=15)
B (n=16)
C (n=14)
surface

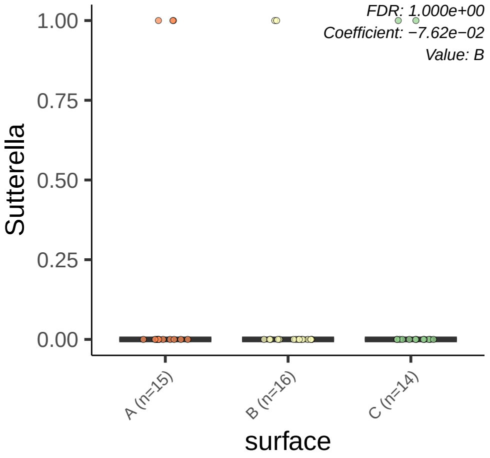

FDR: 1.000e+00
Coefficient: −7.62e−02
Value: B
Sutterella
1.00
0.75
0.50
0.25
0.00
A (n=15)
B (n=16)
C (n=14)
surface

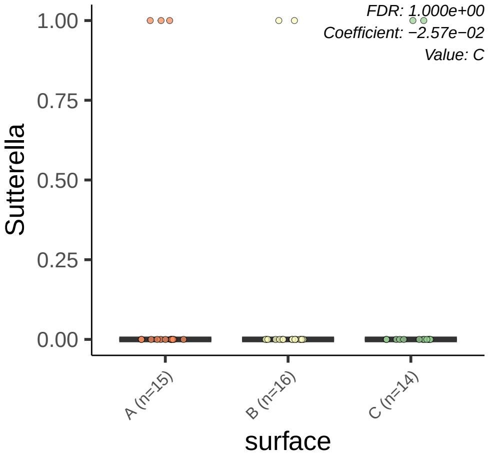

FDR: 1.000e+00
Coefficient: −2.57e−02
Value: C
Sutterella
1.00
0.75
0.50
0.25
0.00
A (n=15)
B (n=16)
C (n=14)
surface

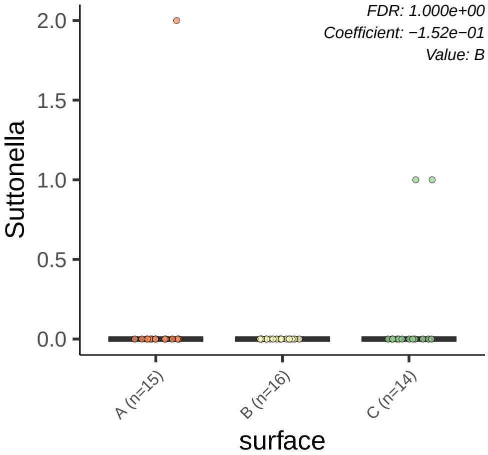
FDR: 1.000e+00
Coefficient: −1.52e−01
Value: B
Suttonella
2.0
1.5
1.0
0.5
0.0
A (n=15)
B (n=16)
C (n=14)
surface

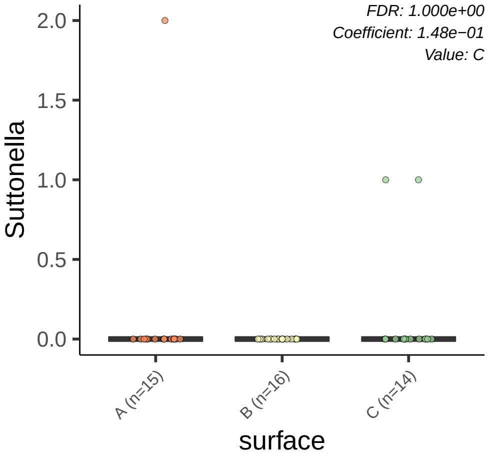

FDR: 1.000e+00
Coefficient: 1.48e−01
Value: C
Suttonella
2.0
1.5
1.0
0.5
0.0
A (n=15)
B (n=16)
C (n=14)
surface

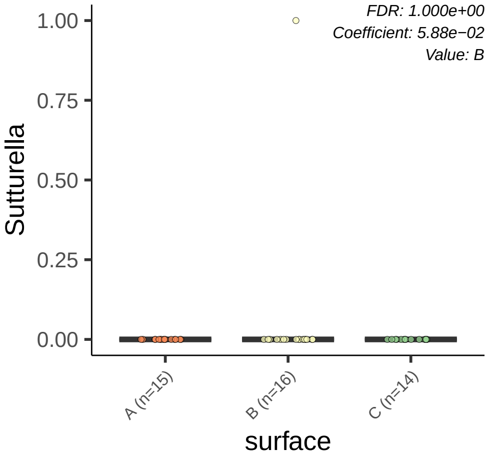
FDR: 1.000e+00
Coefficient: 5.88e−02
Value: B
Sutturella
1.00
0.75
0.50
0.25
0.00
A (n=15)
B (n=16)
C (n=14)
surface

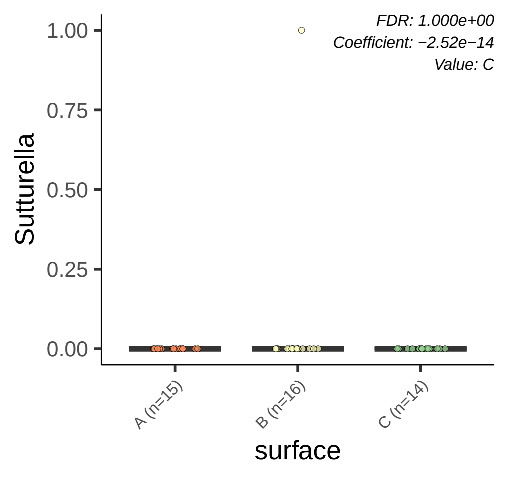

FDR: 1.000e+00
Coefficient: −2.52e−14
Value: C
Sutturella
1.00
0.75
0.50
0.25
0.00
A (n=15)
B (n=16)
C (n=14)
surface

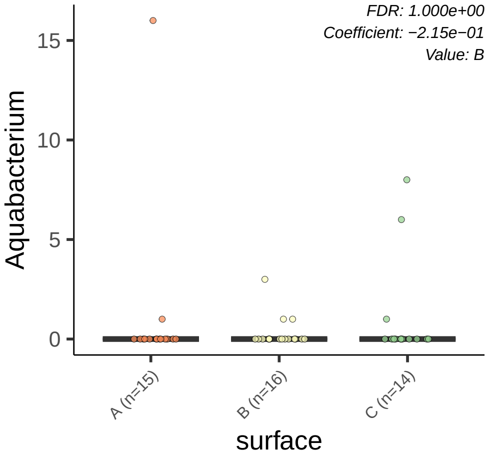
FDR: 1.000e+00
Coefficient: −2.15e−01
Value: B
Aquabacterium
15
10
5
0
A (n=15)
B (n=16)
C (n=14)
surface

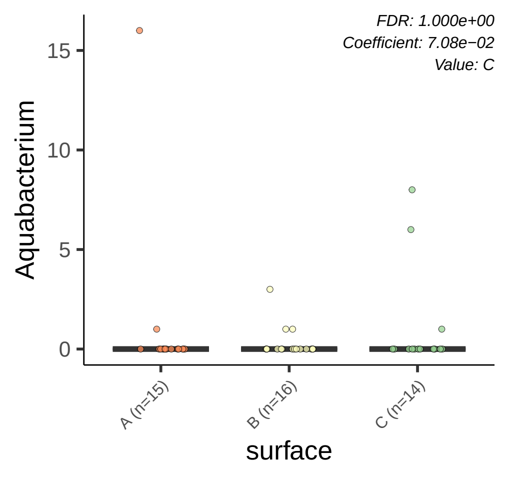

FDR: 1.000e+00
Coefficient: 7.08e−02
Value: C
Aquabacterium
15
10
5
0
A (n=15)
B (n=16)
C (n=14)
surface

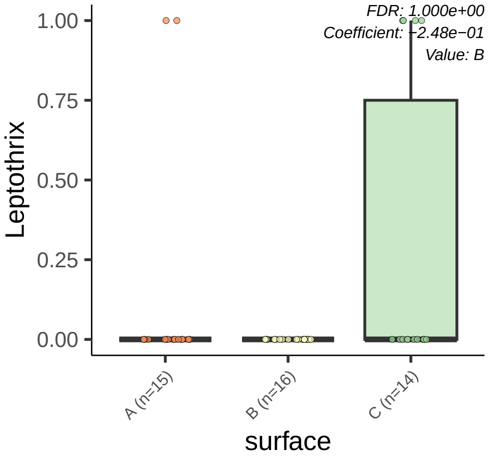
FDR: 1.000e+00
Coefficient: −2.48e−01
Value: B
Leptothrix
1.00
0.75
0.50
0.25
0.00
A (n=15)
B (n=16)
C (n=14)
surface

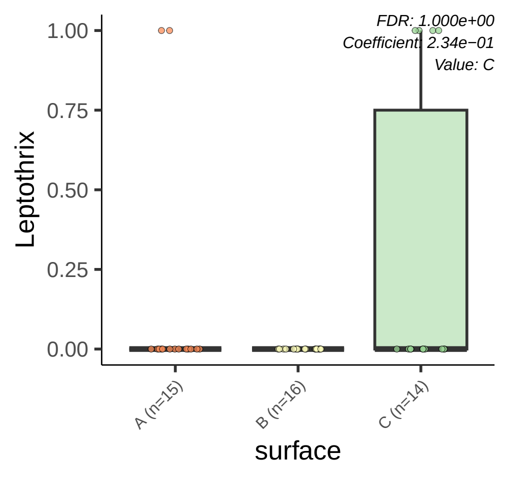
FDR: 1.000e+00
Coefficient: 2.34e−01
Value: C
Leptothrix
1.00
0.75
0.50
0.25
0.00
A (n=15)
B (n=16)
C (n=14)
surface

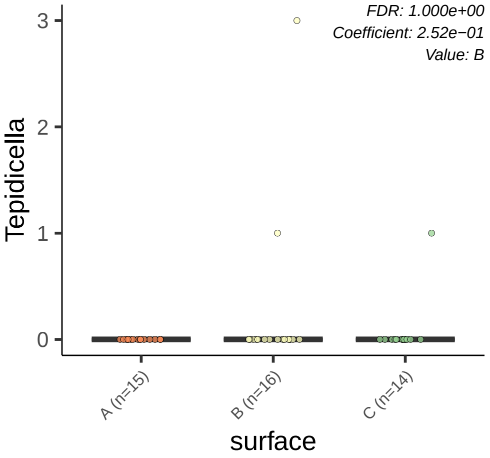

FDR: 1.000e+00
Coefficient: 2.52e−01
Value: B
Tepidicella
0
1
2
3
A (n=15)
B (n=16)
C (n=14)
surface

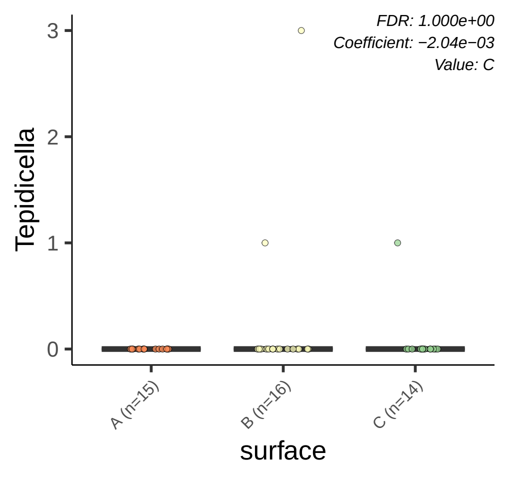
FDR: 1.000e+00
Coefficient: −2.04e−03
Value: C
Tepidicella
0
1
2
3
A (n=15)
B (n=16)
C (n=14)
surface

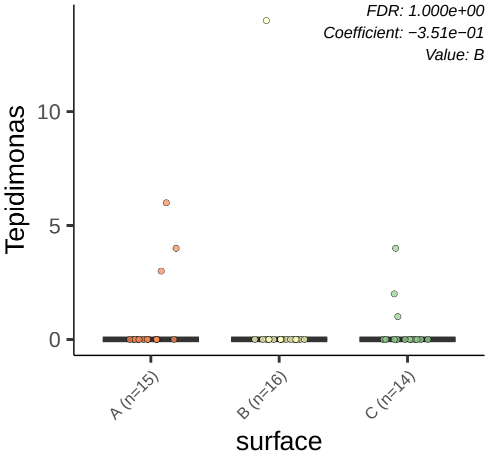
FDR: 1.000e+00
Coefficient: −3.51e−01
Value: B
Tepidimonas
10
5
0
A (n=15)
B (n=16)
C (n=14)
surface

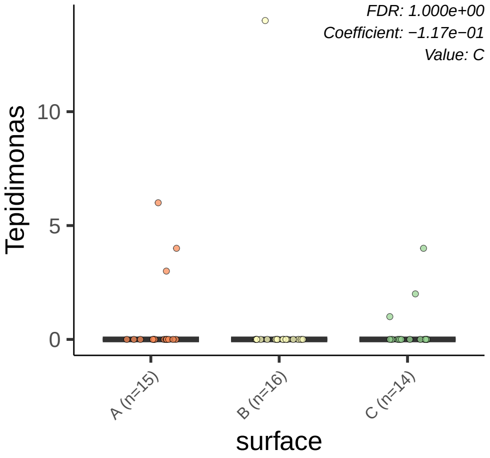

FDR: 1.000e+00
Coefficient: −1.17e−01
Value: C
Tepidimonas
10
5
0
A (n=15)
B (n=16)
C (n=14)
surface

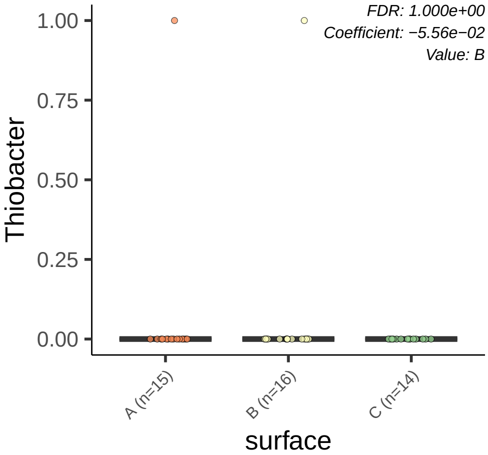
FDR: 1.000e+00
Coefficient: −5.56e−02
Value: B
Thiobacter
1.00
0.75
0.50
0.25
0.00
A (n=15)
B (n=16)
C (n=14)
surface

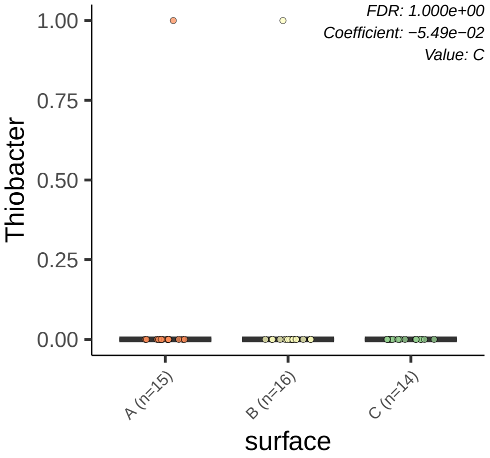

FDR: 1.000e+00
Coefficient: −5.49e−02
Value: C
Thiobacter
1.00
0.75
0.50
0.25
0.00
A (n=15)
B (n=16)
C (n=14)
surface

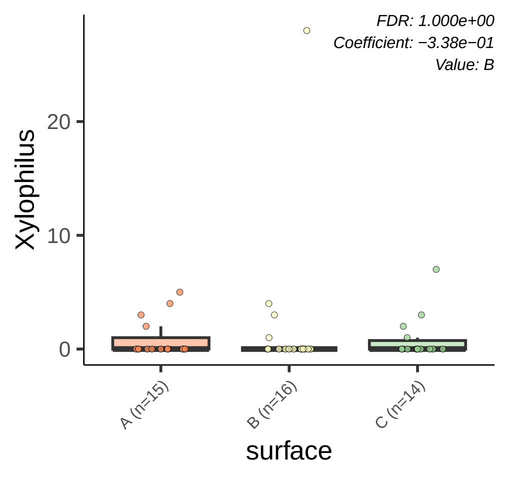
FDR: 1.000e+00
Coefficient: −3.38e−01
Value: B
Xylophilus
0
10
20
A (n=15)
B (n=16)
C (n=14)
surface

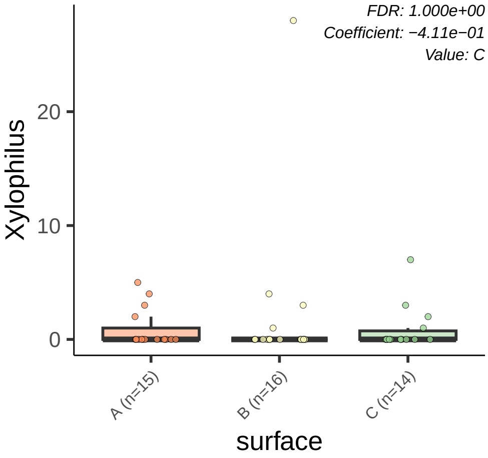

FDR: 1.000e+00
Coefficient: −4.11e−01
Value: C
Xylophilus
0
10
20
A (n=15)
B (n=16)
C (n=14)
surface

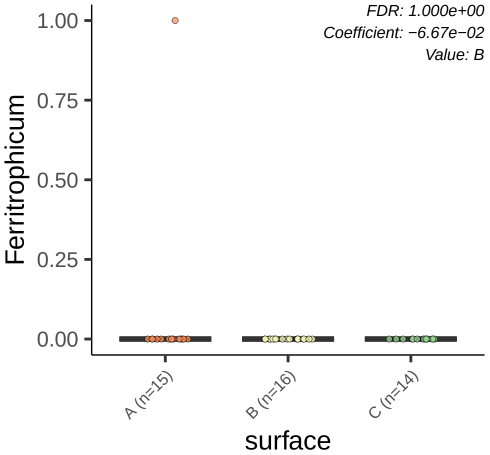

FDR: 1.000e+00
Coefficient: −6.67e−02
Value: B
Ferritrophicum
1.00
0.75
0.50
0.25
0.00
A (n=15)
B (n=16)
C (n=14)
surface

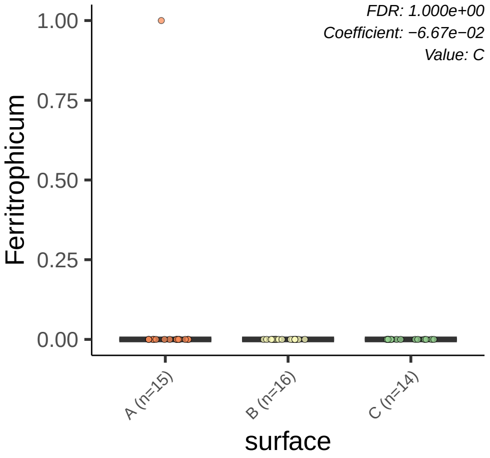

FDR: 1.000e+00
Coefficient: −6.67e−02
Value: C
Ferritrophicum
1.00
0.75
0.50
0.25
0.00
A (n=15)
B (n=16)
C (n=14)
surface

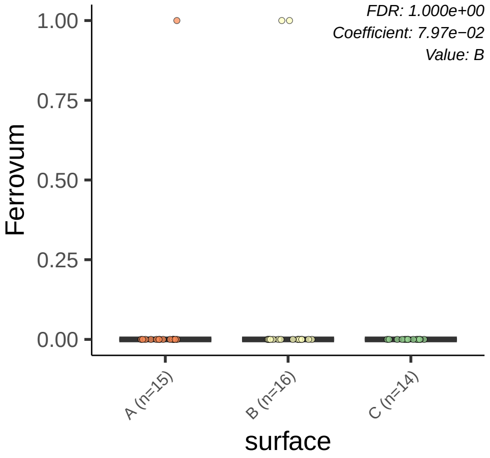

FDR: 1.000e+00
Coefficient: 7.97e−02
Value: B
1.00
0.75
0.50
0.25
0.00
Ferrovum
A (n=15)
B (n=16)
C (n=14)
surface

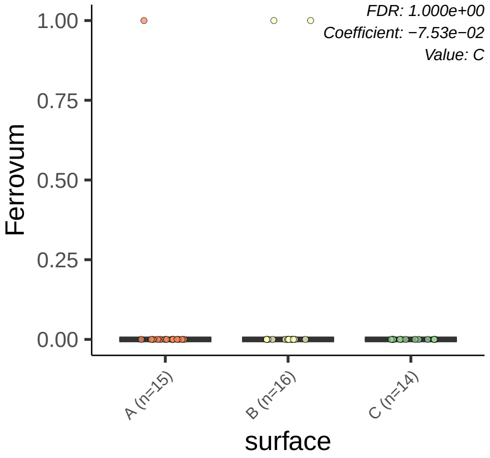

FDR: 1.000e+00
Coefficient: −7.53e−02
Value: C
1.00
0.75
0.50
0.25
0.00
Ferrovum
A (n=15)
B (n=16)
C (n=14)
surface

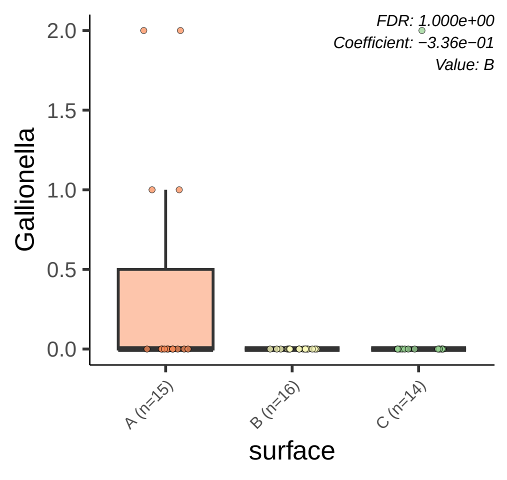
FDR: 1.000e+00
Coefficient: −3.36e−01
Value: B
Gallionella
2.0
1.5
1.0
0.5
0.0
A (n=15)
B (n=16)
C (n=14)
surface

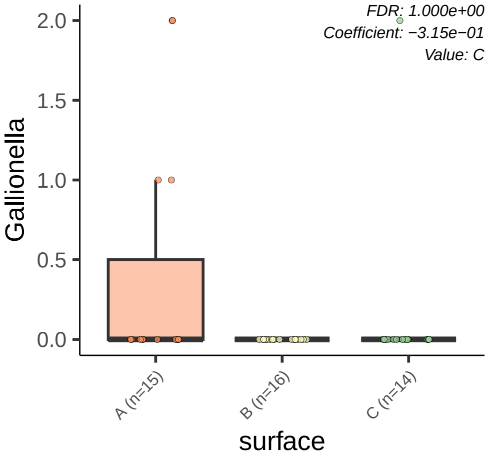

FDR: 1.000e+00
Coefficient: −3.15e−01
Value: C
Gallionella
2.0
1.5
1.0
0.5
0.0
A (n=15)
B (n=16)
C (n=14)
surface

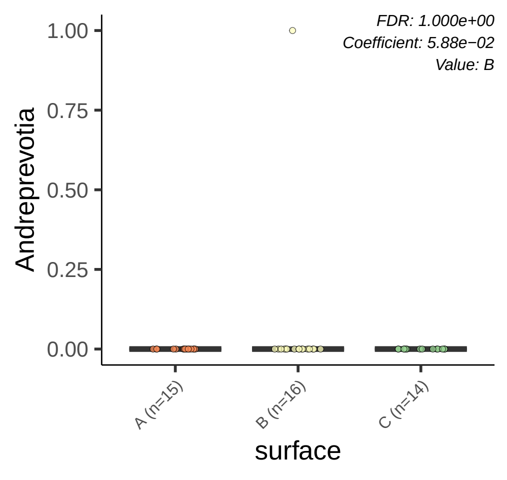
FDR: 1.000e+00
Coefficient: 5.88e−02
Value: B
Andreprevotia
1.00
0.75
0.50
0.25
0.00
A (n=15)
B (n=16)
C (n=14)
surface

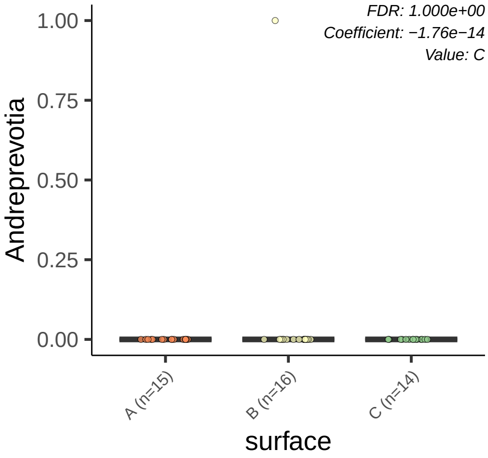

FDR: 1.000e+00
Coefficient: −1.76e−14
Value: C
Andreprevotia
1.00
0.75
0.50
0.25
0.00
A (n=15)
B (n=16)
C (n=14)
surface

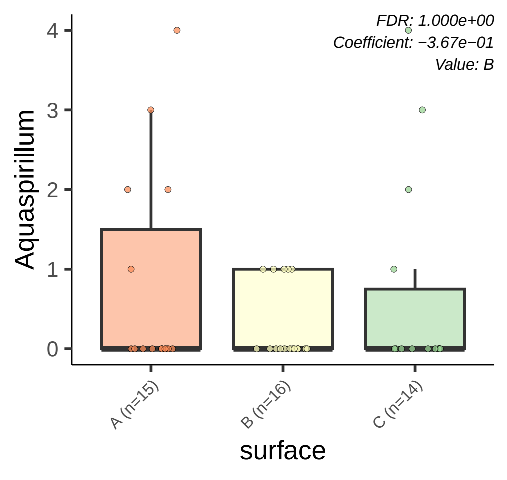
FDR: 1.000e+00
Coefficient: −3.67e−01
Value: B
Aquaspirillum
0
1
2
3
4
A (n=15)
B (n=16)
C (n=14)
surface

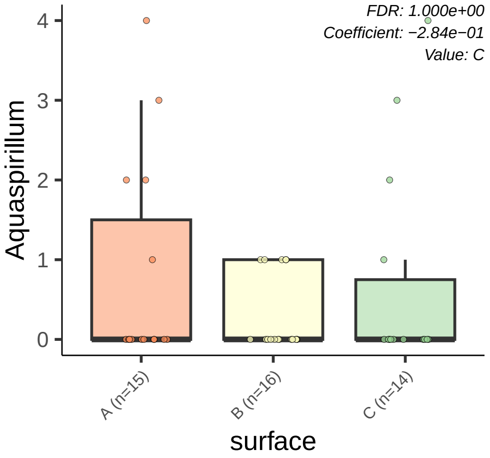

FDR: 1.000e+00
Coefficient: −2.84e−01
Value: C
Aquaspirillum
0
1
2
3
4
A (n=15)
B (n=16)
C (n=14)
surface

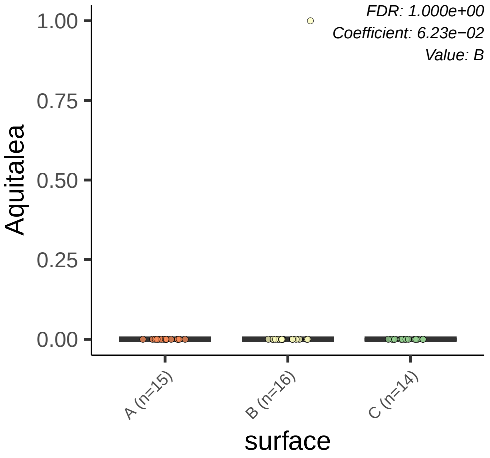

FDR: 1.000e+00
Coefficient: 6.23e−02
Value: B
Aquitalea
1.00
0.75
0.50
0.25
0.00
A (n=15)
B (n=16)
C (n=14)
surface

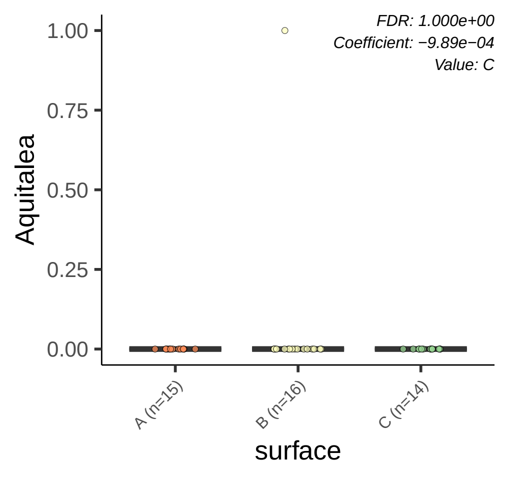
FDR: 1.000e+00
Coefficient: −9.89e−04
Value: C
Aquitalea
1.00
0.75
0.50
0.25
0.00
A (n=15)
B (n=16)
C (n=14)
surface

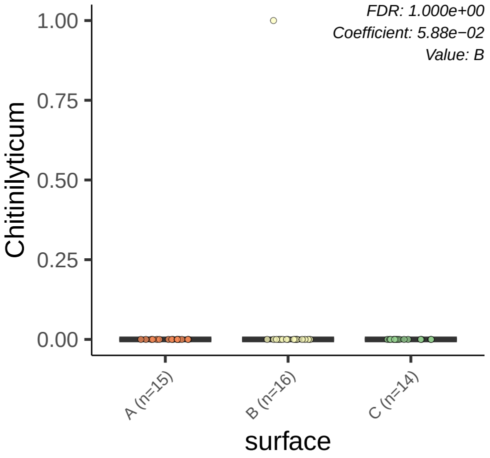

FDR: 1.000e+00
Coefficient: 5.88e−02
Value: B
1.00
0.75
0.50
0.25
0.00
Chitinilyticum
A (n=15)
B (n=16)
C (n=14)
surface

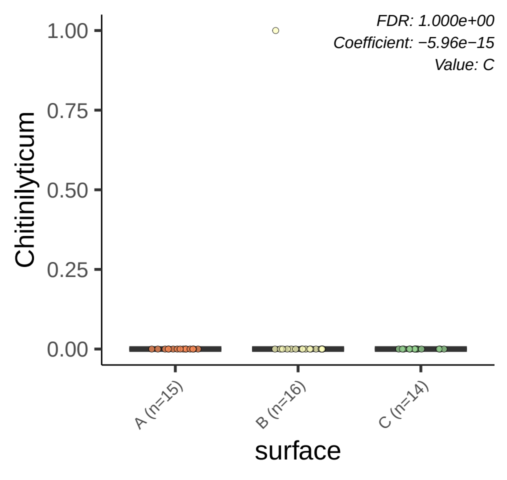

FDR: 1.000e+00
Coefficient: −5.96e−15
Value: C
Chitinilyticum
1.00
0.75
0.50
0.25
0.00
A (n=15)
B (n=16)
C (n=14)
surface

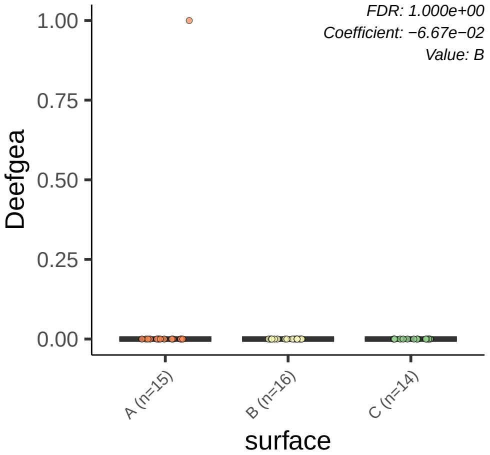
FDR: 1.000e+00
Coefficient: −6.67e−02
Value: B
Deefgea
1.00
0.75
0.50
0.25
0.00
A (n=15)
B (n=16)
C (n=14)
surface

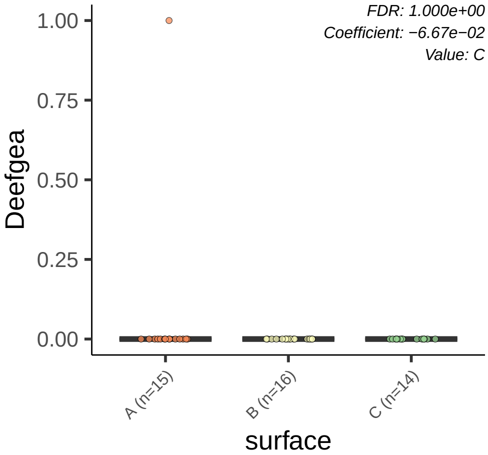
FDR: 1.000e+00
Coefficient: −6.67e−02
Value: C
Deefgea
1.00
0.75
0.50
0.25
0.00
A (n=15)
B (n=16)
C (n=14)
surface

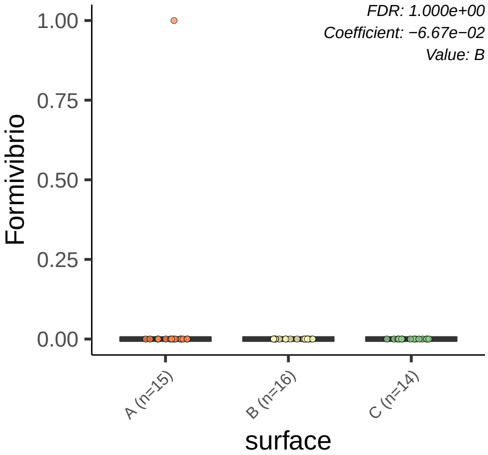
FDR: 1.000e+00
Coefficient: −6.67e−02
Value: B
Formivibrio
1.00
0.75
0.50
0.25
0.00
A (n=15)
B (n=16)
C (n=14)
surface

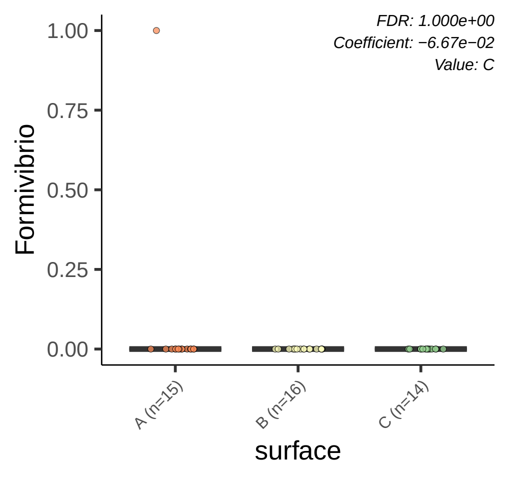
FDR: 1.000e+00
Coefficient: −6.67e−02
Value: C
Formivibrio
1.00
0.75
0.50
0.25
0.00
A (n=15)
B (n=16)
C (n=14)
surface

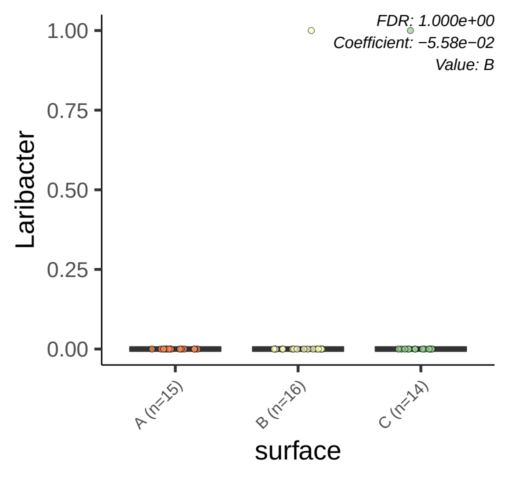

FDR: 1.000e+00
Coefficient: −5.58e−02
Value: B
Laribacter
1.00
0.75
0.50
0.25
0.00
A (n=15)
B (n=16)
C (n=14)
surface

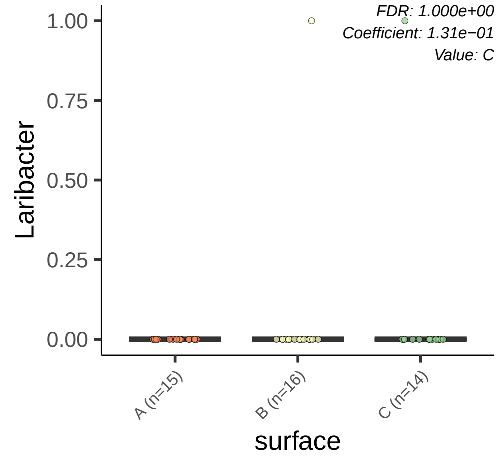

FDR: 1.000e+00
Coefficient: 1.31e−01
Value: C
Laribacter
1.00
0.75
0.50
0.25
0.00
A (n=15)
B (n=16)
C (n=14)
surface

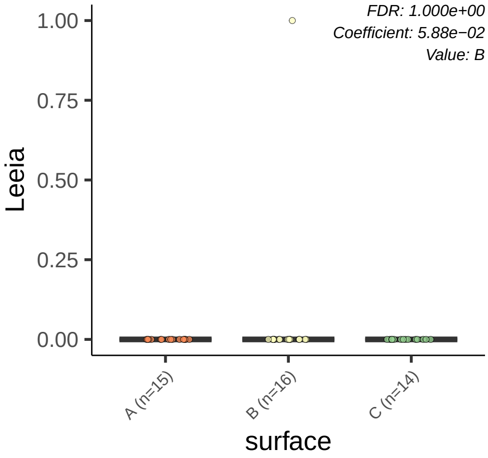
FDR: 1.000e+00
Coefficient: 5.88e−02
Value: B
Leeia
1.00
0.75
0.50
0.25
0.00
A (n=15)
B (n=16)
C (n=14)
surface

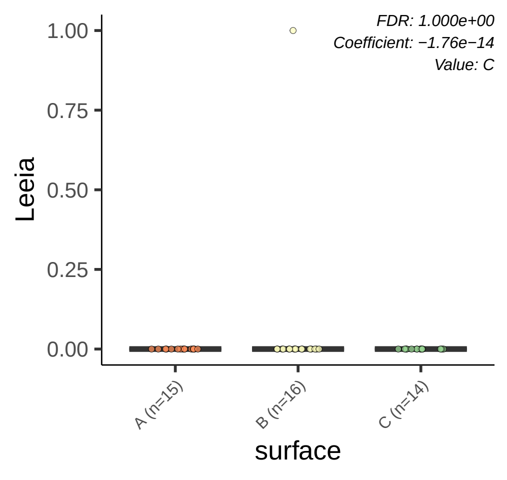
FDR: 1.000e+00
Coefficient: −1.76e−14
Value: C
Leeia
1.00
0.75
0.50
0.25
0.00
A (n=15)
B (n=16)
C (n=14)
surface

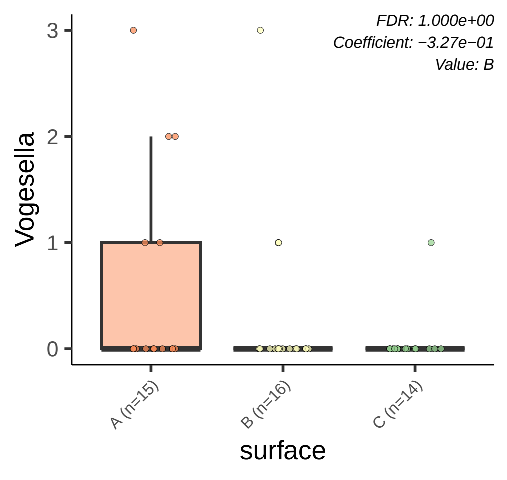
FDR: 1.000e+00
Coefficient: −3.27e−01
Value: B
Vogesella
0
1
2
3
A (n=15)
B (n=16)
C (n=14)
surface

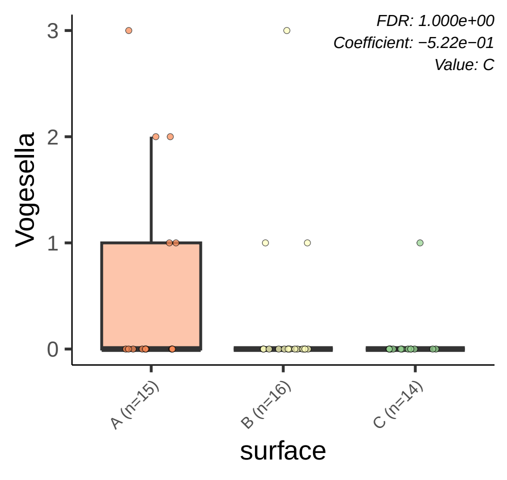
FDR: 1.000e+00
Coefficient: −5.22e−01
Value: C
Vogesella
3
2
1
0
A (n=15)
B (n=16)
C (n=14)
surface

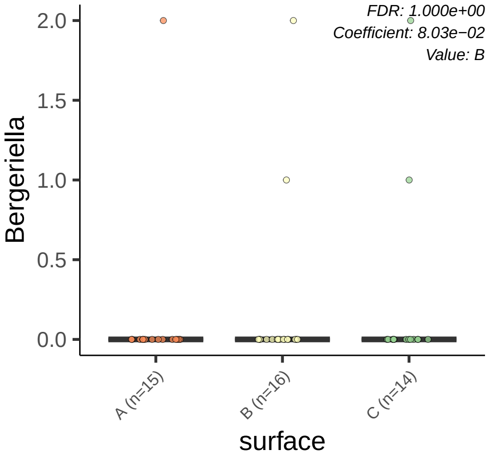

FDR: 1.000e+00
Coefficient: 8.03e−02
Value: B
Bergeriella
2.0
1.5
1.0
0.5
0.0
A (n=15)
B (n=16)
C (n=14)
surface

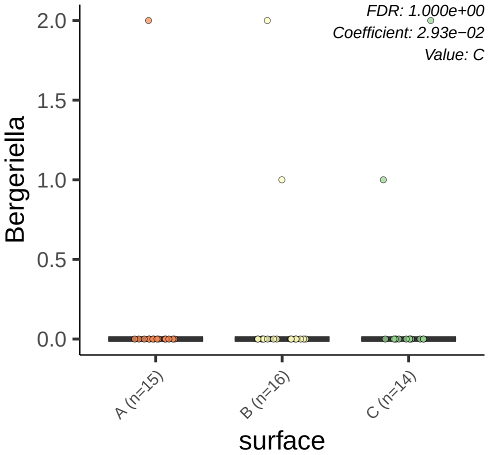
FDR: 1.000e+00
Coefficient: 2.93e−02
Value: C
Bergeriella
2.0
1.5
1.0
0.5
0.0
A (n=15)
B (n=16)
C (n=14)
surface

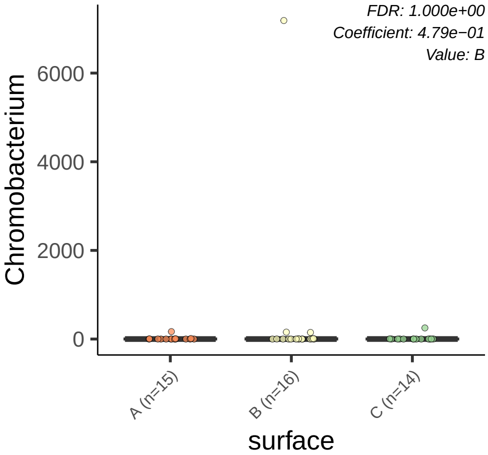

FDR: 1.000e+00
Coefficient: 4.79e−01
Value: B
Chromobacterium
6000
4000
2000
0
A (n=15)
B (n=16)
C (n=14)
surface

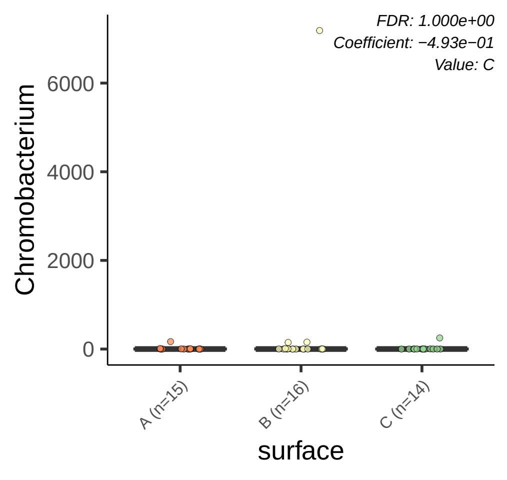

FDR: 1.000e+00
Coefficient: −4.93e−01
Value: C
Chromobacterium
6000
4000
2000
0
A (n=15)
B (n=16)
C (n=14)
surface

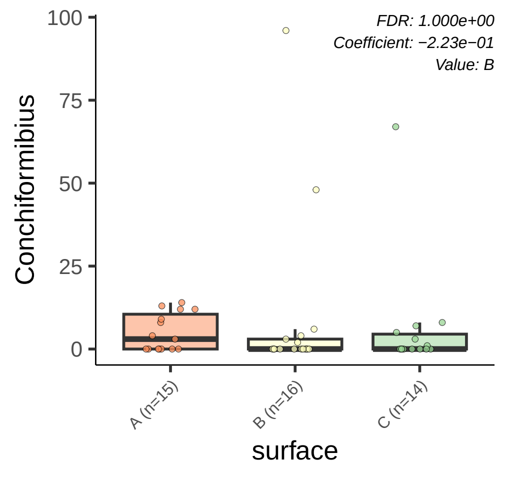
FDR: 1.000e+00
Coefficient: −2.23e−01
Value: B
Conchiformibius
100
75
50
25
0
A (n=15)
B (n=16)
C (n=14)
surface

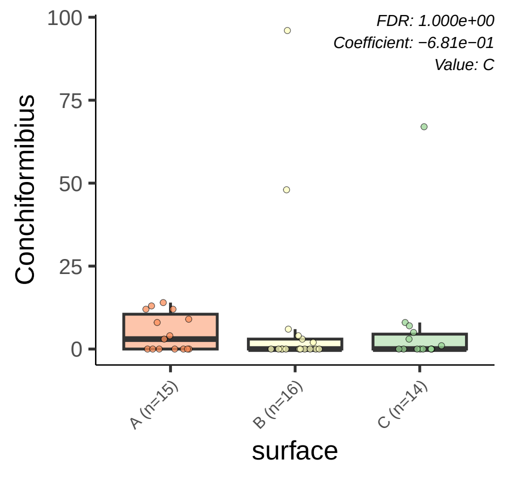
FDR: 1.000e+00
Coefficient: −6.81e−01
Value: C
100
75
50
25
0
Conchiformibius
A (n=15)
B (n=16)
C (n=14)
surface

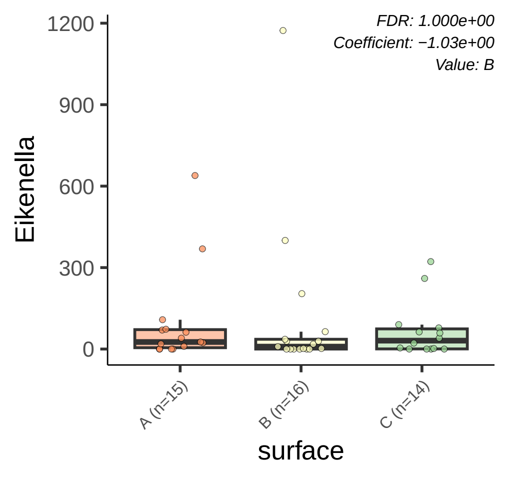
FDR: 1.000e+00
Coefficient: −1.03e+00
Value: B
Eikenella
1200
900
600
300
0
A (n=15)
B (n=16)
C (n=14)
surface

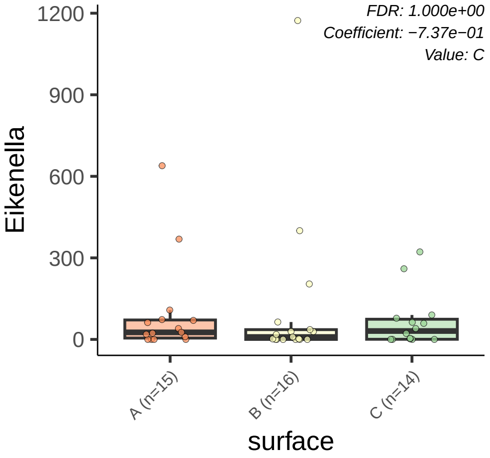
FDR: 1.000e+00
Coefficient: −7.37e−01
Value: C
Eikenella
1200
900
600
300
0
A (n=15)
B (n=16)
C (n=14)
surface

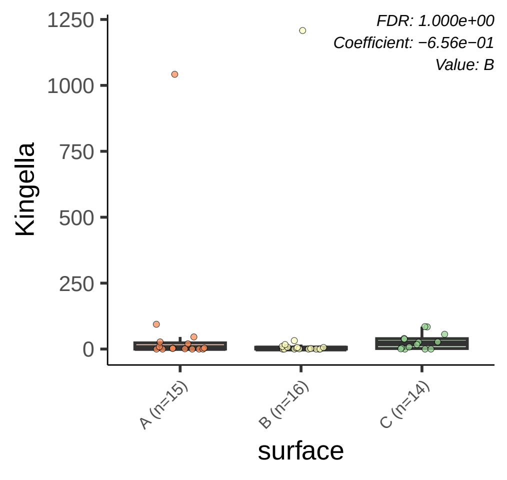
FDR: 1.000e+00
Coefficient: −6.56e−01
Value: B
Kingella
1250
1000
750
500
250
0
A (n=15)
B (n=16)
C (n=14)
surface

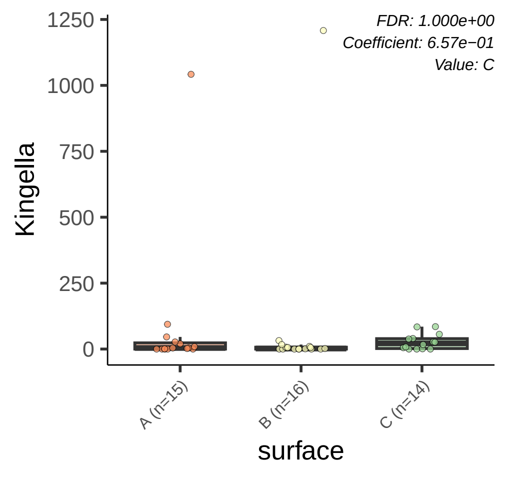
FDR: 1.000e+00
Coefficient: 6.57e−01
Value: C
Kingella
1250
1000
750
500
250
0
A (n=15)
B (n=16)
C (n=14)
surface

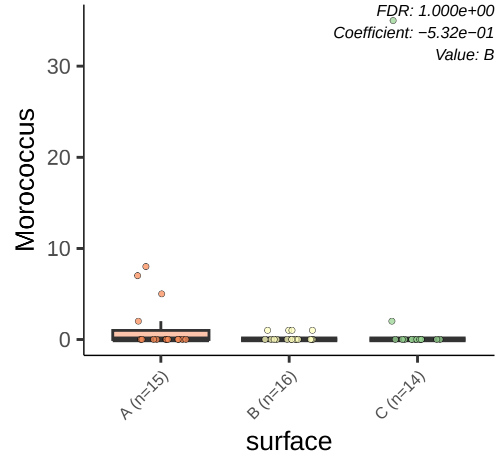
FDR: 1.000e+00
Coefficient: −5.32e−01
Value: B
Morococcus
30
20
10
0
A (n=15)
B (n=16)
C (n=14)
surface

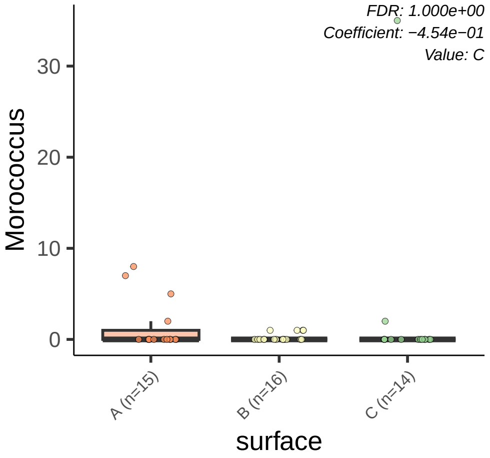
FDR: 1.000e+00
Coefficient: −4.54e−01
Value: C
Morococcus
0
10
20
30
A (n=15)
B (n=16)
C (n=14)
surface

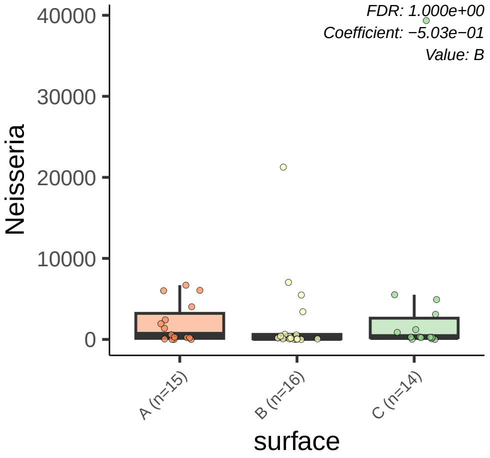
FDR: 1.000e+00
Coefficient: −5.03e−01
Value: B
Neisseria
40000
30000
20000
10000
0
A (n=15)
B (n=16)
C (n=14)
surface

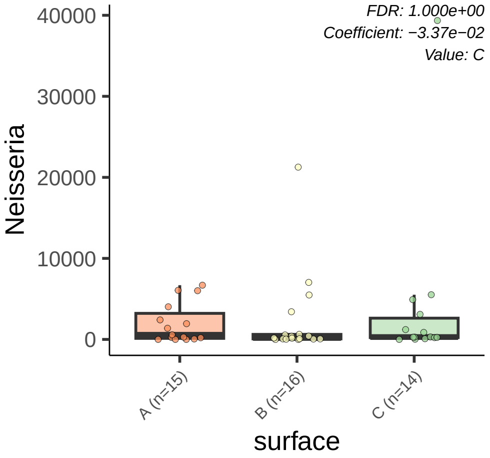
FDR: 1.000e+00
Coefficient: −3.37e−02
Value: C
Neisseria
40000
30000
20000
10000
0
A (n=15)
B (n=16)
C (n=14)
surface

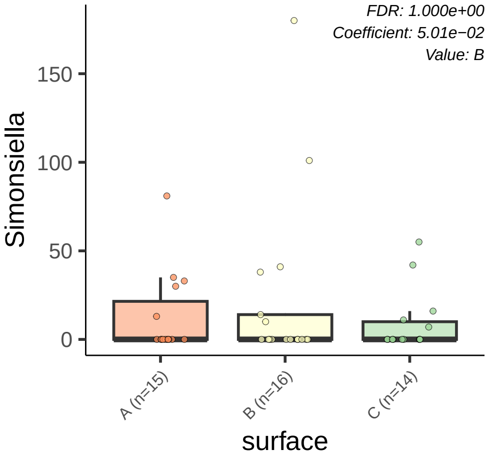
FDR: 1.000e+00
Coefficient: 5.01e−02
Value: B
Simonsiella
150
100
50
0
A (n=15)
B (n=16)
C (n=14)
surface

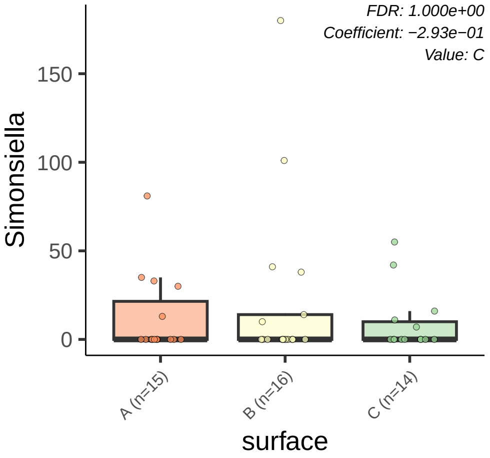
FDR: 1.000e+00
Coefficient: −2.93e−01
Value: C
Simonsiella
150
100
50
0
A (n=15)
B (n=16)
C (n=14)
surface

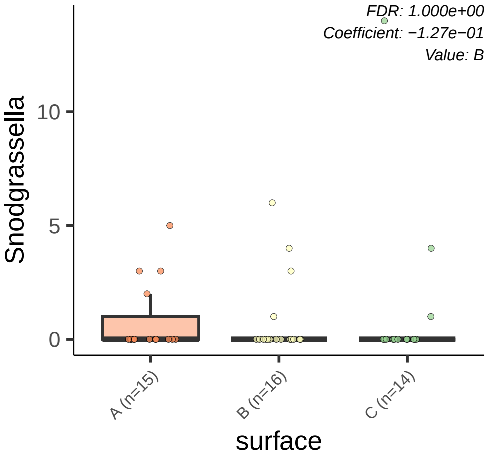
FDR: 1.000e+00
Coefficient: −1.27e−01
Value: B
Snodgrassella
0
5
10
A (n=15)
B (n=16)
C (n=14)
surface

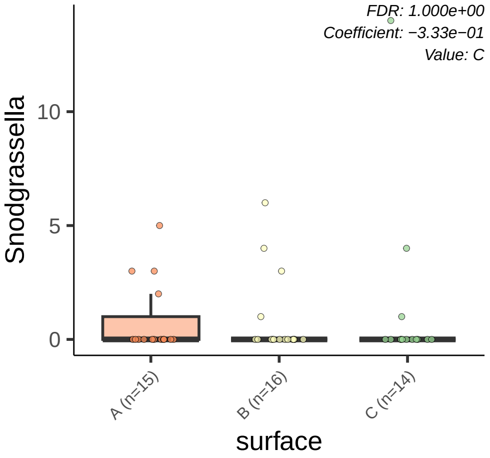

FDR: 1.000e+00
Coefficient: −3.33e−01
Value: C
Snodgrassella
10
5
0
A (n=15)
B (n=16)
C (n=14)
surface

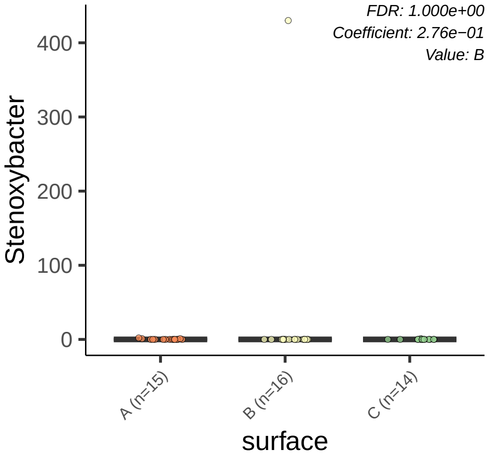
FDR: 1.000e+00
Coefficient: 2.76e−01
Value: B
Stenoxybacter
400
300
200
100
0
A (n=15)
B (n=16)
C (n=14)
surface

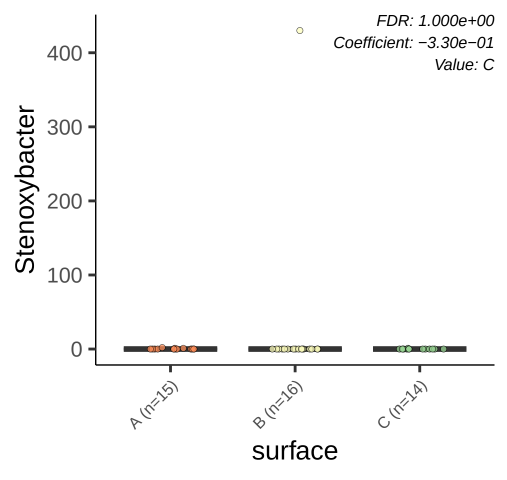
FDR: 1.000e+00
Coefficient: −3.30e−01
Value: C
Stenoxybacter
400
300
200
100
0
A (n=15)
B (n=16)
C (n=14)
surface

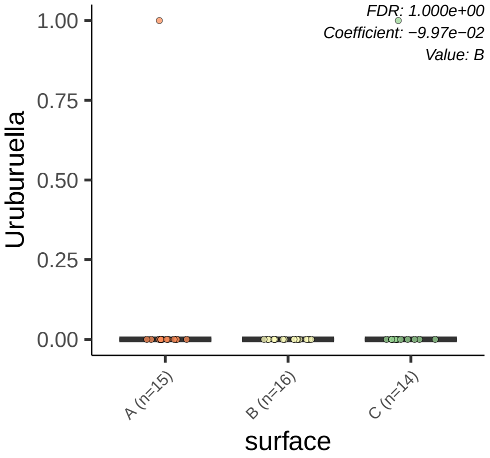

FDR: 1.000e+00
Coefficient: −9.97e−02
Value: B
Uruburuella
1.00
0.75
0.50
0.25
0.00
A (n=15)
B (n=16)
C (n=14)
surface

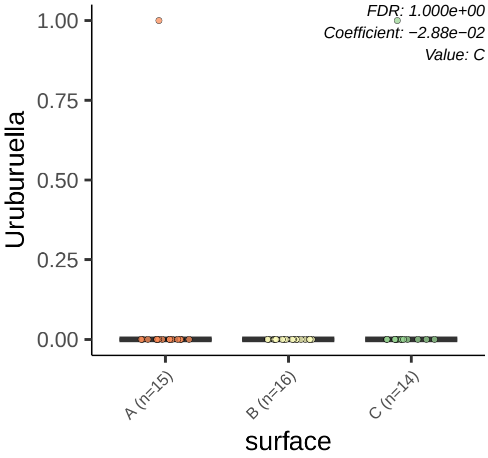
FDR: 1.000e+00
Coefficient: −2.88e−02
Value: C
Uruburuella
1.00
0.75
0.50
0.25
0.00
A (n=15)
B (n=16)
C (n=14)
surface

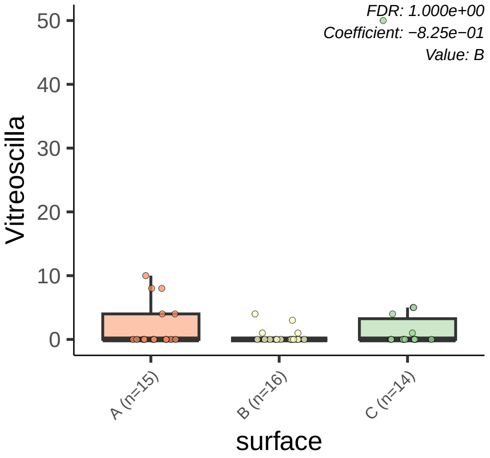

FDR: 1.000e+00
Coefficient: −8.25e−01
Value: B
Vitreoscilla
50
40
30
20
10
0
A (n=15)
B (n=16)
C (n=14)
surface

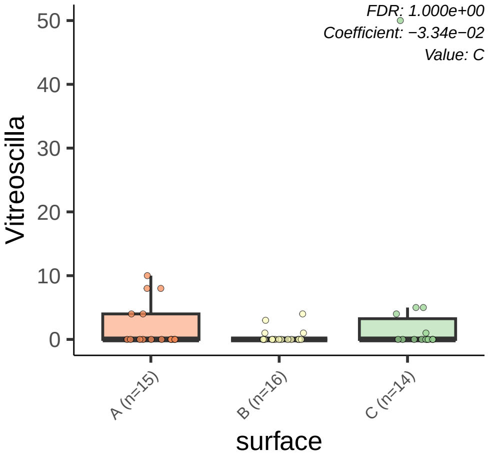

FDR: 1.000e+00
Coefficient: −3.34e−02
Value: C
Vitreoscilla
50
40
30
20
10
0
A (n=15)
B (n=16)
C (n=14)
surface

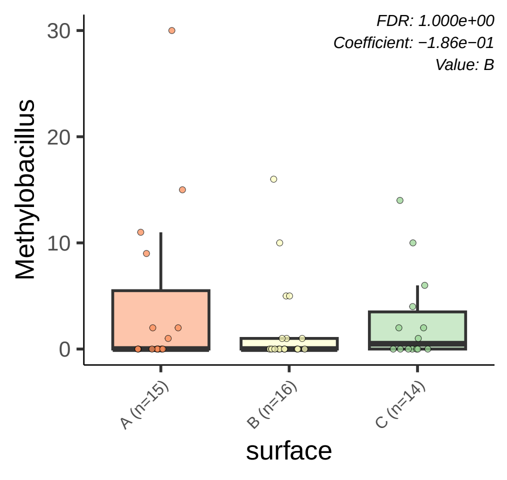

FDR: 1.000e+00
Coefficient: −1.86e−01
Value: B
Methylobacillus
30
20
10
0
A (n=15)
B (n=16)
C (n=14)
surface

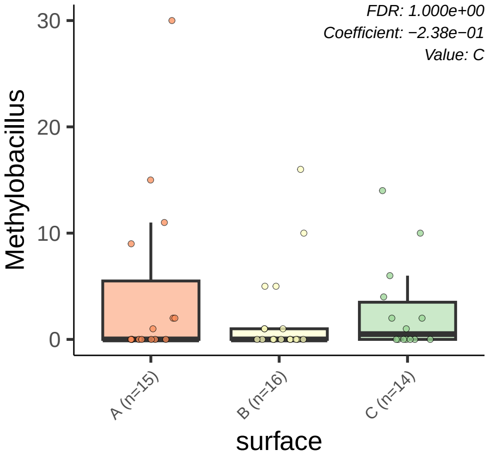
FDR: 1.000e+00
Coefficient: −2.38e−01
Value: C
Methylobacillus
30
20
10
0
A (n=15)
B (n=16)
C (n=14)
surface

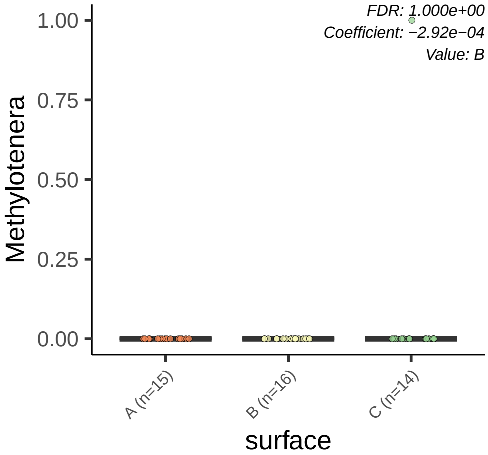

FDR: 1.000e+00
Coefficient: −2.92e−04
Value: B
Methylotenera
1.00
0.75
0.50
0.25
0.00
A (n=15)
B (n=16)
C (n=14)
surface

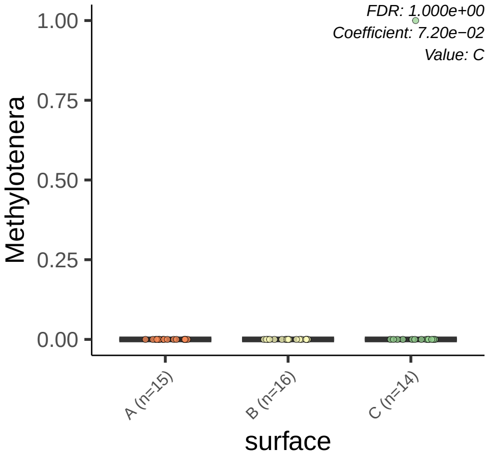

FDR: 1.000e+00
Coefficient: 7.20e−02
Value: C
Methylotenera
1.00
0.75
0.50
0.25
0.00
A (n=15)
B (n=16)
C (n=14)
surface

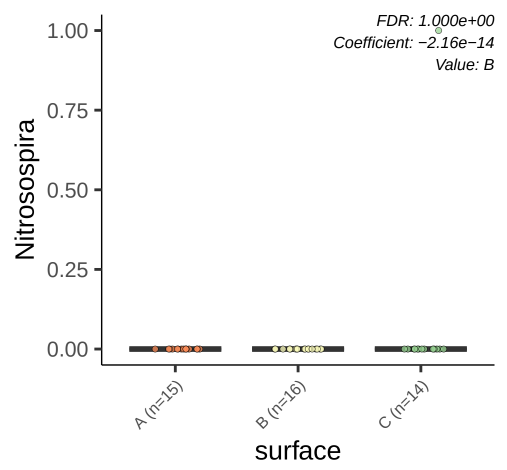

FDR: 1.000e+00
Coefficient: −2.16e−14
Value: B
Nitrosospira
1.00
0.75
0.50
0.25
0.00
A (n=15)
B (n=16)
C (n=14)
surface

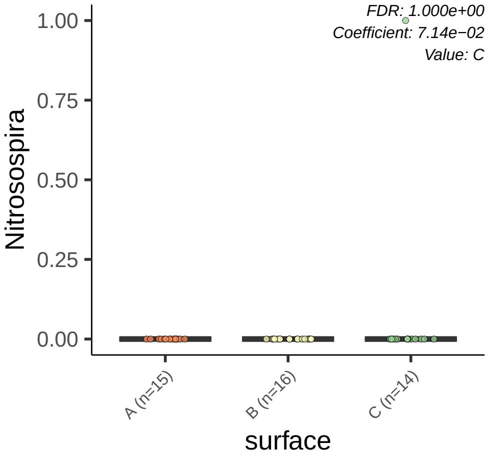
FDR: 1.000e+00
Coefficient: 7.14e−02
Value: C
Nitrosospira
1.00
0.75
0.50
0.25
0.00
A (n=15)
B (n=16)
C (n=14)
surface

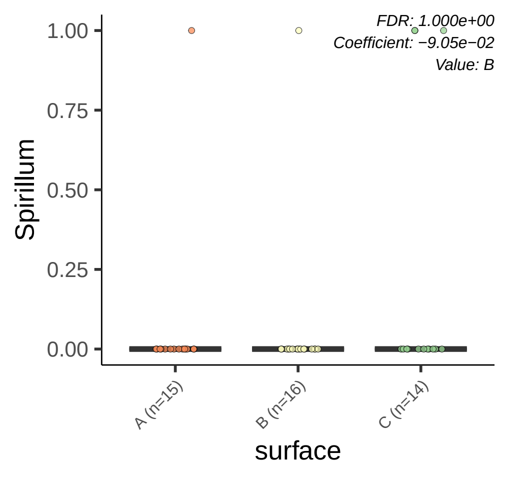

FDR: 1.000e+00
Coefficient: −9.05e−02
Value: B
1.00
0.75
0.50
0.25
0.00
Spirillum
A (n=15)
B (n=16)
C (n=14)
surface

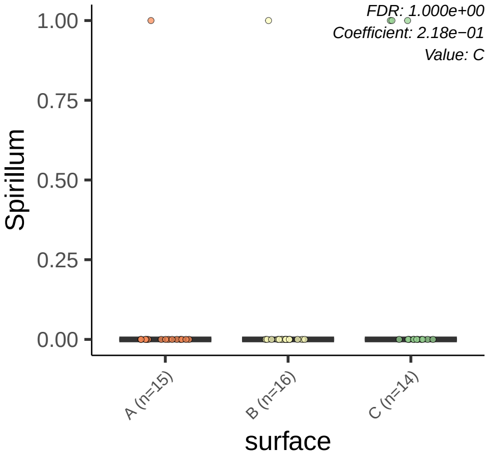

FDR: 1.000e+00
Coefficient: 2.18e−01
Value: C
1.00
0.75
0.50
0.25
0.00
Spirillum
A (n=15)
B (n=16)
C (n=14)
surface

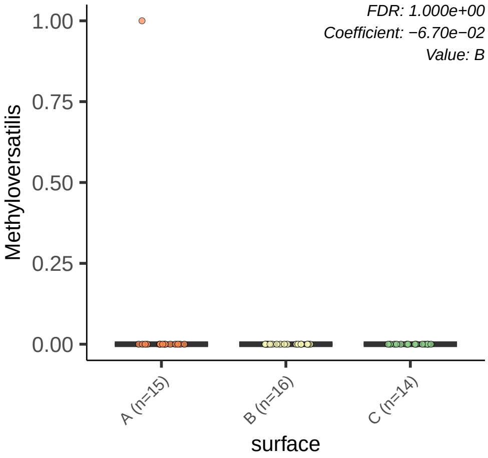
FDR: 1.000e+00
Coefficient: −6.70e−02
Value: B
Methyloversatilis
1.00
0.75
0.50
0.25
0.00
A (n=15)
B (n=16)
C (n=14)
surface

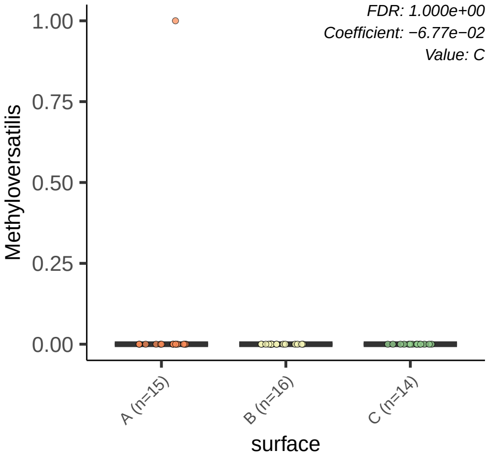

FDR: 1.000e+00
Coefficient: −6.77e−02
Value: C
Methyloversatilis
1.00
0.75
0.50
0.25
0.00
A (n=15)
B (n=16)
C (n=14)
surface

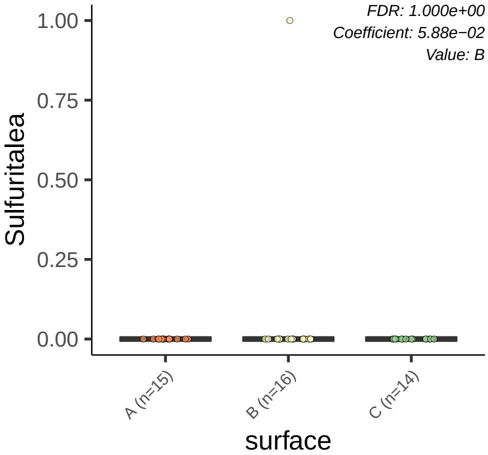
FDR: 1.000e+00
Coefficient: 5.88e−02
Value: B
Sulfuritalea
1.00
0.75
0.50
0.25
0.00
A (n=15)
B (n=16)
C (n=14)
surface

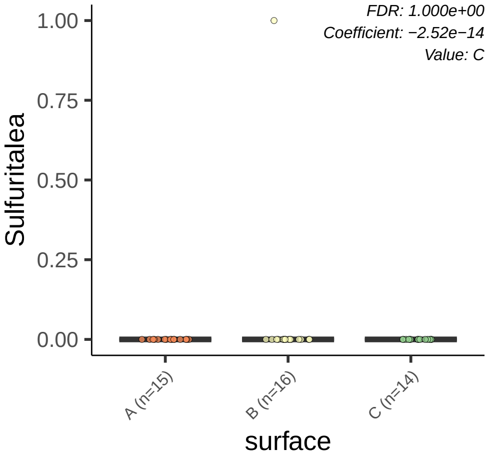

FDR: 1.000e+00
Coefficient: −2.52e−14
Value: C
Sulfuritalea
1.00
0.75
0.50
0.25
0.00
A (n=15)
B (n=16)
C (n=14)
surface

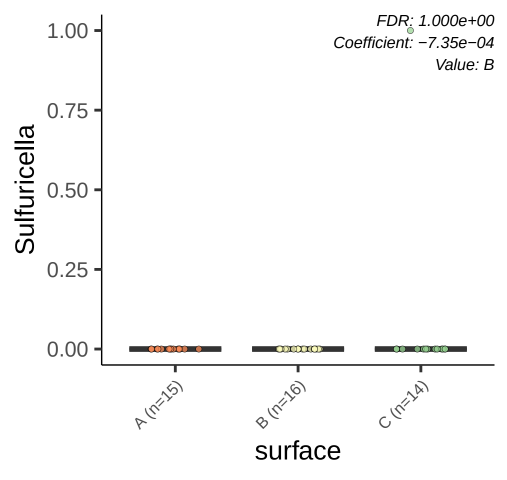
FDR: 1.000e+00
Coefficient: −7.35e−04
Value: B
Sulfuricella
1.00
0.75
0.50
0.25
0.00
A (n=15)
B (n=16)
C (n=14)
surface

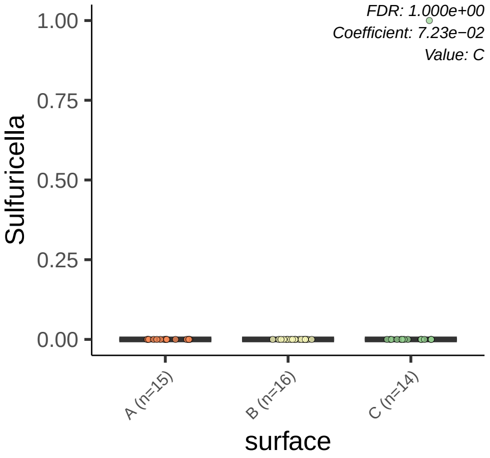

FDR: 1.000e+00
Coefficient: 7.23e−02
Value: C
Sulfuricella
1.00
0.75
0.50
0.25
0.00
A (n=15)
B (n=16)
C (n=14)
surface

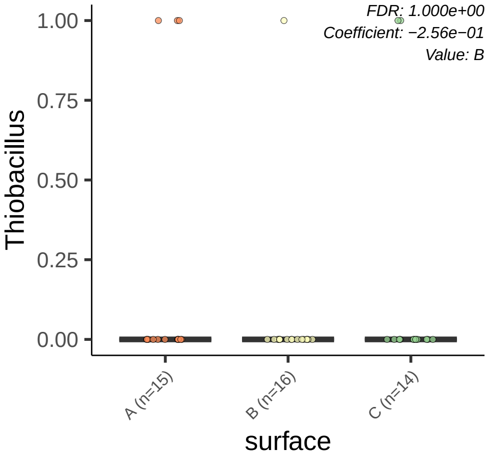
FDR: 1.000e+00
Coefficient: −2.56e−01
Value: B
Thiobacillus
1.00
0.75
0.50
0.25
0.00
A (n=15)
B (n=16)
C (n=14)
surface

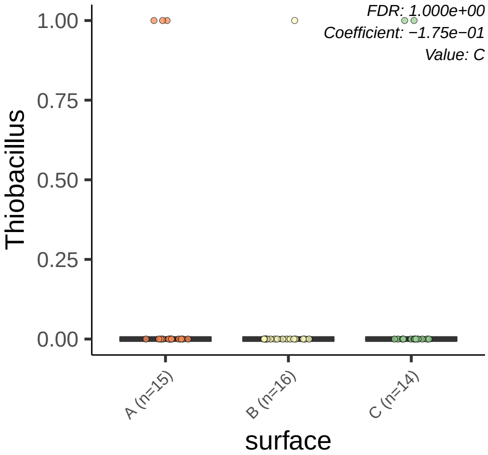
FDR: 1.000e+00
Coefficient: −1.75e−01
Value: C
Thiobacillus
1.00
0.75
0.50
0.25
0.00
A (n=15)
B (n=16)
C (n=14)
surface

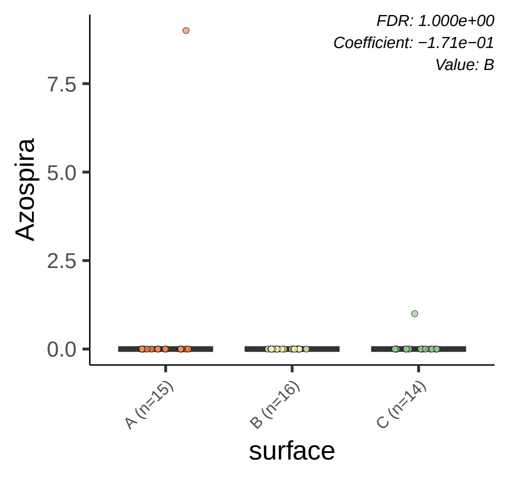

FDR: 1.000e+00
Coefficient: −1.71e−01
Value: B
Azospira
7.5
5.0
2.5
0.0
A (n=15)
B (n=16)
C (n=14)
surface

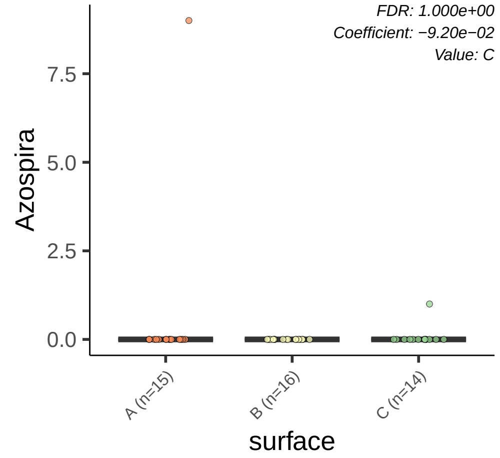
FDR: 1.000e+00
Coefficient: −9.20e−02
Value: C
Azospira
7.5
5.0
2.5
0.0
A (n=15)
B (n=16)
C (n=14)
surface

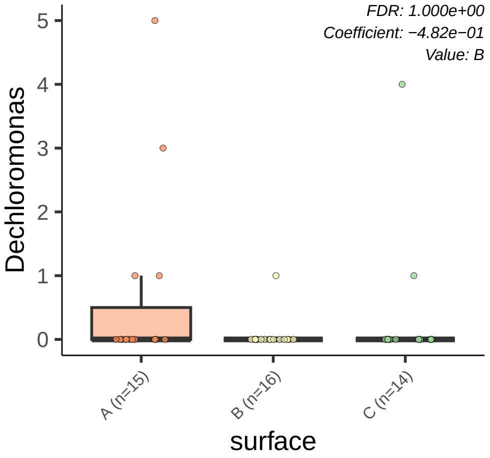
FDR: 1.000e+00
Coefficient: −4.82e−01
Value: B
Dechloromonas
0
1
2
3
4
5
A (n=15)
B (n=16)
C (n=14)
surface

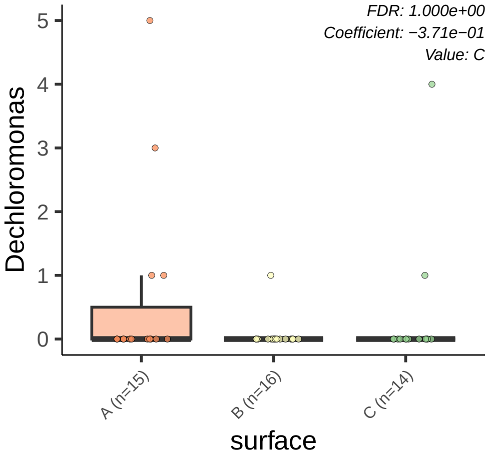
FDR: 1.000e+00
Coefficient: −3.71e−01
Value: C
Dechloromonas
0
1
2
3
4
5
A (n=15)
B (n=16)
C (n=14)
surface

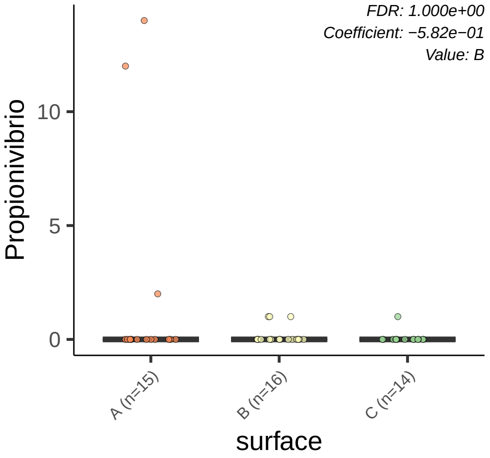

FDR: 1.000e+00
Coefficient: −5.82e−01
Value: B
Propionivibrio
0
5
10
A (n=15)
B (n=16)
C (n=14)
surface

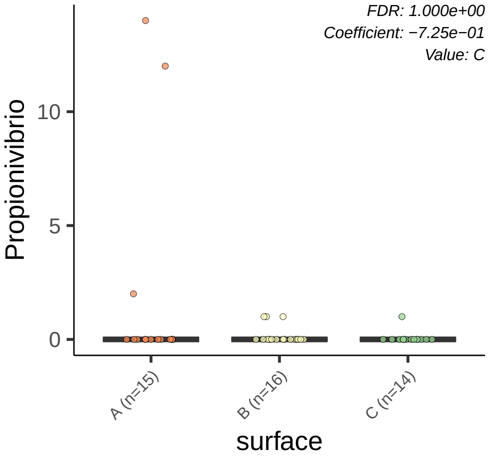
FDR: 1.000e+00
Coefficient: −7.25e−01
Value: C
Propionivibrio
10
5
0
A (n=15)
B (n=16)
C (n=14)
surface

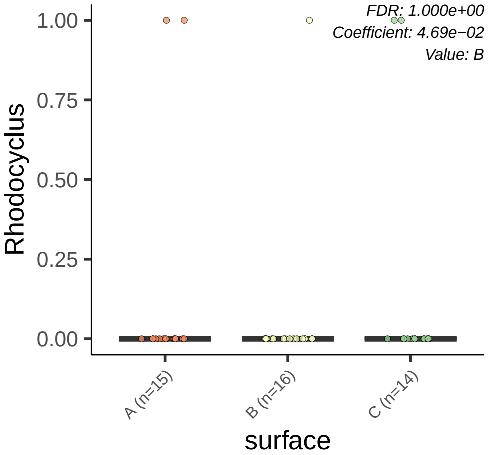
FDR: 1.000e+00
Coefficient: 4.69e−02
Value: B
1.00
0.75
0.50
0.25
0.00
Rhodocyclus
A (n=15)
B (n=16)
C (n=14)
surface

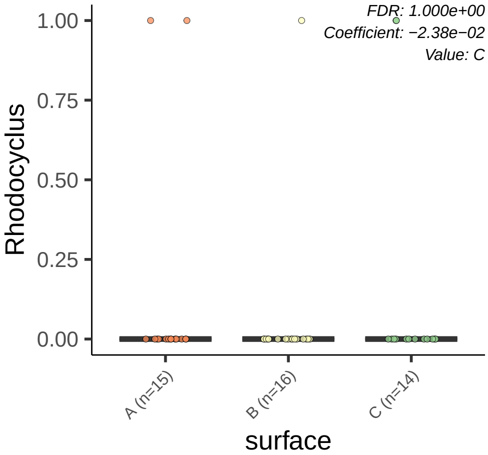

FDR: 1.000e+00
Coefficient: −2.38e−02
Value: C
Rhodocyclus
1.00
0.75
0.50
0.25
0.00
A (n=15)
B (n=16)
C (n=14)
surface

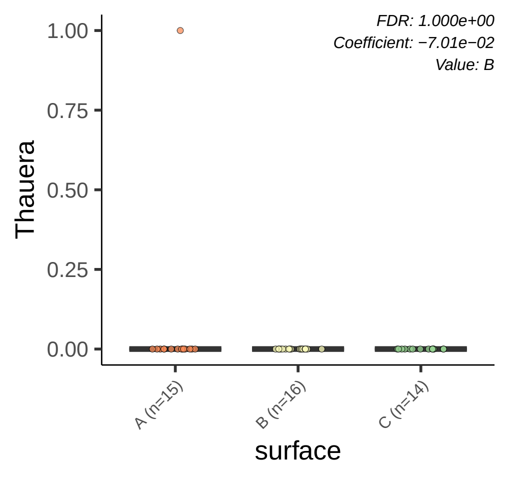
FDR: 1.000e+00
Coefficient: −7.01e−02
Value: B
Thauera
1.00
0.75
0.50
0.25
0.00
A (n=15)
B (n=16)
C (n=14)
surface

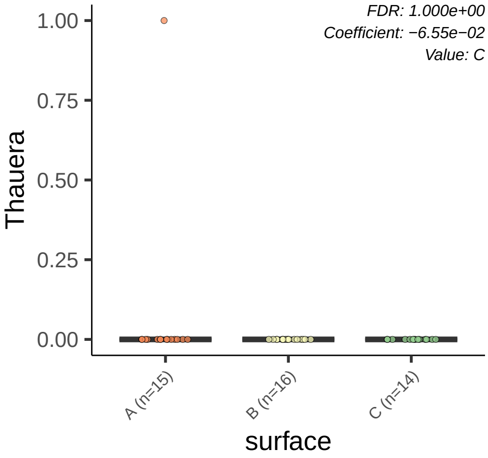
FDR: 1.000e+00
Coefficient: −6.55e−02
Value: C
Thauera
1.00
0.75
0.50
0.25
0.00
A (n=15)
B (n=16)
C (n=14)
surface

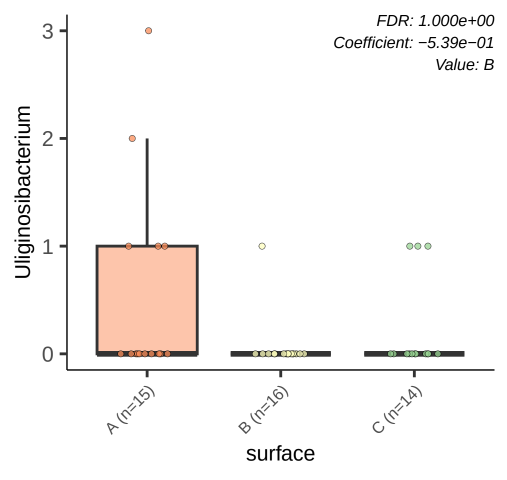

FDR: 1.000e+00
Coefficient: −5.39e−01
Value: B
Uliginosibacterium
3
2
1
0
A (n=15)
B (n=16)
C (n=14)
surface

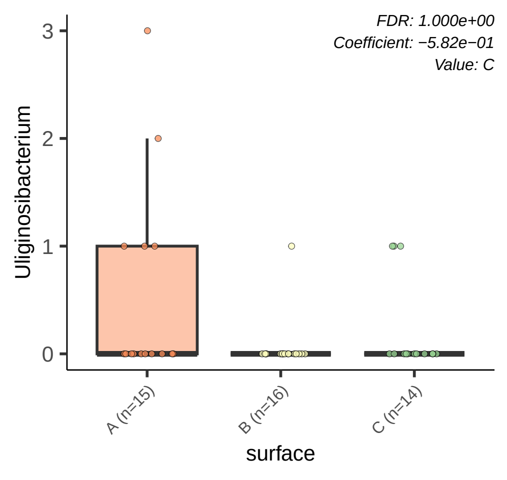
FDR: 1.000e+00
Coefficient: −5.82e−01
Value: C
Uliginosibacterium
3
2
1
0
A (n=15)
B (n=16)
C (n=14)
surface

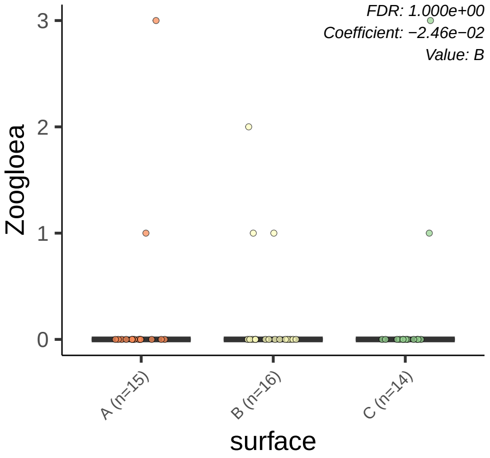

FDR: 1.000e+00
Coefficient: −2.46e−02
Value: B
Zoogloea
3
2
1
0
A (n=15)
B (n=16)
C (n=14)
surface

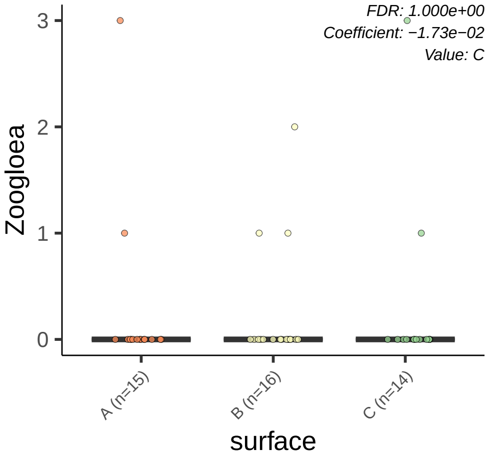

FDR: 1.000e+00
Coefficient: −1.73e−02
Value: C
Zoogloea
0
1
2
3
A (n=15)
B (n=16)
C (n=14)
surface

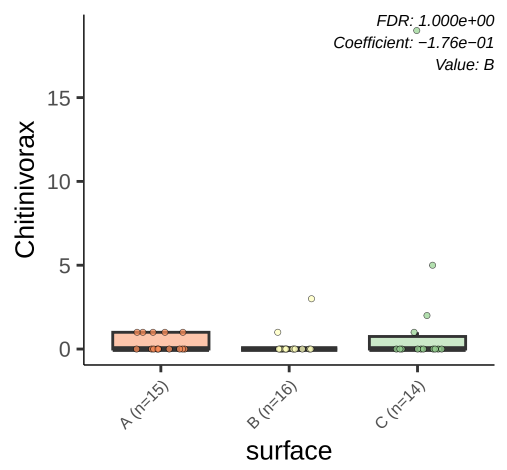
FDR: 1.000e+00
Coefficient: −1.76e−01
Value: B
Chitinivorax
15
10
5
0
A (n=15)
B (n=16)
C (n=14)
surface

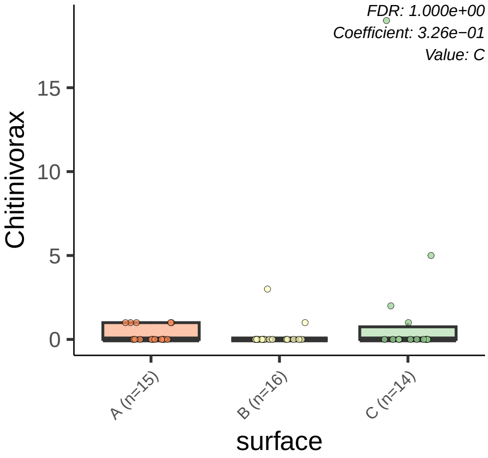
FDR: 1.000e+00
Coefficient: 3.26e−01
Value: C
Chitinivorax
15
10
5
0
A (n=15)
B (n=16)
C (n=14)
surface

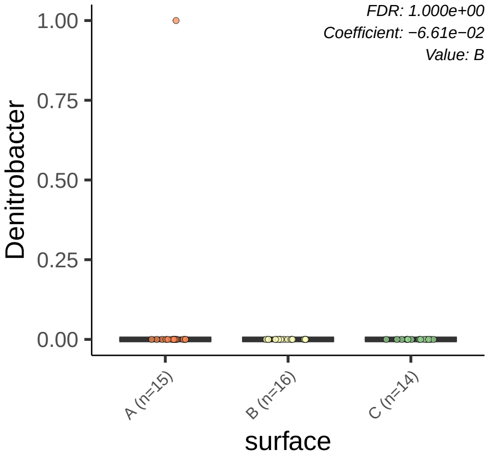

FDR: 1.000e+00
Coefficient: −6.61e−02
Value: B
Denitrobacter
1.00
0.75
0.50
0.25
0.00
A (n=15)
B (n=16)
C (n=14)
surface

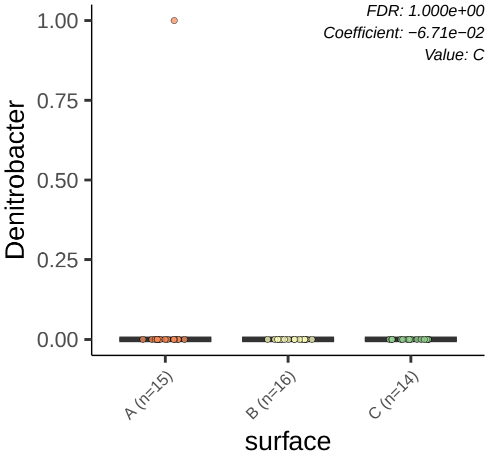
FDR: 1.000e+00
Coefficient: −6.71e−02
Value: C
Denitrobacter
1.00
0.75
0.50
0.25
0.00
A (n=15)
B (n=16)
C (n=14)
surface

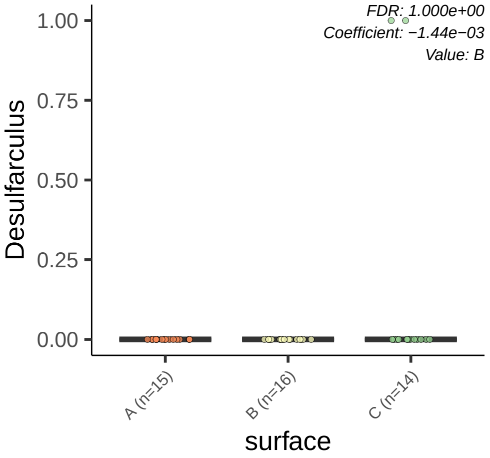

FDR: 1.000e+00
Coefficient: −1.44e−03
Value: B
Desulfarculus
1.00
0.75
0.50
0.25
0.00
A (n=15)
B (n=16)
C (n=14)
surface

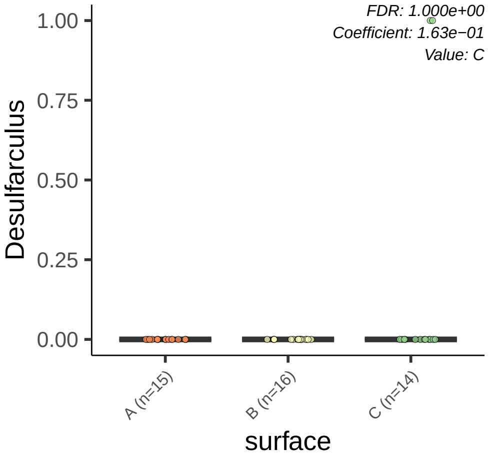
FDR: 1.000e+00
Coefficient: 1.63e−01
Value: C
Desulfarculus
1.00
0.75
0.50
0.25
0.00
A (n=15)
B (n=16)
C (n=14)
surface

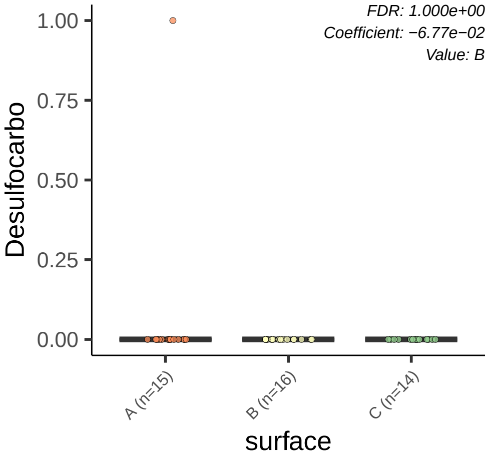
FDR: 1.000e+00
Coefficient: −6.77e−02
Value: B
Desulfocarbo
1.00
0.75
0.50
0.25
0.00
A (n=15)
B (n=16)
C (n=14)
surface

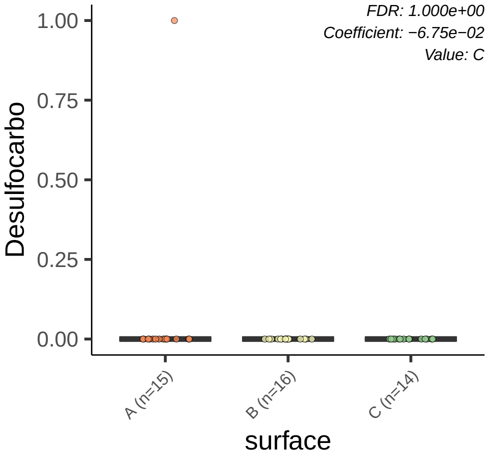
FDR: 1.000e+00
Coefficient: −6.75e−02
Value: C
Desulfocarbo
1.00
0.75
0.50
0.25
0.00
A (n=15)
B (n=16)
C (n=14)
surface

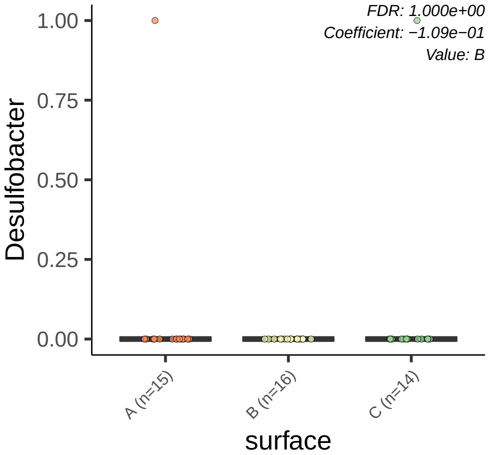
FDR: 1.000e+00
Coefficient: −1.09e−01
Value: B
Desulfobacter
1.00
0.75
0.50
0.25
0.00
A (n=15)
B (n=16)
C (n=14)
surface

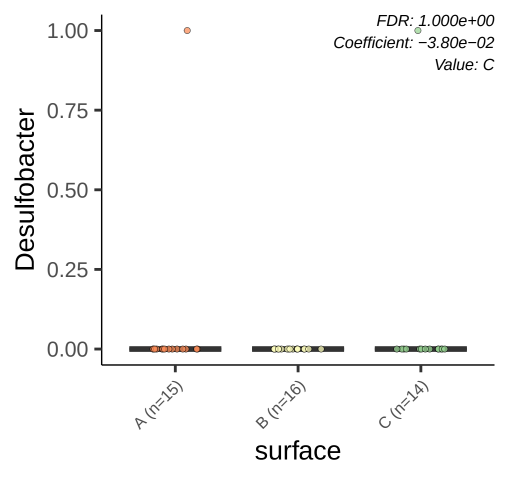
FDR: 1.000e+00
Coefficient: −3.80e−02
Value: C
Desulfobacter
1.00
0.75
0.50
0.25
0.00
A (n=15)
B (n=16)
C (n=14)
surface

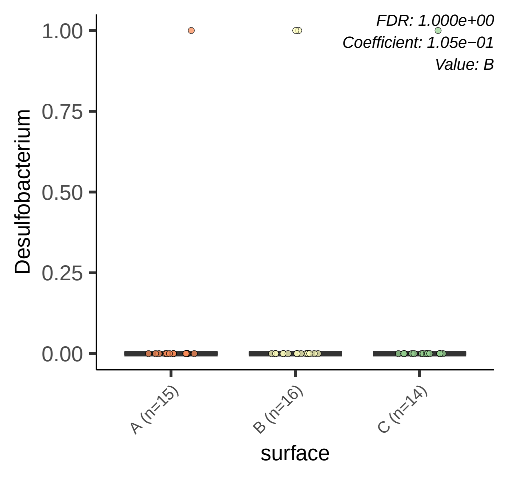
FDR: 1.000e+00
Coefficient: 1.05e−01
Value: B
Desulfobacterium
1.00
0.75
0.50
0.25
0.00
A (n=15)
B (n=16)
C (n=14)
surface

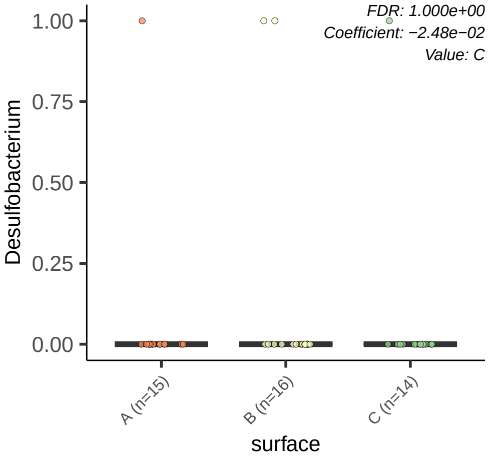

FDR: 1.000e+00
Coefficient: −2.48e−02
Value: C
Desulfobacterium
1.00
0.75
0.50
0.25
0.00
A (n=15)
B (n=16)
C (n=14)
surface

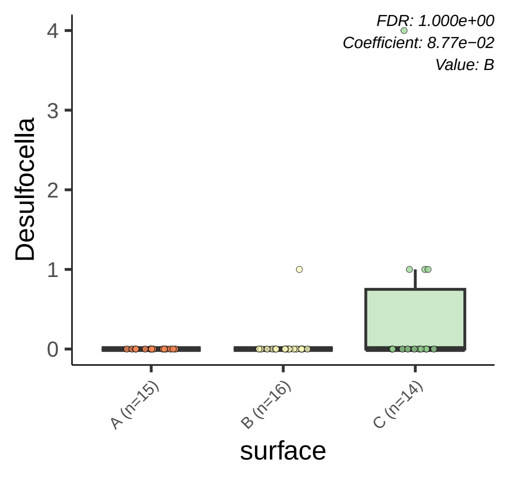
FDR: 1.000e+00
Coefficient: 8.77e−02
Value: B
Desulfocella
4
3
2
1
0
A (n=15)
B (n=16)
C (n=14)
surface

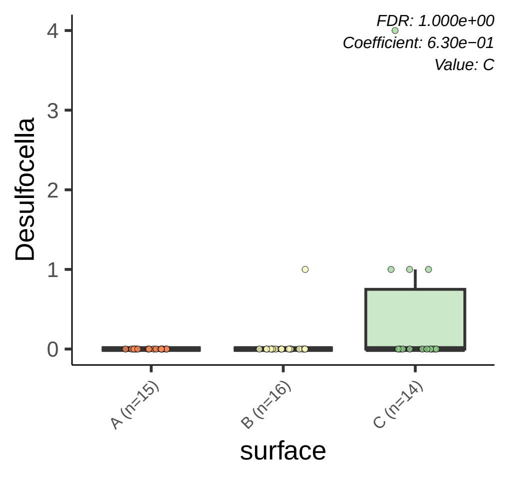

FDR: 1.000e+00
Coefficient: 6.30e−01
Value: C
Desulfocella
4
3
2
1
0
A (n=15)
B (n=16)
C (n=14)
surface

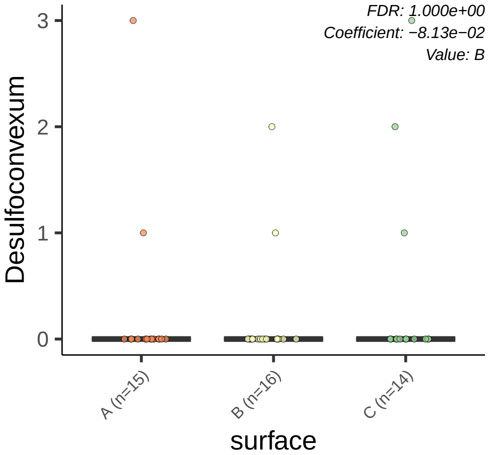
FDR: 1.000e+00
Coefficient: −8.13e−02
Value: B
Desulfoconvexum
3
2
1
0
A (n=15)
B (n=16)
C (n=14)
surface

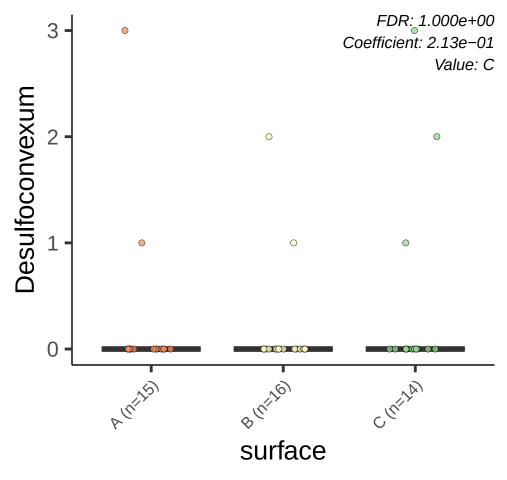
FDR: 1.000e+00
Coefficient: 2.13e−01
Value: C
Desulfoconvexum
0
1
2
3
A (n=15)
B (n=16)
C (n=14)
surface

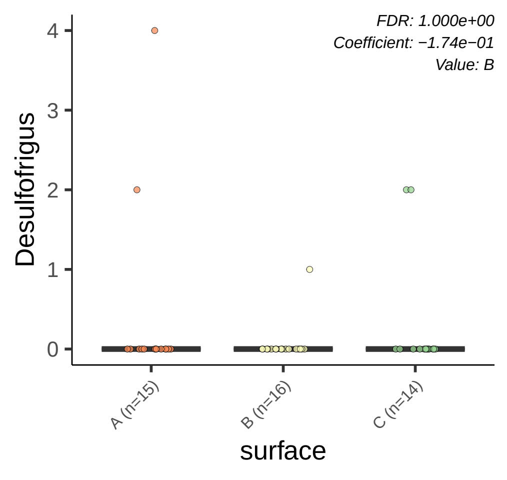
FDR: 1.000e+00
Coefficient: −1.74e−01
Value: B
Desulfofrigus
0
1
2
3
4
A (n=15)
B (n=16)
C (n=14)
surface

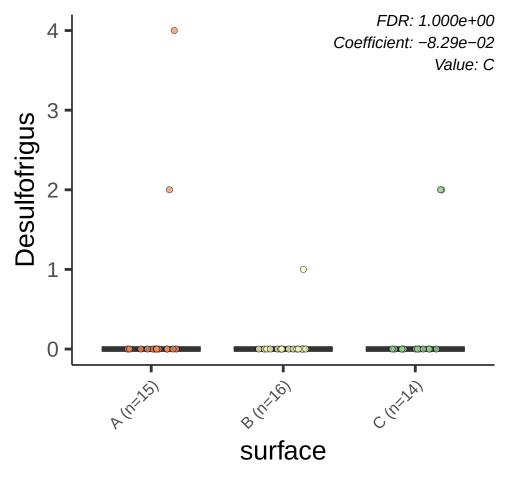
FDR: 1.000e+00
Coefficient: −8.29e−02
Value: C
Desulfofrigus
4
3
2
1
0
A (n=15)
B (n=16)
C (n=14)
surface

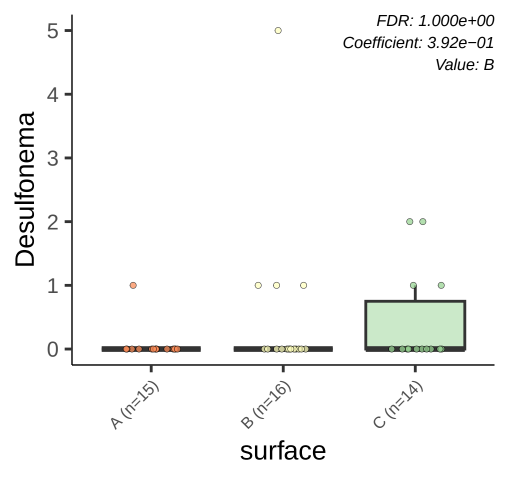
FDR: 1.000e+00
Coefficient: 3.92e−01
Value: B
Desulfonema
5
4
3
2
1
0
A (n=15)
B (n=16)
C (n=14)
surface

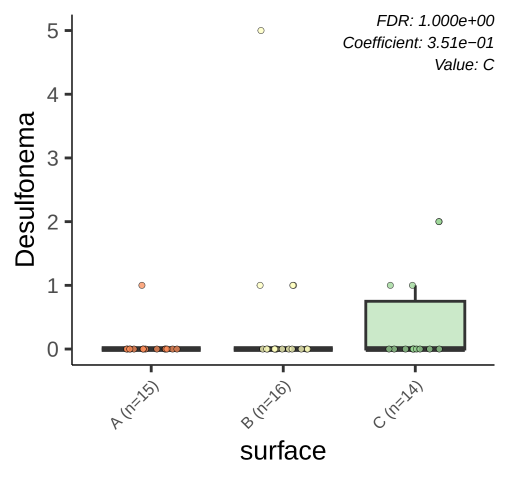
FDR: 1.000e+00
Coefficient: 3.51e−01
Value: C
Desulfonema
0
1
2
3
4
5
A (n=15)
B (n=16)
C (n=14)
surface

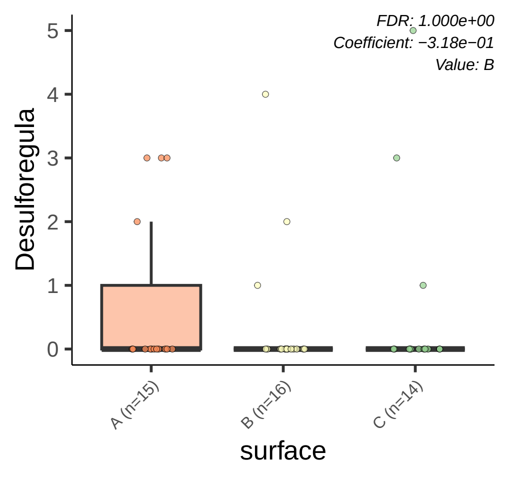

FDR: 1.000e+00
Coefficient: −3.18e−01
Value: B
Desulforegula
0
1
2
3
4
5
A (n=15)
B (n=16)
C (n=14)
surface

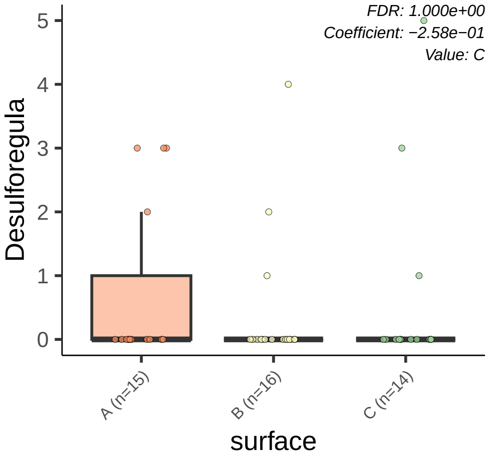

FDR: 1.000e+00
Coefficient: −2.58e−01
Value: C
Desulforegula
0
1
2
3
4
5
A (n=15)
B (n=16)
C (n=14)
surface

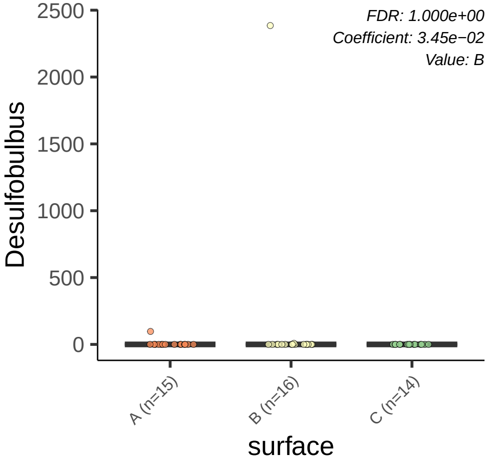

FDR: 1.000e+00
Coefficient: 3.45e−02
Value: B
Desulfobulbus
2500
2000
1500
1000
500
0
A (n=15)
B (n=16)
C (n=14)
surface

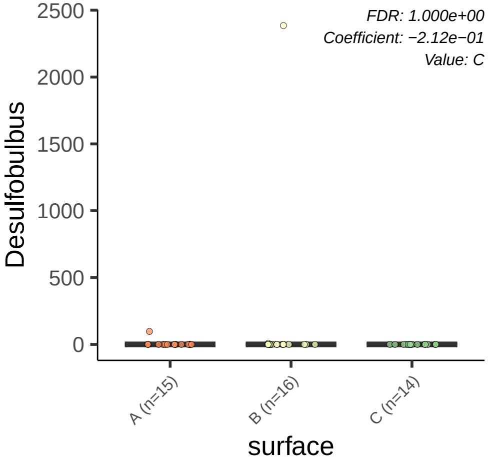
FDR: 1.000e+00
Coefficient: −2.12e−01
Value: C
Desulfobulbus
2500
2000
1500
1000
500
0
A (n=15)
B (n=16)
C (n=14)
surface

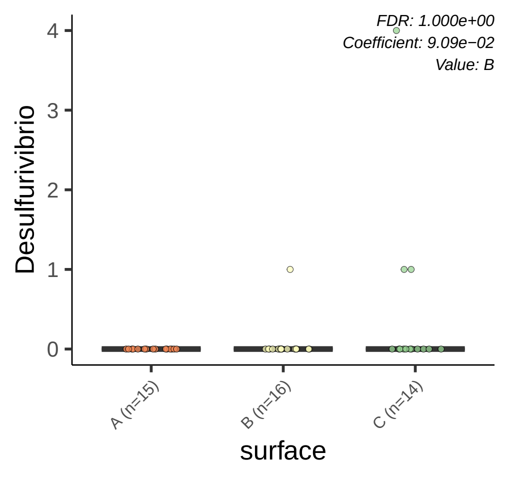
FDR: 1.000e+00
Coefficient: 9.09e−02
Value: B
Desulfurivibrio
4
3
2
1
0
A (n=15)
B (n=16)
C (n=14)
surface

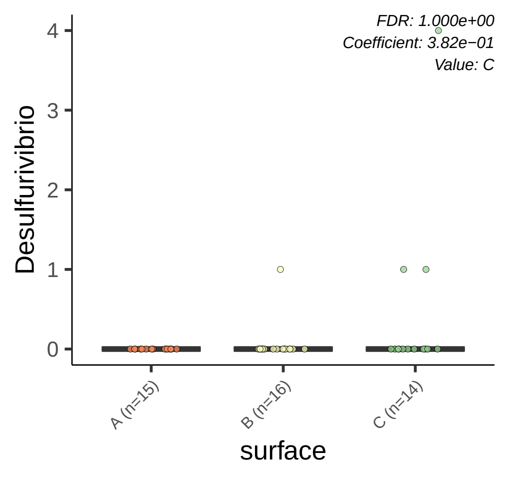
FDR: 1.000e+00
Coefficient: 3.82e−01
Value: C
Desulfurivibrio
0
1
2
3
4
A (n=15)
B (n=16)
C (n=14)
surface

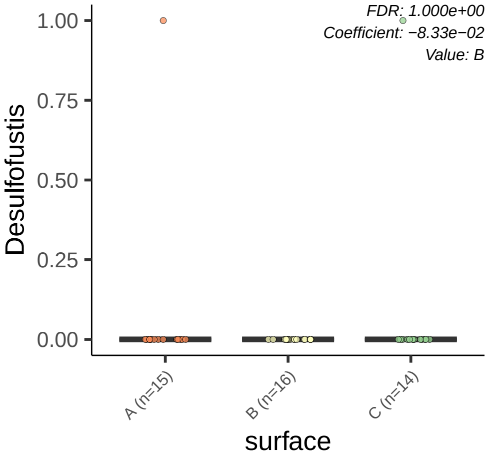
FDR: 1.000e+00
Coefficient: −8.33e−02
Value: B
Desulfofustis
1.00
0.75
0.50
0.25
0.00
A (n=15)
B (n=16)
C (n=14)
surface

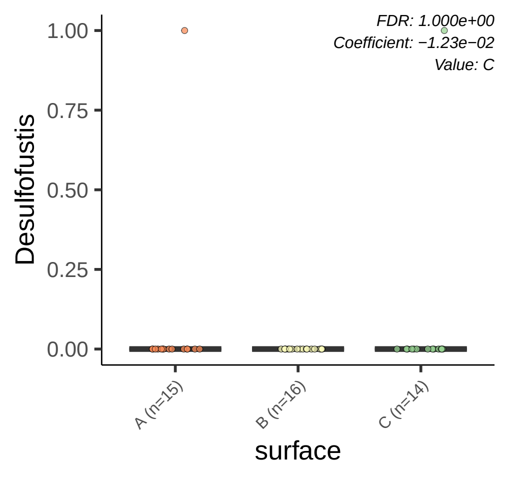
FDR: 1.000e+00
Coefficient: −1.23e−02
Value: C
1.00
0.75
0.50
0.25
0.00
Desulfofustis
A (n=15)
B (n=16)
C (n=14)
surface

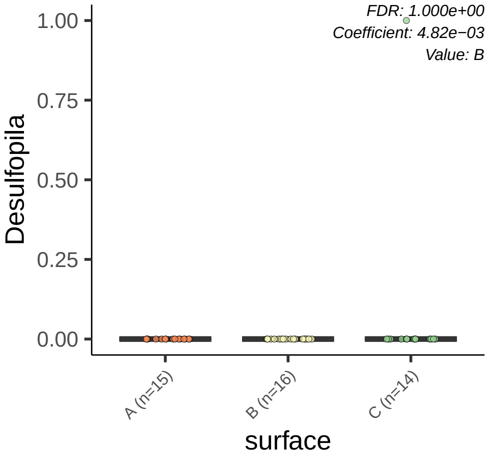
FDR: 1.000e+00
Coefficient: 4.82e−03
Value: B
Desulfopila
1.00
0.75
0.50
0.25
0.00
A (n=15)
B (n=16)
C (n=14)
surface

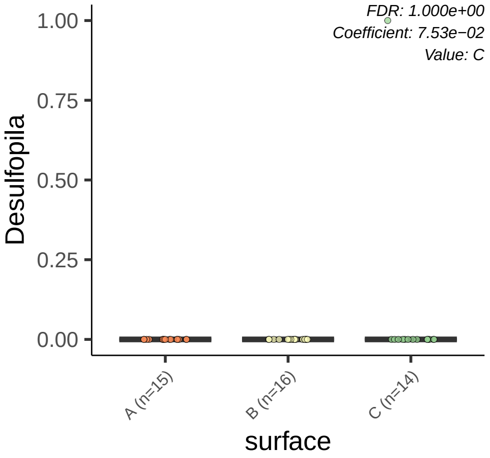
FDR: 1.000e+00
Coefficient: 7.53e−02
Value: C
Desulfopila
1.00
0.75
0.50
0.25
0.00
A (n=15)
B (n=16)
C (n=14)
surface

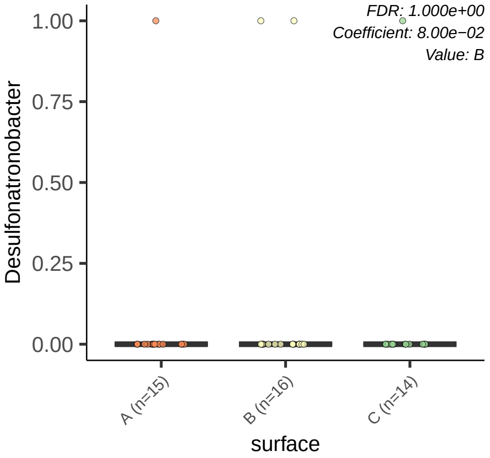
FDR: 1.000e+00
Coefficient: 8.00e−02
Value: B
Desulfonatronobacter
1.00
0.75
0.50
0.25
0.00
A (n=15)
B (n=16)
C (n=14)
surface

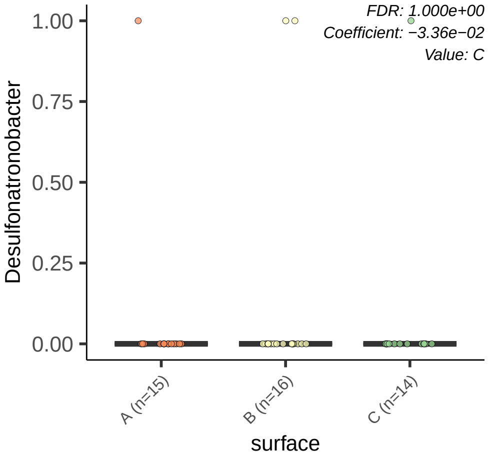

FDR: 1.000e+00
Coefficient: −3.36e−02
Value: C
Desulfonatronobacter
1.00
0.75
0.50
0.25
0.00
A (n=15)
B (n=16)
C (n=14)
surface

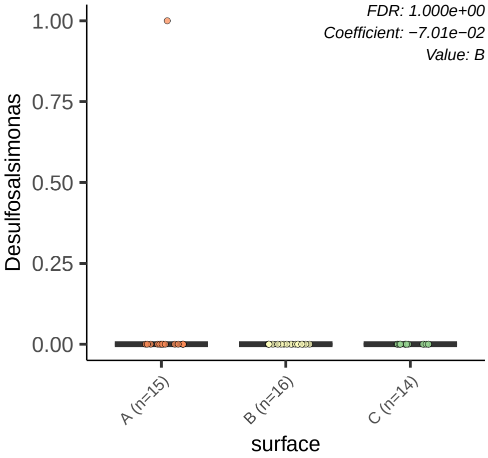
FDR: 1.000e+00
Coefficient: −7.01e−02
Value: B
Desulfosalsimonas
1.00
0.75
0.50
0.25
0.00
A (n=15)
B (n=16)
C (n=14)
surface

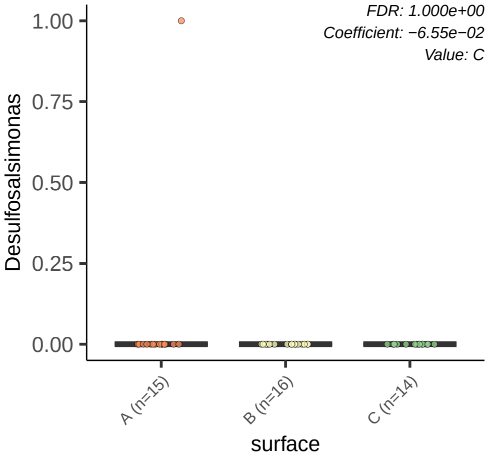

FDR: 1.000e+00
Coefficient: −6.55e−02
Value: C
Desulfosalsimonas
1.00
0.75
0.50
0.25
0.00
A (n=15)
B (n=16)
C (n=14)
surface

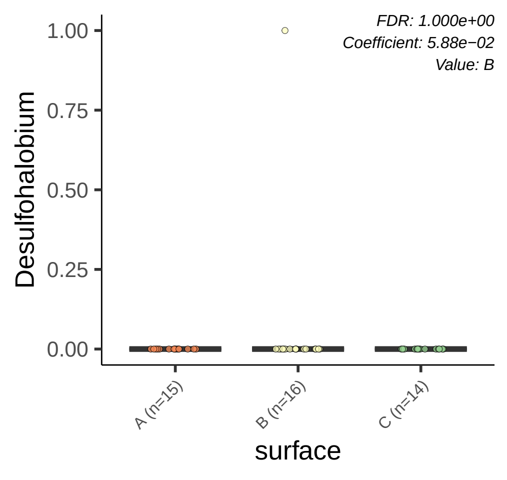
FDR: 1.000e+00
Coefficient: 5.88e−02
Value: B
Desulfohalobium
1.00
0.75
0.50
0.25
0.00
A (n=15)
B (n=16)
C (n=14)
surface

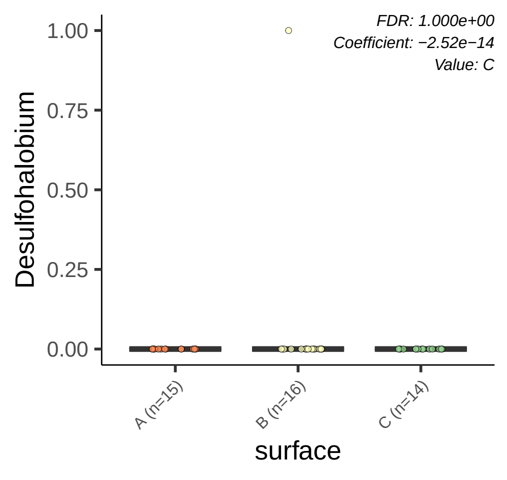
FDR: 1.000e+00
Coefficient: −2.52e−14
Value: C
Desulfohalobium
1.00
0.75
0.50
0.25
0.00
A (n=15)
B (n=16)
C (n=14)
surface

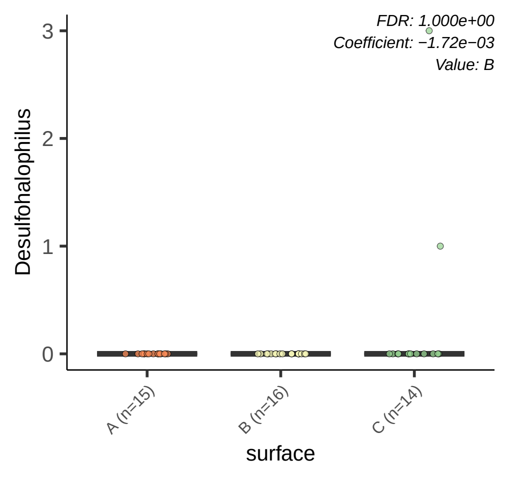
FDR: 1.000e+00
Coefficient: −1.72e−03
Value: B
Desulfohalophilus
3
2
1
0
A (n=15)
B (n=16)
C (n=14)
surface

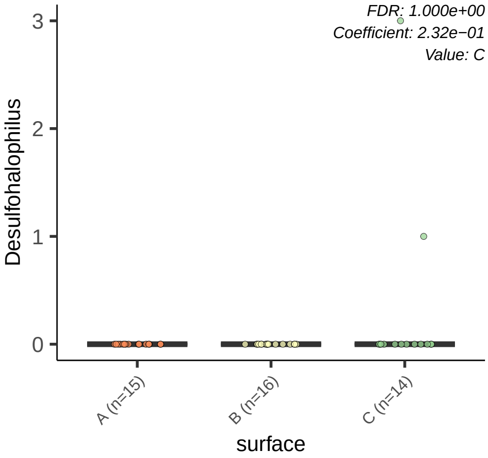
FDR: 1.000e+00
Coefficient: 2.32e−01
Value: C
Desulfohalophilus
3
2
1
0
A (n=15)
B (n=16)
C (n=14)
surface

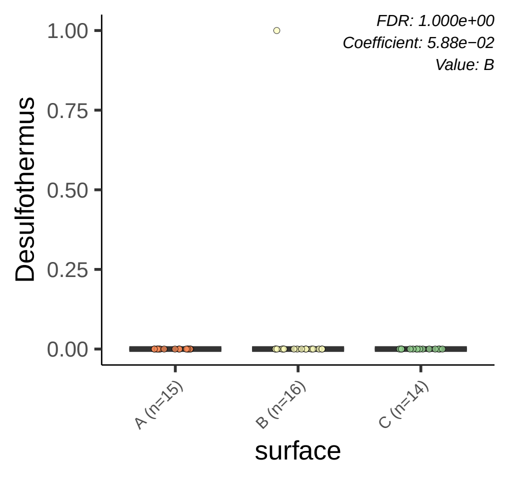
FDR: 1.000e+00
Coefficient: 5.88e−02
Value: B
Desulfothermus
1.00
0.75
0.50
0.25
0.00
A (n=15)
B (n=16)
C (n=14)
surface

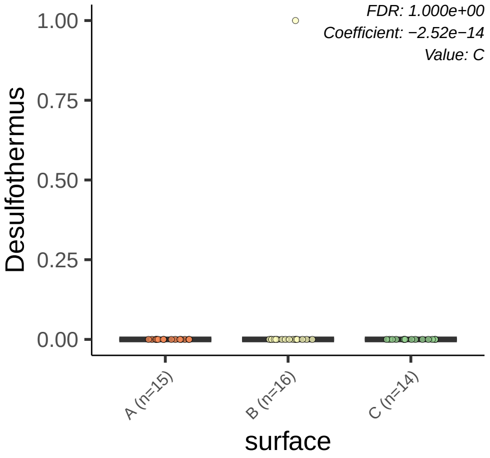

FDR: 1.000e+00
Coefficient: −2.52e−14
Value: C
Desulfothermus
1.00
0.75
0.50
0.25
0.00
A (n=15)
B (n=16)
C (n=14)
surface

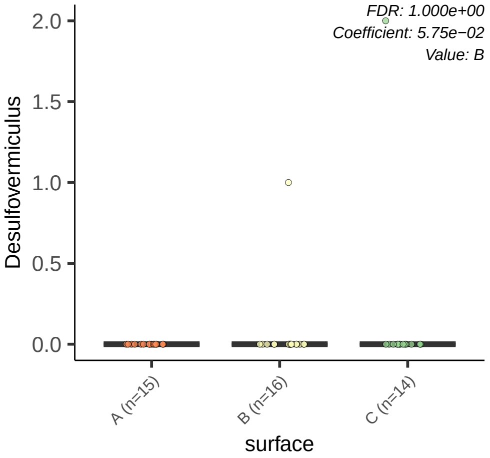

FDR: 1.000e+00
Coefficient: 5.75e−02
Value: B
Desulfovermiculus
2.0
1.5
1.0
0.5
0.0
A (n=15)
B (n=16)
C (n=14)
surface

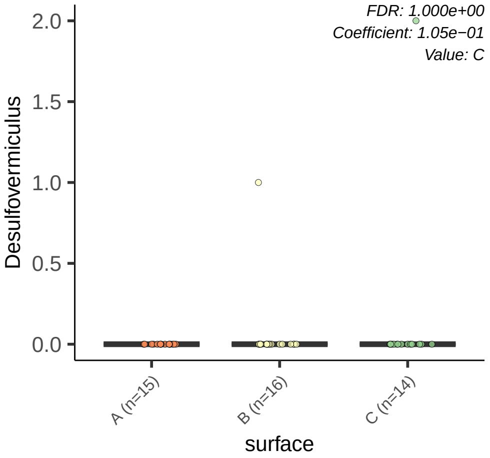

FDR: 1.000e+00
Coefficient: 1.05e−01
Value: C
Desulfovermiculus
2.0
1.5
1.0
0.5
0.0
A (n=15)
B (n=16)
C (n=14)
surface

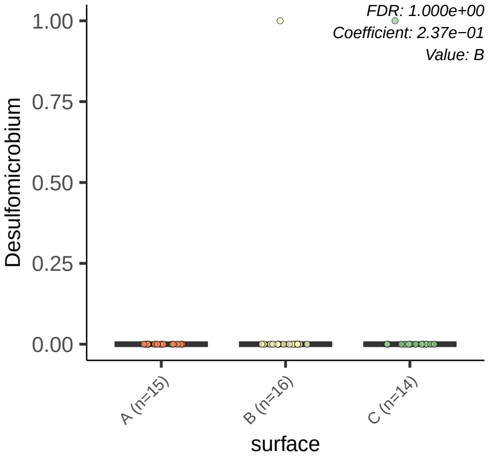
FDR: 1.000e+00
Coefficient: 2.37e−01
Value: B
Desulfomicrobium
1.00
0.75
0.50
0.25
0.00
A (n=15)
B (n=16)
C (n=14)
surface

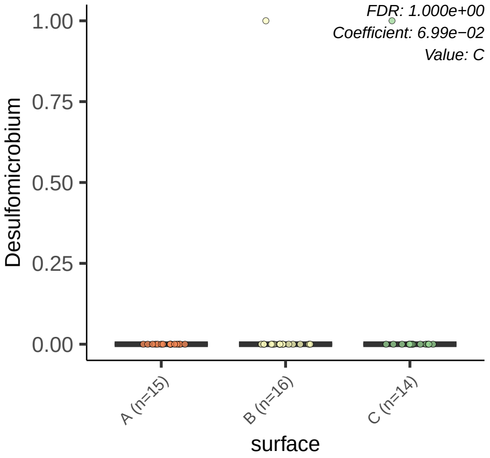
FDR: 1.000e+00
Coefficient: 6.99e−02
Value: C
Desulfomicrobium
1.00
0.75
0.50
0.25
0.00
A (n=15)
B (n=16)
C (n=14)
surface

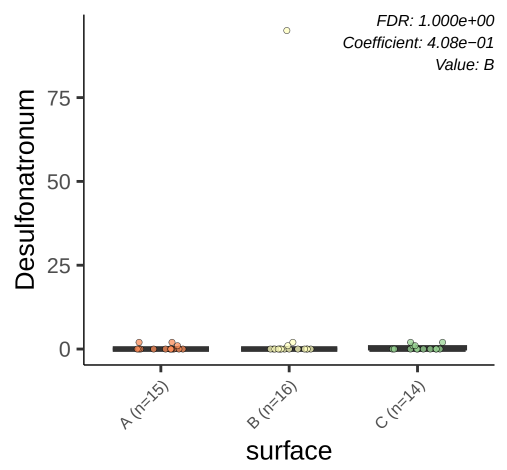

FDR: 1.000e+00
Coefficient: 4.08e−01
Value: B
Desulfonatronum
0
25
50
75
A (n=15)
B (n=16)
C (n=14)
surface

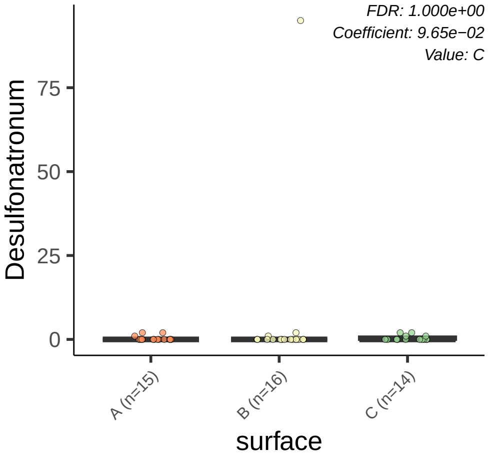

FDR: 1.000e+00
Coefficient: 9.65e−02
Value: C
Desulfonatronum
75
50
25
0
A (n=15)
B (n=16)
C (n=14)
surface

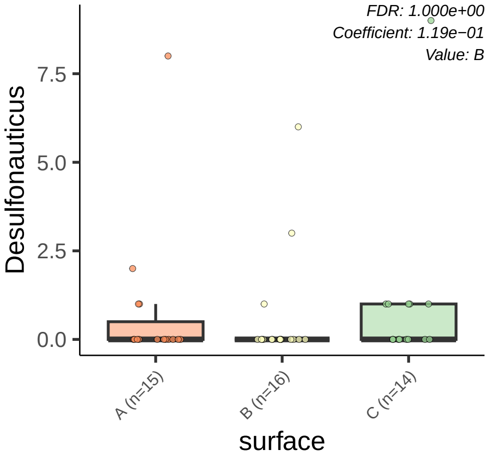

FDR: 1.000e+00
Coefficient: 1.19e−01
Value: B
Desulfonauticus
7.5
5.0
2.5
0.0
A (n=15)
B (n=16)
C (n=14)
surface

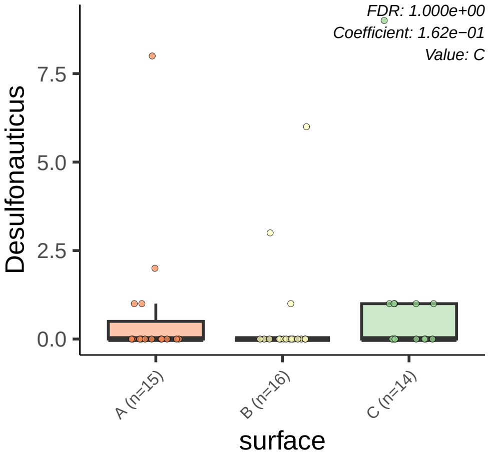

FDR: 1.000e+00
Coefficient: 1.62e−01
Value: C
Desulfonauticus
7.5
5.0
2.5
0.0
A (n=15)
B (n=16)
C (n=14)
surface

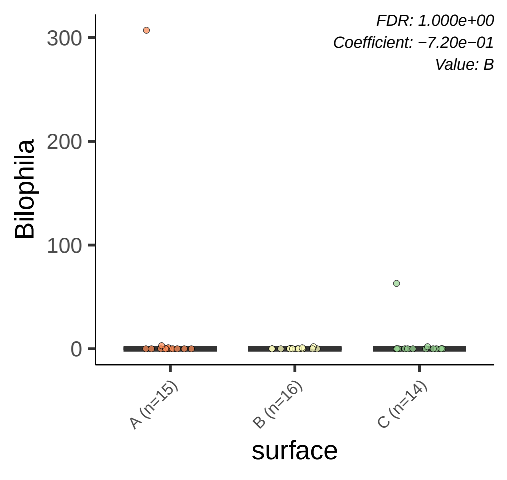

FDR: 1.000e+00
Coefficient: −7.20e−01
Value: B
300
200
100
0
Bilophila
A (n=15)
B (n=16)
C (n=14)
surface

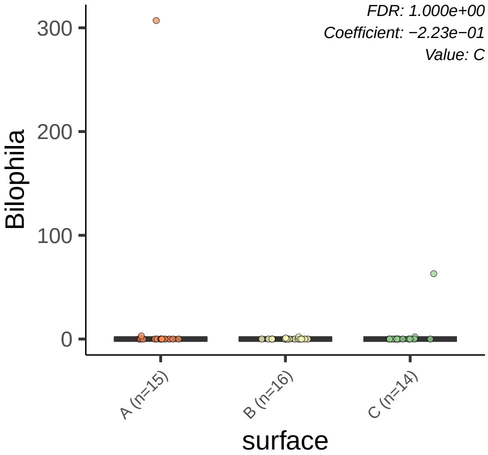
FDR: 1.000e+00
Coefficient: −2.23e−01
Value: C
Bilophila
300
200
100
0
A (n=15)
B (n=16)
C (n=14)
surface

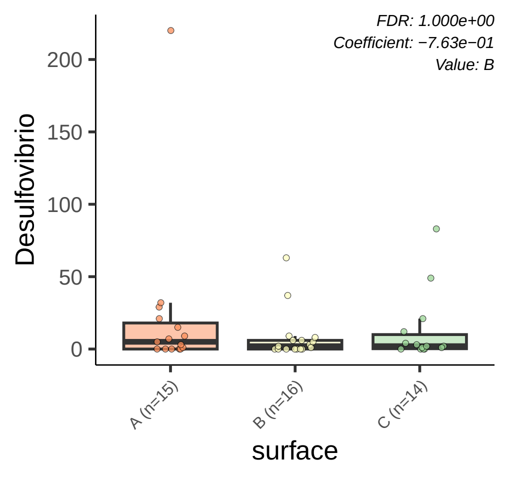
FDR: 1.000e+00
Coefficient: −7.63e−01
Value: B
Desulfovibrio
200
150
100
50
0
A (n=15)
B (n=16)
C (n=14)
surface

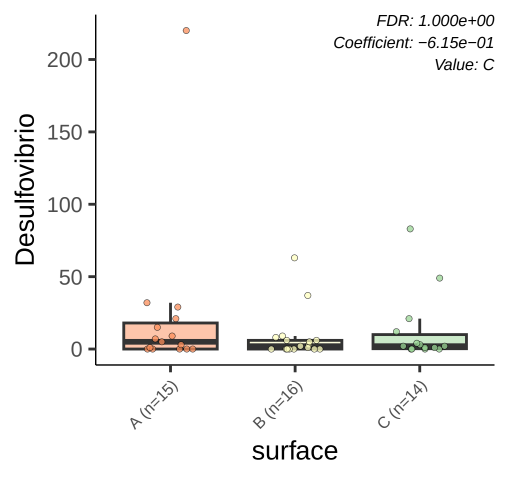
FDR: 1.000e+00
Coefficient: −6.15e−01
Value: C
Desulfovibrio
200
150
100
50
0
A (n=15)
B (n=16)
C (n=14)
surface

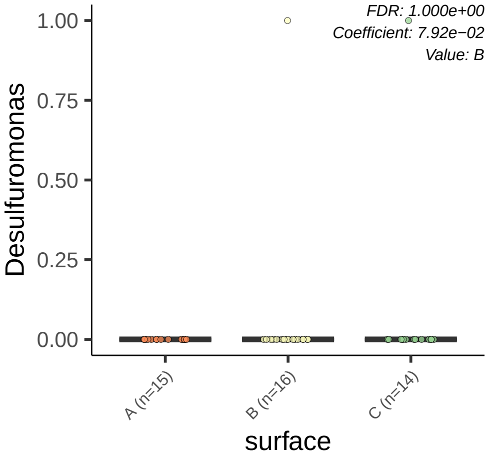
FDR: 1.000e+00
Coefficient: 7.92e−02
Value: B
Desulfuromonas
1.00
0.75
0.50
0.25
0.00
A (n=15)
B (n=16)
C (n=14)
surface

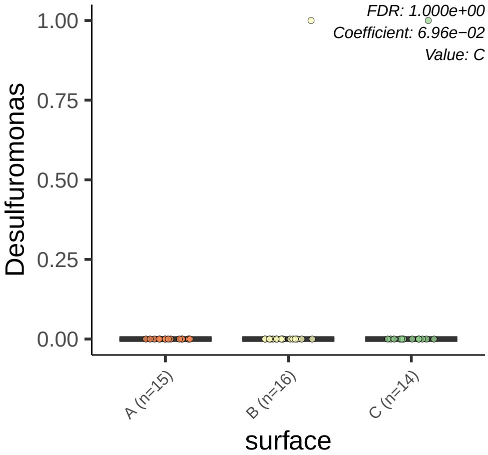

FDR: 1.000e+00
Coefficient: 6.96e−02
Value: C
Desulfuromonas
1.00
0.75
0.50
0.25
0.00
A (n=15)
B (n=16)
C (n=14)
surface

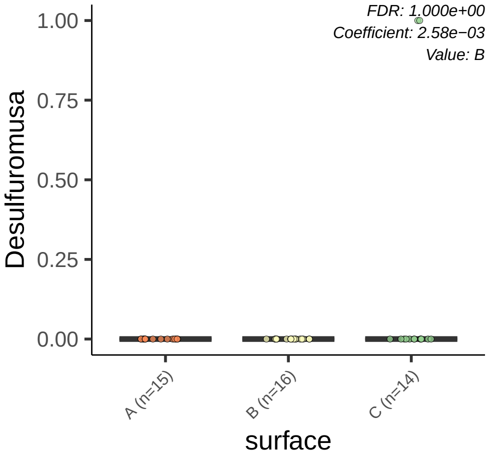
FDR: 1.000e+00
Coefficient: 2.58e−03
Value: B
Desulfuromusa
1.00
0.75
0.50
0.25
0.00
A (n=15)
B (n=16)
C (n=14)
surface

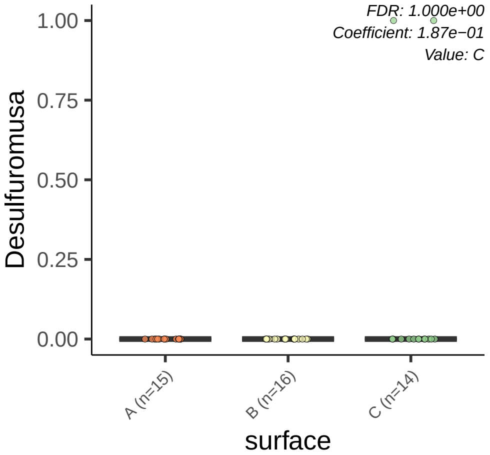
FDR: 1.000e+00
Coefficient: 1.87e−01
Value: C
Desulfuromusa
1.00
0.75
0.50
0.25
0.00
A (n=15)
B (n=16)
C (n=14)
surface

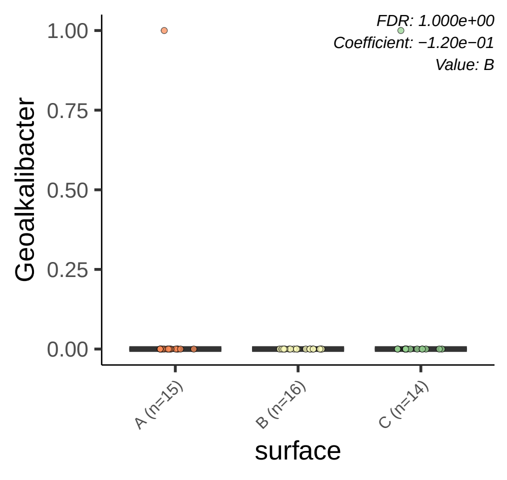

FDR: 1.000e+00
Coefficient: −1.20e−01
Value: B
Geoalkalibacter
1.00
0.75
0.50
0.25
0.00
A (n=15)
B (n=16)
C (n=14)
surface

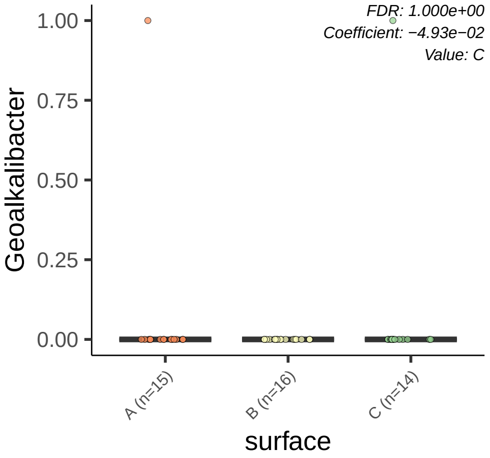
FDR: 1.000e+00
Coefficient: −4.93e−02
Value: C
Geoalkalibacter
1.00
0.75
0.50
0.25
0.00
A (n=15)
B (n=16)
C (n=14)
surface

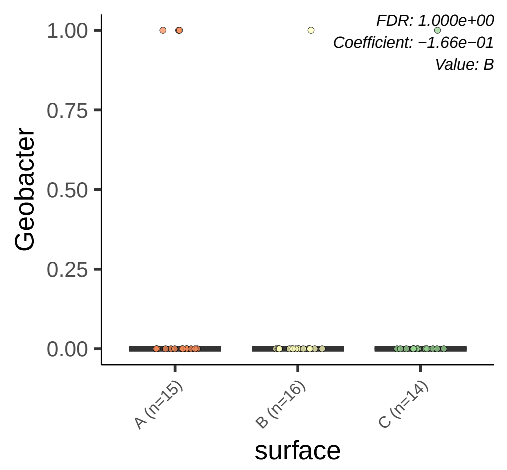
FDR: 1.000e+00
Coefficient: −1.66e−01
Value: B
1.00
0.75
0.50
0.25
0.00
Geobacter
A (n=15)
B (n=16)
C (n=14)
surface

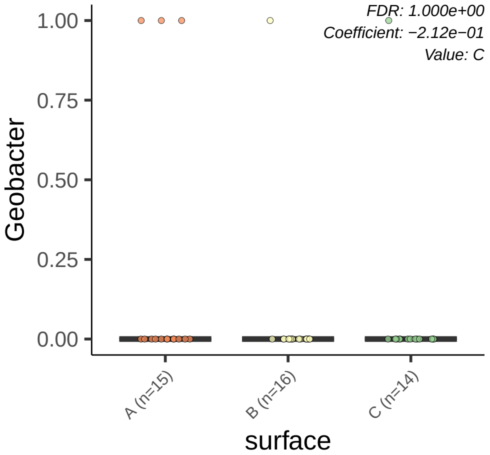
FDR: 1.000e+00
Coefficient: −2.12e−01
Value: C
Geobacter
1.00
0.75
0.50
0.25
0.00
A (n=15)
B (n=16)
C (n=14)
surface

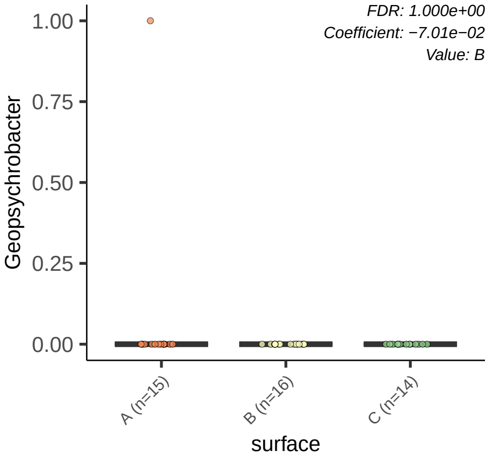
FDR: 1.000e+00
Coefficient: −7.01e−02
Value: B
Geopsychrobacter
1.00
0.75
0.50
0.25
0.00
A (n=15)
B (n=16)
C (n=14)
surface

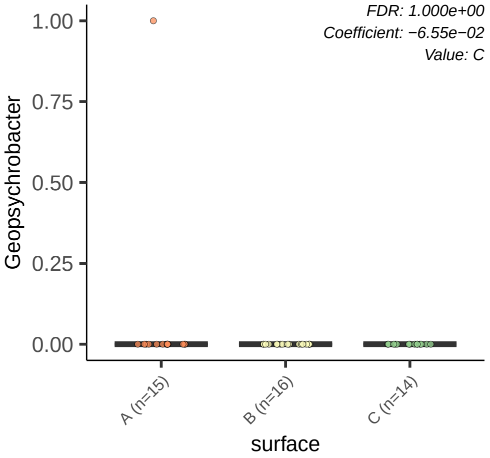

FDR: 1.000e+00
Coefficient: −6.55e−02
Value: C
Geopsychrobacter
1.00
0.75
0.50
0.25
0.00
A (n=15)
B (n=16)
C (n=14)
surface

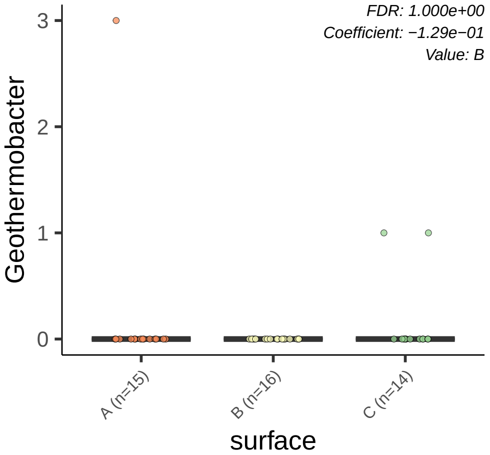
FDR: 1.000e+00
Coefficient: −1.29e−01
Value: B
Geothermobacter
3
2
1
0
A (n=15)
B (n=16)
C (n=14)
surface

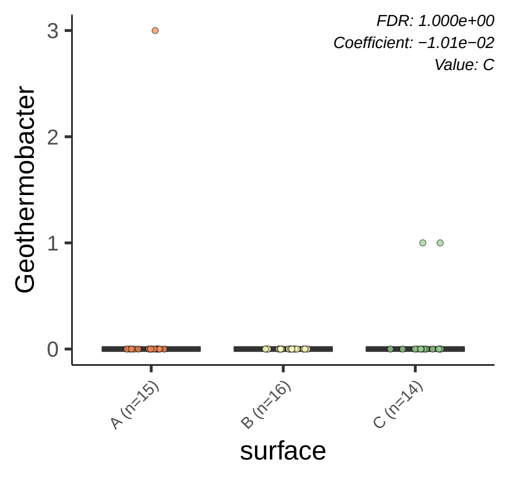
FDR: 1.000e+00
Coefficient: −1.01e−02
Value: C
Geothermobacter
3
2
1
0
A (n=15)
B (n=16)
C (n=14)
surface

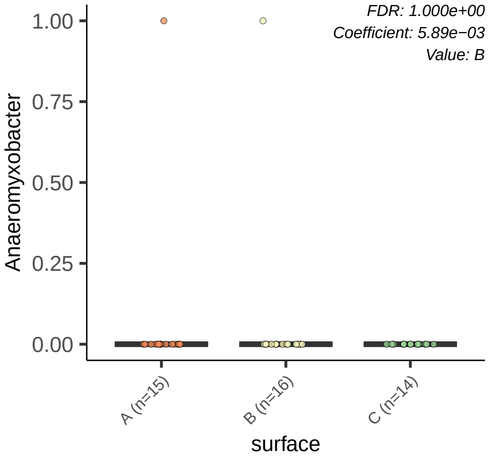
FDR: 1.000e+00
Coefficient: 5.89e−03
Value: B
Anaeromyxobacter
1.00
0.75
0.50
0.25
0.00
A (n=15)
B (n=16)
C (n=14)
surface

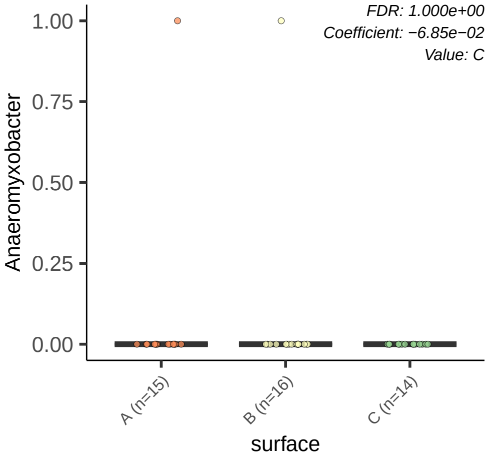

FDR: 1.000e+00
Coefficient: −6.85e−02
Value: C
Anaeromyxobacter
1.00
0.75
0.50
0.25
0.00
A (n=15)
B (n=16)
C (n=14)
surface

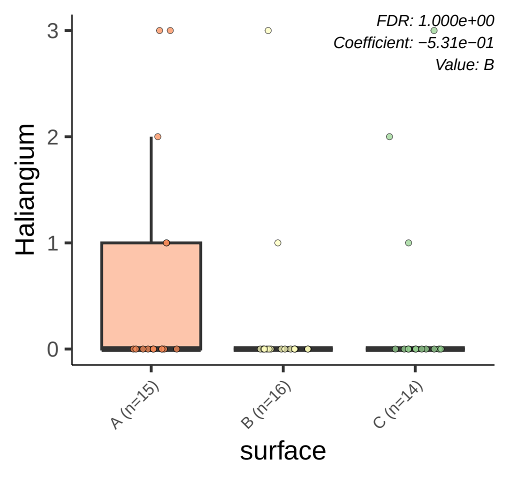
FDR: 1.000e+00
Coefficient: −5.31e−01
Value: B
Haliangium
0
1
2
3
A (n=15)
B (n=16)
C (n=14)
surface

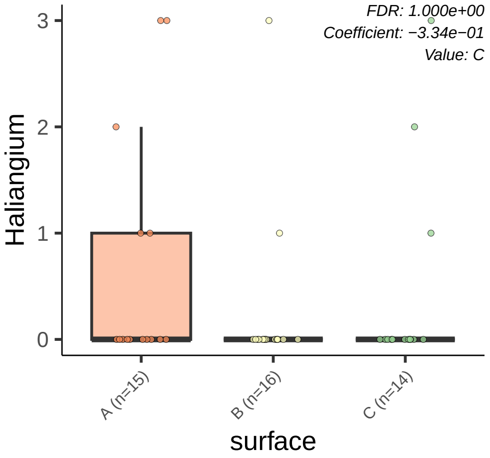
FDR: 1.000e+00
Coefficient: −3.34e−01
Value: C
Haliangium
3
2
1
0
A (n=15)
B (n=16)
C (n=14)
surface

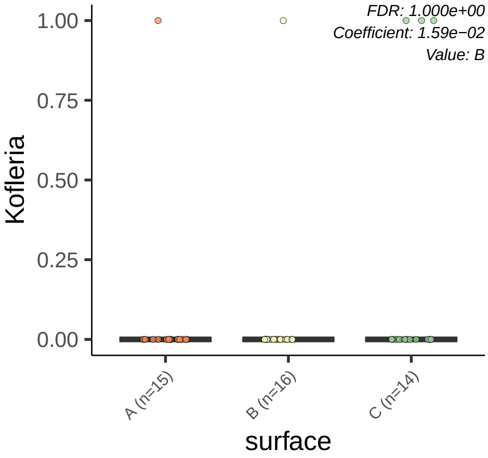
FDR: 1.000e+00
Coefficient: 1.59e−02
Value: B
Kofleria
1.00
0.75
0.50
0.25
0.00
A (n=15)
B (n=16)
C (n=14)
surface

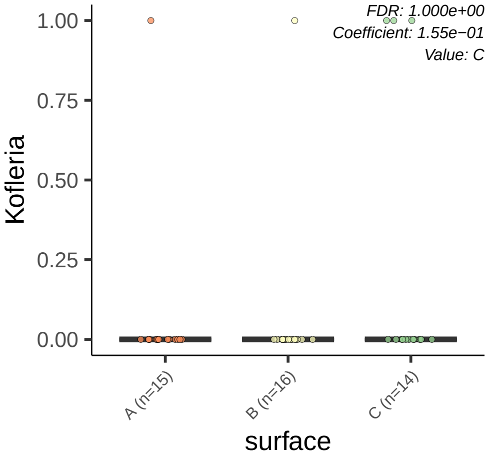

FDR: 1.000e+00
Coefficient: 1.55e−01
Value: C
1.00
0.75
0.50
0.25
0.00
Kofleria
A (n=15)
B (n=16)
C (n=14)
surface

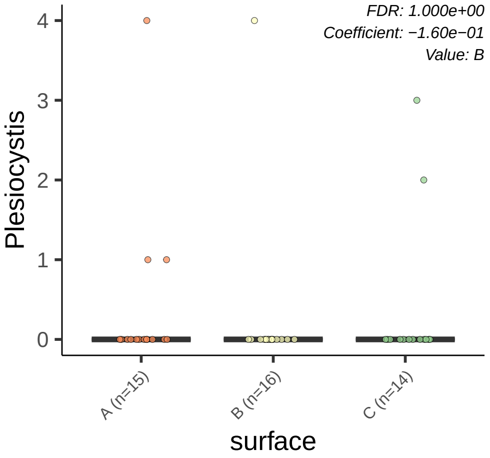

FDR: 1.000e+00
Coefficient: −1.60e−01
Value: B
Plesiocystis
0
1
2
3
4
A (n=15)
B (n=16)
C (n=14)
surface

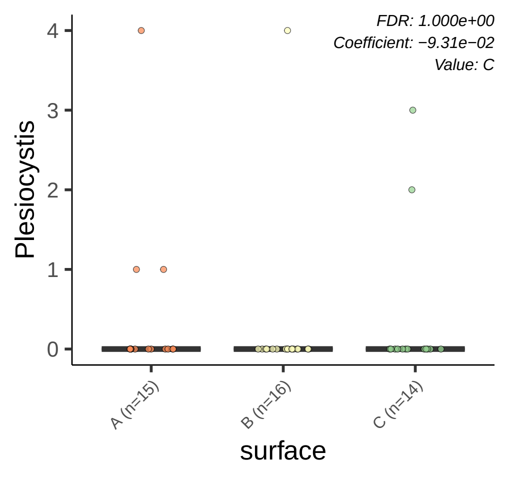

FDR: 1.000e+00
Coefficient: −9.31e−02
Value: C
Plesiocystis
4
3
2
1
0
A (n=15)
B (n=16)
C (n=14)
surface

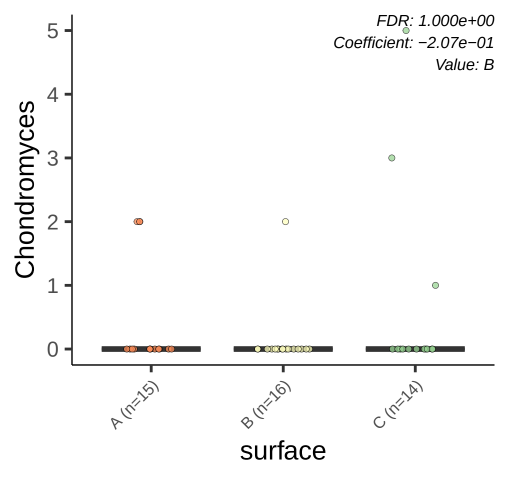
FDR: 1.000e+00
Coefficient: −2.07e−01
Value: B
Chondromyces
0
1
2
3
4
5
A (n=15)
B (n=16)
C (n=14)
surface

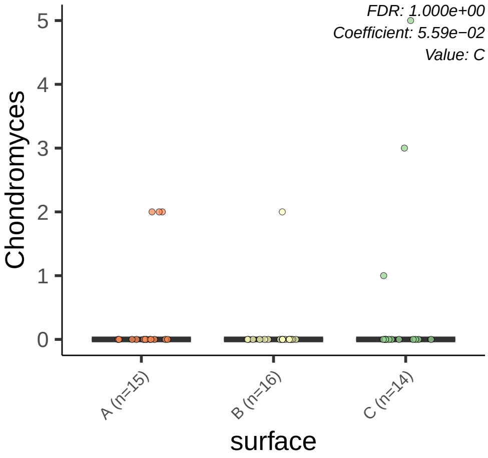

FDR: 1.000e+00
Coefficient: 5.59e−02
Value: C
Chondromyces
0
1
2
3
4
5
A (n=15)
B (n=16)
C (n=14)
surface

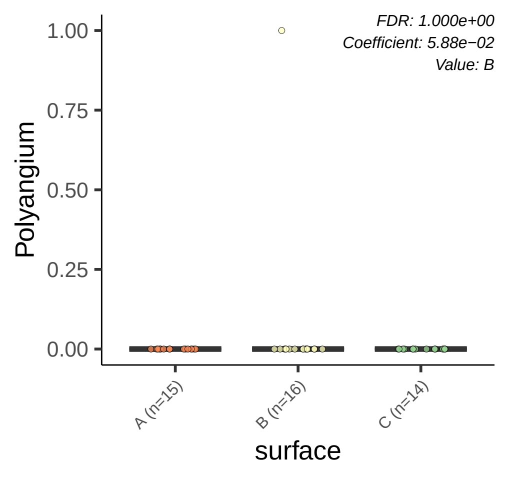

FDR: 1.000e+00
Coefficient: 5.88e−02
Value: B
1.00
0.75
0.50
0.25
0.00
Polyangium
A (n=15)
B (n=16)
C (n=14)
surface

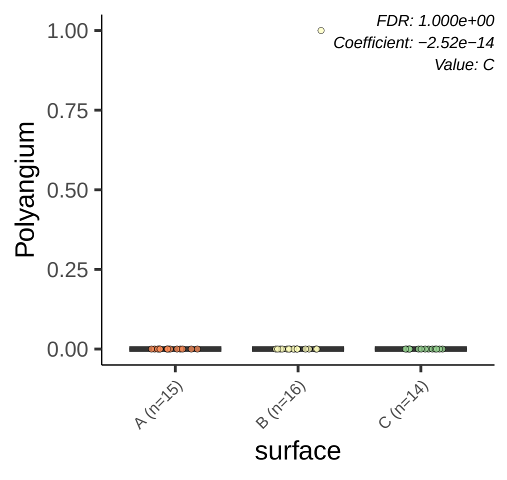

FDR: 1.000e+00
Coefficient: −2.52e−14
Value: C
Polyangium
1.00
0.75
0.50
0.25
0.00
A (n=15)
B (n=16)
C (n=14)
surface

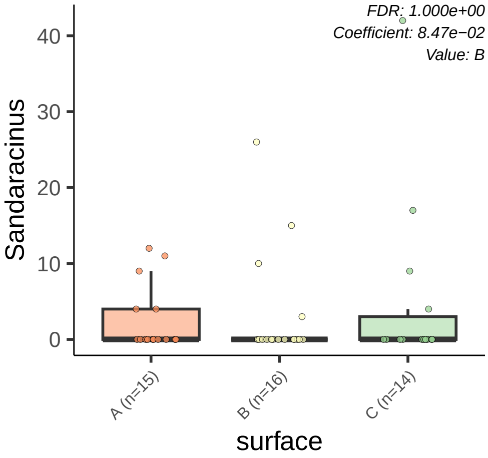

FDR: 1.000e+00
Coefficient: 8.47e−02
Value: B
Sandaracinus
40
30
20
10
0
A (n=15)
B (n=16)
C (n=14)
surface

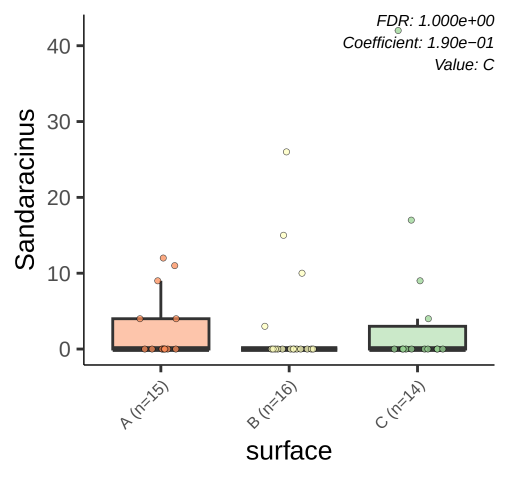
FDR: 1.000e+00
Coefficient: 1.90e−01
Value: C
Sandaracinus
40
30
20
10
0
A (n=15)
B (n=16)
C (n=14)
surface

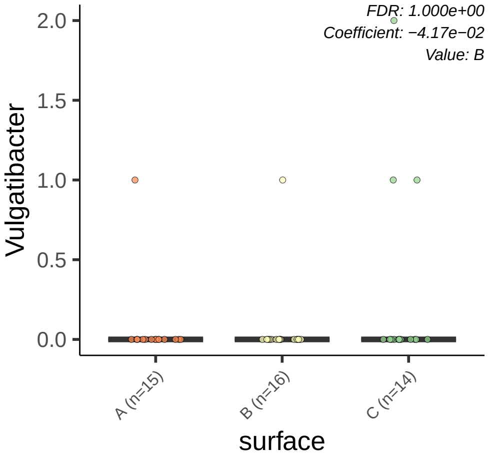

FDR: 1.000e+00
Coefficient: −4.17e−02
Value: B
Vulgatibacter
2.0
1.5
1.0
0.5
0.0
A (n=15)
B (n=16)
C (n=14)
surface

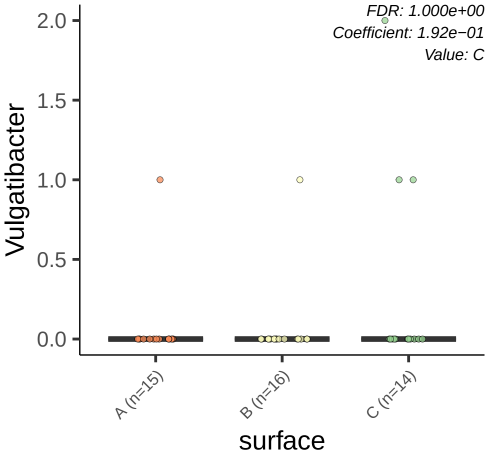
FDR: 1.000e+00
Coefficient: 1.92e−01
Value: C
Vulgatibacter
2.0
1.5
1.0
0.5
0.0
A (n=15)
B (n=16)
C (n=14)
surface

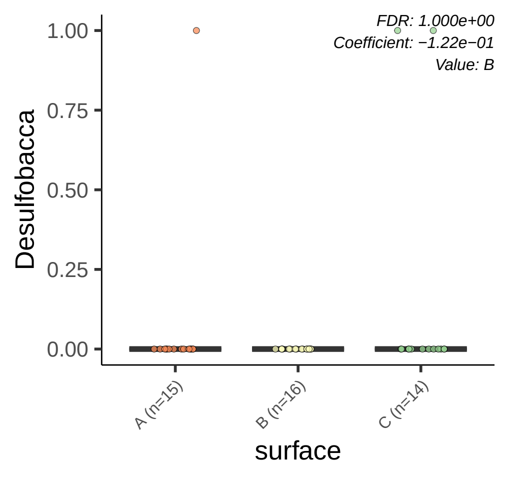
FDR: 1.000e+00
Coefficient: −1.22e−01
Value: B
Desulfobacca
1.00
0.75
0.50
0.25
0.00
A (n=15)
B (n=16)
C (n=14)
surface

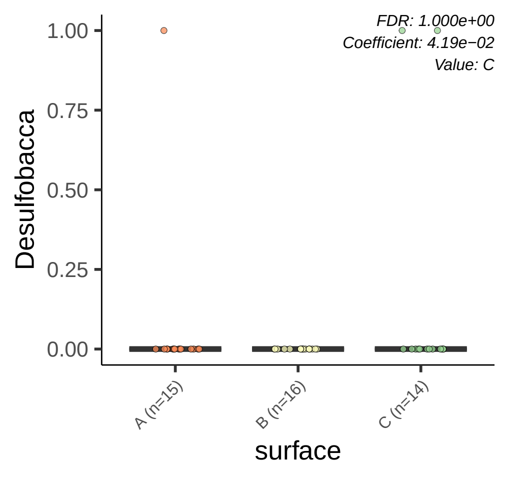

FDR: 1.000e+00
Coefficient: 4.19e−02
Value: C
Desulfobacca
1.00
0.75
0.50
0.25
0.00
A (n=15)
B (n=16)
C (n=14)
surface

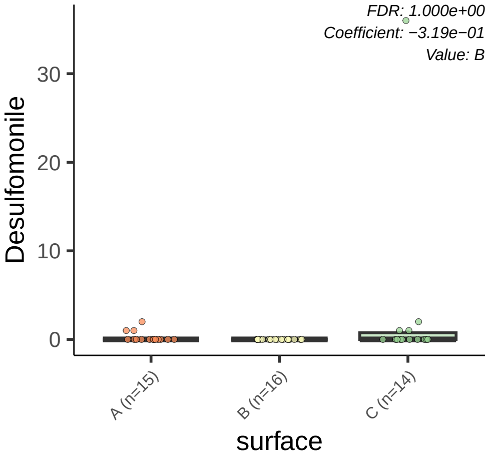
FDR: 1.000e+00
Coefficient: −3.19e−01
Value: B
Desulfomonile
30
20
10
0
A (n=15)
B (n=16)
C (n=14)
surface

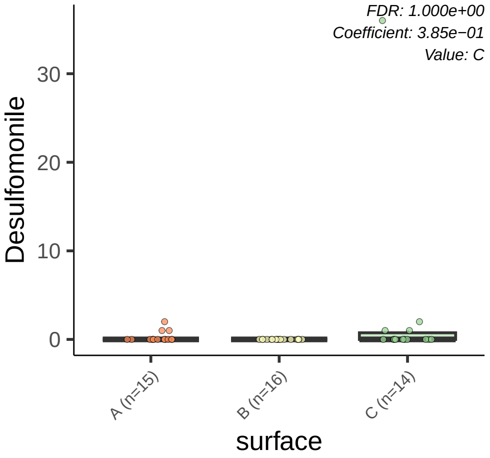

FDR: 1.000e+00
Coefficient: 3.85e−01
Value: C
Desulfomonile
30
20
10
0
A (n=15)
B (n=16)
C (n=14)
surface

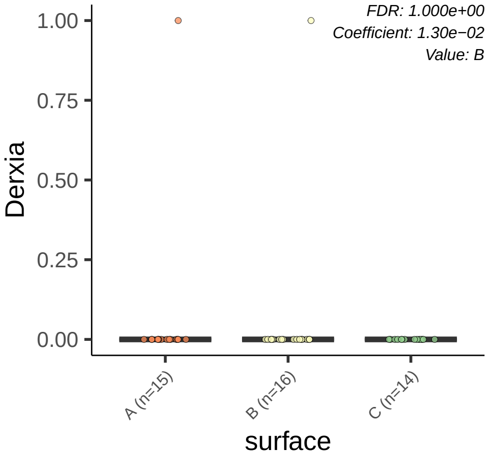
FDR: 1.000e+00
Coefficient: 1.30e−02
Value: B
Derxia
1.00
0.75
0.50
0.25
0.00
A (n=15)
B (n=16)
C (n=14)
surface

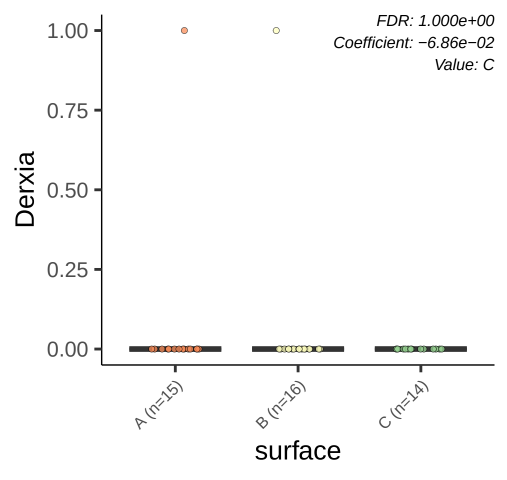
FDR: 1.000e+00
Coefficient: −6.86e−02
Value: C
Derxia
1.00
0.75
0.50
0.25
0.00
A (n=15)
B (n=16)
C (n=14)
surface

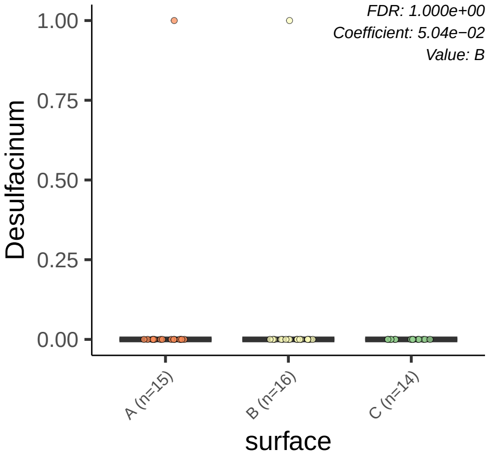
FDR: 1.000e+00
Coefficient: 5.04e−02
Value: B
Desulfacinum
1.00
0.75
0.50
0.25
0.00
A (n=15)
B (n=16)
C (n=14)
surface

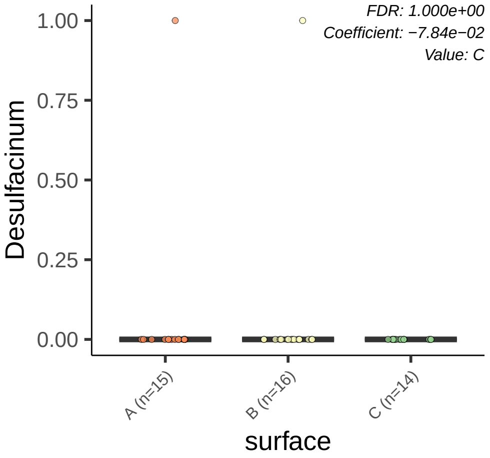
FDR: 1.000e+00
Coefficient: −7.84e−02
Value: C
Desulfacinum
1.00
0.75
0.50
0.25
0.00
A (n=15)
B (n=16)
C (n=14)
surface

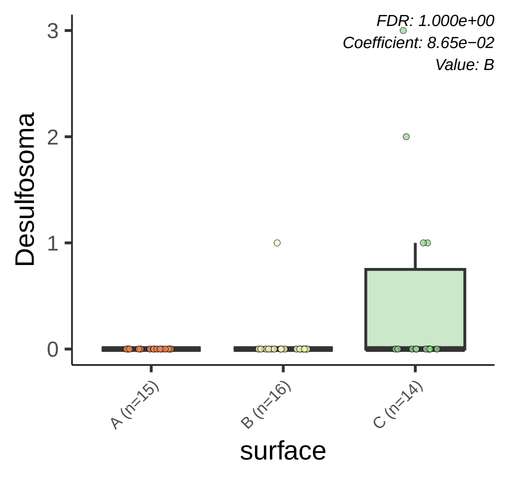

FDR: 1.000e+00
Coefficient: 8.65e−02
Value: B
Desulfosoma
3
2
1
0
A (n=15)
B (n=16)
C (n=14)
surface

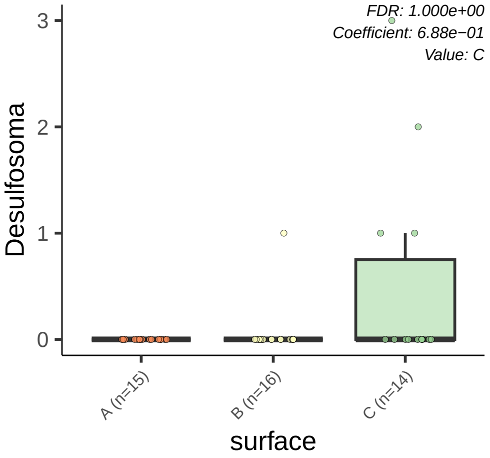
FDR: 1.000e+00
Coefficient: 6.88e−01
Value: C
Desulfosoma
3
2
1
0
A (n=15)
B (n=16)
C (n=14)
surface

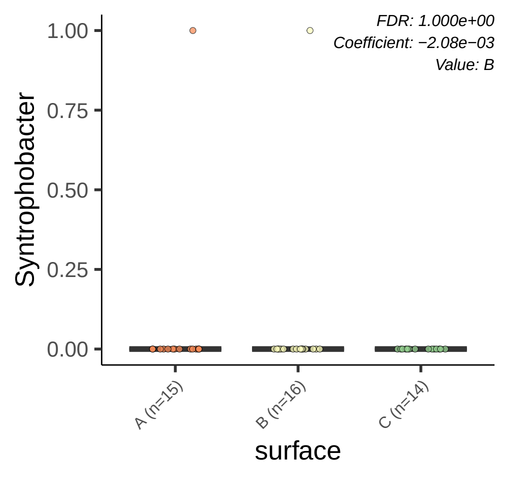

FDR: 1.000e+00
Coefficient: −2.08e−03
Value: B
Syntrophobacter
1.00
0.75
0.50
0.25
0.00
A (n=15)
B (n=16)
C (n=14)
surface

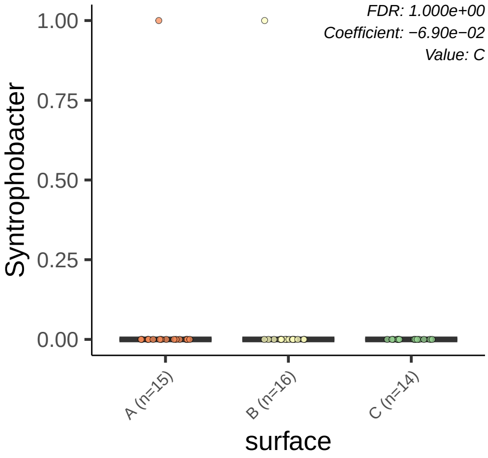

FDR: 1.000e+00
Coefficient: −6.90e−02
Value: C
Syntrophobacter
1.00
0.75
0.50
0.25
0.00
A (n=15)
B (n=16)
C (n=14)
surface

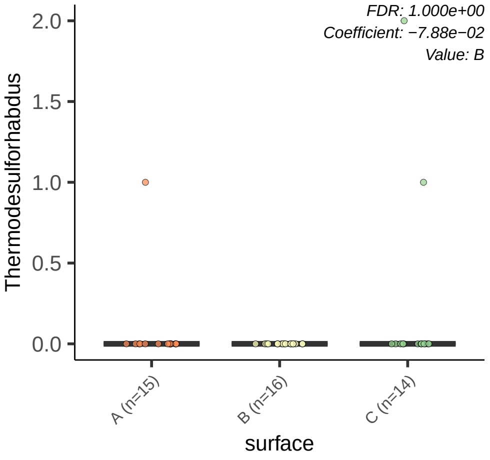

FDR: 1.000e+00
Coefficient: −7.88e−02
Value: B
Thermodesulforhabdus
2.0
1.5
1.0
0.5
0.0
A (n=15)
B (n=16)
C (n=14)
surface

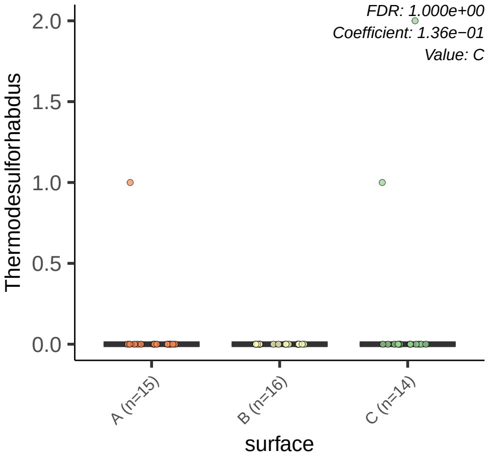
FDR: 1.000e+00
Coefficient: 1.36e−01
Value: C
Thermodesulforhabdus
2.0
1.5
1.0
0.5
0.0
A (n=15)
B (n=16)
C (n=14)
surface

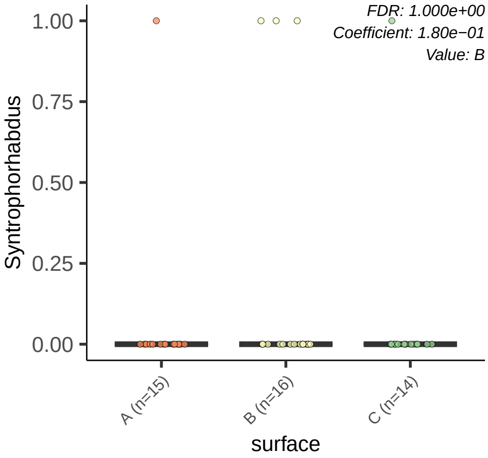
FDR: 1.000e+00
Coefficient: 1.80e−01
Value: B
Syntrophorhabdus
1.00
0.75
0.50
0.25
0.00
A (n=15)
B (n=16)
C (n=14)
surface

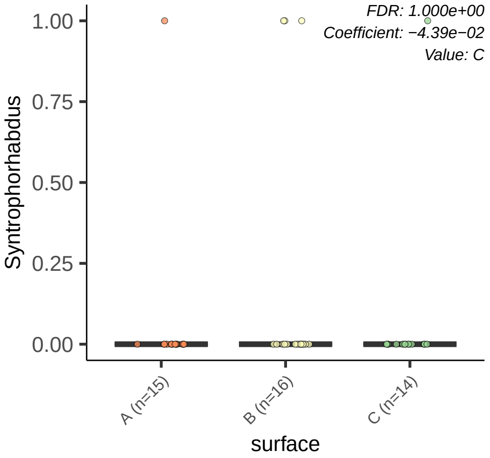
FDR: 1.000e+00
Coefficient: −4.39e−02
Value: C
Syntrophorhabdus
1.00
0.75
0.50
0.25
0.00
A (n=15)
B (n=16)
C (n=14)
surface

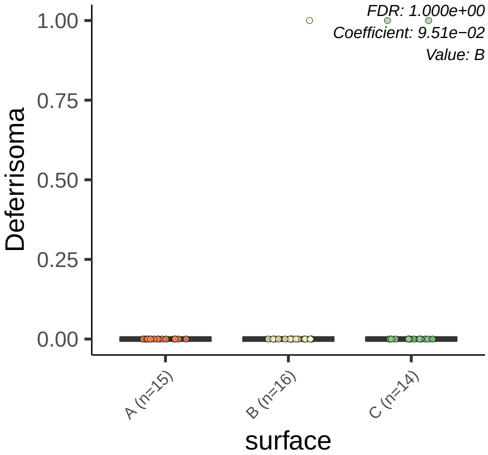
FDR: 1.000e+00
Coefficient: 9.51e−02
Value: B
Deferrisoma
1.00
0.75
0.50
0.25
0.00
A (n=15)
B (n=16)
C (n=14)
surface

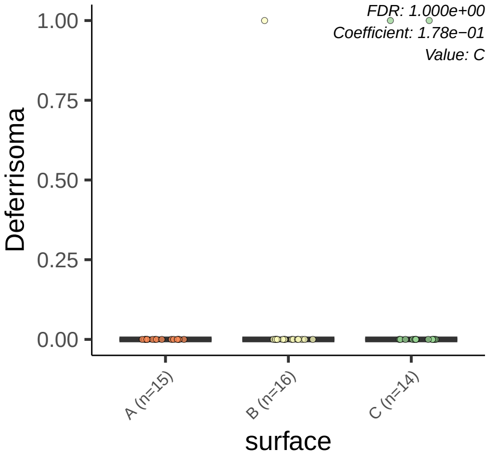
FDR: 1.000e+00
Coefficient: 1.78e−01
Value: C
Deferrisoma
1.00
0.75
0.50
0.25
0.00
A (n=15)
B (n=16)
C (n=14)
surface

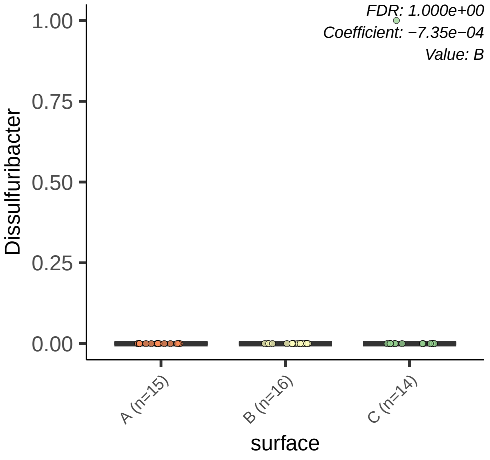

FDR: 1.000e+00
Coefficient: −7.35e−04
Value: B
Dissulfuribacter
1.00
0.75
0.50
0.25
0.00
A (n=15)
B (n=16)
C (n=14)
surface

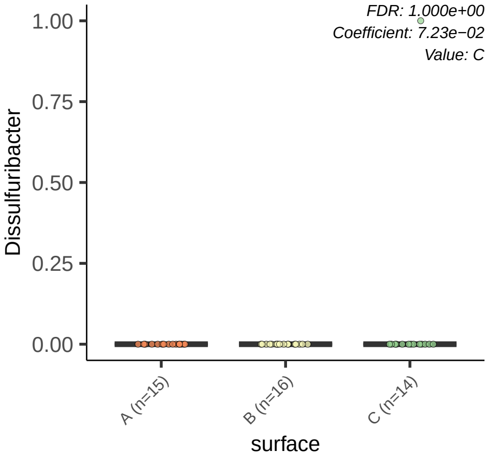
FDR: 1.000e+00
Coefficient: 7.23e−02
Value: C
Dissulfuribacter
1.00
0.75
0.50
0.25
0.00
A (n=15)
B (n=16)
C (n=14)
surface

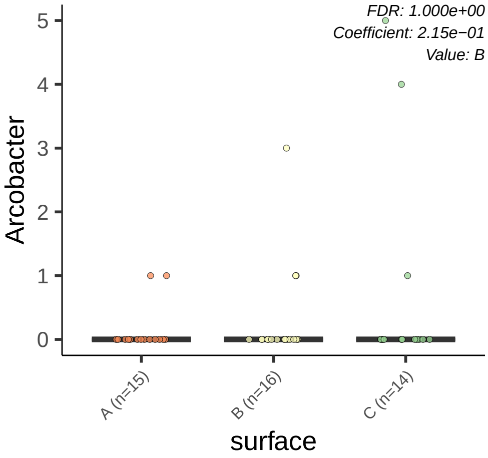

FDR: 1.000e+00
Coefficient: 2.15e−01
Value: B
Arcobacter
5
4
3
2
1
0
A (n=15)
B (n=16)
C (n=14)
surface

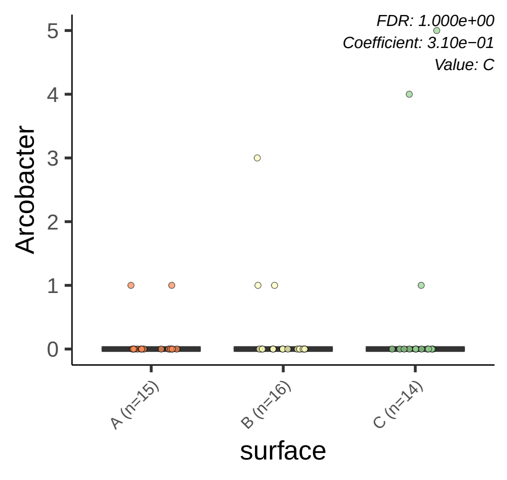
FDR: 1.000e+00
Coefficient: 3.10e−01
Value: C
Arcobacter
0
1
2
3
4
5
A (n=15)
B (n=16)
C (n=14)
surface

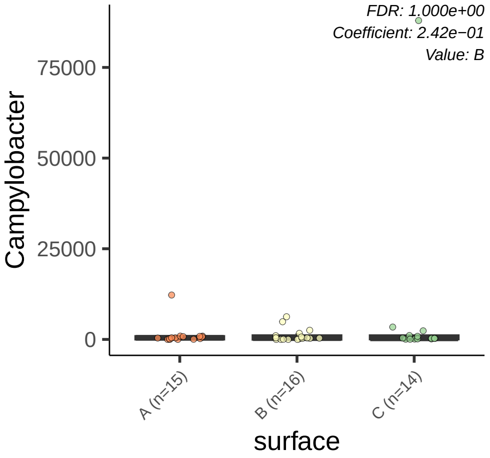

FDR: 1.000e+00
Coefficient: 2.42e−01
Value: B
Campylobacter
75000
50000
25000
0
A (n=15)
B (n=16)
C (n=14)
surface

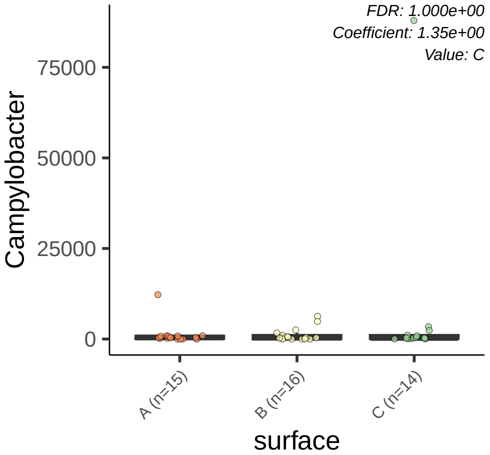

FDR: 1.000e+00
Coefficient: 1.35e+00
Value: C
Campylobacter
75000
50000
25000
0
A (n=15)
B (n=16)
C (n=14)
surface

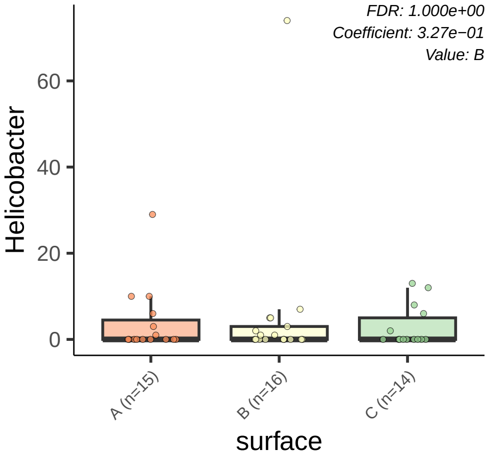

FDR: 1.000e+00
Coefficient: 3.27e−01
Value: B
Helicobacter
60
40
20
0
A (n=15)
B (n=16)
C (n=14)
surface

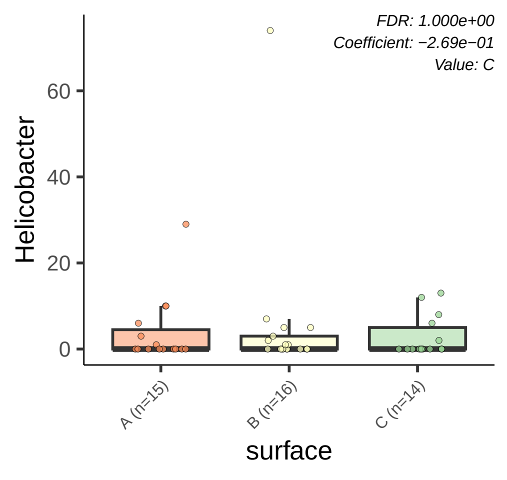

FDR: 1.000e+00
Coefficient: −2.69e−01
Value: C
Helicobacter
60
40
20
0
A (n=15)
B (n=16)
C (n=14)
surface

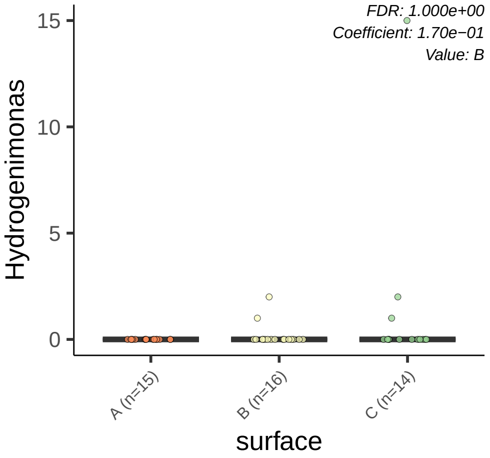
FDR: 1.000e+00
Coefficient: 1.70e−01
Value: B
Hydrogenimonas
15
10
5
0
A (n=15)
B (n=16)
C (n=14)
surface

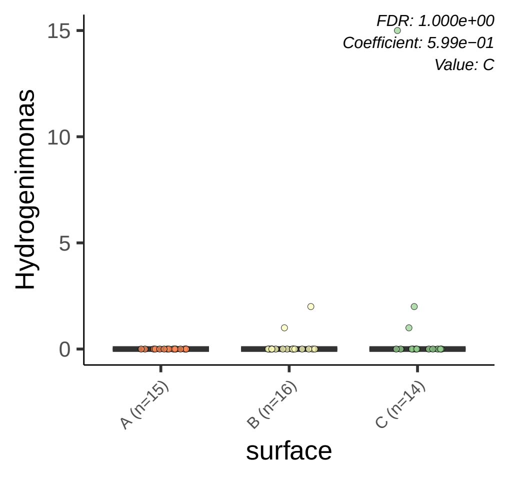

FDR: 1.000e+00
Coefficient: 5.99e−01
Value: C
Hydrogenimonas
15
10
5
0
A (n=15)
B (n=16)
C (n=14)
surface

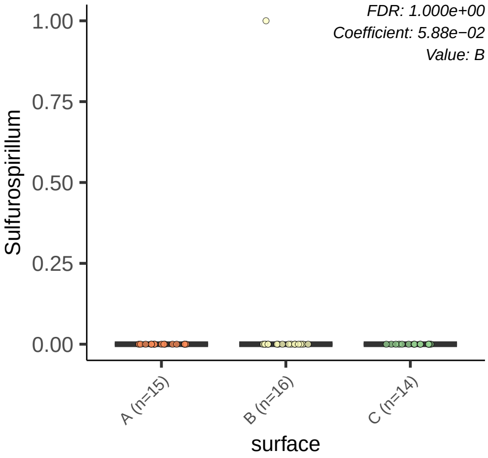

FDR: 1.000e+00
Coefficient: 5.88e−02
Value: B
Sulfurospirillum
1.00
0.75
0.50
0.25
0.00
A (n=15)
B (n=16)
C (n=14)
surface

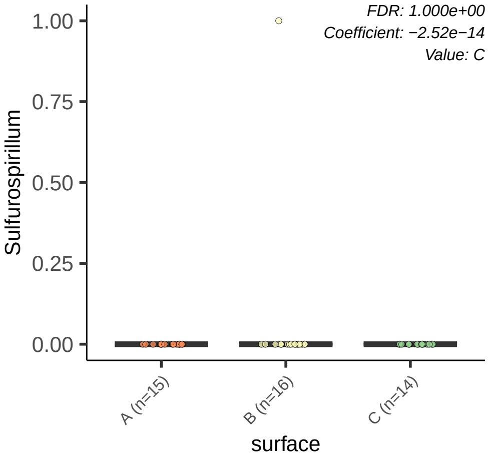
FDR: 1.000e+00
Coefficient: −2.52e−14
Value: C
Sulfurospirillum
1.00
0.75
0.50
0.25
0.00
A (n=15)
B (n=16)
C (n=14)
surface

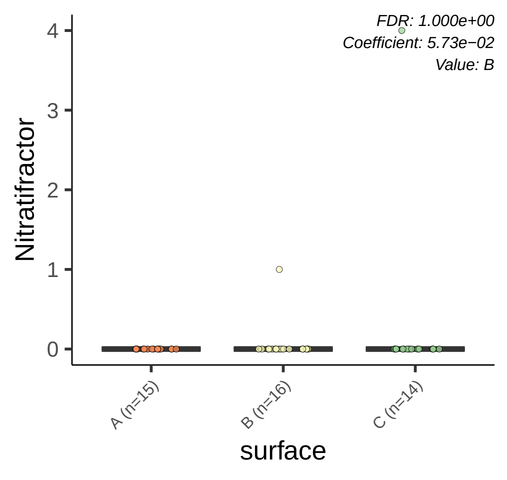
FDR: 1.000e+00
Coefficient: 5.73e−02
Value: B
Nitratifractor
4
3
2
1
0
A (n=15)
B (n=16)
C (n=14)
surface

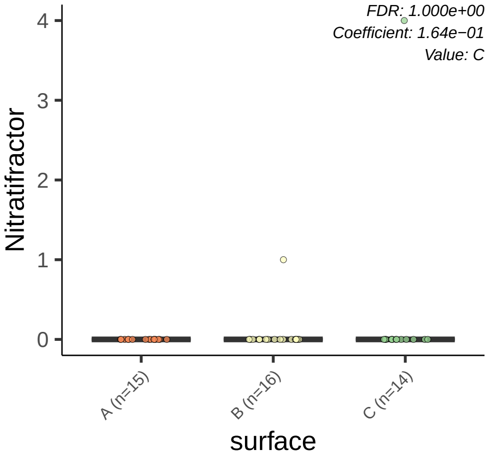
FDR: 1.000e+00
Coefficient: 1.64e−01
Value: C
Nitratifractor
4
3
2
1
0
A (n=15)
B (n=16)
C (n=14)
surface

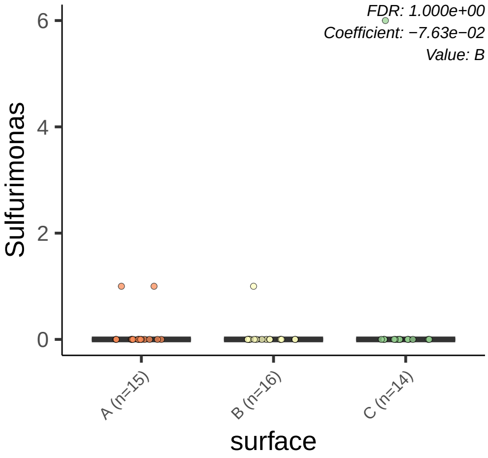
FDR: 1.000e+00
Coefficient: −7.63e−02
Value: B
Sulfurimonas
6
4
2
0
A (n=15)
B (n=16)
C (n=14)
surface

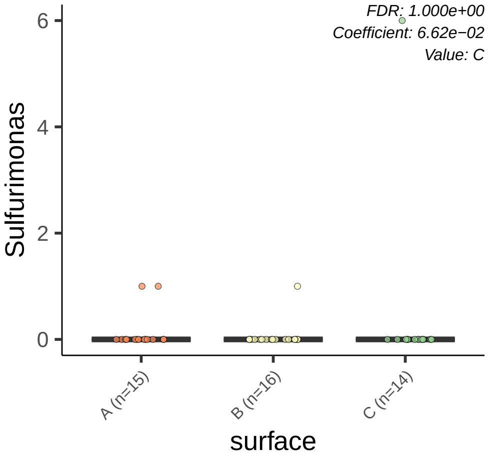

FDR: 1.000e+00
Coefficient: 6.62e−02
Value: C
Sulfurimonas
6
4
2
0
A (n=15)
B (n=16)
C (n=14)
surface

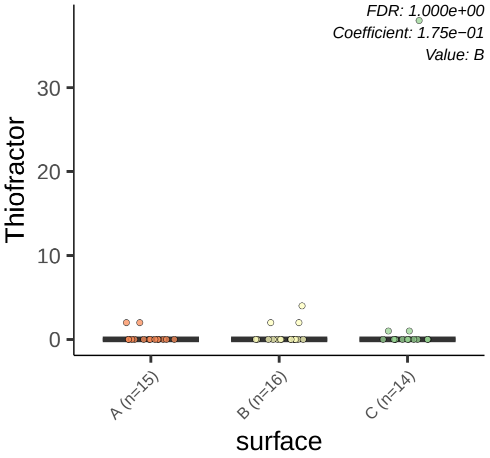

FDR: 1.000e+00
Coefficient: 1.75e−01
Value: B
Thiofractor
30
20
10
0
A (n=15)
B (n=16)
C (n=14)
surface

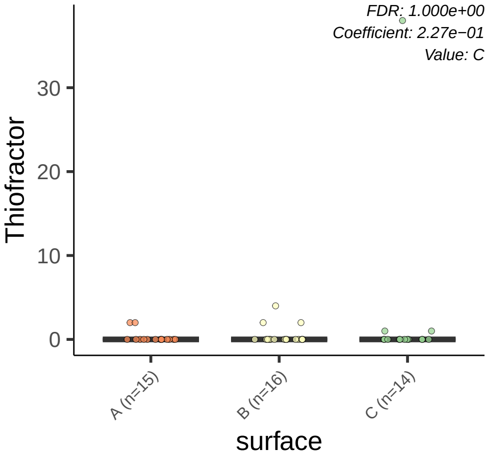
FDR: 1.000e+00
Coefficient: 2.27e−01
Value: C
Thiofractor
0
10
20
30
A (n=15)
B (n=16)
C (n=14)
surface

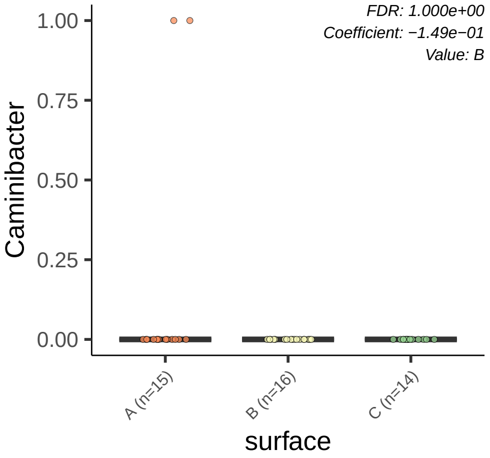

FDR: 1.000e+00
Coefficient: −1.49e−01
Value: B
Caminibacter
1.00
0.75
0.50
0.25
0.00
A (n=15)
B (n=16)
C (n=14)
surface

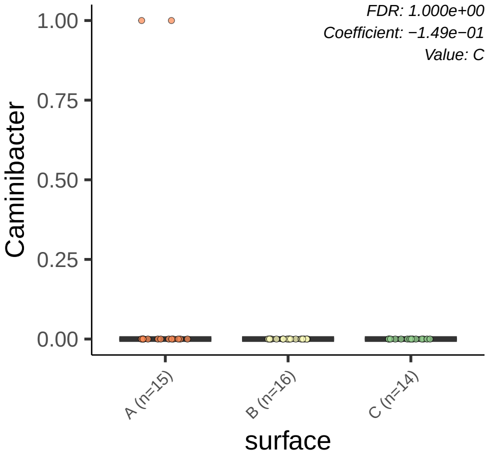
FDR: 1.000e+00
Coefficient: −1.49e−01
Value: C
Caminibacter
1.00
0.75
0.50
0.25
0.00
A (n=15)
B (n=16)
C (n=14)
surface

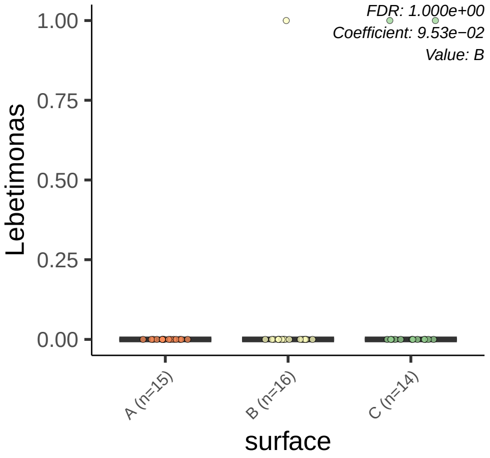

FDR: 1.000e+00
Coefficient: 9.53e−02
Value: B
Lebetimonas
1.00
0.75
0.50
0.25
0.00
A (n=15)
B (n=16)
C (n=14)
surface

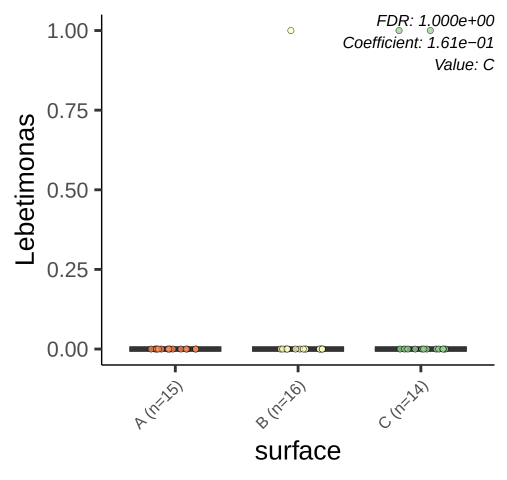

FDR: 1.000e+00
Coefficient: 1.61e−01
Value: C
Lebetimonas
1.00
0.75
0.50
0.25
0.00
A (n=15)
B (n=16)
C (n=14)
surface

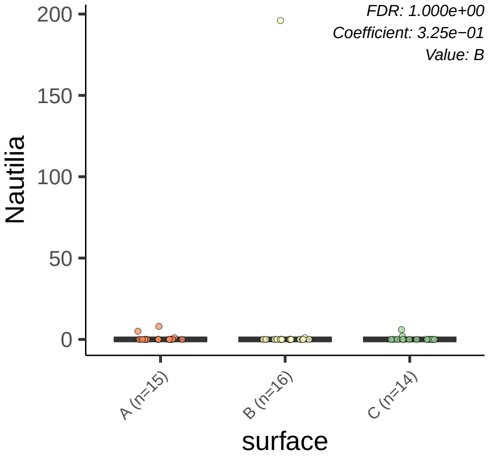
FDR: 1.000e+00
Coefficient: 3.25e−01
Value: B
Nautilia
200
150
100
50
0
A (n=15)
B (n=16)
C (n=14)
surface

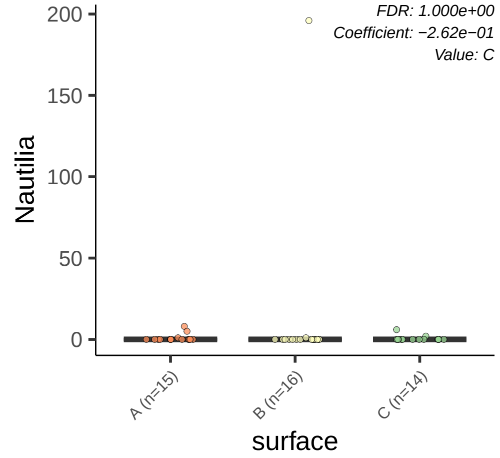
FDR: 1.000e+00
Coefficient: −2.62e−01
Value: C
Nautilia
200
150
100
50
0
A (n=15)
B (n=16)
C (n=14)
surface

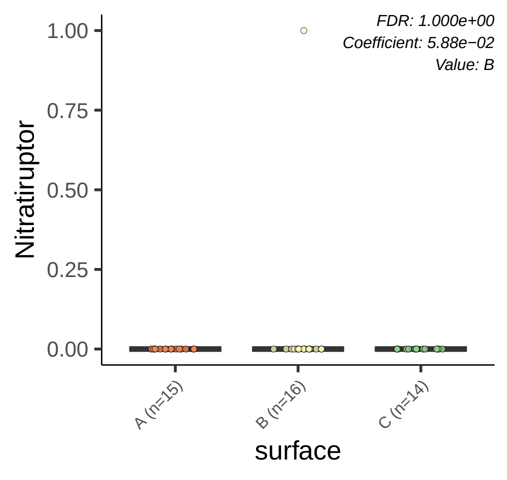
FDR: 1.000e+00
Coefficient: 5.88e−02
Value: B
Nitratiruptor
1.00
0.75
0.50
0.25
0.00
A (n=15)
B (n=16)
C (n=14)
surface

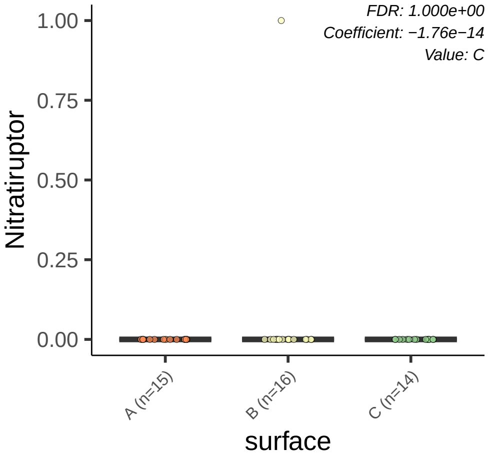
FDR: 1.000e+00
Coefficient: −1.76e−14
Value: C
Nitratiruptor
1.00
0.75
0.50
0.25
0.00
A (n=15)
B (n=16)
C (n=14)
surface

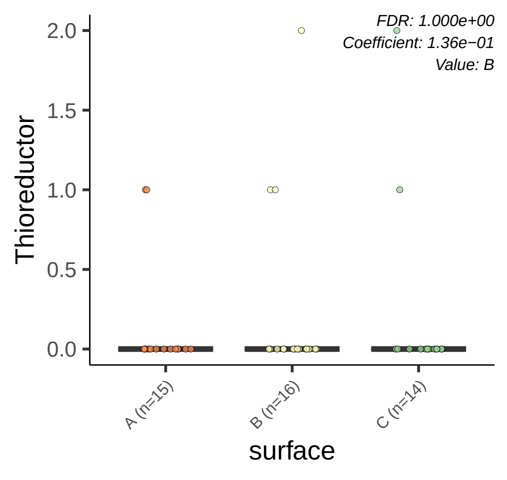
FDR: 1.000e+00
Coefficient: 1.36e−01
Value: B
Thioreductor
2.0
1.5
1.0
0.5
0.0
A (n=15)
B (n=16)
C (n=14)
surface

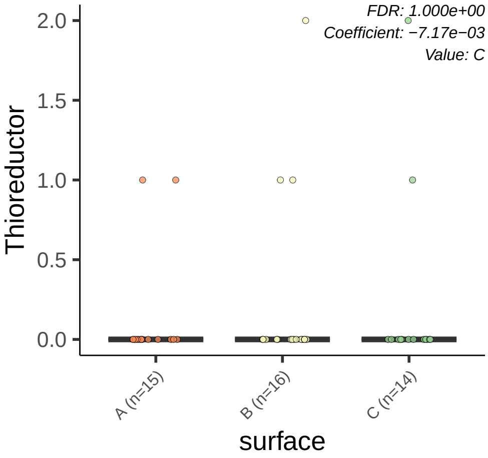
FDR: 1.000e+00
Coefficient: −7.17e−03
Value: C
Thioreductor
2.0
1.5
1.0
0.5
0.0
A (n=15)
B (n=16)
C (n=14)
surface

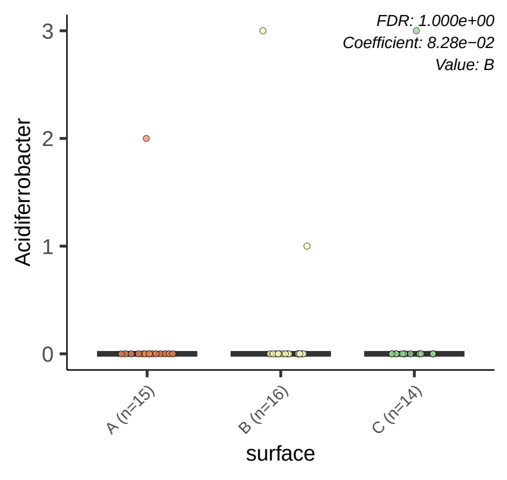
FDR: 1.000e+00
Coefficient: 8.28e−02
Value: B
Acidiferrobacter
3
2
1
0
A (n=15)
B (n=16)
C (n=14)
surface

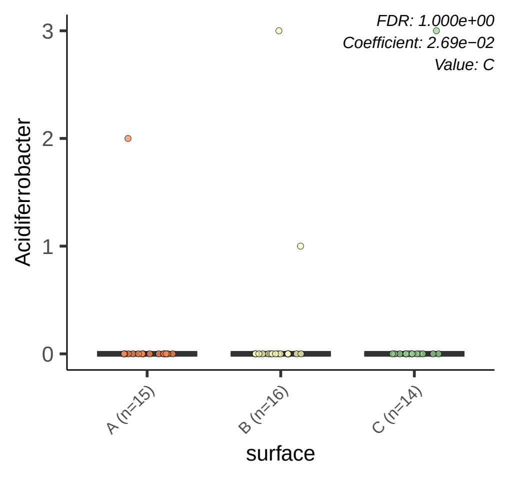
FDR: 1.000e+00
Coefficient: 2.69e−02
Value: C
Acidiferrobacter
3
2
1
0
A (n=15)
B (n=16)
C (n=14)
surface

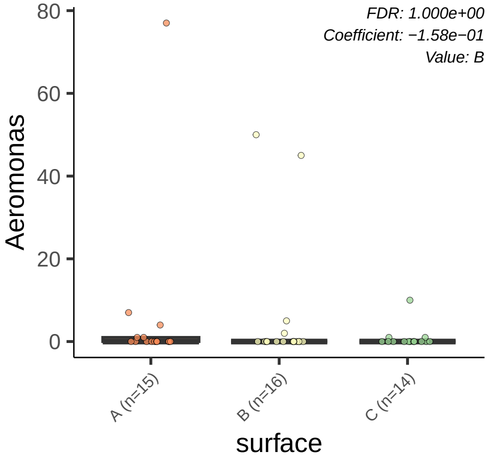
FDR: 1.000e+00
Coefficient: −1.58e−01
Value: B
Aeromonas
0
20
40
60
80
A (n=15)
B (n=16)
C (n=14)
surface

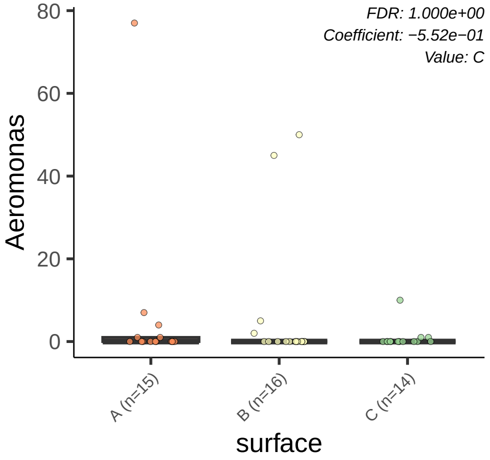
FDR: 1.000e+00
Coefficient: −5.52e−01
Value: C
Aeromonas
80
60
40
20
0
A (n=15)
B (n=16)
C (n=14)
surface

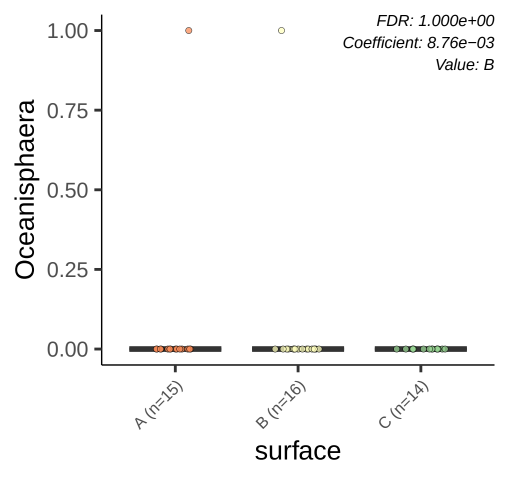
FDR: 1.000e+00
Coefficient: 8.76e−03
Value: B
Oceanisphaera
1.00
0.75
0.50
0.25
0.00
A (n=15)
B (n=16)
C (n=14)
surface

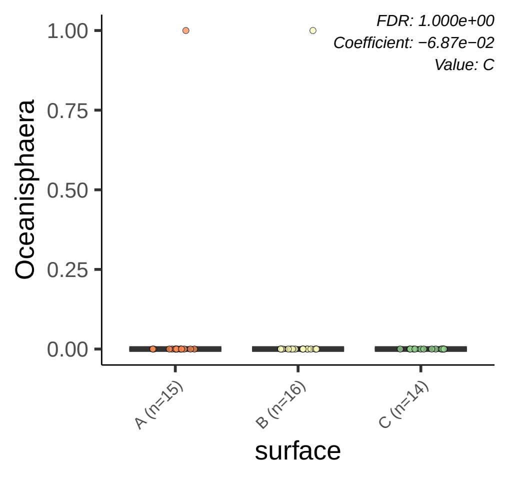

FDR: 1.000e+00
Coefficient: −6.87e−02
Value: C
Oceanisphaera
1.00
0.75
0.50
0.25
0.00
A (n=15)
B (n=16)
C (n=14)
surface

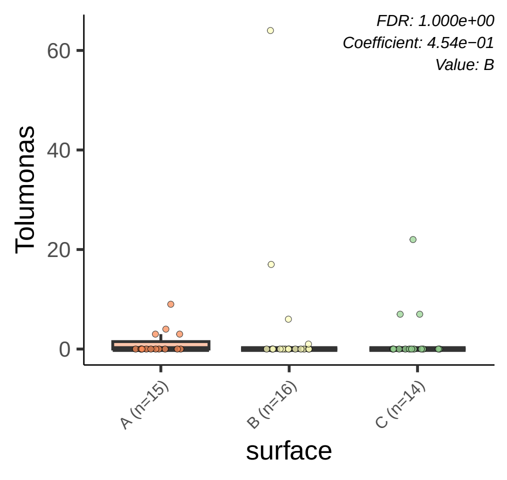

FDR: 1.000e+00
Coefficient: 4.54e−01
Value: B
Tolumonas
60
40
20
0
A (n=15)
B (n=16)
C (n=14)
surface

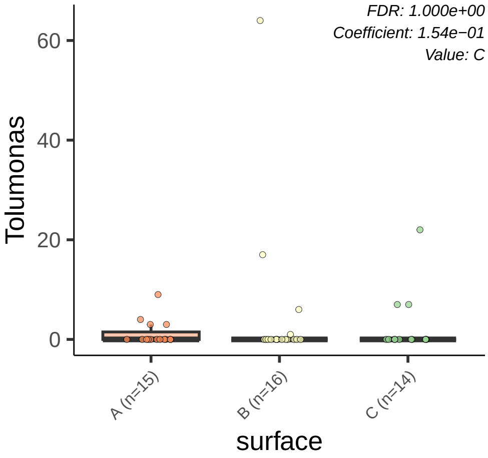

FDR: 1.000e+00
Coefficient: 1.54e−01
Value: C
Tolumonas
0
20
40
60
A (n=15)
B (n=16)
C (n=14)
surface

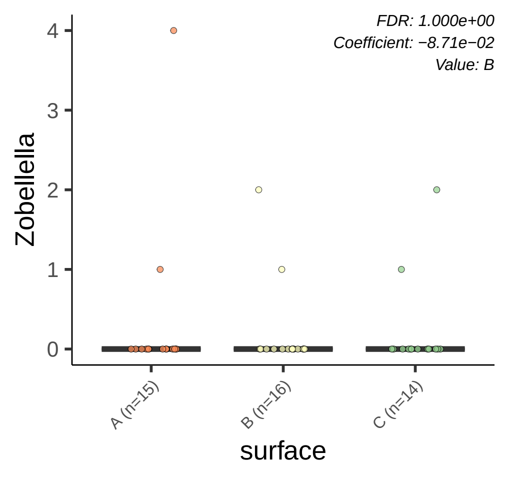
FDR: 1.000e+00
Coefficient: −8.71e−02
Value: B
Zobellella
4
3
2
1
0
A (n=15)
B (n=16)
C (n=14)
surface

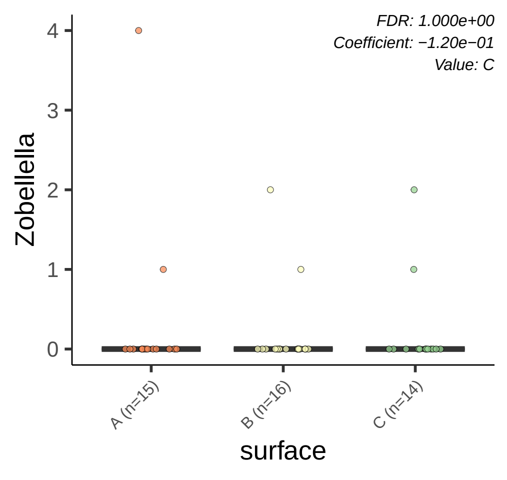

FDR: 1.000e+00
Coefficient: −1.20e−01
Value: C
Zobellella
4
3
2
1
0
A (n=15)
B (n=16)
C (n=14)
surface

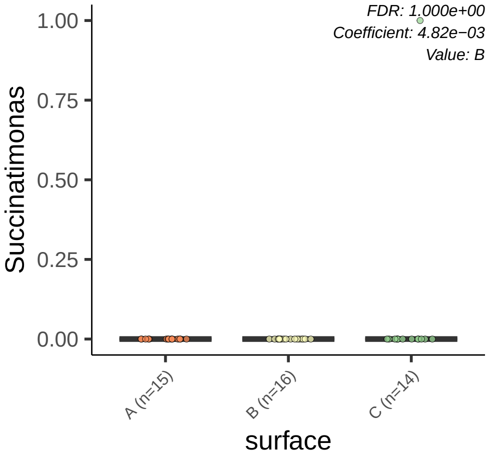
FDR: 1.000e+00
Coefficient: 4.82e−03
Value: B
Succinatimonas
1.00
0.75
0.50
0.25
0.00
A (n=15)
B (n=16)
C (n=14)
surface

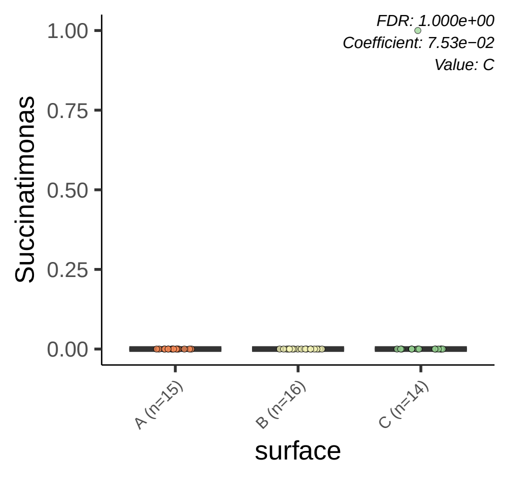
FDR: 1.000e+00
Coefficient: 7.53e−02
Value: C
Succinatimonas
1.00
0.75
0.50
0.25
0.00
A (n=15)
B (n=16)
C (n=14)
surface

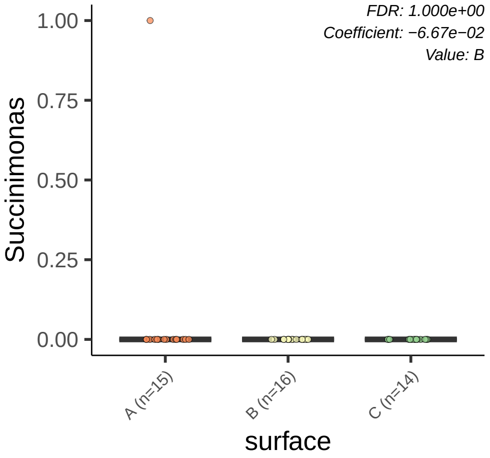
FDR: 1.000e+00
Coefficient: −6.67e−02
Value: B
Succinimonas
1.00
0.75
0.50
0.25
0.00
A (n=15)
B (n=16)
C (n=14)
surface

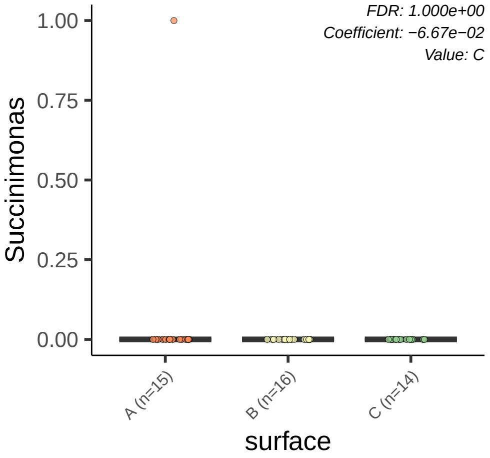
FDR: 1.000e+00
Coefficient: −6.67e−02
Value: C
Succinimonas
1.00
0.75
0.50
0.25
0.00
A (n=15)
B (n=16)
C (n=14)
surface

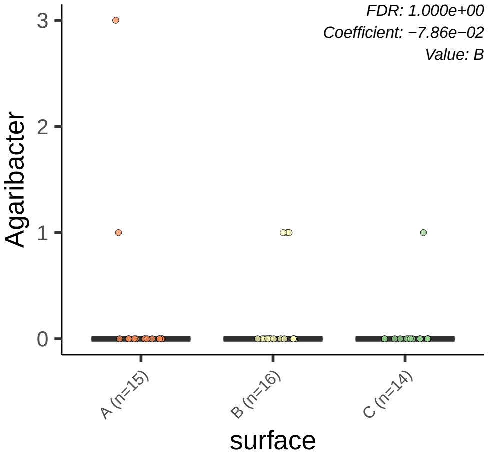
FDR: 1.000e+00
Coefficient: −7.86e−02
Value: B
Agaribacter
0
1
2
3
A (n=15)
B (n=16)
C (n=14)
surface

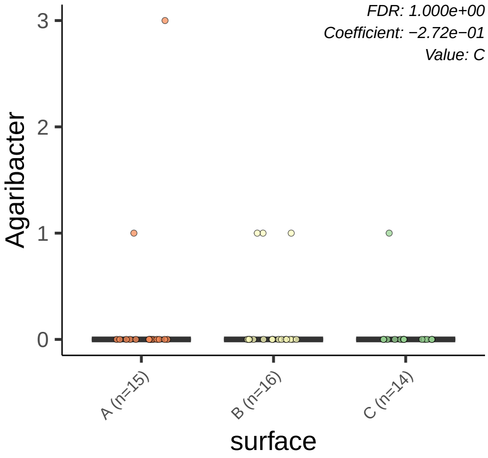
FDR: 1.000e+00
Coefficient: −2.72e−01
Value: C
Agaribacter
3
2
1
0
A (n=15)
B (n=16)
C (n=14)
surface

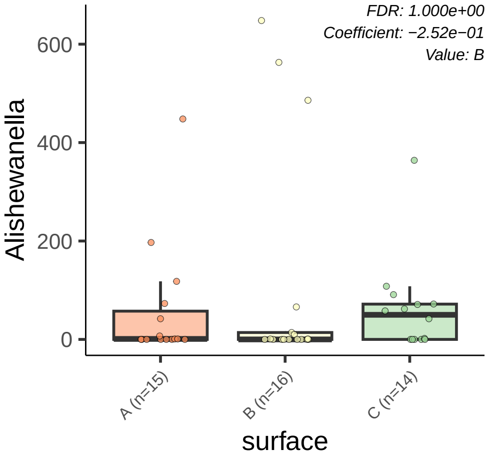

FDR: 1.000e+00
Coefficient: −2.52e−01
Value: B
Alishewanella
600
400
200
0
A (n=15)
B (n=16)
C (n=14)
surface

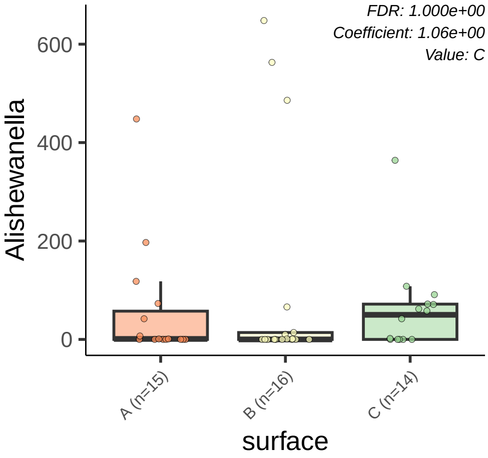

FDR: 1.000e+00
Coefficient: 1.06e+00
Value: C
Alishewanella
600
400
200
0
A (n=15)
B (n=16)
C (n=14)
surface

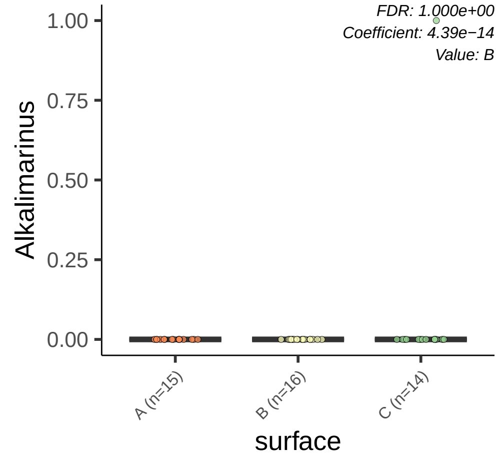

FDR: 1.000e+00
Coefficient: 4.39e−14
Value: B
1.00
0.75
0.50
0.25
0.00
Alkalimarinus
A (n=15)
B (n=16)
C (n=14)
surface

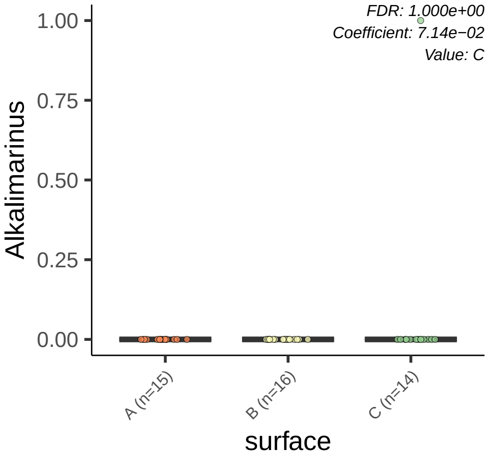

FDR: 1.000e+00
Coefficient: 7.14e−02
Value: C
1.00
0.75
0.50
0.25
0.00
Alkalimarinus
A (n=15)
B (n=16)
C (n=14)
surface

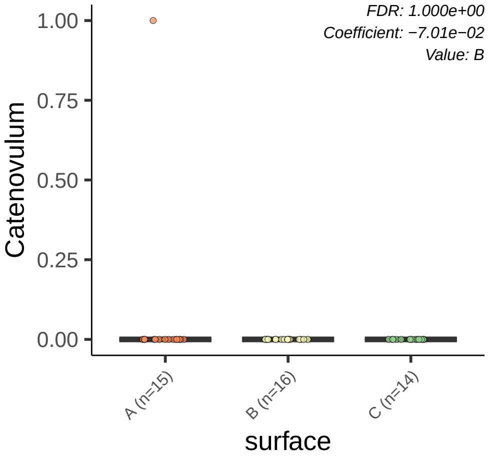
FDR: 1.000e+00
Coefficient: −7.01e−02
Value: B
Catenovulum
1.00
0.75
0.50
0.25
0.00
A (n=15)
B (n=16)
C (n=14)
surface

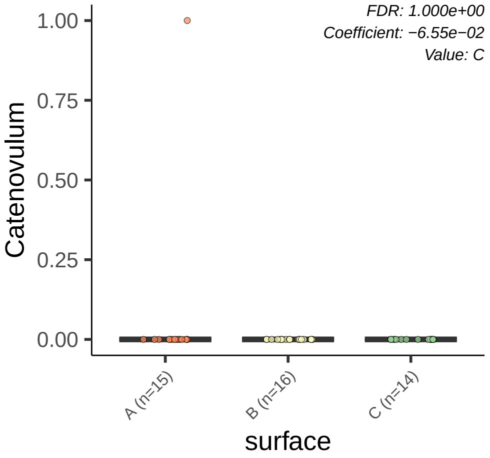
FDR: 1.000e+00
Coefficient: −6.55e−02
Value: C
Catenovulum
1.00
0.75
0.50
0.25
0.00
A (n=15)
B (n=16)
C (n=14)
surface

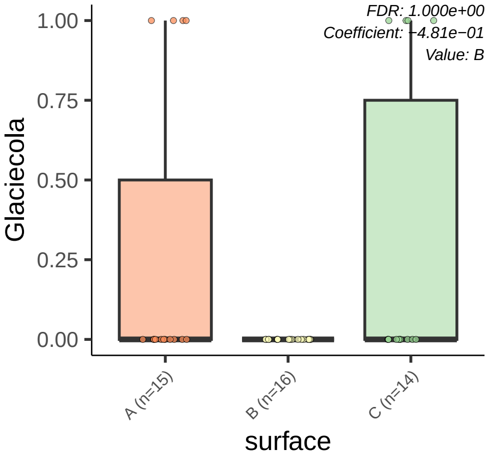

FDR: 1.000e+00
Coefficient: −4.81e−01
Value: B
Glaciecola
1.00
0.75
0.50
0.25
0.00
A (n=15)
B (n=16)
C (n=14)
surface

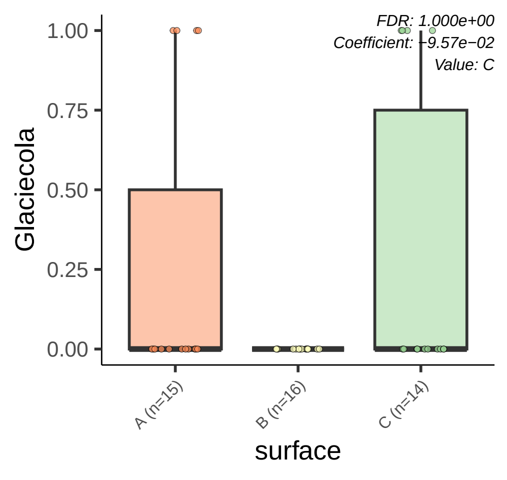
FDR: 1.000e+00
Coefficient: −9.57e−02
Value: C
1.00
0.75
0.50
0.25
0.00
Glaciecola
A (n=15)
B (n=16)
C (n=14)
surface

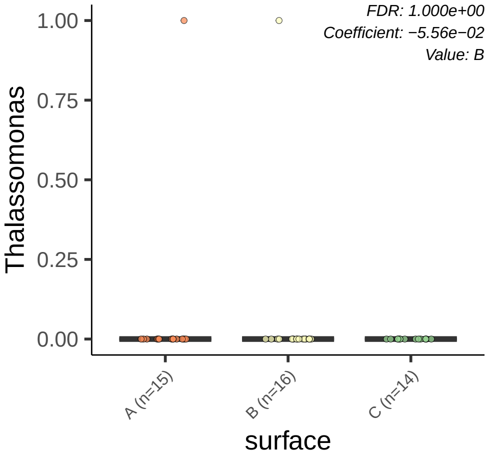
FDR: 1.000e+00
Coefficient: −5.56e−02
Value: B
Thalassomonas
1.00
0.75
0.50
0.25
0.00
A (n=15)
B (n=16)
C (n=14)
surface

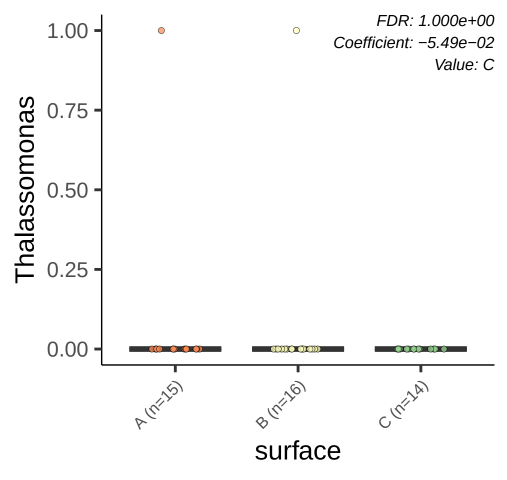
FDR: 1.000e+00
Coefficient: −5.49e−02
Value: C
Thalassomonas
1.00
0.75
0.50
0.25
0.00
A (n=15)
B (n=16)
C (n=14)
surface

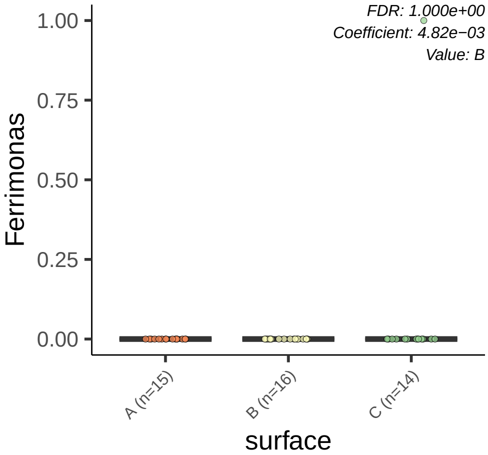
FDR: 1.000e+00
Coefficient: 4.82e−03
Value: B
Ferrimonas
1.00
0.75
0.50
0.25
0.00
A (n=15)
B (n=16)
C (n=14)
surface

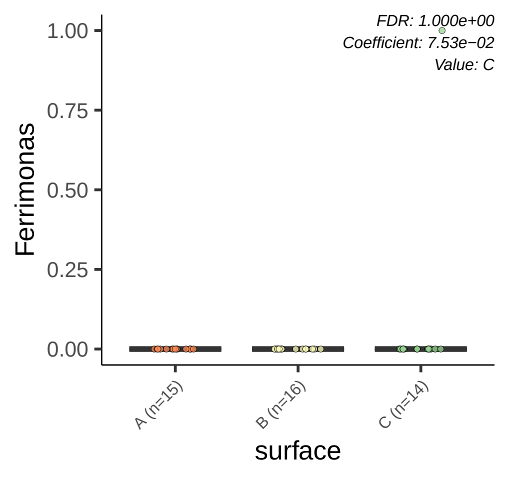
FDR: 1.000e+00
Coefficient: 7.53e−02
Value: C
Ferrimonas
1.00
0.75
0.50
0.25
0.00
A (n=15)
B (n=16)
C (n=14)
surface

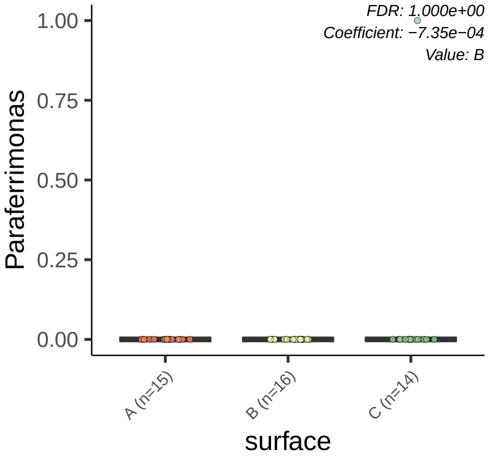

FDR: 1.000e+00
Coefficient: −7.35e−04
Value: B
Paraferrimonas
1.00
0.75
0.50
0.25
0.00
A (n=15)
B (n=16)
C (n=14)
surface

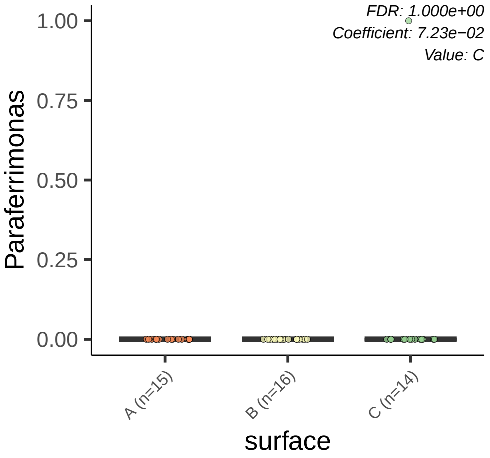
FDR: 1.000e+00
Coefficient: 7.23e−02
Value: C
Paraferrimonas
1.00
0.75
0.50
0.25
0.00
A (n=15)
B (n=16)
C (n=14)
surface

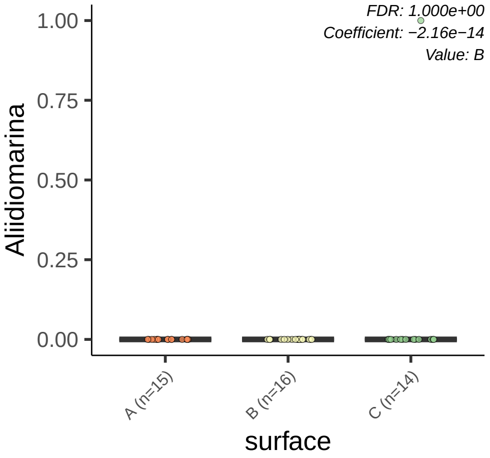

FDR: 1.000e+00
Coefficient: −2.16e−14
Value: B
Aliidiomarina
1.00
0.75
0.50
0.25
0.00
A (n=15)
B (n=16)
C (n=14)
surface

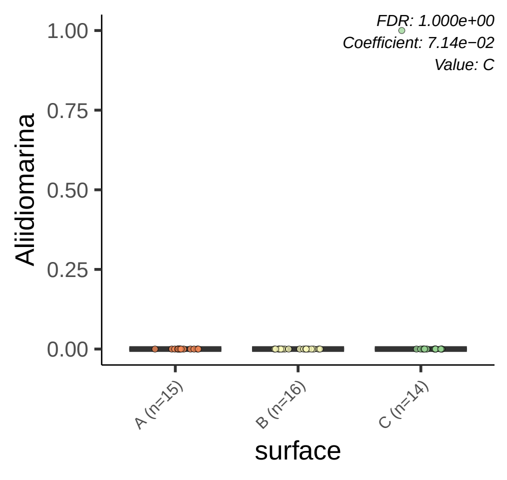
FDR: 1.000e+00
Coefficient: 7.14e−02
Value: C
Aliidiomarina
1.00
0.75
0.50
0.25
0.00
A (n=15)
B (n=16)
C (n=14)
surface

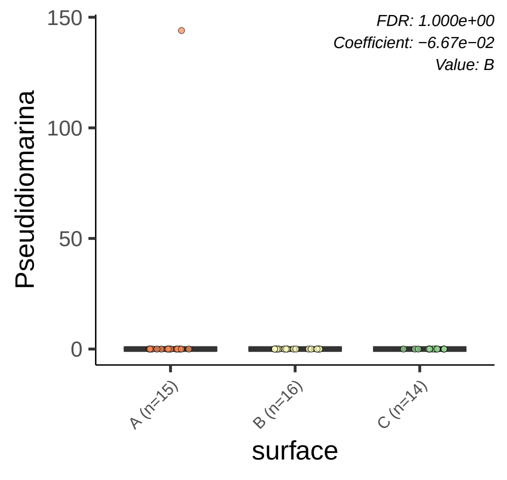
FDR: 1.000e+00
Coefficient: −6.67e−02
Value: B
Pseudidiomarina
150
100
50
0
A (n=15)
B (n=16)
C (n=14)
surface

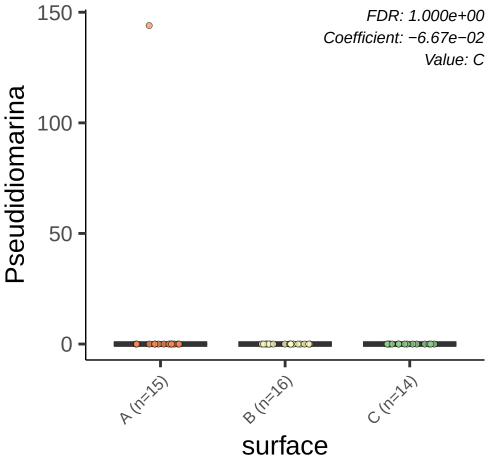
FDR: 1.000e+00
Coefficient: −6.67e−02
Value: C
Pseudidiomarina
150
100
50
0
A (n=15)
B (n=16)
C (n=14)
surface

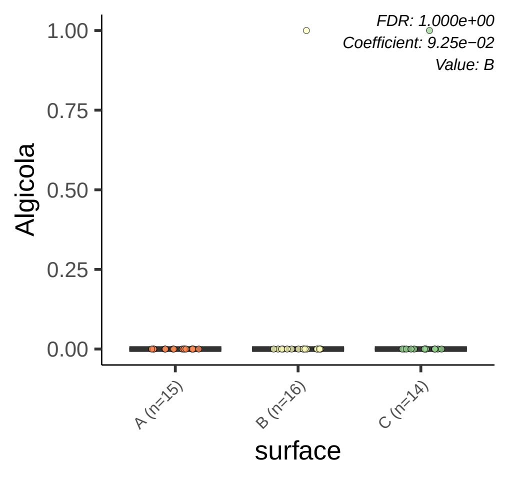

FDR: 1.000e+00
Coefficient: 9.25e−02
Value: B
1.00
0.75
0.50
0.25
0.00
Algicola
A (n=15)
B (n=16)
C (n=14)
surface

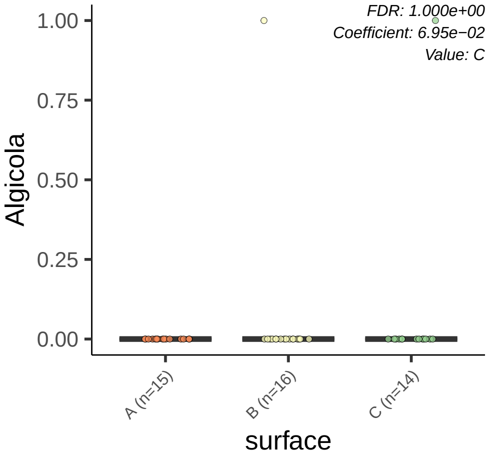
FDR: 1.000e+00
Coefficient: 6.95e−02
Value: C
Algicola
1.00
0.75
0.50
0.25
0.00
A (n=15)
B (n=16)
C (n=14)
surface

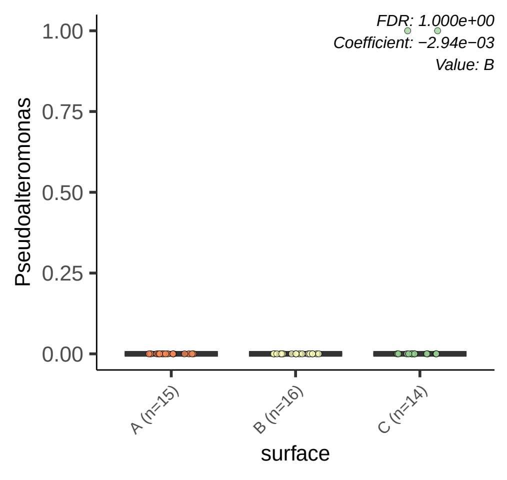
FDR: 1.000e+00
Coefficient: −2.94e−03
Value: B
Pseudoalteromonas
1.00
0.75
0.50
0.25
0.00
A (n=15)
B (n=16)
C (n=14)
surface

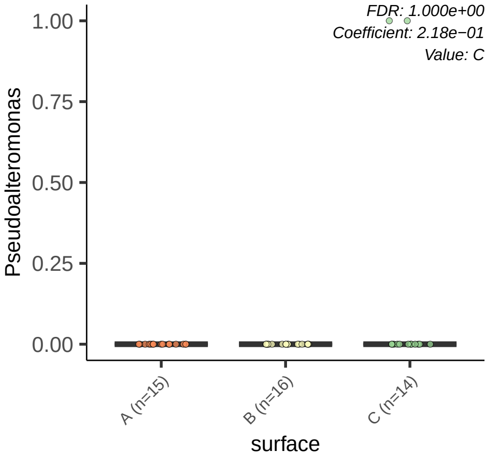
FDR: 1.000e+00
Coefficient: 2.18e−01
Value: C
Pseudoalteromonas
1.00
0.75
0.50
0.25
0.00
A (n=15)
B (n=16)
C (n=14)
surface

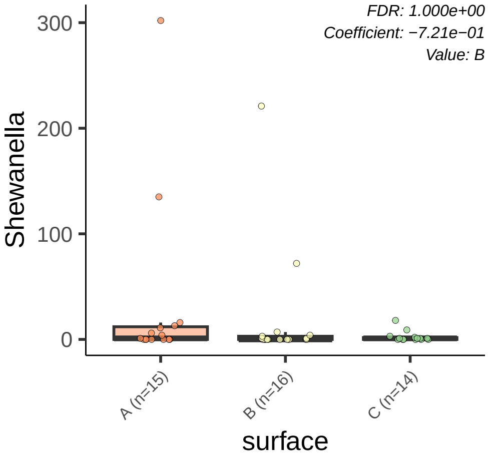
FDR: 1.000e+00
Coefficient: −7.21e−01
Value: B
Shewanella
300
200
100
0
A (n=15)
B (n=16)
C (n=14)
surface

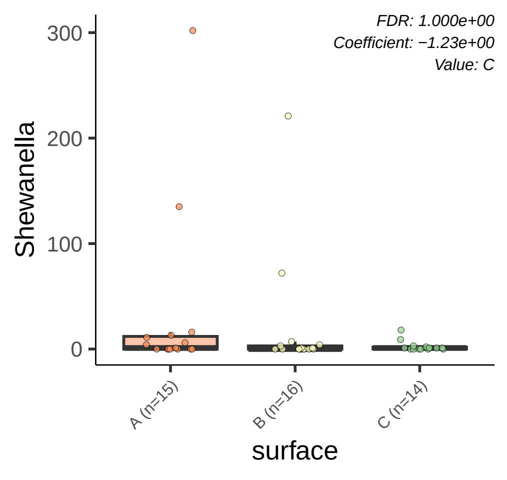
FDR: 1.000e+00
Coefficient: −1.23e+00
Value: C
Shewanella
300
200
100
0
A (n=15)
B (n=16)
C (n=14)
surface

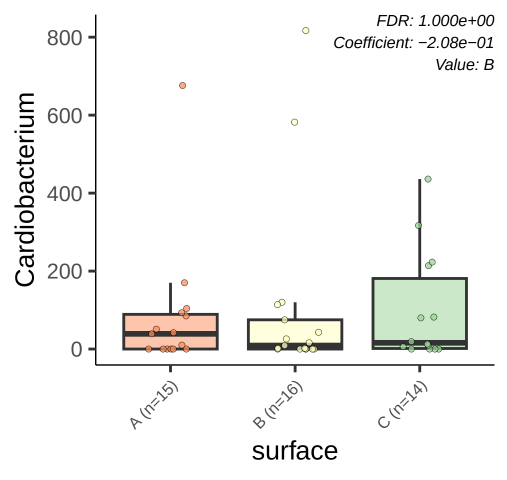

FDR: 1.000e+00
Coefficient: −2.08e−01
Value: B
Cardiobacterium
800
600
400
200
0
A (n=15)
B (n=16)
C (n=14)
surface

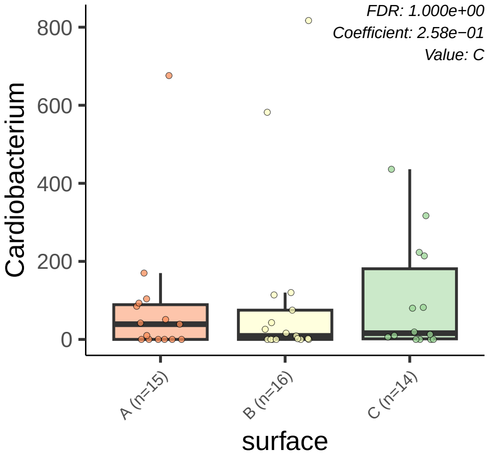
FDR: 1.000e+00
Coefficient: 2.58e−01
Value: C
Cardiobacterium
0
200
400
600
800
A (n=15)
B (n=16)
C (n=14)
surface

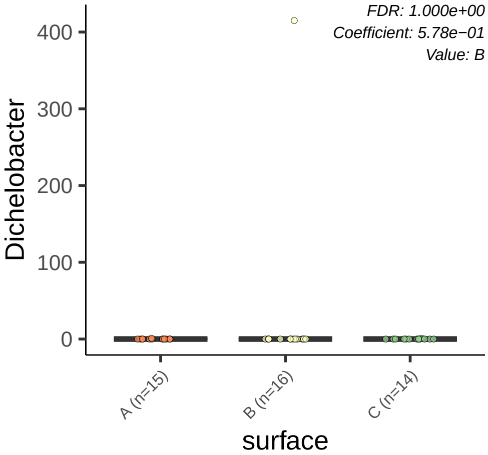

FDR: 1.000e+00
Coefficient: 5.78e−01
Value: B
Dichelobacter
400
300
200
100
0
A (n=15)
B (n=16)
C (n=14)
surface

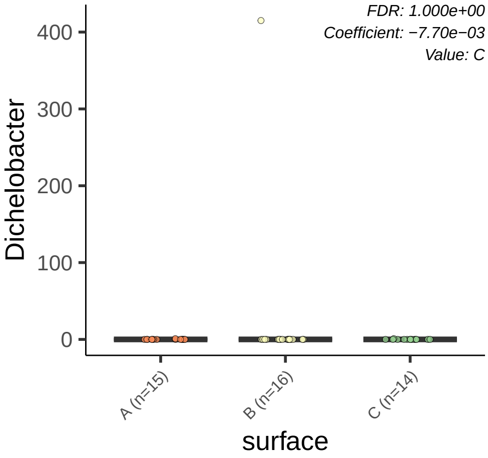
FDR: 1.000e+00
Coefficient: −7.70e−03
Value: C
Dichelobacter
400
300
200
100
0
A (n=15)
B (n=16)
C (n=14)
surface

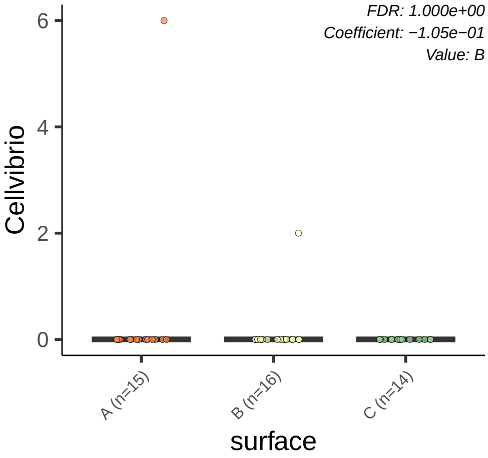
FDR: 1.000e+00
Coefficient: −1.05e−01
Value: B
Cellvibrio
6
4
2
0
A (n=15)
B (n=16)
C (n=14)
surface

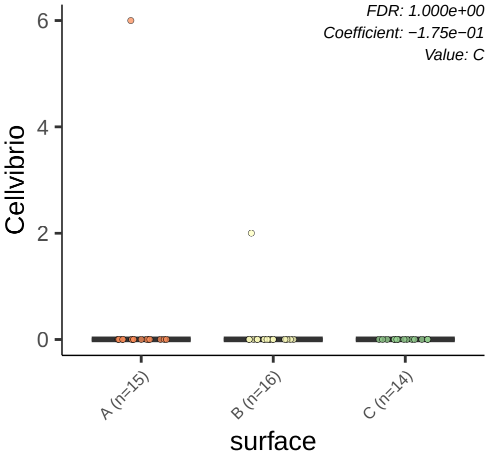

FDR: 1.000e+00
Coefficient: −1.75e−01
Value: C
Cellvibrio
6
4
2
0
A (n=15)
B (n=16)
C (n=14)
surface

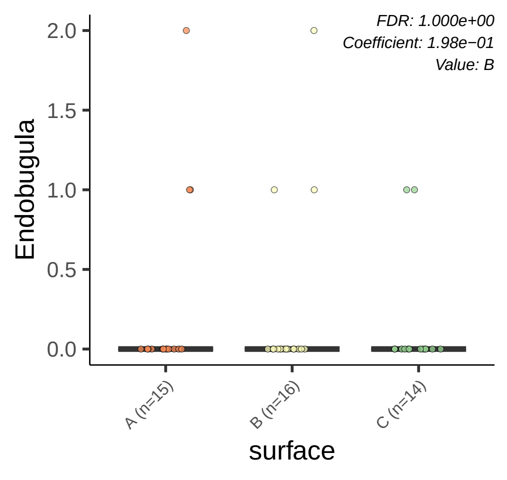
FDR: 1.000e+00
Coefficient: 1.98e−01
Value: B
Endobugula
2.0
1.5
1.0
0.5
0.0
A (n=15)
B (n=16)
C (n=14)
surface

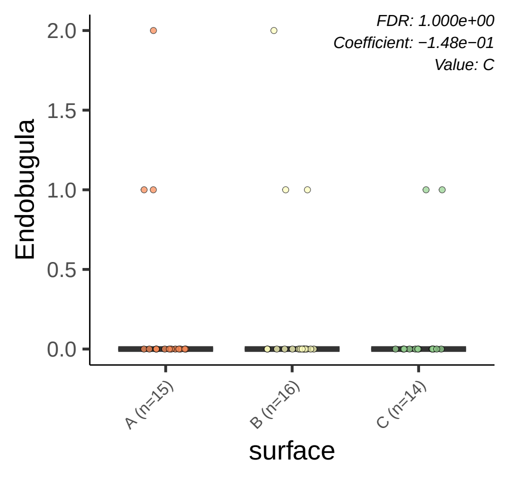
FDR: 1.000e+00
Coefficient: −1.48e−01
Value: C
Endobugula
2.0
1.5
1.0
0.5
0.0
A (n=15)
B (n=16)
C (n=14)
surface

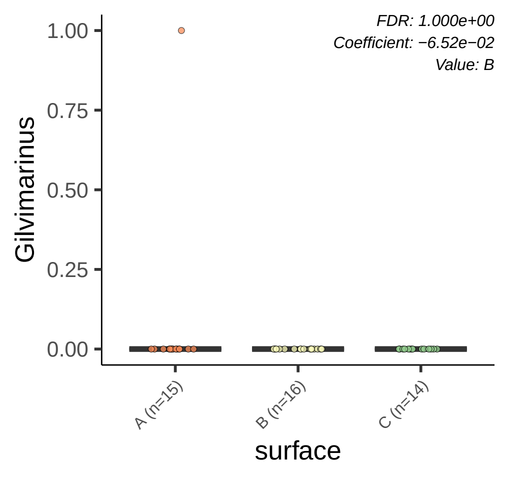

FDR: 1.000e+00
Coefficient: −6.52e−02
Value: B
Gilvimarinus
1.00
0.75
0.50
0.25
0.00
A (n=15)
B (n=16)
C (n=14)
surface

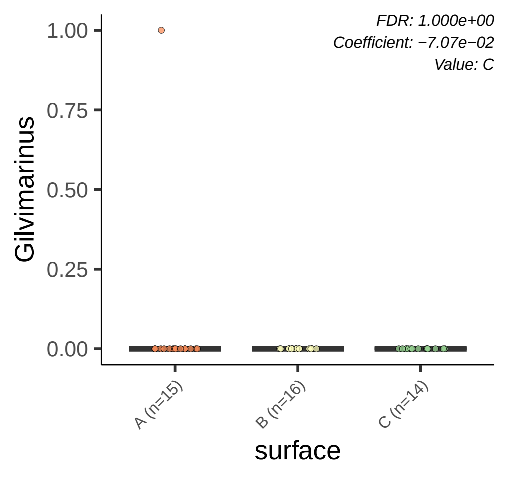
FDR: 1.000e+00
Coefficient: −7.07e−02
Value: C
1.00
0.75
0.50
0.25
0.00
Gilvimarinus
A (n=15)
B (n=16)
C (n=14)
surface

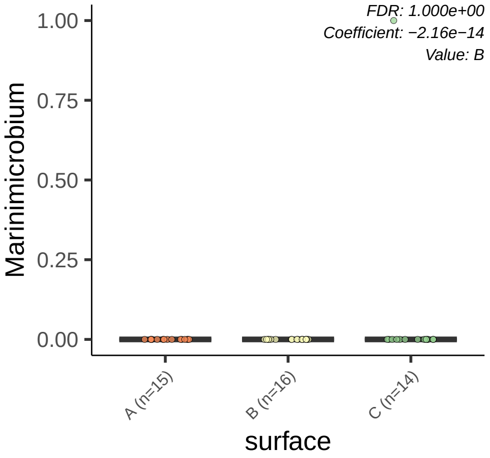
FDR: 1.000e+00
Coefficient: −2.16e−14
Value: B
Marinimicrobium
1.00
0.75
0.50
0.25
0.00
A (n=15)
B (n=16)
C (n=14)
surface

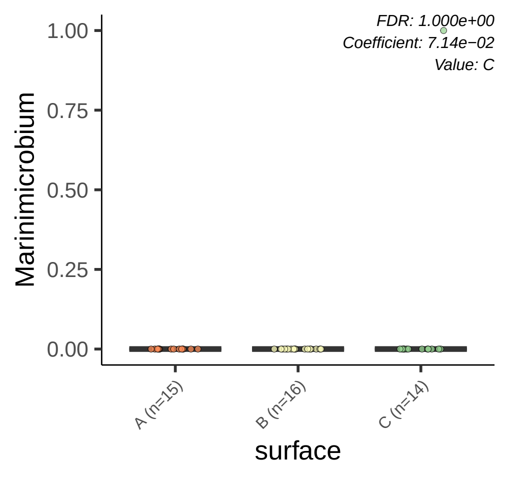

FDR: 1.000e+00
Coefficient: 7.14e−02
Value: C
Marinimicrobium
1.00
0.75
0.50
0.25
0.00
A (n=15)
B (n=16)
C (n=14)
surface

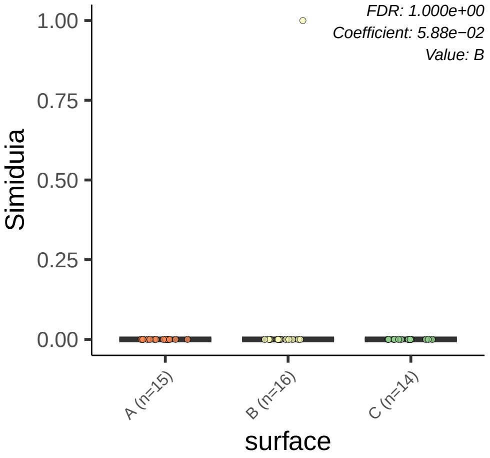
FDR: 1.000e+00
Coefficient: 5.88e−02
Value: B
Simiduia
1.00
0.75
0.50
0.25
0.00
A (n=15)
B (n=16)
C (n=14)
surface

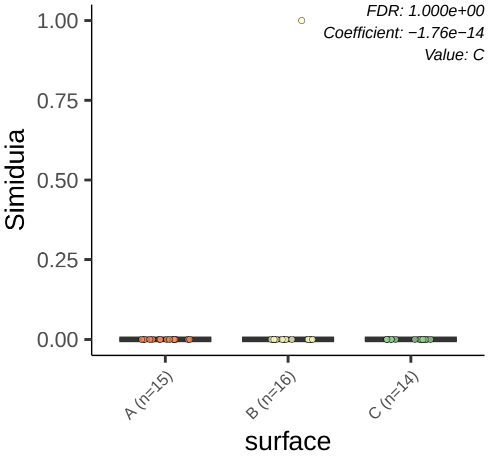
FDR: 1.000e+00
Coefficient: −1.76e−14
Value: C
Simiduia
1.00
0.75
0.50
0.25
0.00
A (n=15)
B (n=16)
C (n=14)
surface

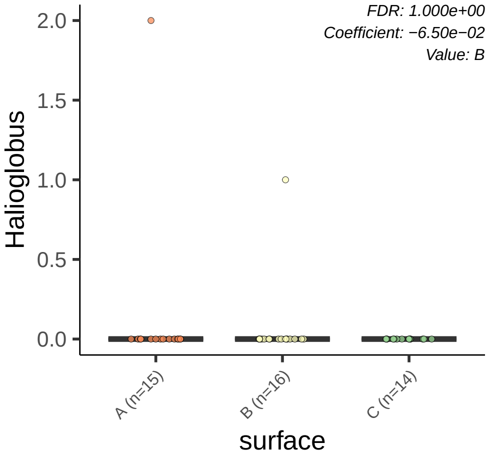
FDR: 1.000e+00
Coefficient: −6.50e−02
Value: B
Halioglobus
2.0
1.5
1.0
0.5
0.0
A (n=15)
B (n=16)
C (n=14)
surface

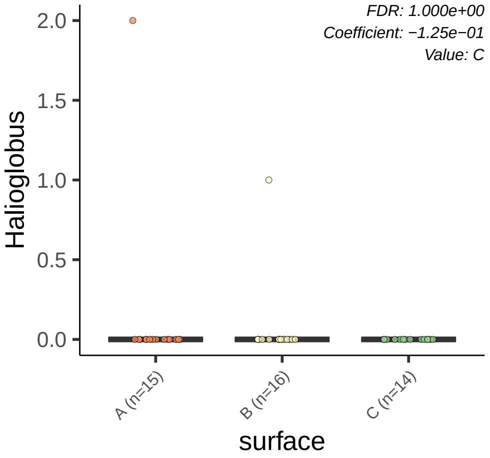
FDR: 1.000e+00
Coefficient: −1.25e−01
Value: C
Halioglobus
2.0
1.5
1.0
0.5
0.0
A (n=15)
B (n=16)
C (n=14)
surface

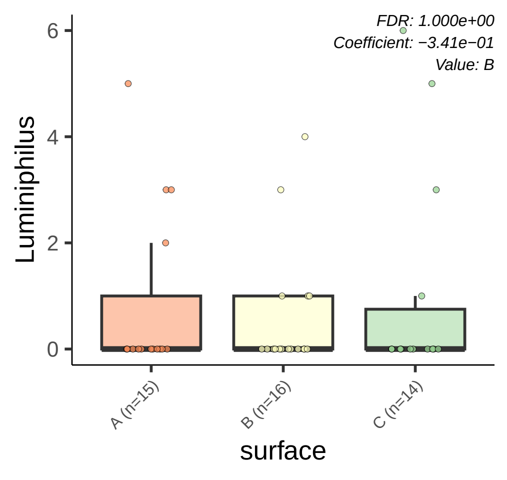
FDR: 1.000e+00
Coefficient: −3.41e−01
Value: B
Luminiphilus
0
2
4
6
A (n=15)
B (n=16)
C (n=14)
surface

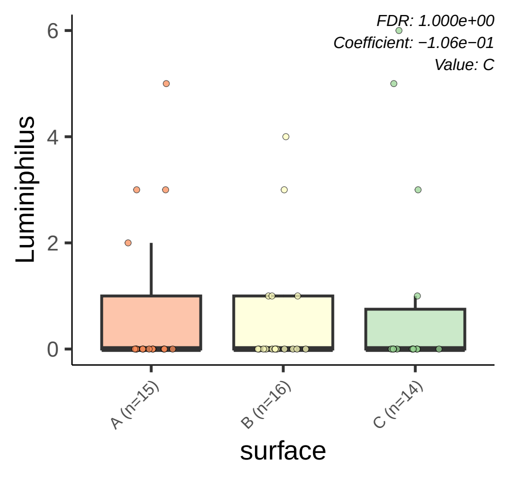
FDR: 1.000e+00
Coefficient: −1.06e−01
Value: C
Luminiphilus
0
2
4
6
A (n=15)
B (n=16)
C (n=14)
surface

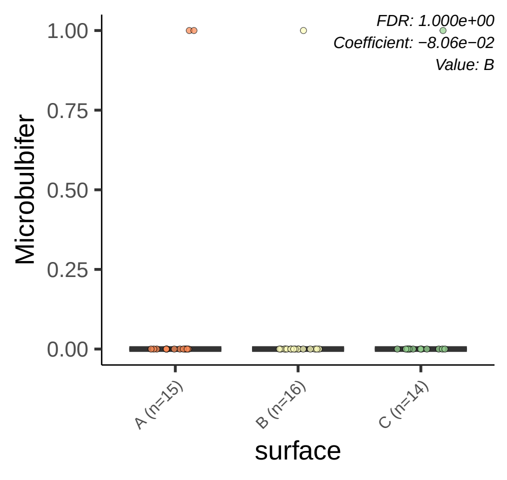
FDR: 1.000e+00
Coefficient: −8.06e−02
Value: B
Microbulbifer
1.00
0.75
0.50
0.25
0.00
A (n=15)
B (n=16)
C (n=14)
surface

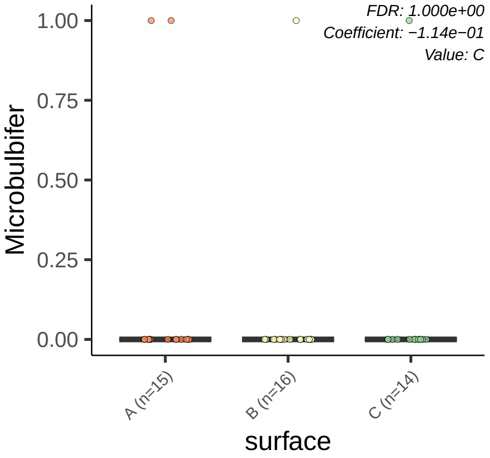
FDR: 1.000e+00
Coefficient: −1.14e−01
Value: C
Microbulbifer
1.00
0.75
0.50
0.25
0.00
A (n=15)
B (n=16)
C (n=14)
surface

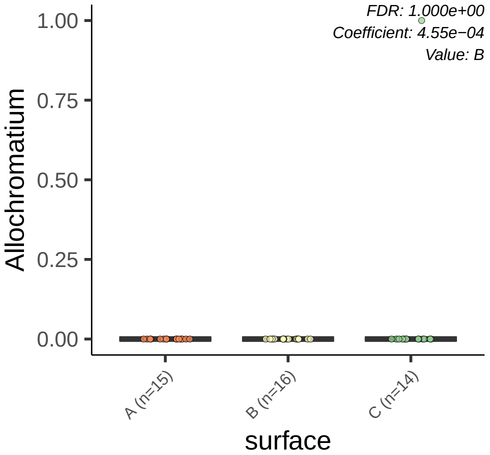

FDR: 1.000e+00
Coefficient: 4.55e−04
Value: B
Allochromatium
1.00
0.75
0.50
0.25
0.00
A (n=15)
B (n=16)
C (n=14)
surface

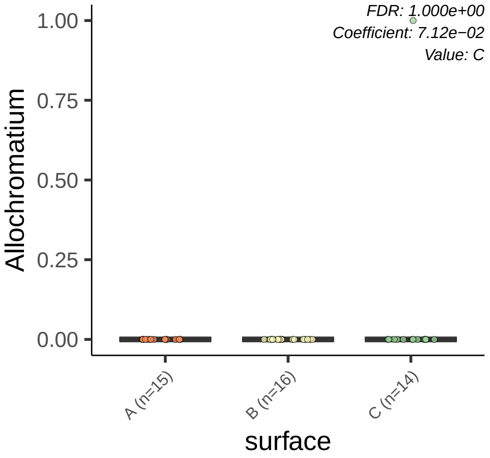
FDR: 1.000e+00
Coefficient: 7.12e−02
Value: C
Allochromatium
1.00
0.75
0.50
0.25
0.00
A (n=15)
B (n=16)
C (n=14)
surface

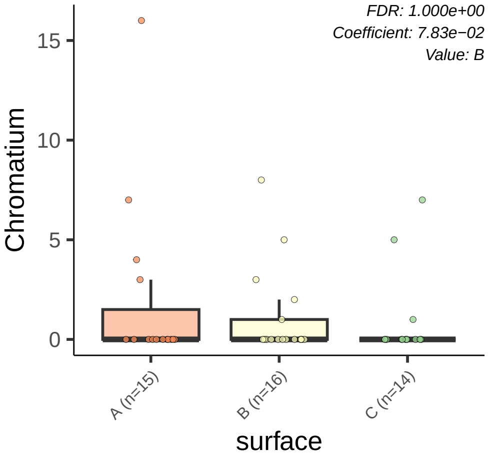

FDR: 1.000e+00
Coefficient: 7.83e−02
Value: B
Chromatium
15
10
5
0
A (n=15)
B (n=16)
C (n=14)
surface

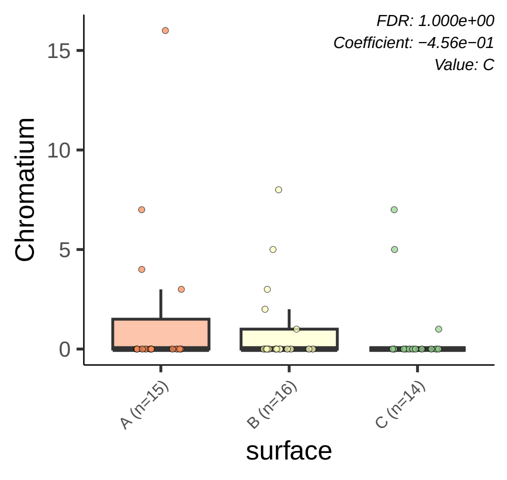

FDR: 1.000e+00
Coefficient: −4.56e−01
Value: C
Chromatium
15
10
5
0
A (n=15)
B (n=16)
C (n=14)
surface

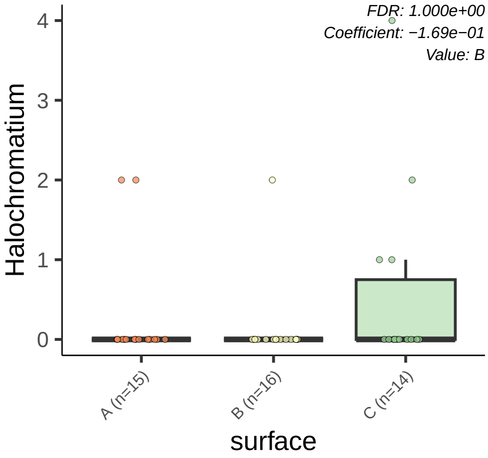

FDR: 1.000e+00
Coefficient: −1.69e−01
Value: B
Halochromatium
0
1
2
3
4
A (n=15)
B (n=16)
C (n=14)
surface

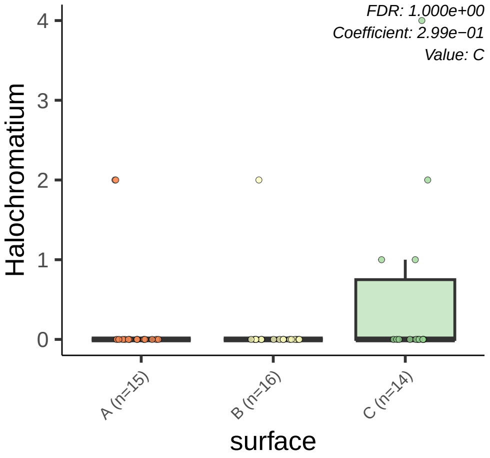

FDR: 1.000e+00
Coefficient: 2.99e−01
Value: C
Halochromatium
0
1
2
3
4
A (n=15)
B (n=16)
C (n=14)
surface

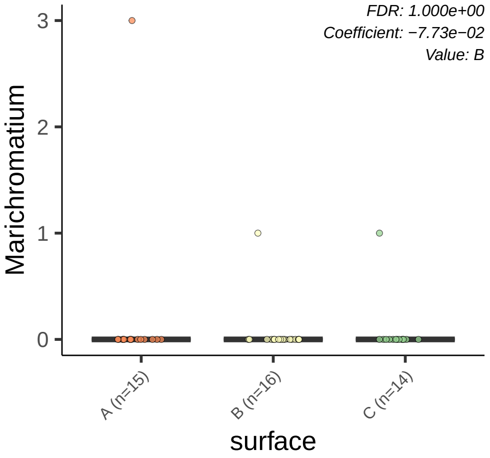

FDR: 1.000e+00
Coefficient: −7.73e−02
Value: B
Marichromatium
3
2
1
0
A (n=15)
B (n=16)
C (n=14)
surface

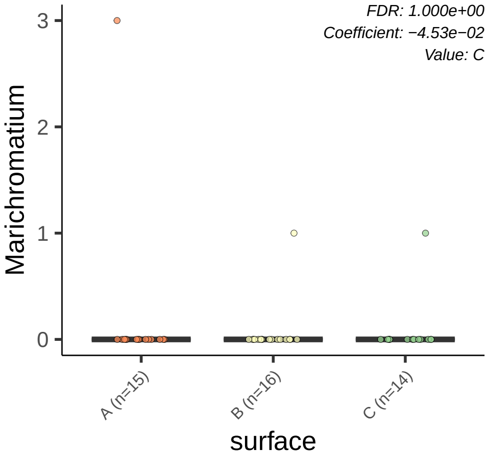

FDR: 1.000e+00
Coefficient: −4.53e−02
Value: C
Marichromatium
3
2
1
0
A (n=15)
B (n=16)
C (n=14)
surface

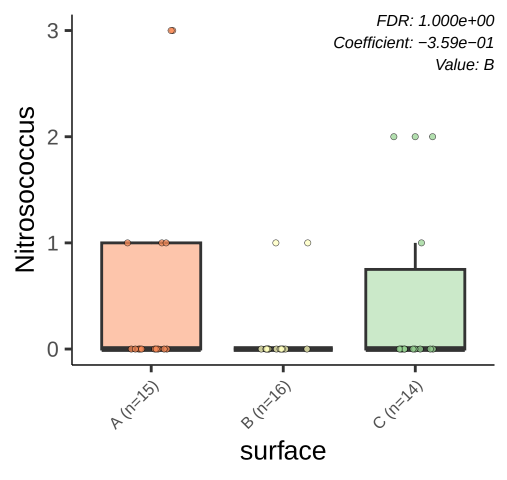
FDR: 1.000e+00
Coefficient: −3.59e−01
Value: B
Nitrosococcus
3
2
1
0
A (n=15)
B (n=16)
C (n=14)
surface

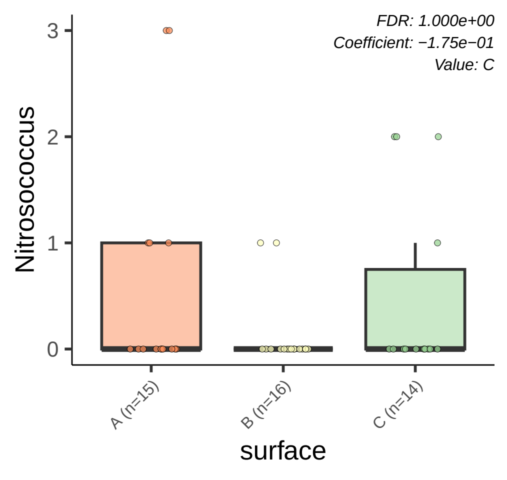
FDR: 1.000e+00
Coefficient: −1.75e−01
Value: C
Nitrosococcus
3
2
1
0
A (n=15)
B (n=16)
C (n=14)
surface

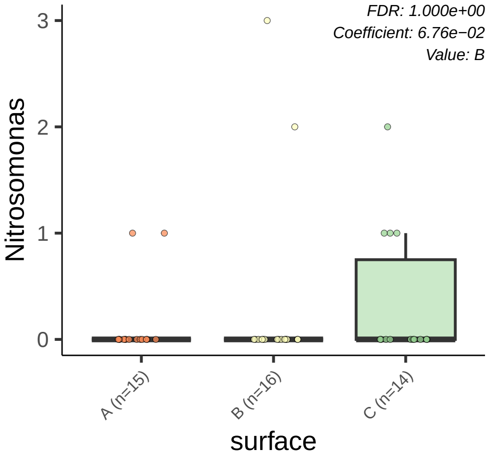
FDR: 1.000e+00
Coefficient: 6.76e−02
Value: B
Nitrosomonas
0
1
2
3
A (n=15)
B (n=16)
C (n=14)
surface

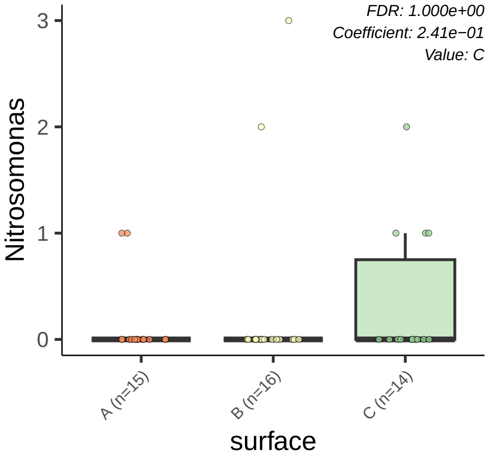
FDR: 1.000e+00
Coefficient: 2.41e−01
Value: C
Nitrosomonas
0
1
2
3
A (n=15)
B (n=16)
C (n=14)
surface

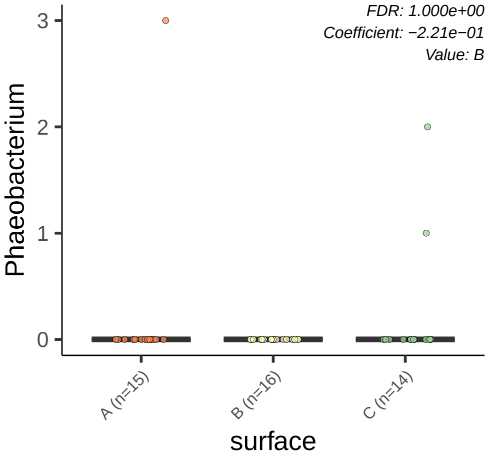
FDR: 1.000e+00
Coefficient: −2.21e−01
Value: B
Phaeobacterium
3
2
1
0
A (n=15)
B (n=16)
C (n=14)
surface

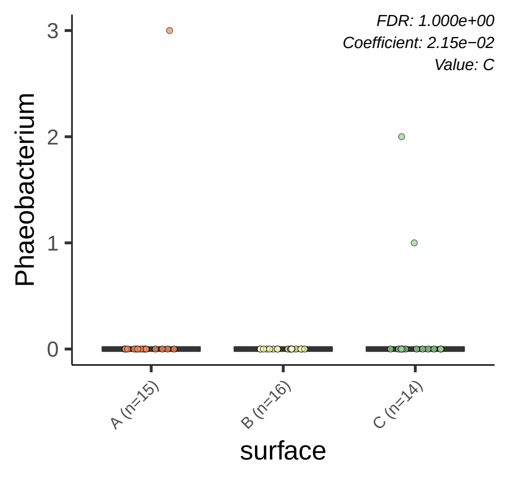

FDR: 1.000e+00
Coefficient: 2.15e−02
Value: C
Phaeobacterium
0
1
2
3
A (n=15)
B (n=16)
C (n=14)
surface

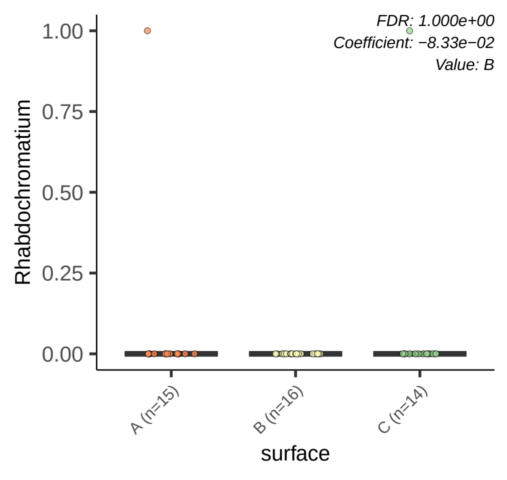
FDR: 1.000e+00
Coefficient: −8.33e−02
Value: B
Rhabdochromatium
1.00
0.75
0.50
0.25
0.00
A (n=15)
B (n=16)
C (n=14)
surface

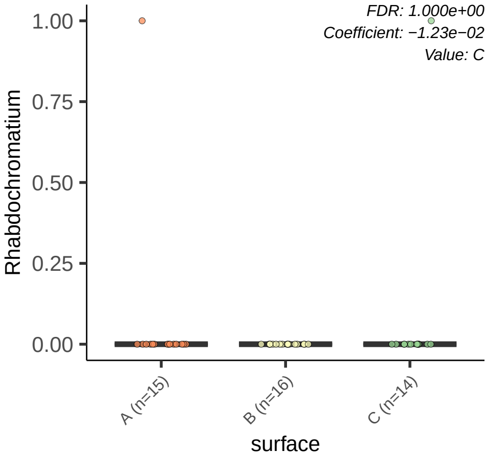
FDR: 1.000e+00
Coefficient: −1.23e−02
Value: C
Rhabdochromatium
1.00
0.75
0.50
0.25
0.00
A (n=15)
B (n=16)
C (n=14)
surface

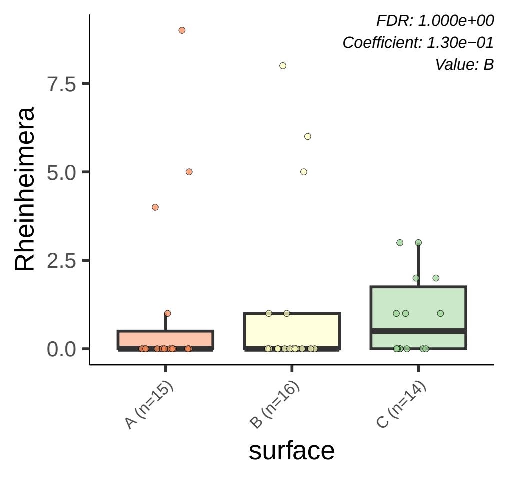

FDR: 1.000e+00
Coefficient: 1.30e−01
Value: B
Rheinheimera
7.5
5.0
2.5
0.0
A (n=15)
B (n=16)
C (n=14)
surface

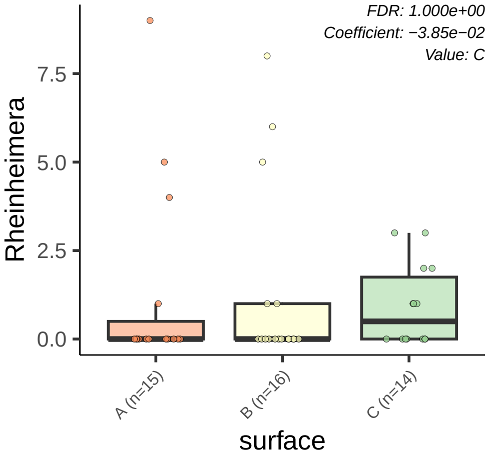

FDR: 1.000e+00
Coefficient: −3.85e−02
Value: C
Rheinheimera
7.5
5.0
2.5
0.0
A (n=15)
B (n=16)
C (n=14)
surface

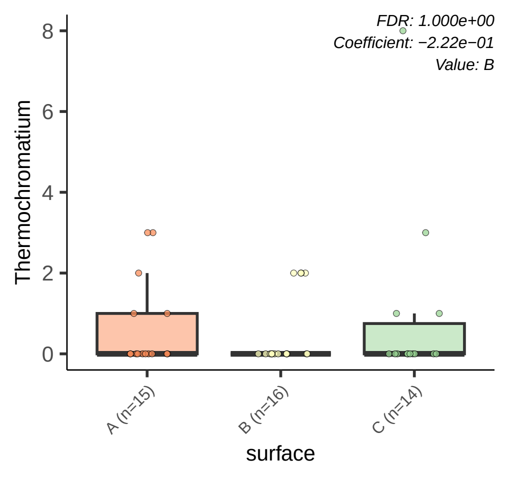
FDR: 1.000e+00
Coefficient: −2.22e−01
Value: B
Thermochromatium
8
6
4
2
0
A (n=15)
B (n=16)
C (n=14)
surface

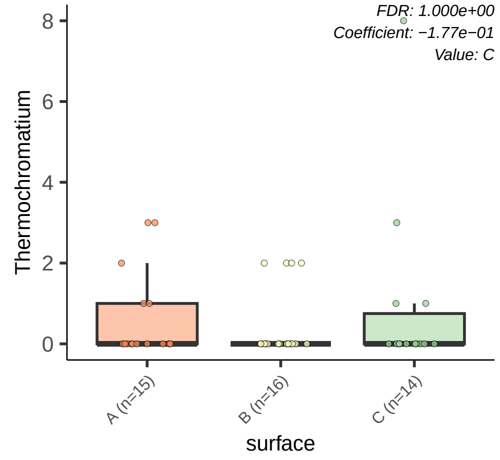
FDR: 1.000e+00
Coefficient: −1.77e−01
Value: C
Thermochromatium
8
6
4
2
0
A (n=15)
B (n=16)
C (n=14)
surface

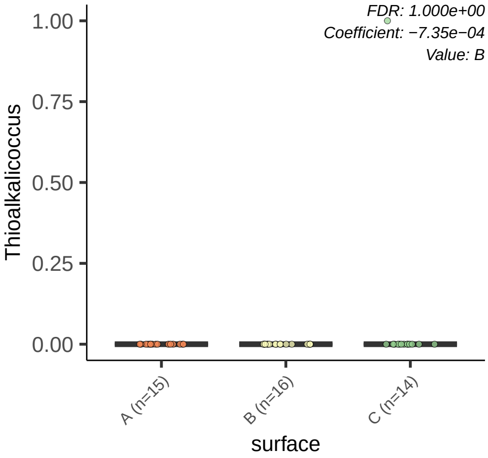
FDR: 1.000e+00
Coefficient: −7.35e−04
Value: B
Thioalkalicoccus
1.00
0.75
0.50
0.25
0.00
A (n=15)
B (n=16)
C (n=14)
surface

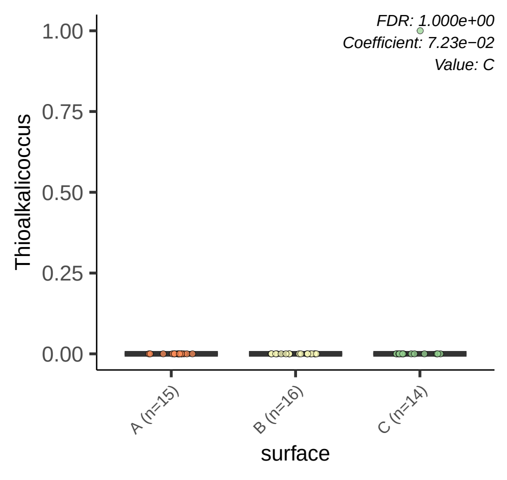
FDR: 1.000e+00
Coefficient: 7.23e−02
Value: C
Thioalkalicoccus
1.00
0.75
0.50
0.25
0.00
A (n=15)
B (n=16)
C (n=14)
surface

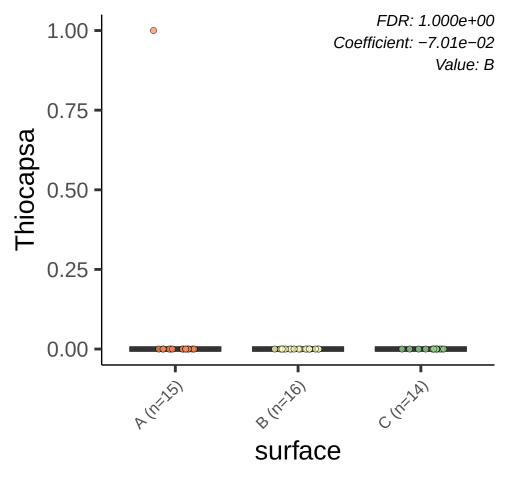

FDR: 1.000e+00
Coefficient: −7.01e−02
Value: B
Thiocapsa
1.00
0.75
0.50
0.25
0.00
A (n=15)
B (n=16)
C (n=14)
surface

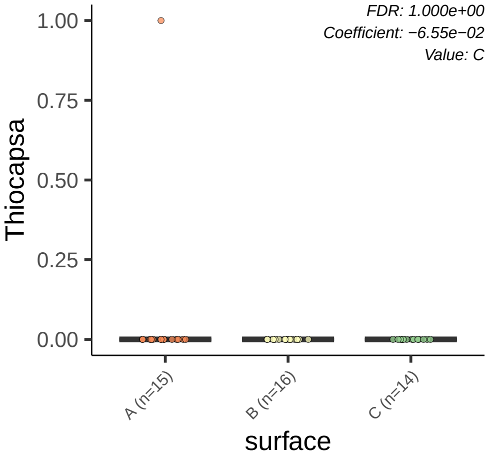

FDR: 1.000e+00
Coefficient: −6.55e−02
Value: C
Thiocapsa
1.00
0.75
0.50
0.25
0.00
A (n=15)
B (n=16)
C (n=14)
surface

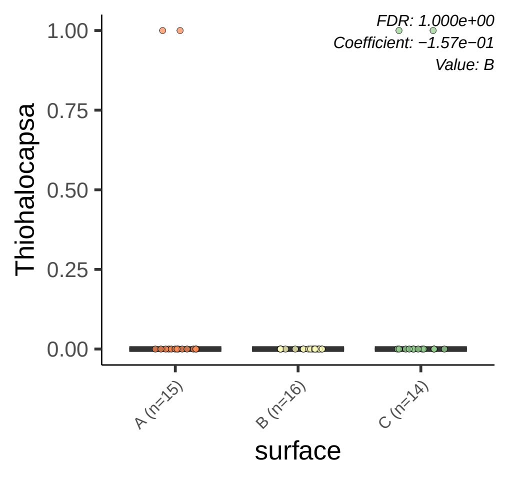
FDR: 1.000e+00
Coefficient: −1.57e−01
Value: B
Thiohalocapsa
1.00
0.75
0.50
0.25
0.00
A (n=15)
B (n=16)
C (n=14)
surface

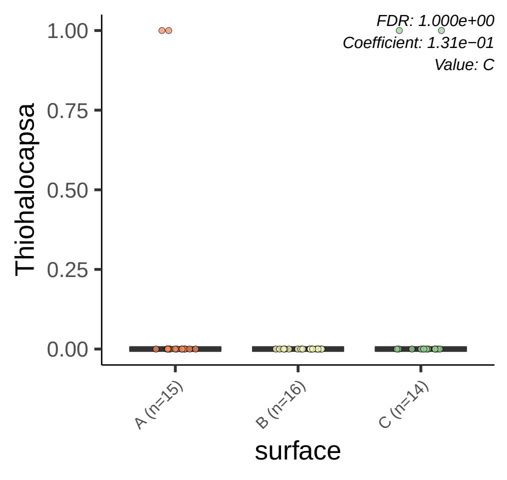

FDR: 1.000e+00
Coefficient: 1.31e−01
Value: C
Thiohalocapsa
1.00
0.75
0.50
0.25
0.00
A (n=15)
B (n=16)
C (n=14)
surface

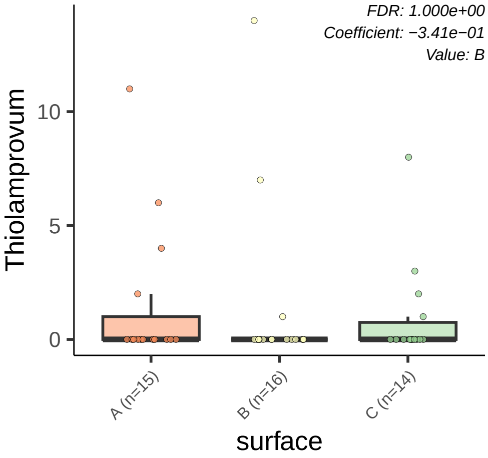
FDR: 1.000e+00
Coefficient: −3.41e−01
Value: B
Thiolamprovum
0
5
10
A (n=15)
B (n=16)
C (n=14)
surface

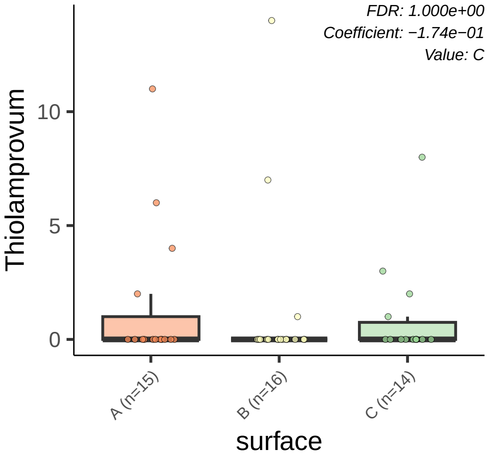

FDR: 1.000e+00
Coefficient: −1.74e−01
Value: C
Thiolamprovum
0
5
10
A (n=15)
B (n=16)
C (n=14)
surface

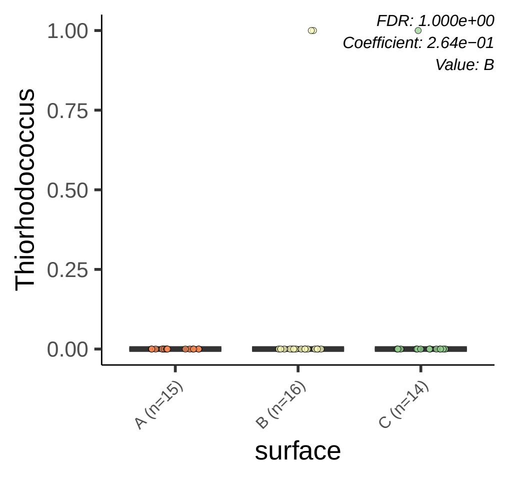
FDR: 1.000e+00
Coefficient: 2.64e−01
Value: B
Thiorhodococcus
1.00
0.75
0.50
0.25
0.00
A (n=15)
B (n=16)
C (n=14)
surface

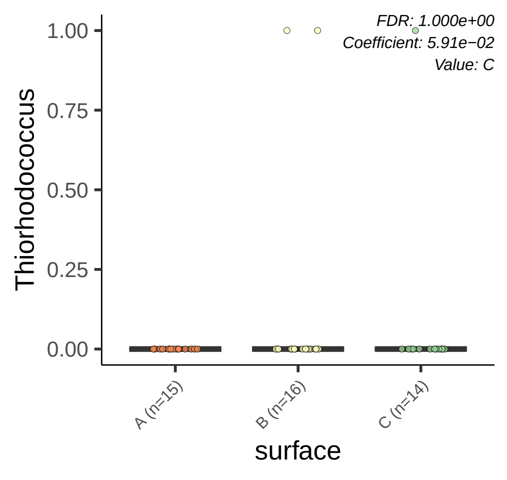

FDR: 1.000e+00
Coefficient: 5.91e−02
Value: C
Thiorhodococcus
1.00
0.75
0.50
0.25
0.00
A (n=15)
B (n=16)
C (n=14)
surface

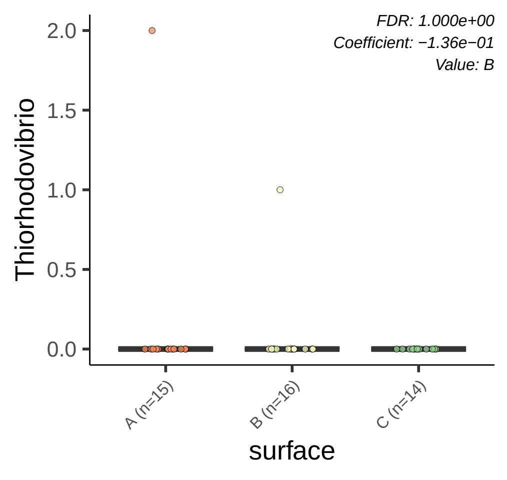
FDR: 1.000e+00
Coefficient: −1.36e−01
Value: B
Thiorhodovibrio
2.0
1.5
1.0
0.5
0.0
A (n=15)
B (n=16)
C (n=14)
surface

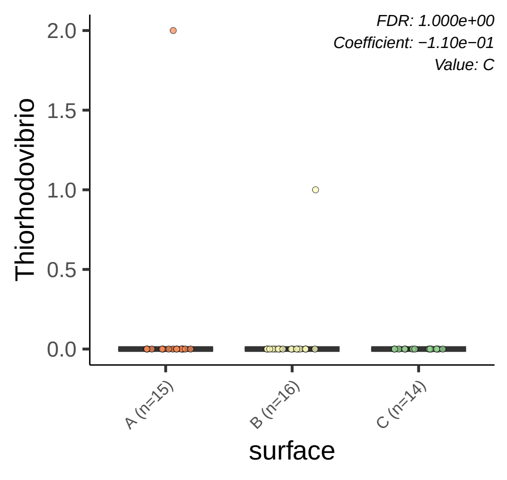

FDR: 1.000e+00
Coefficient: −1.10e−01
Value: C
Thiorhodovibrio
2.0
1.5
1.0
0.5
0.0
A (n=15)
B (n=16)
C (n=14)
surface

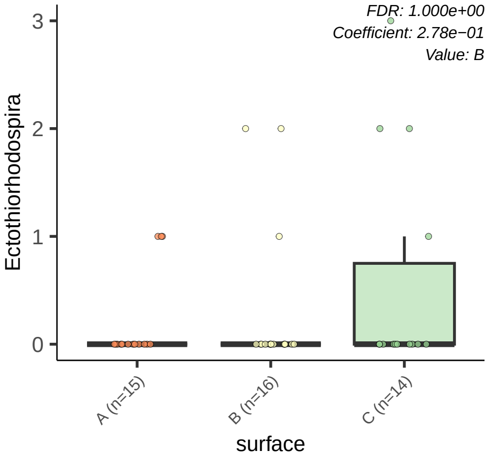

FDR: 1.000e+00
Coefficient: 2.78e−01
Value: B
Ectothiorhodospira
0
1
2
3
A (n=15)
B (n=16)
C (n=14)
surface

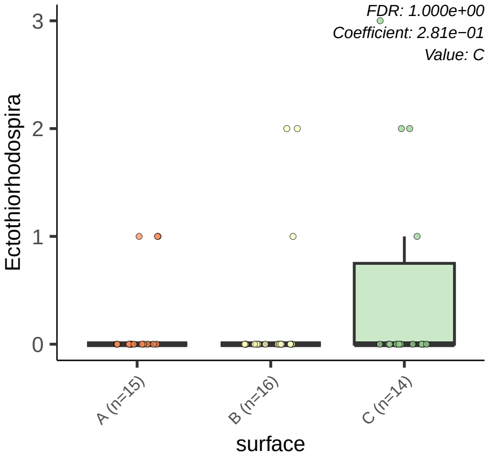
FDR: 1.000e+00
Coefficient: 2.81e−01
Value: C
Ectothiorhodospira
0
1
2
3
A (n=15)
B (n=16)
C (n=14)
surface

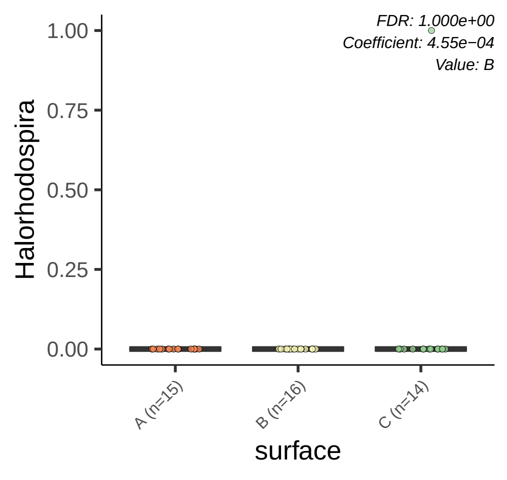
FDR: 1.000e+00
Coefficient: 4.55e−04
Value: B
Halorhodospira
1.00
0.75
0.50
0.25
0.00
A (n=15)
B (n=16)
C (n=14)
surface

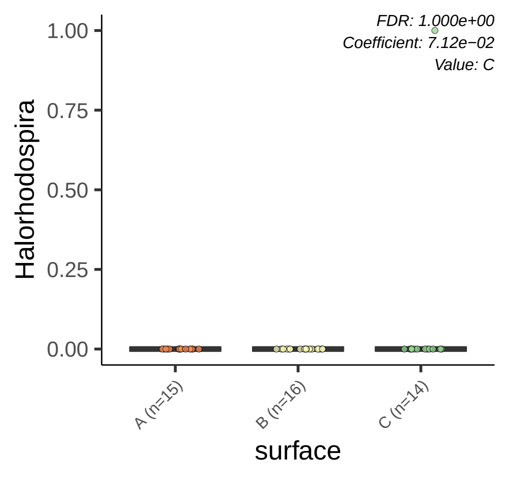
FDR: 1.000e+00
Coefficient: 7.12e−02
Value: C
Halorhodospira
1.00
0.75
0.50
0.25
0.00
A (n=15)
B (n=16)
C (n=14)
surface

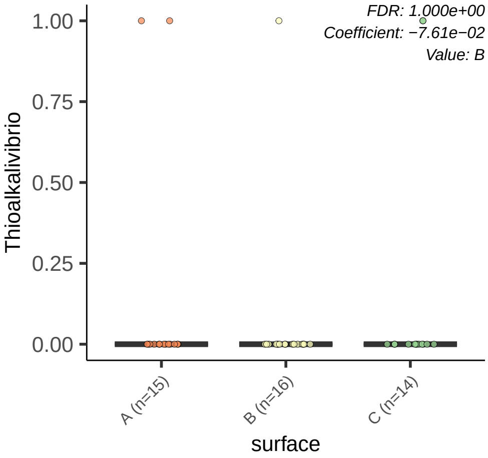

FDR: 1.000e+00
Coefficient: −7.61e−02
Value: B
Thioalkalivibrio
1.00
0.75
0.50
0.25
0.00
A (n=15)
B (n=16)
C (n=14)
surface

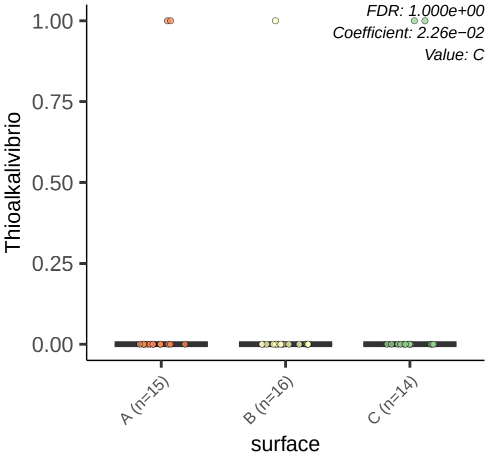
FDR: 1.000e+00
Coefficient: 2.26e−02
Value: C
Thioalkalivibrio
1.00
0.75
0.50
0.25
0.00
A (n=15)
B (n=16)
C (n=14)
surface

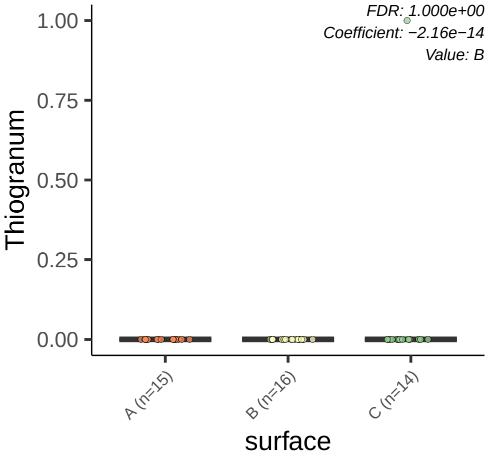

FDR: 1.000e+00
Coefficient: −2.16e−14
Value: B
Thiogranum
1.00
0.75
0.50
0.25
0.00
A (n=15)
B (n=16)
C (n=14)
surface

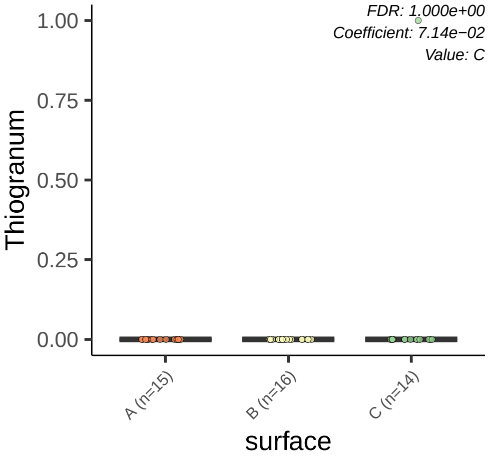

FDR: 1.000e+00
Coefficient: 7.14e−02
Value: C
1.00
0.75
0.50
0.25
0.00
Thiogranum
A (n=15)
B (n=16)
C (n=14)
surface

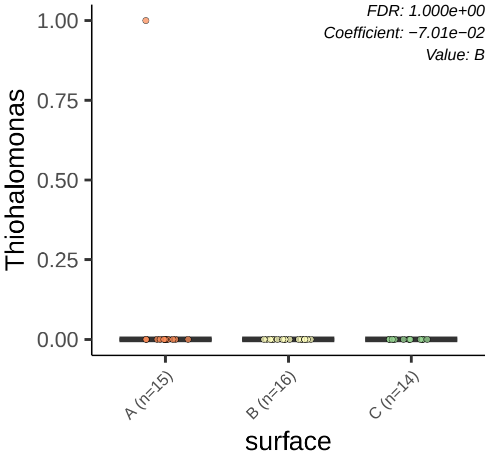

FDR: 1.000e+00
Coefficient: −7.01e−02
Value: B
Thiohalomonas
1.00
0.75
0.50
0.25
0.00
A (n=15)
B (n=16)
C (n=14)
surface

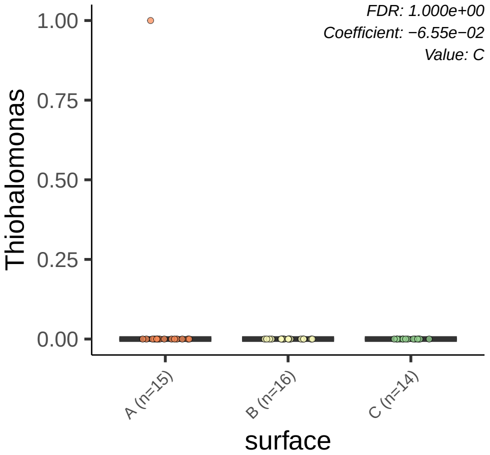
FDR: 1.000e+00
Coefficient: −6.55e−02
Value: C
Thiohalomonas
1.00
0.75
0.50
0.25
0.00
A (n=15)
B (n=16)
C (n=14)
surface

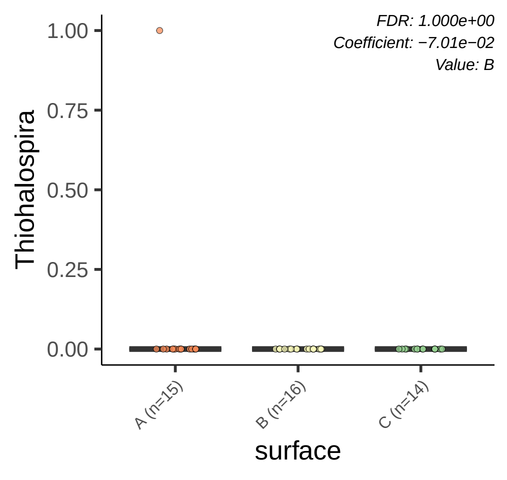

FDR: 1.000e+00
Coefficient: −7.01e−02
Value: B
Thiohalospira
1.00
0.75
0.50
0.25
0.00
A (n=15)
B (n=16)
C (n=14)
surface

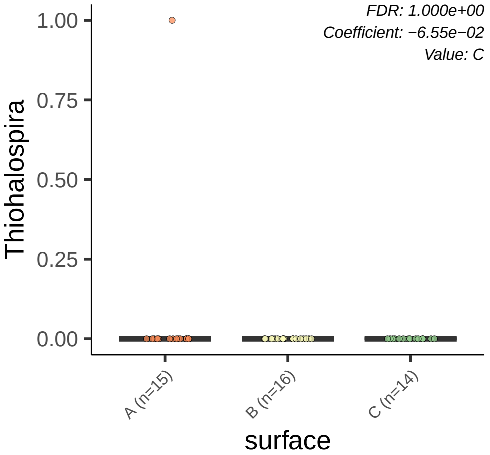

FDR: 1.000e+00
Coefficient: −6.55e−02
Value: C
Thiohalospira
1.00
0.75
0.50
0.25
0.00
A (n=15)
B (n=16)
C (n=14)
surface

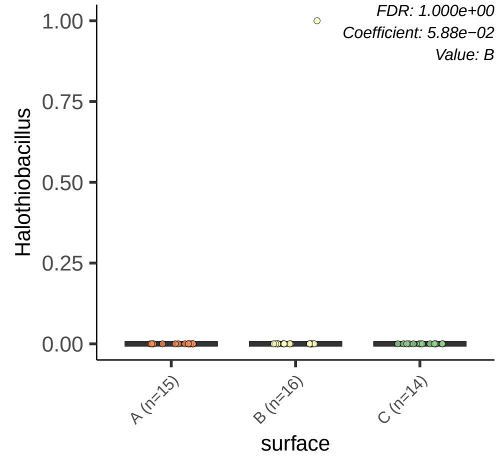
FDR: 1.000e+00
Coefficient: 5.88e−02
Value: B
Halothiobacillus
1.00
0.75
0.50
0.25
0.00
A (n=15)
B (n=16)
C (n=14)
surface

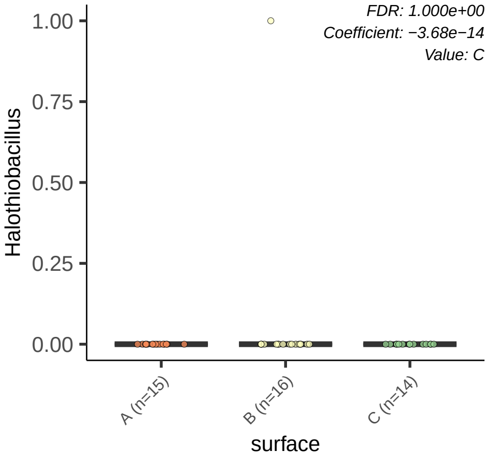
FDR: 1.000e+00
Coefficient: −3.68e−14
Value: C
Halothiobacillus
1.00
0.75
0.50
0.25
0.00
A (n=15)
B (n=16)
C (n=14)
surface

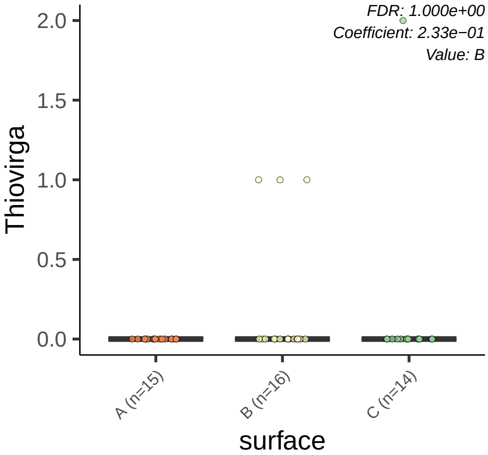

FDR: 1.000e+00
Coefficient: 2.33e−01
Value: B
Thiovirga
2.0
1.5
1.0
0.5
0.0
A (n=15)
B (n=16)
C (n=14)
surface

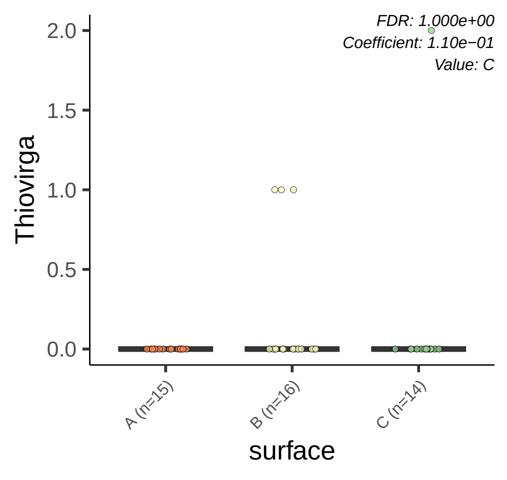
FDR: 1.000e+00
Coefficient: 1.10e−01
Value: C
Thiovirga
2.0
1.5
1.0
0.5
0.0
A (n=15)
B (n=16)
C (n=14)
surface

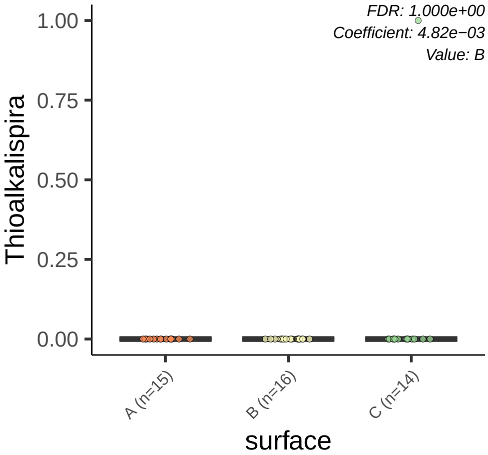
FDR: 1.000e+00
Coefficient: 4.82e−03
Value: B
Thioalkalispira
1.00
0.75
0.50
0.25
0.00
A (n=15)
B (n=16)
C (n=14)
surface

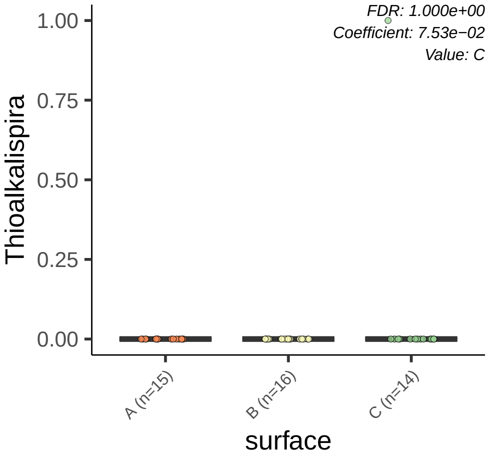
FDR: 1.000e+00
Coefficient: 7.53e−02
Value: C
1.00
0.75
0.50
0.25
0.00
Thioalkalispira
A (n=15)
B (n=16)
C (n=14)
surface

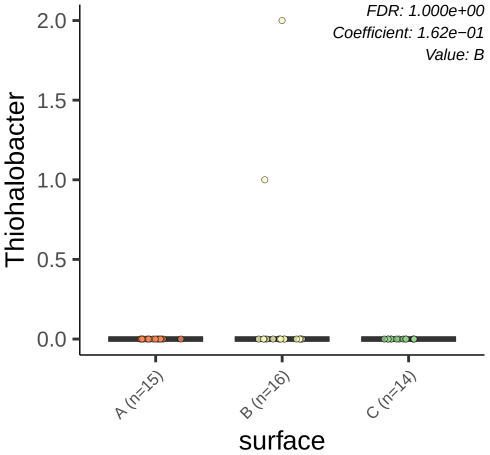
FDR: 1.000e+00
Coefficient: 1.62e−01
Value: B
Thiohalobacter
2.0
1.5
1.0
0.5
0.0
A (n=15)
B (n=16)
C (n=14)
surface

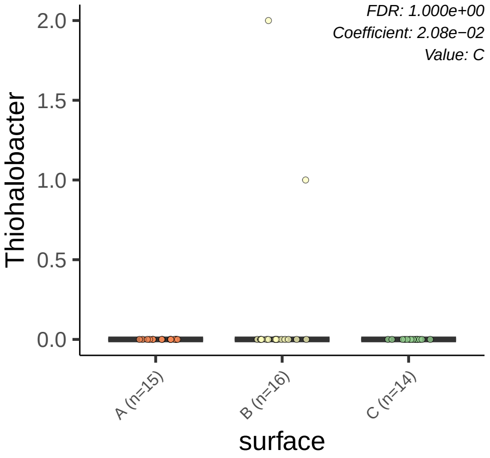
FDR: 1.000e+00
Coefficient: 2.08e−02
Value: C
Thiohalobacter
2.0
1.5
1.0
0.5
0.0
A (n=15)
B (n=16)
C (n=14)
surface

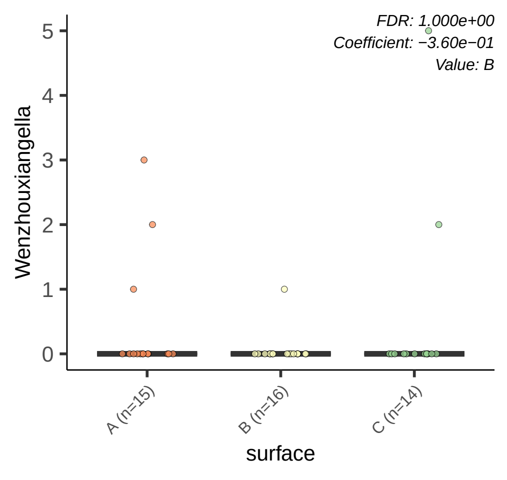

FDR: 1.000e+00
Coefficient: −3.60e−01
Value: B
Wenzhouxiangella
0
1
2
3
4
5
A (n=15)
B (n=16)
C (n=14)
surface

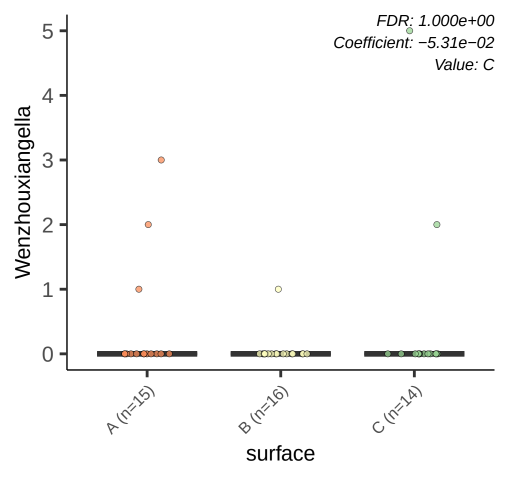

FDR: 1.000e+00
Coefficient: −5.31e−02
Value: C
Wenzhouxiangella
0
1
2
3
4
5
A (n=15)
B (n=16)
C (n=14)
surface

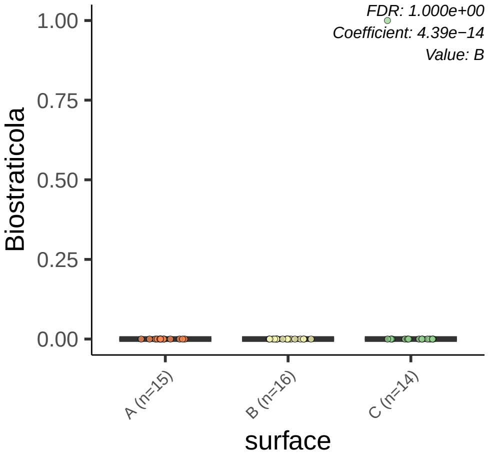
FDR: 1.000e+00
Coefficient: 4.39e−14
Value: B
Biostraticola
1.00
0.75
0.50
0.25
0.00
A (n=15)
B (n=16)
C (n=14)
surface

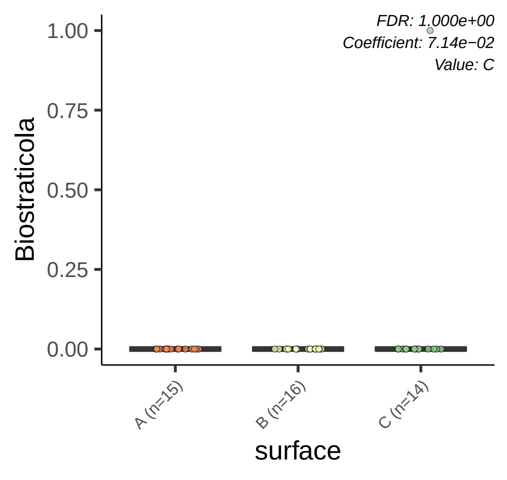
FDR: 1.000e+00
Coefficient: 7.14e−02
Value: C
Biostraticola
1.00
0.75
0.50
0.25
0.00
A (n=15)
B (n=16)
C (n=14)
surface

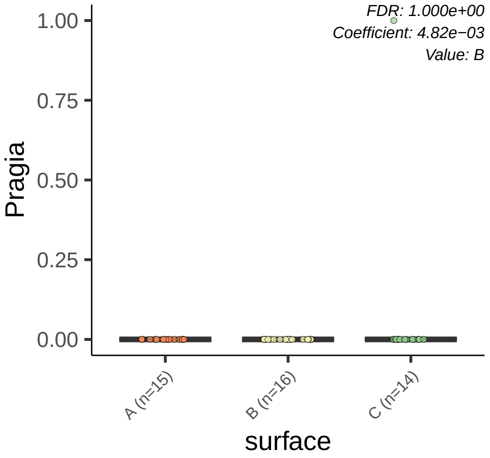
FDR: 1.000e+00
Coefficient: 4.82e−03
Value: B
Pragia
1.00
0.75
0.50
0.25
0.00
A (n=15)
B (n=16)
C (n=14)
surface

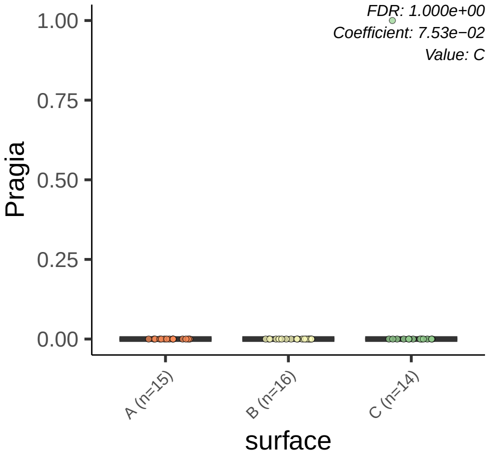
FDR: 1.000e+00
Coefficient: 7.53e−02
Value: C
Pragia
1.00
0.75
0.50
0.25
0.00
A (n=15)
B (n=16)
C (n=14)
surface

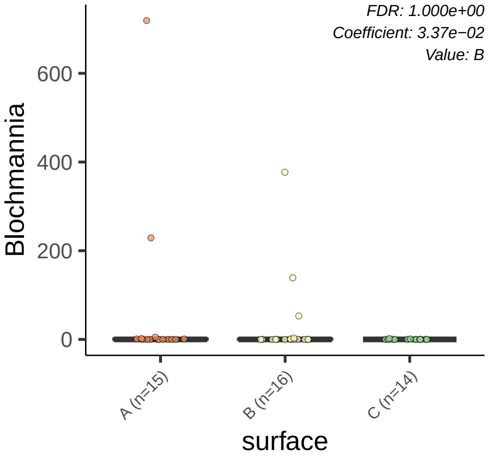

FDR: 1.000e+00
Coefficient: 3.37e−02
Value: B
Blochmannia
600
400
200
0
A (n=15)
B (n=16)
C (n=14)
surface

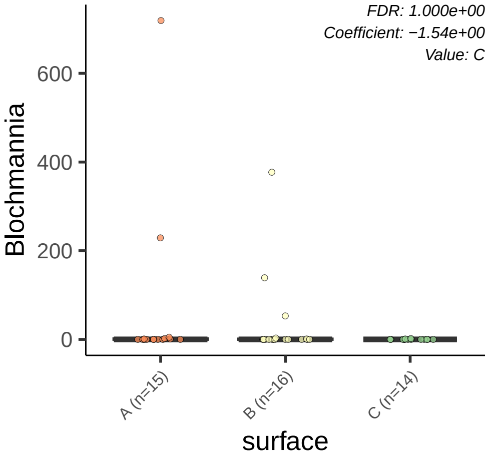
FDR: 1.000e+00
Coefficient: −1.54e+00
Value: C
Blochmannia
600
400
200
0
A (n=15)
B (n=16)
C (n=14)
surface

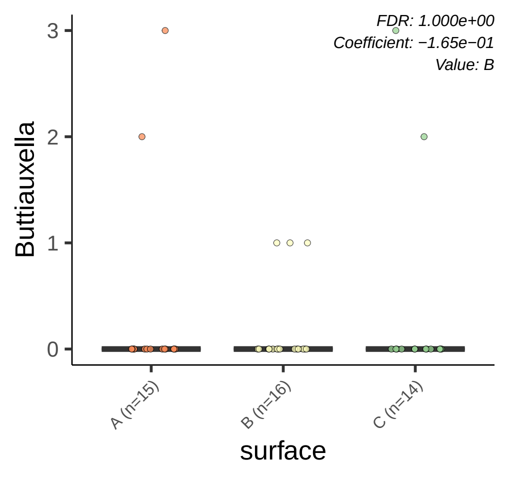
FDR: 1.000e+00
Coefficient: −1.65e−01
Value: B
Butiauxella
3
2
1
0
A (n=15)
B (n=16)
C (n=14)
surface

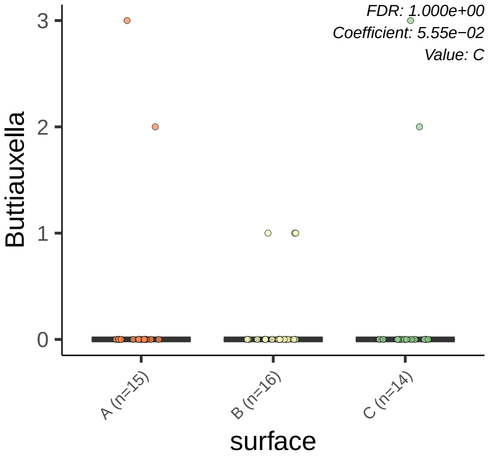
FDR: 1.000e+00
Coefficient: 5.55e−02
Value: C
Buttiauxella
0
1
2
3
A (n=15)
B (n=16)
C (n=14)
surface

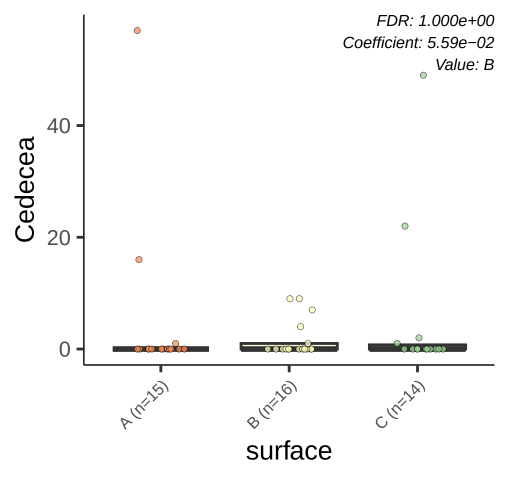

FDR: 1.000e+00
Coefficient: 5.59e−02
Value: B
Cedecea
0
20
40
A (n=15)
B (n=16)
C (n=14)
surface

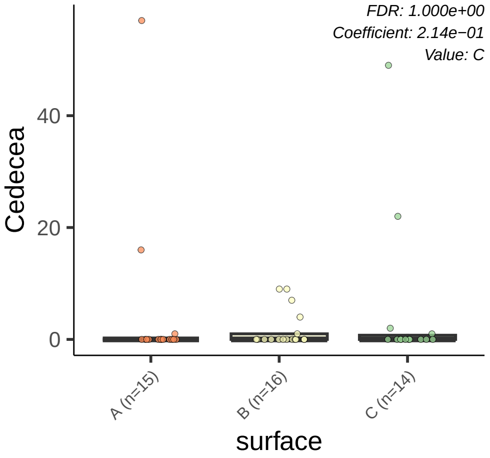

FDR: 1.000e+00
Coefficient: 2.14e−01
Value: C
Cedecea
40
20
0
A (n=15)
B (n=16)
C (n=14)
surface

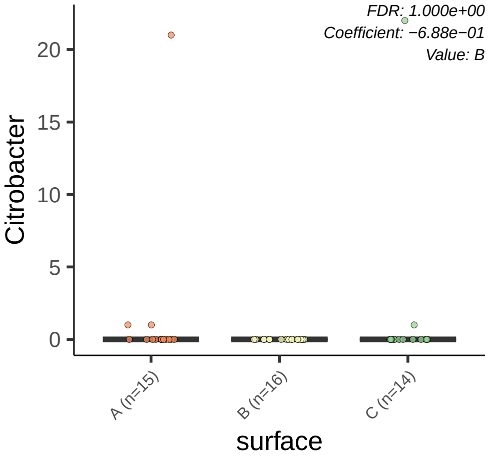

FDR: 1.000e+00
Coefficient: −6.88e−01
Value: B
Citrobacter
20
15
10
5
0
A (n=15)
B (n=16)
C (n=14)
surface

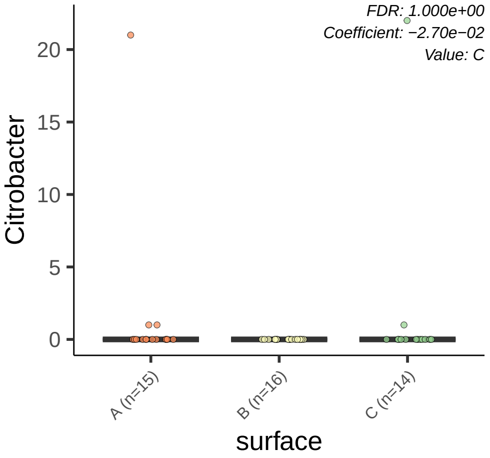
FDR: 1.000e+00
Coefficient: −2.70e−02
Value: C
Citrobacter
0
5
10
15
20
A (n=15)
B (n=16)
C (n=14)
surface

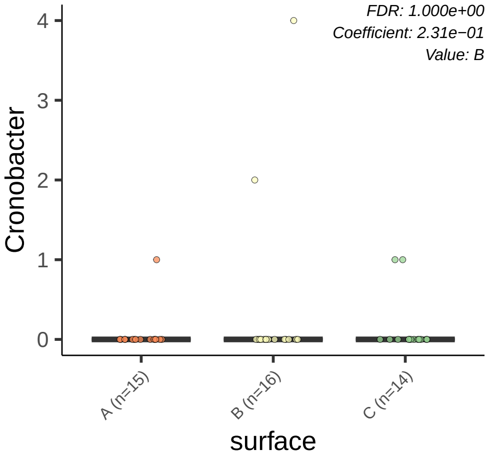
FDR: 1.000e+00
Coefficient: 2.31e−01
Value: B
Cronobacter
4
3
2
1
0
A (n=15)
B (n=16)
C (n=14)
surface

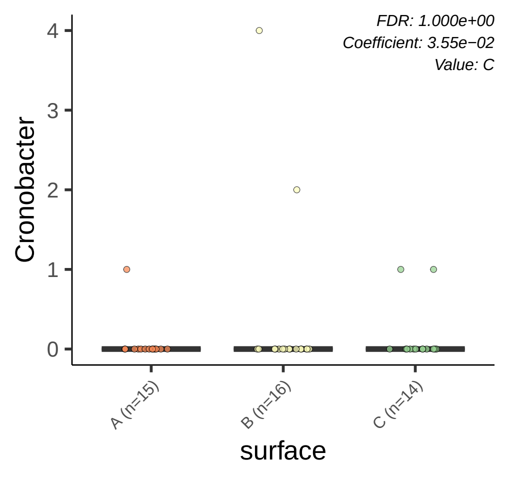

FDR: 1.000e+00
Coefficient: 3.55e−02
Value: C
Cronobacter
4
3
2
1
0
A (n=15)
B (n=16)
C (n=14)
surface

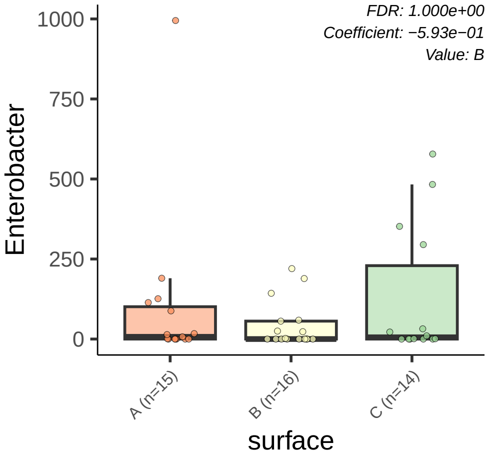

FDR: 1.000e+00
Coefficient: −5.93e−01
Value: B
1000
750
500
250
0
Enterobacter
A (n=15)
B (n=16)
C (n=14)
surface

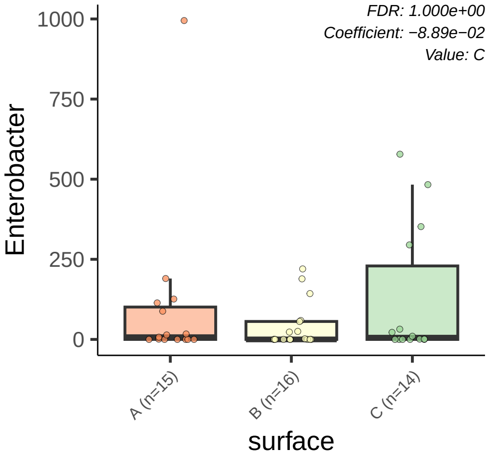
FDR: 1.000e+00
Coefficient: −8.89e−02
Value: C
Enterobacter
1000
750
500
250
0
A (n=15)
B (n=16)
C (n=14)
surface

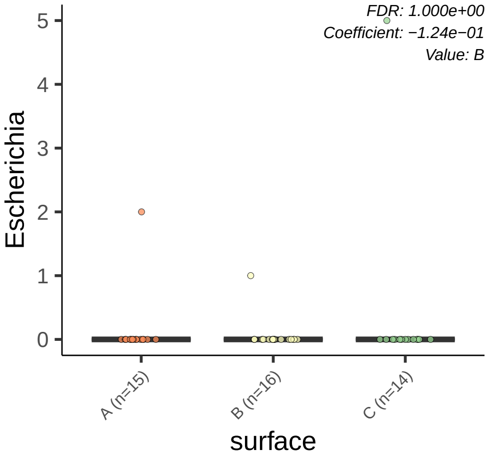

FDR: 1.000e+00
Coefficient: −1.24e−01
Value: B
Escherichia
0
1
2
3
4
5
A (n=15)
B (n=16)
C (n=14)
surface

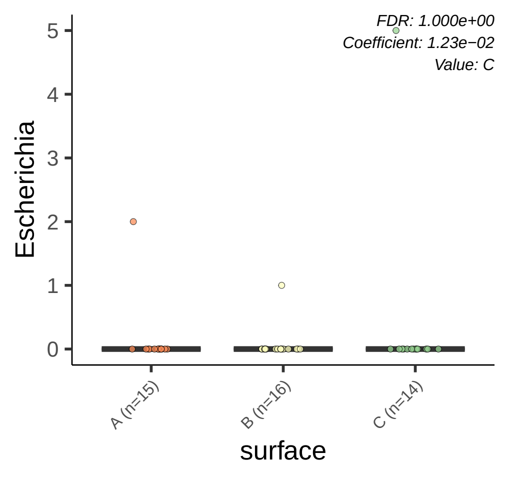

FDR: 1.000e+00
Coefficient: 1.23e−02
Value: C
Escherichia
0
1
2
3
4
5
A (n=15)
B (n=16)
C (n=14)
surface

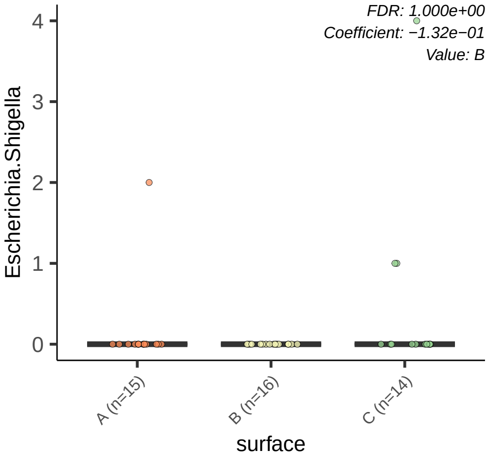
FDR: 1.000e+00
Coefficient: −1.32e−01
Value: B
Escherichia.Shigella
0
1
2
3
4
A (n=15)
B (n=16)
C (n=14)
surface

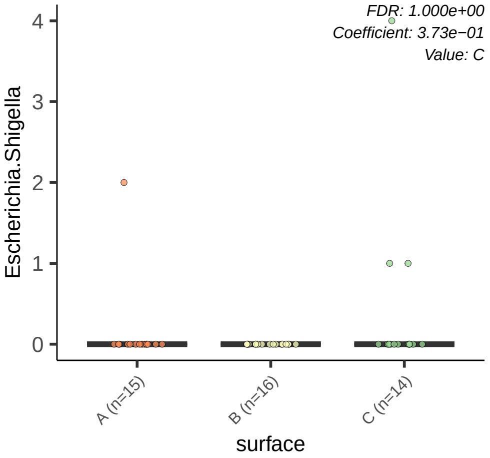

FDR: 1.000e+00
Coefficient: 3.73e−01
Value: C
Escherichia.Shigella
4
3
2
1
0
A (n=15)
B (n=16)
C (n=14)
surface

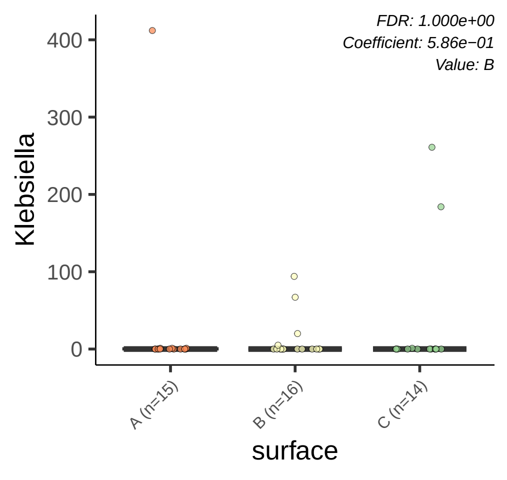

FDR: 1.000e+00
Coefficient: 5.86e−01
Value: B
400
300
200
100
0
Klebsiella
A (n=15)
B (n=16)
C (n=14)
surface

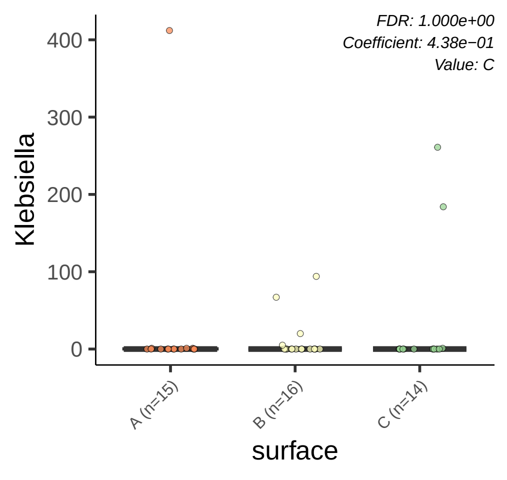

FDR: 1.000e+00
Coefficient: 4.38e−01
Value: C
Klebsiella
400
300
200
100
0
A (n=15)
B (n=16)
C (n=14)
surface

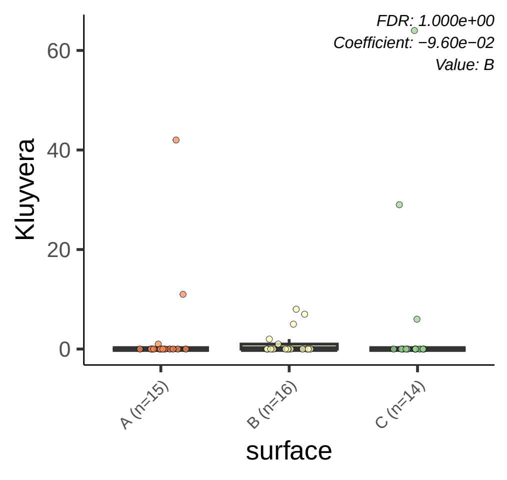

FDR: 1.000e+00
Coefficient: −9.60e−02
Value: B
Kluyvera
60
40
20
0
A (n=15)
B (n=16)
C (n=14)
surface

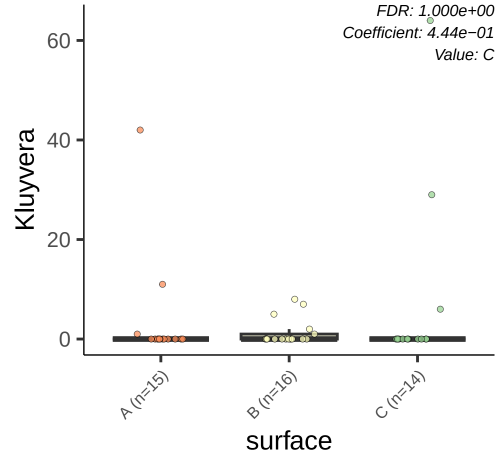
FDR: 1.000e+00
Coefficient: 4.44e−01
Value: C
Kluyvera
60
40
20
0
A (n=15)
B (n=16)
C (n=14)
surface

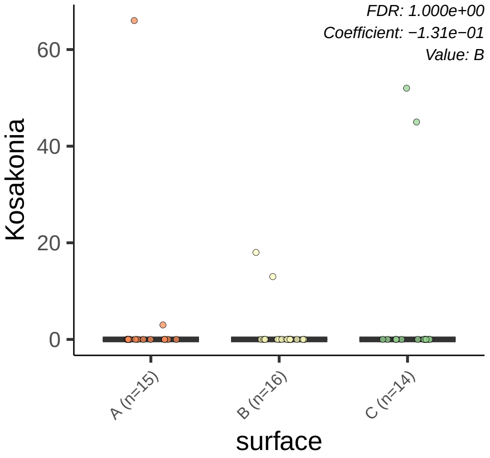
FDR: 1.000e+00
Coefficient: −1.31e−01
Value: B
Kosakonia
60
40
20
0
A (n=15)
B (n=16)
C (n=14)
surface

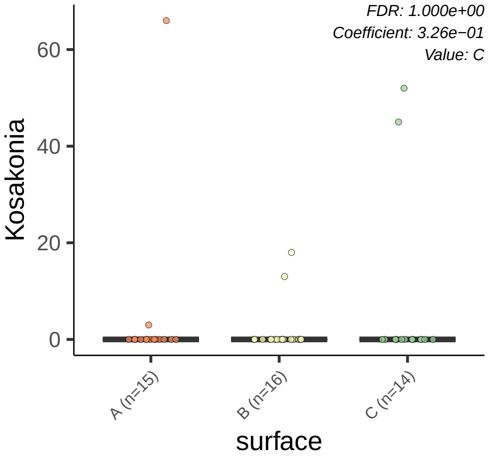

FDR: 1.000e+00
Coefficient: 3.26e−01
Value: C
Kosakonia
60
40
20
0
A (n=15)
B (n=16)
C (n=14)
surface

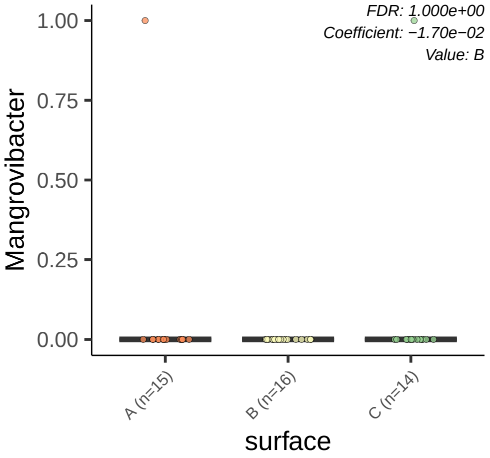
FDR: 1.000e+00
Coefficient: −1.70e−02
Value: B
Mangrovibacter
1.00
0.75
0.50
0.25
0.00
A (n=15)
B (n=16)
C (n=14)
surface

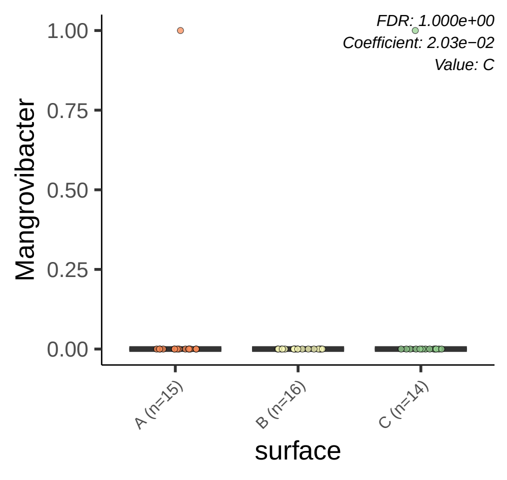

FDR: 1.000e+00
Coefficient: 2.03e−02
Value: C
Mangrovibacter
1.00
0.75
0.50
0.25
0.00
A (n=15)
B (n=16)
C (n=14)
surface

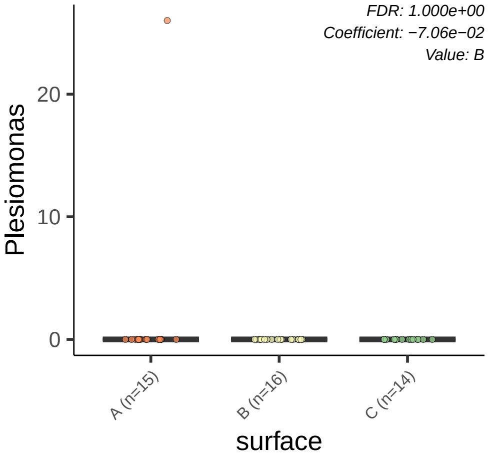
FDR: 1.000e+00
Coefficient: −7.06e−02
Value: B
Plesiomonas
20
10
0
A (n=15)
B (n=16)
C (n=14)
surface

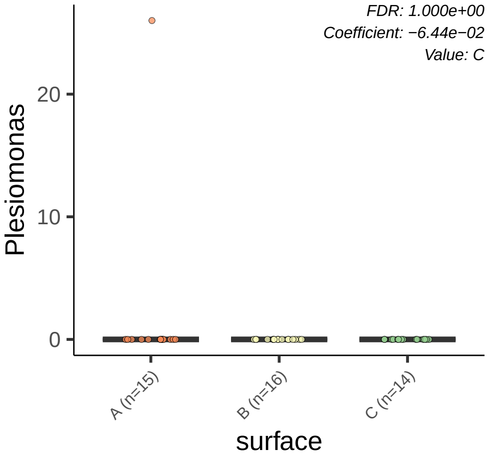

FDR: 1.000e+00
Coefficient: −6.44e−02
Value: C
Plesiomonas
20
10
0
A (n=15)
B (n=16)
C (n=14)
surface

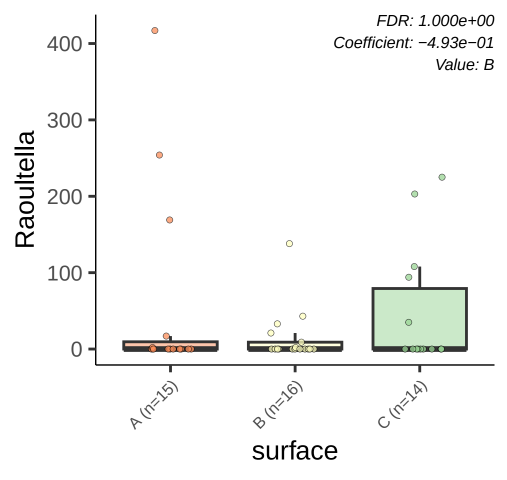
FDR: 1.000e+00
Coefficient: −4.93e−01
Value: B
Raoultella
400
300
200
100
0
A (n=15)
B (n=16)
C (n=14)
surface

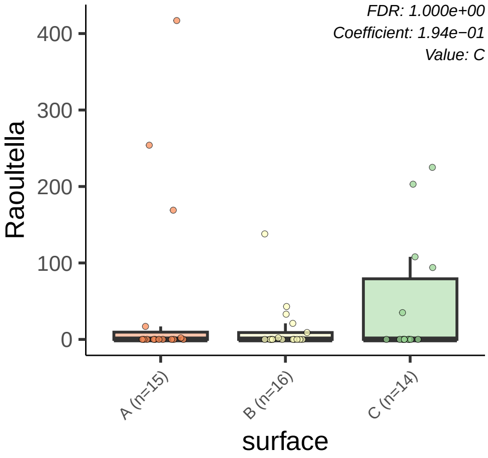
FDR: 1.000e+00
Coefficient: 1.94e−01
Value: C
Raoultella
400
300
200
100
0
A (n=15)
B (n=16)
C (n=14)
surface

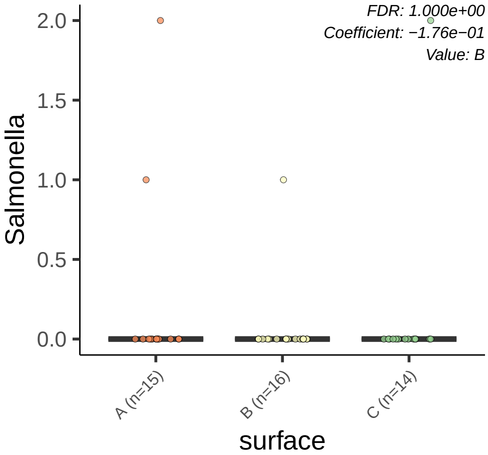
FDR: 1.000e+00
Coefficient: −1.76e−01
Value: B
Salmonella
2.0
1.5
1.0
0.5
0.0
A (n=15)
B (n=16)
C (n=14)
surface

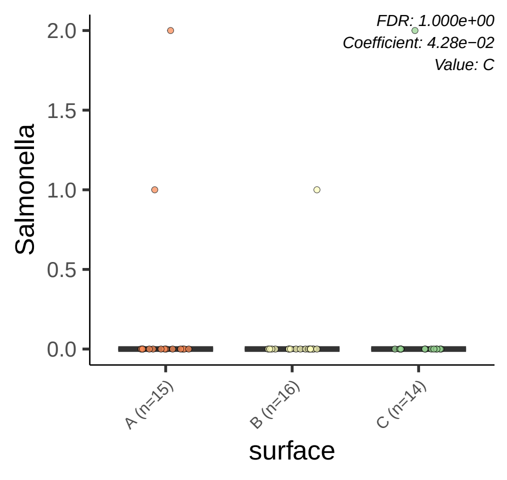

FDR: 1.000e+00
Coefficient: 4.28e−02
Value: C
Salmonella
2.0
1.5
1.0
0.5
0.0
A (n=15)
B (n=16)
C (n=14)
surface

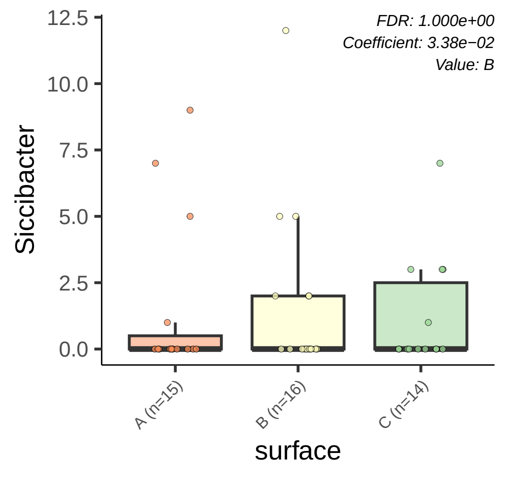
FDR: 1.000e+00
Coefficient: 3.38e−02
Value: B
Siccibacter
12.5
10.0
7.5
5.0
2.5
0.0
A (n=15)
B (n=16)
C (n=14)
surface

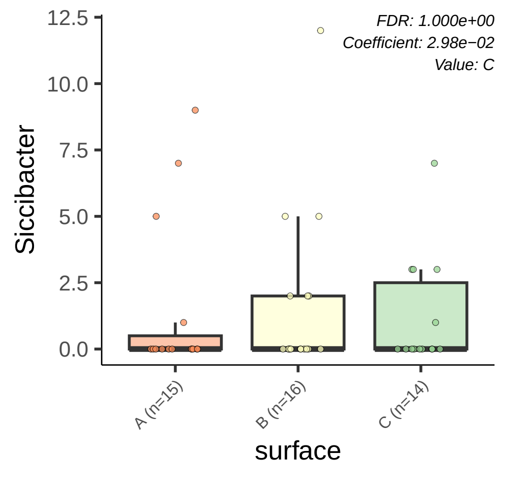

FDR: 1.000e+00
Coefficient: 2.98e−02
Value: C
Siccibacter
12.5
10.0
7.5
5.0
2.5
0.0
A (n=15)
B (n=16)
C (n=14)
surface

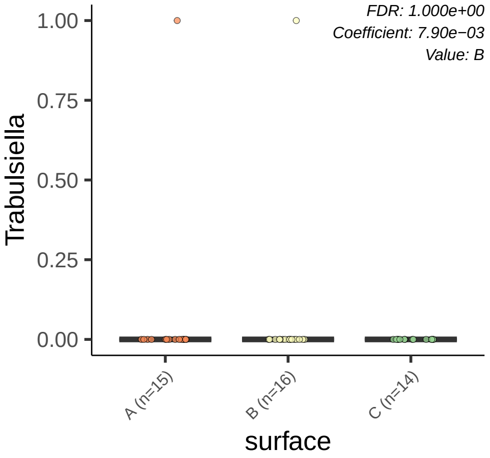
FDR: 1.000e+00
Coefficient: 7.90e−03
Value: B
Trabulsiella
1.00
0.75
0.50
0.25
0.00
A (n=15)
B (n=16)
C (n=14)
surface

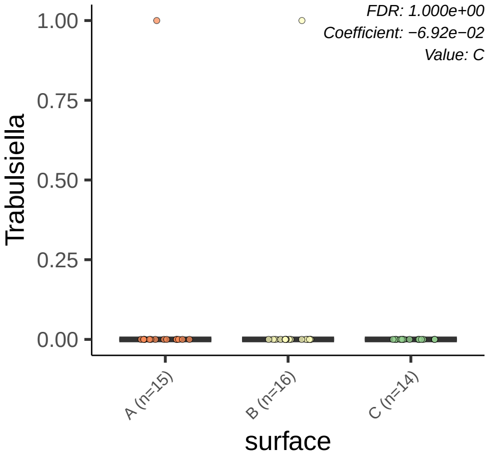
FDR: 1.000e+00
Coefficient: −6.92e−02
Value: C
1.00
0.75
0.50
0.25
0.00
Trabulsiella
A (n=15)
B (n=16)
C (n=14)
surface

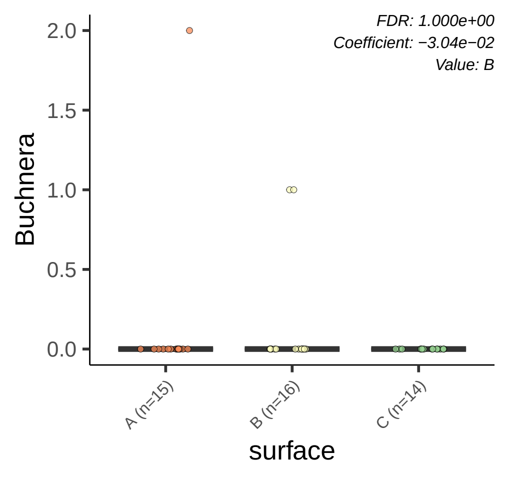
FDR: 1.000e+00
Coefficient: −3.04e−02
Value: B
Buchnera
2.0
1.5
1.0
0.5
0.0
A (n=15)
B (n=16)
C (n=14)
surface

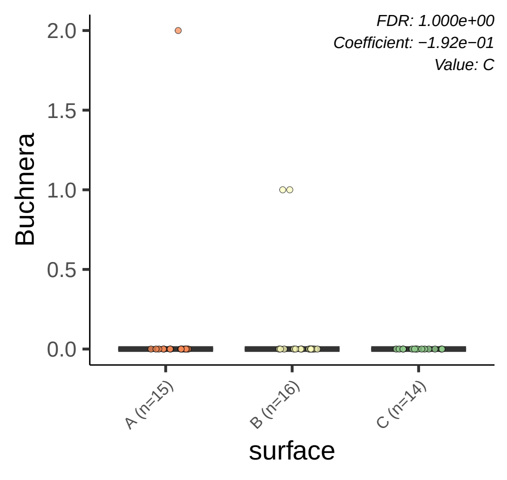

FDR: 1.000e+00
Coefficient: −1.92e−01
Value: C
Buchnera
2.0
1.5
1.0
0.5
0.0
A (n=15)
B (n=16)
C (n=14)
surface

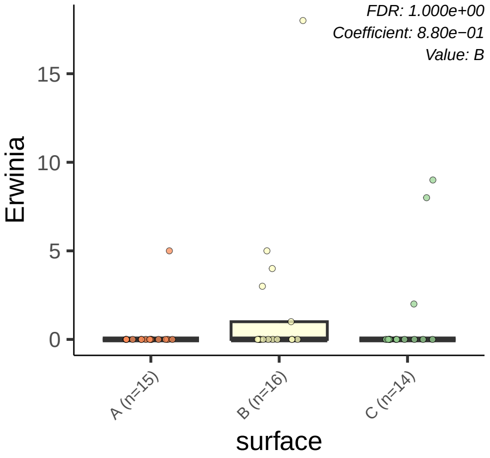
FDR: 1.000e+00
Coefficient: 8.80e−01
Value: B
Erwinia
15
10
5
0
A (n=15)
B (n=16)
C (n=14)
surface

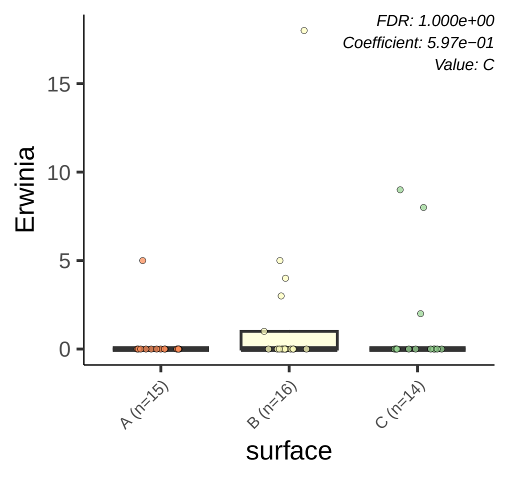
FDR: 1.000e+00
Coefficient: 5.97e−01
Value: C
Erwinia
0
5
10
15
A (n=15)
B (n=16)
C (n=14)
surface

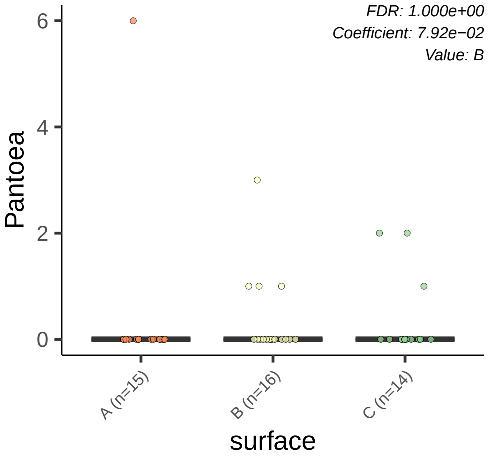

FDR: 1.000e+00
Coefficient: 7.92e−02
Value: B
Pantoea
6
4
2
0
A (n=15)
B (n=16)
C (n=14)
surface

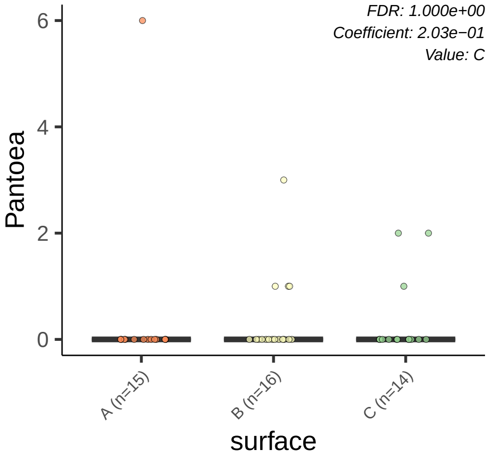

FDR: 1.000e+00
Coefficient: 2.03e−01
Value: C
Pantoea
6
4
2
0
A (n=15)
B (n=16)
C (n=14)
surface

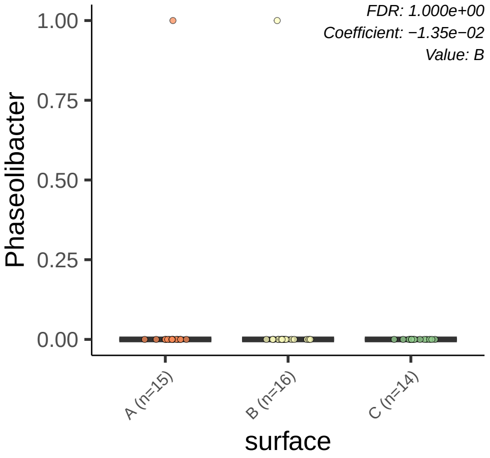
FDR: 1.000e+00
Coefficient: −1.35e−02
Value: B
Phaseolibacter
1.00
0.75
0.50
0.25
0.00
A (n=15)
B (n=16)
C (n=14)
surface

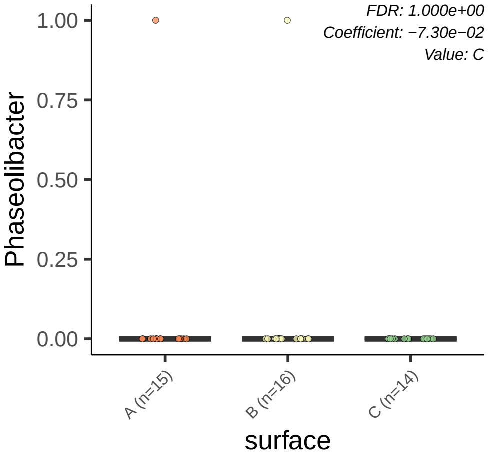

FDR: 1.000e+00
Coefficient: −7.30e−02
Value: C
Phaseolibacter
1.00
0.75
0.50
0.25
0.00
A (n=15)
B (n=16)
C (n=14)
surface

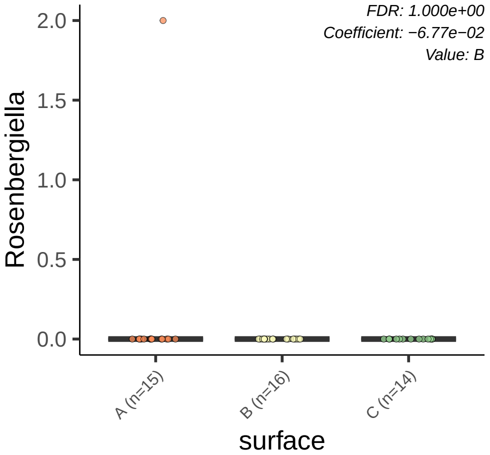
FDR: 1.000e+00
Coefficient: −6.77e−02
Value: B
Rosenbergiella
2.0
1.5
1.0
0.5
0.0
A (n=15)
B (n=16)
C (n=14)
surface

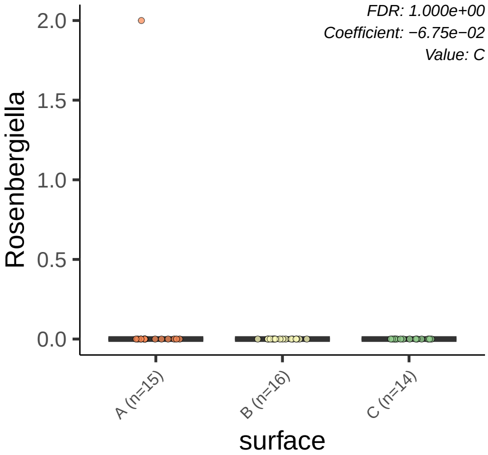

FDR: 1.000e+00
Coefficient: −6.75e−02
Value: C
Rosenbergiella
2.0
1.5
1.0
0.5
0.0
A (n=15)
B (n=16)
C (n=14)
surface

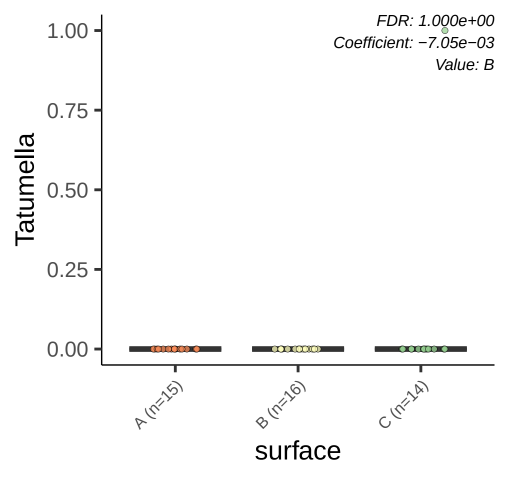

FDR: 1.000e+00
Coefficient: −7.05e−03
Value: B
1.00
0.75
0.50
0.25
0.00
Tatumella
A (n=15)
B (n=16)
C (n=14)
surface

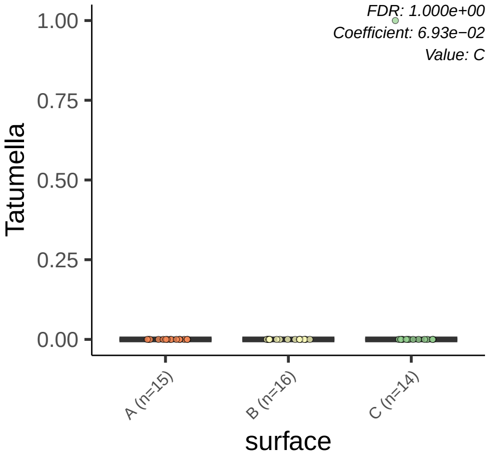
FDR: 1.000e+00
Coefficient: 6.93e−02
Value: C
Tatumella
1.00
0.75
0.50
0.25
0.00
A (n=15)
B (n=16)
C (n=14)
surface

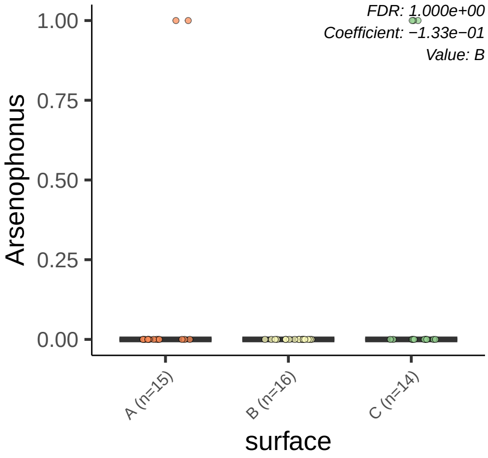

FDR: 1.000e+00
Coefficient: −1.33e−01
Value: B
Arsenophonus
1.00
0.75
0.50
0.25
0.00
A (n=15)
B (n=16)
C (n=14)
surface

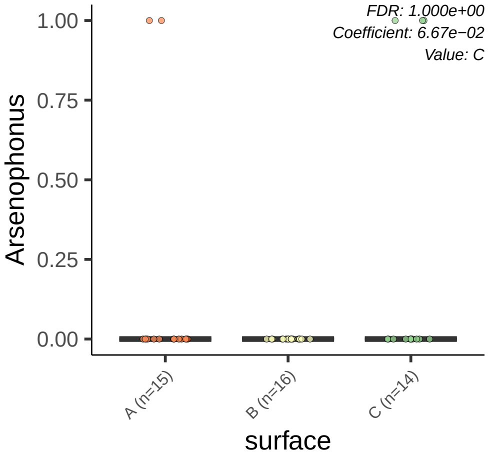
FDR: 1.000e+00
Coefficient: 6.67e−02
Value: C
Arsenophous
1.00
0.75
0.50
0.25
0.00
A (n=15)
B (n=16)
C (n=14)
surface

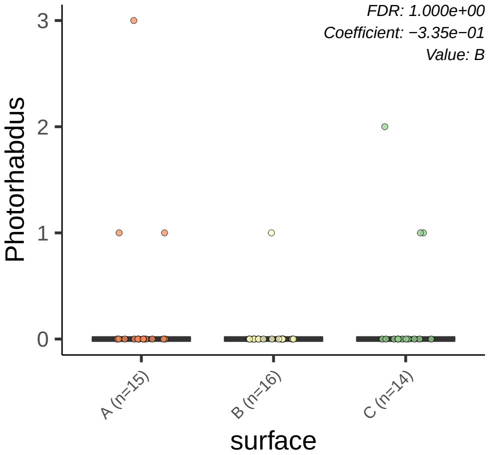
FDR: 1.000e+00
Coefficient: −3.35e−01
Value: B
Photorhabdus
3
2
1
0
A (n=15)
B (n=16)
C (n=14)
surface

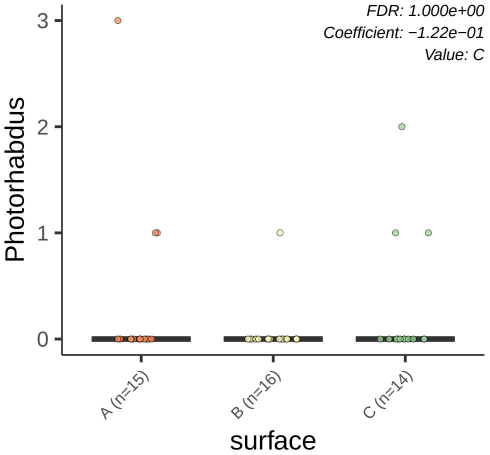
FDR: 1.000e+00
Coefficient: −1.22e−01
Value: C
Photorhabdus
3
2
1
0
A (n=15)
B (n=16)
C (n=14)
surface

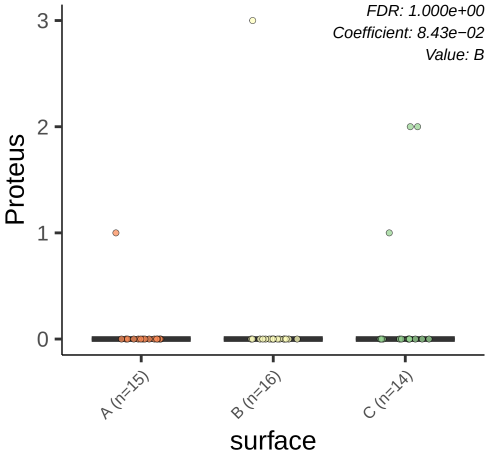
FDR: 1.000e+00
Coefficient: 8.43e−02
Value: B
Proteus
3
2
1
0
A (n=15)
B (n=16)
C (n=14)
surface

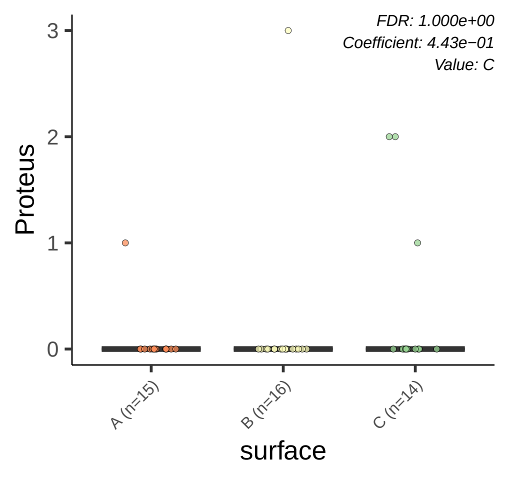
FDR: 1.000e+00
Coefficient: 4.43e−01
Value: C
Proteus
3
2
1
0
A (n=15)
B (n=16)
C (n=14)
surface

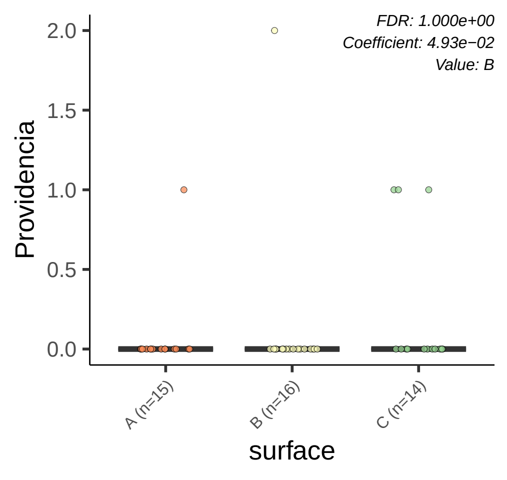
FDR: 1.000e+00
Coefficient: 4.93e−02
Value: B
Providencia
2.0
1.5
1.0
0.5
0.0
A (n=15)
B (n=16)
C (n=14)
surface

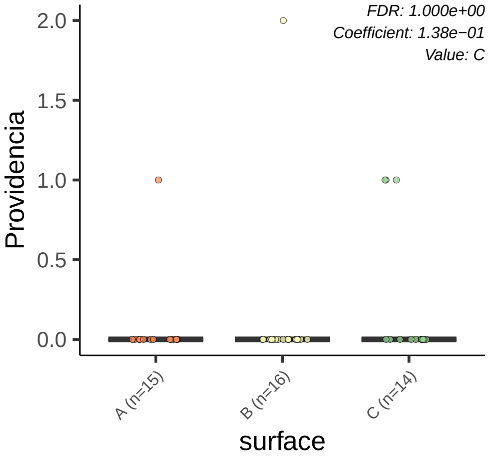

FDR: 1.000e+00
Coefficient: 1.38e−01
Value: C
Providencia
2.0
1.5
1.0
0.5
0.0
A (n=15)
B (n=16)
C (n=14)
surface

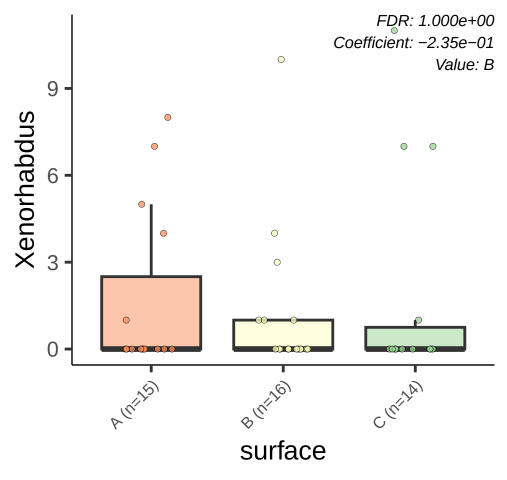
FDR: 1.000e+00
Coefficient: −2.35e−01
Value: B
Xenorhabdus
9
6
3
0
A (n=15)
B (n=16)
C (n=14)
surface

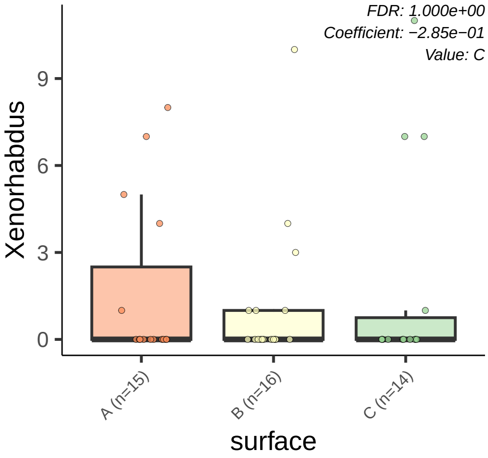
FDR: 1.000e+00
Coefficient: −2.85e−01
Value: C
Xenorhabdus
0
3
6
9
A (n=15)
B (n=16)
C (n=14)
surface

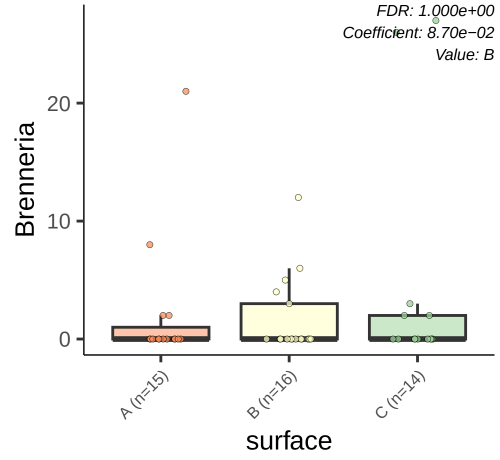

FDR: 1.000e+00
Coefficient: 8.70e−02
Value: B
Brenneria
0
10
20
A (n=15)
B (n=16)
C (n=14)
surface

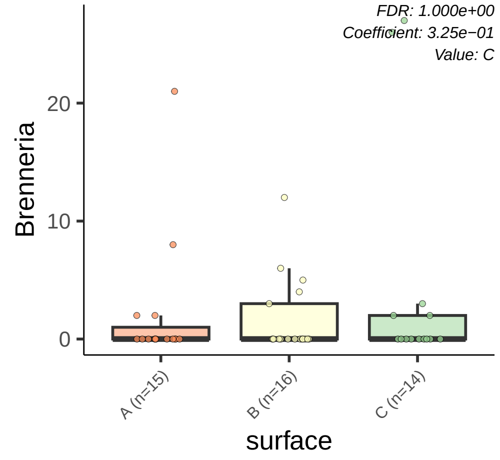
FDR: 1.000e+00
Coefficient: 3.25e−01
Value: C
Brenneria
0
10
20
A (n=15)
B (n=16)
C (n=14)
surface

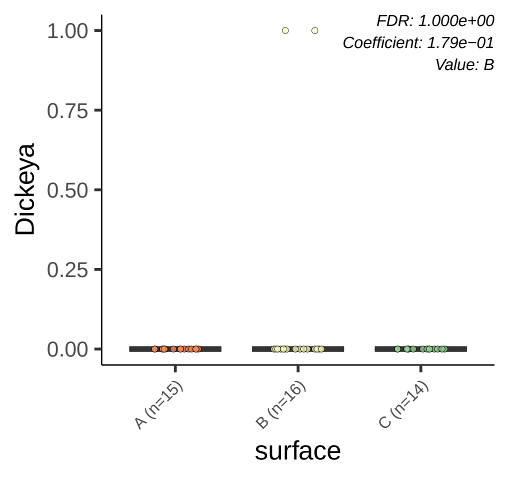

FDR: 1.000e+00
Coefficient: 1.79e−01
Value: B
Dickeya
1.00
0.75
0.50
0.25
0.00
A (n=15)
B (n=16)
C (n=14)
surface

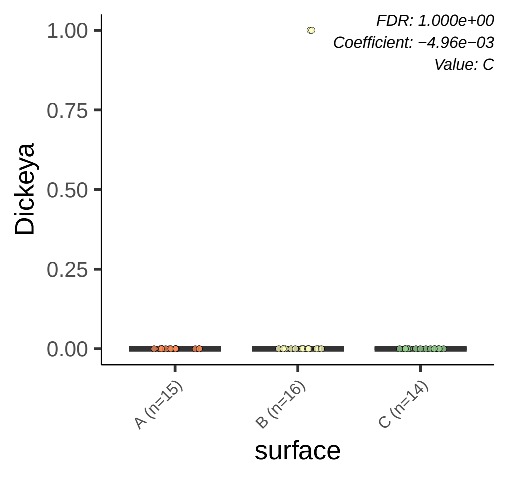
FDR: 1.000e+00
Coefficient: −4.96e−03
Value: C
1.00
0.75
0.50
0.25
0.00
Dickeya
A (n=15)
B (n=16)
C (n=14)
surface

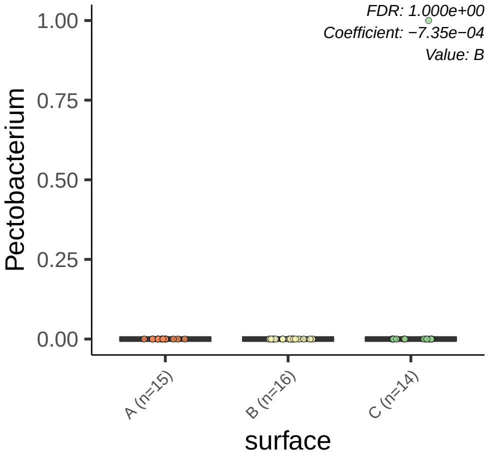
FDR: 1.000e+00
Coefficient: −7.35e−04
Value: B
Pectobacterium
1.00
0.75
0.50
0.25
0.00
A (n=15)
B (n=16)
C (n=14)
surface

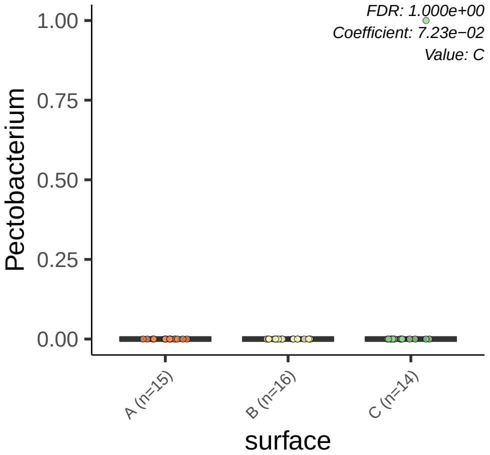
FDR: 1.000e+00
Coefficient: 7.23e−02
Value: C
Pectobacterium
1.00
0.75
0.50
0.25
0.00
A (n=15)
B (n=16)
C (n=14)
surface

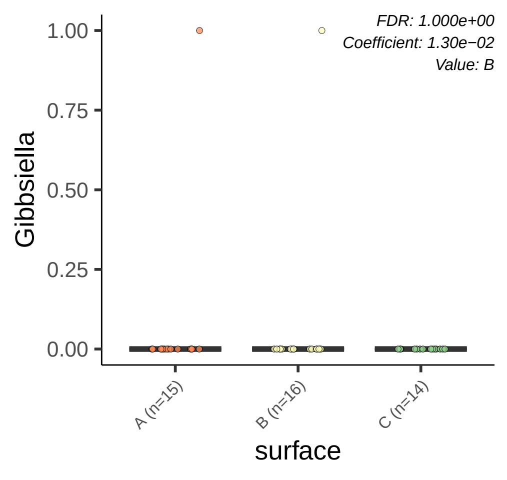
FDR: 1.000e+00
Coefficient: 1.30e−02
Value: B
Gibbsiella
1.00
0.75
0.50
0.25
0.00
A (n=15)
B (n=16)
C (n=14)
surface

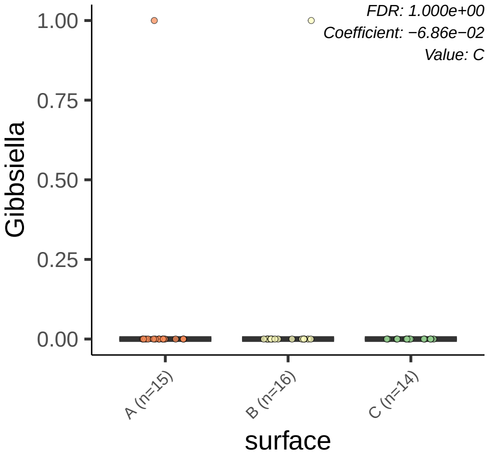
FDR: 1.000e+00
Coefficient: −6.86e−02
Value: C
Gibbsiella
1.00
0.75
0.50
0.25
0.00
A (n=15)
B (n=16)
C (n=14)
surface

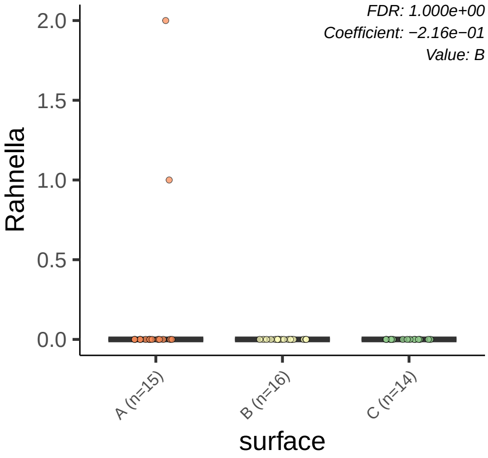
FDR: 1.000e+00
Coefficient: −2.16e−01
Value: B
Rahnella
2.0
1.5
1.0
0.5
0.0
A (n=15)
B (n=16)
C (n=14)
surface

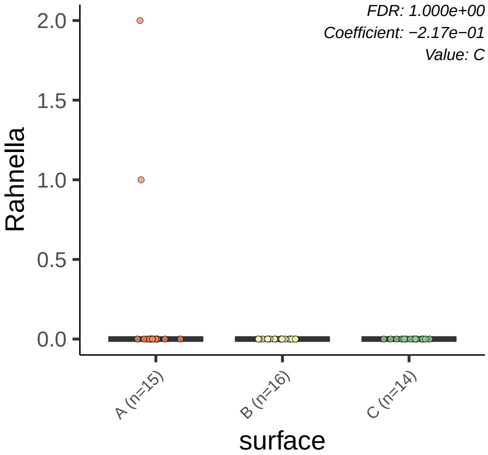
FDR: 1.000e+00
Coefficient: −2.17e−01
Value: C
Rahnella
2.0
1.5
1.0
0.5
0.0
A (n=15)
B (n=16)
C (n=14)
surface

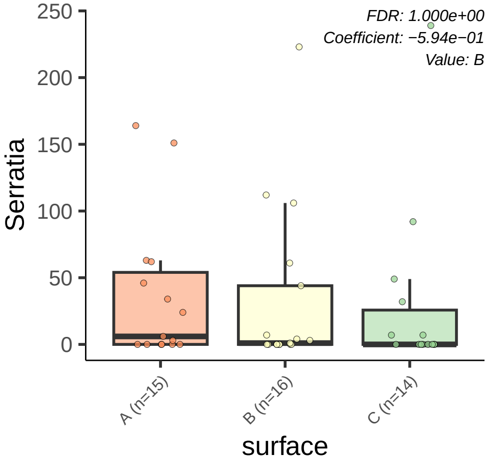
FDR: 1.000e+00
Coefficient: −5.94e−01
Value: B
Serratia
250
200
150
100
50
0
A (n=15)
B (n=16)
C (n=14)
surface

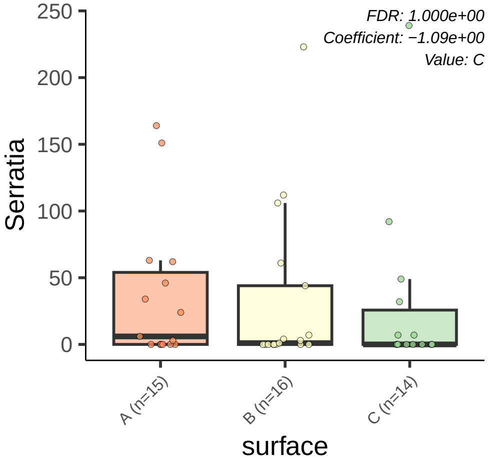
FDR: 1.000e+00
Coefficient: −1.09e+00
Value: C
Serratia
250
200
150
100
50
0
A (n=15)
B (n=16)
C (n=14)
surface

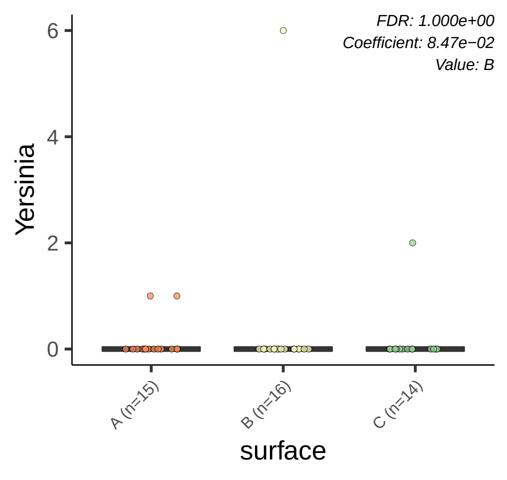
FDR: 1.000e+00
Coefficient: 8.47e−02
Value: B
Yersinia
6
4
2
0
A (n=15)
B (n=16)
C (n=14)
surface

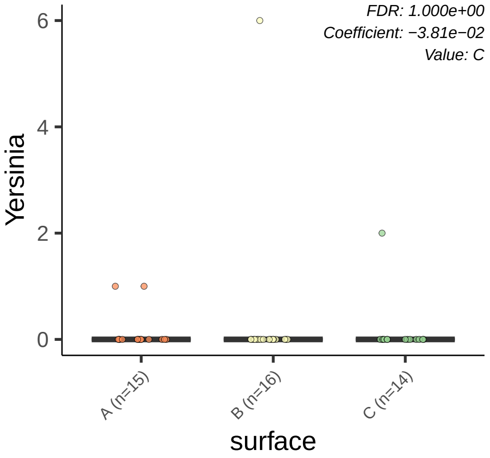
FDR: 1.000e+00
Coefficient: −3.81e−02
Value: C
Yersinia
0
2
4
6
A (n=15)
B (n=16)
C (n=14)
surface

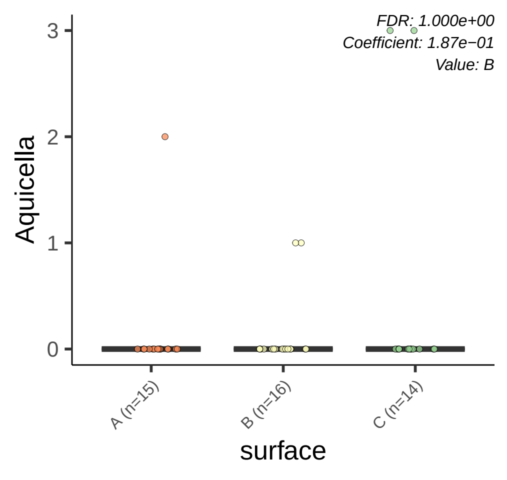
FDR: 1.000e+00
Coefficient: 1.87e−01
Value: B
Aquicella
3
2
1
0
A (n=15)
B (n=16)
C (n=14)
surface

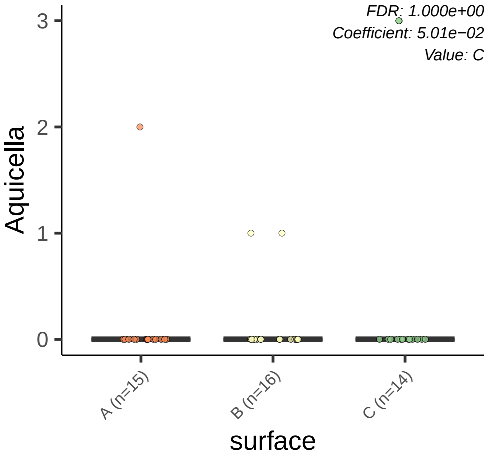

FDR: 1.000e+00
Coefficient: 5.01e−02
Value: C
Aquicella
0
1
2
3
A (n=15)
B (n=16)
C (n=14)
surface

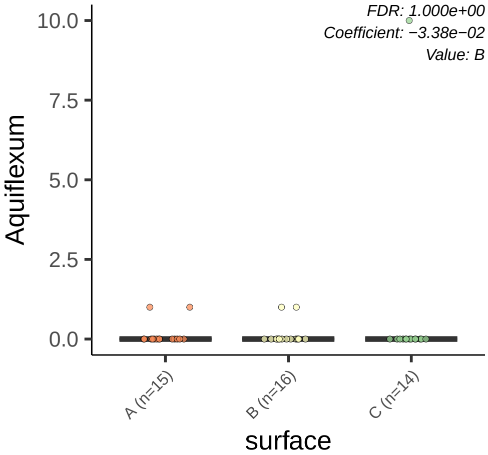

FDR: 1.000e+00
Coefficient: −3.38e−02
Value: B
Aquiflexum
10.0
7.5
5.0
2.5
0.0
A (n=15)
B (n=16)
C (n=14)
surface

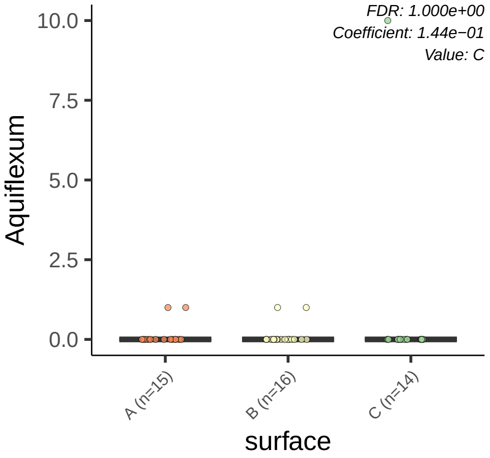
FDR: 1.000e+00
Coefficient: 1.44e−01
Value: C
Aquiflexum
10.0
7.5
5.0
2.5
0.0
A (n=15)
B (n=16)
C (n=14)
surface

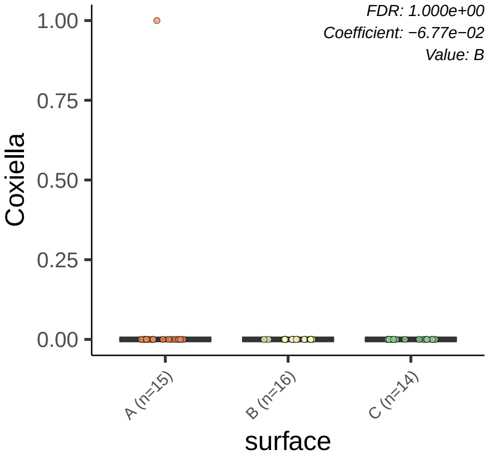
FDR: 1.000e+00
Coefficient: −6.77e−02
Value: B
Coxiella
1.00
0.75
0.50
0.25
0.00
A (n=15)
B (n=16)
C (n=14)
surface

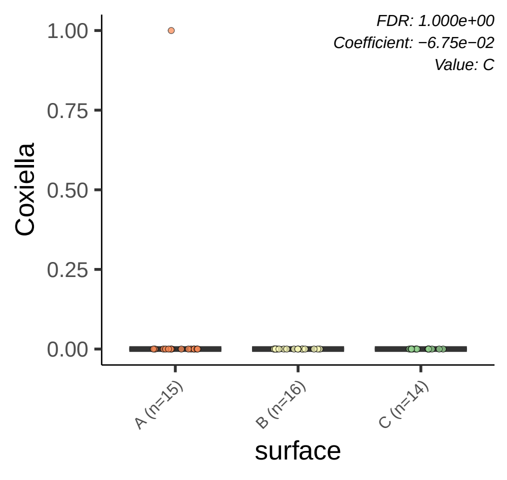
FDR: 1.000e+00
Coefficient: −6.75e−02
Value: C
Coxiella
1.00
0.75
0.50
0.25
0.00
A (n=15)
B (n=16)
C (n=14)
surface

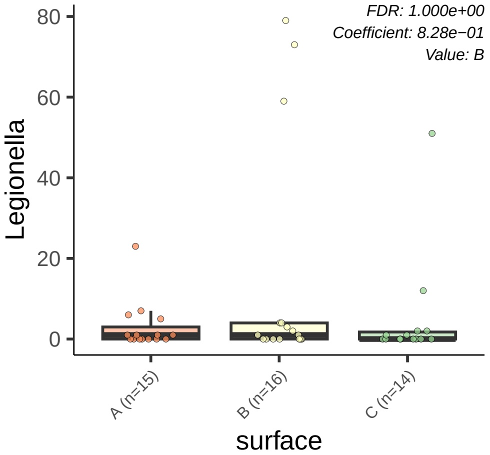
FDR: 1.000e+00
Coefficient: 8.28e−01
Value: B
Legionella
80
60
40
20
0
A (n=15)
B (n=16)
C (n=14)
surface

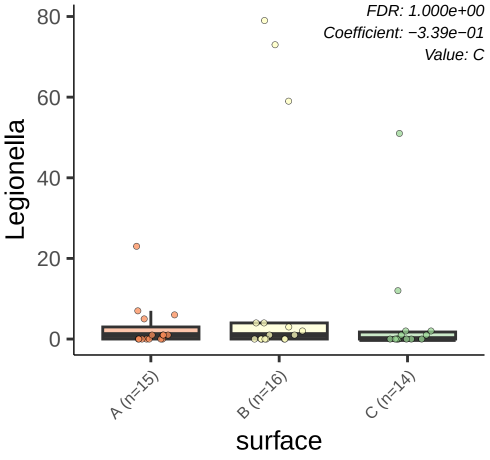

FDR: 1.000e+00
Coefficient: −3.39e−01
Value: C
Legionella
0
20
40
60
80
A (n=15)
B (n=16)
C (n=14)
surface

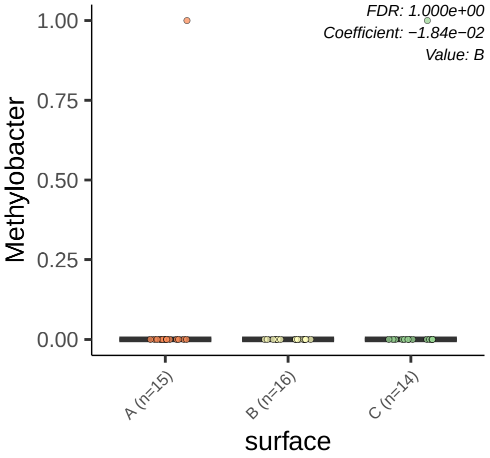
FDR: 1.000e+00
Coefficient: −1.84e−02
Value: B
Methylobacter
1.00
0.75
0.50
0.25
0.00
A (n=15)
B (n=16)
C (n=14)
surface

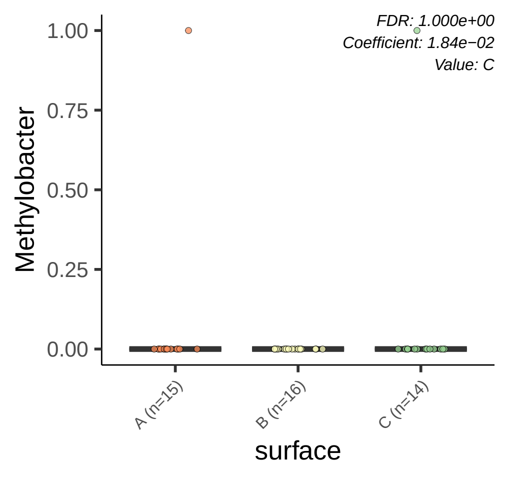
FDR: 1.000e+00
Coefficient: 1.84e−02
Value: C
1.00
0.75
0.50
0.25
0.00
Methylobacter
A (n=15)
B (n=16)
C (n=14)
surface

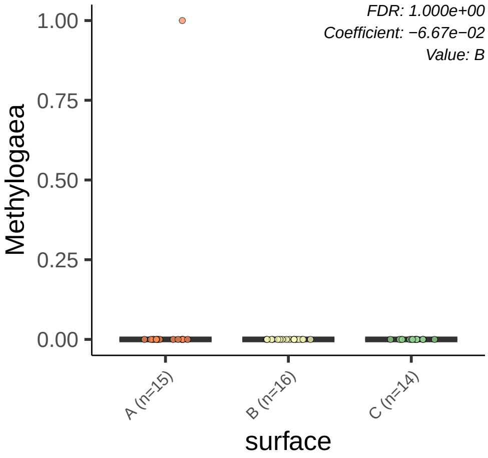

FDR: 1.000e+00
Coefficient: −6.67e−02
Value: B
Methylogaea
1.00
0.75
0.50
0.25
0.00
A (n=15)
B (n=16)
C (n=14)
surface

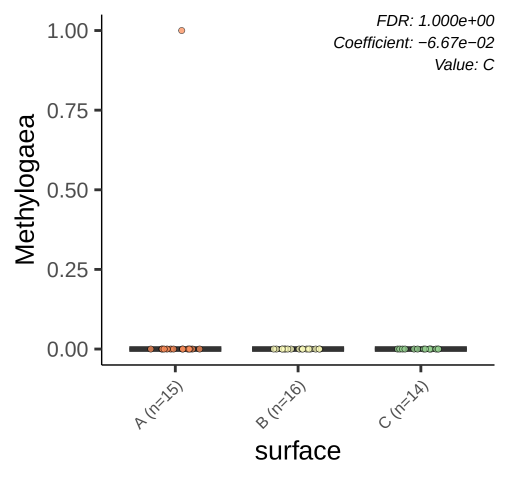

FDR: 1.000e+00
Coefficient: −6.67e−02
Value: C
1.00
0.75
0.50
0.25
0.00
Methylogaea
A (n=15)
B (n=16)
C (n=14)
surface

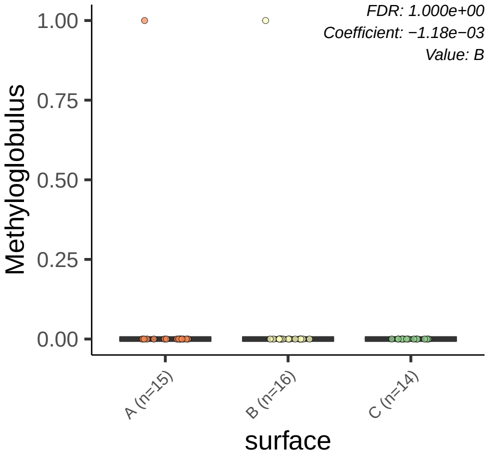

FDR: 1.000e+00
Coefficient: −1.18e−03
Value: B
Methyloglobulus
1.00
0.75
0.50
0.25
0.00
A (n=15)
B (n=16)
C (n=14)
surface

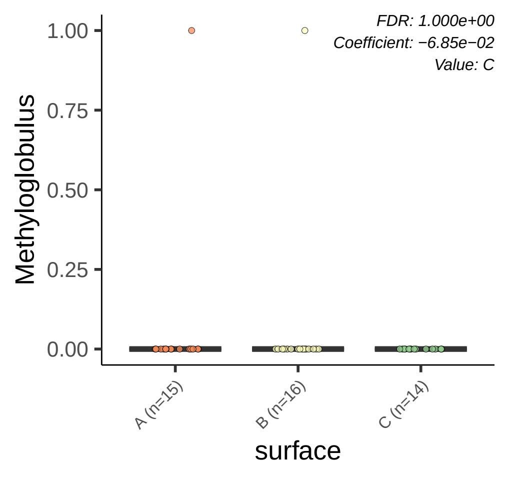
FDR: 1.000e+00
Coefficient: −6.85e−02
Value: C
Methyloglobulus
1.00
0.75
0.50
0.25
0.00
A (n=15)
B (n=16)
C (n=14)
surface

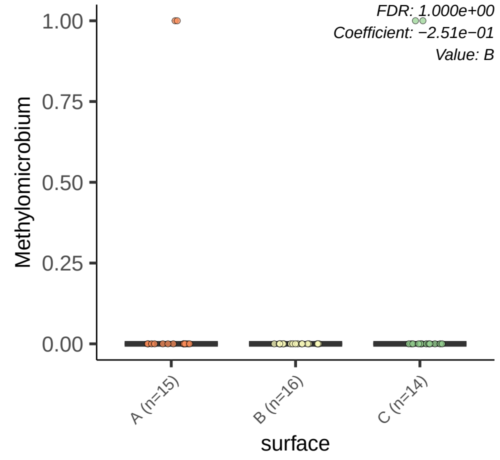

FDR: 1.000e+00
Coefficient: −2.51e−01
Value: B
Methylomicrobium
1.00
0.75
0.50
0.25
0.00
A (n=15)
B (n=16)
C (n=14)
surface

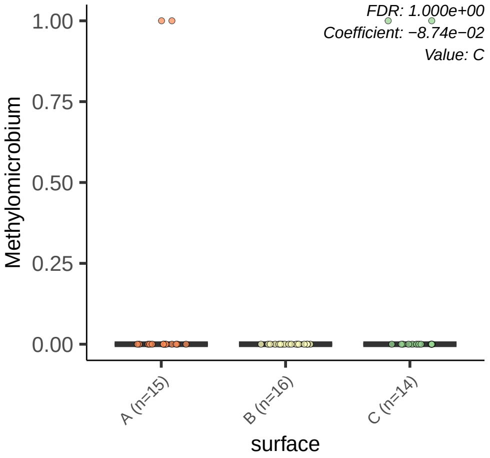
FDR: 1.000e+00
Coefficient: −8.74e−02
Value: C
Methylomicrobium
1.00
0.75
0.50
0.25
0.00
A (n=15)
B (n=16)
C (n=14)
surface

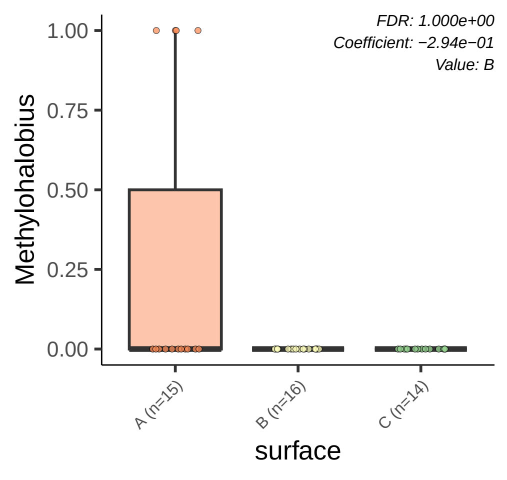

FDR: 1.000e+00
Coefficient: −2.94e−01
Value: B
Methylohalobius
1.00
0.75
0.50
0.25
0.00
A (n=15)
B (n=16)
C (n=14)
surface

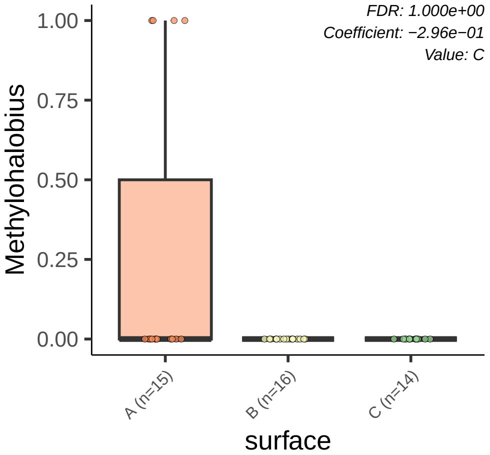

FDR: 1.000e+00
Coefficient: −2.96e−01
Value: C
Methylohalobius
1.00
0.75
0.50
0.25
0.00
A (n=15)
B (n=16)
C (n=14)
surface

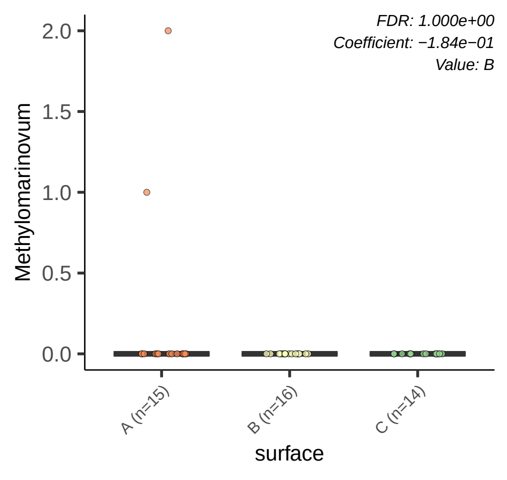
FDR: 1.000e+00
Coefficient: −1.84e−01
Value: B
Methylomarinovum
2.0
1.5
1.0
0.5
0.0
A (n=15)
B (n=16)
C (n=14)
surface

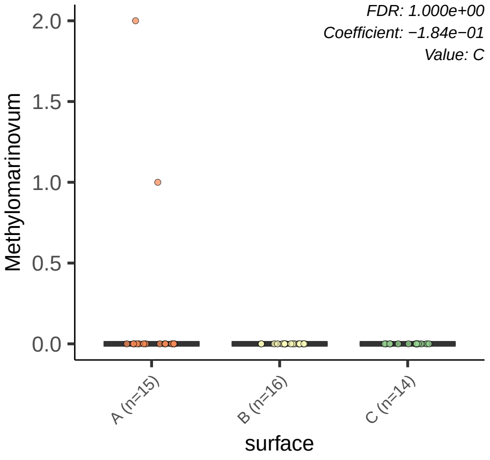

FDR: 1.000e+00
Coefficient: −1.84e−01
Value: C
Methylomarinovum
2.0
1.5
1.0
0.5
0.0
A (n=15)
B (n=16)
C (n=14)
surface

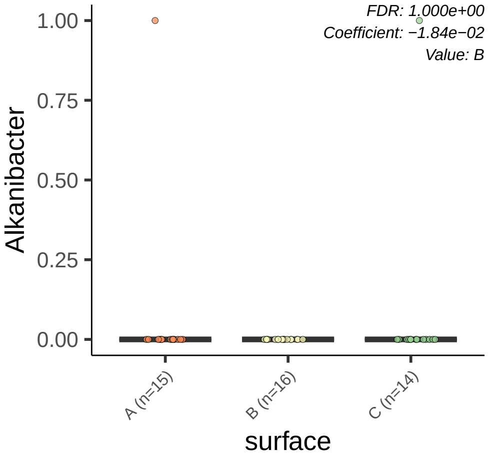
FDR: 1.000e+00
Coefficient: −1.84e−02
Value: B
Alkanibacter
1.00
0.75
0.50
0.25
0.00
A (n=15)
B (n=16)
C (n=14)
surface

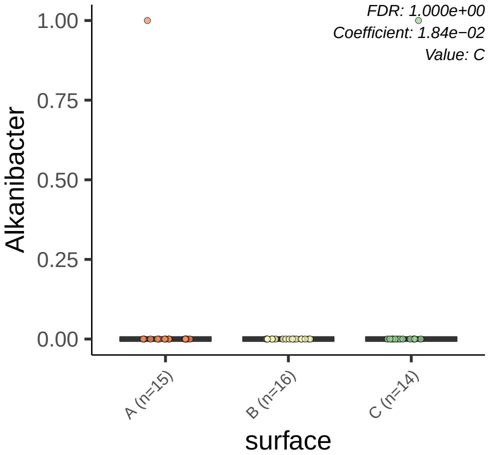
FDR: 1.000e+00
Coefficient: 1.84e−02
Value: C
Alkanibacter
1.00
0.75
0.50
0.25
0.00
A (n=15)
B (n=16)
C (n=14)
surface

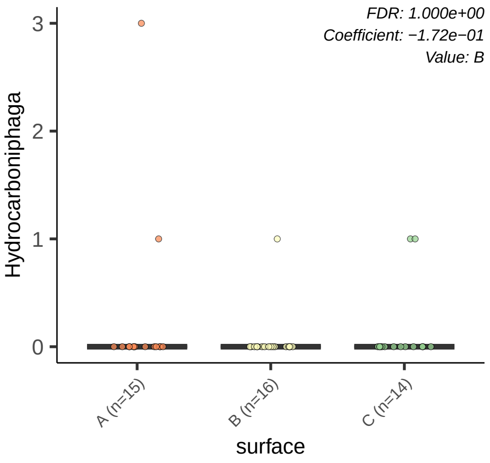
FDR: 1.000e+00
Coefficient: −1.72e−01
Value: B
Hydrocarboniphaga
0
1
2
3
A (n=15)
B (n=16)
C (n=14)
surface

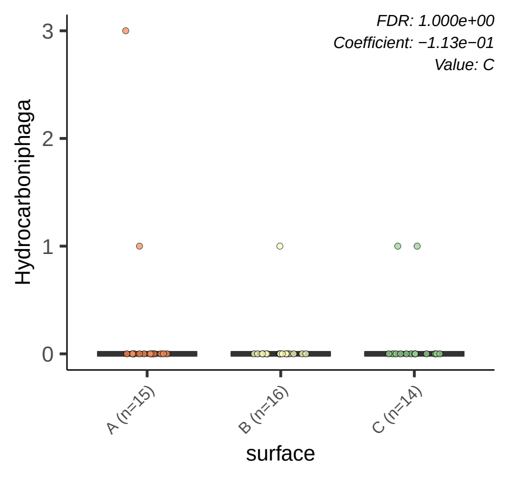
FDR: 1.000e+00
Coefficient: −1.13e−01
Value: C
Hydrocarboniphaga
3
2
1
0
A (n=15)
B (n=16)
C (n=14)
surface

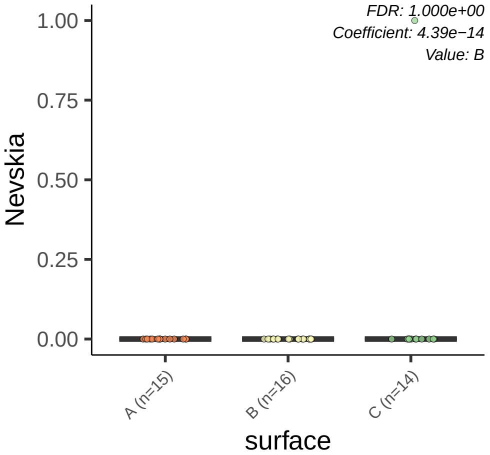

FDR: 1.000e+00
Coefficient: 4.39e−14
Value: B
Nevskia
1.00
0.75
0.50
0.25
0.00
A (n=15)
B (n=16)
C (n=14)
surface

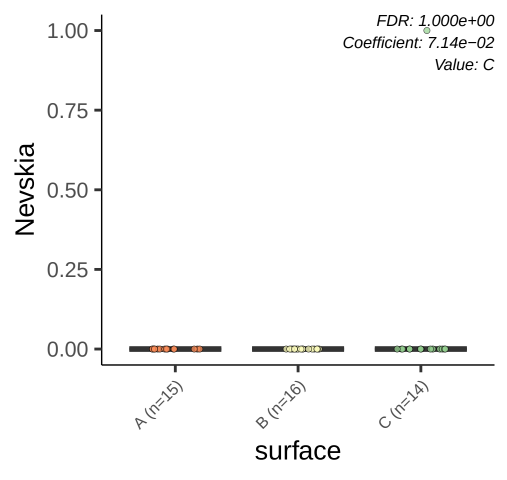
FDR: 1.000e+00
Coefficient: 7.14e−02
Value: C
Nevskia
1.00
0.75
0.50
0.25
0.00
A (n=15)
B (n=16)
C (n=14)
surface

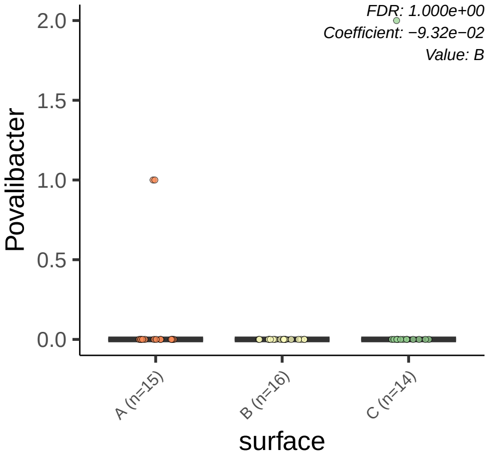
FDR: 1.000e+00
Coefficient: −9.32e−02
Value: B
Povalibacter
2.0
1.5
1.0
0.5
0.0
A (n=15)
B (n=16)
C (n=14)
surface

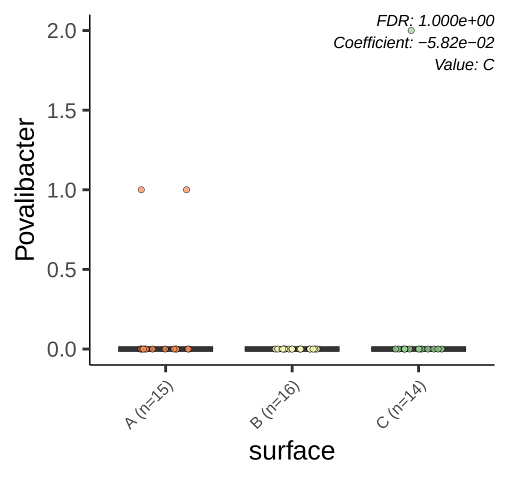
FDR: 1.000e+00
Coefficient: −5.82e−02
Value: C
Povalibacter
2.0
1.5
1.0
0.5
0.0
A (n=15)
B (n=16)
C (n=14)
surface

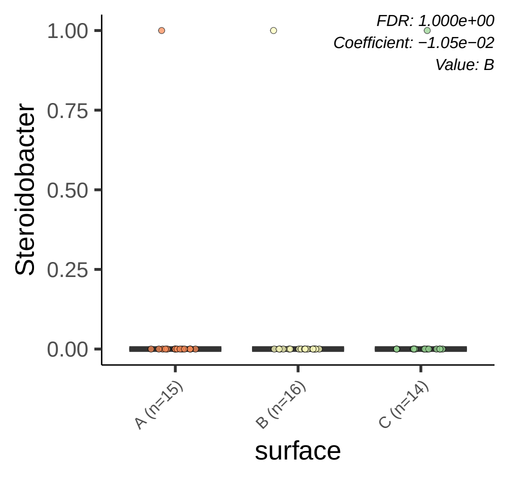
FDR: 1.000e+00
Coefficient: −1.05e−02
Value: B
Steroidobacter
1.00
0.75
0.50
0.25
0.00
A (n=15)
B (n=16)
C (n=14)
surface

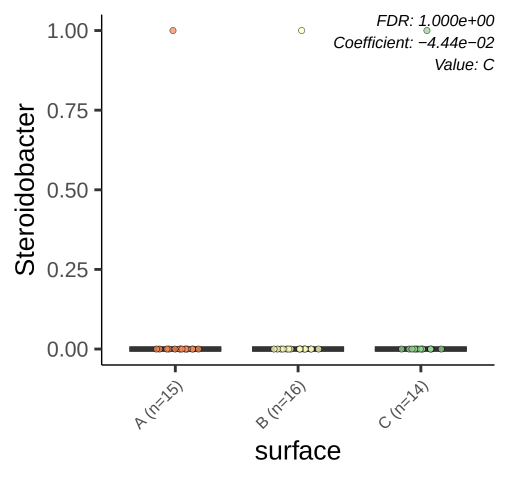
FDR: 1.000e+00
Coefficient: −4.44e−02
Value: C
Steroidobacter
1.00
0.75
0.50
0.25
0.00
A (n=15)
B (n=16)
C (n=14)
surface

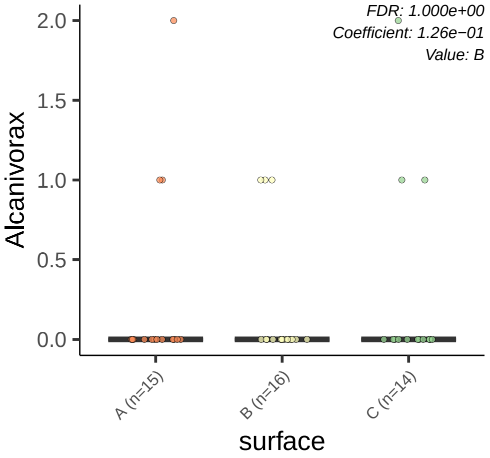
FDR: 1.000e+00
Coefficient: 1.26e−01
Value: B
Alcanivorax
2.0
1.5
1.0
0.5
0.0
A (n=15)
B (n=16)
C (n=14)
surface

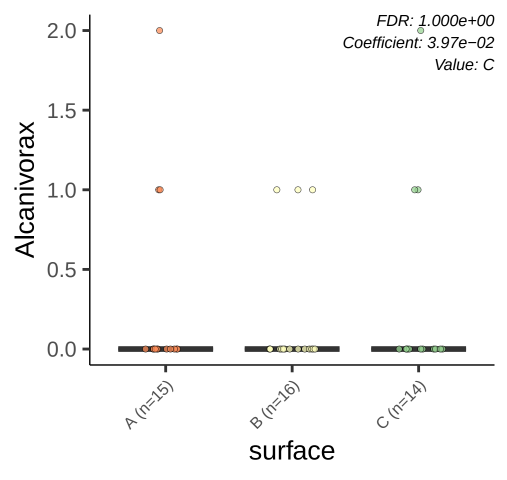
FDR: 1.000e+00
Coefficient: 3.97e−02
Value: C
Alcanivorax
2.0
1.5
1.0
0.5
0.0
A (n=15)
B (n=16)
C (n=14)
surface

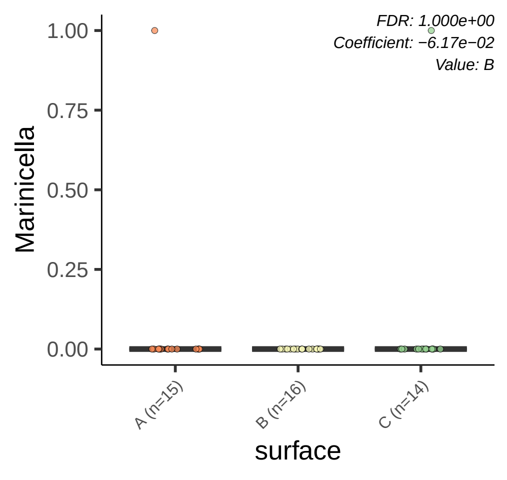

FDR: 1.000e+00
Coefficient: −6.17e−02
Value: B
Marinicella
1.00
0.75
0.50
0.25
0.00
A (n=15)
B (n=16)
C (n=14)
surface

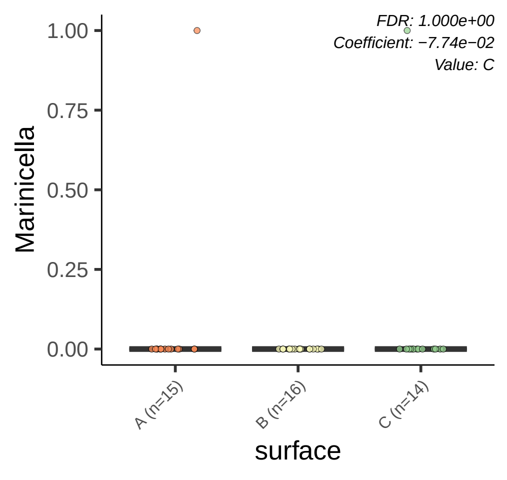

FDR: 1.000e+00
Coefficient: −7.74e−02
Value: C
Marinicella
1.00
0.75
0.50
0.25
0.00
A (n=15)
B (n=16)
C (n=14)
surface

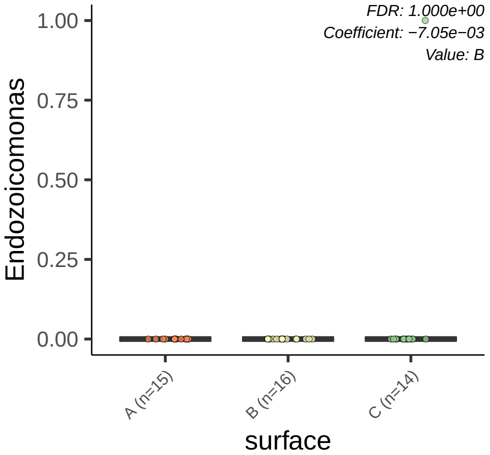

FDR: 1.000e+00
Coefficient: −7.05e−03
Value: B
Endozoicomonas
1.00
0.75
0.50
0.25
0.00
A (n=15)
B (n=16)
C (n=14)
surface

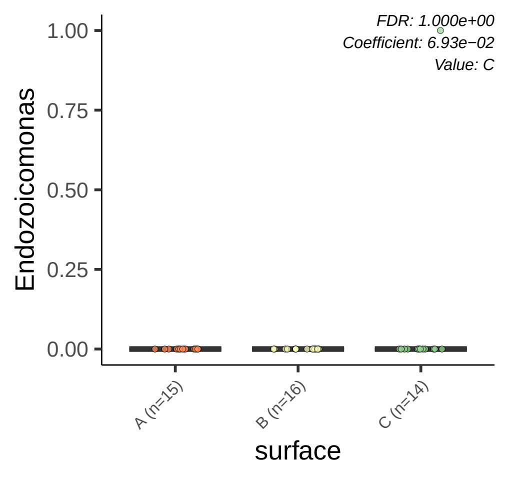
FDR: 1.000e+00
Coefficient: 6.93e−02
Value: C
Endozoicomonas
1.00
0.75
0.50
0.25
0.00
A (n=15)
B (n=16)
C (n=14)
surface

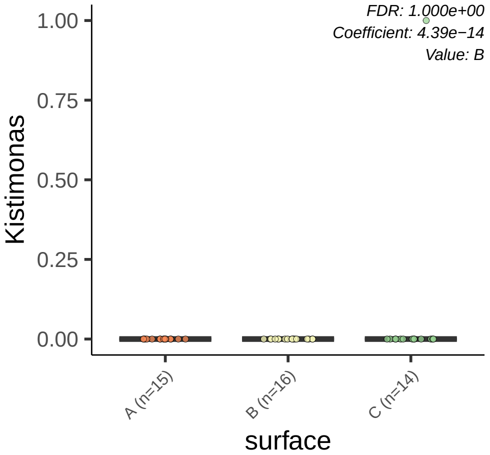
FDR: 1.000e+00
Coefficient: 4.39e−14
Value: B
Kistimonas
1.00
0.75
0.50
0.25
0.00
A (n=15)
B (n=16)
C (n=14)
surface

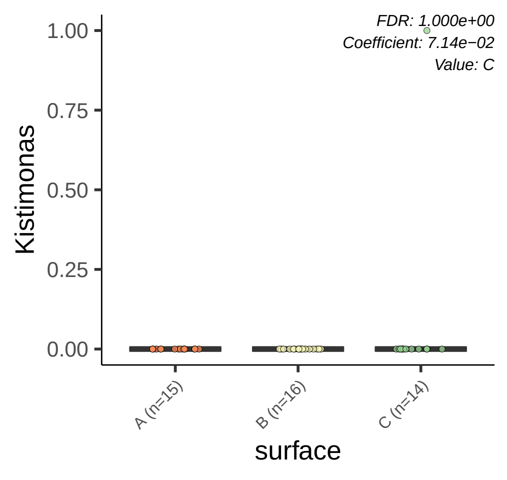

FDR: 1.000e+00
Coefficient: 7.14e−02
Value: C
Kistimonas
1.00
0.75
0.50
0.25
0.00
A (n=15)
B (n=16)
C (n=14)
surface

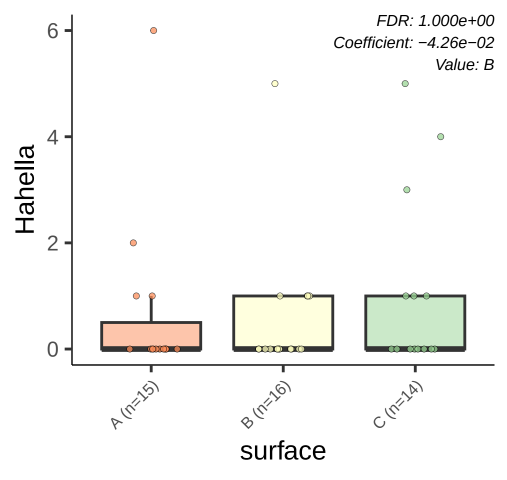
FDR: 1.000e+00
Coefficient: −4.26e−02
Value: B
Hahella
0
2
4
6
A (n=15)
B (n=16)
C (n=14)
surface

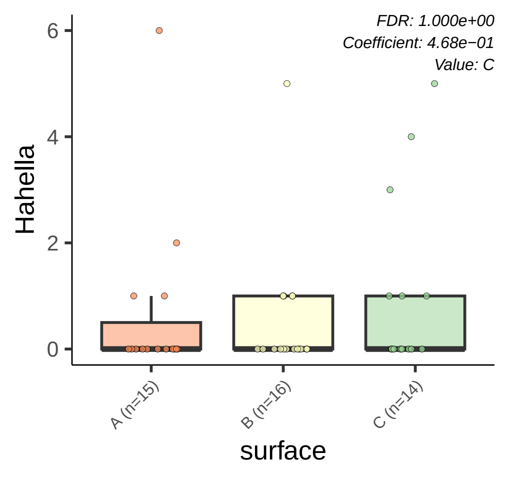

FDR: 1.000e+00
Coefficient: 4.68e−01
Value: C
Hahella
0
2
4
6
A (n=15)
B (n=16)
C (n=14)
surface

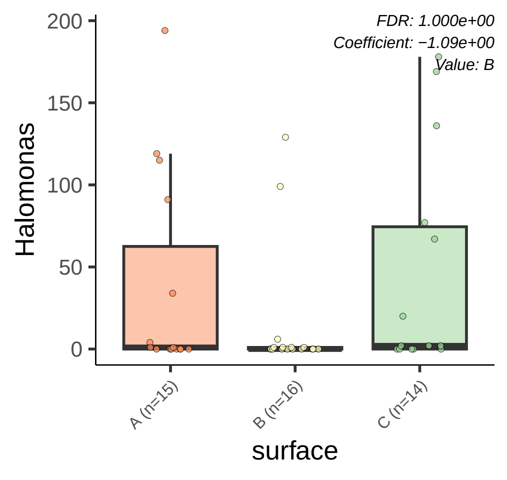

FDR: 1.000e+00
Coefficient: −1.09e+00
Value: B
Halomonas
200
150
100
50
0
A (n=15)
B (n=16)
C (n=14)
surface

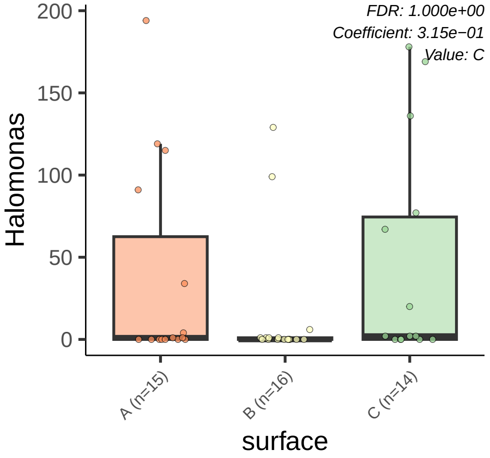

FDR: 1.000e+00
Coefficient: 3.15e−01
Value: C
Halomonas
200
150
100
50
0
A (n=15)
B (n=16)
C (n=14)
surface

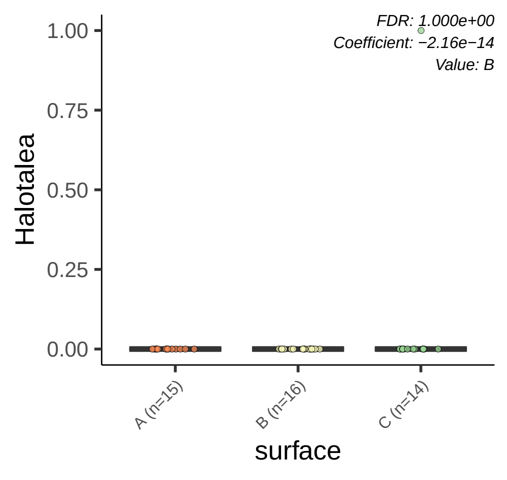

FDR: 1.000e+00
Coefficient: −2.16e−14
Value: B
Halotalea
1.00
0.75
0.50
0.25
0.00
A (n=15)
B (n=16)
C (n=14)
surface

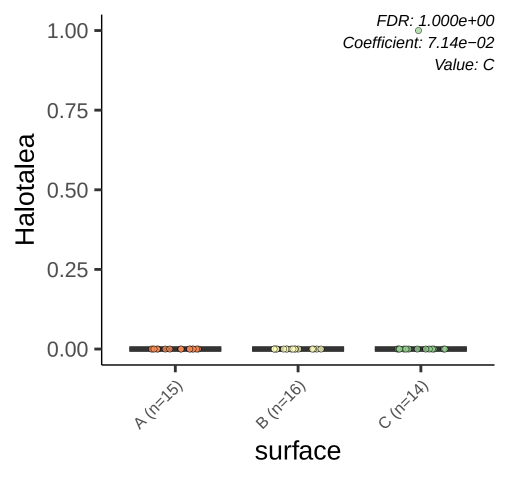
FDR: 1.000e+00
Coefficient: 7.14e−02
Value: C
Halotalea
1.00
0.75
0.50
0.25
0.00
A (n=15)
B (n=16)
C (n=14)
surface

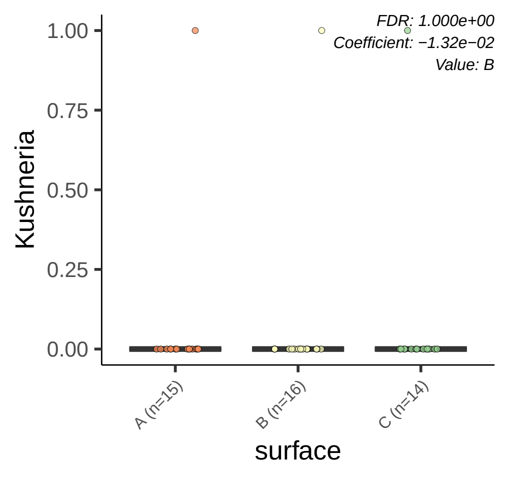
FDR: 1.000e+00
Coefficient: −1.32e−02
Value: B
Kushneria
1.00
0.75
0.50
0.25
0.00
A (n=15)
B (n=16)
C (n=14)
surface

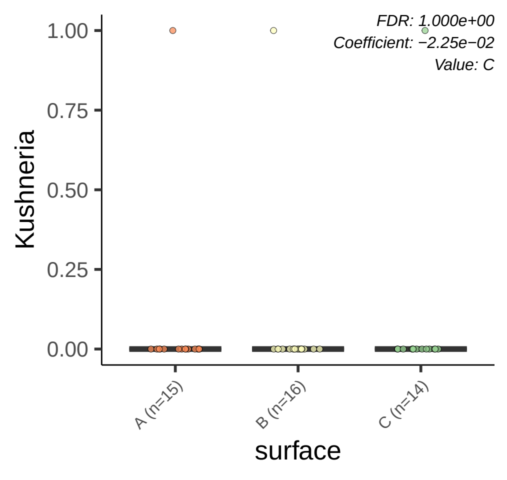

FDR: 1.000e+00
Coefficient: −2.25e−02
Value: C
Kushneria
1.00
0.75
0.50
0.25
0.00
A (n=15)
B (n=16)
C (n=14)
surface

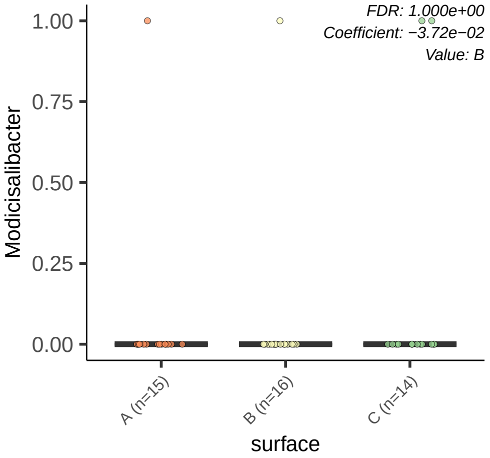
FDR: 1.000e+00
Coefficient: −3.72e−02
Value: B
Modicisalibacter
1.00
0.75
0.50
0.25
0.00
A (n=15)
B (n=16)
C (n=14)
surface

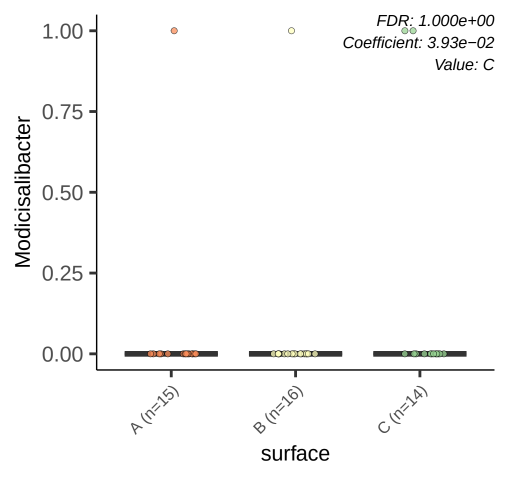

FDR: 1.000e+00
Coefficient: 3.93e−02
Value: C
Modicisalibacter
1.00
0.75
0.50
0.25
0.00
A (n=15)
B (n=16)
C (n=14)
surface

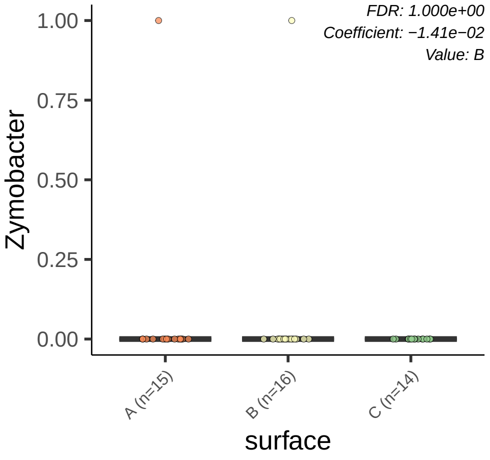

FDR: 1.000e+00
Coefficient: −1.41e−02
Value: B
Zymobacter
1.00
0.75
0.50
0.25
0.00
A (n=15)
B (n=16)
C (n=14)
surface

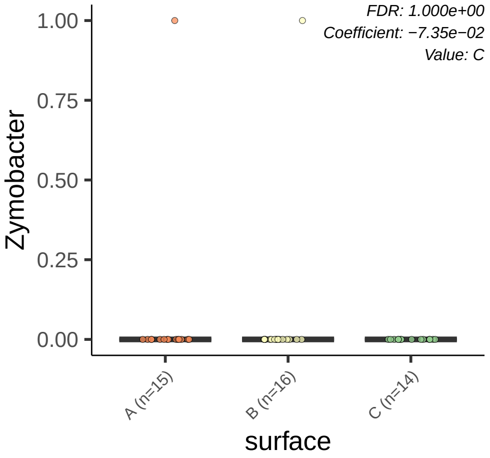

FDR: 1.000e+00
Coefficient: −7.35e−02
Value: C
Zymobacter
1.00
0.75
0.50
0.25
0.00
A (n=15)
B (n=16)
C (n=14)
surface

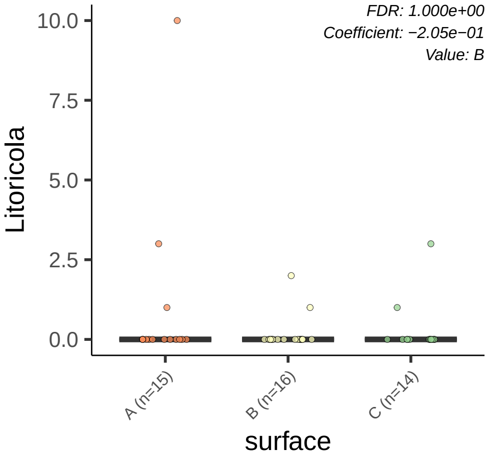
FDR: 1.000e+00
Coefficient: −2.05e−01
Value: B
Litoricola
10.0
7.5
5.0
2.5
0.0
A (n=15)
B (n=16)
C (n=14)
surface

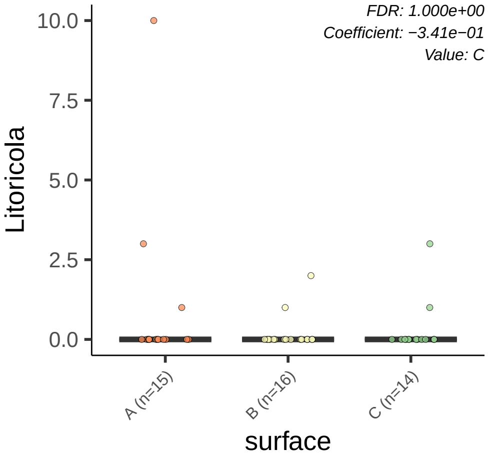

FDR: 1.000e+00
Coefficient: −3.41e−01
Value: C
Litoricola
10.0
7.5
5.0
2.5
0.0
A (n=15)
B (n=16)
C (n=14)
surface

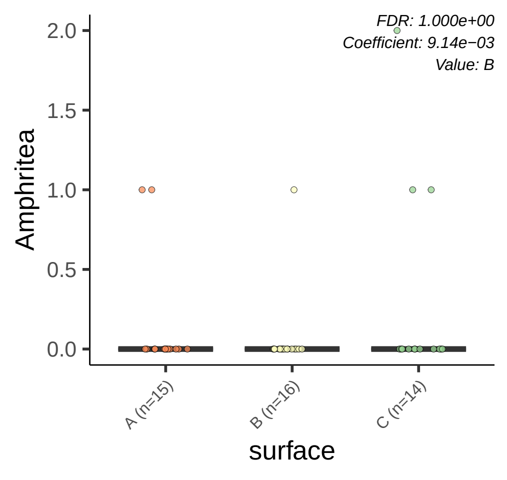

FDR: 1.000e+00
Coefficient: 9.14e−03
Value: B
Amphritea
2.0
1.5
1.0
0.5
0.0
A (n=15)
B (n=16)
C (n=14)
surface

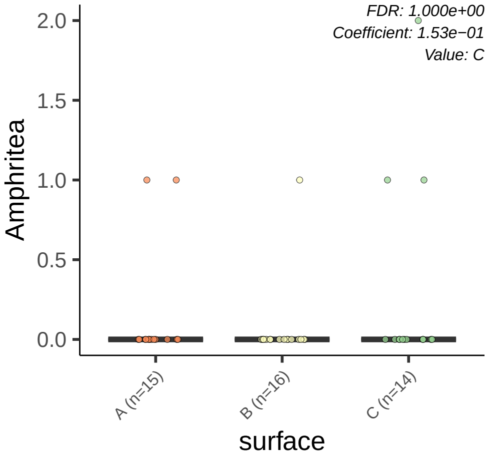

FDR: 1.000e+00
Coefficient: 1.53e−01
Value: C
Amphritea
2.0
1.5
1.0
0.5
0.0
A (n=15)
B (n=16)
C (n=14)
surface

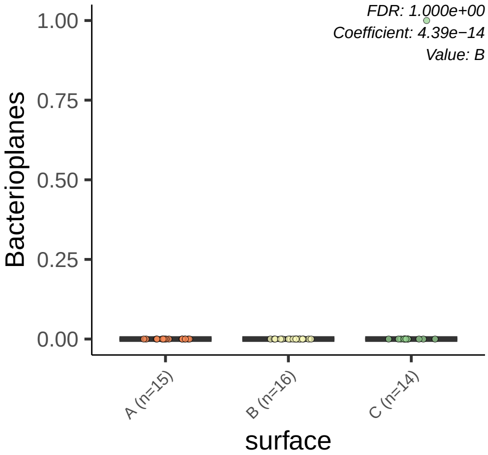
FDR: 1.000e+00
Coefficient: 4.39e−14
Value: B
Bacterioplanes
1.00
0.75
0.50
0.25
0.00
A (n=15)
B (n=16)
C (n=14)
surface

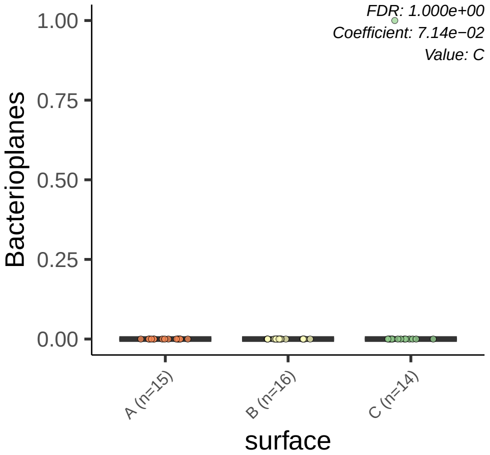
FDR: 1.000e+00
Coefficient: 7.14e−02
Value: C
Bacterioplanes
1.00
0.75
0.50
0.25
0.00
A (n=15)
B (n=16)
C (n=14)
surface

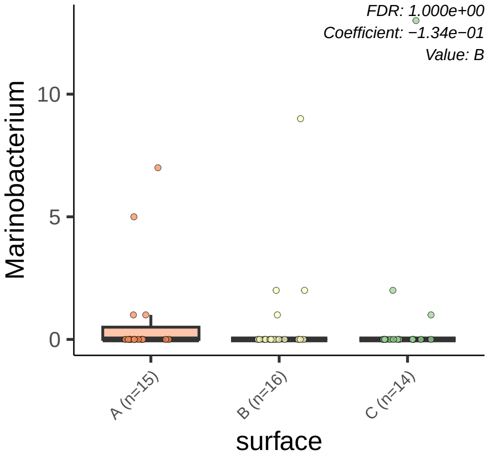
FDR: 1.000e+00
Coefficient: −1.34e−01
Value: B
Marinobacterium
10
5
0
A (n=15)
B (n=16)
C (n=14)
surface

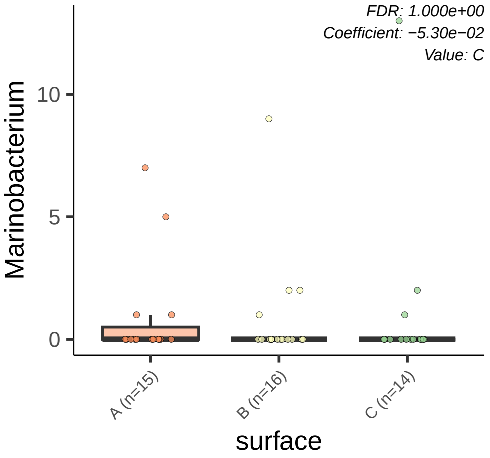
FDR: 1.000e+00
Coefficient: −5.30e−02
Value: C
Marinobacterium
10
5
0
A (n=15)
B (n=16)
C (n=14)
surface

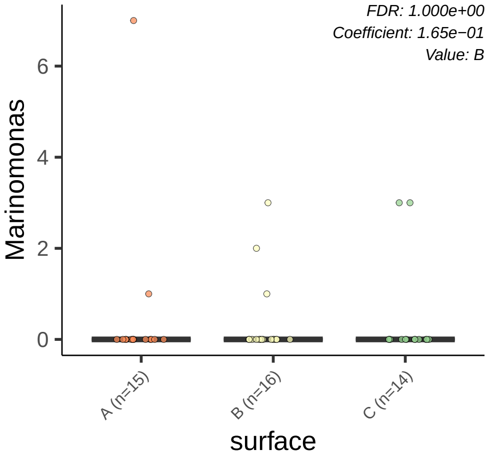
FDR: 1.000e+00
Coefficient: 1.65e−01
Value: B
Marinomonas
0
2
4
6
A (n=15)
B (n=16)
C (n=14)
surface

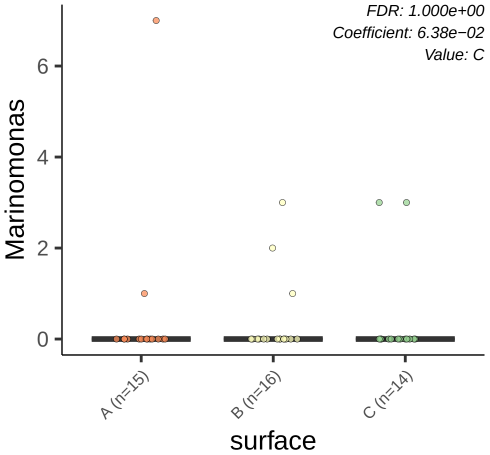
FDR: 1.000e+00
Coefficient: 6.38e−02
Value: C
Marinomonas
0
2
4
6
A (n=15)
B (n=16)
C (n=14)
surface

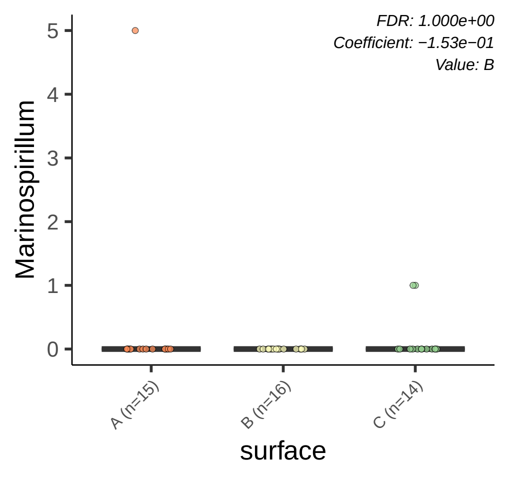

FDR: 1.000e+00
Coefficient: −1.53e−01
Value: B
Marinospirillum
5
4
3
2
1
0
A (n=15)
B (n=16)
C (n=14)
surface

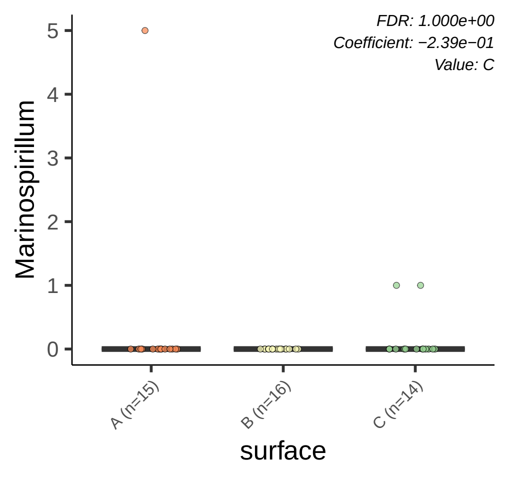

FDR: 1.000e+00
Coefficient: −2.39e−01
Value: C
Marinospirillum
0
1
2
3
4
5
A (n=15)
B (n=16)
C (n=14)
surface

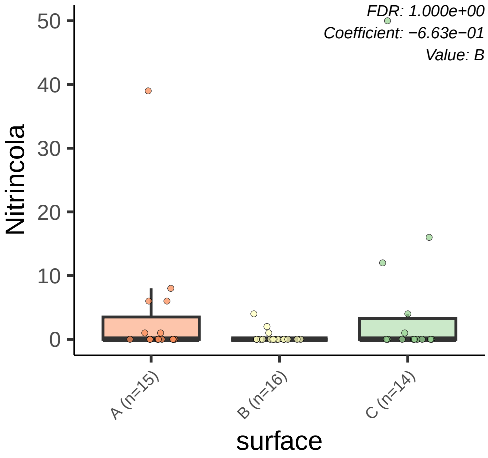
FDR: 1.000e+00
Coefficient: −6.63e−01
Value: B
Nitrincola
50
40
30
20
10
0
A (n=15)
B (n=16)
C (n=14)
surface

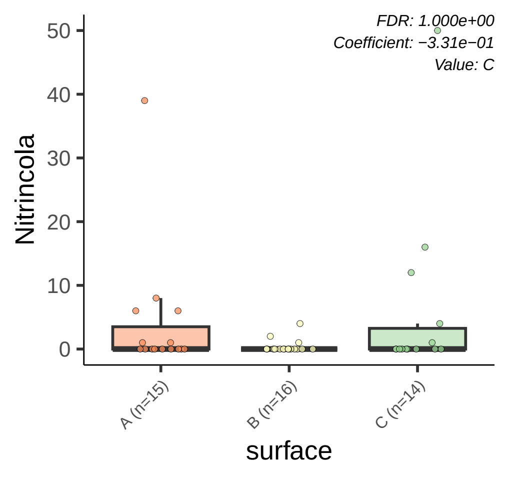
FDR: 1.000e+00
Coefficient: −3.31e−01
Value: C
Nitrincola
0
10
20
30
40
50
A (n=15)
B (n=16)
C (n=14)
surface

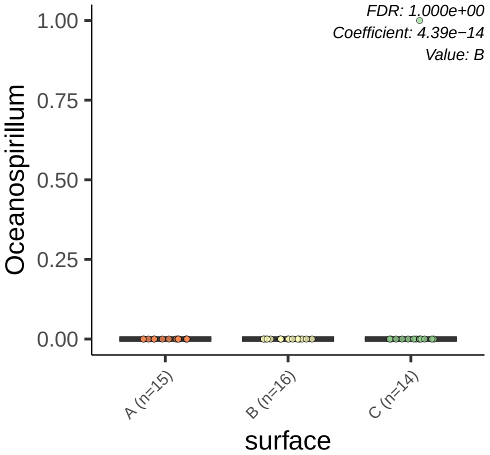
FDR: 1.000e+00
Coefficient: 4.39e−14
Value: B
Oceanospirillum
1.00
0.75
0.50
0.25
0.00
A (n=15)
B (n=16)
C (n=14)
surface

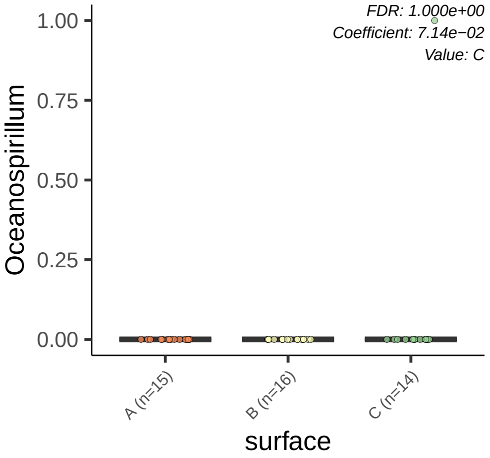
FDR: 1.000e+00
Coefficient: 7.14e−02
Value: C
Oceanospirillum
1.00
0.75
0.50
0.25
0.00
A (n=15)
B (n=16)
C (n=14)
surface

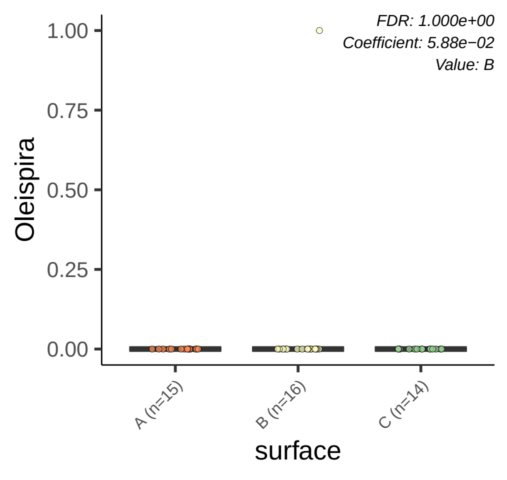

FDR: 1.000e+00
Coefficient: 5.88e−02
Value: B
Oleispira
1.00
0.75
0.50
0.25
0.00
A (n=15)
B (n=16)
C (n=14)
surface

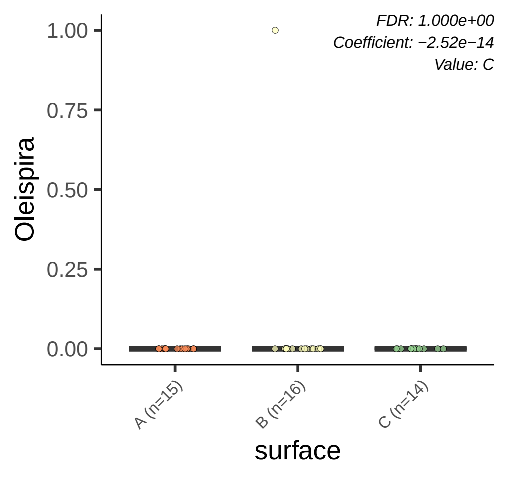
FDR: 1.000e+00
Coefficient: −2.52e−14
Value: C
Oleispira
1.00
0.75
0.50
0.25
0.00
A (n=15)
B (n=16)
C (n=14)
surface

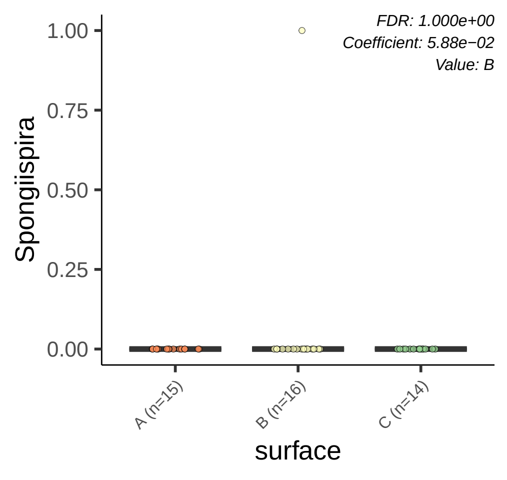
FDR: 1.000e+00
Coefficient: 5.88e−02
Value: B
1.00
0.75
0.50
0.25
0.00
Spongiispira
A (n=15)
B (n=16)
C (n=14)
surface

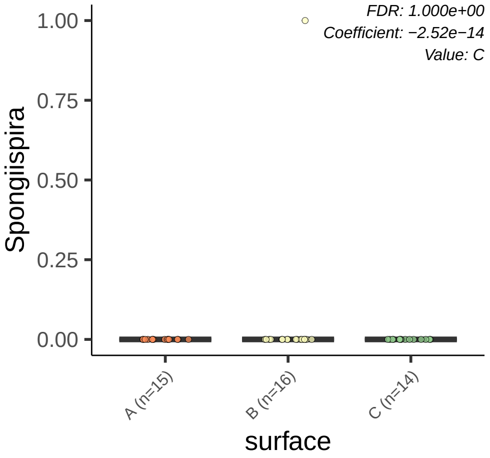

FDR: 1.000e+00
Coefficient: −2.52e−14
Value: C
Spongiispira
1.00
0.75
0.50
0.25
0.00
A (n=15)
B (n=16)
C (n=14)
surface

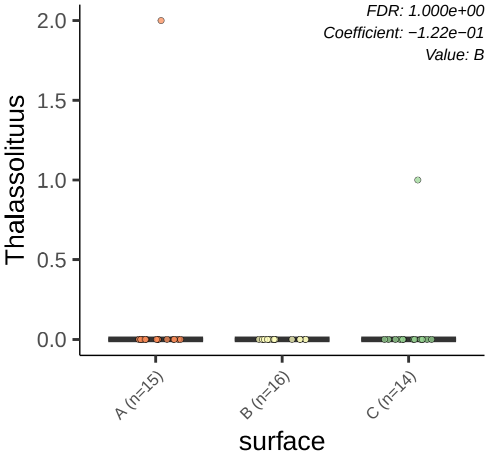
FDR: 1.000e+00
Coefficient: −1.22e−01
Value: B
Thalassolituus
2.0
1.5
1.0
0.5
0.0
A (n=15)
B (n=16)
C (n=14)
surface

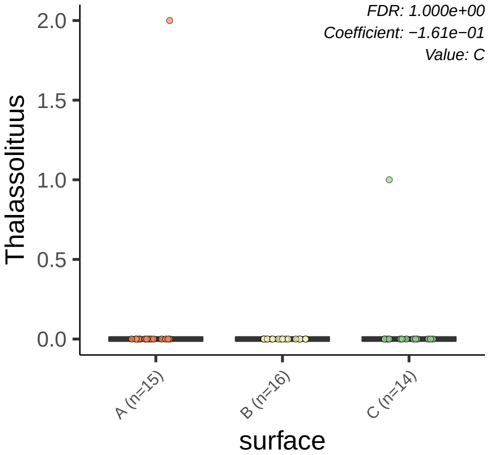
FDR: 1.000e+00
Coefficient: −1.61e−01
Value: C
Thalassolituus
2.0
1.5
1.0
0.5
0.0
A (n=15)
B (n=16)
C (n=14)
surface

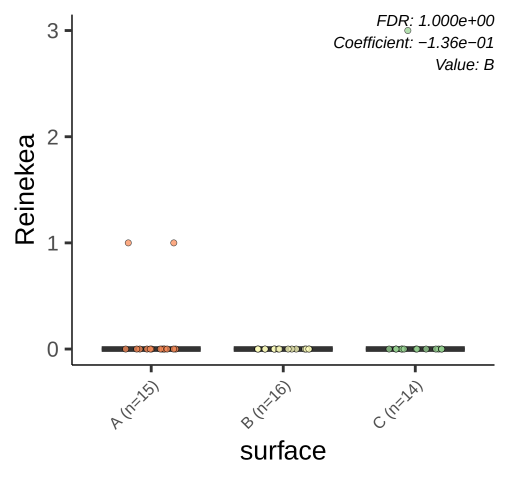
FDR: 1.000e+00
Coefficient: −1.36e−01
Value: B
Reinekea
3
2
1
0
A (n=15)
B (n=16)
C (n=14)
surface

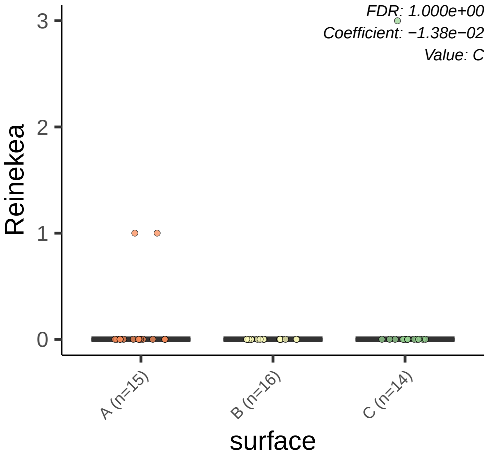
FDR: 1.000e+00
Coefficient: −1.38e−02
Value: C
Reinekea
0
1
2
3
A (n=15)
B (n=16)
C (n=14)
surface

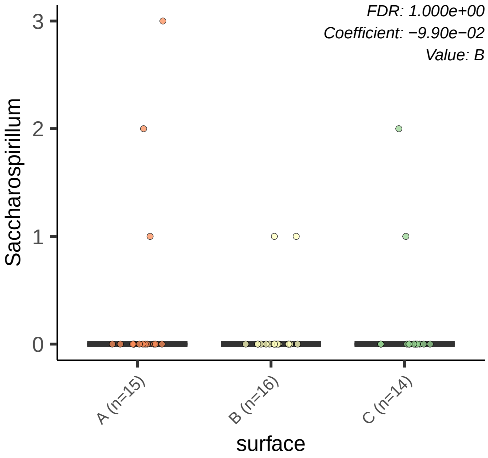

FDR: 1.000e+00
Coefficient: −9.90e−02
Value: B
Saccharospirillum
3
2
1
0
A (n=15)
B (n=16)
C (n=14)
surface

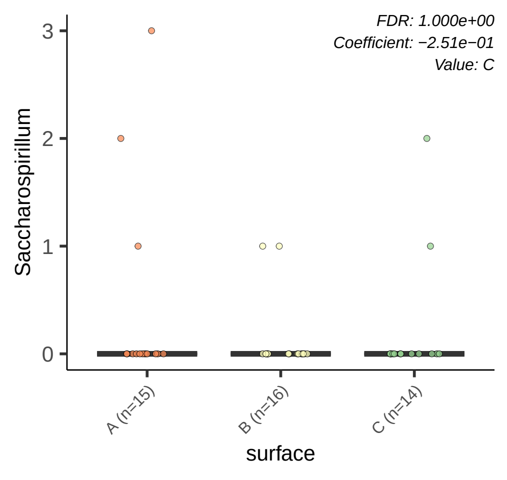
FDR: 1.000e+00
Coefficient: −2.51e−01
Value: C
Saccharospirillum
3
2
1
0
A (n=15)
B (n=16)
C (n=14)
surface

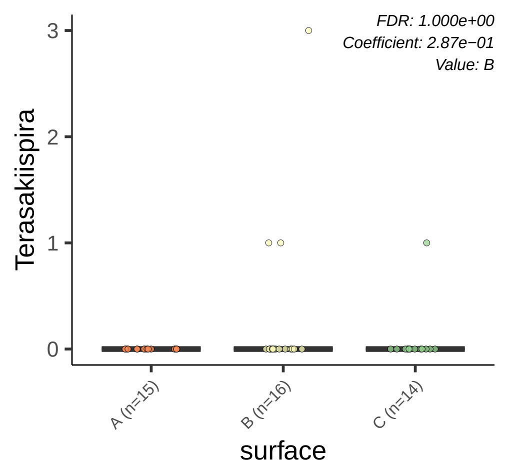
FDR: 1.000e+00
Coefficient: 2.87e−01
Value: B
Terasakiispira
0
1
2
3
A (n=15)
B (n=16)
C (n=14)
surface

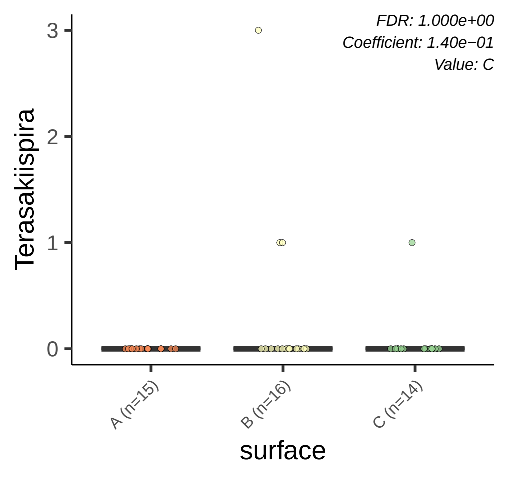
FDR: 1.000e+00
Coefficient: 1.40e−01
Value: C
Terasakiispira
3
2
1
0
A (n=15)
B (n=16)
C (n=14)
surface

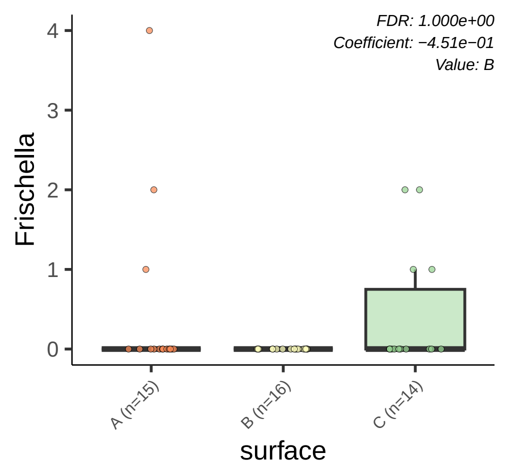
FDR: 1.000e+00
Coefficient: −4.51e−01
Value: B
Frischella
4
3
2
1
0
A (n=15)
B (n=16)
C (n=14)
surface

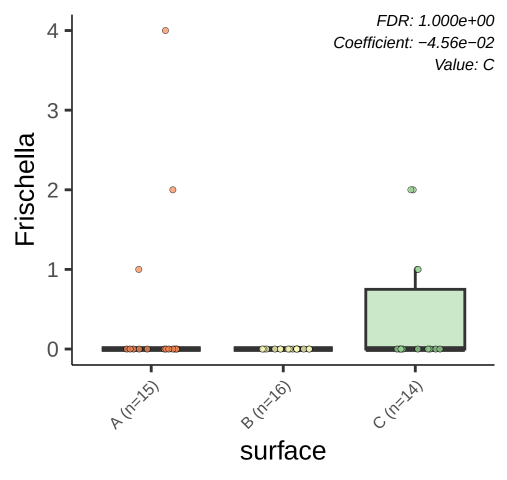

FDR: 1.000e+00
Coefficient: −4.56e−02
Value: C
Frischella
4
3
2
1
0
A (n=15)
B (n=16)
C (n=14)
surface

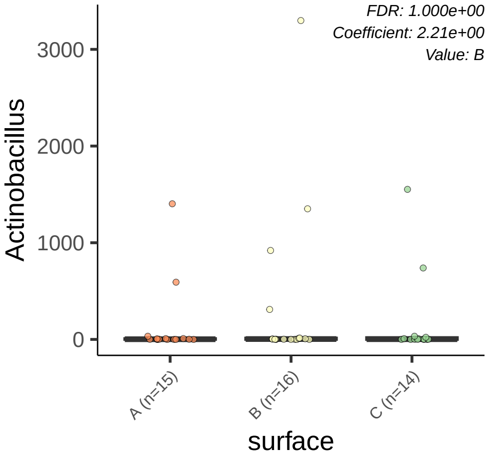
FDR: 1.000e+00
Coefficient: 2.21e+00
Value: B
Actinobacillus
3000
2000
1000
0
A (n=15)
B (n=16)
C (n=14)
surface

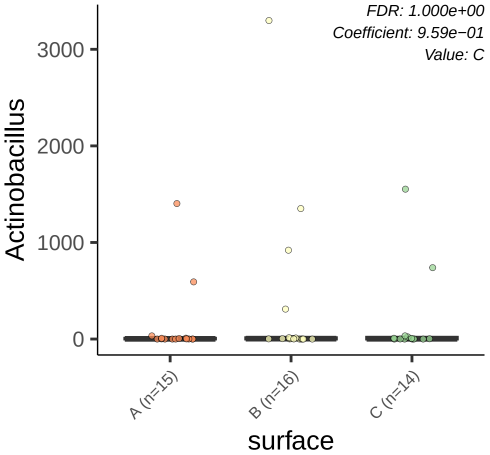
FDR: 1.000e+00
Coefficient: 9.59e−01
Value: C
Actinobacillus
3000
2000
1000
0
A (n=15)
B (n=16)
C (n=14)
surface

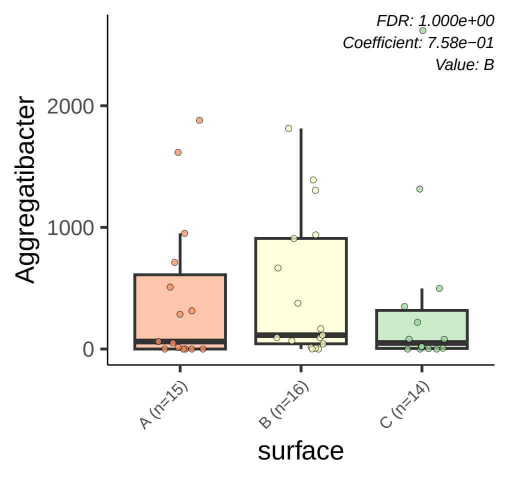

FDR: 1.000e+00
Coefficient: 7.58e−01
Value: B
Aggregatibacter
2000
1000
0
A (n=15)
B (n=16)
C (n=14)
surface

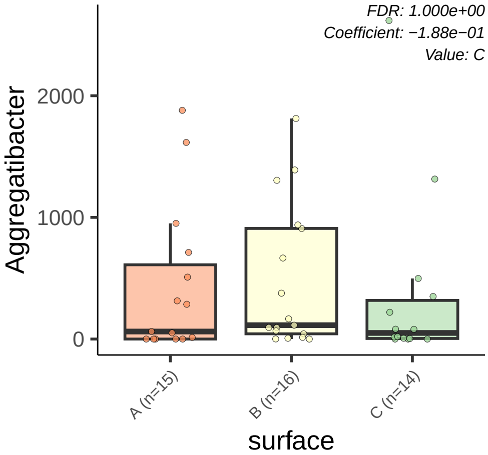

FDR: 1.000e+00
Coefficient: −1.88e−01
Value: C
Aggregatibacter
2000
1000
0
A (n=15)
B (n=16)
C (n=14)
surface

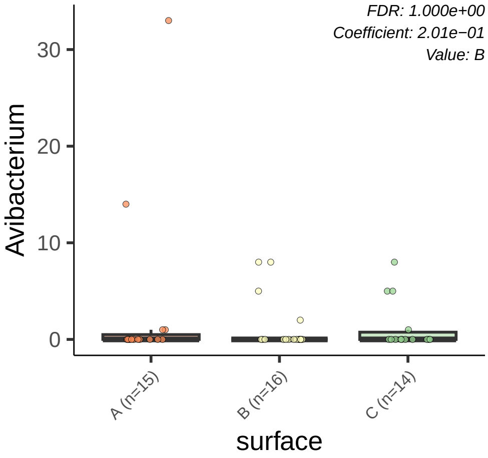
FDR: 1.000e+00
Coefficient: 2.01e−01
Value: B
Avibacterium
30
20
10
0
A (n=15)
B (n=16)
C (n=14)
surface

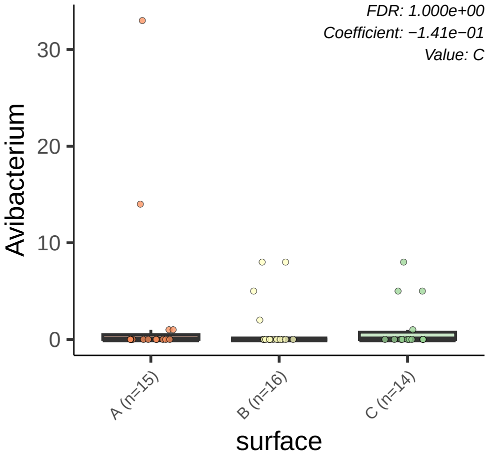
FDR: 1.000e+00
Coefficient: −1.41e−01
Value: C
Avibacterium
30
20
10
0
A (n=15)
B (n=16)
C (n=14)
surface

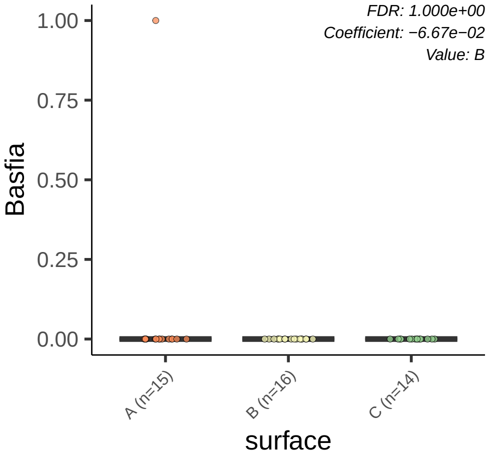

FDR: 1.000e+00
Coefficient: −6.67e−02
Value: B
Basfia
1.00
0.75
0.50
0.25
0.00
A (n=15)
B (n=16)
C (n=14)
surface

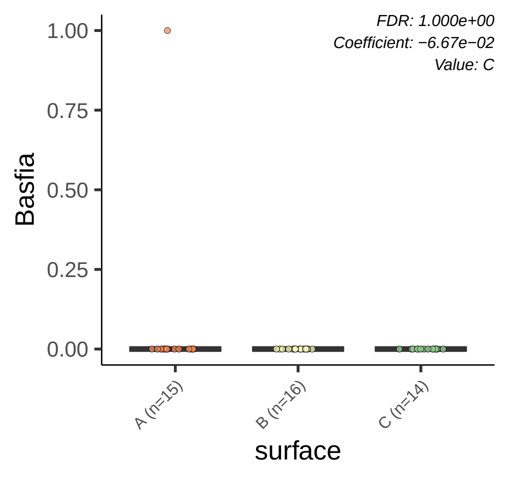
FDR: 1.000e+00
Coefficient: −6.67e−02
Value: C
Basfia
1.00
0.75
0.50
0.25
0.00
A (n=15)
B (n=16)
C (n=14)
surface

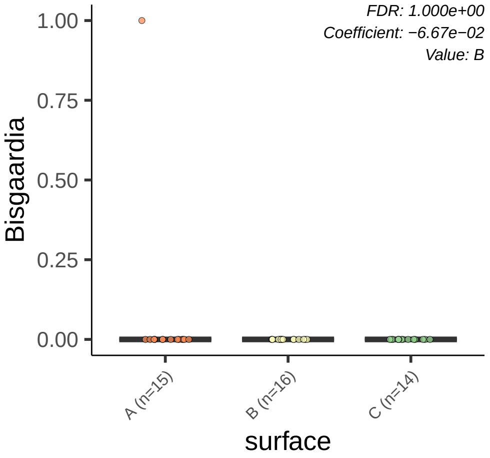
FDR: 1.000e+00
Coefficient: −6.67e−02
Value: B
Bisgaardia
1.00
0.75
0.50
0.25
0.00
A (n=15)
B (n=16)
C (n=14)
surface

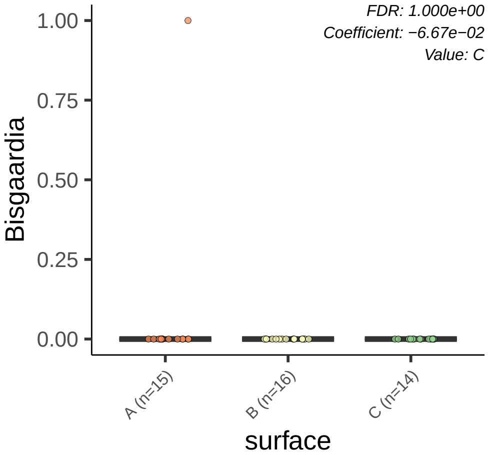

FDR: 1.000e+00
Coefficient: −6.67e−02
Value: C
1.00
0.75
0.50
0.25
0.00
Bisgaardia
A (n=15)
B (n=16)
C (n=14)
surface

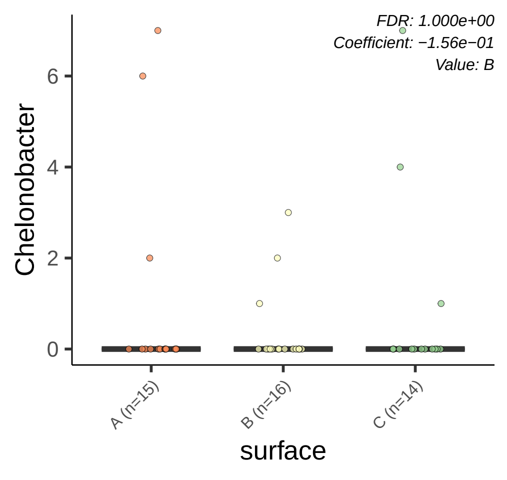
FDR: 1.000e+00
Coefficient: −1.56e−01
Value: B
Chelonobacter
0
2
4
6
A (n=15)
B (n=16)
C (n=14)
surface

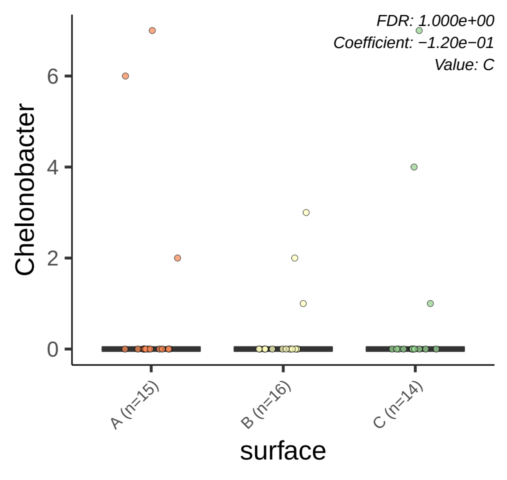
FDR: 1.000e+00
Coefficient: −1.20e−01
Value: C
Chelonobacter
0
2
4
6
A (n=15)
B (n=16)
C (n=14)
surface

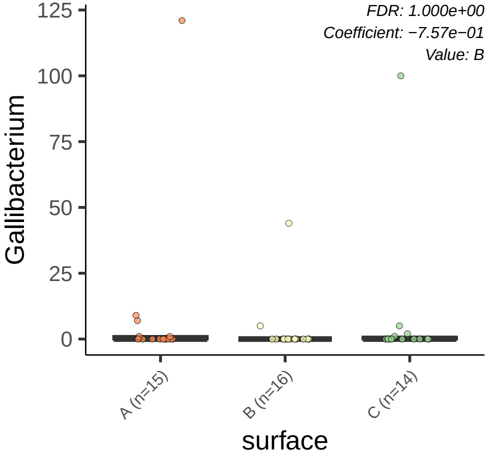
FDR: 1.000e+00
Coefficient: −7.57e−01
Value: B
Gallibacterium
125
100
75
50
25
0
A (n=15)
B (n=16)
C (n=14)
surface

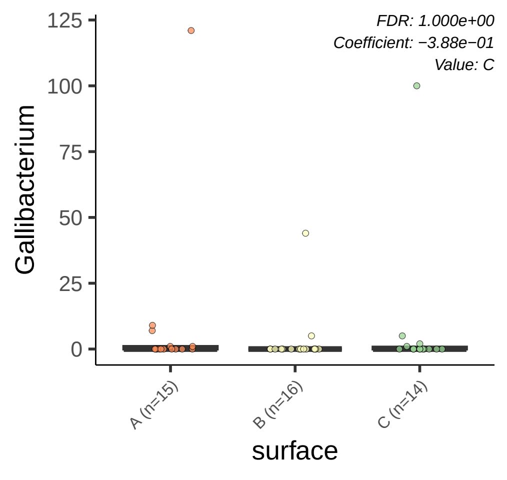
FDR: 1.000e+00
Coefficient: −3.88e−01
Value: C
Gallibacterium
125
100
75
50
25
0
A (n=15)
B (n=16)
C (n=14)
surface

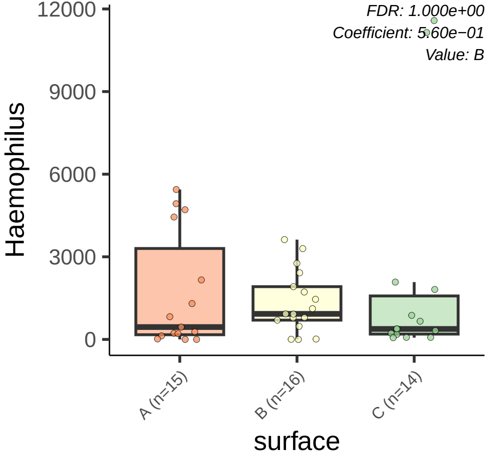

FDR: 1.000e+00
Coefficient: 5.60e−01
Value: B
12000
9000
6000
3000
0
Haemophilus
A (n=15)
B (n=16)
C (n=14)
surface

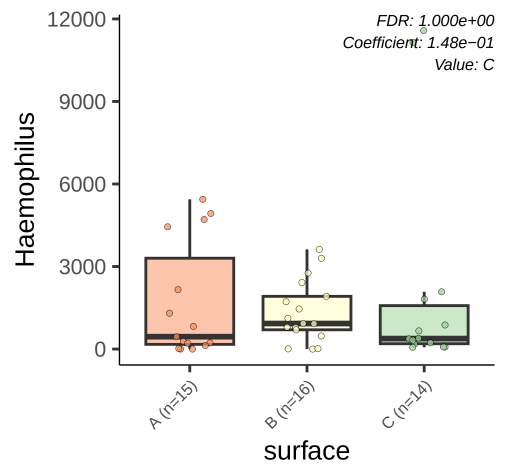

FDR: 1.000e+00
Coefficient: 1.48e−01
Value: C
12000
9000
6000
3000
0
Haemophilus
A (n=15)
B (n=16)
C (n=14)
surface

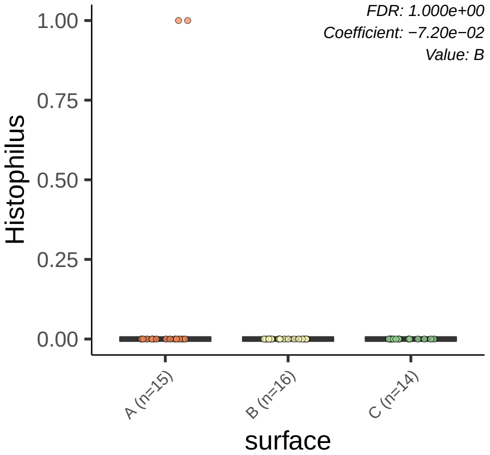
FDR: 1.000e+00
Coefficient: −7.20e−02
Value: B
Histophilus
1.00
0.75
0.50
0.25
0.00
A (n=15)
B (n=16)
C (n=14)
surface

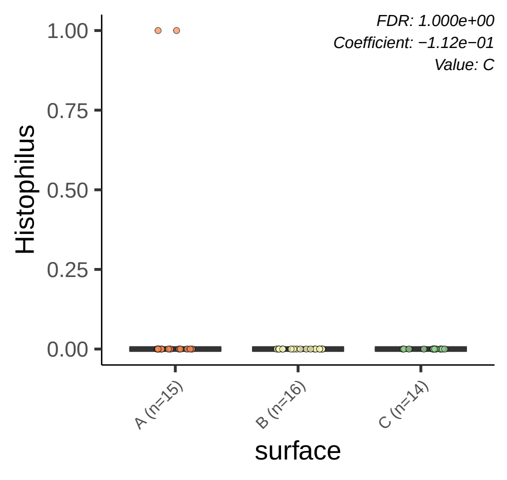
FDR: 1.000e+00
Coefficient: −1.12e−01
Value: C
Histophilus
1.00
0.75
0.50
0.25
0.00
A (n=15)
B (n=16)
C (n=14)
surface

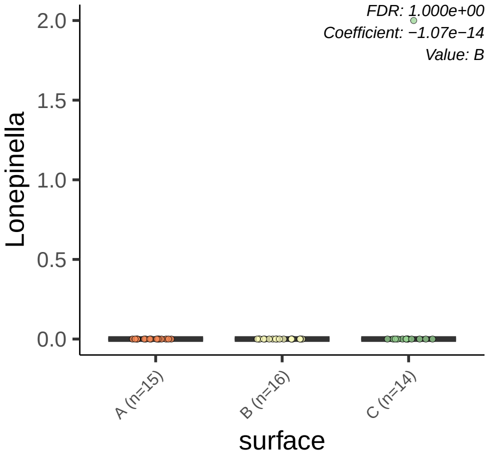
FDR: 1.000e+00
Coefficient: −1.07e−14
Value: B
Lonepinella
2.0
1.5
1.0
0.5
0.0
A (n=15)
B (n=16)
C (n=14)
surface

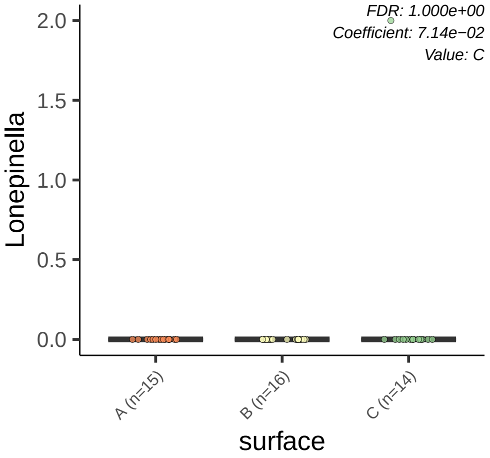

FDR: 1.000e+00
Coefficient: 7.14e−02
Value: C
Lonepinella
2.0
1.5
1.0
0.5
0.0
A (n=15)
B (n=16)
C (n=14)
surface

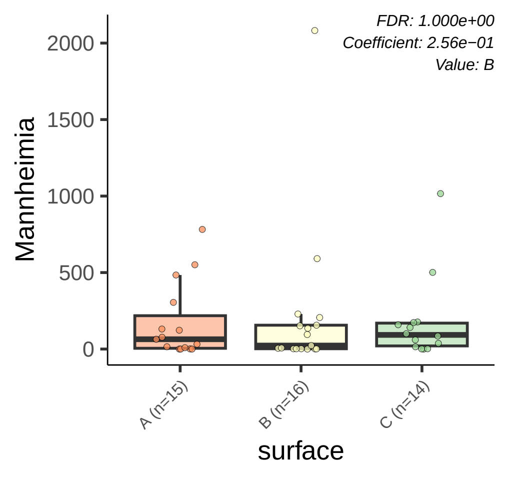
FDR: 1.000e+00
Coefficient: 2.56e−01
Value: B
Mannheimia
2000
1500
1000
500
0
A (n=15)
B (n=16)
C (n=14)
surface

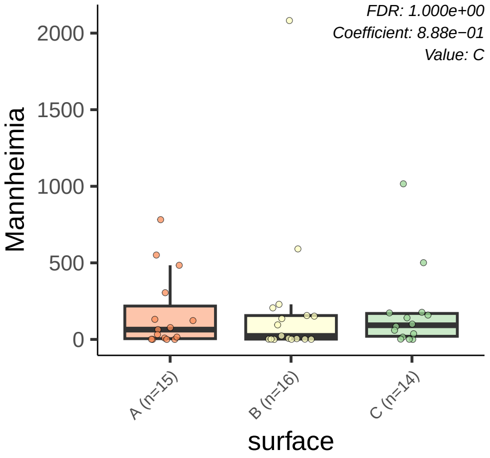

FDR: 1.000e+00
Coefficient: 8.88e−01
Value: C
Mannheimia
2000
1500
1000
500
0
A (n=15)
B (n=16)
C (n=14)
surface

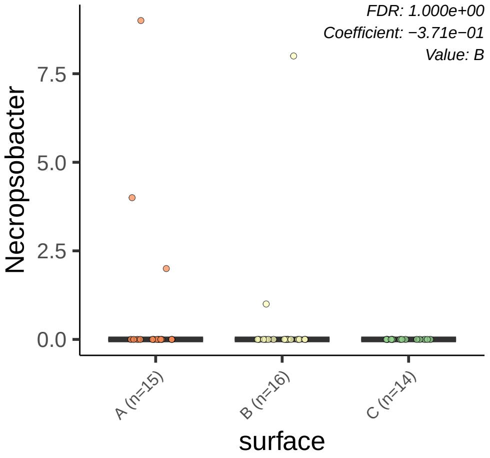
FDR: 1.000e+00
Coefficient: −3.71e−01
Value: B
Necropsobacter
7.5
5.0
2.5
0.0
A (n=15)
B (n=16)
C (n=14)
surface

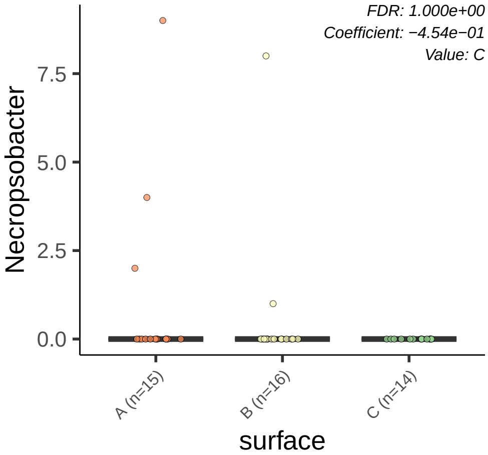
FDR: 1.000e+00
Coefficient: −4.54e−01
Value: C
Necropsobacter
7.5
5.0
2.5
0.0
A (n=15)
B (n=16)
C (n=14)
surface

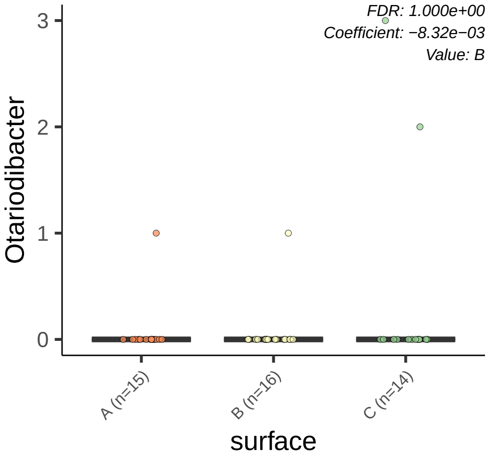
FDR: 1.000e+00
Coefficient: −8.32e−03
Value: B
Otariodibacter
3
2
1
0
A (n=15)
B (n=16)
C (n=14)
surface

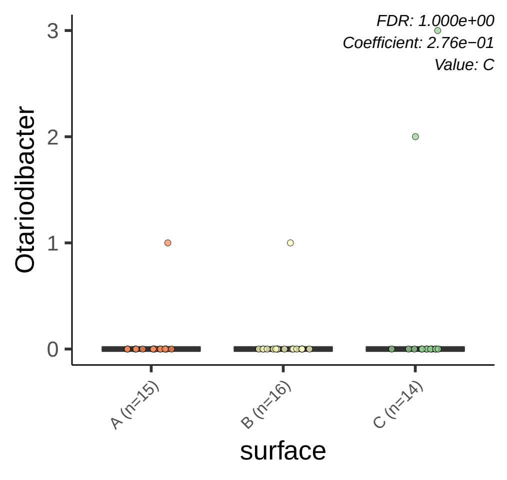
FDR: 1.000e+00
Coefficient: 2.76e−01
Value: C
Otariodibacter
3
2
1
0
A (n=15)
B (n=16)
C (n=14)
surface

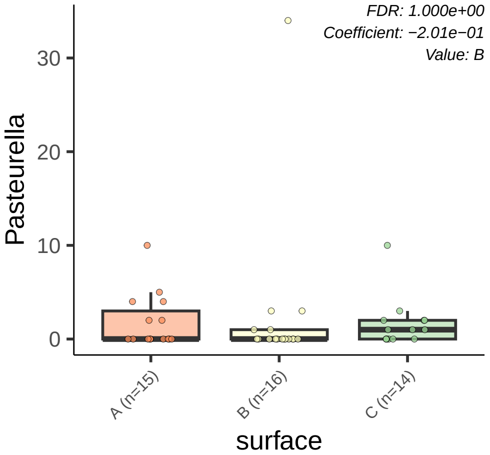

FDR: 1.000e+00
Coefficient: −2.01e−01
Value: B
Pasteurella
0
10
20
30
A (n=15)
B (n=16)
C (n=14)
surface

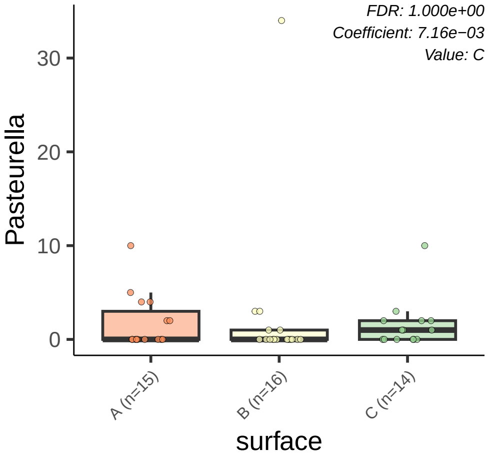

FDR: 1.000e+00
Coefficient: 7.16e−03
Value: C
Pasteurella
0
10
20
30
A (n=15)
B (n=16)
C (n=14)
surface

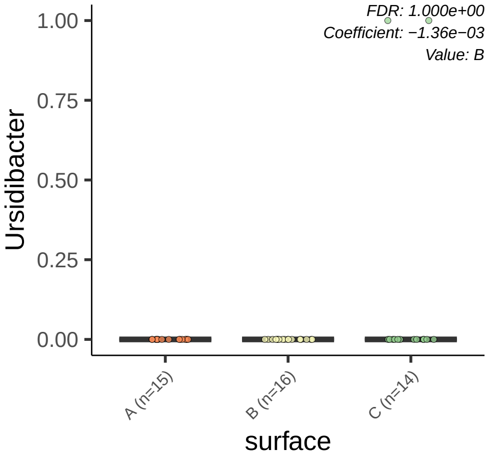

FDR: 1.000e+00
Coefficient: −1.36e−03
Value: B
Ursidibacter
1.00
0.75
0.50
0.25
0.00
A (n=15)
B (n=16)
C (n=14)
surface

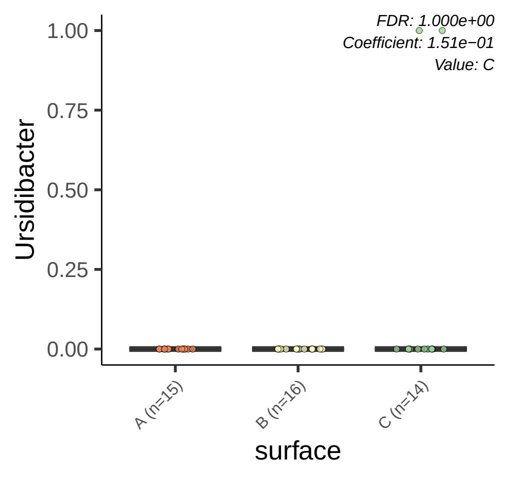

FDR: 1.000e+00
Coefficient: 1.51e−01
Value: C
Ursidibacter
1.00
0.75
0.50
0.25
0.00
A (n=15)
B (n=16)
C (n=14)
surface

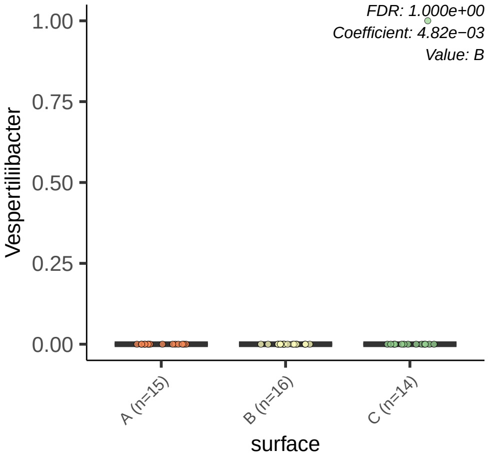
FDR: 1.000e+00
Coefficient: 4.82e−03
Value: B
Vespertiliibacter
1.00
0.75
0.50
0.25
0.00
A (n=15)
B (n=16)
C (n=14)
surface

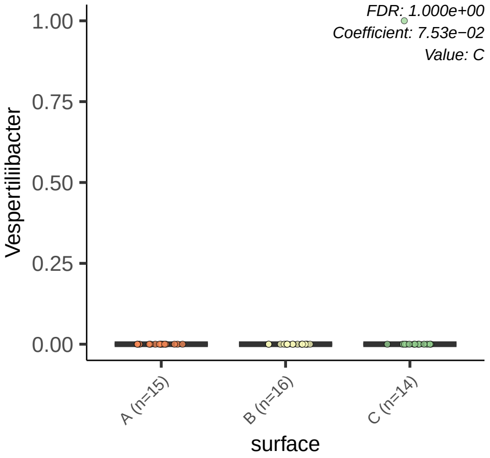
FDR: 1.000e+00
Coefficient: 7.53e−02
Value: C
1.00
0.75
0.50
0.25
0.00
Vespertiliibacter
A (n=15)
B (n=16)
C (n=14)
surface

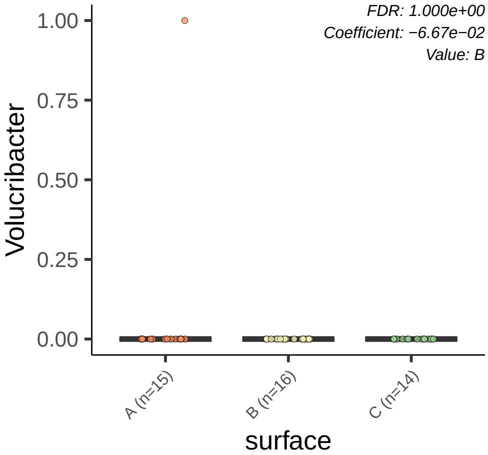
FDR: 1.000e+00
Coefficient: −6.67e−02
Value: B
Volucribacter
1.00
0.75
0.50
0.25
0.00
A (n=15)
B (n=16)
C (n=14)
surface

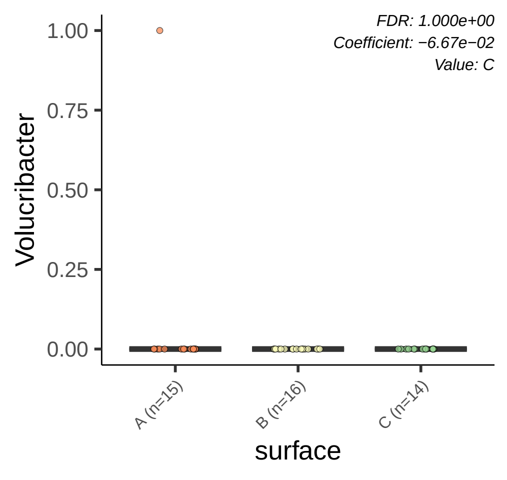
FDR: 1.000e+00
Coefficient: −6.67e−02
Value: C
Volucribacter
1.00
0.75
0.50
0.25
0.00
A (n=15)
B (n=16)
C (n=14)
surface

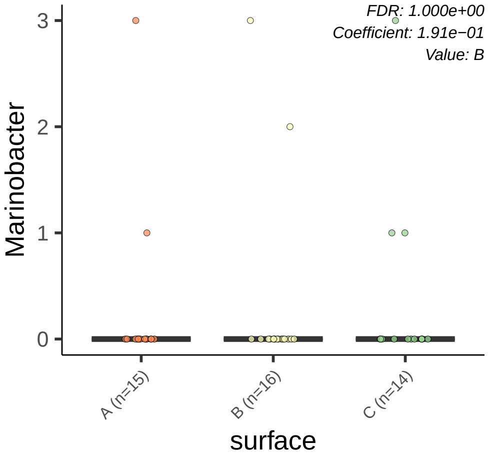
FDR: 1.000e+00
Coefficient: 1.91e−01
Value: B
Marinobacter
0
1
2
3
A (n=15)
B (n=16)
C (n=14)
surface

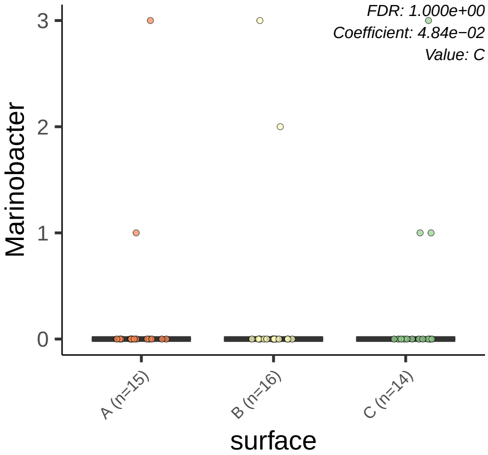
FDR: 1.000e+00
Coefficient: 4.84e−02
Value: C
Marinobacter
3
2
1
0
A (n=15)
B (n=16)
C (n=14)
surface

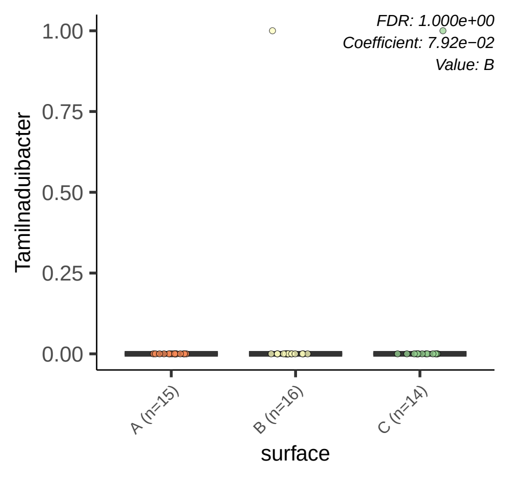

FDR: 1.000e+00
Coefficient: 7.92e−02
Value: B
Tamilnaduibacter
1.00
0.75
0.50
0.25
0.00
A (n=15)
B (n=16)
C (n=14)
surface

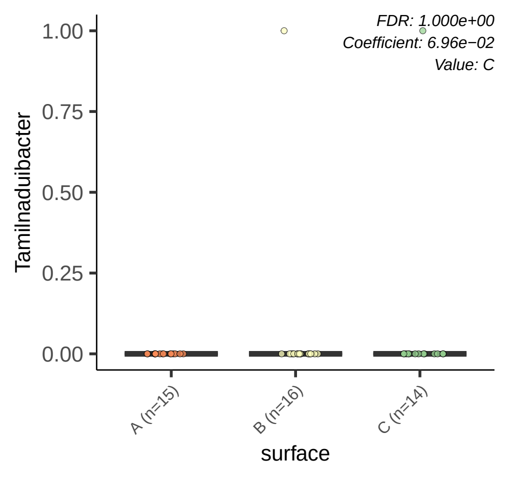
FDR: 1.000e+00
Coefficient: 6.96e−02
Value: C
Tamilnaduibacter
1.00
0.75
0.50
0.25
0.00
A (n=15)
B (n=16)
C (n=14)
surface

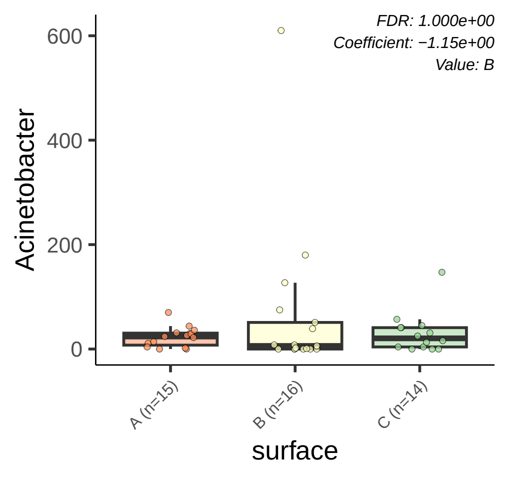

FDR: 1.000e+00
Coefficient: −1.15e+00
Value: B
Acinetobacter
0
200
400
600
A (n=15)
B (n=16)
C (n=14)
surface

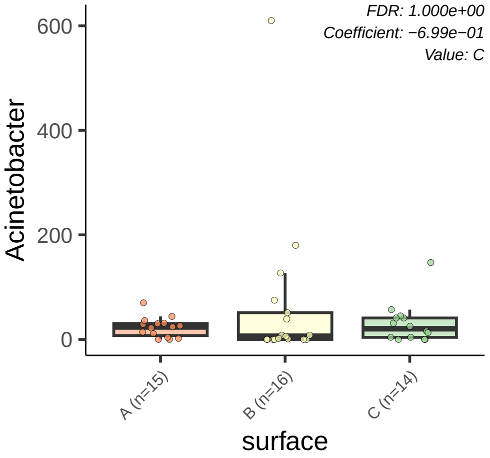
FDR: 1.000e+00
Coefficient: −6.99e−01
Value: C
Acinetobacter
600
400
200
0
A (n=15)
B (n=16)
C (n=14)
surface

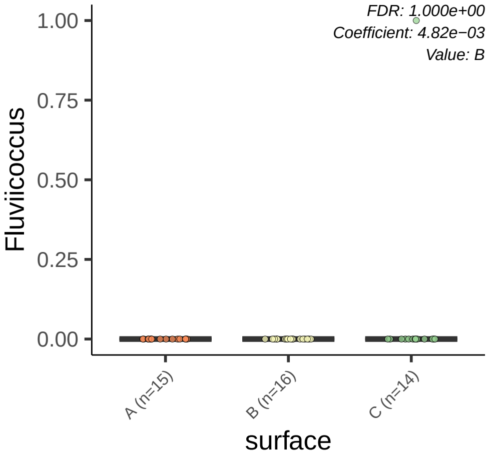

FDR: 1.000e+00
Coefficient: 4.82e−03
Value: B
1.00
0.75
0.50
0.25
0.00
Fluviicoccus
A (n=15)
B (n=16)
C (n=14)
surface

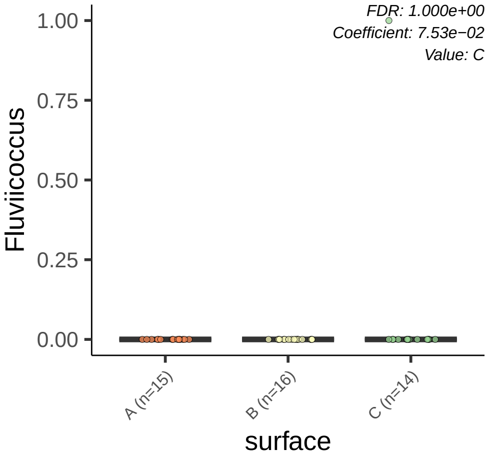
FDR: 1.000e+00
Coefficient: 7.53e−02
Value: C
Fluviicoccus
1.00
0.75
0.50
0.25
0.00
A (n=15)
B (n=16)
C (n=14)
surface

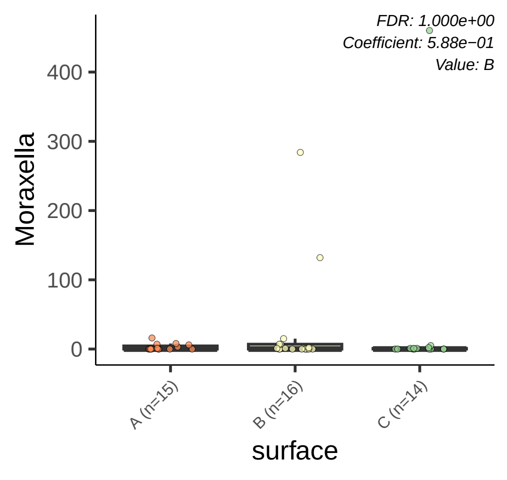

FDR: 1.000e+00
Coefficient: 5.88e−01
Value: B
Moraxella
400
300
200
100
0
A (n=15)
B (n=16)
C (n=14)
surface

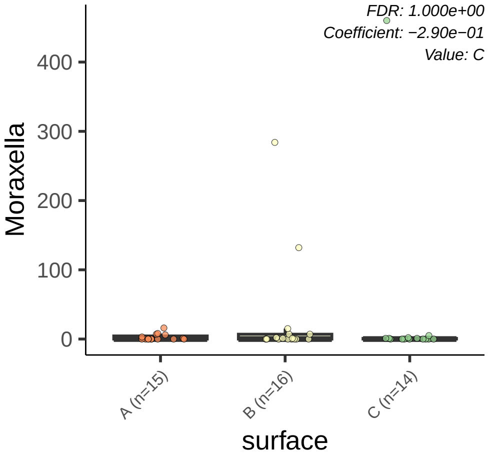
FDR: 1.000e+00
Coefficient: −2.90e−01
Value: C
Moraxella
400
300
200
100
0
A (n=15)
B (n=16)
C (n=14)
surface

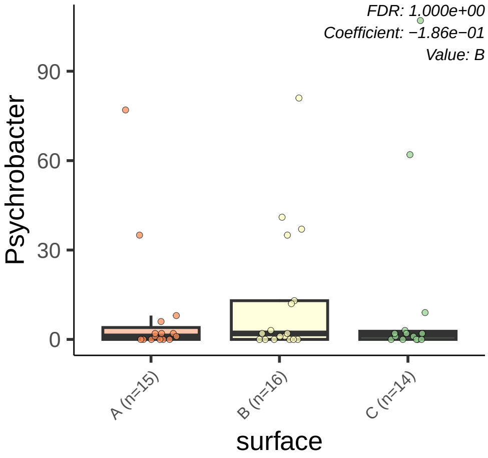

FDR: 1.000e+00
Coefficient: −1.86e−01
Value: B
Psychrobacter
90
60
30
0
A (n=15)
B (n=16)
C (n=14)
surface

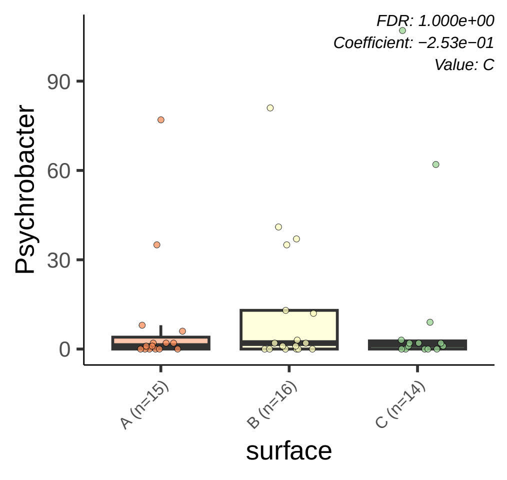

FDR: 1.000e+00
Coefficient: −2.53e−01
Value: C
Psychrobacter
90
60
30
0
A (n=15)
B (n=16)
C (n=14)
surface

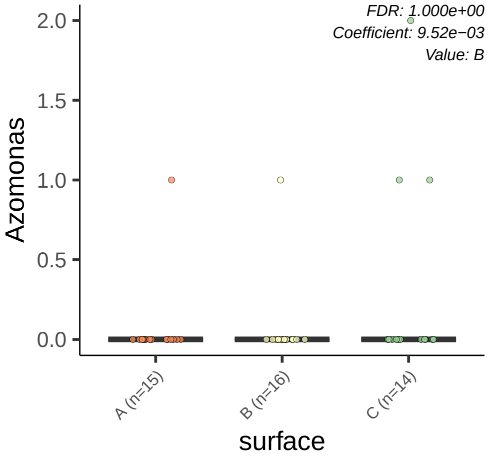

FDR: 1.000e+00
Coefficient: 9.52e−03
Value: B
Azomonas
2.0
1.5
1.0
0.5
0.0
A (n=15)
B (n=16)
C (n=14)
surface

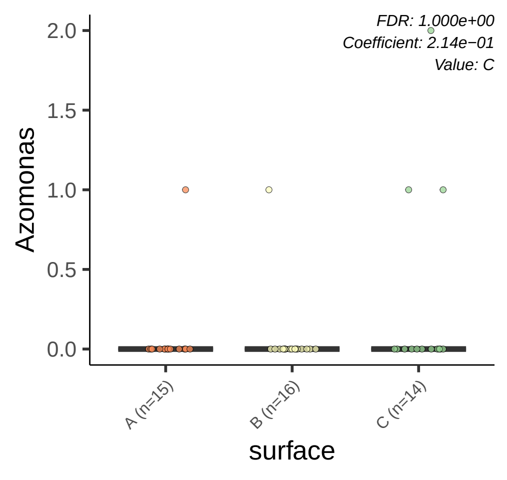
FDR: 1.000e+00
Coefficient: 2.14e−01
Value: C
Azomonas
2.0
1.5
1.0
0.5
0.0
A (n=15)
B (n=16)
C (n=14)
surface

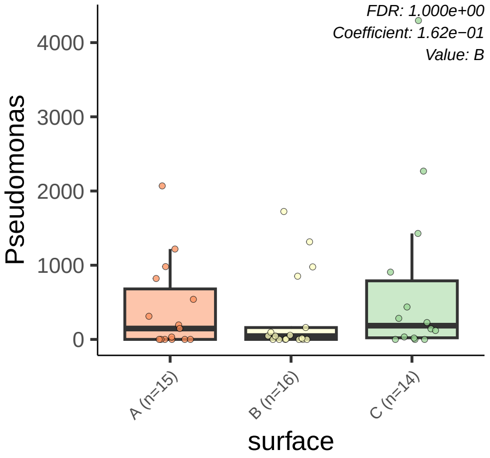

FDR: 1.000e+00
Coefficient: 1.62e−01
Value: B
Pseudomonas
4000
3000
2000
1000
0
A (n=15)
B (n=16)
C (n=14)
surface

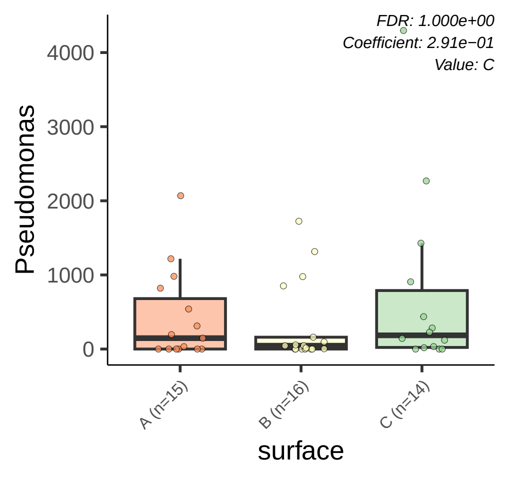

FDR: 1.000e+00
Coefficient: 2.91e−01
Value: C
Pseudomonas
4000
3000
2000
1000
0
A (n=15)
B (n=16)
C (n=14)
surface

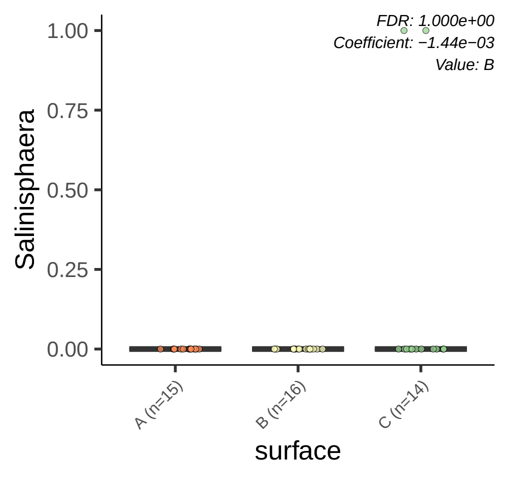

FDR: 1.000e+00
Coefficient: −1.44e−03
Value: B
Salinisphaera
1.00
0.75
0.50
0.25
0.00
A (n=15)
B (n=16)
C (n=14)
surface

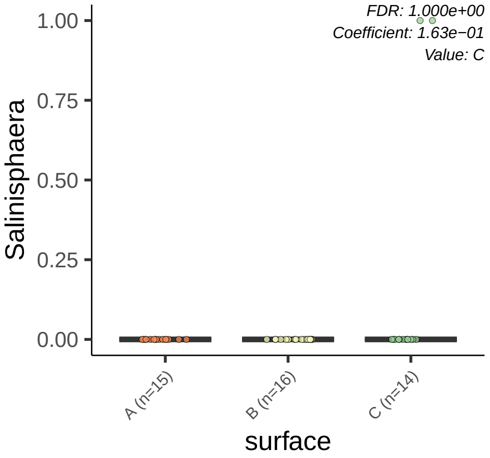
FDR: 1.000e+00
Coefficient: 1.63e−01
Value: C
Salinisphaera
1.00
0.75
0.50
0.25
0.00
A (n=15)
B (n=16)
C (n=14)
surface

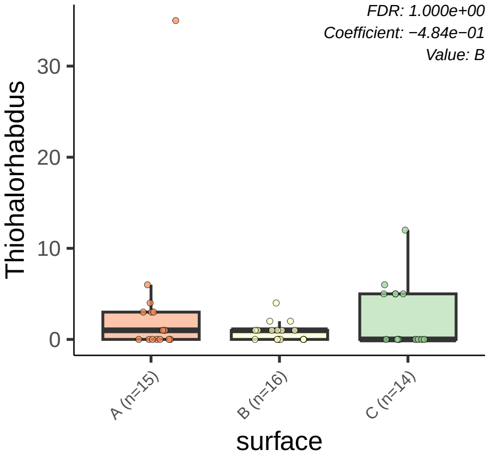

FDR: 1.000e+00
Coefficient: −4.84e−01
Value: B
Thiohalorhabdus
0
10
20
30
A (n=15)
B (n=16)
C (n=14)
surface

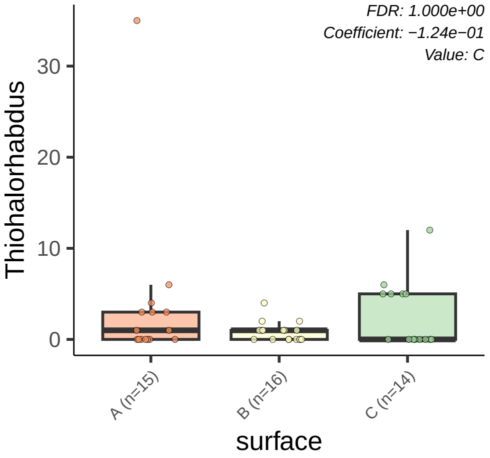
FDR: 1.000e+00
Coefficient: −1.24e−01
Value: C
Thiohalorhabdus
30
20
10
0
A (n=15)
B (n=16)
C (n=14)
surface

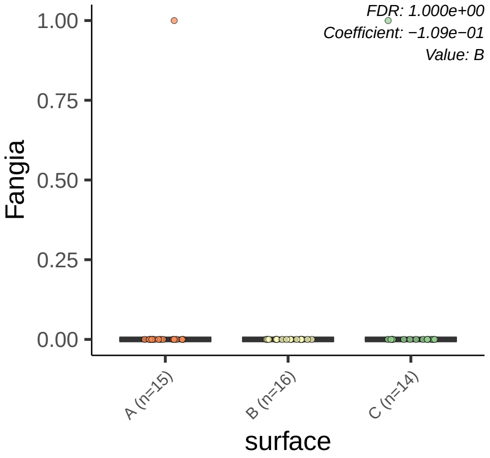

FDR: 1.000e+00
Coefficient: −1.09e−01
Value: B
1.00
0.75
0.50
0.25
0.00
Fangia
A (n=15)
B (n=16)
C (n=14)
surface

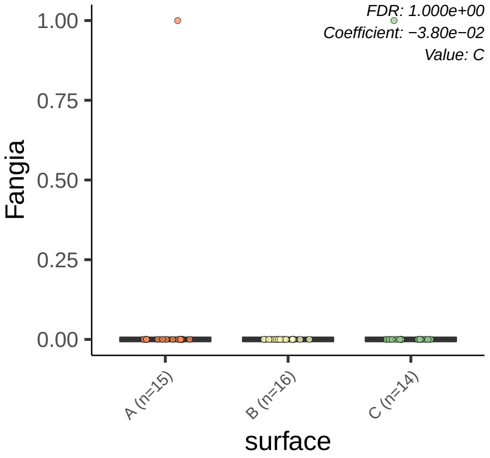

FDR: 1.000e+00
Coefficient: −3.80e−02
Value: C
Fangia
1.00
0.75
0.50
0.25
0.00
A (n=15)
B (n=16)
C (n=14)
surface

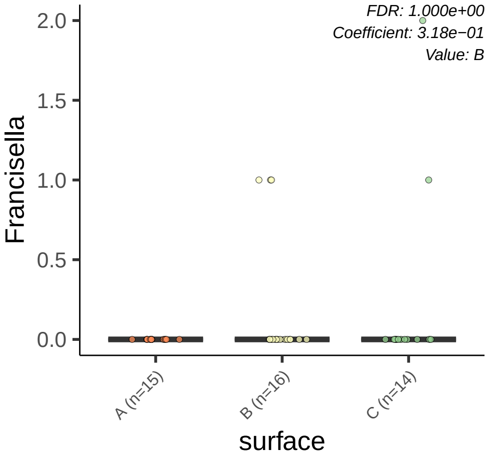

FDR: 1.000e+00
Coefficient: 3.18e−01
Value: B
Francisella
2.0
1.5
1.0
0.5
0.0
A (n=15)
B (n=16)
C (n=14)
surface

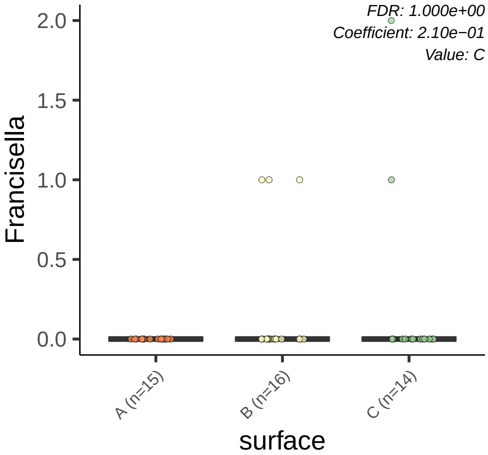
FDR: 1.000e+00
Coefficient: 2.10e−01
Value: C
Francisella
2.0
1.5
1.0
0.5
0.0
A (n=15)
B (n=16)
C (n=14)
surface

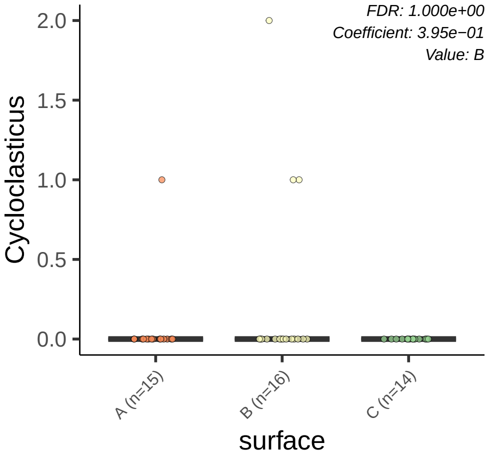
FDR: 1.000e+00
Coefficient: 3.95e−01
Value: B
Cycloclasticus
2.0
1.5
1.0
0.5
0.0
A (n=15)
B (n=16)
C (n=14)
surface

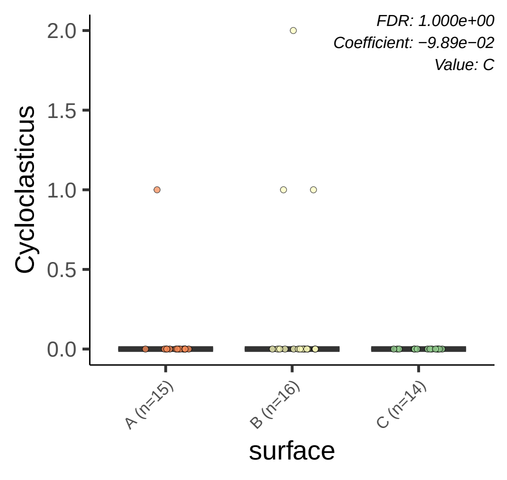

FDR: 1.000e+00
Coefficient: −9.89e−02
Value: C
Cycloclasticus
2.0
1.5
1.0
0.5
0.0
A (n=15)
B (n=16)
C (n=14)
surface

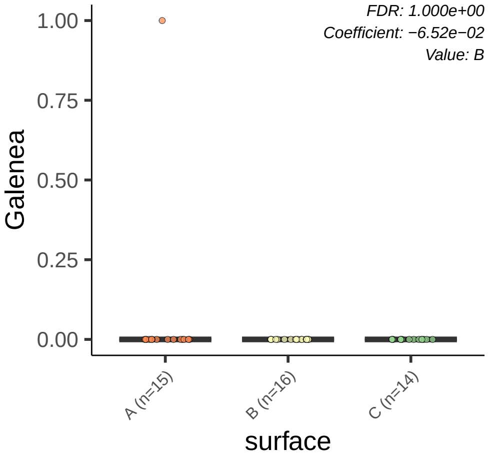

FDR: 1.000e+00
Coefficient: −6.52e−02
Value: B
1.00
0.75
0.50
0.25
0.00
Galenea
A (n=15)
B (n=16)
C (n=14)
surface

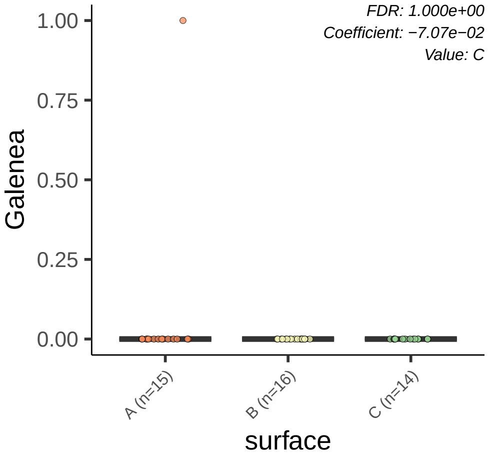
FDR: 1.000e+00
Coefficient: −7.07e−02
Value: C
Galenea
1.00
0.75
0.50
0.25
0.00
A (n=15)
B (n=16)
C (n=14)
surface

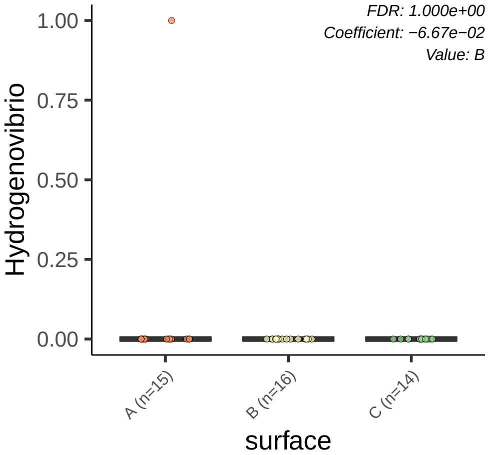

FDR: 1.000e+00
Coefficient: −6.67e−02
Value: B
Hydrogenovibrio
1.00
0.75
0.50
0.25
0.00
A (n=15)
B (n=16)
C (n=14)
surface

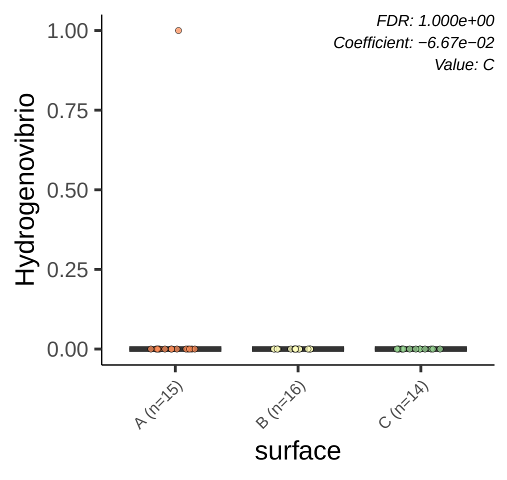
FDR: 1.000e+00
Coefficient: −6.67e−02
Value: C
Hydrogenovibrio
1.00
0.75
0.50
0.25
0.00
A (n=15)
B (n=16)
C (n=14)
surface

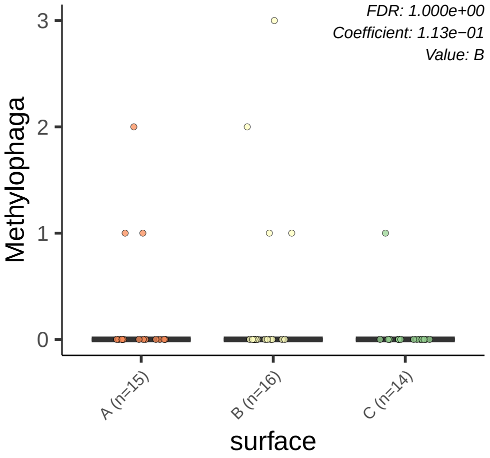
FDR: 1.000e+00
Coefficient: 1.13e−01
Value: B
Methylophaga
3
2
1
0
A (n=15)
B (n=16)
C (n=14)
surface

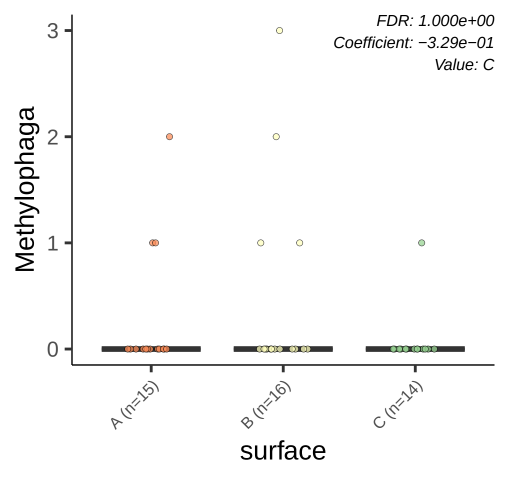

FDR: 1.000e+00
Coefficient: −3.29e−01
Value: C
Methylophaga
3
2
1
0
A (n=15)
B (n=16)
C (n=14)
surface

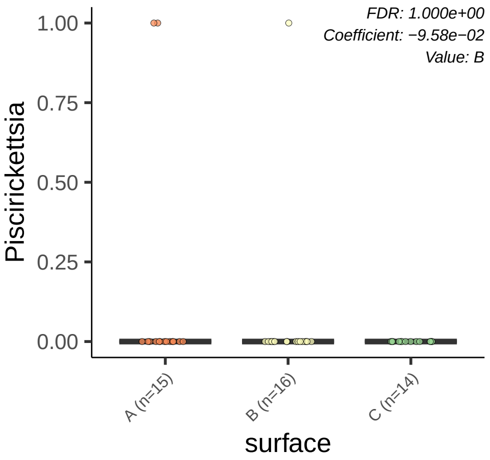
FDR: 1.000e+00
Coefficient: −9.58e−02
Value: B
Piscirickettsia
1.00
0.75
0.50
0.25
0.00
A (n=15)
B (n=16)
C (n=14)
surface

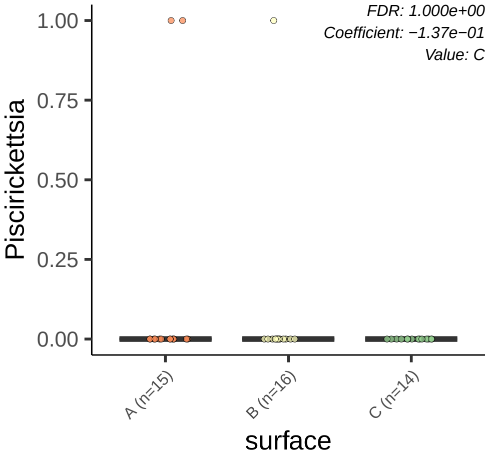
FDR: 1.000e+00
Coefficient: −1.37e−01
Value: C
Piscirickettsia
1.00
0.75
0.50
0.25
0.00
A (n=15)
B (n=16)
C (n=14)
surface

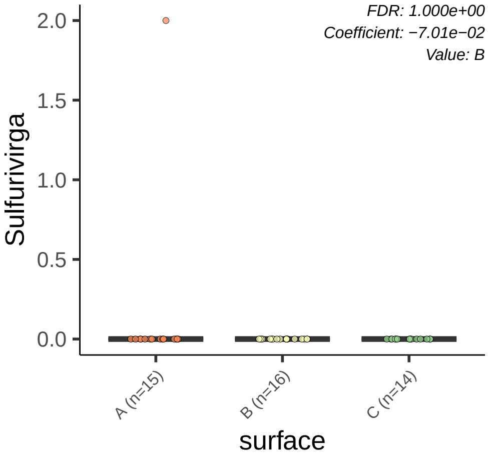
FDR: 1.000e+00
Coefficient: −7.01e−02
Value: B
2.0
1.5
1.0
0.5
0.0
Sulfurivirga
A (n=15)
B (n=16)
C (n=14)
surface

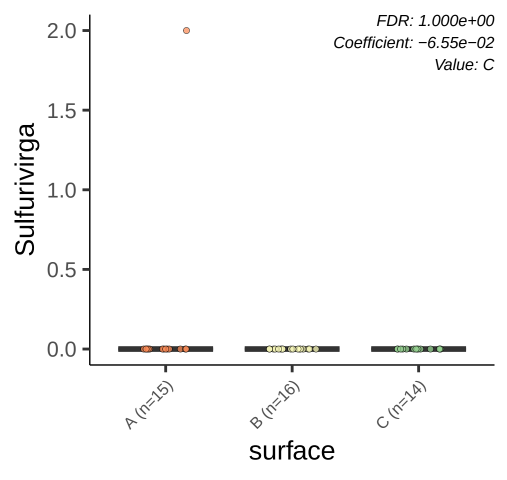
FDR: 1.000e+00
Coefficient: −6.55e−02
Value: C
Sulfurivirga
2.0
1.5
1.0
0.5
0.0
A (n=15)
B (n=16)
C (n=14)
surface

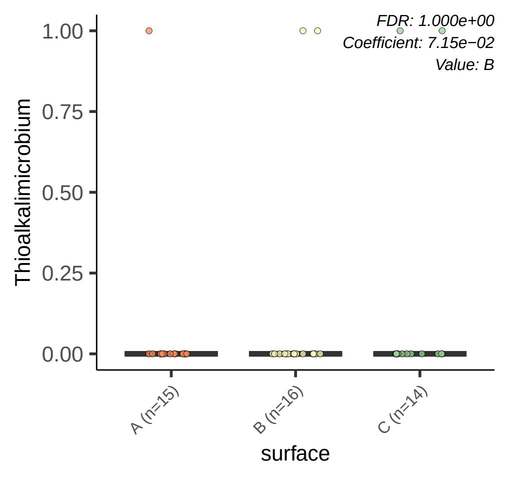
FDR: 1.000e+00
Coefficient: 7.15e−02
Value: B
Thioalkalimicrobium
1.00
0.75
0.50
0.25
0.00
A (n=15)
B (n=16)
C (n=14)
surface

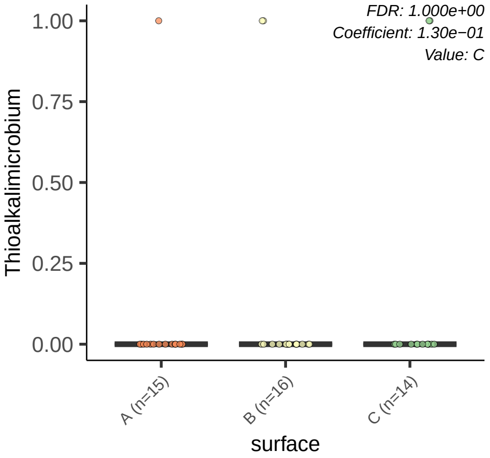

FDR: 1.000e+00
Coefficient: 1.30e−01
Value: C
Thioalkalimicrobium
1.00
0.75
0.50
0.25
0.00
A (n=15)
B (n=16)
C (n=14)
surface

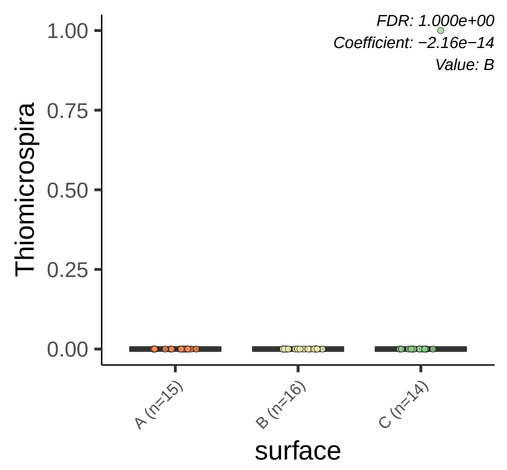

FDR: 1.000e+00
Coefficient: −2.16e−14
Value: B
Thiomicrospira
1.00
0.75
0.50
0.25
0.00
A (n=15)
B (n=16)
C (n=14)
surface

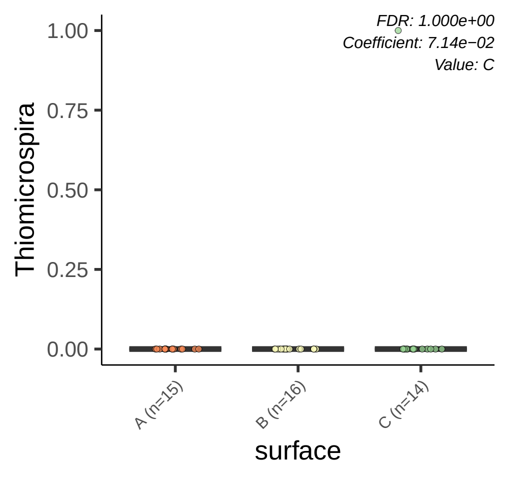

FDR: 1.000e+00
Coefficient: 7.14e−02
Value: C
Thiomicrospira
1.00
0.75
0.50
0.25
0.00
A (n=15)
B (n=16)
C (n=14)
surface

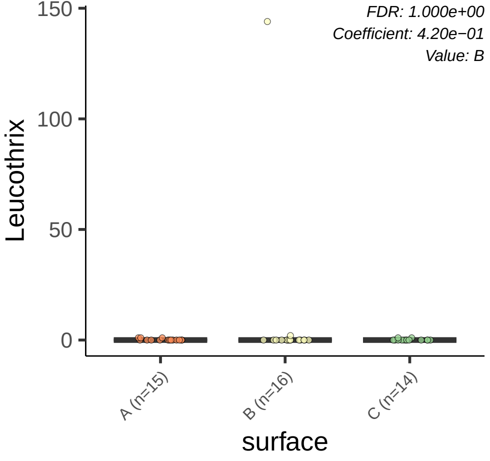

FDR: 1.000e+00
Coefficient: 4.20e−01
Value: B
Leucothrix
150
100
50
0
A (n=15)
B (n=16)
C (n=14)
surface

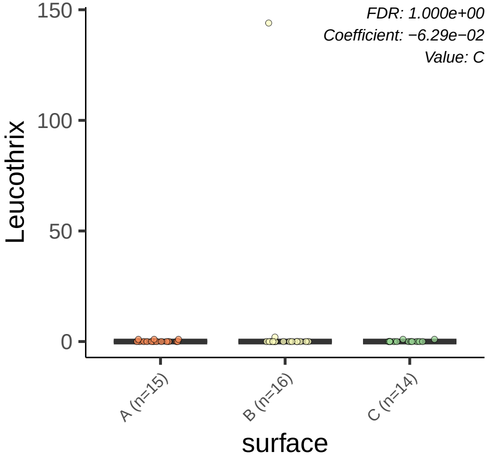

FDR: 1.000e+00
Coefficient: −6.29e−02
Value: C
Leucothrix
150
100
50
0
A (n=15)
B (n=16)
C (n=14)
surface

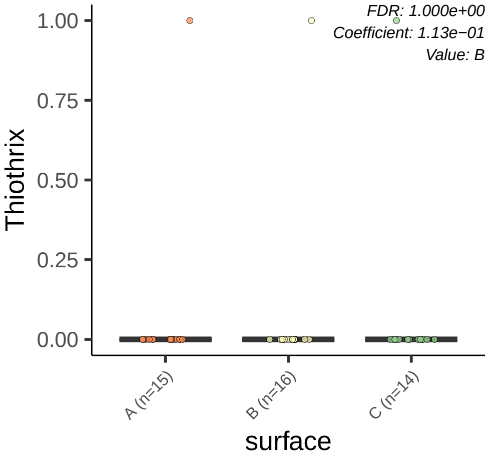
FDR: 1.000e+00
Coefficient: 1.13e−01
Value: B
Thiothrix
1.00
0.75
0.50
0.25
0.00
A (n=15)
B (n=16)
C (n=14)
surface

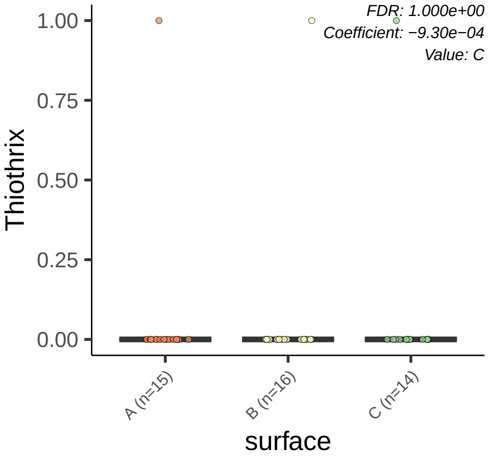

FDR: 1.000e+00
Coefficient: −9.30e−04
Value: C
Thiothrix
1.00
0.75
0.50
0.25
0.00
A (n=15)
B (n=16)
C (n=14)
surface

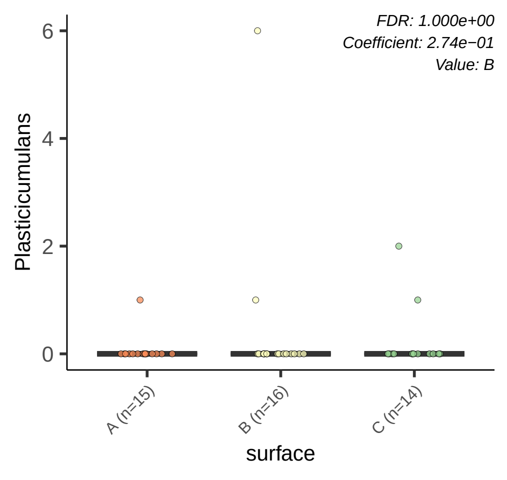

FDR: 1.000e+00
Coefficient: 2.74e−01
Value: B
Plasticicumulans
6
4
2
0
A (n=15)
B (n=16)
C (n=14)
surface

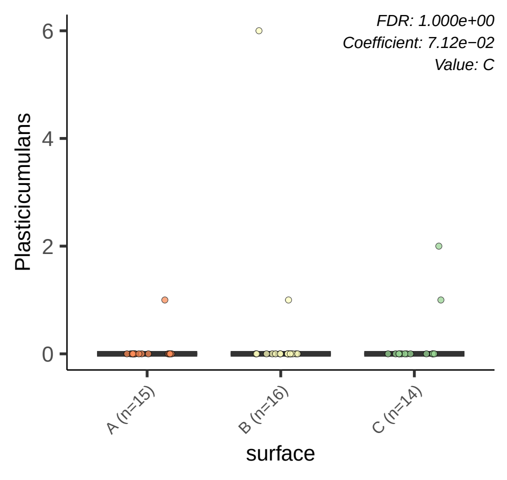

FDR: 1.000e+00
Coefficient: 7.12e−02
Value: C
Plasticicumulans
0
2
4
6
A (n=15)
B (n=16)
C (n=14)
surface

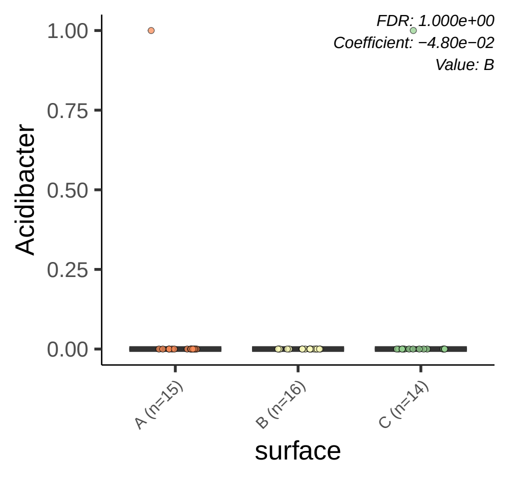

FDR: 1.000e+00
Coefficient: −4.80e−02
Value: B
1.00
0.75
0.50
0.25
0.00
Acidibacter
A (n=15)
B (n=16)
C (n=14)
surface

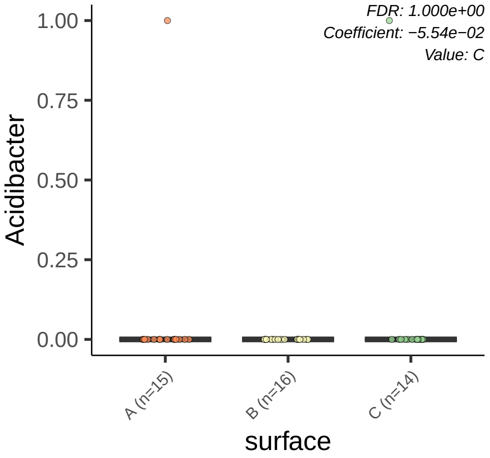

FDR: 1.000e+00
Coefficient: −5.54e−02
Value: C
Acidibacter
1.00
0.75
0.50
0.25
0.00
A (n=15)
B (n=16)
C (n=14)
surface

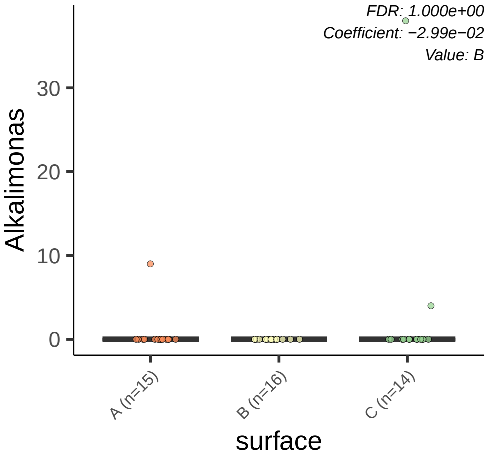
FDR: 1.000e+00
Coefficient: −2.99e−02
Value: B
Alkalimonas
0
10
20
30
A (n=15)
B (n=16)
C (n=14)
surface

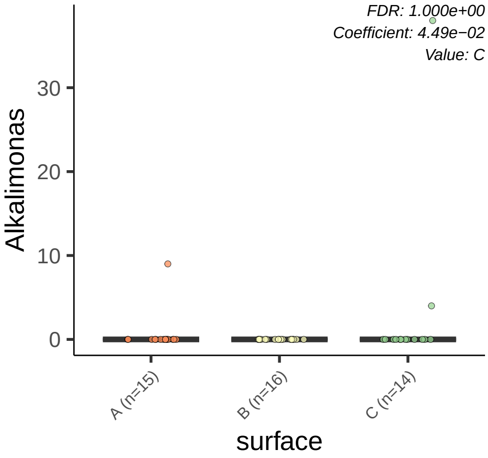
FDR: 1.000e+00
Coefficient: 4.49e−02
Value: C
Alkalimonas
30
20
10
0
A (n=15)
B (n=16)
C (n=14)
surface

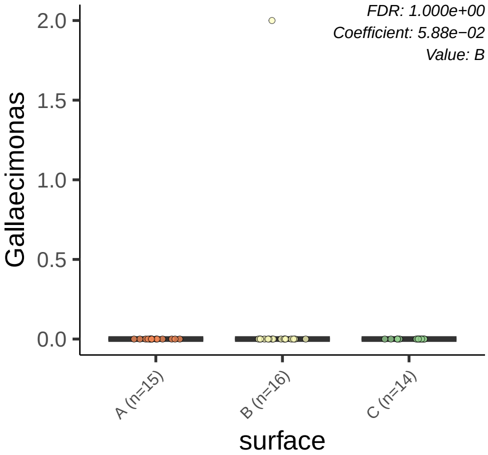
FDR: 1.000e+00
Coefficient: 5.88e−02
Value: B
Gallaecimonas
2.0
1.5
1.0
0.5
0.0
A (n=15)
B (n=16)
C (n=14)
surface

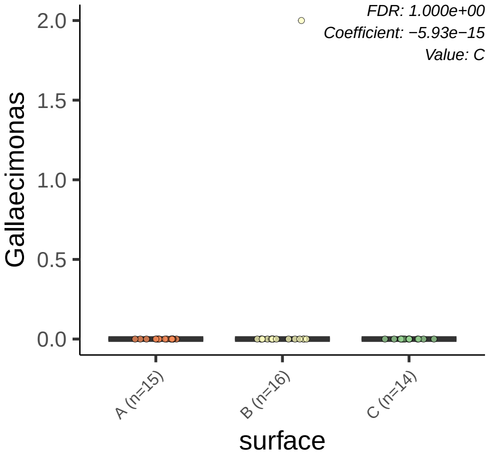
FDR: 1.000e+00
Coefficient: −5.93e−15
Value: C
Gallaecimonas
2.0
1.5
1.0
0.5
0.0
A (n=15)
B (n=16)
C (n=14)
surface

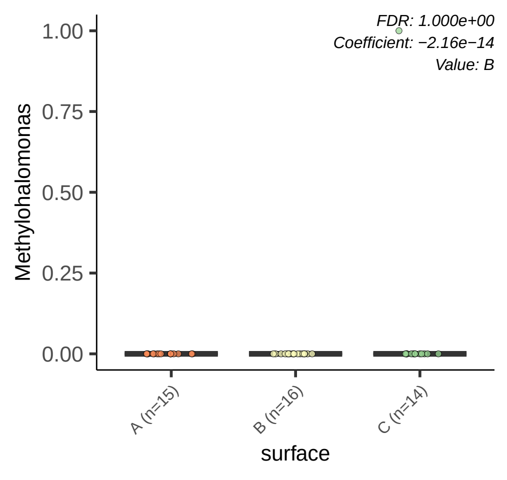
FDR: 1.000e+00
Coefficient: −2.16e−14
Value: B
Methylohalomonas
1.00
0.75
0.50
0.25
0.00
A (n=15)
B (n=16)
C (n=14)
surface

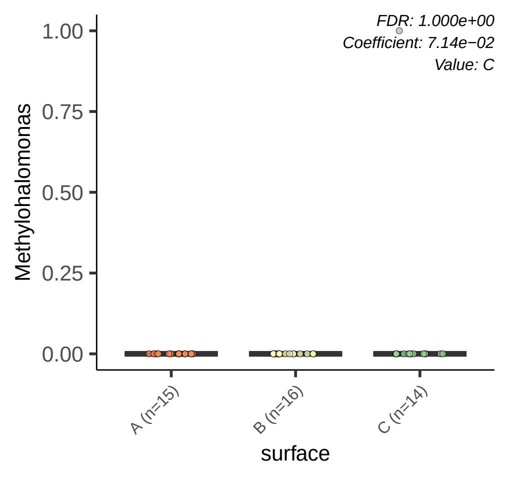

FDR: 1.000e+00
Coefficient: 7.14e−02
Value: C
Methylohalomonas
1.00
0.75
0.50
0.25
0.00
A (n=15)
B (n=16)
C (n=14)
surface

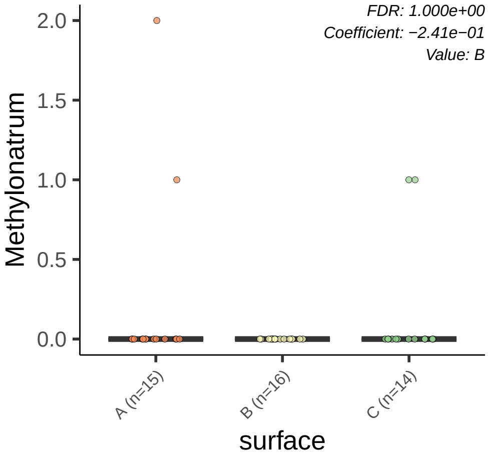

FDR: 1.000e+00
Coefficient: −2.41e−01
Value: B
Methylonatrum
2.0
1.5
1.0
0.5
0.0
A (n=15)
B (n=16)
C (n=14)
surface

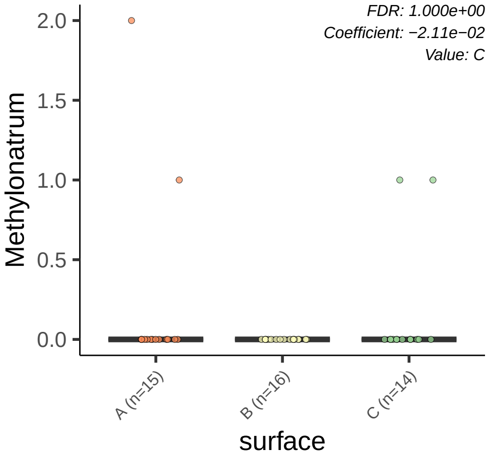

FDR: 1.000e+00
Coefficient: −2.11e−02
Value: C
Methylonatrum
2.0
1.5
1.0
0.5
0.0
A (n=15)
B (n=16)
C (n=14)
surface

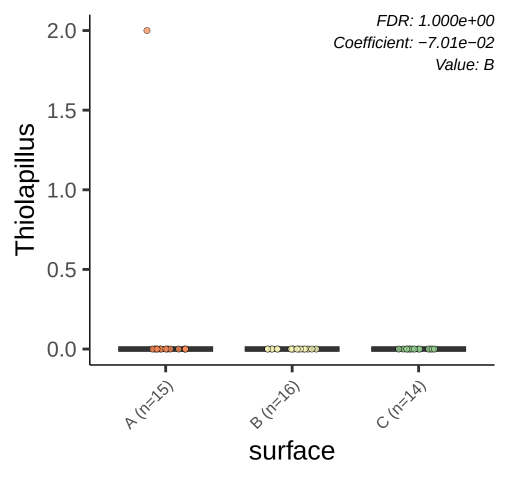
FDR: 1.000e+00
Coefficient: −7.01e−02
Value: B
Thiolapillus
2.0
1.5
1.0
0.5
0.0
A (n=15)
B (n=16)
C (n=14)
surface

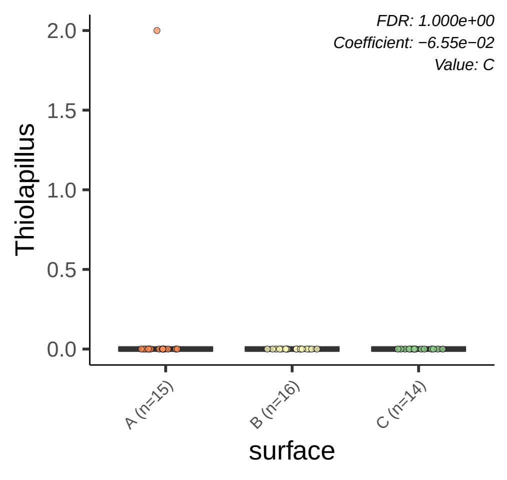
FDR: 1.000e+00
Coefficient: −6.55e−02
Value: C
Thiolapillus
2.0
1.5
1.0
0.5
0.0
A (n=15)
B (n=16)
C (n=14)
surface

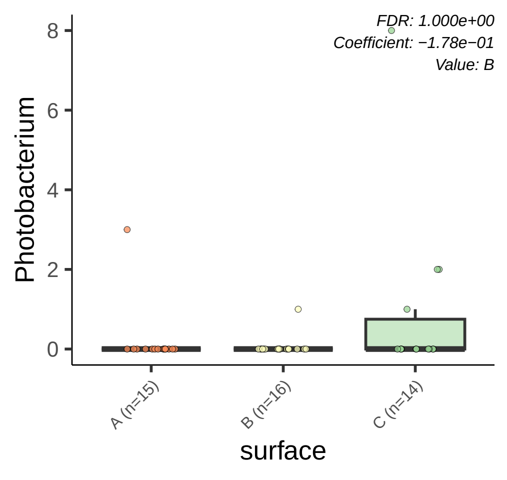
FDR: 1.000e+00
Coefficient: −1.78e−01
Value: B
Photobacterium
8
6
4
2
0
A (n=15)
B (n=16)
C (n=14)
surface

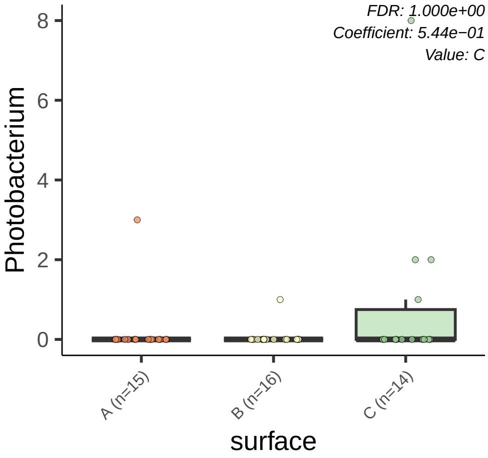
FDR: 1.000e+00
Coefficient: 5.44e−01
Value: C
Photobacterium
8
6
4
2
0
A (n=15)
B (n=16)
C (n=14)
surface

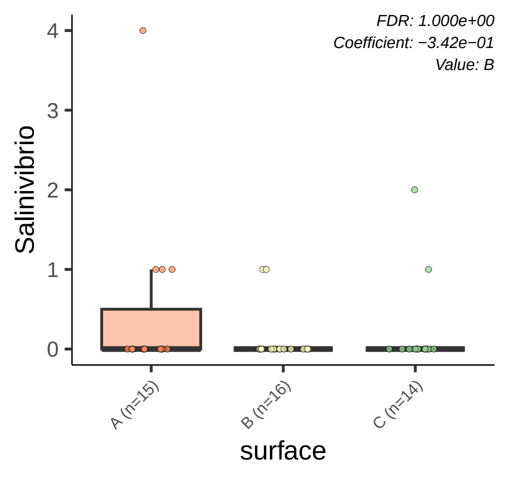
FDR: 1.000e+00
Coefficient: −3.42e−01
Value: B
Salinivibrio
0
1
2
3
4
A (n=15)
B (n=16)
C (n=14)
surface

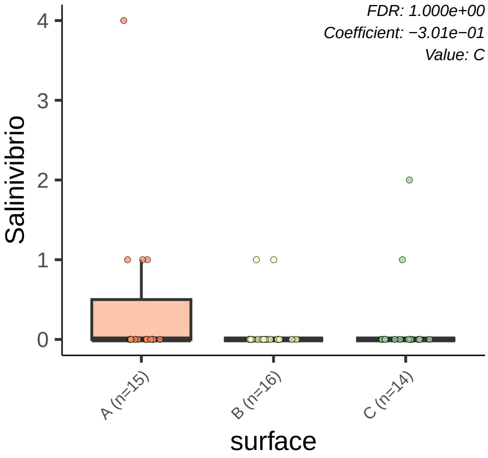

FDR: 1.000e+00
Coefficient: −3.01e−01
Value: C
Salinivibrio
4
3
2
1
0
A (n=15)
B (n=16)
C (n=14)
surface

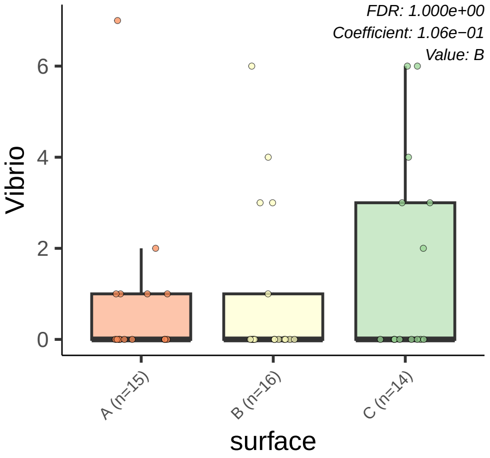

FDR: 1.000e+00
Coefficient: 1.06e−01
Value: B
Vibrio
0
2
4
6
A (n=15)
B (n=16)
C (n=14)
surface

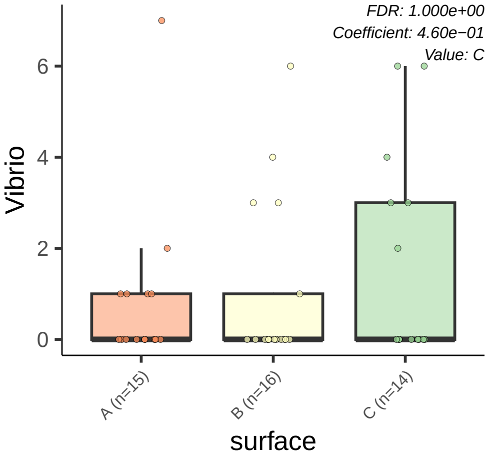

FDR: 1.000e+00
Coefficient: 4.60e−01
Value: C
Vibrio
0
2
4
6
A (n=15)
B (n=16)
C (n=14)
surface

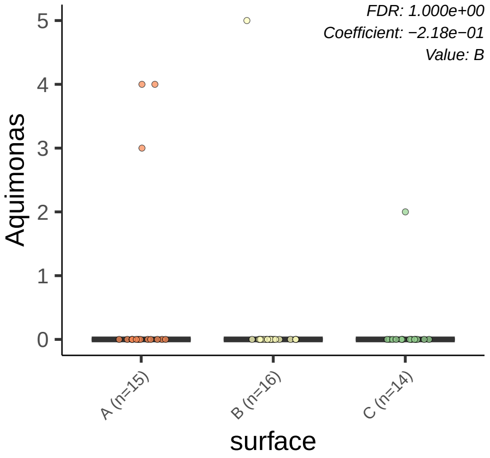
FDR: 1.000e+00
Coefficient: −2.18e−01
Value: B
Aquimonas
5
4
3
2
1
0
A (n=15)
B (n=16)
C (n=14)
surface

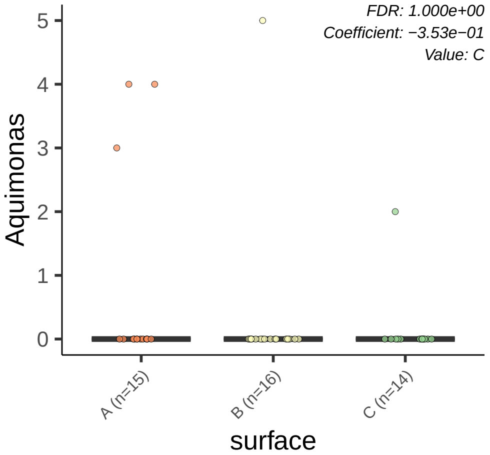

FDR: 1.000e+00
Coefficient: −3.53e−01
Value: C
Aquimonas
5
4
3
2
1
0
A (n=15)
B (n=16)
C (n=14)
surface

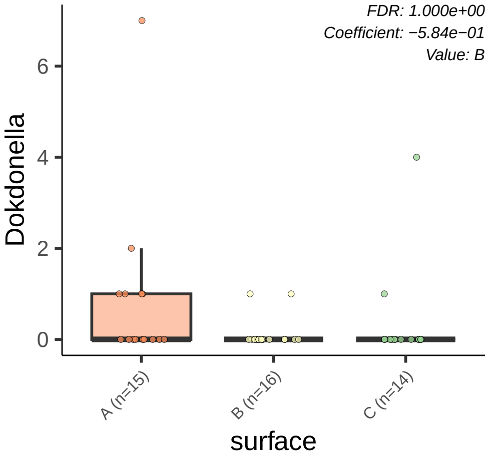
FDR: 1.000e+00
Coefficient: −5.84e−01
Value: B
Dokdonella
0
2
4
6
A (n=15)
B (n=16)
C (n=14)
surface

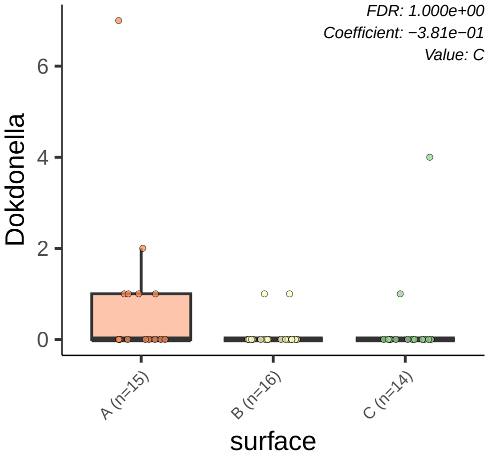
FDR: 1.000e+00
Coefficient: −3.81e−01
Value: C
Dokdonella
0
2
4
6
A (n=15)
B (n=16)
C (n=14)
surface

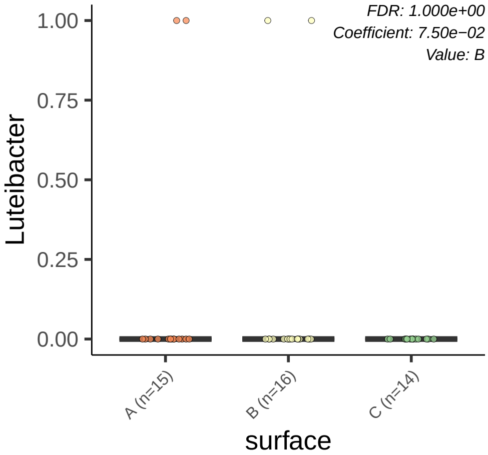

FDR: 1.000e+00
Coefficient: 7.50e−02
Value: B
Luteibacter
1.00
0.75
0.50
0.25
0.00
A (n=15)
B (n=16)
C (n=14)
surface

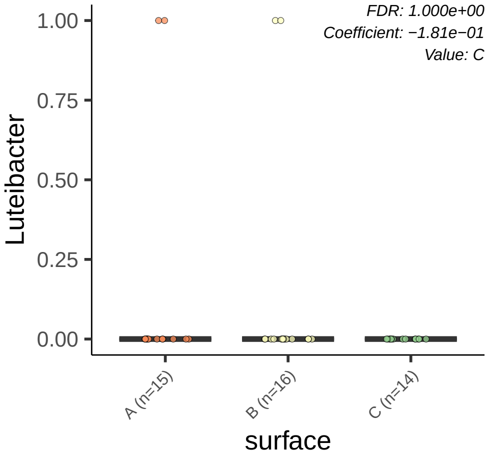

FDR: 1.000e+00
Coefficient: −1.81e−01
Value: C
Luteibacter
1.00
0.75
0.50
0.25
0.00
A (n=15)
B (n=16)
C (n=14)
surface

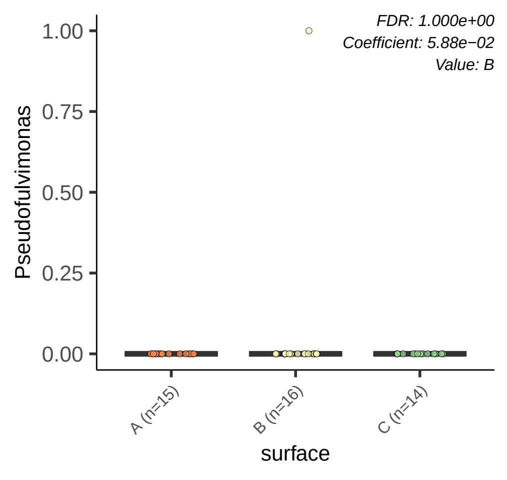
FDR: 1.000e+00
Coefficient: 5.88e−02
Value: B
Pseudofulvimonas
1.00
0.75
0.50
0.25
0.00
A (n=15)
B (n=16)
C (n=14)
surface

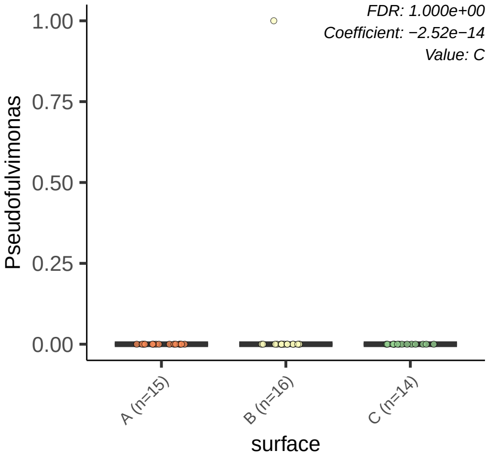

FDR: 1.000e+00
Coefficient: −2.52e−14
Value: C
Pseudofulvimonas
1.00
0.75
0.50
0.25
0.00
A (n=15)
B (n=16)
C (n=14)
surface

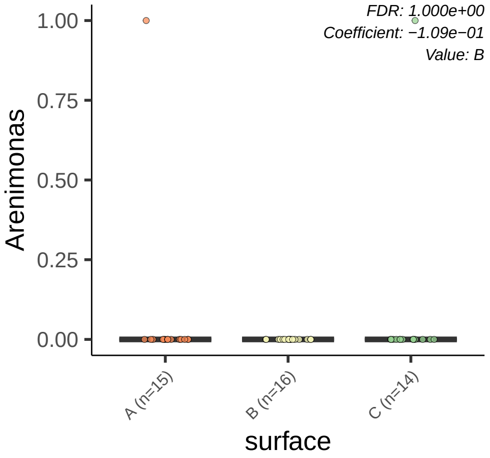
FDR: 1.000e+00
Coefficient: −1.09e−01
Value: B
Arenimonas
1.00
0.75
0.50
0.25
0.00
A (n=15)
B (n=16)
C (n=14)
surface

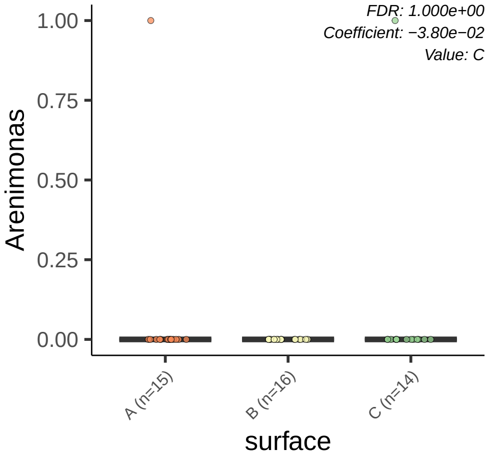

FDR: 1.000e+00
Coefficient: −3.80e−02
Value: C
Arenimonas
1.00
0.75
0.50
0.25
0.00
A (n=15)
B (n=16)
C (n=14)
surface

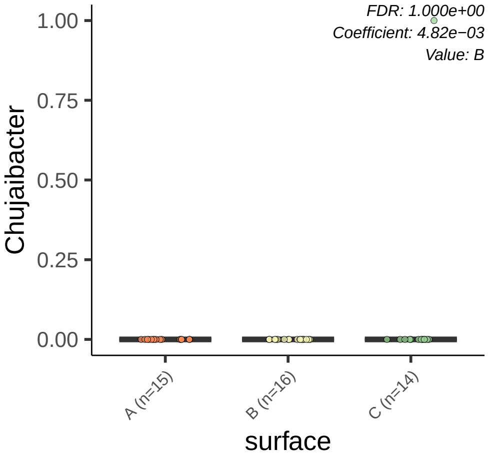

FDR: 1.000e+00
Coefficient: 4.82e−03
Value: B
Chujaibacter
1.00
0.75
0.50
0.25
0.00
A (n=15)
B (n=16)
C (n=14)
surface

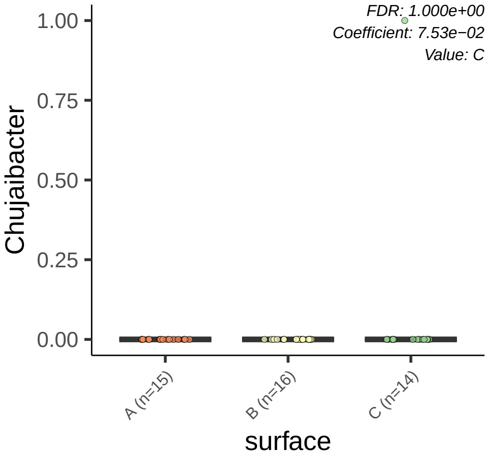

FDR: 1.000e+00
Coefficient: 7.53e−02
Value: C
1.00
0.75
0.50
0.25
0.00
Chujaibacter
A (n=15)
B (n=16)
C (n=14)
surface

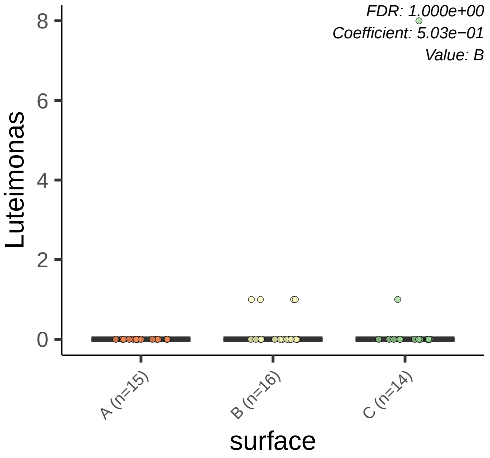
FDR: 1.000e+00
Coefficient: 5.03e−01
Value: B
Luteimonas
8
6
4
2
0
A (n=15)
B (n=16)
C (n=14)
surface

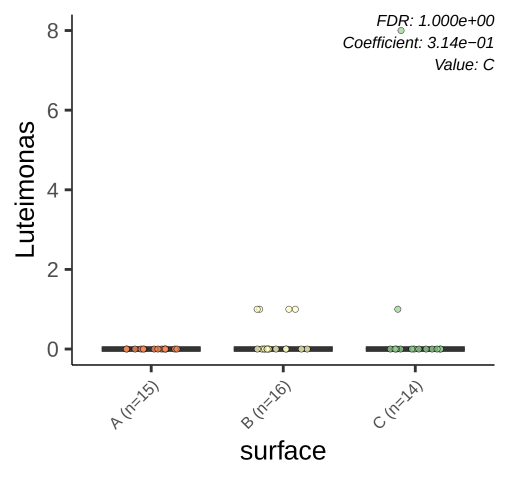
FDR: 1.000e+00
Coefficient: 3.14e−01
Value: C
Luteimonas
8
6
4
2
0
A (n=15)
B (n=16)
C (n=14)
surface

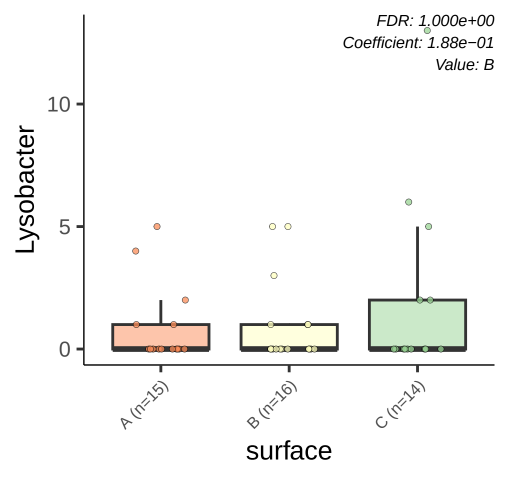

FDR: 1.000e+00
Coefficient: 1.88e−01
Value: B
Lysobacter
10
5
0
A (n=15)
B (n=16)
C (n=14)
surface

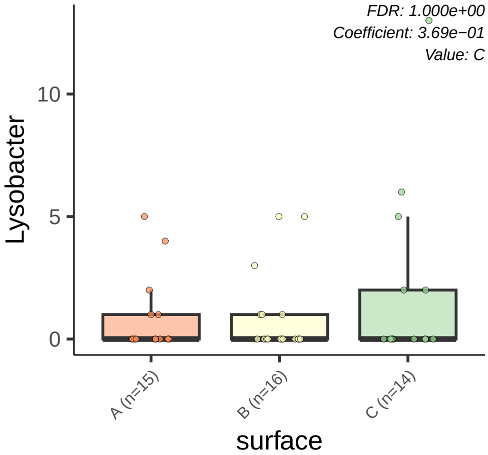

FDR: 1.000e+00
Coefficient: 3.69e−01
Value: C
Lysobacter
10
5
0
A (n=15)
B (n=16)
C (n=14)
surface

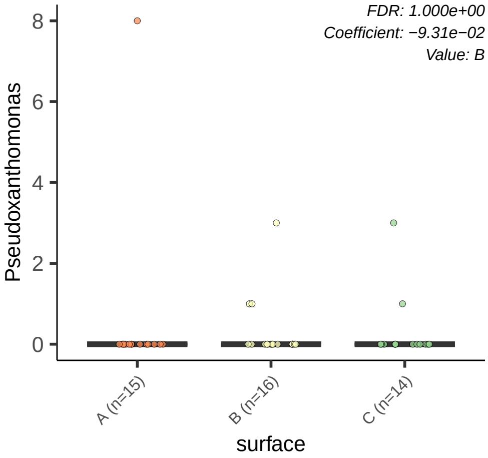
FDR: 1.000e+00
Coefficient: −9.31e−02
Value: B
Pseudoxanthomonas
8
6
4
2
0
A (n=15)
B (n=16)
C (n=14)
surface

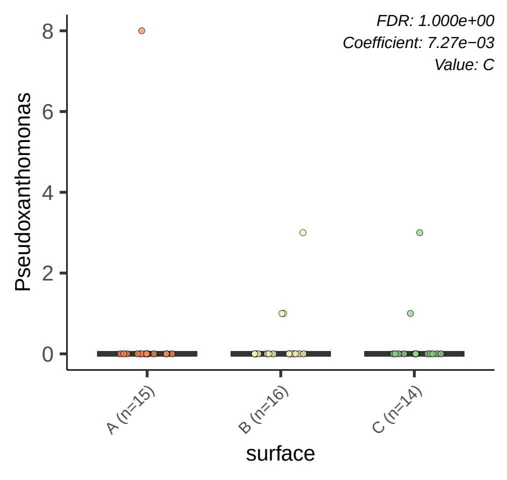
FDR: 1.000e+00
Coefficient: 7.27e−03
Value: C
Pseudoxanthomonas
8
6
4
2
0
A (n=15)
B (n=16)
C (n=14)
surface

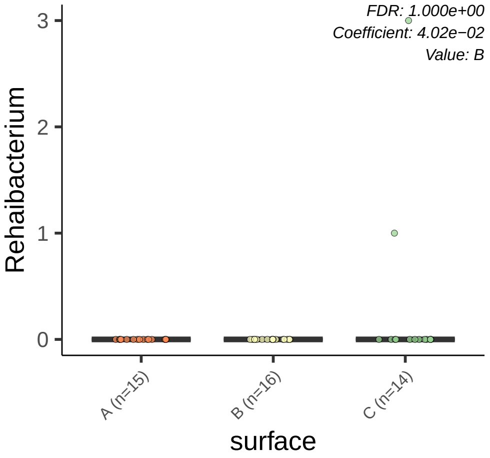

FDR: 1.000e+00
Coefficient: 4.02e−02
Value: B
Rehaibacterium
3
2
1
0
A (n=15)
B (n=16)
C (n=14)
surface

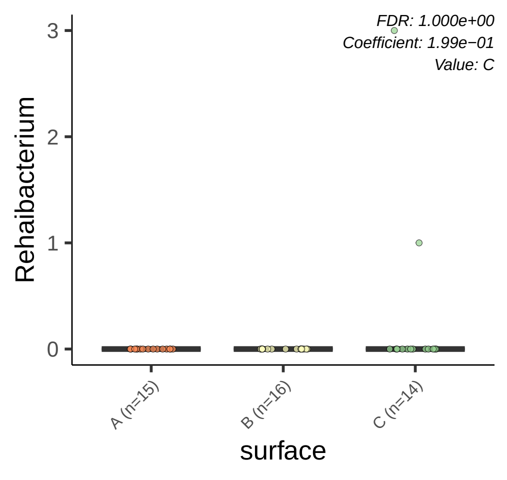

FDR: 1.000e+00
Coefficient: 1.99e−01
Value: C
Rehaibacterium
3
2
1
0
A (n=15)
B (n=16)
C (n=14)
surface

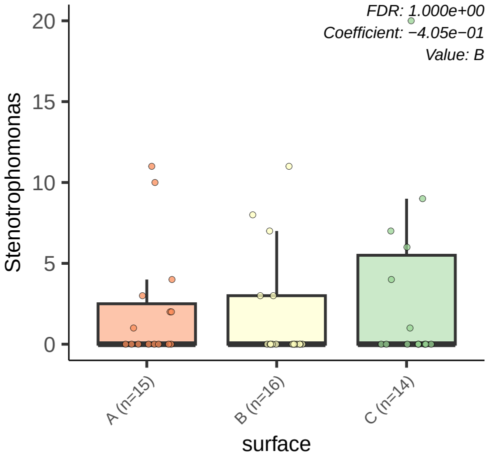
FDR: 1.000e+00
Coefficient: −4.05e−01
Value: B
Stenotrophomonas
20
15
10
5
0
A (n=15)
B (n=16)
C (n=14)
surface

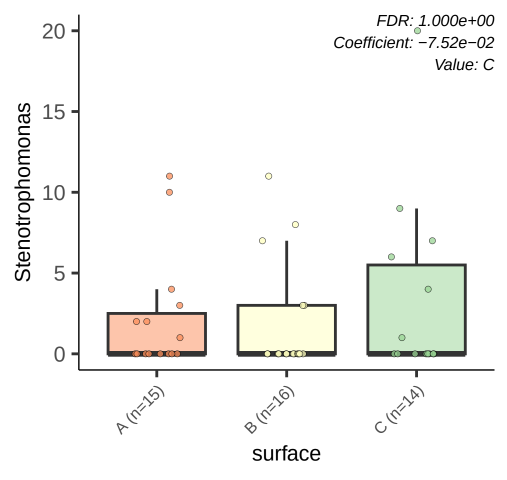
FDR: 1.000e+00
Coefficient: −7.52e−02
Value: C
Stenotrophomonas
20
15
10
5
0
A (n=15)
B (n=16)
C (n=14)
surface

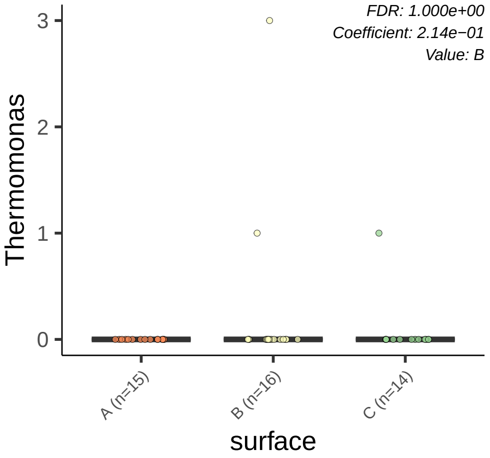

FDR: 1.000e+00
Coefficient: 2.14e−01
Value: B
Thermomonas
3
2
1
0
A (n=15)
B (n=16)
C (n=14)
surface

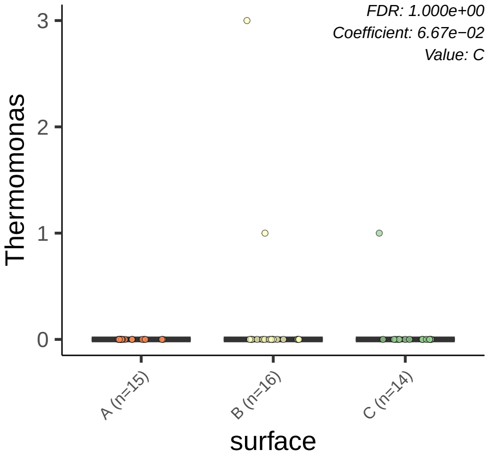
FDR: 1.000e+00
Coefficient: 6.67e−02
Value: C
Thermomonas
3
2
1
0
A (n=15)
B (n=16)
C (n=14)
surface

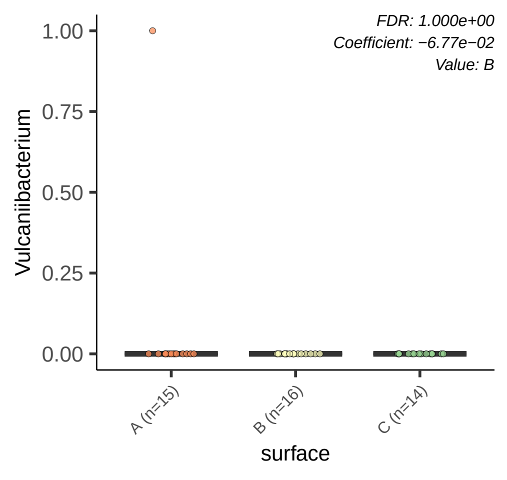
FDR: 1.000e+00
Coefficient: −6.77e−02
Value: B
Vulcaniibacterium
1.00
0.75
0.50
0.25
0.00
A (n=15)
B (n=16)
C (n=14)
surface

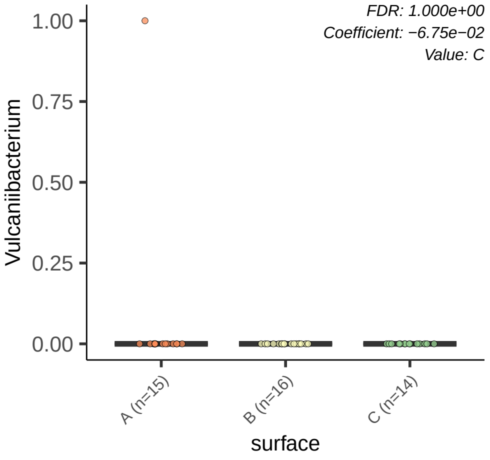
FDR: 1.000e+00
Coefficient: −6.75e−02
Value: C
Vulcaniibacterium
1.00
0.75
0.50
0.25
0.00
A (n=15)
B (n=16)
C (n=14)
surface

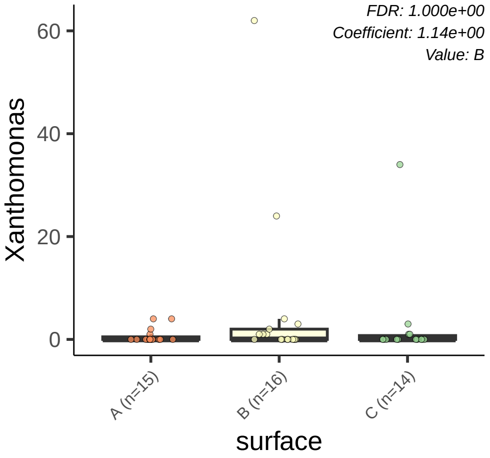

FDR: 1.000e+00
Coefficient: 1.14e+00
Value: B
Xanthomonas
60
40
20
0
A (n=15)
B (n=16)
C (n=14)
surface

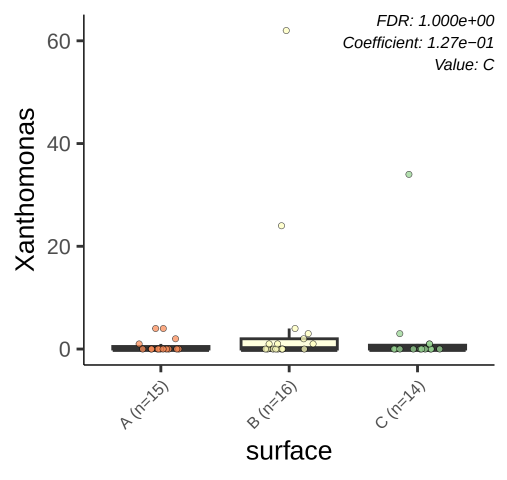

FDR: 1.000e+00
Coefficient: 1.27e−01
Value: C
Xanthomonas
60
40
20
0
A (n=15)
B (n=16)
C (n=14)
surface

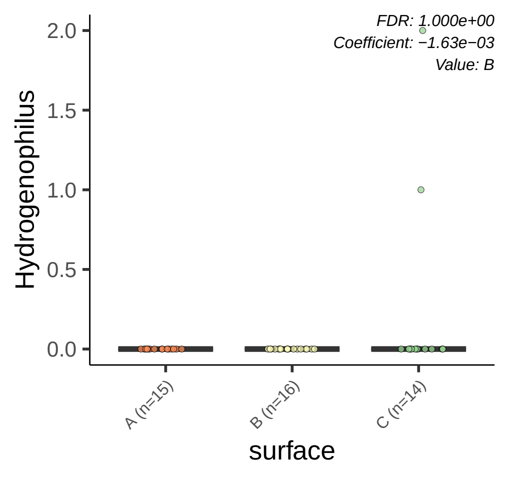
FDR: 1.000e+00
Coefficient: −1.63e−03
Value: B
Hydrogenophilus
2.0
1.5
1.0
0.5
0.0
A (n=15)
B (n=16)
C (n=14)
surface

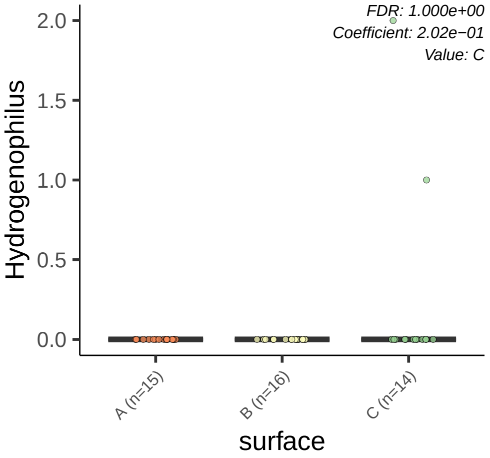
FDR: 1.000e+00
Coefficient: 2.02e−01
Value: C
Hydrogenophilus
2.0
1.5
1.0
0.5
0.0
A (n=15)
B (n=16)
C (n=14)
surface

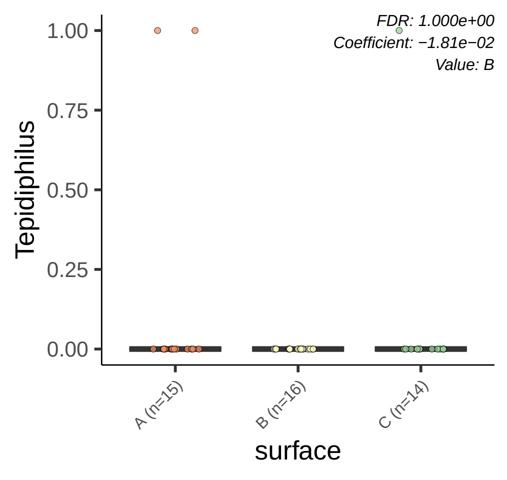

FDR: 1.000e+00
Coefficient: −1.81e−02
Value: B
Tepidiphilus
1.00
0.75
0.50
0.25
0.00
A (n=15)
B (n=16)
C (n=14)
surface

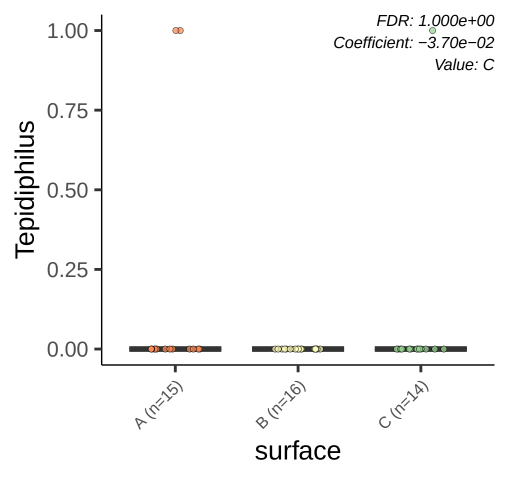

FDR: 1.000e+00
Coefficient: −3.70e−02
Value: C
Tepidiphilus
1.00
0.75
0.50
0.25
0.00
A (n=15)
B (n=16)
C (n=14)
surface

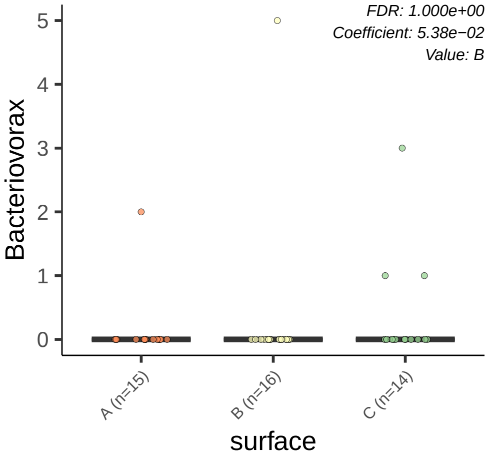
FDR: 1.000e+00
Coefficient: 5.38e−02
Value: B
Bacteriovorax
0
1
2
3
4
5
A (n=15)
B (n=16)
C (n=14)
surface

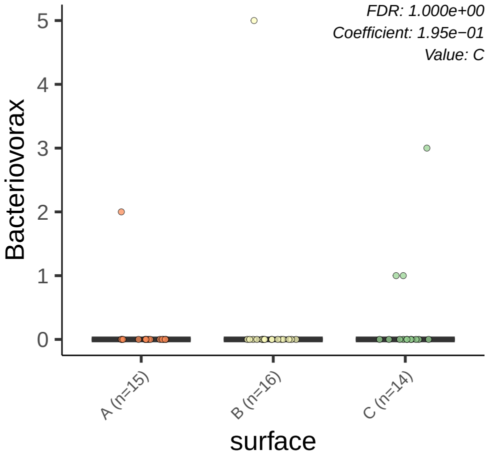
FDR: 1.000e+00
Coefficient: 1.95e−01
Value: C
Bacteriovorax
0
1
2
3
4
5
A (n=15)
B (n=16)
C (n=14)
surface

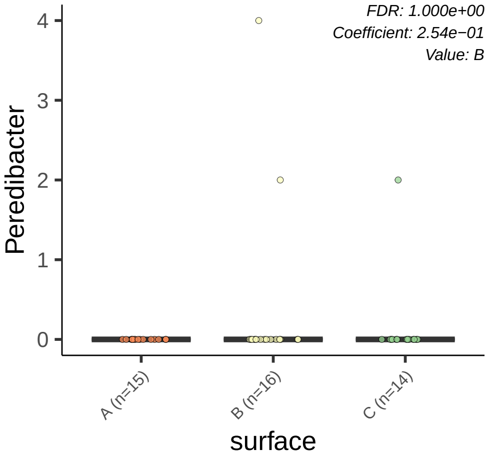
FDR: 1.000e+00
Coefficient: 2.54e−01
Value: B
Peredibacter
4
3
2
1
0
A (n=15)
B (n=16)
C (n=14)
surface

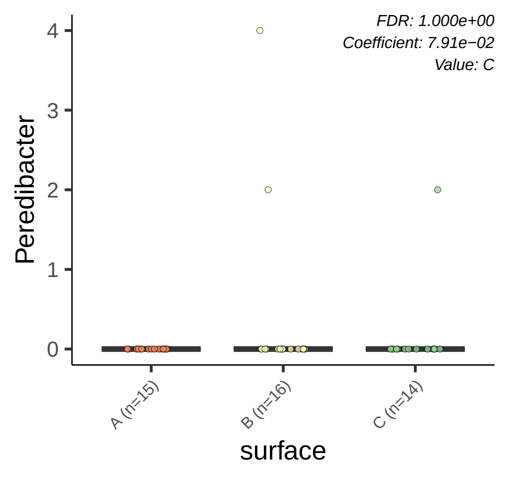
FDR: 1.000e+00
Coefficient: 7.91e−02
Value: C
Peredibacter
0
1
2
3
4
A (n=15)
B (n=16)
C (n=14)
surface

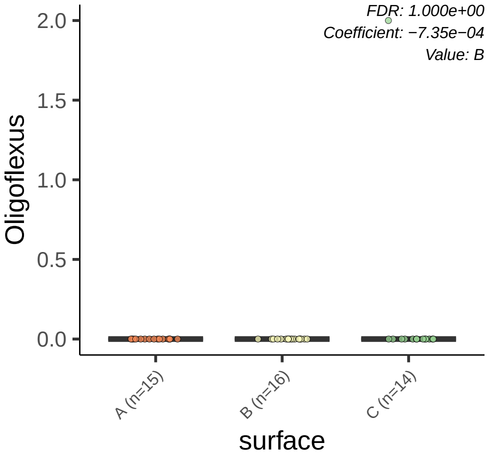
FDR: 1.000e+00
Coefficient: −7.35e−04
Value: B
Oligoflexus
2.0
1.5
1.0
0.5
0.0
A (n=15)
B (n=16)
C (n=14)
surface

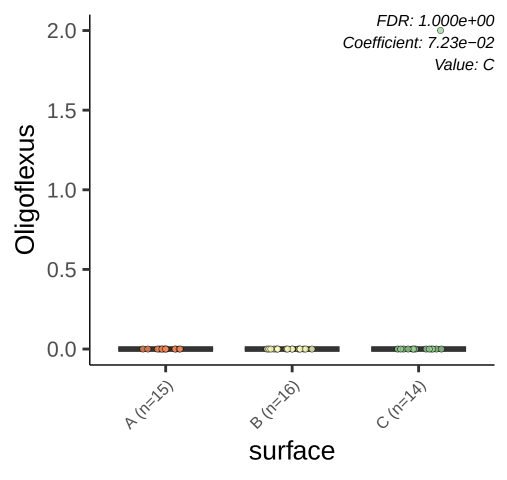
FDR: 1.000e+00
Coefficient: 7.23e−02
Value: C
Oligoflexus
2.0
1.5
1.0
0.5
0.0
A (n=15)
B (n=16)
C (n=14)
surface

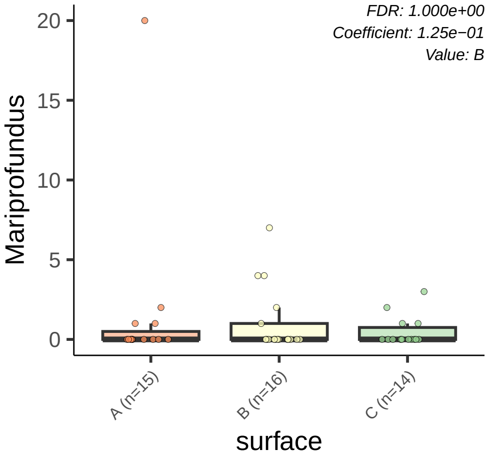

FDR: 1.000e+00
Coefficient: 1.25e−01
Value: B
Mariprofundus
20
15
10
5
0
A (n=15)
B (n=16)
C (n=14)
surface

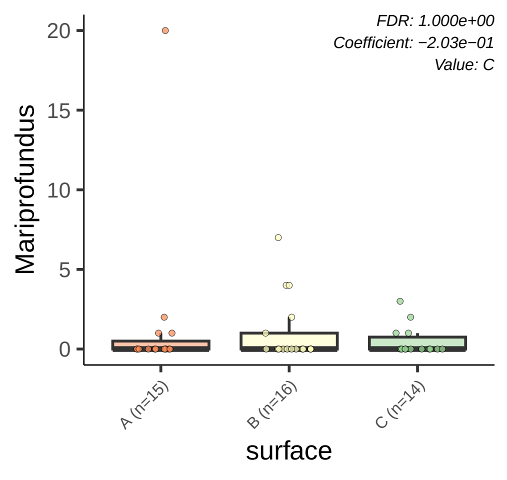
FDR: 1.000e+00
Coefficient: −2.03e−01
Value: C
Mariprofundus
20
15
10
5
0
A (n=15)
B (n=16)
C (n=14)
surface

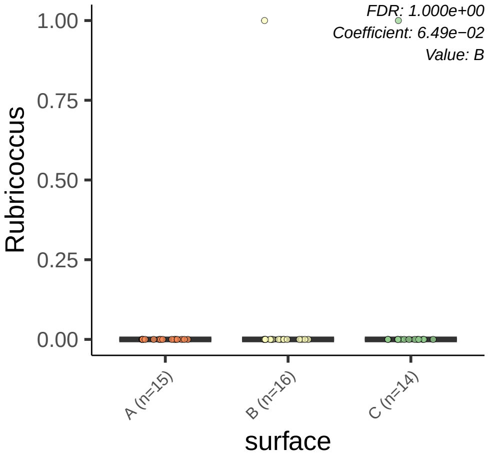

FDR: 1.000e+00
Coefficient: 6.49e−02
Value: B
Rubricoccus
1.00
0.75
0.50
0.25
0.00
A (n=15)
B (n=16)
C (n=14)
surface

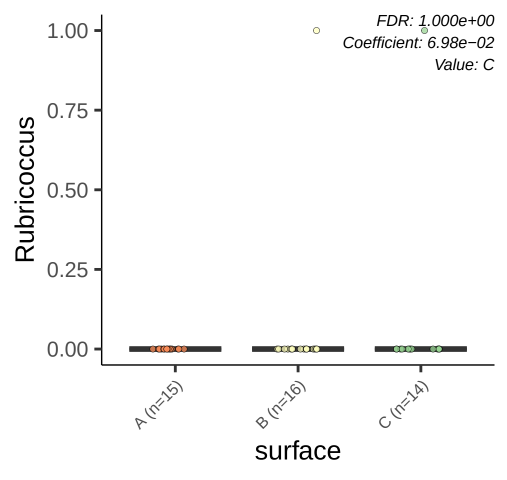
FDR: 1.000e+00
Coefficient: 6.98e−02
Value: C
Rubricoccus
1.00
0.75
0.50
0.25
0.00
A (n=15)
B (n=16)
C (n=14)
surface

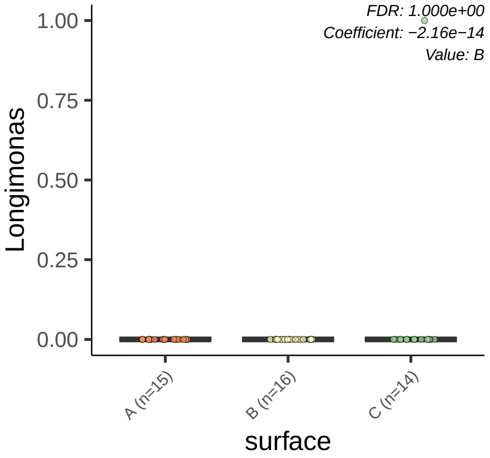
FDR: 1.000e+00
Coefficient: −2.16e−14
Value: B
Longimonas
1.00
0.75
0.50
0.25
0.00
A (n=15)
B (n=16)
C (n=14)
surface

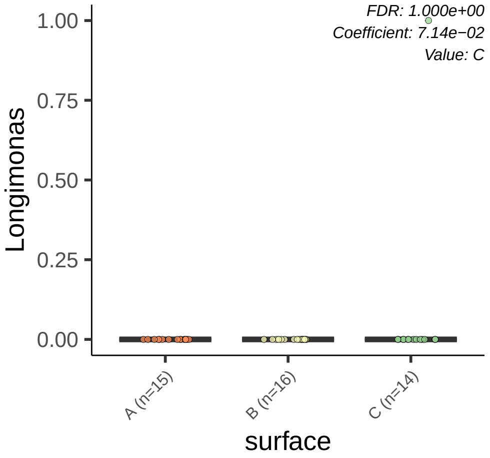

FDR: 1.000e+00
Coefficient: 7.14e−02
Value: C
Longimonas
1.00
0.75
0.50
0.25
0.00
A (n=15)
B (n=16)
C (n=14)
surface

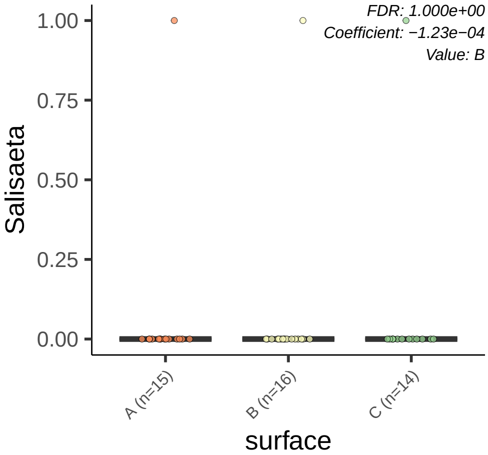
FDR: 1.000e+00
Coefficient: −1.23e−04
Value: B
Salisaeta
1.00
0.75
0.50
0.25
0.00
A (n=15)
B (n=16)
C (n=14)
surface

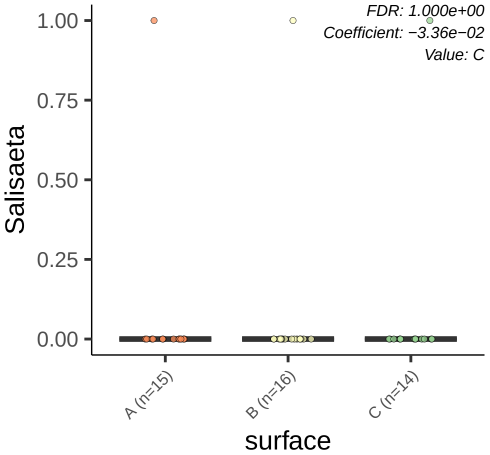

FDR: 1.000e+00
Coefficient: −3.36e−02
Value: C
Salisaeta
1.00
0.75
0.50
0.25
0.00
A (n=15)
B (n=16)
C (n=14)
surface

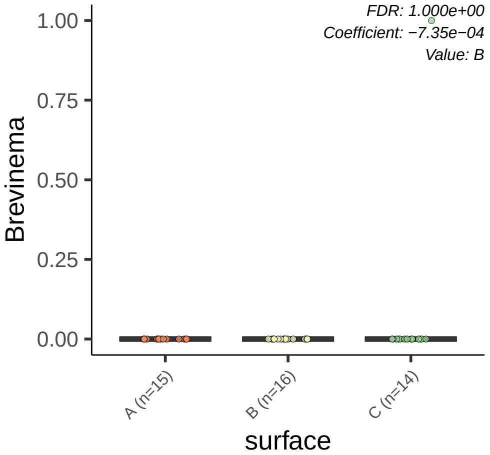
FDR: 1.000e+00
Coefficient: −7.35e−04
Value: B
Brevinema
1.00
0.75
0.50
0.25
0.00
A (n=15)
B (n=16)
C (n=14)
surface

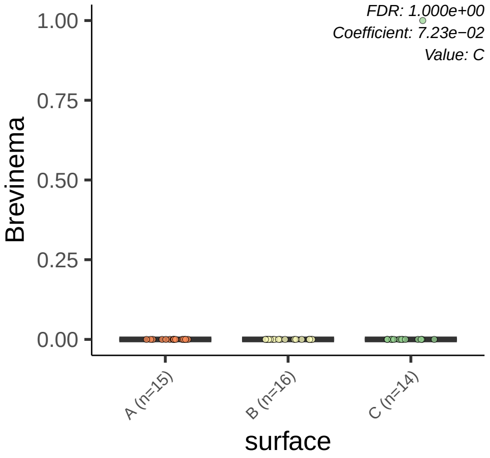
FDR: 1.000e+00
Coefficient: 7.23e−02
Value: C
Brevinema
1.00
0.75
0.50
0.25
0.00
A (n=15)
B (n=16)
C (n=14)
surface

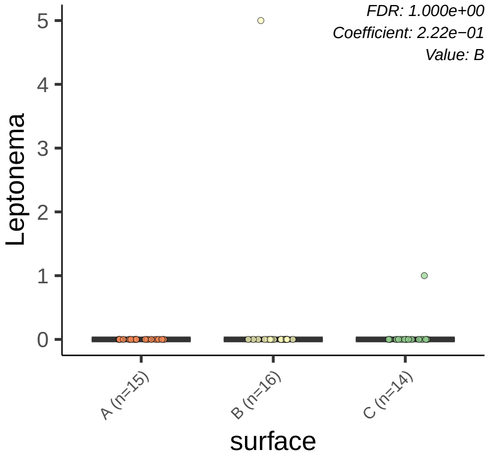
FDR: 1.000e+00
Coefficient: 2.22e−01
Value: B
Leptonema
5
4
3
2
1
0
A (n=15)
B (n=16)
C (n=14)
surface

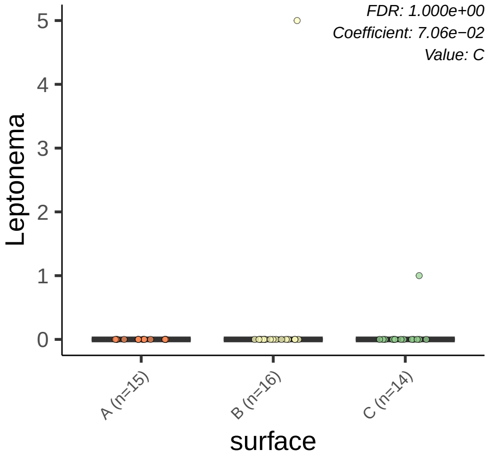
FDR: 1.000e+00
Coefficient: 7.06e−02
Value: C
Leptonema
5
4
3
2
1
0
A (n=15)
B (n=16)
C (n=14)
surface

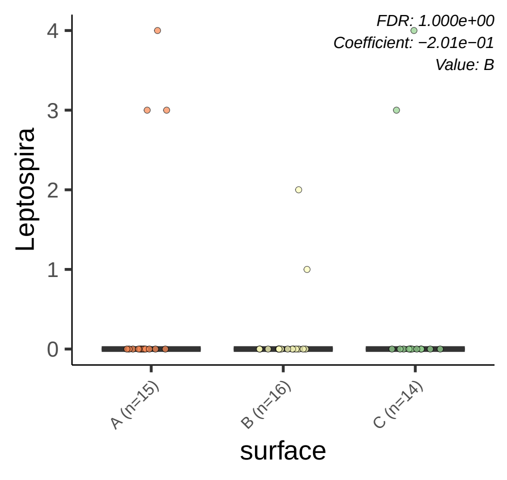

FDR: 1.000e+00
Coefficient: −2.01e−01
Value: B
Leptospira
0
1
2
3
4
A (n=15)
B (n=16)
C (n=14)
surface

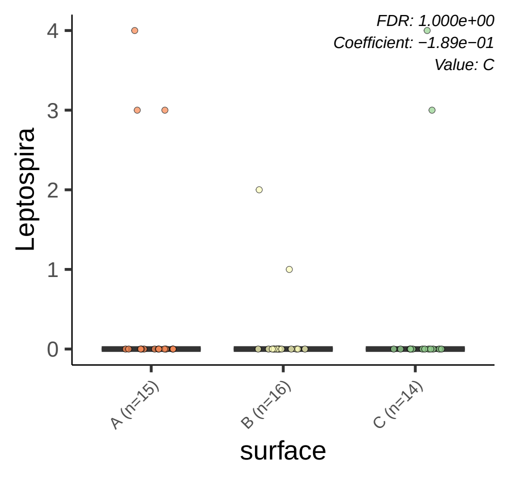
FDR: 1.000e+00
Coefficient: −1.89e−01
Value: C
Leptospira
4
3
2
1
0
A (n=15)
B (n=16)
C (n=14)
surface

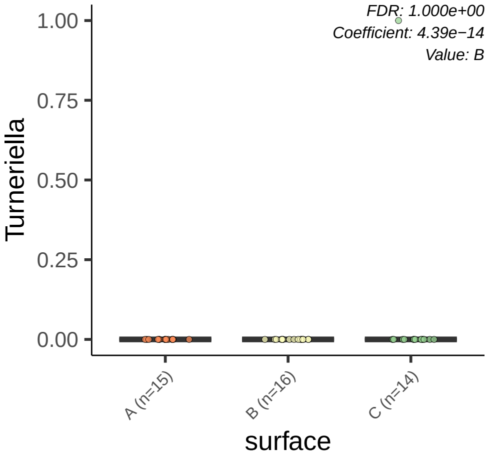
FDR: 1.000e+00
Coefficient: 4.39e−14
Value: B
Turneriella
1.00
0.75
0.50
0.25
0.00
A (n=15)
B (n=16)
C (n=14)
surface

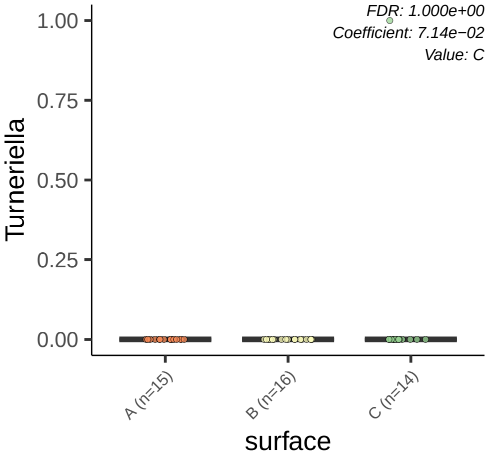

FDR: 1.000e+00
Coefficient: 7.14e−02
Value: C
Turneriella
1.00
0.75
0.50
0.25
0.00
A (n=15)
B (n=16)
C (n=14)
surface

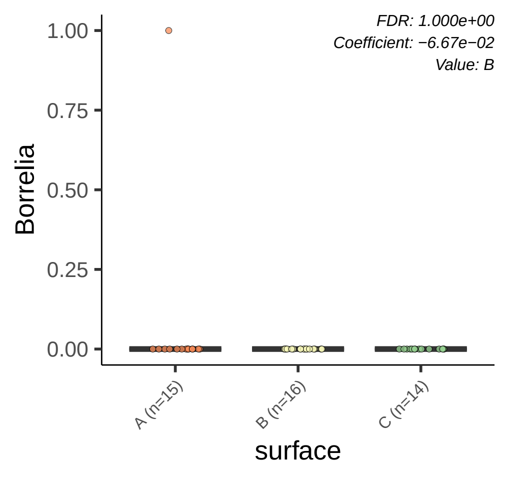

FDR: 1.000e+00
Coefficient: −6.67e−02
Value: B
Borrelia
1.00
0.75
0.50
0.25
0.00
A (n=15)
B (n=16)
C (n=14)
surface

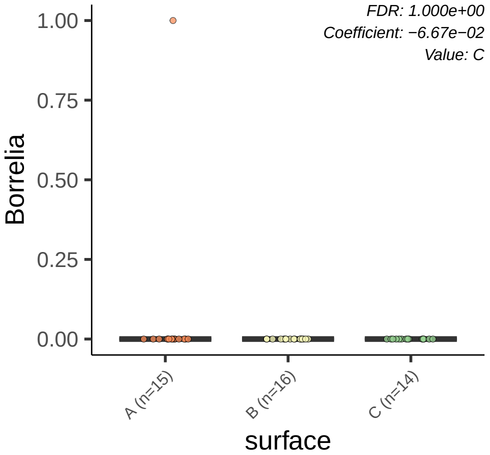
FDR: 1.000e+00
Coefficient: −6.67e−02
Value: C
Borrelia
1.00
0.75
0.50
0.25
0.00
A (n=15)
B (n=16)
C (n=14)
surface

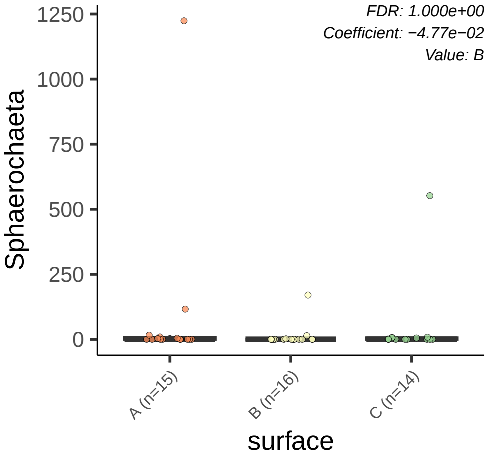
FDR: 1.000e+00
Coefficient: −4.77e−02
Value: B
Sphaerochaeta
1250
1000
750
500
250
0
A (n=15)
B (n=16)
C (n=14)
surface

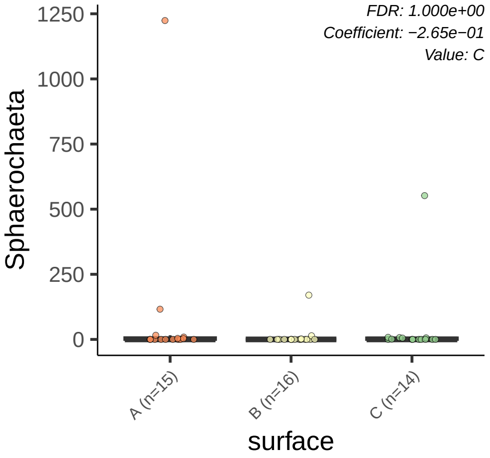

FDR: 1.000e+00
Coefficient: −2.65e−01
Value: C
Sphaerochaeta
1250
1000
750
500
250
0
A (n=15)
B (n=16)
C (n=14)
surface

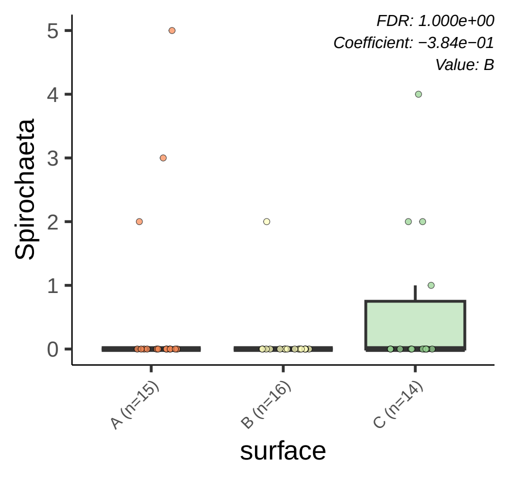
FDR: 1.000e+00
Coefficient: −3.84e−01
Value: B
Spirochaeta
5
4
3
2
1
0
A (n=15)
B (n=16)
C (n=14)
surface

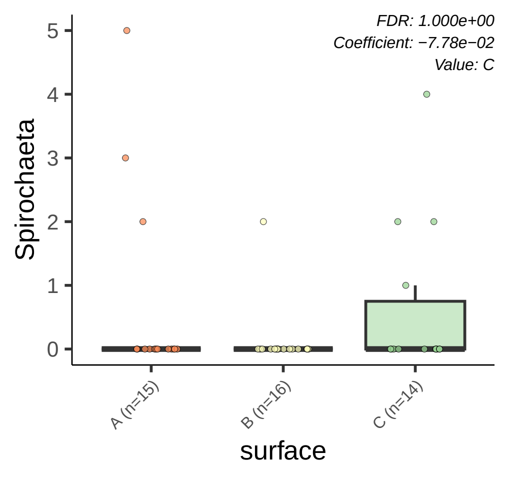

FDR: 1.000e+00
Coefficient: −7.78e−02
Value: C
Spirochaeta
0
1
2
3
4
5
A (n=15)
B (n=16)
C (n=14)
surface

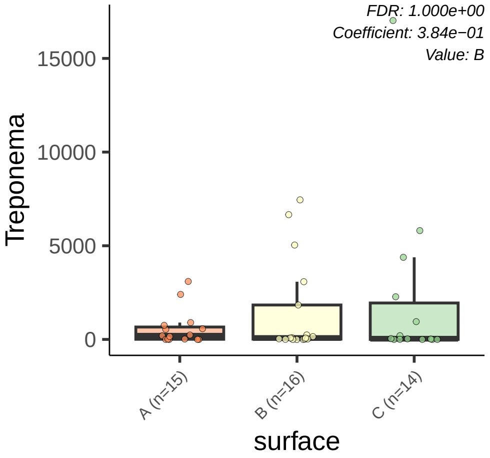
FDR: 1.000e+00
Coefficient: 3.84e−01
Value: B
Treponema
15000
10000
5000
0
A (n=15)
B (n=16)
C (n=14)
surface

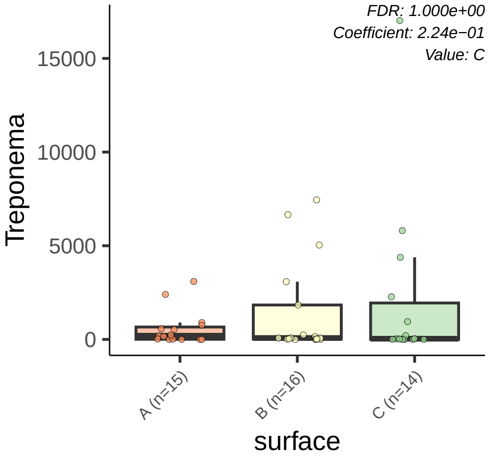
FDR: 1.000e+00
Coefficient: 2.24e−01
Value: C
Treponema
15000
10000
5000
0
A (n=15)
B (n=16)
C (n=14)
surface

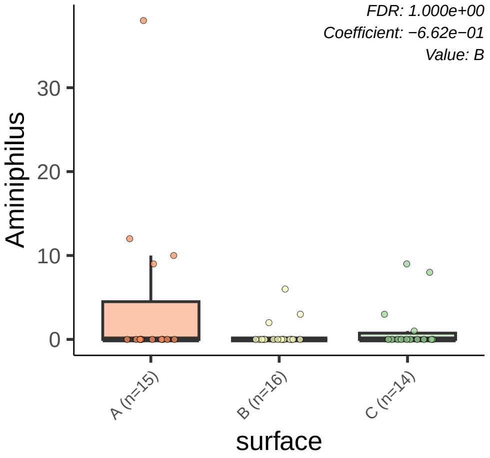
FDR: 1.000e+00
Coefficient: −6.62e−01
Value: B
Aminiphilus
30
20
10
0
A (n=15)
B (n=16)
C (n=14)
surface

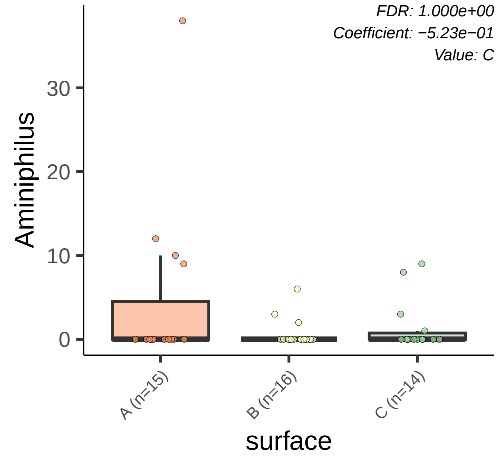
FDR: 1.000e+00
Coefficient: −5.23e−01
Value: C
Aminiphilus
0
10
20
30
A (n=15)
B (n=16)
C (n=14)
surface

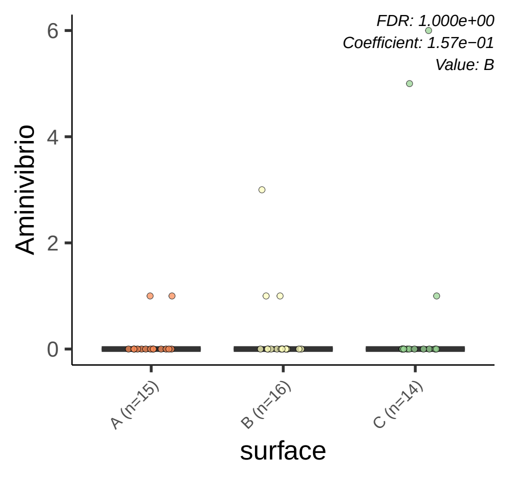
FDR: 1.000e+00
Coefficient: 1.57e−01
Value: B
Aminivibrio
0
2
4
6
A (n=15)
B (n=16)
C (n=14)
surface

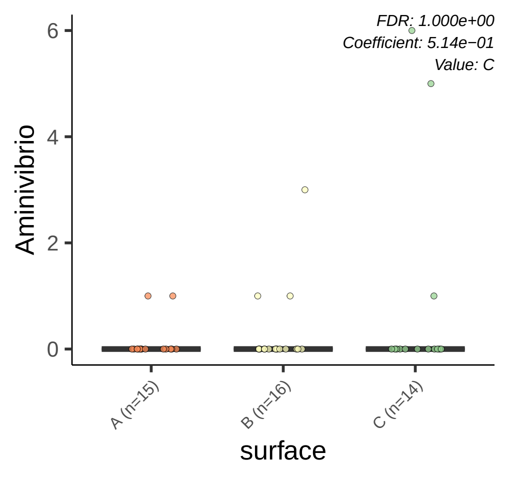
FDR: 1.000e+00
Coefficient: 5.14e−01
Value: C
Aminivibrio
0
2
4
6
A (n=15)
B (n=16)
C (n=14)
surface

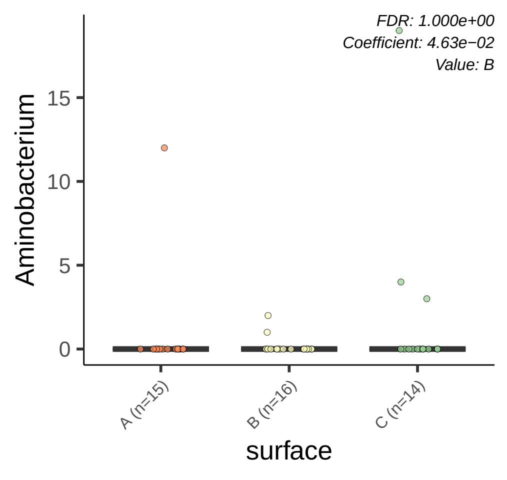

FDR: 1.000e+00
Coefficient: 4.63e−02
Value: B
Aminobacterium
15
10
5
0
A (n=15)
B (n=16)
C (n=14)
surface

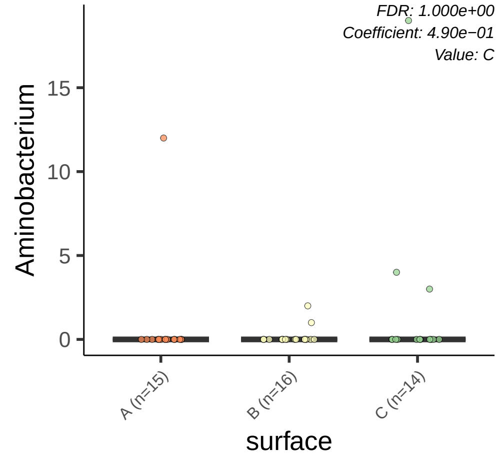
FDR: 1.000e+00
Coefficient: 4.90e−01
Value: C
Aminobacterium
15
10
5
0
A (n=15)
B (n=16)
C (n=14)
surface

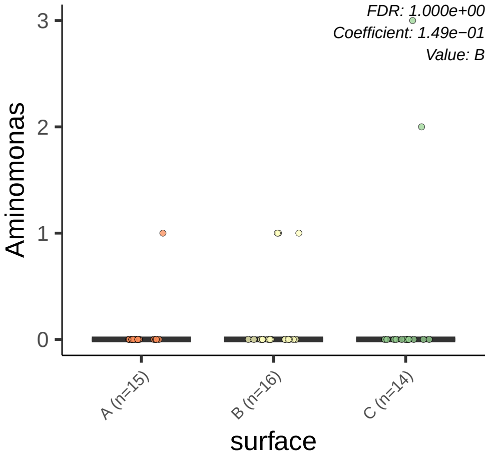
FDR: 1.000e+00
Coefficient: 1.49e−01
Value: B
Aminomonas
0
1
2
3
A (n=15)
B (n=16)
C (n=14)
surface

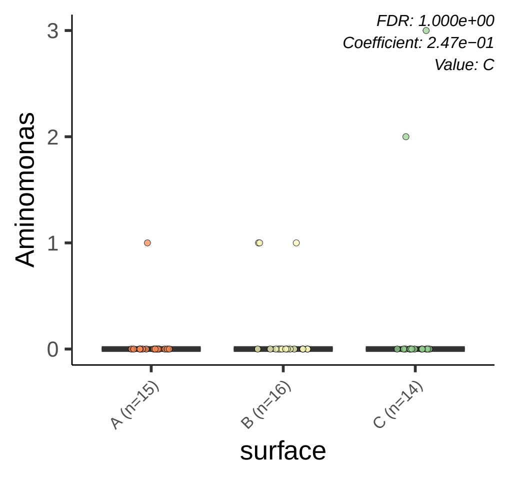
FDR: 1.000e+00
Coefficient: 2.47e−01
Value: C
Aminomonas
0
1
2
3
A (n=15)
B (n=16)
C (n=14)
surface

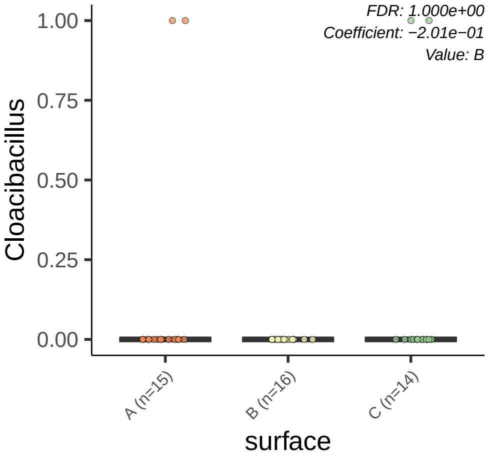

FDR: 1.000e+00
Coefficient: −2.01e−01
Value: B
Cloacibacillus
1.00
0.75
0.50
0.25
0.00
A (n=15)
B (n=16)
C (n=14)
surface

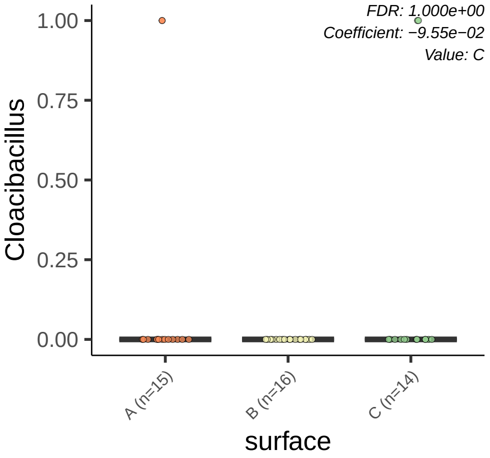
FDR: 1.000e+00
Coefficient: −9.55e−02
Value: C
Cloacibacillus
1.00
0.75
0.50
0.25
0.00
A (n=15)
B (n=16)
C (n=14)
surface

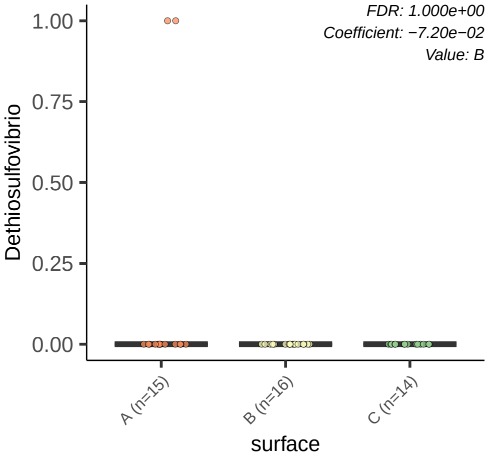

FDR: 1.000e+00
Coefficient: −7.20e−02
Value: B
Dethiosulfovibrio
1.00
0.75
0.50
0.25
0.00
A (n=15)
B (n=16)
C (n=14)
surface

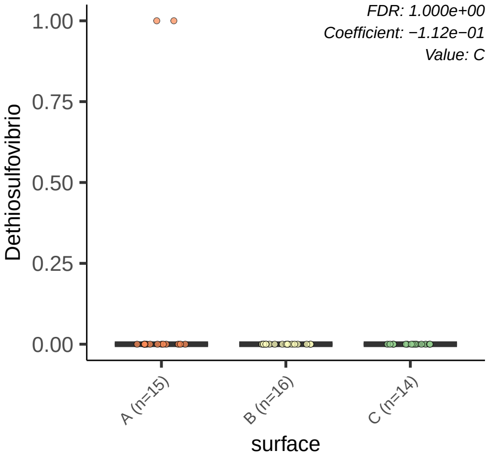

FDR: 1.000e+00
Coefficient: −1.12e−01
Value: C
Dethiosulfovibrio
1.00
0.75
0.50
0.25
0.00
A (n=15)
B (n=16)
C (n=14)
surface

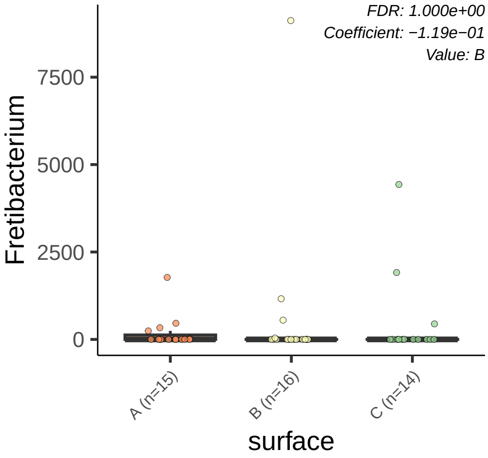
FDR: 1.000e+00
Coefficient: −1.19e−01
Value: B
7500
5000
2500
0
Fretibacterium
A (n=15)
B (n=16)
C (n=14)
surface

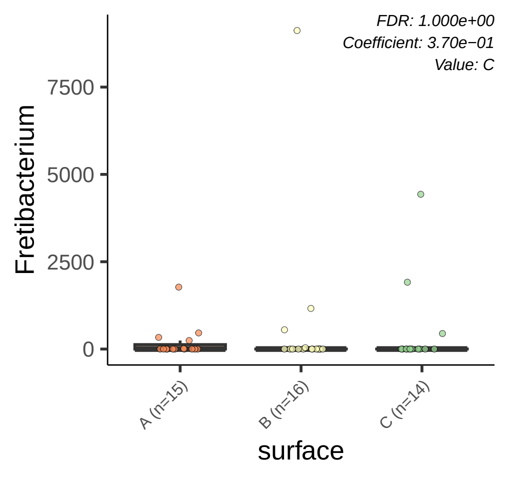
FDR: 1.000e+00
Coefficient: 3.70e−01
Value: C
Fretibacterium
7500
5000
2500
0
A (n=15)
B (n=16)
C (n=14)
surface

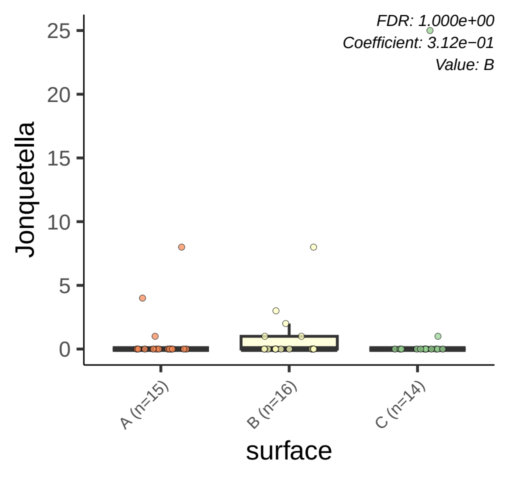
FDR: 1.000e+00
Coefficient: 3.12e−01
Value: B
Jonquetella
0
5
10
15
20
25
A (n=15)
B (n=16)
C (n=14)
surface

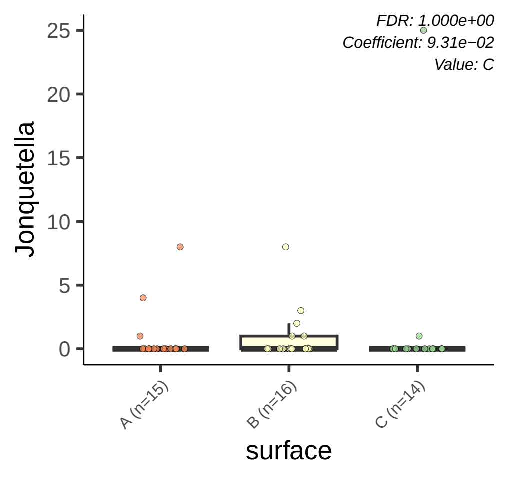
FDR: 1.000e+00
Coefficient: 9.31e−02
Value: C
Jonquetella
0
5
10
15
20
25
A (n=15)
B (n=16)
C (n=14)
surface

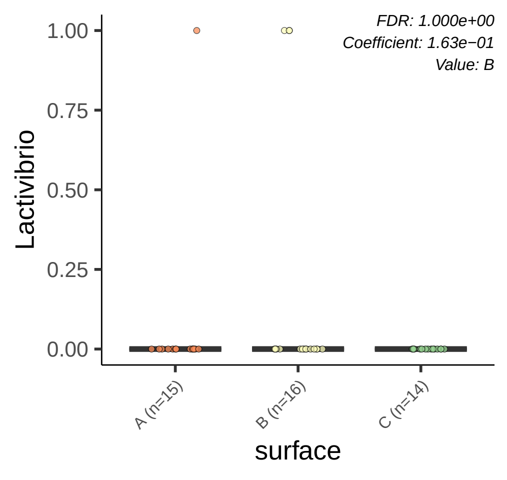
FDR: 1.000e+00
Coefficient: 1.63e−01
Value: B
Lactivibrio
1.00
0.75
0.50
0.25
0.00
A (n=15)
B (n=16)
C (n=14)
surface

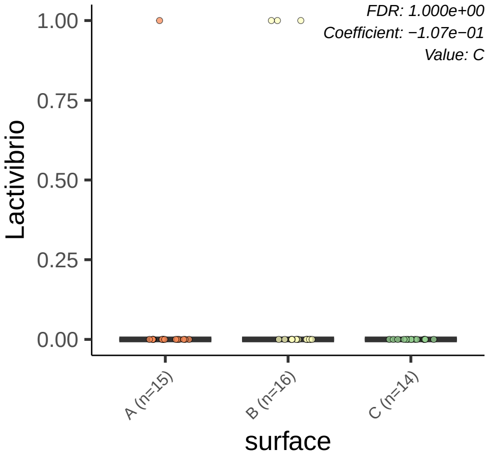

FDR: 1.000e+00
Coefficient: −1.07e−01
Value: C
1.00
0.75
0.50
0.25
0.00
Lactivibrio
A (n=15)
B (n=16)
C (n=14)
surface

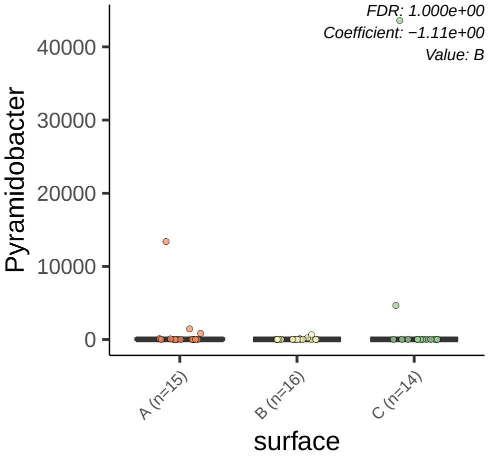

FDR: 1.000e+00
Coefficient: −1.11e+00
Value: B
Pyramidobacter
40000
30000
20000
10000
0
A (n=15)
B (n=16)
C (n=14)
surface

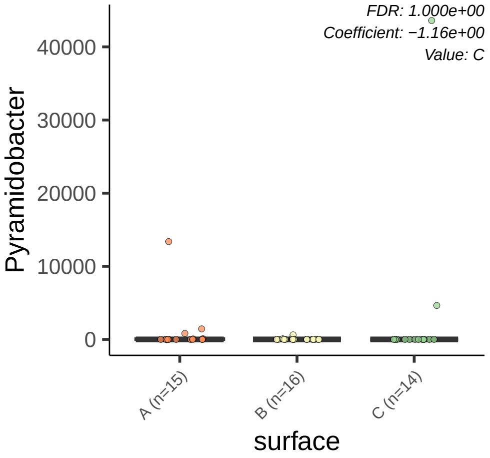

FDR: 1.000e+00
Coefficient: −1.16e+00
Value: C
Pyramidobacter
40000
30000
20000
10000
0
A (n=15)
B (n=16)
C (n=14)
surface

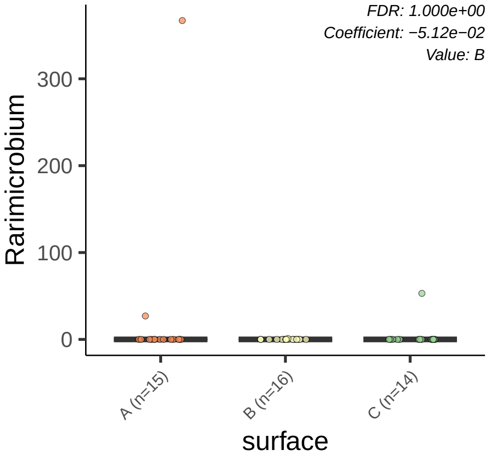

FDR: 1.000e+00
Coefficient: −5.12e−02
Value: B
Rarimicrobium
300
200
100
0
A (n=15)
B (n=16)
C (n=14)
surface

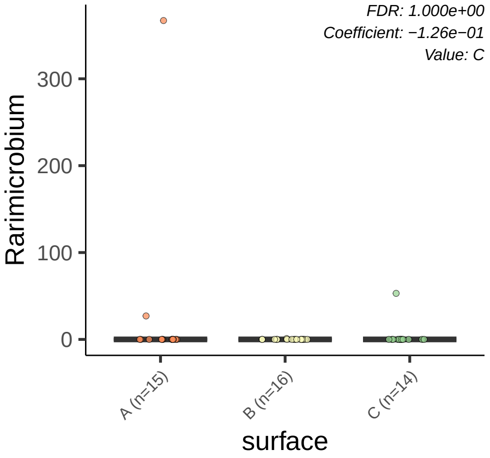
FDR: 1.000e+00
Coefficient: −1.26e−01
Value: C
Rarimicrobium
300
200
100
0
A (n=15)
B (n=16)
C (n=14)
surface

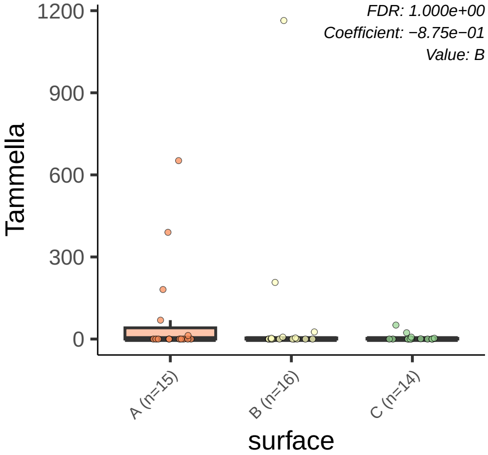

FDR: 1.000e+00
Coefficient: −8.75e−01
Value: B
Tammella
1200
900
600
300
0
A (n=15)
B (n=16)
C (n=14)
surface

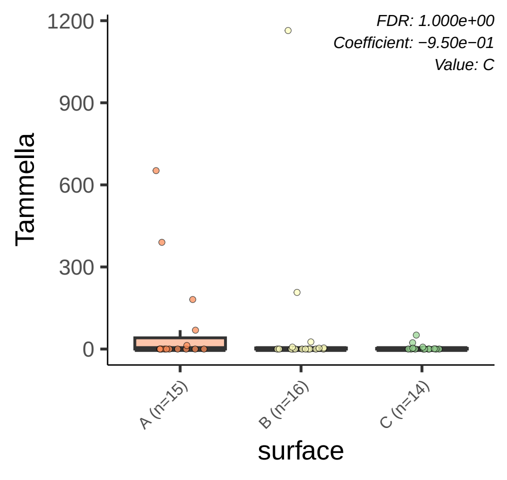

FDR: 1.000e+00
Coefficient: −9.50e−01
Value: C
Tammella
1200
900
600
300
0
A (n=15)
B (n=16)
C (n=14)
surface

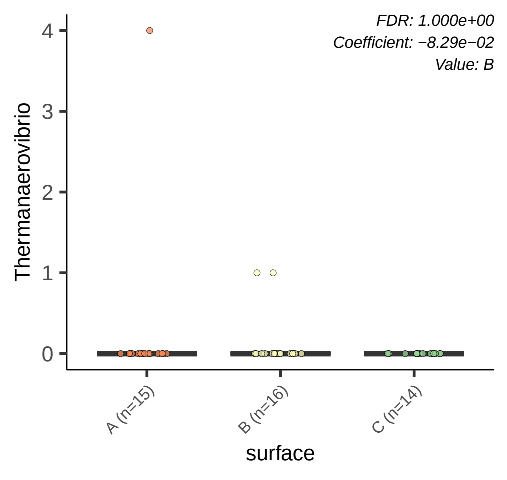

FDR: 1.000e+00
Coefficient: −8.29e−02
Value: B
Thermanaerovibrio
0
1
2
3
4
A (n=15)
B (n=16)
C (n=14)
surface

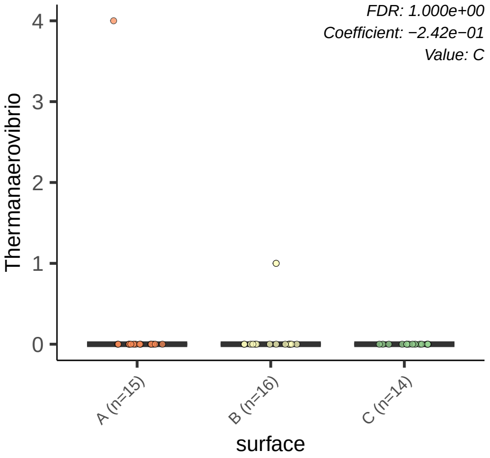
FDR: 1.000e+00
Coefficient: −2.42e−01
Value: C
Thermanaerovibrio
0
1
2
3
4
A (n=15)
B (n=16)
C (n=14)
surface

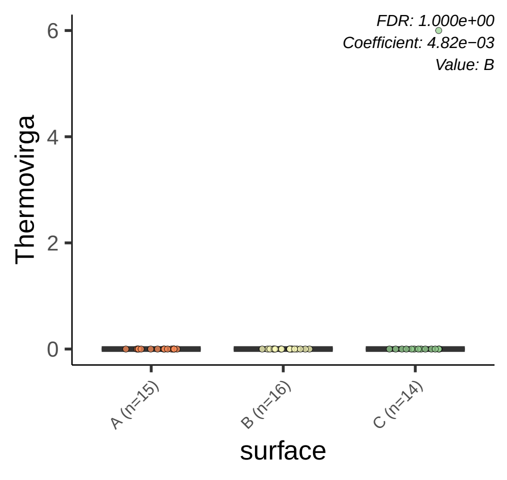
FDR: 1.000e+00
Coefficient: 4.82e−03
Value: B
Thermovirga
6
4
2
0
A (n=15)
B (n=16)
C (n=14)
surface

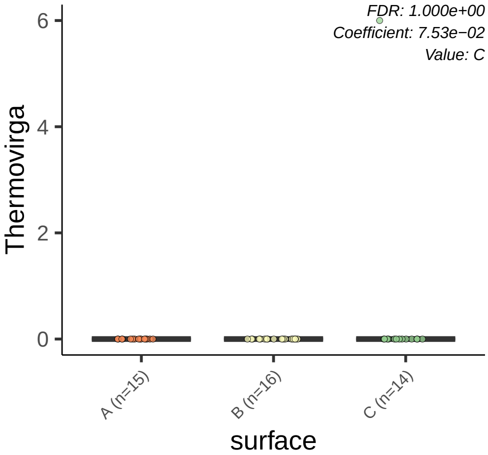
FDR: 1.000e+00
Coefficient: 7.53e−02
Value: C
Thermovirga
6
4
2
0
A (n=15)
B (n=16)
C (n=14)
surface

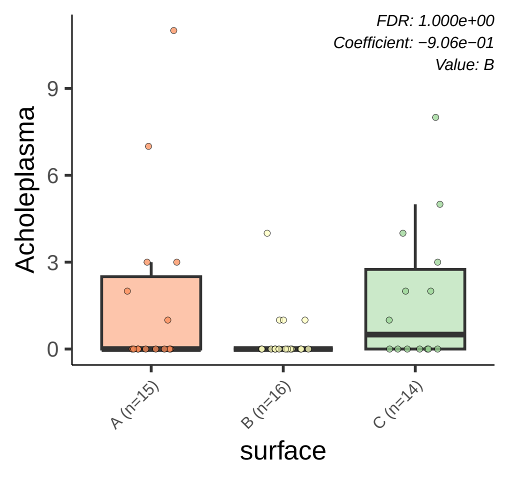
FDR: 1.000e+00
Coefficient: −9.06e−01
Value: B
Acholeplasma
9
6
3
0
A (n=15)
B (n=16)
C (n=14)
surface

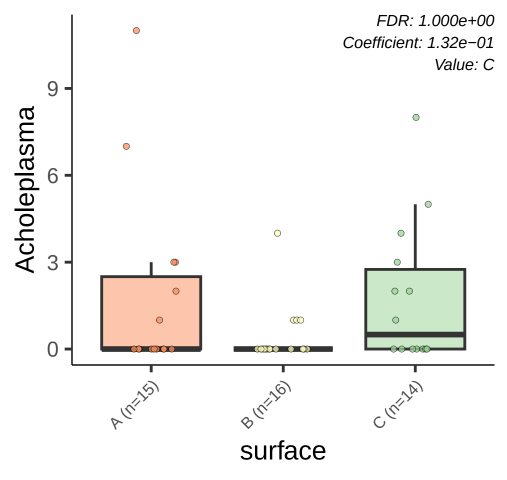

FDR: 1.000e+00
Coefficient: 1.32e−01
Value: C
Acholeplasma
9
6
3
0
A (n=15)
B (n=16)
C (n=14)
surface

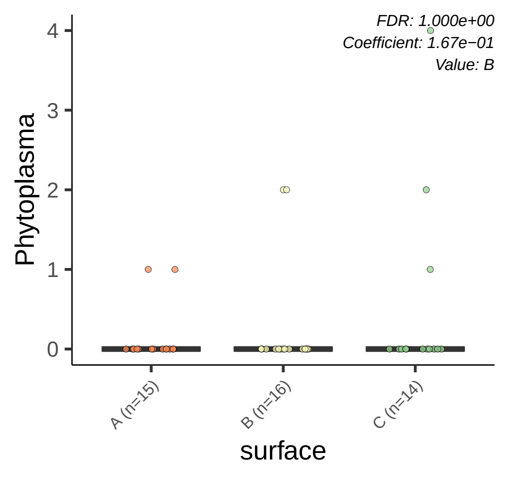

FDR: 1.000e+00
Coefficient: 1.67e−01
Value: B
Phytoplasma
4
3
2
1
0
A (n=15)
B (n=16)
C (n=14)
surface

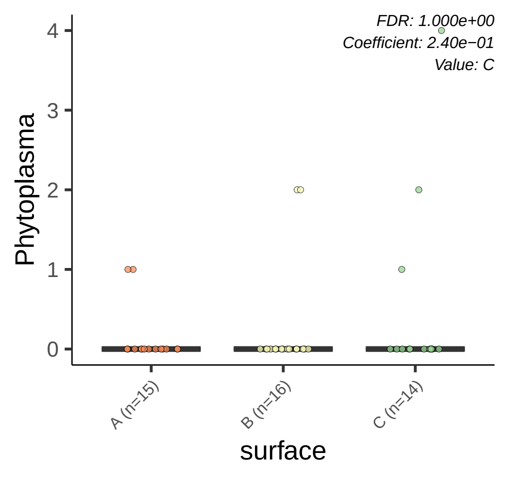
FDR: 1.000e+00
Coefficient: 2.40e−01
Value: C
Phytoplasma
4
3
2
1
0
A (n=15)
B (n=16)
C (n=14)
surface

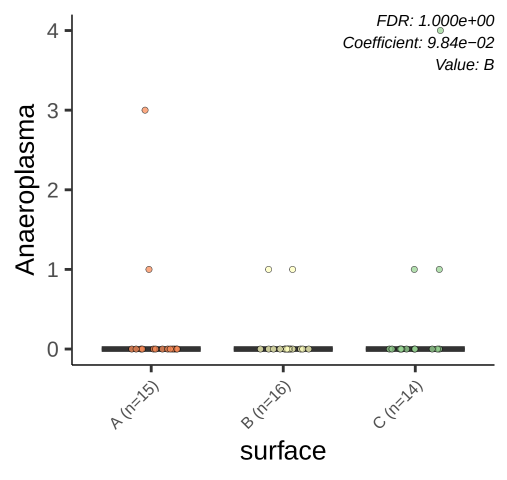
FDR: 1.000e+00
Coefficient: 9.84e−02
Value: B
Anaeroplasma
0
1
2
3
4
A (n=15)
B (n=16)
C (n=14)
surface

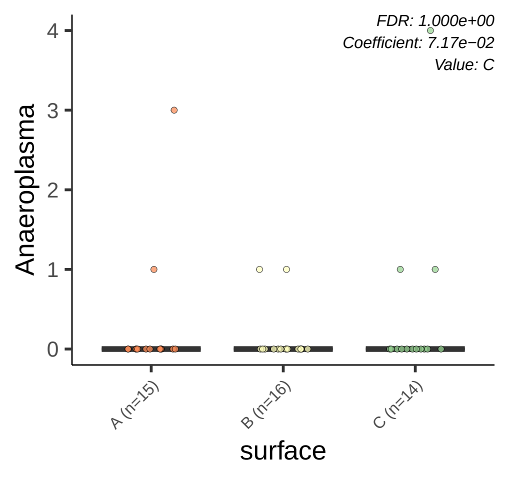

FDR: 1.000e+00
Coefficient: 7.17e−02
Value: C
Anaeroplasma
0
1
2
3
4
A (n=15)
B (n=16)
C (n=14)
surface

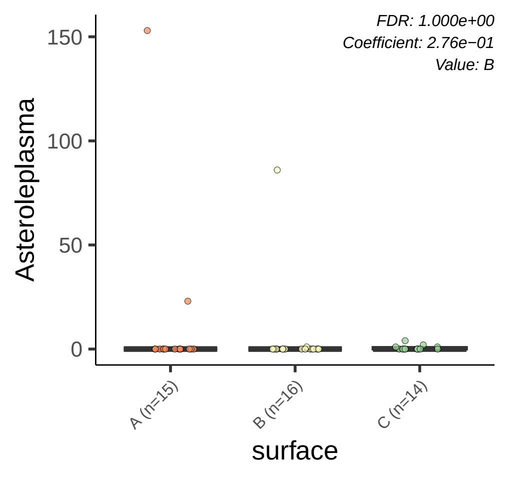

FDR: 1.000e+00
Coefficient: 2.76e−01
Value: B
Asteroleplasma
150
100
50
0
A (n=15)
B (n=16)
C (n=14)
surface

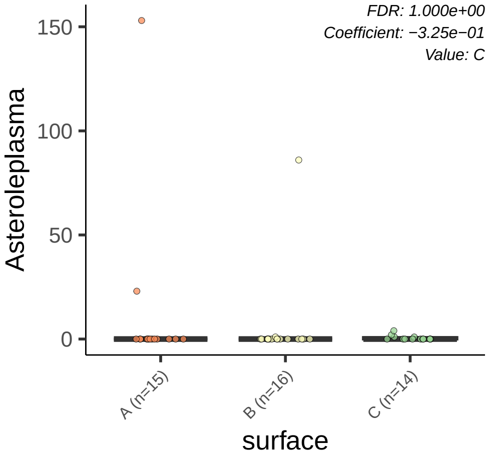

FDR: 1.000e+00
Coefficient: −3.25e−01
Value: C
Asteroleplasma
150
100
50
0
A (n=15)
B (n=16)
C (n=14)
surface

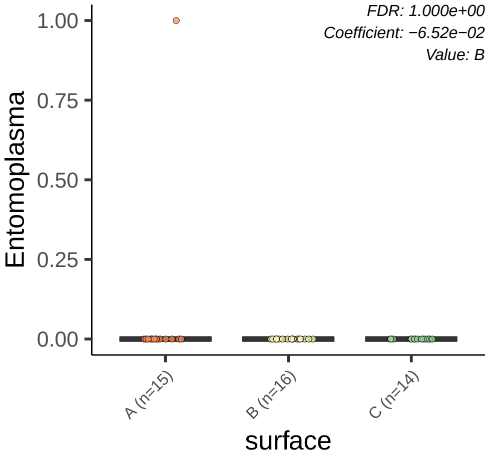

FDR: 1.000e+00
Coefficient: −6.52e−02
Value: B
Entomoplasma
1.00
0.75
0.50
0.25
0.00
A (n=15)
B (n=16)
C (n=14)
surface

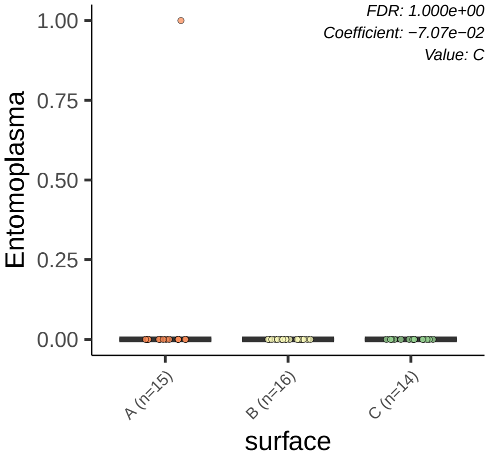
FDR: 1.000e+00
Coefficient: −7.07e−02
Value: C
Entomoplasma
1.00
0.75
0.50
0.25
0.00
A (n=15)
B (n=16)
C (n=14)
surface

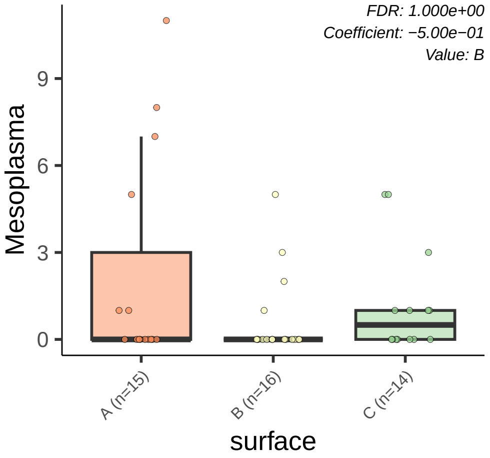
FDR: 1.000e+00
Coefficient: −5.00e−01
Value: B
Mesoplasma
9
6
3
0
A (n=15)
B (n=16)
C (n=14)
surface

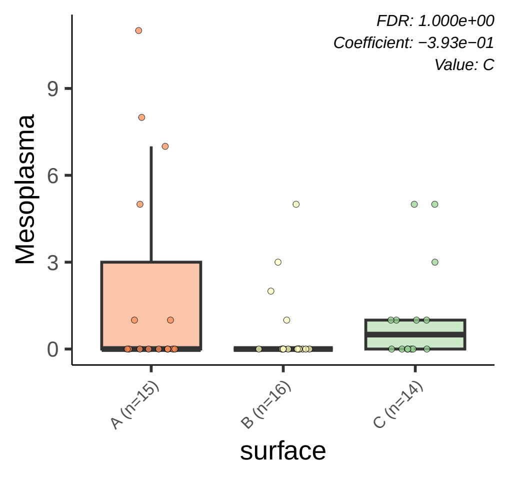
FDR: 1.000e+00
Coefficient: −3.93e−01
Value: C
Mesoplasma
0
3
6
9
A (n=15)
B (n=16)
C (n=14)
surface

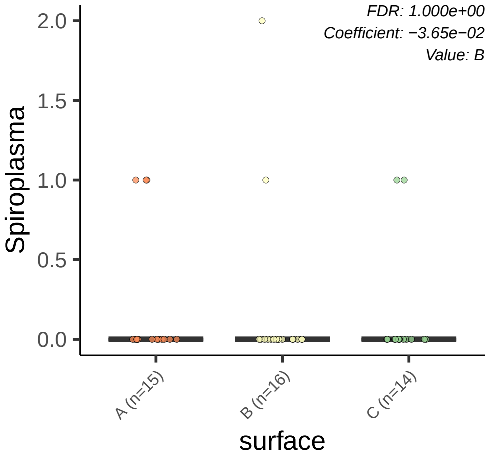
FDR: 1.000e+00
Coefficient: −3.65e−02
Value: B
Spiroplasma
2.0
1.5
1.0
0.5
0.0
A (n=15)
B (n=16)
C (n=14)
surface

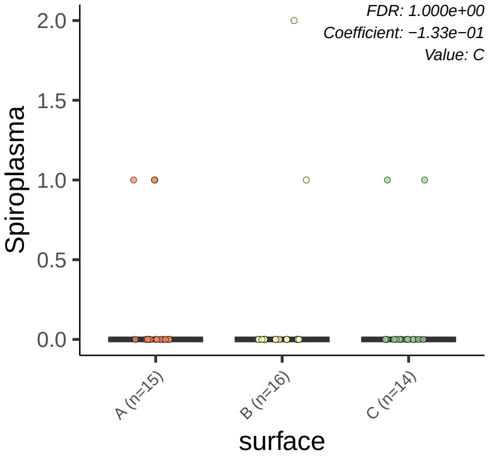
FDR: 1.000e+00
Coefficient: −1.33e−01
Value: C
Spiroplasma
2.0
1.5
1.0
0.5
0.0
A (n=15)
B (n=16)
C (n=14)
surface

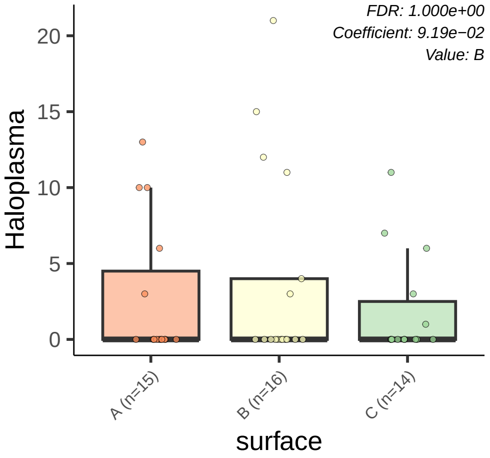
FDR: 1.000e+00
Coefficient: 9.19e−02
Value: B
Haloplasma
20
15
10
5
0
A (n=15)
B (n=16)
C (n=14)
surface

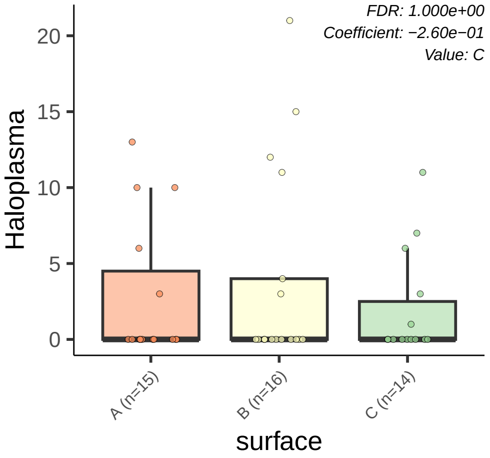
FDR: 1.000e+00
Coefficient: −2.60e−01
Value: C
Haloplasma
20
15
10
5
0
A (n=15)
B (n=16)
C (n=14)
surface

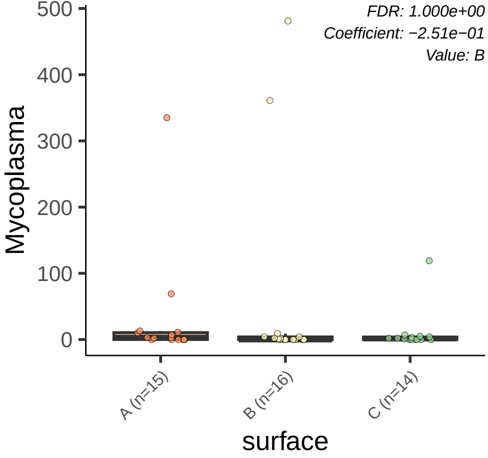

FDR: 1.000e+00
Coefficient: −2.51e−01
Value: B
Mycoplasma
500
400
300
200
100
0
A (n=15)
B (n=16)
C (n=14)
surface

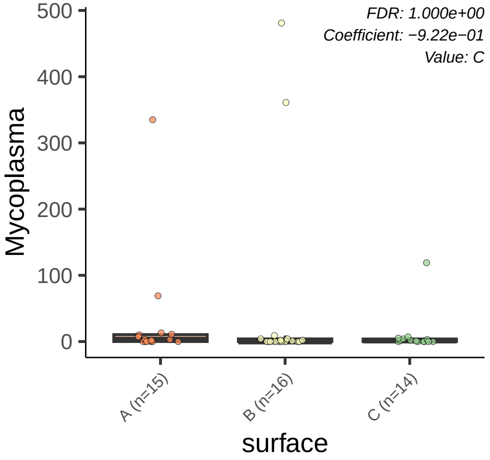
FDR: 1.000e+00
Coefficient: −9.22e−01
Value: C
Mycoplasma
500
400
300
200
100
0
A (n=15)
B (n=16)
C (n=14)
surface

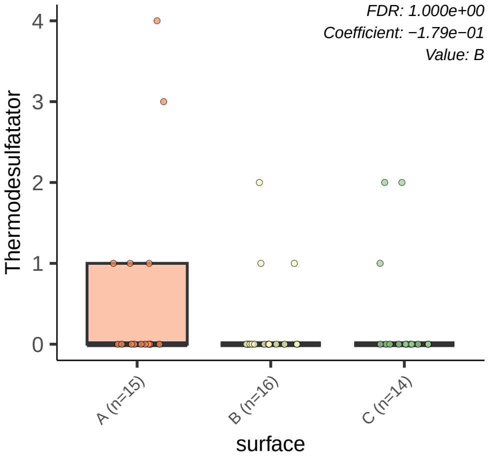

FDR: 1.000e+00
Coefficient: −1.79e−01
Value: B
Thermodesulfatator
0
1
2
3
4
A (n=15)
B (n=16)
C (n=14)
surface

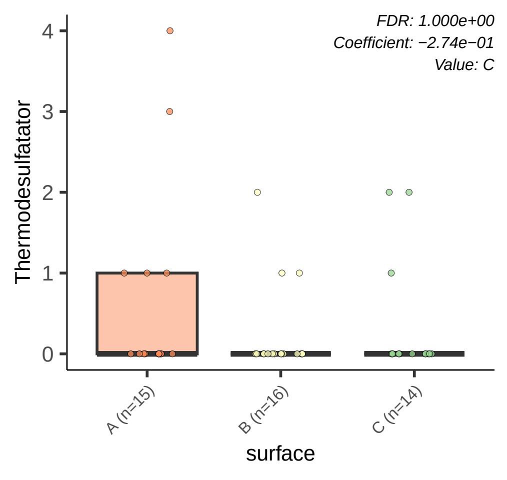

FDR: 1.000e+00
Coefficient: −2.74e−01
Value: C
Thermodesulfatator
4
3
2
1
0
A (n=15)
B (n=16)
C (n=14)
surface

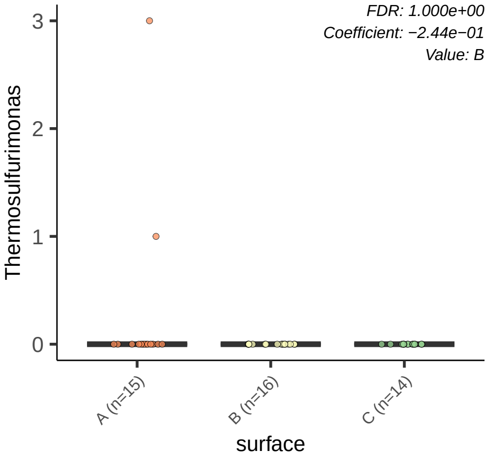
FDR: 1.000e+00
Coefficient: −2.44e−01
Value: B
Thermosulfurimonas
3
2
1
0
A (n=15)
B (n=16)
C (n=14)
surface

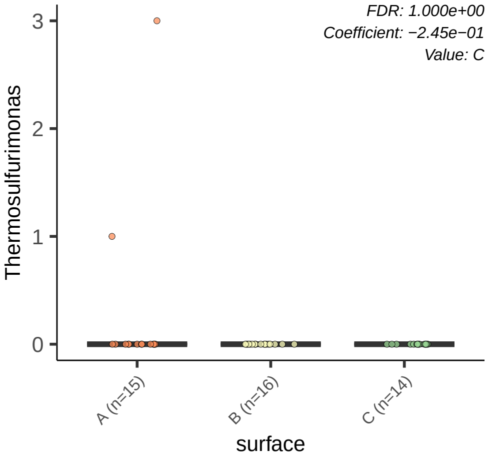
FDR: 1.000e+00
Coefficient: −2.45e−01
Value: C
Thermosulfurimonas
3
2
1
0
A (n=15)
B (n=16)
C (n=14)
surface

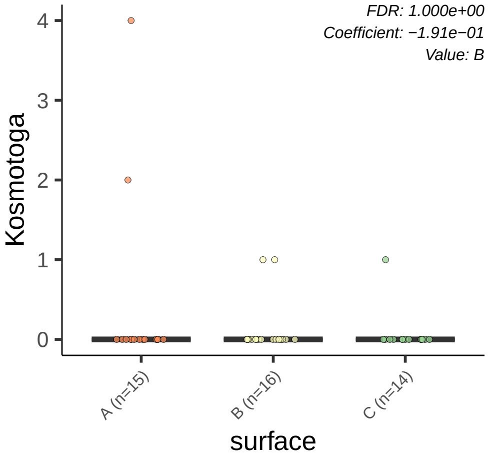
FDR: 1.000e+00
Coefficient: −1.91e−01
Value: B
Kosmotoga
0
1
2
3
4
A (n=15)
B (n=16)
C (n=14)
surface

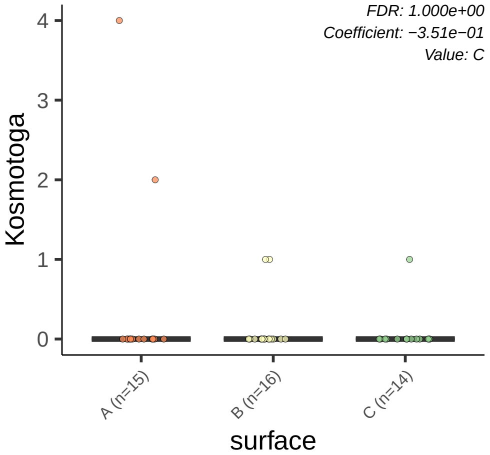
FDR: 1.000e+00
Coefficient: −3.51e−01
Value: C
Kosmotoga
4
3
2
1
0
A (n=15)
B (n=16)
C (n=14)
surface

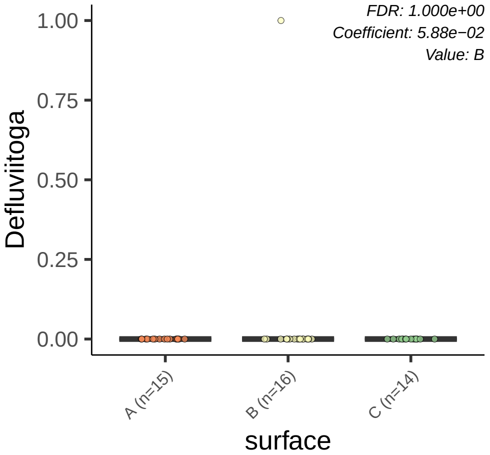
FDR: 1.000e+00
Coefficient: 5.88e−02
Value: B
Defluviitoga
1.00
0.75
0.50
0.25
0.00
A (n=15)
B (n=16)
C (n=14)
surface

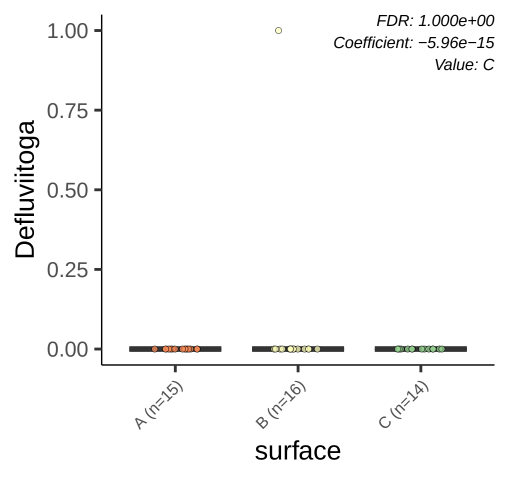
FDR: 1.000e+00
Coefficient: −5.96e−15
Value: C
Defluviitoga
1.00
0.75
0.50
0.25
0.00
A (n=15)
B (n=16)
C (n=14)
surface

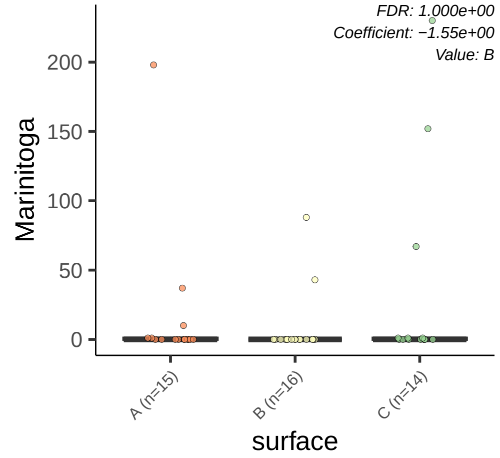

FDR: 1.000e+00
Coefficient: −1.55e+00
Value: B
Marinitoga
200
150
100
50
0
A (n=15)
B (n=16)
C (n=14)
surface

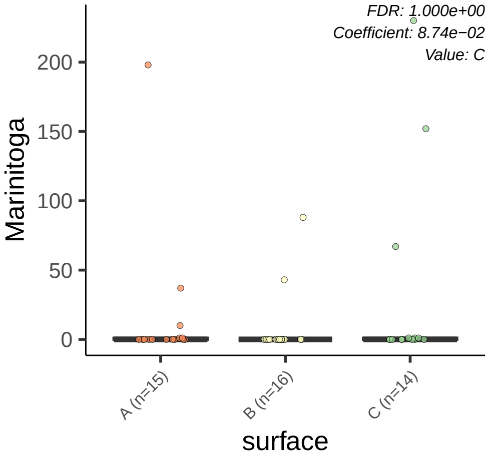

FDR: 1.000e+00
Coefficient: 8.74e−02
Value: C
Marinitoga
200
150
100
50
0
A (n=15)
B (n=16)
C (n=14)
surface

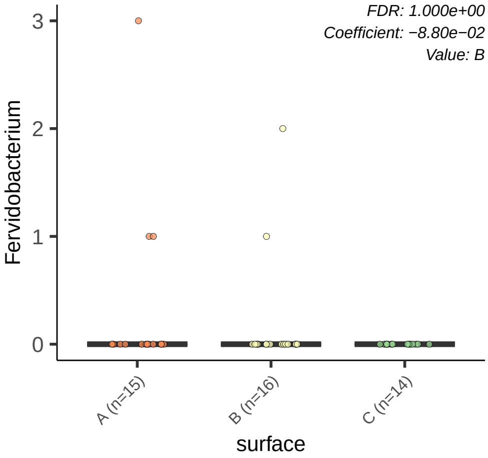
FDR: 1.000e+00
Coefficient: −8.80e−02
Value: B
Fervidobacterium
3
2
1
0
A (n=15)
B (n=16)
C (n=14)
surface

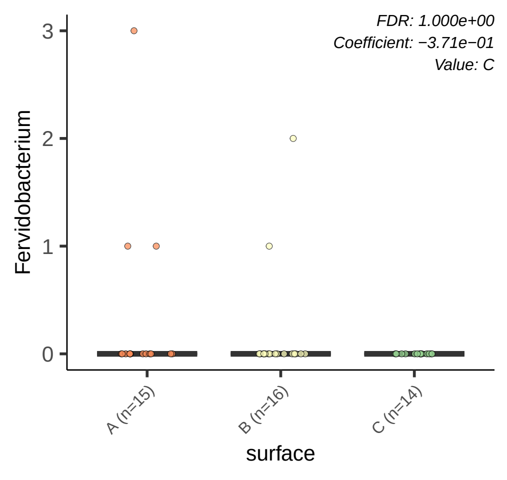

FDR: 1.000e+00
Coefficient: −3.71e−01
Value: C
Fervidobacterium
3
2
1
0
A (n=15)
B (n=16)
C (n=14)
surface

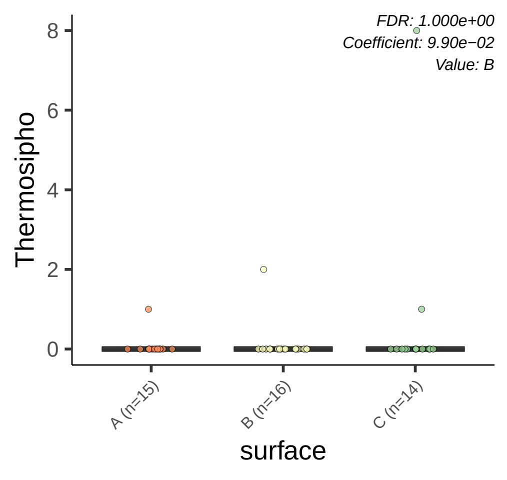

FDR: 1.000e+00
Coefficient: 9.90e−02
Value: B
Thermosipho
0
2
4
6
8
A (n=15)
B (n=16)
C (n=14)
surface

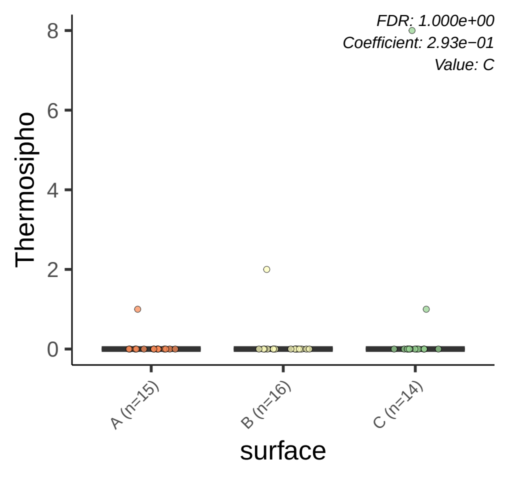
FDR: 1.000e+00
Coefficient: 2.93e−01
Value: C
Thermosipho
0
2
4
6
8
A (n=15)
B (n=16)
C (n=14)
surface

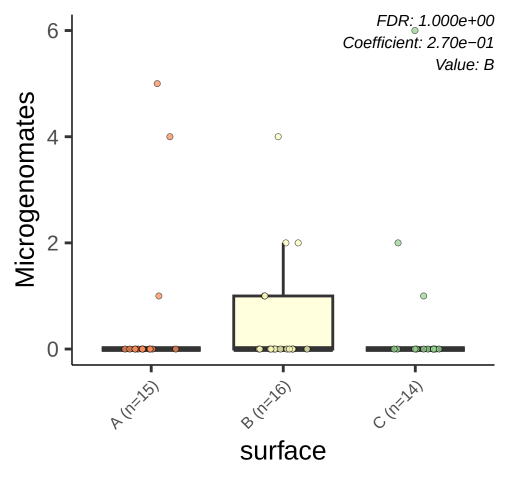

FDR: 1.000e+00
Coefficient: 2.70e−01
Value: B
Microgenomates
0
2
4
6
A (n=15)
B (n=16)
C (n=14)
surface

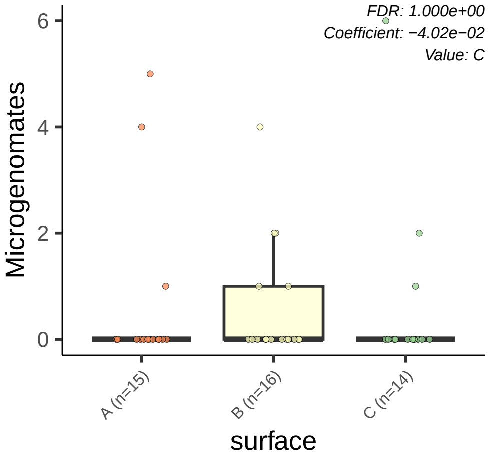
FDR: 1.000e+00
Coefficient: −4.02e−02
Value: C
Microgenomates
0
2
4
6
A (n=15)
B (n=16)
C (n=14)
surface

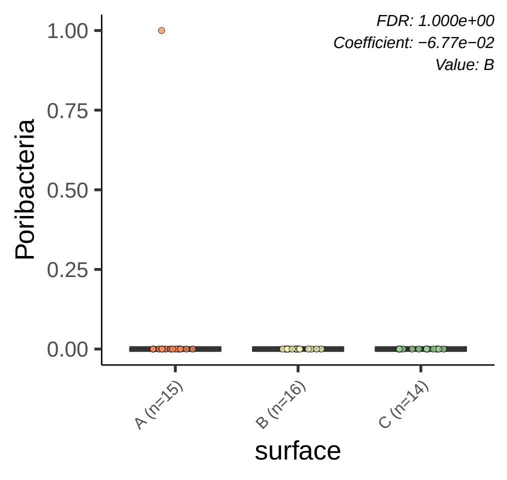

FDR: 1.000e+00
Coefficient: −6.77e−02
Value: B
Poribacteria
1.00
0.75
0.50
0.25
0.00
A (n=15)
B (n=16)
C (n=14)
surface

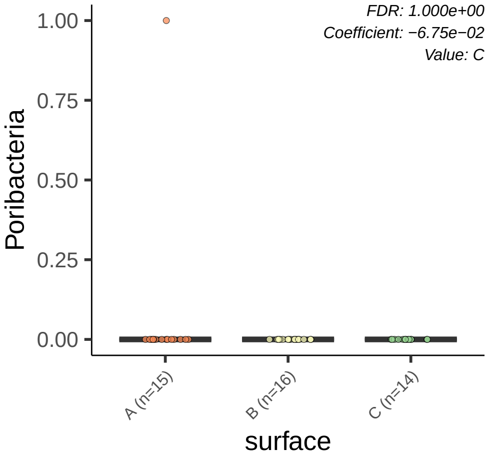

FDR: 1.000e+00
Coefficient: −6.75e−02
Value: C
Poribacteria
1.00
0.75
0.50
0.25
0.00
A (n=15)
B (n=16)
C (n=14)
surface

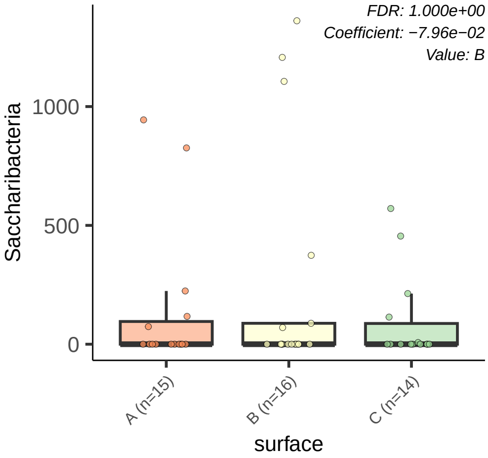
FDR: 1.000e+00
Coefficient: −7.96e−02
Value: B
Saccharibacteria
1000
500
0
A (n=15)
B (n=16)
C (n=14)
surface

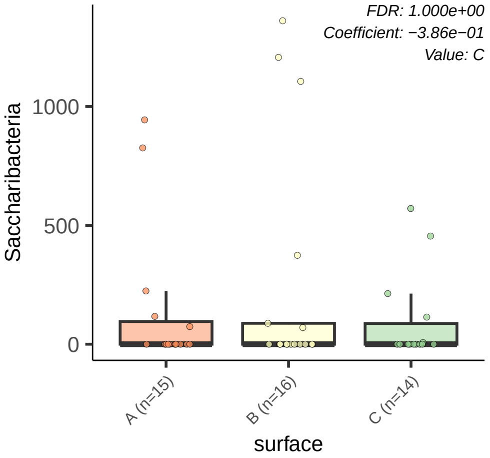
FDR: 1.000e+00
Coefficient: −3.86e−01
Value: C
Saccharibacteria
1000
500
0
A (n=15)
B (n=16)
C (n=14)
surface

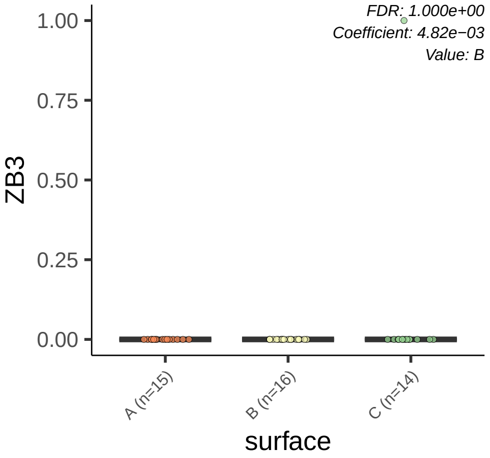
FDR: 1.000e+00
Coefficient: 4.82e−03
Value: B
ZB3
1.00
0.75
0.50
0.25
0.00
A (n=15)
B (n=16)
C (n=14)
surface

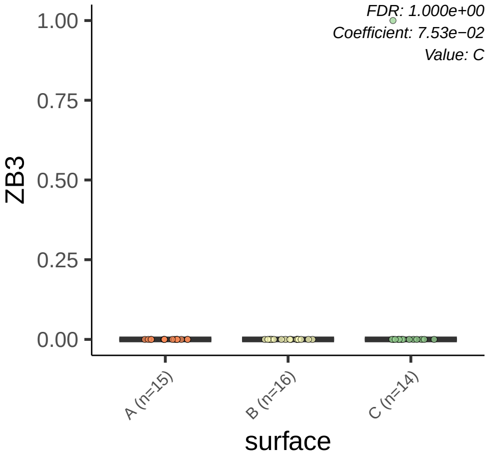

FDR: 1.000e+00
Coefficient: 7.53e−02
Value: C
1.00
0.75
0.50
0.25
0.00
ZB3
A (n=15)
B (n=16)
C (n=14)
surface

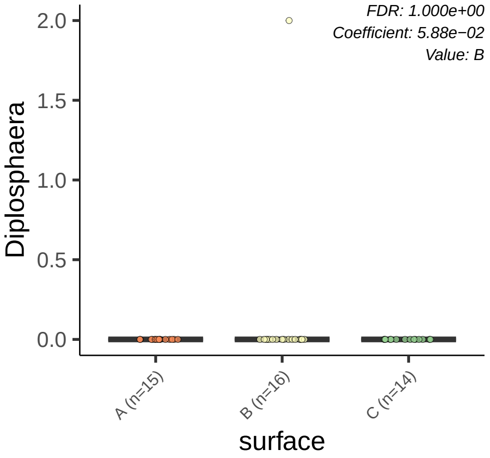
FDR: 1.000e+00
Coefficient: 5.88e−02
Value: B
Diplosphaera
2.0
1.5
1.0
0.5
0.0
A (n=15)
B (n=16)
C (n=14)
surface

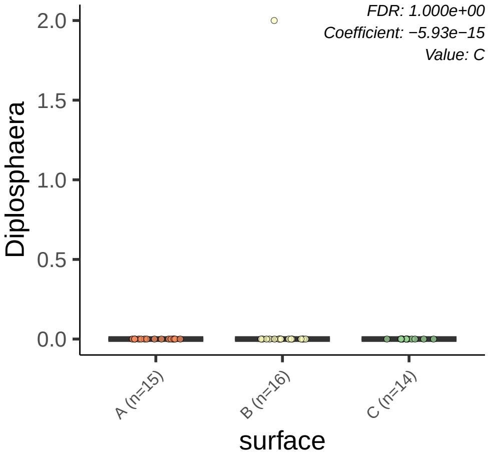
FDR: 1.000e+00
Coefficient: −5.93e−15
Value: C
Diplosphaera
2.0
1.5
1.0
0.5
0.0
A (n=15)
B (n=16)
C (n=14)
surface

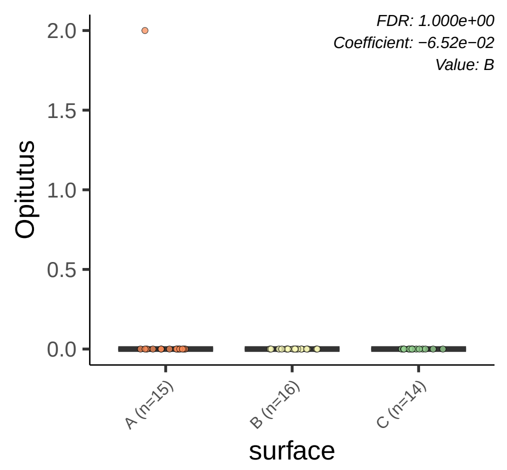
FDR: 1.000e+00
Coefficient: −6.52e−02
Value: B
Opitutus
2.0
1.5
1.0
0.5
0.0
A (n=15)
B (n=16)
C (n=14)
surface

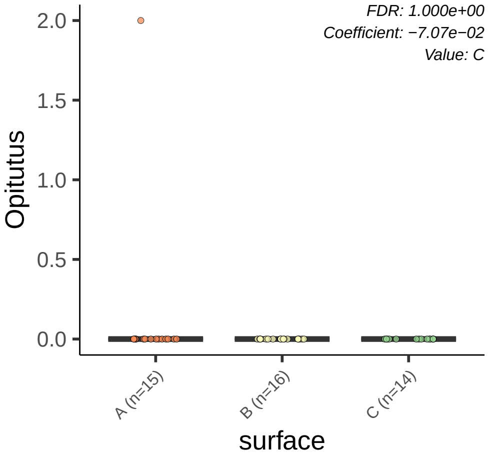
FDR: 1.000e+00
Coefficient: −7.07e−02
Value: C
Opitutus
2.0
1.5
1.0
0.5
0.0
A (n=15)
B (n=16)
C (n=14)
surface

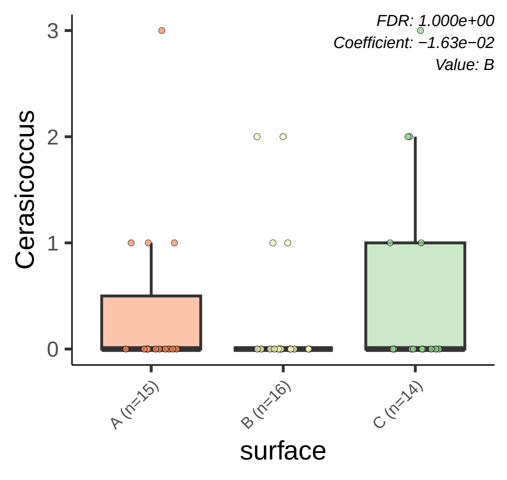
FDR: 1.000e+00
Coefficient: −1.63e−02
Value: B
Cerasicoccus
3
2
1
0
A (n=15)
B (n=16)
C (n=14)
surface

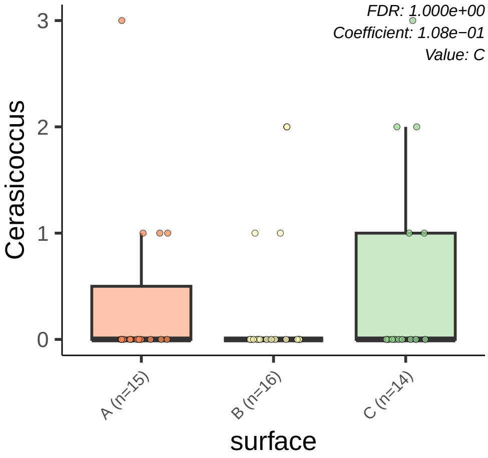
FDR: 1.000e+00
Coefficient: 1.08e−01
Value: C
Cerasicoccus
3
2
1
0
A (n=15)
B (n=16)
C (n=14)
surface

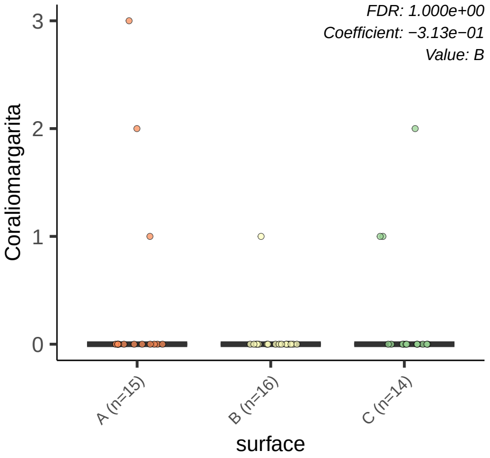

FDR: 1.000e+00
Coefficient: −3.13e−01
Value: B
Coraliomargarita
3
2
1
0
A (n=15)
B (n=16)
C (n=14)
surface

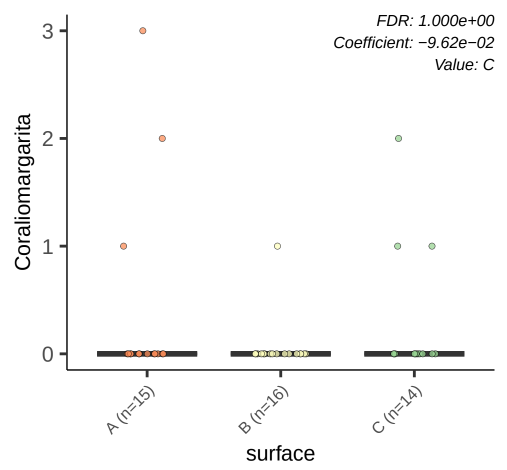

FDR: 1.000e+00
Coefficient: −9.62e−02
Value: C
Coraliomargarita
3
2
1
0
A (n=15)
B (n=16)
C (n=14)
surface

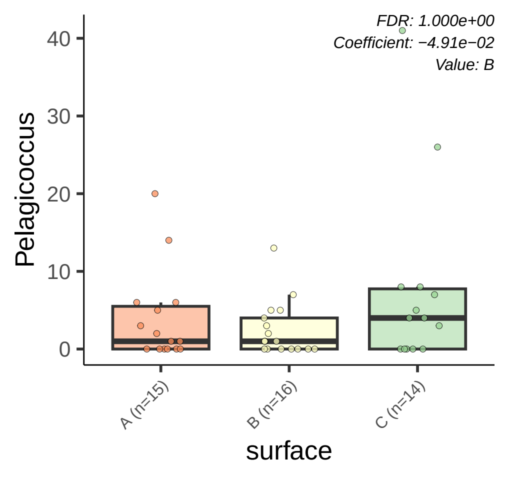
FDR: 1.000e+00
Coefficient: −4.91e−02
Value: B
Pelagicoccus
40
30
20
10
0
A (n=15)
B (n=16)
C (n=14)
surface

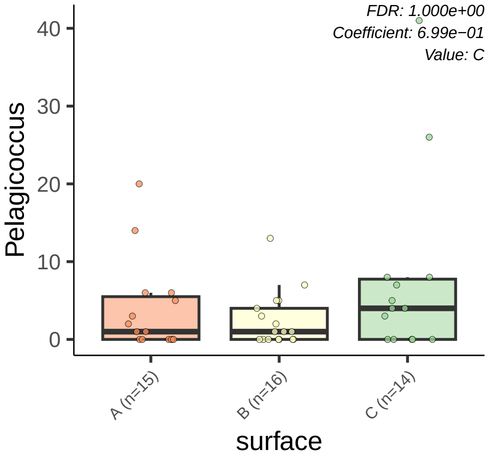

FDR: 1.000e+00
Coefficient: 6.99e−01
Value: C
Pelagicoccus
40
30
20
10
0
A (n=15)
B (n=16)
C (n=14)
surface

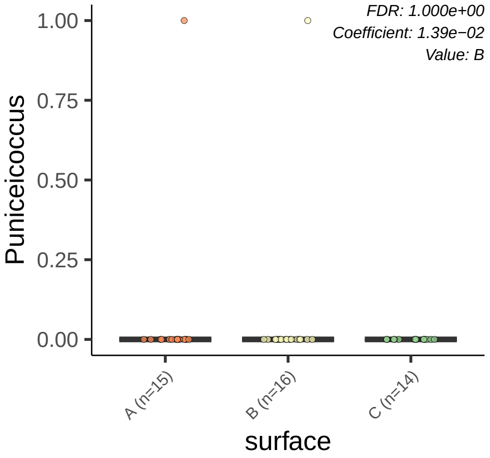
FDR: 1.000e+00
Coefficient: 1.39e−02
Value: B
Puniceicoccus
1.00
0.75
0.50
0.25
0.00
A (n=15)
B (n=16)
C (n=14)
surface

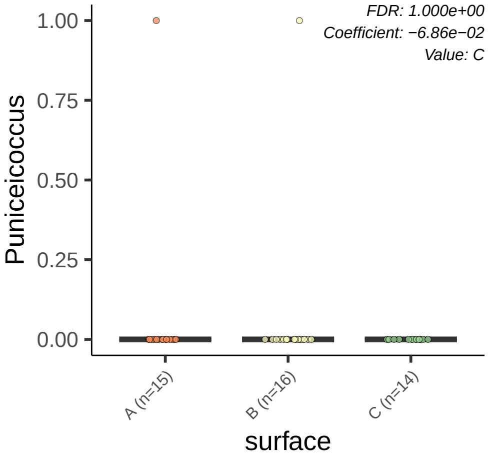

FDR: 1.000e+00
Coefficient: −6.86e−02
Value: C
Puniceicoccus
1.00
0.75
0.50
0.25
0.00
A (n=15)
B (n=16)
C (n=14)
surface

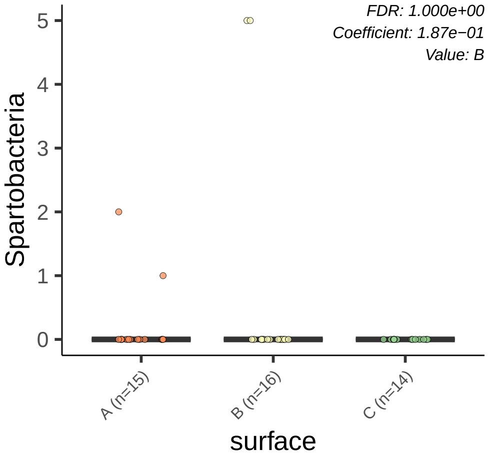

FDR: 1.000e+00
Coefficient: 1.87e−01
Value: B
Spartobacteria
0
1
2
3
4
5
A (n=15)
B (n=16)
C (n=14)
surface

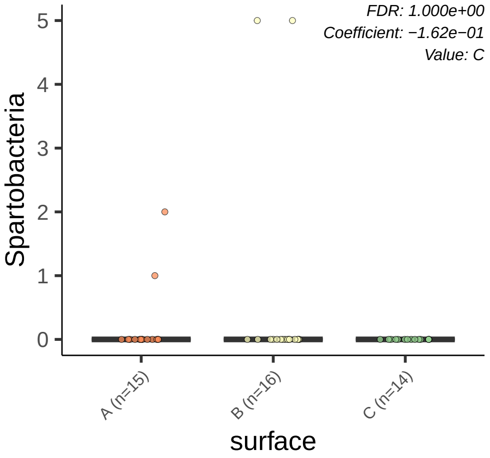
FDR: 1.000e+00
Coefficient: −1.62e−01
Value: C
Spartobacteria
0
1
2
3
4
5
A (n=15)
B (n=16)
C (n=14)
surface

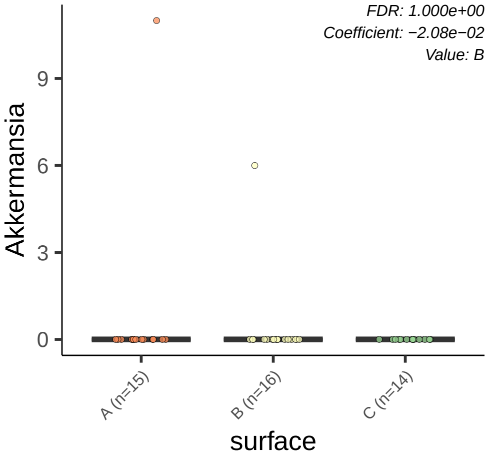
FDR: 1.000e+00
Coefficient: −2.08e−02
Value: B
Akkermansia
0
3
6
9
A (n=15)
B (n=16)
C (n=14)
surface

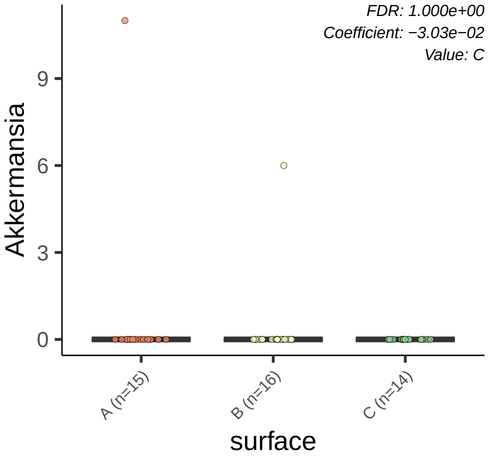

FDR: 1.000e+00
Coefficient: −3.03e−02
Value: C
Akkermansia
9
6
3
0
A (n=15)
B (n=16)
C (n=14)
surface

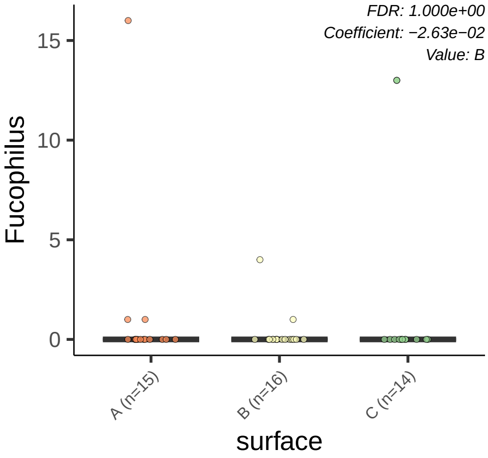
FDR: 1.000e+00
Coefficient: −2.63e−02
Value: B
Fucophilus
0
5
10
15
A (n=15)
B (n=16)
C (n=14)
surface

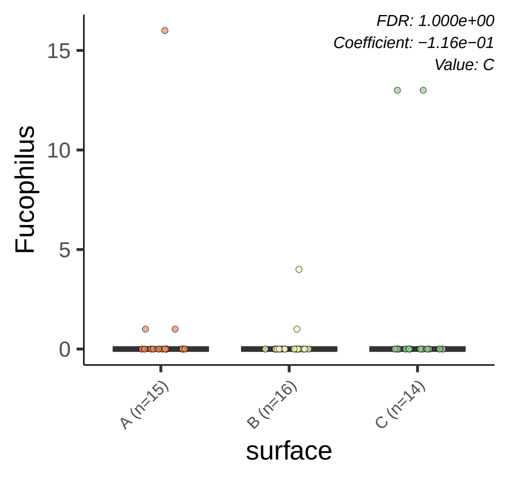
FDR: 1.000e+00
Coefficient: −1.16e−01
Value: C
Fucophilus
0
5
10
15
A (n=15)
B (n=16)
C (n=14)
surface

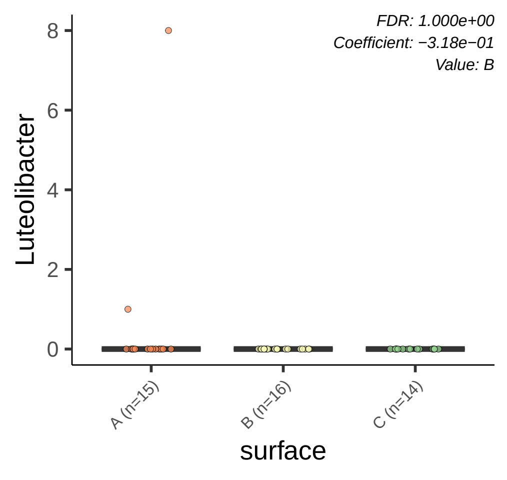
FDR: 1.000e+00
Coefficient: −3.18e−01
Value: B
Luteolibacter
8
6
4
2
0
A (n=15)
B (n=16)
C (n=14)
surface

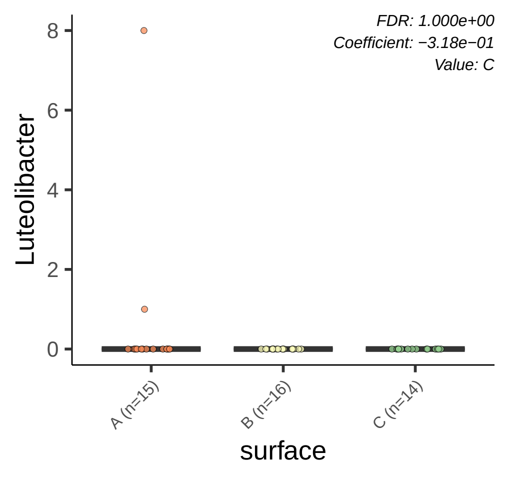
FDR: 1.000e+00
Coefficient: −3.18e−01
Value: C
Luteolibacter
8
6
4
2
0
A (n=15)
B (n=16)
C (n=14)
surface

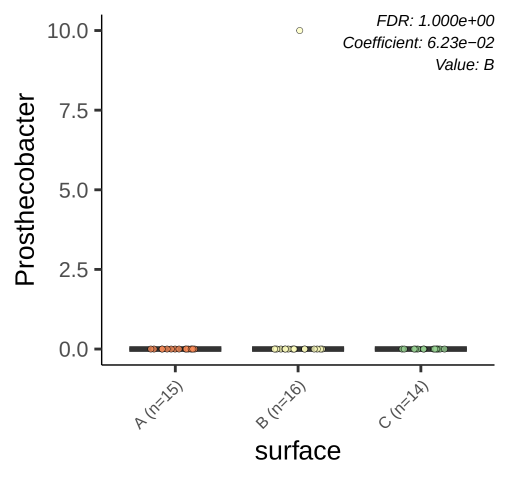
FDR: 1.000e+00
Coefficient: 6.23e−02
Value: B
Prosthecobacter
10.0
7.5
5.0
2.5
0.0
A (n=15)
B (n=16)
C (n=14)
surface

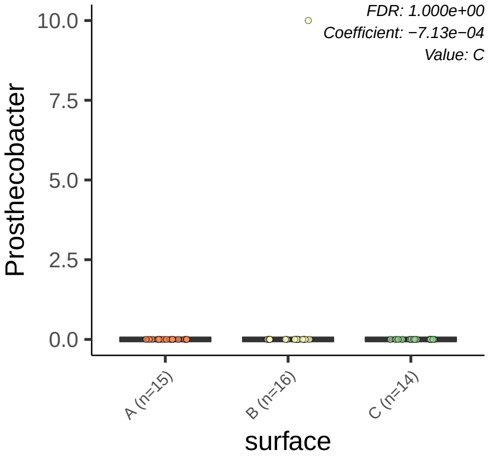

FDR: 1.000e+00
Coefficient: −7.13e−04
Value: C
Prosthecobacter
10.0
7.5
5.0
2.5
0.0
A (n=15)
B (n=16)
C (n=14)
surface

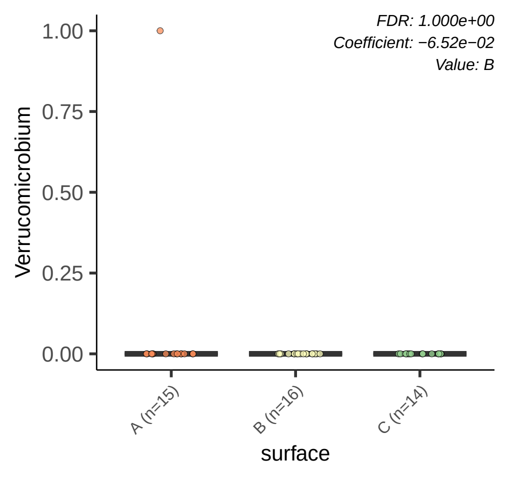
FDR: 1.000e+00
Coefficient: −6.52e−02
Value: B
Verrucomicrobium
1.00
0.75
0.50
0.25
0.00
A (n=15)
B (n=16)
C (n=14)
surface

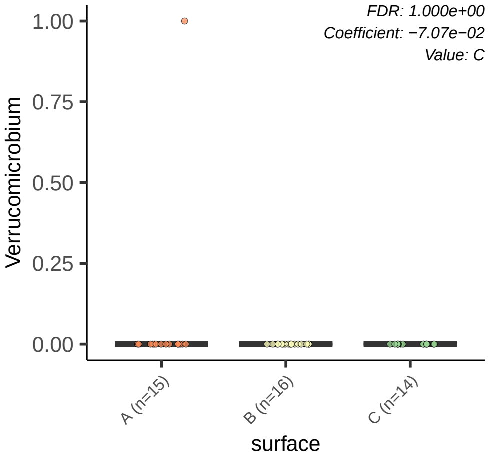
FDR: 1.000e+00
Coefficient: −7.07e−02
Value: C
Verrucomicrobium
1.00
0.75
0.50
0.25
0.00
A (n=15)
B (n=16)
C (n=14)
surface

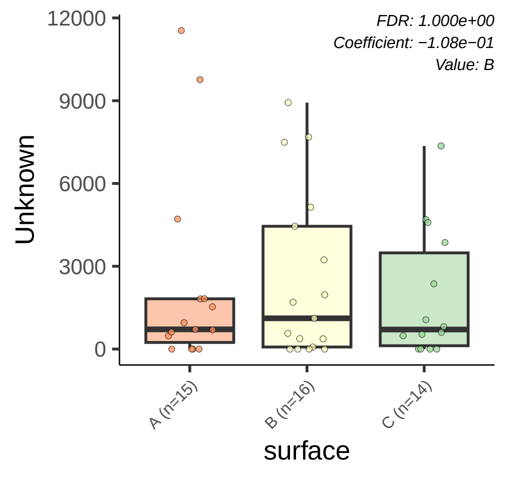

FDR: 1.000e+00
Coefficient: −1.08e−01
Value: B
12000
9000
6000
3000
0
Unknown
A (n=15)
B (n=16)
C (n=14)
surface

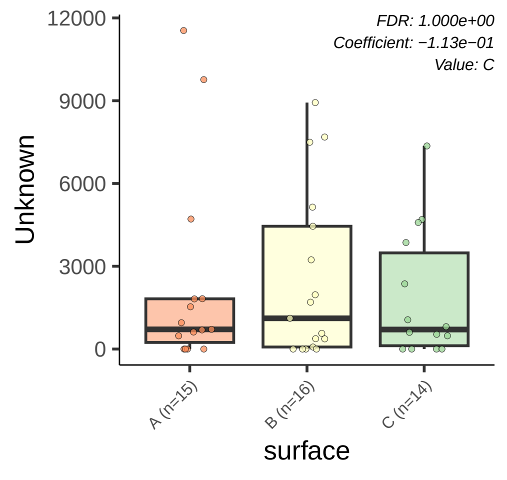

FDR: 1.000e+00
Coefficient: −1.13e−01
Value: C
12000
9000
6000
3000
0
Unknown
A (n=15)
B (n=16)
C (n=14)
surface

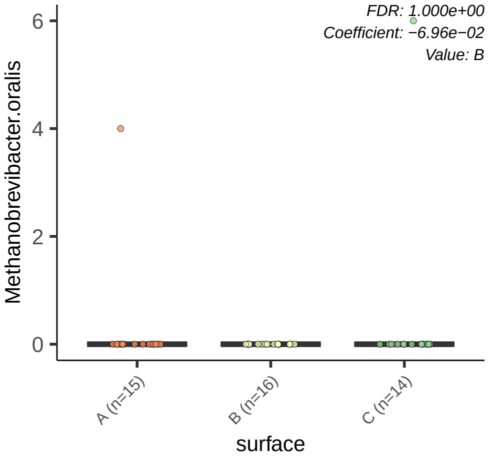

FDR: 1.000e+00
Coefficient: −6.96e−02
Value: B
Methanobrevibacter.oralis
0
2
4
6
A (n=15)
B (n=16)
C (n=14)
surface

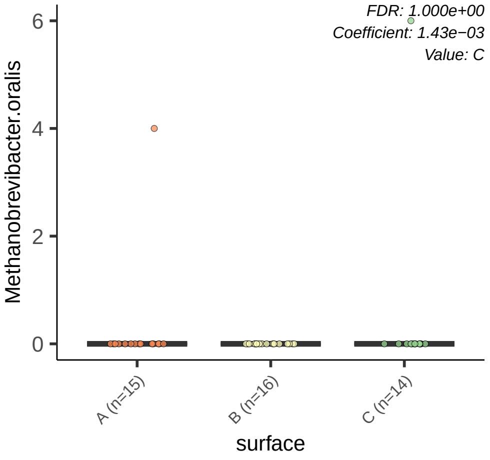

FDR: 1.000e+00
Coefficient: 1.43e−03
Value: C
Methanobrevibacter.oralis
0
2
4
6
A (n=15)
B (n=16)
C (n=14)
surface

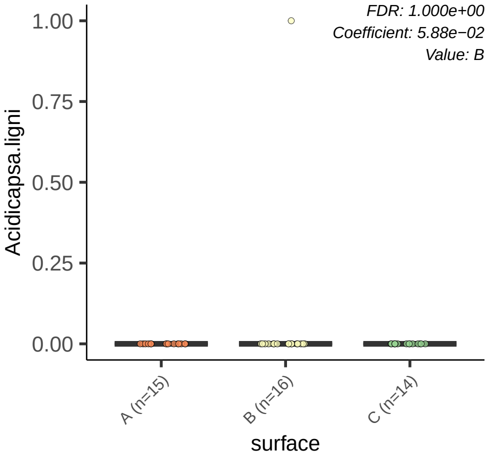
FDR: 1.000e+00
Coefficient: 5.88e−02
Value: B
Acidicapsa.ligni
1.00
0.75
0.50
0.25
0.00
A (n=15)
B (n=16)
C (n=14)
surface

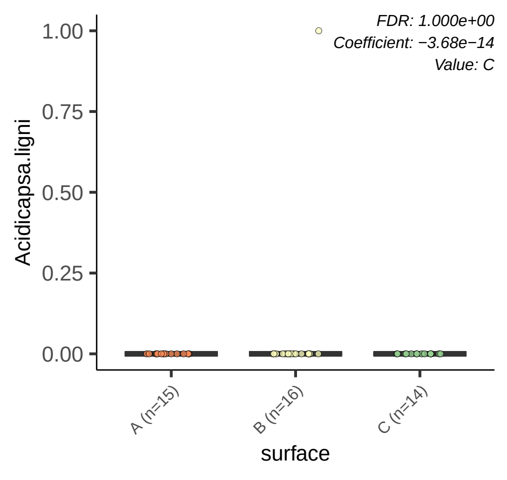
FDR: 1.000e+00
Coefficient: −3.68e−14
Value: C
Acidicapsa.ligni
1.00
0.75
0.50
0.25
0.00
A (n=15)
B (n=16)
C (n=14)
surface

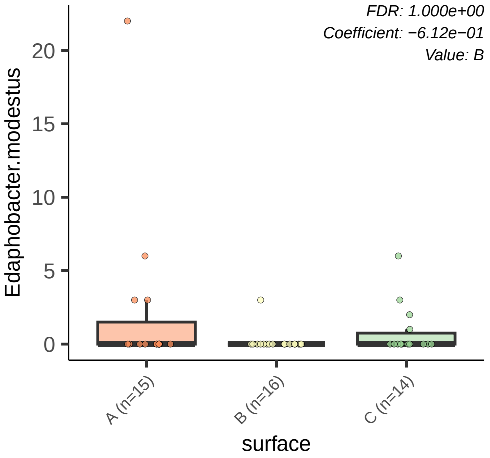

FDR: 1.000e+00
Coefficient: −6.12e−01
Value: B
Edaphobacter.modestus
0
5
10
15
20
A (n=15)
B (n=16)
C (n=14)
surface

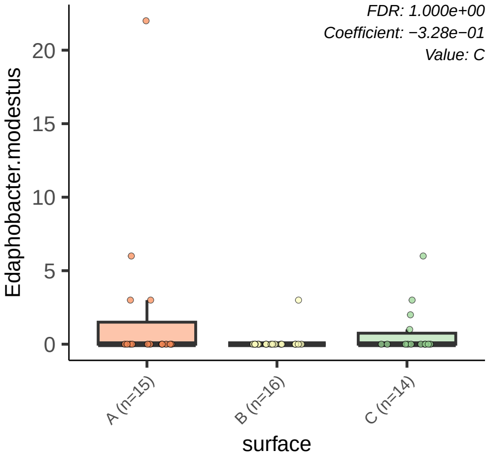
FDR: 1.000e+00
Coefficient: −3.28e−01
Value: C
Edaphobacter.modestus
0
5
10
15
20
A (n=15)
B (n=16)
C (n=14)
surface

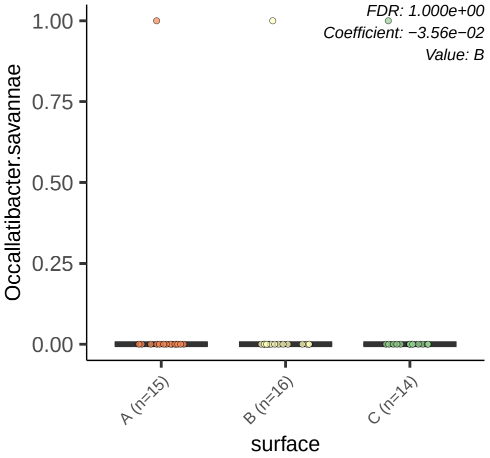

FDR: 1.000e+00
Coefficient: −3.56e−02
Value: B
Occallatibacter.savannae
1.00
0.75
0.50
0.25
0.00
A (n=15)
B (n=16)
C (n=14)
surface

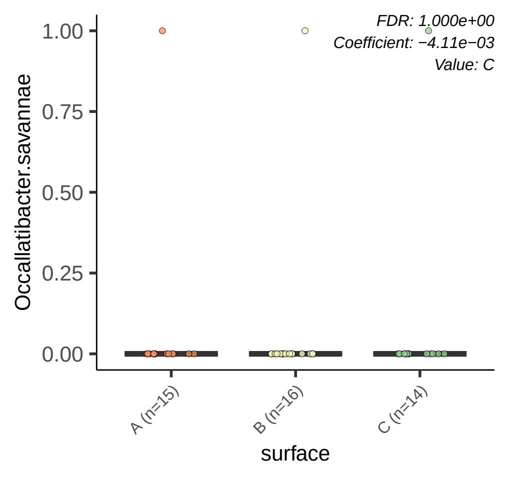
FDR: 1.000e+00
Coefficient: −4.11e−03
Value: C
Occallatibacter.savannae
1.00
0.75
0.50
0.25
0.00
A (n=15)
B (n=16)
C (n=14)
surface

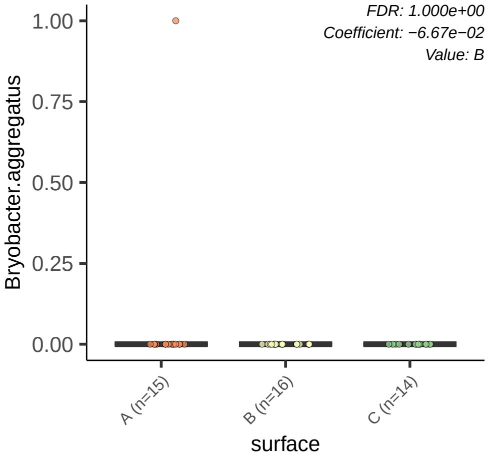
FDR: 1.000e+00
Coefficient: −6.67e−02
Value: B
Bryobacter.aggregatus
1.00
0.75
0.50
0.25
0.00
A (n=15)
B (n=16)
C (n=14)
surface

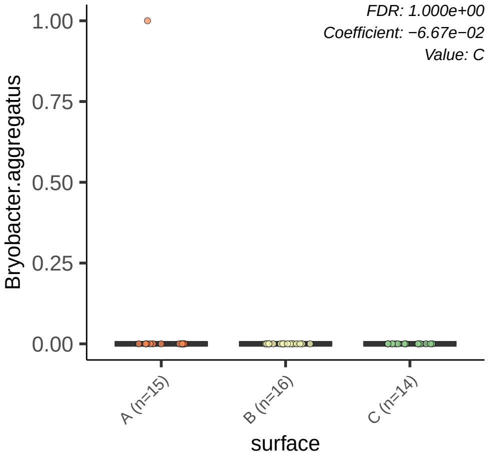
FDR: 1.000e+00
Coefficient: −6.67e−02
Value: C
Bryobacter.aggregatus
1.00
0.75
0.50
0.25
0.00
A (n=15)
B (n=16)
C (n=14)
surface

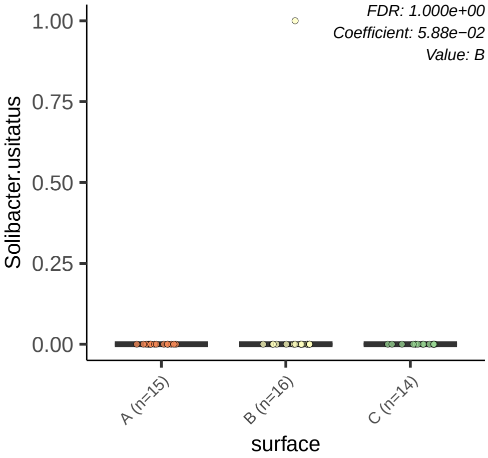
FDR: 1.000e+00
Coefficient: 5.88e−02
Value: B
Solibacter.usitatus
1.00
0.75
0.50
0.25
0.00
A (n=15)
B (n=16)
C (n=14)
surface

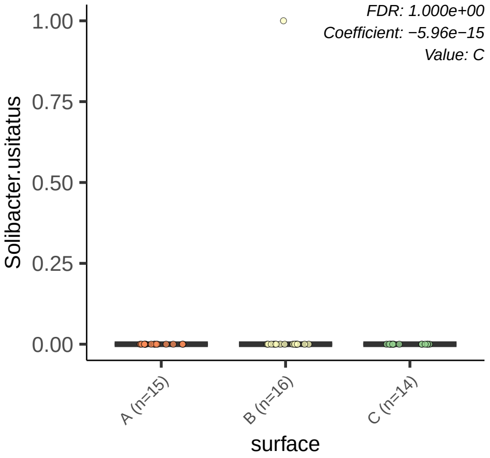

FDR: 1.000e+00
Coefficient: −5.96e−15
Value: C
1.00
0.75
0.50
0.25
0.00
Solibacter.usitatus
A (n=15)
B (n=16)
C (n=14)
surface

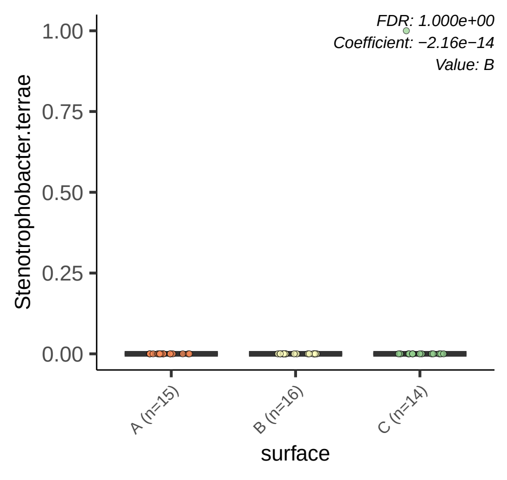
FDR: 1.000e+00
Coefficient: −2.16e−14
Value: B
Stenotrophobacter.terrae
1.00
0.75
0.50
0.25
0.00
A (n=15)
B (n=16)
C (n=14)
surface

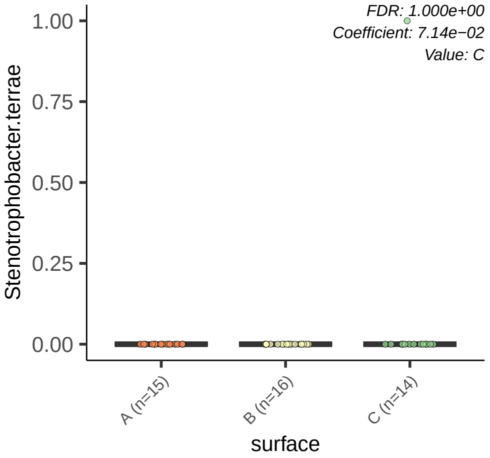

FDR: 1.000e+00
Coefficient: 7.14e−02
Value: C
Stenotrophobacter.terrae
1.00
0.75
0.50
0.25
0.00
A (n=15)
B (n=16)
C (n=14)
surface

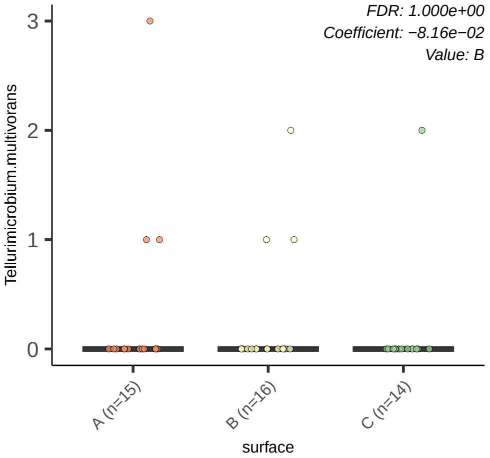

FDR: 1.000e+00
Coefficient: −8.16e−02
Value: B
Tellurimicrobium.multivorans
0
1
2
3
A (n=15)
B (n=16)
C (n=14)
surface

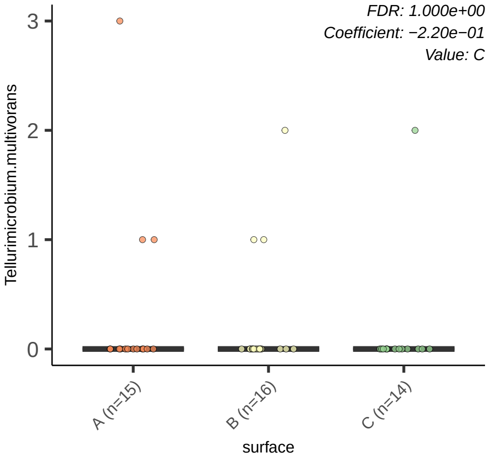
FDR: 1.000e+00
Coefficient: −2.20e−01
Value: C
Tellurimicrobium.multivorans
3
2
1
0
A (n=15)
B (n=16)
C (n=14)
surface

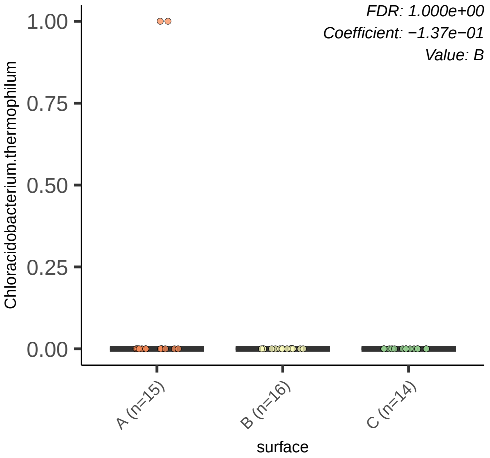
FDR: 1.000e+00
Coefficient: −1.37e−01
Value: B
Chloracidobacterium.thermophilum
1.00
0.75
0.50
0.25
0.00
A (n=15)
B (n=16)
C (n=14)
surface

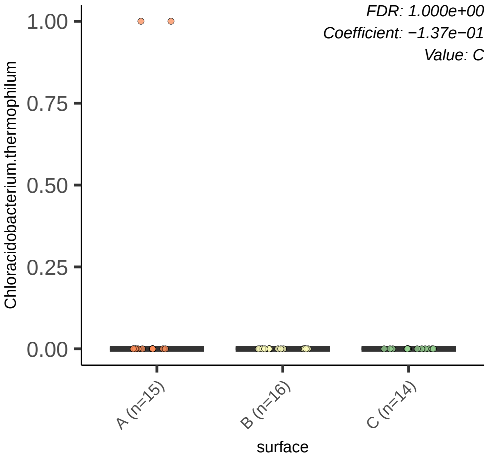

FDR: 1.000e+00
Coefficient: −1.37e−01
Value: C
Chloracidobacterium.thermophilum
1.00
0.75
0.50
0.25
0.00
A (n=15)
B (n=16)
C (n=14)
surface

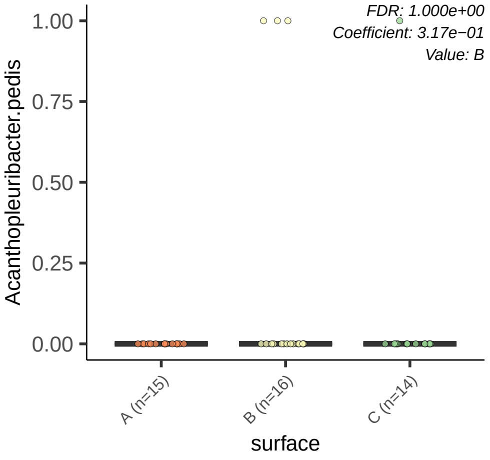

FDR: 1.000e+00
Coefficient: 3.17e−01
Value: B
Acanthopleuribacter.pedis
1.00
0.75
0.50
0.25
0.00
A (n=15)
B (n=16)
C (n=14)
surface

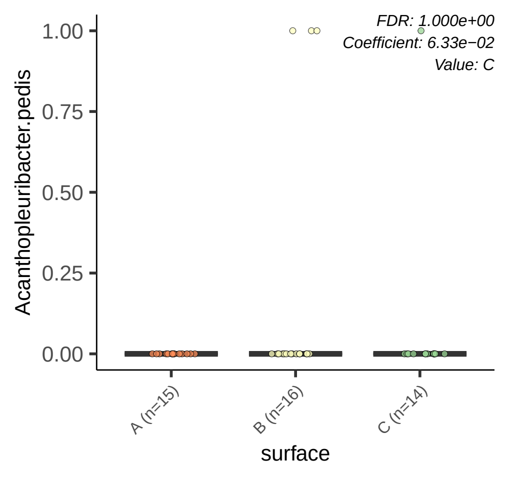
FDR: 1.000e+00
Coefficient: 6.33e−02
Value: C
Acanthopleuribacter.pedis
1.00
0.75
0.50
0.25
0.00
A (n=15)
B (n=16)
C (n=14)
surface

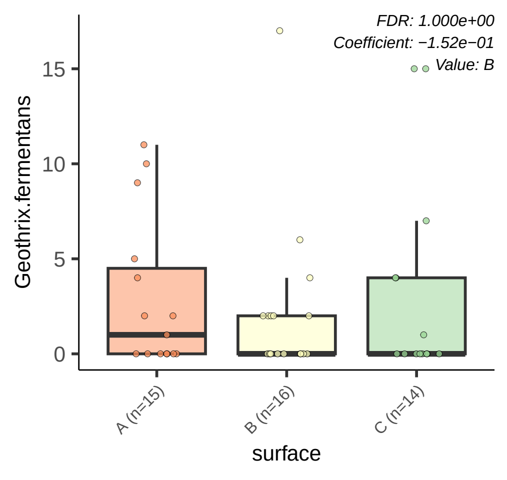

FDR: 1.000e+00
Coefficient: −1.52e−01
Value: B
Geothrix.fermentans
0
5
10
15
A (n=15)
B (n=16)
C (n=14)
surface

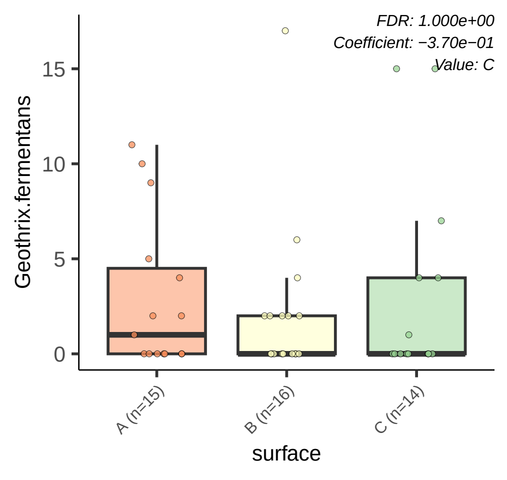

FDR: 1.000e+00
Coefficient: −3.70e−01
Value: C
Geothrix.fermentans
15
10
5
0
A (n=15)
B (n=16)
C (n=14)
surface

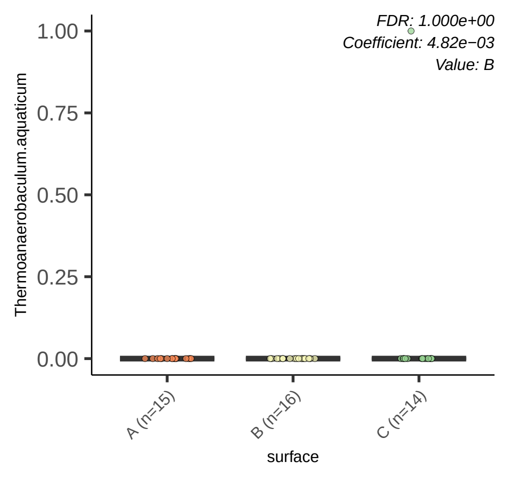

FDR: 1.000e+00
Coefficient: 4.82e−03
Value: B
1.00
0.75
0.50
0.25
0.00
Thermoanaerobaculum.aquaticum
A (n=15)
B (n=16)
C (n=14)
surface

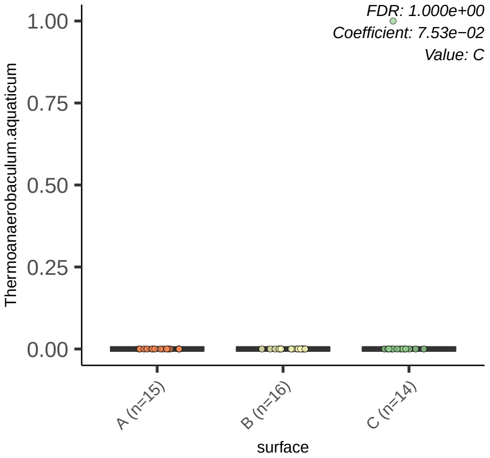
FDR: 1.000e+00
Coefficient: 7.53e−02
Value: C
1.00
0.75
0.50
0.25
0.00
Thermoanaerobaculum.aquaticum
A (n=15)
B (n=16)
C (n=14)
surface

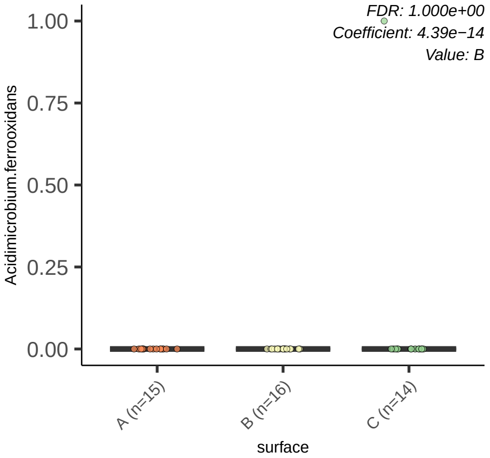

FDR: 1.000e+00
Coefficient: 4.39e−14
Value: B
1.00
0.75
0.50
0.25
0.00
Acidimicrobium.ferrooxidans
A (n=15)
B (n=16)
C (n=14)
surface

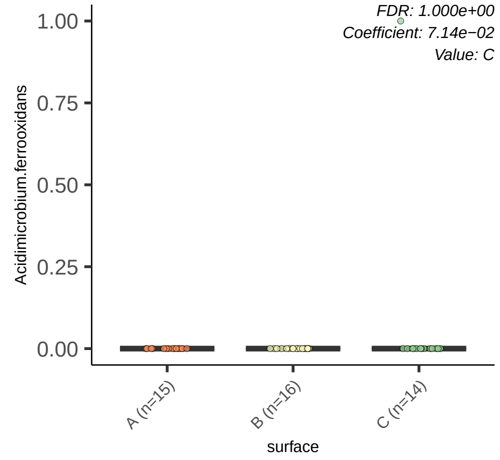
FDR: 1.000e+00
Coefficient: 7.14e−02
Value: C
1.00
0.75
0.50
0.25
0.00
Acidimicrobium.ferrooxidans
A (n=15)
B (n=16)
C (n=14)
surface

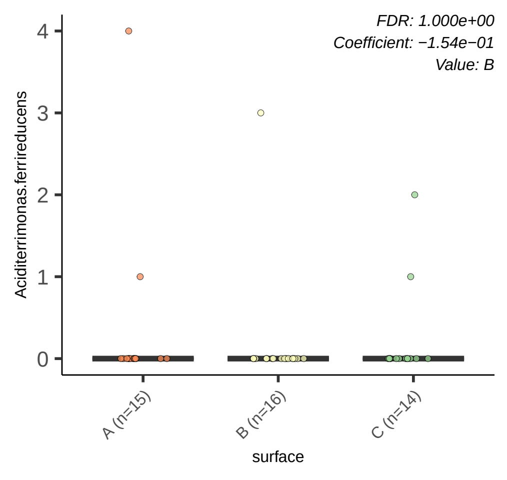
FDR: 1.000e+00
Coefficient: −1.54e−01
Value: B
Aciditerrimonas.ferrireducens
4
3
2
1
0
A (n=15)
B (n=16)
C (n=14)
surface

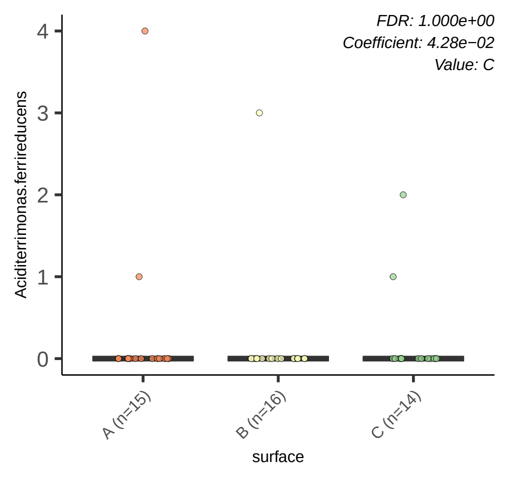

FDR: 1.000e+00
Coefficient: 4.28e−02
Value: C
Aciditerrimonas.ferrireducens
4
3
2
1
0
A (n=15)
B (n=16)
C (n=14)
surface

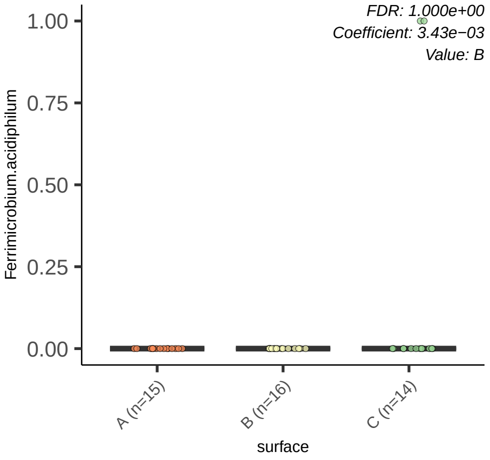
FDR: 1.000e+00
Coefficient: 3.43e−03
Value: B
1.00
0.75
0.50
0.25
0.00
Ferrimicrobium.acidiphilum
A (n=15)
B (n=16)
C (n=14)
surface

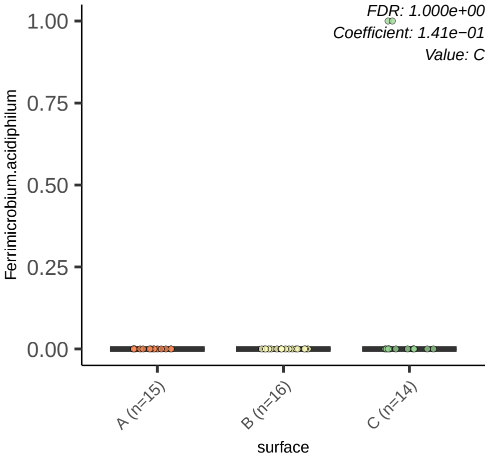
FDR: 1.000e+00
Coefficient: 1.41e−01
Value: C
Ferrimicrobium.acidiphilum
1.00
0.75
0.50
0.25
0.00
A (n=15)
B (n=16)
C (n=14)
surface

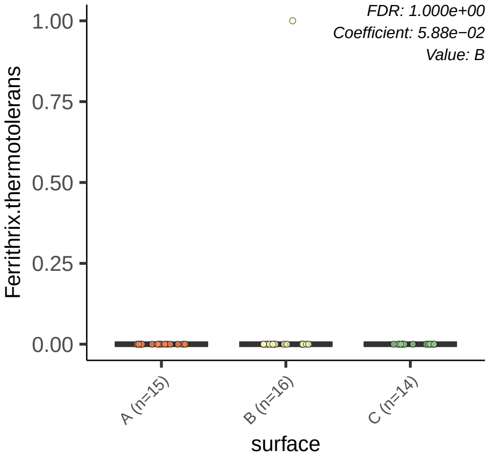
FDR: 1.000e+00
Coefficient: 5.88e−02
Value: B
Ferrithrix.thermotolerans
1.00
0.75
0.50
0.25
0.00
A (n=15)
B (n=16)
C (n=14)
surface

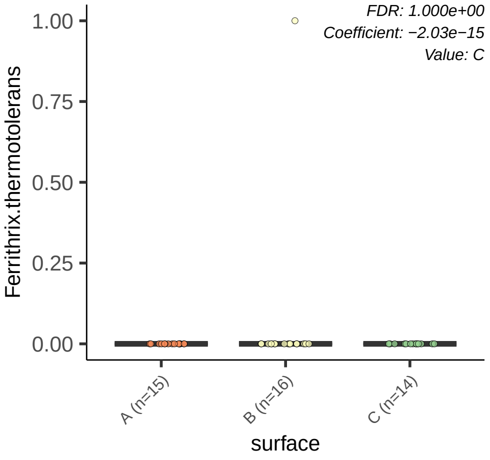
FDR: 1.000e+00
Coefficient: −2.03e−15
Value: C
Ferrithrix.thermotolerans
1.00
0.75
0.50
0.25
0.00
A (n=15)
B (n=16)
C (n=14)
surface

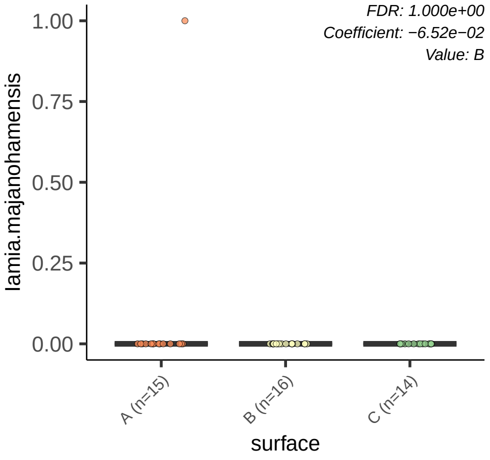
FDR: 1.000e+00
Coefficient: −6.52e−02
Value: B
lamia.majanohamensis
1.00
0.75
0.50
0.25
0.00
A (n=15)
B (n=16)
C (n=14)
surface

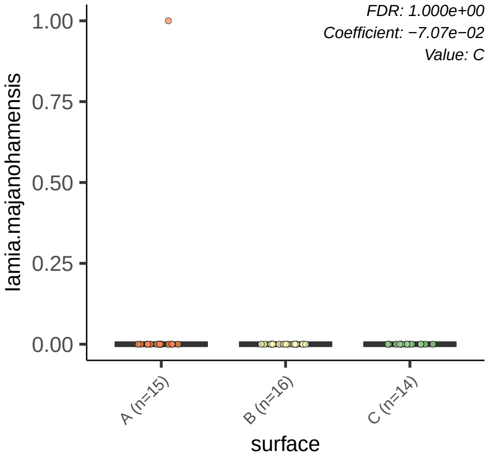

FDR: 1.000e+00
Coefficient: −7.07e−02
Value: C
lamia.majanohamensis
1.00
0.75
0.50
0.25
0.00
A (n=15)
B (n=16)
C (n=14)
surface

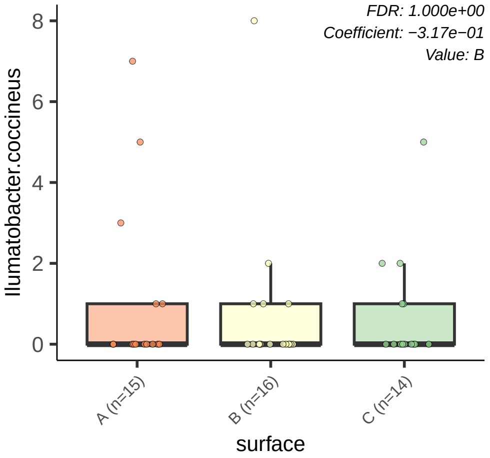
FDR: 1.000e+00
Coefficient: −3.17e−01
Value: B
Ilumatobacter.coccineus
8
6
4
2
0
A (n=15)
B (n=16)
C (n=14)
surface

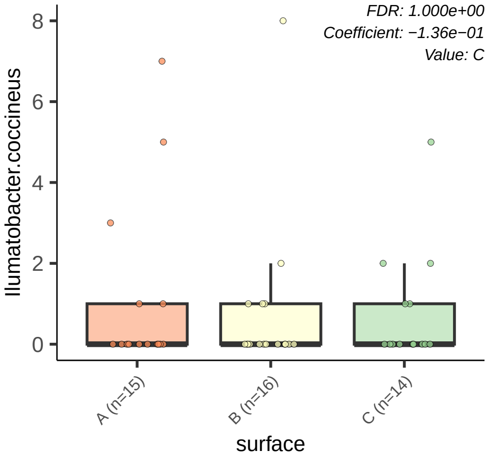

FDR: 1.000e+00
Coefficient: −1.36e−01
Value: C
Ilumatobacter.coccineus
0
2
4
6
8
A (n=15)
B (n=16)
C (n=14)
surface

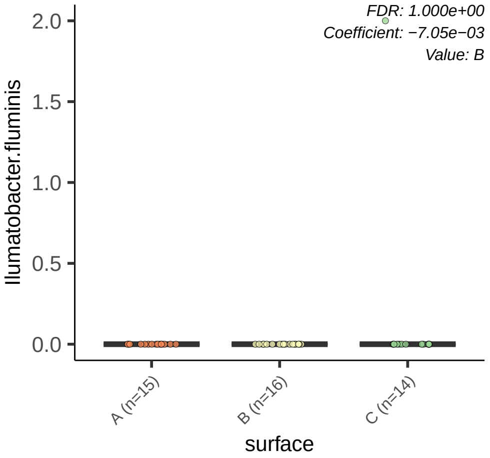
FDR: 1.000e+00
Coefficient: −7.05e−03
Value: B
Ilumatobacter.fluminis
2.0
1.5
1.0
0.5
0.0
A (n=15)
B (n=16)
C (n=14)
surface

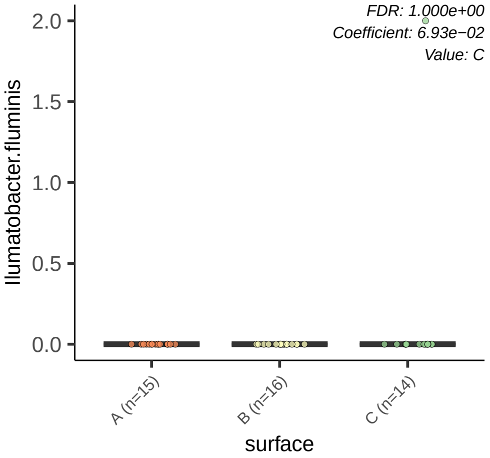

FDR: 1.000e+00
Coefficient: 6.93e−02
Value: C
Ilumatobacter.fluminis
2.0
1.5
1.0
0.5
0.0
A (n=15)
B (n=16)
C (n=14)
surface

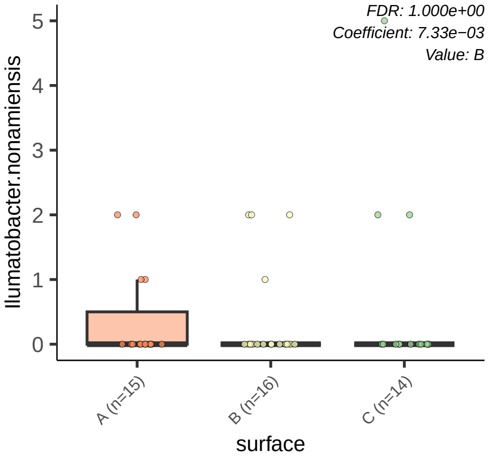
FDR: 1.000e+00
Coefficient: 7.33e−03
Value: B
Ilumatobacter.nonamiensis
0
1
2
3
4
5
A (n=15)
B (n=16)
C (n=14)
surface

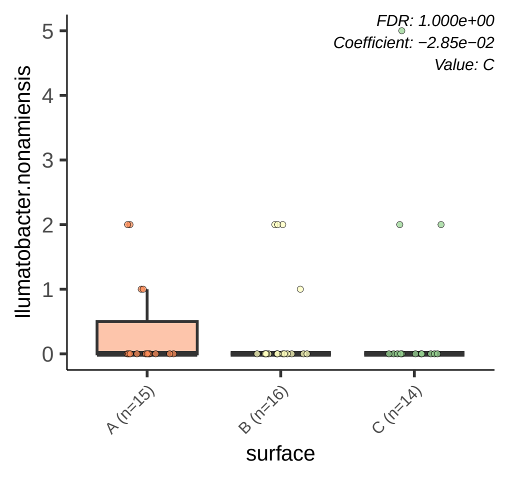
FDR: 1.000e+00
Coefficient: −2.85e−02
Value: C
Ilumatobacter.nonamiensis
5
4
3
2
1
0
A (n=15)
B (n=16)
C (n=14)
surface

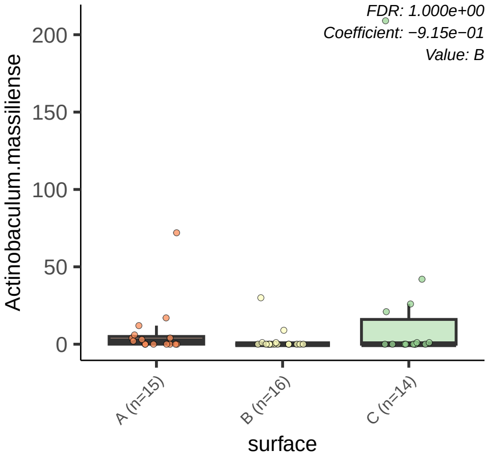
FDR: 1.000e+00
Coefficient: −9.15e−01
Value: B
Actinobaculum.massiliense
200
150
100
50
0
A (n=15)
B (n=16)
C (n=14)
surface

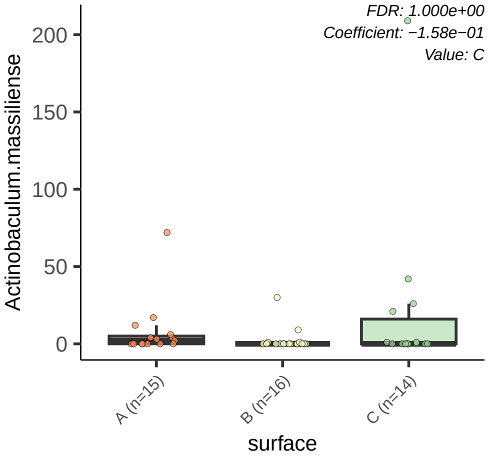
FDR: 1.000e+00
Coefficient: −1.58e−01
Value: C
Actinobaculum.massiliense
200
150
100
50
0
A (n=15)
B (n=16)
C (n=14)
surface

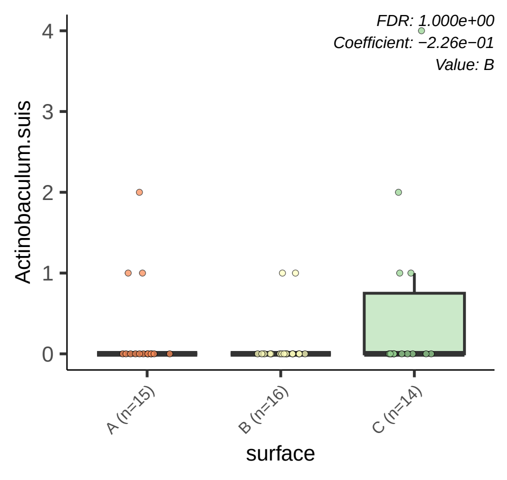

FDR: 1.000e+00
Coefficient: −2.26e−01
Value: B
Actinobaculum.suis
0
1
2
3
4
A (n=15)
B (n=16)
C (n=14)
surface

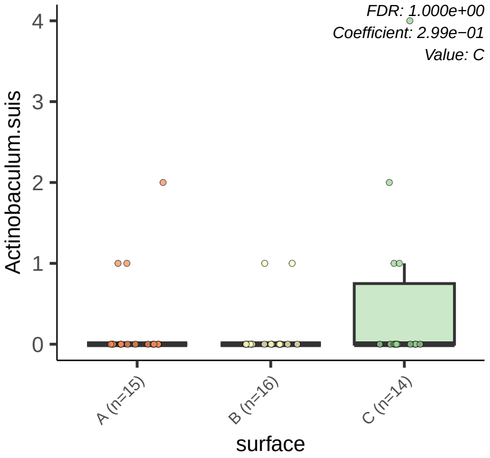
FDR: 1.000e+00
Coefficient: 2.99e−01
Value: C
Actinobaculum.suis
4
3
2
1
0
A (n=15)
B (n=16)
C (n=14)
surface

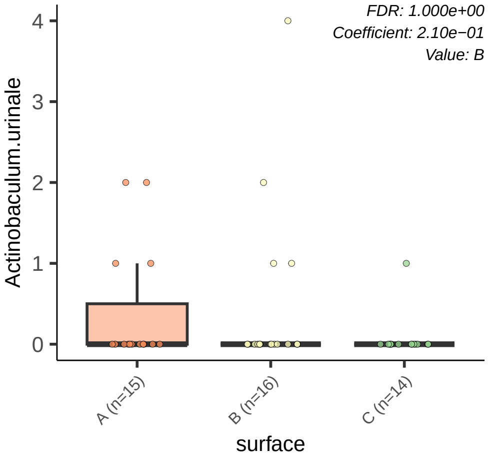
FDR: 1.000e+00
Coefficient: 2.10e−01
Value: B
Actinobaculum.urinale
4
3
2
1
0
A (n=15)
B (n=16)
C (n=14)
surface

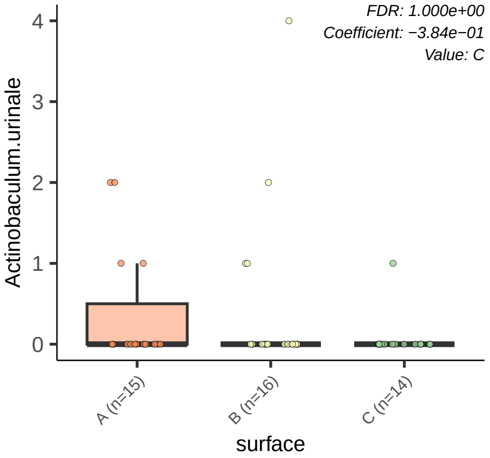
FDR: 1.000e+00
Coefficient: −3.84e−01
Value: C
Actinobaculum.urinale
0
1
2
3
4
A (n=15)
B (n=16)
C (n=14)
surface

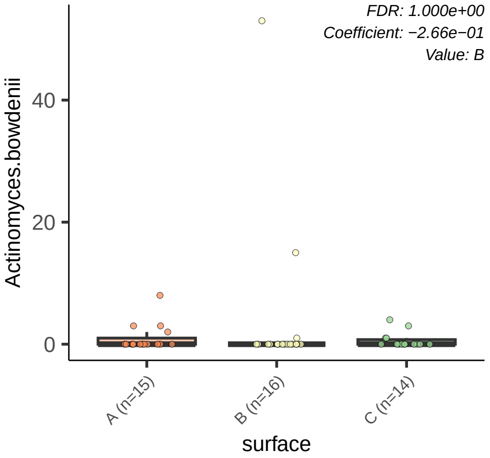

FDR: 1.000e+00
Coefficient: −2.66e−01
Value: B
Actinomyces.bowdenii
0
20
40
A (n=15)
B (n=16)
C (n=14)
surface

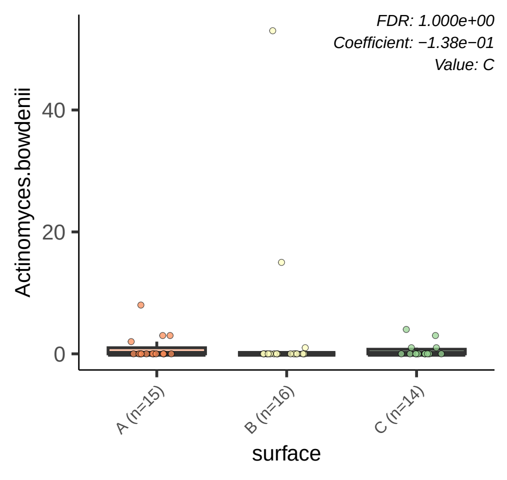

FDR: 1.000e+00
Coefficient: −1.38e−01
Value: C
Actinomyces.bowdenii
40
20
0
A (n=15)
B (n=16)
C (n=14)
surface

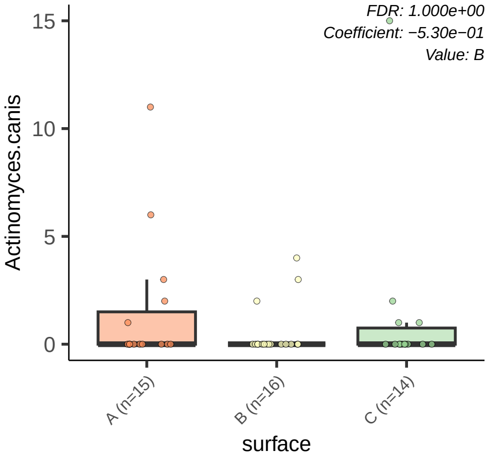
FDR: 1.000e+00
Coefficient: −5.30e−01
Value: B
Actinomyces.canis
15
10
5
0
A (n=15)
B (n=16)
C (n=14)
surface

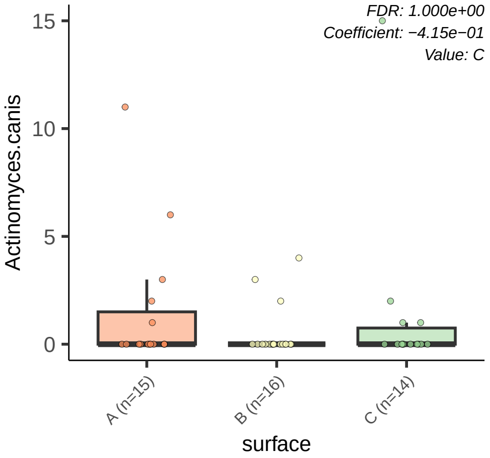

FDR: 1.000e+00
Coefficient: −4.15e−01
Value: C
Actinomyces.canis
15
10
5
0
A (n=15)
B (n=16)
C (n=14)
surface

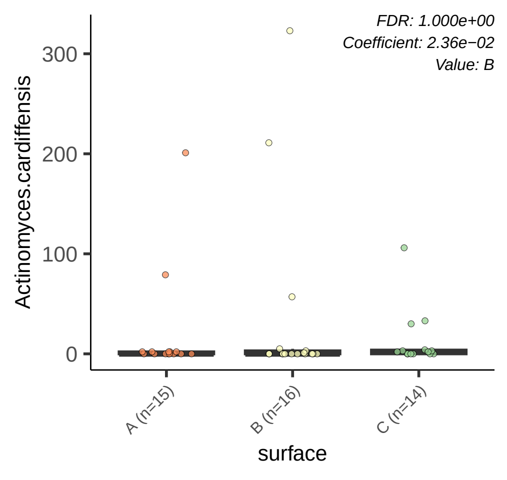

FDR: 1.000e+00
Coefficient: 2.36e−02
Value: B
Actinomyces.cardiffensis
300
200
100
0
A (n=15)
B (n=16)
C (n=14)
surface

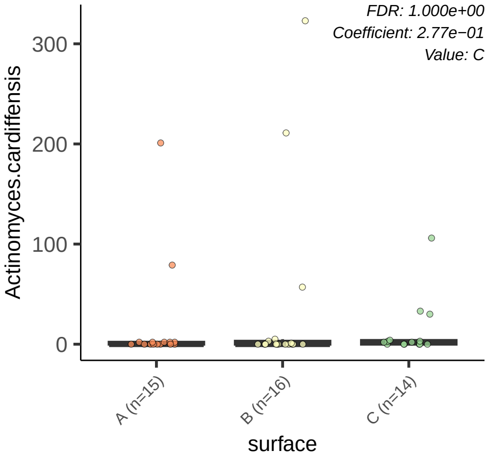
FDR: 1.000e+00
Coefficient: 2.77e−01
Value: C
Actinomyces.cardiffensis
300
200
100
0
A (n=15)
B (n=16)
C (n=14)
surface

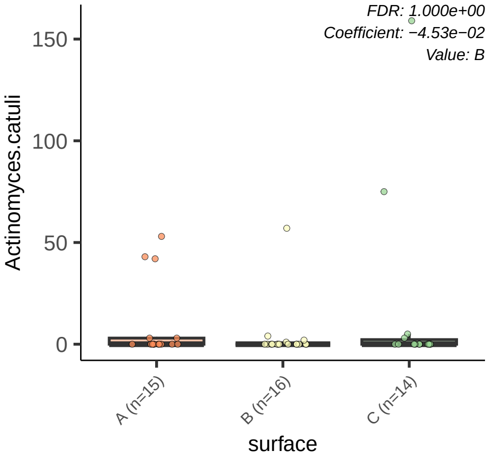
FDR: 1.000e+00
Coefficient: −4.53e−02
Value: B
Actinomyces.catuli
150
100
50
0
A (n=15)
B (n=16)
C (n=14)
surface

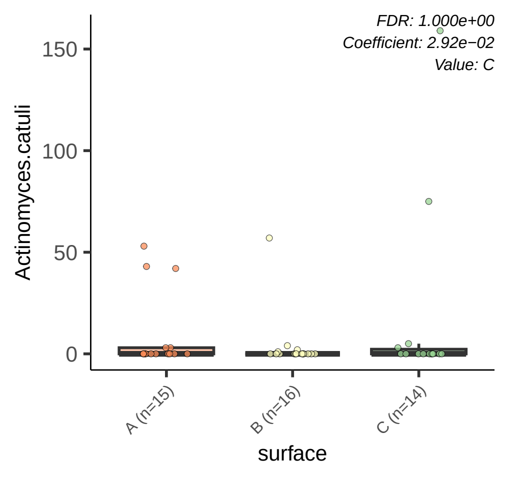
FDR: 1.000e+00
Coefficient: 2.92e−02
Value: C
Actinomyces.catuli
150
100
50
0
A (n=15)
B (n=16)
C (n=14)
surface

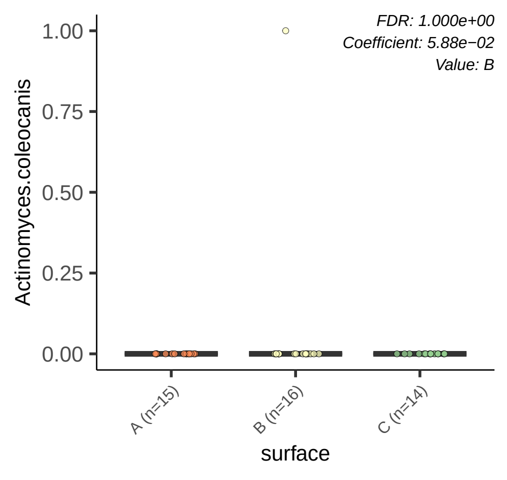

FDR: 1.000e+00
Coefficient: 5.88e−02
Value: B
Actinomyces.coleocanis
1.00
0.75
0.50
0.25
0.00
A (n=15)
B (n=16)
C (n=14)
surface

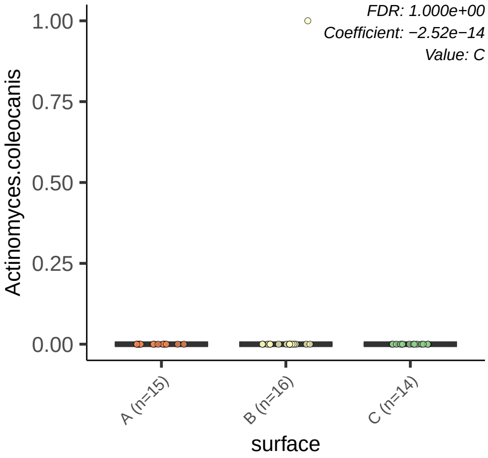

FDR: 1.000e+00
Coefficient: −2.52e−14
Value: C
Actinomyces.coleocanis
1.00
0.75
0.50
0.25
0.00
A (n=15)
B (n=16)
C (n=14)
surface

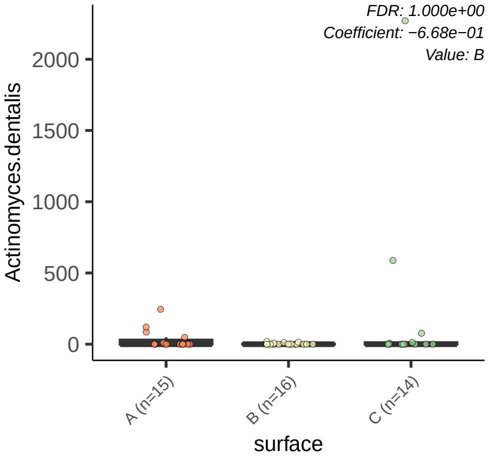

FDR: 1.000e+00
Coefficient: −6.68e−01
Value: B
Actinomyces.dentalis
0
500
1000
1500
2000
A (n=15)
B (n=16)
C (n=14)
surface

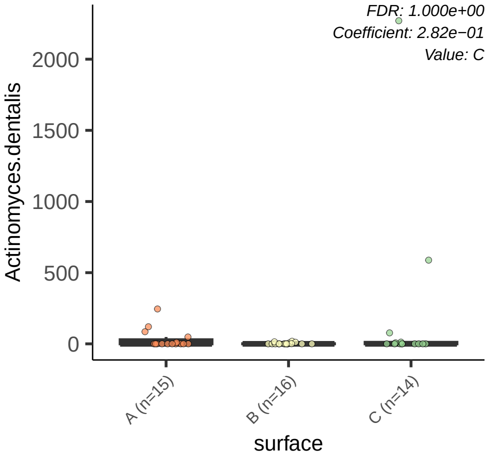
FDR: 1.000e+00
Coefficient: 2.82e−01
Value: C
Actinomyces.dentalis
0
500
1000
1500
2000
A (n=15)
B (n=16)
C (n=14)
surface

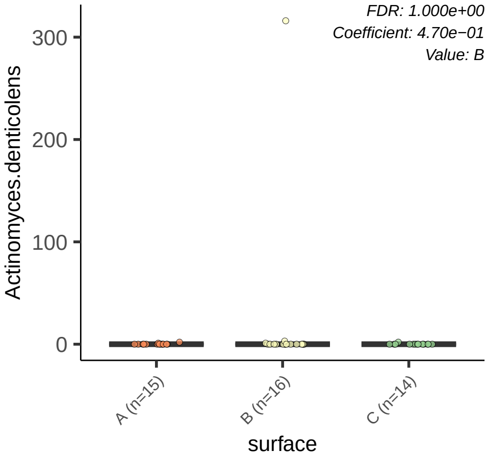
FDR: 1.000e+00
Coefficient: 4.70e−01
Value: B
Actinomyces.denticolens
300
200
100
0
A (n=15)
B (n=16)
C (n=14)
surface

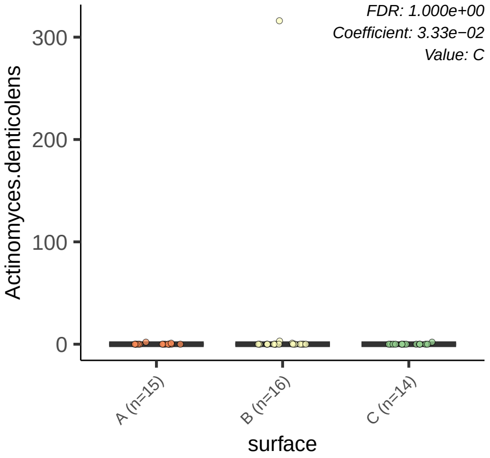

FDR: 1.000e+00
Coefficient: 3.33e−02
Value: C
300
200
100
0
Actinomyces.denticolens
A (n=15)
B (n=16)
C (n=14)
surface

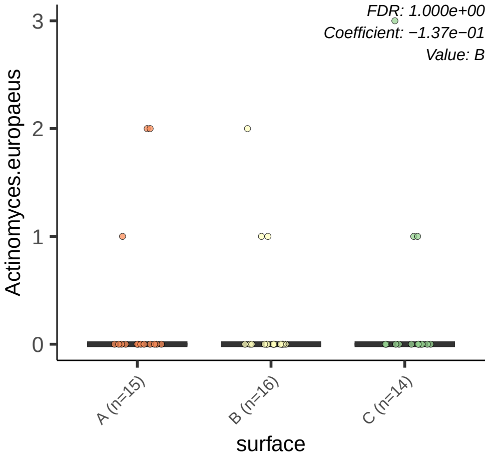
FDR: 1.000e+00
Coefficient: −1.37e−01
Value: B
Actinomyces.europaeus
0
1
2
3
A (n=15)
B (n=16)
C (n=14)
surface

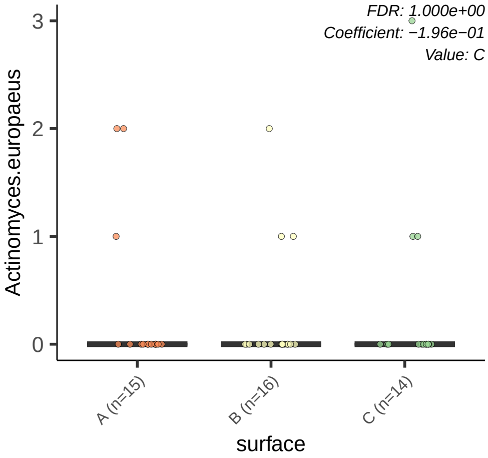
FDR: 1.000e+00
Coefficient: −1.96e−01
Value: C
Actinomyces.europaeus
0
1
2
3
A (n=15)
B (n=16)
C (n=14)
surface

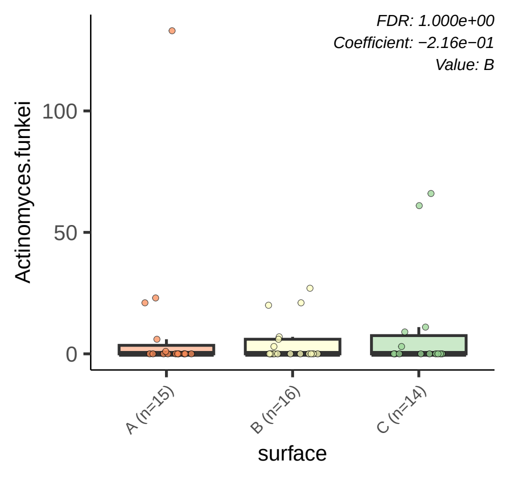
FDR: 1.000e+00
Coefficient: −2.16e−01
Value: B
Actinomyces.funkei
100
50
0
A (n=15)
B (n=16)
C (n=14)
surface

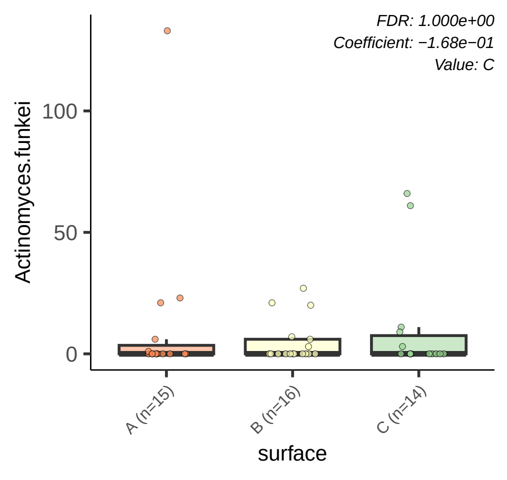

FDR: 1.000e+00
Coefficient: −1.68e−01
Value: C
Actinomyces.funkei
100
50
0
A (n=15)
B (n=16)
C (n=14)
surface

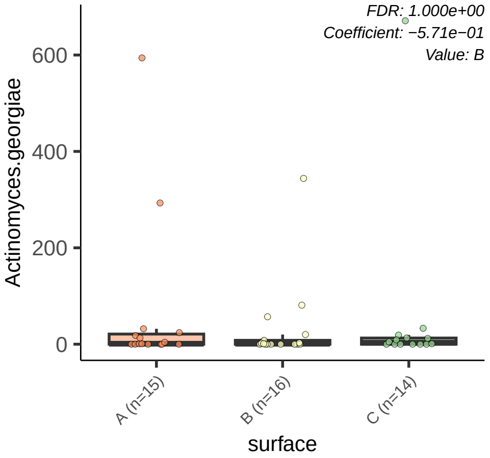

FDR: 1.000e+00
Coefficient: −5.71e−01
Value: B
Actinomyces.georgiae
600
400
200
0
A (n=15)
B (n=16)
C (n=14)
surface

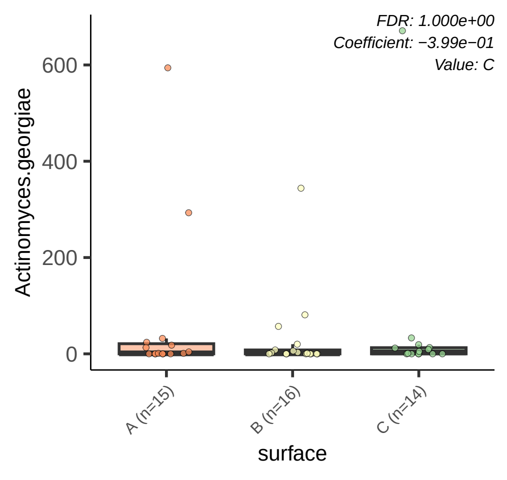
FDR: 1.000e+00
Coefficient: −3.99e−01
Value: C
Actinomyces.georgiae
600
400
200
0
A (n=15)
B (n=16)
C (n=14)
surface

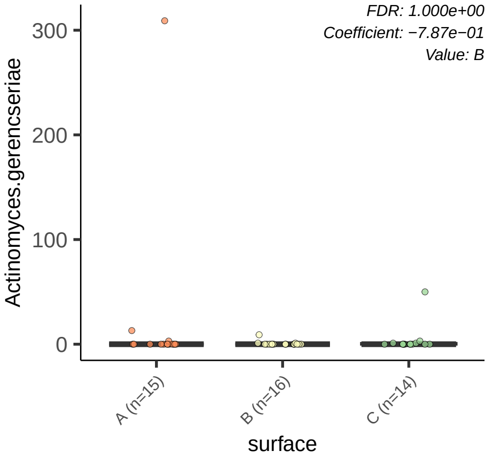

FDR: 1.000e+00
Coefficient: −7.87e−01
Value: B
Actinomyces.gerencseriae
300
200
100
0
A (n=15)
B (n=16)
C (n=14)
surface

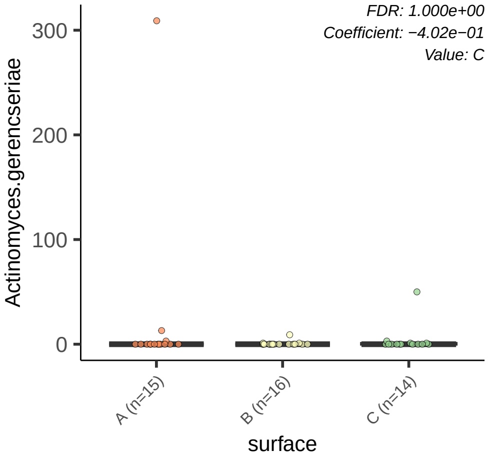
FDR: 1.000e+00
Coefficient: −4.02e−01
Value: C
Actinomyces.gerencseriae
300
200
100
0
A (n=15)
B (n=16)
C (n=14)
surface

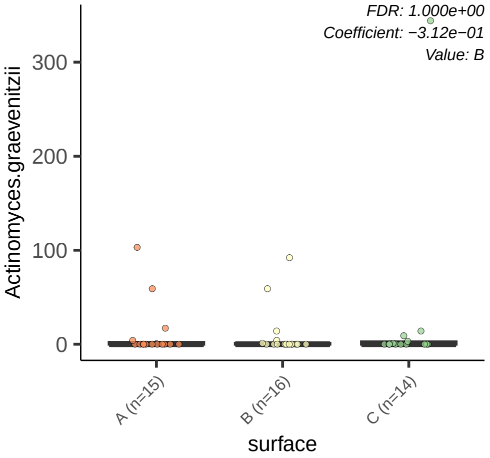

FDR: 1.000e+00
Coefficient: −3.12e−01
Value: B
Actinomyces.graevenitzii
300
200
100
0
A (n=15)
B (n=16)
C (n=14)
surface

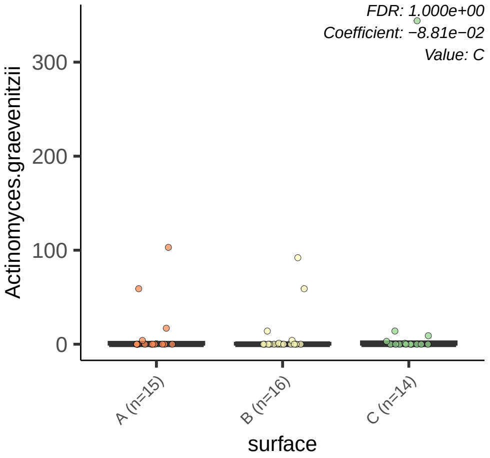

FDR: 1.000e+00
Coefficient: −8.81e−02
Value: C
Actinomyces.graevenitzii
300
200
100
0
A (n=15)
B (n=16)
C (n=14)
surface

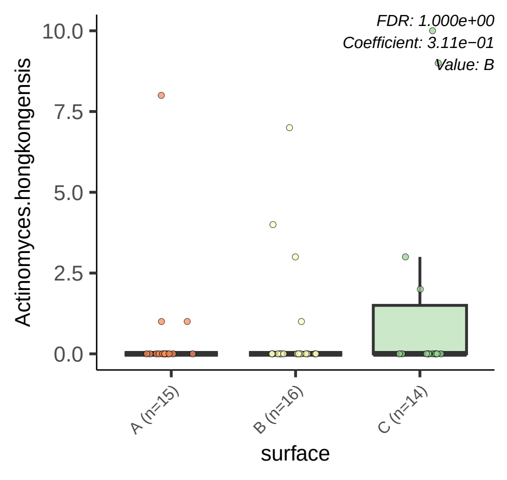
FDR: 1.000e+00
Coefficient: 3.11e−01
Value: B
Actinomyces.hongkongensis
10.0
7.5
5.0
2.5
0.0
A (n=15)
B (n=16)
C (n=14)
surface

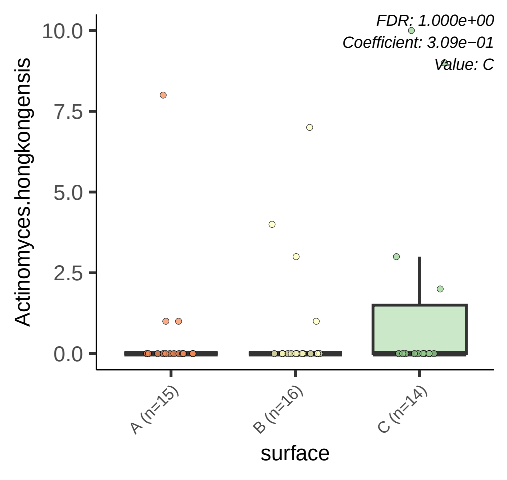
FDR: 1.000e+00
Coefficient: 3.09e−01
Value: C
10.0
7.5
5.0
2.5
0.0
Actinomyces.hongkongensis
A (n=15)
B (n=16)
C (n=14)
surface

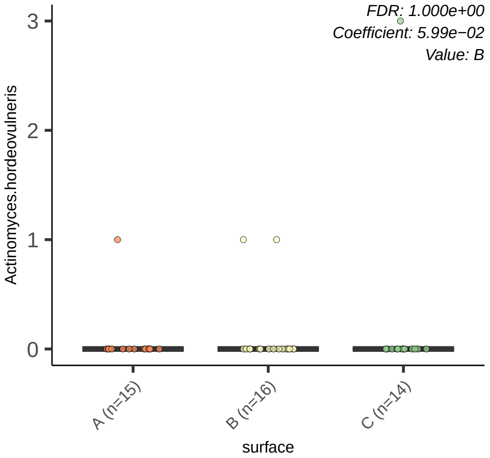
FDR: 1.000e+00
Coefficient: 5.99e−02
Value: B
Actinomyces.hordeovulneris
0
1
2
3
A (n=15)
B (n=16)
C (n=14)
surface

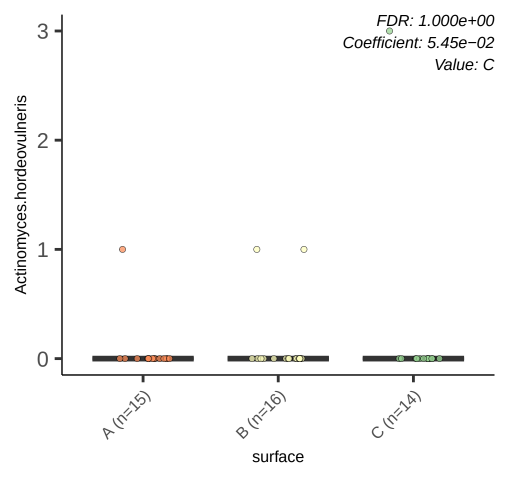

FDR: 1.000e+00
Coefficient: 5.45e−02
Value: C
Actinomyces.hordeovulneris
3
2
1
0
A (n=15)
B (n=16)
C (n=14)
surface

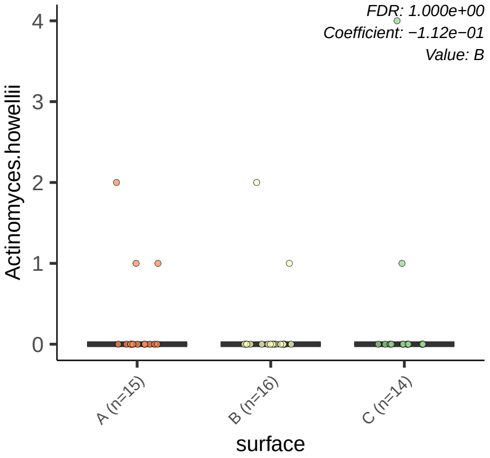

FDR: 1.000e+00
Coefficient: −1.12e−01
Value: B
Actinomyces.howellii
4
3
2
1
0
A (n=15)
B (n=16)
C (n=14)
surface

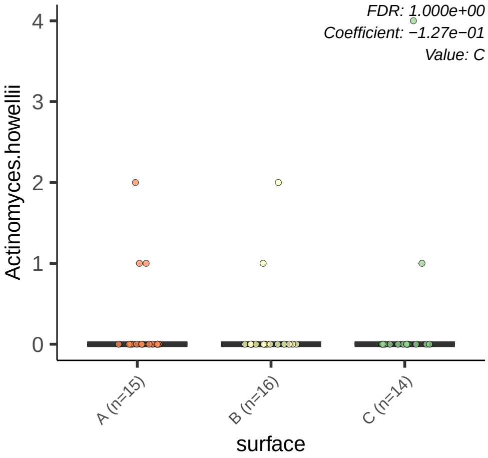

FDR: 1.000e+00
Coefficient: −1.27e−01
Value: C
Actinomyces.howellii
0
1
2
3
4
A (n=15)
B (n=16)
C (n=14)
surface

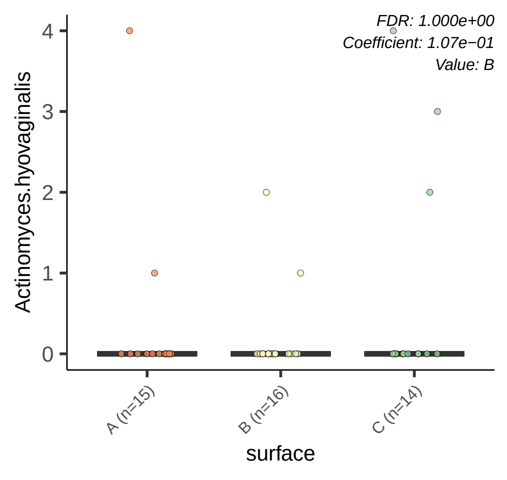
FDR: 1.000e+00
Coefficient: 1.07e−01
Value: B
Actinomyces.hyovaginalis
4
3
2
1
0
A (n=15)
B (n=16)
C (n=14)
surface

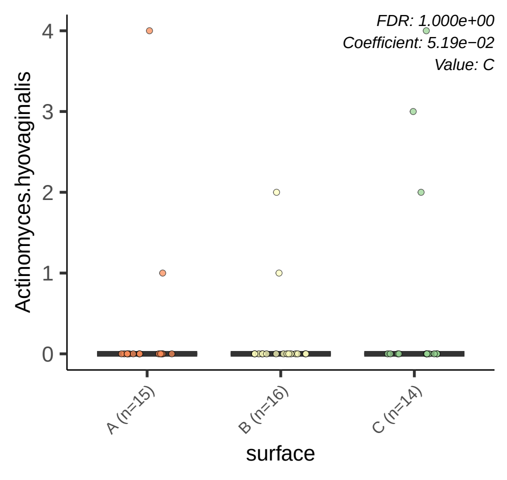
FDR: 1.000e+00
Coefficient: 5.19e−02
Value: C
Actinomyces.hyovaginalis
0
1
2
3
4
A (n=15)
B (n=16)
C (n=14)
surface

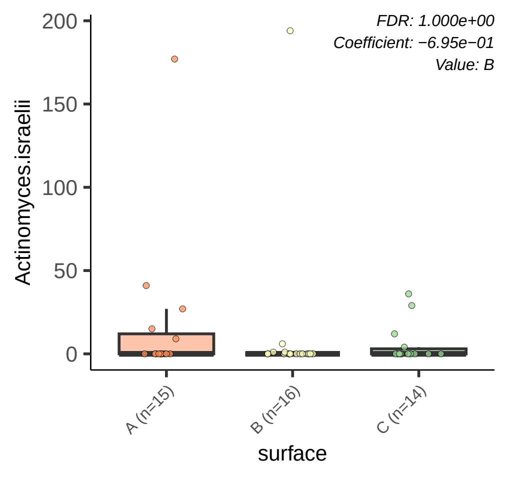
FDR: 1.000e+00
Coefficient: −6.95e−01
Value: B
Actinomyces.israelii
200
150
100
50
0
A (n=15)
B (n=16)
C (n=14)
surface

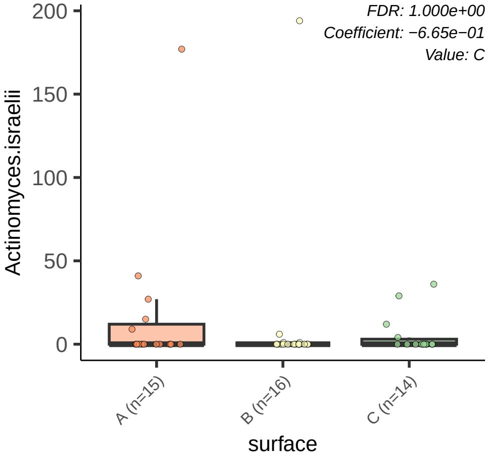

FDR: 1.000e+00
Coefficient: −6.65e−01
Value: C
Actinomyces.israelii
200
150
100
50
0
A (n=15)
B (n=16)
C (n=14)
surface

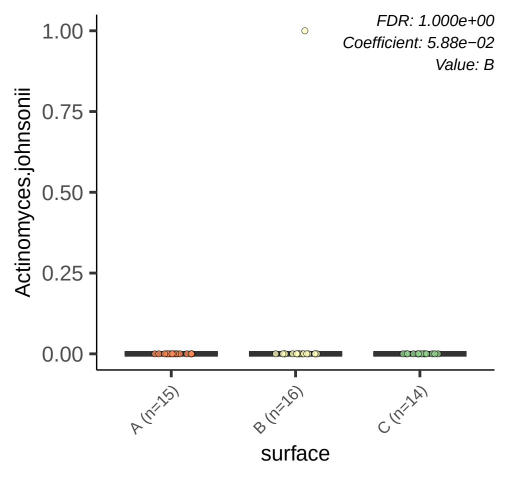

FDR: 1.000e+00
Coefficient: 5.88e−02
Value: B
Actinomyces.johnsonii
1.00
0.75
0.50
0.25
0.00
A (n=15)
B (n=16)
C (n=14)
surface

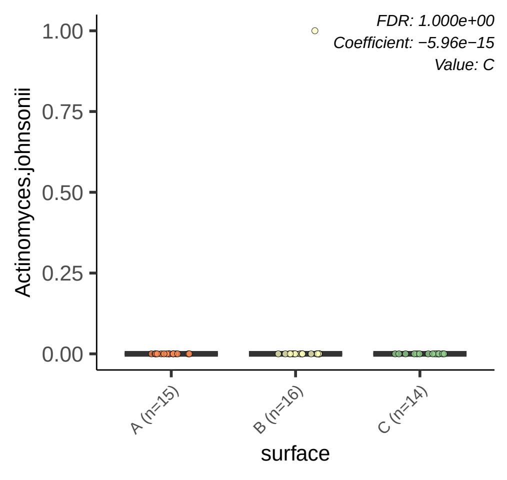
FDR: 1.000e+00
Coefficient: −5.96e−15
Value: C
Actinomyces.johnsonii
1.00
0.75
0.50
0.25
0.00
A (n=15)
B (n=16)
C (n=14)
surface

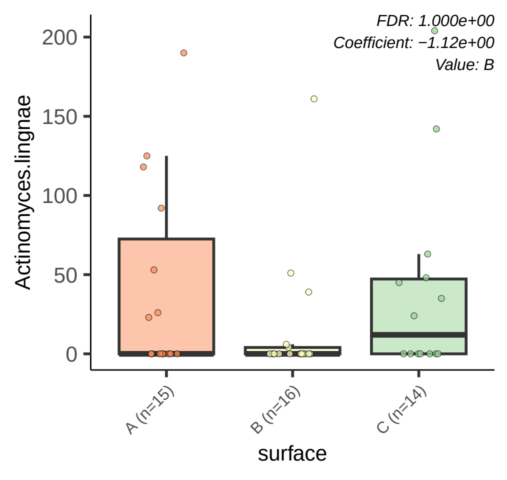
FDR: 1.000e+00
Coefficient: −1.12e+00
Value: B
Actinomyces.lingnae
200
150
100
50
0
A (n=15)
B (n=16)
C (n=14)
surface

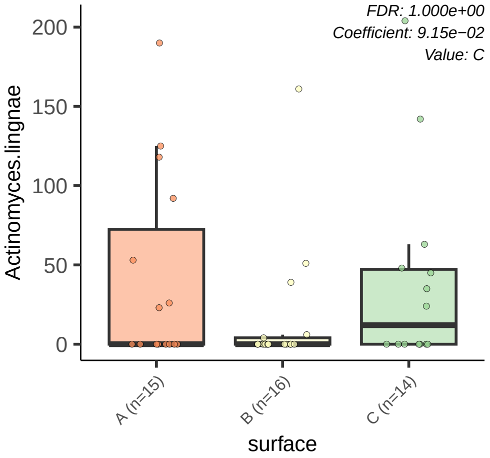
FDR: 1.000e+00
Coefficient: 9.15e−02
Value: C
Actinomyces.lingnae
200
150
100
50
0
A (n=15)
B (n=16)
C (n=14)
surface

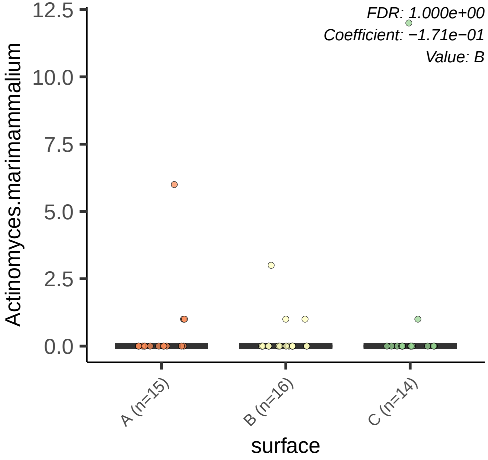
FDR: 1.000e+00
Coefficient: −1.71e−01
Value: B
Actinomyces.marimammalium
12.5
10.0
7.5
5.0
2.5
0.0
A (n=15)
B (n=16)
C (n=14)
surface

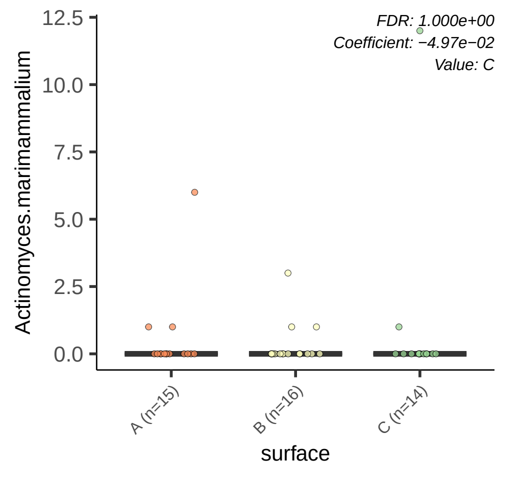
FDR: 1.000e+00
Coefficient: −4.97e−02
Value: C
Actinomyces.marimammalium
12.5
10.0
7.5
5.0
2.5
0.0
A (n=15)
B (n=16)
C (n=14)
surface

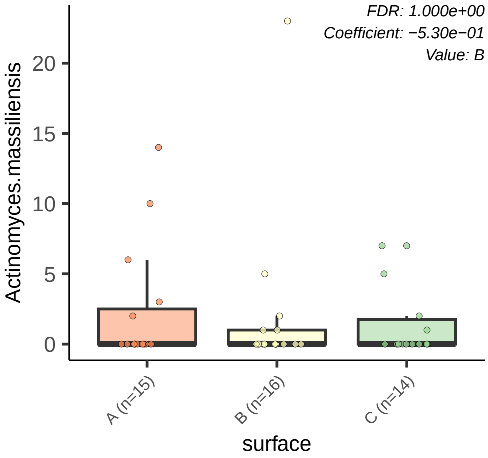

FDR: 1.000e+00
Coefficient: −5.30e−01
Value: B
Actinomyces.massiliensis
0
5
10
15
20
A (n=15)
B (n=16)
C (n=14)
surface

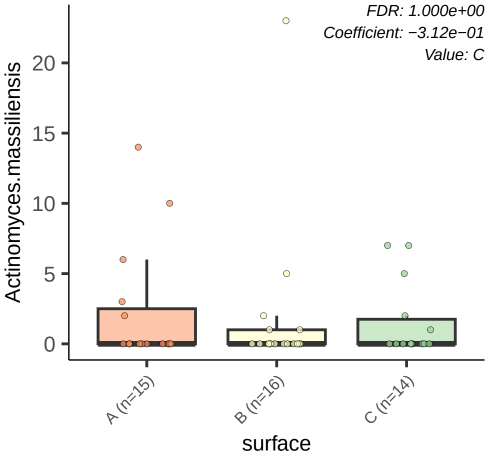
FDR: 1.000e+00
Coefficient: −3.12e−01
Value: C
Actinomyces.massiliensis
0
5
10
15
20
A (n=15)
B (n=16)
C (n=14)
surface

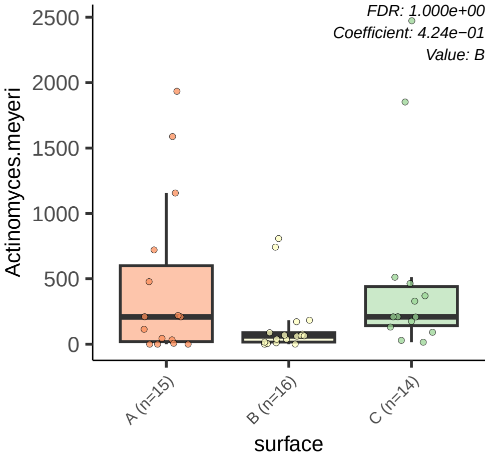

FDR: 1.000e+00
Coefficient: 4.24e−01
Value: B
Actinomyces.meyeri
2500
2000
1500
1000
500
0
A (n=15)
B (n=16)
C (n=14)
surface

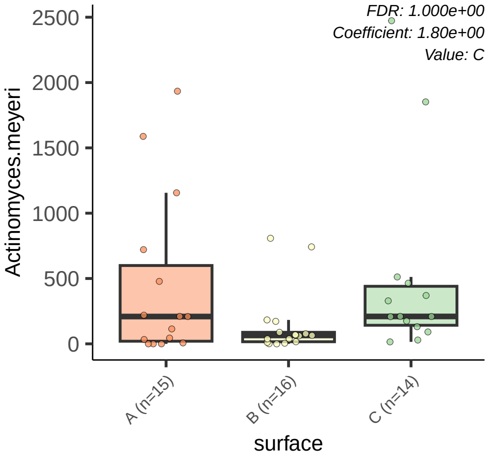

FDR: 1.000e+00
Coefficient: 1.80e+00
Value: C
Actinomyces.meyeri
2500
2000
1500
1000
500
0
A (n=15)
B (n=16)
C (n=14)
surface

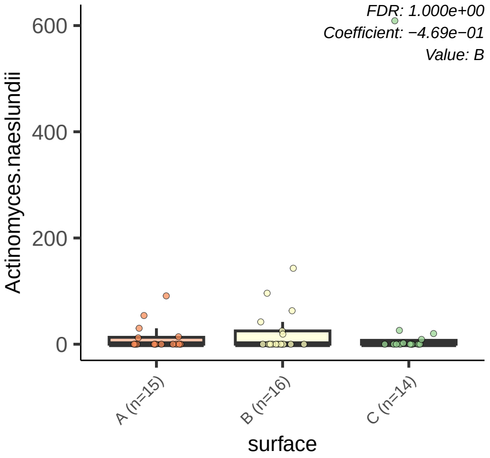

FDR: 1.000e+00
Coefficient: −4.69e−01
Value: B
Actinomyces.naeslundii
0
200
400
600
A (n=15)
B (n=16)
C (n=14)
surface

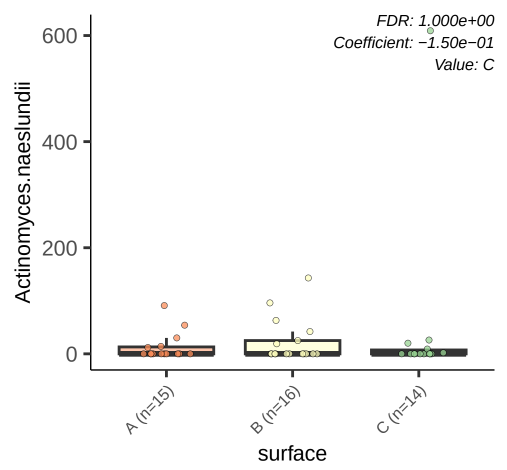
FDR: 1.000e+00
Coefficient: −1.50e−01
Value: C
Actinomyces.naeslundii
600
400
200
0
A (n=15)
B (n=16)
C (n=14)
surface

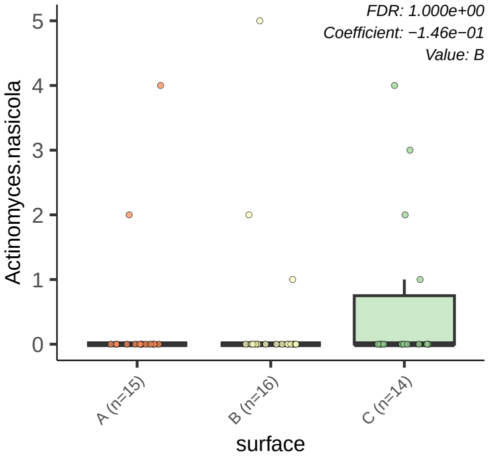

FDR: 1.000e+00
Coefficient: −1.46e−01
Value: B
Actinomyces.nasicola
5
4
3
2
1
0
A (n=15)
B (n=16)
C (n=14)
surface

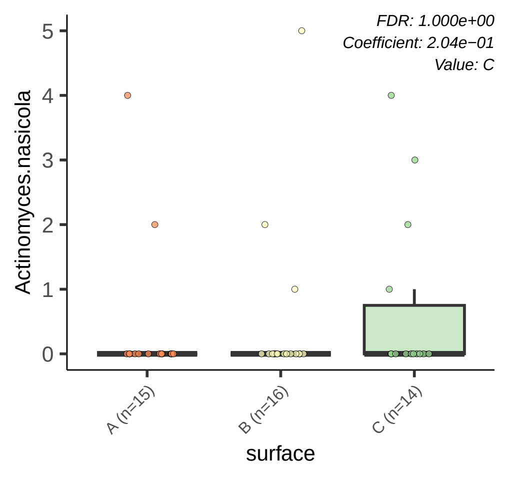
FDR: 1.000e+00
Coefficient: 2.04e−01
Value: C
Actinomyces.nasicola
0
1
2
3
4
5
A (n=15)
B (n=16)
C (n=14)
surface

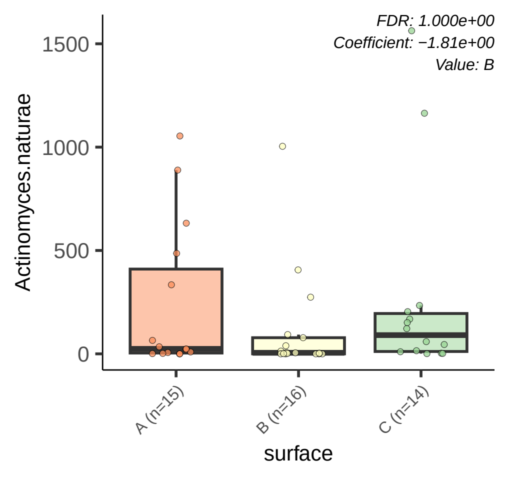

FDR: 1.000e+00
Coefficient: −1.81e+00
Value: B
1500
1000
500
0
Actinomyces.naturae
A (n=15)
B (n=16)
C (n=14)
surface

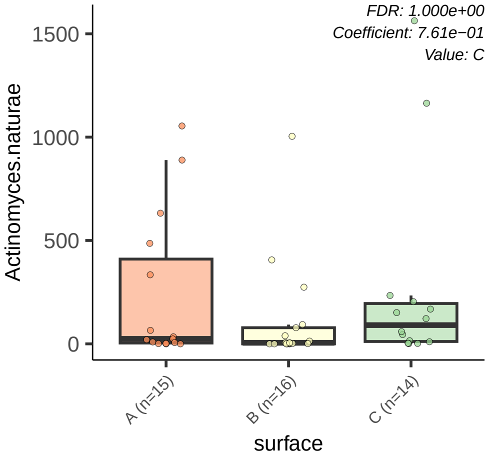
FDR: 1.000e+00
Coefficient: 7.61e−01
Value: C
Actinomyces.naturae
0
500
1000
1500
A (n=15)
B (n=16)
C (n=14)
surface

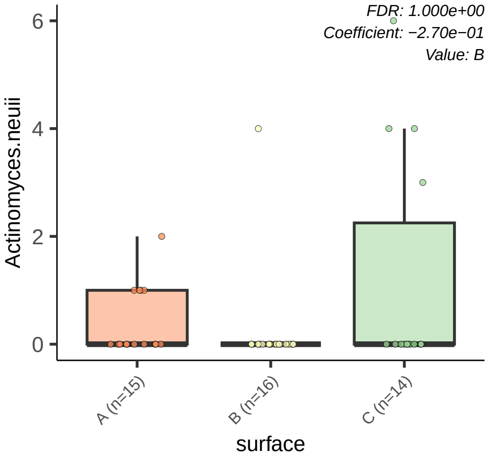
FDR: 1.000e+00
Coefficient: −2.70e−01
Value: B
Actinomyces.neuii
6
4
2
0
A (n=15)
B (n=16)
C (n=14)
surface

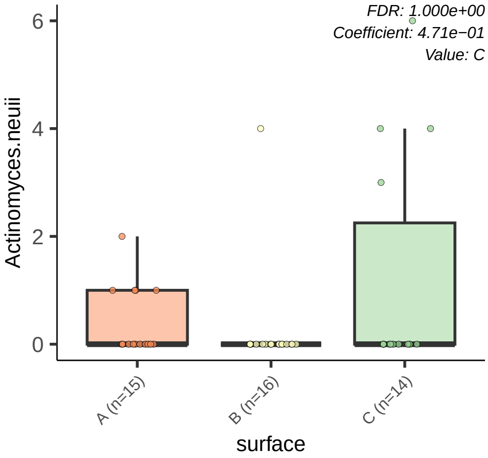

FDR: 1.000e+00
Coefficient: 4.71e−01
Value: C
Actinomyces.neuii
0
2
4
6
A (n=15)
B (n=16)
C (n=14)
surface

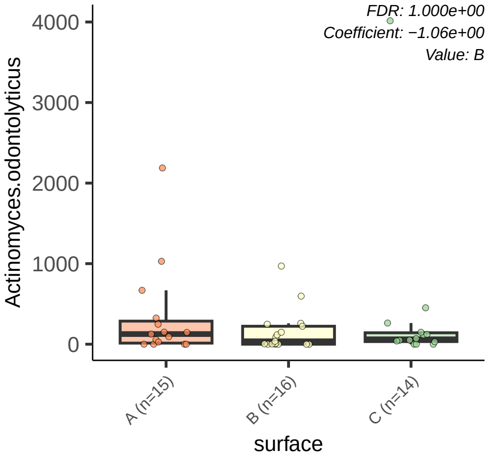

FDR: 1.000e+00
Coefficient: −1.06e+00
Value: B
Actinomyces.odontolyticus
4000
3000
2000
1000
0
A (n=15)
B (n=16)
C (n=14)
surface

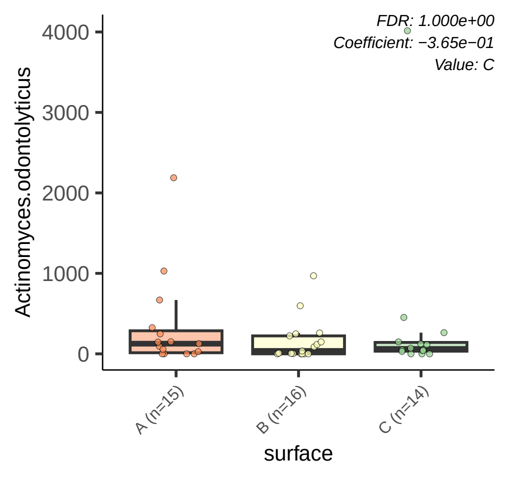

FDR: 1.000e+00
Coefficient: −3.65e−01
Value: C
Actinomyces.odontolyticus
4000
3000
2000
1000
0
A (n=15)
B (n=16)
C (n=14)
surface

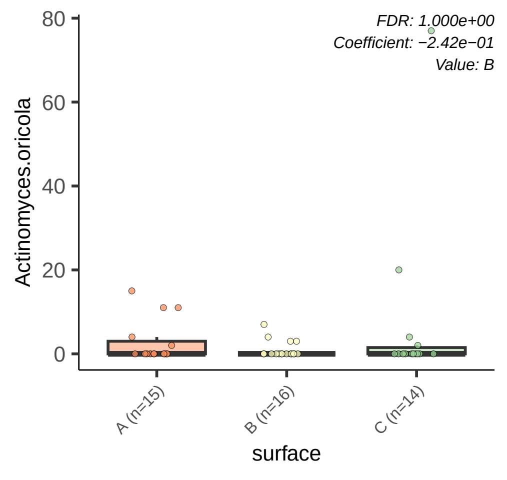
FDR: 1.000e+00
Coefficient: −2.42e−01
Value: B
Actinomyces.oricola
0
20
40
60
80
A (n=15)
B (n=16)
C (n=14)
surface

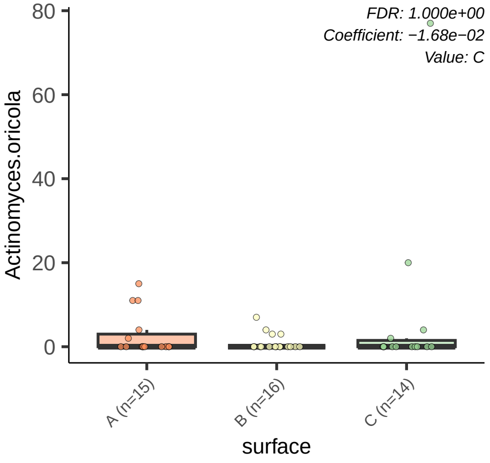

FDR: 1.000e+00
Coefficient: −1.68e−02
Value: C
Actinomyces.oricola
0
20
40
60
80
A (n=15)
B (n=16)
C (n=14)
surface

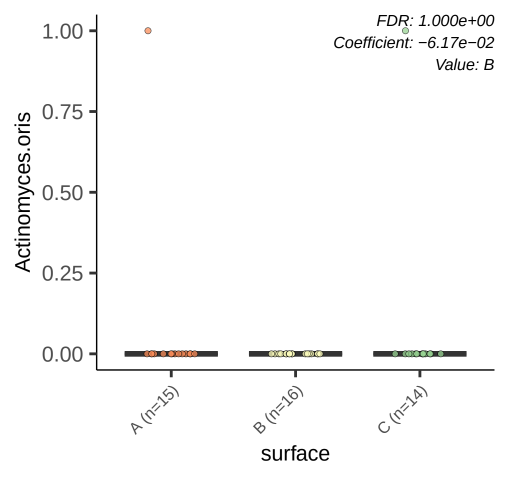
FDR: 1.000e+00
Coefficient: −6.17e−02
Value: B
Actinomyces.oris
1.00
0.75
0.50
0.25
0.00
A (n=15)
B (n=16)
C (n=14)
surface

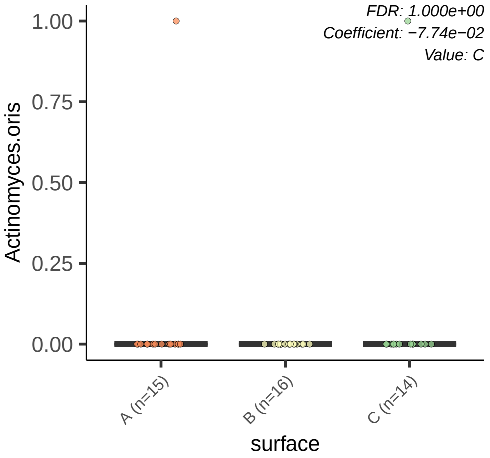

FDR: 1.000e+00
Coefficient: −7.74e−02
Value: C
Actinomyces.oris
1.00
0.75
0.50
0.25
0.00
A (n=15)
B (n=16)
C (n=14)
surface

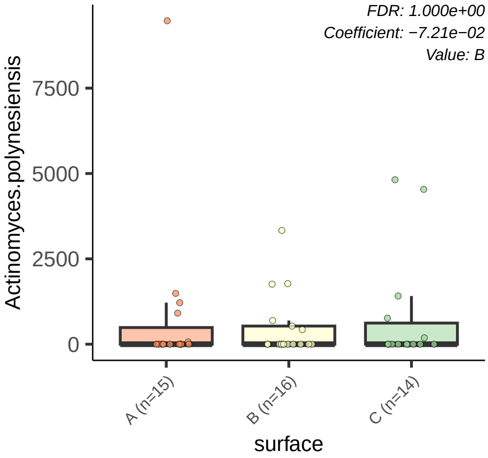

FDR: 1.000e+00
Coefficient: −7.21e−02
Value: B
Actinomyces.polynesiensis
7500
5000
2500
0
A (n=15)
B (n=16)
C (n=14)
surface

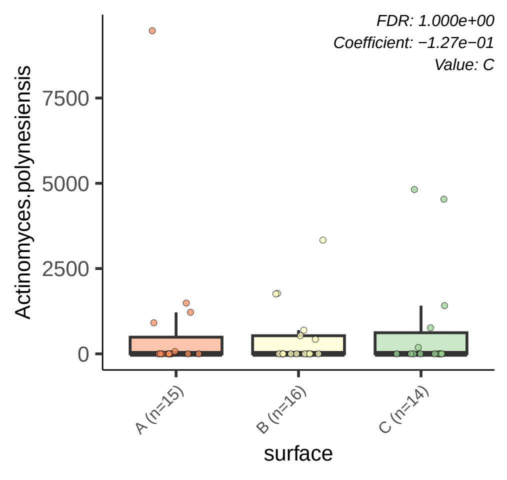
FDR: 1.000e+00
Coefficient: −1.27e−01
Value: C
Actinomyces.polynesiensis
7500
5000
2500
0
A (n=15)
B (n=16)
C (n=14)
surface

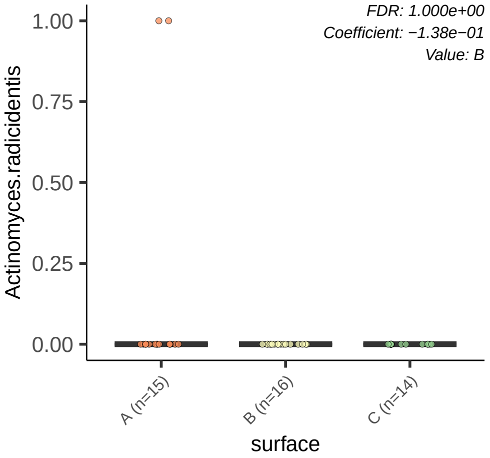

FDR: 1.000e+00
Coefficient: −1.38e−01
Value: B
Actinomyces.radicidentis
1.00
0.75
0.50
0.25
0.00
A (n=15)
B (n=16)
C (n=14)
surface

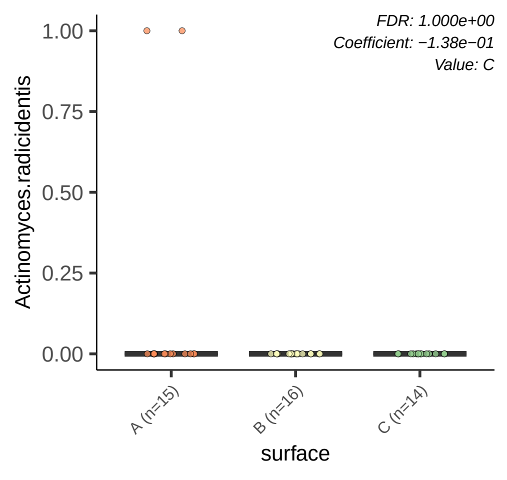

FDR: 1.000e+00
Coefficient: −1.38e−01
Value: C
Actinomyces.radicidentis
1.00
0.75
0.50
0.25
0.00
A (n=15)
B (n=16)
C (n=14)
surface

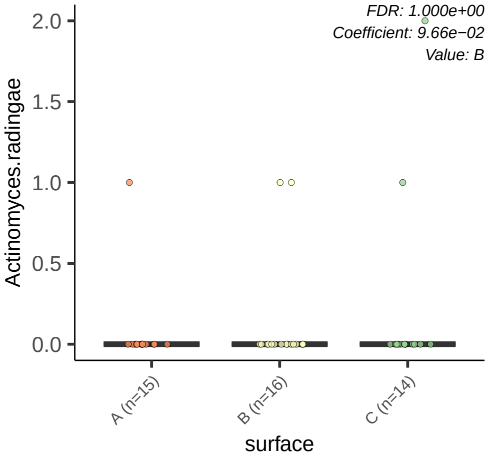
FDR: 1.000e+00
Coefficient: 9.66e−02
Value: B
Actinomyces.radingae
2.0
1.5
1.0
0.5
0.0
A (n=15)
B (n=16)
C (n=14)
surface

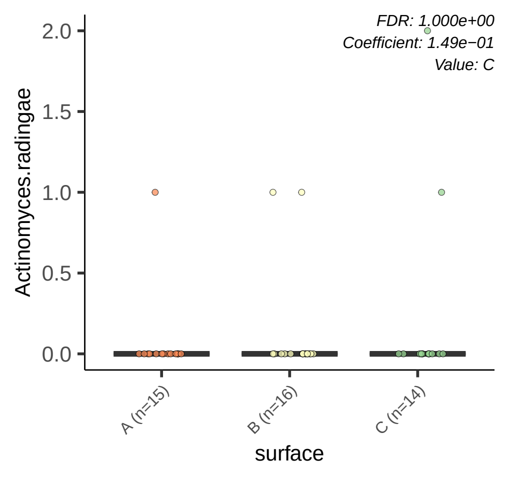
FDR: 1.000e+00
Coefficient: 1.49e−01
Value: C
Actinomyces.radingae
2.0
1.5
1.0
0.5
0.0
A (n=15)
B (n=16)
C (n=14)
surface

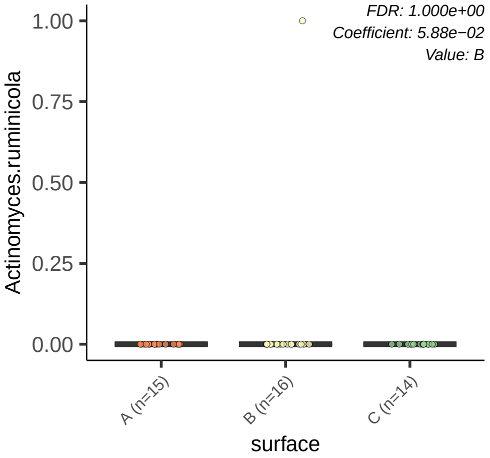

FDR: 1.000e+00
Coefficient: 5.88e−02
Value: B
Actinomyces.ruminicola
1.00
0.75
0.50
0.25
0.00
A (n=15)
B (n=16)
C (n=14)
surface

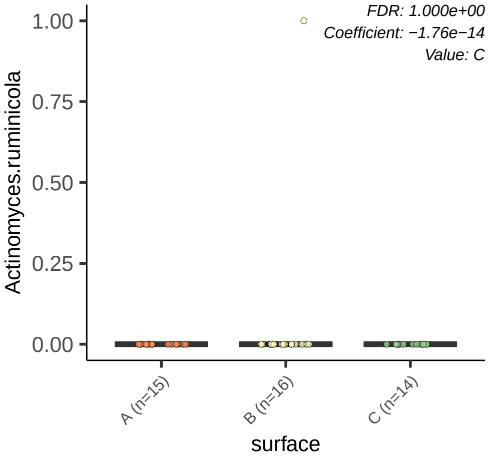
FDR: 1.000e+00
Coefficient: −1.76e−14
Value: C
Actinomyces.ruminicola
1.00
0.75
0.50
0.25
0.00
A (n=15)
B (n=16)
C (n=14)
surface

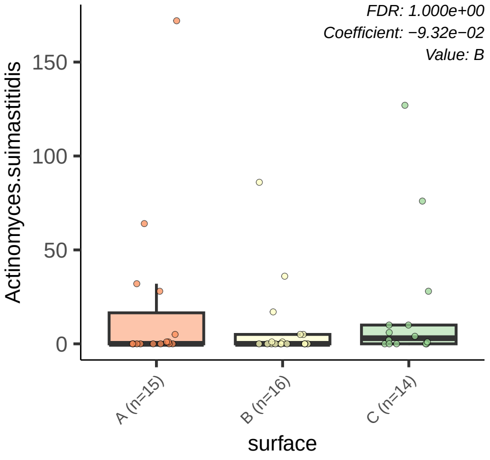

FDR: 1.000e+00
Coefficient: −9.32e−02
Value: B
Actinomyces.suimastitidis
150
100
50
0
A (n=15)
B (n=16)
C (n=14)
surface

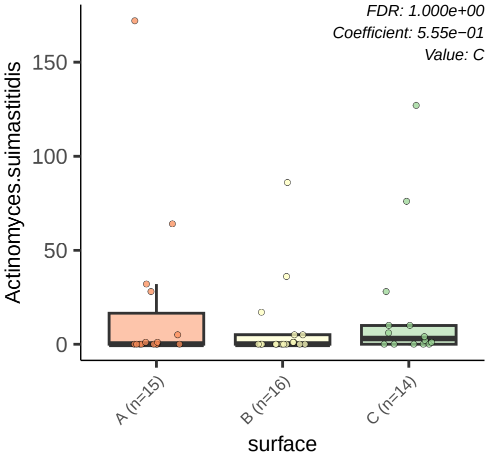

FDR: 1.000e+00
Coefficient: 5.55e−01
Value: C
Actinomyces.suimastitidis
150
100
50
0
A (n=15)
B (n=16)
C (n=14)
surface

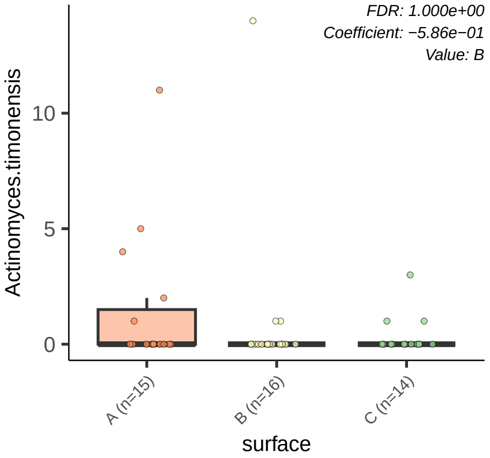
FDR: 1.000e+00
Coefficient: −5.86e−01
Value: B
Actinomyces.timonensis
0
5
10
A (n=15)
B (n=16)
C (n=14)
surface

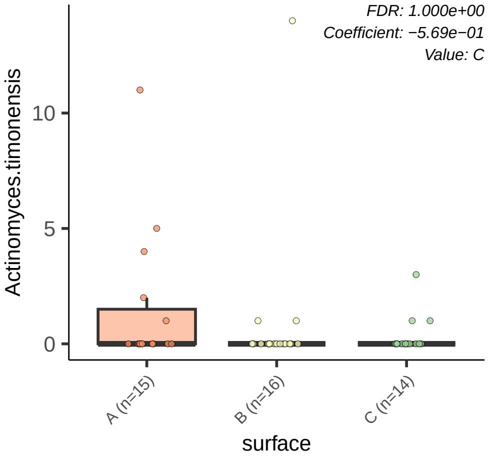

FDR: 1.000e+00
Coefficient: −5.69e−01
Value: C
Actinomyces.timonensis
10
5
0
A (n=15)
B (n=16)
C (n=14)
surface

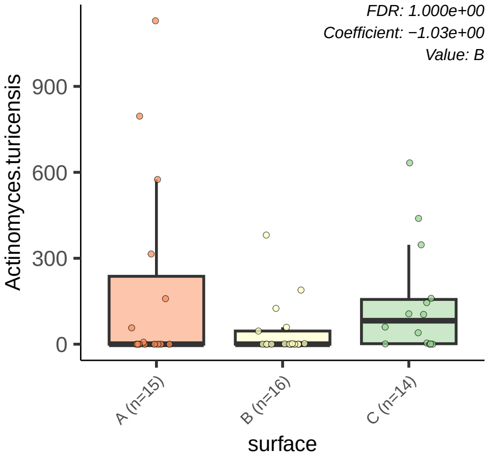

FDR: 1.000e+00
Coefficient: −1.03e+00
Value: B
Actinomyces.turicensis
900
600
300
0
A (n=15)
B (n=16)
C (n=14)
surface

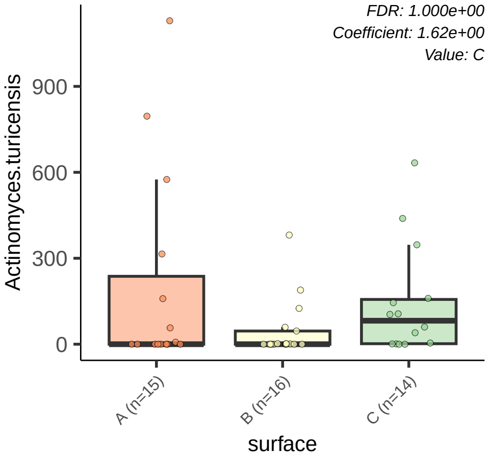

FDR: 1.000e+00
Coefficient: 1.62e+00
Value: C
Actinomyces.turicensis
900
600
300
0
A (n=15)
B (n=16)
C (n=14)
surface

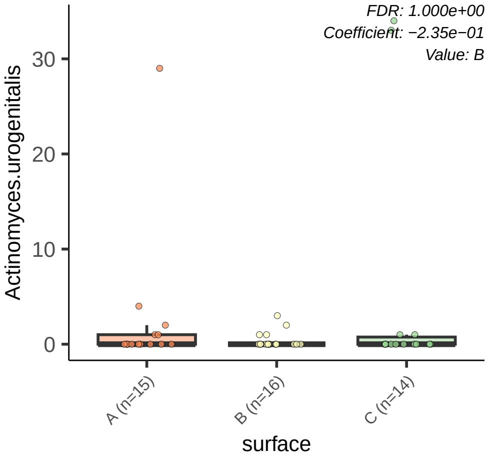
FDR: 1.000e+00
Coefficient: −2.35e−01
Value: B
Actinomyces.urogenitalis
0
10
20
30
A (n=15)
B (n=16)
C (n=14)
surface

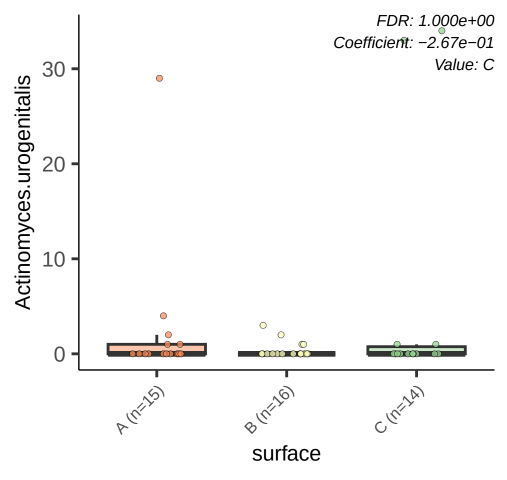
FDR: 1.000e+00
Coefficient: −2.67e−01
Value: C
Actinomyces.urogenitalis
0
10
20
30
A (n=15)
B (n=16)
C (n=14)
surface

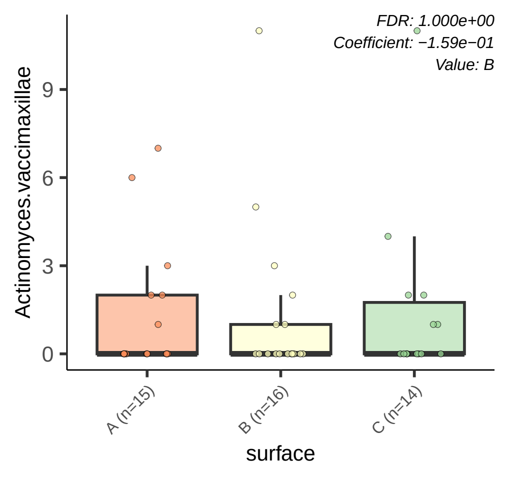

FDR: 1.000e+00
Coefficient: −1.59e−01
Value: B
Actinomyces.vaccimaxillae
0
3
6
9
A (n=15)
B (n=16)
C (n=14)
surface

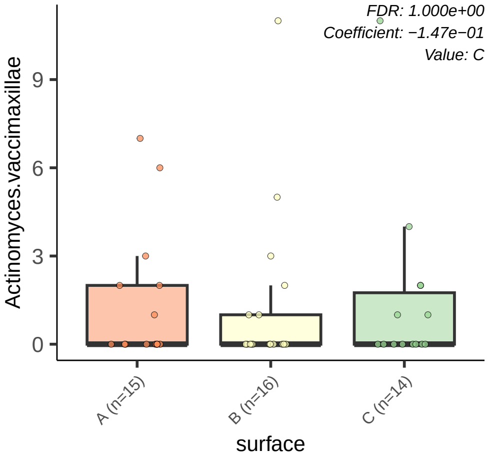
FDR: 1.000e+00
Coefficient: −1.47e−01
Value: C
Actinomyces.vaccimaxillae
0
3
6
9
A (n=15)
B (n=16)
C (n=14)
surface

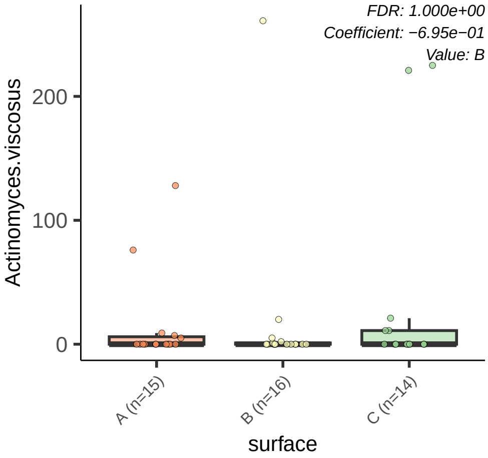

FDR: 1.000e+00
Coefficient: −6.95e−01
Value: B
Actinomyces.viscosus
200
100
0
A (n=15)
B (n=16)
C (n=14)
surface

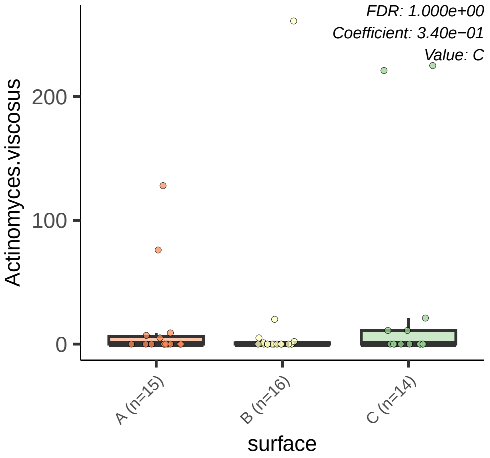
FDR: 1.000e+00
Coefficient: 3.40e−01
Value: C
Actinomyces.viscosus
200
100
0
A (n=15)
B (n=16)
C (n=14)
surface

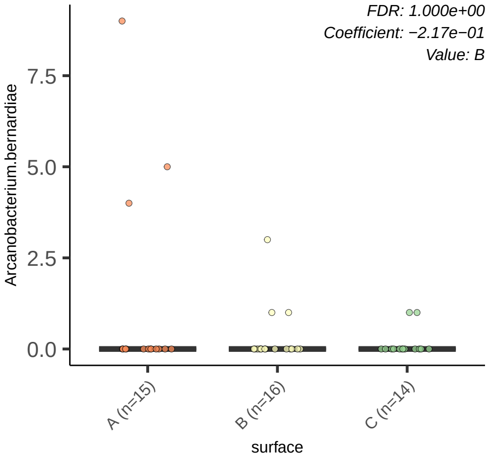

FDR: 1.000e+00
Coefficient: −2.17e−01
Value: B
Arcanobacterium.bernardiae
7.5
5.0
2.5
0.0
A (n=15)
B (n=16)
C (n=14)
surface

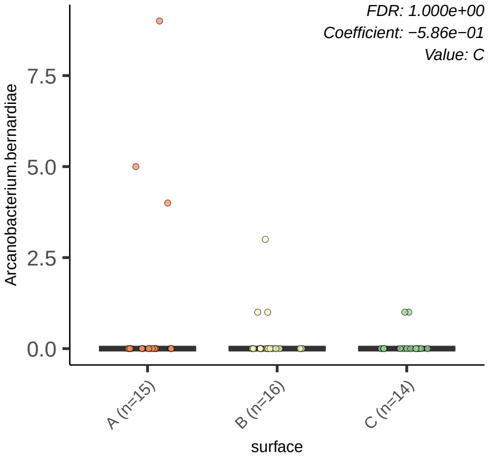
FDR: 1.000e+00
Coefficient: −5.86e−01
Value: C
Arcanobacterium.bernardiae
7.5
5.0
2.5
0.0
A (n=15)
B (n=16)
C (n=14)
surface

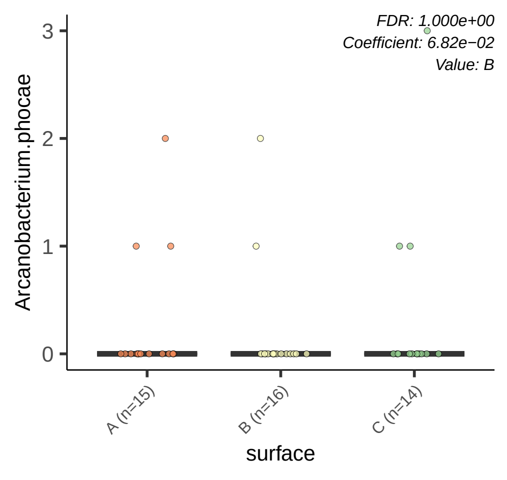
FDR: 1.000e+00
Coefficient: 6.82e−02
Value: B
Arcanobacterium.phocae
3
2
1
0
A (n=15)
B (n=16)
C (n=14)
surface

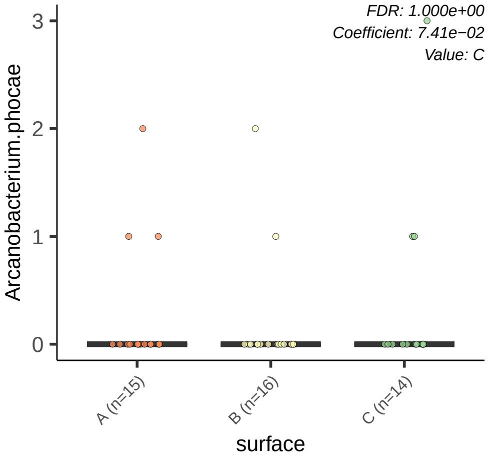
FDR: 1.000e+00
Coefficient: 7.41e−02
Value: C
Arcanobacterium.phocae
0
1
2
3
A (n=15)
B (n=16)
C (n=14)
surface

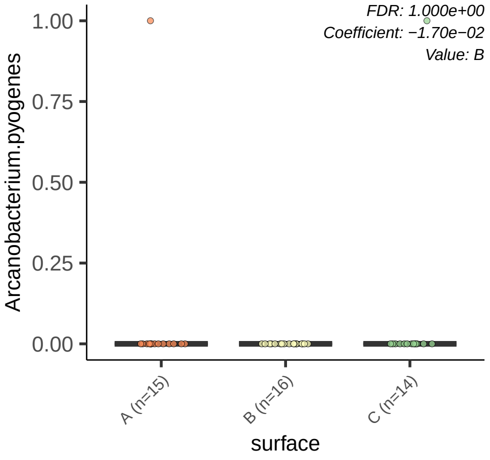

FDR: 1.000e+00
Coefficient: −1.70e−02
Value: B
Arcanobacterium.pyogenes
1.00
0.75
0.50
0.25
0.00
A (n=15)
B (n=16)
C (n=14)
surface

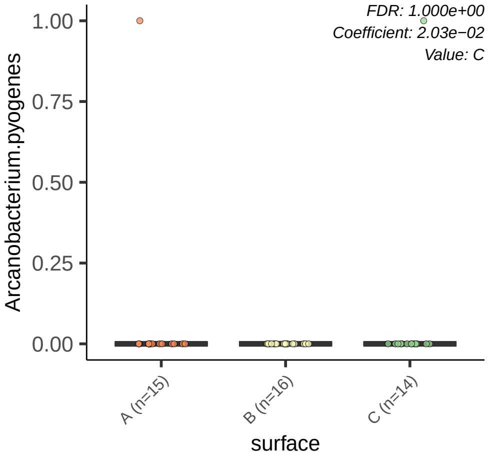
FDR: 1.000e+00
Coefficient: 2.03e−02
Value: C
Arcanobacterium.pyogenes
1.00
0.75
0.50
0.25
0.00
A (n=15)
B (n=16)
C (n=14)
surface

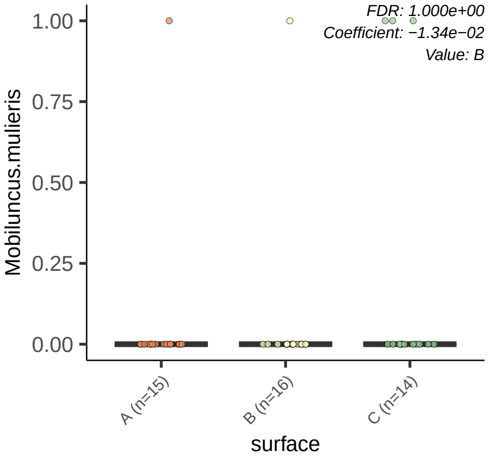
FDR: 1.000e+00
Coefficient: −1.34e−02
Value: B
Mobiluncus.mulieris
1.00
0.75
0.50
0.25
0.00
A (n=15)
B (n=16)
C (n=14)
surface

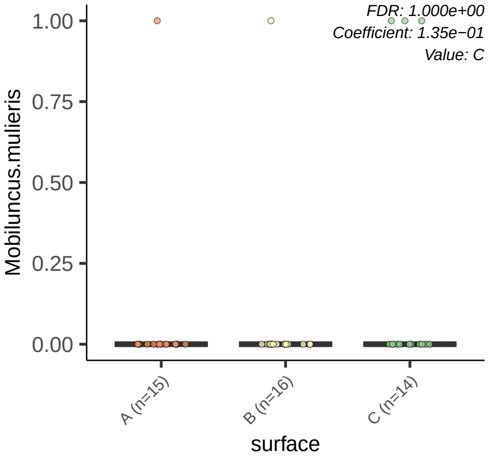
FDR: 1.000e+00
Coefficient: 1.35e−01
Value: C
Mobiluncus.mulieris
1.00
0.75
0.50
0.25
0.00
A (n=15)
B (n=16)
C (n=14)
surface

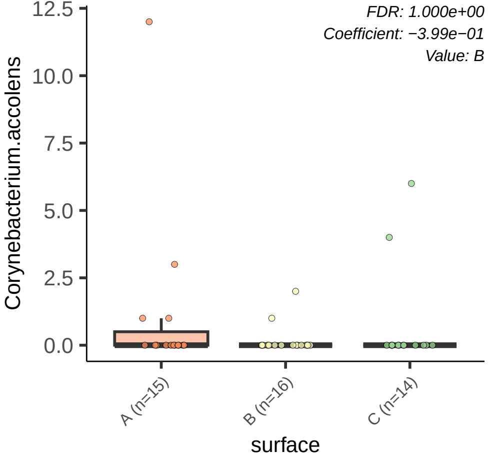
FDR: 1.000e+00
Coefficient: −3.99e−01
Value: B
Corynebacterium.accolens
12.5
10.0
7.5
5.0
2.5
0.0
A (n=15)
B (n=16)
C (n=14)
surface

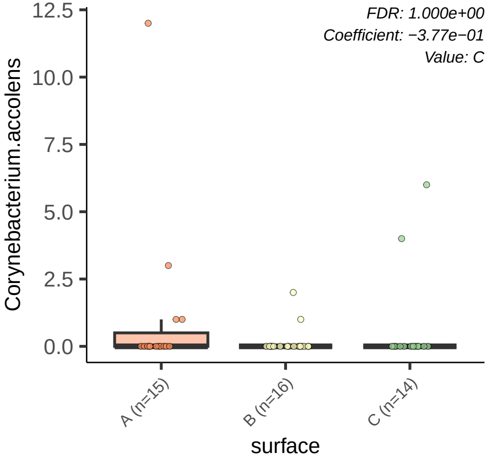

FDR: 1.000e+00
Coefficient: −3.77e−01
Value: C
Corynebacterium.accolens
12.5
10.0
7.5
5.0
2.5
0.0
A (n=15)
B (n=16)
C (n=14)
surface

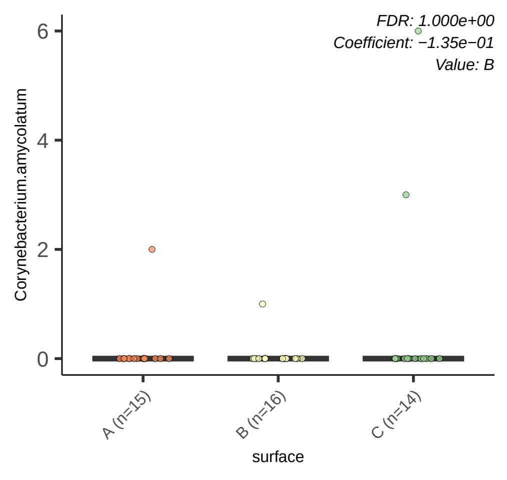
FDR: 1.000e+00
Coefficient: −1.35e−01
Value: B
Corynebacterium.amycolatum
0
2
4
6
A (n=15)
B (n=16)
C (n=14)
surface

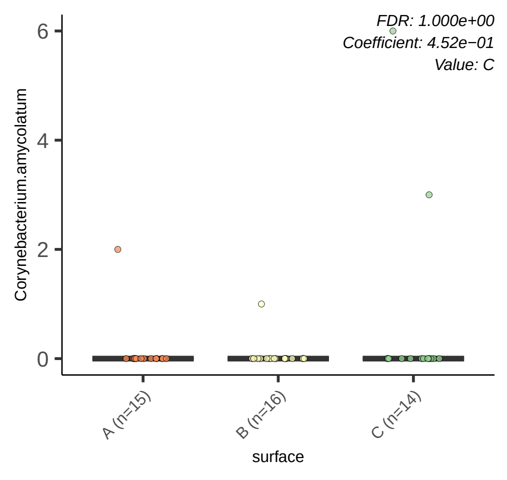
FDR: 1.000e+00
Coefficient: 4.52e−01
Value: C
Corynebacterium.amycolatum
0
2
4
6
A (n=15)
B (n=16)
C (n=14)
surface

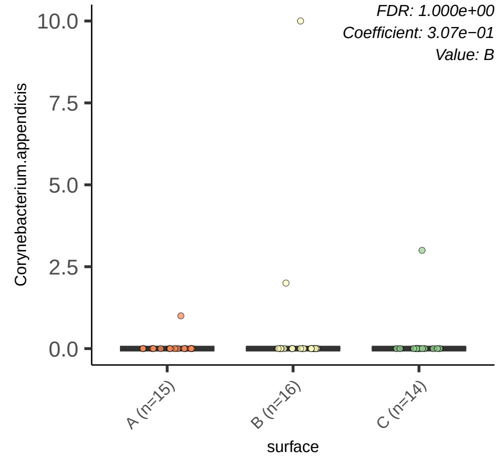

FDR: 1.000e+00
Coefficient: 3.07e−01
Value: B
10.0
7.5
5.0
2.5
0.0
Corynebacterium.appendicis
A (n=15)
B (n=16)
C (n=14)
surface

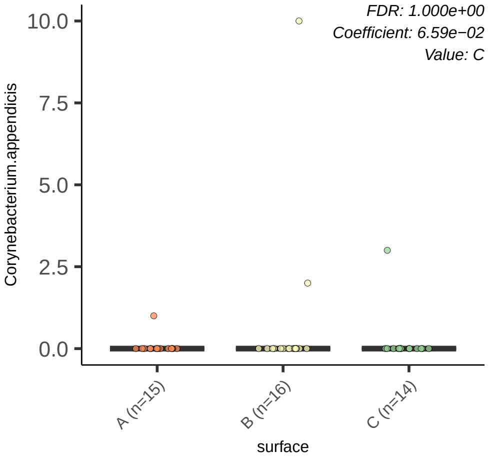
FDR: 1.000e+00
Coefficient: 6.59e−02
Value: C
Corynebacterium.appendicis
10.0
7.5
5.0
2.5
0.0
A (n=15)
B (n=16)
C (n=14)
surface

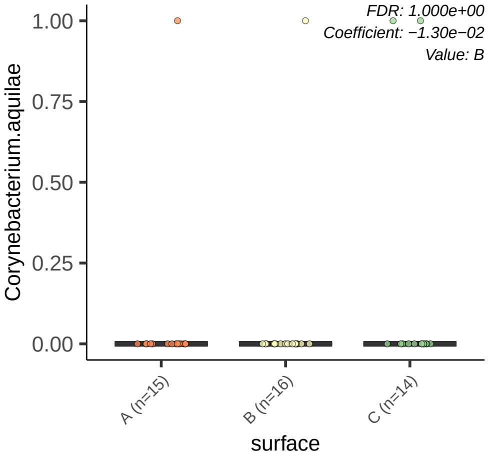
FDR: 1.000e+00
Coefficient: −1.30e−02
Value: B
Corynebacterium.aquilae
1.00
0.75
0.50
0.25
0.00
A (n=15)
B (n=16)
C (n=14)
surface

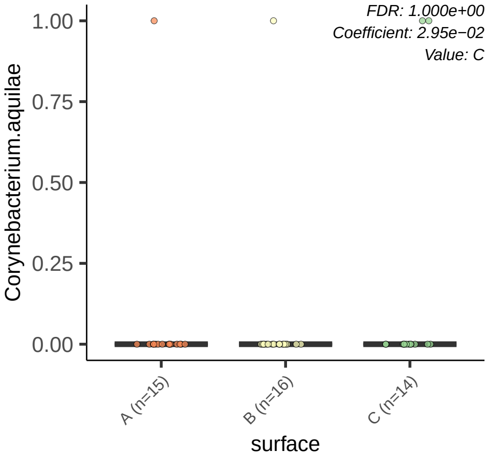

FDR: 1.000e+00
Coefficient: 2.95e−02
Value: C
Corynebacterium.aquilae
1.00
0.75
0.50
0.25
0.00
A (n=15)
B (n=16)
C (n=14)
surface

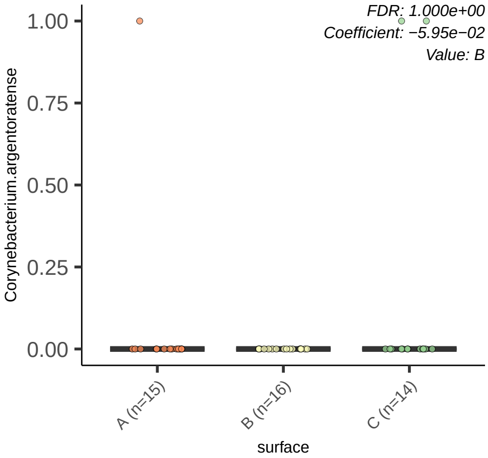

FDR: 1.000e+00
Coefficient: −5.95e−02
Value: B
1.00
0.75
0.50
0.25
0.00
Corynebacterium.argentoratense
A (n=15)
B (n=16)
C (n=14)
surface

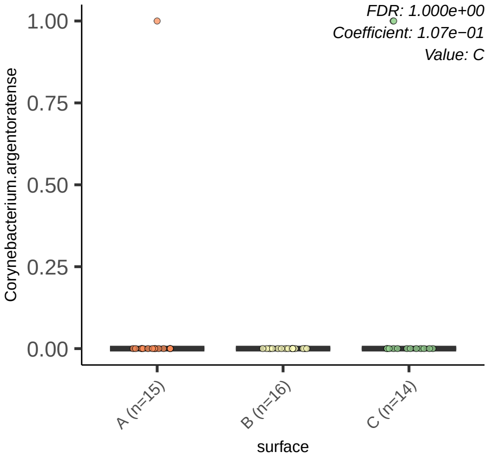
FDR: 1.000e+00
Coefficient: 1.07e−01
Value: C
Corynebacterium.argentoratense
1.00
0.75
0.50
0.25
0.00
A (n=15)
B (n=16)
C (n=14)
surface

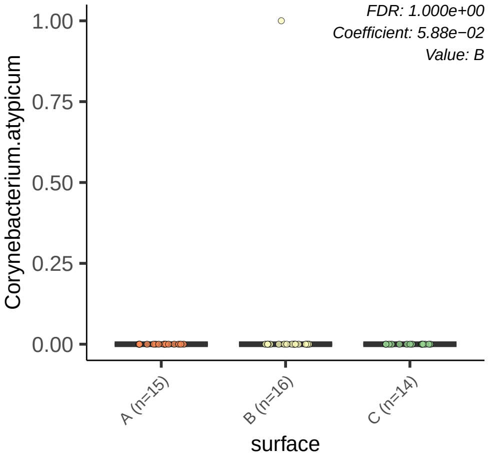

FDR: 1.000e+00
Coefficient: 5.88e−02
Value: B
Corynebacterium.atypicum
1.00
0.75
0.50
0.25
0.00
A (n=15)
B (n=16)
C (n=14)
surface

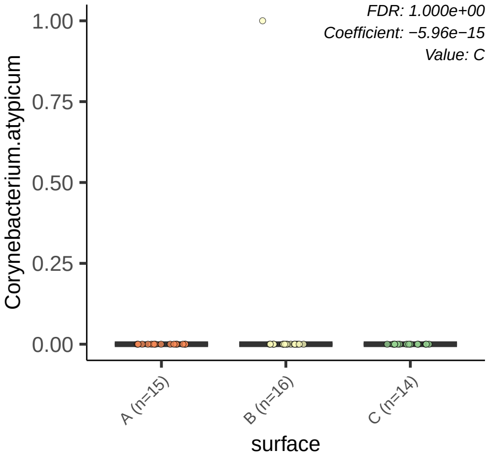
FDR: 1.000e+00
Coefficient: −5.96e−15
Value: C
Corynebacterium.atypicum
1.00
0.75
0.50
0.25
0.00
A (n=15)
B (n=16)
C (n=14)
surface

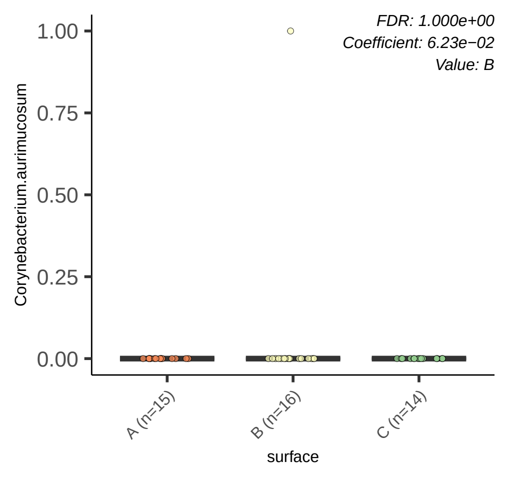

FDR: 1.000e+00
Coefficient: 6.23e−02
Value: B
1.00
0.75
0.50
0.25
0.00
Corynebacterium.aurimucosum
A (n=15)
B (n=16)
C (n=14)
surface

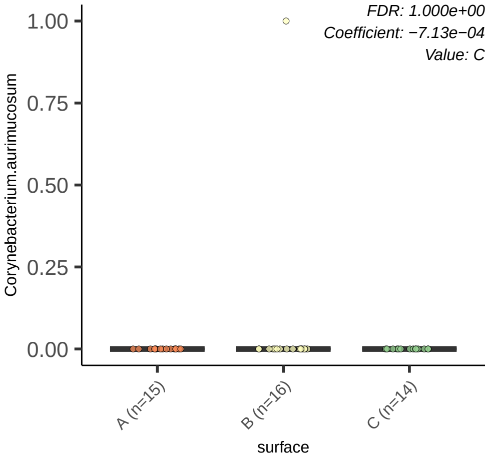
FDR: 1.000e+00
Coefficient: −7.13e−04
Value: C
1.00
0.75
0.50
0.25
0.00
Corynebacterium.aurimucosum
A (n=15)
B (n=16)
C (n=14)
surface

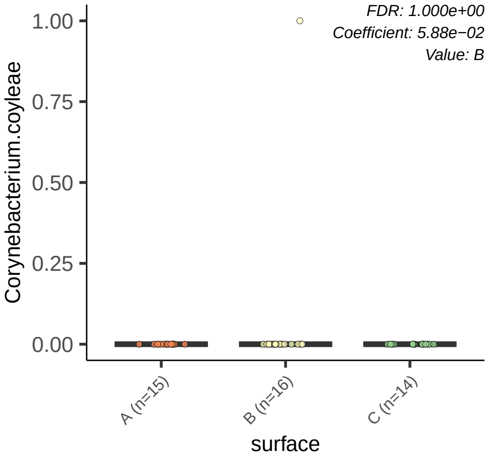

FDR: 1.000e+00
Coefficient: 5.88e−02
Value: B
Corynebacterium.coyleae
1.00
0.75
0.50
0.25
0.00
A (n=15)
B (n=16)
C (n=14)
surface

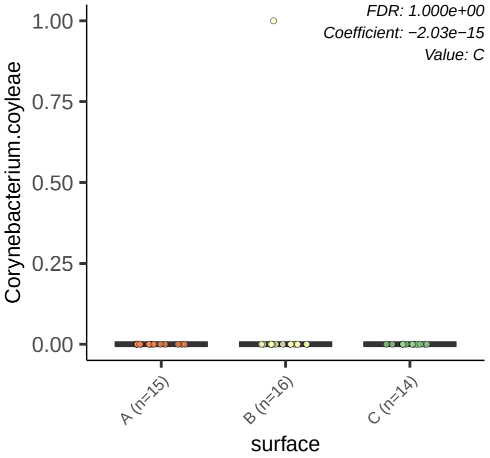
FDR: 1.000e+00
Coefficient: −2.03e−15
Value: C
Corynebacterium.coyleae
1.00
0.75
0.50
0.25
0.00
A (n=15)
B (n=16)
C (n=14)
surface

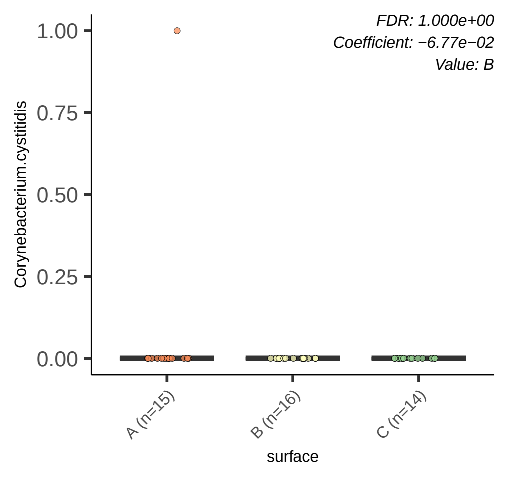
FDR: 1.000e+00
Coefficient: −6.77e−02
Value: B
Corynebacterium.cystitidis
1.00
0.75
0.50
0.25
0.00
A (n=15)
B (n=16)
C (n=14)
surface

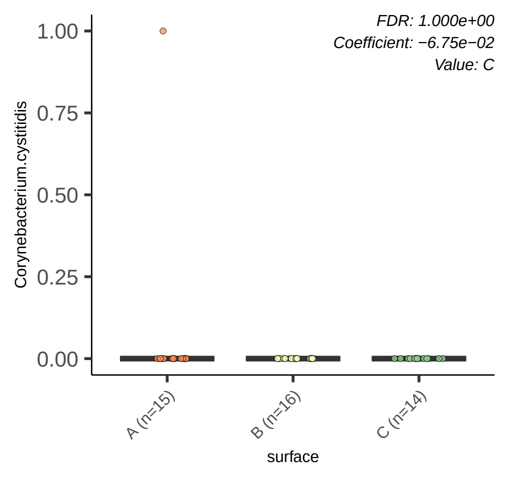
FDR: 1.000e+00
Coefficient: −6.75e−02
Value: C
Corynebacterium.cystitidis
1.00
0.75
0.50
0.25
0.00
A (n=15)
B (n=16)
C (n=14)
surface

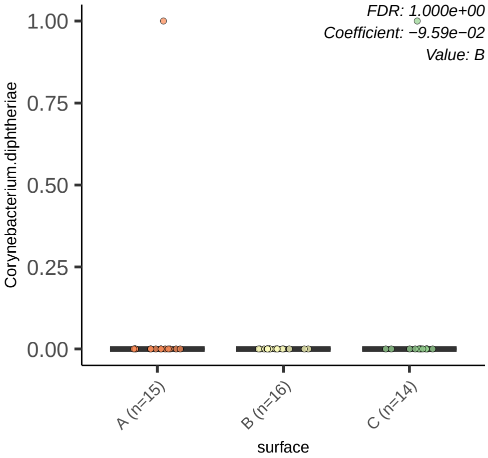
FDR: 1.000e+00
Coefficient: −9.59e−02
Value: B
Corynebacterium.diphtheriae
1.00
0.75
0.50
0.25
0.00
A (n=15)
B (n=16)
C (n=14)
surface

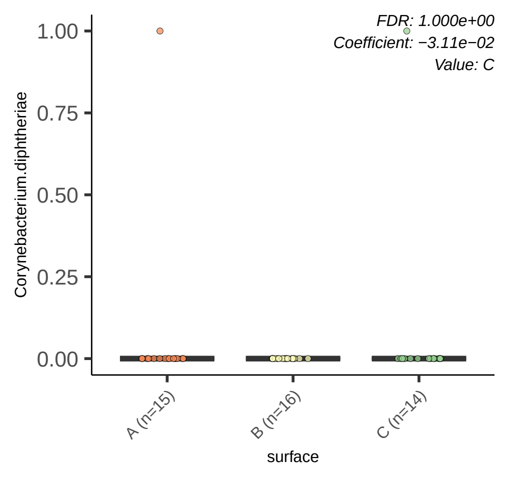

FDR: 1.000e+00
Coefficient: −3.11e−02
Value: C
1.00
0.75
0.50
0.25
0.00
Corynebacterium.diphtheriae
A (n=15)
B (n=16)
C (n=14)
surface

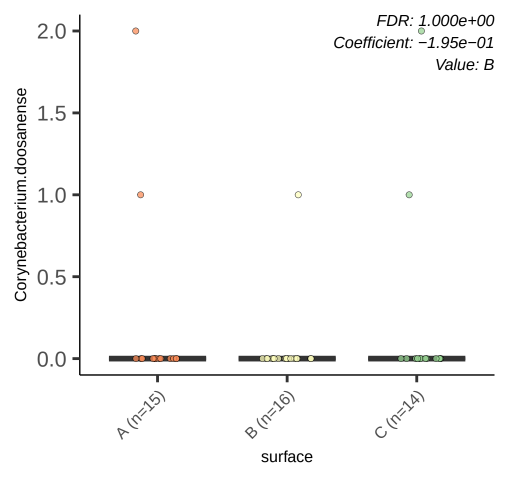
FDR: 1.000e+00
Coefficient: −1.95e−01
Value: B
Corynebacterium.doosanense
2.0
1.5
1.0
0.5
0.0
A (n=15)
B (n=16)
C (n=14)
surface

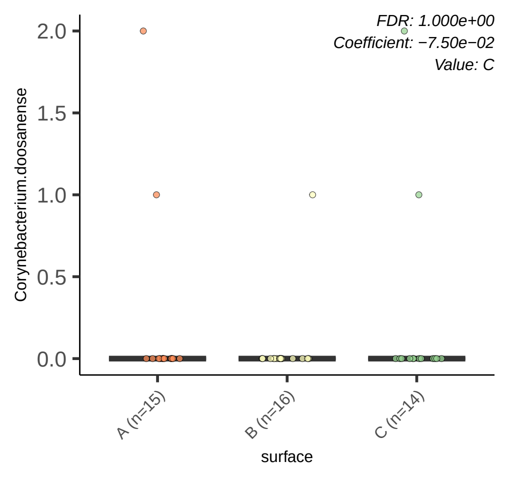

FDR: 1.000e+00
Coefficient: −7.50e−02
Value: C
Corynebacterium.doosanense
2.0
1.5
1.0
0.5
0.0
A (n=15)
B (n=16)
C (n=14)
surface

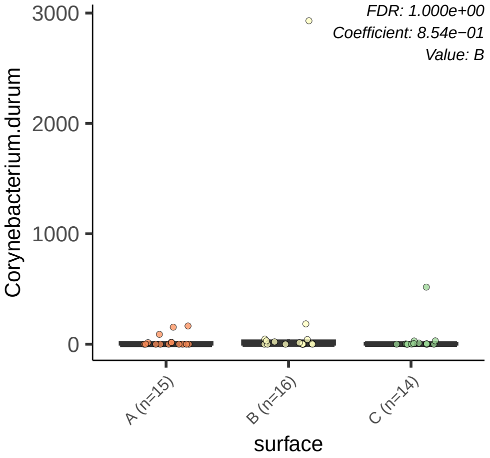

FDR: 1.000e+00
Coefficient: 8.54e−01
Value: B
3000
2000
1000
0
Corynebacterium.durum
A (n=15)
B (n=16)
C (n=14)
surface

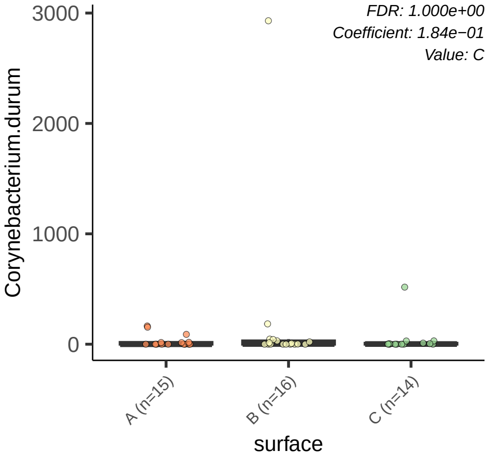
FDR: 1.000e+00
Coefficient: 1.84e−01
Value: C
3000
2000
1000
0
Corynebacterium.durum
A (n=15)
B (n=16)
C (n=14)
surface

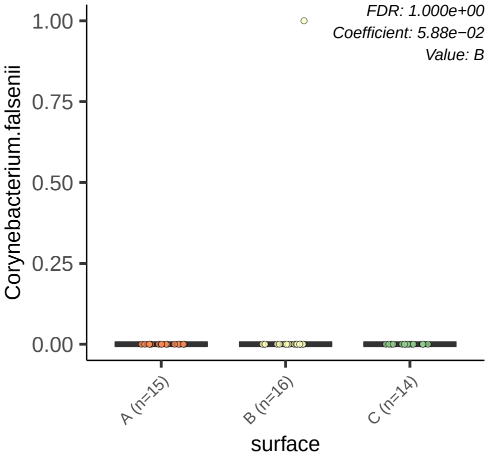
FDR: 1.000e+00
Coefficient: 5.88e−02
Value: B
Corynebacterium.falsenii
1.00
0.75
0.50
0.25
0.00
A (n=15)
B (n=16)
C (n=14)
surface

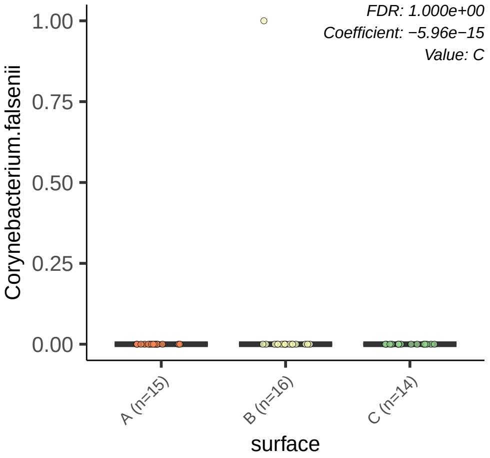
FDR: 1.000e+00
Coefficient: −5.96e−15
Value: C
Corynebacterium.falsenii
1.00
0.75
0.50
0.25
0.00
A (n=15)
B (n=16)
C (n=14)
surface

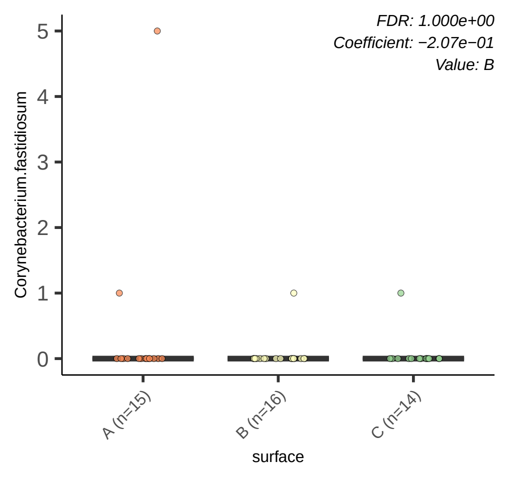
FDR: 1.000e+00
Coefficient: −2.07e−01
Value: B
Corynebacterium.fastidiosum
5
4
3
2
1
0
A (n=15)
B (n=16)
C (n=14)
surface

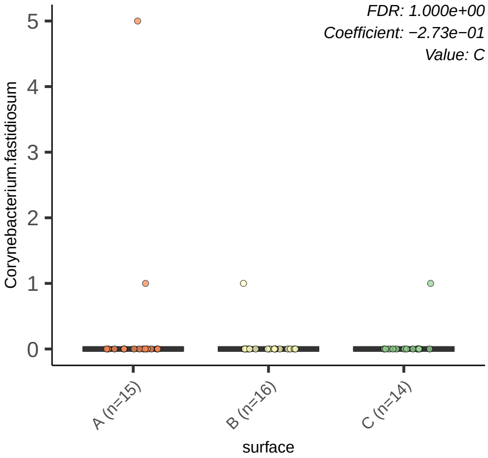
FDR: 1.000e+00
Coefficient: −2.73e−01
Value: C
Corynebacterium.fastidiosum
0
1
2
3
4
5
A (n=15)
B (n=16)
C (n=14)
surface

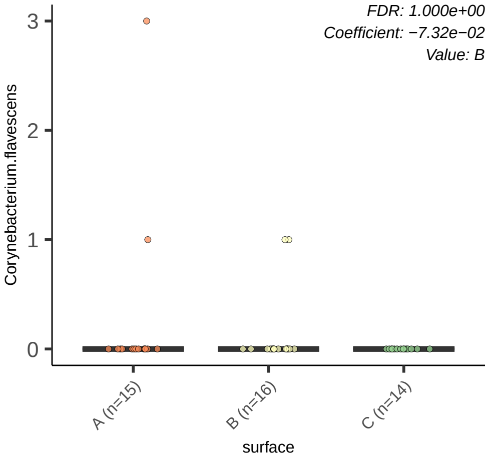
FDR: 1.000e+00
Coefficient: −7.32e−02
Value: B
Corynebacterium.flavescens
3
2
1
0
A (n=15)
B (n=16)
C (n=14)
surface

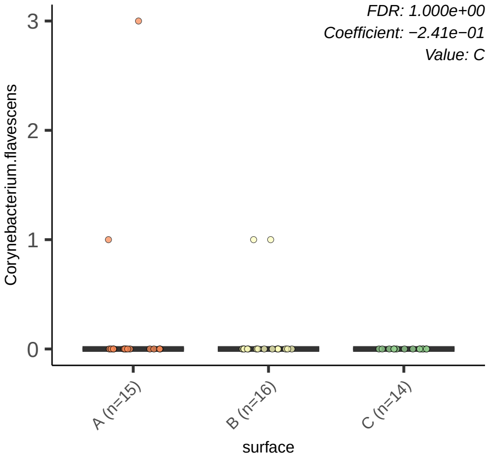
FDR: 1.000e+00
Coefficient: −2.41e−01
Value: C
Corynebacterium.flavescens
3
2
1
0
A (n=15)
B (n=16)
C (n=14)
surface

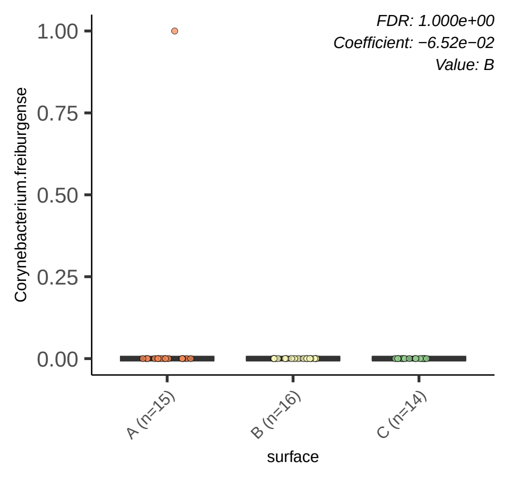
FDR: 1.000e+00
Coefficient: −6.52e−02
Value: B
Corynebacterium.freiburgense
1.00
0.75
0.50
0.25
0.00
A (n=15)
B (n=16)
C (n=14)
surface

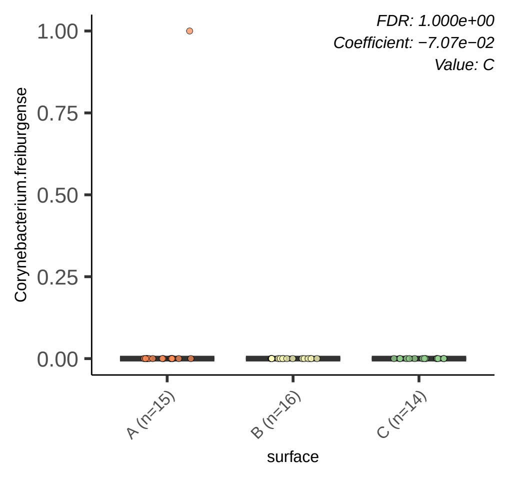
FDR: 1.000e+00
Coefficient: −7.07e−02
Value: C
1.00
0.75
0.50
0.25
0.00
Corynebacterium.freiburgense
A (n=15)
B (n=16)
C (n=14)
surface

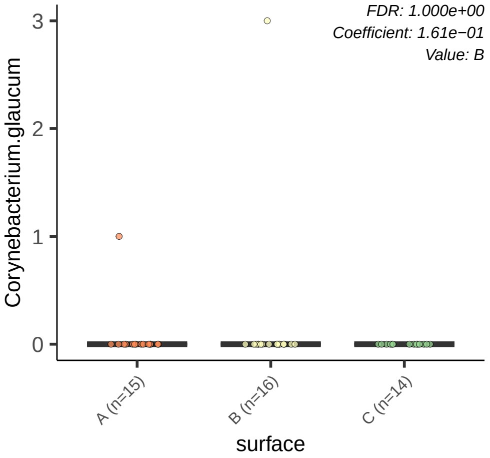

FDR: 1.000e+00
Coefficient: 1.61e−01
Value: B
Corynebacterium.glaucum
0
1
2
3
A (n=15)
B (n=16)
C (n=14)
surface

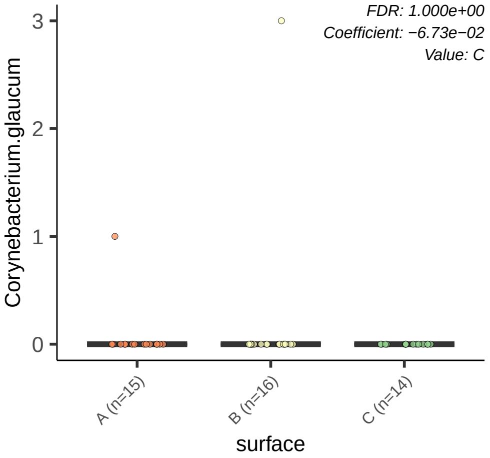

FDR: 1.000e+00
Coefficient: −6.73e−02
Value: C
Corynebacterium.glaucum
3
2
1
0
A (n=15)
B (n=16)
C (n=14)
surface

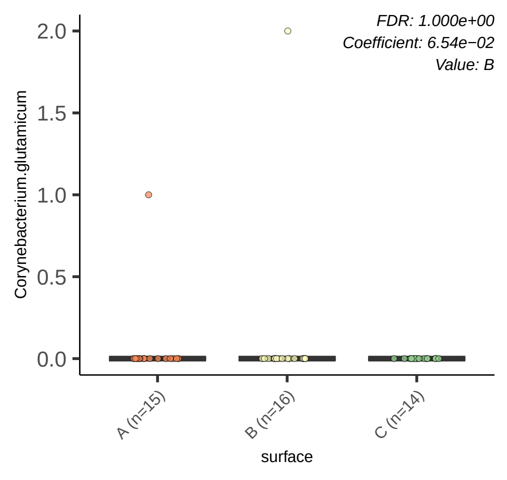
FDR: 1.000e+00
Coefficient: 6.54e−02
Value: B
Corynebacterium.glutamicum
2.0
1.5
1.0
0.5
0.0
A (n=15)
B (n=16)
C (n=14)
surface

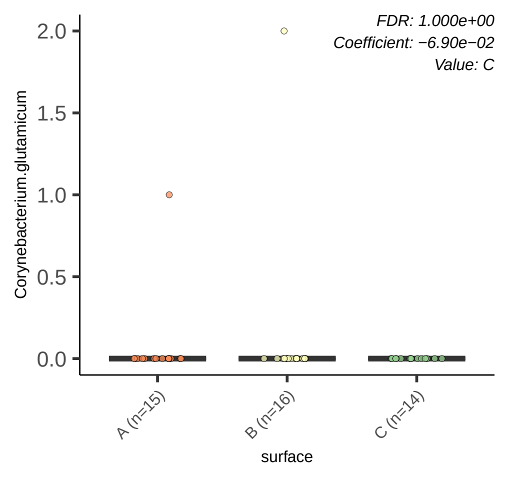

FDR: 1.000e+00
Coefficient: −6.90e−02
Value: C
Corynebacterium.glutamicum
2.0
1.5
1.0
0.5
0.0
A (n=15)
B (n=16)
C (n=14)
surface

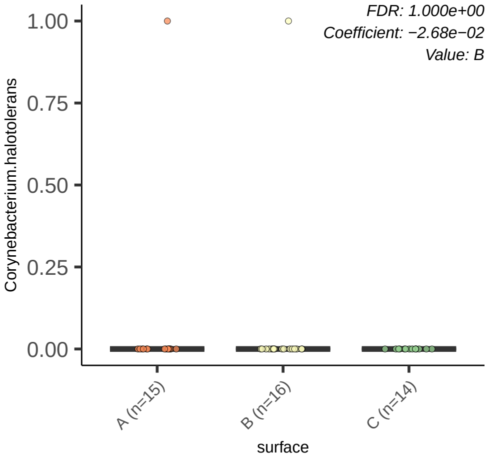
FDR: 1.000e+00
Coefficient: −2.68e−02
Value: B
Corynebacterium.halotolerans
1.00
0.75
0.50
0.25
0.00
A (n=15)
B (n=16)
C (n=14)
surface

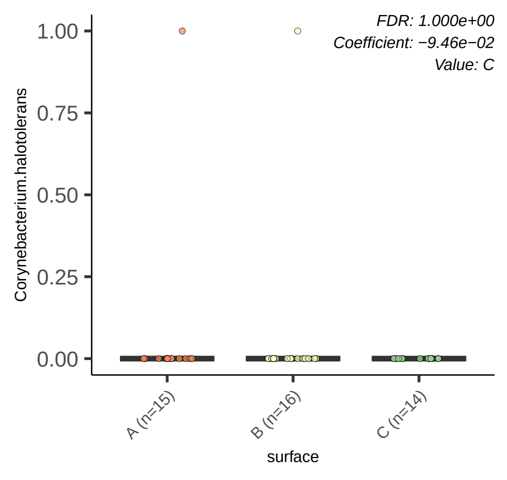

FDR: 1.000e+00
Coefficient: −9.46e−02
Value: C
1.00
0.75
0.50
0.25
0.00
Corynebacterium.halotolerans
A (n=15)
B (n=16)
C (n=14)
surface

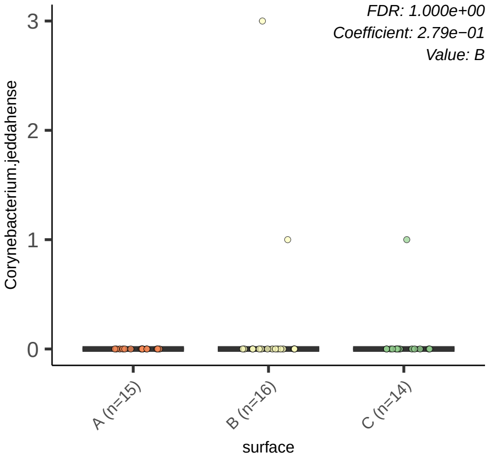
FDR: 1.000e+00
Coefficient: 2.79e−01
Value: B
Corynebacterium.jeddahense
3
2
1
0
A (n=15)
B (n=16)
C (n=14)
surface

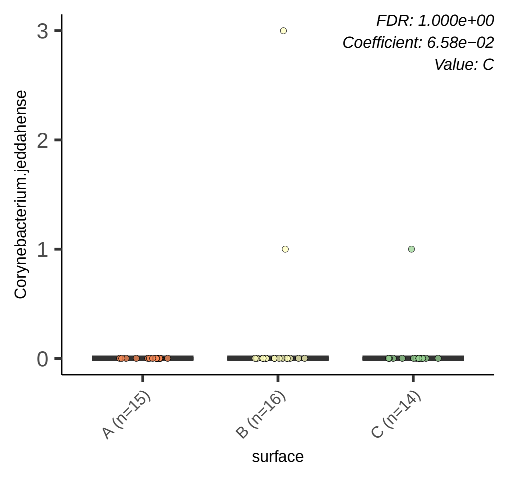
FDR: 1.000e+00
Coefficient: 6.58e−02
Value: C
Corynebacterium.jeddahense
3
2
1
0
A (n=15)
B (n=16)
C (n=14)
surface

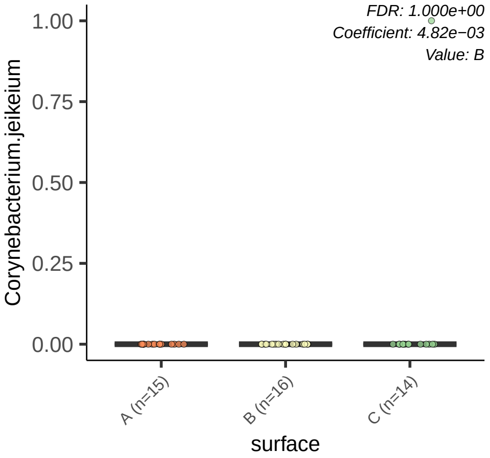

FDR: 1.000e+00
Coefficient: 4.82e−03
Value: B
Corynebacterium.jeikeium
1.00
0.75
0.50
0.25
0.00
A (n=15)
B (n=16)
C (n=14)
surface

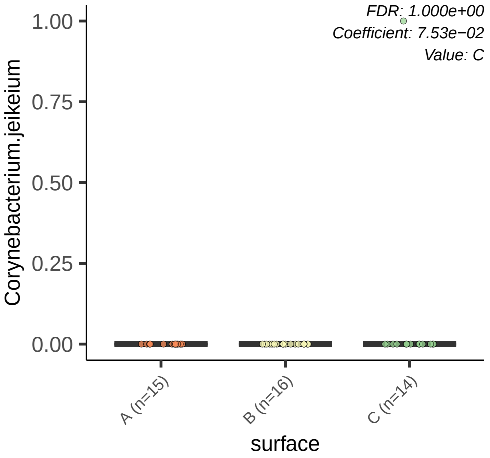

FDR: 1.000e+00
Coefficient: 7.53e−02
Value: C
Corynebacterium.jeikeium
1.00
0.75
0.50
0.25
0.00
A (n=15)
B (n=16)
C (n=14)
surface

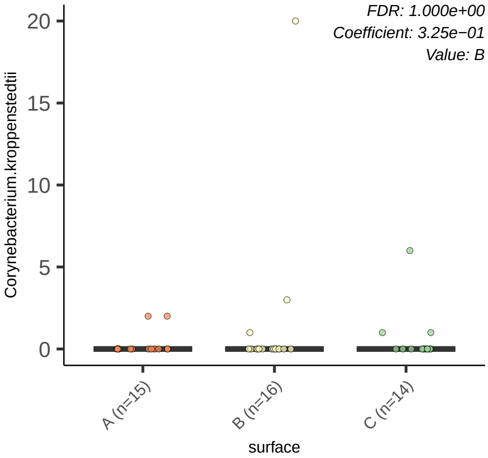

FDR: 1.000e+00
Coefficient: 3.25e−01
Value: B
Corynebacterium.kroppenstedtii
20
15
10
5
0
A (n=15)
B (n=16)
C (n=14)
surface

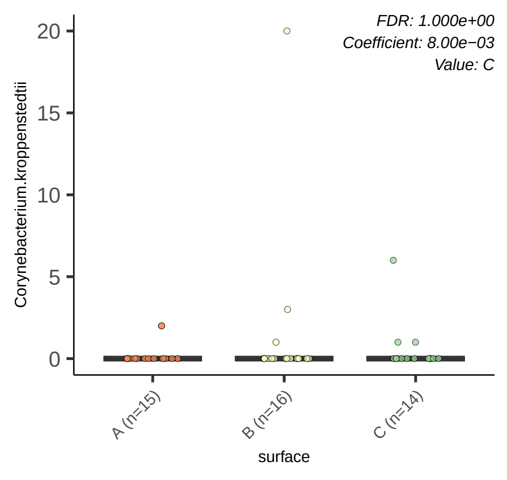

FDR: 1.000e+00
Coefficient: 8.00e−03
Value: C
Corynebacterium.kroppenstedtii
20
15
10
5
0
A (n=15)
B (n=16)
C (n=14)
surface

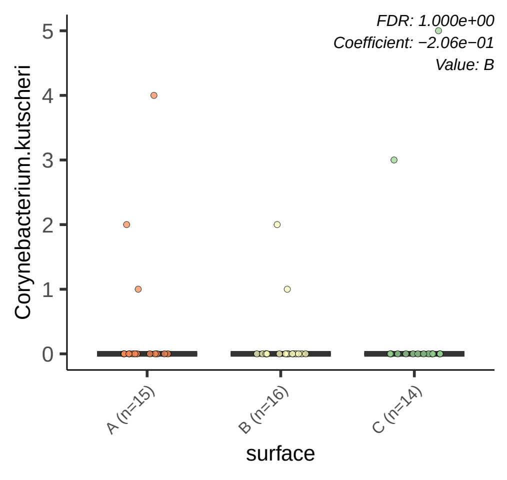
FDR: 1.000e+00
Coefficient: −2.06e−01
Value: B
Corynebacterium.kutscheri
0
1
2
3
4
5
A (n=15)
B (n=16)
C (n=14)
surface

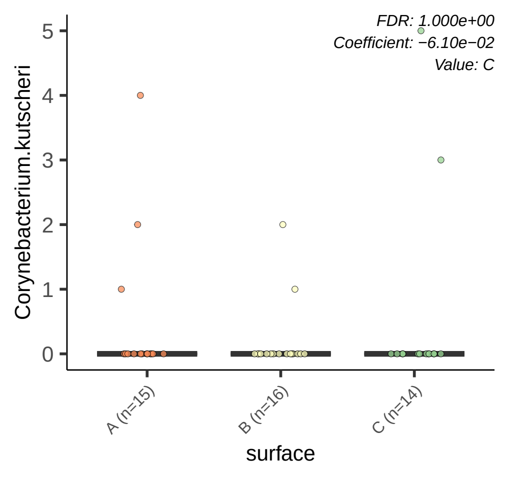
FDR: 1.000e+00
Coefficient: −6.10e−02
Value: C
Corynebacterium.kutscheri
0
1
2
3
4
5
A (n=15)
B (n=16)
C (n=14)
surface

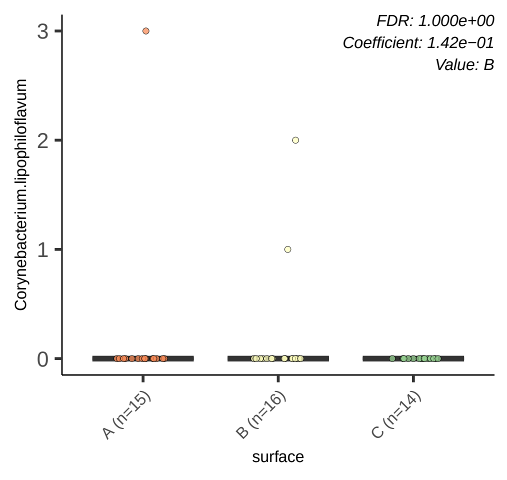
FDR: 1.000e+00
Coefficient: 1.42e−01
Value: B
Corynebacterium.lipophiloflavum
3
2
1
0
A (n=15)
B (n=16)
C (n=14)
surface

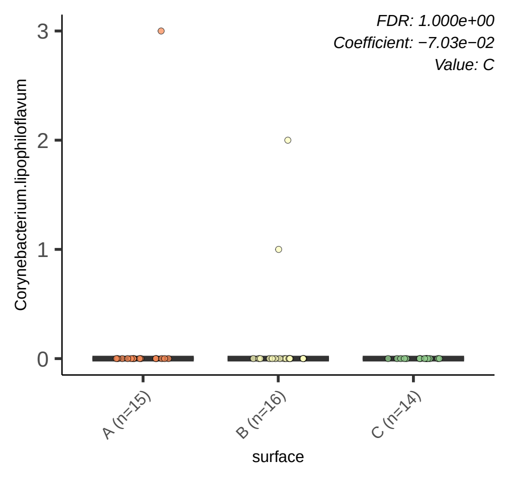
FDR: 1.000e+00
Coefficient: −7.03e−02
Value: C
Corynebacterium.lipophiloflavum
3
2
1
0
A (n=15)
B (n=16)
C (n=14)
surface

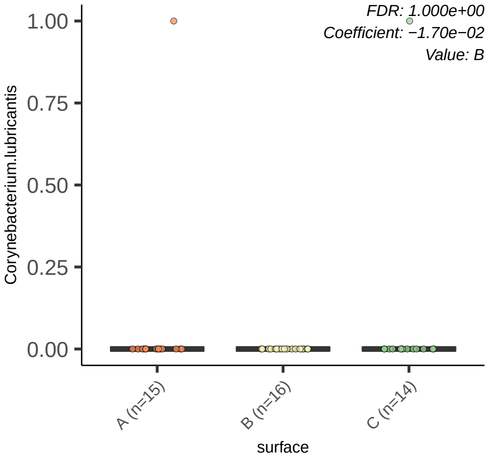
FDR: 1.000e+00
Coefficient: −1.70e−02
Value: B
1.00
0.75
0.50
0.25
0.00
Corynebacterium.lubricantis
A (n=15)
B (n=16)
C (n=14)
surface

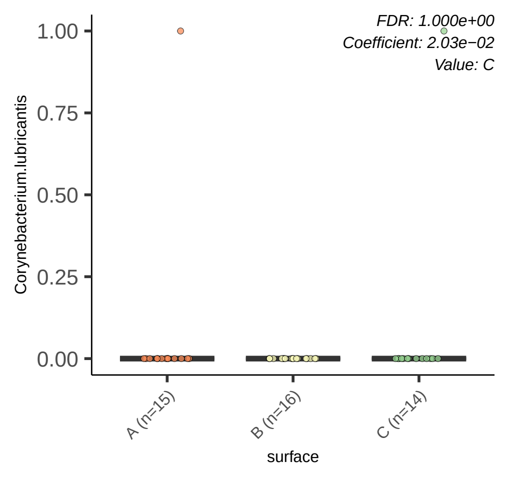
FDR: 1.000e+00
Coefficient: 2.03e−02
Value: C
Corynebacterium.lubricantis
1.00
0.75
0.50
0.25
0.00
A (n=15)
B (n=16)
C (n=14)
surface

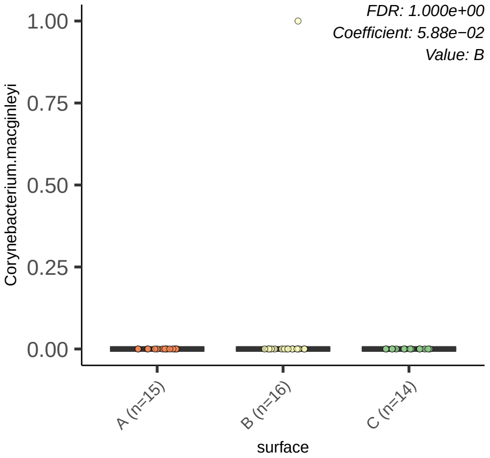
FDR: 1.000e+00
Coefficient: 5.88e−02
Value: B
1.00
0.75
0.50
0.25
0.00
Corynebacterium.macginleyi
A (n=15)
B (n=16)
C (n=14)
surface

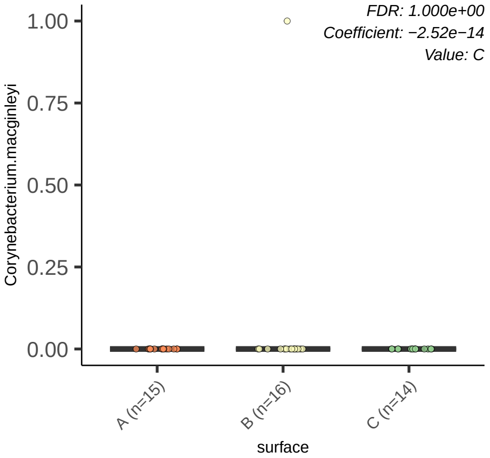

FDR: 1.000e+00
Coefficient: −2.52e−14
Value: C
Corynebacterium.macginleyi
1.00
0.75
0.50
0.25
0.00
A (n=15)
B (n=16)
C (n=14)
surface

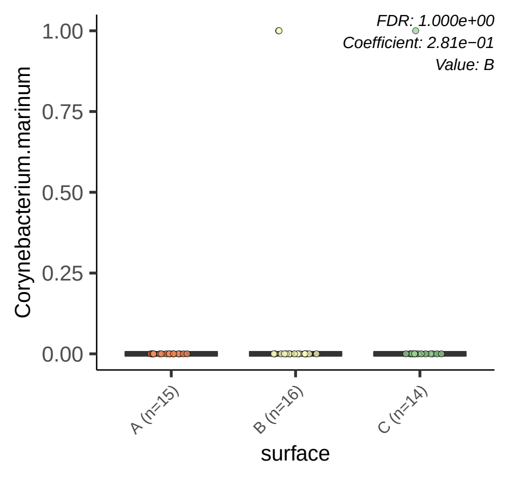

FDR: 1.000e+00
Coefficient: 2.81e−01
Value: B
1.00
0.75
0.50
0.25
0.00
Corynebacterium.marinum
A (n=15)
B (n=16)
C (n=14)
surface

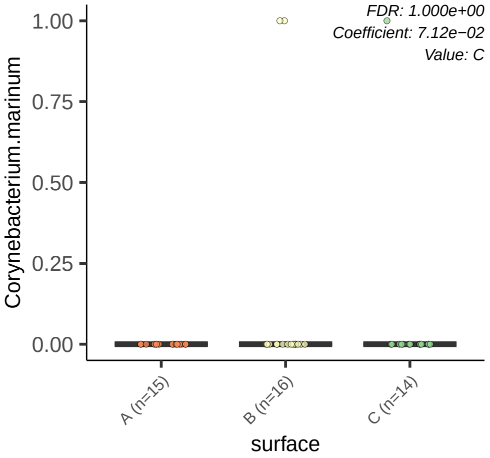
FDR: 1.000e+00
Coefficient: 7.12e−02
Value: C
Corynebacterium.marinum
1.00
0.75
0.50
0.25
0.00
A (n=15)
B (n=16)
C (n=14)
surface

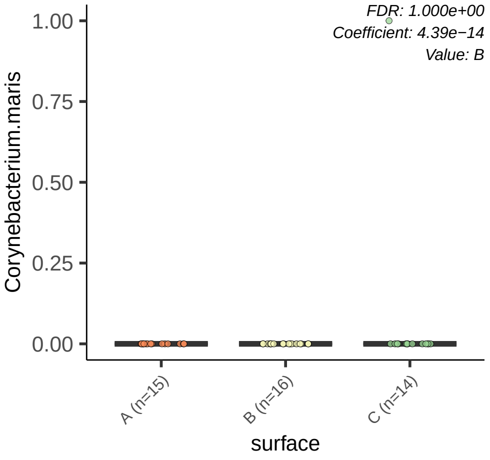

FDR: 1.000e+00
Coefficient: 4.39e−14
Value: B
Corynebacterium.maris
1.00
0.75
0.50
0.25
0.00
A (n=15)
B (n=16)
C (n=14)
surface

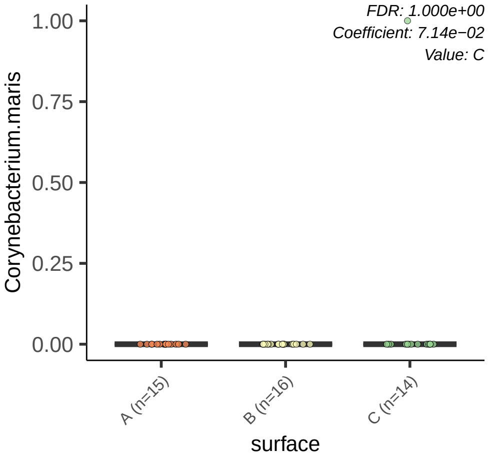
FDR: 1.000e+00
Coefficient: 7.14e−02
Value: C
Corynebacterium.maris
1.00
0.75
0.50
0.25
0.00
A (n=15)
B (n=16)
C (n=14)
surface

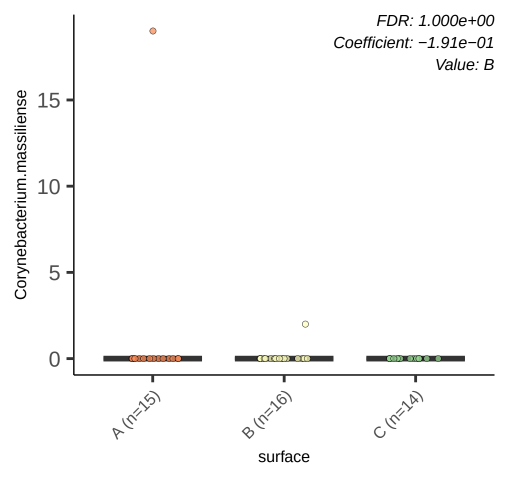

FDR: 1.000e+00
Coefficient: −1.91e−01
Value: B
Corynebacterium.massiliense
0
5
10
15
A (n=15)
B (n=16)
C (n=14)
surface

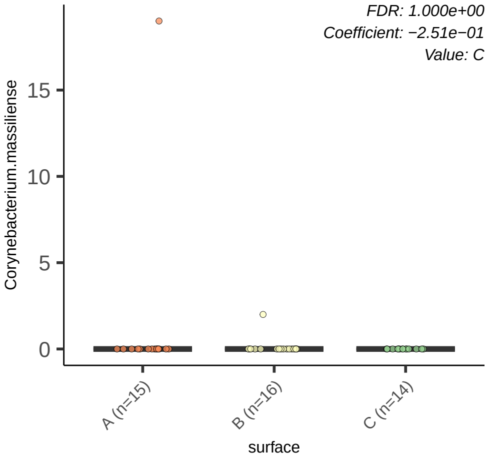
FDR: 1.000e+00
Coefficient: −2.51e−01
Value: C
Corynebacterium.massiliense
15
10
5
0
A (n=15)
B (n=16)
C (n=14)
surface

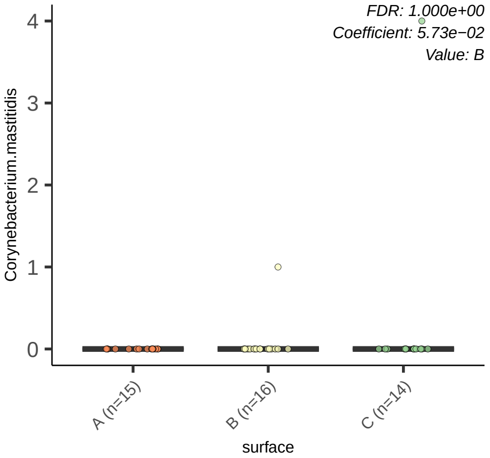

FDR: 1.000e+00
Coefficient: 5.73e−02
Value: B
Corynebacterium.mastitidis
0
1
2
3
4
A (n=15)
B (n=16)
C (n=14)
surface

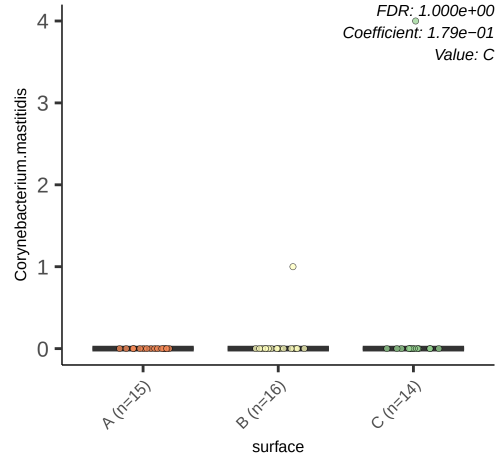
FDR: 1.000e+00
Coefficient: 1.79e−01
Value: C
Corynebacterium.mastitidis
4
3
2
1
0
A (n=15)
B (n=16)
C (n=14)
surface

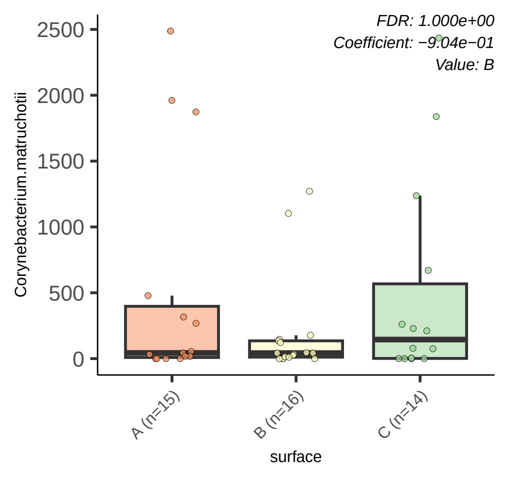

FDR: 1.000e+00
Coefficient: −9.04e−01
Value: B
2500
2000
1500
1000
500
0
Corynebacterium.matruchotii
A (n=15)
B (n=16)
C (n=14)
surface

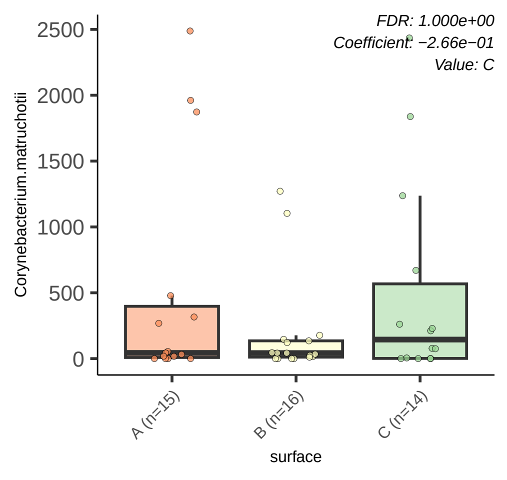
FDR: 1.000e+00
Coefficient: −2.66e−01
Value: C
Corynebacterium.matruchotii
2500
2000
1500
1000
500
0
A (n=15)
B (n=16)
C (n=14)
surface

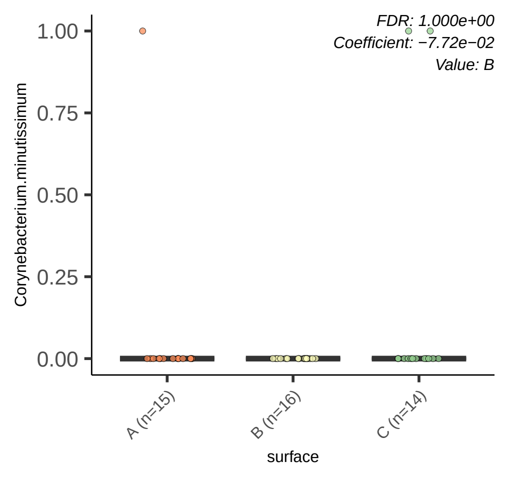

FDR: 1.000e+00
Coefficient: −7.72e−02
Value: B
Corynebacterium.minutissimum
1.00
0.75
0.50
0.25
0.00
A (n=15)
B (n=16)
C (n=14)
surface

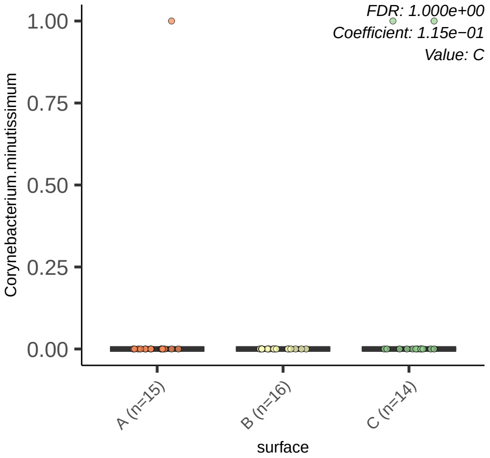
FDR: 1.000e+00
Coefficient: 1.15e−01
Value: C
Corynebacterium.minutissimum
1.00
0.75
0.50
0.25
0.00
A (n=15)
B (n=16)
C (n=14)
surface

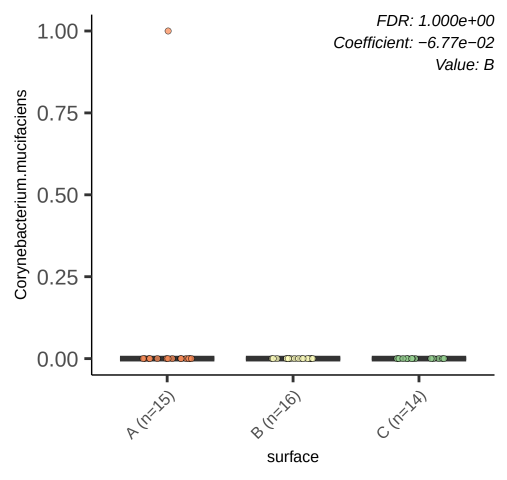

FDR: 1.000e+00
Coefficient: −6.77e−02
Value: B
Corynebacterium.mucifaciens
1.00
0.75
0.50
0.25
0.00
A (n=15)
B (n=16)
C (n=14)
surface

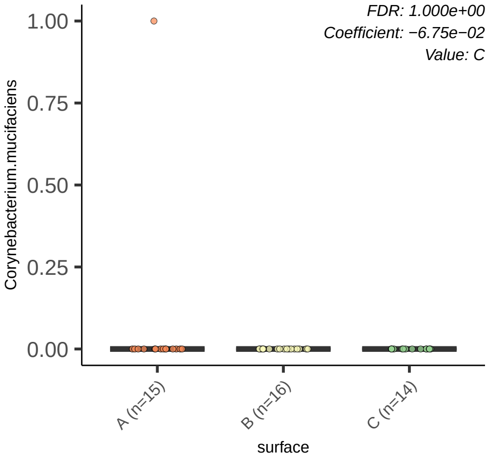

FDR: 1.000e+00
Coefficient: −6.75e−02
Value: C
Corynebacterium.mucifaciens
1.00
0.75
0.50
0.25
0.00
A (n=15)
B (n=16)
C (n=14)
surface

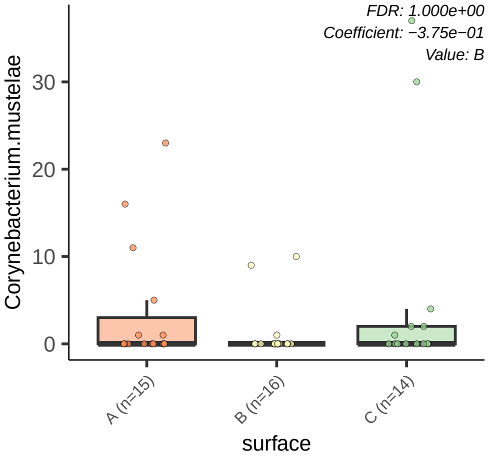

FDR: 1.000e+00
Coefficient: −3.75e−01
Value: B
Corynebacterium.mustelae
30
20
10
0
A (n=15)
B (n=16)
C (n=14)
surface

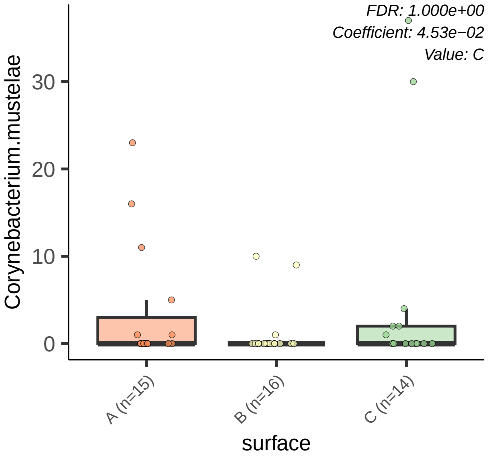

FDR: 1.000e+00
Coefficient: 4.53e−02
Value: C
Corynebacterium.mustelae
0
10
20
30
A (n=15)
B (n=16)
C (n=14)
surface

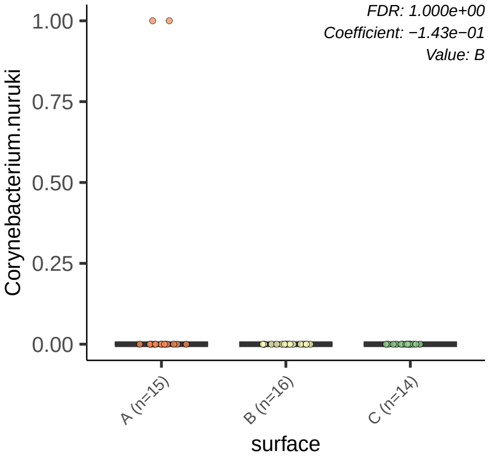
FDR: 1.000e+00
Coefficient: −1.43e−01
Value: B
Corynebacterium.nuruki
1.00
0.75
0.50
0.25
0.00
A (n=15)
B (n=16)
C (n=14)
surface

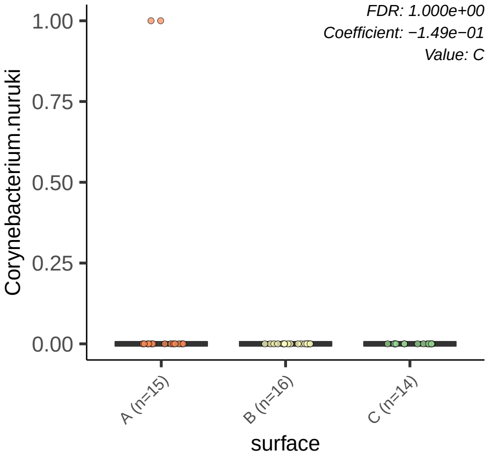

FDR: 1.000e+00
Coefficient: −1.49e−01
Value: C
Corynebacterium.nuruki
1.00
0.75
0.50
0.25
0.00
A (n=15)
B (n=16)
C (n=14)
surface

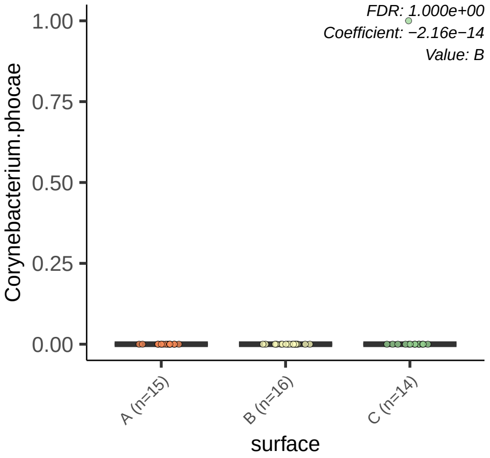
FDR: 1.000e+00
Coefficient: −2.16e−14
Value: B
Corynebacterium.phocae
1.00
0.75
0.50
0.25
0.00
A (n=15)
B (n=16)
C (n=14)
surface

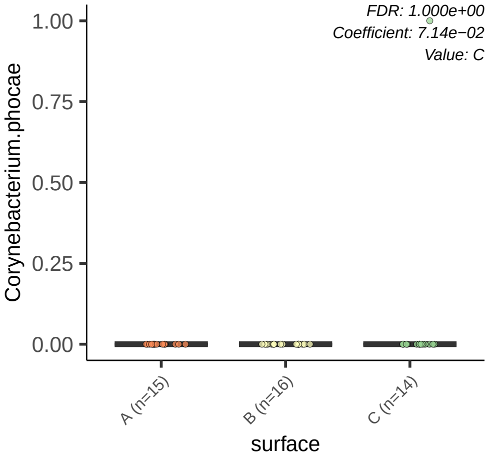
FDR: 1.000e+00
Coefficient: 7.14e−02
Value: C
Corynebacterium.phocae
1.00
0.75
0.50
0.25
0.00
A (n=15)
B (n=16)
C (n=14)
surface

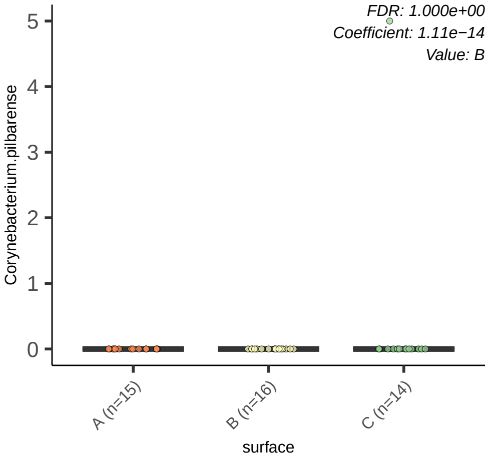
FDR: 1.000e+00
Coefficient: 1.11e−14
Value: B
Corynebacterium.pilbarense
5
4
3
2
1
0
A (n=15)
B (n=16)
C (n=14)
surface

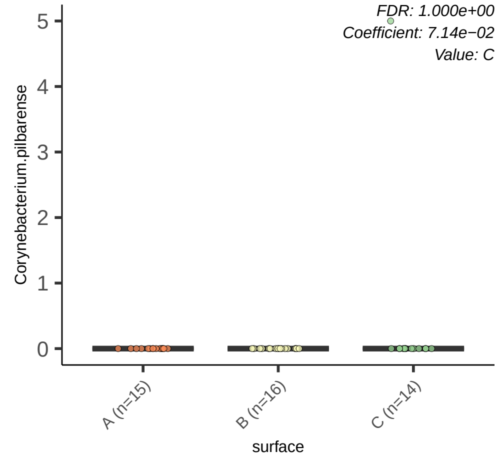

FDR: 1.000e+00
Coefficient: 7.14e−02
Value: C
Corynebacterium.pilbarense
0
1
2
3
4
5
A (n=15)
B (n=16)
C (n=14)
surface

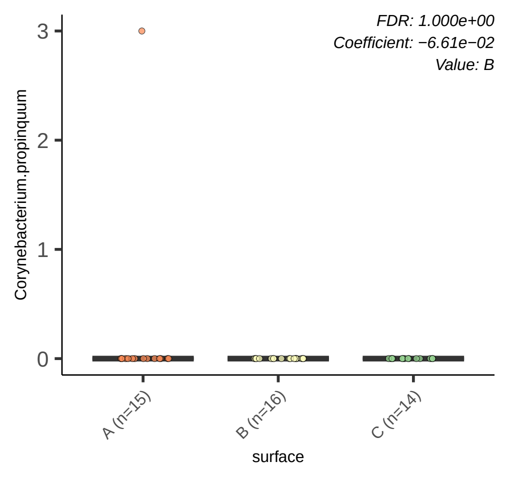

FDR: 1.000e+00
Coefficient: −6.61e−02
Value: B
Corynebacterium.propinquum
3
2
1
0
A (n=15)
B (n=16)
C (n=14)
surface

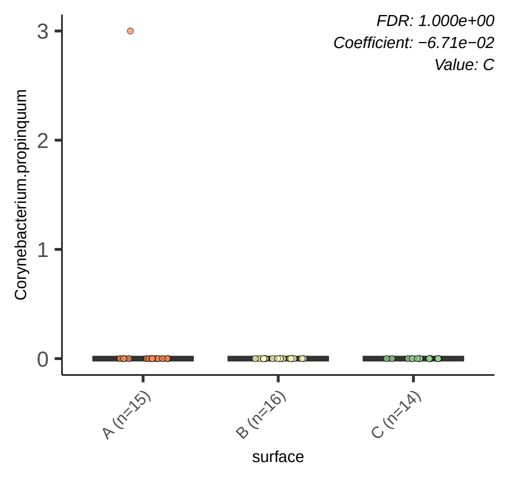
FDR: 1.000e+00
Coefficient: −6.71e−02
Value: C
Corynebacterium.propinquum
3
2
1
0
A (n=15)
B (n=16)
C (n=14)
surface

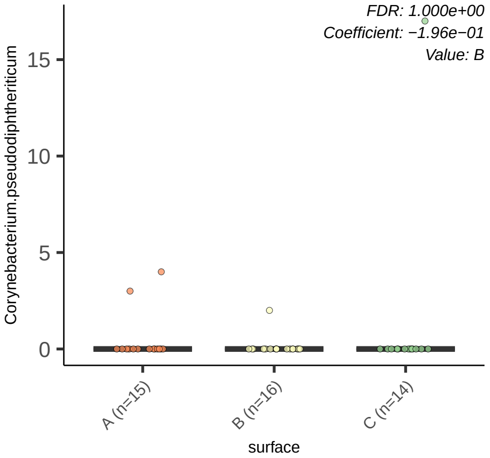
FDR: 1.000e+00
Coefficient: −1.96e−01
Value: B
Corynebacterium.pseudodiphtheriticum
15
10
5
0
A (n=15)
B (n=16)
C (n=14)
surface

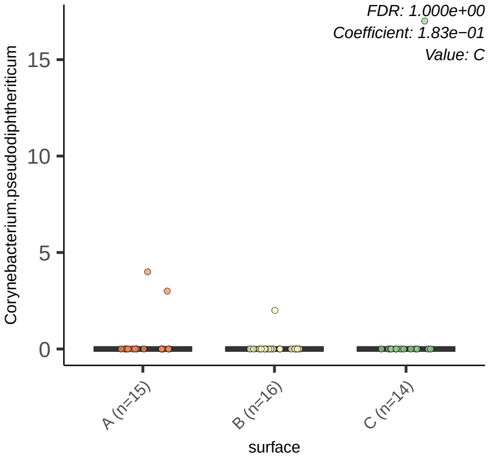
FDR: 1.000e+00
Coefficient: 1.83e−01
Value: C
Corynebacterium.pseudodiphtheriticum
15
10
5
0
A (n=15)
B (n=16)
C (n=14)
surface

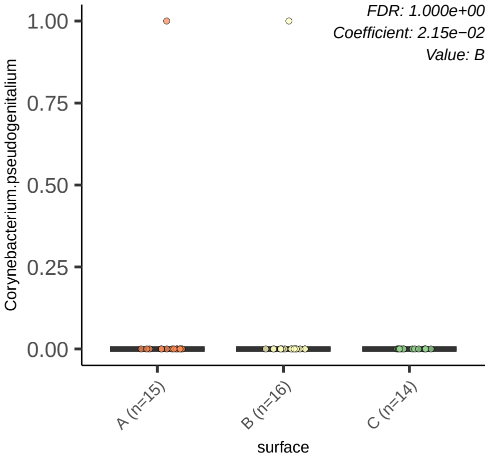

FDR: 1.000e+00
Coefficient: 2.15e−02
Value: B
Corynebacterium.pseudogenitalium
1.00
0.75
0.50
0.25
0.00
A (n=15)
B (n=16)
C (n=14)
surface

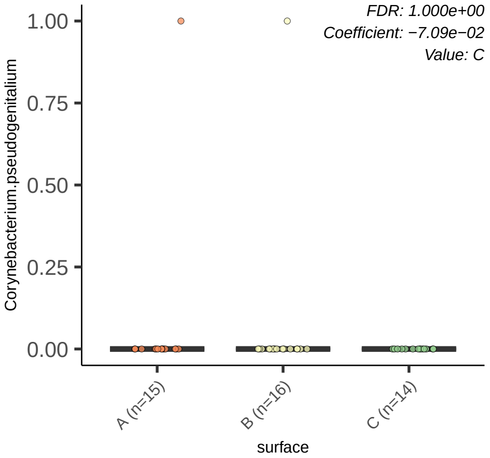
FDR: 1.000e+00
Coefficient: −7.09e−02
Value: C
Corynebacterium.pseudogenitalium
1.00
0.75
0.50
0.25
0.00
A (n=15)
B (n=16)
C (n=14)
surface

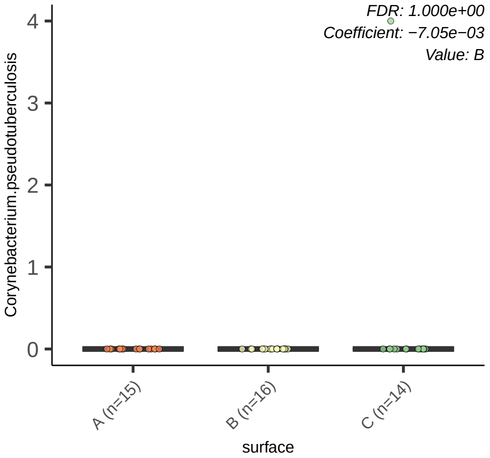
FDR: 1.000e+00
Coefficient: −7.05e−03
Value: B
Corynebacterium.pseudotuberculosis
4
3
2
1
0
A (n=15)
B (n=16)
C (n=14)
surface

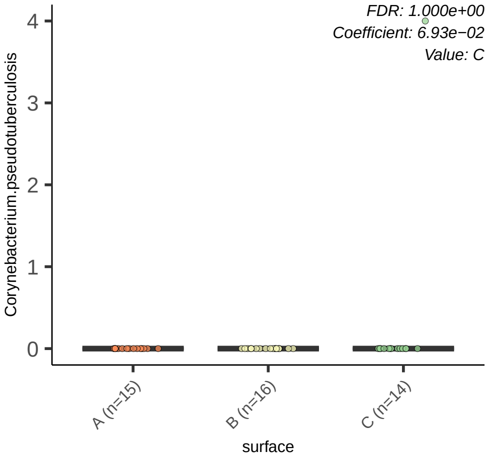

FDR: 1.000e+00
Coefficient: 6.93e−02
Value: C
Corynebacterium.pseudotuberculosis
4
3
2
1
0
A (n=15)
B (n=16)
C (n=14)
surface

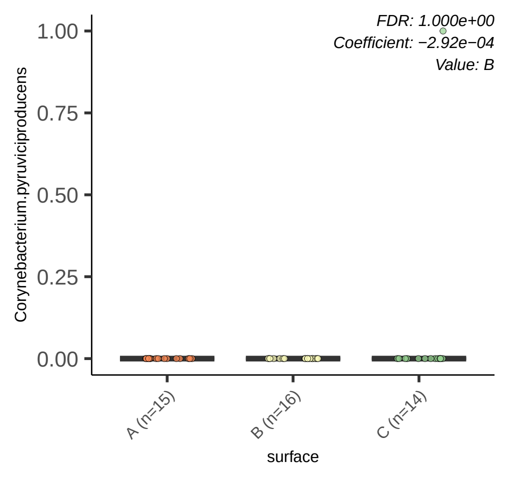
FDR: 1.000e+00
Coefficient: −2.92e−04
Value: B
1.00
0.75
0.50
0.25
0.00
Corynebacterium.pyruviciproducens
A (n=15)
B (n=16)
C (n=14)
surface

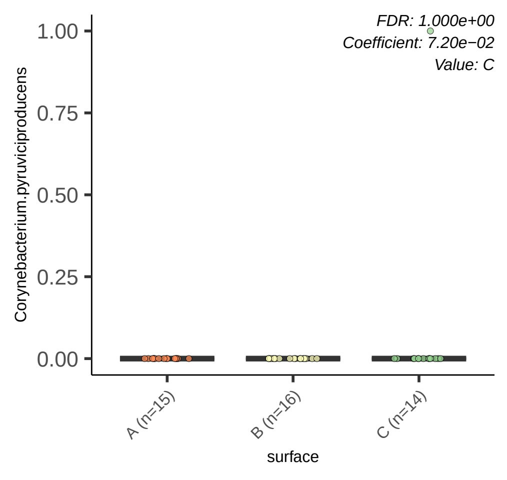

FDR: 1.000e+00
Coefficient: 7.20e−02
Value: C
Corynebacterium.pyruviciproducens
1.00
0.75
0.50
0.25
0.00
A (n=15)
B (n=16)
C (n=14)
surface

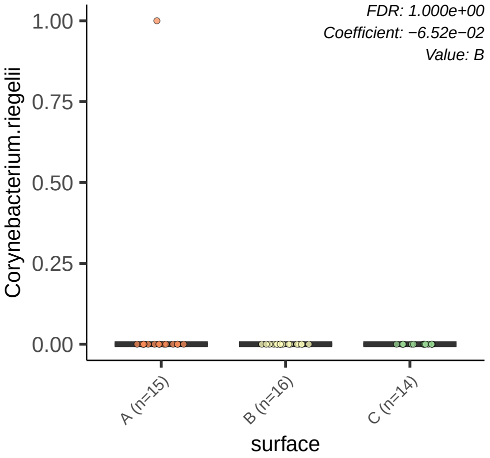

FDR: 1.000e+00
Coefficient: −6.52e−02
Value: B
Corynebacterium.riegelii
1.00
0.75
0.50
0.25
0.00
A (n=15)
B (n=16)
C (n=14)
surface

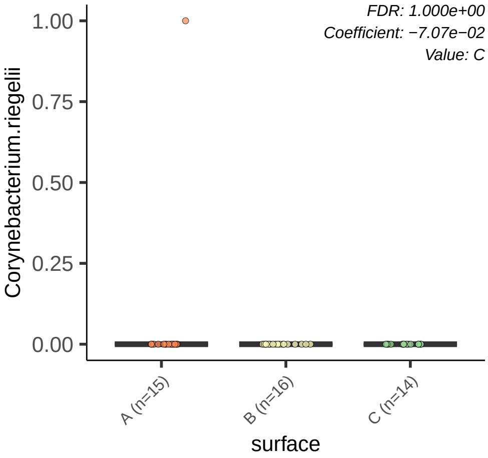
FDR: 1.000e+00
Coefficient: −7.07e−02
Value: C
Corynebacterium.riegelii
1.00
0.75
0.50
0.25
0.00
A (n=15)
B (n=16)
C (n=14)
surface

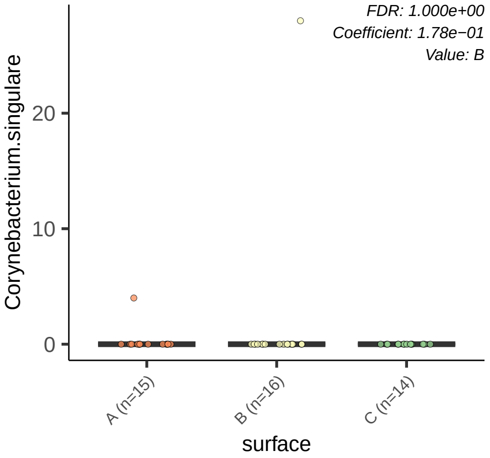

FDR: 1.000e+00
Coefficient: 1.78e−01
Value: B
Corynebacterium.singulare
20
10
0
A (n=15)
B (n=16)
C (n=14)
surface

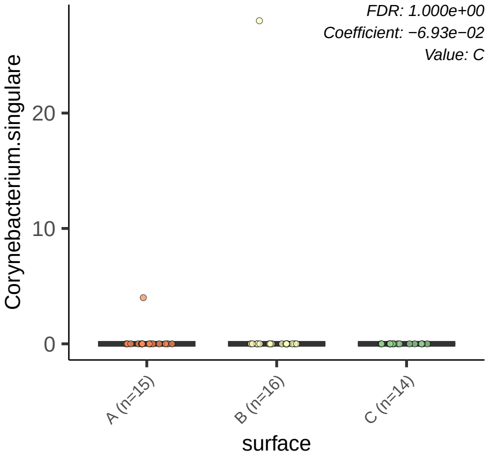
FDR: 1.000e+00
Coefficient: −6.93e−02
Value: C
Corynebacterium.singulare
20
10
0
A (n=15)
B (n=16)
C (n=14)
surface

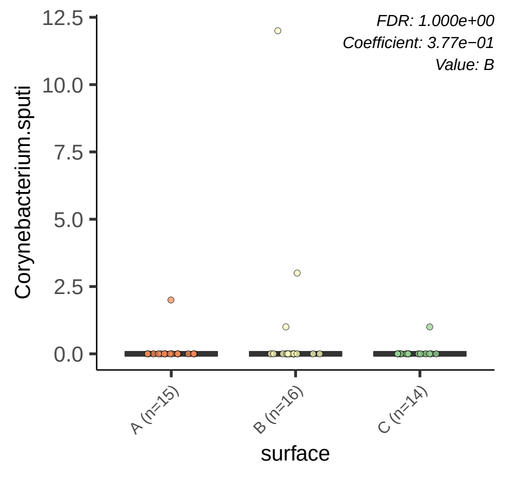
FDR: 1.000e+00
Coefficient: 3.77e−01
Value: B
Corynebacterium.sputi
12.5
10.0
7.5
5.0
2.5
0.0
A (n=15)
B (n=16)
C (n=14)
surface

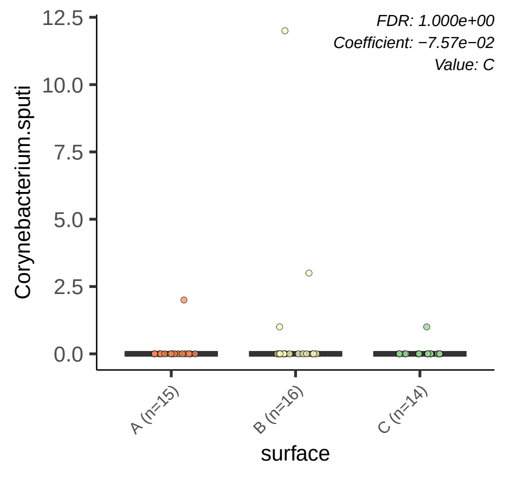
FDR: 1.000e+00
Coefficient: −7.57e−02
Value: C
Corynebacterium.sputi
12.5
10.0
7.5
5.0
2.5
0.0
A (n=15)
B (n=16)
C (n=14)
surface

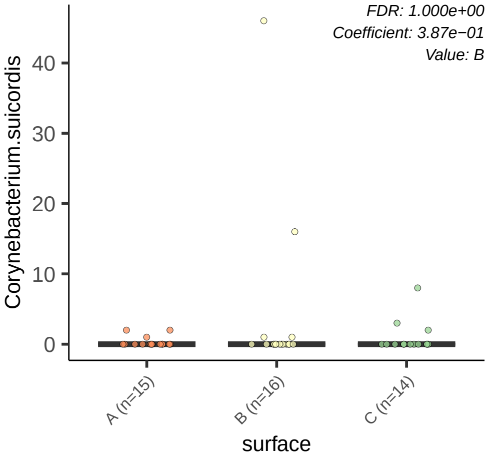
FDR: 1.000e+00
Coefficient: 3.87e−01
Value: B
Corynebacterium.suicordis
0
10
20
30
40
A (n=15)
B (n=16)
C (n=14)
surface

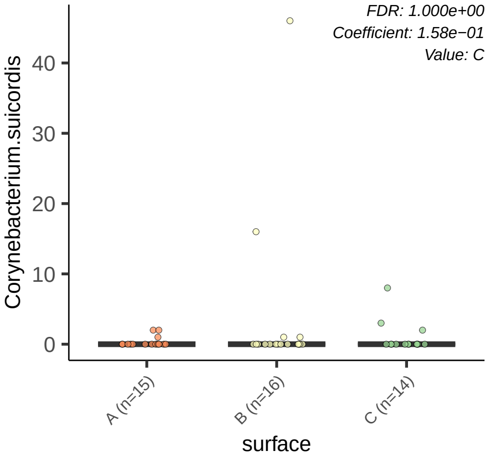

FDR: 1.000e+00
Coefficient: 1.58e−01
Value: C
Corynebacterium.suicordis
0
10
20
30
40
A (n=15)
B (n=16)
C (n=14)
surface

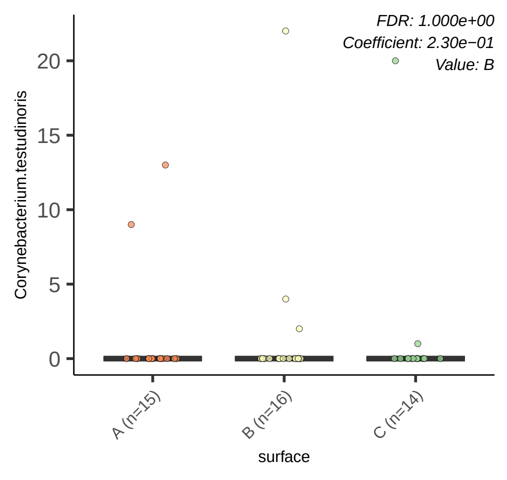

FDR: 1.000e+00
Coefficient: 2.30e−01
Value: B
Corynebacterium.testudinoris
0
5
10
15
20
A (n=15)
B (n=16)
C (n=14)
surface

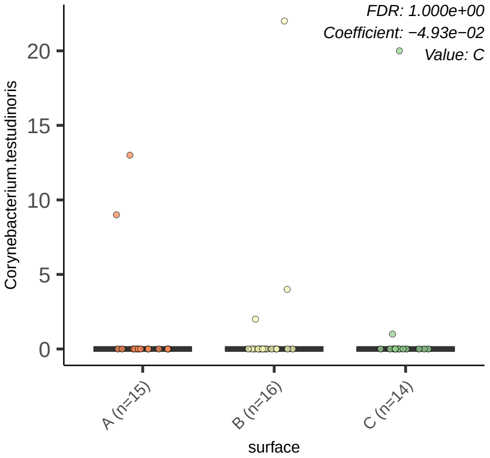
FDR: 1.000e+00
Coefficient: −4.93e−02
Value: C
Corynebacterium.testudinoris
0
5
10
15
20
A (n=15)
B (n=16)
C (n=14)
surface

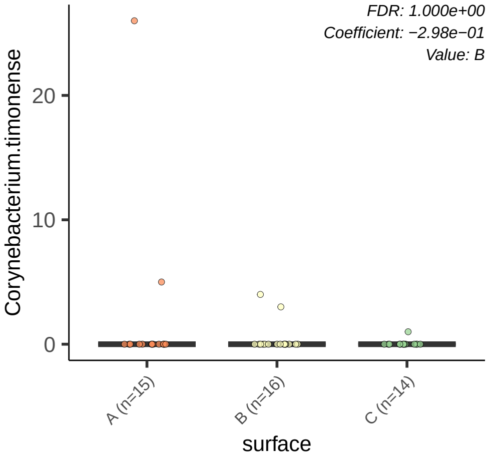

FDR: 1.000e+00
Coefficient: −2.98e−01
Value: B
Corynebacterium.timonense
20
10
0
A (n=15)
B (n=16)
C (n=14)
surface

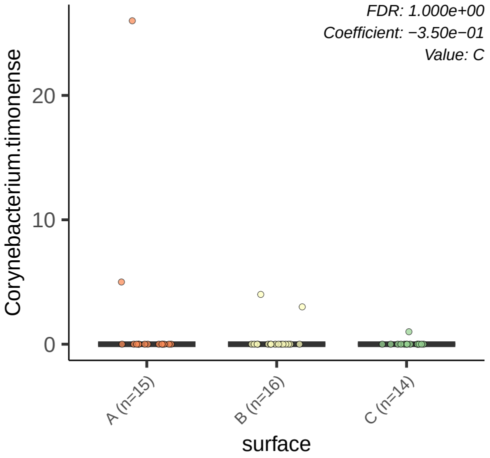

FDR: 1.000e+00
Coefficient: −3.50e−01
Value: C
Corynebacterium.timonense
20
10
0
A (n=15)
B (n=16)
C (n=14)
surface

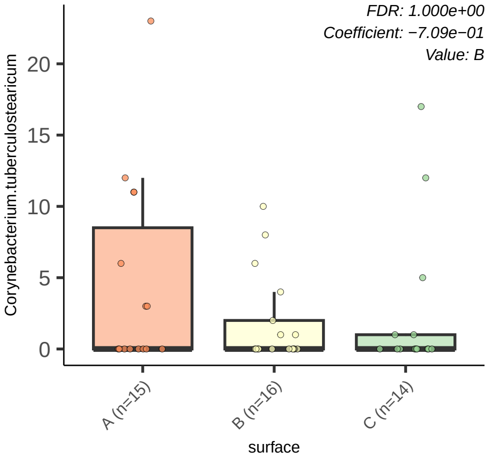
FDR: 1.000e+00
Coefficient: −7.09e−01
Value: B
Corynebacterium.tuberculostearicum
20
15
10
5
0
A (n=15)
B (n=16)
C (n=14)
surface

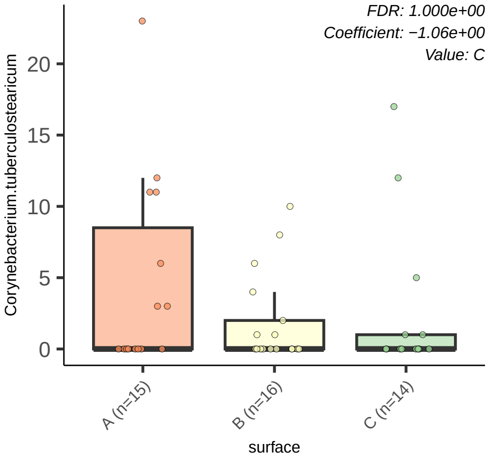

FDR: 1.000e+00
Coefficient: −1.06e+00
Value: C
Corynebacterium.tuberculostearicum
0
5
10
15
20
A (n=15)
B (n=16)
C (n=14)
surface

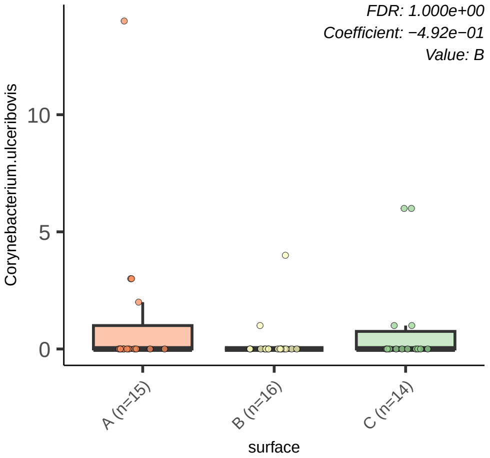
FDR: 1.000e+00
Coefficient: −4.92e−01
Value: B
Corynebacterium.ulceribovis
10
5
0
A (n=15)
B (n=16)
C (n=14)
surface

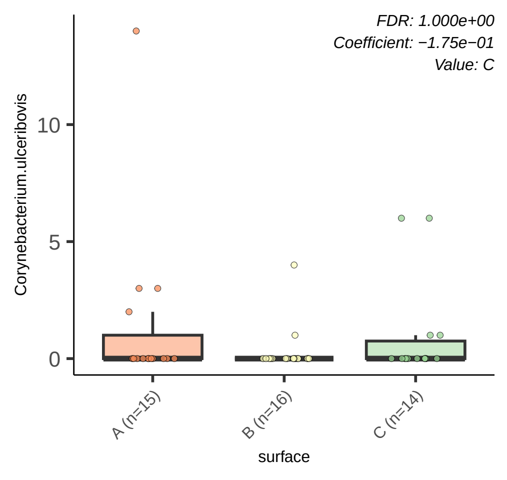

FDR: 1.000e+00
Coefficient: −1.75e−01
Value: C
Corynebacterium.ulceribovis
10
5
0
A (n=15)
B (n=16)
C (n=14)
surface

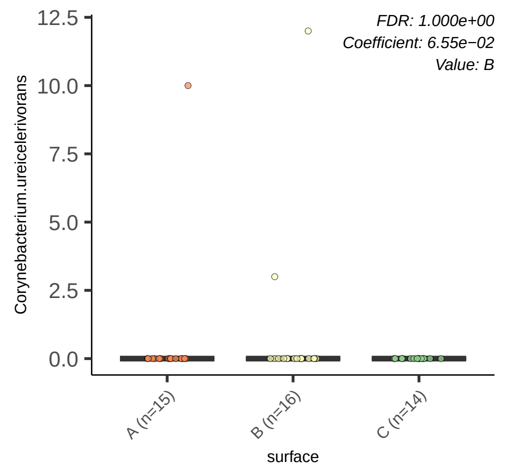

FDR: 1.000e+00
Coefficient: 6.55e−02
Value: B
Corynebacterium.ureicelerivorans
12.5
10.0
7.5
5.0
2.5
0.0
A (n=15)
B (n=16)
C (n=14)
surface

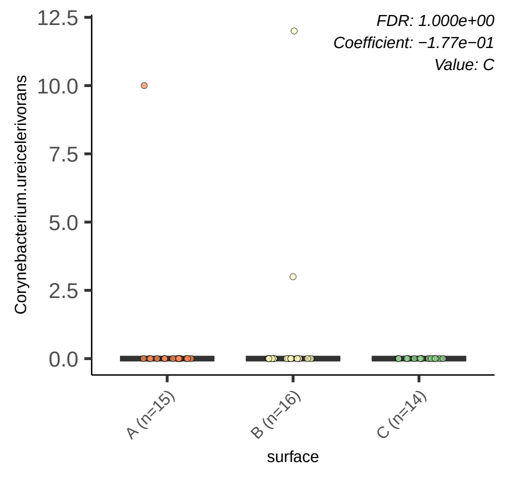
FDR: 1.000e+00
Coefficient: −1.77e−01
Value: C
12.5
10.0
7.5
5.0
2.5
0.0
Corynebacterium.ureicelerivorans
A (n=15)
B (n=16)
C (n=14)
surface

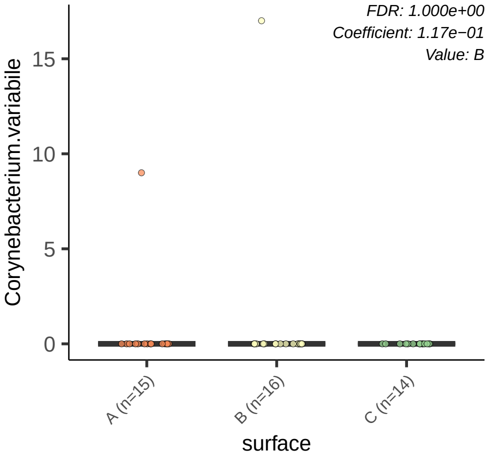
FDR: 1.000e+00
Coefficient: 1.17e−01
Value: B
Corynebacterium.variabile
0
5
10
15
A (n=15)
B (n=16)
C (n=14)
surface

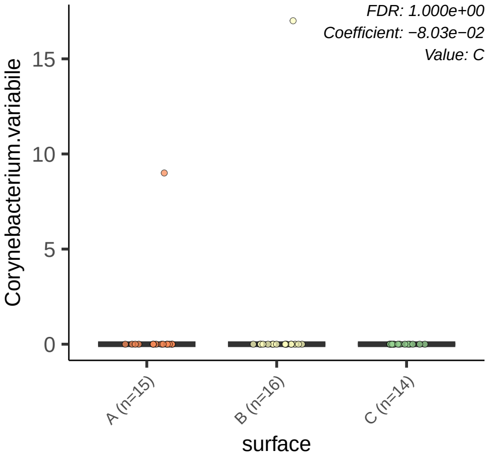

FDR: 1.000e+00
Coefficient: −8.03e−02
Value: C
Corynebacterium.variabile
0
5
10
15
A (n=15)
B (n=16)
C (n=14)
surface

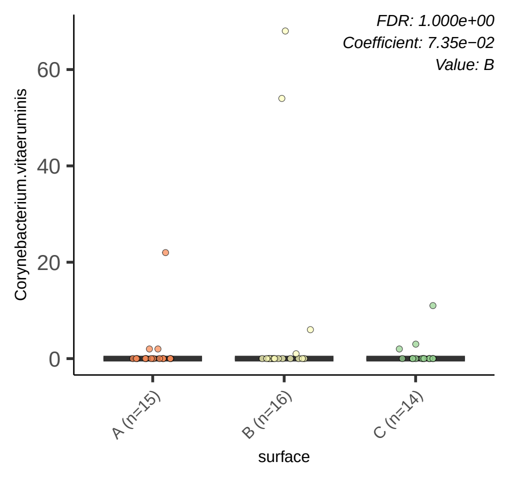

FDR: 1.000e+00
Coefficient: 7.35e−02
Value: B
Corynebacterium.vitaeruminis
60
40
20
0
A (n=15)
B (n=16)
C (n=14)
surface

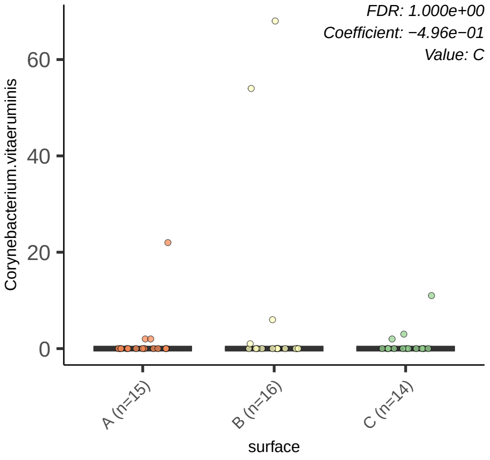

FDR: 1.000e+00
Coefficient: −4.96e−01
Value: C
Corynebacterium.vitaeruminis
60
40
20
0
A (n=15)
B (n=16)
C (n=14)
surface

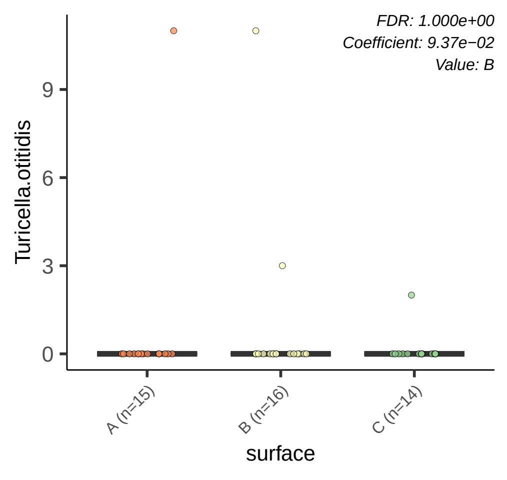

FDR: 1.000e+00
Coefficient: 9.37e−02
Value: B
Turicella.otitidis
0
3
6
9
A (n=15)
B (n=16)
C (n=14)
surface

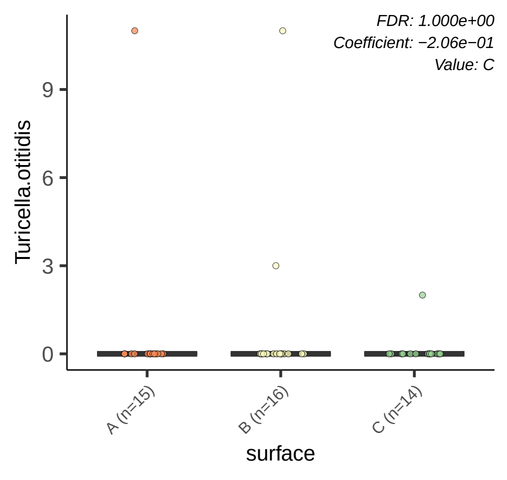
FDR: 1.000e+00
Coefficient: −2.06e−01
Value: C
Turicella.otitidis
9
6
3
0
A (n=15)
B (n=16)
C (n=14)
surface

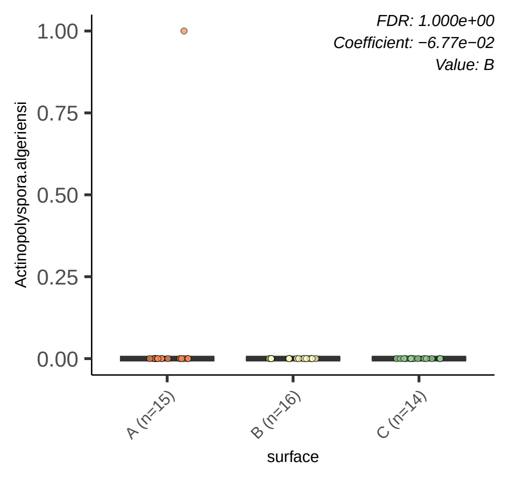
FDR: 1.000e+00
Coefficient: −6.77e−02
Value: B
1.00
0.75
0.50
0.25
0.00
Actinopolyspora.algeriensi
A (n=15)
B (n=16)
C (n=14)
surface

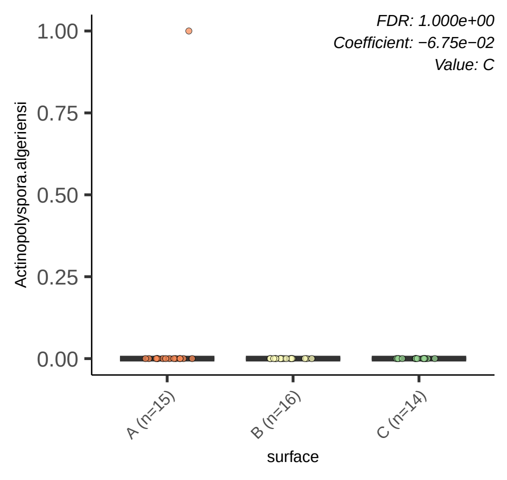
FDR: 1.000e+00
Coefficient: −6.75e−02
Value: C
1.00
0.75
0.50
0.25
0.00
Actinopolyspora.algeriensi
A (n=15)
B (n=16)
C (n=14)
surface

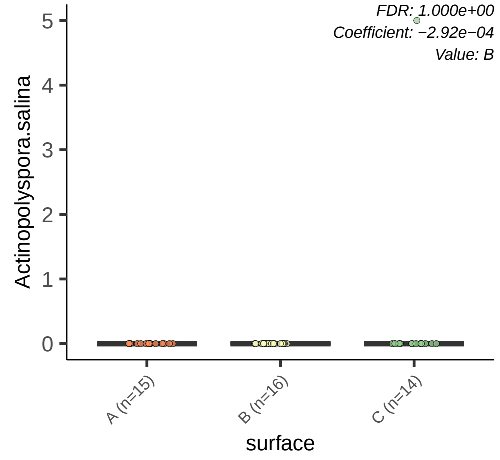
FDR: 1.000e+00
Coefficient: −2.92e−04
Value: B
Actinopolyspora.salina
0
1
2
3
4
5
A (n=15)
B (n=16)
C (n=14)
surface

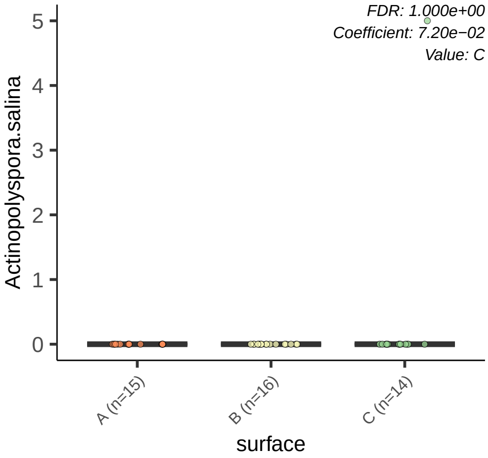
FDR: 1.000e+00
Coefficient: 7.20e−02
Value: C
Actinopolyspora.salina
0
1
2
3
4
5
A (n=15)
B (n=16)
C (n=14)
surface

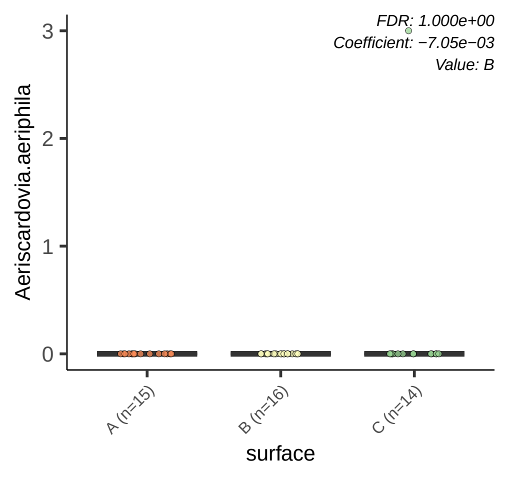
FDR: 1.000e+00
Coefficient: −7.05e−03
Value: B
Aeriscardovia.aeriphila
3
2
1
0
A (n=15)
B (n=16)
C (n=14)
surface

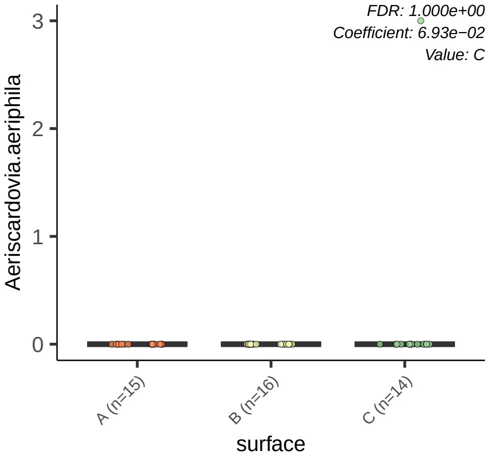
FDR: 1.000e+00
Coefficient: 6.93e−02
Value: C
Aeriscardovia.aeriphila
3
2
1
0
A (n=15)
B (n=16)
C (n=14)
surface

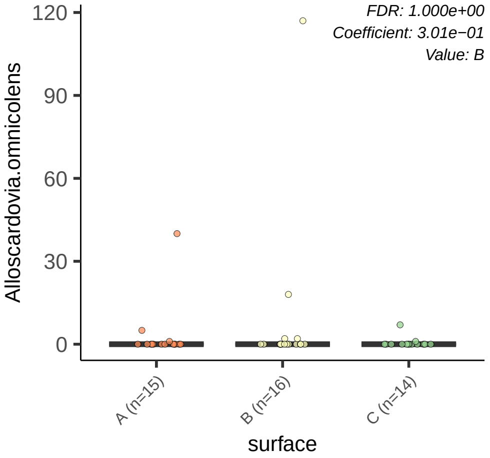

FDR: 1.000e+00
Coefficient: 3.01e−01
Value: B
Alloscardovia.omnicolens
120
90
60
30
0
A (n=15)
B (n=16)
C (n=14)
surface

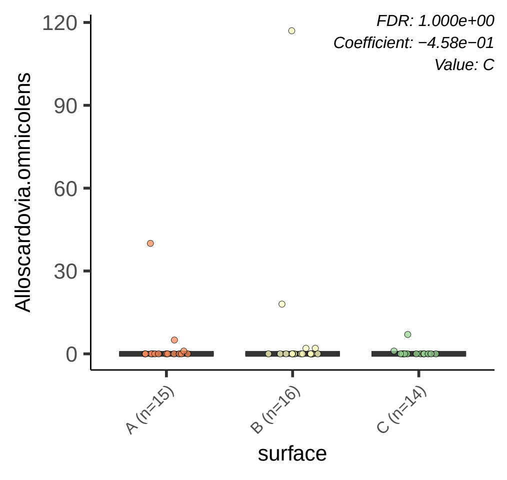
FDR: 1.000e+00
Coefficient: −4.58e−01
Value: C
120
90
60
30
0
Alloscardovia.omnicolens
A (n=15)
B (n=16)
C (n=14)
surface

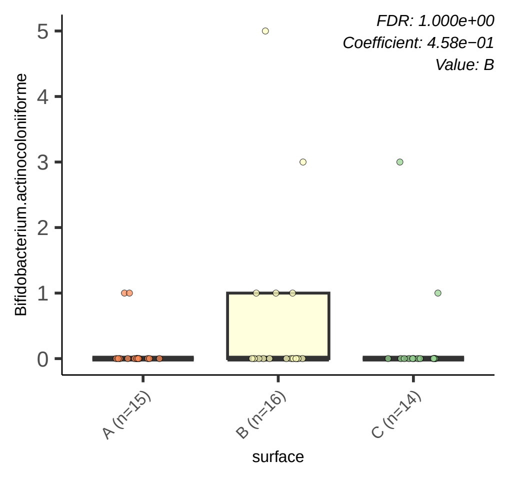

FDR: 1.000e+00
Coefficient: 4.58e−01
Value: B
Bifidobacterium.actinocoloniiforme
0
1
2
3
4
5
A (n=15)
B (n=16)
C (n=14)
surface

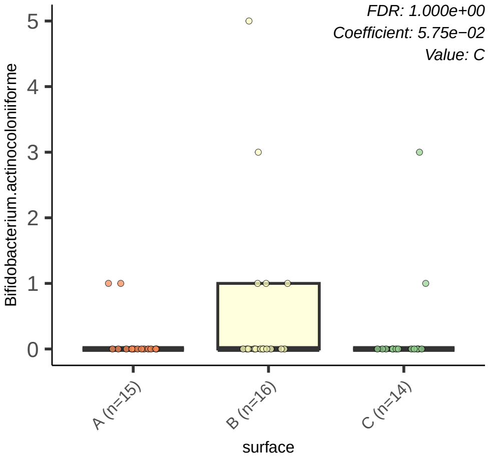

FDR: 1.000e+00
Coefficient: 5.75e−02
Value: C
Bifidobacterium.actinocoloniiforme
0
1
2
3
4
5
A (n=15)
B (n=16)
C (n=14)
surface

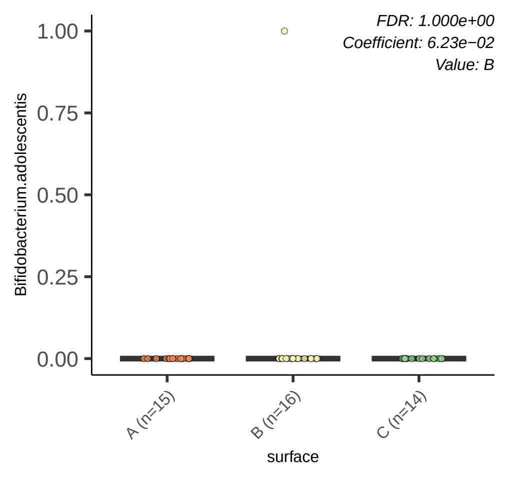
FDR: 1.000e+00
Coefficient: 6.23e−02
Value: B
Bifidobacterium.adolescentis
1.00
0.75
0.50
0.25
0.00
A (n=15)
B (n=16)
C (n=14)
surface

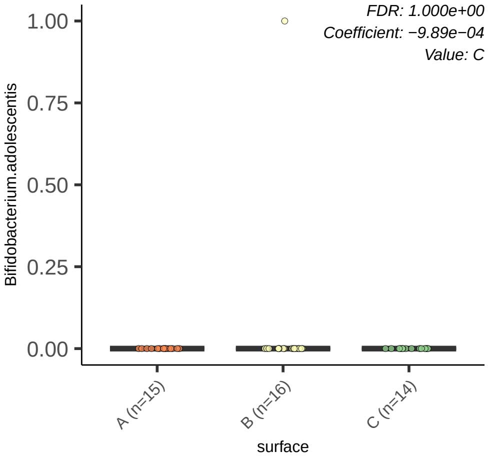

FDR: 1.000e+00
Coefficient: −9.89e−04
Value: C
Bifidobacterium.adolescentis
1.00
0.75
0.50
0.25
0.00
A (n=15)
B (n=16)
C (n=14)
surface

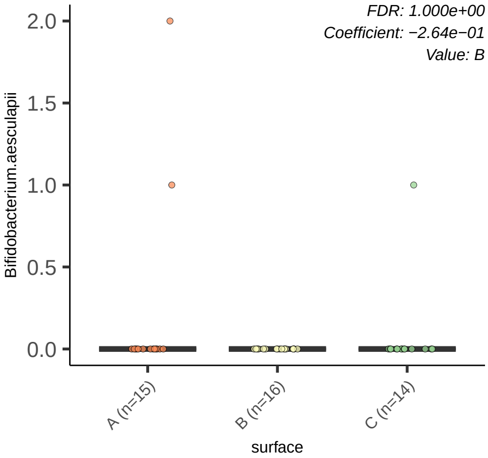

FDR: 1.000e+00
Coefficient: −2.64e−01
Value: B
Bifidobacterium.aesculapii
2.0
1.5
1.0
0.5
0.0
A (n=15)
B (n=16)
C (n=14)
surface

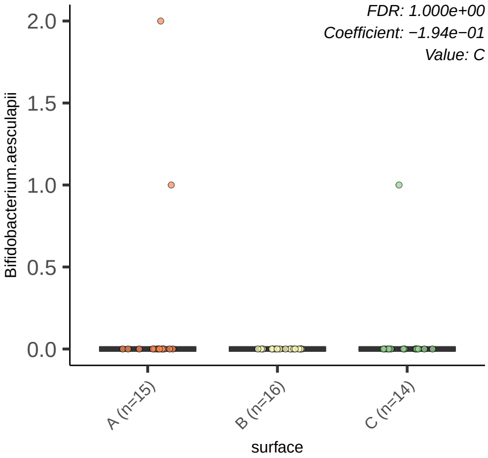
FDR: 1.000e+00
Coefficient: −1.94e−01
Value: C
2.0
1.5
1.0
0.5
0.0
Bifidobacterium.aesculapii
A (n=15)
B (n=16)
C (n=14)
surface

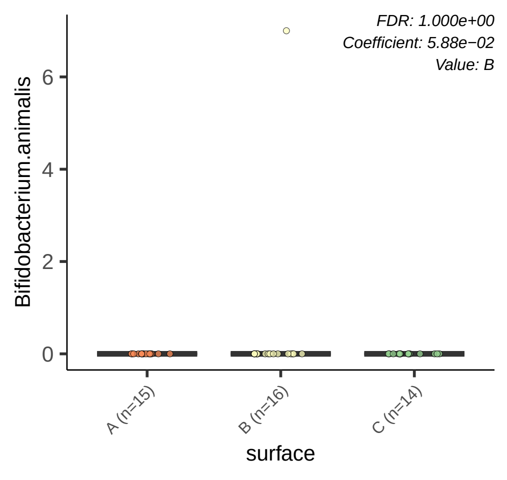

FDR: 1.000e+00
Coefficient: 5.88e−02
Value: B
Bifidobacterium.animalis
0
2
4
6
A (n=15)
B (n=16)
C (n=14)
surface

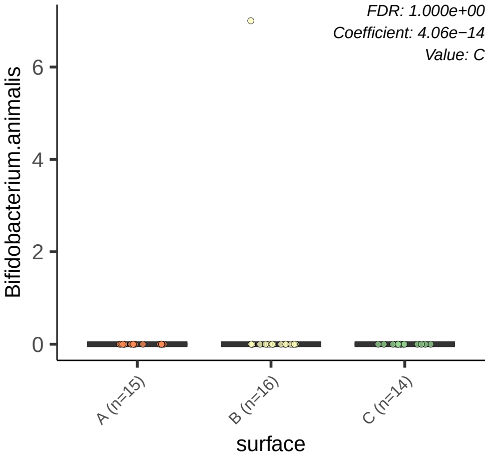
FDR: 1.000e+00
Coefficient: 4.06e−14
Value: C
Bifidobacterium.animalis
0
2
4
6
A (n=15)
B (n=16)
C (n=14)
surface

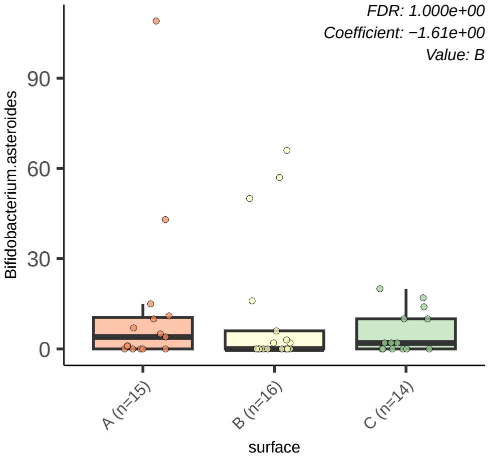
FDR: 1.000e+00
Coefficient: −1.61e+00
Value: B
Bifidobacterium.asteroides
90
60
30
0
A (n=15)
B (n=16)
C (n=14)
surface

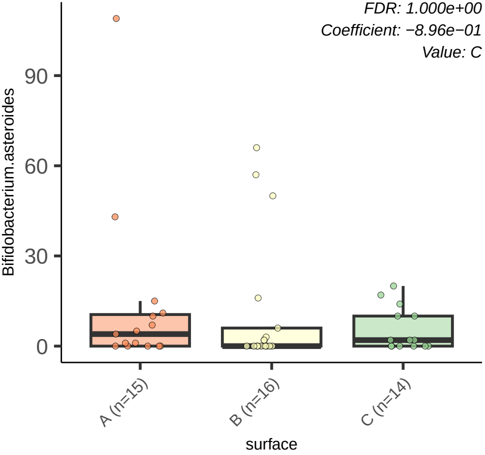
FDR: 1.000e+00
Coefficient: −8.96e−01
Value: C
Bifidobacterium.asteroides
90
60
30
0
A (n=15)
B (n=16)
C (n=14)
surface

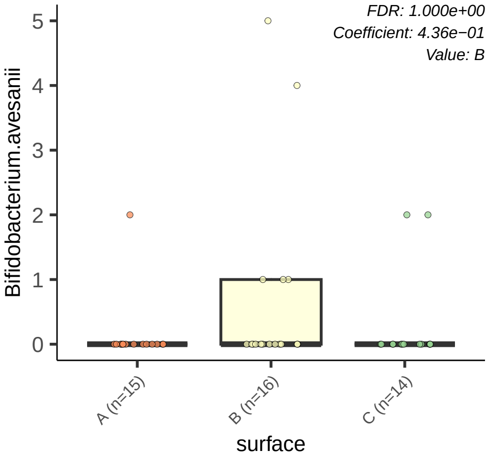

FDR: 1.000e+00
Coefficient: 4.36e−01
Value: B
Bifidobacterium.avesanii
0
1
2
3
4
5
A (n=15)
B (n=16)
C (n=14)
surface

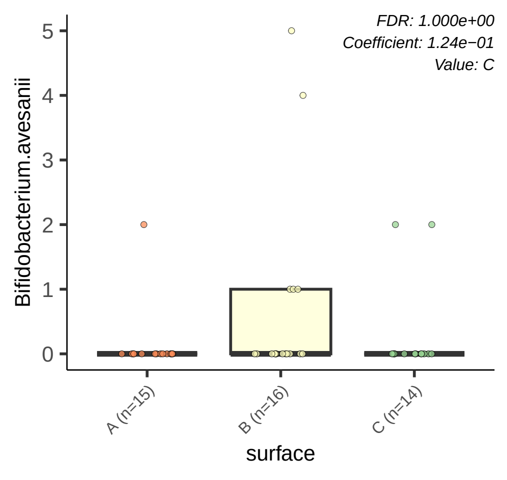
FDR: 1.000e+00
Coefficient: 1.24e−01
Value: C
Bifidobacterium.avesanii
0
1
2
3
4
5
A (n=15)
B (n=16)
C (n=14)
surface

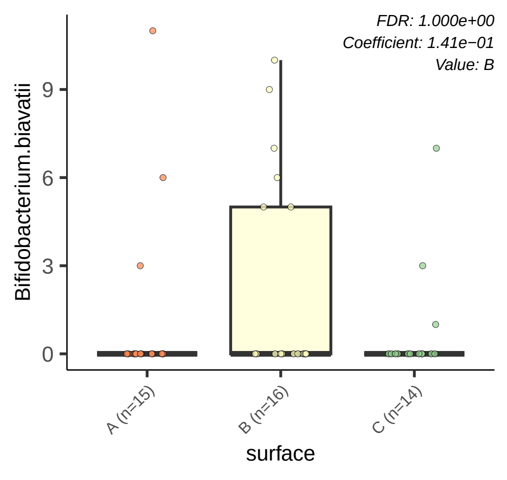

FDR: 1.000e+00
Coefficient: 1.41e−01
Value: B
Bifidobacterium.biavatii
0
3
6
9
A (n=15)
B (n=16)
C (n=14)
surface

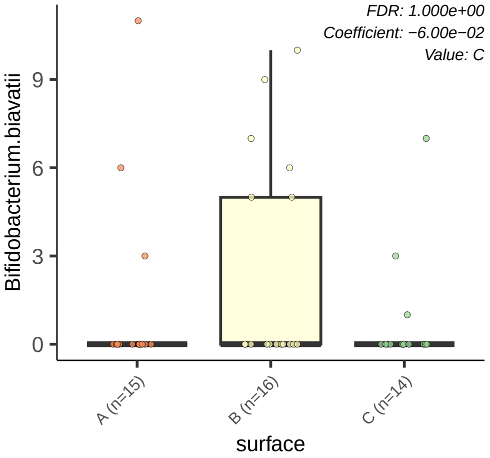

FDR: 1.000e+00
Coefficient: −6.00e−02
Value: C
Bifidobacterium.biavatii
0
3
6
9
A (n=15)
B (n=16)
C (n=14)
surface

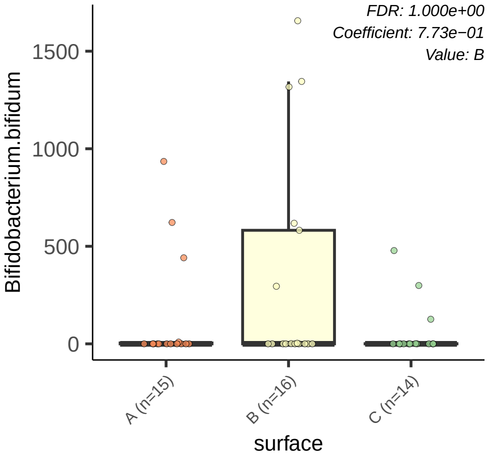

FDR: 1.000e+00
Coefficient: 7.73e−01
Value: B
Bifidobacterium.bifidum
1500
1000
500
0
A (n=15)
B (n=16)
C (n=14)
surface

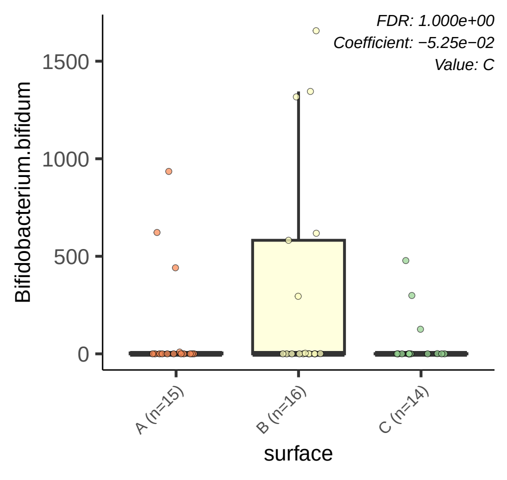

FDR: 1.000e+00
Coefficient: −5.25e−02
Value: C
Bifidobacterium.bifidum
1500
1000
500
0
A (n=15)
B (n=16)
C (n=14)
surface

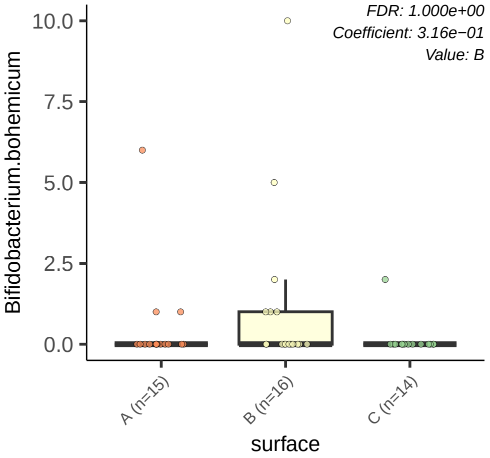

FDR: 1.000e+00
Coefficient: 3.16e−01
Value: B
Bifidobacterium.bohemicum
10.0
7.5
5.0
2.5
0.0
A (n=15)
B (n=16)
C (n=14)
surface

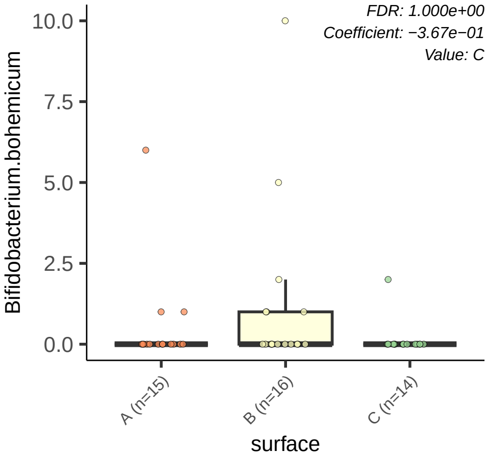
FDR: 1.000e+00
Coefficient: −3.67e−01
Value: C
10.0
7.5
5.0
2.5
0.0
Bifidobacterium.bohemicum
A (n=15)
B (n=16)
C (n=14)
surface

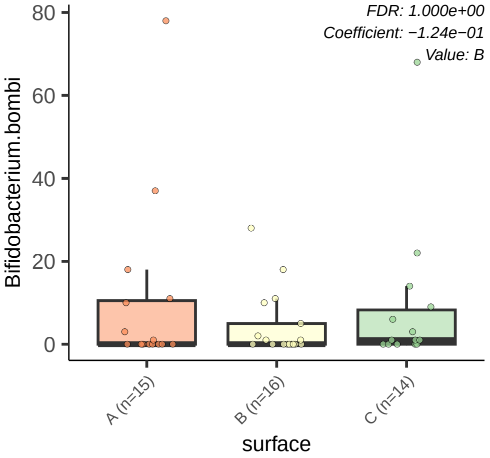

FDR: 1.000e+00
Coefficient: −1.24e−01
Value: B
Bifidobacterium.bombi
80
60
40
20
0
A (n=15)
B (n=16)
C (n=14)
surface

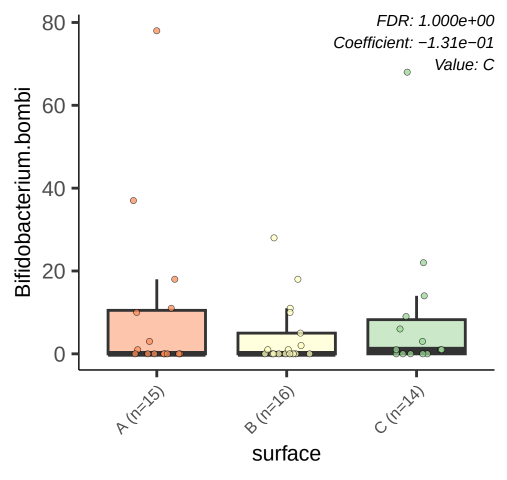
FDR: 1.000e+00
Coefficient: −1.31e−01
Value: C
Bifidobacterium.bombi
80
60
40
20
0
A (n=15)
B (n=16)
C (n=14)
surface

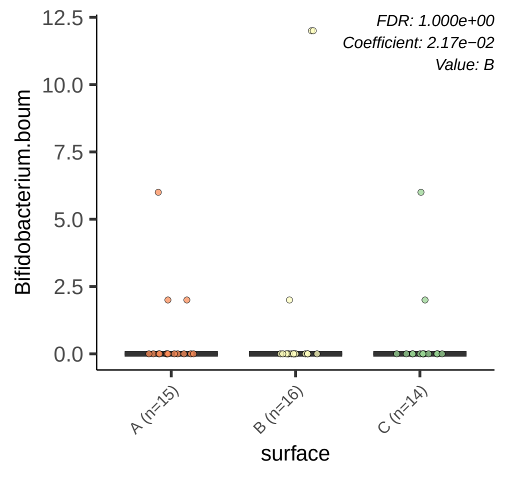

FDR: 1.000e+00
Coefficient: 2.17e−02
Value: B
Bifidobacterium.boum
12.5
10.0
7.5
5.0
2.5
0.0
A (n=15)
B (n=16)
C (n=14)
surface

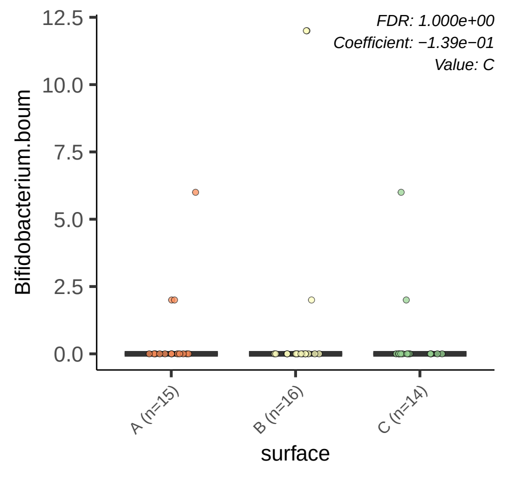
FDR: 1.000e+00
Coefficient: −1.39e−01
Value: C
Bifidobacterium.boum
12.5
10.0
7.5
5.0
2.5
0.0
A (n=15)
B (n=16)
C (n=14)
surface

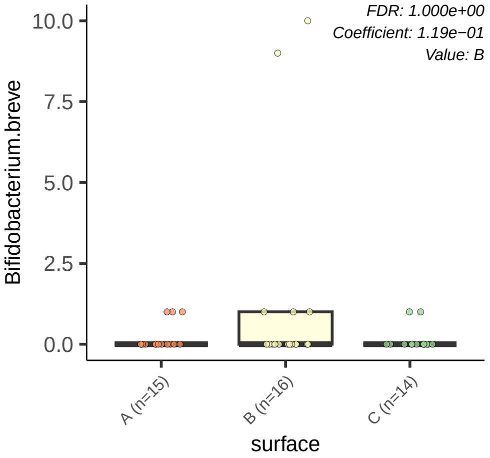

FDR: 1.000e+00
Coefficient: 1.19e−01
Value: B
10.0
7.5
5.0
2.5
0.0
Bifidobacterium.breve
A (n=15)
B (n=16)
C (n=14)
surface

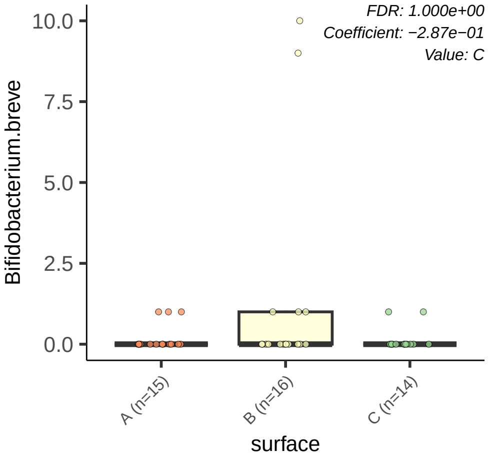
FDR: 1.000e+00
Coefficient: −2.87e−01
Value: C
Bifidobacterium.breve
10.0
7.5
5.0
2.5
0.0
A (n=15)
B (n=16)
C (n=14)
surface

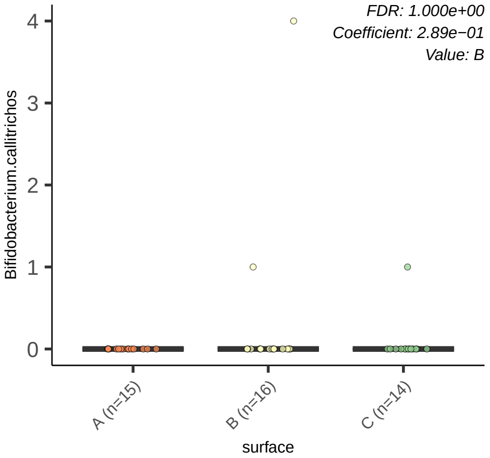

FDR: 1.000e+00
Coefficient: 2.89e−01
Value: B
Bifidobacterium.callitrichos
4
3
2
1
0
A (n=15)
B (n=16)
C (n=14)
surface

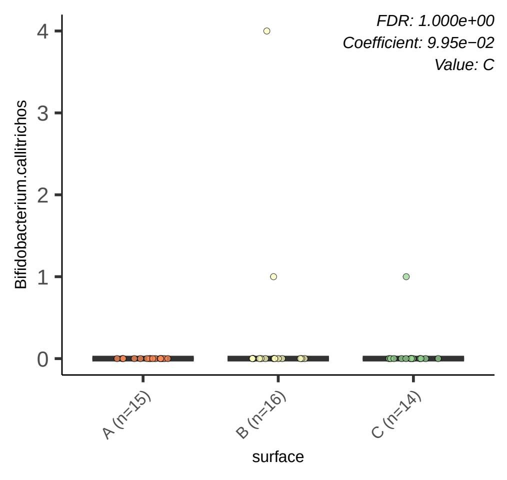

FDR: 1.000e+00
Coefficient: 9.95e−02
Value: C
Bifidobacterium.callitrichos
0
1
2
3
4
A (n=15)
B (n=16)
C (n=14)
surface

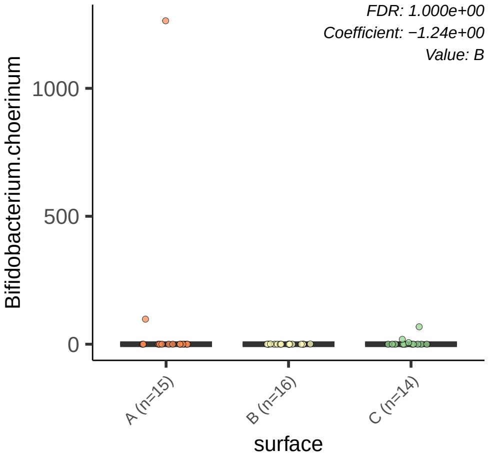
FDR: 1.000e+00
Coefficient: −1.24e+00
Value: B
Bifidobacterium.choerinum
1000
500
0
A (n=15)
B (n=16)
C (n=14)
surface

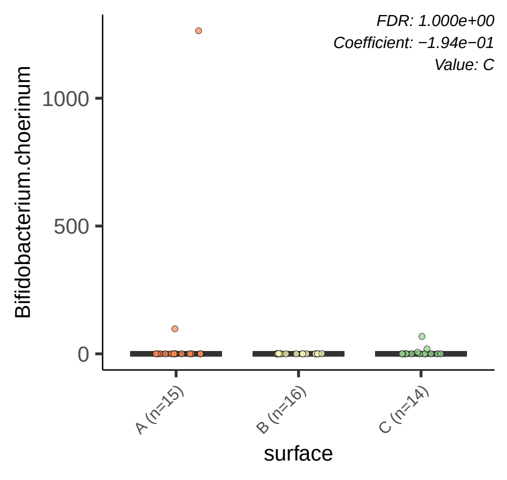
FDR: 1.000e+00
Coefficient: −1.94e−01
Value: C
Bifidobacterium.choerinum
1000
500
0
A (n=15)
B (n=16)
C (n=14)
surface

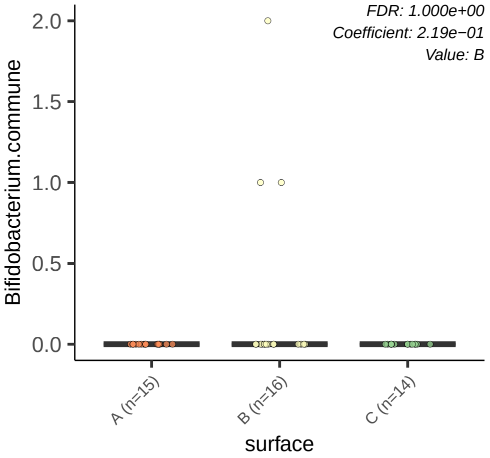
FDR: 1.000e+00
Coefficient: 2.19e−01
Value: B
2.0
1.5
1.0
0.5
0.0
Bifidobacterium.commune
A (n=15)
B (n=16)
C (n=14)
surface

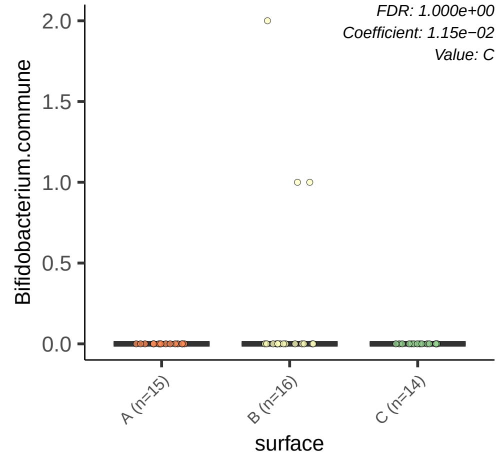
FDR: 1.000e+00
Coefficient: 1.15e−02
Value: C
Bifidobacterium.commune
2.0
1.5
1.0
0.5
0.0
A (n=15)
B (n=16)
C (n=14)
surface

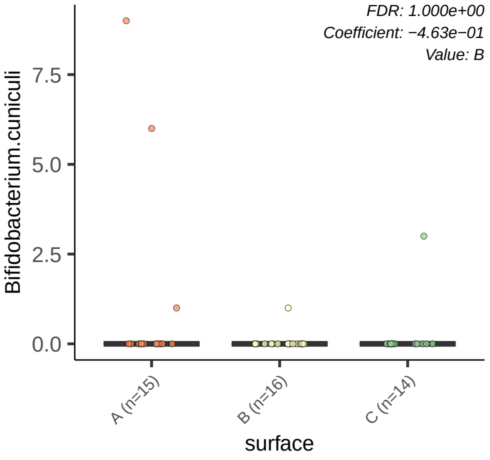
FDR: 1.000e+00
Coefficient: −4.63e−01
Value: B
Bifidobacterium.cuniculi
7.5
5.0
2.5
0.0
A (n=15)
B (n=16)
C (n=14)
surface

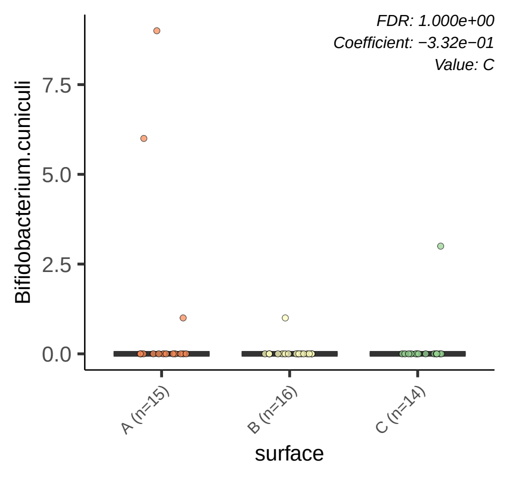
FDR: 1.000e+00
Coefficient: −3.32e−01
Value: C
Bifidobacterium.cunicul i
7.5
5.0
2.5
0.0
A (n=15)
B (n=16)
C (n=14)
surface

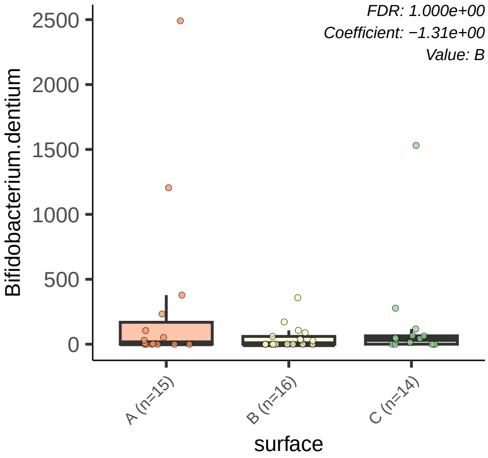

FDR: 1.000e+00
Coefficient: −1.31e+00
Value: B
Bifidobacterium.dentium
2500
2000
1500
1000
500
0
A (n=15)
B (n=16)
C (n=14)
surface

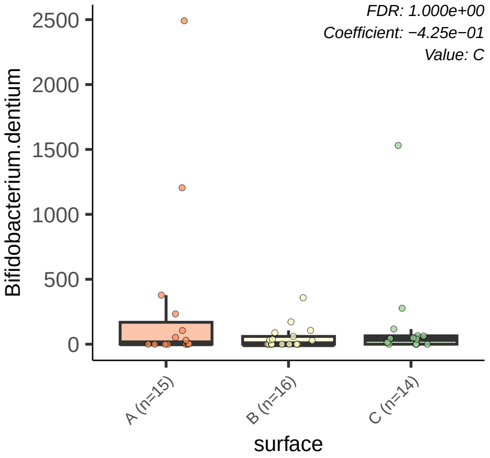
FDR: 1.000e+00
Coefficient: −4.25e−01
Value: C
Bifidobacterium.dentium
2500
2000
1500
1000
500
0
A (n=15)
B (n=16)
C (n=14)
surface

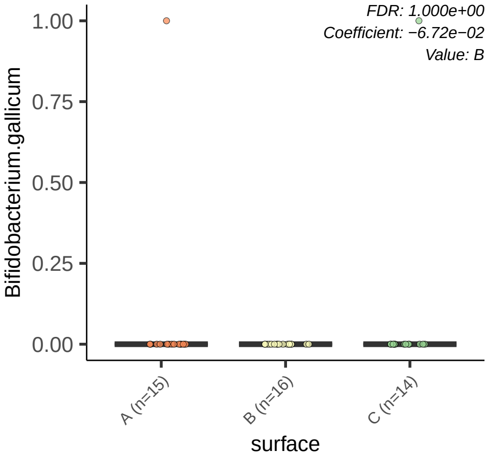
FDR: 1.000e+00
Coefficient: −6.72e−02
Value: B
Bifidobacterium.gallicum
1.00
0.75
0.50
0.25
0.00
A (n=15)
B (n=16)
C (n=14)
surface

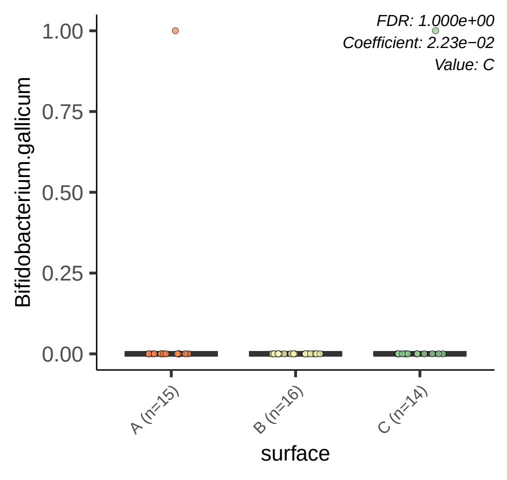
FDR: 1.000e+00
Coefficient: 2.23e−02
Value: C
Bifidobacterium.gallicum
1.00
0.75
0.50
0.25
0.00
A (n=15)
B (n=16)
C (n=14)
surface

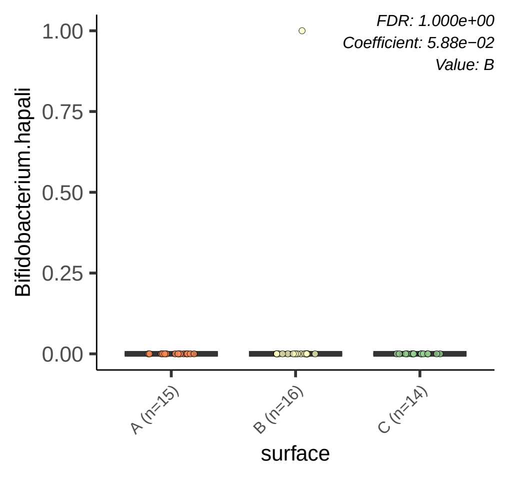

FDR: 1.000e+00
Coefficient: 5.88e−02
Value: B
Bifidobacterium.hapali
1.00
0.75
0.50
0.25
0.00
A (n=15)
B (n=16)
C (n=14)
surface

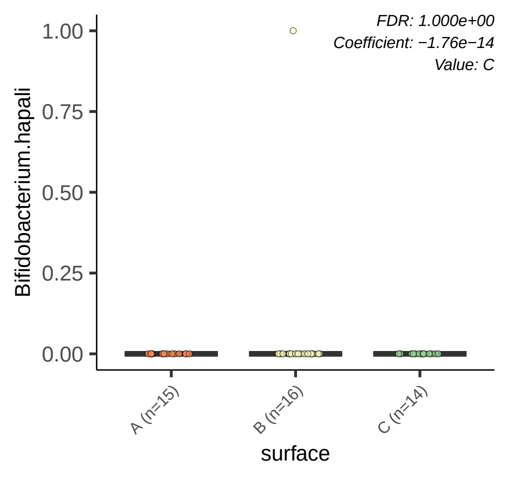
FDR: 1.000e+00
Coefficient: −1.76e−14
Value: C
Bifidobacterium.hapali
1.00
0.75
0.50
0.25
0.00
A (n=15)
B (n=16)
C (n=14)
surface

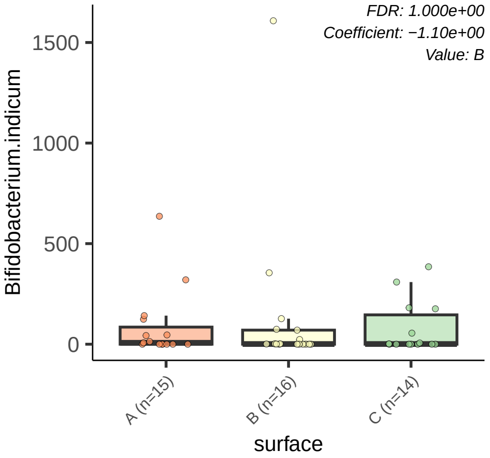
FDR: 1.000e+00
Coefficient: −1.10e+00
Value: B
Bifidobacterium.indicum
1500
1000
500
0
A (n=15)
B (n=16)
C (n=14)
surface

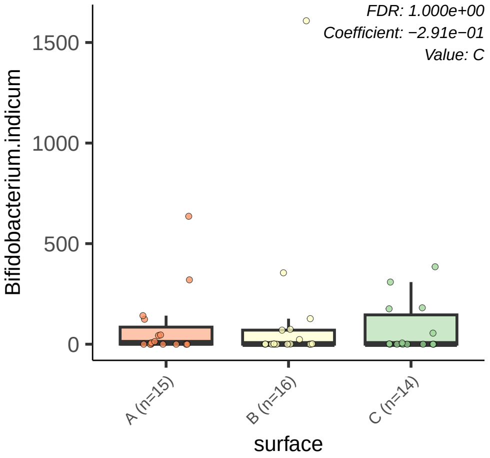

FDR: 1.000e+00
Coefficient: −2.91e−01
Value: C
Bifidobacterium.indicum
1500
1000
500
0
A (n=15)
B (n=16)
C (n=14)
surface

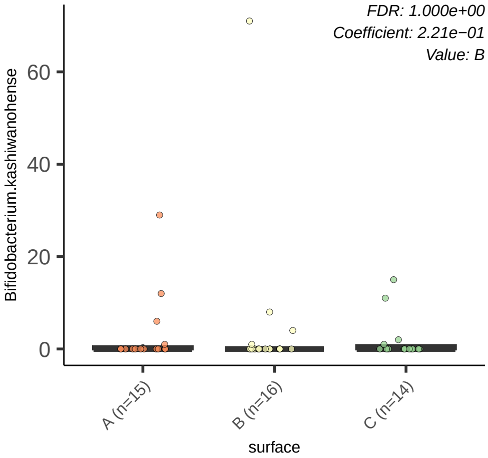

FDR: 1.000e+00
Coefficient: 2.21e−01
Value: B
Bifidobacterium.kashiwanohense
0
20
40
60
A (n=15)
B (n=16)
C (n=14)
surface

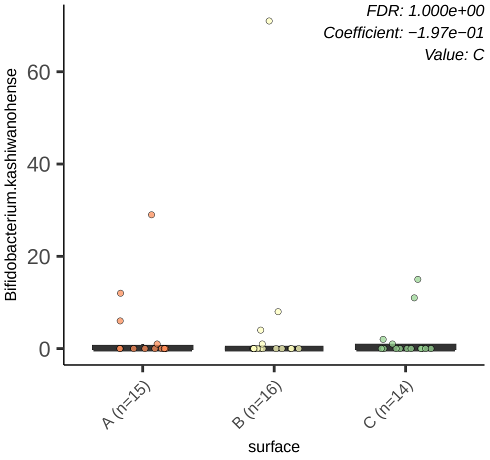

FDR: 1.000e+00
Coefficient: −1.97e−01
Value: C
Bifidobacterium.kashiwanohense
0
20
40
60
A (n=15)
B (n=16)
C (n=14)
surface

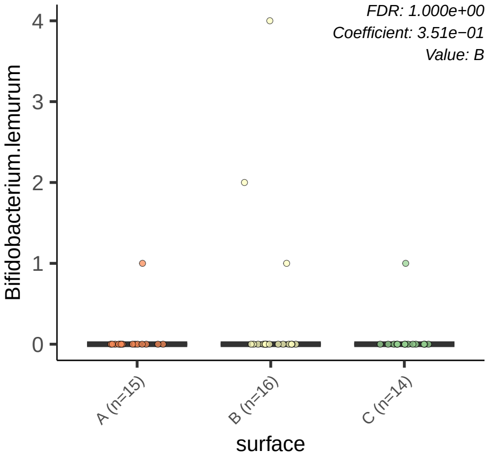
FDR: 1.000e+00
Coefficient: 3.51e−01
Value: B
Bifidobacterium.lemurum
0
1
2
3
4
A (n=15)
B (n=16)
C (n=14)
surface

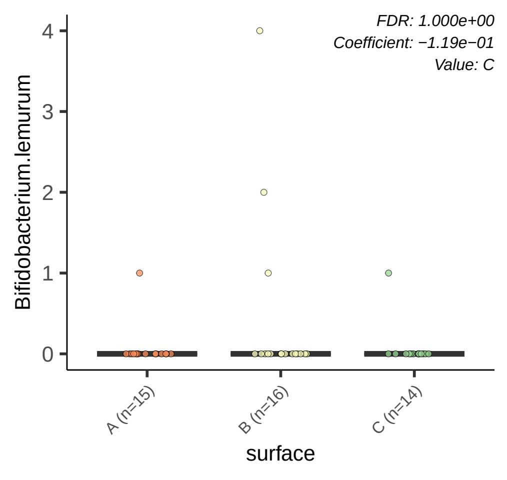

FDR: 1.000e+00
Coefficient: −1.19e−01
Value: C
Bifidobacterium.lemurum
0
1
2
3
4
A (n=15)
B (n=16)
C (n=14)
surface

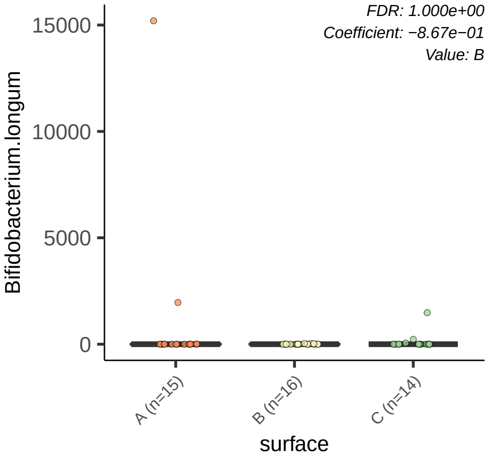
FDR: 1.000e+00
Coefficient: −8.67e−01
Value: B
15000
10000
5000
0
Bifidobacterium.longum
A (n=15)
B (n=16)
C (n=14)
surface

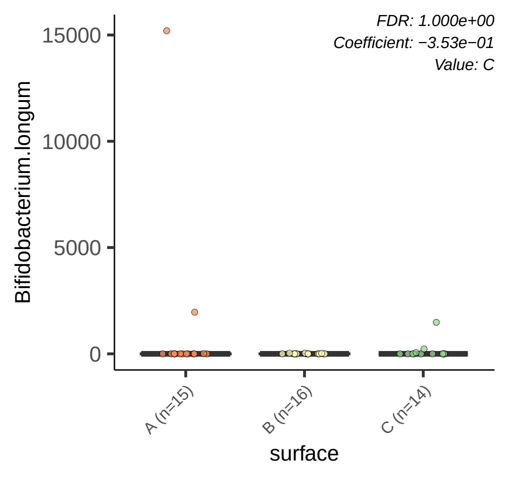

FDR: 1.000e+00
Coefficient: −3.53e−01
Value: C
Bifidobacterium.longum
15000
10000
5000
0
A (n=15)
B (n=16)
C (n=14)
surface

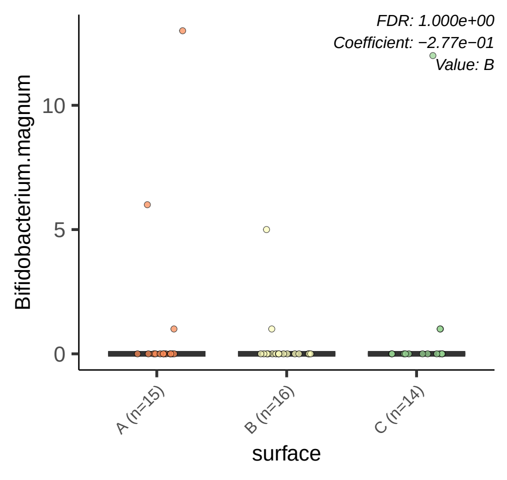
FDR: 1.000e+00
Coefficient: −2.77e−01
Value: B
Bifidobacterium.magnum
0
5
10
A (n=15)
B (n=16)
C (n=14)
surface

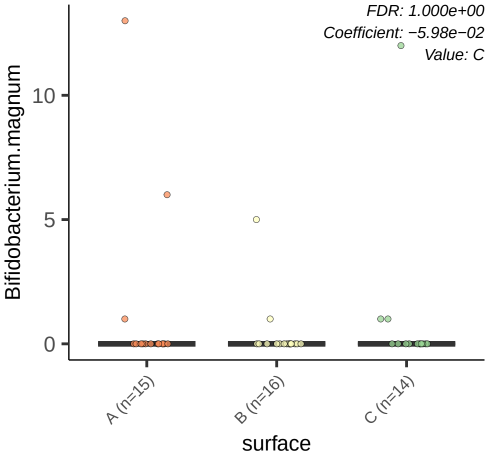
FDR: 1.000e+00
Coefficient: −5.98e−02
Value: C
Bifidobacterium.magnum
0
5
10
A (n=15)
B (n=16)
C (n=14)
surface

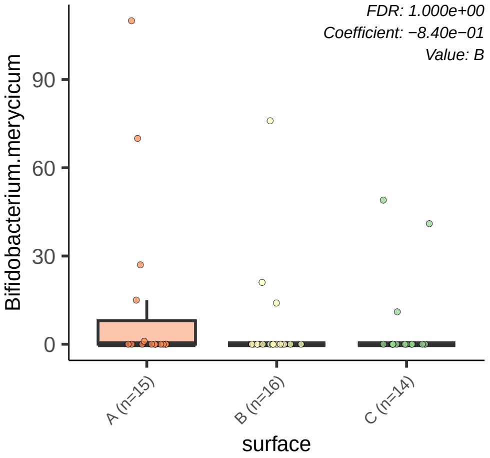

FDR: 1.000e+00
Coefficient: −8.40e−01
Value: B
Bifidobacterium.merycicum
90
60
30
0
A (n=15)
B (n=16)
C (n=14)
surface

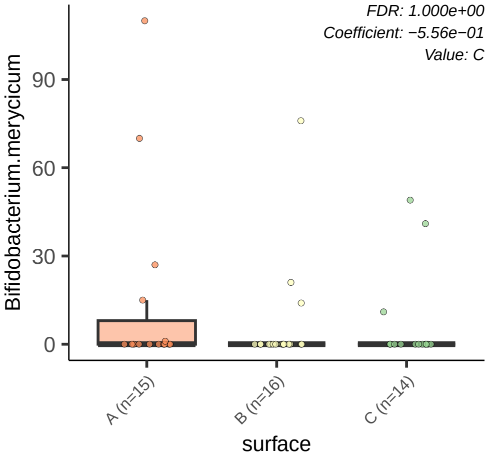
FDR: 1.000e+00
Coefficient: −5.56e−01
Value: C
Bifidobacterium.merycicum
0
30
60
90
A (n=15)
B (n=16)
C (n=14)
surface

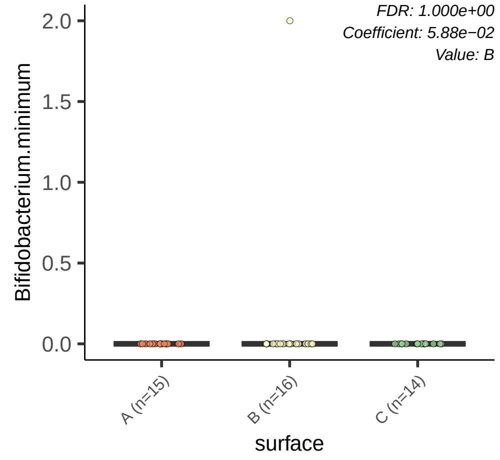
FDR: 1.000e+00
Coefficient: 5.88e−02
Value: B
Bifidobacterium.minimum
2.0
1.5
1.0
0.5
0.0
A (n=15)
B (n=16)
C (n=14)
surface

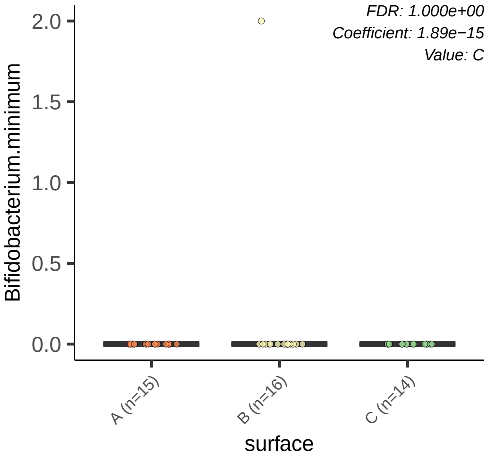
FDR: 1.000e+00
Coefficient: 1.89e−15
Value: C
2.0
1.5
1.0
0.5
0.0
Bifidobacterium.minimum
A (n=15)
B (n=16)
C (n=14)
surface

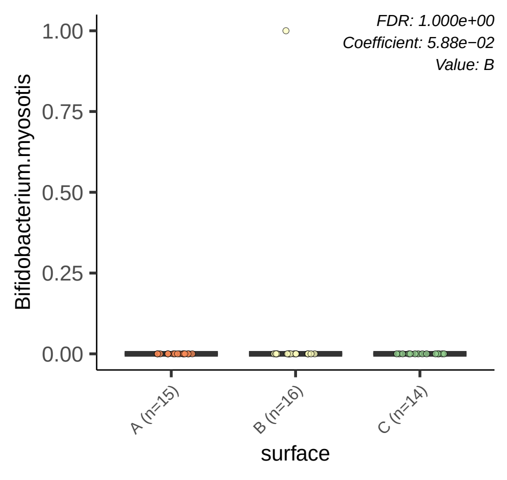
FDR: 1.000e+00
Coefficient: 5.88e−02
Value: B
Bifidobacterium.myosotis
1.00
0.75
0.50
0.25
0.00
A (n=15)
B (n=16)
C (n=14)
surface

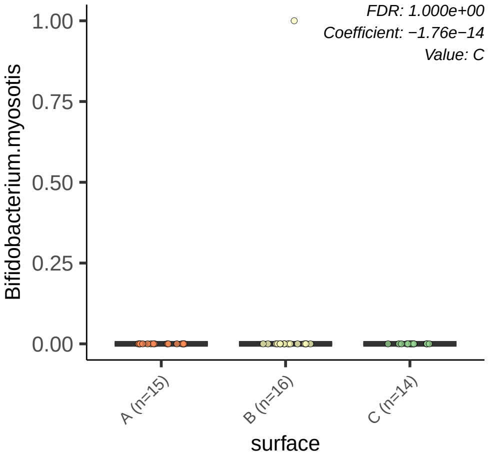
FDR: 1.000e+00
Coefficient: −1.76e−14
Value: C
Bifidobacterium.myosotis
1.00
0.75
0.50
0.25
0.00
A (n=15)
B (n=16)
C (n=14)
surface

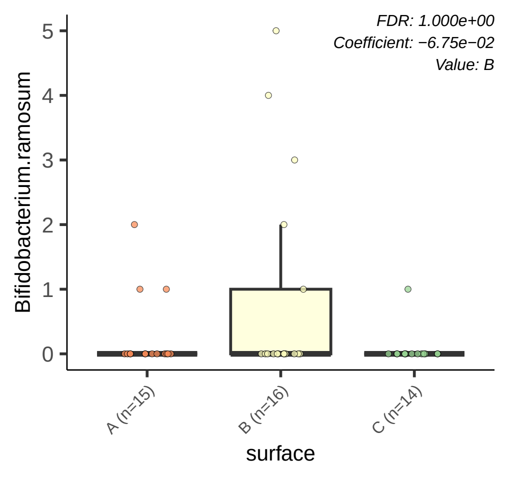
FDR: 1.000e+00
Coefficient: −6.75e−02
Value: B
Bifidobacterium.ramosum
0
1
2
3
4
5
A (n=15)
B (n=16)
C (n=14)
surface

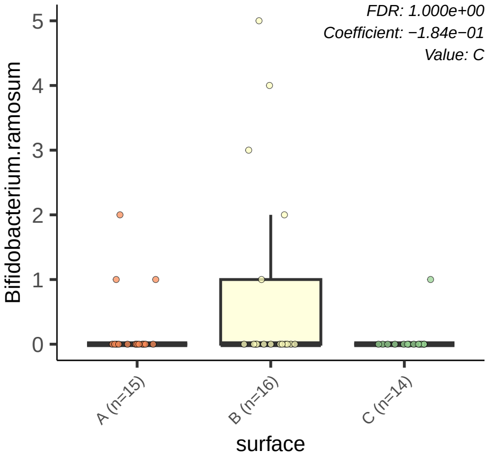
FDR: 1.000e+00
Coefficient: −1.84e−01
Value: C
Bifidobacterium.ramosum
0
1
2
3
4
5
A (n=15)
B (n=16)
C (n=14)
surface

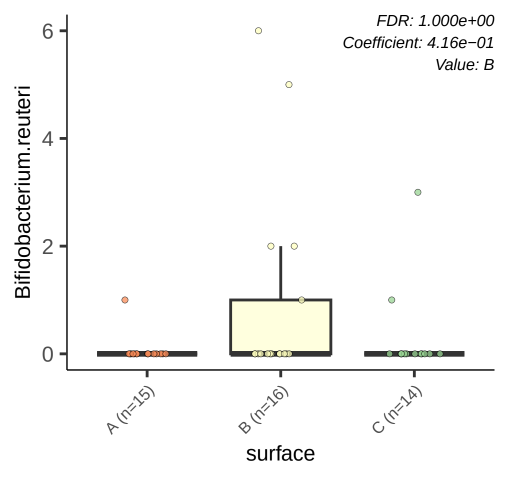

FDR: 1.000e+00
Coefficient: 4.16e−01
Value: B
Bifidobacterium.reuteri
6
4
2
0
A (n=15)
B (n=16)
C (n=14)
surface

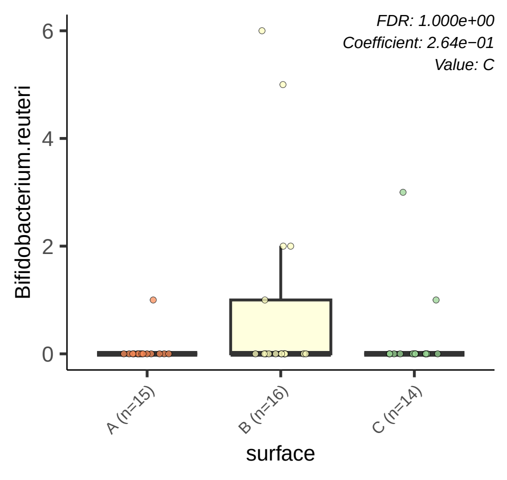

FDR: 1.000e+00
Coefficient: 2.64e−01
Value: C
Bifidobacterium.reuteri
0
2
4
6
A (n=15)
B (n=16)
C (n=14)
surface

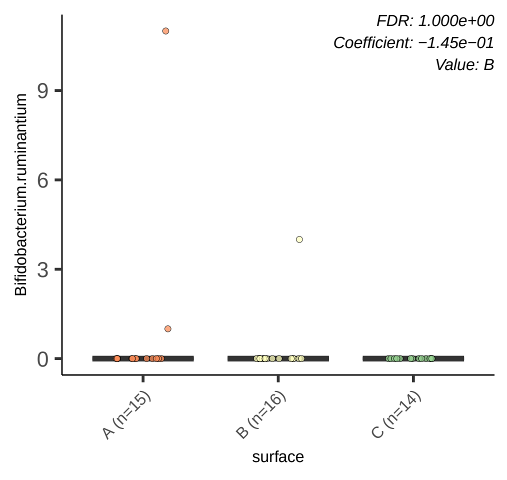
FDR: 1.000e+00
Coefficient: −1.45e−01
Value: B
Bifidobacterium.ruminantium
0
3
6
9
A (n=15)
B (n=16)
C (n=14)
surface

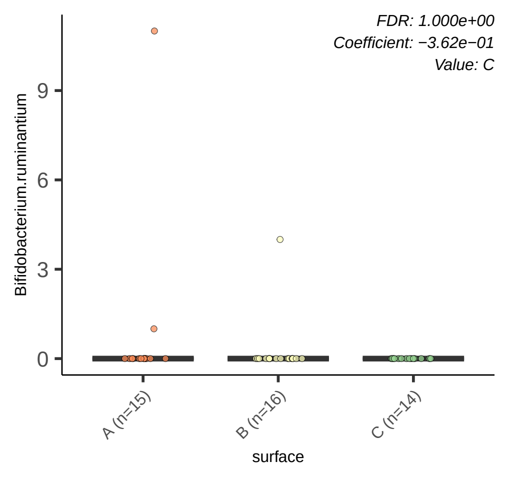

FDR: 1.000e+00
Coefficient: −3.62e−01
Value: C
Bifidobacterium.ruminantium
9
6
3
0
A (n=15)
B (n=16)
C (n=14)
surface

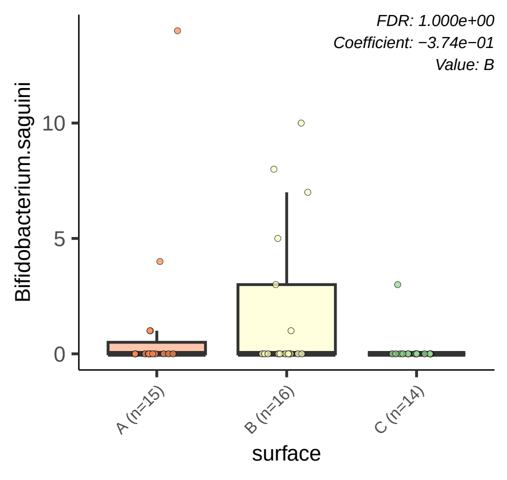
FDR: 1.000e+00
Coefficient: −3.74e−01
Value: B
Bifidobacterium.saguini
10
5
0
A (n=15)
B (n=16)
C (n=14)
surface

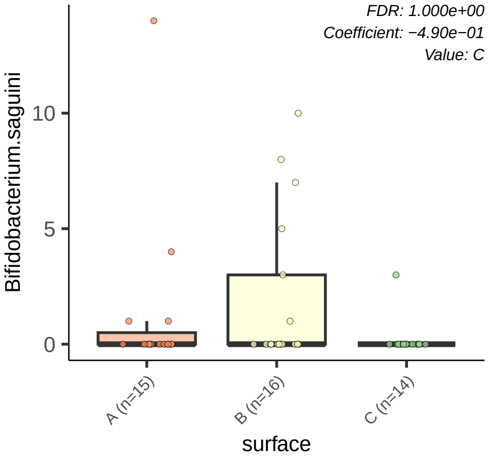

FDR: 1.000e+00
Coefficient: −4.90e−01
Value: C
Bifidobacterium.saguini
10
5
0
A (n=15)
B (n=16)
C (n=14)
surface

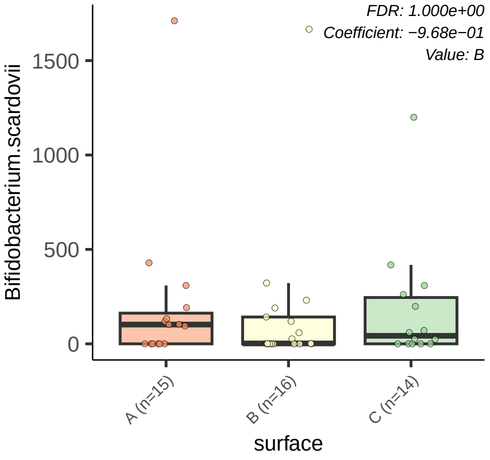
FDR: 1.000e+00
Coefficient: −9.68e−01
Value: B
Bifidobacterium.scardovii
1500
1000
500
0
A (n=15)
B (n=16)
C (n=14)
surface

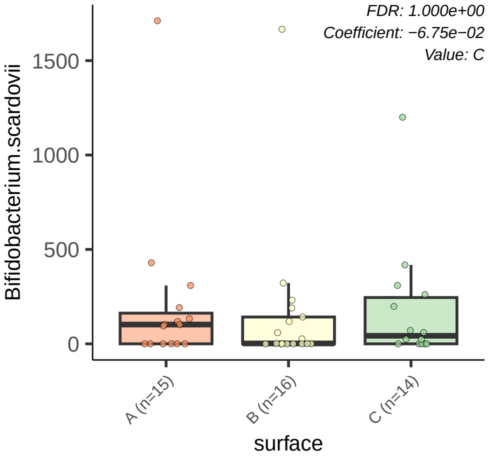
FDR: 1.000e+00
Coefficient: −6.75e−02
Value: C
Bifidobacterium.scardovii
1500
1000
500
0
A (n=15)
B (n=16)
C (n=14)
surface

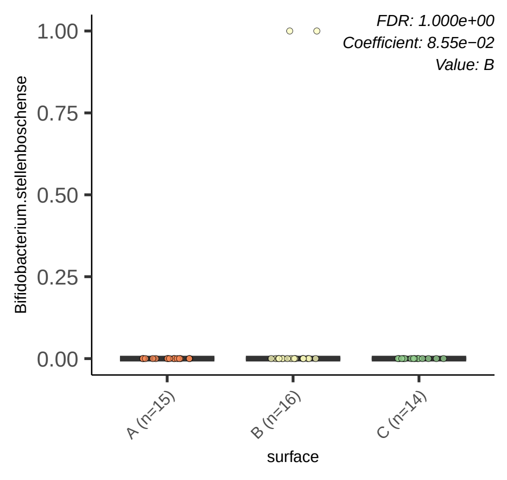
FDR: 1.000e+00
Coefficient: 8.55e−02
Value: B
1.00
0.75
0.50
0.25
0.00
Bifidobacterium.stellenboschense
A (n=15)
B (n=16)
C (n=14)
surface

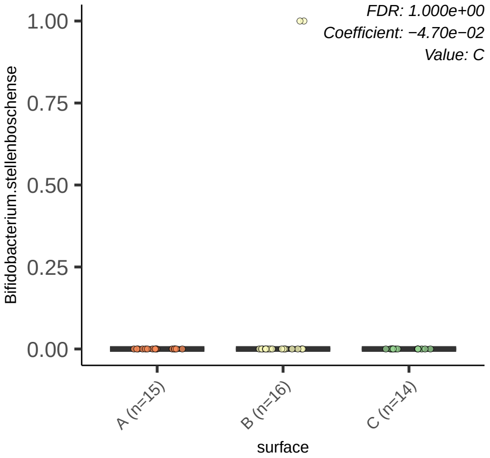
FDR: 1.000e+00
Coefficient: −4.70e−02
Value: C
Bifidobacterium.stellenboschense
1.00
0.75
0.50
0.25
0.00
A (n=15)
B (n=16)
C (n=14)
surface

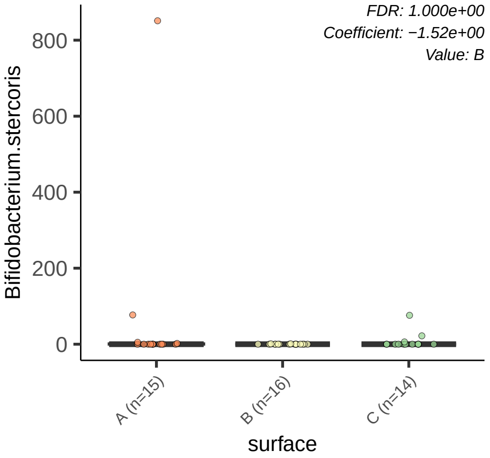

FDR: 1.000e+00
Coefficient: −1.52e+00
Value: B
Bifidobacterium.stercoris
800
600
400
200
0
A (n=15)
B (n=16)
C (n=14)
surface

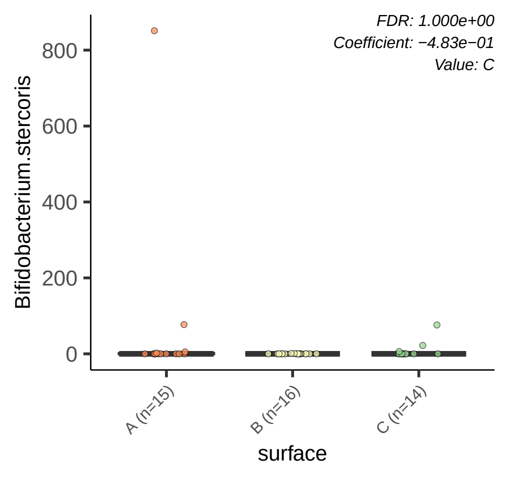
FDR: 1.000e+00
Coefficient: −4.83e−01
Value: C
Bifidobacterium.stercoris
800
600
400
200
0
A (n=15)
B (n=16)
C (n=14)
surface

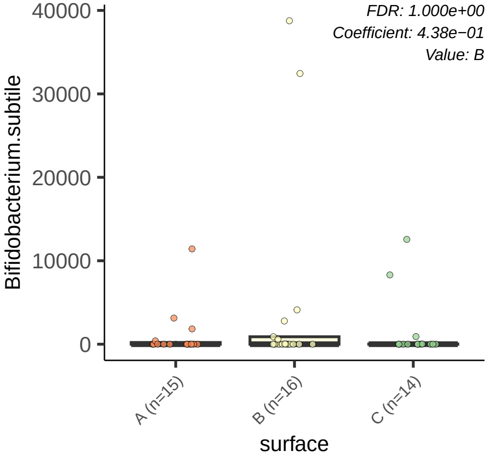
FDR: 1.000e+00
Coefficient: 4.38e−01
Value: B
40000
30000
20000
10000
0
Bifidobacterium.subtile
A (n=15)
B (n=16)
C (n=14)
surface

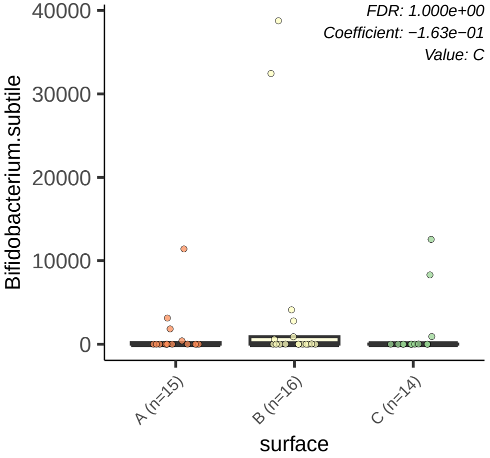
FDR: 1.000e+00
Coefficient: −1.63e−01
Value: C
Bifidobacterium.subtile
40000
30000
20000
10000
0
A (n=15)
B (n=16)
C (n=14)
surface

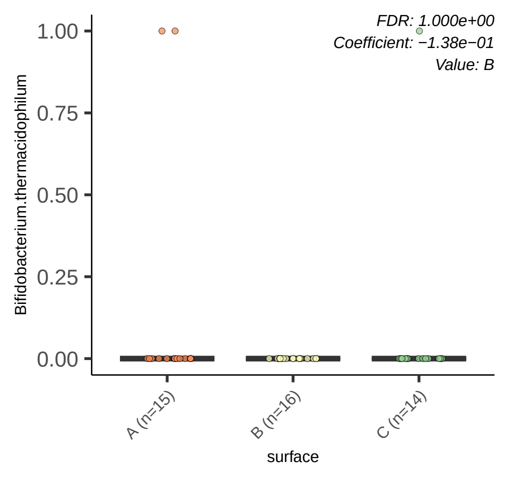
FDR: 1.000e+00
Coefficient: −1.38e−01
Value: B
Bifidobacterium.thermacidophilum
1.00
0.75
0.50
0.25
0.00
A (n=15)
B (n=16)
C (n=14)
surface

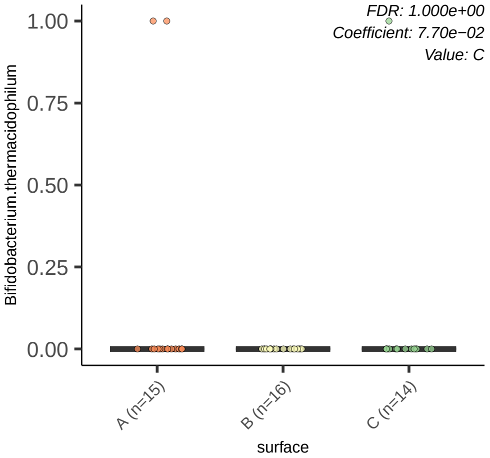
FDR: 1.000e+00
Coefficient: 7.70e−02
Value: C
Bifidobacterium.thermacidophilum
1.00
0.75
0.50
0.25
0.00
A (n=15)
B (n=16)
C (n=14)
surface

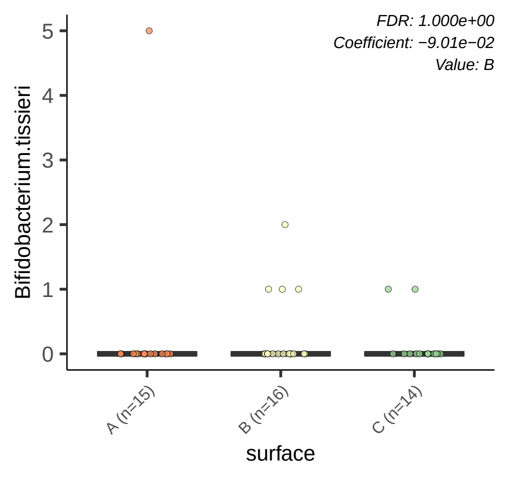
FDR: 1.000e+00
Coefficient: −9.01e−02
Value: B
Bifidobacterium.tissieri
5
4
3
2
1
0
A (n=15)
B (n=16)
C (n=14)
surface

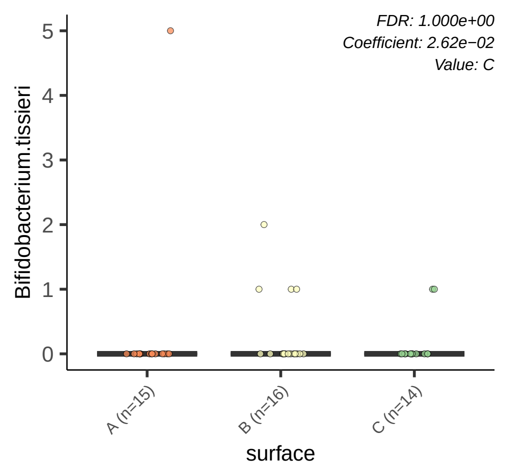

FDR: 1.000e+00
Coefficient: 2.62e−02
Value: C
Bifidobacterium.tissieri
0
1
2
3
4
5
A (n=15)
B (n=16)
C (n=14)
surface

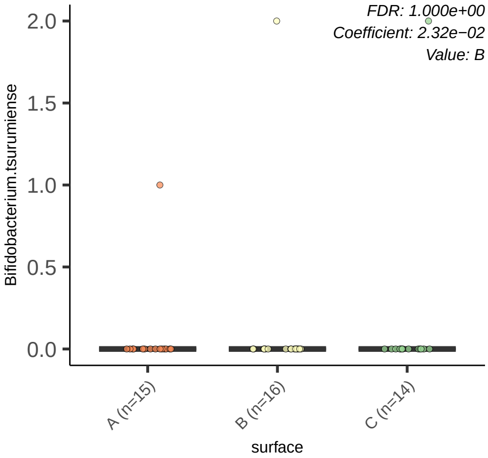
FDR: 1.000e+00
Coefficient: 2.32e−02
Value: B
Bifidobacterium.tsurumiense
2.0
1.5
1.0
0.5
0.0
A (n=15)
B (n=16)
C (n=14)
surface

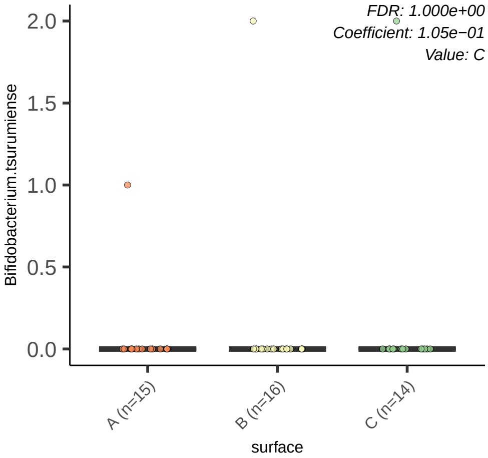
FDR: 1.000e+00
Coefficient: 1.05e−01
Value: C
Bifidobacterium.tsurumiense
2.0
1.5
1.0
0.5
0.0
A (n=15)
B (n=16)
C (n=14)
surface

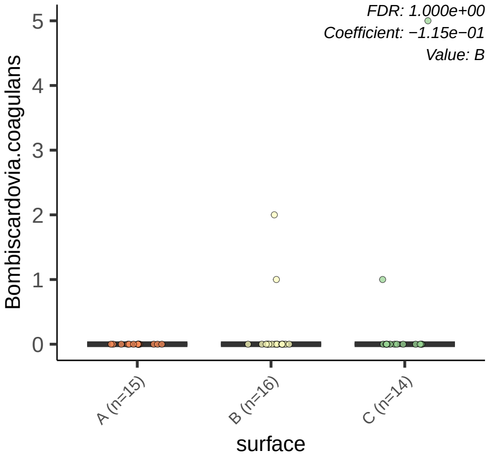

FDR: 1.000e+00
Coefficient: −1.15e−01
Value: B
Bombiscardovia.coagulans
0
1
2
3
4
5
A (n=15)
B (n=16)
C (n=14)
surface

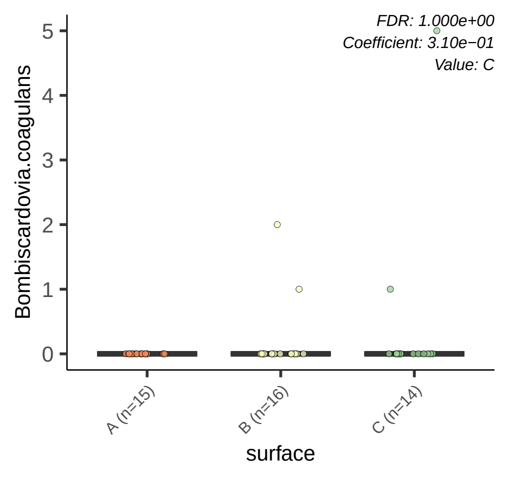
FDR: 1.000e+00
Coefficient: 3.10e−01
Value: C
Bombiscardovia.coagulans
0
1
2
3
4
5
A (n=15)
B (n=16)
C (n=14)
surface

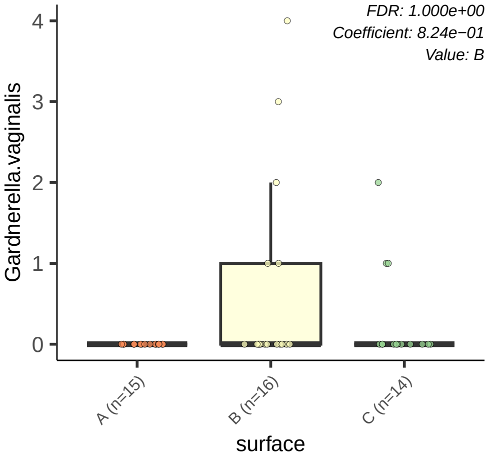
FDR: 1.000e+00
Coefficient: 8.24e−01
Value: B
Gardnerella.vaginalis
4
3
2
1
0
A (n=15)
B (n=16)
C (n=14)
surface

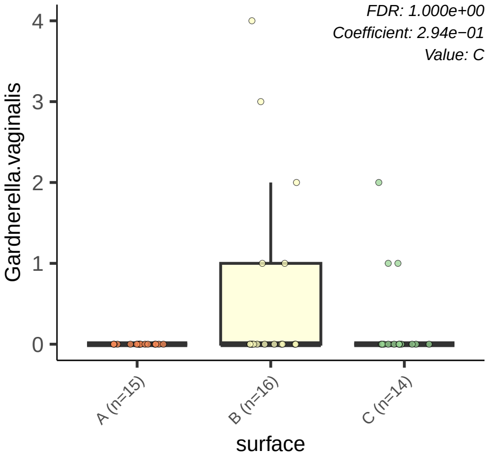
FDR: 1.000e+00
Coefficient: 2.94e−01
Value: C
Gardnerella.vaginalis
4
3
2
1
0
A (n=15)
B (n=16)
C (n=14)
surface

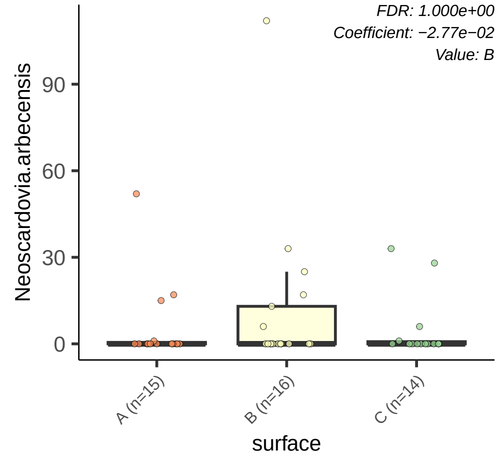

FDR: 1.000e+00
Coefficient: −2.77e−02
Value: B
Neoscardovia.arbecensis
90
60
30
0
A (n=15)
B (n=16)
C (n=14)
surface

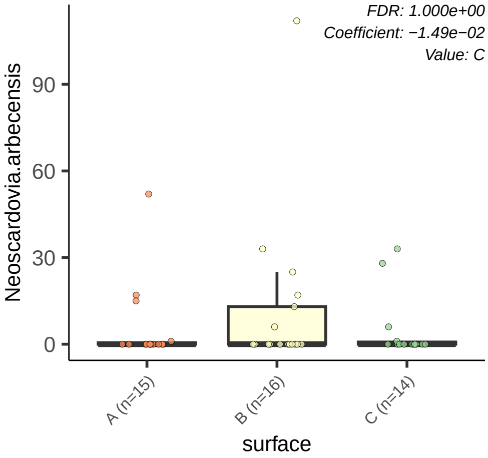
FDR: 1.000e+00
Coefficient: −1.49e−02
Value: C
Neoscardovia.arbecensis
90
60
30
0
A (n=15)
B (n=16)
C (n=14)
surface

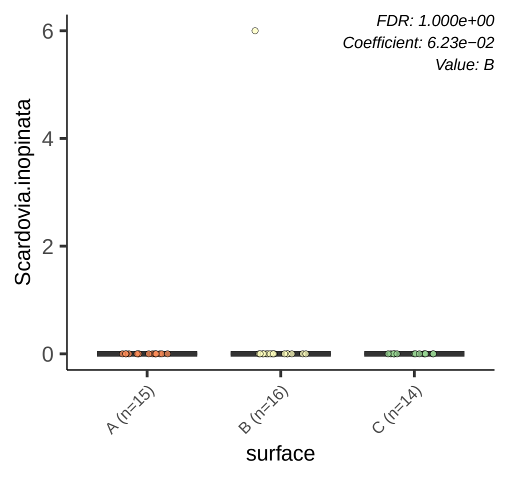
FDR: 1.000e+00
Coefficient: 6.23e−02
Value: B
Scardovia.inopinata
6
4
2
0
A (n=15)
B (n=16)
C (n=14)
surface

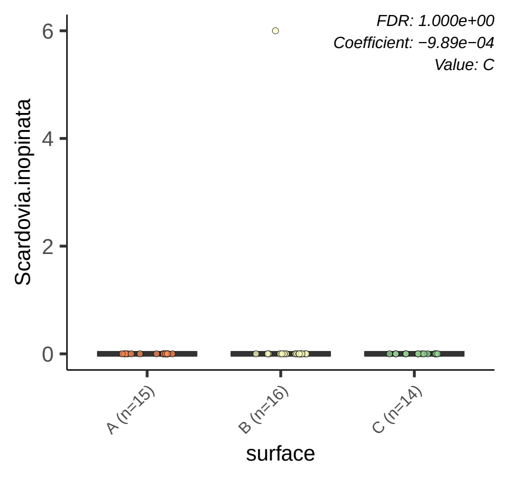
FDR: 1.000e+00
Coefficient: −9.89e−04
Value: C
Scardovia.inopinata
0
2
4
6
A (n=15)
B (n=16)
C (n=14)
surface

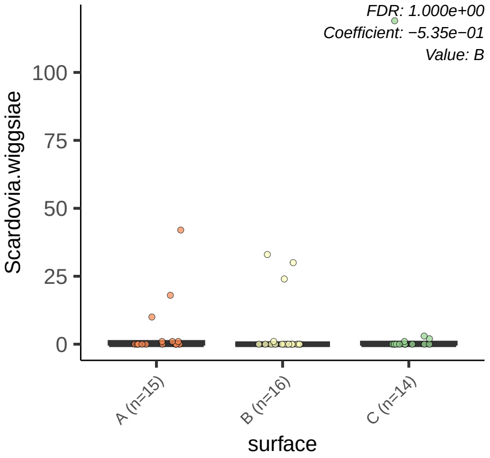
FDR: 1.000e+00
Coefficient: −5.35e−01
Value: B
Scardovia.wiggsiae
100
75
50
25
0
A (n=15)
B (n=16)
C (n=14)
surface

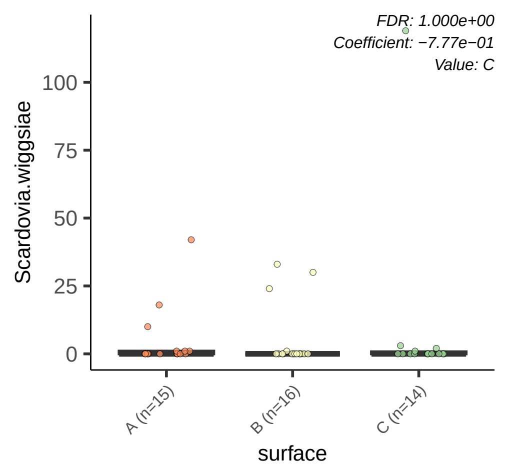

FDR: 1.000e+00
Coefficient: −7.77e−01
Value: C
Scardovia.wiggsiae
100
75
50
25
0
A (n=15)
B (n=16)
C (n=14)
surface

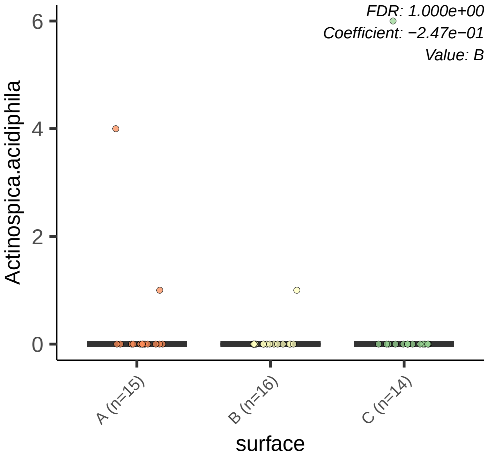
FDR: 1.000e+00
Coefficient: −2.47e−01
Value: B
Actinospica.acidiphila
6
4
2
0
A (n=15)
B (n=16)
C (n=14)
surface

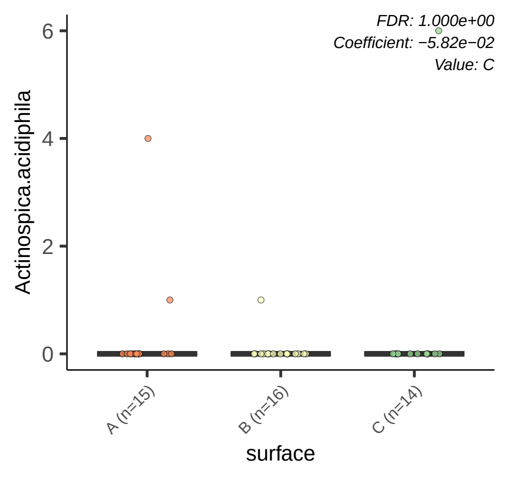
FDR: 1.000e+00
Coefficient: −5.82e−02
Value: C
Actinospica.acidiphila
6
4
2
0
A (n=15)
B (n=16)
C (n=14)
surface

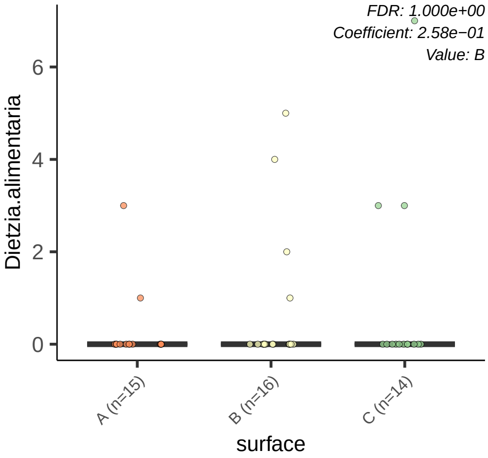
FDR: 1.000e+00
Coefficient: 2.58e−01
Value: B
Dietzia.alimentaria
0
2
4
6
A (n=15)
B (n=16)
C (n=14)
surface

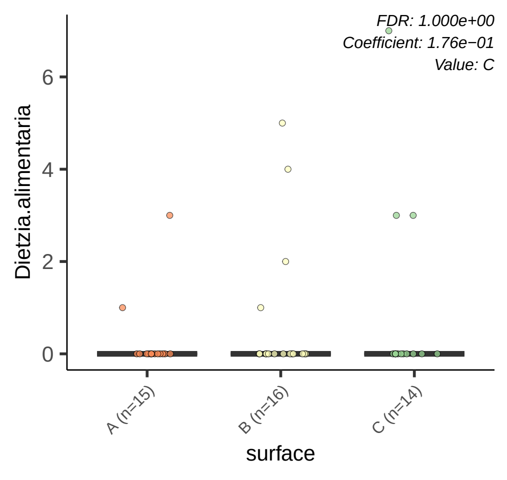
FDR: 1.000e+00
Coefficient: 1.76e−01
Value: C
Dietzia.alimentaria
0
2
4
6
A (n=15)
B (n=16)
C (n=14)
surface

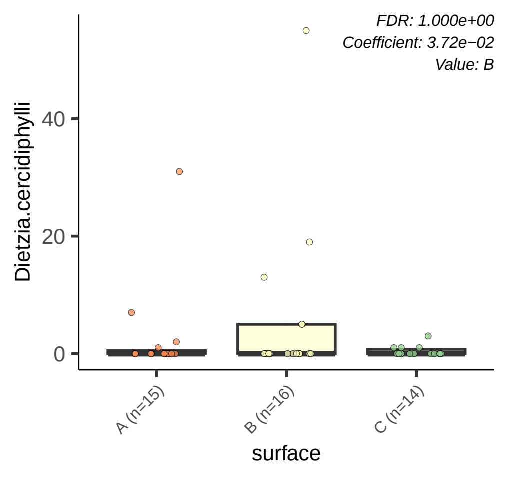

FDR: 1.000e+00
Coefficient: 3.72e−02
Value: B
Dietzia.cercidiphylli
40
20
0
A (n=15)
B (n=16)
C (n=14)
surface

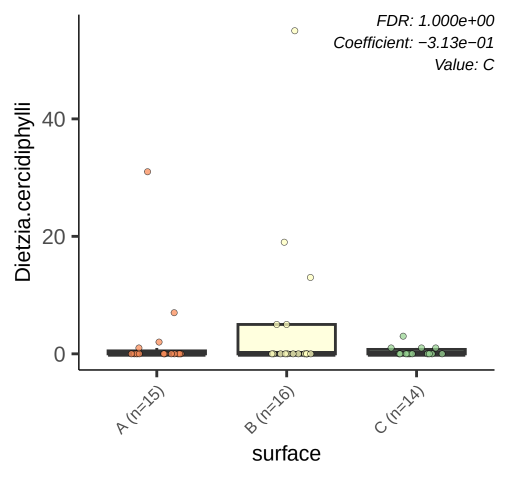
FDR: 1.000e+00
Coefficient: −3.13e−01
Value: C
Dietzia.cercidiphylli
0
20
40
A (n=15)
B (n=16)
C (n=14)
surface

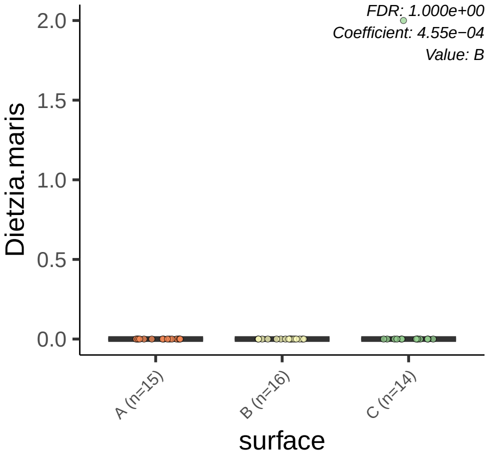
FDR: 1.000e+00
Coefficient: 4.55e−04
Value: B
Dietzia.maris
2.0
1.5
1.0
0.5
0.0
A (n=15)
B (n=16)
C (n=14)
surface

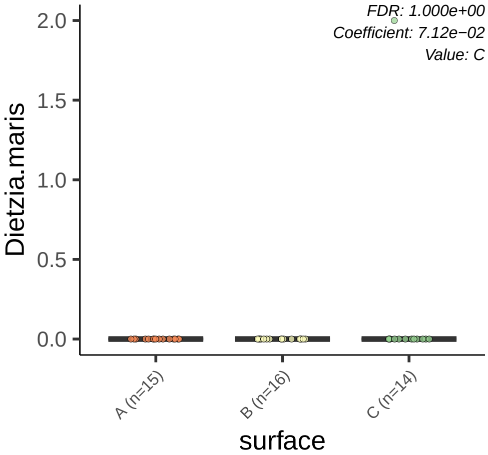
FDR: 1.000e+00
Coefficient: 7.12e−02
Value: C
Dietzia.maris
2.0
1.5
1.0
0.5
0.0
A (n=15)
B (n=16)
C (n=14)
surface

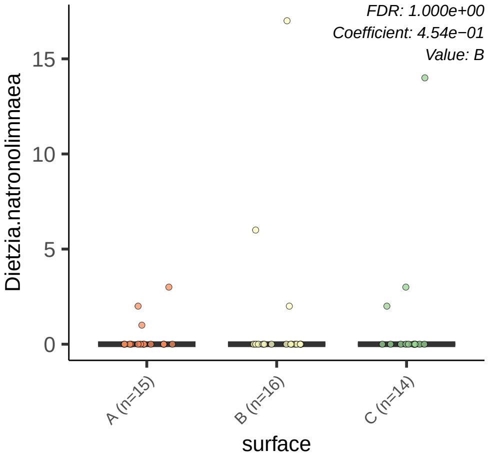

FDR: 1.000e+00
Coefficient: 4.54e−01
Value: B
Dietzia.natronolimnaea
0
5
10
15
A (n=15)
B (n=16)
C (n=14)
surface

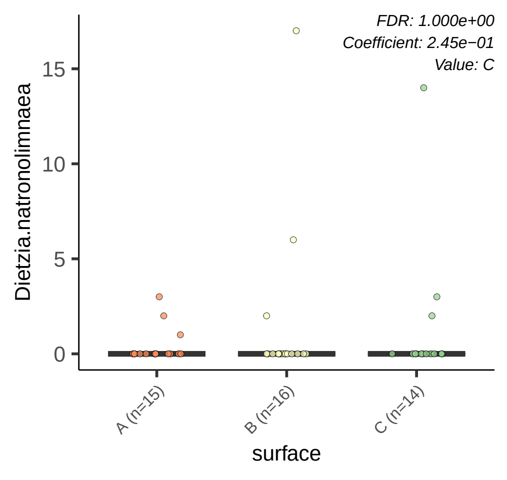
FDR: 1.000e+00
Coefficient: 2.45e−01
Value: C
Dietzia.natronolimnaea
0
5
10
15
A (n=15)
B (n=16)
C (n=14)
surface

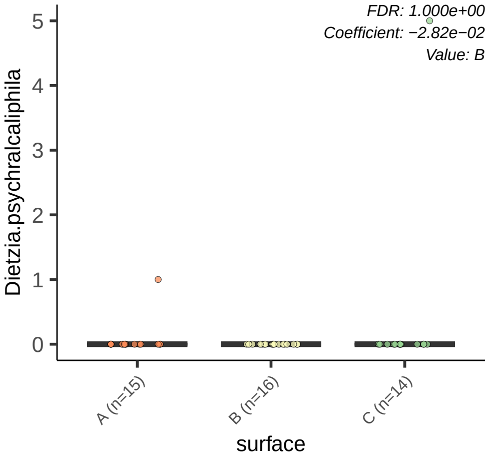

FDR: 1.000e+00
Coefficient: −2.82e−02
Value: B
Dietzia.psychralcaliphila
0
1
2
3
4
5
A (n=15)
B (n=16)
C (n=14)
surface

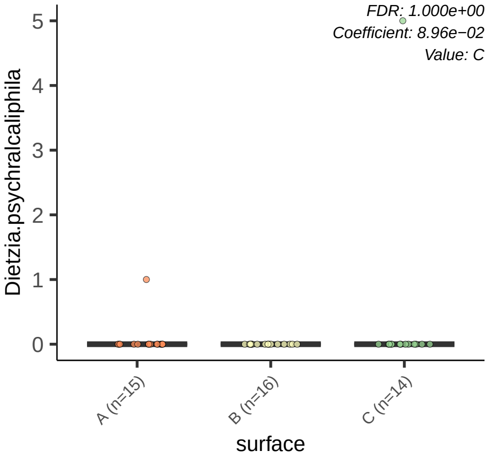
FDR: 1.000e+00
Coefficient: 8.96e−02
Value: C
Dietzia.psychralcaliphila
0
1
2
3
4
5
A (n=15)
B (n=16)
C (n=14)
surface

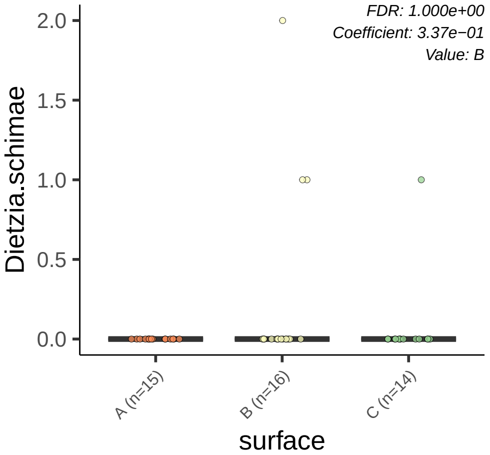
FDR: 1.000e+00
Coefficient: 3.37e−01
Value: B
Dietzia.schimae
2.0
1.5
1.0
0.5
0.0
A (n=15)
B (n=16)
C (n=14)
surface

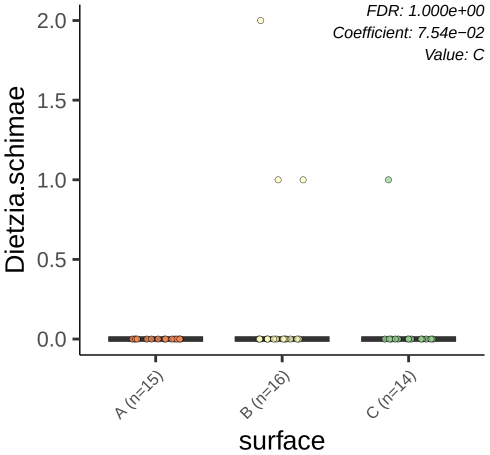
FDR: 1.000e+00
Coefficient: 7.54e−02
Value: C
Dietzia.schimae
2.0
1.5
1.0
0.5
0.0
A (n=15)
B (n=16)
C (n=14)
surface

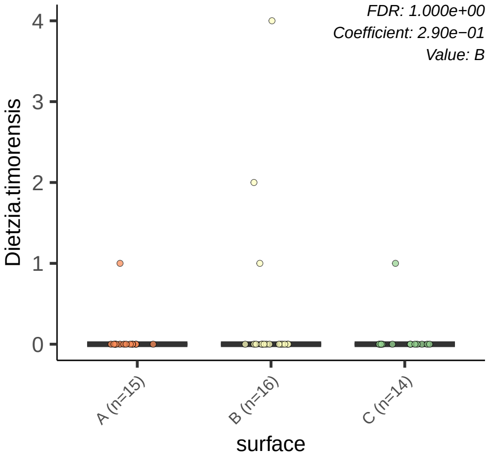
FDR: 1.000e+00
Coefficient: 2.90e−01
Value: B
Dietzia.timorensis
0
1
2
3
4
A (n=15)
B (n=16)
C (n=14)
surface

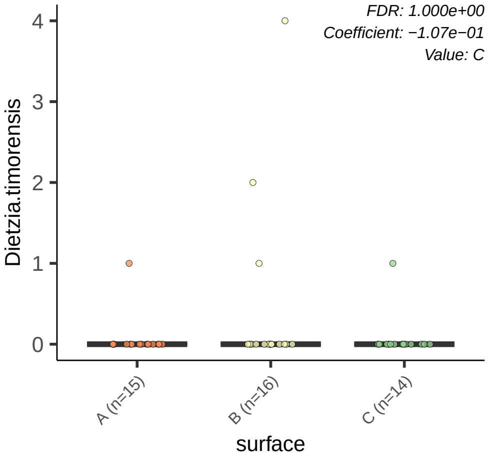
FDR: 1.000e+00
Coefficient: −1.07e−01
Value: C
Dietzia.timorensis
0
1
2
3
4
A (n=15)
B (n=16)
C (n=14)
surface

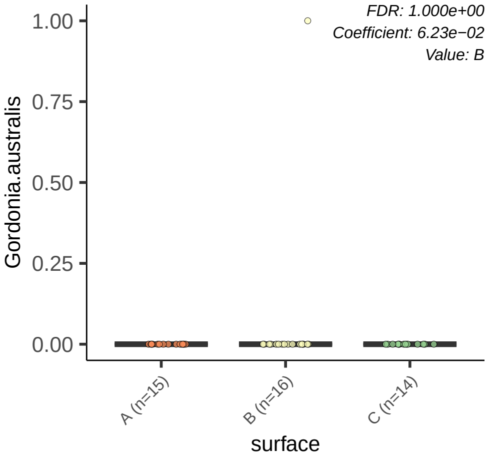

FDR: 1.000e+00
Coefficient: 6.23e−02
Value: B
Gordonia.australis
1.00
0.75
0.50
0.25
0.00
A (n=15)
B (n=16)
C (n=14)
surface

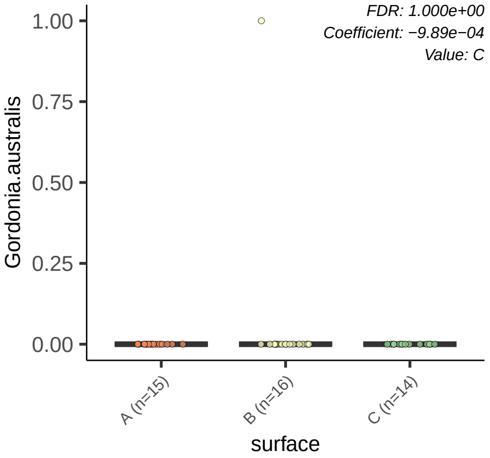
FDR: 1.000e+00
Coefficient: −9.89e−04
Value: C
Gordonia.australis
1.00
0.75
0.50
0.25
0.00
A (n=15)
B (n=16)
C (n=14)
surface

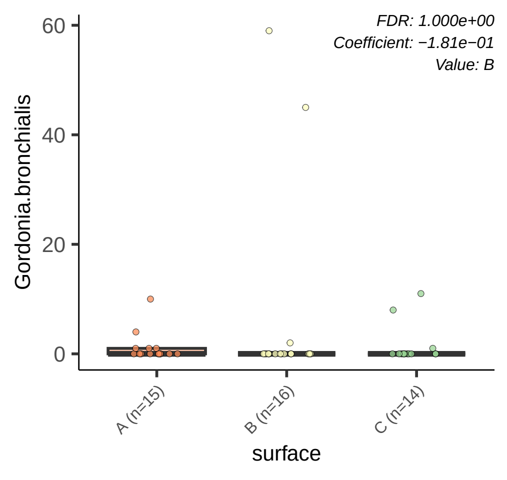
FDR: 1.000e+00
Coefficient: −1.81e−01
Value: B
Gordonia.bronchialis
60
40
20
0
A (n=15)
B (n=16)
C (n=14)
surface

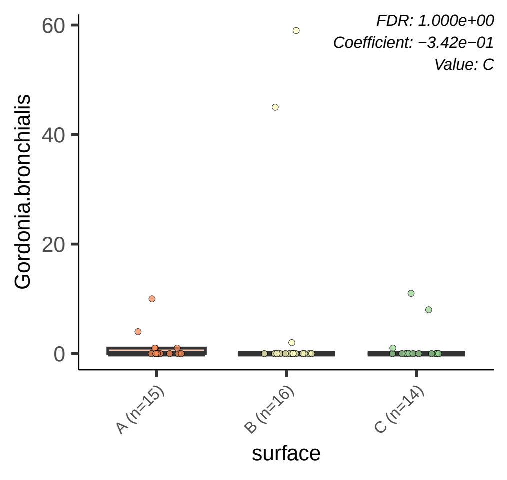
FDR: 1.000e+00
Coefficient: −3.42e−01
Value: C
Gordonia.bronchialis
0
20
40
60
A (n=15)
B (n=16)
C (n=14)
surface

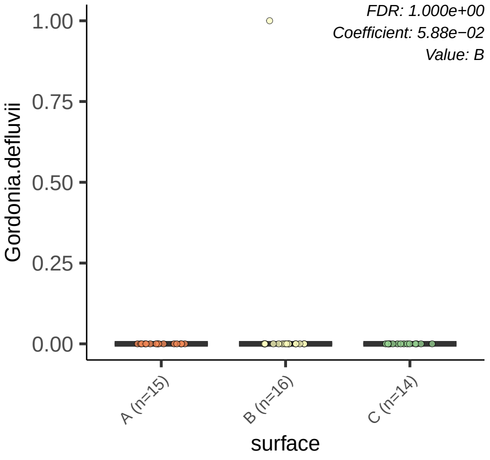
FDR: 1.000e+00
Coefficient: 5.88e−02
Value: B
Gordonia.defluvii
1.00
0.75
0.50
0.25
0.00
A (n=15)
B (n=16)
C (n=14)
surface

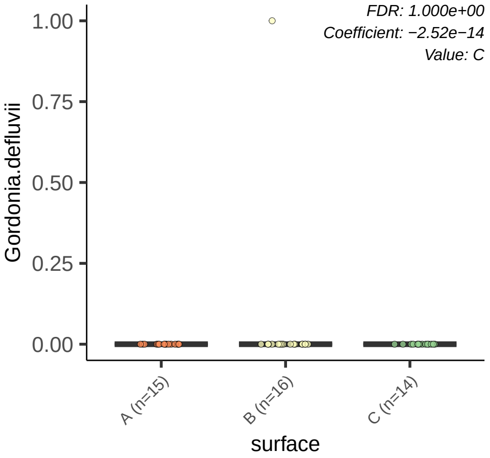
FDR: 1.000e+00
Coefficient: −2.52e−14
Value: C
Gordonia.defluvii
1.00
0.75
0.50
0.25
0.00
A (n=15)
B (n=16)
C (n=14)
surface

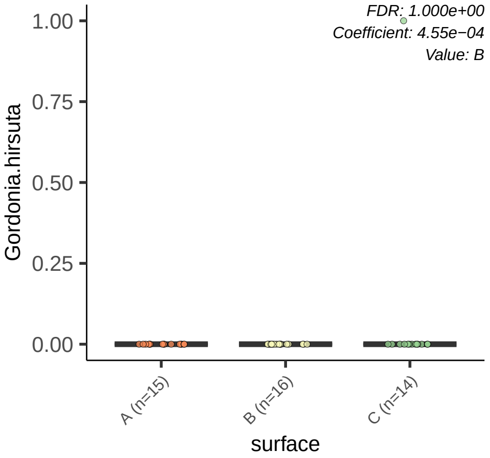

FDR: 1.000e+00
Coefficient: 4.55e−04
Value: B
Gordonia.hirsuta
1.00
0.75
0.50
0.25
0.00
A (n=15)
B (n=16)
C (n=14)
surface

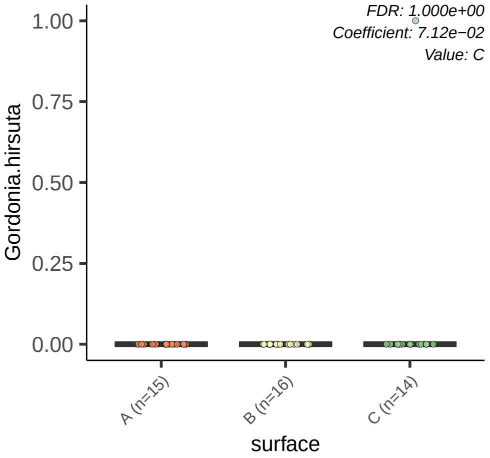
FDR: 1.000e+00
Coefficient: 7.12e−02
Value: C
Gordonia.hirsuta
1.00
0.75
0.50
0.25
0.00
A (n=15)
B (n=16)
C (n=14)
surface

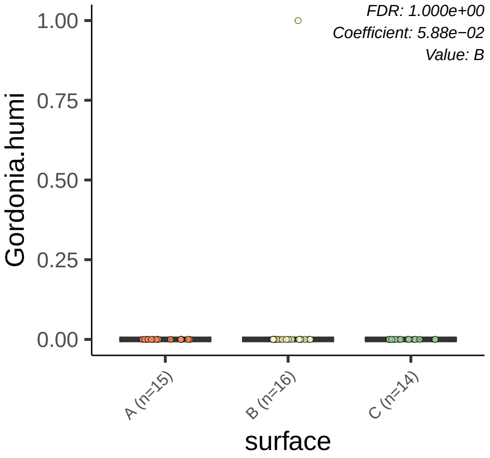
FDR: 1.000e+00
Coefficient: 5.88e−02
Value: B
Gordonia.humi
1.00
0.75
0.50
0.25
0.00
A (n=15)
B (n=16)
C (n=14)
surface

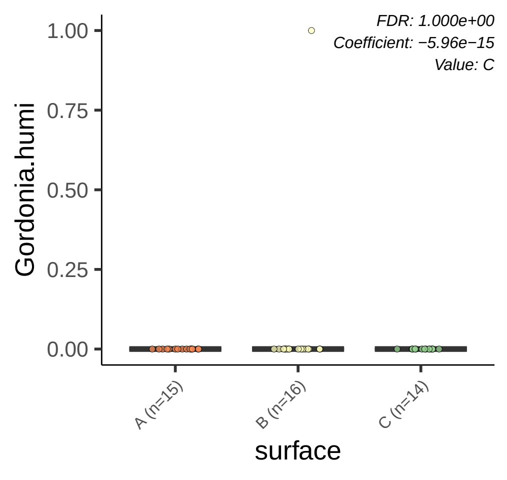
FDR: 1.000e+00
Coefficient: −5.96e−15
Value: C
Gordonia.humi
1.00
0.75
0.50
0.25
0.00
A (n=15)
B (n=16)
C (n=14)
surface

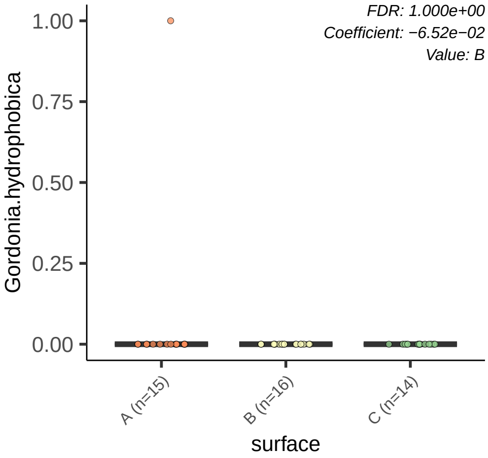
FDR: 1.000e+00
Coefficient: −6.52e−02
Value: B
Gordonia.hydrophobica
1.00
0.75
0.50
0.25
0.00
A (n=15)
B (n=16)
C (n=14)
surface

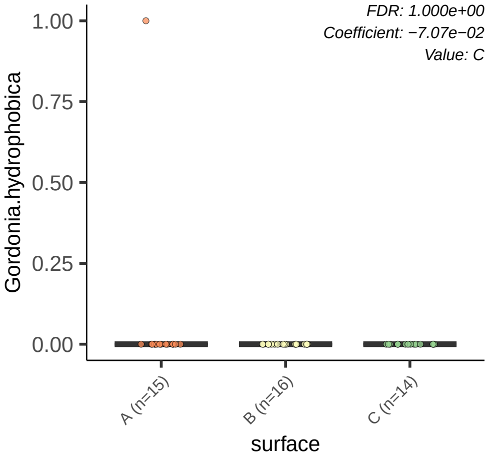

FDR: 1.000e+00
Coefficient: −7.07e−02
Value: C
Gordonia.hydrophobica
1.00
0.75
0.50
0.25
0.00
A (n=15)
B (n=16)
C (n=14)
surface

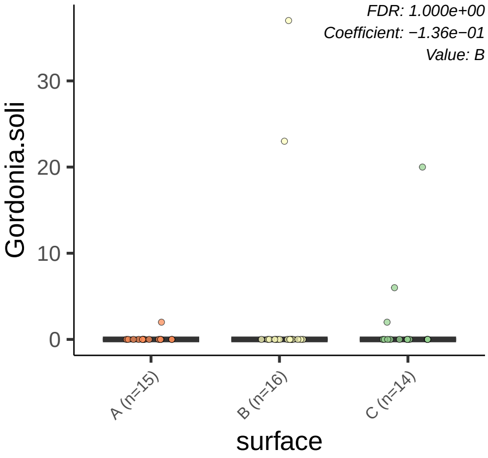

FDR: 1.000e+00
Coefficient: −1.36e−01
Value: B
Gordonia.soli
30
20
10
0
A (n=15)
B (n=16)
C (n=14)
surface

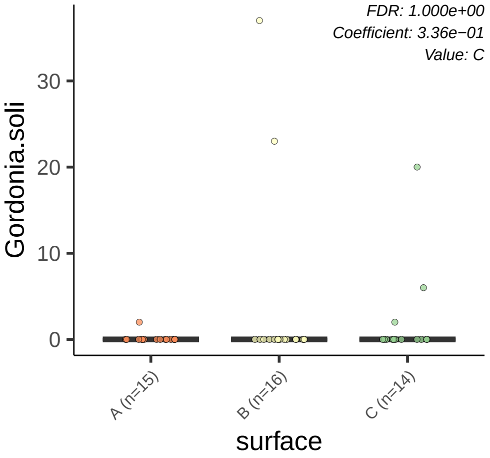

FDR: 1.000e+00
Coefficient: 3.36e−01
Value: C
Gordonia.soli
0
10
20
30
A (n=15)
B (n=16)
C (n=14)
surface

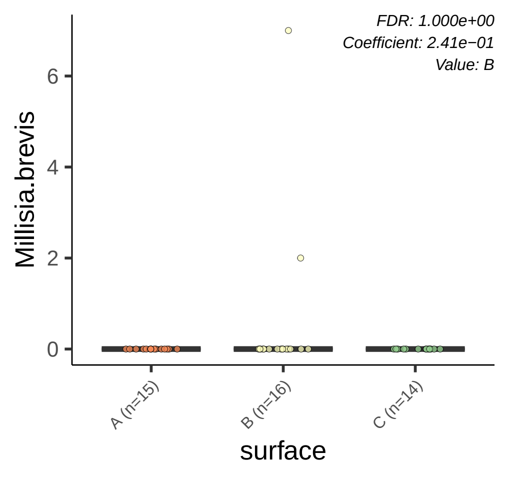
FDR: 1.000e+00
Coefficient: 2.41e−01
Value: B
Millisia.brevis
0
2
4
6
A (n=15)
B (n=16)
C (n=14)
surface

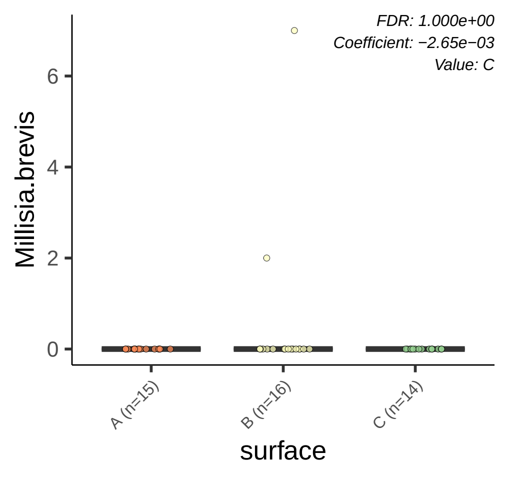

FDR: 1.000e+00
Coefficient: −2.65e−03
Value: C
Millisia.brevis
0
2
4
6
A (n=15)
B (n=16)
C (n=14)
surface

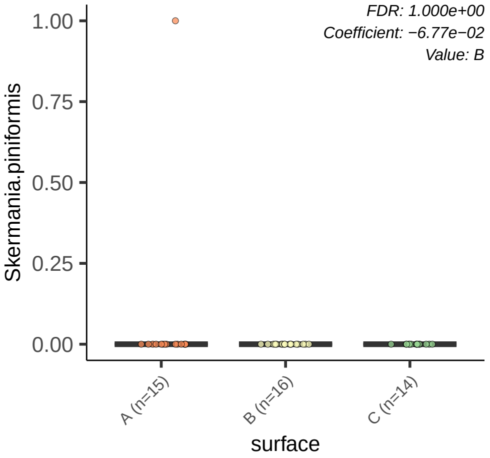
FDR: 1.000e+00
Coefficient: −6.77e−02
Value: B
Skermania.piniformis
1.00
0.75
0.50
0.25
0.00
A (n=15)
B (n=16)
C (n=14)
surface

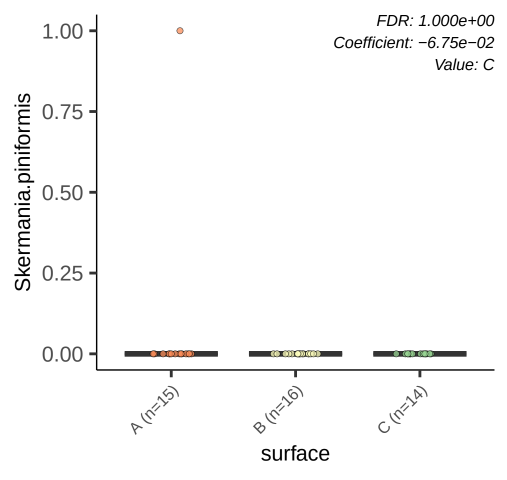
FDR: 1.000e+00
Coefficient: −6.75e−02
Value: C
Skermania.piniformis
1.00
0.75
0.50
0.25
0.00
A (n=15)
B (n=16)
C (n=14)
surface

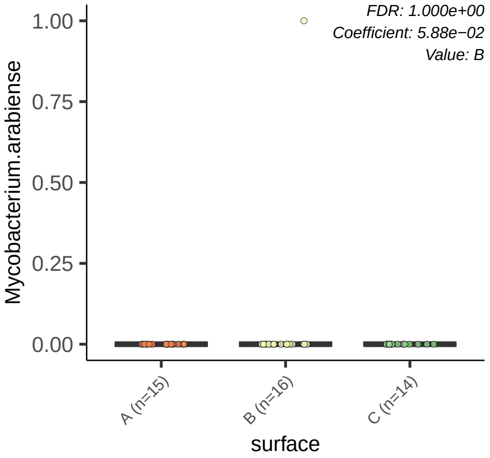

FDR: 1.000e+00
Coefficient: 5.88e−02
Value: B
Mycobacterium.arabiense
1.00
0.75
0.50
0.25
0.00
A (n=15)
B (n=16)
C (n=14)
surface

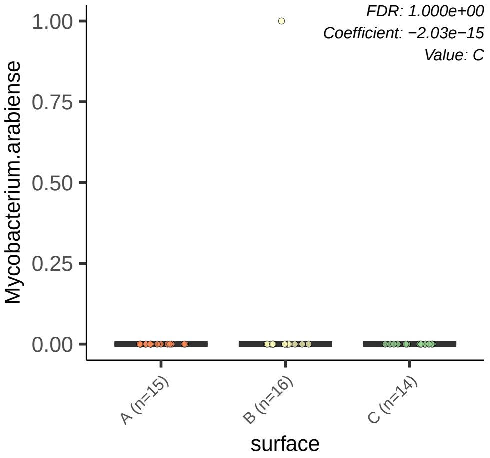

FDR: 1.000e+00
Coefficient: −2.03e−15
Value: C
Mycobacterium.arabiense
1.00
0.75
0.50
0.25
0.00
A (n=15)
B (n=16)
C (n=14)
surface

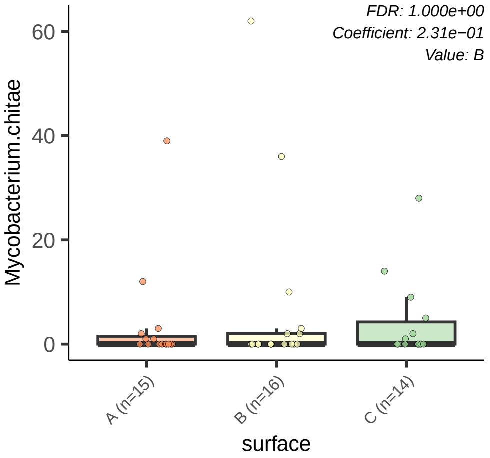

FDR: 1.000e+00
Coefficient: 2.31e−01
Value: B
Mycobacterium.chitae
0
20
40
60
A (n=15)
B (n=16)
C (n=14)
surface

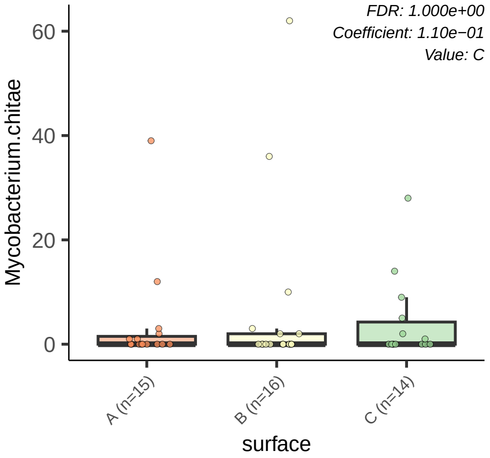

FDR: 1.000e+00
Coefficient: 1.10e−01
Value: C
Mycobacterium.chitae
60
40
20
0
A (n=15)
B (n=16)
C (n=14)
surface

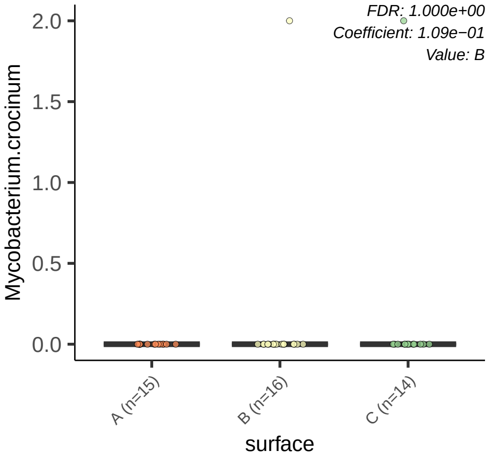

FDR: 1.000e+00
Coefficient: 1.09e−01
Value: B
Mycobacterium.crocinum
2.0
1.5
1.0
0.5
0.0
A (n=15)
B (n=16)
C (n=14)
surface

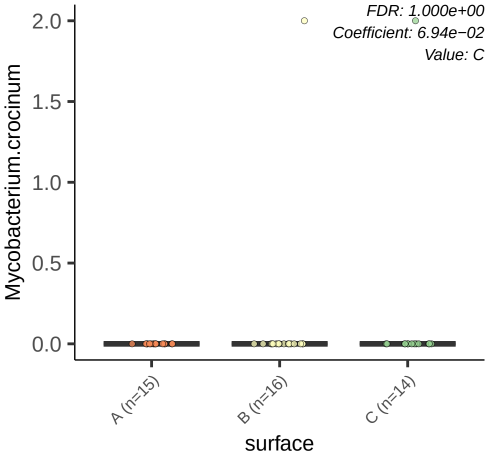
FDR: 1.000e+00
Coefficient: 6.94e−02
Value: C
Mycobacterium.crocinum
2.0
1.5
1.0
0.5
0.0
A (n=15)
B (n=16)
C (n=14)
surface

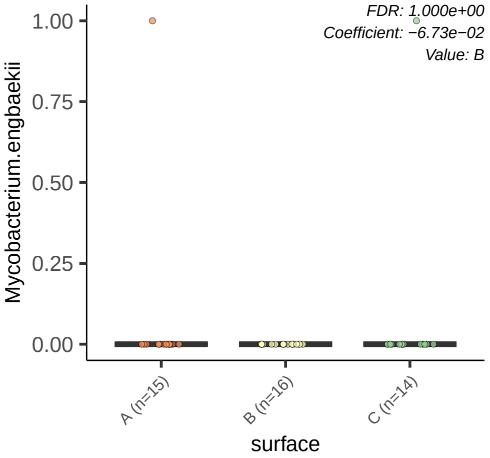
FDR: 1.000e+00
Coefficient: −6.73e−02
Value: B
Mycobacterium.engbaekii
1.00
0.75
0.50
0.25
0.00
A (n=15)
B (n=16)
C (n=14)
surface

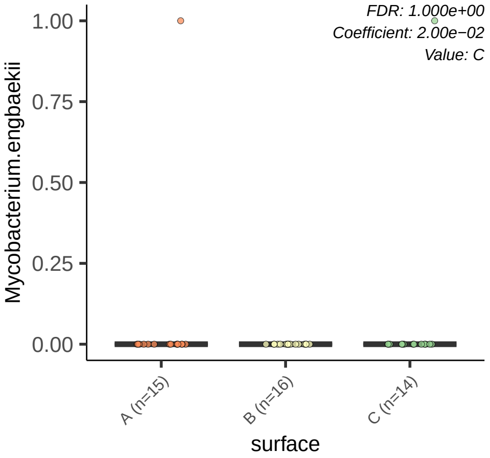
FDR: 1.000e+00
Coefficient: 2.00e−02
Value: C
Mycobacterium.engbaekii
1.00
0.75
0.50
0.25
0.00
A (n=15)
B (n=16)
C (n=14)
surface

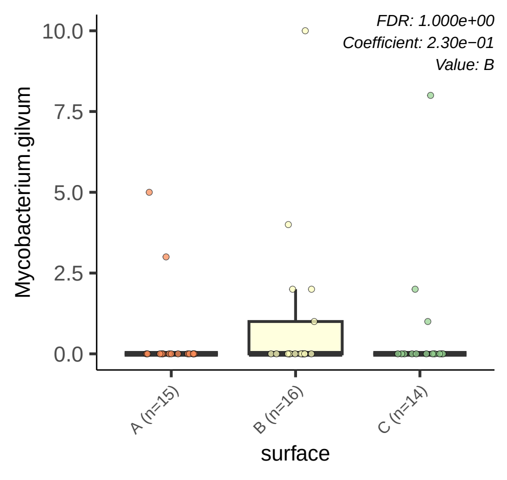

FDR: 1.000e+00
Coefficient: 2.30e−01
Value: B
Mycobacterium.gilvum
10.0
7.5
5.0
2.5
0.0
A (n=15)
B (n=16)
C (n=14)
surface

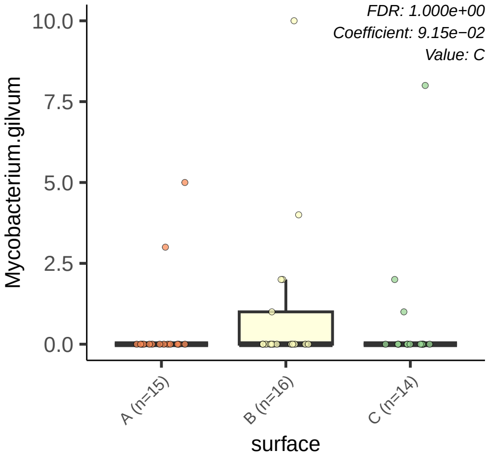
FDR: 1.000e+00
Coefficient: 9.15e−02
Value: C
Mycobacterium.gilvum
10.0
7.5
5.0
2.5
0.0
A (n=15)
B (n=16)
C (n=14)
surface

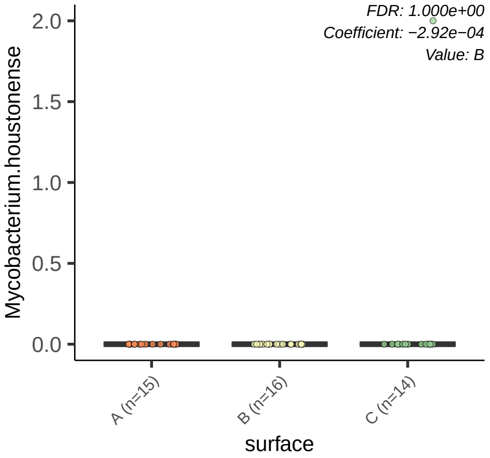

FDR: 1.000e+00
Coefficient: −2.92e−04
Value: B
Mycobacterium.houstonense
2.0
1.5
1.0
0.5
0.0
A (n=15)
B (n=16)
C (n=14)
surface

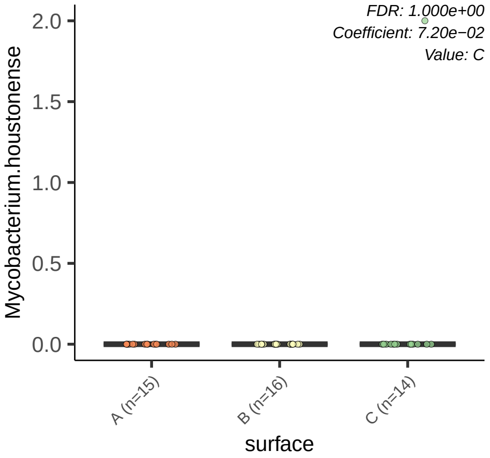
FDR: 1.000e+00
Coefficient: 7.20e−02
Value: C
Mycobacterium.houstonense
2.0
1.5
1.0
0.5
0.0
A (n=15)
B (n=16)
C (n=14)
surface

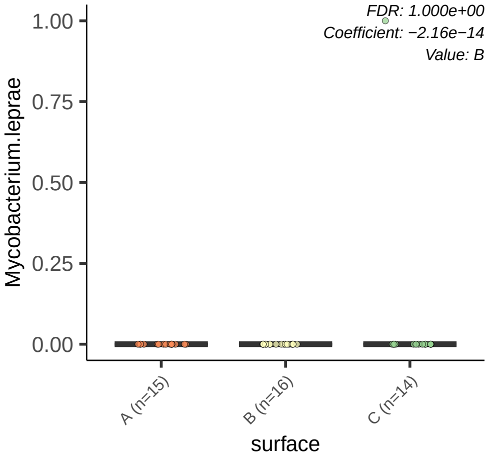

FDR: 1.000e+00
Coefficient: −2.16e−14
Value: B
Mycobacterium.leprae
1.00
0.75
0.50
0.25
0.00
A (n=15)
B (n=16)
C (n=14)
surface

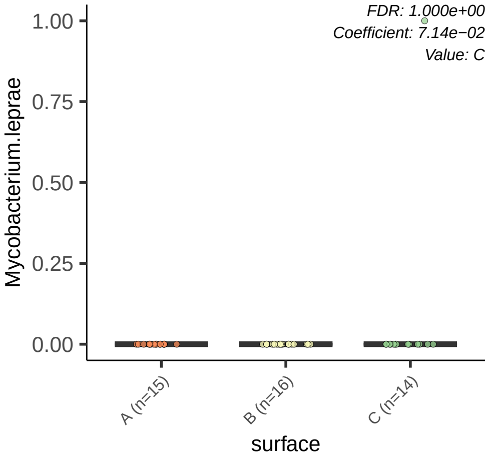
FDR: 1.000e+00
Coefficient: 7.14e−02
Value: C
Mycobacterium.leprae
1.00
0.75
0.50
0.25
0.00
A (n=15)
B (n=16)
C (n=14)
surface

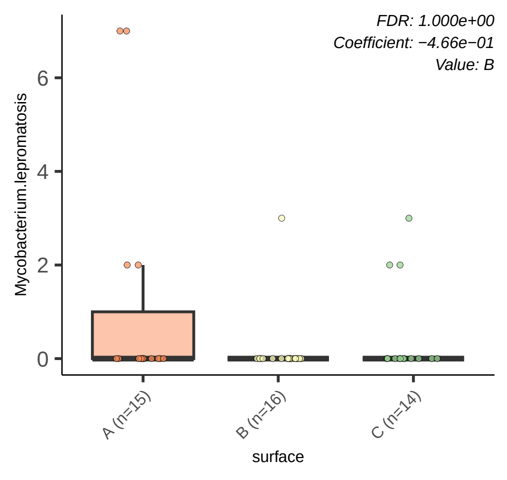

FDR: 1.000e+00
Coefficient: −4.66e−01
Value: B
Mycobacterium.lepromatosis
0
2
4
6
A (n=15)
B (n=16)
C (n=14)
surface

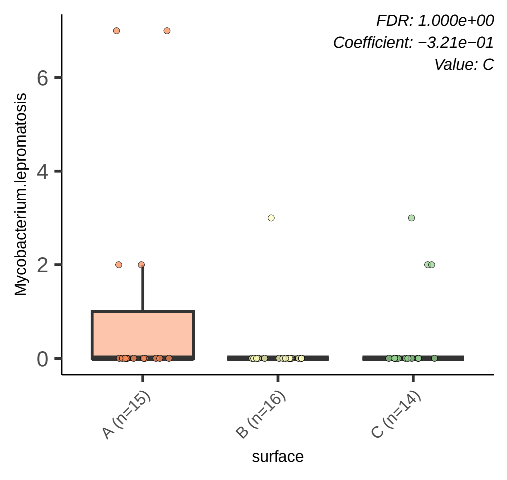

FDR: 1.000e+00
Coefficient: −3.21e−01
Value: C
Mycobacterium.lepromatosis
6
4
2
0
A (n=15)
B (n=16)
C (n=14)
surface

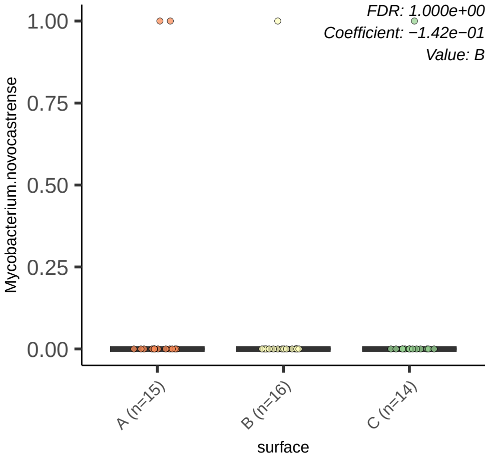
FDR: 1.000e+00
Coefficient: −1.42e−01
Value: B
Mycobacterium.novocastrense
1.00
0.75
0.50
0.25
0.00
A (n=15)
B (n=16)
C (n=14)
surface

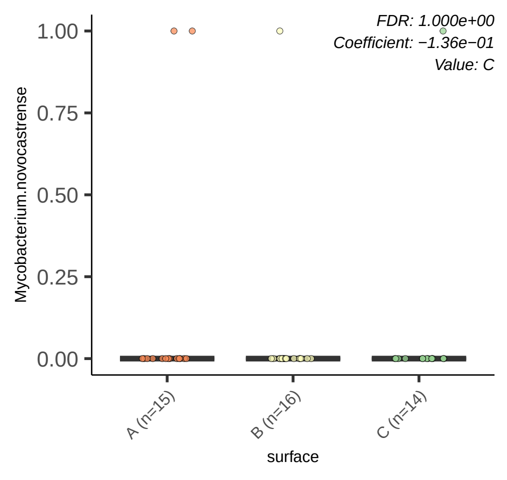
FDR: 1.000e+00
Coefficient: −1.36e−01
Value: C
1.00
0.75
0.50
0.25
0.00
Mycobacterium.novocastrense
A (n=15)
B (n=16)
C (n=14)
surface

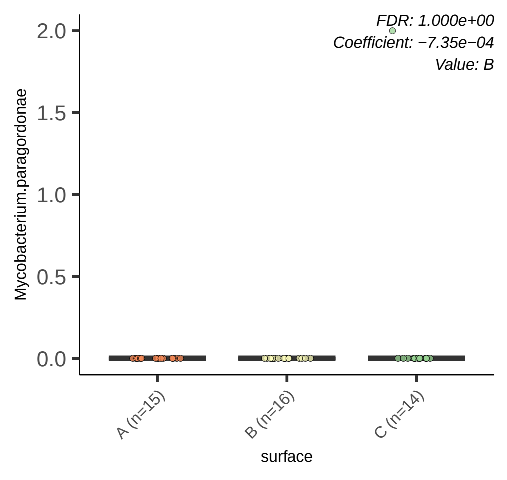

FDR: 1.000e+00
Coefficient: −7.35e−04
Value: B
Mycobacterium.paragordonae
2.0
1.5
1.0
0.5
0.0
A (n=15)
B (n=16)
C (n=14)
surface

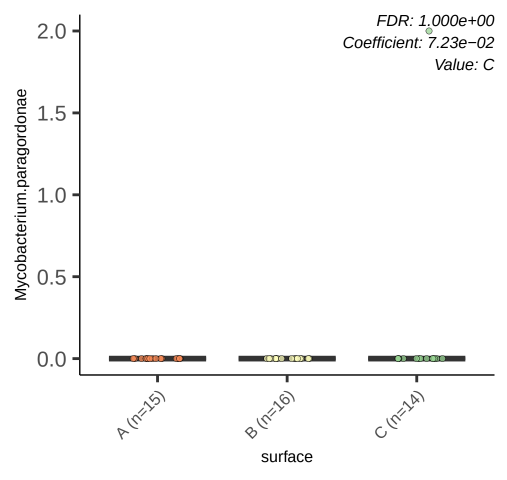

FDR: 1.000e+00
Coefficient: 7.23e−02
Value: C
Mycobacterium.paragordonae
2.0
1.5
1.0
0.5
0.0
A (n=15)
B (n=16)
C (n=14)
surface

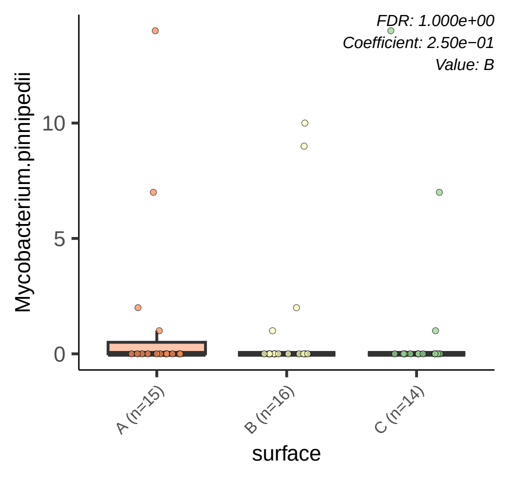
FDR: 1.000e+00
Coefficient: 2.50e−01
Value: B
Mycobacterium.pinnipedii
0
5
10
A (n=15)
B (n=16)
C (n=14)
surface

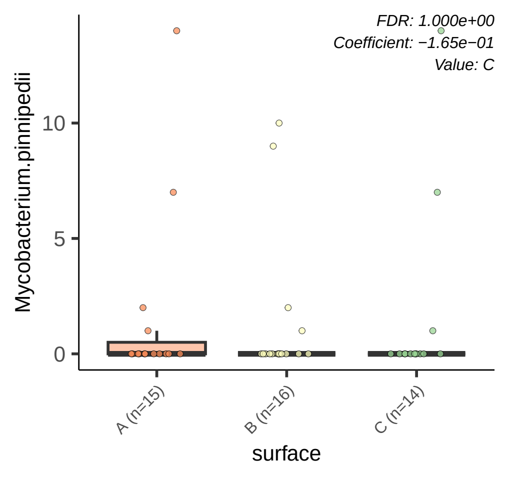
FDR: 1.000e+00
Coefficient: −1.65e−01
Value: C
Mycobacterium.pinnipedii
0
5
10
A (n=15)
B (n=16)
C (n=14)
surface

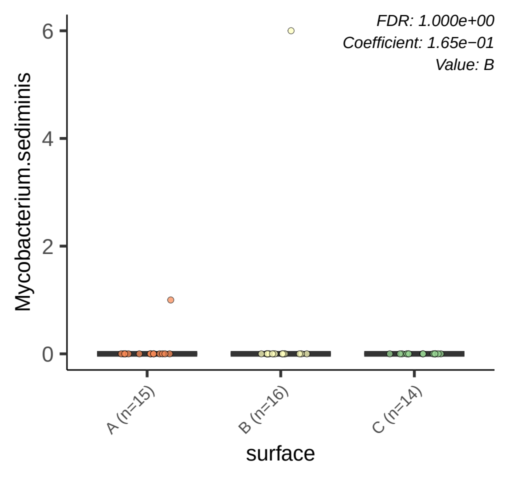

FDR: 1.000e+00
Coefficient: 1.65e−01
Value: B
Mycobacterium.sedimininis
6
4
2
0
A (n=15)
B (n=16)
C (n=14)
surface

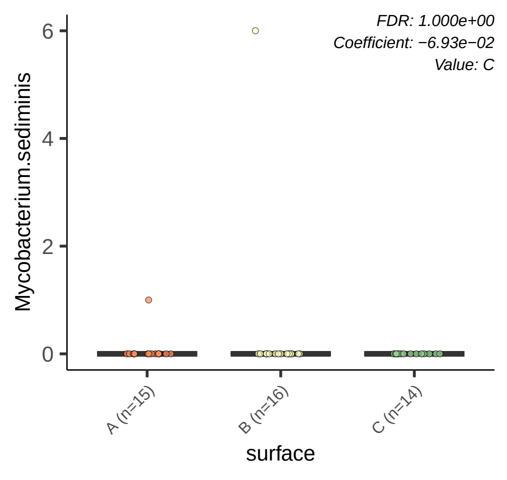
FDR: 1.000e+00
Coefficient: −6.93e−02
Value: C
Mycobacterium.sediminis
6
4
2
0
A (n=15)
B (n=16)
C (n=14)
surface

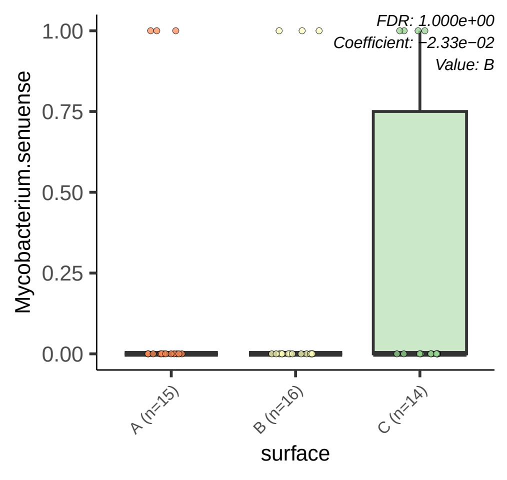

FDR: 1.000e+00
Coefficient: −2.33e−02
Value: B
Mycobacterium.senuense
1.00
0.75
0.50
0.25
0.00
A (n=15)
B (n=16)
C (n=14)
surface

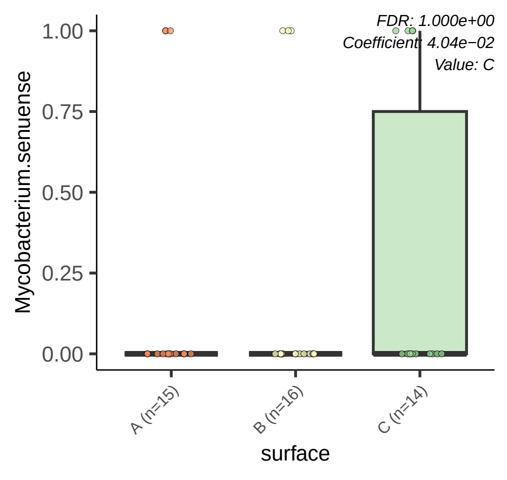
FDR: 1.000e+00
Coefficient: 4.04e−02
Value: C
Mycobacterium.senuense
1.00
0.75
0.50
0.25
0.00
A (n=15)
B (n=16)
C (n=14)
surface

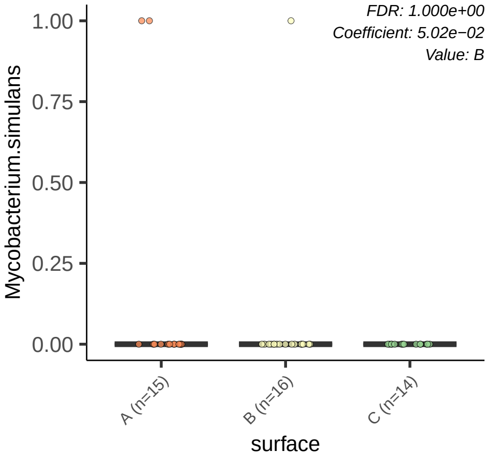

FDR: 1.000e+00
Coefficient: 5.02e−02
Value: B
Mycobacterium.simulans
1.00
0.75
0.50
0.25
0.00
A (n=15)
B (n=16)
C (n=14)
surface

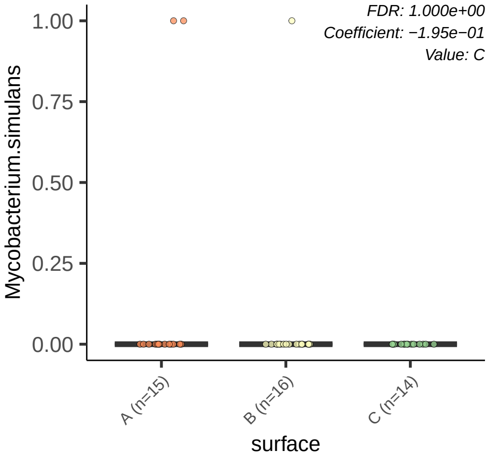
FDR: 1.000e+00
Coefficient: −1.95e−01
Value: C
Mycobacterium.simulans
1.00
0.75
0.50
0.25
0.00
A (n=15)
B (n=16)
C (n=14)
surface

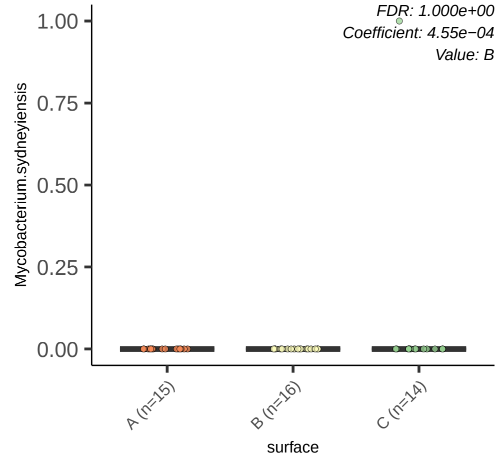

FDR: 1.000e+00
Coefficient: 4.55e−04
Value: B
1.00
0.75
0.50
0.25
0.00
Mycobacterium.sydneyiensis
A (n=15)
B (n=16)
C (n=14)
surface

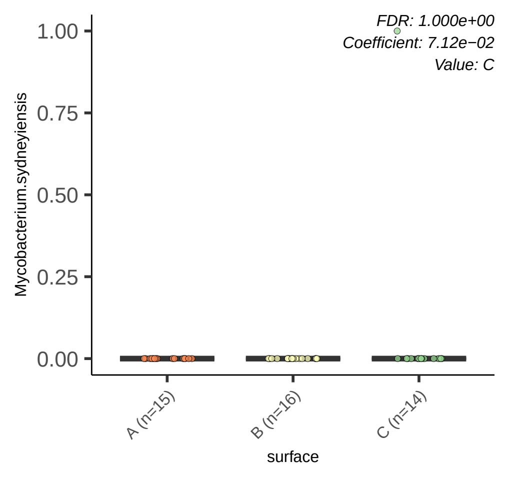
FDR: 1.000e+00
Coefficient: 7.12e−02
Value: C
Mycobacterium.sydneyiensis
1.00
0.75
0.50
0.25
0.00
A (n=15)
B (n=16)
C (n=14)
surface

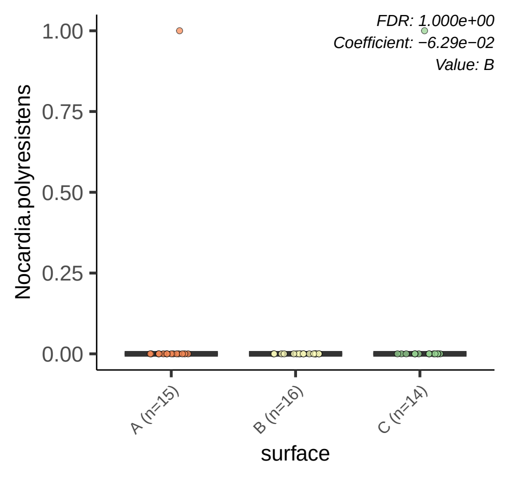
FDR: 1.000e+00
Coefficient: −6.29e−02
Value: B
Nocardia.polyresistens
1.00
0.75
0.50
0.25
0.00
A (n=15)
B (n=16)
C (n=14)
surface

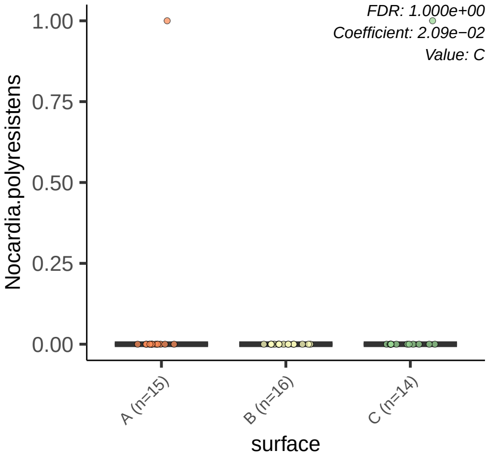

FDR: 1.000e+00
Coefficient: 2.09e−02
Value: C
Nocardia.polyresistens
1.00
0.75
0.50
0.25
0.00
A (n=15)
B (n=16)
C (n=14)
surface

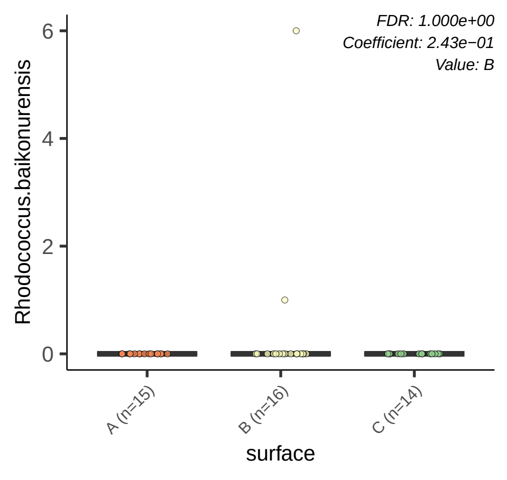
FDR: 1.000e+00
Coefficient: 2.43e−01
Value: B
Rhodococcus.baikonurensis
0
2
4
6
A (n=15)
B (n=16)
C (n=14)
surface

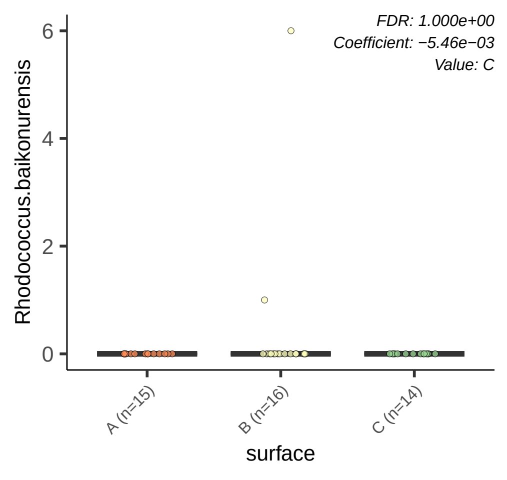
FDR: 1.000e+00
Coefficient: −5.46e−03
Value: C
Rhodococcus.baikonurensis
0
2
4
6
A (n=15)
B (n=16)
C (n=14)
surface

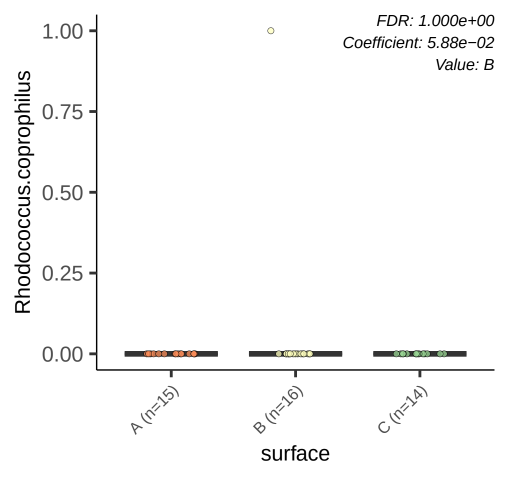
FDR: 1.000e+00
Coefficient: 5.88e−02
Value: B
Rhodococcus.coprophilus
1.00
0.75
0.50
0.25
0.00
A (n=15)
B (n=16)
C (n=14)
surface

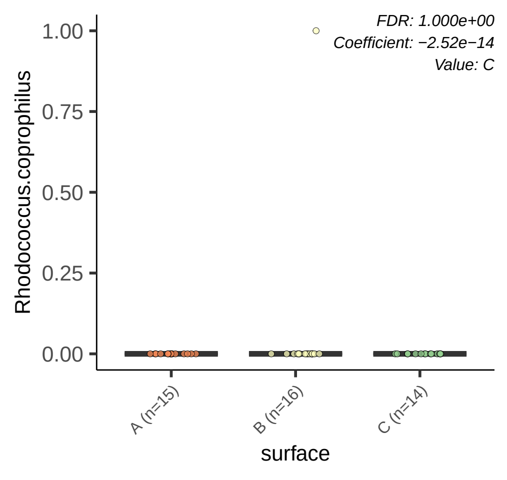

FDR: 1.000e+00
Coefficient: −2.52e−14
Value: C
Rhodococcus.coprophilus
1.00
0.75
0.50
0.25
0.00
A (n=15)
B (n=16)
C (n=14)
surface

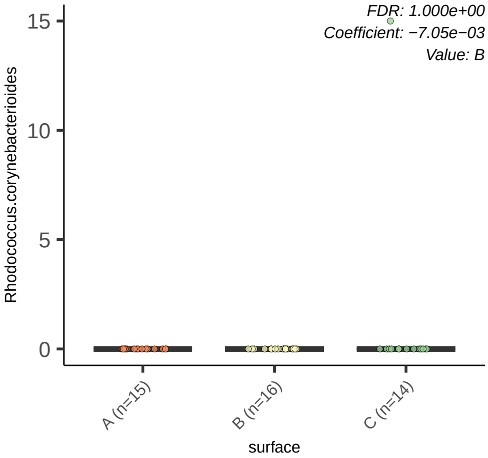
FDR: 1.000e+00
Coefficient: −7.05e−03
Value: B
Rhodococcus.corynebacterioides
15
10
5
0
A (n=15)
B (n=16)
C (n=14)
surface

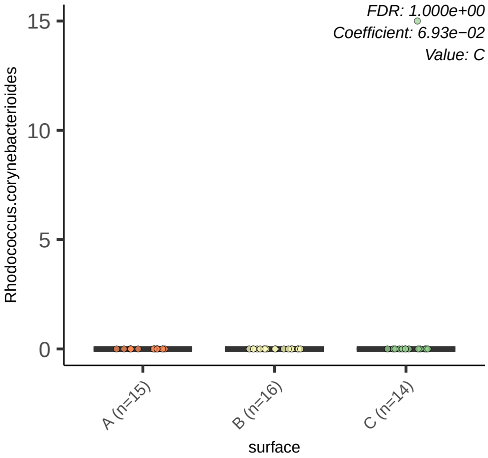
FDR: 1.000e+00
Coefficient: 6.93e−02
Value: C
Rhodococcus.corynebacterioides
15
10
5
0
A (n=15)
B (n=16)
C (n=14)
surface

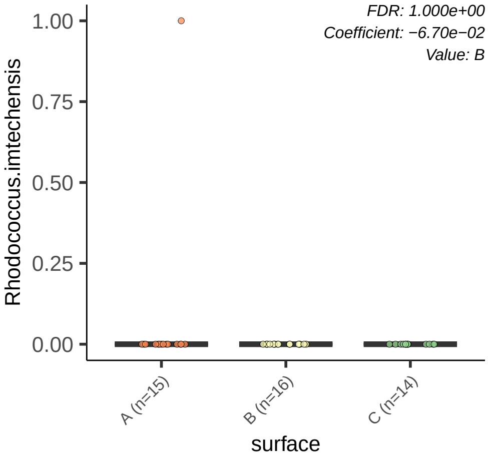

FDR: 1.000e+00
Coefficient: −6.70e−02
Value: B
Rhodococcus.imtechensis
1.00
0.75
0.50
0.25
0.00
A (n=15)
B (n=16)
C (n=14)
surface

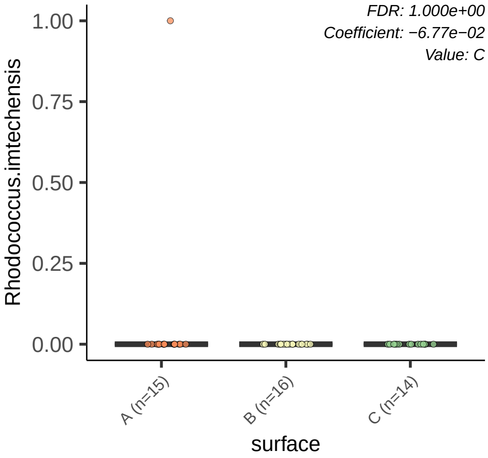

FDR: 1.000e+00
Coefficient: −6.77e−02
Value: C
Rhodococcus.imtechensis
1.00
0.75
0.50
0.25
0.00
A (n=15)
B (n=16)
C (n=14)
surface

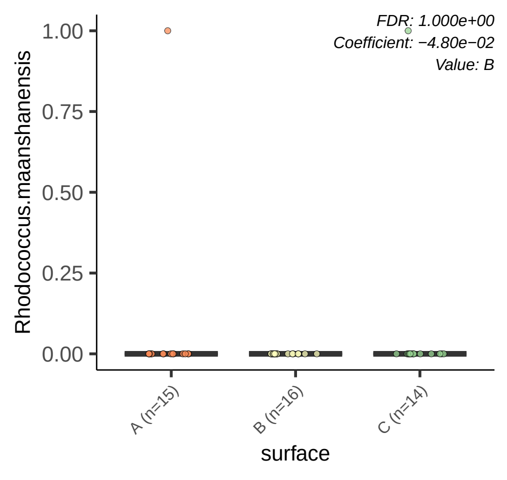
FDR: 1.000e+00
Coefficient: −4.80e−02
Value: B
Rhodococcus.maanshanensis
1.00
0.75
0.50
0.25
0.00
A (n=15)
B (n=16)
C (n=14)
surface

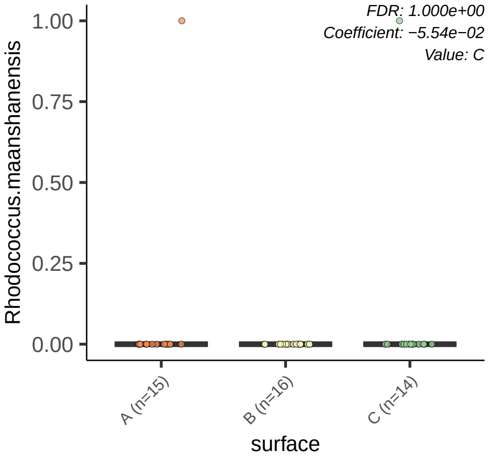

FDR: 1.000e+00
Coefficient: −5.54e−02
Value: C
Rhodococcus.maanshanensis
1.00
0.75
0.50
0.25
0.00
A (n=15)
B (n=16)
C (n=14)
surface

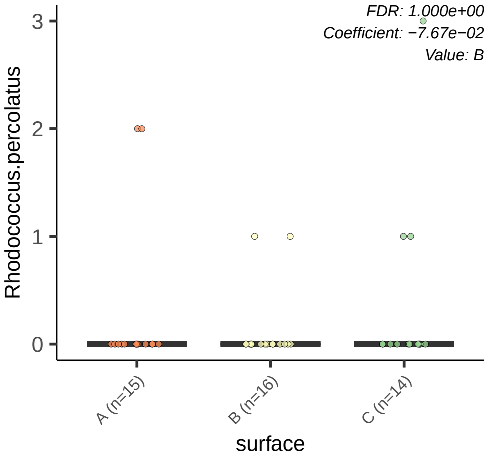
FDR: 1.000e+00
Coefficient: −7.67e−02
Value: B
Rhodococcus.percolatus
3
2
1
0
A (n=15)
B (n=16)
C (n=14)
surface

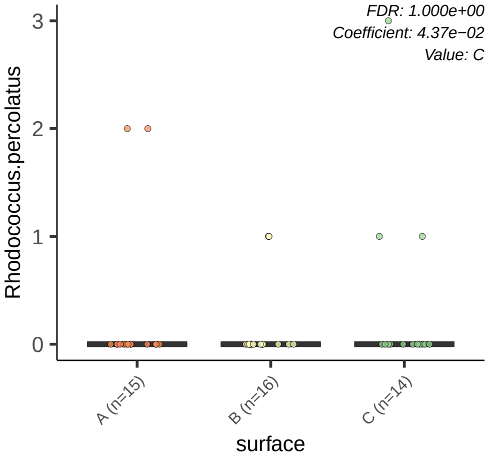
FDR: 1.000e+00
Coefficient: 4.37e−02
Value: C
Rhodococcus.percolatus
3
2
1
0
A (n=15)
B (n=16)
C (n=14)
surface

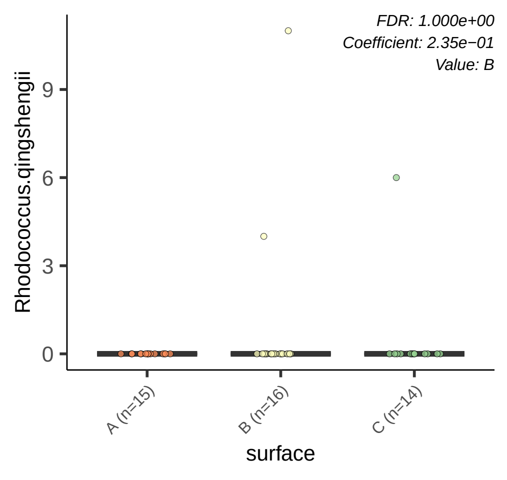
FDR: 1.000e+00
Coefficient: 2.35e−01
Value: B
Rhodococcus.qingshengii
0
3
6
9
A (n=15)
B (n=16)
C (n=14)
surface

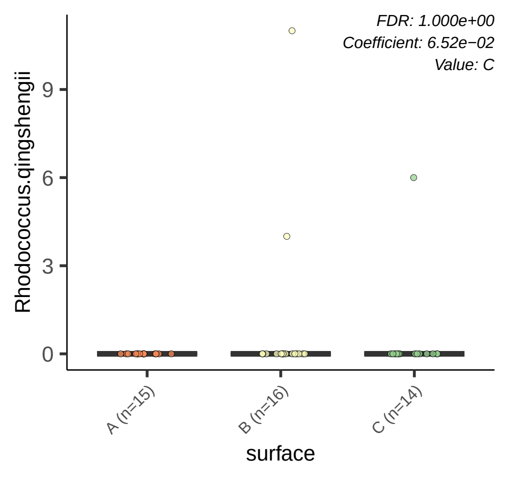
FDR: 1.000e+00
Coefficient: 6.52e−02
Value: C
Rhodococcus.qingshengii
0
3
6
9
A (n=15)
B (n=16)
C (n=14)
surface

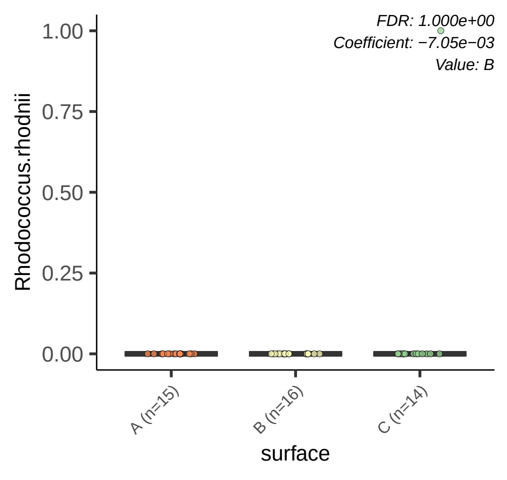
FDR: 1.000e+00
Coefficient: −7.05e−03
Value: B
Rhodococcus.rhodnii
1.00
0.75
0.50
0.25
0.00
A (n=15)
B (n=16)
C (n=14)
surface

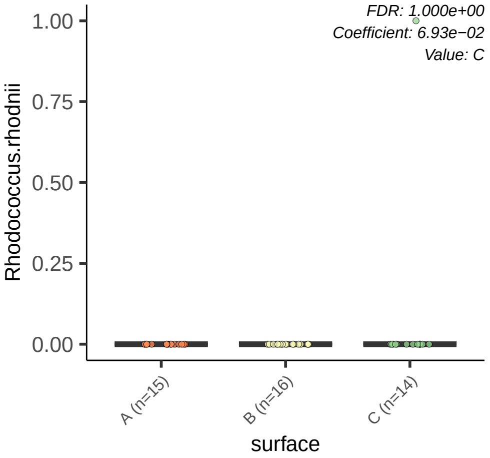

FDR: 1.000e+00
Coefficient: 6.93e−02
Value: C
Rhodococcus.rhodnii
1.00
0.75
0.50
0.25
0.00
A (n=15)
B (n=16)
C (n=14)
surface

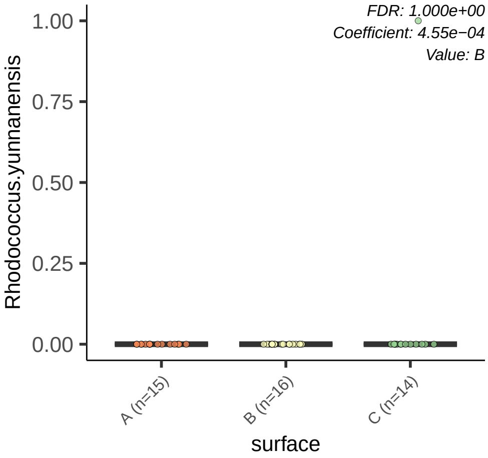

FDR: 1.000e+00
Coefficient: 4.55e−04
Value: B
Rhodococcus.yunnanensis
1.00
0.75
0.50
0.25
0.00
A (n=15)
B (n=16)
C (n=14)
surface

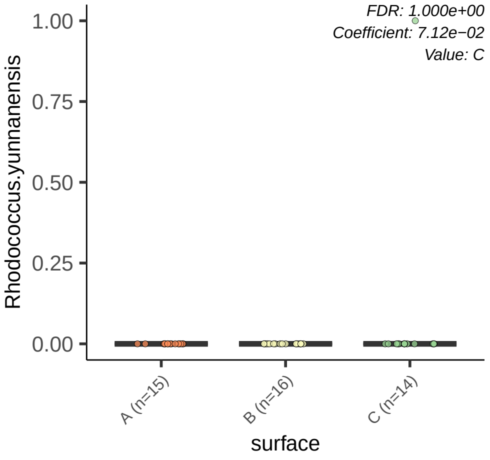

FDR: 1.000e+00
Coefficient: 7.12e−02
Value: C
Rhodococcus.yunnanensis
1.00
0.75
0.50
0.25
0.00
A (n=15)
B (n=16)
C (n=14)
surface

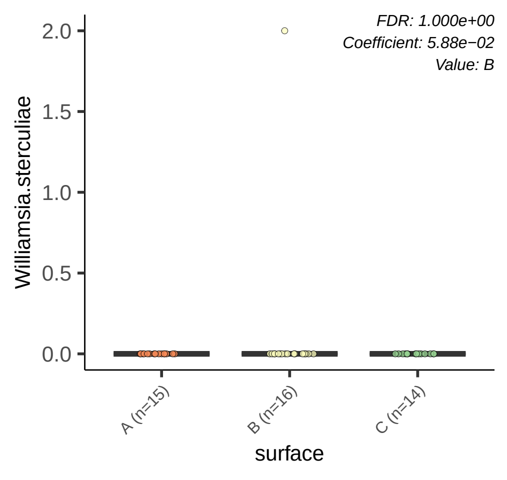

FDR: 1.000e+00
Coefficient: 5.88e−02
Value: B
Williamsia.sterculiae
2.0
1.5
1.0
0.5
0.0
A (n=15)
B (n=16)
C (n=14)
surface

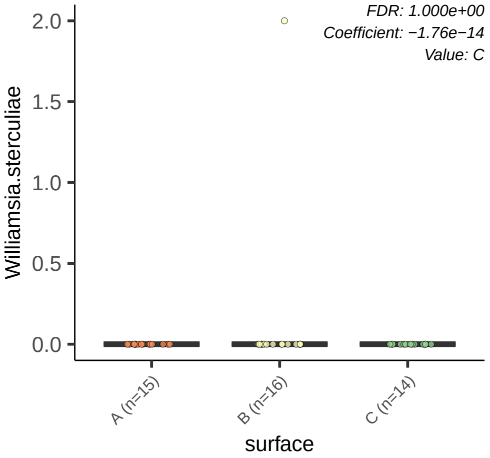

FDR: 1.000e+00
Coefficient: −1.76e−14
Value: C
Williamsia.sterculiae
2.0
1.5
1.0
0.5
0.0
A (n=15)
B (n=16)
C (n=14)
surface

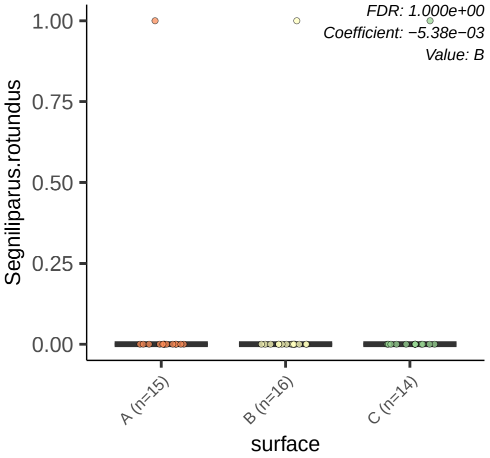

FDR: 1.000e+00
Coefficient: −5.38e−03
Value: B
Segniliparus.rotundus
1.00
0.75
0.50
0.25
0.00
A (n=15)
B (n=16)
C (n=14)
surface

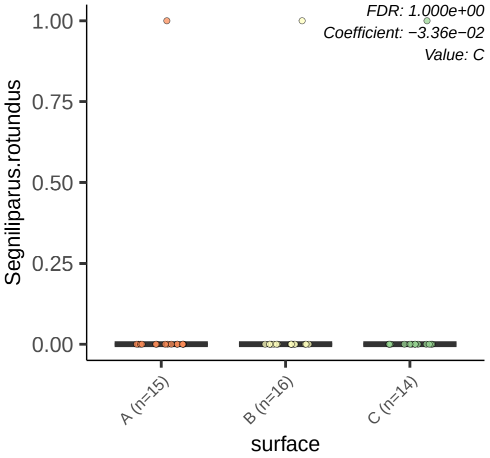
FDR: 1.000e+00
Coefficient: −3.36e−02
Value: C
Segniliparus.rotundus
1.00
0.75
0.50
0.25
0.00
A (n=15)
B (n=16)
C (n=14)
surface

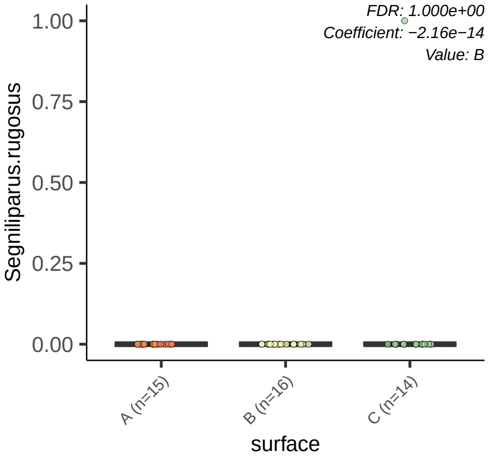
FDR: 1.000e+00
Coefficient: −2.16e−14
Value: B
Segniliparus.rugosus
1.00
0.75
0.50
0.25
0.00
A (n=15)
B (n=16)
C (n=14)
surface

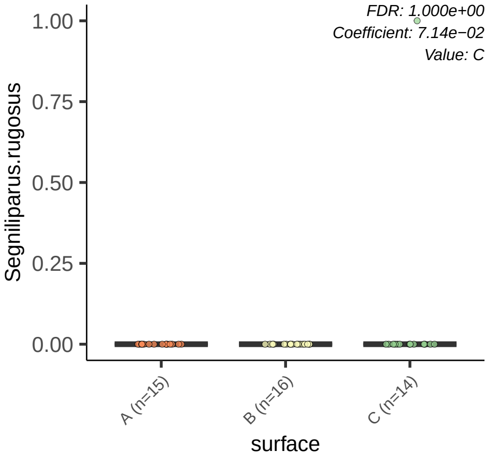
FDR: 1.000e+00
Coefficient: 7.14e−02
Value: C
Segniliparus.rugosus
1.00
0.75
0.50
0.25
0.00
A (n=15)
B (n=16)
C (n=14)
surface

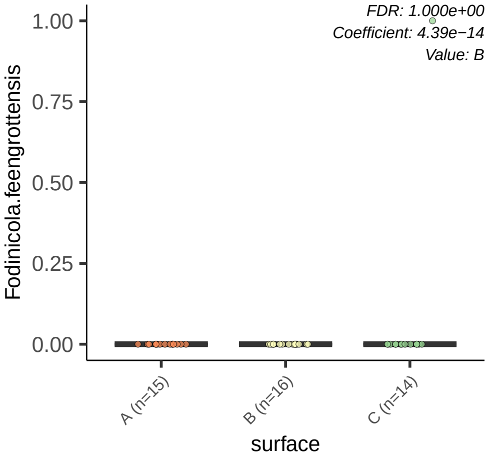
FDR: 1.000e+00
Coefficient: 4.39e−14
Value: B
Fodinicola.feengrottensis
1.00
0.75
0.50
0.25
0.00
A (n=15)
B (n=16)
C (n=14)
surface

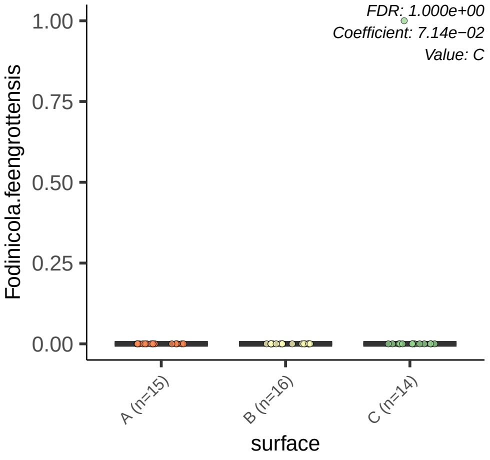
FDR: 1.000e+00
Coefficient: 7.14e−02
Value: C
Fodinicola.feengrottensis
1.00
0.75
0.50
0.25
0.00
A (n=15)
B (n=16)
C (n=14)
surface

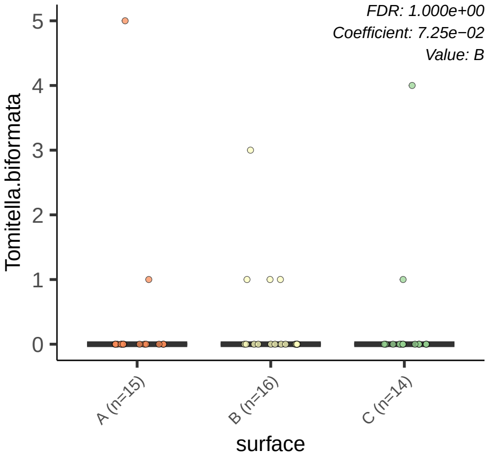

FDR: 1.000e+00
Coefficient: 7.25e−02
Value: B
Tomitella.biformata
0
1
2
3
4
5
A (n=15)
B (n=16)
C (n=14)
surface

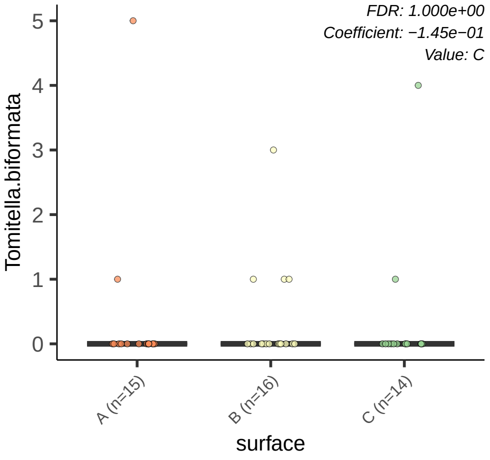
FDR: 1.000e+00
Coefficient: −1.45e−01
Value: C
Tomitella.biformata
0
1
2
3
4
5
A (n=15)
B (n=16)
C (n=14)
surface

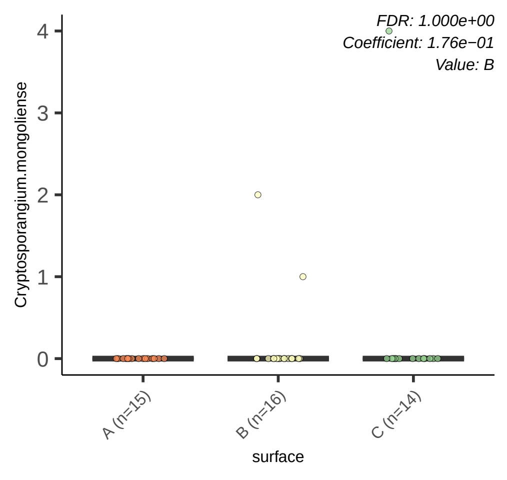
FDR: 1.000e+00
Coefficient: 1.76e−01
Value: B
Cryptosporangium.mongoliense
0
1
2
3
4
A (n=15)
B (n=16)
C (n=14)
surface

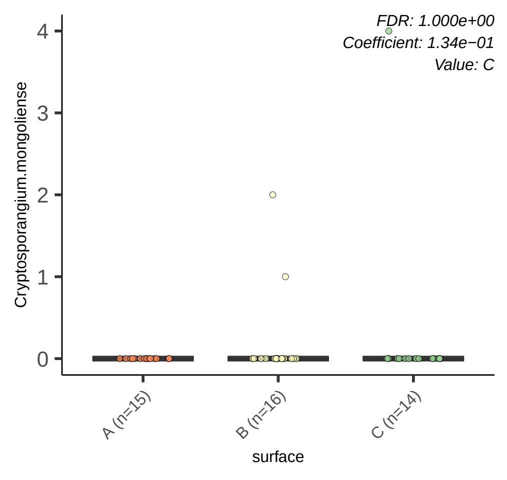
FDR: 1.000e+00
Coefficient: 1.34e−01
Value: C
Cryptosporangium.mongoliense
0
1
2
3
4
A (n=15)
B (n=16)
C (n=14)
surface

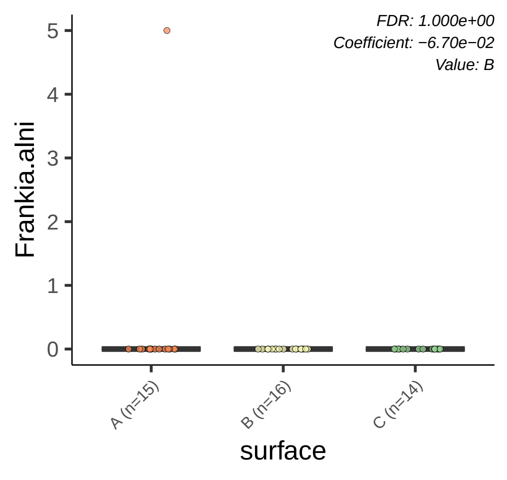
FDR: 1.000e+00
Coefficient: −6.70e−02
Value: B
Frankia.alni
5
4
3
2
1
0
A (n=15)
B (n=16)
C (n=14)
surface

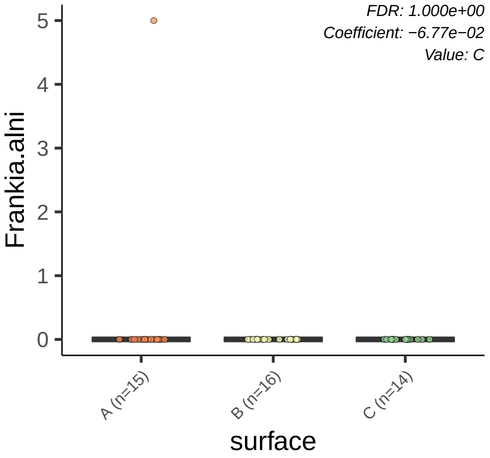
FDR: 1.000e+00
Coefficient: −6.77e−02
Value: C
Frankia.alni
5
4
3
2
1
0
A (n=15)
B (n=16)
C (n=14)
surface

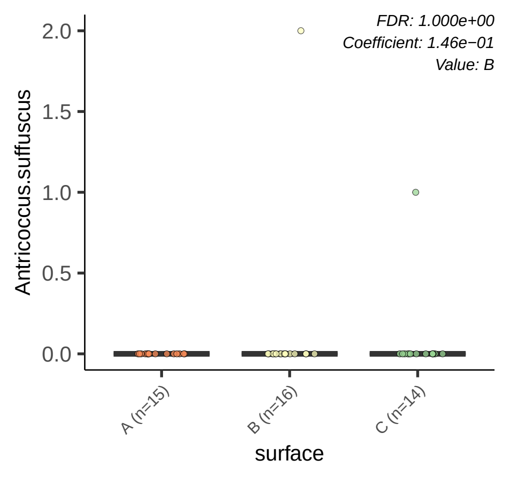

FDR: 1.000e+00
Coefficient: 1.46e−01
Value: B
2.0
1.5
1.0
0.5
0.0
Antricoccus.suffuscus
A (n=15)
B (n=16)
C (n=14)
surface

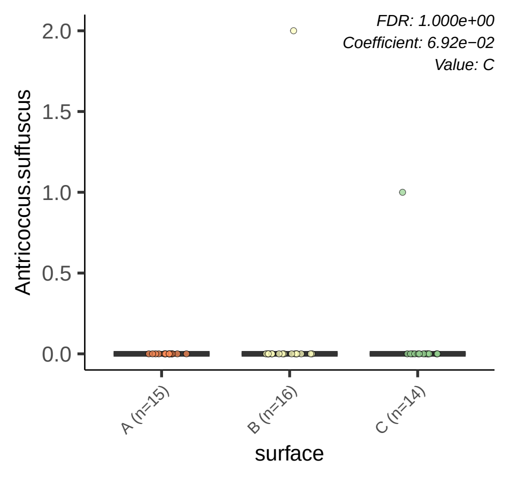
FDR: 1.000e+00
Coefficient: 6.92e−02
Value: C
Antricoccus.suffuscus
2.0
1.5
1.0
0.5
0.0
A (n=15)
B (n=16)
C (n=14)
surface

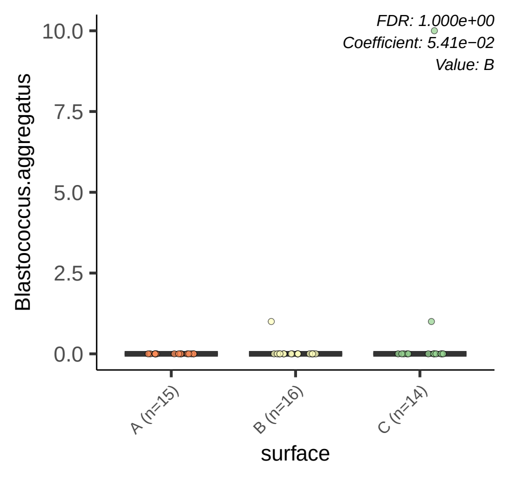
FDR: 1.000e+00
Coefficient: 5.41e−02
Value: B
Blastococcus.aggregatus
10.0
7.5
5.0
2.5
0.0
A (n=15)
B (n=16)
C (n=14)
surface

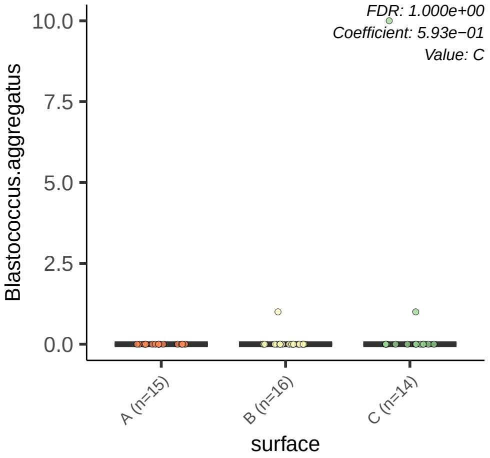
FDR: 1.000e+00
Coefficient: 5.93e−01
Value: C
Blastococcus.aggregatus
10.0
7.5
5.0
2.5
0.0
A (n=15)
B (n=16)
C (n=14)
surface

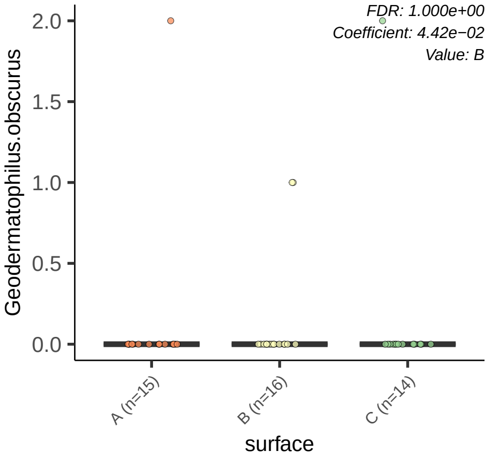

FDR: 1.000e+00
Coefficient: 4.42e−02
Value: B
Geodermatophilus.obscurus
2.0
1.5
1.0
0.5
0.0
A (n=15)
B (n=16)
C (n=14)
surface

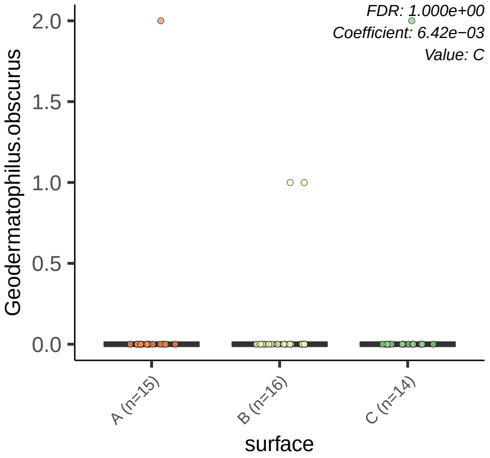
FDR: 1.000e+00
Coefficient: 6.42e−03
Value: C
Geodermatophilus.obscurus
2.0
1.5
1.0
0.5
0.0
A (n=15)
B (n=16)
C (n=14)
surface

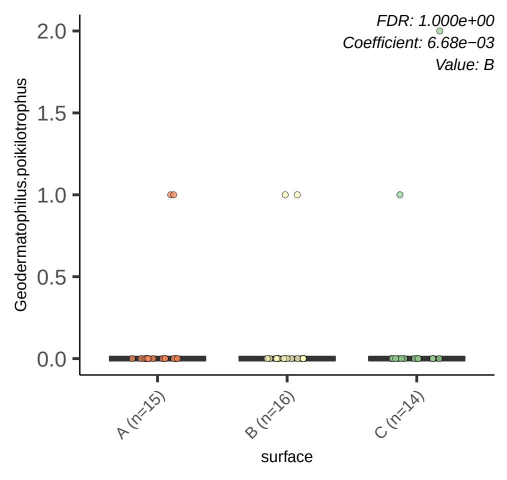
FDR: 1.000e+00
Coefficient: 6.68e−03
Value: B
Geodermatophilus.poikilotrophus
2.0
1.5
1.0
0.5
0.0
A (n=15)
B (n=16)
C (n=14)
surface

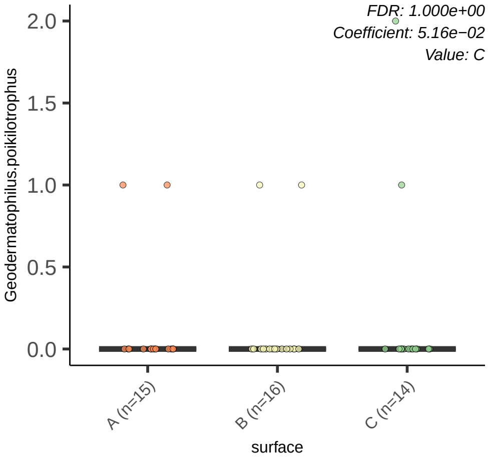
FDR: 1.000e+00
Coefficient: 5.16e−02
Value: C
Geodermatophilus.poikilotrophus
2.0
1.5
1.0
0.5
0.0
A (n=15)
B (n=16)
C (n=14)
surface

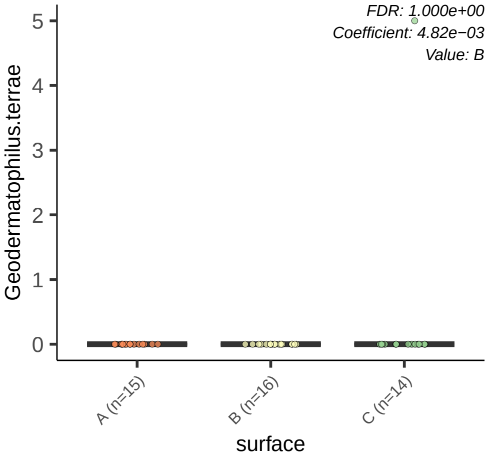

FDR: 1.000e+00
Coefficient: 4.82e−03
Value: B
Geodermatophilus.terrae
0
1
2
3
4
5
A (n=15)
B (n=16)
C (n=14)
surface

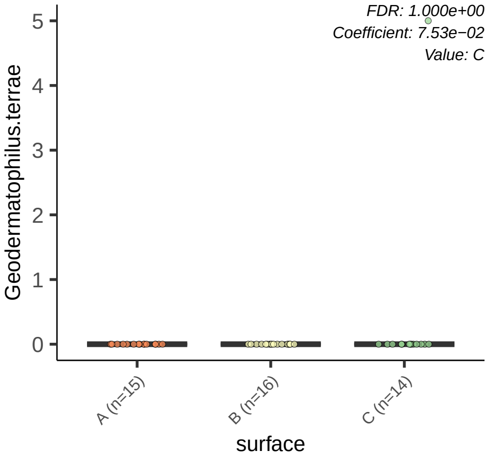
FDR: 1.000e+00
Coefficient: 7.53e−02
Value: C
Geodermatophilus.terrae
0
1
2
3
4
5
A (n=15)
B (n=16)
C (n=14)
surface

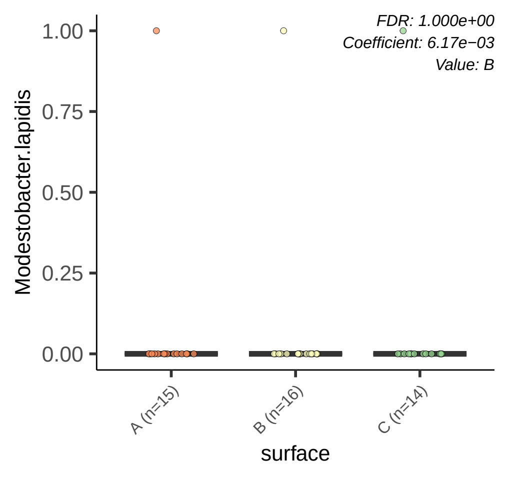

FDR: 1.000e+00
Coefficient: 6.17e−03
Value: B
Modestobacter.lapidis
1.00
0.75
0.50
0.25
0.00
A (n=15)
B (n=16)
C (n=14)
surface

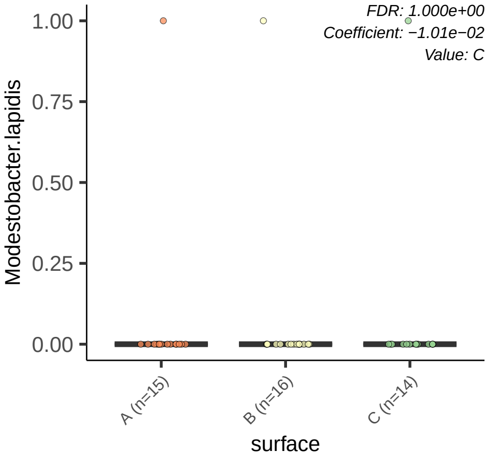
FDR: 1.000e+00
Coefficient: −1.01e−02
Value: C
Modestobacter.lapidis
1.00
0.75
0.50
0.25
0.00
A (n=15)
B (n=16)
C (n=14)
surface

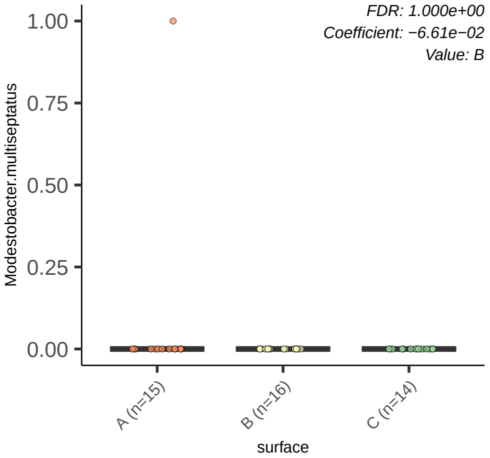
FDR: 1.000e+00
Coefficient: −6.61e−02
Value: B
Modestobacter.multiseptatus
1.00
0.75
0.50
0.25
0.00
A (n=15)
B (n=16)
C (n=14)
surface

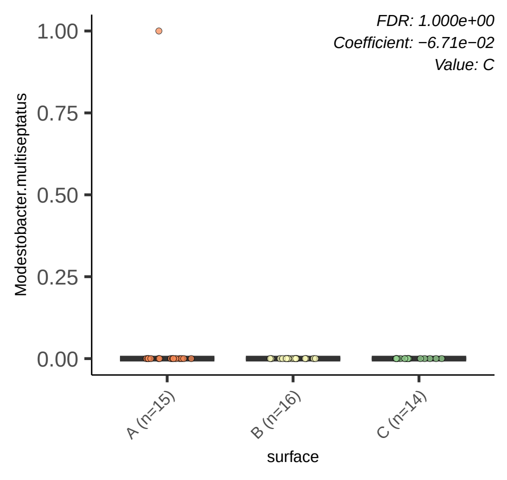
FDR: 1.000e+00
Coefficient: −6.71e−02
Value: C
Modestobacter.multiseptatus
1.00
0.75
0.50
0.25
0.00
A (n=15)
B (n=16)
C (n=14)
surface

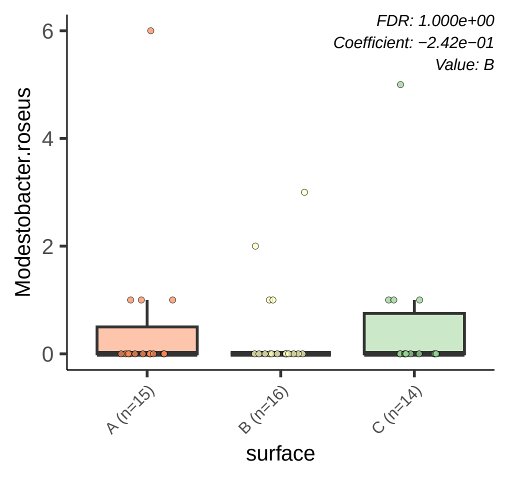
FDR: 1.000e+00
Coefficient: −2.42e−01
Value: B
Modestobacter.roseus
0
2
4
6
A (n=15)
B (n=16)
C (n=14)
surface

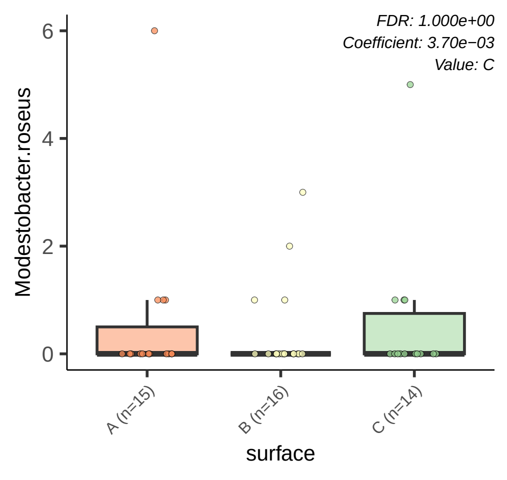

FDR: 1.000e+00
Coefficient: 3.70e−03
Value: C
Modestobacter.roseus
0
2
4
6
A (n=15)
B (n=16)
C (n=14)
surface

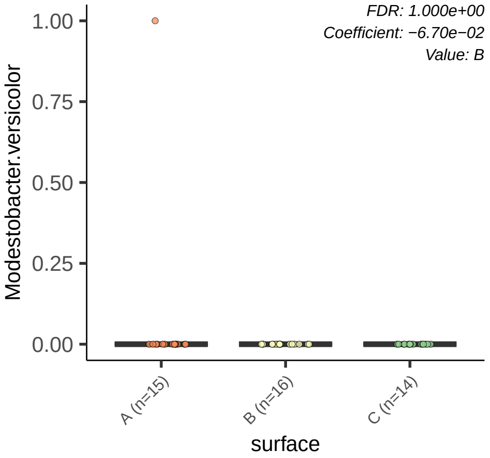

FDR: 1.000e+00
Coefficient: −6.70e−02
Value: B
Modestobacter.versicolor
1.00
0.75
0.50
0.25
0.00
A (n=15)
B (n=16)
C (n=14)
surface

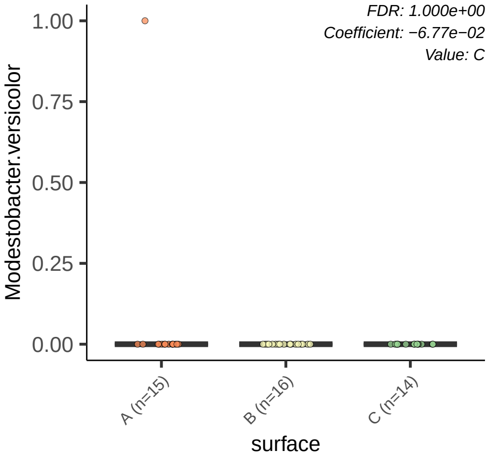
FDR: 1.000e+00
Coefficient: −6.77e−02
Value: C
Modestobacter.versicolor
1.00
0.75
0.50
0.25
0.00
A (n=15)
B (n=16)
C (n=14)
surface

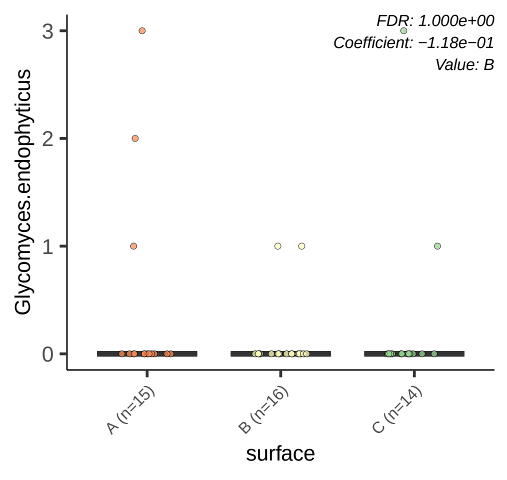
FDR: 1.000e+00
Coefficient: −1.18e−01
Value: B
Glycomyces.endophyticus
3
2
1
0
A (n=15)
B (n=16)
C (n=14)
surface

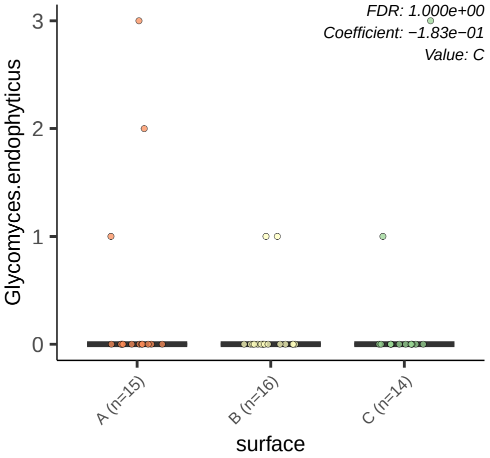
FDR: 1.000e+00
Coefficient: −1.83e−01
Value: C
Glycomyces.endophyticus
0
1
2
3
A (n=15)
B (n=16)
C (n=14)
surface

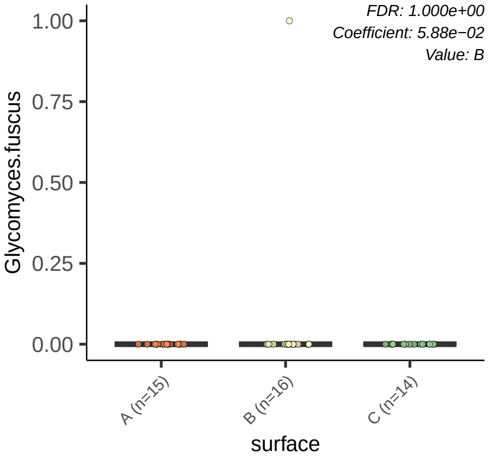
FDR: 1.000e+00
Coefficient: 5.88e−02
Value: B
Glycomyces.fuscus
1.00
0.75
0.50
0.25
0.00
A (n=15)
B (n=16)
C (n=14)
surface

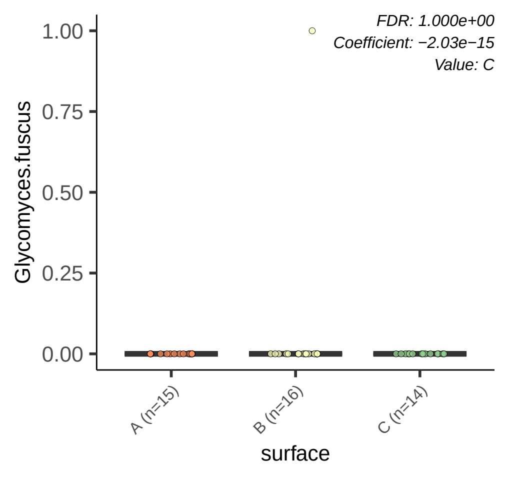
FDR: 1.000e+00
Coefficient: −2.03e−15
Value: C
Glycomyces.fuscus
1.00
0.75
0.50
0.25
0.00
A (n=15)
B (n=16)
C (n=14)
surface

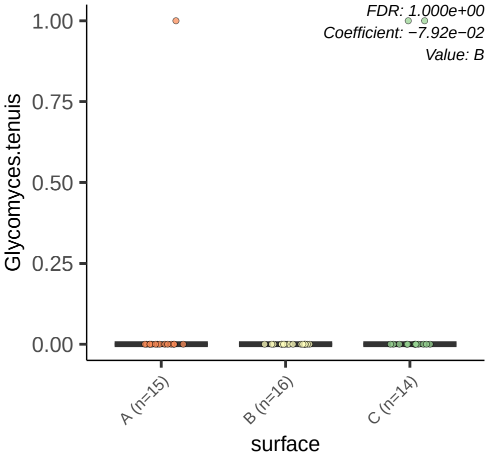

FDR: 1.000e+00
Coefficient: −7.92e−02
Value: B
Glycomyces.tenuis
1.00
0.75
0.50
0.25
0.00
A (n=15)
B (n=16)
C (n=14)
surface

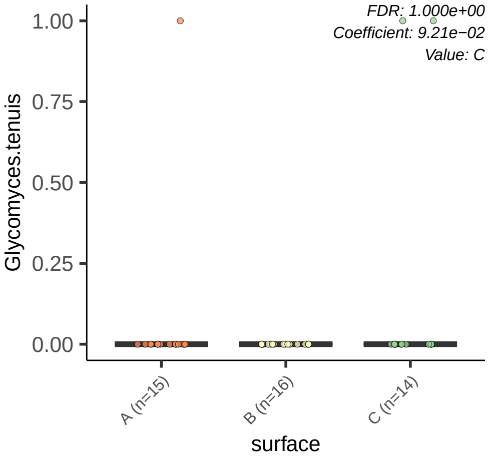

FDR: 1.000e+00
Coefficient: 9.21e−02
Value: C
Glycomyces.tenuis
1.00
0.75
0.50
0.25
0.00
A (n=15)
B (n=16)
C (n=14)
surface

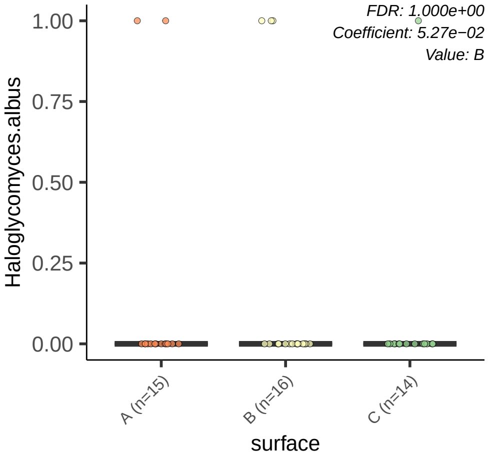

FDR: 1.000e+00
Coefficient: 5.27e−02
Value: B
Haloglycomyces.albus
1.00
0.75
0.50
0.25
0.00
A (n=15)
B (n=16)
C (n=14)
surface

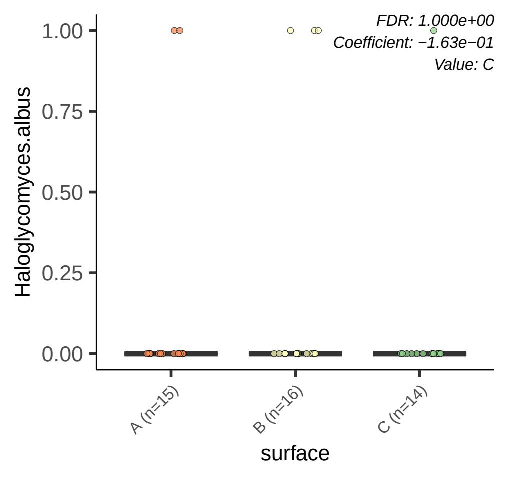

FDR: 1.000e+00
Coefficient: −1.63e−01
Value: C
Haloglycomyces.albus
1.00
0.75
0.50
0.25
0.00
A (n=15)
B (n=16)
C (n=14)
surface

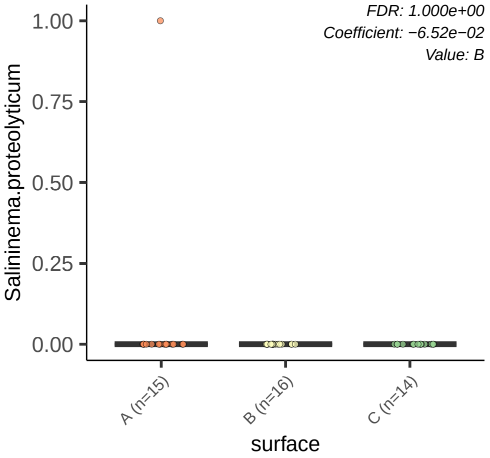

FDR: 1.000e+00
Coefficient: −6.52e−02
Value: B
Salininema.proteolyticum
1.00
0.75
0.50
0.25
0.00
A (n=15)
B (n=16)
C (n=14)
surface

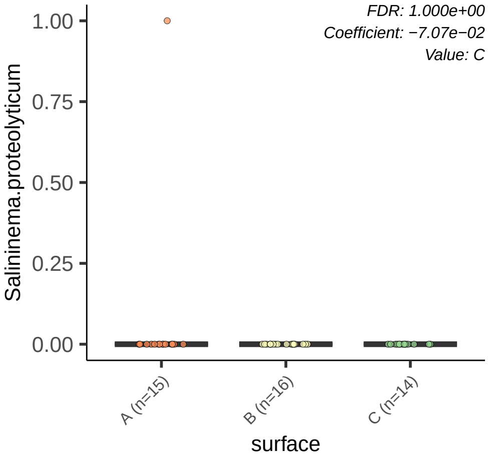
FDR: 1.000e+00
Coefficient: −7.07e−02
Value: C
Salininema.proteolyticum
1.00
0.75
0.50
0.25
0.00
A (n=15)
B (n=16)
C (n=14)
surface

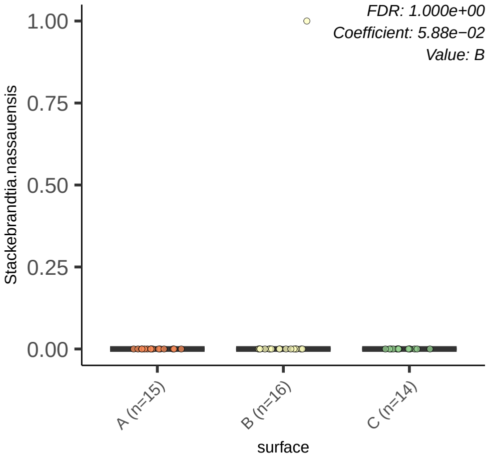
FDR: 1.000e+00
Coefficient: 5.88e−02
Value: B
Stackebrandtia.nassauensis
1.00
0.75
0.50
0.25
0.00
A (n=15)
B (n=16)
C (n=14)
surface

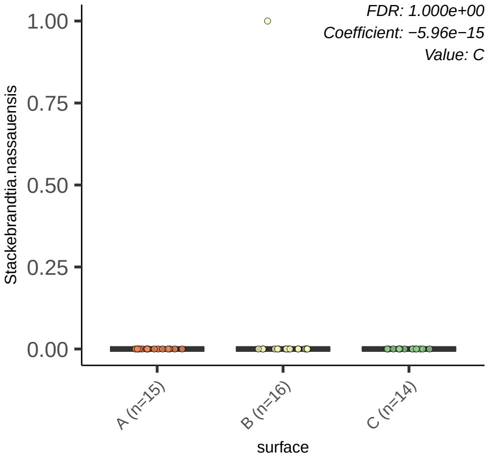
FDR: 1.000e+00
Coefficient: −5.96e−15
Value: C
Stackebrandtia.nassauensis
1.00
0.75
0.50
0.25
0.00
A (n=15)
B (n=16)
C (n=14)
surface

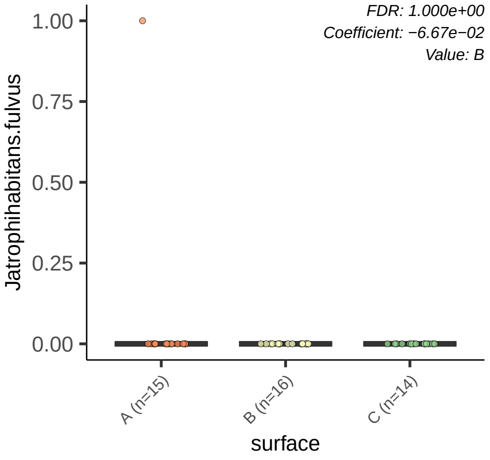

FDR: 1.000e+00
Coefficient: −6.67e−02
Value: B
Jatrophihabitans.fulvus
1.00
0.75
0.50
0.25
0.00
A (n=15)
B (n=16)
C (n=14)
surface

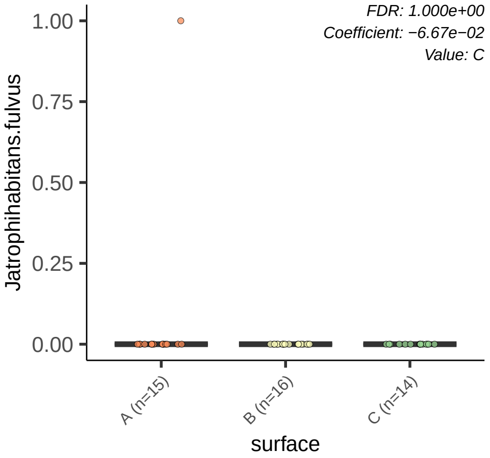

FDR: 1.000e+00
Coefficient: −6.67e−02
Value: C
1.00
0.75
0.50
0.25
0.00
Jatrophihabitans.fulvus
A (n=15)
B (n=16)
C (n=14)
surface

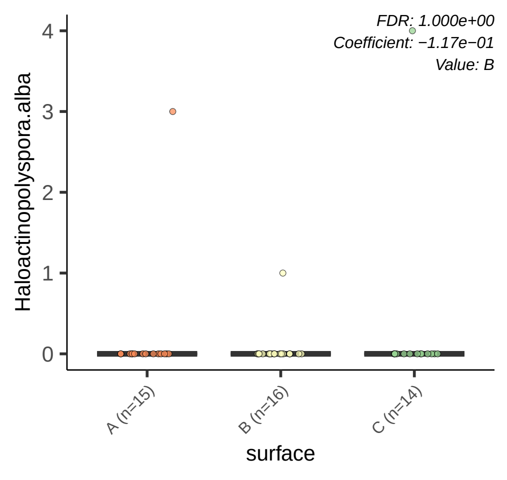
FDR: 1.000e+00
Coefficient: −1.17e−01
Value: B
Haloactinopolyspora.alba
0
1
2
3
4
A (n=15)
B (n=16)
C (n=14)
surface

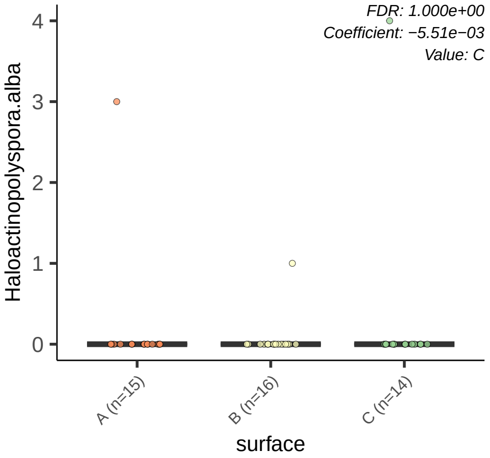

FDR: 1.000e+00
Coefficient: −5.51e−03
Value: C
Haloactinopolyspora.alba
4
3
2
1
0
A (n=15)
B (n=16)
C (n=14)
surface

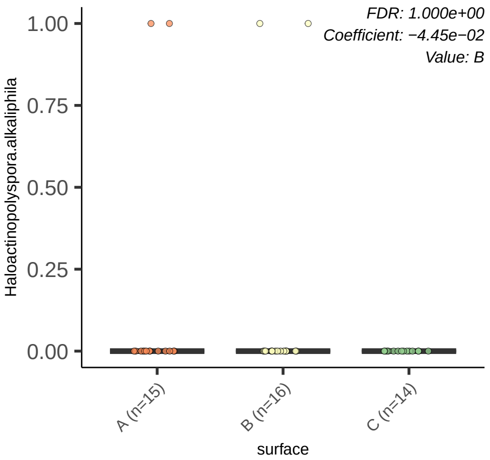
FDR: 1.000e+00
Coefficient: −4.45e−02
Value: B
Haloactinopolyspora.alkaliphila
1.00
0.75
0.50
0.25
0.00
A (n=15)
B (n=16)
C (n=14)
surface

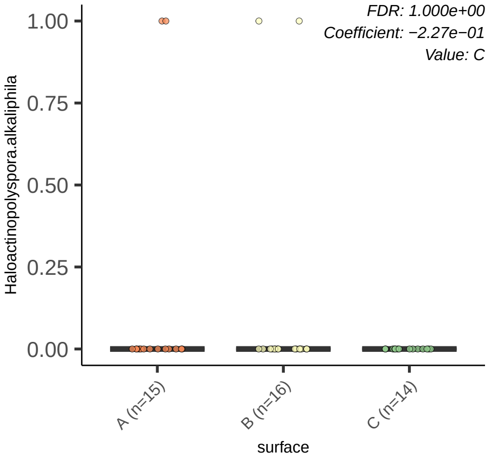

FDR: 1.000e+00
Coefficient: −2.27e−01
Value: C
Haloactinopolyspora.alkaliphila
1.00
0.75
0.50
0.25
0.00
A (n=15)
B (n=16)
C (n=14)
surface

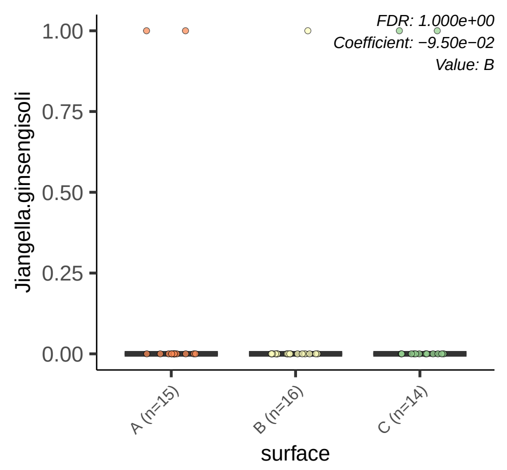
FDR: 1.000e+00
Coefficient: −9.50e−02
Value: B
Jiangella.ginsengisoli
1.00
0.75
0.50
0.25
0.00
A (n=15)
B (n=16)
C (n=14)
surface

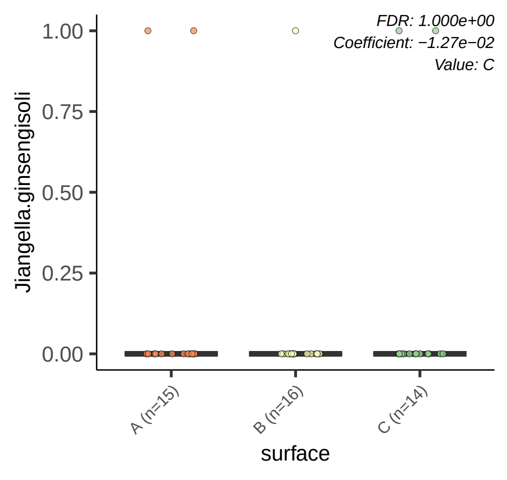
FDR: 1.000e+00
Coefficient: −1.27e−02
Value: C
Jiangella.ginsengisoli
1.00
0.75
0.50
0.25
0.00
A (n=15)
B (n=16)
C (n=14)
surface

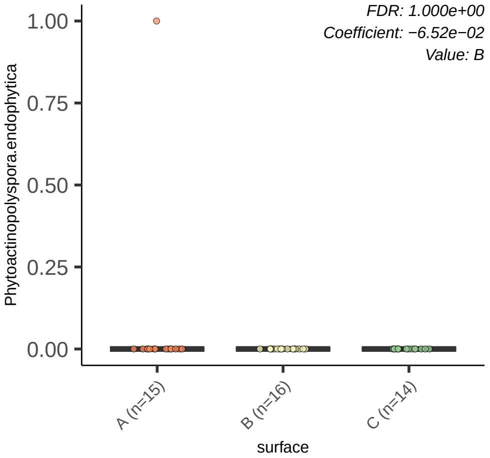

FDR: 1.000e+00
Coefficient: −6.52e−02
Value: B
Phytoactinopolyspora.endophytica
1.00
0.75
0.50
0.25
0.00
A (n=15)
B (n=16)
C (n=14)
surface

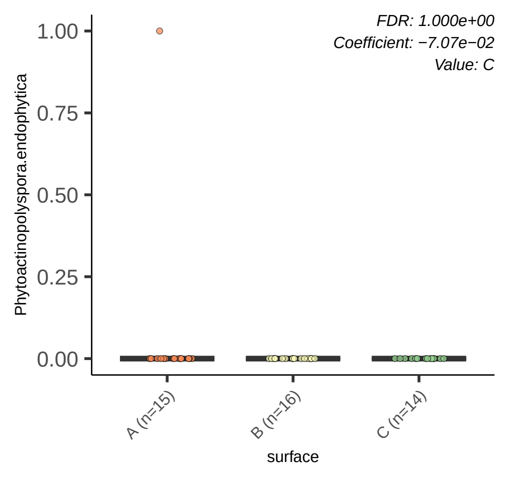
FDR: 1.000e+00
Coefficient: −7.07e−02
Value: C
Phytoactinopolyspora.endophytica
1.00
0.75
0.50
0.25
0.00
A (n=15)
B (n=16)
C (n=14)
surface

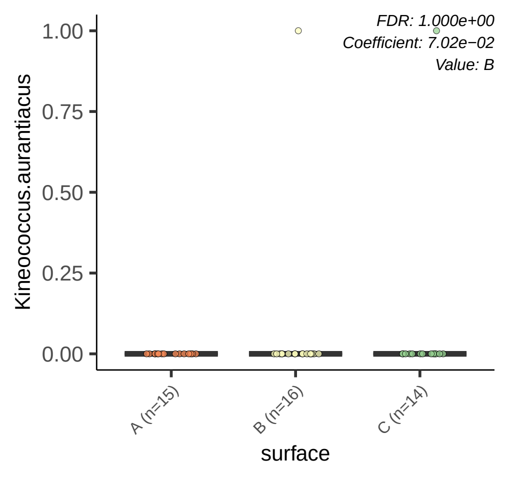

FDR: 1.000e+00
Coefficient: 7.02e−02
Value: B
1.00
0.75
0.50
0.25
0.00
Kineococcus.aurantiacus
A (n=15)
B (n=16)
C (n=14)
surface

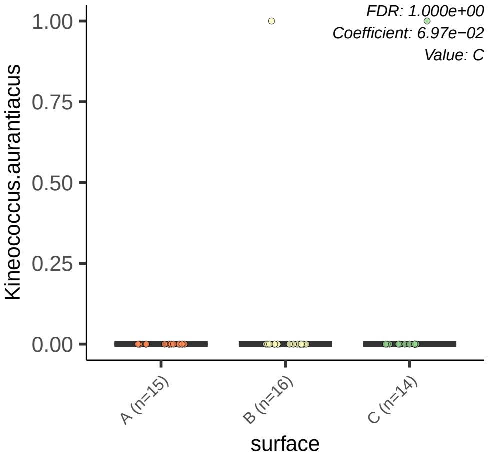
FDR: 1.000e+00
Coefficient: 6.97e−02
Value: C
Kineococcus.aurantiacus
1.00
0.75
0.50
0.25
0.00
A (n=15)
B (n=16)
C (n=14)
surface

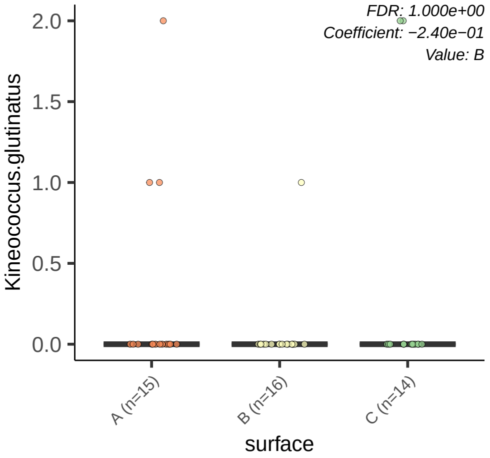

FDR: 1.000e+00
Coefficient: −2.40e−01
Value: B
Kineococcus.glutinatus
2.0
1.5
1.0
0.5
0.0
A (n=15)
B (n=16)
C (n=14)
surface

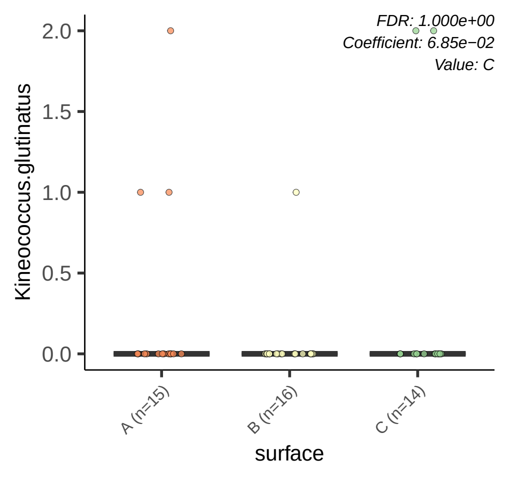
FDR: 1.000e+00
Coefficient: 6.85e−02
Value: C
Kineococcus.glutinatus
2.0
1.5
1.0
0.5
0.0
A (n=15)
B (n=16)
C (n=14)
surface

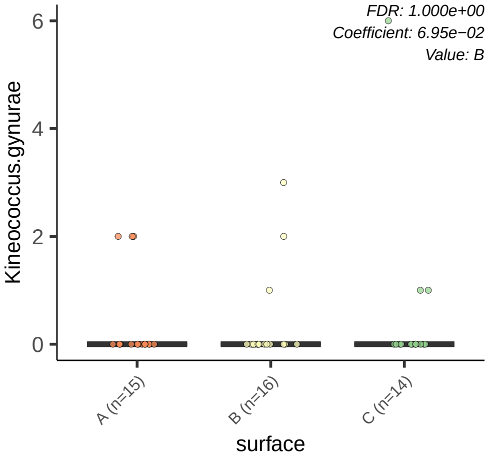
FDR: 1.000e+00
Coefficient: 6.95e−02
Value: B
Kineococcus.gynurae
6
4
2
0
A (n=15)
B (n=16)
C (n=14)
surface

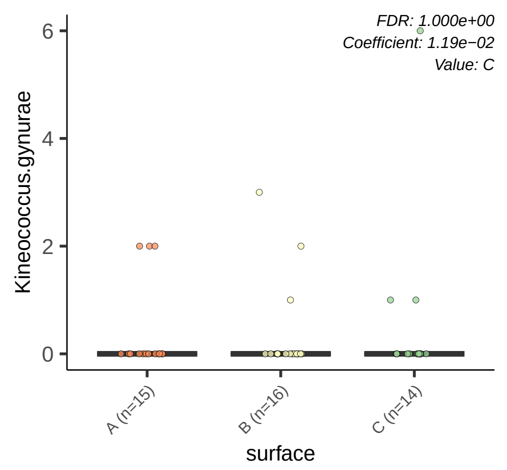

FDR: 1.000e+00
Coefficient: 1.19e−02
Value: C
Kineococcus.gynurae
6
4
2
0
A (n=15)
B (n=16)
C (n=14)
surface

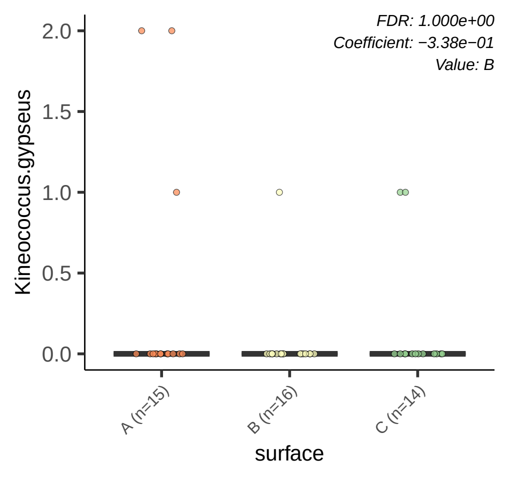
FDR: 1.000e+00
Coefficient: −3.38e−01
Value: B
Kineococcus.gypseus
2.0
1.5
1.0
0.5
0.0
A (n=15)
B (n=16)
C (n=14)
surface

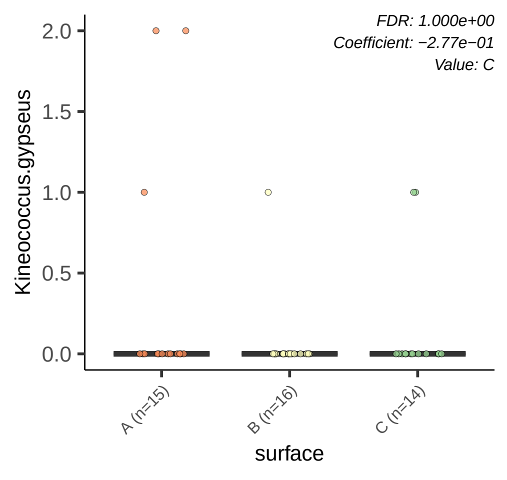

FDR: 1.000e+00
Coefficient: −2.77e−01
Value: C
Kineococcus.gypseus
2.0
1.5
1.0
0.5
0.0
A (n=15)
B (n=16)
C (n=14)
surface

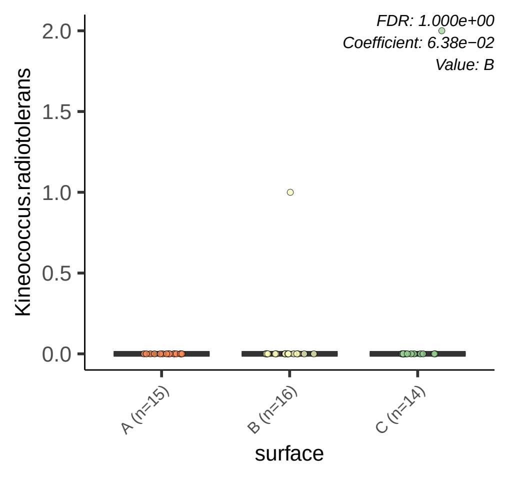
FDR: 1.000e+00
Coefficient: 6.38e−02
Value: B
Kineococcus.radiotolerans
2.0
1.5
1.0
0.5
0.0
A (n=15)
B (n=16)
C (n=14)
surface

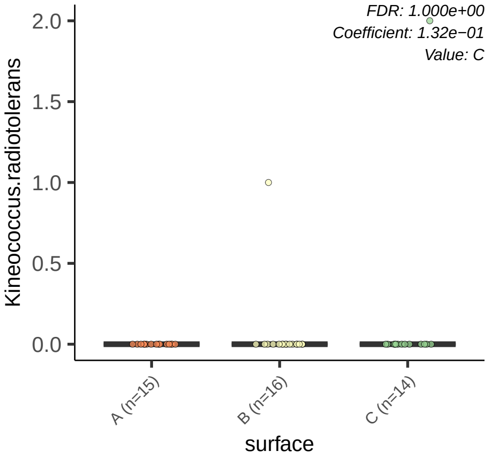
FDR: 1.000e+00
Coefficient: 1.32e−01
Value: C
2.0
1.5
1.0
0.5
0.0
Kineococcus.radiotolerans
A (n=15)
B (n=16)
C (n=14)
surface

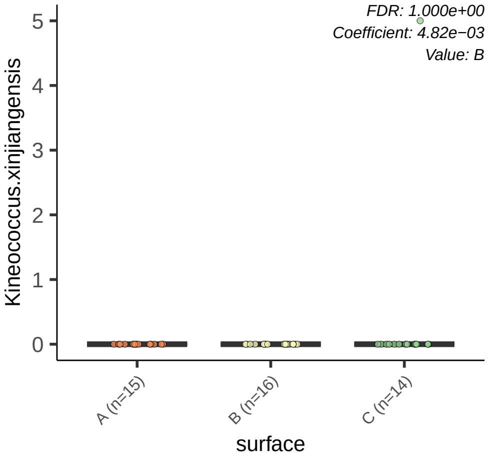

FDR: 1.000e+00
Coefficient: 4.82e−03
Value: B
Kineococcus.xinjiangensis
5
4
3
2
1
0
A (n=15)
B (n=16)
C (n=14)
surface

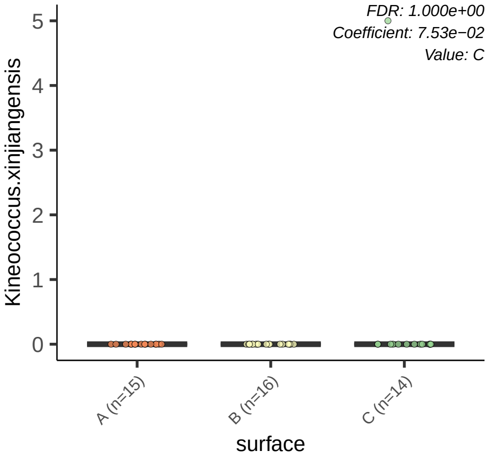

FDR: 1.000e+00
Coefficient: 7.53e−02
Value: C
Kineococcus.xinjiangensis
0
1
2
3
4
5
A (n=15)
B (n=16)
C (n=14)
surface

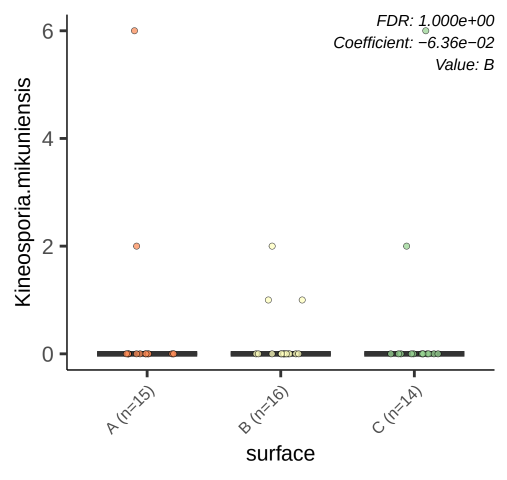
FDR: 1.000e+00
Coefficient: −6.36e−02
Value: B
Kineosporia.mikuniensis
0
2
4
6
A (n=15)
B (n=16)
C (n=14)
surface

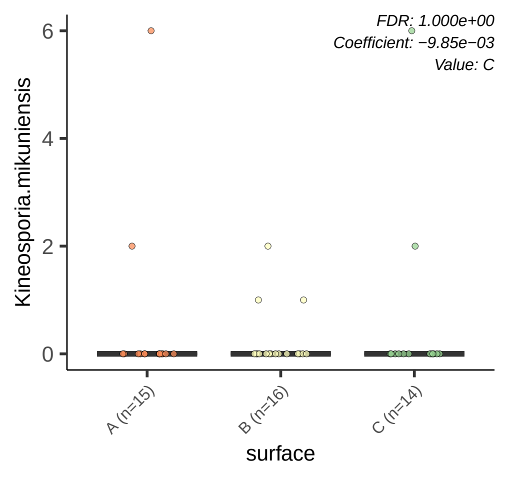
FDR: 1.000e+00
Coefficient: −9.85e−03
Value: C
Kineosporia.mikuniensis
0
2
4
6
A (n=15)
B (n=16)
C (n=14)
surface

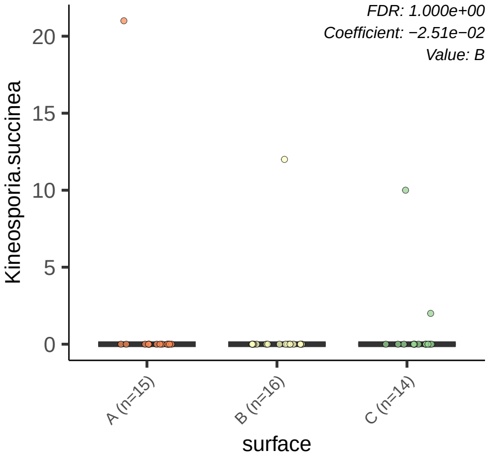

FDR: 1.000e+00
Coefficient: −2.51e−02
Value: B
Kineosporia.succinea
0
5
10
15
20
A (n=15)
B (n=16)
C (n=14)
surface

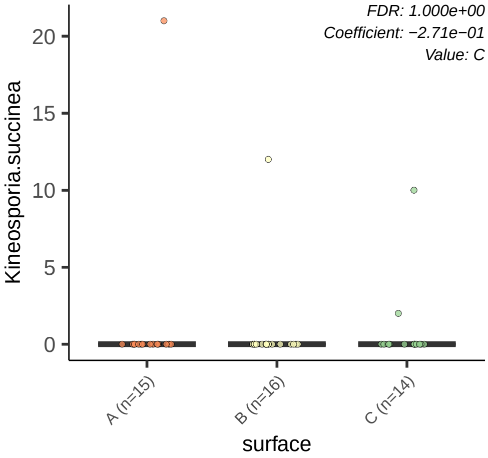
FDR: 1.000e+00
Coefficient: −2.71e−01
Value: C
Kineosporia.succinea
0
5
10
15
20
A (n=15)
B (n=16)
C (n=14)
surface

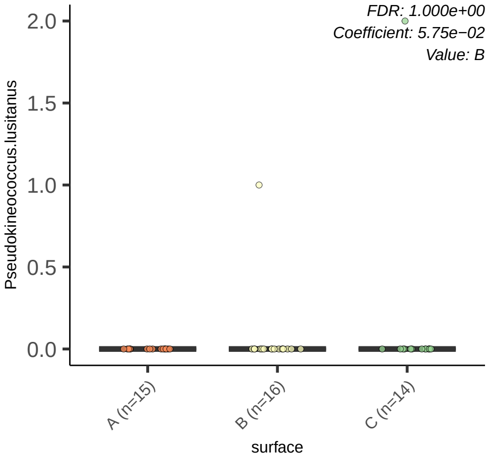
FDR: 1.000e+00
Coefficient: 5.75e−02
Value: B
Pseudokineococcus.lusitanus
2.0
1.5
1.0
0.5
0.0
A (n=15)
B (n=16)
C (n=14)
surface

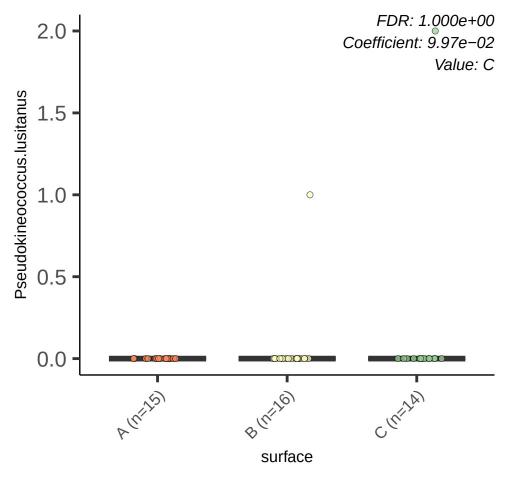

FDR: 1.000e+00
Coefficient: 9.97e−02
Value: C
Pseudokineococcus.lusitanus
2.0
1.5
1.0
0.5
0.0
A (n=15)
B (n=16)
C (n=14)
surface

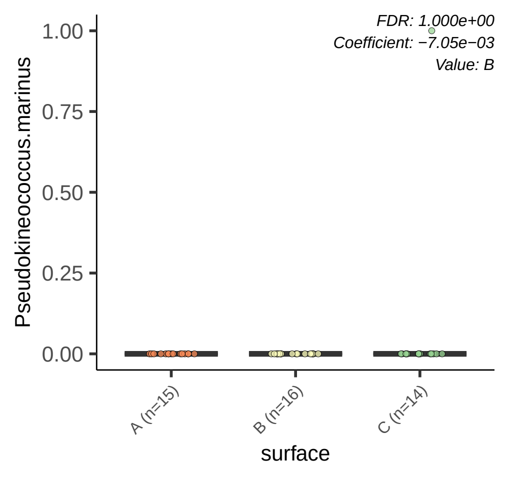
FDR: 1.000e+00
Coefficient: −7.05e−03
Value: B
Pseudokineococcus.marinus
1.00
0.75
0.50
0.25
0.00
A (n=15)
B (n=16)
C (n=14)
surface

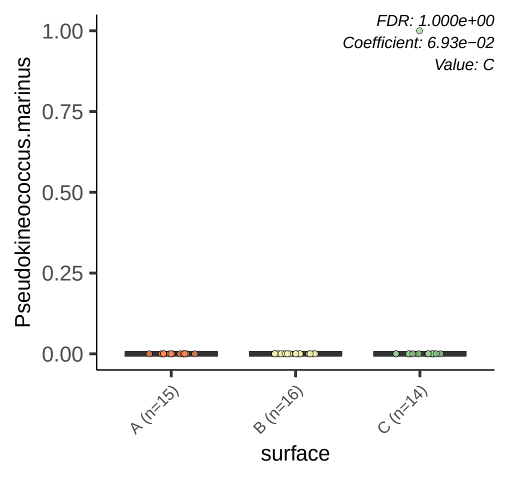
FDR: 1.000e+00
Coefficient: 6.93e−02
Value: C
Pseudokineococcus.marinus
1.00
0.75
0.50
0.25
0.00
A (n=15)
B (n=16)
C (n=14)
surface

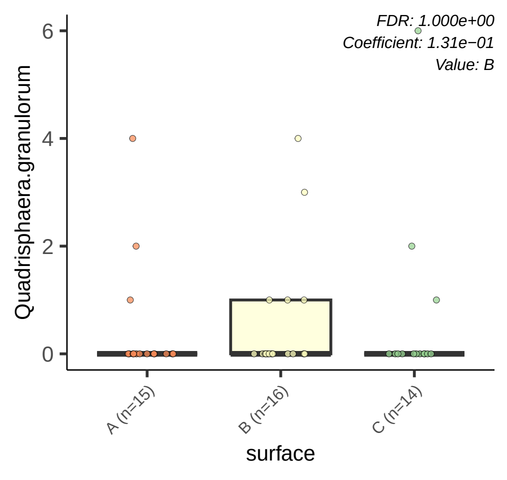

FDR: 1.000e+00
Coefficient: 1.31e−01
Value: B
Quadrisphaera.granulorum
6
4
2
0
A (n=15)
B (n=16)
C (n=14)
surface

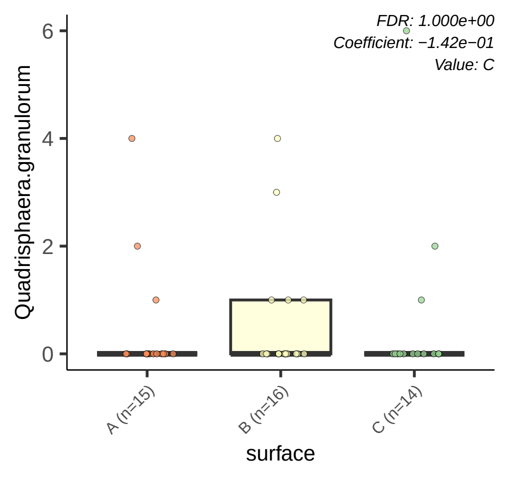
FDR: 1.000e+00
Coefficient: −1.42e−01
Value: C
Quadrisphaera.granulorum
6
4
2
0
A (n=15)
B (n=16)
C (n=14)
surface

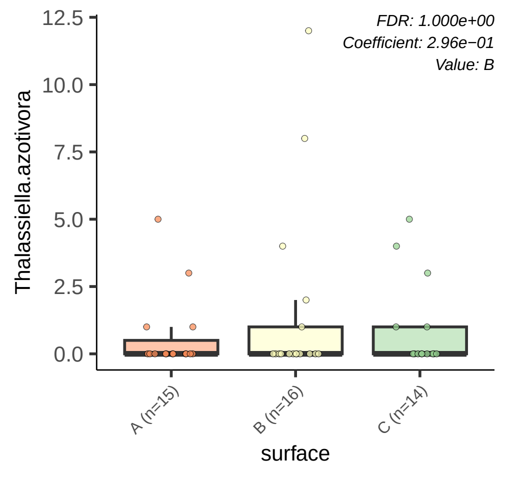
FDR: 1.000e+00
Coefficient: 2.96e−01
Value: B
Thalassiella.azotivora
12.5
10.0
7.5
5.0
2.5
0.0
A (n=15)
B (n=16)
C (n=14)
surface

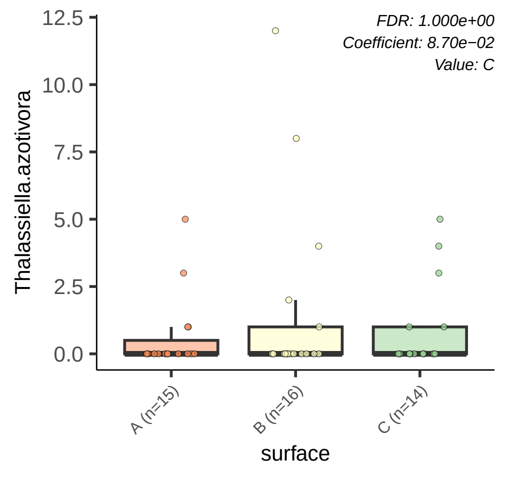

FDR: 1.000e+00
Coefficient: 8.70e−02
Value: C
Thalassiella.azotivora
0.0
2.5
5.0
7.5
10.0
12.5
A (n=15)
B (n=16)
C (n=14)
surface

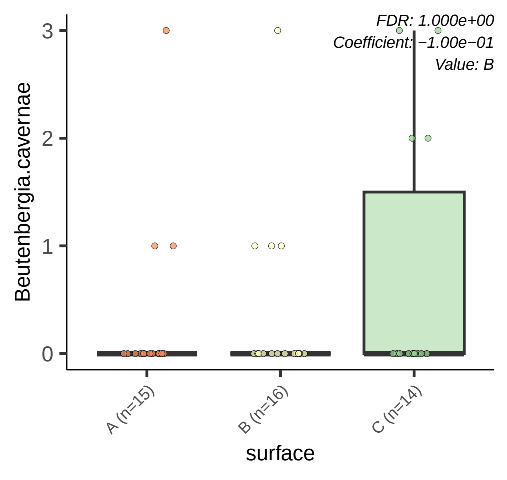
FDR: 1.000e+00
Coefficient: −1.00e−01
Value: B
Beutenbergia.cavernae
3
2
1
0
A (n=15)
B (n=16)
C (n=14)
surface

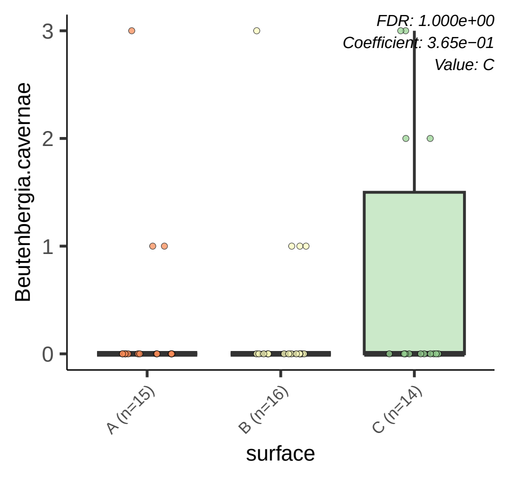

FDR: 1.000e+00
Coefficient: 3.65e−01
Value: C
Beutenbergia.cavernae
0
1
2
3
A (n=15)
B (n=16)
C (n=14)
surface

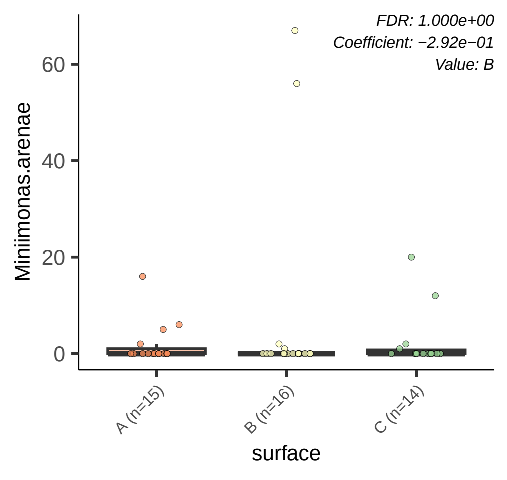

FDR: 1.000e+00
Coefficient: −2.92e−01
Value: B
Miniimonas.arenae
0
20
40
60
A (n=15)
B (n=16)
C (n=14)
surface

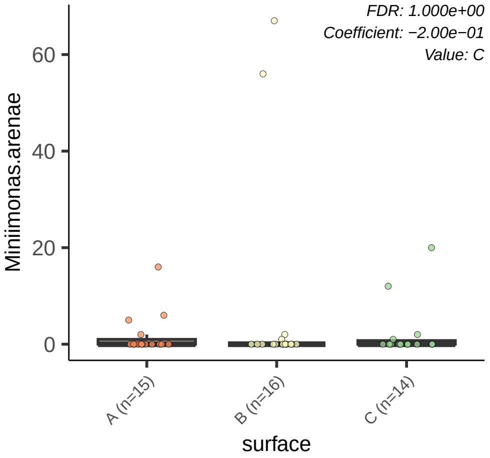
FDR: 1.000e+00
Coefficient: −2.00e−01
Value: C
Miniimonas.arenae
0
20
40
60
A (n=15)
B (n=16)
C (n=14)
surface

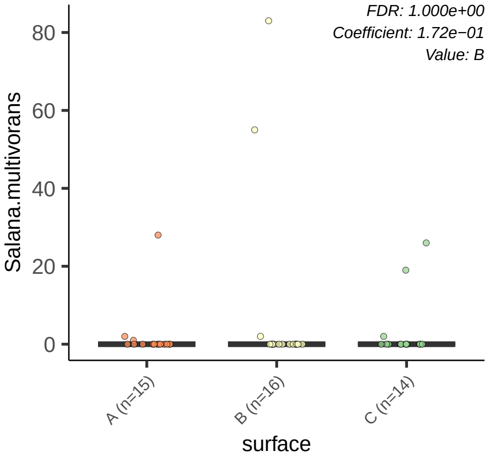

FDR: 1.000e+00
Coefficient: 1.72e−01
Value: B
80
60
40
20
0
Salana.multivorans
A (n=15)
B (n=16)
C (n=14)
surface

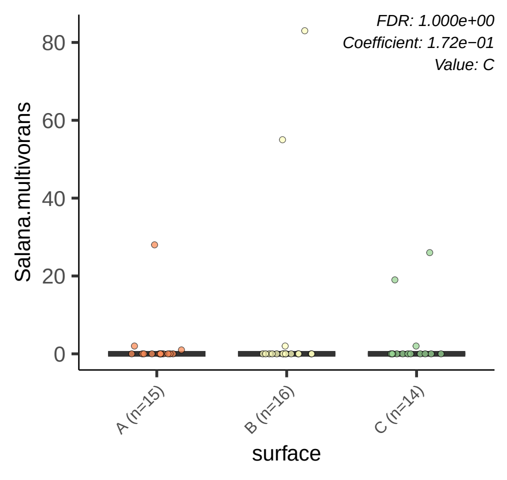
FDR: 1.000e+00
Coefficient: 1.72e−01
Value: C
80
60
40
20
0
Salana.multivorans
A (n=15)
B (n=16)
C (n=14)
surface

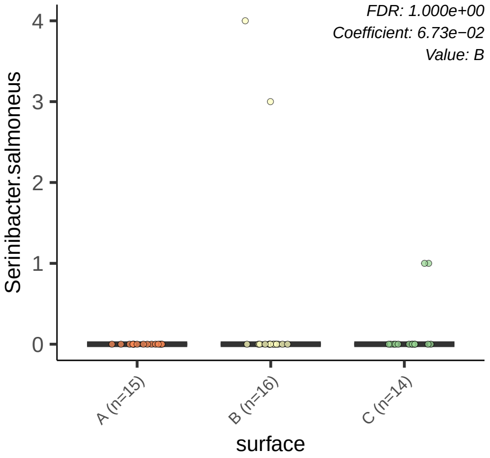

FDR: 1.000e+00
Coefficient: 6.73e−02
Value: B
Serinibacter.salmoneus
0
1
2
3
4
A (n=15)
B (n=16)
C (n=14)
surface

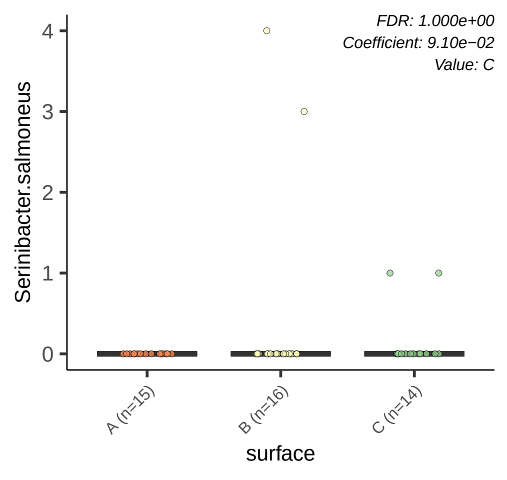
FDR: 1.000e+00
Coefficient: 9.10e−02
Value: C
Serinibacter.salmoneus
0
1
2
3
4
A (n=15)
B (n=16)
C (n=14)
surface

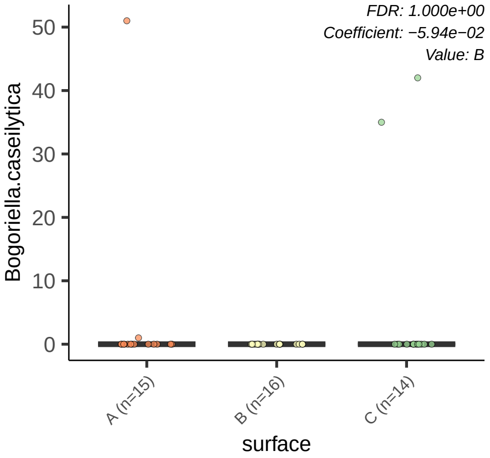
FDR: 1.000e+00
Coefficient: −5.94e−02
Value: B
Bogoriella.caseilytica
0
10
20
30
40
50
A (n=15)
B (n=16)
C (n=14)
surface

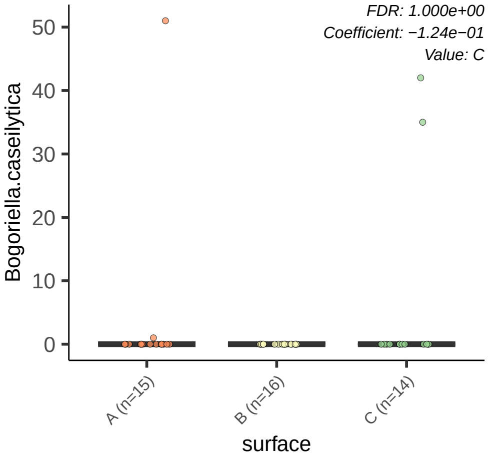
FDR: 1.000e+00
Coefficient: −1.24e−01
Value: C
Bogoriella.caseilytica
50
40
30
20
10
0
A (n=15)
B (n=16)
C (n=14)
surface

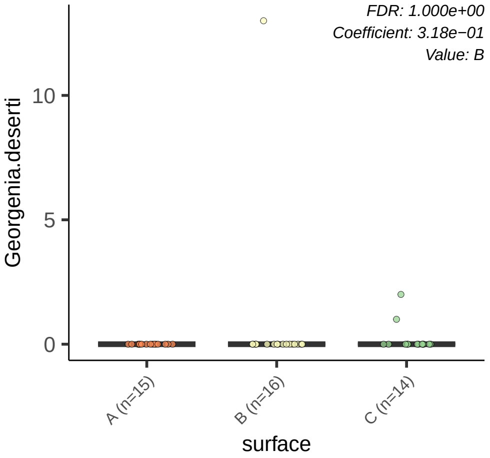

FDR: 1.000e+00
Coefficient: 3.18e−01
Value: B
Georgenia.deserti
10
5
0
A (n=15)
B (n=16)
C (n=14)
surface

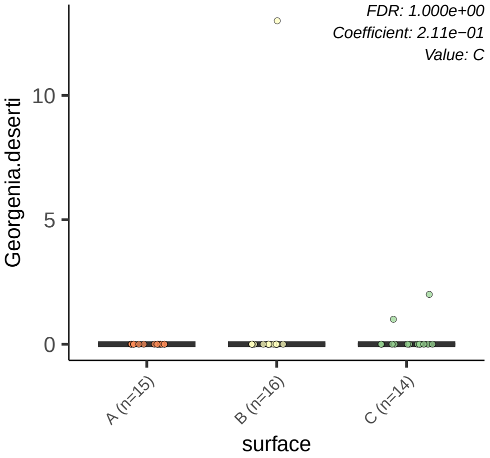
FDR: 1.000e+00
Coefficient: 2.11e−01
Value: C
Georgenia.deserti
0
5
10
A (n=15)
B (n=16)
C (n=14)
surface

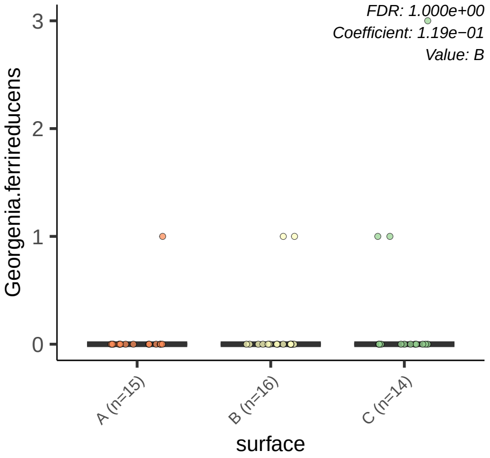
FDR: 1.000e+00
Coefficient: 1.19e−01
Value: B
Georgenia.ferrireducens
3
2
1
0
A (n=15)
B (n=16)
C (n=14)
surface

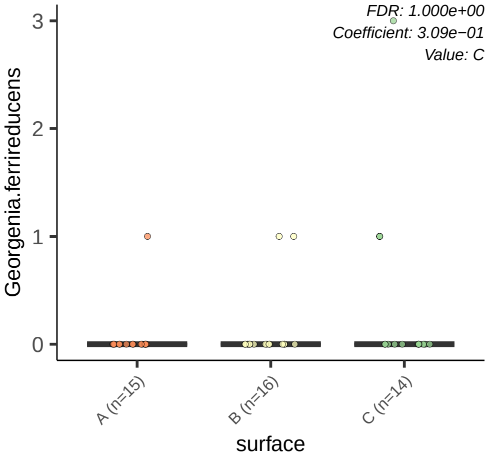
FDR: 1.000e+00
Coefficient: 3.09e−01
Value: C
Georgenia.ferrireducens
0
1
2
3
A (n=15)
B (n=16)
C (n=14)
surface

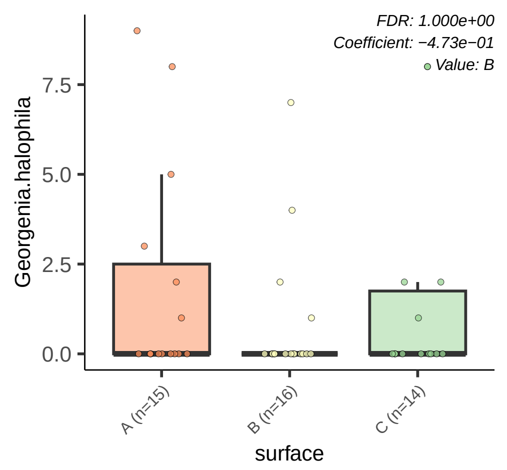

FDR: 1.000e+00
Coefficient: −4.73e−01
Value: B
Georgenia.halophila
7.5
5.0
2.5
0.0
A (n=15)
B (n=16)
C (n=14)
surface

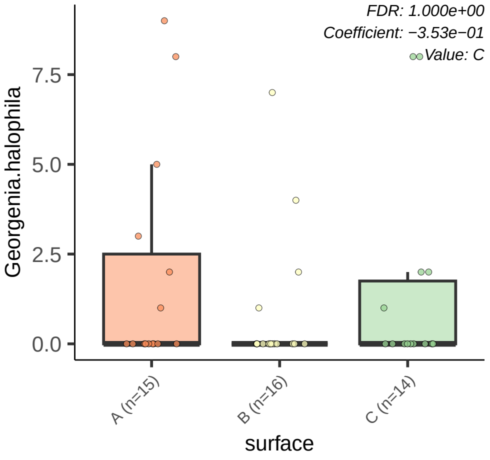
FDR: 1.000e+00
Coefficient: −3.53e−01
Value: C
Georgenia.halophila
0.0
2.5
5.0
7.5
A (n=15)
B (n=16)
C (n=14)
surface

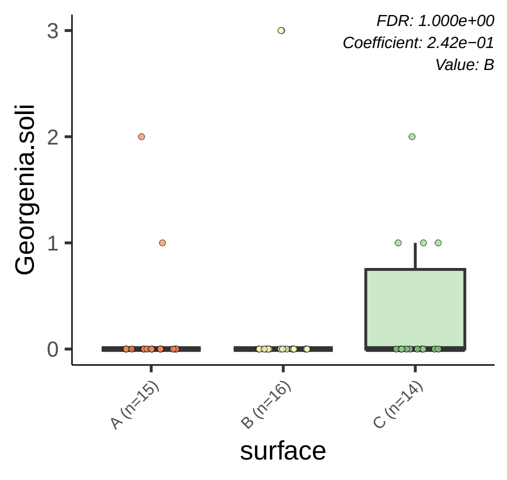
FDR: 1.000e+00
Coefficient: 2.42e−01
Value: B
Georgenia.soli
0
1
2
3
A (n=15)
B (n=16)
C (n=14)
surface

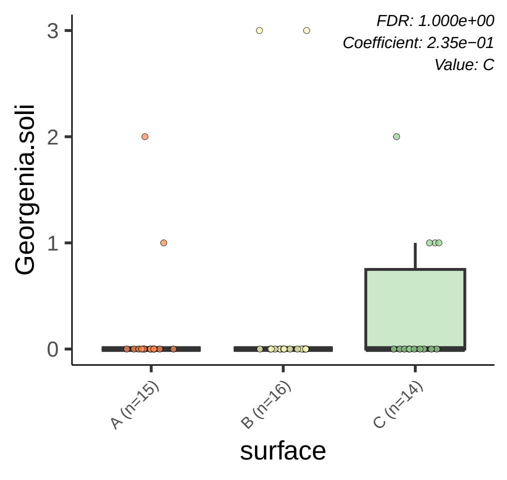
FDR: 1.000e+00
Coefficient: 2.35e−01
Value: C
Georgenia.soli
0
1
2
3
A (n=15)
B (n=16)
C (n=14)
surface

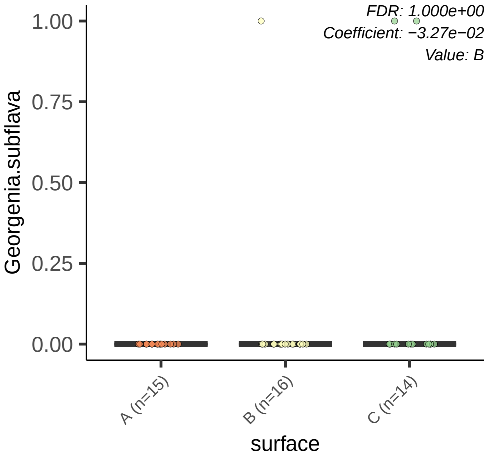
FDR: 1.000e+00
Coefficient: −3.27e−02
Value: B
Georgenia.subflava
1.00
0.75
0.50
0.25
0.00
A (n=15)
B (n=16)
C (n=14)
surface

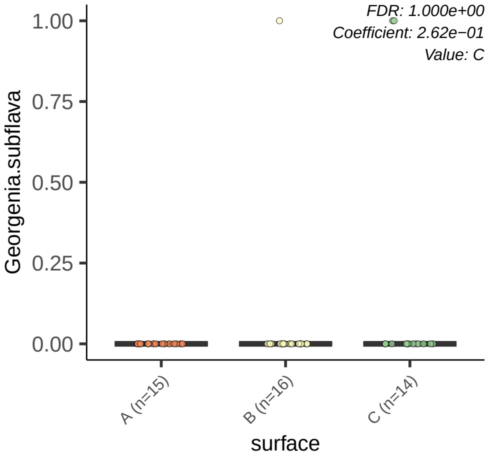
FDR: 1.000e+00
Coefficient: 2.62e−01
Value: C
Georgenia.subflava
1.00
0.75
0.50
0.25
0.00
A (n=15)
B (n=16)
C (n=14)
surface

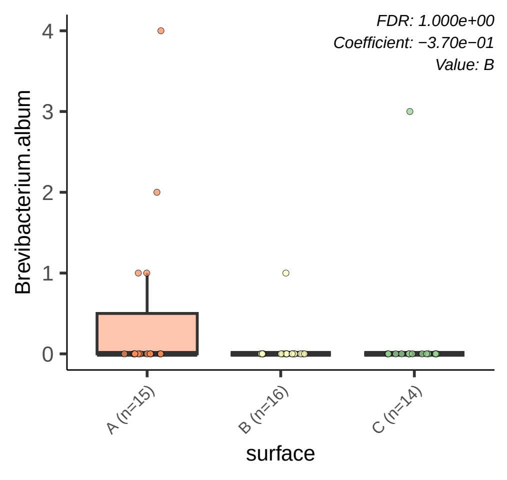

FDR: 1.000e+00
Coefficient: −3.70e−01
Value: B
Brevibacterium.album
4
3
2
1
0
A (n=15)
B (n=16)
C (n=14)
surface

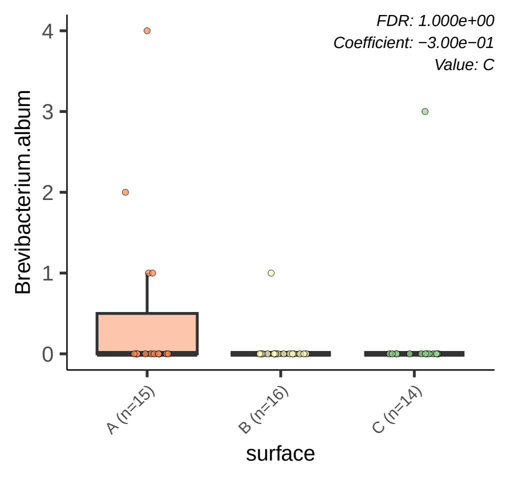

FDR: 1.000e+00
Coefficient: −3.00e−01
Value: C
Brevibacterium.album
0
1
2
3
4
A (n=15)
B (n=16)
C (n=14)
surface

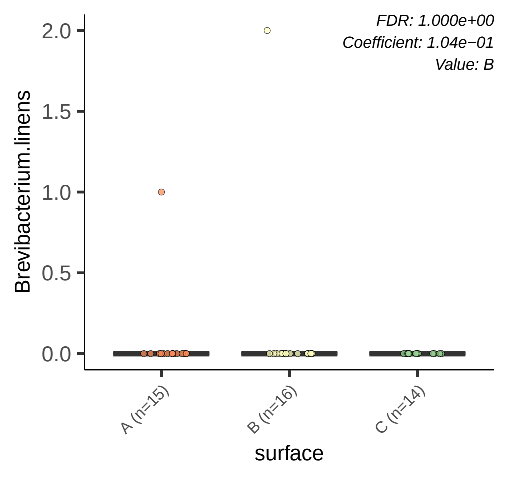

FDR: 1.000e+00
Coefficient: 1.04e−01
Value: B
Brevibacterium.linens
2.0
1.5
1.0
0.5
0.0
A (n=15)
B (n=16)
C (n=14)
surface

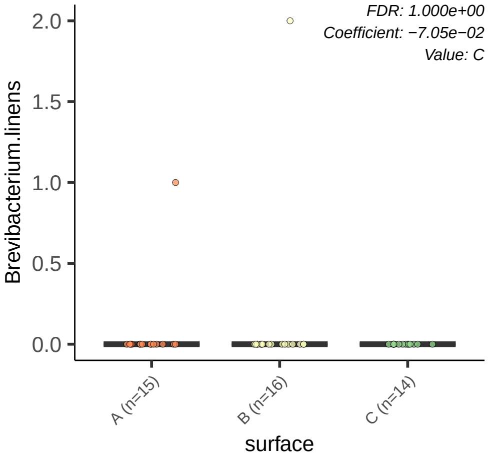

FDR: 1.000e+00
Coefficient: −7.05e−02
Value: C
Brevibacterium.linens
2.0
1.5
1.0
0.5
0.0
A (n=15)
B (n=16)
C (n=14)
surface

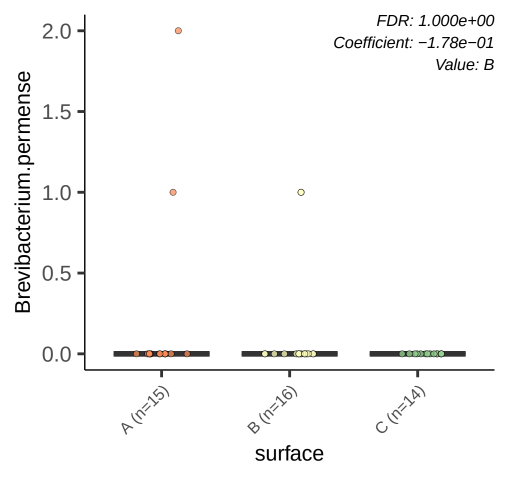

FDR: 1.000e+00
Coefficient: −1.78e−01
Value: B
Brevibacterium.permense
2.0
1.5
1.0
0.5
0.0
A (n=15)
B (n=16)
C (n=14)
surface

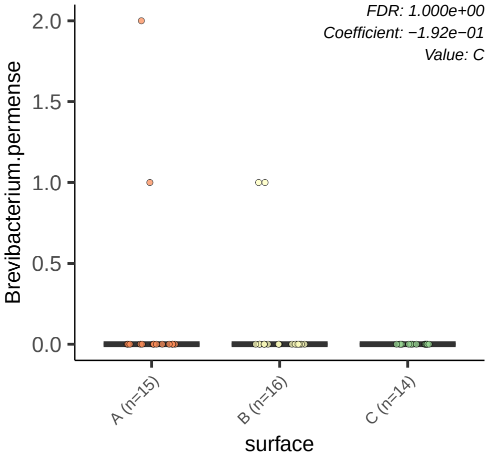
FDR: 1.000e+00
Coefficient: −1.92e−01
Value: C
Brevibacterium.permense
2.0
1.5
1.0
0.5
0.0
A (n=15)
B (n=16)
C (n=14)
surface

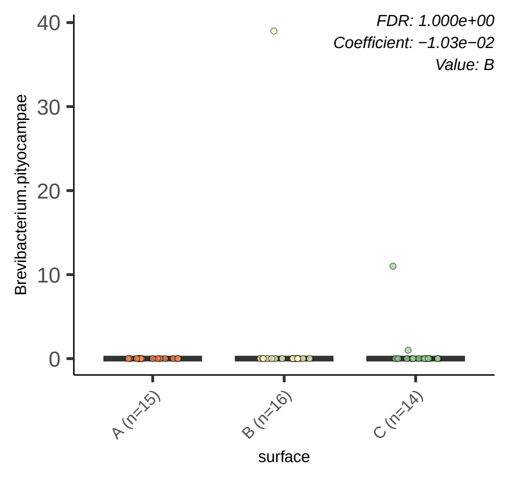

FDR: 1.000e+00
Coefficient: −1.03e−02
Value: B
Brevibacterium.pityocampae
40
30
20
10
0
A (n=15)
B (n=16)
C (n=14)
surface

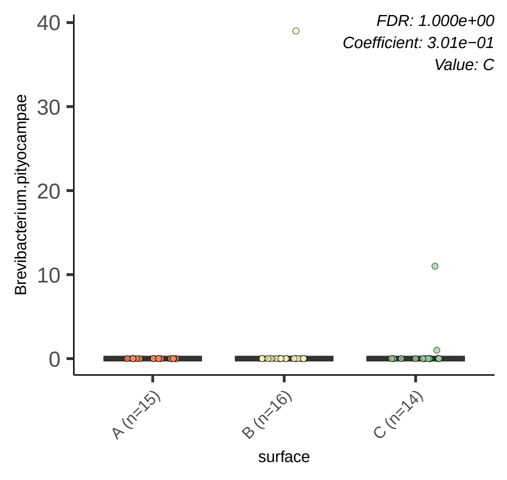

FDR: 1.000e+00
Coefficient: 3.01e−01
Value: C
Brevibacterium.pityocampae
0
10
20
30
40
A (n=15)
B (n=16)
C (n=14)
surface

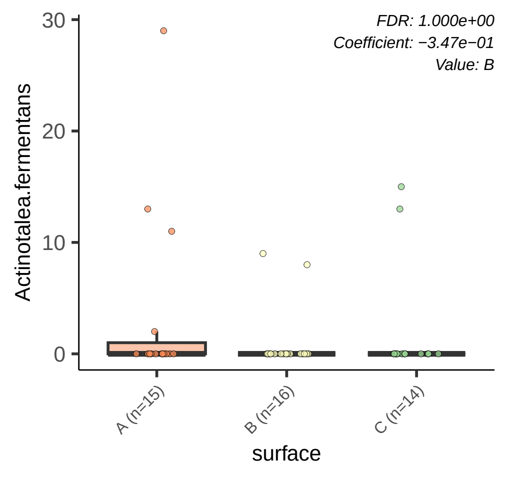
FDR: 1.000e+00
Coefficient: −3.47e−01
Value: B
Actinotalea.fermentans
30
20
10
0
A (n=15)
B (n=16)
C (n=14)
surface

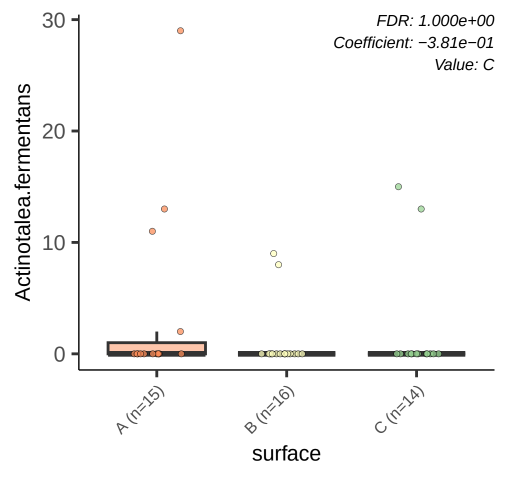
FDR: 1.000e+00
Coefficient: −3.81e−01
Value: C
Actinotalea.fermentans
30
20
10
0
A (n=15)
B (n=16)
C (n=14)
surface

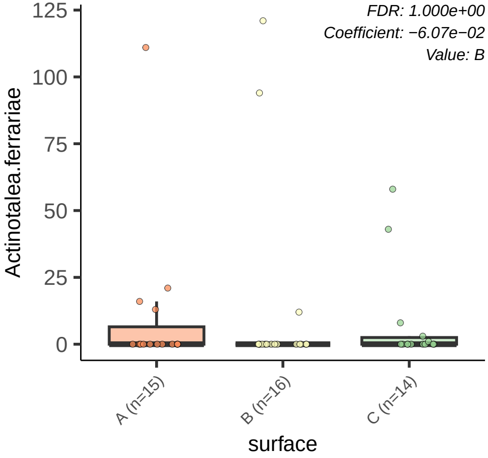
FDR: 1.000e+00
Coefficient: −6.07e−02
Value: B
125
100
75
50
25
0
Actinotalea.ferrariae
A (n=15)
B (n=16)
C (n=14)
surface

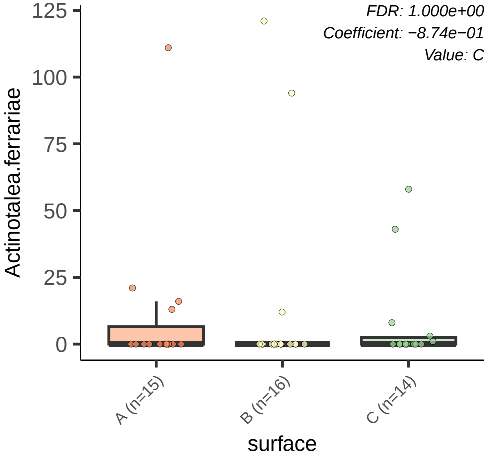
FDR: 1.000e+00
Coefficient: −8.74e−01
Value: C
Actinotalea.ferrariae
125
100
75
50
25
0
A (n=15)
B (n=16)
C (n=14)
surface

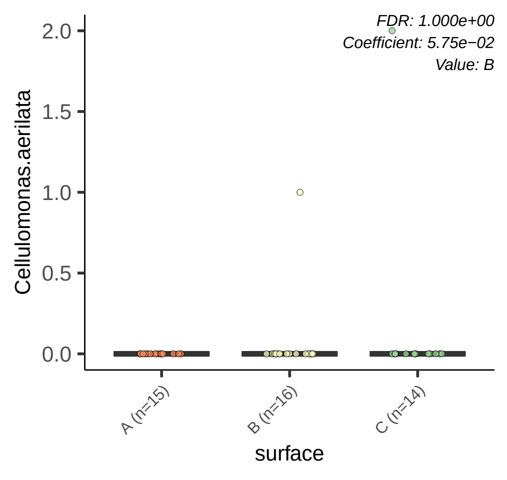

FDR: 1.000e+00
Coefficient: 5.75e−02
Value: B
2.0
1.5
1.0
0.5
0.0
Cellulomonas.aerilata
A (n=15)
B (n=16)
C (n=14)
surface

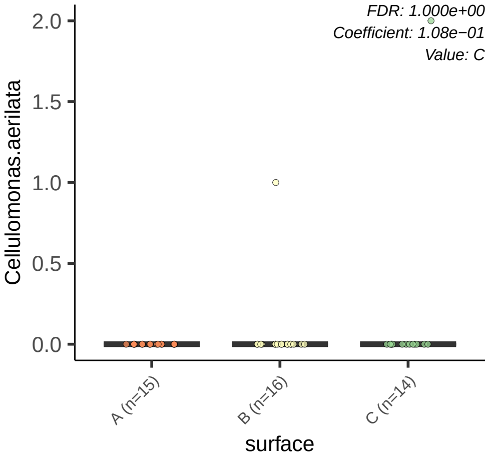
FDR: 1.000e+00
Coefficient: 1.08e−01
Value: C
Cellulomonas.aerilata
2.0
1.5
1.0
0.5
0.0
A (n=15)
B (n=16)
C (n=14)
surface

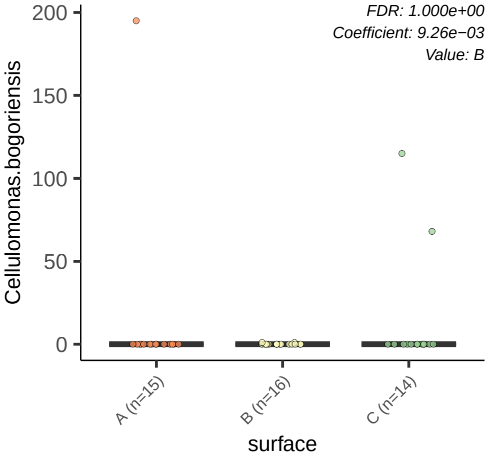
FDR: 1.000e+00
Coefficient: 9.26e−03
Value: B
Cellulomonas.bogoriensis
200
150
100
50
0
A (n=15)
B (n=16)
C (n=14)
surface

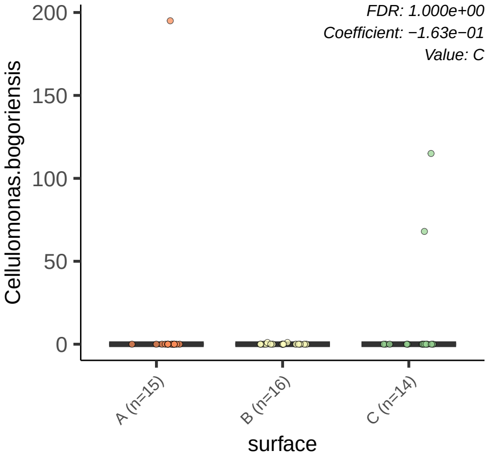
FDR: 1.000e+00
Coefficient: −1.63e−01
Value: C
Cellulomonas.bogoriensis
200
150
100
50
0
A (n=15)
B (n=16)
C (n=14)
surface

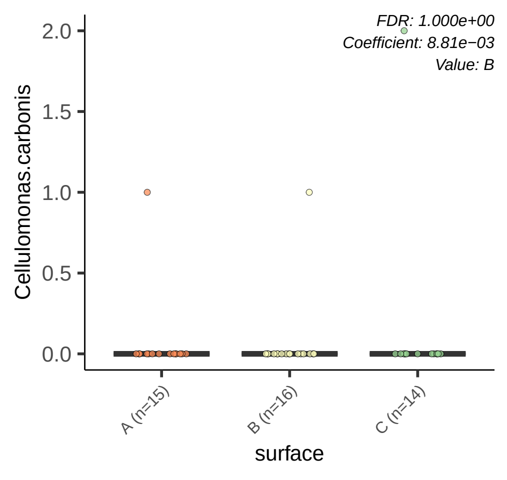
FDR: 1.000e+00
Coefficient: 8.81e−03
Value: B
Cellulomonas.carbonis
2.0
1.5
1.0
0.5
0.0
A (n=15)
B (n=16)
C (n=14)
surface

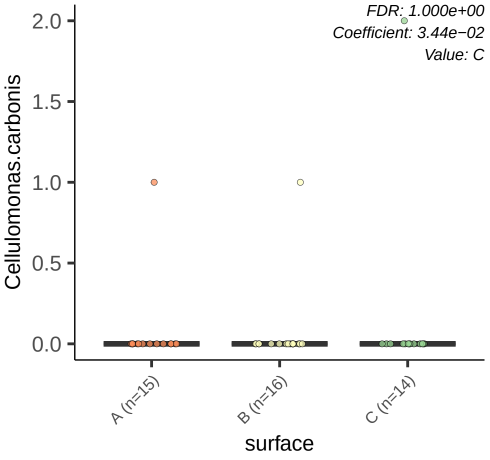
FDR: 1.000e+00
Coefficient: 3.44e−02
Value: C
Cellulomonas.carbonis
2.0
1.5
1.0
0.5
0.0
A (n=15)
B (n=16)
C (n=14)
surface

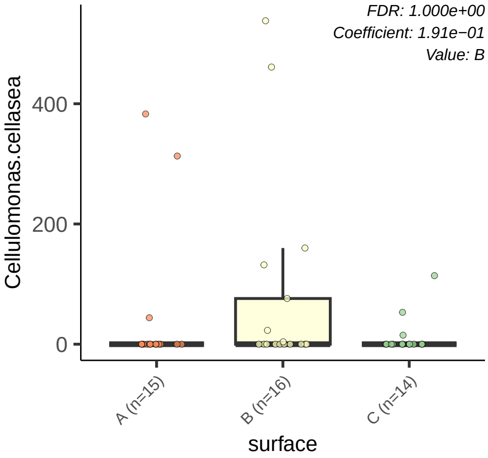

FDR: 1.000e+00
Coefficient: 1.91e−01
Value: B
Cellulomonas.cellasea
400
200
0
A (n=15)
B (n=16)
C (n=14)
surface

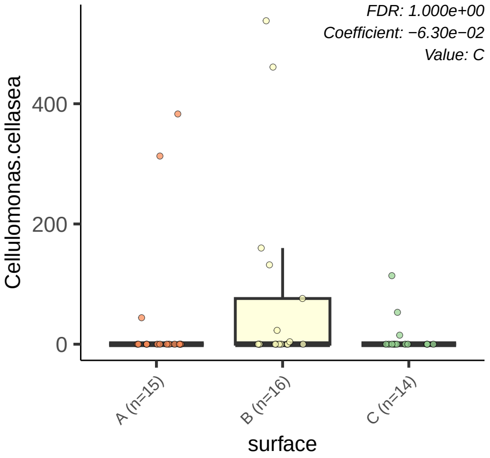
FDR: 1.000e+00
Coefficient: −6.30e−02
Value: C
Cellulomonas.cellasea
400
200
0
A (n=15)
B (n=16)
C (n=14)
surface

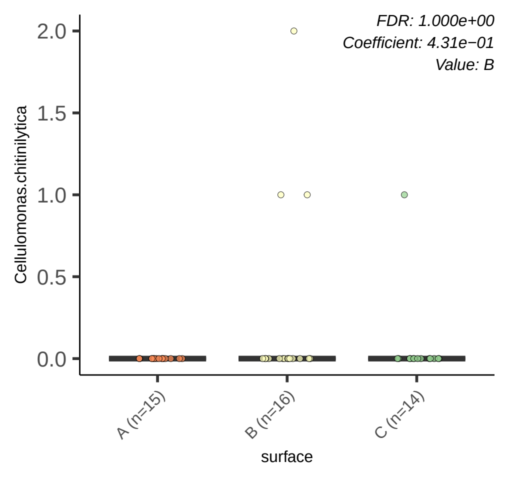

FDR: 1.000e+00
Coefficient: 4.31e−01
Value: B
Cellulomonas.chitinilytica
2.0
1.5
1.0
0.5
0.0
A (n=15)
B (n=16)
C (n=14)
surface

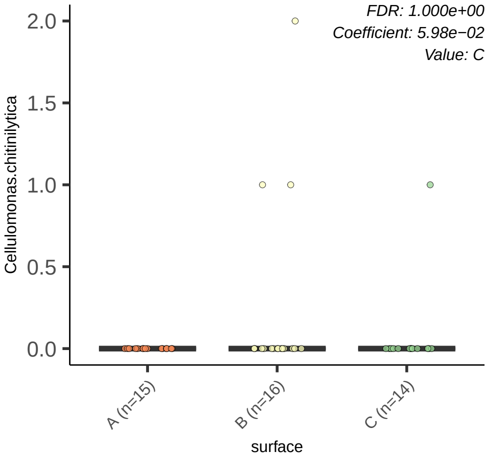
FDR: 1.000e+00
Coefficient: 5.98e−02
Value: C
Cellulomonas.chitinilytica
2.0
1.5
1.0
0.5
0.0
A (n=15)
B (n=16)
C (n=14)
surface

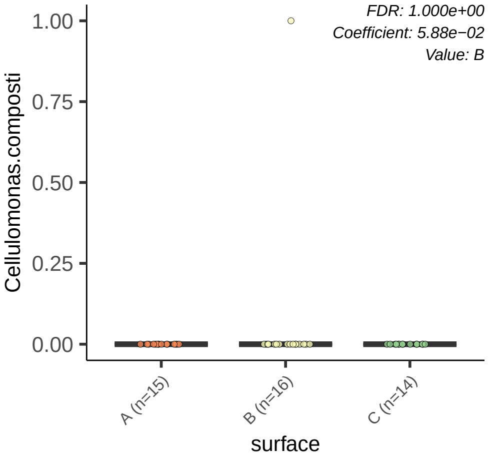
FDR: 1.000e+00
Coefficient: 5.88e−02
Value: B
Cellulomonas.composti
1.00
0.75
0.50
0.25
0.00
A (n=15)
B (n=16)
C (n=14)
surface

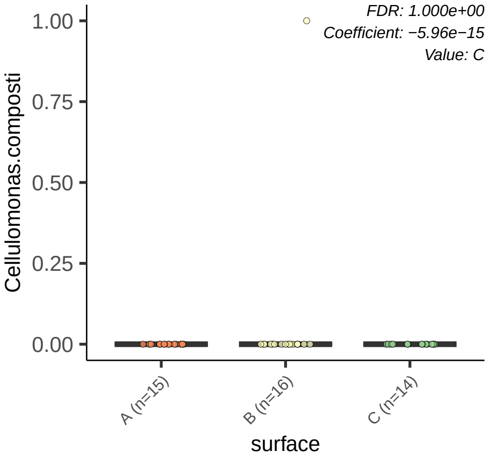
FDR: 1.000e+00
Coefficient: −5.96e−15
Value: C
Cellulomonas.composti
1.00
0.75
0.50
0.25
0.00
A (n=15)
B (n=16)
C (n=14)
surface

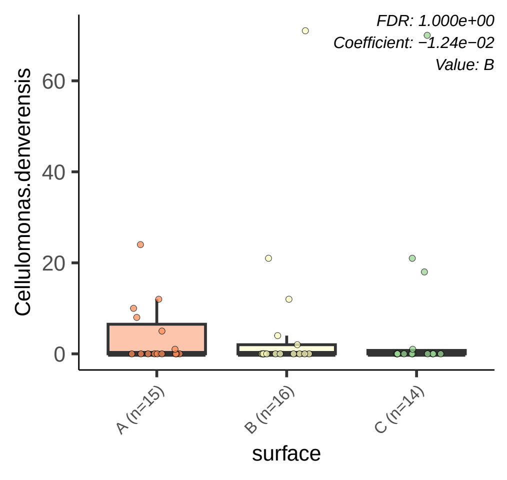

FDR: 1.000e+00
Coefficient: −1.24e−02
Value: B
Cellulomonas.denverensis
0
20
40
60
A (n=15)
B (n=16)
C (n=14)
surface

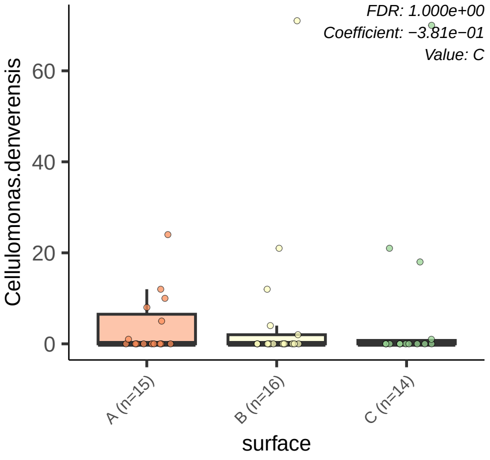

FDR: 1.000e+00
Coefficient: −3.81e−01
Value: C
Cellulomonas.denverensis
0
20
40
60
A (n=15)
B (n=16)
C (n=14)
surface

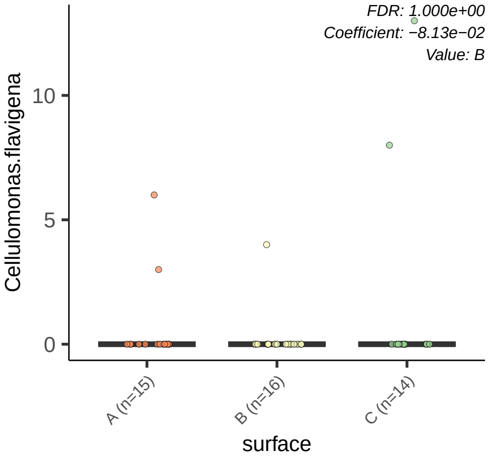
FDR: 1.000e+00
Coefficient: −8.13e−02
Value: B
Cellulomonas.flavigena
10
5
0
A (n=15)
B (n=16)
C (n=14)
surface

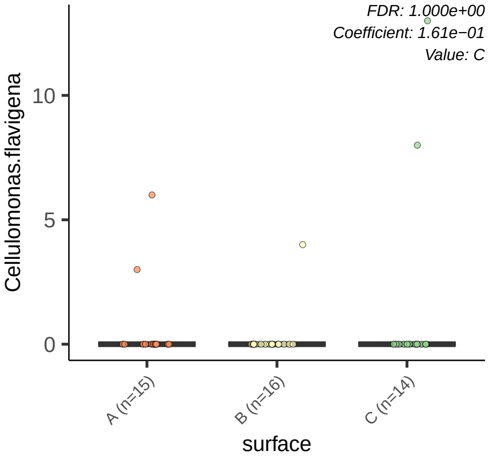
FDR: 1.000e+00
Coefficient: 1.61e−01
Value: C
Cellulomonas.flavigena
10
5
0
A (n=15)
B (n=16)
C (n=14)
surface

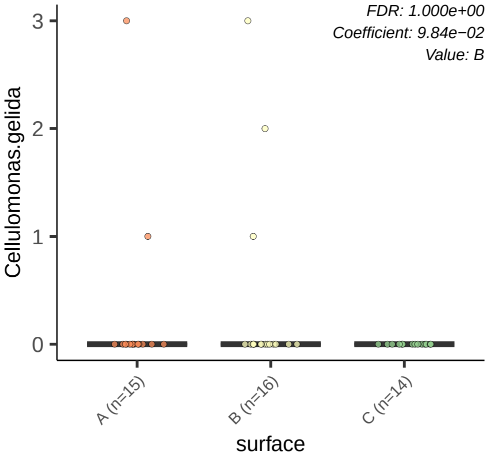

FDR: 1.000e+00
Coefficient: 9.84e−02
Value: B
Cellulomonas.gelida
3
2
1
0
A (n=15)
B (n=16)
C (n=14)
surface

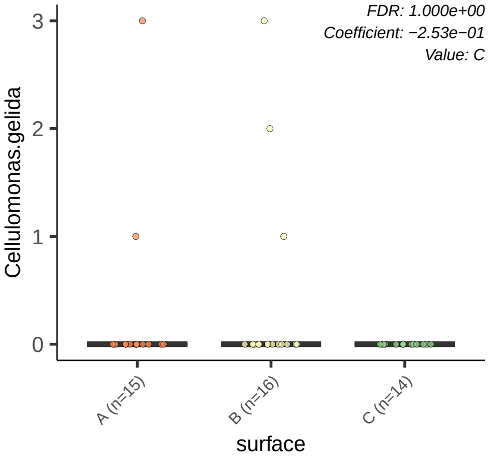

FDR: 1.000e+00
Coefficient: −2.53e−01
Value: C
Cellulomonas.gelida
3
2
1
0
A (n=15)
B (n=16)
C (n=14)
surface

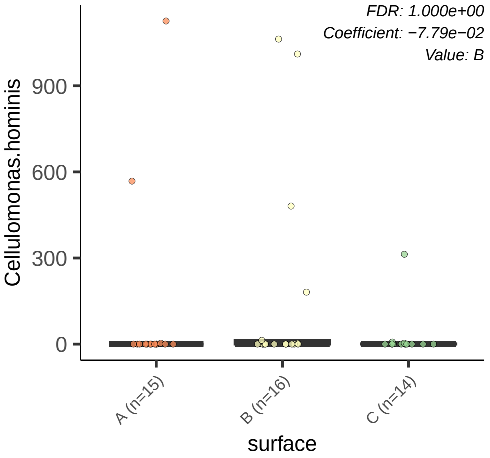
FDR: 1.000e+00
Coefficient: −7.79e−02
Value: B
Cellulomonas.hominis
900
600
300
0
A (n=15)
B (n=16)
C (n=14)
surface

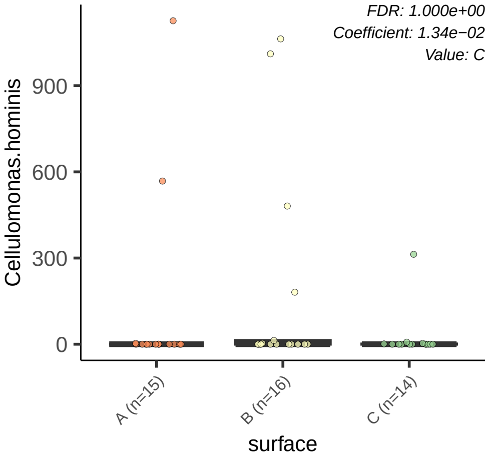

FDR: 1.000e+00
Coefficient: 1.34e−02
Value: C
Cellulomonas.hominis
900
600
300
0
A (n=15)
B (n=16)
C (n=14)
surface

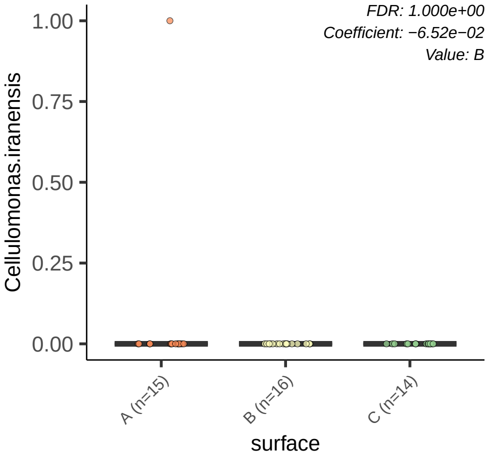
FDR: 1.000e+00
Coefficient: −6.52e−02
Value: B
Cellulomonas.iranensis
1.00
0.75
0.50
0.25
0.00
A (n=15)
B (n=16)
C (n=14)
surface

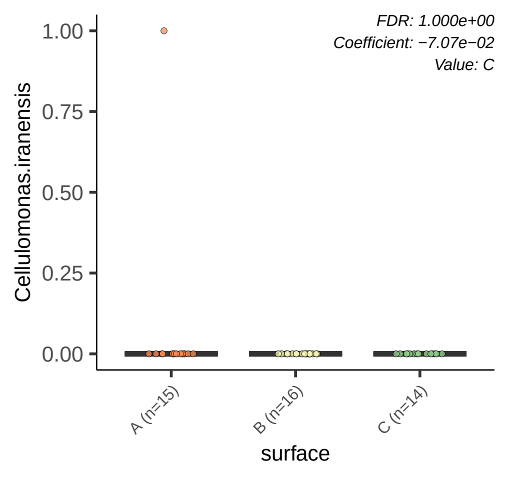
FDR: 1.000e+00
Coefficient: −7.07e−02
Value: C
Cellulomonas.iranensis
1.00
0.75
0.50
0.25
0.00
A (n=15)
B (n=16)
C (n=14)
surface

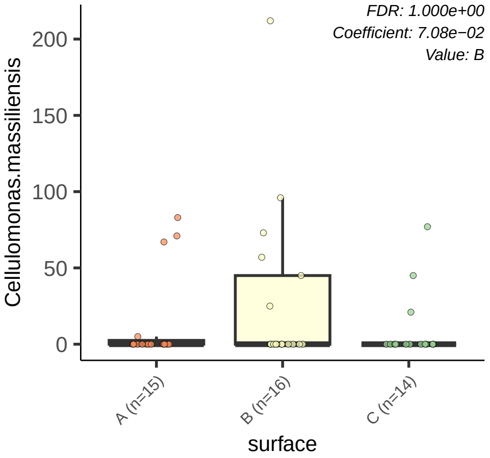

FDR: 1.000e+00
Coefficient: 7.08e−02
Value: B
Cellulomonas.massiliensis
200
150
100
50
0
A (n=15)
B (n=16)
C (n=14)
surface

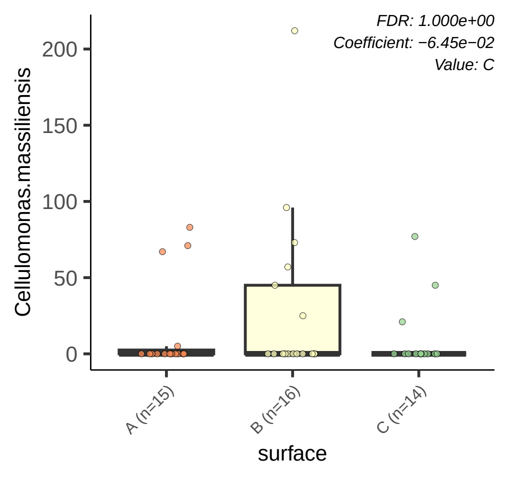
FDR: 1.000e+00
Coefficient: −6.45e−02
Value: C
Cellulomonas.massiliensis
200
150
100
50
0
A (n=15)
B (n=16)
C (n=14)
surface

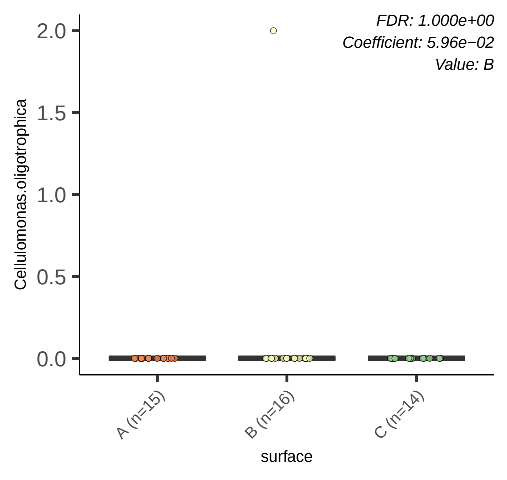

FDR: 1.000e+00
Coefficient: 5.96e−02
Value: B
Cellulomonas.oligotrophica
2.0
1.5
1.0
0.5
0.0
A (n=15)
B (n=16)
C (n=14)
surface

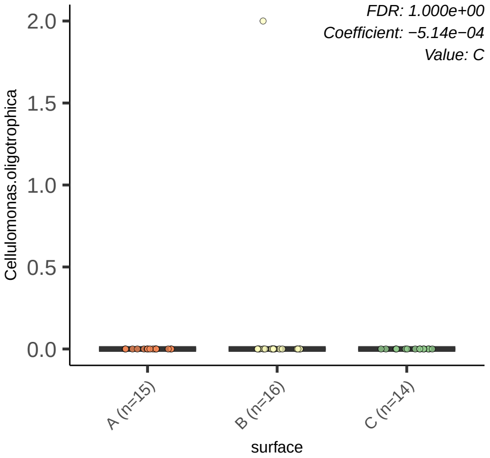
FDR: 1.000e+00
Coefficient: −5.14e−04
Value: C
Cellulomonas.oligotrophica
2.0
1.5
1.0
0.5
0.0
A (n=15)
B (n=16)
C (n=14)
surface

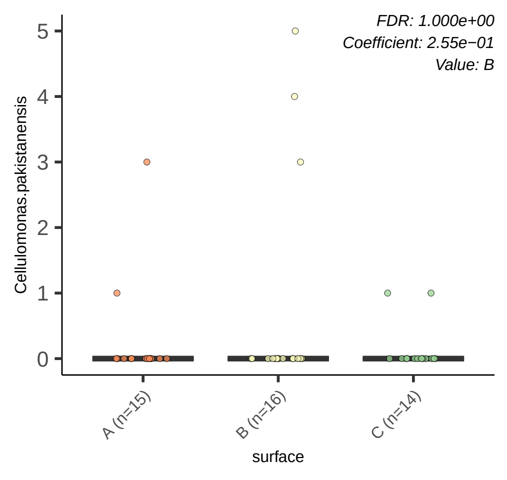

FDR: 1.000e+00
Coefficient: 2.55e−01
Value: B
Cellulomonas.pakistanensis
5
4
3
2
1
0
A (n=15)
B (n=16)
C (n=14)
surface

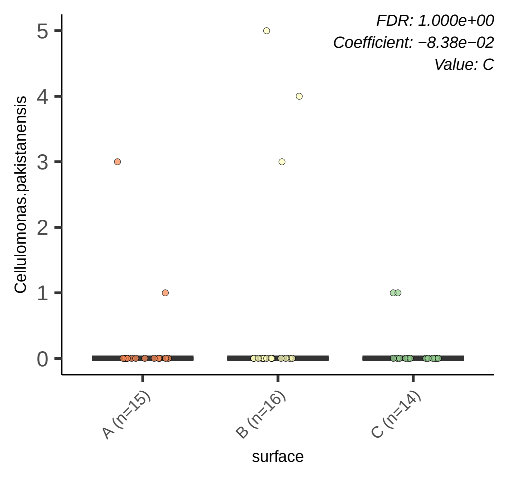

FDR: 1.000e+00
Coefficient: −8.38e−02
Value: C
Cellulomonas.pakistanensis
5
4
3
2
1
0
A (n=15)
B (n=16)
C (n=14)
surface

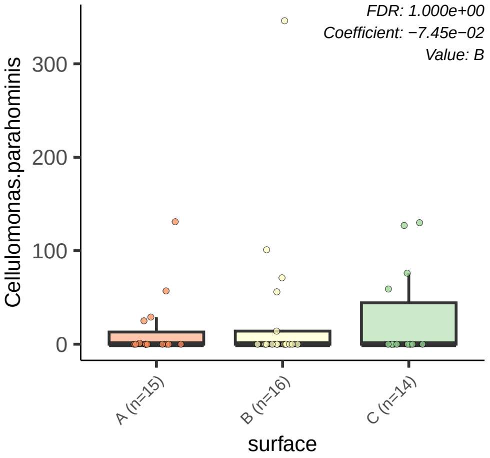
FDR: 1.000e+00
Coefficient: −7.45e−02
Value: B
Cellulomonas.parahominis
300
200
100
0
A (n=15)
B (n=16)
C (n=14)
surface

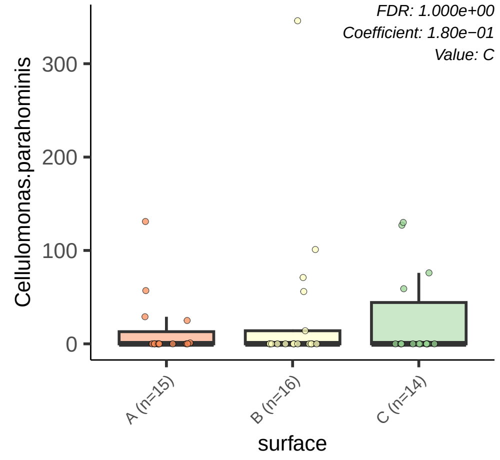
FDR: 1.000e+00
Coefficient: 1.80e−01
Value: C
Cellulomonas.parahominis
300
200
100
0
A (n=15)
B (n=16)
C (n=14)
surface

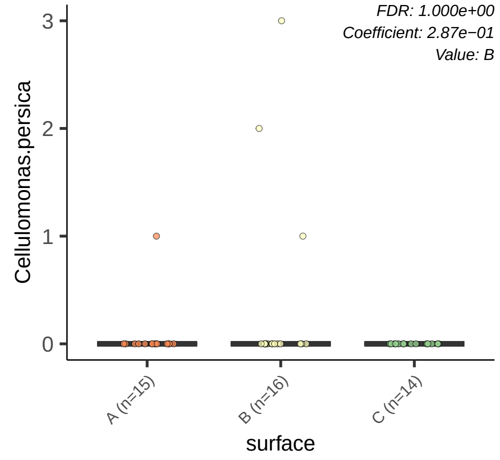

FDR: 1.000e+00
Coefficient: 2.87e−01
Value: B
Cellulomonas.persica
0
1
2
3
A (n=15)
B (n=16)
C (n=14)
surface

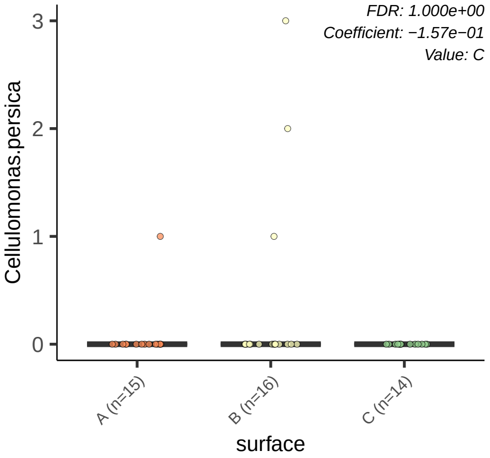
FDR: 1.000e+00
Coefficient: −1.57e−01
Value: C
Cellulomonas.persica
3
2
1
0
A (n=15)
B (n=16)
C (n=14)
surface

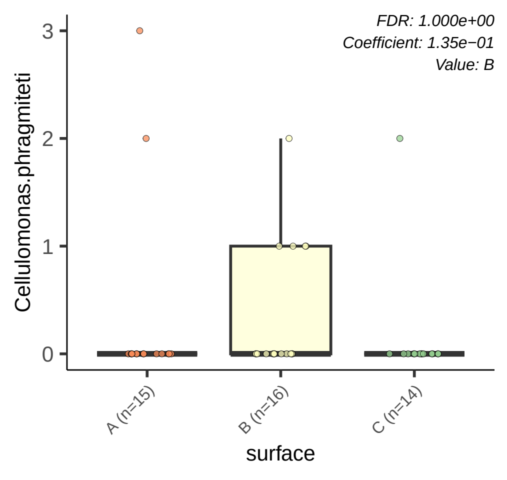
FDR: 1.000e+00
Coefficient: 1.35e−01
Value: B
Cellulomonas.phragmiteti
0
1
2
3
A (n=15)
B (n=16)
C (n=14)
surface

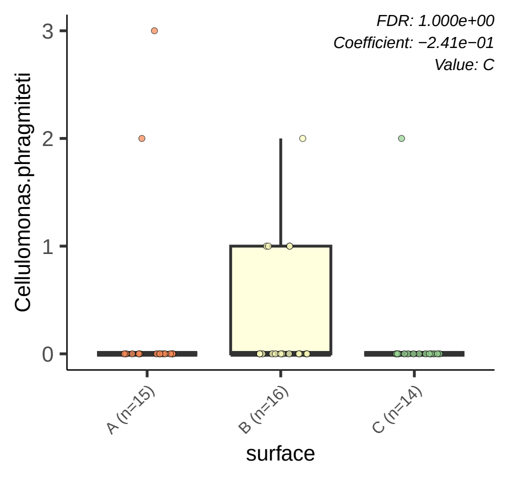

FDR: 1.000e+00
Coefficient: −2.41e−01
Value: C
Cellulomonas.phragmiteti
3
2
1
0
A (n=15)
B (n=16)
C (n=14)
surface

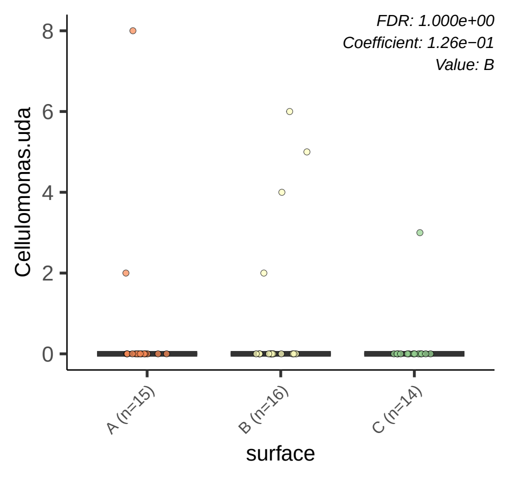
FDR: 1.000e+00
Coefficient: 1.26e−01
Value: B
Cellulomonas.uda
0
2
4
6
8
A (n=15)
B (n=16)
C (n=14)
surface

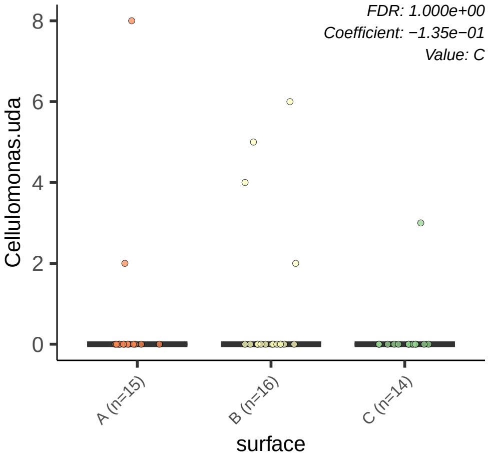

FDR: 1.000e+00
Coefficient: −1.35e−01
Value: C
Cellulomonas.uda
8
6
4
2
0
A (n=15)
B (n=16)
C (n=14)
surface

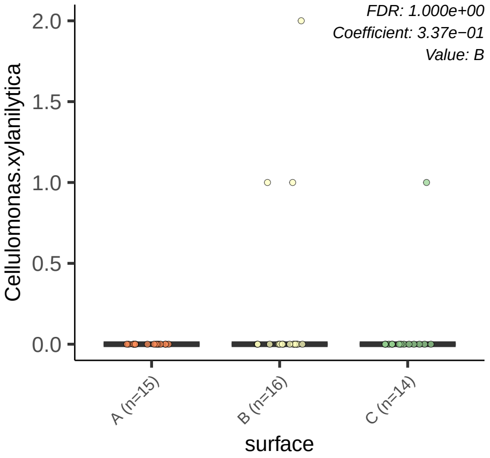

FDR: 1.000e+00
Coefficient: 3.37e−01
Value: B
Cellulomonas.xylanilytica
2.0
1.5
1.0
0.5
0.0
A (n=15)
B (n=16)
C (n=14)
surface

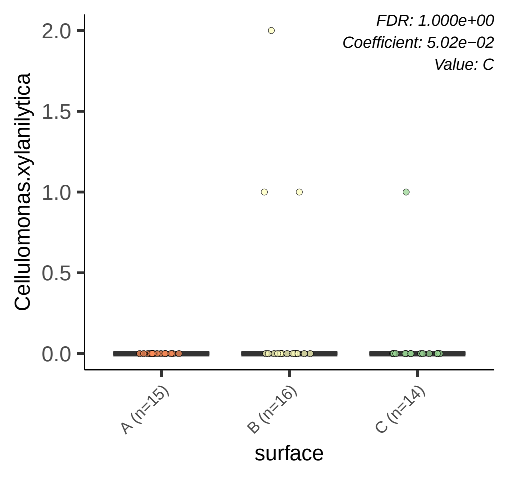

FDR: 1.000e+00
Coefficient: 5.02e−02
Value: C
Cellulomonas.xylanilytica
2.0
1.5
1.0
0.5
0.0
A (n=15)
B (n=16)
C (n=14)
surface

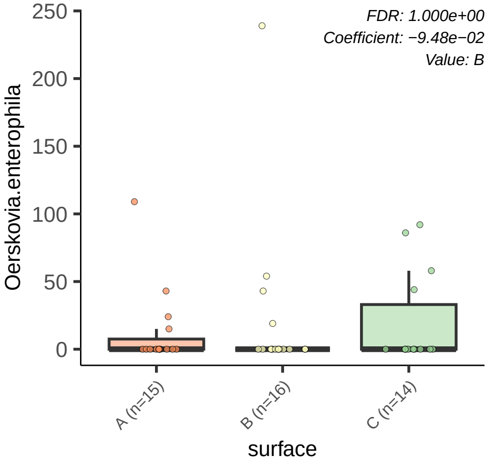

FDR: 1.000e+00
Coefficient: −9.48e−02
Value: B
Oerskovia.enterophila
250
200
150
100
50
0
A (n=15)
B (n=16)
C (n=14)
surface

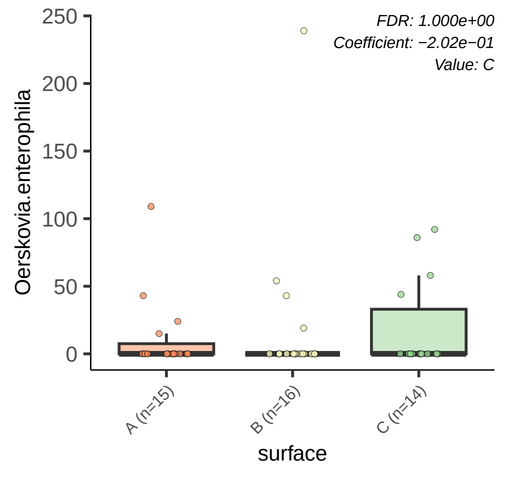

FDR: 1.000e+00
Coefficient: −2.02e−01
Value: C
Oerskovia.enterophila
250
200
150
100
50
0
A (n=15)
B (n=16)
C (n=14)
surface

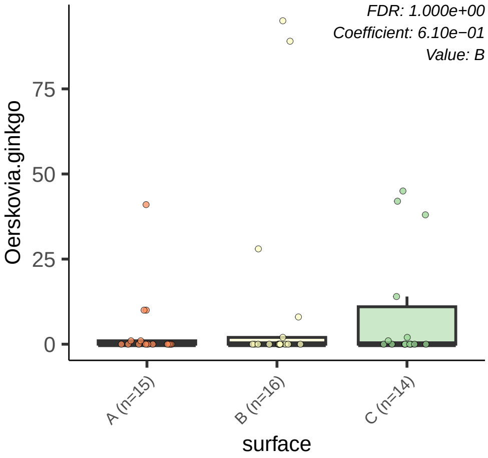

FDR: 1.000e+00
Coefficient: 6.10e−01
Value: B
Oerskovia.ginkgo
75
50
25
0
A (n=15)
B (n=16)
C (n=14)
surface

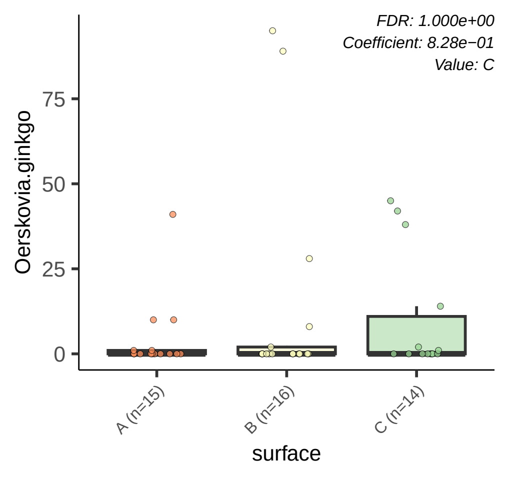
FDR: 1.000e+00
Coefficient: 8.28e−01
Value: C
Oerskovia.ginkgo
0
25
50
75
A (n=15)
B (n=16)
C (n=14)
surface

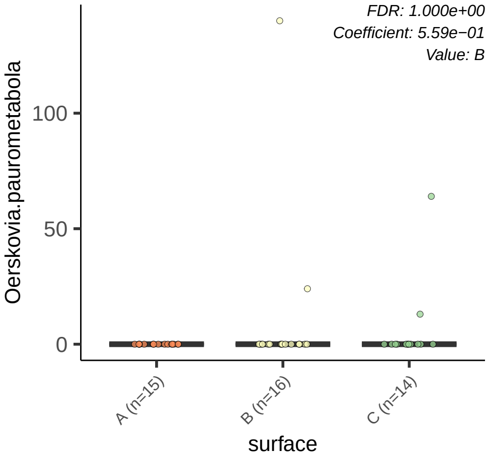
FDR: 1.000e+00
Coefficient: 5.59e−01
Value: B
Oerskovia.paurometabola
100
50
0
A (n=15)
B (n=16)
C (n=14)
surface

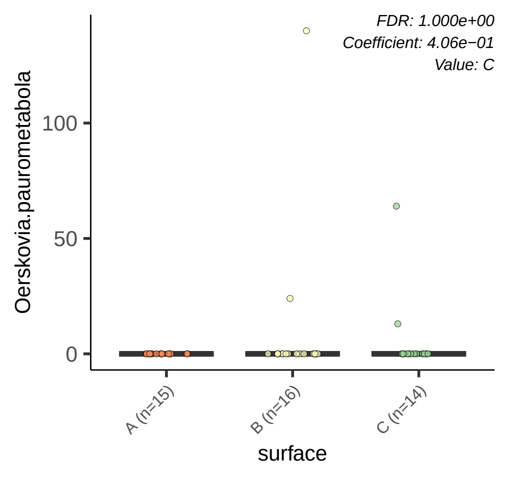
FDR: 1.000e+00
Coefficient: 4.06e−01
Value: C
Oerskovia.paurometabola
100
50
0
A (n=15)
B (n=16)
C (n=14)
surface

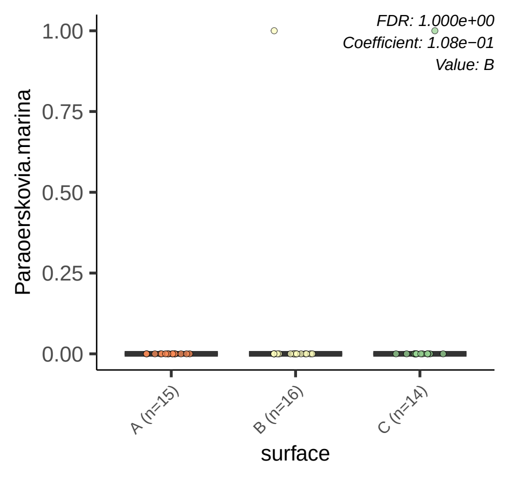
FDR: 1.000e+00
Coefficient: 1.08e−01
Value: B
Paraoerskovia.marina
1.00
0.75
0.50
0.25
0.00
A (n=15)
B (n=16)
C (n=14)
surface

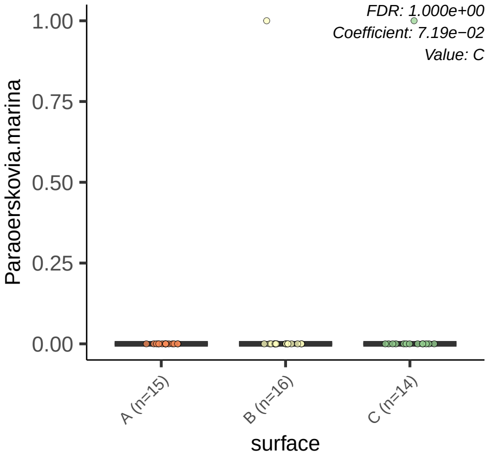
FDR: 1.000e+00
Coefficient: 7.19e−02
Value: C
Paraoerskovia.marina
1.00
0.75
0.50
0.25
0.00
A (n=15)
B (n=16)
C (n=14)
surface

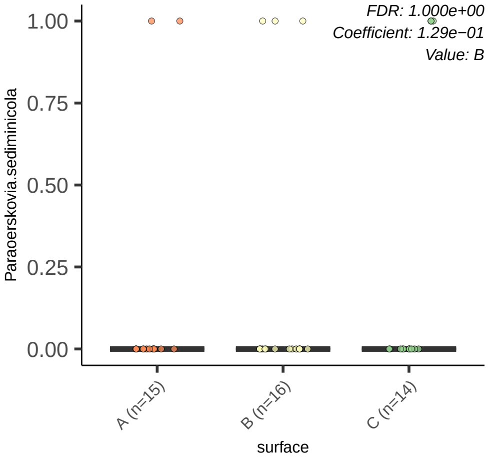
FDR: 1.000e+00
Coefficient: 1.29e−01
Value: B
Paraoerskovia.sedimenticola
1.00
0.75
0.50
0.25
0.00
A (n=15)
B (n=16)
C (n=14)
surface

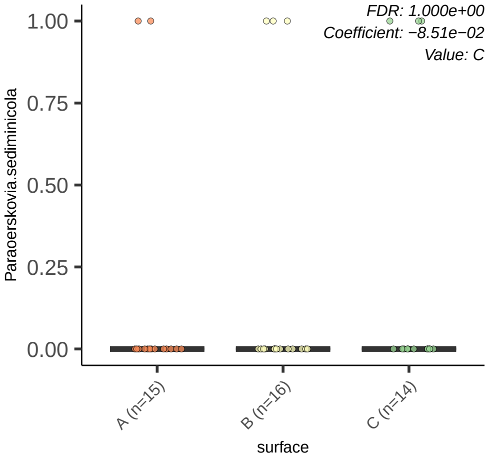
FDR: 1.000e+00
Coefficient: −8.51e−02
Value: C
Paraoerskovia.sedimінicola
1.00
0.75
0.50
0.25
0.00
A (n=15)
B (n=16)
C (n=14)
surface

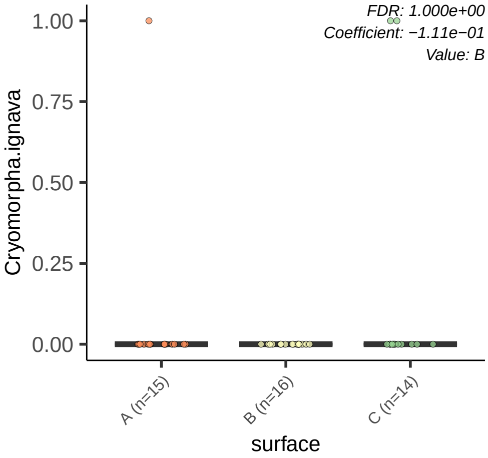

FDR: 1.000e+00
Coefficient: −1.11e−01
Value: B
Cryomorpha.ignava
1.00
0.75
0.50
0.25
0.00
A (n=15)
B (n=16)
C (n=14)
surface

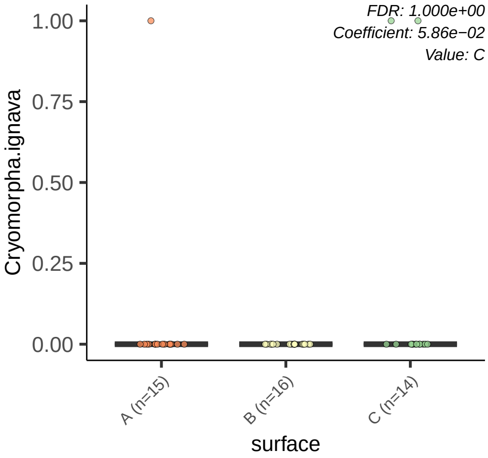

FDR: 1.000e+00
Coefficient: 5.86e−02
Value: C
Cryomorpha.ignava
1.00
0.75
0.50
0.25
0.00
A (n=15)
B (n=16)
C (n=14)
surface

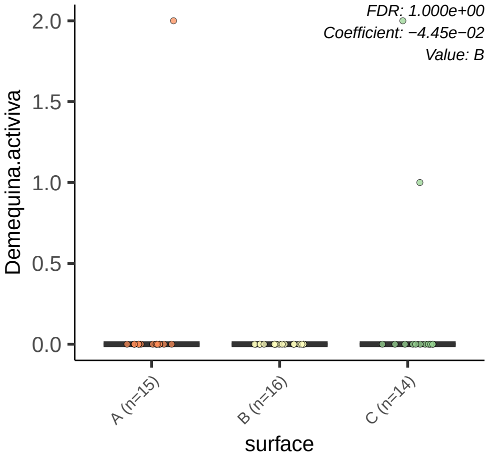

FDR: 1.000e+00
Coefficient: −4.45e−02
Value: B
Demequina.activiva
2.0
1.5
1.0
0.5
0.0
A (n=15)
B (n=16)
C (n=14)
surface

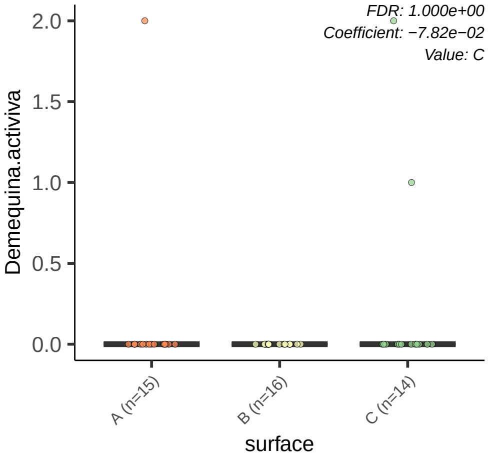
FDR: 1.000e+00
Coefficient: −7.82e−02
Value: C
Demequina.activiva
2.0
1.5
1.0
0.5
0.0
A (n=15)
B (n=16)
C (n=14)
surface

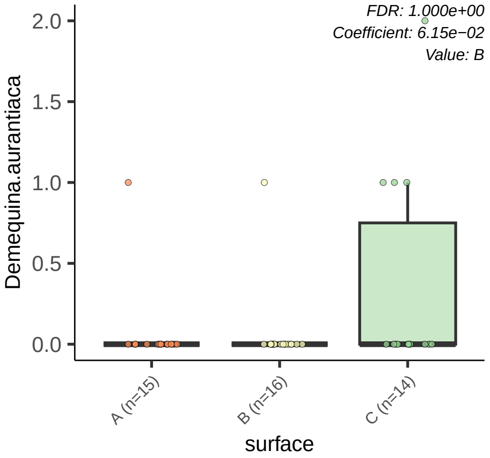

FDR: 1.000e+00
Coefficient: 6.15e−02
Value: B
Demequina.aurantiaca
2.0
1.5
1.0
0.5
0.0
A (n=15)
B (n=16)
C (n=14)
surface

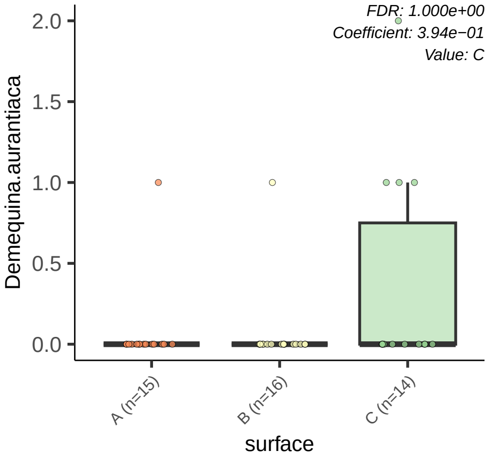
FDR: 1.000e+00
Coefficient: 3.94e−01
Value: C
Demequina.aurantiaca
2.0
1.5
1.0
0.5
0.0
A (n=15)
B (n=16)
C (n=14)
surface

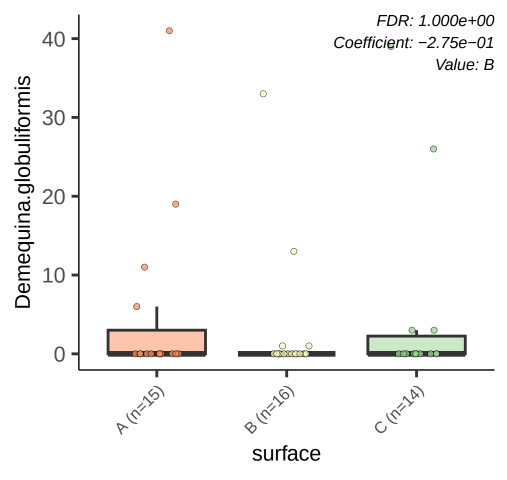

FDR: 1.000e+00
Coefficient: −2.75e−01
Value: B
Demequina.globuliformis
40
30
20
10
0
A (n=15)
B (n=16)
C (n=14)
surface

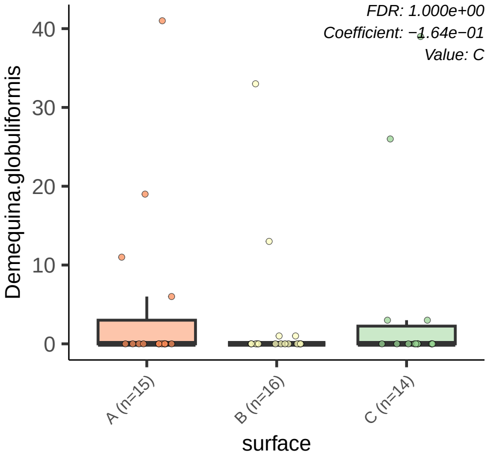
FDR: 1.000e+00
Coefficient: −1.64e−01
Value: C
Demequina.globuliformis
40
30
20
10
0
A (n=15)
B (n=16)
C (n=14)
surface

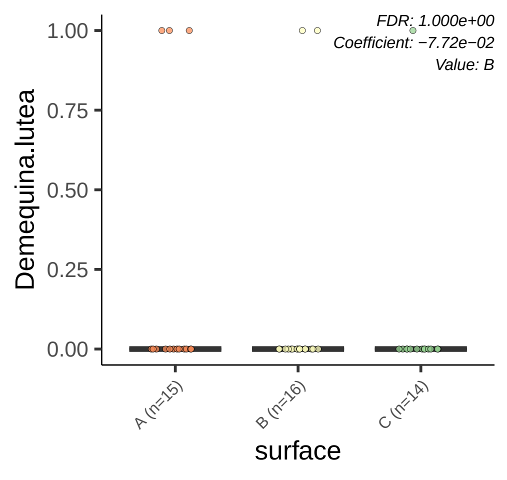
FDR: 1.000e+00
Coefficient: −7.72e−02
Value: B
Demequina.lutea
1.00
0.75
0.50
0.25
0.00
A (n=15)
B (n=16)
C (n=14)
surface

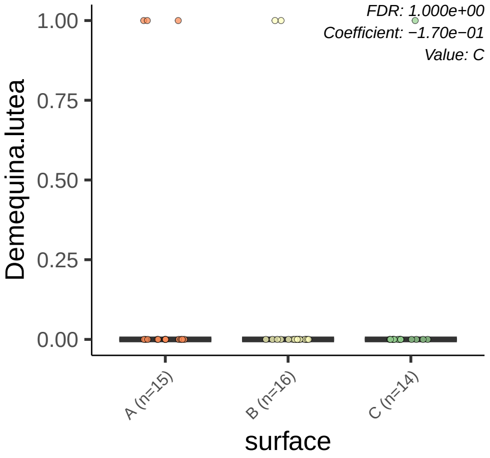

FDR: 1.000e+00
Coefficient: −1.70e−01
Value: C
Demequina.lutea
1.00
0.75
0.50
0.25
0.00
A (n=15)
B (n=16)
C (n=14)
surface

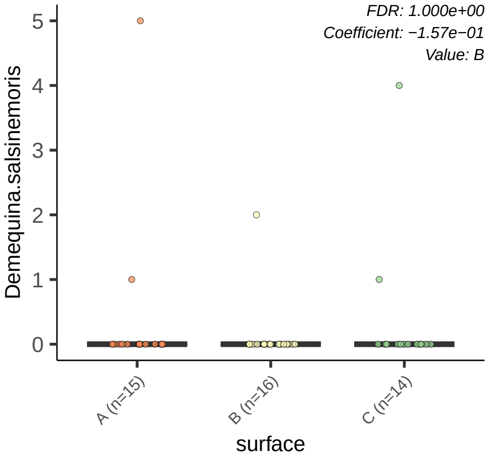

FDR: 1.000e+00
Coefficient: −1.57e−01
Value: B
Demequina.salsinemoris
0
1
2
3
4
5
A (n=15)
B (n=16)
C (n=14)
surface

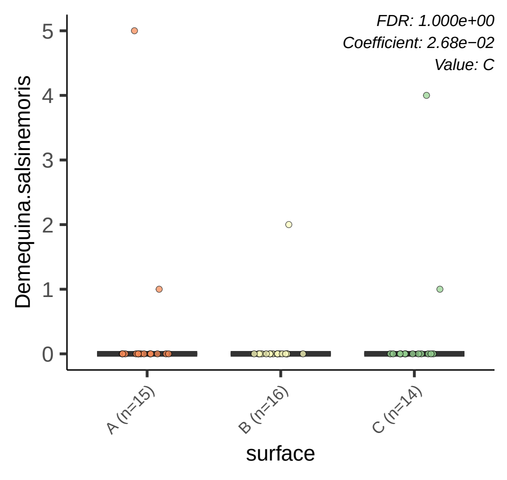
FDR: 1.000e+00
Coefficient: 2.68e−02
Value: C
Demequina.salsinemoris
0
1
2
3
4
5
A (n=15)
B (n=16)
C (n=14)
surface

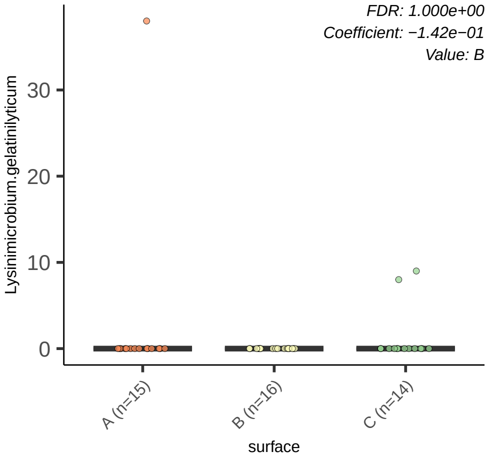

FDR: 1.000e+00
Coefficient: −1.42e−01
Value: B
Lysinimicrobium.gelatinilyticum
0
10
20
30
A (n=15)
B (n=16)
C (n=14)
surface

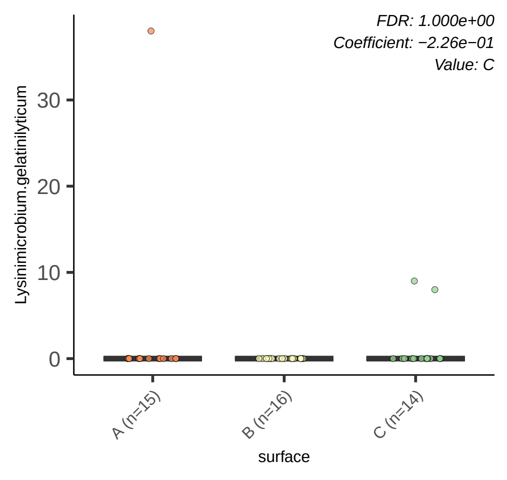
FDR: 1.000e+00
Coefficient: −2.26e−01
Value: C
Lysinimicrobium.gelatinilyticum
30
20
10
0
A (n=15)
B (n=16)
C (n=14)
surface

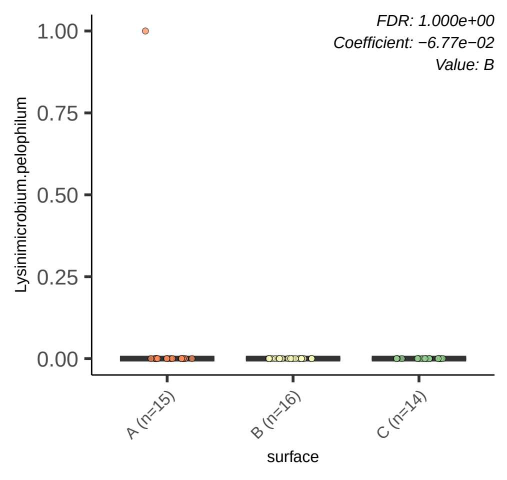

FDR: 1.000e+00
Coefficient: −6.77e−02
Value: B
Lysinimicrobium.pelophilum
1.00
0.75
0.50
0.25
0.00
A (n=15)
B (n=16)
C (n=14)
surface

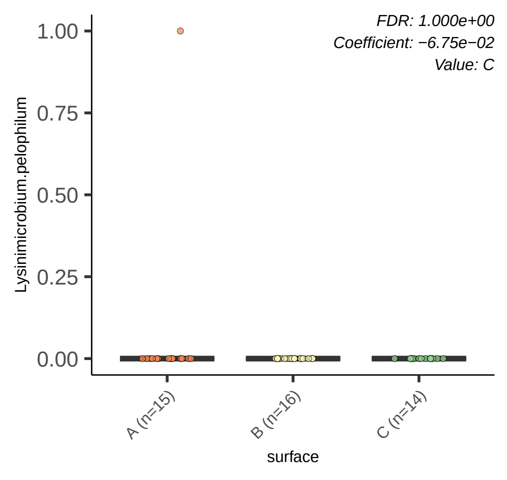
FDR: 1.000e+00
Coefficient: −6.75e−02
Value: C
1.00
0.75
0.50
0.25
0.00
Lysinimicrobium.pelophilum
A (n=15)
B (n=16)
C (n=14)
surface

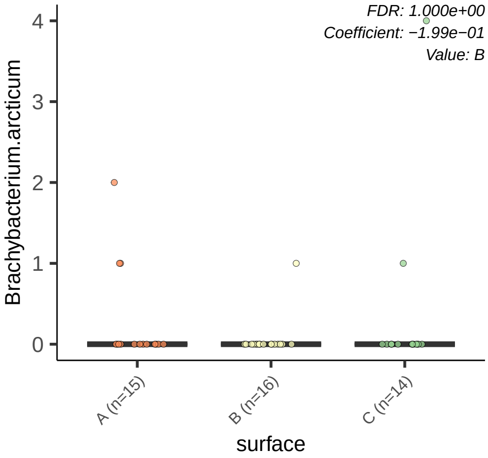
FDR: 1.000e+00
Coefficient: −1.99e−01
Value: B
Brachybacterium.arcticum
4
3
2
1
0
A (n=15)
B (n=16)
C (n=14)
surface

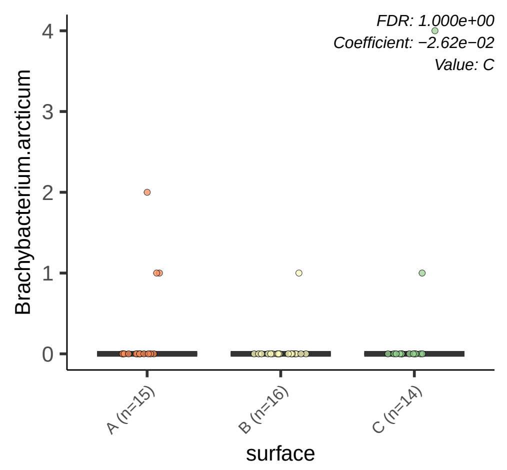
FDR: 1.000e+00
Coefficient: −2.62e−02
Value: C
Brachybacterium.arcticum
0
1
2
3
4
A (n=15)
B (n=16)
C (n=14)
surface

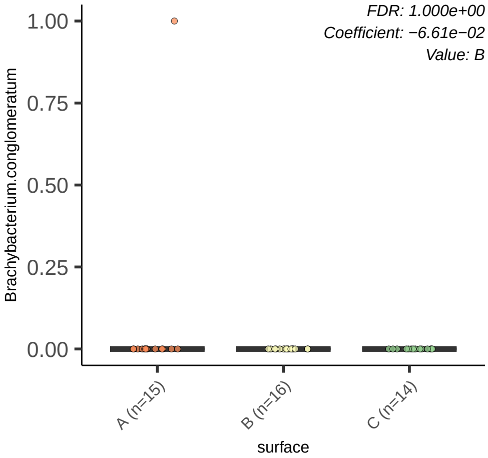
FDR: 1.000e+00
Coefficient: −6.61e−02
Value: B
1.00
0.75
0.50
0.25
0.00
Brachybacterium.conglomeratum
A (n=15)
B (n=16)
C (n=14)
surface

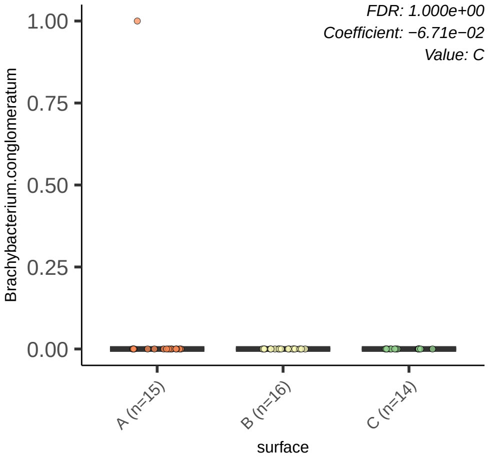
FDR: 1.000e+00
Coefficient: −6.71e−02
Value: C
Brachybacterium.conglomeratum
1.00
0.75
0.50
0.25
0.00
A (n=15)
B (n=16)
C (n=14)
surface

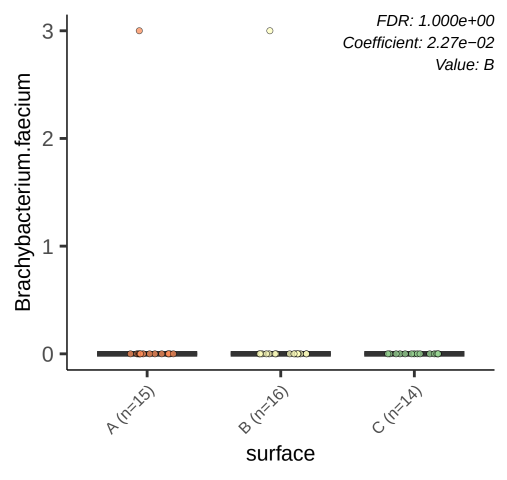
FDR: 1.000e+00
Coefficient: 2.27e−02
Value: B
Brachybacterium.faecium
0
1
2
3
A (n=15)
B (n=16)
C (n=14)
surface

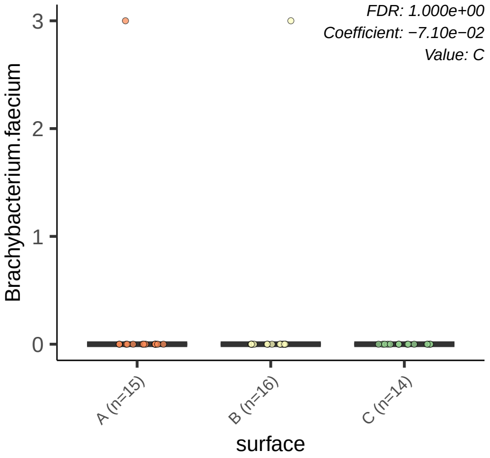

FDR: 1.000e+00
Coefficient: −7.10e−02
Value: C
Brachybacterium.faecium
0
1
2
3
A (n=15)
B (n=16)
C (n=14)
surface

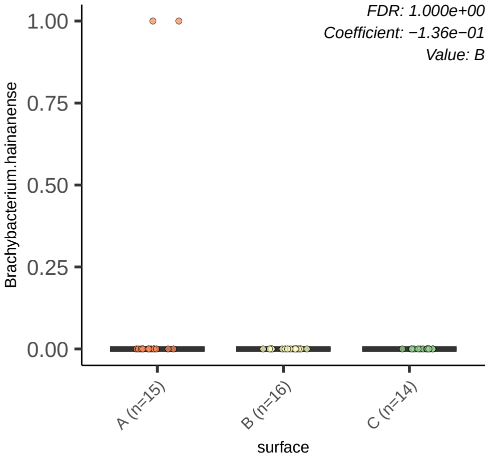
FDR: 1.000e+00
Coefficient: −1.36e−01
Value: B
Brachybacterium.hainanense
1.00
0.75
0.50
0.25
0.00
A (n=15)
B (n=16)
C (n=14)
surface

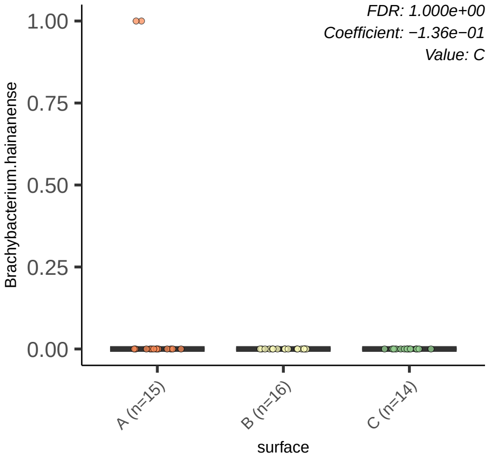
FDR: 1.000e+00
Coefficient: −1.36e−01
Value: C
Brachybacterium.hainanense
1.00
0.75
0.50
0.25
0.00
A (n=15)
B (n=16)
C (n=14)
surface

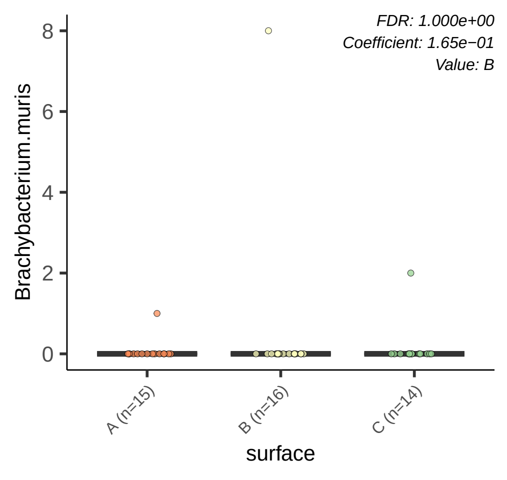
FDR: 1.000e+00
Coefficient: 1.65e−01
Value: B
Brachybacterium.muris
0
2
4
6
8
A (n=15)
B (n=16)
C (n=14)
surface

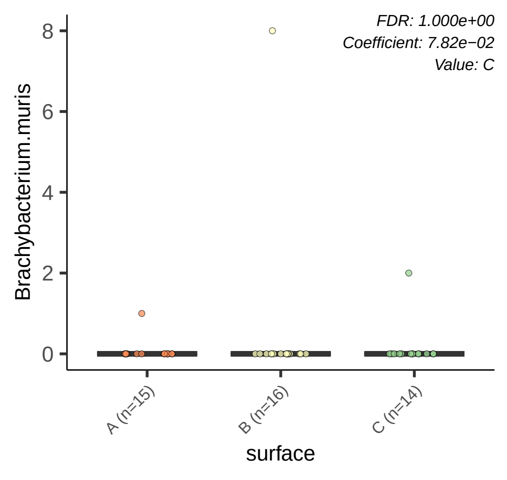
FDR: 1.000e+00
Coefficient: 7.82e−02
Value: C
Brachybacterium.muris
0
2
4
6
8
A (n=15)
B (n=16)
C (n=14)
surface

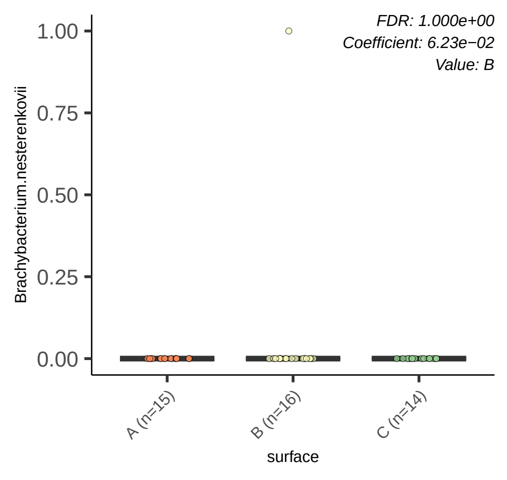

FDR: 1.000e+00
Coefficient: 6.23e−02
Value: B
1.00
0.75
0.50
0.25
0.00
Brachybacterium.nesterenkovii
A (n=15)
B (n=16)
C (n=14)
surface

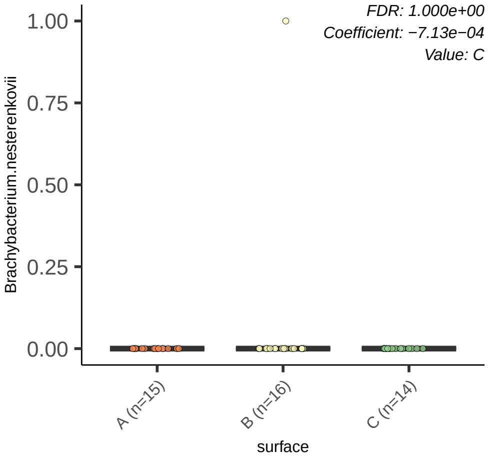
FDR: 1.000e+00
Coefficient: −7.13e−04
Value: C
Brachybacterium.nesterenkovii
1.00
0.75
0.50
0.25
0.00
A (n=15)
B (n=16)
C (n=14)
surface

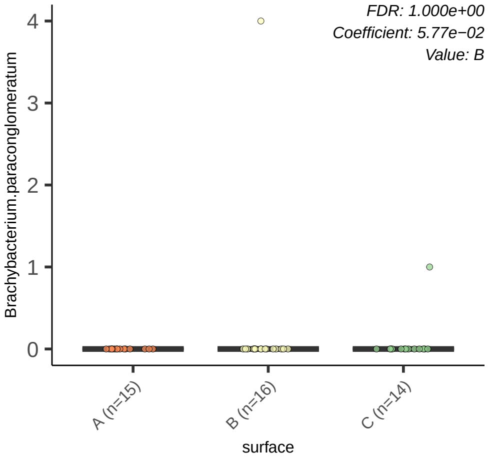

FDR: 1.000e+00
Coefficient: 5.77e−02
Value: B
Brachybacterium.paraconglomeratum
4
3
2
1
0
A (n=15)
B (n=16)
C (n=14)
surface

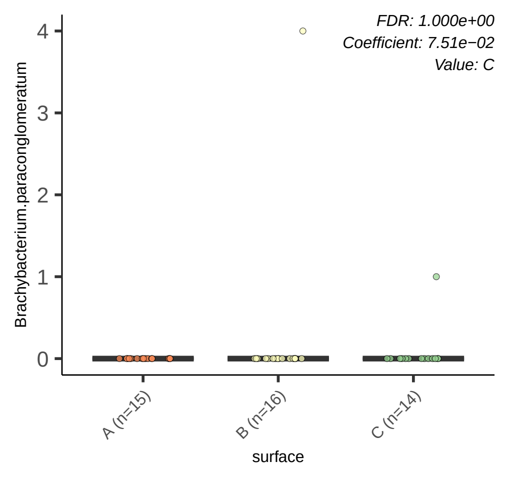

FDR: 1.000e+00
Coefficient: 7.51e−02
Value: C
Brachybacterium.paraconglomeratum
0
1
2
3
4
A (n=15)
B (n=16)
C (n=14)
surface

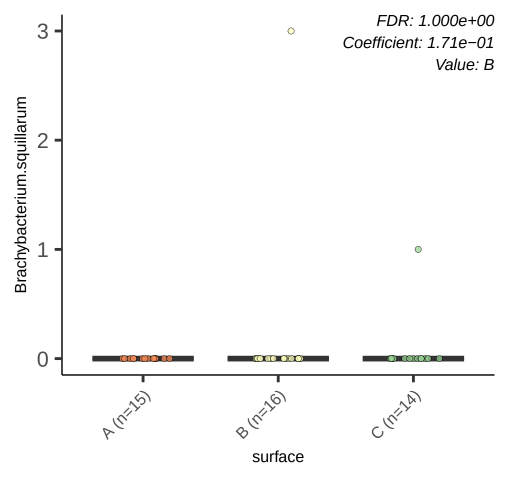
FDR: 1.000e+00
Coefficient: 1.71e−01
Value: B
Brachybacterium.squillarum
0
1
2
3
A (n=15)
B (n=16)
C (n=14)
surface

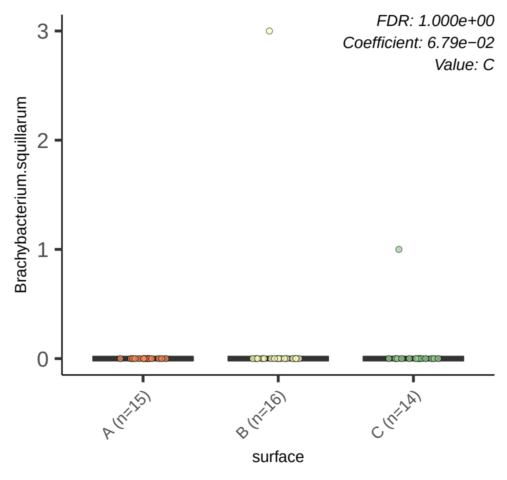
FDR: 1.000e+00
Coefficient: 6.79e−02
Value: C
Brachybacterium.squillarum
3
2
1
0
A (n=15)
B (n=16)
C (n=14)
surface

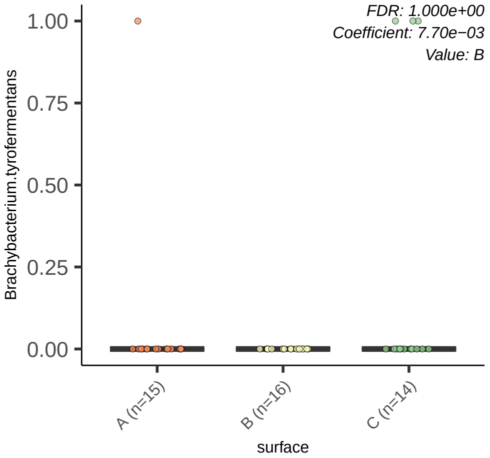

FDR: 1.000e+00
Coefficient: 7.70e−03
Value: B
Brachybacterium.tyrofermentans
1.00
0.75
0.50
0.25
0.00
A (n=15)
B (n=16)
C (n=14)
surface

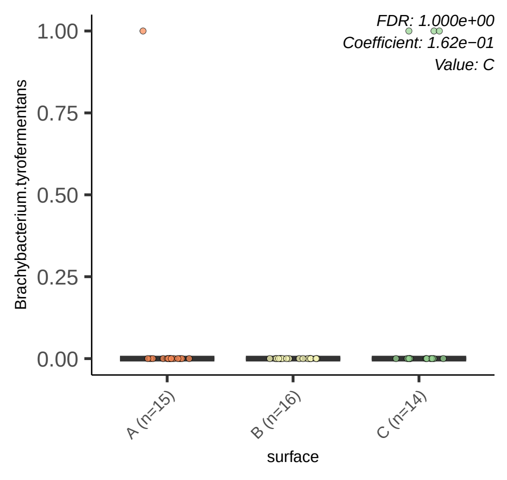
FDR: 1.000e+00
Coefficient: 1.62e−01
Value: C
Brachybacterium.tyrofermentans
1.00
0.75
0.50
0.25
0.00
A (n=15)
B (n=16)
C (n=14)
surface

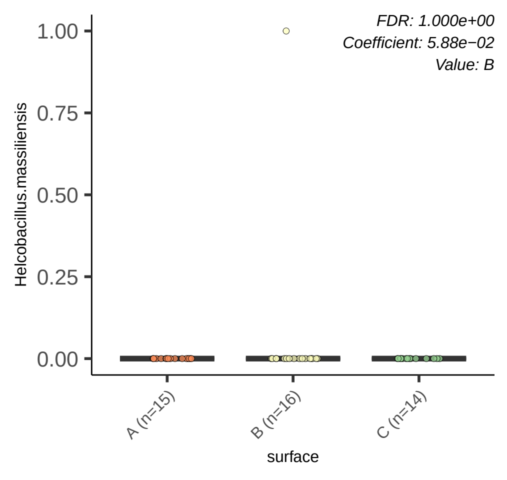

FDR: 1.000e+00
Coefficient: 5.88e−02
Value: B
1.00
0.75
0.50
0.25
0.00
Helcobacillus.massiliensis
A (n=15)
B (n=16)
C (n=14)
surface

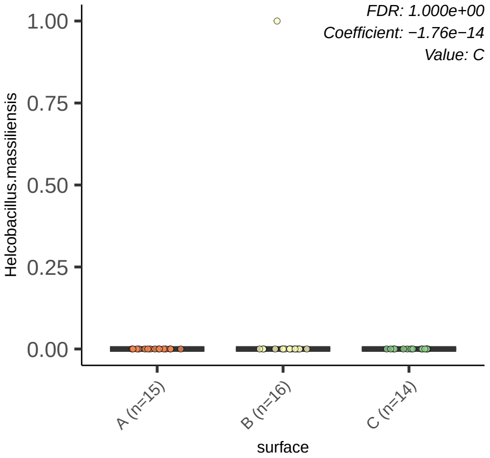
FDR: 1.000e+00
Coefficient: −1.76e−14
Value: C
Helcobacillus.massiliensis
1.00
0.75
0.50
0.25
0.00
A (n=15)
B (n=16)
C (n=14)
surface

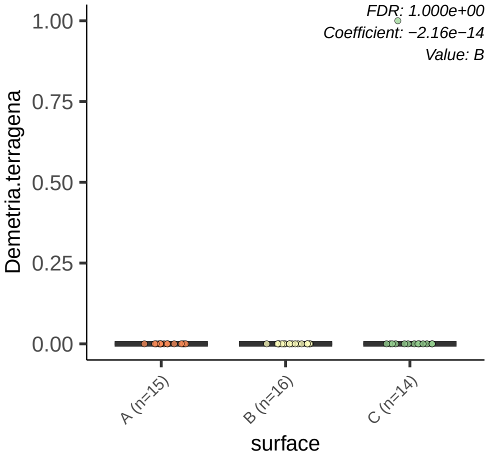

FDR: 1.000e+00
Coefficient: −2.16e−14
Value: B
Demetria.terragena
1.00
0.75
0.50
0.25
0.00
A (n=15)
B (n=16)
C (n=14)
surface

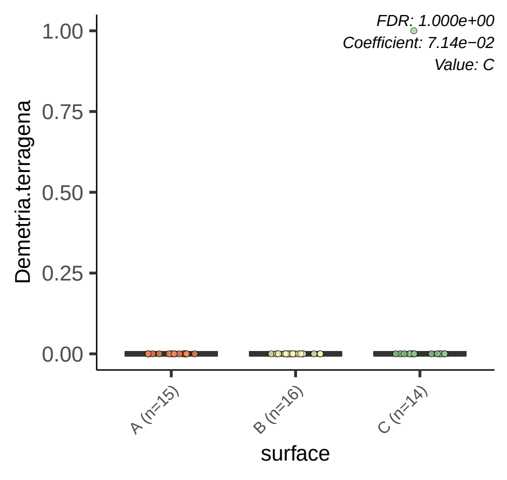

FDR: 1.000e+00
Coefficient: 7.14e−02
Value: C
Demetria.terragena
1.00
0.75
0.50
0.25
0.00
A (n=15)
B (n=16)
C (n=14)
surface

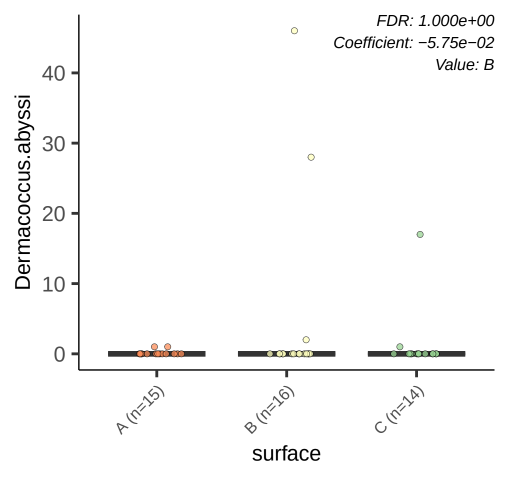

FDR: 1.000e+00
Coefficient: −5.75e−02
Value: B
Dermacoccus.abyssi
40
30
20
10
0
A (n=15)
B (n=16)
C (n=14)
surface

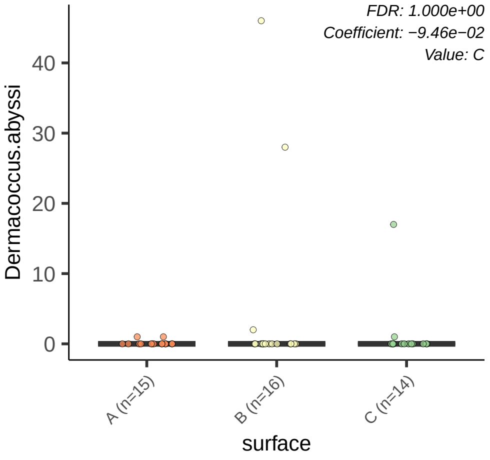

FDR: 1.000e+00
Coefficient: −9.46e−02
Value: C
Dermacoccus.abyssi
40
30
20
10
0
A (n=15)
B (n=16)
C (n=14)
surface

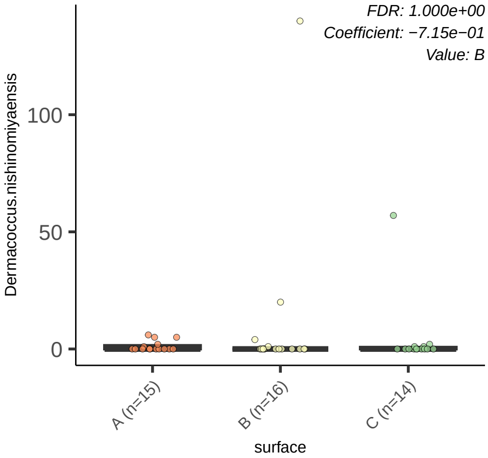
FDR: 1.000e+00
Coefficient: −7.15e−01
Value: B
Dermacoccus.nishinomiyaensis
100
50
0
A (n=15)
B (n=16)
C (n=14)
surface

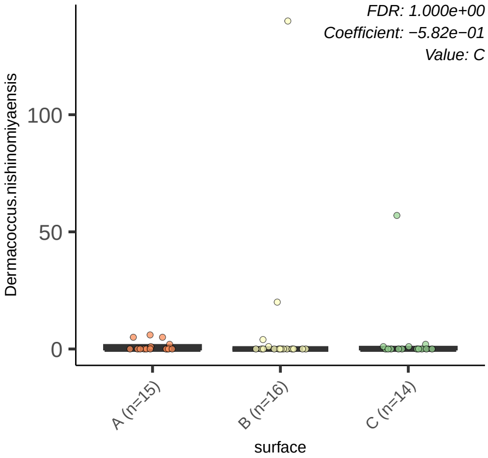
FDR: 1.000e+00
Coefficient: −5.82e−01
Value: C
Dermacoccus.nishinomiyaensis
100
50
0
A (n=15)
B (n=16)
C (n=14)
surface

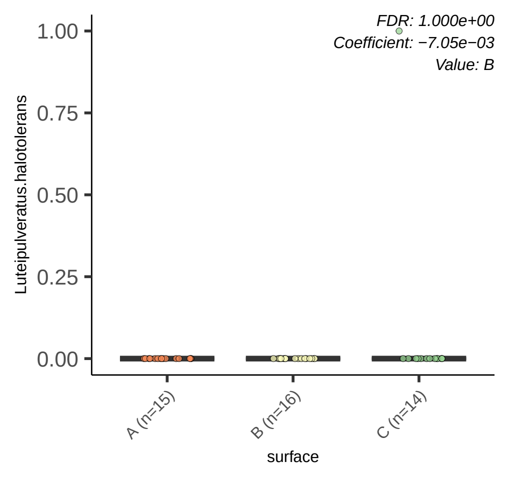

FDR: 1.000e+00
Coefficient: −7.05e−03
Value: B
Luteipulveratus.halotolerans
1.00
0.75
0.50
0.25
0.00
A (n=15)
B (n=16)
C (n=14)
surface

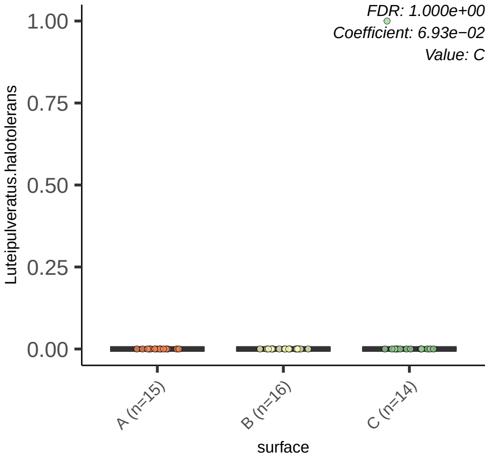
FDR: 1.000e+00
Coefficient: 6.93e−02
Value: C
Luteipulveratus.halotolerans
1.00
0.75
0.50
0.25
0.00
A (n=15)
B (n=16)
C (n=14)
surface

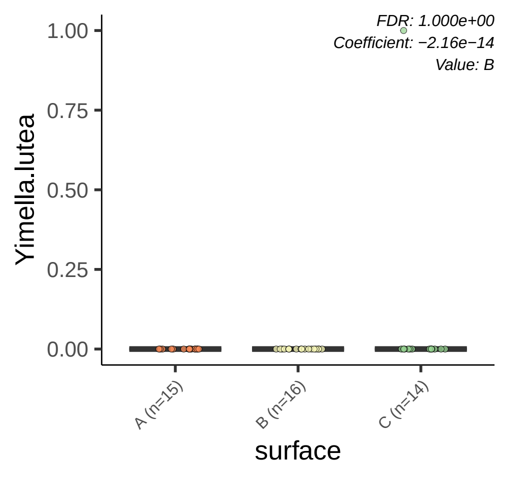
FDR: 1.000e+00
Coefficient: −2.16e−14
Value: B
Yimella.lutea
1.00
0.75
0.50
0.25
0.00
A (n=15)
B (n=16)
C (n=14)
surface

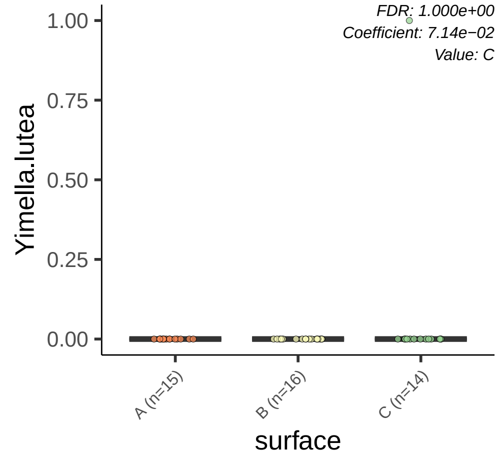

FDR: 1.000e+00
Coefficient: 7.14e−02
Value: C
Yimella.lutea
1.00
0.75
0.50
0.25
0.00
A (n=15)
B (n=16)
C (n=14)
surface

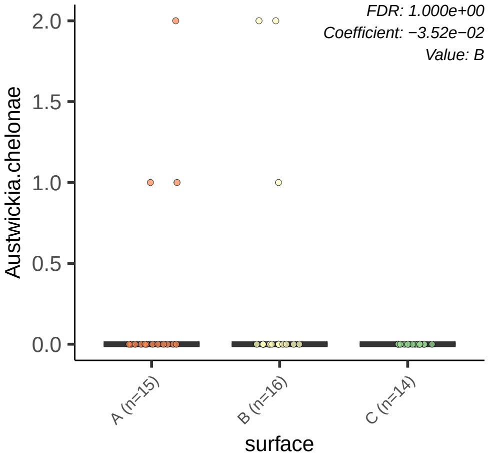
FDR: 1.000e+00
Coefficient: −3.52e−02
Value: B
Austwickia.chelonae
2.0
1.5
1.0
0.5
0.0
A (n=15)
B (n=16)
C (n=14)
surface

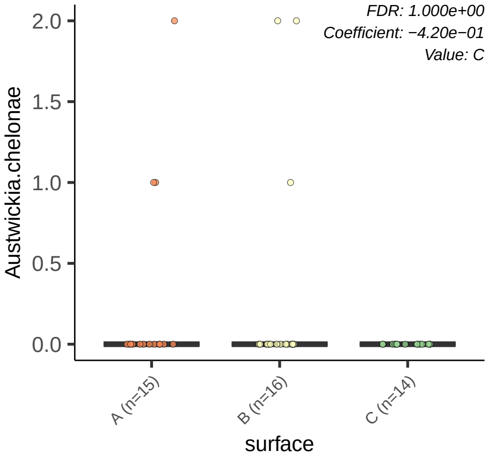

FDR: 1.000e+00
Coefficient: −4.20e−01
Value: C
Austwickia.chelonae
2.0
1.5
1.0
0.5
0.0
A (n=15)
B (n=16)
C (n=14)
surface

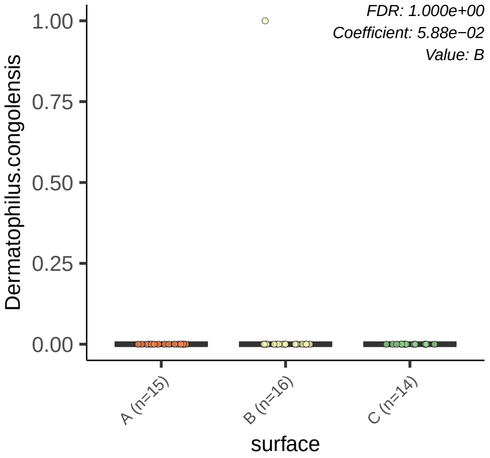
FDR: 1.000e+00
Coefficient: 5.88e−02
Value: B
Dermatophilus.congolensis
1.00
0.75
0.50
0.25
0.00
A (n=15)
B (n=16)
C (n=14)
surface

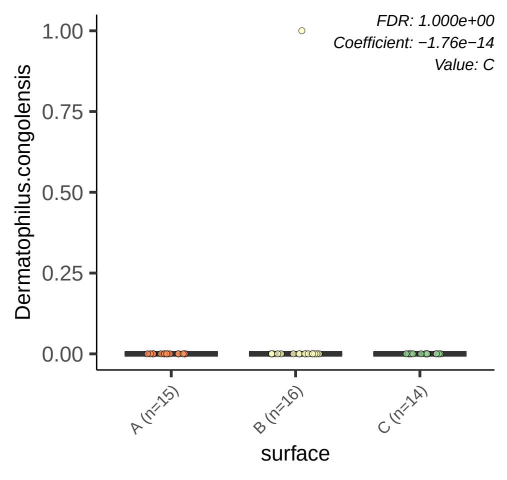

FDR: 1.000e+00
Coefficient: −1.76e−14
Value: C
Dermatophilus.congolensis
1.00
0.75
0.50
0.25
0.00
A (n=15)
B (n=16)
C (n=14)
surface

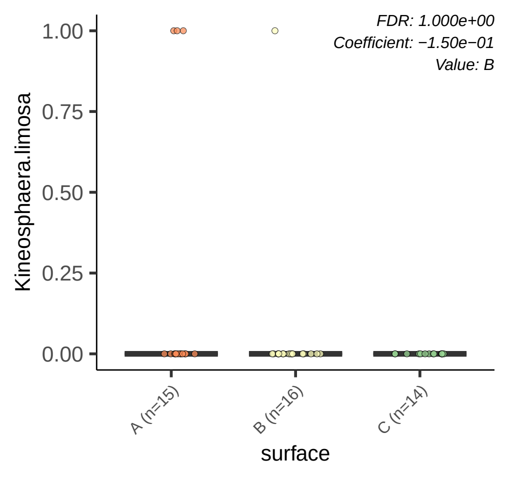
FDR: 1.000e+00
Coefficient: −1.50e−01
Value: B
Kineosphaera.limosa
1.00
0.75
0.50
0.25
0.00
A (n=15)
B (n=16)
C (n=14)
surface

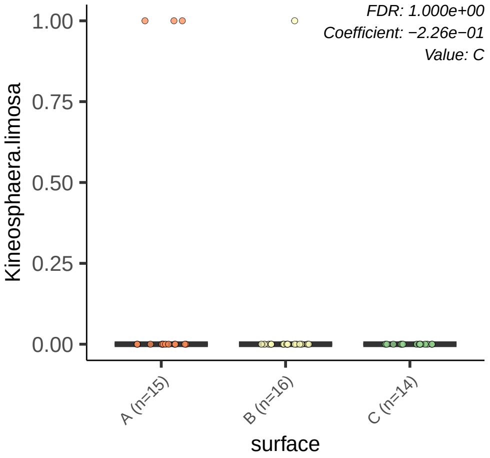
FDR: 1.000e+00
Coefficient: −2.26e−01
Value: C
Kineosphaera.limosa
1.00
0.75
0.50
0.25
0.00
A (n=15)
B (n=16)
C (n=14)
surface

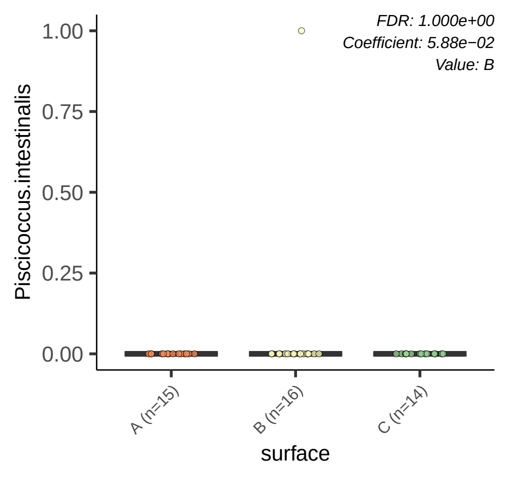

FDR: 1.000e+00
Coefficient: 5.88e−02
Value: B
Piscicoccus.intestinalis
1.00
0.75
0.50
0.25
0.00
A (n=15)
B (n=16)
C (n=14)
surface

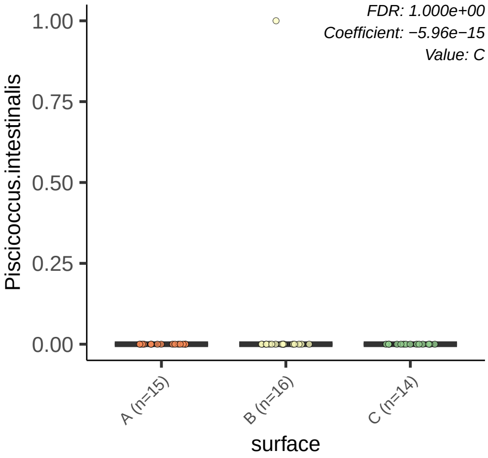
FDR: 1.000e+00
Coefficient: −5.96e−15
Value: C
Piscicoccus.intestinalis
1.00
0.75
0.50
0.25
0.00
A (n=15)
B (n=16)
C (n=14)
surface

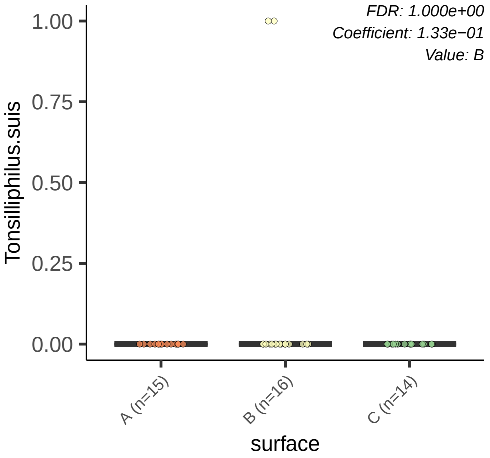
FDR: 1.000e+00
Coefficient: 1.33e−01
Value: B
Tonsilliphilus.suis
1.00
0.75
0.50
0.25
0.00
A (n=15)
B (n=16)
C (n=14)
surface

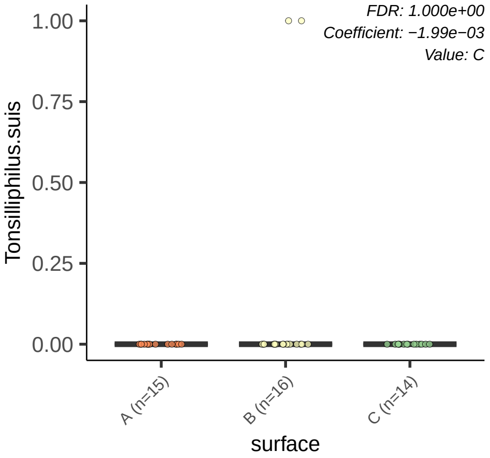
FDR: 1.000e+00
Coefficient: −1.99e−03
Value: C
Tonsilliphilus.suis
1.00
0.75
0.50
0.25
0.00
A (n=15)
B (n=16)
C (n=14)
surface

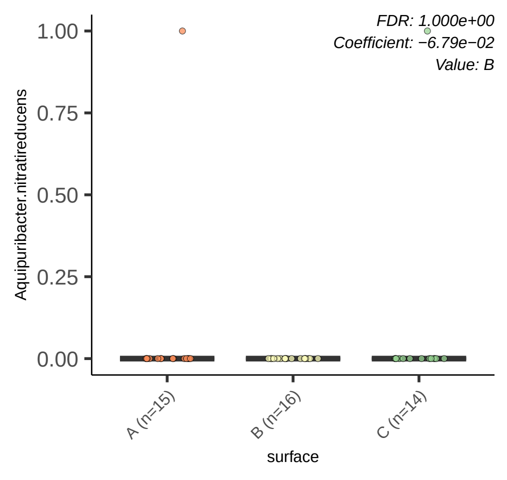
FDR: 1.000e+00
Coefficient: −6.79e−02
Value: B
Aquipuribacter.nitratireducens
1.00
0.75
0.50
0.25
0.00
A (n=15)
B (n=16)
C (n=14)
surface

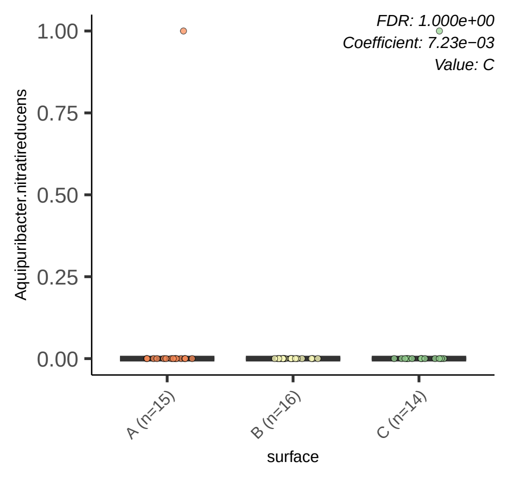
FDR: 1.000e+00
Coefficient: 7.23e−03
Value: C
Aquipuribacter.nitratireducens
1.00
0.75
0.50
0.25
0.00
A (n=15)
B (n=16)
C (n=14)
surface

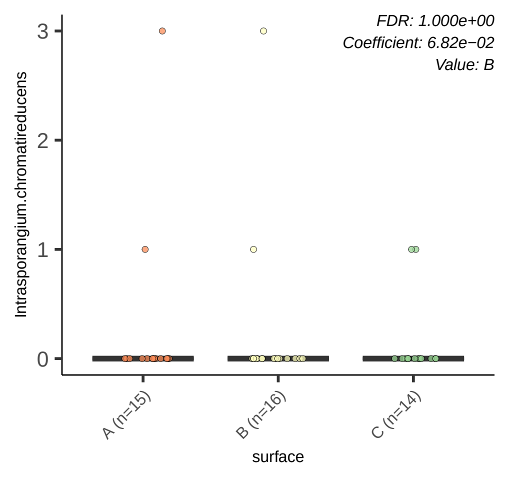

FDR: 1.000e+00
Coefficient: 6.82e−02
Value: B
Intrasporangium.chromatireducens
0
1
2
3
A (n=15)
B (n=16)
C (n=14)
surface

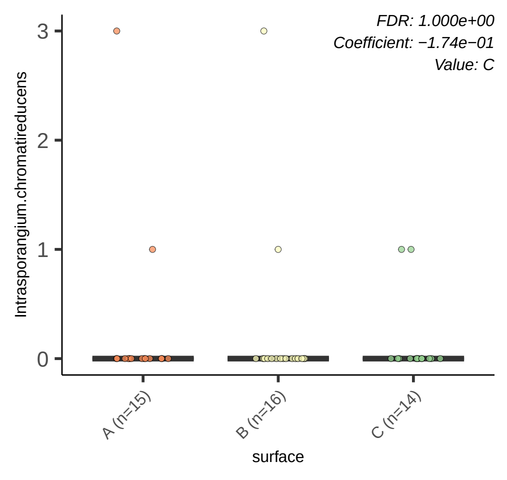

FDR: 1.000e+00
Coefficient: −1.74e−01
Value: C
Intrasporangium.chromatireducens
0
1
2
3
A (n=15)
B (n=16)
C (n=14)
surface

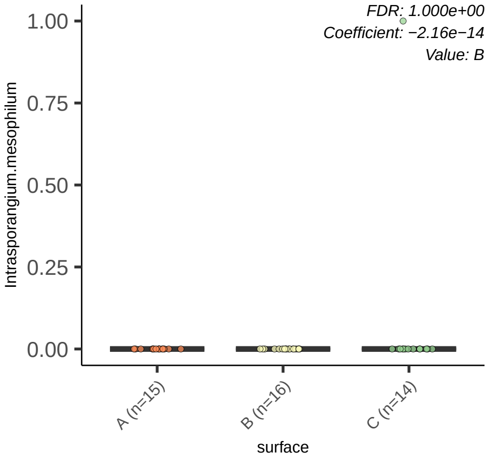
FDR: 1.000e+00
Coefficient: −2.16e−14
Value: B
Intrasporangium.mesophilum
1.00
0.75
0.50
0.25
0.00
A (n=15)
B (n=16)
C (n=14)
surface

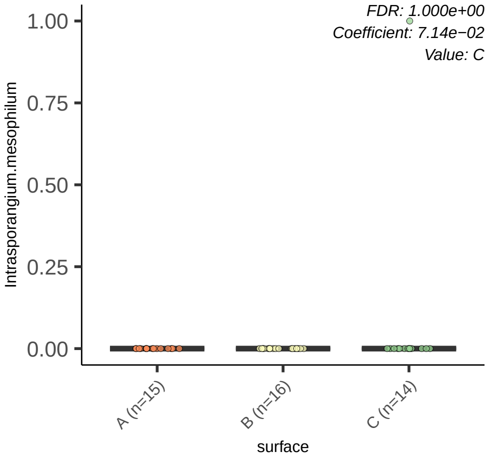
FDR: 1.000e+00
Coefficient: 7.14e−02
Value: C
1.00
0.75
0.50
0.25
0.00
Intrasporangium.mesophilum
A (n=15)
B (n=16)
C (n=14)
surface

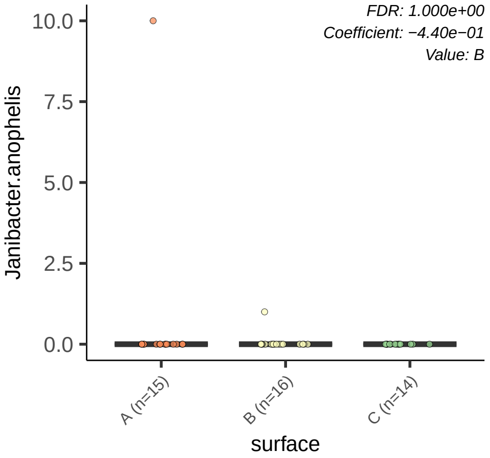
FDR: 1.000e+00
Coefficient: −4.40e−01
Value: B
Janibacter.anophelis
10.0
7.5
5.0
2.5
0.0
A (n=15)
B (n=16)
C (n=14)
surface

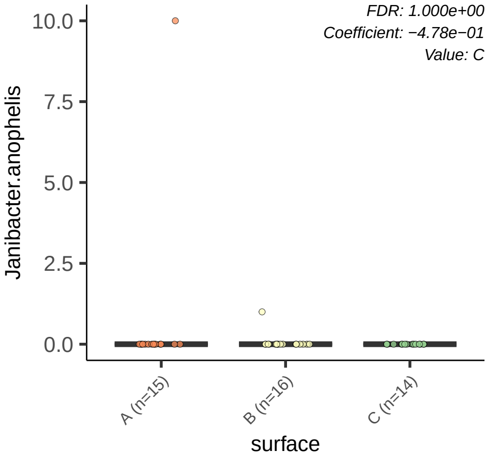

FDR: 1.000e+00
Coefficient: −4.78e−01
Value: C
Janibacter.anophelis
10.0
7.5
5.0
2.5
0.0
A (n=15)
B (n=16)
C (n=14)
surface

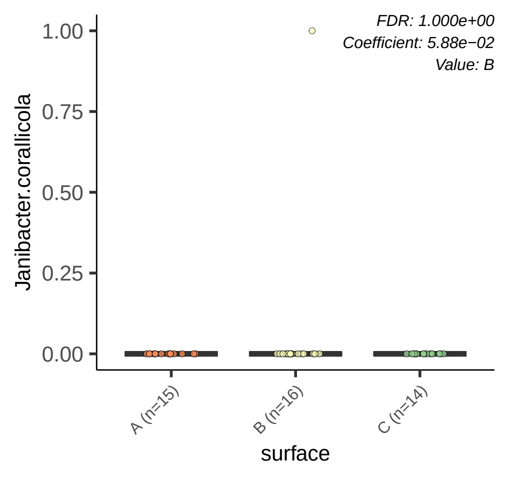
FDR: 1.000e+00
Coefficient: 5.88e−02
Value: B
Janibacter.corallicola
1.00
0.75
0.50
0.25
0.00
A (n=15)
B (n=16)
C (n=14)
surface

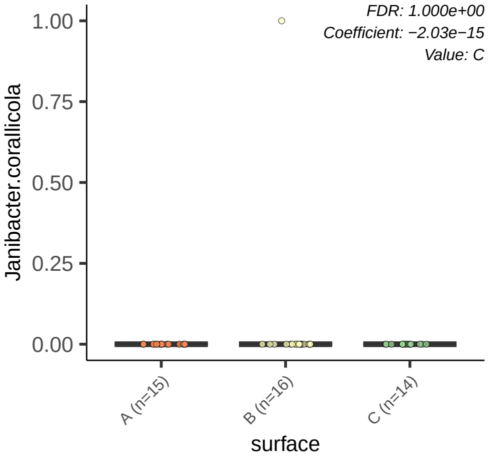
FDR: 1.000e+00
Coefficient: −2.03e−15
Value: C
Janibacter.corallicola
1.00
0.75
0.50
0.25
0.00
A (n=15)
B (n=16)
C (n=14)
surface

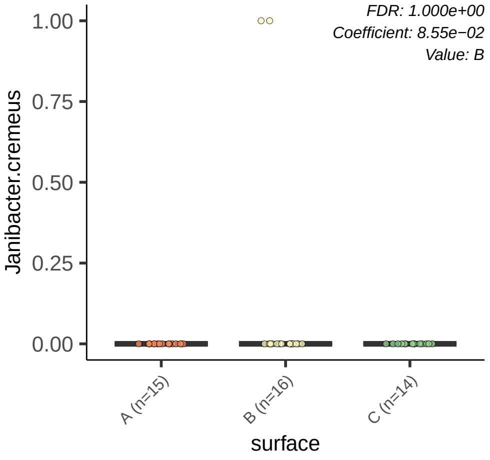

FDR: 1.000e+00
Coefficient: 8.55e−02
Value: B
Janibacter.cremeus
1.00
0.75
0.50
0.25
0.00
A (n=15)
B (n=16)
C (n=14)
surface

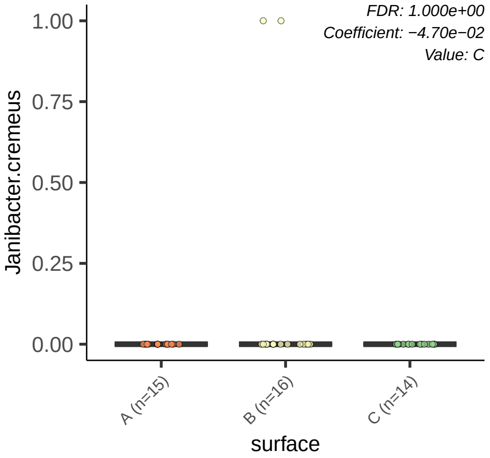
FDR: 1.000e+00
Coefficient: −4.70e−02
Value: C
Janibacter.cremeus
1.00
0.75
0.50
0.25
0.00
A (n=15)
B (n=16)
C (n=14)
surface

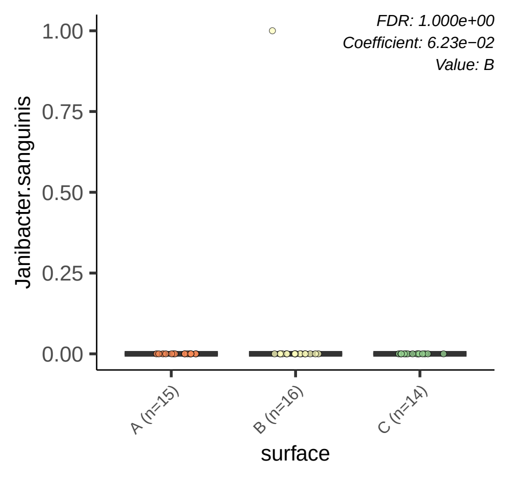

FDR: 1.000e+00
Coefficient: 6.23e−02
Value: B
Janibacter.sanguinis
1.00
0.75
0.50
0.25
0.00
A (n=15)
B (n=16)
C (n=14)
surface

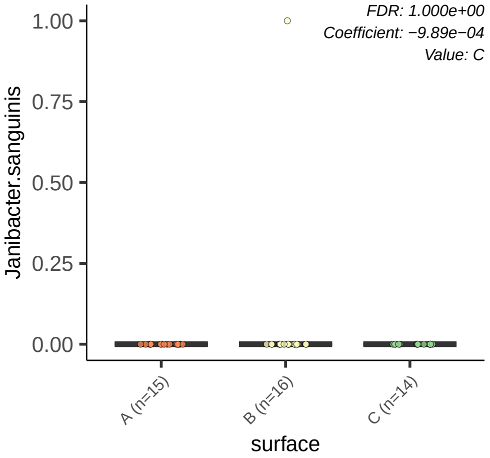

FDR: 1.000e+00
Coefficient: −9.89e−04
Value: C
Janibacter.sanguinis
1.00
0.75
0.50
0.25
0.00
A (n=15)
B (n=16)
C (n=14)
surface

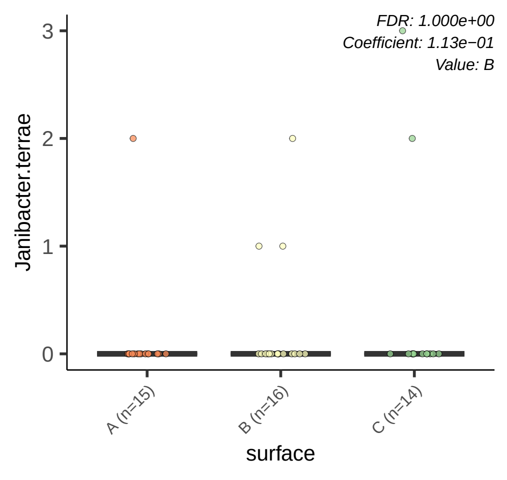
FDR: 1.000e+00
Coefficient: 1.13e−01
Value: B
Janibacter.terrae
0
1
2
3
A (n=15)
B (n=16)
C (n=14)
surface

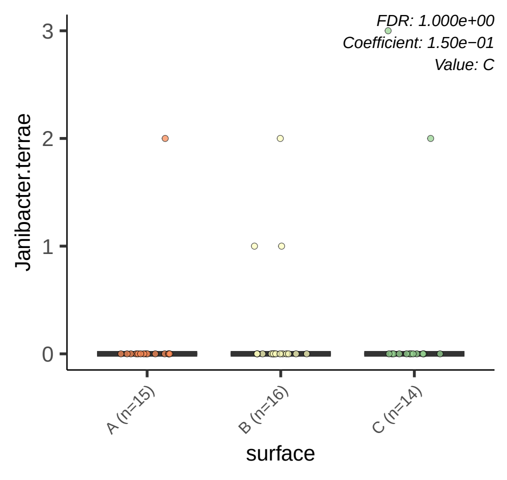
FDR: 1.000e+00
Coefficient: 1.50e−01
Value: C
Janibacter.terrae
0
1
2
3
A (n=15)
B (n=16)
C (n=14)
surface

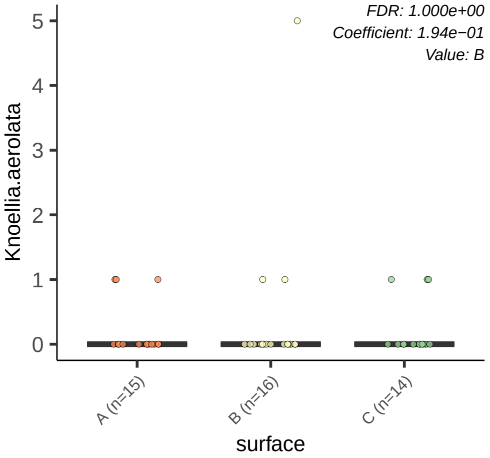

FDR: 1.000e+00
Coefficient: 1.94e−01
Value: B
Knoellia.aerolata
0
1
2
3
4
5
A (n=15)
B (n=16)
C (n=14)
surface

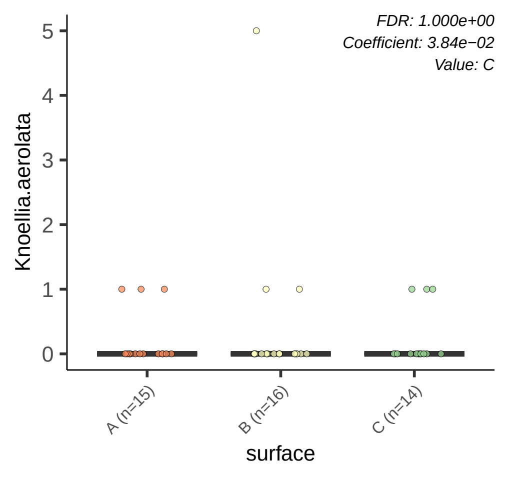

FDR: 1.000e+00
Coefficient: 3.84e−02
Value: C
Knoellia.aerolata
0
1
2
3
4
5
A (n=15)
B (n=16)
C (n=14)
surface

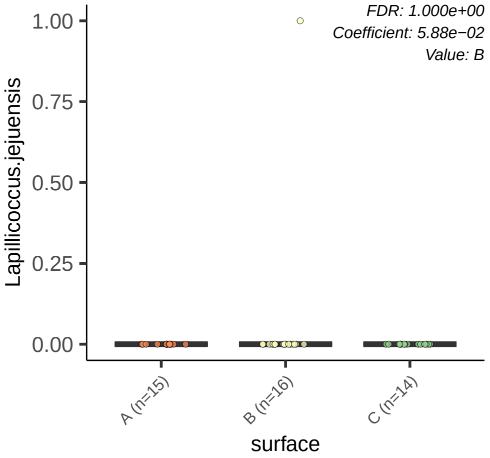

FDR: 1.000e+00
Coefficient: 5.88e−02
Value: B
Lapillicoccus.jejuensis
1.00
0.75
0.50
0.25
0.00
A (n=15)
B (n=16)
C (n=14)
surface

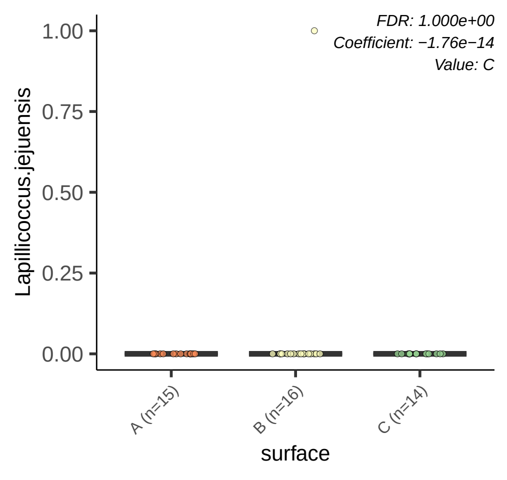

FDR: 1.000e+00
Coefficient: −1.76e−14
Value: C
Lapillicoccus.jejuensis
1.00
0.75
0.50
0.25
0.00
A (n=15)
B (n=16)
C (n=14)
surface

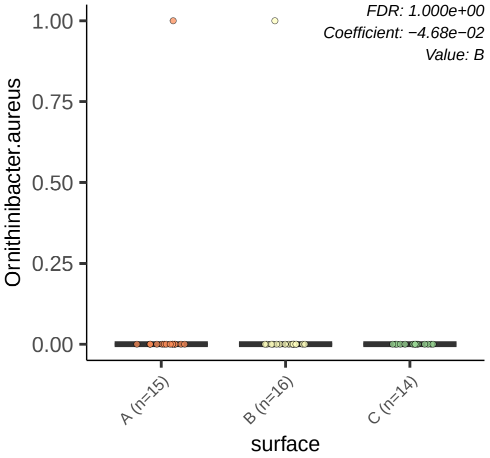
FDR: 1.000e+00
Coefficient: −4.68e−02
Value: B
Ornithinibacter.aureus
1.00
0.75
0.50
0.25
0.00
A (n=15)
B (n=16)
C (n=14)
surface

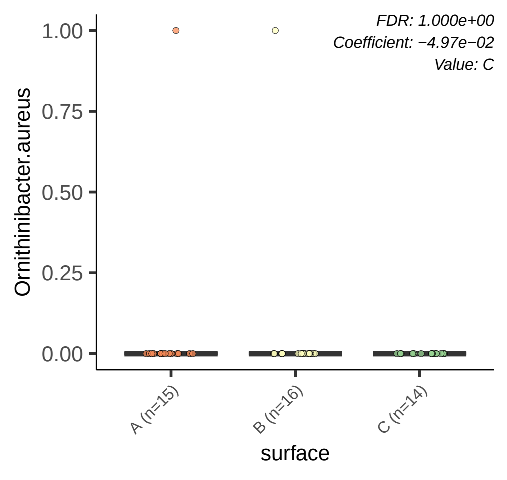
FDR: 1.000e+00
Coefficient: −4.97e−02
Value: C
Ornithinibacter.aureus
1.00
0.75
0.50
0.25
0.00
A (n=15)
B (n=16)
C (n=14)
surface

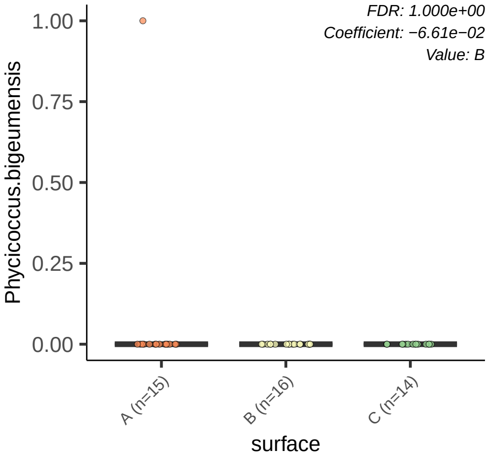

FDR: 1.000e+00
Coefficient: −6.61e−02
Value: B
Phycicoccus.bigeumensis
1.00
0.75
0.50
0.25
0.00
A (n=15)
B (n=16)
C (n=14)
surface

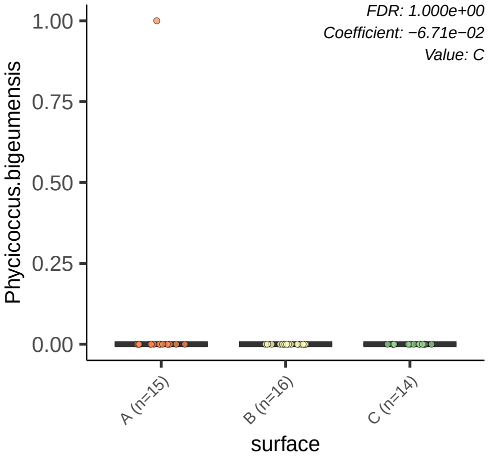

FDR: 1.000e+00
Coefficient: −6.71e−02
Value: C
Phycicoccus.bigeumensis
1.00
0.75
0.50
0.25
0.00
A (n=15)
B (n=16)
C (n=14)
surface

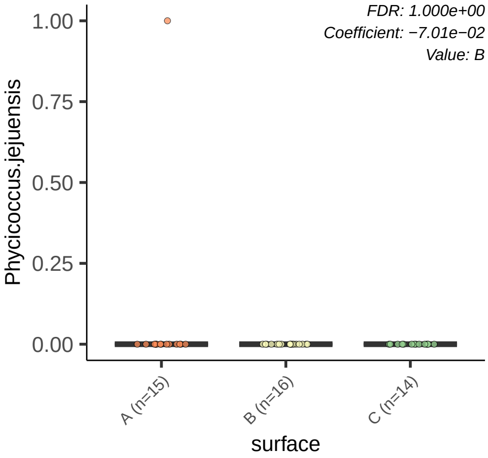
FDR: 1.000e+00
Coefficient: −7.01e−02
Value: B
Phycicoccus.jejuensis
1.00
0.75
0.50
0.25
0.00
A (n=15)
B (n=16)
C (n=14)
surface

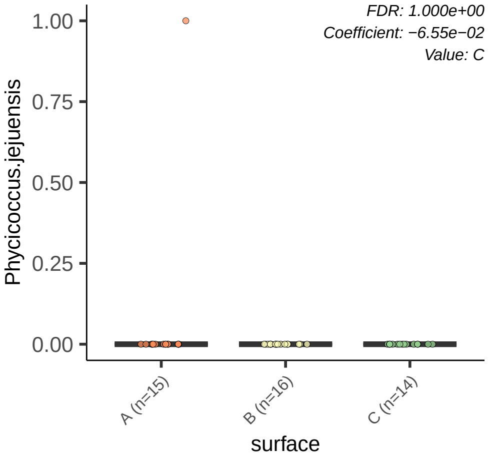
FDR: 1.000e+00
Coefficient: −6.55e−02
Value: C
Phycicoccus.jejuensis
1.00
0.75
0.50
0.25
0.00
A (n=15)
B (n=16)
C (n=14)
surface

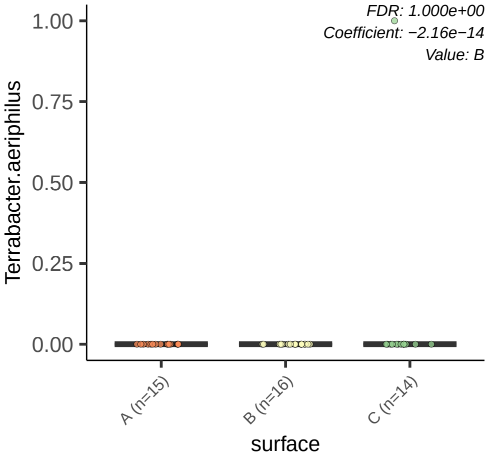
FDR: 1.000e+00
Coefficient: −2.16e−14
Value: B
Terrabacter.aeriphilus
1.00
0.75
0.50
0.25
0.00
A (n=15)
B (n=16)
C (n=14)
surface

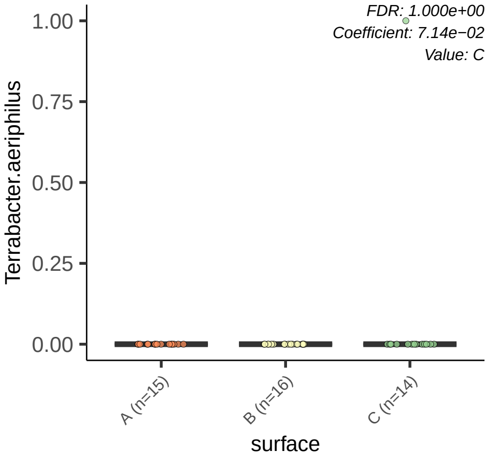

FDR: 1.000e+00
Coefficient: 7.14e−02
Value: C
Terrabacter.aeriphilus
1.00
0.75
0.50
0.25
0.00
A (n=15)
B (n=16)
C (n=14)
surface

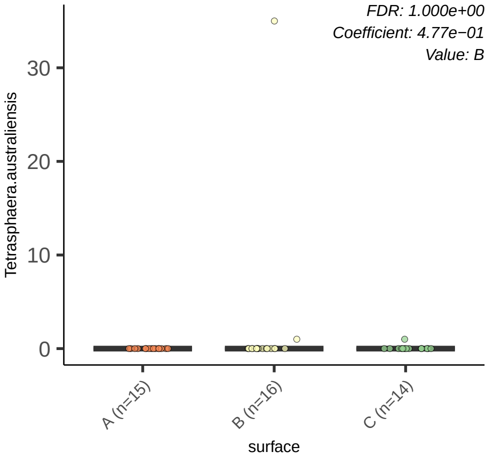

FDR: 1.000e+00
Coefficient: 4.77e−01
Value: B
Tetrasphaera.australiensis
30
20
10
0
A (n=15)
B (n=16)
C (n=14)
surface

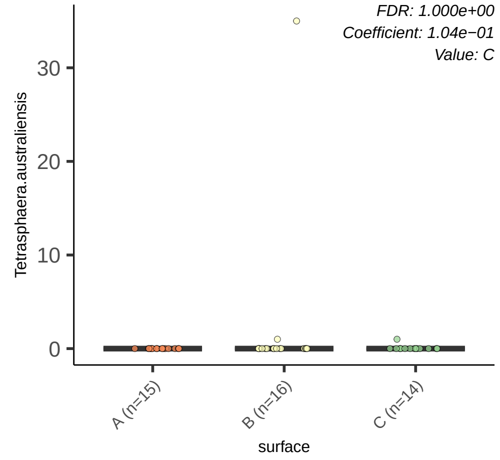

FDR: 1.000e+00
Coefficient: 1.04e−01
Value: C
Tetrasphaera.australiensis
30
20
10
0
A (n=15)
B (n=16)
C (n=14)
surface

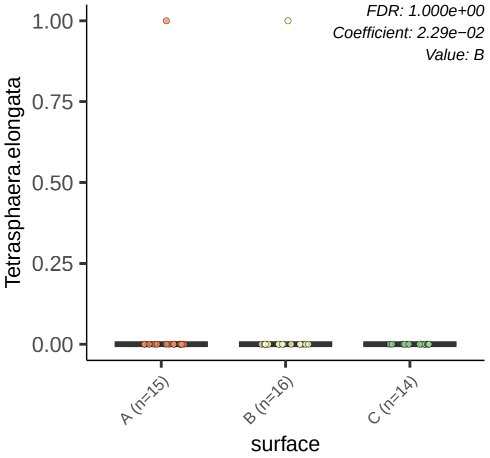

FDR: 1.000e+00
Coefficient: 2.29e−02
Value: B
Tetrasphaera.elongata
1.00
0.75
0.50
0.25
0.00
A (n=15)
B (n=16)
C (n=14)
surface

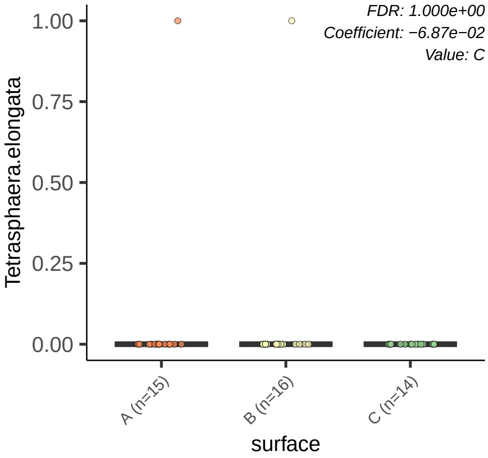

FDR: 1.000e+00
Coefficient: −6.87e−02
Value: C
Tetrasphaera.elongata
1.00
0.75
0.50
0.25
0.00
A (n=15)
B (n=16)
C (n=14)
surface

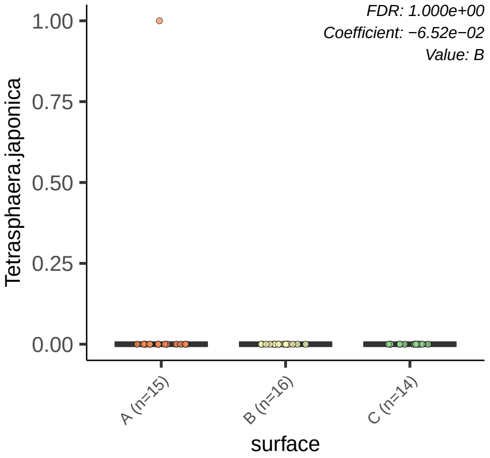

FDR: 1.000e+00
Coefficient: −6.52e−02
Value: B
Tetrasphaera.japonica
1.00
0.75
0.50
0.25
0.00
A (n=15)
B (n=16)
C (n=14)
surface

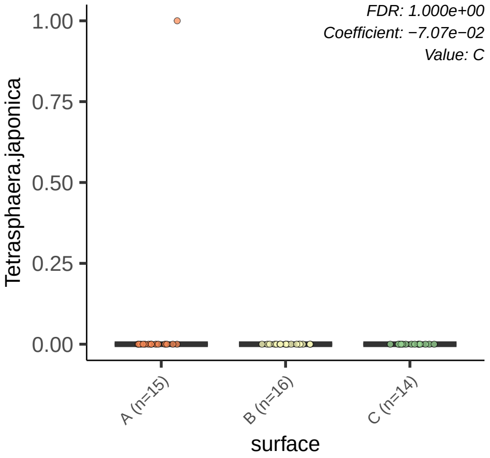
FDR: 1.000e+00
Coefficient: −7.07e−02
Value: C
Tetrasphaera.japonica
1.00
0.75
0.50
0.25
0.00
A (n=15)
B (n=16)
C (n=14)
surface

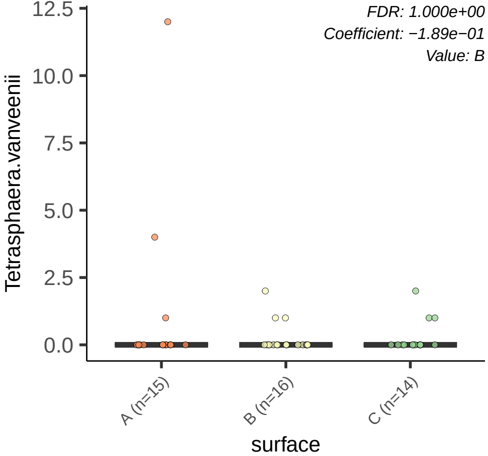

FDR: 1.000e+00
Coefficient: −1.89e−01
Value: B
Tetrasphaera.vanveenii
12.5
10.0
7.5
5.0
2.5
0.0
A (n=15)
B (n=16)
C (n=14)
surface

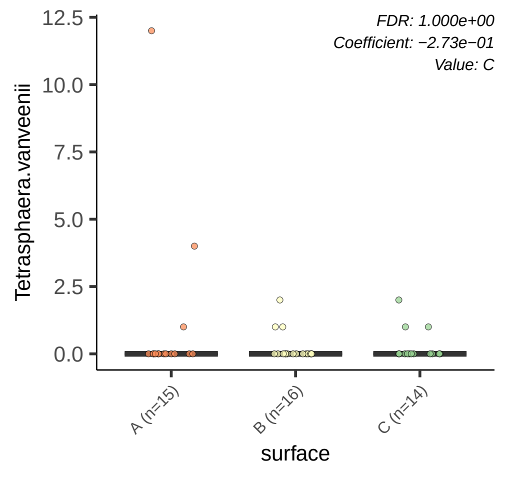
FDR: 1.000e+00
Coefficient: −2.73e−01
Value: C
Tetrasphaera.vanveenii
12.5
10.0
7.5
5.0
2.5
0.0
A (n=15)
B (n=16)
C (n=14)
surface

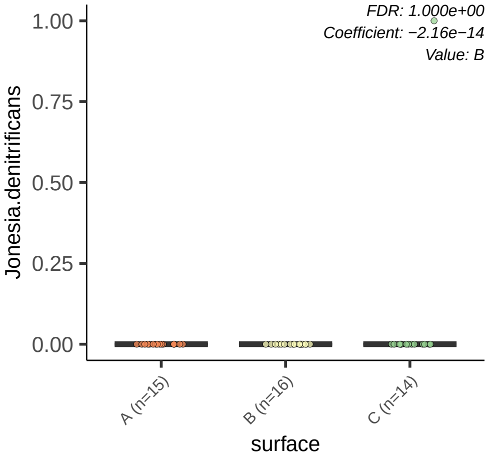

FDR: 1.000e+00
Coefficient: −2.16e−14
Value: B
Jonesia.denitrificans
1.00
0.75
0.50
0.25
0.00
A (n=15)
B (n=16)
C (n=14)
surface

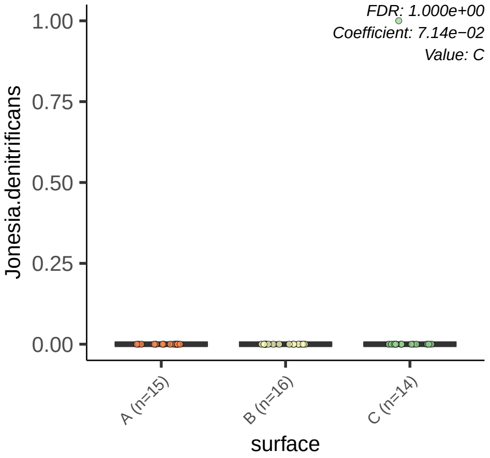

FDR: 1.000e+00
Coefficient: 7.14e−02
Value: C
Jonesia.denitrificans
1.00
0.75
0.50
0.25
0.00
A (n=15)
B (n=16)
C (n=14)
surface

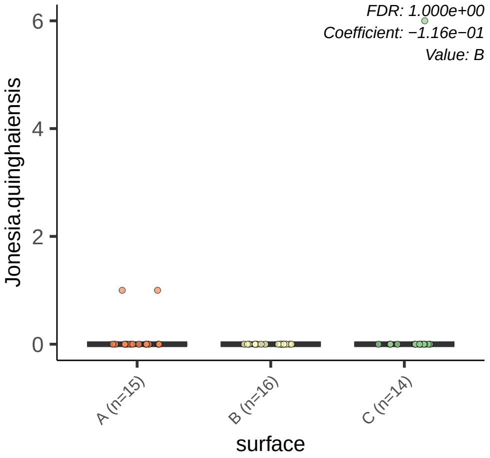

FDR: 1.000e+00
Coefficient: −1.16e−01
Value: B
6
4
2
0
Jonesia.quinghaiensis
A (n=15)
B (n=16)
C (n=14)
surface

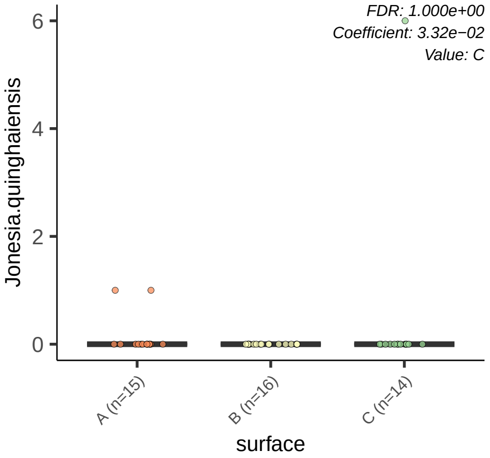
FDR: 1.000e+00
Coefficient: 3.32e−02
Value: C
Jonesia.quinghaiensis
0
2
4
6
A (n=15)
B (n=16)
C (n=14)
surface

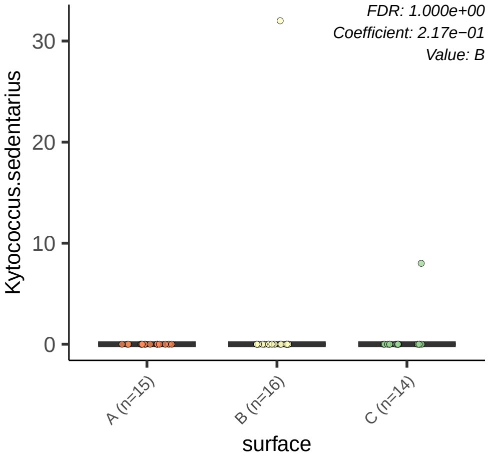
FDR: 1.000e+00
Coefficient: 2.17e−01
Value: B
Kytococcus.sedentarius
0
10
20
30
A (n=15)
B (n=16)
C (n=14)
surface

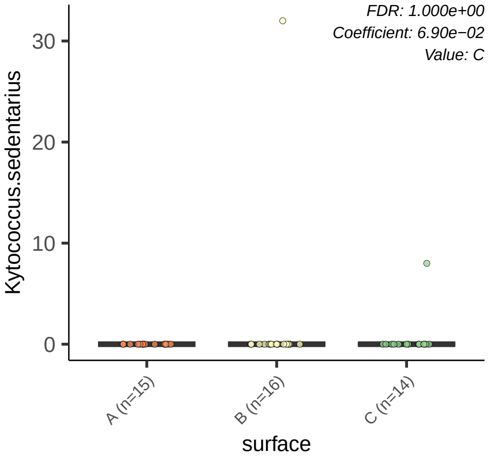

FDR: 1.000e+00
Coefficient: 6.90e−02
Value: C
Kytococcus.sedentarius
30
20
10
0
A (n=15)
B (n=16)
C (n=14)
surface

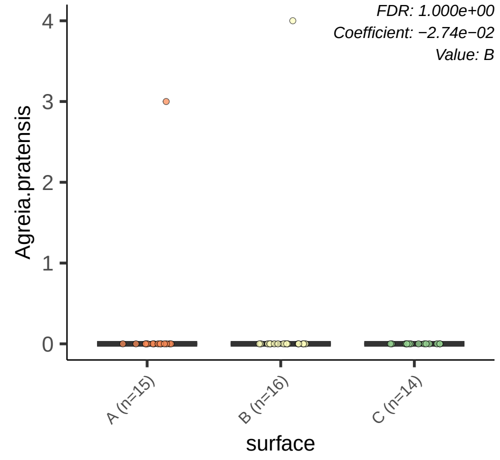
FDR: 1.000e+00
Coefficient: −2.74e−02
Value: B
Agreia.pratensis
4
3
2
1
0
A (n=15)
B (n=16)
C (n=14)
surface

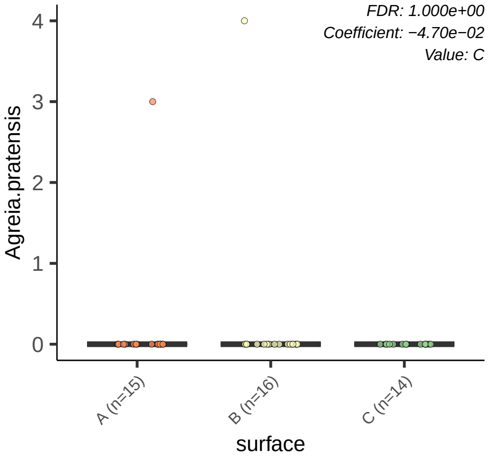
FDR: 1.000e+00
Coefficient: −4.70e−02
Value: C
Agreia.pratensis
0
1
2
3
4
A (n=15)
B (n=16)
C (n=14)
surface

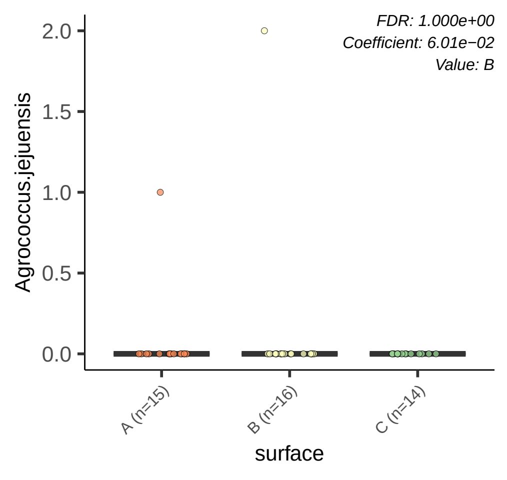
FDR: 1.000e+00
Coefficient: 6.01e−02
Value: B
Agrococcus.jejuensis
2.0
1.5
1.0
0.5
0.0
A (n=15)
B (n=16)
C (n=14)
surface

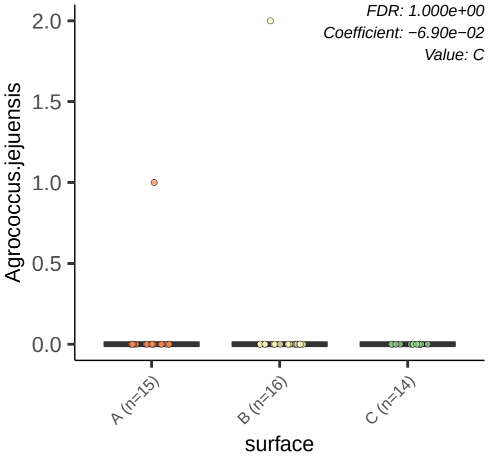
FDR: 1.000e+00
Coefficient: −6.90e−02
Value: C
Agrococcus.jejuensis
2.0
1.5
1.0
0.5
0.0
A (n=15)
B (n=16)
C (n=14)
surface

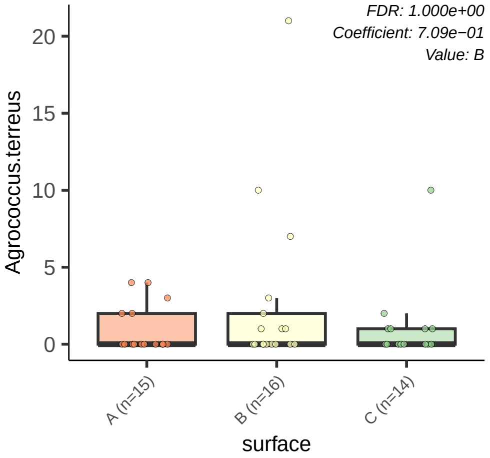

FDR: 1.000e+00
Coefficient: 7.09e−01
Value: B
Agrococcus.terreus
20
15
10
5
0
A (n=15)
B (n=16)
C (n=14)
surface

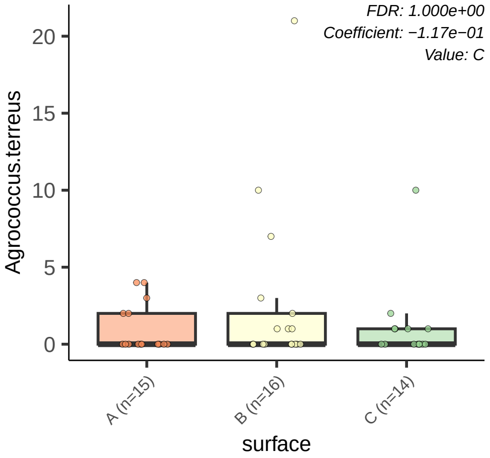

FDR: 1.000e+00
Coefficient: −1.17e−01
Value: C
Agrococcus.terreus
20
15
10
5
0
A (n=15)
B (n=16)
C (n=14)
surface

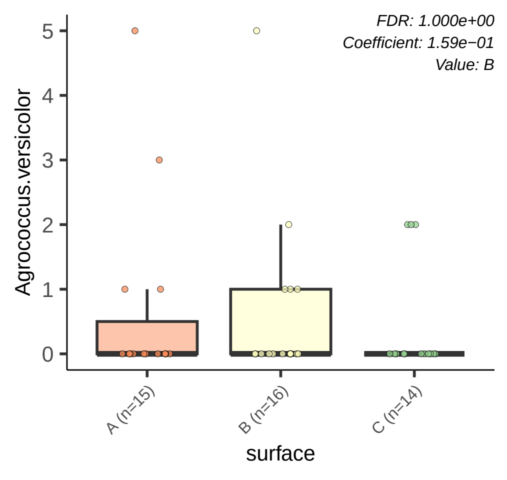
FDR: 1.000e+00
Coefficient: 1.59e−01
Value: B
Agrococcus.versicolor
0
1
2
3
4
5
A (n=15)
B (n=16)
C (n=14)
surface

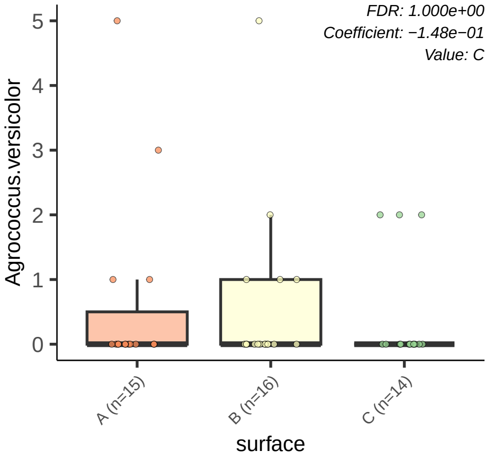
FDR: 1.000e+00
Coefficient: −1.48e−01
Value: C
Agrococcus.versicolor
0
1
2
3
4
5
A (n=15)
B (n=16)
C (n=14)
surface

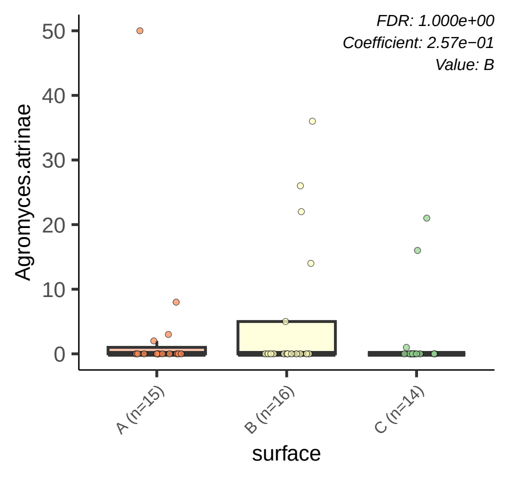

FDR: 1.000e+00
Coefficient: 2.57e−01
Value: B
Agromyces.atrinae
50
40
30
20
10
0
A (n=15)
B (n=16)
C (n=14)
surface

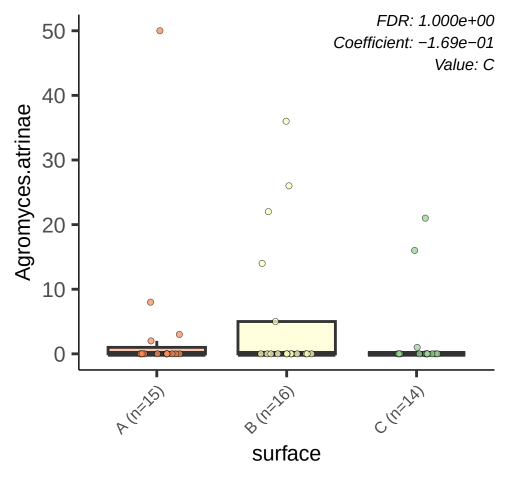
FDR: 1.000e+00
Coefficient: −1.69e−01
Value: C
Agromyces.atrinae
50
40
30
20
10
0
A (n=15)
B (n=16)
C (n=14)
surface

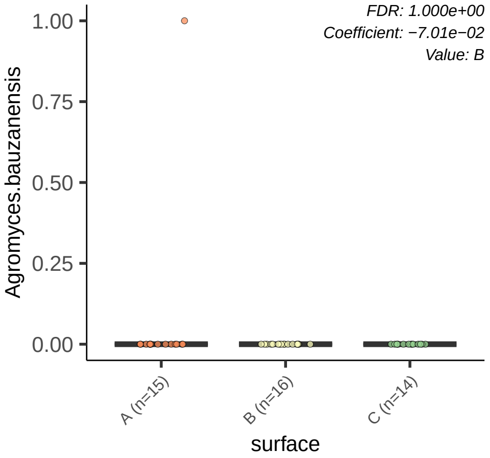
FDR: 1.000e+00
Coefficient: −7.01e−02
Value: B
Agromyces.bauzanensis
1.00
0.75
0.50
0.25
0.00
A (n=15)
B (n=16)
C (n=14)
surface

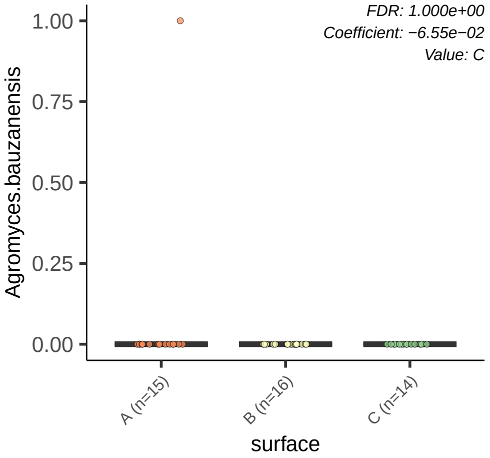
FDR: 1.000e+00
Coefficient: −6.55e−02
Value: C
Agromyces.bauzanensis
1.00
0.75
0.50
0.25
0.00
A (n=15)
B (n=16)
C (n=14)
surface

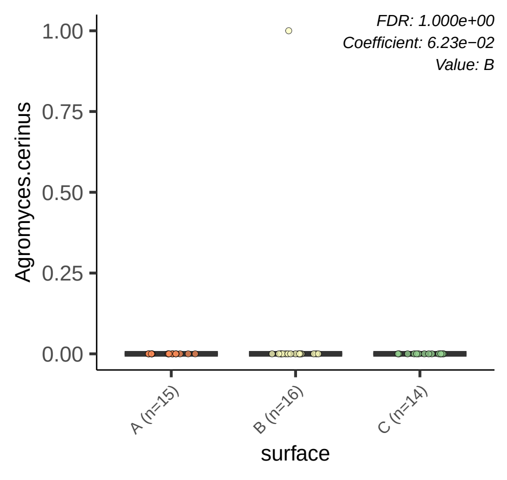

FDR: 1.000e+00
Coefficient: 6.23e−02
Value: B
1.00
0.75
0.50
0.25
0.00
Agromyces.cerinus
A (n=15)
B (n=16)
C (n=14)
surface

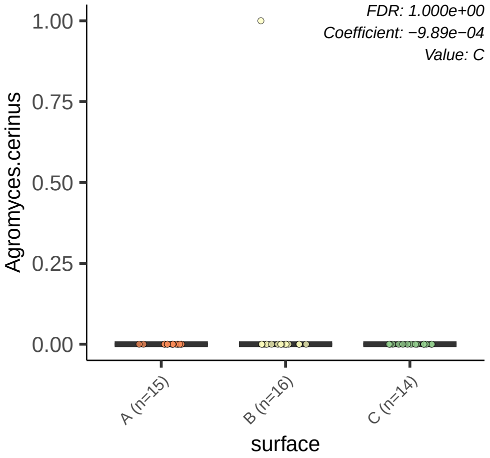

FDR: 1.000e+00
Coefficient: −9.89e−04
Value: C
Agromyces.cerinus
1.00
0.75
0.50
0.25
0.00
A (n=15)
B (n=16)
C (n=14)
surface

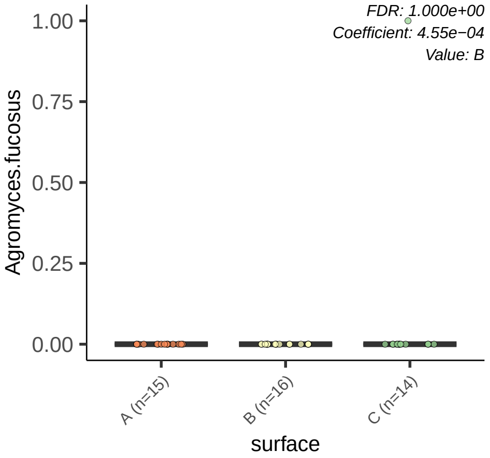

FDR: 1.000e+00
Coefficient: 4.55e−04
Value: B
Agromyces.fucosus
1.00
0.75
0.50
0.25
0.00
A (n=15)
B (n=16)
C (n=14)
surface

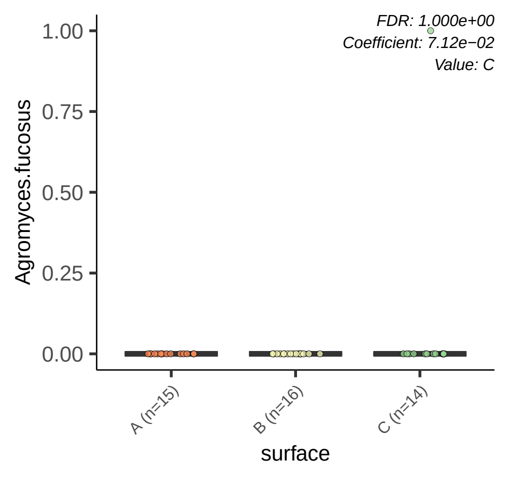
FDR: 1.000e+00
Coefficient: 7.12e−02
Value: C
Agromyces.fucosus
1.00
0.75
0.50
0.25
0.00
A (n=15)
B (n=16)
C (n=14)
surface

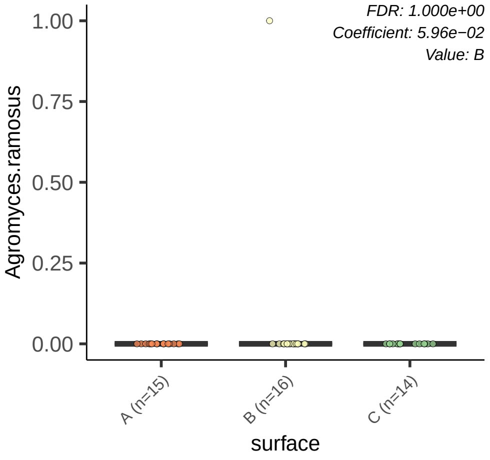
FDR: 1.000e+00
Coefficient: 5.96e−02
Value: B
1.00
0.75
0.50
0.25
0.00
Agromyces.ramosus
A (n=15)
B (n=16)
C (n=14)
surface

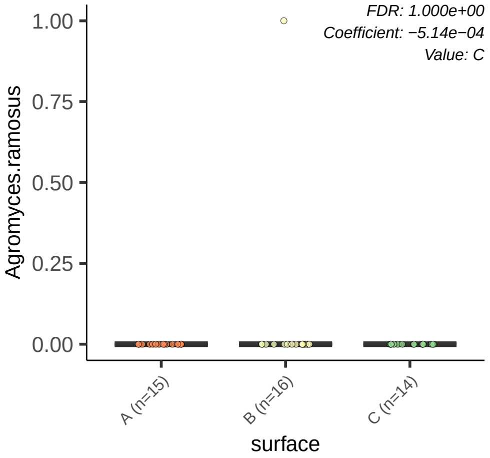
FDR: 1.000e+00
Coefficient: −5.14e−04
Value: C
Agromyces.ramosus
1.00
0.75
0.50
0.25
0.00
A (n=15)
B (n=16)
C (n=14)
surface

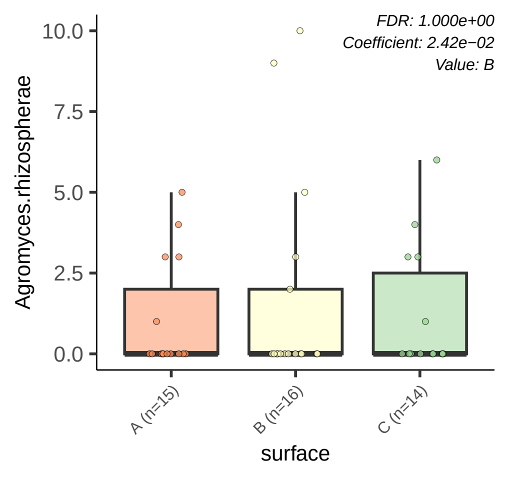
FDR: 1.000e+00
Coefficient: 2.42e−02
Value: B
Agromyces.rhizospherae
10.0
7.5
5.0
2.5
0.0
A (n=15)
B (n=16)
C (n=14)
surface

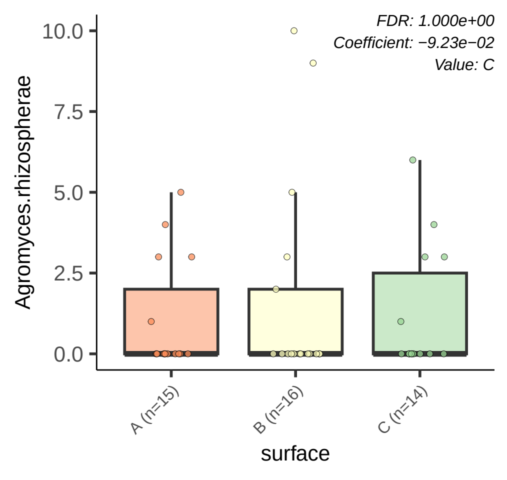

FDR: 1.000e+00
Coefficient: −9.23e−02
Value: C
Agromyces.rhizospherae
10.0
7.5
5.0
2.5
0.0
A (n=15)
B (n=16)
C (n=14)
surface

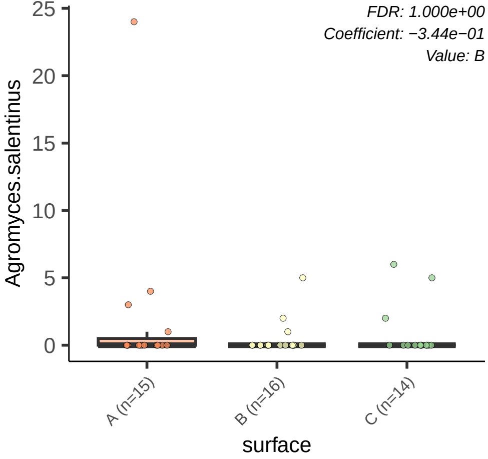
FDR: 1.000e+00
Coefficient: −3.44e−01
Value: B
Agromyces.salentinus
0
5
10
15
20
25
A (n=15)
B (n=16)
C (n=14)
surface

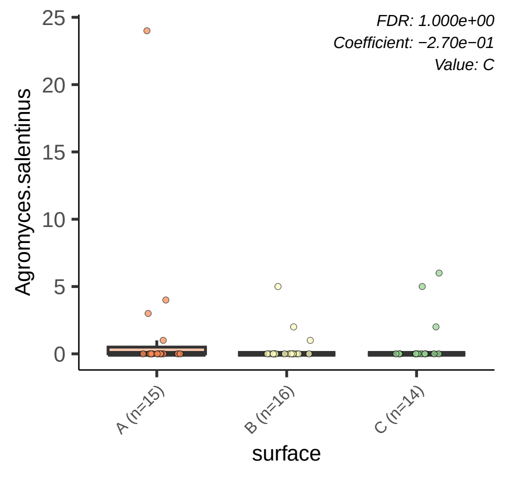
FDR: 1.000e+00
Coefficient: −2.70e−01
Value: C
Agromyces.salentinus
25
20
15
10
5
0
A (n=15)
B (n=16)
C (n=14)
surface

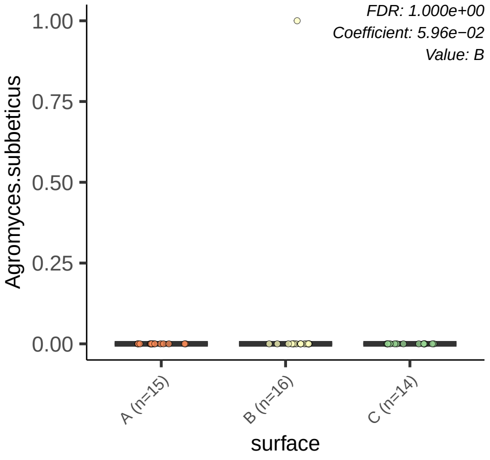
FDR: 1.000e+00
Coefficient: 5.96e−02
Value: B
Agromyces.subbeticus
1.00
0.75
0.50
0.25
0.00
A (n=15)
B (n=16)
C (n=14)
surface

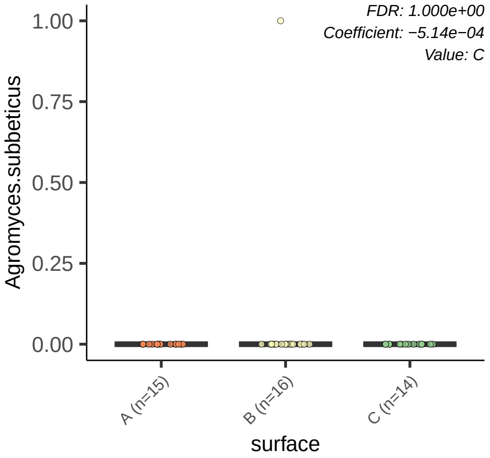

FDR: 1.000e+00
Coefficient: −5.14e−04
Value: C
Agromyces.subbeticus
1.00
0.75
0.50
0.25
0.00
A (n=15)
B (n=16)
C (n=14)
surface

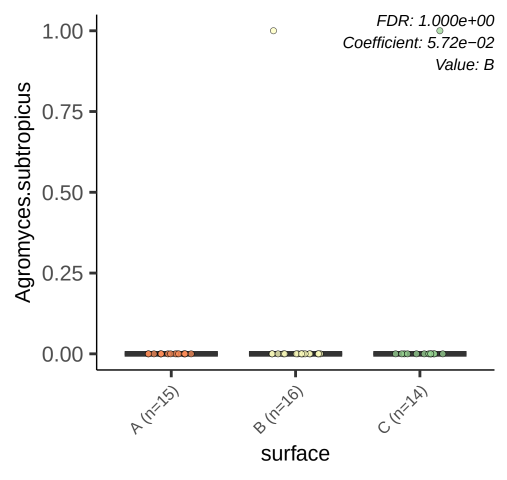

FDR: 1.000e+00
Coefficient: 5.72e−02
Value: B
Agromyces.subtropicus
1.00
0.75
0.50
0.25
0.00
A (n=15)
B (n=16)
C (n=14)
surface

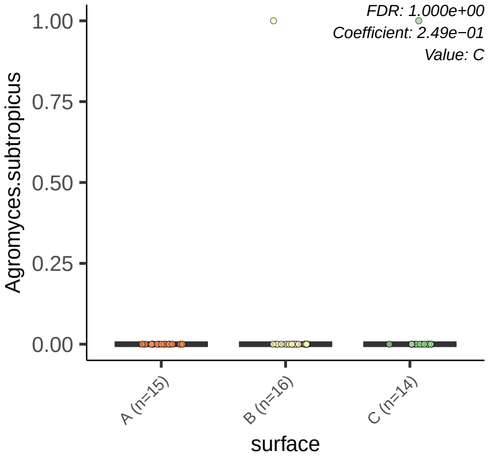
FDR: 1.000e+00
Coefficient: 2.49e−01
Value: C
Agromyces.subtropicus
1.00
0.75
0.50
0.25
0.00
A (n=15)
B (n=16)
C (n=14)
surface

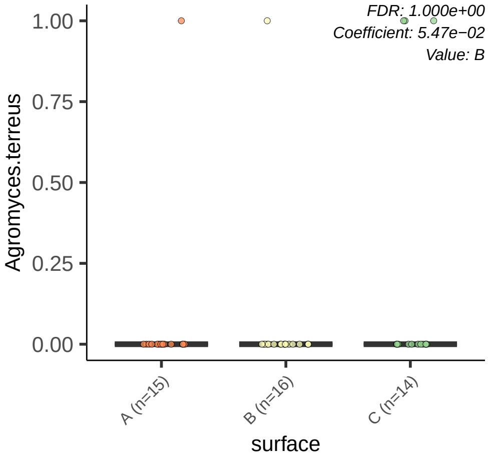
FDR: 1.000e+00
Coefficient: 5.47e−02
Value: B
Agromyces.terreus
1.00
0.75
0.50
0.25
0.00
A (n=15)
B (n=16)
C (n=14)
surface

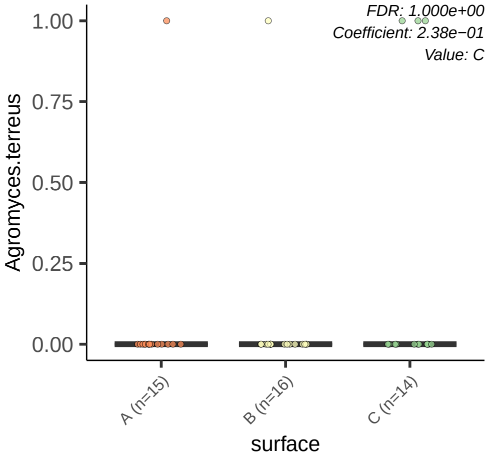
FDR: 1.000e+00
Coefficient: 2.38e−01
Value: C
Agromyces.terreus
1.00
0.75
0.50
0.25
0.00
A (n=15)
B (n=16)
C (n=14)
surface

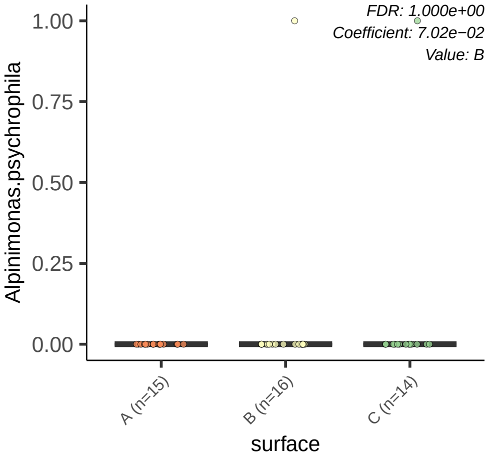

FDR: 1.000e+00
Coefficient: 7.02e−02
Value: B
Alpinimonas.psychrophila
1.00
0.75
0.50
0.25
0.00
A (n=15)
B (n=16)
C (n=14)
surface

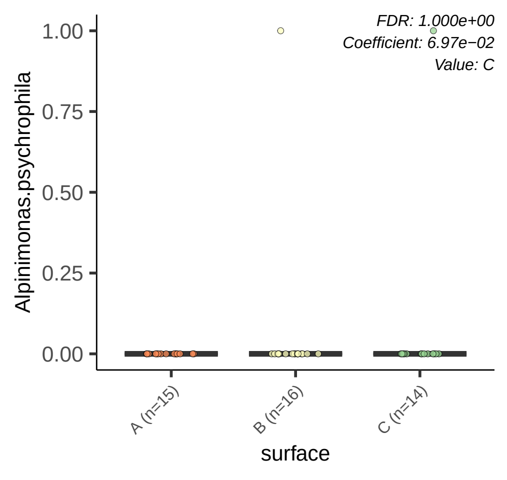
FDR: 1.000e+00
Coefficient: 6.97e−02
Value: C
Alpinimonas.psychrophila
1.00
0.75
0.50
0.25
0.00
A (n=15)
B (n=16)
C (n=14)
surface

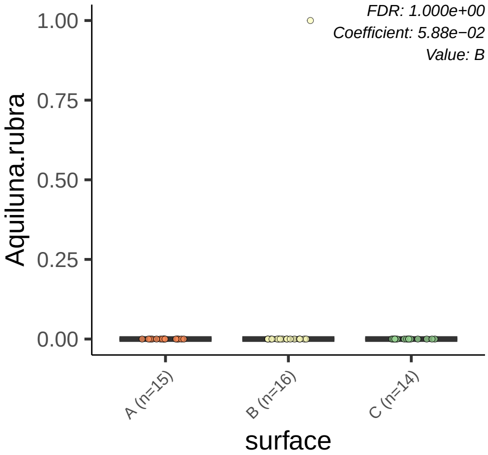
FDR: 1.000e+00
Coefficient: 5.88e−02
Value: B
Aquiluna.rubra
1.00
0.75
0.50
0.25
0.00
A (n=15)
B (n=16)
C (n=14)
surface

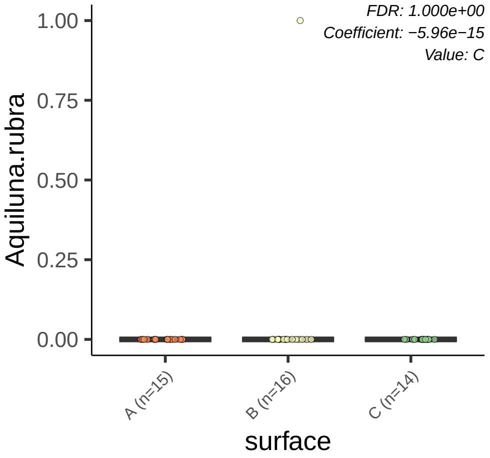
FDR: 1.000e+00
Coefficient: −5.96e−15
Value: C
Aquiluna.rubra
1.00
0.75
0.50
0.25
0.00
A (n=15)
B (n=16)
C (n=14)
surface

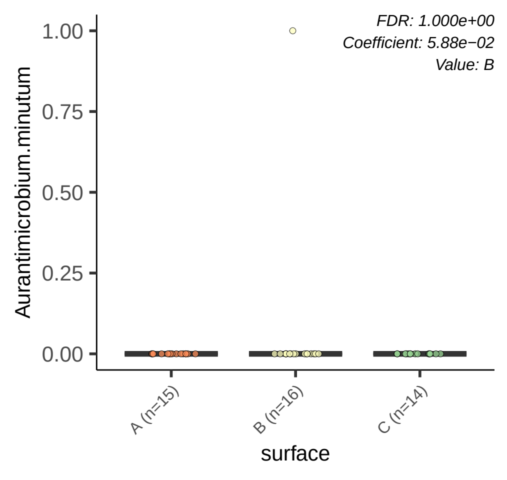
FDR: 1.000e+00
Coefficient: 5.88e−02
Value: B
Aurantimicrobium.minutum
1.00
0.75
0.50
0.25
0.00
A (n=15)
B (n=16)
C (n=14)
surface

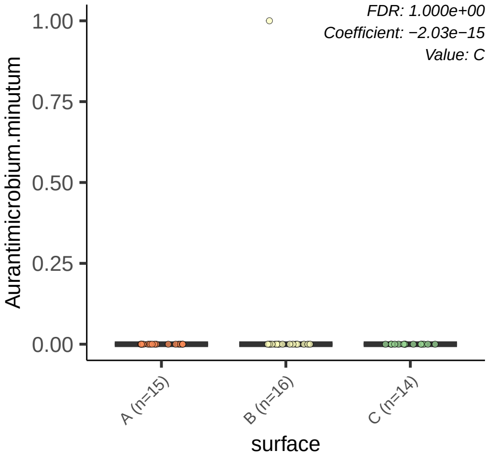

FDR: 1.000e+00
Coefficient: −2.03e−15
Value: C
Aurantimicrobium.minutum
1.00
0.75
0.50
0.25
0.00
A (n=15)
B (n=16)
C (n=14)
surface

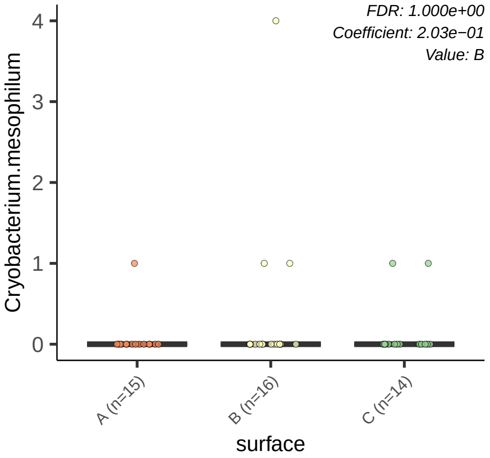

FDR: 1.000e+00
Coefficient: 2.03e−01
Value: B
Cryobacterium.mesophilum
0
1
2
3
4
A (n=15)
B (n=16)
C (n=14)
surface

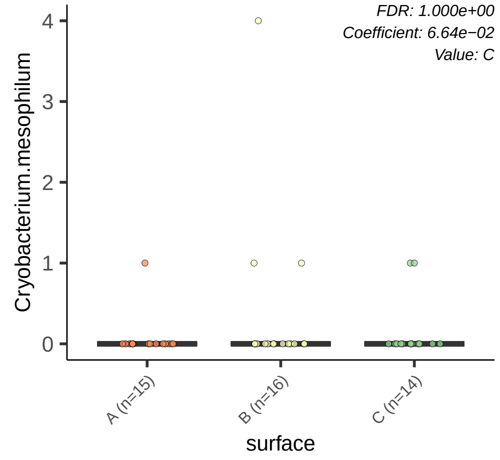
FDR: 1.000e+00
Coefficient: 6.64e−02
Value: C
Cryobacterium.mesophilum
0
1
2
3
4
A (n=15)
B (n=16)
C (n=14)
surface

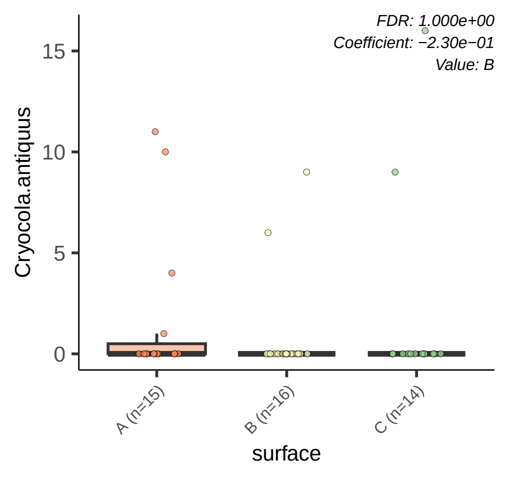
FDR: 1.000e+00
Coefficient: −2.30e−01
Value: B
Cryocola.antiquus
0
5
10
15
A (n=15)
B (n=16)
C (n=14)
surface

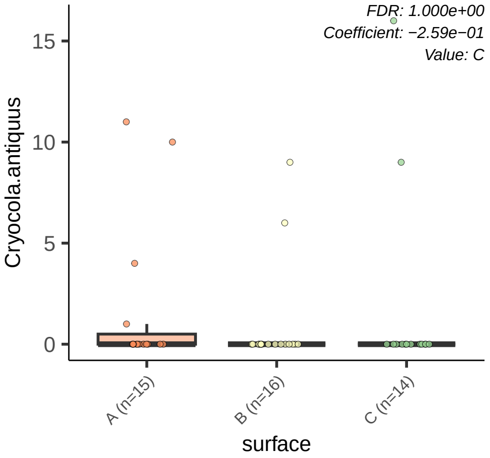

FDR: 1.000e+00
Coefficient: −2.59e−01
Value: C
Cryocola.antiquus
15
10
5
0
A (n=15)
B (n=16)
C (n=14)
surface

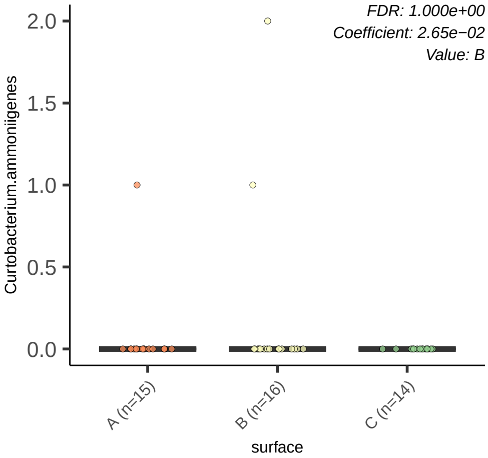

FDR: 1.000e+00
Coefficient: 2.65e−02
Value: B
Curtobacterium.ammoniigenes
2.0
1.5
1.0
0.5
0.0
A (n=15)
B (n=16)
C (n=14)
surface

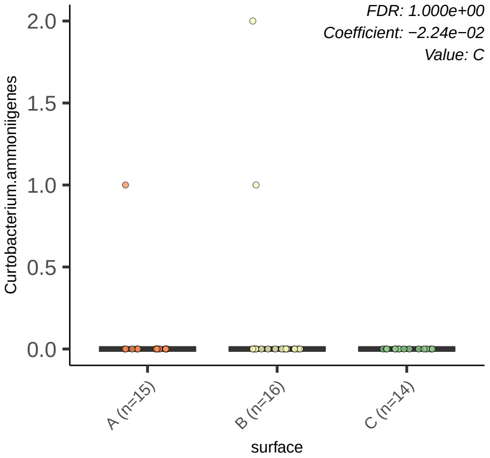

FDR: 1.000e+00
Coefficient: −2.24e−02
Value: C
Curtobacterium.ammoniigenes
2.0
1.5
1.0
0.5
0.0
A (n=15)
B (n=16)
C (n=14)
surface

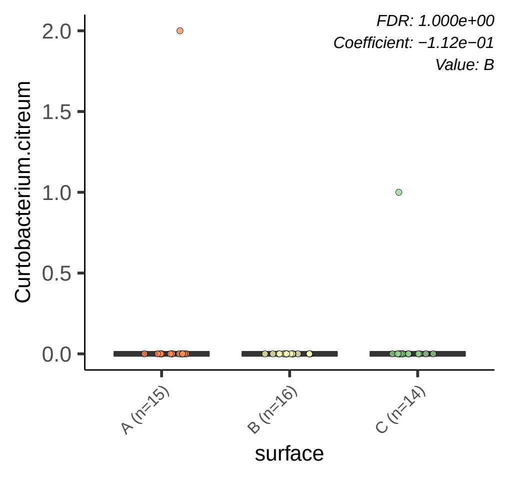
FDR: 1.000e+00
Coefficient: −1.12e−01
Value: B
Curtobacterium.citreum
2.0
1.5
1.0
0.5
0.0
A (n=15)
B (n=16)
C (n=14)
surface

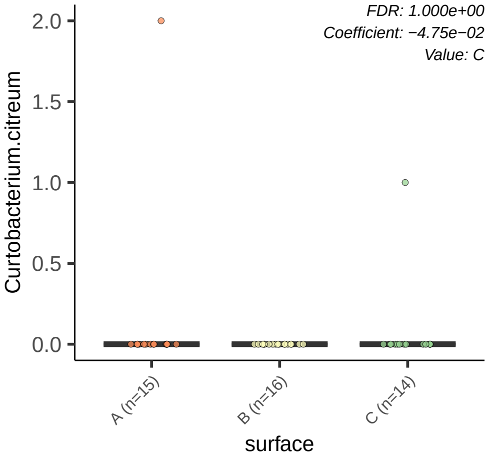

FDR: 1.000e+00
Coefficient: −4.75e−02
Value: C
2.0
1.5
1.0
0.5
0.0
Curtobacterium.citreum
A (n=15)
B (n=16)
C (n=14)
surface

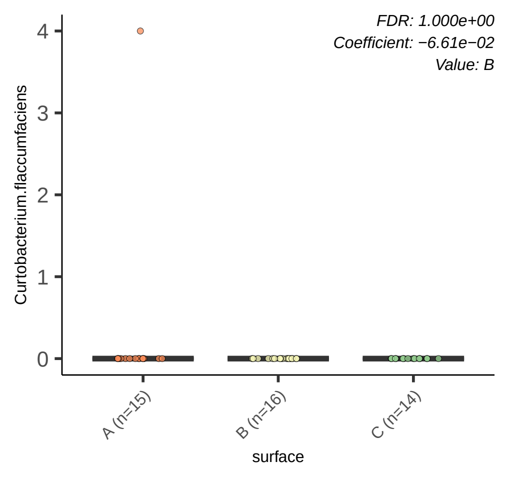

FDR: 1.000e+00
Coefficient: −6.61e−02
Value: B
Curtobacterium.flaccumfaciens
0
1
2
3
4
A (n=15)
B (n=16)
C (n=14)
surface

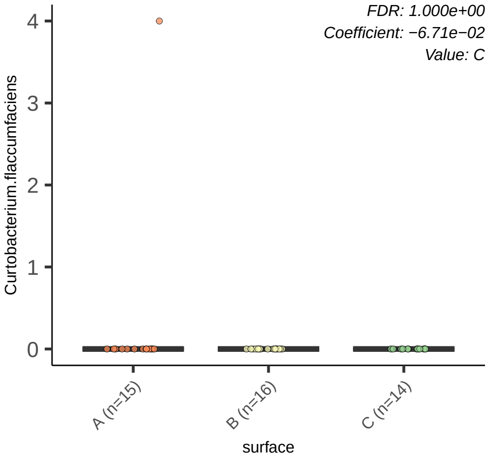
FDR: 1.000e+00
Coefficient: −6.71e−02
Value: C
Curtobacterium.flaccumfaciens
4
3
2
1
0
A (n=15)
B (n=16)
C (n=14)
surface

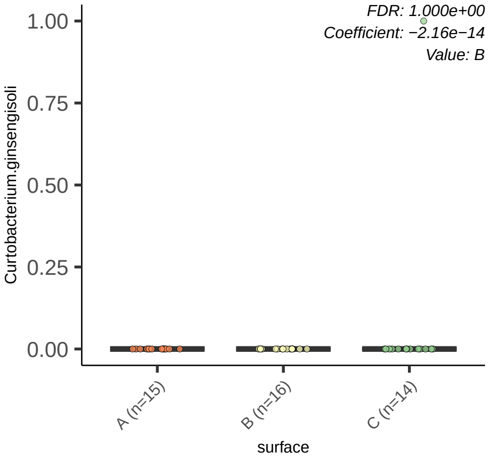

FDR: 1.000e+00
Coefficient: −2.16e−14
Value: B
Curtobacterium.ginsengisoli
1.00
0.75
0.50
0.25
0.00
A (n=15)
B (n=16)
C (n=14)
surface

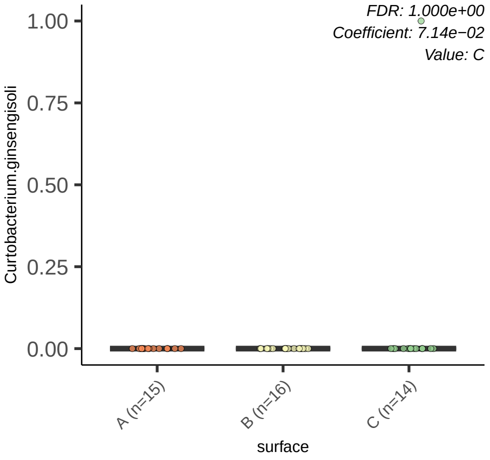

FDR: 1.000e+00
Coefficient: 7.14e−02
Value: C
Curtobacterium.ginsengisoli
1.00
0.75
0.50
0.25
0.00
A (n=15)
B (n=16)
C (n=14)
surface

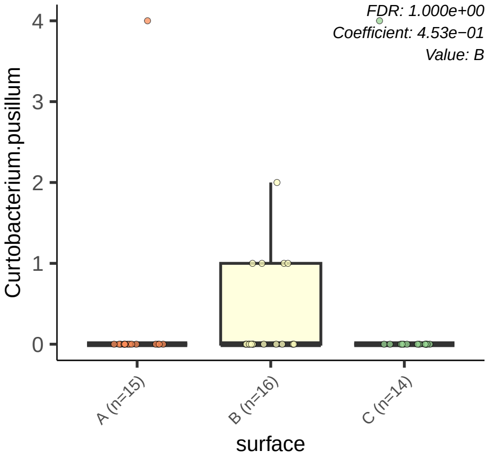
FDR: 1.000e+00
Coefficient: 4.53e−01
Value: B
Curtobacterium.pusillum
0
1
2
3
4
A (n=15)
B (n=16)
C (n=14)
surface

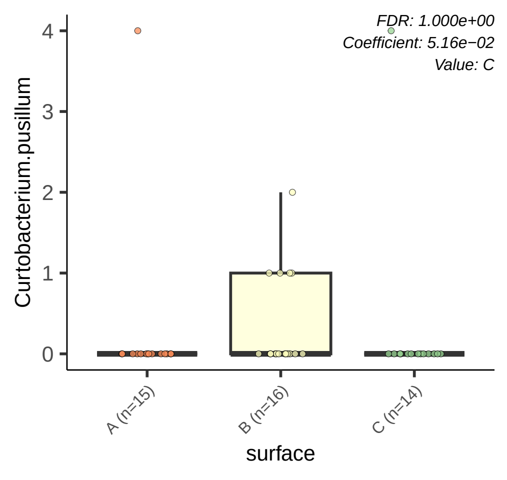
FDR: 1.000e+00
Coefficient: 5.16e−02
Value: C
Curtobacterium.pusillum
0
1
2
3
4
A (n=15)
B (n=16)
C (n=14)
surface

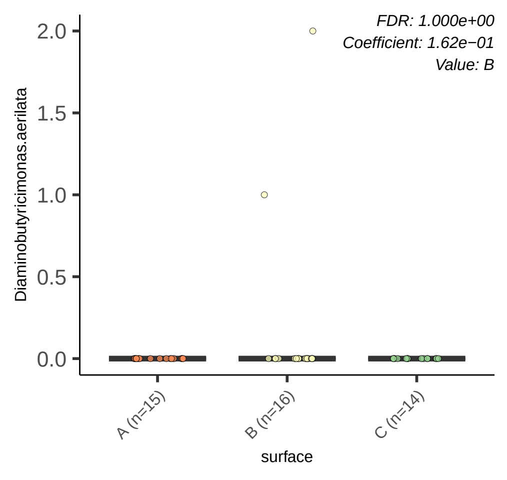

FDR: 1.000e+00
Coefficient: 1.62e−01
Value: B
Diaminobutyricimonas.aerilata
2.0
1.5
1.0
0.5
0.0
A (n=15)
B (n=16)
C (n=14)
surface

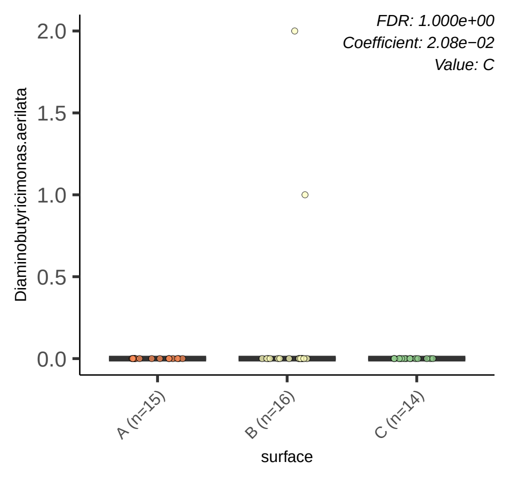
FDR: 1.000e+00
Coefficient: 2.08e−02
Value: C
Diaminobutyricimonas.aerilata
2.0
1.5
1.0
0.5
0.0
A (n=15)
B (n=16)
C (n=14)
surface

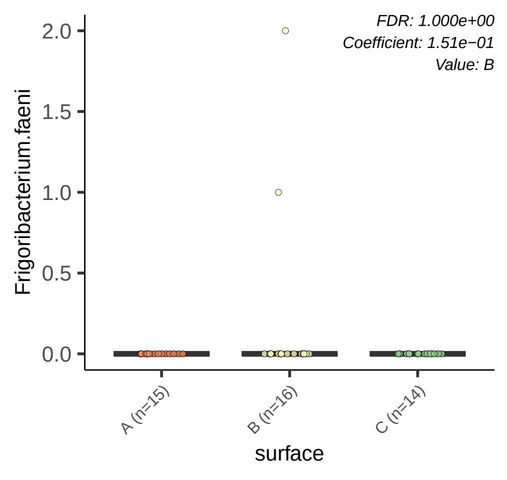
FDR: 1.000e+00
Coefficient: 1.51e−01
Value: B
Frigoribacterium.faeni
2.0
1.5
1.0
0.5
0.0
A (n=15)
B (n=16)
C (n=14)
surface

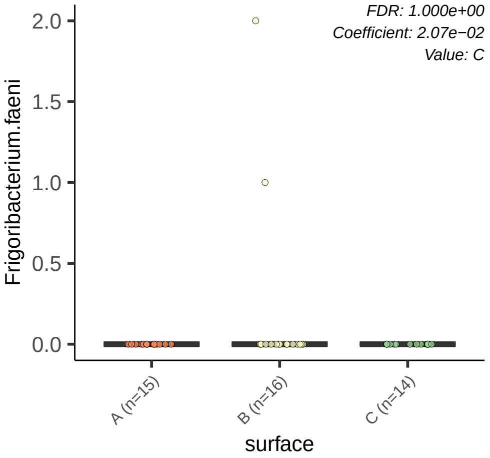
FDR: 1.000e+00
Coefficient: 2.07e−02
Value: C
Frigoribacterium.faeni
2.0
1.5
1.0
0.5
0.0
A (n=15)
B (n=16)
C (n=14)
surface

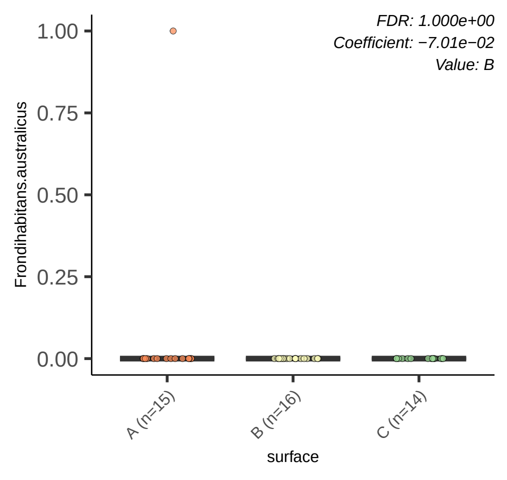

FDR: 1.000e+00
Coefficient: −7.01e−02
Value: B
Frondihabitans.australicus
1.00
0.75
0.50
0.25
0.00
A (n=15)
B (n=16)
C (n=14)
surface

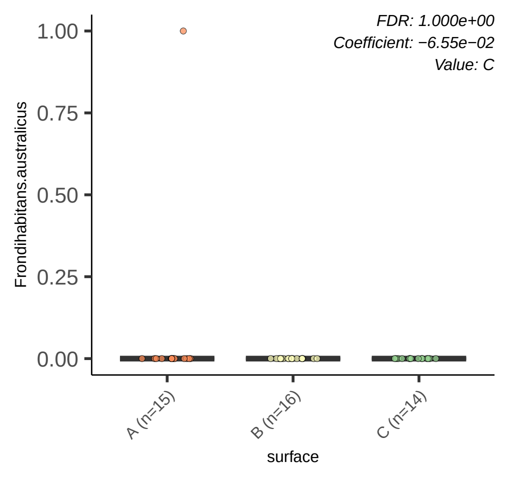

FDR: 1.000e+00
Coefficient: −6.55e−02
Value: C
Frondihabitans.australicus
1.00
0.75
0.50
0.25
0.00
A (n=15)
B (n=16)
C (n=14)
surface

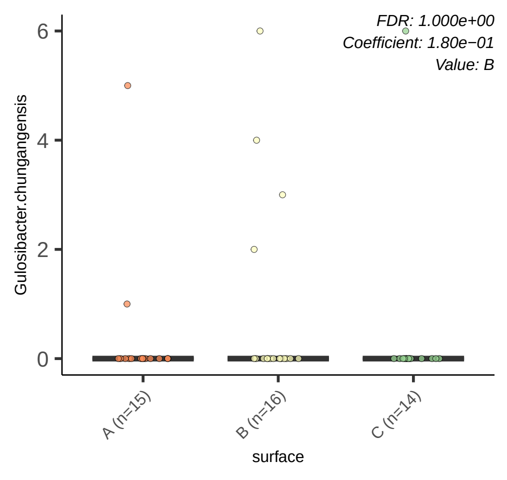
FDR: 1.000e+00
Coefficient: 1.80e−01
Value: B
Gulosibacter.chungangensis
0
2
4
6
A (n=15)
B (n=16)
C (n=14)
surface

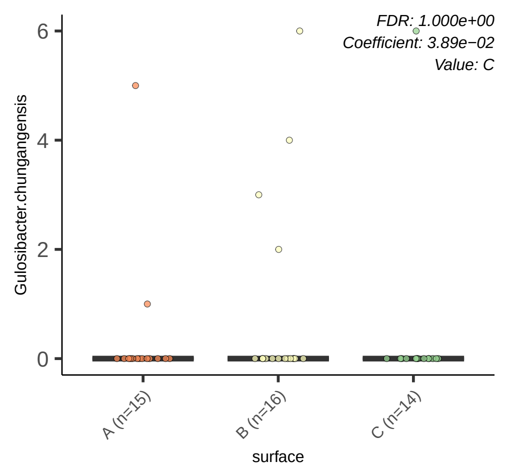
FDR: 1.000e+00
Coefficient: 3.89e−02
Value: C
Gulosibacter.chungangensis
6
4
2
0
A (n=15)
B (n=16)
C (n=14)
surface

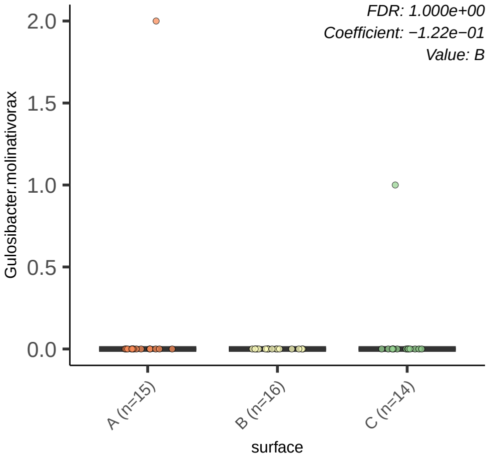
FDR: 1.000e+00
Coefficient: −1.22e−01
Value: B
Gulosibacter.molinativorax
2.0
1.5
1.0
0.5
0.0
A (n=15)
B (n=16)
C (n=14)
surface

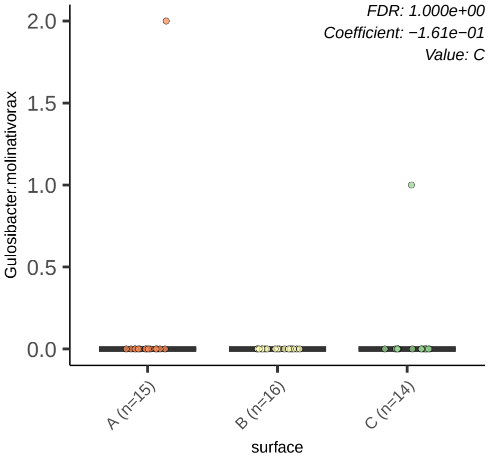

FDR: 1.000e+00
Coefficient: −1.61e−01
Value: C
Gulosibacter.molinativorax
2.0
1.5
1.0
0.5
0.0
A (n=15)
B (n=16)
C (n=14)
surface

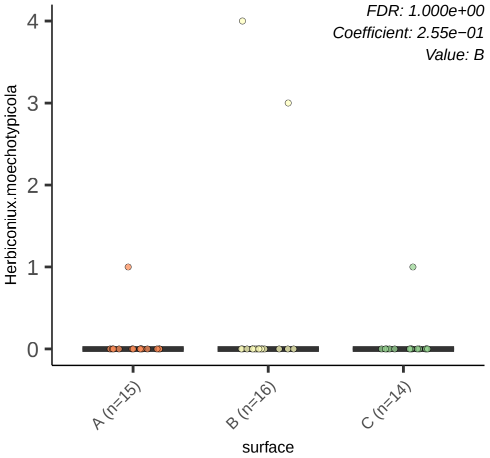
FDR: 1.000e+00
Coefficient: 2.55e−01
Value: B
Herbiconiux.moechotypicola
4
3
2
1
0
A (n=15)
B (n=16)
C (n=14)
surface

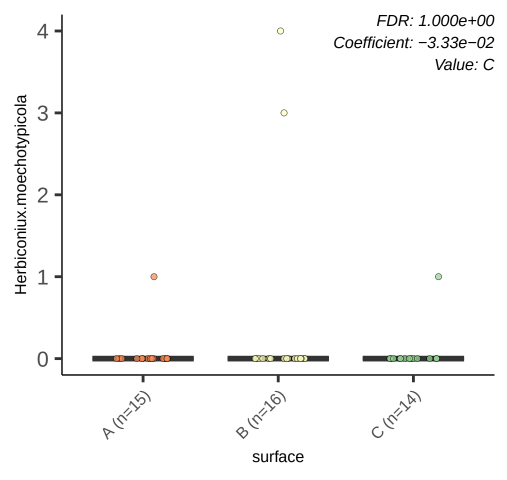
FDR: 1.000e+00
Coefficient: −3.33e−02
Value: C
Herbiconiux.moechotypicola
0
1
2
3
4
A (n=15)
B (n=16)
C (n=14)
surface

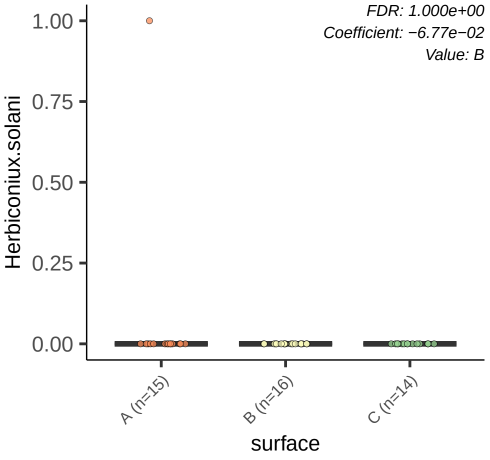

FDR: 1.000e+00
Coefficient: −6.77e−02
Value: B
Herbiconiux.solani
1.00
0.75
0.50
0.25
0.00
A (n=15)
B (n=16)
C (n=14)
surface

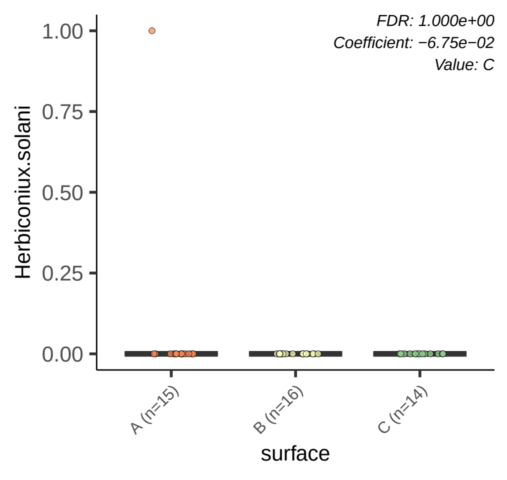

FDR: 1.000e+00
Coefficient: −6.75e−02
Value: C
Herbiconiux.solani
1.00
0.75
0.50
0.25
0.00
A (n=15)
B (n=16)
C (n=14)
surface

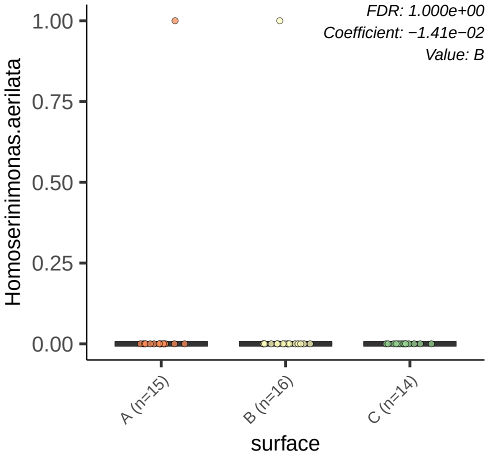

FDR: 1.000e+00
Coefficient: −1.41e−02
Value: B
Homoserinimonas.aerilata
1.00
0.75
0.50
0.25
0.00
A (n=15)
B (n=16)
C (n=14)
surface

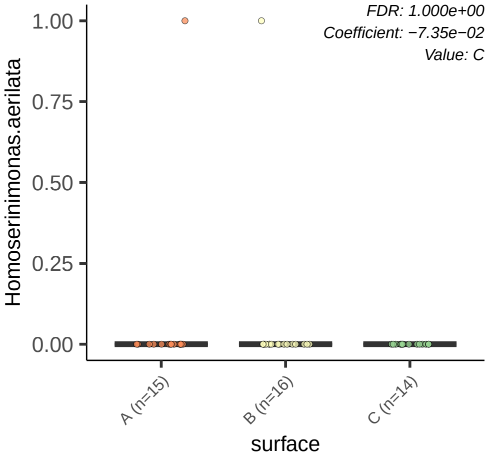
FDR: 1.000e+00
Coefficient: −7.35e−02
Value: C
Homoserinimonas.aerilata
1.00
0.75
0.50
0.25
0.00
A (n=15)
B (n=16)
C (n=14)
surface

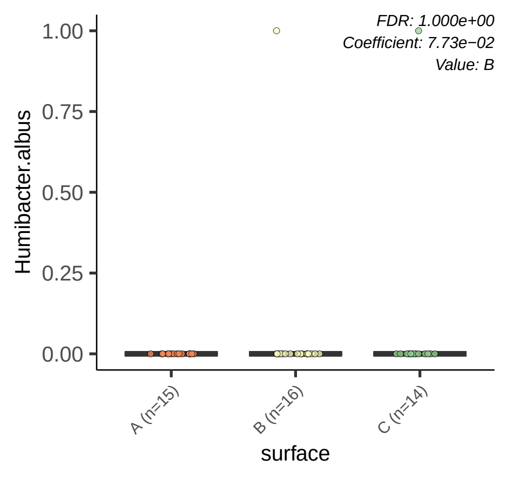
FDR: 1.000e+00
Coefficient: 7.73e−02
Value: B
Humibacter.albus
1.00
0.75
0.50
0.25
0.00
A (n=15)
B (n=16)
C (n=14)
surface

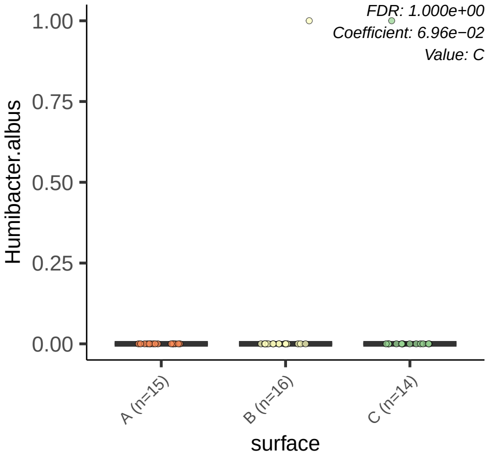
FDR: 1.000e+00
Coefficient: 6.96e−02
Value: C
Humibacter.albus
1.00
0.75
0.50
0.25
0.00
A (n=15)
B (n=16)
C (n=14)
surface

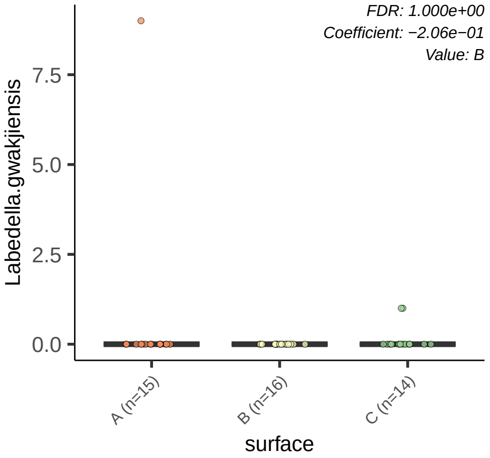

FDR: 1.000e+00
Coefficient: −2.06e−01
Value: B
Labedella.gwakjiensis
7.5
5.0
2.5
0.0
A (n=15)
B (n=16)
C (n=14)
surface

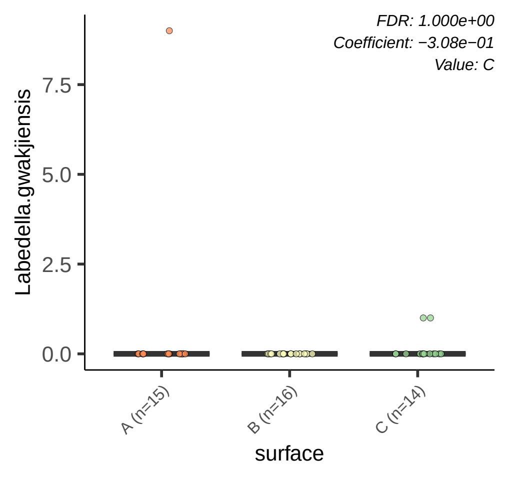
FDR: 1.000e+00
Coefficient: −3.08e−01
Value: C
Labedella.gwakjiensis
7.5
5.0
2.5
0.0
A (n=15)
B (n=16)
C (n=14)
surface

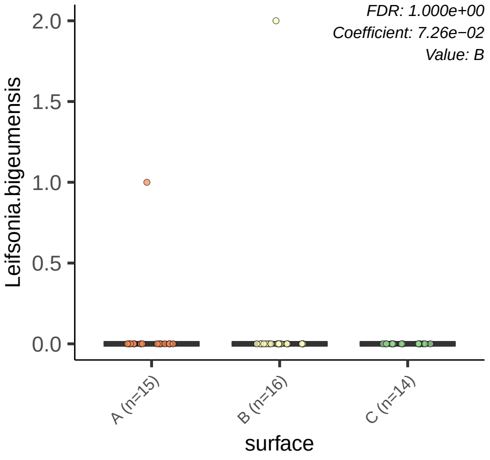

FDR: 1.000e+00
Coefficient: 7.26e−02
Value: B
Leifsonia.bigeumensis
2.0
1.5
1.0
0.5
0.0
A (n=15)
B (n=16)
C (n=14)
surface

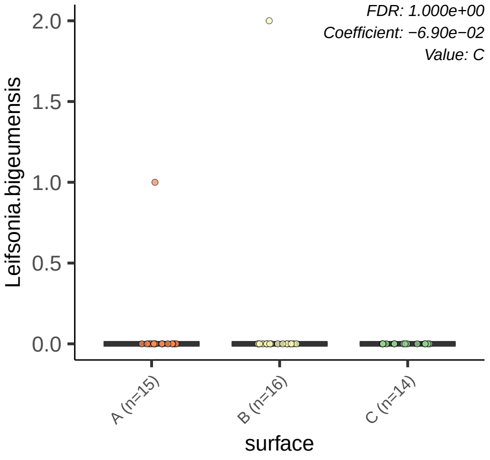

FDR: 1.000e+00
Coefficient: −6.90e−02
Value: C
Leifsonia.bigeumensis
2.0
1.5
1.0
0.5
0.0
A (n=15)
B (n=16)
C (n=14)
surface

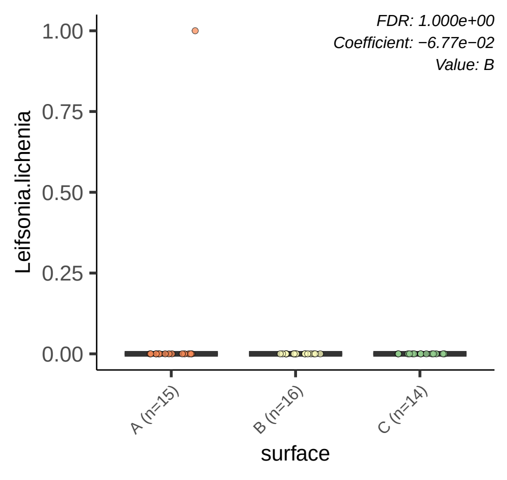
FDR: 1.000e+00
Coefficient: −6.77e−02
Value: B
Leifsonia.lichenia
1.00
0.75
0.50
0.25
0.00
A (n=15)
B (n=16)
C (n=14)
surface

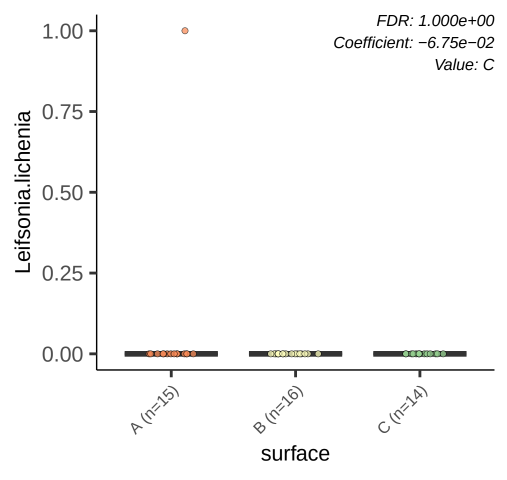
FDR: 1.000e+00
Coefficient: −6.75e−02
Value: C
Leifsonia.lichenia
1.00
0.75
0.50
0.25
0.00
A (n=15)
B (n=16)
C (n=14)
surface

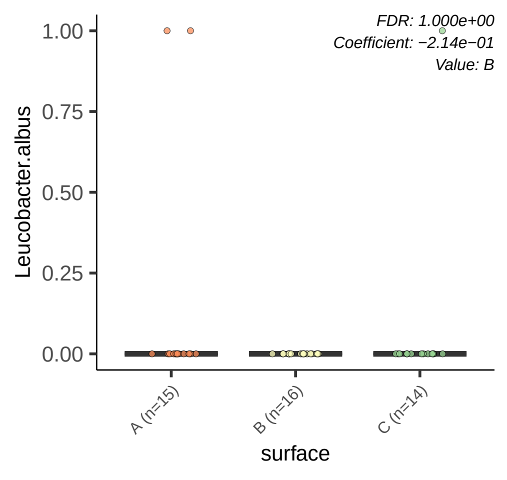

FDR: 1.000e+00
Coefficient: −2.14e−01
Value: B
Leucobacter.albus
1.00
0.75
0.50
0.25
0.00
A (n=15)
B (n=16)
C (n=14)
surface

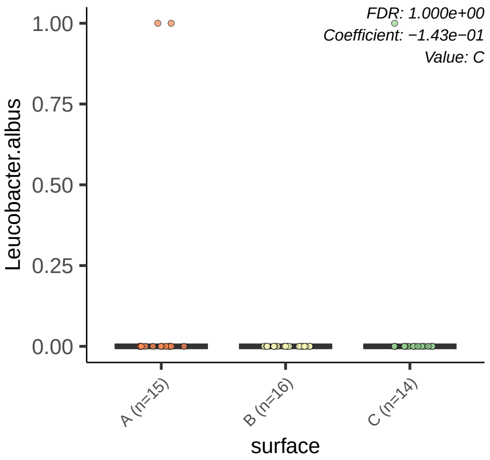
FDR: 1.000e+00
Coefficient: −1.43e−01
Value: C
Leucobacter.albus
1.00
0.75
0.50
0.25
0.00
A (n=15)
B (n=16)
C (n=14)
surface

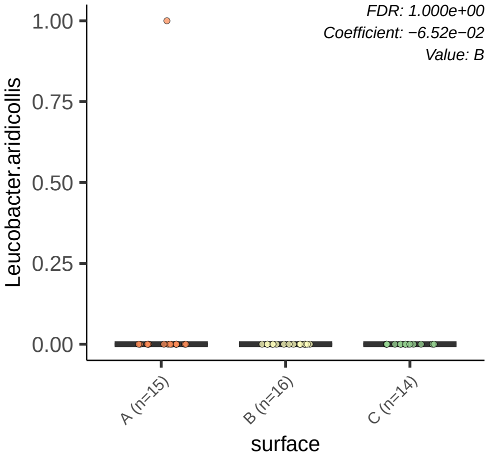
FDR: 1.000e+00
Coefficient: −6.52e−02
Value: B
Leucobacter.aridicollis
1.00
0.75
0.50
0.25
0.00
A (n=15)
B (n=16)
C (n=14)
surface

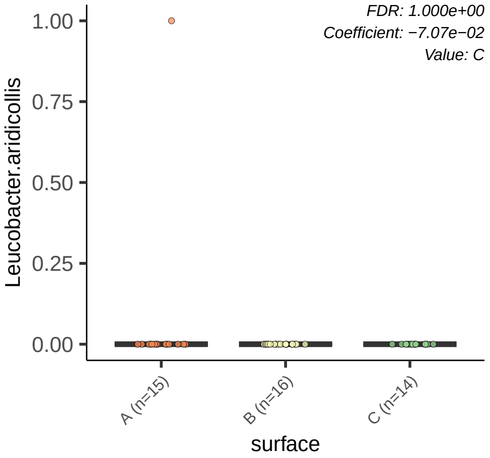

FDR: 1.000e+00
Coefficient: −7.07e−02
Value: C
Leucobacter.aridicollis
1.00
0.75
0.50
0.25
0.00
A (n=15)
B (n=16)
C (n=14)
surface

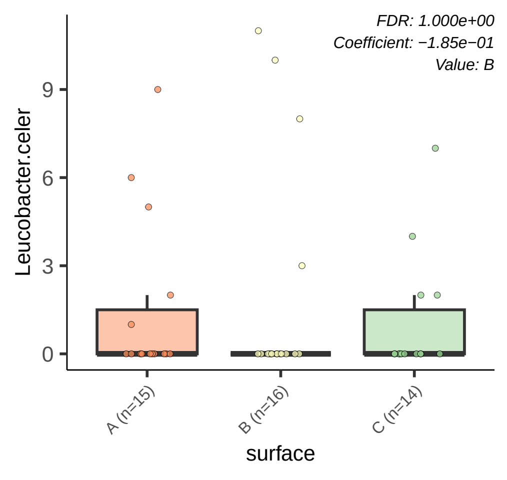

FDR: 1.000e+00
Coefficient: −1.85e−01
Value: B
Leucobacter.celer
0
3
6
9
A (n=15)
B (n=16)
C (n=14)
surface

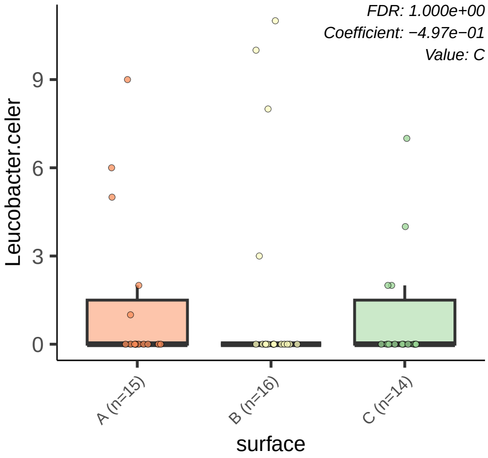
FDR: 1.000e+00
Coefficient: −4.97e−01
Value: C
Leucobacter.celer
0
3
6
9
A (n=15)
B (n=16)
C (n=14)
surface

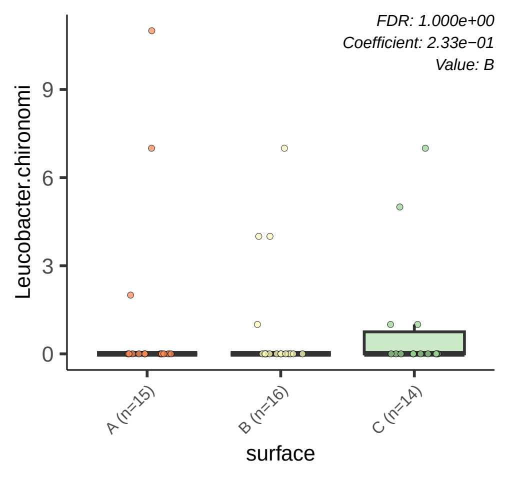
FDR: 1.000e+00
Coefficient: 2.33e−01
Value: B
Leucobacter.chironomi
9
6
3
0
A (n=15)
B (n=16)
C (n=14)
surface

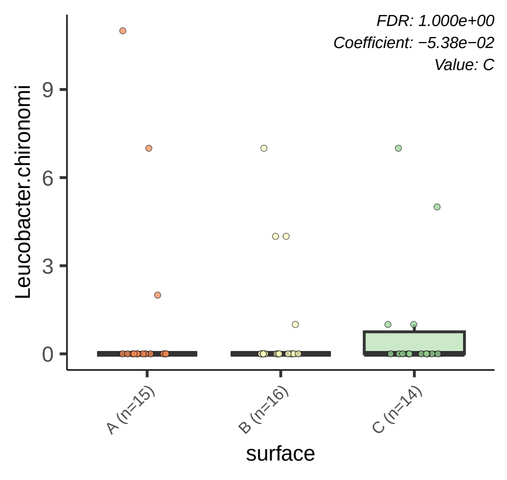
FDR: 1.000e+00
Coefficient: −5.38e−02
Value: C
Leucobacter.chironomi
0
3
6
9
A (n=15)
B (n=16)
C (n=14)
surface

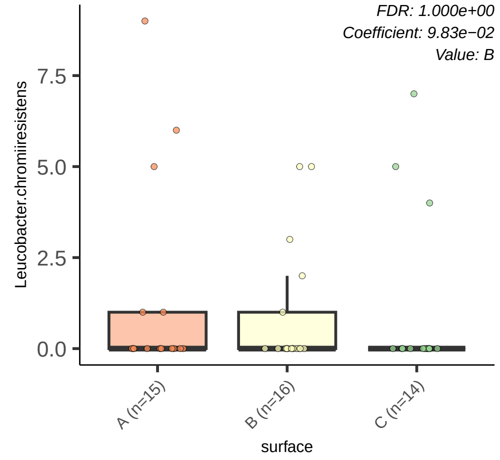

FDR: 1.000e+00
Coefficient: 9.83e−02
Value: B
Leucobacter.chromiiresistens
0.0
2.5
5.0
7.5
A (n=15)
B (n=16)
C (n=14)
surface

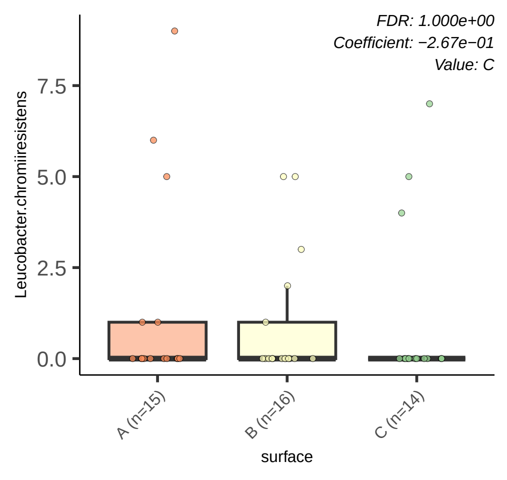
FDR: 1.000e+00
Coefficient: −2.67e−01
Value: C
Leucobacter.chromiiresistens
7.5
5.0
2.5
0.0
A (n=15)
B (n=16)
C (n=14)
surface

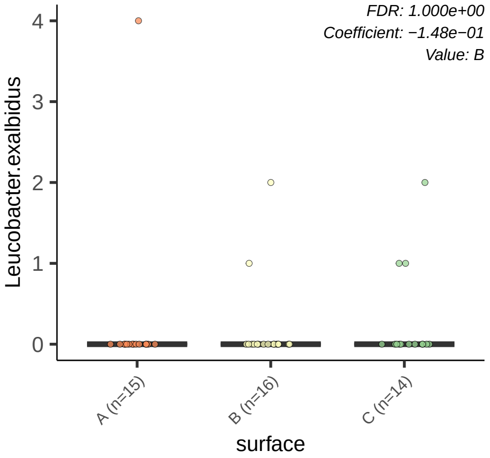

FDR: 1.000e+00
Coefficient: −1.48e−01
Value: B
Leucobacter.exalbidus
4
3
2
1
0
A (n=15)
B (n=16)
C (n=14)
surface

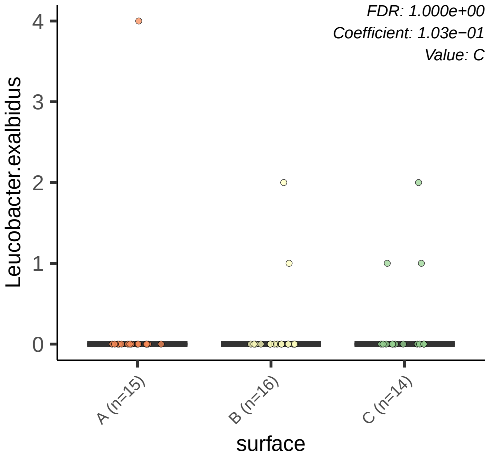

FDR: 1.000e+00
Coefficient: 1.03e−01
Value: C
Leucobacter.exalbidus
0
1
2
3
4
A (n=15)
B (n=16)
C (n=14)
surface

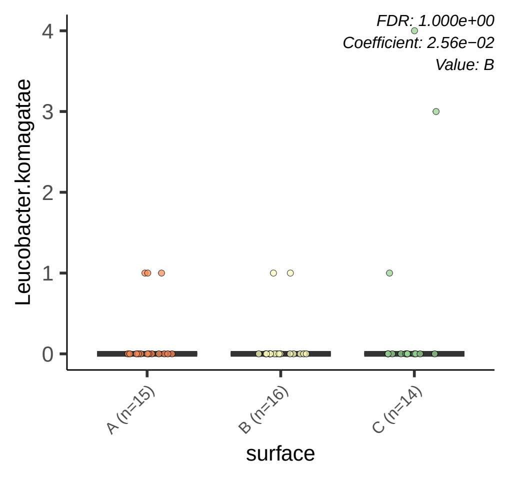

FDR: 1.000e+00
Coefficient: 2.56e−02
Value: B
Leucobacter.komagatae
0
1
2
3
4
A (n=15)
B (n=16)
C (n=14)
surface

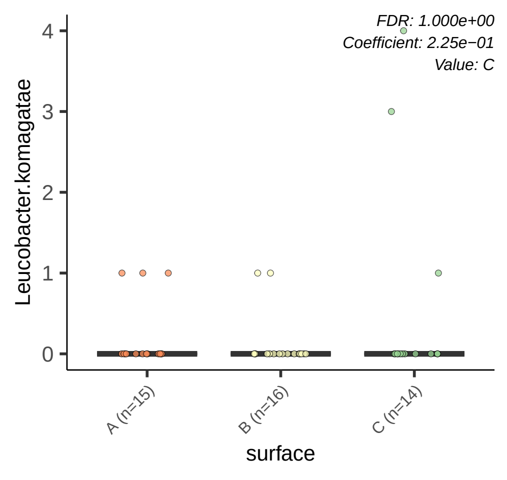
FDR: 1.000e+00
Coefficient: 2.25e−01
Value: C
Leucobacter.komagatae
0
1
2
3
4
A (n=15)
B (n=16)
C (n=14)
surface

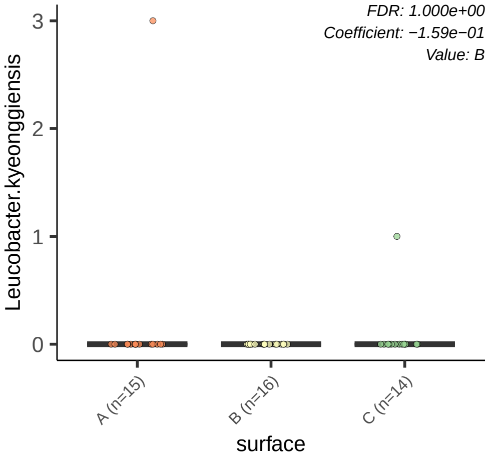
FDR: 1.000e+00
Coefficient: −1.59e−01
Value: B
Leucobacter.kyeonggiensis
3
2
1
0
A (n=15)
B (n=16)
C (n=14)
surface

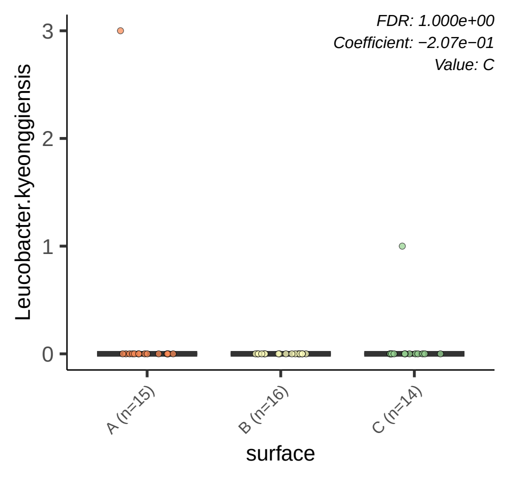

FDR: 1.000e+00
Coefficient: −2.07e−01
Value: C
Leucobacter.kyeonggiensis
0
1
2
3
A (n=15)
B (n=16)
C (n=14)
surface

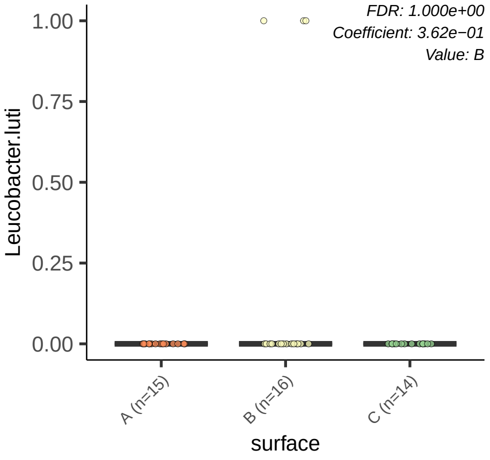

FDR: 1.000e+00
Coefficient: 3.62e−01
Value: B
Leucobacter.luti
1.00
0.75
0.50
0.25
0.00
A (n=15)
B (n=16)
C (n=14)
surface

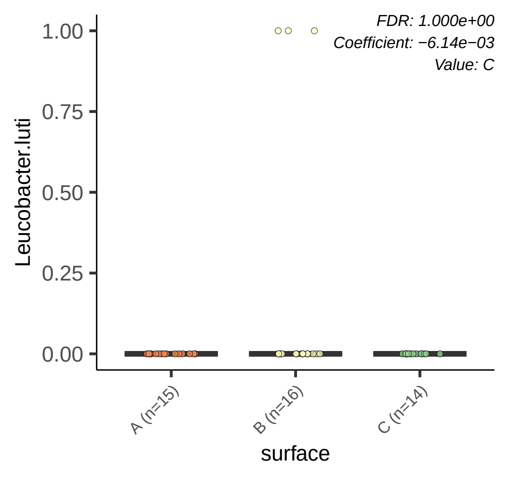

FDR: 1.000e+00
Coefficient: −6.14e−03
Value: C
Leucobacter.luti
1.00
0.75
0.50
0.25
0.00
A (n=15)
B (n=16)
C (n=14)
surface

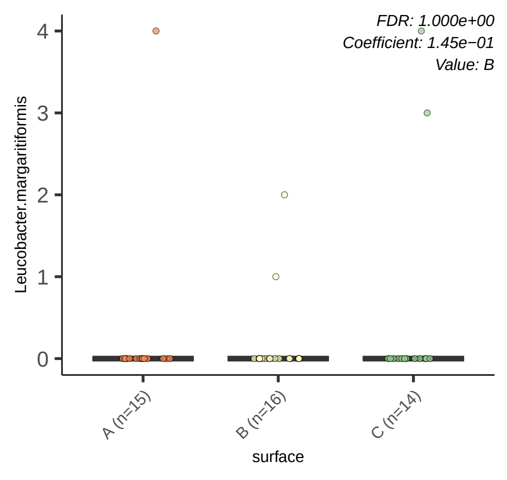

FDR: 1.000e+00
Coefficient: 1.45e−01
Value: B
Leucobacter.margaritiformis
0
1
2
3
4
A (n=15)
B (n=16)
C (n=14)
surface

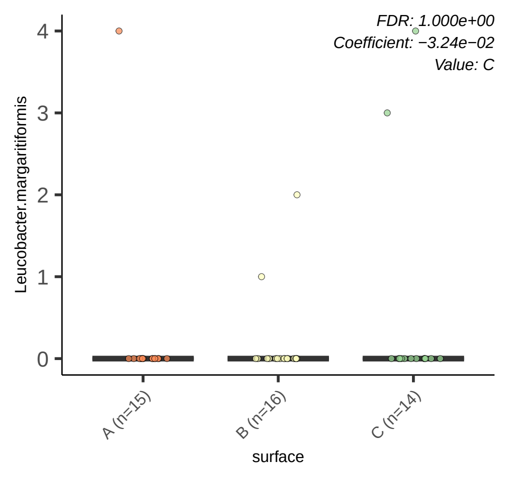
FDR: 1.000e+00
Coefficient: −3.24e−02
Value: C
Leucobacter.margaritiformis
0
1
2
3
4
A (n=15)
B (n=16)
C (n=14)
surface

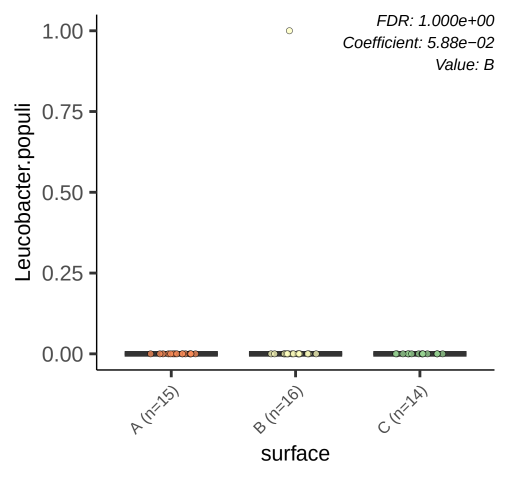
FDR: 1.000e+00
Coefficient: 5.88e−02
Value: B
Leucobacter.populi
1.00
0.75
0.50
0.25
0.00
A (n=15)
B (n=16)
C (n=14)
surface

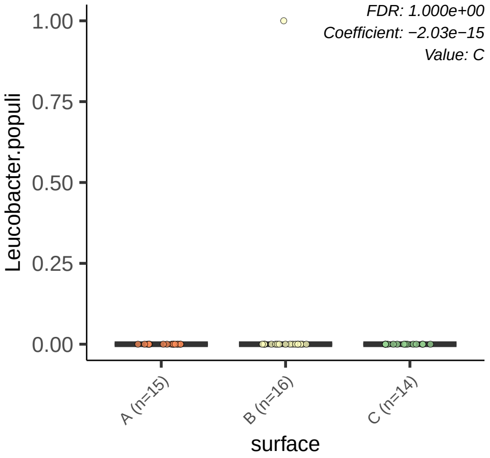

FDR: 1.000e+00
Coefficient: −2.03e−15
Value: C
Leucobacter.populi
1.00
0.75
0.50
0.25
0.00
A (n=15)
B (n=16)
C (n=14)
surface

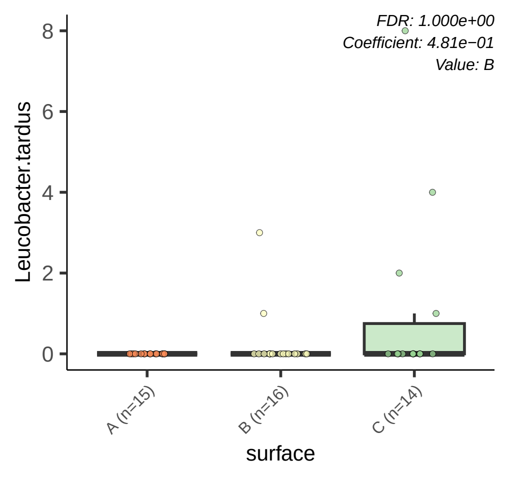
FDR: 1.000e+00
Coefficient: 4.81e−01
Value: B
Leucobacter.tardus
0
2
4
6
8
A (n=15)
B (n=16)
C (n=14)
surface

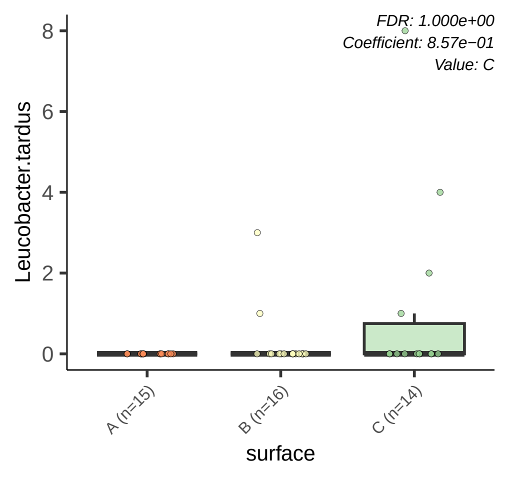
FDR: 1.000e+00
Coefficient: 8.57e−01
Value: C
Leucobacter.tardus
0
2
4
6
8
A (n=15)
B (n=16)
C (n=14)
surface

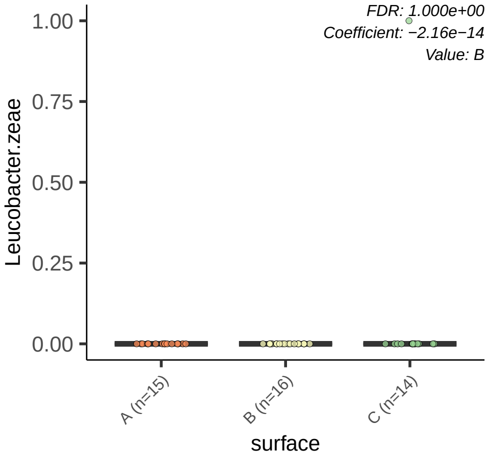

FDR: 1.000e+00
Coefficient: −2.16e−14
Value: B
Leucobacter.zeae
1.00
0.75
0.50
0.25
0.00
A (n=15)
B (n=16)
C (n=14)
surface

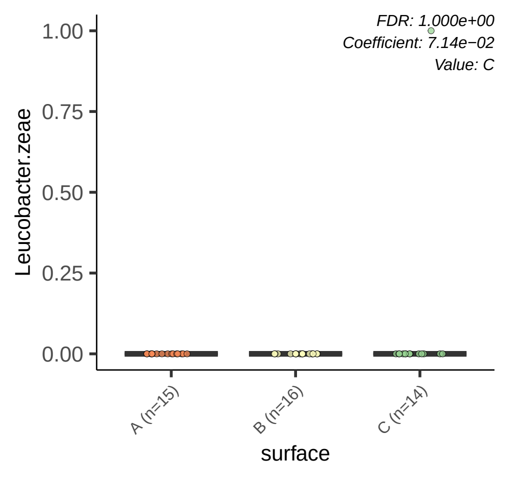
FDR: 1.000e+00
Coefficient: 7.14e−02
Value: C
1.00
0.75
0.50
0.25
0.00
Leucobacter.zeae
A (n=15)
B (n=16)
C (n=14)
surface

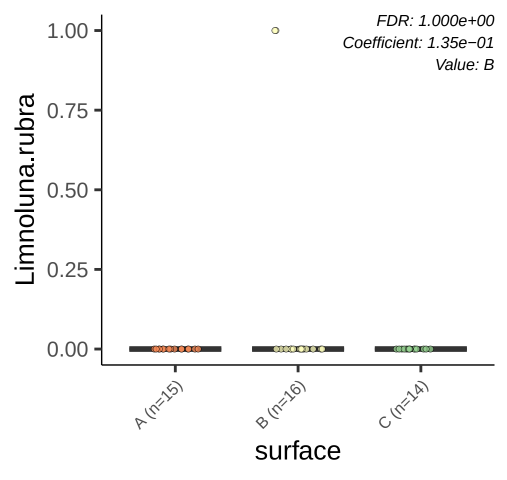
FDR: 1.000e+00
Coefficient: 1.35e−01
Value: B
Limnoluna.rubra
1.00
0.75
0.50
0.25
0.00
A (n=15)
B (n=16)
C (n=14)
surface

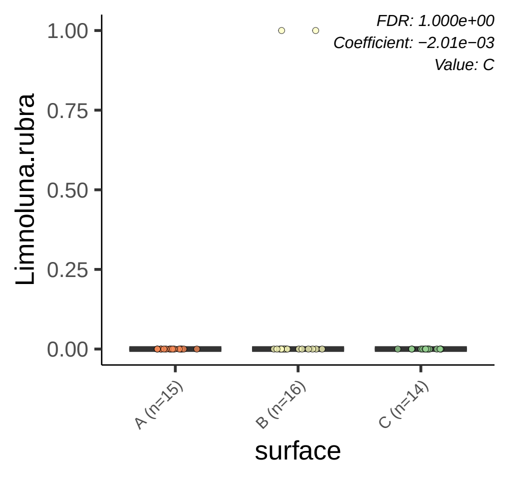
FDR: 1.000e+00
Coefficient: −2.01e−03
Value: C
Limnoluna.rubra
1.00
0.75
0.50
0.25
0.00
A (n=15)
B (n=16)
C (n=14)
surface

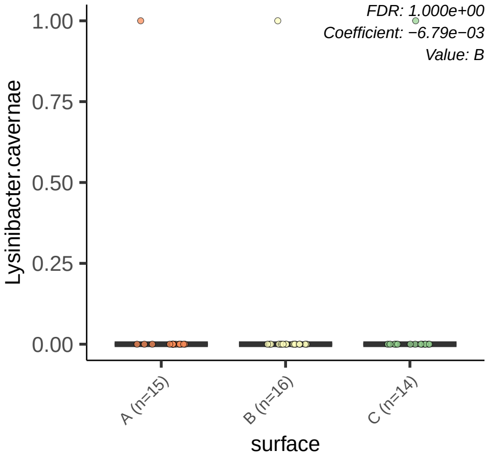
FDR: 1.000e+00
Coefficient: −6.79e−03
Value: B
Lysinibacter.cavernae
1.00
0.75
0.50
0.25
0.00
A (n=15)
B (n=16)
C (n=14)
surface

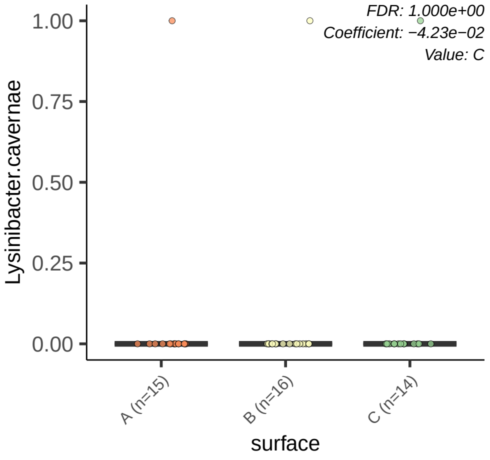
FDR: 1.000e+00
Coefficient: −4.23e−02
Value: C
1.00
0.75
0.50
0.25
0.00
Lysinibacter.cavernae
A (n=15)
B (n=16)
C (n=14)
surface

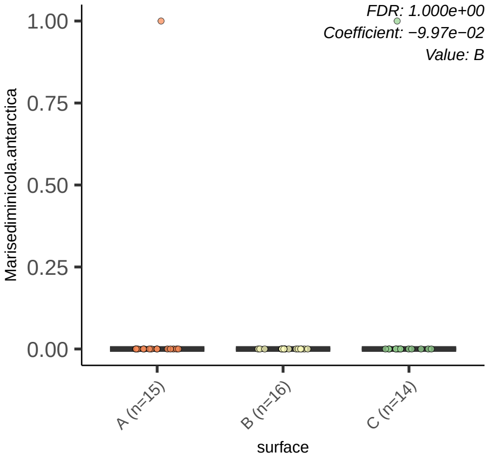

FDR: 1.000e+00
Coefficient: −9.97e−02
Value: B
1.00
0.75
0.50
0.25
0.00
Marisediminicola.antarctica
A (n=15)
B (n=16)
C (n=14)
surface

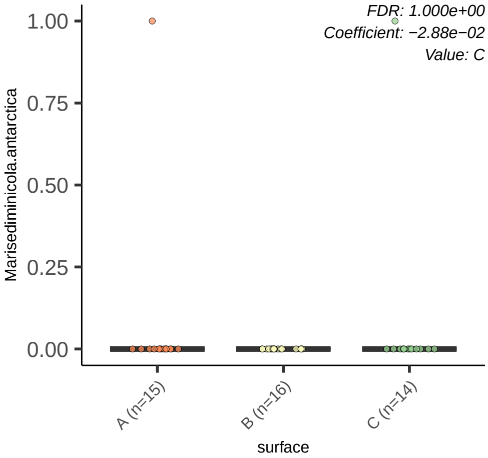
FDR: 1.000e+00
Coefficient: −2.88e−02
Value: C
Marisediminicola.antarctica
1.00
0.75
0.50
0.25
0.00
A (n=15)
B (n=16)
C (n=14)
surface

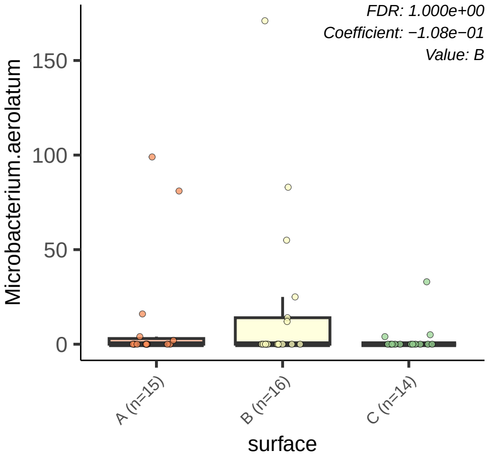

FDR: 1.000e+00
Coefficient: −1.08e−01
Value: B
Microbacterium.aerolatum
150
100
50
0
A (n=15)
B (n=16)
C (n=14)
surface

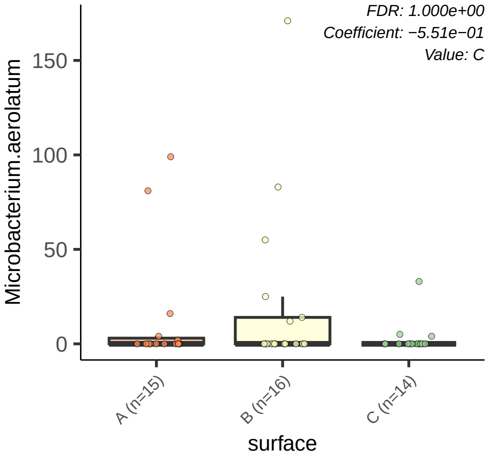
FDR: 1.000e+00
Coefficient: −5.51e−01
Value: C
Microbacterium.aerolatum
150
100
50
0
A (n=15)
B (n=16)
C (n=14)
surface

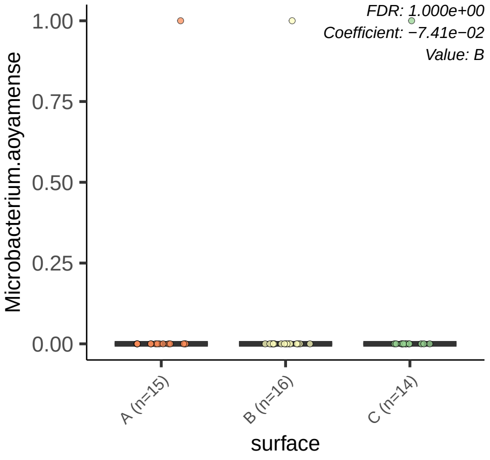
FDR: 1.000e+00
Coefficient: −7.41e−02
Value: B
Microbacterium.aoyamense
1.00
0.75
0.50
0.25
0.00
A (n=15)
B (n=16)
C (n=14)
surface

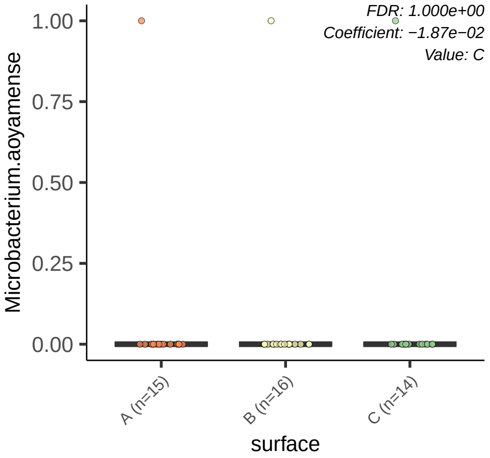
FDR: 1.000e+00
Coefficient: −1.87e−02
Value: C
Microbacterium.aoyamense
1.00
0.75
0.50
0.25
0.00
A (n=15)
B (n=16)
C (n=14)
surface

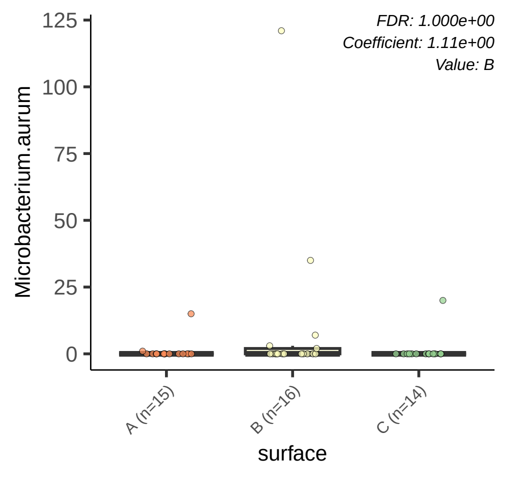
FDR: 1.000e+00
Coefficient: 1.11e+00
Value: B
Microbacterium.aurum
125
100
75
50
25
0
A (n=15)
B (n=16)
C (n=14)
surface

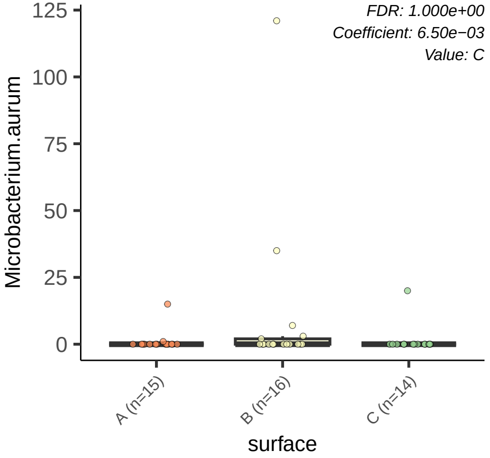
FDR: 1.000e+00
Coefficient: 6.50e−03
Value: C
Microbacterium.aurum
125
100
75
50
25
0
A (n=15)
B (n=16)
C (n=14)
surface

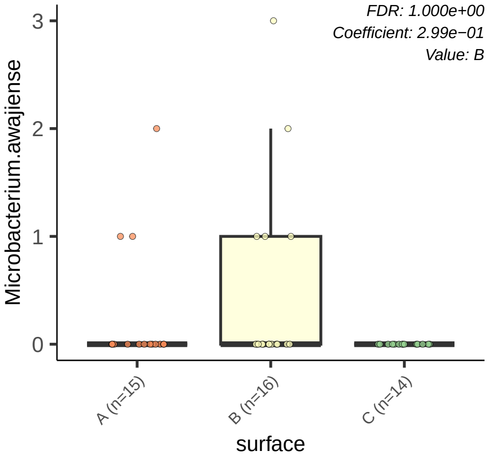

FDR: 1.000e+00
Coefficient: 2.99e−01
Value: B
Microbacterium.awajiense
3
2
1
0
A (n=15)
B (n=16)
C (n=14)
surface

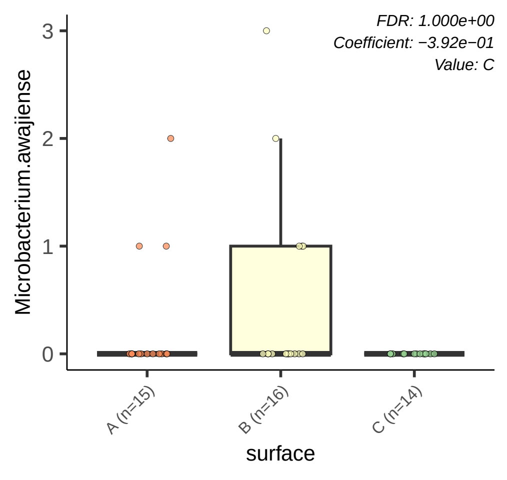

FDR: 1.000e+00
Coefficient: −3.92e−01
Value: C
Microbacterium.awajiense
3
2
1
0
A (n=15)
B (n=16)
C (n=14)
surface

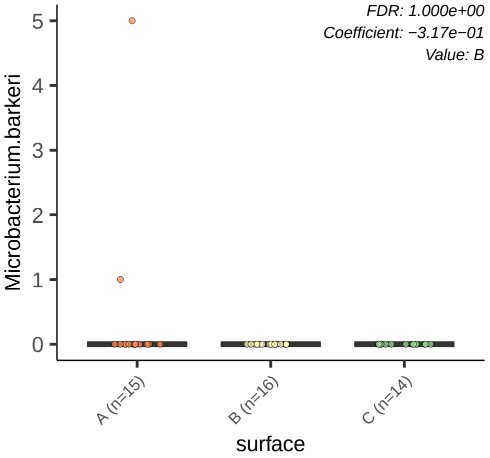

FDR: 1.000e+00
Coefficient: −3.17e−01
Value: B
Microbacterium.barkeri
5
4
3
2
1
0
A (n=15)
B (n=16)
C (n=14)
surface

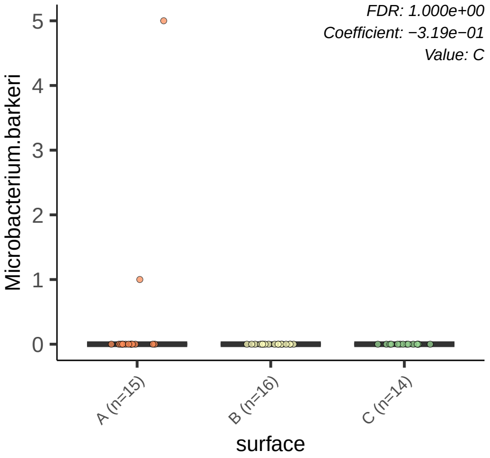
FDR: 1.000e+00
Coefficient: −3.19e−01
Value: C
Microbacterium.barkeri
0
1
2
3
4
5
A (n=15)
B (n=16)
C (n=14)
surface

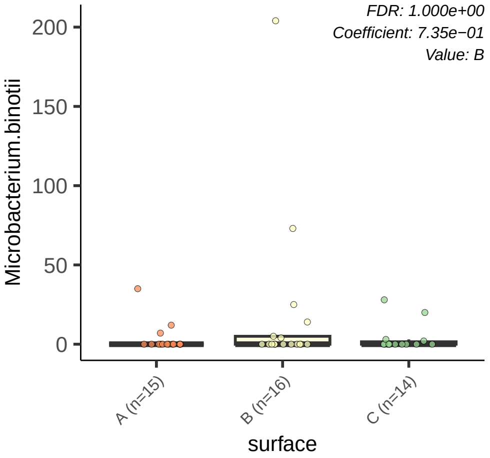
FDR: 1.000e+00
Coefficient: 7.35e−01
Value: B
Microbacterium.binotii
200
150
100
50
0
A (n=15)
B (n=16)
C (n=14)
surface

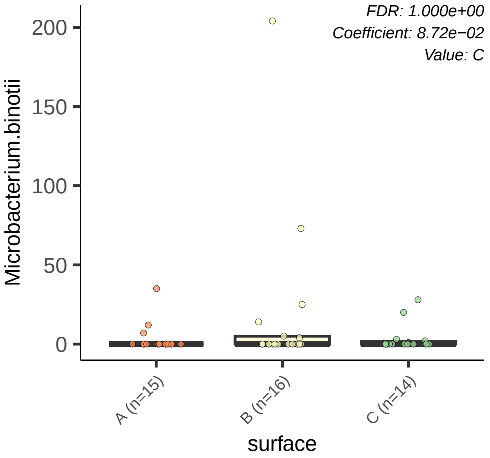

FDR: 1.000e+00
Coefficient: 8.72e−02
Value: C
Microbacterium.binotii
200
150
100
50
0
A (n=15)
B (n=16)
C (n=14)
surface

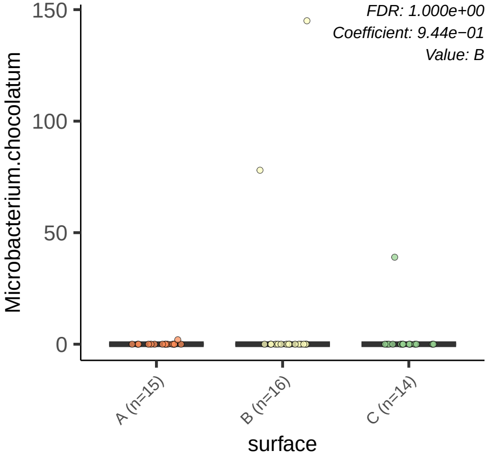
FDR: 1.000e+00
Coefficient: 9.44e−01
Value: B
Microbacterium.chocolatum
150
100
50
0
A (n=15)
B (n=16)
C (n=14)
surface

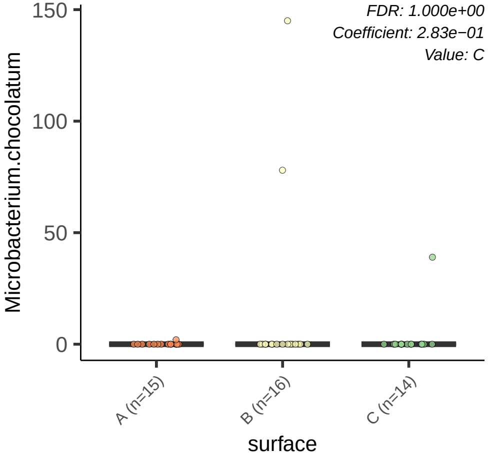

FDR: 1.000e+00
Coefficient: 2.83e−01
Value: C
Microbacterium.chocolatum
150
100
50
0
A (n=15)
B (n=16)
C (n=14)
surface

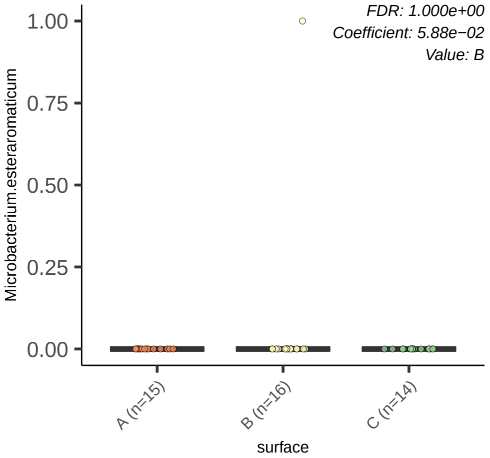
FDR: 1.000e+00
Coefficient: 5.88e−02
Value: B
Microbacterium.esteraromaticum
1.00
0.75
0.50
0.25
0.00
A (n=15)
B (n=16)
C (n=14)
surface

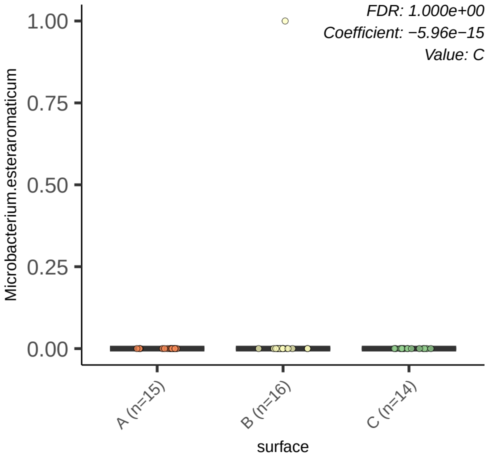

FDR: 1.000e+00
Coefficient: −5.96e−15
Value: C
1.00
0.75
0.50
0.25
0.00
Microbacterium.esteraromaticum
A (n=15)
B (n=16)
C (n=14)
surface

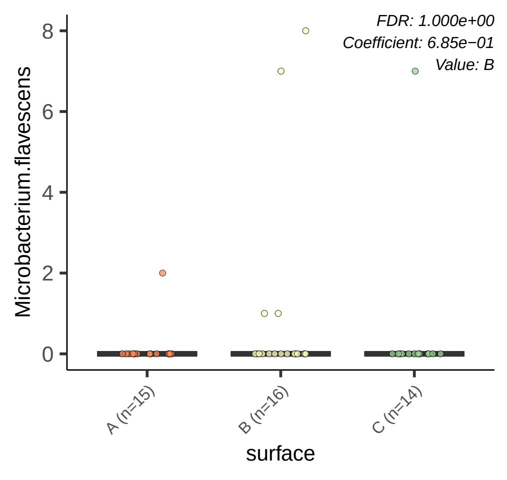

FDR: 1.000e+00
Coefficient: 6.85e−01
Value: B
Microbacterium.flavescens
0
2
4
6
8
A (n=15)
B (n=16)
C (n=14)
surface

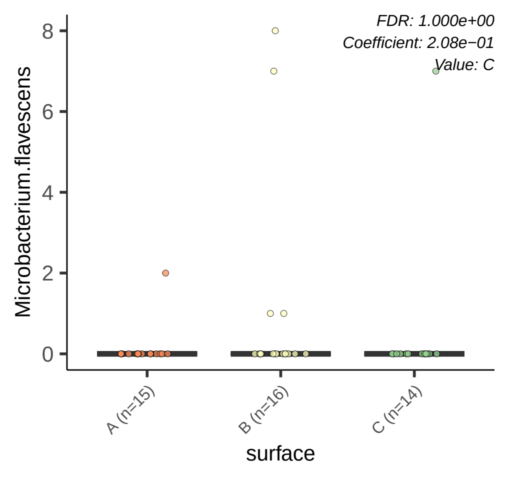

FDR: 1.000e+00
Coefficient: 2.08e−01
Value: C
Microbacterium.flavescens
0
2
4
6
8
A (n=15)
B (n=16)
C (n=14)
surface

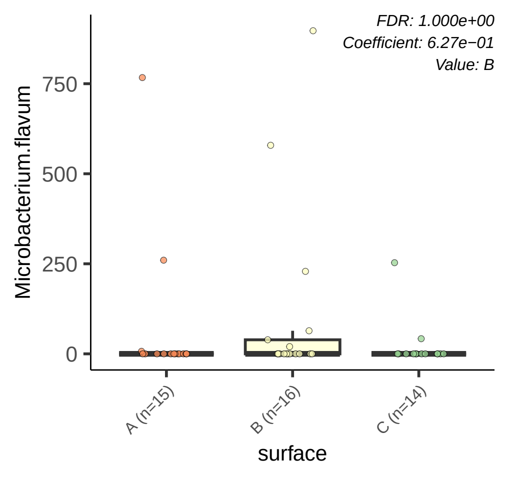
FDR: 1.000e+00
Coefficient: 6.27e−01
Value: B
Microbacterium.flavum
750
500
250
0
A (n=15)
B (n=16)
C (n=14)
surface

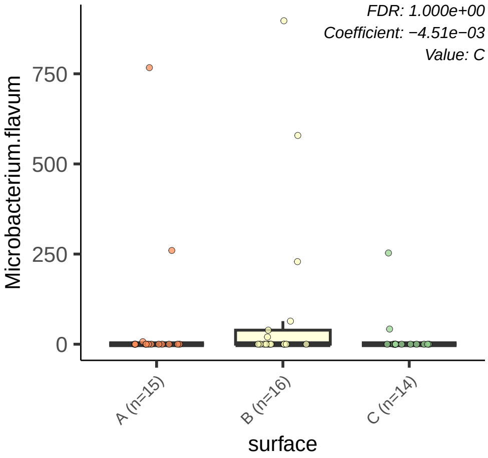
FDR: 1.000e+00
Coefficient: −4.51e−03
Value: C
Microbacterium.flavum
750
500
250
0
A (n=15)
B (n=16)
C (n=14)
surface

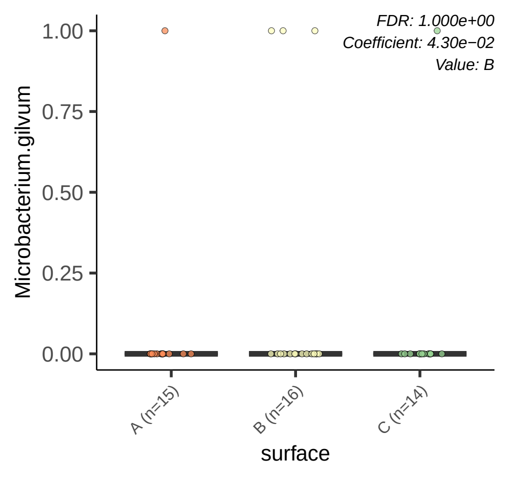

FDR: 1.000e+00
Coefficient: 4.30e−02
Value: B
Microbacterium.gilvum
1.00
0.75
0.50
0.25
0.00
A (n=15)
B (n=16)
C (n=14)
surface

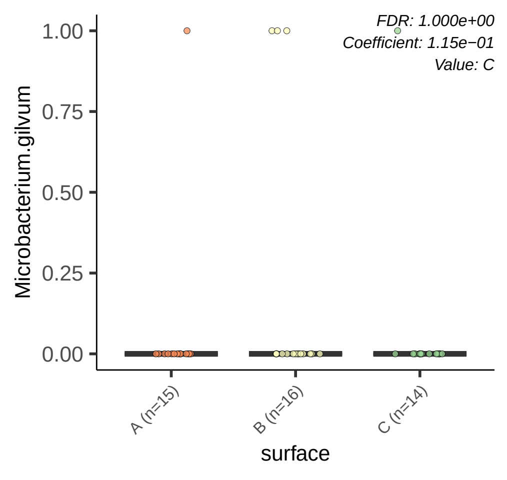
FDR: 1.000e+00
Coefficient: 1.15e−01
Value: C
Microbacterium.gilvum
1.00
0.75
0.50
0.25
0.00
A (n=15)
B (n=16)
C (n=14)
surface

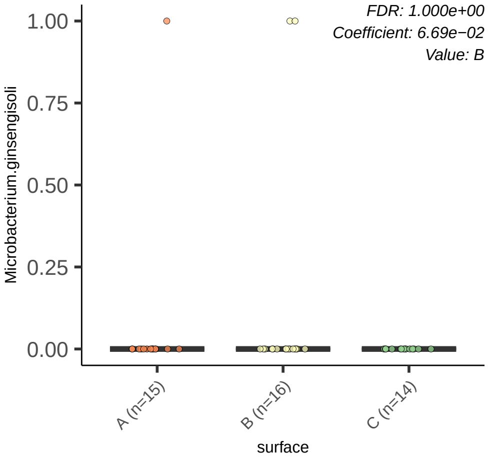
FDR: 1.000e+00
Coefficient: 6.69e−02
Value: B
Microbacterium.ginsengisoli
1.00
0.75
0.50
0.25
0.00
A (n=15)
B (n=16)
C (n=14)
surface

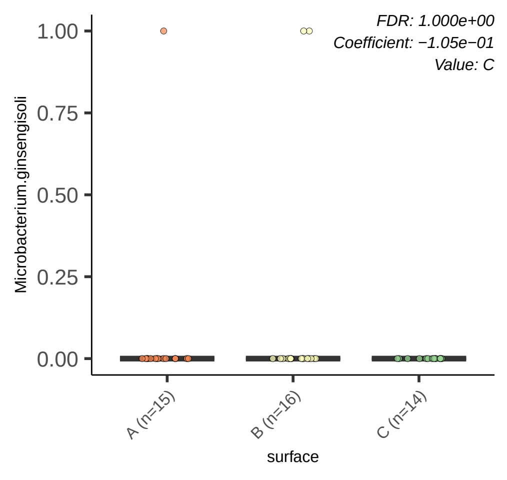

FDR: 1.000e+00
Coefficient: −1.05e−01
Value: C
Microbacterium.ginsengisoli
1.00
0.75
0.50
0.25
0.00
A (n=15)
B (n=16)
C (n=14)
surface

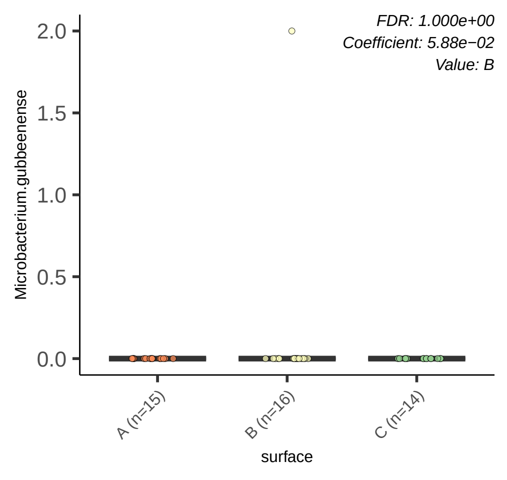
FDR: 1.000e+00
Coefficient: 5.88e−02
Value: B
Microbacterium.gubbeenense
2.0
1.5
1.0
0.5
0.0
A (n=15)
B (n=16)
C (n=14)
surface

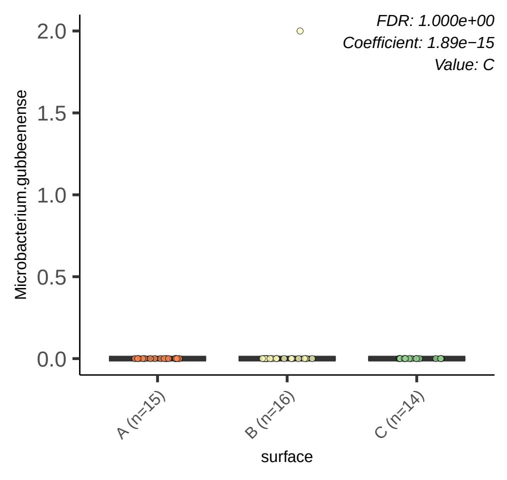
FDR: 1.000e+00
Coefficient: 1.89e−15
Value: C
2.0
1.5
1.0
0.5
0.0
Microbacterium.gubbeenense
A (n=15)
B (n=16)
C (n=14)
surface

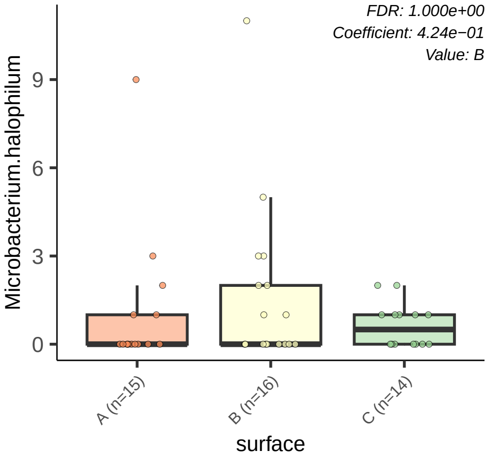

FDR: 1.000e+00
Coefficient: 4.24e−01
Value: B
Microbacterium.halophilum
0
3
6
9
A (n=15)
B (n=16)
C (n=14)
surface

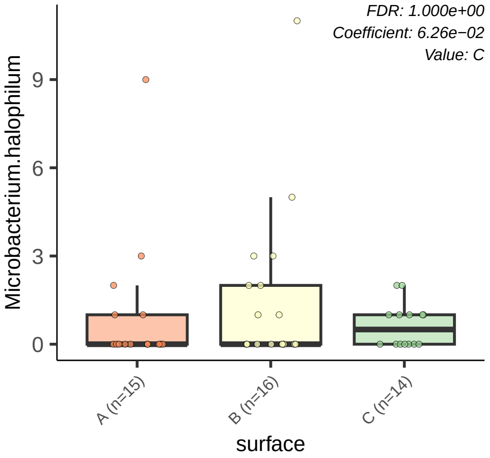
FDR: 1.000e+00
Coefficient: 6.26e−02
Value: C
Microbacterium.halophilum
0
3
6
9
A (n=15)
B (n=16)
C (n=14)
surface

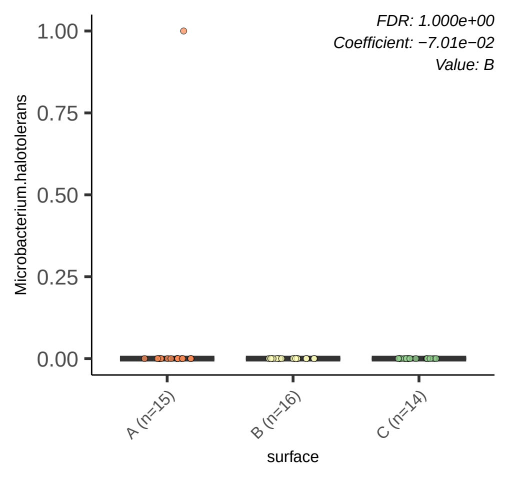

FDR: 1.000e+00
Coefficient: −7.01e−02
Value: B
1.00
0.75
0.50
0.25
0.00
Microbacterium.halotolerans
A (n=15)
B (n=16)
C (n=14)
surface

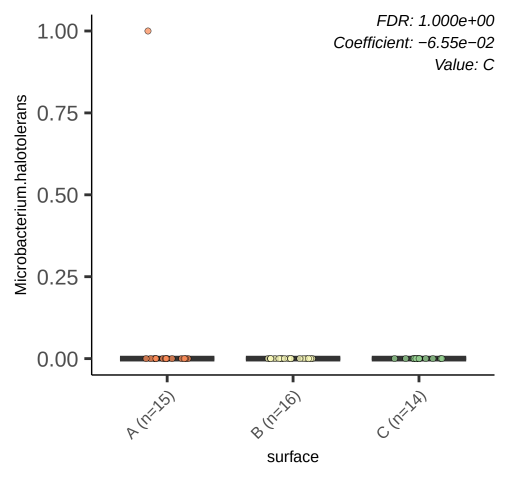

FDR: 1.000e+00
Coefficient: −6.55e−02
Value: C
Microbacterium.halotolerans
1.00
0.75
0.50
0.25
0.00
A (n=15)
B (n=16)
C (n=14)
surface

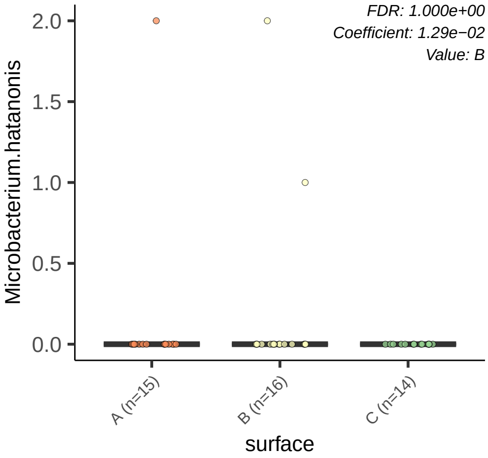

FDR: 1.000e+00
Coefficient: 1.29e−02
Value: B
Microbacterium.hatanonis
2.0
1.5
1.0
0.5
0.0
A (n=15)
B (n=16)
C (n=14)
surface

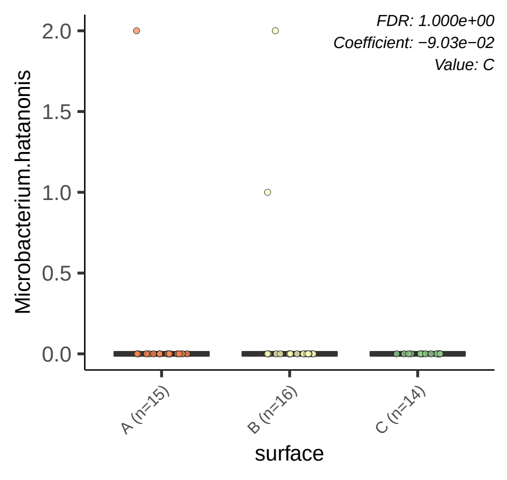
FDR: 1.000e+00
Coefficient: −9.03e−02
Value: C
Microbacterium.hatanonis
2.0
1.5
1.0
0.5
0.0
A (n=15)
B (n=16)
C (n=14)
surface

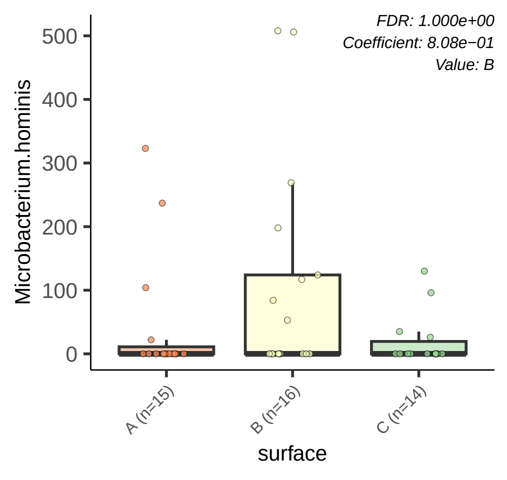

FDR: 1.000e+00
Coefficient: 8.08e−01
Value: B
Microbacterium.hominis
500
400
300
200
100
0
A (n=15)
B (n=16)
C (n=14)
surface

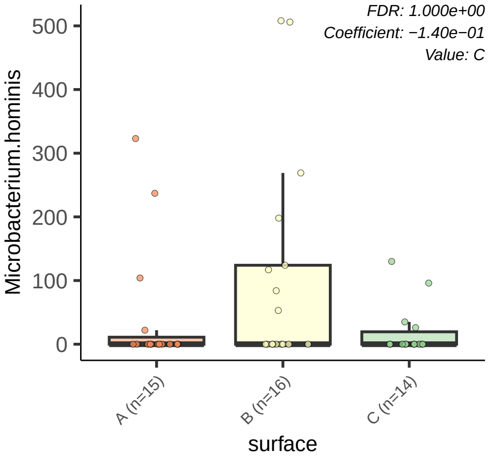
FDR: 1.000e+00
Coefficient: −1.40e−01
Value: C
Microbacterium.hominis
500
400
300
200
100
0
A (n=15)
B (n=16)
C (n=14)
surface

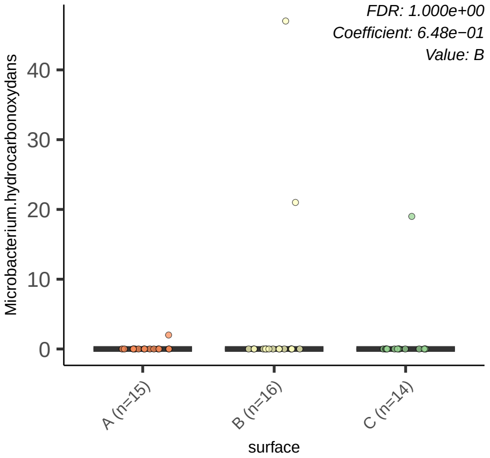

FDR: 1.000e+00
Coefficient: 6.48e−01
Value: B
Microbacterium.hydrocarbonoxydans
40
30
20
10
0
A (n=15)
B (n=16)
C (n=14)
surface

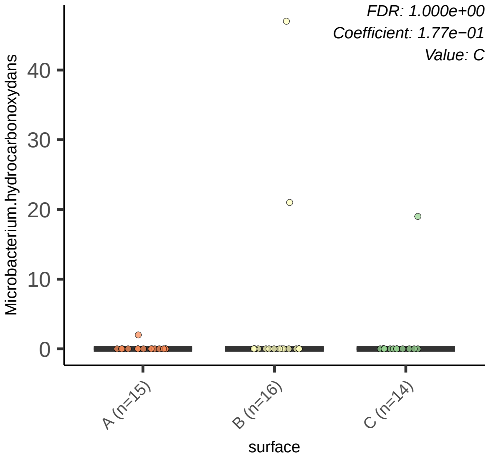

FDR: 1.000e+00
Coefficient: 1.77e−01
Value: C
Microbacterium.hydrocarbonoxydans
40
30
20
10
0
A (n=15)
B (n=16)
C (n=14)
surface

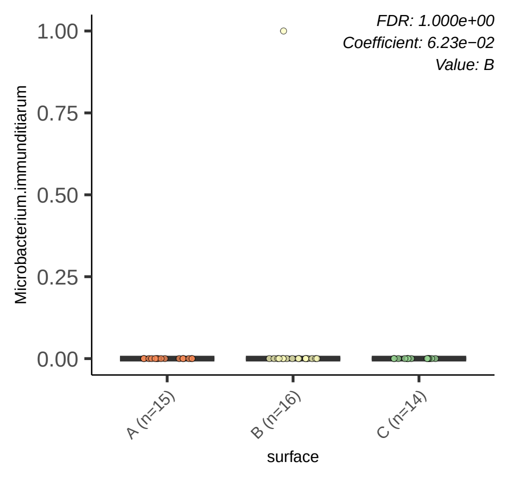
FDR: 1.000e+00
Coefficient: 6.23e−02
Value: B
Microbacterium.immunditiarum
1.00
0.75
0.50
0.25
0.00
A (n=15)
B (n=16)
C (n=14)
surface

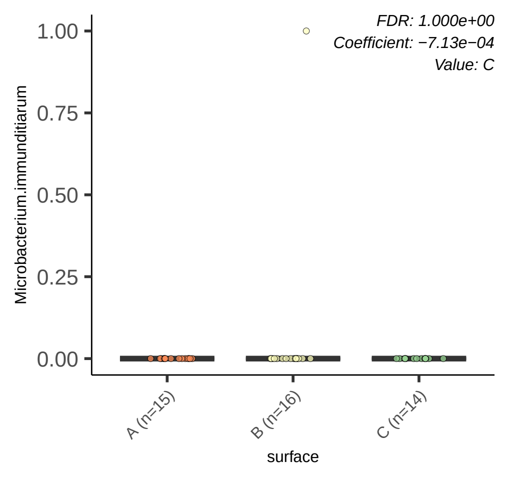
FDR: 1.000e+00
Coefficient: −7.13e−04
Value: C
Microbacterium.immunditiarum
1.00
0.75
0.50
0.25
0.00
A (n=15)
B (n=16)
C (n=14)
surface

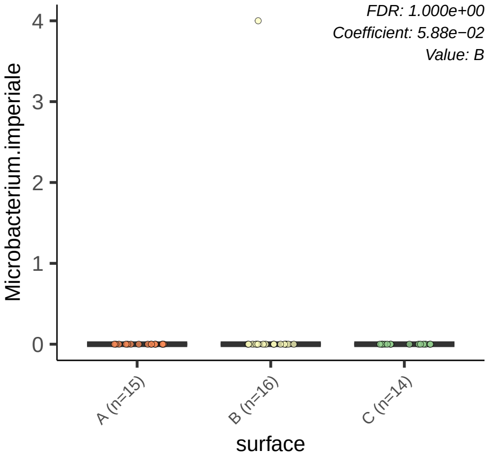
FDR: 1.000e+00
Coefficient: 5.88e−02
Value: B
Microbacterium.imperiale
0
1
2
3
4
A (n=15)
B (n=16)
C (n=14)
surface

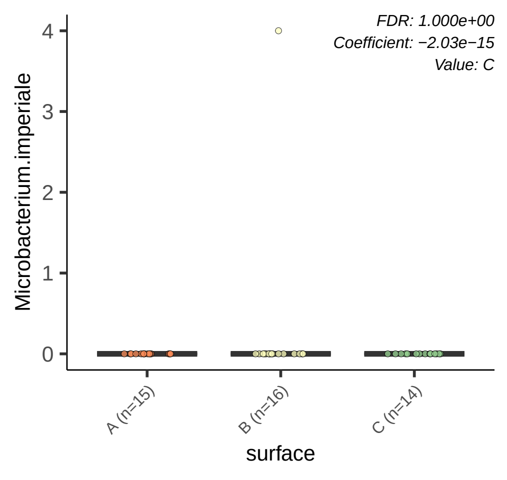
FDR: 1.000e+00
Coefficient: −2.03e−15
Value: C
Microbacterium.imperiale
0
1
2
3
4
A (n=15)
B (n=16)
C (n=14)
surface

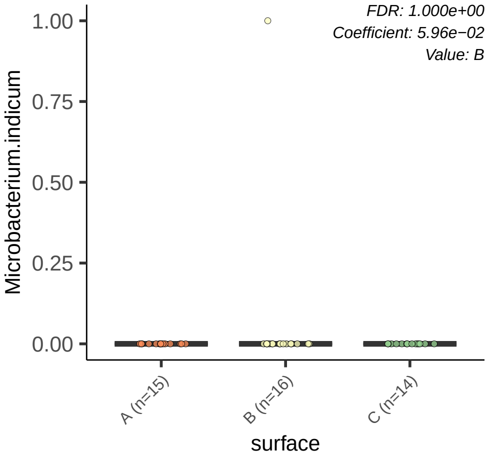
FDR: 1.000e+00
Coefficient: 5.96e−02
Value: B
Microbacterium.indicum
1.00
0.75
0.50
0.25
0.00
A (n=15)
B (n=16)
C (n=14)
surface

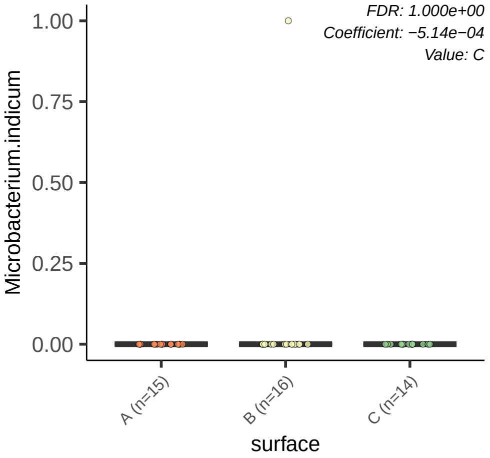

FDR: 1.000e+00
Coefficient: −5.14e−04
Value: C
Microbacterium.indicum
1.00
0.75
0.50
0.25
0.00
A (n=15)
B (n=16)
C (n=14)
surface

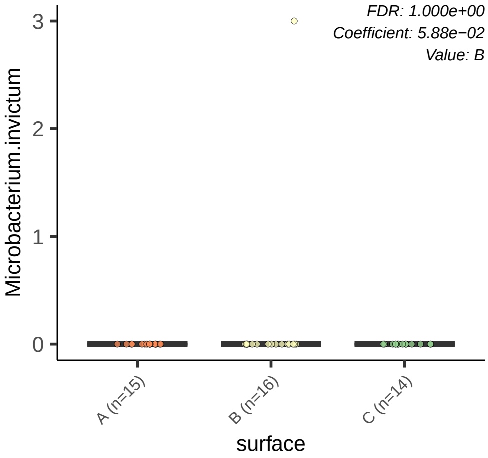
FDR: 1.000e+00
Coefficient: 5.88e−02
Value: B
Microbacterium.invictum
3
2
1
0
A (n=15)
B (n=16)
C (n=14)
surface

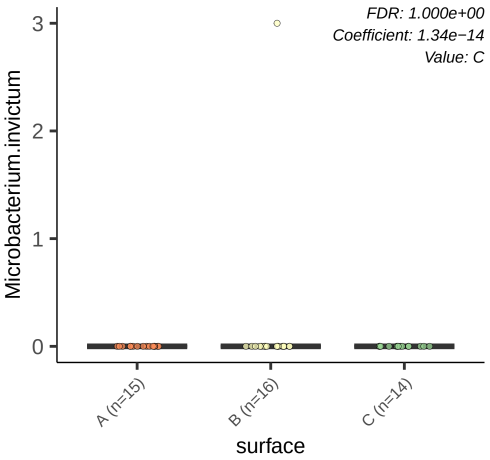
FDR: 1.000e+00
Coefficient: 1.34e−14
Value: C
Microbacterium.invictum
3
2
1
0
A (n=15)
B (n=16)
C (n=14)
surface

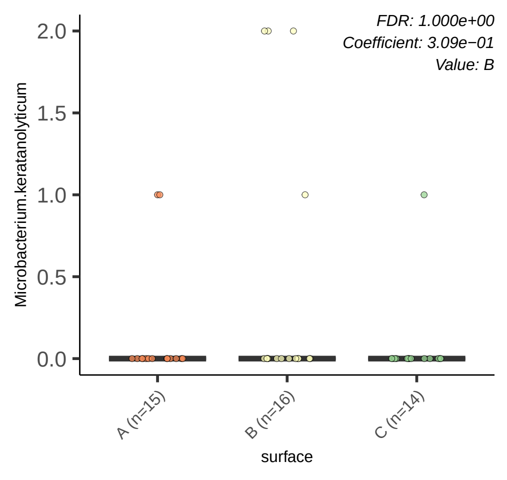
FDR: 1.000e+00
Coefficient: 3.09e−01
Value: B
Microbacterium.keratanolyticum
2.0
1.5
1.0
0.5
0.0
A (n=15)
B (n=16)
C (n=14)
surface

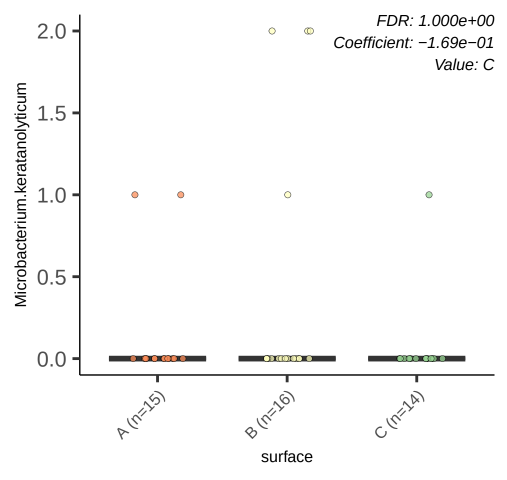
FDR: 1.000e+00
Coefficient: −1.69e−01
Value: C
Microbacterium.keratanolyticum
2.0
1.5
1.0
0.5
0.0
A (n=15)
B (n=16)
C (n=14)
surface

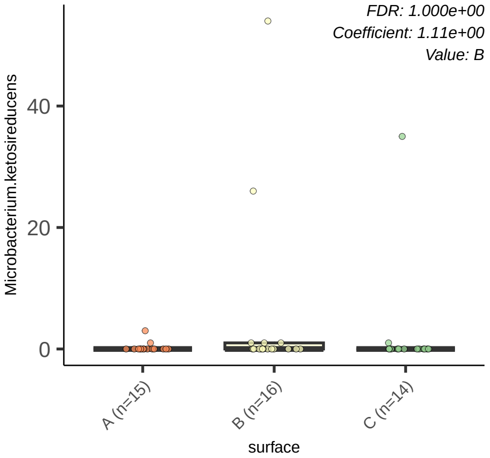
FDR: 1.000e+00
Coefficient: 1.11e+00
Value: B
Microbacterium.ketosireducens
40
20
0
A (n=15)
B (n=16)
C (n=14)
surface

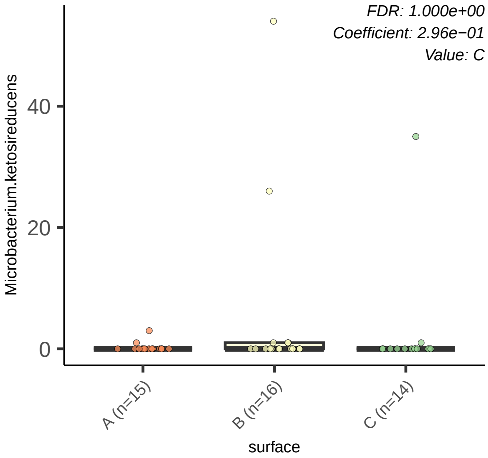
FDR: 1.000e+00
Coefficient: 2.96e−01
Value: C
Microbacterium.ketosireducens
0
20
40
A (n=15)
B (n=16)
C (n=14)
surface

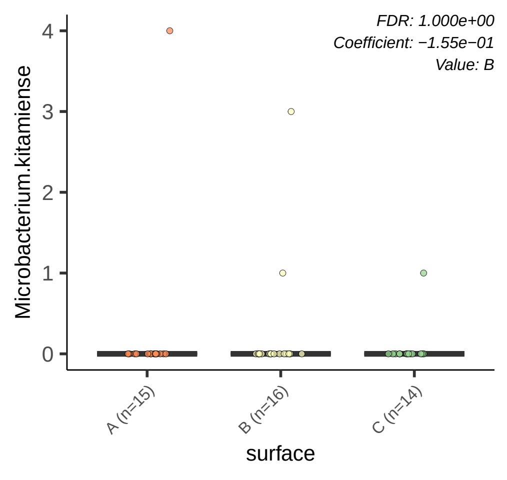
FDR: 1.000e+00
Coefficient: −1.55e−01
Value: B
Microbacterium.kitamiense
4
3
2
1
0
A (n=15)
B (n=16)
C (n=14)
surface

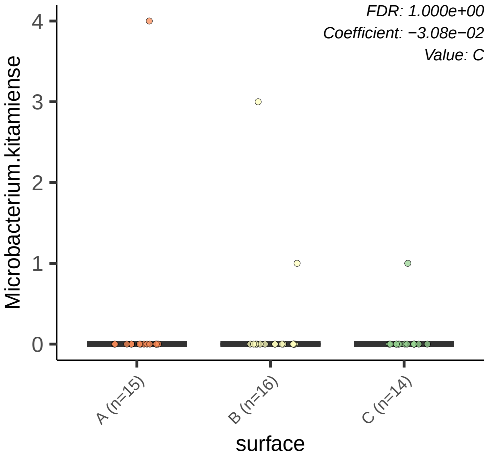
FDR: 1.000e+00
Coefficient: −3.08e−02
Value: C
Microbacterium.kitamiense
4
3
2
1
0
A (n=15)
B (n=16)
C (n=14)
surface

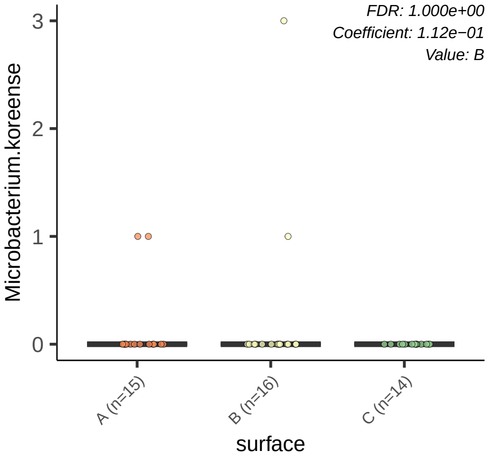
FDR: 1.000e+00
Coefficient: 1.12e−01
Value: B
Microbacterium.koreense
3
2
1
0
A (n=15)
B (n=16)
C (n=14)
surface

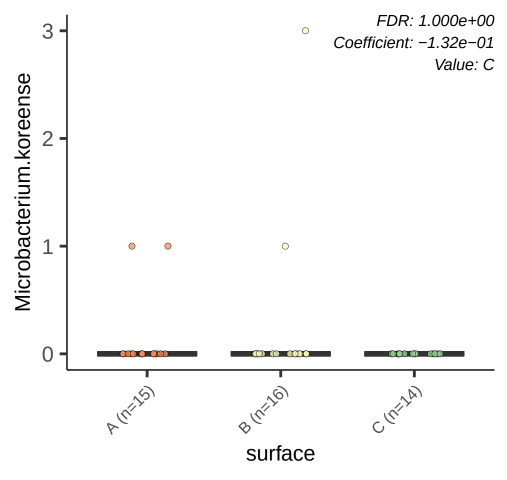

FDR: 1.000e+00
Coefficient: −1.32e−01
Value: C
Microbacterium.koreense
0
1
2
3
A (n=15)
B (n=16)
C (n=14)
surface

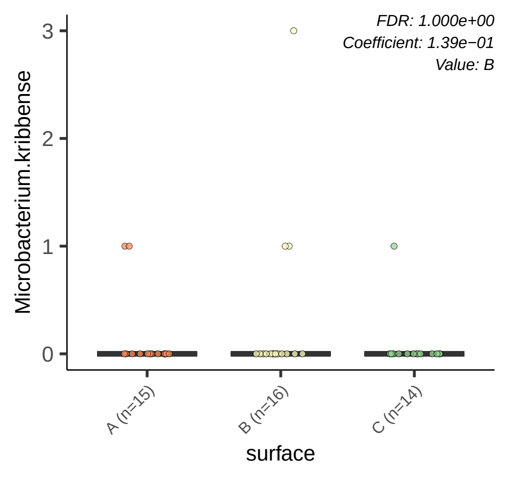
FDR: 1.000e+00
Coefficient: 1.39e−01
Value: B
Microbacterium.kribbense
3
2
1
0
A (n=15)
B (n=16)
C (n=14)
surface

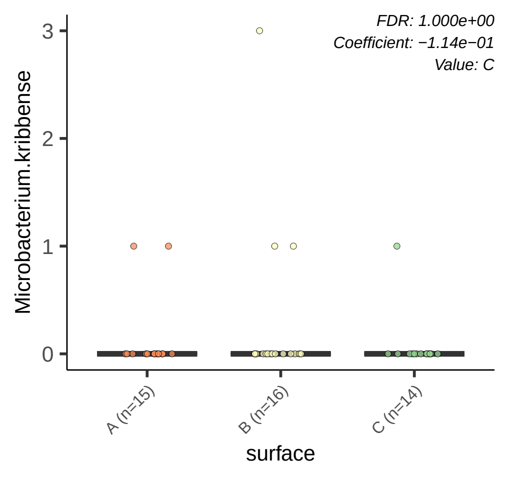
FDR: 1.000e+00
Coefficient: −1.14e−01
Value: C
Microbacterium.kribbense
3
2
1
0
A (n=15)
B (n=16)
C (n=14)
surface

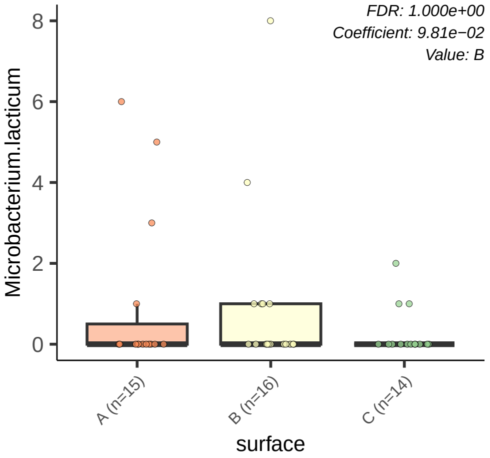
FDR: 1.000e+00
Coefficient: 9.81e−02
Value: B
Microbacterium.lacticum
0
2
4
6
8
A (n=15)
B (n=16)
C (n=14)
surface

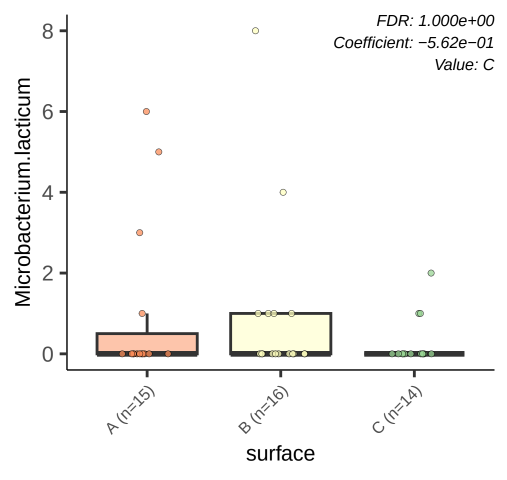

FDR: 1.000e+00
Coefficient: −5.62e−01
Value: C
Microbacterium.lacticum
0
2
4
6
8
A (n=15)
B (n=16)
C (n=14)
surface

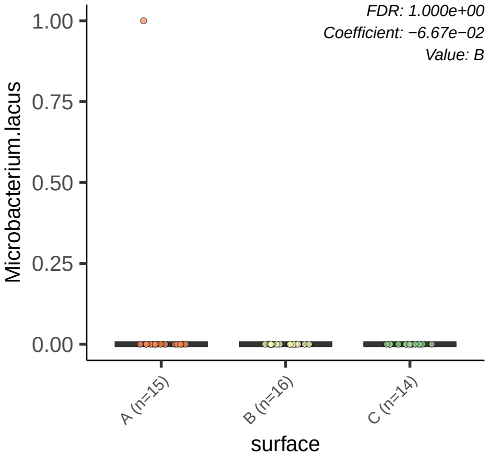

FDR: 1.000e+00
Coefficient: −6.67e−02
Value: B
Microbacterium.lacus
1.00
0.75
0.50
0.25
0.00
A (n=15)
B (n=16)
C (n=14)
surface

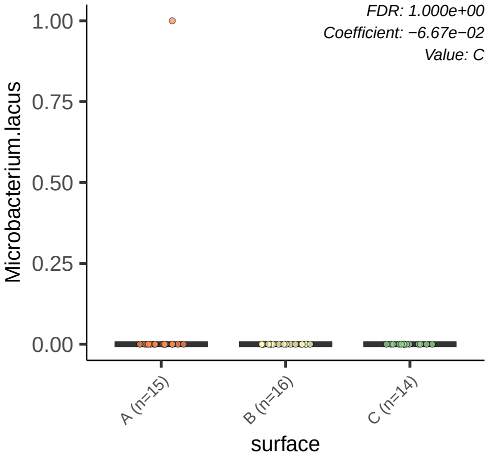
FDR: 1.000e+00
Coefficient: −6.67e−02
Value: C
Microbacterium.lacus
1.00
0.75
0.50
0.25
0.00
A (n=15)
B (n=16)
C (n=14)
surface

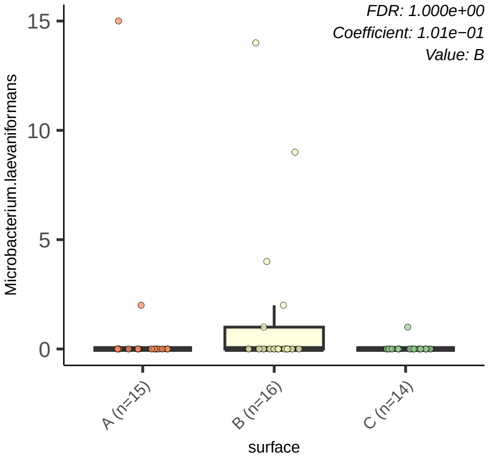
FDR: 1.000e+00
Coefficient: 1.01e−01
Value: B
Microbacterium.laevaniformans
15
10
5
0
A (n=15)
B (n=16)
C (n=14)
surface

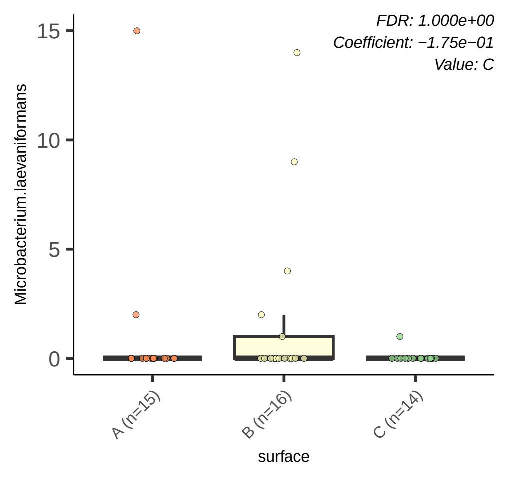

FDR: 1.000e+00
Coefficient: −1.75e−01
Value: C
Microbacterium.laevaniformans
15
10
5
0
A (n=15)
B (n=16)
C (n=14)
surface

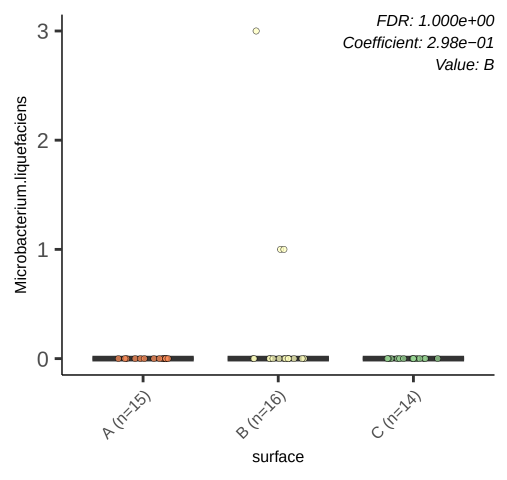

FDR: 1.000e+00
Coefficient: 2.98e−01
Value: B
Microbacterium.liquefaciens
3
2
1
0
A (n=15)
B (n=16)
C (n=14)
surface

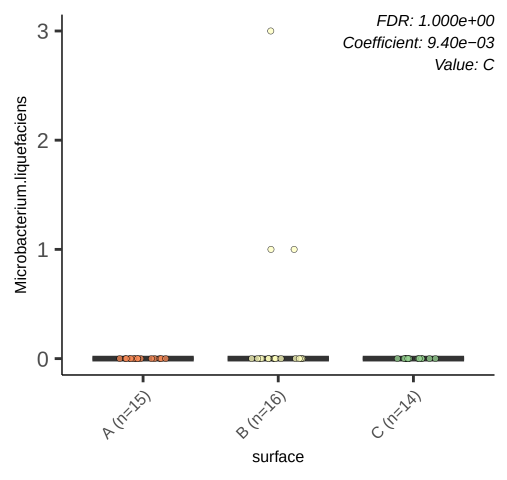
FDR: 1.000e+00
Coefficient: 9.40e−03
Value: C
Microbacterium.liquefaciens
0
1
2
3
A (n=15)
B (n=16)
C (n=14)
surface

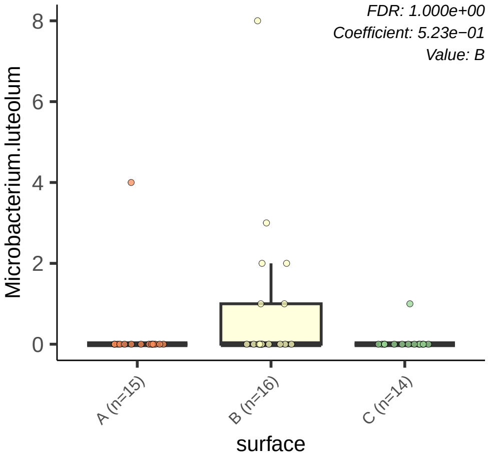

FDR: 1.000e+00
Coefficient: 5.23e−01
Value: B
Microbacterium.luteolum
0
2
4
6
8
A (n=15)
B (n=16)
C (n=14)
surface

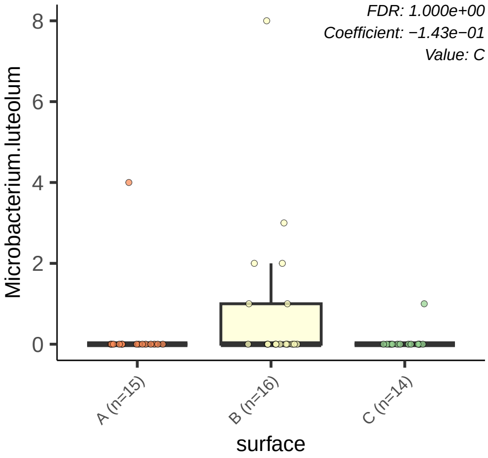

FDR: 1.000e+00
Coefficient: −1.43e−01
Value: C
Microbacterium.luteolum
8
6
4
2
0
A (n=15)
B (n=16)
C (n=14)
surface

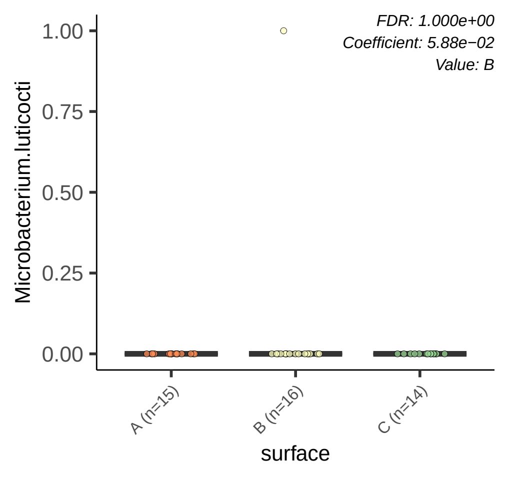
FDR: 1.000e+00
Coefficient: 5.88e−02
Value: B
Microbacterium.luticocti
1.00
0.75
0.50
0.25
0.00
A (n=15)
B (n=16)
C (n=14)
surface

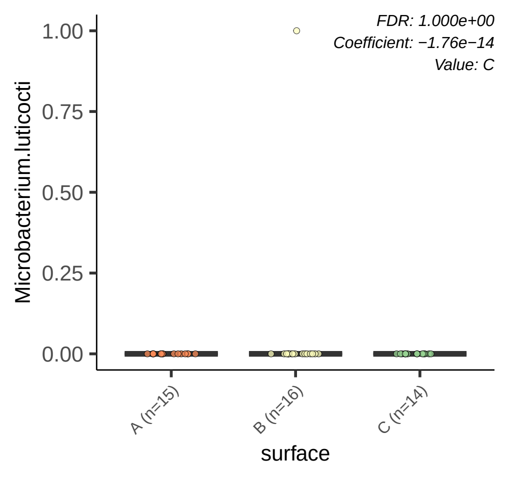

FDR: 1.000e+00
Coefficient: −1.76e−14
Value: C
Microbacterium.luticocti
1.00
0.75
0.50
0.25
0.00
A (n=15)
B (n=16)
C (n=14)
surface

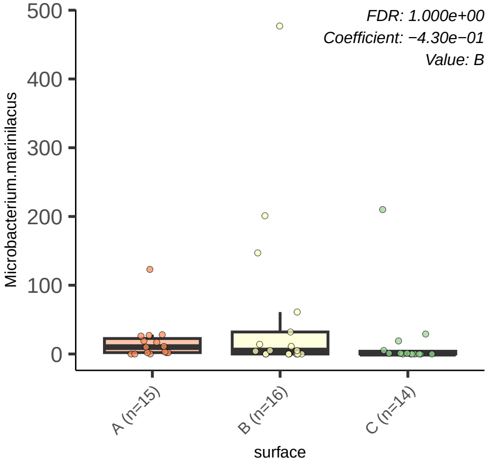
FDR: 1.000e+00
Coefficient: −4.30e−01
Value: B
Microbacterium.marinilacus
500
400
300
200
100
0
A (n=15)
B (n=16)
C (n=14)
surface

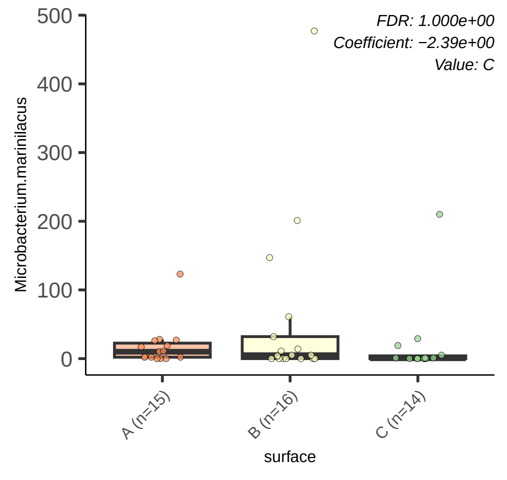
FDR: 1.000e+00
Coefficient: −2.39e+00
Value: C
Microbacterium.marinilacus
500
400
300
200
100
0
A (n=15)
B (n=16)
C (n=14)
surface

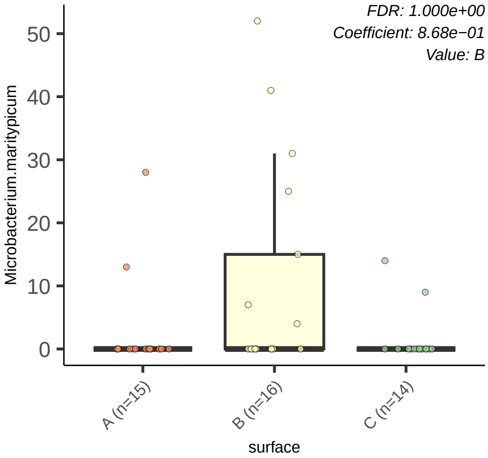
FDR: 1.000e+00
Coefficient: 8.68e−01
Value: B
Microbacterium.maritypicum
0
10
20
30
40
50
A (n=15)
B (n=16)
C (n=14)
surface

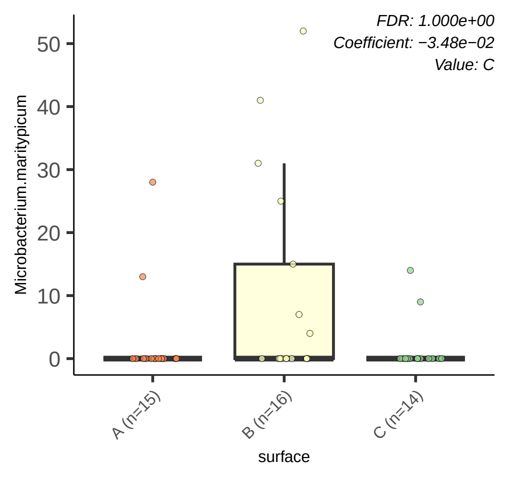

FDR: 1.000e+00
Coefficient: −3.48e−02
Value: C
Microbacterium.maritypicum
0
10
20
30
40
50
A (n=15)
B (n=16)
C (n=14)
surface

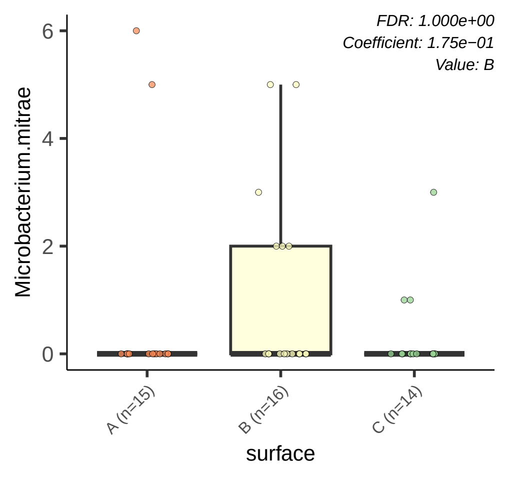

FDR: 1.000e+00
Coefficient: 1.75e−01
Value: B
Microbacterium.mitrae
0
2
4
6
A (n=15)
B (n=16)
C (n=14)
surface

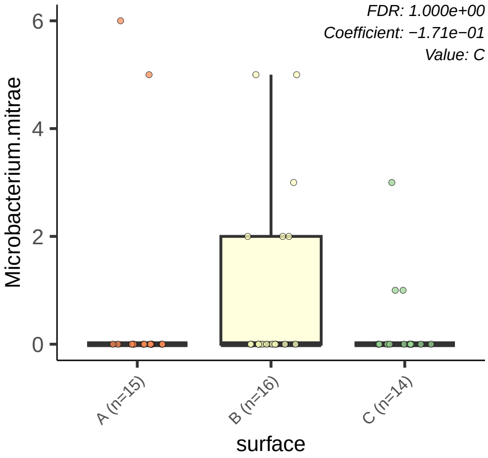

FDR: 1.000e+00
Coefficient: −1.71e−01
Value: C
Microbacterium.mitrae
0
2
4
6
A (n=15)
B (n=16)
C (n=14)
surface

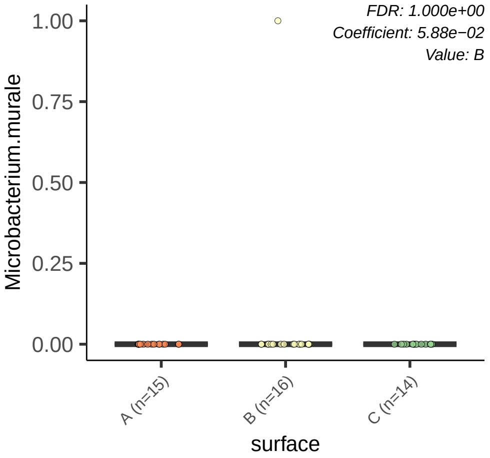
FDR: 1.000e+00
Coefficient: 5.88e−02
Value: B
Microbacterium.murale
1.00
0.75
0.50
0.25
0.00
A (n=15)
B (n=16)
C (n=14)
surface

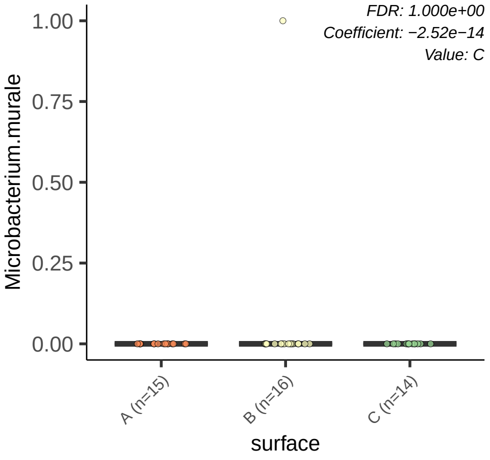
FDR: 1.000e+00
Coefficient: −2.52e−14
Value: C
Microbacterium.murale
1.00
0.75
0.50
0.25
0.00
A (n=15)
B (n=16)
C (n=14)
surface

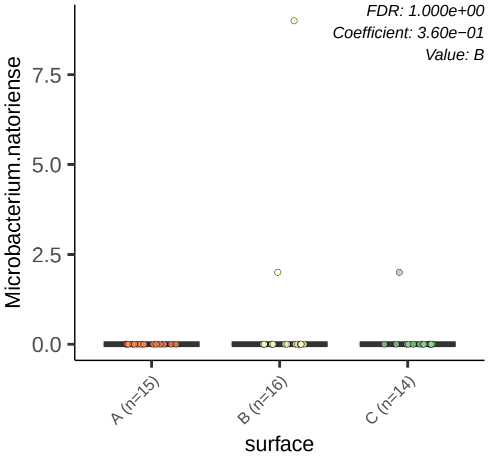

FDR: 1.000e+00
Coefficient: 3.60e−01
Value: B
Microbacterium.natoriense
7.5
5.0
2.5
0.0
A (n=15)
B (n=16)
C (n=14)
surface

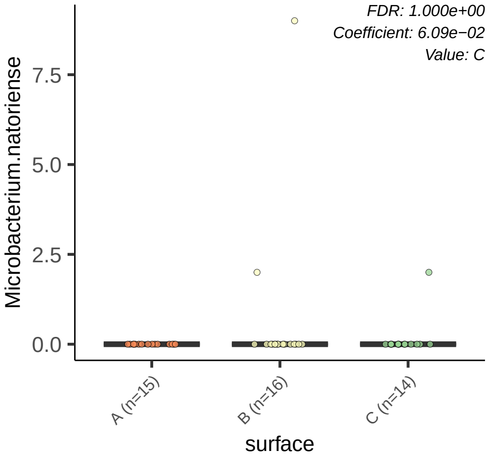

FDR: 1.000e+00
Coefficient: 6.09e−02
Value: C
Microbacterium.natoriense
7.5
5.0
2.5
0.0
A (n=15)
B (n=16)
C (n=14)
surface

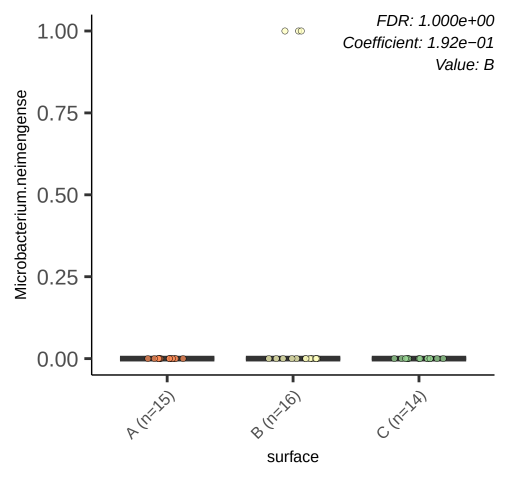
FDR: 1.000e+00
Coefficient: 1.92e−01
Value: B
Microbacterium.neimengense
1.00
0.75
0.50
0.25
0.00
A (n=15)
B (n=16)
C (n=14)
surface

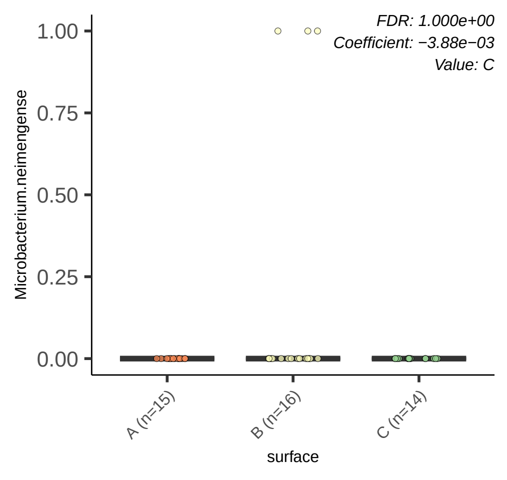
FDR: 1.000e+00
Coefficient: −3.88e−03
Value: C
Microbacterium.neimengense
1.00
0.75
0.50
0.25
0.00
A (n=15)
B (n=16)
C (n=14)
surface

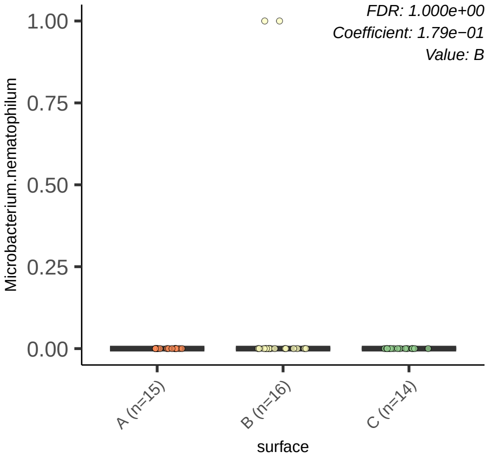

FDR: 1.000e+00
Coefficient: 1.79e−01
Value: B
Microbacterium.nematophilum
1.00
0.75
0.50
0.25
0.00
A (n=15)
B (n=16)
C (n=14)
surface

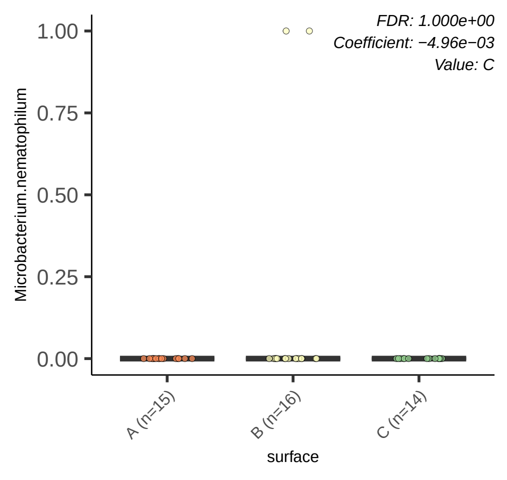
FDR: 1.000e+00
Coefficient: −4.96e−03
Value: C
Microbacterium.nematophilum
1.00
0.75
0.50
0.25
0.00
A (n=15)
B (n=16)
C (n=14)
surface

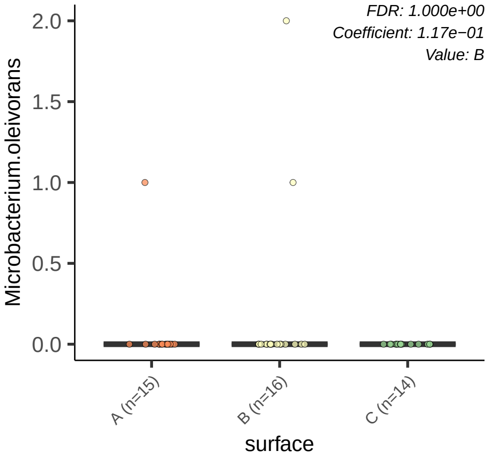

FDR: 1.000e+00
Coefficient: 1.17e−01
Value: B
Microbacterium.oleivorans
2.0
1.5
1.0
0.5
0.0
A (n=15)
B (n=16)
C (n=14)
surface

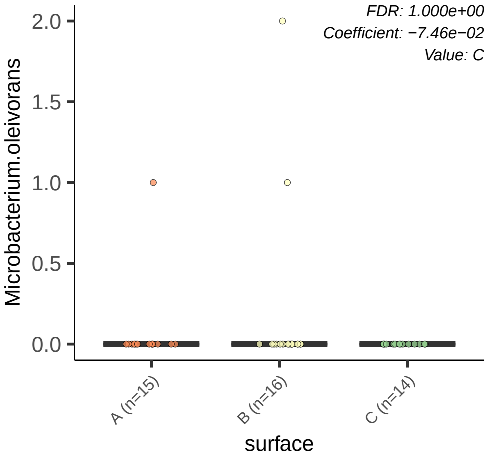
FDR: 1.000e+00
Coefficient: −7.46e−02
Value: C
Microbacterium.oleivorans
2.0
1.5
1.0
0.5
0.0
A (n=15)
B (n=16)
C (n=14)
surface

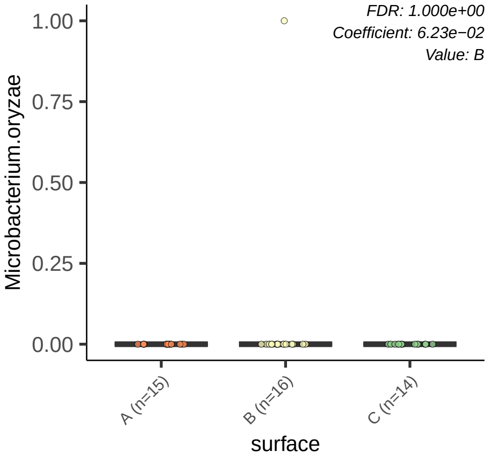
FDR: 1.000e+00
Coefficient: 6.23e−02
Value: B
Microbacterium.oryzae
1.00
0.75
0.50
0.25
0.00
A (n=15)
B (n=16)
C (n=14)
surface

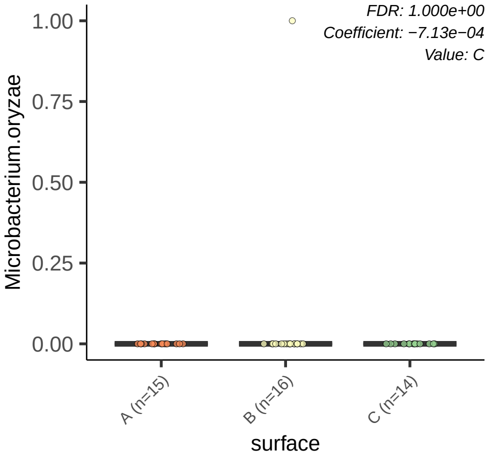

FDR: 1.000e+00
Coefficient: −7.13e−04
Value: C
Microbacterium.oryzae
1.00
0.75
0.50
0.25
0.00
A (n=15)
B (n=16)
C (n=14)
surface

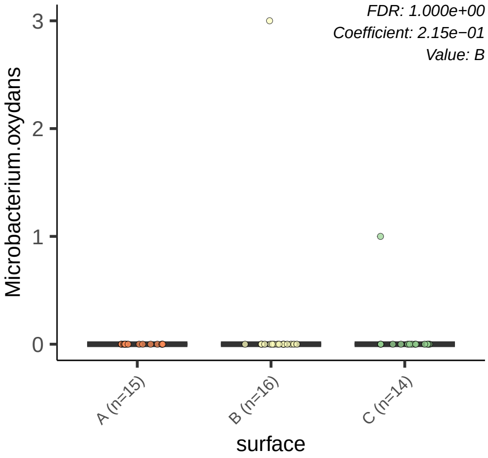

FDR: 1.000e+00
Coefficient: 2.15e−01
Value: B
Microbacterium.oxydans
0
1
2
3
A (n=15)
B (n=16)
C (n=14)
surface

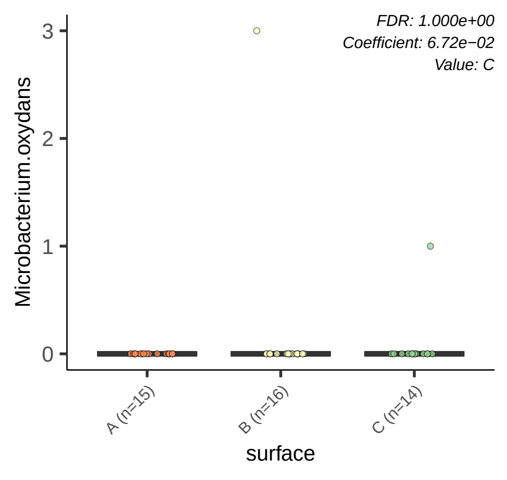

FDR: 1.000e+00
Coefficient: 6.72e−02
Value: C
Microbacterium.oxydans
0
1
2
3
A (n=15)
B (n=16)
C (n=14)
surface

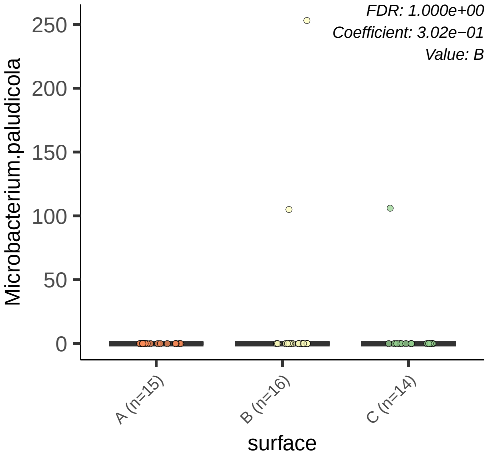
FDR: 1.000e+00
Coefficient: 3.02e−01
Value: B
Microbacterium.paludicola
250
200
150
100
50
0
A (n=15)
B (n=16)
C (n=14)
surface

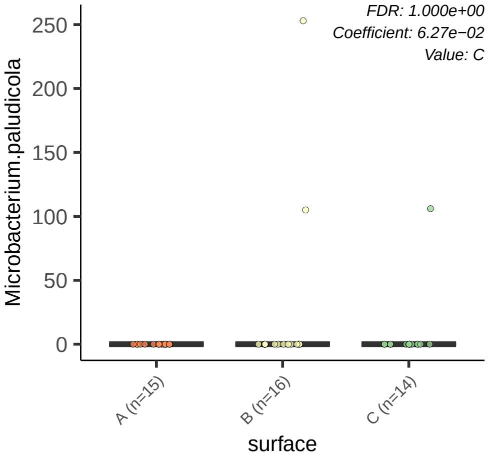
FDR: 1.000e+00
Coefficient: 6.27e−02
Value: C
Microbacterium.paludicola
250
200
150
100
50
0
A (n=15)
B (n=16)
C (n=14)
surface

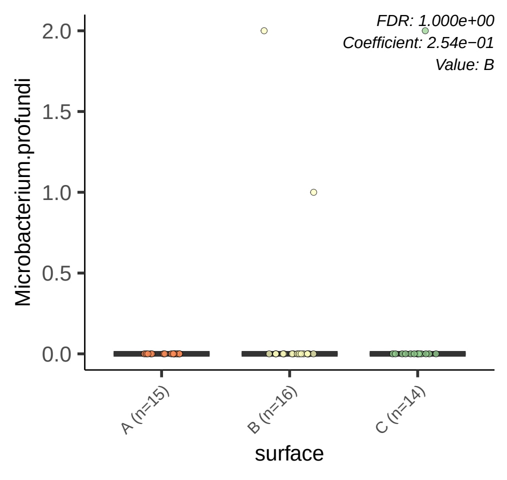

FDR: 1.000e+00
Coefficient: 2.54e−01
Value: B
Microbacterium.profundi
2.0
1.5
1.0
0.5
0.0
A (n=15)
B (n=16)
C (n=14)
surface

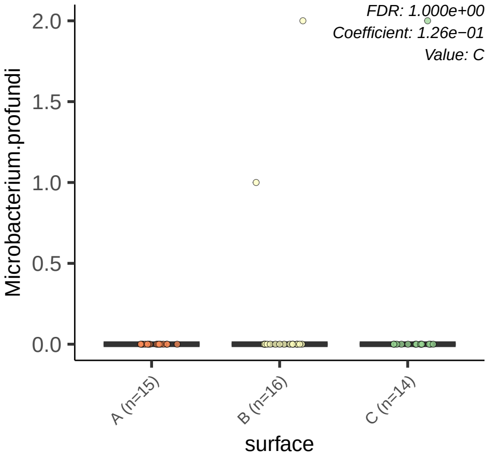
FDR: 1.000e+00
Coefficient: 1.26e−01
Value: C
Microbacterium.profundi
2.0
1.5
1.0
0.5
0.0
A (n=15)
B (n=16)
C (n=14)
surface

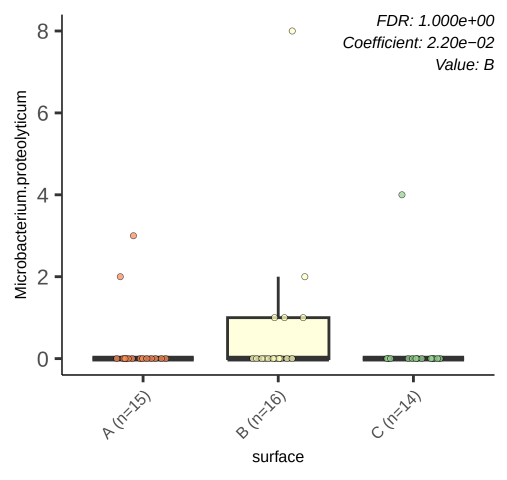

FDR: 1.000e+00
Coefficient: 2.20e−02
Value: B
Microbacterium.proteolyticum
0
2
4
6
8
A (n=15)
B (n=16)
C (n=14)
surface

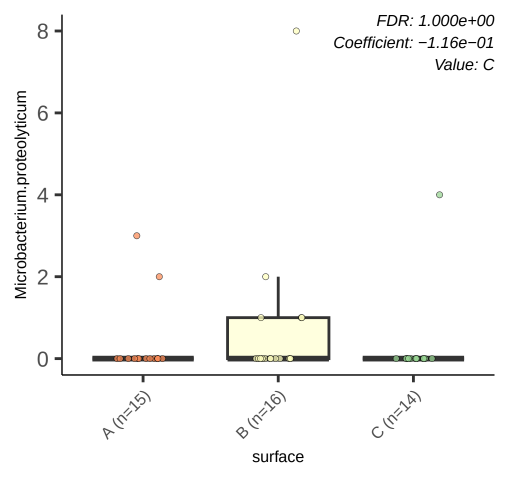

FDR: 1.000e+00
Coefficient: −1.16e−01
Value: C
Microbacterium.proteolyticum
0
2
4
6
8
A (n=15)
B (n=16)
C (n=14)
surface

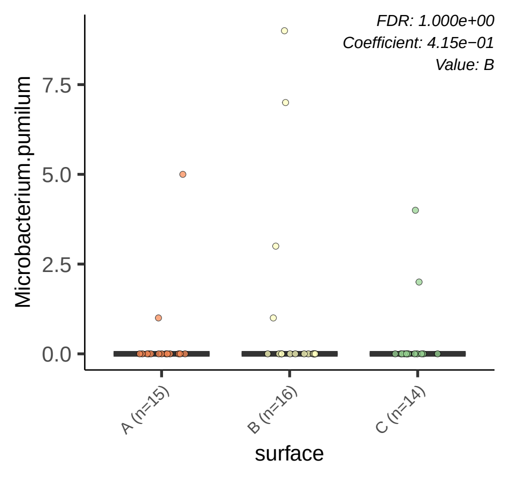
FDR: 1.000e+00
Coefficient: 4.15e−01
Value: B
Microbacterium.pumilum
7.5
5.0
2.5
0.0
A (n=15)
B (n=16)
C (n=14)
surface

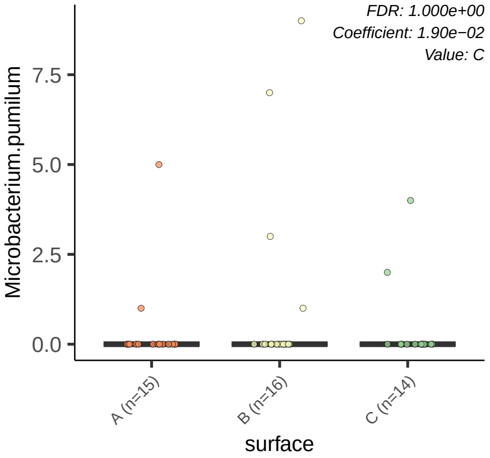

FDR: 1.000e+00
Coefficient: 1.90e−02
Value: C
Microbacterium.pumilum
7.5
5.0
2.5
0.0
A (n=15)
B (n=16)
C (n=14)
surface

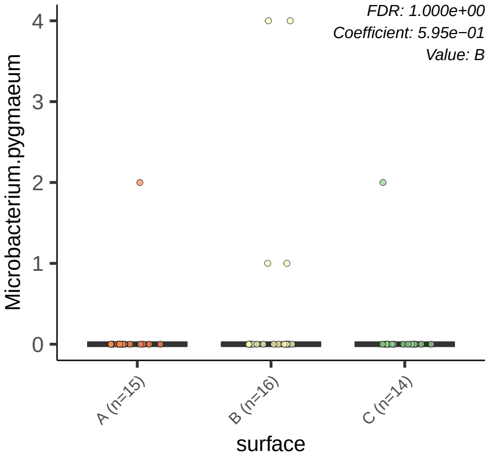
FDR: 1.000e+00
Coefficient: 5.95e−01
Value: B
Microbacterium.pygmaeum
0
1
2
3
4
A (n=15)
B (n=16)
C (n=14)
surface

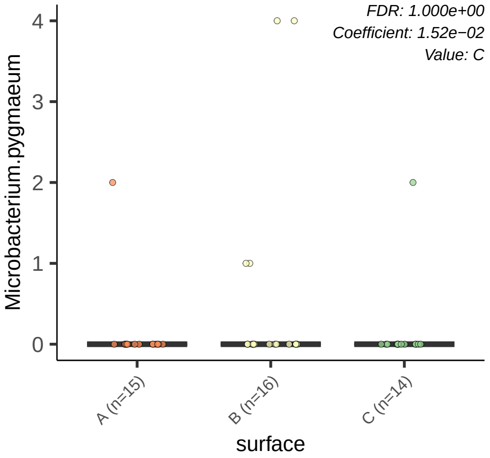

FDR: 1.000e+00
Coefficient: 1.52e−02
Value: C
Microbacterium.pygmaeum
0
1
2
3
4
A (n=15)
B (n=16)
C (n=14)
surface

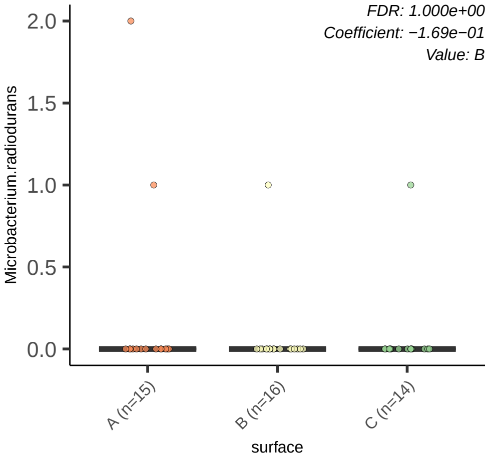

FDR: 1.000e+00
Coefficient: −1.69e−01
Value: B
Microbacterium.radiodurans
2.0
1.5
1.0
0.5
0.0
A (n=15)
B (n=16)
C (n=14)
surface

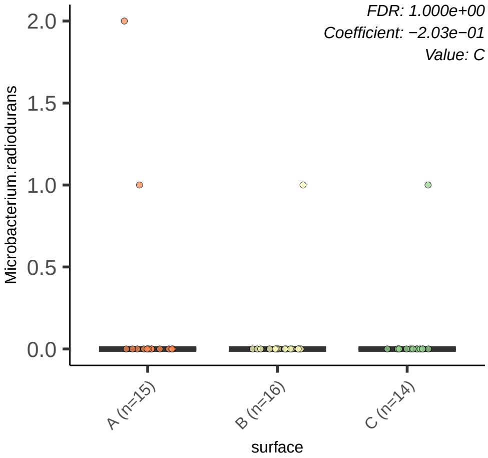

FDR: 1.000e+00
Coefficient: −2.03e−01
Value: C
Microbacterium.radiodurans
2.0
1.5
1.0
0.5
0.0
A (n=15)
B (n=16)
C (n=14)
surface

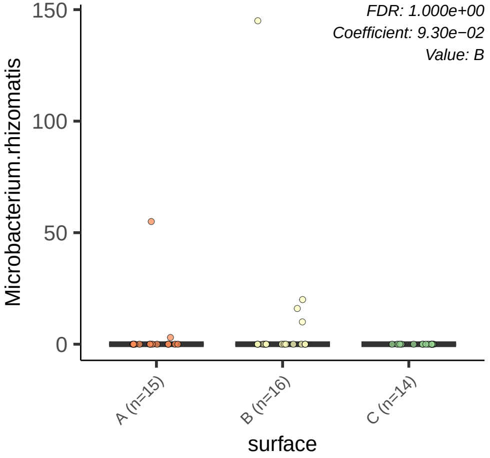
FDR: 1.000e+00
Coefficient: 9.30e−02
Value: B
Microbacterium.rhizomatis
150
100
50
0
A (n=15)
B (n=16)
C (n=14)
surface

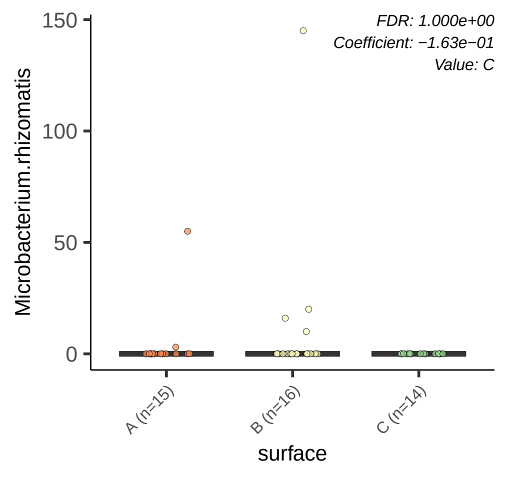

FDR: 1.000e+00
Coefficient: −1.63e−01
Value: C
150
100
50
0
Microbacterium.rhizomatis
A (n=15)
B (n=16)
C (n=14)
surface

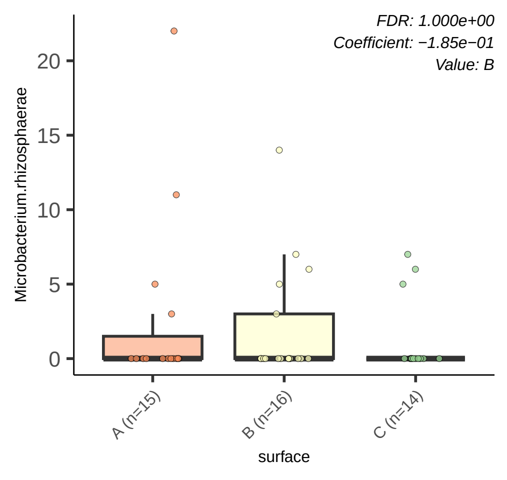
FDR: 1.000e+00
Coefficient: −1.85e−01
Value: B
Microbacterium.rhizosphaerae
0
5
10
15
20
A (n=15)
B (n=16)
C (n=14)
surface

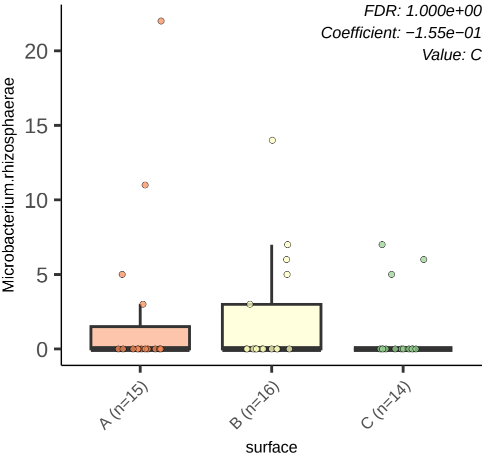
FDR: 1.000e+00
Coefficient: −1.55e−01
Value: C
Microbacterium.rhizosphaerae
20
15
10
5
0
A (n=15)
B (n=16)
C (n=14)
surface

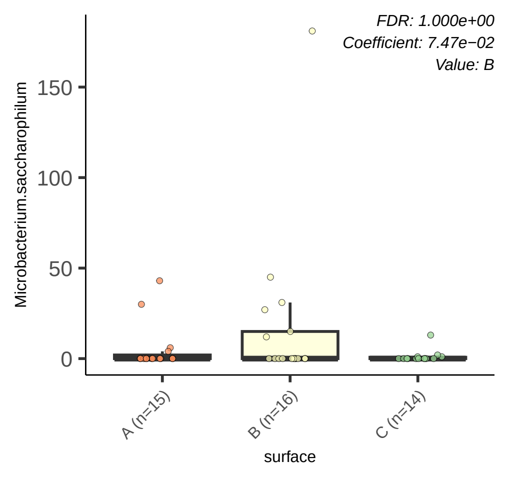

FDR: 1.000e+00
Coefficient: 7.47e−02
Value: B
Microbacterium.saccharophilum
150
100
50
0
A (n=15)
B (n=16)
C (n=14)
surface

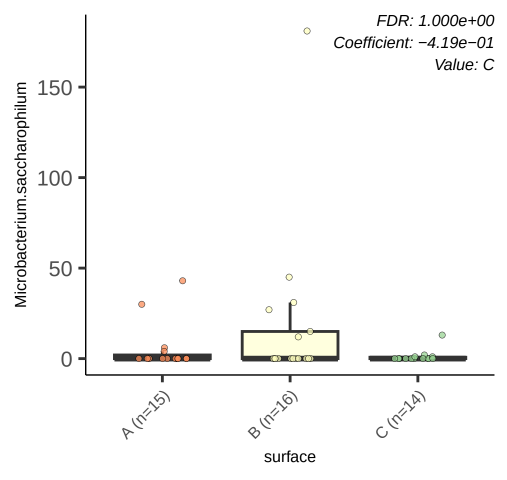

FDR: 1.000e+00
Coefficient: −4.19e−01
Value: C
Microbacterium.saccharophilum
150
100
50
0
A (n=15)
B (n=16)
C (n=14)
surface

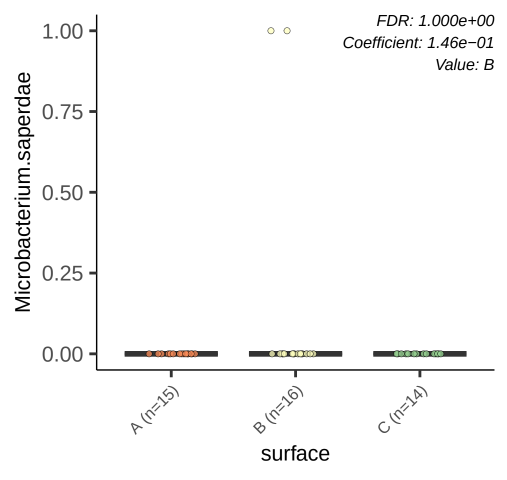
FDR: 1.000e+00
Coefficient: 1.46e−01
Value: B
Microbacterium.saperdae
1.00
0.75
0.50
0.25
0.00
A (n=15)
B (n=16)
C (n=14)
surface

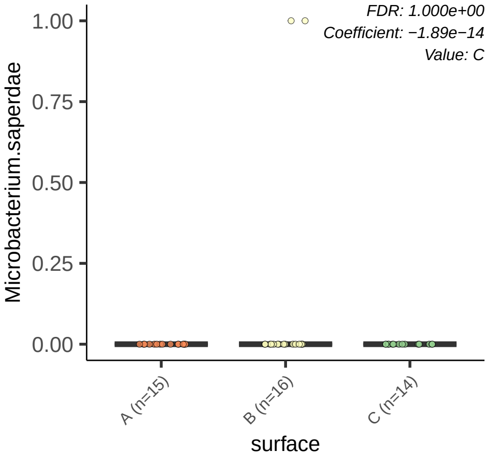

FDR: 1.000e+00
Coefficient: −1.89e−14
Value: C
Microbacterium.saperdae
1.00
0.75
0.50
0.25
0.00
A (n=15)
B (n=16)
C (n=14)
surface

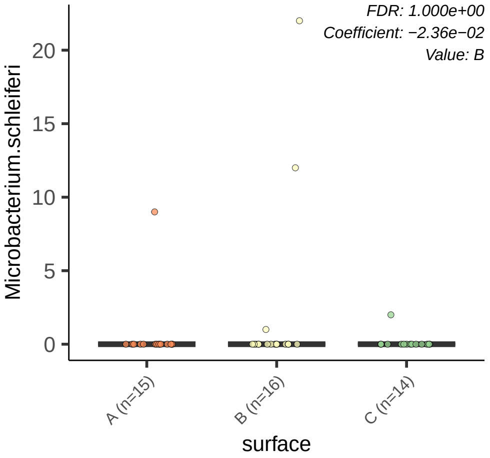

FDR: 1.000e+00
Coefficient: −2.36e−02
Value: B
Microbacterium.schleiferi
20
15
10
5
0
A (n=15)
B (n=16)
C (n=14)
surface

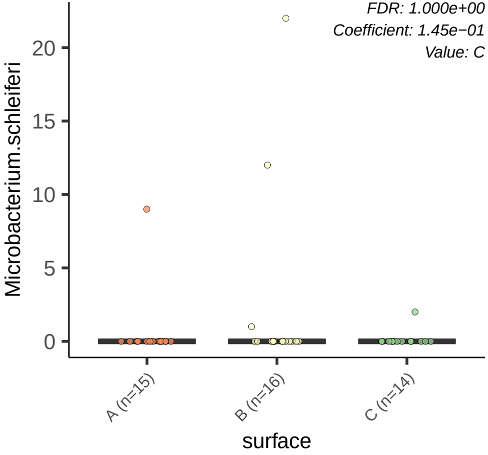

FDR: 1.000e+00
Coefficient: 1.45e−01
Value: C
Microbacterium.schleiferi
20
15
10
5
0
A (n=15)
B (n=16)
C (n=14)
surface

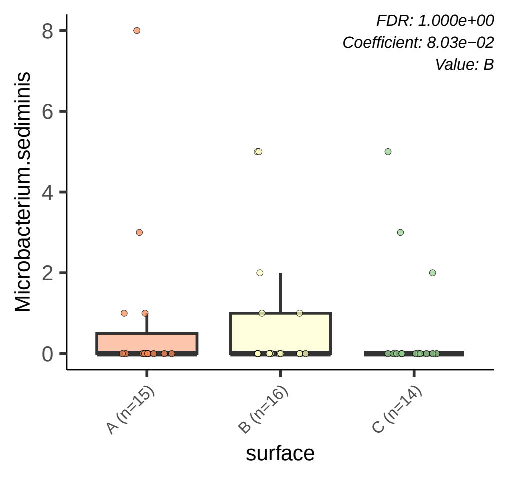
FDR: 1.000e+00
Coefficient: 8.03e−02
Value: B
Microbacterium.sedimінis
8
6
4
2
0
A (n=15)
B (n=16)
C (n=14)
surface

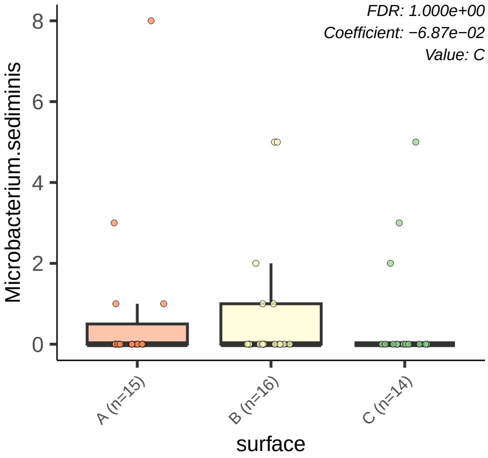
FDR: 1.000e+00
Coefficient: −6.87e−02
Value: C
Microbacterium.sedimінis
0
2
4
6
8
A (n=15)
B (n=16)
C (n=14)
surface

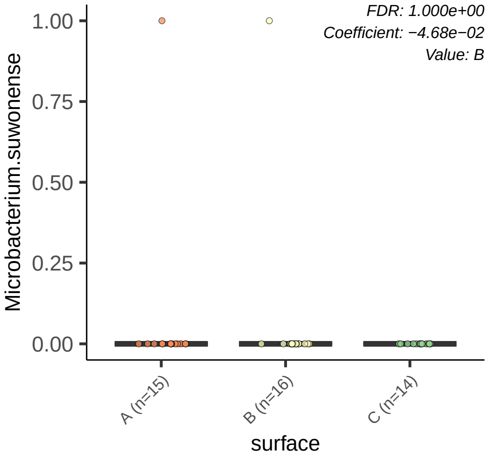
FDR: 1.000e+00
Coefficient: −4.68e−02
Value: B
Microbacterium.suwonense
1.00
0.75
0.50
0.25
0.00
A (n=15)
B (n=16)
C (n=14)
surface

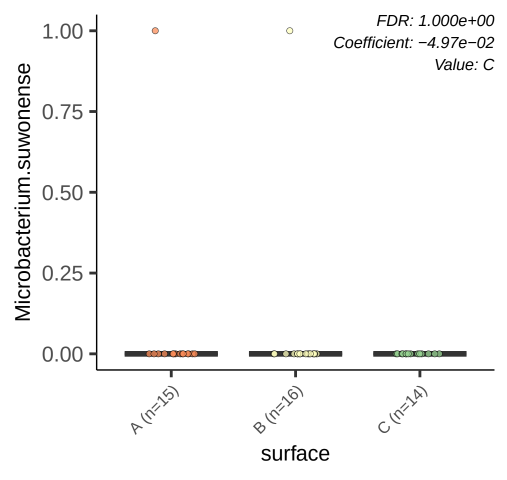
FDR: 1.000e+00
Coefficient: −4.97e−02
Value: C
Microbacterium.suwonense
1.00
0.75
0.50
0.25
0.00
A (n=15)
B (n=16)
C (n=14)
surface

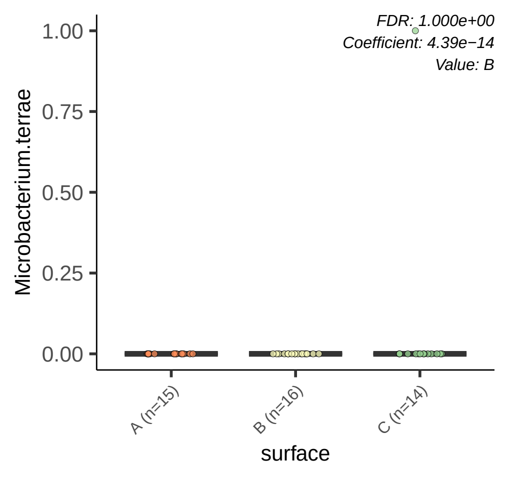
FDR: 1.000e+00
Coefficient: 4.39e−14
Value: B
Microbacterium.terrae
1.00
0.75
0.50
0.25
0.00
A (n=15)
B (n=16)
C (n=14)
surface

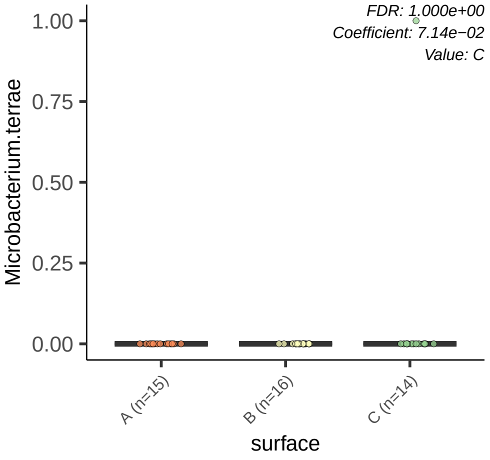

FDR: 1.000e+00
Coefficient: 7.14e−02
Value: C
Microbacterium.terrae
1.00
0.75
0.50
0.25
0.00
A (n=15)
B (n=16)
C (n=14)
surface

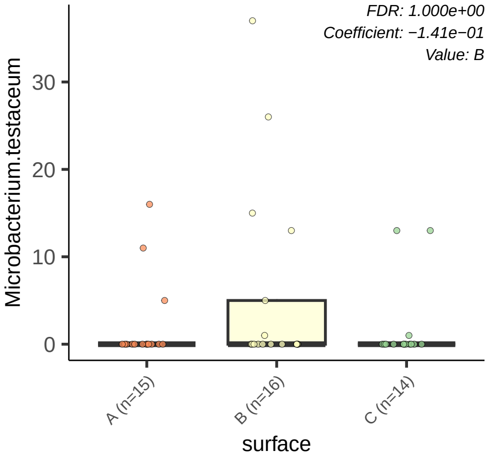

FDR: 1.000e+00
Coefficient: −1.41e−01
Value: B
Microbacterium.testaceum
0
10
20
30
A (n=15)
B (n=16)
C (n=14)
surface

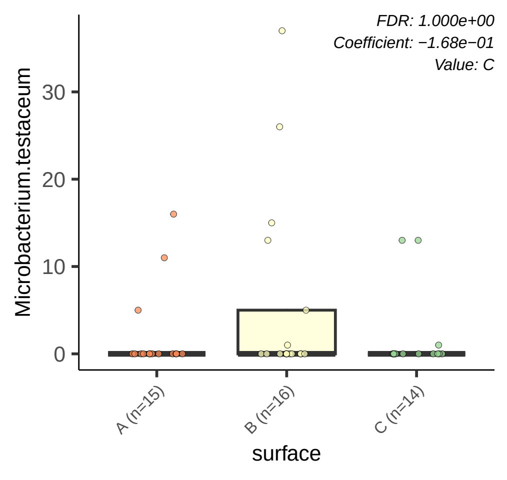
FDR: 1.000e+00
Coefficient: −1.68e−01
Value: C
Microbacterium.testaceum
0
10
20
30
A (n=15)
B (n=16)
C (n=14)
surface

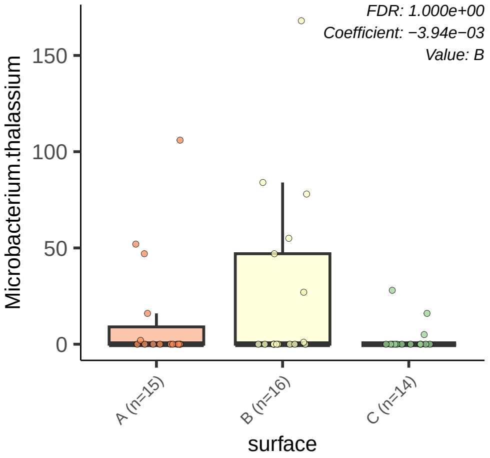

FDR: 1.000e+00
Coefficient: −3.94e−03
Value: B
Microbacterium.thalassium
150
100
50
0
A (n=15)
B (n=16)
C (n=14)
surface

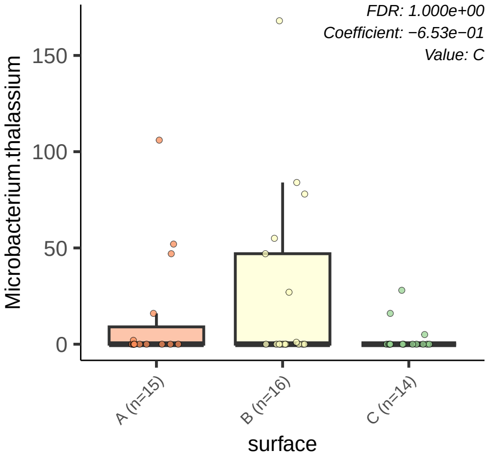
FDR: 1.000e+00
Coefficient: −6.53e−01
Value: C
Microbacterium.thalassium
150
100
50
0
A (n=15)
B (n=16)
C (n=14)
surface

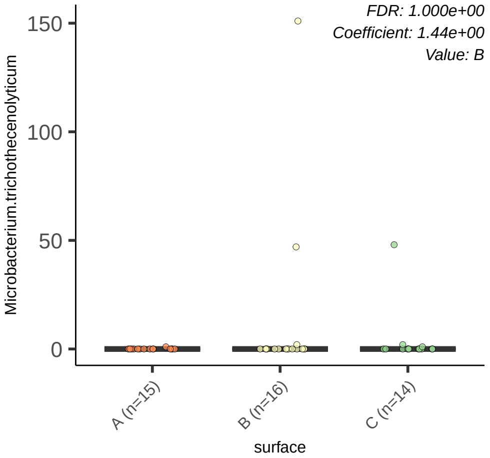
FDR: 1.000e+00
Coefficient: 1.44e+00
Value: B
Microbacterium.trichothecenolyticum
150
100
50
0
A (n=15)
B (n=16)
C (n=14)
surface

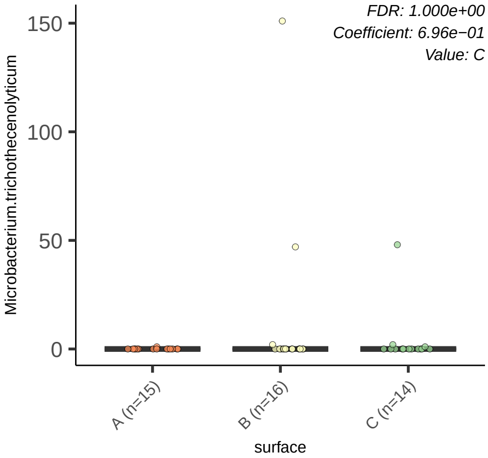

FDR: 1.000e+00
Coefficient: 6.96e−01
Value: C
Microbacterium.trichothecenolyticum
150
100
50
0
A (n=15)
B (n=16)
C (n=14)
surface

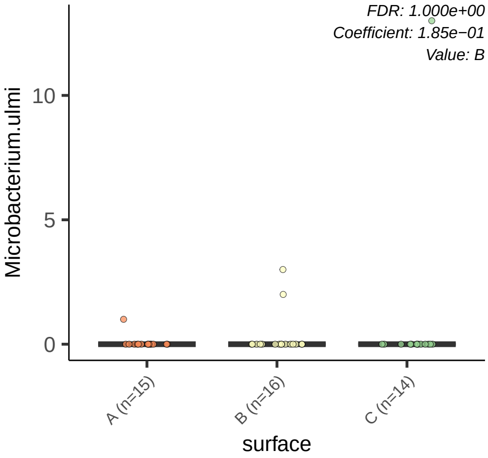

FDR: 1.000e+00
Coefficient: 1.85e−01
Value: B
Microbacterium.ulmi
10
5
0
A (n=15)
B (n=16)
C (n=14)
surface

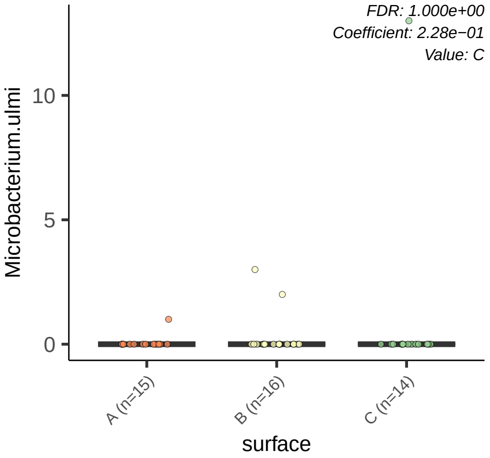
FDR: 1.000e+00
Coefficient: 2.28e−01
Value: C
Microbacterium.ulmi
10
5
0
A (n=15)
B (n=16)
C (n=14)
surface

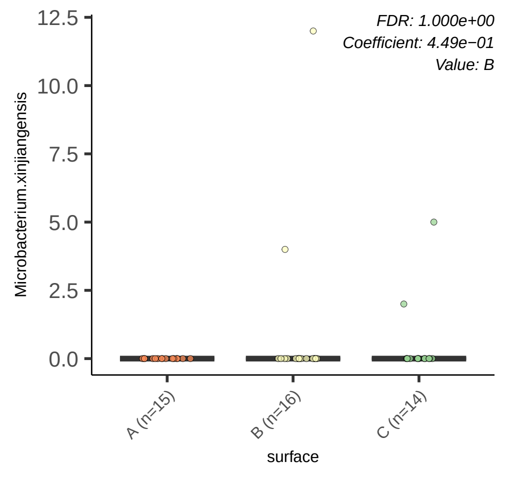

FDR: 1.000e+00
Coefficient: 4.49e−01
Value: B
Microbacterium.xinjiangensis
12.5
10.0
7.5
5.0
2.5
0.0
A (n=15)
B (n=16)
C (n=14)
surface

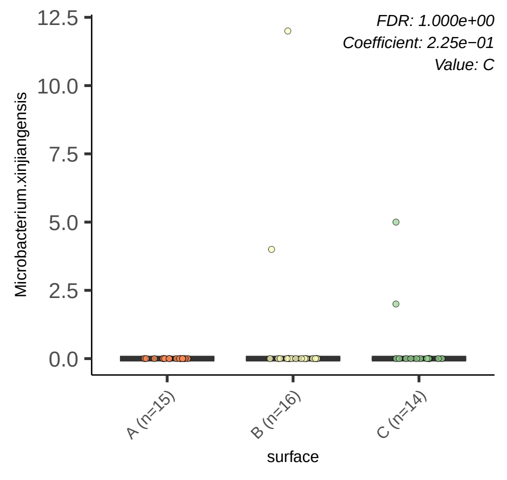
FDR: 1.000e+00
Coefficient: 2.25e−01
Value: C
Microbacterium.xinjiangensis
12.5
10.0
7.5
5.0
2.5
0.0
A (n=15)
B (n=16)
C (n=14)
surface

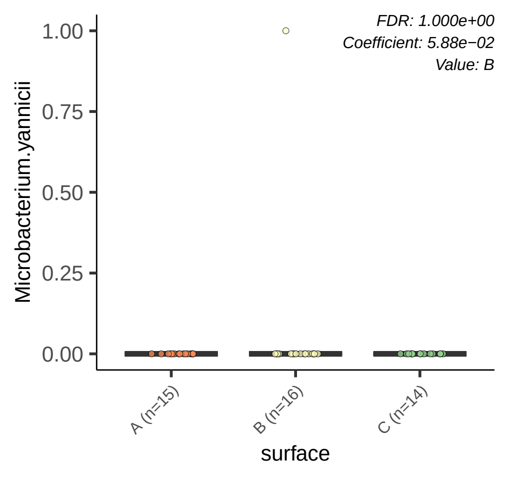

FDR: 1.000e+00
Coefficient: 5.88e−02
Value: B
Microbacterium.yannicii
1.00
0.75
0.50
0.25
0.00
A (n=15)
B (n=16)
C (n=14)
surface

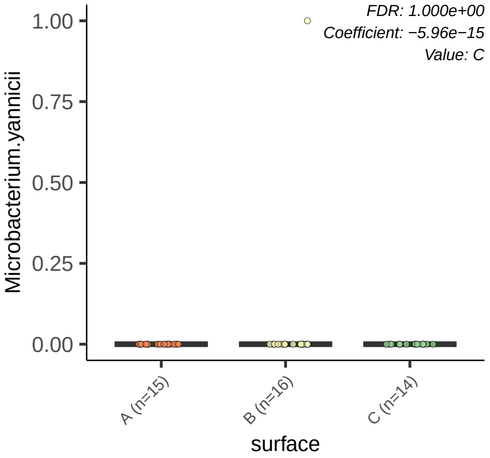
FDR: 1.000e+00
Coefficient: −5.96e−15
Value: C
Microbacterium.yannicii
1.00
0.75
0.50
0.25
0.00
A (n=15)
B (n=16)
C (n=14)
surface

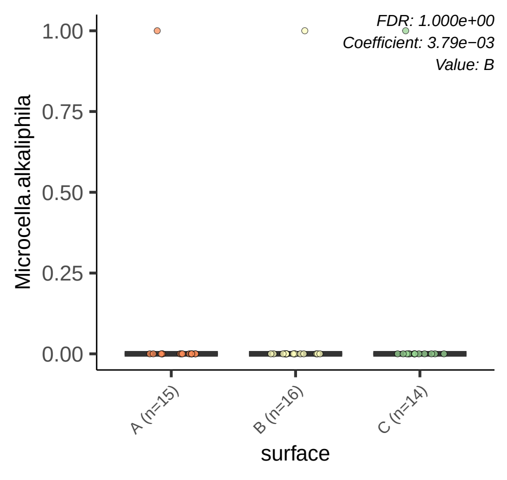

FDR: 1.000e+00
Coefficient: 3.79e−03
Value: B
Microcella.alkaliphila
1.00
0.75
0.50
0.25
0.00
A (n=15)
B (n=16)
C (n=14)
surface

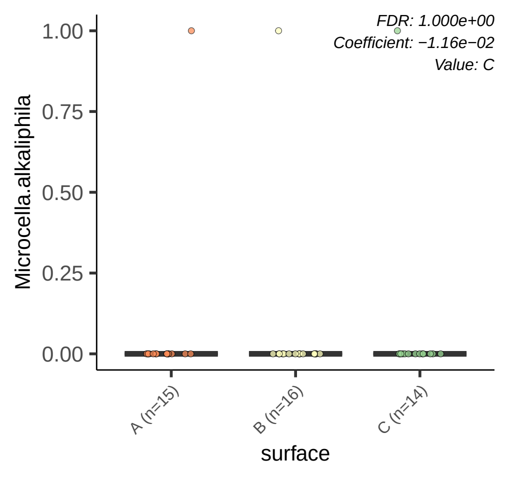
FDR: 1.000e+00
Coefficient: −1.16e−02
Value: C
Microcella.alkaliphila
1.00
0.75
0.50
0.25
0.00
A (n=15)
B (n=16)
C (n=14)
surface

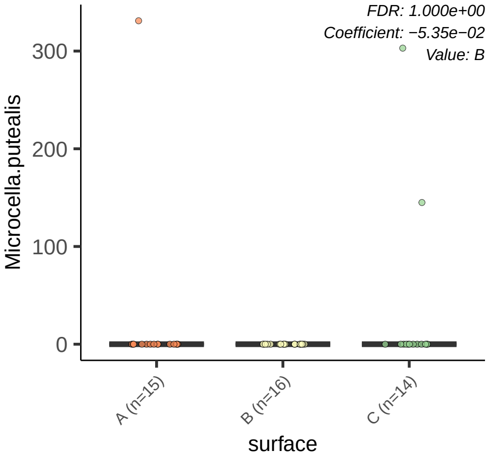
FDR: 1.000e+00
Coefficient: −5.35e−02
Value: B
Microcella.putealis
300
200
100
0
A (n=15)
B (n=16)
C (n=14)
surface

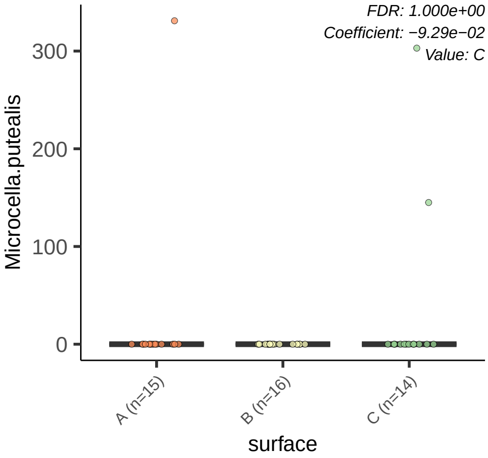
FDR: 1.000e+00
Coefficient: −9.29e−02
Value: C
Microcella.putealis
300
200
100
0
A (n=15)
B (n=16)
C (n=14)
surface

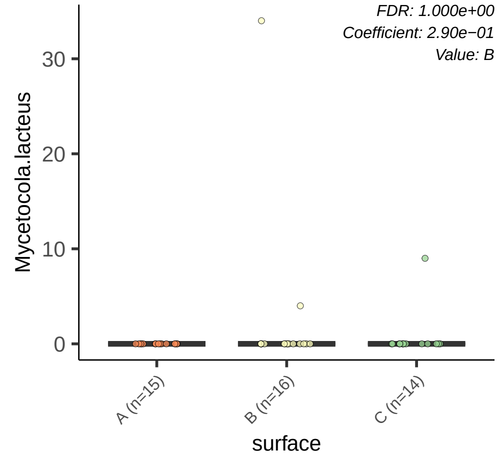

FDR: 1.000e+00
Coefficient: 2.90e−01
Value: B
Mycetocola.lacteus
30
20
10
0
A (n=15)
B (n=16)
C (n=14)
surface

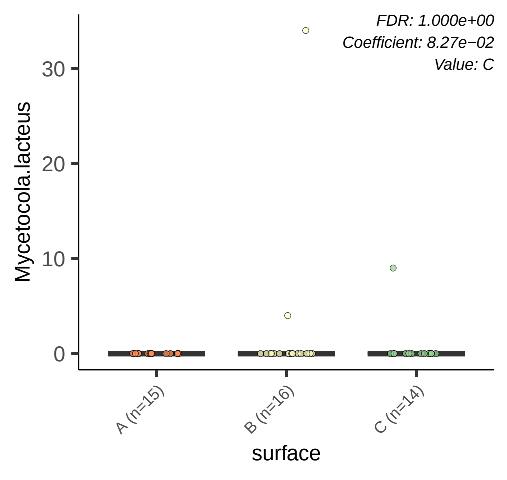

FDR: 1.000e+00
Coefficient: 8.27e−02
Value: C
Mycetocola.lacteus
30
20
10
0
A (n=15)
B (n=16)
C (n=14)
surface

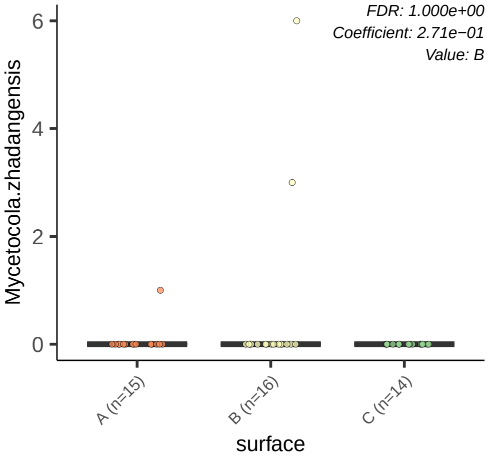

FDR: 1.000e+00
Coefficient: 2.71e−01
Value: B
Mycetocola.zhadangensis
6
4
2
0
A (n=15)
B (n=16)
C (n=14)
surface

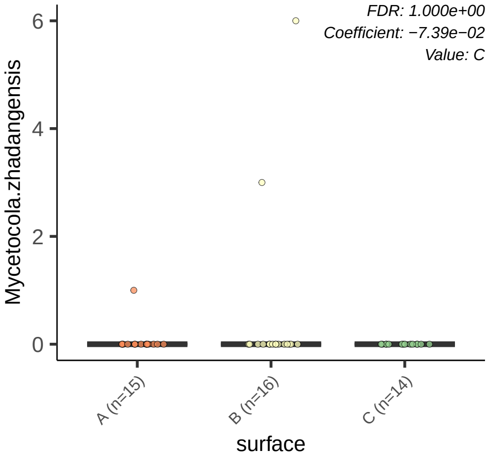
FDR: 1.000e+00
Coefficient: −7.39e−02
Value: C
Mycetocola.zhadangensis
0
2
4
6
A (n=15)
B (n=16)
C (n=14)
surface

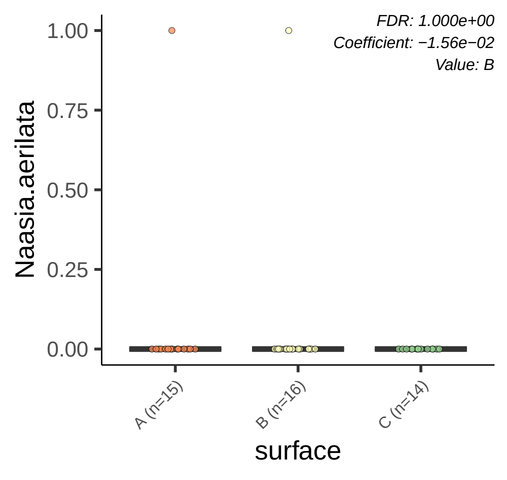
FDR: 1.000e+00
Coefficient: −1.56e−02
Value: B
Naasia.aerilata
1.00
0.75
0.50
0.25
0.00
A (n=15)
B (n=16)
C (n=14)
surface

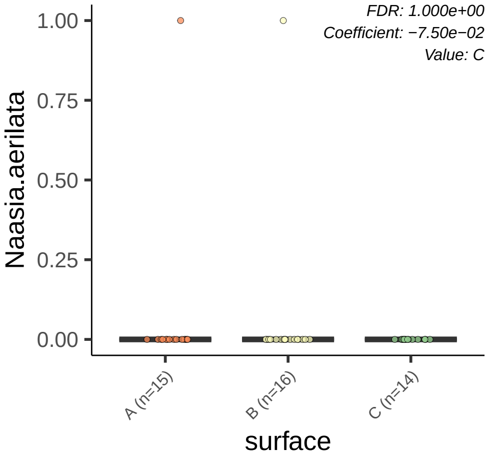
FDR: 1.000e+00
Coefficient: −7.50e−02
Value: C
Naasia.aerilata
1.00
0.75
0.50
0.25
0.00
A (n=15)
B (n=16)
C (n=14)
surface

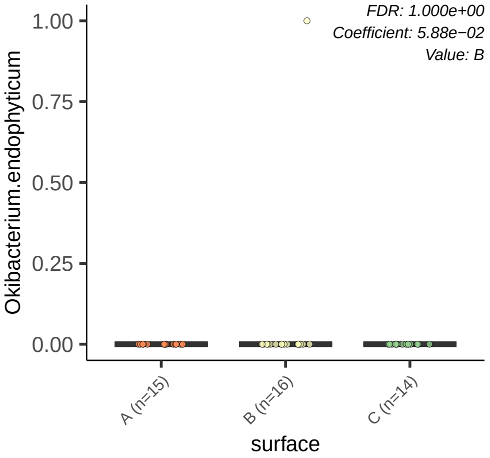
FDR: 1.000e+00
Coefficient: 5.88e−02
Value: B
Okibacterium.endophyticum
1.00
0.75
0.50
0.25
0.00
A (n=15)
B (n=16)
C (n=14)
surface

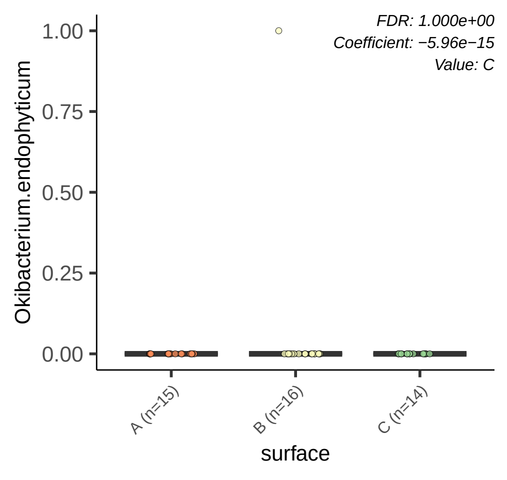
FDR: 1.000e+00
Coefficient: −5.96e−15
Value: C
Okibacterium.endophyticum
1.00
0.75
0.50
0.25
0.00
A (n=15)
B (n=16)
C (n=14)
surface

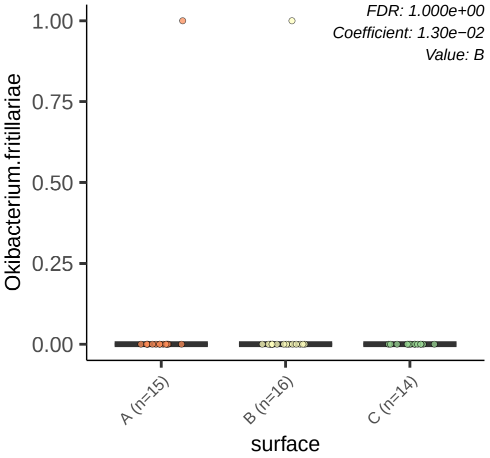

FDR: 1.000e+00
Coefficient: 1.30e−02
Value: B
Okibacterium.fritillariae
1.00
0.75
0.50
0.25
0.00
A (n=15)
B (n=16)
C (n=14)
surface

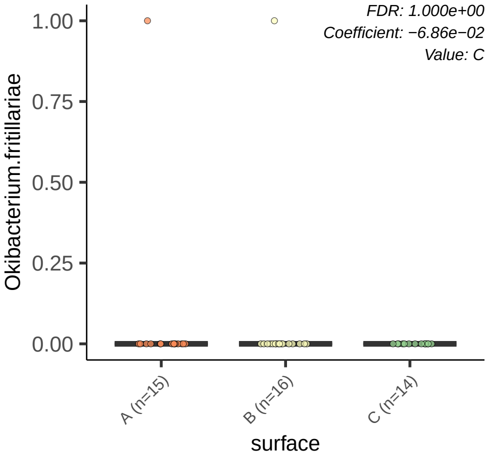
FDR: 1.000e+00
Coefficient: −6.86e−02
Value: C
Okibacterium.fritillariae
1.00
0.75
0.50
0.25
0.00
A (n=15)
B (n=16)
C (n=14)
surface

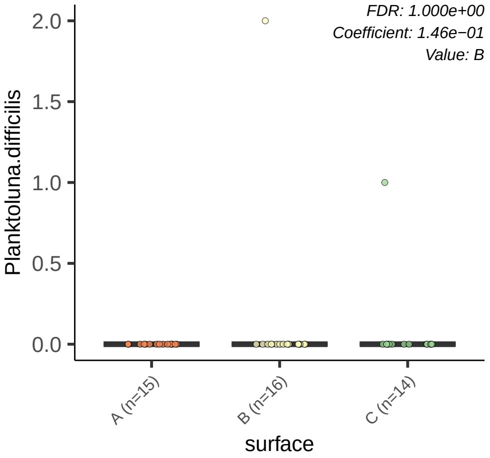

FDR: 1.000e+00
Coefficient: 1.46e−01
Value: B
Planktoluna.difficilis
2.0
1.5
1.0
0.5
0.0
A (n=15)
B (n=16)
C (n=14)
surface

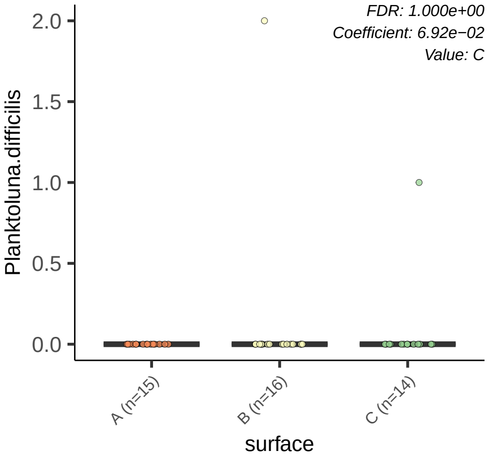
FDR: 1.000e+00
Coefficient: 6.92e−02
Value: C
Planktoluna.difficilis
2.0
1.5
1.0
0.5
0.0
A (n=15)
B (n=16)
C (n=14)
surface

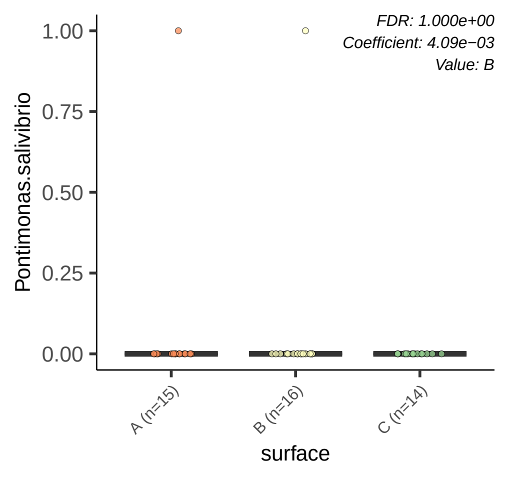
FDR: 1.000e+00
Coefficient: 4.09e−03
Value: B
Pontimonas.salivibrio
1.00
0.75
0.50
0.25
0.00
A (n=15)
B (n=16)
C (n=14)
surface

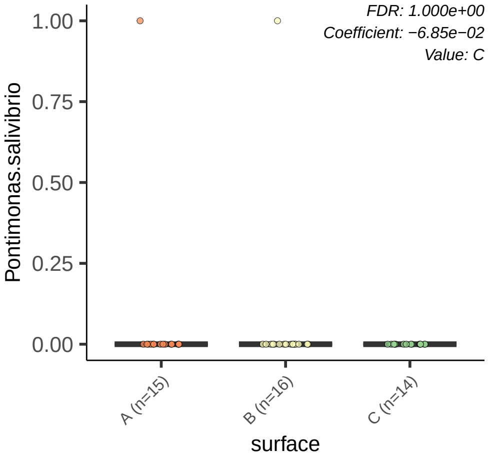
FDR: 1.000e+00
Coefficient: −6.85e−02
Value: C
Pontimonas.salivibrio
1.00
0.75
0.50
0.25
0.00
A (n=15)
B (n=16)
C (n=14)
surface

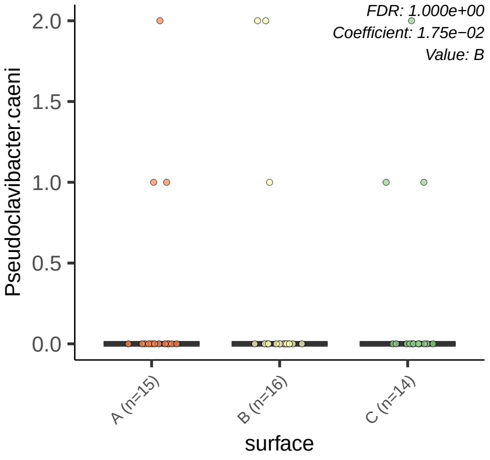

FDR: 1.000e+00
Coefficient: 1.75e−02
Value: B
Pseudoclavibacter.caeni
2.0
1.5
1.0
0.5
0.0
A (n=15)
B (n=16)
C (n=14)
surface

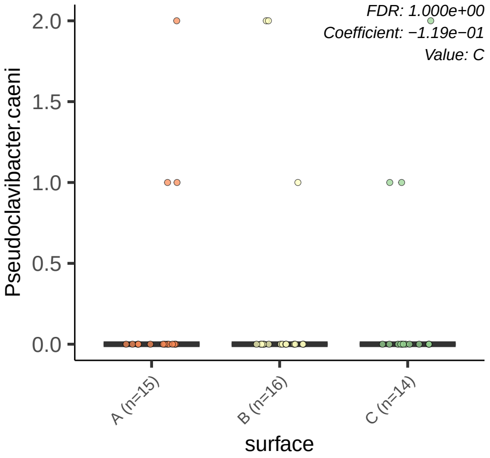
FDR: 1.000e+00
Coefficient: −1.19e−01
Value: C
Pseudoclavibacter.caeni
2.0
1.5
1.0
0.5
0.0
A (n=15)
B (n=16)
C (n=14)
surface

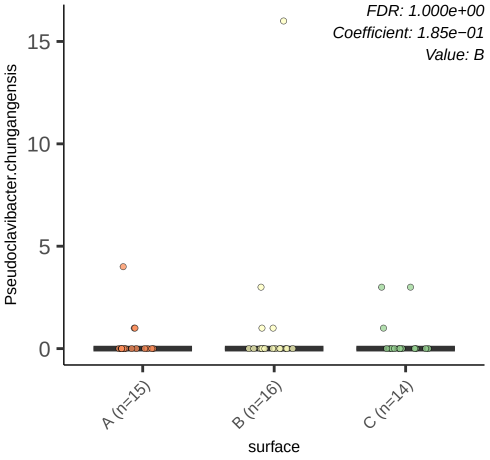

FDR: 1.000e+00
Coefficient: 1.85e−01
Value: B
Pseudoclavibacter.chungangensis
0
5
10
15
A (n=15)
B (n=16)
C (n=14)
surface

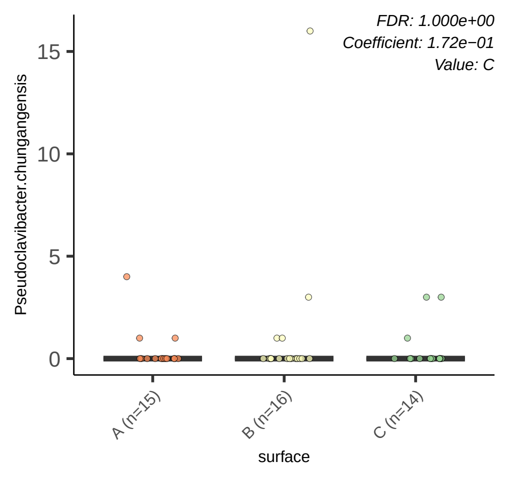

FDR: 1.000e+00
Coefficient: 1.72e−01
Value: C
Pseudoclavibacter.chungangensis
0
5
10
15
A (n=15)
B (n=16)
C (n=14)
surface

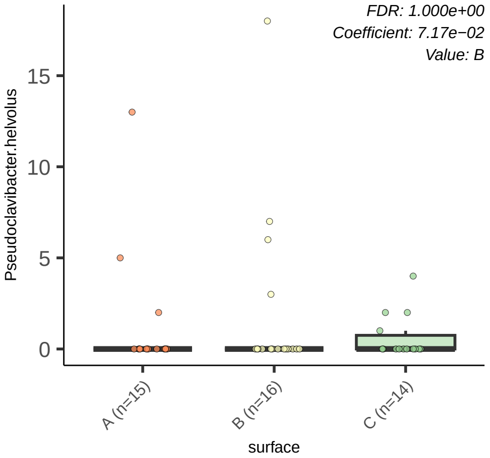
FDR: 1.000e+00
Coefficient: 7.17e−02
Value: B
Pseudoclavibacter.helvolus
0
5
10
15
A (n=15)
B (n=16)
C (n=14)
surface

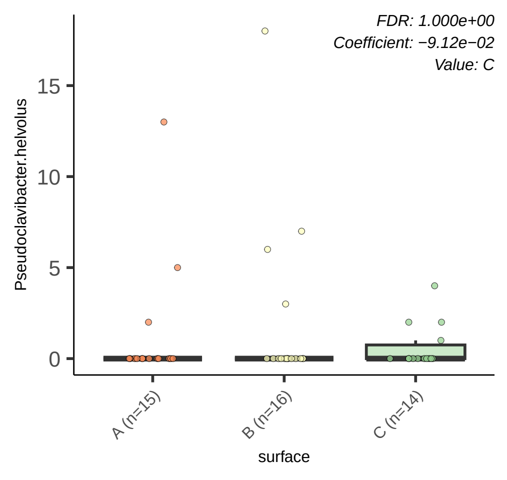
FDR: 1.000e+00
Coefficient: −9.12e−02
Value: C
Pseudoclavibacter.helvolus
15
10
5
0
A (n=15)
B (n=16)
C (n=14)
surface

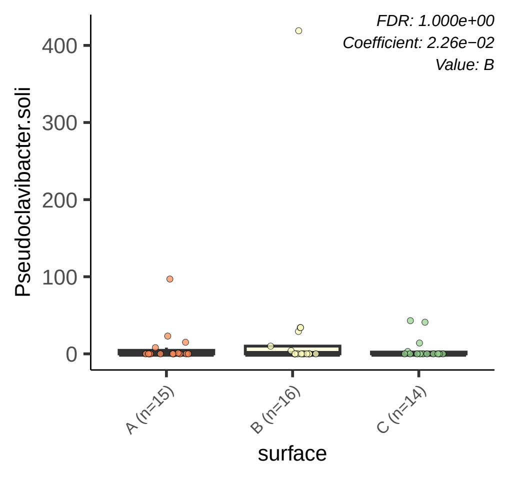
FDR: 1.000e+00
Coefficient: 2.26e−02
Value: B
Pseudoclavibacter.soli
400
300
200
100
0
A (n=15)
B (n=16)
C (n=14)
surface

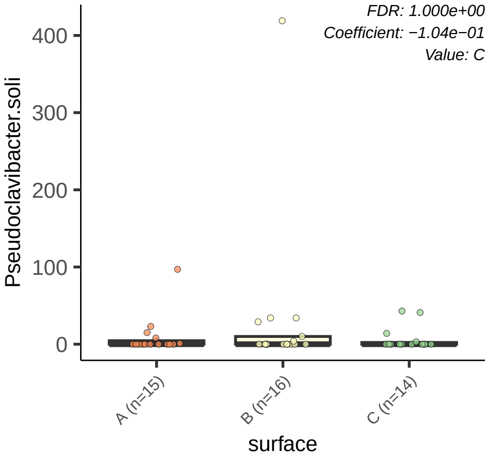

FDR: 1.000e+00
Coefficient: −1.04e−01
Value: C
Pseudoclavibacter.soli
400
300
200
100
0
A (n=15)
B (n=16)
C (n=14)
surface

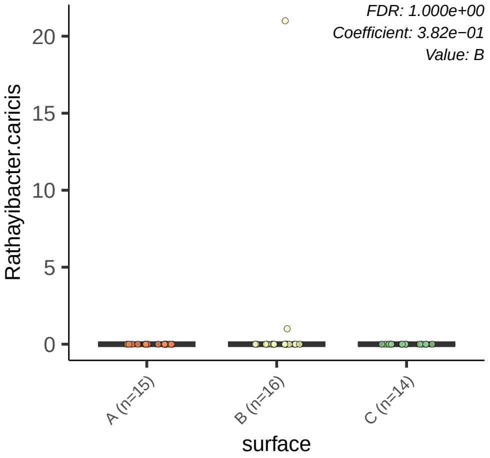

FDR: 1.000e+00
Coefficient: 3.82e−01
Value: B
Rathayibacter.caricis
20
15
10
5
0
A (n=15)
B (n=16)
C (n=14)
surface

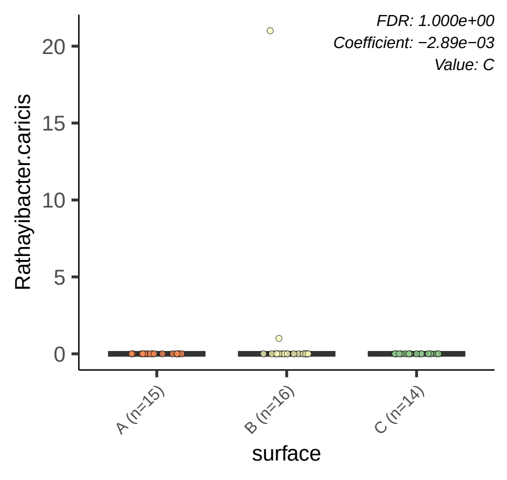

FDR: 1.000e+00
Coefficient: −2.89e−03
Value: C
Rathayibacter.caricis
20
15
10
5
0
A (n=15)
B (n=16)
C (n=14)
surface

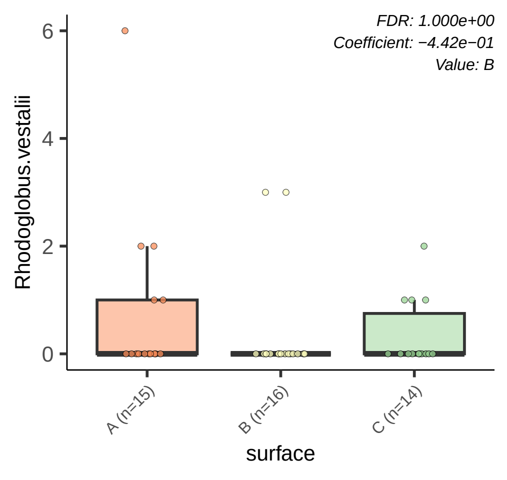

FDR: 1.000e+00
Coefficient: −4.42e−01
Value: B
Rhodoglobus.vestalii
6
4
2
0
A (n=15)
B (n=16)
C (n=14)
surface

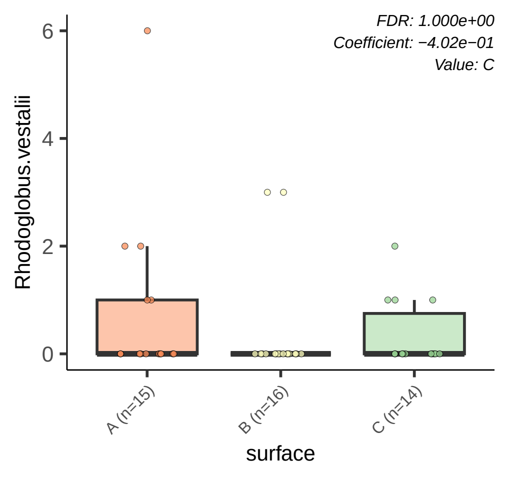
FDR: 1.000e+00
Coefficient: −4.02e−01
Value: C
Rhodoglobus.vestalii
6
4
2
0
A (n=15)
B (n=16)
C (n=14)
surface

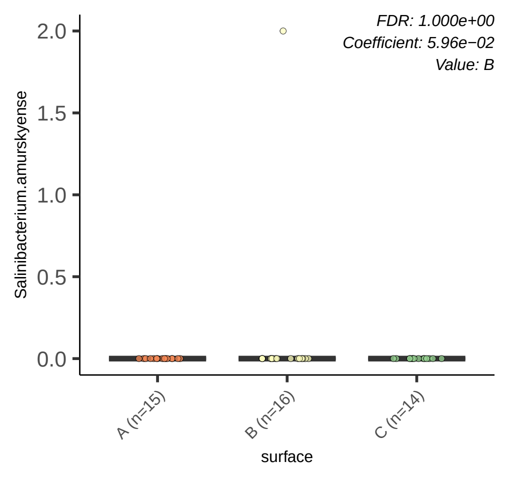
FDR: 1.000e+00
Coefficient: 5.96e−02
Value: B
Salinibacterium.amurskyense
2.0
1.5
1.0
0.5
0.0
A (n=15)
B (n=16)
C (n=14)
surface

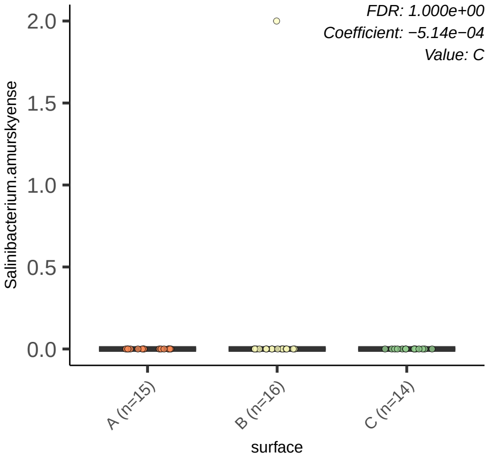

FDR: 1.000e+00
Coefficient: −5.14e−04
Value: C
Salinibacterium.amurskyense
2.0
1.5
1.0
0.5
0.0
A (n=15)
B (n=16)
C (n=14)
surface

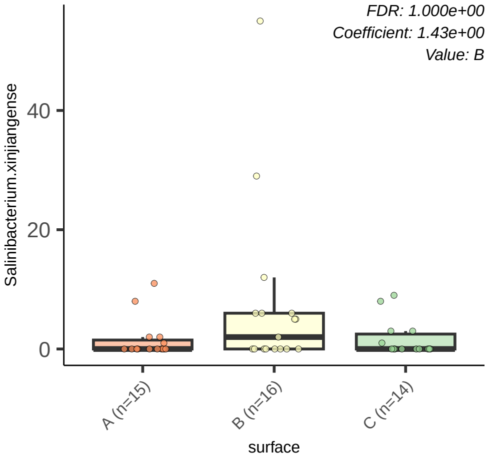

FDR: 1.000e+00
Coefficient: 1.43e+00
Value: B
Salinibacterium.xinjiangense
40
20
0
A (n=15)
B (n=16)
C (n=14)
surface

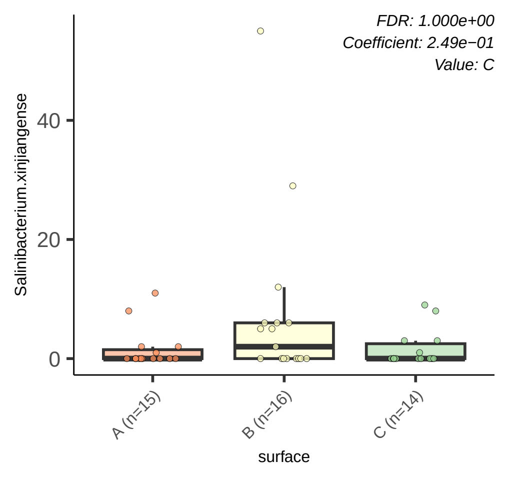
FDR: 1.000e+00
Coefficient: 2.49e−01
Value: C
Salinibacterium.xinjiangense
40
20
0
A (n=15)
B (n=16)
C (n=14)
surface

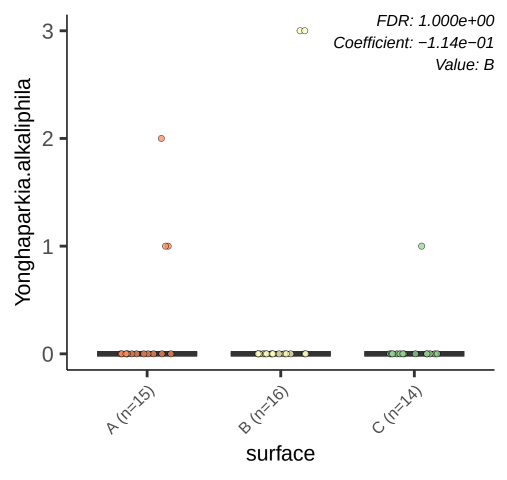
FDR: 1.000e+00
Coefficient: −1.14e−01
Value: B
Yonghaparkia.alkaliphila
0
1
2
3
A (n=15)
B (n=16)
C (n=14)
surface

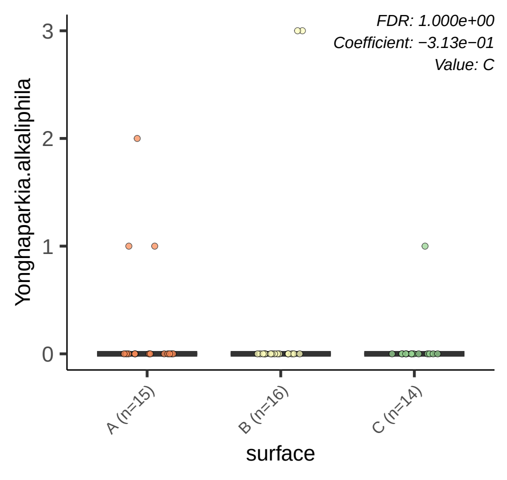
FDR: 1.000e+00
Coefficient: −3.13e−01
Value: C
Yonghaparkia.alkaliphila
0
1
2
3
A (n=15)
B (n=16)
C (n=14)
surface

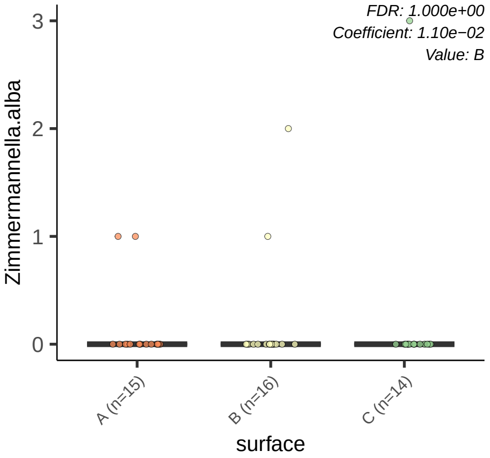
FDR: 1.000e+00
Coefficient: 1.10e−02
Value: B
Zimmermannella.alba
0
1
2
3
A (n=15)
B (n=16)
C (n=14)
surface

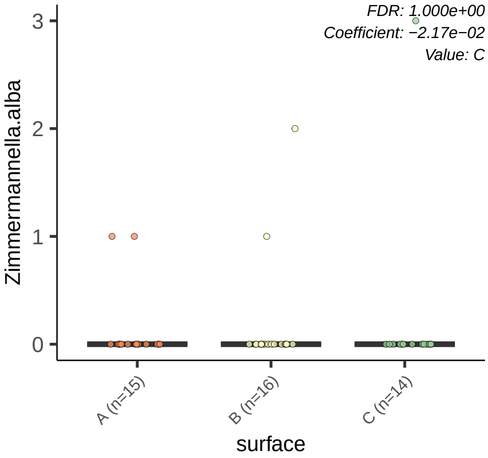
FDR: 1.000e+00
Coefficient: −2.17e−02
Value: C
Zimmermannella.alba
0
1
2
3
A (n=15)
B (n=16)
C (n=14)
surface

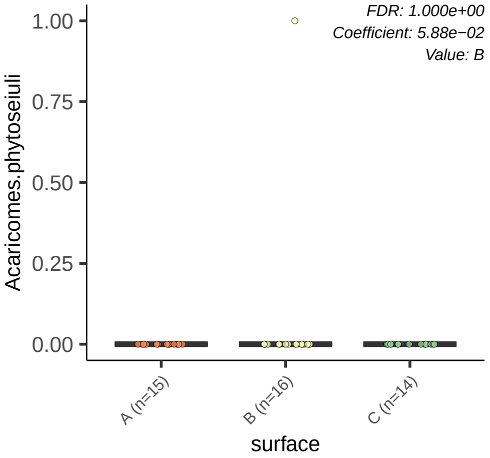
FDR: 1.000e+00
Coefficient: 5.88e−02
Value: B
Acaricomes.phytoseiuli
1.00
0.75
0.50
0.25
0.00
A (n=15)
B (n=16)
C (n=14)
surface

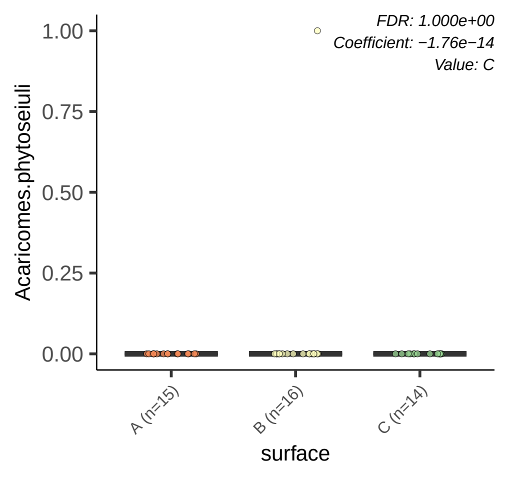
FDR: 1.000e+00
Coefficient: −1.76e−14
Value: C
Acaricomes.phytoseiuli
1.00
0.75
0.50
0.25
0.00
A (n=15)
B (n=16)
C (n=14)
surface

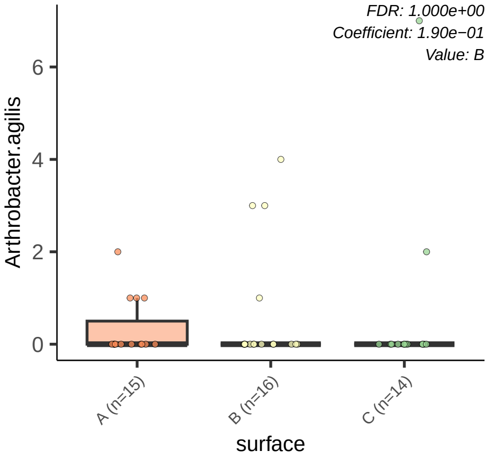

FDR: 1.000e+00
Coefficient: 1.90e−01
Value: B
Arthrobacter.agilis
0
2
4
6
A (n=15)
B (n=16)
C (n=14)
surface

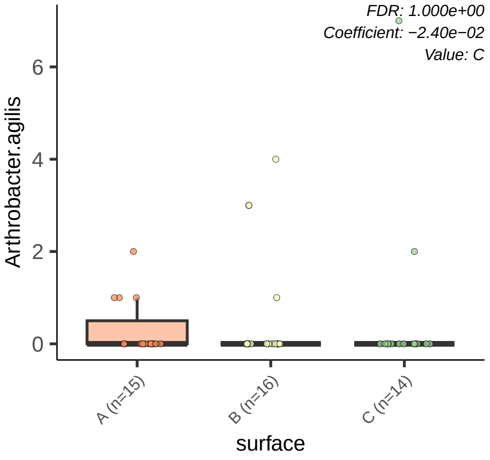
FDR: 1.000e+00
Coefficient: −2.40e−02
Value: C
Arthrobacter.agilis
0
2
4
6
A (n=15)
B (n=16)
C (n=14)
surface

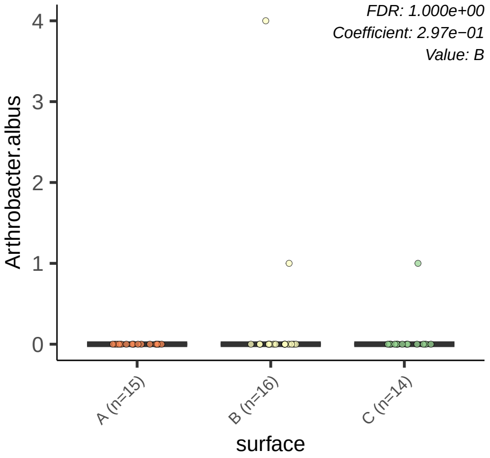
FDR: 1.000e+00
Coefficient: 2.97e−01
Value: B
Arthrobacter.albus
4
3
2
1
0
A (n=15)
B (n=16)
C (n=14)
surface

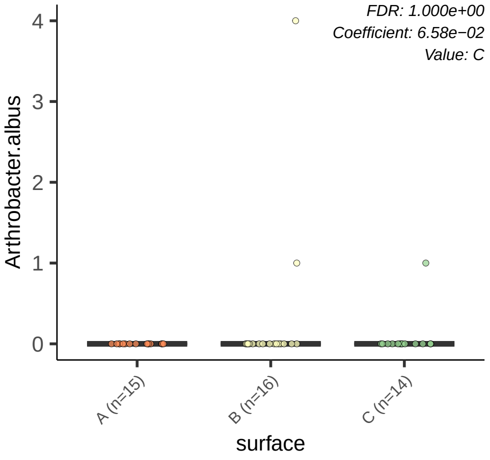

FDR: 1.000e+00
Coefficient: 6.58e−02
Value: C
Arthrobacter.albus
0
1
2
3
4
A (n=15)
B (n=16)
C (n=14)
surface

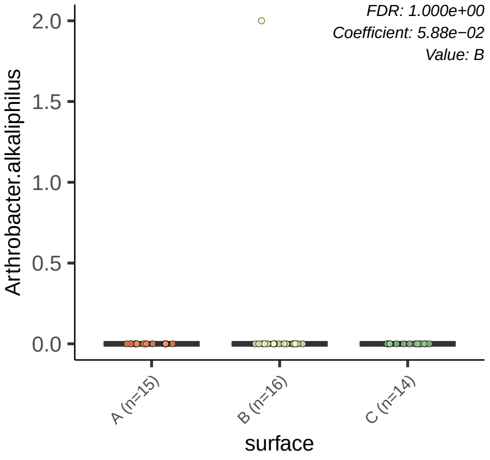
FDR: 1.000e+00
Coefficient: 5.88e−02
Value: B
Arthrobacter.alkaliphilus
2.0
1.5
1.0
0.5
0.0
A (n=15)
B (n=16)
C (n=14)
surface

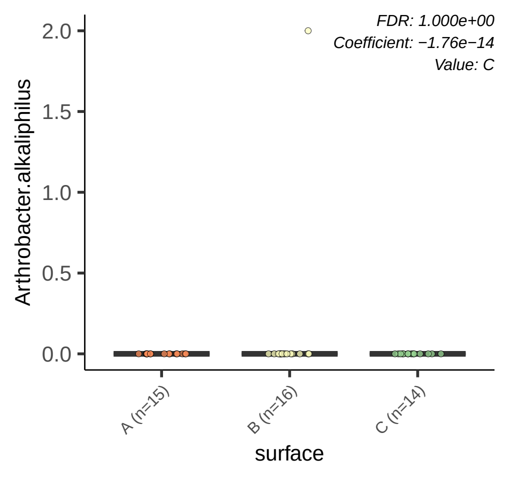

FDR: 1.000e+00
Coefficient: −1.76e−14
Value: C
Arthrobacter.alkaliphilus
2.0
1.5
1.0
0.5
0.0
A (n=15)
B (n=16)
C (n=14)
surface

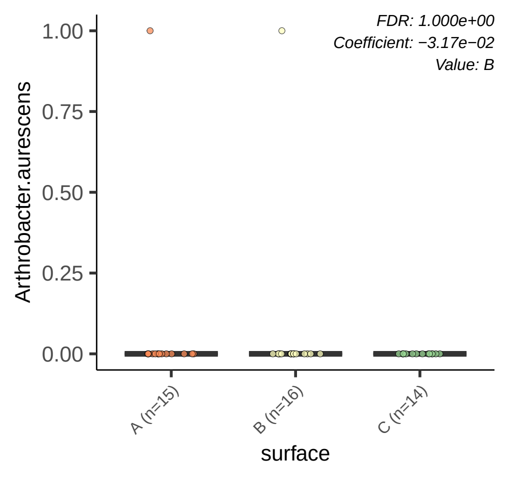
FDR: 1.000e+00
Coefficient: −3.17e−02
Value: B
Arthrobacter.aurescens
1.00
0.75
0.50
0.25
0.00
A (n=15)
B (n=16)
C (n=14)
surface

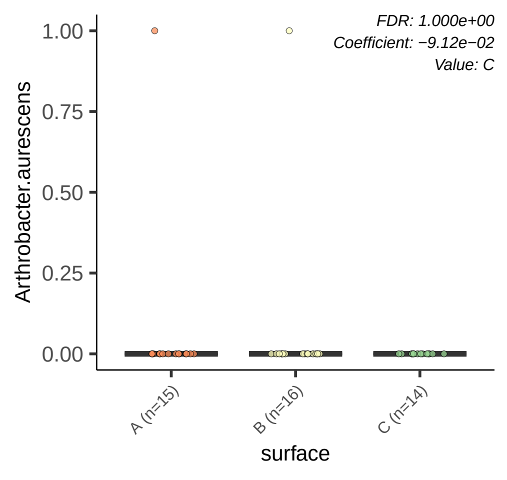
FDR: 1.000e+00
Coefficient: −9.12e−02
Value: C
Arthrobacter.aurescens
1.00
0.75
0.50
0.25
0.00
A (n=15)
B (n=16)
C (n=14)
surface

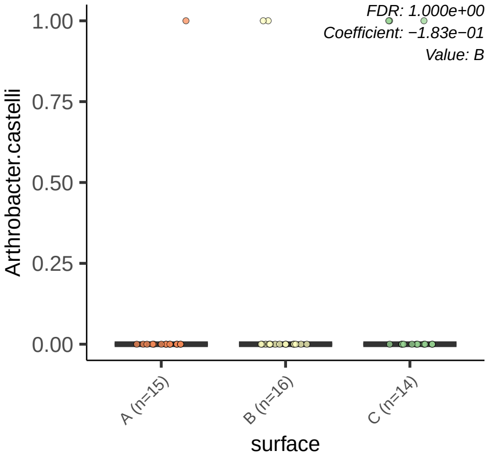
FDR: 1.000e+00
Coefficient: −1.83e−01
Value: B
Arthrobacter.castelli
1.00
0.75
0.50
0.25
0.00
A (n=15)
B (n=16)
C (n=14)
surface

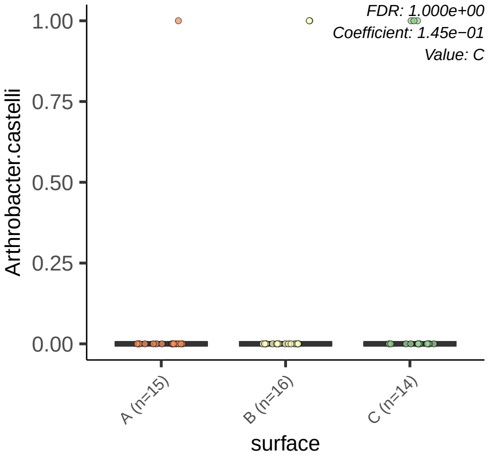

FDR: 1.000e+00
Coefficient: 1.45e−01
Value: C
1.00
0.75
0.50
0.25
0.00
Arthrobacter.castelli
A (n=15)
B (n=16)
C (n=14)
surface

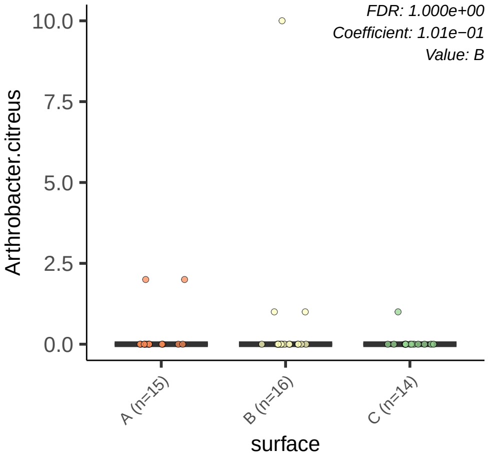
FDR: 1.000e+00
Coefficient: 1.01e−01
Value: B
Arthrobacter.citreus
10.0
7.5
5.0
2.5
0.0
A (n=15)
B (n=16)
C (n=14)
surface

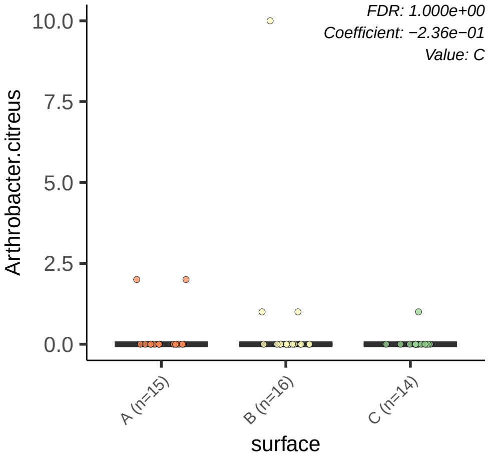
FDR: 1.000e+00
Coefficient: −2.36e−01
Value: C
10.0
7.5
5.0
2.5
0.0
Arthrobacter.citreus
A (n=15)
B (n=16)
C (n=14)
surface

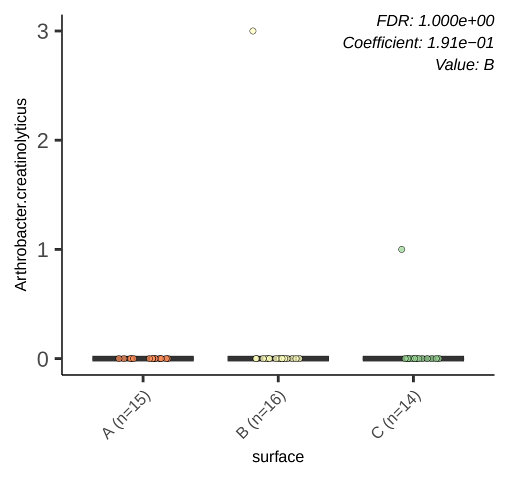
FDR: 1.000e+00
Coefficient: 1.91e−01
Value: B
Arthrobacter.creatinolyticus
3
2
1
0
A (n=15)
B (n=16)
C (n=14)
surface

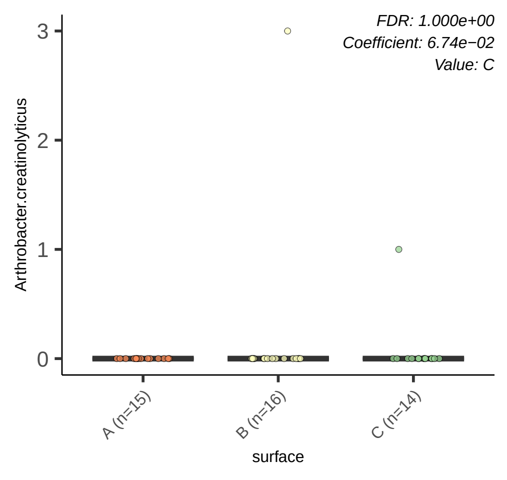
FDR: 1.000e+00
Coefficient: 6.74e−02
Value: C
Arthrobacter.creatinolyticus
3
2
1
0
A (n=15)
B (n=16)
C (n=14)
surface

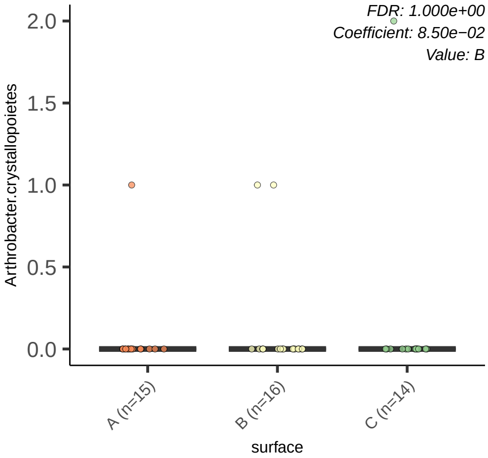
FDR: 1.000e+00
Coefficient: 8.50e−02
Value: B
Arthrobacter.crystallopoietes
2.0
1.5
1.0
0.5
0.0
A (n=15)
B (n=16)
C (n=14)
surface

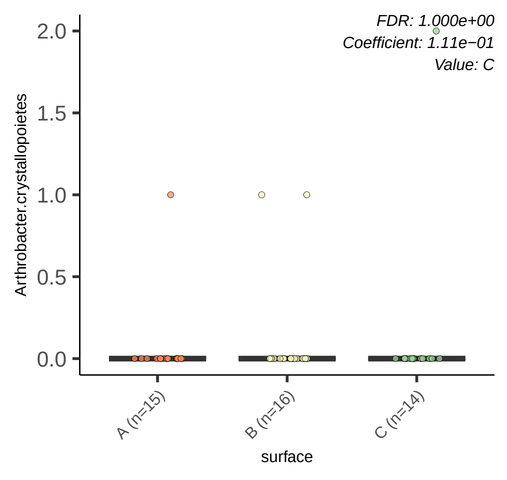
FDR: 1.000e+00
Coefficient: 1.11e−01
Value: C
Arthrobacter.crystallopoietes
2.0
1.5
1.0
0.5
0.0
A (n=15)
B (n=16)
C (n=14)
surface

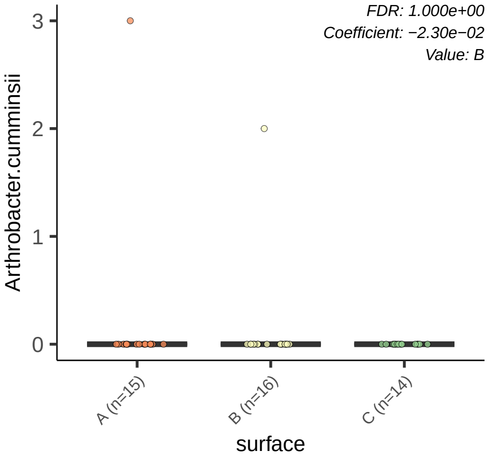
FDR: 1.000e+00
Coefficient: −2.30e−02
Value: B
Arthrobacter.cumminsii
3
2
1
0
A (n=15)
B (n=16)
C (n=14)
surface

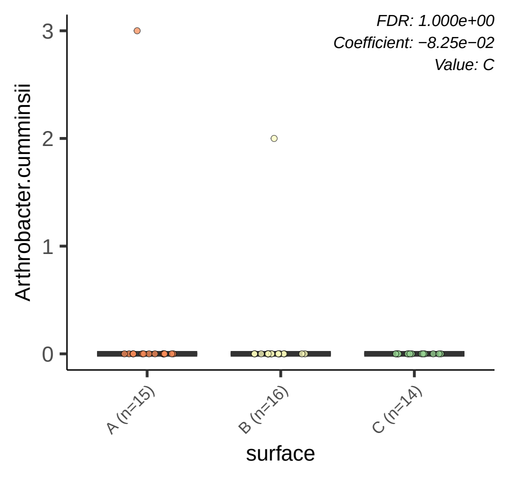
FDR: 1.000e+00
Coefficient: −8.25e−02
Value: C
Arthrobacter.cumminsii
0
1
2
3
A (n=15)
B (n=16)
C (n=14)
surface

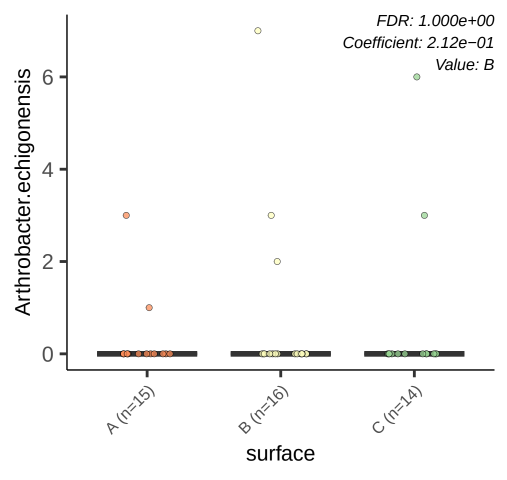

FDR: 1.000e+00
Coefficient: 2.12e−01
Value: B
Arthrobacter.echigonensis
0
2
4
6
A (n=15)
B (n=16)
C (n=14)
surface

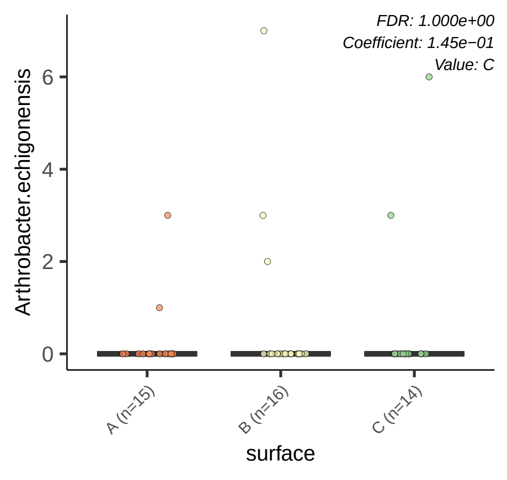
FDR: 1.000e+00
Coefficient: 1.45e−01
Value: C
Arthrobacter.echigonensis
0
2
4
6
A (n=15)
B (n=16)
C (n=14)
surface

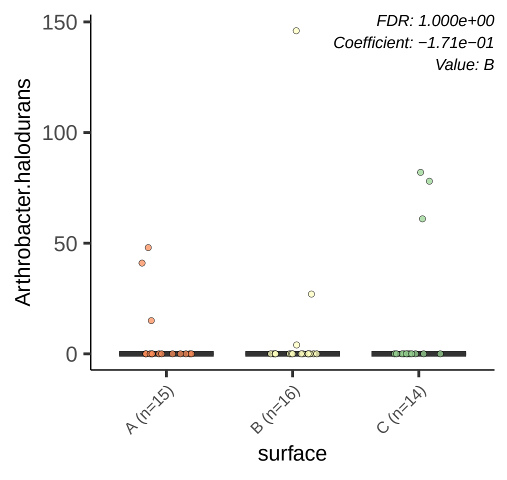

FDR: 1.000e+00
Coefficient: −1.71e−01
Value: B
Arthrobacter.halodurans
150
100
50
0
A (n=15)
B (n=16)
C (n=14)
surface

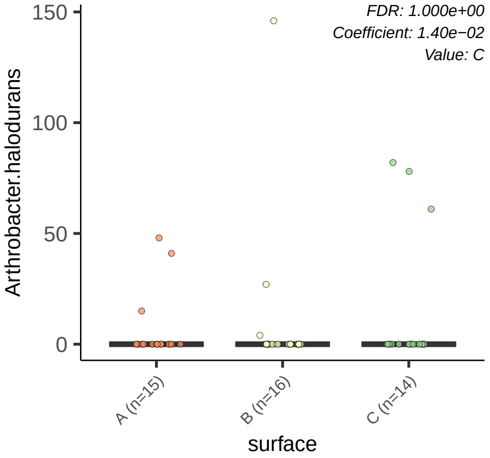
FDR: 1.000e+00
Coefficient: 1.40e−02
Value: C
Arthrobacter.halodurans
150
100
50
0
A (n=15)
B (n=16)
C (n=14)
surface

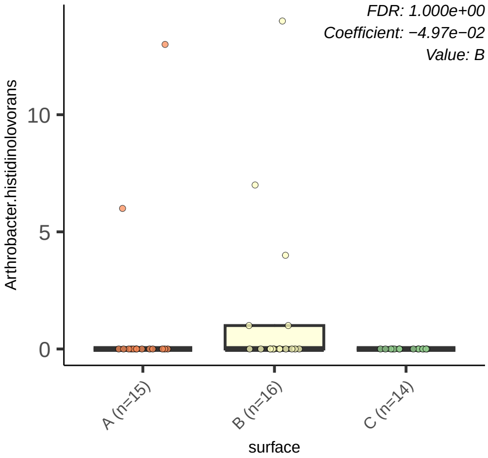

FDR: 1.000e+00
Coefficient: −4.97e−02
Value: B
Arthrobacter.histidinolovorans
10
5
0
A (n=15)
B (n=16)
C (n=14)
surface

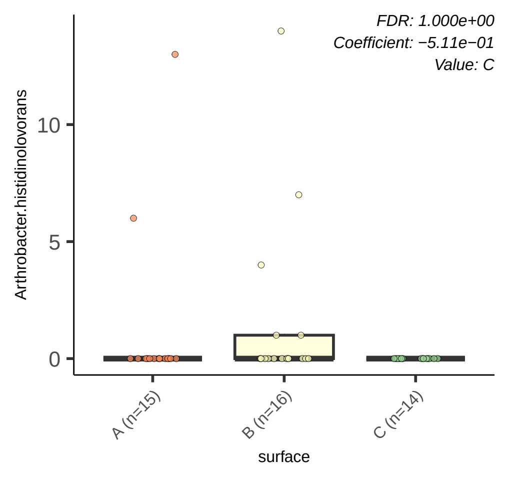
FDR: 1.000e+00
Coefficient: −5.11e−01
Value: C
Arthrobacter.histidinolovorans
10
5
0
A (n=15)
B (n=16)
C (n=14)
surface

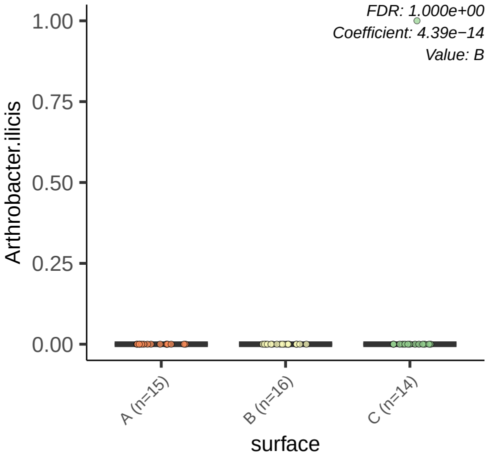
FDR: 1.000e+00
Coefficient: 4.39e−14
Value: B
Arthrobacter.ilicis
1.00
0.75
0.50
0.25
0.00
A (n=15)
B (n=16)
C (n=14)
surface

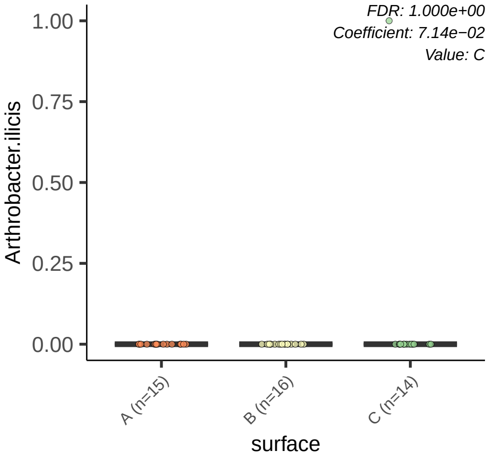
FDR: 1.000e+00
Coefficient: 7.14e−02
Value: C
1.00
0.75
0.50
0.25
0.00
Arthrobacter.ilicis
A (n=15)
B (n=16)
C (n=14)
surface

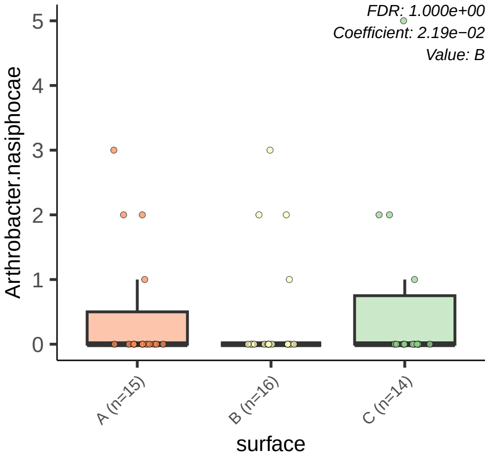

FDR: 1.000e+00
Coefficient: 2.19e−02
Value: B
Arthrobacter.nasiphocae
0
1
2
3
4
5
A (n=15)
B (n=16)
C (n=14)
surface

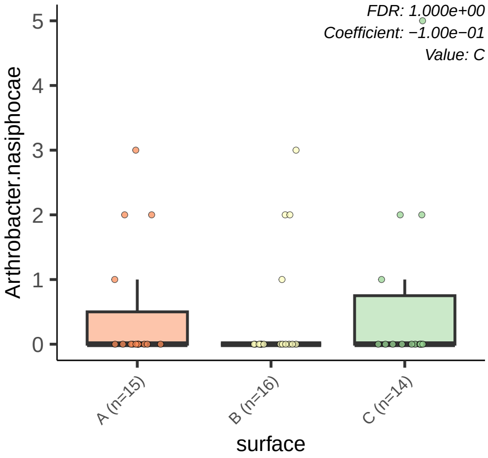

FDR: 1.000e+00
Coefficient: −1.00e−01
Value: C
Arthrobacter.nasiphocae
0
1
2
3
4
5
A (n=15)
B (n=16)
C (n=14)
surface

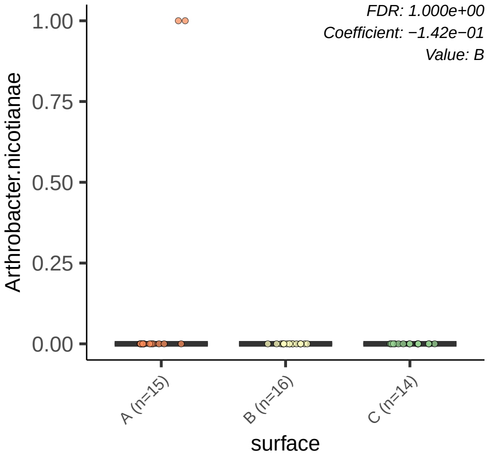
FDR: 1.000e+00
Coefficient: −1.42e−01
Value: B
Arthrobacter.nicotianae
1.00
0.75
0.50
0.25
0.00
A (n=15)
B (n=16)
C (n=14)
surface

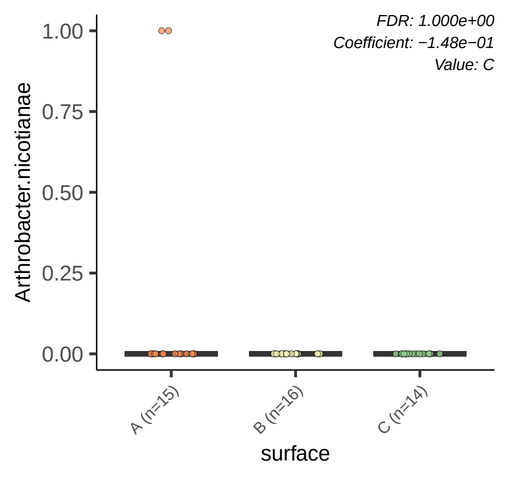

FDR: 1.000e+00
Coefficient: −1.48e−01
Value: C
Arthrobacter.nicotianae
1.00
0.75
0.50
0.25
0.00
A (n=15)
B (n=16)
C (n=14)
surface

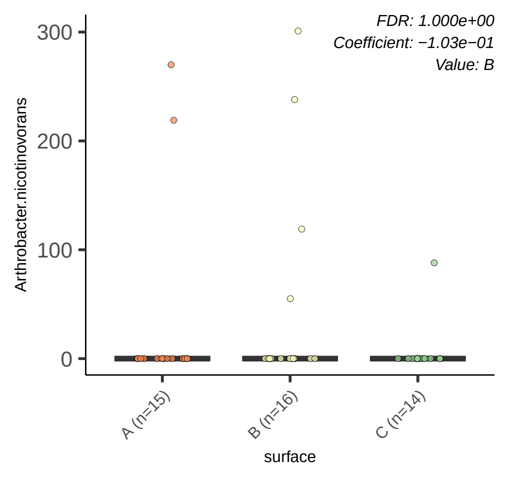

FDR: 1.000e+00
Coefficient: −1.03e−01
Value: B
Arthrobacter.nicotinovorans
300
200
100
0
A (n=15)
B (n=16)
C (n=14)
surface

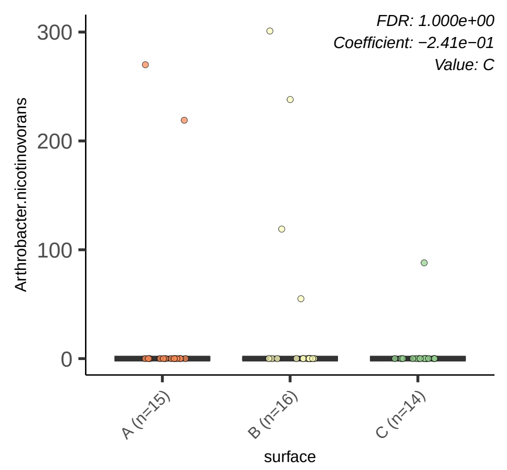
FDR: 1.000e+00
Coefficient: −2.41e−01
Value: C
Arthrobacter.nicotinovorans
300
200
100
0
A (n=15)
B (n=16)
C (n=14)
surface

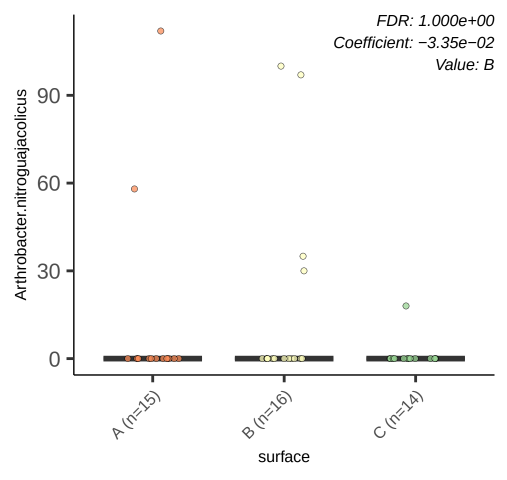
FDR: 1.000e+00
Coefficient: −3.35e−02
Value: B
Arthrobacter.nitroguajacolicus
90
60
30
0
A (n=15)
B (n=16)
C (n=14)
surface

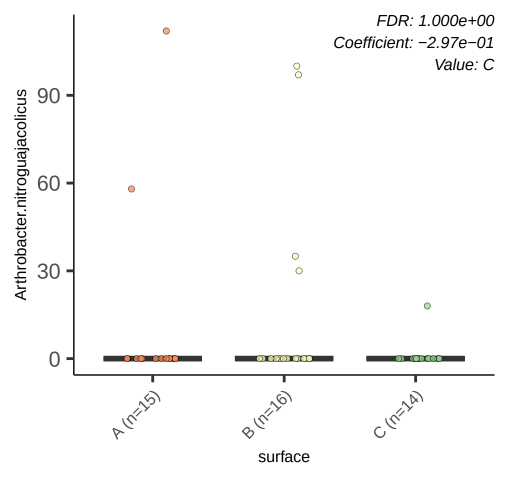
FDR: 1.000e+00
Coefficient: −2.97e−01
Value: C
Arthrobacter.nitroguajacolicus
90
60
30
0
A (n=15)
B (n=16)
C (n=14)
surface

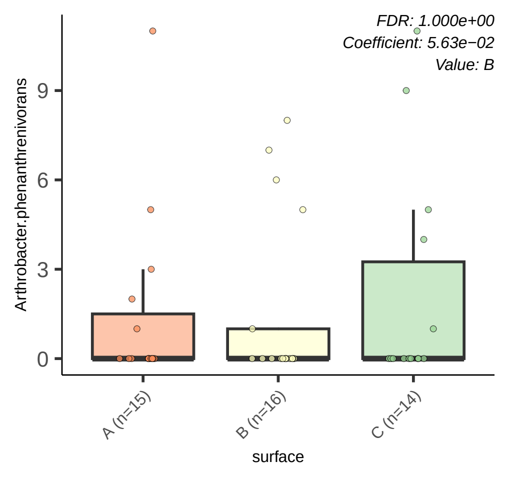
FDR: 1.000e+00
Coefficient: 5.63e−02
Value: B
Arthrobacter.phenanthrenivorans
0
3
6
9
A (n=15)
B (n=16)
C (n=14)
surface

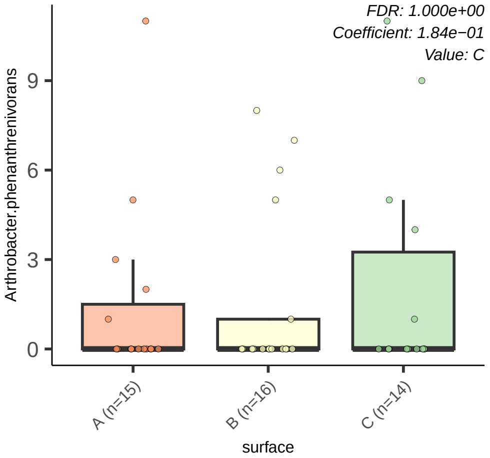
FDR: 1.000e+00
Coefficient: 1.84e−01
Value: C
Arthrobacter.phenanthrenivorans
0
3
6
9
A (n=15)
B (n=16)
C (n=14)
surface

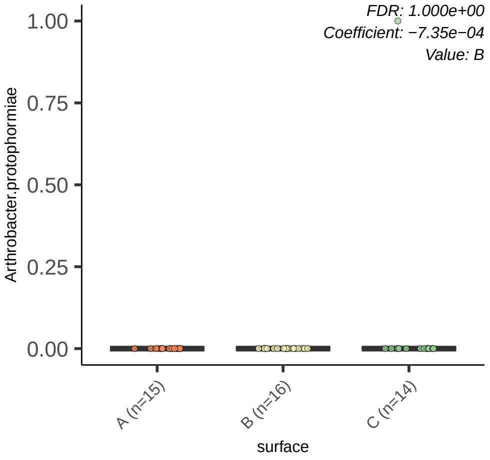
FDR: 1.000e+00
Coefficient: −7.35e−04
Value: B
Arthrobacter.protophormiae
1.00
0.75
0.50
0.25
0.00
A (n=15)
B (n=16)
C (n=14)
surface

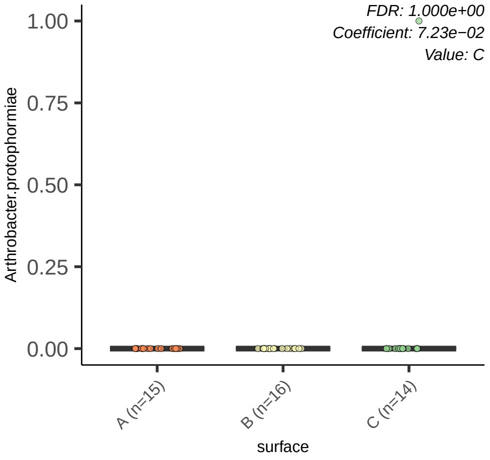
FDR: 1.000e+00
Coefficient: 7.23e−02
Value: C
1.00
0.75
0.50
0.25
0.00
Arthrobacter.protophormiae
A (n=15)
B (n=16)
C (n=14)
surface

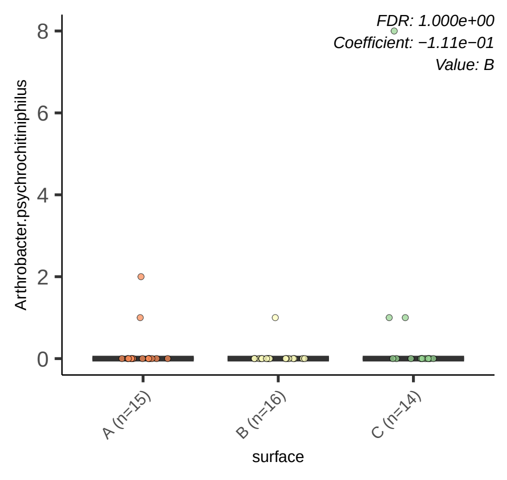
FDR: 1.000e+00
Coefficient: −1.11e−01
Value: B
Arthrobacter.psychrochitiniphilus
0
2
4
6
8
A (n=15)
B (n=16)
C (n=14)
surface

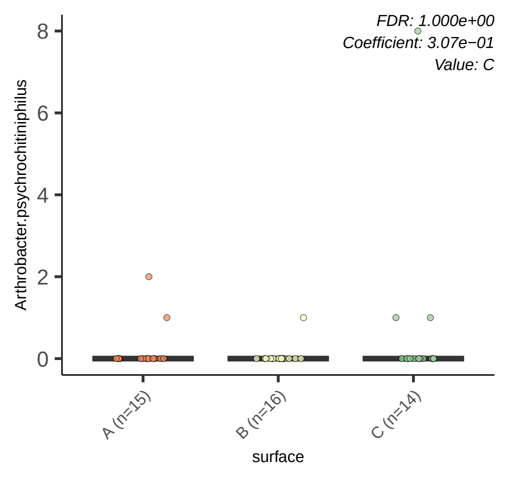
FDR: 1.000e+00
Coefficient: 3.07e−01
Value: C
Arthrobacter.psychrochitiniphilus
8
6
4
2
0
A (n=15)
B (n=16)
C (n=14)
surface

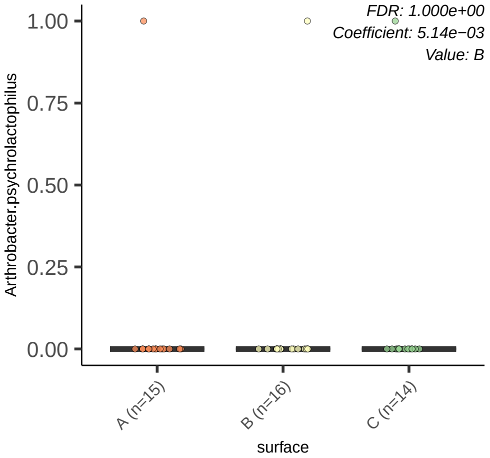

FDR: 1.000e+00
Coefficient: 5.14e−03
Value: B
Arthrobacter.psychrolactophilus
1.00
0.75
0.50
0.25
0.00
A (n=15)
B (n=16)
C (n=14)
surface

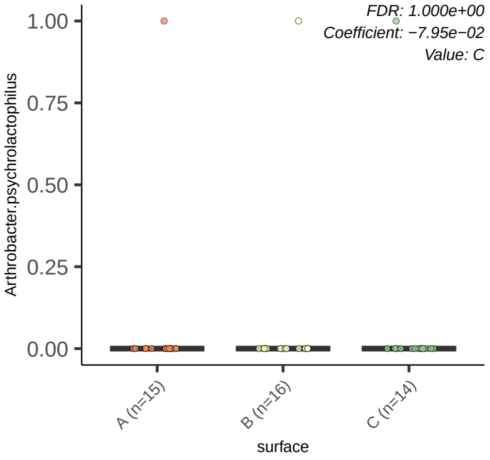
FDR: 1.000e+00
Coefficient: −7.95e−02
Value: C
Arthrobacter.psychrolactophilus
1.00
0.75
0.50
0.25
0.00
A (n=15)
B (n=16)
C (n=14)
surface

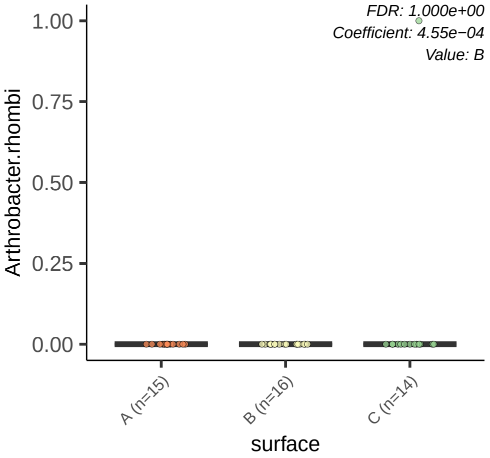

FDR: 1.000e+00
Coefficient: 4.55e−04
Value: B
Arthrobacter.rhombi
1.00
0.75
0.50
0.25
0.00
A (n=15)
B (n=16)
C (n=14)
surface

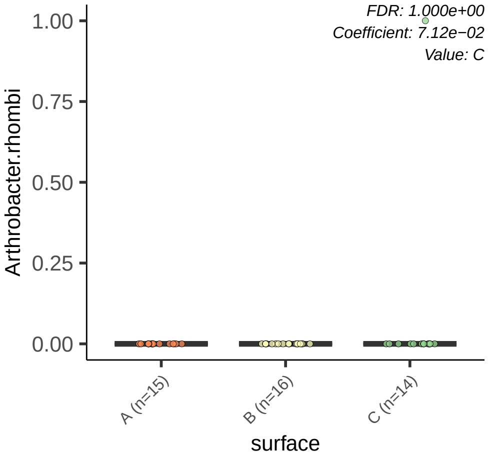

FDR: 1.000e+00
Coefficient: 7.12e−02
Value: C
1.00
0.75
0.50
0.25
0.00
Arthrobacter.rhombi
A (n=15)
B (n=16)
C (n=14)
surface

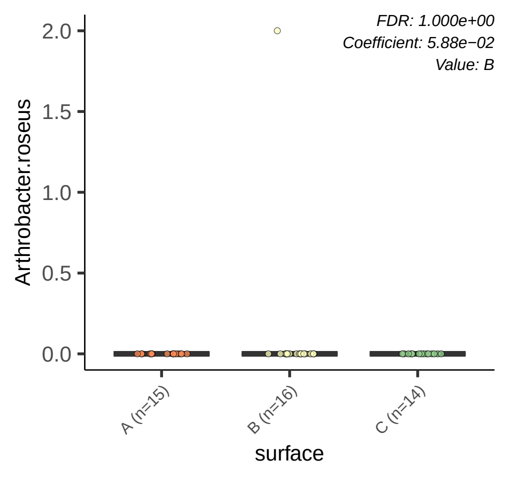
FDR: 1.000e+00
Coefficient: 5.88e−02
Value: B
Arthrobacter.roseus
2.0
1.5
1.0
0.5
0.0
A (n=15)
B (n=16)
C (n=14)
surface

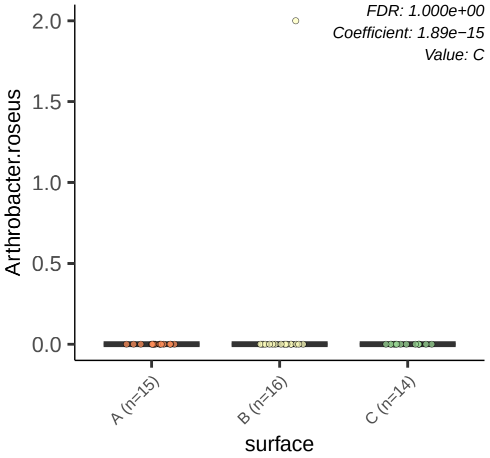
FDR: 1.000e+00
Coefficient: 1.89e−15
Value: C
Arthrobacter.roseus
2.0
1.5
1.0
0.5
0.0
A (n=15)
B (n=16)
C (n=14)
surface

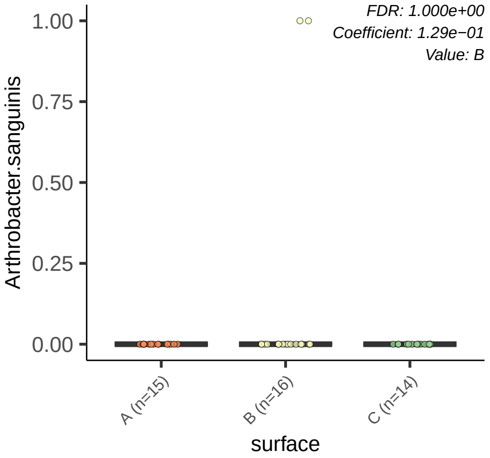

FDR: 1.000e+00
Coefficient: 1.29e−01
Value: B
Arthrobacter.sanguinis
1.00
0.75
0.50
0.25
0.00
A (n=15)
B (n=16)
C (n=14)
surface

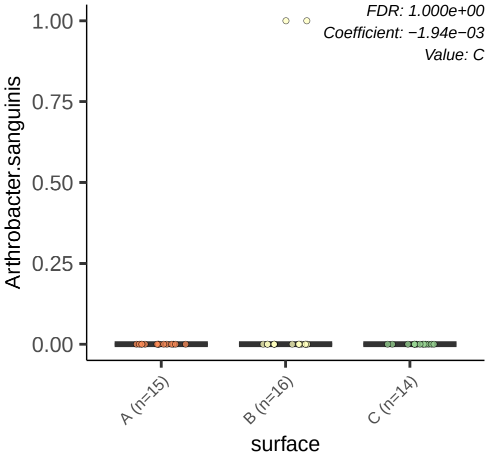
FDR: 1.000e+00
Coefficient: −1.94e−03
Value: C
Arthrobacter.sanguinis
1.00
0.75
0.50
0.25
0.00
A (n=15)
B (n=16)
C (n=14)
surface

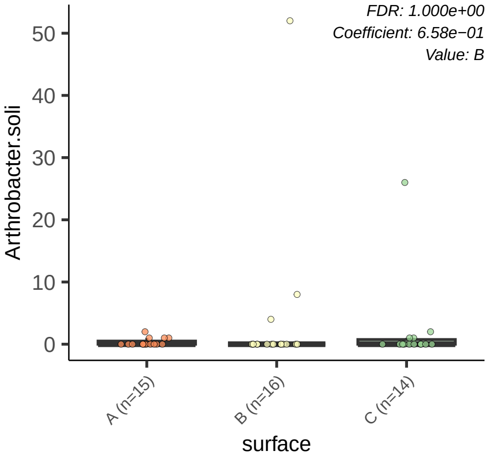
FDR: 1.000e+00
Coefficient: 6.58e−01
Value: B
Arthrobacter.soli
0
10
20
30
40
50
A (n=15)
B (n=16)
C (n=14)
surface

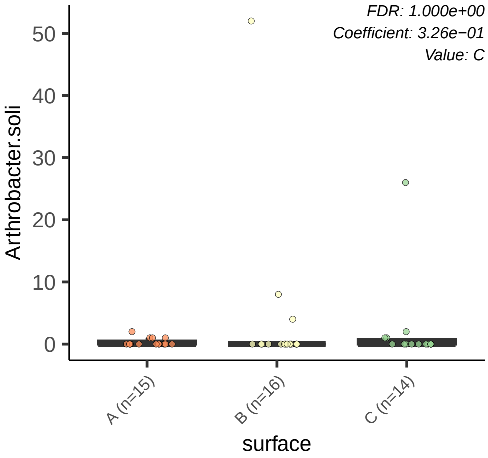

FDR: 1.000e+00
Coefficient: 3.26e−01
Value: C
Arthrobacter.soli
50
40
30
20
10
0
A (n=15)
B (n=16)
C (n=14)
surface

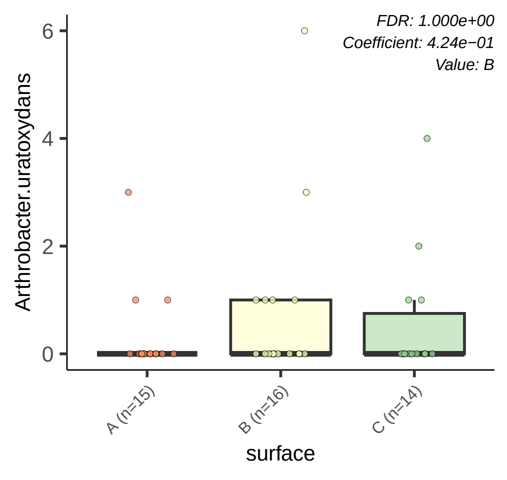
FDR: 1.000e+00
Coefficient: 4.24e−01
Value: B
Arthrobacter.uratoxydans
0
2
4
6
A (n=15)
B (n=16)
C (n=14)
surface

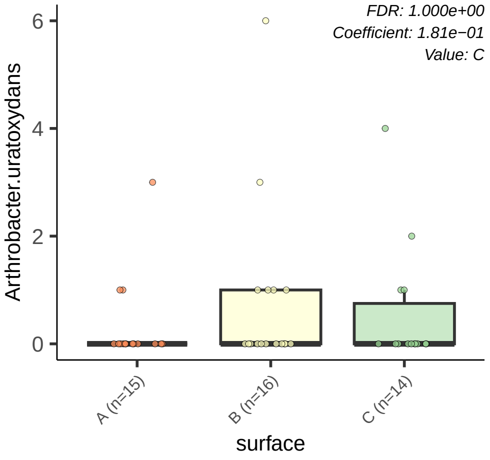

FDR: 1.000e+00
Coefficient: 1.81e−01
Value: C
Arthrobacter.uratoxydans
0
2
4
6
A (n=15)
B (n=16)
C (n=14)
surface

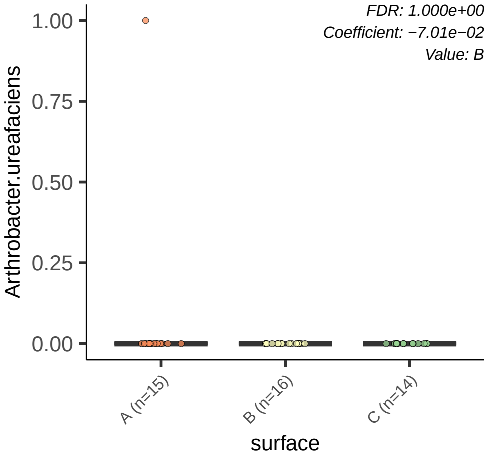
FDR: 1.000e+00
Coefficient: −7.01e−02
Value: B
Arthrobacter.ureafaciens
1.00
0.75
0.50
0.25
0.00
A (n=15)
B (n=16)
C (n=14)
surface

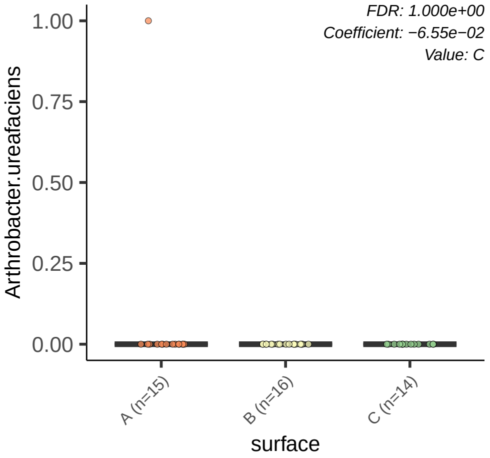

FDR: 1.000e+00
Coefficient: −6.55e−02
Value: C
Arthrobacter.ureafaciens
1.00
0.75
0.50
0.25
0.00
A (n=15)
B (n=16)
C (n=14)
surface

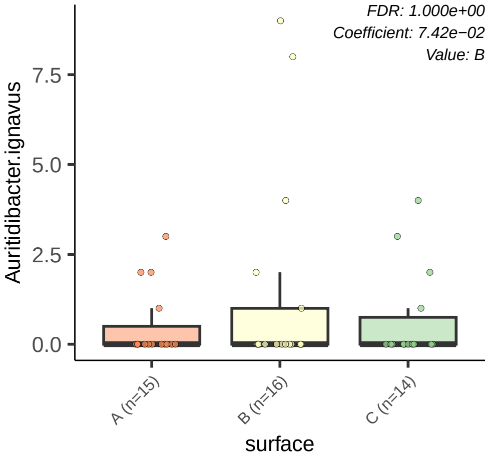

FDR: 1.000e+00
Coefficient: 7.42e−02
Value: B
Auritidibacter.ignavus
7.5
5.0
2.5
0.0
A (n=15)
B (n=16)
C (n=14)
surface

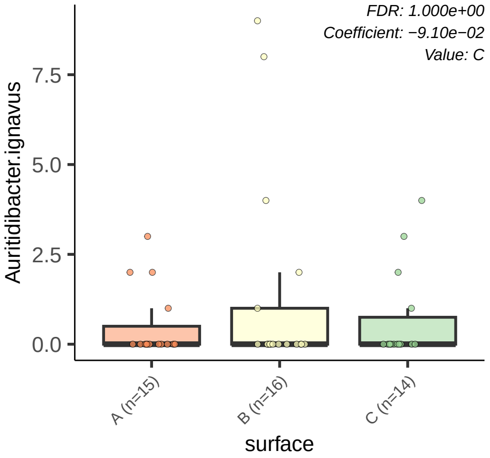

FDR: 1.000e+00
Coefficient: −9.10e−02
Value: C
Auritidibacter.ignavus
7.5
5.0
2.5
0.0
A (n=15)
B (n=16)
C (n=14)
surface

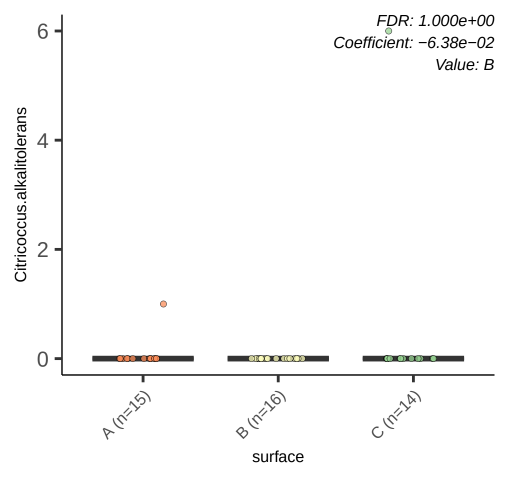

FDR: 1.000e+00
Coefficient: −6.38e−02
Value: B
Citricoccus.alkalitolerans
0
2
4
6
A (n=15)
B (n=16)
C (n=14)
surface

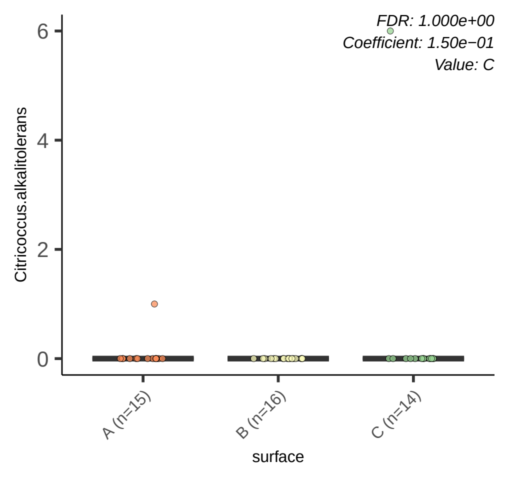
FDR: 1.000e+00
Coefficient: 1.50e−01
Value: C
Citricoccus.alkalitolerans
6
4
2
0
A (n=15)
B (n=16)
C (n=14)
surface

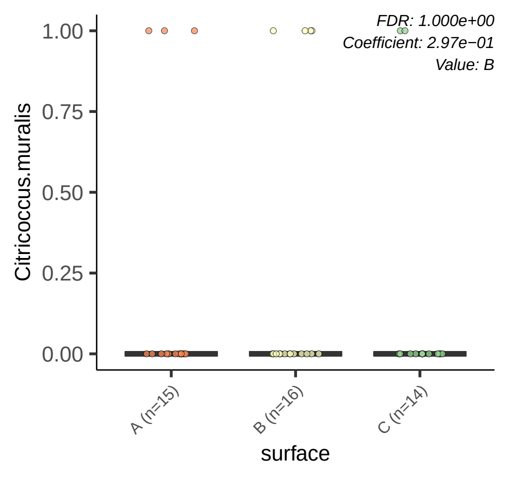
FDR: 1.000e+00
Coefficient: 2.97e−01
Value: B
Citricoccus.muralis
1.00
0.75
0.50
0.25
0.00
A (n=15)
B (n=16)
C (n=14)
surface

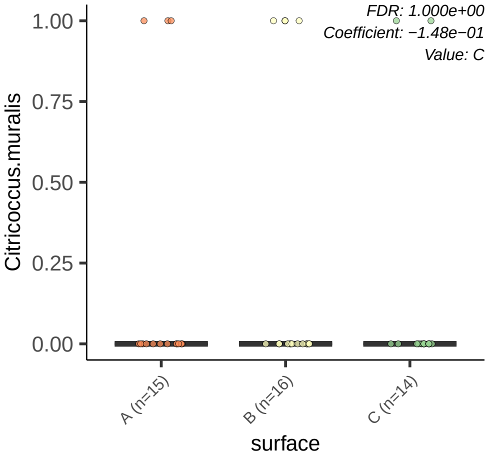

FDR: 1.000e+00
Coefficient: −1.48e−01
Value: C
Citricoccus.muralis
1.00
0.75
0.50
0.25
0.00
A (n=15)
B (n=16)
C (n=14)
surface

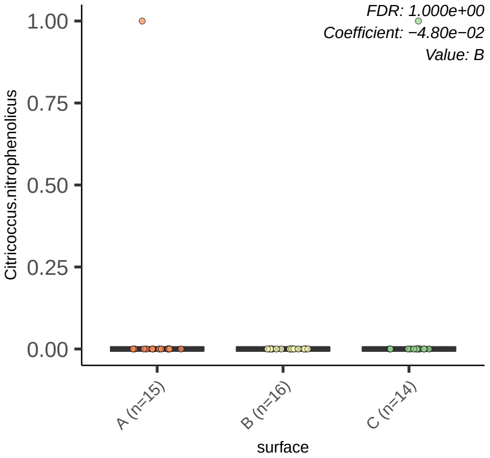

FDR: 1.000e+00
Coefficient: −4.80e−02
Value: B
Citricoccus.nitrophenolicus
1.00
0.75
0.50
0.25
0.00
A (n=15)
B (n=16)
C (n=14)
surface

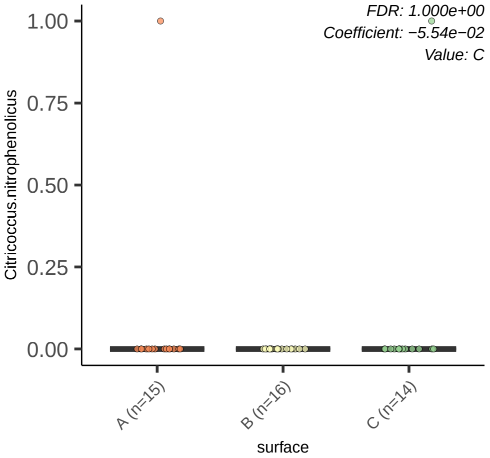
FDR: 1.000e+00
Coefficient: −5.54e−02
Value: C
Citricoccus.nitrophenolicus
1.00
0.75
0.50
0.25
0.00
A (n=15)
B (n=16)
C (n=14)
surface

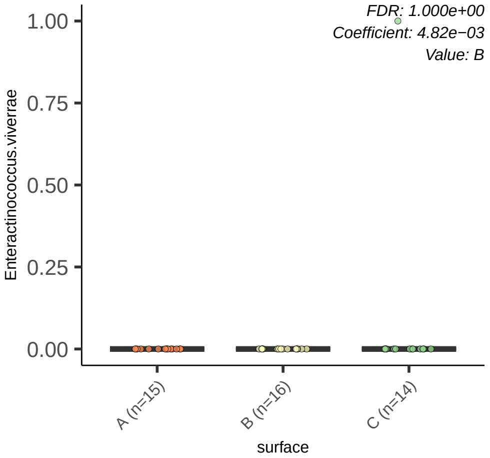

FDR: 1.000e+00
Coefficient: 4.82e−03
Value: B
Enteractinococcus.viverrae
1.00
0.75
0.50
0.25
0.00
A (n=15)
B (n=16)
C (n=14)
surface

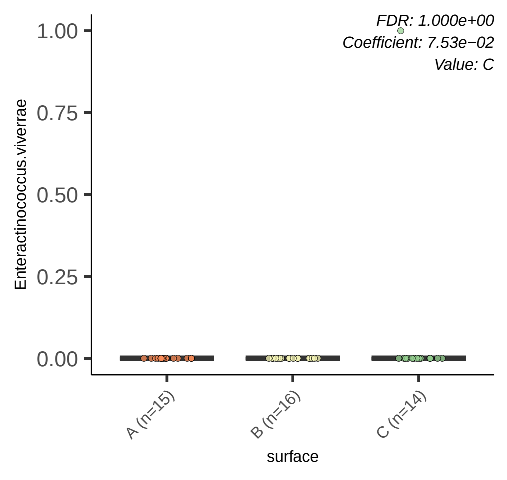
FDR: 1.000e+00
Coefficient: 7.53e−02
Value: C
1.00
0.75
0.50
0.25
0.00
Enteractinococcus.viverrae
A (n=15)
B (n=16)
C (n=14)
surface

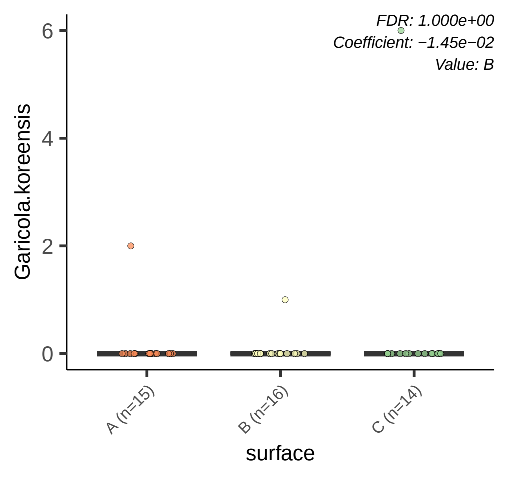
FDR: 1.000e+00
Coefficient: −1.45e−02
Value: B
Garicola.koreensis
6
4
2
0
A (n=15)
B (n=16)
C (n=14)
surface

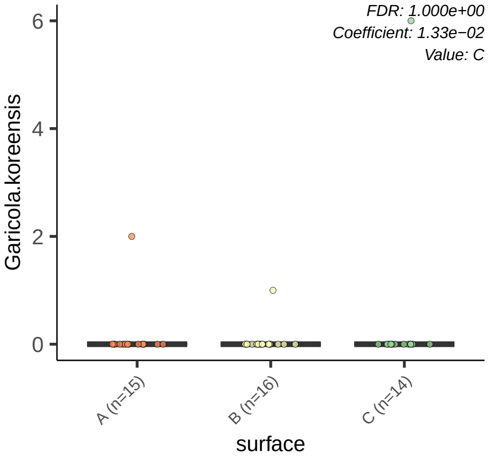

FDR: 1.000e+00
Coefficient: 1.33e−02
Value: C
Garicola.koreensis
0
2
4
6
A (n=15)
B (n=16)
C (n=14)
surface

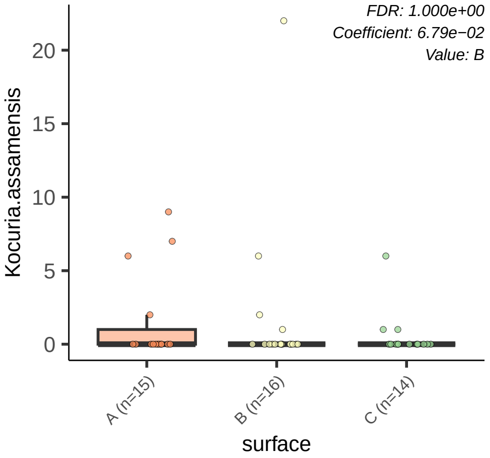
FDR: 1.000e+00
Coefficient: 6.79e−02
Value: B
Kocuria.assamensis
0
5
10
15
20
A (n=15)
B (n=16)
C (n=14)
surface

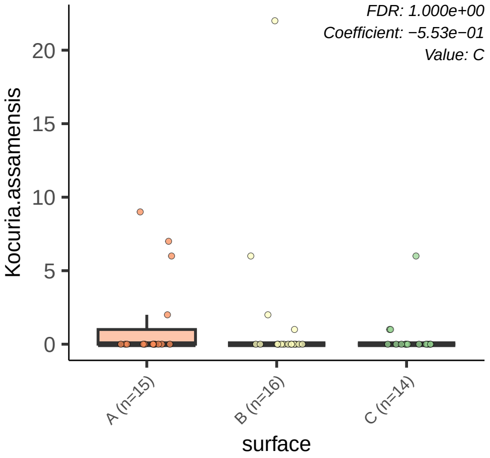
FDR: 1.000e+00
Coefficient: −5.53e−01
Value: C
Kocuria.assamensis
0
5
10
15
20
A (n=15)
B (n=16)
C (n=14)
surface

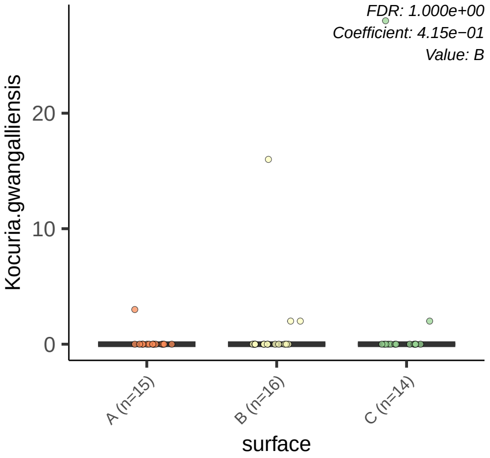
FDR: 1.000e+00
Coefficient: 4.15e−01
Value: B
Kocuria.gwangalliensis
20
10
0
A (n=15)
B (n=16)
C (n=14)
surface

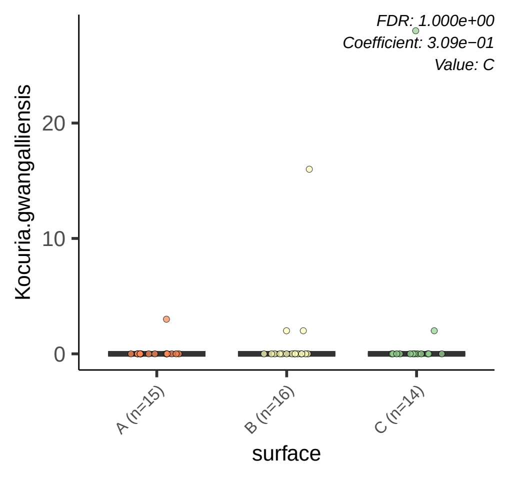
FDR: 1.000e+00
Coefficient: 3.09e−01
Value: C
Kocuria.gwangalliensis
20
10
0
A (n=15)
B (n=16)
C (n=14)
surface

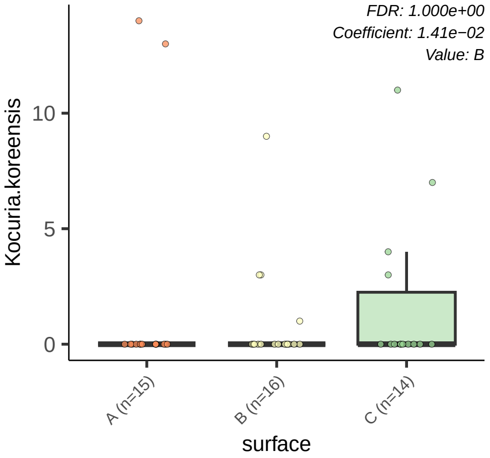

FDR: 1.000e+00
Coefficient: 1.41e−02
Value: B
Kocuria.koreensis
10
5
0
A (n=15)
B (n=16)
C (n=14)
surface

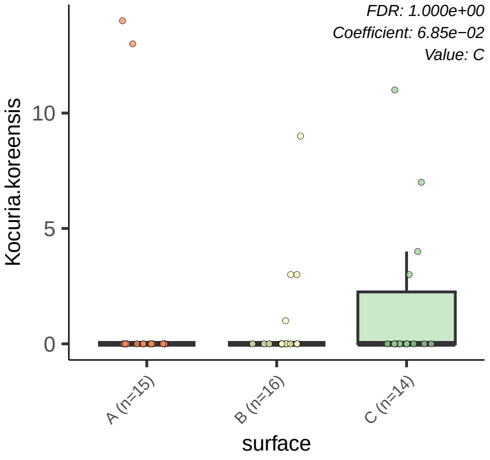
FDR: 1.000e+00
Coefficient: 6.85e−02
Value: C
Kocuria.koreensis
10
5
0
A (n=15)
B (n=16)
C (n=14)
surface

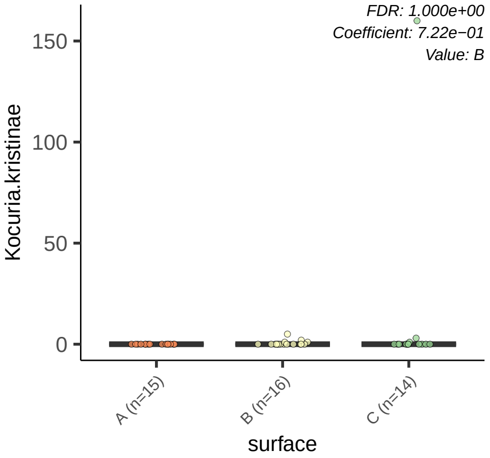
FDR: 1.000e+00
Coefficient: 7.22e−01
Value: B
Kocuria.kristinae
150
100
50
0
A (n=15)
B (n=16)
C (n=14)
surface

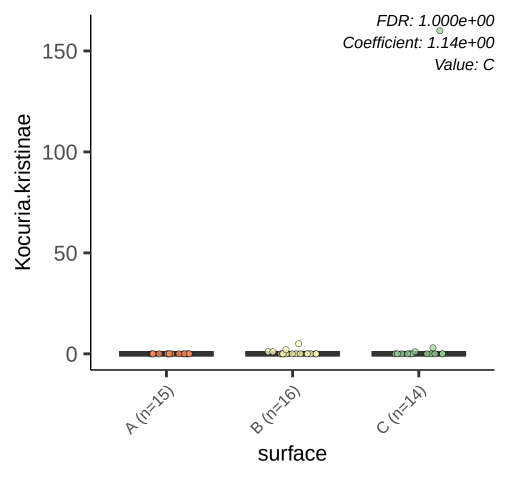
FDR: 1.000e+00
Coefficient: 1.14e+00
Value: C
Kocuria.kristinae
150
100
50
0
A (n=15)
B (n=16)
C (n=14)
surface

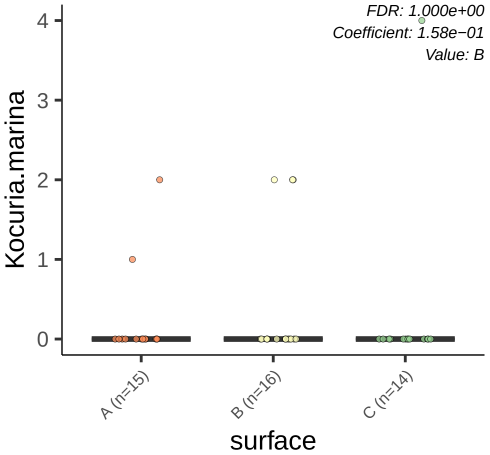

FDR: 1.000e+00
Coefficient: 1.58e−01
Value: B
Kocuria.marina
0
1
2
3
4
A (n=15)
B (n=16)
C (n=14)
surface

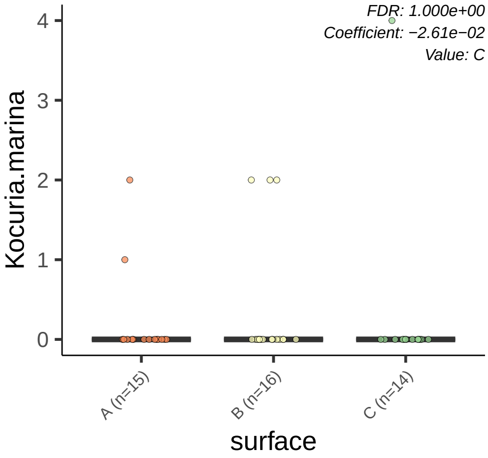

FDR: 1.000e+00
Coefficient: −2.61e−02
Value: C
Kocuria.marina
4
3
2
1
0
A (n=15)
B (n=16)
C (n=14)
surface

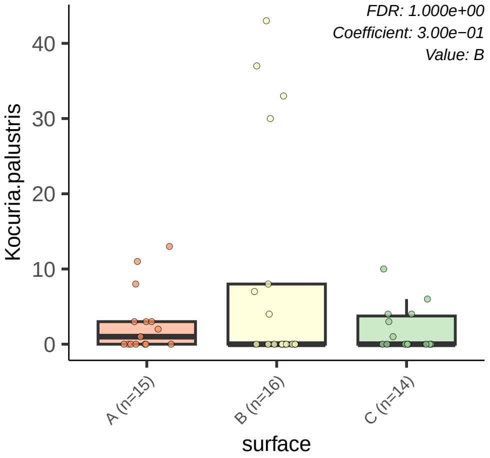

FDR: 1.000e+00
Coefficient: 3.00e−01
Value: B
Kocuria.palustris
0
10
20
30
40
A (n=15)
B (n=16)
C (n=14)
surface

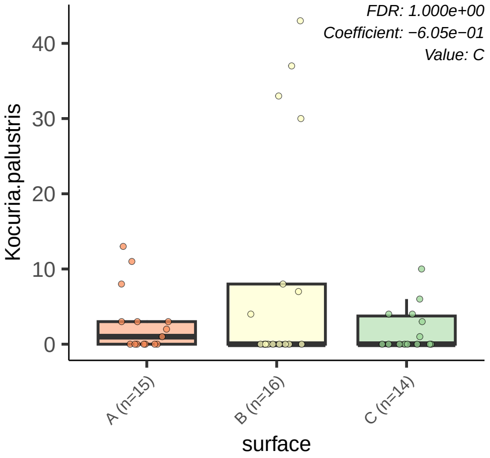

FDR: 1.000e+00
Coefficient: −6.05e−01
Value: C
Kocuria.palustris
40
30
20
10
0
A (n=15)
B (n=16)
C (n=14)
surface

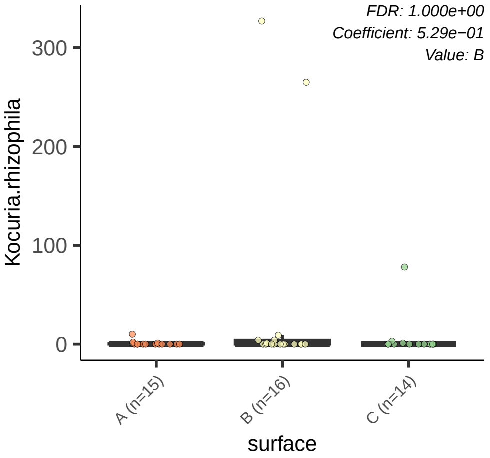
FDR: 1.000e+00
Coefficient: 5.29e−01
Value: B
Kocuria.rhizophila
300
200
100
0
A (n=15)
B (n=16)
C (n=14)
surface

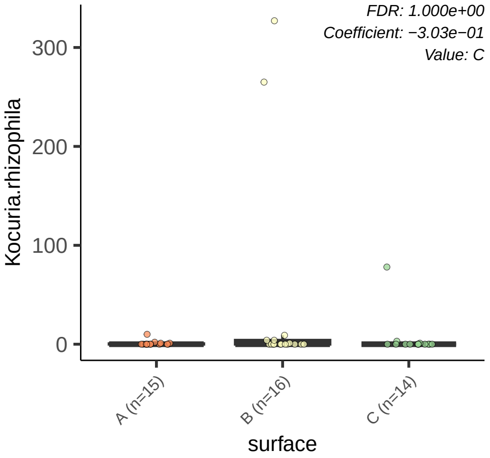

FDR: 1.000e+00
Coefficient: −3.03e−01
Value: C
Kocuria.rhizophila
300
200
100
0
A (n=15)
B (n=16)
C (n=14)
surface

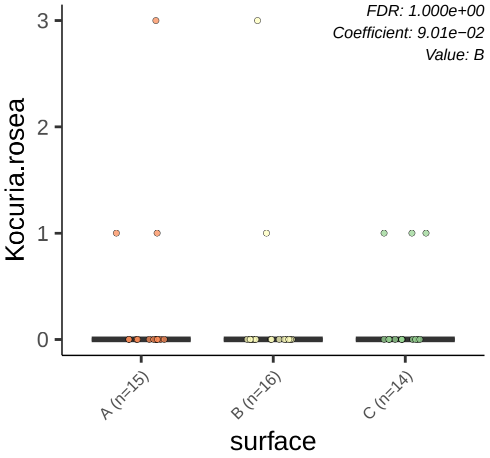

FDR: 1.000e+00
Coefficient: 9.01e−02
Value: B
Kocuria.rosea
0
1
2
3
A (n=15)
B (n=16)
C (n=14)
surface

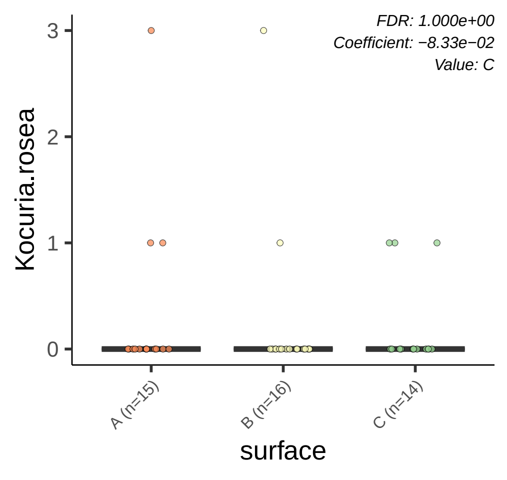
FDR: 1.000e+00
Coefficient: −8.33e−02
Value: C
Kocuria.rosea
3
2
1
0
A (n=15)
B (n=16)
C (n=14)
surface

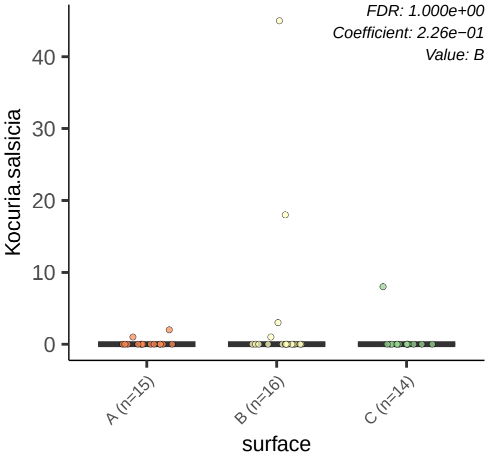
FDR: 1.000e+00
Coefficient: 2.26e−01
Value: B
Kocuria.salsicia
0
10
20
30
40
A (n=15)
B (n=16)
C (n=14)
surface

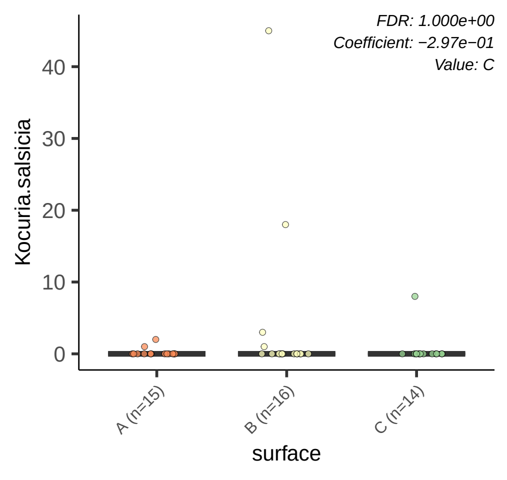

FDR: 1.000e+00
Coefficient: −2.97e−01
Value: C
Kocuria.salsicia
40
30
20
10
0
A (n=15)
B (n=16)
C (n=14)
surface

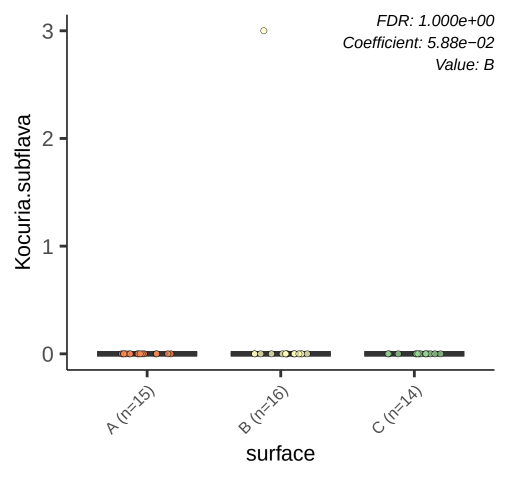
FDR: 1.000e+00
Coefficient: 5.88e−02
Value: B
Kocuria.subflava
0
1
2
3
A (n=15)
B (n=16)
C (n=14)
surface

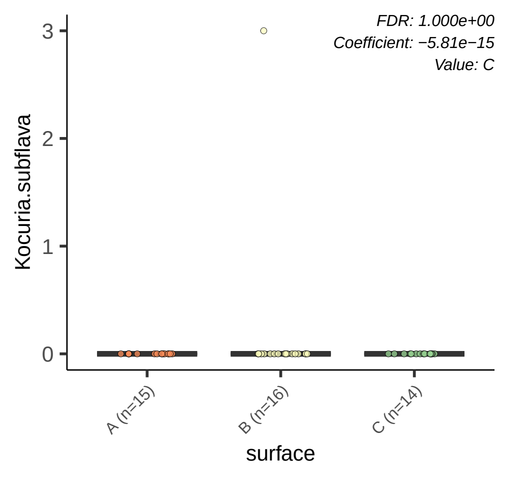
FDR: 1.000e+00
Coefficient: −5.81e−15
Value: C
Kocuria.subflava
3
2
1
0
A (n=15)
B (n=16)
C (n=14)
surface

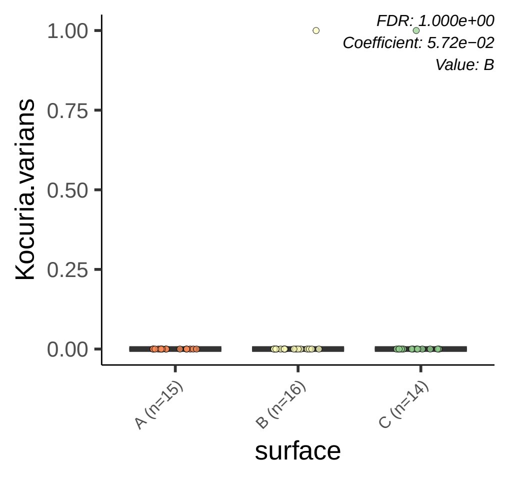
FDR: 1.000e+00
Coefficient: 5.72e−02
Value: B
Kocuria.varians
1.00
0.75
0.50
0.25
0.00
A (n=15)
B (n=16)
C (n=14)
surface

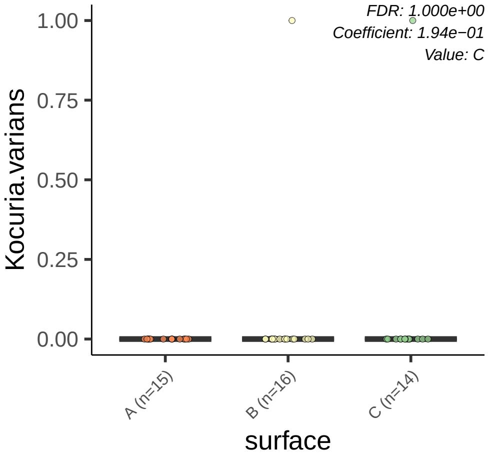

FDR: 1.000e+00
Coefficient: 1.94e−01
Value: C
Kocuria.varians
1.00
0.75
0.50
0.25
0.00
A (n=15)
B (n=16)
C (n=14)
surface

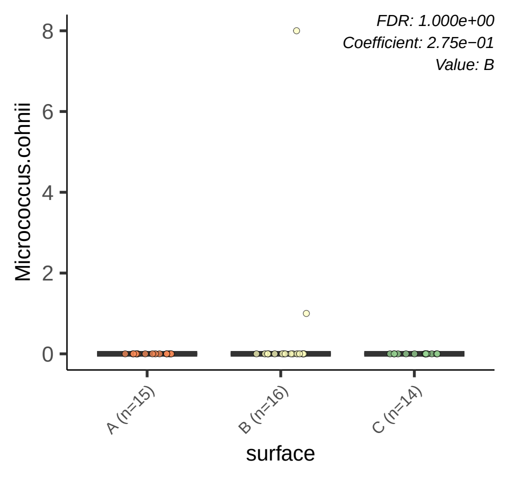
FDR: 1.000e+00
Coefficient: 2.75e−01
Value: B
Micrococcus.cohnii
0
2
4
6
8
A (n=15)
B (n=16)
C (n=14)
surface

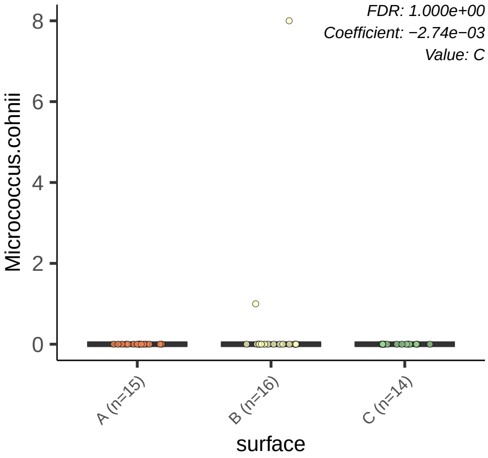
FDR: 1.000e+00
Coefficient: −2.74e−03
Value: C
Micrococcus.cohnii
8
6
4
2
0
A (n=15)
B (n=16)
C (n=14)
surface

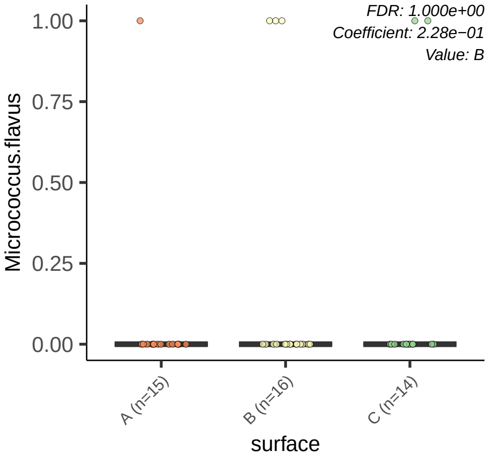

FDR: 1.000e+00
Coefficient: 2.28e−01
Value: B
Micrococcus.flavus
1.00
0.75
0.50
0.25
0.00
A (n=15)
B (n=16)
C (n=14)
surface

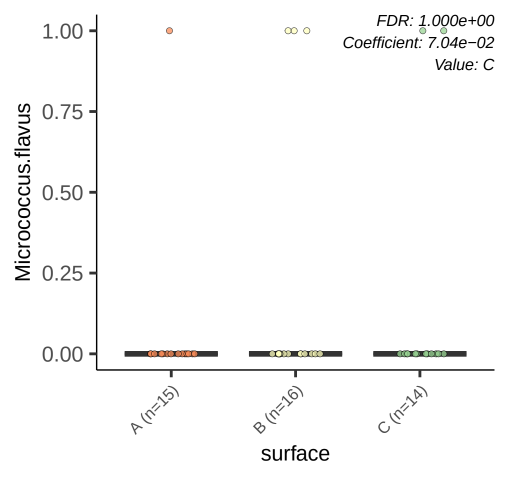

FDR: 1.000e+00
Coefficient: 7.04e−02
Value: C
Micrococcus.flavus
1.00
0.75
0.50
0.25
0.00
A (n=15)
B (n=16)
C (n=14)
surface

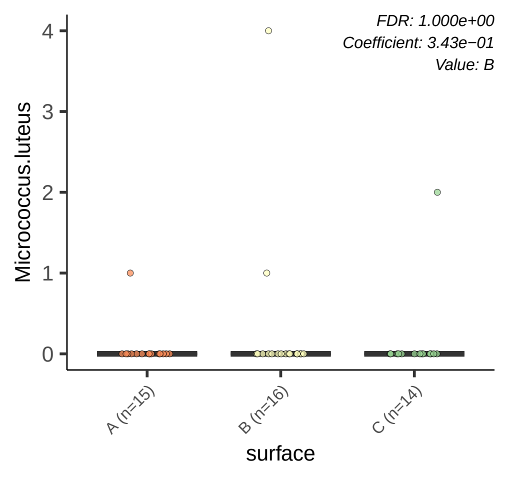
FDR: 1.000e+00
Coefficient: 3.43e−01
Value: B
Micrococcus.luteus
0
1
2
3
4
A (n=15)
B (n=16)
C (n=14)
surface

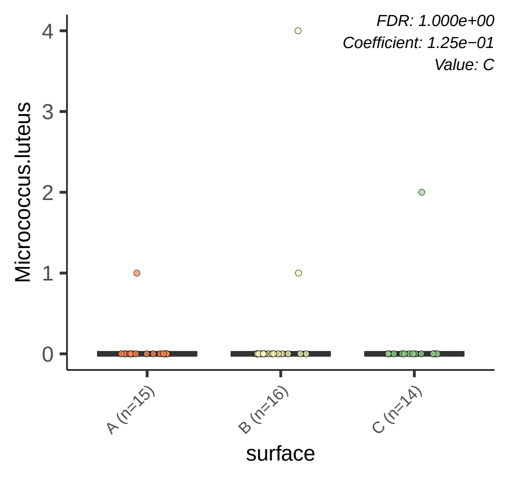
FDR: 1.000e+00
Coefficient: 1.25e−01
Value: C
Micrococcus.luteus
0
1
2
3
4
A (n=15)
B (n=16)
C (n=14)
surface

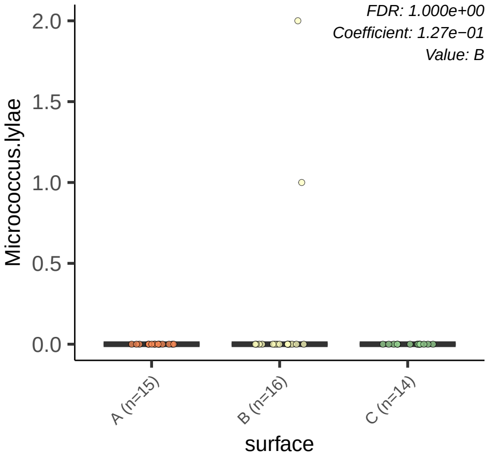

FDR: 1.000e+00
Coefficient: 1.27e−01
Value: B
Micrococcus.lylae
2.0
1.5
1.0
0.5
0.0
A (n=15)
B (n=16)
C (n=14)
surface

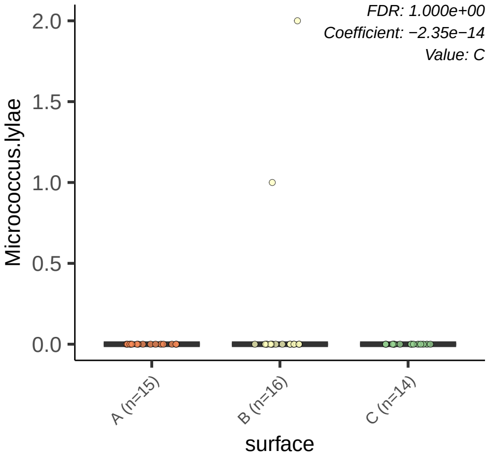
FDR: 1.000e+00
Coefficient: −2.35e−14
Value: C
Micrococcus.lylae
2.0
1.5
1.0
0.5
0.0
A (n=15)
B (n=16)
C (n=14)
surface

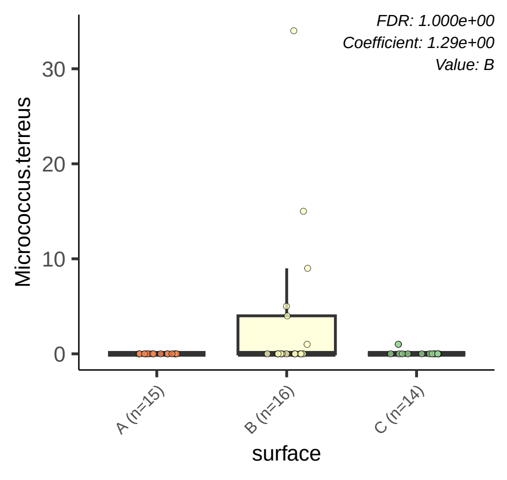
FDR: 1.000e+00
Coefficient: 1.29e+00
Value: B
Micrococcus.terreus
0
10
20
30
A (n=15)
B (n=16)
C (n=14)
surface

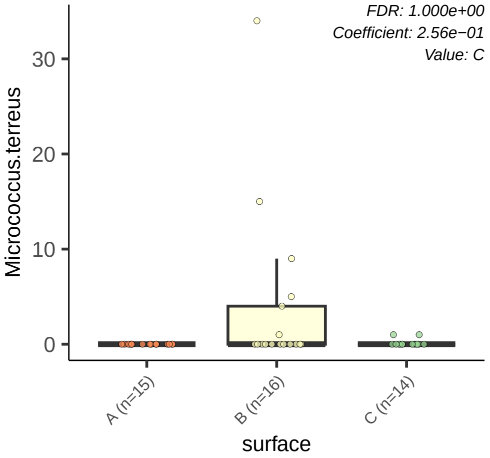
FDR: 1.000e+00
Coefficient: 2.56e−01
Value: C
Micrococcus.terreus
30
20
10
0
A (n=15)
B (n=16)
C (n=14)
surface

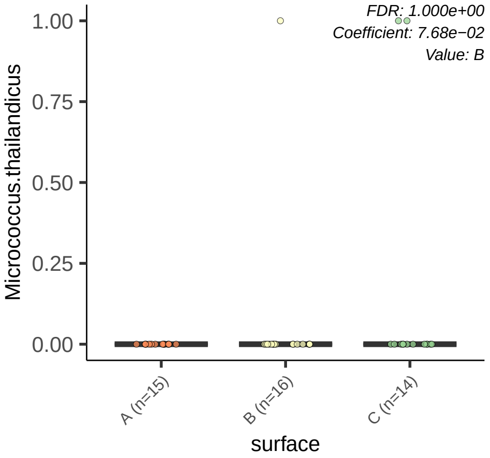
FDR: 1.000e+00
Coefficient: 7.68e−02
Value: B
Micrococcus.thailandicus
1.00
0.75
0.50
0.25
0.00
A (n=15)
B (n=16)
C (n=14)
surface

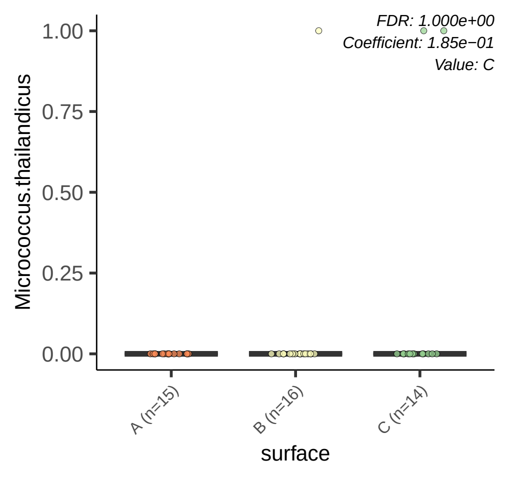
FDR: 1.000e+00
Coefficient: 1.85e−01
Value: C
Micrococcus.thailandicus
1.00
0.75
0.50
0.25
0.00
A (n=15)
B (n=16)
C (n=14)
surface

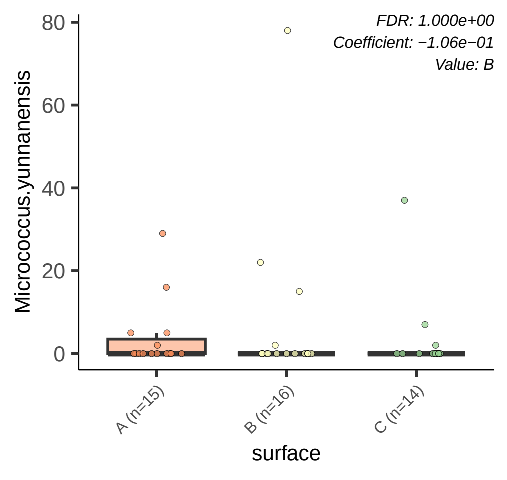
FDR: 1.000e+00
Coefficient: −1.06e−01
Value: B
Micrococcus.yunnanensis
80
60
40
20
0
A (n=15)
B (n=16)
C (n=14)
surface

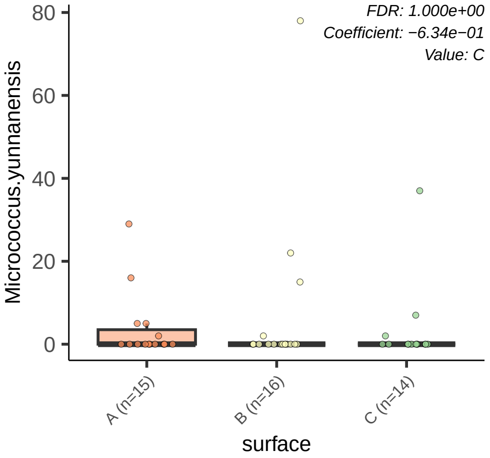

FDR: 1.000e+00
Coefficient: −6.34e−01
Value: C
Micrococcus.yunnanensis
80
60
40
20
0
A (n=15)
B (n=16)
C (n=14)
surface

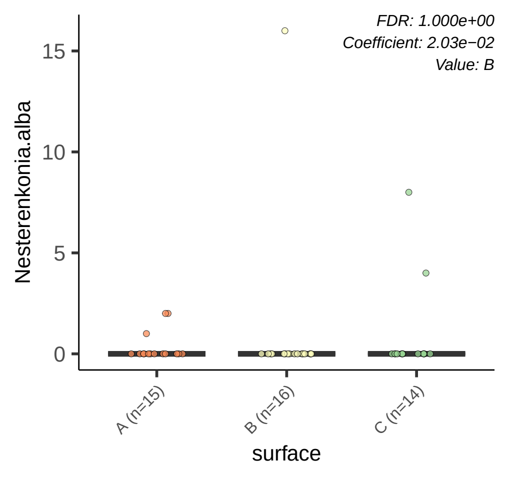
FDR: 1.000e+00
Coefficient: 2.03e−02
Value: B
Nesterenkonia.alba
15
10
5
0
A (n=15)
B (n=16)
C (n=14)
surface

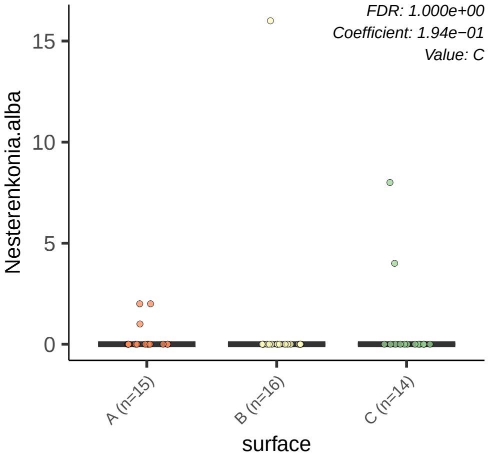

FDR: 1.000e+00
Coefficient: 1.94e−01
Value: C
Nesterenkonia.alba
15
10
5
0
A (n=15)
B (n=16)
C (n=14)
surface

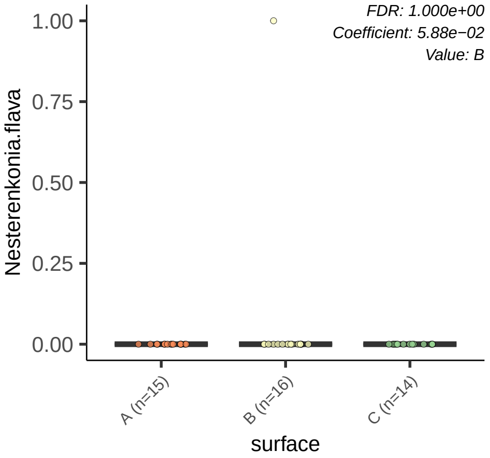

FDR: 1.000e+00
Coefficient: 5.88e−02
Value: B
Nesterenkonia.flava
1.00
0.75
0.50
0.25
0.00
A (n=15)
B (n=16)
C (n=14)
surface

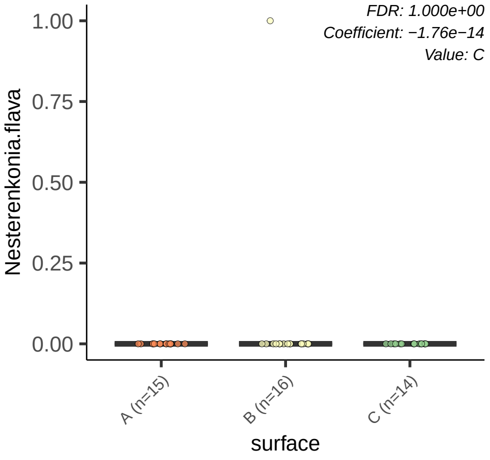
FDR: 1.000e+00
Coefficient: −1.76e−14
Value: C
Nesterenkonia.flava
1.00
0.75
0.50
0.25
0.00
A (n=15)
B (n=16)
C (n=14)
surface

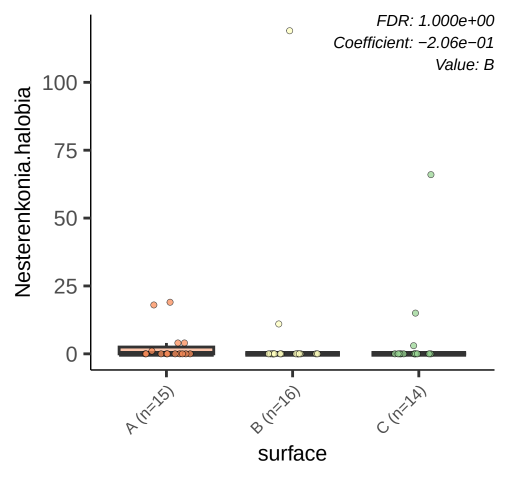
FDR: 1.000e+00
Coefficient: −2.06e−01
Value: B
Nesterenkonia.halobia
100
75
50
25
0
A (n=15)
B (n=16)
C (n=14)
surface

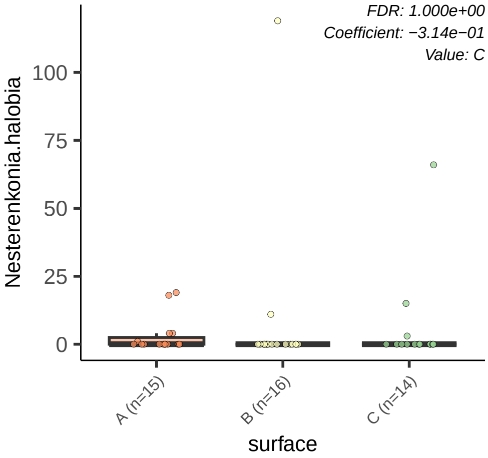

FDR: 1.000e+00
Coefficient: −3.14e−01
Value: C
Nesterenkonia.halobia
100
75
50
25
0
A (n=15)
B (n=16)
C (n=14)
surface

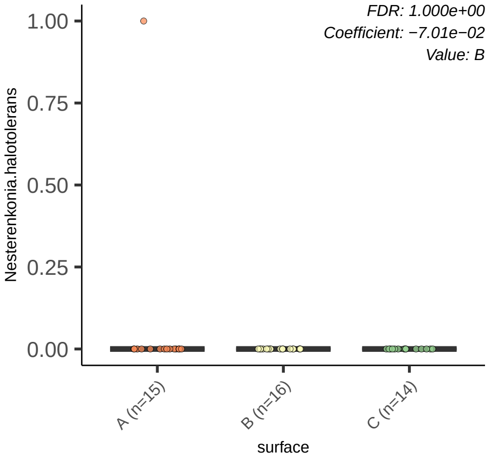

FDR: 1.000e+00
Coefficient: −7.01e−02
Value: B
Nesterenkonia.halotolerans
1.00
0.75
0.50
0.25
0.00
A (n=15)
B (n=16)
C (n=14)
surface

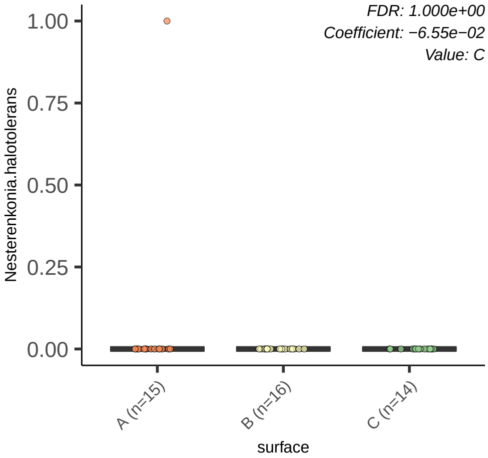
FDR: 1.000e+00
Coefficient: −6.55e−02
Value: C
Nesterenkonia.halotolerans
1.00
0.75
0.50
0.25
0.00
A (n=15)
B (n=16)
C (n=14)
surface

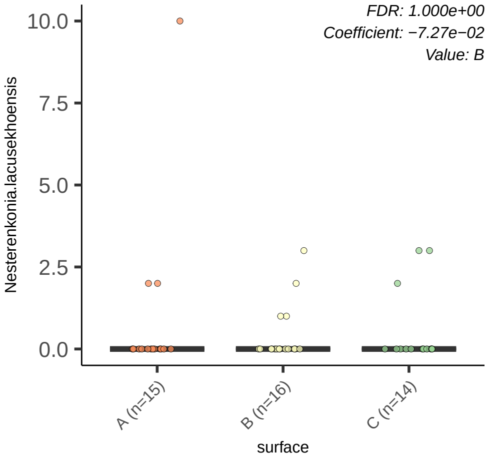

FDR: 1.000e+00
Coefficient: −7.27e−02
Value: B
Nesterenkonia.lacusekhoensis
10.0
7.5
5.0
2.5
0.0
A (n=15)
B (n=16)
C (n=14)
surface

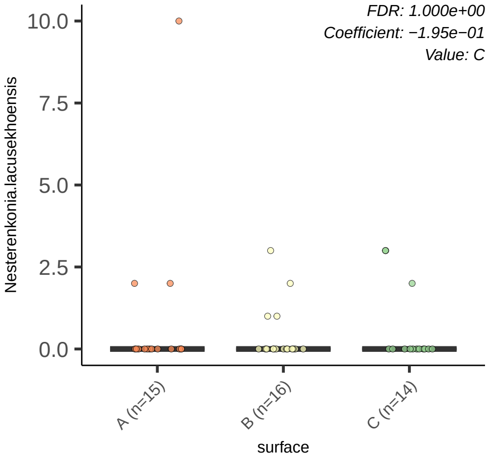
FDR: 1.000e+00
Coefficient: −1.95e−01
Value: C
Nesterenkonia.lacusekhoensis
10.0
7.5
5.0
2.5
0.0
A (n=15)
B (n=16)
C (n=14)
surface

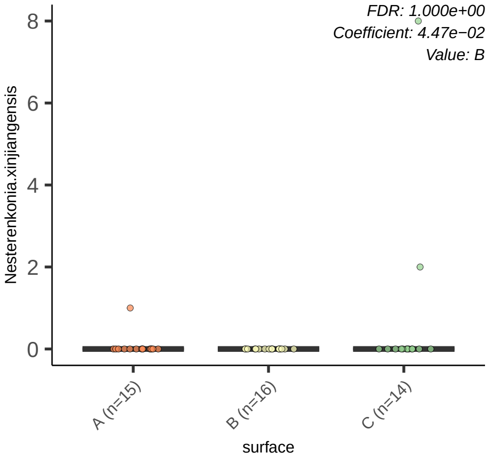
FDR: 1.000e+00
Coefficient: 4.47e−02
Value: B
Nesterenkonia.xinjiangensis
8
6
4
2
0
A (n=15)
B (n=16)
C (n=14)
surface

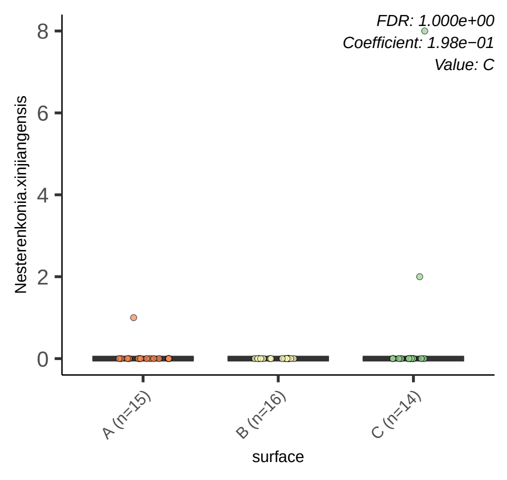
FDR: 1.000e+00
Coefficient: 1.98e−01
Value: C
Nesterenkonia.xinjiangensis
0
2
4
6
8
A (n=15)
B (n=16)
C (n=14)
surface

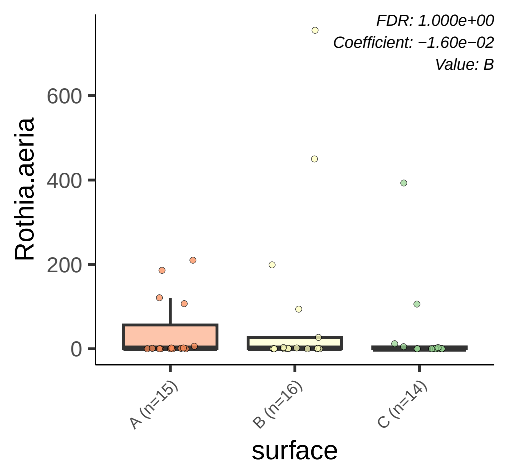
FDR: 1.000e+00
Coefficient: −1.60e−02
Value: B
Rothia.aeria
600
400
200
0
A (n=15)
B (n=16)
C (n=14)
surface

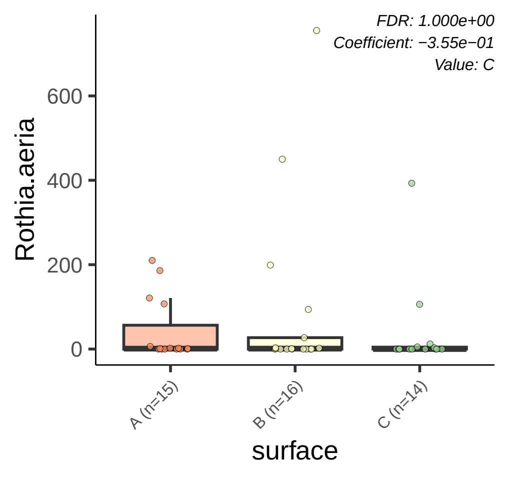

FDR: 1.000e+00
Coefficient: −3.55e−01
Value: C
Rothia.aeria
600
400
200
0
A (n=15)
B (n=16)
C (n=14)
surface

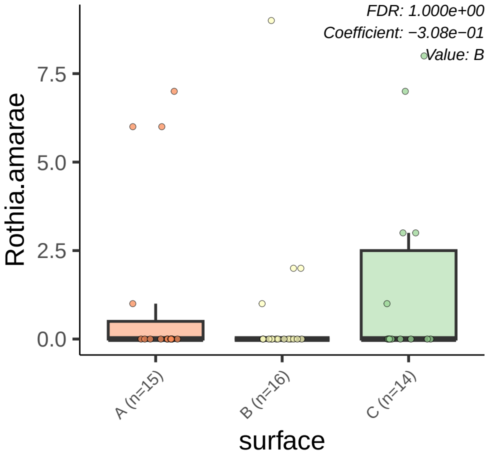
FDR: 1.000e+00
Coefficient: −3.08e−01
Value: B
Rothia.amarae
7.5
5.0
2.5
0.0
A (n=15)
B (n=16)
C (n=14)
surface

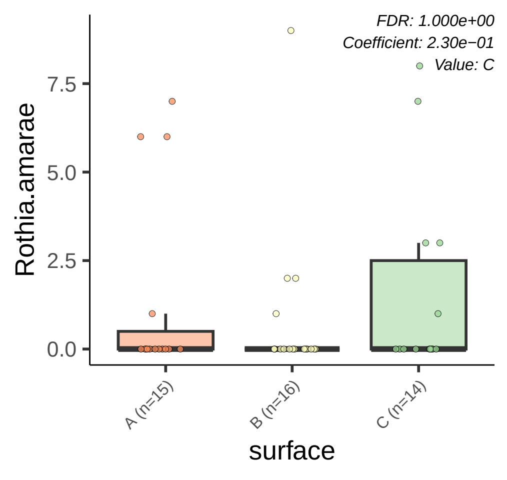
FDR: 1.000e+00
Coefficient: 2.30e−01
Value: C
Rothia.amarae
0.0
2.5
5.0
7.5
A (n=15)
B (n=16)
C (n=14)
surface

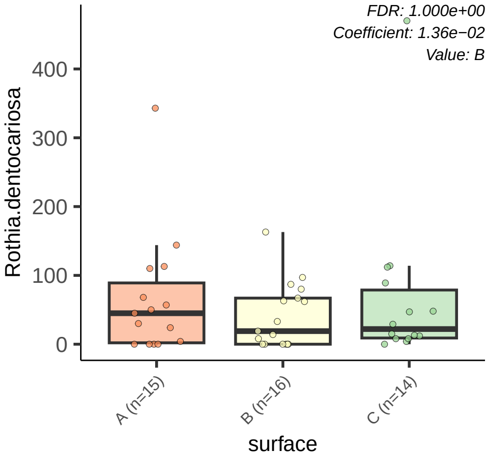

FDR: 1.000e+00
Coefficient: 1.36e−02
Value: B
Rothia.dentocariosa
400
300
200
100
0
A (n=15)
B (n=16)
C (n=14)
surface

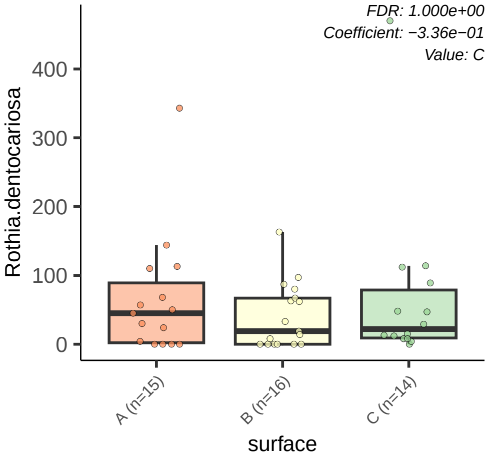
FDR: 1.000e+00
Coefficient: −3.36e−01
Value: C
Rothia.dentocariosa
400
300
200
100
0
A (n=15)
B (n=16)
C (n=14)
surface

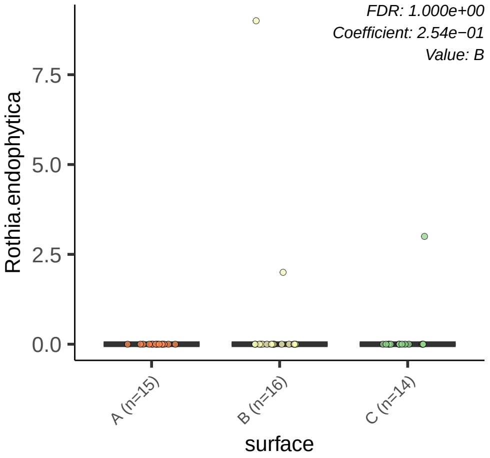
FDR: 1.000e+00
Coefficient: 2.54e−01
Value: B
Rothia.endophytica
7.5
5.0
2.5
0.0
A (n=15)
B (n=16)
C (n=14)
surface

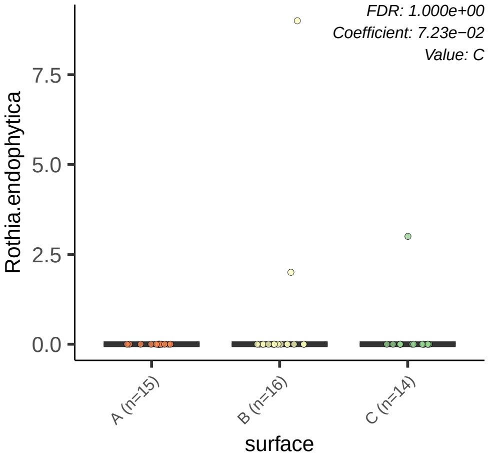
FDR: 1.000e+00
Coefficient: 7.23e−02
Value: C
Rothia.endophytica
7.5
5.0
2.5
0.0
A (n=15)
B (n=16)
C (n=14)
surface

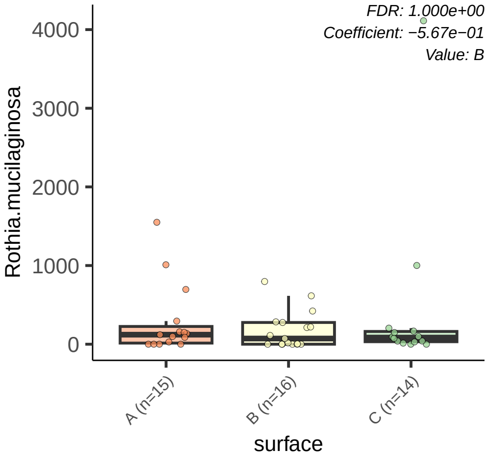

FDR: 1.000e+00
Coefficient: −5.67e−01
Value: B
Rothia.mucilaginosa
4000
3000
2000
1000
0
A (n=15)
B (n=16)
C (n=14)
surface

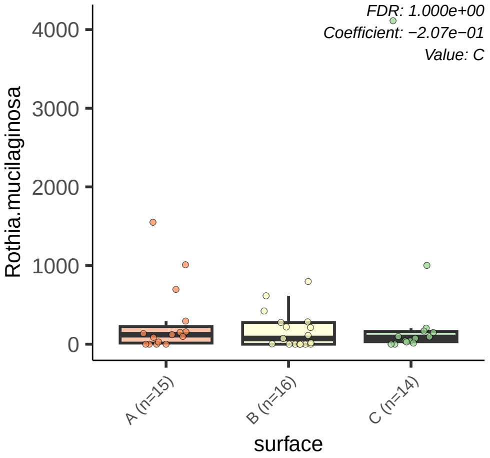
FDR: 1.000e+00
Coefficient: −2.07e−01
Value: C
Rothia.mucilaginosa
4000
3000
2000
1000
0
A (n=15)
B (n=16)
C (n=14)
surface

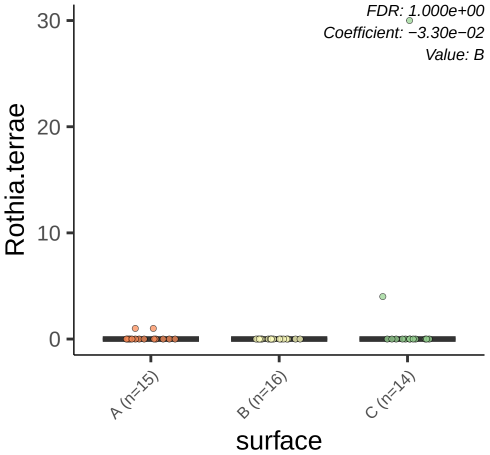
FDR: 1.000e+00
Coefficient: −3.30e−02
Value: B
Rothia.terrae
30
20
10
0
A (n=15)
B (n=16)
C (n=14)
surface

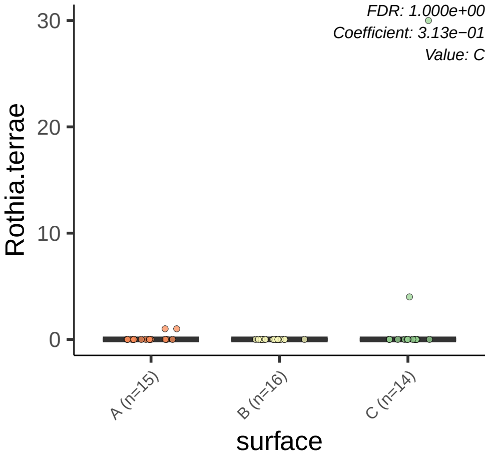

FDR: 1.000e+00
Coefficient: 3.13e−01
Value: C
Rothia.terrae
30
20
10
0
A (n=15)
B (n=16)
C (n=14)
surface

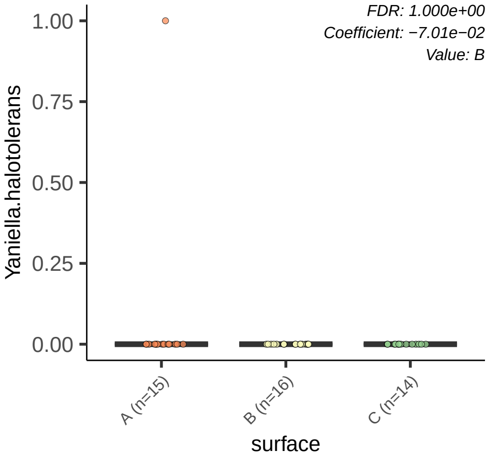
FDR: 1.000e+00
Coefficient: −7.01e−02
Value: B
Yaniella.halotolerans
1.00
0.75
0.50
0.25
0.00
A (n=15)
B (n=16)
C (n=14)
surface

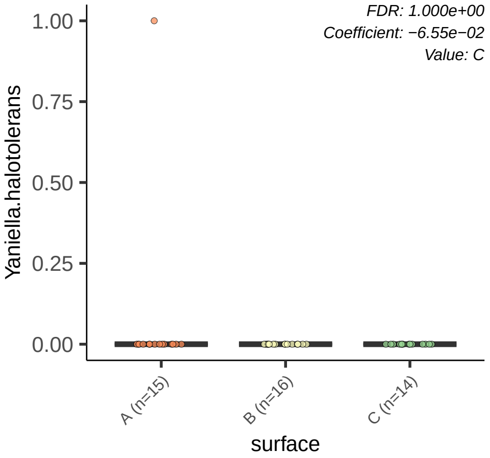

FDR: 1.000e+00
Coefficient: −6.55e−02
Value: C
Yaniella.halotolerans
1.00
0.75
0.50
0.25
0.00
A (n=15)
B (n=16)
C (n=14)
surface

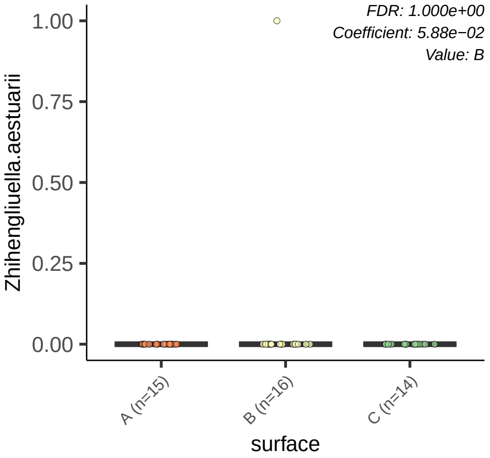

FDR: 1.000e+00
Coefficient: 5.88e−02
Value: B
Zhihengliuella.aestuarii
1.00
0.75
0.50
0.25
0.00
A (n=15)
B (n=16)
C (n=14)
surface

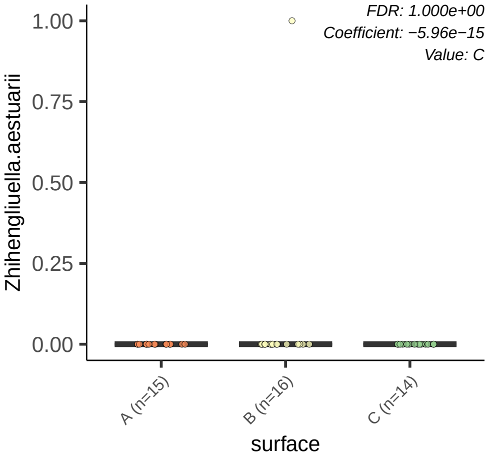
FDR: 1.000e+00
Coefficient: −5.96e−15
Value: C
Zhihengliuella.aestuarii
1.00
0.75
0.50
0.25
0.00
A (n=15)
B (n=16)
C (n=14)
surface

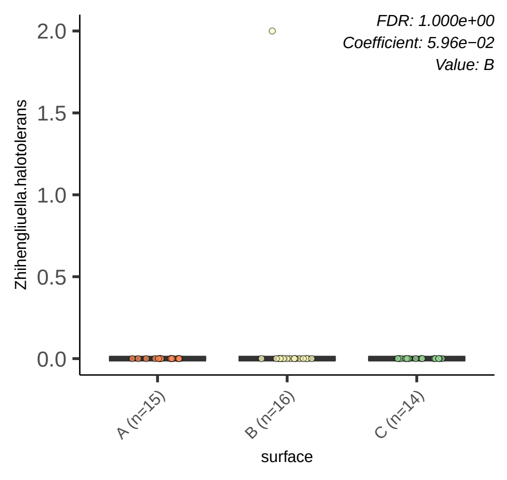
FDR: 1.000e+00
Coefficient: 5.96e−02
Value: B
Zhihengliuella.halotolerans
2.0
1.5
1.0
0.5
0.0
A (n=15)
B (n=16)
C (n=14)
surface

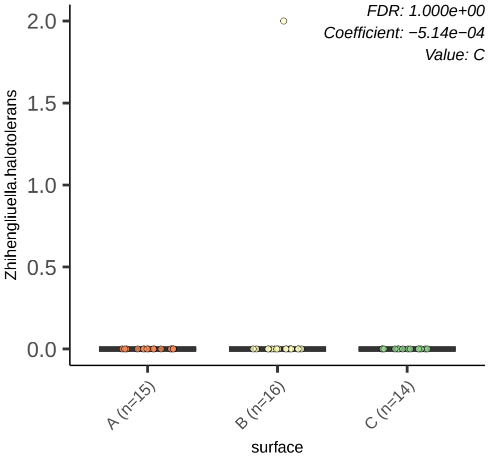

FDR: 1.000e+00
Coefficient: −5.14e−04
Value: C
2.0
1.5
1.0
0.5
0.0
Zhihengliuella.halotolerans
A (n=15)
B (n=16)
C (n=14)
surface

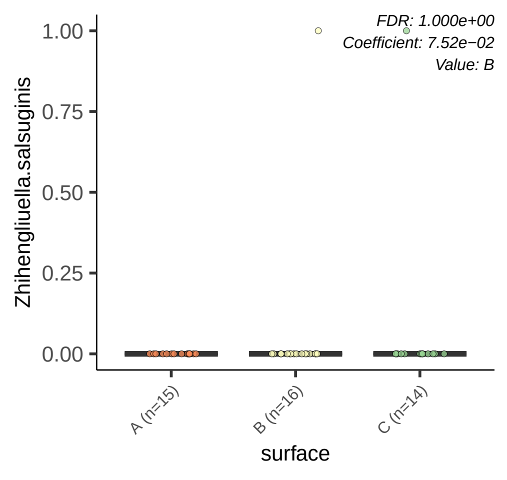
FDR: 1.000e+00
Coefficient: 7.52e−02
Value: B
Zhihengliuella.salsuginis
1.00
0.75
0.50
0.25
0.00
A (n=15)
B (n=16)
C (n=14)
surface

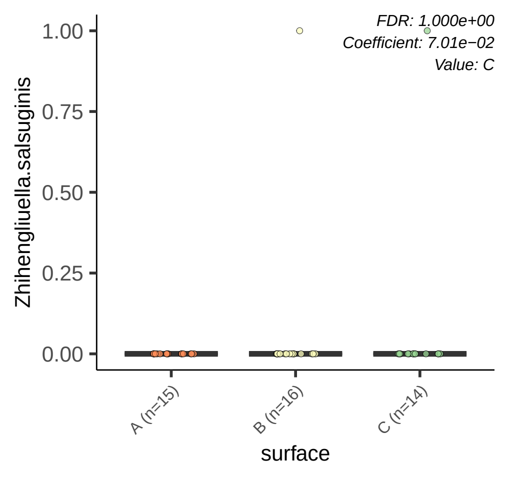

FDR: 1.000e+00
Coefficient: 7.01e−02
Value: C
Zhihengliuella.salsuginis
1.00
0.75
0.50
0.25
0.00
A (n=15)
B (n=16)
C (n=14)
surface

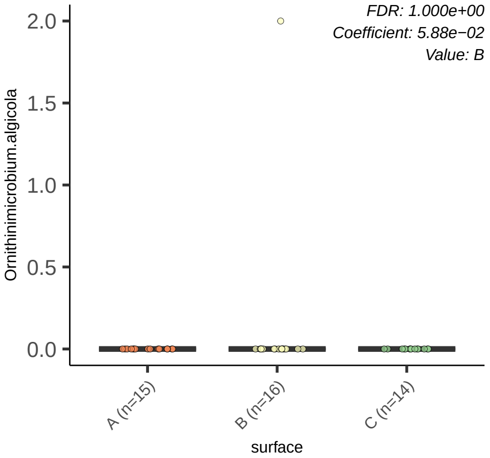

FDR: 1.000e+00
Coefficient: 5.88e−02
Value: B
Ornithinimicrobium.algicola
2.0
1.5
1.0
0.5
0.0
A (n=15)
B (n=16)
C (n=14)
surface

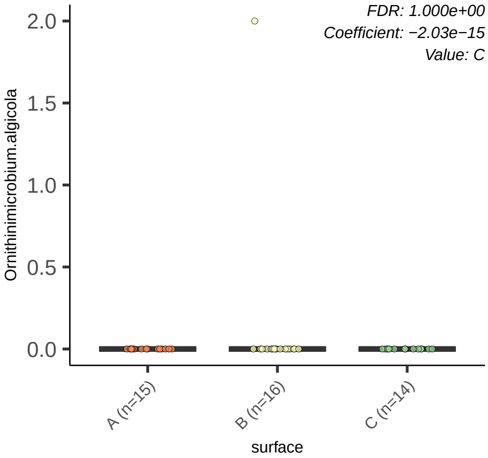
FDR: 1.000e+00
Coefficient: −2.03e−15
Value: C
Ornithinimicrobium.algicola
2.0
1.5
1.0
0.5
0.0
A (n=15)
B (n=16)
C (n=14)
surface

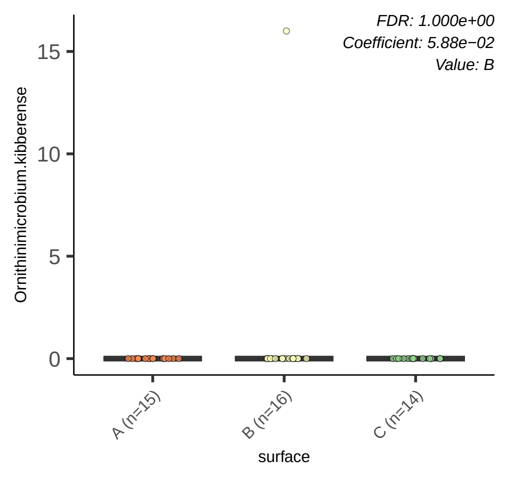

FDR: 1.000e+00
Coefficient: 5.88e−02
Value: B
Ornithinimicrobium.kibberense
15
10
5
0
A (n=15)
B (n=16)
C (n=14)
surface

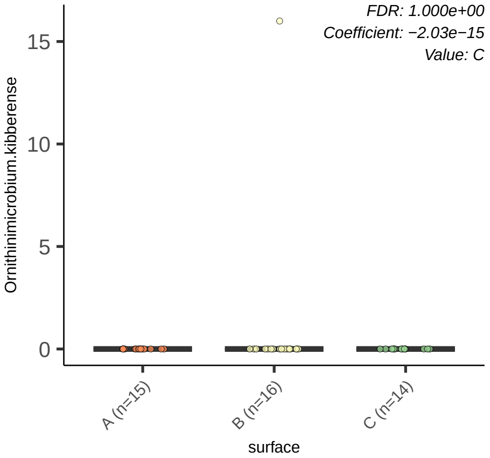
FDR: 1.000e+00
Coefficient: −2.03e−15
Value: C
Ornithinimicrobium.kibberense
15
10
5
0
A (n=15)
B (n=16)
C (n=14)
surface

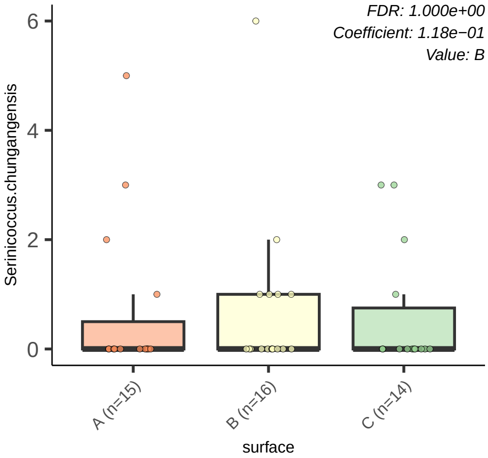
FDR: 1.000e+00
Coefficient: 1.18e−01
Value: B
Serinicoccus.chungangensis
0
2
4
6
A (n=15)
B (n=16)
C (n=14)
surface

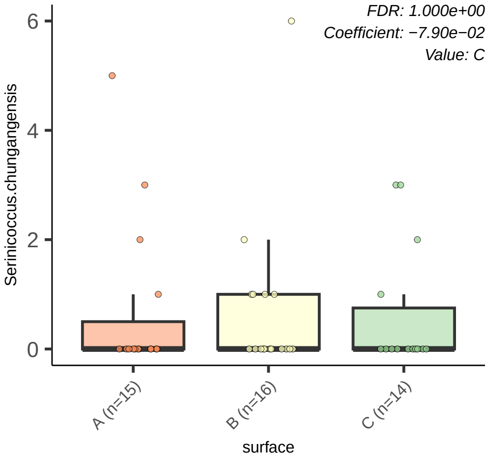
FDR: 1.000e+00
Coefficient: −7.90e−02
Value: C
Serinicoccus.chungangensis
6
4
2
0
A (n=15)
B (n=16)
C (n=14)
surface

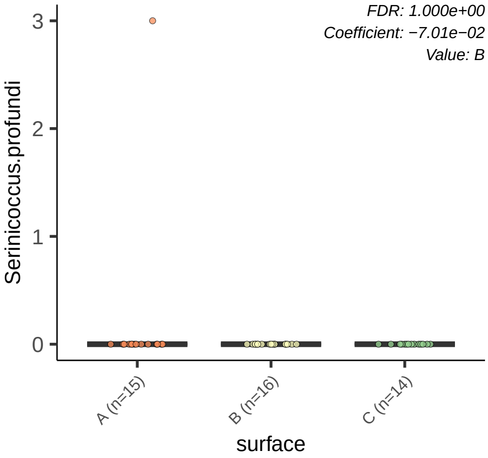
FDR: 1.000e+00
Coefficient: −7.01e−02
Value: B
Serinicoccus.profundi
3
2
1
0
A (n=15)
B (n=16)
C (n=14)
surface

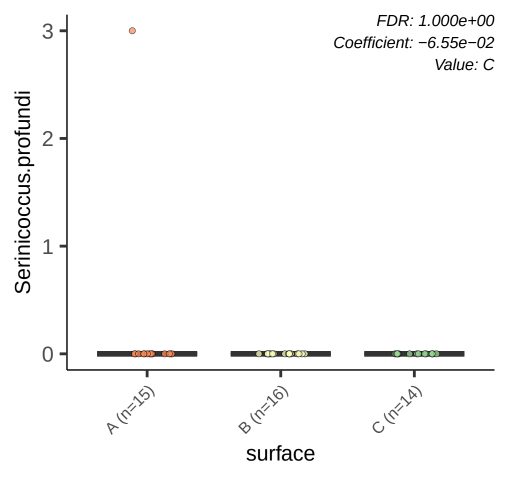
FDR: 1.000e+00
Coefficient: −6.55e−02
Value: C
Serinicoccus.profundi
3
2
1
0
A (n=15)
B (n=16)
C (n=14)
surface

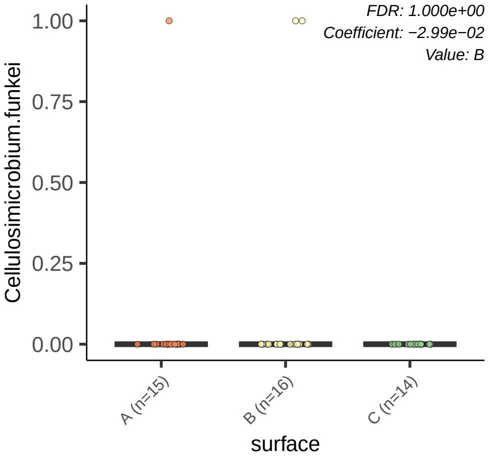
FDR: 1.000e+00
Coefficient: −2.99e−02
Value: B
Cellulosimicrobium.funkei
1.00
0.75
0.50
0.25
0.00
A (n=15)
B (n=16)
C (n=14)
surface

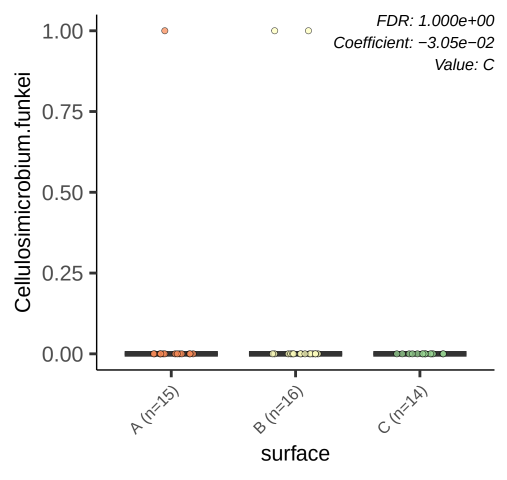
FDR: 1.000e+00
Coefficient: −3.05e−02
Value: C
Cellulosimicrobium.funkei
1.00
0.75
0.50
0.25
0.00
A (n=15)
B (n=16)
C (n=14)
surface

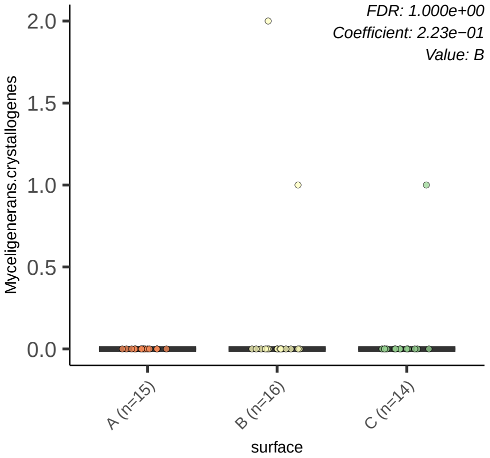
FDR: 1.000e+00
Coefficient: 2.23e−01
Value: B
Myceligenerans.crystallogenes
2.0
1.5
1.0
0.5
0.0
A (n=15)
B (n=16)
C (n=14)
surface

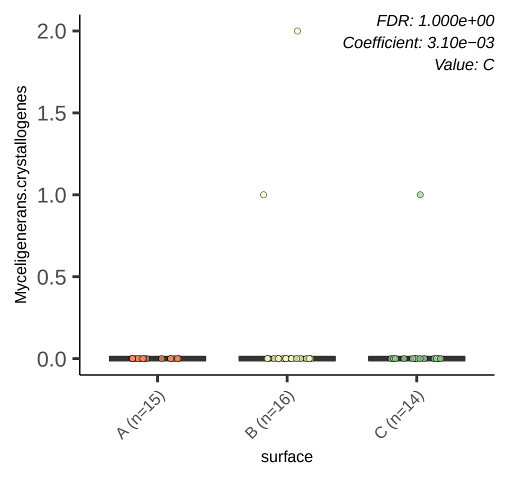

FDR: 1.000e+00
Coefficient: 3.10e−03
Value: C
Myceligenerans.crystallogenes
2.0
1.5
1.0
0.5
0.0
A (n=15)
B (n=16)
C (n=14)
surface

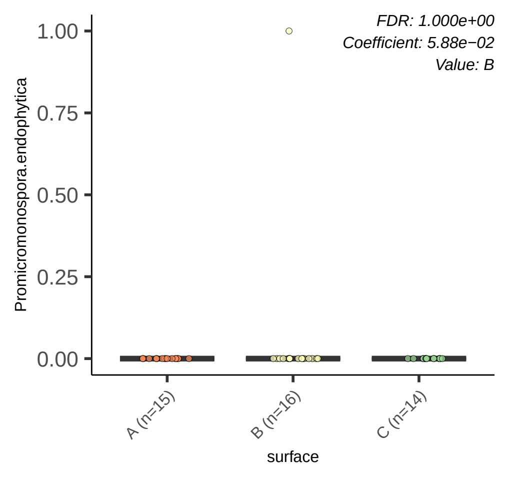
FDR: 1.000e+00
Coefficient: 5.88e−02
Value: B
Promicromonospora.endophytica
1.00
0.75
0.50
0.25
0.00
A (n=15)
B (n=16)
C (n=14)
surface

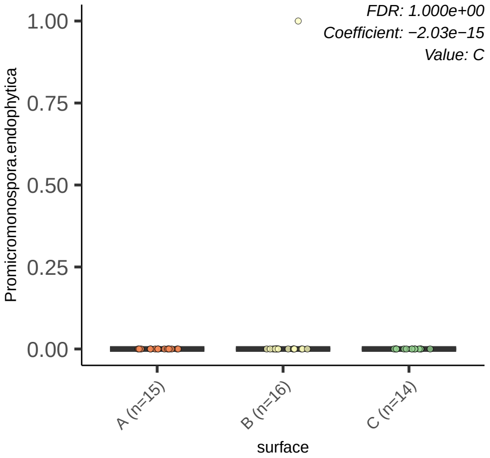
FDR: 1.000e+00
Coefficient: −2.03e−15
Value: C
Promicromonospora.endophytica
1.00
0.75
0.50
0.25
0.00
A (n=15)
B (n=16)
C (n=14)
surface

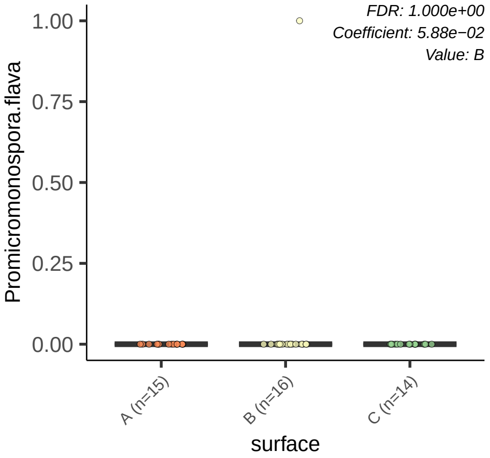
FDR: 1.000e+00
Coefficient: 5.88e−02
Value: B
Promicromonospora.flava
1.00
0.75
0.50
0.25
0.00
A (n=15)
B (n=16)
C (n=14)
surface

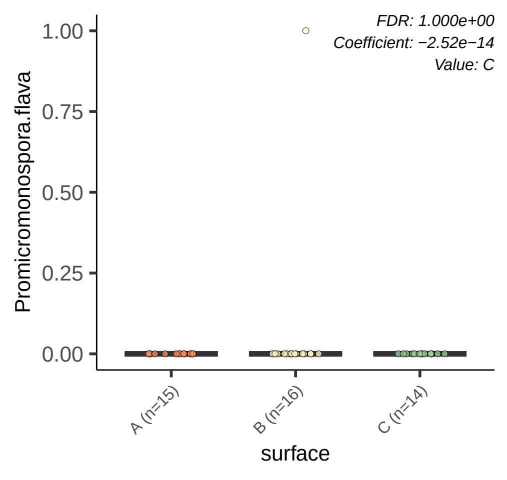
FDR: 1.000e+00
Coefficient: −2.52e−14
Value: C
Promicromonospora.flava
1.00
0.75
0.50
0.25
0.00
A (n=15)
B (n=16)
C (n=14)
surface

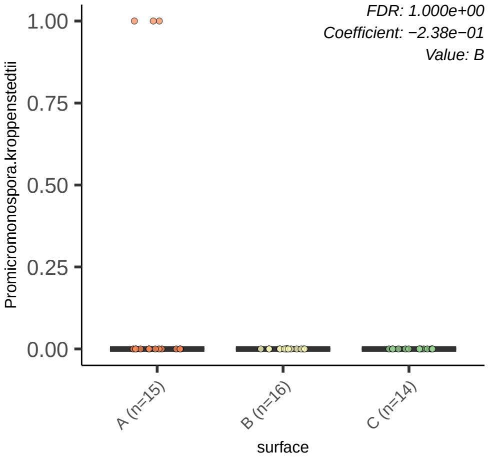
FDR: 1.000e+00
Coefficient: −2.38e−01
Value: B
Promicromonospora.kroppenstedtii
1.00
0.75
0.50
0.25
0.00
A (n=15)
B (n=16)
C (n=14)
surface

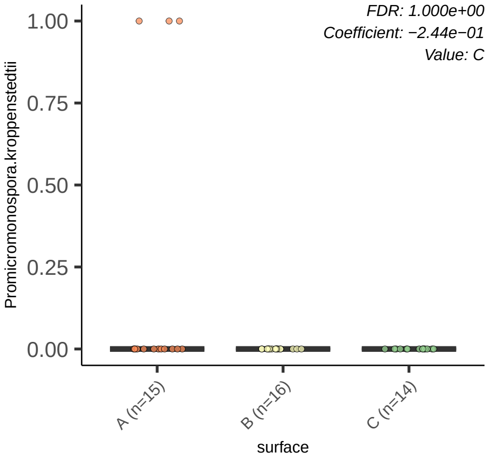
FDR: 1.000e+00
Coefficient: −2.44e−01
Value: C
Promicromonospora.kroppenstedtii
1.00
0.75
0.50
0.25
0.00
A (n=15)
B (n=16)
C (n=14)
surface

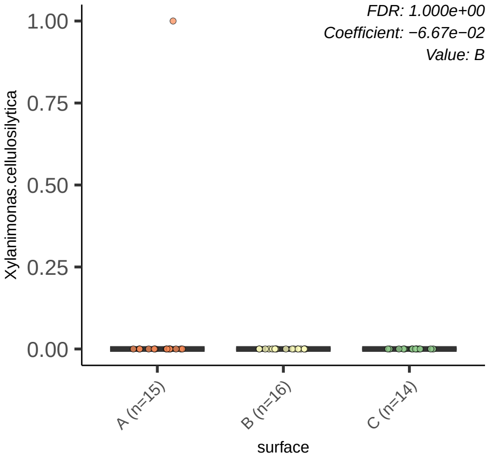

FDR: 1.000e+00
Coefficient: −6.67e−02
Value: B
1.00
0.75
0.50
0.25
0.00
Xylanimonas.cellulosilytica
A (n=15)
B (n=16)
C (n=14)
surface

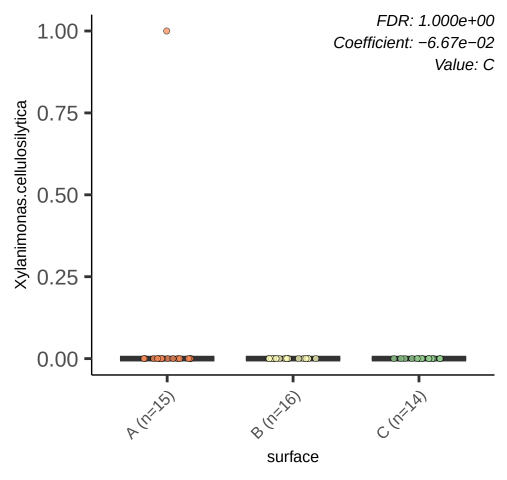
FDR: 1.000e+00
Coefficient: −6.67e−02
Value: C
Xylanimonas.cellulosilytica
1.00
0.75
0.50
0.25
0.00
A (n=15)
B (n=16)
C (n=14)
surface

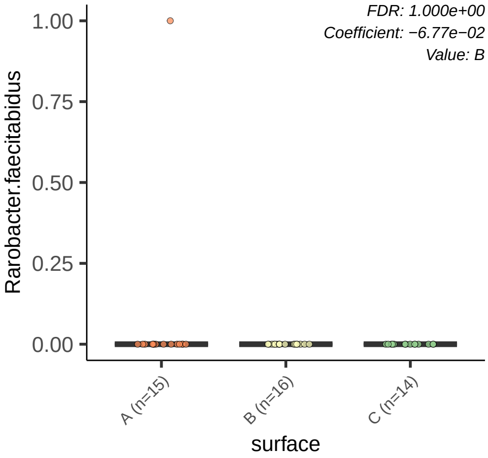
FDR: 1.000e+00
Coefficient: −6.77e−02
Value: B
Rarobacter.faecitabidus
1.00
0.75
0.50
0.25
0.00
A (n=15)
B (n=16)
C (n=14)
surface

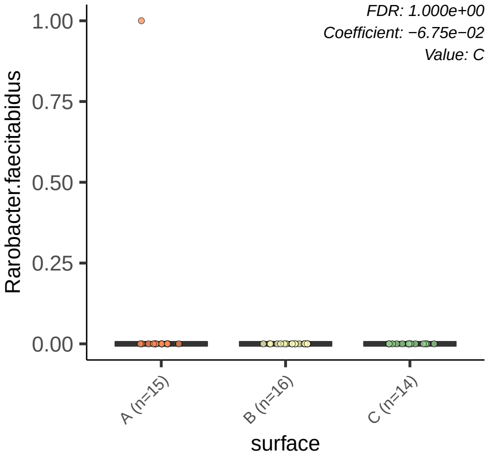

FDR: 1.000e+00
Coefficient: −6.75e−02
Value: C
Rarobacter.faecitabidus
1.00
0.75
0.50
0.25
0.00
A (n=15)
B (n=16)
C (n=14)
surface

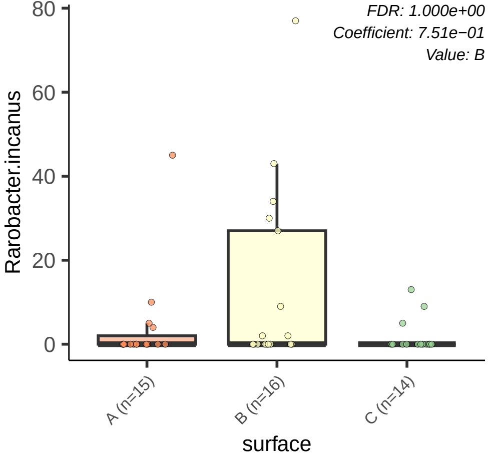

FDR: 1.000e+00
Coefficient: 7.51e−01
Value: B
Rarobacter.incanus
0
20
40
60
80
A (n=15)
B (n=16)
C (n=14)
surface

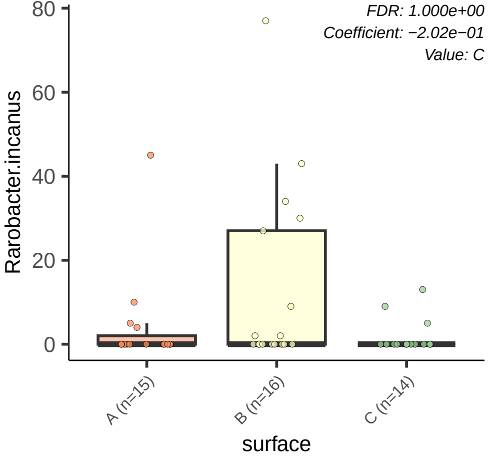

FDR: 1.000e+00
Coefficient: −2.02e−01
Value: C
Rarobacter.incanus
80
60
40
20
0
A (n=15)
B (n=16)
C (n=14)
surface

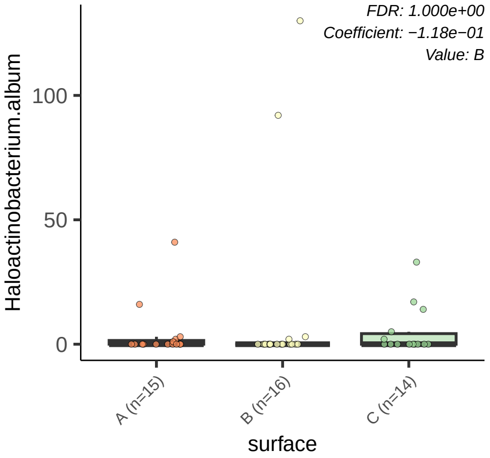

FDR: 1.000e+00
Coefficient: −1.18e−01
Value: B
Haloactinobacterium.album
100
50
0
A (n=15)
B (n=16)
C (n=14)
surface

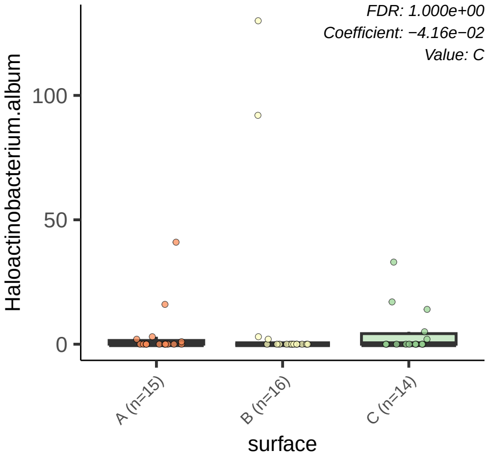
FDR: 1.000e+00
Coefficient: −4.16e−02
Value: C
Haloactinobacterium.album
100
50
0
A (n=15)
B (n=16)
C (n=14)
surface

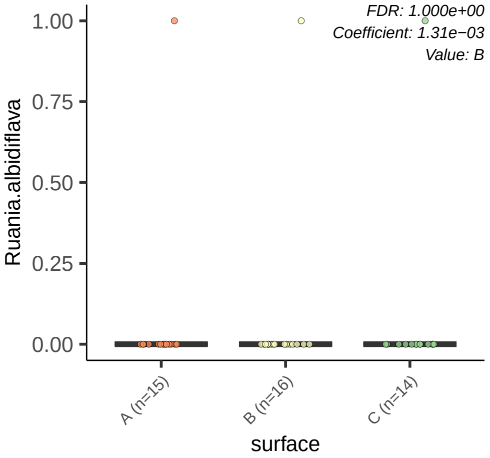
FDR: 1.000e+00
Coefficient: 1.31e−03
Value: B
Ruania.albidiflava
1.00
0.75
0.50
0.25
0.00
A (n=15)
B (n=16)
C (n=14)
surface

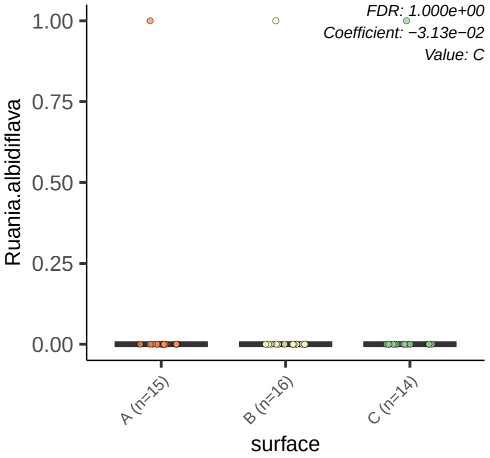
FDR: 1.000e+00
Coefficient: −3.13e−02
Value: C
Ruania.albidiflava
1.00
0.75
0.50
0.25
0.00
A (n=15)
B (n=16)
C (n=14)
surface

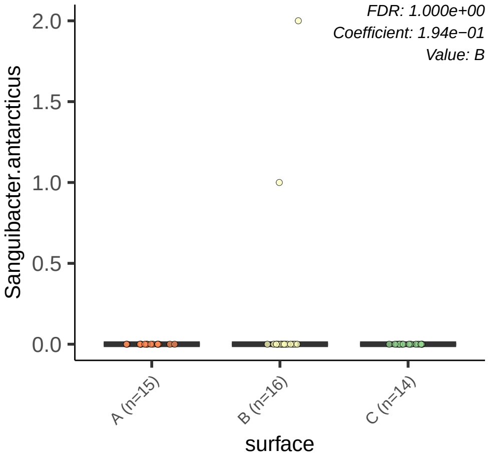
FDR: 1.000e+00
Coefficient: 1.94e−01
Value: B
Sanguibacter.antarcticus
2.0
1.5
1.0
0.5
0.0
A (n=15)
B (n=16)
C (n=14)
surface

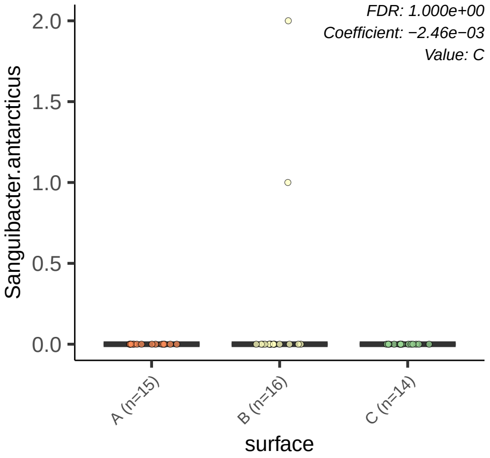
FDR: 1.000e+00
Coefficient: −2.46e−03
Value: C
Sanguibacter.antarcticus
2.0
1.5
1.0
0.5
0.0
A (n=15)
B (n=16)
C (n=14)
surface

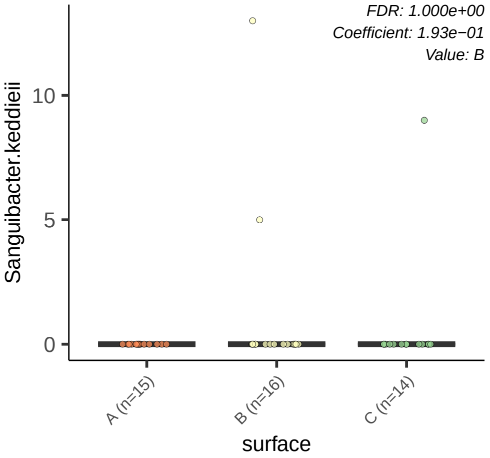
FDR: 1.000e+00
Coefficient: 1.93e−01
Value: B
Sanguibacter.keddieii
0
5
10
A (n=15)
B (n=16)
C (n=14)
surface

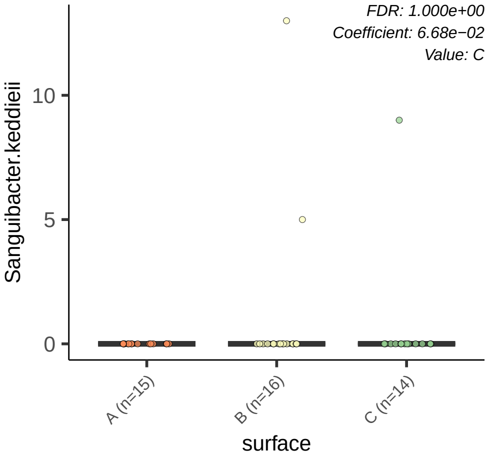
FDR: 1.000e+00
Coefficient: 6.68e−02
Value: C
Sanguibacter.keddieii
10
5
0
A (n=15)
B (n=16)
C (n=14)
surface

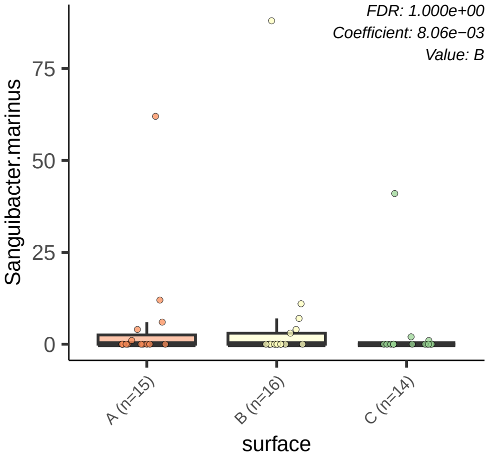

FDR: 1.000e+00
Coefficient: 8.06e−03
Value: B
Sanguibacter.marinus
0
25
50
75
A (n=15)
B (n=16)
C (n=14)
surface

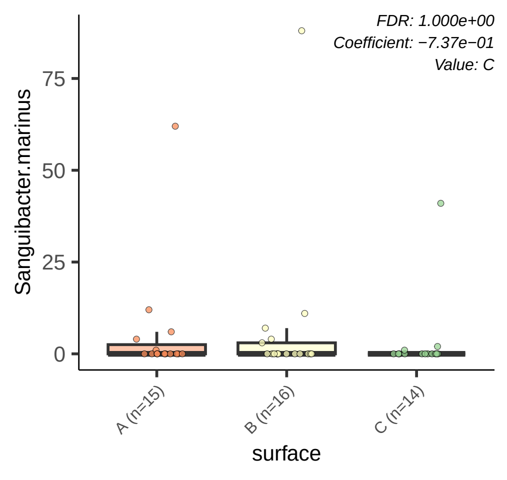
FDR: 1.000e+00
Coefficient: −7.37e−01
Value: C
Sanguibacter.marinus
0
25
50
75
A (n=15)
B (n=16)
C (n=14)
surface

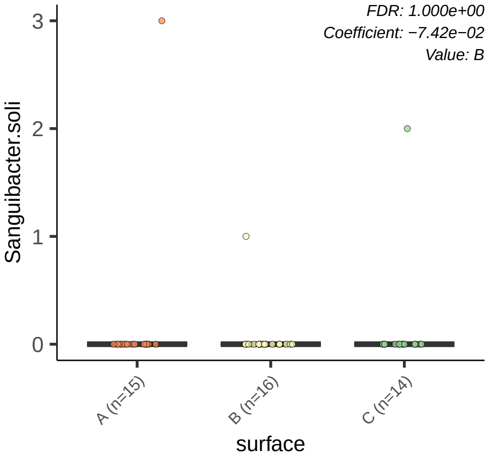
FDR: 1.000e+00
Coefficient: −7.42e−02
Value: B
Sanguibacter.soli
3
2
1
0
A (n=15)
B (n=16)
C (n=14)
surface

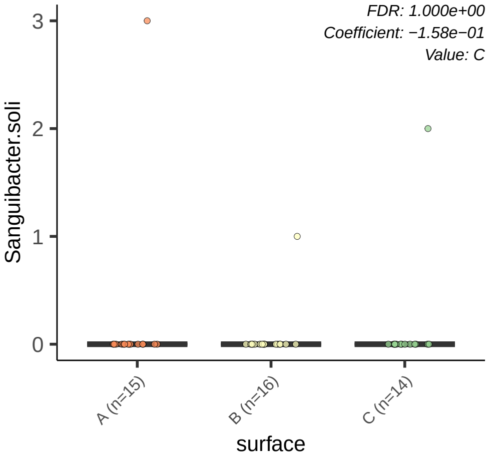

FDR: 1.000e+00
Coefficient: −1.58e−01
Value: C
Sanguibacter.soli
0
1
2
3
A (n=15)
B (n=16)
C (n=14)
surface

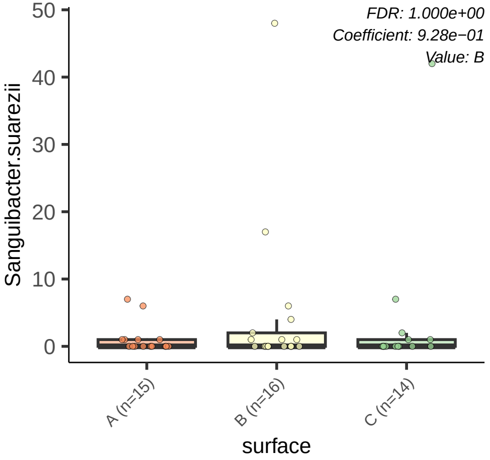
FDR: 1.000e+00
Coefficient: 9.28e−01
Value: B
Sanguibacter.suarezii
50
40
30
20
10
0
A (n=15)
B (n=16)
C (n=14)
surface

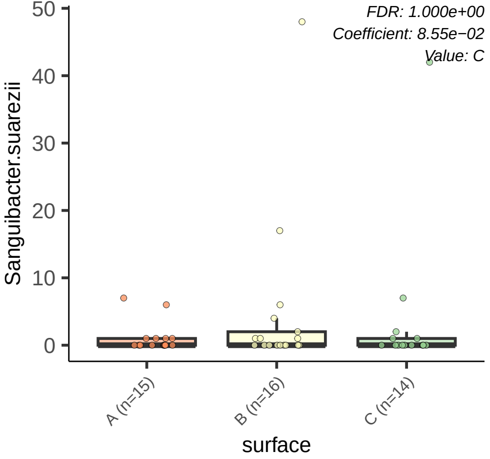

FDR: 1.000e+00
Coefficient: 8.55e−02
Value: C
Sanguibacter.suarezii
50
40
30
20
10
0
A (n=15)
B (n=16)
C (n=14)
surface

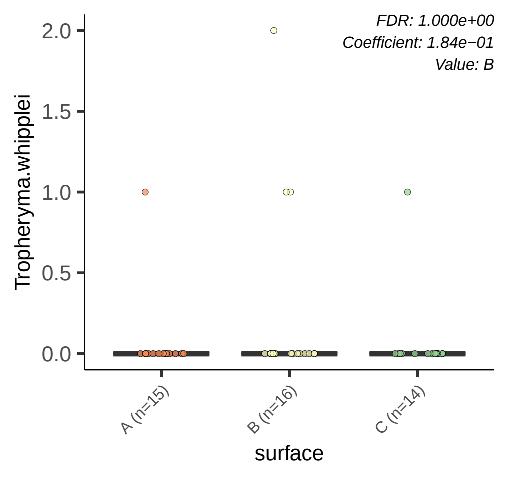
FDR: 1.000e+00
Coefficient: 1.84e−01
Value: B
Tropheryma.whipplei
2.0
1.5
1.0
0.5
0.0
A (n=15)
B (n=16)
C (n=14)
surface

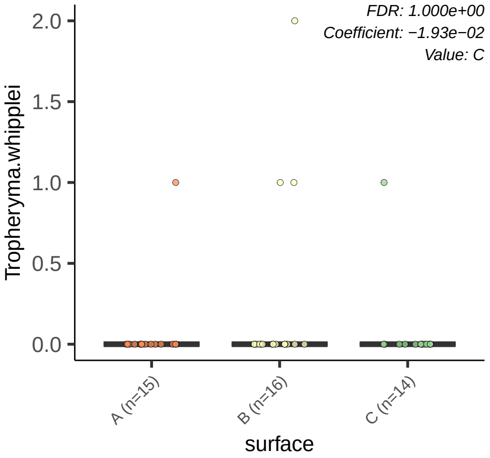
FDR: 1.000e+00
Coefficient: −1.93e−02
Value: C
Tropheryma.whipplei
2.0
1.5
1.0
0.5
0.0
A (n=15)
B (n=16)
C (n=14)
surface

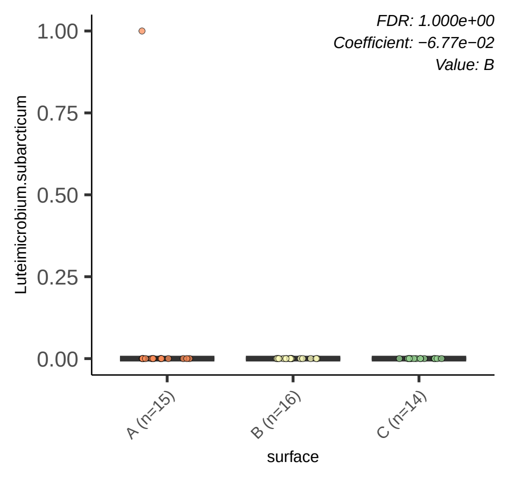

FDR: 1.000e+00
Coefficient: −6.77e−02
Value: B
Luteimicrobium.subarcticum
1.00
0.75
0.50
0.25
0.00
A (n=15)
B (n=16)
C (n=14)
surface

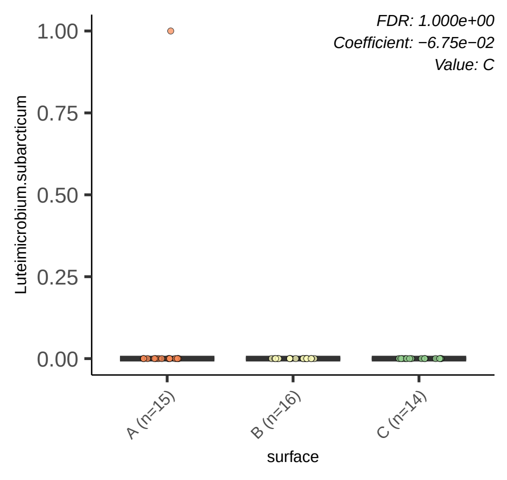
FDR: 1.000e+00
Coefficient: −6.75e−02
Value: C
Luteimicrobium.subarcticum
1.00
0.75
0.50
0.25
0.00
A (n=15)
B (n=16)
C (n=14)
surface

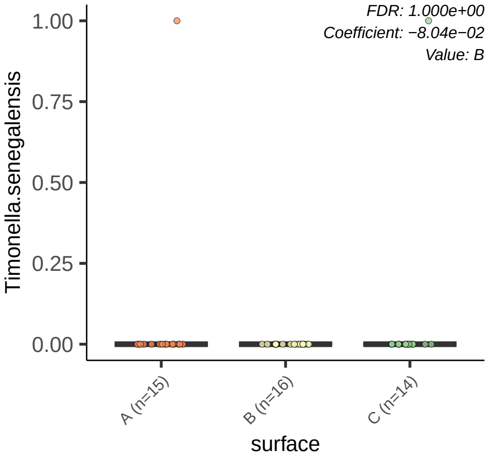

FDR: 1.000e+00
Coefficient: −8.04e−02
Value: B
Timonella.senegalensis
1.00
0.75
0.50
0.25
0.00
A (n=15)
B (n=16)
C (n=14)
surface

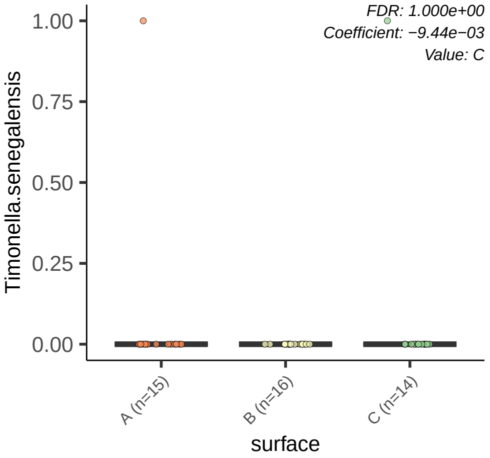
FDR: 1.000e+00
Coefficient: −9.44e−03
Value: C
Timonella.senegalensis
1.00
0.75
0.50
0.25
0.00
A (n=15)
B (n=16)
C (n=14)
surface

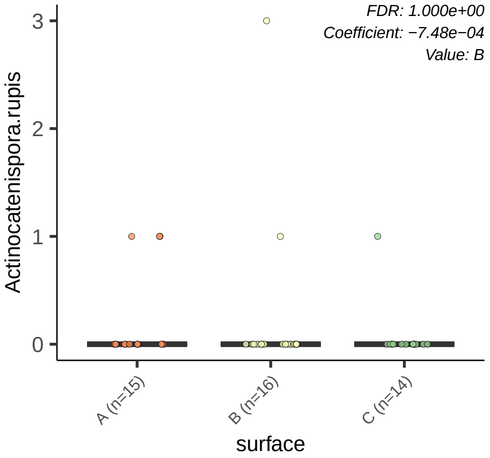
FDR: 1.000e+00
Coefficient: −7.48e−04
Value: B
Actinocatenispora.rupis
3
2
1
0
A (n=15)
B (n=16)
C (n=14)
surface

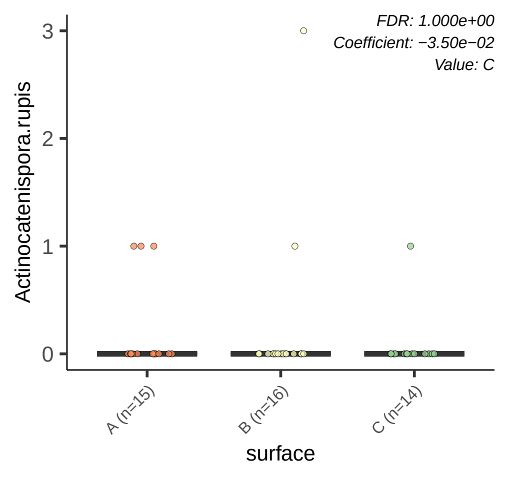
FDR: 1.000e+00
Coefficient: −3.50e−02
Value: C
Actinocatenispora.rupis
3
2
1
0
A (n=15)
B (n=16)
C (n=14)
surface

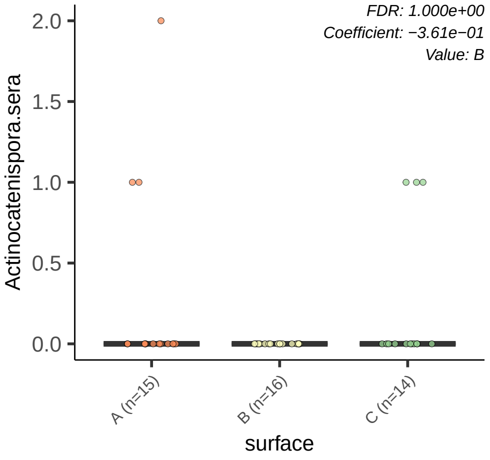
FDR: 1.000e+00
Coefficient: −3.61e−01
Value: B
Actinocatenispora.sera
2.0
1.5
1.0
0.5
0.0
A (n=15)
B (n=16)
C (n=14)
surface

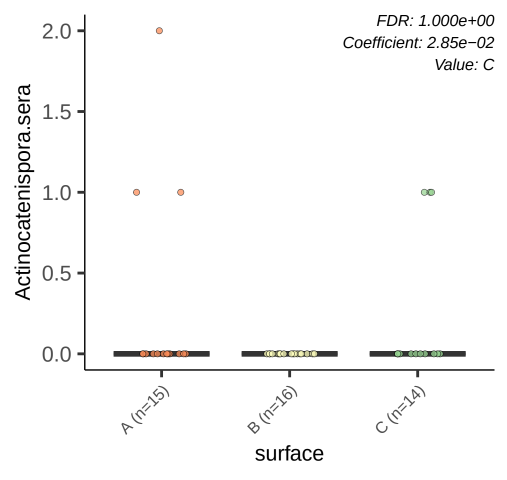
FDR: 1.000e+00
Coefficient: 2.85e−02
Value: C
Actinocatenispora.sera
2.0
1.5
1.0
0.5
0.0
A (n=15)
B (n=16)
C (n=14)
surface

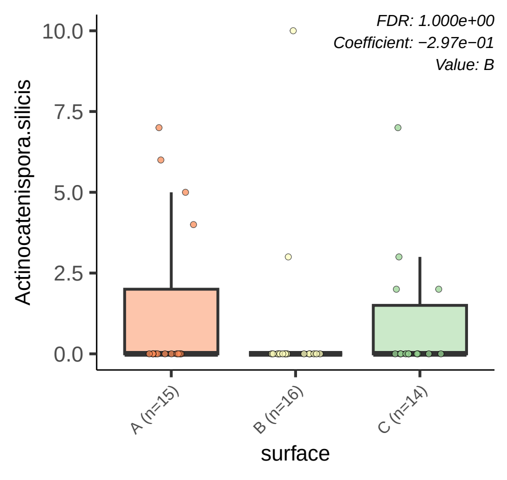
FDR: 1.000e+00
Coefficient: −2.97e−01
Value: B
Actinocatenispora.silicis
10.0
7.5
5.0
2.5
0.0
A (n=15)
B (n=16)
C (n=14)
surface

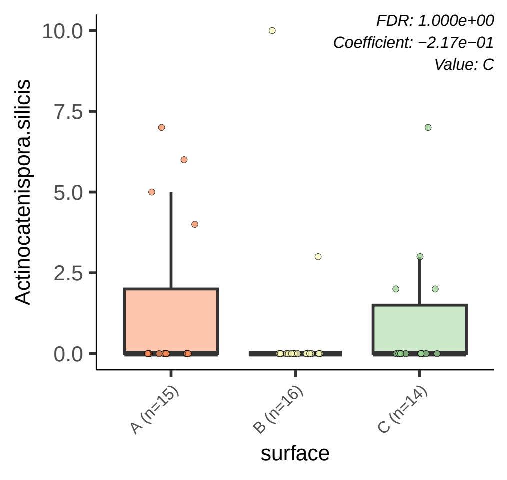
FDR: 1.000e+00
Coefficient: −2.17e−01
Value: C
Actinocatenispora.silicis
10.0
7.5
5.0
2.5
0.0
A (n=15)
B (n=16)
C (n=14)
surface

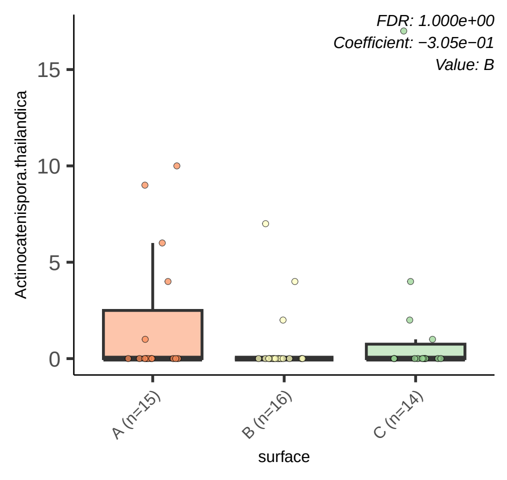

FDR: 1.000e+00
Coefficient: −3.05e−01
Value: B
Actinocatenispora.thailandica
0
5
10
15
A (n=15)
B (n=16)
C (n=14)
surface

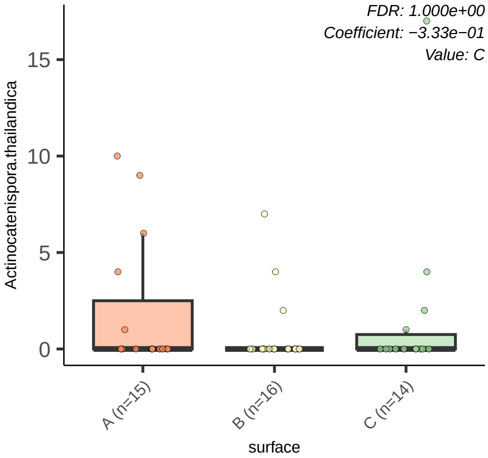
FDR: 1.000e+00
Coefficient: −3.33e−01
Value: C
Actinocatenispora.thailandica
0
5
10
15
A (n=15)
B (n=16)
C (n=14)
surface

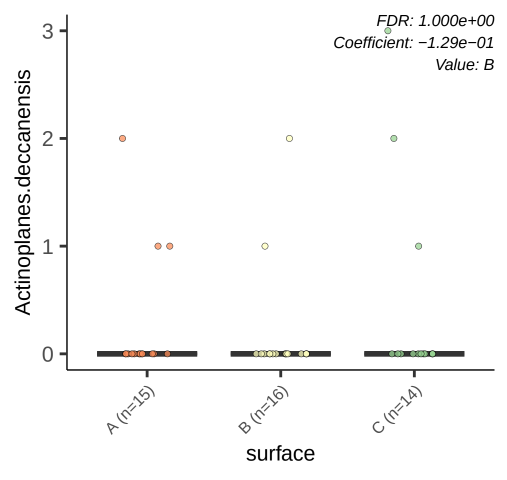
FDR: 1.000e+00
Coefficient: −1.29e−01
Value: B
Actinoplanes.deccanensis
3
2
1
0
A (n=15)
B (n=16)
C (n=14)
surface

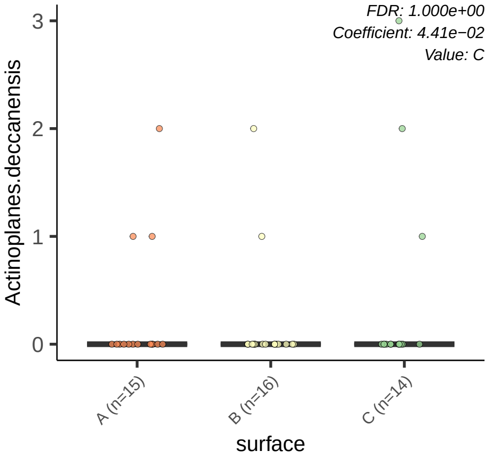
FDR: 1.000e+00
Coefficient: 4.41e−02
Value: C
Actinoplanes.deccanensis
0
1
2
3
A (n=15)
B (n=16)
C (n=14)
surface

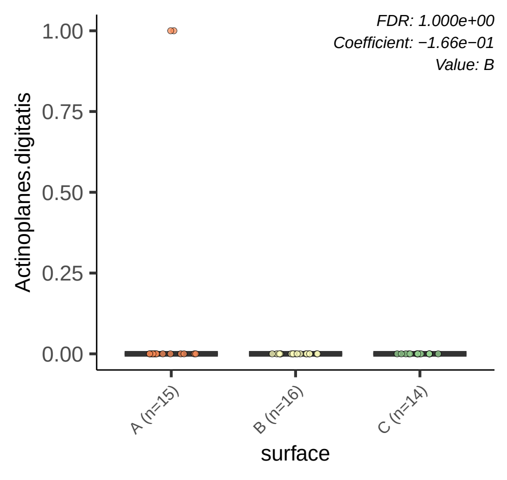

FDR: 1.000e+00
Coefficient: −1.66e−01
Value: B
Actinoplanes.digitatis
1.00
0.75
0.50
0.25
0.00
A (n=15)
B (n=16)
C (n=14)
surface

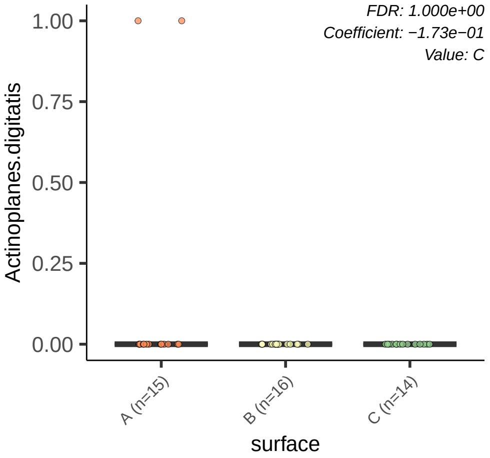

FDR: 1.000e+00
Coefficient: −1.73e−01
Value: C
Actinoplanes.digitatis
1.00
0.75
0.50
0.25
0.00
A (n=15)
B (n=16)
C (n=14)
surface

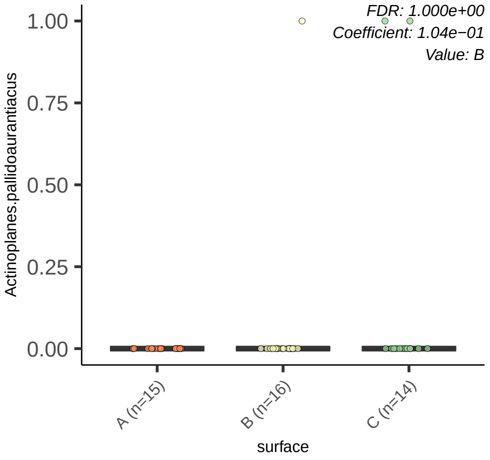

FDR: 1.000e+00
Coefficient: 1.04e−01
Value: B
Actinoplanes.pallidoaurantiacus
1.00
0.75
0.50
0.25
0.00
A (n=15)
B (n=16)
C (n=14)
surface

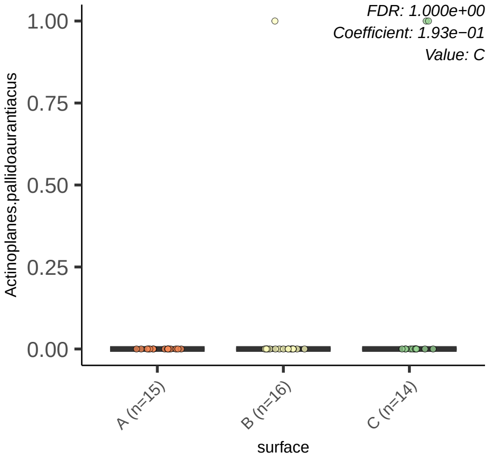
FDR: 1.000e+00
Coefficient: 1.93e−01
Value: C
Actinoplanes.pallidoaurantiacus
1.00
0.75
0.50
0.25
0.00
A (n=15)
B (n=16)
C (n=14)
surface

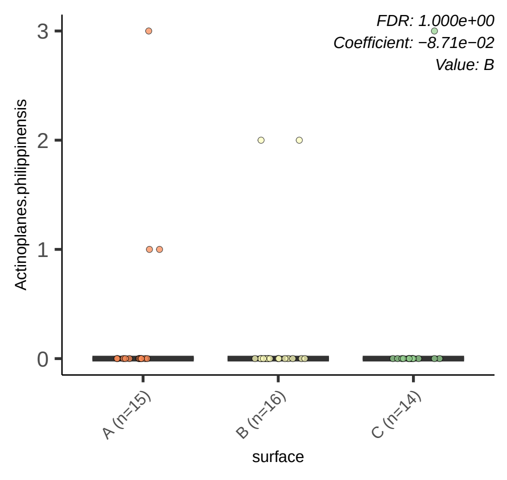
FDR: 1.000e+00
Coefficient: −8.71e−02
Value: B
Actinoplanes.philippinensis
3
2
1
0
A (n=15)
B (n=16)
C (n=14)
surface

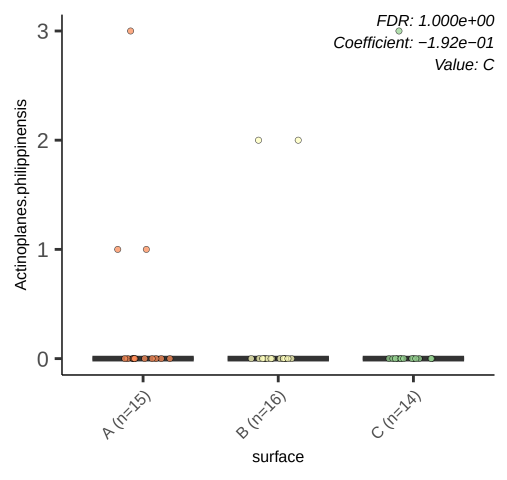

FDR: 1.000e+00
Coefficient: −1.92e−01
Value: C
Actinoplanes.philippinensis
0
1
2
3
A (n=15)
B (n=16)
C (n=14)
surface

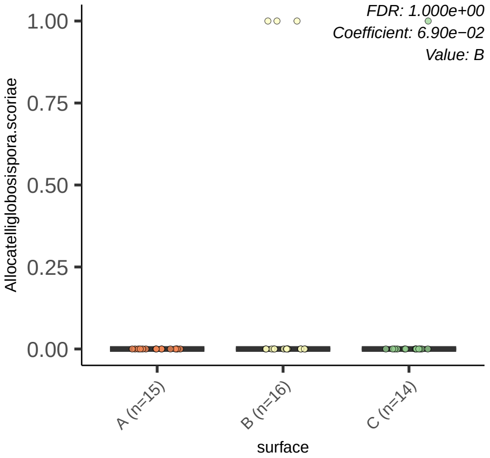
FDR: 1.000e+00
Coefficient: 6.90e−02
Value: B
Allocatelliglobosispora.scoriae
1.00
0.75
0.50
0.25
0.00
A (n=15)
B (n=16)
C (n=14)
surface

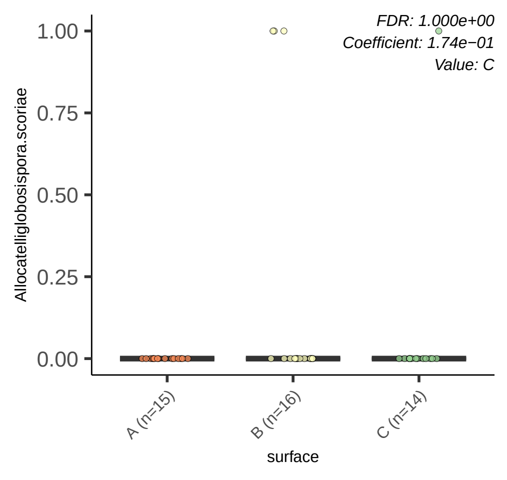
FDR: 1.000e+00
Coefficient: 1.74e−01
Value: C
Allocatelliglobosispora.scoriae
1.00
0.75
0.50
0.25
0.00
A (n=15)
B (n=16)
C (n=14)
surface

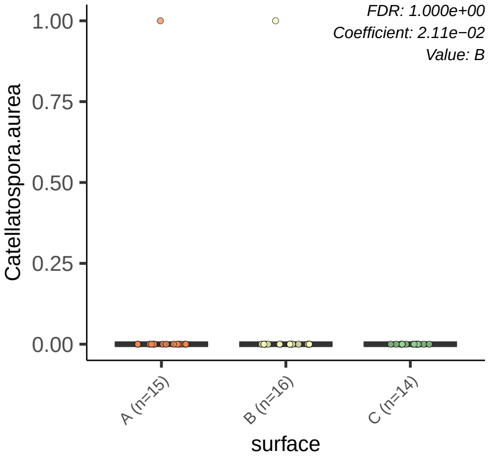
FDR: 1.000e+00
Coefficient: 2.11e−02
Value: B
Catellatospora.aurea
1.00
0.75
0.50
0.25
0.00
A (n=15)
B (n=16)
C (n=14)
surface

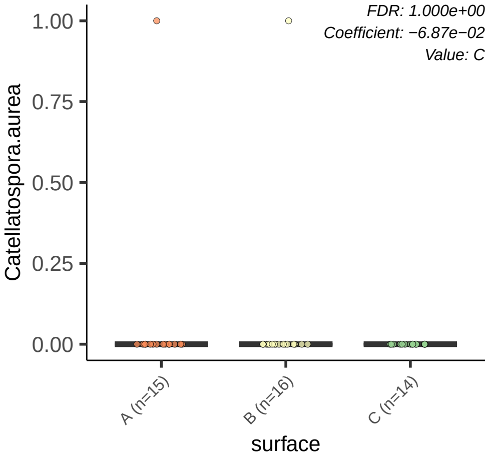
FDR: 1.000e+00
Coefficient: −6.87e−02
Value: C
Catellatospora.aurea
1.00
0.75
0.50
0.25
0.00
A (n=15)
B (n=16)
C (n=14)
surface

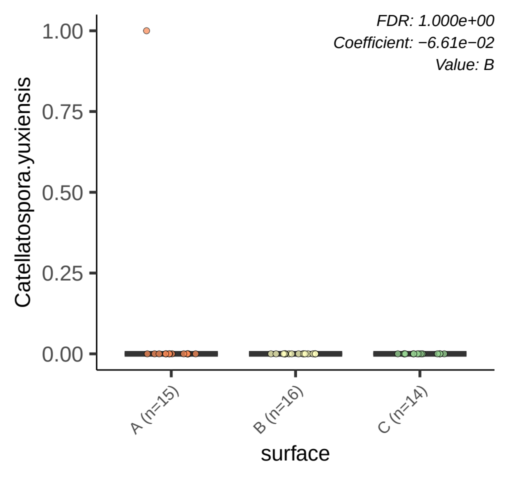

FDR: 1.000e+00
Coefficient: −6.61e−02
Value: B
Catellatospora.yuxiensis
1.00
0.75
0.50
0.25
0.00
A (n=15)
B (n=16)
C (n=14)
surface

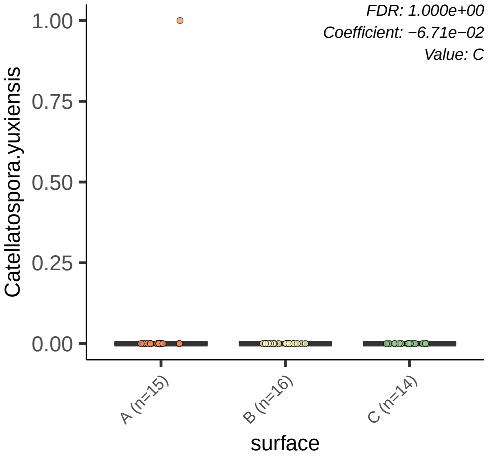
FDR: 1.000e+00
Coefficient: −6.71e−02
Value: C
Catellatospora.yuxiensis
1.00
0.75
0.50
0.25
0.00
A (n=15)
B (n=16)
C (n=14)
surface

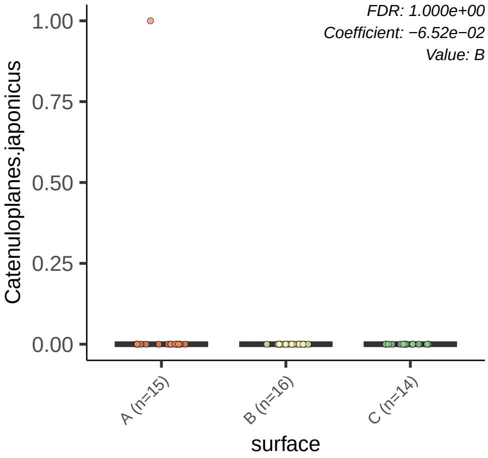
FDR: 1.000e+00
Coefficient: −6.52e−02
Value: B
Catenuloplanes.japonicus
1.00
0.75
0.50
0.25
0.00
A (n=15)
B (n=16)
C (n=14)
surface

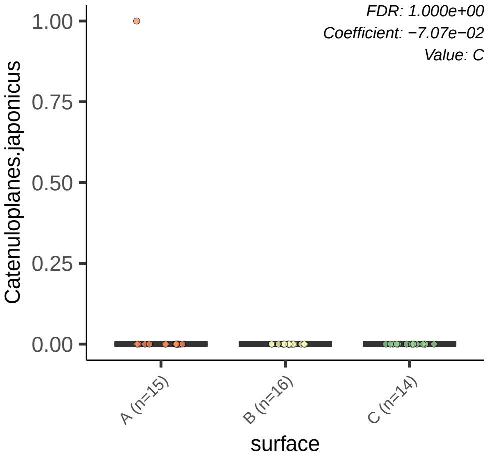

FDR: 1.000e+00
Coefficient: −7.07e−02
Value: C
Catenuloplanes.japonicus
1.00
0.75
0.50
0.25
0.00
A (n=15)
B (n=16)
C (n=14)
surface

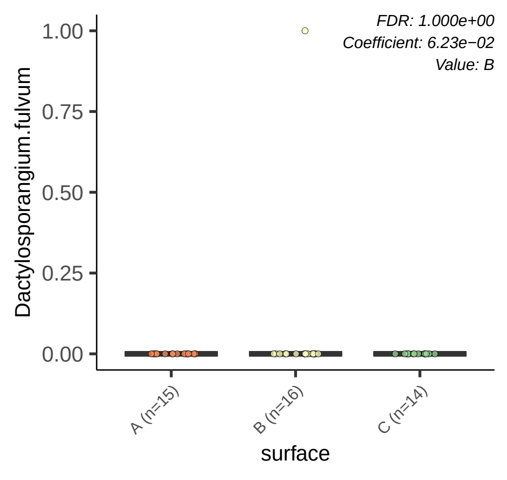

FDR: 1.000e+00
Coefficient: 6.23e−02
Value: B
Dactylosporangium.fulvum
1.00
0.75
0.50
0.25
0.00
A (n=15)
B (n=16)
C (n=14)
surface

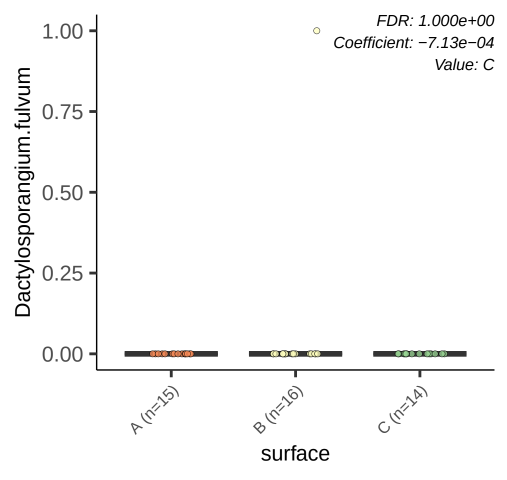
FDR: 1.000e+00
Coefficient: −7.13e−04
Value: C
Dactylosporangium.fulvum
1.00
0.75
0.50
0.25
0.00
A (n=15)
B (n=16)
C (n=14)
surface

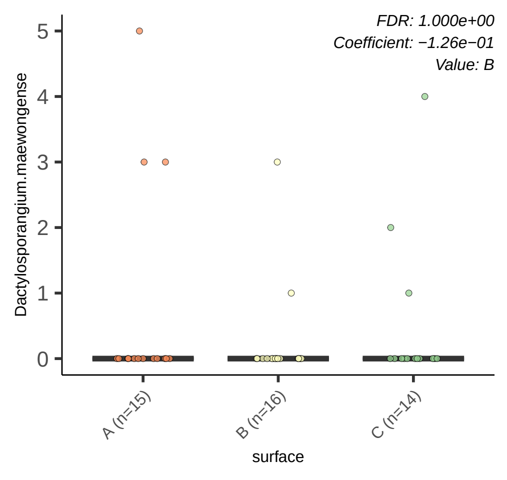
FDR: 1.000e+00
Coefficient: −1.26e−01
Value: B
Dactylosporangium.maewongense
0
1
2
3
4
5
A (n=15)
B (n=16)
C (n=14)
surface

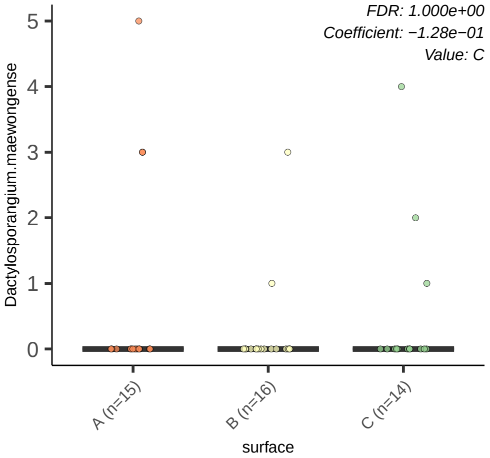

FDR: 1.000e+00
Coefficient: −1.28e−01
Value: C
Dactylosporangium.maewongense
0
1
2
3
4
5
A (n=15)
B (n=16)
C (n=14)
surface

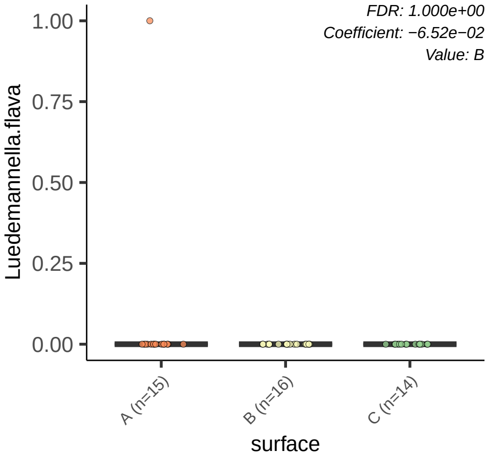

FDR: 1.000e+00
Coefficient: −6.52e−02
Value: B
Luedemannella.flava
1.00
0.75
0.50
0.25
0.00
A (n=15)
B (n=16)
C (n=14)
surface

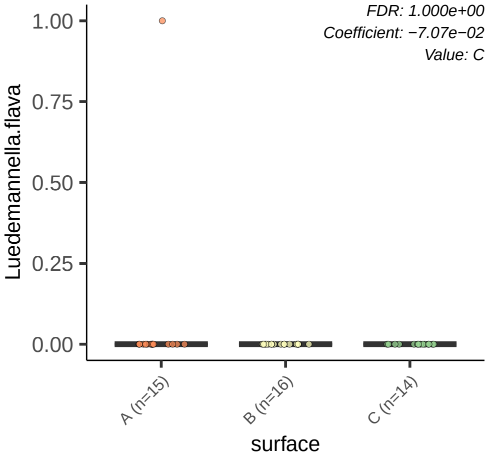
FDR: 1.000e+00
Coefficient: −7.07e−02
Value: C
Luedemannella.flava
1.00
0.75
0.50
0.25
0.00
A (n=15)
B (n=16)
C (n=14)
surface

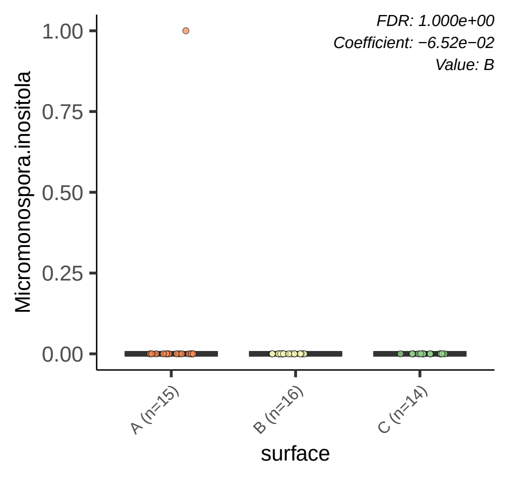
FDR: 1.000e+00
Coefficient: −6.52e−02
Value: B
Micromonospora.inositola
1.00
0.75
0.50
0.25
0.00
A (n=15)
B (n=16)
C (n=14)
surface

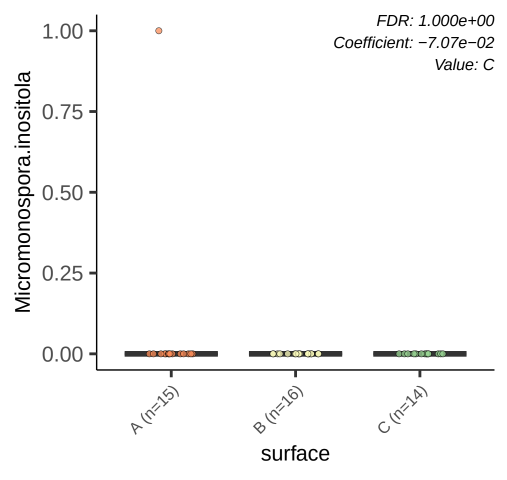

FDR: 1.000e+00
Coefficient: −7.07e−02
Value: C
Micromonospora.inositola
1.00
0.75
0.50
0.25
0.00
A (n=15)
B (n=16)
C (n=14)
surface

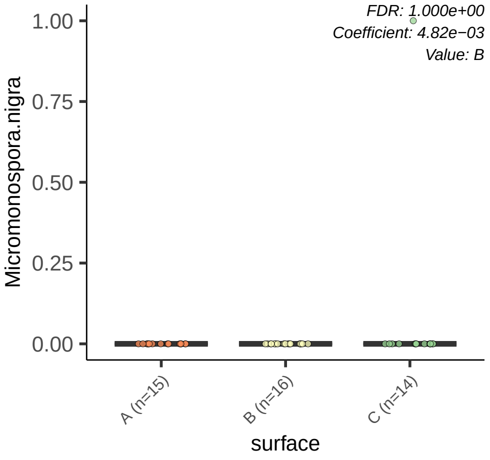
FDR: 1.000e+00
Coefficient: 4.82e−03
Value: B
Micromonospora.nigra
1.00
0.75
0.50
0.25
0.00
A (n=15)
B (n=16)
C (n=14)
surface

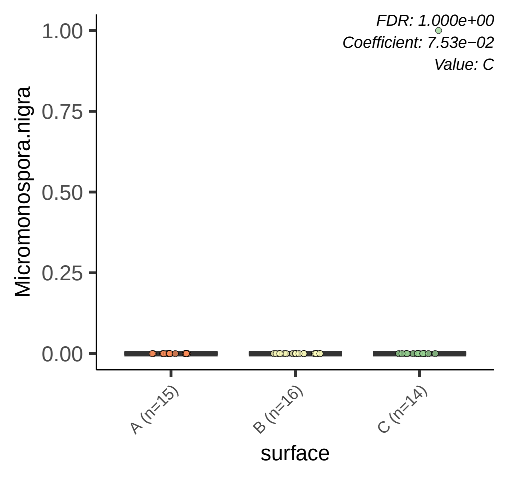

FDR: 1.000e+00
Coefficient: 7.53e−02
Value: C
Micromonospora.nigra
1.00
0.75
0.50
0.25
0.00
A (n=15)
B (n=16)
C (n=14)
surface

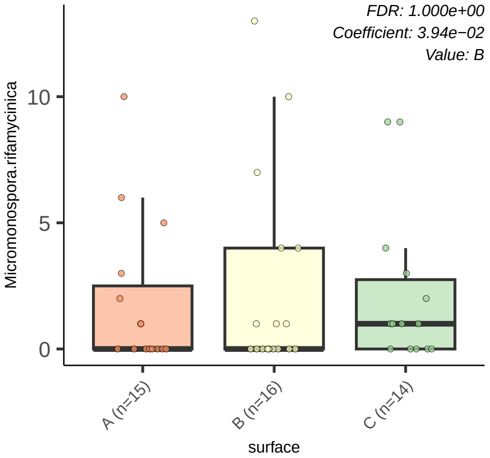

FDR: 1.000e+00
Coefficient: 3.94e−02
Value: B
Micromonospora.rifamycinica
10
5
0
A (n=15)
B (n=16)
C (n=14)
surface

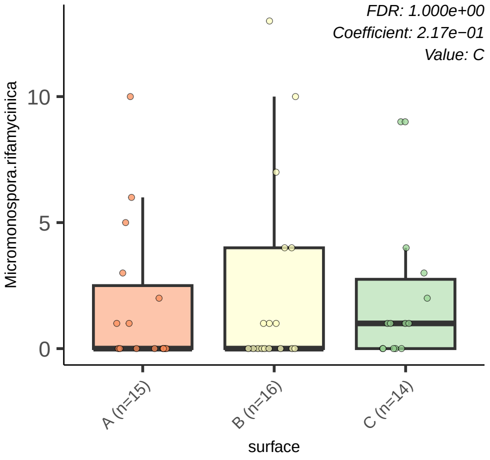
FDR: 1.000e+00
Coefficient: 2.17e−01
Value: C
Micromonospora.rifamycinica
10
5
0
A (n=15)
B (n=16)
C (n=14)
surface

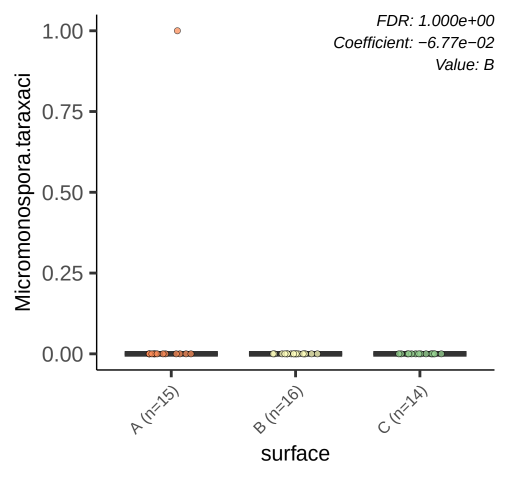
FDR: 1.000e+00
Coefficient: −6.77e−02
Value: B
Micromonospora.taraxaci
1.00
0.75
0.50
0.25
0.00
A (n=15)
B (n=16)
C (n=14)
surface

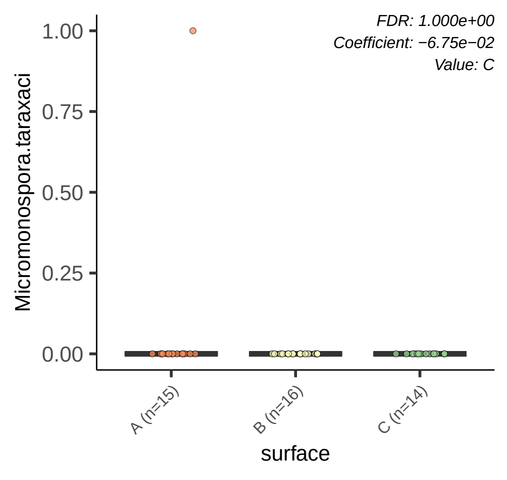

FDR: 1.000e+00
Coefficient: −6.75e−02
Value: C
Micromonospora.taraxaci
1.00
0.75
0.50
0.25
0.00
A (n=15)
B (n=16)
C (n=14)
surface

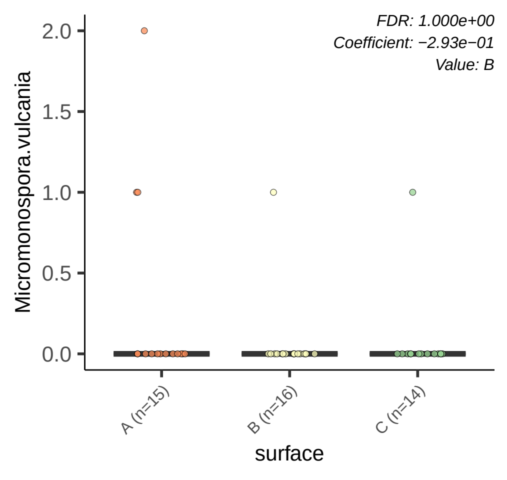

FDR: 1.000e+00
Coefficient: −2.93e−01
Value: B
Micromonospora.vulcania
2.0
1.5
1.0
0.5
0.0
A (n=15)
B (n=16)
C (n=14)
surface

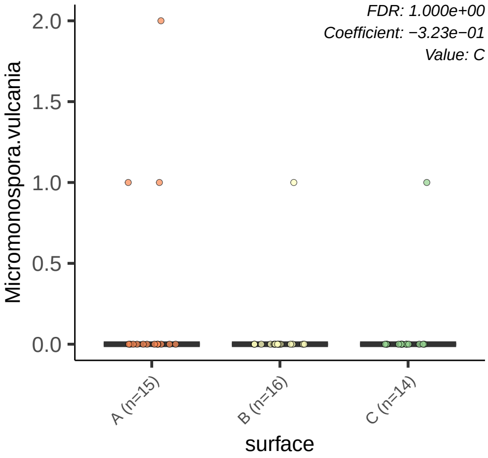
FDR: 1.000e+00
Coefficient: −3.23e−01
Value: C
Micromonospora.vulcania
2.0
1.5
1.0
0.5
0.0
A (n=15)
B (n=16)
C (n=14)
surface

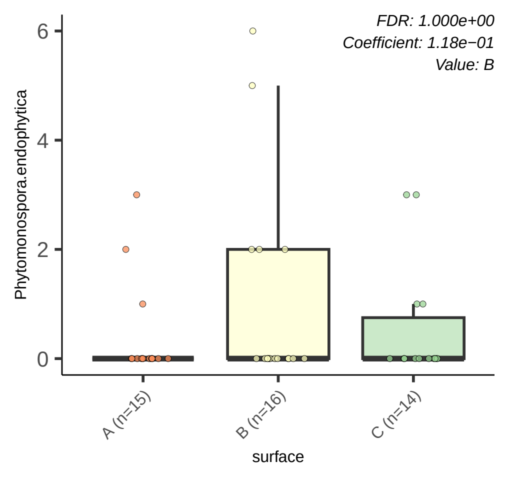

FDR: 1.000e+00
Coefficient: 1.18e−01
Value: B
Phytomonospora.endophytica
0
2
4
6
A (n=15)
B (n=16)
C (n=14)
surface

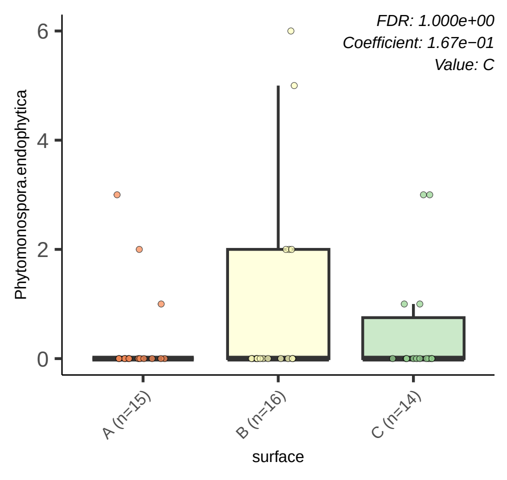

FDR: 1.000e+00
Coefficient: 1.67e−01
Value: C
Phytomonospora.endophytica
0
2
4
6
A (n=15)
B (n=16)
C (n=14)
surface

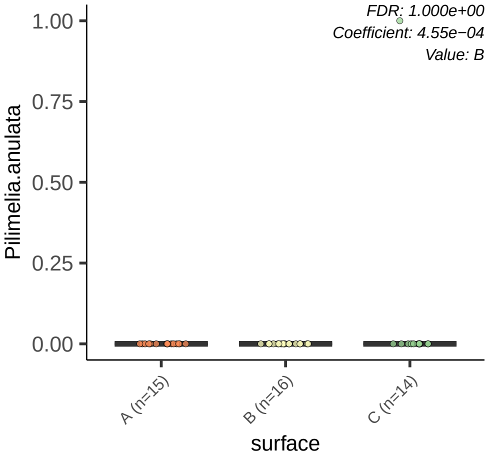
FDR: 1.000e+00
Coefficient: 4.55e−04
Value: B
Pilimelia.anulata
1.00
0.75
0.50
0.25
0.00
A (n=15)
B (n=16)
C (n=14)
surface

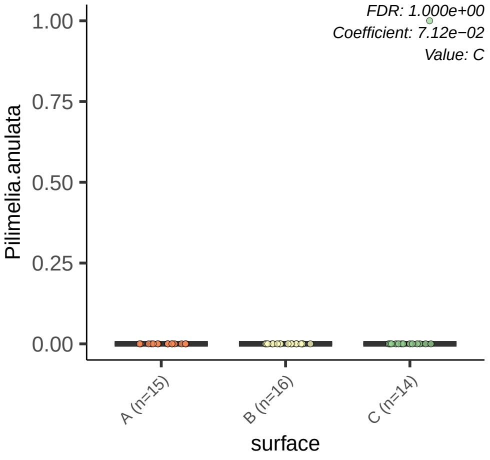
FDR: 1.000e+00
Coefficient: 7.12e−02
Value: C
Pilimelia.anulata
1.00
0.75
0.50
0.25
0.00
A (n=15)
B (n=16)
C (n=14)
surface

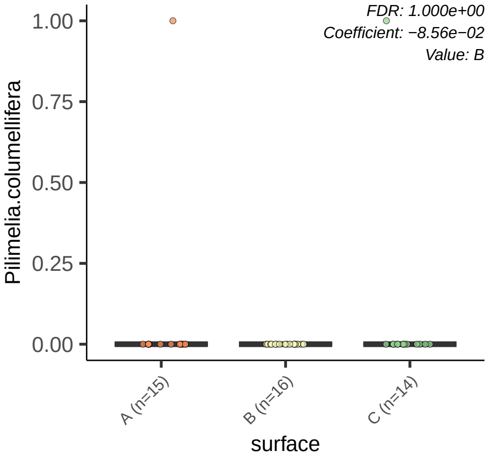
FDR: 1.000e+00
Coefficient: −8.56e−02
Value: B
Pilimelia.columellifera
1.00
0.75
0.50
0.25
0.00
A (n=15)
B (n=16)
C (n=14)
surface

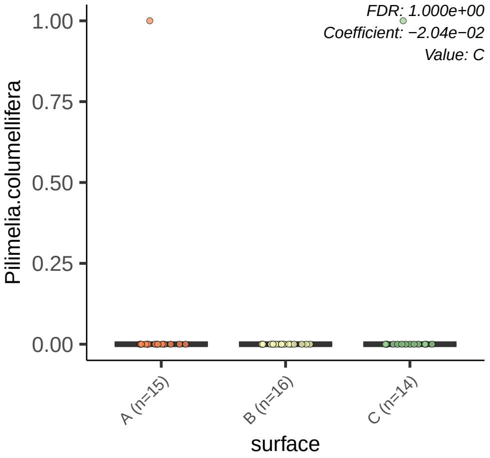
FDR: 1.000e+00
Coefficient: −2.04e−02
Value: C
Pilimelia.columellifera
1.00
0.75
0.50
0.25
0.00
A (n=15)
B (n=16)
C (n=14)
surface

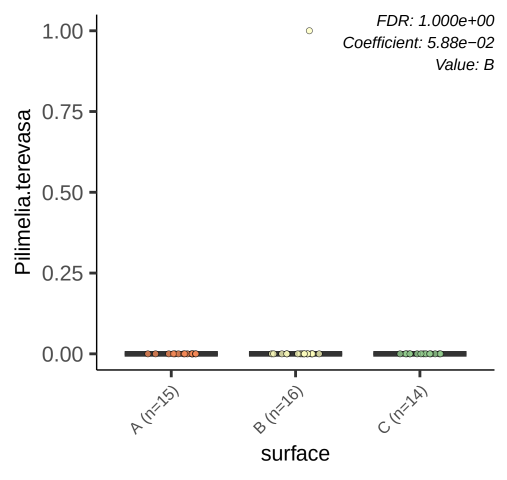

FDR: 1.000e+00
Coefficient: 5.88e−02
Value: B
Pilimelia.terevasa
1.00
0.75
0.50
0.25
0.00
A (n=15)
B (n=16)
C (n=14)
surface

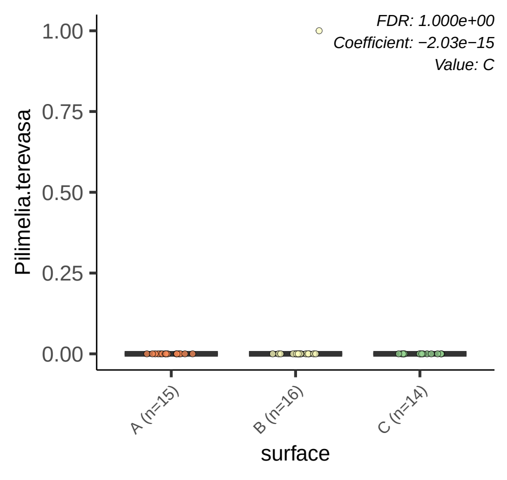

FDR: 1.000e+00
Coefficient: −2.03e−15
Value: C
Pilimelia.terevasa
1.00
0.75
0.50
0.25
0.00
A (n=15)
B (n=16)
C (n=14)
surface

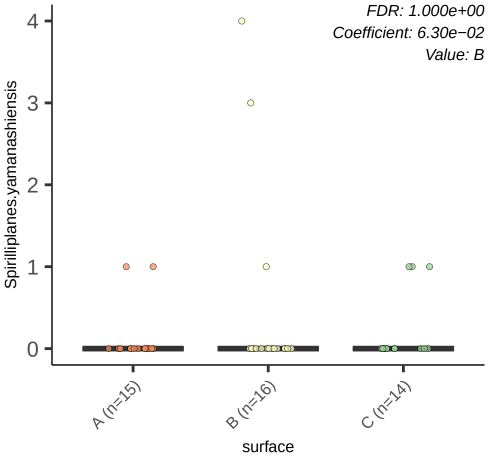
FDR: 1.000e+00
Coefficient: 6.30e−02
Value: B
Spirilliplanes.yamanashiensis
0
1
2
3
4
A (n=15)
B (n=16)
C (n=14)
surface

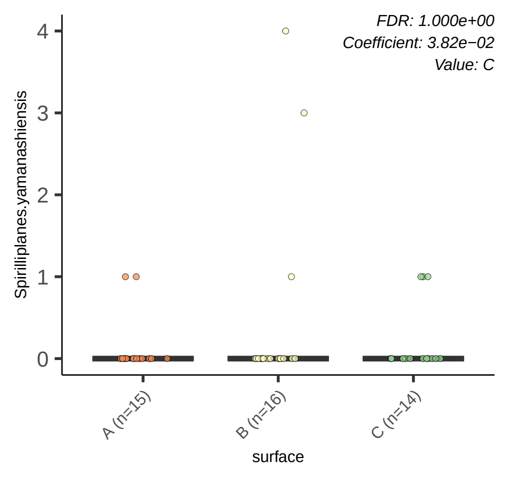
FDR: 1.000e+00
Coefficient: 3.82e−02
Value: C
Spirilliplanes.yamanashiensis
4
3
2
1
0
A (n=15)
B (n=16)
C (n=14)
surface

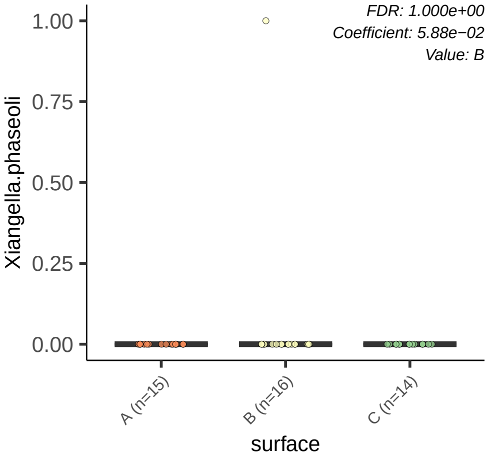

FDR: 1.000e+00
Coefficient: 5.88e−02
Value: B
Xiangella.phaseoli
1.00
0.75
0.50
0.25
0.00
A (n=15)
B (n=16)
C (n=14)
surface

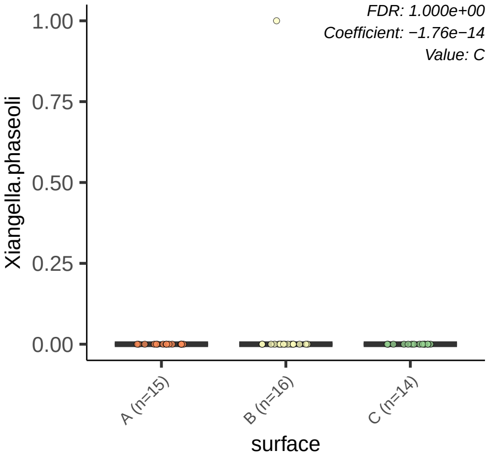
FDR: 1.000e+00
Coefficient: −1.76e−14
Value: C
Xiangella.phaseoli
1.00
0.75
0.50
0.25
0.00
A (n=15)
B (n=16)
C (n=14)
surface

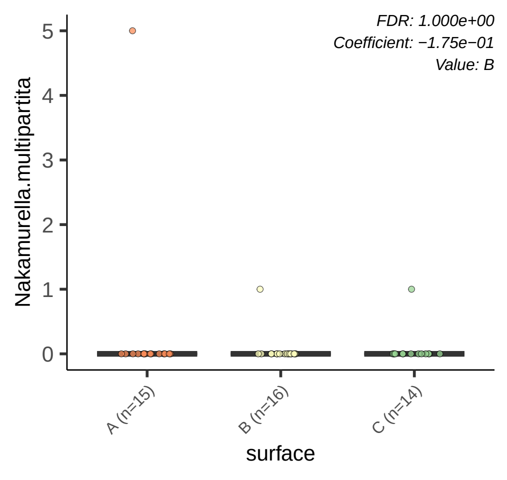
FDR: 1.000e+00
Coefficient: −1.75e−01
Value: B
Nakamurella.multipartita
0
1
2
3
4
5
A (n=15)
B (n=16)
C (n=14)
surface

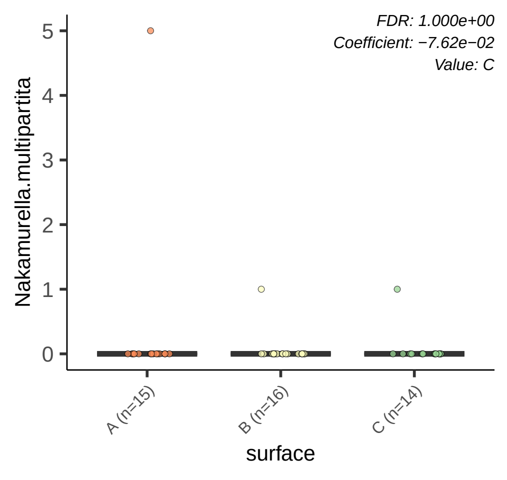

FDR: 1.000e+00
Coefficient: −7.62e−02
Value: C
Nakamurella.multipartita
0
1
2
3
4
5
A (n=15)
B (n=16)
C (n=14)
surface

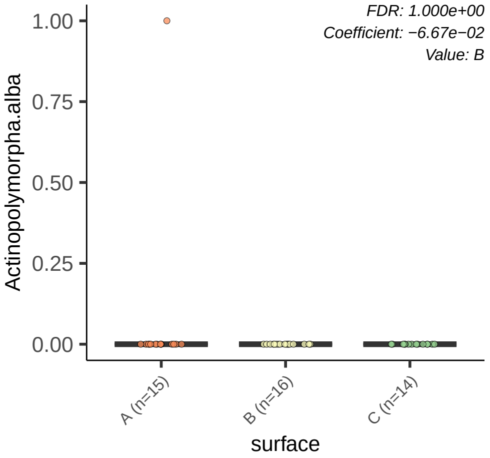
FDR: 1.000e+00
Coefficient: −6.67e−02
Value: B
Actinopolymorpha.alba
1.00
0.75
0.50
0.25
0.00
A (n=15)
B (n=16)
C (n=14)
surface

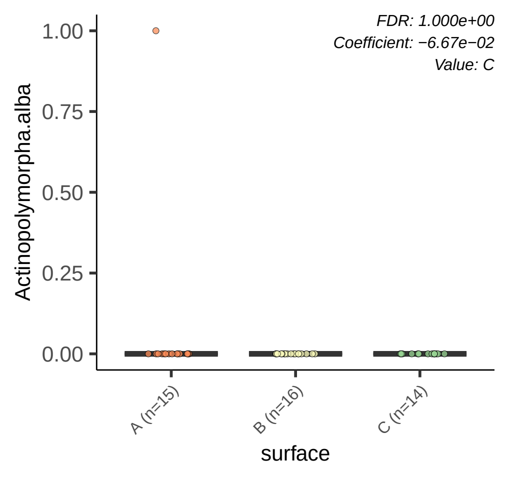
FDR: 1.000e+00
Coefficient: −6.67e−02
Value: C
Actinopolymorpha.alba
1.00
0.75
0.50
0.25
0.00
A (n=15)
B (n=16)
C (n=14)
surface

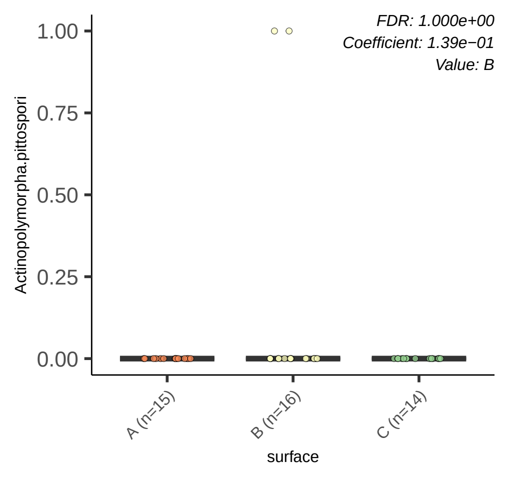
FDR: 1.000e+00
Coefficient: 1.39e−01
Value: B
1.00
0.75
0.50
0.25
0.00
Actinopolymorpha.pittospori
A (n=15)
B (n=16)
C (n=14)
surface

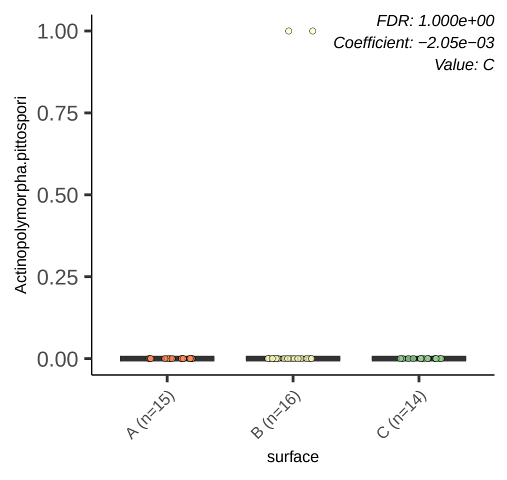

FDR: 1.000e+00
Coefficient: −2.05e−03
Value: C
Actinopolymorpha.pittospori
1.00
0.75
0.50
0.25
0.00
A (n=15)
B (n=16)
C (n=14)
surface

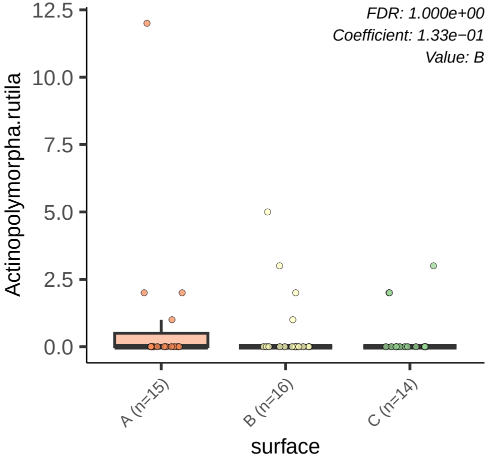

FDR: 1.000e+00
Coefficient: 1.33e−01
Value: B
Actinopolymorpha.rutila
12.5
10.0
7.5
5.0
2.5
0.0
A (n=15)
B (n=16)
C (n=14)
surface

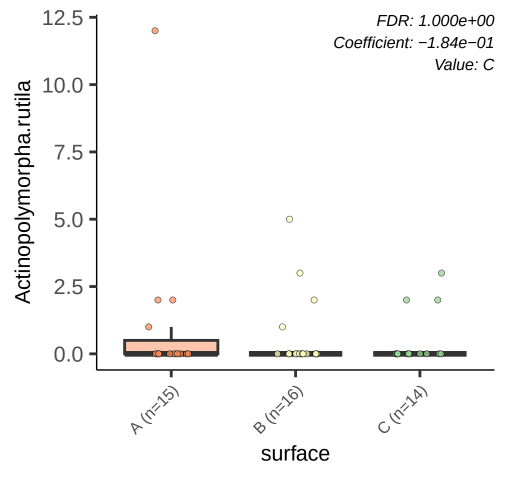
FDR: 1.000e+00
Coefficient: −1.84e−01
Value: C
Actinopolymorpha.rutila
12.5
10.0
7.5
5.0
2.5
0.0
A (n=15)
B (n=16)
C (n=14)
surface

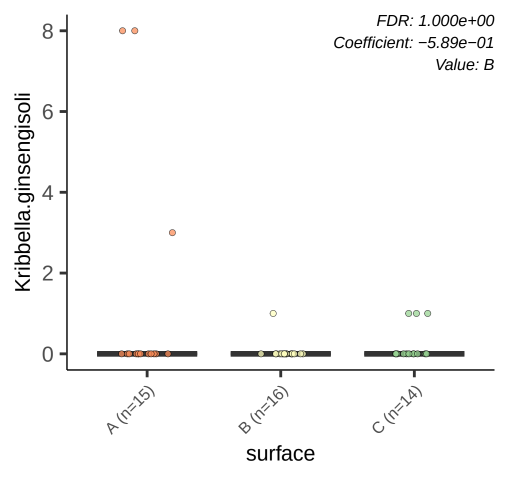

FDR: 1.000e+00
Coefficient: −5.89e−01
Value: B
Kribbella.ginsengisoli
8
6
4
2
0
A (n=15)
B (n=16)
C (n=14)
surface

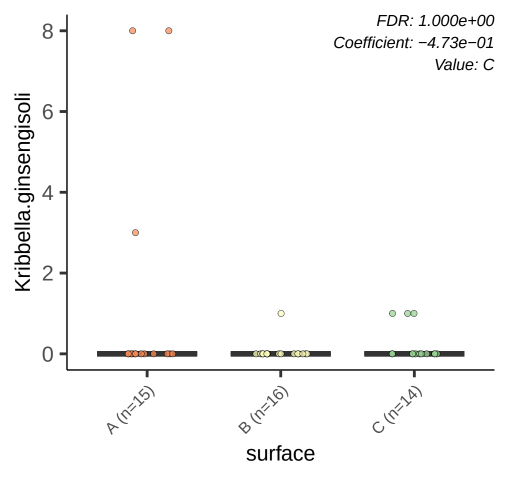

FDR: 1.000e+00
Coefficient: −4.73e−01
Value: C
Kribbella.ginsengisoli
0
2
4
6
8
A (n=15)
B (n=16)
C (n=14)
surface

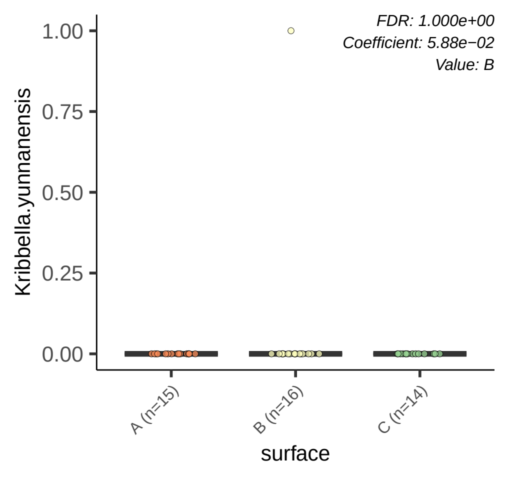

FDR: 1.000e+00
Coefficient: 5.88e−02
Value: B
Kribbella.yunnanensis
1.00
0.75
0.50
0.25
0.00
A (n=15)
B (n=16)
C (n=14)
surface

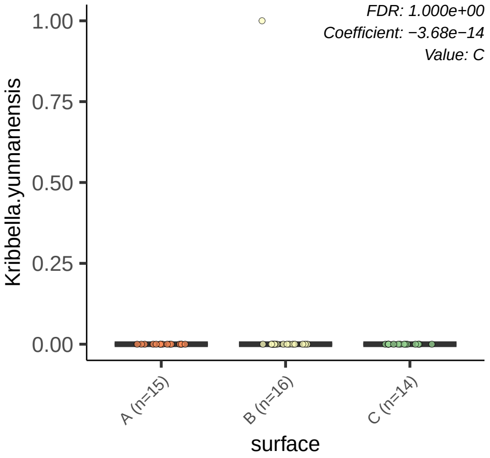
FDR: 1.000e+00
Coefficient: −3.68e−14
Value: C
Kribbella.yunnanensis
1.00
0.75
0.50
0.25
0.00
A (n=15)
B (n=16)
C (n=14)
surface

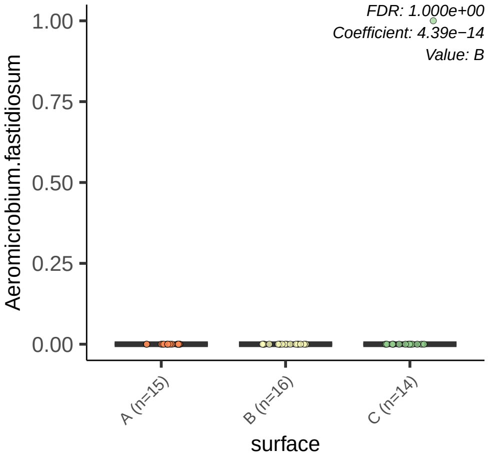
FDR: 1.000e+00
Coefficient: 4.39e−14
Value: B
Aeromicrobium.fastidiosum
1.00
0.75
0.50
0.25
0.00
A (n=15)
B (n=16)
C (n=14)
surface

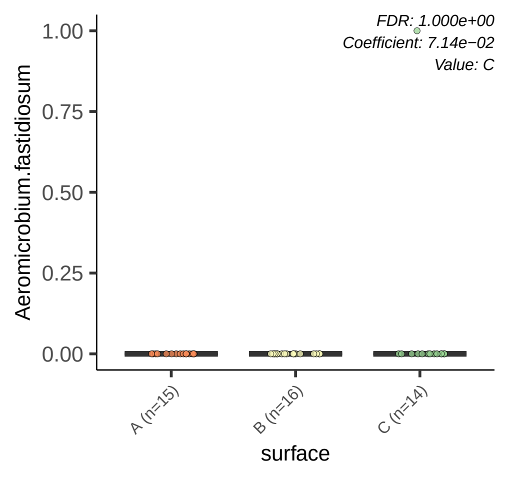
FDR: 1.000e+00
Coefficient: 7.14e−02
Value: C
Aeromicrobium.fastidiosum
1.00
0.75
0.50
0.25
0.00
A (n=15)
B (n=16)
C (n=14)
surface

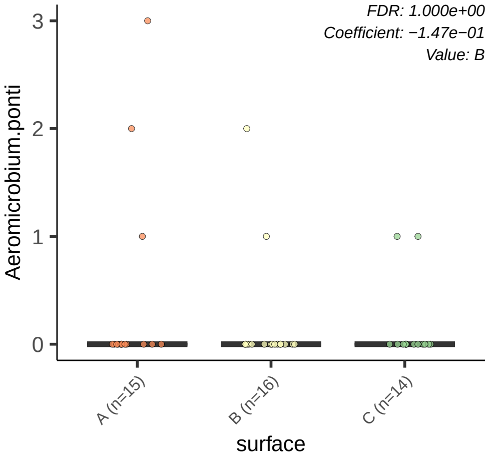

FDR: 1.000e+00
Coefficient: −1.47e−01
Value: B
Aeromicrobium.ponti
3
2
1
0
A (n=15)
B (n=16)
C (n=14)
surface

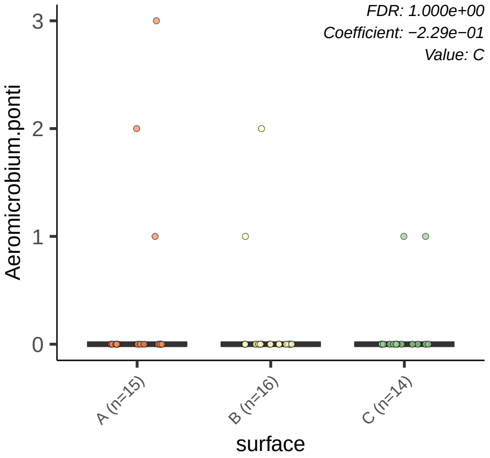

FDR: 1.000e+00
Coefficient: −2.29e−01
Value: C
Aeromicrobium.ponti
3
2
1
0
A (n=15)
B (n=16)
C (n=14)
surface

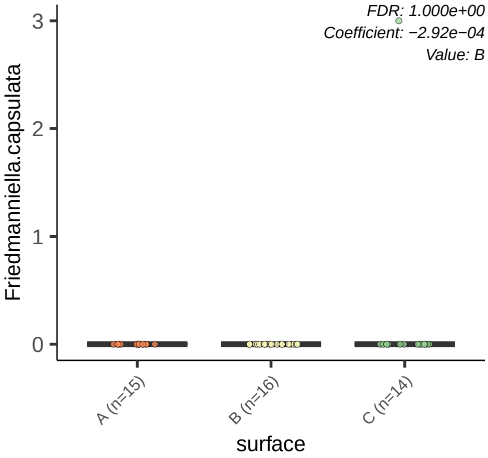
FDR: 1.000e+00
Coefficient: −2.92e−04
Value: B
Friedmanniella.capsulata
3
2
1
0
A (n=15)
B (n=16)
C (n=14)
surface

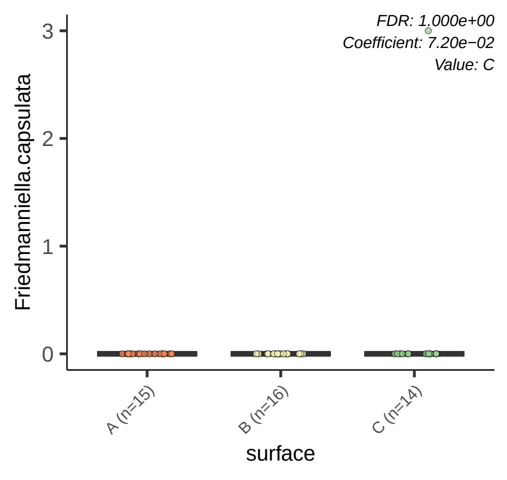
FDR: 1.000e+00
Coefficient: 7.20e−02
Value: C
Friedmanniella.capsulata
3
2
1
0
A (n=15)
B (n=16)
C (n=14)
surface

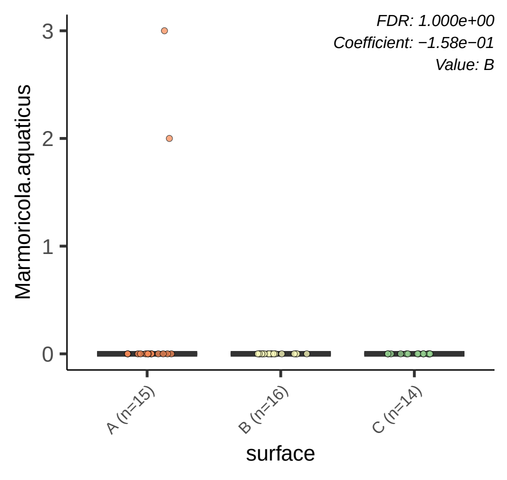

FDR: 1.000e+00
Coefficient: −1.58e−01
Value: B
Marmoricola.aquaticus
3
2
1
0
A (n=15)
B (n=16)
C (n=14)
surface

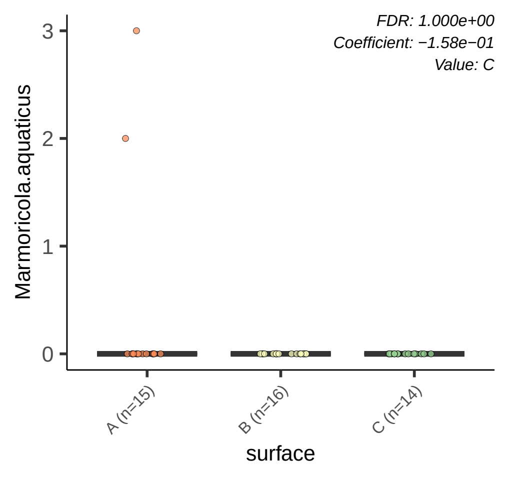
FDR: 1.000e+00
Coefficient: −1.58e−01
Value: C
Marmoricola.aquaticus
3
2
1
0
A (n=15)
B (n=16)
C (n=14)
surface

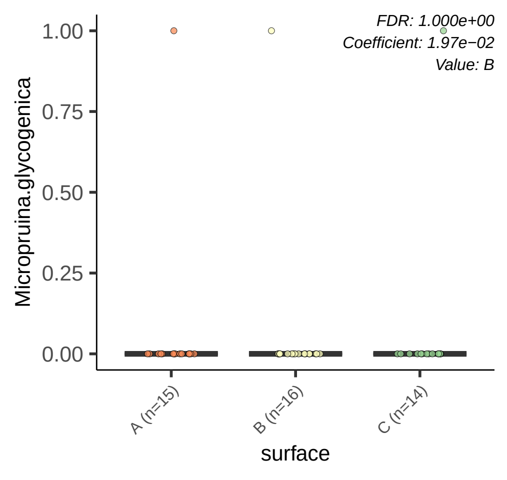

FDR: 1.000e+00
Coefficient: 1.97e−02
Value: B
Micropruina.glycogenica
1.00
0.75
0.50
0.25
0.00
A (n=15)
B (n=16)
C (n=14)
surface

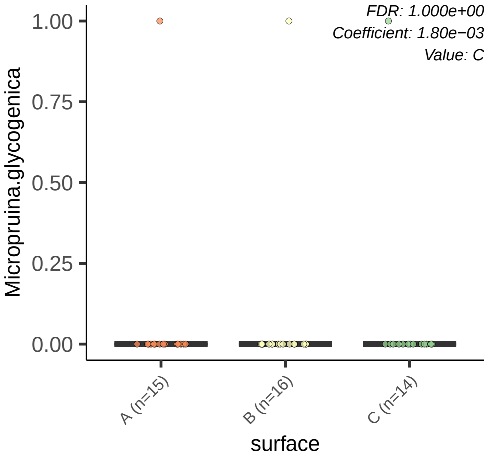
FDR: 1.000e+00
Coefficient: 1.80e−03
Value: C
Micropruina.glycogenica
1.00
0.75
0.50
0.25
0.00
A (n=15)
B (n=16)
C (n=14)
surface

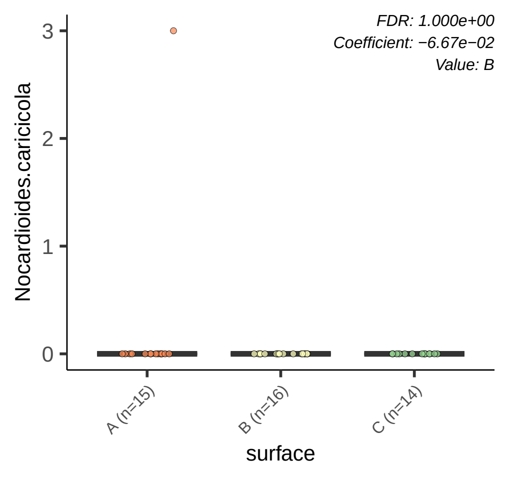
FDR: 1.000e+00
Coefficient: −6.67e−02
Value: B
Nocardioides.caricicola
3
2
1
0
A (n=15)
B (n=16)
C (n=14)
surface

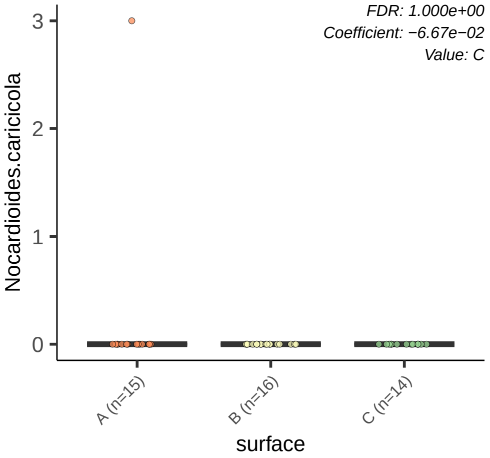
FDR: 1.000e+00
Coefficient: −6.67e−02
Value: C
Nocardioides.caricicola
3
2
1
0
A (n=15)
B (n=16)
C (n=14)
surface

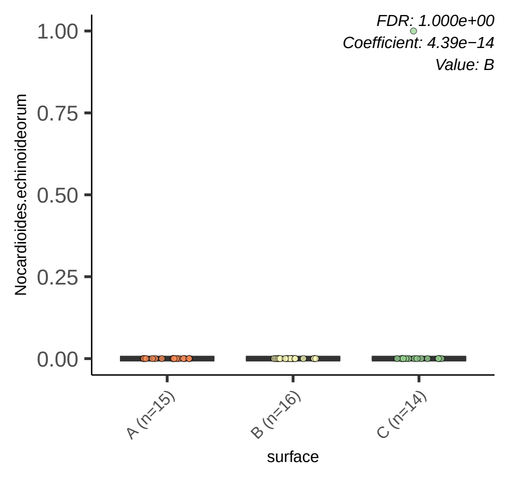
FDR: 1.000e+00
Coefficient: 4.39e−14
Value: B
Nocardioides.echinoideorum
1.00
0.75
0.50
0.25
0.00
A (n=15)
B (n=16)
C (n=14)
surface

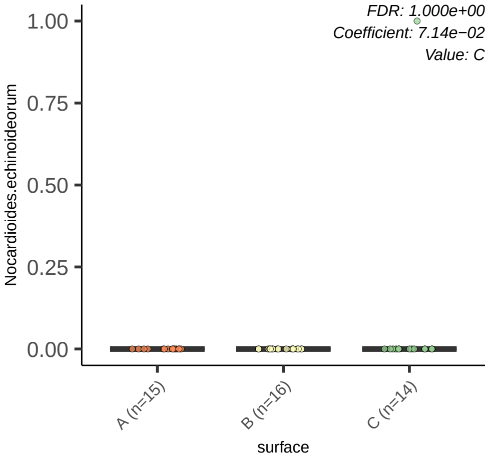
FDR: 1.000e+00
Coefficient: 7.14e−02
Value: C
1.00
0.75
0.50
0.25
0.00
Nocardioides.echinoideorum
A (n=15)
B (n=16)
C (n=14)
surface

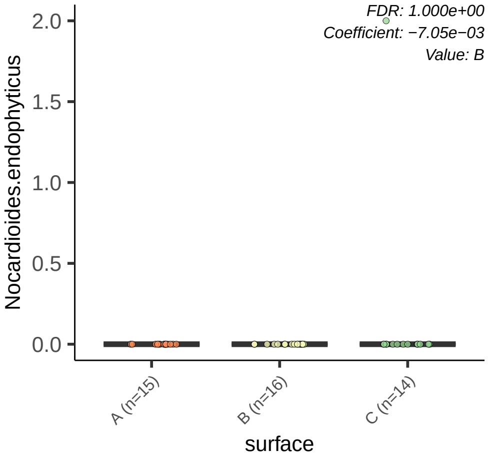
FDR: 1.000e+00
Coefficient: −7.05e−03
Value: B
Nocardioides.endophyticus
2.0
1.5
1.0
0.5
0.0
A (n=15)
B (n=16)
C (n=14)
surface

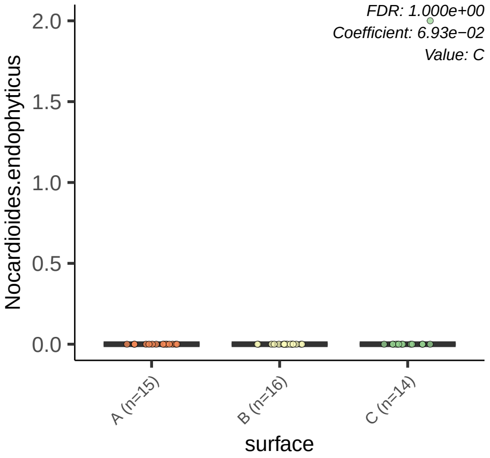
FDR: 1.000e+00
Coefficient: 6.93e−02
Value: C
2.0
1.5
1.0
0.5
0.0
Nocardioides.endophyticus
A (n=15)
B (n=16)
C (n=14)
surface

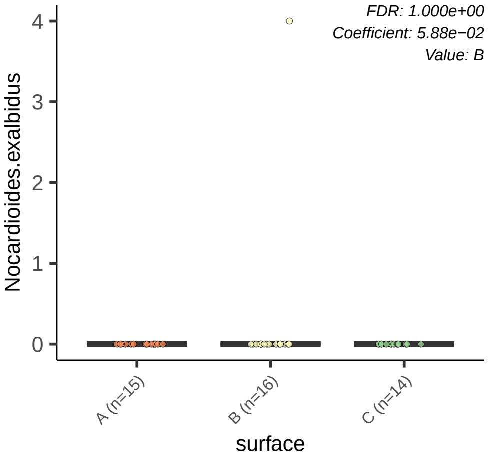

FDR: 1.000e+00
Coefficient: 5.88e−02
Value: B
Nocardioides.exalbidus
0
1
2
3
4
A (n=15)
B (n=16)
C (n=14)
surface

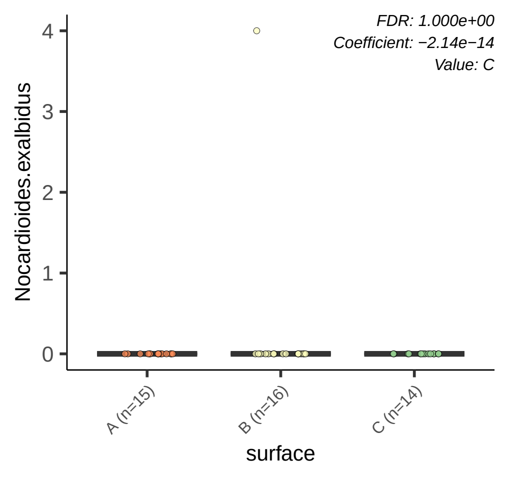
FDR: 1.000e+00
Coefficient: −2.14e−14
Value: C
Nocardioides.exalbidus
0
1
2
3
4
A (n=15)
B (n=16)
C (n=14)
surface

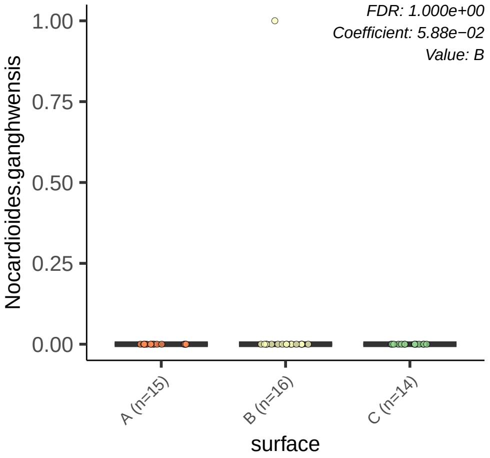
FDR: 1.000e+00
Coefficient: 5.88e−02
Value: B
Nocardioides.ganghwensis
1.00
0.75
0.50
0.25
0.00
A (n=15)
B (n=16)
C (n=14)
surface

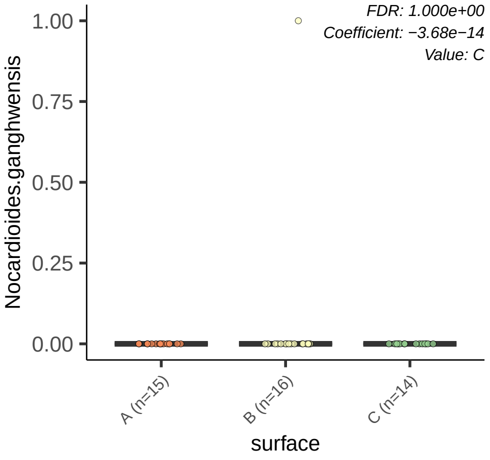

FDR: 1.000e+00
Coefficient: −3.68e−14
Value: C
Nocardioides.ganghwensis
1.00
0.75
0.50
0.25
0.00
A (n=15)
B (n=16)
C (n=14)
surface

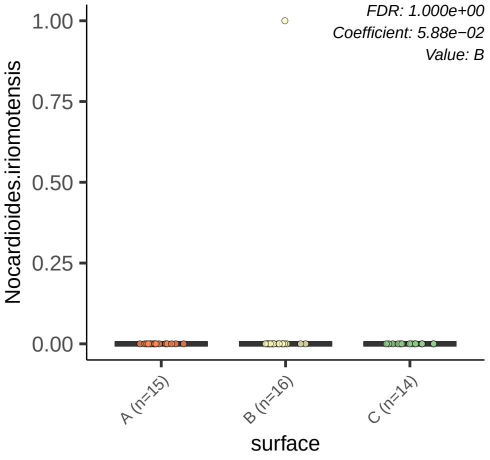
FDR: 1.000e+00
Coefficient: 5.88e−02
Value: B
Nocardioides.iriomotensis
1.00
0.75
0.50
0.25
0.00
A (n=15)
B (n=16)
C (n=14)
surface

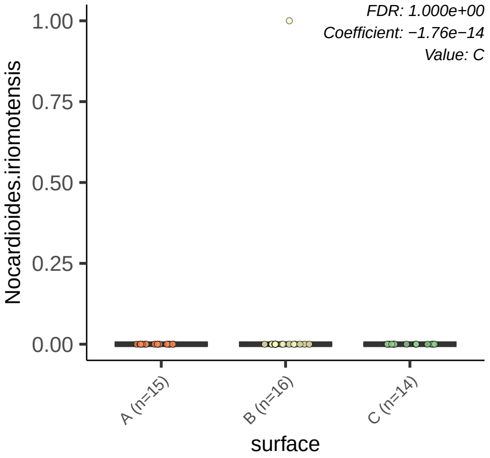
FDR: 1.000e+00
Coefficient: −1.76e−14
Value: C
Nocardioides.iriomotensis
1.00
0.75
0.50
0.25
0.00
A (n=15)
B (n=16)
C (n=14)
surface

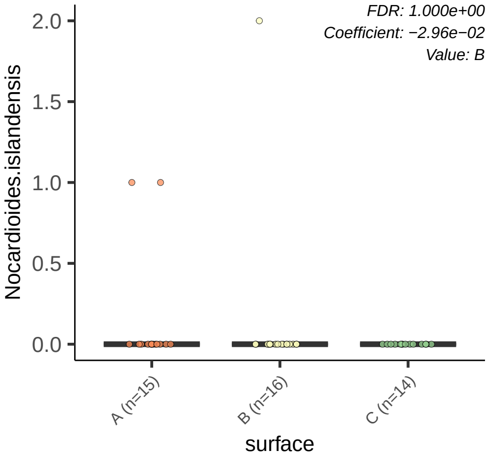
FDR: 1.000e+00
Coefficient: −2.96e−02
Value: B
Nocardioides.islandensis
2.0
1.5
1.0
0.5
0.0
A (n=15)
B (n=16)
C (n=14)
surface

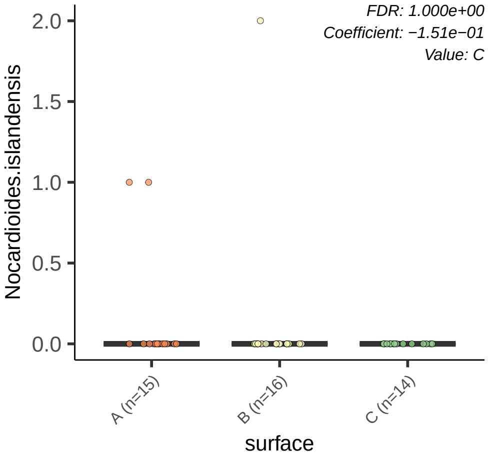
FDR: 1.000e+00
Coefficient: −1.51e−01
Value: C
Nocardioides.islandensis
2.0
1.5
1.0
0.5
0.0
A (n=15)
B (n=16)
C (n=14)
surface

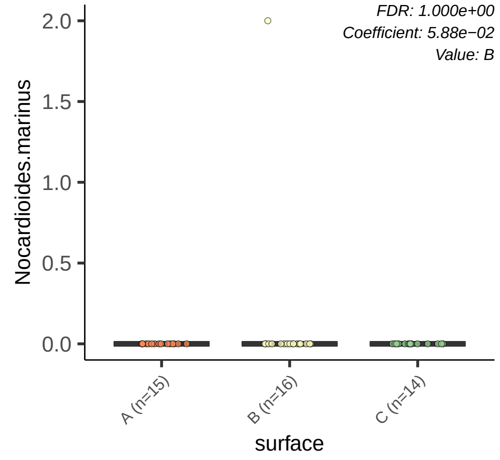
FDR: 1.000e+00
Coefficient: 5.88e−02
Value: B
Nocardioides.marinus
2.0
1.5
1.0
0.5
0.0
A (n=15)
B (n=16)
C (n=14)
surface

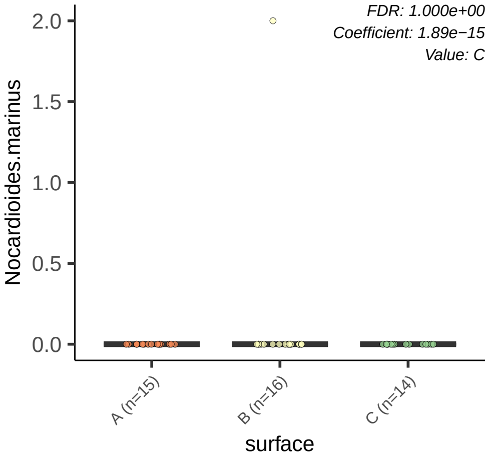
FDR: 1.000e+00
Coefficient: 1.89e−15
Value: C
Nocardioides.marinus
2.0
1.5
1.0
0.5
0.0
A (n=15)
B (n=16)
C (n=14)
surface

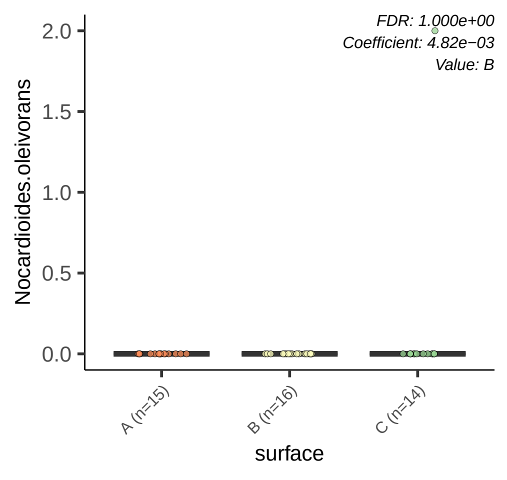
FDR: 1.000e+00
Coefficient: 4.82e−03
Value: B
Nocardioides.oleivorans
2.0
1.5
1.0
0.5
0.0
A (n=15)
B (n=16)
C (n=14)
surface

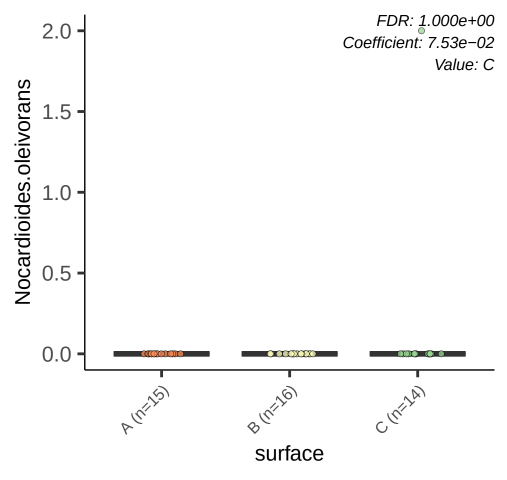
FDR: 1.000e+00
Coefficient: 7.53e−02
Value: C
Nocardioides.oleivorans
2.0
1.5
1.0
0.5
0.0
A (n=15)
B (n=16)
C (n=14)
surface

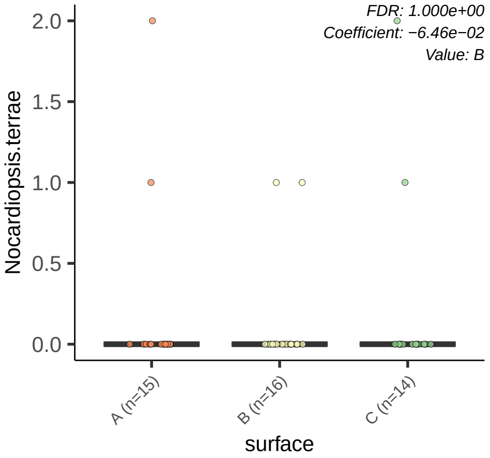

FDR: 1.000e+00
Coefficient: −6.46e−02
Value: B
Nocardiopsis.terrae
2.0
1.5
1.0
0.5
0.0
A (n=15)
B (n=16)
C (n=14)
surface

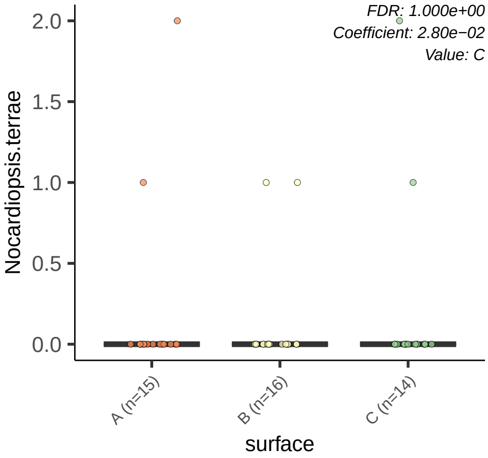
FDR: 1.000e+00
Coefficient: 2.80e−02
Value: C
Nocardiopsis.terrae
2.0
1.5
1.0
0.5
0.0
A (n=15)
B (n=16)
C (n=14)
surface

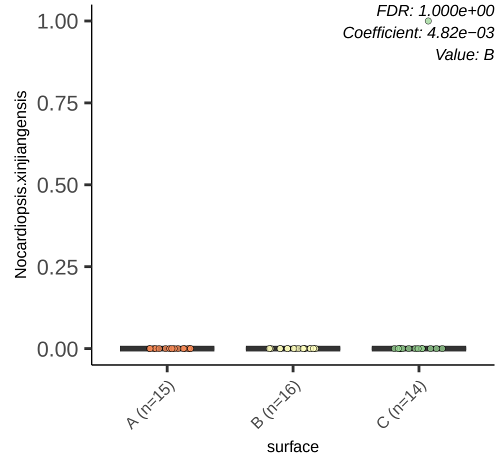
FDR: 1.000e+00
Coefficient: 4.82e−03
Value: B
Nocardiopsis.xinjiangensis
1.00
0.75
0.50
0.25
0.00
A (n=15)
B (n=16)
C (n=14)
surface

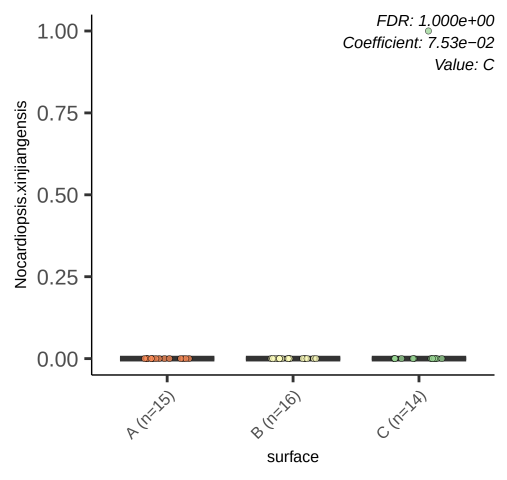
FDR: 1.000e+00
Coefficient: 7.53e−02
Value: C
Nocardiopsis.xinjiangensis
1.00
0.75
0.50
0.25
0.00
A (n=15)
B (n=16)
C (n=14)
surface

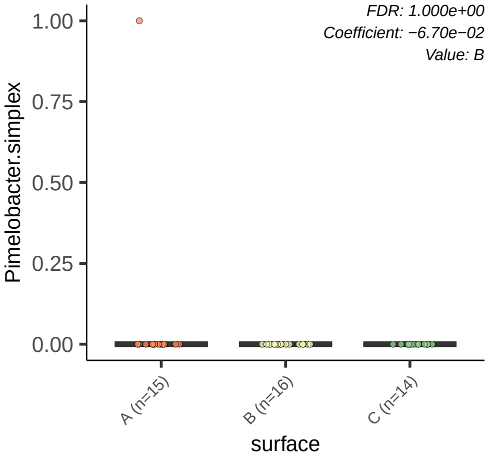
FDR: 1.000e+00
Coefficient: −6.70e−02
Value: B
Pimelobacter.simplex
1.00
0.75
0.50
0.25
0.00
A (n=15)
B (n=16)
C (n=14)
surface

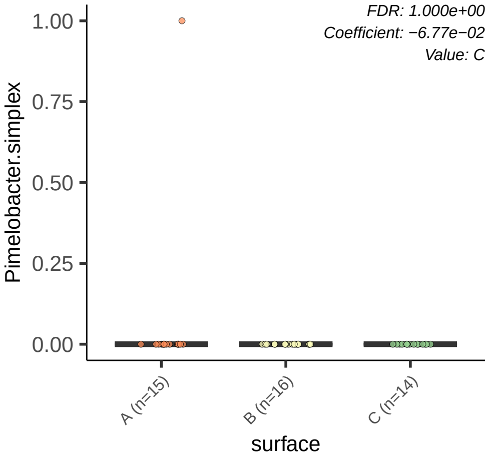

FDR: 1.000e+00
Coefficient: −6.77e−02
Value: C
Pimelobacter.simplex
1.00
0.75
0.50
0.25
0.00
A (n=15)
B (n=16)
C (n=14)
surface

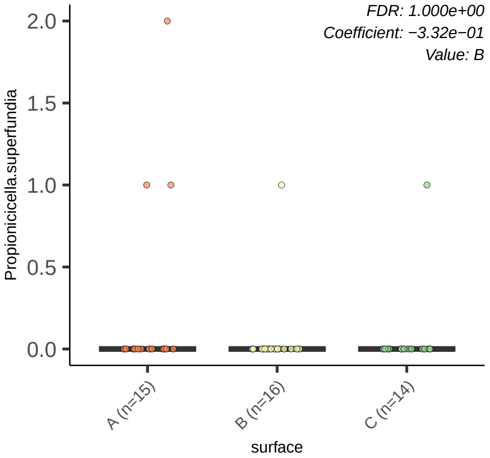

FDR: 1.000e+00
Coefficient: −3.32e−01
Value: B
Propionicicella.superfundia
2.0
1.5
1.0
0.5
0.0
A (n=15)
B (n=16)
C (n=14)
surface

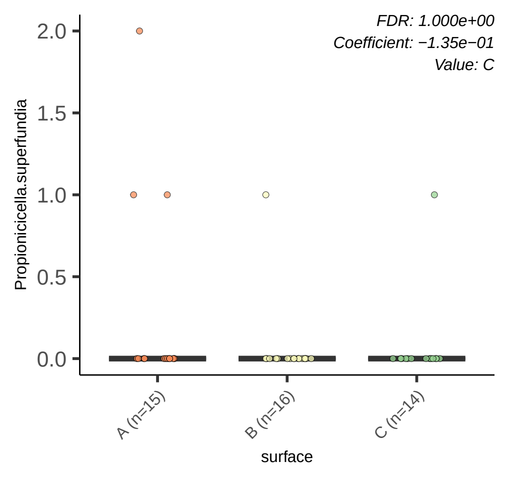
FDR: 1.000e+00
Coefficient: −1.35e−01
Value: C
Propionicicella.superfundia
2.0
1.5
1.0
0.5
0.0
A (n=15)
B (n=16)
C (n=14)
surface

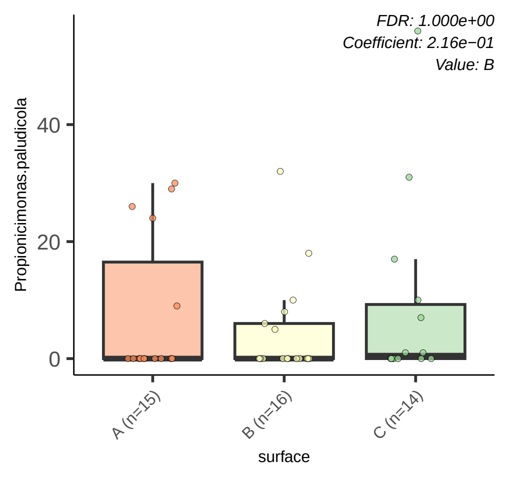

FDR: 1.000e+00
Coefficient: 2.16e−01
Value: B
Propionicimonas.paludicola
0
20
40
A (n=15)
B (n=16)
C (n=14)
surface

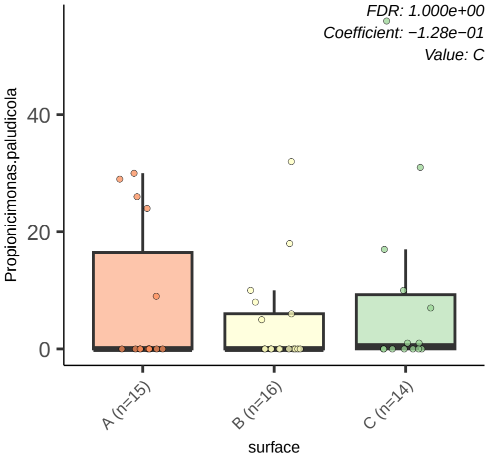

FDR: 1.000e+00
Coefficient: −1.28e−01
Value: C
Propionicimonas.paludicola
40
20
0
A (n=15)
B (n=16)
C (n=14)
surface

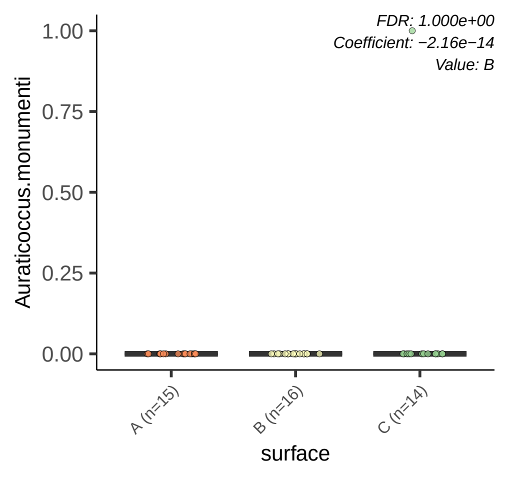

FDR: 1.000e+00
Coefficient: −2.16e−14
Value: B
Auraticoccus.monumenti
1.00
0.75
0.50
0.25
0.00
A (n=15)
B (n=16)
C (n=14)
surface

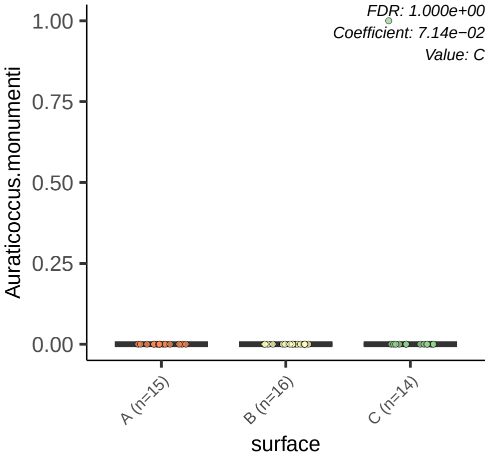
FDR: 1.000e+00
Coefficient: 7.14e−02
Value: C
Auraticoccus.monumenti
1.00
0.75
0.50
0.25
0.00
A (n=15)
B (n=16)
C (n=14)
surface

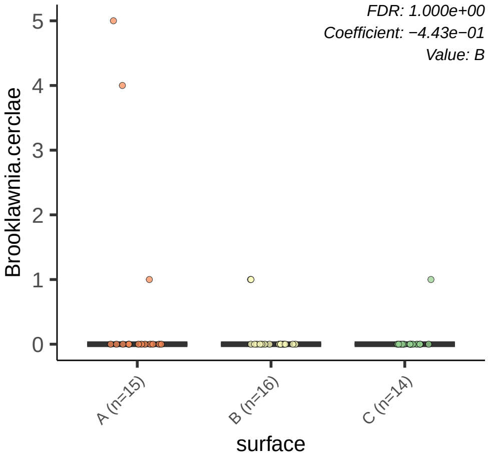

FDR: 1.000e+00
Coefficient: −4.43e−01
Value: B
Brooklawnia.cerclae
0
1
2
3
4
5
A (n=15)
B (n=16)
C (n=14)
surface

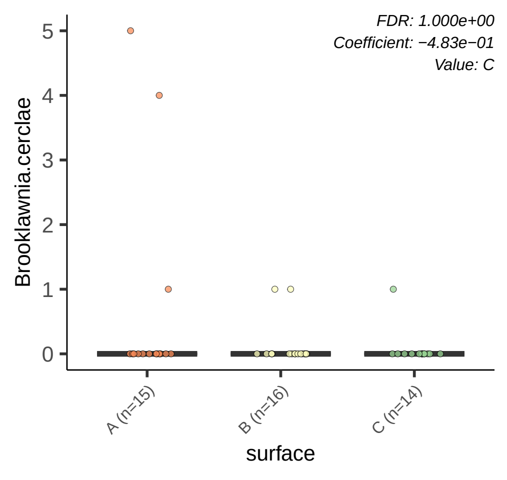

FDR: 1.000e+00
Coefficient: −4.83e−01
Value: C
Brooklawnia.cerclae
0
1
2
3
4
5
A (n=15)
B (n=16)
C (n=14)
surface

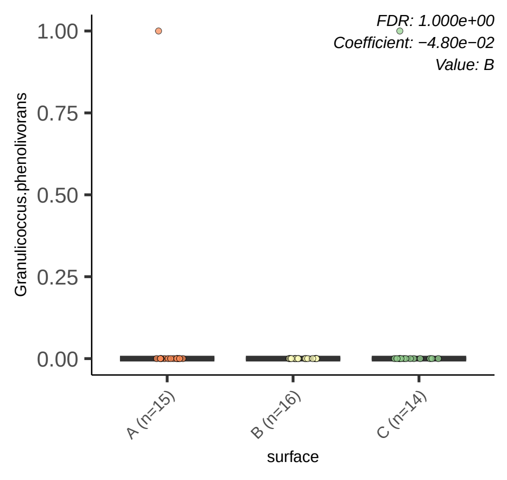
FDR: 1.000e+00
Coefficient: −4.80e−02
Value: B
Granulicoccus.phenolivorans
1.00
0.75
0.50
0.25
0.00
A (n=15)
B (n=16)
C (n=14)
surface

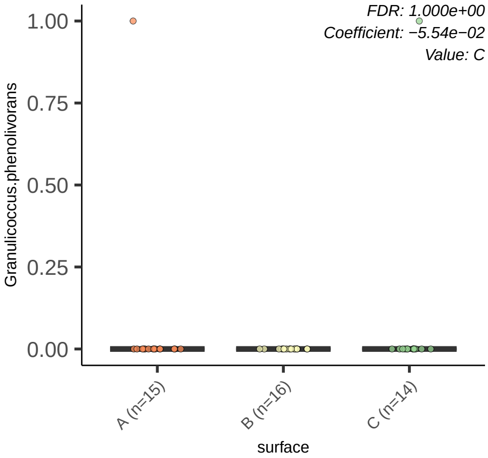

FDR: 1.000e+00
Coefficient: −5.54e−02
Value: C
Granulicoccus.phenolivorans
1.00
0.75
0.50
0.25
0.00
A (n=15)
B (n=16)
C (n=14)
surface

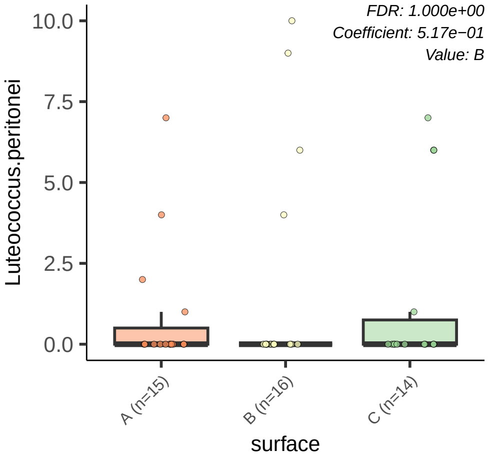
FDR: 1.000e+00
Coefficient: 5.17e−01
Value: B
Luteococcus.peritonei
10.0
7.5
5.0
2.5
0.0
A (n=15)
B (n=16)
C (n=14)
surface

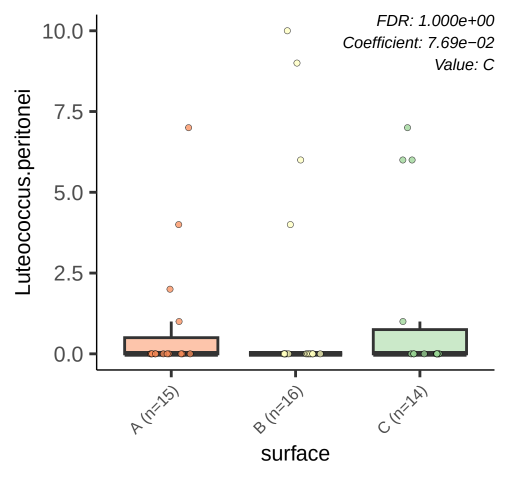
FDR: 1.000e+00
Coefficient: 7.69e−02
Value: C
Luteococcus.peritonei
10.0
7.5
5.0
2.5
0.0
A (n=15)
B (n=16)
C (n=14)
surface

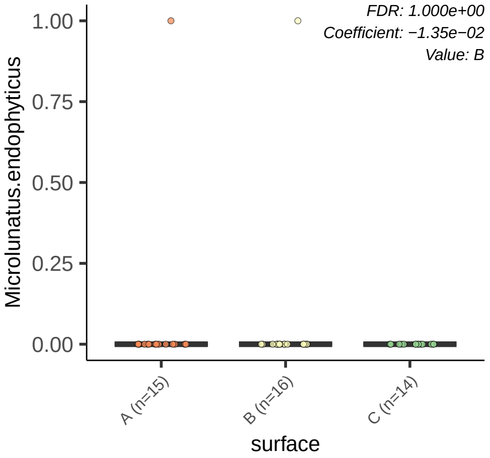

FDR: 1.000e+00
Coefficient: −1.35e−02
Value: B
Microlunatus.endophyticus
1.00
0.75
0.50
0.25
0.00
A (n=15)
B (n=16)
C (n=14)
surface

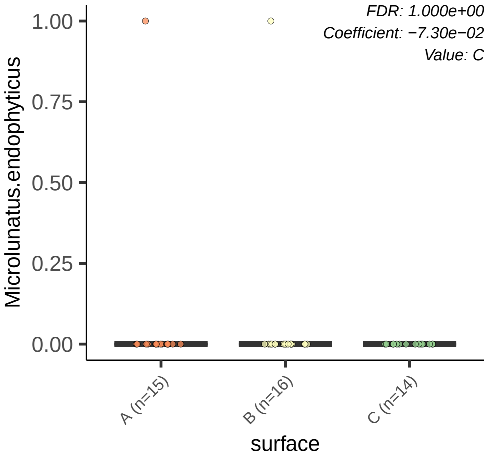
FDR: 1.000e+00
Coefficient: −7.30e−02
Value: C
Microlunatus.endophyticus
1.00
0.75
0.50
0.25
0.00
A (n=15)
B (n=16)
C (n=14)
surface

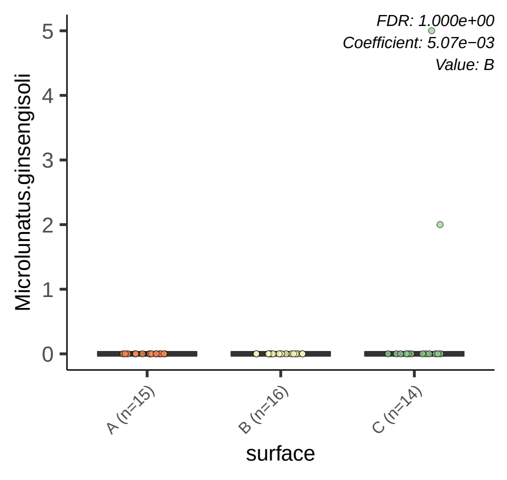
FDR: 1.000e+00
Coefficient: 5.07e−03
Value: B
Microlunatus.ginsengisoli
0
1
2
3
4
5
A (n=15)
B (n=16)
C (n=14)
surface

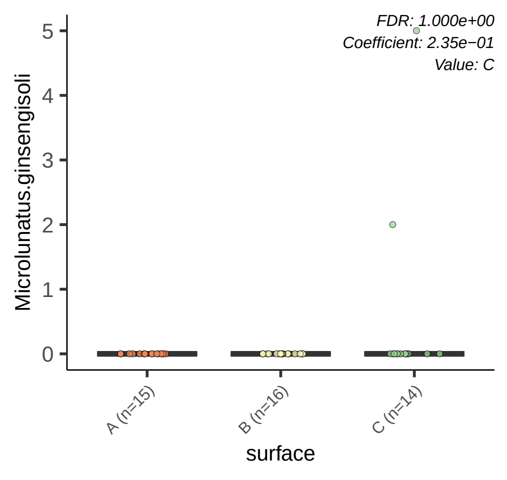

FDR: 1.000e+00
Coefficient: 2.35e−01
Value: C
Microlunatus.ginsengisoli
5
4
3
2
1
0
A (n=15)
B (n=16)
C (n=14)
surface

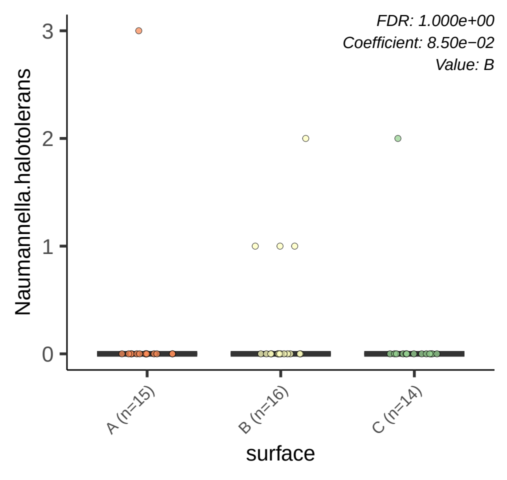
FDR: 1.000e+00
Coefficient: 8.50e−02
Value: B
Naumannella.halotolerans
3
2
1
0
A (n=15)
B (n=16)
C (n=14)
surface

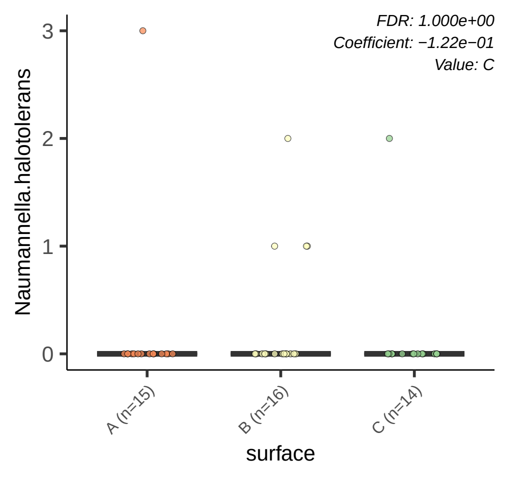
FDR: 1.000e+00
Coefficient: −1.22e−01
Value: C
Naumannella.halotolerans
3
2
1
0
A (n=15)
B (n=16)
C (n=14)
surface

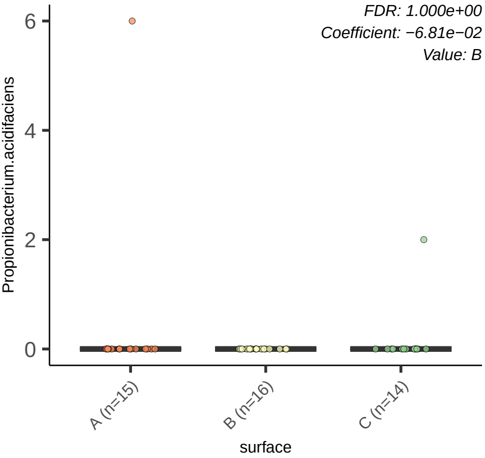
FDR: 1.000e+00
Coefficient: −6.81e−02
Value: B
Propionibacterium.acidifaciens
6
4
2
0
A (n=15)
B (n=16)
C (n=14)
surface

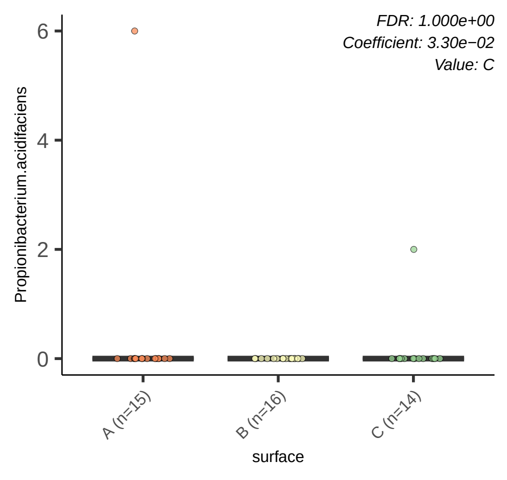
FDR: 1.000e+00
Coefficient: 3.30e−02
Value: C
Propionibacterium.acidifaciens
0
2
4
6
A (n=15)
B (n=16)
C (n=14)
surface

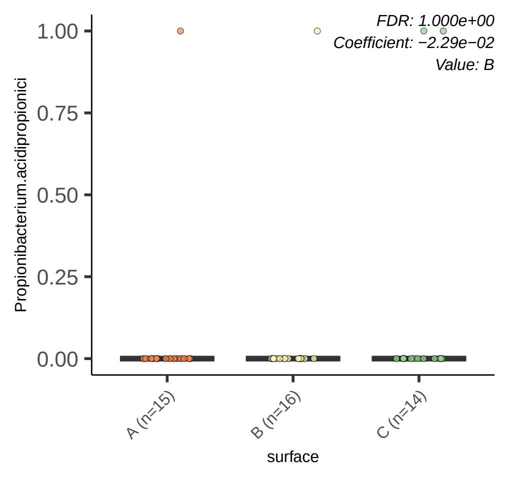
FDR: 1.000e+00
Coefficient: −2.29e−02
Value: B
Propionibacterium.acidipropionici
1.00
0.75
0.50
0.25
0.00
A (n=15)
B (n=16)
C (n=14)
surface

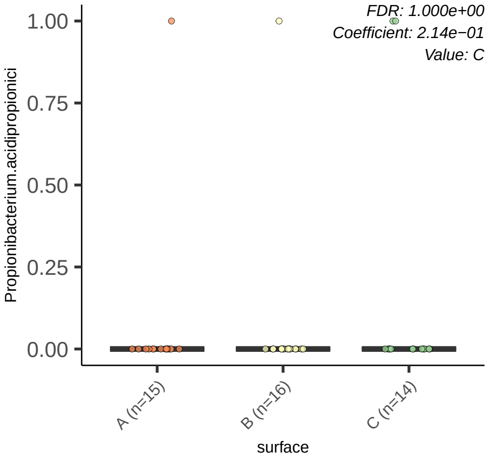

FDR: 1.000e+00
Coefficient: 2.14e−01
Value: C
Propionibacterium.acidipropionici
1.00
0.75
0.50
0.25
0.00
A (n=15)
B (n=16)
C (n=14)
surface

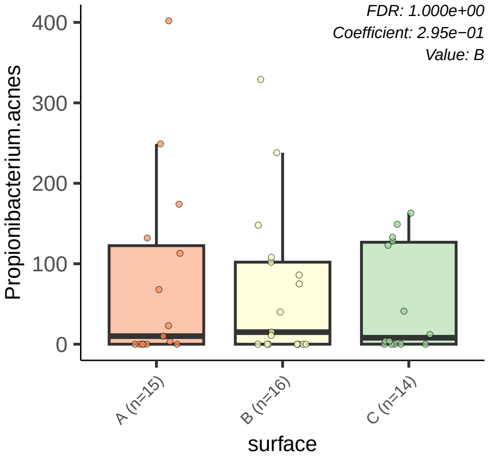

FDR: 1.000e+00
Coefficient: 2.95e−01
Value: B
Propionibacterium.acnes
0
100
200
300
400
A (n=15)
B (n=16)
C (n=14)
surface

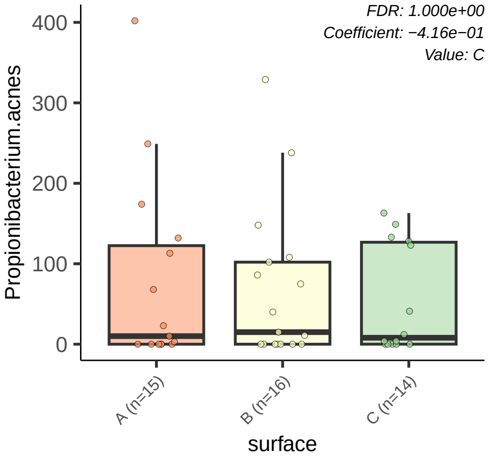
FDR: 1.000e+00
Coefficient: −4.16e−01
Value: C
Propionibacterium.acnes
400
300
200
100
0
A (n=15)
B (n=16)
C (n=14)
surface

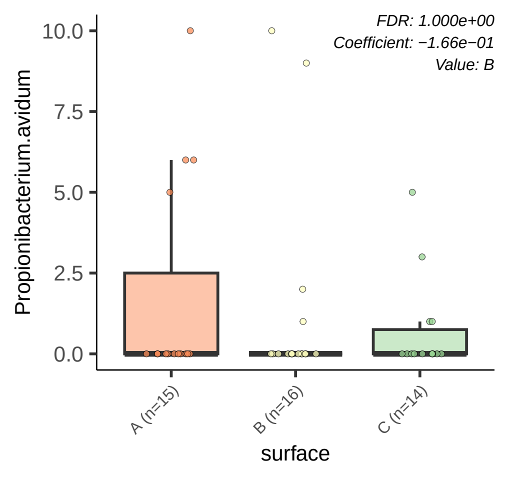
FDR: 1.000e+00
Coefficient: −1.66e−01
Value: B
Propionibacterium.avidum
10.0
7.5
5.0
2.5
0.0
A (n=15)
B (n=16)
C (n=14)
surface

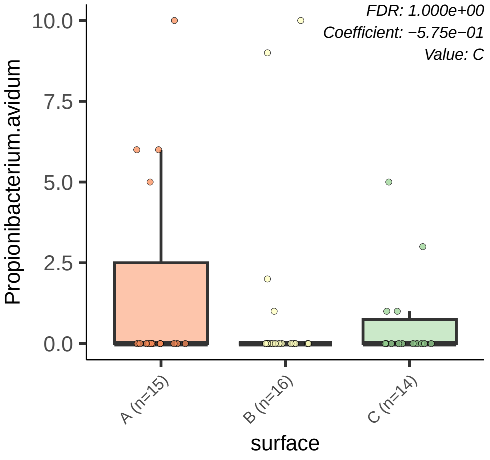
FDR: 1.000e+00
Coefficient: −5.75e−01
Value: C
Propionibacterium.avidum
10.0
7.5
5.0
2.5
0.0
A (n=15)
B (n=16)
C (n=14)
surface

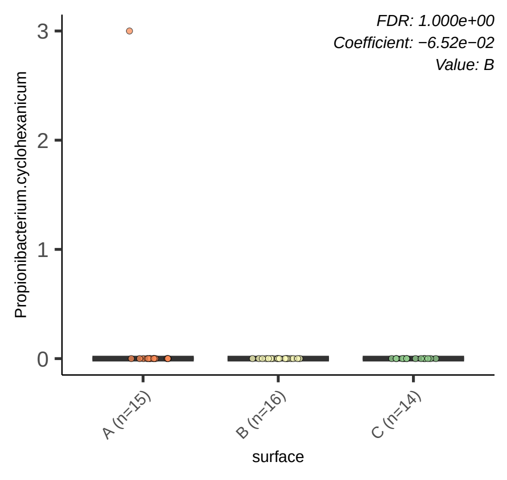

FDR: 1.000e+00
Coefficient: −6.52e−02
Value: B
Propionibacterium.cyclohexanicum
3
2
1
0
A (n=15)
B (n=16)
C (n=14)
surface

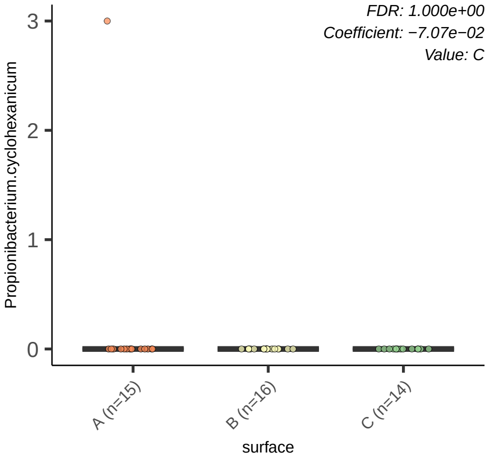
FDR: 1.000e+00
Coefficient: −7.07e−02
Value: C
Propionibacterium.cyclohexanicum
3
2
1
0
A (n=15)
B (n=16)
C (n=14)
surface

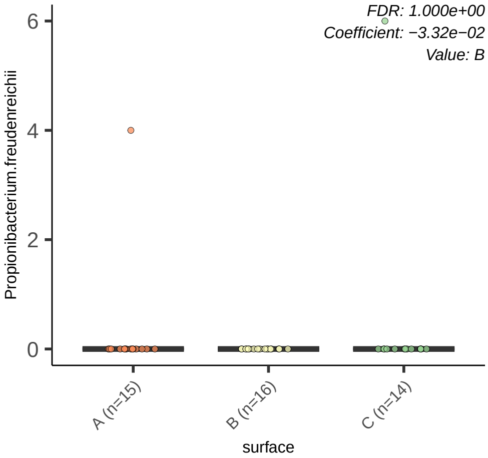
FDR: 1.000e+00
Coefficient: −3.32e−02
Value: B
Propionibacterium.freudenreichii
6
4
2
0
A (n=15)
B (n=16)
C (n=14)
surface

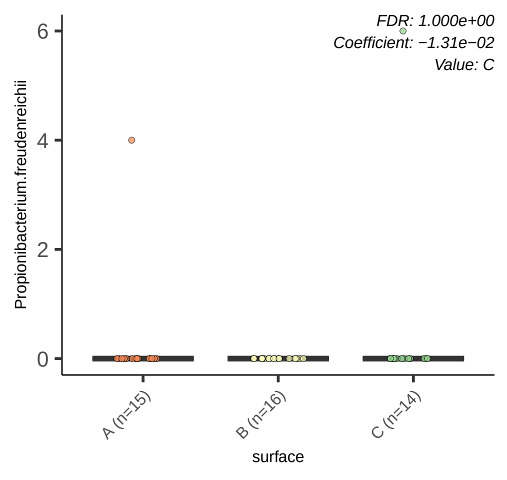
FDR: 1.000e+00
Coefficient: −1.31e−02
Value: C
Propionibacterium.freudenreichii
0
2
4
6
A (n=15)
B (n=16)
C (n=14)
surface

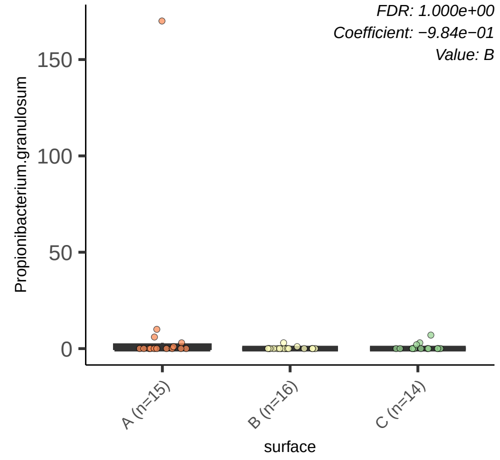
FDR: 1.000e+00
Coefficient: −9.84e−01
Value: B
Propionibacterium.granulosum
150
100
50
0
A (n=15)
B (n=16)
C (n=14)
surface

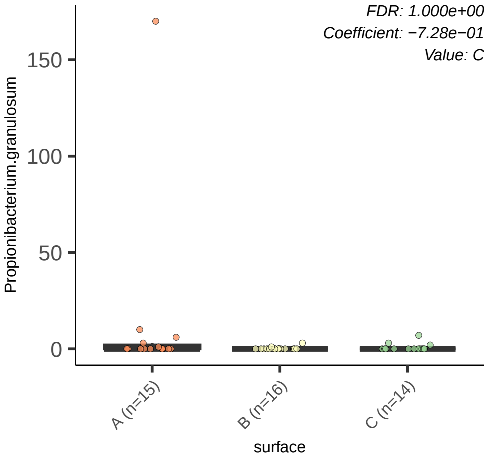
FDR: 1.000e+00
Coefficient: −7.28e−01
Value: C
Propionibacterium.granulosum
150
100
50
0
A (n=15)
B (n=16)
C (n=14)
surface

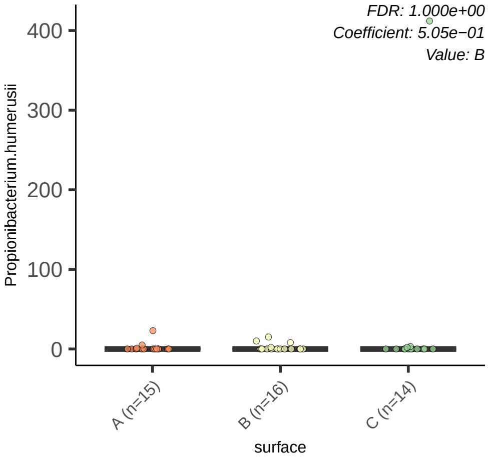

FDR: 1.000e+00
Coefficient: 5.05e−01
Value: B
400
300
200
100
0
Propionibacterium.humerusii
A (n=15)
B (n=16)
C (n=14)
surface

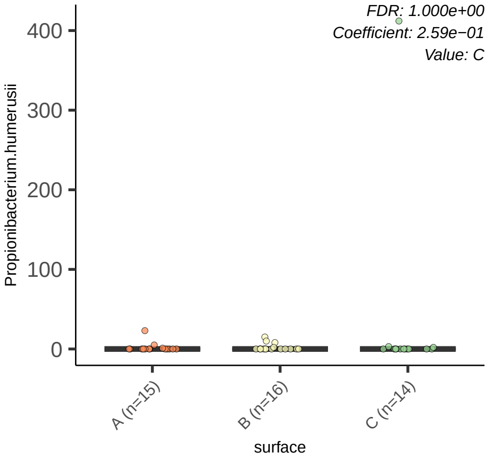
FDR: 1.000e+00
Coefficient: 2.59e−01
Value: C
Propionibacterium.humerusii
400
300
200
100
0
A (n=15)
B (n=16)
C (n=14)
surface

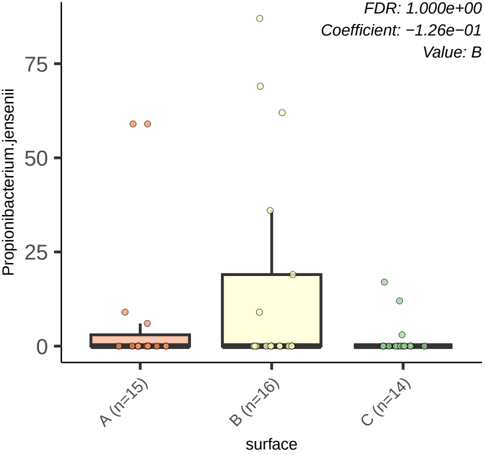

FDR: 1.000e+00
Coefficient: −1.26e−01
Value: B
Propionibacterium.jensenii
75
50
25
0
A (n=15)
B (n=16)
C (n=14)
surface

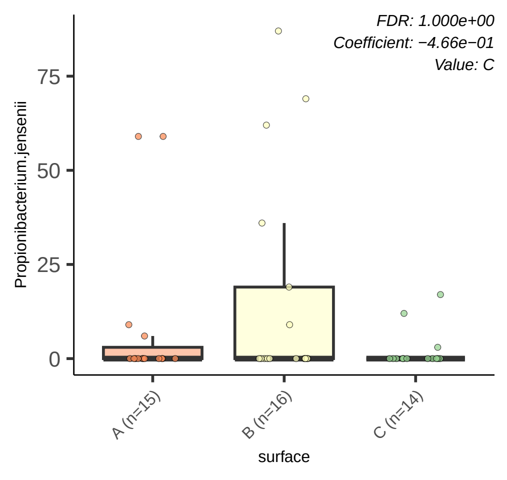
FDR: 1.000e+00
Coefficient: −4.66e−01
Value: C
Propionibacterium.jensenii
75
50
25
0
A (n=15)
B (n=16)
C (n=14)
surface

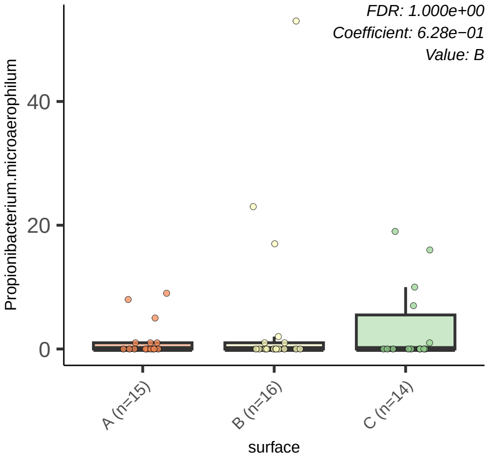

FDR: 1.000e+00
Coefficient: 6.28e−01
Value: B
Propionibacterium.microaerophilum
40
20
0
A (n=15)
B (n=16)
C (n=14)
surface

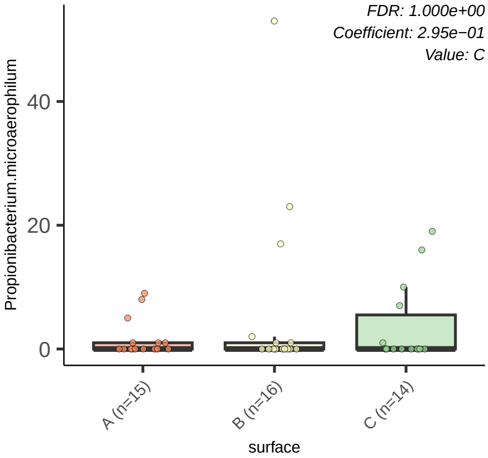
FDR: 1.000e+00
Coefficient: 2.95e−01
Value: C
Propionibacterium.microaerophilum
40
20
0
A (n=15)
B (n=16)
C (n=14)
surface

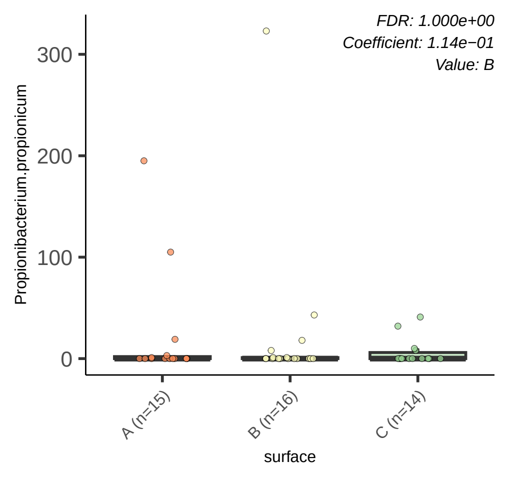
FDR: 1.000e+00
Coefficient: 1.14e−01
Value: B
300
200
100
0
Propionibacterium.propionicum
A (n=15)
B (n=16)
C (n=14)
surface

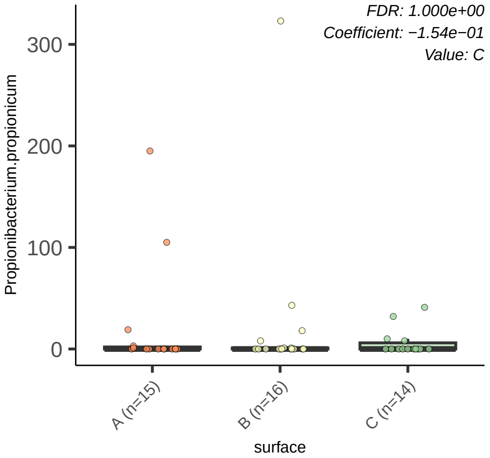

FDR: 1.000e+00
Coefficient: −1.54e−01
Value: C
Propionibacterium.propionicum
300
200
100
0
A (n=15)
B (n=16)
C (n=14)
surface

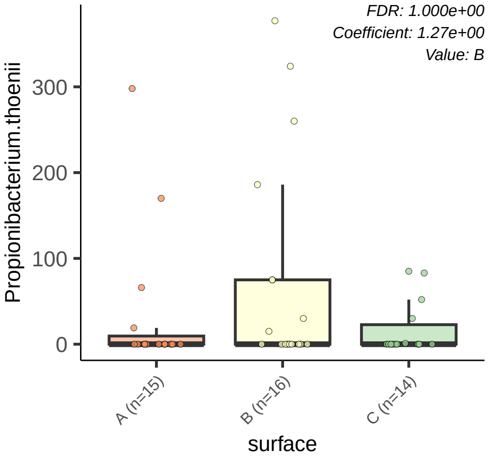
FDR: 1.000e+00
Coefficient: 1.27e+00
Value: B
Propionibacterium.thoenii
300
200
100
0
A (n=15)
B (n=16)
C (n=14)
surface

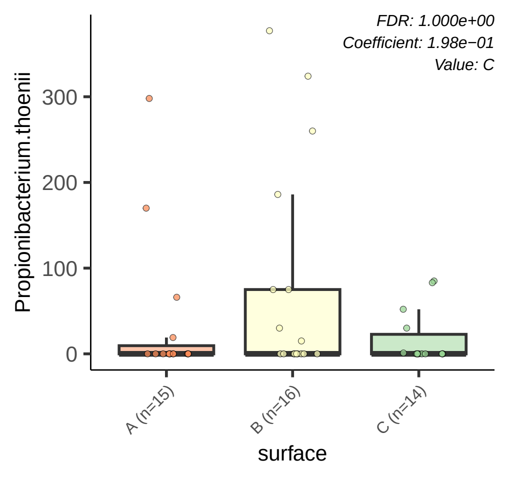
FDR: 1.000e+00
Coefficient: 1.98e−01
Value: C
Propionibacterium.thoenii
0
100
200
300
A (n=15)
B (n=16)
C (n=14)
surface

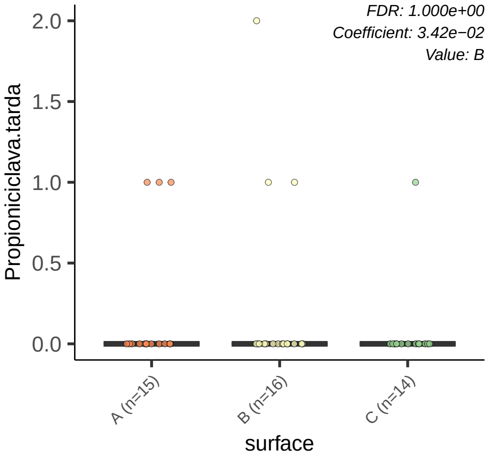
FDR: 1.000e+00
Coefficient: 3.42e−02
Value: B
Propioniciclava.tarda
2.0
1.5
1.0
0.5
0.0
A (n=15)
B (n=16)
C (n=14)
surface

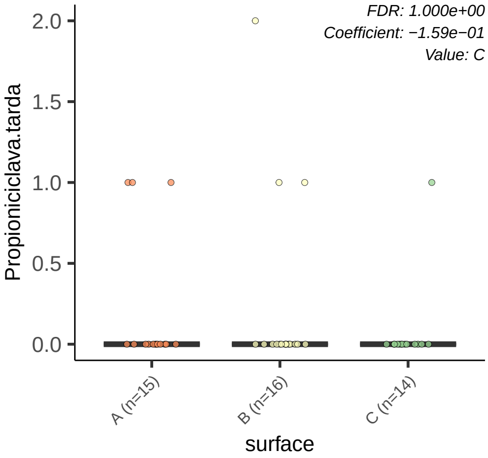

FDR: 1.000e+00
Coefficient: −1.59e−01
Value: C
Propioniciclava.tarda
2.0
1.5
1.0
0.5
0.0
A (n=15)
B (n=16)
C (n=14)
surface

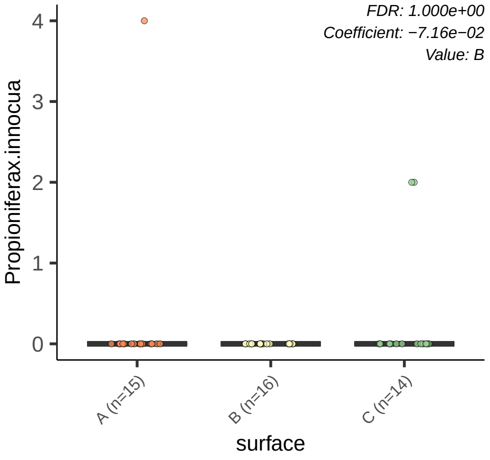

FDR: 1.000e+00
Coefficient: −7.16e−02
Value: B
Propioniferax.innocua
0
1
2
3
4
A (n=15)
B (n=16)
C (n=14)
surface

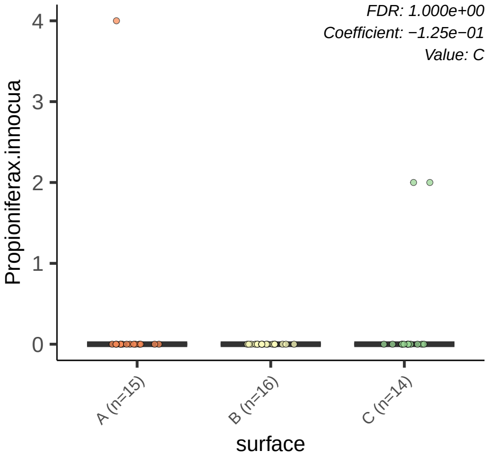

FDR: 1.000e+00
Coefficient: −1.25e−01
Value: C
Propioniferax.innocua
0
1
2
3
4
A (n=15)
B (n=16)
C (n=14)
surface

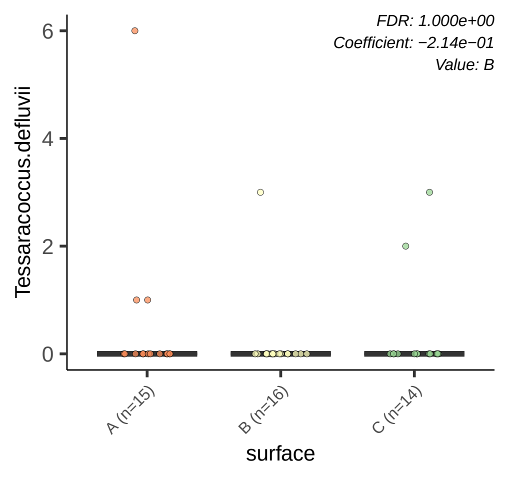

FDR: 1.000e+00
Coefficient: −2.14e−01
Value: B
Tessaracoccus.defluvii
0
2
4
6
A (n=15)
B (n=16)
C (n=14)
surface

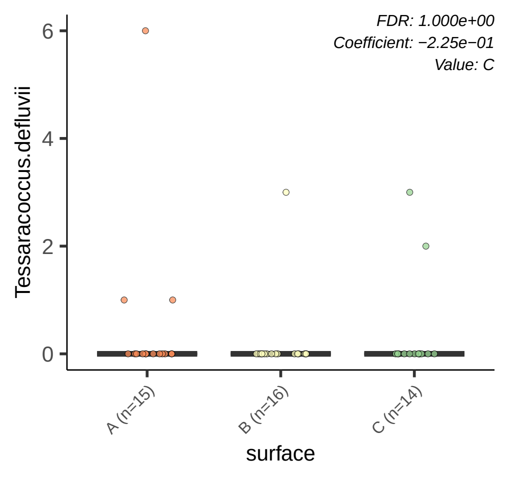
FDR: 1.000e+00
Coefficient: −2.25e−01
Value: C
Tessaracoccus.defluvii
6
4
2
0
A (n=15)
B (n=16)
C (n=14)
surface

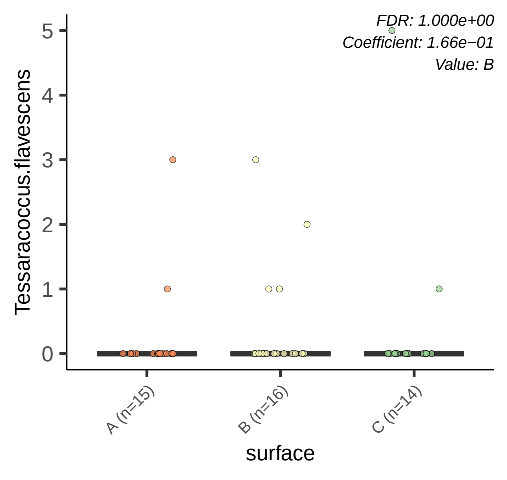

FDR: 1.000e+00
Coefficient: 1.66e−01
Value: B
Tessaracoccus.flavescens
0
1
2
3
4
5
A (n=15)
B (n=16)
C (n=14)
surface

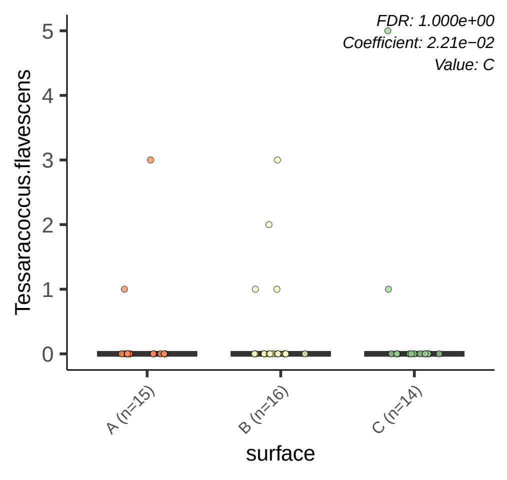

FDR: 1.000e+00
Coefficient: 2.21e−02
Value: C
Tessaracoccus.flavescens
0
1
2
3
4
5
A (n=15)
B (n=16)
C (n=14)
surface

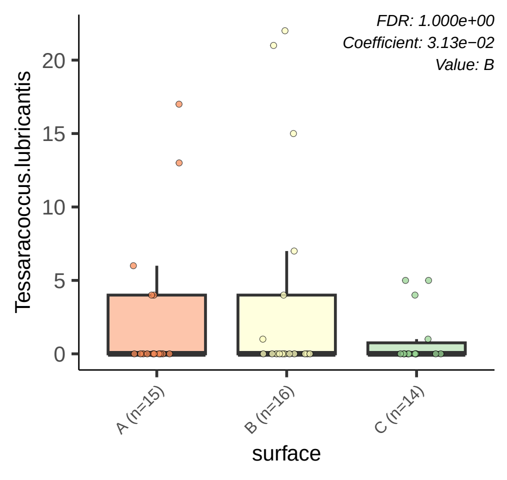

FDR: 1.000e+00
Coefficient: 3.13e−02
Value: B
Tessaracoccus.lubricantis
0
5
10
15
20
A (n=15)
B (n=16)
C (n=14)
surface

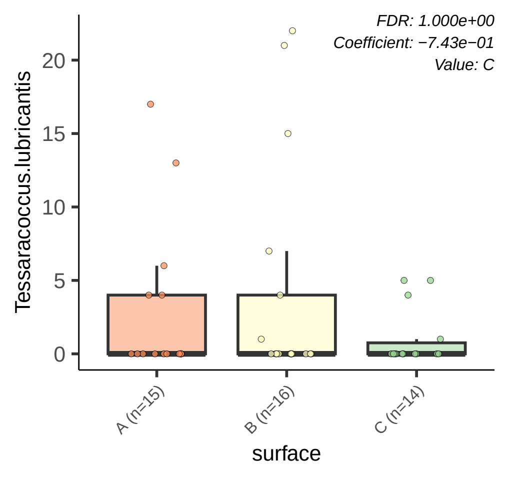

FDR: 1.000e+00
Coefficient: −7.43e−01
Value: C
Tessaracoccus.lubricantis
0
5
10
15
20
A (n=15)
B (n=16)
C (n=14)
surface

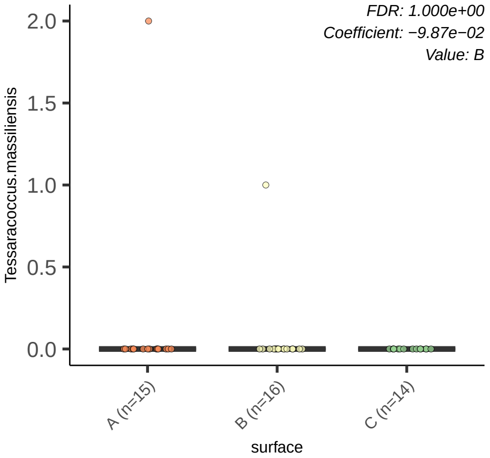
FDR: 1.000e+00
Coefficient: −9.87e−02
Value: B
Tessaracoccus.massiliensis
2.0
1.5
1.0
0.5
0.0
A (n=15)
B (n=16)
C (n=14)
surface

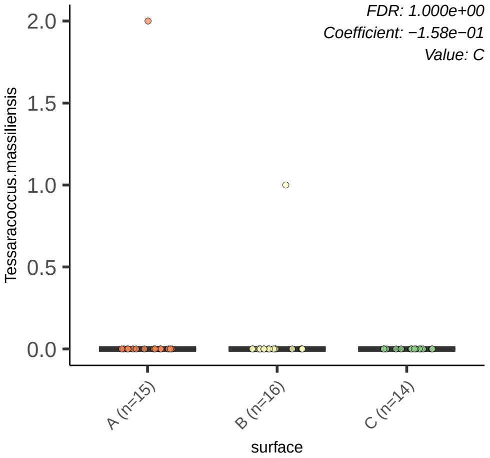
FDR: 1.000e+00
Coefficient: −1.58e−01
Value: C
Tessaracoccus.massiliensis
2.0
1.5
1.0
0.5
0.0
A (n=15)
B (n=16)
C (n=14)
surface

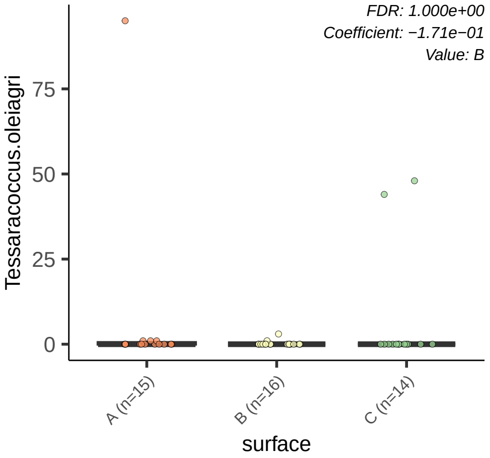

FDR: 1.000e+00
Coefficient: −1.71e−01
Value: B
Tessaracoccus.oleiagri
75
50
25
0
A (n=15)
B (n=16)
C (n=14)
surface

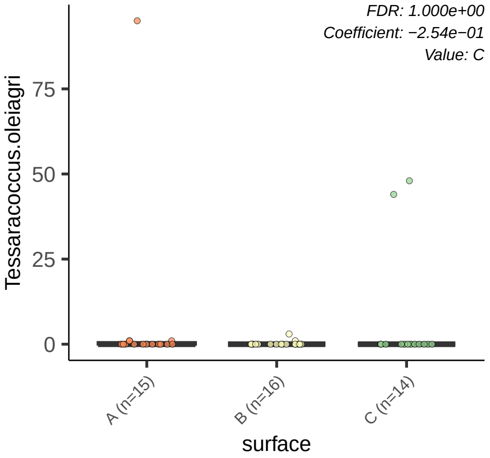

FDR: 1.000e+00
Coefficient: −2.54e−01
Value: C
Tessaracoccus.oleiagri
0
25
50
75
A (n=15)
B (n=16)
C (n=14)
surface

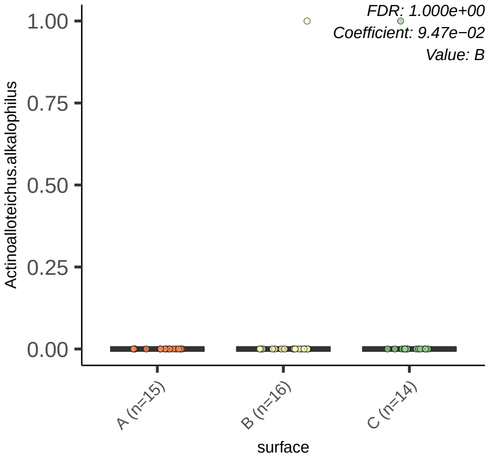

FDR: 1.000e+00
Coefficient: 9.47e−02
Value: B
Actinoalloteichus.alkalophilus
1.00
0.75
0.50
0.25
0.00
A (n=15)
B (n=16)
C (n=14)
surface

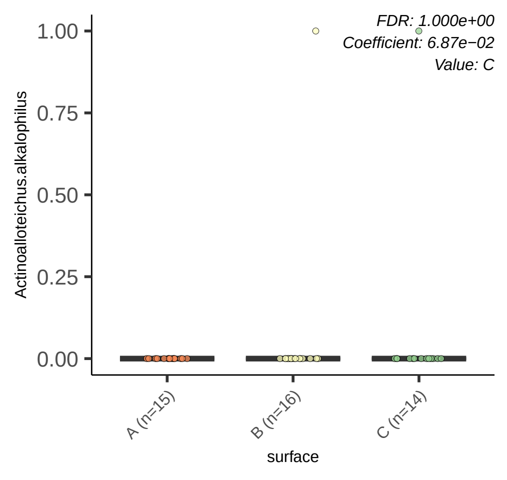
FDR: 1.000e+00
Coefficient: 6.87e−02
Value: C
Actinoalloteichus.alkalophilus
1.00
0.75
0.50
0.25
0.00
A (n=15)
B (n=16)
C (n=14)
surface

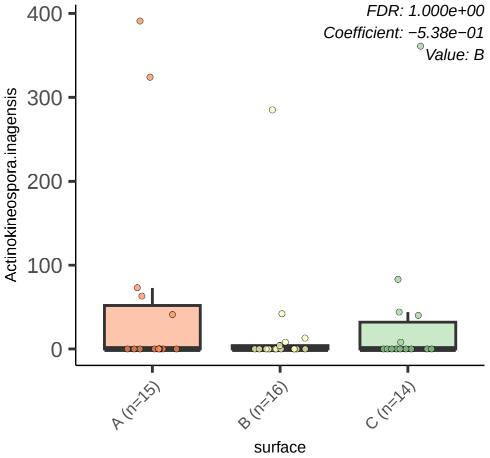

FDR: 1.000e+00
Coefficient: −5.38e−01
Value: B
400
300
200
100
0
Actinokineospora.inagensis
A (n=15)
B (n=16)
C (n=14)
surface

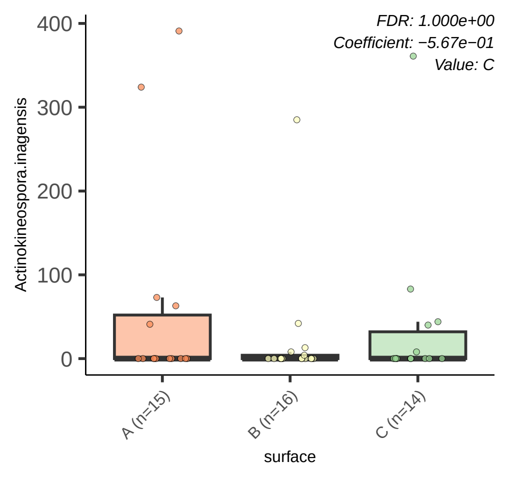

FDR: 1.000e+00
Coefficient: −5.67e−01
Value: C
Actinokineospora.inagensis
400
300
200
100
0
A (n=15)
B (n=16)
C (n=14)
surface

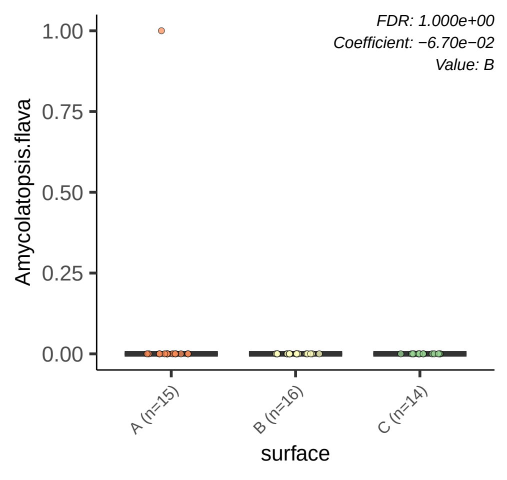

FDR: 1.000e+00
Coefficient: −6.70e−02
Value: B
Amycolatopsis.flava
1.00
0.75
0.50
0.25
0.00
A (n=15)
B (n=16)
C (n=14)
surface

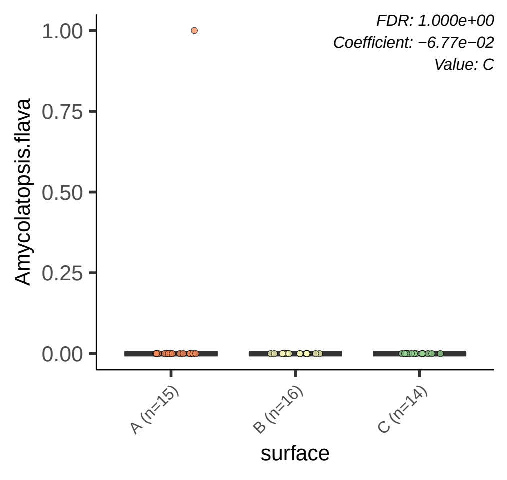
FDR: 1.000e+00
Coefficient: −6.77e−02
Value: C
Amycolatopsis.flava
1.00
0.75
0.50
0.25
0.00
A (n=15)
B (n=16)
C (n=14)
surface

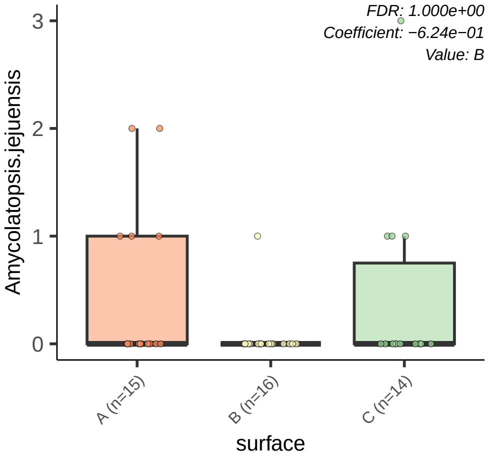
FDR: 1.000e+00
Coefficient: −6.24e−01
Value: B
Amycolatopsis.jejuensis
3
2
1
0
A (n=15)
B (n=16)
C (n=14)
surface

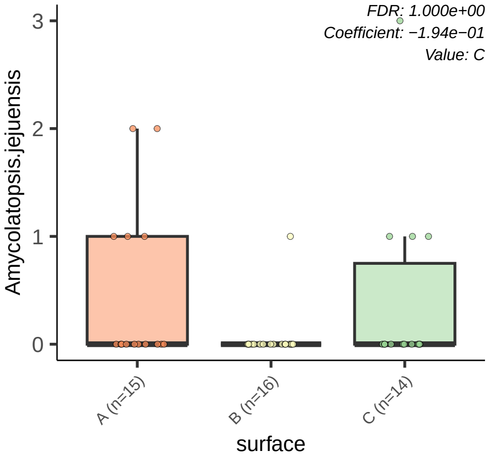
FDR: 1.000e+00
Coefficient: −1.94e−01
Value: C
Amycolatopsis.jejuensis
0
1
2
3
A (n=15)
B (n=16)
C (n=14)
surface

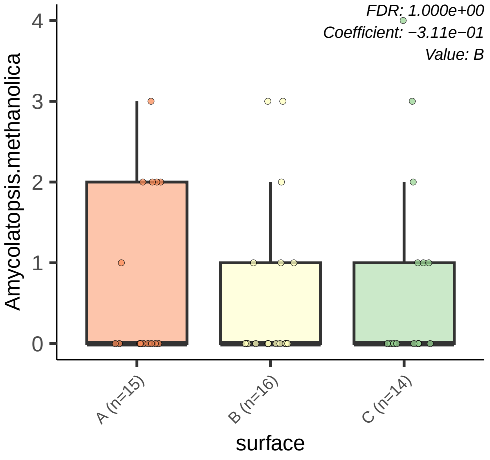
FDR: 1.000e+00
Coefficient: −3.11e−01
Value: B
Amycolatopsis.methanolica
0
1
2
3
4
A (n=15)
B (n=16)
C (n=14)
surface

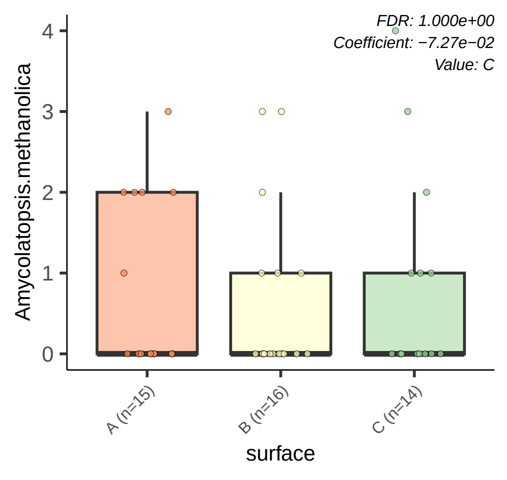
FDR: 1.000e+00
Coefficient: −7.27e−02
Value: C
Amycolatopsis.methanolica
0
1
2
3
4
A (n=15)
B (n=16)
C (n=14)
surface

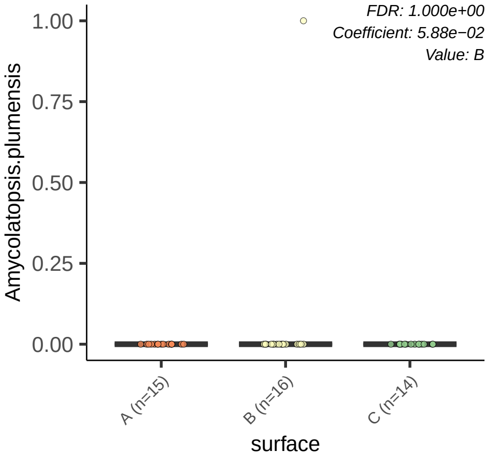
FDR: 1.000e+00
Coefficient: 5.88e−02
Value: B
Amycolatopsis.plumensis
1.00
0.75
0.50
0.25
0.00
A (n=15)
B (n=16)
C (n=14)
surface

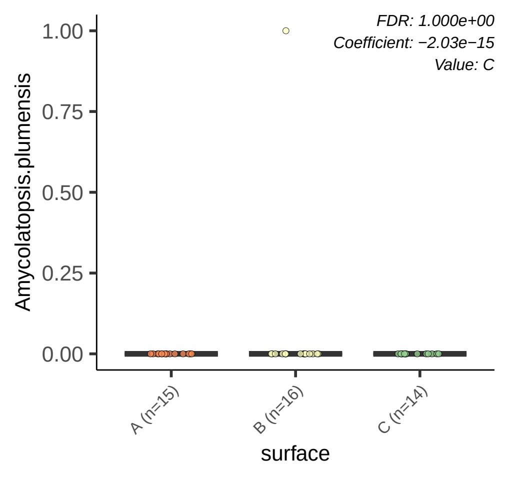

FDR: 1.000e+00
Coefficient: −2.03e−15
Value: C
Amycolatopsis.plumensis
1.00
0.75
0.50
0.25
0.00
A (n=15)
B (n=16)
C (n=14)
surface

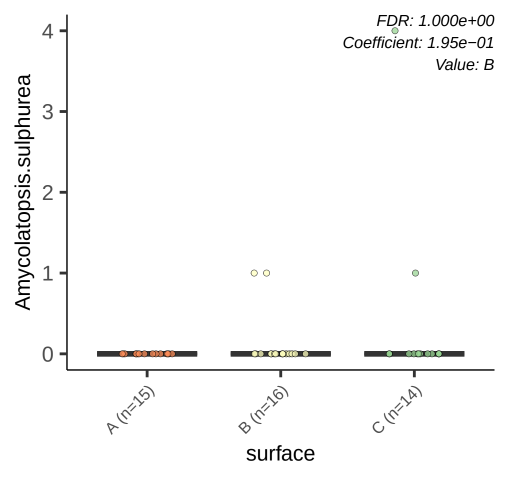

FDR: 1.000e+00
Coefficient: 1.95e−01
Value: B
Amycolatopsis.sulphurea
0
1
2
3
4
A (n=15)
B (n=16)
C (n=14)
surface

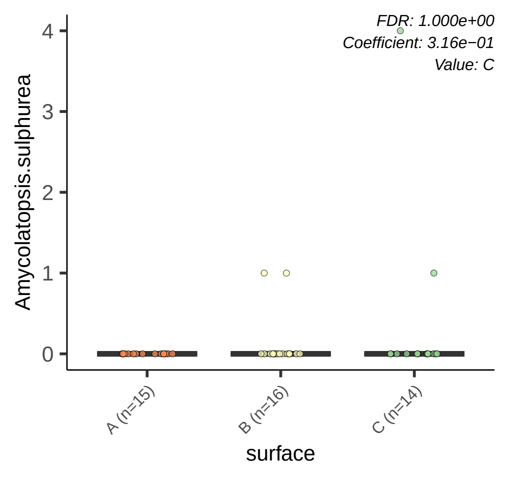
FDR: 1.000e+00
Coefficient: 3.16e−01
Value: C
Amycolatopsis.sulphurea
0
1
2
3
4
A (n=15)
B (n=16)
C (n=14)
surface

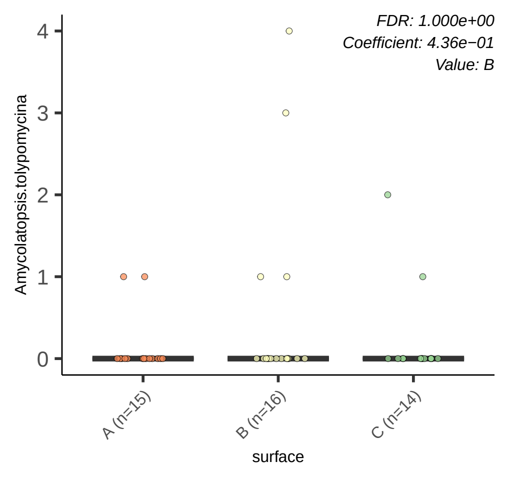

FDR: 1.000e+00
Coefficient: 4.36e−01
Value: B
Amycolatopsis.tolypomycina
4
3
2
1
0
A (n=15)
B (n=16)
C (n=14)
surface

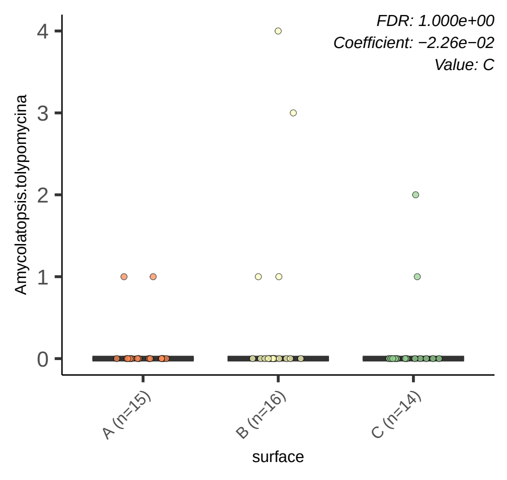
FDR: 1.000e+00
Coefficient: −2.26e−02
Value: C
Amycolatopsis.tolypomycina
4
3
2
1
0
A (n=15)
B (n=16)
C (n=14)
surface

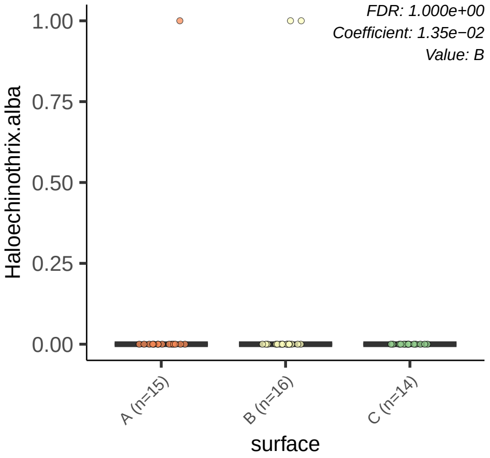

FDR: 1.000e+00
Coefficient: 1.35e−02
Value: B
Haloechinothrix.alba
1.00
0.75
0.50
0.25
0.00
A (n=15)
B (n=16)
C (n=14)
surface

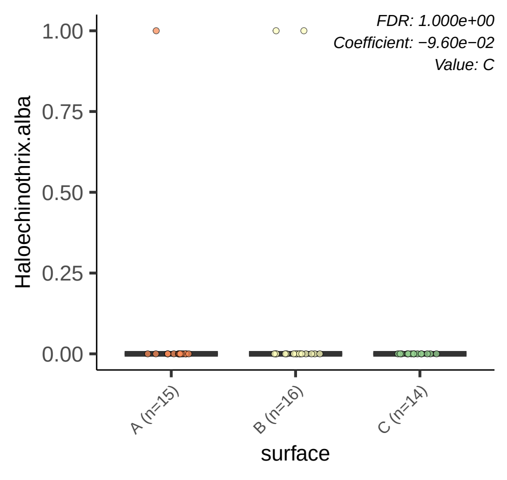
FDR: 1.000e+00
Coefficient: −9.60e−02
Value: C
Haloechinothrix.alba
1.00
0.75
0.50
0.25
0.00
A (n=15)
B (n=16)
C (n=14)
surface

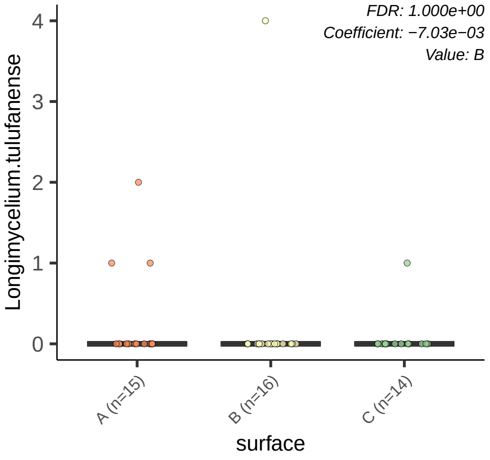
FDR: 1.000e+00
Coefficient: −7.03e−03
Value: B
Longimycelium.tulufanense
0
1
2
3
4
A (n=15)
B (n=16)
C (n=14)
surface

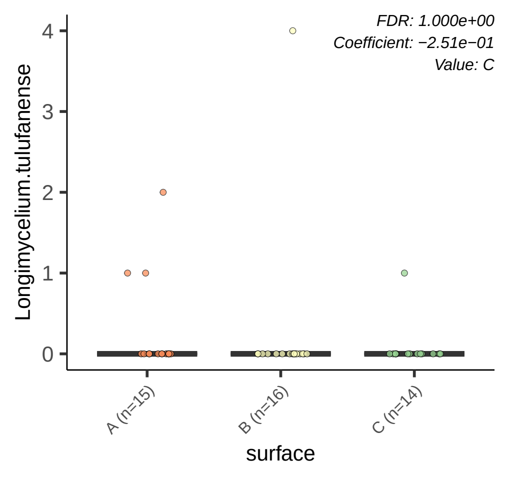
FDR: 1.000e+00
Coefficient: −2.51e−01
Value: C
Longimycelium.tulufanense
0
1
2
3
4
A (n=15)
B (n=16)
C (n=14)
surface

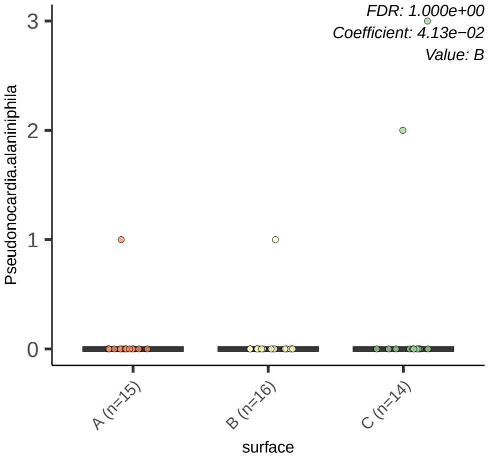

FDR: 1.000e+00
Coefficient: 4.13e−02
Value: B
Pseudonocardia.alaniniphila
3
2
1
0
A (n=15)
B (n=16)
C (n=14)
surface

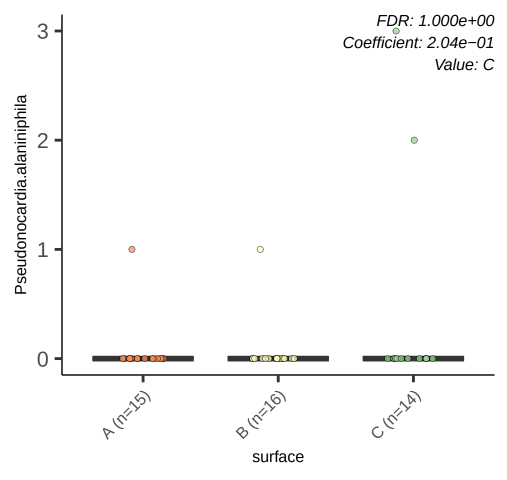
FDR: 1.000e+00
Coefficient: 2.04e−01
Value: C
Pseudonocardia.alaniniphila
3
2
1
0
A (n=15)
B (n=16)
C (n=14)
surface

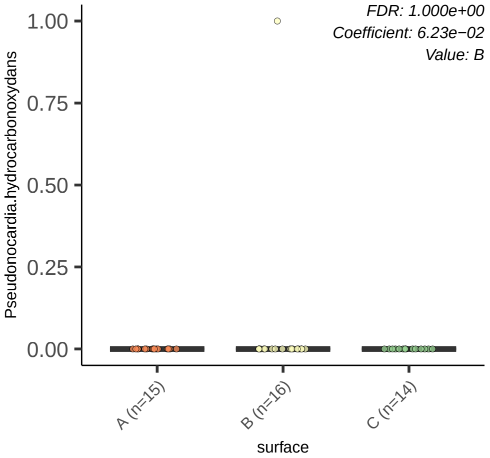
FDR: 1.000e+00
Coefficient: 6.23e−02
Value: B
Pseudonocardia.hydrocarbonoxydans
1.00
0.75
0.50
0.25
0.00
A (n=15)
B (n=16)
C (n=14)
surface

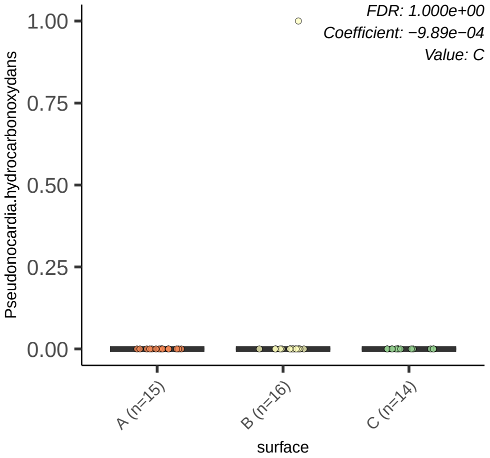
FDR: 1.000e+00
Coefficient: −9.89e−04
Value: C
1.00
0.75
0.50
0.25
0.00
Pseudonocardia.hydrocarbonoxydans
A (n=15)
B (n=16)
C (n=14)
surface

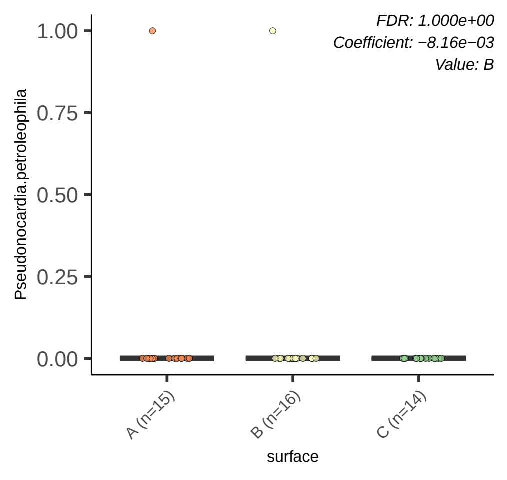

FDR: 1.000e+00
Coefficient: −8.16e−03
Value: B
Pseudonocardia.petroleophila
1.00
0.75
0.50
0.25
0.00
A (n=15)
B (n=16)
C (n=14)
surface

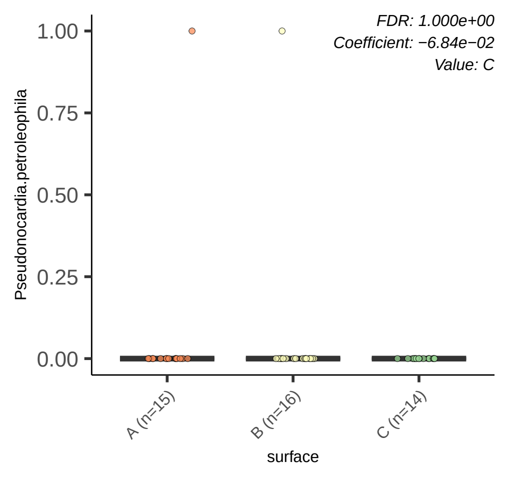
FDR: 1.000e+00
Coefficient: −6.84e−02
Value: C
Pseudonocardia.petroleophila
1.00
0.75
0.50
0.25
0.00
A (n=15)
B (n=16)
C (n=14)
surface

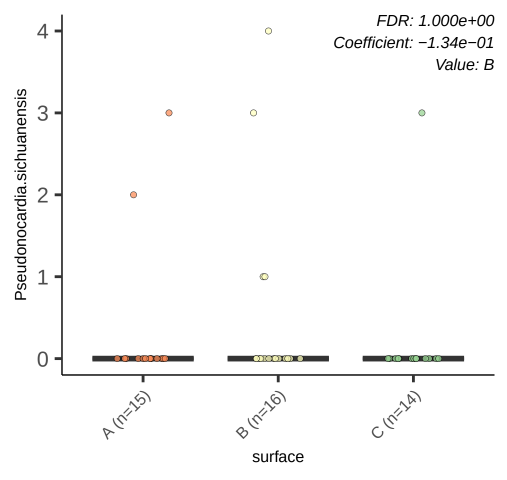
FDR: 1.000e+00
Coefficient: −1.34e−01
Value: B
Pseudonocardia.sichuanensis
4
3
2
1
0
A (n=15)
B (n=16)
C (n=14)
surface

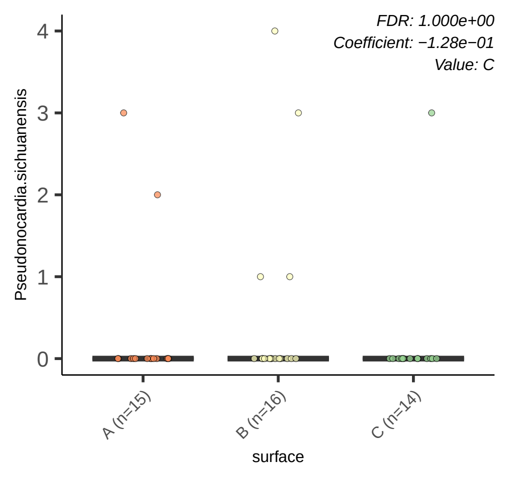
FDR: 1.000e+00
Coefficient: −1.28e−01
Value: C
Pseudonocardia.sichuanensis
0
1
2
3
4
A (n=15)
B (n=16)
C (n=14)
surface

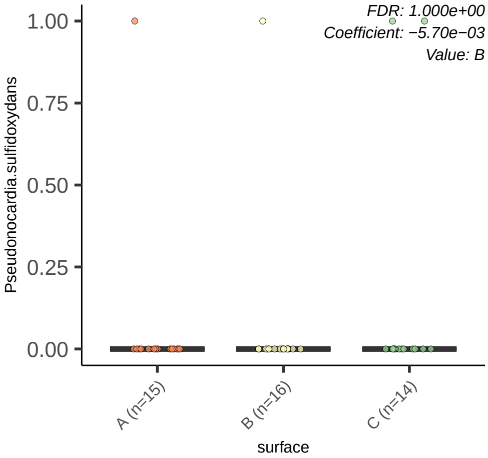

FDR: 1.000e+00
Coefficient: −5.70e−03
Value: B
Pseudonocardia.sulfidoxydans
1.00
0.75
0.50
0.25
0.00
A (n=15)
B (n=16)
C (n=14)
surface

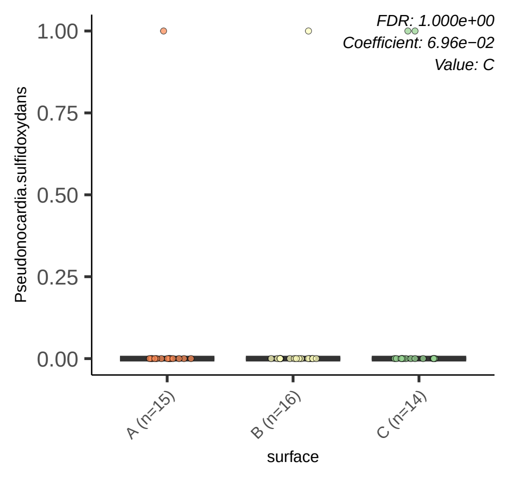
FDR: 1.000e+00
Coefficient: 6.96e−02
Value: C
Pseudonocardia.sulfidoxydans
1.00
0.75
0.50
0.25
0.00
A (n=15)
B (n=16)
C (n=14)
surface

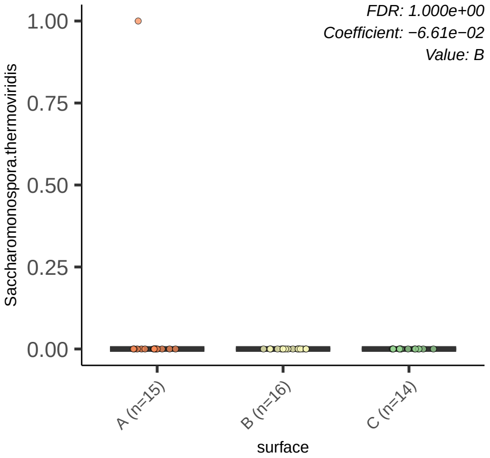
FDR: 1.000e+00
Coefficient: −6.61e−02
Value: B
Saccharomonospora.thermoviridis
1.00
0.75
0.50
0.25
0.00
A (n=15)
B (n=16)
C (n=14)
surface

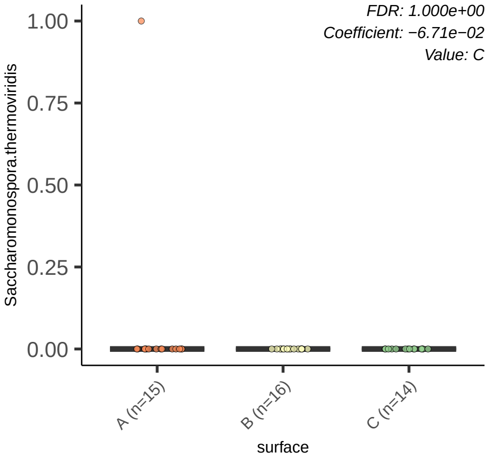

FDR: 1.000e+00
Coefficient: −6.71e−02
Value: C
Saccharomonospora.thermoviridis
1.00
0.75
0.50
0.25
0.00
A (n=15)
B (n=16)
C (n=14)
surface

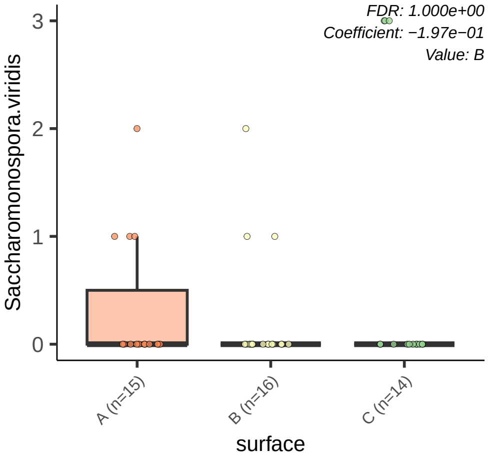

FDR: 1.000e+00
Coefficient: −1.97e−01
Value: B
Saccharomonospora.viridis
0
1
2
3
A (n=15)
B (n=16)
C (n=14)
surface

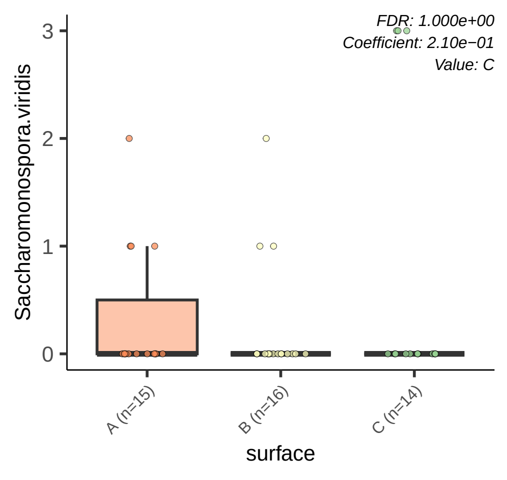

FDR: 1.000e+00
Coefficient: 2.10e−01
Value: C
Saccharomonospora.viridis
0
1
2
3
A (n=15)
B (n=16)
C (n=14)
surface

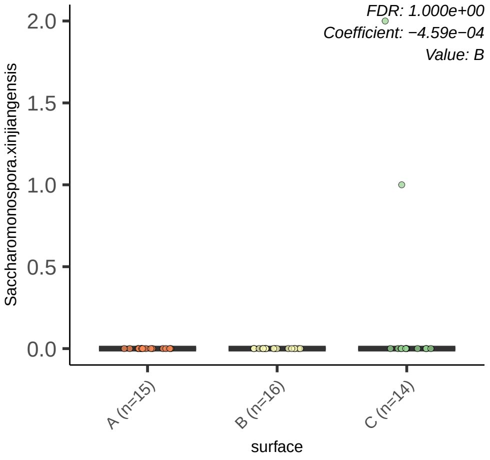

FDR: 1.000e+00
Coefficient: −4.59e−04
Value: B
Saccharomonospora.xinjiangensis
2.0
1.5
1.0
0.5
0.0
A (n=15)
B (n=16)
C (n=14)
surface

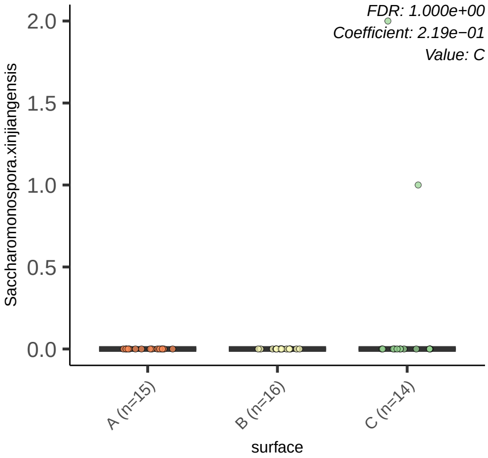

FDR: 1.000e+00
Coefficient: 2.19e−01
Value: C
Saccharomonospora.xinjiangensis
2.0
1.5
1.0
0.5
0.0
A (n=15)
B (n=16)
C (n=14)
surface

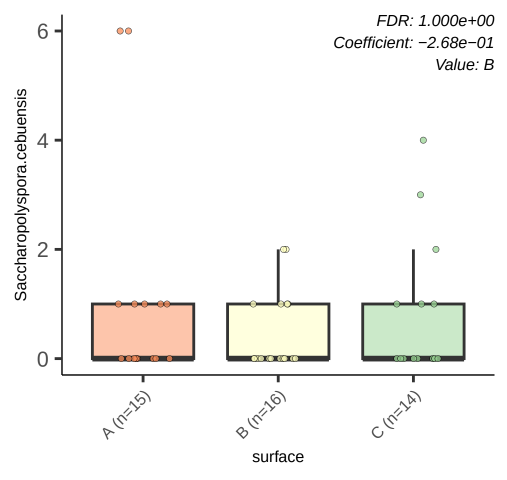
FDR: 1.000e+00
Coefficient: −2.68e−01
Value: B
Saccharopolyspora.cebuensis
0
2
4
6
A (n=15)
B (n=16)
C (n=14)
surface

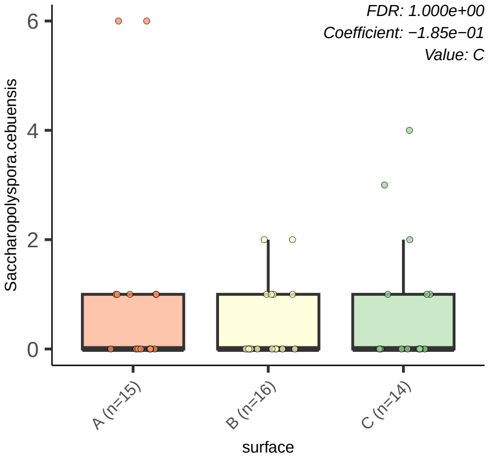
FDR: 1.000e+00
Coefficient: −1.85e−01
Value: C
Saccharopolyspora.cebuensis
6
4
2
0
A (n=15)
B (n=16)
C (n=14)
surface

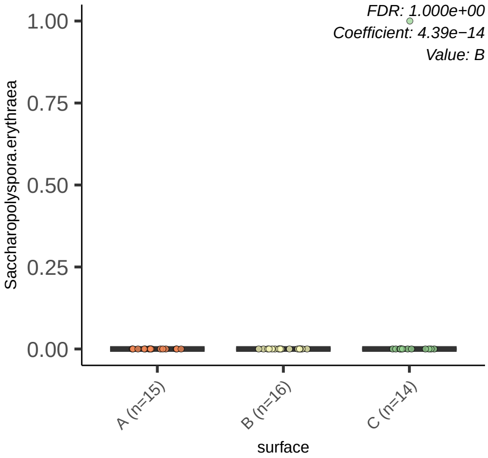
FDR: 1.000e+00
Coefficient: 4.39e−14
Value: B
Saccharopolyspora.erythraea
1.00
0.75
0.50
0.25
0.00
A (n=15)
B (n=16)
C (n=14)
surface

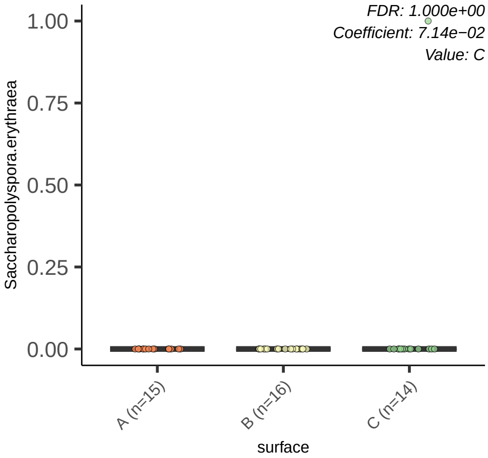
FDR: 1.000e+00
Coefficient: 7.14e−02
Value: C
Saccharopolyspora.erythraea
1.00
0.75
0.50
0.25
0.00
A (n=15)
B (n=16)
C (n=14)
surface

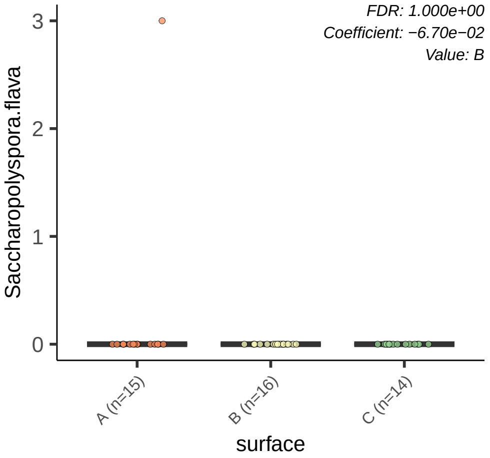

FDR: 1.000e+00
Coefficient: −6.70e−02
Value: B
Saccharopolyspora.flava
3
2
1
0
A (n=15)
B (n=16)
C (n=14)
surface

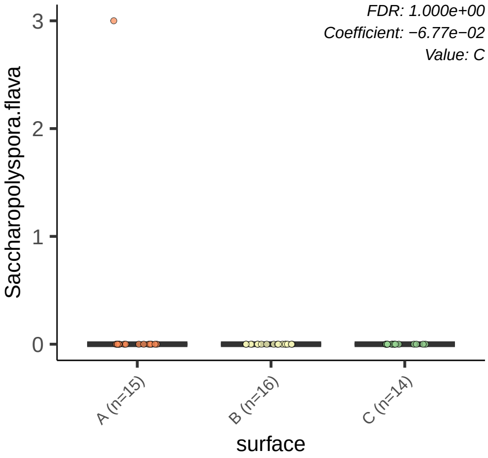
FDR: 1.000e+00
Coefficient: −6.77e−02
Value: C
Saccharopolyspora.flava
3
2
1
0
A (n=15)
B (n=16)
C (n=14)
surface

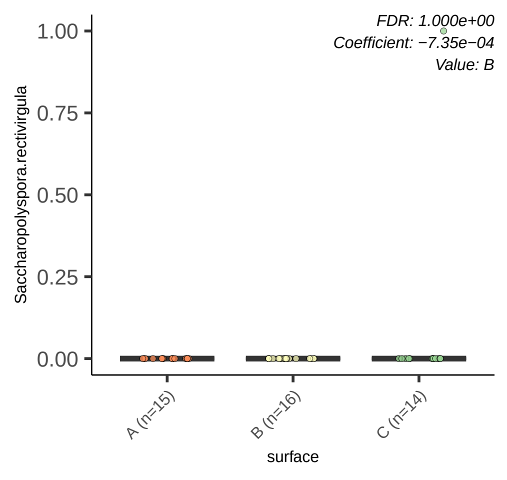

FDR: 1.000e+00
Coefficient: −7.35e−04
Value: B
1.00
0.75
0.50
0.25
0.00
Saccharopolyspora.rectivirgula
A (n=15)
B (n=16)
C (n=14)
surface

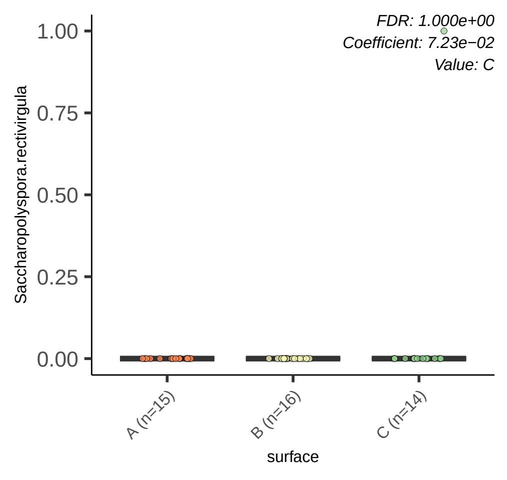
FDR: 1.000e+00
Coefficient: 7.23e−02
Value: C
1.00
0.75
0.50
0.25
0.00
Saccharopolyspora.rectivirgula
A (n=15)
B (n=16)
C (n=14)
surface

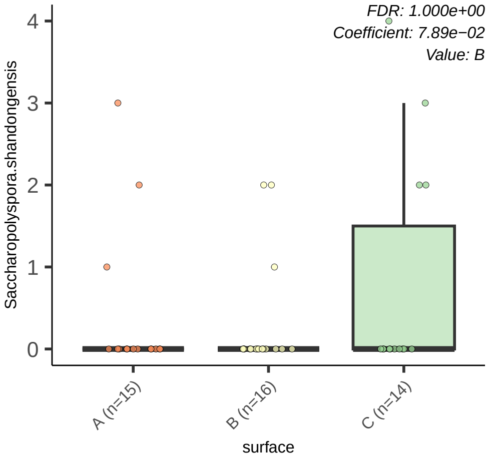
FDR: 1.000e+00
Coefficient: 7.89e−02
Value: B
Saccharopolyspora.shandongensis
0
1
2
3
4
A (n=15)
B (n=16)
C (n=14)
surface

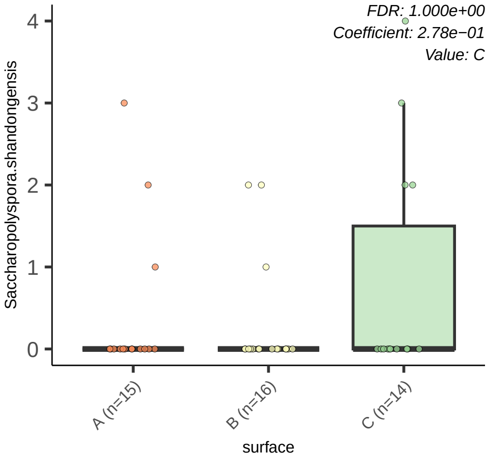

FDR: 1.000e+00
Coefficient: 2.78e−01
Value: C
Saccharopolyspora.shandongensis
0
1
2
3
4
A (n=15)
B (n=16)
C (n=14)
surface

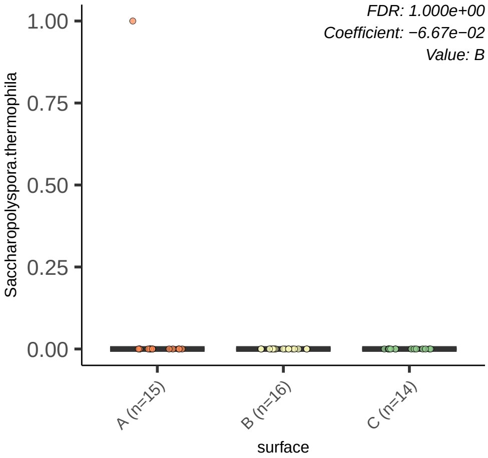
FDR: 1.000e+00
Coefficient: −6.67e−02
Value: B
Saccharopolyspora.thermophila
1.00
0.75
0.50
0.25
0.00
A (n=15)
B (n=16)
C (n=14)
surface

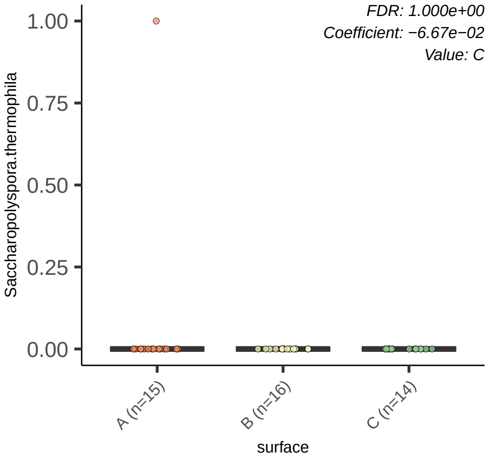

FDR: 1.000e+00
Coefficient: −6.67e−02
Value: C
1.00
0.75
0.50
0.25
0.00
Saccharopolyspora.thermophila
A (n=15)
B (n=16)
C (n=14)
surface

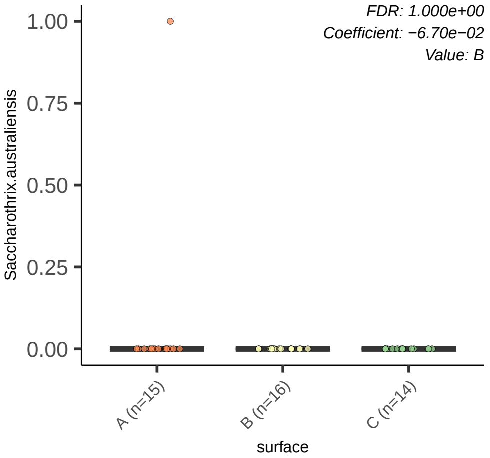

FDR: 1.000e+00
Coefficient: −6.70e−02
Value: B
Saccharothrix.australiensis
1.00
0.75
0.50
0.25
0.00
A (n=15)
B (n=16)
C (n=14)
surface

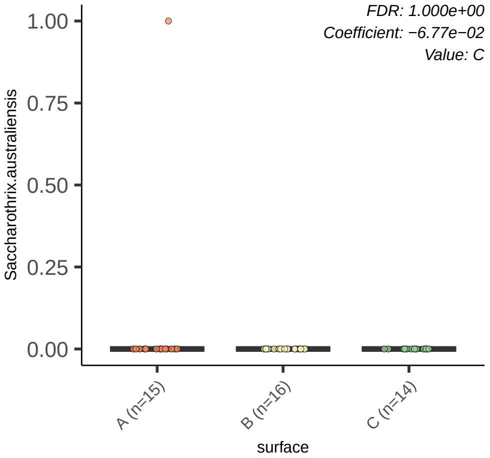
FDR: 1.000e+00
Coefficient: −6.77e−02
Value: C
Saccharothrix.australiensis
1.00
0.75
0.50
0.25
0.00
A (n=15)
B (n=16)
C (n=14)
surface

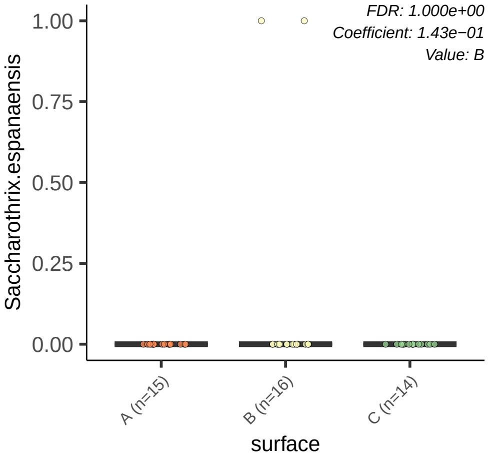
FDR: 1.000e+00
Coefficient: 1.43e−01
Value: B
1.00
0.75
0.50
0.25
0.00
Saccharothrix.espanaensis
A (n=15)
B (n=16)
C (n=14)
surface

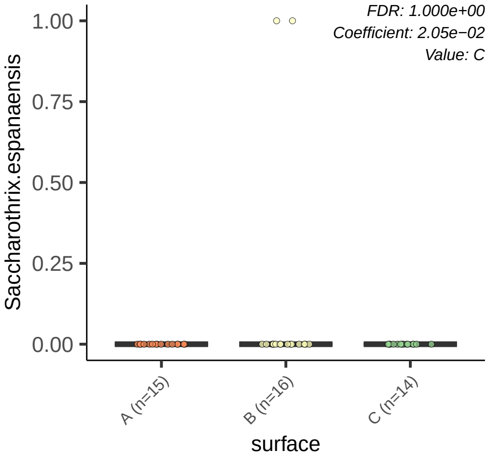

FDR: 1.000e+00
Coefficient: 2.05e−02
Value: C
Saccharothrix.espanaensis
1.00
0.75
0.50
0.25
0.00
A (n=15)
B (n=16)
C (n=14)
surface

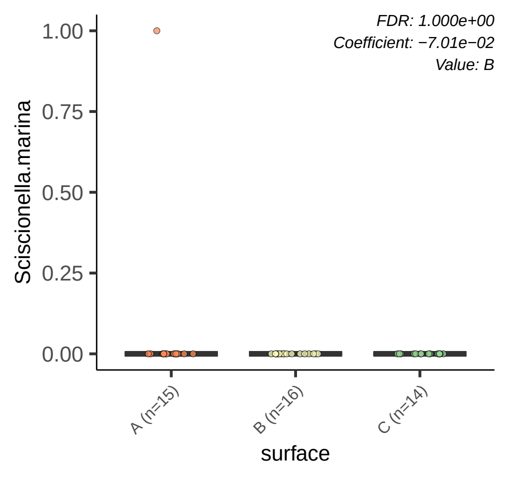
FDR: 1.000e+00
Coefficient: −7.01e−02
Value: B
Sciscionella.marina
1.00
0.75
0.50
0.25
0.00
A (n=15)
B (n=16)
C (n=14)
surface

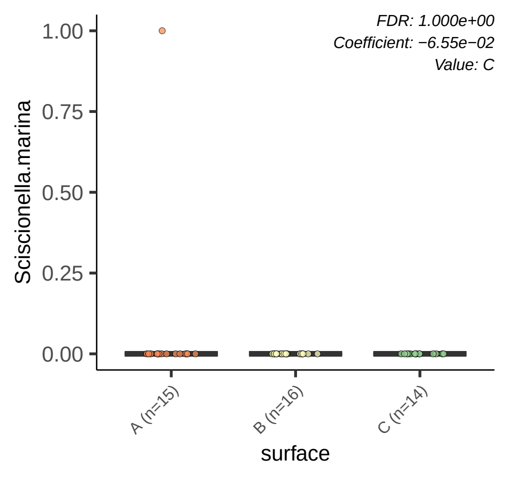
FDR: 1.000e+00
Coefficient: −6.55e−02
Value: C
Sciscionella.marina
1.00
0.75
0.50
0.25
0.00
A (n=15)
B (n=16)
C (n=14)
surface

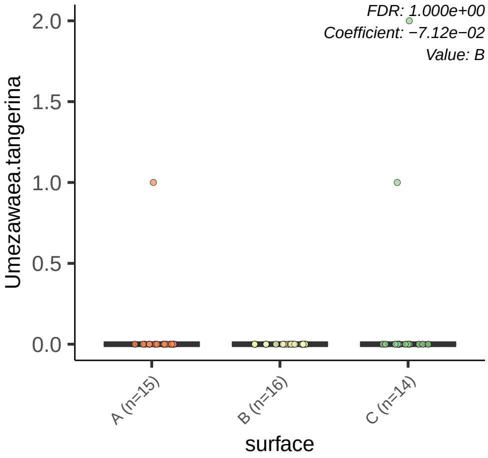

FDR: 1.000e+00
Coefficient: −7.12e−02
Value: B
Umezawaea.tangerina
2.0
1.5
1.0
0.5
0.0
A (n=15)
B (n=16)
C (n=14)
surface

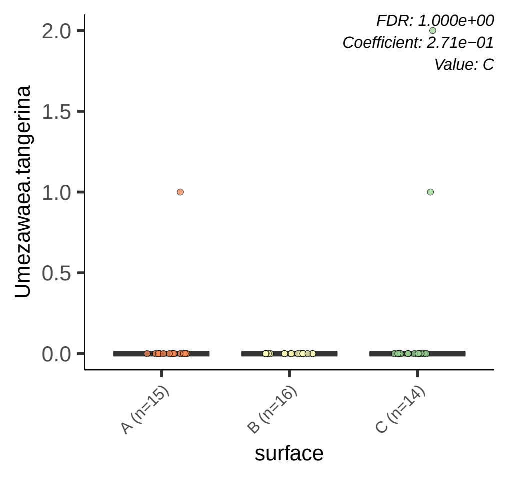

FDR: 1.000e+00
Coefficient: 2.71e−01
Value: C
Umezawaea.tangerina
2.0
1.5
1.0
0.5
0.0
A (n=15)
B (n=16)
C (n=14)
surface

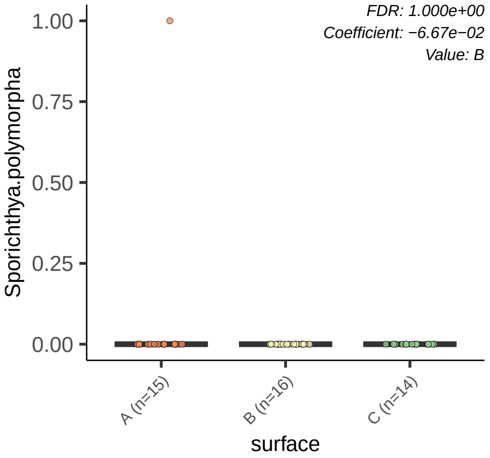
FDR: 1.000e+00
Coefficient: −6.67e−02
Value: B
Sporichthya.polymorpha
1.00
0.75
0.50
0.25
0.00
A (n=15)
B (n=16)
C (n=14)
surface

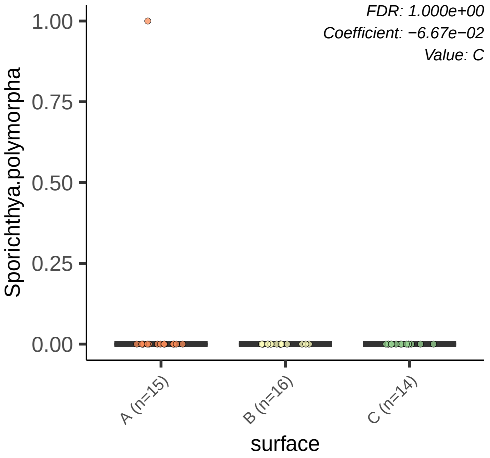
FDR: 1.000e+00
Coefficient: −6.67e−02
Value: C
Sporichthya.polymorpha
1.00
0.75
0.50
0.25
0.00
A (n=15)
B (n=16)
C (n=14)
surface

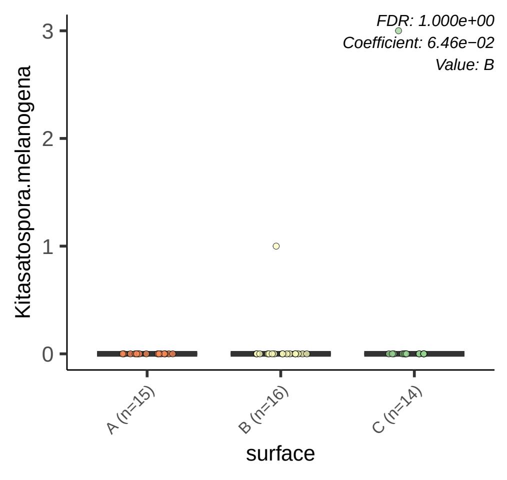
FDR: 1.000e+00
Coefficient: 6.46e−02
Value: B
Kitasatospora.melanogena
3
2
1
0
A (n=15)
B (n=16)
C (n=14)
surface

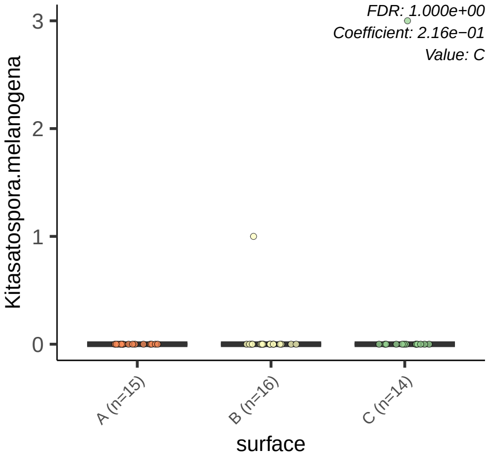
FDR: 1.000e+00
Coefficient: 2.16e−01
Value: C
Kitasatospora.melanogena
0
1
2
3
A (n=15)
B (n=16)
C (n=14)
surface

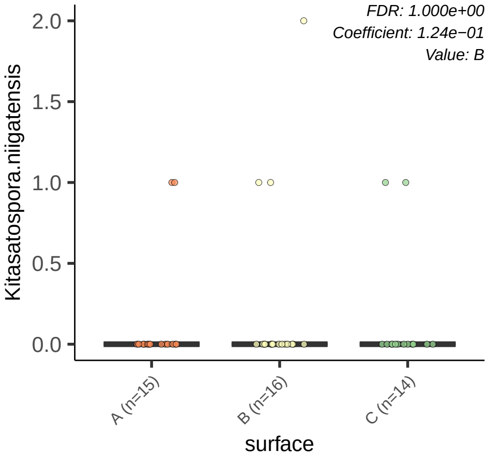

FDR: 1.000e+00
Coefficient: 1.24e−01
Value: B
2.0
1.5
1.0
0.5
0.0
Kitasatospora.niigatensis
A (n=15)
B (n=16)
C (n=14)
surface

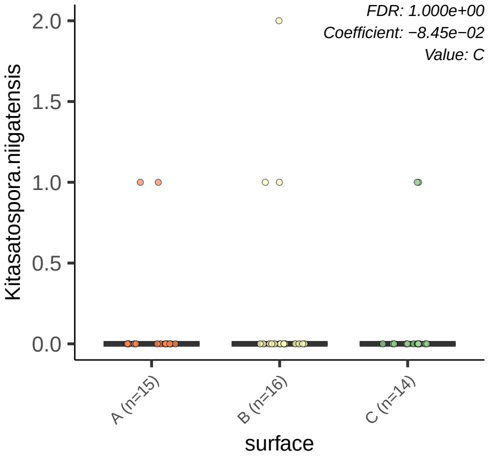
FDR: 1.000e+00
Coefficient: −8.45e−02
Value: C
Kitasatospora.niigatensis
2.0
1.5
1.0
0.5
0.0
A (n=15)
B (n=16)
C (n=14)
surface

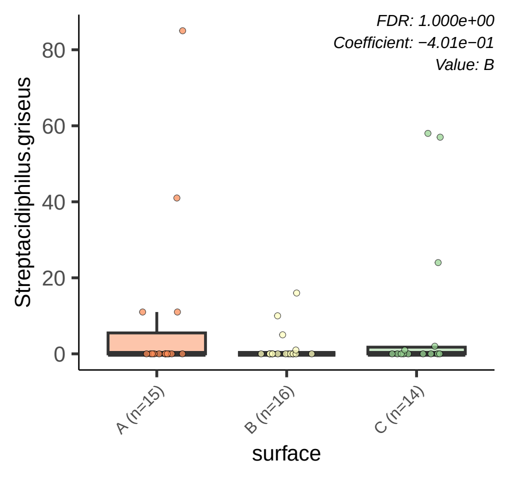

FDR: 1.000e+00
Coefficient: −4.01e−01
Value: B
Streptacidiphilus.griseus
0
20
40
60
80
A (n=15)
B (n=16)
C (n=14)
surface

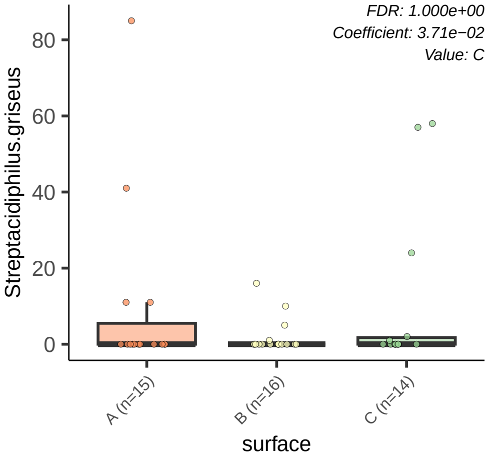

FDR: 1.000e+00
Coefficient: 3.71e−02
Value: C
Streptacidiphilus.griseus
0
20
40
60
80
A (n=15)
B (n=16)
C (n=14)
surface

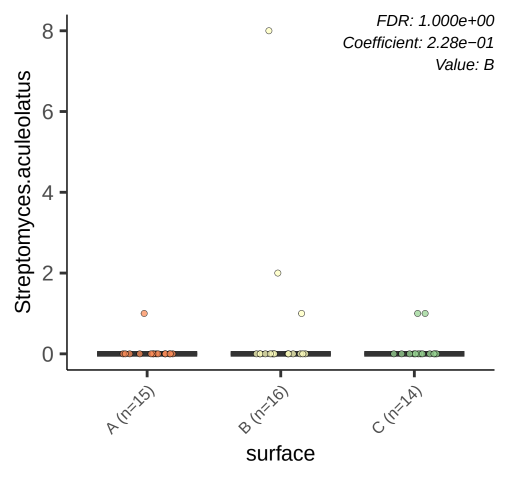

FDR: 1.000e+00
Coefficient: 2.28e−01
Value: B
Streptomyces.aculeolatus
8
6
4
2
0
A (n=15)
B (n=16)
C (n=14)
surface

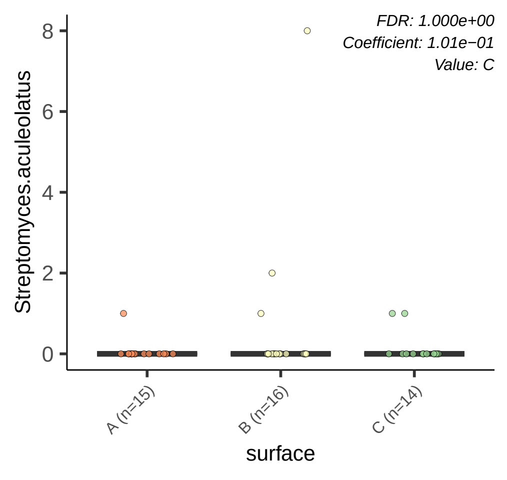
FDR: 1.000e+00
Coefficient: 1.01e−01
Value: C
Streptomyces.aculeolatus
0
2
4
6
8
A (n=15)
B (n=16)
C (n=14)
surface

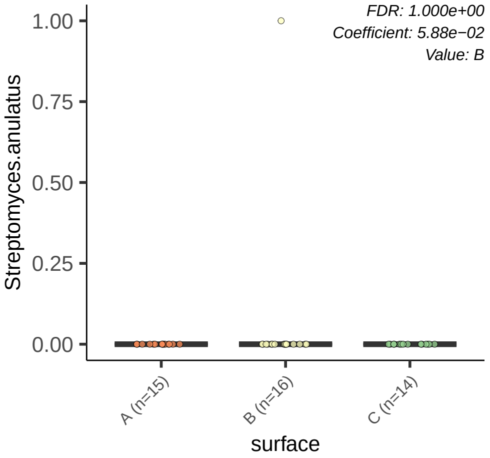
FDR: 1.000e+00
Coefficient: 5.88e−02
Value: B
Streptomyces.anulatus
1.00
0.75
0.50
0.25
0.00
A (n=15)
B (n=16)
C (n=14)
surface

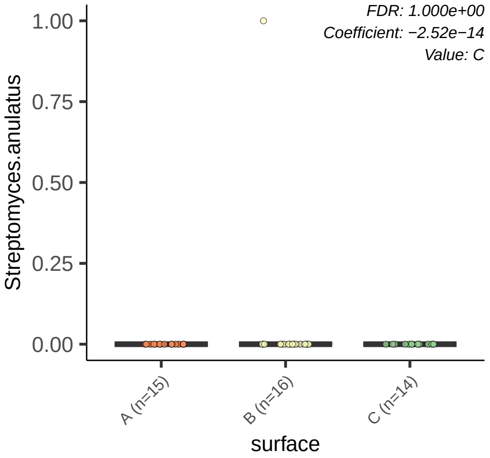

FDR: 1.000e+00
Coefficient: −2.52e−14
Value: C
Streptomyces.anulatus
1.00
0.75
0.50
0.25
0.00
A (n=15)
B (n=16)
C (n=14)
surface

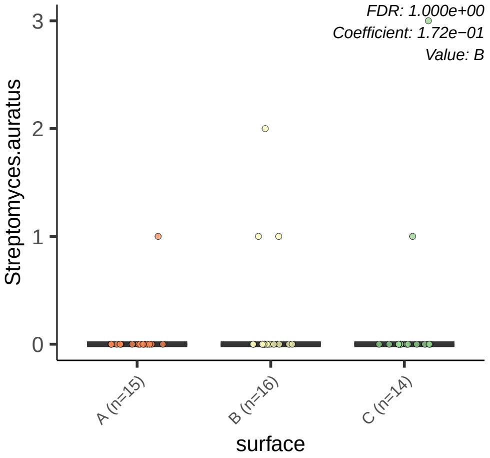
FDR: 1.000e+00
Coefficient: 1.72e−01
Value: B
Streptomyces.auratus
3
2
1
0
A (n=15)
B (n=16)
C (n=14)
surface

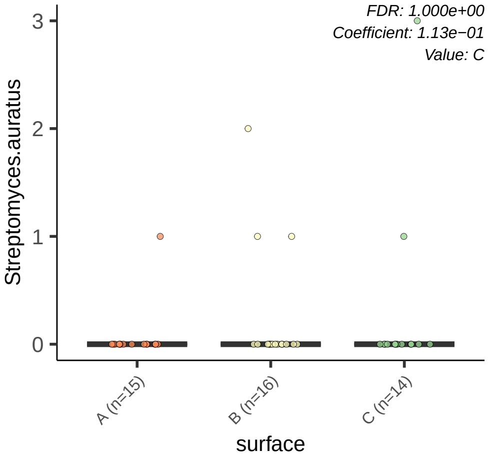
FDR: 1.000e+00
Coefficient: 1.13e−01
Value: C
Streptomyces.auratus
0
1
2
3
A (n=15)
B (n=16)
C (n=14)
surface

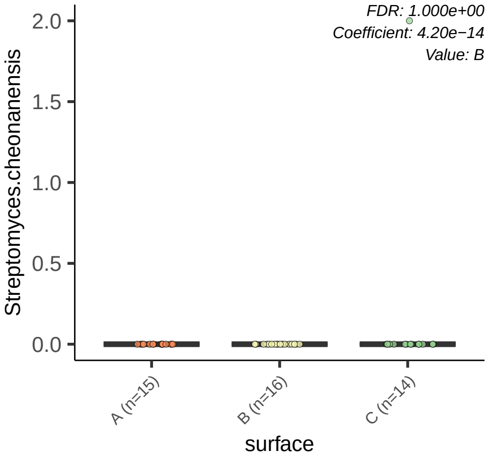

FDR: 1.000e+00
Coefficient: 4.20e−14
Value: B
Streptomyces.cheonanensis
2.0
1.5
1.0
0.5
0.0
A (n=15)
B (n=16)
C (n=14)
surface

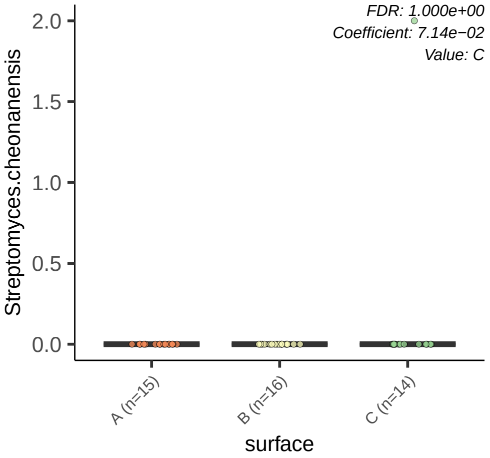
FDR: 1.000e+00
Coefficient: 7.14e−02
Value: C
Streptomyces.cheonanensis
2.0
1.5
1.0
0.5
0.0
A (n=15)
B (n=16)
C (n=14)
surface

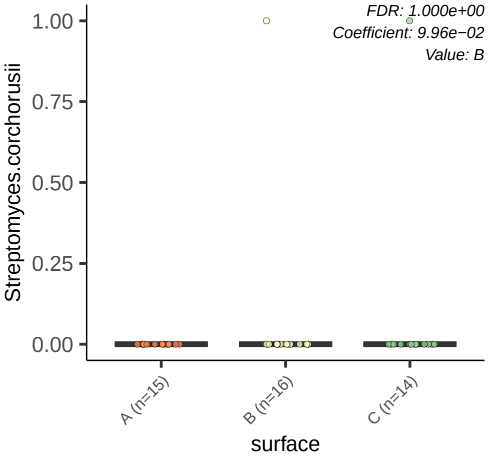
FDR: 1.000e+00
Coefficient: 9.96e−02
Value: B
Streptomyces.corchorusii
1.00
0.75
0.50
0.25
0.00
A (n=15)
B (n=16)
C (n=14)
surface

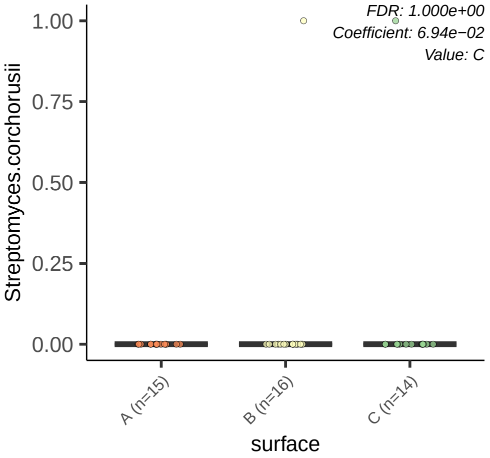

FDR: 1.000e+00
Coefficient: 6.94e−02
Value: C
Streptomyces.corchorusii
1.00
0.75
0.50
0.25
0.00
A (n=15)
B (n=16)
C (n=14)
surface

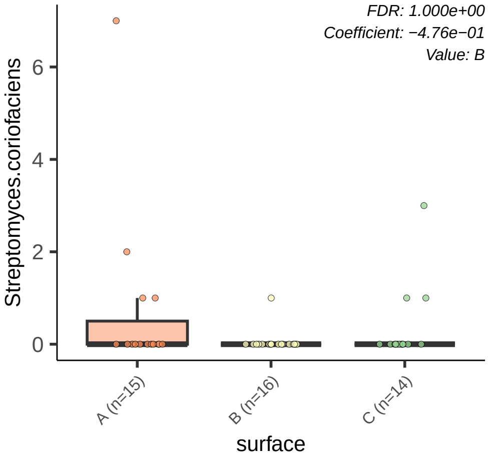

FDR: 1.000e+00
Coefficient: −4.76e−01
Value: B
Streptomyces.coriofaciens
0
2
4
6
A (n=15)
B (n=16)
C (n=14)
surface

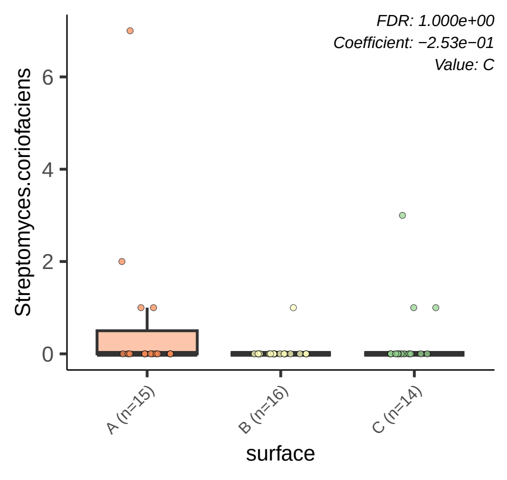

FDR: 1.000e+00
Coefficient: −2.53e−01
Value: C
Streptomyces.coriofaciens
0
2
4
6
A (n=15)
B (n=16)
C (n=14)
surface

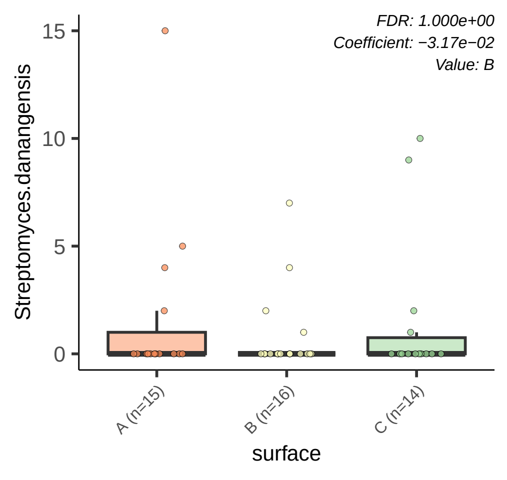
FDR: 1.000e+00
Coefficient: −3.17e−02
Value: B
Streptomyces.danangensis
15
10
5
0
A (n=15)
B (n=16)
C (n=14)
surface

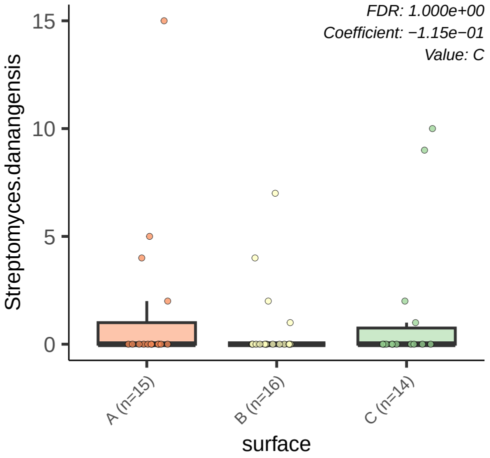

FDR: 1.000e+00
Coefficient: −1.15e−01
Value: C
Streptomyces.danangensis
15
10
5
0
A (n=15)
B (n=16)
C (n=14)
surface

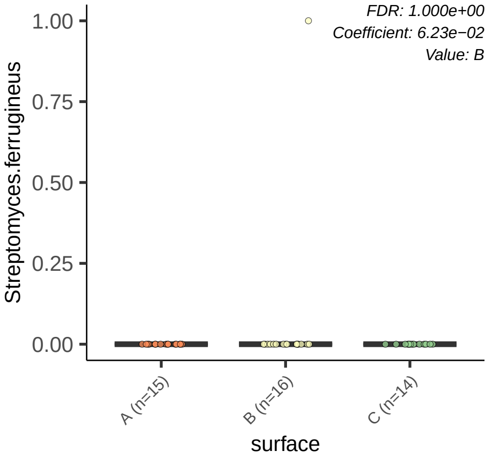
FDR: 1.000e+00
Coefficient: 6.23e−02
Value: B
Streptomyces.ferrugineus
1.00
0.75
0.50
0.25
0.00
A (n=15)
B (n=16)
C (n=14)
surface

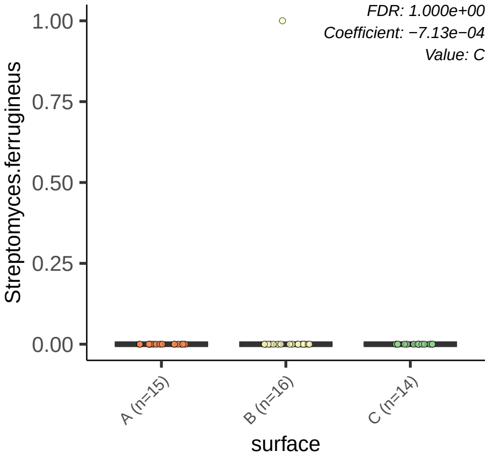
FDR: 1.000e+00
Coefficient: −7.13e−04
Value: C
Streptomyces.ferrugineus
1.00
0.75
0.50
0.25
0.00
A (n=15)
B (n=16)
C (n=14)
surface

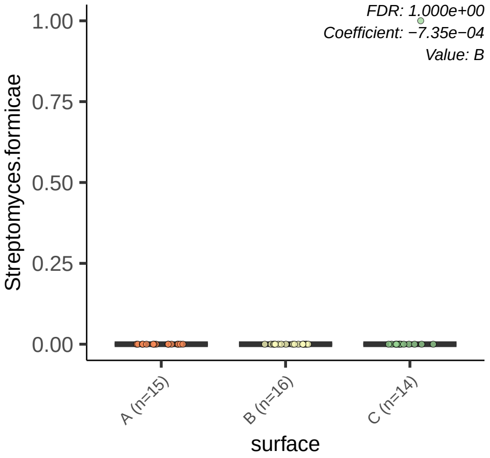
FDR: 1.000e+00
Coefficient: −7.35e−04
Value: B
Streptomyces.formicae
1.00
0.75
0.50
0.25
0.00
A (n=15)
B (n=16)
C (n=14)
surface

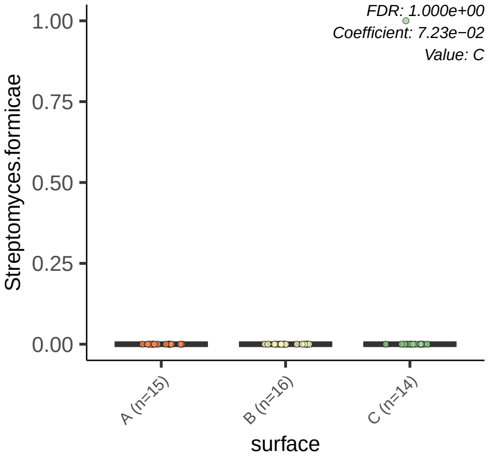
FDR: 1.000e+00
Coefficient: 7.23e−02
Value: C
Streptomyces.formicae
1.00
0.75
0.50
0.25
0.00
A (n=15)
B (n=16)
C (n=14)
surface

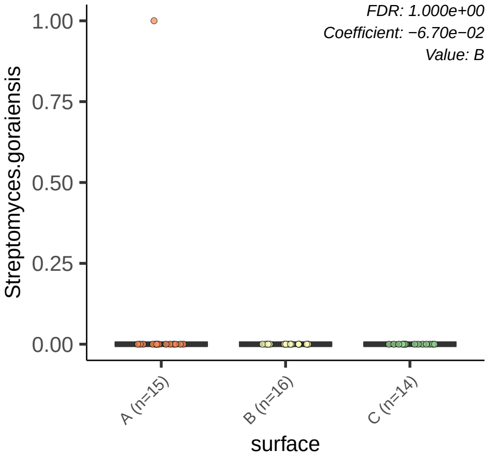

FDR: 1.000e+00
Coefficient: −6.70e−02
Value: B
Streptomyces.goraiensis
1.00
0.75
0.50
0.25
0.00
A (n=15)
B (n=16)
C (n=14)
surface

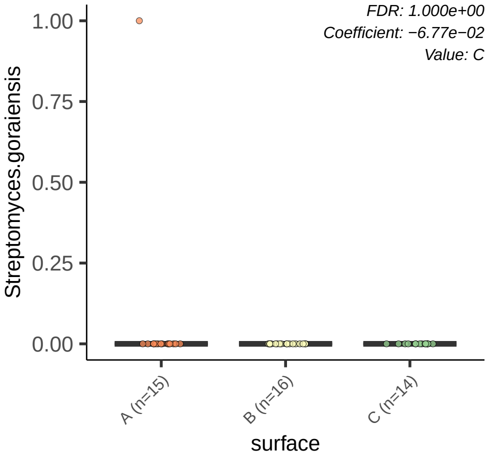
FDR: 1.000e+00
Coefficient: −6.77e−02
Value: C
Streptomyces.goraiensis
1.00
0.75
0.50
0.25
0.00
A (n=15)
B (n=16)
C (n=14)
surface

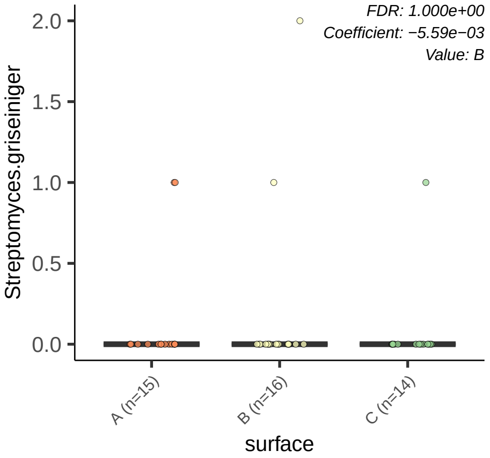

FDR: 1.000e+00
Coefficient: −5.59e−03
Value: B
Streptomyces.griseiniger
2.0
1.5
1.0
0.5
0.0
A (n=15)
B (n=16)
C (n=14)
surface

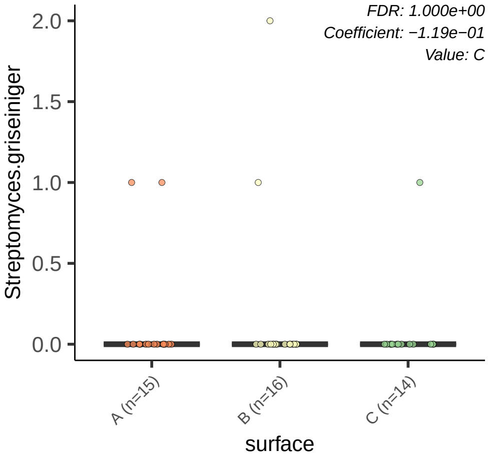
FDR: 1.000e+00
Coefficient: −1.19e−01
Value: C
Streptomyces.griseiniger
2.0
1.5
1.0
0.5
0.0
A (n=15)
B (n=16)
C (n=14)
surface

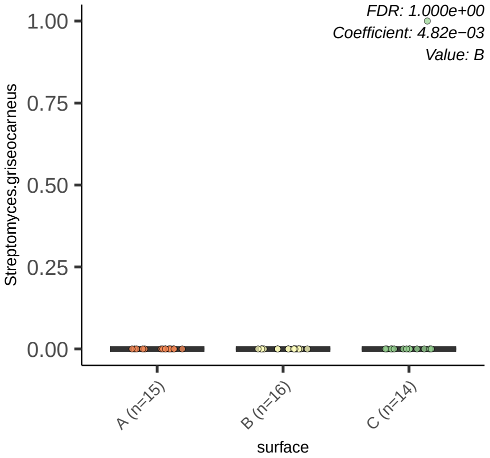
FDR: 1.000e+00
Coefficient: 4.82e−03
Value: B
Streptomyces.griseocarneus
1.00
0.75
0.50
0.25
0.00
A (n=15)
B (n=16)
C (n=14)
surface

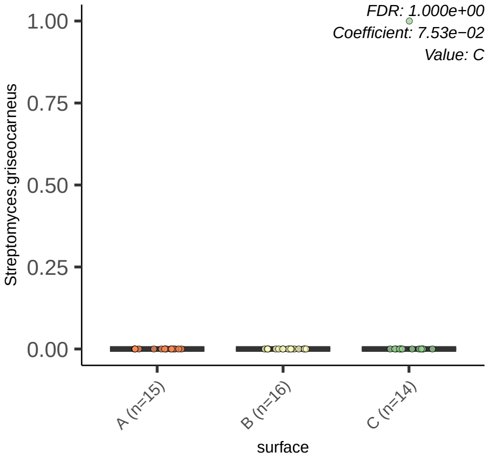

FDR: 1.000e+00
Coefficient: 7.53e−02
Value: C
1.00
0.75
0.50
0.25
0.00
Streptomyces.griseocarneus
A (n=15)
B (n=16)
C (n=14)
surface

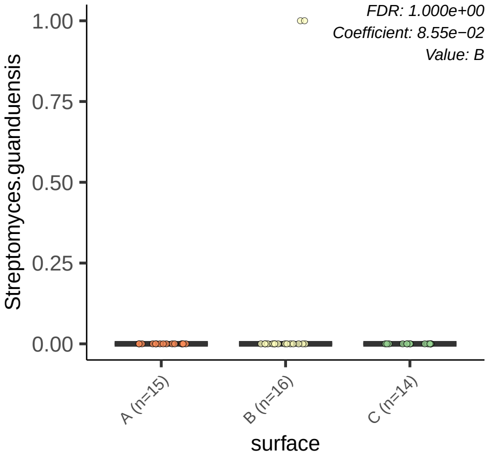

FDR: 1.000e+00
Coefficient: 8.55e−02
Value: B
Streptomyces.guanduensis
1.00
0.75
0.50
0.25
0.00
A (n=15)
B (n=16)
C (n=14)
surface

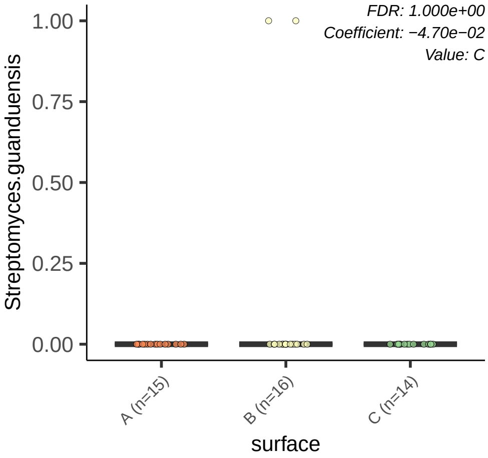

FDR: 1.000e+00
Coefficient: −4.70e−02
Value: C
Streptomyces.guanduensis
1.00
0.75
0.50
0.25
0.00
A (n=15)
B (n=16)
C (n=14)
surface

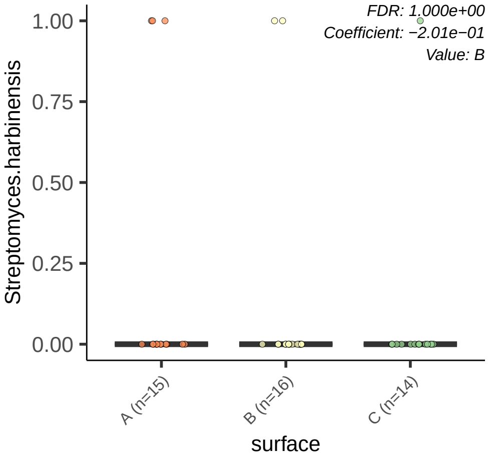

FDR: 1.000e+00
Coefficient: −2.01e−01
Value: B
Streptomyces.harbinensis
1.00
0.75
0.50
0.25
0.00
A (n=15)
B (n=16)
C (n=14)
surface

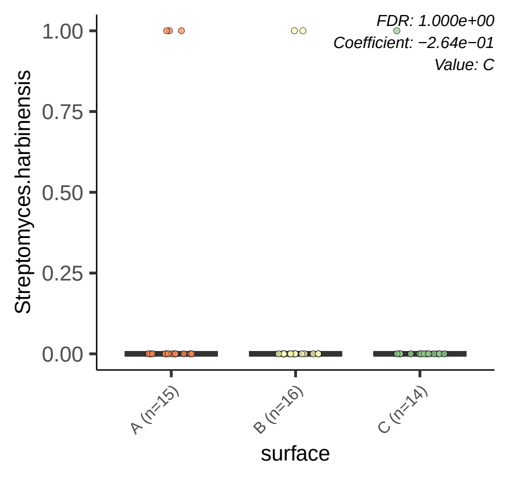
FDR: 1.000e+00
Coefficient: −2.64e−01
Value: C
Streptomyces.harbinensis
1.00
0.75
0.50
0.25
0.00
A (n=15)
B (n=16)
C (n=14)
surface

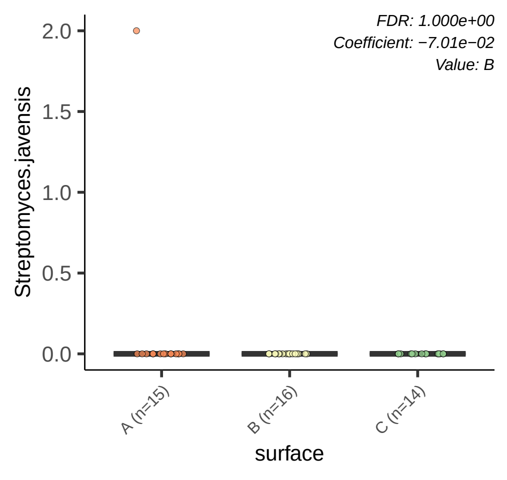
FDR: 1.000e+00
Coefficient: −7.01e−02
Value: B
Streptomyces.javensis
2.0
1.5
1.0
0.5
0.0
A (n=15)
B (n=16)
C (n=14)
surface

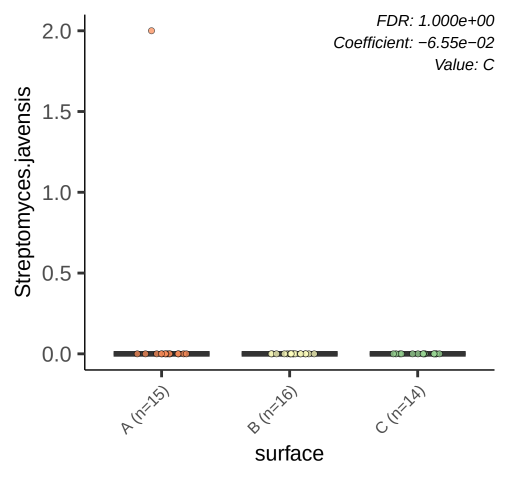
FDR: 1.000e+00
Coefficient: −6.55e−02
Value: C
Streptomyces.javensis
2.0
1.5
1.0
0.5
0.0
A (n=15)
B (n=16)
C (n=14)
surface

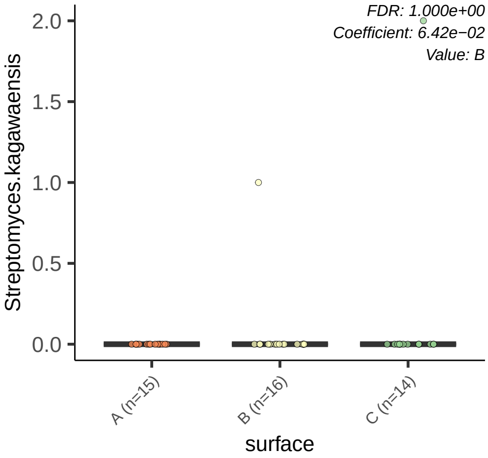

FDR: 1.000e+00
Coefficient: 6.42e−02
Value: B
Streptomyces.kagawaensis
2.0
1.5
1.0
0.5
0.0
A (n=15)
B (n=16)
C (n=14)
surface

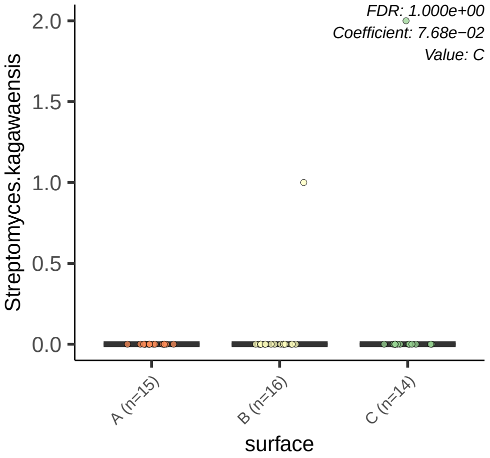
FDR: 1.000e+00
Coefficient: 7.68e−02
Value: C
Streptomyces.kagawaensis
2.0
1.5
1.0
0.5
0.0
A (n=15)
B (n=16)
C (n=14)
surface

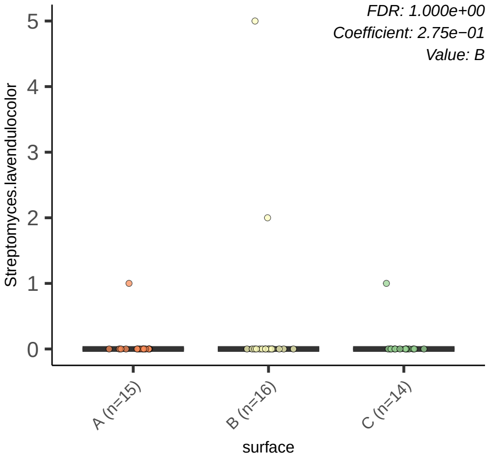

FDR: 1.000e+00
Coefficient: 2.75e−01
Value: B
Streptomyces.lavendulocolor
5
4
3
2
1
0
A (n=15)
B (n=16)
C (n=14)
surface

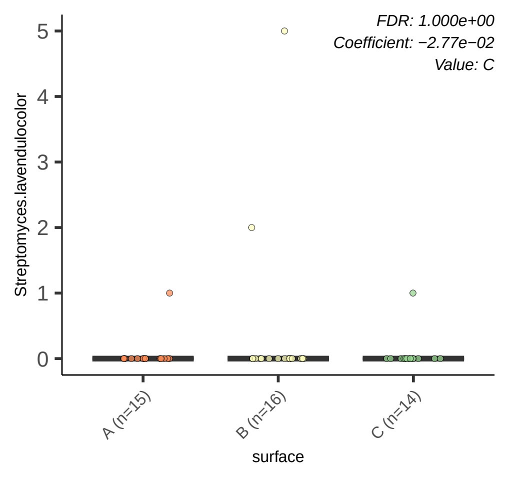

FDR: 1.000e+00
Coefficient: −2.77e−02
Value: C
Streptomyces.lavendulocolor
5
4
3
2
1
0
A (n=15)
B (n=16)
C (n=14)
surface

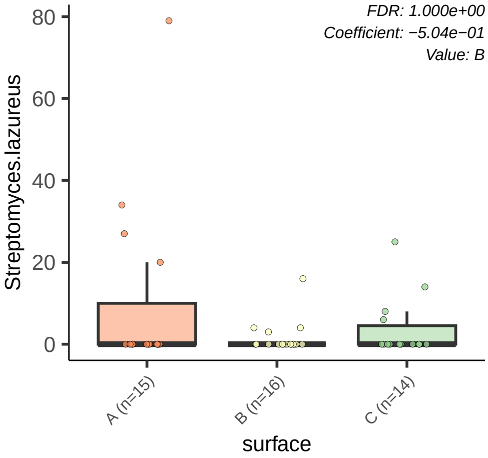

FDR: 1.000e+00
Coefficient: −5.04e−01
Value: B
Streptomyces.lazureus
0
20
40
60
80
A (n=15)
B (n=16)
C (n=14)
surface

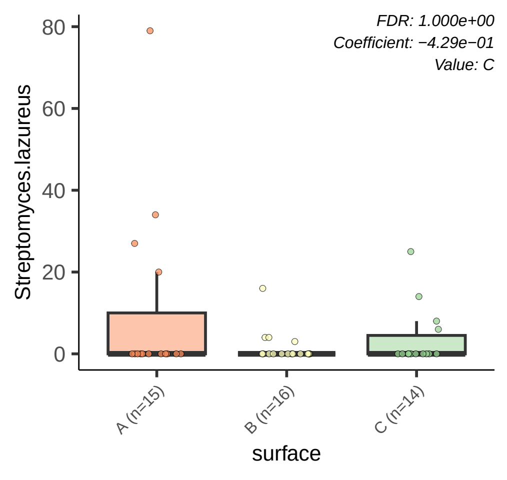
FDR: 1.000e+00
Coefficient: −4.29e−01
Value: C
Streptomyces.lazureus
80
60
40
20
0
A (n=15)
B (n=16)
C (n=14)
surface

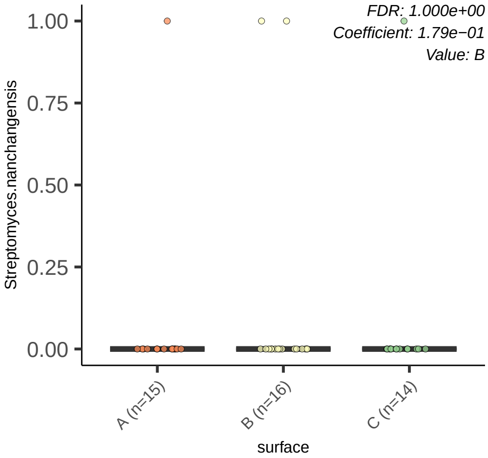

FDR: 1.000e+00
Coefficient: 1.79e−01
Value: B
1.00
0.75
0.50
0.25
0.00
Streptomyces.nanchangensis
A (n=15)
B (n=16)
C (n=14)
surface

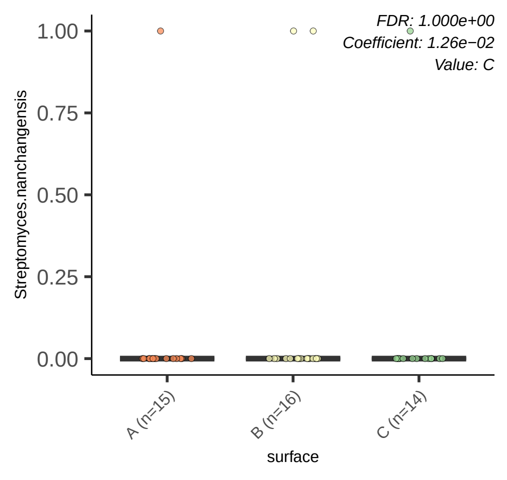
FDR: 1.000e+00
Coefficient: 1.26e−02
Value: C
1.00
0.75
0.50
0.25
0.00
Streptomyces.nanchangensis
A (n=15)
B (n=16)
C (n=14)
surface

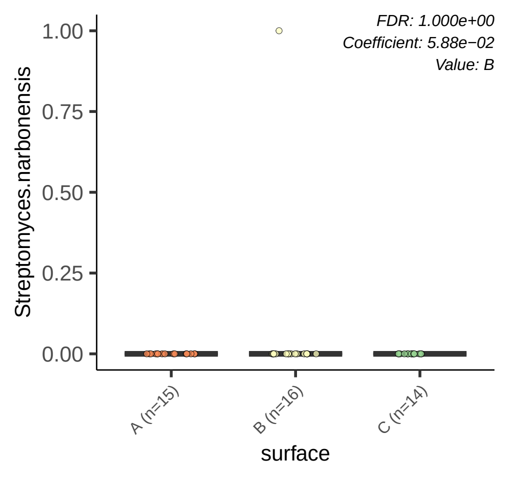

FDR: 1.000e+00
Coefficient: 5.88e−02
Value: B
Streptomyces.narbonensis
1.00
0.75
0.50
0.25
0.00
A (n=15)
B (n=16)
C (n=14)
surface

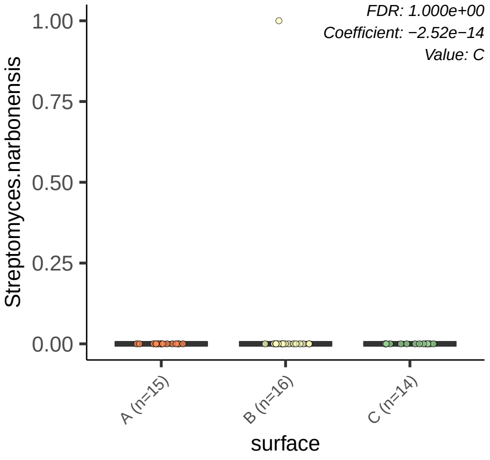

FDR: 1.000e+00
Coefficient: −2.52e−14
Value: C
Streptomyces.narbonensis
1.00
0.75
0.50
0.25
0.00
A (n=15)
B (n=16)
C (n=14)
surface

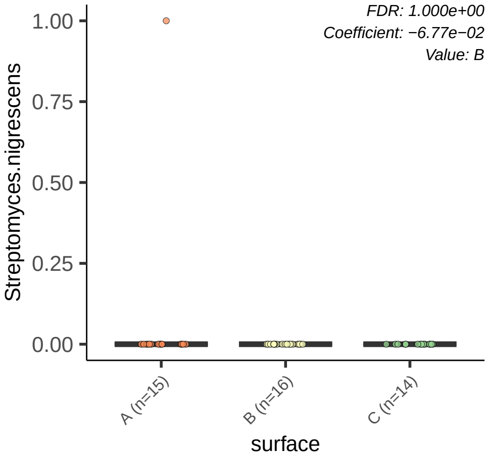
FDR: 1.000e+00
Coefficient: −6.77e−02
Value: B
Streptomyces.nigrescens
1.00
0.75
0.50
0.25
0.00
A (n=15)
B (n=16)
C (n=14)
surface

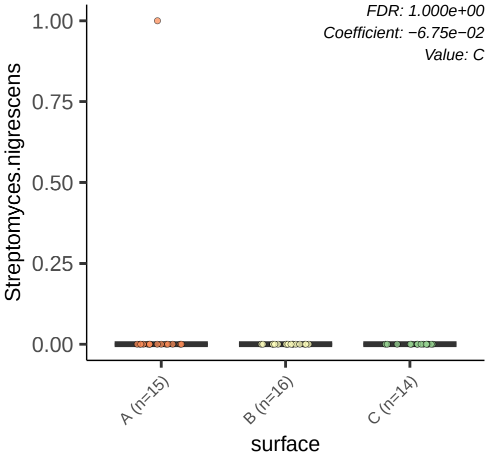
FDR: 1.000e+00
Coefficient: −6.75e−02
Value: C
1.00
0.75
0.50
0.25
0.00
Streptomyces.nigrescens
A (n=15)
B (n=16)
C (n=14)
surface

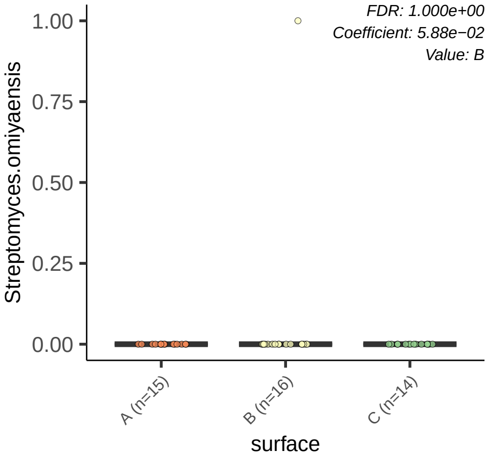

FDR: 1.000e+00
Coefficient: 5.88e−02
Value: B
Streptomyces.omiyaensis
1.00
0.75
0.50
0.25
0.00
A (n=15)
B (n=16)
C (n=14)
surface

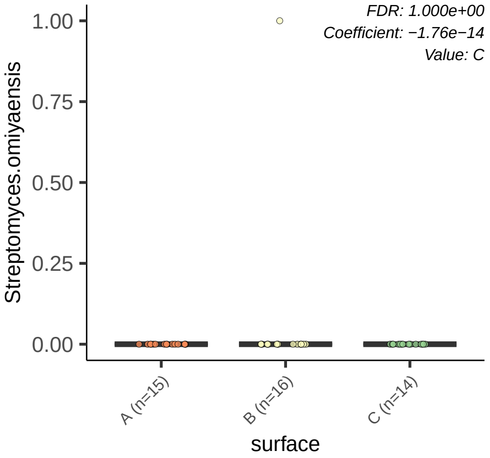
FDR: 1.000e+00
Coefficient: −1.76e−14
Value: C
Streptomyces.omiyaensis
1.00
0.75
0.50
0.25
0.00
A (n=15)
B (n=16)
C (n=14)
surface

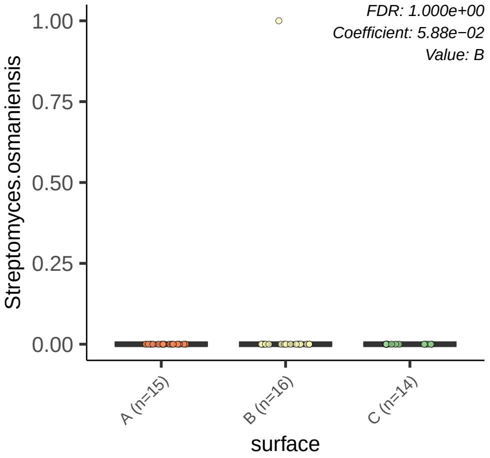
FDR: 1.000e+00
Coefficient: 5.88e−02
Value: B
Streptomyces.osmaniensis
1.00
0.75
0.50
0.25
0.00
A (n=15)
B (n=16)
C (n=14)
surface

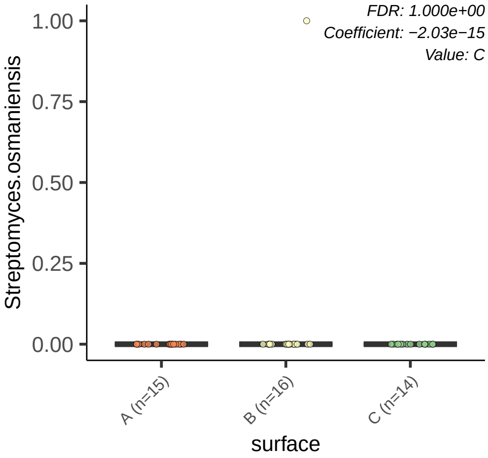
FDR: 1.000e+00
Coefficient: −2.03e−15
Value: C
Streptomyces.osmaniensis
1.00
0.75
0.50
0.25
0.00
A (n=15)
B (n=16)
C (n=14)
surface

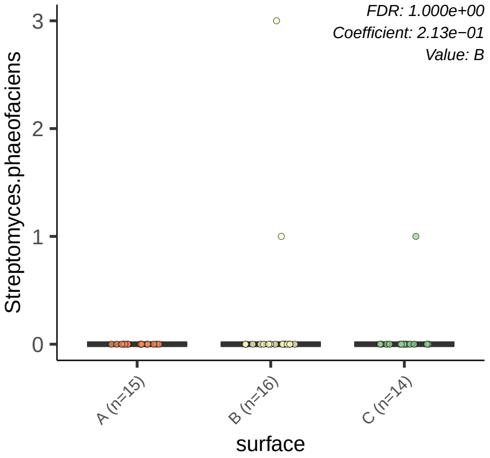
FDR: 1.000e+00
Coefficient: 2.13e−01
Value: B
Streptomyces.phaeofaciens
3
2
1
0
A (n=15)
B (n=16)
C (n=14)
surface

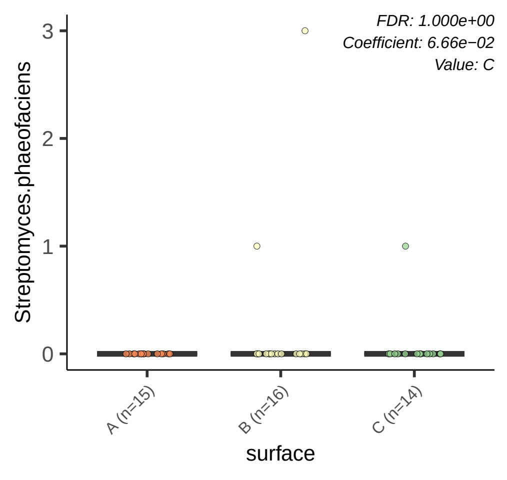
FDR: 1.000e+00
Coefficient: 6.66e−02
Value: C
Streptomyces.phaeofaciens
3
2
1
0
A (n=15)
B (n=16)
C (n=14)
surface

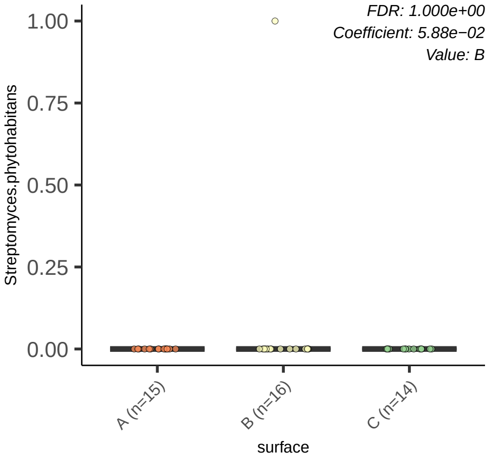

FDR: 1.000e+00
Coefficient: 5.88e−02
Value: B
Streptomyces.phytohabitans
1.00
0.75
0.50
0.25
0.00
A (n=15)
B (n=16)
C (n=14)
surface

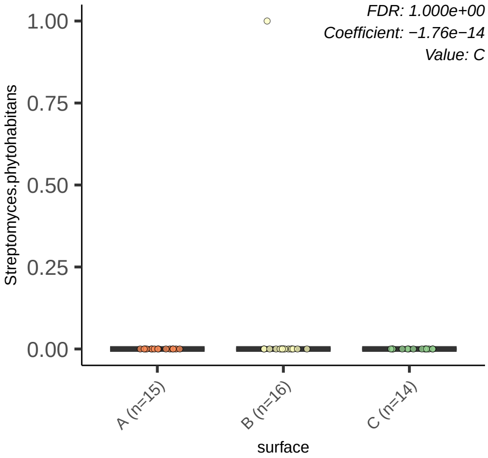
FDR: 1.000e+00
Coefficient: −1.76e−14
Value: C
Streptomyces.phytohabitans
1.00
0.75
0.50
0.25
0.00
A (n=15)
B (n=16)
C (n=14)
surface

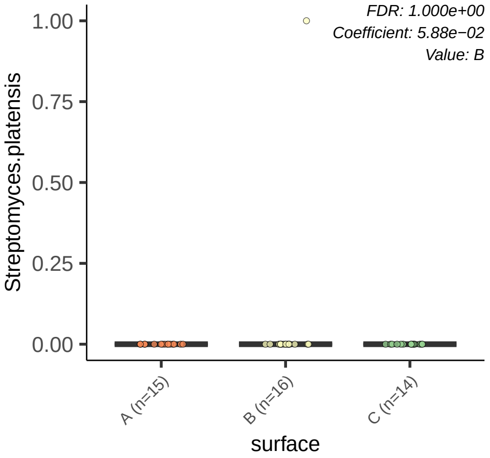

FDR: 1.000e+00
Coefficient: 5.88e−02
Value: B
Streptomyces.platensis
1.00
0.75
0.50
0.25
0.00
A (n=15)
B (n=16)
C (n=14)
surface

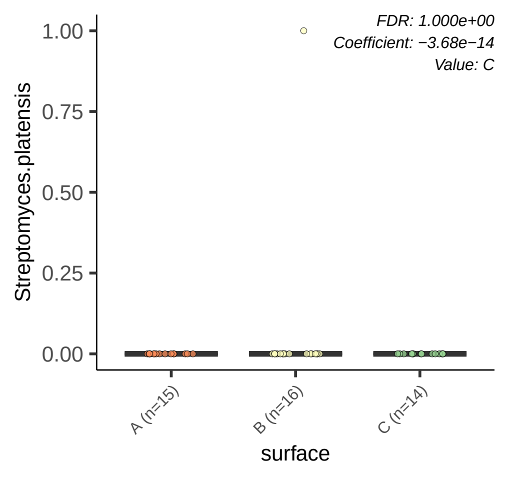
FDR: 1.000e+00
Coefficient: −3.68e−14
Value: C
Streptomyces.platensis
1.00
0.75
0.50
0.25
0.00
A (n=15)
B (n=16)
C (n=14)
surface

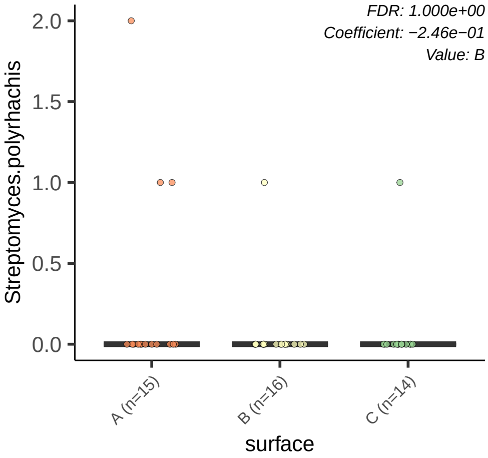

FDR: 1.000e+00
Coefficient: −2.46e−01
Value: B
Streptomyces.polyrhachis
2.0
1.5
1.0
0.5
0.0
A (n=15)
B (n=16)
C (n=14)
surface

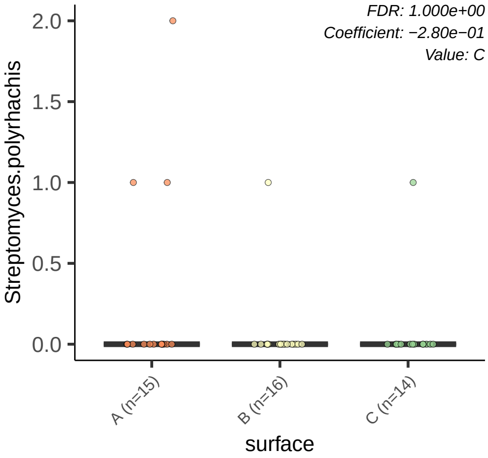

FDR: 1.000e+00
Coefficient: −2.80e−01
Value: C
Streptomyces.polyrhachis
2.0
1.5
1.0
0.5
0.0
A (n=15)
B (n=16)
C (n=14)
surface

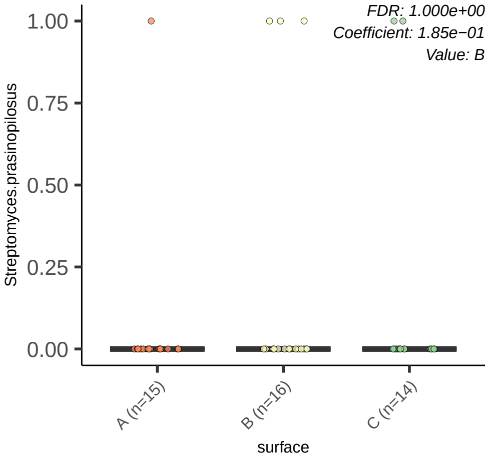
FDR: 1.000e+00
Coefficient: 1.85e−01
Value: B
Streptomyces.prasinopilosus
1.00
0.75
0.50
0.25
0.00
A (n=15)
B (n=16)
C (n=14)
surface

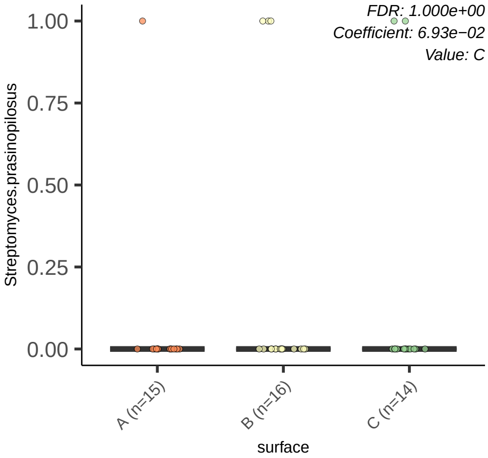
FDR: 1.000e+00
Coefficient: 6.93e−02
Value: C
1.00
0.75
0.50
0.25
0.00
Streptomyces.prasinopilosus
A (n=15)
B (n=16)
C (n=14)
surface

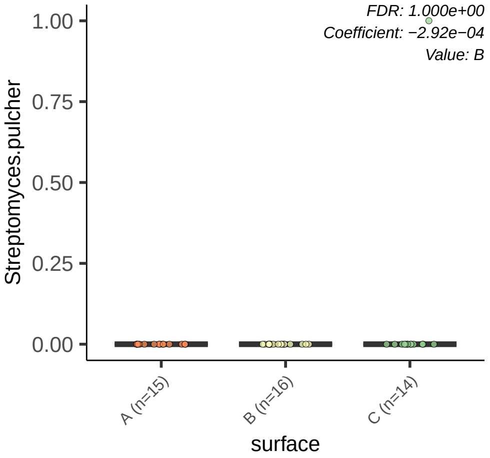

FDR: 1.000e+00
Coefficient: −2.92e−04
Value: B
Streptomyces.pulcher
1.00
0.75
0.50
0.25
0.00
A (n=15)
B (n=16)
C (n=14)
surface

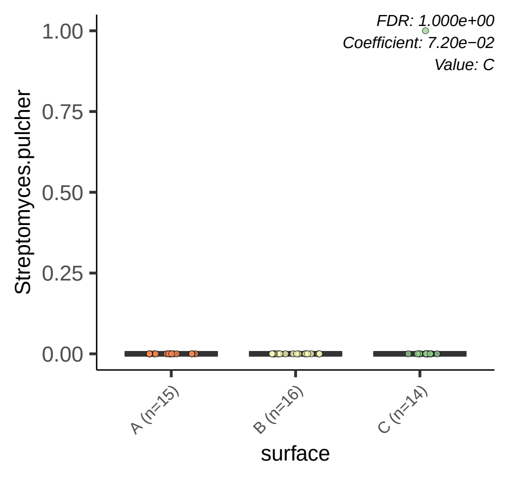
FDR: 1.000e+00
Coefficient: 7.20e−02
Value: C
1.00
0.75
0.50
0.25
0.00
Streptomyces.pulcher
A (n=15)
B (n=16)
C (n=14)
surface

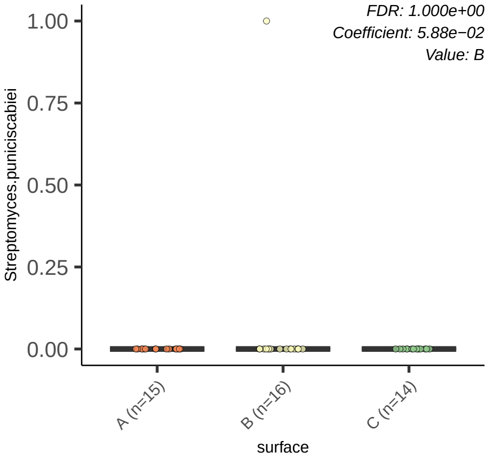
FDR: 1.000e+00
Coefficient: 5.88e−02
Value: B
Streptomyces.puniciscabiei
1.00
0.75
0.50
0.25
0.00
A (n=15)
B (n=16)
C (n=14)
surface

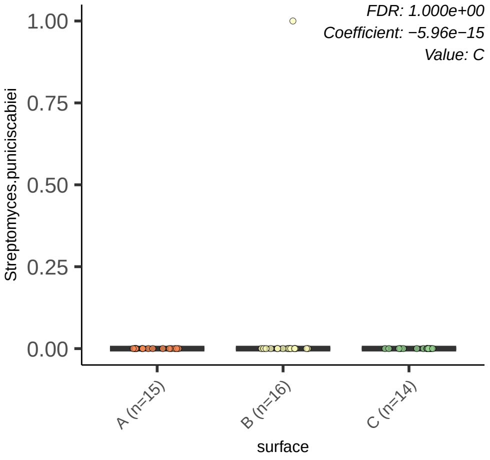

FDR: 1.000e+00
Coefficient: −5.96e−15
Value: C
Streptomyces.puniciscabiei
1.00
0.75
0.50
0.25
0.00
A (n=15)
B (n=16)
C (n=14)
surface

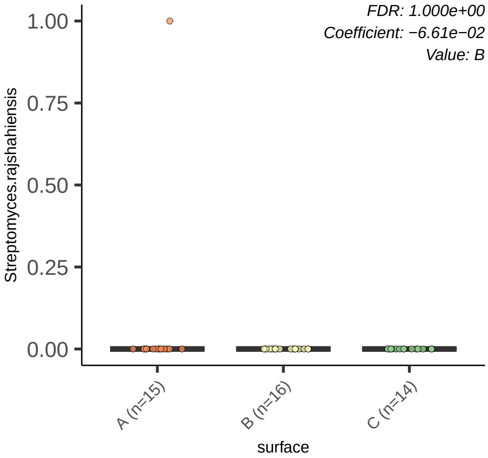

FDR: 1.000e+00
Coefficient: −6.61e−02
Value: B
Streptomyces.rajshahiensis
1.00
0.75
0.50
0.25
0.00
A (n=15)
B (n=16)
C (n=14)
surface

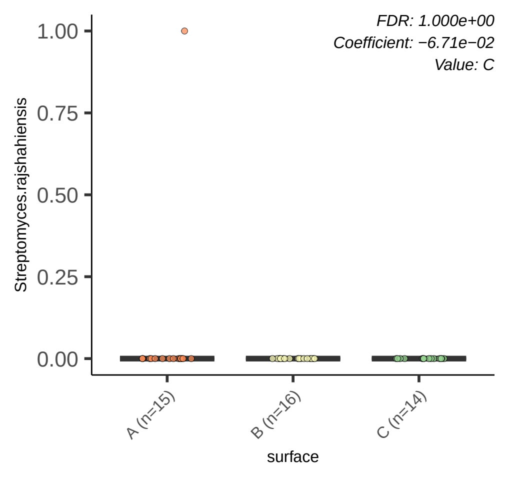

FDR: 1.000e+00
Coefficient: −6.71e−02
Value: C
1.00
0.75
0.50
0.25
0.00
Streptomyces.rajshahiensis
A (n=15)
B (n=16)
C (n=14)
surface

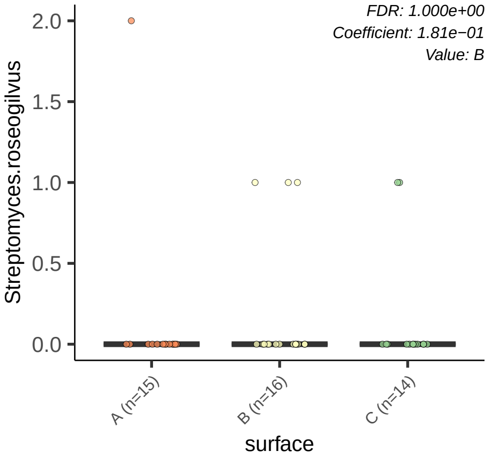
FDR: 1.000e+00
Coefficient: 1.81e−01
Value: B
Streptomyces.roseogilvus
2.0
1.5
1.0
0.5
0.0
A (n=15)
B (n=16)
C (n=14)
surface

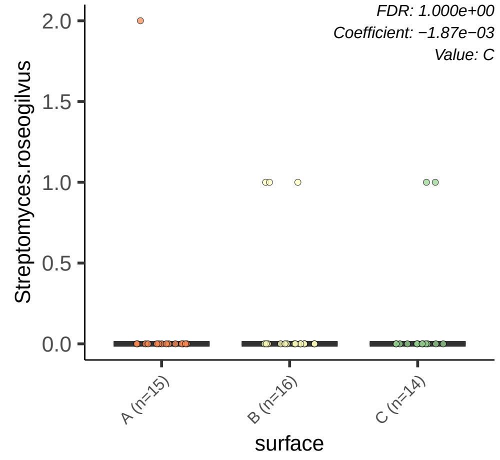

FDR: 1.000e+00
Coefficient: −1.87e−03
Value: C
Streptomyces.roseogilvus
2.0
1.5
1.0
0.5
0.0
A (n=15)
B (n=16)
C (n=14)
surface

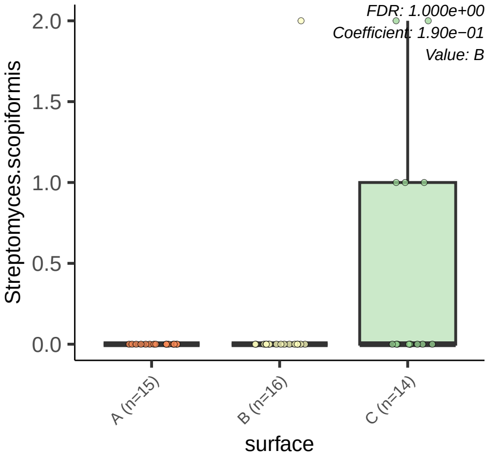

FDR: 1.000e+00
Coefficient: 1.90e−01
Value: B
Streptomyces.scopiformis
2.0
1.5
1.0
0.5
0.0
A (n=15)
B (n=16)
C (n=14)
surface

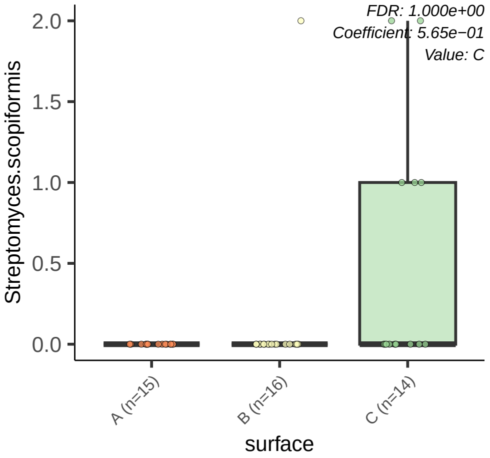
FDR: 1.000e+00
Coefficient: 5.65e−01
Value: C
Streptomyces.scopiformis
2.0
1.5
1.0
0.5
0.0
A (n=15)
B (n=16)
C (n=14)
surface

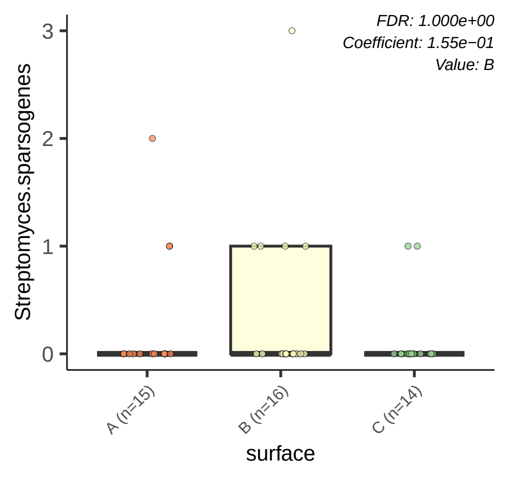
FDR: 1.000e+00
Coefficient: 1.55e−01
Value: B
Streptomyces.sparsogenes
0
1
2
3
A (n=15)
B (n=16)
C (n=14)
surface

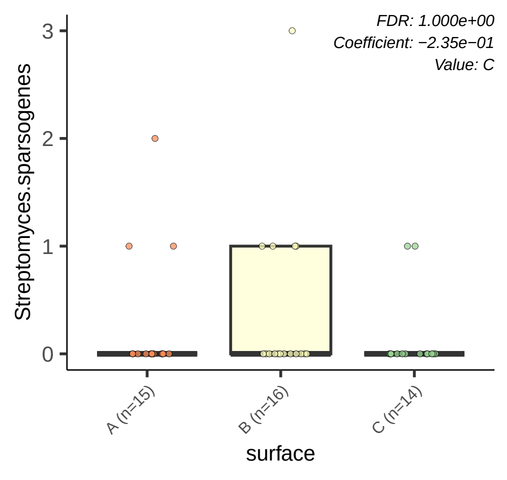

FDR: 1.000e+00
Coefficient: −2.35e−01
Value: C
Streptomyces.sparsogenes
0
1
2
3
A (n=15)
B (n=16)
C (n=14)
surface

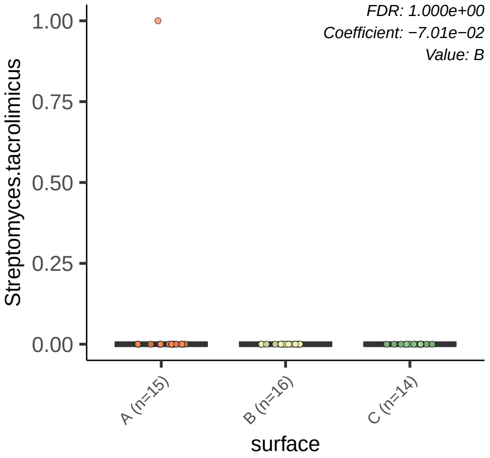
FDR: 1.000e+00
Coefficient: −7.01e−02
Value: B
Streptomyces.tacrolimicus
1.00
0.75
0.50
0.25
0.00
A (n=15)
B (n=16)
C (n=14)
surface

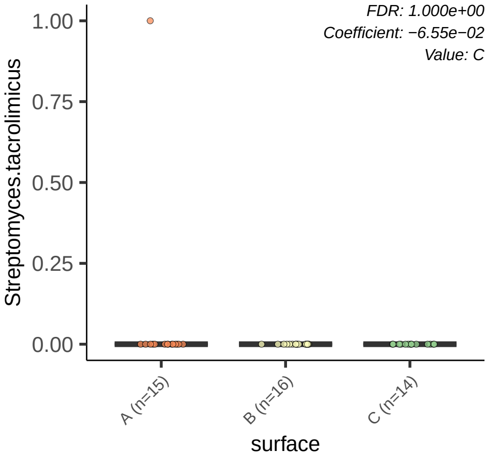

FDR: 1.000e+00
Coefficient: −6.55e−02
Value: C
Streptomyces.tacrolimicus
1.00
0.75
0.50
0.25
0.00
A (n=15)
B (n=16)
C (n=14)
surface

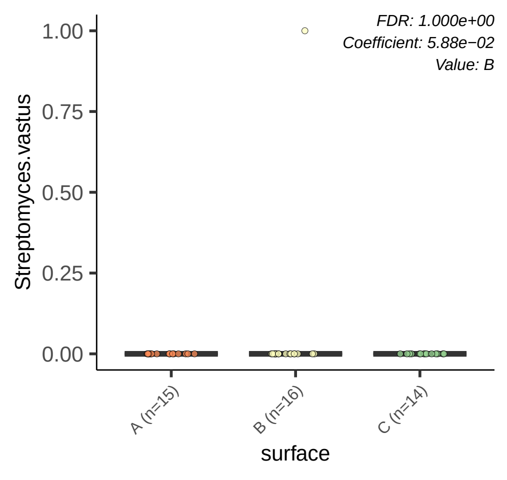
FDR: 1.000e+00
Coefficient: 5.88e−02
Value: B
Streptomyces.vastus
1.00
0.75
0.50
0.25
0.00
A (n=15)
B (n=16)
C (n=14)
surface

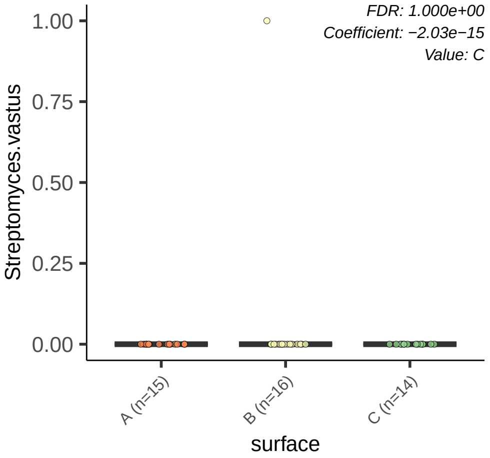
FDR: 1.000e+00
Coefficient: −2.03e−15
Value: C
Streptomyces.vastus
1.00
0.75
0.50
0.25
0.00
A (n=15)
B (n=16)
C (n=14)
surface

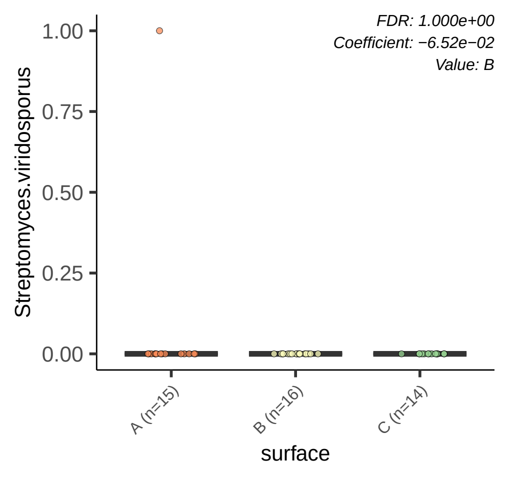
FDR: 1.000e+00
Coefficient: −6.52e−02
Value: B
Streptomyces.viridosporus
1.00
0.75
0.50
0.25
0.00
A (n=15)
B (n=16)
C (n=14)
surface

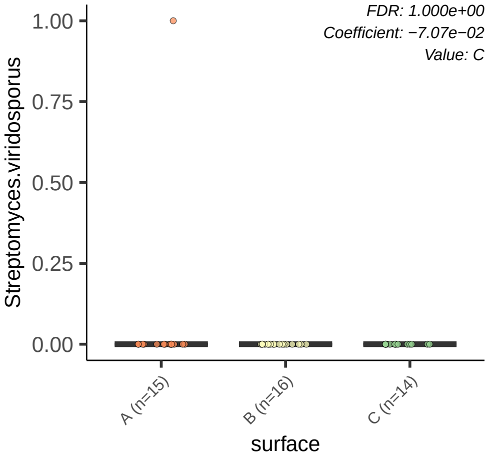
FDR: 1.000e+00
Coefficient: −7.07e−02
Value: C
Streptomyces.viridosporus
1.00
0.75
0.50
0.25
0.00
A (n=15)
B (n=16)
C (n=14)
surface

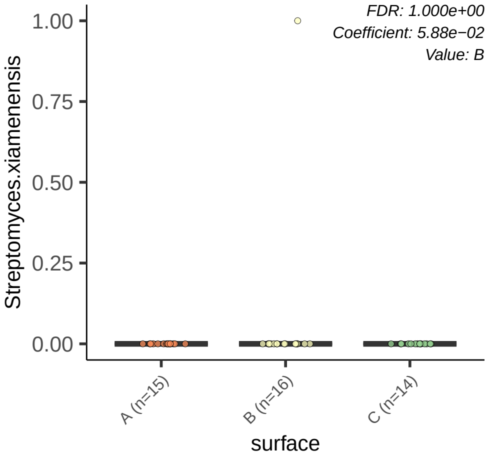

FDR: 1.000e+00
Coefficient: 5.88e−02
Value: B
Streptomyces.xiamenensis
1.00
0.75
0.50
0.25
0.00
A (n=15)
B (n=16)
C (n=14)
surface

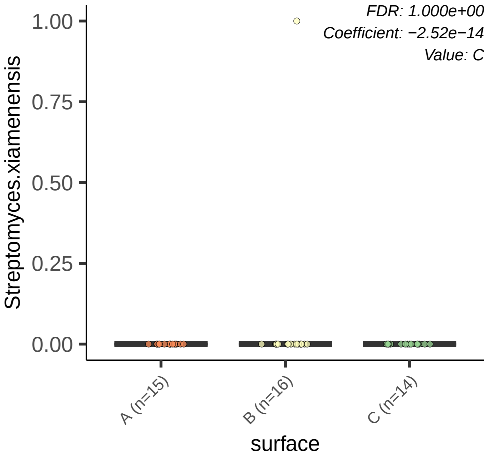

FDR: 1.000e+00
Coefficient: −2.52e−14
Value: C
Streptomyces.xiamenensis
1.00
0.75
0.50
0.25
0.00
A (n=15)
B (n=16)
C (n=14)
surface

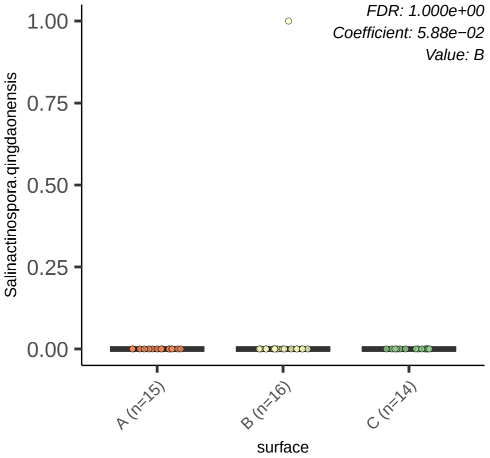
FDR: 1.000e+00
Coefficient: 5.88e−02
Value: B
Salinactinospora.qingdaonensis
1.00
0.75
0.50
0.25
0.00
A (n=15)
B (n=16)
C (n=14)
surface

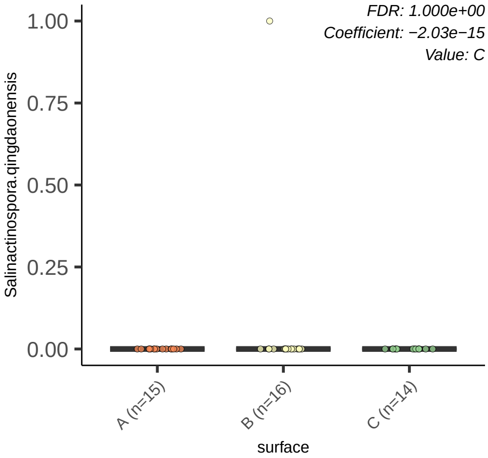
FDR: 1.000e+00
Coefficient: −2.03e−15
Value: C
1.00
0.75
0.50
0.25
0.00
Salinactinospora.qingdaonensis
A (n=15)
B (n=16)
C (n=14)
surface

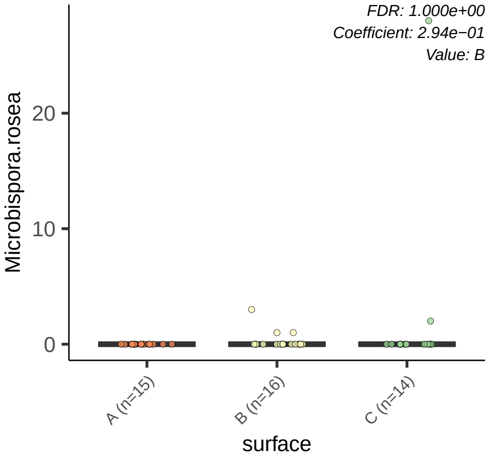

FDR: 1.000e+00
Coefficient: 2.94e−01
Value: B
Microbispora.rosea
20
10
0
A (n=15)
B (n=16)
C (n=14)
surface

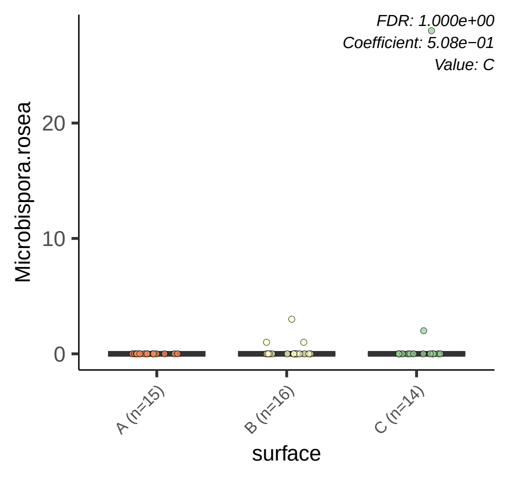

FDR: 1.000e+00
Coefficient: 5.08e−01
Value: C
Microbispora.rosea
20
10
0
A (n=15)
B (n=16)
C (n=14)
surface

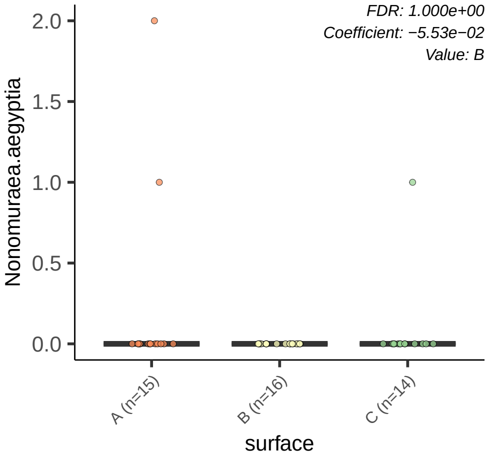
FDR: 1.000e+00
Coefficient: −5.53e−02
Value: B
Nonomuraea.aegyptia
2.0
1.5
1.0
0.5
0.0
A (n=15)
B (n=16)
C (n=14)
surface

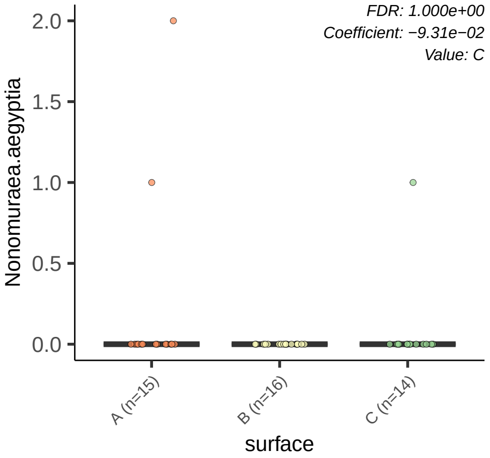
FDR: 1.000e+00
Coefficient: −9.31e−02
Value: C
Nonomuraea.aegyptia
2.0
1.5
1.0
0.5
0.0
A (n=15)
B (n=16)
C (n=14)
surface

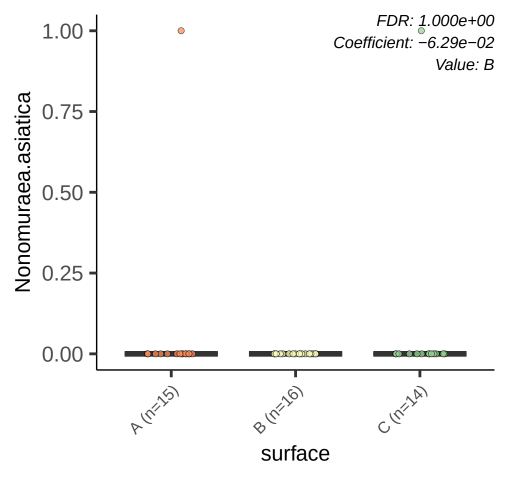

FDR: 1.000e+00
Coefficient: −6.29e−02
Value: B
Nonomuraea.asiatica
1.00
0.75
0.50
0.25
0.00
A (n=15)
B (n=16)
C (n=14)
surface

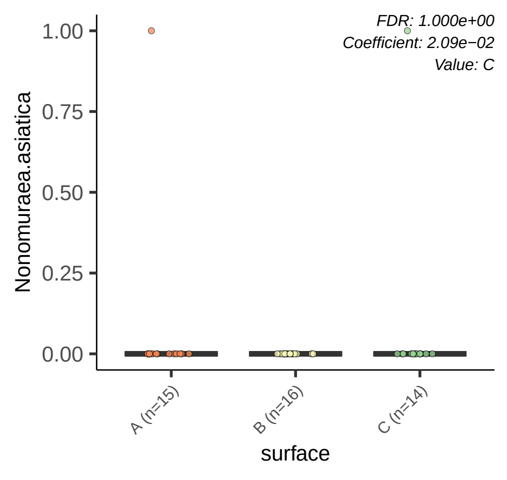
FDR: 1.000e+00
Coefficient: 2.09e−02
Value: C
Nonomuraea.asiatica
1.00
0.75
0.50
0.25
0.00
A (n=15)
B (n=16)
C (n=14)
surface

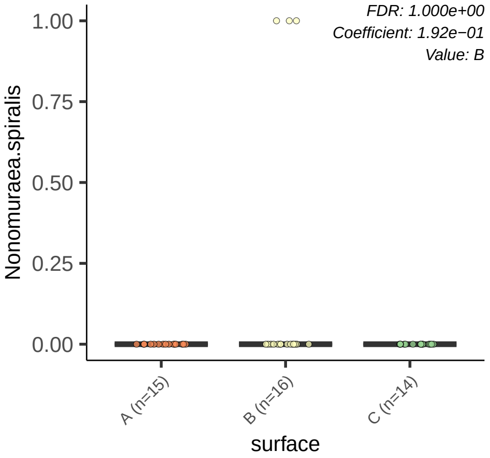
FDR: 1.000e+00
Coefficient: 1.92e−01
Value: B
Nonomuraea.spiralis
1.00
0.75
0.50
0.25
0.00
A (n=15)
B (n=16)
C (n=14)
surface

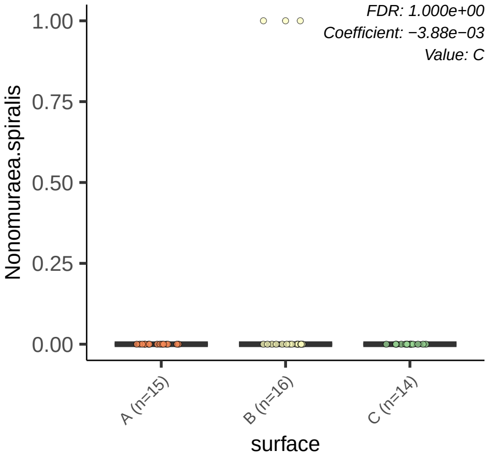
FDR: 1.000e+00
Coefficient: −3.88e−03
Value: C
Nonomuraea.spiralis
1.00
0.75
0.50
0.25
0.00
A (n=15)
B (n=16)
C (n=14)
surface

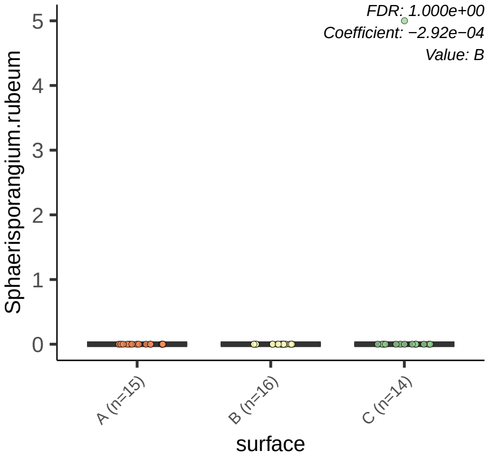

FDR: 1.000e+00
Coefficient: −2.92e−04
Value: B
Sphaerisporangium.rubeum
0
1
2
3
4
5
A (n=15)
B (n=16)
C (n=14)
surface

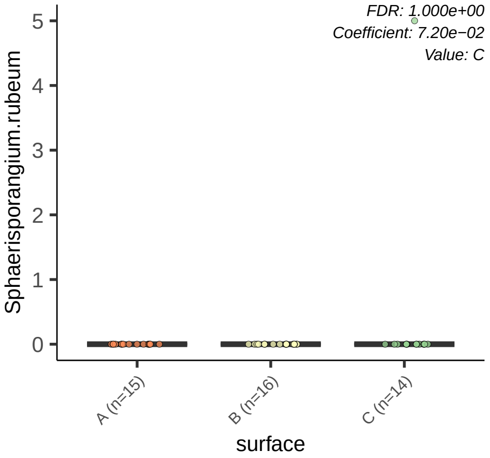
FDR: 1.000e+00
Coefficient: 7.20e−02
Value: C
Sphaerisporangium.rubeum
5
4
3
2
1
0
A (n=15)
B (n=16)
C (n=14)
surface

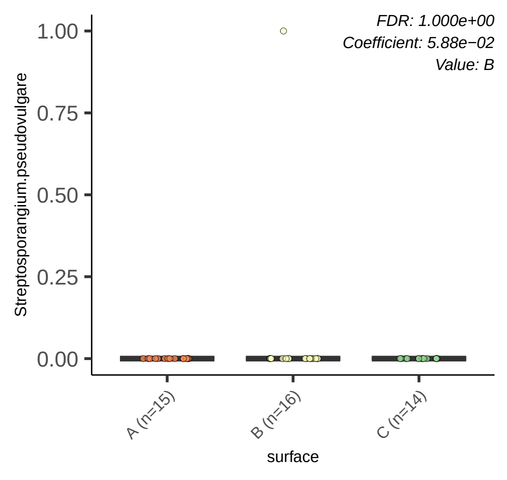
FDR: 1.000e+00
Coefficient: 5.88e−02
Value: B
Streptosporangium.pseudovulgare
1.00
0.75
0.50
0.25
0.00
A (n=15)
B (n=16)
C (n=14)
surface

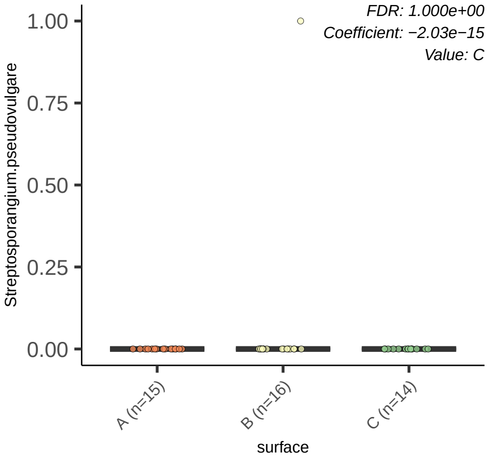

FDR: 1.000e+00
Coefficient: −2.03e−15
Value: C
1.00
0.75
0.50
0.25
0.00
Streptosporangium.pseudovulgare
A (n=15)
B (n=16)
C (n=14)
surface

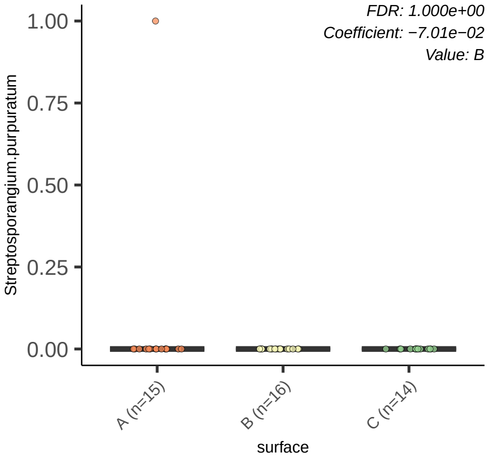

FDR: 1.000e+00
Coefficient: −7.01e−02
Value: B
1.00
0.75
0.50
0.25
0.00
Streptosporangium.purpuratum
A (n=15)
B (n=16)
C (n=14)
surface

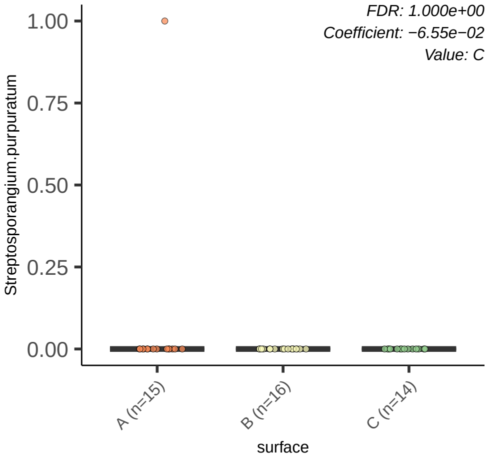
FDR: 1.000e+00
Coefficient: −6.55e−02
Value: C
Streptosporangium.purpuratum
1.00
0.75
0.50
0.25
0.00
A (n=15)
B (n=16)
C (n=14)
surface

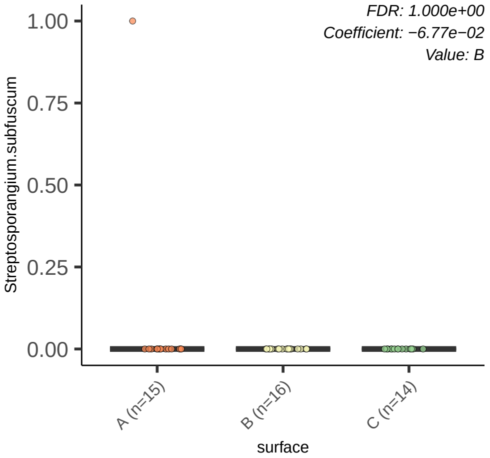

FDR: 1.000e+00
Coefficient: −6.77e−02
Value: B
Streptosporangium.subfuscum
1.00
0.75
0.50
0.25
0.00
A (n=15)
B (n=16)
C (n=14)
surface

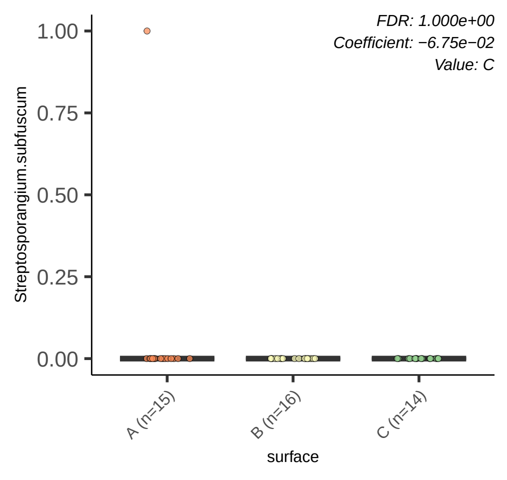
FDR: 1.000e+00
Coefficient: −6.75e−02
Value: C
Streptosporangium.subfuscum
1.00
0.75
0.50
0.25
0.00
A (n=15)
B (n=16)
C (n=14)
surface

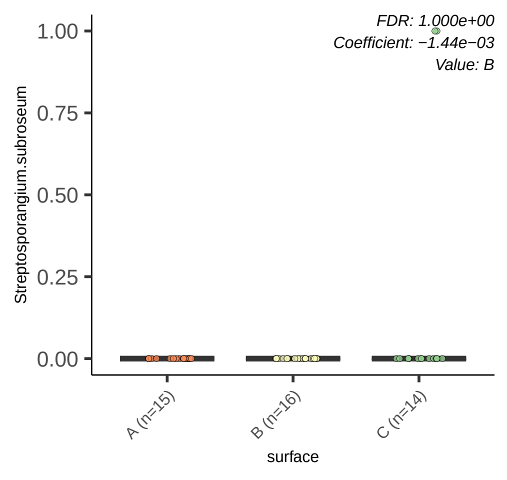

FDR: 1.000e+00
Coefficient: −1.44e−03
Value: B
Streptosporangium.subroseum
1.00
0.75
0.50
0.25
0.00
A (n=15)
B (n=16)
C (n=14)
surface

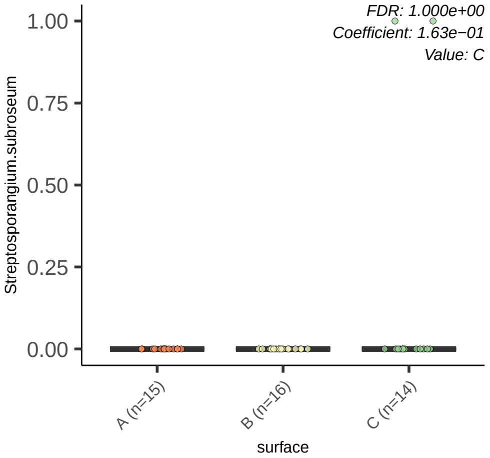

FDR: 1.000e+00
Coefficient: 1.63e−01
Value: C
1.00
0.75
0.50
0.25
0.00
Streptosporangium.subroseum
A (n=15)
B (n=16)
C (n=14)
surface

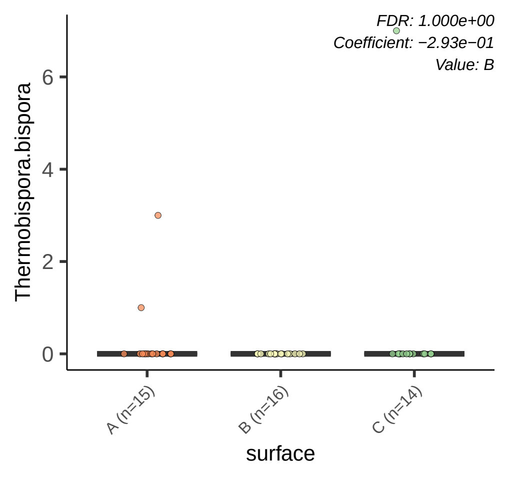

FDR: 1.000e+00
Coefficient: −2.93e−01
Value: B
Thermobispora.bispora
0
2
4
6
A (n=15)
B (n=16)
C (n=14)
surface

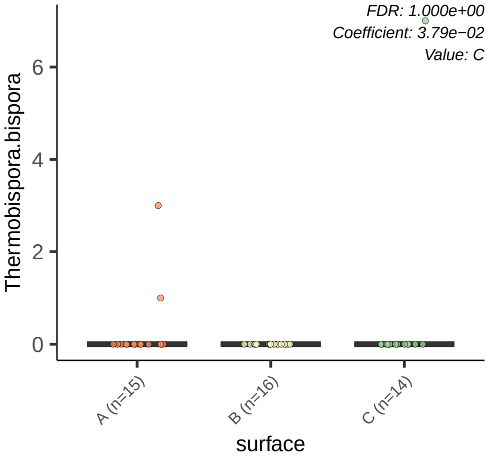

FDR: 1.000e+00
Coefficient: 3.79e−02
Value: C
Thermobispora.bispora
0
2
4
6
A (n=15)
B (n=16)
C (n=14)
surface

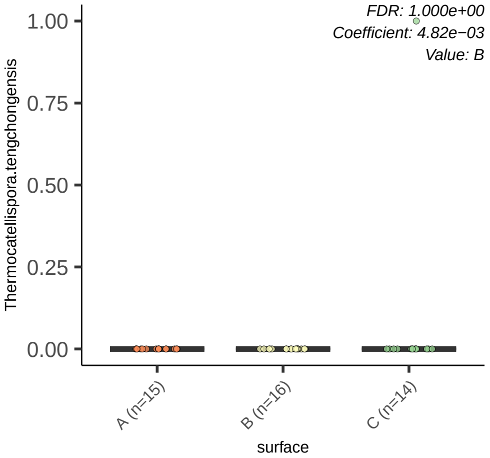
FDR: 1.000e+00
Coefficient: 4.82e−03
Value: B
Thermocatellispora.tengchongensis
1.00
0.75
0.50
0.25
0.00
A (n=15)
B (n=16)
C (n=14)
surface

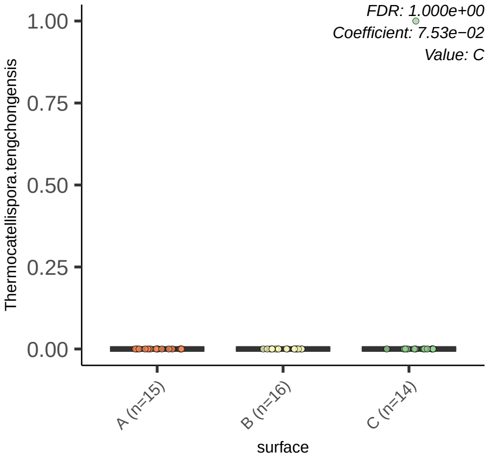
FDR: 1.000e+00
Coefficient: 7.53e−02
Value: C
Thermocatellispora.tengchongensis
1.00
0.75
0.50
0.25
0.00
A (n=15)
B (n=16)
C (n=14)
surface

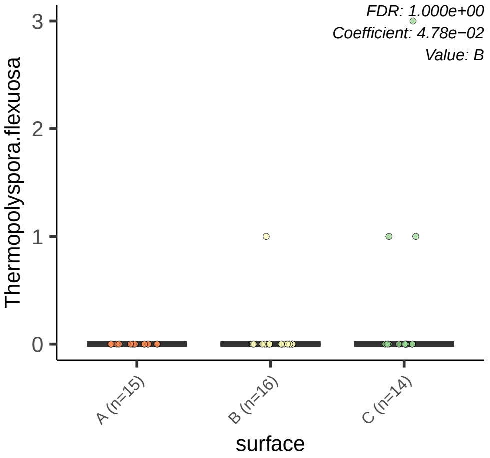

FDR: 1.000e+00
Coefficient: 4.78e−02
Value: B
Thermopolyspora.flexuosa
0
1
2
3
A (n=15)
B (n=16)
C (n=14)
surface

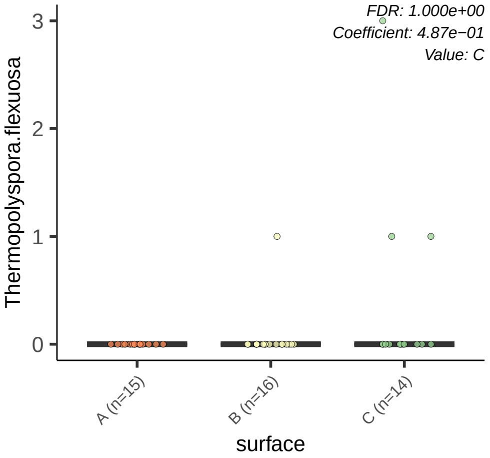

FDR: 1.000e+00
Coefficient: 4.87e−01
Value: C
Thermopolyspora.flexuosa
3
2
1
0
A (n=15)
B (n=16)
C (n=14)
surface

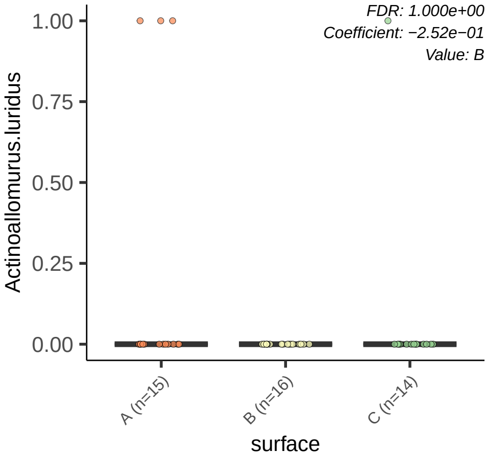
FDR: 1.000e+00
Coefficient: −2.52e−01
Value: B
Actinoallomurus.luridus
1.00
0.75
0.50
0.25
0.00
A (n=15)
B (n=16)
C (n=14)
surface

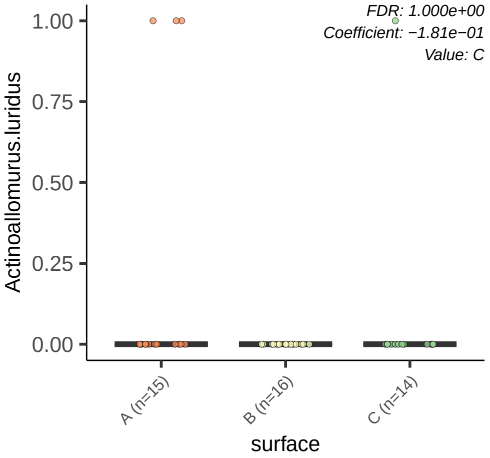
FDR: 1.000e+00
Coefficient: −1.81e−01
Value: C
Actinoallomurus.luridus
1.00
0.75
0.50
0.25
0.00
A (n=15)
B (n=16)
C (n=14)
surface

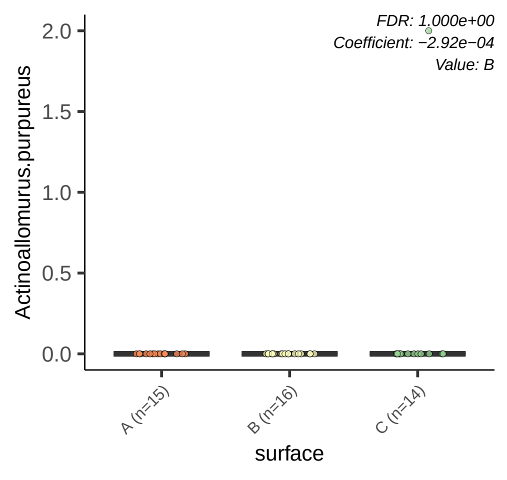
FDR: 1.000e+00
Coefficient: −2.92e−04
Value: B
Actinoallomurus.purpureus
2.0
1.5
1.0
0.5
0.0
A (n=15)
B (n=16)
C (n=14)
surface

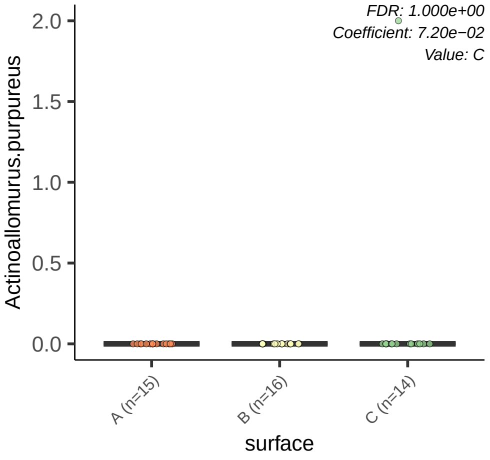
FDR: 1.000e+00
Coefficient: 7.20e−02
Value: C
Actinoallomurus.purpureus
2.0
1.5
1.0
0.5
0.0
A (n=15)
B (n=16)
C (n=14)
surface

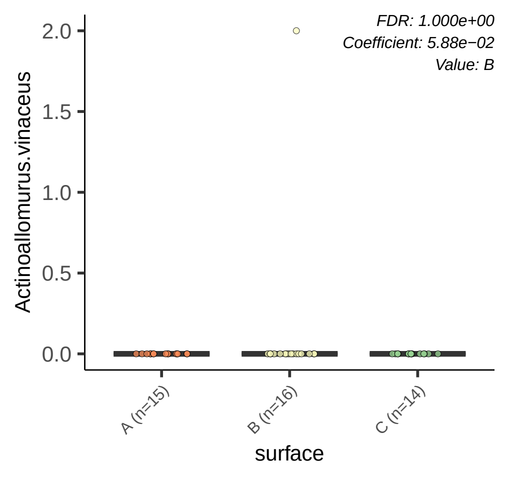
FDR: 1.000e+00
Coefficient: 5.88e−02
Value: B
Actinoallomurus.vinaceus
2.0
1.5
1.0
0.5
0.0
A (n=15)
B (n=16)
C (n=14)
surface

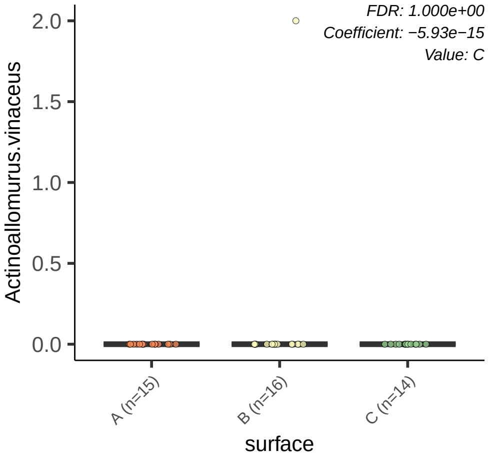

FDR: 1.000e+00
Coefficient: −5.93e−15
Value: C
Actinoallomurus.vinaceus
2.0
1.5
1.0
0.5
0.0
A (n=15)
B (n=16)
C (n=14)
surface

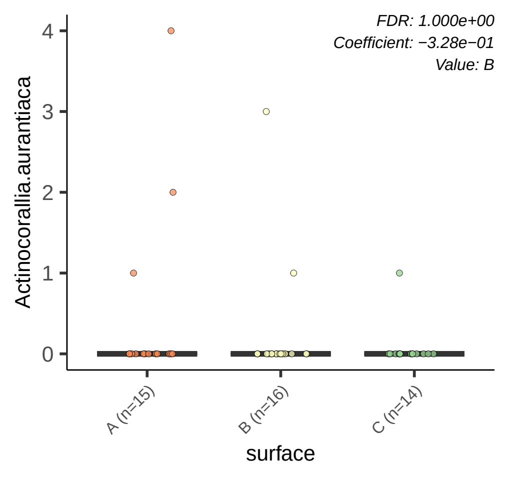

FDR: 1.000e+00
Coefficient: −3.28e−01
Value: B
Actinocorallia.aurantiaca
0
1
2
3
4
A (n=15)
B (n=16)
C (n=14)
surface

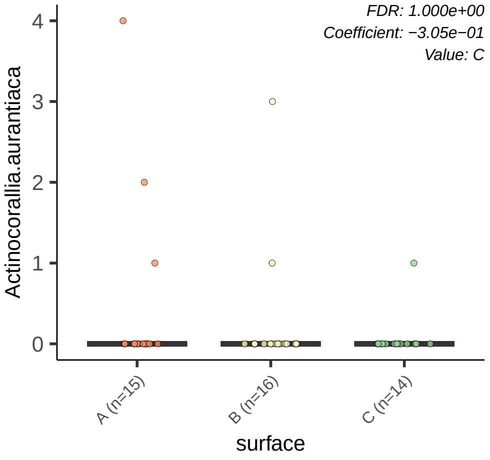
FDR: 1.000e+00
Coefficient: −3.05e−01
Value: C
Actinocorallia.aurantiaca
0
1
2
3
4
A (n=15)
B (n=16)
C (n=14)
surface

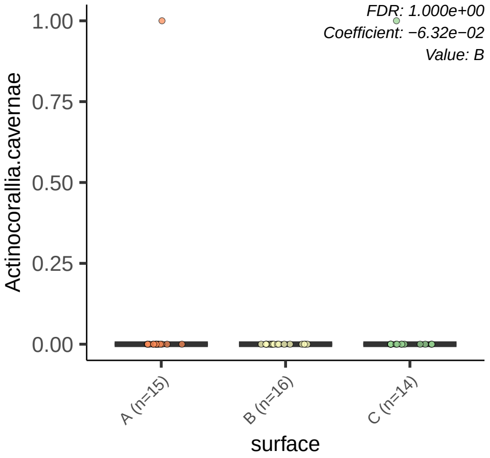
FDR: 1.000e+00
Coefficient: −6.32e−02
Value: B
Actinocorallia.cavernae
1.00
0.75
0.50
0.25
0.00
A (n=15)
B (n=16)
C (n=14)
surface

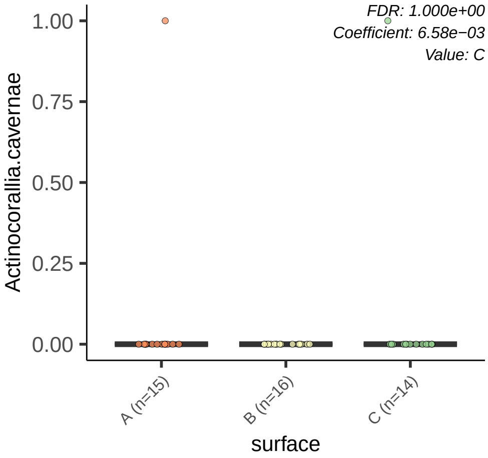
FDR: 1.000e+00
Coefficient: 6.58e−03
Value: C
Actinocorallia.cavernae
1.00
0.75
0.50
0.25
0.00
A (n=15)
B (n=16)
C (n=14)
surface

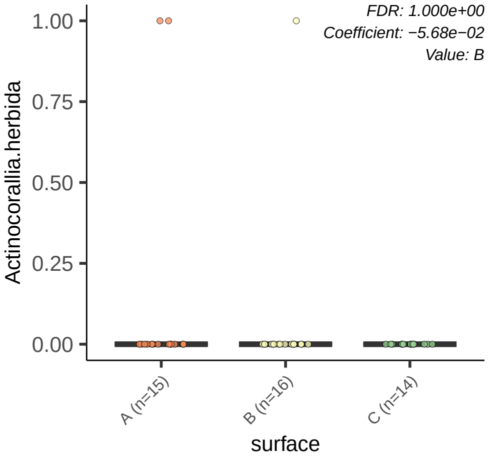
FDR: 1.000e+00
Coefficient: −5.68e−02
Value: B
Actinocorallia.herbida
1.00
0.75
0.50
0.25
0.00
A (n=15)
B (n=16)
C (n=14)
surface

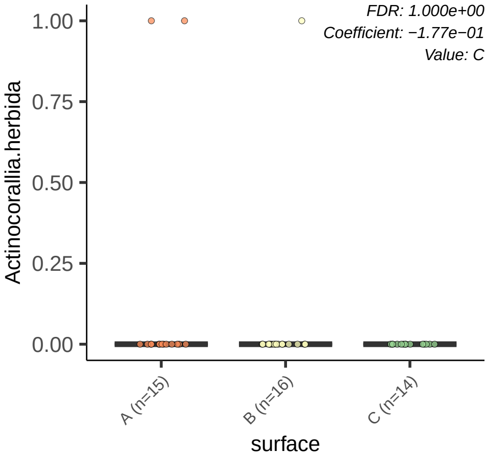
FDR: 1.000e+00
Coefficient: −1.77e−01
Value: C
Actinocorallia.herbida
1.00
0.75
0.50
0.25
0.00
A (n=15)
B (n=16)
C (n=14)
surface

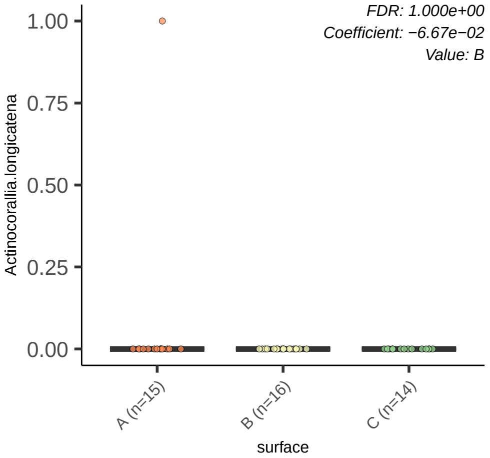
FDR: 1.000e+00
Coefficient: −6.67e−02
Value: B
1.00
0.75
0.50
0.25
0.00
Actinocorallia.longicatena
A (n=15)
B (n=16)
C (n=14)
surface

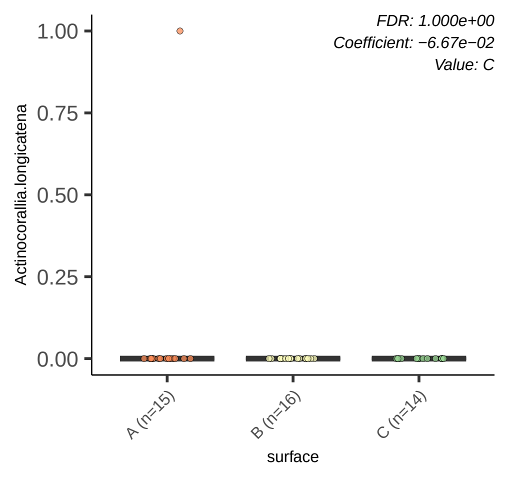
FDR: 1.000e+00
Coefficient: −6.67e−02
Value: C
Actinocorallia.longicatena
1.00
0.75
0.50
0.25
0.00
A (n=15)
B (n=16)
C (n=14)
surface

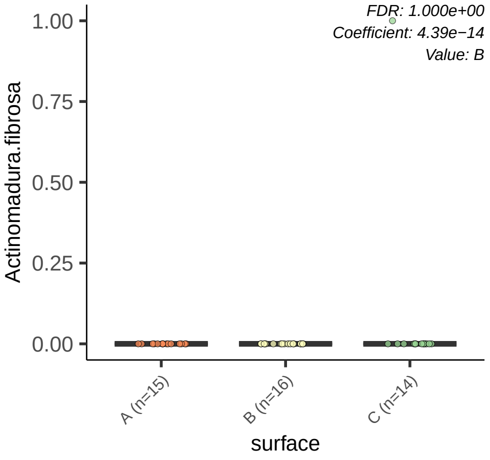
FDR: 1.000e+00
Coefficient: 4.39e−14
Value: B
Actinomadura.fibrosa
1.00
0.75
0.50
0.25
0.00
A (n=15)
B (n=16)
C (n=14)
surface

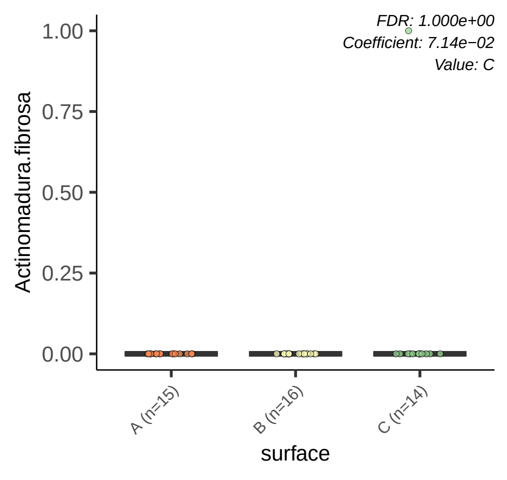
FDR: 1.000e+00
Coefficient: 7.14e−02
Value: C
Actinomadura.fibrosa
1.00
0.75
0.50
0.25
0.00
A (n=15)
B (n=16)
C (n=14)
surface

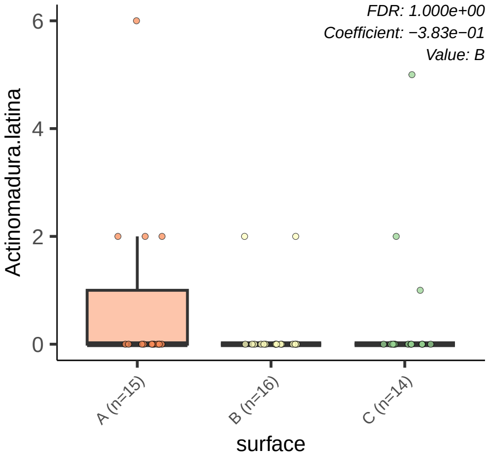
FDR: 1.000e+00
Coefficient: −3.83e−01
Value: B
Actinomadura.latina
0
2
4
6
A (n=15)
B (n=16)
C (n=14)
surface

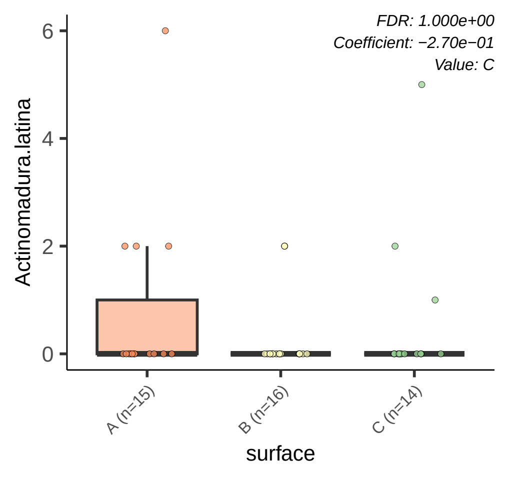
FDR: 1.000e+00
Coefficient: −2.70e−01
Value: C
Actinomadura.latina
6
4
2
0
A (n=15)
B (n=16)
C (n=14)
surface

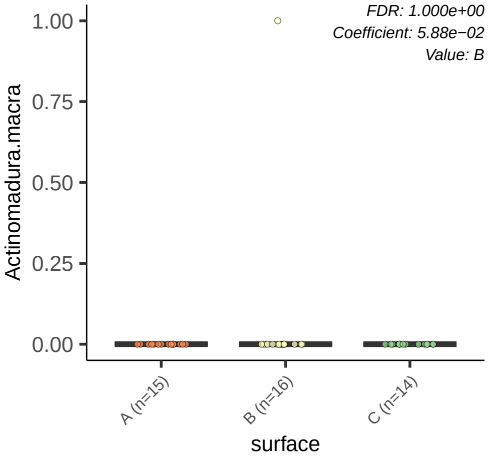
FDR: 1.000e+00
Coefficient: 5.88e−02
Value: B
Actinomadura.macra
1.00
0.75
0.50
0.25
0.00
A (n=15)
B (n=16)
C (n=14)
surface

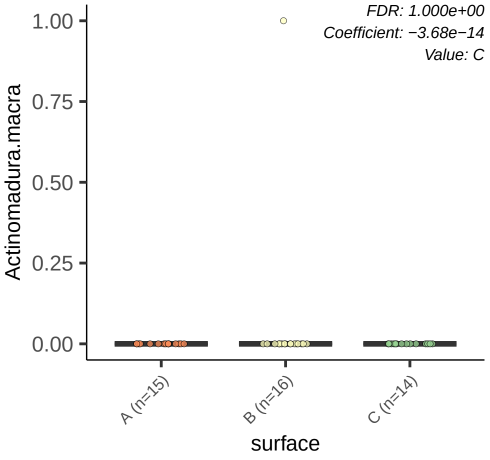

FDR: 1.000e+00
Coefficient: −3.68e−14
Value: C
Actinomadura.macra
1.00
0.75
0.50
0.25
0.00
A (n=15)
B (n=16)
C (n=14)
surface

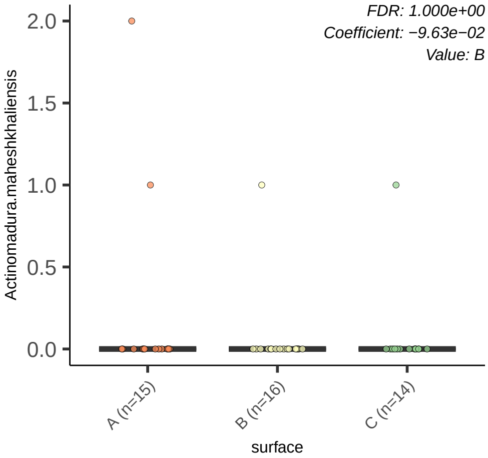

FDR: 1.000e+00
Coefficient: −9.63e−02
Value: B
Actinomadura.maheshkhaliensis
2.0
1.5
1.0
0.5
0.0
A (n=15)
B (n=16)
C (n=14)
surface

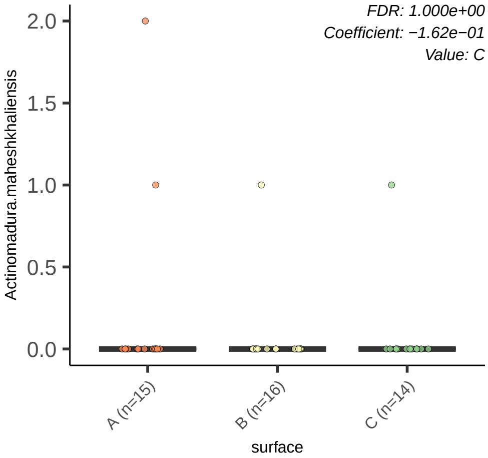

FDR: 1.000e+00
Coefficient: −1.62e−01
Value: C
Actinomadura.maheshkhaliensis
2.0
1.5
1.0
0.5
0.0
A (n=15)
B (n=16)
C (n=14)
surface

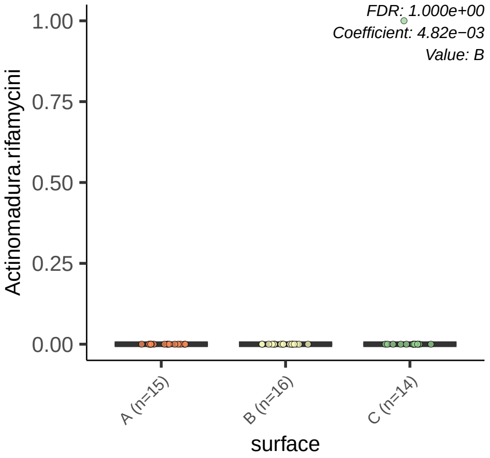
FDR: 1.000e+00
Coefficient: 4.82e−03
Value: B
Actinomadura.rifamycini
1.00
0.75
0.50
0.25
0.00
A (n=15)
B (n=16)
C (n=14)
surface

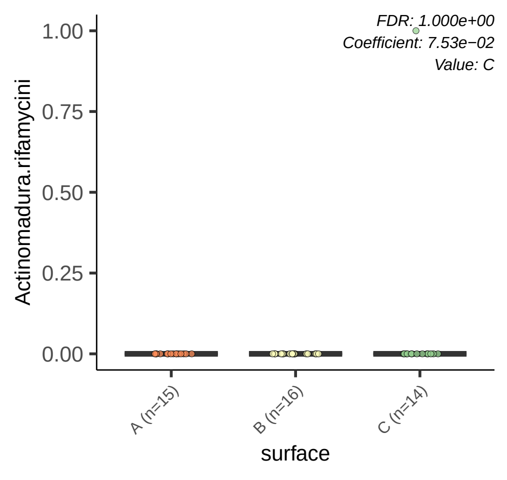

FDR: 1.000e+00
Coefficient: 7.53e−02
Value: C
Actinomadura.rifamycini
1.00
0.75
0.50
0.25
0.00
A (n=15)
B (n=16)
C (n=14)
surface

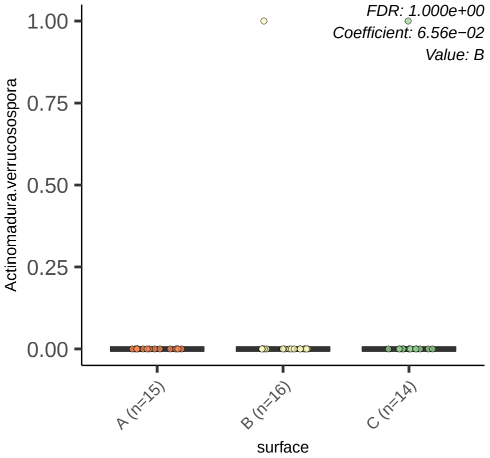

FDR: 1.000e+00
Coefficient: 6.56e−02
Value: B
Actinomadura.verrucosospora
1.00
0.75
0.50
0.25
0.00
A (n=15)
B (n=16)
C (n=14)
surface

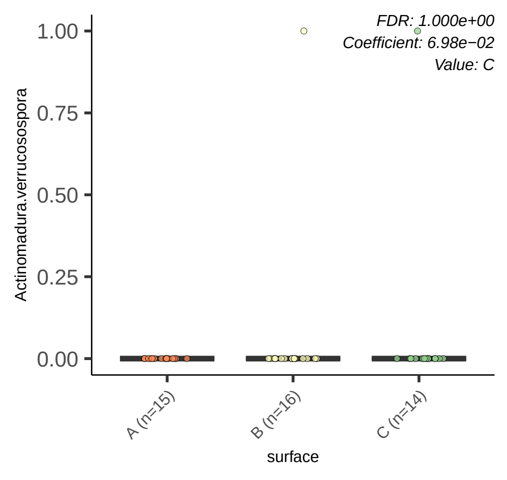

FDR: 1.000e+00
Coefficient: 6.98e−02
Value: C
Actinomadura.verrucosospora
1.00
0.75
0.50
0.25
0.00
A (n=15)
B (n=16)
C (n=14)
surface

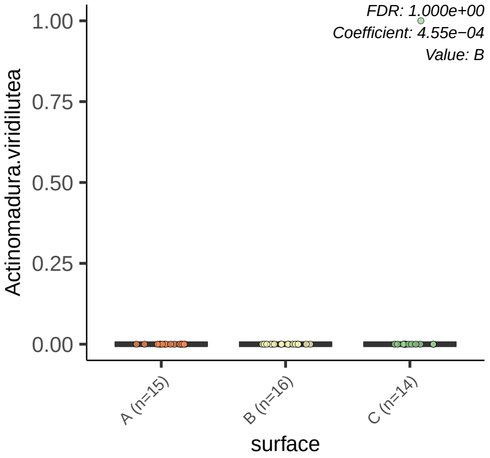
FDR: 1.000e+00
Coefficient: 4.55e−04
Value: B
Actinomadura.viridilutea
1.00
0.75
0.50
0.25
0.00
A (n=15)
B (n=16)
C (n=14)
surface

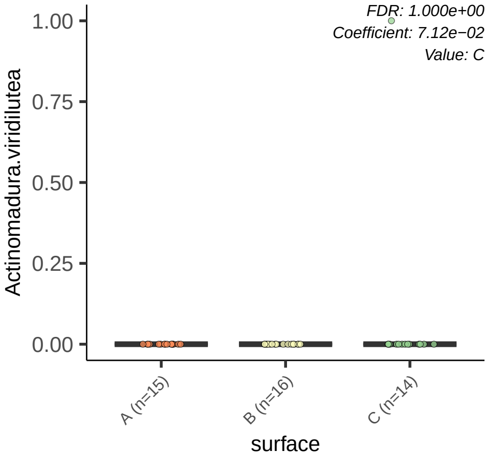

FDR: 1.000e+00
Coefficient: 7.12e−02
Value: C
Actinomadura.viridilutea
1.00
0.75
0.50
0.25
0.00
A (n=15)
B (n=16)
C (n=14)
surface

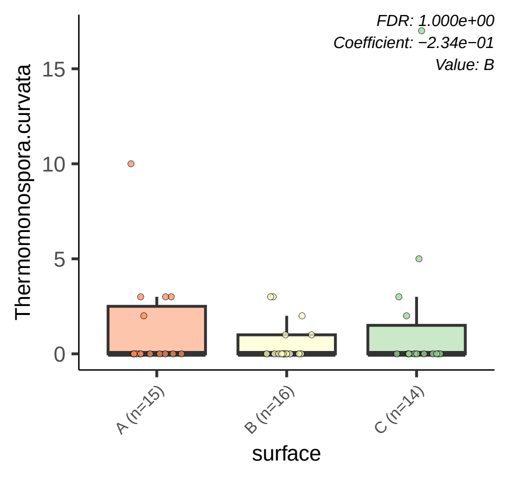
FDR: 1.000e+00
Coefficient: −2.34e−01
Value: B
Thermomonospora.curvata
0
5
10
15
A (n=15)
B (n=16)
C (n=14)
surface

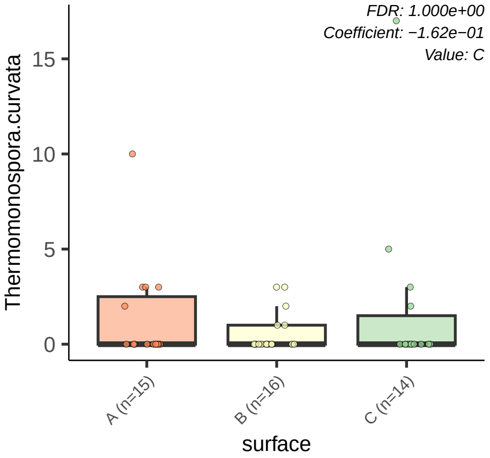

FDR: 1.000e+00
Coefficient: −1.62e−01
Value: C
Thermomonospora.curvata
15
10
5
0
A (n=15)
B (n=16)
C (n=14)
surface

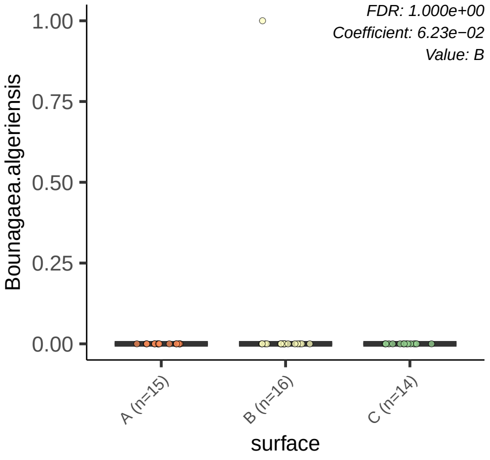
FDR: 1.000e+00
Coefficient: 6.23e−02
Value: B
Bounagaea.algeriensis
1.00
0.75
0.50
0.25
0.00
A (n=15)
B (n=16)
C (n=14)
surface

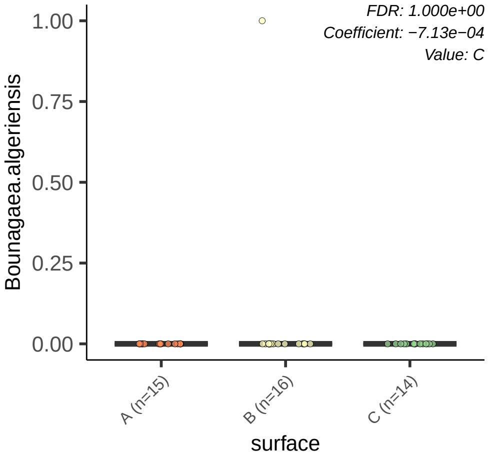
FDR: 1.000e+00
Coefficient: −7.13e−04
Value: C
Bounagaea.algeriensis
1.00
0.75
0.50
0.25
0.00
A (n=15)
B (n=16)
C (n=14)
surface

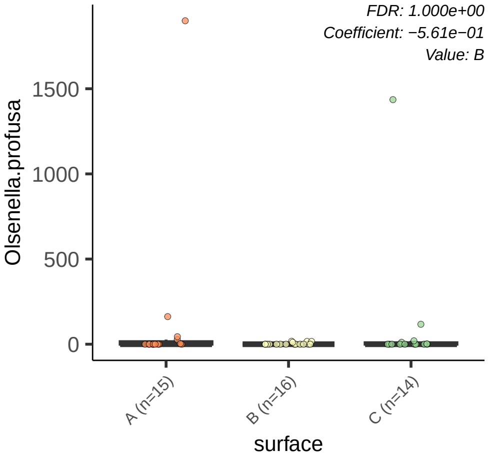
FDR: 1.000e+00
Coefficient: −5.61e−01
Value: B
Olsenella.profusa
1500
1000
500
0
A (n=15)
B (n=16)
C (n=14)
surface

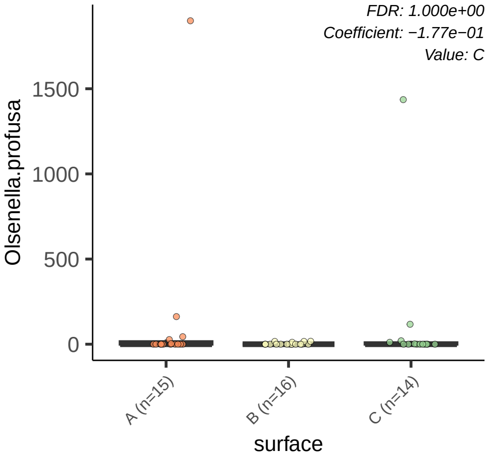
FDR: 1.000e+00
Coefficient: −1.77e−01
Value: C
Olsenella.profusa
1500
1000
500
0
A (n=15)
B (n=16)
C (n=14)
surface

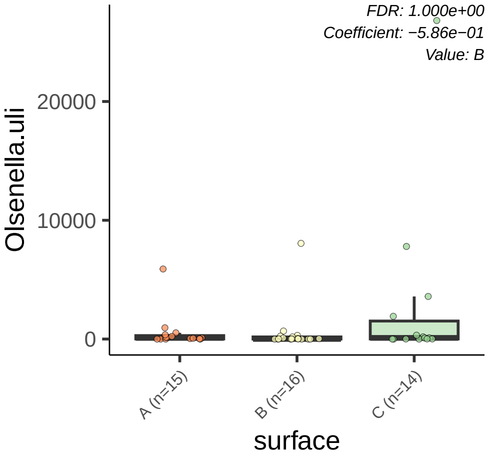
FDR: 1.000e+00
Coefficient: −5.86e−01
Value: B
Olsenella.uli
20000
10000
0
A (n=15)
B (n=16)
C (n=14)
surface

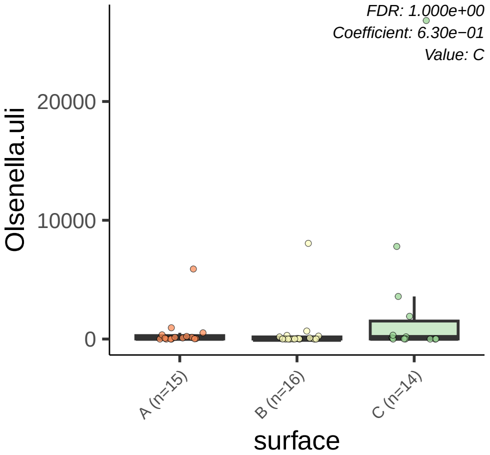
FDR: 1.000e+00
Coefficient: 6.30e−01
Value: C
Olsenella.uli
20000
10000
0
A (n=15)
B (n=16)
C (n=14)
surface

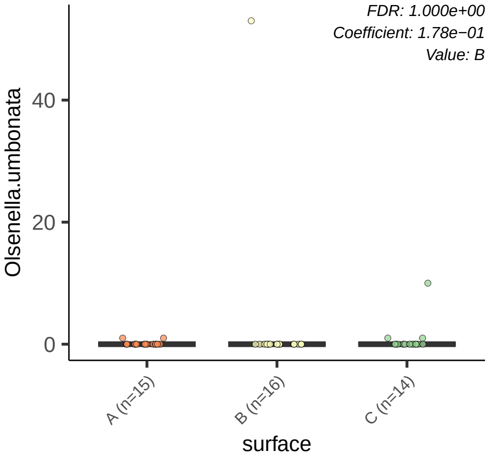

FDR: 1.000e+00
Coefficient: 1.78e−01
Value: B
Olsenella.umbonata
40
20
0
A (n=15)
B (n=16)
C (n=14)
surface

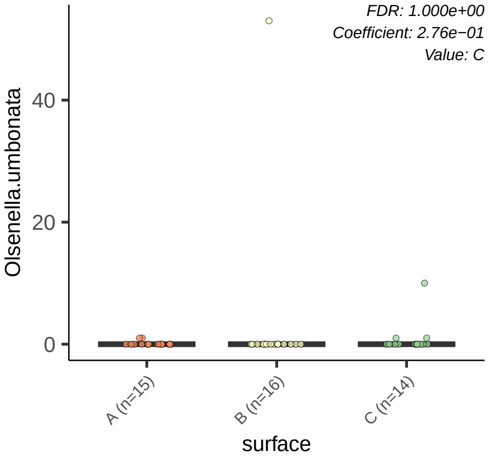

FDR: 1.000e+00
Coefficient: 2.76e−01
Value: C
Olsenella.umbonata
40
20
0
A (n=15)
B (n=16)
C (n=14)
surface

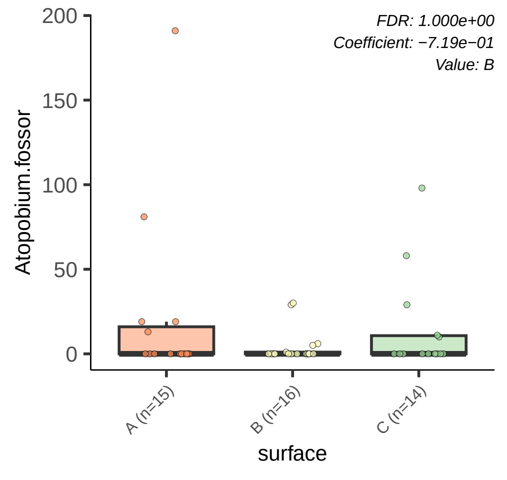

FDR: 1.000e+00
Coefficient: −7.19e−01
Value: B
Atopobium.fossor
200
150
100
50
0
A (n=15)
B (n=16)
C (n=14)
surface

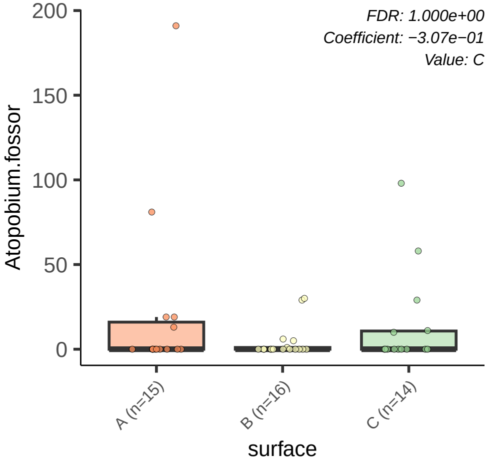

FDR: 1.000e+00
Coefficient: −3.07e−01
Value: C
Atopobium.fossor
200
150
100
50
0
A (n=15)
B (n=16)
C (n=14)
surface

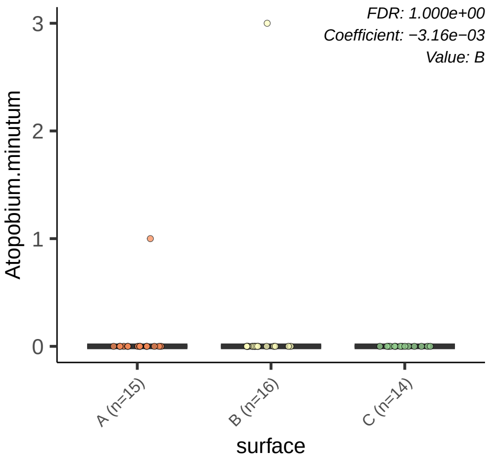

FDR: 1.000e+00
Coefficient: −3.16e−03
Value: B
Atopobium.minutum
3
2
1
0
A (n=15)
B (n=16)
C (n=14)
surface

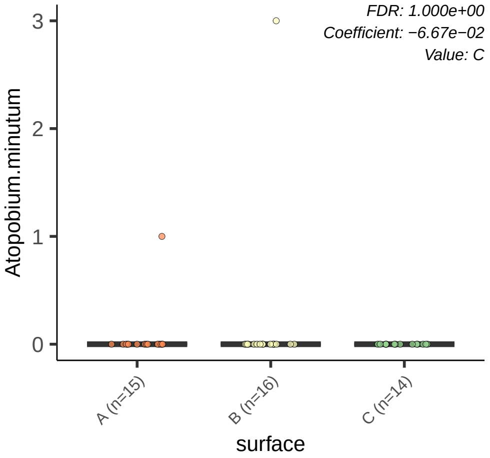
FDR: 1.000e+00
Coefficient: −6.67e−02
Value: C
Atopobium.minutum
3
2
1
0
A (n=15)
B (n=16)
C (n=14)
surface

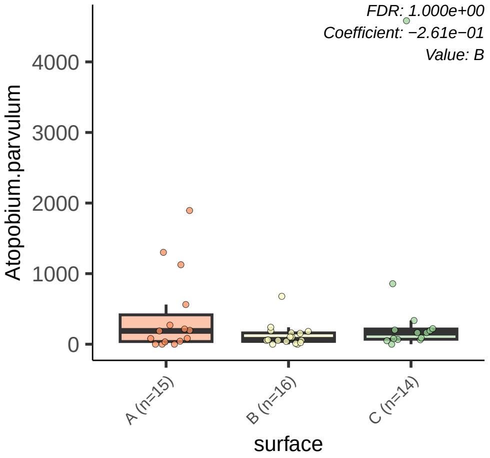

FDR: 1.000e+00
Coefficient: −2.61e−01
Value: B
Atopobium.parvulum
4000
3000
2000
1000
0
A (n=15)
B (n=16)
C (n=14)
surface

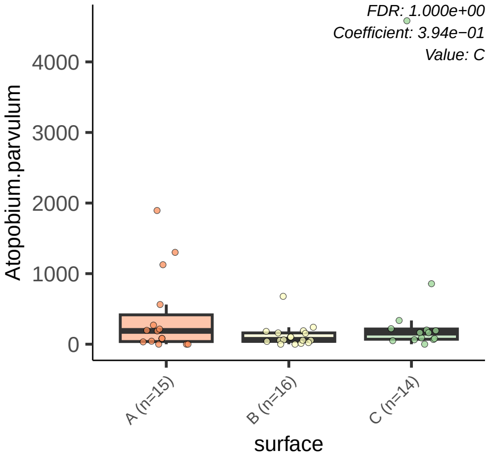
FDR: 1.000e+00
Coefficient: 3.94e−01
Value: C
Atopobium.parvulum
4000
3000
2000
1000
0
A (n=15)
B (n=16)
C (n=14)
surface

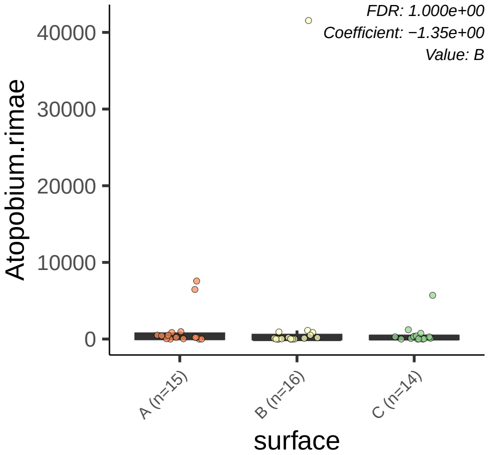

FDR: 1.000e+00
Coefficient: −1.35e+00
Value: B
40000
30000
20000
10000
0
Atopobium.rimae
A (n=15)
B (n=16)
C (n=14)
surface

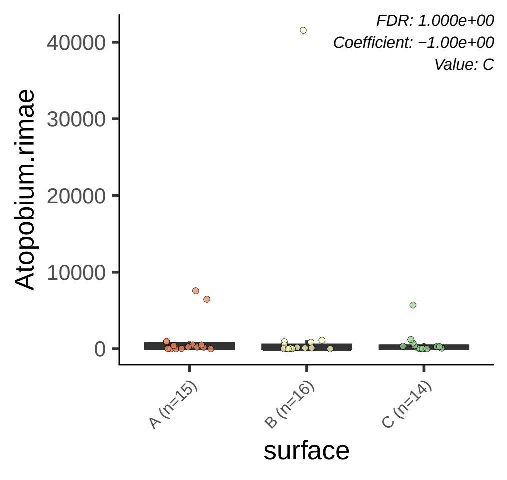

FDR: 1.000e+00
Coefficient: −1.00e+00
Value: C
40000
30000
20000
10000
0
Atopobium.rimae
A (n=15)
B (n=16)
C (n=14)
surface

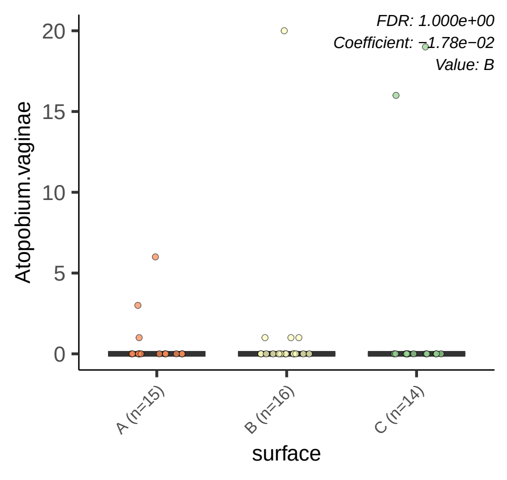

FDR: 1.000e+00
Coefficient: −1.78e−02
Value: B
Atopobium.vaginae
0
5
10
15
20
A (n=15)
B (n=16)
C (n=14)
surface

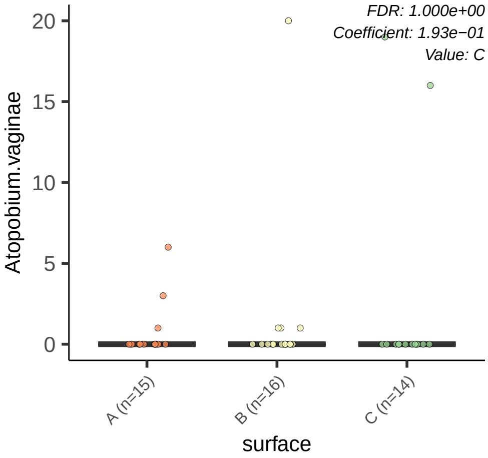
FDR: 1.000e+00
Coefficient: 1.93e−01
Value: C
Atopobium.vaginae
20
15
10
5
0
A (n=15)
B (n=16)
C (n=14)
surface

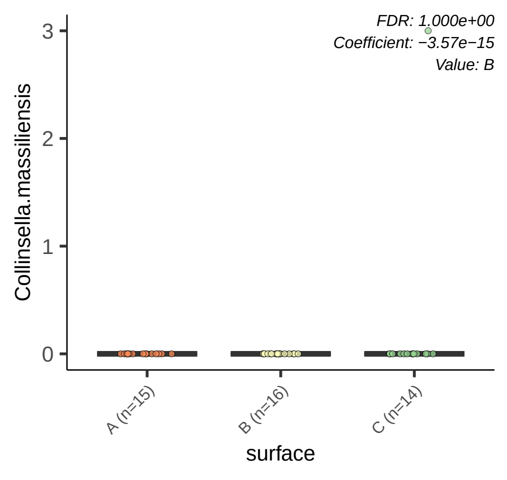
FDR: 1.000e+00
Coefficient: −3.57e−15
Value: B
Collinsella.massiliensis
3
2
1
0
A (n=15)
B (n=16)
C (n=14)
surface

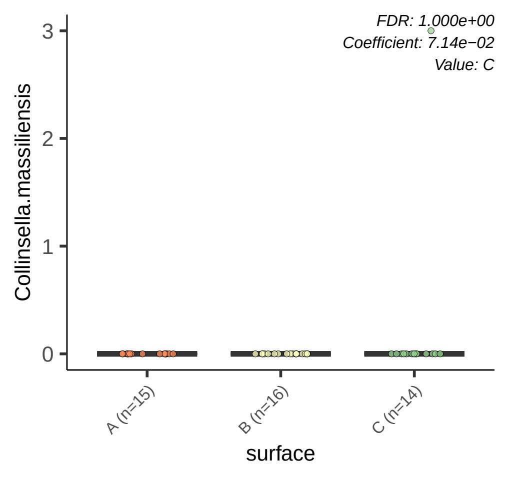

FDR: 1.000e+00
Coefficient: 7.14e−02
Value: C
Collinsella.massiliensis
3
2
1
0
A (n=15)
B (n=16)
C (n=14)
surface

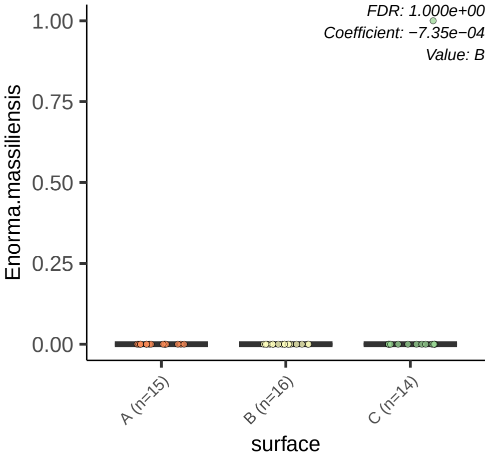
FDR: 1.000e+00
Coefficient: −7.35e−04
Value: B
Enorma.massiliensis
1.00
0.75
0.50
0.25
0.00
A (n=15)
B (n=16)
C (n=14)
surface

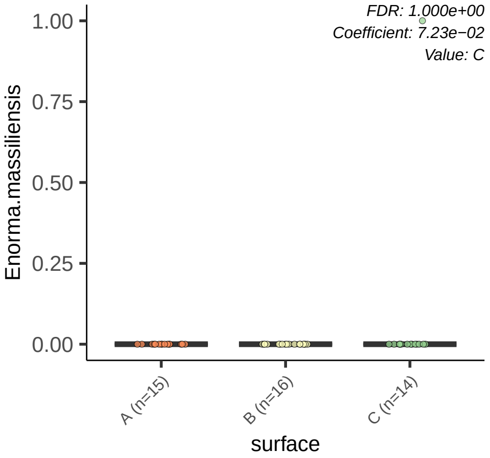
FDR: 1.000e+00
Coefficient: 7.23e−02
Value: C
Enorma.massiliensis
1.00
0.75
0.50
0.25
0.00
A (n=15)
B (n=16)
C (n=14)
surface

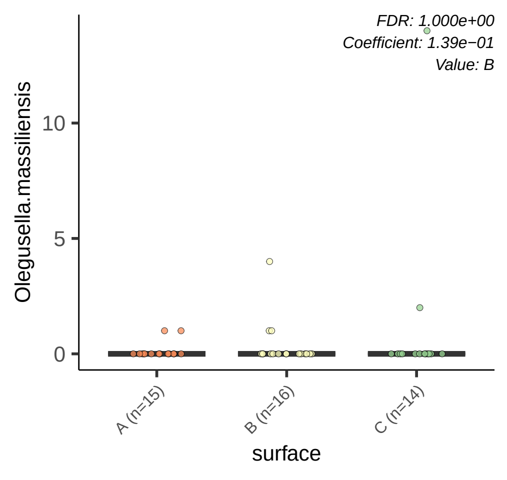
FDR: 1.000e+00
Coefficient: 1.39e−01
Value: B
Olegusella.massiliensis
10
5
0
A (n=15)
B (n=16)
C (n=14)
surface

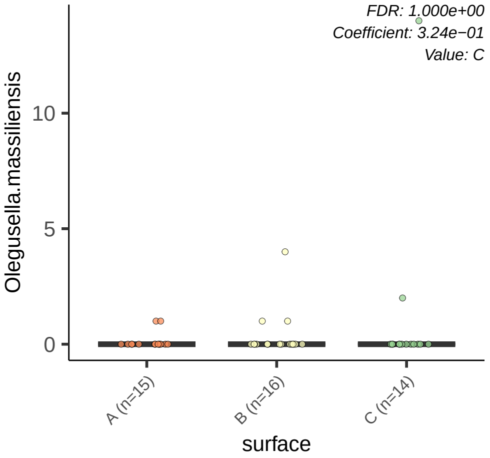

FDR: 1.000e+00
Coefficient: 3.24e−01
Value: C
Olegusella.massiliensis
0
5
10
A (n=15)
B (n=16)
C (n=14)
surface

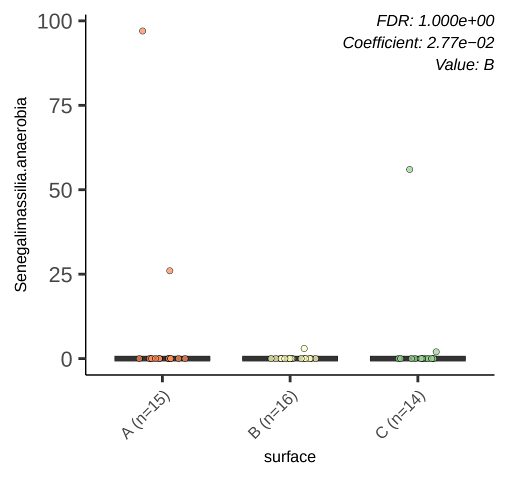

FDR: 1.000e+00
Coefficient: 2.77e−02
Value: B
100
75
50
25
0
Senegalimassilia.anaerobia
A (n=15)
B (n=16)
C (n=14)
surface

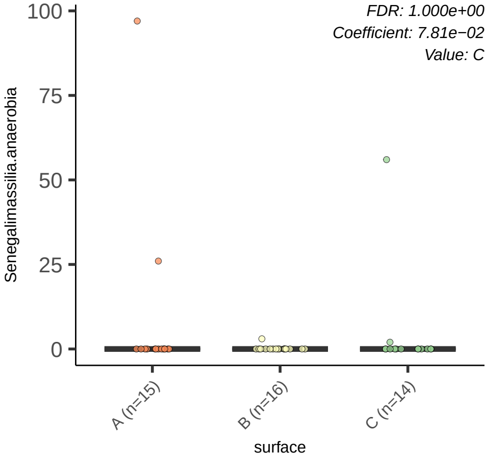
FDR: 1.000e+00
Coefficient: 7.81e−02
Value: C
Senegalimassilia.anaerobia
100
75
50
25
0
A (n=15)
B (n=16)
C (n=14)
surface

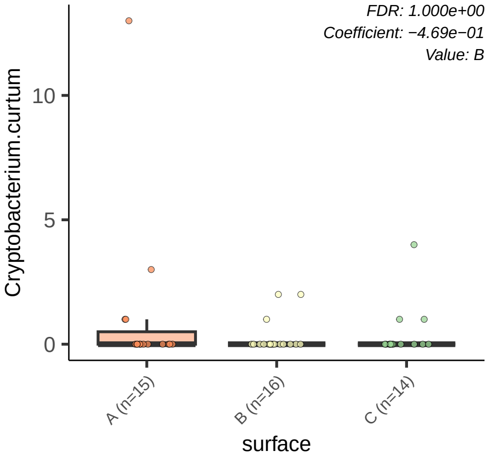

FDR: 1.000e+00
Coefficient: −4.69e−01
Value: B
Cryptobacterium.curtum
10
5
0
A (n=15)
B (n=16)
C (n=14)
surface

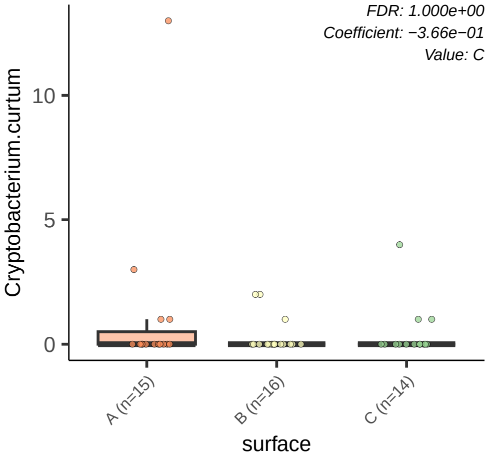
FDR: 1.000e+00
Coefficient: −3.66e−01
Value: C
Cryptobacterium.curtum
0
5
10
A (n=15)
B (n=16)
C (n=14)
surface

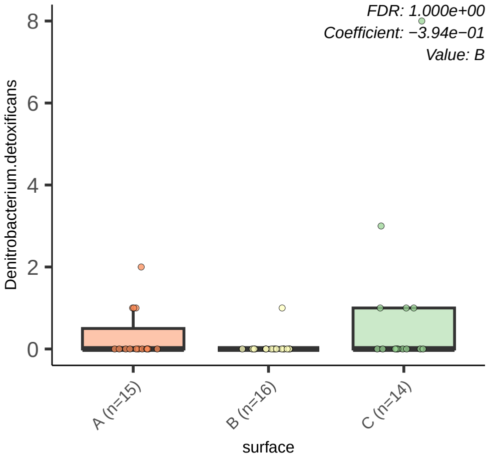
FDR: 1.000e+00
Coefficient: −3.94e−01
Value: B
Denitrobacterium.detoxificans
8
6
4
2
0
A (n=15)
B (n=16)
C (n=14)
surface

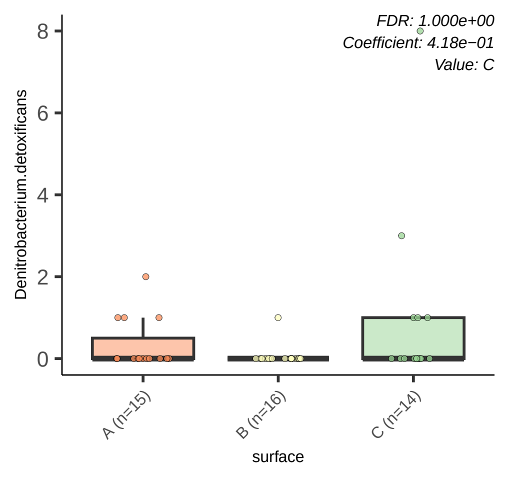

FDR: 1.000e+00
Coefficient: 4.18e−01
Value: C
Denitrobacterium.detoxificans
8
6
4
2
0
A (n=15)
B (n=16)
C (n=14)
surface

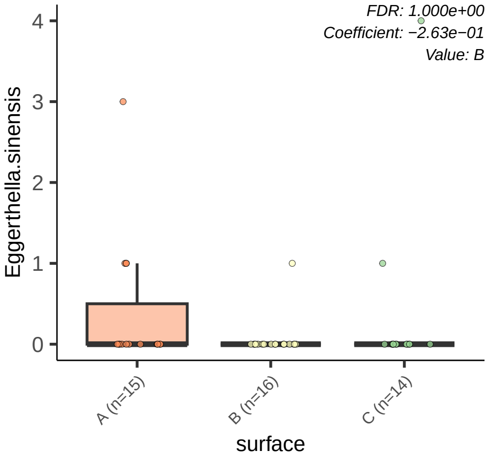

FDR: 1.000e+00
Coefficient: −2.63e−01
Value: B
Eggerthella.sinensis
0
1
2
3
4
A (n=15)
B (n=16)
C (n=14)
surface

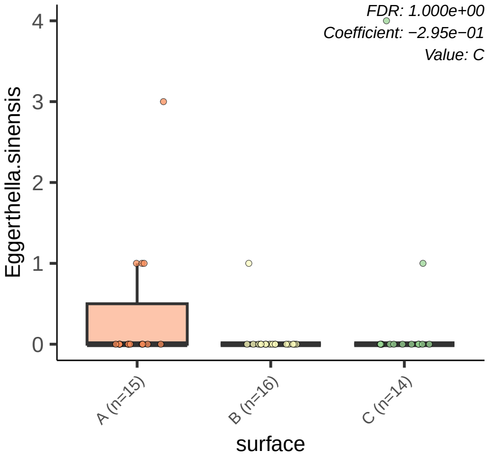
FDR: 1.000e+00
Coefficient: −2.95e−01
Value: C
Eggerthella.sinensis
0
1
2
3
4
A (n=15)
B (n=16)
C (n=14)
surface

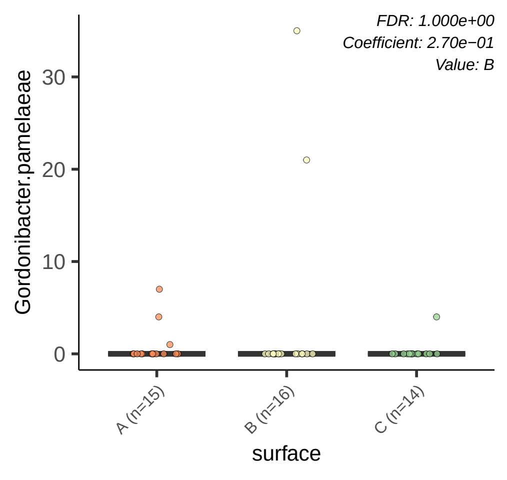
FDR: 1.000e+00
Coefficient: 2.70e−01
Value: B
Gordonibacter.pamelaeae
0
10
20
30
A (n=15)
B (n=16)
C (n=14)
surface

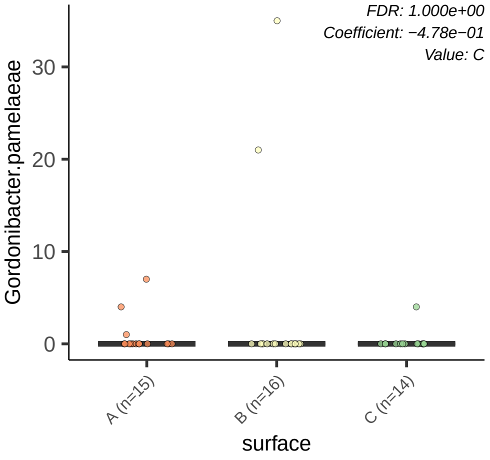

FDR: 1.000e+00
Coefficient: −4.78e−01
Value: C
Gordonibacter.pamelaeae
0
10
20
30
A (n=15)
B (n=16)
C (n=14)
surface

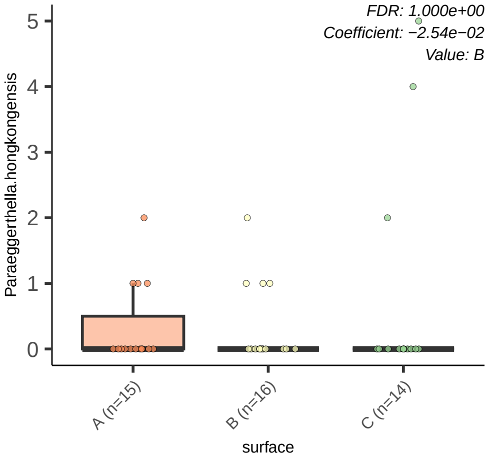

FDR: 1.000e+00
Coefficient: −2.54e−02
Value: B
Paraeggerthella.hongkongensis
5
4
3
2
1
0
A (n=15)
B (n=16)
C (n=14)
surface

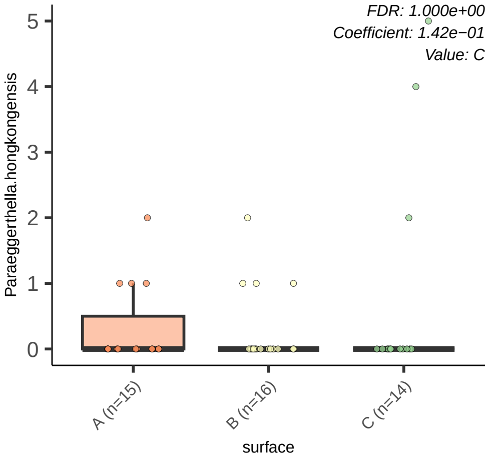

FDR: 1.000e+00
Coefficient: 1.42e−01
Value: C
Paraeggerthella.hongkongensis
5
4
3
2
1
0
A (n=15)
B (n=16)
C (n=14)
surface

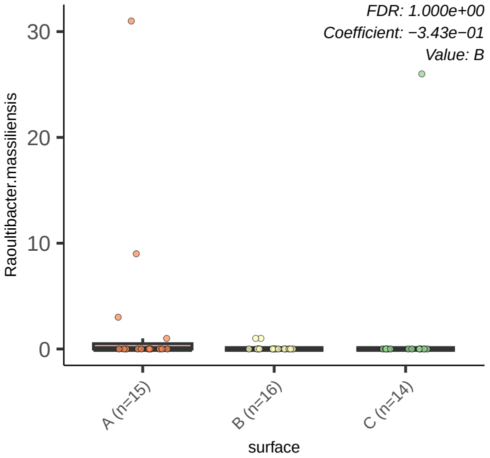
FDR: 1.000e+00
Coefficient: −3.43e−01
Value: B
30
20
10
0
Raoultibacter.massiliensis
A (n=15)
B (n=16)
C (n=14)
surface

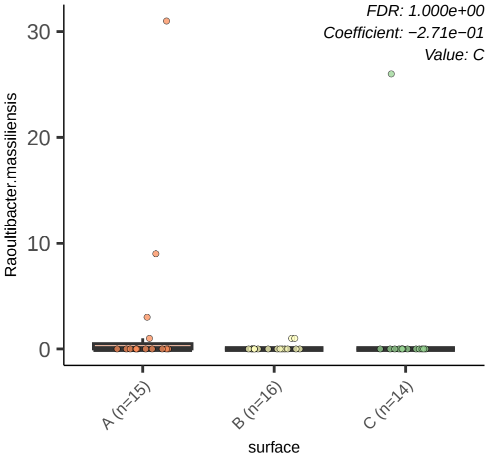

FDR: 1.000e+00
Coefficient: −2.71e−01
Value: C
Raoultibacter.massiliensis
30
20
10
0
A (n=15)
B (n=16)
C (n=14)
surface

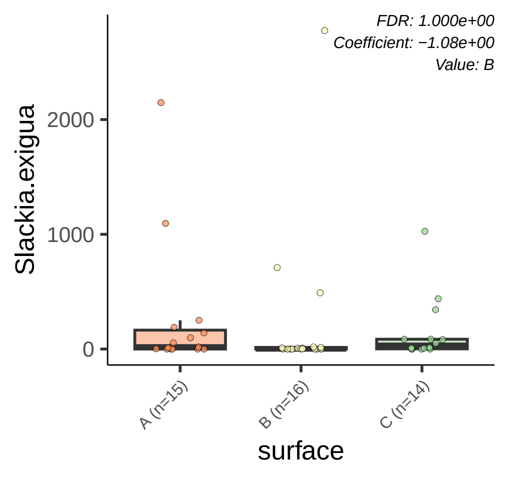

FDR: 1.000e+00
Coefficient: −1.08e+00
Value: B
Slackia.exigua
2000
1000
0
A (n=15)
B (n=16)
C (n=14)
surface

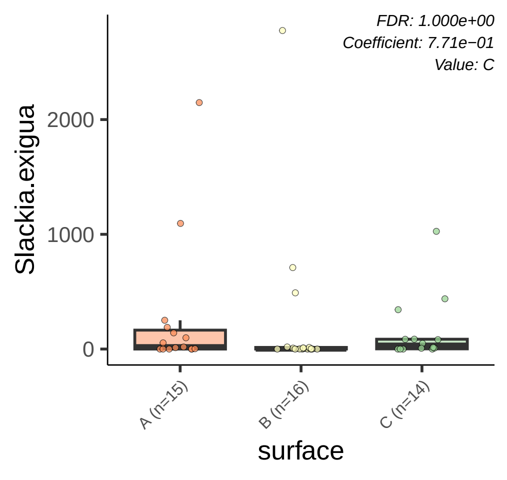

FDR: 1.000e+00
Coefficient: 7.71e−01
Value: C
Slackia.exigua
2000
1000
0
A (n=15)
B (n=16)
C (n=14)
surface

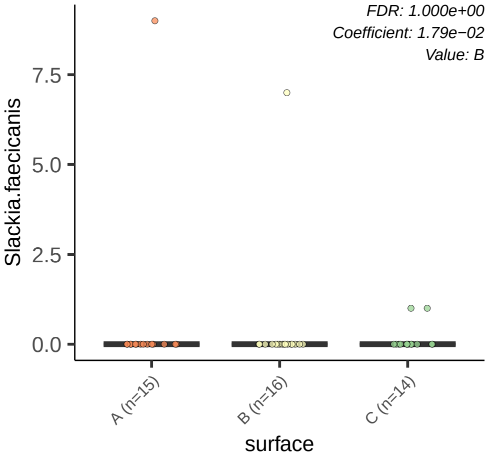

FDR: 1.000e+00
Coefficient: 1.79e−02
Value: B
Slackia.faecicanis
7.5
5.0
2.5
0.0
A (n=15)
B (n=16)
C (n=14)
surface

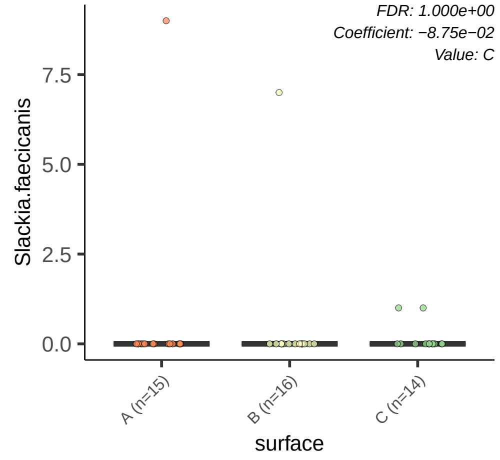
FDR: 1.000e+00
Coefficient: −8.75e−02
Value: C
Slackia.faecicanis
7.5
5.0
2.5
0.0
A (n=15)
B (n=16)
C (n=14)
surface

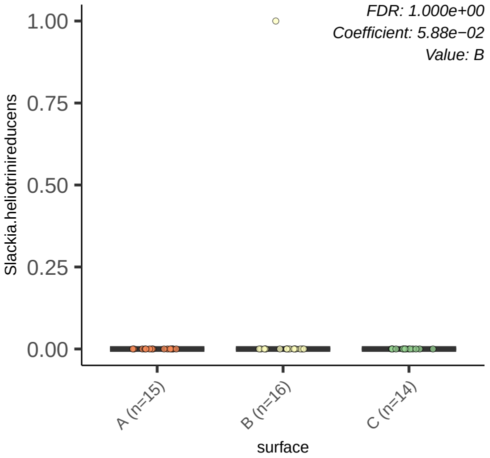

FDR: 1.000e+00
Coefficient: 5.88e−02
Value: B
Slackia.heliotrinireducens
1.00
0.75
0.50
0.25
0.00
A (n=15)
B (n=16)
C (n=14)
surface

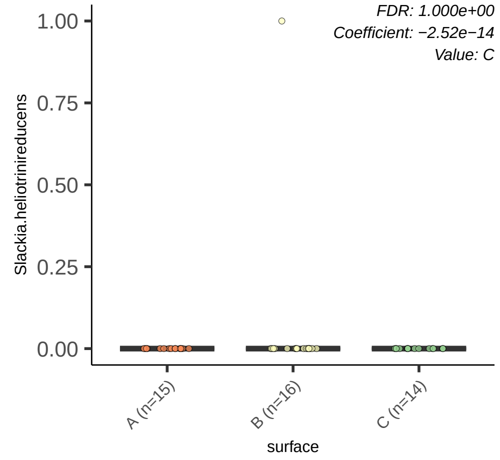

FDR: 1.000e+00
Coefficient: −2.52e−14
Value: C
1.00
0.75
0.50
0.25
0.00
Slackia.heliotriniredducens
A (n=15)
B (n=16)
C (n=14)
surface

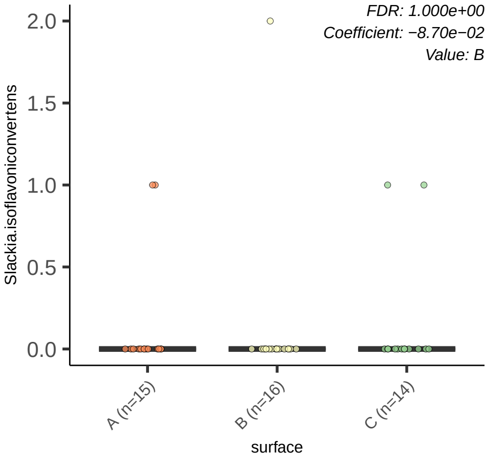
FDR: 1.000e+00
Coefficient: −8.70e−02
Value: B
Slackia.isoflavoniconvertens
2.0
1.5
1.0
0.5
0.0
A (n=15)
B (n=16)
C (n=14)
surface

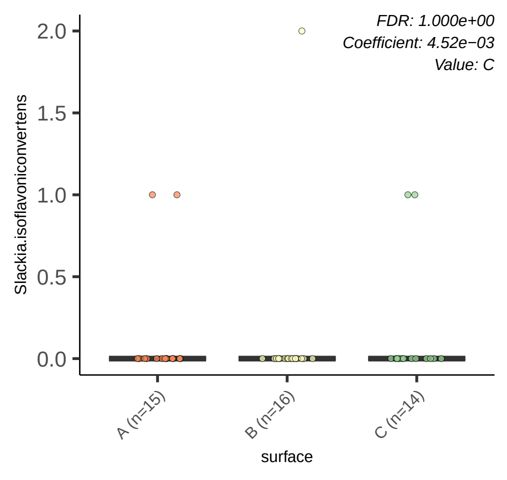

FDR: 1.000e+00
Coefficient: 4.52e−03
Value: C
Slackia.isoflavoniconvertens
2.0
1.5
1.0
0.5
0.0
A (n=15)
B (n=16)
C (n=14)
surface

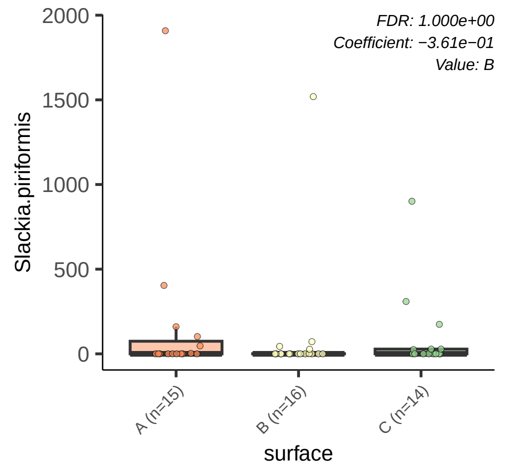
FDR: 1.000e+00
Coefficient: −3.61e−01
Value: B
2000
1500
1000
500
0
Slackia.piriformis
A (n=15)
B (n=16)
C (n=14)
surface

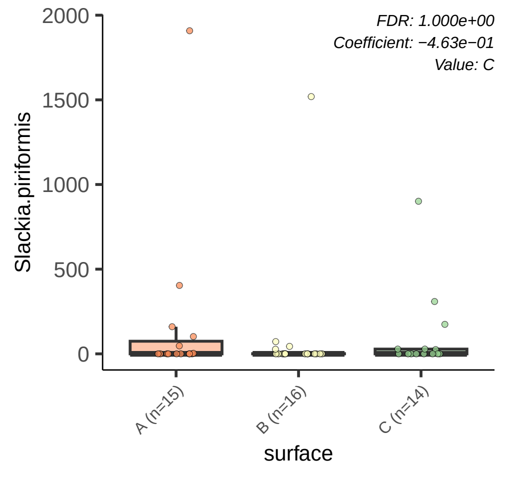

FDR: 1.000e+00
Coefficient: −4.63e−01
Value: C
2000
1500
1000
500
0
Slackia.piriformis
A (n=15)
B (n=16)
C (n=14)
surface

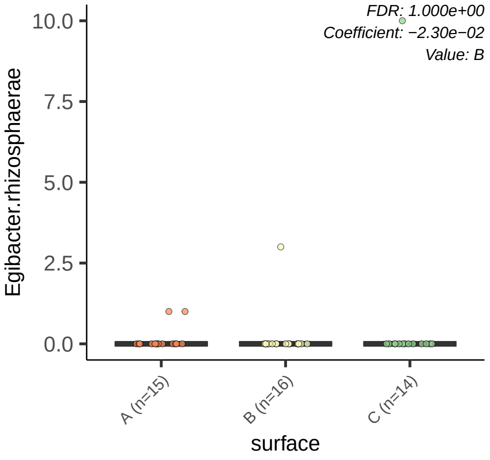

FDR: 1.000e+00
Coefficient: −2.30e−02
Value: B
Egibacter.rhizosphaerae
10.0
7.5
5.0
2.5
0.0
A (n=15)
B (n=16)
C (n=14)
surface

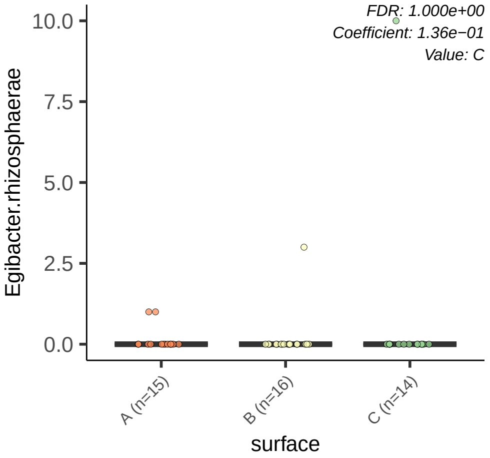
FDR: 1.000e+00
Coefficient: 1.36e−01
Value: C
Egibacter.rhizosphaerae
10.0
7.5
5.0
2.5
0.0
A (n=15)
B (n=16)
C (n=14)
surface

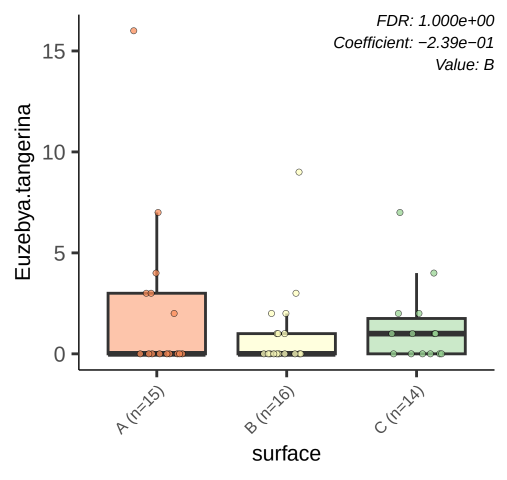

FDR: 1.000e+00
Coefficient: −2.39e−01
Value: B
Euzebya.tangerina
0
5
10
15
A (n=15)
B (n=16)
C (n=14)
surface

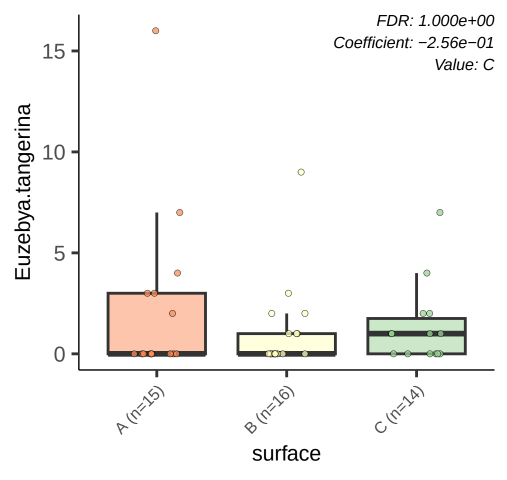
FDR: 1.000e+00
Coefficient: −2.56e−01
Value: C
Euzebya.tangerina
15
10
5
0
A (n=15)
B (n=16)
C (n=14)
surface

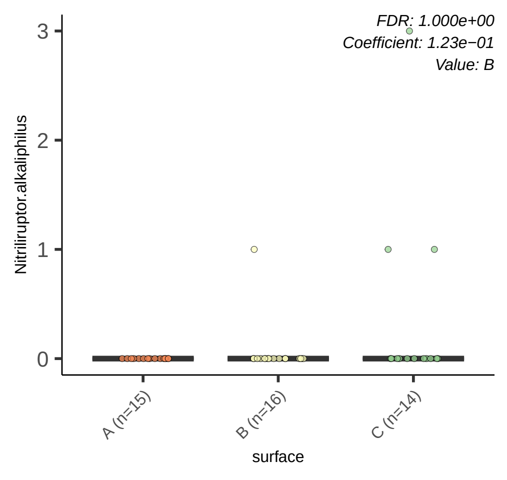
FDR: 1.000e+00
Coefficient: 1.23e−01
Value: B
Nitriliruptor.alkaliphilus
3
2
1
0
A (n=15)
B (n=16)
C (n=14)
surface

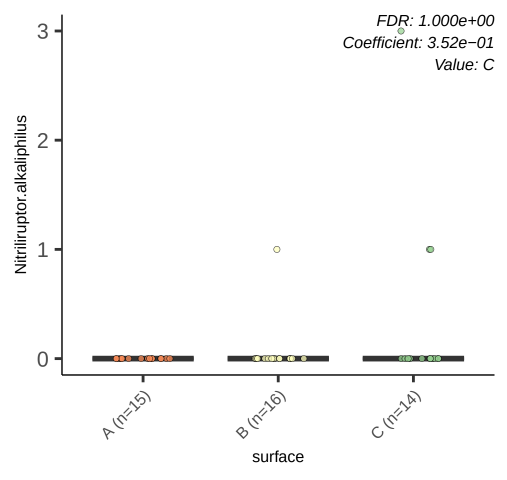
FDR: 1.000e+00
Coefficient: 3.52e−01
Value: C
Nitriliruptor.alkaliphilus
3
2
1
0
A (n=15)
B (n=16)
C (n=14)
surface

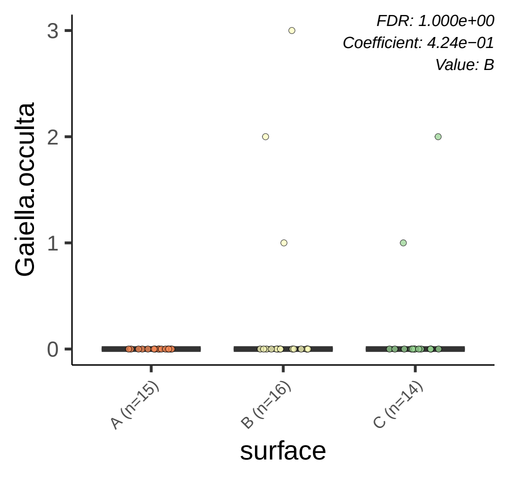
FDR: 1.000e+00
Coefficient: 4.24e−01
Value: B
Gaiella.occulta
3
2
1
0
A (n=15)
B (n=16)
C (n=14)
surface

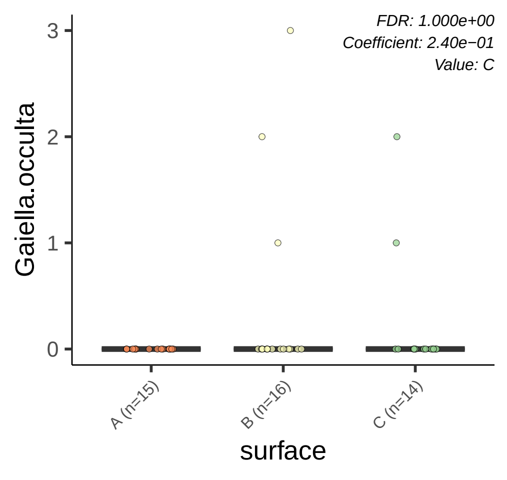
FDR: 1.000e+00
Coefficient: 2.40e−01
Value: C
Gaiella.occulta
3
2
1
0
A (n=15)
B (n=16)
C (n=14)
surface

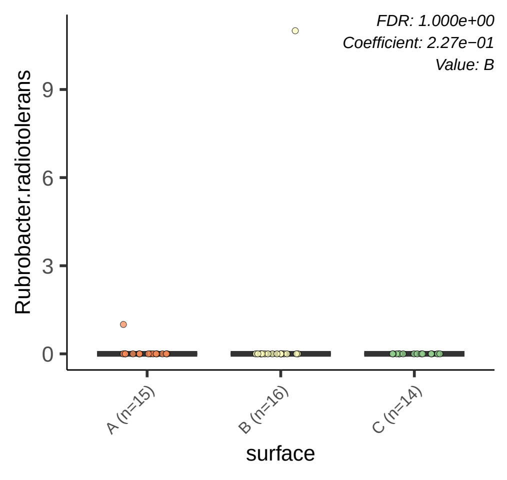
FDR: 1.000e+00
Coefficient: 2.27e−01
Value: B
Rubrobacter.radiotolerans
0
3
6
9
A (n=15)
B (n=16)
C (n=14)
surface

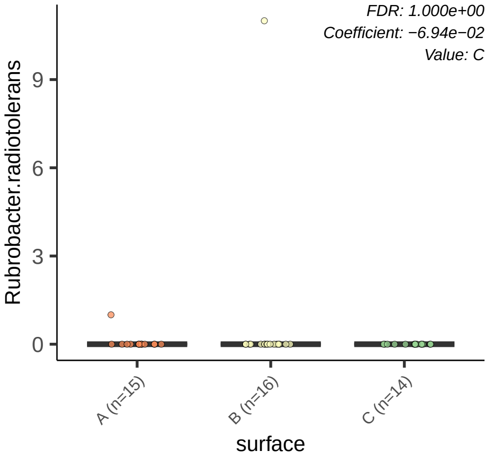
FDR: 1.000e+00
Coefficient: −6.94e−02
Value: C
Rubrobacter.radiotolerans
0
3
6
9
A (n=15)
B (n=16)
C (n=14)
surface

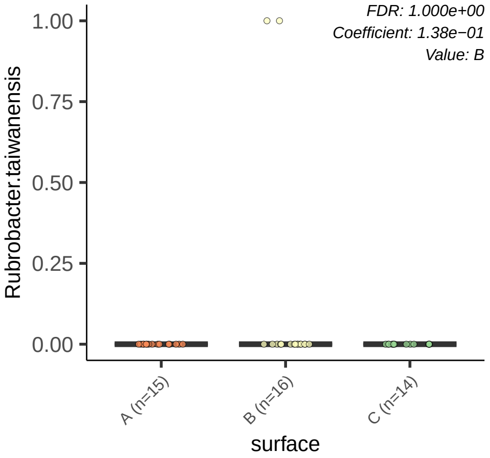
FDR: 1.000e+00
Coefficient: 1.38e−01
Value: B
Rubrobacter.taiwanensis
1.00
0.75
0.50
0.25
0.00
A (n=15)
B (n=16)
C (n=14)
surface

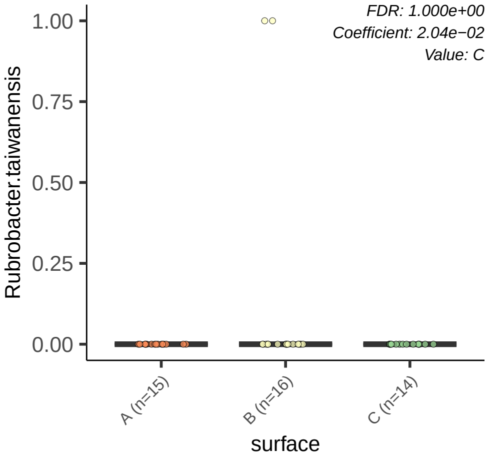
FDR: 1.000e+00
Coefficient: 2.04e−02
Value: C
Rubrobacter.taiwanensis
1.00
0.75
0.50
0.25
0.00
A (n=15)
B (n=16)
C (n=14)
surface

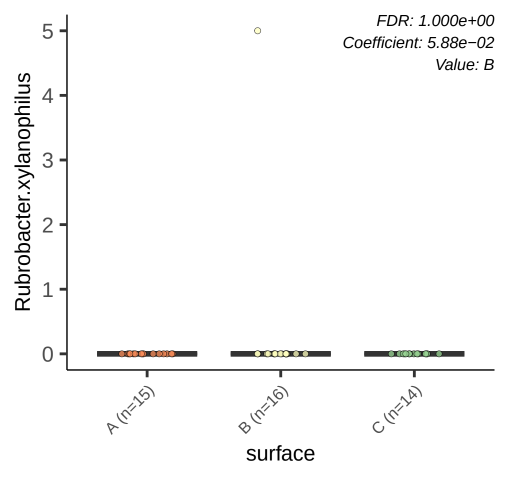

FDR: 1.000e+00
Coefficient: 5.88e−02
Value: B
Rubrobacter.xylanophilus
0
1
2
3
4
5
A (n=15)
B (n=16)
C (n=14)
surface

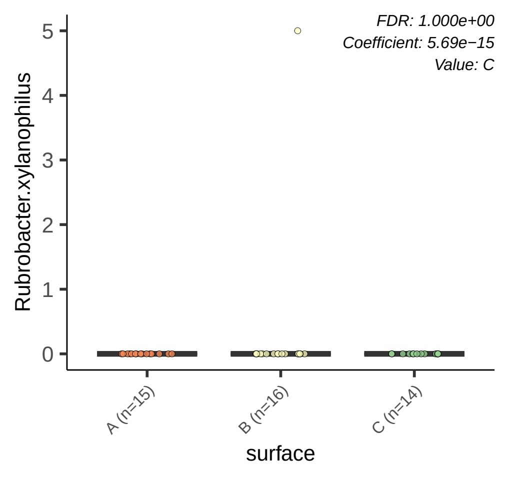
FDR: 1.000e+00
Coefficient: 5.69e−15
Value: C
Rubrobacter.xylanophilus
0
1
2
3
4
5
A (n=15)
B (n=16)
C (n=14)
surface

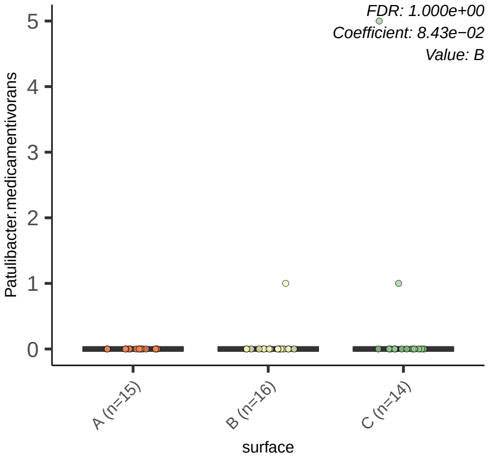

FDR: 1.000e+00
Coefficient: 8.43e−02
Value: B
Patulibacter.medicamentivorans
0
1
2
3
4
5
A (n=15)
B (n=16)
C (n=14)
surface

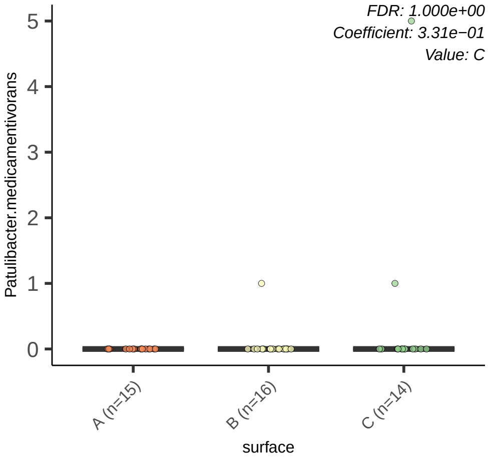

FDR: 1.000e+00
Coefficient: 3.31e−01
Value: C
Patulibacter.medicamentivorans
0
1
2
3
4
5
A (n=15)
B (n=16)
C (n=14)
surface

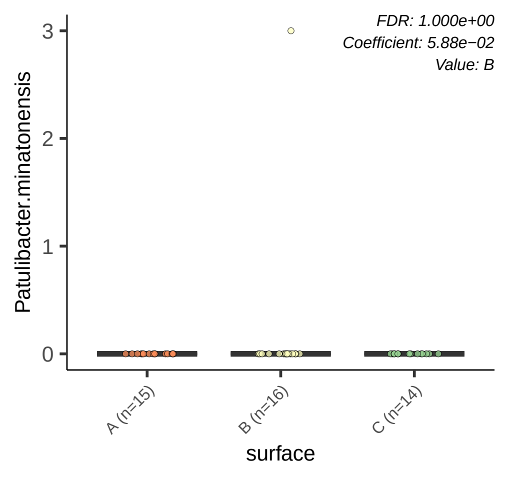
FDR: 1.000e+00
Coefficient: 5.88e−02
Value: B
Patulibacter.minatonensis
0
1
2
3
A (n=15)
B (n=16)
C (n=14)
surface

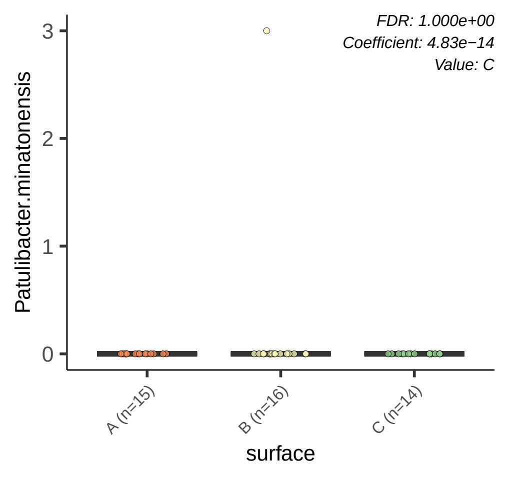

FDR: 1.000e+00
Coefficient: 4.83e−14
Value: C
Patulibacter.minatonensis
3
2
1
0
A (n=15)
B (n=16)
C (n=14)
surface

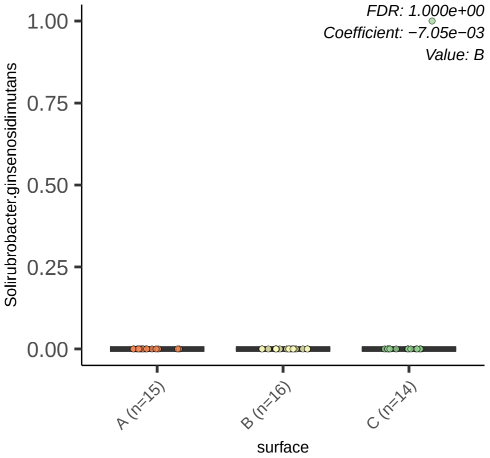
FDR: 1.000e+00
Coefficient: −7.05e−03
Value: B
1.00
0.75
0.50
0.25
0.00
Solirubrobacter.ginsenosidimutans
A (n=15)
B (n=16)
C (n=14)
surface

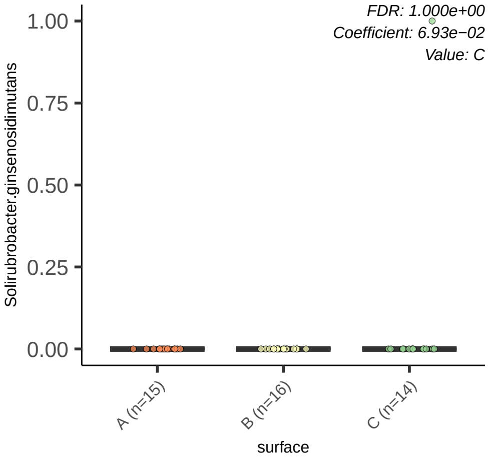

FDR: 1.000e+00
Coefficient: 6.93e−02
Value: C
1.00
0.75
0.50
0.25
0.00
Solirubrobacter.ginsenosidimutans
A (n=15)
B (n=16)
C (n=14)
surface

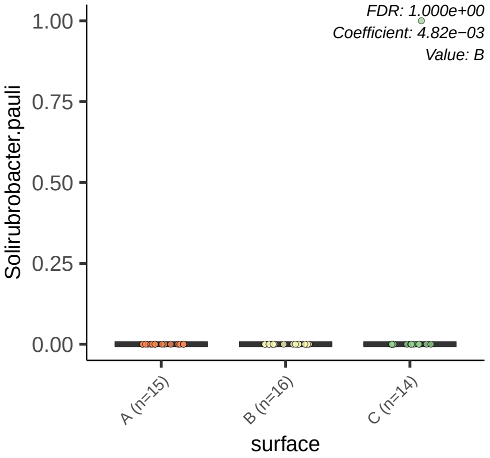
FDR: 1.000e+00
Coefficient: 4.82e−03
Value: B
Solirubrobacter.pauli
1.00
0.75
0.50
0.25
0.00
A (n=15)
B (n=16)
C (n=14)
surface

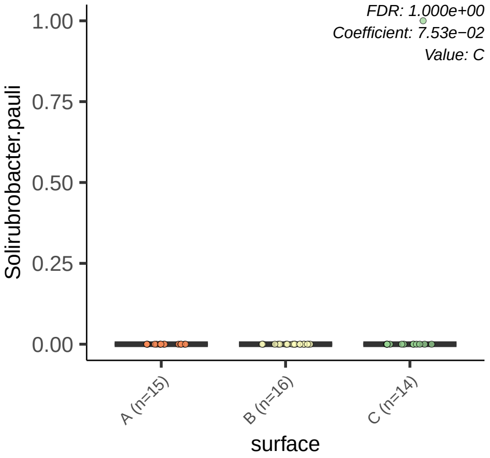
FDR: 1.000e+00
Coefficient: 7.53e−02
Value: C
Solirubrobacter.pauli
1.00
0.75
0.50
0.25
0.00
A (n=15)
B (n=16)
C (n=14)
surface

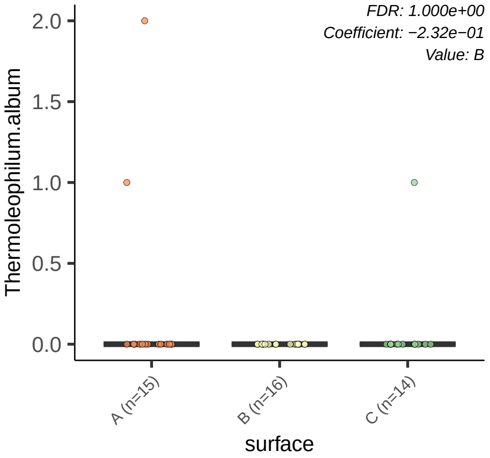

FDR: 1.000e+00
Coefficient: −2.32e−01
Value: B
Thermoleophilum.album
2.0
1.5
1.0
0.5
0.0
A (n=15)
B (n=16)
C (n=14)
surface

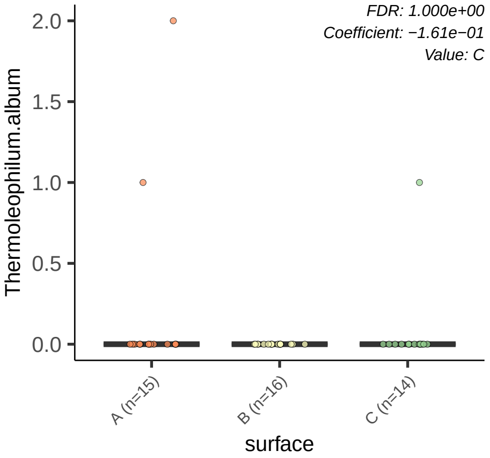
FDR: 1.000e+00
Coefficient: −1.61e−01
Value: C
Thermoleophilum.album
2.0
1.5
1.0
0.5
0.0
A (n=15)
B (n=16)
C (n=14)
surface

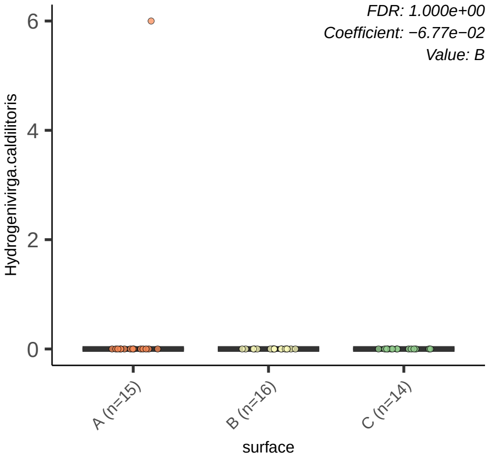

FDR: 1.000e+00
Coefficient: −6.77e−02
Value: B
Hydrogenivirga.caldilitoris
6
4
2
0
A (n=15)
B (n=16)
C (n=14)
surface

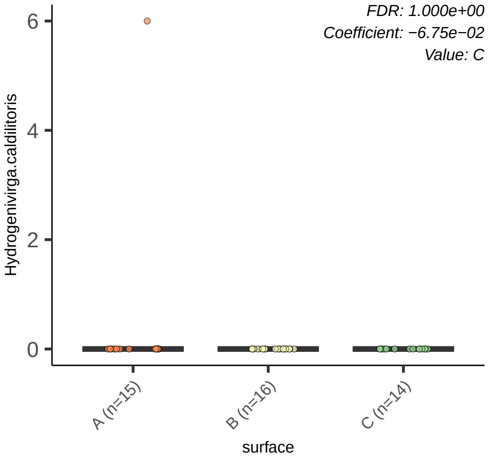
FDR: 1.000e+00
Coefficient: −6.75e−02
Value: C
Hydrogenivirga.caldilitoris
6
4
2
0
A (n=15)
B (n=16)
C (n=14)
surface

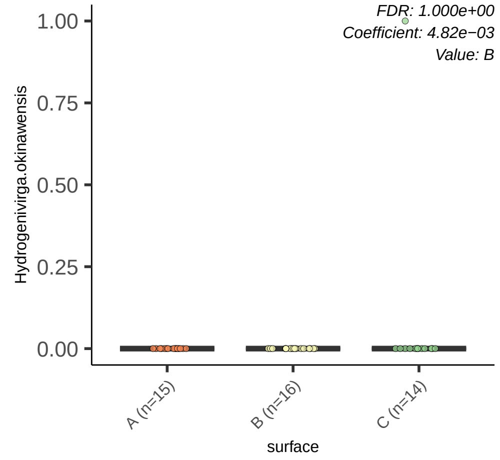

FDR: 1.000e+00
Coefficient: 4.82e−03
Value: B
Hydrogenivirga.okinawensis
1.00
0.75
0.50
0.25
0.00
A (n=15)
B (n=16)
C (n=14)
surface

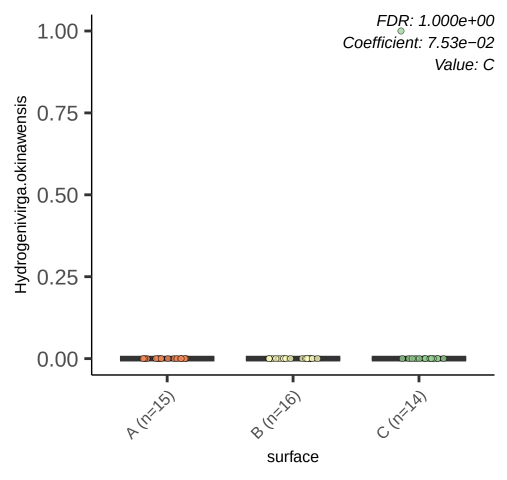
FDR: 1.000e+00
Coefficient: 7.53e−02
Value: C
Hydrogenivirga.okinawensis
1.00
0.75
0.50
0.25
0.00
A (n=15)
B (n=16)
C (n=14)
surface

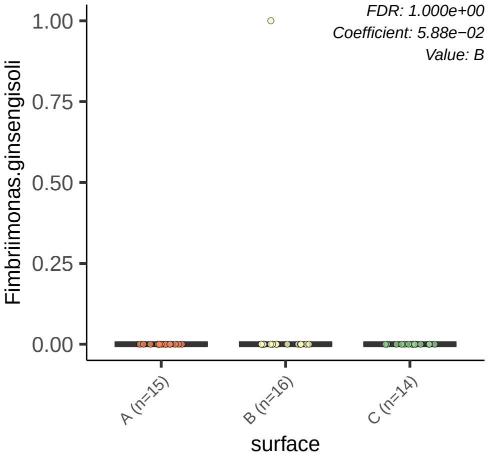

FDR: 1.000e+00
Coefficient: 5.88e−02
Value: B
Fimbriimonas.ginsengisoli
1.00
0.75
0.50
0.25
0.00
A (n=15)
B (n=16)
C (n=14)
surface

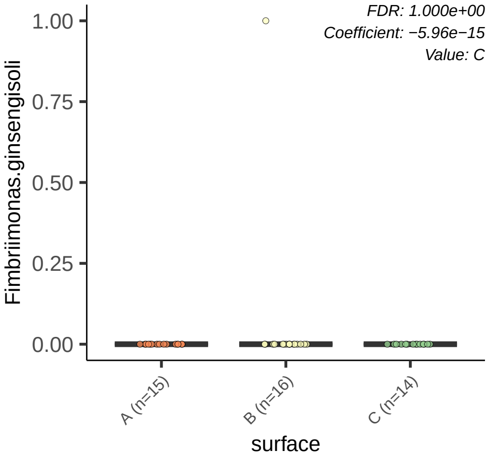
FDR: 1.000e+00
Coefficient: −5.96e−15
Value: C
Fimbriimonas.ginsengisoli
1.00
0.75
0.50
0.25
0.00
A (n=15)
B (n=16)
C (n=14)
surface

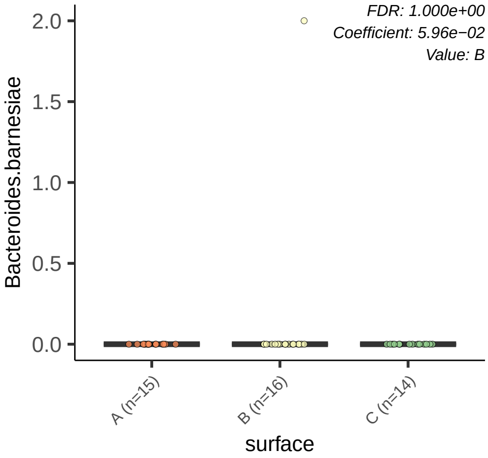

FDR: 1.000e+00
Coefficient: 5.96e−02
Value: B
Bacteroides.barnesiae
2.0
1.5
1.0
0.5
0.0
A (n=15)
B (n=16)
C (n=14)
surface

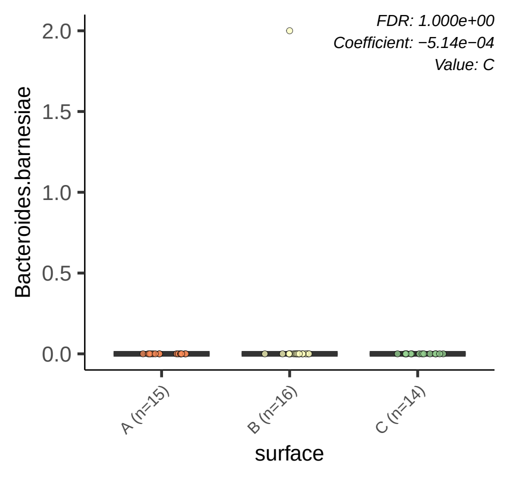
FDR: 1.000e+00
Coefficient: −5.14e−04
Value: C
Bacteroides.barnesiae
2.0
1.5
1.0
0.5
0.0
A (n=15)
B (n=16)
C (n=14)
surface

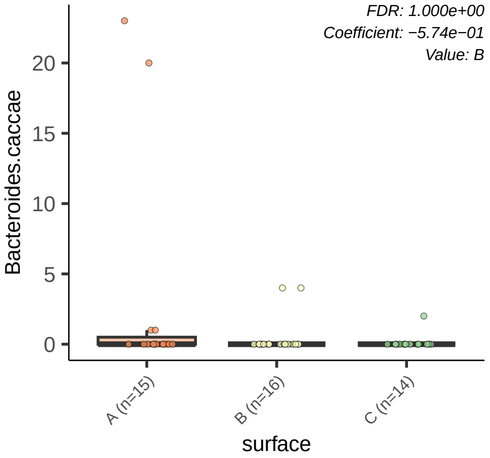

FDR: 1.000e+00
Coefficient: −5.74e−01
Value: B
Bacteroides.caccae
20
15
10
5
0
A (n=15)
B (n=16)
C (n=14)
surface

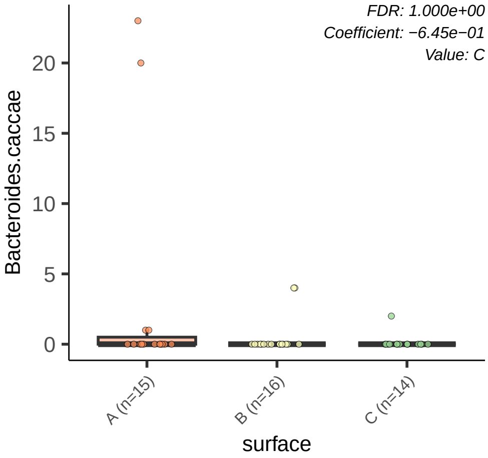
FDR: 1.000e+00
Coefficient: −6.45e−01
Value: C
Bacteroides.caccae
0
5
10
15
20
A (n=15)
B (n=16)
C (n=14)
surface

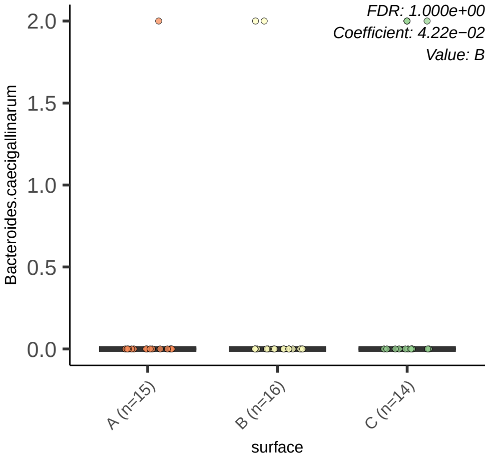

FDR: 1.000e+00
Coefficient: 4.22e−02
Value: B
Bacteroides.caecigallinarum
2.0
1.5
1.0
0.5
0.0
A (n=15)
B (n=16)
C (n=14)
surface

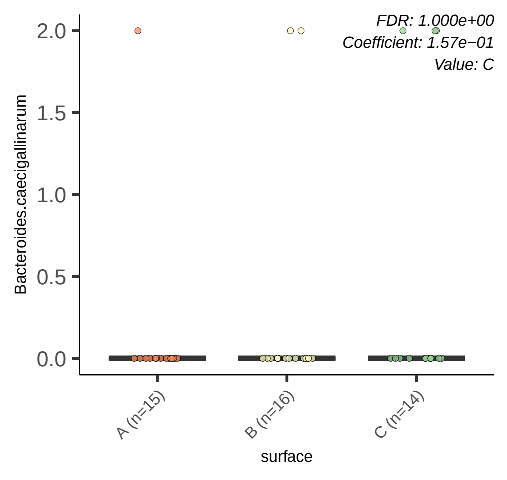

FDR: 1.000e+00
Coefficient: 1.57e−01
Value: C
Bacteroides.caecigallinarum
2.0
1.5
1.0
0.5
0.0
A (n=15)
B (n=16)
C (n=14)
surface

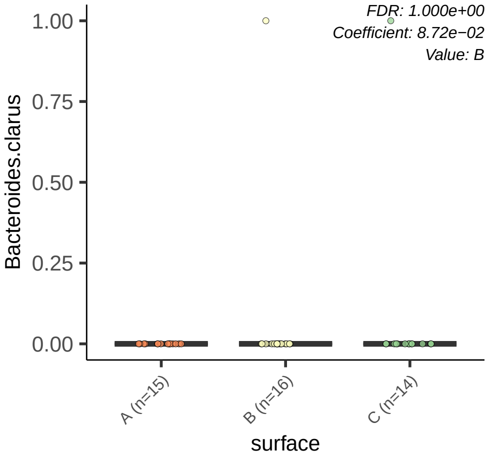
FDR: 1.000e+00
Coefficient: 8.72e−02
Value: B
Bacteroides.clarus
1.00
0.75
0.50
0.25
0.00
A (n=15)
B (n=16)
C (n=14)
surface

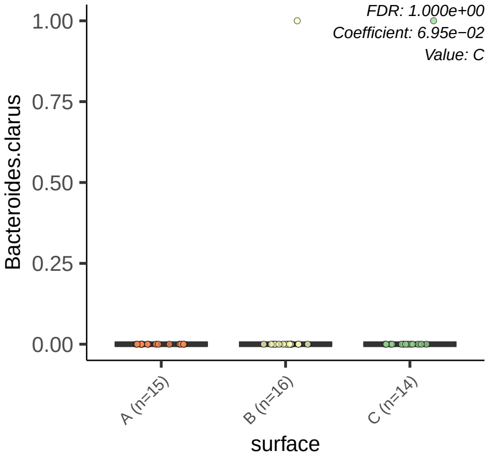
FDR: 1.000e+00
Coefficient: 6.95e−02
Value: C
Bacteroides.clarus
1.00
0.75
0.50
0.25
0.00
A (n=15)
B (n=16)
C (n=14)
surface

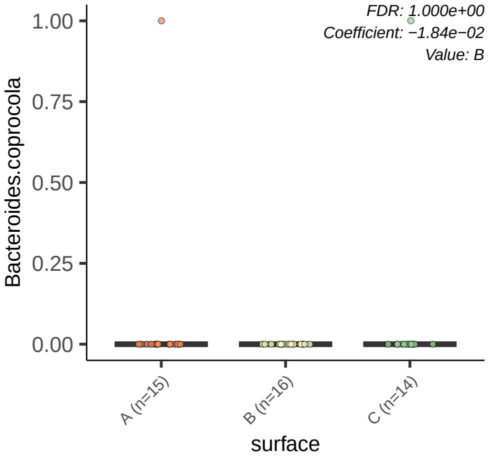
FDR: 1.000e+00
Coefficient: −1.84e−02
Value: B
Bacteroides.coprocola
1.00
0.75
0.50
0.25
0.00
A (n=15)
B (n=16)
C (n=14)
surface

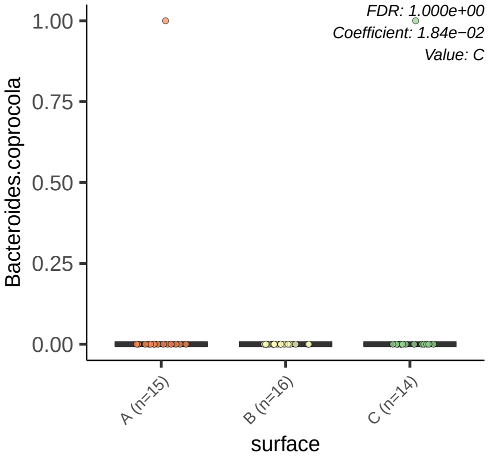

FDR: 1.000e+00
Coefficient: 1.84e−02
Value: C
Bacteroides.coprocola
1.00
0.75
0.50
0.25
0.00
A (n=15)
B (n=16)
C (n=14)
surface

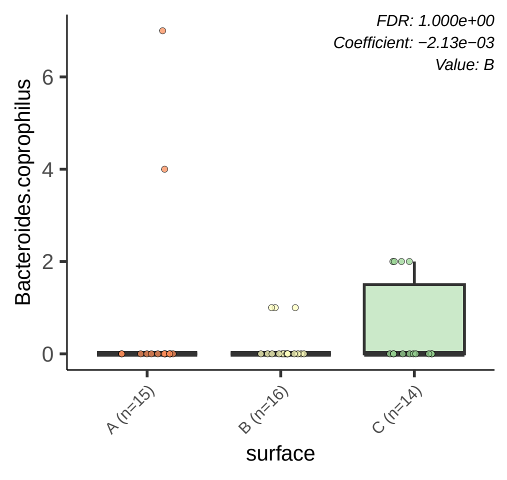

FDR: 1.000e+00
Coefficient: −2.13e−03
Value: B
Bacteroides.coprophilus
0
2
4
6
A (n=15)
B (n=16)
C (n=14)
surface

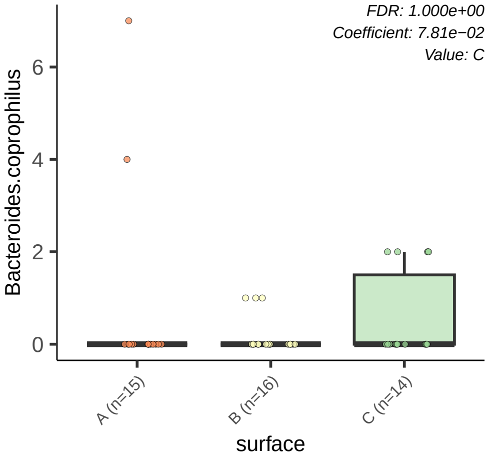
FDR: 1.000e+00
Coefficient: 7.81e−02
Value: C
Bacteroides.coprophilus
0
2
4
6
A (n=15)
B (n=16)
C (n=14)
surface

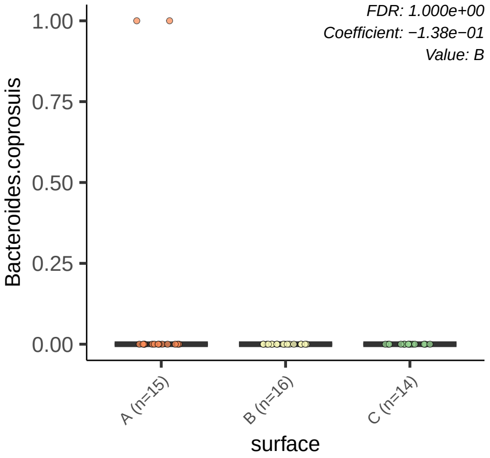

FDR: 1.000e+00
Coefficient: −1.38e−01
Value: B
Bacteroides.coprosuis
1.00
0.75
0.50
0.25
0.00
A (n=15)
B (n=16)
C (n=14)
surface

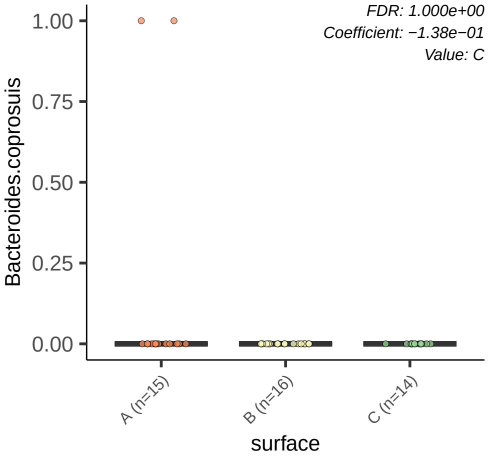

FDR: 1.000e+00
Coefficient: −1.38e−01
Value: C
Bacteroides.coprosuis
1.00
0.75
0.50
0.25
0.00
A (n=15)
B (n=16)
C (n=14)
surface

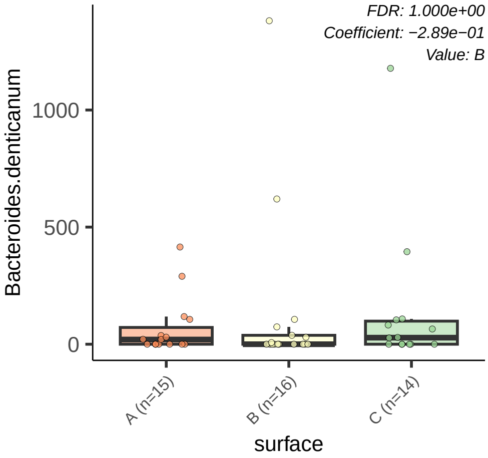

FDR: 1.000e+00
Coefficient: −2.89e−01
Value: B
Bacteroides.denticanum
1000
500
0
A (n=15)
B (n=16)
C (n=14)
surface

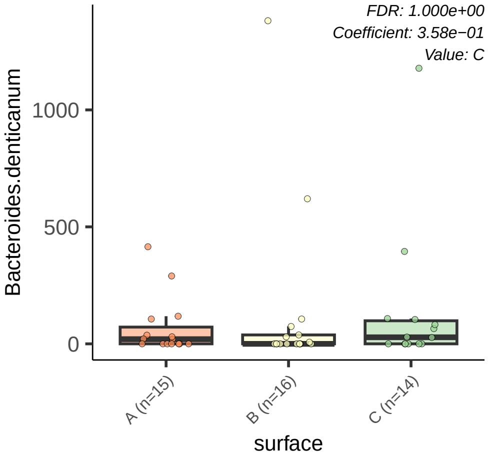

FDR: 1.000e+00
Coefficient: 3.58e−01
Value: C
Bacteroides.denticanum
1000
500
0
A (n=15)
B (n=16)
C (n=14)
surface

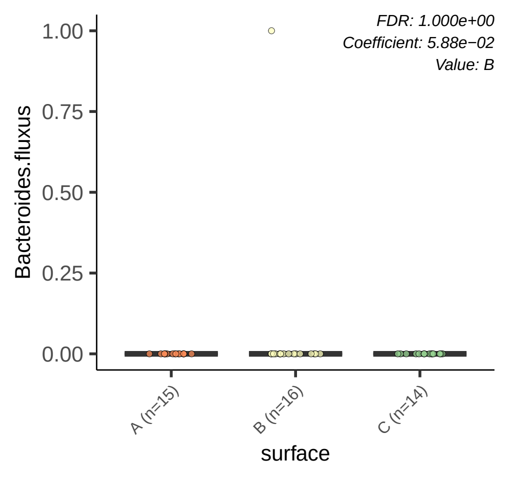
FDR: 1.000e+00
Coefficient: 5.88e−02
Value: B
Bacteroides.fluxus
1.00
0.75
0.50
0.25
0.00
A (n=15)
B (n=16)
C (n=14)
surface

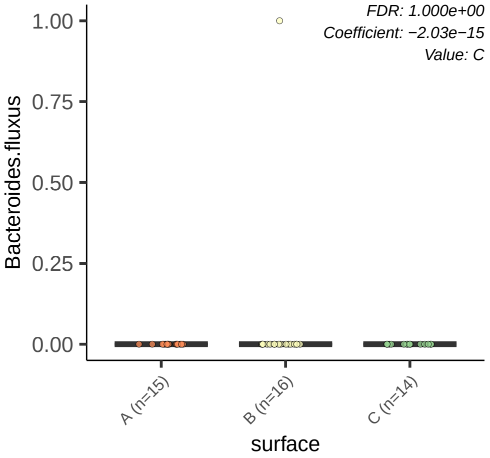

FDR: 1.000e+00
Coefficient: −2.03e−15
Value: C
Bacteroides.fluxus
1.00
0.75
0.50
0.25
0.00
A (n=15)
B (n=16)
C (n=14)
surface

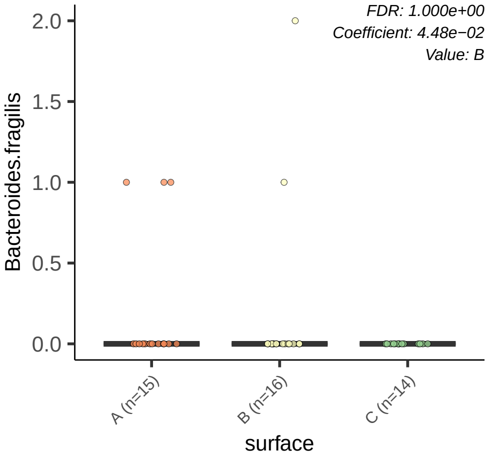

FDR: 1.000e+00
Coefficient: 4.48e−02
Value: B
Bacteroides.fragilis
2.0
1.5
1.0
0.5
0.0
A (n=15)
B (n=16)
C (n=14)
surface

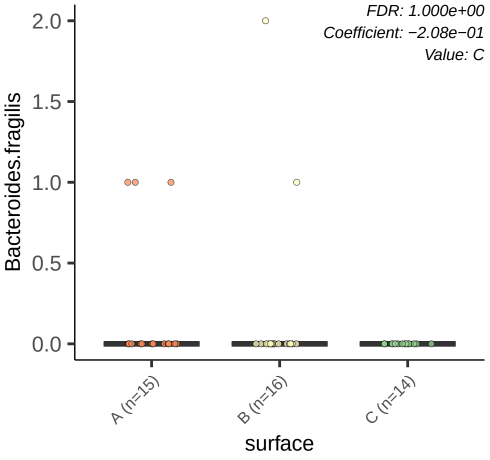
FDR: 1.000e+00
Coefficient: −2.08e−01
Value: C
Bacteroides.fragilis
2.0
1.5
1.0
0.5
0.0
A (n=15)
B (n=16)
C (n=14)
surface

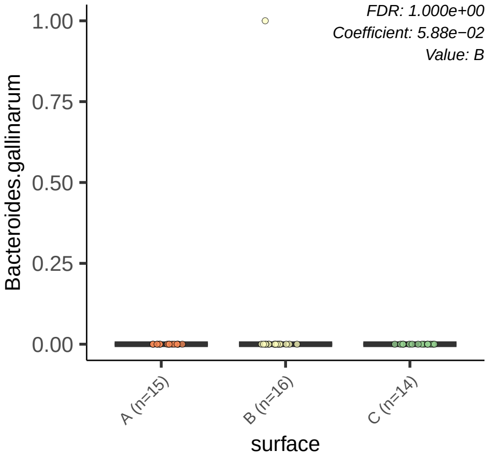
FDR: 1.000e+00
Coefficient: 5.88e−02
Value: B
Bacteroides.gallinarum
1.00
0.75
0.50
0.25
0.00
A (n=15)
B (n=16)
C (n=14)
surface

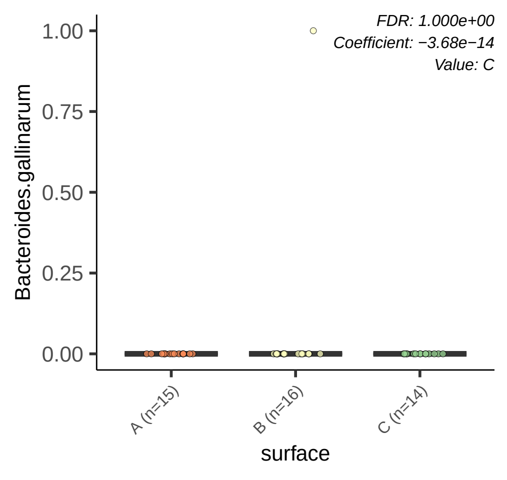
FDR: 1.000e+00
Coefficient: −3.68e−14
Value: C
Bacteroides.gallinarum
1.00
0.75
0.50
0.25
0.00
A (n=15)
B (n=16)
C (n=14)
surface

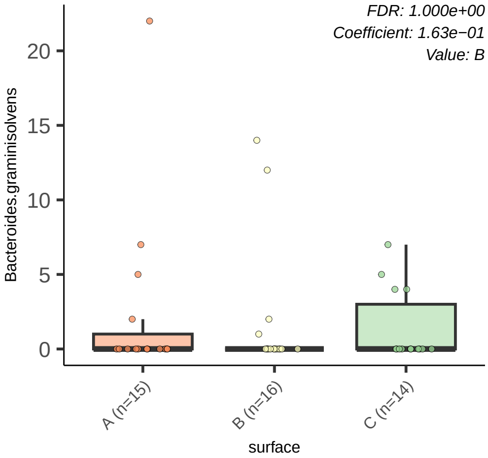

FDR: 1.000e+00
Coefficient: 1.63e−01
Value: B
Bacteroides.graminisolvens
20
15
10
5
0
A (n=15)
B (n=16)
C (n=14)
surface

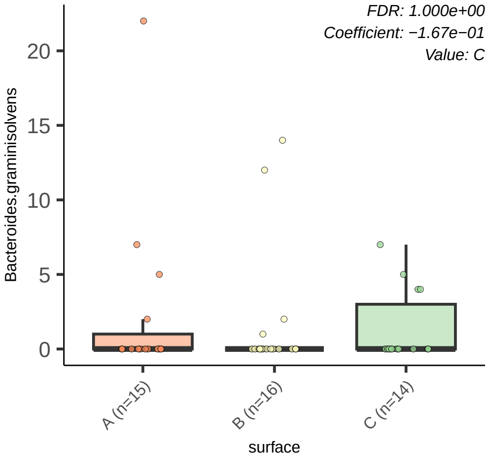
FDR: 1.000e+00
Coefficient: −1.67e−01
Value: C
Bacteroides.graminisolvens
0
5
10
15
20
A (n=15)
B (n=16)
C (n=14)
surface

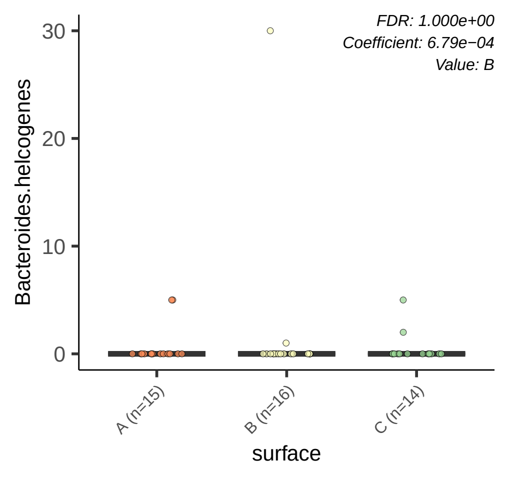
FDR: 1.000e+00
Coefficient: 6.79e−04
Value: B
Bacteroides.helcogenes
0
10
20
30
A (n=15)
B (n=16)
C (n=14)
surface

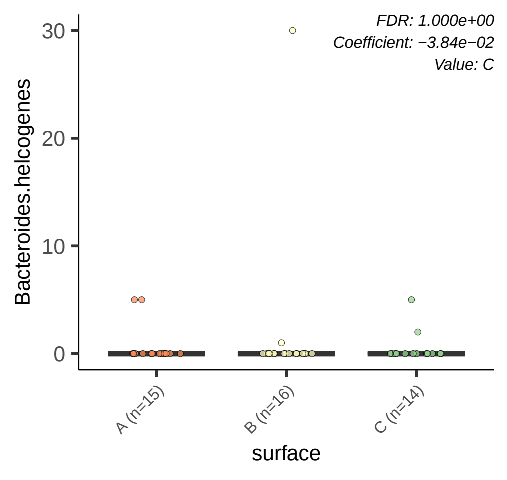
FDR: 1.000e+00
Coefficient: −3.84e−02
Value: C
Bacteroides.helcogenes
30
20
10
0
A (n=15)
B (n=16)
C (n=14)
surface

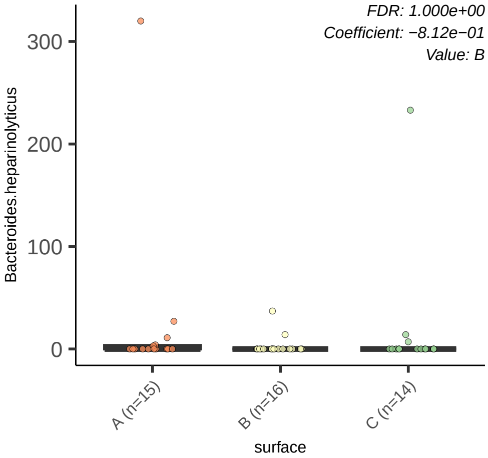
FDR: 1.000e+00
Coefficient: −8.12e−01
Value: B
300
200
100
0
Bacteroides.heparinolyticus
A (n=15)
B (n=16)
C (n=14)
surface

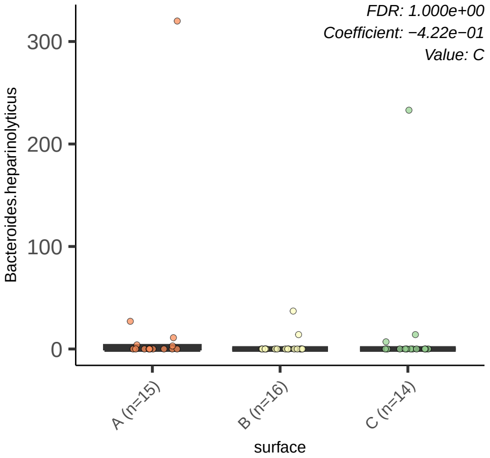

FDR: 1.000e+00
Coefficient: −4.22e−01
Value: C
300
200
100
0
Bacteroides.heparinolyticus
A (n=15)
B (n=16)
C (n=14)
surface

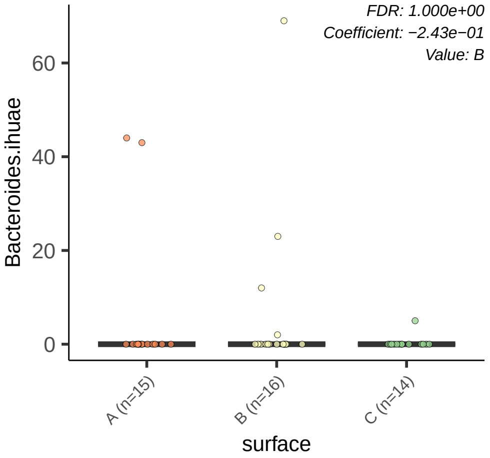

FDR: 1.000e+00
Coefficient: −2.43e−01
Value: B
Bacteroides.ihuae
0
20
40
60
A (n=15)
B (n=16)
C (n=14)
surface

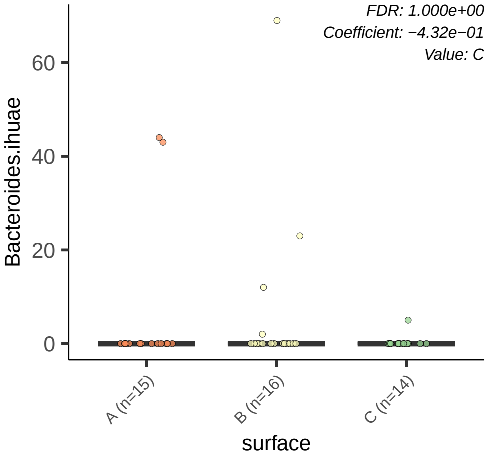
FDR: 1.000e+00
Coefficient: −4.32e−01
Value: C
Bacteroides.ihuae
0
20
40
60
A (n=15)
B (n=16)
C (n=14)
surface

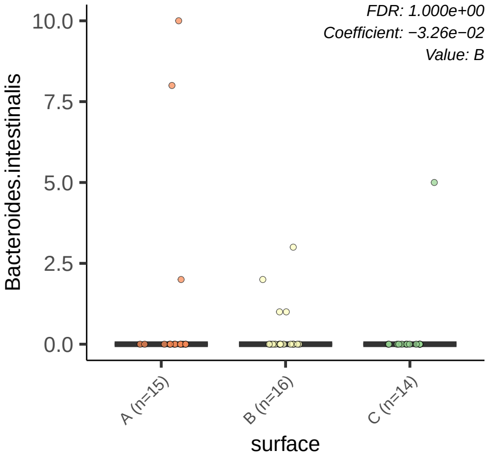
FDR: 1.000e+00
Coefficient: −3.26e−02
Value: B
Bacteroides.intestinalis
10.0
7.5
5.0
2.5
0.0
A (n=15)
B (n=16)
C (n=14)
surface

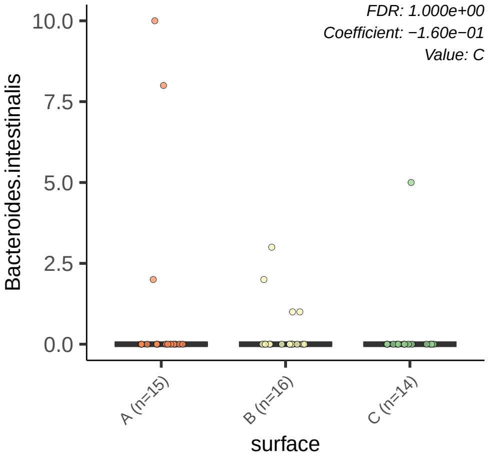
FDR: 1.000e+00
Coefficient: −1.60e−01
Value: C
Bacteroides.intestinalis
10.0
7.5
5.0
2.5
0.0
A (n=15)
B (n=16)
C (n=14)
surface

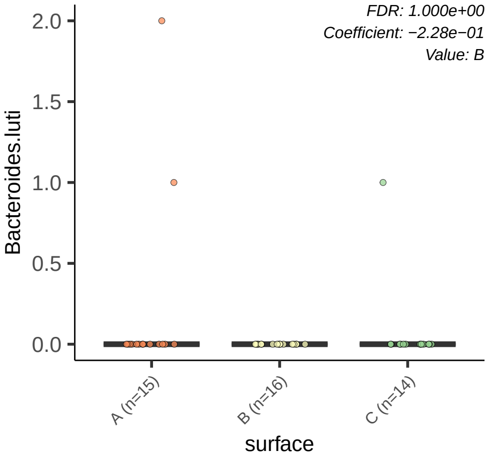
FDR: 1.000e+00
Coefficient: −2.28e−01
Value: B
Bacteroides.luti
2.0
1.5
1.0
0.5
0.0
A (n=15)
B (n=16)
C (n=14)
surface

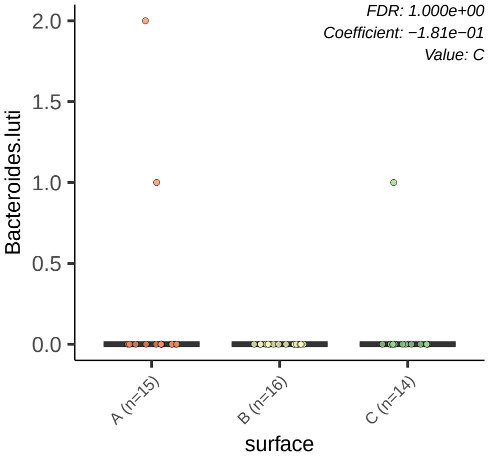

FDR: 1.000e+00
Coefficient: −1.81e−01
Value: C
Bacteroides.luti
2.0
1.5
1.0
0.5
0.0
A (n=15)
B (n=16)
C (n=14)
surface

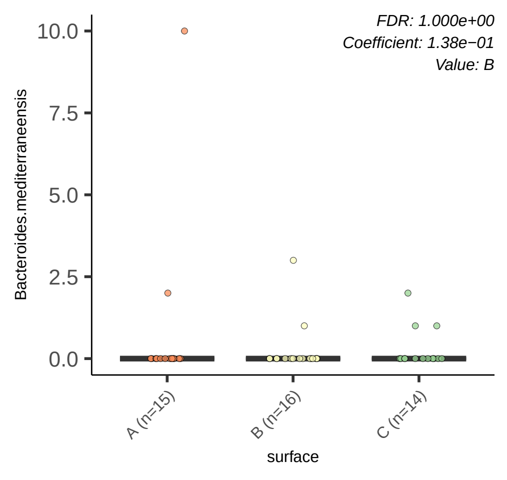

FDR: 1.000e+00
Coefficient: 1.38e−01
Value: B
Bacteroides.mediterraneensis
10.0
7.5
5.0
2.5
0.0
A (n=15)
B (n=16)
C (n=14)
surface

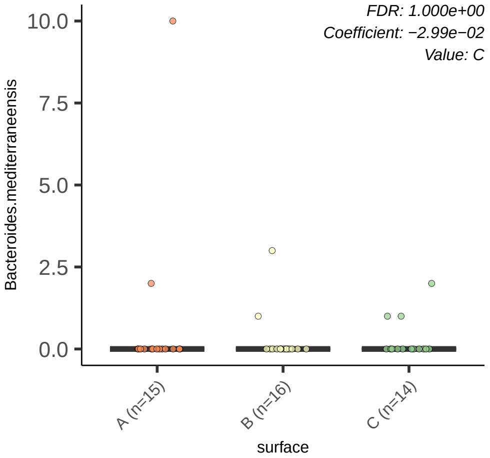
FDR: 1.000e+00
Coefficient: −2.99e−02
Value: C
Bacteroides.mediterraneensis
10.0
7.5
5.0
2.5
0.0
A (n=15)
B (n=16)
C (n=14)
surface

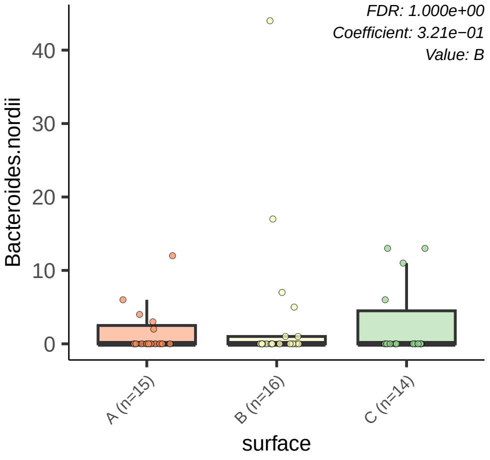
FDR: 1.000e+00
Coefficient: 3.21e−01
Value: B
Bacteroides.nordii
0
10
20
30
40
A (n=15)
B (n=16)
C (n=14)
surface

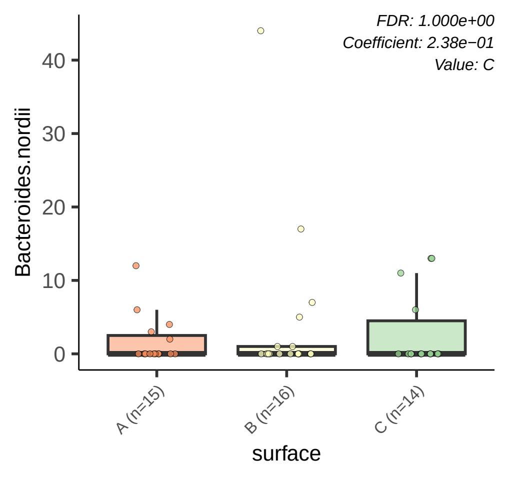
FDR: 1.000e+00
Coefficient: 2.38e−01
Value: C
Bacteroides.nordii
0
10
20
30
40
A (n=15)
B (n=16)
C (n=14)
surface

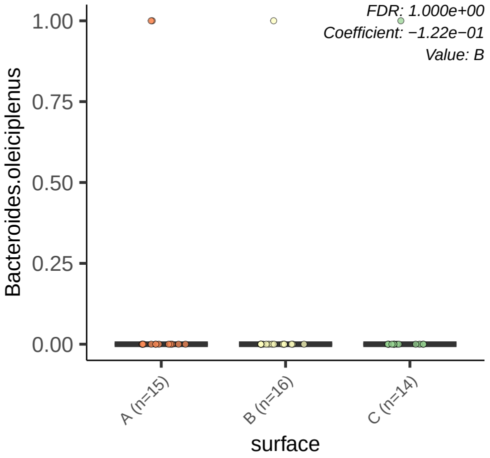
FDR: 1.000e+00
Coefficient: −1.22e−01
Value: B
Bacteroides.oleiciplenus
1.00
0.75
0.50
0.25
0.00
A (n=15)
B (n=16)
C (n=14)
surface

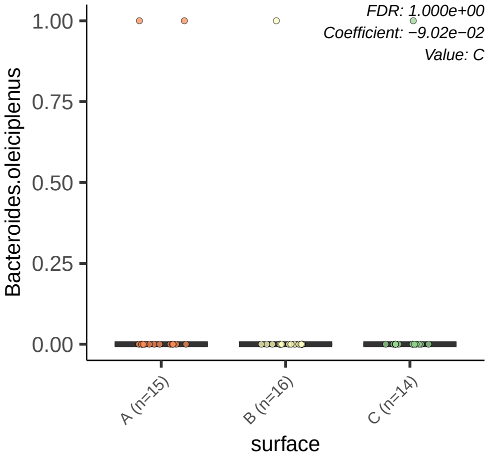
FDR: 1.000e+00
Coefficient: −9.02e−02
Value: C
Bacteroides.oleiciplenus
1.00
0.75
0.50
0.25
0.00
A (n=15)
B (n=16)
C (n=14)
surface

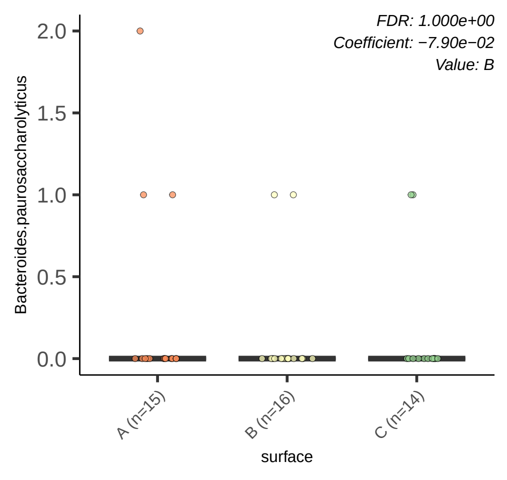

FDR: 1.000e+00
Coefficient: −7.90e−02
Value: B
Bacteroides.paurosaccharolyticus
2.0
1.5
1.0
0.5
0.0
A (n=15)
B (n=16)
C (n=14)
surface

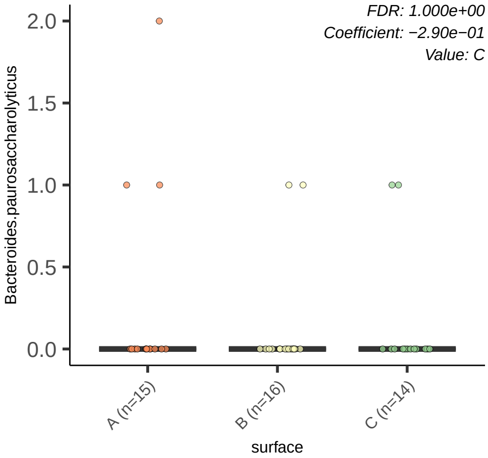

FDR: 1.000e+00
Coefficient: −2.90e−01
Value: C
Bacteroides.paurosaccharolyticus
2.0
1.5
1.0
0.5
0.0
A (n=15)
B (n=16)
C (n=14)
surface

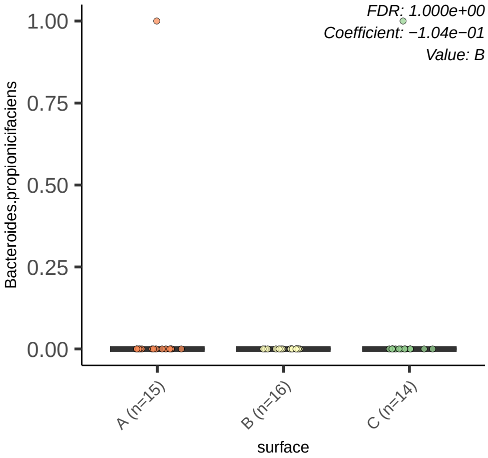
FDR: 1.000e+00
Coefficient: −1.04e−01
Value: B
1.00
0.75
0.50
0.25
0.00
Bacteroides.propionicifaciens
A (n=15)
B (n=16)
C (n=14)
surface

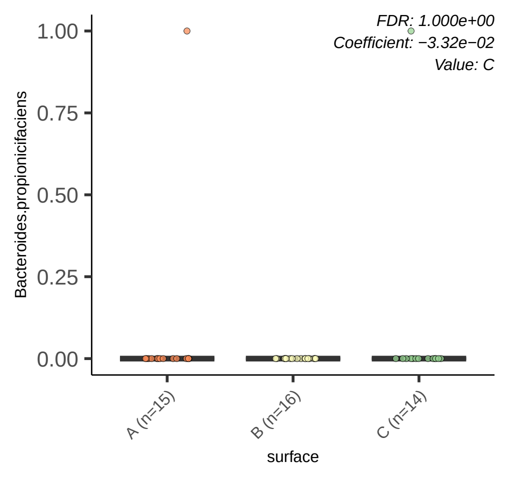
FDR: 1.000e+00
Coefficient: −3.32e−02
Value: C
1.00
0.75
0.50
0.25
0.00
Bacteroides.propionicifaciens
A (n=15)
B (n=16)
C (n=14)
surface

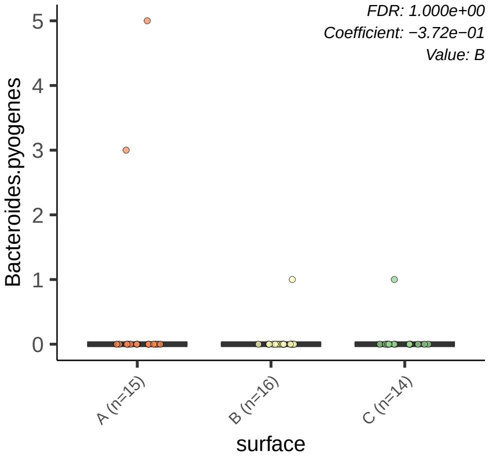

FDR: 1.000e+00
Coefficient: −3.72e−01
Value: B
Bacteroides.pyogenes
0
1
2
3
4
5
A (n=15)
B (n=16)
C (n=14)
surface

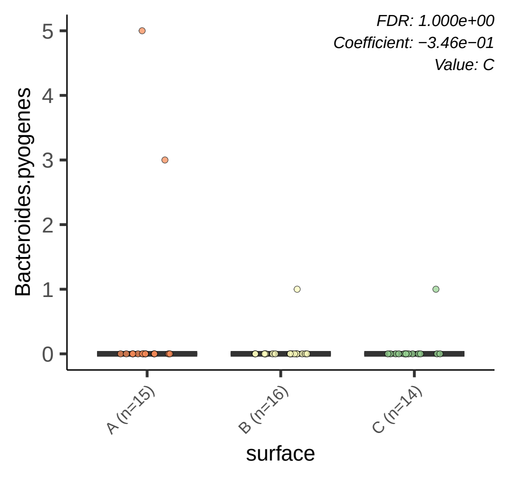
FDR: 1.000e+00
Coefficient: −3.46e−01
Value: C
Bacteroides.pyogenes
5
4
3
2
1
0
A (n=15)
B (n=16)
C (n=14)
surface

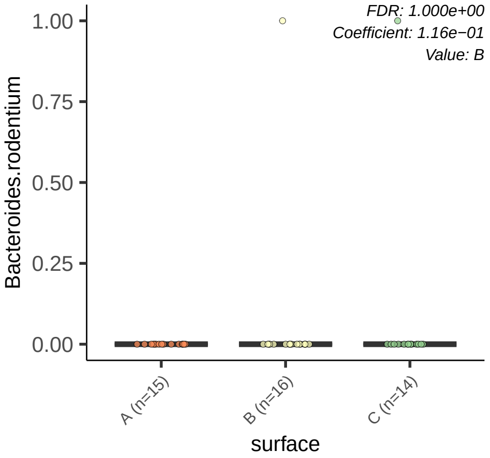

FDR: 1.000e+00
Coefficient: 1.16e−01
Value: B
Bacteroides.rodentium
1.00
0.75
0.50
0.25
0.00
A (n=15)
B (n=16)
C (n=14)
surface

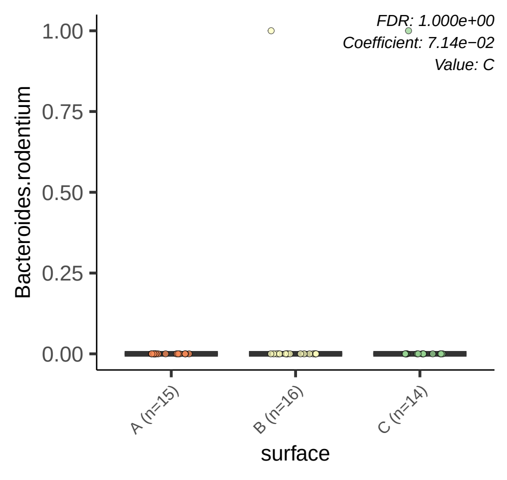

FDR: 1.000e+00
Coefficient: 7.14e−02
Value: C
Bacteroides.rodentium
1.00
0.75
0.50
0.25
0.00
A (n=15)
B (n=16)
C (n=14)
surface

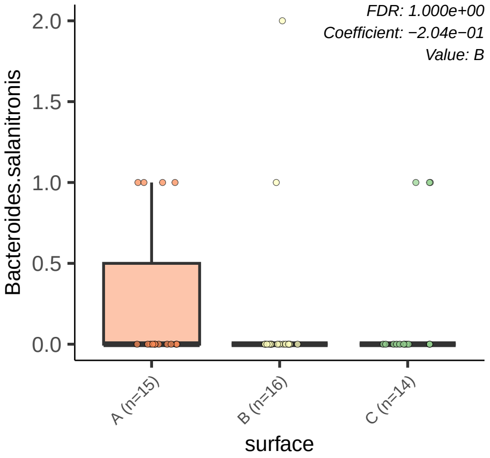
FDR: 1.000e+00
Coefficient: −2.04e−01
Value: B
Bacteroides.salanitronis
2.0
1.5
1.0
0.5
0.0
A (n=15)
B (n=16)
C (n=14)
surface

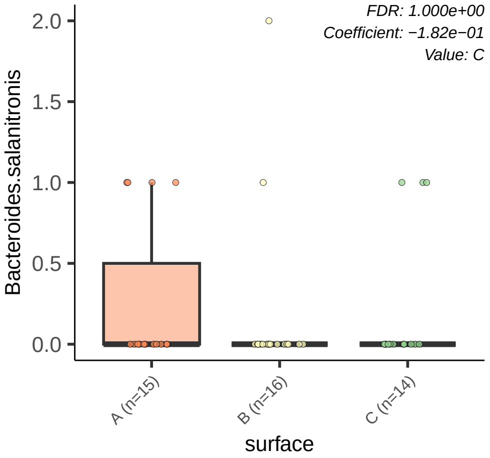
FDR: 1.000e+00
Coefficient: −1.82e−01
Value: C
Bacteroides.salanitronis
2.0
1.5
1.0
0.5
0.0
A (n=15)
B (n=16)
C (n=14)
surface

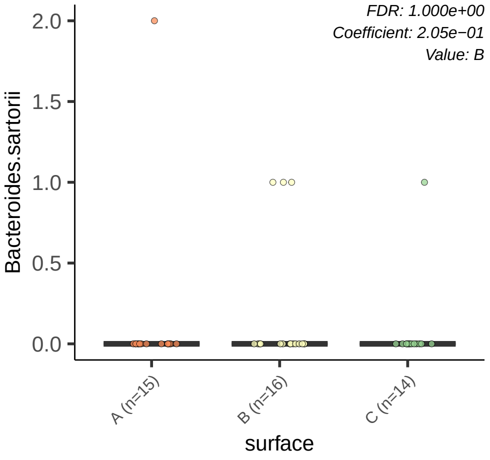

FDR: 1.000e+00
Coefficient: 2.05e−01
Value: B
Bacteroides.sartorii
2.0
1.5
1.0
0.5
0.0
A (n=15)
B (n=16)
C (n=14)
surface

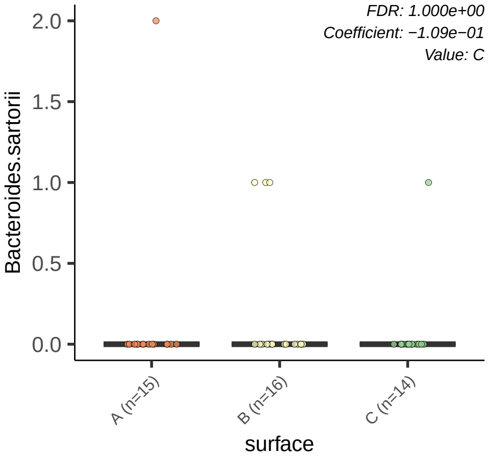

FDR: 1.000e+00
Coefficient: −1.09e−01
Value: C
Bacteroides.sartorii
2.0
1.5
1.0
0.5
0.0
A (n=15)
B (n=16)
C (n=14)
surface

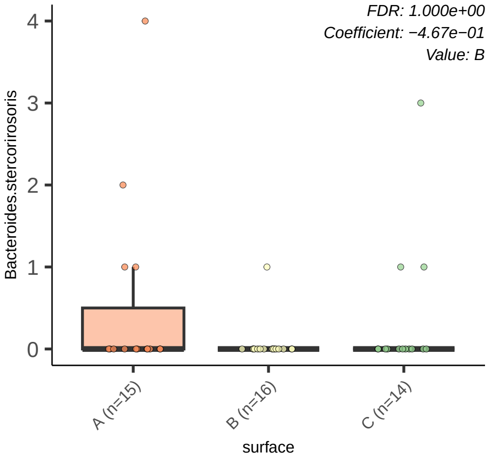
FDR: 1.000e+00
Coefficient: −4.67e−01
Value: B
Bacteroides.stercorirosoris
0
1
2
3
4
A (n=15)
B (n=16)
C (n=14)
surface

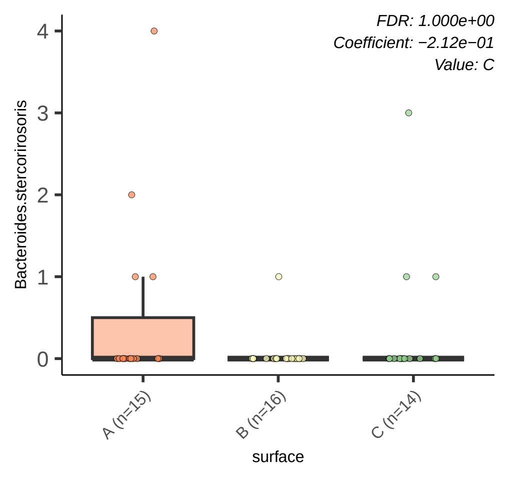
FDR: 1.000e+00
Coefficient: −2.12e−01
Value: C
Bacteroides.stercorirosoris
0
1
2
3
4
A (n=15)
B (n=16)
C (n=14)
surface

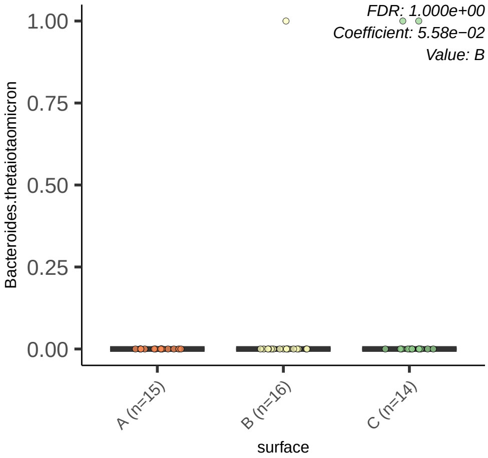
FDR: 1.000e+00
Coefficient: 5.58e−02
Value: B
1.00
0.75
0.50
0.25
0.00
Bacteroides.thetaiotaomicron
A (n=15)
B (n=16)
C (n=14)
surface

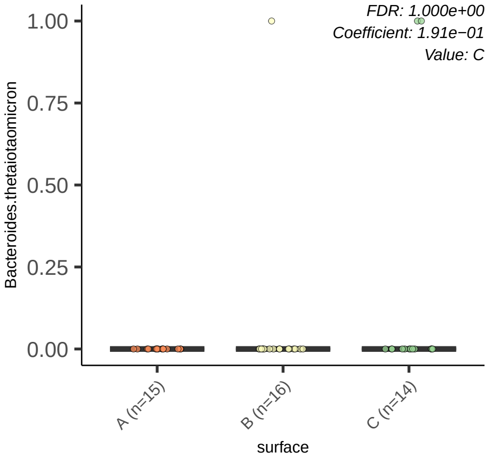
FDR: 1.000e+00
Coefficient: 1.91e−01
Value: C
1.00
0.75
0.50
0.25
0.00
Bacteroides.thetaiotaomicron
A (n=15)
B (n=16)
C (n=14)
surface

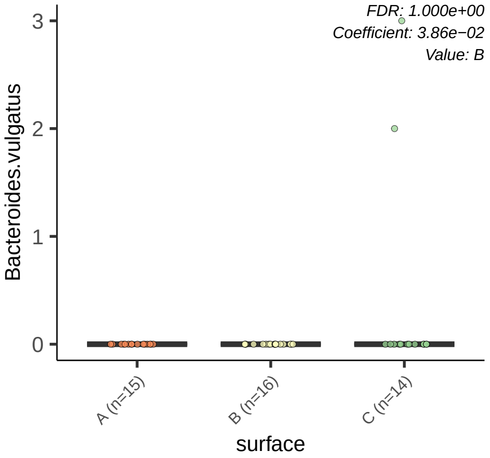

FDR: 1.000e+00
Coefficient: 3.86e−02
Value: B
Bacteroides.vulgatus
3
2
1
0
A (n=15)
B (n=16)
C (n=14)
surface

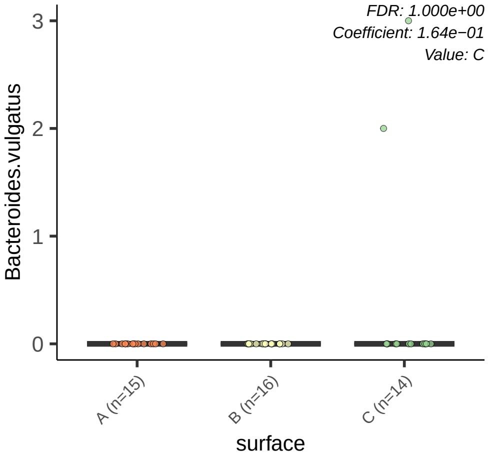
FDR: 1.000e+00
Coefficient: 1.64e−01
Value: C
Bacteroides.vulgatus
0
1
2
3
A (n=15)
B (n=16)
C (n=14)
surface

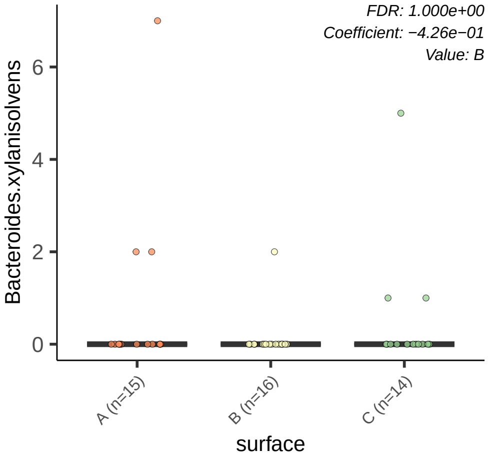
FDR: 1.000e+00
Coefficient: −4.26e−01
Value: B
Bacteroides.xylanisolvens
0
2
4
6
A (n=15)
B (n=16)
C (n=14)
surface

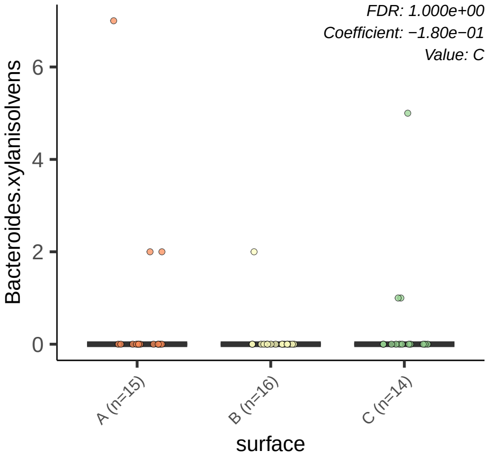
FDR: 1.000e+00
Coefficient: −1.80e−01
Value: C
Bacteroides.xylanisolvens
0
2
4
6
A (n=15)
B (n=16)
C (n=14)
surface

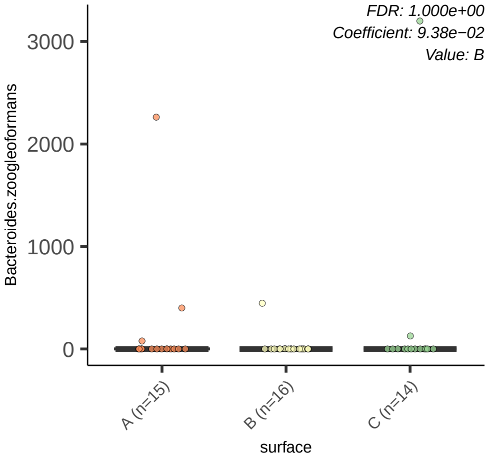

FDR: 1.000e+00
Coefficient: 9.38e−02
Value: B
3000
2000
1000
0
Bacteroides.zoogleoformans
A (n=15)
B (n=16)
C (n=14)
surface

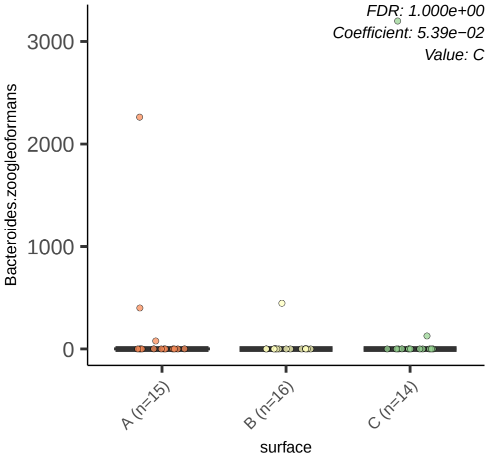

FDR: 1.000e+00
Coefficient: 5.39e−02
Value: C
3000
2000
1000
0
Bacteroides.zoogleoformans
A (n=15)
B (n=16)
C (n=14)
surface

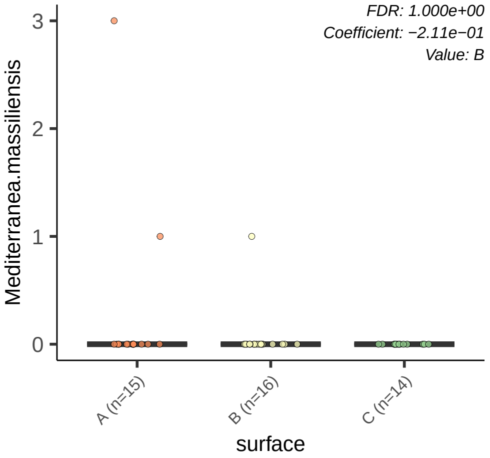

FDR: 1.000e+00
Coefficient: −2.11e−01
Value: B
Mediterranea.massiliensis
0
1
2
3
A (n=15)
B (n=16)
C (n=14)
surface

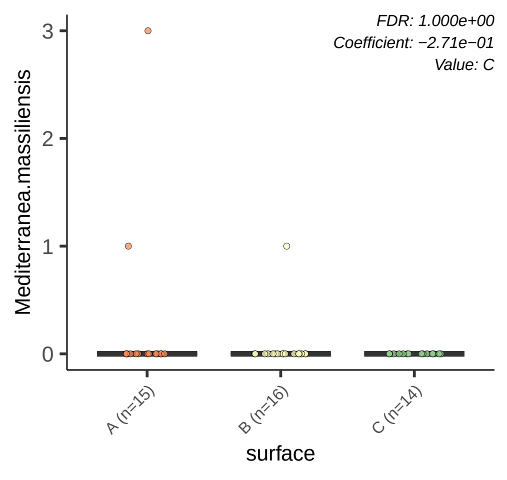
FDR: 1.000e+00
Coefficient: −2.71e−01
Value: C
Mediterranea.massiliensis
3
2
1
0
A (n=15)
B (n=16)
C (n=14)
surface

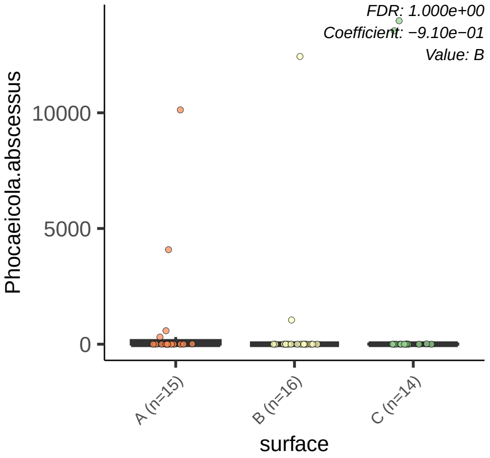

FDR: 1.000e+00
Coefficient: −9.10e−01
Value: B
Phocaeicola.abscessus
10000
5000
0
A (n=15)
B (n=16)
C (n=14)
surface

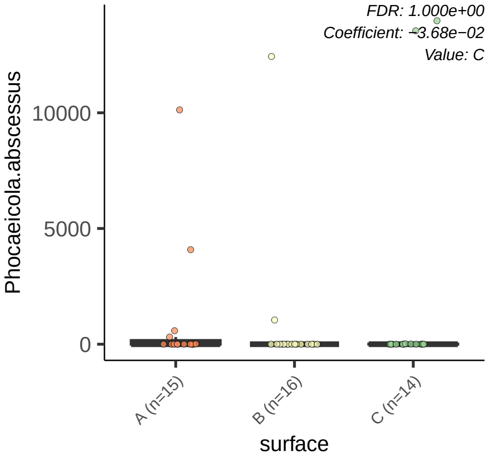
FDR: 1.000e+00
Coefficient: −3.68e−02
Value: C
Phocaeicola.abscessus
10000
5000
0
A (n=15)
B (n=16)
C (n=14)
surface

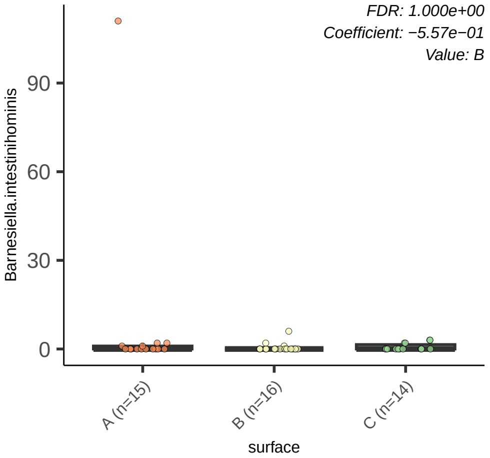
FDR: 1.000e+00
Coefficient: −5.57e−01
Value: B
Barnesiella.intestinihominis
90
60
30
0
A (n=15)
B (n=16)
C (n=14)
surface

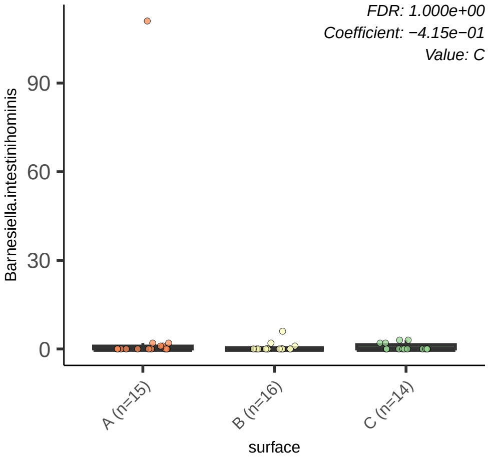
FDR: 1.000e+00
Coefficient: −4.15e−01
Value: C
Barnesiella.intestinihominis
90
60
30
0
A (n=15)
B (n=16)
C (n=14)
surface

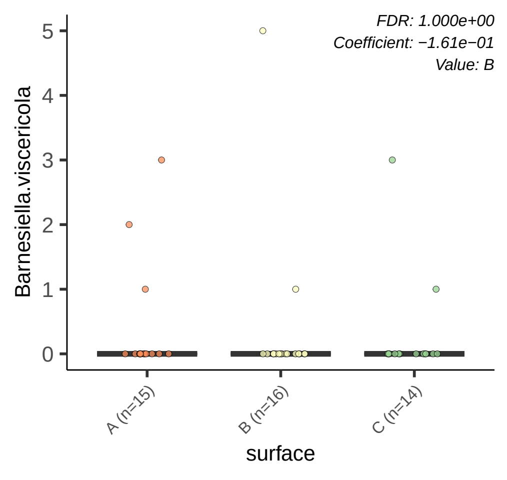

FDR: 1.000e+00
Coefficient: −1.61e−01
Value: B
Barnesiella.viscericola
0
1
2
3
4
5
A (n=15)
B (n=16)
C (n=14)
surface

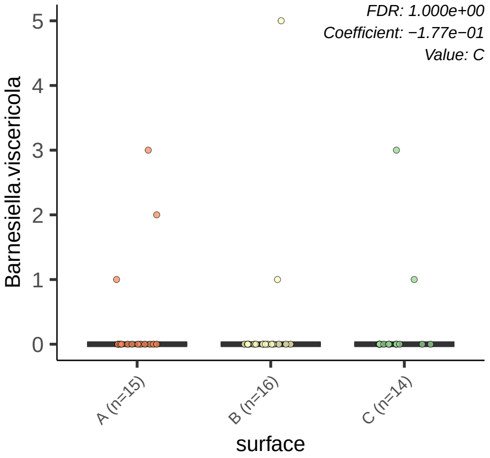
FDR: 1.000e+00
Coefficient: −1.77e−01
Value: C
Barnesiella.viscericola
0
1
2
3
4
5
A (n=15)
B (n=16)
C (n=14)
surface

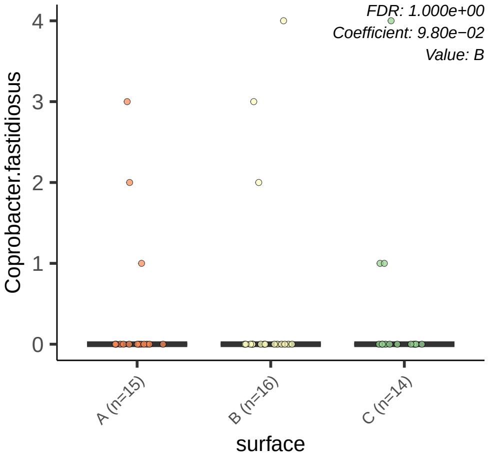

FDR: 1.000e+00
Coefficient: 9.80e−02
Value: B
Coprobacter.fastidiosus
0
1
2
3
4
A (n=15)
B (n=16)
C (n=14)
surface

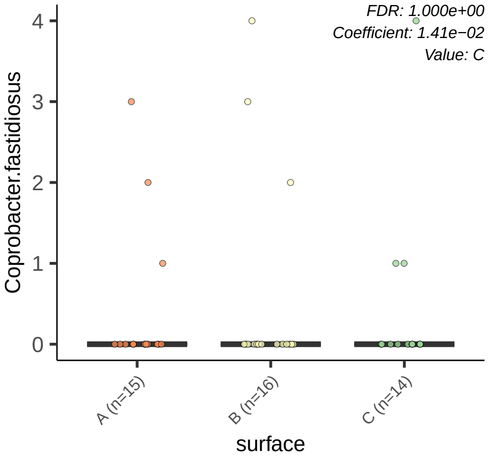

FDR: 1.000e+00
Coefficient: 1.41e−02
Value: C
Coprobacter.fastidiosus
0
1
2
3
4
A (n=15)
B (n=16)
C (n=14)
surface

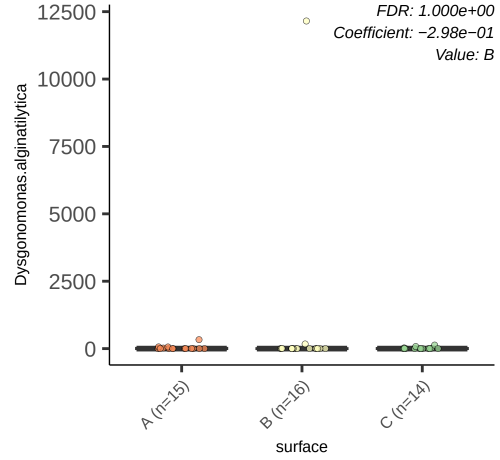

FDR: 1.000e+00
Coefficient: −2.98e−01
Value: B
12500
10000
7500
5000
2500
0
Dysgonomonas.alginatilytica
A (n=15)
B (n=16)
C (n=14)
surface

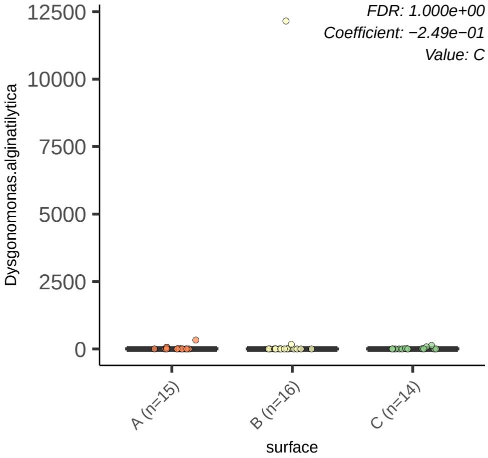
FDR: 1.000e+00
Coefficient: −2.49e−01
Value: C
Dysgonomonas.alginatilytica
12500
10000
7500
5000
2500
0
A (n=15)
B (n=16)
C (n=14)
surface

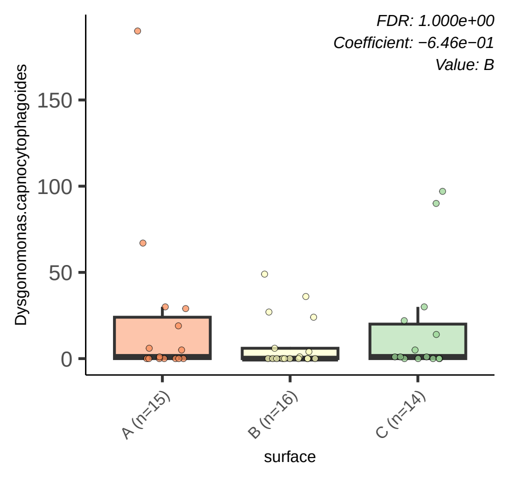

FDR: 1.000e+00
Coefficient: −6.46e−01
Value: B
Dysgonomonas.capnocytophagoides
0
50
100
150
A (n=15)
B (n=16)
C (n=14)
surface

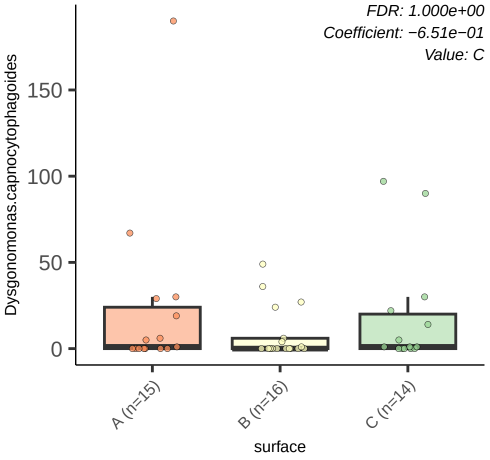
FDR: 1.000e+00
Coefficient: −6.51e−01
Value: C
Dysgonomonas.capnocytophagoides
150
100
50
0
A (n=15)
B (n=16)
C (n=14)
surface

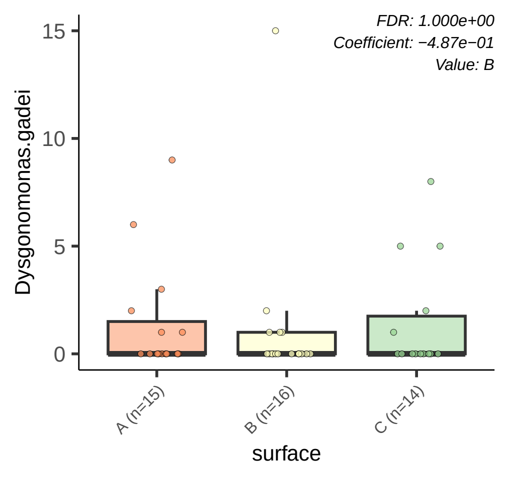

FDR: 1.000e+00
Coefficient: −4.87e−01
Value: B
Dysgonomonas.gadei
15
10
5
0
A (n=15)
B (n=16)
C (n=14)
surface

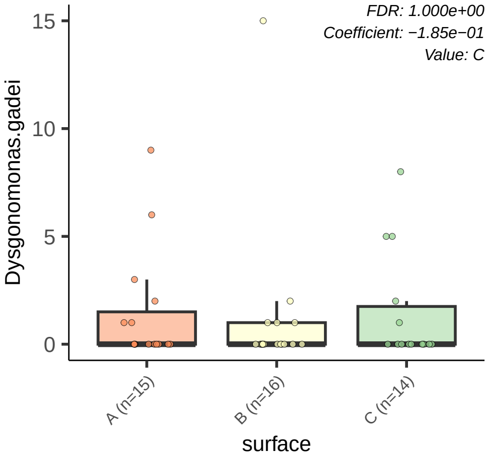
FDR: 1.000e+00
Coefficient: −1.85e−01
Value: C
Dysgonomonas.gadei
15
10
5
0
A (n=15)
B (n=16)
C (n=14)
surface

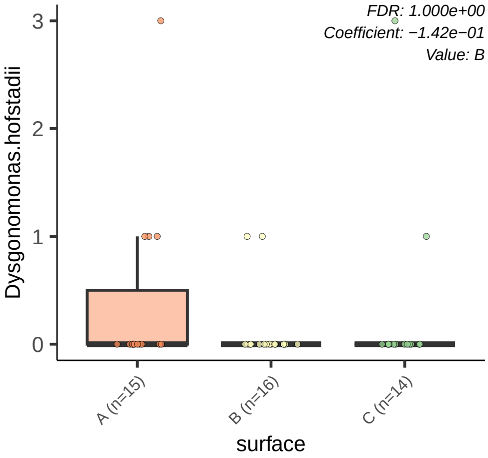

FDR: 1.000e+00
Coefficient: −1.42e−01
Value: B
Dysgonomonas.hofstadii
3
2
1
0
A (n=15)
B (n=16)
C (n=14)
surface

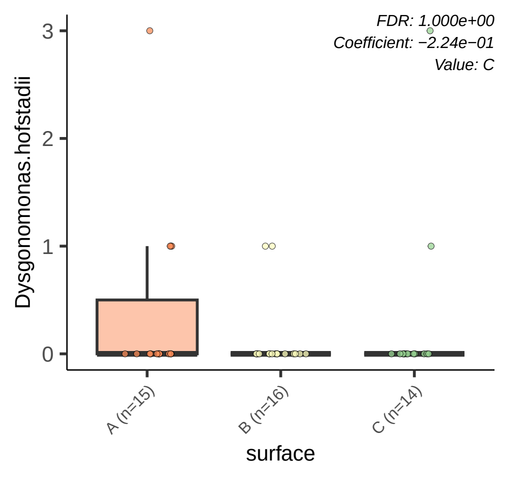

FDR: 1.000e+00
Coefficient: −2.24e−01
Value: C
Dysgonomonas.hofstadii
3
2
1
0
A (n=15)
B (n=16)
C (n=14)
surface

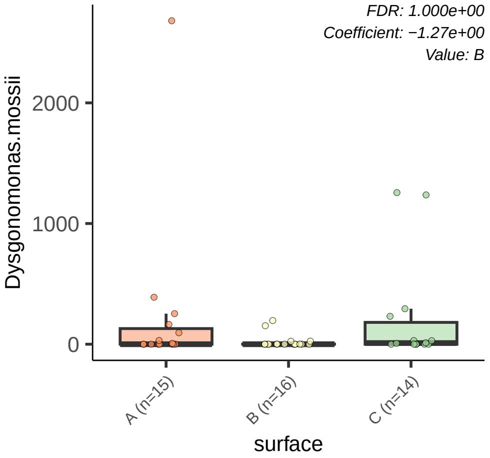
FDR: 1.000e+00
Coefficient: −1.27e+00
Value: B
Dysgonomonas.mossii
2000
1000
0
A (n=15)
B (n=16)
C (n=14)
surface

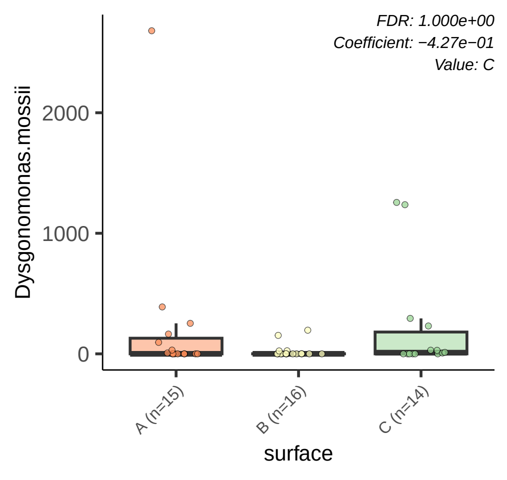
FDR: 1.000e+00
Coefficient: −4.27e−01
Value: C
Dysgonomonas.mossii
2000
1000
0
A (n=15)
B (n=16)
C (n=14)
surface

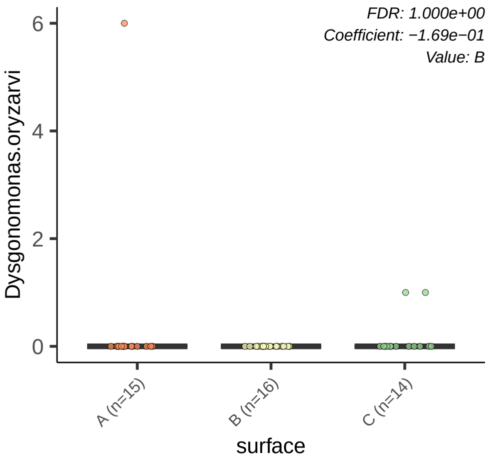

FDR: 1.000e+00
Coefficient: −1.69e−01
Value: B
Dysgonomonas.oryzarvi
0
2
4
6
A (n=15)
B (n=16)
C (n=14)
surface

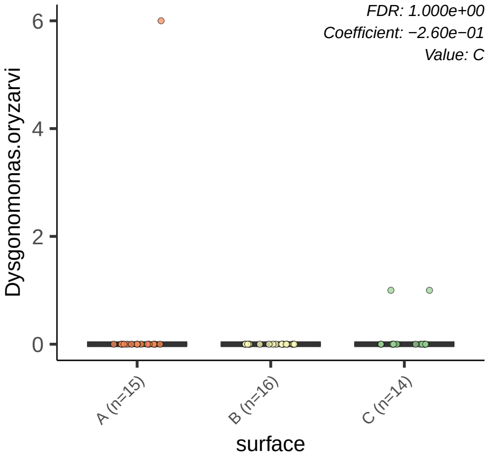

FDR: 1.000e+00
Coefficient: −2.60e−01
Value: C
Dysgonomonas.oryzarvi
0
2
4
6
A (n=15)
B (n=16)
C (n=14)
surface

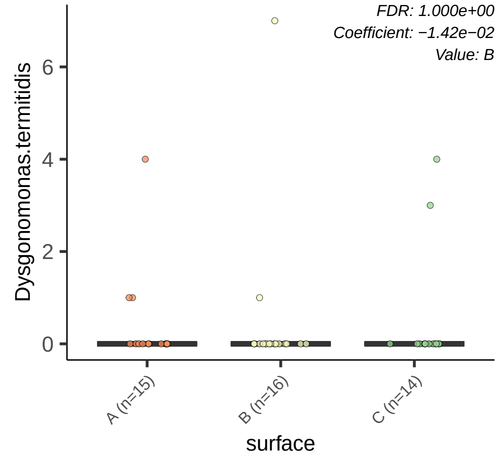

FDR: 1.000e+00
Coefficient: −1.42e−02
Value: B
Dysgonomonas.termitidis
0
2
4
6
A (n=15)
B (n=16)
C (n=14)
surface

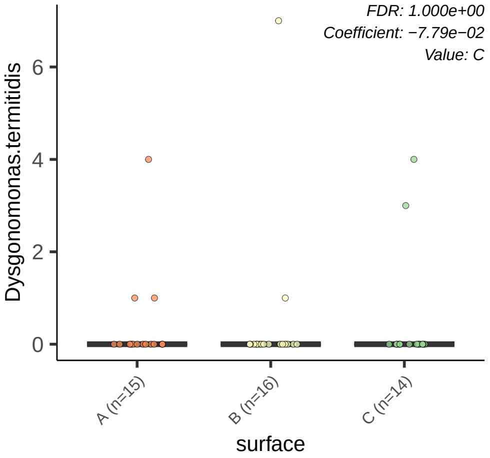
FDR: 1.000e+00
Coefficient: −7.79e−02
Value: C
Dysgonomonas.termitidis
0
2
4
6
A (n=15)
B (n=16)
C (n=14)
surface

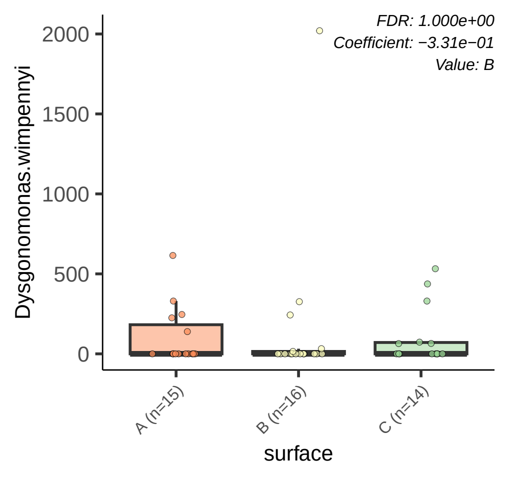

FDR: 1.000e+00
Coefficient: −3.31e−01
Value: B
Dysgonomonas.wimpennyi
0
500
1000
1500
2000
A (n=15)
B (n=16)
C (n=14)
surface

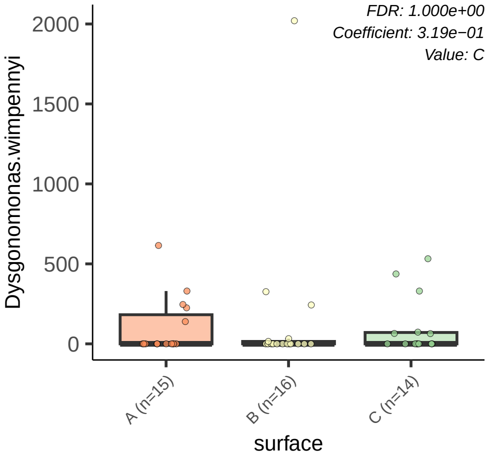
FDR: 1.000e+00
Coefficient: 3.19e−01
Value: C
Dysgonomonas.wimpennyi
2000
1500
1000
500
0
A (n=15)
B (n=16)
C (n=14)
surface

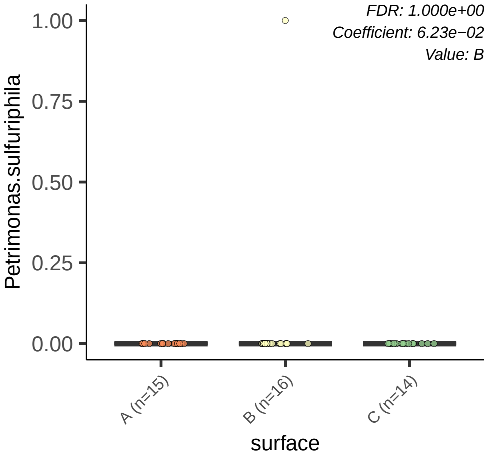
FDR: 1.000e+00
Coefficient: 6.23e−02
Value: B
Petrimonas.sulfuriphila
1.00
0.75
0.50
0.25
0.00
A (n=15)
B (n=16)
C (n=14)
surface

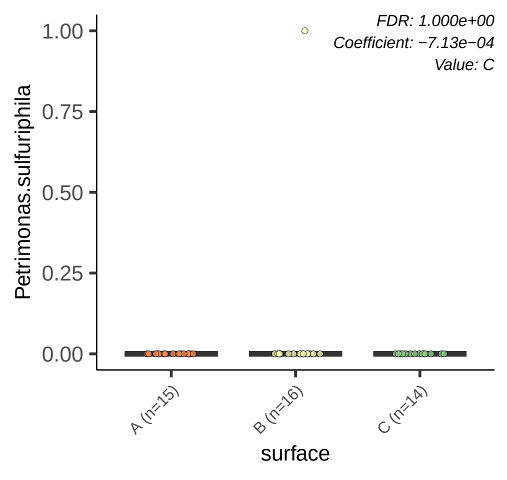
FDR: 1.000e+00
Coefficient: −7.13e−04
Value: C
Petrimonas.sulfuriphila
1.00
0.75
0.50
0.25
0.00
A (n=15)
B (n=16)
C (n=14)
surface

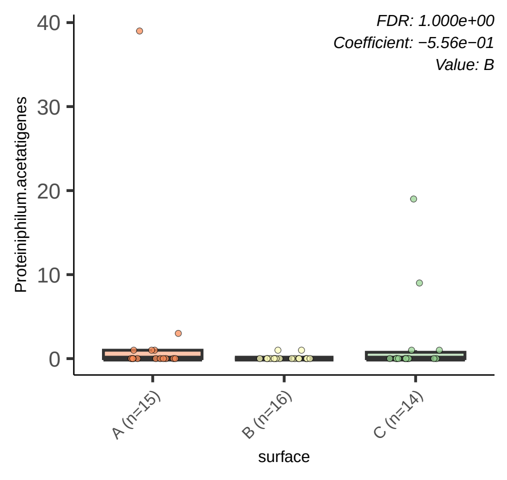
FDR: 1.000e+00
Coefficient: −5.56e−01
Value: B
Proteiniphilum.acetatigenes
0
10
20
30
40
A (n=15)
B (n=16)
C (n=14)
surface

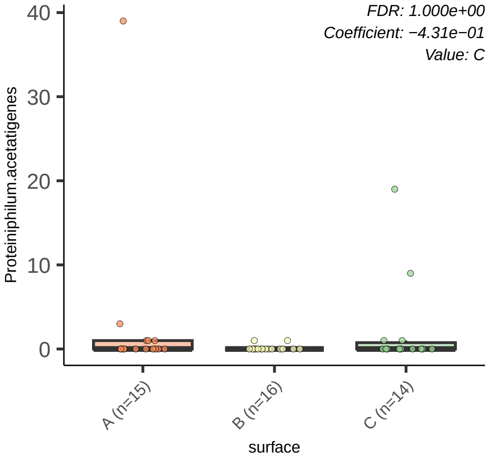

FDR: 1.000e+00
Coefficient: −4.31e−01
Value: C
Proteiniphilum.acetatigenes
40
30
20
10
0
A (n=15)
B (n=16)
C (n=14)
surface

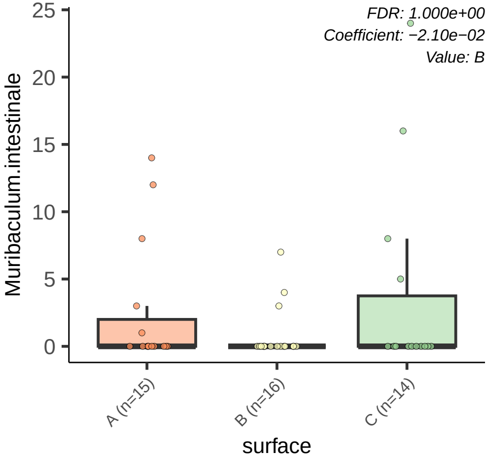
FDR: 1.000e+00
Coefficient: −2.10e−02
Value: B
Muribaculum.intestinale
25
20
15
10
5
0
A (n=15)
B (n=16)
C (n=14)
surface

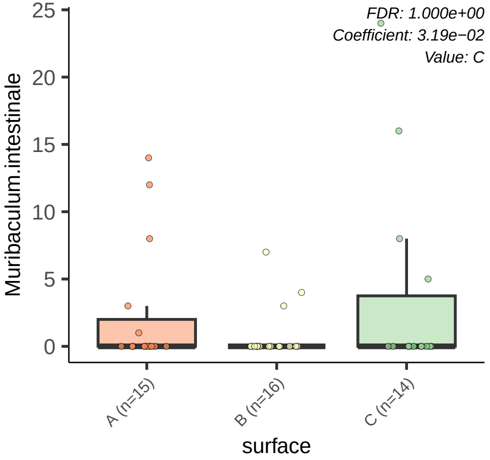

FDR: 1.000e+00
Coefficient: 3.19e−02
Value: C
Muribaculum.intestinale
0
5
10
15
20
25
A (n=15)
B (n=16)
C (n=14)
surface

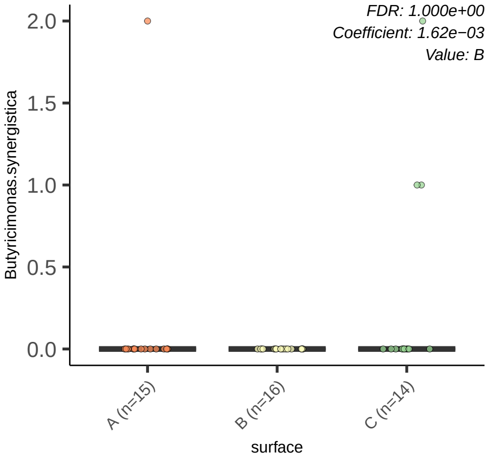

FDR: 1.000e+00
Coefficient: 1.62e−03
Value: B
Butyricimonas.synergistica
2.0
1.5
1.0
0.5
0.0
A (n=15)
B (n=16)
C (n=14)
surface

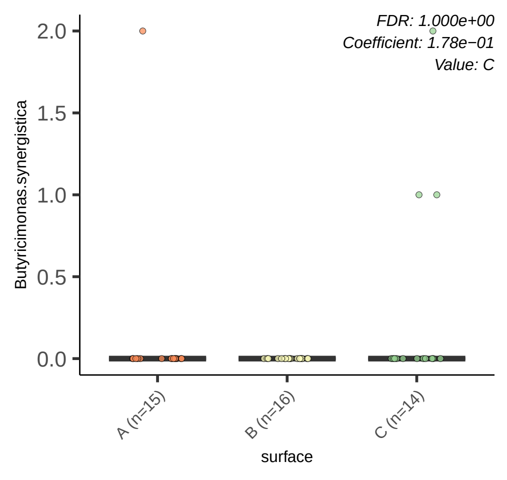

FDR: 1.000e+00
Coefficient: 1.78e−01
Value: C
Butyricimonas.synergistica
2.0
1.5
1.0
0.5
0.0
A (n=15)
B (n=16)
C (n=14)
surface

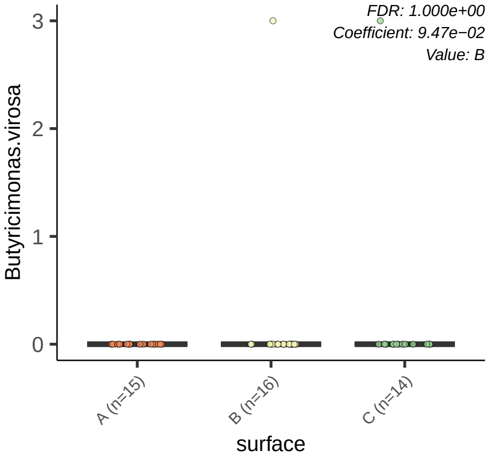
FDR: 1.000e+00
Coefficient: 9.47e−02
Value: B
Butyricimonas.virosa
3
2
1
0
A (n=15)
B (n=16)
C (n=14)
surface

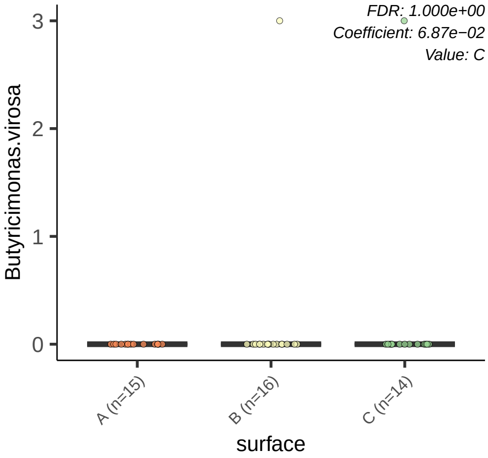
FDR: 1.000e+00
Coefficient: 6.87e−02
Value: C
Butyricimonas.virosa
3
2
1
0
A (n=15)
B (n=16)
C (n=14)
surface

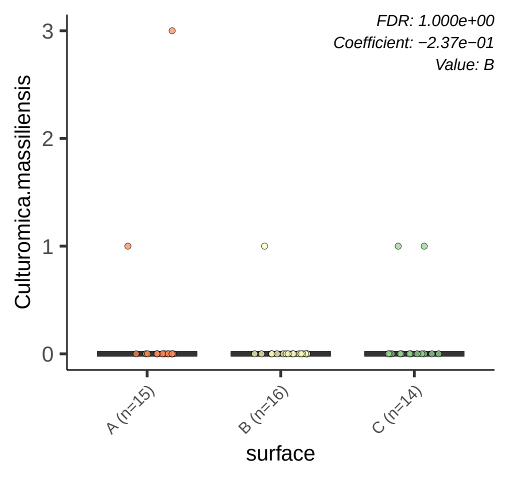
FDR: 1.000e+00
Coefficient: −2.37e−01
Value: B
Culturomica.massiliensis
3
2
1
0
A (n=15)
B (n=16)
C (n=14)
surface

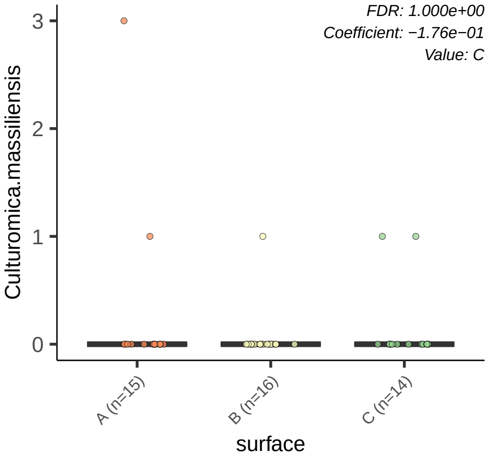

FDR: 1.000e+00
Coefficient: −1.76e−01
Value: C
Culturomica.massiliensis
0
1
2
3
A (n=15)
B (n=16)
C (n=14)
surface

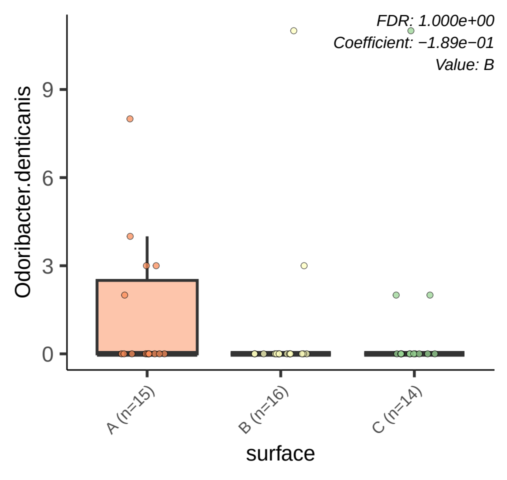
FDR: 1.000e+00
Coefficient: −1.89e−01
Value: B
Odoribacter.denticanis
0
3
6
9
A (n=15)
B (n=16)
C (n=14)
surface

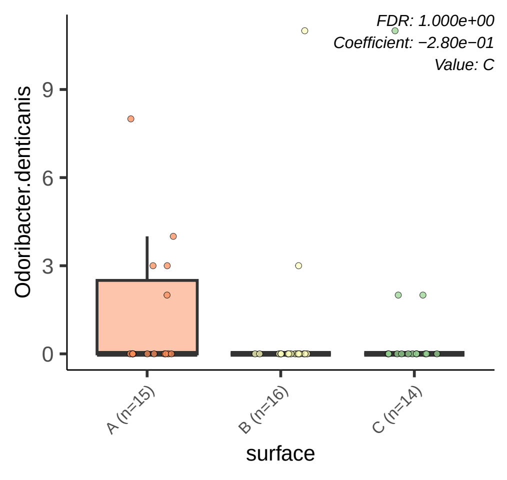

FDR: 1.000e+00
Coefficient: −2.80e−01
Value: C
Odoribacter.denticanis
0
3
6
9
A (n=15)
B (n=16)
C (n=14)
surface

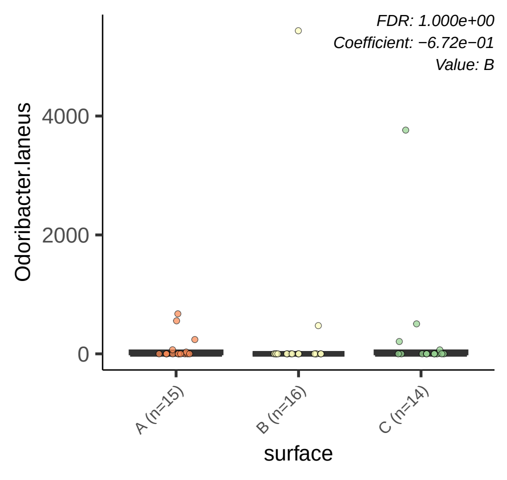
FDR: 1.000e+00
Coefficient: −6.72e−01
Value: B
Odoribacter.laneus
4000
2000
0
A (n=15)
B (n=16)
C (n=14)
surface

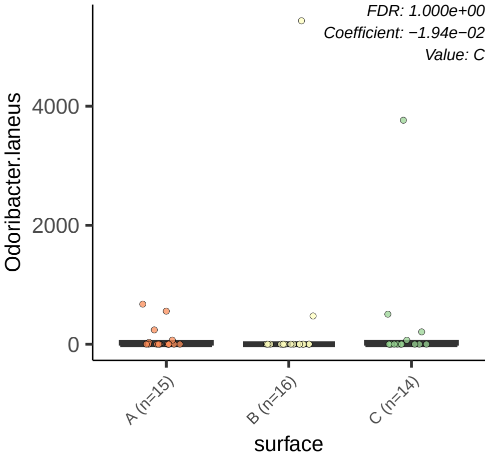

FDR: 1.000e+00
Coefficient: −1.94e−02
Value: C
Odoribacter.laneus
4000
2000
0
A (n=15)
B (n=16)
C (n=14)
surface

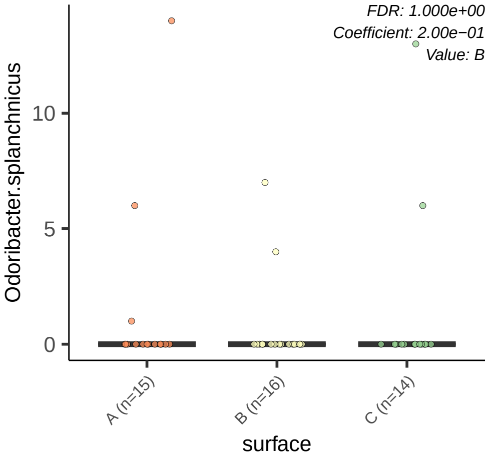

FDR: 1.000e+00
Coefficient: 2.00e−01
Value: B
Odoribacter.splanchnicus
0
5
10
A (n=15)
B (n=16)
C (n=14)
surface

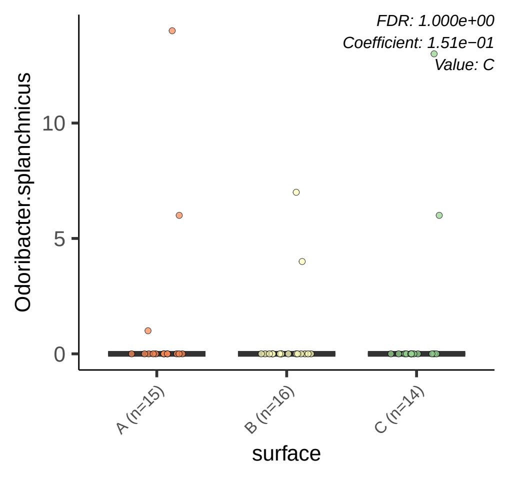

FDR: 1.000e+00
Coefficient: 1.51e−01
Value: C
Odoribacter.splanchnicus
0
5
10
A (n=15)
B (n=16)
C (n=14)
surface

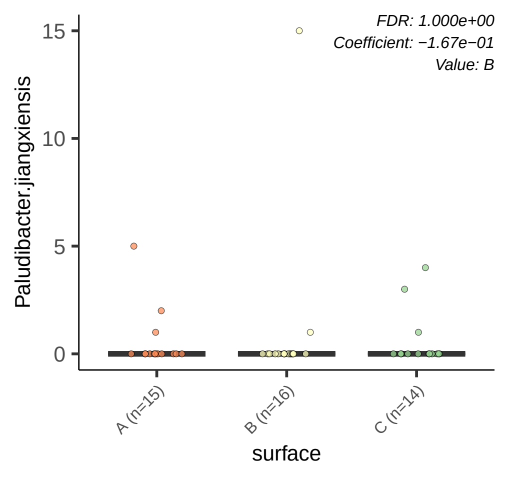

FDR: 1.000e+00
Coefficient: −1.67e−01
Value: B
Paludibacter.jiangxiensis
15
10
5
0
A (n=15)
B (n=16)
C (n=14)
surface

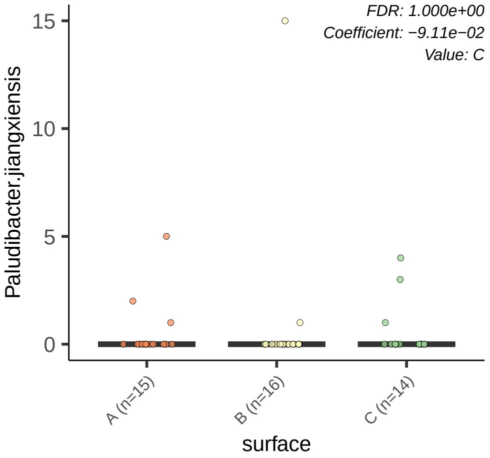
FDR: 1.000e+00
Coefficient: −9.11e−02
Value: C
Paludibacter.jiangxiensis
15
10
5
0
A (n=15)
B (n=16)
C (n=14)
surface

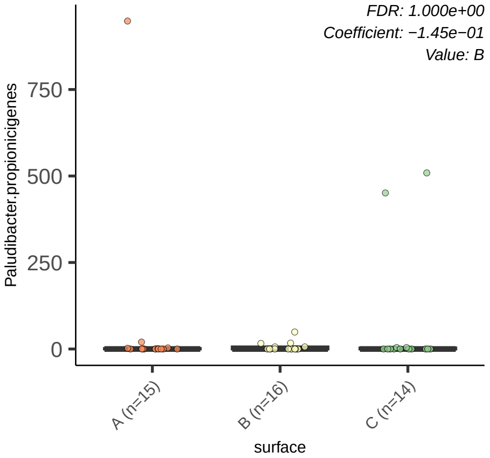
FDR: 1.000e+00
Coefficient: −1.45e−01
Value: B
Paludibacter.propionicigenes
750
500
250
0
A (n=15)
B (n=16)
C (n=14)
surface

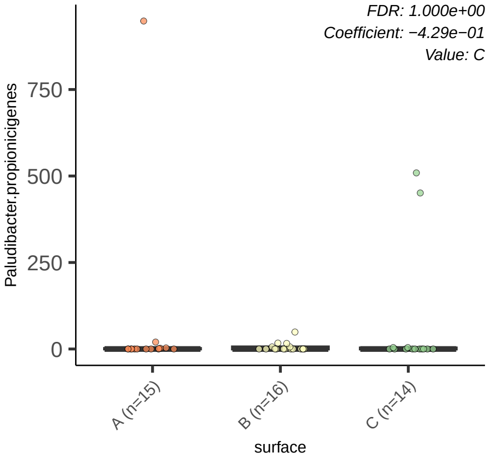
FDR: 1.000e+00
Coefficient: −4.29e−01
Value: C
Paludibacter.propionicigenes
750
500
250
0
A (n=15)
B (n=16)
C (n=14)
surface

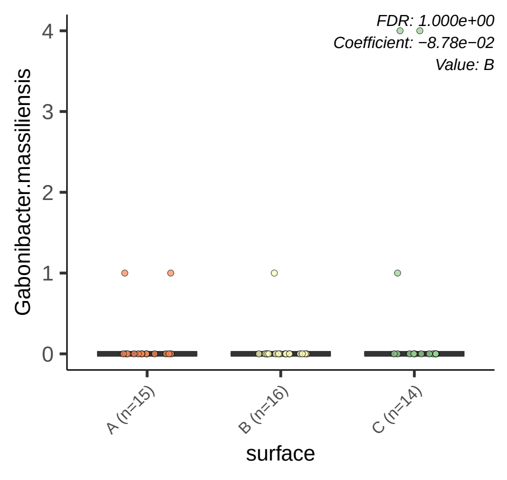
FDR: 1.000e+00
Coefficient: −8.78e−02
Value: B
Gabonibacter.massiliensis
4
3
2
1
0
A (n=15)
B (n=16)
C (n=14)
surface

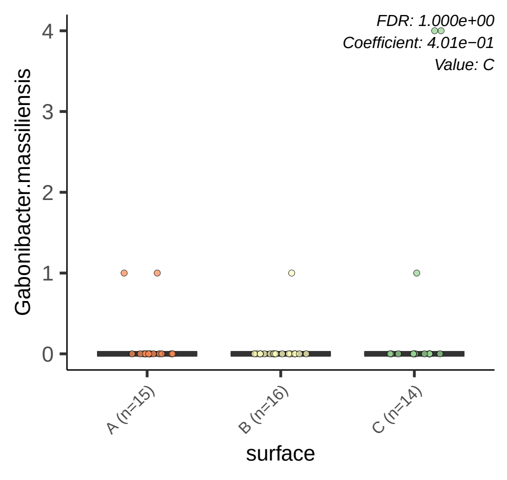
FDR: 1.000e+00
Coefficient: 4.01e−01
Value: C
Gabonibacter.massiliensis
4
3
2
1
0
A (n=15)
B (n=16)
C (n=14)
surface

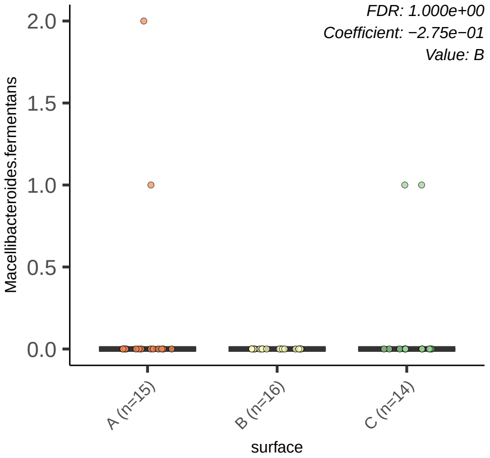

FDR: 1.000e+00
Coefficient: −2.75e−01
Value: B
Macellibacteroides.fermentans
2.0
1.5
1.0
0.5
0.0
A (n=15)
B (n=16)
C (n=14)
surface

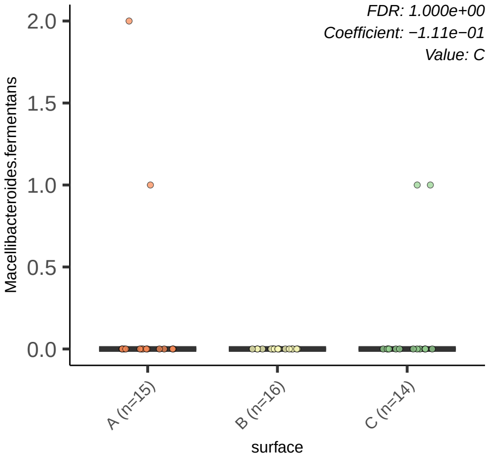

FDR: 1.000e+00
Coefficient: −1.11e−01
Value: C
Macellibacteroides.fermentans
2.0
1.5
1.0
0.5
0.0
A (n=15)
B (n=16)
C (n=14)
surface

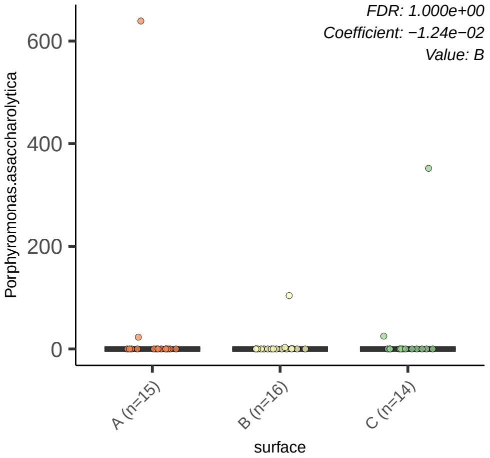

FDR: 1.000e+00
Coefficient: −1.24e−02
Value: B
600
400
200
0
Porphyromonas.asaccharolytica
A (n=15)
B (n=16)
C (n=14)
surface

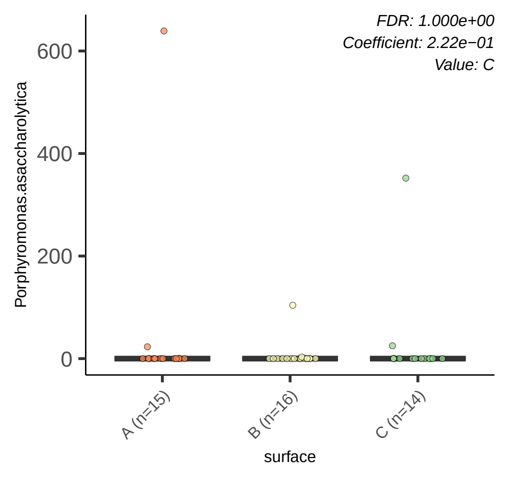
FDR: 1.000e+00
Coefficient: 2.22e−01
Value: C
Porphyromonas.asaccharolytica
600
400
200
0
A (n=15)
B (n=16)
C (n=14)
surface

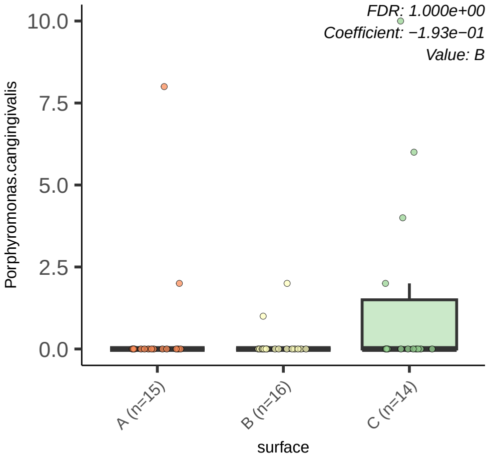

FDR: 1.000e+00
Coefficient: −1.93e−01
Value: B
Porphyromonas.cangingivalis
10.0
7.5
5.0
2.5
0.0
A (n=15)
B (n=16)
C (n=14)
surface

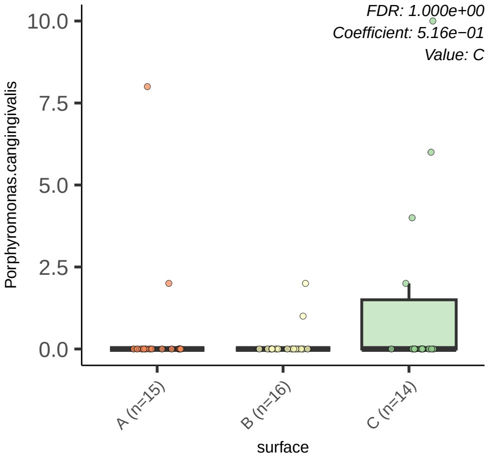
FDR: 1.000e+00
Coefficient: 5.16e−01
Value: C
Porphyromonas.cangingivalis
10.0
7.5
5.0
2.5
0.0
A (n=15)
B (n=16)
C (n=14)
surface

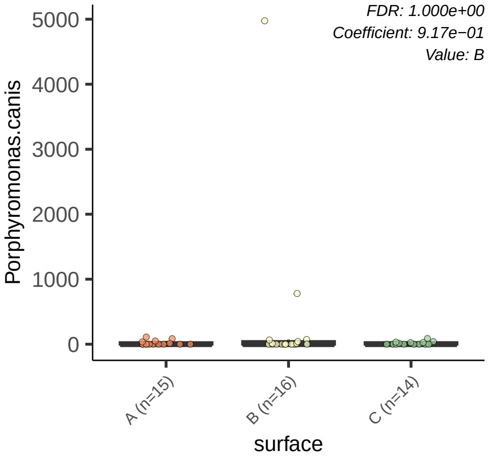

FDR: 1.000e+00
Coefficient: 9.17e−01
Value: B
Porphyromonas.canis
5000
4000
3000
2000
1000
0
A (n=15)
B (n=16)
C (n=14)
surface

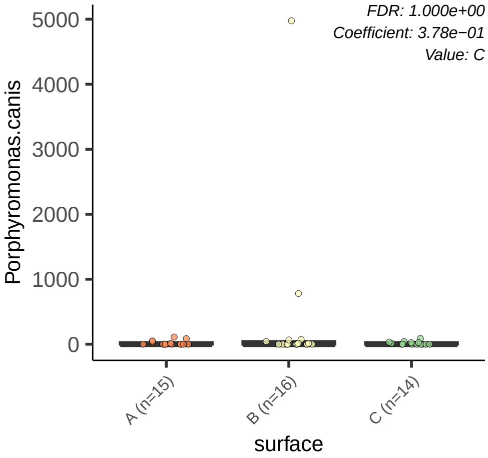

FDR: 1.000e+00
Coefficient: 3.78e−01
Value: C
Porphyromonas.canis
5000
4000
3000
2000
1000
0
A (n=15)
B (n=16)
C (n=14)
surface

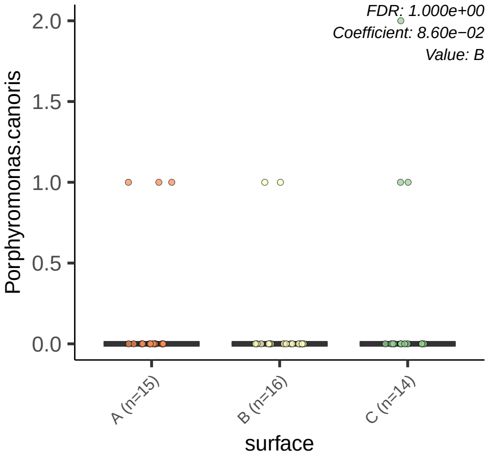
FDR: 1.000e+00
Coefficient: 8.60e−02
Value: B
Porphyromonas.canoris
2.0
1.5
1.0
0.5
0.0
A (n=15)
B (n=16)
C (n=14)
surface

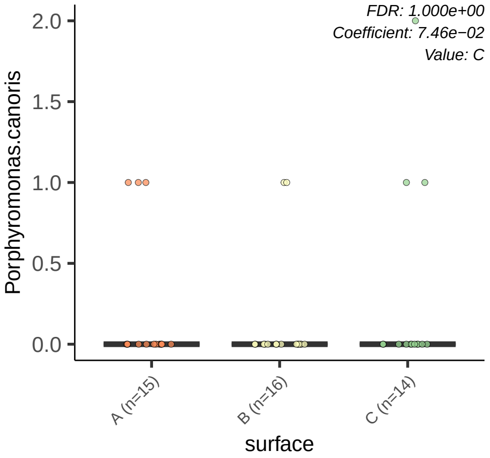
FDR: 1.000e+00
Coefficient: 7.46e−02
Value: C
Porphyromonas.canoris
2.0
1.5
1.0
0.5
0.0
A (n=15)
B (n=16)
C (n=14)
surface

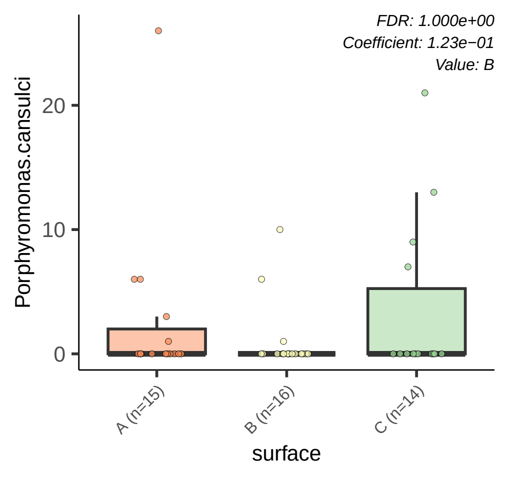
FDR: 1.000e+00
Coefficient: 1.23e−01
Value: B
Porphyromonas.cansulci
0
10
20
A (n=15)
B (n=16)
C (n=14)
surface

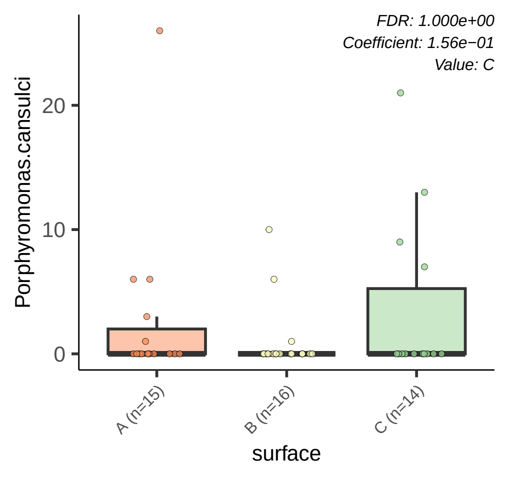

FDR: 1.000e+00
Coefficient: 1.56e−01
Value: C
Porphyromonas.cansulci
0
10
20
A (n=15)
B (n=16)
C (n=14)
surface

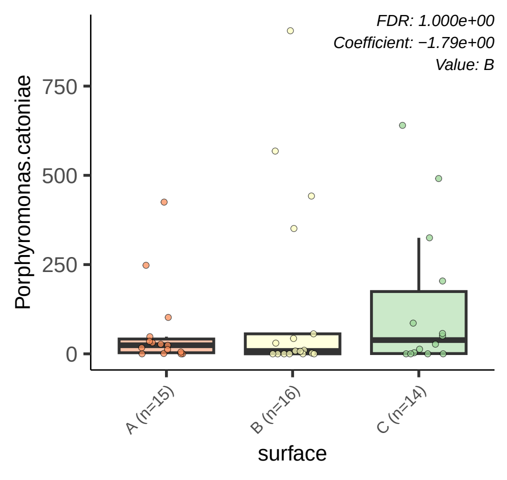

FDR: 1.000e+00
Coefficient: −1.79e+00
Value: B
Porphyromonas.catoniae
750
500
250
0
A (n=15)
B (n=16)
C (n=14)
surface

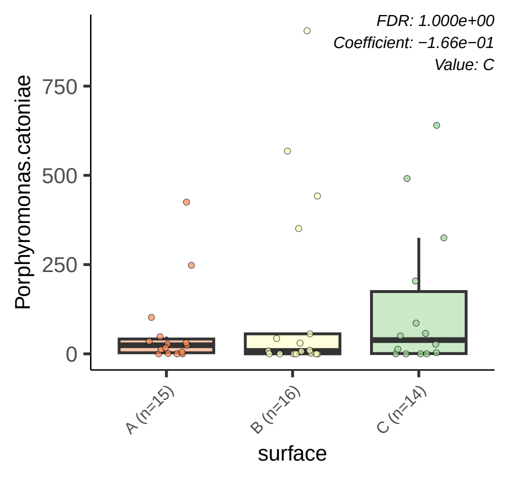
FDR: 1.000e+00
Coefficient: −1.66e−01
Value: C
Porphyromonas.catoniae
750
500
250
0
A (n=15)
B (n=16)
C (n=14)
surface

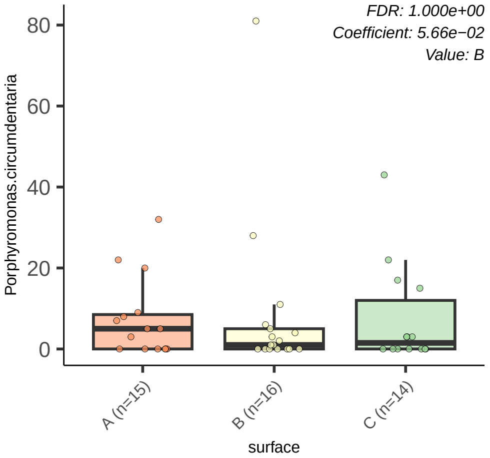
FDR: 1.000e+00
Coefficient: 5.66e−02
Value: B
Porphyromonas.circumdentaria
0
20
40
60
80
A (n=15)
B (n=16)
C (n=14)
surface

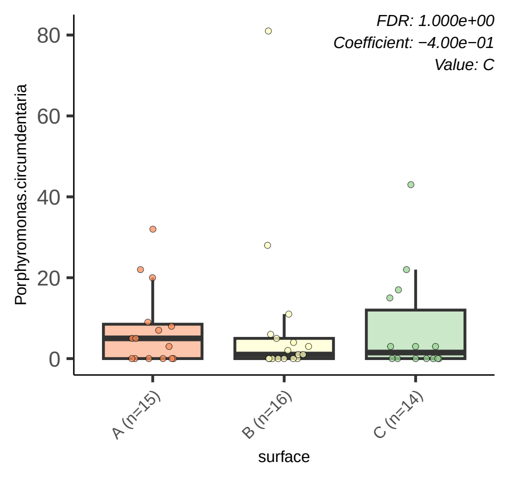

FDR: 1.000e+00
Coefficient: −4.00e−01
Value: C
Porphyromonas.circumdentaria
0
20
40
60
80
A (n=15)
B (n=16)
C (n=14)
surface

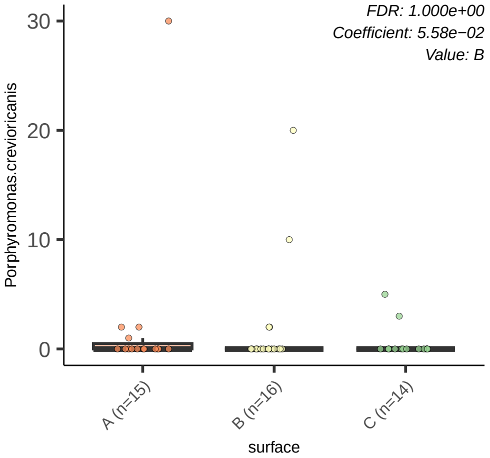
FDR: 1.000e+00
Coefficient: 5.58e−02
Value: B
Porphyromonas.crevioricanis
30
20
10
0
A (n=15)
B (n=16)
C (n=14)
surface

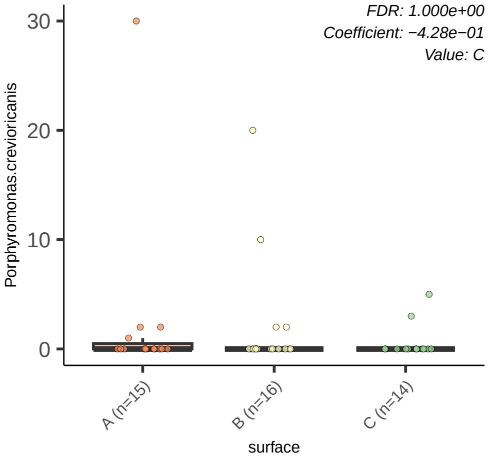
FDR: 1.000e+00
Coefficient: −4.28e−01
Value: C
Porphyromonas.crevioricanis
30
20
10
0
A (n=15)
B (n=16)
C (n=14)
surface

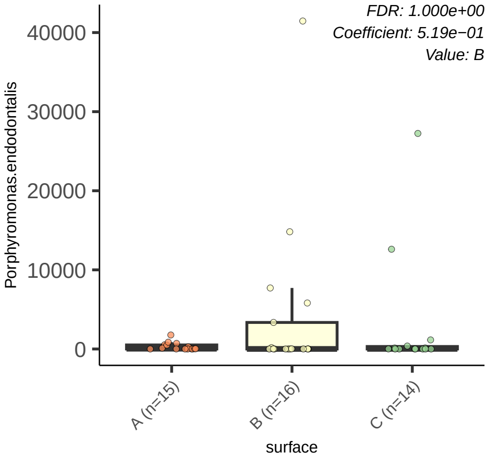
FDR: 1.000e+00
Coefficient: 5.19e−01
Value: B
Porphyromonas.endodontalis
40000
30000
20000
10000
0
A (n=15)
B (n=16)
C (n=14)
surface

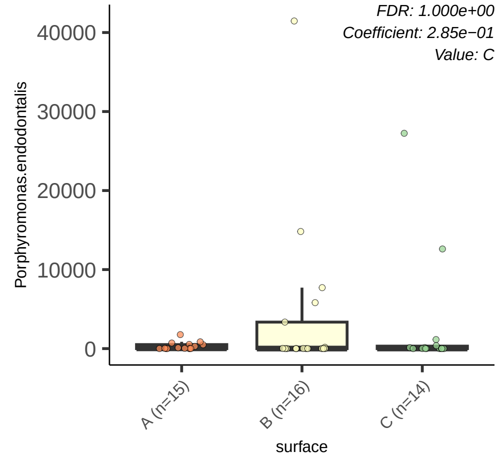

FDR: 1.000e+00
Coefficient: 2.85e−01
Value: C
40000
30000
20000
10000
0
Porphyromonas.endodontalis
A (n=15)
B (n=16)
C (n=14)
surface

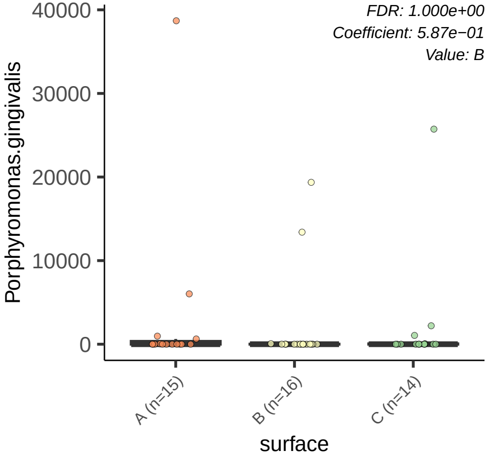
FDR: 1.000e+00
Coefficient: 5.87e−01
Value: B
Porphyromonas.gingivalis
40000
30000
20000
10000
0
A (n=15)
B (n=16)
C (n=14)
surface

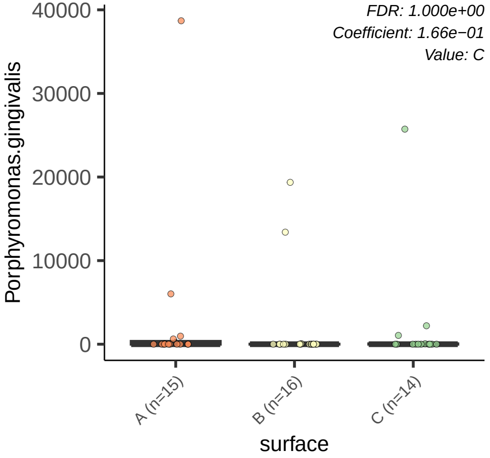

FDR: 1.000e+00
Coefficient: 1.66e−01
Value: C
Porphyromonas.gingivalis
40000
30000
20000
10000
0
A (n=15)
B (n=16)
C (n=14)
surface

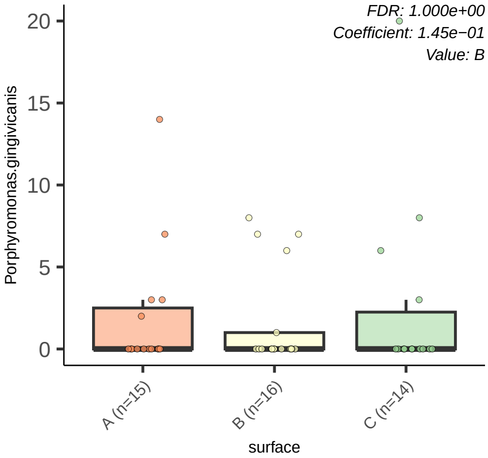
FDR: 1.000e+00
Coefficient: 1.45e−01
Value: B
Porphyromonas.gingivicanis
20
15
10
5
0
A (n=15)
B (n=16)
C (n=14)
surface

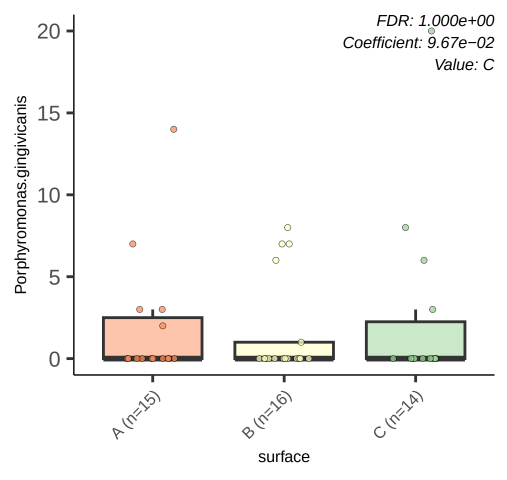
FDR: 1.000e+00
Coefficient: 9.67e−02
Value: C
Porphyromonas.gingivicanis
20
15
10
5
0
A (n=15)
B (n=16)
C (n=14)
surface

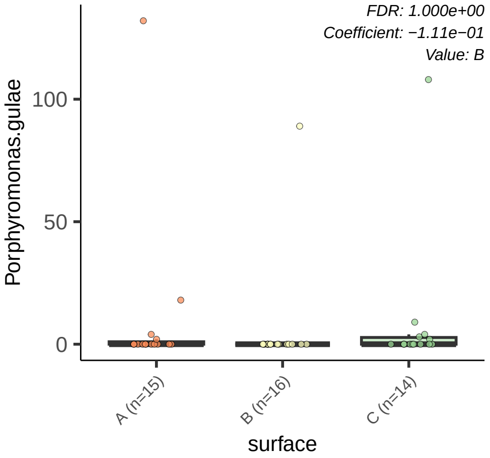

FDR: 1.000e+00
Coefficient: −1.11e−01
Value: B
Porphyromonas.gulae
100
50
0
A (n=15)
B (n=16)
C (n=14)
surface

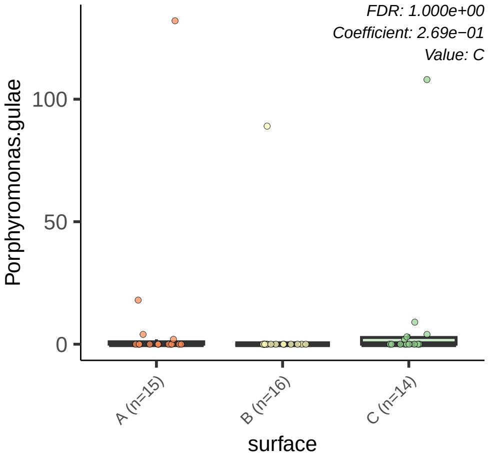
FDR: 1.000e+00
Coefficient: 2.69e−01
Value: C
Porphyromonas.gulae
100
50
0
A (n=15)
B (n=16)
C (n=14)
surface

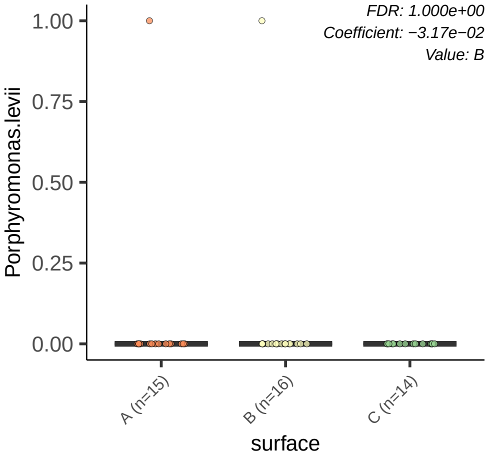
FDR: 1.000e+00
Coefficient: −3.17e−02
Value: B
Porphyromonas.levii
1.00
0.75
0.50
0.25
0.00
A (n=15)
B (n=16)
C (n=14)
surface

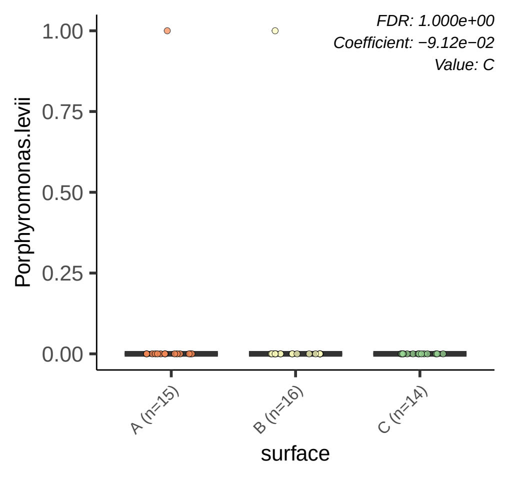
FDR: 1.000e+00
Coefficient: −9.12e−02
Value: C
Porphyromonas.levii
1.00
0.75
0.50
0.25
0.00
A (n=15)
B (n=16)
C (n=14)
surface

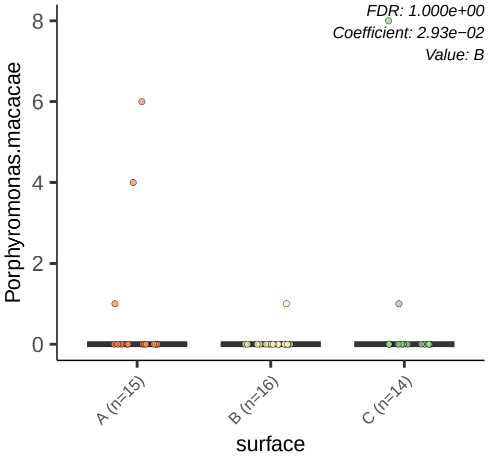
FDR: 1.000e+00
Coefficient: 2.93e−02
Value: B
Porphyromonas.macacae
0
2
4
6
8
A (n=15)
B (n=16)
C (n=14)
surface

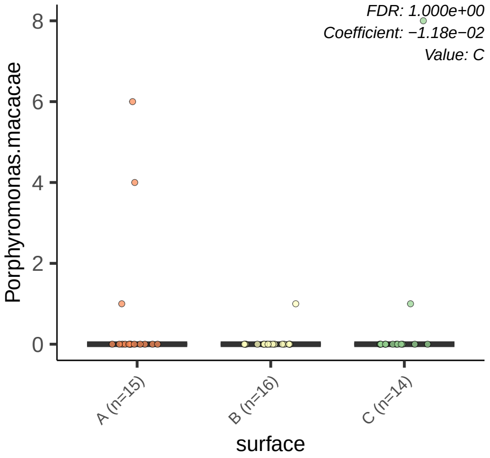
FDR: 1.000e+00
Coefficient: −1.18e−02
Value: C
Porphyromonas.macacae
0
2
4
6
8
A (n=15)
B (n=16)
C (n=14)
surface

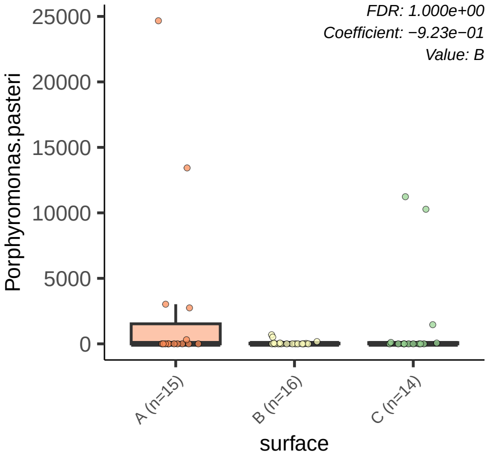
FDR: 1.000e+00
Coefficient: −9.23e−01
Value: B
Porphyromonas.pasteri
25000
20000
15000
10000
5000
0
A (n=15)
B (n=16)
C (n=14)
surface

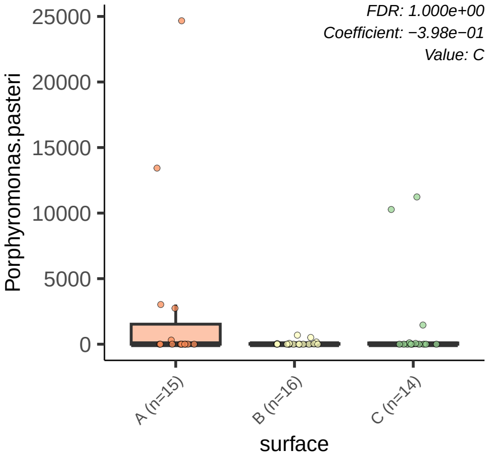
FDR: 1.000e+00
Coefficient: −3.98e−01
Value: C
Porphyromonas.pasteri
25000
20000
15000
10000
5000
0
A (n=15)
B (n=16)
C (n=14)
surface

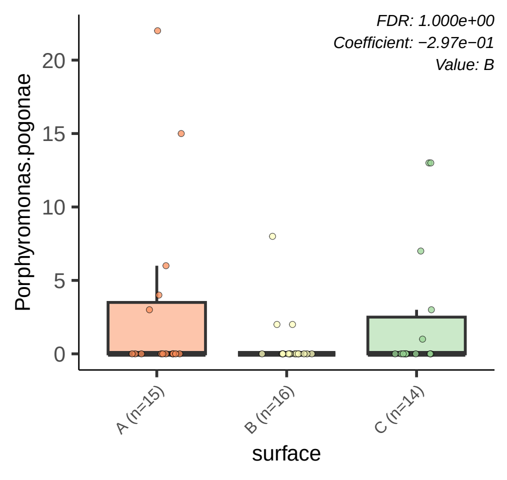
FDR: 1.000e+00
Coefficient: −2.97e−01
Value: B
Porphyromonas.pogonae
0
5
10
15
20
A (n=15)
B (n=16)
C (n=14)
surface

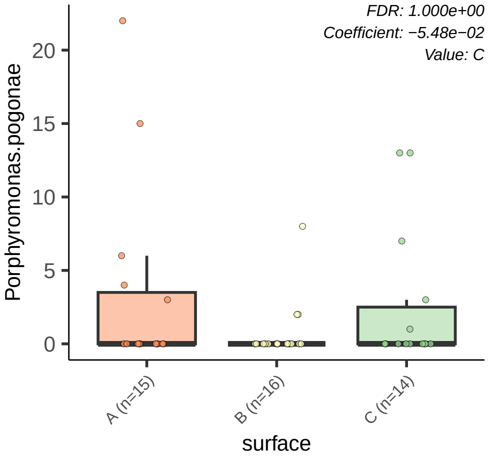
FDR: 1.000e+00
Coefficient: −5.48e−02
Value: C
Porphyromonas.pogonae
0
5
10
15
20
A (n=15)
B (n=16)
C (n=14)
surface

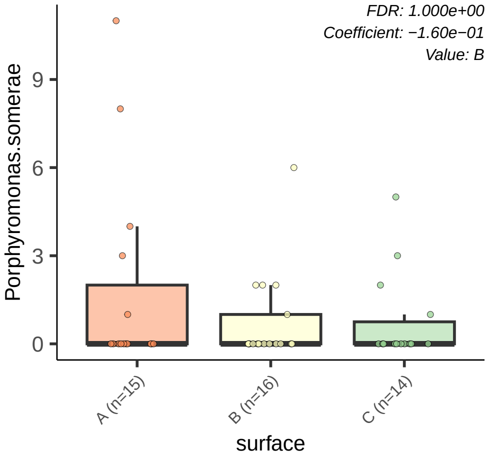

FDR: 1.000e+00
Coefficient: −1.60e−01
Value: B
Porphyromonas.somerae
0
3
6
9
A (n=15)
B (n=16)
C (n=14)
surface

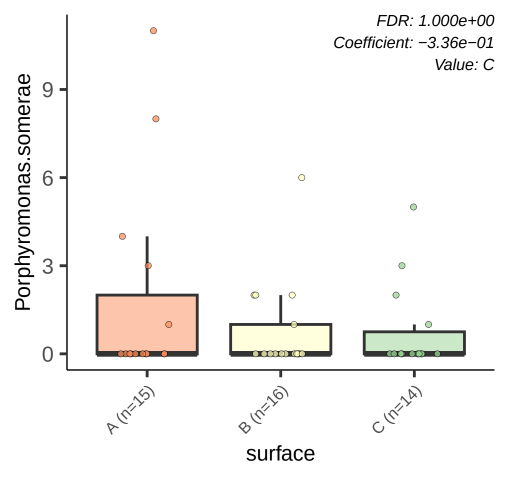
FDR: 1.000e+00
Coefficient: −3.36e−01
Value: C
Porphyromonas.somerae
0
3
6
9
A (n=15)
B (n=16)
C (n=14)
surface

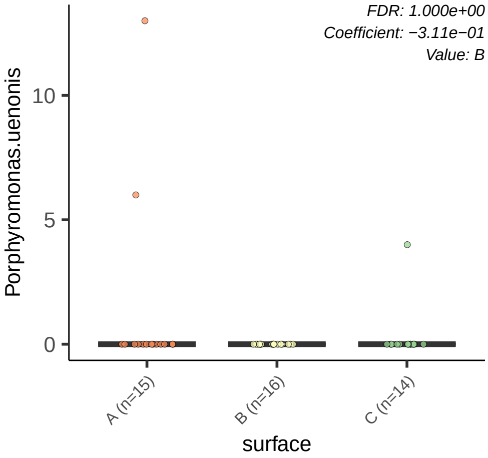
FDR: 1.000e+00
Coefficient: −3.11e−01
Value: B
Porphyromonas.uenonis
10
5
0
A (n=15)
B (n=16)
C (n=14)
surface

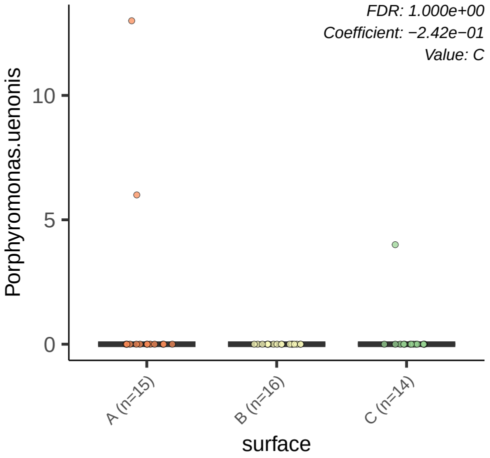

FDR: 1.000e+00
Coefficient: −2.42e−01
Value: C
Porphyromonas.uenonis
10
5
0
A (n=15)
B (n=16)
C (n=14)
surface

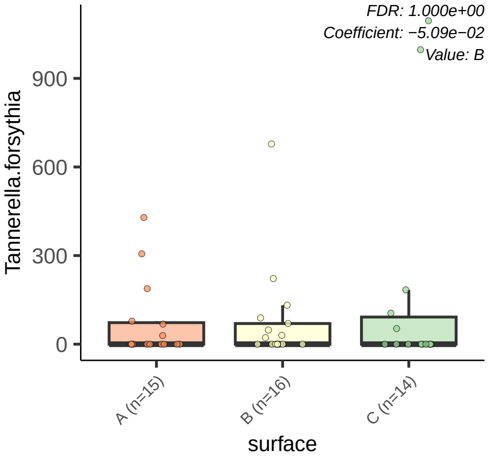

FDR: 1.000e+00
Coefficient: −5.09e−02
Value: B
Tannerella.forsythia
900
600
300
0
A (n=15)
B (n=16)
C (n=14)
surface

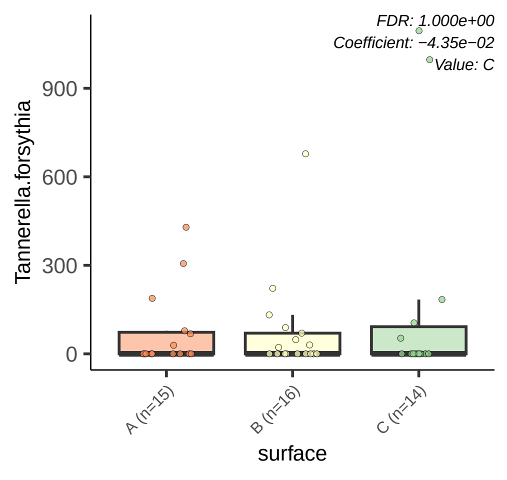

FDR: 1.000e+00
Coefficient: −4.35e−02
Value: C
Tannerella.forsythia
900
600
300
0
A (n=15)
B (n=16)
C (n=14)
surface

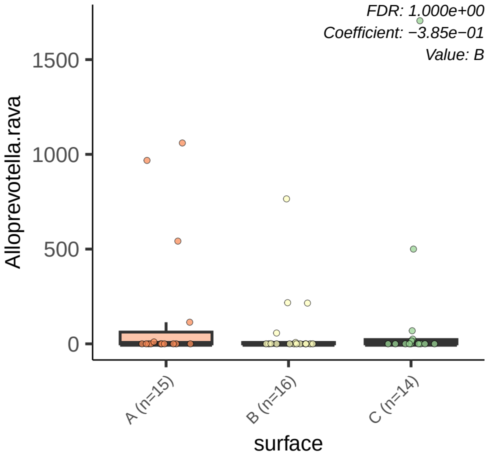
FDR: 1.000e+00
Coefficient: −3.85e−01
Value: B
Alloprevotella.rava
1500
1000
500
0
A (n=15)
B (n=16)
C (n=14)
surface

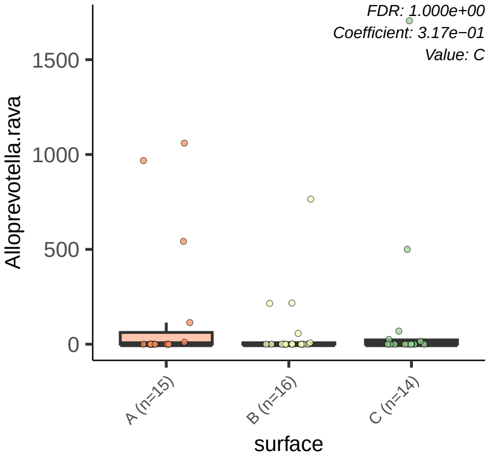

FDR: 1.000e+00
Coefficient: 3.17e−01
Value: C
1500
1000
500
0
Alloprevotella.rava
A (n=15)
B (n=16)
C (n=14)
surface

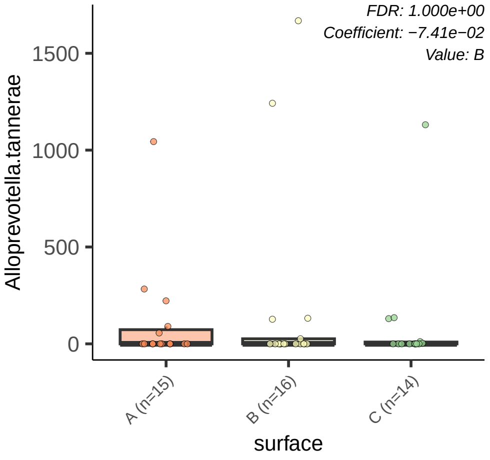
FDR: 1.000e+00
Coefficient: −7.41e−02
Value: B
Alloprevotella.tannerae
1500
1000
500
0
A (n=15)
B (n=16)
C (n=14)
surface

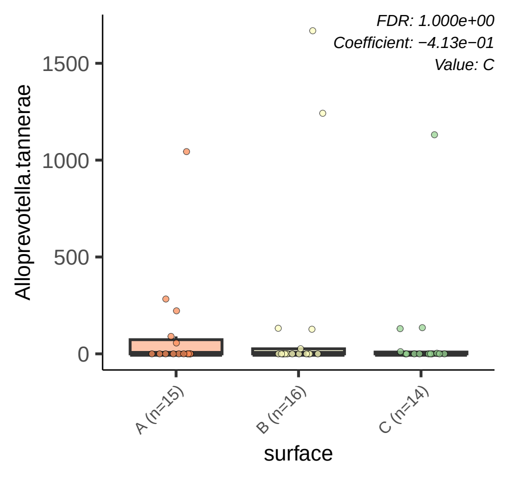
FDR: 1.000e+00
Coefficient: −4.13e−01
Value: C
Alloprevotella.tannerae
1500
1000
500
0
A (n=15)
B (n=16)
C (n=14)
surface

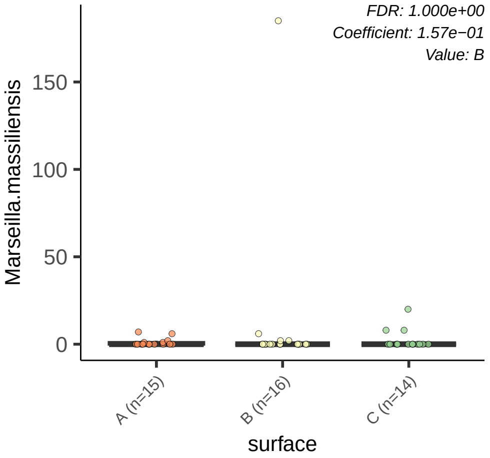
FDR: 1.000e+00
Coefficient: 1.57e−01
Value: B
Marseilla.massiliensis
150
100
50
0
A (n=15)
B (n=16)
C (n=14)
surface

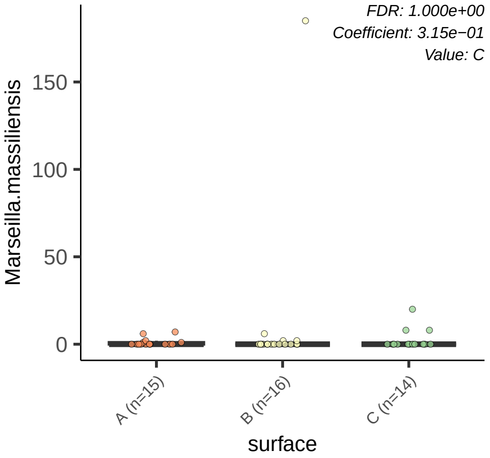
FDR: 1.000e+00
Coefficient: 3.15e−01
Value: C
Marseilla.massiliensis
150
100
50
0
A (n=15)
B (n=16)
C (n=14)
surface

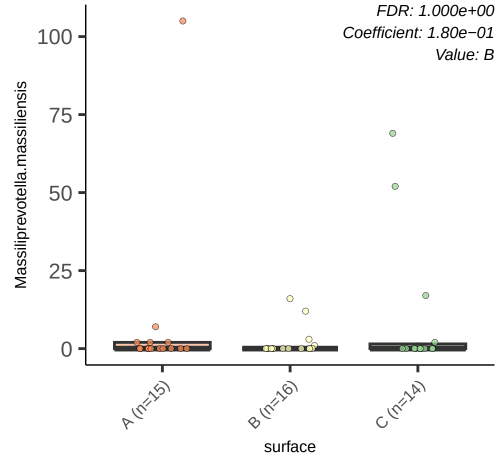
FDR: 1.000e+00
Coefficient: 1.80e−01
Value: B
Massiliprevotella.massiliensis
100
75
50
25
0
A (n=15)
B (n=16)
C (n=14)
surface

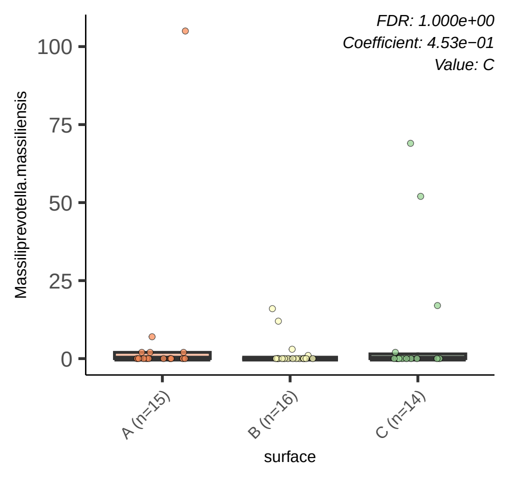
FDR: 1.000e+00
Coefficient: 4.53e−01
Value: C
Massiliprevotella.massiliensis
100
75
50
25
0
A (n=15)
B (n=16)
C (n=14)
surface

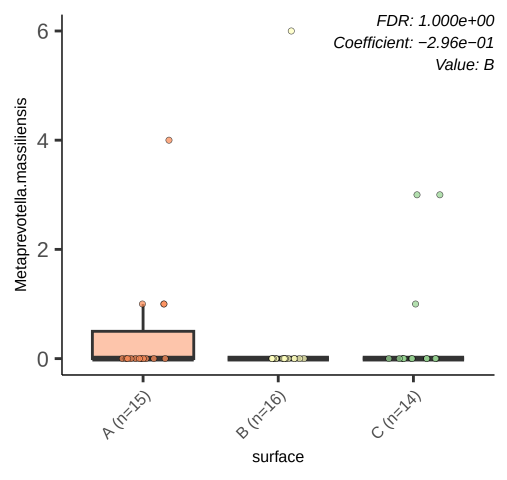
FDR: 1.000e+00
Coefficient: −2.96e−01
Value: B
Metaprevotella.massiliensis
0
2
4
6
A (n=15)
B (n=16)
C (n=14)
surface

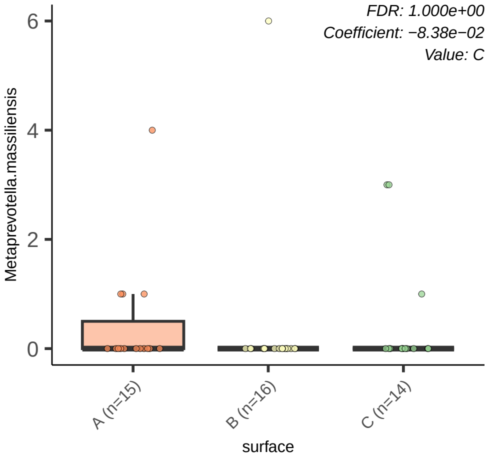

FDR: 1.000e+00
Coefficient: −8.38e−02
Value: C
Metaprevotella.massiliensis
0
2
4
6
A (n=15)
B (n=16)
C (n=14)
surface

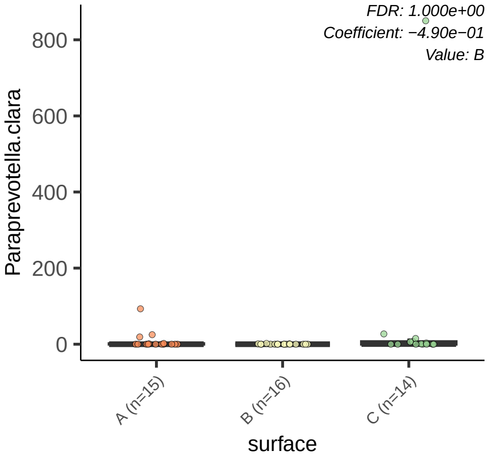
FDR: 1.000e+00
Coefficient: −4.90e−01
Value: B
Paraprevotella.clara
800
600
400
200
0
A (n=15)
B (n=16)
C (n=14)
surface

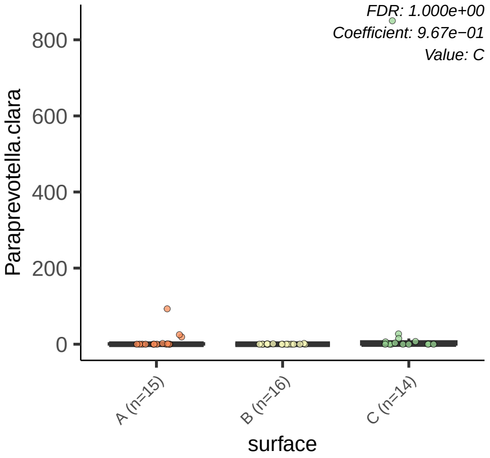
FDR: 1.000e+00
Coefficient: 9.67e−01
Value: C
Paraprevotella.clara
800
600
400
200
0
A (n=15)
B (n=16)
C (n=14)
surface

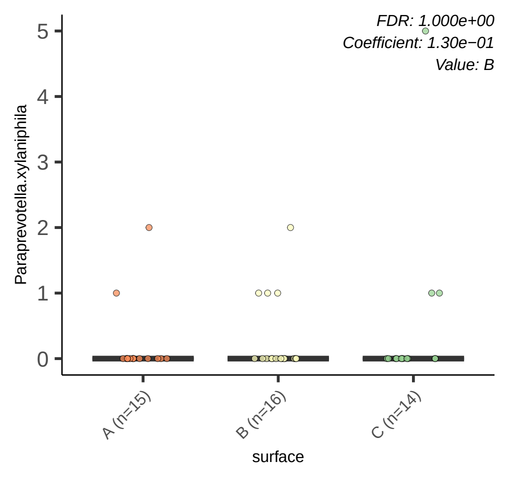
FDR: 1.000e+00
Coefficient: 1.30e−01
Value: B
Paraprevotella.xylaniphila
0
1
2
3
4
5
A (n=15)
B (n=16)
C (n=14)
surface

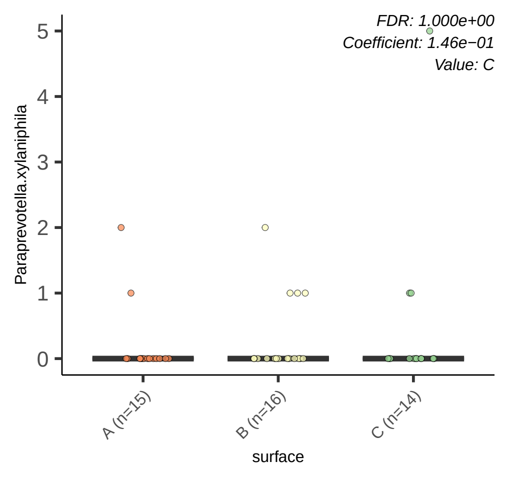
FDR: 1.000e+00
Coefficient: 1.46e−01
Value: C
Paraprevotella.xylaniphila
0
1
2
3
4
5
A (n=15)
B (n=16)
C (n=14)
surface

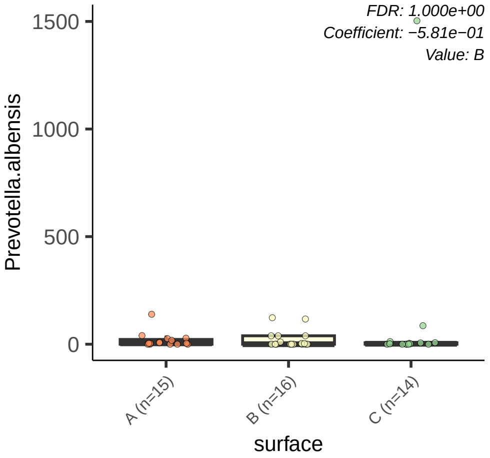
FDR: 1.000e+00
Coefficient: −5.81e−01
Value: B
1500
1000
500
0
Prevotella.albensis
A (n=15)
B (n=16)
C (n=14)
surface

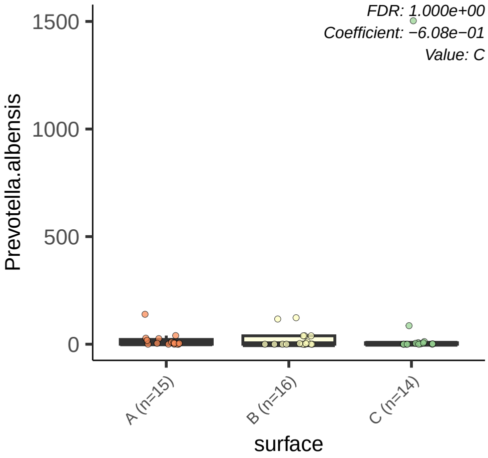
FDR: 1.000e+00
Coefficient: −6.08e−01
Value: C
Prevotella.albensis
1500
1000
500
0
A (n=15)
B (n=16)
C (n=14)
surface

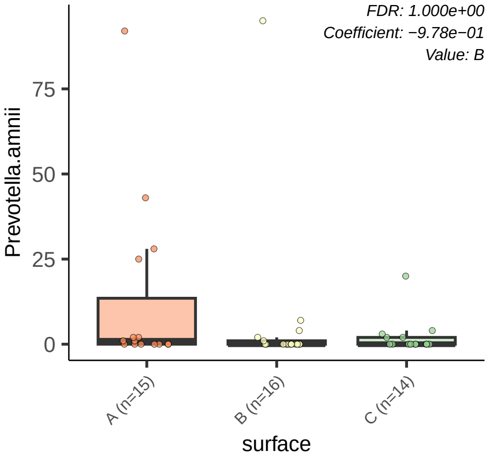
FDR: 1.000e+00
Coefficient: −9.78e−01
Value: B
Prevotella.amnii
75
50
25
0
A (n=15)
B (n=16)
C (n=14)
surface

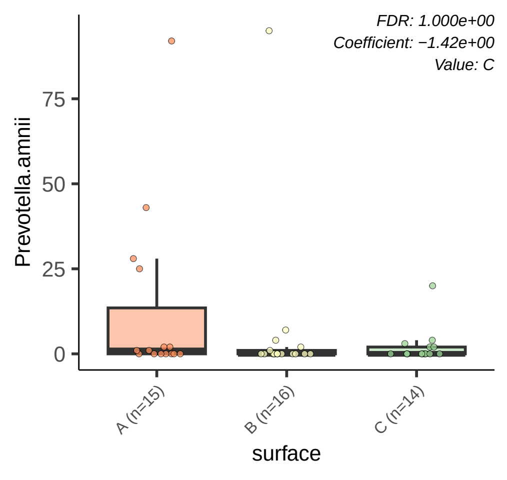

FDR: 1.000e+00
Coefficient: −1.42e+00
Value: C
Prevotella.amnii
75
50
25
0
A (n=15)
B (n=16)
C (n=14)
surface

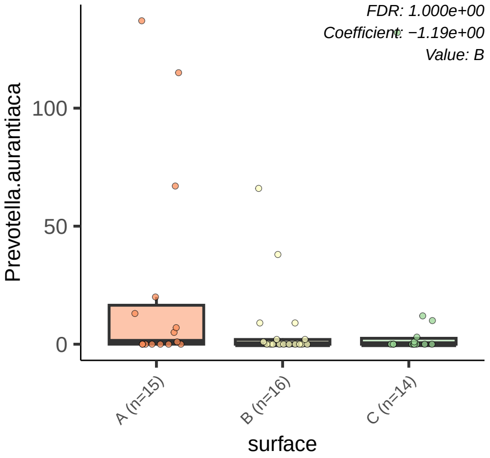

FDR: 1.000e+00
Coefficient: −1.19e+00
Value: B
Prevotella.aurantiaca
100
50
0
A (n=15)
B (n=16)
C (n=14)
surface

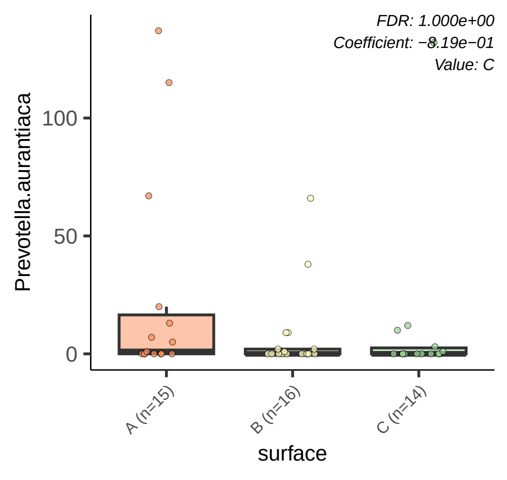
FDR: 1.000e+00
Coefficient: −8.19e−01
Value: C
Prevotella.aurantiaca
100
50
0
A (n=15)
B (n=16)
C (n=14)
surface

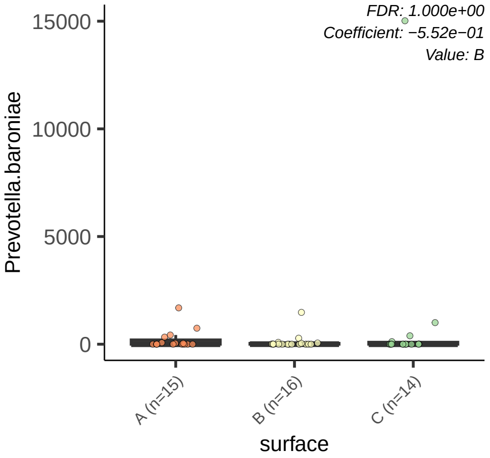
FDR: 1.000e+00
Coefficient: −5.52e−01
Value: B
Prevotella.baroniae
15000
10000
5000
0
A (n=15)
B (n=16)
C (n=14)
surface

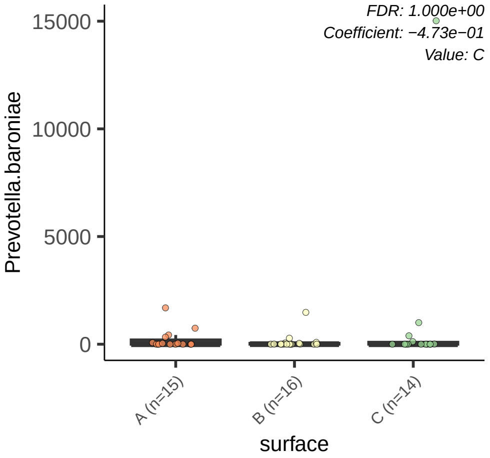

FDR: 1.000e+00
Coefficient: −4.73e−01
Value: C
Prevotella.baroniae
15000
10000
5000
0
A (n=15)
B (n=16)
C (n=14)
surface

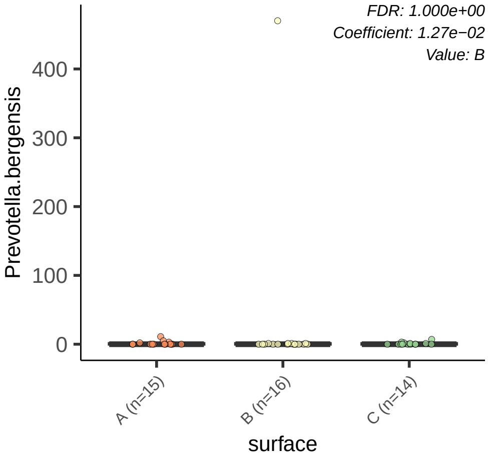

FDR: 1.000e+00
Coefficient: 1.27e−02
Value: B
Prevotella.bergensis
400
300
200
100
0
A (n=15)
B (n=16)
C (n=14)
surface

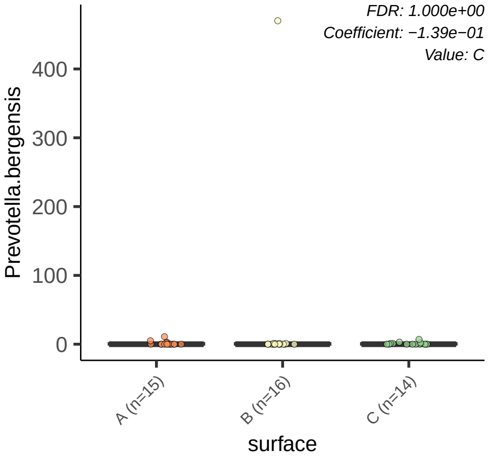
FDR: 1.000e+00
Coefficient: −1.39e−01
Value: C
Prevotella.bergensis
400
300
200
100
0
A (n=15)
B (n=16)
C (n=14)
surface

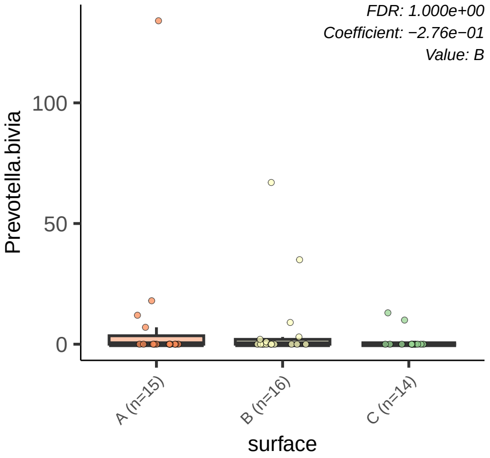
FDR: 1.000e+00
Coefficient: −2.76e−01
Value: B
Prevotella.bivia
100
50
0
A (n=15)
B (n=16)
C (n=14)
surface

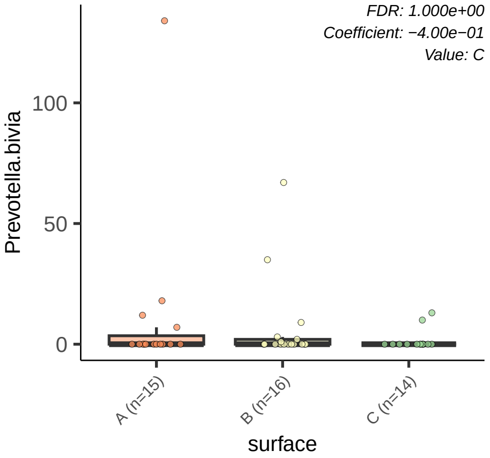
FDR: 1.000e+00
Coefficient: −4.00e−01
Value: C
Prevotella.bivia
100
50
0
A (n=15)
B (n=16)
C (n=14)
surface

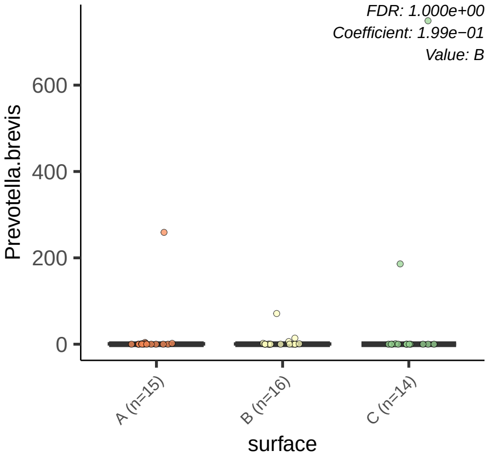
FDR: 1.000e+00
Coefficient: 1.99e−01
Value: B
Prevotella.brevis
600
400
200
0
A (n=15)
B (n=16)
C (n=14)
surface

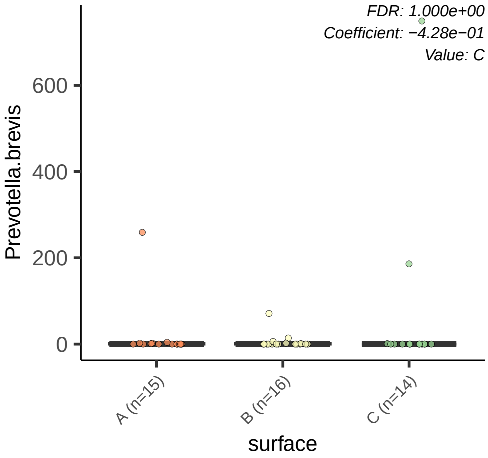
FDR: 1.000e+00
Coefficient: −4.28e−01
Value: C
Prevotella.brevis
600
400
200
0
A (n=15)
B (n=16)
C (n=14)
surface

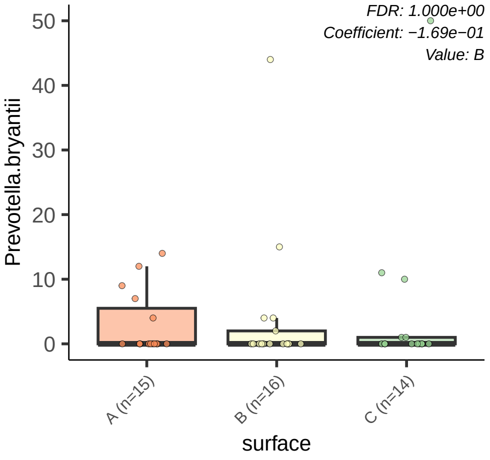

FDR: 1.000e+00
Coefficient: −1.69e−01
Value: B
Prevotella.bryantii
50
40
30
20
10
0
A (n=15)
B (n=16)
C (n=14)
surface

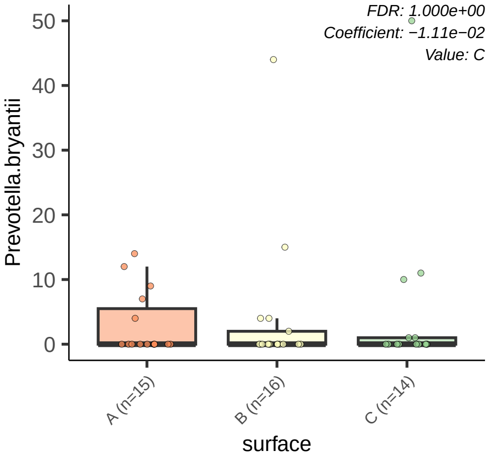

FDR: 1.000e+00
Coefficient: −1.11e−02
Value: C
Prevotella.bryantii
0
10
20
30
40
50
A (n=15)
B (n=16)
C (n=14)
surface

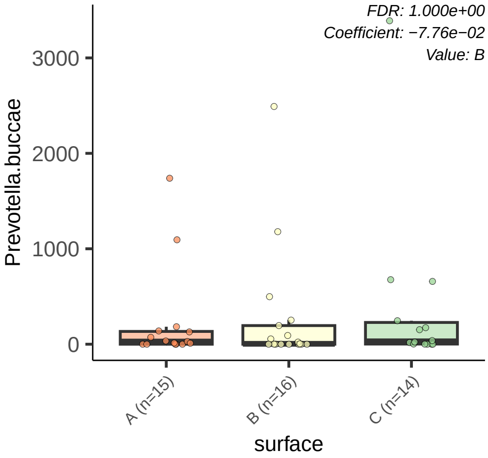

FDR: 1.000e+00
Coefficient: −7.76e−02
Value: B
Prevotella.buccae
3000
2000
1000
0
A (n=15)
B (n=16)
C (n=14)
surface

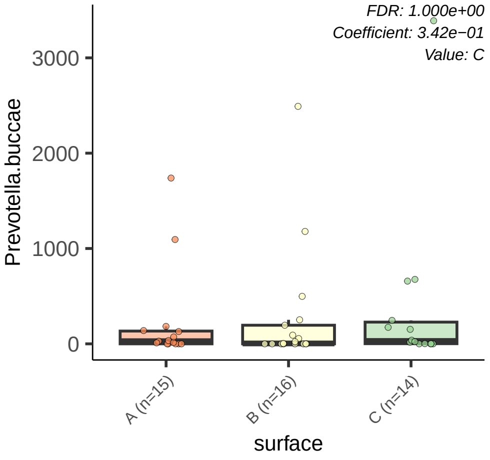

FDR: 1.000e+00
Coefficient: 3.42e−01
Value: C
Prevotella.buccae
3000
2000
1000
0
A (n=15)
B (n=16)
C (n=14)
surface

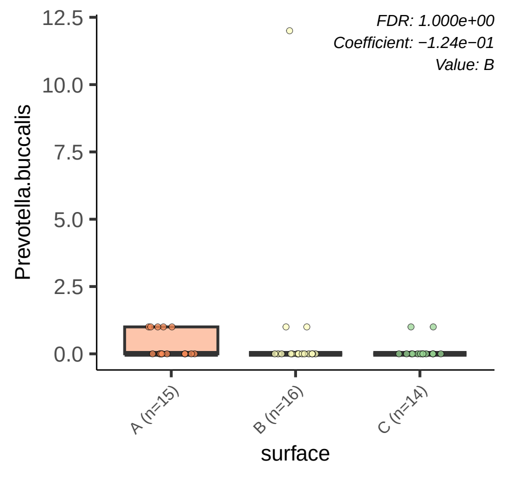
FDR: 1.000e+00
Coefficient: −1.24e−01
Value: B
Prevotella.buccalis
12.5
10.0
7.5
5.0
2.5
0.0
A (n=15)
B (n=16)
C (n=14)
surface

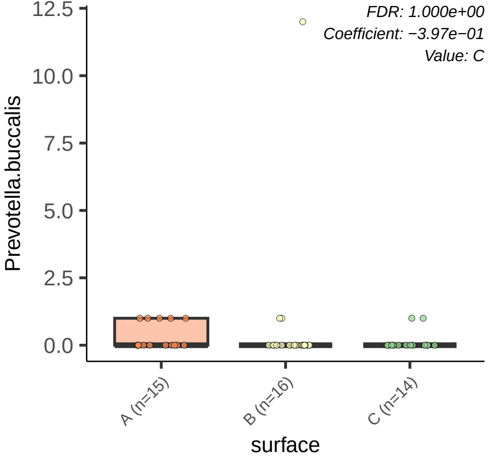

FDR: 1.000e+00
Coefficient: −3.97e−01
Value: C
Prevotella.buccalis
12.5
10.0
7.5
5.0
2.5
0.0
A (n=15)
B (n=16)
C (n=14)
surface

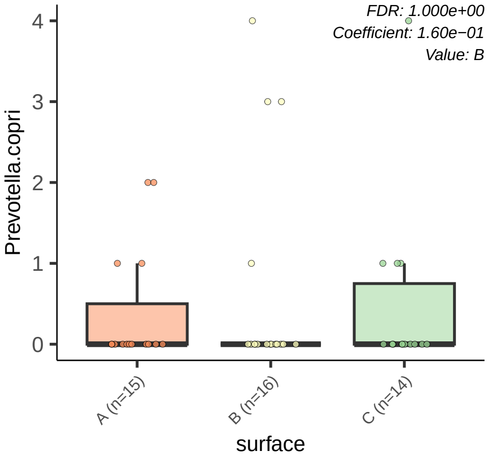
FDR: 1.000e+00
Coefficient: 1.60e−01
Value: B
Prevotella.copri
0
1
2
3
4
A (n=15)
B (n=16)
C (n=14)
surface

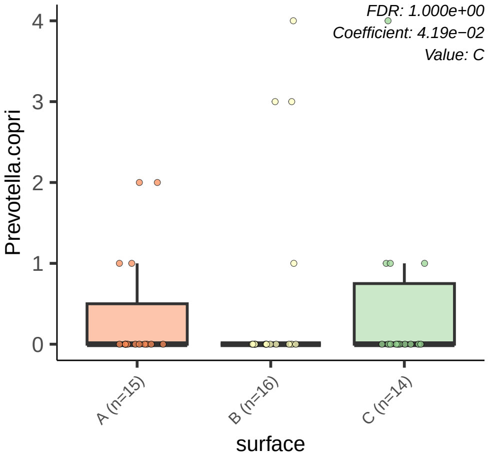

FDR: 1.000e+00
Coefficient: 4.19e−02
Value: C
Prevotella.copri
0
1
2
3
4
A (n=15)
B (n=16)
C (n=14)
surface

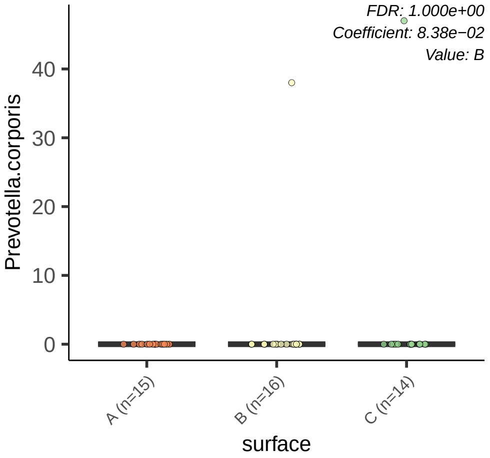

FDR: 1.000e+00
Coefficient: 8.38e−02
Value: B
Prevotella.corporis
40
30
20
10
0
A (n=15)
B (n=16)
C (n=14)
surface

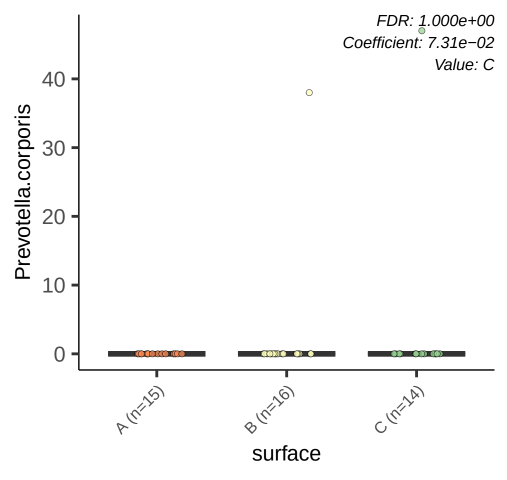
FDR: 1.000e+00
Coefficient: 7.31e−02
Value: C
Prevotella.corporis
40
30
20
10
0
A (n=15)
B (n=16)
C (n=14)
surface

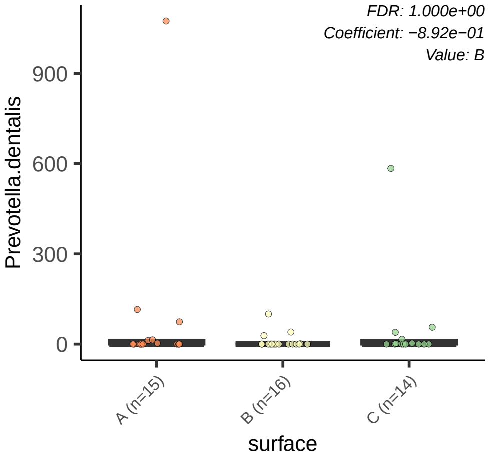

FDR: 1.000e+00
Coefficient: −8.92e−01
Value: B
Prevotella.dentalis
900
600
300
0
A (n=15)
B (n=16)
C (n=14)
surface

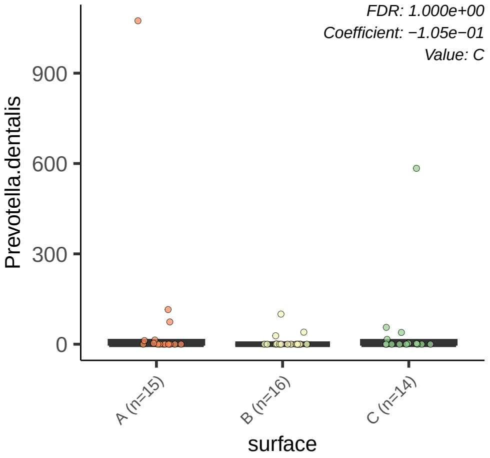
FDR: 1.000e+00
Coefficient: −1.05e−01
Value: C
Prevotella.dentalis
900
600
300
0
A (n=15)
B (n=16)
C (n=14)
surface

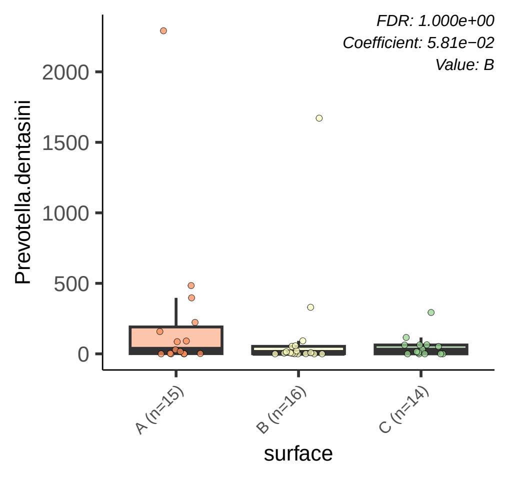
FDR: 1.000e+00
Coefficient: 5.81e−02
Value: B
Prevotella.dentasini
2000
1500
1000
500
0
A (n=15)
B (n=16)
C (n=14)
surface

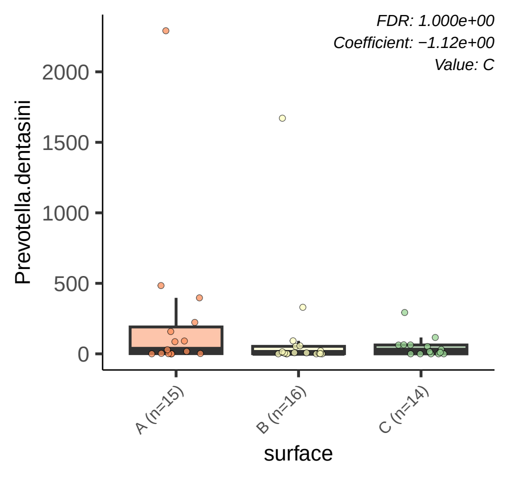
FDR: 1.000e+00
Coefficient: −1.12e+00
Value: C
Prevotella.dentasini
2000
1500
1000
500
0
A (n=15)
B (n=16)
C (n=14)
surface

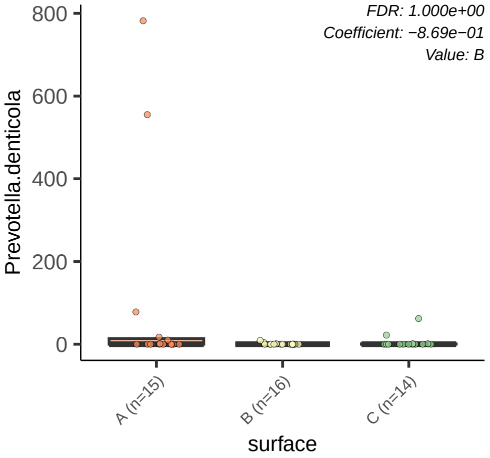

FDR: 1.000e+00
Coefficient: −8.69e−01
Value: B
Prevotella.denticola
800
600
400
200
0
A (n=15)
B (n=16)
C (n=14)
surface

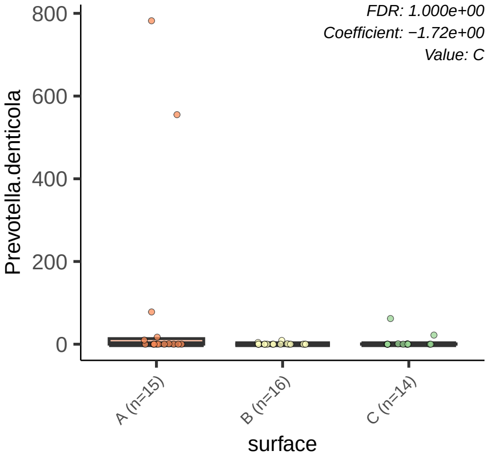

FDR: 1.000e+00
Coefficient: −1.72e+00
Value: C
Prevotella.denticola
800
600
400
200
0
A (n=15)
B (n=16)
C (n=14)
surface

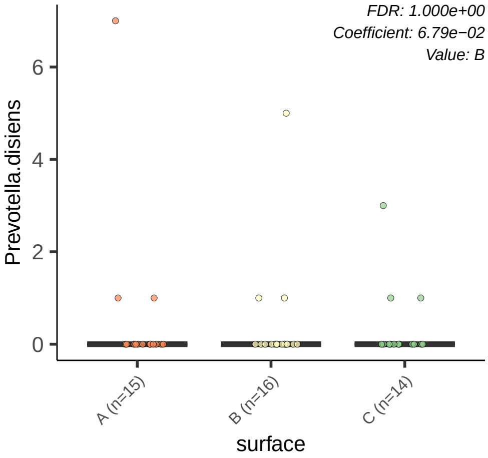
FDR: 1.000e+00
Coefficient: 6.79e−02
Value: B
Prevotella.disiens
0
2
4
6
A (n=15)
B (n=16)
C (n=14)
surface

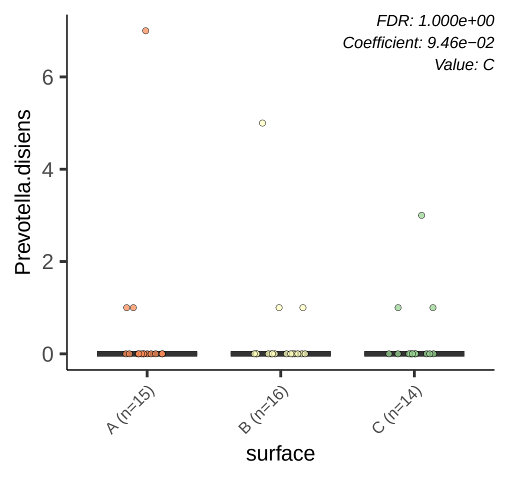
FDR: 1.000e+00
Coefficient: 9.46e−02
Value: C
Prevotella.disiens
0
2
4
6
A (n=15)
B (n=16)
C (n=14)
surface

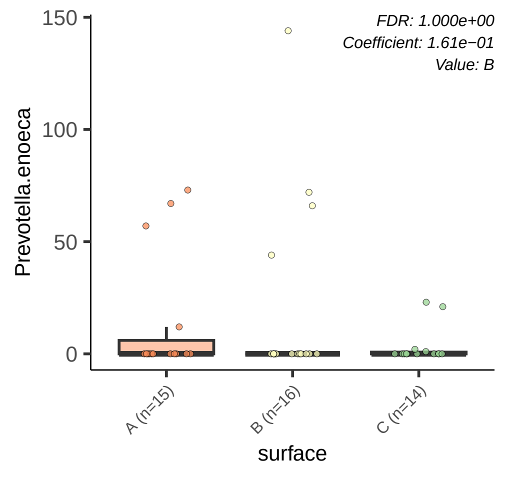
FDR: 1.000e+00
Coefficient: 1.61e−01
Value: B
Prevotella.enoeca
150
100
50
0
A (n=15)
B (n=16)
C (n=14)
surface

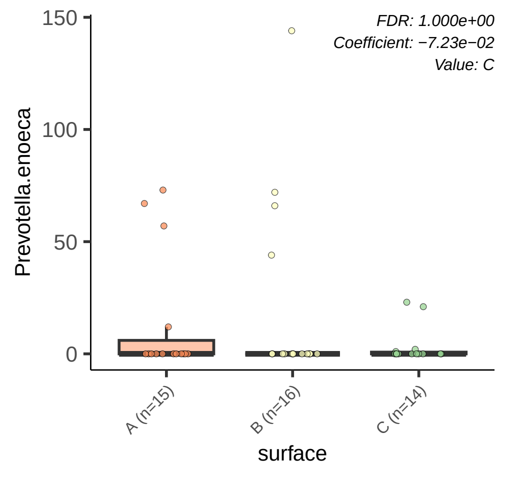

FDR: 1.000e+00
Coefficient: −7.23e−02
Value: C
Prevotella.enoeca
150
100
50
0
A (n=15)
B (n=16)
C (n=14)
surface

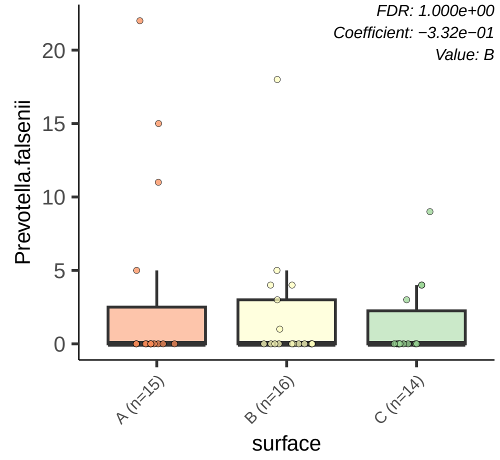
FDR: 1.000e+00
Coefficient: −3.32e−01
Value: B
Prevotella.falsenii
0
5
10
15
20
A (n=15)
B (n=16)
C (n=14)
surface

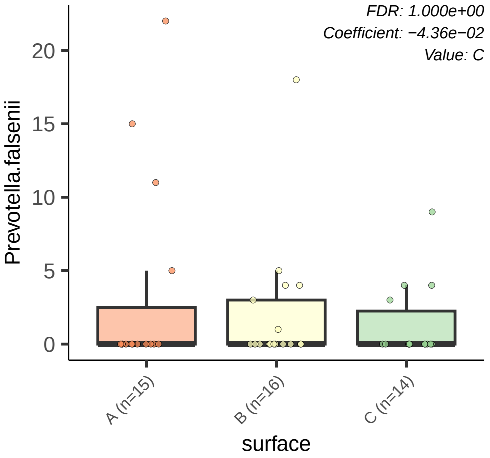
FDR: 1.000e+00
Coefficient: −4.36e−02
Value: C
Prevotella.falsenii
20
15
10
5
0
A (n=15)
B (n=16)
C (n=14)
surface

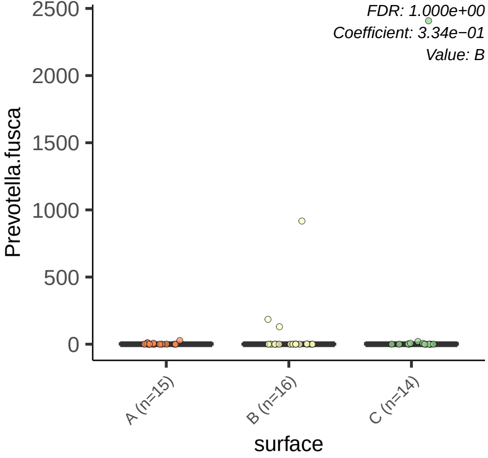
FDR: 1.000e+00
Coefficient: 3.34e−01
Value: B
Prevotella.fusca
2500
2000
1500
1000
500
0
A (n=15)
B (n=16)
C (n=14)
surface

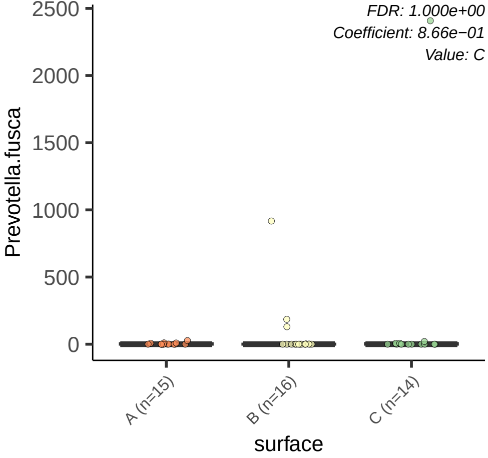

FDR: 1.000e+00
Coefficient: 8.66e−01
Value: C
Prevotella.fusca
2500
2000
1500
1000
500
0
A (n=15)
B (n=16)
C (n=14)
surface

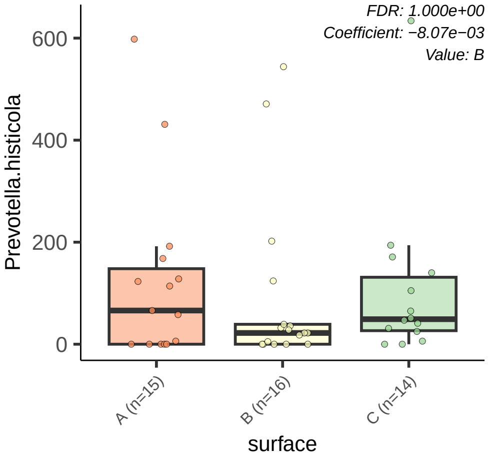
FDR: 1.000e+00
Coefficient: −8.07e−03
Value: B
Prevotella.histicola
600
400
200
0
A (n=15)
B (n=16)
C (n=14)
surface

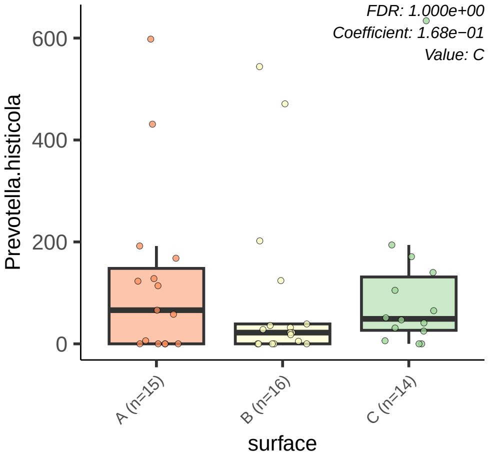

FDR: 1.000e+00
Coefficient: 1.68e−01
Value: C
Prevotella.histicola
600
400
200
0
A (n=15)
B (n=16)
C (n=14)
surface

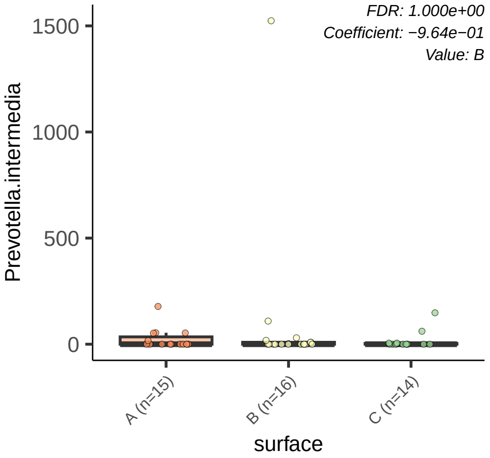

FDR: 1.000e+00
Coefficient: −9.64e−01
Value: B
Prevotella.intermedia
1500
1000
500
0
A (n=15)
B (n=16)
C (n=14)
surface

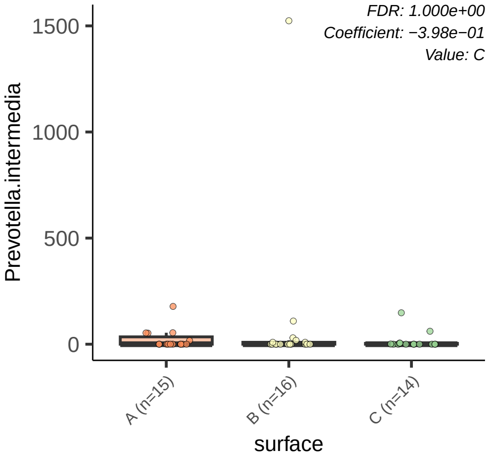
FDR: 1.000e+00
Coefficient: −3.98e−01
Value: C
Prevotella.intermedia
1500
1000
500
0
A (n=15)
B (n=16)
C (n=14)
surface

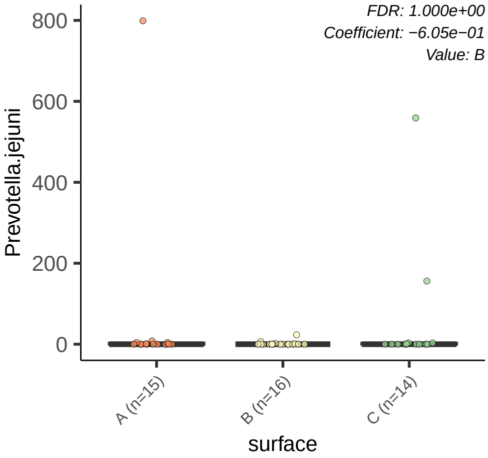
FDR: 1.000e+00
Coefficient: −6.05e−01
Value: B
Prevotella.jejuni
800
600
400
200
0
A (n=15)
B (n=16)
C (n=14)
surface

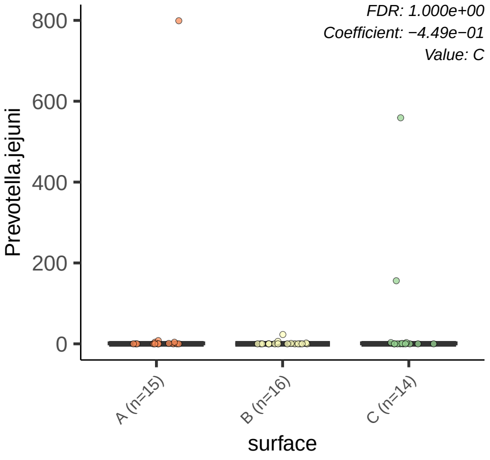

FDR: 1.000e+00
Coefficient: −4.49e−01
Value: C
Prevotella.jejuni
800
600
400
200
0
A (n=15)
B (n=16)
C (n=14)
surface

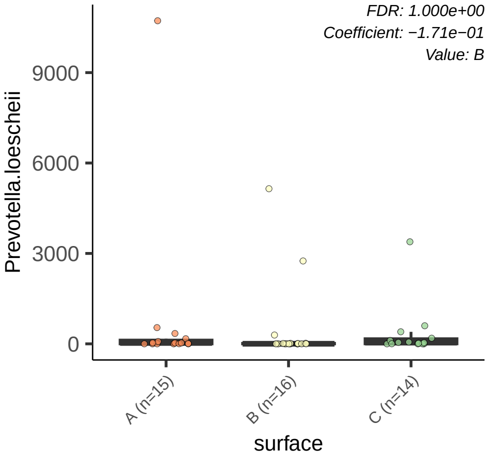

FDR: 1.000e+00
Coefficient: −1.71e−01
Value: B
Prevotella.loescheii
9000
6000
3000
0
A (n=15)
B (n=16)
C (n=14)
surface

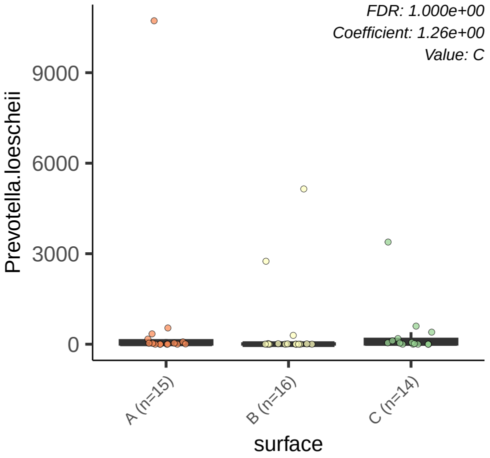

FDR: 1.000e+00
Coefficient: 1.26e+00
Value: C
Prevotella.loescheii
9000
6000
3000
0
A (n=15)
B (n=16)
C (n=14)
surface

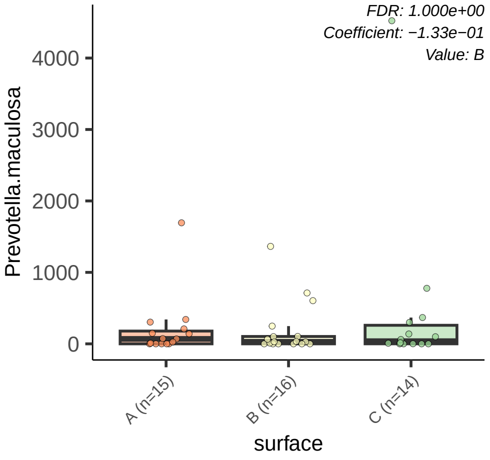

FDR: 1.000e+00
Coefficient: −1.33e−01
Value: B
Prevotella.maculosa
4000
3000
2000
1000
0
A (n=15)
B (n=16)
C (n=14)
surface

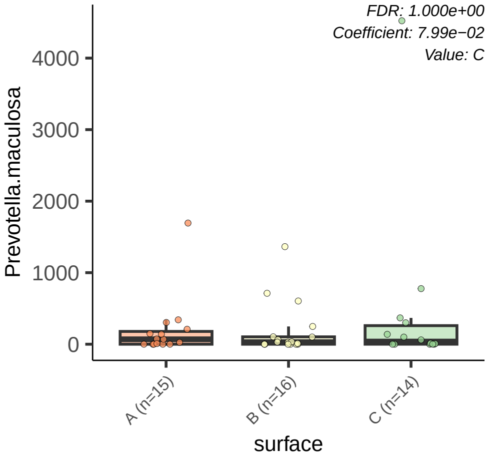
FDR: 1.000e+00
Coefficient: 7.99e−02
Value: C
Prevotella.maculosa
4000
3000
2000
1000
0
A (n=15)
B (n=16)
C (n=14)
surface

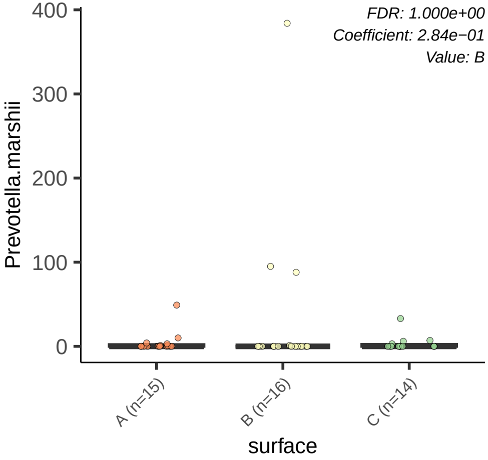
FDR: 1.000e+00
Coefficient: 2.84e−01
Value: B
400
300
200
100
0
Prevotella.marshii
A (n=15)
B (n=16)
C (n=14)
surface

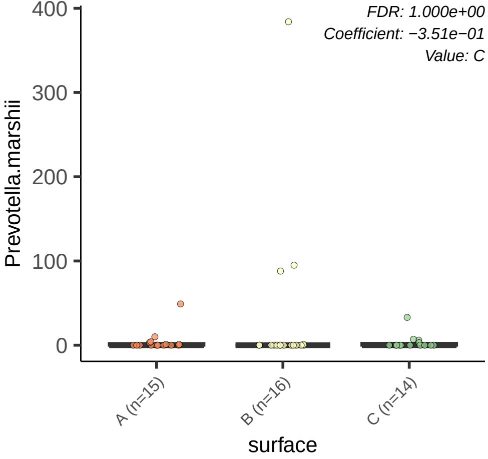

FDR: 1.000e+00
Coefficient: −3.51e−01
Value: C
Prevotella.marshii
400
300
200
100
0
A (n=15)
B (n=16)
C (n=14)
surface

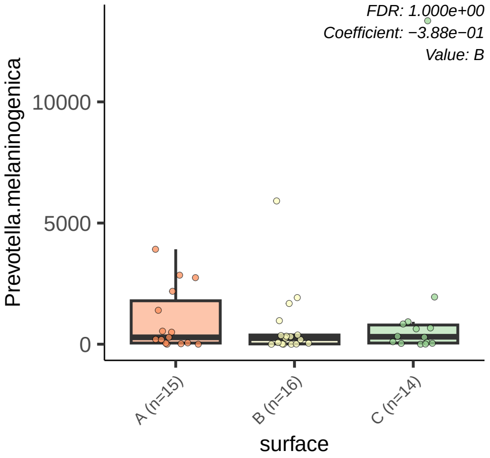

FDR: 1.000e+00
Coefficient: −3.88e−01
Value: B
Prevotella.melaninogenica
10000
5000
0
A (n=15)
B (n=16)
C (n=14)
surface

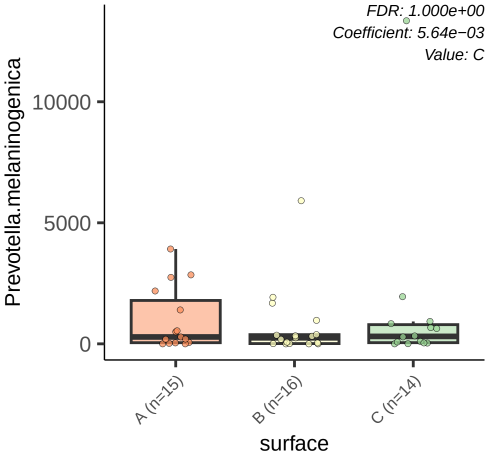
FDR: 1.000e+00
Coefficient: 5.64e−03
Value: C
Prevotella.melaninogenica
10000
5000
0
A (n=15)
B (n=16)
C (n=14)
surface

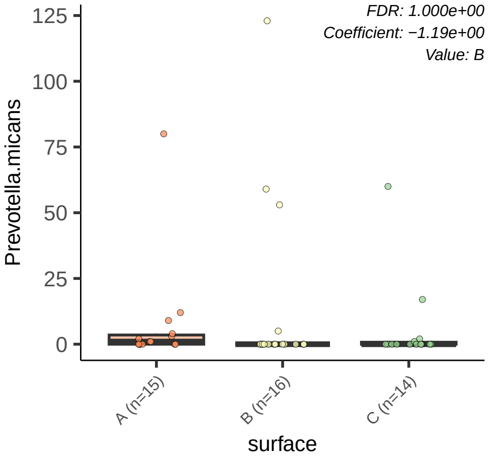

FDR: 1.000e+00
Coefficient: −1.19e+00
Value: B
Prevotella.micans
125
100
75
50
25
0
A (n=15)
B (n=16)
C (n=14)
surface

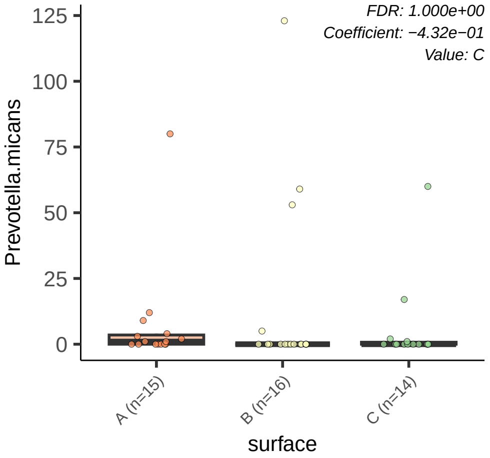

FDR: 1.000e+00
Coefficient: −4.32e−01
Value: C
Prevotella.micans
125
100
75
50
25
0
A (n=15)
B (n=16)
C (n=14)
surface

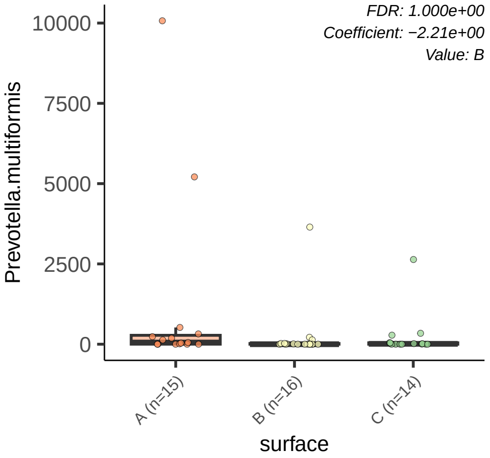
FDR: 1.000e+00
Coefficient: −2.21e+00
Value: B
10000
7500
5000
2500
0
Prevotella.multiformis
A (n=15)
B (n=16)
C (n=14)
surface

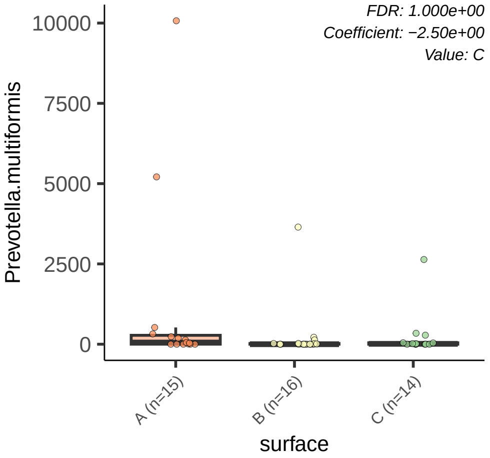
FDR: 1.000e+00
Coefficient: −2.50e+00
Value: C
Prevotella.multiformis
10000
7500
5000
2500
0
A (n=15)
B (n=16)
C (n=14)
surface

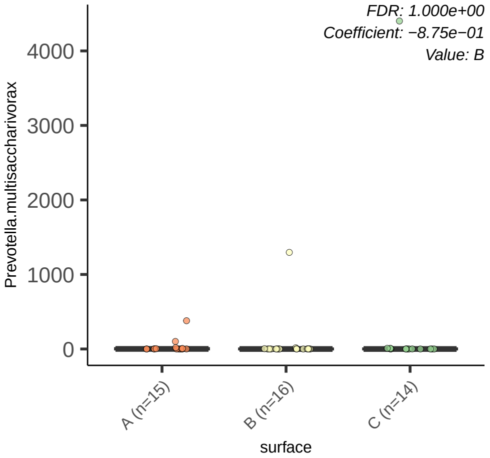
FDR: 1.000e+00
Coefficient: −8.75e−01
Value: B
Prevotella.multisaccharivorax
4000
3000
2000
1000
0
A (n=15)
B (n=16)
C (n=14)
surface

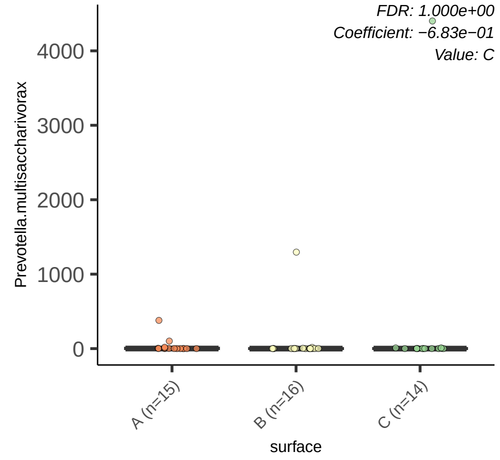

FDR: 1.000e+00
Coefficient: −6.83e−01
Value: C
Prevotella.multisaccharivorax
4000
3000
2000
1000
0
A (n=15)
B (n=16)
C (n=14)
surface

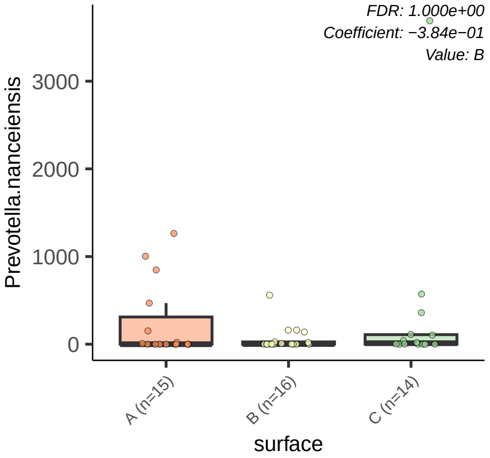
FDR: 1.000e+00
Coefficient: −3.84e−01
Value: B
Prevotella.nanceiensis
3000
2000
1000
0
A (n=15)
B (n=16)
C (n=14)
surface

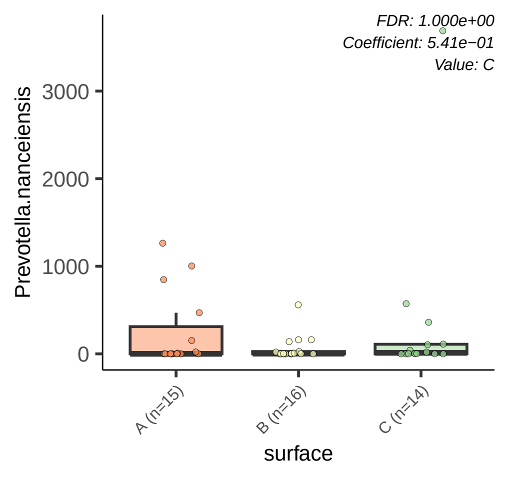
FDR: 1.000e+00
Coefficient: 5.41e−01
Value: C
3000
2000
1000
0
Prevotella.nanceiensis
A (n=15)
B (n=16)
C (n=14)
surface

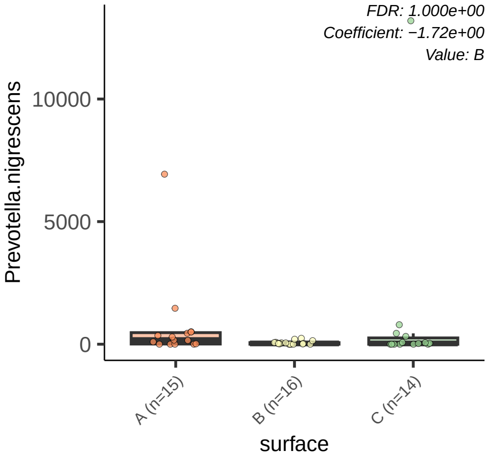
FDR: 1.000e+00
Coefficient: −1.72e+00
Value: B
Prevotella.nigrescens
10000
5000
0
A (n=15)
B (n=16)
C (n=14)
surface

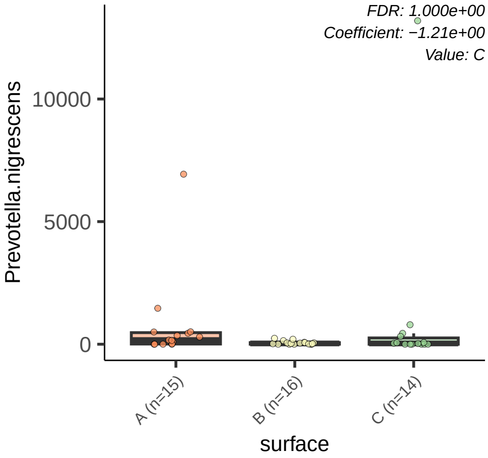
FDR: 1.000e+00
Coefficient: −1.21e+00
Value: C
Prevotella.nigrescens
10000
5000
0
A (n=15)
B (n=16)
C (n=14)
surface

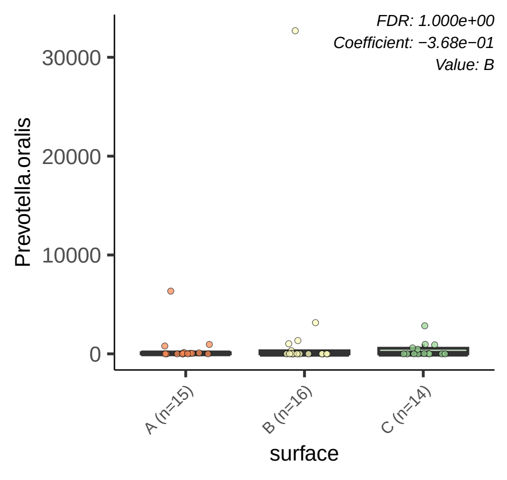

FDR: 1.000e+00
Coefficient: −3.68e−01
Value: B
Prevotella.oralis
30000
20000
10000
0
A (n=15)
B (n=16)
C (n=14)
surface

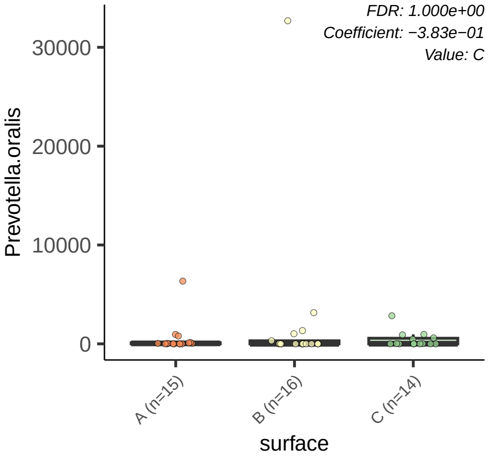
FDR: 1.000e+00
Coefficient: −3.83e−01
Value: C
Prevotella.oralis
30000
20000
10000
0
A (n=15)
B (n=16)
C (n=14)
surface

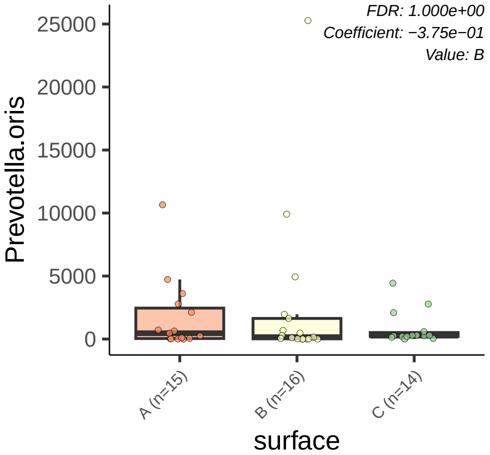

FDR: 1.000e+00
Coefficient: −3.75e−01
Value: B
Prevotella.oris
25000
20000
15000
10000
5000
0
A (n=15)
B (n=16)
C (n=14)
surface

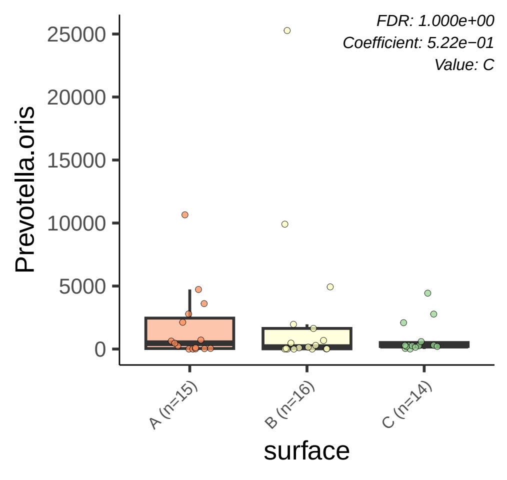
FDR: 1.000e+00
Coefficient: 5.22e−01
Value: C
Prevotella.oris
25000
20000
15000
10000
5000
0
A (n=15)
B (n=16)
C (n=14)
surface

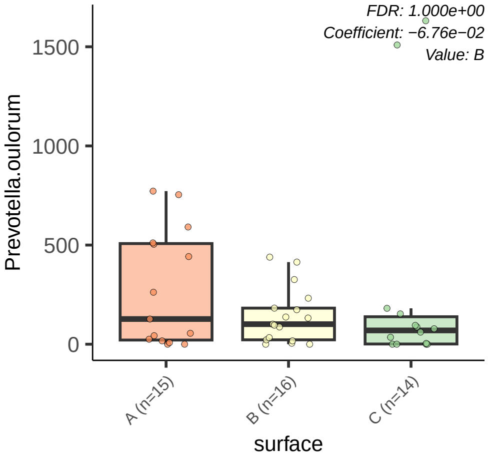
FDR: 1.000e+00
Coefficient: −6.76e−02
Value: B
Prevotella.oulorum
1500
1000
500
0
A (n=15)
B (n=16)
C (n=14)
surface

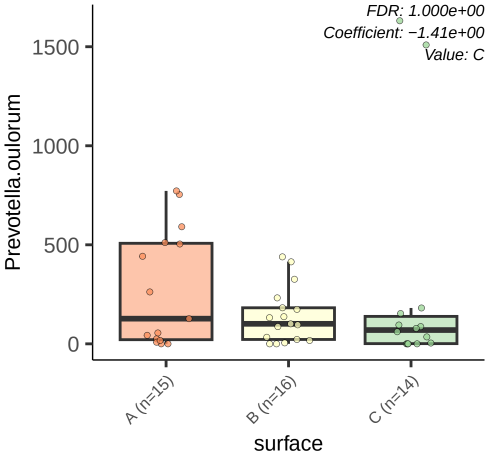

FDR: 1.000e+00
Coefficient: −1.41e+00
Value: C
Prevotella.oulorum
1500
1000
500
0
A (n=15)
B (n=16)
C (n=14)
surface

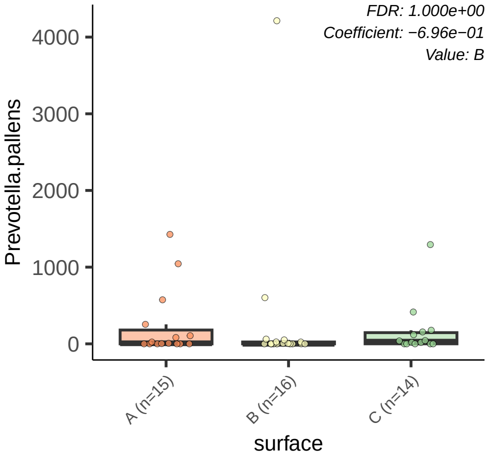

FDR: 1.000e+00
Coefficient: −6.96e−01
Value: B
Prevotella.pallens
4000
3000
2000
1000
0
A (n=15)
B (n=16)
C (n=14)
surface

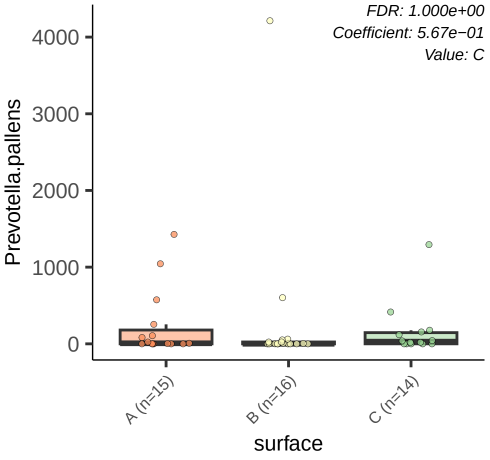
FDR: 1.000e+00
Coefficient: 5.67e−01
Value: C
Prevotella.pallens
4000
3000
2000
1000
0
A (n=15)
B (n=16)
C (n=14)
surface

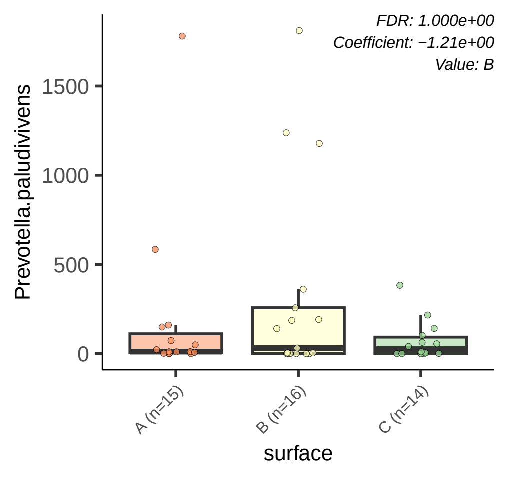
FDR: 1.000e+00
Coefficient: −1.21e+00
Value: B
Prevotella.paludivivens
1500
1000
500
0
A (n=15)
B (n=16)
C (n=14)
surface

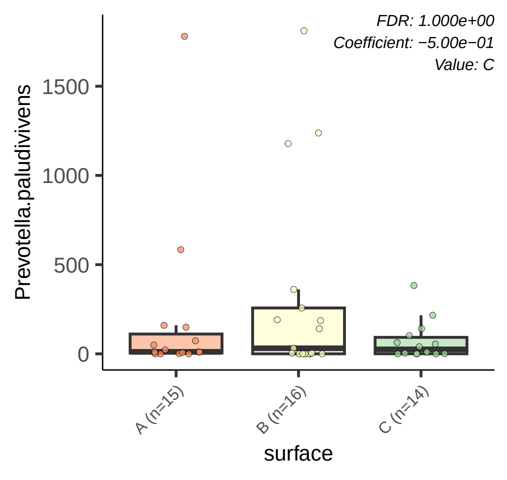
FDR: 1.000e+00
Coefficient: −5.00e−01
Value: C
Prevotella.paludivivens
1500
1000
500
0
A (n=15)
B (n=16)
C (n=14)
surface

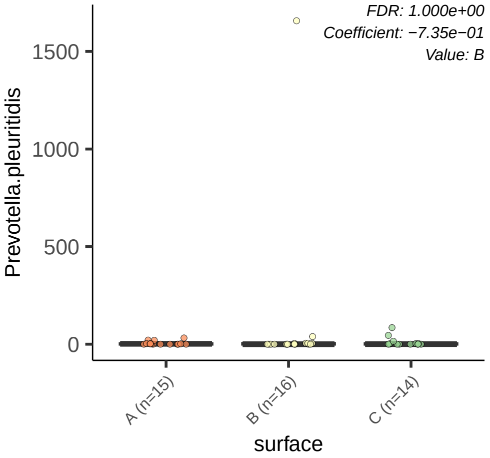
FDR: 1.000e+00
Coefficient: −7.35e−01
Value: B
Prevotella.pleuritidis
1500
1000
500
0
A (n=15)
B (n=16)
C (n=14)
surface

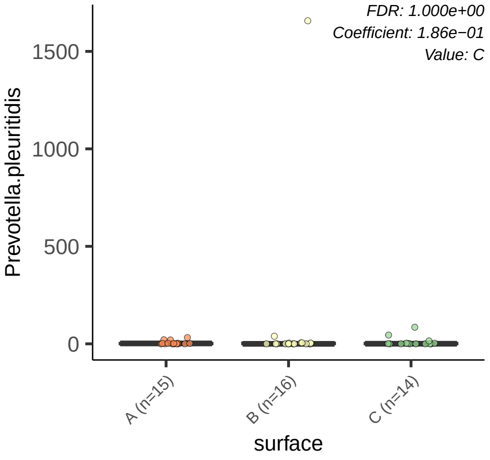

FDR: 1.000e+00
Coefficient: 1.86e−01
Value: C
Prevotella.pleuritidis
1500
1000
500
0
A (n=15)
B (n=16)
C (n=14)
surface

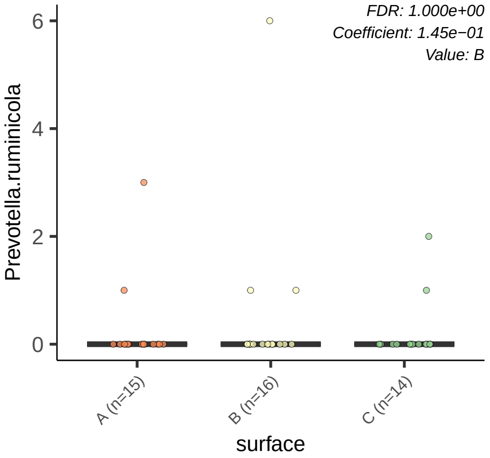
FDR: 1.000e+00
Coefficient: 1.45e−01
Value: B
Prevotella.ruminicola
6
4
2
0
A (n=15)
B (n=16)
C (n=14)
surface

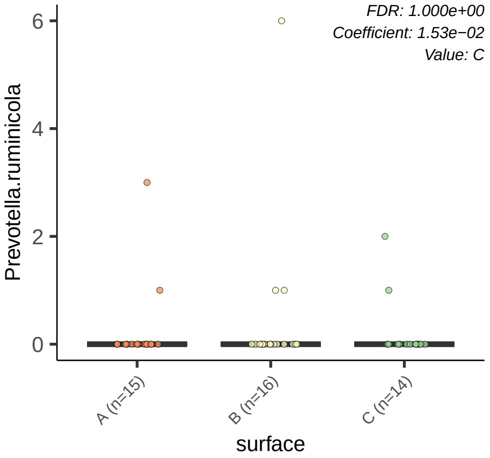

FDR: 1.000e+00
Coefficient: 1.53e−02
Value: C
Prevotella.ruminicola
6
4
2
0
A (n=15)
B (n=16)
C (n=14)
surface

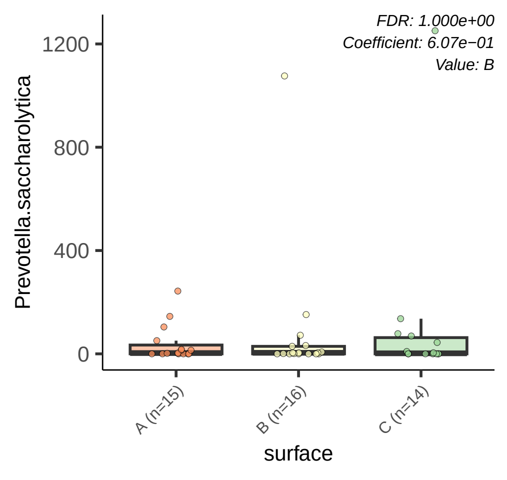
FDR: 1.000e+00
Coefficient: 6.07e−01
Value: B
Prevotella.saccharolytica
1200
800
400
0
A (n=15)
B (n=16)
C (n=14)
surface

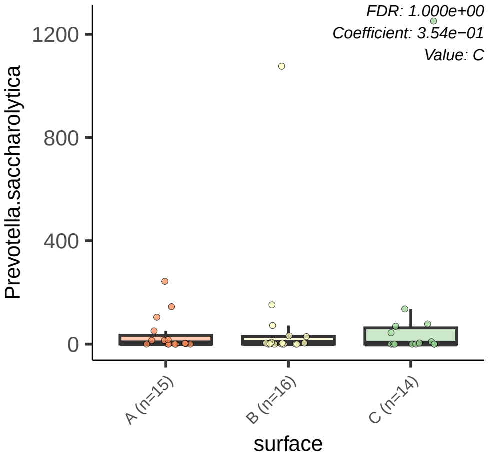
FDR: 1.000e+00
Coefficient: 3.54e−01
Value: C
Prevotella.saccharolytica
1200
800
400
0
A (n=15)
B (n=16)
C (n=14)
surface

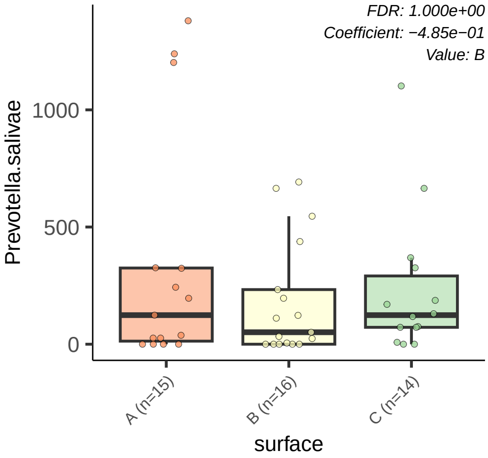

FDR: 1.000e+00
Coefficient: −4.85e−01
Value: B
Prevotella.salivae
1000
500
0
A (n=15)
B (n=16)
C (n=14)
surface

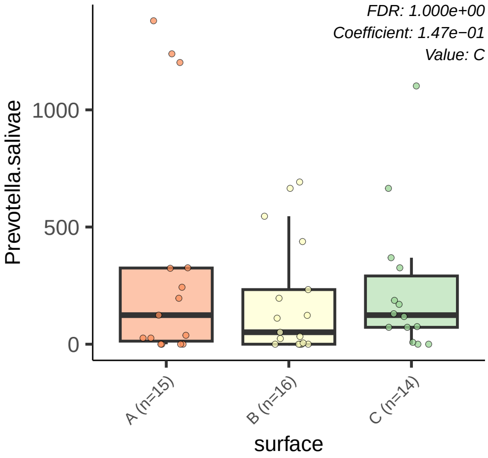
FDR: 1.000e+00
Coefficient: 1.47e−01
Value: C
Prevotella.salivae
1000
500
0
A (n=15)
B (n=16)
C (n=14)
surface

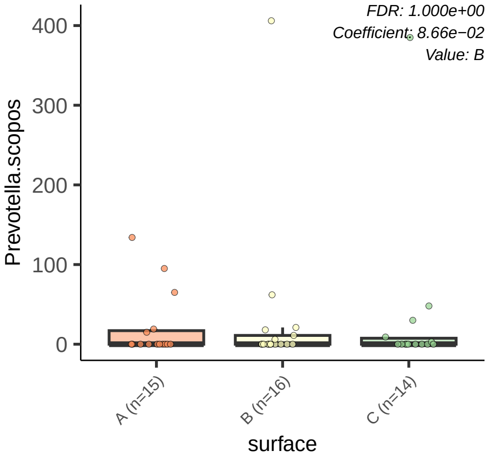
FDR: 1.000e+00
Coefficient: 8.66e−02
Value: B
Prevotella.scopos
400
300
200
100
0
A (n=15)
B (n=16)
C (n=14)
surface

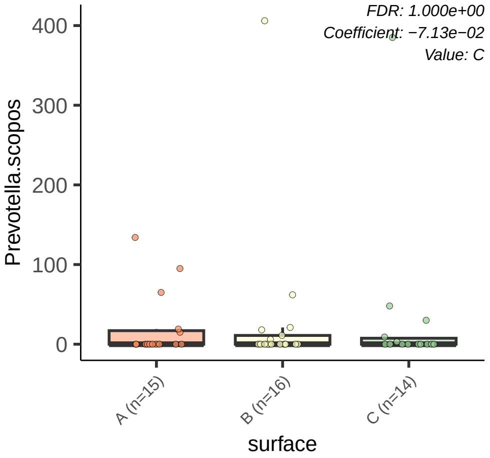

FDR: 1.000e+00
Coefficient: −7.13e−02
Value: C
Prevotella.scopos
400
300
200
100
0
A (n=15)
B (n=16)
C (n=14)
surface

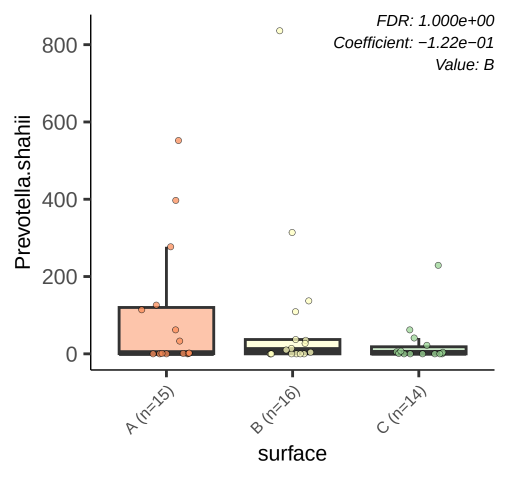

FDR: 1.000e+00
Coefficient: −1.22e−01
Value: B
Prevotella.shahii
800
600
400
200
0
A (n=15)
B (n=16)
C (n=14)
surface

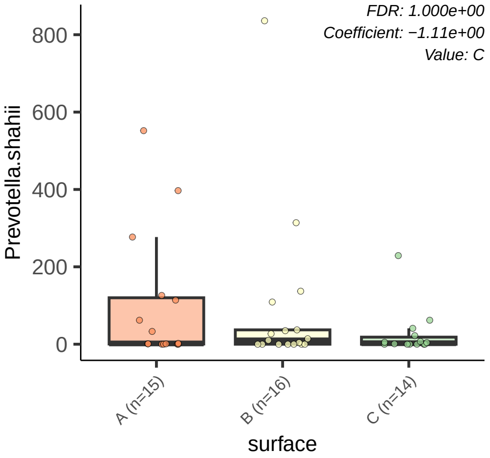

FDR: 1.000e+00
Coefficient: −1.11e+00
Value: C
Prevotella.shahii
800
600
400
200
0
A (n=15)
B (n=16)
C (n=14)
surface

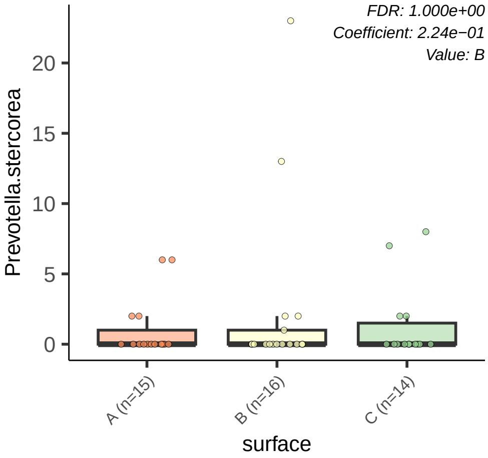

FDR: 1.000e+00
Coefficient: 2.24e−01
Value: B
Prevotella.stercorea
20
15
10
5
0
A (n=15)
B (n=16)
C (n=14)
surface

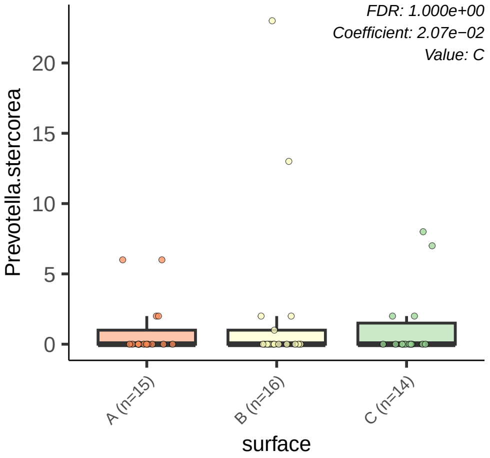
FDR: 1.000e+00
Coefficient: 2.07e−02
Value: C
Prevotella.stercorea
0
5
10
15
20
A (n=15)
B (n=16)
C (n=14)
surface

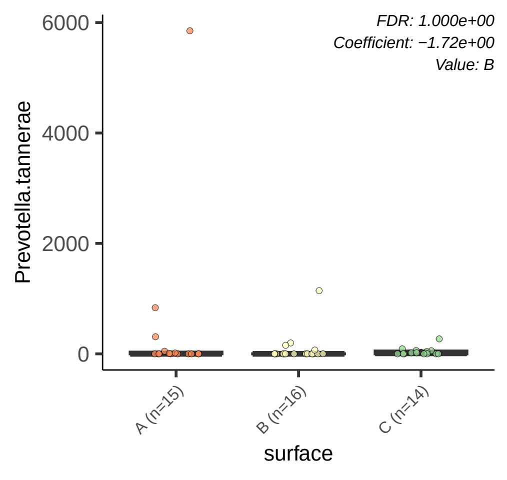

FDR: 1.000e+00
Coefficient: −1.72e+00
Value: B
Prevotella.tannerae
6000
4000
2000
0
A (n=15)
B (n=16)
C (n=14)
surface

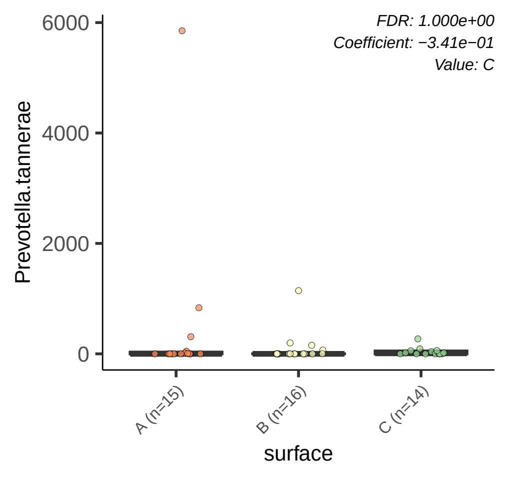
FDR: 1.000e+00
Coefficient: −3.41e−01
Value: C
Prevotella.tannerae
6000
4000
2000
0
A (n=15)
B (n=16)
C (n=14)
surface

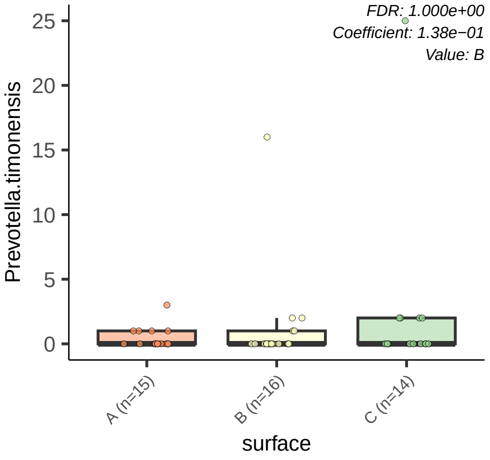
FDR: 1.000e+00
Coefficient: 1.38e−01
Value: B
Prevotella.timonensis
0
5
10
15
20
25
A (n=15)
B (n=16)
C (n=14)
surface

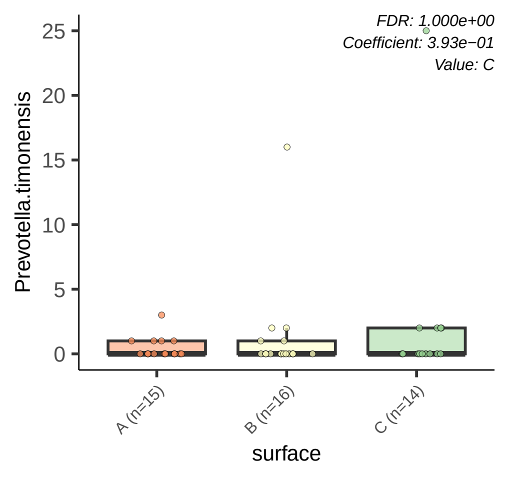
FDR: 1.000e+00
Coefficient: 3.93e−01
Value: C
Prevotella.timonensis
25
20
15
10
5
0
A (n=15)
B (n=16)
C (n=14)
surface

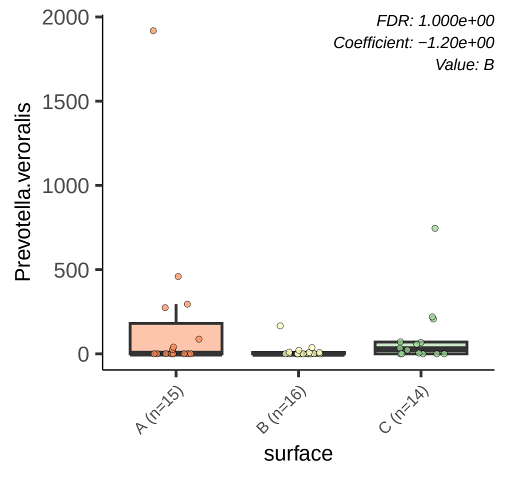

FDR: 1.000e+00
Coefficient: −1.20e+00
Value: B
Prevotella.veroralis
2000
1500
1000
500
0
A (n=15)
B (n=16)
C (n=14)
surface

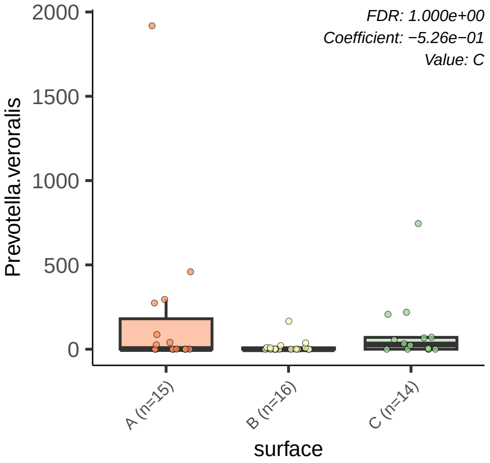

FDR: 1.000e+00
Coefficient: −5.26e−01
Value: C
Prevotella.veroralis
2000
1500
1000
500
0
A (n=15)
B (n=16)
C (n=14)
surface

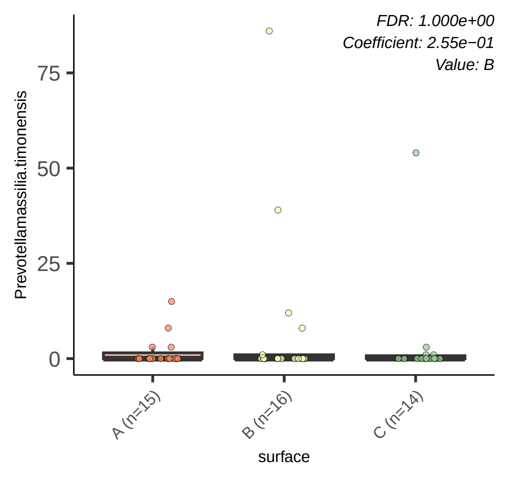

FDR: 1.000e+00
Coefficient: 2.55e−01
Value: B
Prevotellamassilia.timonensis
75
50
25
0
A (n=15)
B (n=16)
C (n=14)
surface

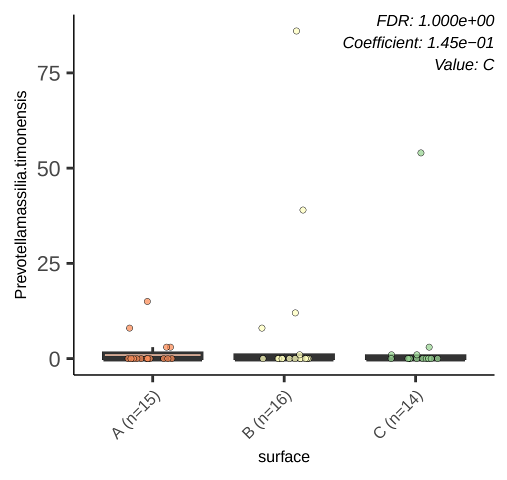
FDR: 1.000e+00
Coefficient: 1.45e−01
Value: C
Prevotellamassilia.timonensis
75
50
25
0
A (n=15)
B (n=16)
C (n=14)
surface

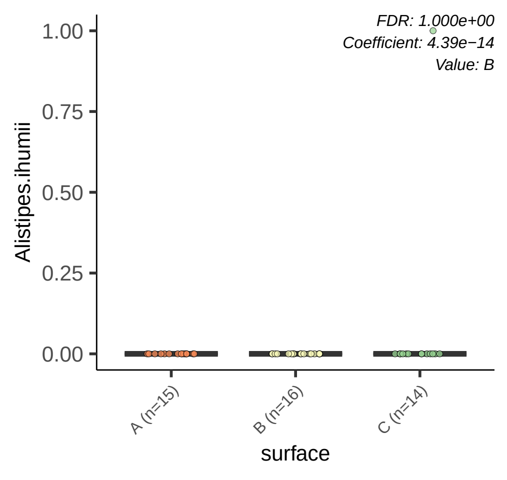
FDR: 1.000e+00
Coefficient: 4.39e−14
Value: B
1.00
0.75
0.50
0.25
0.00
Alistipes.ihumii
A (n=15)
B (n=16)
C (n=14)
surface

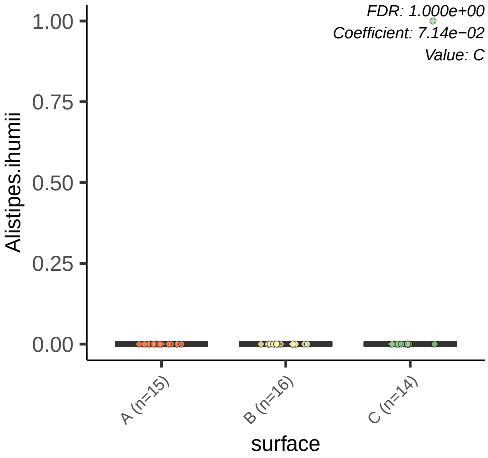
FDR: 1.000e+00
Coefficient: 7.14e−02
Value: C
Alistipes.ihumii
1.00
0.75
0.50
0.25
0.00
A (n=15)
B (n=16)
C (n=14)
surface

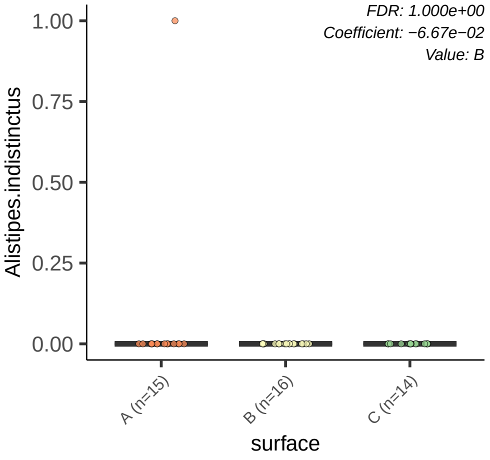

FDR: 1.000e+00
Coefficient: −6.67e−02
Value: B
Alistipes.indistinctus
1.00
0.75
0.50
0.25
0.00
A (n=15)
B (n=16)
C (n=14)
surface

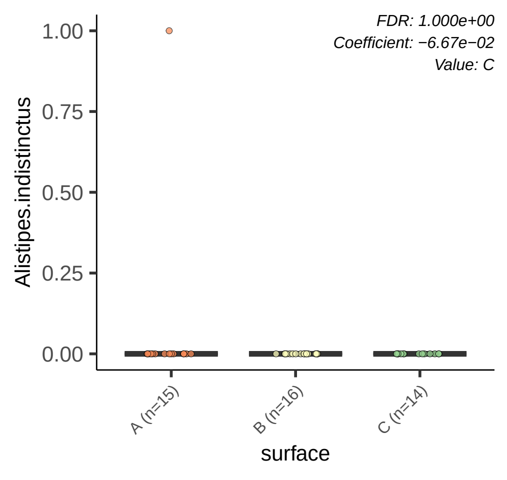
FDR: 1.000e+00
Coefficient: −6.67e−02
Value: C
Alistipes.indistinctus
1.00
0.75
0.50
0.25
0.00
A (n=15)
B (n=16)
C (n=14)
surface

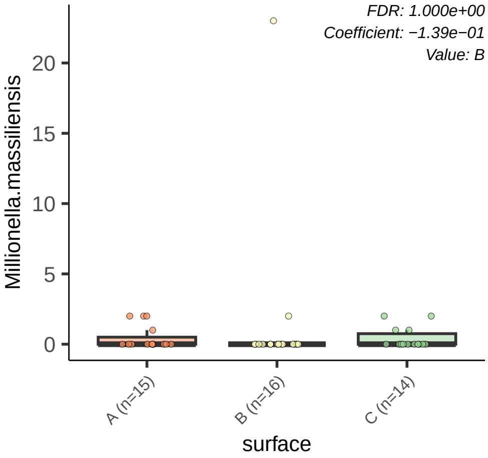

FDR: 1.000e+00
Coefficient: −1.39e−01
Value: B
Millionella.massiliensis
0
5
10
15
20
A (n=15)
B (n=16)
C (n=14)
surface

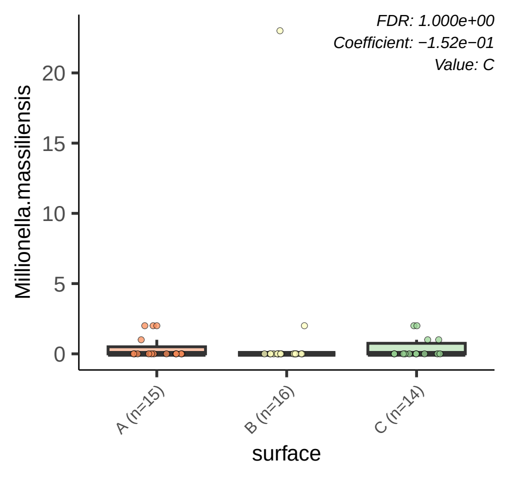
FDR: 1.000e+00
Coefficient: −1.52e−01
Value: C
Millionella.massiliensis
0
5
10
15
20
A (n=15)
B (n=16)
C (n=14)
surface

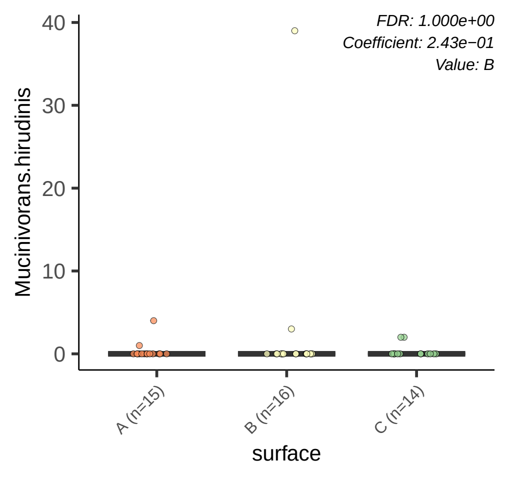

FDR: 1.000e+00
Coefficient: 2.43e−01
Value: B
Mucinivorans.hirudinis
40
30
20
10
0
A (n=15)
B (n=16)
C (n=14)
surface

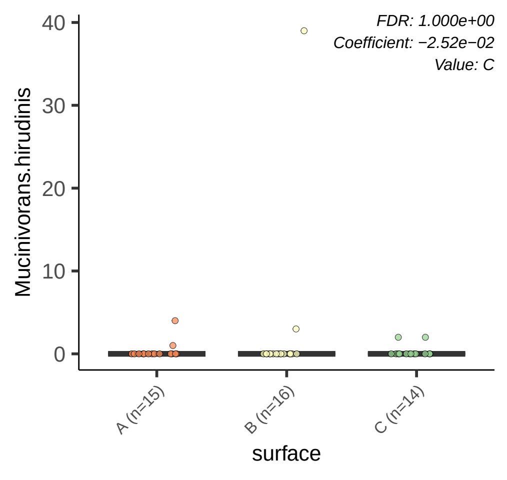
FDR: 1.000e+00
Coefficient: −2.52e−02
Value: C
Mucinivorans.hirudinis
40
30
20
10
0
A (n=15)
B (n=16)
C (n=14)
surface

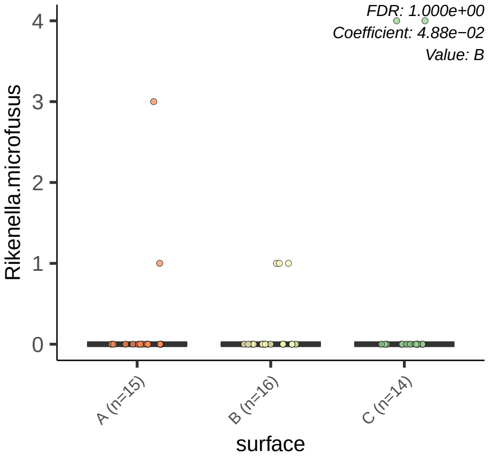
FDR: 1.000e+00
Coefficient: 4.88e−02
Value: B
Rikenella.microfusus
0
1
2
3
4
A (n=15)
B (n=16)
C (n=14)
surface

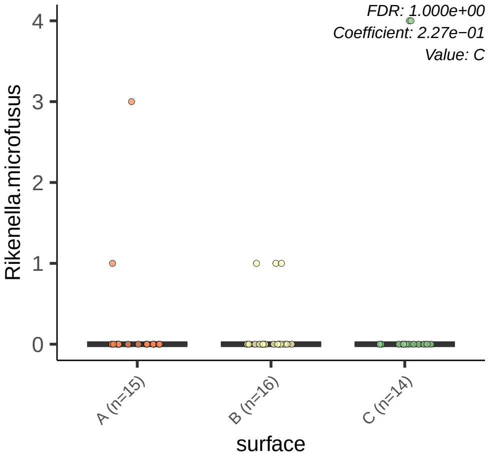
FDR: 1.000e+00
Coefficient: 2.27e−01
Value: C
Rikenella.microfusus
0
1
2
3
4
A (n=15)
B (n=16)
C (n=14)
surface

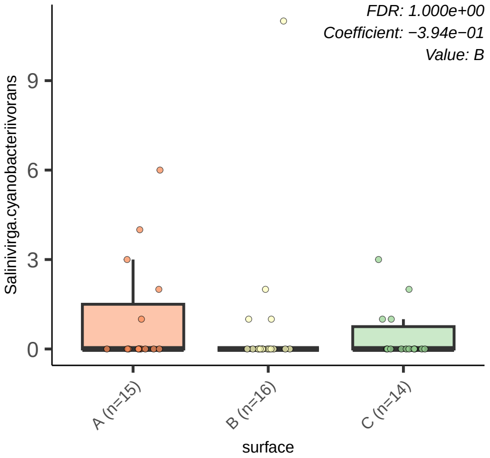
FDR: 1.000e+00
Coefficient: −3.94e−01
Value: B
Saliniviirga.cyanobacteriivorans
0
3
6
9
A (n=15)
B (n=16)
C (n=14)
surface

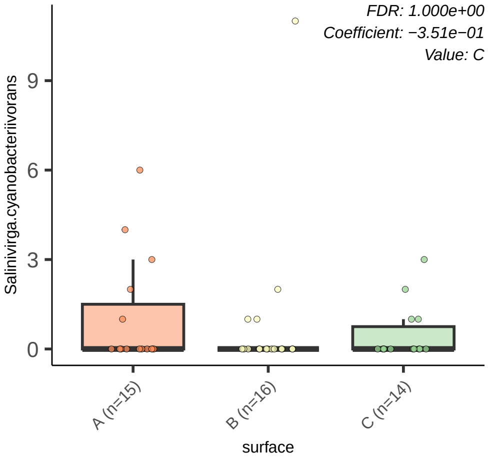
FDR: 1.000e+00
Coefficient: −3.51e−01
Value: C
Saliniviirga.cyanobacteriivorans
0
3
6
9
A (n=15)
B (n=16)
C (n=14)
surface

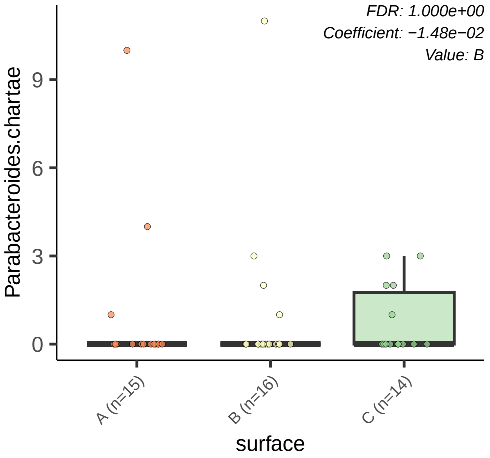

FDR: 1.000e+00
Coefficient: −1.48e−02
Value: B
Parabacteroides.chartae
0
3
6
9
A (n=15)
B (n=16)
C (n=14)
surface

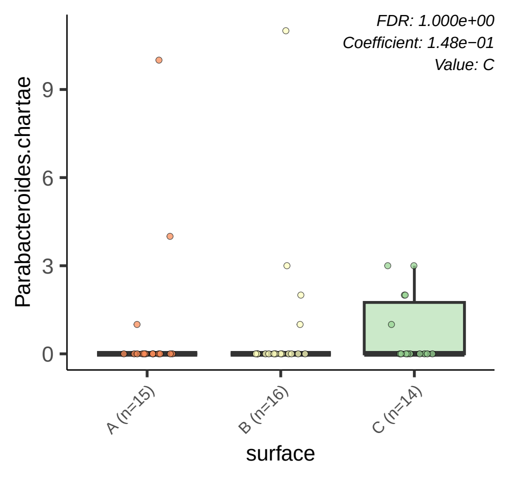

FDR: 1.000e+00
Coefficient: 1.48e−01
Value: C
Parabacteroides.chartae
0
3
6
9
A (n=15)
B (n=16)
C (n=14)
surface

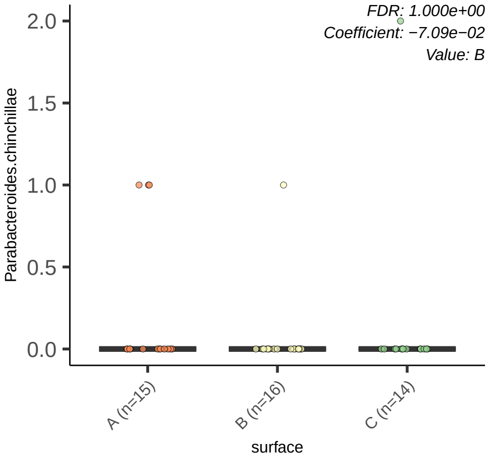

FDR: 1.000e+00
Coefficient: −7.09e−02
Value: B
Parabacteroides.chinchillae
2.0
1.5
1.0
0.5
0.0
A (n=15)
B (n=16)
C (n=14)
surface

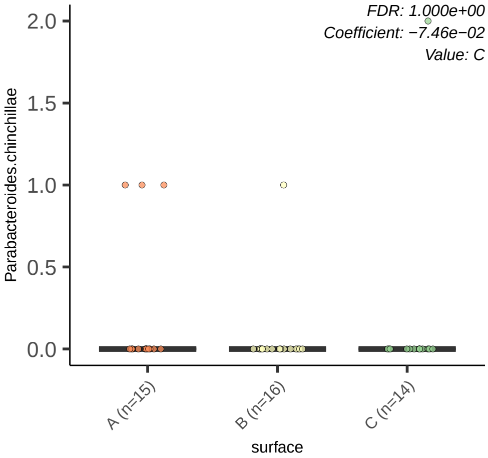
FDR: 1.000e+00
Coefficient: −7.46e−02
Value: C
Parabacteroides.chinchillae
2.0
1.5
1.0
0.5
0.0
A (n=15)
B (n=16)
C (n=14)
surface

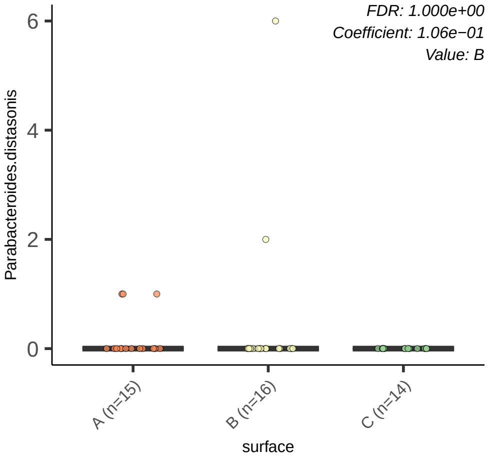
FDR: 1.000e+00
Coefficient: 1.06e−01
Value: B
Parabacteroides.distasonis
0
2
4
6
A (n=15)
B (n=16)
C (n=14)
surface

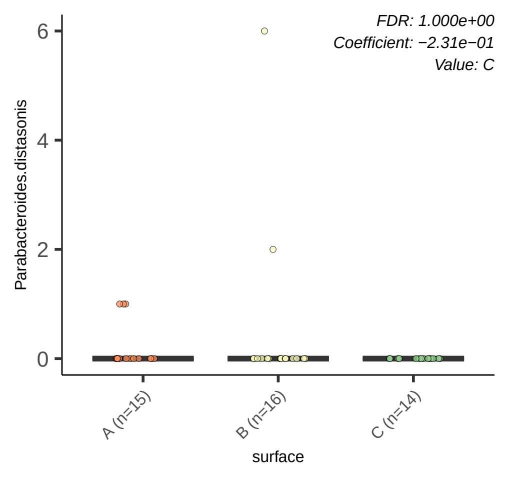
FDR: 1.000e+00
Coefficient: −2.31e−01
Value: C
Parabacteroides.distasonis
0
2
4
6
A (n=15)
B (n=16)
C (n=14)
surface

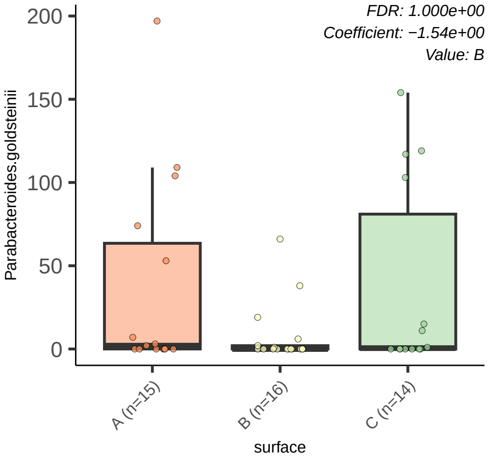

FDR: 1.000e+00
Coefficient: −1.54e+00
Value: B
Parabacteroides.goldsteinii
200
150
100
50
0
A (n=15)
B (n=16)
C (n=14)
surface

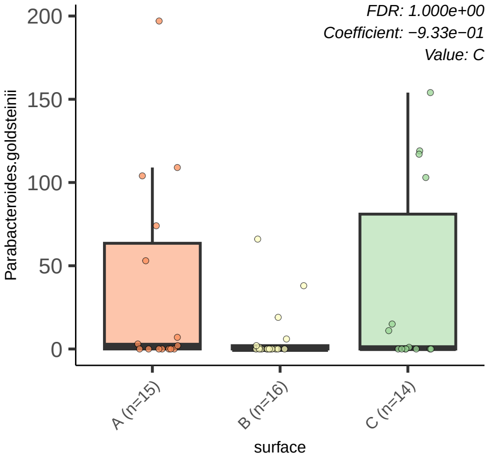

FDR: 1.000e+00
Coefficient: −9.33e−01
Value: C
Parabacteroides.goldsteinii
200
150
100
50
0
A (n=15)
B (n=16)
C (n=14)
surface

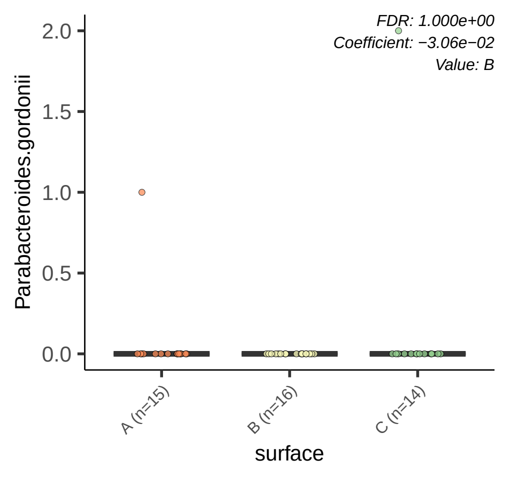
FDR: 1.000e+00
Coefficient: −3.06e−02
Value: B
Parabacteroides.gordonii
2.0
1.5
1.0
0.5
0.0
A (n=15)
B (n=16)
C (n=14)
surface

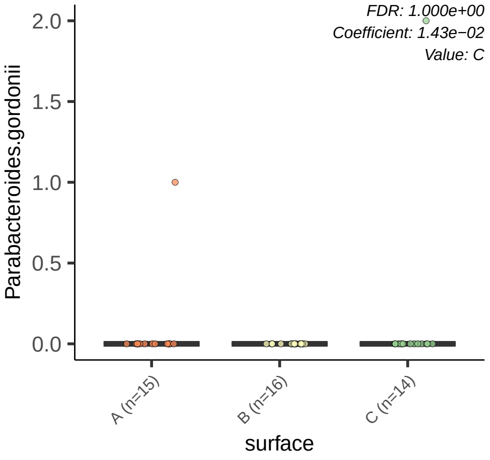

FDR: 1.000e+00
Coefficient: 1.43e−02
Value: C
Parabacteroides.gordonii
2.0
1.5
1.0
0.5
0.0
A (n=15)
B (n=16)
C (n=14)
surface

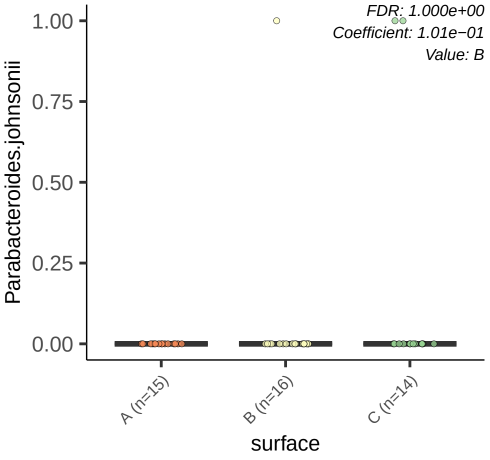

FDR: 1.000e+00
Coefficient: 1.01e−01
Value: B
Parabacteroides.johnsonii
1.00
0.75
0.50
0.25
0.00
A (n=15)
B (n=16)
C (n=14)
surface

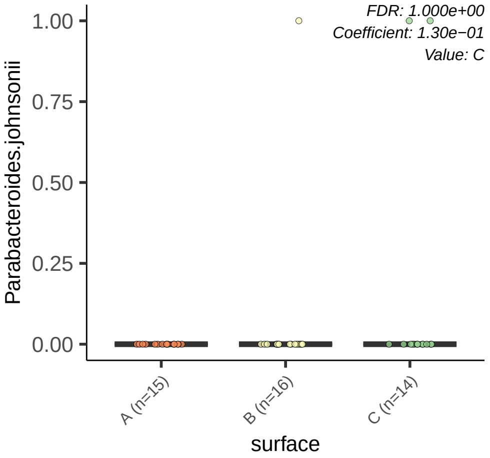
FDR: 1.000e+00
Coefficient: 1.30e−01
Value: C
Parabacteroides.johnsonii
1.00
0.75
0.50
0.25
0.00
A (n=15)
B (n=16)
C (n=14)
surface

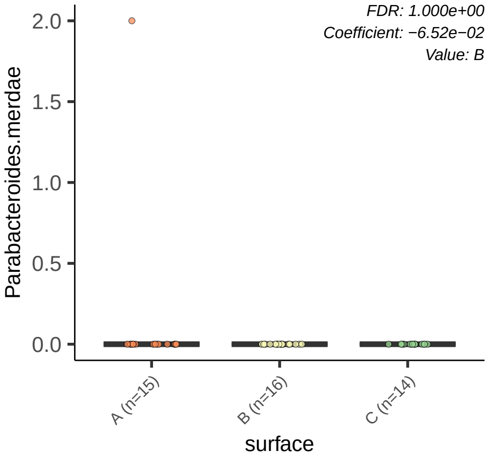
FDR: 1.000e+00
Coefficient: −6.52e−02
Value: B
Parabacteroides.merdae
2.0
1.5
1.0
0.5
0.0
A (n=15)
B (n=16)
C (n=14)
surface

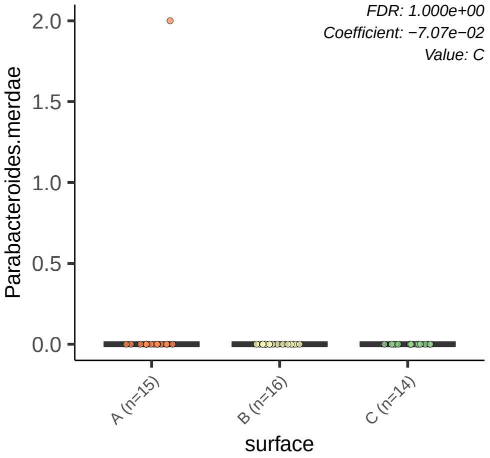
FDR: 1.000e+00
Coefficient: −7.07e−02
Value: C
Parabacteroides.merdae
2.0
1.5
1.0
0.5
0.0
A (n=15)
B (n=16)
C (n=14)
surface

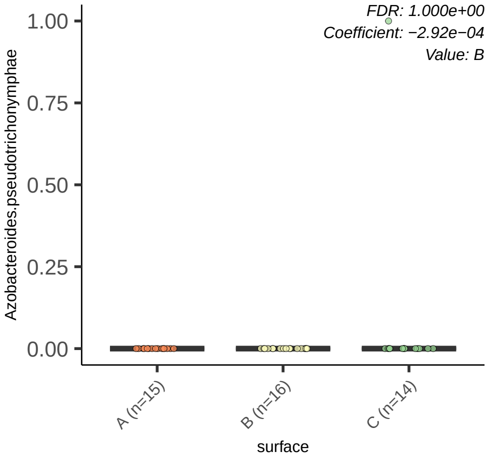
FDR: 1.000e+00
Coefficient: −2.92e−04
Value: B
Azobacteroides.pseudotrichonymphae
1.00
0.75
0.50
0.25
0.00
A (n=15)
B (n=16)
C (n=14)
surface

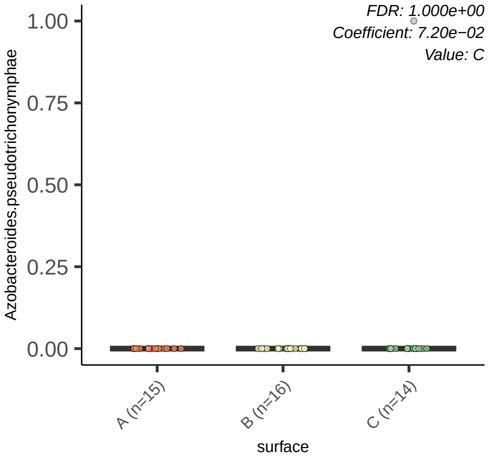

FDR: 1.000e+00
Coefficient: 7.20e−02
Value: C
Azobacteroides.pseudotrichonymphae
1.00
0.75
0.50
0.25
0.00
A (n=15)
B (n=16)
C (n=14)
surface

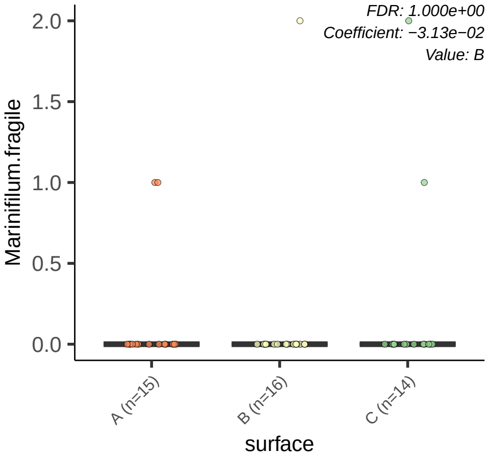
FDR: 1.000e+00
Coefficient: −3.13e−02
Value: B
Marinifilum.fragile
2.0
1.5
1.0
0.5
0.0
A (n=15)
B (n=16)
C (n=14)
surface

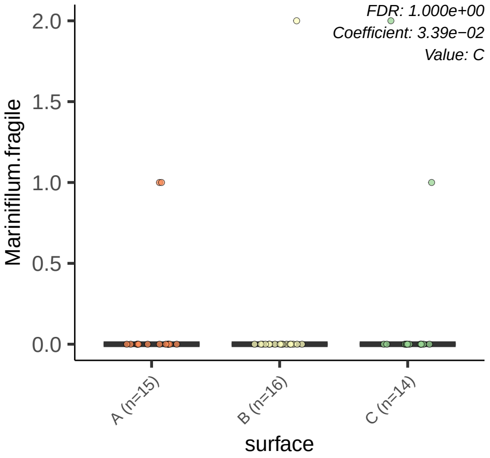
FDR: 1.000e+00
Coefficient: 3.39e−02
Value: C
Marinifilum.fragile
2.0
1.5
1.0
0.5
0.0
A (n=15)
B (n=16)
C (n=14)
surface

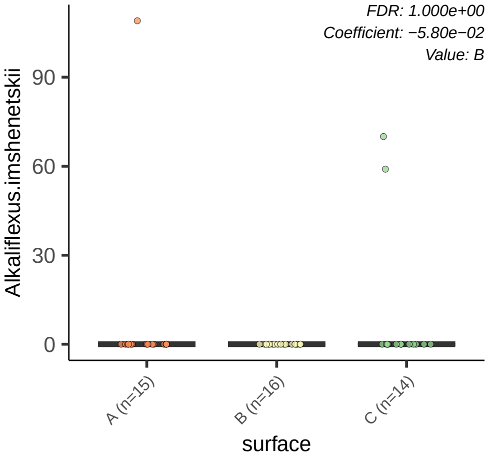

FDR: 1.000e+00
Coefficient: −5.80e−02
Value: B
Alkaliflexus.imshenetskii
90
60
30
0
A (n=15)
B (n=16)
C (n=14)
surface

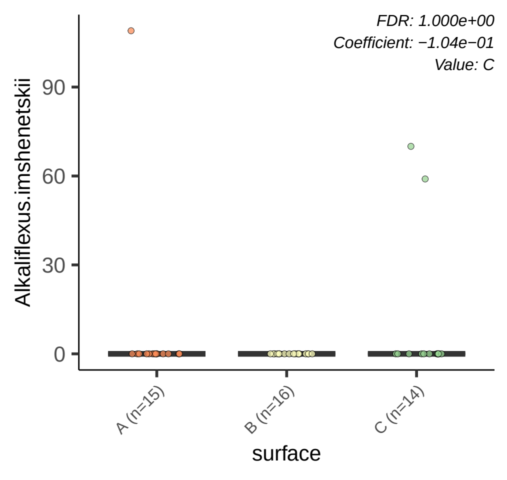

FDR: 1.000e+00
Coefficient: −1.04e−01
Value: C
Alkaliflexus.imshenetskii
90
60
30
0
A (n=15)
B (n=16)
C (n=14)
surface

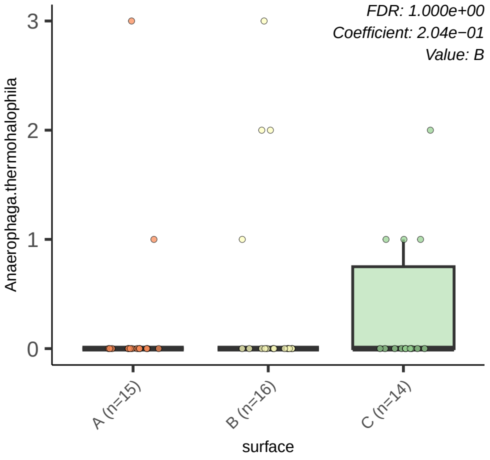

FDR: 1.000e+00
Coefficient: 2.04e−01
Value: B
Anaerophaga.thermohalophila
3
2
1
0
A (n=15)
B (n=16)
C (n=14)
surface

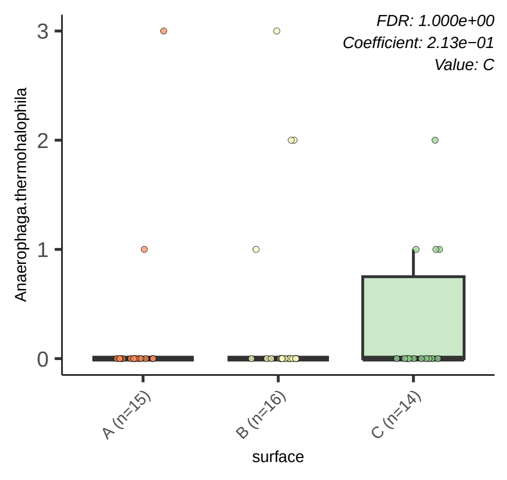
FDR: 1.000e+00
Coefficient: 2.13e−01
Value: C
Anaerophaga.thermohalophila
3
2
1
0
A (n=15)
B (n=16)
C (n=14)
surface

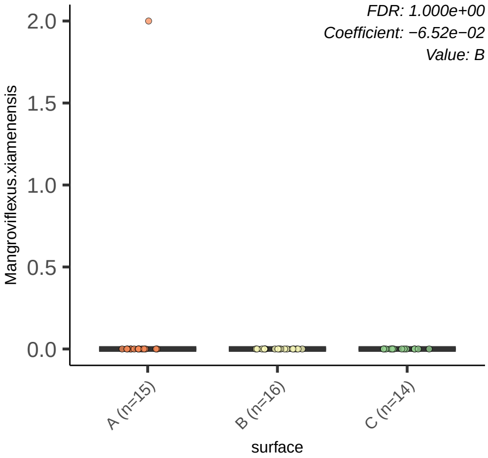

FDR: 1.000e+00
Coefficient: −6.52e−02
Value: B
Mangroviflexus.xiamenensis
2.0
1.5
1.0
0.5
0.0
A (n=15)
B (n=16)
C (n=14)
surface

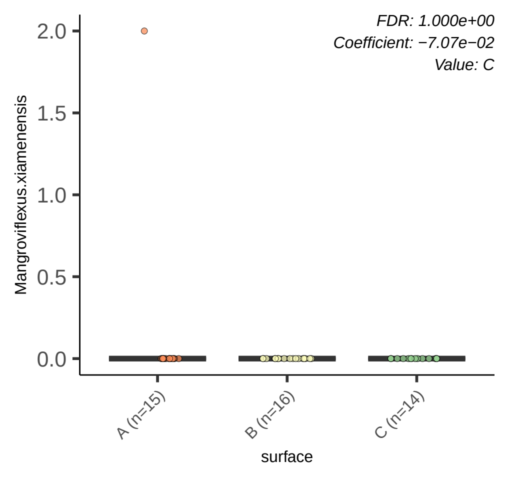
FDR: 1.000e+00
Coefficient: −7.07e−02
Value: C
Mangroviflexus.xiamenensis
2.0
1.5
1.0
0.5
0.0
A (n=15)
B (n=16)
C (n=14)
surface

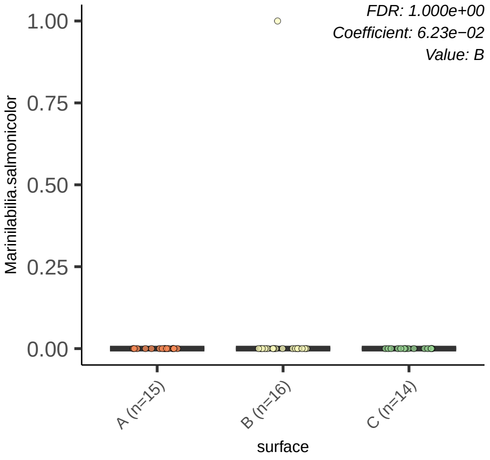

FDR: 1.000e+00
Coefficient: 6.23e−02
Value: B
Marinilabilia.salmonicolor
1.00
0.75
0.50
0.25
0.00
A (n=15)
B (n=16)
C (n=14)
surface

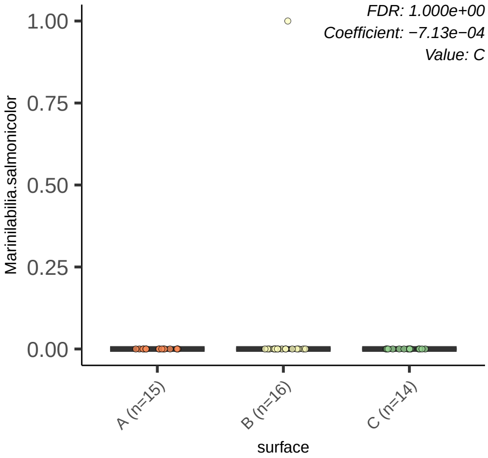

FDR: 1.000e+00
Coefficient: −7.13e−04
Value: C
Marinilabilia.salmonicolor
1.00
0.75
0.50
0.25
0.00
A (n=15)
B (n=16)
C (n=14)
surface

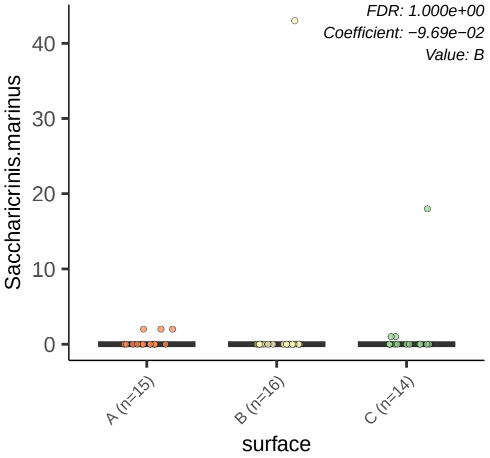
FDR: 1.000e+00
Coefficient: −9.69e−02
Value: B
Saccharicrinis.marinus
40
30
20
10
0
A (n=15)
B (n=16)
C (n=14)
surface

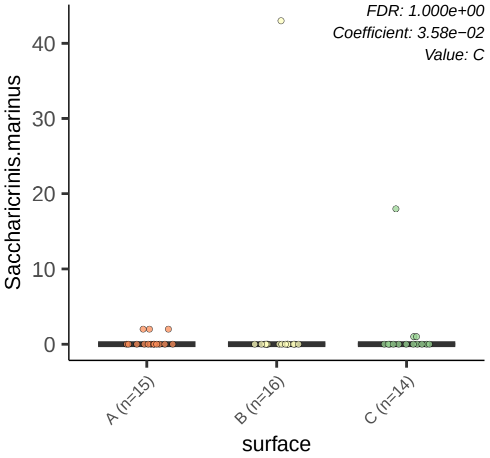

FDR: 1.000e+00
Coefficient: 3.58e−02
Value: C
Saccharicrinis.marinus
40
30
20
10
0
A (n=15)
B (n=16)
C (n=14)
surface

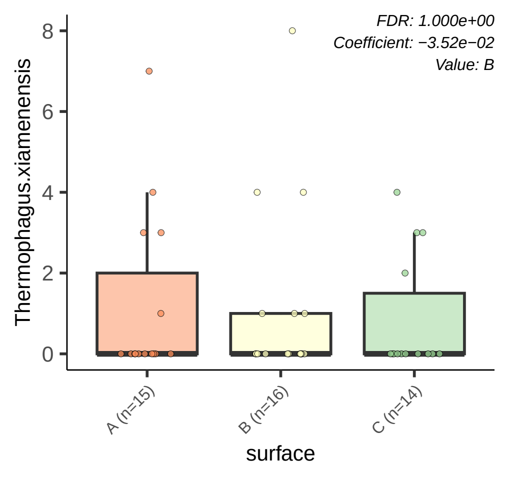

FDR: 1.000e+00
Coefficient: −3.52e−02
Value: B
Thermophagus.xiamenensis
0
2
4
6
8
A (n=15)
B (n=16)
C (n=14)
surface

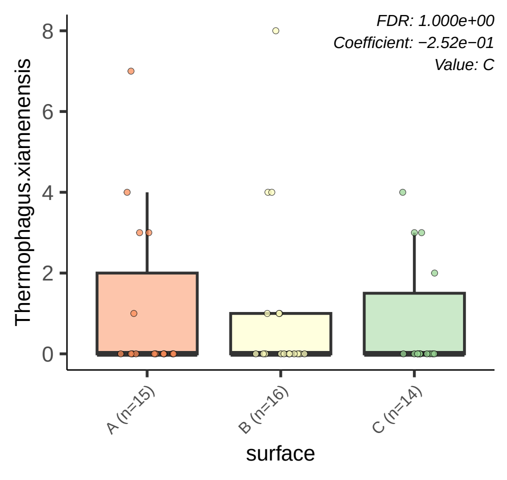

FDR: 1.000e+00
Coefficient: −2.52e−01
Value: C
Thermophagus.xiamenensis
0
2
4
6
8
A (n=15)
B (n=16)
C (n=14)
surface

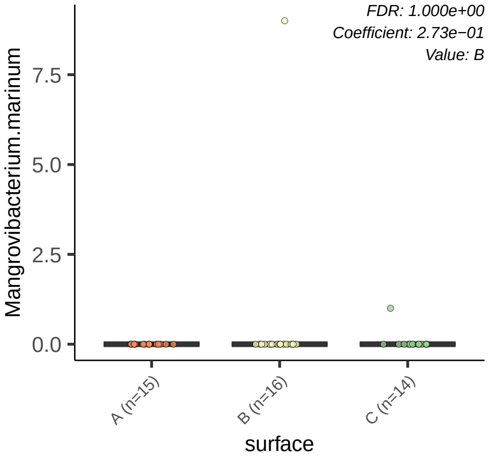

FDR: 1.000e+00
Coefficient: 2.73e−01
Value: B
Mangrovibacterium.marinum
7.5
5.0
2.5
0.0
A (n=15)
B (n=16)
C (n=14)
surface

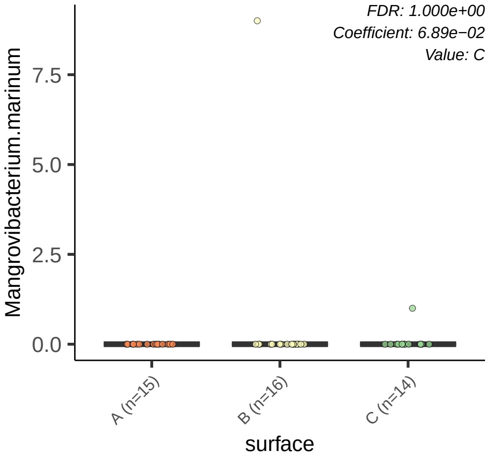

FDR: 1.000e+00
Coefficient: 6.89e−02
Value: C
Mangrovibacterium.marinum
7.5
5.0
2.5
0.0
A (n=15)
B (n=16)
C (n=14)
surface

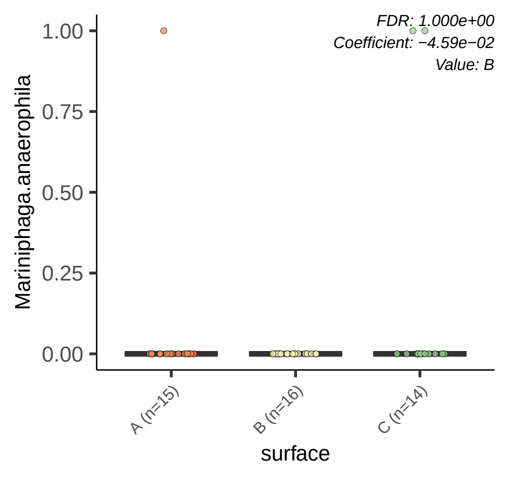

FDR: 1.000e+00
Coefficient: −4.59e−02
Value: B
Mariniphaga.anaerophila
1.00
0.75
0.50
0.25
0.00
A (n=15)
B (n=16)
C (n=14)
surface

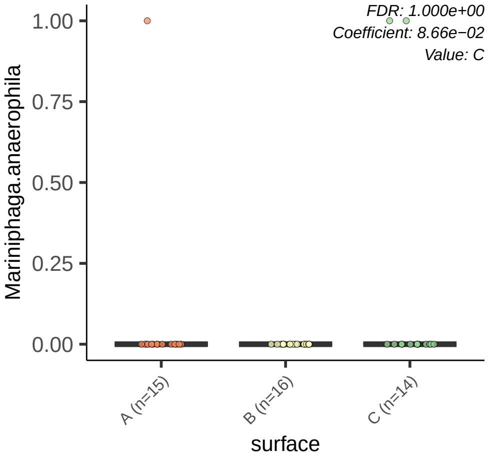

FDR: 1.000e+00
Coefficient: 8.66e−02
Value: C
Mariniphaga.anaerophila
1.00
0.75
0.50
0.25
0.00
A (n=15)
B (n=16)
C (n=14)
surface

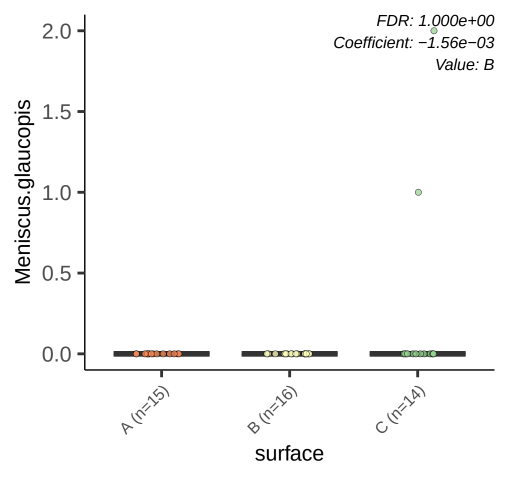
FDR: 1.000e+00
Coefficient: −1.56e−03
Value: B
Meniscus.glaucopis
2.0
1.5
1.0
0.5
0.0
A (n=15)
B (n=16)
C (n=14)
surface

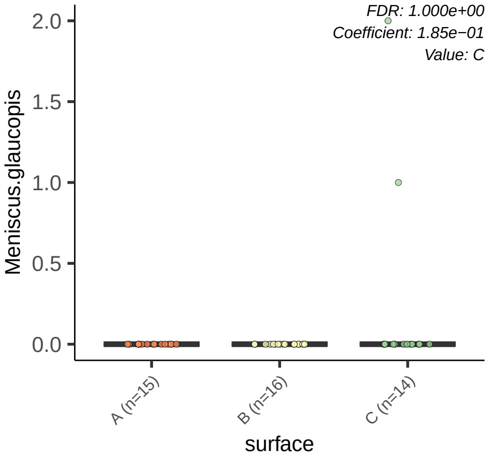

FDR: 1.000e+00
Coefficient: 1.85e−01
Value: C
Meniscus.glaucopis
2.0
1.5
1.0
0.5
0.0
A (n=15)
B (n=16)
C (n=14)
surface

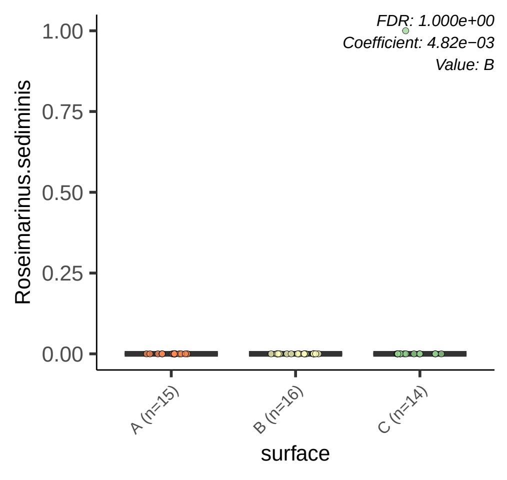
FDR: 1.000e+00
Coefficient: 4.82e−03
Value: B
1.00
0.75
0.50
0.25
0.00
Roseimarinus.sedimin is
A (n=15)
B (n=16)
C (n=14)
surface

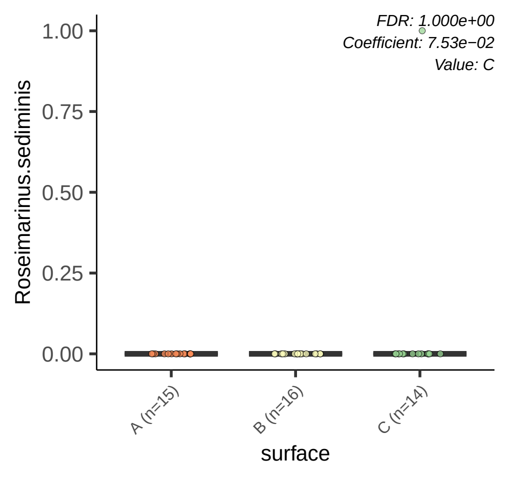
FDR: 1.000e+00
Coefficient: 7.53e−02
Value: C
Roseimarinus.sedimini s
1.00
0.75
0.50
0.25
0.00
A (n=15)
B (n=16)
C (n=14)
surface

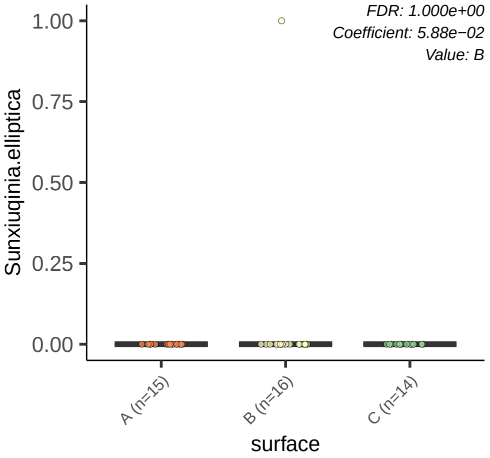
FDR: 1.000e+00
Coefficient: 5.88e−02
Value: B
Sunxiuqinia.elliptica
1.00
0.75
0.50
0.25
0.00
A (n=15)
B (n=16)
C (n=14)
surface

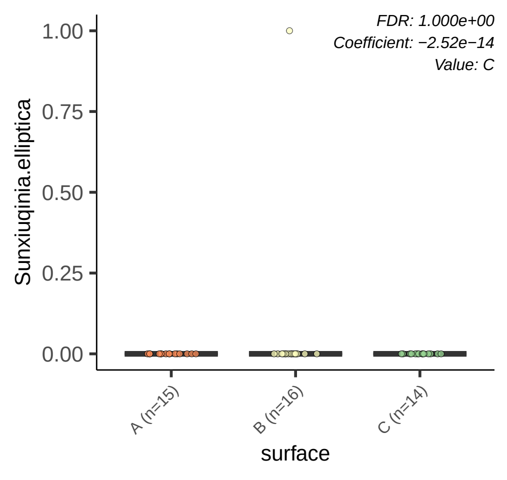
FDR: 1.000e+00
Coefficient: −2.52e−14
Value: C
Sunxiuqinia.elliptica
1.00
0.75
0.50
0.25
0.00
A (n=15)
B (n=16)
C (n=14)
surface

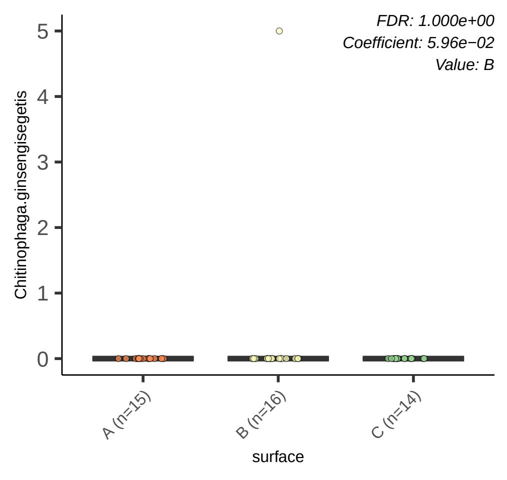

FDR: 1.000e+00
Coefficient: 5.96e−02
Value: B
Chitinophaga.ginsengisegetis
5
4
3
2
1
0
A (n=15)
B (n=16)
C (n=14)
surface

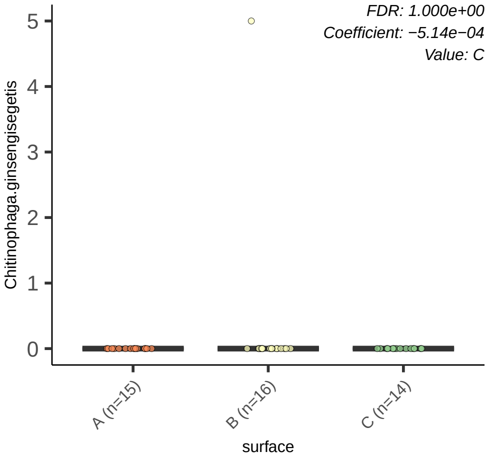

FDR: 1.000e+00
Coefficient: −5.14e−04
Value: C
Chitinophaga.ginsengisegetis
0
1
2
3
4
5
A (n=15)
B (n=16)
C (n=14)
surface

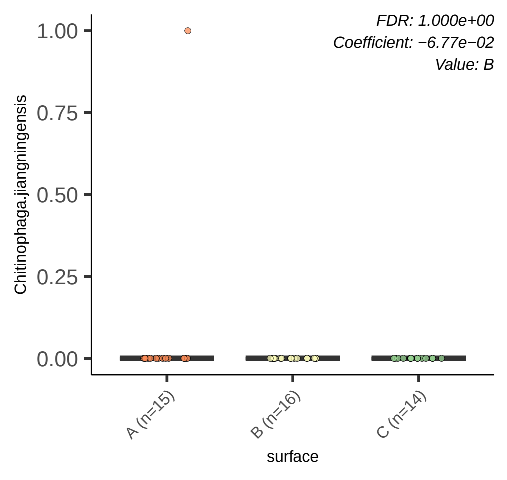
FDR: 1.000e+00
Coefficient: −6.77e−02
Value: B
1.00
0.75
0.50
0.25
0.00
Chitinophaga.jiangningensis
A (n=15)
B (n=16)
C (n=14)
surface

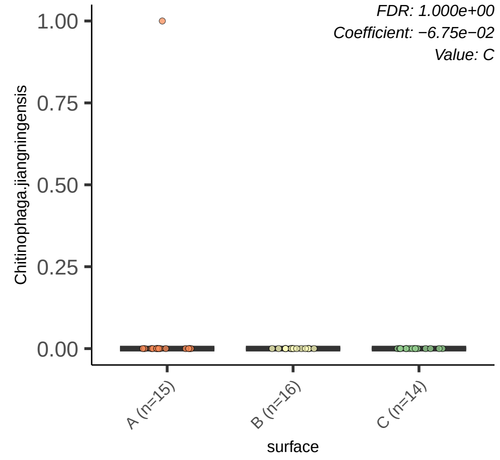
FDR: 1.000e+00
Coefficient: −6.75e−02
Value: C
1.00
0.75
0.50
0.25
0.00
Chitinophaga.jiangningensis
A (n=15)
B (n=16)
C (n=14)
surface

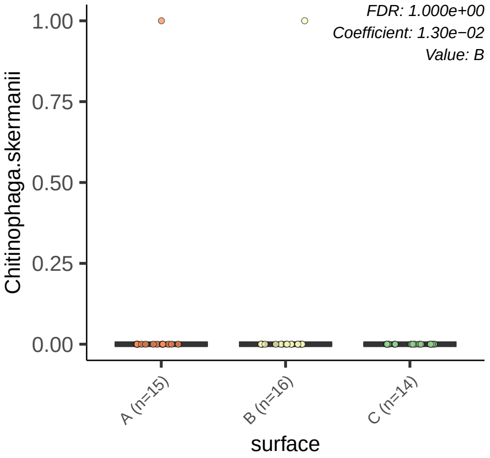
FDR: 1.000e+00
Coefficient: 1.30e−02
Value: B
Chitinophaga.skermanii
1.00
0.75
0.50
0.25
0.00
A (n=15)
B (n=16)
C (n=14)
surface

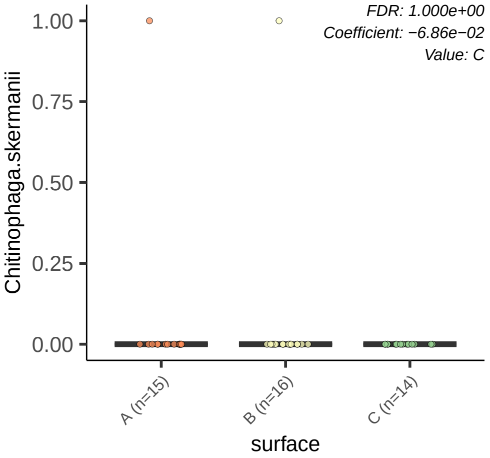

FDR: 1.000e+00
Coefficient: −6.86e−02
Value: C
Chitinophaga.skermanii
1.00
0.75
0.50
0.25
0.00
A (n=15)
B (n=16)
C (n=14)
surface

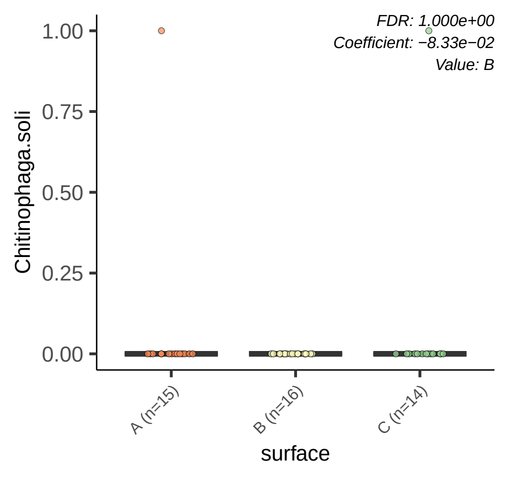

FDR: 1.000e+00
Coefficient: −8.33e−02
Value: B
Chitinophaga.soli
1.00
0.75
0.50
0.25
0.00
A (n=15)
B (n=16)
C (n=14)
surface

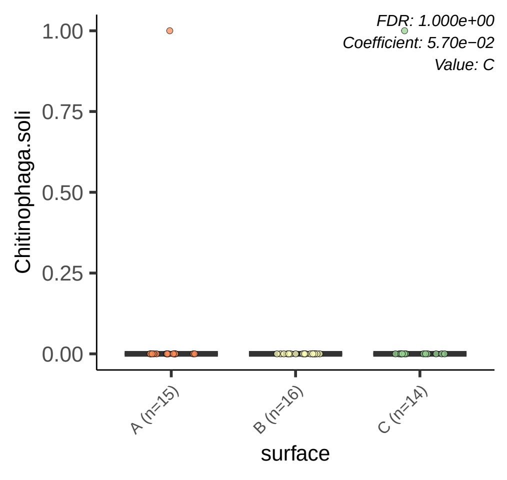

FDR: 1.000e+00
Coefficient: 5.70e−02
Value: C
Chitinophaga.soli
1.00
0.75
0.50
0.25
0.00
A (n=15)
B (n=16)
C (n=14)
surface

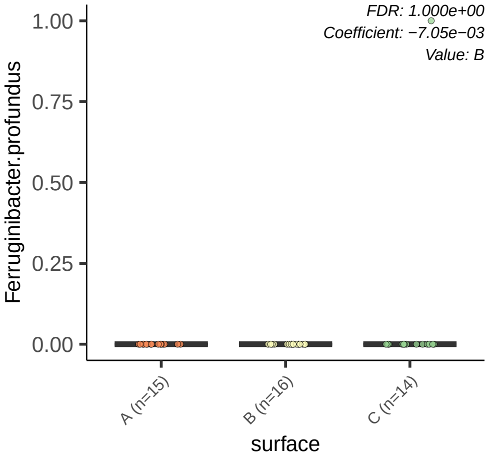
FDR: 1.000e+00
Coefficient: −7.05e−03
Value: B
Ferruginibacter.profundus
1.00
0.75
0.50
0.25
0.00
A (n=15)
B (n=16)
C (n=14)
surface

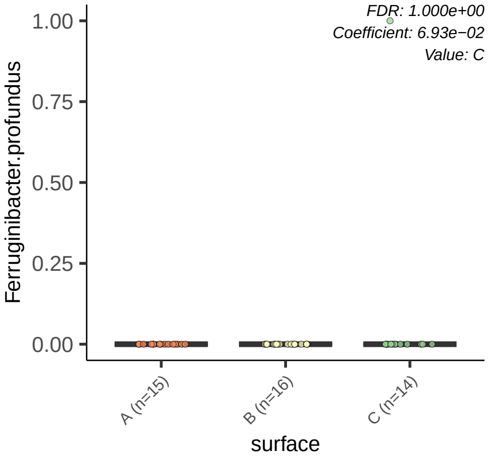
FDR: 1.000e+00
Coefficient: 6.93e−02
Value: C
Ferruginibacter.profundus
1.00
0.75
0.50
0.25
0.00
A (n=15)
B (n=16)
C (n=14)
surface

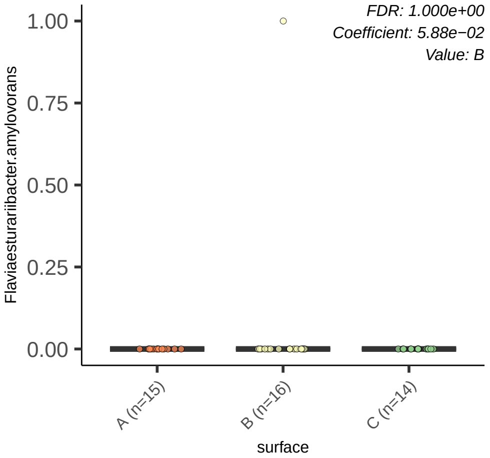

FDR: 1.000e+00
Coefficient: 5.88e−02
Value: B
Flaviaesturariibacter.amylovorans
1.00
0.75
0.50
0.25
0.00
A (n=15)
B (n=16)
C (n=14)
surface

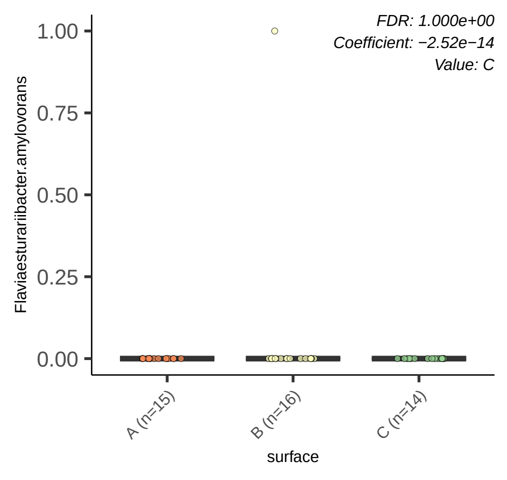
FDR: 1.000e+00
Coefficient: −2.52e−14
Value: C
1.00
0.75
0.50
0.25
0.00
Flaviaesturariibacter.amylovorans
A (n=15)
B (n=16)
C (n=14)
surface

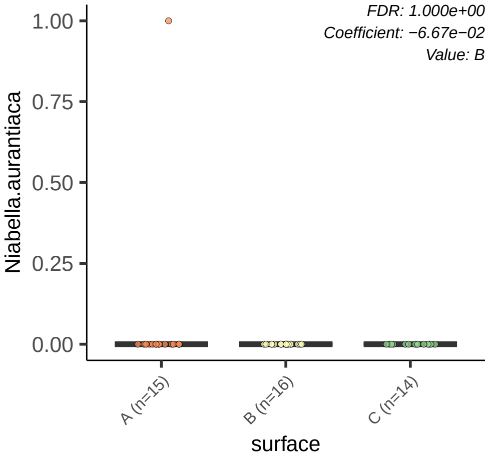
FDR: 1.000e+00
Coefficient: −6.67e−02
Value: B
Niabella.aurantiaca
1.00
0.75
0.50
0.25
0.00
A (n=15)
B (n=16)
C (n=14)
surface

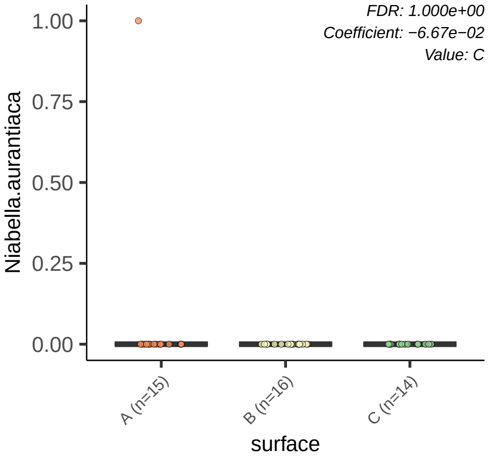
FDR: 1.000e+00
Coefficient: −6.67e−02
Value: C
Niabella.aurantiaca
1.00
0.75
0.50
0.25
0.00
A (n=15)
B (n=16)
C (n=14)
surface

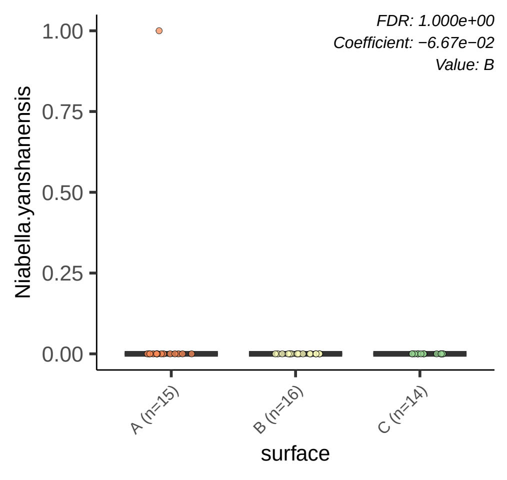
FDR: 1.000e+00
Coefficient: −6.67e−02
Value: B
Niabella.yanshanensis
1.00
0.75
0.50
0.25
0.00
A (n=15)
B (n=16)
C (n=14)
surface

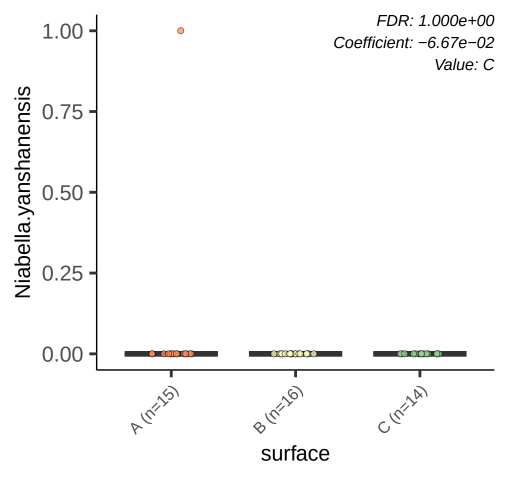
FDR: 1.000e+00
Coefficient: −6.67e−02
Value: C
Niabella.yanshanensis
1.00
0.75
0.50
0.25
0.00
A (n=15)
B (n=16)
C (n=14)
surface

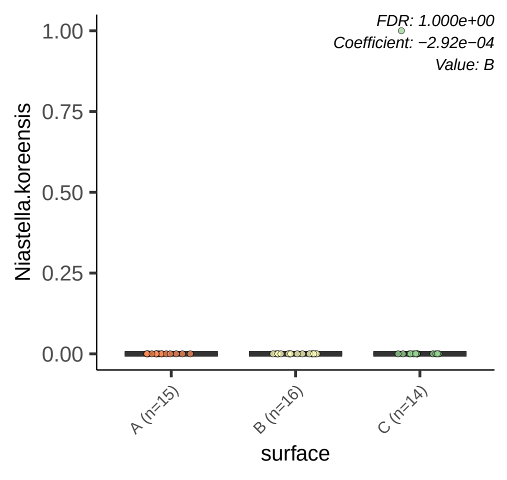
FDR: 1.000e+00
Coefficient: −2.92e−04
Value: B
1.00
0.75
0.50
0.25
0.00
Niastella.koreensis
A (n=15)
B (n=16)
C (n=14)
surface

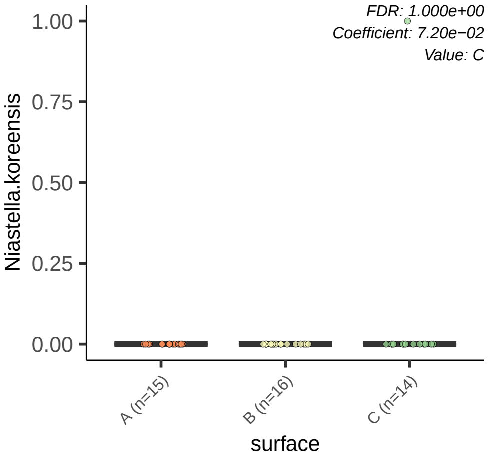

FDR: 1.000e+00
Coefficient: 7.20e−02
Value: C
Niastella.koreensis
1.00
0.75
0.50
0.25
0.00
A (n=15)
B (n=16)
C (n=14)
surface

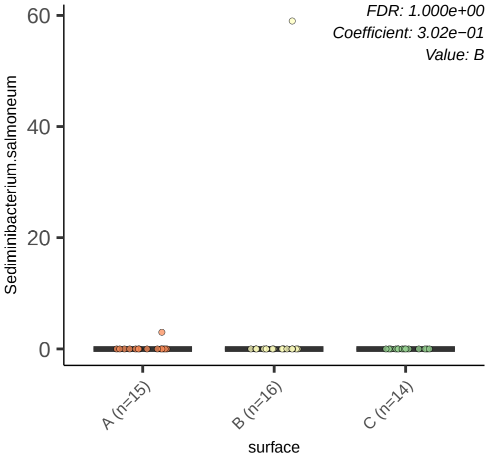
FDR: 1.000e+00
Coefficient: 3.02e−01
Value: B
Sediminibacterium.salmoneum
0
20
40
60
A (n=15)
B (n=16)
C (n=14)
surface

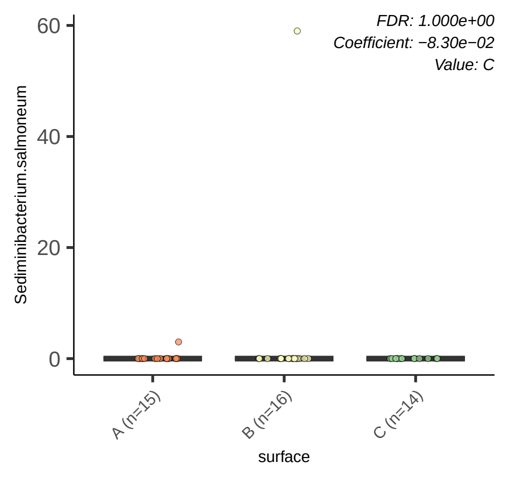

FDR: 1.000e+00
Coefficient: −8.30e−02
Value: C
Sediminibacterium.salmoneum
60
40
20
0
A (n=15)
B (n=16)
C (n=14)
surface

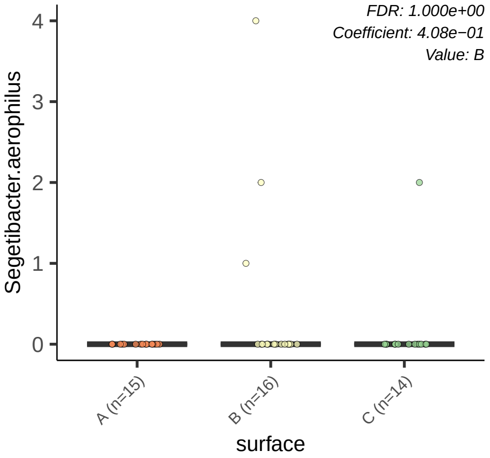
FDR: 1.000e+00
Coefficient: 4.08e−01
Value: B
Segetibacter.aerophilus
4
3
2
1
0
A (n=15)
B (n=16)
C (n=14)
surface

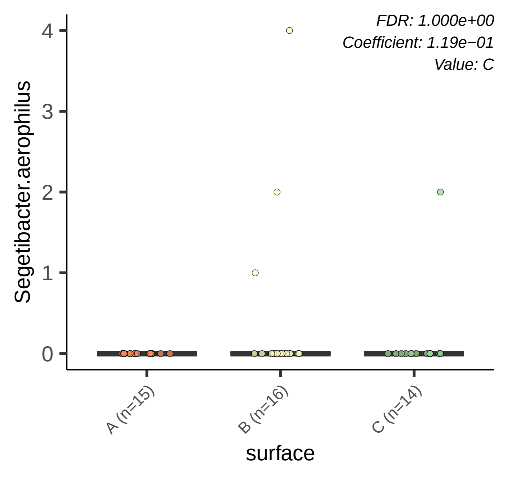

FDR: 1.000e+00
Coefficient: 1.19e−01
Value: C
Segetibacter.aerophilus
4
3
2
1
0
A (n=15)
B (n=16)
C (n=14)
surface

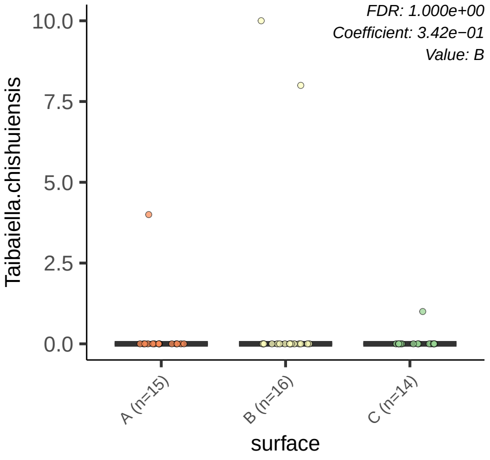
FDR: 1.000e+00
Coefficient: 3.42e−01
Value: B
Taibaiella.chishuiensis
10.0
7.5
5.0
2.5
0.0
A (n=15)
B (n=16)
C (n=14)
surface

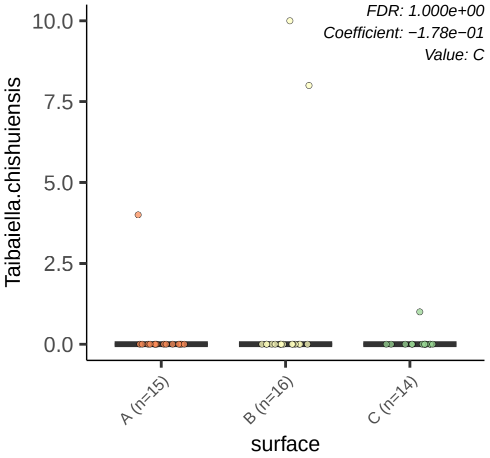

FDR: 1.000e+00
Coefficient: −1.78e−01
Value: C
Taibaiella.chishuiensis
10.0
7.5
5.0
2.5
0.0
A (n=15)
B (n=16)
C (n=14)
surface

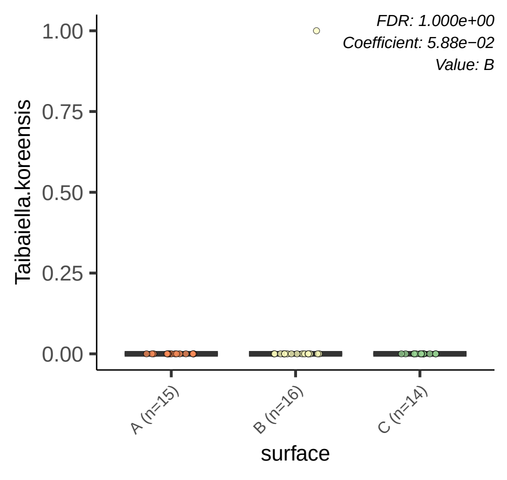
FDR: 1.000e+00
Coefficient: 5.88e−02
Value: B
Taibaiella.koreensis
1.00
0.75
0.50
0.25
0.00
A (n=15)
B (n=16)
C (n=14)
surface

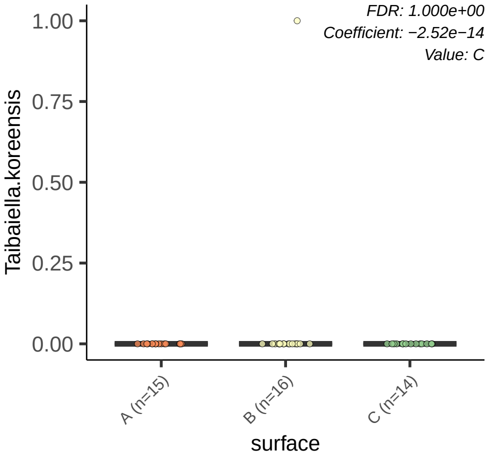

FDR: 1.000e+00
Coefficient: −2.52e−14
Value: C
Taibaiella.koreensis
1.00
0.75
0.50
0.25
0.00
A (n=15)
B (n=16)
C (n=14)
surface

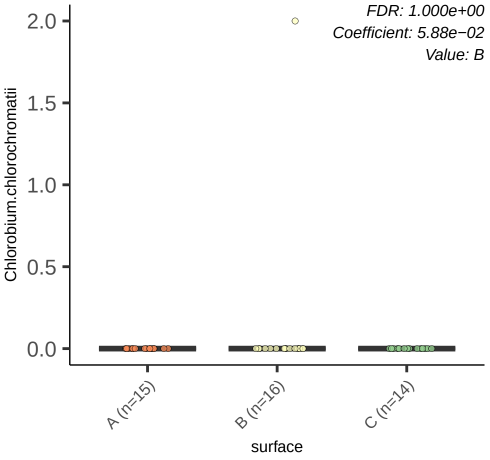
FDR: 1.000e+00
Coefficient: 5.88e−02
Value: B
Chlorobium.chlorochromatii
2.0
1.5
1.0
0.5
0.0
A (n=15)
B (n=16)
C (n=14)
surface

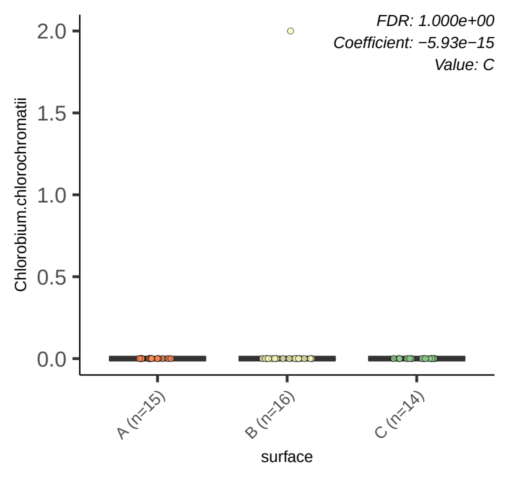

FDR: 1.000e+00
Coefficient: −5.93e−15
Value: C
Chlorobium.chlorochromatii
2.0
1.5
1.0
0.5
0.0
A (n=15)
B (n=16)
C (n=14)
surface

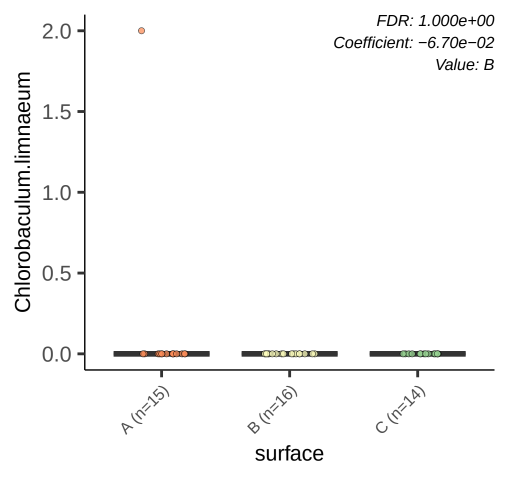
FDR: 1.000e+00
Coefficient: −6.70e−02
Value: B
Chlorobaculum.limnaeum
2.0
1.5
1.0
0.5
0.0
A (n=15)
B (n=16)
C (n=14)
surface

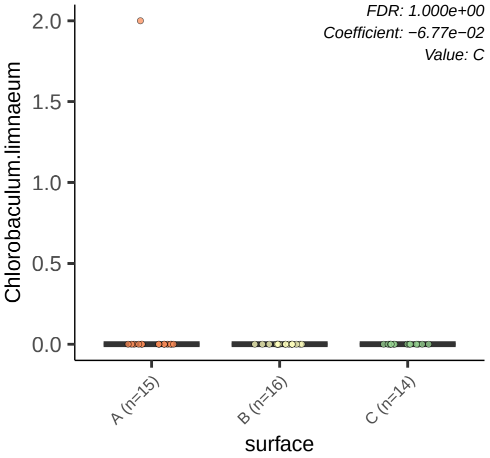

FDR: 1.000e+00
Coefficient: −6.77e−02
Value: C
Chlorobaculum.limnaeum
2.0
1.5
1.0
0.5
0.0
A (n=15)
B (n=16)
C (n=14)
surface

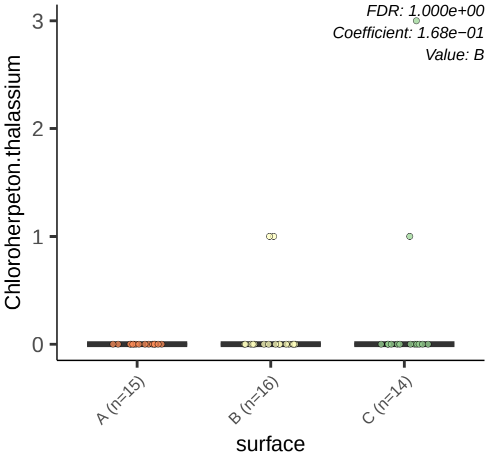
FDR: 1.000e+00
Coefficient: 1.68e−01
Value: B
Chloroherpeton.thalassium
0
1
2
3
A (n=15)
B (n=16)
C (n=14)
surface

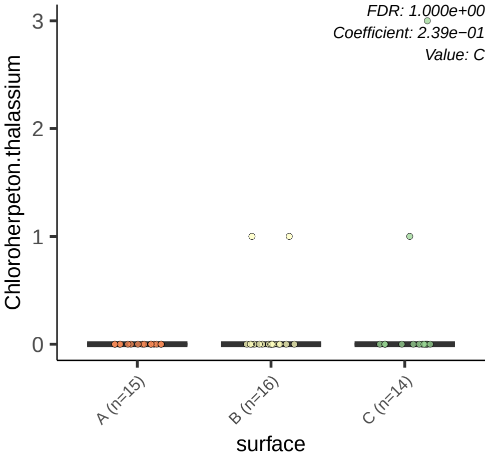
FDR: 1.000e+00
Coefficient: 2.39e−01
Value: C
Chloroherpeton.thalassium
0
1
2
3
A (n=15)
B (n=16)
C (n=14)
surface

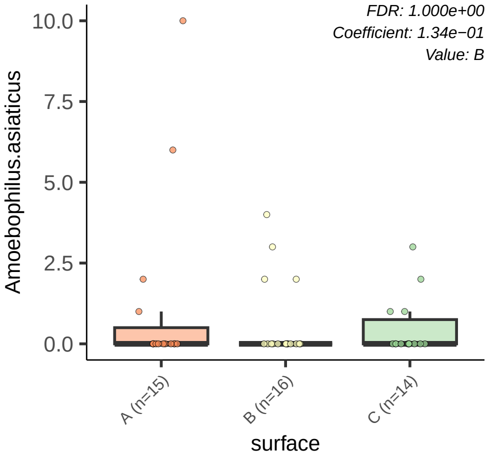
FDR: 1.000e+00
Coefficient: 1.34e−01
Value: B
Amoebophilus.asiaticus
10.0
7.5
5.0
2.5
0.0
A (n=15)
B (n=16)
C (n=14)
surface

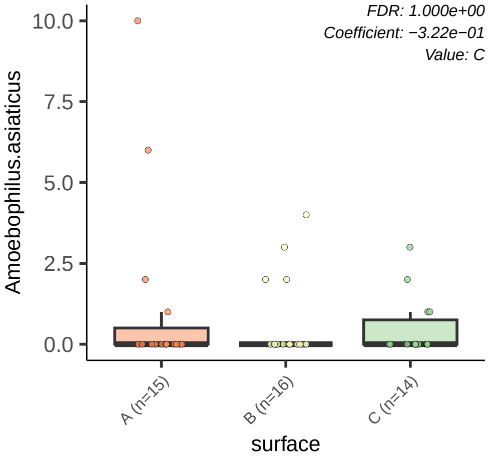

FDR: 1.000e+00
Coefficient: −3.22e−01
Value: C
Amoebophilus.asiaticus
10.0
7.5
5.0
2.5
0.0
A (n=15)
B (n=16)
C (n=14)
surface

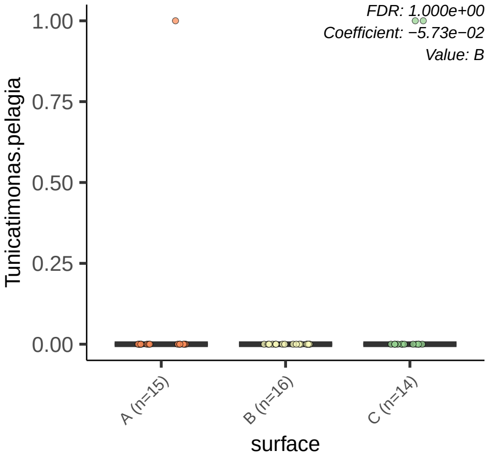
FDR: 1.000e+00
Coefficient: −5.73e−02
Value: B
Tunicatimonas.pelagia
1.00
0.75
0.50
0.25
0.00
A (n=15)
B (n=16)
C (n=14)
surface

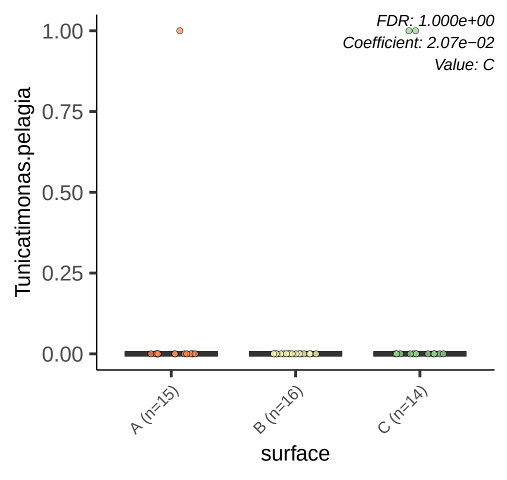

FDR: 1.000e+00
Coefficient: 2.07e−02
Value: C
Tunicatimonas.pelagia
1.00
0.75
0.50
0.25
0.00
A (n=15)
B (n=16)
C (n=14)
surface

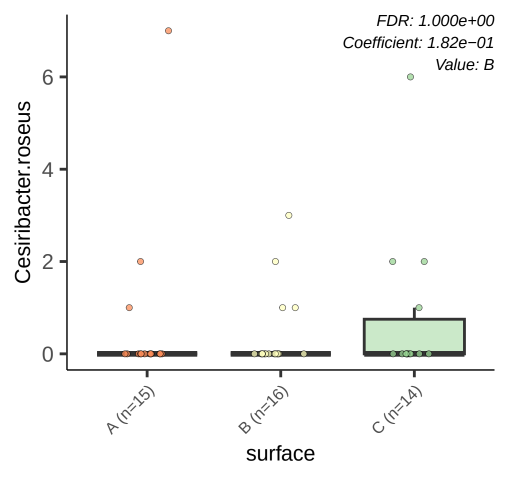
FDR: 1.000e+00
Coefficient: 1.82e−01
Value: B
Cesiribacter.roseus
0
2
4
6
A (n=15)
B (n=16)
C (n=14)
surface

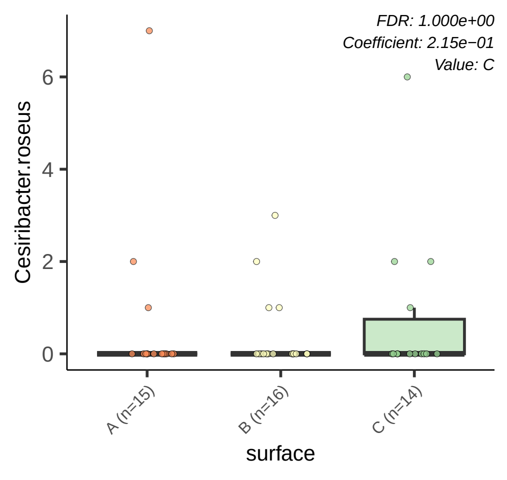

FDR: 1.000e+00
Coefficient: 2.15e−01
Value: C
Cesiribacter.roseus
0
2
4
6
A (n=15)
B (n=16)
C (n=14)
surface

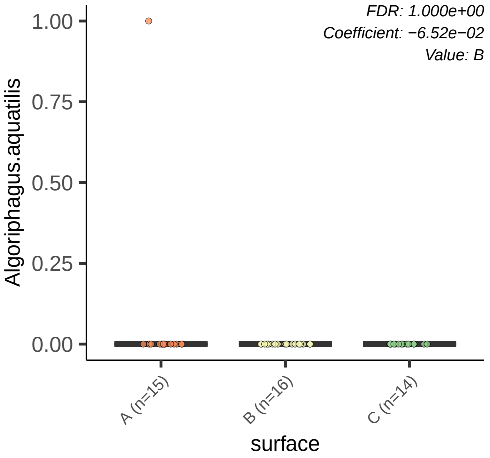
FDR: 1.000e+00
Coefficient: −6.52e−02
Value: B
Algoriphagus.aquatilis
1.00
0.75
0.50
0.25
0.00
A (n=15)
B (n=16)
C (n=14)
surface

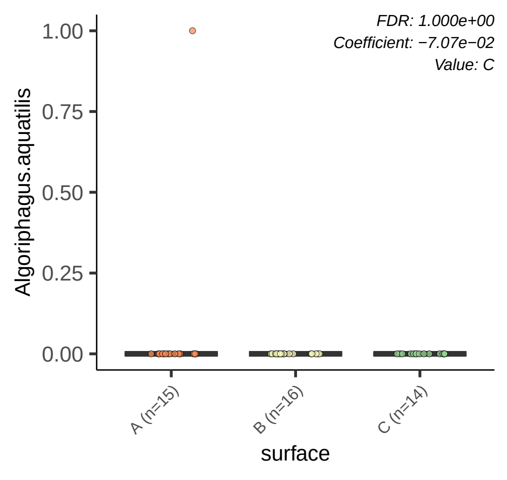

FDR: 1.000e+00
Coefficient: −7.07e−02
Value: C
Algoriphagus.aquatilis
1.00
0.75
0.50
0.25
0.00
A (n=15)
B (n=16)
C (n=14)
surface

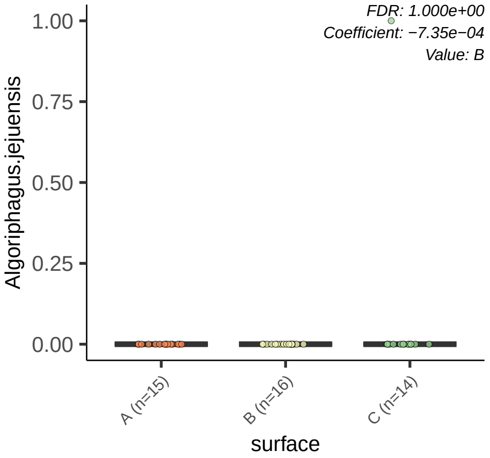
FDR: 1.000e+00
Coefficient: −7.35e−04
Value: B
Algoriphagus.jejuensis
1.00
0.75
0.50
0.25
0.00
A (n=15)
B (n=16)
C (n=14)
surface

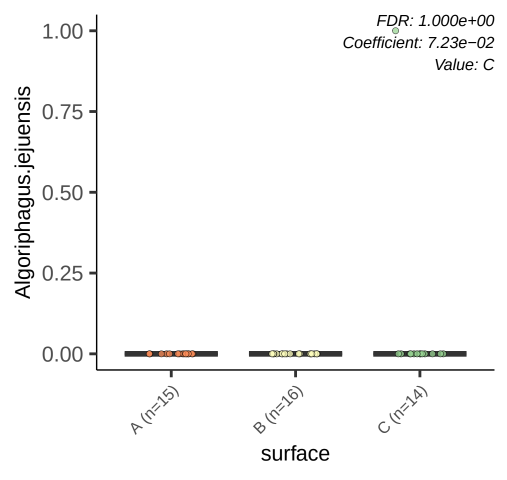

FDR: 1.000e+00
Coefficient: 7.23e−02
Value: C
Algoriphagus.jejuensis
1.00
0.75
0.50
0.25
0.00
A (n=15)
B (n=16)
C (n=14)
surface

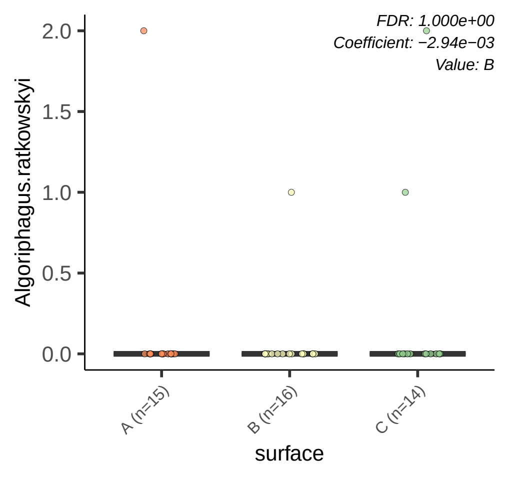

FDR: 1.000e+00
Coefficient: −2.94e−03
Value: B
Algoriphagus.ratkowskyi
2.0
1.5
1.0
0.5
0.0
A (n=15)
B (n=16)
C (n=14)
surface

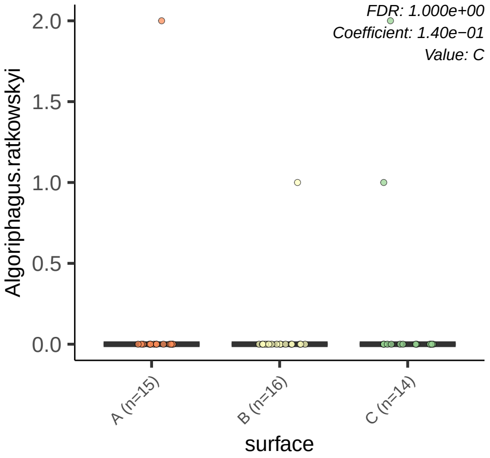

FDR: 1.000e+00
Coefficient: 1.40e−01
Value: C
Algoriphagus.ratkowskyi
2.0
1.5
1.0
0.5
0.0
A (n=15)
B (n=16)
C (n=14)
surface

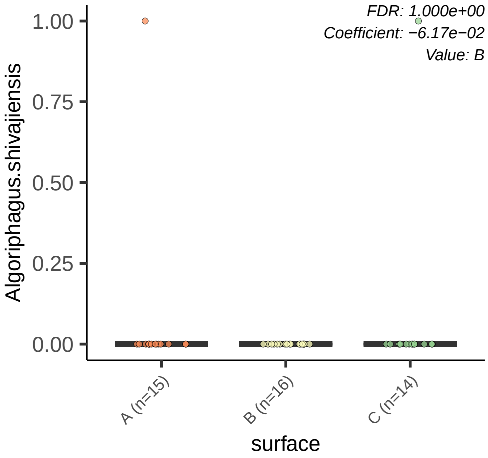
FDR: 1.000e+00
Coefficient: −6.17e−02
Value: B
Algoriphagus.shivajiensis
1.00
0.75
0.50
0.25
0.00
A (n=15)
B (n=16)
C (n=14)
surface

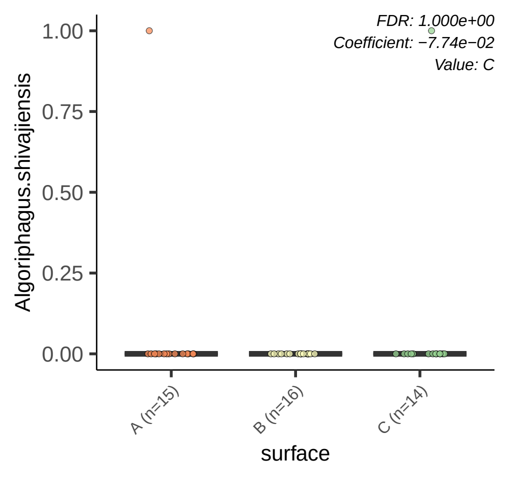
FDR: 1.000e+00
Coefficient: −7.74e−02
Value: C
Algoriphagus.shivajiensis
1.00
0.75
0.50
0.25
0.00
A (n=15)
B (n=16)
C (n=14)
surface

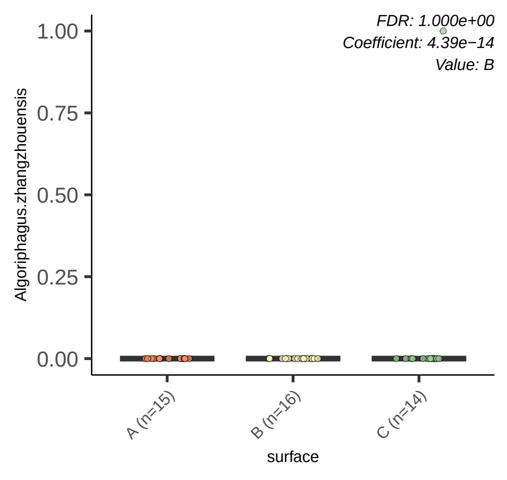
FDR: 1.000e+00
Coefficient: 4.39e−14
Value: B
1.00
0.75
0.50
0.25
0.00
Algoriphagus.zhangzhouensis
A (n=15)
B (n=16)
C (n=14)
surface

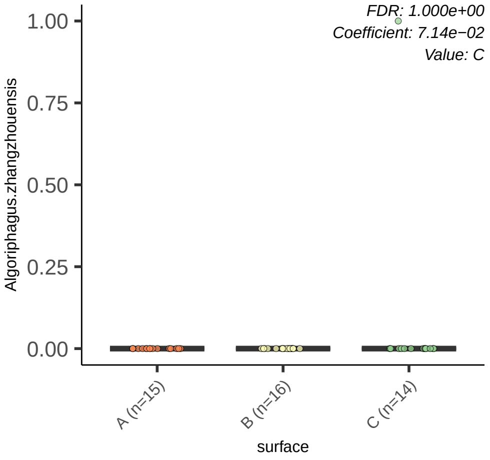

FDR: 1.000e+00
Coefficient: 7.14e−02
Value: C
Algoriphagus.zhangzhouensis
1.00
0.75
0.50
0.25
0.00
A (n=15)
B (n=16)
C (n=14)
surface

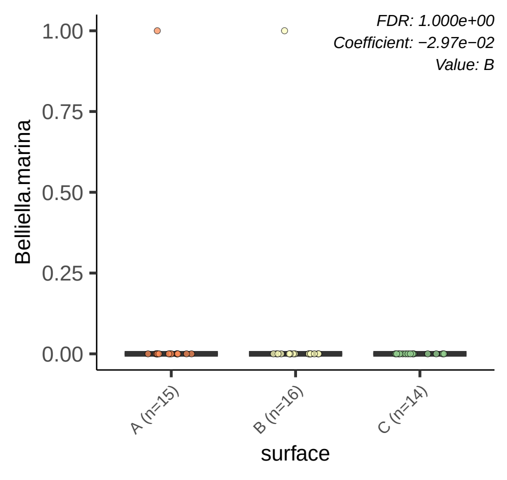

FDR: 1.000e+00
Coefficient: −2.97e−02
Value: B
Belliella.marina
1.00
0.75
0.50
0.25
0.00
A (n=15)
B (n=16)
C (n=14)
surface

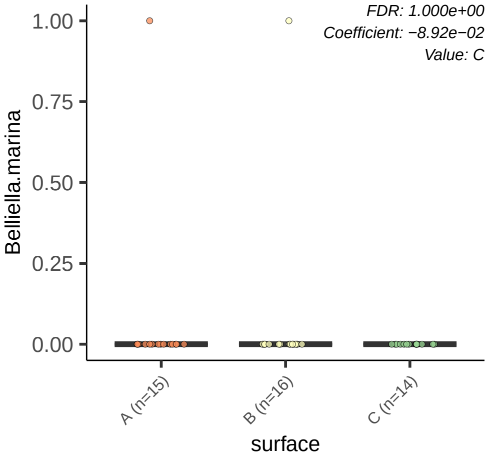
FDR: 1.000e+00
Coefficient: −8.92e−02
Value: C
Belliella.marina
1.00
0.75
0.50
0.25
0.00
A (n=15)
B (n=16)
C (n=14)
surface

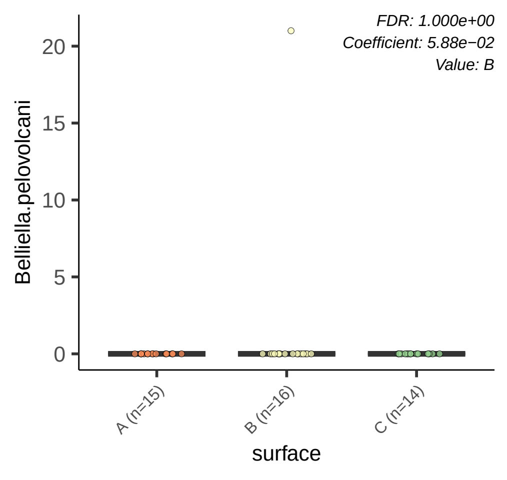
FDR: 1.000e+00
Coefficient: 5.88e−02
Value: B
Belliella.pelovolcani
20
15
10
5
0
A (n=15)
B (n=16)
C (n=14)
surface

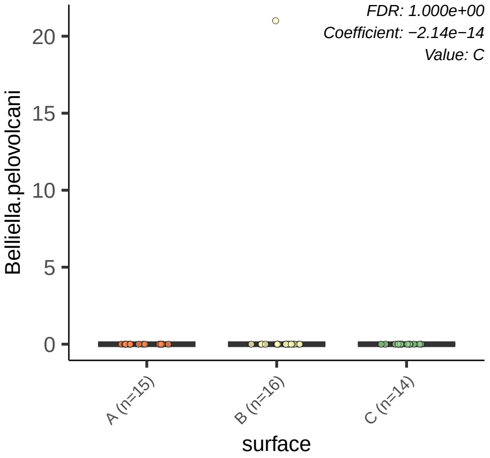
FDR: 1.000e+00
Coefficient: −2.14e−14
Value: C
Belliella.pelovolcani
20
15
10
5
0
A (n=15)
B (n=16)
C (n=14)
surface

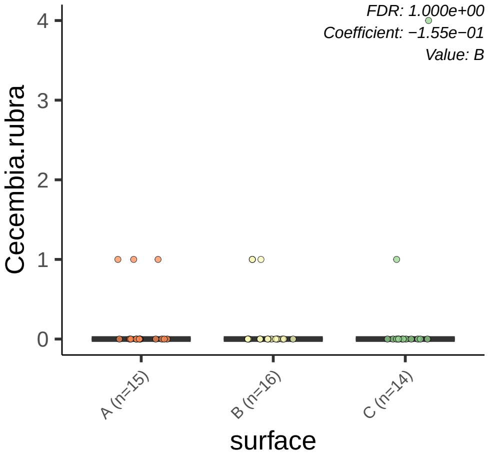
FDR: 1.000e+00
Coefficient: −1.55e−01
Value: B
Cecembia.rubra
0
1
2
3
4
A (n=15)
B (n=16)
C (n=14)
surface

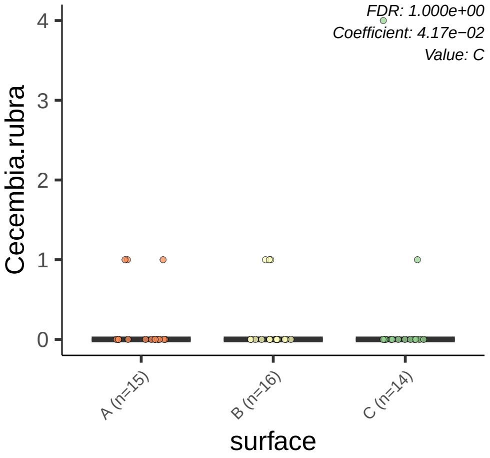
FDR: 1.000e+00
Coefficient: 4.17e−02
Value: C
Cecembia.rubra
0
1
2
3
4
A (n=15)
B (n=16)
C (n=14)
surface

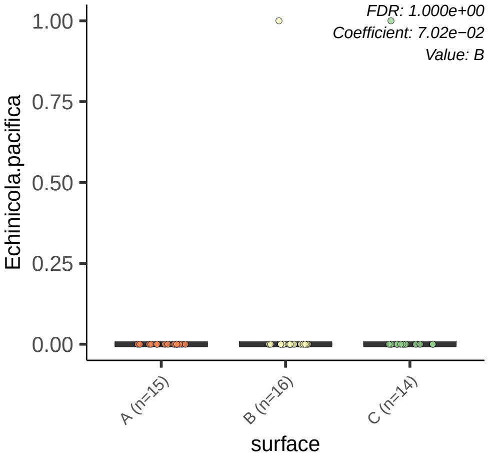

FDR: 1.000e+00
Coefficient: 7.02e−02
Value: B
Echinicola.pacifica
1.00
0.75
0.50
0.25
0.00
A (n=15)
B (n=16)
C (n=14)
surface

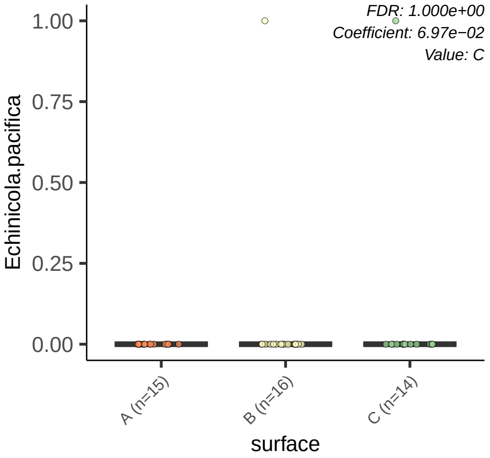
FDR: 1.000e+00
Coefficient: 6.97e−02
Value: C
Echinicola.pacifica
1.00
0.75
0.50
0.25
0.00
A (n=15)
B (n=16)
C (n=14)
surface

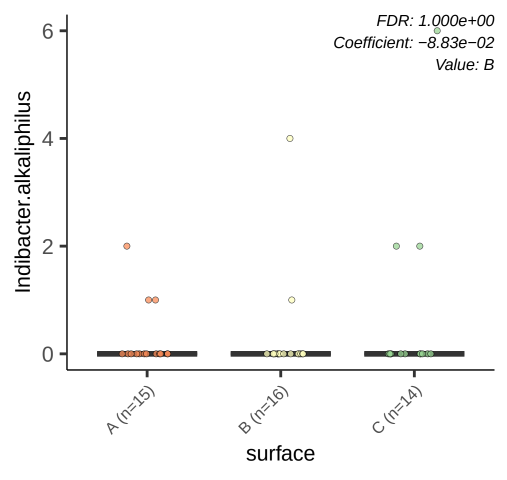
FDR: 1.000e+00
Coefficient: −8.83e−02
Value: B
Indibacter.alkaliphilus
0
2
4
6
A (n=15)
B (n=16)
C (n=14)
surface

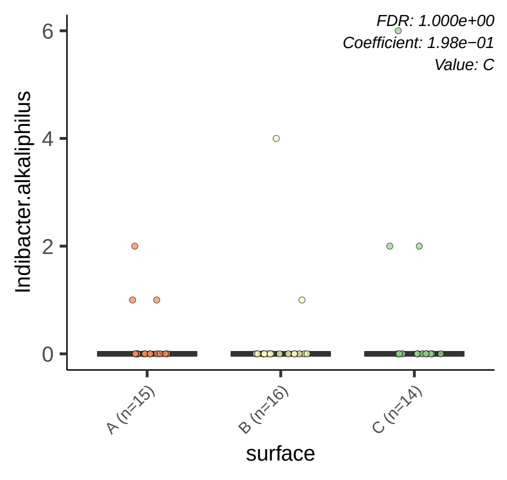
FDR: 1.000e+00
Coefficient: 1.98e−01
Value: C
Indibacter.alkaliphilus
0
2
4
6
A (n=15)
B (n=16)
C (n=14)
surface

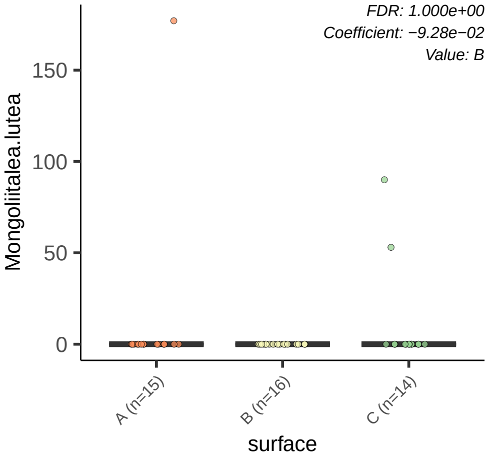
FDR: 1.000e+00
Coefficient: −9.28e−02
Value: B
Mongoliitalea.lutea
150
100
50
0
A (n=15)
B (n=16)
C (n=14)
surface

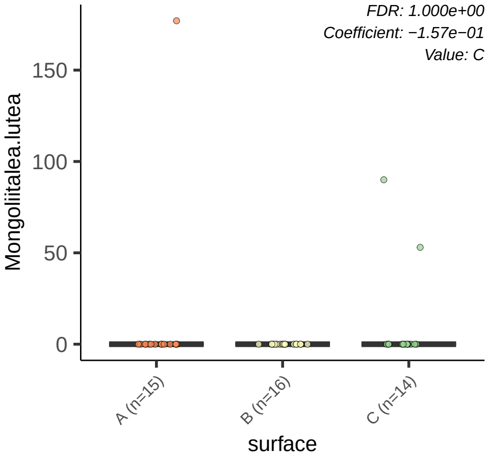
FDR: 1.000e+00
Coefficient: −1.57e−01
Value: C
Mongoliitalea.lutea
150
100
50
0
A (n=15)
B (n=16)
C (n=14)
surface

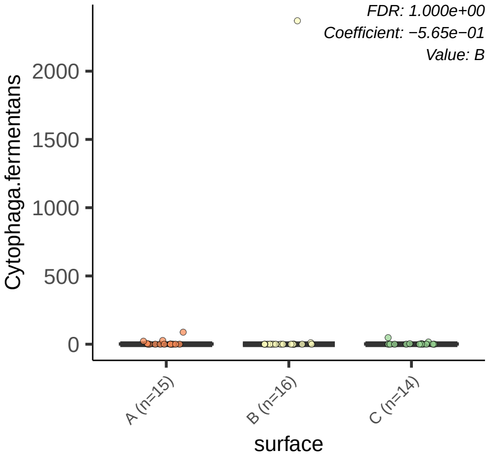

FDR: 1.000e+00
Coefficient: −5.65e−01
Value: B
Cytophaga.fermentans
2000
1500
1000
500
0
A (n=15)
B (n=16)
C (n=14)
surface

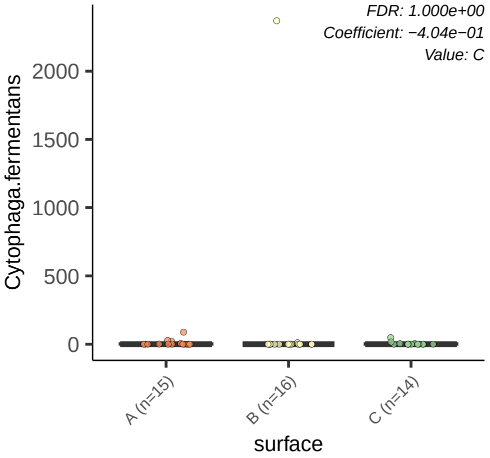
FDR: 1.000e+00
Coefficient: −4.04e−01
Value: C
Cytophaga.fermentans
2000
1500
1000
500
0
A (n=15)
B (n=16)
C (n=14)
surface

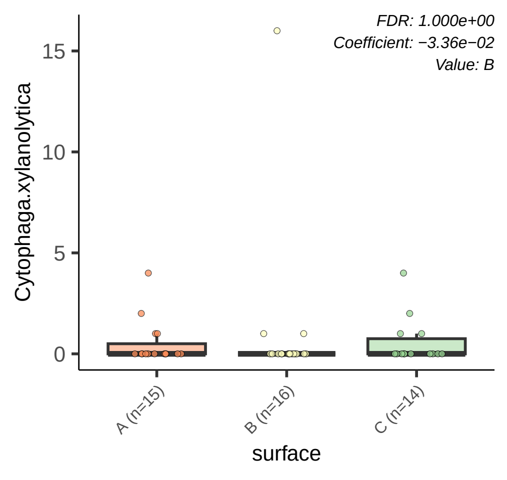

FDR: 1.000e+00
Coefficient: −3.36e−02
Value: B
Cytophaga.xylanolytica
15
10
5
0
A (n=15)
B (n=16)
C (n=14)
surface

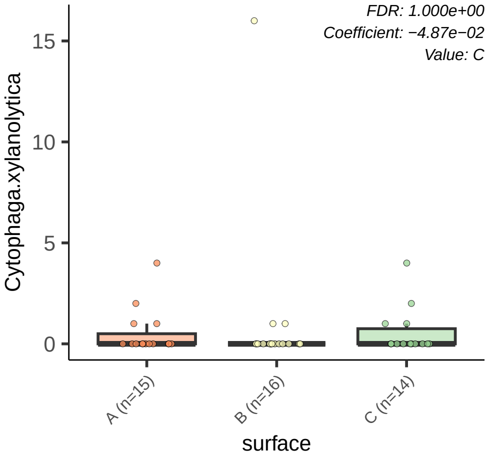

FDR: 1.000e+00
Coefficient: −4.87e−02
Value: C
Cytophaga.xylanolytica
15
10
5
0
A (n=15)
B (n=16)
C (n=14)
surface

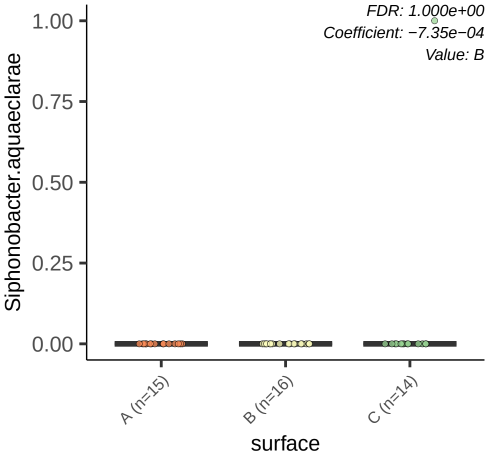
FDR: 1.000e+00
Coefficient: −7.35e−04
Value: B
Siphonobacter.aquaeclarae
1.00
0.75
0.50
0.25
0.00
A (n=15)
B (n=16)
C (n=14)
surface

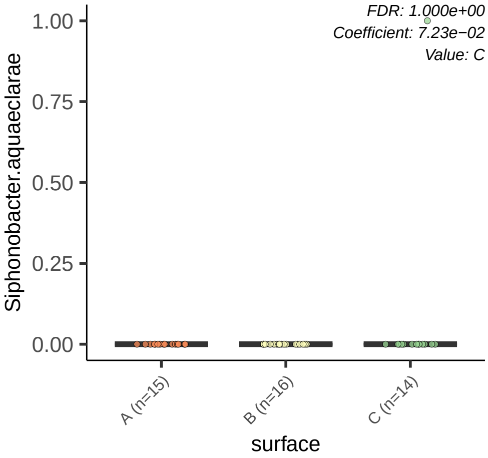
FDR: 1.000e+00
Coefficient: 7.23e−02
Value: C
Siphonobacter.aquaeclarae
1.00
0.75
0.50
0.25
0.00
A (n=15)
B (n=16)
C (n=14)
surface

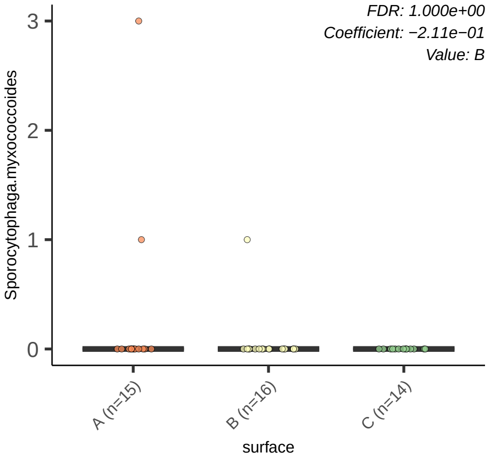

FDR: 1.000e+00
Coefficient: −2.11e−01
Value: B
Sporocytophaga.myxococcoides
3
2
1
0
A (n=15)
B (n=16)
C (n=14)
surface

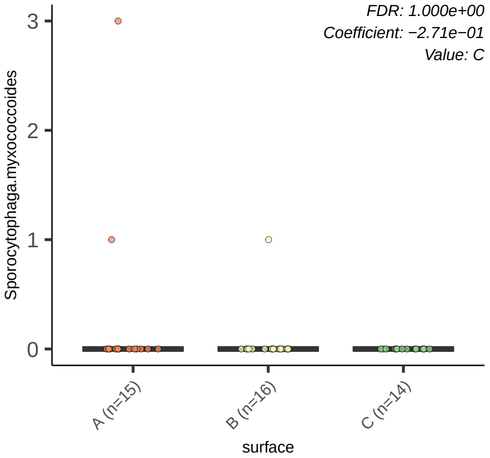
FDR: 1.000e+00
Coefficient: −2.71e−01
Value: C
Sporocytophaga.myxococcoides
0
1
2
3
A (n=15)
B (n=16)
C (n=14)
surface

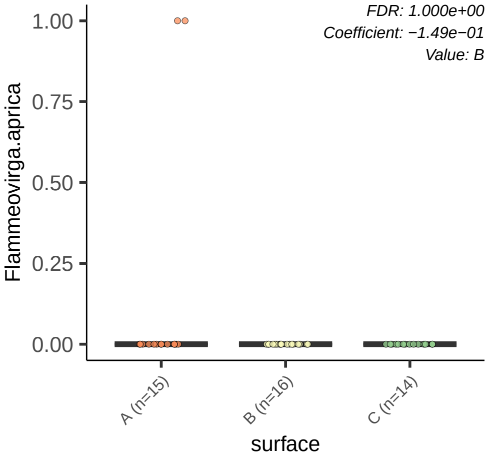
FDR: 1.000e+00
Coefficient: −1.49e−01
Value: B
Flammeovirga.aprica
1.00
0.75
0.50
0.25
0.00
A (n=15)
B (n=16)
C (n=14)
surface

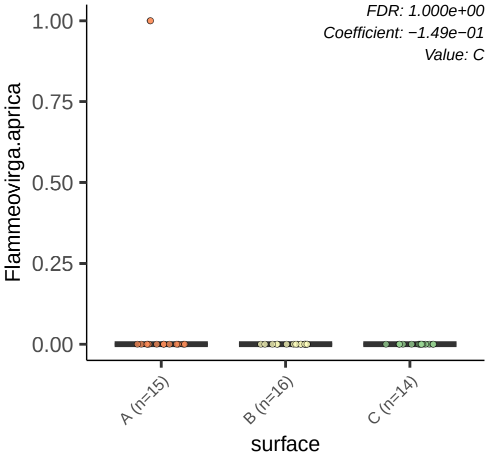
FDR: 1.000e+00
Coefficient: −1.49e−01
Value: C
Flammeovirga.aprica
1.00
0.75
0.50
0.25
0.00
A (n=15)
B (n=16)
C (n=14)
surface

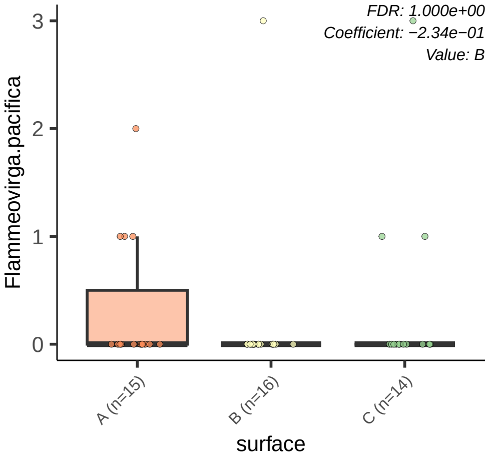

FDR: 1.000e+00
Coefficient: −2.34e−01
Value: B
Flammeovirga.pacifica
0
1
2
3
A (n=15)
B (n=16)
C (n=14)
surface

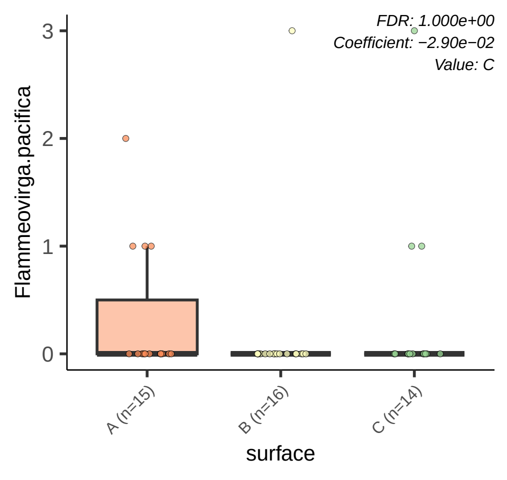

FDR: 1.000e+00
Coefficient: −2.90e−02
Value: C
Flammeovirga.pacifica
0
1
2
3
A (n=15)
B (n=16)
C (n=14)
surface

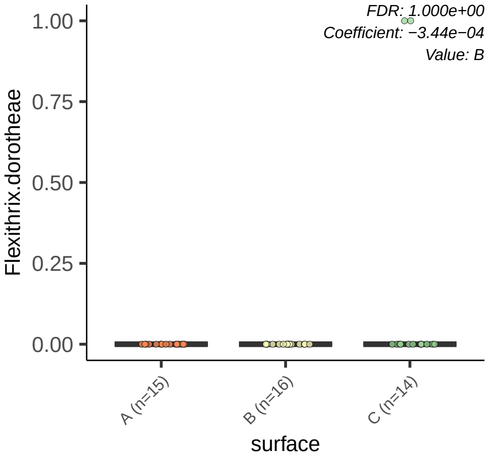
FDR: 1.000e+00
Coefficient: −3.44e−04
Value: B
Flexithrix.dorotheae
1.00
0.75
0.50
0.25
0.00
A (n=15)
B (n=16)
C (n=14)
surface

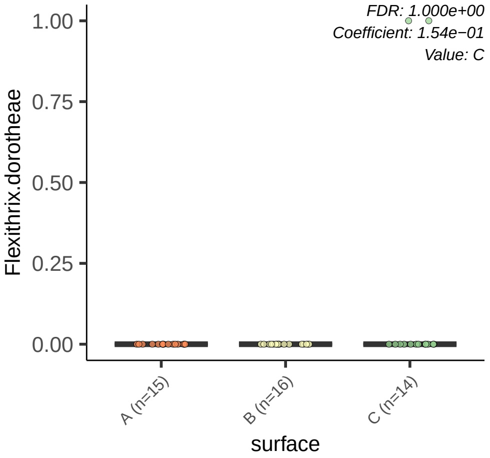

FDR: 1.000e+00
Coefficient: 1.54e−01
Value: C
Flexithrix.dorotheae
1.00
0.75
0.50
0.25
0.00
A (n=15)
B (n=16)
C (n=14)
surface

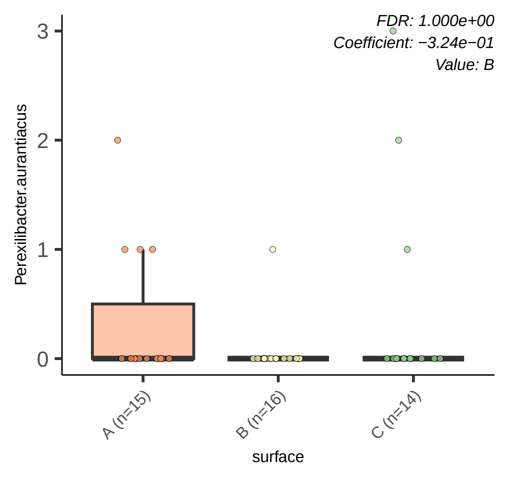

FDR: 1.000e+00
Coefficient: −3.24e−01
Value: B
Perexilibacter.aurantiacus
0
1
2
3
A (n=15)
B (n=16)
C (n=14)
surface

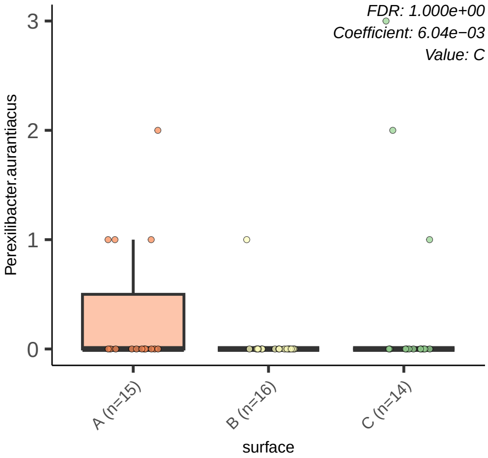
FDR: 1.000e+00
Coefficient: 6.04e−03
Value: C
Perexilibacter.aurantiacus
0
1
2
3
A (n=15)
B (n=16)
C (n=14)
surface

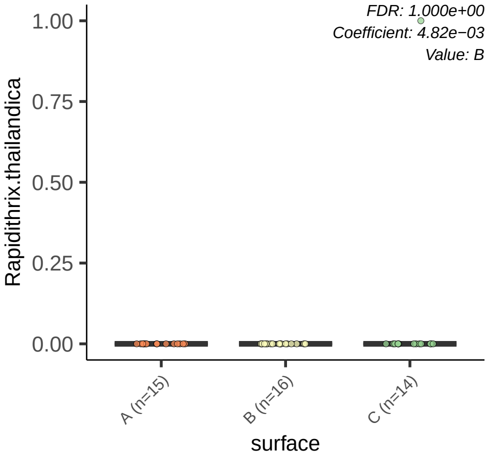
FDR: 1.000e+00
Coefficient: 4.82e−03
Value: B
Rapidithrix.thailandica
1.00
0.75
0.50
0.25
0.00
A (n=15)
B (n=16)
C (n=14)
surface

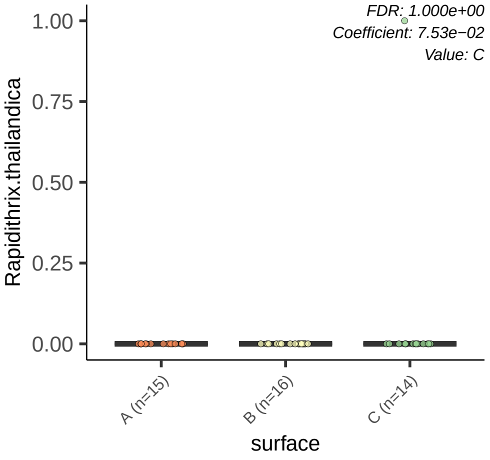
FDR: 1.000e+00
Coefficient: 7.53e−02
Value: C
1.00
0.75
0.50
0.25
0.00
Rapidithrix.thailandica
A (n=15)
B (n=16)
C (n=14)
surface

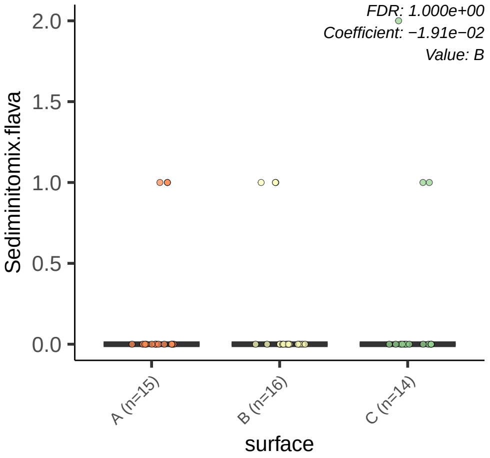

FDR: 1.000e+00
Coefficient: −1.91e−02
Value: B
Sediminitomix.flava
2.0
1.5
1.0
0.5
0.0
A (n=15)
B (n=16)
C (n=14)
surface

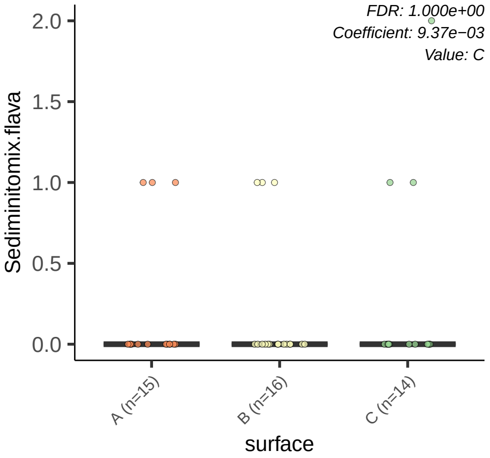
FDR: 1.000e+00
Coefficient: 9.37e−03
Value: C
Sedimintomix.flava
2.0
1.5
1.0
0.5
0.0
A (n=15)
B (n=16)
C (n=14)
surface

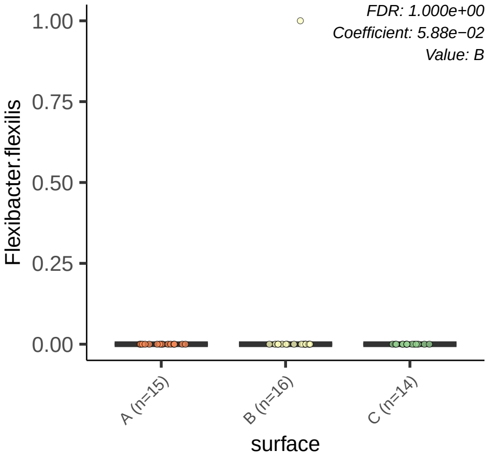
FDR: 1.000e+00
Coefficient: 5.88e−02
Value: B
Flexibacter.flexilis
1.00
0.75
0.50
0.25
0.00
A (n=15)
B (n=16)
C (n=14)
surface

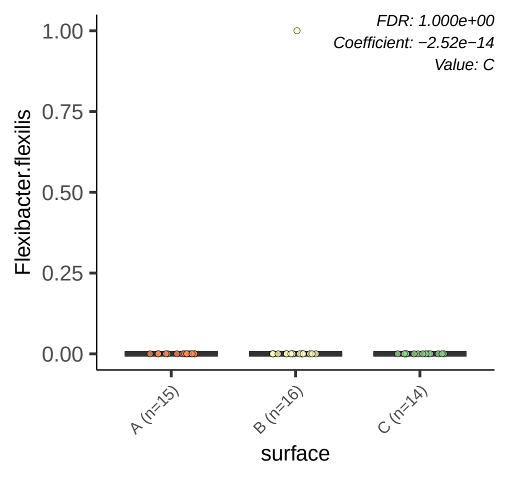

FDR: 1.000e+00
Coefficient: −2.52e−14
Value: C
Flexibacter.flexilis
1.00
0.75
0.50
0.25
0.00
A (n=15)
B (n=16)
C (n=14)
surface

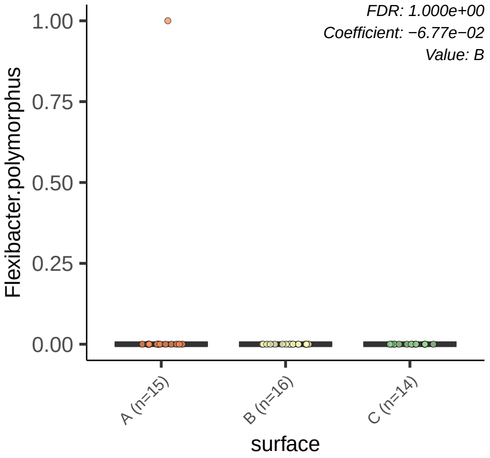

FDR: 1.000e+00
Coefficient: −6.77e−02
Value: B
Flexibacter.polymorphus
1.00
0.75
0.50
0.25
0.00
A (n=15)
B (n=16)
C (n=14)
surface

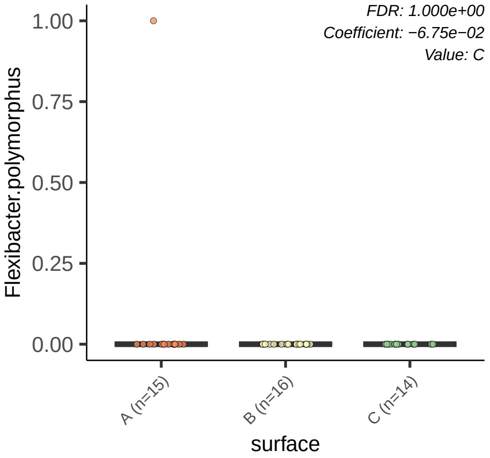
FDR: 1.000e+00
Coefficient: −6.75e−02
Value: C
Flexibacter.polymorphus
1.00
0.75
0.50
0.25
0.00
A (n=15)
B (n=16)
C (n=14)
surface

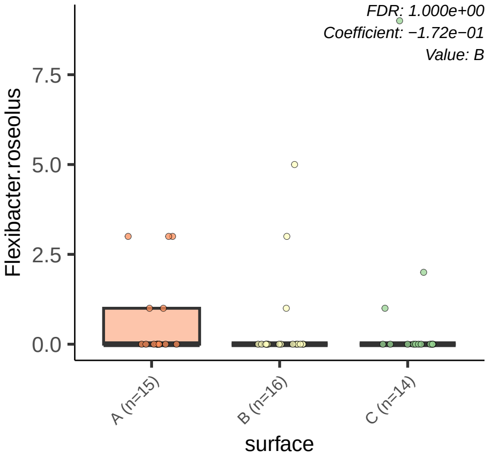
FDR: 1.000e+00
Coefficient: −1.72e−01
Value: B
Flexibacter.roseolus
7.5
5.0
2.5
0.0
A (n=15)
B (n=16)
C (n=14)
surface

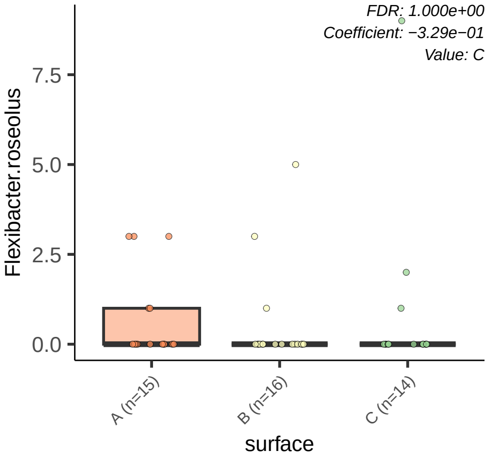
FDR: 1.000e+00
Coefficient: −3.29e−01
Value: C
Flexibacter.roseolus
7.5
5.0
2.5
0.0
A (n=15)
B (n=16)
C (n=14)
surface

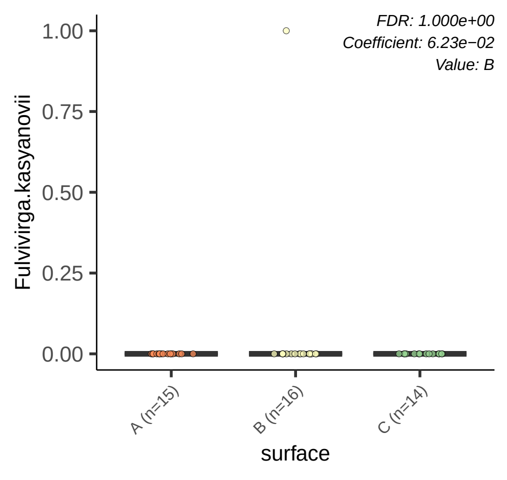

FDR: 1.000e+00
Coefficient: 6.23e−02
Value: B
Fulvivirga.kasyanovii
1.00
0.75
0.50
0.25
0.00
A (n=15)
B (n=16)
C (n=14)
surface

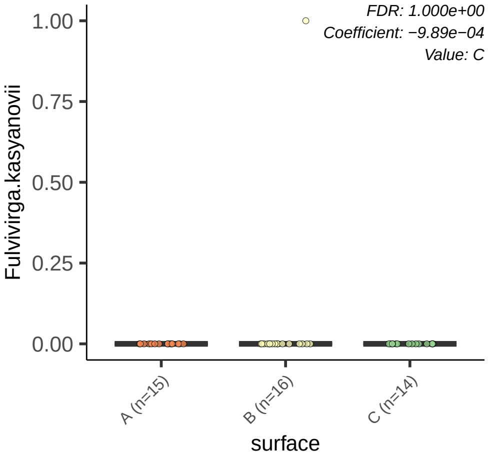

FDR: 1.000e+00
Coefficient: −9.89e−04
Value: C
Fulvivirga.kasyanovii
1.00
0.75
0.50
0.25
0.00
A (n=15)
B (n=16)
C (n=14)
surface

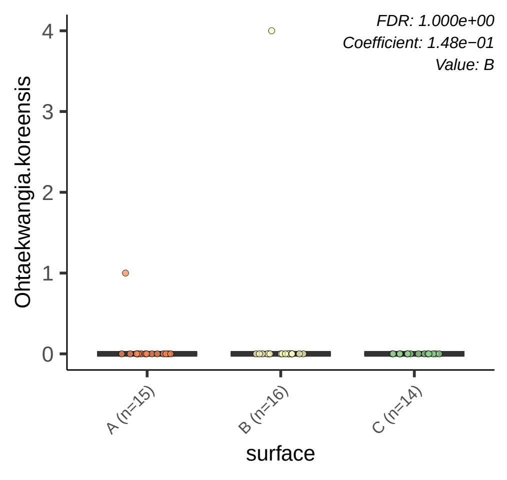
FDR: 1.000e+00
Coefficient: 1.48e−01
Value: B
Ohtaekwangia.koreensis
0
1
2
3
4
A (n=15)
B (n=16)
C (n=14)
surface

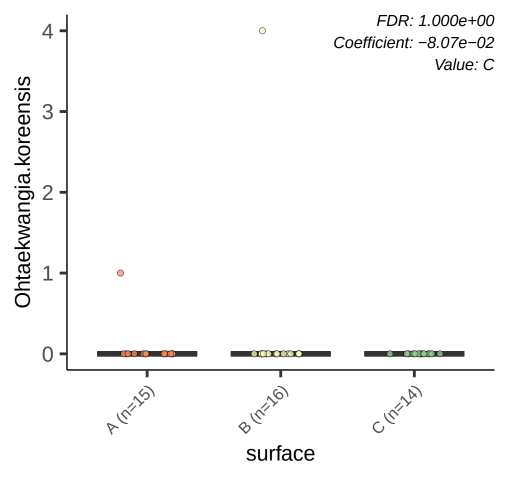

FDR: 1.000e+00
Coefficient: −8.07e−02
Value: C
Ohtaekwangia.koreensis
0
1
2
3
4
A (n=15)
B (n=16)
C (n=14)
surface

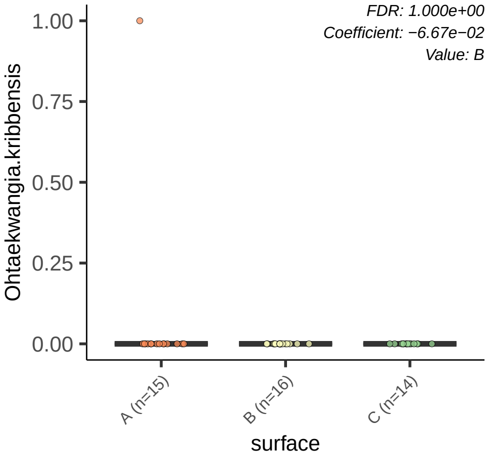
FDR: 1.000e+00
Coefficient: −6.67e−02
Value: B
Ohtaekwangia.kribbensis
1.00
0.75
0.50
0.25
0.00
A (n=15)
B (n=16)
C (n=14)
surface

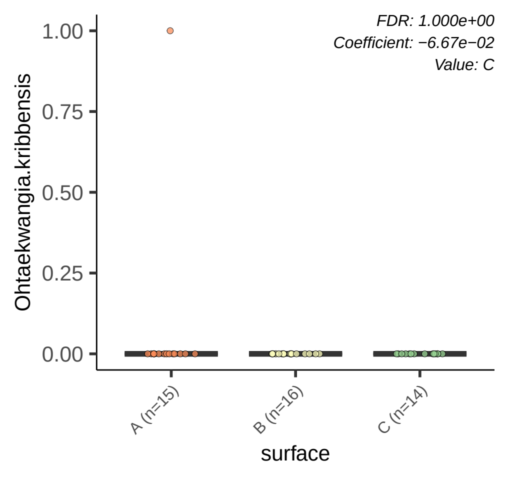
FDR: 1.000e+00
Coefficient: −6.67e−02
Value: C
1.00
0.75
0.50
0.25
0.00
Ohtaekwangia.kribbensis
A (n=15)
B (n=16)
C (n=14)
surface

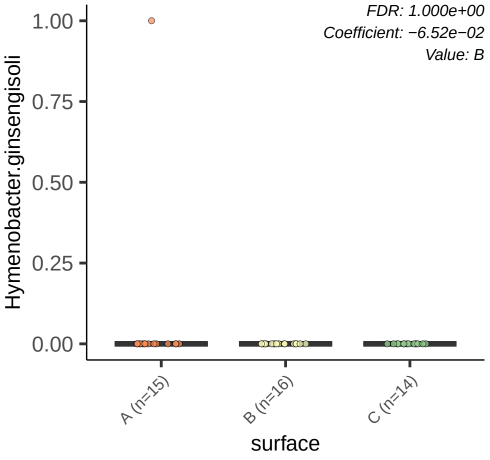
FDR: 1.000e+00
Coefficient: −6.52e−02
Value: B
Hymenobacter.ginsengisoli
1.00
0.75
0.50
0.25
0.00
A (n=15)
B (n=16)
C (n=14)
surface

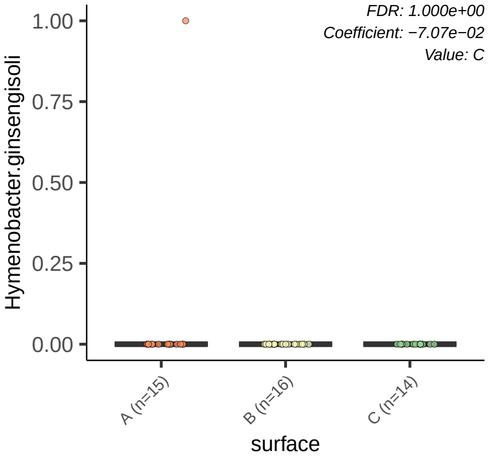
FDR: 1.000e+00
Coefficient: −7.07e−02
Value: C
Hymenobacter.ginsengisoli
1.00
0.75
0.50
0.25
0.00
A (n=15)
B (n=16)
C (n=14)
surface

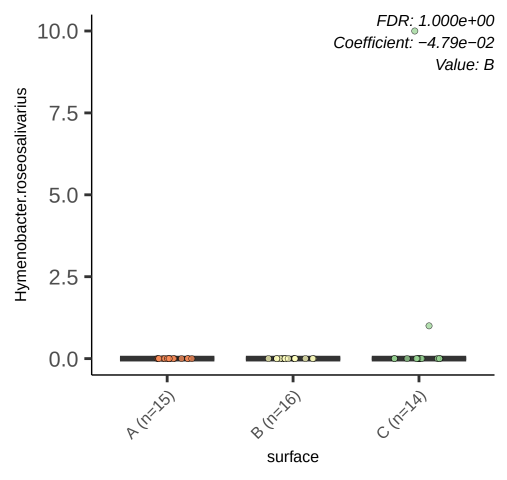
FDR: 1.000e+00
Coefficient: −4.79e−02
Value: B
Hymenobacter.roseosalivarius
10.0
7.5
5.0
2.5
0.0
A (n=15)
B (n=16)
C (n=14)
surface

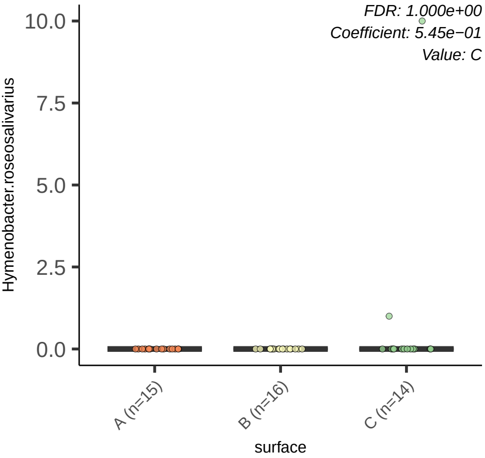

FDR: 1.000e+00
Coefficient: 5.45e−01
Value: C
10.0
7.5
5.0
2.5
0.0
Hymenobacter.roseosalivarius
A (n=15)
B (n=16)
C (n=14)
surface

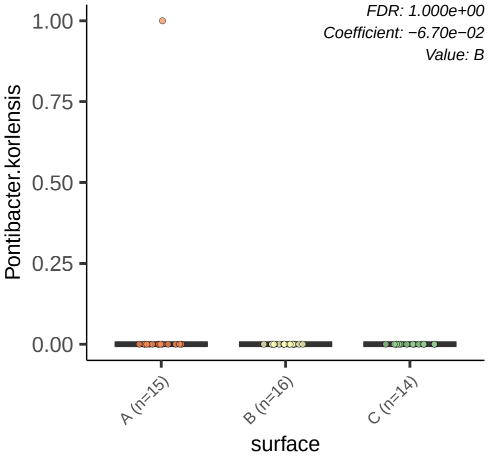

FDR: 1.000e+00
Coefficient: −6.70e−02
Value: B
Pontibacter.korlensis
1.00
0.75
0.50
0.25
0.00
A (n=15)
B (n=16)
C (n=14)
surface

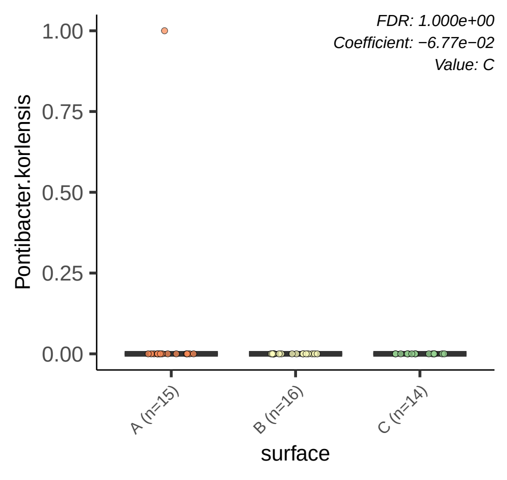

FDR: 1.000e+00
Coefficient: −6.77e−02
Value: C
Pontibacter.korlensis
1.00
0.75
0.50
0.25
0.00
A (n=15)
B (n=16)
C (n=14)
surface

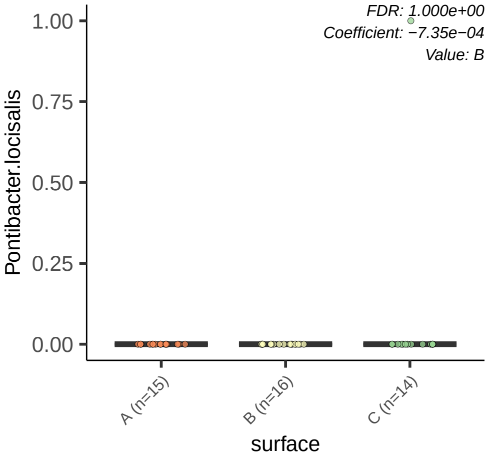
FDR: 1.000e+00
Coefficient: −7.35e−04
Value: B
Pontibacter.locisalis
1.00
0.75
0.50
0.25
0.00
A (n=15)
B (n=16)
C (n=14)
surface

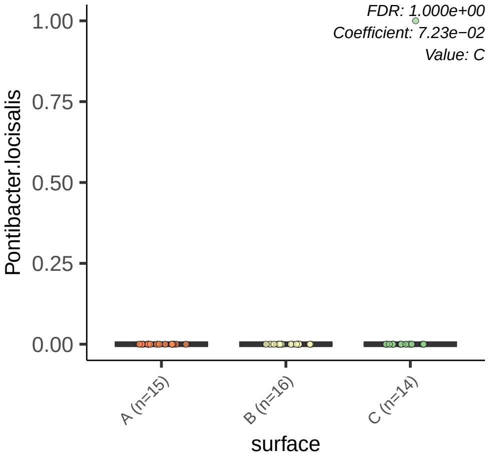
FDR: 1.000e+00
Coefficient: 7.23e−02
Value: C
Pontibacter.locisalis
1.00
0.75
0.50
0.25
0.00
A (n=15)
B (n=16)
C (n=14)
surface

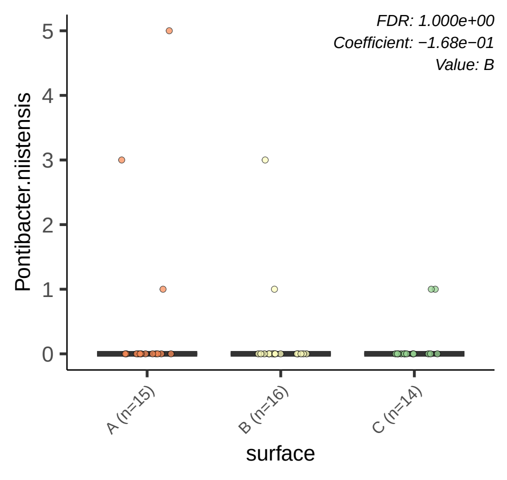
FDR: 1.000e+00
Coefficient: −1.68e−01
Value: B
Pontibacter.niistensis
0
1
2
3
4
5
A (n=15)
B (n=16)
C (n=14)
surface

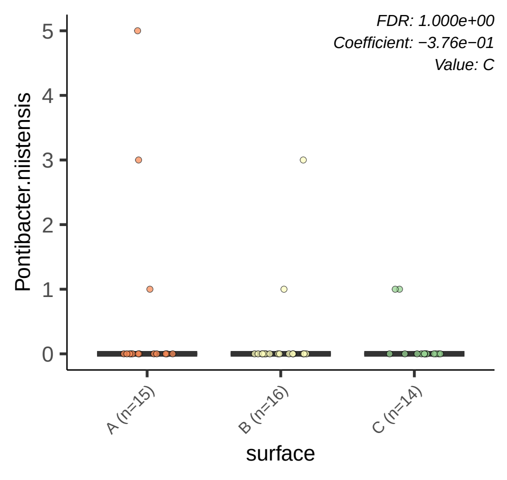

FDR: 1.000e+00
Coefficient: −3.76e−01
Value: C
Pontibacter.niistensis
0
1
2
3
4
5
A (n=15)
B (n=16)
C (n=14)
surface

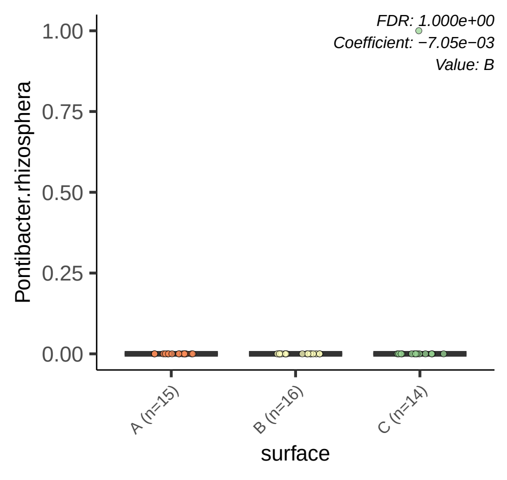
FDR: 1.000e+00
Coefficient: −7.05e−03
Value: B
Pontibacter.rhizosphera
1.00
0.75
0.50
0.25
0.00
A (n=15)
B (n=16)
C (n=14)
surface

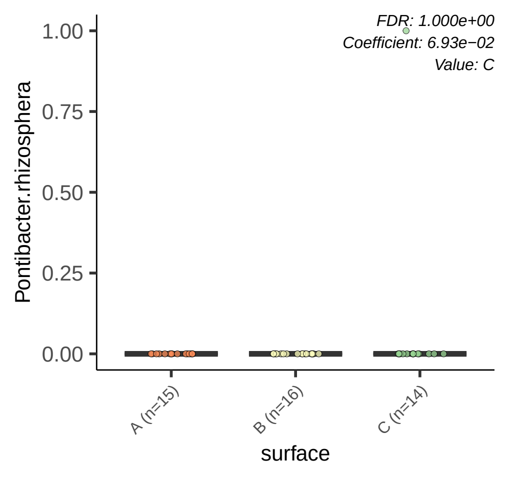
FDR: 1.000e+00
Coefficient: 6.93e−02
Value: C
Pontibacter.rhizosphera
1.00
0.75
0.50
0.25
0.00
A (n=15)
B (n=16)
C (n=14)
surface

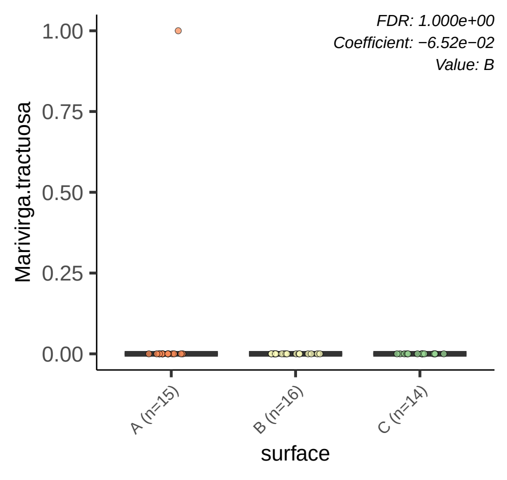
FDR: 1.000e+00
Coefficient: −6.52e−02
Value: B
Marivirga.tractuosa
1.00
0.75
0.50
0.25
0.00
A (n=15)
B (n=16)
C (n=14)
surface

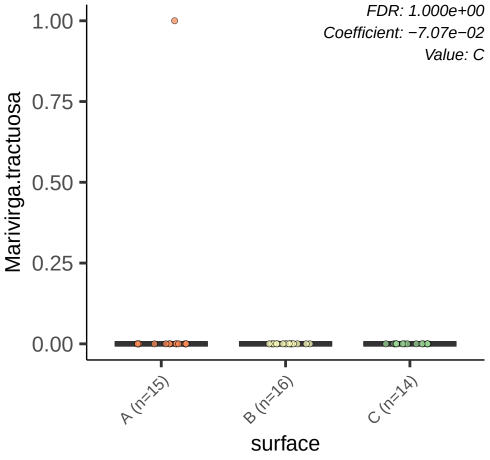
FDR: 1.000e+00
Coefficient: −7.07e−02
Value: C
Marivirga.tractuosa
1.00
0.75
0.50
0.25
0.00
A (n=15)
B (n=16)
C (n=14)
surface

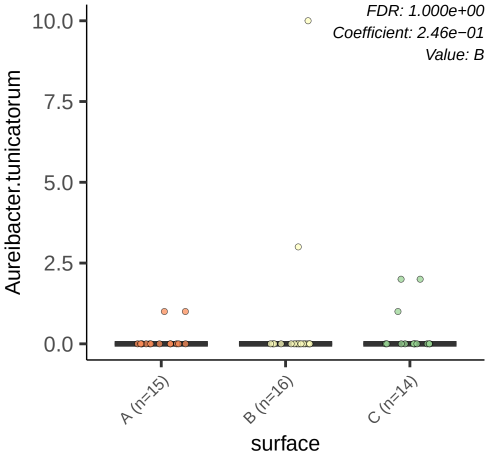

FDR: 1.000e+00
Coefficient: 2.46e−01
Value: B
10.0
7.5
5.0
2.5
0.0
Aureibacter.tunicatorum
A (n=15)
B (n=16)
C (n=14)
surface

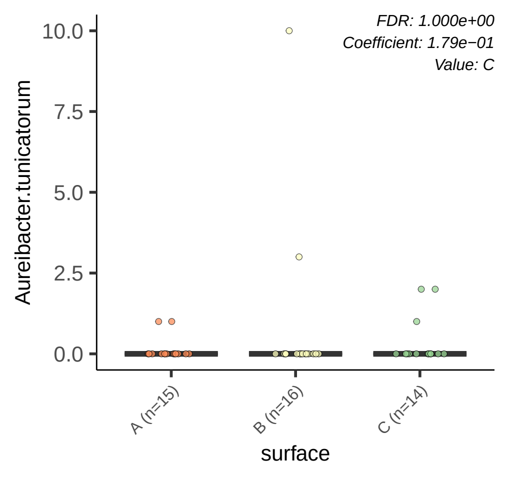
FDR: 1.000e+00
Coefficient: 1.79e−01
Value: C
Aureibacter.tunicatorum
10.0
7.5
5.0
2.5
0.0
A (n=15)
B (n=16)
C (n=14)
surface

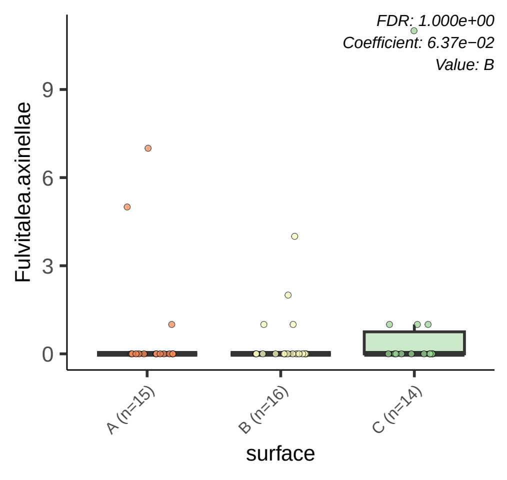
FDR: 1.000e+00
Coefficient: 6.37e−02
Value: B
Fulvitalea.axinellae
0
3
6
9
A (n=15)
B (n=16)
C (n=14)
surface

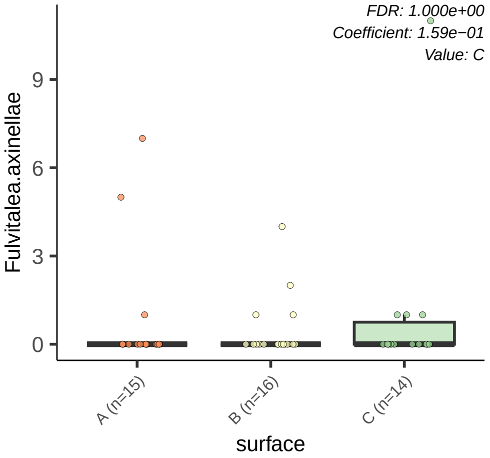
FDR: 1.000e+00
Coefficient: 1.59e−01
Value: C
Fulvitalea.axinellae
9
6
3
0
A (n=15)
B (n=16)
C (n=14)
surface

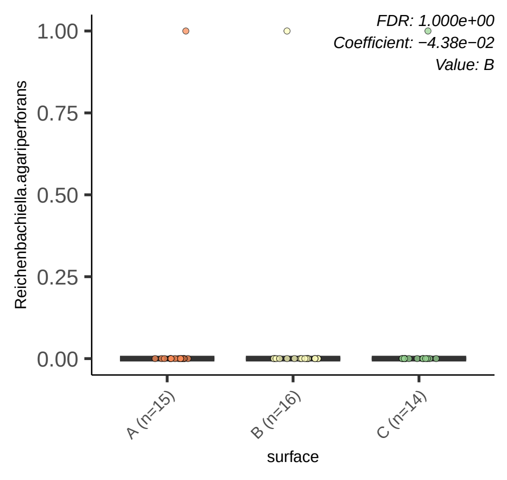

FDR: 1.000e+00
Coefficient: −4.38e−02
Value: B
1.00
0.75
0.50
0.25
0.00
Reichenbachiella.agariperforans
A (n=15)
B (n=16)
C (n=14)
surface

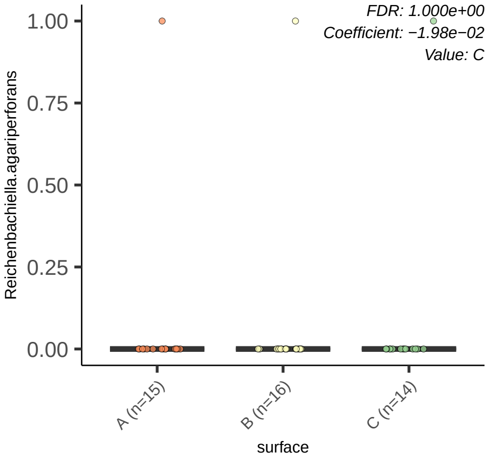

FDR: 1.000e+00
Coefficient: −1.98e−02
Value: C
Reichenbachiella.agariperforans
1.00
0.75
0.50
0.25
0.00
A (n=15)
B (n=16)
C (n=14)
surface

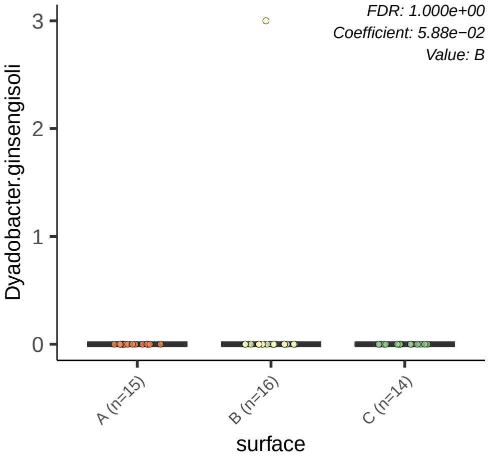
FDR: 1.000e+00
Coefficient: 5.88e−02
Value: B
Dyadobacter.ginsengisoli
3
2
1
0
A (n=15)
B (n=16)
C (n=14)
surface

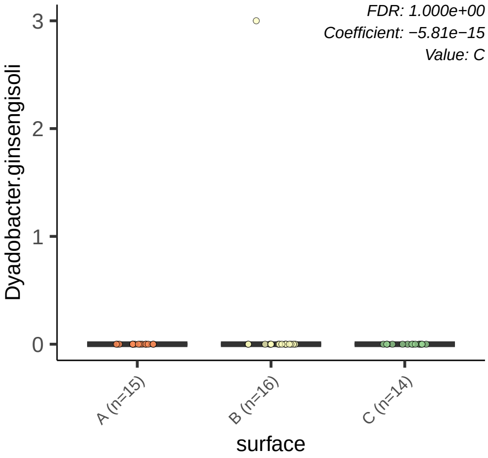

FDR: 1.000e+00
Coefficient: −5.81e−15
Value: C
Dyadobacter.ginsengisoli
0
1
2
3
A (n=15)
B (n=16)
C (n=14)
surface

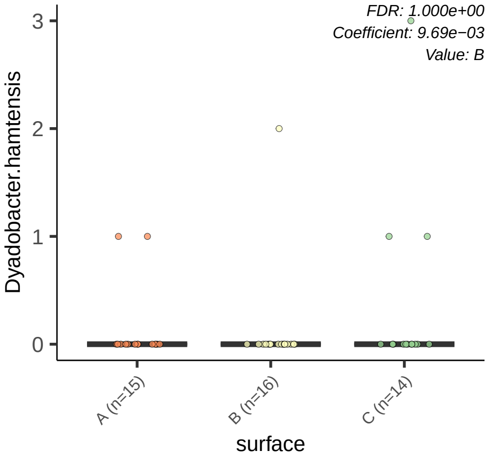

FDR: 1.000e+00
Coefficient: 9.69e−03
Value: B
Dyadobacter.hamtensis
0
1
2
3
A (n=15)
B (n=16)
C (n=14)
surface

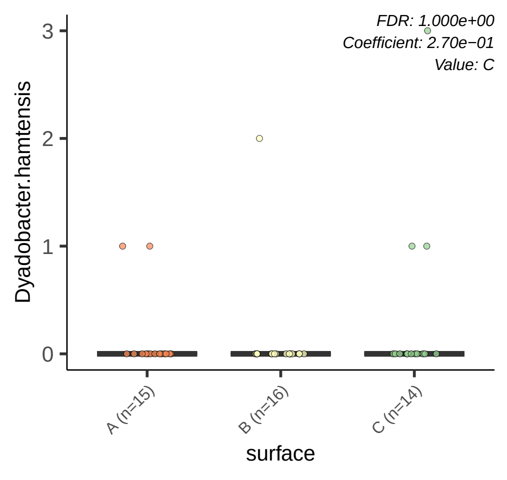

FDR: 1.000e+00
Coefficient: 2.70e−01
Value: C
Dyadobacter.hamtensis
3
2
1
0
A (n=15)
B (n=16)
C (n=14)
surface

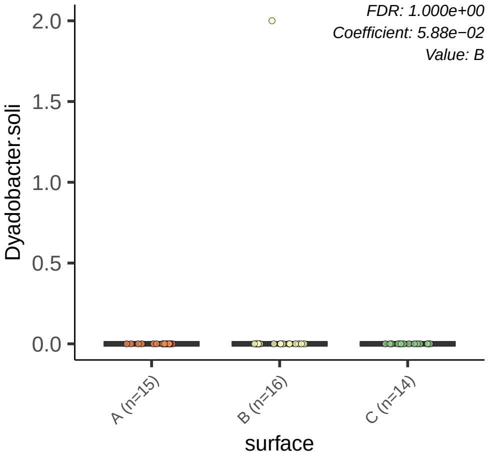
FDR: 1.000e+00
Coefficient: 5.88e−02
Value: B
Dyadobacter.soli
2.0
1.5
1.0
0.5
0.0
A (n=15)
B (n=16)
C (n=14)
surface

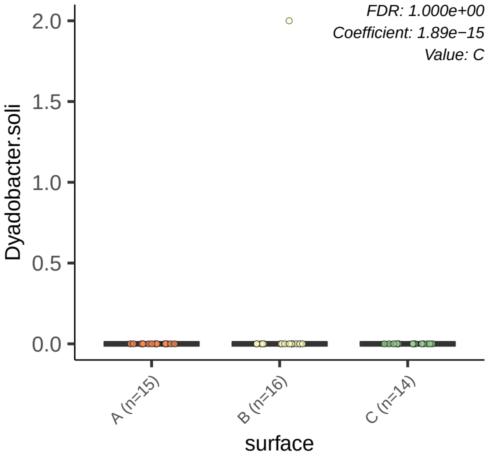
FDR: 1.000e+00
Coefficient: 1.89e−15
Value: C
Dyadobacter.soli
2.0
1.5
1.0
0.5
0.0
A (n=15)
B (n=16)
C (n=14)
surface

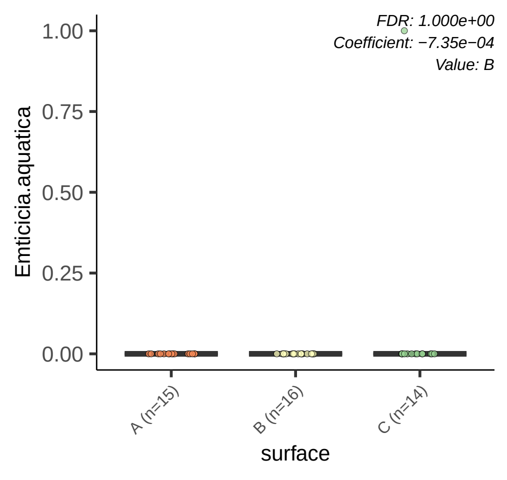
FDR: 1.000e+00
Coefficient: −7.35e−04
Value: B
Emticicia.aquatica
1.00
0.75
0.50
0.25
0.00
A (n=15)
B (n=16)
C (n=14)
surface

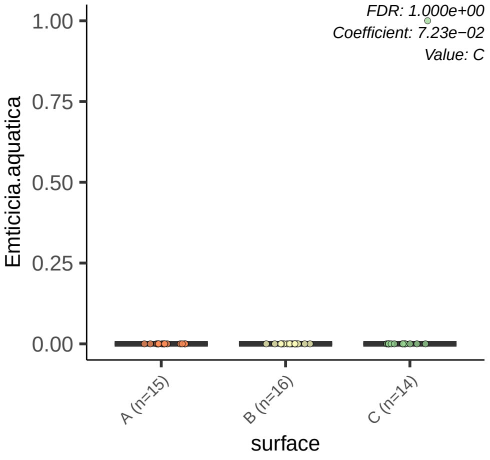
FDR: 1.000e+00
Coefficient: 7.23e−02
Value: C
Emticicia.aquatica
1.00
0.75
0.50
0.25
0.00
A (n=15)
B (n=16)
C (n=14)
surface

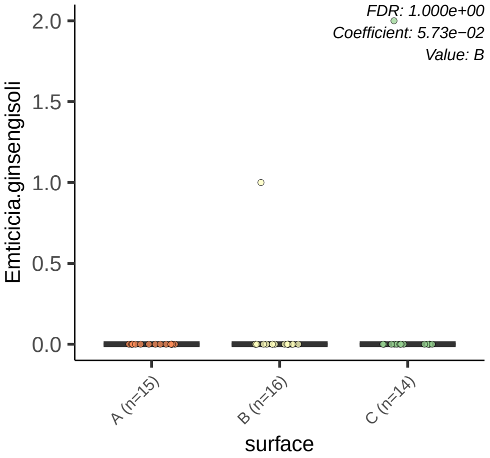
FDR: 1.000e+00
Coefficient: 5.73e−02
Value: B
Emticicia.ginsengisoli
2.0
1.5
1.0
0.5
0.0
A (n=15)
B (n=16)
C (n=14)
surface

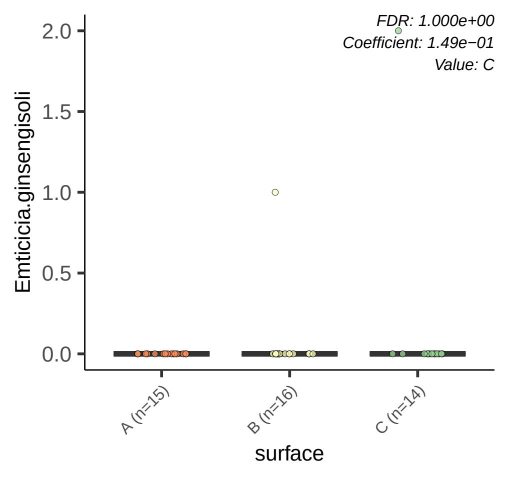

FDR: 1.000e+00
Coefficient: 1.49e−01
Value: C
Emticicia.ginsengisoli
2.0
1.5
1.0
0.5
0.0
A (n=15)
B (n=16)
C (n=14)
surface

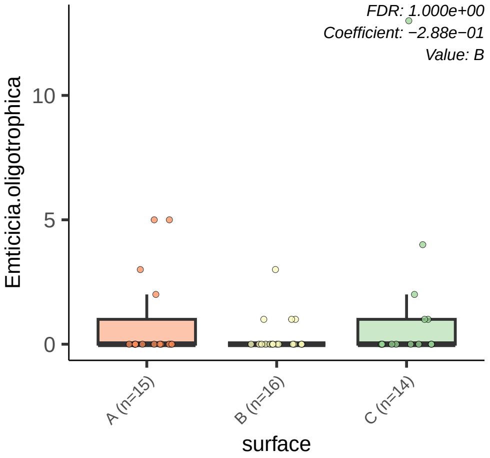
FDR: 1.000e+00
Coefficient: −2.88e−01
Value: B
Emticicia.oligotrophica
10
5
0
A (n=15)
B (n=16)
C (n=14)
surface

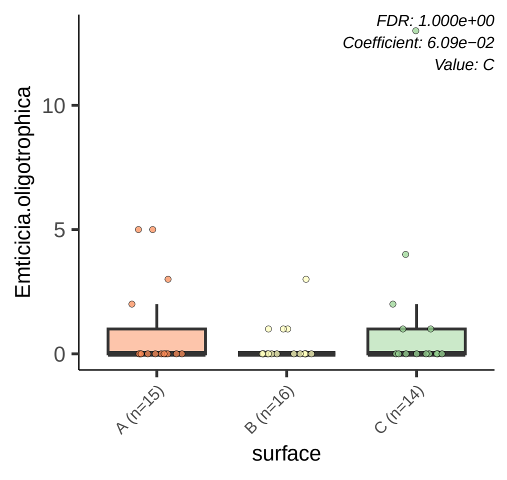

FDR: 1.000e+00
Coefficient: 6.09e−02
Value: C
Emticicia.oligotrophica
0
5
10
A (n=15)
B (n=16)
C (n=14)
surface

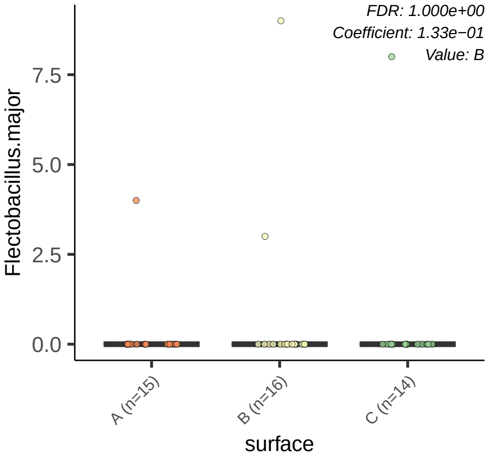

FDR: 1.000e+00
Coefficient: 1.33e−01
Value: B
Flectobacillus.major
7.5
5.0
2.5
0.0
A (n=15)
B (n=16)
C (n=14)
surface

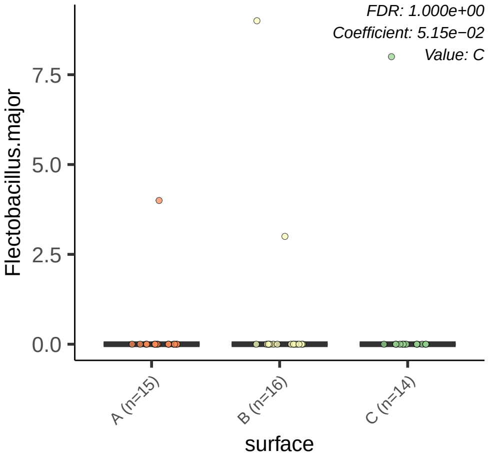

FDR: 1.000e+00
Coefficient: 5.15e−02
Value: C
Flectobacillus.major
7.5
5.0
2.5
0.0
A (n=15)
B (n=16)
C (n=14)
surface

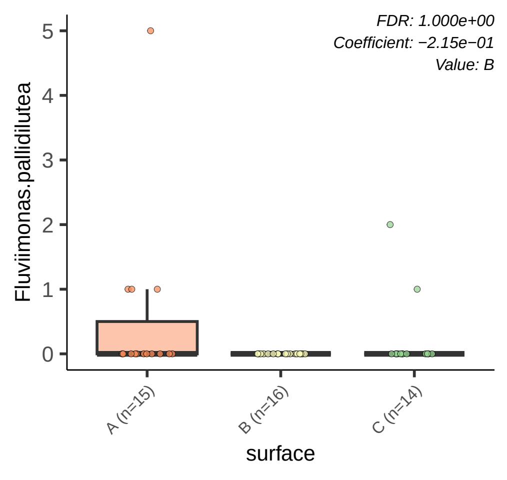
FDR: 1.000e+00
Coefficient: −2.15e−01
Value: B
Fluviimonas.pallidilutea
0
1
2
3
4
5
A (n=15)
B (n=16)
C (n=14)
surface

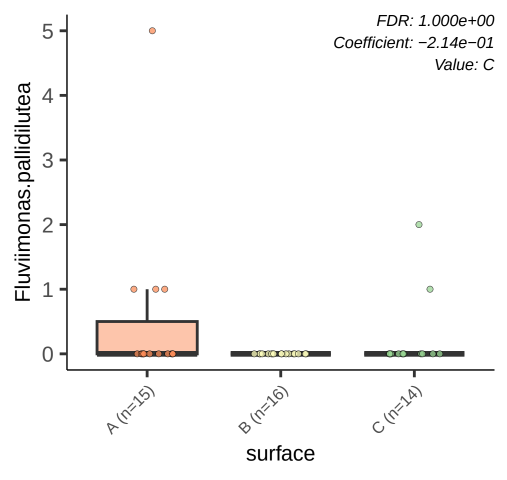
FDR: 1.000e+00
Coefficient: −2.14e−01
Value: C
Fluviimonas.pallidilutea
0
1
2
3
4
5
A (n=15)
B (n=16)
C (n=14)
surface

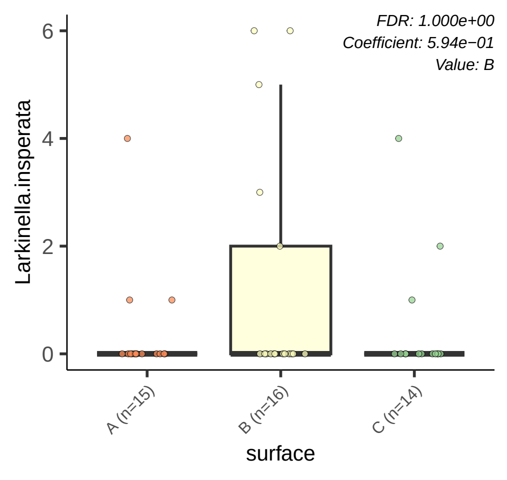

FDR: 1.000e+00
Coefficient: 5.94e−01
Value: B
Larkinella.insperata
0
2
4
6
A (n=15)
B (n=16)
C (n=14)
surface

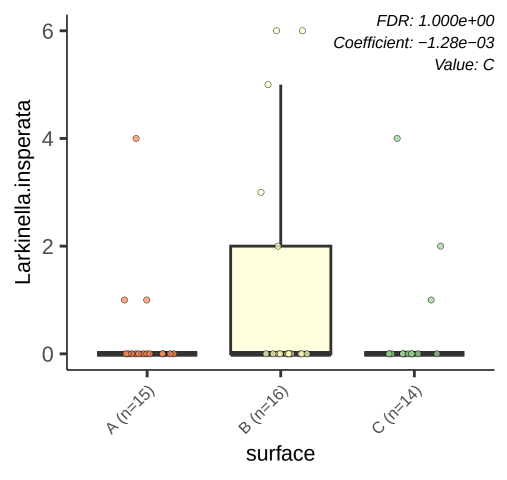

FDR: 1.000e+00
Coefficient: −1.28e−03
Value: C
Larkinella.insperata
0
2
4
6
A (n=15)
B (n=16)
C (n=14)
surface

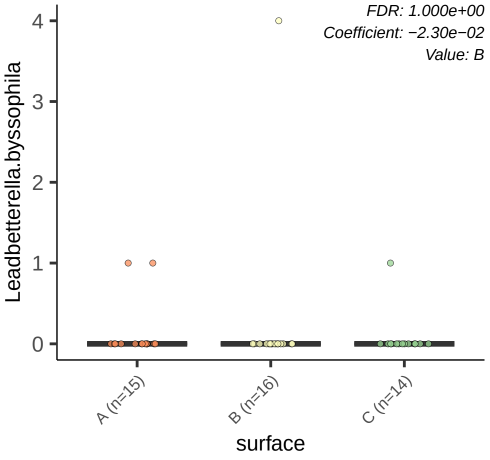

FDR: 1.000e+00
Coefficient: −2.30e−02
Value: B
Leadbetterella.byssophila
0
1
2
3
4
A (n=15)
B (n=16)
C (n=14)
surface

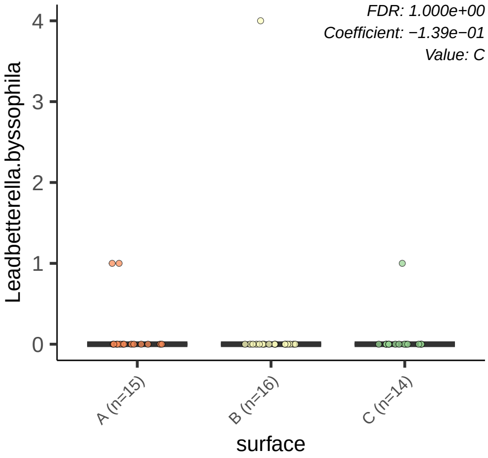
FDR: 1.000e+00
Coefficient: −1.39e−01
Value: C
Leadbetterella.byssophila
0
1
2
3
4
A (n=15)
B (n=16)
C (n=14)
surface

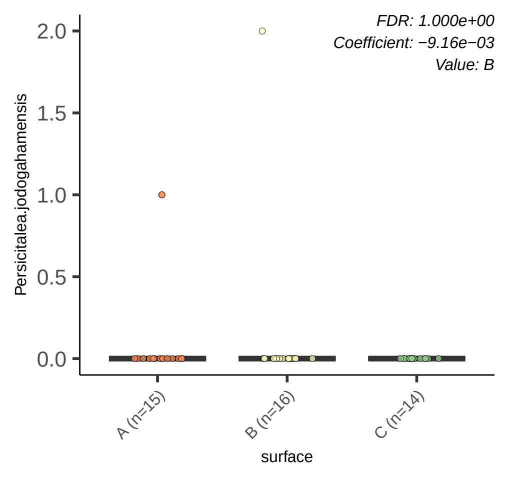

FDR: 1.000e+00
Coefficient: −9.16e−03
Value: B
Persicitalea.jodogahamensis
2.0
1.5
1.0
0.5
0.0
A (n=15)
B (n=16)
C (n=14)
surface

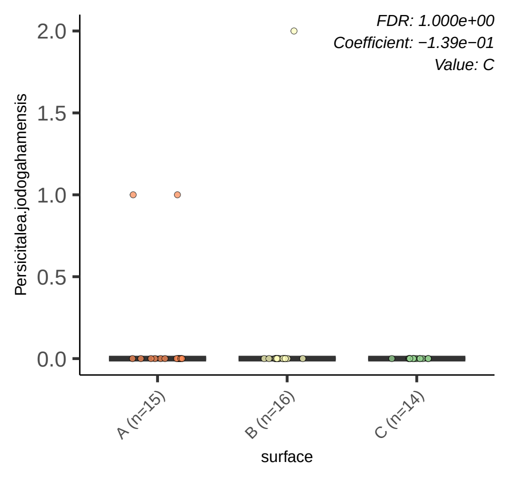
FDR: 1.000e+00
Coefficient: −1.39e−01
Value: C
Persicitalea.jodogahamensis
2.0
1.5
1.0
0.5
0.0
A (n=15)
B (n=16)
C (n=14)
surface

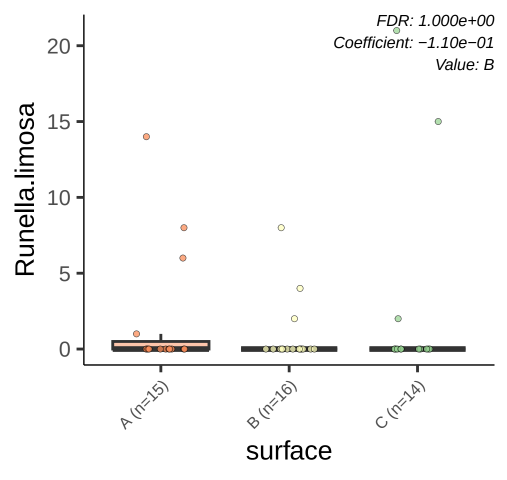
FDR: 1.000e+00
Coefficient: −1.10e−01
Value: B
Runella.limosa
20
15
10
5
0
A (n=15)
B (n=16)
C (n=14)
surface

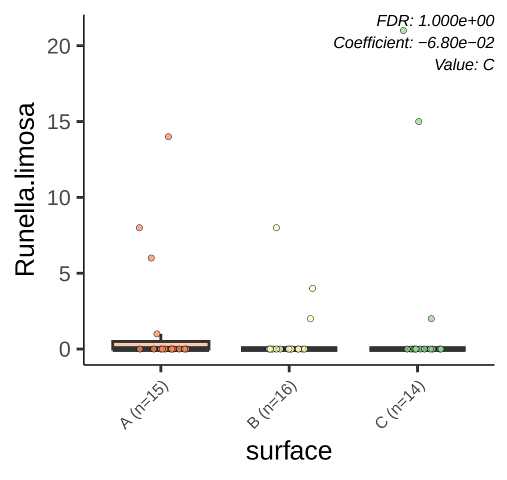

FDR: 1.000e+00
Coefficient: −6.80e−02
Value: C
Runella.limosa
0
5
10
15
20
A (n=15)
B (n=16)
C (n=14)
surface

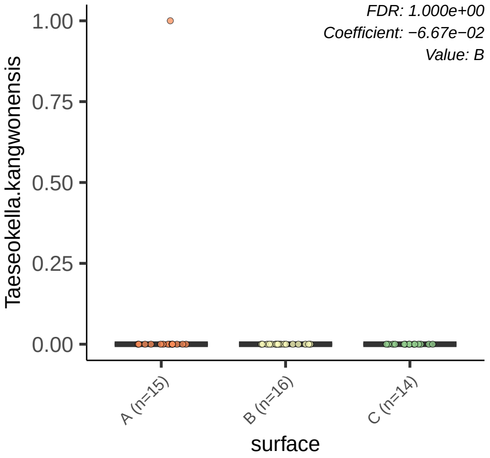
FDR: 1.000e+00
Coefficient: −6.67e−02
Value: B
Taeseokella.kangwonensis
1.00
0.75
0.50
0.25
0.00
A (n=15)
B (n=16)
C (n=14)
surface

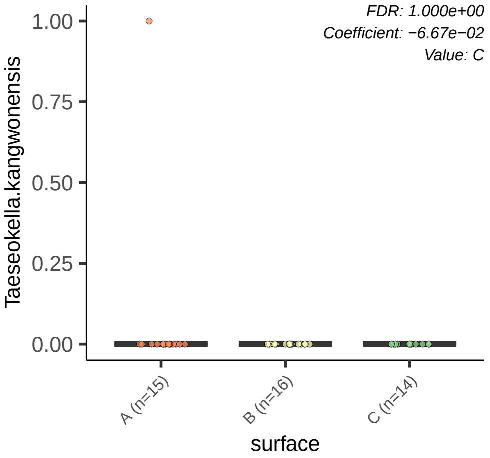
FDR: 1.000e+00
Coefficient: −6.67e−02
Value: C
Taeseokella.kangwonensis
1.00
0.75
0.50
0.25
0.00
A (n=15)
B (n=16)
C (n=14)
surface

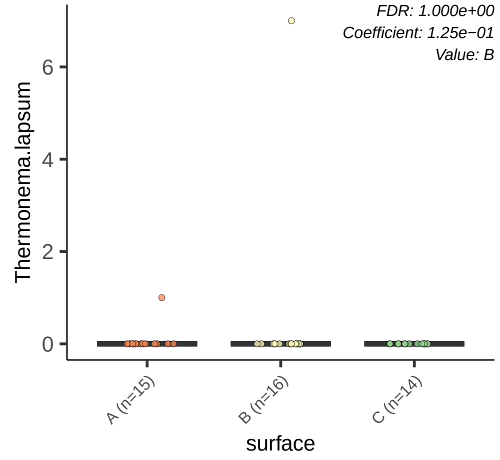

FDR: 1.000e+00
Coefficient: 1.25e−01
Value: B
Thermonema.lapsum
0
2
4
6
A (n=15)
B (n=16)
C (n=14)
surface

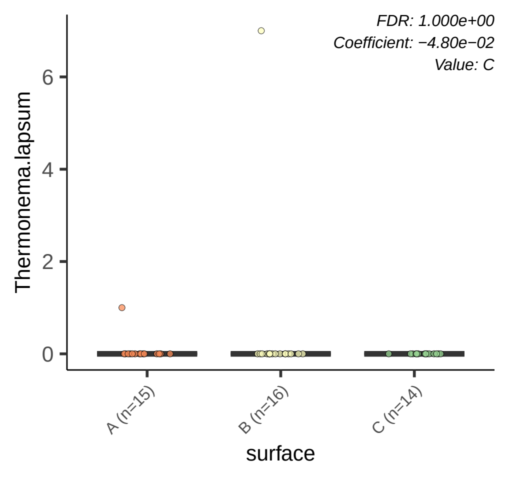
FDR: 1.000e+00
Coefficient: −4.80e−02
Value: C
Thermonema.lapsum
0
2
4
6
A (n=15)
B (n=16)
C (n=14)
surface

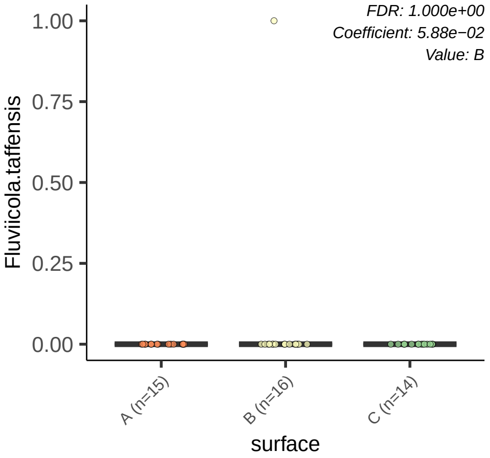
FDR: 1.000e+00
Coefficient: 5.88e−02
Value: B
Fluviicola.taffensis
1.00
0.75
0.50
0.25
0.00
A (n=15)
B (n=16)
C (n=14)
surface

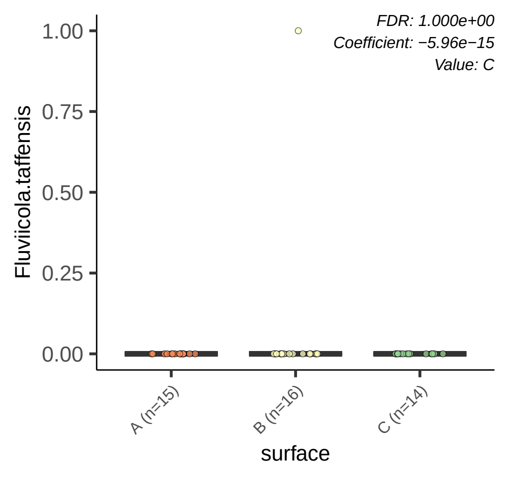
FDR: 1.000e+00
Coefficient: −5.96e−15
Value: C
Fluviicola.taffensis
1.00
0.75
0.50
0.25
0.00
A (n=15)
B (n=16)
C (n=14)
surface

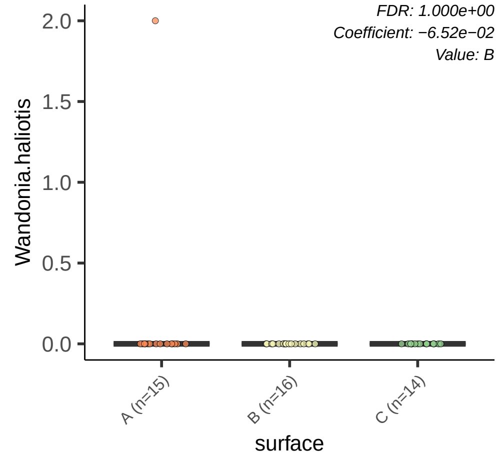
FDR: 1.000e+00
Coefficient: −6.52e−02
Value: B
Wandonia.haliotis
2.0
1.5
1.0
0.5
0.0
A (n=15)
B (n=16)
C (n=14)
surface

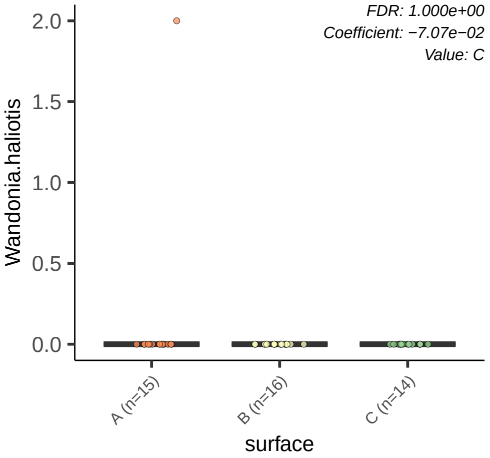
FDR: 1.000e+00
Coefficient: −7.07e−02
Value: C
Wandonia.haliotis
2.0
1.5
1.0
0.5
0.0
A (n=15)
B (n=16)
C (n=14)
surface

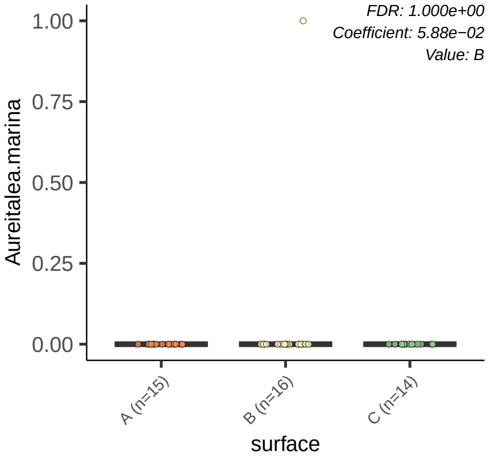
FDR: 1.000e+00
Coefficient: 5.88e−02
Value: B
Aureitalea.marina
1.00
0.75
0.50
0.25
0.00
A (n=15)
B (n=16)
C (n=14)
surface

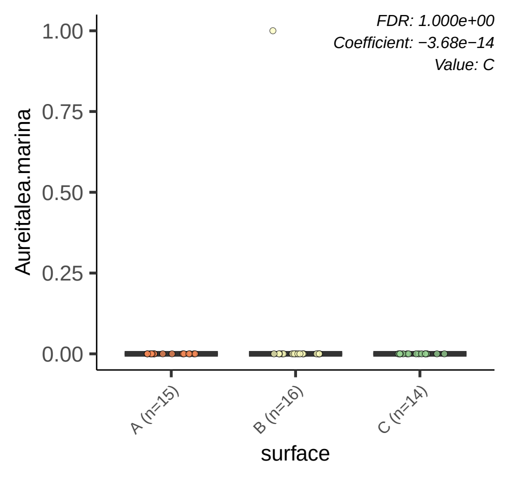
FDR: 1.000e+00
Coefficient: −3.68e−14
Value: C
1.00
0.75
0.50
0.25
0.00
Aureitalea.marina
A (n=15)
B (n=16)
C (n=14)
surface

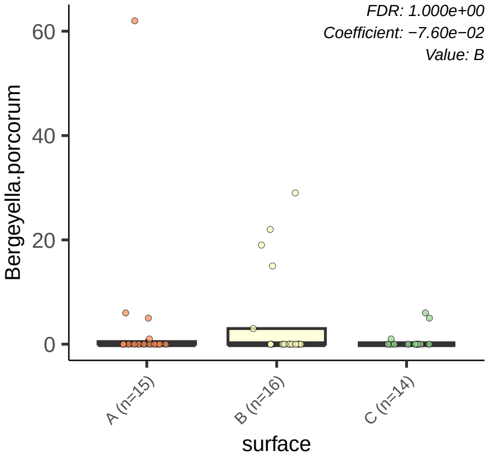

FDR: 1.000e+00
Coefficient: −7.60e−02
Value: B
Bergeyella.porcorum
60
40
20
0
A (n=15)
B (n=16)
C (n=14)
surface

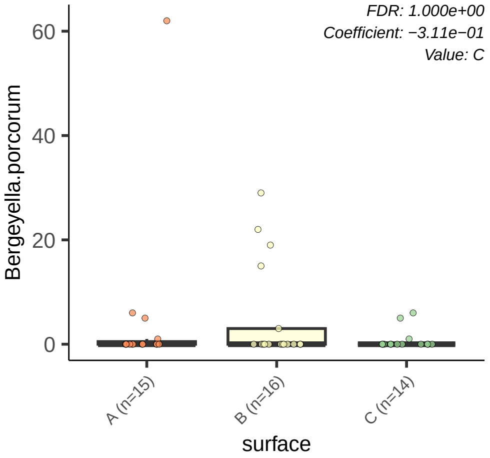
FDR: 1.000e+00
Coefficient: −3.11e−01
Value: C
Bergeyella.porcorum
60
40
20
0
A (n=15)
B (n=16)
C (n=14)
surface

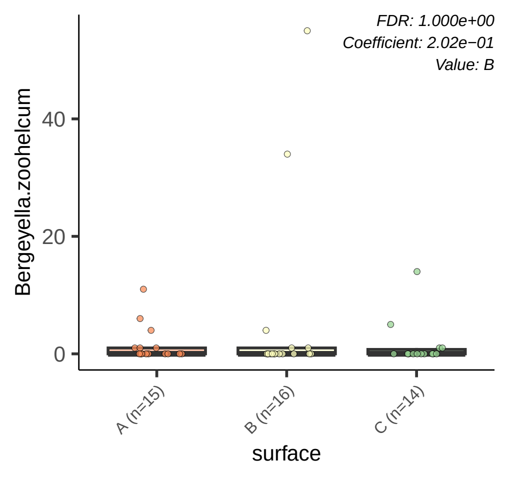
FDR: 1.000e+00
Coefficient: 2.02e−01
Value: B
Bergeyella.zoohelcum
40
20
0
A (n=15)
B (n=16)
C (n=14)
surface

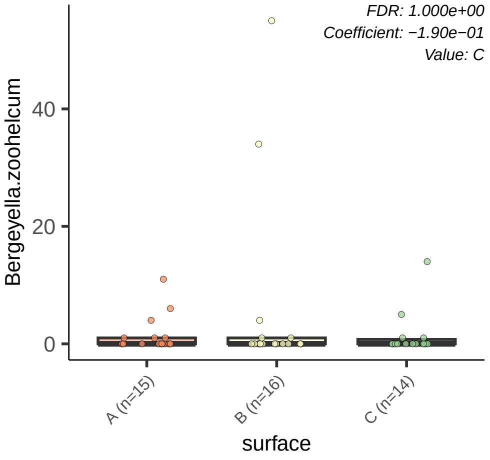

FDR: 1.000e+00
Coefficient: −1.90e−01
Value: C
Bergeyella.zoohelcum
40
20
0
A (n=15)
B (n=16)
C (n=14)
surface

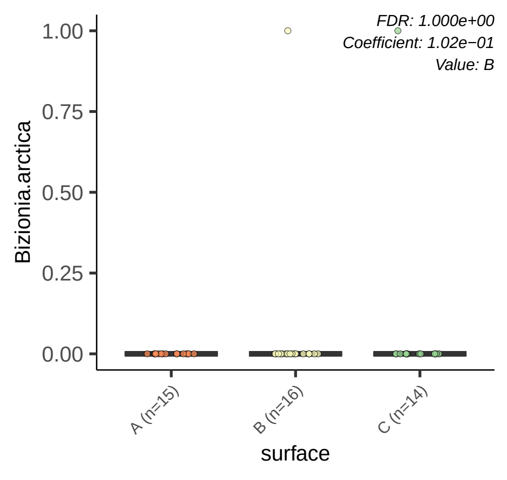

FDR: 1.000e+00
Coefficient: 1.02e−01
Value: B
Bizionia.arctica
1.00
0.75
0.50
0.25
0.00
A (n=15)
B (n=16)
C (n=14)
surface

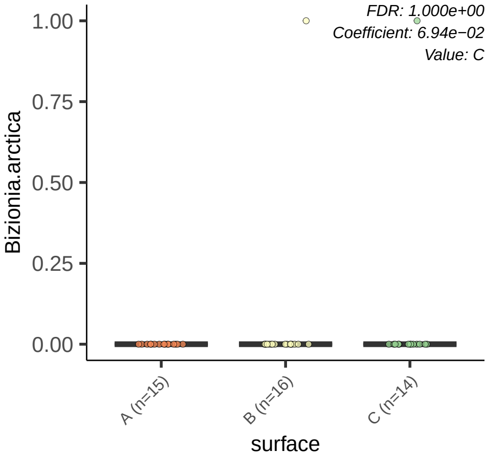
FDR: 1.000e+00
Coefficient: 6.94e−02
Value: C
Bizionia.arctica
1.00
0.75
0.50
0.25
0.00
A (n=15)
B (n=16)
C (n=14)
surface

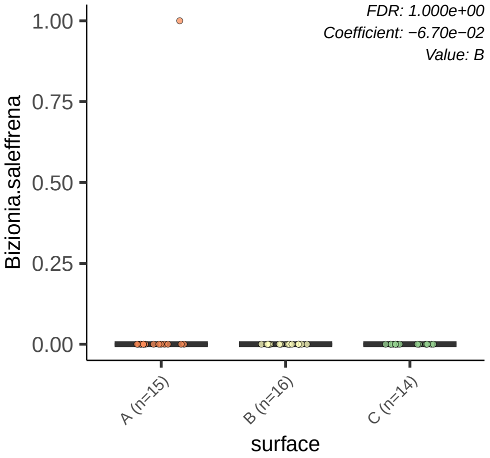

FDR: 1.000e+00
Coefficient: −6.70e−02
Value: B
Bizionia.saleffrena
1.00
0.75
0.50
0.25
0.00
A (n=15)
B (n=16)
C (n=14)
surface

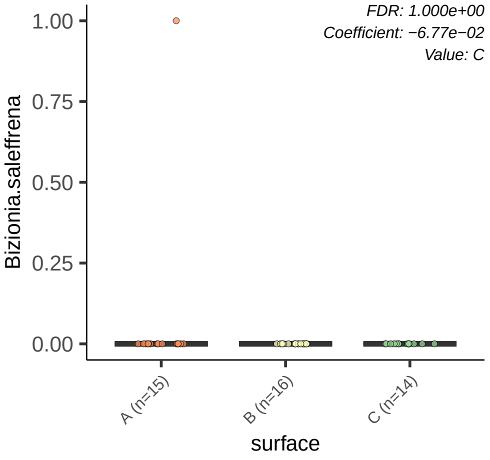

FDR: 1.000e+00
Coefficient: −6.77e−02
Value: C
1.00
0.75
0.50
0.25
0.00
Bizionia.saleffrena
A (n=15)
B (n=16)
C (n=14)
surface

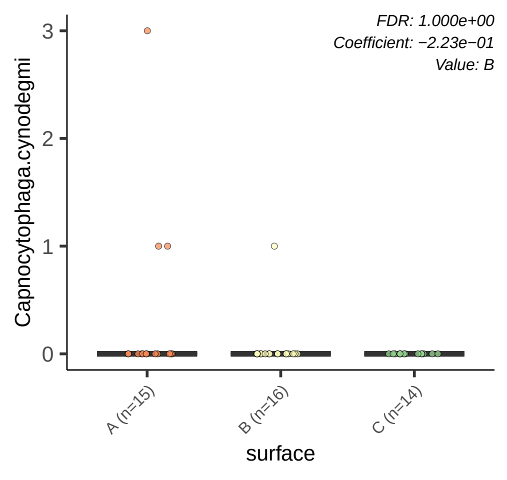

FDR: 1.000e+00
Coefficient: −2.23e−01
Value: B
Capnocytophaga.cynodegmi
3
2
1
0
A (n=15)
B (n=16)
C (n=14)
surface

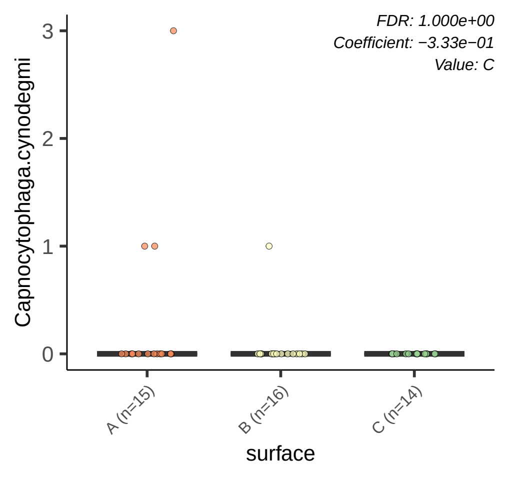
FDR: 1.000e+00
Coefficient: −3.33e−01
Value: C
Capnocytophaga.cynodegmi
3
2
1
0
A (n=15)
B (n=16)
C (n=14)
surface

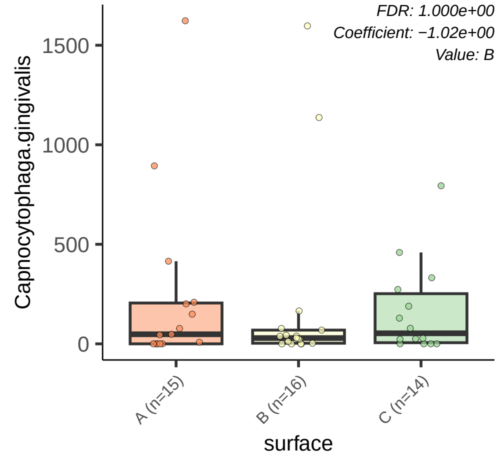
FDR: 1.000e+00
Coefficient: −1.02e+00
Value: B
Capnocytophaga.gingivalis
1500
1000
500
0
A (n=15)
B (n=16)
C (n=14)
surface

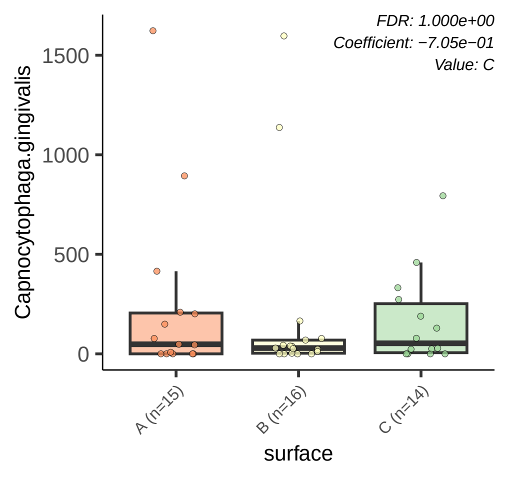
FDR: 1.000e+00
Coefficient: −7.05e−01
Value: C
Capnocytophaga.gingivalis
1500
1000
500
0
A (n=15)
B (n=16)
C (n=14)
surface

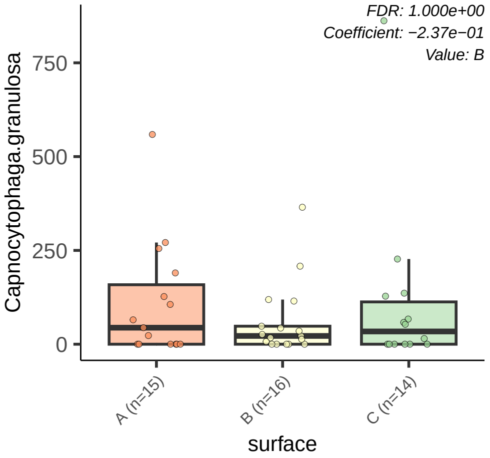
FDR: 1.000e+00
Coefficient: −2.37e−01
Value: B
Capnocytophaga.granulosa
750
500
250
0
A (n=15)
B (n=16)
C (n=14)
surface

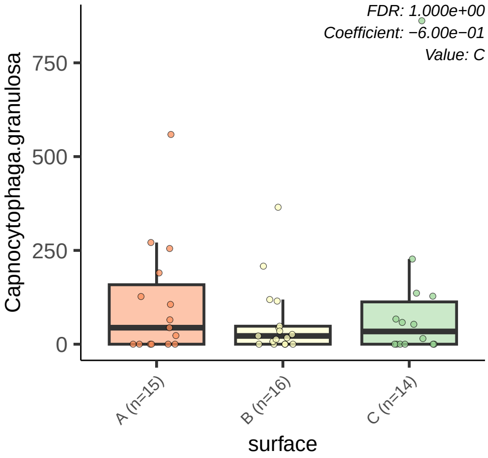

FDR: 1.000e+00
Coefficient: −6.00e−01
Value: C
Capnocytophaga.granulosa
750
500
250
0
A (n=15)
B (n=16)
C (n=14)
surface

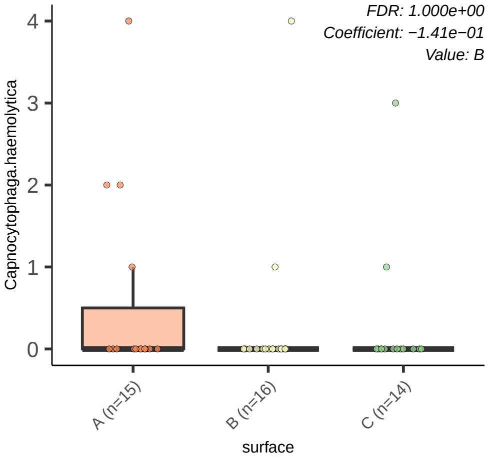

FDR: 1.000e+00
Coefficient: −1.41e−01
Value: B
Capnocytophaga.haemolytica
4
3
2
1
0
A (n=15)
B (n=16)
C (n=14)
surface

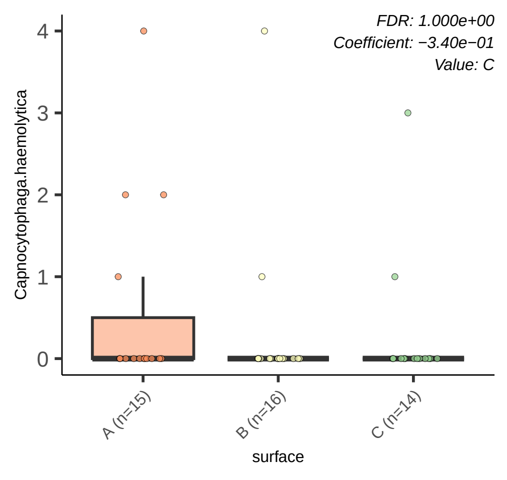

FDR: 1.000e+00
Coefficient: −3.40e−01
Value: C
Capnocytophaga.haemolytica
0
1
2
3
4
A (n=15)
B (n=16)
C (n=14)
surface

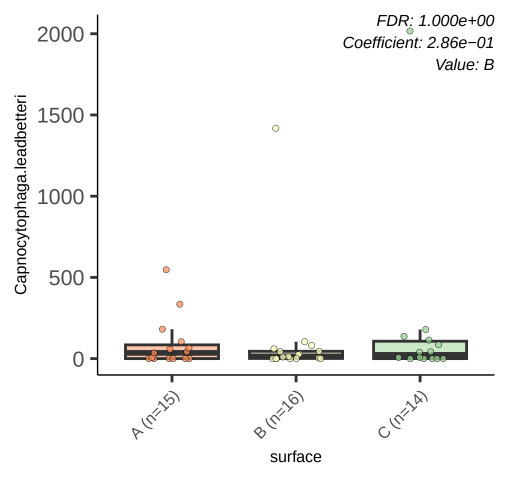
FDR: 1.000e+00
Coefficient: 2.86e−01
Value: B
2000
1500
1000
500
0
Capnocytophaga.leadbetteri
A (n=15)
B (n=16)
C (n=14)
surface

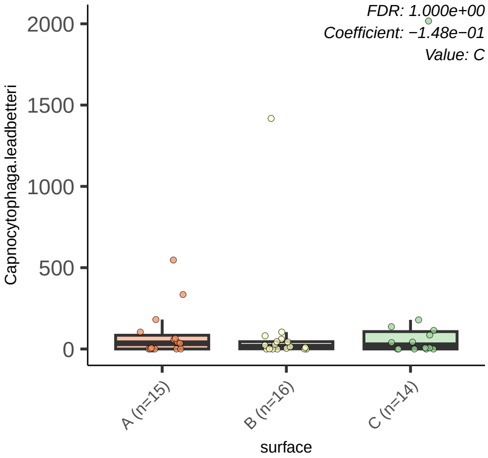

FDR: 1.000e+00
Coefficient: −1.48e−01
Value: C
Capnocytophaga.leadbetteri
2000
1500
1000
500
0
A (n=15)
B (n=16)
C (n=14)
surface

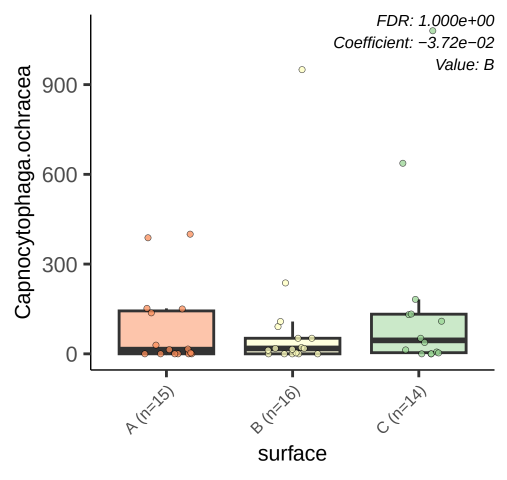

FDR: 1.000e+00
Coefficient: −3.72e−02
Value: B
Capnocytophaga.ochracea
900
600
300
0
A (n=15)
B (n=16)
C (n=14)
surface

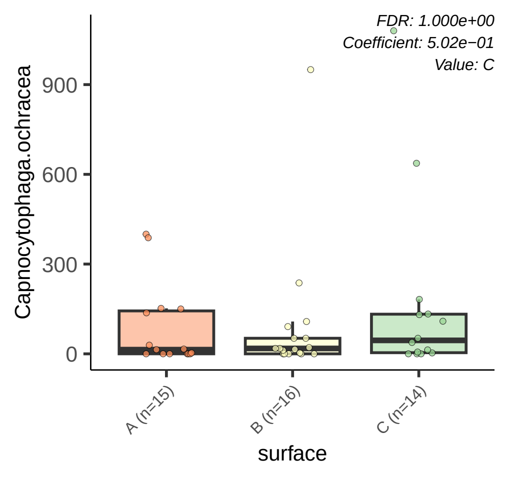

FDR: 1.000e+00
Coefficient: 5.02e−01
Value: C
Capnocytophaga.ochracea
900
600
300
0
A (n=15)
B (n=16)
C (n=14)
surface

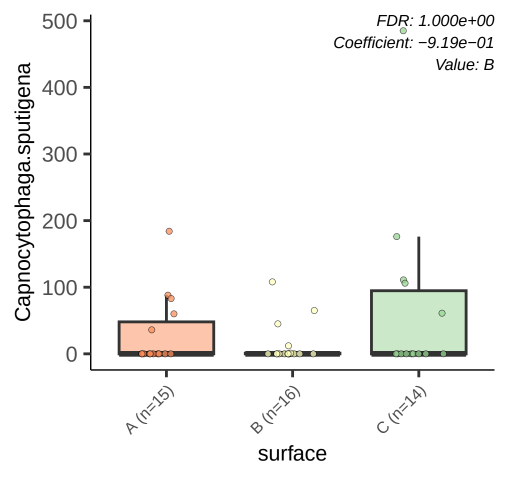

FDR: 1.000e+00
Coefficient: −9.19e−01
Value: B
Capnocytophaga.sputigena
500
400
300
200
100
0
A (n=15)
B (n=16)
C (n=14)
surface

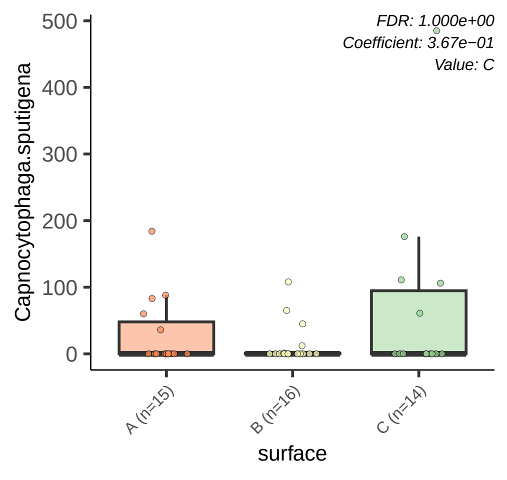
FDR: 1.000e+00
Coefficient: 3.67e−01
Value: C
Capnocytophaga.sputigena
500
400
300
200
100
0
A (n=15)
B (n=16)
C (n=14)
surface

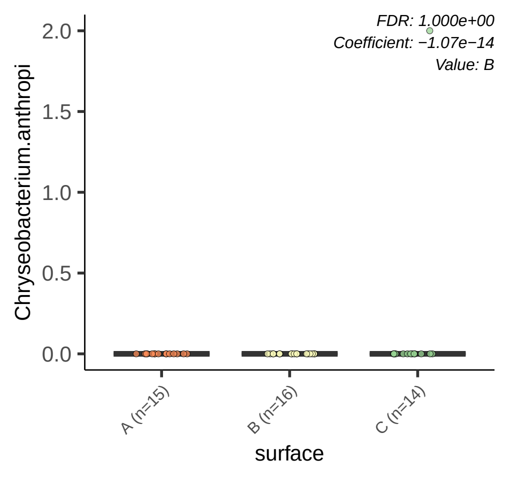
FDR: 1.000e+00
Coefficient: −1.07e−14
Value: B
Chryseobacterium.anthropi
2.0
1.5
1.0
0.5
0.0
A (n=15)
B (n=16)
C (n=14)
surface

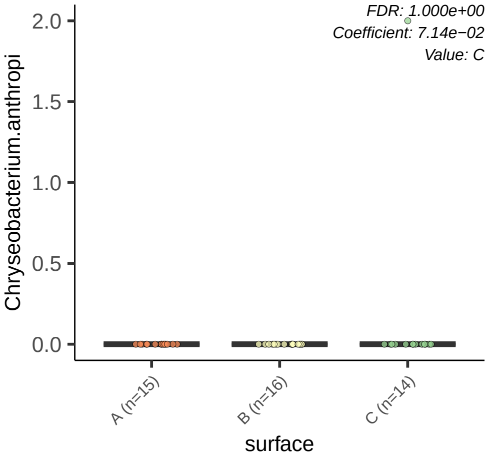
FDR: 1.000e+00
Coefficient: 7.14e−02
Value: C
Chryseobacterium.anthropi
2.0
1.5
1.0
0.5
0.0
A (n=15)
B (n=16)
C (n=14)
surface

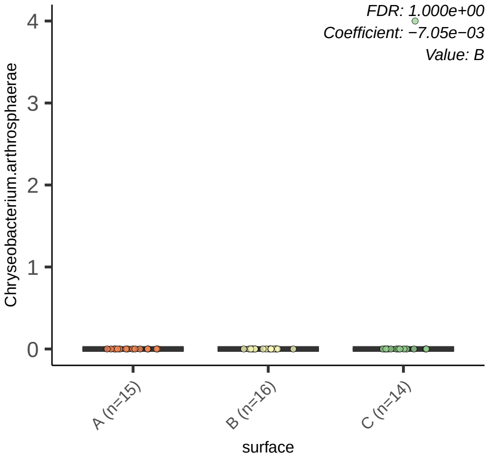
FDR: 1.000e+00
Coefficient: −7.05e−03
Value: B
Chryseobacterium.arthrosphaerae
4
3
2
1
0
A (n=15)
B (n=16)
C (n=14)
surface

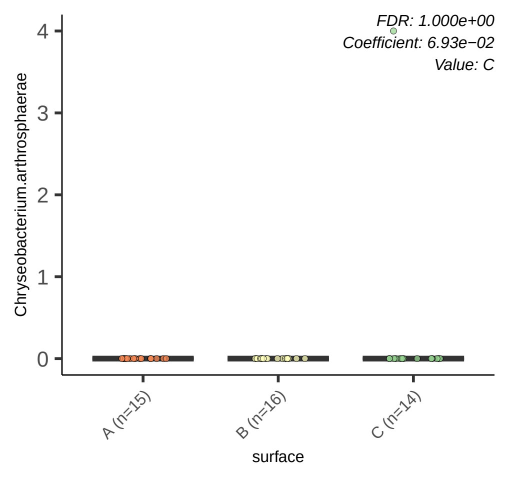
FDR: 1.000e+00
Coefficient: 6.93e−02
Value: C
Chryseobacterium.arthrosphaerae
0
1
2
3
4
A (n=15)
B (n=16)
C (n=14)
surface

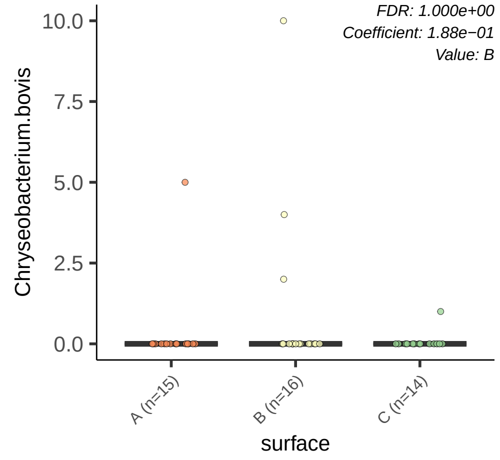

FDR: 1.000e+00
Coefficient: 1.88e−01
Value: B
Chryseobacterium.bovis
10.0
7.5
5.0
2.5
0.0
A (n=15)
B (n=16)
C (n=14)
surface

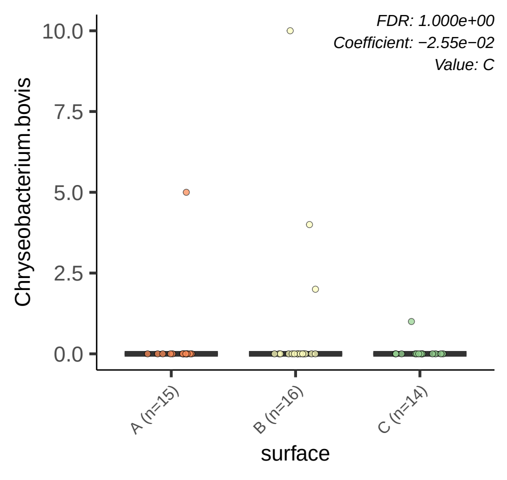
FDR: 1.000e+00
Coefficient: −2.55e−02
Value: C
Chryseobacterium.bovis
10.0
7.5
5.0
2.5
0.0
A (n=15)
B (n=16)
C (n=14)
surface

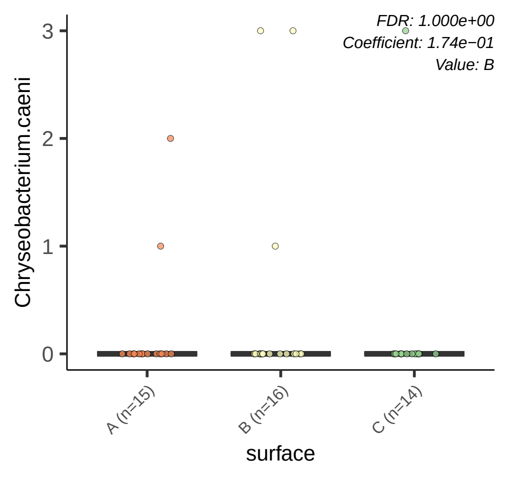
FDR: 1.000e+00
Coefficient: 1.74e−01
Value: B
Chryseobacterium.caeni
0
1
2
3
A (n=15)
B (n=16)
C (n=14)
surface

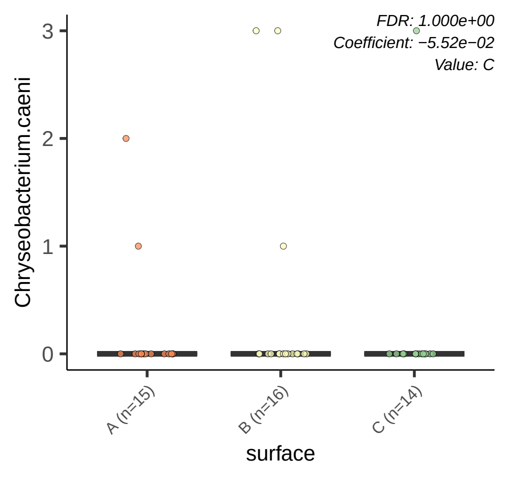
FDR: 1.000e+00
Coefficient: −5.52e−02
Value: C
Chryseobacterium.caeni
3
2
1
0
A (n=15)
B (n=16)
C (n=14)
surface

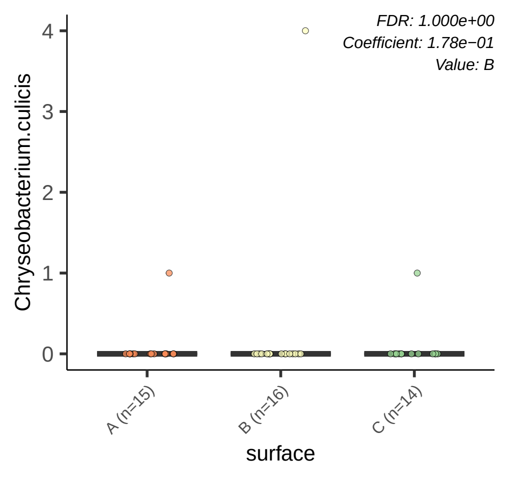

FDR: 1.000e+00
Coefficient: 1.78e−01
Value: B
Chryseobacterium.culicis
0
1
2
3
4
A (n=15)
B (n=16)
C (n=14)
surface

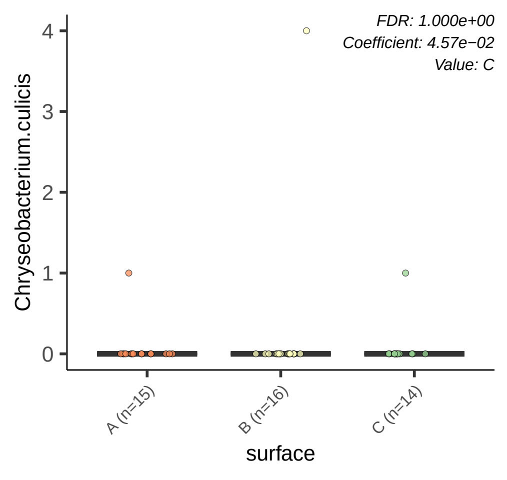
FDR: 1.000e+00
Coefficient: 4.57e−02
Value: C
Chryseobacterium.culicis
0
1
2
3
4
A (n=15)
B (n=16)
C (n=14)
surface

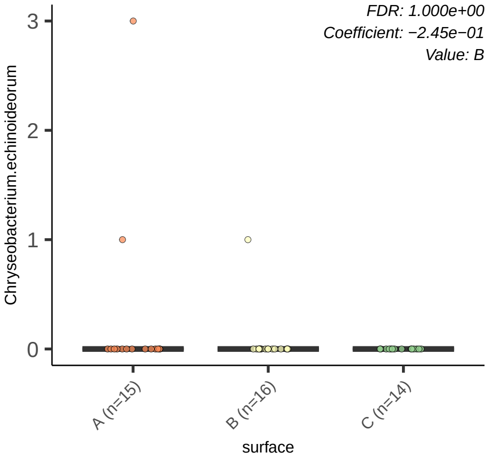
FDR: 1.000e+00
Coefficient: −2.45e−01
Value: B
Chryseobacterium.echinoideorum
3
2
1
0
A (n=15)
B (n=16)
C (n=14)
surface

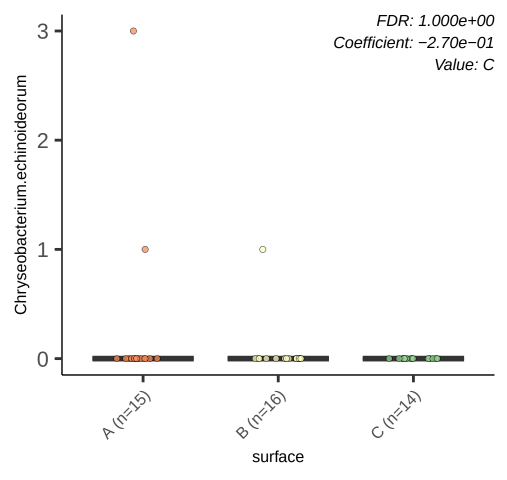
FDR: 1.000e+00
Coefficient: −2.70e−01
Value: C
Chryseobacterium.echinoideorum
3
2
1
0
A (n=15)
B (n=16)
C (n=14)
surface

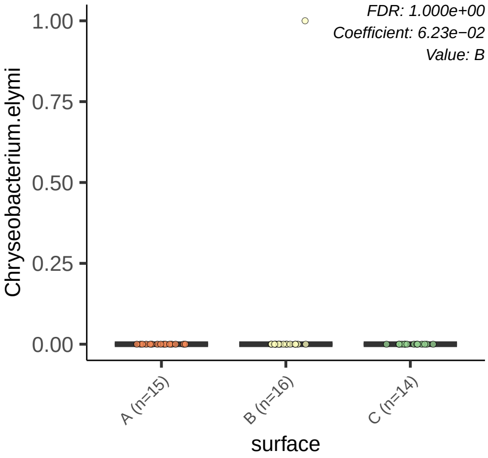
FDR: 1.000e+00
Coefficient: 6.23e−02
Value: B
Chryseobacterium.elymi
1.00
0.75
0.50
0.25
0.00
A (n=15)
B (n=16)
C (n=14)
surface

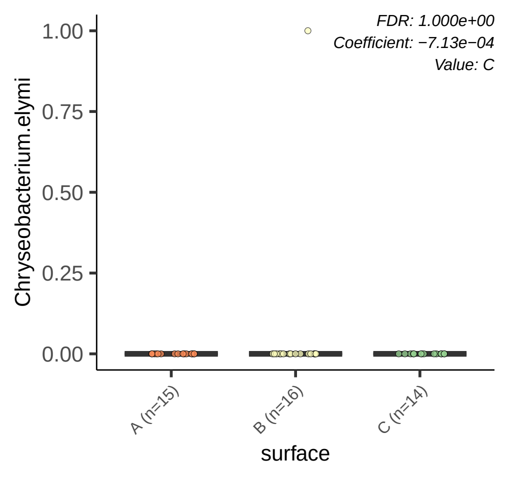

FDR: 1.000e+00
Coefficient: −7.13e−04
Value: C
Chryseobacterium.elymi
1.00
0.75
0.50
0.25
0.00
A (n=15)
B (n=16)
C (n=14)
surface

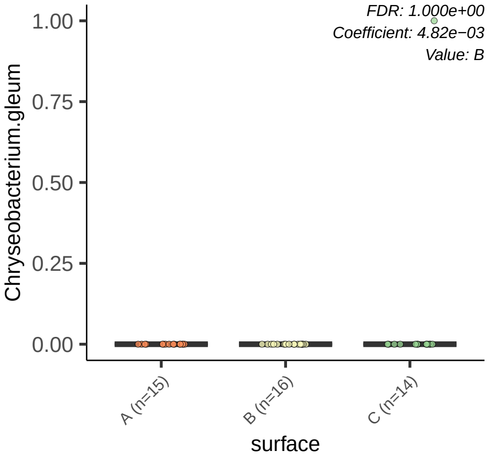

FDR: 1.000e+00
Coefficient: 4.82e−03
Value: B
Chryseobacterium.gleum
1.00
0.75
0.50
0.25
0.00
A (n=15)
B (n=16)
C (n=14)
surface

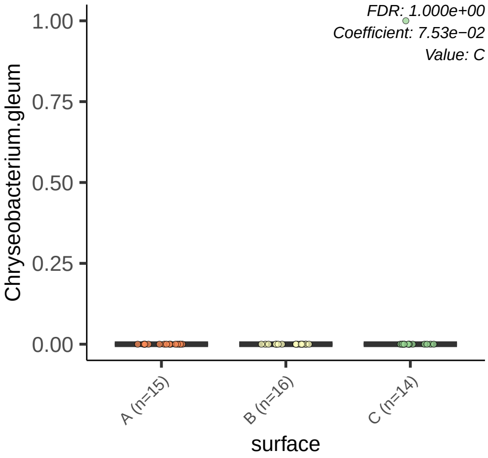

FDR: 1.000e+00
Coefficient: 7.53e−02
Value: C
Chryseobacterium.gleum
1.00
0.75
0.50
0.25
0.00
A (n=15)
B (n=16)
C (n=14)
surface

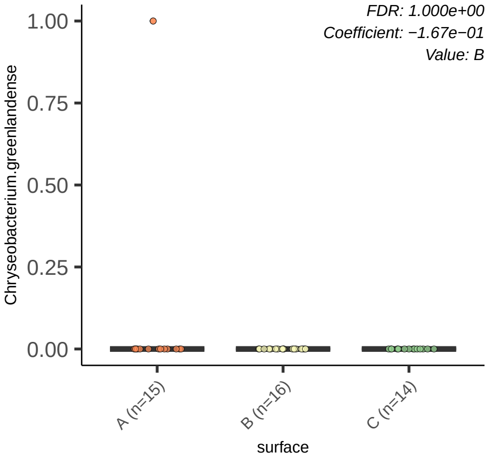

FDR: 1.000e+00
Coefficient: −1.67e−01
Value: B
Chryseobacterium.greenlandense
1.00
0.75
0.50
0.25
0.00
A (n=15)
B (n=16)
C (n=14)
surface

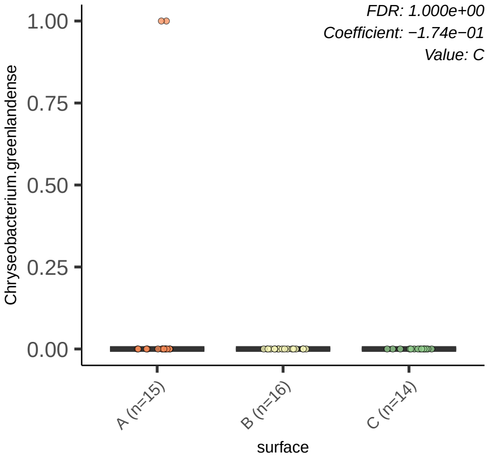
FDR: 1.000e+00
Coefficient: −1.74e−01
Value: C
Chryseobacterium.greenlandense
1.00
0.75
0.50
0.25
0.00
A (n=15)
B (n=16)
C (n=14)
surface

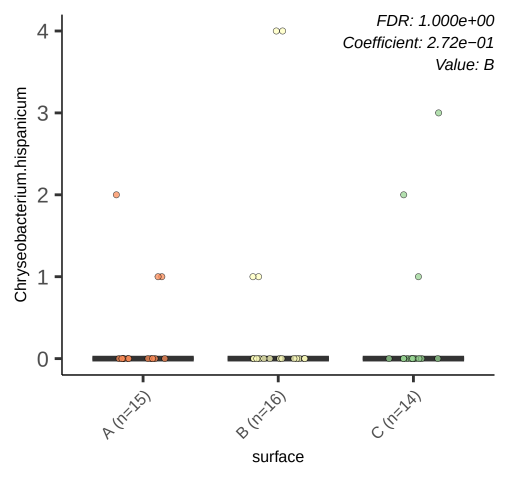
FDR: 1.000e+00
Coefficient: 2.72e−01
Value: B
Chryseobacterium.hispanicum
4
3
2
1
0
A (n=15)
B (n=16)
C (n=14)
surface

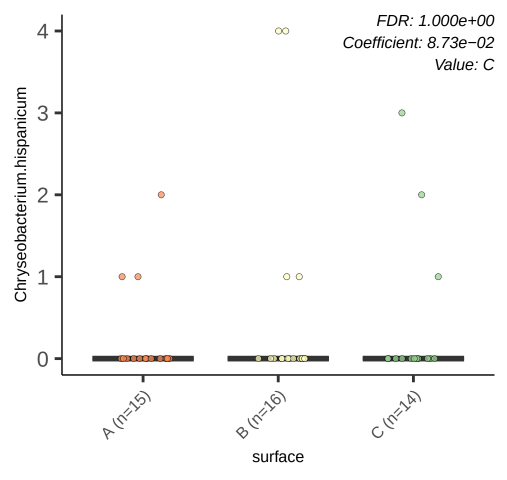

FDR: 1.000e+00
Coefficient: 8.73e−02
Value: C
Chryseobacterium.hispanicum
0
1
2
3
4
A (n=15)
B (n=16)
C (n=14)
surface

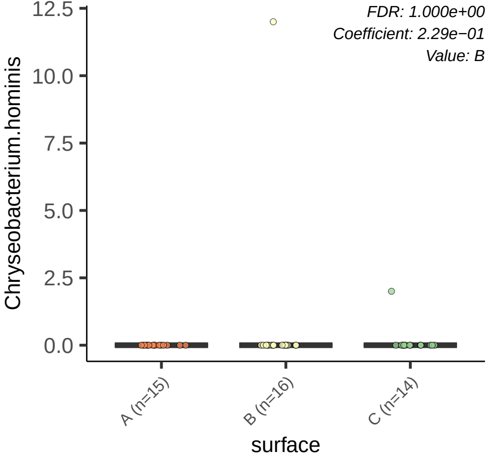

FDR: 1.000e+00
Coefficient: 2.29e−01
Value: B
Chryseobacterium.hominis
12.5
10.0
7.5
5.0
2.5
0.0
A (n=15)
B (n=16)
C (n=14)
surface

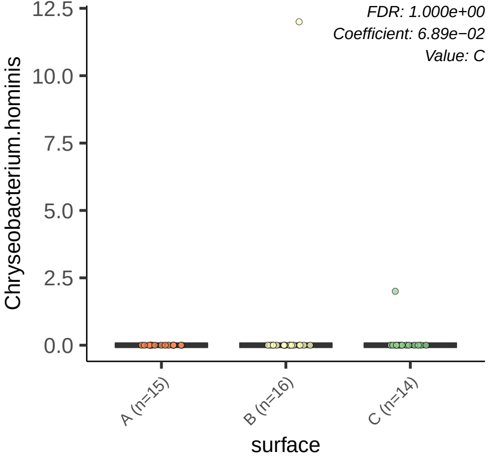

FDR: 1.000e+00
Coefficient: 6.89e−02
Value: C
Chryseobacterium.hominis
12.5
10.0
7.5
5.0
2.5
0.0
A (n=15)
B (n=16)
C (n=14)
surface

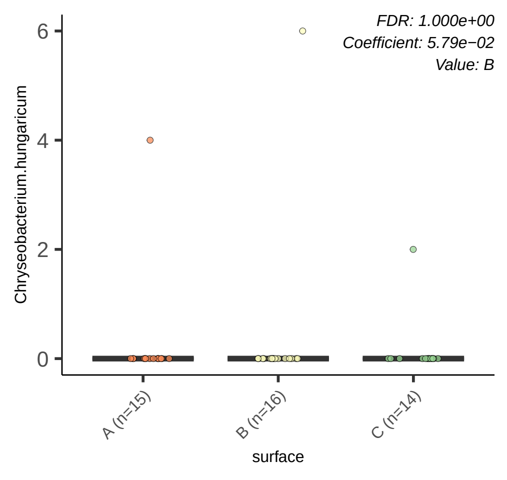

FDR: 1.000e+00
Coefficient: 5.79e−02
Value: B
Chryseobacterium.hungaricum
0
2
4
6
A (n=15)
B (n=16)
C (n=14)
surface

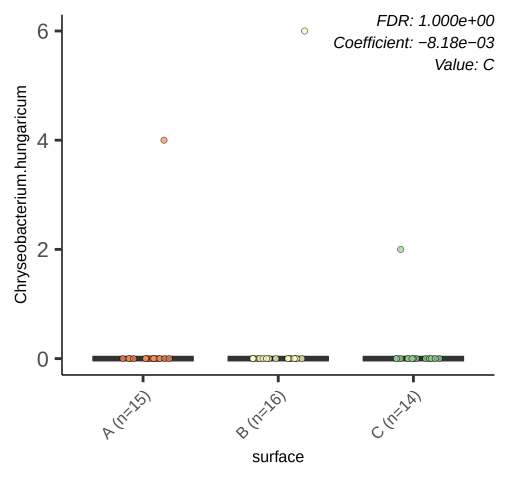
FDR: 1.000e+00
Coefficient: −8.18e−03
Value: C
Chryseobacterium.hungaricum
0
2
4
6
A (n=15)
B (n=16)
C (n=14)
surface

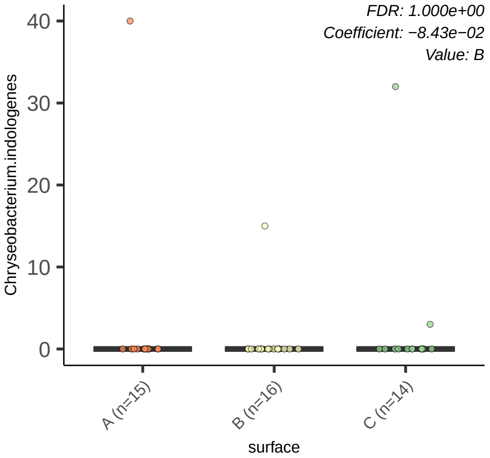
FDR: 1.000e+00
Coefficient: −8.43e−02
Value: B
Chryseobacterium.indologenes
40
30
20
10
0
A (n=15)
B (n=16)
C (n=14)
surface

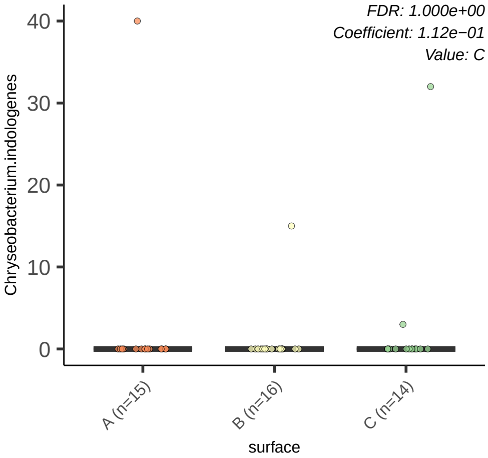
FDR: 1.000e+00
Coefficient: 1.12e−01
Value: C
Chryseobacterium.indologenes
40
30
20
10
0
A (n=15)
B (n=16)
C (n=14)
surface

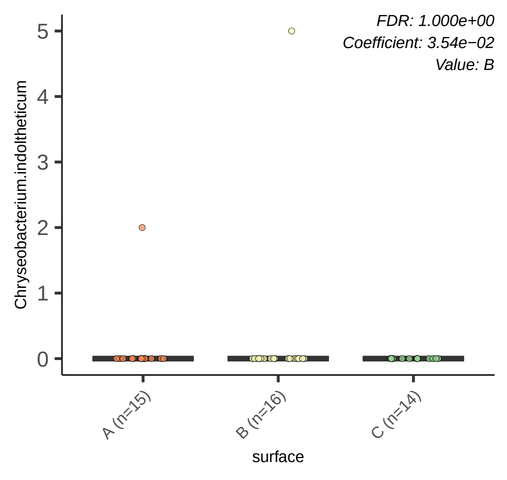
FDR: 1.000e+00
Coefficient: 3.54e−02
Value: B
Chryseobacterium.indoltheticum
0
1
2
3
4
5
A (n=15)
B (n=16)
C (n=14)
surface

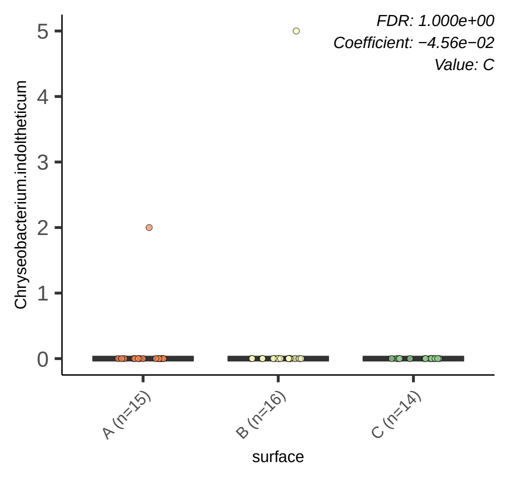
FDR: 1.000e+00
Coefficient: −4.56e−02
Value: C
Chryseobacterium.indoltheticum
0
1
2
3
4
5
A (n=15)
B (n=16)
C (n=14)
surface

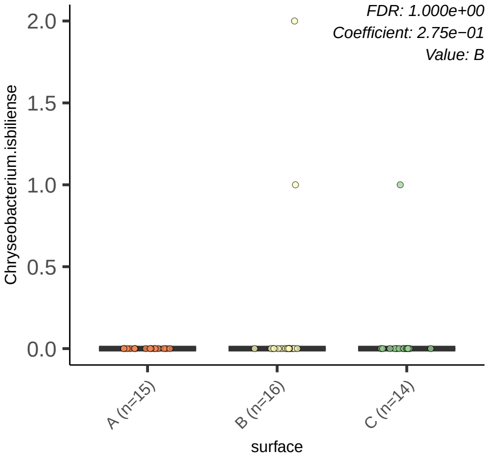
FDR: 1.000e+00
Coefficient: 2.75e−01
Value: B
Chryseobacterium.isbiliense
2.0
1.5
1.0
0.5
0.0
A (n=15)
B (n=16)
C (n=14)
surface

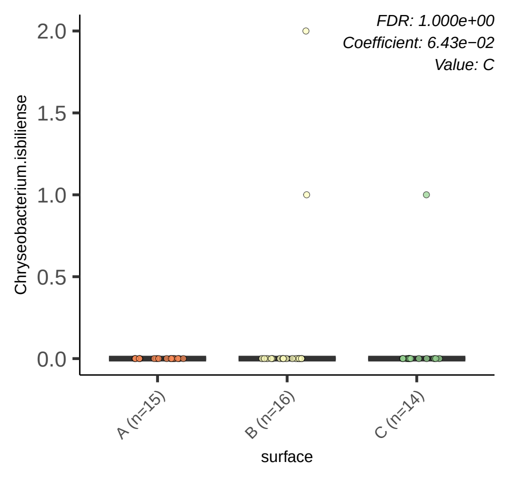
FDR: 1.000e+00
Coefficient: 6.43e−02
Value: C
Chryseobacterium.isbiliense
2.0
1.5
1.0
0.5
0.0
A (n=15)
B (n=16)
C (n=14)
surface

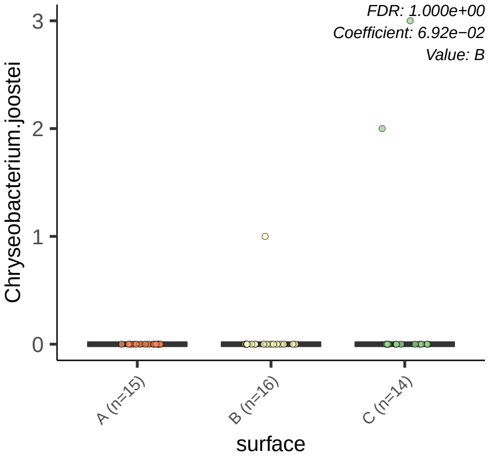

FDR: 1.000e+00
Coefficient: 6.92e−02
Value: B
Chryseobacterium.joostei
0
1
2
3
A (n=15)
B (n=16)
C (n=14)
surface

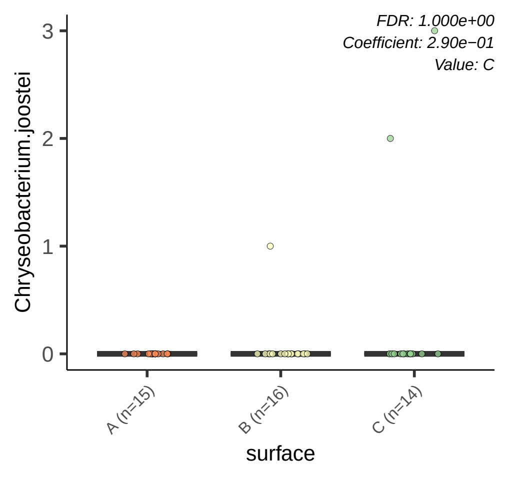
FDR: 1.000e+00
Coefficient: 2.90e−01
Value: C
Chryseobacterium.joostei
3
2
1
0
A (n=15)
B (n=16)
C (n=14)
surface

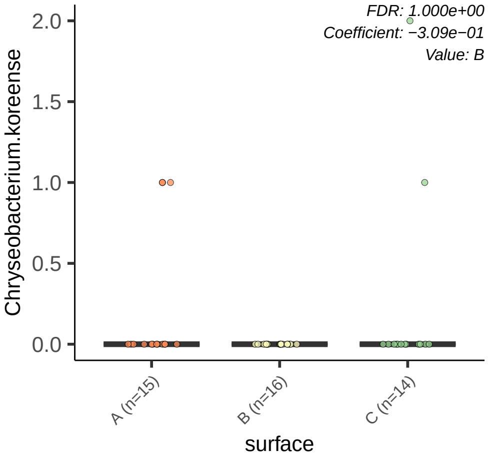
FDR: 1.000e+00
Coefficient: −3.09e−01
Value: B
Chryseobacterium.koreense
2.0
1.5
1.0
0.5
0.0
A (n=15)
B (n=16)
C (n=14)
surface

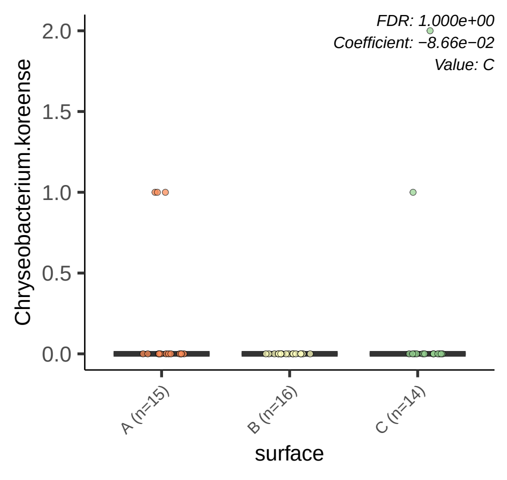
FDR: 1.000e+00
Coefficient: −8.66e−02
Value: C
Chryseobacterium.koreense
2.0
1.5
1.0
0.5
0.0
A (n=15)
B (n=16)
C (n=14)
surface

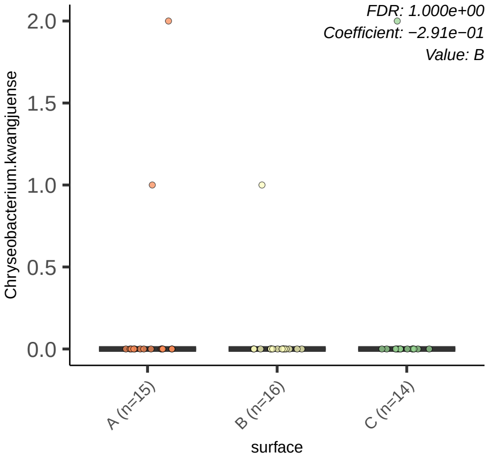

FDR: 1.000e+00
Coefficient: −2.91e−01
Value: B
Chryseobacterium.kwangjuense
2.0
1.5
1.0
0.5
0.0
A (n=15)
B (n=16)
C (n=14)
surface

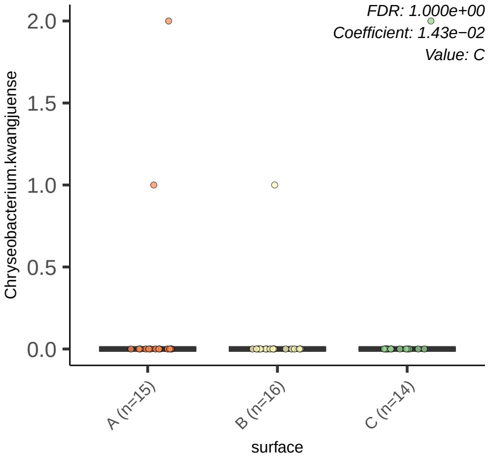
FDR: 1.000e+00
Coefficient: 1.43e−02
Value: C
2.0
1.5
1.0
0.5
0.0
Chryseobacterium.kwangjuense
A (n=15)
B (n=16)
C (n=14)
surface

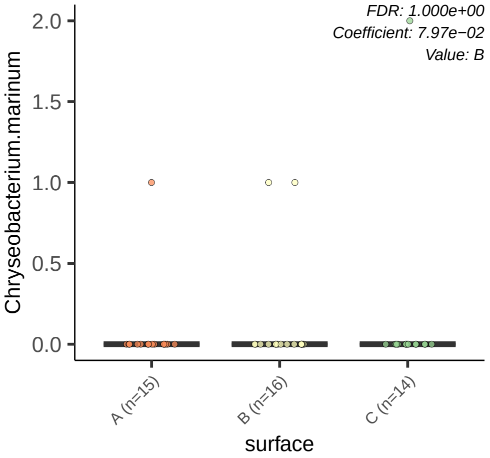

FDR: 1.000e+00
Coefficient: 7.97e−02
Value: B
Chryseobacterium.marinum
2.0
1.5
1.0
0.5
0.0
A (n=15)
B (n=16)
C (n=14)
surface

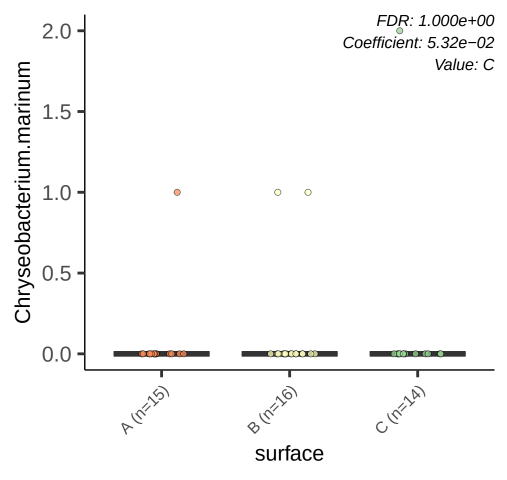
FDR: 1.000e+00
Coefficient: 5.32e−02
Value: C
Chryseobacterium.marinum
2.0
1.5
1.0
0.5
0.0
A (n=15)
B (n=16)
C (n=14)
surface

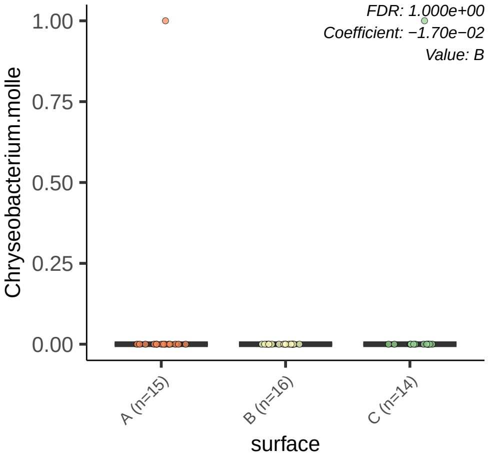
FDR: 1.000e+00
Coefficient: −1.70e−02
Value: B
Chryseobacterium.molle
1.00
0.75
0.50
0.25
0.00
A (n=15)
B (n=16)
C (n=14)
surface

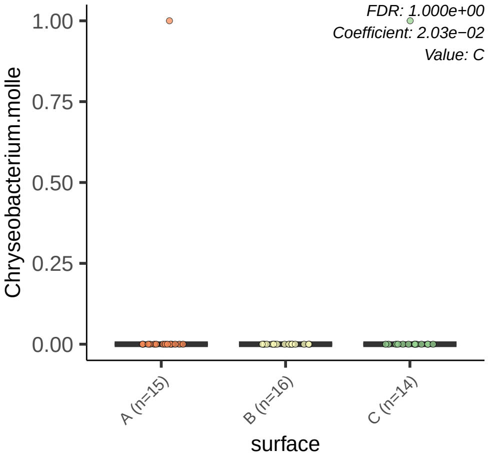
FDR: 1.000e+00
Coefficient: 2.03e−02
Value: C
Chryseobacterium.molle
1.00
0.75
0.50
0.25
0.00
A (n=15)
B (n=16)
C (n=14)
surface

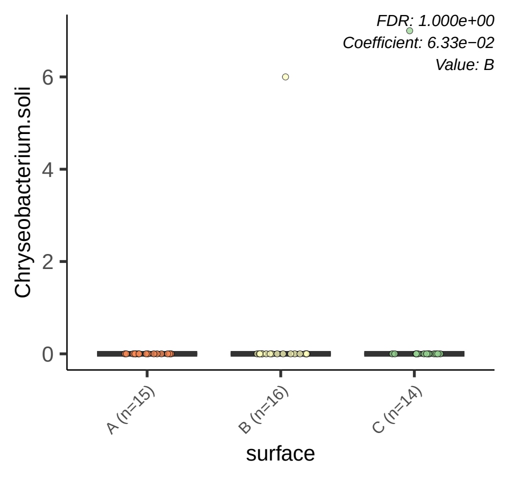
FDR: 1.000e+00
Coefficient: 6.33e−02
Value: B
Chryseobacterium.soli
0
2
4
6
A (n=15)
B (n=16)
C (n=14)
surface

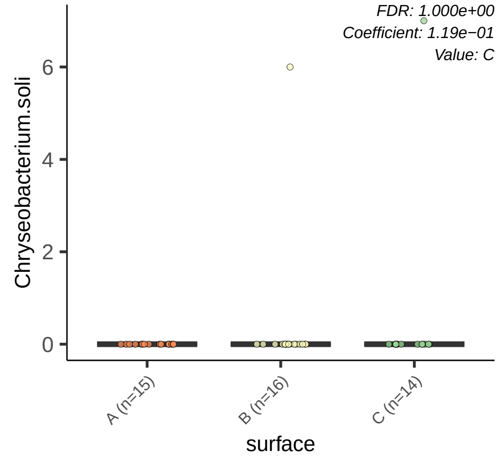

FDR: 1.000e+00
Coefficient: 1.19e−01
Value: C
Chryseobacterium.soli
0
2
4
6
A (n=15)
B (n=16)
C (n=14)
surface

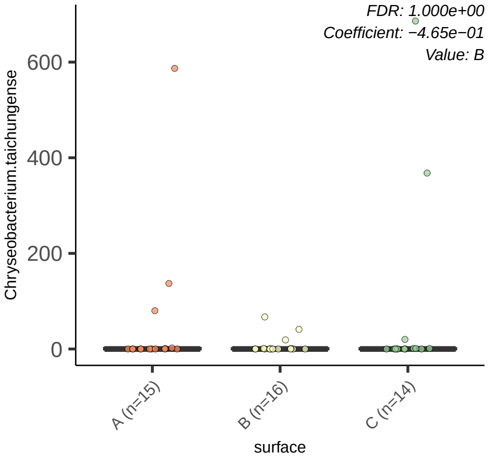

FDR: 1.000e+00
Coefficient: −4.65e−01
Value: B
Chryseobacterium.taichungense
600
400
200
0
A (n=15)
B (n=16)
C (n=14)
surface

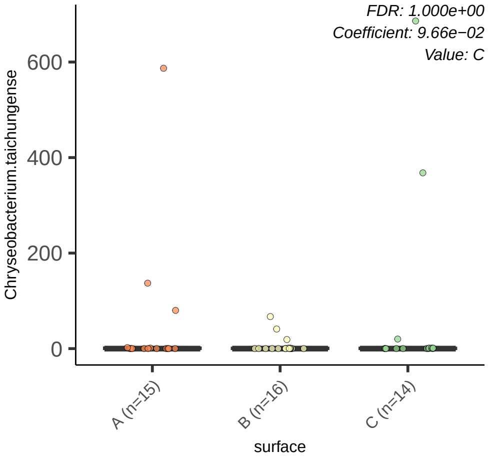

FDR: 1.000e+00
Coefficient: 9.66e−02
Value: C
600
400
200
0
Chryseobacterium.taichungense
A (n=15)
B (n=16)
C (n=14)
surface

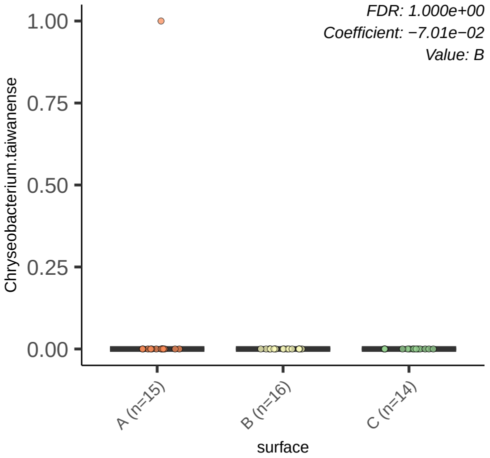
FDR: 1.000e+00
Coefficient: −7.01e−02
Value: B
1.00
0.75
0.50
0.25
0.00
Chryseobacterium.taiwanense
A (n=15)
B (n=16)
C (n=14)
surface

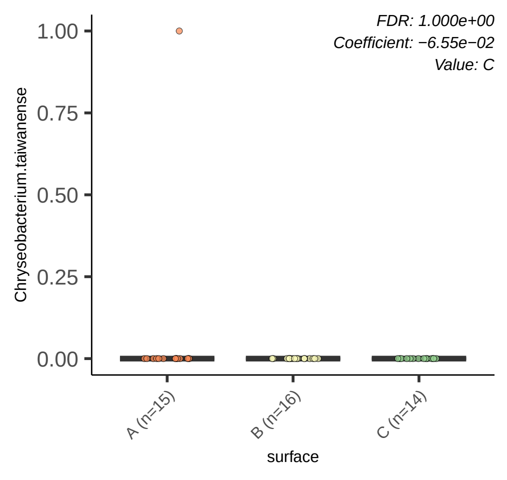

FDR: 1.000e+00
Coefficient: −6.55e−02
Value: C
Chryseobacterium.taiwanense
1.00
0.75
0.50
0.25
0.00
A (n=15)
B (n=16)
C (n=14)
surface

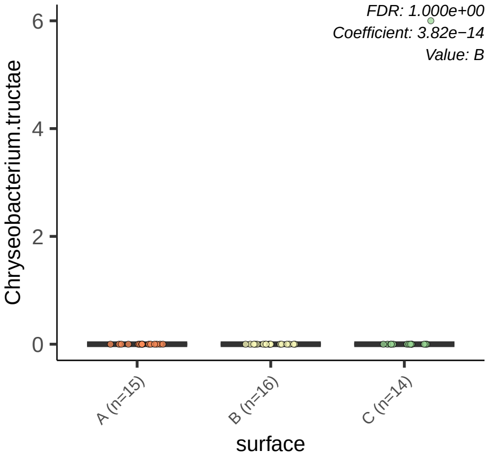

FDR: 1.000e+00
Coefficient: 3.82e−14
Value: B
Chryseobacterium.tructae
6
4
2
0
A (n=15)
B (n=16)
C (n=14)
surface

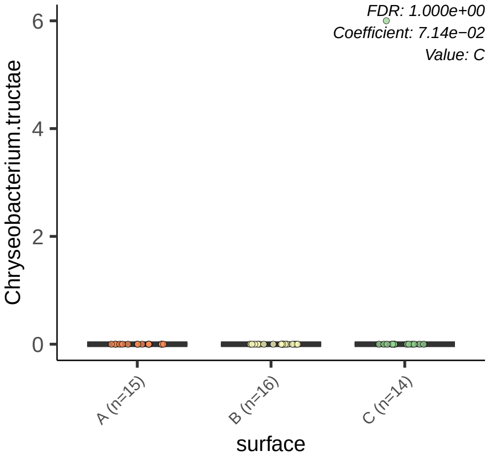

FDR: 1.000e+00
Coefficient: 7.14e−02
Value: C
Chryseobacterium.tructae
0
2
4
6
A (n=15)
B (n=16)
C (n=14)
surface

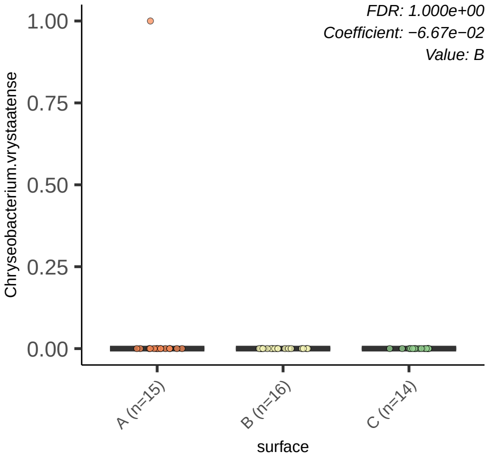
FDR: 1.000e+00
Coefficient: −6.67e−02
Value: B
Chryseobacterium.vrystaatense
1.00
0.75
0.50
0.25
0.00
A (n=15)
B (n=16)
C (n=14)
surface

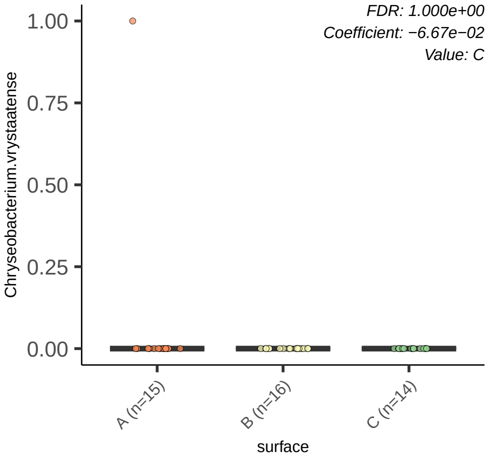
FDR: 1.000e+00
Coefficient: −6.67e−02
Value: C
1.00
0.75
0.50
0.25
0.00
Chryseobacterium.vrystaatense
A (n=15)
B (n=16)
C (n=14)
surface

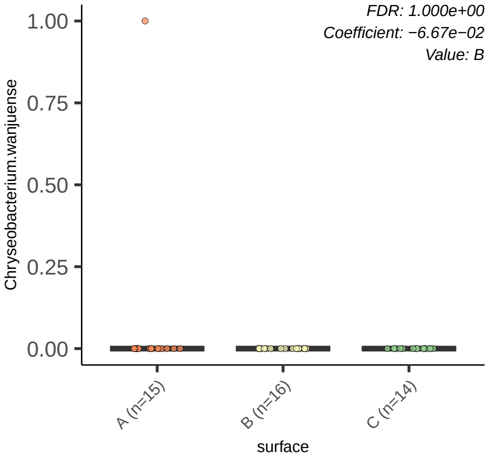
FDR: 1.000e+00
Coefficient: −6.67e−02
Value: B
1.00
0.75
0.50
0.25
0.00
Chryseobacterium.wanjuense
A (n=15)
B (n=16)
C (n=14)
surface

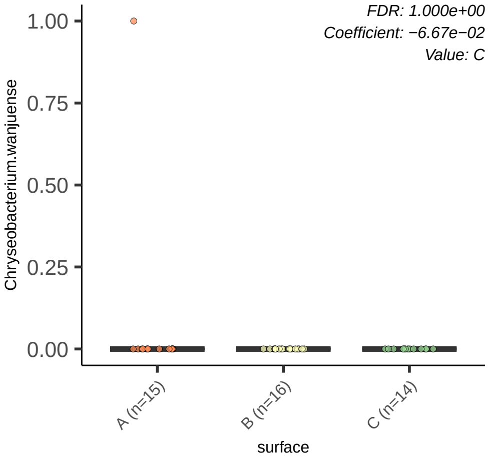
FDR: 1.000e+00
Coefficient: −6.67e−02
Value: C
1.00
0.75
0.50
0.25
0.00
Chryseobacterium.wanjuense
A (n=15)
B (n=16)
C (n=14)
surface

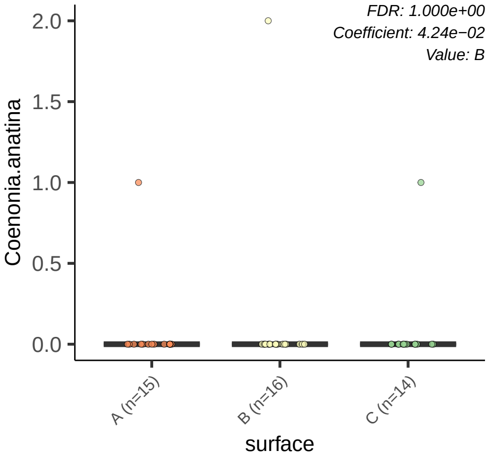

FDR: 1.000e+00
Coefficient: 4.24e−02
Value: B
Coenonia.anatina
2.0
1.5
1.0
0.5
0.0
A (n=15)
B (n=16)
C (n=14)
surface

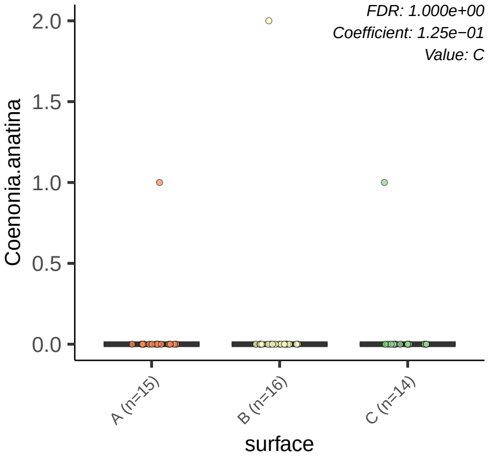

FDR: 1.000e+00
Coefficient: 1.25e−01
Value: C
2.0
1.5
1.0
0.5
0.0
Coenonia.anatina
A (n=15)
B (n=16)
C (n=14)
surface

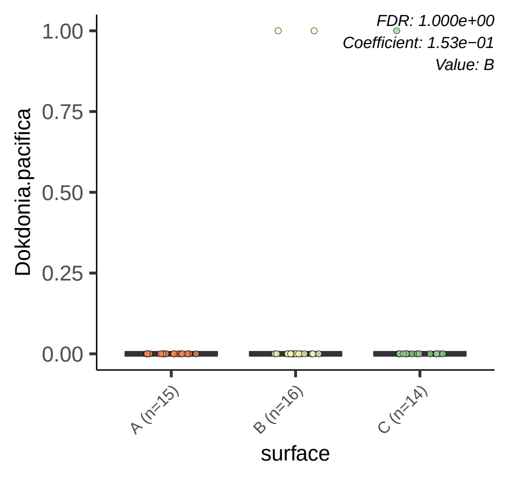
FDR: 1.000e+00
Coefficient: 1.53e−01
Value: B
Dokdonia.pacifica
1.00
0.75
0.50
0.25
0.00
A (n=15)
B (n=16)
C (n=14)
surface

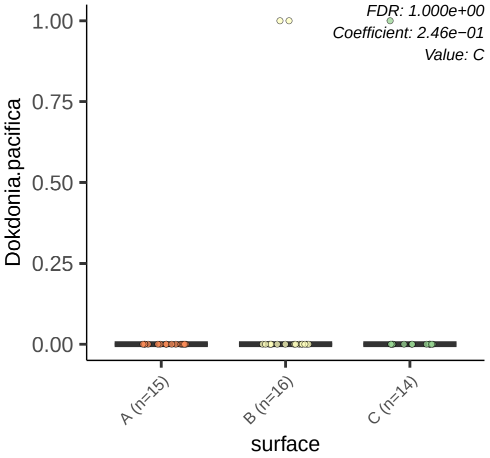
FDR: 1.000e+00
Coefficient: 2.46e−01
Value: C
1.00
0.75
0.50
0.25
0.00
Dokdonia.pacifica
A (n=15)
B (n=16)
C (n=14)
surface

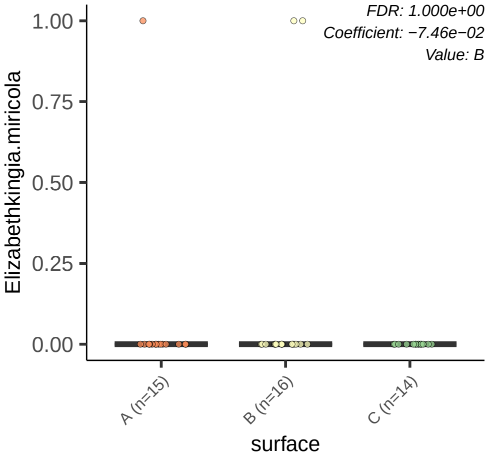
FDR: 1.000e+00
Coefficient: −7.46e−02
Value: B
Elizabethkingia.miricola
1.00
0.75
0.50
0.25
0.00
A (n=15)
B (n=16)
C (n=14)
surface

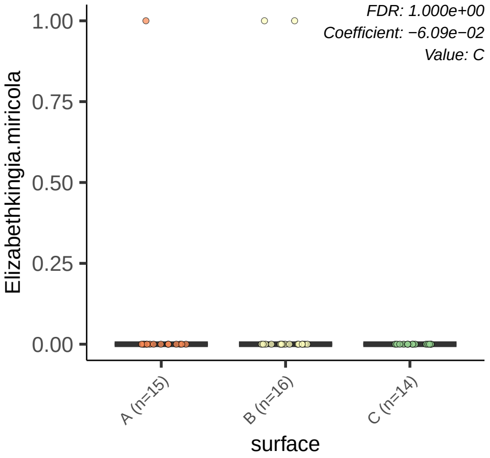
FDR: 1.000e+00
Coefficient: −6.09e−02
Value: C
Elizabethkingia.miricola
1.00
0.75
0.50
0.25
0.00
A (n=15)
B (n=16)
C (n=14)
surface

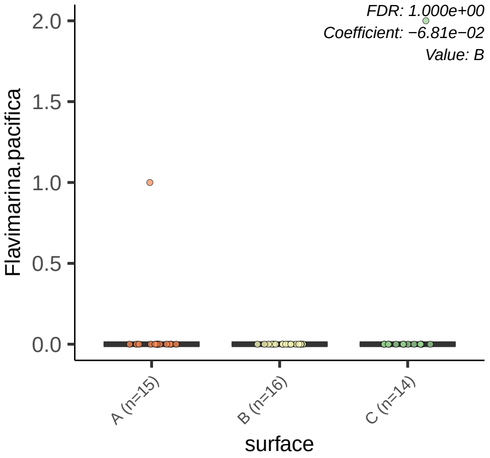
FDR: 1.000e+00
Coefficient: −6.81e−02
Value: B
2.0
1.5
1.0
0.5
0.0
Flavimarina.pacifica
A (n=15)
B (n=16)
C (n=14)
surface

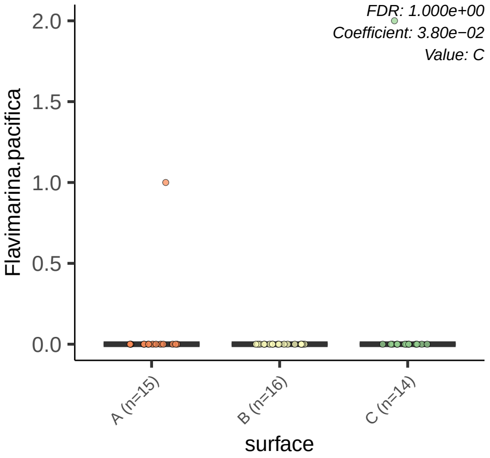

FDR: 1.000e+00
Coefficient: 3.80e−02
Value: C
Flavimarina.pacifica
2.0
1.5
1.0
0.5
0.0
A (n=15)
B (n=16)
C (n=14)
surface

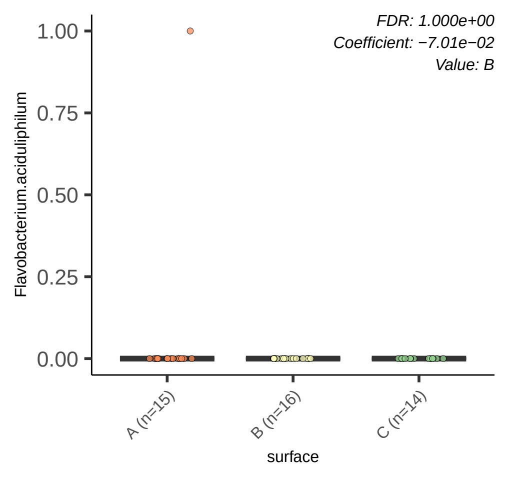

FDR: 1.000e+00
Coefficient: −7.01e−02
Value: B
1.00
0.75
0.50
0.25
0.00
Flavobacterium.aciduliphilum
A (n=15)
B (n=16)
C (n=14)
surface

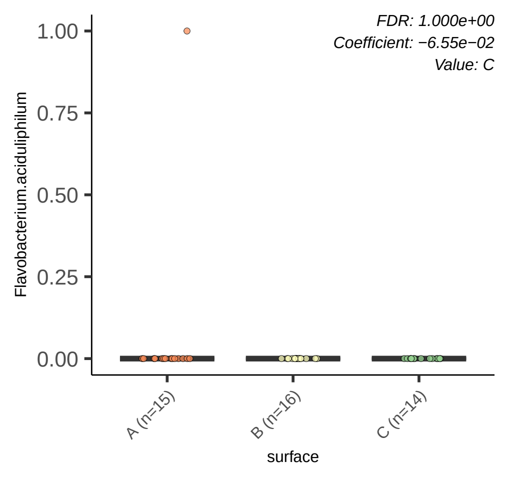
FDR: 1.000e+00
Coefficient: −6.55e−02
Value: C
Flavobacterium.aciduliphilum
1.00
0.75
0.50
0.25
0.00
A (n=15)
B (n=16)
C (n=14)
surface

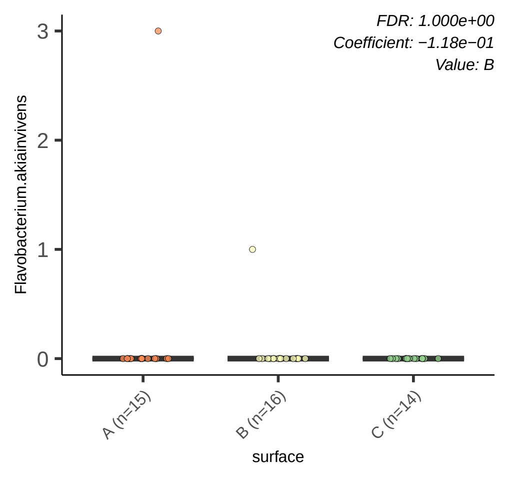
FDR: 1.000e+00
Coefficient: −1.18e−01
Value: B
Flavobacterium.akiainvivens
3
2
1
0
A (n=15)
B (n=16)
C (n=14)
surface

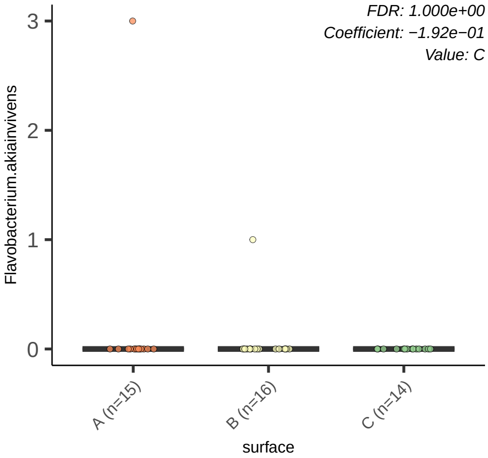

FDR: 1.000e+00
Coefficient: −1.92e−01
Value: C
Flavobacterium.akiainvivens
3
2
1
0
A (n=15)
B (n=16)
C (n=14)
surface

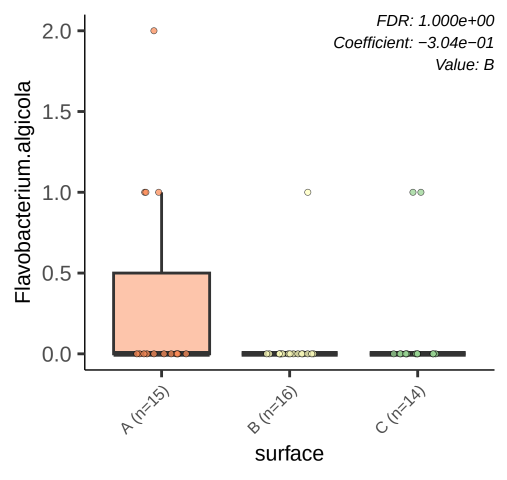

FDR: 1.000e+00
Coefficient: −3.04e−01
Value: B
Flavobacterium.algicola
2.0
1.5
1.0
0.5
0.0
A (n=15)
B (n=16)
C (n=14)
surface

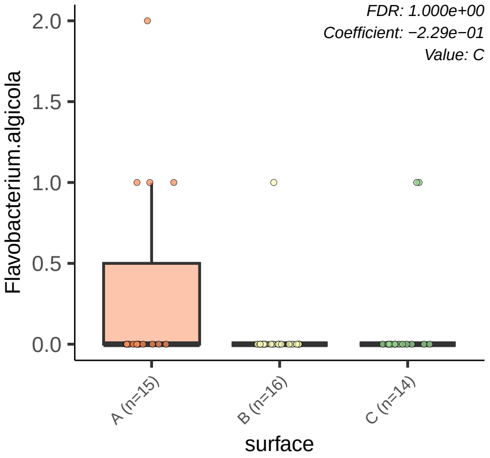

FDR: 1.000e+00
Coefficient: −2.29e−01
Value: C
Flavobacterium.algicola
2.0
1.5
1.0
0.5
0.0
A (n=15)
B (n=16)
C (n=14)
surface

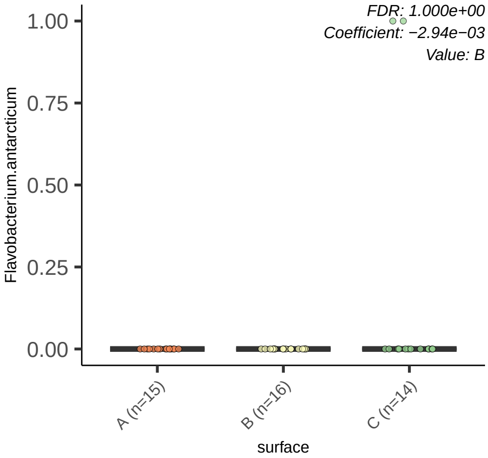
FDR: 1.000e+00
Coefficient: −2.94e−03
Value: B
Flavobacterium.antarcticum
1.00
0.75
0.50
0.25
0.00
A (n=15)
B (n=16)
C (n=14)
surface

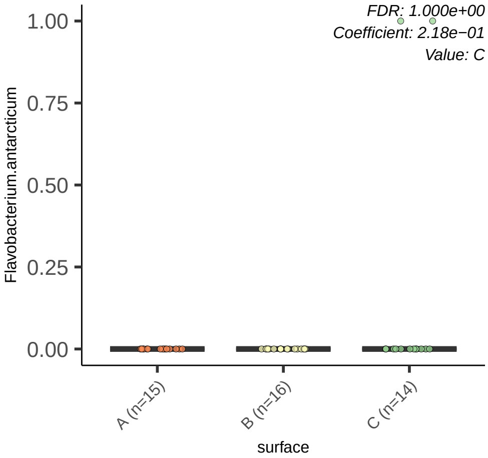
FDR: 1.000e+00
Coefficient: 2.18e−01
Value: C
Flavobacterium.antarcticum
1.00
0.75
0.50
0.25
0.00
A (n=15)
B (n=16)
C (n=14)
surface

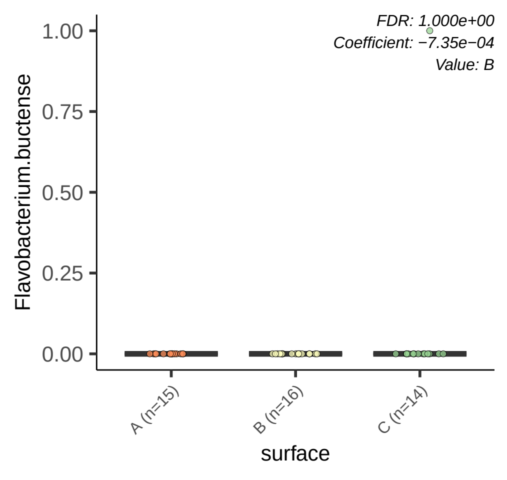
FDR: 1.000e+00
Coefficient: −7.35e−04
Value: B
Flavobacterium.buctense
1.00
0.75
0.50
0.25
0.00
A (n=15)
B (n=16)
C (n=14)
surface

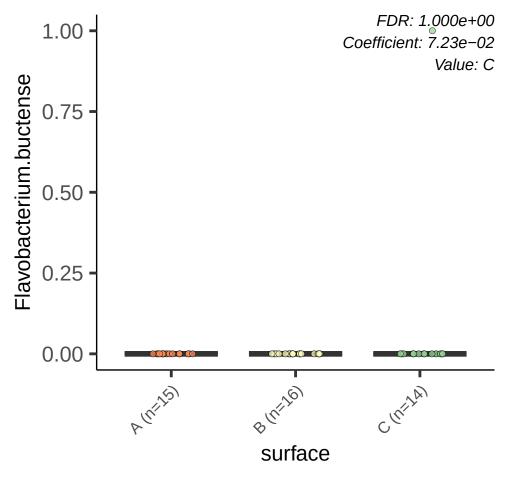

FDR: 1.000e+00
Coefficient: 7.23e−02
Value: C
Flavobacterium.buctense
1.00
0.75
0.50
0.25
0.00
A (n=15)
B (n=16)
C (n=14)
surface

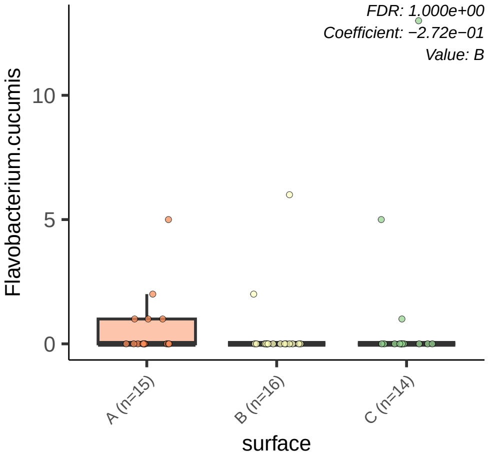

FDR: 1.000e+00
Coefficient: −2.72e−01
Value: B
Flavobacterium.cucumis
10
5
0
A (n=15)
B (n=16)
C (n=14)
surface

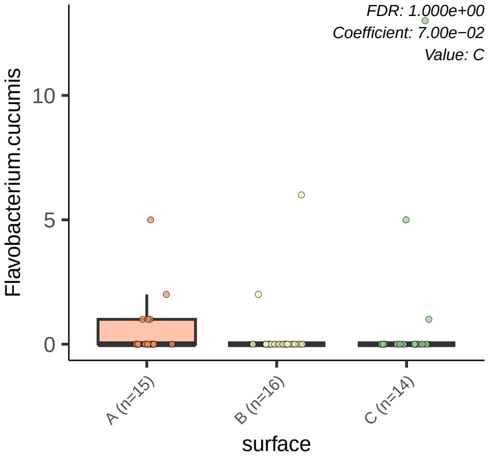

FDR: 1.000e+00
Coefficient: 7.00e−02
Value: C
Flavobacterium.cucumis
10
5
0
A (n=15)
B (n=16)
C (n=14)
surface

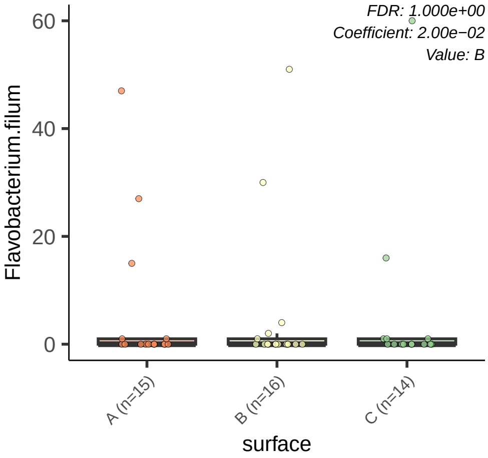
FDR: 1.000e+00
Coefficient: 2.00e−02
Value: B
Flavobacterium.filum
0
20
40
60
A (n=15)
B (n=16)
C (n=14)
surface

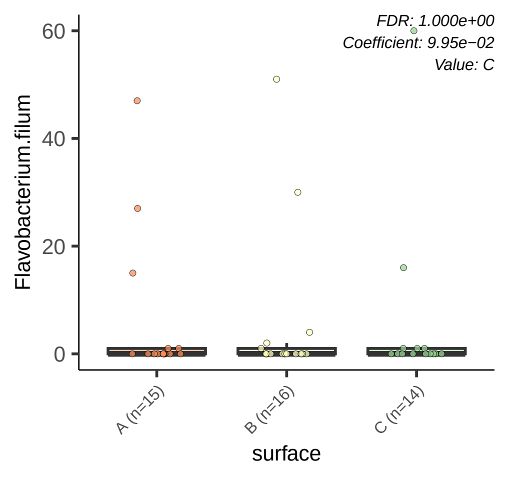

FDR: 1.000e+00
Coefficient: 9.95e−02
Value: C
60
40
20
0
Flavobacterium.filum
A (n=15)
B (n=16)
C (n=14)
surface

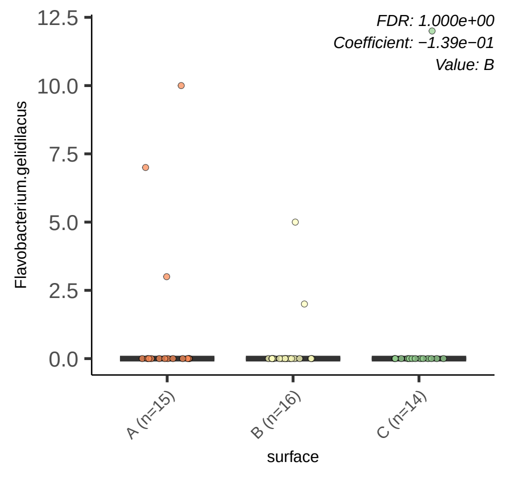

FDR: 1.000e+00
Coefficient: −1.39e−01
Value: B
Flavobacterium.gelidilacus
12.5
10.0
7.5
5.0
2.5
0.0
A (n=15)
B (n=16)
C (n=14)
surface

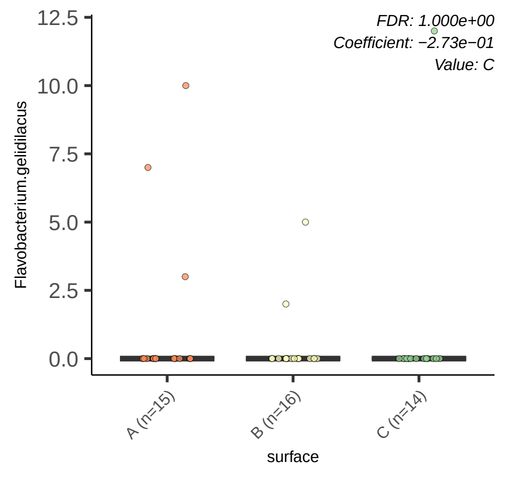
FDR: 1.000e+00
Coefficient: −2.73e−01
Value: C
12.5
10.0
7.5
5.0
2.5
0.0
Flavobacterium.gelidilacus
A (n=15)
B (n=16)
C (n=14)
surface

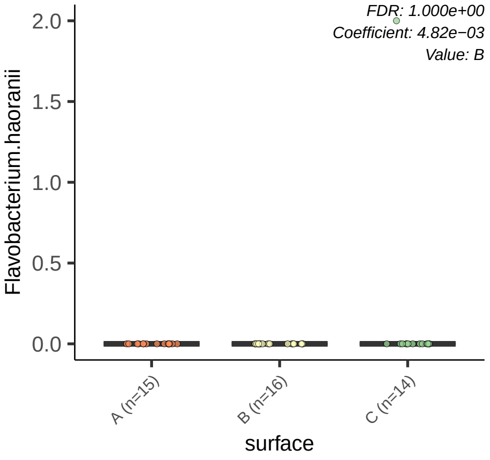

FDR: 1.000e+00
Coefficient: 4.82e−03
Value: B
Flavobacterium.haoranii
2.0
1.5
1.0
0.5
0.0
A (n=15)
B (n=16)
C (n=14)
surface

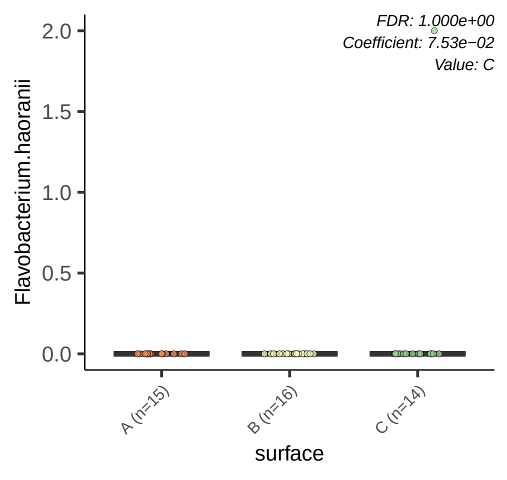

FDR: 1.000e+00
Coefficient: 7.53e−02
Value: C
Flavobacterium.haoranii
2.0
1.5
1.0
0.5
0.0
A (n=15)
B (n=16)
C (n=14)
surface

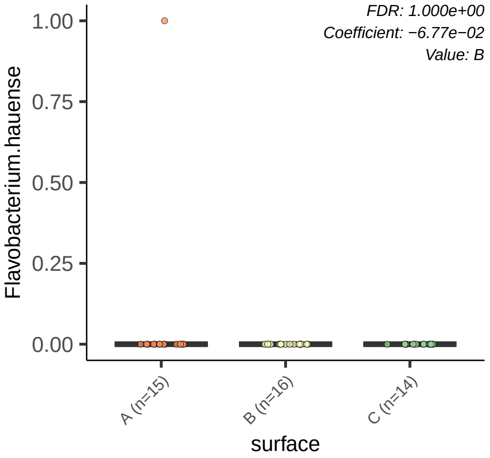
FDR: 1.000e+00
Coefficient: −6.77e−02
Value: B
Flavobacterium.hauense
1.00
0.75
0.50
0.25
0.00
A (n=15)
B (n=16)
C (n=14)
surface

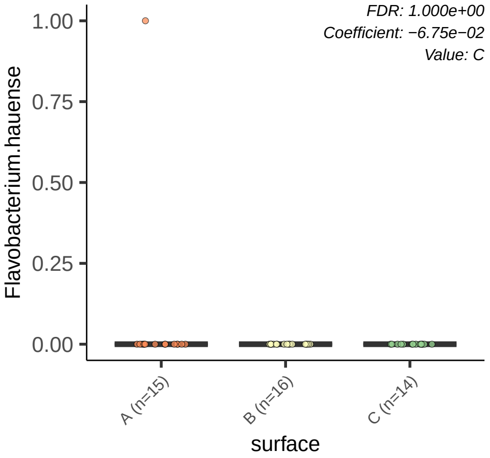

FDR: 1.000e+00
Coefficient: −6.75e−02
Value: C
Flavobacterium.hauense
1.00
0.75
0.50
0.25
0.00
A (n=15)
B (n=16)
C (n=14)
surface

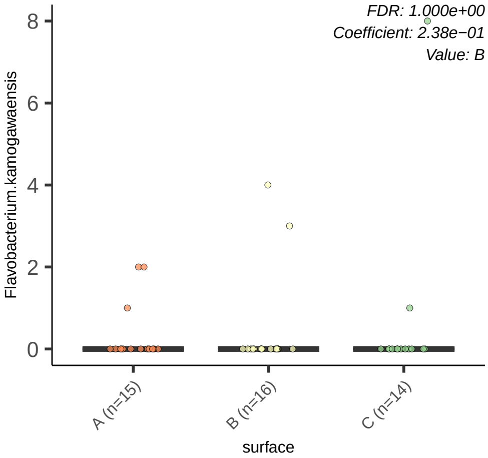

FDR: 1.000e+00
Coefficient: 2.38e−01
Value: B
Flavobacterium.kamogawaensis
0
2
4
6
8
A (n=15)
B (n=16)
C (n=14)
surface

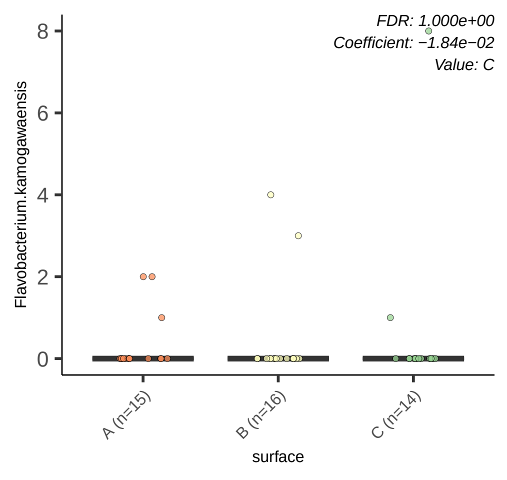
FDR: 1.000e+00
Coefficient: −1.84e−02
Value: C
Flavobacterium.kamogawaensis
0
2
4
6
8
A (n=15)
B (n=16)
C (n=14)
surface

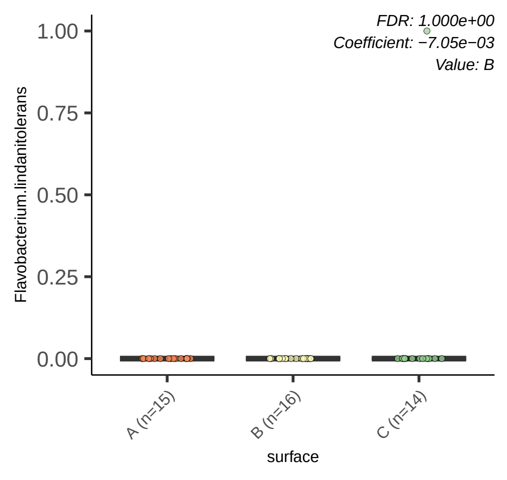
FDR: 1.000e+00
Coefficient: −7.05e−03
Value: B
Flavobacterium.lindanitolerans
1.00
0.75
0.50
0.25
0.00
A (n=15)
B (n=16)
C (n=14)
surface

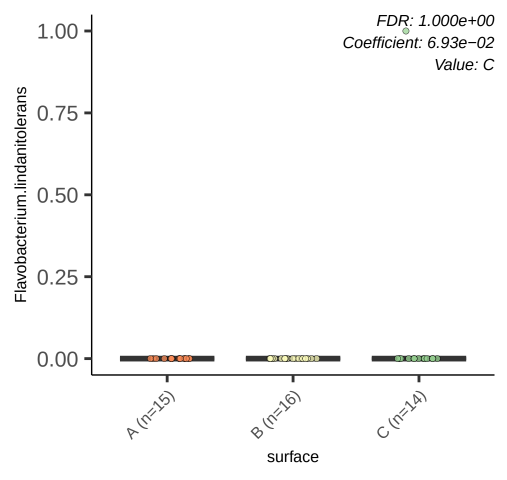

FDR: 1.000e+00
Coefficient: 6.93e−02
Value: C
1.00
0.75
0.50
0.25
0.00
Flavobacterium.lindanitolerans
A (n=15)
B (n=16)
C (n=14)
surface

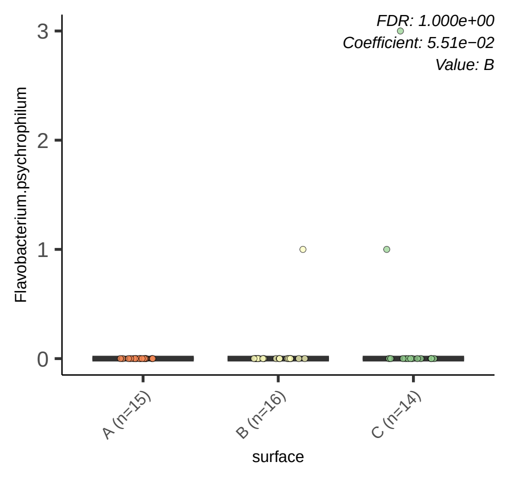

FDR: 1.000e+00
Coefficient: 5.51e−02
Value: B
Flavobacterium.psychrophilum
3
2
1
0
A (n=15)
B (n=16)
C (n=14)
surface

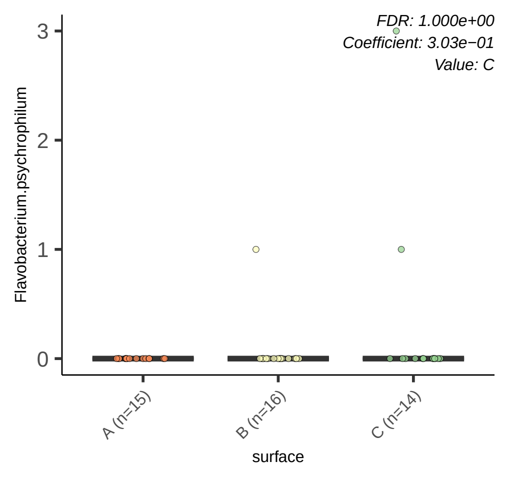
FDR: 1.000e+00
Coefficient: 3.03e−01
Value: C
Flavobacterium.psychrophilum
3
2
1
0
A (n=15)
B (n=16)
C (n=14)
surface

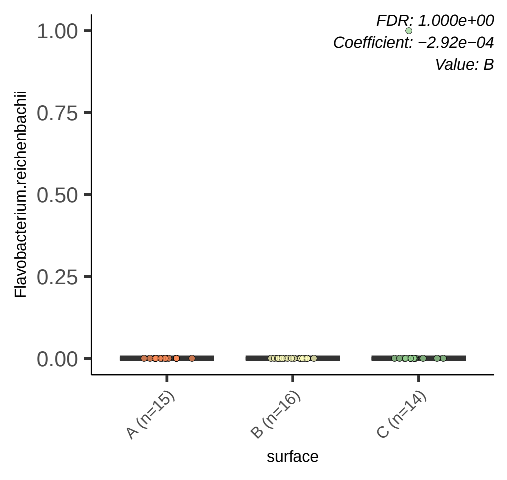
FDR: 1.000e+00
Coefficient: −2.92e−04
Value: B
1.00
0.75
0.50
0.25
0.00
Flavobacterium.reichenbachii
A (n=15)
B (n=16)
C (n=14)
surface

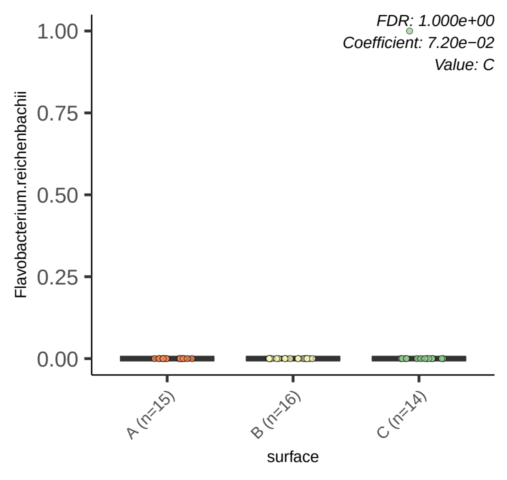

FDR: 1.000e+00
Coefficient: 7.20e−02
Value: C
Flavobacterium.reichenbachii
1.00
0.75
0.50
0.25
0.00
A (n=15)
B (n=16)
C (n=14)
surface

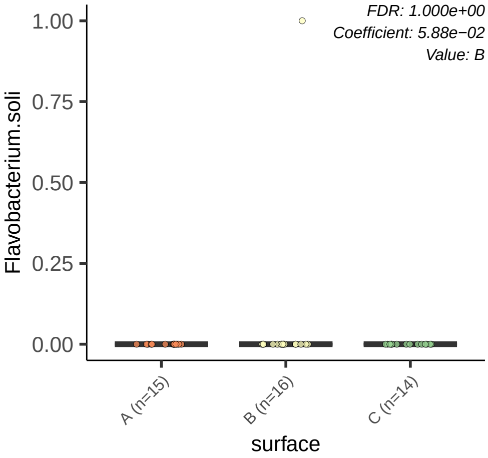
FDR: 1.000e+00
Coefficient: 5.88e−02
Value: B
Flavobacterium.soli
1.00
0.75
0.50
0.25
0.00
A (n=15)
B (n=16)
C (n=14)
surface

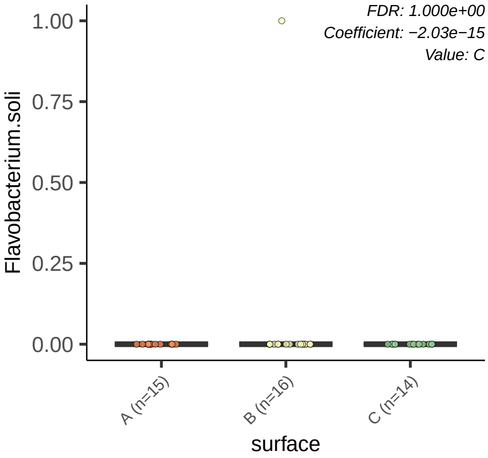
FDR: 1.000e+00
Coefficient: −2.03e−15
Value: C
Flavobacterium.soli
1.00
0.75
0.50
0.25
0.00
A (n=15)
B (n=16)
C (n=14)
surface

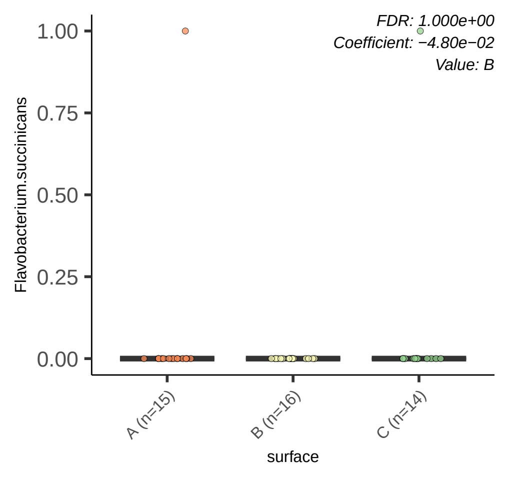
FDR: 1.000e+00
Coefficient: −4.80e−02
Value: B
Flavobacterium.succinicans
1.00
0.75
0.50
0.25
0.00
A (n=15)
B (n=16)
C (n=14)
surface

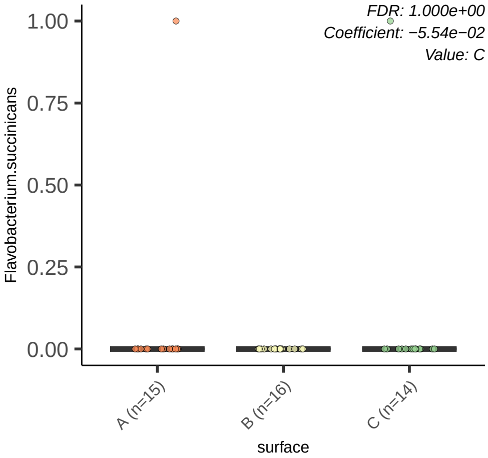

FDR: 1.000e+00
Coefficient: −5.54e−02
Value: C
Flavobacterium.succinicans
1.00
0.75
0.50
0.25
0.00
A (n=15)
B (n=16)
C (n=14)
surface

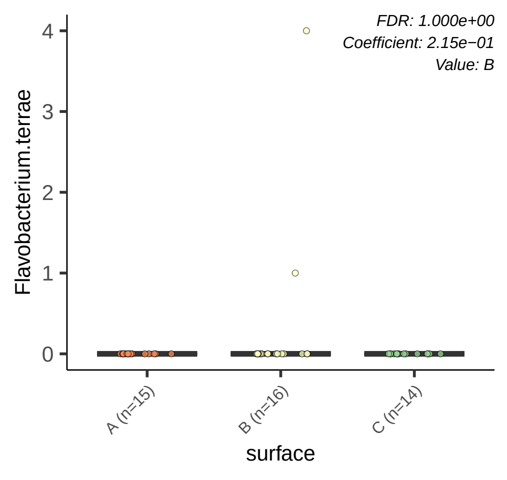

FDR: 1.000e+00
Coefficient: 2.15e−01
Value: B
Flavobacterium.terrae
0
1
2
3
4
A (n=15)
B (n=16)
C (n=14)
surface

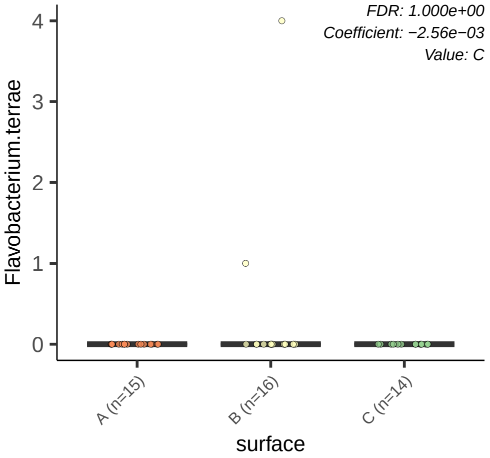
FDR: 1.000e+00
Coefficient: −2.56e−03
Value: C
Flavobacterium.terrae
4
3
2
1
0
A (n=15)
B (n=16)
C (n=14)
surface

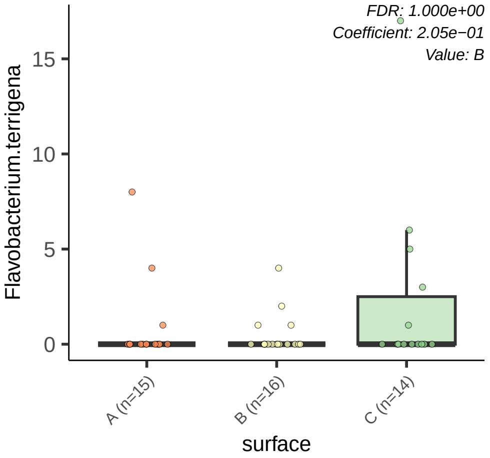

FDR: 1.000e+00
Coefficient: 2.05e−01
Value: B
Flavobacterium.terrigena
0
5
10
15
A (n=15)
B (n=16)
C (n=14)
surface

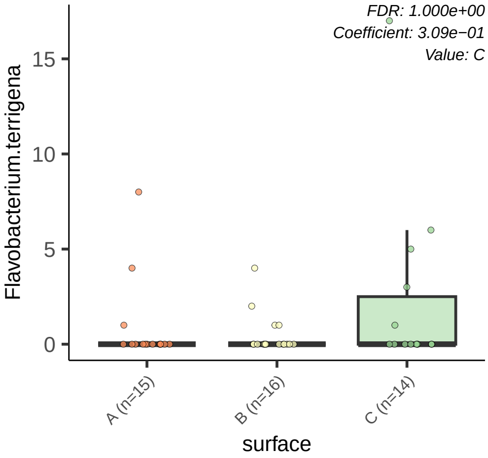
FDR: 1.000e+00
Coefficient: 3.09e−01
Value: C
Flavobacterium.terrigena
15
10
5
0
A (n=15)
B (n=16)
C (n=14)
surface

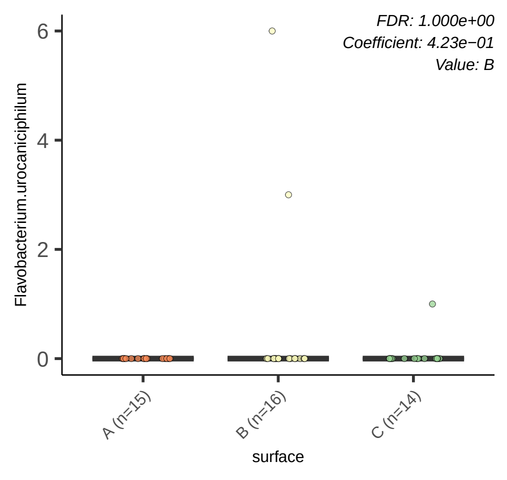
FDR: 1.000e+00
Coefficient: 4.23e−01
Value: B
Flavobacterium.urocaniciphilum
6
4
2
0
A (n=15)
B (n=16)
C (n=14)
surface

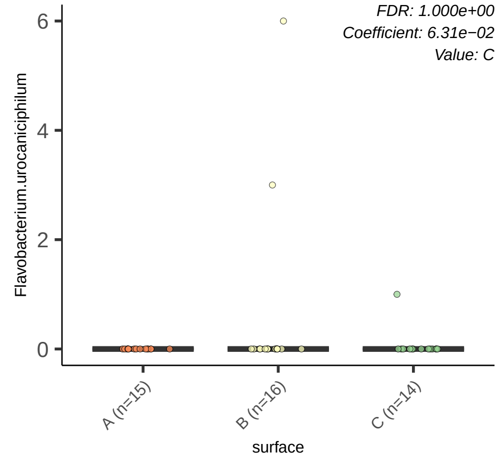
FDR: 1.000e+00
Coefficient: 6.31e−02
Value: C
Flavobacterium.urocaniciphilum
6
4
2
0
A (n=15)
B (n=16)
C (n=14)
surface

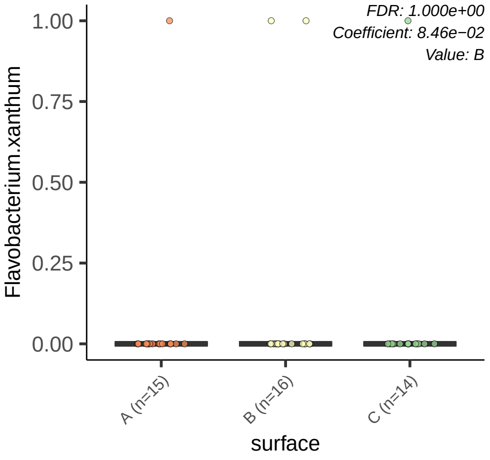

FDR: 1.000e+00
Coefficient: 8.46e−02
Value: B
Flavobacterium.xanthum
1.00
0.75
0.50
0.25
0.00
A (n=15)
B (n=16)
C (n=14)
surface

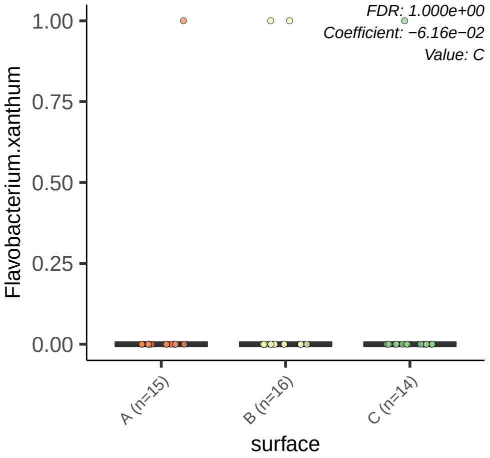

FDR: 1.000e+00
Coefficient: −6.16e−02
Value: C
Flavobacterium.xanthum
1.00
0.75
0.50
0.25
0.00
A (n=15)
B (n=16)
C (n=14)
surface

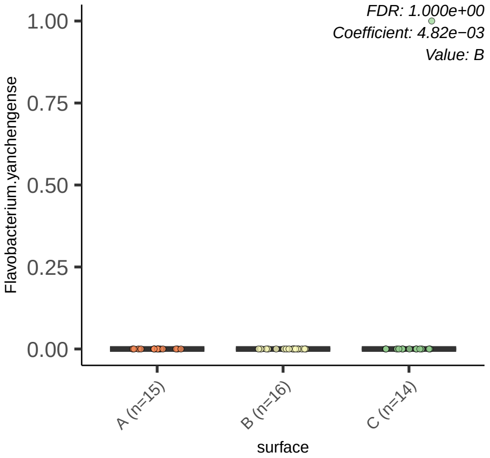
FDR: 1.000e+00
Coefficient: 4.82e−03
Value: B
1.00
0.75
0.50
0.25
0.00
Flavobacterium.yanchengense
A (n=15)
B (n=16)
C (n=14)
surface

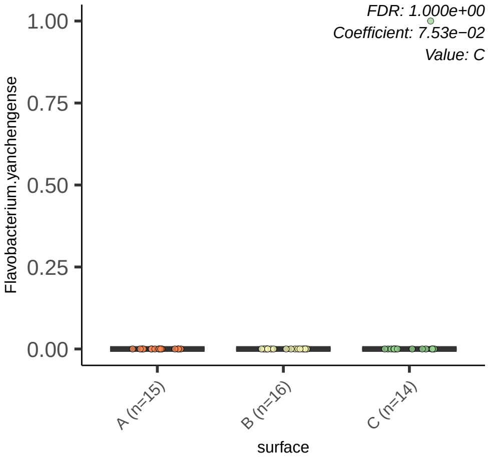

FDR: 1.000e+00
Coefficient: 7.53e−02
Value: C
Flavobacterium.yanchengense
1.00
0.75
0.50
0.25
0.00
A (n=15)
B (n=16)
C (n=14)
surface

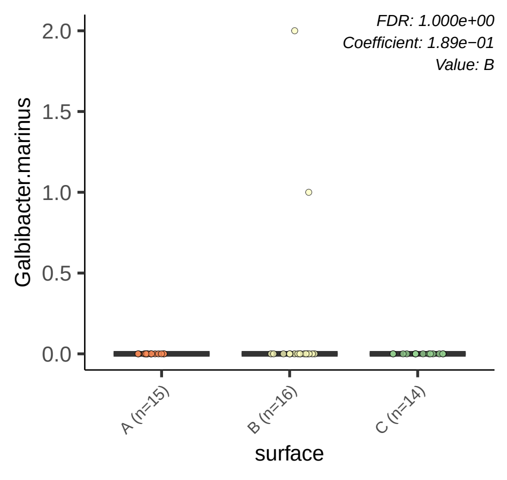

FDR: 1.000e+00
Coefficient: 1.89e−01
Value: B
Galbibacter.marinus
2.0
1.5
1.0
0.5
0.0
A (n=15)
B (n=16)
C (n=14)
surface

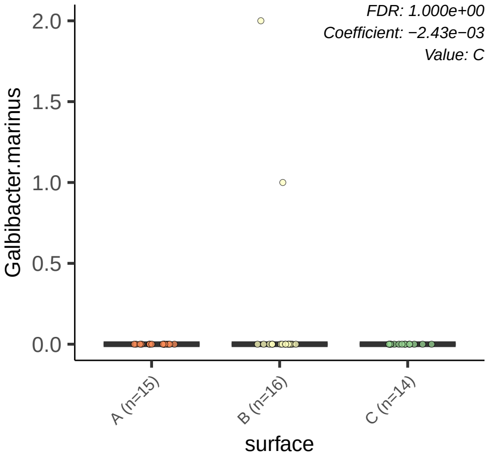
FDR: 1.000e+00
Coefficient: −2.43e−03
Value: C
Galbibacter.marinus
2.0
1.5
1.0
0.5
0.0
A (n=15)
B (n=16)
C (n=14)
surface

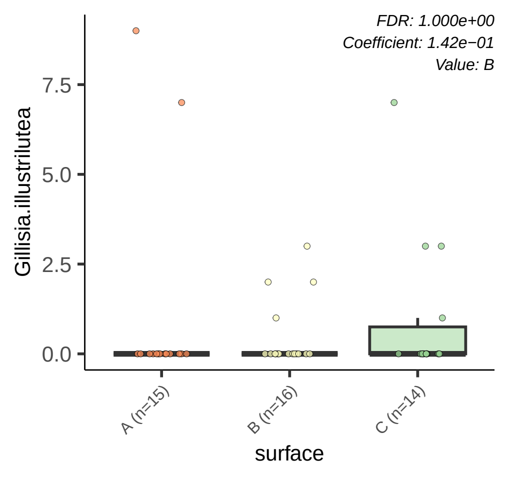
FDR: 1.000e+00
Coefficient: 1.42e−01
Value: B
Gillisia.illustrilutea
7.5
5.0
2.5
0.0
A (n=15)
B (n=16)
C (n=14)
surface

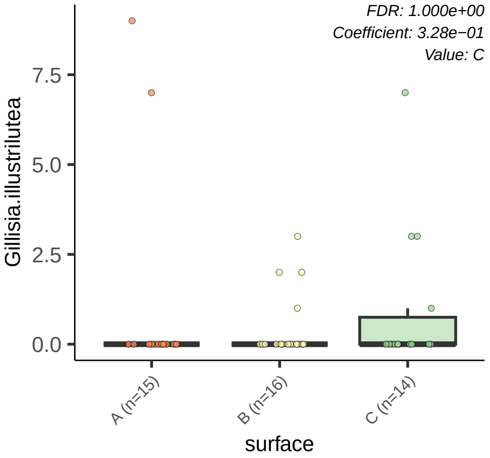
FDR: 1.000e+00
Coefficient: 3.28e−01
Value: C
Gillisia.illustrilutea
7.5
5.0
2.5
0.0
A (n=15)
B (n=16)
C (n=14)
surface

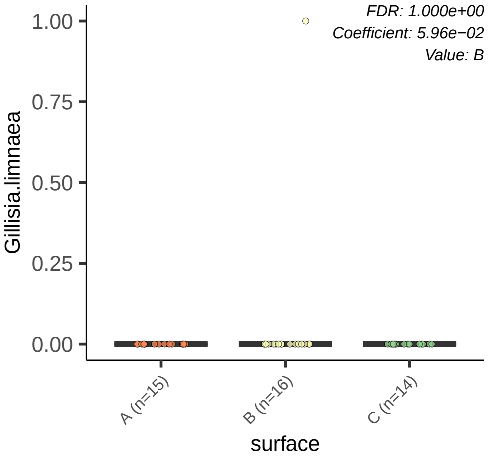
FDR: 1.000e+00
Coefficient: 5.96e−02
Value: B
Gillisia.limnaea
1.00
0.75
0.50
0.25
0.00
A (n=15)
B (n=16)
C (n=14)
surface

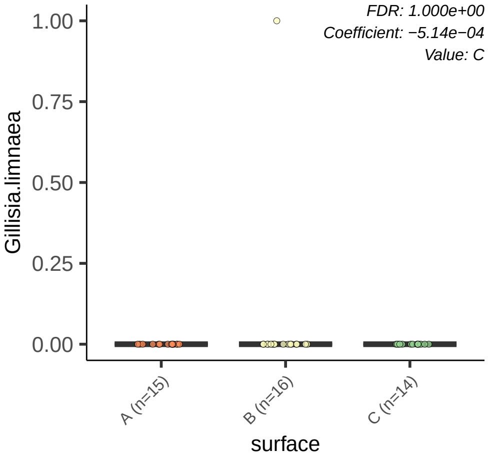
FDR: 1.000e+00
Coefficient: −5.14e−04
Value: C
Gillisia.limnaea
1.00
0.75
0.50
0.25
0.00
A (n=15)
B (n=16)
C (n=14)
surface

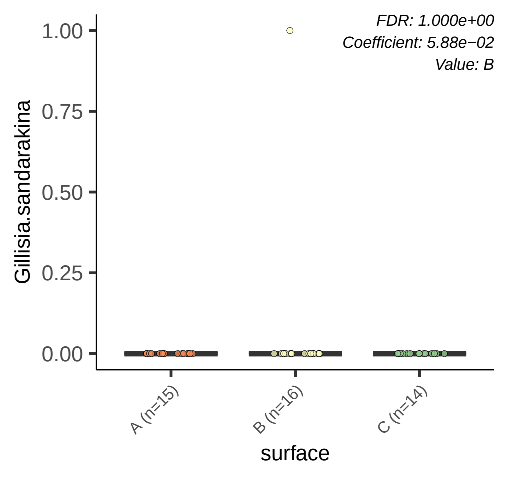
FDR: 1.000e+00
Coefficient: 5.88e−02
Value: B
Gillisia.sandarakina
1.00
0.75
0.50
0.25
0.00
A (n=15)
B (n=16)
C (n=14)
surface

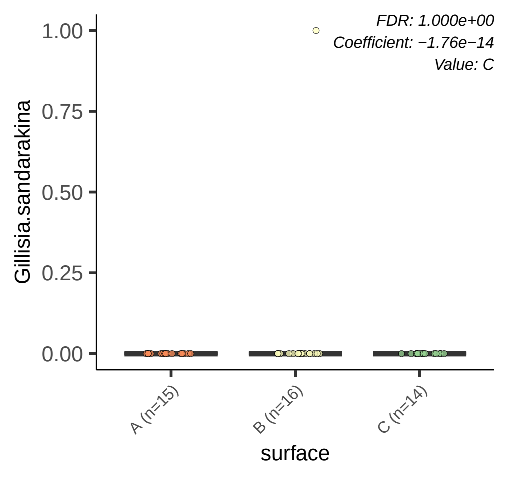
FDR: 1.000e+00
Coefficient: −1.76e−14
Value: C
Gillisia.sandarakina
1.00
0.75
0.50
0.25
0.00
A (n=15)
B (n=16)
C (n=14)
surface

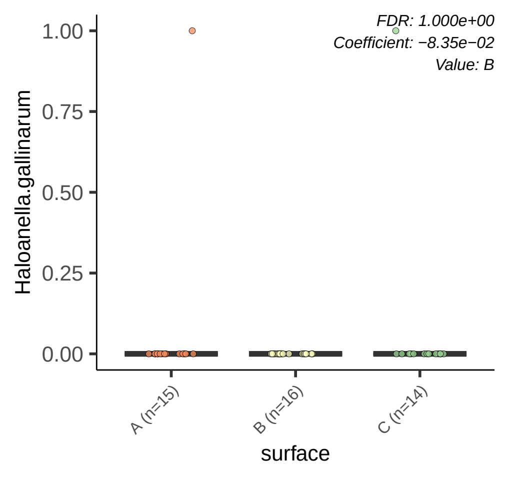
FDR: 1.000e+00
Coefficient: −8.35e−02
Value: B
Haloanella.gallinarum
1.00
0.75
0.50
0.25
0.00
A (n=15)
B (n=16)
C (n=14)
surface

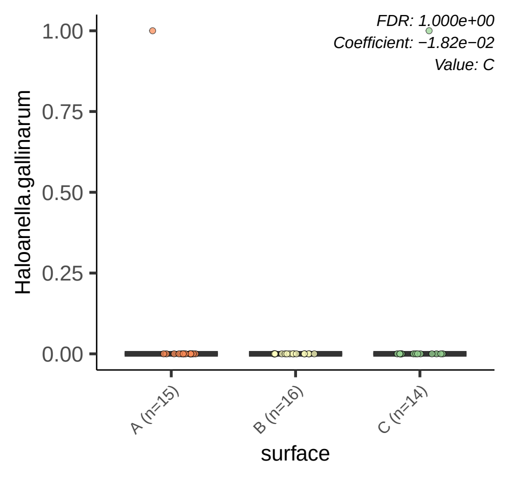
FDR: 1.000e+00
Coefficient: −1.82e−02
Value: C
Haloanella.gallinarum
1.00
0.75
0.50
0.25
0.00
A (n=15)
B (n=16)
C (n=14)
surface

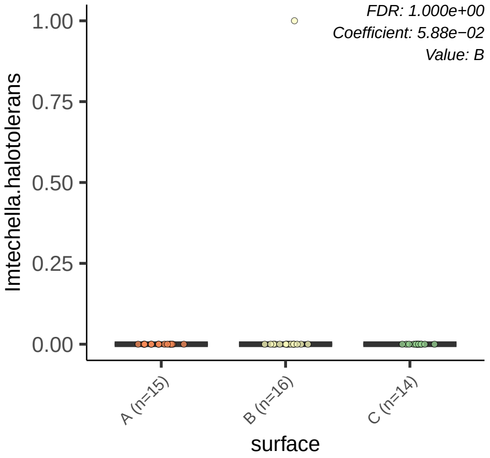

FDR: 1.000e+00
Coefficient: 5.88e−02
Value: B
Imtechella.halotolerans
1.00
0.75
0.50
0.25
0.00
A (n=15)
B (n=16)
C (n=14)
surface

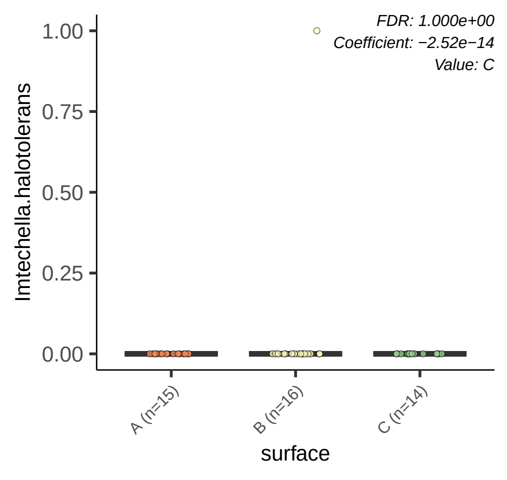
FDR: 1.000e+00
Coefficient: −2.52e−14
Value: C
Imtechella.halotolerans
1.00
0.75
0.50
0.25
0.00
A (n=15)
B (n=16)
C (n=14)
surface

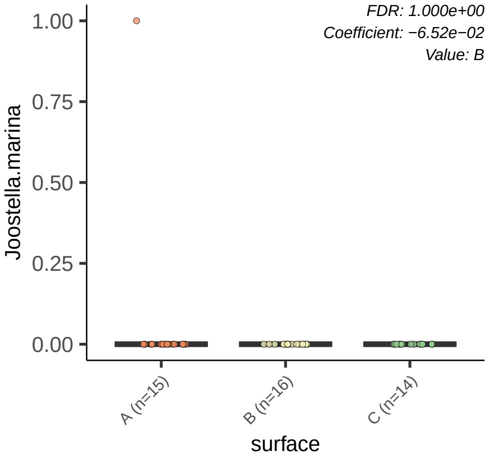
FDR: 1.000e+00
Coefficient: −6.52e−02
Value: B
Joostella.marina
1.00
0.75
0.50
0.25
0.00
A (n=15)
B (n=16)
C (n=14)
surface

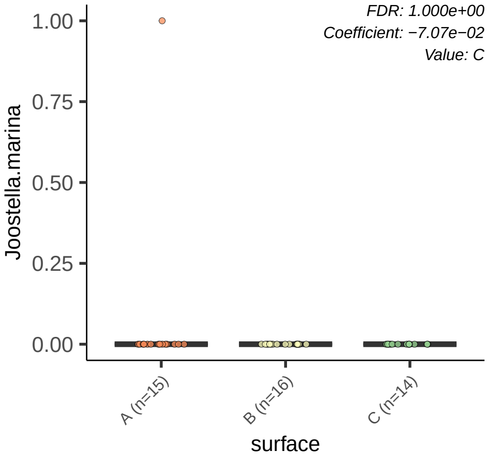

FDR: 1.000e+00
Coefficient: −7.07e−02
Value: C
Joostella.marina
1.00
0.75
0.50
0.25
0.00
A (n=15)
B (n=16)
C (n=14)
surface

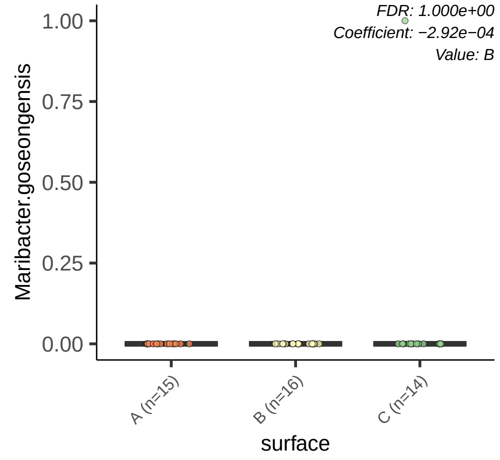

FDR: 1.000e+00
Coefficient: −2.92e−04
Value: B
Maribacter.goseongensis
1.00
0.75
0.50
0.25
0.00
A (n=15)
B (n=16)
C (n=14)
surface

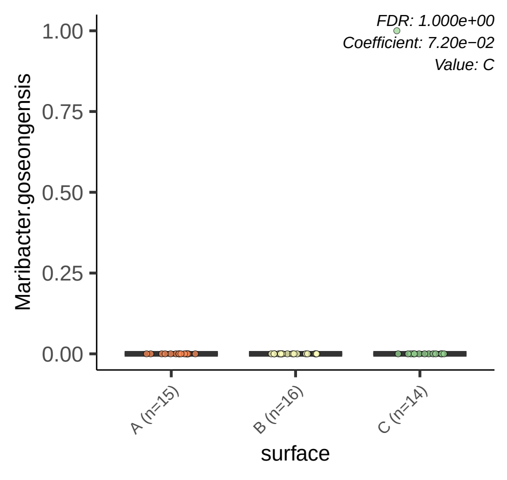
FDR: 1.000e+00
Coefficient: 7.20e−02
Value: C
Maribacter.goseongensis
1.00
0.75
0.50
0.25
0.00
A (n=15)
B (n=16)
C (n=14)
surface

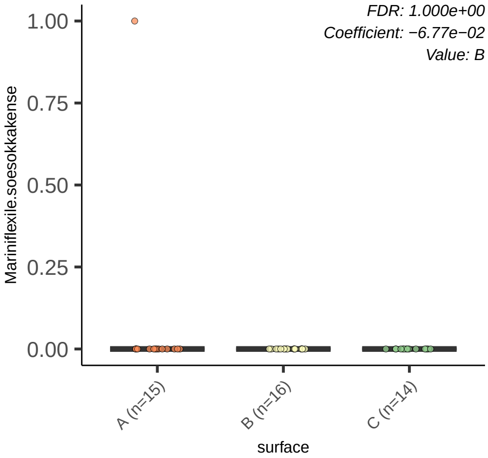
FDR: 1.000e+00
Coefficient: −6.77e−02
Value: B
Mariniflexile.soesokkakense
1.00
0.75
0.50
0.25
0.00
A (n=15)
B (n=16)
C (n=14)
surface

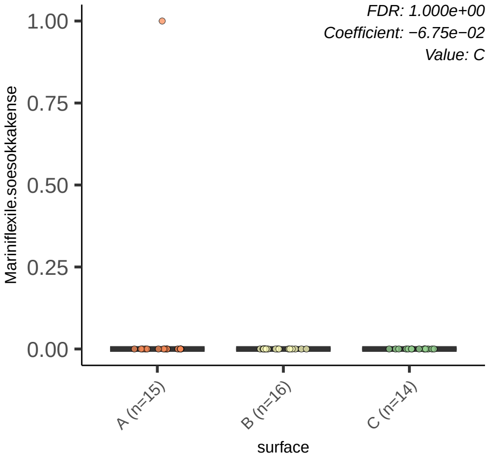
FDR: 1.000e+00
Coefficient: −6.75e−02
Value: C
Mariniflexile.soesokkakense
1.00
0.75
0.50
0.25
0.00
A (n=15)
B (n=16)
C (n=14)
surface

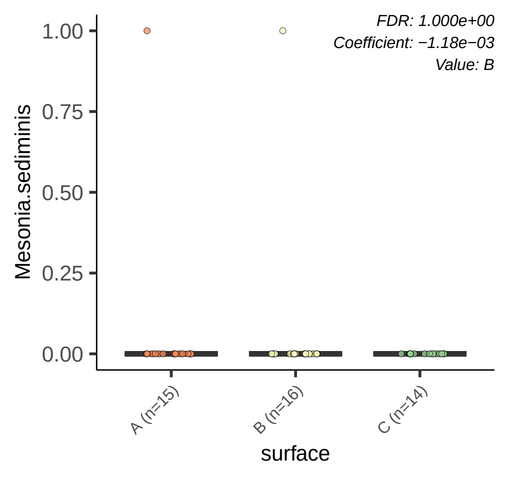
FDR: 1.000e+00
Coefficient: −1.18e−03
Value: B
Mesonia.sedeminis
1.00
0.75
0.50
0.25
0.00
A (n=15)
B (n=16)
C (n=14)
surface

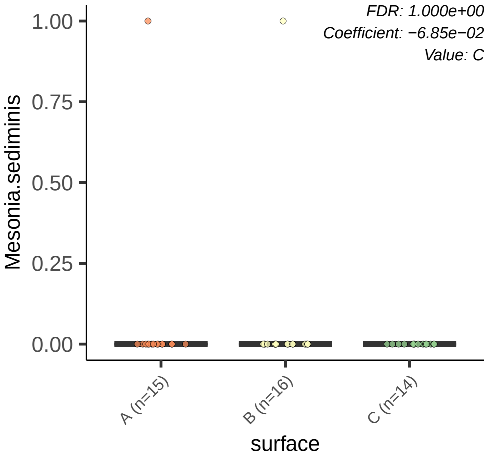
FDR: 1.000e+00
Coefficient: −6.85e−02
Value: C
Mesonia.sedimini
1.00
0.75
0.50
0.25
0.00
A (n=15)
B (n=16)
C (n=14)
surface

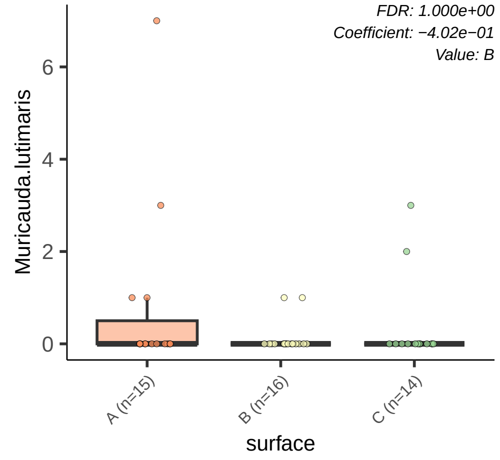
FDR: 1.000e+00
Coefficient: −4.02e−01
Value: B
Muricauda.lutimaris
0
2
4
6
A (n=15)
B (n=16)
C (n=14)
surface

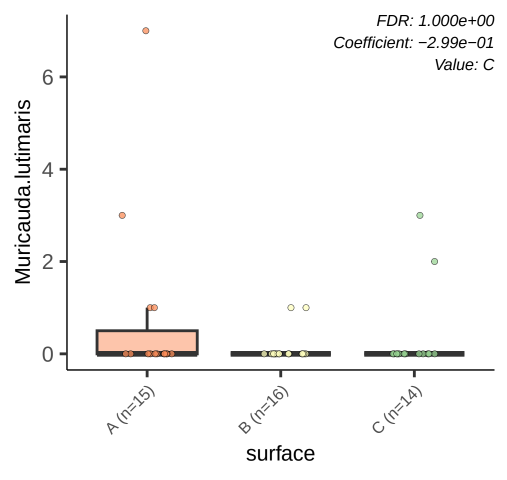
FDR: 1.000e+00
Coefficient: −2.99e−01
Value: C
Muricauda.lutimaris
0
2
4
6
A (n=15)
B (n=16)
C (n=14)
surface

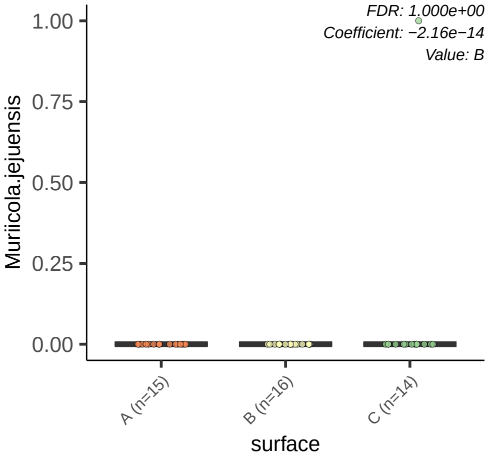
FDR: 1.000e+00
Coefficient: −2.16e−14
Value: B
Muriicola.jejuensis
1.00
0.75
0.50
0.25
0.00
A (n=15)
B (n=16)
C (n=14)
surface

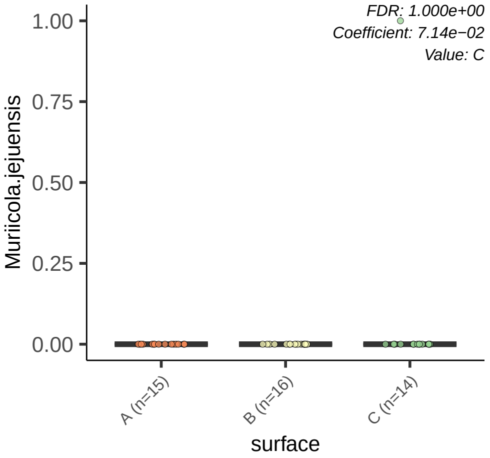
FDR: 1.000e+00
Coefficient: 7.14e−02
Value: C
Muriicola.jejuensis
1.00
0.75
0.50
0.25
0.00
A (n=15)
B (n=16)
C (n=14)
surface

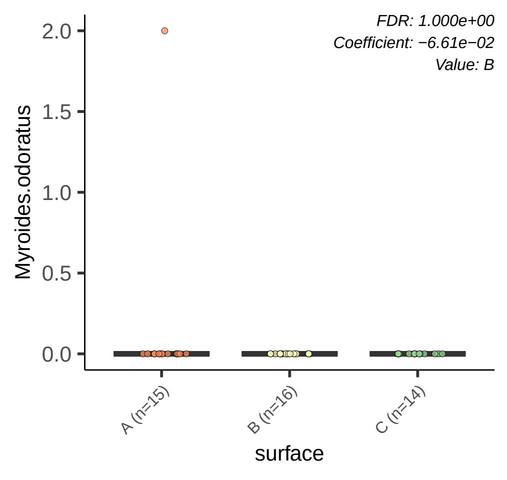

FDR: 1.000e+00
Coefficient: −6.61e−02
Value: B
Myroides.odoratus
2.0
1.5
1.0
0.5
0.0
A (n=15)
B (n=16)
C (n=14)
surface

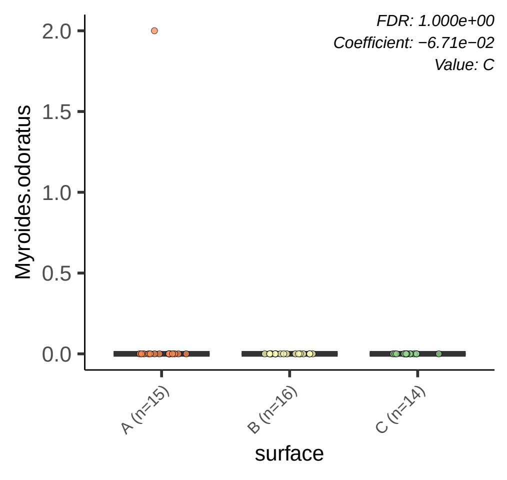
FDR: 1.000e+00
Coefficient: −6.71e−02
Value: C
Myroides.odoratus
2.0
1.5
1.0
0.5
0.0
A (n=15)
B (n=16)
C (n=14)
surface

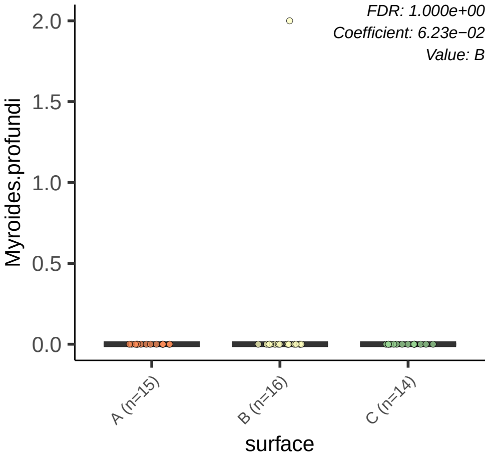
FDR: 1.000e+00
Coefficient: 6.23e−02
Value: B
Myroides.profundi
2.0
1.5
1.0
0.5
0.0
A (n=15)
B (n=16)
C (n=14)
surface

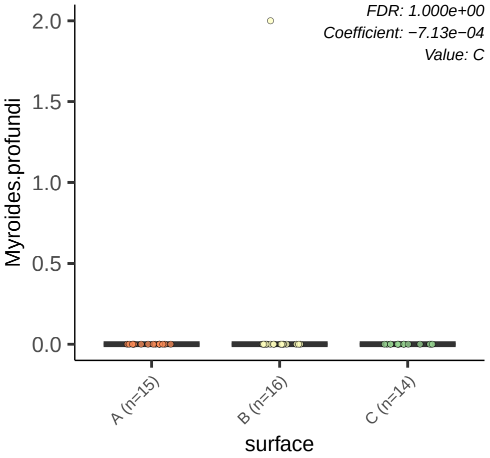

FDR: 1.000e+00
Coefficient: −7.13e−04
Value: C
Myroides.profundi
2.0
1.5
1.0
0.5
0.0
A (n=15)
B (n=16)
C (n=14)
surface

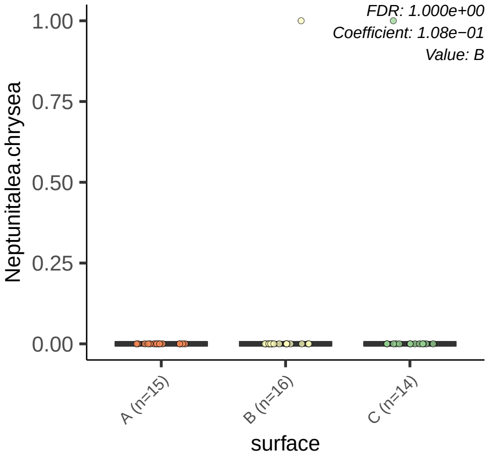
FDR: 1.000e+00
Coefficient: 1.08e−01
Value: B
Neptunitalea.chrysea
1.00
0.75
0.50
0.25
0.00
A (n=15)
B (n=16)
C (n=14)
surface

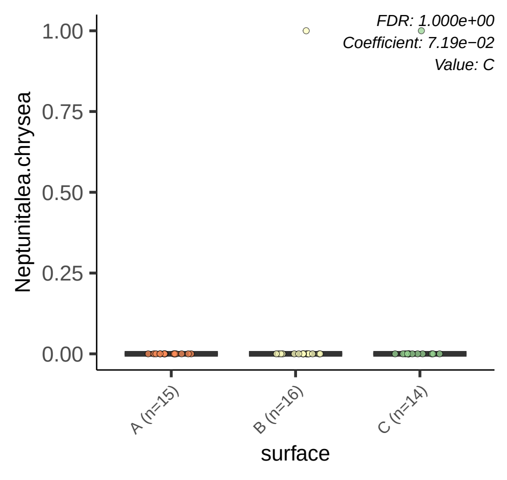
FDR: 1.000e+00
Coefficient: 7.19e−02
Value: C
Neptunitalea.chrysea
1.00
0.75
0.50
0.25
0.00
A (n=15)
B (n=16)
C (n=14)
surface

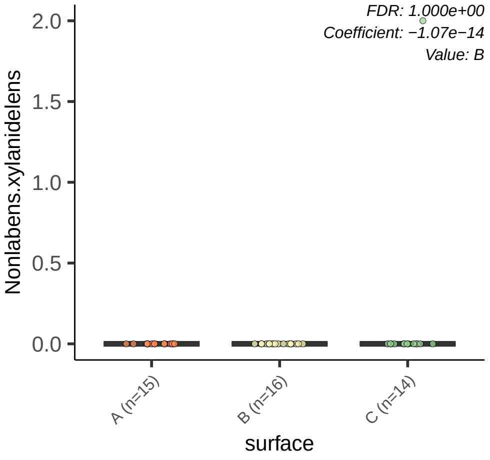
FDR: 1.000e+00
Coefficient: −1.07e−14
Value: B
Nonlabens.xylanidelens
2.0
1.5
1.0
0.5
0.0
A (n=15)
B (n=16)
C (n=14)
surface

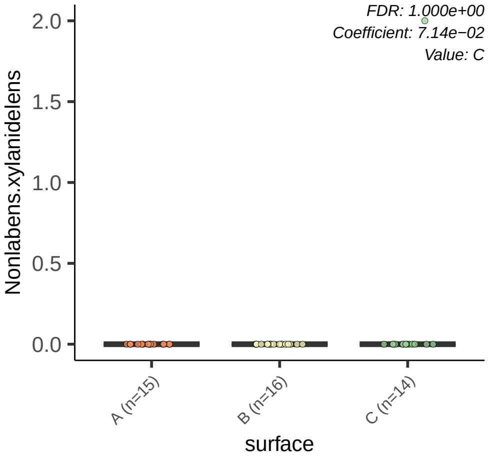
FDR: 1.000e+00
Coefficient: 7.14e−02
Value: C
Nonlabens.xylanidelens
2.0
1.5
1.0
0.5
0.0
A (n=15)
B (n=16)
C (n=14)
surface

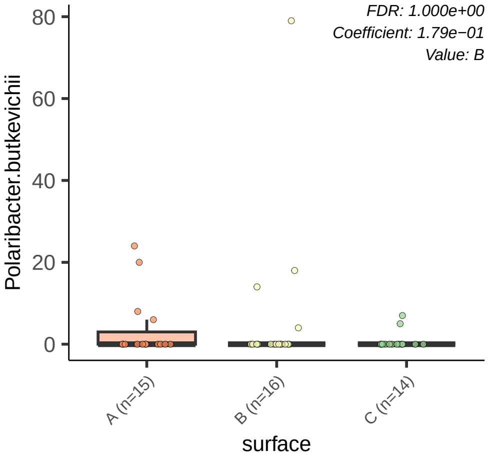

FDR: 1.000e+00
Coefficient: 1.79e−01
Value: B
Polaribacter.butkevichii
0
20
40
60
80
A (n=15)
B (n=16)
C (n=14)
surface

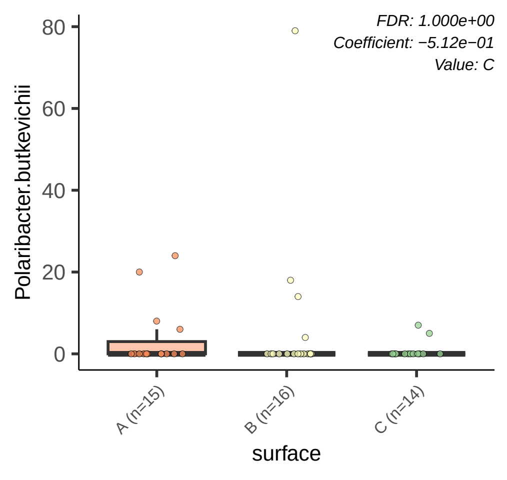

FDR: 1.000e+00
Coefficient: −5.12e−01
Value: C
Polaribacter.butkevichii
0
20
40
60
80
A (n=15)
B (n=16)
C (n=14)
surface

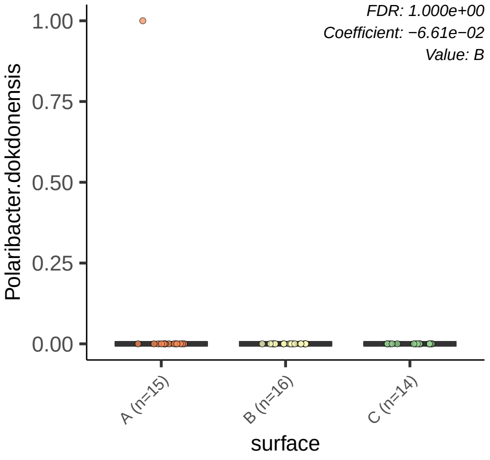
FDR: 1.000e+00
Coefficient: −6.61e−02
Value: B
Polaribacter.dokdonensis
1.00
0.75
0.50
0.25
0.00
A (n=15)
B (n=16)
C (n=14)
surface

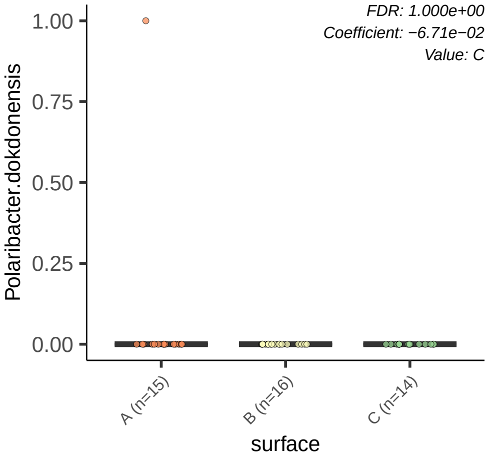
FDR: 1.000e+00
Coefficient: −6.71e−02
Value: C
Polaribacter.dokdonensis
1.00
0.75
0.50
0.25
0.00
A (n=15)
B (n=16)
C (n=14)
surface

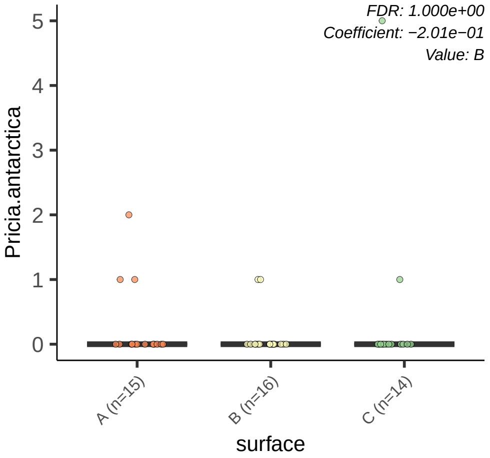
FDR: 1.000e+00
Coefficient: −2.01e−01
Value: B
Pricia.antarctica
0
1
2
3
4
5
A (n=15)
B (n=16)
C (n=14)
surface

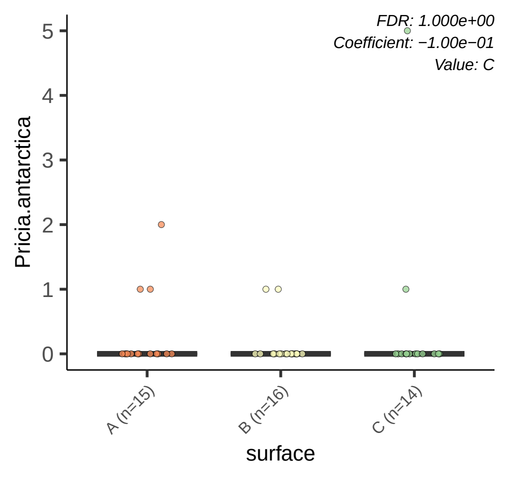
FDR: 1.000e+00
Coefficient: −1.00e−01
Value: C
Pricia.antarctica
0
1
2
3
4
5
A (n=15)
B (n=16)
C (n=14)
surface

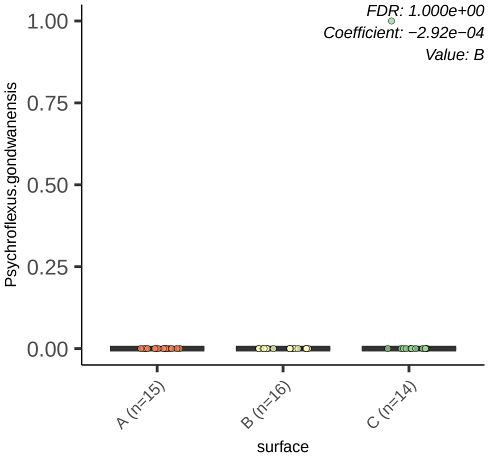
FDR: 1.000e+00
Coefficient: −2.92e−04
Value: B
Psychroflexus.gondwanensis
1.00
0.75
0.50
0.25
0.00
A (n=15)
B (n=16)
C (n=14)
surface

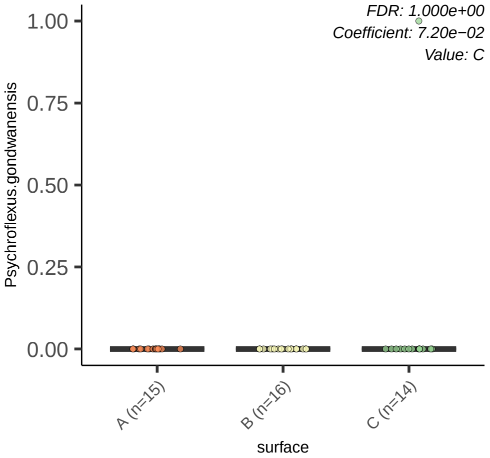

FDR: 1.000e+00
Coefficient: 7.20e−02
Value: C
Psychroflexus.gondwanensis
1.00
0.75
0.50
0.25
0.00
A (n=15)
B (n=16)
C (n=14)
surface

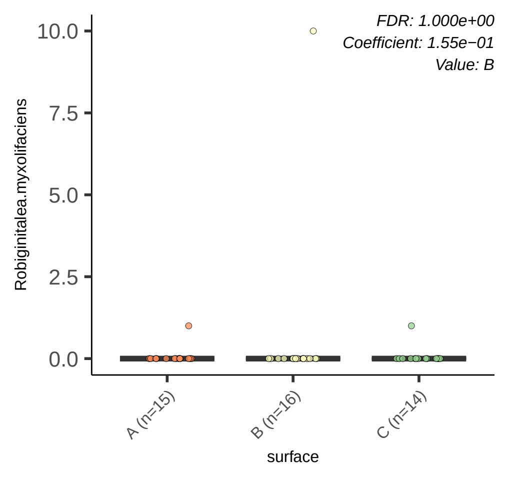
FDR: 1.000e+00
Coefficient: 1.55e−01
Value: B
10.0
7.5
5.0
2.5
0.0
Robiginitalea.myxolifaciens
A (n=15)
B (n=16)
C (n=14)
surface

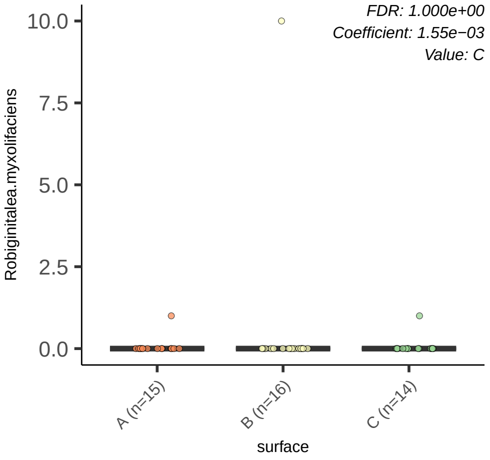

FDR: 1.000e+00
Coefficient: 1.55e−03
Value: C
Robiginitalea.myxolifaciens
10.0
7.5
5.0
2.5
0.0
A (n=15)
B (n=16)
C (n=14)
surface

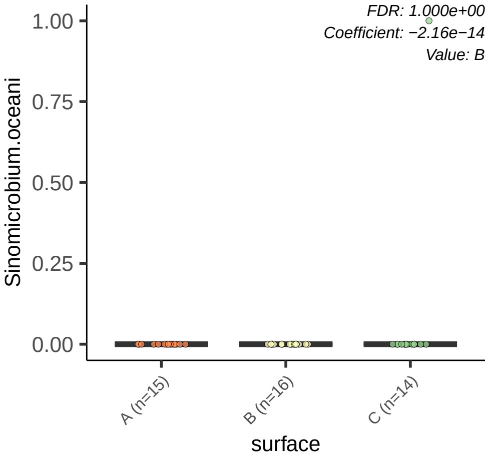

FDR: 1.000e+00
Coefficient: −2.16e−14
Value: B
Sinomicrobium.oceani
1.00
0.75
0.50
0.25
0.00
A (n=15)
B (n=16)
C (n=14)
surface

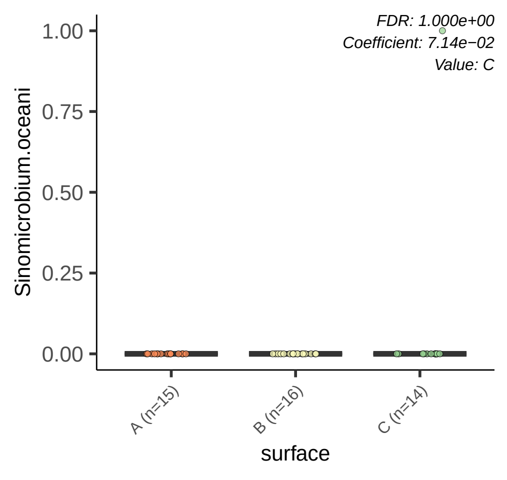

FDR: 1.000e+00
Coefficient: 7.14e−02
Value: C
Sinomicrobium.oceani
1.00
0.75
0.50
0.25
0.00
A (n=15)
B (n=16)
C (n=14)
surface

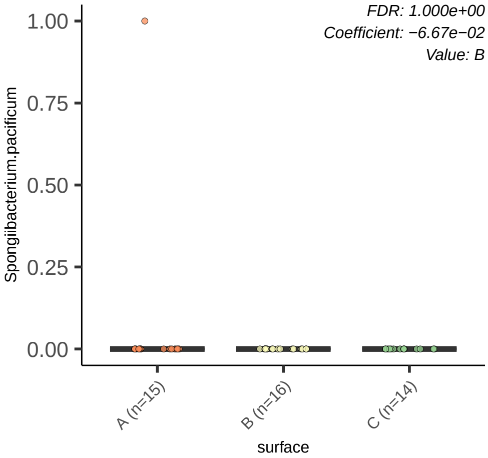

FDR: 1.000e+00
Coefficient: −6.67e−02
Value: B
1.00
0.75
0.50
0.25
0.00
Spongiibacterium.pacificum
A (n=15)
B (n=16)
C (n=14)
surface

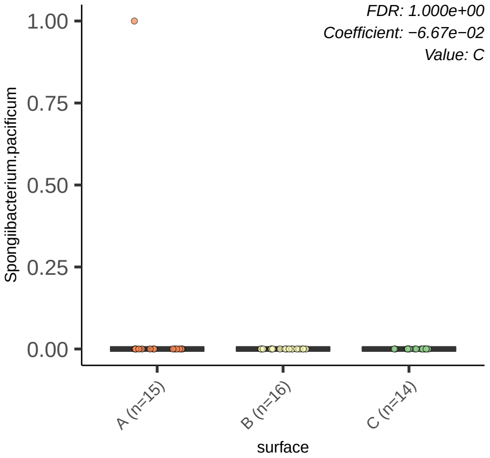
FDR: 1.000e+00
Coefficient: −6.67e−02
Value: C
Spongiibacterium.pacificum
1.00
0.75
0.50
0.25
0.00
A (n=15)
B (n=16)
C (n=14)
surface

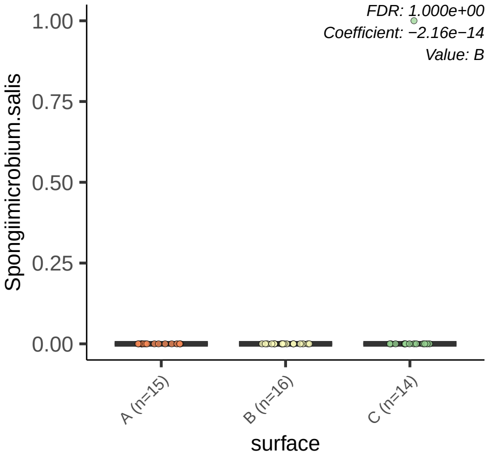
FDR: 1.000e+00
Coefficient: −2.16e−14
Value: B
Spongiimicrobium.salis
1.00
0.75
0.50
0.25
0.00
A (n=15)
B (n=16)
C (n=14)
surface

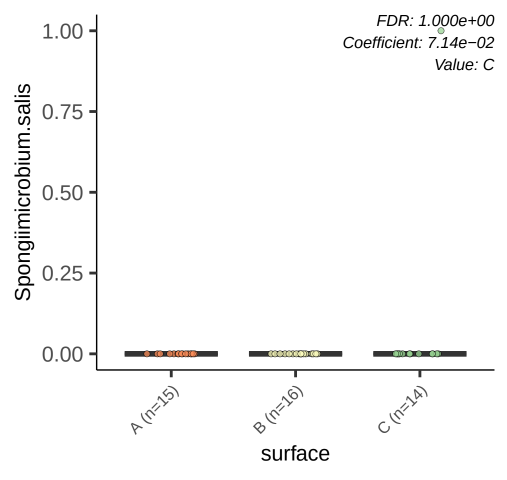
FDR: 1.000e+00
Coefficient: 7.14e−02
Value: C
Spongiimicrobium.salis
1.00
0.75
0.50
0.25
0.00
A (n=15)
B (n=16)
C (n=14)
surface

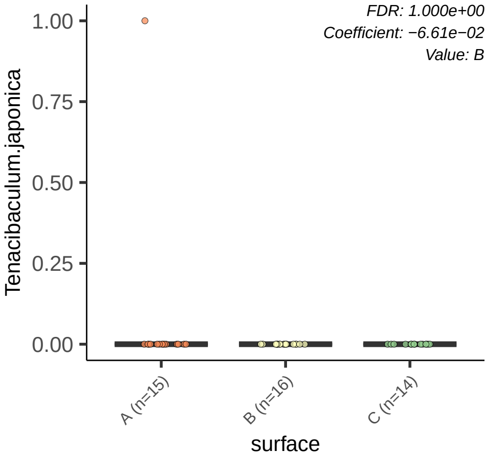

FDR: 1.000e+00
Coefficient: −6.61e−02
Value: B
Tenacibaculum.japonica
1.00
0.75
0.50
0.25
0.00
A (n=15)
B (n=16)
C (n=14)
surface

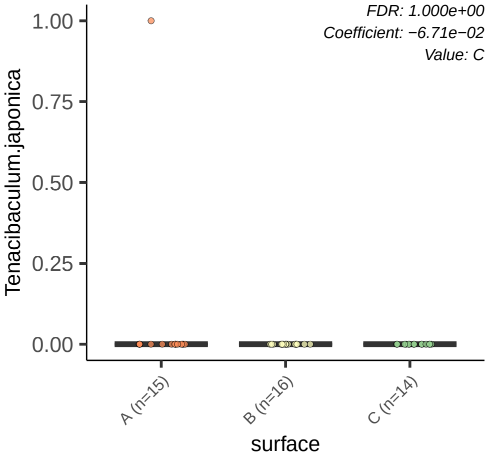
FDR: 1.000e+00
Coefficient: −6.71e−02
Value: C
Tenacibaculum.japonica
1.00
0.75
0.50
0.25
0.00
A (n=15)
B (n=16)
C (n=14)
surface

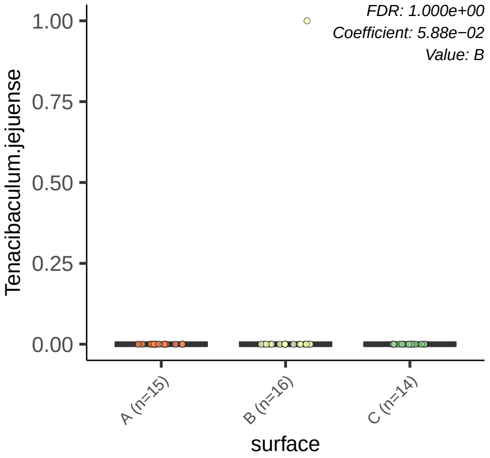
FDR: 1.000e+00
Coefficient: 5.88e−02
Value: B
Tenacibaculum.jejuense
1.00
0.75
0.50
0.25
0.00
A (n=15)
B (n=16)
C (n=14)
surface

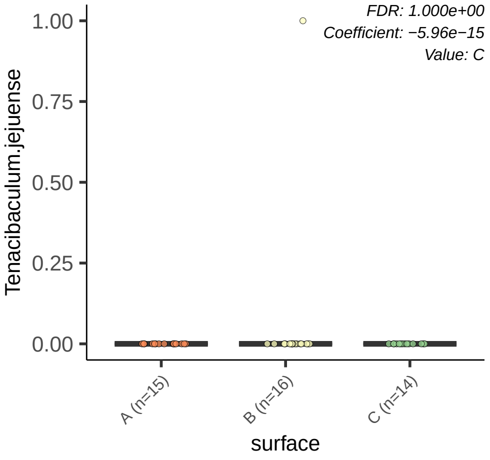
FDR: 1.000e+00
Coefficient: −5.96e−15
Value: C
Tenacibaculum.jejuense
1.00
0.75
0.50
0.25
0.00
A (n=15)
B (n=16)
C (n=14)
surface

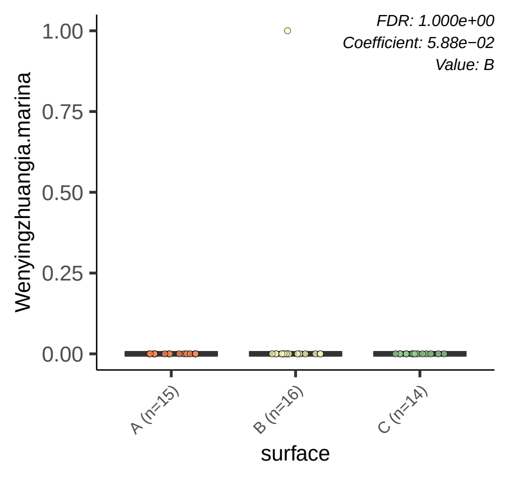

FDR: 1.000e+00
Coefficient: 5.88e−02
Value: B
Wenyingzhuangia.marina
1.00
0.75
0.50
0.25
0.00
A (n=15)
B (n=16)
C (n=14)
surface

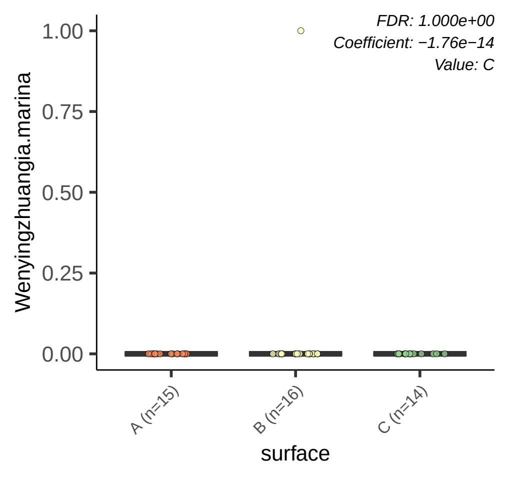
FDR: 1.000e+00
Coefficient: −1.76e−14
Value: C
Wenyingzhuangia.marina
1.00
0.75
0.50
0.25
0.00
A (n=15)
B (n=16)
C (n=14)
surface

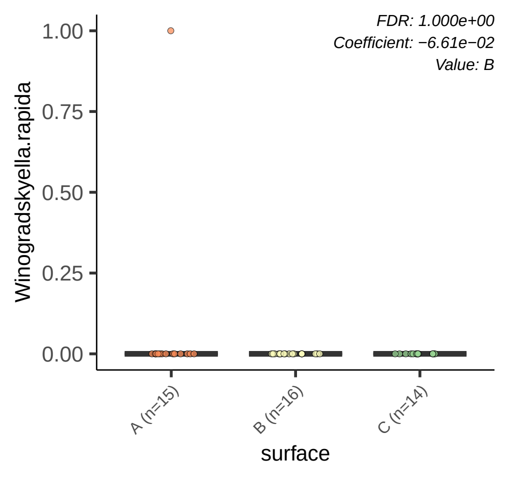

FDR: 1.000e+00
Coefficient: −6.61e−02
Value: B
Winogradskyella.rapida
1.00
0.75
0.50
0.25
0.00
A (n=15)
B (n=16)
C (n=14)
surface

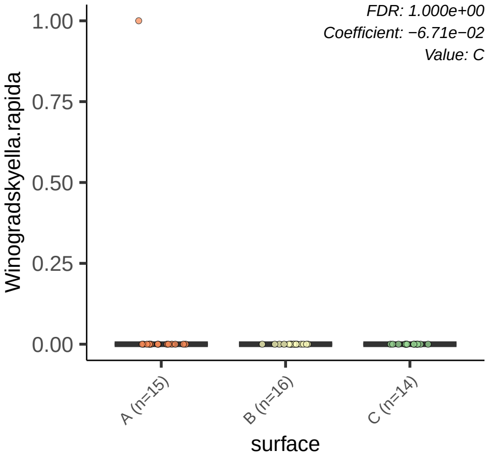

FDR: 1.000e+00
Coefficient: −6.71e−02
Value: C
Winogradskyella.rapida
1.00
0.75
0.50
0.25
0.00
A (n=15)
B (n=16)
C (n=14)
surface

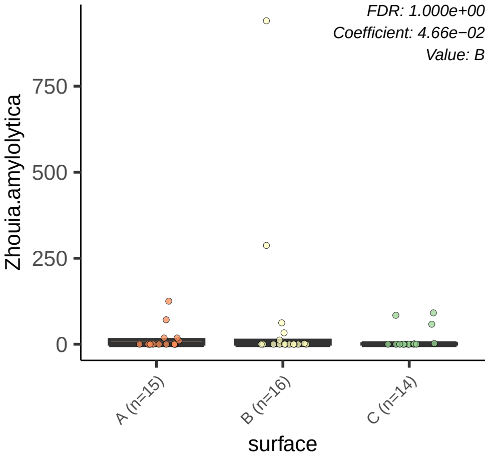

FDR: 1.000e+00
Coefficient: 4.66e−02
Value: B
Zhouia.amylolytica
750
500
250
0
A (n=15)
B (n=16)
C (n=14)
surface

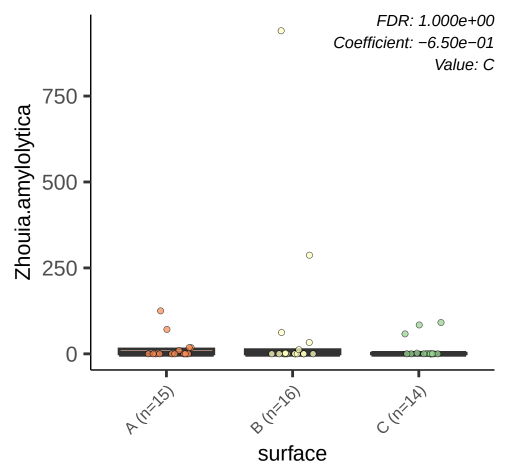

FDR: 1.000e+00
Coefficient: −6.50e−01
Value: C
Zhouia.amylolytica
750
500
250
0
A (n=15)
B (n=16)
C (n=14)
surface

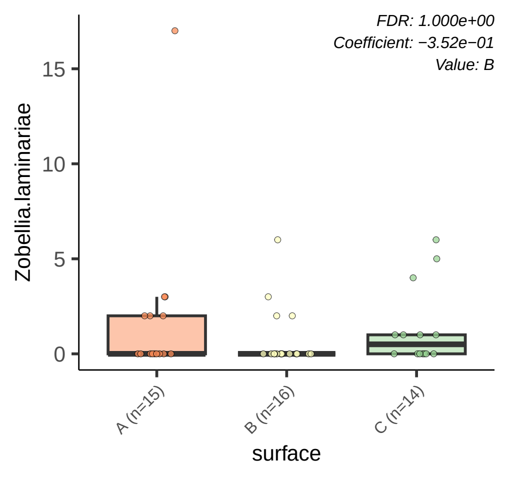

FDR: 1.000e+00
Coefficient: −3.52e−01
Value: B
Zobellia.laminariae
15
10
5
0
A (n=15)
B (n=16)
C (n=14)
surface

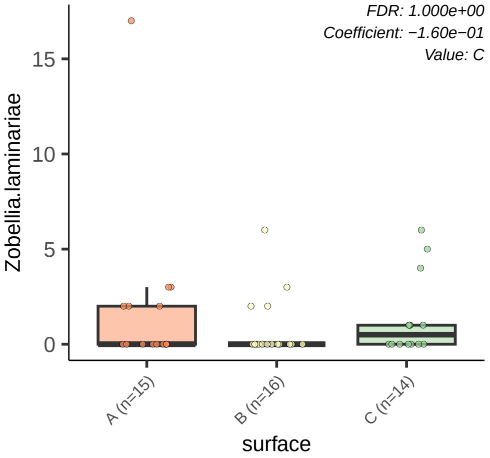

FDR: 1.000e+00
Coefficient: −1.60e−01
Value: C
Zobellia.laminariae
15
10
5
0
A (n=15)
B (n=16)
C (n=14)
surface

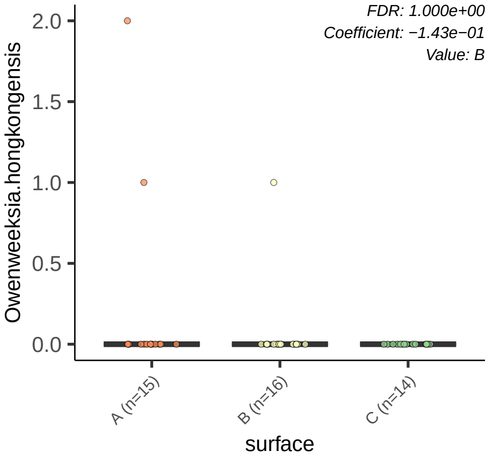
FDR: 1.000e+00
Coefficient: −1.43e−01
Value: B
Owenweeksia.hongkongensis
2.0
1.5
1.0
0.5
0.0
A (n=15)
B (n=16)
C (n=14)
surface

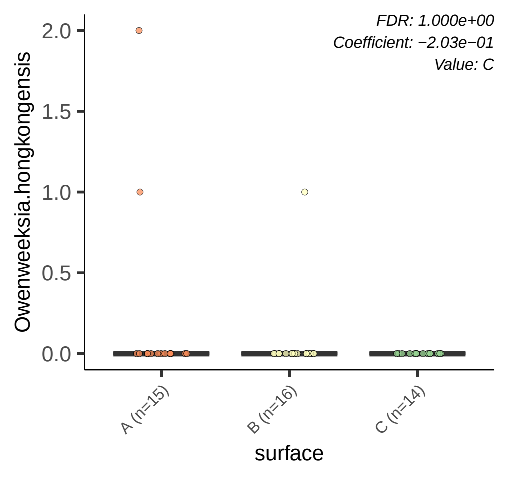
FDR: 1.000e+00
Coefficient: −2.03e−01
Value: C
Owenweeksia.hongkongensis
2.0
1.5
1.0
0.5
0.0
A (n=15)
B (n=16)
C (n=14)
surface

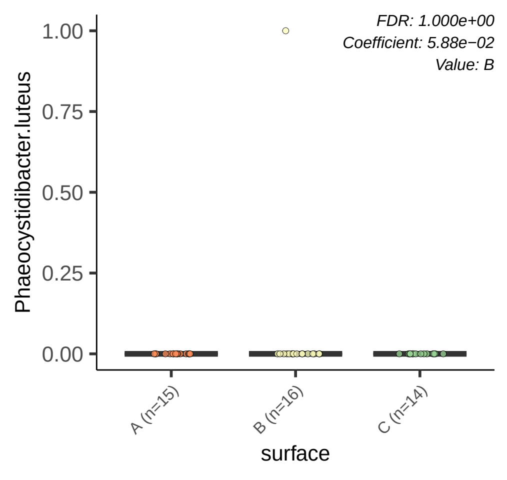

FDR: 1.000e+00
Coefficient: 5.88e−02
Value: B
Phaeocystidibacter.luteus
1.00
0.75
0.50
0.25
0.00
A (n=15)
B (n=16)
C (n=14)
surface

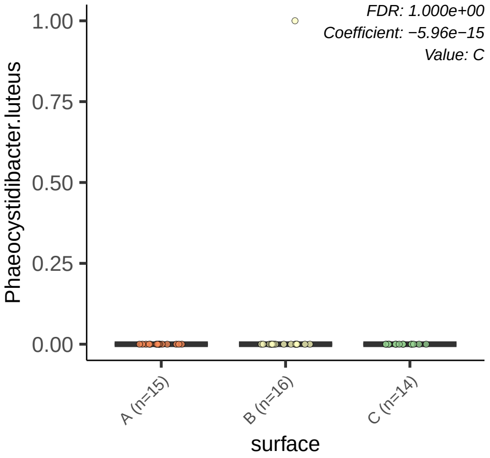

FDR: 1.000e+00
Coefficient: −5.96e−15
Value: C
Phaeocystidibacter.luteus
1.00
0.75
0.50
0.25
0.00
A (n=15)
B (n=16)
C (n=14)
surface

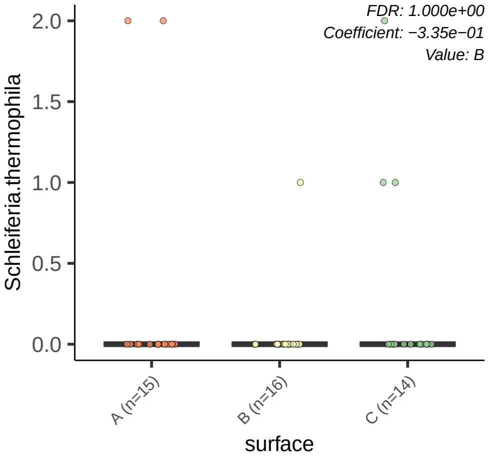
FDR: 1.000e+00
Coefficient: −3.35e−01
Value: B
Schleiferia.thermophila
2.0
1.5
1.0
0.5
0.0
A (n=15)
B (n=16)
C (n=14)
surface

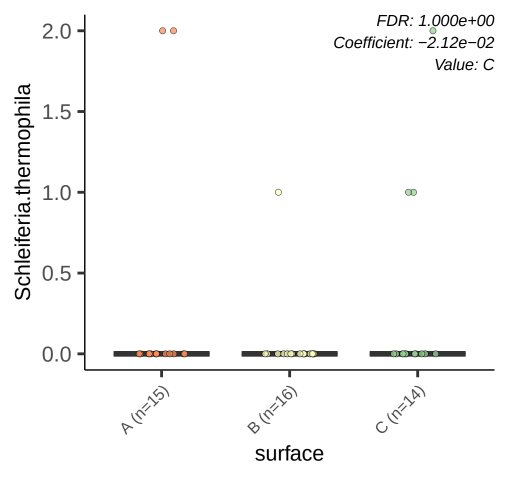

FDR: 1.000e+00
Coefficient: −2.12e−02
Value: C
Schleiferia.thermophila
2.0
1.5
1.0
0.5
0.0
A (n=15)
B (n=16)
C (n=14)
surface

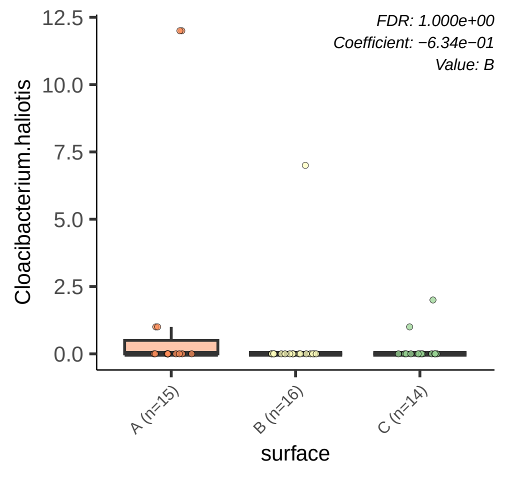

FDR: 1.000e+00
Coefficient: −6.34e−01
Value: B
Cloacibacterium.haliotis
12.5
10.0
7.5
5.0
2.5
0.0
A (n=15)
B (n=16)
C (n=14)
surface

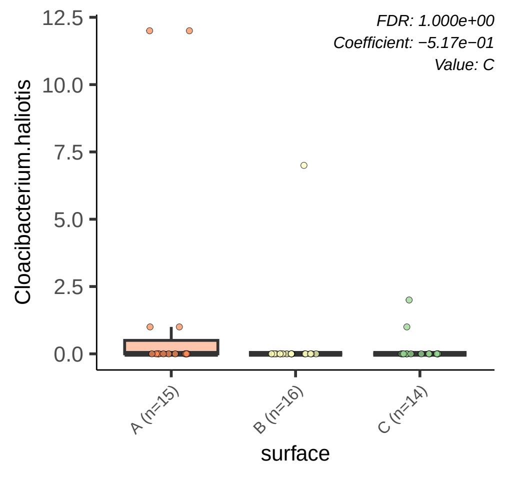

FDR: 1.000e+00
Coefficient: −5.17e−01
Value: C
Cloacibacterium.haliotis
12.5
10.0
7.5
5.0
2.5
0.0
A (n=15)
B (n=16)
C (n=14)
surface

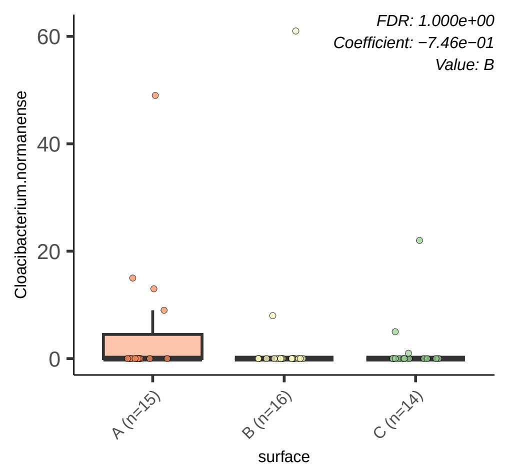
FDR: 1.000e+00
Coefficient: −7.46e−01
Value: B
Cloacibacterium.normanense
0
20
40
60
A (n=15)
B (n=16)
C (n=14)
surface

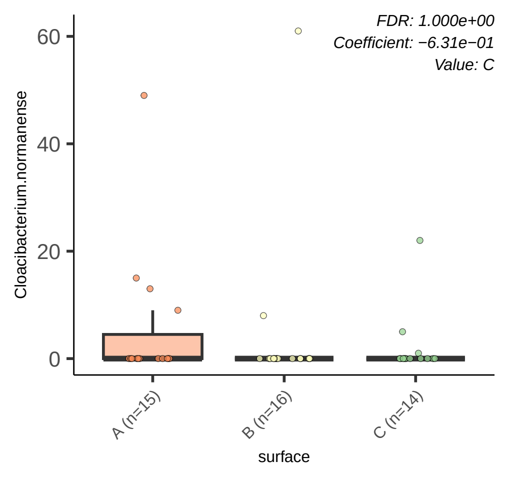
FDR: 1.000e+00
Coefficient: −6.31e−01
Value: C
Cloacibacterium.normanense
60
40
20
0
A (n=15)
B (n=16)
C (n=14)
surface

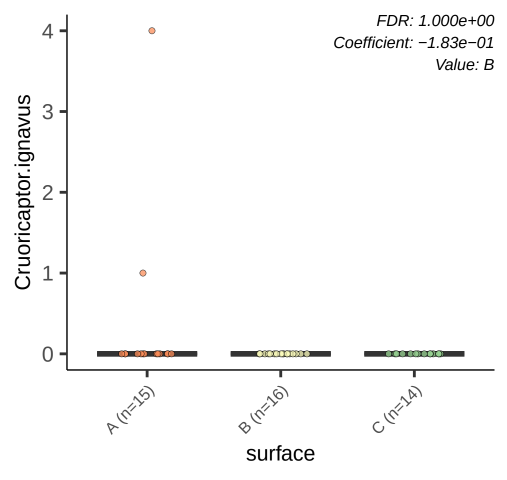
FDR: 1.000e+00
Coefficient: −1.83e−01
Value: B
Cruoricaptor.ignavus
4
3
2
1
0
A (n=15)
B (n=16)
C (n=14)
surface

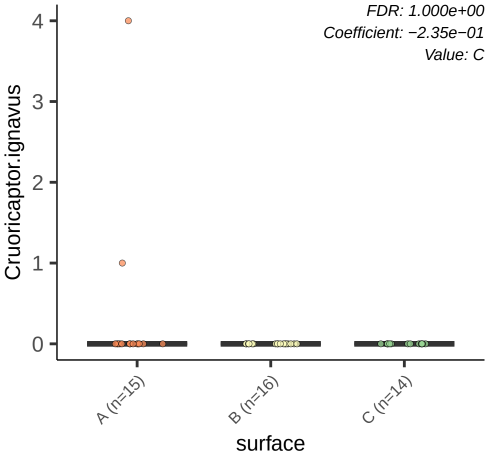
FDR: 1.000e+00
Coefficient: −2.35e−01
Value: C
Cruoricaptor.ignavus
0
1
2
3
4
A (n=15)
B (n=16)
C (n=14)
surface

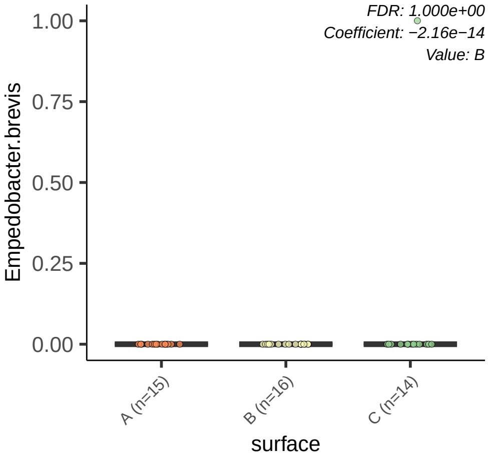
FDR: 1.000e+00
Coefficient: −2.16e−14
Value: B
Empedobacter.brevis
1.00
0.75
0.50
0.25
0.00
A (n=15)
B (n=16)
C (n=14)
surface

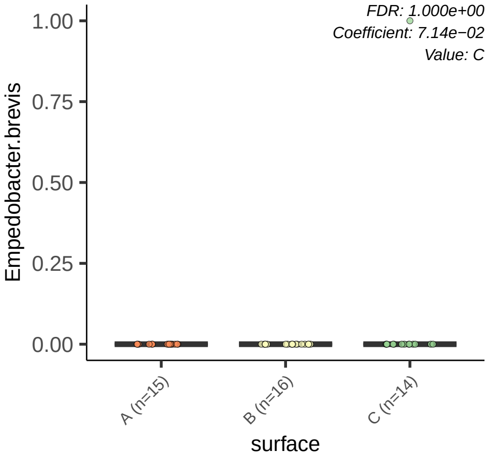
FDR: 1.000e+00
Coefficient: 7.14e−02
Value: C
Empedobacter.brevis
1.00
0.75
0.50
0.25
0.00
A (n=15)
B (n=16)
C (n=14)
surface

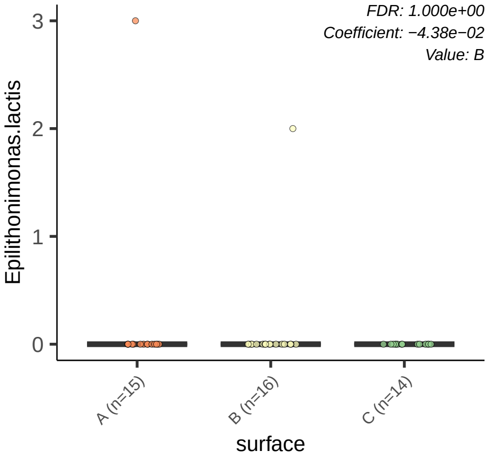
FDR: 1.000e+00
Coefficient: −4.38e−02
Value: B
Epilithonimonas.lactis
3
2
1
0
A (n=15)
B (n=16)
C (n=14)
surface

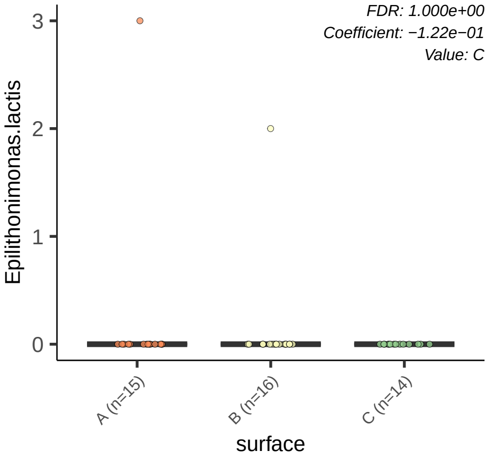

FDR: 1.000e+00
Coefficient: −1.22e−01
Value: C
Epilithonimonas.lactis
3
2
1
0
A (n=15)
B (n=16)
C (n=14)
surface

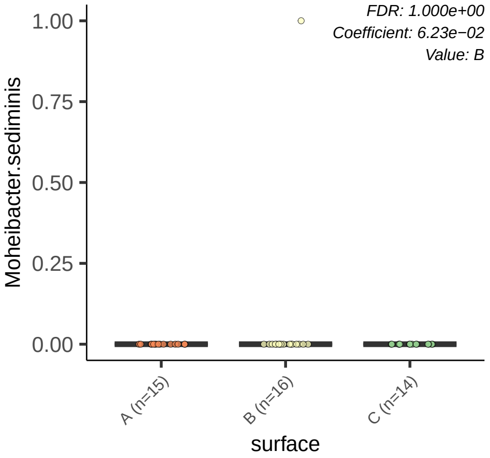

FDR: 1.000e+00
Coefficient: 6.23e−02
Value: B
Moheibacter.sedimінis
1.00
0.75
0.50
0.25
0.00
A (n=15)
B (n=16)
C (n=14)
surface

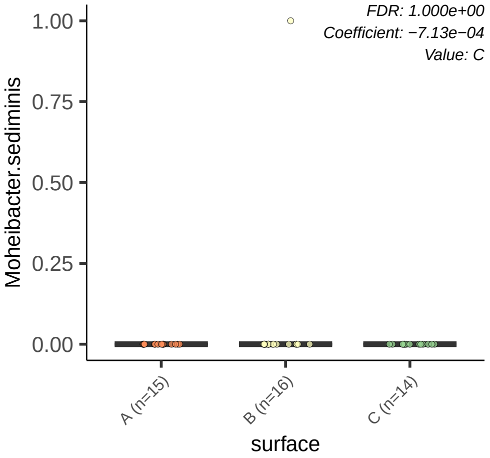

FDR: 1.000e+00
Coefficient: −7.13e−04
Value: C
Moheibacter.sedimini
1.00
0.75
0.50
0.25
0.00
A (n=15)
B (n=16)
C (n=14)
surface

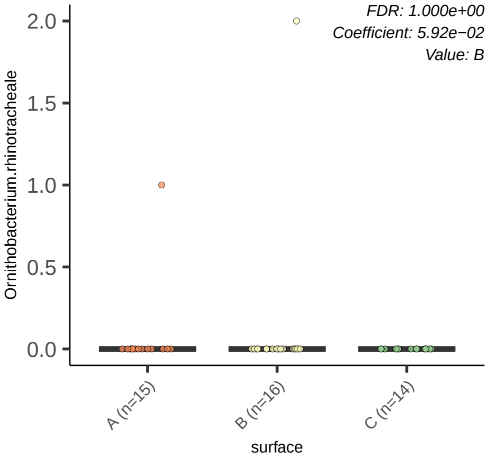
FDR: 1.000e+00
Coefficient: 5.92e−02
Value: B
Ornithobacterium.rhinotracheale
2.0
1.5
1.0
0.5
0.0
A (n=15)
B (n=16)
C (n=14)
surface

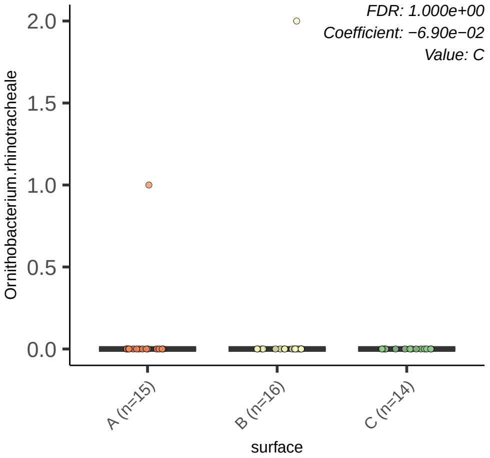

FDR: 1.000e+00
Coefficient: −6.90e−02
Value: C
Ornithobacterium.rhinotracheale
2.0
1.5
1.0
0.5
0.0
A (n=15)
B (n=16)
C (n=14)
surface

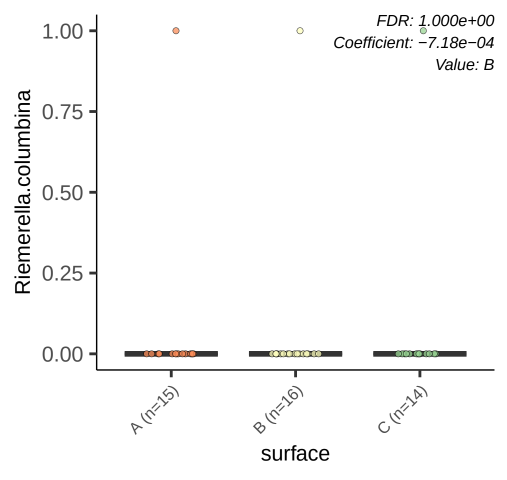
FDR: 1.000e+00
Coefficient: −7.18e−04
Value: B
Riemerella.columbina
1.00
0.75
0.50
0.25
0.00
A (n=15)
B (n=16)
C (n=14)
surface

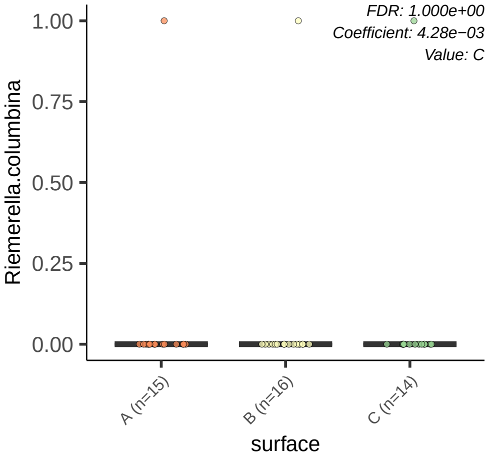
FDR: 1.000e+00
Coefficient: 4.28e−03
Value: C
Riemerella.columbina
1.00
0.75
0.50
0.25
0.00
A (n=15)
B (n=16)
C (n=14)
surface

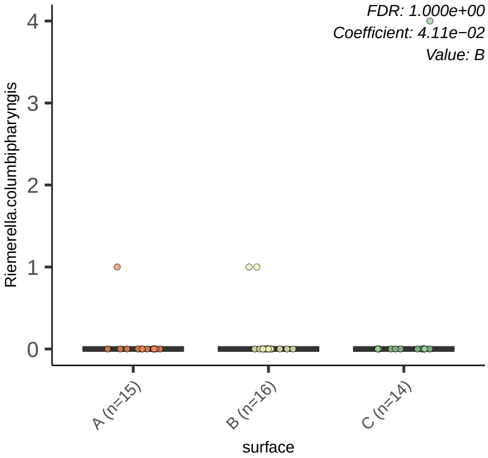

FDR: 1.000e+00
Coefficient: 4.11e−02
Value: B
Riemerella.columbipharyngis
4
3
2
1
0
A (n=15)
B (n=16)
C (n=14)
surface

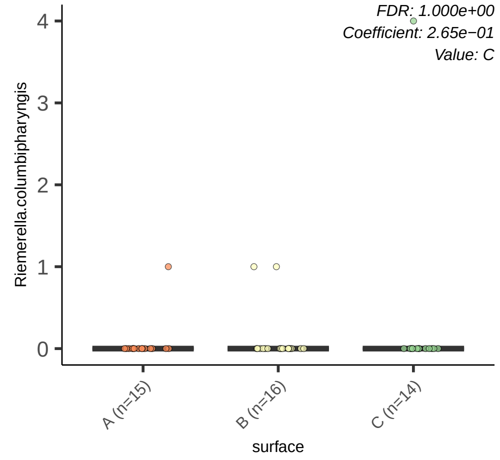

FDR: 1.000e+00
Coefficient: 2.65e−01
Value: C
Riemerella.columbipharyngis
4
3
2
1
0
A (n=15)
B (n=16)
C (n=14)
surface

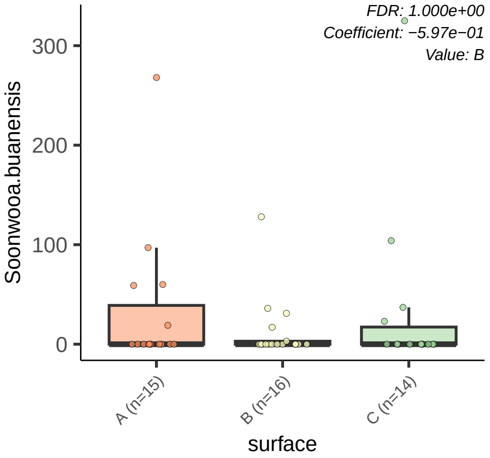

FDR: 1.000e+00
Coefficient: −5.97e−01
Value: B
Soonwooa.buanensis
300
200
100
0
A (n=15)
B (n=16)
C (n=14)
surface

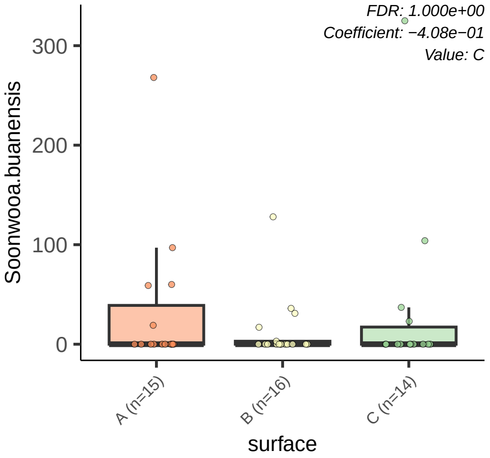

FDR: 1.000e+00
Coefficient: −4.08e−01
Value: C
Soonwooa.buanensis
300
200
100
0
A (n=15)
B (n=16)
C (n=14)
surface

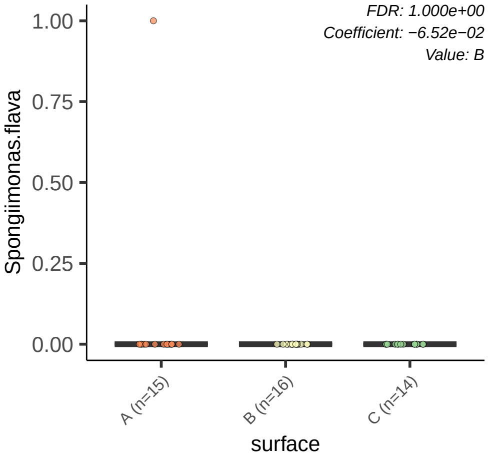

FDR: 1.000e+00
Coefficient: −6.52e−02
Value: B
Spongiimonas.flava
1.00
0.75
0.50
0.25
0.00
A (n=15)
B (n=16)
C (n=14)
surface

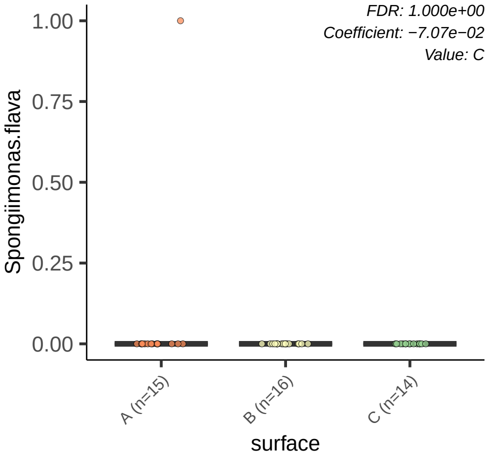
FDR: 1.000e+00
Coefficient: −7.07e−02
Value: C
Spongiimonas.flava
1.00
0.75
0.50
0.25
0.00
A (n=15)
B (n=16)
C (n=14)
surface

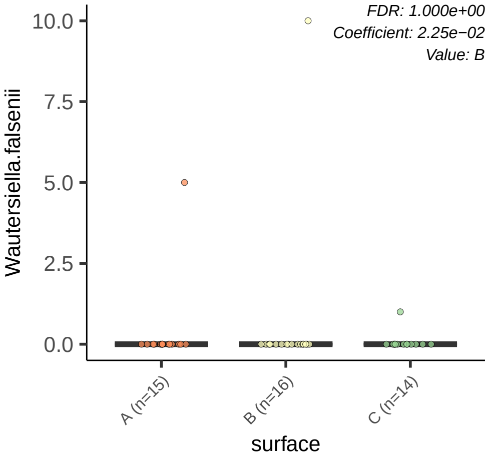
FDR: 1.000e+00
Coefficient: 2.25e−02
Value: B
Wautersiella.falsenii
10.0
7.5
5.0
2.5
0.0
A (n=15)
B (n=16)
C (n=14)
surface

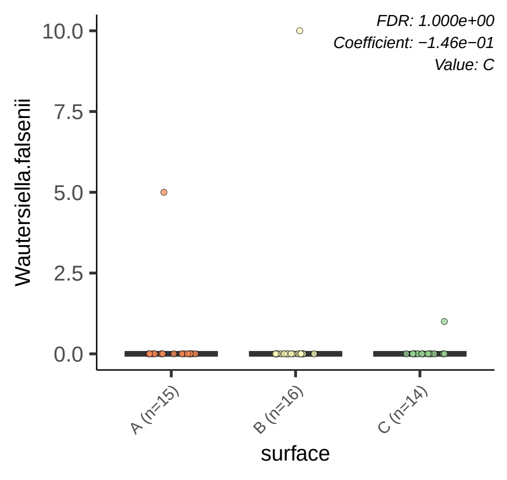
FDR: 1.000e+00
Coefficient: −1.46e−01
Value: C
10.0
7.5
5.0
2.5
0.0
Wautersiella.falsenii
A (n=15)
B (n=16)
C (n=14)
surface

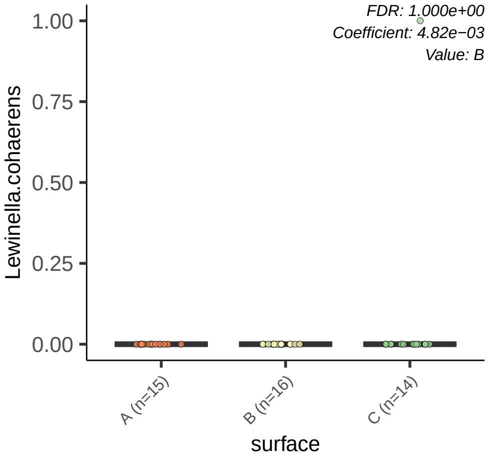
FDR: 1.000e+00
Coefficient: 4.82e−03
Value: B
Lewinella.cohaerens
1.00
0.75
0.50
0.25
0.00
A (n=15)
B (n=16)
C (n=14)
surface

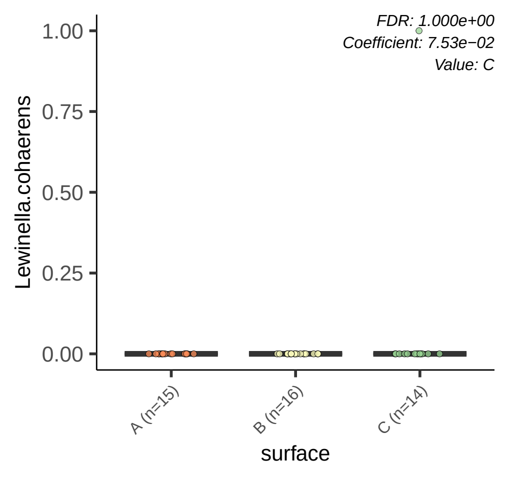

FDR: 1.000e+00
Coefficient: 7.53e−02
Value: C
Lewinella.cohaerens
1.00
0.75
0.50
0.25
0.00
A (n=15)
B (n=16)
C (n=14)
surface

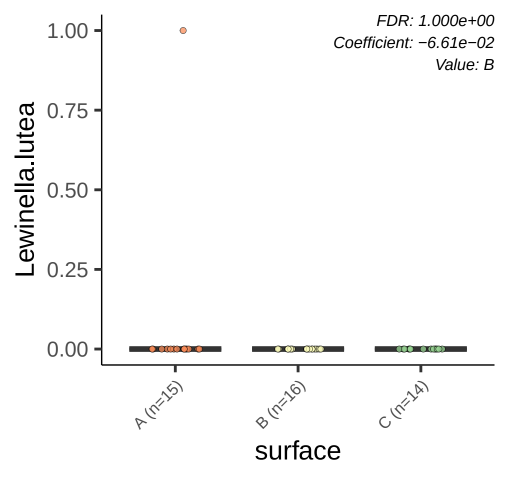

FDR: 1.000e+00
Coefficient: −6.61e−02
Value: B
Lewinella.lutea
1.00
0.75
0.50
0.25
0.00
A (n=15)
B (n=16)
C (n=14)
surface

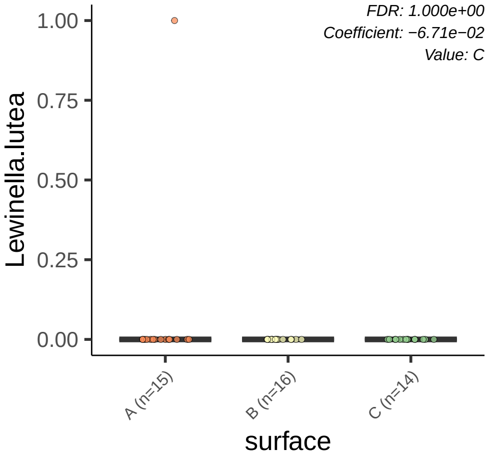

FDR: 1.000e+00
Coefficient: −6.71e−02
Value: C
Lewinella.lutea
1.00
0.75
0.50
0.25
0.00
A (n=15)
B (n=16)
C (n=14)
surface

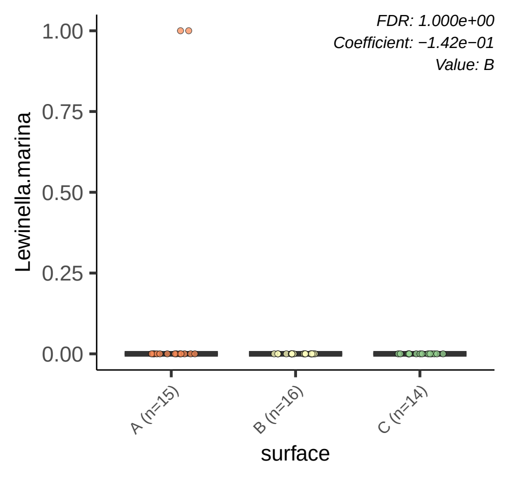
FDR: 1.000e+00
Coefficient: −1.42e−01
Value: B
Lewinella.marina
1.00
0.75
0.50
0.25
0.00
A (n=15)
B (n=16)
C (n=14)
surface

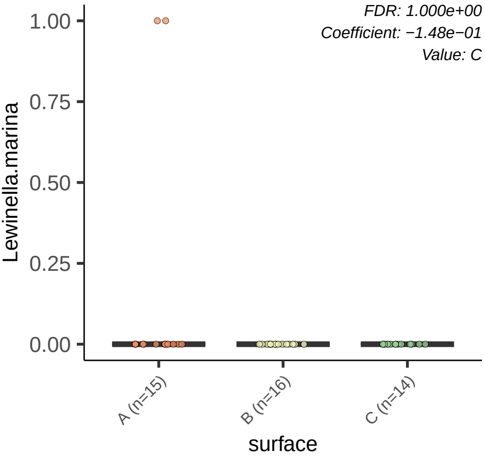
FDR: 1.000e+00
Coefficient: −1.48e−01
Value: C
Lewinella.marina
1.00
0.75
0.50
0.25
0.00
A (n=15)
B (n=16)
C (n=14)
surface

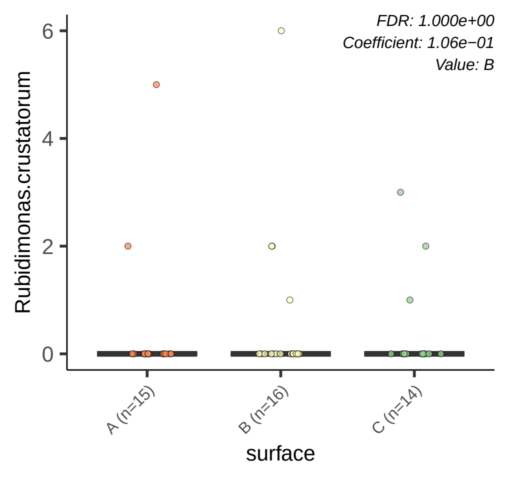

FDR: 1.000e+00
Coefficient: 1.06e−01
Value: B
Rubidimonas.crustatorum
0
2
4
6
A (n=15)
B (n=16)
C (n=14)
surface

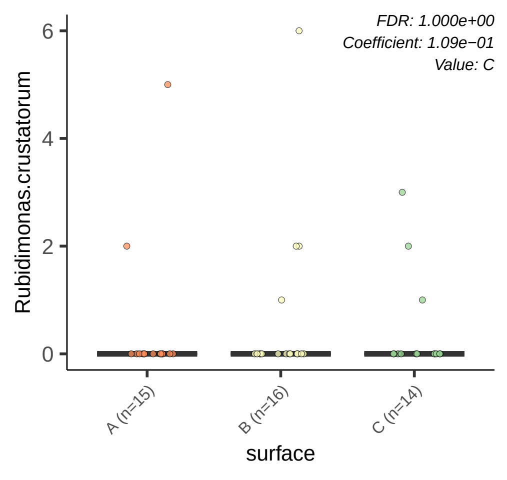

FDR: 1.000e+00
Coefficient: 1.09e−01
Value: C
Rubidimonas.crustatorum
0
2
4
6
A (n=15)
B (n=16)
C (n=14)
surface

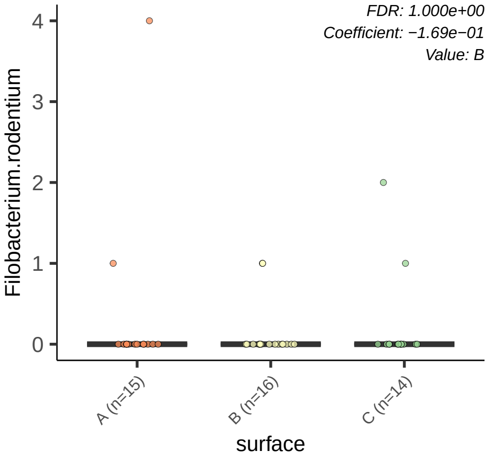

FDR: 1.000e+00
Coefficient: −1.69e−01
Value: B
Filobacterium.rodentium
0
1
2
3
4
A (n=15)
B (n=16)
C (n=14)
surface

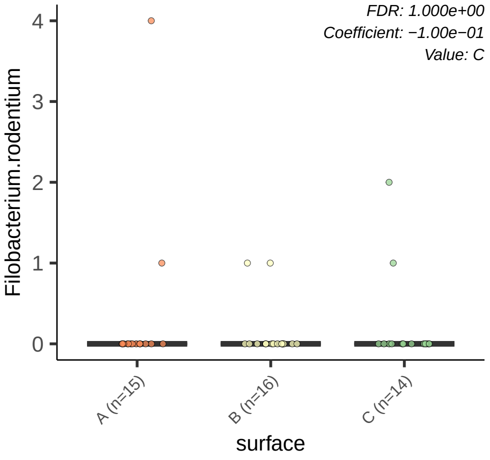
FDR: 1.000e+00
Coefficient: −1.00e−01
Value: C
Filobacterium.rodentium
0
1
2
3
4
A (n=15)
B (n=16)
C (n=14)
surface

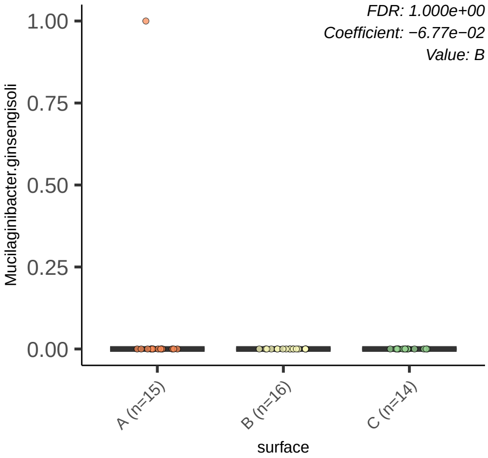
FDR: 1.000e+00
Coefficient: −6.77e−02
Value: B
1.00
0.75
0.50
0.25
0.00
Mucilaginibacter.ginsengisoli
A (n=15)
B (n=16)
C (n=14)
surface

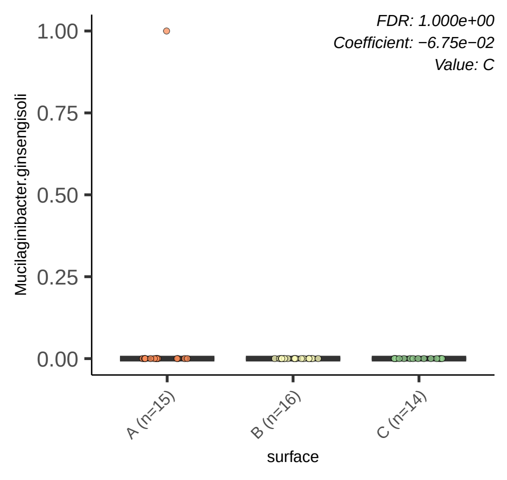
FDR: 1.000e+00
Coefficient: −6.75e−02
Value: C
Mucilaginibacter.ginsengisoli
1.00
0.75
0.50
0.25
0.00
A (n=15)
B (n=16)
C (n=14)
surface

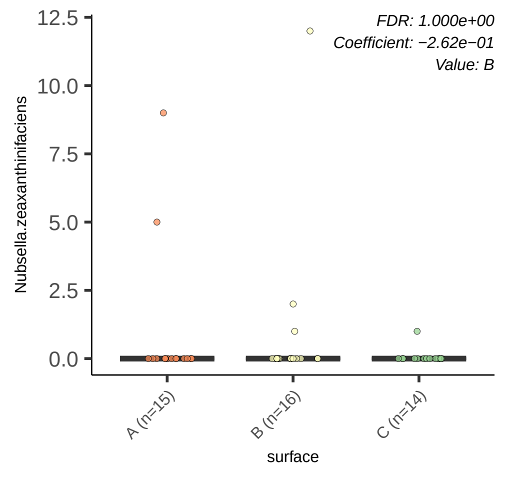

FDR: 1.000e+00
Coefficient: −2.62e−01
Value: B
Nubsella.zeaxanthinifaciens
12.5
10.0
7.5
5.0
2.5
0.0
A (n=15)
B (n=16)
C (n=14)
surface

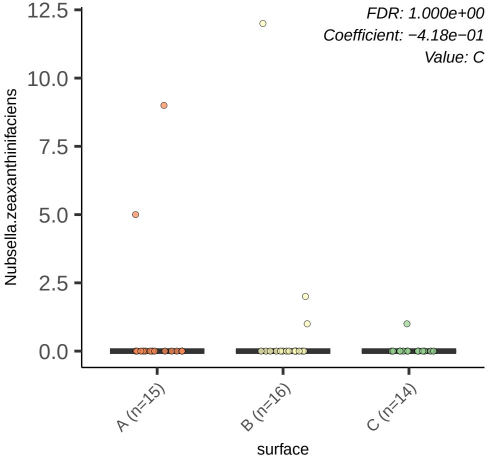

FDR: 1.000e+00
Coefficient: −4.18e−01
Value: C
Nubsella.zeaxanthinifaciens
12.5
10.0
7.5
5.0
2.5
0.0
A (n=15)
B (n=16)
C (n=14)
surface

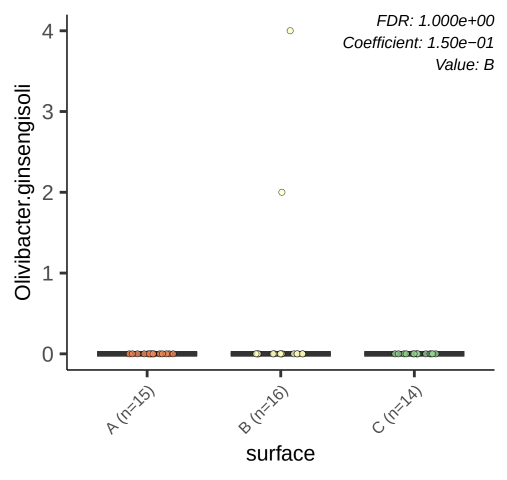
FDR: 1.000e+00
Coefficient: 1.50e−01
Value: B
Olivibacter.ginsengisoli
0
1
2
3
4
A (n=15)
B (n=16)
C (n=14)
surface

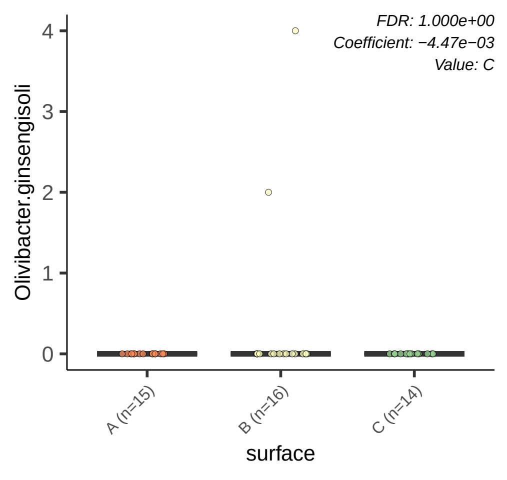

FDR: 1.000e+00
Coefficient: −4.47e−03
Value: C
Olivibacter.ginsengisoli
0
1
2
3
4
A (n=15)
B (n=16)
C (n=14)
surface

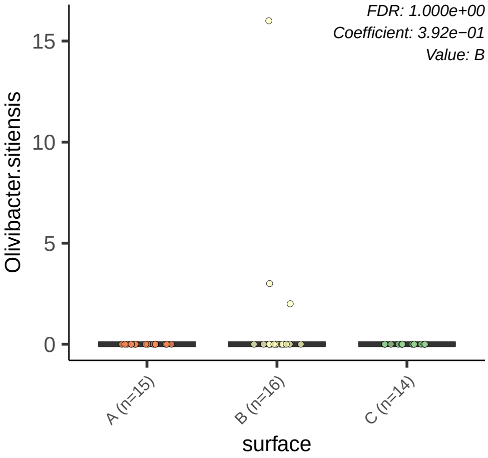
FDR: 1.000e+00
Coefficient: 3.92e−01
Value: B
Olivibacter.sitiensis
15
10
5
0
A (n=15)
B (n=16)
C (n=14)
surface

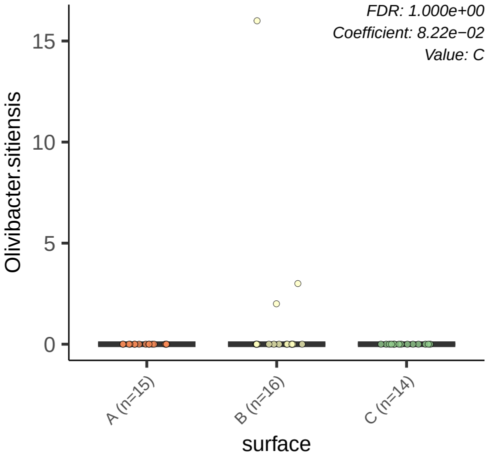

FDR: 1.000e+00
Coefficient: 8.22e−02
Value: C
Olivibacter.sitiensis
15
10
5
0
A (n=15)
B (n=16)
C (n=14)
surface

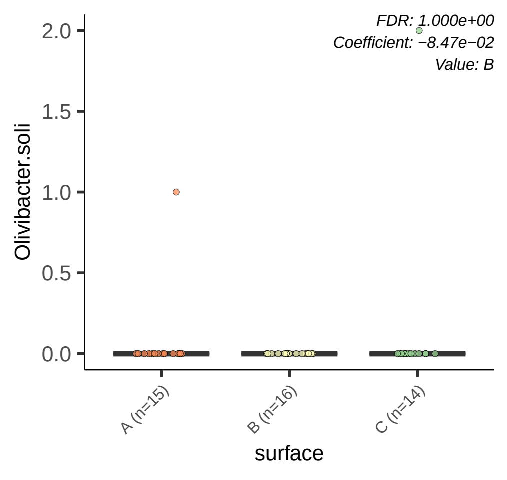
FDR: 1.000e+00
Coefficient: −8.47e−02
Value: B
Olivibacter.soli
2.0
1.5
1.0
0.5
0.0
A (n=15)
B (n=16)
C (n=14)
surface

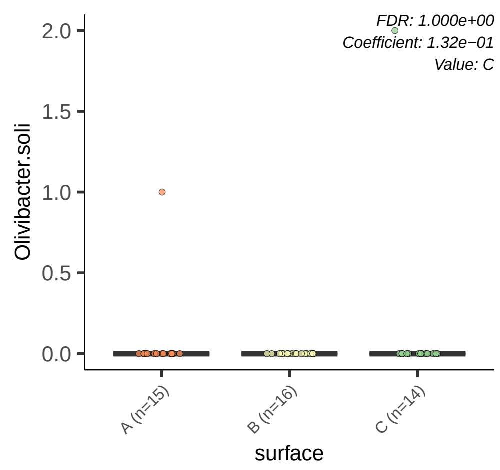
FDR: 1.000e+00
Coefficient: 1.32e−01
Value: C
Olivibacter.soli
2.0
1.5
1.0
0.5
0.0
A (n=15)
B (n=16)
C (n=14)
surface

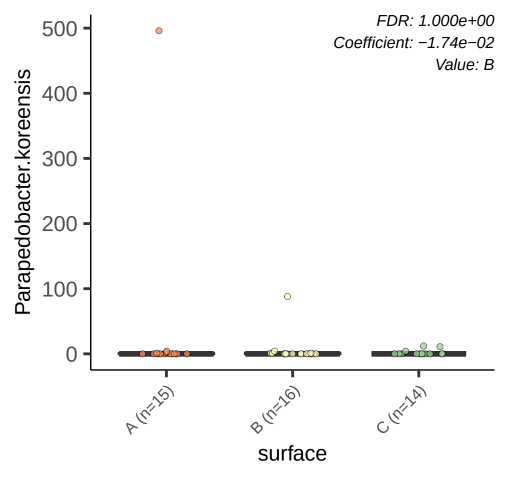

FDR: 1.000e+00
Coefficient: −1.74e−02
Value: B
Parapedobacter.koreensis
500
400
300
200
100
0
A (n=15)
B (n=16)
C (n=14)
surface

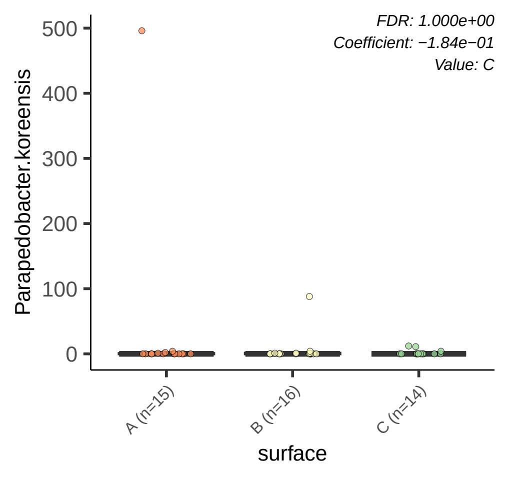
FDR: 1.000e+00
Coefficient: −1.84e−01
Value: C
Parapedobacter.koreensis
500
400
300
200
100
0
A (n=15)
B (n=16)
C (n=14)
surface

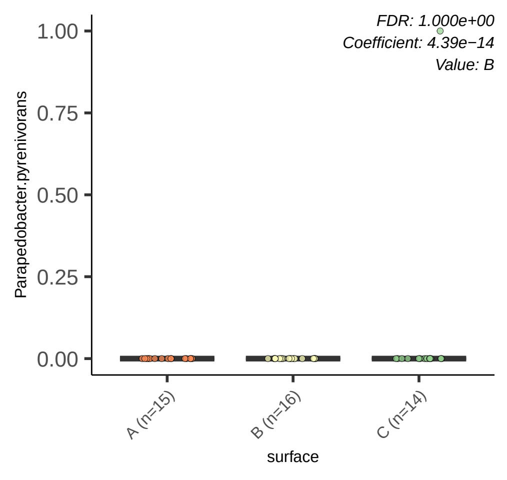
FDR: 1.000e+00
Coefficient: 4.39e−14
Value: B
Parapedobacter.pyrenivorans
1.00
0.75
0.50
0.25
0.00
A (n=15)
B (n=16)
C (n=14)
surface

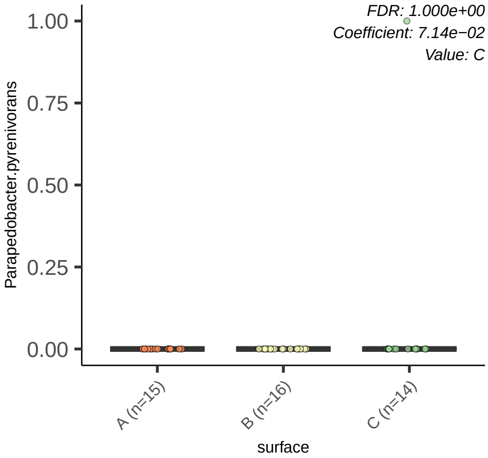
FDR: 1.000e+00
Coefficient: 7.14e−02
Value: C
1.00
0.75
0.50
0.25
0.00
Parapedobacter.pyrenivorans
A (n=15)
B (n=16)
C (n=14)
surface

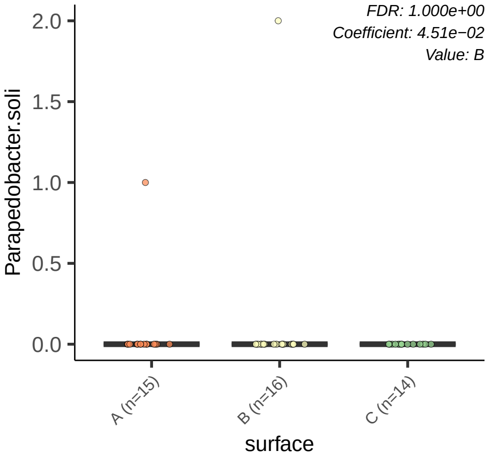

FDR: 1.000e+00
Coefficient: 4.51e−02
Value: B
Parapedobacter.soli
2.0
1.5
1.0
0.5
0.0
A (n=15)
B (n=16)
C (n=14)
surface

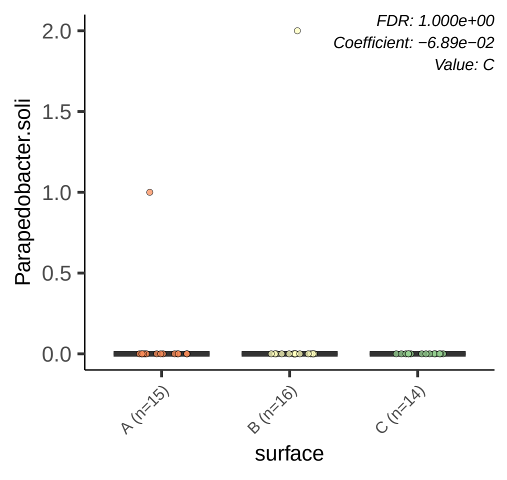

FDR: 1.000e+00
Coefficient: −6.89e−02
Value: C
Parapedobacter.soli
2.0
1.5
1.0
0.5
0.0
A (n=15)
B (n=16)
C (n=14)
surface

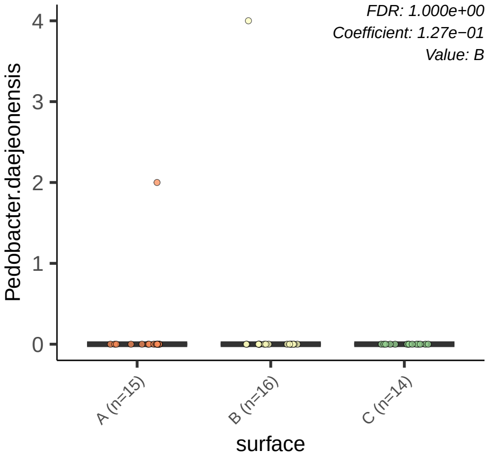
FDR: 1.000e+00
Coefficient: 1.27e−01
Value: B
Pedobacter.daejeonensis
4
3
2
1
0
A (n=15)
B (n=16)
C (n=14)
surface

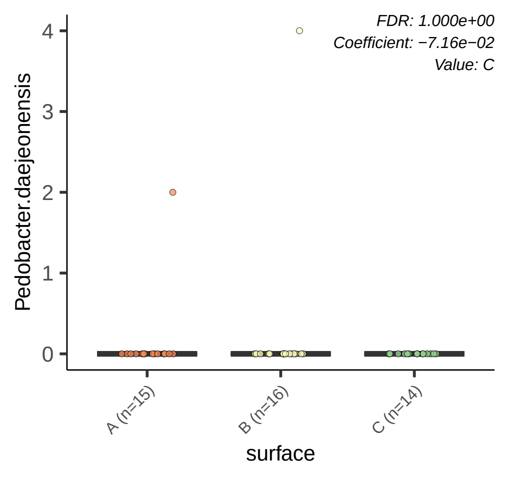
FDR: 1.000e+00
Coefficient: −7.16e−02
Value: C
Pedobacter.daejeonensis
0
1
2
3
4
A (n=15)
B (n=16)
C (n=14)
surface

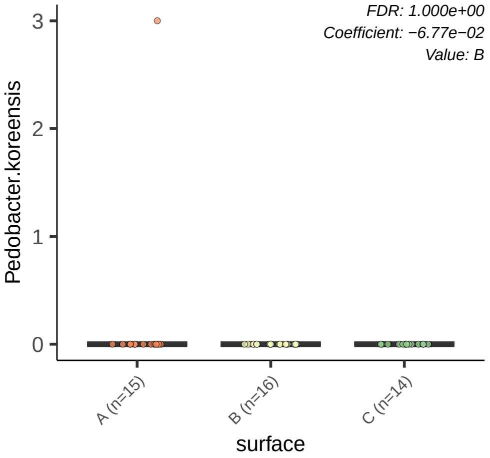
FDR: 1.000e+00
Coefficient: −6.77e−02
Value: B
Pedobacter.koreensis
3
2
1
0
A (n=15)
B (n=16)
C (n=14)
surface

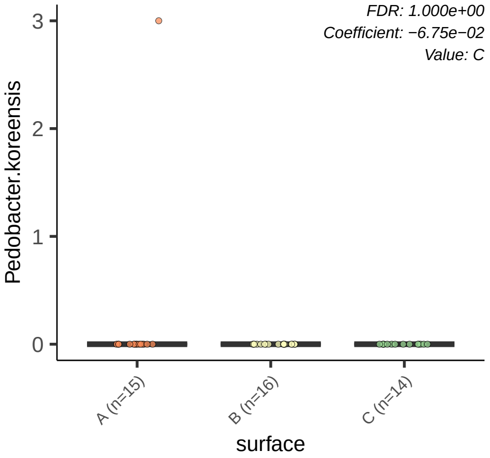

FDR: 1.000e+00
Coefficient: −6.75e−02
Value: C
Pedobacter.koreensis
3
2
1
0
A (n=15)
B (n=16)
C (n=14)
surface

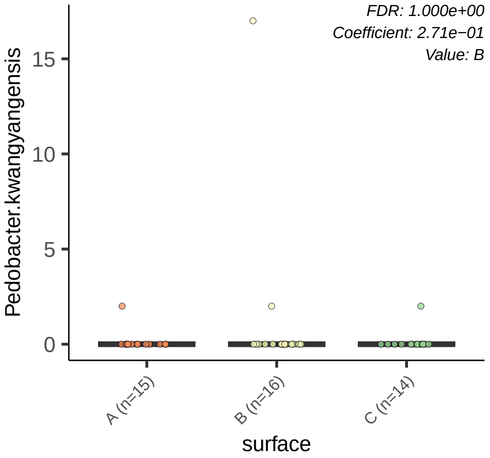

FDR: 1.000e+00
Coefficient: 2.71e−01
Value: B
Pedobacter.kwangyangensis
15
10
5
0
A (n=15)
B (n=16)
C (n=14)
surface

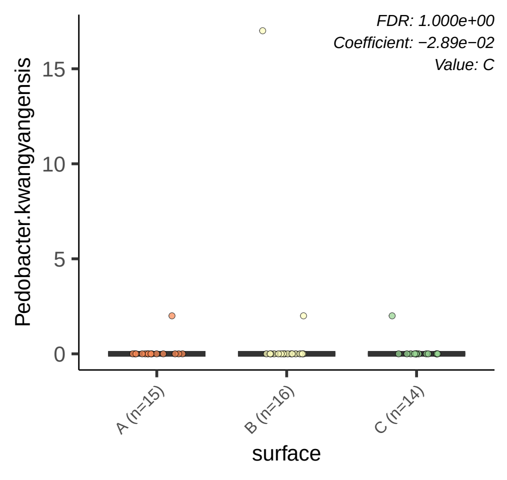

FDR: 1.000e+00
Coefficient: −2.89e−02
Value: C
Pedobacter.kwangyangensis
0
5
10
15
A (n=15)
B (n=16)
C (n=14)
surface

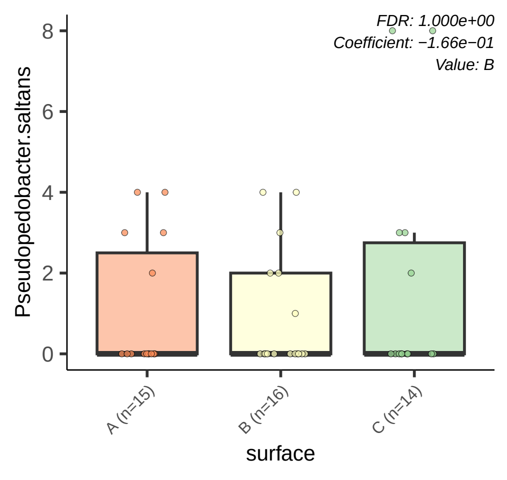
FDR: 1.000e+00
Coefficient: −1.66e−01
Value: B
Pseudopedobacter.saltans
8
6
4
2
0
A (n=15)
B (n=16)
C (n=14)
surface

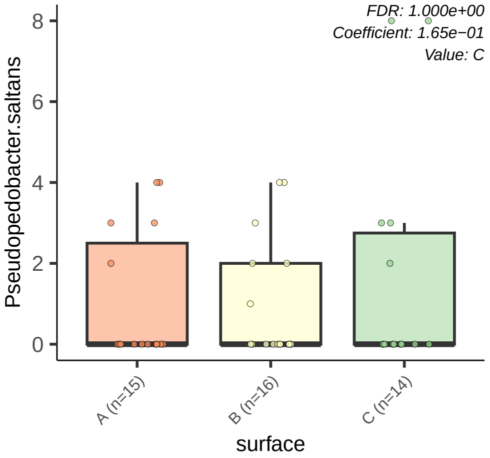

FDR: 1.000e+00
Coefficient: 1.65e−01
Value: C
Pseudopedobacter.saltans
0
2
4
6
8
A (n=15)
B (n=16)
C (n=14)
surface

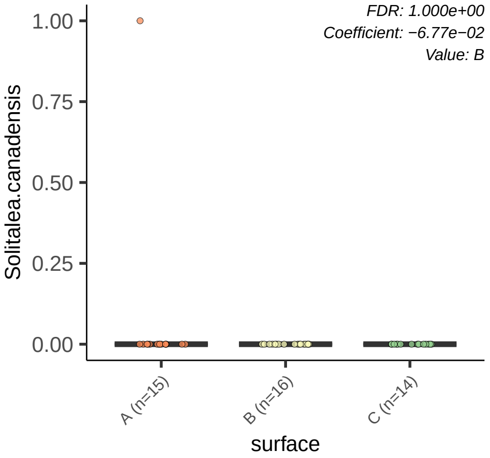
FDR: 1.000e+00
Coefficient: −6.77e−02
Value: B
Solitalea.canadensis
1.00
0.75
0.50
0.25
0.00
A (n=15)
B (n=16)
C (n=14)
surface

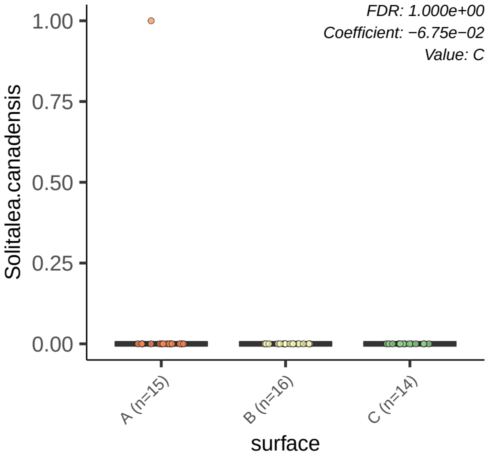
FDR: 1.000e+00
Coefficient: −6.75e−02
Value: C
Solitalea.canadensis
1.00
0.75
0.50
0.25
0.00
A (n=15)
B (n=16)
C (n=14)
surface

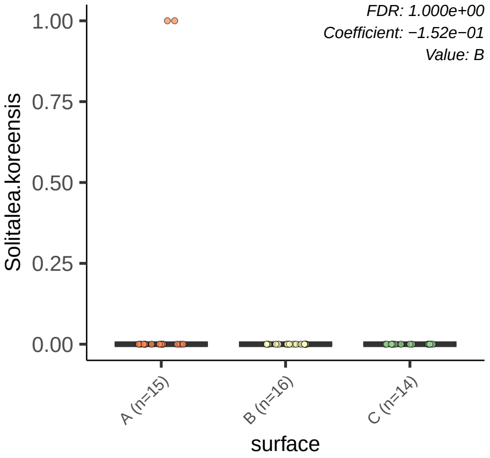

FDR: 1.000e+00
Coefficient: −1.52e−01
Value: B
Solitalea.koreensis
1.00
0.75
0.50
0.25
0.00
A (n=15)
B (n=16)
C (n=14)
surface

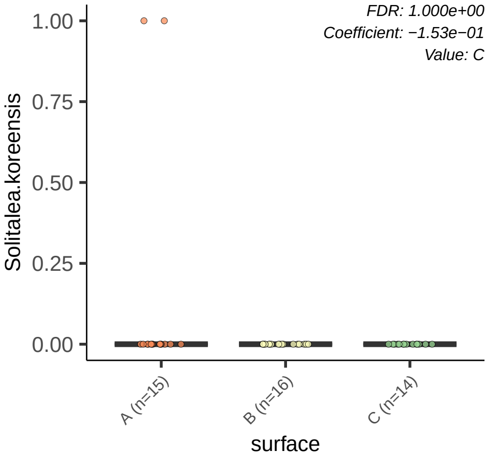
FDR: 1.000e+00
Coefficient: −1.53e−01
Value: C
Solitalea.koreensis
1.00
0.75
0.50
0.25
0.00
A (n=15)
B (n=16)
C (n=14)
surface

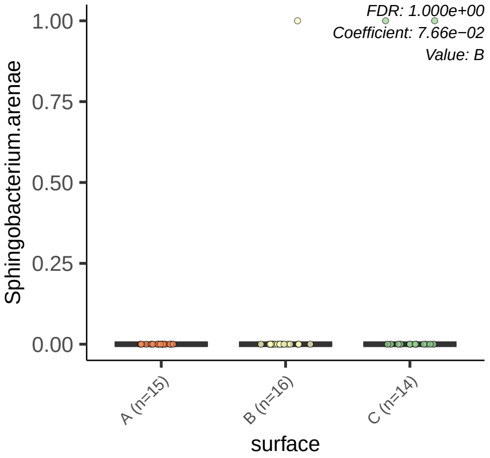
FDR: 1.000e+00
Coefficient: 7.66e−02
Value: B
Sphingobacterium.arenae
1.00
0.75
0.50
0.25
0.00
A (n=15)
B (n=16)
C (n=14)
surface

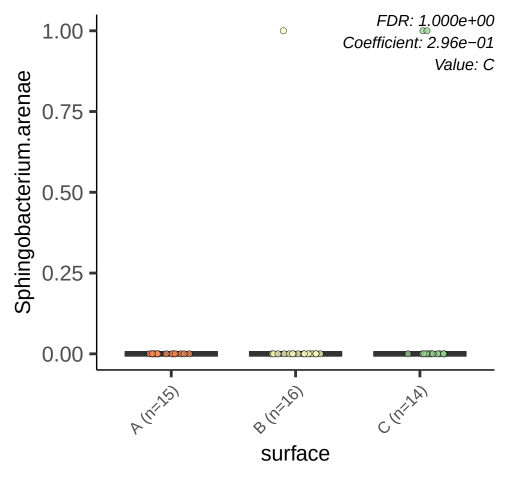
FDR: 1.000e+00
Coefficient: 2.96e−01
Value: C
Sphingobacterium.arenae
1.00
0.75
0.50
0.25
0.00
A (n=15)
B (n=16)
C (n=14)
surface

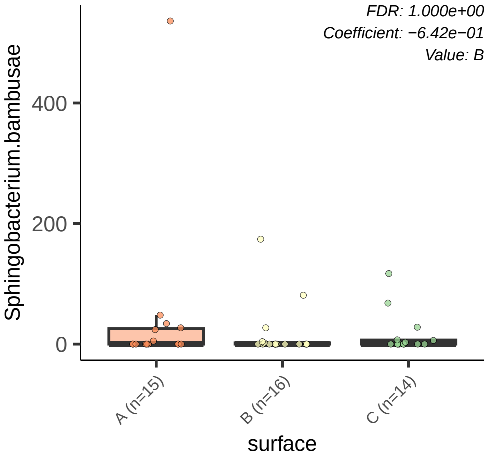

FDR: 1.000e+00
Coefficient: −6.42e−01
Value: B
Sphingobacterium.bambusae
400
200
0
A (n=15)
B (n=16)
C (n=14)
surface

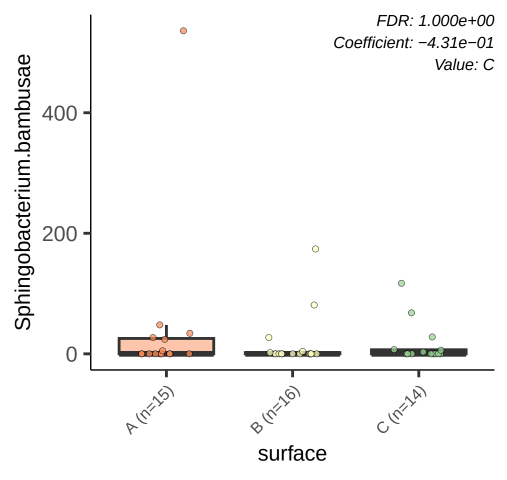

FDR: 1.000e+00
Coefficient: −4.31e−01
Value: C
Sphingobacterium.bambusae
400
200
0
A (n=15)
B (n=16)
C (n=14)
surface

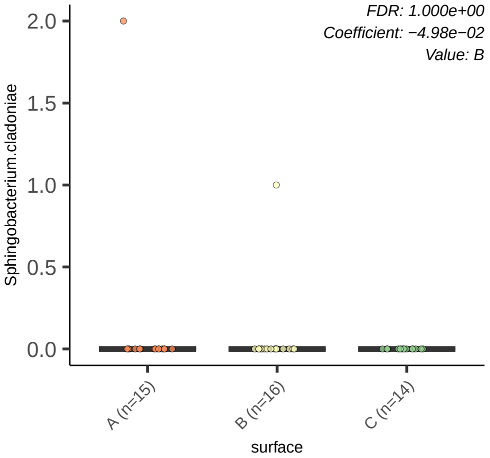

FDR: 1.000e+00
Coefficient: −4.98e−02
Value: B
Sphingobacterium.cladoniae
2.0
1.5
1.0
0.5
0.0
A (n=15)
B (n=16)
C (n=14)
surface

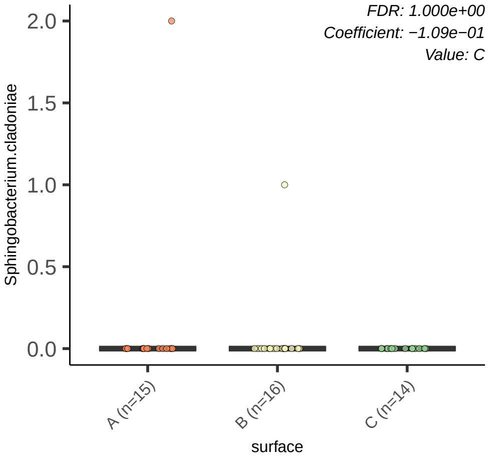
FDR: 1.000e+00
Coefficient: −1.09e−01
Value: C
Sphingobacterium.cladoniae
2.0
1.5
1.0
0.5
0.0
A (n=15)
B (n=16)
C (n=14)
surface

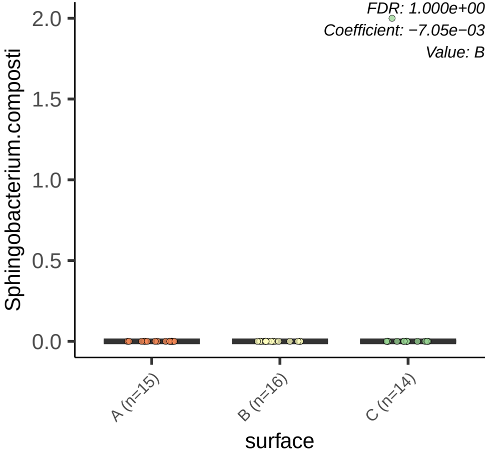
FDR: 1.000e+00
Coefficient: −7.05e−03
Value: B
Sphingobacterium.composti
2.0
1.5
1.0
0.5
0.0
A (n=15)
B (n=16)
C (n=14)
surface

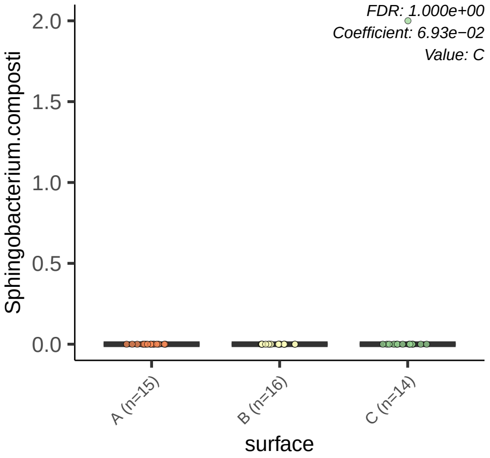

FDR: 1.000e+00
Coefficient: 6.93e−02
Value: C
2.0
1.5
1.0
0.5
0.0
Sphingobacterium.composti
A (n=15)
B (n=16)
C (n=14)
surface

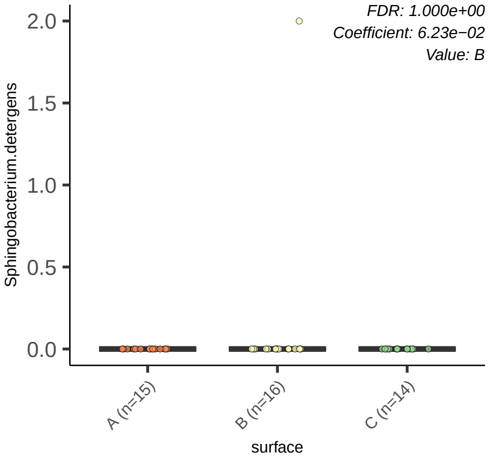

FDR: 1.000e+00
Coefficient: 6.23e−02
Value: B
Sphingobacterium.detergens
2.0
1.5
1.0
0.5
0.0
A (n=15)
B (n=16)
C (n=14)
surface

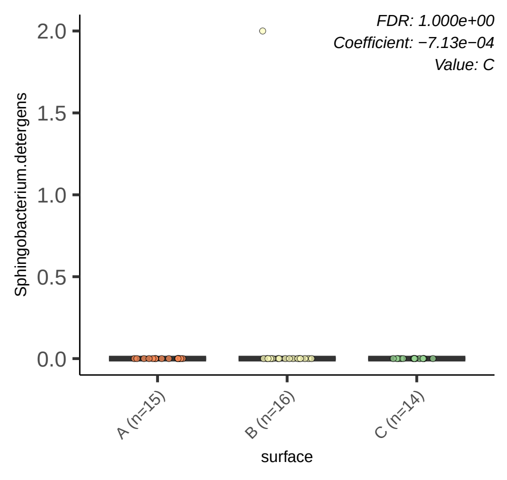

FDR: 1.000e+00
Coefficient: −7.13e−04
Value: C
Sphingobacterium.detergens
2.0
1.5
1.0
0.5
0.0
A (n=15)
B (n=16)
C (n=14)
surface

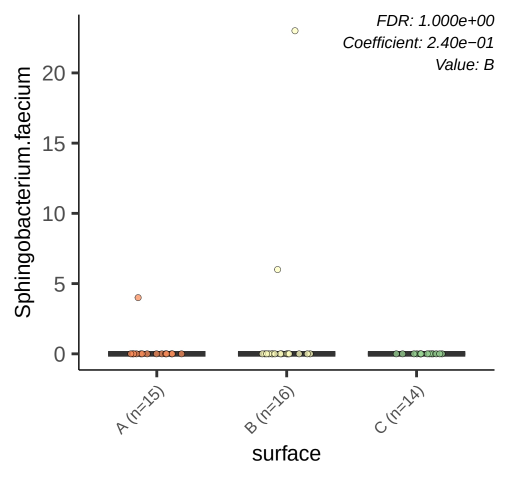

FDR: 1.000e+00
Coefficient: 2.40e−01
Value: B
Sphingobacterium.faecium
0
5
10
15
20
A (n=15)
B (n=16)
C (n=14)
surface

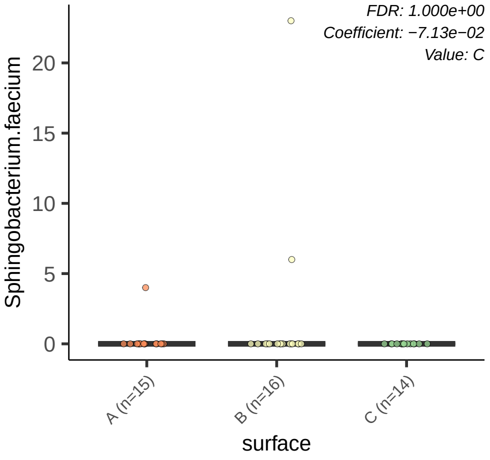
FDR: 1.000e+00
Coefficient: −7.13e−02
Value: C
Sphingobacterium.faecium
20
15
10
5
0
A (n=15)
B (n=16)
C (n=14)
surface

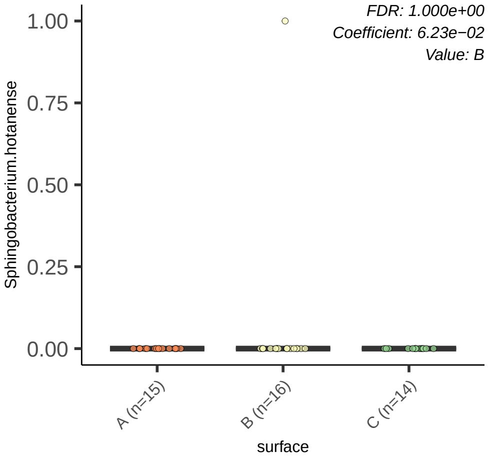

FDR: 1.000e+00
Coefficient: 6.23e−02
Value: B
1.00
0.75
0.50
0.25
0.00
Sphingobacterium.hotanense
A (n=15)
B (n=16)
C (n=14)
surface

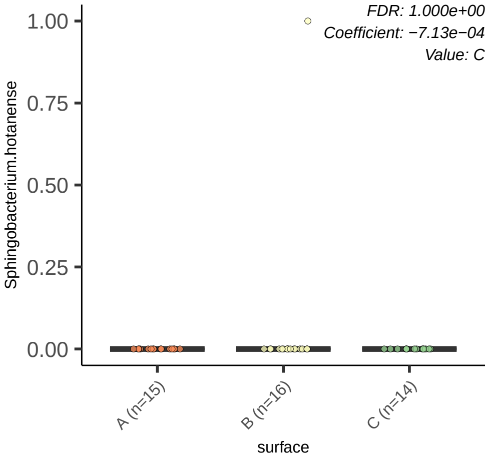
FDR: 1.000e+00
Coefficient: −7.13e−04
Value: C
1.00
0.75
0.50
0.25
0.00
Sphingobacterium.hotanense
A (n=15)
B (n=16)
C (n=14)
surface

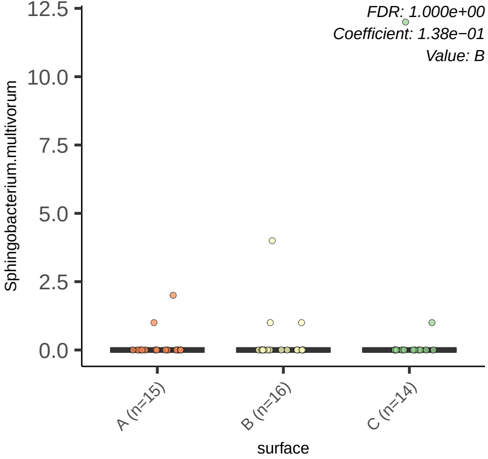

FDR: 1.000e+00
Coefficient: 1.38e−01
Value: B
Sphingobacterium.multivorum
12.5
10.0
7.5
5.0
2.5
0.0
A (n=15)
B (n=16)
C (n=14)
surface

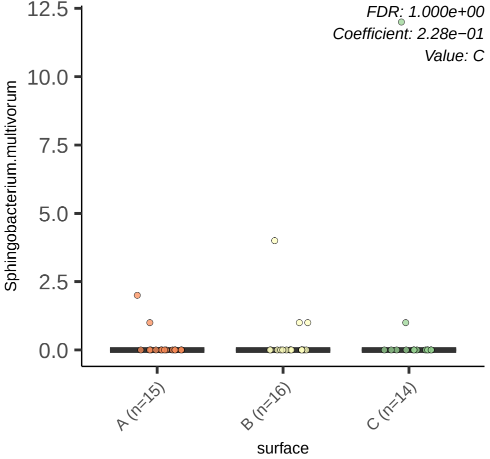

FDR: 1.000e+00
Coefficient: 2.28e−01
Value: C
Sphingobacterium.multivorum
12.5
10.0
7.5
5.0
2.5
0.0
A (n=15)
B (n=16)
C (n=14)
surface

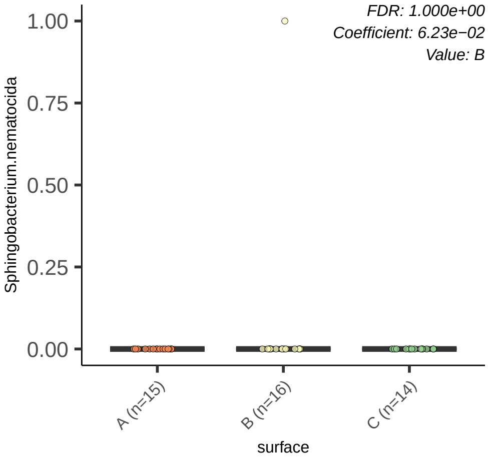
FDR: 1.000e+00
Coefficient: 6.23e−02
Value: B
Sphingobacterium.nematocida
1.00
0.75
0.50
0.25
0.00
A (n=15)
B (n=16)
C (n=14)
surface

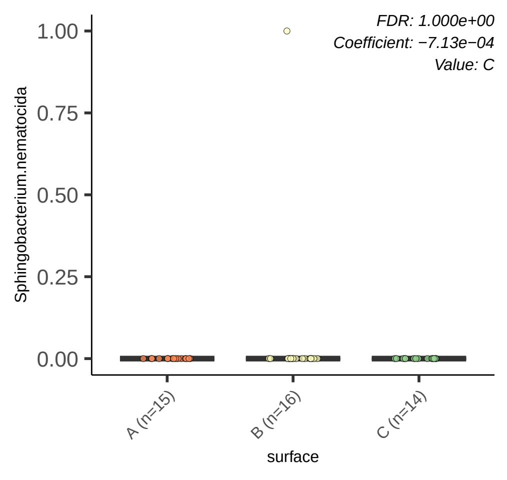
FDR: 1.000e+00
Coefficient: −7.13e−04
Value: C
1.00
0.75
0.50
0.25
0.00
Sphingobacterium.nematocida
A (n=15)
B (n=16)
C (n=14)
surface

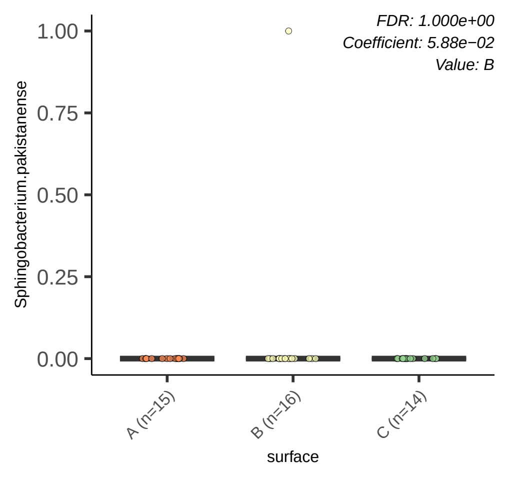
FDR: 1.000e+00
Coefficient: 5.88e−02
Value: B
1.00
0.75
0.50
0.25
0.00
Sphingobacterium.pakistanense
A (n=15)
B (n=16)
C (n=14)
surface

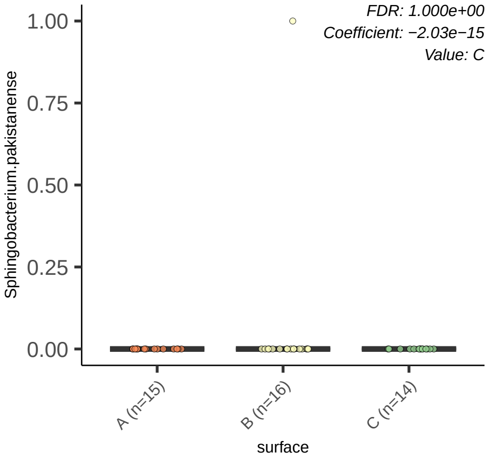
FDR: 1.000e+00
Coefficient: −2.03e−15
Value: C
1.00
0.75
0.50
0.25
0.00
Sphingobacterium.pakistanense
A (n=15)
B (n=16)
C (n=14)
surface

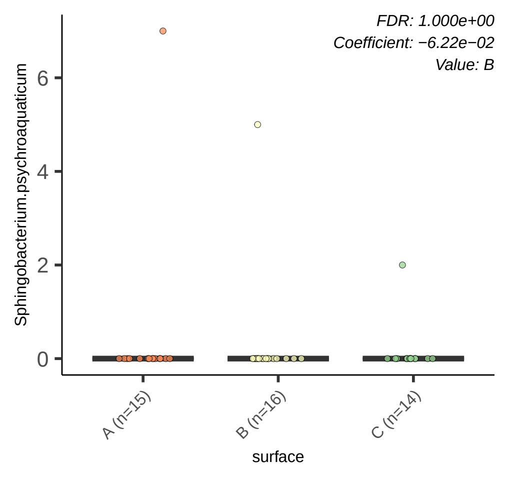
FDR: 1.000e+00
Coefficient: −6.22e−02
Value: B
Sphingobacterium.psychroaquaticum
0
2
4
6
A (n=15)
B (n=16)
C (n=14)
surface

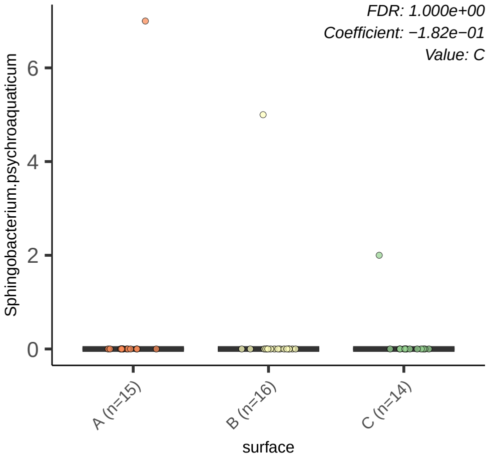
FDR: 1.000e+00
Coefficient: −1.82e−01
Value: C
Sphingobacterium.psychroaquaticum
0
2
4
6
A (n=15)
B (n=16)
C (n=14)
surface

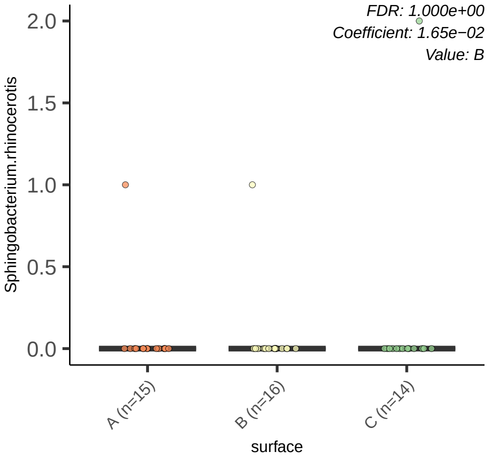
FDR: 1.000e+00
Coefficient: 1.65e−02
Value: B
Sphingobacterium.rhinocerotis
2.0
1.5
1.0
0.5
0.0
A (n=15)
B (n=16)
C (n=14)
surface

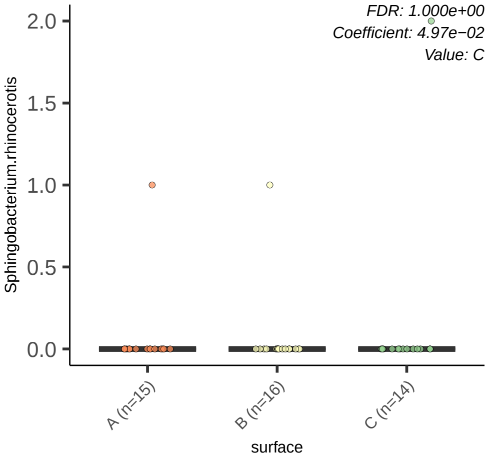

FDR: 1.000e+00
Coefficient: 4.97e−02
Value: C
Sphingobacterium.rhinocerotis
2.0
1.5
1.0
0.5
0.0
A (n=15)
B (n=16)
C (n=14)
surface

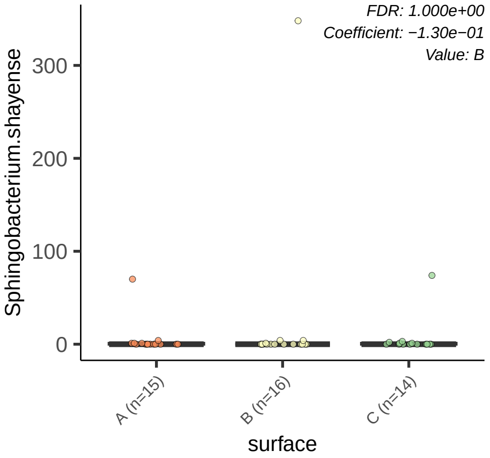
FDR: 1.000e+00
Coefficient: −1.30e−01
Value: B
Sphingobacterium.shayense
300
200
100
0
A (n=15)
B (n=16)
C (n=14)
surface

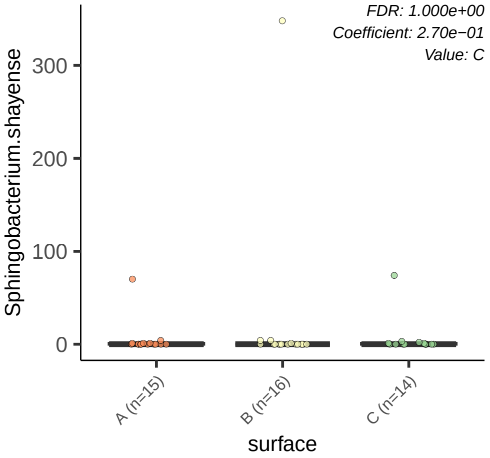
FDR: 1.000e+00
Coefficient: 2.70e−01
Value: C
Sphingobacterium.shayense
300
200
100
0
A (n=15)
B (n=16)
C (n=14)
surface

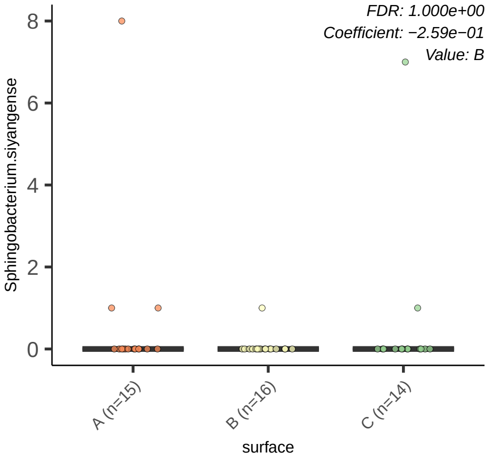

FDR: 1.000e+00
Coefficient: −2.59e−01
Value: B
Sphingobacterium.siyangense
8
6
4
2
0
A (n=15)
B (n=16)
C (n=14)
surface

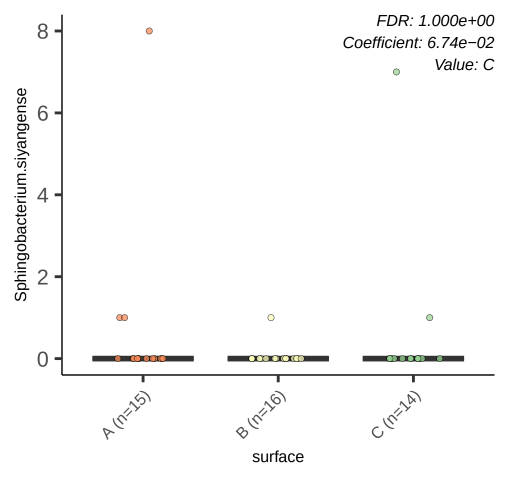

FDR: 1.000e+00
Coefficient: 6.74e−02
Value: C
Sphingobacterium.siyangense
8
6
4
2
0
A (n=15)
B (n=16)
C (n=14)
surface

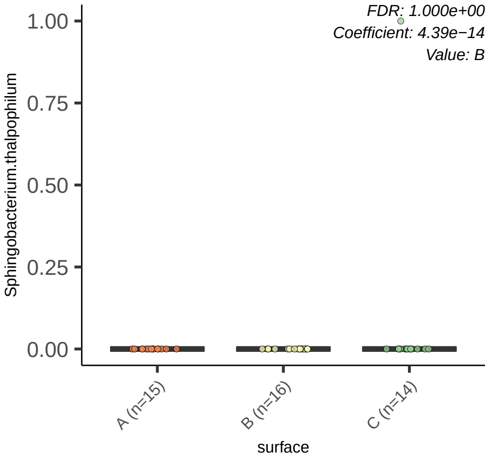

FDR: 1.000e+00
Coefficient: 4.39e−14
Value: B
Sphingobacterium.thalpophilum
1.00
0.75
0.50
0.25
0.00
A (n=15)
B (n=16)
C (n=14)
surface

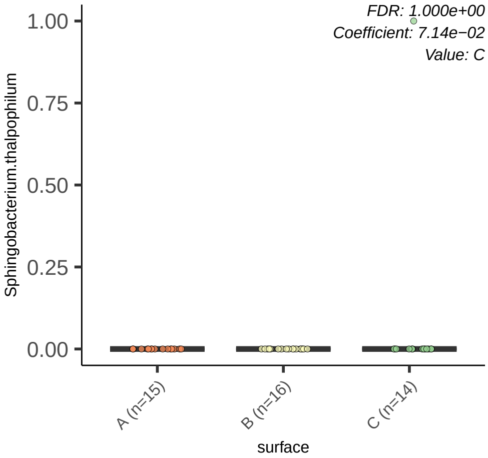

FDR: 1.000e+00
Coefficient: 7.14e−02
Value: C
1.00
0.75
0.50
0.25
0.00
Sphingobacterium.thalpophilum
A (n=15)
B (n=16)
C (n=14)
surface

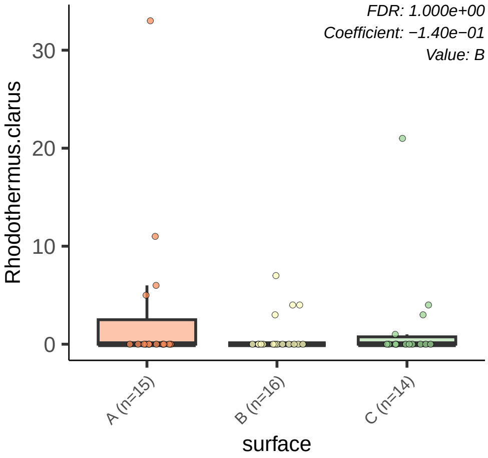
FDR: 1.000e+00
Coefficient: −1.40e−01
Value: B
Rhodothermus.clarus
30
20
10
0
A (n=15)
B (n=16)
C (n=14)
surface

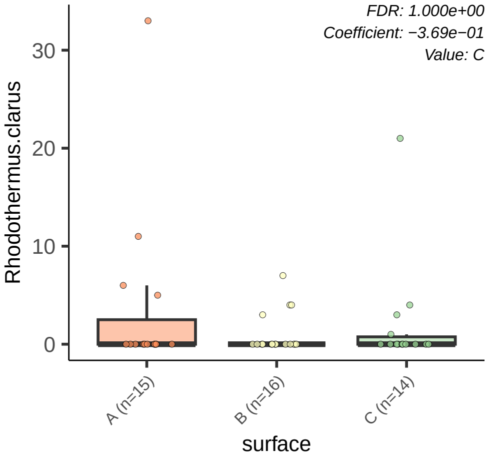

FDR: 1.000e+00
Coefficient: −3.69e−01
Value: C
Rhodothermus.clarus
0
10
20
30
A (n=15)
B (n=16)
C (n=14)
surface

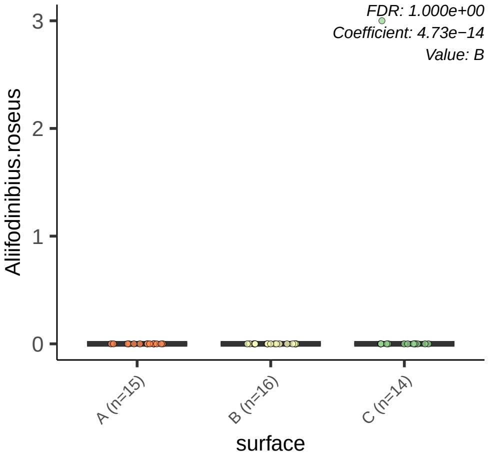
FDR: 1.000e+00
Coefficient: 4.73e−14
Value: B
Aliifodinibius.roseus
0
1
2
3
A (n=15)
B (n=16)
C (n=14)
surface

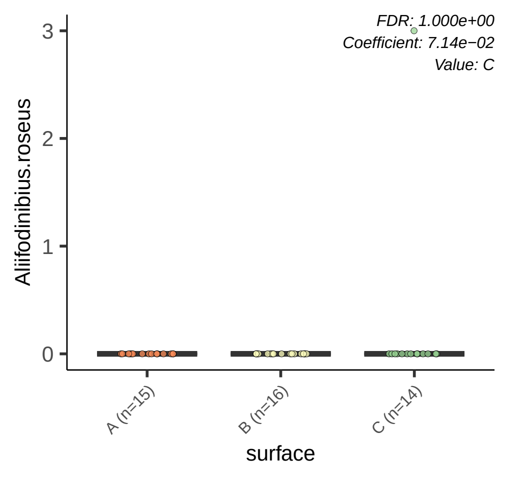

FDR: 1.000e+00
Coefficient: 7.14e−02
Value: C
Aliifodinibius.roseus
3
2
1
0
A (n=15)
B (n=16)
C (n=14)
surface

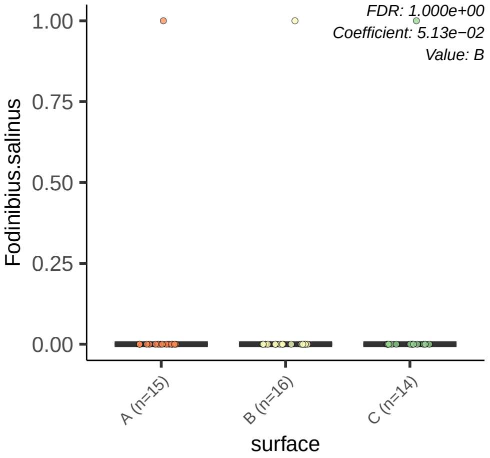
FDR: 1.000e+00
Coefficient: 5.13e−02
Value: B
Fodinibius.salinus
1.00
0.75
0.50
[truncated: 663,614 more chars]
